# Periodic oscillations of atmospheric electric field during snowfall in the Tokyo metropolitan area

**DOI:** 10.1038/s41598-021-82091-w

**Published:** 2021-01-26

**Authors:** Hiroyo Ohya, Kota Nakamori, Toshiaki Takano, Masashi Kamogawa, Tomoyuki Suzuki, Kazuomi Morotomi

**Affiliations:** 1 Graduate School of Engineering, Chiba University, Chiba, 263-8522 Japan; 2 Global Center for Asian and Regional Research, University of Shizuoka, Shizuoka, 420-0839 Japan; 3 Department of Physics, Tokyo Gakugei University, Koganei, 184-8501 Japan; 4 Water Infrastructure Department, Japan Radio Co., Ltd., Fujimino, 356-8580 Japan

**Keywords:** Environmental sciences, Natural hazards

## Abstract

We report the first observations of periodic oscillations of an atmospheric electric field simultaneously derived by field mills at four observation sites at a distance of 50–65 km in metropolitan Tokyo. Oscillations were detected during a snowfall event on 23–24 November, 2016. The main period of the oscillations of the atmospheric electric field at CHB was 78 min, which was similar to those at other sites. The periods of 39.0, 54.6, and 78.0 min observed at Chiba (CHB) were similar to those observed by W-band cloud radar (FALCON-I) reflectivity below a height of 5 km. High coherence of the 78-min period between the atmospheric electric field at CHB and the X-band phased array weather radar reflectivity suggest that the periodic oscillations of the atmospheric electric field during snowfall were caused by vertically convective cells in snow clouds with a radius of 60 km centered on CHB.

## Introduction

An atmospheric electric field or potential gradient oscillation observed at ground level with long periods (60 ~ 80 min) during sleet and snowfall events without wind turbulence was first reported^1^. The pattern of the oscillation was clear sinusoidal waves, and the oscillation was observed at Kew Observatory, England, on 26 February, 1946. Traditionally, the atmospheric electric field is defined as positive (negative) when it is downward (upward) from the ionosphere to the Earth surface. During periodic oscillations of the atmospheric electric field during snowfall events, when the atmospheric electric field is positive or negative, the polarity of snowfall particles measured at the ground is negative or positive, respectively. The relationship between the two polarities of the atmospheric electric field and rain or snow particles at the ground is called “mirror image relation” or “inverse relation”^2–4^. Previous studies compared the atmospheric electric field with the polarity and number density of snow/rain particles on the ground using thermionic electrometers^2^. Most periodic oscillations of the atmospheric electric field occurred during snowfall from nimbostratus clouds^5^, which are typical rain or snow clouds that are widely spread and close to the ground. A wave pattern of the atmospheric electric field, with periods of 10–30 min, during snowfall in Hokkaido, Japan, was revealed from cumulonimbus clouds^6^. Oscillations of the atmospheric electric field with periods of about 20 min and inverse relation were also observed in Hokkaido, Japan, during snowfall on 16 January, 1978^7^. In seven out of eight oscillation events during snowfall in Japan, graupel particles were charged positively, and the surface of the graupel particles was dry. A number of small snow particles were present with the graupel particles. The mirror image relation was not always observed at heights of 100–200 m, although it was always observed at 0–22 m (where 22 m corresponded to the building rooftop), based on balloon observations^8^. At a cloud height of 100–200 m, the charge strength and polarity of snow particles were weak and positive, respectively, although they were intense and negative near the ground, suggesting that snow particles became negatively charged while falling to the ground. The previous studies proposed a mechanism in which the atmospheric electric field at ground level was affected by ions due to corona discharges^8^. These studies revealed a relationship between oscillations of the atmospheric electric field and the polarity of snow/rain particles, based on observations obtained with Bendorf and thermionic electrometers. However, the studies did not investigate the internal structure of snow clouds during the oscillations, nor the variation in the electric field. Moreover, the following questions remain: why did the atmospheric electric field oscillate during snowfall, and what underlies the long period of the oscillations, of several tens of minutes?

End-of-storm oscillations (EOSO) below thunderstorms in the dissipating stage are associated with long-period (several tens of minutes) oscillations of the atmospheric electric field^9,10^. This study investigated the relationship between oscillations in the atmospheric electric field and snow clouds. In this study, we investigate periodic oscillations of the atmospheric electric field during a snowfall event on 23–24 November, 2016, using a field mill and 95-GHz cloud radar, FMCW radar for cloud observations (FALCON-I). FALCON-I is a cloud radar with high spatial and sampling resolution developed at Chiba University, Japan^11^. This study first reports the relationship between long oscillations of the atmospheric electric field and temporal variation in the internal microstructure of snow clouds using FALCON-I. The periodic oscillations of the atmospheric electric field during this event did not apply to the EOSO, because thunderstorms did not occur.

## Results and discussion

Snowfall occurred due to an intense cold air mass and low atmospheric pressure in the Tokyo metropolitan area from 15:00 UT on 23 November to 07:00 UT on 24 November, 2016. The accumulation of snow in November was the first observation ever in both Tokyo and Chiba (CHB), and the snowfall was the first in Tokyo in 54 years. On 24 November, 2016, the maximum accumulation of wet snow at CHB was 2 cm according to the Japan Meteorology Agency (JMA).

**Figure 1:**
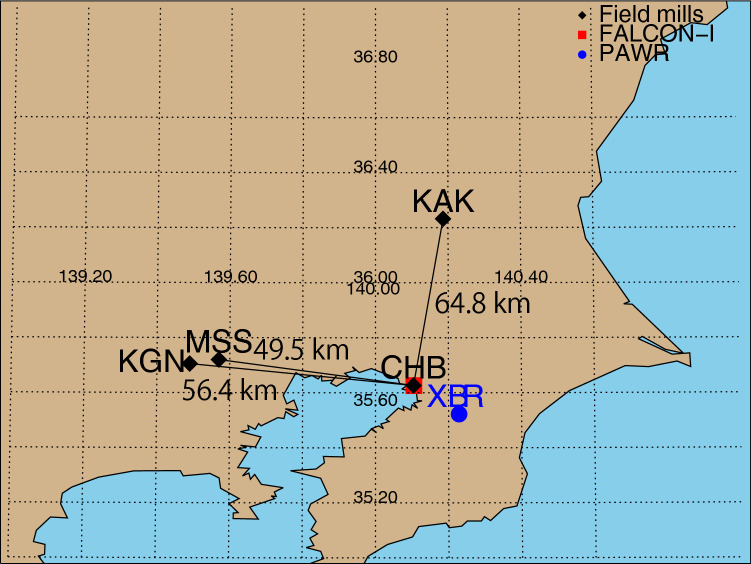
Location of field mills at Chiba University (CHB, 35.63° N, 140.10° E), Kakioka (KAK, 36.23° N, 140.19° E), Koganei (KGN, 35.71° N, 139.49° E), and Musashino (MSS, 35.72° N, 139.57° E); the FALCON-I at CHB; and a PAWR operated by Japan Radio at Chiba (XBR, 35.52° N, 140.23° E), Japan. This map was drawn using the Interactive Data Language (IDL) ver. 8.3 (https://www.harrisgeospatial.co.jp/).

The locations of field mills at Chiba University (CHB, 35.63° N, 140.10° E), Kakioka, Ibaraki (KAK, 36.23° N, 140.19° E), Koganei, Tokyo (KGN, 35.71° N, 139.49° E), and Musashino, Tokyo (MSS, 35.72° N, 139.57° E); the FALCON-I at CHB; and a X-band phased array weather radar (PAWR) operated by Japan Radio Co., Ltd. (XBR, 35.52° N, 140.23° E), Japan are shown (Fig. 1). The distances between CHB and KAK, KGN, and MSS are 64.8, 56.4, and 49.5 km, respectively.

**Figure 2:**
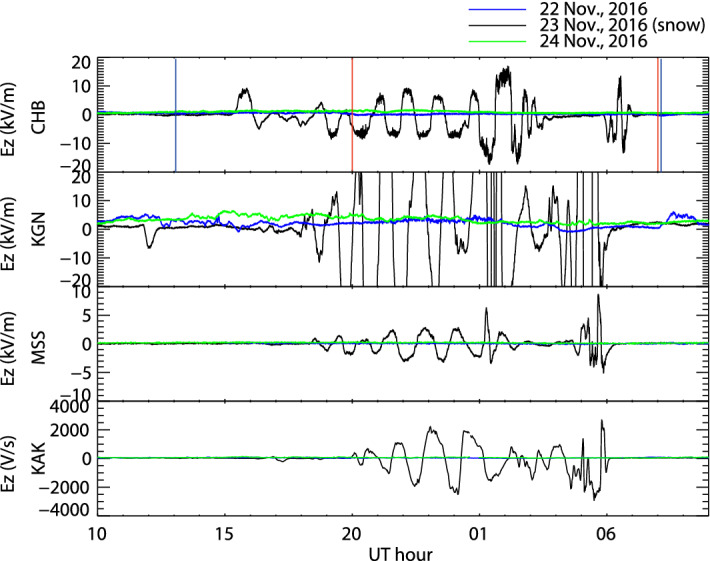
Variation in the atmospheric electric field at CHB, KGN, MSS, and KAK during 23–24 November, 2016. The blue and green lines indicate the atmospheric electric field on the reference days before and after the snow day. On the CHB panel, the vertical blue and red lines indicate the onset of precipitation and time interval of snowfall, respectively, based on weather data from the Japan Meteorological Agency.

The variation in the atmospheric electric fields at CHB, KGN, MSS, and KAK during 23–24 November, 2016 is shown (Fig. 2). The blue and green lines indicate the atmospheric electric field on the reference days before and after the snowfall. On the CHB panel, the vertical blue and red lines indicate the onset of precipitation and the time interval of snowfall at CHB (35.60° N, 140.10° E, distance from CHB: 2.8 km), respectively, based on weather data operated by the JMA^12^. At the other stations, observation data for precipitation and snowfall by JMA were scant. However, snow was observed across a wide area of Tokyo, CHB, and Ibaraki using cameras smartphones in a citizen science project, including all study sites, at 15:00 UT on 23 November^13^. Only KAK data were calibrated; the data at other three stations are relative values. At all sites, the atmospheric electric fields oscillated greatly in the positive and negative direction during snowfall compared with the reference days. The KGN data of the snow day were saturated. The output voltage range of the field mill is − 20 to + 20 kV/m. The sensitivity of the field mill was set to detect very small variations in the atmospheric electric fields at KGN, which explains why the output voltage at KGN exceeded the limit of the field mill during the snowfall event. We could not anticipate the large changes in atmospheric electric field during the snowfall event because these were the first observations of oscillations. The periodic oscillations of the atmospheric electric field started at CHB at 15:11 UT on 23 November. Next, the atmospheric electric field at KGN began to vary at 16:17 UT on 23 November, and oscillated from 17:59 UT. The atmospheric electric field at MSS began to oscillate at 18:12 UT on 23 November. Finally, there were oscillations at KAK at 19:55 UT on 23 November, although the variation in the atmospheric electric field started at 16:18 UT. These are the first observations of similarly periodic oscillations of the atmospheric electric field at four observation sites within a distance of 50–65 km. The oscillations among the four sites were not in phase.

**Figure 3:**
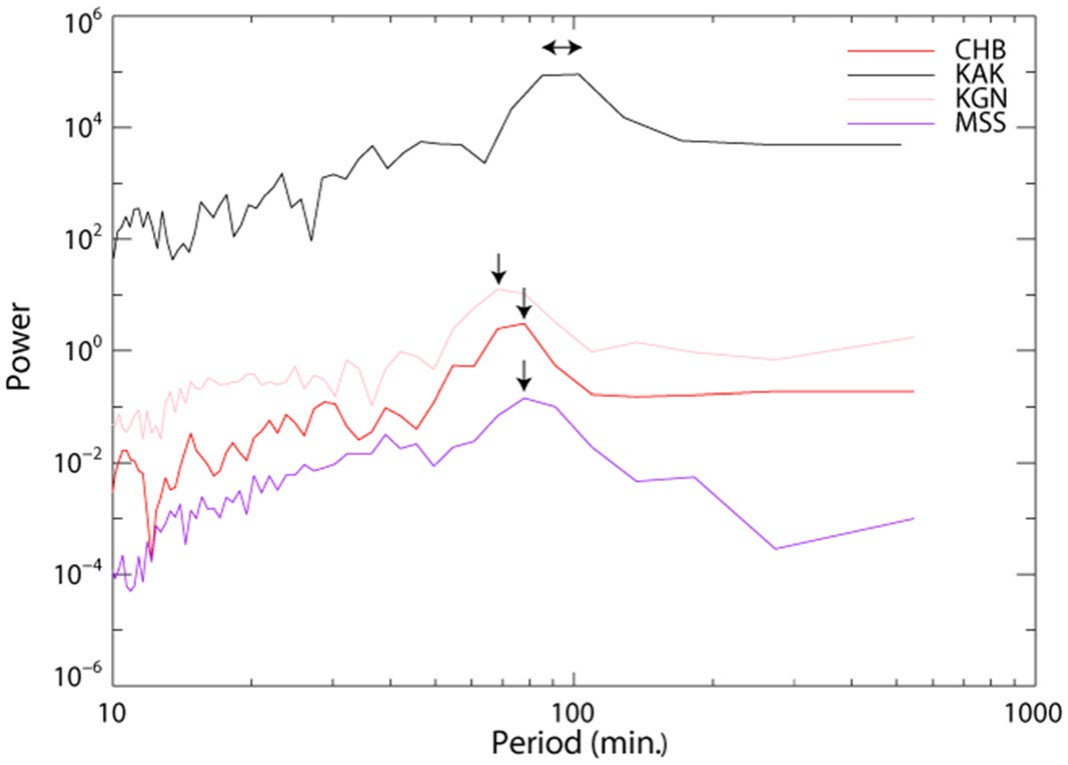
A periodogram of the oscillations of the atmospheric electric fields at the four sites during snowfall. The arrows indicate the maxima of the power spectra for each site.

A periodogram of the oscillations of the atmospheric electric fields based on fast Fourier transform (FFT) at the four sites during snowfall is shown (Fig. 3). The arrows indicate the maxima of the power spectra for each site. The maxima of the power spectra of the atmospheric electric fields at CHB, KGN, MSS, and KAK were observed at 78.0, 68.3, 78.0, and 85.3–102.4 min, respectively. The periods of the oscillations at CHB, KGN, and MSS were 70–80 min, although the periods at KAK were slightly longer than those at other sites. At CHB, the sub-periods were 39.0 and 54.6 min.

**Figure 4:**
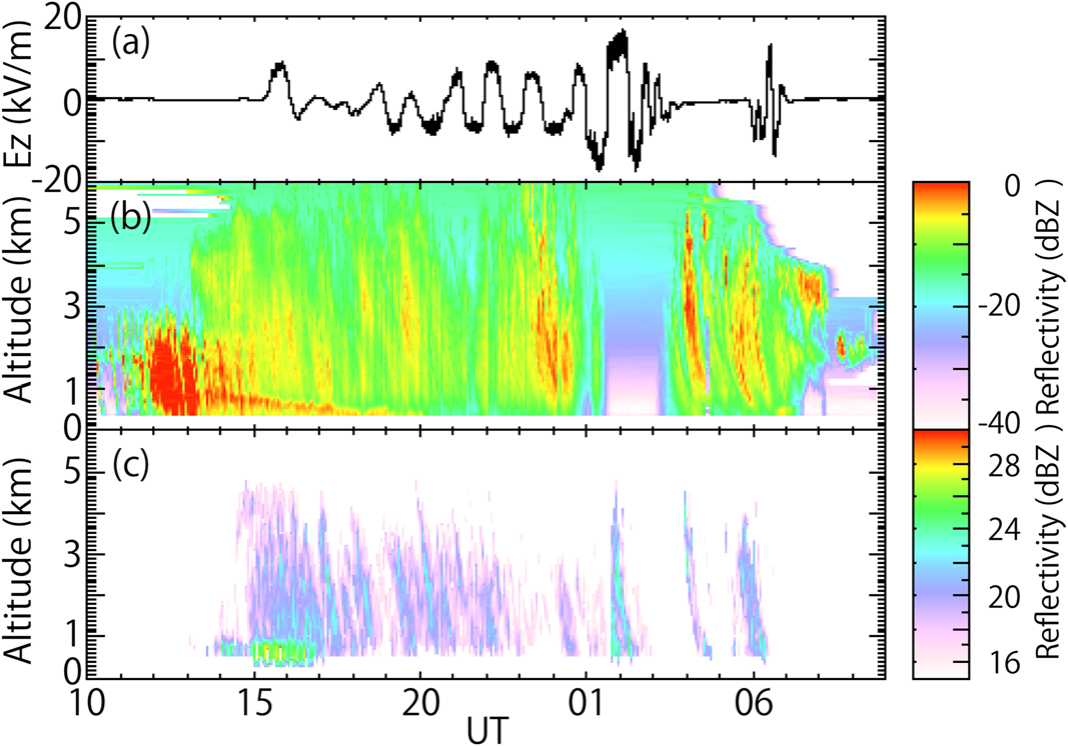
(**a**) Variation in the atmospheric electric field at CHB, (**b**) FALCON-I radar reflectivity, and (**c**) PAWR reflectivity.

Variation in the atmospheric electric field at CHB, FALCON-I radar reflectivity, and PAWR reflectivity is shown (Fig. 4). Many vertical curves of intense radar reflectivity were observed from heights of 5 km down to 0.3 km, in particular at 23:00–01:00 UT, 04:00–05:00 UT, and 05:00–07:00 UT (Fig. 4b). This suggested that the diameter of cloud particles and the number or density of the cloud particles was large, because the radar reflectivity theoretically depends on the sixth power of diameter and number/density of cloud and snow particles^14^. The intense period also started at a height of 800 m at 13:00 UT, and gradually fell to a height of 300 m at 18:00 UT on 23 November, 2016, corresponding to a bright band^15^. The bright band is a radar signature of the layer in which snow transitions to rain. Such detailed internal structures of the snow cloud could be seen by the FALCON-I (Fig. 4b), although there was no clear one-to-one correspondence between the atmospheric electric field and radar reflectivity. The PAWR reflectivity was similar to the FALCON-I reflectivity during the snowfall period, in particular with respect to the intense periods of radar reflectivity below a height of 5 km at 04:00–05:00 UT and 05:00–07:00 UT on 23 November (Fig. 4c). However, at 01:30–03:20 UT, FALCON-I did not observe snow clouds or snow particles (Fig. 4b). The FALCON-I beam might have been attenuated by melted water pooling on the roof of the radar container. Around 03:20 UT (12:20 LT, about noon), the water on the roof ran off naturally. Similar blackout of radar reflectivity due to melted water on the roofs of other weather radars during other snowfall events has occurred^16^. At 23:00–00:30 UT, no intense periods of radar reflectivity were observed by the PAWR, although there were intense periods of radar reflectivity observed by FALCON-I. This could be caused by differences in transmission frequency (95 GHz and 9.4 GHz for FALCON-I and PAWR, respectively). Another factor may be differences in location among the snow clouds. FALCON-I monitors clouds overhead with a narrow beam (0.18-degree full width at half maximum, FWHM)^11^, which is about ~ 100 m from the location of the PAWR data. The bright band was observed at heights of 0.5–1.0 km between 15:00 UT and 17:00 UT, which was consistent with the FALCON-I reflectivity (Fig. 4c). The spatial resolution of the PAWR is lower than that of the FALCON-I. The polarity of the intense radar reflectivity might be positive, because it was reported that larger snowflakes have positive charges based on microscopic photography of snow crystals and atmospheric electric field observations conducted in Hokkaido, Japan^17^.

**Figure 5:**
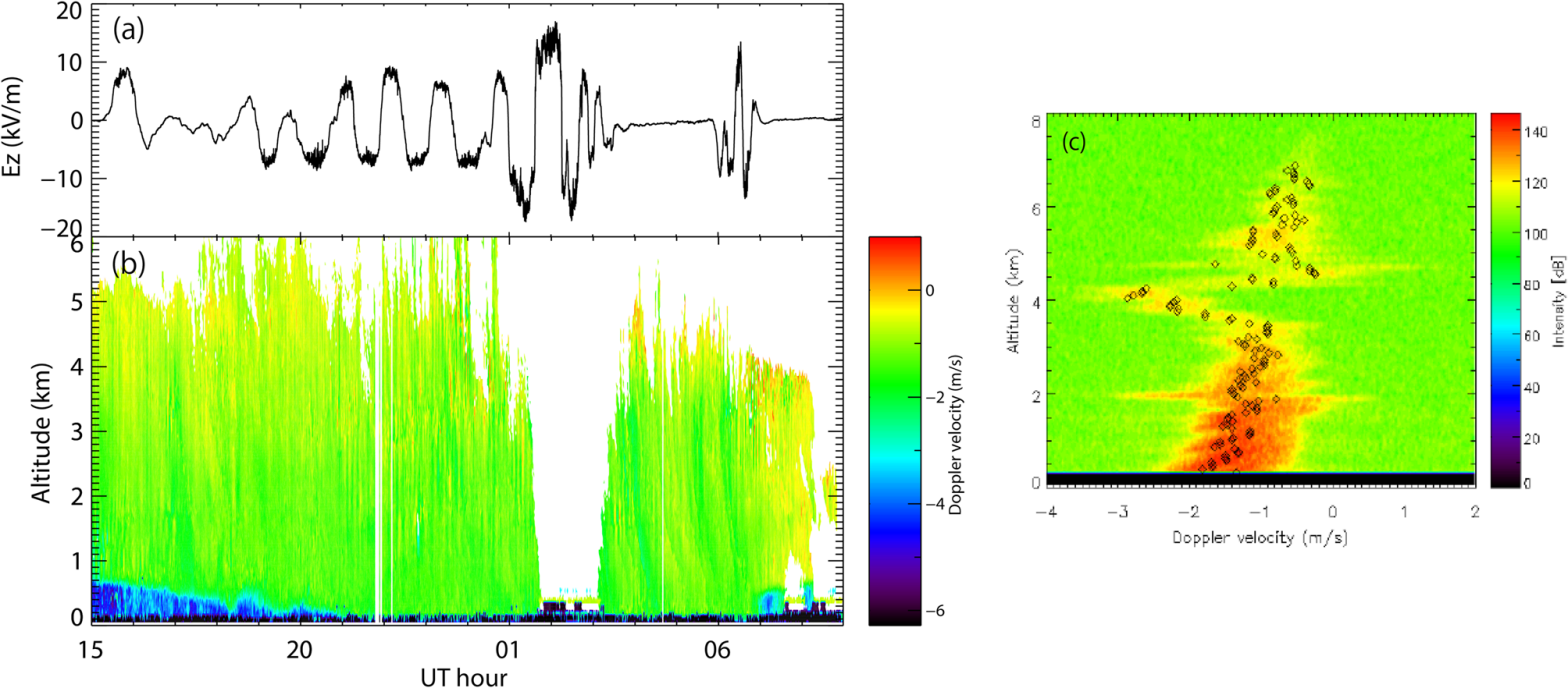
(**a**) Variation in the atmospheric electric field, (**b**) vertical Doppler velocity for each height observed by FALCON-I, and (**c**) typical radar Doppler map by FALCON-I at 23:00 UT on 23 December, 2016, during the snowfall event. The direction of positive (negative) Doppler velocity corresponds to vertical upward (downward) movement. The diamonds in (**c**) indicate the maximum intensity for each height.

Variation in the atmospheric electric field, vertical Doppler velocity for each height observed by FALCON-I, and a typical radar Doppler profile map at 23:00 UT on 23 December, 2016, during the snowfall event is shown (Fig. 5). The direction of positive (negative) Doppler velocity corresponds to vertical upward (downward) movement. The diamonds in Fig. 5c indicate the maximum intensity for each height. Snow clouds were seen in the Doppler velocity range from − 4.0 to 1.0 m/s at heights of 0.3–7.5 km (Fig. 5c). The cloud base height may have been less than 0.3 km; however, FALCON-I data below 0.3 km are unreliable, because parallax correction of radar reflectivity is difficult at heights of less than 0.3 km. Large downward velocities up to − 4 m/s were seen under the bright band at 700 m height at 15:00 UT, which corresponded to rain drops. Changes in the Doppler velocity at heights of 4–5 km might have been a stable layer base height equal to the upper limit of the convective mixed layer^15^. The potential temperature is consistent on the vertical axis due to strong convective mixture of the atmosphere below the stable layer base height. The Doppler velocities in Fig. 5b correspond to those with maximum radar intensity (the diamonds) for each height. The bright band was observed at a height of 700 m at 15:00 UT, and gradually fell 300 m at 19:00 UT on 23 November, 2016 (Fig. 5b), which corresponded to the intense period of radar reflectivity in Fig. 4b. After 19:00 UT, the bright band disappeared, indicating that raindrops were not being produced. Both snow cloud and snow particles exhibited downward movement below 4 km. During the snowfall events, wind velocity at ground level was 2.5–4.4 m/s at CHB based on the JMA weather data^12^, indicating that a blizzard or drifting/blowing snow (> 10 m/s) did not occur due to the slow speed of the wet snow, although the snow particles might move horizontally.

**Figure 6:**
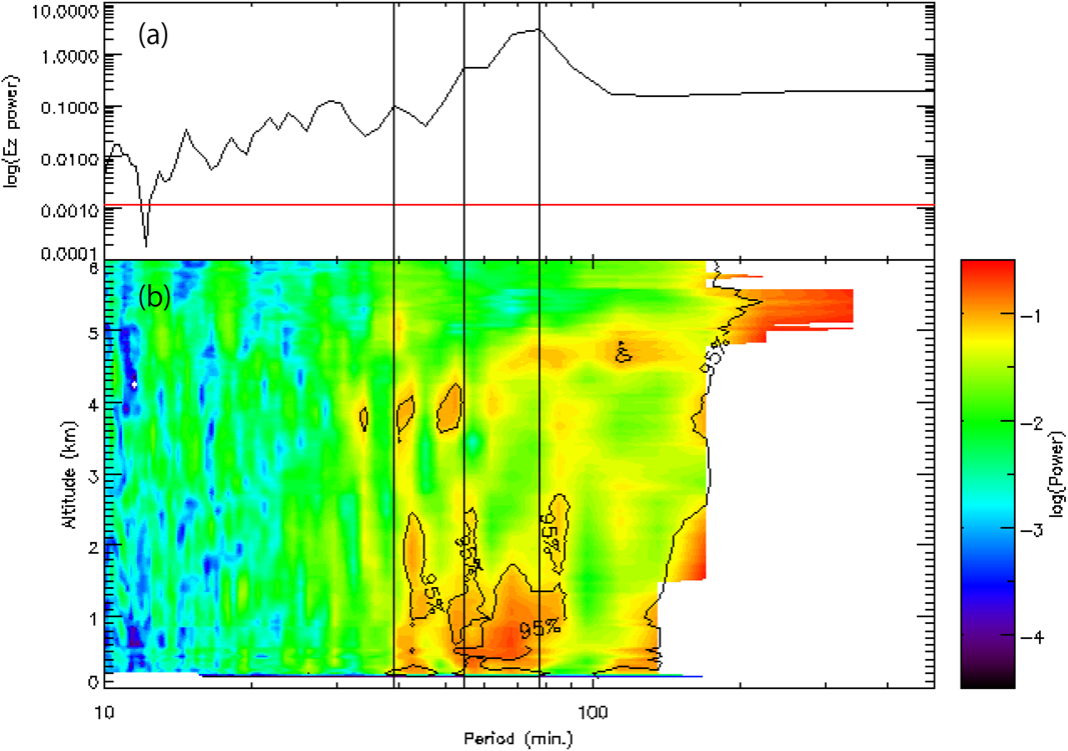
(**a**) Periodogram of the atmospheric electric field at CHB, and (**b**) period of the FALCON-I radar reflectivity for each height by the FFT. The red line in (**a**) indicates the 95% significance threshold. The three vertical black lines indicate the periods of 39.0, 54.6, and 78.0 min of the atmospheric electric field.

A periodogram of the atmospheric electric field at CHB, and the period of the FALCON-I radar reflectivity for each height based on FFT, are shown (Fig. 6). The main period of the atmospheric electric field was 78.0, and the subperiods were 39.0 and 54.6 min, respectively. In Fig. 6b, periods of intense radar reflectivity can be seen at 44, 57, and 85 min below a height of 2.5 km, and 42, 50, and 78 min at heights of 3.5–4.6 km. The main and sub-periods of atmospheric electric fields were similar to those of FALCON-I radar reflectivity below a height of 5 km.

**Figure 7:**
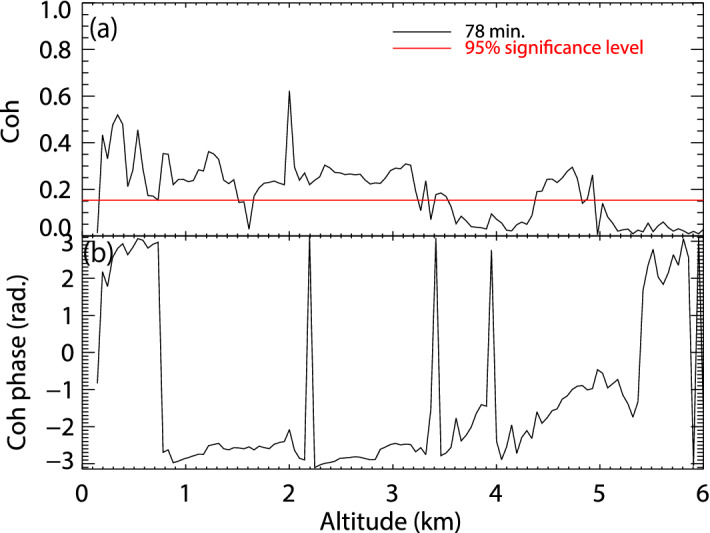
(**a**) Coherence of the main period (78.0 min) between the atmospheric electric fields at CHB and radar reflectivity of the FALCON-I for each height, and (**b**) the phase difference.

The coherence between the atmospheric electric fields at CHB and KAK peaked at 0.82 after 73.1 min. The coherence calculation is described in the Methods. The maximum coherence values of the atmospheric electric fields for CHB-KGN, CHB-MSN, KAK-KGN, KAK-MSN, and MSN-KGN were calculated to be 0.62 (period 51.2 min), 0.45 (113.8 min), 0.58 (53.9 min), 0.52 (64.0 min), and 0.99 (78.8 min), respectively. These coherence values were all significant at the 95% confidence level. The coherence values were particularly high at 73.1 and 78.8 min. Only the coherence of the 78-min period between the atmospheric electric field at CHB and FALCON-I radar reflectivity was significantly large below a height of 3 km (Fig. 7a). The phase difference was close to an anti-phase (− π) between the heights of 0.8 and 3.3 km, except for at 2.2 km (+ π) (Fig. 7b). If the intense radar reflectivity corresponds to large cloud/snow particles with positive charges, as seen in Fig. 4, the anti-phase is consistent with the mirror image relation between the atmospheric electric field and polarity of snow particles on the ground. The novelty of this study is that it found that the source of the oscillation in the atmospheric electric field was the internal structure of the snow clouds.

**Figure 8:**
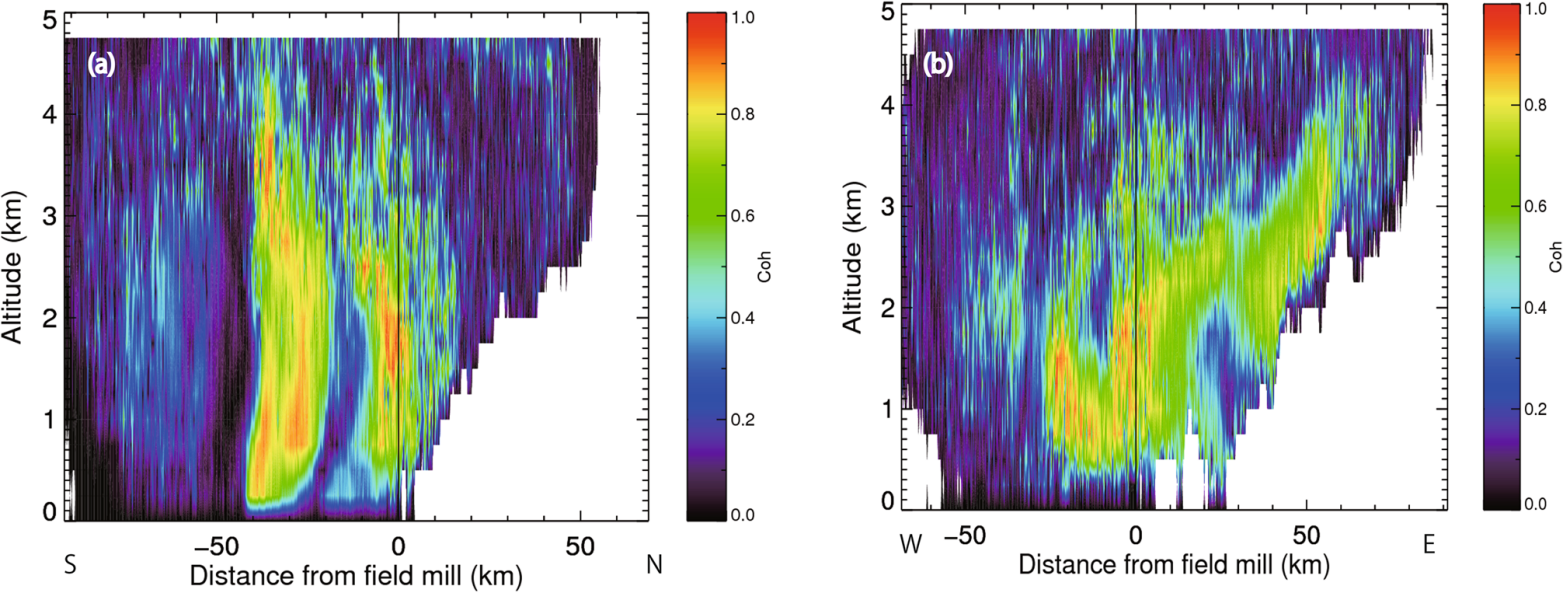
(**a**) Coherence of the main period between the atmospheric electric fields at CHB and radar reflectivity of the PAWR in the North–South direction, and (**b**) the same format but in the East–West direction centered at CHB.

Coherence of only the 78-min period between the atmospheric electric field at CHB and radar reflectivity of the PAWR in the (a) North–South and (b) East–West directions, centered at CHB (Fig. 8) (see the coherence calculation in the Methods). The high coherence for the 78-min period was within a radius of 60 km and height of 4 km (Fig. 8a,b), suggesting that the atmospheric electric field near the ground was affected by snow clouds within a 60-km radius. The similar periodic oscillations of the atmospheric electric field at the four sites were presumably caused by the same snow clouds.

This study showed that the periodic oscillations with a 78-min period in the atmospheric electric field during snowfall were strongly associated with vertical structures in the lower snow clouds within a radius of 60 km from the field mill based on FALCON-I and PAWR observations. From 23:00 UT on 23 November to 03:00 UT on 24 November, 2016, snowflakes with dendrite crystals without cloud particles, fan-like crystals snowflakes with needle crystals and dense cloud particles, and soft hail were observed over a wide area in more than 5,000 images of snow crystals in Tokyo and CHB^13^. These various kinds of snow crystal indicate that the snow clouds were convective^13^, which is consistent with our results. This study demonstrated convective cells in snow clouds based on FALCON-I and PAWR radar reflectivity data.

## Methods

### Observations

The field mill of Boltek Corporation, EFM100, and FALCON-I are located at a distance of 62.7 m. The distance between FALCON-I at CHB and PAWR at XBR is 16.2 km.

The atmospheric electric field has been observed with an EFM-100 field mill (Boltek) on the roof of a building on the campus of Chiba University, Japan, since 1 June, 2016. The dynamic voltage range is ± 20 kV/m with a 0.5-s sampling time. The cut-off frequency of the low-pass filter is 11 Hz. Field mills made by Boltek Corporation were also used at KGN and MSS. We discuss the variation in the atmospheric electric field, because the three field mills at CHB, KGN, and MSS have not been calibrated. At KAK, the atmospheric electric field has been observed using a water dropper inside the observatory^18^. The atmospheric electric field values inside the room were calibrated by multiplying by the calibration coefficient (2.17), which was determined by comparing the values observed inside the room to those on the ground outside, and used as the reference for two observation systems.

The 95-GHz (W-band) cloud radar, FALCON-I, was originally developed by our research group at Chiba University, Japan. FALCON-I is shown (Fig. 8); it consists of two 1-m-diameter of, a transmitter and a receiver. FALCON-I has high sensitivity (− 34 dBZ at a height of 5 km) and high spatial and sampling (10 s) resolution. The horizontal and vertical spatial resolutions are 1 m at a height of 300 m (47 m at a height of 15 km) and 48.8 m, respectively. The FALCON-I can observe the reflectivity of cloud/rain and Doppler velocity only in the vertical direction at heights of up to 15 km. As a portable radar, FALCON-I can be transported on a truck to observe cloud/rain at different locations. It is useful for simultaneous observations between satellites and ground-based instruments and observations of local weather phenomena.

We also used a PAWR developed by Japan Radio, for which the transmitted frequency is 9.4 GHz. The PAWR can observe the radar reflectivity of cloud/rain particles and radial Doppler velocity every 30 s with a grid spacing of 75 m and a radius of 80 km at heights of up to 15 km. The PAWR can cover the FALCON-I location, since the distance between them is 16.2 km. The atmospheric electric field, FALCON-I, and PAWR data are provided in the Supplementary Information.

### Coherence calculation

Coherence, *coh*(*ω*), is defined by the following formula:

1 $$coh\left( \omega \right) = \frac{{\left| {S_{xy} \left( \omega \right)} \right|^{2} }}{{S_{xx} \left( \omega \right)S_{yy} \left( \omega \right)}} = \frac{{K_{xy}^{2} \left( \omega \right) + Q_{xy}^{2} \left( \omega \right)}}{{S_{xx} \left( \omega \right)S_{yy} \left( \omega \right)}},$$

where *ω* is the angular frequency; *S*_*xy*_(*ω*) is the cross-spectra of two discrete time signals; *S*_*xx*_(*ω*) and *S*_*yy*_(*ω*) are the spectra of *x*(*t*) and *y*(*t*), respectively; and *K*_*xy*_(*ω*) and *Q*_*xy*_(*ω*) are co- and quad-spectra, respectively^19^. Here, *x*(*t*) and *y*(*t*) denote the variation in the atmospheric electric field between two observation sites, or the variation in the atmospheric electric field and radar reflectivity for each height, respectively.

K_xy_ (*ω*) and Q_xy_ (*ω*) are the real and imaginary parts of S_xy_ (*ω*), respectively, and are calculated as follows:

2 $$S_{xy} \left( \omega \right) = K_{xy} \left( \omega \right) - iQ_{xy} \left( \omega \right).$$

The phase difference, $\theta_{xy} \left( \omega \right)$, is defined by the following equation:

3 $$\theta_{xy} \left( \omega \right) = tan^{ - 1} \left( {\frac{{Q_{xy} \left( \omega \right)}}{{K_{xy} \left( \omega \right)}}} \right).$$

## Supplementary Information

Supplementary Information 1.

Supplementary Information 2.

Supplementary Information 3.

Supplementary Information 4.

Supplementary Information 5.

Supplementary Information 6.

Supplementary Information 7.

Supplementary Information 8.

## Data Availability

Observational data for the atmospheric electric field collected from Chiba (H. O.), Koganei (M. K. and T. S.), and Musashino (M. K. and T. S.), FALCON-I (T. T.), and X-band phased array weather radar (K. M.) data, are provided in the Supplementary Information.

## References

[1] Simpson GC (1948). Atmospheric electricity during disturbed weather. Terr. Magn. Atmos. Electr..

[2] Orikasa K (1961). On the disturbance of the surface electric field caused by snow fall. J. Jpn. Soc. Snow Ice..

[3] Chalmers JA (1965). The relation between precipitation current and potential gradient. J. Atmos. Terr. Phys..

[4] Magono C, Orikasa K (1966). On the disturbance of surface electric field caused by snowfall. J. Meteorol. Soc. Jpn..

[5] Whitlock WS, Chalmers JA (1956). Short-period variations in the atmospheric electric potential gradient. Q. J. R. Meteorol. Soc..

[6] Orikasa K (1962). On the disturbance of the surface electric field caused by rain and snowfall. Geophys. Bull. Hokkaido Univ..

[7] Kikuchi K, Inatsu K (1979). Electric polarity of graupel particles. J. Fac. Sci. Hokkaido Univ. (Geophys.).

[8] Asuma Y, Kikuchi K, Taniguchi T, Fujii S (1988). Vertical structures of the atmospheric electrical potential gradient and the behavior of the precipitation charges during snowfalls near the ground surface. J. Meteorol. Soc. Jpn..

[9] Williams ER, Zhang R (1996). Density of rime in laboratory simulations of thunderstorm microphysics and electrification. J. Geophys. Res..

[10] Pawar SD, Kamra AK (2007). End-of-storm oscillation in tropical air mass thunderstorms. J. Geophys. Res..

[11] Takano T (2010). Development and performance of millimeter-wave cloud profiling radar at 95 GHz: Sensitivity and spatial resolution. Electron. Commun. Jpn..

[12] Japan Meteorological Agency, Weather datasets. figshare https://www.jma.go.jp/jma/indexe.html (2016).

[13] Araki K (2018). Ultra-dense ground observation of snow crystals by citizen science “#KantoSnowCrystal project”. Seppyo.

[14] Atlas D (2015). Radar in Meteorology: Battan Memorial and 40th Anniversary Radar Meteorology Conference.

[15] Yamagishi Y (1980). Characteristics of stable layer over the Sea of Japan in winter. Tenki.

[16] Suitz T (1981). The measurement of rain attenuation of waves by FM-CW radar method using large scale rainfall facilities. Rev. Radio Res. Lab..

[17] Kikuchi K (1975). Atmospheric electrical properties of snow clouds with precipitation. J. Meteorol. Soc. Jpn..

[18] Nagamachi S, Toya T, Yoshikake Y (2013). Influence of environmental change near the atmospheric electric field observation hut at Kakioka—numerical calculations to estimate the effect of a nearby bamboo grove. Tech. Rep. Kakioka Magn. Observ..

[19] Hino M (1977). Spectral Analysis.

