# Supplementary material for: Periodic oscillations of atmospheric electric field during snowfall in the Tokyo metropolitan area
Source: Sci Rep. 2021 Jan 26;11:2280. doi: 10.1038/s41598-021-82091-w (PMC7838252; doi:10.1038/s41598-021-82091-w)
Supplement: Supplementary file 6 — Supplementary Information 6. [file 41598_2021_82091_MOESM6_ESM.docx]

*Scientific Reports*

Supplementary Information for

Periodic Oscillations of Atmospheric Electric Field during Snowfall in the Tokyo Metropolitan Area

Hiroyo Ohya^1, *^, Kota Nakamori^1^, Toshiaki Takano^1^, Masashi Kamogawa^2^, Tomoyuki Suzuki^3^, and Kazuomi Morotomi^4^

^1^ Chiba University, Graduate School of Engineering, Chiba, 263-8522, Japan

^2^ University of Shizuoka, Global Center for Asian and Regional Research, Shizuoka, 420-0839, Japan

^3^ Tokyo Gakugei University, Department of Physics, Koganei, 184-8501, Japan

^4^ Japan Radio Co., Ltd., Water Infrastructure Department, Fujimino, 356-8580, Japan

**Contents**

Supplementary Information contains three datasets of atmospheric electric field at CHB, KGN, and MSS, three datasets of FALCON-I, and one dataset of PAWR.

CHB.pdf

There are two columns in the data file.

1. UT (s) from 00:00 UT on 23 November, 2016
2. Atmospheric electric field at CHB (kV/m)

KGN.pdf

There are two columns in the data file.

1. UT (s) from 00:00 UT on 23 November, 2016
2. Atmospheric electric field at KGN (kV/m)

MSS.pdf

There are two columns in the data file.

1. UT (s) from 00:00 UT on 23 November, 2016
2. Atmospheric electric field at MSS (kV/m)

FALCONreflectivity.pdf

There are three columns in the data file.

1. UT (s) from 00:00 UT on 23 November, 2016
2. Altitude (km)
3. FALCON-I reflectivity at CHB (dBz)

FALCONDoppelervFig5b.pdf

There are three columns in the data file.

1. UT (hour) from 15:00 UT on 23 November, 2016
2. Altitude (km)
3. FALCON-I Doppler velocity at CHB (m/s) (positive: vertical, negative: vertical downward)

FALCONDoppelervFig5c.pdf

There are three columns in the data file.

1. FALCON-I Doppler velocity at CHB (m/s) (positive: vertical, negative: vertical downward)
2. Altitude (km)
3. FALCON-I intensity at CHB (dB)

PAWRreflectivity.pdf

There are three columns in the data file.

1. UT (s) from 10:00 UT on 23 November, 2016
2. Altitude (km)
3. PAWR reflectivity at CHB (dBz)
