# Supplementary material for: Periodic oscillations of atmospheric electric field during snowfall in the Tokyo metropolitan area
Source: Sci Rep. 2021 Jan 26;11:2280. doi: 10.1038/s41598-021-82091-w (PMC7838252; doi:10.1038/s41598-021-82091-w)
Supplement: Supplementary file 1 — Supplementary Information 1. [file 41598_2021_82091_MOESM1_ESM.pdf]

0.0000000	0.2450000
1.0000000	0.2500000
2.0000000	0.2500000
3.0000000	0.2600000
4.0000000	0.2500000
5.0000000	0.2550000
6.0000000	0.2550000
7.0000000	0.2650000
8.0000000	0.2533333
9.0000000	0.2450000
10.0000000	0.2550000
11.0000000	0.2600000
12.0000000	0.2600000
13.0000000	0.2600000
14.0000000	0.2550000
15.0000000	0.2500000
16.0000000	0.2500000
17.0000000	0.2550000
18.0000000	0.2600000
19.0000000	0.2600000
20.0000000	0.2600000
21.0000000	0.2600000
22.0000000	0.2600000
23.0000000	0.2700000
24.0000000	0.2500000
25.0000000	0.2700000
26.0000000	0.2450000
27.0000000	0.2550000
28.0000000	0.2550000
29.0000000	0.2600000
30.0000000	0.2600000
31.0000000	0.2550000
32.0000000	0.2600000
33.0000000	0.2600000
34.0000000	0.2650000
35.0000000	0.2600000
36.0000000	0.2550000
37.0000000	0.2550000
38.0000000	0.2550000
39.0000000	0.2666667
40.0000000	0.2650000
41.0000000	0.2700000
42.0000000	0.2550000
43.0000000	0.2650000
44.0000000	0.2550000
45.0000000	0.2650000
46.0000000	0.2650000
47.0000000	0.2650000
48.0000000	0.2700000
49.0000000	0.2600000
50.0000000	0.2600000
51.0000000	0.2600000
52.0000000	0.2650000
53.0000000	0.2700000
54.0000000	0.2700000
55.0000000	0.2600000
56.0000000	0.2700000

57.0000000	0.2650000
58.0000000	0.2750000
59.0000000	0.2650000
60.0000000	0.2650000
61.0000000	0.2650000
62.0000000	0.2600000
63.0000000	0.2650000
64.0000000	0.2650000
65.0000000	0.2650000
66.0000000	0.2700000
67.0000000	0.2700000
68.0000000	0.2600000
69.0000000	0.2666667
70.0000000	0.2650000
71.0000000	0.2650000
72.0000000	0.2600000
73.0000000	0.2600000
74.0000000	0.2600000
75.0000000	0.2500000
76.0000000	0.2550000
77.0000000	0.2550000
78.0000000	0.2600000
79.0000000	0.2600000
80.0000000	0.2600000
81.0000000	0.2550000
82.0000000	0.2450000
83.0000000	0.2550000
84.0000000	0.2550000
85.0000000	0.2700000
86.0000000	0.2600000
87.0000000	0.2600000
88.0000000	0.2500000
89.0000000	0.2700000
90.0000000	0.2700000
91.0000000	0.2600000
92.0000000	0.2650000
93.0000000	0.2550000
94.0000000	0.2700000
95.0000000	0.2650000
96.0000000	0.2800000
97.0000000	0.2700000
98.0000000	0.2700000
99.0000000	0.2666667
100.0000000	0.2650000
101.0000000	0.2750000
102.0000000	0.2650000
103.0000000	0.2700000
104.0000000	0.2750000
105.0000000	0.2700000
106.0000000	0.2750000
107.0000000	0.2700000
108.0000000	0.2700000
109.0000000	0.2750000
110.0000000	0.2700000
111.0000000	0.2750000
112.0000000	0.2800000
113.0000000	0.2700000

114.0000000	0.2850000
115.0000000	0.2800000
116.0000000	0.2800000
117.0000000	0.2850000
118.0000000	0.2750000
119.0000000	0.2750000
120.0000000	0.2750000
121.0000000	0.2850000
122.0000000	0.2850000
123.0000000	0.2800000
124.0000000	0.2800000
125.0000000	0.2800000
126.0000000	0.2800000
127.0000000	0.2900000
128.0000000	0.2800000
129.0000000	0.2833333
130.0000000	0.2750000
131.0000000	0.2800000
132.0000000	0.2950000
133.0000000	0.2750000
134.0000000	0.2850000
135.0000000	0.2700000
136.0000000	0.2750000
137.0000000	0.2750000
138.0000000	0.2800000
139.0000000	0.2850000
140.0000000	0.2800000
141.0000000	0.2800000
142.0000000	0.2800000
143.0000000	0.2850000
144.0000000	0.2750000
145.0000000	0.2750000
146.0000000	0.2800000
147.0000000	0.2800000
148.0000000	0.2800000
149.0000000	0.2700000
150.0000000	0.2700000
151.0000000	0.2700000
152.0000000	0.2750000
153.0000000	0.2750000
154.0000000	0.2700000
155.0000000	0.2700000
156.0000000	0.2600000
157.0000000	0.2650000
158.0000000	0.2600000
159.0000000	0.2650000
160.0000000	0.2600000
161.0000000	0.2650000
162.0000000	0.2550000
163.0000000	0.2450000
164.0000000	0.2700000
165.0000000	0.2500000
166.0000000	0.2500000
167.0000000	0.2550000
168.0000000	0.2500000
169.0000000	0.2600000
170.0000000	0.2600000

171.0000000	0.2450000
172.0000000	0.2500000
173.0000000	0.2450000
174.0000000	0.2400000
175.0000000	0.2450000
176.0000000	0.2400000
177.0000000	0.2400000
178.0000000	0.2400000
179.0000000	0.2550000
180.0000000	0.2400000
181.0000000	0.2400000
182.0000000	0.2450000
183.0000000	0.2400000
184.0000000	0.2400000
185.0000000	0.2450000
186.0000000	0.2450000
187.0000000	0.2450000
188.0000000	0.2500000
189.0000000	0.2450000
190.0000000	0.2500000
191.0000000	0.2400000
192.0000000	0.2350000
193.0000000	0.2550000
194.0000000	0.2550000
195.0000000	0.2450000
196.0000000	0.2500000
197.0000000	0.2550000
198.0000000	0.2500000
199.0000000	0.2600000
200.0000000	0.2550000
201.0000000	0.2500000
202.0000000	0.2600000
203.0000000	0.2650000
204.0000000	0.2500000
205.0000000	0.2600000
206.0000000	0.2650000
207.0000000	0.2650000
208.0000000	0.2700000
209.0000000	0.2550000
210.0000000	0.2650000
211.0000000	0.2750000
212.0000000	0.2750000
213.0000000	0.2750000
214.0000000	0.2600000
215.0000000	0.2700000
216.0000000	0.2650000
217.0000000	0.2750000
218.0000000	0.2800000
219.0000000	0.2750000
220.0000000	0.2700000
221.0000000	0.2700000
222.0000000	0.2700000
223.0000000	0.2700000
224.0000000	0.2700000
225.0000000	0.2750000
226.0000000	0.2700000
227.0000000	0.2750000

228.0000000	0.2700000
229.0000000	0.2700000
230.0000000	0.2650000
231.0000000	0.2750000
232.0000000	0.2700000
233.0000000	0.2700000
234.0000000	0.2800000
235.0000000	0.2700000
236.0000000	0.2750000
237.0000000	0.2650000
238.0000000	0.2700000
239.0000000	0.2800000
240.0000000	0.2650000
241.0000000	0.2800000
242.0000000	0.2650000
243.0000000	0.2800000
244.0000000	0.2750000
245.0000000	0.2750000
246.0000000	0.2750000
247.0000000	0.2750000
248.0000000	0.2800000
249.0000000	0.2800000
250.0000000	0.2733333
251.0000000	0.2900000
252.0000000	0.2800000
253.0000000	0.2750000
254.0000000	0.2700000
255.0000000	0.2850000
256.0000000	0.2850000
257.0000000	0.2750000
258.0000000	0.2950000
259.0000000	0.2850000
260.0000000	0.2950000
261.0000000	0.2900000
262.0000000	0.2850000
263.0000000	0.2950000
264.0000000	0.2900000
265.0000000	0.3000000
266.0000000	0.3000000
267.0000000	0.2950000
268.0000000	0.2950000
269.0000000	0.2900000
270.0000000	0.2900000
271.0000000	0.3050000
272.0000000	0.2900000
273.0000000	0.3050000
274.0000000	0.2950000
275.0000000	0.3100000
276.0000000	0.3000000
277.0000000	0.3000000
278.0000000	0.3000000
279.0000000	0.2950000
280.0000000	0.3000000
281.0000000	0.3000000
282.0000000	0.3050000
283.0000000	0.3000000
284.0000000	0.3050000

285.0000000	0.3050000
286.0000000	0.2950000
287.0000000	0.3050000
288.0000000	0.2950000
289.0000000	0.3000000
290.0000000	0.3050000
291.0000000	0.3050000
292.0000000	0.3100000
293.0000000	0.3050000
294.0000000	0.3000000
295.0000000	0.3000000
296.0000000	0.3050000
297.0000000	0.3100000
298.0000000	0.3050000
299.0000000	0.3000000
300.0000000	0.3050000
301.0000000	0.3000000
302.0000000	0.3150000
303.0000000	0.3050000
304.0000000	0.3150000
305.0000000	0.3000000
306.0000000	0.3000000
307.0000000	0.3100000
308.0000000	0.3100000
309.0000000	0.3150000
310.0000000	0.3066667
311.0000000	0.3150000
312.0000000	0.3100000
313.0000000	0.3150000
314.0000000	0.3150000
315.0000000	0.3100000
316.0000000	0.3100000
317.0000000	0.3100000
318.0000000	0.3150000
319.0000000	0.3100000
320.0000000	0.3150000
321.0000000	0.3100000
322.0000000	0.3150000
323.0000000	0.3150000
324.0000000	0.3100000
325.0000000	0.3100000
326.0000000	0.3100000
327.0000000	0.3100000
328.0000000	0.3150000
329.0000000	0.3100000
330.0000000	0.3100000
331.0000000	0.3100000
332.0000000	0.3150000
333.0000000	0.3100000
334.0000000	0.3100000
335.0000000	0.3150000
336.0000000	0.3050000
337.0000000	0.3100000
338.0000000	0.3100000
339.0000000	0.3000000
340.0000000	0.3000000
341.0000000	0.3100000

342.0000000	0.3100000
343.0000000	0.3050000
344.0000000	0.3050000
345.0000000	0.3000000
346.0000000	0.2950000
347.0000000	0.3000000
348.0000000	0.3000000
349.0000000	0.3000000
350.0000000	0.2900000
351.0000000	0.2950000
352.0000000	0.2850000
353.0000000	0.2900000
354.0000000	0.2800000
355.0000000	0.2850000
356.0000000	0.2850000
357.0000000	0.2850000
358.0000000	0.2800000
359.0000000	0.2750000
360.0000000	0.2750000
361.0000000	0.2750000
362.0000000	0.2800000
363.0000000	0.2800000
364.0000000	0.2800000
365.0000000	0.2800000
366.0000000	0.2750000
367.0000000	0.2850000
368.0000000	0.2850000
369.0000000	0.2750000
370.0000000	0.2800000
371.0000000	0.2600000
372.0000000	0.2700000
373.0000000	0.2750000
374.0000000	0.2600000
375.0000000	0.2600000
376.0000000	0.2500000
377.0000000	0.2550000
378.0000000	0.2500000
379.0000000	0.2650000
380.0000000	0.2450000
381.0000000	0.2550000
382.0000000	0.2450000
383.0000000	0.2600000
384.0000000	0.2500000
385.0000000	0.2550000
386.0000000	0.2450000
387.0000000	0.2500000
388.0000000	0.2350000
389.0000000	0.2450000
390.0000000	0.2400000
391.0000000	0.2450000
392.0000000	0.2350000
393.0000000	0.2300000
394.0000000	0.2200000
395.0000000	0.2300000
396.0000000	0.2350000
397.0000000	0.2350000
398.0000000	0.2250000

399.0000000	0.2200000
400.0000000	0.2250000
401.0000000	0.2200000
402.0000000	0.2300000
403.0000000	0.2300000
404.0000000	0.2350000
405.0000000	0.2350000
406.0000000	0.2200000
407.0000000	0.2300000
408.0000000	0.2250000
409.0000000	0.2300000
410.0000000	0.2350000
411.0000000	0.2150000
412.0000000	0.2300000
413.0000000	0.2250000
414.0000000	0.2200000
415.0000000	0.2150000
416.0000000	0.2100000
417.0000000	0.2100000
418.0000000	0.2200000
419.0000000	0.2100000
420.0000000	0.2100000
421.0000000	0.2200000
422.0000000	0.2200000
423.0000000	0.2200000
424.0000000	0.2050000
425.0000000	0.2050000
426.0000000	0.2100000
427.0000000	0.2200000
428.0000000	0.2050000
429.0000000	0.2100000
430.0000000	0.2150000
431.0000000	0.2133333
432.0000000	0.2100000
433.0000000	0.2150000
434.0000000	0.2050000
435.0000000	0.2150000
436.0000000	0.2100000
437.0000000	0.2100000
438.0000000	0.2150000
439.0000000	0.2100000
440.0000000	0.2000000
441.0000000	0.2200000
442.0000000	0.2100000
443.0000000	0.2100000
444.0000000	0.2100000
445.0000000	0.2150000
446.0000000	0.2050000
447.0000000	0.2150000
448.0000000	0.2050000
449.0000000	0.2050000
450.0000000	0.2150000
451.0000000	0.2050000
452.0000000	0.2150000
453.0000000	0.2050000
454.0000000	0.2100000
455.0000000	0.2200000

456.0000000	0.2100000
457.0000000	0.2050000
458.0000000	0.2150000
459.0000000	0.2200000
460.0000000	0.2150000
461.0000000	0.2150000
462.0000000	0.2200000
463.0000000	0.2250000
464.0000000	0.2150000
465.0000000	0.2150000
466.0000000	0.2150000
467.0000000	0.2200000
468.0000000	0.2100000
469.0000000	0.2150000
470.0000000	0.2100000
471.0000000	0.2200000
472.0000000	0.2100000
473.0000000	0.2100000
474.0000000	0.2100000
475.0000000	0.2150000
476.0000000	0.2200000
477.0000000	0.2200000
478.0000000	0.2150000
479.0000000	0.2200000
480.0000000	0.2150000
481.0000000	0.2200000
482.0000000	0.2050000
483.0000000	0.2150000
484.0000000	0.2100000
485.0000000	0.2200000
486.0000000	0.2100000
487.0000000	0.2150000
488.0000000	0.2050000
489.0000000	0.2150000
490.0000000	0.2100000
491.0000000	0.2100000
492.0000000	0.2133333
493.0000000	0.2150000
494.0000000	0.2100000
495.0000000	0.2050000
496.0000000	0.2050000
497.0000000	0.2200000
498.0000000	0.2200000
499.0000000	0.2100000
500.0000000	0.2150000
501.0000000	0.2050000
502.0000000	0.2150000
503.0000000	0.2150000
504.0000000	0.2200000
505.0000000	0.2100000
506.0000000	0.2150000
507.0000000	0.2050000
508.0000000	0.2100000
509.0000000	0.2100000
510.0000000	0.2200000
511.0000000	0.2050000
512.0000000	0.2100000

513.0000000	0.2050000
514.0000000	0.2100000
515.0000000	0.2100000
516.0000000	0.2200000
517.0000000	0.2100000
518.0000000	0.2050000
519.0000000	0.2100000
520.0000000	0.2100000
521.0000000	0.2150000
522.0000000	0.2133333
523.0000000	0.2150000
524.0000000	0.2100000
525.0000000	0.2150000
526.0000000	0.2050000
527.0000000	0.2200000
528.0000000	0.2200000
529.0000000	0.2200000
530.0000000	0.2100000
531.0000000	0.2000000
532.0000000	0.2100000
533.0000000	0.2050000
534.0000000	0.2200000
535.0000000	0.2100000
536.0000000	0.2100000
537.0000000	0.2050000
538.0000000	0.2100000
539.0000000	0.2100000
540.0000000	0.2100000
541.0000000	0.2150000
542.0000000	0.2050000
543.0000000	0.2150000
544.0000000	0.2050000
545.0000000	0.2100000
546.0000000	0.2000000
547.0000000	0.2100000
548.0000000	0.2100000
549.0000000	0.2200000
550.0000000	0.2200000
551.0000000	0.2100000
552.0000000	0.2066667
553.0000000	0.2100000
554.0000000	0.2100000
555.0000000	0.2050000
556.0000000	0.2100000
557.0000000	0.2100000
558.0000000	0.2100000
559.0000000	0.2100000
560.0000000	0.2100000
561.0000000	0.2150000
562.0000000	0.2150000
563.0000000	0.2150000
564.0000000	0.2000000
565.0000000	0.2050000
566.0000000	0.2050000
567.0000000	0.2200000
568.0000000	0.2250000
569.0000000	0.2150000

570.0000000	0.2150000
571.0000000	0.2050000
572.0000000	0.2200000
573.0000000	0.2150000
574.0000000	0.2100000
575.0000000	0.2150000
576.0000000	0.2150000
577.0000000	0.2200000
578.0000000	0.2250000
579.0000000	0.2100000
580.0000000	0.2100000
581.0000000	0.2100000
582.0000000	0.2166667
583.0000000	0.2100000
584.0000000	0.2200000
585.0000000	0.2150000
586.0000000	0.2100000
587.0000000	0.2150000
588.0000000	0.2200000
589.0000000	0.2200000
590.0000000	0.2200000
591.0000000	0.2100000
592.0000000	0.2100000
593.0000000	0.2150000
594.0000000	0.2200000
595.0000000	0.2200000
596.0000000	0.2100000
597.0000000	0.2250000
598.0000000	0.2050000
599.0000000	0.2250000
600.0000000	0.2200000
601.0000000	0.2150000
602.0000000	0.2150000
603.0000000	0.2100000
604.0000000	0.2250000
605.0000000	0.2200000
606.0000000	0.2200000
607.0000000	0.2150000
608.0000000	0.2150000
609.0000000	0.2200000
610.0000000	0.2200000
611.0000000	0.2250000
612.0000000	0.2050000
613.0000000	0.2200000
614.0000000	0.2000000
615.0000000	0.2050000
616.0000000	0.2150000
617.0000000	0.2100000
618.0000000	0.2150000
619.0000000	0.2150000
620.0000000	0.2250000
621.0000000	0.2150000
622.0000000	0.2150000
623.0000000	0.2100000
624.0000000	0.2100000
625.0000000	0.2200000
626.0000000	0.2150000

627.0000000	0.2200000
628.0000000	0.2250000
629.0000000	0.2100000
630.0000000	0.2200000
631.0000000	0.2150000
632.0000000	0.2100000
633.0000000	0.2200000
634.0000000	0.2150000
635.0000000	0.2150000
636.0000000	0.2200000
637.0000000	0.2150000
638.0000000	0.2050000
639.0000000	0.2050000
640.0000000	0.2050000
641.0000000	0.2100000
642.0000000	0.2133333
643.0000000	0.2150000
644.0000000	0.2050000
645.0000000	0.2100000
646.0000000	0.2250000
647.0000000	0.2150000
648.0000000	0.2050000
649.0000000	0.2100000
650.0000000	0.2250000
651.0000000	0.2150000
652.0000000	0.2150000
653.0000000	0.2250000
654.0000000	0.2100000
655.0000000	0.2200000
656.0000000	0.2250000
657.0000000	0.2100000
658.0000000	0.2150000
659.0000000	0.2150000
660.0000000	0.2150000
661.0000000	0.2200000
662.0000000	0.2150000
663.0000000	0.2150000
664.0000000	0.2300000
665.0000000	0.2150000
666.0000000	0.2250000
667.0000000	0.2100000
668.0000000	0.2150000
669.0000000	0.2300000
670.0000000	0.2250000
671.0000000	0.2200000
672.0000000	0.2150000
673.0000000	0.2200000
674.0000000	0.2150000
675.0000000	0.2200000
676.0000000	0.2200000
677.0000000	0.2250000
678.0000000	0.2250000
679.0000000	0.2200000
680.0000000	0.2150000
681.0000000	0.2250000
682.0000000	0.2250000
683.0000000	0.2250000

684.0000000	0.2200000
685.0000000	0.2200000
686.0000000	0.2200000
687.0000000	0.2200000
688.0000000	0.2250000
689.0000000	0.2300000
690.0000000	0.2250000
691.0000000	0.2200000
692.0000000	0.2250000
693.0000000	0.2250000
694.0000000	0.2250000
695.0000000	0.2300000
696.0000000	0.2250000
697.0000000	0.2250000
698.0000000	0.2250000
699.0000000	0.2200000
700.0000000	0.2250000
701.0000000	0.2200000
702.0000000	0.2250000
703.0000000	0.2200000
704.0000000	0.2200000
705.0000000	0.2200000
706.0000000	0.2200000
707.0000000	0.2250000
708.0000000	0.2150000
709.0000000	0.2100000
710.0000000	0.2150000
711.0000000	0.2200000
712.0000000	0.2150000
713.0000000	0.2150000
714.0000000	0.2200000
715.0000000	0.2150000
716.0000000	0.2150000
717.0000000	0.2150000
718.0000000	0.2100000
719.0000000	0.2250000
720.0000000	0.2200000
721.0000000	0.2200000
722.0000000	0.2200000
723.0000000	0.2150000
724.0000000	0.2150000
725.0000000	0.2200000
726.0000000	0.2200000
727.0000000	0.2200000
728.0000000	0.2150000
729.0000000	0.2200000
730.0000000	0.2250000
731.0000000	0.2300000
732.0000000	0.2200000
733.0000000	0.2266667
734.0000000	0.2250000
735.0000000	0.2300000
736.0000000	0.2300000
737.0000000	0.2300000
738.0000000	0.2250000
739.0000000	0.2300000
740.0000000	0.2350000

741.0000000	0.2350000
742.0000000	0.2250000
743.0000000	0.2350000
744.0000000	0.2300000
745.0000000	0.2400000
746.0000000	0.2300000
747.0000000	0.2400000
748.0000000	0.2250000
749.0000000	0.2350000
750.0000000	0.2250000
751.0000000	0.2350000
752.0000000	0.2250000
753.0000000	0.2300000
754.0000000	0.2350000
755.0000000	0.2350000
756.0000000	0.2300000
757.0000000	0.2300000
758.0000000	0.2350000
759.0000000	0.2350000
760.0000000	0.2400000
761.0000000	0.2400000
762.0000000	0.2300000
763.0000000	0.2300000
764.0000000	0.2250000
765.0000000	0.2300000
766.0000000	0.2400000
767.0000000	0.2400000
768.0000000	0.2400000
769.0000000	0.2300000
770.0000000	0.2300000
771.0000000	0.2300000
772.0000000	0.2300000
773.0000000	0.2350000
774.0000000	0.2250000
775.0000000	0.2400000
776.0000000	0.2300000
777.0000000	0.2300000
778.0000000	0.2450000
779.0000000	0.2300000
780.0000000	0.2300000
781.0000000	0.2400000
782.0000000	0.2450000
783.0000000	0.2350000
784.0000000	0.2400000
785.0000000	0.2350000
786.0000000	0.2350000
787.0000000	0.2400000
788.0000000	0.2450000
789.0000000	0.2450000
790.0000000	0.2350000
791.0000000	0.2350000
792.0000000	0.2400000
793.0000000	0.2400000
794.0000000	0.2433333
795.0000000	0.2500000
796.0000000	0.2450000
797.0000000	0.2300000

798.0000000	0.2400000
799.0000000	0.2300000
800.0000000	0.2350000
801.0000000	0.2450000
802.0000000	0.2300000
803.0000000	0.2300000
804.0000000	0.2400000
805.0000000	0.2350000
806.0000000	0.2350000
807.0000000	0.2300000
808.0000000	0.2250000
809.0000000	0.2450000
810.0000000	0.2450000
811.0000000	0.2300000
812.0000000	0.2400000
813.0000000	0.2400000
814.0000000	0.2400000
815.0000000	0.2350000
816.0000000	0.2350000
817.0000000	0.2400000
818.0000000	0.2350000
819.0000000	0.2550000
820.0000000	0.2450000
821.0000000	0.2450000
822.0000000	0.2500000
823.0000000	0.2500000
824.0000000	0.2400000
825.0000000	0.2450000
826.0000000	0.2350000
827.0000000	0.2500000
828.0000000	0.2350000
829.0000000	0.2500000
830.0000000	0.2550000
831.0000000	0.2500000
832.0000000	0.2500000
833.0000000	0.2450000
834.0000000	0.2550000
835.0000000	0.2600000
836.0000000	0.2600000
837.0000000	0.2550000
838.0000000	0.2500000
839.0000000	0.2600000
840.0000000	0.2600000
841.0000000	0.2550000
842.0000000	0.2550000
843.0000000	0.2600000
844.0000000	0.2700000
845.0000000	0.2600000
846.0000000	0.2650000
847.0000000	0.2550000
848.0000000	0.2550000
849.0000000	0.2600000
850.0000000	0.2650000
851.0000000	0.2600000
852.0000000	0.2550000
853.0000000	0.2500000
854.0000000	0.2533333

855.0000000	0.2550000
856.0000000	0.2550000
857.0000000	0.2500000
858.0000000	0.2600000
859.0000000	0.2500000
860.0000000	0.2500000
861.0000000	0.2500000
862.0000000	0.2500000
863.0000000	0.2500000
864.0000000	0.2450000
865.0000000	0.2400000
866.0000000	0.2500000
867.0000000	0.2450000
868.0000000	0.2400000
869.0000000	0.2350000
870.0000000	0.2350000
871.0000000	0.2450000
872.0000000	0.2400000
873.0000000	0.2400000
874.0000000	0.2500000
875.0000000	0.2350000
876.0000000	0.2450000
877.0000000	0.2400000
878.0000000	0.2400000
879.0000000	0.2450000
880.0000000	0.2400000
881.0000000	0.2450000
882.0000000	0.2500000
883.0000000	0.2400000
884.0000000	0.2466667
885.0000000	0.2450000
886.0000000	0.2450000
887.0000000	0.2400000
888.0000000	0.2500000
889.0000000	0.2500000
890.0000000	0.2550000
891.0000000	0.2450000
892.0000000	0.2500000
893.0000000	0.2550000
894.0000000	0.2600000
895.0000000	0.2600000
896.0000000	0.2500000
897.0000000	0.2550000
898.0000000	0.2500000
899.0000000	0.2600000
900.0000000	0.2550000
901.0000000	0.2600000
902.0000000	0.2500000
903.0000000	0.2500000
904.0000000	0.2500000
905.0000000	0.2550000
906.0000000	0.2550000
907.0000000	0.2550000
908.0000000	0.2500000
909.0000000	0.2450000
910.0000000	0.2500000
911.0000000	0.2500000

912.0000000	0.2600000
913.0000000	0.2500000
914.0000000	0.2466667
915.0000000	0.2550000
916.0000000	0.2500000
917.0000000	0.2500000
918.0000000	0.2450000
919.0000000	0.2500000
920.0000000	0.2500000
921.0000000	0.2500000
922.0000000	0.2450000
923.0000000	0.2500000
924.0000000	0.2400000
925.0000000	0.2500000
926.0000000	0.2400000
927.0000000	0.2450000
928.0000000	0.2400000
929.0000000	0.2400000
930.0000000	0.2300000
931.0000000	0.2400000
932.0000000	0.2400000
933.0000000	0.2250000
934.0000000	0.2350000
935.0000000	0.2250000
936.0000000	0.2350000
937.0000000	0.2350000
938.0000000	0.2450000
939.0000000	0.2250000
940.0000000	0.2300000
941.0000000	0.2250000
942.0000000	0.2250000
943.0000000	0.2400000
944.0000000	0.2350000
945.0000000	0.2300000
946.0000000	0.2300000
947.0000000	0.2300000
948.0000000	0.2250000
949.0000000	0.2350000
950.0000000	0.2300000
951.0000000	0.2450000
952.0000000	0.2400000
953.0000000	0.2350000
954.0000000	0.2350000
955.0000000	0.2300000
956.0000000	0.2450000
957.0000000	0.2350000
958.0000000	0.2400000
959.0000000	0.2300000
960.0000000	0.2300000
961.0000000	0.2300000
962.0000000	0.2400000
963.0000000	0.2300000
964.0000000	0.2350000
965.0000000	0.2300000
966.0000000	0.2300000
967.0000000	0.2300000
968.0000000	0.2450000

969.0000000	0.2400000
970.0000000	0.2450000
971.0000000	0.2300000
972.0000000	0.2300000
973.0000000	0.2350000
974.0000000	0.2366667
975.0000000	0.2350000
976.0000000	0.2450000
977.0000000	0.2400000
978.0000000	0.2400000
979.0000000	0.2300000
980.0000000	0.2350000
981.0000000	0.2400000
982.0000000	0.2350000
983.0000000	0.2350000
984.0000000	0.2250000
985.0000000	0.2250000
986.0000000	0.2250000
987.0000000	0.2300000
988.0000000	0.2250000
989.0000000	0.2300000
990.0000000	0.2250000
991.0000000	0.2250000
992.0000000	0.2200000
993.0000000	0.2200000
994.0000000	0.2300000
995.0000000	0.2150000
996.0000000	0.2250000
997.0000000	0.2250000
998.0000000	0.2250000
999.0000000	0.2300000
1000.0000000	0.2150000
1001.0000000	0.2300000
1002.0000000	0.2250000
1003.0000000	0.2450000
1004.0000000	0.2200000
1005.0000000	0.2233333
1006.0000000	0.2200000
1007.0000000	0.2350000
1008.0000000	0.2250000
1009.0000000	0.2250000
1010.0000000	0.2200000
1011.0000000	0.2300000
1012.0000000	0.2250000
1013.0000000	0.2200000
1014.0000000	0.2200000
1015.0000000	0.2250000
1016.0000000	0.2300000
1017.0000000	0.2300000
1018.0000000	0.2200000
1019.0000000	0.2250000
1020.0000000	0.2350000
1021.0000000	0.2300000
1022.0000000	0.2350000
1023.0000000	0.2300000
1024.0000000	0.2300000
1025.0000000	0.2250000

1026.0000000	0.2350000
1027.0000000	0.2350000
1028.0000000	0.2400000
1029.0000000	0.2350000
1030.0000000	0.2300000
1031.0000000	0.2250000
1032.0000000	0.2350000
1033.0000000	0.2450000
1034.0000000	0.2500000
1035.0000000	0.2300000
1036.0000000	0.2400000
1037.0000000	0.2400000
1038.0000000	0.2450000
1039.0000000	0.2400000
1040.0000000	0.2350000
1041.0000000	0.2400000
1042.0000000	0.2400000
1043.0000000	0.2450000
1044.0000000	0.2400000
1045.0000000	0.2500000
1046.0000000	0.2400000
1047.0000000	0.2400000
1048.0000000	0.2350000
1049.0000000	0.2500000
1050.0000000	0.2500000
1051.0000000	0.2400000
1052.0000000	0.2300000
1053.0000000	0.2400000
1054.0000000	0.2450000
1055.0000000	0.2350000
1056.0000000	0.2500000
1057.0000000	0.2350000
1058.0000000	0.2450000
1059.0000000	0.2400000
1060.0000000	0.2550000
1061.0000000	0.2500000
1062.0000000	0.2550000
1063.0000000	0.2350000
1064.0000000	0.2450000
1065.0000000	0.2433333
1066.0000000	0.2400000
1067.0000000	0.2500000
1068.0000000	0.2450000
1069.0000000	0.2400000
1070.0000000	0.2450000
1071.0000000	0.2500000
1072.0000000	0.2550000
1073.0000000	0.2450000
1074.0000000	0.2450000
1075.0000000	0.2400000
1076.0000000	0.2500000
1077.0000000	0.2450000
1078.0000000	0.2500000
1079.0000000	0.2500000
1080.0000000	0.2600000
1081.0000000	0.2550000
1082.0000000	0.2550000

1083.0000000	0.2450000
1084.0000000	0.2500000
1085.0000000	0.2500000
1086.0000000	0.2550000
1087.0000000	0.2550000
1088.0000000	0.2600000
1089.0000000	0.2450000
1090.0000000	0.2550000
1091.0000000	0.2700000
1092.0000000	0.2550000
1093.0000000	0.2550000
1094.0000000	0.2600000
1095.0000000	0.2600000
1096.0000000	0.2550000
1097.0000000	0.2650000
1098.0000000	0.2550000
1099.0000000	0.2400000
1100.0000000	0.2550000
1101.0000000	0.2600000
1102.0000000	0.2600000
1103.0000000	0.2500000
1104.0000000	0.2550000
1105.0000000	0.2650000
1106.0000000	0.2500000
1107.0000000	0.2400000
1108.0000000	0.2600000
1109.0000000	0.2600000
1110.0000000	0.2500000
1111.0000000	0.2450000
1112.0000000	0.2500000
1113.0000000	0.2550000
1114.0000000	0.2400000
1115.0000000	0.2400000
1116.0000000	0.2450000
1117.0000000	0.2450000
1118.0000000	0.2400000
1119.0000000	0.2400000
1120.0000000	0.2400000
1121.0000000	0.2400000
1122.0000000	0.2400000
1123.0000000	0.2500000
1124.0000000	0.2350000
1125.0000000	0.2300000
1126.0000000	0.2433333
1127.0000000	0.2450000
1128.0000000	0.2350000
1129.0000000	0.2400000
1130.0000000	0.2400000
1131.0000000	0.2400000
1132.0000000	0.2500000
1133.0000000	0.2350000
1134.0000000	0.2400000
1135.0000000	0.2400000
1136.0000000	0.2500000
1137.0000000	0.2400000
1138.0000000	0.2400000
1139.0000000	0.2400000

1140.0000000	0.2350000
1141.0000000	0.2400000
1142.0000000	0.2400000
1143.0000000	0.2350000
1144.0000000	0.2400000
1145.0000000	0.2500000
1146.0000000	0.2350000
1147.0000000	0.2350000
1148.0000000	0.2450000
1149.0000000	0.2400000
1150.0000000	0.2350000
1151.0000000	0.2300000
1152.0000000	0.2300000
1153.0000000	0.2350000
1154.0000000	0.2350000
1155.0000000	0.2350000
1156.0000000	0.2333333
1157.0000000	0.2300000
1158.0000000	0.2200000
1159.0000000	0.2300000
1160.0000000	0.2350000
1161.0000000	0.2350000
1162.0000000	0.2300000
1163.0000000	0.2300000
1164.0000000	0.2300000
1165.0000000	0.2300000
1166.0000000	0.2200000
1167.0000000	0.2250000
1168.0000000	0.2200000
1169.0000000	0.2300000
1170.0000000	0.2150000
1171.0000000	0.2200000
1172.0000000	0.2200000
1173.0000000	0.2100000
1174.0000000	0.2250000
1175.0000000	0.2200000
1176.0000000	0.2250000
1177.0000000	0.2200000
1178.0000000	0.2200000
1179.0000000	0.2250000
1180.0000000	0.2250000
1181.0000000	0.2250000
1182.0000000	0.2150000
1183.0000000	0.2250000
1184.0000000	0.2200000
1185.0000000	0.2300000
1186.0000000	0.2266666
1187.0000000	0.2250000
1188.0000000	0.2250000
1189.0000000	0.2350000
1190.0000000	0.2300000
1191.0000000	0.2200000
1192.0000000	0.2250000
1193.0000000	0.2250000
1194.0000000	0.2300000
1195.0000000	0.2250000
1196.0000000	0.2300000

1197.0000000	0.2250000
1198.0000000	0.2250000
1199.0000000	0.2200000
1200.0000000	0.2250000
1201.0000000	0.2250000
1202.0000000	0.2300000
1203.0000000	0.2300000
1204.0000000	0.2200000
1205.0000000	0.2250000
1206.0000000	0.2200000
1207.0000000	0.2200000
1208.0000000	0.2250000
1209.0000000	0.2250000
1210.0000000	0.2150000
1211.0000000	0.2200000
1212.0000000	0.2250000
1213.0000000	0.2350000
1214.0000000	0.2200000
1215.0000000	0.2300000
1216.0000000	0.2166667
1217.0000000	0.2350000
1218.0000000	0.2250000
1219.0000000	0.2300000
1220.0000000	0.2100000
1221.0000000	0.2250000
1222.0000000	0.2200000
1223.0000000	0.2300000
1224.0000000	0.2300000
1225.0000000	0.2300000
1226.0000000	0.2250000
1227.0000000	0.2300000
1228.0000000	0.2250000
1229.0000000	0.2300000
1230.0000000	0.2300000
1231.0000000	0.2250000
1232.0000000	0.2250000
1233.0000000	0.2200000
1234.0000000	0.2250000
1235.0000000	0.2250000
1236.0000000	0.2250000
1237.0000000	0.2200000
1238.0000000	0.2250000
1239.0000000	0.2250000
1240.0000000	0.2250000
1241.0000000	0.2200000
1242.0000000	0.2300000
1243.0000000	0.2200000
1244.0000000	0.2300000
1245.0000000	0.2200000
1246.0000000	0.2250000
1247.0000000	0.2233333
1248.0000000	0.2250000
1249.0000000	0.2300000
1250.0000000	0.2250000
1251.0000000	0.2250000
1252.0000000	0.2200000
1253.0000000	0.2250000

1254.0000000	0.2350000
1255.0000000	0.2250000
1256.0000000	0.2400000
1257.0000000	0.2200000
1258.0000000	0.2300000
1259.0000000	0.2300000
1260.0000000	0.2300000
1261.0000000	0.2250000
1262.0000000	0.2400000
1263.0000000	0.2300000
1264.0000000	0.2300000
1265.0000000	0.2300000
1266.0000000	0.2350000
1267.0000000	0.2300000
1268.0000000	0.2350000
1269.0000000	0.2400000
1270.0000000	0.2300000
1271.0000000	0.2400000
1272.0000000	0.2300000
1273.0000000	0.2400000
1274.0000000	0.2350000
1275.0000000	0.2400000
1276.0000000	0.2400000
1277.0000000	0.2300000
1278.0000000	0.2400000
1279.0000000	0.2400000
1280.0000000	0.2450000
1281.0000000	0.2400000
1282.0000000	0.2300000
1283.0000000	0.2300000
1284.0000000	0.2350000
1285.0000000	0.2350000
1286.0000000	0.2400000
1287.0000000	0.2400000
1288.0000000	0.2350000
1289.0000000	0.2400000
1290.0000000	0.2350000
1291.0000000	0.2400000
1292.0000000	0.2350000
1293.0000000	0.2400000
1294.0000000	0.2400000
1295.0000000	0.2400000
1296.0000000	0.2350000
1297.0000000	0.2400000
1298.0000000	0.2350000
1299.0000000	0.2400000
1300.0000000	0.2350000
1301.0000000	0.2400000
1302.0000000	0.2350000
1303.0000000	0.2450000
1304.0000000	0.2400000
1305.0000000	0.2500000
1306.0000000	0.2366667
1307.0000000	0.2550000
1308.0000000	0.2400000
1309.0000000	0.2450000
1310.0000000	0.2450000

1311.0000000	0.2350000
1312.0000000	0.2500000
1313.0000000	0.2450000
1314.0000000	0.2450000
1315.0000000	0.2400000
1316.0000000	0.2500000
1317.0000000	0.2500000
1318.0000000	0.2500000
1319.0000000	0.2500000
1320.0000000	0.2450000
1321.0000000	0.2450000
1322.0000000	0.2550000
1323.0000000	0.2550000
1324.0000000	0.2500000
1325.0000000	0.2450000
1326.0000000	0.2500000
1327.0000000	0.2450000
1328.0000000	0.2550000
1329.0000000	0.2550000
1330.0000000	0.2550000
1331.0000000	0.2500000
1332.0000000	0.2500000
1333.0000000	0.2550000
1334.0000000	0.2600000
1335.0000000	0.2550000
1336.0000000	0.2600000
1337.0000000	0.2400000
1338.0000000	0.2550000
1339.0000000	0.2500000
1340.0000000	0.2450000
1341.0000000	0.2500000
1342.0000000	0.2500000
1343.0000000	0.2500000
1344.0000000	0.2450000
1345.0000000	0.2500000
1346.0000000	0.2450000
1347.0000000	0.2500000
1348.0000000	0.2450000
1349.0000000	0.2450000
1350.0000000	0.2450000
1351.0000000	0.2350000
1352.0000000	0.2350000
1353.0000000	0.2400000
1354.0000000	0.2450000
1355.0000000	0.2450000
1356.0000000	0.2350000
1357.0000000	0.2300000
1358.0000000	0.2350000
1359.0000000	0.2350000
1360.0000000	0.2550000
1361.0000000	0.2350000
1362.0000000	0.2300000
1363.0000000	0.2300000
1364.0000000	0.2400000
1365.0000000	0.2400000
1366.0000000	0.2400000
1367.0000000	0.2250000

1368.0000000	0.2266667
1369.0000000	0.2400000
1370.0000000	0.2350000
1371.0000000	0.2350000
1372.0000000	0.2250000
1373.0000000	0.2400000
1374.0000000	0.2300000
1375.0000000	0.2450000
1376.0000000	0.2350000
1377.0000000	0.2400000
1378.0000000	0.2300000
1379.0000000	0.2350000
1380.0000000	0.2350000
1381.0000000	0.2400000
1382.0000000	0.2400000
1383.0000000	0.2450000
1384.0000000	0.2300000
1385.0000000	0.2350000
1386.0000000	0.2400000
1387.0000000	0.2400000
1388.0000000	0.2400000
1389.0000000	0.2350000
1390.0000000	0.2300000
1391.0000000	0.2300000
1392.0000000	0.2350000
1393.0000000	0.2400000
1394.0000000	0.2400000
1395.0000000	0.2350000
1396.0000000	0.2300000
1397.0000000	0.2333333
1398.0000000	0.2300000
1399.0000000	0.2400000
1400.0000000	0.2300000
1401.0000000	0.2300000
1402.0000000	0.2250000
1403.0000000	0.2350000
1404.0000000	0.2300000
1405.0000000	0.2350000
1406.0000000	0.2250000
1407.0000000	0.2250000
1408.0000000	0.2200000
1409.0000000	0.2450000
1410.0000000	0.2400000
1411.0000000	0.2250000
1412.0000000	0.2300000
1413.0000000	0.2250000
1414.0000000	0.2250000
1415.0000000	0.2350000
1416.0000000	0.2400000
1417.0000000	0.2350000
1418.0000000	0.2250000
1419.0000000	0.2300000
1420.0000000	0.2300000
1421.0000000	0.2350000
1422.0000000	0.2400000
1423.0000000	0.2400000
1424.0000000	0.2300000

1425.0000000	0.2350000
1426.0000000	0.2300000
1427.0000000	0.2366667
1428.0000000	0.2350000
1429.0000000	0.2250000
1430.0000000	0.2400000
1431.0000000	0.2300000
1432.0000000	0.2300000
1433.0000000	0.2350000
1434.0000000	0.2250000
1435.0000000	0.2350000
1436.0000000	0.2250000
1437.0000000	0.2400000
1438.0000000	0.2300000
1439.0000000	0.2350000
1440.0000000	0.2300000
1441.0000000	0.2300000
1442.0000000	0.2350000
1443.0000000	0.2400000
1444.0000000	0.2450000
1445.0000000	0.2400000
1446.0000000	0.2350000
1447.0000000	0.2300000
1448.0000000	0.2350000
1449.0000000	0.2500000
1450.0000000	0.2300000
1451.0000000	0.2450000
1452.0000000	0.2400000
1453.0000000	0.2350000
1454.0000000	0.2400000
1455.0000000	0.2400000
1456.0000000	0.2450000
1457.0000000	0.2400000
1458.0000000	0.2433333
1459.0000000	0.2400000
1460.0000000	0.2450000
1461.0000000	0.2450000
1462.0000000	0.2400000
1463.0000000	0.2400000
1464.0000000	0.2400000
1465.0000000	0.2500000
1466.0000000	0.2500000
1467.0000000	0.2450000
1468.0000000	0.2400000
1469.0000000	0.2450000
1470.0000000	0.2450000
1471.0000000	0.2500000
1472.0000000	0.2500000
1473.0000000	0.2450000
1474.0000000	0.2450000
1475.0000000	0.2400000
1476.0000000	0.2500000
1477.0000000	0.2400000
1478.0000000	0.2500000
1479.0000000	0.2400000
1480.0000000	0.2350000
1481.0000000	0.2450000

1482.0000000	0.2350000
1483.0000000	0.2450000
1484.0000000	0.2400000
1485.0000000	0.2400000
1486.0000000	0.2550000
1487.0000000	0.2450000
1488.0000000	0.2400000
1489.0000000	0.2400000
1490.0000000	0.2500000
1491.0000000	0.2400000
1492.0000000	0.2450000
1493.0000000	0.2500000
1494.0000000	0.2550000
1495.0000000	0.2500000
1496.0000000	0.2350000
1497.0000000	0.2500000
1498.0000000	0.2350000
1499.0000000	0.2550000
1500.0000000	0.2450000
1501.0000000	0.2500000
1502.0000000	0.2400000
1503.0000000	0.2400000
1504.0000000	0.2550000
1505.0000000	0.2500000
1506.0000000	0.2400000
1507.0000000	0.2450000
1508.0000000	0.2450000
1509.0000000	0.2550000
1510.0000000	0.2550000
1511.0000000	0.2500000
1512.0000000	0.2450000
1513.0000000	0.2400000
1514.0000000	0.2600000
1515.0000000	0.2500000
1516.0000000	0.2500000
1517.0000000	0.2500000
1518.0000000	0.2433333
1519.0000000	0.2500000
1520.0000000	0.2500000
1521.0000000	0.2550000
1522.0000000	0.2600000
1523.0000000	0.2500000
1524.0000000	0.2500000
1525.0000000	0.2600000
1526.0000000	0.2450000
1527.0000000	0.2600000
1528.0000000	0.2450000
1529.0000000	0.2450000
1530.0000000	0.2600000
1531.0000000	0.2550000
1532.0000000	0.2450000
1533.0000000	0.2400000
1534.0000000	0.2500000
1535.0000000	0.2550000
1536.0000000	0.2500000
1537.0000000	0.2500000
1538.0000000	0.2500000

1539.0000000	0.2500000
1540.0000000	0.2600000
1541.0000000	0.2500000
1542.0000000	0.2500000
1543.0000000	0.2600000
1544.0000000	0.2600000
1545.0000000	0.2550000
1546.0000000	0.2500000
1547.0000000	0.2500000
1548.0000000	0.2600000
1549.0000000	0.2550000
1550.0000000	0.2500000
1551.0000000	0.2600000
1552.0000000	0.2450000
1553.0000000	0.2400000
1554.0000000	0.2550000
1555.0000000	0.2600000
1556.0000000	0.2500000
1557.0000000	0.2650000
1558.0000000	0.2500000
1559.0000000	0.2600000
1560.0000000	0.2550000
1561.0000000	0.2450000
1562.0000000	0.2550000
1563.0000000	0.2550000
1564.0000000	0.2550000
1565.0000000	0.2550000
1566.0000000	0.2500000
1567.0000000	0.2450000
1568.0000000	0.2500000
1569.0000000	0.2550000
1570.0000000	0.2600000
1571.0000000	0.2550000
1572.0000000	0.2550000
1573.0000000	0.2450000
1574.0000000	0.2450000
1575.0000000	0.2450000
1576.0000000	0.2600000
1577.0000000	0.2450000
1578.0000000	0.2500000
1579.0000000	0.2500000
1580.0000000	0.2500000
1581.0000000	0.2450000
1582.0000000	0.2500000
1583.0000000	0.2500000
1584.0000000	0.2550000
1585.0000000	0.2500000
1586.0000000	0.2450000
1587.0000000	0.2450000
1588.0000000	0.2550000
1589.0000000	0.2500000
1590.0000000	0.2550000
1591.0000000	0.2600000
1592.0000000	0.2550000
1593.0000000	0.2600000
1594.0000000	0.2550000
1595.0000000	0.2550000

1596.0000000	0.2550000
1597.0000000	0.2550000
1598.0000000	0.2650000
1599.0000000	0.2550000
1600.0000000	0.2600000
1601.0000000	0.2500000
1602.0000000	0.2600000
1603.0000000	0.2550000
1604.0000000	0.2600000
1605.0000000	0.2600000
1606.0000000	0.2600000
1607.0000000	0.2450000
1608.0000000	0.2500000
1609.0000000	0.2566667
1610.0000000	0.2550000
1611.0000000	0.2550000
1612.0000000	0.2550000
1613.0000000	0.2550000
1614.0000000	0.2500000
1615.0000000	0.2500000
1616.0000000	0.2500000
1617.0000000	0.2500000
1618.0000000	0.2400000
1619.0000000	0.2500000
1620.0000000	0.2600000
1621.0000000	0.2600000
1622.0000000	0.2500000
1623.0000000	0.2550000
1624.0000000	0.2550000
1625.0000000	0.2600000
1626.0000000	0.2550000
1627.0000000	0.2600000
1628.0000000	0.2600000
1629.0000000	0.2650000
1630.0000000	0.2550000
1631.0000000	0.2550000
1632.0000000	0.2600000
1633.0000000	0.2550000
1634.0000000	0.2500000
1635.0000000	0.2550000
1636.0000000	0.2550000
1637.0000000	0.2600000
1638.0000000	0.2533333
1639.0000000	0.2500000
1640.0000000	0.2550000
1641.0000000	0.2500000
1642.0000000	0.2450000
1643.0000000	0.2500000
1644.0000000	0.2500000
1645.0000000	0.2500000
1646.0000000	0.2400000
1647.0000000	0.2450000
1648.0000000	0.2350000
1649.0000000	0.2550000
1650.0000000	0.2500000
1651.0000000	0.2500000
1652.0000000	0.2400000

1653.0000000	0.2500000
1654.0000000	0.2500000
1655.0000000	0.2550000
1656.0000000	0.2500000
1657.0000000	0.2400000
1658.0000000	0.2450000
1659.0000000	0.2500000
1660.0000000	0.2500000
1661.0000000	0.2500000
1662.0000000	0.2500000
1663.0000000	0.2450000
1664.0000000	0.2500000
1665.0000000	0.2500000
1666.0000000	0.2600000
1667.0000000	0.2550000
1668.0000000	0.2550000
1669.0000000	0.2600000
1670.0000000	0.2550000
1671.0000000	0.2550000
1672.0000000	0.2550000
1673.0000000	0.2600000
1674.0000000	0.2650000
1675.0000000	0.2650000
1676.0000000	0.2600000
1677.0000000	0.2500000
1678.0000000	0.2600000
1679.0000000	0.2650000
1680.0000000	0.2550000
1681.0000000	0.2650000
1682.0000000	0.2500000
1683.0000000	0.2700000
1684.0000000	0.2550000
1685.0000000	0.2700000
1686.0000000	0.2550000
1687.0000000	0.2650000
1688.0000000	0.2500000
1689.0000000	0.2600000
1690.0000000	0.2550000
1691.0000000	0.2600000
1692.0000000	0.2500000
1693.0000000	0.2550000
1694.0000000	0.2600000
1695.0000000	0.2550000
1696.0000000	0.2550000
1697.0000000	0.2650000
1698.0000000	0.2550000
1699.0000000	0.2600000
1700.0000000	0.2533333
1701.0000000	0.2600000
1702.0000000	0.2600000
1703.0000000	0.2550000
1704.0000000	0.2550000
1705.0000000	0.2500000
1706.0000000	0.2600000
1707.0000000	0.2650000
1708.0000000	0.2550000
1709.0000000	0.2500000

1710.0000000	0.2450000
1711.0000000	0.2500000
1712.0000000	0.2450000
1713.0000000	0.2500000
1714.0000000	0.2450000
1715.0000000	0.2500000
1716.0000000	0.2300000
1717.0000000	0.2350000
1718.0000000	0.2400000
1719.0000000	0.2500000
1720.0000000	0.2450000
1721.0000000	0.2350000
1722.0000000	0.2400000
1723.0000000	0.2450000
1724.0000000	0.2400000
1725.0000000	0.2400000
1726.0000000	0.2450000
1727.0000000	0.2450000
1728.0000000	0.2400000
1729.0000000	0.2366667
1730.0000000	0.2350000
1731.0000000	0.2400000
1732.0000000	0.2300000
1733.0000000	0.2400000
1734.0000000	0.2400000
1735.0000000	0.2450000
1736.0000000	0.2450000
1737.0000000	0.2400000
1738.0000000	0.2400000
1739.0000000	0.2350000
1740.0000000	0.2450000
1741.0000000	0.2500000
1742.0000000	0.2500000
1743.0000000	0.2450000
1744.0000000	0.2450000
1745.0000000	0.2400000
1746.0000000	0.2500000
1747.0000000	0.2500000
1748.0000000	0.2450000
1749.0000000	0.2500000
1750.0000000	0.2400000
1751.0000000	0.2500000
1752.0000000	0.2400000
1753.0000000	0.2550000
1754.0000000	0.2500000
1755.0000000	0.2400000
1756.0000000	0.2500000
1757.0000000	0.2550000
1758.0000000	0.2450000
1759.0000000	0.2500000
1760.0000000	0.2500000
1761.0000000	0.2400000
1762.0000000	0.2500000
1763.0000000	0.2550000
1764.0000000	0.2500000
1765.0000000	0.2500000
1766.0000000	0.2450000

1767.0000000	0.2500000
1768.0000000	0.2600000
1769.0000000	0.2550000
1770.0000000	0.2500000
1771.0000000	0.2650000
1772.0000000	0.2550000
1773.0000000	0.2650000
1774.0000000	0.2600000
1775.0000000	0.2450000
1776.0000000	0.2600000
1777.0000000	0.2600000
1778.0000000	0.2650000
1779.0000000	0.2600000
1780.0000000	0.2600000
1781.0000000	0.2600000
1782.0000000	0.2750000
1783.0000000	0.2700000
1784.0000000	0.2500000
1785.0000000	0.2600000
1786.0000000	0.2550000
1787.0000000	0.2650000
1788.0000000	0.2650000
1789.0000000	0.2650000
1790.0000000	0.2666667
1791.0000000	0.2550000
1792.0000000	0.2600000
1793.0000000	0.2550000
1794.0000000	0.2700000
1795.0000000	0.2600000
1796.0000000	0.2650000
1797.0000000	0.2450000
1798.0000000	0.2500000
1799.0000000	0.2550000
1800.0000000	0.2550000
1801.0000000	0.2650000
1802.0000000	0.2550000
1803.0000000	0.2550000
1804.0000000	0.2550000
1805.0000000	0.2600000
1806.0000000	0.2550000
1807.0000000	0.2500000
1808.0000000	0.2550000
1809.0000000	0.2550000
1810.0000000	0.2600000
1811.0000000	0.2600000
1812.0000000	0.2450000
1813.0000000	0.2550000
1814.0000000	0.2500000
1815.0000000	0.2650000
1816.0000000	0.2450000
1817.0000000	0.2550000
1818.0000000	0.2600000
1819.0000000	0.2500000
1820.0000000	0.2600000
1821.0000000	0.2600000
1822.0000000	0.2700000
1823.0000000	0.2600000

1824.0000000	0.2600000
1825.0000000	0.2650000
1826.0000000	0.2550000
1827.0000000	0.2650000
1828.0000000	0.2550000
1829.0000000	0.2500000
1830.0000000	0.2650000
1831.0000000	0.2550000
1832.0000000	0.2750000
1833.0000000	0.2600000
1834.0000000	0.2550000
1835.0000000	0.2500000
1836.0000000	0.2550000
1837.0000000	0.2700000
1838.0000000	0.2600000
1839.0000000	0.2550000
1840.0000000	0.2550000
1841.0000000	0.2550000
1842.0000000	0.2650000
1843.0000000	0.2600000
1844.0000000	0.2650000
1845.0000000	0.2550000
1846.0000000	0.2550000
1847.0000000	0.2650000
1848.0000000	0.2600000
1849.0000000	0.2650000
1850.0000000	0.2533333
1851.0000000	0.2700000
1852.0000000	0.2650000
1853.0000000	0.2700000
1854.0000000	0.2550000
1855.0000000	0.2650000
1856.0000000	0.2600000
1857.0000000	0.2550000
1858.0000000	0.2600000
1859.0000000	0.2650000
1860.0000000	0.2550000
1861.0000000	0.2650000
1862.0000000	0.2500000
1863.0000000	0.2600000
1864.0000000	0.2650000
1865.0000000	0.2550000
1866.0000000	0.2600000
1867.0000000	0.2500000
1868.0000000	0.2650000
1869.0000000	0.2550000
1870.0000000	0.2650000
1871.0000000	0.2500000
1872.0000000	0.2450000
1873.0000000	0.2500000
1874.0000000	0.2450000
1875.0000000	0.2550000
1876.0000000	0.2500000
1877.0000000	0.2400000
1878.0000000	0.2350000
1879.0000000	0.2400000
1880.0000000	0.2433333

1881.0000000	0.2450000
1882.0000000	0.2400000
1883.0000000	0.2450000
1884.0000000	0.2400000
1885.0000000	0.2450000
1886.0000000	0.2350000
1887.0000000	0.2450000
1888.0000000	0.2400000
1889.0000000	0.2400000
1890.0000000	0.2400000
1891.0000000	0.2350000
1892.0000000	0.2400000
1893.0000000	0.2400000
1894.0000000	0.2500000
1895.0000000	0.2400000
1896.0000000	0.2400000
1897.0000000	0.2400000
1898.0000000	0.2400000
1899.0000000	0.2450000
1900.0000000	0.2450000
1901.0000000	0.2400000
1902.0000000	0.2450000
1903.0000000	0.2350000
1904.0000000	0.2450000
1905.0000000	0.2400000
1906.0000000	0.2500000
1907.0000000	0.2350000
1908.0000000	0.2350000
1909.0000000	0.2300000
1910.0000000	0.2450000
1911.0000000	0.2466667
1912.0000000	0.2450000
1913.0000000	0.2400000
1914.0000000	0.2550000
1915.0000000	0.2350000
1916.0000000	0.2400000
1917.0000000	0.2300000
1918.0000000	0.2450000
1919.0000000	0.2350000
1920.0000000	0.2500000
1921.0000000	0.2250000
1922.0000000	0.2350000
1923.0000000	0.2250000
1924.0000000	0.2450000
1925.0000000	0.2400000
1926.0000000	0.2350000
1927.0000000	0.2250000
1928.0000000	0.2350000
1929.0000000	0.2350000
1930.0000000	0.2350000
1931.0000000	0.2300000
1932.0000000	0.2200000
1933.0000000	0.2250000
1934.0000000	0.2250000
1935.0000000	0.2350000
1936.0000000	0.2300000
1937.0000000	0.2450000

1938.0000000	0.2300000
1939.0000000	0.2300000
1940.0000000	0.2300000
1941.0000000	0.2366667
1942.0000000	0.2350000
1943.0000000	0.2400000
1944.0000000	0.2400000
1945.0000000	0.2350000
1946.0000000	0.2300000
1947.0000000	0.2350000
1948.0000000	0.2300000
1949.0000000	0.2350000
1950.0000000	0.2400000
1951.0000000	0.2300000
1952.0000000	0.2300000
1953.0000000	0.2300000
1954.0000000	0.2300000
1955.0000000	0.2350000
1956.0000000	0.2300000
1957.0000000	0.2300000
1958.0000000	0.2150000
1959.0000000	0.2150000
1960.0000000	0.2250000
1961.0000000	0.2250000
1962.0000000	0.2300000
1963.0000000	0.2100000
1964.0000000	0.2200000
1965.0000000	0.2150000
1966.0000000	0.2150000
1967.0000000	0.2250000
1968.0000000	0.2100000
1969.0000000	0.2200000
1970.0000000	0.2200000
1971.0000000	0.2200000
1972.0000000	0.2300000
1973.0000000	0.2200000
1974.0000000	0.2200000
1975.0000000	0.2200000
1976.0000000	0.2200000
1977.0000000	0.2350000
1978.0000000	0.2250000
1979.0000000	0.2200000
1980.0000000	0.2250000
1981.0000000	0.2250000
1982.0000000	0.2250000
1983.0000000	0.2300000
1984.0000000	0.2300000
1985.0000000	0.2200000
1986.0000000	0.2400000
1987.0000000	0.2300000
1988.0000000	0.2150000
1989.0000000	0.2300000
1990.0000000	0.2300000
1991.0000000	0.2300000
1992.0000000	0.2400000
1993.0000000	0.2250000
1994.0000000	0.2300000

1995.0000000	0.2450000
1996.0000000	0.2300000
1997.0000000	0.2300000
1998.0000000	0.2400000
1999.0000000	0.2300000
2000.0000000	0.2250000
2001.0000000	0.2300000
2002.0000000	0.2250000
2003.0000000	0.2200000
2004.0000000	0.2350000
2005.0000000	0.2300000
2006.0000000	0.2250000
2007.0000000	0.2150000
2008.0000000	0.2300000
2009.0000000	0.2200000
2010.0000000	0.2150000
2011.0000000	0.2200000
2012.0000000	0.2200000
2013.0000000	0.2300000
2014.0000000	0.2350000
2015.0000000	0.2250000
2016.0000000	0.2200000
2017.0000000	0.2250000
2018.0000000	0.2200000
2019.0000000	0.2250000
2020.0000000	0.2200000
2021.0000000	0.2250000
2022.0000000	0.2150000
2023.0000000	0.2200000
2024.0000000	0.2150000
2025.0000000	0.2250000
2026.0000000	0.2150000
2027.0000000	0.2150000
2028.0000000	0.2050000
2029.0000000	0.2100000
2030.0000000	0.2200000
2031.0000000	0.2100000
2032.0000000	0.2066667
2033.0000000	0.2200000
2034.0000000	0.2100000
2035.0000000	0.2100000
2036.0000000	0.2000000
2037.0000000	0.2150000
2038.0000000	0.2100000
2039.0000000	0.2200000
2040.0000000	0.2050000
2041.0000000	0.2050000
2042.0000000	0.2050000
2043.0000000	0.2050000
2044.0000000	0.2150000
2045.0000000	0.2100000
2046.0000000	0.2100000
2047.0000000	0.2050000
2048.0000000	0.2050000
2049.0000000	0.2000000
2050.0000000	0.2050000
2051.0000000	0.2050000

2052.0000000	0.2100000
2053.0000000	0.2100000
2054.0000000	0.2100000
2055.0000000	0.2050000
2056.0000000	0.2000000
2057.0000000	0.2100000
2058.0000000	0.2050000
2059.0000000	0.2100000
2060.0000000	0.2100000
2061.0000000	0.2033333
2062.0000000	0.2100000
2063.0000000	0.2100000
2064.0000000	0.2000000
2065.0000000	0.2050000
2066.0000000	0.2000000
2067.0000000	0.2050000
2068.0000000	0.2000000
2069.0000000	0.2100000
2070.0000000	0.2100000
2071.0000000	0.2000000
2072.0000000	0.2000000
2073.0000000	0.2000000
2074.0000000	0.2050000
2075.0000000	0.2100000
2076.0000000	0.2100000
2077.0000000	0.2100000
2078.0000000	0.2000000
2079.0000000	0.2100000
2080.0000000	0.2100000
2081.0000000	0.2150000
2082.0000000	0.2050000
2083.0000000	0.2100000
2084.0000000	0.1950000
2085.0000000	0.2100000
2086.0000000	0.2050000
2087.0000000	0.2250000
2088.0000000	0.2200000
2089.0000000	0.2150000
2090.0000000	0.2000000
2091.0000000	0.2200000
2092.0000000	0.2050000
2093.0000000	0.2200000
2094.0000000	0.2100000
2095.0000000	0.2250000
2096.0000000	0.2050000
2097.0000000	0.2150000
2098.0000000	0.2150000
2099.0000000	0.2150000
2100.0000000	0.2150000
2101.0000000	0.2200000
2102.0000000	0.2150000
2103.0000000	0.2200000
2104.0000000	0.2150000
2105.0000000	0.2150000
2106.0000000	0.2250000
2107.0000000	0.2200000
2108.0000000	0.2100000

2109.0000000	0.2150000
2110.0000000	0.2150000
2111.0000000	0.2200000
2112.0000000	0.2200000
2113.0000000	0.2150000
2114.0000000	0.2100000
2115.0000000	0.2100000
2116.0000000	0.2150000
2117.0000000	0.2150000
2118.0000000	0.2100000
2119.0000000	0.2100000
2120.0000000	0.2250000
2121.0000000	0.2200000
2122.0000000	0.2166667
2123.0000000	0.2200000
2124.0000000	0.2100000
2125.0000000	0.2150000
2126.0000000	0.2150000
2127.0000000	0.2200000
2128.0000000	0.2250000
2129.0000000	0.2200000
2130.0000000	0.2200000
2131.0000000	0.2300000
2132.0000000	0.2200000
2133.0000000	0.2200000
2134.0000000	0.2250000
2135.0000000	0.2300000
2136.0000000	0.2300000
2137.0000000	0.2300000
2138.0000000	0.2250000
2139.0000000	0.2250000
2140.0000000	0.2400000
2141.0000000	0.2300000
2142.0000000	0.2250000
2143.0000000	0.2200000
2144.0000000	0.2400000
2145.0000000	0.2300000
2146.0000000	0.2200000
2147.0000000	0.2300000
2148.0000000	0.2350000
2149.0000000	0.2350000
2150.0000000	0.2300000
2151.0000000	0.2300000
2152.0000000	0.2300000
2153.0000000	0.2250000
2154.0000000	0.2350000
2155.0000000	0.2300000
2156.0000000	0.2300000
2157.0000000	0.2300000
2158.0000000	0.2350000
2159.0000000	0.2350000
2160.0000000	0.2350000
2161.0000000	0.2250000
2162.0000000	0.2250000
2163.0000000	0.2350000
2164.0000000	0.2300000
2165.0000000	0.2300000

2166.0000000	0.2350000
2167.0000000	0.2350000
2168.0000000	0.2450000
2169.0000000	0.2400000
2170.0000000	0.2250000
2171.0000000	0.2400000
2172.0000000	0.2350000
2173.0000000	0.2450000
2174.0000000	0.2450000
2175.0000000	0.2350000
2176.0000000	0.2350000
2177.0000000	0.2500000
2178.0000000	0.2450000
2179.0000000	0.2350000
2180.0000000	0.2300000
2181.0000000	0.2350000
2182.0000000	0.2466667
2183.0000000	0.2400000
2184.0000000	0.2450000
2185.0000000	0.2500000
2186.0000000	0.2450000
2187.0000000	0.2300000
2188.0000000	0.2450000
2189.0000000	0.2350000
2190.0000000	0.2400000
2191.0000000	0.2400000
2192.0000000	0.2350000
2193.0000000	0.2300000
2194.0000000	0.2350000
2195.0000000	0.2450000
2196.0000000	0.2400000
2197.0000000	0.2350000
2198.0000000	0.2300000
2199.0000000	0.2350000
2200.0000000	0.2300000
2201.0000000	0.2450000
2202.0000000	0.2400000
2203.0000000	0.2450000
2204.0000000	0.2200000
2205.0000000	0.2250000
2206.0000000	0.2200000
2207.0000000	0.2350000
2208.0000000	0.2300000
2209.0000000	0.2300000
2210.0000000	0.2300000
2211.0000000	0.2200000
2212.0000000	0.2333333
2213.0000000	0.2250000
2214.0000000	0.2250000
2215.0000000	0.2200000
2216.0000000	0.2250000
2217.0000000	0.2300000
2218.0000000	0.2250000
2219.0000000	0.2300000
2220.0000000	0.2150000
2221.0000000	0.2200000
2222.0000000	0.2200000

2223.0000000	0.2250000
2224.0000000	0.2250000
2225.0000000	0.2250000
2226.0000000	0.2250000
2227.0000000	0.2200000
2228.0000000	0.2250000
2229.0000000	0.2200000
2230.0000000	0.2300000
2231.0000000	0.2250000
2232.0000000	0.2300000
2233.0000000	0.2250000
2234.0000000	0.2300000
2235.0000000	0.2300000
2236.0000000	0.2350000
2237.0000000	0.2300000
2238.0000000	0.2300000
2239.0000000	0.2300000
2240.0000000	0.2400000
2241.0000000	0.2350000
2242.0000000	0.2400000
2243.0000000	0.2366667
2244.0000000	0.2350000
2245.0000000	0.2400000
2246.0000000	0.2450000
2247.0000000	0.2400000
2248.0000000	0.2400000
2249.0000000	0.2350000
2250.0000000	0.2400000
2251.0000000	0.2400000
2252.0000000	0.2450000
2253.0000000	0.2400000
2254.0000000	0.2500000
2255.0000000	0.2400000
2256.0000000	0.2450000
2257.0000000	0.2400000
2258.0000000	0.2350000
2259.0000000	0.2450000
2260.0000000	0.2450000
2261.0000000	0.2550000
2262.0000000	0.2350000
2263.0000000	0.2350000
2264.0000000	0.2400000
2265.0000000	0.2400000
2266.0000000	0.2400000
2267.0000000	0.2500000
2268.0000000	0.2400000
2269.0000000	0.2400000
2270.0000000	0.2400000
2271.0000000	0.2350000
2272.0000000	0.2450000
2273.0000000	0.2333333
2274.0000000	0.2450000
2275.0000000	0.2400000
2276.0000000	0.2300000
2277.0000000	0.2450000
2278.0000000	0.2400000
2279.0000000	0.2300000

2280.0000000	0.2350000
2281.0000000	0.2300000
2282.0000000	0.2550000
2283.0000000	0.2550000
2284.0000000	0.2450000
2285.0000000	0.2550000
2286.0000000	0.2450000
2287.0000000	0.2550000
2288.0000000	0.2550000
2289.0000000	0.2500000
2290.0000000	0.2500000
2291.0000000	0.2600000
2292.0000000	0.2600000
2293.0000000	0.2600000
2294.0000000	0.2450000
2295.0000000	0.2400000
2296.0000000	0.2650000
2297.0000000	0.2550000
2298.0000000	0.2500000
2299.0000000	0.2550000
2300.0000000	0.2650000
2301.0000000	0.2650000
2302.0000000	0.2600000
2303.0000000	0.2600000
2304.0000000	0.2550000
2305.0000000	0.2600000
2306.0000000	0.2600000
2307.0000000	0.2600000
2308.0000000	0.2650000
2309.0000000	0.2500000
2310.0000000	0.2500000
2311.0000000	0.2600000
2312.0000000	0.2500000
2313.0000000	0.2650000
2314.0000000	0.2400000
2315.0000000	0.2450000
2316.0000000	0.2450000
2317.0000000	0.2500000
2318.0000000	0.2450000
2319.0000000	0.2350000
2320.0000000	0.2400000
2321.0000000	0.2500000
2322.0000000	0.2450000
2323.0000000	0.2450000
2324.0000000	0.2400000
2325.0000000	0.2450000
2326.0000000	0.2600000
2327.0000000	0.2450000
2328.0000000	0.2450000
2329.0000000	0.2400000
2330.0000000	0.2450000
2331.0000000	0.2550000
2332.0000000	0.2500000
2333.0000000	0.2533333
2334.0000000	0.2550000
2335.0000000	0.2550000
2336.0000000	0.2500000

2337.0000000	0.2550000
2338.0000000	0.2550000
2339.0000000	0.2600000
2340.0000000	0.2550000
2341.0000000	0.2600000
2342.0000000	0.2550000
2343.0000000	0.2500000
2344.0000000	0.2600000
2345.0000000	0.2650000
2346.0000000	0.2700000
2347.0000000	0.2550000
2348.0000000	0.2650000
2349.0000000	0.2550000
2350.0000000	0.2700000
2351.0000000	0.2700000
2352.0000000	0.2850000
2353.0000000	0.2650000
2354.0000000	0.2700000
2355.0000000	0.2650000
2356.0000000	0.2700000
2357.0000000	0.2700000
2358.0000000	0.2700000
2359.0000000	0.2650000
2360.0000000	0.2650000
2361.0000000	0.2600000
2362.0000000	0.2650000
2363.0000000	0.2650000
2364.0000000	0.2600000
2365.0000000	0.2750000
2366.0000000	0.2600000
2367.0000000	0.2600000
2368.0000000	0.2700000
2369.0000000	0.2650000
2370.0000000	0.2700000
2371.0000000	0.2700000
2372.0000000	0.2600000
2373.0000000	0.2750000
2374.0000000	0.2750000
2375.0000000	0.2650000
2376.0000000	0.2650000
2377.0000000	0.2650000
2378.0000000	0.2750000
2379.0000000	0.2850000
2380.0000000	0.2750000
2381.0000000	0.2650000
2382.0000000	0.2750000
2383.0000000	0.2700000
2384.0000000	0.2800000
2385.0000000	0.2700000
2386.0000000	0.2700000
2387.0000000	0.2700000
2388.0000000	0.2850000
2389.0000000	0.2800000
2390.0000000	0.2800000
2391.0000000	0.2800000
2392.0000000	0.2750000
2393.0000000	0.2866667

2394.0000000	0.2750000
2395.0000000	0.2800000
2396.0000000	0.2800000
2397.0000000	0.2800000
2398.0000000	0.2800000
2399.0000000	0.2750000
2400.0000000	0.2750000
2401.0000000	0.2850000
2402.0000000	0.2950000
2403.0000000	0.2800000
2404.0000000	0.2800000
2405.0000000	0.2800000
2406.0000000	0.2800000
2407.0000000	0.2850000
2408.0000000	0.2800000
2409.0000000	0.2850000
2410.0000000	0.2750000
2411.0000000	0.2800000
2412.0000000	0.2850000
2413.0000000	0.2850000
2414.0000000	0.2750000
2415.0000000	0.2850000
2416.0000000	0.2800000
2417.0000000	0.3000000
2418.0000000	0.2900000
2419.0000000	0.2750000
2420.0000000	0.2900000
2421.0000000	0.2900000
2422.0000000	0.2900000
2423.0000000	0.2866667
2424.0000000	0.2800000
2425.0000000	0.2800000
2426.0000000	0.2700000
2427.0000000	0.2900000
2428.0000000	0.2850000
2429.0000000	0.2800000
2430.0000000	0.2750000
2431.0000000	0.2750000
2432.0000000	0.2750000
2433.0000000	0.2800000
2434.0000000	0.2800000
2435.0000000	0.2700000
2436.0000000	0.2750000
2437.0000000	0.2700000
2438.0000000	0.2750000
2439.0000000	0.2850000
2440.0000000	0.2650000
2441.0000000	0.2700000
2442.0000000	0.2750000
2443.0000000	0.2700000
2444.0000000	0.2650000
2445.0000000	0.2700000
2446.0000000	0.2750000
2447.0000000	0.2800000
2448.0000000	0.2700000
2449.0000000	0.2800000
2450.0000000	0.2750000

2451.0000000	0.2750000
2452.0000000	0.2950000
2453.0000000	0.2850000
2454.0000000	0.2766667
2455.0000000	0.2850000
2456.0000000	0.2950000
2457.0000000	0.2800000
2458.0000000	0.2900000
2459.0000000	0.2900000
2460.0000000	0.2850000
2461.0000000	0.2900000
2462.0000000	0.2800000
2463.0000000	0.2900000
2464.0000000	0.2850000
2465.0000000	0.2850000
2466.0000000	0.2800000
2467.0000000	0.2850000
2468.0000000	0.2800000
2469.0000000	0.2800000
2470.0000000	0.2800000
2471.0000000	0.2800000
2472.0000000	0.2700000
2473.0000000	0.2750000
2474.0000000	0.2750000
2475.0000000	0.2700000
2476.0000000	0.2700000
2477.0000000	0.2700000
2478.0000000	0.2800000
2479.0000000	0.2750000
2480.0000000	0.2750000
2481.0000000	0.2700000
2482.0000000	0.2600000
2483.0000000	0.2800000
2484.0000000	0.2800000
2485.0000000	0.2750000
2486.0000000	0.2850000
2487.0000000	0.2800000
2488.0000000	0.2800000
2489.0000000	0.2850000
2490.0000000	0.2800000
2491.0000000	0.2750000
2492.0000000	0.2900000
2493.0000000	0.2750000
2494.0000000	0.2800000
2495.0000000	0.2800000
2496.0000000	0.2750000
2497.0000000	0.2700000
2498.0000000	0.2650000
2499.0000000	0.2700000
2500.0000000	0.2700000
2501.0000000	0.2750000
2502.0000000	0.2600000
2503.0000000	0.2700000
2504.0000000	0.2600000
2505.0000000	0.2750000
2506.0000000	0.2600000
2507.0000000	0.2650000

2508.0000000	0.2700000
2509.0000000	0.2700000
2510.0000000	0.2750000
2511.0000000	0.2700000
2512.0000000	0.2700000
2513.0000000	0.2650000
2514.0000000	0.2700000
2515.0000000	0.2700000
2516.0000000	0.2600000
2517.0000000	0.2800000
2518.0000000	0.2550000
2519.0000000	0.2500000
2520.0000000	0.2500000
2521.0000000	0.2500000
2522.0000000	0.2600000
2523.0000000	0.2650000
2524.0000000	0.2550000
2525.0000000	0.2650000
2526.0000000	0.2650000
2527.0000000	0.2600000
2528.0000000	0.2700000
2529.0000000	0.2500000
2530.0000000	0.2650000
2531.0000000	0.2650000
2532.0000000	0.2700000
2533.0000000	0.2750000
2534.0000000	0.2550000
2535.0000000	0.2650000
2536.0000000	0.2700000
2537.0000000	0.2650000
2538.0000000	0.2750000
2539.0000000	0.2700000
2540.0000000	0.2750000
2541.0000000	0.2800000
2542.0000000	0.2750000
2543.0000000	0.2750000
2544.0000000	0.2666667
2545.0000000	0.2700000
2546.0000000	0.2650000
2547.0000000	0.2700000
2548.0000000	0.2750000
2549.0000000	0.2750000
2550.0000000	0.2600000
2551.0000000	0.2650000
2552.0000000	0.2700000
2553.0000000	0.2600000
2554.0000000	0.2650000
2555.0000000	0.2750000
2556.0000000	0.2800000
2557.0000000	0.2700000
2558.0000000	0.2700000
2559.0000000	0.2800000
2560.0000000	0.2750000
2561.0000000	0.2800000
2562.0000000	0.2750000
2563.0000000	0.2800000
2564.0000000	0.2800000

2565.0000000	0.2900000
2566.0000000	0.2850000
2567.0000000	0.2800000
2568.0000000	0.2850000
2569.0000000	0.2950000
2570.0000000	0.2750000
2571.0000000	0.2750000
2572.0000000	0.2750000
2573.0000000	0.2750000
2574.0000000	0.2900000
2575.0000000	0.2800000
2576.0000000	0.2900000
2577.0000000	0.2850000
2578.0000000	0.2850000
2579.0000000	0.2750000
2580.0000000	0.2800000
2581.0000000	0.2850000
2582.0000000	0.2850000
2583.0000000	0.2900000
2584.0000000	0.2750000
2585.0000000	0.2800000
2586.0000000	0.2800000
2587.0000000	0.2800000
2588.0000000	0.2850000
2589.0000000	0.2750000
2590.0000000	0.2750000
2591.0000000	0.2850000
2592.0000000	0.2750000
2593.0000000	0.2850000
2594.0000000	0.2750000
2595.0000000	0.2750000
2596.0000000	0.2800000
2597.0000000	0.2800000
2598.0000000	0.2900000
2599.0000000	0.2700000
2600.0000000	0.2850000
2601.0000000	0.2800000
2602.0000000	0.2850000
2603.0000000	0.2850000
2604.0000000	0.2850000
2605.0000000	0.2900000
2606.0000000	0.2750000
2607.0000000	0.2850000
2608.0000000	0.2850000
2609.0000000	0.2900000
2610.0000000	0.2900000
2611.0000000	0.2800000
2612.0000000	0.2850000
2613.0000000	0.2800000
2614.0000000	0.2950000
2615.0000000	0.2900000
2616.0000000	0.2850000
2617.0000000	0.2900000
2618.0000000	0.2900000
2619.0000000	0.2950000
2620.0000000	0.2950000
2621.0000000	0.2850000

2622.0000000	0.3000000
2623.0000000	0.2850000
2624.0000000	0.2900000
2625.0000000	0.2950000
2626.0000000	0.2850000
2627.0000000	0.3000000
2628.0000000	0.2850000
2629.0000000	0.2900000
2630.0000000	0.2950000
2631.0000000	0.2900000
2632.0000000	0.2950000
2633.0000000	0.3000000
2634.0000000	0.2966667
2635.0000000	0.3050000
2636.0000000	0.2950000
2637.0000000	0.2900000
2638.0000000	0.2900000
2639.0000000	0.2950000
2640.0000000	0.3000000
2641.0000000	0.3050000
2642.0000000	0.3000000
2643.0000000	0.3000000
2644.0000000	0.2950000
2645.0000000	0.2950000
2646.0000000	0.3050000
2647.0000000	0.2950000
2648.0000000	0.3100000
2649.0000000	0.2900000
2650.0000000	0.3150000
2651.0000000	0.3050000
2652.0000000	0.3000000
2653.0000000	0.3000000
2654.0000000	0.2950000
2655.0000000	0.3050000
2656.0000000	0.3100000
2657.0000000	0.3000000
2658.0000000	0.3000000
2659.0000000	0.3100000
2660.0000000	0.3050000
2661.0000000	0.3150000
2662.0000000	0.3050000
2663.0000000	0.3050000
2664.0000000	0.3050000
2665.0000000	0.3066667
2666.0000000	0.2950000
2667.0000000	0.3050000
2668.0000000	0.3100000
2669.0000000	0.3100000
2670.0000000	0.3100000
2671.0000000	0.3000000
2672.0000000	0.3100000
2673.0000000	0.3050000
2674.0000000	0.3000000
2675.0000000	0.3000000
2676.0000000	0.2950000
2677.0000000	0.3050000
2678.0000000	0.3000000

2679.0000000	0.3150000
2680.0000000	0.2900000
2681.0000000	0.2900000
2682.0000000	0.3000000
2683.0000000	0.2950000
2684.0000000	0.3000000
2685.0000000	0.3000000
2686.0000000	0.2950000
2687.0000000	0.3000000
2688.0000000	0.3050000
2689.0000000	0.2850000
2690.0000000	0.2900000
2691.0000000	0.2950000
2692.0000000	0.2900000
2693.0000000	0.3050000
2694.0000000	0.2900000
2695.0000000	0.2850000
2696.0000000	0.2966667
2697.0000000	0.2800000
2698.0000000	0.2900000
2699.0000000	0.2900000
2700.0000000	0.2800000
2701.0000000	0.2900000
2702.0000000	0.2950000
2703.0000000	0.2900000
2704.0000000	0.2950000
2705.0000000	0.3000000
2706.0000000	0.2800000
2707.0000000	0.2900000
2708.0000000	0.3000000
2709.0000000	0.2900000
2710.0000000	0.3000000
2711.0000000	0.2900000
2712.0000000	0.3050000
2713.0000000	0.3000000
2714.0000000	0.3050000
2715.0000000	0.3000000
2716.0000000	0.3000000
2717.0000000	0.3050000
2718.0000000	0.3050000
2719.0000000	0.3150000
2720.0000000	0.3050000
2721.0000000	0.3050000
2722.0000000	0.3000000
2723.0000000	0.3050000
2724.0000000	0.3100000
2725.0000000	0.3100000
2726.0000000	0.3050000
2727.0000000	0.3150000
2728.0000000	0.3150000
2729.0000000	0.3100000
2730.0000000	0.3050000
2731.0000000	0.3100000
2732.0000000	0.3000000
2733.0000000	0.3050000
2734.0000000	0.3000000
2735.0000000	0.2950000

2736.0000000	0.2800000
2737.0000000	0.2900000
2738.0000000	0.2850000
2739.0000000	0.2850000
2740.0000000	0.2750000
2741.0000000	0.2700000
2742.0000000	0.2800000
2743.0000000	0.2700000
2744.0000000	0.2800000
2745.0000000	0.2750000
2746.0000000	0.2700000
2747.0000000	0.2650000
2748.0000000	0.2650000
2749.0000000	0.2700000
2750.0000000	0.2700000
2751.0000000	0.2700000
2752.0000000	0.2600000
2753.0000000	0.2600000
2754.0000000	0.2600000
2755.0000000	0.2633333
2756.0000000	0.2700000
2757.0000000	0.2600000
2758.0000000	0.2650000
2759.0000000	0.2650000
2760.0000000	0.2700000
2761.0000000	0.2600000
2762.0000000	0.2700000
2763.0000000	0.2550000
2764.0000000	0.2650000
2765.0000000	0.2700000
2766.0000000	0.2700000
2767.0000000	0.2700000
2768.0000000	0.2600000
2769.0000000	0.2650000
2770.0000000	0.2650000
2771.0000000	0.2700000
2772.0000000	0.2700000
2773.0000000	0.2650000
2774.0000000	0.2700000
2775.0000000	0.2700000
2776.0000000	0.2700000
2777.0000000	0.2550000
2778.0000000	0.2600000
2779.0000000	0.2650000
2780.0000000	0.2700000
2781.0000000	0.2650000
2782.0000000	0.2550000
2783.0000000	0.2600000
2784.0000000	0.2600000
2785.0000000	0.2650000
2786.0000000	0.2666667
2787.0000000	0.2750000
2788.0000000	0.2750000
2789.0000000	0.2600000
2790.0000000	0.2550000
2791.0000000	0.2700000
2792.0000000	0.2700000

2793.0000000	0.2800000
2794.0000000	0.2700000
2795.0000000	0.2700000
2796.0000000	0.2700000
2797.0000000	0.2700000
2798.0000000	0.2700000
2799.0000000	0.2700000
2800.0000000	0.2600000
2801.0000000	0.2650000
2802.0000000	0.2700000
2803.0000000	0.2700000
2804.0000000	0.2700000
2805.0000000	0.2700000
2806.0000000	0.2700000
2807.0000000	0.2650000
2808.0000000	0.2750000
2809.0000000	0.2650000
2810.0000000	0.2700000
2811.0000000	0.2700000
2812.0000000	0.2700000
2813.0000000	0.2750000
2814.0000000	0.2700000
2815.0000000	0.2750000
2816.0000000	0.2766666
2817.0000000	0.2750000
2818.0000000	0.2700000
2819.0000000	0.2800000
2820.0000000	0.2800000
2821.0000000	0.2800000
2822.0000000	0.2800000
2823.0000000	0.2750000
2824.0000000	0.2800000
2825.0000000	0.2800000
2826.0000000	0.2850000
2827.0000000	0.2700000
2828.0000000	0.2800000
2829.0000000	0.2750000
2830.0000000	0.2850000
2831.0000000	0.2700000
2832.0000000	0.2800000
2833.0000000	0.2750000
2834.0000000	0.2700000
2835.0000000	0.2800000
2836.0000000	0.2850000
2837.0000000	0.2850000
2838.0000000	0.2800000
2839.0000000	0.2800000
2840.0000000	0.2750000
2841.0000000	0.2850000
2842.0000000	0.2850000
2843.0000000	0.2850000
2844.0000000	0.2800000
2845.0000000	0.2800000
2846.0000000	0.2800000
2847.0000000	0.2850000
2848.0000000	0.2850000
2849.0000000	0.2800000

2850.0000000	0.2850000
2851.0000000	0.2850000
2852.0000000	0.2800000
2853.0000000	0.2850000
2854.0000000	0.2800000
2855.0000000	0.2900000
2856.0000000	0.2900000
2857.0000000	0.2850000
2858.0000000	0.2850000
2859.0000000	0.2800000
2860.0000000	0.2900000
2861.0000000	0.2900000
2862.0000000	0.3000000
2863.0000000	0.2950000
2864.0000000	0.2800000
2865.0000000	0.2950000
2866.0000000	0.3000000
2867.0000000	0.3050000
2868.0000000	0.2900000
2869.0000000	0.2950000
2870.0000000	0.2900000
2871.0000000	0.3000000
2872.0000000	0.2950000
2873.0000000	0.3050000
2874.0000000	0.3000000
2875.0000000	0.3000000
2876.0000000	0.3066667
2877.0000000	0.2950000
2878.0000000	0.3050000
2879.0000000	0.3050000
2880.0000000	0.3150000
2881.0000000	0.3100000
2882.0000000	0.3150000
2883.0000000	0.3000000
2884.0000000	0.3100000
2885.0000000	0.3050000
2886.0000000	0.3200000
2887.0000000	0.3150000
2888.0000000	0.3100000
2889.0000000	0.3100000
2890.0000000	0.3050000
2891.0000000	0.3200000
2892.0000000	0.3150000
2893.0000000	0.3050000
2894.0000000	0.3050000
2895.0000000	0.3250000
2896.0000000	0.3200000
2897.0000000	0.3200000
2898.0000000	0.3150000
2899.0000000	0.3100000
2900.0000000	0.3150000
2901.0000000	0.3150000
2902.0000000	0.3200000
2903.0000000	0.3050000
2904.0000000	0.3050000
2905.0000000	0.3000000
2906.0000000	0.3050000

2907.0000000	0.2966667
2908.0000000	0.3000000
2909.0000000	0.3000000
2910.0000000	0.2950000
2911.0000000	0.3000000
2912.0000000	0.2950000
2913.0000000	0.3000000
2914.0000000	0.2950000
2915.0000000	0.3050000
2916.0000000	0.3000000
2917.0000000	0.2950000
2918.0000000	0.2950000
2919.0000000	0.2950000
2920.0000000	0.3050000
2921.0000000	0.3050000
2922.0000000	0.2900000
2923.0000000	0.3000000
2924.0000000	0.2900000
2925.0000000	0.3000000
2926.0000000	0.2950000
2927.0000000	0.3000000
2928.0000000	0.2950000
2929.0000000	0.2950000
2930.0000000	0.3000000
2931.0000000	0.3000000
2932.0000000	0.3100000
2933.0000000	0.2950000
2934.0000000	0.3050000
2935.0000000	0.2900000
2936.0000000	0.3050000
2937.0000000	0.2966667
2938.0000000	0.3100000
2939.0000000	0.3050000
2940.0000000	0.3000000
2941.0000000	0.2950000
2942.0000000	0.2950000
2943.0000000	0.3050000
2944.0000000	0.3050000
2945.0000000	0.3050000
2946.0000000	0.3000000
2947.0000000	0.3050000
2948.0000000	0.3050000
2949.0000000	0.3100000
2950.0000000	0.3050000
2951.0000000	0.3000000
2952.0000000	0.3100000
2953.0000000	0.3100000
2954.0000000	0.3200000
2955.0000000	0.3050000
2956.0000000	0.3050000
2957.0000000	0.3100000
2958.0000000	0.3100000
2959.0000000	0.3200000
2960.0000000	0.3100000
2961.0000000	0.3150000
2962.0000000	0.3150000
2963.0000000	0.3100000

2964.0000000	0.3100000
2965.0000000	0.3100000
2966.0000000	0.3100000
2967.0000000	0.3200000
2968.0000000	0.3050000
2969.0000000	0.3250000
2970.0000000	0.3200000
2971.0000000	0.3100000
2972.0000000	0.3100000
2973.0000000	0.3000000
2974.0000000	0.3150000
2975.0000000	0.3200000
2976.0000000	0.3050000
2977.0000000	0.3100000
2978.0000000	0.3050000
2979.0000000	0.3050000
2980.0000000	0.3150000
2981.0000000	0.3000000
2982.0000000	0.3100000
2983.0000000	0.3100000
2984.0000000	0.3150000
2985.0000000	0.3050000
2986.0000000	0.3050000
2987.0000000	0.3050000
2988.0000000	0.3050000
2989.0000000	0.3050000
2990.0000000	0.3100000
2991.0000000	0.2950000
2992.0000000	0.3050000
2993.0000000	0.3100000
2994.0000000	0.3000000
2995.0000000	0.3150000
2996.0000000	0.3000000
2997.0000000	0.3133333
2998.0000000	0.3000000
2999.0000000	0.3150000
3000.0000000	0.3100000
3001.0000000	0.3100000
3002.0000000	0.3100000
3003.0000000	0.3050000
3004.0000000	0.3150000
3005.0000000	0.3100000
3006.0000000	0.3200000
3007.0000000	0.3200000
3008.0000000	0.3100000
3009.0000000	0.3200000
3010.0000000	0.3200000
3011.0000000	0.3300000
3012.0000000	0.3300000
3013.0000000	0.3100000
3014.0000000	0.3250000
3015.0000000	0.3200000
3016.0000000	0.3200000
3017.0000000	0.3150000
3018.0000000	0.3050000
3019.0000000	0.3150000
3020.0000000	0.3250000

3021.0000000	0.3250000
3022.0000000	0.3200000
3023.0000000	0.3100000
3024.0000000	0.3150000
3025.0000000	0.3100000
3026.0000000	0.3150000
3027.0000000	0.3150000
3028.0000000	0.3133334
3029.0000000	0.3100000
3030.0000000	0.3100000
3031.0000000	0.3150000
3032.0000000	0.3200000
3033.0000000	0.3150000
3034.0000000	0.3050000
3035.0000000	0.3250000
3036.0000000	0.3250000
3037.0000000	0.3200000
3038.0000000	0.3100000
3039.0000000	0.3100000
3040.0000000	0.3150000
3041.0000000	0.3200000
3042.0000000	0.3100000
3043.0000000	0.3150000
3044.0000000	0.3200000
3045.0000000	0.3200000
3046.0000000	0.3250000
3047.0000000	0.3150000
3048.0000000	0.3100000
3049.0000000	0.3300000
3050.0000000	0.3150000
3051.0000000	0.3250000
3052.0000000	0.3250000
3053.0000000	0.3200000
3054.0000000	0.3300000
3055.0000000	0.3350000
3056.0000000	0.3250000
3057.0000000	0.3333333
3058.0000000	0.3150000
3059.0000000	0.3250000
3060.0000000	0.3250000
3061.0000000	0.3300000
3062.0000000	0.3300000
3063.0000000	0.3200000
3064.0000000	0.3200000
3065.0000000	0.3300000
3066.0000000	0.3400000
3067.0000000	0.3200000
3068.0000000	0.3200000
3069.0000000	0.3250000
3070.0000000	0.3350000
3071.0000000	0.3250000
3072.0000000	0.3200000
3073.0000000	0.3150000
3074.0000000	0.3250000
3075.0000000	0.3250000
3076.0000000	0.3300000
3077.0000000	0.3150000

3078.0000000	0.3200000
3079.0000000	0.3250000
3080.0000000	0.3250000
3081.0000000	0.3350000
3082.0000000	0.3250000
3083.0000000	0.3300000
3084.0000000	0.3200000
3085.0000000	0.3350000
3086.0000000	0.3200000
3087.0000000	0.3266667
3088.0000000	0.3250000
3089.0000000	0.3300000
3090.0000000	0.3250000
3091.0000000	0.3250000
3092.0000000	0.3200000
3093.0000000	0.3200000
3094.0000000	0.3250000
3095.0000000	0.3150000
3096.0000000	0.3200000
3097.0000000	0.3150000
3098.0000000	0.3250000
3099.0000000	0.3150000
3100.0000000	0.3200000
3101.0000000	0.3150000
3102.0000000	0.3150000
3103.0000000	0.3100000
3104.0000000	0.3100000
3105.0000000	0.3150000
3106.0000000	0.3250000
3107.0000000	0.3250000
3108.0000000	0.3200000
3109.0000000	0.3200000
3110.0000000	0.3250000
3111.0000000	0.3350000
3112.0000000	0.3200000
3113.0000000	0.3250000
3114.0000000	0.3300000
3115.0000000	0.3300000
3116.0000000	0.3300000
3117.0000000	0.3350000
3118.0000000	0.3333333
3119.0000000	0.3450000
3120.0000000	0.3450000
3121.0000000	0.3350000
3122.0000000	0.3450000
3123.0000000	0.3350000
3124.0000000	0.3450000
3125.0000000	0.3450000
3126.0000000	0.3450000
3127.0000000	0.3500000
3128.0000000	0.3450000
3129.0000000	0.3450000
3130.0000000	0.3450000
3131.0000000	0.3550000
3132.0000000	0.3450000
3133.0000000	0.3450000
3134.0000000	0.3400000

3135.0000000	0.3450000
3136.0000000	0.3400000
3137.0000000	0.3400000
3138.0000000	0.3400000
3139.0000000	0.3400000
3140.0000000	0.3350000
3141.0000000	0.3350000
3142.0000000	0.3250000
3143.0000000	0.3300000
3144.0000000	0.3350000
3145.0000000	0.3300000
3146.0000000	0.3300000
3147.0000000	0.3350000
3148.0000000	0.3233334
3149.0000000	0.3300000
3150.0000000	0.3250000
3151.0000000	0.3150000
3152.0000000	0.3200000
3153.0000000	0.3150000
3154.0000000	0.3300000
3155.0000000	0.3200000
3156.0000000	0.3200000
3157.0000000	0.3100000
3158.0000000	0.3200000
3159.0000000	0.3250000
3160.0000000	0.3200000
3161.0000000	0.3200000
3162.0000000	0.3150000
3163.0000000	0.3150000
3164.0000000	0.3200000
3165.0000000	0.3300000
3166.0000000	0.3200000
3167.0000000	0.3150000
3168.0000000	0.3250000
3169.0000000	0.3200000
3170.0000000	0.3250000
3171.0000000	0.3200000
3172.0000000	0.3150000
3173.0000000	0.3250000
3174.0000000	0.3300000
3175.0000000	0.3150000
3176.0000000	0.3150000
3177.0000000	0.3350000
3178.0000000	0.3166667
3179.0000000	0.3300000
3180.0000000	0.3250000
3181.0000000	0.3200000
3182.0000000	0.3200000
3183.0000000	0.3350000
3184.0000000	0.3250000
3185.0000000	0.3250000
3186.0000000	0.3250000
3187.0000000	0.3250000
3188.0000000	0.3300000
3189.0000000	0.3300000
3190.0000000	0.3250000
3191.0000000	0.3250000

3192.0000000	0.3300000
3193.0000000	0.3450000
3194.0000000	0.3200000
3195.0000000	0.3400000
3196.0000000	0.3400000
3197.0000000	0.3400000
3198.0000000	0.3250000
3199.0000000	0.3300000
3200.0000000	0.3450000
3201.0000000	0.3300000
3202.0000000	0.3400000
3203.0000000	0.3400000
3204.0000000	0.3450000
3205.0000000	0.3450000
3206.0000000	0.3500000
3207.0000000	0.3400000
3208.0000000	0.3500000
3209.0000000	0.3350000
3210.0000000	0.3400000
3211.0000000	0.3450000
3212.0000000	0.3500000
3213.0000000	0.3400000
3214.0000000	0.3300000
3215.0000000	0.3400000
3216.0000000	0.3250000
3217.0000000	0.3300000
3218.0000000	0.3300000
3219.0000000	0.3300000
3220.0000000	0.3250000
3221.0000000	0.3200000
3222.0000000	0.3250000
3223.0000000	0.3350000
3224.0000000	0.3250000
3225.0000000	0.3200000
3226.0000000	0.3300000
3227.0000000	0.3350000
3228.0000000	0.3150000
3229.0000000	0.3200000
3230.0000000	0.3250000
3231.0000000	0.3150000
3232.0000000	0.3200000
3233.0000000	0.3250000
3234.0000000	0.3200000
3235.0000000	0.3250000
3236.0000000	0.3300000
3237.0000000	0.3150000
3238.0000000	0.3150000
3239.0000000	0.3200000
3240.0000000	0.3200000
3241.0000000	0.3150000
3242.0000000	0.3300000
3243.0000000	0.3150000
3244.0000000	0.3100000
3245.0000000	0.3200000
3246.0000000	0.3150000
3247.0000000	0.3150000
3248.0000000	0.3250000

3249.0000000	0.3150000
3250.0000000	0.3150000
3251.0000000	0.3100000
3252.0000000	0.3150000
3253.0000000	0.3150000
3254.0000000	0.3250000
3255.0000000	0.3150000
3256.0000000	0.3250000
3257.0000000	0.3100000
3258.0000000	0.3150000
3259.0000000	0.3150000
3260.0000000	0.3250000
3261.0000000	0.3100000
3262.0000000	0.3200000
3263.0000000	0.3150000
3264.0000000	0.3200000
3265.0000000	0.3200000
3266.0000000	0.3250000
3267.0000000	0.3200000
3268.0000000	0.3200000
3269.0000000	0.3200000
3270.0000000	0.3200000
3271.0000000	0.3200000
3272.0000000	0.3250000
3273.0000000	0.3250000
3274.0000000	0.3300000
3275.0000000	0.3100000
3276.0000000	0.3200000
3277.0000000	0.3200000
3278.0000000	0.3300000
3279.0000000	0.3200000
3280.0000000	0.3350000
3281.0000000	0.3150000
3282.0000000	0.3250000
3283.0000000	0.3250000
3284.0000000	0.3200000
3285.0000000	0.3250000
3286.0000000	0.3150000
3287.0000000	0.3300000
3288.0000000	0.3300000
3289.0000000	0.3300000
3290.0000000	0.3300000
3291.0000000	0.3150000
3292.0000000	0.3250000
3293.0000000	0.3300000
3294.0000000	0.3300000
3295.0000000	0.3300000
3296.0000000	0.3250000
3297.0000000	0.3250000
3298.0000000	0.3266667
3299.0000000	0.3400000
3300.0000000	0.3300000
3301.0000000	0.3450000
3302.0000000	0.3350000
3303.0000000	0.3350000
3304.0000000	0.3350000
3305.0000000	0.3300000

3306.0000000	0.3450000
3307.0000000	0.3350000
3308.0000000	0.3400000
3309.0000000	0.3400000
3310.0000000	0.3450000
3311.0000000	0.3450000
3312.0000000	0.3450000
3313.0000000	0.3350000
3314.0000000	0.3550000
3315.0000000	0.3500000
3316.0000000	0.3500000
3317.0000000	0.3450000
3318.0000000	0.3400000
3319.0000000	0.3450000
3320.0000000	0.3500000
3321.0000000	0.3400000
3322.0000000	0.3400000
3323.0000000	0.3450000
3324.0000000	0.3500000
3325.0000000	0.3450000
3326.0000000	0.3500000
3327.0000000	0.3450000
3328.0000000	0.3550000
3329.0000000	0.3433333
3330.0000000	0.3550000
3331.0000000	0.3550000
3332.0000000	0.3500000
3333.0000000	0.3500000
3334.0000000	0.3400000
3335.0000000	0.3500000
3336.0000000	0.3450000
3337.0000000	0.3450000
3338.0000000	0.3600000
3339.0000000	0.3500000
3340.0000000	0.3650000
3341.0000000	0.3400000
3342.0000000	0.3550000
3343.0000000	0.3450000
3344.0000000	0.3550000
3345.0000000	0.3550000
3346.0000000	0.3600000
3347.0000000	0.3550000
3348.0000000	0.3500000
3349.0000000	0.3450000
3350.0000000	0.3550000
3351.0000000	0.3600000
3352.0000000	0.3600000
3353.0000000	0.3500000
3354.0000000	0.3450000
3355.0000000	0.3600000
3356.0000000	0.3550000
3357.0000000	0.3500000
3358.0000000	0.3500000
3359.0000000	0.3400000
3360.0000000	0.3500000
3361.0000000	0.3500000
3362.0000000	0.3550000

3363.0000000	0.3550000
3364.0000000	0.3400000
3365.0000000	0.3450000
3366.0000000	0.3450000
3367.0000000	0.3300000
3368.0000000	0.3350000
3369.0000000	0.3400000
3370.0000000	0.3400000
3371.0000000	0.3400000
3372.0000000	0.3500000
3373.0000000	0.3400000
3374.0000000	0.3400000
3375.0000000	0.3400000
3376.0000000	0.3400000
3377.0000000	0.3350000
3378.0000000	0.3400000
3379.0000000	0.3500000
3380.0000000	0.3550000
3381.0000000	0.3350000
3382.0000000	0.3400000
3383.0000000	0.3500000
3384.0000000	0.3400000
3385.0000000	0.3300000
3386.0000000	0.3350000
3387.0000000	0.3350000
3388.0000000	0.3450000
3389.0000000	0.3400000
3390.0000000	0.3500000
3391.0000000	0.3500000
3392.0000000	0.3350000
3393.0000000	0.3350000
3394.0000000	0.3350000
3395.0000000	0.3350000
3396.0000000	0.3300000
3397.0000000	0.3300000
3398.0000000	0.3300000
3399.0000000	0.3450000
3400.0000000	0.3300000
3401.0000000	0.3250000
3402.0000000	0.3350000
3403.0000000	0.3250000
3404.0000000	0.3350000
3405.0000000	0.3300000
3406.0000000	0.3250000
3407.0000000	0.3250000
3408.0000000	0.3350000
3409.0000000	0.3200000
3410.0000000	0.3350000
3411.0000000	0.3250000
3412.0000000	0.3300000
3413.0000000	0.3250000
3414.0000000	0.3350000
3415.0000000	0.3350000
3416.0000000	0.3150000
3417.0000000	0.3250000
3418.0000000	0.3200000
3419.0000000	0.3233333

3420.0000000	0.3250000
3421.0000000	0.3250000
3422.0000000	0.3150000
3423.0000000	0.3150000
3424.0000000	0.3000000
3425.0000000	0.3100000
3426.0000000	0.3150000
3427.0000000	0.3200000
3428.0000000	0.3150000
3429.0000000	0.3000000
3430.0000000	0.3100000
3431.0000000	0.3050000
3432.0000000	0.3150000
3433.0000000	0.3100000
3434.0000000	0.3150000
3435.0000000	0.3000000
3436.0000000	0.3100000
3437.0000000	0.3100000
3438.0000000	0.3200000
3439.0000000	0.3050000
3440.0000000	0.3100000
3441.0000000	0.3000000
3442.0000000	0.3000000
3443.0000000	0.3050000
3444.0000000	0.3150000
3445.0000000	0.3150000
3446.0000000	0.3050000
3447.0000000	0.3100000
3448.0000000	0.3100000
3449.0000000	0.3050000
3450.0000000	0.3100000
3451.0000000	0.3050000
3452.0000000	0.3050000
3453.0000000	0.3200000
3454.0000000	0.3200000
3455.0000000	0.3100000
3456.0000000	0.3150000
3457.0000000	0.3100000
3458.0000000	0.3200000
3459.0000000	0.3150000
3460.0000000	0.3150000
3461.0000000	0.3200000
3462.0000000	0.3100000
3463.0000000	0.3250000
3464.0000000	0.3200000
3465.0000000	0.3250000
3466.0000000	0.3200000
3467.0000000	0.3200000
3468.0000000	0.3150000
3469.0000000	0.3250000
3470.0000000	0.3300000
3471.0000000	0.3200000
3472.0000000	0.3200000
3473.0000000	0.3200000
3474.0000000	0.3250000
3475.0000000	0.3200000
3476.0000000	0.3250000

3477.0000000	0.3200000
3478.0000000	0.3200000
3479.0000000	0.3100000
3480.0000000	0.3133333
3481.0000000	0.3150000
3482.0000000	0.3250000
3483.0000000	0.3150000
3484.0000000	0.3200000
3485.0000000	0.3100000
3486.0000000	0.3150000
3487.0000000	0.3200000
3488.0000000	0.3200000
3489.0000000	0.3300000
3490.0000000	0.3150000
3491.0000000	0.3250000
3492.0000000	0.3200000
3493.0000000	0.3200000
3494.0000000	0.3150000
3495.0000000	0.3250000
3496.0000000	0.3250000
3497.0000000	0.3300000
3498.0000000	0.3200000
3499.0000000	0.3300000
3500.0000000	0.3150000
3501.0000000	0.3150000
3502.0000000	0.3250000
3503.0000000	0.3200000
3504.0000000	0.3250000
3505.0000000	0.3300000
3506.0000000	0.3350000
3507.0000000	0.3400000
3508.0000000	0.3300000
3509.0000000	0.3250000
3510.0000000	0.3300000
3511.0000000	0.3250000
3512.0000000	0.3300000
3513.0000000	0.3300000
3514.0000000	0.3350000
3515.0000000	0.3300000
3516.0000000	0.3250000
3517.0000000	0.3400000
3518.0000000	0.3450000
3519.0000000	0.3350000
3520.0000000	0.3350000
3521.0000000	0.3350000
3522.0000000	0.3400000
3523.0000000	0.3500000
3524.0000000	0.3450000
3525.0000000	0.3300000
3526.0000000	0.3450000
3527.0000000	0.3400000
3528.0000000	0.3400000
3529.0000000	0.3400000
3530.0000000	0.3600000
3531.0000000	0.3550000
3532.0000000	0.3450000
3533.0000000	0.3550000

3534.0000000	0.3600000
3535.0000000	0.3550000
3536.0000000	0.3600000
3537.0000000	0.3650000
3538.0000000	0.3500000
3539.0000000	0.3650000
3540.0000000	0.3633333
3541.0000000	0.3750000
3542.0000000	0.3650000
3543.0000000	0.3750000
3544.0000000	0.3800000
3545.0000000	0.3700000
3546.0000000	0.3700000
3547.0000000	0.3700000
3548.0000000	0.3700000
3549.0000000	0.3750000
3550.0000000	0.3750000
3551.0000000	0.3800000
3552.0000000	0.3700000
3553.0000000	0.3850000
3554.0000000	0.3900000
3555.0000000	0.3750000
3556.0000000	0.3750000
3557.0000000	0.3750000
3558.0000000	0.3750000
3559.0000000	0.3900000
3560.0000000	0.3850000
3561.0000000	0.3800000
3562.0000000	0.3850000
3563.0000000	0.3800000
3564.0000000	0.3950000
3565.0000000	0.3850000
3566.0000000	0.3700000
3567.0000000	0.3850000
3568.0000000	0.3850000
3569.0000000	0.3850000
3570.0000000	0.3850000
3571.0000000	0.3800000
3572.0000000	0.3750000
3573.0000000	0.3750000
3574.0000000	0.3850000
3575.0000000	0.3800000
3576.0000000	0.3850000
3577.0000000	0.3750000
3578.0000000	0.3800000
3579.0000000	0.3850000
3580.0000000	0.3750000
3581.0000000	0.3900000
3582.0000000	0.3800000
3583.0000000	0.3950000
3584.0000000	0.3800000
3585.0000000	0.3950000
3586.0000000	0.3850000
3587.0000000	0.3900000
3588.0000000	0.3900000
3589.0000000	0.3900000
3590.0000000	0.3950000

3591.0000000	0.3900000
3592.0000000	0.3950000
3593.0000000	0.3850000
3594.0000000	0.3850000
3595.0000000	0.3900000
3596.0000000	0.3850000
3597.0000000	0.3950000
3598.0000000	0.3800000
3599.0000000	0.3850000
3600.0000000	0.3800000
3601.0000000	0.3933333
3602.0000000	0.3850000
3603.0000000	0.3900000
3604.0000000	0.3850000
3605.0000000	0.3950000
3606.0000000	0.3800000
3607.0000000	0.3850000
3608.0000000	0.3800000
3609.0000000	0.3850000
3610.0000000	0.3900000
3611.0000000	0.3900000
3612.0000000	0.3900000
3613.0000000	0.3900000
3614.0000000	0.3850000
3615.0000000	0.3950000
3616.0000000	0.3850000
3617.0000000	0.4000000
3618.0000000	0.3900000
3619.0000000	0.4000000
3620.0000000	0.3850000
3621.0000000	0.3950000
3622.0000000	0.3850000
3623.0000000	0.4000000
3624.0000000	0.3900000
3625.0000000	0.3900000
3626.0000000	0.3950000
3627.0000000	0.3900000
3628.0000000	0.3900000
3629.0000000	0.3950000
3630.0000000	0.3966667
3631.0000000	0.3900000
3632.0000000	0.3900000
3633.0000000	0.3900000
3634.0000000	0.4000000
3635.0000000	0.3900000
3636.0000000	0.3950000
3637.0000000	0.3900000
3638.0000000	0.3950000
3639.0000000	0.3850000
3640.0000000	0.3950000
3641.0000000	0.3900000
3642.0000000	0.3900000
3643.0000000	0.3900000
3644.0000000	0.3900000
3645.0000000	0.3850000
3646.0000000	0.3950000
3647.0000000	0.4000000

3648.0000000	0.3950000
3649.0000000	0.3900000
3650.0000000	0.3900000
3651.0000000	0.3950000
3652.0000000	0.3950000
3653.0000000	0.4000000
3654.0000000	0.3850000
3655.0000000	0.3800000
3656.0000000	0.3800000
3657.0000000	0.3800000
3658.0000000	0.3850000
3659.0000000	0.3900000
3660.0000000	0.3950000
3661.0000000	0.3833333
3662.0000000	0.3750000
3663.0000000	0.3700000
3664.0000000	0.3800000
3665.0000000	0.3800000
3666.0000000	0.3850000
3667.0000000	0.3800000
3668.0000000	0.3650000
3669.0000000	0.3800000
3670.0000000	0.3650000
3671.0000000	0.3600000
3672.0000000	0.3700000
3673.0000000	0.3550000
3674.0000000	0.3650000
3675.0000000	0.3750000
3676.0000000	0.3600000
3677.0000000	0.3500000
3678.0000000	0.3500000
3679.0000000	0.3500000
3680.0000000	0.3550000
3681.0000000	0.3650000
3682.0000000	0.3450000
3683.0000000	0.3550000
3684.0000000	0.3550000
3685.0000000	0.3450000
3686.0000000	0.3400000
3687.0000000	0.3400000
3688.0000000	0.3550000
3689.0000000	0.3450000
3690.0000000	0.3550000
3691.0000000	0.3500000
3692.0000000	0.3466667
3693.0000000	0.3500000
3694.0000000	0.3550000
3695.0000000	0.3450000
3696.0000000	0.3550000
3697.0000000	0.3550000
3698.0000000	0.3550000
3699.0000000	0.3600000
3700.0000000	0.3550000
3701.0000000	0.3650000
3702.0000000	0.3650000
3703.0000000	0.3650000
3704.0000000	0.3650000

3705.0000000	0.3650000
3706.0000000	0.3650000
3707.0000000	0.3650000
3708.0000000	0.3650000
3709.0000000	0.3600000
3710.0000000	0.3600000
3711.0000000	0.3650000
3712.0000000	0.3650000
3713.0000000	0.3650000
3714.0000000	0.3550000
3715.0000000	0.3650000
3716.0000000	0.3600000
3717.0000000	0.3600000
3718.0000000	0.3600000
3719.0000000	0.3650000
3720.0000000	0.3700000
3721.0000000	0.3633333
3722.0000000	0.3600000
3723.0000000	0.3700000
3724.0000000	0.3750000
3725.0000000	0.3700000
3726.0000000	0.3550000
3727.0000000	0.3800000
3728.0000000	0.3750000
3729.0000000	0.3650000
3730.0000000	0.3650000
3731.0000000	0.3700000
3732.0000000	0.3800000
3733.0000000	0.3650000
3734.0000000	0.3650000
3735.0000000	0.3650000
3736.0000000	0.3700000
3737.0000000	0.3700000
3738.0000000	0.3650000
3739.0000000	0.3700000
3740.0000000	0.3800000
3741.0000000	0.3700000
3742.0000000	0.3700000
3743.0000000	0.3750000
3744.0000000	0.3850000
3745.0000000	0.3700000
3746.0000000	0.3800000
3747.0000000	0.3850000
3748.0000000	0.3900000
3749.0000000	0.3900000
3750.0000000	0.3900000
3751.0000000	0.3833333
3752.0000000	0.4000000
3753.0000000	0.3900000
3754.0000000	0.3850000
3755.0000000	0.3950000
3756.0000000	0.4000000
3757.0000000	0.3850000
3758.0000000	0.3900000
3759.0000000	0.4050000
3760.0000000	0.3950000
3761.0000000	0.3800000

3762.0000000	0.4000000
3763.0000000	0.4000000
3764.0000000	0.3950000
3765.0000000	0.3900000
3766.0000000	0.4100000
3767.0000000	0.4000000
3768.0000000	0.4100000
3769.0000000	0.4050000
3770.0000000	0.4000000
3771.0000000	0.4100000
3772.0000000	0.4000000
3773.0000000	0.4050000
3774.0000000	0.4100000
3775.0000000	0.4050000
3776.0000000	0.4050000
3777.0000000	0.4250000
3778.0000000	0.4050000
3779.0000000	0.4100000
3780.0000000	0.4050000
3781.0000000	0.4200000
3782.0000000	0.4100000
3783.0000000	0.4250000
3784.0000000	0.4250000
3785.0000000	0.4200000
3786.0000000	0.4150000
3787.0000000	0.4050000
3788.0000000	0.4200000
3789.0000000	0.4300000
3790.0000000	0.4250000
3791.0000000	0.4250000
3792.0000000	0.4150000
3793.0000000	0.4200000
3794.0000000	0.4150000
3795.0000000	0.4300000
3796.0000000	0.4200000
3797.0000000	0.4300000
3798.0000000	0.4200000
3799.0000000	0.4200000
3800.0000000	0.4150000
3801.0000000	0.4300000
3802.0000000	0.4200000
3803.0000000	0.4200000
3804.0000000	0.4150000
3805.0000000	0.4100000
3806.0000000	0.4200000
3807.0000000	0.4200000
3808.0000000	0.4300000
3809.0000000	0.4150000
3810.0000000	0.4200000
3811.0000000	0.4200000
3812.0000000	0.4133333
3813.0000000	0.4200000
3814.0000000	0.4150000
3815.0000000	0.4100000
3816.0000000	0.4100000
3817.0000000	0.4200000
3818.0000000	0.4200000

3819.0000000	0.4200000
3820.0000000	0.4250000
3821.0000000	0.4050000
3822.0000000	0.4150000
3823.0000000	0.4250000
3824.0000000	0.4200000
3825.0000000	0.4150000
3826.0000000	0.4150000
3827.0000000	0.4150000
3828.0000000	0.4200000
3829.0000000	0.4150000
3830.0000000	0.4200000
3831.0000000	0.4150000
3832.0000000	0.4200000
3833.0000000	0.4300000
3834.0000000	0.4050000
3835.0000000	0.4200000
3836.0000000	0.4050000
3837.0000000	0.4050000
3838.0000000	0.4200000
3839.0000000	0.4100000
3840.0000000	0.4050000
3841.0000000	0.4000000
3842.0000000	0.4033333
3843.0000000	0.3900000
3844.0000000	0.3950000
3845.0000000	0.4000000
3846.0000000	0.3950000
3847.0000000	0.3950000
3848.0000000	0.3900000
3849.0000000	0.4050000
3850.0000000	0.3950000
3851.0000000	0.3900000
3852.0000000	0.3800000
3853.0000000	0.3700000
3854.0000000	0.3900000
3855.0000000	0.3850000
3856.0000000	0.3750000
3857.0000000	0.3700000
3858.0000000	0.3750000
3859.0000000	0.3800000
3860.0000000	0.3800000
3861.0000000	0.3800000
3862.0000000	0.3700000
3863.0000000	0.3800000
3864.0000000	0.3800000
3865.0000000	0.3600000
3866.0000000	0.3700000
3867.0000000	0.3700000
3868.0000000	0.3700000
3869.0000000	0.3600000
3870.0000000	0.3700000
3871.0000000	0.3650000
3872.0000000	0.3666667
3873.0000000	0.3800000
3874.0000000	0.3700000
3875.0000000	0.3550000

3876.0000000	0.3700000
3877.0000000	0.3650000
3878.0000000	0.3650000
3879.0000000	0.3650000
3880.0000000	0.3650000
3881.0000000	0.3750000
3882.0000000	0.3650000
3883.0000000	0.3700000
3884.0000000	0.3700000
3885.0000000	0.3700000
3886.0000000	0.3650000
3887.0000000	0.3600000
3888.0000000	0.3700000
3889.0000000	0.3700000
3890.0000000	0.3650000
3891.0000000	0.3600000
3892.0000000	0.3700000
3893.0000000	0.3750000
3894.0000000	0.3550000
3895.0000000	0.3550000
3896.0000000	0.3650000
3897.0000000	0.3550000
3898.0000000	0.3500000
3899.0000000	0.3450000
3900.0000000	0.3550000
3901.0000000	0.3550000
3902.0000000	0.3400000
3903.0000000	0.3500000
3904.0000000	0.3500000
3905.0000000	0.3500000
3906.0000000	0.3500000
3907.0000000	0.3550000
3908.0000000	0.3450000
3909.0000000	0.3400000
3910.0000000	0.3450000
3911.0000000	0.3400000
3912.0000000	0.3450000
3913.0000000	0.3450000
3914.0000000	0.3550000
3915.0000000	0.3550000
3916.0000000	0.3450000
3917.0000000	0.3600000
3918.0000000	0.3550000
3919.0000000	0.3550000
3920.0000000	0.3500000
3921.0000000	0.3450000
3922.0000000	0.3500000
3923.0000000	0.3550000
3924.0000000	0.3500000
3925.0000000	0.3500000
3926.0000000	0.3350000
3927.0000000	0.3400000
3928.0000000	0.3400000
3929.0000000	0.3450000
3930.0000000	0.3450000
3931.0000000	0.3450000
3932.0000000	0.3500000

3933.0000000	0.3400000
3934.0000000	0.3350000
3935.0000000	0.3500000
3936.0000000	0.3500000
3937.0000000	0.3600000
3938.0000000	0.3350000
3939.0000000	0.3500000
3940.0000000	0.3350000
3941.0000000	0.3550000
3942.0000000	0.3400000
3943.0000000	0.3550000
3944.0000000	0.3400000
3945.0000000	0.3400000
3946.0000000	0.3350000
3947.0000000	0.3500000
3948.0000000	0.3500000
3949.0000000	0.3550000
3950.0000000	0.3500000
3951.0000000	0.3450000
3952.0000000	0.3500000
3953.0000000	0.3450000
3954.0000000	0.3500000
3955.0000000	0.3400000
3956.0000000	0.3400000
3957.0000000	0.3500000
3958.0000000	0.3500000
3959.0000000	0.3450000
3960.0000000	0.3500000
3961.0000000	0.3400000
3962.0000000	0.3600000
3963.0000000	0.3450000
3964.0000000	0.3400000
3965.0000000	0.3450000
3966.0000000	0.3450000
3967.0000000	0.3500000
3968.0000000	0.3500000
3969.0000000	0.3400000
3970.0000000	0.3400000
3971.0000000	0.3350000
3972.0000000	0.3450000
3973.0000000	0.3450000
3974.0000000	0.3400000
3975.0000000	0.3350000
3976.0000000	0.3350000
3977.0000000	0.3350000
3978.0000000	0.3350000
3979.0000000	0.3500000
3980.0000000	0.3400000
3981.0000000	0.3400000
3982.0000000	0.3400000
3983.0000000	0.3400000
3984.0000000	0.3450000
3985.0000000	0.3550000
3986.0000000	0.3550000
3987.0000000	0.3450000
3988.0000000	0.3450000
3989.0000000	0.3500000

3990.0000000	0.3600000
3991.0000000	0.3550000
3992.0000000	0.3500000
3993.0000000	0.3566667
3994.0000000	0.3500000
3995.0000000	0.3500000
3996.0000000	0.3500000
3997.0000000	0.3600000
3998.0000000	0.3550000
3999.0000000	0.3650000
4000.0000000	0.3500000
4001.0000000	0.3650000
4002.0000000	0.3600000
4003.0000000	0.3550000
4004.0000000	0.3700000
4005.0000000	0.3550000
4006.0000000	0.3600000
4007.0000000	0.3650000
4008.0000000	0.3650000
4009.0000000	0.3600000
4010.0000000	0.3550000
4011.0000000	0.3650000
4012.0000000	0.3650000
4013.0000000	0.3650000
4014.0000000	0.3700000
4015.0000000	0.3600000
4016.0000000	0.3700000
4017.0000000	0.3650000
4018.0000000	0.3700000
4019.0000000	0.3750000
4020.0000000	0.3700000
4021.0000000	0.3750000
4022.0000000	0.3750000
4023.0000000	0.3700000
4024.0000000	0.3666667
4025.0000000	0.3750000
4026.0000000	0.3700000
4027.0000000	0.3700000
4028.0000000	0.3650000
4029.0000000	0.3750000
4030.0000000	0.3750000
4031.0000000	0.3600000
4032.0000000	0.3600000
4033.0000000	0.3700000
4034.0000000	0.3700000
4035.0000000	0.3500000
4036.0000000	0.3500000
4037.0000000	0.3650000
4038.0000000	0.3650000
4039.0000000	0.3550000
4040.0000000	0.3600000
4041.0000000	0.3700000
4042.0000000	0.3550000
4043.0000000	0.3450000
4044.0000000	0.3600000
4045.0000000	0.3600000
4046.0000000	0.3550000

4047.0000000	0.3550000
4048.0000000	0.3700000
4049.0000000	0.3750000
4050.0000000	0.3700000
4051.0000000	0.3700000
4052.0000000	0.3650000
4053.0000000	0.3700000
4054.0000000	0.3800000
4055.0000000	0.3800000
4056.0000000	0.3650000
4057.0000000	0.3650000
4058.0000000	0.3700000
4059.0000000	0.3700000
4060.0000000	0.3600000
4061.0000000	0.3550000
4062.0000000	0.3700000
4063.0000000	0.3700000
4064.0000000	0.3550000
4065.0000000	0.3600000
4066.0000000	0.3650000
4067.0000000	0.3600000
4068.0000000	0.3650000
4069.0000000	0.3500000
4070.0000000	0.3550000
4071.0000000	0.3750000
4072.0000000	0.3550000
4073.0000000	0.3500000
4074.0000000	0.3550000
4075.0000000	0.3550000
4076.0000000	0.3600000
4077.0000000	0.3650000
4078.0000000	0.3550000
4079.0000000	0.3600000
4080.0000000	0.3550000
4081.0000000	0.3700000
4082.0000000	0.3650000
4083.0000000	0.3600000
4084.0000000	0.3650000
4085.0000000	0.3600000
4086.0000000	0.3650000
4087.0000000	0.3550000
4088.0000000	0.3700000
4089.0000000	0.3600000
4090.0000000	0.3700000
4091.0000000	0.3650000
4092.0000000	0.3700000
4093.0000000	0.3750000
4094.0000000	0.3700000
4095.0000000	0.3700000
4096.0000000	0.3600000
4097.0000000	0.3700000
4098.0000000	0.3750000
4099.0000000	0.3700000
4100.0000000	0.3700000
4101.0000000	0.3650000
4102.0000000	0.3700000
4103.0000000	0.3750000

4104.0000000	0.3850000
4105.0000000	0.3750000
4106.0000000	0.3800000
4107.0000000	0.3650000
4108.0000000	0.3750000
4109.0000000	0.3750000
4110.0000000	0.3800000
4111.0000000	0.3700000
4112.0000000	0.3800000
4113.0000000	0.3700000
4114.0000000	0.3766667
4115.0000000	0.3700000
4116.0000000	0.3650000
4117.0000000	0.3700000
4118.0000000	0.3700000
4119.0000000	0.3650000
4120.0000000	0.3650000
4121.0000000	0.3600000
4122.0000000	0.3600000
4123.0000000	0.3600000
4124.0000000	0.3600000
4125.0000000	0.3500000
4126.0000000	0.3550000
4127.0000000	0.3500000
4128.0000000	0.3600000
4129.0000000	0.3500000
4130.0000000	0.3600000
4131.0000000	0.3450000
4132.0000000	0.3550000
4133.0000000	0.3450000
4134.0000000	0.3500000
4135.0000000	0.3400000
4136.0000000	0.3550000
4137.0000000	0.3450000
4138.0000000	0.3450000
4139.0000000	0.3450000
4140.0000000	0.3450000
4141.0000000	0.3450000
4142.0000000	0.3400000
4143.0000000	0.3500000
4144.0000000	0.3433333
4145.0000000	0.3550000
4146.0000000	0.3450000
4147.0000000	0.3550000
4148.0000000	0.3550000
4149.0000000	0.3500000
4150.0000000	0.3550000
4151.0000000	0.3500000
4152.0000000	0.3550000
4153.0000000	0.3450000
4154.0000000	0.3600000
4155.0000000	0.3600000
4156.0000000	0.3550000
4157.0000000	0.3500000
4158.0000000	0.3500000
4159.0000000	0.3550000
4160.0000000	0.3600000

4161.0000000	0.3650000
4162.0000000	0.3550000
4163.0000000	0.3550000
4164.0000000	0.3450000
4165.0000000	0.3650000
4166.0000000	0.3700000
4167.0000000	0.3700000
4168.0000000	0.3700000
4169.0000000	0.3650000
4170.0000000	0.3650000
4171.0000000	0.3700000
4172.0000000	0.3800000
4173.0000000	0.3750000
4174.0000000	0.3700000
4175.0000000	0.3700000
4176.0000000	0.3700000
4177.0000000	0.3750000
4178.0000000	0.3650000
4179.0000000	0.3600000
4180.0000000	0.3750000
4181.0000000	0.3750000
4182.0000000	0.3750000
4183.0000000	0.3700000
4184.0000000	0.3650000
4185.0000000	0.3650000
4186.0000000	0.3750000
4187.0000000	0.3700000
4188.0000000	0.3700000
4189.0000000	0.3600000
4190.0000000	0.3700000
4191.0000000	0.3600000
4192.0000000	0.3700000
4193.0000000	0.3700000
4194.0000000	0.3600000
4195.0000000	0.3650000
4196.0000000	0.3700000
4197.0000000	0.3650000
4198.0000000	0.3700000
4199.0000000	0.3650000
4200.0000000	0.3600000
4201.0000000	0.3600000
4202.0000000	0.3700000
4203.0000000	0.3650000
4204.0000000	0.3600000
4205.0000000	0.3650000
4206.0000000	0.3700000
4207.0000000	0.3650000
4208.0000000	0.3650000
4209.0000000	0.3600000
4210.0000000	0.3750000
4211.0000000	0.3650000
4212.0000000	0.3750000
4213.0000000	0.3600000
4214.0000000	0.3700000
4215.0000000	0.3700000
4216.0000000	0.3900000
4217.0000000	0.3750000

4218.0000000	0.3700000
4219.0000000	0.3650000
4220.0000000	0.3750000
4221.0000000	0.3800000
4222.0000000	0.3800000
4223.0000000	0.3750000
4224.0000000	0.3600000
4225.0000000	0.3700000
4226.0000000	0.3750000
4227.0000000	0.3650000
4228.0000000	0.3700000
4229.0000000	0.3600000
4230.0000000	0.3550000
4231.0000000	0.3650000
4232.0000000	0.3600000
4233.0000000	0.3500000
4234.0000000	0.3400000
4235.0000000	0.3533334
4236.0000000	0.3500000
4237.0000000	0.3500000
4238.0000000	0.3400000
4239.0000000	0.3550000
4240.0000000	0.3450000
4241.0000000	0.3500000
4242.0000000	0.3500000
4243.0000000	0.3550000
4244.0000000	0.3550000
4245.0000000	0.3450000
4246.0000000	0.3450000
4247.0000000	0.3400000
4248.0000000	0.3450000
4249.0000000	0.3500000
4250.0000000	0.3500000
4251.0000000	0.3500000
4252.0000000	0.3400000
4253.0000000	0.3400000
4254.0000000	0.3550000
4255.0000000	0.3400000
4256.0000000	0.3400000
4257.0000000	0.3500000
4258.0000000	0.3450000
4259.0000000	0.3450000
4260.0000000	0.3450000
4261.0000000	0.3400000
4262.0000000	0.3450000
4263.0000000	0.3500000
4264.0000000	0.3450000
4265.0000000	0.3500000
4266.0000000	0.3400000
4267.0000000	0.3500000
4268.0000000	0.3500000
4269.0000000	0.3500000
4270.0000000	0.3400000
4271.0000000	0.3450000
4272.0000000	0.3450000
4273.0000000	0.3600000
4274.0000000	0.3500000

4275.0000000	0.3400000
4276.0000000	0.3550000
4277.0000000	0.3500000
4278.0000000	0.3550000
4279.0000000	0.3500000
4280.0000000	0.3500000
4281.0000000	0.3550000
4282.0000000	0.3650000
4283.0000000	0.3600000
4284.0000000	0.3650000
4285.0000000	0.3500000
4286.0000000	0.3600000
4287.0000000	0.3550000
4288.0000000	0.3700000
4289.0000000	0.3700000
4290.0000000	0.3800000
4291.0000000	0.3650000
4292.0000000	0.3700000
4293.0000000	0.3700000
4294.0000000	0.3766667
4295.0000000	0.3700000
4296.0000000	0.3850000
4297.0000000	0.3750000
4298.0000000	0.3700000
4299.0000000	0.3700000
4300.0000000	0.3750000
4301.0000000	0.3800000
4302.0000000	0.3900000
4303.0000000	0.3750000
4304.0000000	0.3800000
4305.0000000	0.3800000
4306.0000000	0.3850000
4307.0000000	0.3850000
4308.0000000	0.3750000
4309.0000000	0.3800000
4310.0000000	0.3800000
4311.0000000	0.3950000
4312.0000000	0.3900000
4313.0000000	0.3850000
4314.0000000	0.3800000
4315.0000000	0.3950000
4316.0000000	0.3850000
4317.0000000	0.3950000
4318.0000000	0.3850000
4319.0000000	0.3950000
4320.0000000	0.3850000
4321.0000000	0.3950000
4322.0000000	0.3950000
4323.0000000	0.3950000
4324.0000000	0.3900000
4325.0000000	0.3833333
4326.0000000	0.4000000
4327.0000000	0.3900000
4328.0000000	0.3900000
4329.0000000	0.3800000
4330.0000000	0.3850000
4331.0000000	0.3800000

4332.0000000	0.3950000
4333.0000000	0.3850000
4334.0000000	0.3950000
4335.0000000	0.3750000
4336.0000000	0.3850000
4337.0000000	0.3850000
4338.0000000	0.3950000
4339.0000000	0.3950000
4340.0000000	0.3950000
4341.0000000	0.3800000
4342.0000000	0.3850000
4343.0000000	0.3900000
4344.0000000	0.3950000
4345.0000000	0.3900000
4346.0000000	0.3900000
4347.0000000	0.3800000
4348.0000000	0.3900000
4349.0000000	0.3900000
4350.0000000	0.3950000
4351.0000000	0.3950000
4352.0000000	0.3900000
4353.0000000	0.3950000
4354.0000000	0.3950000
4355.0000000	0.4000000
4356.0000000	0.3966666
4357.0000000	0.4000000
4358.0000000	0.4050000
4359.0000000	0.4050000
4360.0000000	0.3850000
4361.0000000	0.3950000
4362.0000000	0.4000000
4363.0000000	0.3900000
4364.0000000	0.4000000
4365.0000000	0.3950000
4366.0000000	0.4000000
4367.0000000	0.4100000
4368.0000000	0.3900000
4369.0000000	0.4100000
4370.0000000	0.3950000
4371.0000000	0.4000000
4372.0000000	0.4150000
4373.0000000	0.4050000
4374.0000000	0.4000000
4375.0000000	0.4100000
4376.0000000	0.4000000
4377.0000000	0.4050000
4378.0000000	0.4100000
4379.0000000	0.3950000
4380.0000000	0.4150000
4381.0000000	0.4100000
4382.0000000	0.4150000
4383.0000000	0.4150000
4384.0000000	0.3950000
4385.0000000	0.4033333
4386.0000000	0.3950000
4387.0000000	0.4100000
4388.0000000	0.4150000

4389.0000000	0.4050000
4390.0000000	0.4150000
4391.0000000	0.4050000
4392.0000000	0.4050000
4393.0000000	0.4150000
4394.0000000	0.4050000
4395.0000000	0.4100000
4396.0000000	0.4100000
4397.0000000	0.4150000
4398.0000000	0.4100000
4399.0000000	0.4100000
4400.0000000	0.4100000
4401.0000000	0.3950000
4402.0000000	0.4050000
4403.0000000	0.4100000
4404.0000000	0.4050000
4405.0000000	0.4050000
4406.0000000	0.3950000
4407.0000000	0.4000000
4408.0000000	0.4050000
4409.0000000	0.4100000
4410.0000000	0.4050000
4411.0000000	0.4000000
4412.0000000	0.3950000
4413.0000000	0.4000000
4414.0000000	0.3950000
4415.0000000	0.4000000
4416.0000000	0.3900000
4417.0000000	0.3900000
4418.0000000	0.3950000
4419.0000000	0.3950000
4420.0000000	0.3950000
4421.0000000	0.3900000
4422.0000000	0.3800000
4423.0000000	0.3900000
4424.0000000	0.3900000
4425.0000000	0.4000000
4426.0000000	0.3900000
4427.0000000	0.3800000
4428.0000000	0.3800000
4429.0000000	0.3900000
4430.0000000	0.3850000
4431.0000000	0.3850000
4432.0000000	0.3800000
4433.0000000	0.3850000
4434.0000000	0.3850000
4435.0000000	0.3950000
4436.0000000	0.3850000
4437.0000000	0.3900000
4438.0000000	0.3800000
4439.0000000	0.3850000
4440.0000000	0.3850000
4441.0000000	0.4000000
4442.0000000	0.3900000
4443.0000000	0.3900000
4444.0000000	0.3800000
4445.0000000	0.3900000

4446.0000000	0.3900000
4447.0000000	0.4000000
4448.0000000	0.3900000
4449.0000000	0.4050000
4450.0000000	0.3850000
4451.0000000	0.3950000
4452.0000000	0.3950000
4453.0000000	0.4000000
4454.0000000	0.3900000
4455.0000000	0.4000000
4456.0000000	0.3850000
4457.0000000	0.3900000
4458.0000000	0.3850000
4459.0000000	0.4000000
4460.0000000	0.3850000
4461.0000000	0.3950000
4462.0000000	0.3900000
4463.0000000	0.3900000
4464.0000000	0.3900000
4465.0000000	0.3950000
4466.0000000	0.3900000
4467.0000000	0.3900000
4468.0000000	0.3950000
4469.0000000	0.3800000
4470.0000000	0.3900000
4471.0000000	0.3800000
4472.0000000	0.3900000
4473.0000000	0.3900000
4474.0000000	0.3950000
4475.0000000	0.3900000
4476.0000000	0.3833333
4477.0000000	0.3950000
4478.0000000	0.3850000
4479.0000000	0.3850000
4480.0000000	0.3850000
4481.0000000	0.3950000
4482.0000000	0.3850000
4483.0000000	0.3850000
4484.0000000	0.3800000
4485.0000000	0.3850000
4486.0000000	0.3850000
4487.0000000	0.3850000
4488.0000000	0.3900000
4489.0000000	0.3750000
4490.0000000	0.3750000
4491.0000000	0.3750000
4492.0000000	0.3850000
4493.0000000	0.3850000
4494.0000000	0.3850000
4495.0000000	0.3750000
4496.0000000	0.3750000
4497.0000000	0.3700000
4498.0000000	0.3700000
4499.0000000	0.3750000
4500.0000000	0.3750000
4501.0000000	0.3800000
4502.0000000	0.3600000

4503.0000000	0.3750000
4504.0000000	0.3750000
4505.0000000	0.3700000
4506.0000000	0.3733333
4507.0000000	0.3800000
4508.0000000	0.3700000
4509.0000000	0.3600000
4510.0000000	0.3700000
4511.0000000	0.3700000
4512.0000000	0.3700000
4513.0000000	0.3650000
4514.0000000	0.3700000
4515.0000000	0.3750000
4516.0000000	0.3650000
4517.0000000	0.3650000
4518.0000000	0.3700000
4519.0000000	0.3550000
4520.0000000	0.3750000
4521.0000000	0.3700000
4522.0000000	0.3650000
4523.0000000	0.3650000
4524.0000000	0.3550000
4525.0000000	0.3600000
4526.0000000	0.3650000
4527.0000000	0.3550000
4528.0000000	0.3600000
4529.0000000	0.3650000
4530.0000000	0.3600000
4531.0000000	0.3700000
4532.0000000	0.3550000
4533.0000000	0.3550000
4534.0000000	0.3600000
4535.0000000	0.3600000
4536.0000000	0.3533333
4537.0000000	0.3700000
4538.0000000	0.3600000
4539.0000000	0.3650000
4540.0000000	0.3650000
4541.0000000	0.3650000
4542.0000000	0.3500000
4543.0000000	0.3600000
4544.0000000	0.3700000
4545.0000000	0.3500000
4546.0000000	0.3450000
4547.0000000	0.3600000
4548.0000000	0.3600000
4549.0000000	0.3550000
4550.0000000	0.3500000
4551.0000000	0.3600000
4552.0000000	0.3550000
4553.0000000	0.3600000
4554.0000000	0.3500000
4555.0000000	0.3500000
4556.0000000	0.3600000
4557.0000000	0.3650000
4558.0000000	0.3550000
4559.0000000	0.3500000

4560.0000000	0.3600000
4561.0000000	0.3650000
4562.0000000	0.3550000
4563.0000000	0.3500000
4564.0000000	0.3600000
4565.0000000	0.3550000
4566.0000000	0.3450000
4567.0000000	0.3566667
4568.0000000	0.3600000
4569.0000000	0.3600000
4570.0000000	0.3700000
4571.0000000	0.3750000
4572.0000000	0.3600000
4573.0000000	0.3600000
4574.0000000	0.3750000
4575.0000000	0.3650000
4576.0000000	0.3650000
4577.0000000	0.3650000
4578.0000000	0.3750000
4579.0000000	0.3700000
4580.0000000	0.3750000
4581.0000000	0.3750000
4582.0000000	0.3650000
4583.0000000	0.3800000
4584.0000000	0.3700000
4585.0000000	0.3650000
4586.0000000	0.3750000
4587.0000000	0.3750000
4588.0000000	0.3750000
4589.0000000	0.3800000
4590.0000000	0.3600000
4591.0000000	0.3700000
4592.0000000	0.3600000
4593.0000000	0.3700000
4594.0000000	0.3700000
4595.0000000	0.3650000
4596.0000000	0.3750000
4597.0000000	0.3766667
4598.0000000	0.3650000
4599.0000000	0.3700000
4600.0000000	0.3800000
4601.0000000	0.3700000
4602.0000000	0.3700000
4603.0000000	0.3650000
4604.0000000	0.3700000
4605.0000000	0.3750000
4606.0000000	0.3700000
4607.0000000	0.3700000
4608.0000000	0.3650000
4609.0000000	0.3700000
4610.0000000	0.3750000
4611.0000000	0.3600000
4612.0000000	0.3650000
4613.0000000	0.3550000
4614.0000000	0.3550000
4615.0000000	0.3650000
4616.0000000	0.3600000

4617.0000000	0.3650000
4618.0000000	0.3600000
4619.0000000	0.3600000
4620.0000000	0.3650000
4621.0000000	0.3550000
4622.0000000	0.3550000
4623.0000000	0.3500000
4624.0000000	0.3550000
4625.0000000	0.3600000
4626.0000000	0.3450000
4627.0000000	0.3566667
4628.0000000	0.3400000
4629.0000000	0.3500000
4630.0000000	0.3500000
4631.0000000	0.3500000
4632.0000000	0.3600000
4633.0000000	0.3500000
4634.0000000	0.3450000
4635.0000000	0.3500000
4636.0000000	0.3450000
4637.0000000	0.3550000
4638.0000000	0.3500000
4639.0000000	0.3450000
4640.0000000	0.3550000
4641.0000000	0.3550000
4642.0000000	0.3600000
4643.0000000	0.3500000
4644.0000000	0.3600000
4645.0000000	0.3550000
4646.0000000	0.3650000
4647.0000000	0.3650000
4648.0000000	0.3600000
4649.0000000	0.3550000
4650.0000000	0.3600000
4651.0000000	0.3600000
4652.0000000	0.3700000
4653.0000000	0.3700000
4654.0000000	0.3600000
4655.0000000	0.3500000
4656.0000000	0.3400000
4657.0000000	0.3566667
4658.0000000	0.3550000
4659.0000000	0.3550000
4660.0000000	0.3600000
4661.0000000	0.3550000
4662.0000000	0.3450000
4663.0000000	0.3600000
4664.0000000	0.3550000
4665.0000000	0.3500000
4666.0000000	0.3450000
4667.0000000	0.3550000
4668.0000000	0.3450000
4669.0000000	0.3500000
4670.0000000	0.3500000
4671.0000000	0.3450000
4672.0000000	0.3400000
4673.0000000	0.3350000

4674.0000000	0.3400000
4675.0000000	0.3350000
4676.0000000	0.3450000
4677.0000000	0.3300000
4678.0000000	0.3450000
4679.0000000	0.3350000
4680.0000000	0.3350000
4681.0000000	0.3350000
4682.0000000	0.3350000
4683.0000000	0.3300000
4684.0000000	0.3250000
4685.0000000	0.3250000
4686.0000000	0.3250000
4687.0000000	0.3300000
4688.0000000	0.3200000
4689.0000000	0.3250000
4690.0000000	0.3250000
4691.0000000	0.3300000
4692.0000000	0.3200000
4693.0000000	0.3200000
4694.0000000	0.3250000
4695.0000000	0.3200000
4696.0000000	0.3300000
4697.0000000	0.3150000
4698.0000000	0.3200000
4699.0000000	0.3100000
4700.0000000	0.3150000
4701.0000000	0.3150000
4702.0000000	0.3150000
4703.0000000	0.3100000
4704.0000000	0.3050000
4705.0000000	0.3050000
4706.0000000	0.3050000
4707.0000000	0.3150000
4708.0000000	0.3050000
4709.0000000	0.3100000
4710.0000000	0.3100000
4711.0000000	0.3150000
4712.0000000	0.3050000
4713.0000000	0.3100000
4714.0000000	0.3050000
4715.0000000	0.3100000
4716.0000000	0.3100000
4717.0000000	0.3100000
4718.0000000	0.3150000
4719.0000000	0.3150000
4720.0000000	0.3050000
4721.0000000	0.3150000
4722.0000000	0.3100000
4723.0000000	0.3100000
4724.0000000	0.3200000
4725.0000000	0.3100000
4726.0000000	0.3150000
4727.0000000	0.3200000
4728.0000000	0.3100000
4729.0000000	0.3100000
4730.0000000	0.3000000

4731.0000000	0.3100000
4732.0000000	0.3100000
4733.0000000	0.3200000
4734.0000000	0.3100000
4735.0000000	0.3100000
4736.0000000	0.3050000
4737.0000000	0.3050000
4738.0000000	0.3200000
4739.0000000	0.3200000
4740.0000000	0.3100000
4741.0000000	0.3000000
4742.0000000	0.3100000
4743.0000000	0.3150000
4744.0000000	0.3050000
4745.0000000	0.3100000
4746.0000000	0.3050000
4747.0000000	0.3150000
4748.0000000	0.3000000
4749.0000000	0.3150000
4750.0000000	0.3100000
4751.0000000	0.3050000
4752.0000000	0.3100000
4753.0000000	0.3000000
4754.0000000	0.3050000
4755.0000000	0.3200000
4756.0000000	0.3000000
4757.0000000	0.3100000
4758.0000000	0.3000000
4759.0000000	0.3000000
4760.0000000	0.3100000
4761.0000000	0.3050000
4762.0000000	0.3000000
4763.0000000	0.3150000
4764.0000000	0.3150000
4765.0000000	0.3100000
4766.0000000	0.3150000
4767.0000000	0.3100000
4768.0000000	0.3150000
4769.0000000	0.3200000
4770.0000000	0.3050000
4771.0000000	0.3100000
4772.0000000	0.3150000
4773.0000000	0.3100000
4774.0000000	0.3000000
4775.0000000	0.2950000
4776.0000000	0.3100000
4777.0000000	0.3100000
4778.0000000	0.3100000
4779.0000000	0.3100000
4780.0000000	0.3000000
4781.0000000	0.3100000
4782.0000000	0.3000000
4783.0000000	0.3000000
4784.0000000	0.3000000
4785.0000000	0.2950000
4786.0000000	0.3050000
4787.0000000	0.3000000

4788.0000000	0.3100000
4789.0000000	0.2950000
4790.0000000	0.2950000
4791.0000000	0.2850000
4792.0000000	0.2850000
4793.0000000	0.3000000
4794.0000000	0.2950000
4795.0000000	0.2950000
4796.0000000	0.2950000
4797.0000000	0.2900000
4798.0000000	0.2950000
4799.0000000	0.2950000
4800.0000000	0.2900000
4801.0000000	0.2950000
4802.0000000	0.2950000
4803.0000000	0.3100000
4804.0000000	0.2950000
4805.0000000	0.3000000
4806.0000000	0.3050000
4807.0000000	0.3000000
4808.0000000	0.3000000
4809.0000000	0.3000000
4810.0000000	0.3050000
4811.0000000	0.3000000
4812.0000000	0.2900000
4813.0000000	0.3000000
4814.0000000	0.2950000
4815.0000000	0.3050000
4816.0000000	0.2950000
4817.0000000	0.3000000
4818.0000000	0.3000000
4819.0000000	0.2950000
4820.0000000	0.3050000
4821.0000000	0.2950000
4822.0000000	0.2950000
4823.0000000	0.2950000
4824.0000000	0.3150000
4825.0000000	0.3150000
4826.0000000	0.3050000
4827.0000000	0.3050000
4828.0000000	0.3050000
4829.0000000	0.3150000
4830.0000000	0.3200000
4831.0000000	0.3150000
4832.0000000	0.3100000
4833.0000000	0.3050000
4834.0000000	0.3200000
4835.0000000	0.3050000
4836.0000000	0.3100000
4837.0000000	0.3050000
4838.0000000	0.3100000
4839.0000000	0.3050000
4840.0000000	0.3000000
4841.0000000	0.3050000
4842.0000000	0.3000000
4843.0000000	0.3000000
4844.0000000	0.2850000

4845.0000000	0.3050000
4846.0000000	0.3000000
4847.0000000	0.2900000
4848.0000000	0.2950000
4849.0000000	0.3000000
4850.0000000	0.3000000
4851.0000000	0.2900000
4852.0000000	0.2950000
4853.0000000	0.2900000
4854.0000000	0.3000000
4855.0000000	0.3050000
4856.0000000	0.2950000
4857.0000000	0.2950000
4858.0000000	0.3050000
4859.0000000	0.3000000
4860.0000000	0.2900000
4861.0000000	0.2950000
4862.0000000	0.2950000
4863.0000000	0.3050000
4864.0000000	0.3000000
4865.0000000	0.3050000
4866.0000000	0.2950000
4867.0000000	0.3050000
4868.0000000	0.3033333
4869.0000000	0.3000000
4870.0000000	0.3050000
4871.0000000	0.3100000
4872.0000000	0.3100000
4873.0000000	0.3050000
4874.0000000	0.3000000
4875.0000000	0.3050000
4876.0000000	0.3100000
4877.0000000	0.3150000
4878.0000000	0.3100000
4879.0000000	0.3100000
4880.0000000	0.3050000
4881.0000000	0.3200000
4882.0000000	0.3150000
4883.0000000	0.3050000
4884.0000000	0.3050000
4885.0000000	0.3100000
4886.0000000	0.3200000
4887.0000000	0.3150000
4888.0000000	0.3150000
4889.0000000	0.3000000
4890.0000000	0.3150000
4891.0000000	0.3050000
4892.0000000	0.3150000
4893.0000000	0.3150000
4894.0000000	0.3000000
4895.0000000	0.3050000
4896.0000000	0.3100000
4897.0000000	0.3150000
4898.0000000	0.3050000
4899.0000000	0.2933334
4900.0000000	0.3000000
4901.0000000	0.2950000

4902.0000000	0.2900000
4903.0000000	0.2900000
4904.0000000	0.3050000
4905.0000000	0.2950000
4906.0000000	0.2900000
4907.0000000	0.3000000
4908.0000000	0.2900000
4909.0000000	0.3100000
4910.0000000	0.2950000
4911.0000000	0.3000000
4912.0000000	0.3000000
4913.0000000	0.3000000
4914.0000000	0.3000000
4915.0000000	0.3000000
4916.0000000	0.2950000
4917.0000000	0.2950000
4918.0000000	0.2950000
4919.0000000	0.3050000
4920.0000000	0.3050000
4921.0000000	0.3100000
4922.0000000	0.3000000
4923.0000000	0.3050000
4924.0000000	0.3200000
4925.0000000	0.3100000
4926.0000000	0.3200000
4927.0000000	0.3000000
4928.0000000	0.3200000
4929.0000000	0.3166667
4930.0000000	0.3150000
4931.0000000	0.3050000
4932.0000000	0.3150000
4933.0000000	0.3150000
4934.0000000	0.3100000
4935.0000000	0.2950000
4936.0000000	0.3050000
4937.0000000	0.3000000
4938.0000000	0.3050000
4939.0000000	0.3100000
4940.0000000	0.3000000
4941.0000000	0.2950000
4942.0000000	0.3000000
4943.0000000	0.3050000
4944.0000000	0.2950000
4945.0000000	0.2950000
4946.0000000	0.2950000
4947.0000000	0.2950000
4948.0000000	0.3000000
4949.0000000	0.2800000
4950.0000000	0.2850000
4951.0000000	0.2900000
4952.0000000	0.2800000
4953.0000000	0.3000000
4954.0000000	0.2800000
4955.0000000	0.2850000
4956.0000000	0.2850000
4957.0000000	0.2850000
4958.0000000	0.2850000

4959.0000000	0.2766667
4960.0000000	0.2950000
4961.0000000	0.2900000
4962.0000000	0.2900000
4963.0000000	0.2850000
4964.0000000	0.2900000
4965.0000000	0.2900000
4966.0000000	0.2950000
4967.0000000	0.2850000
4968.0000000	0.2900000
4969.0000000	0.2950000
4970.0000000	0.2900000
4971.0000000	0.2950000
4972.0000000	0.2850000
4973.0000000	0.2900000
4974.0000000	0.2800000
4975.0000000	0.3000000
4976.0000000	0.2850000
4977.0000000	0.2900000
4978.0000000	0.2850000
4979.0000000	0.2850000
4980.0000000	0.2950000
4981.0000000	0.2900000
4982.0000000	0.2850000
4983.0000000	0.2850000
4984.0000000	0.2900000
4985.0000000	0.2850000
4986.0000000	0.2950000
4987.0000000	0.2850000
4988.0000000	0.2950000
4989.0000000	0.2833333
4990.0000000	0.3000000
4991.0000000	0.2900000
4992.0000000	0.2850000
4993.0000000	0.2850000
4994.0000000	0.2700000
4995.0000000	0.2850000
4996.0000000	0.2900000
4997.0000000	0.2900000
4998.0000000	0.2850000
4999.0000000	0.2750000
5000.0000000	0.2800000
5001.0000000	0.2750000
5002.0000000	0.2850000
5003.0000000	0.2800000
5004.0000000	0.2750000
5005.0000000	0.2850000
5006.0000000	0.2650000
5007.0000000	0.2850000
5008.0000000	0.2650000
5009.0000000	0.2700000
5010.0000000	0.2750000
5011.0000000	0.2800000
5012.0000000	0.2850000
5013.0000000	0.2800000
5014.0000000	0.2700000
5015.0000000	0.2800000

5016.0000000	0.2750000
5017.0000000	0.2700000
5018.0000000	0.2750000
5019.0000000	0.2700000
5020.0000000	0.2733333
5021.0000000	0.2750000
5022.0000000	0.2800000
5023.0000000	0.2600000
5024.0000000	0.2700000
5025.0000000	0.2800000
5026.0000000	0.2700000
5027.0000000	0.2750000
5028.0000000	0.2700000
5029.0000000	0.2700000
5030.0000000	0.2750000
5031.0000000	0.2650000
5032.0000000	0.2750000
5033.0000000	0.2700000
5034.0000000	0.2700000
5035.0000000	0.2750000
5036.0000000	0.2800000
5037.0000000	0.2800000
5038.0000000	0.2600000
5039.0000000	0.2600000
5040.0000000	0.2800000
5041.0000000	0.2700000
5042.0000000	0.2650000
5043.0000000	0.2700000
5044.0000000	0.2800000
5045.0000000	0.2700000
5046.0000000	0.2700000
5047.0000000	0.2800000
5048.0000000	0.2800000
5049.0000000	0.2766667
5050.0000000	0.2750000
5051.0000000	0.2750000
5052.0000000	0.2750000
5053.0000000	0.2750000
5054.0000000	0.2850000
5055.0000000	0.2800000
5056.0000000	0.2750000
5057.0000000	0.2750000
5058.0000000	0.2900000
5059.0000000	0.2700000
5060.0000000	0.2700000
5061.0000000	0.2800000
5062.0000000	0.2800000
5063.0000000	0.2750000
5064.0000000	0.2800000
5065.0000000	0.2750000
5066.0000000	0.2800000
5067.0000000	0.2800000
5068.0000000	0.2800000
5069.0000000	0.2750000
5070.0000000	0.2750000
5071.0000000	0.2900000
5072.0000000	0.2850000

5073.0000000	0.2900000
5074.0000000	0.2850000
5075.0000000	0.2950000
5076.0000000	0.2750000
5077.0000000	0.2850000
5078.0000000	0.2850000
5079.0000000	0.2900000
5080.0000000	0.2850000
5081.0000000	0.2850000
5082.0000000	0.2900000
5083.0000000	0.2900000
5084.0000000	0.2850000
5085.0000000	0.2750000
5086.0000000	0.2800000
5087.0000000	0.2850000
5088.0000000	0.2900000
5089.0000000	0.2900000
5090.0000000	0.2750000
5091.0000000	0.2900000
5092.0000000	0.2750000
5093.0000000	0.2850000
5094.0000000	0.2850000
5095.0000000	0.2850000
5096.0000000	0.2850000
5097.0000000	0.2700000
5098.0000000	0.2800000
5099.0000000	0.2700000
5100.0000000	0.2800000
5101.0000000	0.2800000
5102.0000000	0.2800000
5103.0000000	0.2900000
5104.0000000	0.2800000
5105.0000000	0.2850000
5106.0000000	0.2800000
5107.0000000	0.2800000
5108.0000000	0.2800000
5109.0000000	0.2850000
5110.0000000	0.2833333
5111.0000000	0.2850000
5112.0000000	0.2850000
5113.0000000	0.2800000
5114.0000000	0.2850000
5115.0000000	0.2850000
5116.0000000	0.2900000
5117.0000000	0.2850000
5118.0000000	0.2900000
5119.0000000	0.2900000
5120.0000000	0.2900000
5121.0000000	0.2900000
5122.0000000	0.2800000
5123.0000000	0.2900000
5124.0000000	0.2850000
5125.0000000	0.2900000
5126.0000000	0.2800000
5127.0000000	0.2800000
5128.0000000	0.2800000
5129.0000000	0.2800000

5130.0000000	0.2850000
5131.0000000	0.2850000
5132.0000000	0.2750000
5133.0000000	0.2750000
5134.0000000	0.2750000
5135.0000000	0.2750000
5136.0000000	0.2700000
5137.0000000	0.2750000
5138.0000000	0.2750000
5139.0000000	0.2750000
5140.0000000	0.2633333
5141.0000000	0.2650000
5142.0000000	0.2600000
5143.0000000	0.2650000
5144.0000000	0.2750000
5145.0000000	0.2600000
5146.0000000	0.2650000
5147.0000000	0.2550000
5148.0000000	0.2450000
5149.0000000	0.2700000
5150.0000000	0.2650000
5151.0000000	0.2650000
5152.0000000	0.2650000
5153.0000000	0.2600000
5154.0000000	0.2500000
5155.0000000	0.2500000
5156.0000000	0.2500000
5157.0000000	0.2600000
5158.0000000	0.2650000
5159.0000000	0.2600000
5160.0000000	0.2500000
5161.0000000	0.2600000
5162.0000000	0.2650000
5163.0000000	0.2500000
5164.0000000	0.2600000
5165.0000000	0.2550000
5166.0000000	0.2500000
5167.0000000	0.2650000
5168.0000000	0.2600000
5169.0000000	0.2550000
5170.0000000	0.2566667
5171.0000000	0.2500000
5172.0000000	0.2450000
5173.0000000	0.2650000
5174.0000000	0.2550000
5175.0000000	0.2450000
5176.0000000	0.2500000
5177.0000000	0.2600000
5178.0000000	0.2600000
5179.0000000	0.2550000
5180.0000000	0.2600000
5181.0000000	0.2550000
5182.0000000	0.2600000
5183.0000000	0.2550000
5184.0000000	0.2550000
5185.0000000	0.2550000
5186.0000000	0.2650000

5187.0000000	0.2650000
5188.0000000	0.2450000
5189.0000000	0.2600000
5190.0000000	0.2600000
5191.0000000	0.2600000
5192.0000000	0.2450000
5193.0000000	0.2550000
5194.0000000	0.2650000
5195.0000000	0.2500000
5196.0000000	0.2450000
5197.0000000	0.2600000
5198.0000000	0.2700000
5199.0000000	0.2600000
5200.0000000	0.2566667
5201.0000000	0.2550000
5202.0000000	0.2600000
5203.0000000	0.2650000
5204.0000000	0.2450000
5205.0000000	0.2550000
5206.0000000	0.2650000
5207.0000000	0.2550000
5208.0000000	0.2500000
5209.0000000	0.2500000
5210.0000000	0.2650000
5211.0000000	0.2600000
5212.0000000	0.2500000
5213.0000000	0.2600000
5214.0000000	0.2550000
5215.0000000	0.2550000
5216.0000000	0.2500000
5217.0000000	0.2500000
5218.0000000	0.2500000
5219.0000000	0.2600000
5220.0000000	0.2650000
5221.0000000	0.2450000
5222.0000000	0.2500000
5223.0000000	0.2600000
5224.0000000	0.2550000
5225.0000000	0.2450000
5226.0000000	0.2450000
5227.0000000	0.2600000
5228.0000000	0.2500000
5229.0000000	0.2450000
5230.0000000	0.2550000
5231.0000000	0.2600000
5232.0000000	0.2500000
5233.0000000	0.2500000
5234.0000000	0.2550000
5235.0000000	0.2550000
5236.0000000	0.2500000
5237.0000000	0.2600000
5238.0000000	0.2500000
5239.0000000	0.2500000
5240.0000000	0.2550000
5241.0000000	0.2450000
5242.0000000	0.2500000
5243.0000000	0.2500000

5244.0000000	0.2400000
5245.0000000	0.2500000
5246.0000000	0.2500000
5247.0000000	0.2400000
5248.0000000	0.2500000
5249.0000000	0.2500000
5250.0000000	0.2500000
5251.0000000	0.2600000
5252.0000000	0.2400000
5253.0000000	0.2550000
5254.0000000	0.2400000
5255.0000000	0.2550000
5256.0000000	0.2550000
5257.0000000	0.2550000
5258.0000000	0.2500000
5259.0000000	0.2500000
5260.0000000	0.2550000
5261.0000000	0.2533333
5262.0000000	0.2550000
5263.0000000	0.2600000
5264.0000000	0.2600000
5265.0000000	0.2550000
5266.0000000	0.2500000
5267.0000000	0.2550000
5268.0000000	0.2500000
5269.0000000	0.2600000
5270.0000000	0.2600000
5271.0000000	0.2550000
5272.0000000	0.2450000
5273.0000000	0.2600000
5274.0000000	0.2550000
5275.0000000	0.2600000
5276.0000000	0.2550000
5277.0000000	0.2600000
5278.0000000	0.2450000
5279.0000000	0.2500000
5280.0000000	0.2550000
5281.0000000	0.2550000
5282.0000000	0.2500000
5283.0000000	0.2500000
5284.0000000	0.2550000
5285.0000000	0.2550000
5286.0000000	0.2550000
5287.0000000	0.2500000
5288.0000000	0.2500000
5289.0000000	0.2600000
5290.0000000	0.2600000
5291.0000000	0.2500000
5292.0000000	0.2600000
5293.0000000	0.2500000
5294.0000000	0.2600000
5295.0000000	0.2550000
5296.0000000	0.2500000
5297.0000000	0.2500000
5298.0000000	0.2500000
5299.0000000	0.2550000
5300.0000000	0.2500000

5301.0000000	0.2550000
5302.0000000	0.2450000
5303.0000000	0.2500000
5304.0000000	0.2450000
5305.0000000	0.2600000
5306.0000000	0.2600000
5307.0000000	0.2500000
5308.0000000	0.2500000
5309.0000000	0.2500000
5310.0000000	0.2600000
5311.0000000	0.2600000
5312.0000000	0.2550000
5313.0000000	0.2650000
5314.0000000	0.2550000
5315.0000000	0.2500000
5316.0000000	0.2500000
5317.0000000	0.2600000
5318.0000000	0.2600000
5319.0000000	0.2550000
5320.0000000	0.2550000
5321.0000000	0.2600000
5322.0000000	0.2600000
5323.0000000	0.2600000
5324.0000000	0.2700000
5325.0000000	0.2600000
5326.0000000	0.2550000
5327.0000000	0.2500000
5328.0000000	0.2500000
5329.0000000	0.2550000
5330.0000000	0.2550000
5331.0000000	0.2650000
5332.0000000	0.2550000
5333.0000000	0.2500000
5334.0000000	0.2600000
5335.0000000	0.2600000
5336.0000000	0.2600000
5337.0000000	0.2600000
5338.0000000	0.2600000
5339.0000000	0.2700000
5340.0000000	0.2700000
5341.0000000	0.2550000
5342.0000000	0.2550000
5343.0000000	0.2700000
5344.0000000	0.2550000
5345.0000000	0.2650000
5346.0000000	0.2600000
5347.0000000	0.2700000
5348.0000000	0.2700000
5349.0000000	0.2650000
5350.0000000	0.2700000
5351.0000000	0.2600000
5352.0000000	0.2666667
5353.0000000	0.2650000
5354.0000000	0.2700000
5355.0000000	0.2800000
5356.0000000	0.2650000
5357.0000000	0.2750000

5358.0000000	0.2550000
5359.0000000	0.2550000
5360.0000000	0.2650000
5361.0000000	0.2650000
5362.0000000	0.2700000
5363.0000000	0.2650000
5364.0000000	0.2650000
5365.0000000	0.2650000
5366.0000000	0.2650000
5367.0000000	0.2600000
5368.0000000	0.2750000
5369.0000000	0.2650000
5370.0000000	0.2750000
5371.0000000	0.2750000
5372.0000000	0.2650000
5373.0000000	0.2650000
5374.0000000	0.2700000
5375.0000000	0.2650000
5376.0000000	0.2650000
5377.0000000	0.2700000
5378.0000000	0.2600000
5379.0000000	0.2800000
5380.0000000	0.2700000
5381.0000000	0.2733333
5382.0000000	0.2650000
5383.0000000	0.2650000
5384.0000000	0.2750000
5385.0000000	0.2750000
5386.0000000	0.2750000
5387.0000000	0.2750000
5388.0000000	0.2650000
5389.0000000	0.2800000
5390.0000000	0.2800000
5391.0000000	0.2700000
5392.0000000	0.2800000
5393.0000000	0.2800000
5394.0000000	0.2800000
5395.0000000	0.2700000
5396.0000000	0.2750000
5397.0000000	0.2800000
5398.0000000	0.2800000
5399.0000000	0.2800000
5400.0000000	0.2800000
5401.0000000	0.2750000
5402.0000000	0.2800000
5403.0000000	0.2950000
5404.0000000	0.2800000
5405.0000000	0.2850000
5406.0000000	0.2950000
5407.0000000	0.2950000
5408.0000000	0.2900000
5409.0000000	0.2850000
5410.0000000	0.2850000
5411.0000000	0.2950000
5412.0000000	0.2966667
5413.0000000	0.3050000
5414.0000000	0.3050000

5415.0000000	0.2900000
5416.0000000	0.3000000
5417.0000000	0.2950000
5418.0000000	0.2950000
5419.0000000	0.2950000
5420.0000000	0.2900000
5421.0000000	0.3050000
5422.0000000	0.2950000
5423.0000000	0.3000000
5424.0000000	0.3050000
5425.0000000	0.2950000
5426.0000000	0.3000000
5427.0000000	0.2950000
5428.0000000	0.3050000
5429.0000000	0.3000000
5430.0000000	0.3000000
5431.0000000	0.2950000
5432.0000000	0.3000000
5433.0000000	0.3000000
5434.0000000	0.3050000
5435.0000000	0.3050000
5436.0000000	0.3000000
5437.0000000	0.2950000
5438.0000000	0.3000000
5439.0000000	0.3050000
5440.0000000	0.3100000
5441.0000000	0.3100000
5442.0000000	0.3033333
5443.0000000	0.3100000
5444.0000000	0.3050000
5445.0000000	0.3100000
5446.0000000	0.3000000
5447.0000000	0.3150000
5448.0000000	0.3100000
5449.0000000	0.3200000
5450.0000000	0.3050000
5451.0000000	0.3200000
5452.0000000	0.3100000
5453.0000000	0.3200000
5454.0000000	0.3100000
5455.0000000	0.3100000
5456.0000000	0.3000000
5457.0000000	0.3150000
5458.0000000	0.3000000
5459.0000000	0.3200000
5460.0000000	0.3100000
5461.0000000	0.3200000
5462.0000000	0.2950000
5463.0000000	0.3050000
5464.0000000	0.3100000
5465.0000000	0.3100000
5466.0000000	0.3050000
5467.0000000	0.3050000
5468.0000000	0.3050000
5469.0000000	0.2950000
5470.0000000	0.3150000
5471.0000000	0.3100000

5472.0000000	0.3033333
5473.0000000	0.3050000
5474.0000000	0.3050000
5475.0000000	0.3000000
5476.0000000	0.3100000
5477.0000000	0.3100000
5478.0000000	0.3150000
5479.0000000	0.3150000
5480.0000000	0.3100000
5481.0000000	0.3050000
5482.0000000	0.3100000
5483.0000000	0.3100000
5484.0000000	0.3050000
5485.0000000	0.3150000
5486.0000000	0.3100000
5487.0000000	0.3200000
5488.0000000	0.3050000
5489.0000000	0.3100000
5490.0000000	0.3150000
5491.0000000	0.3200000
5492.0000000	0.3150000
5493.0000000	0.3200000
5494.0000000	0.3150000
5495.0000000	0.3150000
5496.0000000	0.3150000
5497.0000000	0.3100000
5498.0000000	0.3250000
5499.0000000	0.3150000
5500.0000000	0.3150000
5501.0000000	0.3150000
5502.0000000	0.3233334
5503.0000000	0.3150000
5504.0000000	0.3200000
5505.0000000	0.3150000
5506.0000000	0.3250000
5507.0000000	0.3100000
5508.0000000	0.3150000
5509.0000000	0.3100000
5510.0000000	0.3250000
5511.0000000	0.3200000
5512.0000000	0.3350000
5513.0000000	0.3200000
5514.0000000	0.3250000
5515.0000000	0.3150000
5516.0000000	0.3200000
5517.0000000	0.3200000
5518.0000000	0.3350000
5519.0000000	0.3200000
5520.0000000	0.3200000
5521.0000000	0.3200000
5522.0000000	0.3150000
5523.0000000	0.3300000
5524.0000000	0.3350000
5525.0000000	0.3200000
5526.0000000	0.3200000
5527.0000000	0.3200000
5528.0000000	0.3250000

5529.0000000	0.3350000
5530.0000000	0.3350000
5531.0000000	0.3250000
5532.0000000	0.3250000
5533.0000000	0.3266667
5534.0000000	0.3250000
5535.0000000	0.3150000
5536.0000000	0.3350000
5537.0000000	0.3250000
5538.0000000	0.3250000
5539.0000000	0.3200000
5540.0000000	0.3200000
5541.0000000	0.3250000
5542.0000000	0.3300000
5543.0000000	0.3250000
5544.0000000	0.3200000
5545.0000000	0.3200000
5546.0000000	0.3200000
5547.0000000	0.3250000
5548.0000000	0.3250000
5549.0000000	0.3300000
5550.0000000	0.3200000
5551.0000000	0.3200000
5552.0000000	0.3250000
5553.0000000	0.3300000
5554.0000000	0.3250000
5555.0000000	0.3300000
5556.0000000	0.3200000
5557.0000000	0.3250000
5558.0000000	0.3300000
5559.0000000	0.3300000
5560.0000000	0.3300000
5561.0000000	0.3250000
5562.0000000	0.3250000
5563.0000000	0.3266667
5564.0000000	0.3250000
5565.0000000	0.3300000
5566.0000000	0.3250000
5567.0000000	0.3350000
5568.0000000	0.3300000
5569.0000000	0.3300000
5570.0000000	0.3300000
5571.0000000	0.3300000
5572.0000000	0.3350000
5573.0000000	0.3150000
5574.0000000	0.3300000
5575.0000000	0.3250000
5576.0000000	0.3400000
5577.0000000	0.3350000
5578.0000000	0.3200000
5579.0000000	0.3200000
5580.0000000	0.3300000
5581.0000000	0.3350000
5582.0000000	0.3300000
5583.0000000	0.3350000
5584.0000000	0.3250000
5585.0000000	0.3250000

5586.0000000	0.3200000
5587.0000000	0.3350000
5588.0000000	0.3300000
5589.0000000	0.3250000
5590.0000000	0.3350000
5591.0000000	0.3250000
5592.0000000	0.3350000
5593.0000000	0.3300000
5594.0000000	0.3400000
5595.0000000	0.3350000
5596.0000000	0.3350000
5597.0000000	0.3250000
5598.0000000	0.3200000
5599.0000000	0.3200000
5600.0000000	0.3300000
5601.0000000	0.3300000
5602.0000000	0.3250000
5603.0000000	0.3250000
5604.0000000	0.3200000
5605.0000000	0.3200000
5606.0000000	0.3250000
5607.0000000	0.3150000
5608.0000000	0.3050000
5609.0000000	0.3100000
5610.0000000	0.3200000
5611.0000000	0.3200000
5612.0000000	0.3050000
5613.0000000	0.3050000
5614.0000000	0.2950000
5615.0000000	0.3100000
5616.0000000	0.3100000
5617.0000000	0.3050000
5618.0000000	0.3050000
5619.0000000	0.2950000
5620.0000000	0.3050000
5621.0000000	0.3050000
5622.0000000	0.2900000
5623.0000000	0.3066667
5624.0000000	0.2900000
5625.0000000	0.3000000
5626.0000000	0.3100000
5627.0000000	0.3050000
5628.0000000	0.3000000
5629.0000000	0.3000000
5630.0000000	0.3000000
5631.0000000	0.3100000
5632.0000000	0.3000000
5633.0000000	0.3050000
5634.0000000	0.3000000
5635.0000000	0.2950000
5636.0000000	0.3050000
5637.0000000	0.2950000
5638.0000000	0.2900000
5639.0000000	0.2950000
5640.0000000	0.2950000
5641.0000000	0.3000000
5642.0000000	0.2950000

5643.0000000	0.2900000
5644.0000000	0.2900000
5645.0000000	0.2900000
5646.0000000	0.2950000
5647.0000000	0.2950000
5648.0000000	0.3000000
5649.0000000	0.2850000
5650.0000000	0.2950000
5651.0000000	0.2800000
5652.0000000	0.3000000
5653.0000000	0.2833333
5654.0000000	0.3000000
5655.0000000	0.2900000
5656.0000000	0.2950000
5657.0000000	0.2850000
5658.0000000	0.2950000
5659.0000000	0.2900000
5660.0000000	0.3000000
5661.0000000	0.2850000
5662.0000000	0.2850000
5663.0000000	0.2900000
5664.0000000	0.3000000
5665.0000000	0.2950000
5666.0000000	0.2850000
5667.0000000	0.2900000
5668.0000000	0.2850000
5669.0000000	0.2900000
5670.0000000	0.2850000
5671.0000000	0.3000000
5672.0000000	0.2900000
5673.0000000	0.2900000
5674.0000000	0.2850000
5675.0000000	0.2950000
5676.0000000	0.2950000
5677.0000000	0.3000000
5678.0000000	0.2900000
5679.0000000	0.3000000
5680.0000000	0.2850000
5681.0000000	0.2950000
5682.0000000	0.2950000
5683.0000000	0.3050000
5684.0000000	0.3000000
5685.0000000	0.3000000
5686.0000000	0.2950000
5687.0000000	0.2950000
5688.0000000	0.3000000
5689.0000000	0.2900000
5690.0000000	0.3050000
5691.0000000	0.2950000
5692.0000000	0.3000000
5693.0000000	0.2950000
5694.0000000	0.2900000
5695.0000000	0.3000000
5696.0000000	0.3050000
5697.0000000	0.3000000
5698.0000000	0.3050000
5699.0000000	0.2950000

5700.0000000	0.2900000
5701.0000000	0.3050000
5702.0000000	0.2950000
5703.0000000	0.2950000
5704.0000000	0.2950000
5705.0000000	0.3000000
5706.0000000	0.3000000
5707.0000000	0.3050000
5708.0000000	0.3000000
5709.0000000	0.3000000
5710.0000000	0.3000000
5711.0000000	0.3000000
5712.0000000	0.2950000
5713.0000000	0.3000000
5714.0000000	0.2900000
5715.0000000	0.2900000
5716.0000000	0.3000000
5717.0000000	0.3050000
5718.0000000	0.3000000
5719.0000000	0.3050000
5720.0000000	0.3000000
5721.0000000	0.3050000
5722.0000000	0.2950000
5723.0000000	0.3000000
5724.0000000	0.2950000
5725.0000000	0.2950000
5726.0000000	0.3000000
5727.0000000	0.3000000
5728.0000000	0.3000000
5729.0000000	0.2900000
5730.0000000	0.2950000
5731.0000000	0.2900000
5732.0000000	0.3000000
5733.0000000	0.3000000
5734.0000000	0.2950000
5735.0000000	0.2900000
5736.0000000	0.2950000
5737.0000000	0.2900000
5738.0000000	0.3050000
5739.0000000	0.3000000
5740.0000000	0.2900000
5741.0000000	0.3050000
5742.0000000	0.2900000
5743.0000000	0.2950000
5744.0000000	0.2966667
5745.0000000	0.2950000
5746.0000000	0.2950000
5747.0000000	0.3000000
5748.0000000	0.3000000
5749.0000000	0.3050000
5750.0000000	0.3000000
5751.0000000	0.2850000
5752.0000000	0.3100000
5753.0000000	0.3000000
5754.0000000	0.2850000
5755.0000000	0.2950000
5756.0000000	0.3000000

5757.0000000	0.3000000
5758.0000000	0.2950000
5759.0000000	0.3000000
5760.0000000	0.3000000
5761.0000000	0.3000000
5762.0000000	0.2850000
5763.0000000	0.2900000
5764.0000000	0.2950000
5765.0000000	0.2950000
5766.0000000	0.3000000
5767.0000000	0.2900000
5768.0000000	0.2950000
5769.0000000	0.3050000
5770.0000000	0.3000000
5771.0000000	0.2850000
5772.0000000	0.2900000
5773.0000000	0.3000000
5774.0000000	0.2933333
5775.0000000	0.2950000
5776.0000000	0.2950000
5777.0000000	0.2950000
5778.0000000	0.2850000
5779.0000000	0.2850000
5780.0000000	0.2900000
5781.0000000	0.2850000
5782.0000000	0.2850000
5783.0000000	0.2850000
5784.0000000	0.2950000
5785.0000000	0.2900000
5786.0000000	0.2850000
5787.0000000	0.2800000
5788.0000000	0.2850000
5789.0000000	0.2850000
5790.0000000	0.2850000
5791.0000000	0.2900000
5792.0000000	0.2850000
5793.0000000	0.2850000
5794.0000000	0.2800000
5795.0000000	0.2900000
5796.0000000	0.2850000
5797.0000000	0.2900000
5798.0000000	0.2850000
5799.0000000	0.2800000
5800.0000000	0.2800000
5801.0000000	0.2850000
5802.0000000	0.2850000
5803.0000000	0.2850000
5804.0000000	0.2800000
5805.0000000	0.2750000
5806.0000000	0.2850000
5807.0000000	0.2700000
5808.0000000	0.2850000
5809.0000000	0.2750000
5810.0000000	0.2800000
5811.0000000	0.2800000
5812.0000000	0.2800000
5813.0000000	0.2800000

5814.0000000	0.2750000
5815.0000000	0.2800000
5816.0000000	0.2800000
5817.0000000	0.2750000
5818.0000000	0.2800000
5819.0000000	0.2750000
5820.0000000	0.2850000
5821.0000000	0.2800000
5822.0000000	0.2900000
5823.0000000	0.2750000
5824.0000000	0.2750000
5825.0000000	0.2750000
5826.0000000	0.2800000
5827.0000000	0.2800000
5828.0000000	0.2750000
5829.0000000	0.2850000
5830.0000000	0.2750000
5831.0000000	0.2800000
5832.0000000	0.2800000
5833.0000000	0.2850000
5834.0000000	0.2766667
5835.0000000	0.2750000
5836.0000000	0.2700000
5837.0000000	0.2850000
5838.0000000	0.2650000
5839.0000000	0.2850000
5840.0000000	0.2700000
5841.0000000	0.2800000
5842.0000000	0.2750000
5843.0000000	0.2800000
5844.0000000	0.2900000
5845.0000000	0.2850000
5846.0000000	0.2900000
5847.0000000	0.2800000
5848.0000000	0.2800000
5849.0000000	0.2750000
5850.0000000	0.2900000
5851.0000000	0.2950000
5852.0000000	0.2750000
5853.0000000	0.2750000
5854.0000000	0.2750000
5855.0000000	0.2700000
5856.0000000	0.2750000
5857.0000000	0.2600000
5858.0000000	0.2700000
5859.0000000	0.2650000
5860.0000000	0.2750000
5861.0000000	0.2750000
5862.0000000	0.2700000
5863.0000000	0.2700000
5864.0000000	0.2750000
5865.0000000	0.2700000
5866.0000000	0.2700000
5867.0000000	0.2750000
5868.0000000	0.2750000
5869.0000000	0.2650000
5870.0000000	0.2650000

5871.0000000	0.2750000
5872.0000000	0.2750000
5873.0000000	0.2750000
5874.0000000	0.2650000
5875.0000000	0.2700000
5876.0000000	0.2750000
5877.0000000	0.2700000
5878.0000000	0.2700000
5879.0000000	0.2650000
5880.0000000	0.2750000
5881.0000000	0.2750000
5882.0000000	0.2750000
5883.0000000	0.2800000
5884.0000000	0.2700000
5885.0000000	0.2800000
5886.0000000	0.2700000
5887.0000000	0.2850000
5888.0000000	0.2750000
5889.0000000	0.2750000
5890.0000000	0.2750000
5891.0000000	0.2700000
5892.0000000	0.2700000
5893.0000000	0.2700000
5894.0000000	0.2750000
5895.0000000	0.2666667
5896.0000000	0.2650000
5897.0000000	0.2550000
5898.0000000	0.2600000
5899.0000000	0.2700000
5900.0000000	0.2600000
5901.0000000	0.2500000
5902.0000000	0.2500000
5903.0000000	0.2600000
5904.0000000	0.2650000
5905.0000000	0.2550000
5906.0000000	0.2550000
5907.0000000	0.2550000
5908.0000000	0.2650000
5909.0000000	0.2600000
5910.0000000	0.2500000
5911.0000000	0.2650000
5912.0000000	0.2600000
5913.0000000	0.2550000
5914.0000000	0.2600000
5915.0000000	0.2600000
5916.0000000	0.2600000
5917.0000000	0.2600000
5918.0000000	0.2650000
5919.0000000	0.2550000
5920.0000000	0.2550000
5921.0000000	0.2650000
5922.0000000	0.2600000
5923.0000000	0.2550000
5924.0000000	0.2650000
5925.0000000	0.2700000
5926.0000000	0.2650000
5927.0000000	0.2650000

5928.0000000	0.2600000
5929.0000000	0.2600000
5930.0000000	0.2700000
5931.0000000	0.2750000
5932.0000000	0.2700000
5933.0000000	0.2600000
5934.0000000	0.2700000
5935.0000000	0.2750000
5936.0000000	0.2650000
5937.0000000	0.2600000
5938.0000000	0.2700000
5939.0000000	0.2700000
5940.0000000	0.2750000
5941.0000000	0.2600000
5942.0000000	0.2650000
5943.0000000	0.2800000
5944.0000000	0.2650000
5945.0000000	0.2800000
5946.0000000	0.2650000
5947.0000000	0.2650000
5948.0000000	0.2650000
5949.0000000	0.2750000
5950.0000000	0.2650000
5951.0000000	0.2650000
5952.0000000	0.2600000
5953.0000000	0.2700000
5954.0000000	0.2700000
5955.0000000	0.2666667
5956.0000000	0.2700000
5957.0000000	0.2750000
5958.0000000	0.2700000
5959.0000000	0.2700000
5960.0000000	0.2700000
5961.0000000	0.2650000
5962.0000000	0.2750000
5963.0000000	0.2650000
5964.0000000	0.2650000
5965.0000000	0.2700000
5966.0000000	0.2700000
5967.0000000	0.2650000
5968.0000000	0.2800000
5969.0000000	0.2600000
5970.0000000	0.2700000
5971.0000000	0.2550000
5972.0000000	0.2700000
5973.0000000	0.2650000
5974.0000000	0.2700000
5975.0000000	0.2600000
5976.0000000	0.2700000
5977.0000000	0.2700000
5978.0000000	0.2700000
5979.0000000	0.2750000
5980.0000000	0.2650000
5981.0000000	0.2700000
5982.0000000	0.2600000
5983.0000000	0.2650000
5984.0000000	0.2650000

5985.0000000	0.2666667
5986.0000000	0.2750000
5987.0000000	0.2700000
5988.0000000	0.2700000
5989.0000000	0.2650000
5990.0000000	0.2700000
5991.0000000	0.2650000
5992.0000000	0.2700000
5993.0000000	0.2650000
5994.0000000	0.2700000
5995.0000000	0.2700000
5996.0000000	0.2650000
5997.0000000	0.2650000
5998.0000000	0.2600000
5999.0000000	0.2700000
6000.0000000	0.2650000
6001.0000000	0.2700000
6002.0000000	0.2650000
6003.0000000	0.2650000
6004.0000000	0.2550000
6005.0000000	0.2700000
6006.0000000	0.2650000
6007.0000000	0.2800000
6008.0000000	0.2800000
6009.0000000	0.2750000
6010.0000000	0.2700000
6011.0000000	0.2750000
6012.0000000	0.2750000
6013.0000000	0.2850000
6014.0000000	0.2850000
6015.0000000	0.2750000
6016.0000000	0.2900000
6017.0000000	0.2750000
6018.0000000	0.2800000
6019.0000000	0.2850000
6020.0000000	0.2800000
6021.0000000	0.2850000
6022.0000000	0.2800000
6023.0000000	0.2800000
6024.0000000	0.2750000
6025.0000000	0.2750000
6026.0000000	0.2800000
6027.0000000	0.2800000
6028.0000000	0.2800000
6029.0000000	0.2800000
6030.0000000	0.2800000
6031.0000000	0.2850000
6032.0000000	0.2750000
6033.0000000	0.2850000
6034.0000000	0.2850000
6035.0000000	0.2850000
6036.0000000	0.2800000
6037.0000000	0.2800000
6038.0000000	0.2900000
6039.0000000	0.2900000
6040.0000000	0.2950000
6041.0000000	0.2800000

6042.0000000	0.2750000
6043.0000000	0.2950000
6044.0000000	0.2900000
6045.0000000	0.2800000
6046.0000000	0.2850000
6047.0000000	0.2900000
6048.0000000	0.2800000
6049.0000000	0.2700000
6050.0000000	0.2800000
6051.0000000	0.2750000
6052.0000000	0.2850000
6053.0000000	0.2700000
6054.0000000	0.2750000
6055.0000000	0.2750000
6056.0000000	0.2700000
6057.0000000	0.2800000
6058.0000000	0.2650000
6059.0000000	0.2700000
6060.0000000	0.2700000
6061.0000000	0.2750000
6062.0000000	0.2700000
6063.0000000	0.2700000
6064.0000000	0.2700000
6065.0000000	0.2800000
6066.0000000	0.2750000
6067.0000000	0.2750000
6068.0000000	0.2750000
6069.0000000	0.2650000
6070.0000000	0.2900000
6071.0000000	0.2750000
6072.0000000	0.2800000
6073.0000000	0.2800000
6074.0000000	0.2800000
6075.0000000	0.2800000
6076.0000000	0.2733333
6077.0000000	0.2750000
6078.0000000	0.2750000
6079.0000000	0.2600000
6080.0000000	0.2700000
6081.0000000	0.2750000
6082.0000000	0.2750000
6083.0000000	0.2750000
6084.0000000	0.2750000
6085.0000000	0.2750000
6086.0000000	0.2750000
6087.0000000	0.2850000
6088.0000000	0.2750000
6089.0000000	0.2900000
6090.0000000	0.2900000
6091.0000000	0.2850000
6092.0000000	0.2900000
6093.0000000	0.2850000
6094.0000000	0.2850000
6095.0000000	0.2900000
6096.0000000	0.3000000
6097.0000000	0.2800000
6098.0000000	0.2900000

6099.0000000	0.2900000
6100.0000000	0.3000000
6101.0000000	0.2900000
6102.0000000	0.2900000
6103.0000000	0.2850000
6104.0000000	0.2900000
6105.0000000	0.3000000
6106.0000000	0.2900000
6107.0000000	0.2850000
6108.0000000	0.2850000
6109.0000000	0.2800000
6110.0000000	0.2900000
6111.0000000	0.2800000
6112.0000000	0.2750000
6113.0000000	0.2800000
6114.0000000	0.2750000
6115.0000000	0.2850000
6116.0000000	0.2850000
6117.0000000	0.2700000
6118.0000000	0.2750000
6119.0000000	0.2700000
6120.0000000	0.2850000
6121.0000000	0.2700000
6122.0000000	0.2750000
6123.0000000	0.2800000
6124.0000000	0.2800000
6125.0000000	0.2750000
6126.0000000	0.2850000
6127.0000000	0.2650000
6128.0000000	0.2700000
6129.0000000	0.2750000
6130.0000000	0.2650000
6131.0000000	0.2800000
6132.0000000	0.2650000
6133.0000000	0.2800000
6134.0000000	0.2700000
6135.0000000	0.2850000
6136.0000000	0.2733333
6137.0000000	0.2800000
6138.0000000	0.2800000
6139.0000000	0.2800000
6140.0000000	0.2700000
6141.0000000	0.2800000
6142.0000000	0.2850000
6143.0000000	0.2900000
6144.0000000	0.2850000
6145.0000000	0.2800000
6146.0000000	0.2850000
6147.0000000	0.2850000
6148.0000000	0.2850000
6149.0000000	0.2900000
6150.0000000	0.2900000
6151.0000000	0.2900000
6152.0000000	0.2900000
6153.0000000	0.2800000
6154.0000000	0.2900000
6155.0000000	0.2850000

6156.0000000	0.2900000
6157.0000000	0.2850000
6158.0000000	0.2850000
6159.0000000	0.2850000
6160.0000000	0.2850000
6161.0000000	0.2900000
6162.0000000	0.2900000
6163.0000000	0.2800000
6164.0000000	0.2750000
6165.0000000	0.2850000
6166.0000000	0.2800000
6167.0000000	0.2800000
6168.0000000	0.2900000
6169.0000000	0.2750000
6170.0000000	0.2900000
6171.0000000	0.2750000
6172.0000000	0.2850000
6173.0000000	0.2750000
6174.0000000	0.2750000
6175.0000000	0.2750000
6176.0000000	0.2750000
6177.0000000	0.2850000
6178.0000000	0.2850000
6179.0000000	0.2850000
6180.0000000	0.2800000
6181.0000000	0.2750000
6182.0000000	0.2850000
6183.0000000	0.2900000
6184.0000000	0.2850000
6185.0000000	0.2950000
6186.0000000	0.2800000
6187.0000000	0.2850000
6188.0000000	0.2850000
6189.0000000	0.2850000
6190.0000000	0.2900000
6191.0000000	0.2800000
6192.0000000	0.3000000
6193.0000000	0.2900000
6194.0000000	0.3000000
6195.0000000	0.2950000
6196.0000000	0.2900000
6197.0000000	0.2966667
6198.0000000	0.2900000
6199.0000000	0.3000000
6200.0000000	0.2950000
6201.0000000	0.3000000
6202.0000000	0.3000000
6203.0000000	0.3000000
6204.0000000	0.2900000
6205.0000000	0.2850000
6206.0000000	0.2850000
6207.0000000	0.2900000
6208.0000000	0.2900000
6209.0000000	0.2850000
6210.0000000	0.2850000
6211.0000000	0.2800000
6212.0000000	0.2800000

6213.0000000	0.2800000
6214.0000000	0.2900000
6215.0000000	0.2850000
6216.0000000	0.2800000
6217.0000000	0.2800000
6218.0000000	0.2750000
6219.0000000	0.2850000
6220.0000000	0.2850000
6221.0000000	0.2850000
6222.0000000	0.2850000
6223.0000000	0.2750000
6224.0000000	0.2850000
6225.0000000	0.2750000
6226.0000000	0.2850000
6227.0000000	0.2833333
6228.0000000	0.2900000
6229.0000000	0.2950000
6230.0000000	0.2900000
6231.0000000	0.2800000
6232.0000000	0.2900000
6233.0000000	0.2950000
6234.0000000	0.2950000
6235.0000000	0.2900000
6236.0000000	0.2900000
6237.0000000	0.3000000
6238.0000000	0.2900000
6239.0000000	0.2950000
6240.0000000	0.2900000
6241.0000000	0.2900000
6242.0000000	0.3050000
6243.0000000	0.3000000
6244.0000000	0.3050000
6245.0000000	0.2950000
6246.0000000	0.3050000
6247.0000000	0.2950000
6248.0000000	0.3050000
6249.0000000	0.3050000
6250.0000000	0.3000000
6251.0000000	0.2900000
6252.0000000	0.2950000
6253.0000000	0.3050000
6254.0000000	0.3050000
6255.0000000	0.3000000
6256.0000000	0.3000000
6257.0000000	0.3033333
6258.0000000	0.3100000
6259.0000000	0.3100000
6260.0000000	0.3050000
6261.0000000	0.3100000
6262.0000000	0.3000000
6263.0000000	0.2950000
6264.0000000	0.3000000
6265.0000000	0.3100000
6266.0000000	0.3000000
6267.0000000	0.3000000
6268.0000000	0.3100000
6269.0000000	0.3050000

6270.0000000	0.3100000
6271.0000000	0.3000000
6272.0000000	0.2950000
6273.0000000	0.3050000
6274.0000000	0.3050000
6275.0000000	0.2950000
6276.0000000	0.3050000
6277.0000000	0.3100000
6278.0000000	0.3050000
6279.0000000	0.3050000
6280.0000000	0.3100000
6281.0000000	0.3000000
6282.0000000	0.3050000
6283.0000000	0.3100000
6284.0000000	0.3050000
6285.0000000	0.3000000
6286.0000000	0.2950000
6287.0000000	0.3100000
6288.0000000	0.3000000
6289.0000000	0.3000000
6290.0000000	0.3150000
6291.0000000	0.3000000
6292.0000000	0.2950000
6293.0000000	0.3000000
6294.0000000	0.3050000
6295.0000000	0.2950000
6296.0000000	0.3050000
6297.0000000	0.2900000
6298.0000000	0.2950000
6299.0000000	0.3050000
6300.0000000	0.3000000
6301.0000000	0.2900000
6302.0000000	0.2950000
6303.0000000	0.3150000
6304.0000000	0.3000000
6305.0000000	0.2950000
6306.0000000	0.3100000
6307.0000000	0.3050000
6308.0000000	0.3100000
6309.0000000	0.3100000
6310.0000000	0.3050000
6311.0000000	0.3100000
6312.0000000	0.3100000
6313.0000000	0.3050000
6314.0000000	0.3150000
6315.0000000	0.3000000
6316.0000000	0.3050000
6317.0000000	0.3050000
6318.0000000	0.3133333
6319.0000000	0.3050000
6320.0000000	0.3150000
6321.0000000	0.2950000
6322.0000000	0.3100000
6323.0000000	0.3050000
6324.0000000	0.3050000
6325.0000000	0.3050000
6326.0000000	0.3050000

6327.0000000	0.3100000
6328.0000000	0.2900000
6329.0000000	0.3050000
6330.0000000	0.3000000
6331.0000000	0.3050000
6332.0000000	0.3050000
6333.0000000	0.2950000
6334.0000000	0.2950000
6335.0000000	0.2950000
6336.0000000	0.3000000
6337.0000000	0.3000000
6338.0000000	0.3100000
6339.0000000	0.2900000
6340.0000000	0.3000000
6341.0000000	0.2950000
6342.0000000	0.3100000
6343.0000000	0.3000000
6344.0000000	0.3000000
6345.0000000	0.2900000
6346.0000000	0.2900000
6347.0000000	0.3050000
6348.0000000	0.3000000
6349.0000000	0.2900000
6350.0000000	0.3000000
6351.0000000	0.2900000
6352.0000000	0.3000000
6353.0000000	0.2800000
6354.0000000	0.2900000
6355.0000000	0.2950000
6356.0000000	0.2950000
6357.0000000	0.2950000
6358.0000000	0.2800000
6359.0000000	0.2850000
6360.0000000	0.2800000
6361.0000000	0.2900000
6362.0000000	0.2950000
6363.0000000	0.2900000
6364.0000000	0.2900000
6365.0000000	0.2900000
6366.0000000	0.2900000
6367.0000000	0.2850000
6368.0000000	0.2950000
6369.0000000	0.2800000
6370.0000000	0.2900000
6371.0000000	0.2750000
6372.0000000	0.2950000
6373.0000000	0.2800000
6374.0000000	0.2950000
6375.0000000	0.2850000
6376.0000000	0.2850000
6377.0000000	0.2800000
6378.0000000	0.2800000
6379.0000000	0.2800000
6380.0000000	0.2700000
6381.0000000	0.2700000
6382.0000000	0.2700000
6383.0000000	0.2750000

6384.0000000	0.2700000
6385.0000000	0.2750000
6386.0000000	0.2700000
6387.0000000	0.2650000
6388.0000000	0.2700000
6389.0000000	0.2750000
6390.0000000	0.2800000
6391.0000000	0.2750000
6392.0000000	0.2750000
6393.0000000	0.2650000
6394.0000000	0.2750000
6395.0000000	0.2750000
6396.0000000	0.2750000
6397.0000000	0.2700000
6398.0000000	0.2750000
6399.0000000	0.2650000
6400.0000000	0.2750000
6401.0000000	0.2750000
6402.0000000	0.2750000
6403.0000000	0.2700000
6404.0000000	0.2650000
6405.0000000	0.2650000
6406.0000000	0.2700000
6407.0000000	0.2700000
6408.0000000	0.2666667
6409.0000000	0.2650000
6410.0000000	0.2600000
6411.0000000	0.2700000
6412.0000000	0.2700000
6413.0000000	0.2650000
6414.0000000	0.2650000
6415.0000000	0.2700000
6416.0000000	0.2700000
6417.0000000	0.2700000
6418.0000000	0.2750000
6419.0000000	0.2700000
6420.0000000	0.2700000
6421.0000000	0.2700000
6422.0000000	0.2700000
6423.0000000	0.2700000
6424.0000000	0.2700000
6425.0000000	0.2700000
6426.0000000	0.2700000
6427.0000000	0.2750000
6428.0000000	0.2700000
6429.0000000	0.2700000
6430.0000000	0.2650000
6431.0000000	0.2800000
6432.0000000	0.2700000
6433.0000000	0.2800000
6434.0000000	0.2650000
6435.0000000	0.2750000
6436.0000000	0.2600000
6437.0000000	0.2750000
6438.0000000	0.2700000
6439.0000000	0.2800000
6440.0000000	0.2750000

6441.0000000	0.2650000
6442.0000000	0.2650000
6443.0000000	0.2550000
6444.0000000	0.2650000
6445.0000000	0.2650000
6446.0000000	0.2650000
6447.0000000	0.2700000
6448.0000000	0.2600000
6449.0000000	0.2700000
6450.0000000	0.2650000
6451.0000000	0.2650000
6452.0000000	0.2600000
6453.0000000	0.2550000
6454.0000000	0.2750000
6455.0000000	0.2650000
6456.0000000	0.2650000
6457.0000000	0.2650000
6458.0000000	0.2600000
6459.0000000	0.2700000
6460.0000000	0.2650000
6461.0000000	0.2700000
6462.0000000	0.2600000
6463.0000000	0.2600000
6464.0000000	0.2700000
6465.0000000	0.2600000
6466.0000000	0.2650000
6467.0000000	0.2500000
6468.0000000	0.2633333
6469.0000000	0.2500000
6470.0000000	0.2550000
6471.0000000	0.2550000
6472.0000000	0.2550000
6473.0000000	0.2600000
6474.0000000	0.2450000
6475.0000000	0.2550000
6476.0000000	0.2600000
6477.0000000	0.2600000
6478.0000000	0.2600000
6479.0000000	0.2550000
6480.0000000	0.2550000
6481.0000000	0.2550000
6482.0000000	0.2650000
6483.0000000	0.2600000
6484.0000000	0.2450000
6485.0000000	0.2550000
6486.0000000	0.2550000
6487.0000000	0.2650000
6488.0000000	0.2600000
6489.0000000	0.2550000
6490.0000000	0.2650000
6491.0000000	0.2500000
6492.0000000	0.2550000
6493.0000000	0.2500000
6494.0000000	0.2500000
6495.0000000	0.2450000
6496.0000000	0.2600000
6497.0000000	0.2550000

6498.0000000	0.2566667
6499.0000000	0.2500000
6500.0000000	0.2450000
6501.0000000	0.2500000
6502.0000000	0.2500000
6503.0000000	0.2550000
6504.0000000	0.2450000
6505.0000000	0.2600000
6506.0000000	0.2550000
6507.0000000	0.2550000
6508.0000000	0.2600000
6509.0000000	0.2550000
6510.0000000	0.2500000
6511.0000000	0.2600000
6512.0000000	0.2550000
6513.0000000	0.2400000
6514.0000000	0.2600000
6515.0000000	0.2650000
6516.0000000	0.2600000
6517.0000000	0.2600000
6518.0000000	0.2500000
6519.0000000	0.2500000
6520.0000000	0.2500000
6521.0000000	0.2500000
6522.0000000	0.2450000
6523.0000000	0.2450000
6524.0000000	0.2550000
6525.0000000	0.2600000
6526.0000000	0.2500000
6527.0000000	0.2500000
6528.0000000	0.2500000
6529.0000000	0.2533333
6530.0000000	0.2550000
6531.0000000	0.2600000
6532.0000000	0.2550000
6533.0000000	0.2500000
6534.0000000	0.2450000
6535.0000000	0.2500000
6536.0000000	0.2500000
6537.0000000	0.2600000
6538.0000000	0.2500000
6539.0000000	0.2600000
6540.0000000	0.2450000
6541.0000000	0.2450000
6542.0000000	0.2450000
6543.0000000	0.2600000
6544.0000000	0.2550000
6545.0000000	0.2550000
6546.0000000	0.2450000
6547.0000000	0.2450000
6548.0000000	0.2500000
6549.0000000	0.2600000
6550.0000000	0.2550000
6551.0000000	0.2500000
6552.0000000	0.2500000
6553.0000000	0.2500000
6554.0000000	0.2600000

6555.0000000	0.2550000
6556.0000000	0.2550000
6557.0000000	0.2500000
6558.0000000	0.2500000
6559.0000000	0.2500000
6560.0000000	0.2500000
6561.0000000	0.2550000
6562.0000000	0.2400000
6563.0000000	0.2500000
6564.0000000	0.2550000
6565.0000000	0.2550000
6566.0000000	0.2500000
6567.0000000	0.2500000
6568.0000000	0.2400000
6569.0000000	0.2500000
6570.0000000	0.2500000
6571.0000000	0.2550000
6572.0000000	0.2550000
6573.0000000	0.2600000
6574.0000000	0.2550000
6575.0000000	0.2550000
6576.0000000	0.2500000
6577.0000000	0.2550000
6578.0000000	0.2550000
6579.0000000	0.2550000
6580.0000000	0.2500000
6581.0000000	0.2550000
6582.0000000	0.2500000
6583.0000000	0.2600000
6584.0000000	0.2600000
6585.0000000	0.2450000
6586.0000000	0.2450000
6587.0000000	0.2550000
6588.0000000	0.2450000
6589.0000000	0.2500000
6590.0000000	0.2500000
6591.0000000	0.2550000
6592.0000000	0.2450000
6593.0000000	0.2450000
6594.0000000	0.2400000
6595.0000000	0.2400000
6596.0000000	0.2600000
6597.0000000	0.2450000
6598.0000000	0.2500000
6599.0000000	0.2500000
6600.0000000	0.2450000
6601.0000000	0.2450000
6602.0000000	0.2450000
6603.0000000	0.2400000
6604.0000000	0.2450000
6605.0000000	0.2550000
6606.0000000	0.2450000
6607.0000000	0.2400000
6608.0000000	0.2300000
6609.0000000	0.2350000
6610.0000000	0.2550000
6611.0000000	0.2350000

6612.0000000	0.2550000
6613.0000000	0.2400000
6614.0000000	0.2550000
6615.0000000	0.2500000
6616.0000000	0.2450000
6617.0000000	0.2450000
6618.0000000	0.2500000
6619.0000000	0.2400000
6620.0000000	0.2550000
6621.0000000	0.2600000
6622.0000000	0.2550000
6623.0000000	0.2450000
6624.0000000	0.2550000
6625.0000000	0.2600000
6626.0000000	0.2500000
6627.0000000	0.2400000
6628.0000000	0.2550000
6629.0000000	0.2500000
6630.0000000	0.2500000
6631.0000000	0.2350000
6632.0000000	0.2450000
6633.0000000	0.2500000
6634.0000000	0.2400000
6635.0000000	0.2400000
6636.0000000	0.2450000
6637.0000000	0.2450000
6638.0000000	0.2300000
6639.0000000	0.2400000
6640.0000000	0.2300000
6641.0000000	0.2250000
6642.0000000	0.2350000
6643.0000000	0.2350000
6644.0000000	0.2300000
6645.0000000	0.2250000
6646.0000000	0.2450000
6647.0000000	0.2300000
6648.0000000	0.2300000
6649.0000000	0.2350000
6650.0000000	0.2266666
6651.0000000	0.2300000
6652.0000000	0.2250000
6653.0000000	0.2350000
6654.0000000	0.2300000
6655.0000000	0.2350000
6656.0000000	0.2400000
6657.0000000	0.2250000
6658.0000000	0.2250000
6659.0000000	0.2300000
6660.0000000	0.2250000
6661.0000000	0.2350000
6662.0000000	0.2300000
6663.0000000	0.2250000
6664.0000000	0.2250000
6665.0000000	0.2300000
6666.0000000	0.2350000
6667.0000000	0.2250000
6668.0000000	0.2300000

6669.0000000	0.2250000
6670.0000000	0.2350000
6671.0000000	0.2250000
6672.0000000	0.2350000
6673.0000000	0.2250000
6674.0000000	0.2200000
6675.0000000	0.2200000
6676.0000000	0.2400000
6677.0000000	0.2250000
6678.0000000	0.2300000
6679.0000000	0.2200000
6680.0000000	0.2366667
6681.0000000	0.2300000
6682.0000000	0.2300000
6683.0000000	0.2200000
6684.0000000	0.2350000
6685.0000000	0.2300000
6686.0000000	0.2250000
6687.0000000	0.2250000
6688.0000000	0.2300000
6689.0000000	0.2300000
6690.0000000	0.2400000
6691.0000000	0.2400000
6692.0000000	0.2300000
6693.0000000	0.2350000
6694.0000000	0.2300000
6695.0000000	0.2400000
6696.0000000	0.2350000
6697.0000000	0.2300000
6698.0000000	0.2400000
6699.0000000	0.2300000
6700.0000000	0.2350000
6701.0000000	0.2300000
6702.0000000	0.2400000
6703.0000000	0.2250000
6704.0000000	0.2200000
6705.0000000	0.2150000
6706.0000000	0.2300000
6707.0000000	0.2250000
6708.0000000	0.2300000
6709.0000000	0.2266667
6710.0000000	0.2250000
6711.0000000	0.2350000
6712.0000000	0.2350000
6713.0000000	0.2250000
6714.0000000	0.2250000
6715.0000000	0.2200000
6716.0000000	0.2250000
6717.0000000	0.2250000
6718.0000000	0.2300000
6719.0000000	0.2250000
6720.0000000	0.2350000
6721.0000000	0.2300000
6722.0000000	0.2250000
6723.0000000	0.2250000
6724.0000000	0.2200000
6725.0000000	0.2300000

6726.0000000	0.2350000
6727.0000000	0.2250000
6728.0000000	0.2250000
6729.0000000	0.2250000
6730.0000000	0.2300000
6731.0000000	0.2400000
6732.0000000	0.2350000
6733.0000000	0.2350000
6734.0000000	0.2250000
6735.0000000	0.2250000
6736.0000000	0.2250000
6737.0000000	0.2250000
6738.0000000	0.2300000
6739.0000000	0.2100000
6740.0000000	0.2233333
6741.0000000	0.2100000
6742.0000000	0.2150000
6743.0000000	0.2100000
6744.0000000	0.2300000
6745.0000000	0.2200000
6746.0000000	0.2200000
6747.0000000	0.2050000
6748.0000000	0.2100000
6749.0000000	0.2150000
6750.0000000	0.2250000
6751.0000000	0.2150000
6752.0000000	0.2250000
6753.0000000	0.2100000
6754.0000000	0.2150000
6755.0000000	0.2250000
6756.0000000	0.2250000
6757.0000000	0.2200000
6758.0000000	0.2250000
6759.0000000	0.2200000
6760.0000000	0.2200000
6761.0000000	0.2150000
6762.0000000	0.2150000
6763.0000000	0.2200000
6764.0000000	0.2150000
6765.0000000	0.2200000
6766.0000000	0.2100000
6767.0000000	0.2200000
6768.0000000	0.2050000
6769.0000000	0.2150000
6770.0000000	0.2100000
6771.0000000	0.2200000
6772.0000000	0.2100000
6773.0000000	0.2100000
6774.0000000	0.2200000
6775.0000000	0.2150000
6776.0000000	0.2150000
6777.0000000	0.2150000
6778.0000000	0.2250000
6779.0000000	0.2250000
6780.0000000	0.2200000
6781.0000000	0.2150000
6782.0000000	0.2050000

6783.0000000	0.2250000
6784.0000000	0.2200000
6785.0000000	0.2250000
6786.0000000	0.2100000
6787.0000000	0.2150000
6788.0000000	0.2200000
6789.0000000	0.2150000
6790.0000000	0.2200000
6791.0000000	0.2200000
6792.0000000	0.2300000
6793.0000000	0.2150000
6794.0000000	0.2200000
6795.0000000	0.2250000
6796.0000000	0.2200000
6797.0000000	0.2300000
6798.0000000	0.2200000
6799.0000000	0.2300000
6800.0000000	0.2233333
6801.0000000	0.2200000
6802.0000000	0.2350000
6803.0000000	0.2300000
6804.0000000	0.2350000
6805.0000000	0.2300000
6806.0000000	0.2300000
6807.0000000	0.2350000
6808.0000000	0.2300000
6809.0000000	0.2200000
6810.0000000	0.2250000
6811.0000000	0.2200000
6812.0000000	0.2200000
6813.0000000	0.2250000
6814.0000000	0.2200000
6815.0000000	0.2300000
6816.0000000	0.2350000
6817.0000000	0.2300000
6818.0000000	0.2200000
6819.0000000	0.2300000
6820.0000000	0.2300000
6821.0000000	0.2200000
6822.0000000	0.2350000
6823.0000000	0.2300000
6824.0000000	0.2350000
6825.0000000	0.2400000
6826.0000000	0.2400000
6827.0000000	0.2250000
6828.0000000	0.2350000
6829.0000000	0.2500000
6830.0000000	0.2333333
6831.0000000	0.2450000
6832.0000000	0.2400000
6833.0000000	0.2450000
6834.0000000	0.2400000
6835.0000000	0.2400000
6836.0000000	0.2400000
6837.0000000	0.2450000
6838.0000000	0.2300000
6839.0000000	0.2400000

6840.0000000	0.2550000
6841.0000000	0.2400000
6842.0000000	0.2450000
6843.0000000	0.2450000
6844.0000000	0.2450000
6845.0000000	0.2350000
6846.0000000	0.2500000
6847.0000000	0.2400000
6848.0000000	0.2350000
6849.0000000	0.2500000
6850.0000000	0.2400000
6851.0000000	0.2350000
6852.0000000	0.2400000
6853.0000000	0.2350000
6854.0000000	0.2400000
6855.0000000	0.2400000
6856.0000000	0.2200000
6857.0000000	0.2450000
6858.0000000	0.2450000
6859.0000000	0.2400000
6860.0000000	0.2400000
6861.0000000	0.2333333
6862.0000000	0.2350000
6863.0000000	0.2250000
6864.0000000	0.2350000
6865.0000000	0.2400000
6866.0000000	0.2350000
6867.0000000	0.2400000
6868.0000000	0.2300000
6869.0000000	0.2350000
6870.0000000	0.2400000
6871.0000000	0.2300000
6872.0000000	0.2350000
6873.0000000	0.2300000
6874.0000000	0.2400000
6875.0000000	0.2300000
6876.0000000	0.2350000
6877.0000000	0.2350000
6878.0000000	0.2350000
6879.0000000	0.2350000
6880.0000000	0.2350000
6881.0000000	0.2400000
6882.0000000	0.2350000
6883.0000000	0.2250000
6884.0000000	0.2250000
6885.0000000	0.2250000
6886.0000000	0.2400000
6887.0000000	0.2250000
6888.0000000	0.2300000
6889.0000000	0.2200000
6890.0000000	0.2200000
6891.0000000	0.2233333
6892.0000000	0.2300000
6893.0000000	0.2100000
6894.0000000	0.2300000
6895.0000000	0.2150000
6896.0000000	0.2250000

6897.0000000	0.2150000
6898.0000000	0.2250000
6899.0000000	0.2250000
6900.0000000	0.2300000
6901.0000000	0.2150000
6902.0000000	0.2300000
6903.0000000	0.2150000
6904.0000000	0.2250000
6905.0000000	0.2150000
6906.0000000	0.2300000
6907.0000000	0.2150000
6908.0000000	0.2250000
6909.0000000	0.2200000
6910.0000000	0.2200000
6911.0000000	0.2200000
6912.0000000	0.2250000
6913.0000000	0.2250000
6914.0000000	0.2250000
6915.0000000	0.2200000
6916.0000000	0.2300000
6917.0000000	0.2250000
6918.0000000	0.2250000
6919.0000000	0.2150000
6920.0000000	0.2200000
6921.0000000	0.2200000
6922.0000000	0.2150000
6923.0000000	0.2250000
6924.0000000	0.2150000
6925.0000000	0.2100000
6926.0000000	0.2200000
6927.0000000	0.2200000
6928.0000000	0.2300000
6929.0000000	0.2200000
6930.0000000	0.2150000
6931.0000000	0.2150000
6932.0000000	0.2200000
6933.0000000	0.2250000
6934.0000000	0.2150000
6935.0000000	0.2300000
6936.0000000	0.2150000
6937.0000000	0.2200000
6938.0000000	0.2150000
6939.0000000	0.2350000
6940.0000000	0.2250000
6941.0000000	0.2350000
6942.0000000	0.2300000
6943.0000000	0.2350000
6944.0000000	0.2350000
6945.0000000	0.2400000
6946.0000000	0.2350000
6947.0000000	0.2450000
6948.0000000	0.2350000
6949.0000000	0.2500000
6950.0000000	0.2400000
6951.0000000	0.2500000
6952.0000000	0.2450000
6953.0000000	0.2400000

6954.0000000	0.2550000
6955.0000000	0.2450000
6956.0000000	0.2550000
6957.0000000	0.2500000
6958.0000000	0.2450000
6959.0000000	0.2450000
6960.0000000	0.2450000
6961.0000000	0.2450000
6962.0000000	0.2450000
6963.0000000	0.2400000
6964.0000000	0.2550000
6965.0000000	0.2450000
6966.0000000	0.2450000
6967.0000000	0.2350000
6968.0000000	0.2400000
6969.0000000	0.2350000
6970.0000000	0.2350000
6971.0000000	0.2350000
6972.0000000	0.2350000
6973.0000000	0.2350000
6974.0000000	0.2350000
6975.0000000	0.2350000
6976.0000000	0.2400000
6977.0000000	0.2400000
6978.0000000	0.2350000
6979.0000000	0.2350000
6980.0000000	0.2300000
6981.0000000	0.2400000
6982.0000000	0.2333333
6983.0000000	0.2400000
6984.0000000	0.2400000
6985.0000000	0.2450000
6986.0000000	0.2350000
6987.0000000	0.2300000
6988.0000000	0.2450000
6989.0000000	0.2250000
6990.0000000	0.2350000
6991.0000000	0.2350000
6992.0000000	0.2250000
6993.0000000	0.2350000
6994.0000000	0.2400000
6995.0000000	0.2150000
6996.0000000	0.2150000
6997.0000000	0.2350000
6998.0000000	0.2300000
6999.0000000	0.2150000
7000.0000000	0.2250000
7001.0000000	0.2350000
7002.0000000	0.2250000
7003.0000000	0.2300000
7004.0000000	0.2300000
7005.0000000	0.2300000
7006.0000000	0.2300000
7007.0000000	0.2300000
7008.0000000	0.2250000
7009.0000000	0.2300000
7010.0000000	0.2400000

7011.0000000	0.2450000
7012.0000000	0.2300000
7013.0000000	0.2350000
7014.0000000	0.2250000
7015.0000000	0.2250000
7016.0000000	0.2400000
7017.0000000	0.2300000
7018.0000000	0.2450000
7019.0000000	0.2400000
7020.0000000	0.2350000
7021.0000000	0.2500000
7022.0000000	0.2350000
7023.0000000	0.2450000
7024.0000000	0.2350000
7025.0000000	0.2400000
7026.0000000	0.2300000
7027.0000000	0.2550000
7028.0000000	0.2300000
7029.0000000	0.2400000
7030.0000000	0.2300000
7031.0000000	0.2350000
7032.0000000	0.2350000
7033.0000000	0.2500000
7034.0000000	0.2400000
7035.0000000	0.2400000
7036.0000000	0.2350000
7037.0000000	0.2350000
7038.0000000	0.2400000
7039.0000000	0.2450000
7040.0000000	0.2500000
7041.0000000	0.2366667
7042.0000000	0.2400000
7043.0000000	0.2300000
7044.0000000	0.2450000
7045.0000000	0.2400000
7046.0000000	0.2450000
7047.0000000	0.2450000
7048.0000000	0.2300000
7049.0000000	0.2400000
7050.0000000	0.2350000
7051.0000000	0.2500000
7052.0000000	0.2450000
7053.0000000	0.2400000
7054.0000000	0.2350000
7055.0000000	0.2300000
7056.0000000	0.2400000
7057.0000000	0.2400000
7058.0000000	0.2450000
7059.0000000	0.2350000
7060.0000000	0.2350000
7061.0000000	0.2500000
7062.0000000	0.2350000
7063.0000000	0.2350000
7064.0000000	0.2400000
7065.0000000	0.2350000
7066.0000000	0.2450000
7067.0000000	0.2450000

7068.0000000	0.2450000
7069.0000000	0.2350000
7070.0000000	0.2400000
7071.0000000	0.2350000
7072.0000000	0.2433333
7073.0000000	0.2300000
7074.0000000	0.2500000
7075.0000000	0.2400000
7076.0000000	0.2350000
7077.0000000	0.2350000
7078.0000000	0.2400000
7079.0000000	0.2400000
7080.0000000	0.2450000
7081.0000000	0.2400000
7082.0000000	0.2300000
7083.0000000	0.2350000
7084.0000000	0.2300000
7085.0000000	0.2450000
7086.0000000	0.2400000
7087.0000000	0.2400000
7088.0000000	0.2300000
7089.0000000	0.2250000
7090.0000000	0.2350000
7091.0000000	0.2350000
7092.0000000	0.2450000
7093.0000000	0.2300000
7094.0000000	0.2300000
7095.0000000	0.2250000
7096.0000000	0.2250000
7097.0000000	0.2250000
7098.0000000	0.2350000
7099.0000000	0.2250000
7100.0000000	0.2350000
7101.0000000	0.2300000
7102.0000000	0.2300000
7103.0000000	0.2300000
7104.0000000	0.2350000
7105.0000000	0.2400000
7106.0000000	0.2350000
7107.0000000	0.2350000
7108.0000000	0.2200000
7109.0000000	0.2300000
7110.0000000	0.2400000
7111.0000000	0.2350000
7112.0000000	0.2350000
7113.0000000	0.2300000
7114.0000000	0.2250000
7115.0000000	0.2400000
7116.0000000	0.2350000
7117.0000000	0.2450000
7118.0000000	0.2350000
7119.0000000	0.2400000
7120.0000000	0.2300000
7121.0000000	0.2400000
7122.0000000	0.2400000
7123.0000000	0.2300000
7124.0000000	0.2400000

7125.0000000	0.2350000
7126.0000000	0.2500000
7127.0000000	0.2450000
7128.0000000	0.2450000
7129.0000000	0.2350000
7130.0000000	0.2400000
7131.0000000	0.2400000
7132.0000000	0.2433333
7133.0000000	0.2400000
7134.0000000	0.2450000
7135.0000000	0.2450000
7136.0000000	0.2400000
7137.0000000	0.2500000
7138.0000000	0.2400000
7139.0000000	0.2600000
7140.0000000	0.2500000
7141.0000000	0.2400000
7142.0000000	0.2400000
7143.0000000	0.2400000
7144.0000000	0.2450000
7145.0000000	0.2500000
7146.0000000	0.2400000
7147.0000000	0.2450000
7148.0000000	0.2350000
7149.0000000	0.2400000
7150.0000000	0.2400000
7151.0000000	0.2500000
7152.0000000	0.2350000
7153.0000000	0.2450000
7154.0000000	0.2400000
7155.0000000	0.2400000
7156.0000000	0.2400000
7157.0000000	0.2450000
7158.0000000	0.2400000
7159.0000000	0.2400000
7160.0000000	0.2450000
7161.0000000	0.2400000
7162.0000000	0.2366667
7163.0000000	0.2500000
7164.0000000	0.2450000
7165.0000000	0.2500000
7166.0000000	0.2300000
7167.0000000	0.2450000
7168.0000000	0.2300000
7169.0000000	0.2450000
7170.0000000	0.2350000
7171.0000000	0.2500000
7172.0000000	0.2300000
7173.0000000	0.2400000
7174.0000000	0.2350000
7175.0000000	0.2350000
7176.0000000	0.2450000
7177.0000000	0.2550000
7178.0000000	0.2450000
7179.0000000	0.2350000
7180.0000000	0.2350000
7181.0000000	0.2450000

7182.0000000	0.2450000
7183.0000000	0.2350000
7184.0000000	0.2350000
7185.0000000	0.2400000
7186.0000000	0.2350000
7187.0000000	0.2400000
7188.0000000	0.2400000
7189.0000000	0.2350000
7190.0000000	0.2250000
7191.0000000	0.2250000
7192.0000000	0.2350000
7193.0000000	0.2400000
7194.0000000	0.2350000
7195.0000000	0.2400000
7196.0000000	0.2400000
7197.0000000	0.2300000
7198.0000000	0.2400000
7199.0000000	0.2300000
7200.0000000	0.2400000
7201.0000000	0.2450000
7202.0000000	0.2400000
7203.0000000	0.2300000
7204.0000000	0.2300000
7205.0000000	0.2250000
7206.0000000	0.2350000
7207.0000000	0.2400000
7208.0000000	0.2400000
7209.0000000	0.2300000
7210.0000000	0.2300000
7211.0000000	0.2300000
7212.0000000	0.2400000
7213.0000000	0.2400000
7214.0000000	0.2350000
7215.0000000	0.2300000
7216.0000000	0.2300000
7217.0000000	0.2350000
7218.0000000	0.2300000
7219.0000000	0.2400000
7220.0000000	0.2350000
7221.0000000	0.2350000
7222.0000000	0.2250000
7223.0000000	0.2333333
7224.0000000	0.2300000
7225.0000000	0.2400000
7226.0000000	0.2350000
7227.0000000	0.2450000
7228.0000000	0.2300000
7229.0000000	0.2350000
7230.0000000	0.2300000
7231.0000000	0.2350000
7232.0000000	0.2300000
7233.0000000	0.2400000
7234.0000000	0.2250000
7235.0000000	0.2300000
7236.0000000	0.2250000
7237.0000000	0.2400000
7238.0000000	0.2300000

7239.0000000	0.2450000
7240.0000000	0.2250000
7241.0000000	0.2250000
7242.0000000	0.2300000
7243.0000000	0.2450000
7244.0000000	0.2400000
7245.0000000	0.2450000
7246.0000000	0.2300000
7247.0000000	0.2300000
7248.0000000	0.2250000
7249.0000000	0.2350000
7250.0000000	0.2300000
7251.0000000	0.2400000
7252.0000000	0.2350000
7253.0000000	0.2266666
7254.0000000	0.2400000
7255.0000000	0.2350000
7256.0000000	0.2300000
7257.0000000	0.2250000
7258.0000000	0.2300000
7259.0000000	0.2350000
7260.0000000	0.2300000
7261.0000000	0.2350000
7262.0000000	0.2300000
7263.0000000	0.2300000
7264.0000000	0.2200000
7265.0000000	0.2350000
7266.0000000	0.2300000
7267.0000000	0.2300000
7268.0000000	0.2300000
7269.0000000	0.2300000
7270.0000000	0.2200000
7271.0000000	0.2300000
7272.0000000	0.2350000
7273.0000000	0.2300000
7274.0000000	0.2350000
7275.0000000	0.2200000
7276.0000000	0.2200000
7277.0000000	0.2300000
7278.0000000	0.2300000
7279.0000000	0.2350000
7280.0000000	0.2350000
7281.0000000	0.2250000
7282.0000000	0.2300000
7283.0000000	0.2333333
7284.0000000	0.2250000
7285.0000000	0.2300000
7286.0000000	0.2350000
7287.0000000	0.2300000
7288.0000000	0.2300000
7289.0000000	0.2300000
7290.0000000	0.2300000
7291.0000000	0.2350000
7292.0000000	0.2400000
7293.0000000	0.2300000
7294.0000000	0.2350000
7295.0000000	0.2250000

7296.0000000	0.2350000
7297.0000000	0.2250000
7298.0000000	0.2450000
7299.0000000	0.2250000
7300.0000000	0.2300000
7301.0000000	0.2250000
7302.0000000	0.2400000
7303.0000000	0.2250000
7304.0000000	0.2500000
7305.0000000	0.2300000
7306.0000000	0.2300000
7307.0000000	0.2300000
7308.0000000	0.2350000
7309.0000000	0.2450000
7310.0000000	0.2350000
7311.0000000	0.2350000
7312.0000000	0.2300000
7313.0000000	0.2300000
7314.0000000	0.2300000
7315.0000000	0.2400000
7316.0000000	0.2350000
7317.0000000	0.2400000
7318.0000000	0.2400000
7319.0000000	0.2300000
7320.0000000	0.2400000
7321.0000000	0.2350000
7322.0000000	0.2500000
7323.0000000	0.2300000
7324.0000000	0.2400000
7325.0000000	0.2400000
7326.0000000	0.2350000
7327.0000000	0.2400000
7328.0000000	0.2300000
7329.0000000	0.2300000
7330.0000000	0.2400000
7331.0000000	0.2350000
7332.0000000	0.2300000
7333.0000000	0.2350000
7334.0000000	0.2250000
7335.0000000	0.2300000
7336.0000000	0.2400000
7337.0000000	0.2250000
7338.0000000	0.2300000
7339.0000000	0.2350000
7340.0000000	0.2400000
7341.0000000	0.2300000
7342.0000000	0.2300000
7343.0000000	0.2200000
7344.0000000	0.2300000
7345.0000000	0.2200000
7346.0000000	0.2350000
7347.0000000	0.2300000
7348.0000000	0.2300000
7349.0000000	0.2300000
7350.0000000	0.2250000
7351.0000000	0.2300000
7352.0000000	0.2350000

7353.0000000	0.2350000
7354.0000000	0.2300000
7355.0000000	0.2300000
7356.0000000	0.2250000
7357.0000000	0.2400000
7358.0000000	0.2200000
7359.0000000	0.2200000
7360.0000000	0.2250000
7361.0000000	0.2300000
7362.0000000	0.2350000
7363.0000000	0.2350000
7364.0000000	0.2350000
7365.0000000	0.2350000
7366.0000000	0.2300000
7367.0000000	0.2350000
7368.0000000	0.2300000
7369.0000000	0.2250000
7370.0000000	0.2350000
7371.0000000	0.2300000
7372.0000000	0.2500000
7373.0000000	0.2266667
7374.0000000	0.2450000
7375.0000000	0.2350000
7376.0000000	0.2400000
7377.0000000	0.2300000
7378.0000000	0.2450000
7379.0000000	0.2350000
7380.0000000	0.2400000
7381.0000000	0.2400000
7382.0000000	0.2350000
7383.0000000	0.2400000
7384.0000000	0.2400000
7385.0000000	0.2450000
7386.0000000	0.2400000
7387.0000000	0.2300000
7388.0000000	0.2400000
7389.0000000	0.2350000
7390.0000000	0.2400000
7391.0000000	0.2350000
7392.0000000	0.2350000
7393.0000000	0.2350000
7394.0000000	0.2300000
7395.0000000	0.2450000
7396.0000000	0.2250000
7397.0000000	0.2300000
7398.0000000	0.2300000
7399.0000000	0.2350000
7400.0000000	0.2300000
7401.0000000	0.2350000
7402.0000000	0.2250000
7403.0000000	0.2350000
7404.0000000	0.2366667
7405.0000000	0.2300000
7406.0000000	0.2350000
7407.0000000	0.2450000
7408.0000000	0.2350000
7409.0000000	0.2400000

7410.0000000	0.2300000
7411.0000000	0.2400000
7412.0000000	0.2350000
7413.0000000	0.2450000
7414.0000000	0.2350000
7415.0000000	0.2400000
7416.0000000	0.2300000
7417.0000000	0.2400000
7418.0000000	0.2350000
7419.0000000	0.2400000
7420.0000000	0.2400000
7421.0000000	0.2250000
7422.0000000	0.2350000
7423.0000000	0.2350000
7424.0000000	0.2450000
7425.0000000	0.2400000
7426.0000000	0.2350000
7427.0000000	0.2350000
7428.0000000	0.2350000
7429.0000000	0.2450000
7430.0000000	0.2450000
7431.0000000	0.2500000
7432.0000000	0.2500000
7433.0000000	0.2450000
7434.0000000	0.2500000
7435.0000000	0.2466667
7436.0000000	0.2550000
7437.0000000	0.2450000
7438.0000000	0.2550000
7439.0000000	0.2500000
7440.0000000	0.2450000
7441.0000000	0.2400000
7442.0000000	0.2400000
7443.0000000	0.2500000
7444.0000000	0.2450000
7445.0000000	0.2450000
7446.0000000	0.2400000
7447.0000000	0.2400000
7448.0000000	0.2350000
7449.0000000	0.2400000
7450.0000000	0.2350000
7451.0000000	0.2400000
7452.0000000	0.2500000
7453.0000000	0.2400000
7454.0000000	0.2400000
7455.0000000	0.2400000
7456.0000000	0.2350000
7457.0000000	0.2350000
7458.0000000	0.2400000
7459.0000000	0.2350000
7460.0000000	0.2350000
7461.0000000	0.2350000
7462.0000000	0.2300000
7463.0000000	0.2400000
7464.0000000	0.2500000
7465.0000000	0.2350000
7466.0000000	0.2350000

7467.0000000	0.2400000
7468.0000000	0.2400000
7469.0000000	0.2400000
7470.0000000	0.2400000
7471.0000000	0.2350000
7472.0000000	0.2350000
7473.0000000	0.2350000
7474.0000000	0.2400000
7475.0000000	0.2400000
7476.0000000	0.2350000
7477.0000000	0.2400000
7478.0000000	0.2400000
7479.0000000	0.2300000
7480.0000000	0.2350000
7481.0000000	0.2200000
7482.0000000	0.2400000
7483.0000000	0.2300000
7484.0000000	0.2450000
7485.0000000	0.2450000
7486.0000000	0.2400000
7487.0000000	0.2400000
7488.0000000	0.2400000
7489.0000000	0.2450000
7490.0000000	0.2500000
7491.0000000	0.2400000
7492.0000000	0.2400000
7493.0000000	0.2450000
7494.0000000	0.2400000
7495.0000000	0.2450000
7496.0000000	0.2300000
7497.0000000	0.2450000
7498.0000000	0.2350000
7499.0000000	0.2350000
7500.0000000	0.2450000
7501.0000000	0.2450000
7502.0000000	0.2300000
7503.0000000	0.2350000
7504.0000000	0.2400000
7505.0000000	0.2350000
7506.0000000	0.2400000
7507.0000000	0.2350000
7508.0000000	0.2400000
7509.0000000	0.2400000
7510.0000000	0.2550000
7511.0000000	0.2250000
7512.0000000	0.2300000
7513.0000000	0.2450000
7514.0000000	0.2350000
7515.0000000	0.2350000
7516.0000000	0.2400000
7517.0000000	0.2350000
7518.0000000	0.2350000
7519.0000000	0.2450000
7520.0000000	0.2300000
7521.0000000	0.2300000
7522.0000000	0.2400000
7523.0000000	0.2400000

7524.0000000	0.2300000
7525.0000000	0.2400000
7526.0000000	0.2300000
7527.0000000	0.2350000
7528.0000000	0.2450000
7529.0000000	0.2300000
7530.0000000	0.2300000
7531.0000000	0.2300000
7532.0000000	0.2300000
7533.0000000	0.2300000
7534.0000000	0.2350000
7535.0000000	0.2350000
7536.0000000	0.2200000
7537.0000000	0.2350000
7538.0000000	0.2350000
7539.0000000	0.2250000
7540.0000000	0.2250000
7541.0000000	0.2300000
7542.0000000	0.2300000
7543.0000000	0.2300000
7544.0000000	0.2300000
7545.0000000	0.2250000
7546.0000000	0.2350000
7547.0000000	0.2350000
7548.0000000	0.2250000
7549.0000000	0.2200000
7550.0000000	0.2300000
7551.0000000	0.2250000
7552.0000000	0.2300000
7553.0000000	0.2250000
7554.0000000	0.2300000
7555.0000000	0.2333333
7556.0000000	0.2350000
7557.0000000	0.2350000
7558.0000000	0.2250000
7559.0000000	0.2350000
7560.0000000	0.2300000
7561.0000000	0.2300000
7562.0000000	0.2350000
7563.0000000	0.2350000
7564.0000000	0.2300000
7565.0000000	0.2250000
7566.0000000	0.2350000
7567.0000000	0.2400000
7568.0000000	0.2350000
7569.0000000	0.2450000
7570.0000000	0.2250000
7571.0000000	0.2350000
7572.0000000	0.2200000
7573.0000000	0.2400000
7574.0000000	0.2450000
7575.0000000	0.2350000
7576.0000000	0.2300000
7577.0000000	0.2200000
7578.0000000	0.2350000
7579.0000000	0.2250000
7580.0000000	0.2400000

7581.0000000	0.2300000
7582.0000000	0.2300000
7583.0000000	0.2300000
7584.0000000	0.2300000
7585.0000000	0.2266667
7586.0000000	0.2400000
7587.0000000	0.2300000
7588.0000000	0.2400000
7589.0000000	0.2300000
7590.0000000	0.2200000
7591.0000000	0.2250000
7592.0000000	0.2400000
7593.0000000	0.2400000
7594.0000000	0.2350000
7595.0000000	0.2300000
7596.0000000	0.2250000
7597.0000000	0.2250000
7598.0000000	0.2350000
7599.0000000	0.2400000
7600.0000000	0.2300000
7601.0000000	0.2350000
7602.0000000	0.2300000
7603.0000000	0.2300000
7604.0000000	0.2350000
7605.0000000	0.2400000
7606.0000000	0.2300000
7607.0000000	0.2350000
7608.0000000	0.2250000
7609.0000000	0.2350000
7610.0000000	0.2300000
7611.0000000	0.2400000
7612.0000000	0.2400000
7613.0000000	0.2300000
7614.0000000	0.2300000
7615.0000000	0.2366667
7616.0000000	0.2350000
7617.0000000	0.2300000
7618.0000000	0.2350000
7619.0000000	0.2350000
7620.0000000	0.2350000
7621.0000000	0.2350000
7622.0000000	0.2400000
7623.0000000	0.2350000
7624.0000000	0.2350000
7625.0000000	0.2400000
7626.0000000	0.2400000
7627.0000000	0.2450000
7628.0000000	0.2450000
7629.0000000	0.2500000
7630.0000000	0.2350000
7631.0000000	0.2450000
7632.0000000	0.2450000
7633.0000000	0.2500000
7634.0000000	0.2500000
7635.0000000	0.2400000
7636.0000000	0.2400000
7637.0000000	0.2350000

7638.0000000	0.2400000
7639.0000000	0.2400000
7640.0000000	0.2550000
7641.0000000	0.2500000
7642.0000000	0.2500000
7643.0000000	0.2550000
7644.0000000	0.2400000
7645.0000000	0.2500000
7646.0000000	0.2400000
7647.0000000	0.2450000
7648.0000000	0.2450000
7649.0000000	0.2450000
7650.0000000	0.2550000
7651.0000000	0.2500000
7652.0000000	0.2500000
7653.0000000	0.2500000
7654.0000000	0.2500000
7655.0000000	0.2500000
7656.0000000	0.2500000
7657.0000000	0.2550000
7658.0000000	0.2550000
7659.0000000	0.2450000
7660.0000000	0.2450000
7661.0000000	0.2550000
7662.0000000	0.2500000
7663.0000000	0.2600000
7664.0000000	0.2400000
7665.0000000	0.2500000
7666.0000000	0.2450000
7667.0000000	0.2500000
7668.0000000	0.2550000
7669.0000000	0.2550000
7670.0000000	0.2450000
7671.0000000	0.2400000
7672.0000000	0.2500000
7673.0000000	0.2450000
7674.0000000	0.2500000
7675.0000000	0.2550000
7676.0000000	0.2466667
7677.0000000	0.2550000
7678.0000000	0.2400000
7679.0000000	0.2450000
7680.0000000	0.2550000
7681.0000000	0.2500000
7682.0000000	0.2450000
7683.0000000	0.2450000
7684.0000000	0.2500000
7685.0000000	0.2450000
7686.0000000	0.2500000
7687.0000000	0.2550000
7688.0000000	0.2350000
7689.0000000	0.2450000
7690.0000000	0.2500000
7691.0000000	0.2450000
7692.0000000	0.2550000
7693.0000000	0.2400000
7694.0000000	0.2450000

7695.0000000	0.2450000
7696.0000000	0.2450000
7697.0000000	0.2600000
7698.0000000	0.2450000
7699.0000000	0.2400000
7700.0000000	0.2450000
7701.0000000	0.2450000
7702.0000000	0.2450000
7703.0000000	0.2300000
7704.0000000	0.2350000
7705.0000000	0.2400000
7706.0000000	0.2366667
7707.0000000	0.2400000
7708.0000000	0.2500000
7709.0000000	0.2600000
7710.0000000	0.2450000
7711.0000000	0.2500000
7712.0000000	0.2400000
7713.0000000	0.2400000
7714.0000000	0.2500000
7715.0000000	0.2450000
7716.0000000	0.2450000
7717.0000000	0.2350000
7718.0000000	0.2450000
7719.0000000	0.2500000
7720.0000000	0.2500000
7721.0000000	0.2500000
7722.0000000	0.2400000
7723.0000000	0.2450000
7724.0000000	0.2450000
7725.0000000	0.2550000
7726.0000000	0.2450000
7727.0000000	0.2550000
7728.0000000	0.2400000
7729.0000000	0.2500000
7730.0000000	0.2450000
7731.0000000	0.2500000
7732.0000000	0.2550000
7733.0000000	0.2500000
7734.0000000	0.2500000
7735.0000000	0.2400000
7736.0000000	0.2433333
7737.0000000	0.2400000
7738.0000000	0.2450000
7739.0000000	0.2500000
7740.0000000	0.2500000
7741.0000000	0.2450000
7742.0000000	0.2400000
7743.0000000	0.2500000
7744.0000000	0.2500000
7745.0000000	0.2500000
7746.0000000	0.2500000
7747.0000000	0.2500000
7748.0000000	0.2500000
7749.0000000	0.2350000
7750.0000000	0.2400000
7751.0000000	0.2450000

7752.0000000	0.2400000
7753.0000000	0.2500000
7754.0000000	0.2400000
7755.0000000	0.2450000
7756.0000000	0.2400000
7757.0000000	0.2450000
7758.0000000	0.2400000
7759.0000000	0.2450000
7760.0000000	0.2400000
7761.0000000	0.2450000
7762.0000000	0.2400000
7763.0000000	0.2400000
7764.0000000	0.2450000
7765.0000000	0.2450000
7766.0000000	0.2400000
7767.0000000	0.2300000
7768.0000000	0.2400000
7769.0000000	0.2250000
7770.0000000	0.2300000
7771.0000000	0.2250000
7772.0000000	0.2300000
7773.0000000	0.2300000
7774.0000000	0.2300000
7775.0000000	0.2250000
7776.0000000	0.2300000
7777.0000000	0.2300000
7778.0000000	0.2250000
7779.0000000	0.2300000
7780.0000000	0.2350000
7781.0000000	0.2250000
7782.0000000	0.2350000
7783.0000000	0.2250000
7784.0000000	0.2300000
7785.0000000	0.2350000
7786.0000000	0.2300000
7787.0000000	0.2350000
7788.0000000	0.2300000
7789.0000000	0.2350000
7790.0000000	0.2200000
7791.0000000	0.2350000
7792.0000000	0.2200000
7793.0000000	0.2250000
7794.0000000	0.2350000
7795.0000000	0.2350000
7796.0000000	0.2266667
7797.0000000	0.2300000
7798.0000000	0.2250000
7799.0000000	0.2400000
7800.0000000	0.2350000
7801.0000000	0.2400000
7802.0000000	0.2200000
7803.0000000	0.2350000
7804.0000000	0.2300000
7805.0000000	0.2450000
7806.0000000	0.2400000
7807.0000000	0.2400000
7808.0000000	0.2400000

7809.0000000	0.2350000
7810.0000000	0.2450000
7811.0000000	0.2550000
7812.0000000	0.2600000
7813.0000000	0.2400000
7814.0000000	0.2500000
7815.0000000	0.2450000
7816.0000000	0.2550000
7817.0000000	0.2500000
7818.0000000	0.2500000
7819.0000000	0.2500000
7820.0000000	0.2400000
7821.0000000	0.2450000
7822.0000000	0.2500000
7823.0000000	0.2500000
7824.0000000	0.2550000
7825.0000000	0.2350000
7826.0000000	0.2366667
7827.0000000	0.2450000
7828.0000000	0.2350000
7829.0000000	0.2400000
7830.0000000	0.2250000
7831.0000000	0.2350000
7832.0000000	0.2300000
7833.0000000	0.2350000
7834.0000000	0.2350000
7835.0000000	0.2350000
7836.0000000	0.2300000
7837.0000000	0.2350000
7838.0000000	0.2250000
7839.0000000	0.2450000
7840.0000000	0.2400000
7841.0000000	0.2400000
7842.0000000	0.2300000
7843.0000000	0.2250000
7844.0000000	0.2300000
7845.0000000	0.2400000
7846.0000000	0.2400000
7847.0000000	0.2300000
7848.0000000	0.2250000
7849.0000000	0.2250000
7850.0000000	0.2250000
7851.0000000	0.2400000
7852.0000000	0.2250000
7853.0000000	0.2350000
7854.0000000	0.2150000
7855.0000000	0.2200000
7856.0000000	0.2250000
7857.0000000	0.2300000
7858.0000000	0.2200000
7859.0000000	0.2250000
7860.0000000	0.2300000
7861.0000000	0.2300000
7862.0000000	0.2250000
7863.0000000	0.2250000
7864.0000000	0.2200000
7865.0000000	0.2350000

7866.0000000	0.2250000
7867.0000000	0.2300000
7868.0000000	0.2150000
7869.0000000	0.2400000
7870.0000000	0.2200000
7871.0000000	0.2300000
7872.0000000	0.2200000
7873.0000000	0.2350000
7874.0000000	0.2250000
7875.0000000	0.2350000
7876.0000000	0.2300000
7877.0000000	0.2350000
7878.0000000	0.2300000
7879.0000000	0.2300000
7880.0000000	0.2250000
7881.0000000	0.2200000
7882.0000000	0.2350000
7883.0000000	0.2300000
7884.0000000	0.2350000
7885.0000000	0.2250000
7886.0000000	0.2200000
7887.0000000	0.2233333
7888.0000000	0.2300000
7889.0000000	0.2200000
7890.0000000	0.2250000
7891.0000000	0.2300000
7892.0000000	0.2300000
7893.0000000	0.2300000
7894.0000000	0.2350000
7895.0000000	0.2350000
7896.0000000	0.2300000
7897.0000000	0.2300000
7898.0000000	0.2350000
7899.0000000	0.2400000
7900.0000000	0.2300000
7901.0000000	0.2300000
7902.0000000	0.2350000
7903.0000000	0.2300000
7904.0000000	0.2250000
7905.0000000	0.2400000
7906.0000000	0.2400000
7907.0000000	0.2400000
7908.0000000	0.2250000
7909.0000000	0.2350000
7910.0000000	0.2400000
7911.0000000	0.2350000
7912.0000000	0.2400000
7913.0000000	0.2350000
7914.0000000	0.2400000
7915.0000000	0.2400000
7916.0000000	0.2400000
7917.0000000	0.2433333
7918.0000000	0.2450000
7919.0000000	0.2500000
7920.0000000	0.2450000
7921.0000000	0.2450000
7922.0000000	0.2450000

7923.0000000	0.2500000
7924.0000000	0.2450000
7925.0000000	0.2500000
7926.0000000	0.2500000
7927.0000000	0.2500000
7928.0000000	0.2500000
7929.0000000	0.2500000
7930.0000000	0.2500000
7931.0000000	0.2550000
7932.0000000	0.2550000
7933.0000000	0.2500000
7934.0000000	0.2400000
7935.0000000	0.2400000
7936.0000000	0.2500000
7937.0000000	0.2450000
7938.0000000	0.2550000
7939.0000000	0.2400000
7940.0000000	0.2450000
7941.0000000	0.2250000
7942.0000000	0.2500000
7943.0000000	0.2350000
7944.0000000	0.2400000
7945.0000000	0.2400000
7946.0000000	0.2350000
7947.0000000	0.2366667
7948.0000000	0.2400000
7949.0000000	0.2300000
7950.0000000	0.2400000
7951.0000000	0.2400000
7952.0000000	0.2450000
7953.0000000	0.2400000
7954.0000000	0.2400000
7955.0000000	0.2350000
7956.0000000	0.2400000
7957.0000000	0.2400000
7958.0000000	0.2350000
7959.0000000	0.2400000
7960.0000000	0.2400000
7961.0000000	0.2400000
7962.0000000	0.2550000
7963.0000000	0.2500000
7964.0000000	0.2450000
7965.0000000	0.2500000
7966.0000000	0.2400000
7967.0000000	0.2600000
7968.0000000	0.2450000
7969.0000000	0.2500000
7970.0000000	0.2500000
7971.0000000	0.2500000
7972.0000000	0.2550000
7973.0000000	0.2600000
7974.0000000	0.2500000
7975.0000000	0.2550000
7976.0000000	0.2400000
7977.0000000	0.2600000
7978.0000000	0.2466667
7979.0000000	0.2550000

7980.0000000	0.2400000
7981.0000000	0.2450000
7982.0000000	0.2450000
7983.0000000	0.2450000
7984.0000000	0.2450000
7985.0000000	0.2600000
7986.0000000	0.2550000
7987.0000000	0.2450000
7988.0000000	0.2450000
7989.0000000	0.2450000
7990.0000000	0.2550000
7991.0000000	0.2550000
7992.0000000	0.2500000
7993.0000000	0.2500000
7994.0000000	0.2450000
7995.0000000	0.2500000
7996.0000000	0.2550000
7997.0000000	0.2500000
7998.0000000	0.2500000
7999.0000000	0.2500000
8000.0000000	0.2650000
8001.0000000	0.2600000
8002.0000000	0.2550000
8003.0000000	0.2550000
8004.0000000	0.2600000
8005.0000000	0.2600000
8006.0000000	0.2750000
8007.0000000	0.2650000
8008.0000000	0.2666667
8009.0000000	0.2550000
8010.0000000	0.2600000
8011.0000000	0.2600000
8012.0000000	0.2650000
8013.0000000	0.2700000
8014.0000000	0.2700000
8015.0000000	0.2600000
8016.0000000	0.2550000
8017.0000000	0.2500000
8018.0000000	0.2500000
8019.0000000	0.2550000
8020.0000000	0.2600000
8021.0000000	0.2550000
8022.0000000	0.2550000
8023.0000000	0.2550000
8024.0000000	0.2650000
8025.0000000	0.2550000
8026.0000000	0.2600000
8027.0000000	0.2550000
8028.0000000	0.2600000
8029.0000000	0.2550000
8030.0000000	0.2600000
8031.0000000	0.2500000
8032.0000000	0.2600000
8033.0000000	0.2500000
8034.0000000	0.2550000
8035.0000000	0.2550000
8036.0000000	0.2600000

8037.0000000	0.2600000
8038.0000000	0.2533333
8039.0000000	0.2600000
8040.0000000	0.2700000
8041.0000000	0.2500000
8042.0000000	0.2700000
8043.0000000	0.2600000
8044.0000000	0.2650000
8045.0000000	0.2650000
8046.0000000	0.2700000
8047.0000000	0.2650000
8048.0000000	0.2700000
8049.0000000	0.2650000
8050.0000000	0.2700000
8051.0000000	0.2700000
8052.0000000	0.2700000
8053.0000000	0.2600000
8054.0000000	0.2550000
8055.0000000	0.2700000
8056.0000000	0.2650000
8057.0000000	0.2700000
8058.0000000	0.2650000
8059.0000000	0.2650000
8060.0000000	0.2600000
8061.0000000	0.2650000
8062.0000000	0.2650000
8063.0000000	0.2700000
8064.0000000	0.2700000
8065.0000000	0.2700000
8066.0000000	0.2700000
8067.0000000	0.2700000
8068.0000000	0.2666667
8069.0000000	0.2600000
8070.0000000	0.2600000
8071.0000000	0.2600000
8072.0000000	0.2700000
8073.0000000	0.2650000
8074.0000000	0.2600000
8075.0000000	0.2600000
8076.0000000	0.2550000
8077.0000000	0.2500000
8078.0000000	0.2650000
8079.0000000	0.2650000
8080.0000000	0.2650000
8081.0000000	0.2600000
8082.0000000	0.2600000
8083.0000000	0.2550000
8084.0000000	0.2650000
8085.0000000	0.2650000
8086.0000000	0.2650000
8087.0000000	0.2600000
8088.0000000	0.2600000
8089.0000000	0.2600000
8090.0000000	0.2600000
8091.0000000	0.2700000
8092.0000000	0.2700000
8093.0000000	0.2700000

8094.0000000	0.2550000
8095.0000000	0.2650000
8096.0000000	0.2650000
8097.0000000	0.2650000
8098.0000000	0.2650000
8099.0000000	0.2733333
8100.0000000	0.2700000
8101.0000000	0.2700000
8102.0000000	0.2700000
8103.0000000	0.2700000
8104.0000000	0.2800000
8105.0000000	0.2700000
8106.0000000	0.2700000
8107.0000000	0.2650000
8108.0000000	0.2750000
8109.0000000	0.2800000
8110.0000000	0.2850000
8111.0000000	0.2800000
8112.0000000	0.2800000
8113.0000000	0.2750000
8114.0000000	0.2750000
8115.0000000	0.2850000
8116.0000000	0.2750000
8117.0000000	0.2800000
8118.0000000	0.2800000
8119.0000000	0.2800000
8120.0000000	0.2800000
8121.0000000	0.2800000
8122.0000000	0.2850000
8123.0000000	0.2800000
8124.0000000	0.2850000
8125.0000000	0.2900000
8126.0000000	0.2950000
8127.0000000	0.2850000
8128.0000000	0.2900000
8129.0000000	0.2850000
8130.0000000	0.2800000
8131.0000000	0.2850000
8132.0000000	0.2900000
8133.0000000	0.2950000
8134.0000000	0.2850000
8135.0000000	0.2850000
8136.0000000	0.2850000
8137.0000000	0.2900000
8138.0000000	0.2950000
8139.0000000	0.2900000
8140.0000000	0.2950000
8141.0000000	0.2850000
8142.0000000	0.2850000
8143.0000000	0.3000000
8144.0000000	0.2950000
8145.0000000	0.2850000
8146.0000000	0.2900000
8147.0000000	0.2900000
8148.0000000	0.2950000
8149.0000000	0.3050000
8150.0000000	0.2800000

8151.0000000	0.2900000
8152.0000000	0.3000000
8153.0000000	0.2900000
8154.0000000	0.3000000
8155.0000000	0.2950000
8156.0000000	0.3000000
8157.0000000	0.3050000
8158.0000000	0.2966667
8159.0000000	0.2950000
8160.0000000	0.3050000
8161.0000000	0.2900000
8162.0000000	0.2900000
8163.0000000	0.3100000
8164.0000000	0.3100000
8165.0000000	0.3000000
8166.0000000	0.3050000
8167.0000000	0.3100000
8168.0000000	0.3000000
8169.0000000	0.3050000
8170.0000000	0.2950000
8171.0000000	0.3050000
8172.0000000	0.3050000
8173.0000000	0.3150000
8174.0000000	0.3000000
8175.0000000	0.2950000
8176.0000000	0.3100000
8177.0000000	0.3050000
8178.0000000	0.3050000
8179.0000000	0.3000000
8180.0000000	0.3000000
8181.0000000	0.3000000
8182.0000000	0.3100000
8183.0000000	0.3100000
8184.0000000	0.2950000
8185.0000000	0.3050000
8186.0000000	0.3050000
8187.0000000	0.3000000
8188.0000000	0.3050000
8189.0000000	0.3066667
8190.0000000	0.3050000
8191.0000000	0.3150000
8192.0000000	0.3050000
8193.0000000	0.3200000
8194.0000000	0.3050000
8195.0000000	0.3150000
8196.0000000	0.3050000
8197.0000000	0.3100000
8198.0000000	0.3150000
8199.0000000	0.3150000
8200.0000000	0.3100000
8201.0000000	0.3100000
8202.0000000	0.3150000
8203.0000000	0.3000000
8204.0000000	0.3100000
8205.0000000	0.3150000
8206.0000000	0.3250000
8207.0000000	0.3100000

8208.0000000	0.3100000
8209.0000000	0.3100000
8210.0000000	0.3200000
8211.0000000	0.3150000
8212.0000000	0.3150000
8213.0000000	0.3050000
8214.0000000	0.3100000
8215.0000000	0.3050000
8216.0000000	0.3150000
8217.0000000	0.3200000
8218.0000000	0.3200000
8219.0000000	0.3133333
8220.0000000	0.3100000
8221.0000000	0.3100000
8222.0000000	0.3100000
8223.0000000	0.3100000
8224.0000000	0.3150000
8225.0000000	0.3100000
8226.0000000	0.3200000
8227.0000000	0.3200000
8228.0000000	0.3200000
8229.0000000	0.3200000
8230.0000000	0.3150000
8231.0000000	0.3150000
8232.0000000	0.3150000
8233.0000000	0.3250000
8234.0000000	0.3250000
8235.0000000	0.3300000
8236.0000000	0.3250000
8237.0000000	0.3250000
8238.0000000	0.3250000
8239.0000000	0.3150000
8240.0000000	0.3200000
8241.0000000	0.3100000
8242.0000000	0.3250000
8243.0000000	0.3150000
8244.0000000	0.3350000
8245.0000000	0.3200000
8246.0000000	0.3300000
8247.0000000	0.3300000
8248.0000000	0.3250000
8249.0000000	0.3233333
8250.0000000	0.3200000
8251.0000000	0.3300000
8252.0000000	0.3250000
8253.0000000	0.3250000
8254.0000000	0.3300000
8255.0000000	0.3300000
8256.0000000	0.3250000
8257.0000000	0.3350000
8258.0000000	0.3250000
8259.0000000	0.3300000
8260.0000000	0.3300000
8261.0000000	0.3250000
8262.0000000	0.3250000
8263.0000000	0.3300000
8264.0000000	0.3250000

8265.0000000	0.3400000
8266.0000000	0.3350000
8267.0000000	0.3300000
8268.0000000	0.3300000
8269.0000000	0.3250000
8270.0000000	0.3400000
8271.0000000	0.3400000
8272.0000000	0.3400000
8273.0000000	0.3350000
8274.0000000	0.3200000
8275.0000000	0.3250000
8276.0000000	0.3300000
8277.0000000	0.3250000
8278.0000000	0.3300000
8279.0000000	0.3233333
8280.0000000	0.3200000
8281.0000000	0.3350000
8282.0000000	0.3250000
8283.0000000	0.3350000
8284.0000000	0.3150000
8285.0000000	0.3250000
8286.0000000	0.3200000
8287.0000000	0.3250000
8288.0000000	0.3250000
8289.0000000	0.3250000
8290.0000000	0.3250000
8291.0000000	0.3250000
8292.0000000	0.3250000
8293.0000000	0.3300000
8294.0000000	0.3150000
8295.0000000	0.3300000
8296.0000000	0.3250000
8297.0000000	0.3250000
8298.0000000	0.3250000
8299.0000000	0.3250000
8300.0000000	0.3100000
8301.0000000	0.3200000
8302.0000000	0.3200000
8303.0000000	0.3200000
8304.0000000	0.3250000
8305.0000000	0.3150000
8306.0000000	0.3200000
8307.0000000	0.3150000
8308.0000000	0.3300000
8309.0000000	0.3250000
8310.0000000	0.3233334
8311.0000000	0.3200000
8312.0000000	0.3150000
8313.0000000	0.3300000
8314.0000000	0.3150000
8315.0000000	0.3200000
8316.0000000	0.3150000
8317.0000000	0.3200000
8318.0000000	0.3200000
8319.0000000	0.3200000
8320.0000000	0.3150000
8321.0000000	0.3200000

8322.0000000	0.3150000
8323.0000000	0.3200000
8324.0000000	0.3200000
8325.0000000	0.3250000
8326.0000000	0.3250000
8327.0000000	0.3150000
8328.0000000	0.3150000
8329.0000000	0.3150000
8330.0000000	0.3200000
8331.0000000	0.3150000
8332.0000000	0.3100000
8333.0000000	0.3150000
8334.0000000	0.3250000
8335.0000000	0.3150000
8336.0000000	0.3250000
8337.0000000	0.3250000
8338.0000000	0.3200000
8339.0000000	0.3150000
8340.0000000	0.3200000
8341.0000000	0.3150000
8342.0000000	0.3150000
8343.0000000	0.3150000
8344.0000000	0.3150000
8345.0000000	0.3250000
8346.0000000	0.3150000
8347.0000000	0.3300000
8348.0000000	0.3150000
8349.0000000	0.3250000
8350.0000000	0.3150000
8351.0000000	0.3300000
8352.0000000	0.3250000
8353.0000000	0.3300000
8354.0000000	0.3150000
8355.0000000	0.3200000
8356.0000000	0.3200000
8357.0000000	0.3250000
8358.0000000	0.3200000
8359.0000000	0.3300000
8360.0000000	0.3250000
8361.0000000	0.3200000
8362.0000000	0.3150000
8363.0000000	0.3250000
8364.0000000	0.3100000
8365.0000000	0.3250000
8366.0000000	0.3150000
8367.0000000	0.3250000
8368.0000000	0.3150000
8369.0000000	0.3300000
8370.0000000	0.3166667
8371.0000000	0.3100000
8372.0000000	0.3200000
8373.0000000	0.3200000
8374.0000000	0.3350000
8375.0000000	0.3250000
8376.0000000	0.3200000
8377.0000000	0.3150000
8378.0000000	0.3200000

8379.0000000	0.3250000
8380.0000000	0.3250000
8381.0000000	0.3250000
8382.0000000	0.3250000
8383.0000000	0.3250000
8384.0000000	0.3200000
8385.0000000	0.3250000
8386.0000000	0.3250000
8387.0000000	0.3250000
8388.0000000	0.3250000
8389.0000000	0.3150000
8390.0000000	0.3250000
8391.0000000	0.3250000
8392.0000000	0.3300000
8393.0000000	0.3150000
8394.0000000	0.3200000
8395.0000000	0.3300000
8396.0000000	0.3200000
8397.0000000	0.3300000
8398.0000000	0.3250000
8399.0000000	0.3300000
8400.0000000	0.3333333
8401.0000000	0.3200000
8402.0000000	0.3250000
8403.0000000	0.3200000
8404.0000000	0.3300000
8405.0000000	0.3300000
8406.0000000	0.3250000
8407.0000000	0.3300000
8408.0000000	0.3300000
8409.0000000	0.3250000
8410.0000000	0.3250000
8411.0000000	0.3350000
8412.0000000	0.3300000
8413.0000000	0.3300000
8414.0000000	0.3250000
8415.0000000	0.3300000
8416.0000000	0.3300000
8417.0000000	0.3350000
8418.0000000	0.3300000
8419.0000000	0.3300000
8420.0000000	0.3350000
8421.0000000	0.3300000
8422.0000000	0.3350000
8423.0000000	0.3350000
8424.0000000	0.3450000
8425.0000000	0.3350000
8426.0000000	0.3350000
8427.0000000	0.3350000
8428.0000000	0.3400000
8429.0000000	0.3350000
8430.0000000	0.3400000
8431.0000000	0.3333333
8432.0000000	0.3450000
8433.0000000	0.3350000
8434.0000000	0.3300000
8435.0000000	0.3350000

8436.0000000	0.3350000
8437.0000000	0.3400000
8438.0000000	0.3400000
8439.0000000	0.3350000
8440.0000000	0.3350000
8441.0000000	0.3350000
8442.0000000	0.3350000
8443.0000000	0.3500000
8444.0000000	0.3400000
8445.0000000	0.3450000
8446.0000000	0.3350000
8447.0000000	0.3450000
8448.0000000	0.3350000
8449.0000000	0.3400000
8450.0000000	0.3350000
8451.0000000	0.3300000
8452.0000000	0.3350000
8453.0000000	0.3300000
8454.0000000	0.3400000
8455.0000000	0.3350000
8456.0000000	0.3350000
8457.0000000	0.3350000
8458.0000000	0.3350000
8459.0000000	0.3300000
8460.0000000	0.3350000
8461.0000000	0.3333333
8462.0000000	0.3350000
8463.0000000	0.3300000
8464.0000000	0.3350000
8465.0000000	0.3250000
8466.0000000	0.3250000
8467.0000000	0.3300000
8468.0000000	0.3400000
8469.0000000	0.3300000
8470.0000000	0.3300000
8471.0000000	0.3200000
8472.0000000	0.3300000
8473.0000000	0.3200000
8474.0000000	0.3350000
8475.0000000	0.3350000
8476.0000000	0.3350000
8477.0000000	0.3200000
8478.0000000	0.3300000
8479.0000000	0.3200000
8480.0000000	0.3350000
8481.0000000	0.3250000
8482.0000000	0.3300000
8483.0000000	0.3200000
8484.0000000	0.3200000
8485.0000000	0.3150000
8486.0000000	0.3250000
8487.0000000	0.3200000
8488.0000000	0.3250000
8489.0000000	0.3150000
8490.0000000	0.3166667
8491.0000000	0.3050000
8492.0000000	0.3150000

8493.0000000	0.3150000
8494.0000000	0.3150000
8495.0000000	0.3050000
8496.0000000	0.3000000
8497.0000000	0.3000000
8498.0000000	0.2900000
8499.0000000	0.3000000
8500.0000000	0.2950000
8501.0000000	0.2950000
8502.0000000	0.2900000
8503.0000000	0.2850000
8504.0000000	0.2650000
8505.0000000	0.2800000
8506.0000000	0.2850000
8507.0000000	0.2750000
8508.0000000	0.2700000
8509.0000000	0.2700000
8510.0000000	0.2650000
8511.0000000	0.2650000
8512.0000000	0.2700000
8513.0000000	0.2600000
8514.0000000	0.2650000
8515.0000000	0.2500000
8516.0000000	0.2500000
8517.0000000	0.2500000
8518.0000000	0.2600000
8519.0000000	0.2500000
8520.0000000	0.2550000
8521.0000000	0.2466667
8522.0000000	0.2550000
8523.0000000	0.2500000
8524.0000000	0.2450000
8525.0000000	0.2350000
8526.0000000	0.2450000
8527.0000000	0.2450000
8528.0000000	0.2500000
8529.0000000	0.2400000
8530.0000000	0.2450000
8531.0000000	0.2400000
8532.0000000	0.2400000
8533.0000000	0.2400000
8534.0000000	0.2400000
8535.0000000	0.2450000
8536.0000000	0.2400000
8537.0000000	0.2350000
8538.0000000	0.2350000
8539.0000000	0.2400000
8540.0000000	0.2350000
8541.0000000	0.2400000
8542.0000000	0.2350000
8543.0000000	0.2400000
8544.0000000	0.2400000
8545.0000000	0.2450000
8546.0000000	0.2400000
8547.0000000	0.2400000
8548.0000000	0.2350000
8549.0000000	0.2400000

8550.0000000	0.2450000
8551.0000000	0.2400000
8552.0000000	0.2350000
8553.0000000	0.2400000
8554.0000000	0.2450000
8555.0000000	0.2300000
8556.0000000	0.2500000
8557.0000000	0.2350000
8558.0000000	0.2400000
8559.0000000	0.2300000
8560.0000000	0.2350000
8561.0000000	0.2400000
8562.0000000	0.2350000
8563.0000000	0.2450000
8564.0000000	0.2300000
8565.0000000	0.2300000
8566.0000000	0.2350000
8567.0000000	0.2300000
8568.0000000	0.2400000
8569.0000000	0.2400000
8570.0000000	0.2350000
8571.0000000	0.2400000
8572.0000000	0.2350000
8573.0000000	0.2300000
8574.0000000	0.2300000
8575.0000000	0.2250000
8576.0000000	0.2300000
8577.0000000	0.2450000
8578.0000000	0.2300000
8579.0000000	0.2250000
8580.0000000	0.2400000
8581.0000000	0.2200000
8582.0000000	0.2300000
8583.0000000	0.2300000
8584.0000000	0.2300000
8585.0000000	0.2300000
8586.0000000	0.2350000
8587.0000000	0.2300000
8588.0000000	0.2400000
8589.0000000	0.2350000
8590.0000000	0.2300000
8591.0000000	0.2300000
8592.0000000	0.2400000
8593.0000000	0.2300000
8594.0000000	0.2150000
8595.0000000	0.2250000
8596.0000000	0.2250000
8597.0000000	0.2200000
8598.0000000	0.2200000
8599.0000000	0.2250000
8600.0000000	0.2250000
8601.0000000	0.2200000
8602.0000000	0.2200000
8603.0000000	0.2100000
8604.0000000	0.2100000
8605.0000000	0.2200000
8606.0000000	0.2200000

8607.0000000	0.2150000
8608.0000000	0.2200000
8609.0000000	0.2200000
8610.0000000	0.2200000
8611.0000000	0.2166667
8612.0000000	0.2300000
8613.0000000	0.2150000
8614.0000000	0.2250000
8615.0000000	0.2250000
8616.0000000	0.2150000
8617.0000000	0.2200000
8618.0000000	0.2100000
8619.0000000	0.2150000
8620.0000000	0.2150000
8621.0000000	0.2200000
8622.0000000	0.2150000
8623.0000000	0.2200000
8624.0000000	0.2050000
8625.0000000	0.2150000
8626.0000000	0.2100000
8627.0000000	0.2200000
8628.0000000	0.2150000
8629.0000000	0.2100000
8630.0000000	0.2100000
8631.0000000	0.2050000
8632.0000000	0.2100000
8633.0000000	0.2100000
8634.0000000	0.1950000
8635.0000000	0.2050000
8636.0000000	0.2100000
8637.0000000	0.2100000
8638.0000000	0.2100000
8639.0000000	0.1950000
8640.0000000	0.2100000
8641.0000000	0.2050000
8642.0000000	0.2000000
8643.0000000	0.2100000
8644.0000000	0.2100000
8645.0000000	0.2000000
8646.0000000	0.1900000
8647.0000000	0.2000000
8648.0000000	0.2100000
8649.0000000	0.2050000
8650.0000000	0.2100000
8651.0000000	0.2000000
8652.0000000	0.2050000
8653.0000000	0.2150000
8654.0000000	0.2100000
8655.0000000	0.2100000
8656.0000000	0.1950000
8657.0000000	0.2100000
8658.0000000	0.2000000
8659.0000000	0.2250000
8660.0000000	0.2200000
8661.0000000	0.2100000
8662.0000000	0.2100000
8663.0000000	0.2150000

8664.0000000	0.2250000
8665.0000000	0.2200000
8666.0000000	0.2100000
8667.0000000	0.2200000
8668.0000000	0.2150000
8669.0000000	0.2150000
8670.0000000	0.2200000
8671.0000000	0.2100000
8672.0000000	0.2233333
8673.0000000	0.2150000
8674.0000000	0.2150000
8675.0000000	0.2250000
8676.0000000	0.2250000
8677.0000000	0.2150000
8678.0000000	0.2150000
8679.0000000	0.2200000
8680.0000000	0.2200000
8681.0000000	0.2200000
8682.0000000	0.2200000
8683.0000000	0.2150000
8684.0000000	0.2150000
8685.0000000	0.2100000
8686.0000000	0.2200000
8687.0000000	0.2150000
8688.0000000	0.2200000
8689.0000000	0.2100000
8690.0000000	0.2100000
8691.0000000	0.2200000
8692.0000000	0.2050000
8693.0000000	0.2200000
8694.0000000	0.2150000
8695.0000000	0.2100000
8696.0000000	0.2150000
8697.0000000	0.2250000
8698.0000000	0.2100000
8699.0000000	0.2150000
8700.0000000	0.2150000
8701.0000000	0.2150000
8702.0000000	0.2133333
8703.0000000	0.2200000
8704.0000000	0.2150000
8705.0000000	0.2100000
8706.0000000	0.2150000
8707.0000000	0.2150000
8708.0000000	0.2200000
8709.0000000	0.2300000
8710.0000000	0.2150000
8711.0000000	0.2200000
8712.0000000	0.2200000
8713.0000000	0.2200000
8714.0000000	0.2300000
8715.0000000	0.2300000
8716.0000000	0.2200000
8717.0000000	0.2250000
8718.0000000	0.2250000
8719.0000000	0.2250000
8720.0000000	0.2300000

8721.0000000	0.2200000
8722.0000000	0.2300000
8723.0000000	0.2300000
8724.0000000	0.2200000
8725.0000000	0.2250000
8726.0000000	0.2250000
8727.0000000	0.2300000
8728.0000000	0.2300000
8729.0000000	0.2300000
8730.0000000	0.2150000
8731.0000000	0.2250000
8732.0000000	0.2233333
8733.0000000	0.2300000
8734.0000000	0.2250000
8735.0000000	0.2350000
8736.0000000	0.2350000
8737.0000000	0.2250000
8738.0000000	0.2250000
8739.0000000	0.2250000
8740.0000000	0.2300000
8741.0000000	0.2400000
8742.0000000	0.2300000
8743.0000000	0.2300000
8744.0000000	0.2300000
8745.0000000	0.2350000
8746.0000000	0.2250000
8747.0000000	0.2350000
8748.0000000	0.2250000
8749.0000000	0.2300000
8750.0000000	0.2400000
8751.0000000	0.2350000
8752.0000000	0.2450000
8753.0000000	0.2250000
8754.0000000	0.2300000
8755.0000000	0.2300000
8756.0000000	0.2450000
8757.0000000	0.2300000
8758.0000000	0.2350000
8759.0000000	0.2200000
8760.0000000	0.2300000
8761.0000000	0.2300000
8762.0000000	0.2300000
8763.0000000	0.2300000
8764.0000000	0.2250000
8765.0000000	0.2350000
8766.0000000	0.2200000
8767.0000000	0.2200000
8768.0000000	0.2300000
8769.0000000	0.2300000
8770.0000000	0.2350000
8771.0000000	0.2200000
8772.0000000	0.2250000
8773.0000000	0.2100000
8774.0000000	0.2250000
8775.0000000	0.2200000
8776.0000000	0.2250000
8777.0000000	0.2200000

8778.0000000	0.2200000
8779.0000000	0.2250000
8780.0000000	0.2150000
8781.0000000	0.2250000
8782.0000000	0.2250000
8783.0000000	0.2200000
8784.0000000	0.2150000
8785.0000000	0.2200000
8786.0000000	0.2200000
8787.0000000	0.2250000
8788.0000000	0.2200000
8789.0000000	0.2250000
8790.0000000	0.2200000
8791.0000000	0.2300000
8792.0000000	0.2150000
8793.0000000	0.2266666
8794.0000000	0.2250000
8795.0000000	0.2250000
8796.0000000	0.2200000
8797.0000000	0.2150000
8798.0000000	0.2150000
8799.0000000	0.2050000
8800.0000000	0.2150000
8801.0000000	0.2050000
8802.0000000	0.2150000
8803.0000000	0.2150000
8804.0000000	0.2050000
8805.0000000	0.2100000
8806.0000000	0.2150000
8807.0000000	0.2150000
8808.0000000	0.2150000
8809.0000000	0.2000000
8810.0000000	0.2100000
8811.0000000	0.2150000
8812.0000000	0.2200000
8813.0000000	0.2150000
8814.0000000	0.2150000
8815.0000000	0.2100000
8816.0000000	0.2150000
8817.0000000	0.2150000
8818.0000000	0.2250000
8819.0000000	0.2200000
8820.0000000	0.2150000
8821.0000000	0.2200000
8822.0000000	0.2200000
8823.0000000	0.2150000
8824.0000000	0.2150000
8825.0000000	0.2250000
8826.0000000	0.2250000
8827.0000000	0.2200000
8828.0000000	0.2200000
8829.0000000	0.2150000
8830.0000000	0.2200000
8831.0000000	0.2250000
8832.0000000	0.2300000
8833.0000000	0.2200000
8834.0000000	0.2250000

8835.0000000	0.2250000
8836.0000000	0.2300000
8837.0000000	0.2300000
8838.0000000	0.2300000
8839.0000000	0.2250000
8840.0000000	0.2350000
8841.0000000	0.2300000
8842.0000000	0.2350000
8843.0000000	0.2350000
8844.0000000	0.2300000
8845.0000000	0.2250000
8846.0000000	0.2350000
8847.0000000	0.2400000
8848.0000000	0.2250000
8849.0000000	0.2300000
8850.0000000	0.2250000
8851.0000000	0.2250000
8852.0000000	0.2350000
8853.0000000	0.2466667
8854.0000000	0.2250000
8855.0000000	0.2450000
8856.0000000	0.2300000
8857.0000000	0.2450000
8858.0000000	0.2300000
8859.0000000	0.2450000
8860.0000000	0.2300000
8861.0000000	0.2450000
8862.0000000	0.2400000
8863.0000000	0.2450000
8864.0000000	0.2400000
8865.0000000	0.2500000
8866.0000000	0.2550000
8867.0000000	0.2500000
8868.0000000	0.2450000
8869.0000000	0.2450000
8870.0000000	0.2500000
8871.0000000	0.2550000
8872.0000000	0.2550000
8873.0000000	0.2550000
8874.0000000	0.2500000
8875.0000000	0.2500000
8876.0000000	0.2450000
8877.0000000	0.2650000
8878.0000000	0.2550000
8879.0000000	0.2650000
8880.0000000	0.2550000
8881.0000000	0.2600000
8882.0000000	0.2600000
8883.0000000	0.2700000
8884.0000000	0.2566667
8885.0000000	0.2750000
8886.0000000	0.2700000
8887.0000000	0.2650000
8888.0000000	0.2650000
8889.0000000	0.2600000
8890.0000000	0.2600000
8891.0000000	0.2650000

8892.0000000	0.2550000
8893.0000000	0.2600000
8894.0000000	0.2450000
8895.0000000	0.2600000
8896.0000000	0.2500000
8897.0000000	0.2650000
8898.0000000	0.2600000
8899.0000000	0.2600000
8900.0000000	0.2600000
8901.0000000	0.2650000
8902.0000000	0.2700000
8903.0000000	0.2650000
8904.0000000	0.2650000
8905.0000000	0.2550000
8906.0000000	0.2650000
8907.0000000	0.2700000
8908.0000000	0.2700000
8909.0000000	0.2700000
8910.0000000	0.2700000
8911.0000000	0.2650000
8912.0000000	0.2700000
8913.0000000	0.2700000
8914.0000000	0.2650000
8915.0000000	0.2700000
8916.0000000	0.2750000
8917.0000000	0.2750000
8918.0000000	0.2800000
8919.0000000	0.2700000
8920.0000000	0.2700000
8921.0000000	0.2800000
8922.0000000	0.2700000
8923.0000000	0.2800000
8924.0000000	0.2700000
8925.0000000	0.2700000
8926.0000000	0.2750000
8927.0000000	0.2700000
8928.0000000	0.2850000
8929.0000000	0.2750000
8930.0000000	0.2700000
8931.0000000	0.2650000
8932.0000000	0.2750000
8933.0000000	0.2750000
8934.0000000	0.2750000
8935.0000000	0.2800000
8936.0000000	0.2700000
8937.0000000	0.2800000
8938.0000000	0.2800000
8939.0000000	0.2700000
8940.0000000	0.2750000
8941.0000000	0.2650000
8942.0000000	0.2750000
8943.0000000	0.2733333
8944.0000000	0.2750000
8945.0000000	0.2750000
8946.0000000	0.2850000
8947.0000000	0.2700000
8948.0000000	0.2800000

8949.0000000	0.2750000
8950.0000000	0.2800000
8951.0000000	0.2800000
8952.0000000	0.2850000
8953.0000000	0.2800000
8954.0000000	0.2700000
8955.0000000	0.2750000
8956.0000000	0.2900000
8957.0000000	0.2750000
8958.0000000	0.2800000
8959.0000000	0.2700000
8960.0000000	0.2800000
8961.0000000	0.2800000
8962.0000000	0.2900000
8963.0000000	0.2900000
8964.0000000	0.2750000
8965.0000000	0.2800000
8966.0000000	0.2700000
8967.0000000	0.2850000
8968.0000000	0.2750000
8969.0000000	0.2750000
8970.0000000	0.2700000
8971.0000000	0.2700000
8972.0000000	0.2800000
8973.0000000	0.2700000
8974.0000000	0.2633333
8975.0000000	0.2700000
8976.0000000	0.2800000
8977.0000000	0.2700000
8978.0000000	0.2750000
8979.0000000	0.2650000
8980.0000000	0.2700000
8981.0000000	0.2750000
8982.0000000	0.2750000
8983.0000000	0.2750000
8984.0000000	0.2700000
8985.0000000	0.2800000
8986.0000000	0.2650000
8987.0000000	0.2850000
8988.0000000	0.2650000
8989.0000000	0.2800000
8990.0000000	0.2800000
8991.0000000	0.2750000
8992.0000000	0.2850000
8993.0000000	0.2750000
8994.0000000	0.2700000
8995.0000000	0.2750000
8996.0000000	0.2700000
8997.0000000	0.2800000
8998.0000000	0.2850000
8999.0000000	0.2750000
9000.0000000	0.2800000
9001.0000000	0.2800000
9002.0000000	0.2750000
9003.0000000	0.2800000
9004.0000000	0.2766667
9005.0000000	0.2800000

9006.0000000	0.2750000
9007.0000000	0.2850000
9008.0000000	0.2850000
9009.0000000	0.2850000
9010.0000000	0.2850000
9011.0000000	0.2750000
9012.0000000	0.2850000
9013.0000000	0.2850000
9014.0000000	0.2900000
9015.0000000	0.2800000
9016.0000000	0.2750000
9017.0000000	0.2800000
9018.0000000	0.2750000
9019.0000000	0.2850000
9020.0000000	0.2800000
9021.0000000	0.2750000
9022.0000000	0.2750000
9023.0000000	0.2800000
9024.0000000	0.2800000
9025.0000000	0.2850000
9026.0000000	0.2850000
9027.0000000	0.2800000
9028.0000000	0.2800000
9029.0000000	0.2800000
9030.0000000	0.2800000
9031.0000000	0.2850000
9032.0000000	0.2800000
9033.0000000	0.2800000
9034.0000000	0.2900000
9035.0000000	0.2750000
9036.0000000	0.2850000
9037.0000000	0.2850000
9038.0000000	0.2850000
9039.0000000	0.2850000
9040.0000000	0.2800000
9041.0000000	0.2950000
9042.0000000	0.2850000
9043.0000000	0.2900000
9044.0000000	0.2850000
9045.0000000	0.2850000
9046.0000000	0.2850000
9047.0000000	0.2850000
9048.0000000	0.2900000
9049.0000000	0.2950000
9050.0000000	0.2800000
9051.0000000	0.2850000
9052.0000000	0.2850000
9053.0000000	0.2900000
9054.0000000	0.2850000
9055.0000000	0.2750000
9056.0000000	0.2800000
9057.0000000	0.2850000
9058.0000000	0.2850000
9059.0000000	0.2800000
9060.0000000	0.2750000
9061.0000000	0.2700000
9062.0000000	0.2950000

9063.0000000	0.2850000
9064.0000000	0.2866667
9065.0000000	0.2850000
9066.0000000	0.2800000
9067.0000000	0.2800000
9068.0000000	0.2850000
9069.0000000	0.2850000
9070.0000000	0.3000000
9071.0000000	0.2850000
9072.0000000	0.2900000
9073.0000000	0.2850000
9074.0000000	0.2850000
9075.0000000	0.3000000
9076.0000000	0.2850000
9077.0000000	0.2850000
9078.0000000	0.2900000
9079.0000000	0.2900000
9080.0000000	0.2850000
9081.0000000	0.2900000
9082.0000000	0.2850000
9083.0000000	0.2850000
9084.0000000	0.2900000
9085.0000000	0.2950000
9086.0000000	0.2950000
9087.0000000	0.2900000
9088.0000000	0.2850000
9089.0000000	0.2900000
9090.0000000	0.2850000
9091.0000000	0.3000000
9092.0000000	0.2850000
9093.0000000	0.2950000
9094.0000000	0.2900000
9095.0000000	0.3000000
9096.0000000	0.2850000
9097.0000000	0.2950000
9098.0000000	0.2950000
9099.0000000	0.3000000
9100.0000000	0.2950000
9101.0000000	0.2850000
9102.0000000	0.2850000
9103.0000000	0.2900000
9104.0000000	0.2900000
9105.0000000	0.2950000
9106.0000000	0.2900000
9107.0000000	0.2800000
9108.0000000	0.2850000
9109.0000000	0.2850000
9110.0000000	0.2850000
9111.0000000	0.2900000
9112.0000000	0.2800000
9113.0000000	0.2850000
9114.0000000	0.2800000
9115.0000000	0.2800000
9116.0000000	0.2800000
9117.0000000	0.2750000
9118.0000000	0.2850000
9119.0000000	0.2850000

9120.0000000	0.2950000
9121.0000000	0.2750000
9122.0000000	0.2850000
9123.0000000	0.2750000
9124.0000000	0.2850000
9125.0000000	0.2800000
9126.0000000	0.2850000
9127.0000000	0.2700000
9128.0000000	0.2900000
9129.0000000	0.2800000
9130.0000000	0.2850000
9131.0000000	0.2750000
9132.0000000	0.2800000
9133.0000000	0.2800000
9134.0000000	0.2800000
9135.0000000	0.2750000
9136.0000000	0.2750000
9137.0000000	0.2750000
9138.0000000	0.2800000
9139.0000000	0.2800000
9140.0000000	0.2850000
9141.0000000	0.2700000
9142.0000000	0.2750000
9143.0000000	0.2750000
9144.0000000	0.2750000
9145.0000000	0.2750000
9146.0000000	0.2700000
9147.0000000	0.2700000
9148.0000000	0.2700000
9149.0000000	0.2800000
9150.0000000	0.2800000
9151.0000000	0.2750000
9152.0000000	0.2800000
9153.0000000	0.2800000
9154.0000000	0.2800000
9155.0000000	0.2750000
9156.0000000	0.2700000
9157.0000000	0.2750000
9158.0000000	0.2750000
9159.0000000	0.2750000
9160.0000000	0.2850000
9161.0000000	0.2900000
9162.0000000	0.2850000
9163.0000000	0.2750000
9164.0000000	0.2750000
9165.0000000	0.2900000
9166.0000000	0.2800000
9167.0000000	0.2850000
9168.0000000	0.2900000
9169.0000000	0.2750000
9170.0000000	0.2850000
9171.0000000	0.2900000
9172.0000000	0.2850000
9173.0000000	0.2850000
9174.0000000	0.2750000
9175.0000000	0.2850000
9176.0000000	0.2900000

9177.0000000	0.3000000
9178.0000000	0.2900000
9179.0000000	0.2850000
9180.0000000	0.2800000
9181.0000000	0.2900000
9182.0000000	0.2900000
9183.0000000	0.2950000
9184.0000000	0.2900000
9185.0000000	0.2866667
9186.0000000	0.2900000
9187.0000000	0.2800000
9188.0000000	0.3000000
9189.0000000	0.2900000
9190.0000000	0.3000000
9191.0000000	0.3000000
9192.0000000	0.2850000
9193.0000000	0.2900000
9194.0000000	0.2950000
9195.0000000	0.2900000
9196.0000000	0.2900000
9197.0000000	0.3000000
9198.0000000	0.2900000
9199.0000000	0.3050000
9200.0000000	0.3000000
9201.0000000	0.2800000
9202.0000000	0.2900000
9203.0000000	0.3000000
9204.0000000	0.2900000
9205.0000000	0.2850000
9206.0000000	0.3000000
9207.0000000	0.2950000
9208.0000000	0.3000000
9209.0000000	0.3000000
9210.0000000	0.2900000
9211.0000000	0.2950000
9212.0000000	0.2950000
9213.0000000	0.2850000
9214.0000000	0.3000000
9215.0000000	0.3050000
9216.0000000	0.3066667
9217.0000000	0.3000000
9218.0000000	0.2950000
9219.0000000	0.3050000
9220.0000000	0.2950000
9221.0000000	0.2900000
9222.0000000	0.3000000
9223.0000000	0.3000000
9224.0000000	0.3050000
9225.0000000	0.2950000
9226.0000000	0.2950000
9227.0000000	0.2950000
9228.0000000	0.2950000
9229.0000000	0.3050000
9230.0000000	0.3050000
9231.0000000	0.3000000
9232.0000000	0.2900000
9233.0000000	0.3000000

9234.0000000	0.2950000
9235.0000000	0.3050000
9236.0000000	0.3000000
9237.0000000	0.3050000
9238.0000000	0.2900000
9239.0000000	0.2950000
9240.0000000	0.2950000
9241.0000000	0.3000000
9242.0000000	0.3050000
9243.0000000	0.3050000
9244.0000000	0.3000000
9245.0000000	0.2966667
9246.0000000	0.3050000
9247.0000000	0.2950000
9248.0000000	0.3000000
9249.0000000	0.3000000
9250.0000000	0.2950000
9251.0000000	0.3050000
9252.0000000	0.3050000
9253.0000000	0.2950000
9254.0000000	0.3000000
9255.0000000	0.2950000
9256.0000000	0.3000000
9257.0000000	0.3000000
9258.0000000	0.3100000
9259.0000000	0.3050000
9260.0000000	0.3000000
9261.0000000	0.3000000
9262.0000000	0.3000000
9263.0000000	0.3050000
9264.0000000	0.3100000
9265.0000000	0.3100000
9266.0000000	0.3050000
9267.0000000	0.3000000
9268.0000000	0.3000000
9269.0000000	0.3000000
9270.0000000	0.3050000
9271.0000000	0.3000000
9272.0000000	0.3050000
9273.0000000	0.3050000
9274.0000000	0.2950000
9275.0000000	0.3033333
9276.0000000	0.3050000
9277.0000000	0.3100000
9278.0000000	0.3100000
9279.0000000	0.3100000
9280.0000000	0.3000000
9281.0000000	0.3100000
9282.0000000	0.3000000
9283.0000000	0.3100000
9284.0000000	0.3100000
9285.0000000	0.3050000
9286.0000000	0.3150000
9287.0000000	0.3000000
9288.0000000	0.3150000
9289.0000000	0.3050000
9290.0000000	0.3100000

9291.0000000	0.3100000
9292.0000000	0.3150000
9293.0000000	0.3100000
9294.0000000	0.3050000
9295.0000000	0.3150000
9296.0000000	0.3050000
9297.0000000	0.3150000
9298.0000000	0.3100000
9299.0000000	0.3200000
9300.0000000	0.3100000
9301.0000000	0.3150000
9302.0000000	0.3100000
9303.0000000	0.3150000
9304.0000000	0.3150000
9305.0000000	0.3200000
9306.0000000	0.3100000
9307.0000000	0.3200000
9308.0000000	0.3050000
9309.0000000	0.3150000
9310.0000000	0.3000000
9311.0000000	0.3150000
9312.0000000	0.3050000
9313.0000000	0.3150000
9314.0000000	0.2950000
9315.0000000	0.2950000
9316.0000000	0.2950000
9317.0000000	0.3050000
9318.0000000	0.2850000
9319.0000000	0.3000000
9320.0000000	0.2900000
9321.0000000	0.2850000
9322.0000000	0.2950000
9323.0000000	0.2850000
9324.0000000	0.3000000
9325.0000000	0.3000000
9326.0000000	0.3000000
9327.0000000	0.2900000
9328.0000000	0.3000000
9329.0000000	0.3000000
9330.0000000	0.3000000
9331.0000000	0.3050000
9332.0000000	0.3000000
9333.0000000	0.3050000
9334.0000000	0.3050000
9335.0000000	0.3050000
9336.0000000	0.2966667
9337.0000000	0.3100000
9338.0000000	0.3000000
9339.0000000	0.3000000
9340.0000000	0.3100000
9341.0000000	0.2950000
9342.0000000	0.2950000
9343.0000000	0.2900000
9344.0000000	0.3050000
9345.0000000	0.2900000
9346.0000000	0.2950000
9347.0000000	0.2950000

9348.0000000	0.2900000
9349.0000000	0.2850000
9350.0000000	0.2900000
9351.0000000	0.2900000
9352.0000000	0.2950000
9353.0000000	0.2850000
9354.0000000	0.2950000
9355.0000000	0.2750000
9356.0000000	0.2800000
9357.0000000	0.2750000
9358.0000000	0.2900000
9359.0000000	0.2800000
9360.0000000	0.2850000
9361.0000000	0.2700000
9362.0000000	0.2800000
9363.0000000	0.2800000
9364.0000000	0.2850000
9365.0000000	0.2750000
9366.0000000	0.2800000
9367.0000000	0.2850000
9368.0000000	0.2750000
9369.0000000	0.2750000
9370.0000000	0.2800000
9371.0000000	0.2850000
9372.0000000	0.2800000
9373.0000000	0.2800000
9374.0000000	0.2800000
9375.0000000	0.2750000
9376.0000000	0.2800000
9377.0000000	0.2800000
9378.0000000	0.2800000
9379.0000000	0.2700000
9380.0000000	0.2700000
9381.0000000	0.2800000
9382.0000000	0.2700000
9383.0000000	0.2700000
9384.0000000	0.2650000
9385.0000000	0.2750000
9386.0000000	0.2750000
9387.0000000	0.2850000
9388.0000000	0.2700000
9389.0000000	0.2750000
9390.0000000	0.2650000
9391.0000000	0.2750000
9392.0000000	0.2750000
9393.0000000	0.2800000
9394.0000000	0.2600000
9395.0000000	0.2700000
9396.0000000	0.2733333
9397.0000000	0.2700000
9398.0000000	0.2700000
9399.0000000	0.2750000
9400.0000000	0.2750000
9401.0000000	0.2850000
9402.0000000	0.2750000
9403.0000000	0.2800000
9404.0000000	0.2750000

9405.0000000	0.2800000
9406.0000000	0.2700000
9407.0000000	0.2800000
9408.0000000	0.2700000
9409.0000000	0.2750000
9410.0000000	0.2750000
9411.0000000	0.2800000
9412.0000000	0.2800000
9413.0000000	0.2800000
9414.0000000	0.2750000
9415.0000000	0.2800000
9416.0000000	0.2750000
9417.0000000	0.2800000
9418.0000000	0.2750000
9419.0000000	0.2750000
9420.0000000	0.2850000
9421.0000000	0.2700000
9422.0000000	0.2850000
9423.0000000	0.2750000
9424.0000000	0.2900000
9425.0000000	0.2850000
9426.0000000	0.2900000
9427.0000000	0.2733333
9428.0000000	0.2850000
9429.0000000	0.2750000
9430.0000000	0.2850000
9431.0000000	0.2800000
9432.0000000	0.2800000
9433.0000000	0.2750000
9434.0000000	0.2900000
9435.0000000	0.2700000
9436.0000000	0.2750000
9437.0000000	0.2800000
9438.0000000	0.2850000
9439.0000000	0.2750000
9440.0000000	0.2850000
9441.0000000	0.2800000
9442.0000000	0.2750000
9443.0000000	0.2800000
9444.0000000	0.2850000
9445.0000000	0.2850000
9446.0000000	0.2900000
9447.0000000	0.2750000
9448.0000000	0.2800000
9449.0000000	0.2850000
9450.0000000	0.2800000
9451.0000000	0.3000000
9452.0000000	0.2850000
9453.0000000	0.2850000
9454.0000000	0.2750000
9455.0000000	0.2850000
9456.0000000	0.2850000
9457.0000000	0.2833333
9458.0000000	0.2900000
9459.0000000	0.2850000
9460.0000000	0.2900000
9461.0000000	0.2800000

9462.0000000	0.3000000
9463.0000000	0.2850000
9464.0000000	0.2900000
9465.0000000	0.2900000
9466.0000000	0.3000000
9467.0000000	0.2900000
9468.0000000	0.2900000
9469.0000000	0.2850000
9470.0000000	0.2950000
9471.0000000	0.2850000
9472.0000000	0.3050000
9473.0000000	0.2950000
9474.0000000	0.2950000
9475.0000000	0.2950000
9476.0000000	0.3050000
9477.0000000	0.2950000
9478.0000000	0.3050000
9479.0000000	0.3000000
9480.0000000	0.2950000
9481.0000000	0.3000000
9482.0000000	0.3050000
9483.0000000	0.2950000
9484.0000000	0.3050000
9485.0000000	0.2950000
9486.0000000	0.3000000
9487.0000000	0.3100000
9488.0000000	0.2950000
9489.0000000	0.3050000
9490.0000000	0.3000000
9491.0000000	0.2950000
9492.0000000	0.3050000
9493.0000000	0.3100000
9494.0000000	0.3050000
9495.0000000	0.3100000
9496.0000000	0.3000000
9497.0000000	0.3100000
9498.0000000	0.3150000
9499.0000000	0.3200000
9500.0000000	0.3050000
9501.0000000	0.3150000
9502.0000000	0.3000000
9503.0000000	0.3200000
9504.0000000	0.3100000
9505.0000000	0.3150000
9506.0000000	0.3050000
9507.0000000	0.3100000
9508.0000000	0.3150000
9509.0000000	0.3200000
9510.0000000	0.3050000
9511.0000000	0.3150000
9512.0000000	0.3150000
9513.0000000	0.3100000
9514.0000000	0.3150000
9515.0000000	0.3100000
9516.0000000	0.3150000
9517.0000000	0.3133333
9518.0000000	0.3050000

9519.0000000	0.3050000
9520.0000000	0.3050000
9521.0000000	0.2950000
9522.0000000	0.3050000
9523.0000000	0.2950000
9524.0000000	0.3050000
9525.0000000	0.3100000
9526.0000000	0.2950000
9527.0000000	0.2950000
9528.0000000	0.2950000
9529.0000000	0.3000000
9530.0000000	0.2950000
9531.0000000	0.3100000
9532.0000000	0.2950000
9533.0000000	0.3100000
9534.0000000	0.2900000
9535.0000000	0.3050000
9536.0000000	0.2950000
9537.0000000	0.3050000
9538.0000000	0.3050000
9539.0000000	0.3050000
9540.0000000	0.3000000
9541.0000000	0.3050000
9542.0000000	0.3100000
9543.0000000	0.3050000
9544.0000000	0.3100000
9545.0000000	0.3050000
9546.0000000	0.3100000
9547.0000000	0.3050000
9548.0000000	0.3100000
9549.0000000	0.3050000
9550.0000000	0.3000000
9551.0000000	0.3050000
9552.0000000	0.3100000
9553.0000000	0.3050000
9554.0000000	0.3100000
9555.0000000	0.2950000
9556.0000000	0.3050000
9557.0000000	0.2900000
9558.0000000	0.3050000
9559.0000000	0.2950000
9560.0000000	0.3000000
9561.0000000	0.3100000
9562.0000000	0.3050000
9563.0000000	0.3000000
9564.0000000	0.3000000
9565.0000000	0.2950000
9566.0000000	0.2950000
9567.0000000	0.3050000
9568.0000000	0.3000000
9569.0000000	0.3100000
9570.0000000	0.2950000
9571.0000000	0.3000000
9572.0000000	0.3050000
9573.0000000	0.3000000
9574.0000000	0.3100000
9575.0000000	0.3050000

9576.0000000	0.3050000
9577.0000000	0.3066667
9578.0000000	0.2950000
9579.0000000	0.3050000
9580.0000000	0.3000000
9581.0000000	0.3100000
9582.0000000	0.3100000
9583.0000000	0.3050000
9584.0000000	0.3000000
9585.0000000	0.3000000
9586.0000000	0.3100000
9587.0000000	0.3050000
9588.0000000	0.3100000
9589.0000000	0.3050000
9590.0000000	0.3000000
9591.0000000	0.3100000
9592.0000000	0.3150000
9593.0000000	0.3150000
9594.0000000	0.3150000
9595.0000000	0.3200000
9596.0000000	0.3150000
9597.0000000	0.3150000
9598.0000000	0.3150000
9599.0000000	0.3200000
9600.0000000	0.3100000
9601.0000000	0.3000000
9602.0000000	0.3200000
9603.0000000	0.3150000
9604.0000000	0.3200000
9605.0000000	0.3150000
9606.0000000	0.3150000
9607.0000000	0.3233334
9608.0000000	0.3200000
9609.0000000	0.3100000
9610.0000000	0.3250000
9611.0000000	0.3250000
9612.0000000	0.3250000
9613.0000000	0.3350000
9614.0000000	0.3300000
9615.0000000	0.3300000
9616.0000000	0.3150000
9617.0000000	0.3300000
9618.0000000	0.3250000
9619.0000000	0.3400000
9620.0000000	0.3250000
9621.0000000	0.3300000
9622.0000000	0.3150000
9623.0000000	0.3300000
9624.0000000	0.3200000
9625.0000000	0.3300000
9626.0000000	0.3200000
9627.0000000	0.3400000
9628.0000000	0.3150000
9629.0000000	0.3250000
9630.0000000	0.3200000
9631.0000000	0.3300000
9632.0000000	0.3200000

9633.0000000	0.3300000
9634.0000000	0.3300000
9635.0000000	0.3300000
9636.0000000	0.3300000
9637.0000000	0.3300000
9638.0000000	0.3333333
9639.0000000	0.3200000
9640.0000000	0.3250000
9641.0000000	0.3300000
9642.0000000	0.3350000
9643.0000000	0.3250000
9644.0000000	0.3300000
9645.0000000	0.3200000
9646.0000000	0.3300000
9647.0000000	0.3300000
9648.0000000	0.3350000
9649.0000000	0.3250000
9650.0000000	0.3200000
9651.0000000	0.3300000
9652.0000000	0.3250000
9653.0000000	0.3200000
9654.0000000	0.3300000
9655.0000000	0.3250000
9656.0000000	0.3200000
9657.0000000	0.3300000
9658.0000000	0.3150000
9659.0000000	0.3250000
9660.0000000	0.3150000
9661.0000000	0.3100000
9662.0000000	0.3350000
9663.0000000	0.3250000
9664.0000000	0.3250000
9665.0000000	0.3200000
9666.0000000	0.3200000
9667.0000000	0.3200000
9668.0000000	0.3166667
9669.0000000	0.3350000
9670.0000000	0.3300000
9671.0000000	0.3150000
9672.0000000	0.3250000
9673.0000000	0.3100000
9674.0000000	0.3250000
9675.0000000	0.3300000
9676.0000000	0.3150000
9677.0000000	0.3200000
9678.0000000	0.3250000
9679.0000000	0.3200000
9680.0000000	0.3250000
9681.0000000	0.3100000
9682.0000000	0.3200000
9683.0000000	0.3150000
9684.0000000	0.3250000
9685.0000000	0.3200000
9686.0000000	0.3200000
9687.0000000	0.3150000
9688.0000000	0.3100000
9689.0000000	0.3150000

9690.0000000	0.3200000
9691.0000000	0.3300000
9692.0000000	0.3150000
9693.0000000	0.3200000
9694.0000000	0.3150000
9695.0000000	0.3150000
9696.0000000	0.3150000
9697.0000000	0.3050000
9698.0000000	0.3166667
9699.0000000	0.3100000
9700.0000000	0.3050000
9701.0000000	0.3050000
9702.0000000	0.3100000
9703.0000000	0.3000000
9704.0000000	0.2950000
9705.0000000	0.3000000
9706.0000000	0.2950000
9707.0000000	0.3100000
9708.0000000	0.3000000
9709.0000000	0.2950000
9710.0000000	0.3000000
9711.0000000	0.2900000
9712.0000000	0.3000000
9713.0000000	0.3000000
9714.0000000	0.2950000
9715.0000000	0.3000000
9716.0000000	0.2900000
9717.0000000	0.3000000
9718.0000000	0.2950000
9719.0000000	0.3050000
9720.0000000	0.2950000
9721.0000000	0.2900000
9722.0000000	0.2900000
9723.0000000	0.3050000
9724.0000000	0.3100000
9725.0000000	0.3050000
9726.0000000	0.3000000
9727.0000000	0.3000000
9728.0000000	0.3033333
9729.0000000	0.3000000
9730.0000000	0.2950000
9731.0000000	0.3000000
9732.0000000	0.3100000
9733.0000000	0.3000000
9734.0000000	0.3100000
9735.0000000	0.2950000
9736.0000000	0.3050000
9737.0000000	0.2900000
9738.0000000	0.3000000
9739.0000000	0.3000000
9740.0000000	0.3000000
9741.0000000	0.2900000
9742.0000000	0.2950000
9743.0000000	0.2950000
9744.0000000	0.3000000
9745.0000000	0.3000000
9746.0000000	0.2900000

9747.0000000	0.2950000
9748.0000000	0.2900000
9749.0000000	0.2950000
9750.0000000	0.2900000
9751.0000000	0.2800000
9752.0000000	0.2900000
9753.0000000	0.3000000
9754.0000000	0.2950000
9755.0000000	0.2900000
9756.0000000	0.2900000
9757.0000000	0.2850000
9758.0000000	0.2900000
9759.0000000	0.2800000
9760.0000000	0.2950000
9761.0000000	0.2850000
9762.0000000	0.2900000
9763.0000000	0.2850000
9764.0000000	0.2750000
9765.0000000	0.2800000
9766.0000000	0.2850000
9767.0000000	0.2750000
9768.0000000	0.2850000
9769.0000000	0.2750000
9770.0000000	0.2850000
9771.0000000	0.2900000
9772.0000000	0.2800000
9773.0000000	0.2850000
9774.0000000	0.2750000
9775.0000000	0.2900000
9776.0000000	0.2900000
9777.0000000	0.2900000
9778.0000000	0.2800000
9779.0000000	0.2750000
9780.0000000	0.2900000
9781.0000000	0.2900000
9782.0000000	0.2900000
9783.0000000	0.2900000
9784.0000000	0.2850000
9785.0000000	0.2900000
9786.0000000	0.2850000
9787.0000000	0.2900000
9788.0000000	0.2900000
9789.0000000	0.2900000
9790.0000000	0.2900000
9791.0000000	0.2850000
9792.0000000	0.2850000
9793.0000000	0.2800000
9794.0000000	0.2900000
9795.0000000	0.2850000
9796.0000000	0.2950000
9797.0000000	0.2800000
9798.0000000	0.2950000
9799.0000000	0.2800000
9800.0000000	0.2850000
9801.0000000	0.2900000
9802.0000000	0.2750000
9803.0000000	0.2850000

9804.0000000	0.2850000
9805.0000000	0.2850000
9806.0000000	0.2850000
9807.0000000	0.2850000
9808.0000000	0.2800000
9809.0000000	0.2850000
9810.0000000	0.2950000
9811.0000000	0.2800000
9812.0000000	0.2900000
9813.0000000	0.2800000
9814.0000000	0.2800000
9815.0000000	0.2850000
9816.0000000	0.2850000
9817.0000000	0.2950000
9818.0000000	0.2850000
9819.0000000	0.2866667
9820.0000000	0.2800000
9821.0000000	0.2800000
9822.0000000	0.2850000
9823.0000000	0.2850000
9824.0000000	0.2950000
9825.0000000	0.2800000
9826.0000000	0.2800000
9827.0000000	0.2900000
9828.0000000	0.2800000
9829.0000000	0.2800000
9830.0000000	0.2800000
9831.0000000	0.2750000
9832.0000000	0.2900000
9833.0000000	0.2950000
9834.0000000	0.2900000
9835.0000000	0.2950000
9836.0000000	0.2850000
9837.0000000	0.2900000
9838.0000000	0.2950000
9839.0000000	0.2850000
9840.0000000	0.2950000
9841.0000000	0.2900000
9842.0000000	0.3000000
9843.0000000	0.2950000
9844.0000000	0.2950000
9845.0000000	0.2850000
9846.0000000	0.2850000
9847.0000000	0.2900000
9848.0000000	0.2900000
9849.0000000	0.2966667
9850.0000000	0.2900000
9851.0000000	0.2850000
9852.0000000	0.2900000
9853.0000000	0.2950000
9854.0000000	0.2900000
9855.0000000	0.3000000
9856.0000000	0.2900000
9857.0000000	0.2900000
9858.0000000	0.3000000
9859.0000000	0.3000000
9860.0000000	0.2900000

9861.0000000	0.2950000
9862.0000000	0.2900000
9863.0000000	0.3050000
9864.0000000	0.3000000
9865.0000000	0.3000000
9866.0000000	0.2950000
9867.0000000	0.2950000
9868.0000000	0.3050000
9869.0000000	0.3050000
9870.0000000	0.3000000
9871.0000000	0.2950000
9872.0000000	0.3050000
9873.0000000	0.2950000
9874.0000000	0.3050000
9875.0000000	0.3000000
9876.0000000	0.3050000
9877.0000000	0.2950000
9878.0000000	0.3050000
9879.0000000	0.3100000
9880.0000000	0.3100000
9881.0000000	0.3050000
9882.0000000	0.3100000
9883.0000000	0.3100000
9884.0000000	0.3100000
9885.0000000	0.3150000
9886.0000000	0.3050000
9887.0000000	0.3050000
9888.0000000	0.3050000
9889.0000000	0.3050000
9890.0000000	0.3200000
9891.0000000	0.3100000
9892.0000000	0.3050000
9893.0000000	0.3100000
9894.0000000	0.3100000
9895.0000000	0.3100000
9896.0000000	0.3150000
9897.0000000	0.3150000
9898.0000000	0.3150000
9899.0000000	0.3100000
9900.0000000	0.3100000
9901.0000000	0.3150000
9902.0000000	0.3150000
9903.0000000	0.3200000
9904.0000000	0.3200000
9905.0000000	0.3100000
9906.0000000	0.3100000
9907.0000000	0.3050000
9908.0000000	0.3100000
9909.0000000	0.3100000
9910.0000000	0.3050000
9911.0000000	0.3100000
9912.0000000	0.3100000
9913.0000000	0.2950000
9914.0000000	0.3050000
9915.0000000	0.3050000
9916.0000000	0.3050000
9917.0000000	0.3050000

9918.0000000	0.2950000
9919.0000000	0.3000000
9920.0000000	0.2950000
9921.0000000	0.3000000
9922.0000000	0.2900000
9923.0000000	0.3050000
9924.0000000	0.3000000
9925.0000000	0.3100000
9926.0000000	0.3050000
9927.0000000	0.3050000
9928.0000000	0.2950000
9929.0000000	0.2950000
9930.0000000	0.2950000
9931.0000000	0.3050000
9932.0000000	0.2950000
9933.0000000	0.3050000
9934.0000000	0.2950000
9935.0000000	0.2950000
9936.0000000	0.3050000
9937.0000000	0.3000000
9938.0000000	0.2900000
9939.0000000	0.3000000
9940.0000000	0.2950000
9941.0000000	0.2900000
9942.0000000	0.3000000
9943.0000000	0.3000000
9944.0000000	0.3050000
9945.0000000	0.3050000
9946.0000000	0.3000000
9947.0000000	0.3200000
9948.0000000	0.3050000
9949.0000000	0.3050000
9950.0000000	0.3000000
9951.0000000	0.3050000
9952.0000000	0.3100000
9953.0000000	0.3150000
9954.0000000	0.3150000
9955.0000000	0.3050000
9956.0000000	0.3100000
9957.0000000	0.3100000
9958.0000000	0.3150000
9959.0000000	0.3200000
9960.0000000	0.3150000
9961.0000000	0.3200000
9962.0000000	0.3100000
9963.0000000	0.3250000
9964.0000000	0.3200000
9965.0000000	0.3300000
9966.0000000	0.3150000
9967.0000000	0.3250000
9968.0000000	0.3300000
9969.0000000	0.3250000
9970.0000000	0.3200000
9971.0000000	0.3400000
9972.0000000	0.3300000
9973.0000000	0.3400000
9974.0000000	0.3300000

9975.0000000	0.3250000
9976.0000000	0.3300000
9977.0000000	0.3300000
9978.0000000	0.3300000
9979.0000000	0.3350000
9980.0000000	0.3400000
9981.0000000	0.3250000
9982.0000000	0.3450000
9983.0000000	0.3300000
9984.0000000	0.3400000
9985.0000000	0.3400000
9986.0000000	0.3350000
9987.0000000	0.3400000
9988.0000000	0.3350000
9989.0000000	0.3450000
9990.0000000	0.3350000
9991.0000000	0.3350000
9992.0000000	0.3450000
9993.0000000	0.3450000
9994.0000000	0.3450000
9995.0000000	0.3400000
9996.0000000	0.3400000
9997.0000000	0.3350000
9998.0000000	0.3250000
9999.0000000	0.3450000
10000.0000000	0.3333333
10001.0000000	0.3350000
10002.0000000	0.3400000
10003.0000000	0.3350000
10004.0000000	0.3350000
10005.0000000	0.3350000
10006.0000000	0.3400000
10007.0000000	0.3350000
10008.0000000	0.3450000
10009.0000000	0.3250000
10010.0000000	0.3400000
10011.0000000	0.3350000
10012.0000000	0.3400000
10013.0000000	0.3400000
10014.0000000	0.3350000
10015.0000000	0.3300000
10016.0000000	0.3350000
10017.0000000	0.3400000
10018.0000000	0.3400000
10019.0000000	0.3400000
10020.0000000	0.3350000
10021.0000000	0.3300000
10022.0000000	0.3350000
10023.0000000	0.3250000
10024.0000000	0.3450000
10025.0000000	0.3400000
10026.0000000	0.3450000
10027.0000000	0.3350000
10028.0000000	0.3350000
10029.0000000	0.3350000
10030.0000000	0.3400000
10031.0000000	0.3350000

10032.0000000	0.3450000
10033.0000000	0.3450000
10034.0000000	0.3350000
10035.0000000	0.3400000
10036.0000000	0.3350000
10037.0000000	0.3500000
10038.0000000	0.3400000
10039.0000000	0.3450000
10040.0000000	0.3400000
10041.0000000	0.3400000
10042.0000000	0.3500000
10043.0000000	0.3400000
10044.0000000	0.3500000
10045.0000000	0.3450000
10046.0000000	0.3550000
10047.0000000	0.3550000
10048.0000000	0.3350000
10049.0000000	0.3500000
10050.0000000	0.3400000
10051.0000000	0.3350000
10052.0000000	0.3400000
10053.0000000	0.3350000
10054.0000000	0.3400000
10055.0000000	0.3300000
10056.0000000	0.3300000
10057.0000000	0.3300000
10058.0000000	0.3300000
10059.0000000	0.3350000
10060.0000000	0.3233334
10061.0000000	0.3400000
10062.0000000	0.3200000
10063.0000000	0.3250000
10064.0000000	0.3300000
10065.0000000	0.3250000
10066.0000000	0.3300000
10067.0000000	0.3250000
10068.0000000	0.3250000
10069.0000000	0.3350000
10070.0000000	0.3250000
10071.0000000	0.3200000
10072.0000000	0.3250000
10073.0000000	0.3150000
10074.0000000	0.3250000
10075.0000000	0.3250000
10076.0000000	0.3250000
10077.0000000	0.3250000
10078.0000000	0.3150000
10079.0000000	0.3300000
10080.0000000	0.3200000
10081.0000000	0.3300000
10082.0000000	0.3300000
10083.0000000	0.3300000
10084.0000000	0.3300000
10085.0000000	0.3250000
10086.0000000	0.3200000
10087.0000000	0.3300000
10088.0000000	0.3300000

10089.0000000	0.3450000
10090.0000000	0.3350000
10091.0000000	0.3366667
10092.0000000	0.3350000
10093.0000000	0.3300000
10094.0000000	0.3300000
10095.0000000	0.3400000
10096.0000000	0.3350000
10097.0000000	0.3300000
10098.0000000	0.3250000
10099.0000000	0.3350000
10100.0000000	0.3400000
10101.0000000	0.3400000
10102.0000000	0.3400000
10103.0000000	0.3250000
10104.0000000	0.3350000
10105.0000000	0.3300000
10106.0000000	0.3250000
10107.0000000	0.3350000
10108.0000000	0.3300000
10109.0000000	0.3350000
10110.0000000	0.3350000
10111.0000000	0.3300000
10112.0000000	0.3300000
10113.0000000	0.3400000
10114.0000000	0.3350000
10115.0000000	0.3250000
10116.0000000	0.3450000
10117.0000000	0.3300000
10118.0000000	0.3450000
10119.0000000	0.3450000
10120.0000000	0.3250000
10121.0000000	0.3400000
10122.0000000	0.3300000
10123.0000000	0.3300000
10124.0000000	0.3400000
10125.0000000	0.3400000
10126.0000000	0.3500000
10127.0000000	0.3350000
10128.0000000	0.3350000
10129.0000000	0.3400000
10130.0000000	0.3450000
10131.0000000	0.3550000
10132.0000000	0.3400000
10133.0000000	0.3450000
10134.0000000	0.3350000
10135.0000000	0.3500000
10136.0000000	0.3350000
10137.0000000	0.3500000
10138.0000000	0.3400000
10139.0000000	0.3550000
10140.0000000	0.3450000
10141.0000000	0.3600000
10142.0000000	0.3450000
10143.0000000	0.3600000
10144.0000000	0.3500000
10145.0000000	0.3550000

10146.0000000	0.3450000
10147.0000000	0.3550000
10148.0000000	0.3500000
10149.0000000	0.3550000
10150.0000000	0.3550000
10151.0000000	0.3533334
10152.0000000	0.3550000
10153.0000000	0.3650000
10154.0000000	0.3550000
10155.0000000	0.3600000
10156.0000000	0.3550000
10157.0000000	0.3500000
10158.0000000	0.3650000
10159.0000000	0.3650000
10160.0000000	0.3700000
10161.0000000	0.3650000
10162.0000000	0.3600000
10163.0000000	0.3700000
10164.0000000	0.3650000
10165.0000000	0.3800000
10166.0000000	0.3650000
10167.0000000	0.3750000
10168.0000000	0.3700000
10169.0000000	0.3700000
10170.0000000	0.3750000
10171.0000000	0.3700000
10172.0000000	0.3750000
10173.0000000	0.3750000
10174.0000000	0.3800000
10175.0000000	0.3700000
10176.0000000	0.3800000
10177.0000000	0.3700000
10178.0000000	0.3750000
10179.0000000	0.3800000
10180.0000000	0.3850000
10181.0000000	0.3766667
10182.0000000	0.3850000
10183.0000000	0.3700000
10184.0000000	0.3800000
10185.0000000	0.3650000
10186.0000000	0.3800000
10187.0000000	0.3800000
10188.0000000	0.3800000
10189.0000000	0.3650000
10190.0000000	0.3750000
10191.0000000	0.3750000
10192.0000000	0.3800000
10193.0000000	0.3850000
10194.0000000	0.3800000
10195.0000000	0.3750000
10196.0000000	0.3700000
10197.0000000	0.3850000
10198.0000000	0.3850000
10199.0000000	0.3800000
10200.0000000	0.3900000
10201.0000000	0.3800000
10202.0000000	0.3700000

10203.0000000	0.3850000
10204.0000000	0.3800000
10205.0000000	0.3850000
10206.0000000	0.3850000
10207.0000000	0.3850000
10208.0000000	0.3800000
10209.0000000	0.3800000
10210.0000000	0.3950000
10211.0000000	0.3800000
10212.0000000	0.3833333
10213.0000000	0.3800000
10214.0000000	0.3850000
10215.0000000	0.3750000
10216.0000000	0.3800000
10217.0000000	0.3850000
10218.0000000	0.3850000
10219.0000000	0.3800000
10220.0000000	0.3700000
10221.0000000	0.3650000
10222.0000000	0.3700000
10223.0000000	0.3800000
10224.0000000	0.3750000
10225.0000000	0.3800000
10226.0000000	0.3700000
10227.0000000	0.3650000
10228.0000000	0.3700000
10229.0000000	0.3750000
10230.0000000	0.3750000
10231.0000000	0.3650000
10232.0000000	0.3650000
10233.0000000	0.3750000
10234.0000000	0.3750000
10235.0000000	0.3800000
10236.0000000	0.3700000
10237.0000000	0.3750000
10238.0000000	0.3650000
10239.0000000	0.3750000
10240.0000000	0.3550000
10241.0000000	0.3633333
10242.0000000	0.3650000
10243.0000000	0.3700000
10244.0000000	0.3750000
10245.0000000	0.3550000
10246.0000000	0.3600000
10247.0000000	0.3550000
10248.0000000	0.3600000
10249.0000000	0.3650000
10250.0000000	0.3650000
10251.0000000	0.3650000
10252.0000000	0.3650000
10253.0000000	0.3650000
10254.0000000	0.3650000
10255.0000000	0.3600000
10256.0000000	0.3650000
10257.0000000	0.3650000
10258.0000000	0.3750000
10259.0000000	0.3650000

10260.0000000	0.3650000
10261.0000000	0.3600000
10262.0000000	0.3650000
10263.0000000	0.3700000
10264.0000000	0.3800000
10265.0000000	0.3800000
10266.0000000	0.3750000
10267.0000000	0.3600000
10268.0000000	0.3650000
10269.0000000	0.3750000
10270.0000000	0.3700000
10271.0000000	0.3733333
10272.0000000	0.3650000
10273.0000000	0.3900000
10274.0000000	0.3650000
10275.0000000	0.3750000
10276.0000000	0.3750000
10277.0000000	0.3800000
10278.0000000	0.3850000
10279.0000000	0.3700000
10280.0000000	0.3800000
10281.0000000	0.3800000
10282.0000000	0.3850000
10283.0000000	0.3850000
10284.0000000	0.3900000
10285.0000000	0.3850000
10286.0000000	0.3800000
10287.0000000	0.3850000
10288.0000000	0.3800000
10289.0000000	0.3900000
10290.0000000	0.3900000
10291.0000000	0.3850000
10292.0000000	0.3900000
10293.0000000	0.3900000
10294.0000000	0.3950000
10295.0000000	0.3950000
10296.0000000	0.3950000
10297.0000000	0.3900000
10298.0000000	0.3950000
10299.0000000	0.3900000
10300.0000000	0.4050000
10301.0000000	0.3900000
10302.0000000	0.3900000
10303.0000000	0.3950000
10304.0000000	0.3950000
10305.0000000	0.3900000
10306.0000000	0.3950000
10307.0000000	0.3950000
10308.0000000	0.3900000
10309.0000000	0.3950000
10310.0000000	0.4000000
10311.0000000	0.4050000
10312.0000000	0.4050000
10313.0000000	0.3950000
10314.0000000	0.3950000
10315.0000000	0.3850000
10316.0000000	0.3950000

10317.0000000	0.3900000
10318.0000000	0.4000000
10319.0000000	0.4050000
10320.0000000	0.3850000
10321.0000000	0.3900000
10322.0000000	0.3950000
10323.0000000	0.3950000
10324.0000000	0.3950000
10325.0000000	0.3900000
10326.0000000	0.3800000
10327.0000000	0.3800000
10328.0000000	0.3800000
10329.0000000	0.3800000
10330.0000000	0.3850000
10331.0000000	0.3750000
10332.0000000	0.3833333
10333.0000000	0.3700000
10334.0000000	0.3750000
10335.0000000	0.3750000
10336.0000000	0.3850000
10337.0000000	0.3750000
10338.0000000	0.3800000
10339.0000000	0.3750000
10340.0000000	0.3650000
10341.0000000	0.3850000
10342.0000000	0.3750000
10343.0000000	0.3750000
10344.0000000	0.3700000
10345.0000000	0.3750000
10346.0000000	0.3850000
10347.0000000	0.3750000
10348.0000000	0.3750000
10349.0000000	0.3700000
10350.0000000	0.3700000
10351.0000000	0.3800000
10352.0000000	0.3650000
10353.0000000	0.3750000
10354.0000000	0.3650000
10355.0000000	0.3750000
10356.0000000	0.3650000
10357.0000000	0.3750000
10358.0000000	0.3650000
10359.0000000	0.3750000
10360.0000000	0.3650000
10361.0000000	0.3550000
10362.0000000	0.3633333
10363.0000000	0.3750000
10364.0000000	0.3700000
10365.0000000	0.3750000
10366.0000000	0.3700000
10367.0000000	0.3600000
10368.0000000	0.3650000
10369.0000000	0.3600000
10370.0000000	0.3700000
10371.0000000	0.3600000
10372.0000000	0.3700000
10373.0000000	0.3550000

10374.0000000	0.3600000
10375.0000000	0.3550000
10376.0000000	0.3650000
10377.0000000	0.3600000
10378.0000000	0.3500000
10379.0000000	0.3550000
10380.0000000	0.3500000
10381.0000000	0.3600000
10382.0000000	0.3550000
10383.0000000	0.3550000
10384.0000000	0.3550000
10385.0000000	0.3500000
10386.0000000	0.3700000
10387.0000000	0.3550000
10388.0000000	0.3650000
10389.0000000	0.3500000
10390.0000000	0.3600000
10391.0000000	0.3650000
10392.0000000	0.3600000
10393.0000000	0.3600000
10394.0000000	0.3550000
10395.0000000	0.3550000
10396.0000000	0.3450000
10397.0000000	0.3500000
10398.0000000	0.3550000
10399.0000000	0.3600000
10400.0000000	0.3550000
10401.0000000	0.3500000
10402.0000000	0.3450000
10403.0000000	0.3550000
10404.0000000	0.3550000
10405.0000000	0.3550000
10406.0000000	0.3600000
10407.0000000	0.3450000
10408.0000000	0.3450000
10409.0000000	0.3450000
10410.0000000	0.3600000
10411.0000000	0.3500000
10412.0000000	0.3600000
10413.0000000	0.3450000
10414.0000000	0.3550000
10415.0000000	0.3600000
10416.0000000	0.3600000
10417.0000000	0.3650000
10418.0000000	0.3500000
10419.0000000	0.3550000
10420.0000000	0.3550000
10421.0000000	0.3650000
10422.0000000	0.3550000
10423.0000000	0.3533334
10424.0000000	0.3600000
10425.0000000	0.3500000
10426.0000000	0.3550000
10427.0000000	0.3500000
10428.0000000	0.3550000
10429.0000000	0.3550000
10430.0000000	0.3550000

10431.0000000	0.3500000
10432.0000000	0.3500000
10433.0000000	0.3550000
10434.0000000	0.3450000
10435.0000000	0.3550000
10436.0000000	0.3550000
10437.0000000	0.3400000
10438.0000000	0.3450000
10439.0000000	0.3450000
10440.0000000	0.3550000
10441.0000000	0.3450000
10442.0000000	0.3500000
10443.0000000	0.3450000
10444.0000000	0.3450000
10445.0000000	0.3500000
10446.0000000	0.3500000
10447.0000000	0.3500000
10448.0000000	0.3500000
10449.0000000	0.3450000
10450.0000000	0.3550000
10451.0000000	0.3550000
10452.0000000	0.3550000
10453.0000000	0.3533334
10454.0000000	0.3600000
10455.0000000	0.3550000
10456.0000000	0.3600000
10457.0000000	0.3550000
10458.0000000	0.3550000
10459.0000000	0.3450000
10460.0000000	0.3550000
10461.0000000	0.3550000
10462.0000000	0.3500000
10463.0000000	0.3450000
10464.0000000	0.3450000
10465.0000000	0.3500000
10466.0000000	0.3450000
10467.0000000	0.3600000
10468.0000000	0.3500000
10469.0000000	0.3450000
10470.0000000	0.3400000
10471.0000000	0.3450000
10472.0000000	0.3350000
10473.0000000	0.3450000
10474.0000000	0.3350000
10475.0000000	0.3350000
10476.0000000	0.3250000
10477.0000000	0.3400000
10478.0000000	0.3350000
10479.0000000	0.3350000
10480.0000000	0.3350000
10481.0000000	0.3400000
10482.0000000	0.3350000
10483.0000000	0.3300000
10484.0000000	0.3400000
10485.0000000	0.3300000
10486.0000000	0.3300000
10487.0000000	0.3300000

10488.0000000	0.3300000
10489.0000000	0.3300000
10490.0000000	0.3250000
10491.0000000	0.3250000
10492.0000000	0.3200000
10493.0000000	0.3100000
10494.0000000	0.3300000
10495.0000000	0.3200000
10496.0000000	0.3150000
10497.0000000	0.3200000
10498.0000000	0.3100000
10499.0000000	0.3250000
10500.0000000	0.3200000
10501.0000000	0.3250000
10502.0000000	0.3200000
10503.0000000	0.3200000
10504.0000000	0.3200000
10505.0000000	0.3200000
10506.0000000	0.3250000
10507.0000000	0.3200000
10508.0000000	0.3200000
10509.0000000	0.3150000
10510.0000000	0.3300000
10511.0000000	0.3250000
10512.0000000	0.3250000
10513.0000000	0.3266667
10514.0000000	0.3250000
10515.0000000	0.3200000
10516.0000000	0.3150000
10517.0000000	0.3200000
10518.0000000	0.3200000
10519.0000000	0.3200000
10520.0000000	0.3150000
10521.0000000	0.3150000
10522.0000000	0.3200000
10523.0000000	0.3100000
10524.0000000	0.3300000
10525.0000000	0.3100000
10526.0000000	0.3150000
10527.0000000	0.3100000
10528.0000000	0.3200000
10529.0000000	0.3200000
10530.0000000	0.3150000
10531.0000000	0.3200000
10532.0000000	0.3200000
10533.0000000	0.3150000
10534.0000000	0.3200000
10535.0000000	0.3100000
10536.0000000	0.3200000
10537.0000000	0.3200000
10538.0000000	0.3150000
10539.0000000	0.3300000
10540.0000000	0.3050000
10541.0000000	0.3150000
10542.0000000	0.3100000
10543.0000000	0.3100000
10544.0000000	0.3200000

10545.0000000	0.3250000
10546.0000000	0.3150000
10547.0000000	0.3150000
10548.0000000	0.3150000
10549.0000000	0.3150000
10550.0000000	0.3150000
10551.0000000	0.3200000
10552.0000000	0.3200000
10553.0000000	0.3150000
10554.0000000	0.3150000
10555.0000000	0.3100000
10556.0000000	0.3150000
10557.0000000	0.3200000
10558.0000000	0.3150000
10559.0000000	0.3150000
10560.0000000	0.3150000
10561.0000000	0.3200000
10562.0000000	0.3150000
10563.0000000	0.3250000
10564.0000000	0.3100000
10565.0000000	0.3150000
10566.0000000	0.3250000
10567.0000000	0.3150000
10568.0000000	0.3200000
10569.0000000	0.3250000
10570.0000000	0.3150000
10571.0000000	0.3200000
10572.0000000	0.3200000
10573.0000000	0.3150000
10574.0000000	0.3100000
10575.0000000	0.3100000
10576.0000000	0.3100000
10577.0000000	0.3250000
10578.0000000	0.3050000
10579.0000000	0.3100000
10580.0000000	0.3000000
10581.0000000	0.3200000
10582.0000000	0.3100000
10583.0000000	0.3200000
10584.0000000	0.3050000
10585.0000000	0.3150000
10586.0000000	0.3050000
10587.0000000	0.3100000
10588.0000000	0.3050000
10589.0000000	0.3200000
10590.0000000	0.3050000
10591.0000000	0.3000000
10592.0000000	0.3150000
10593.0000000	0.3100000
10594.0000000	0.3150000
10595.0000000	0.3150000
10596.0000000	0.3200000
10597.0000000	0.3000000
10598.0000000	0.3150000
10599.0000000	0.3100000
10600.0000000	0.3250000
10601.0000000	0.3150000

10602.0000000	0.3250000
10603.0000000	0.3200000
10604.0000000	0.3150000
10605.0000000	0.3150000
10606.0000000	0.3050000
10607.0000000	0.3250000
10608.0000000	0.3200000
10609.0000000	0.3200000
10610.0000000	0.3150000
10611.0000000	0.3200000
10612.0000000	0.3150000
10613.0000000	0.3150000
10614.0000000	0.3250000
10615.0000000	0.3200000
10616.0000000	0.3300000
10617.0000000	0.3200000
10618.0000000	0.3250000
10619.0000000	0.3100000
10620.0000000	0.3200000
10621.0000000	0.3250000
10622.0000000	0.3250000
10623.0000000	0.3250000
10624.0000000	0.3250000
10625.0000000	0.3150000
10626.0000000	0.3250000
10627.0000000	0.3100000
10628.0000000	0.3250000
10629.0000000	0.3200000
10630.0000000	0.3300000
10631.0000000	0.3200000
10632.0000000	0.3300000
10633.0000000	0.3050000
10634.0000000	0.3133333
10635.0000000	0.3050000
10636.0000000	0.3000000
10637.0000000	0.3100000
10638.0000000	0.3100000
10639.0000000	0.3200000
10640.0000000	0.3150000
10641.0000000	0.3200000
10642.0000000	0.3100000
10643.0000000	0.3150000
10644.0000000	0.3200000
10645.0000000	0.3150000
10646.0000000	0.3200000
10647.0000000	0.3050000
10648.0000000	0.3050000
10649.0000000	0.3100000
10650.0000000	0.3100000
10651.0000000	0.3100000
10652.0000000	0.3100000
10653.0000000	0.3100000
10654.0000000	0.3050000
10655.0000000	0.3150000
10656.0000000	0.3100000
10657.0000000	0.3200000
10658.0000000	0.3100000

10659.0000000	0.3200000
10660.0000000	0.3200000
10661.0000000	0.3150000
10662.0000000	0.3250000
10663.0000000	0.3100000
10664.0000000	0.3133333
10665.0000000	0.3200000
10666.0000000	0.3150000
10667.0000000	0.3100000
10668.0000000	0.3200000
10669.0000000	0.3150000
10670.0000000	0.3100000
10671.0000000	0.3150000
10672.0000000	0.3100000
10673.0000000	0.3000000
10674.0000000	0.3100000
10675.0000000	0.3000000
10676.0000000	0.3150000
10677.0000000	0.3000000
10678.0000000	0.3100000
10679.0000000	0.3000000
10680.0000000	0.3100000
10681.0000000	0.3150000
10682.0000000	0.3200000
10683.0000000	0.3100000
10684.0000000	0.3100000
10685.0000000	0.3050000
10686.0000000	0.3100000
10687.0000000	0.3150000
10688.0000000	0.3150000
10689.0000000	0.3050000
10690.0000000	0.3100000
10691.0000000	0.3000000
10692.0000000	0.3200000
10693.0000000	0.3050000
10694.0000000	0.3166667
10695.0000000	0.3200000
10696.0000000	0.3150000
10697.0000000	0.3150000
10698.0000000	0.3150000
10699.0000000	0.3200000
10700.0000000	0.3150000
10701.0000000	0.3200000
10702.0000000	0.3050000
10703.0000000	0.3100000
10704.0000000	0.3150000
10705.0000000	0.3150000
10706.0000000	0.3150000
10707.0000000	0.3050000
10708.0000000	0.3100000
10709.0000000	0.3100000
10710.0000000	0.3150000
10711.0000000	0.3100000
10712.0000000	0.3050000
10713.0000000	0.3150000
10714.0000000	0.3100000
10715.0000000	0.3200000

10716.0000000	0.3150000
10717.0000000	0.3100000
10718.0000000	0.3100000
10719.0000000	0.3050000
10720.0000000	0.3100000
10721.0000000	0.3200000
10722.0000000	0.3050000
10723.0000000	0.3150000
10724.0000000	0.3100000
10725.0000000	0.3150000
10726.0000000	0.3050000
10727.0000000	0.3150000
10728.0000000	0.3100000
10729.0000000	0.3100000
10730.0000000	0.3100000
10731.0000000	0.3100000
10732.0000000	0.3150000
10733.0000000	0.3200000
10734.0000000	0.3000000
10735.0000000	0.3150000
10736.0000000	0.3050000
10737.0000000	0.3150000
10738.0000000	0.3200000
10739.0000000	0.3200000
10740.0000000	0.3150000
10741.0000000	0.3050000
10742.0000000	0.3150000
10743.0000000	0.3150000
10744.0000000	0.3150000
10745.0000000	0.3150000
10746.0000000	0.3100000
10747.0000000	0.3200000
10748.0000000	0.3200000
10749.0000000	0.3050000
10750.0000000	0.3150000
10751.0000000	0.3150000
10752.0000000	0.3200000
10753.0000000	0.3250000
10754.0000000	0.3150000
10755.0000000	0.3166667
10756.0000000	0.3150000
10757.0000000	0.3100000
10758.0000000	0.3150000
10759.0000000	0.3200000
10760.0000000	0.3150000
10761.0000000	0.3250000
10762.0000000	0.3100000
10763.0000000	0.3200000
10764.0000000	0.3300000
10765.0000000	0.3250000
10766.0000000	0.3150000
10767.0000000	0.3150000
10768.0000000	0.3250000
10769.0000000	0.3250000
10770.0000000	0.3250000
10771.0000000	0.3200000
10772.0000000	0.3150000

10773.0000000	0.3150000
10774.0000000	0.3150000
10775.0000000	0.3250000
10776.0000000	0.3200000
10777.0000000	0.3200000
10778.0000000	0.3200000
10779.0000000	0.3250000
10780.0000000	0.3250000
10781.0000000	0.3250000
10782.0000000	0.3300000
10783.0000000	0.3300000
10784.0000000	0.3300000
10785.0000000	0.3300000
10786.0000000	0.3300000
10787.0000000	0.3200000
10788.0000000	0.3200000
10789.0000000	0.3250000
10790.0000000	0.3250000
10791.0000000	0.3350000
10792.0000000	0.3300000
10793.0000000	0.3300000
10794.0000000	0.3200000
10795.0000000	0.3200000
10796.0000000	0.3300000
10797.0000000	0.3200000
10798.0000000	0.3300000
10799.0000000	0.3200000
10800.0000000	0.3250000
10801.0000000	0.3300000
10802.0000000	0.3200000
10803.0000000	0.3250000
10804.0000000	0.3250000
10805.0000000	0.3250000
10806.0000000	0.3300000
10807.0000000	0.3300000
10808.0000000	0.3200000
10809.0000000	0.3200000
10810.0000000	0.3150000
10811.0000000	0.3200000
10812.0000000	0.3200000
10813.0000000	0.3250000
10814.0000000	0.3200000
10815.0000000	0.3166667
10816.0000000	0.3250000
10817.0000000	0.3200000
10818.0000000	0.3200000
10819.0000000	0.3150000
10820.0000000	0.3100000
10821.0000000	0.3100000
10822.0000000	0.3200000
10823.0000000	0.3100000
10824.0000000	0.3100000
10825.0000000	0.3250000
10826.0000000	0.3150000
10827.0000000	0.3200000
10828.0000000	0.3150000
10829.0000000	0.3200000

10830.0000000	0.3250000
10831.0000000	0.3150000
10832.0000000	0.3200000
10833.0000000	0.3200000
10834.0000000	0.3250000
10835.0000000	0.3200000
10836.0000000	0.3250000
10837.0000000	0.3150000
10838.0000000	0.3200000
10839.0000000	0.3300000
10840.0000000	0.3250000
10841.0000000	0.3300000
10842.0000000	0.3250000
10843.0000000	0.3300000
10844.0000000	0.3200000
10845.0000000	0.3266667
10846.0000000	0.3250000
10847.0000000	0.3250000
10848.0000000	0.3250000
10849.0000000	0.3200000
10850.0000000	0.3200000
10851.0000000	0.3150000
10852.0000000	0.3200000
10853.0000000	0.3300000
10854.0000000	0.3350000
10855.0000000	0.3150000
10856.0000000	0.3250000
10857.0000000	0.3300000
10858.0000000	0.3300000
10859.0000000	0.3300000
10860.0000000	0.3250000
10861.0000000	0.3200000
10862.0000000	0.3250000
10863.0000000	0.3200000
10864.0000000	0.3250000
10865.0000000	0.3300000
10866.0000000	0.3250000
10867.0000000	0.3200000
10868.0000000	0.3250000
10869.0000000	0.3300000
10870.0000000	0.3200000
10871.0000000	0.3250000
10872.0000000	0.3250000
10873.0000000	0.3300000
10874.0000000	0.3200000
10875.0000000	0.3200000
10876.0000000	0.3200000
10877.0000000	0.3250000
10878.0000000	0.3150000
10879.0000000	0.3150000
10880.0000000	0.3200000
10881.0000000	0.3200000
10882.0000000	0.3100000
10883.0000000	0.3000000
10884.0000000	0.3100000
10885.0000000	0.3150000
10886.0000000	0.3150000

10887.0000000	0.3100000
10888.0000000	0.3150000
10889.0000000	0.3150000
10890.0000000	0.3100000
10891.0000000	0.3100000
10892.0000000	0.3050000
10893.0000000	0.3050000
10894.0000000	0.3100000
10895.0000000	0.3050000
10896.0000000	0.3150000
10897.0000000	0.3150000
10898.0000000	0.3150000
10899.0000000	0.3100000
10900.0000000	0.3150000
10901.0000000	0.3050000
10902.0000000	0.3100000
10903.0000000	0.3150000
10904.0000000	0.3150000
10905.0000000	0.3150000
10906.0000000	0.3133333
10907.0000000	0.3200000
10908.0000000	0.3150000
10909.0000000	0.3200000
10910.0000000	0.3100000
10911.0000000	0.3050000
10912.0000000	0.3100000
10913.0000000	0.3050000
10914.0000000	0.3100000
10915.0000000	0.3100000
10916.0000000	0.3050000
10917.0000000	0.3050000
10918.0000000	0.3050000
10919.0000000	0.3000000
10920.0000000	0.2900000
10921.0000000	0.2950000
10922.0000000	0.3000000
10923.0000000	0.2950000
10924.0000000	0.3050000
10925.0000000	0.2900000
10926.0000000	0.2900000
10927.0000000	0.2800000
10928.0000000	0.2950000
10929.0000000	0.2850000
10930.0000000	0.2950000
10931.0000000	0.2800000
10932.0000000	0.2750000
10933.0000000	0.2850000
10934.0000000	0.2850000
10935.0000000	0.2833333
10936.0000000	0.2850000
10937.0000000	0.2750000
10938.0000000	0.2800000
10939.0000000	0.2800000
10940.0000000	0.2750000
10941.0000000	0.2800000
10942.0000000	0.2850000
10943.0000000	0.2900000

10944.0000000	0.2750000
10945.0000000	0.2750000
10946.0000000	0.2700000
10947.0000000	0.2700000
10948.0000000	0.2850000
10949.0000000	0.2700000
10950.0000000	0.2700000
10951.0000000	0.2600000
10952.0000000	0.2650000
10953.0000000	0.2700000
10954.0000000	0.2750000
10955.0000000	0.2650000
10956.0000000	0.2700000
10957.0000000	0.2650000
10958.0000000	0.2600000
10959.0000000	0.2700000
10960.0000000	0.2650000
10961.0000000	0.2750000
10962.0000000	0.2650000
10963.0000000	0.2700000
10964.0000000	0.2600000
10965.0000000	0.2750000
10966.0000000	0.2633333
10967.0000000	0.2650000
10968.0000000	0.2800000
10969.0000000	0.2700000
10970.0000000	0.2650000
10971.0000000	0.2600000
10972.0000000	0.2700000
10973.0000000	0.2700000
10974.0000000	0.2750000
10975.0000000	0.2700000
10976.0000000	0.2750000
10977.0000000	0.2750000
10978.0000000	0.2700000
10979.0000000	0.2700000
10980.0000000	0.2650000
10981.0000000	0.2650000
10982.0000000	0.2700000
10983.0000000	0.2750000
10984.0000000	0.2700000
10985.0000000	0.2700000
10986.0000000	0.2650000
10987.0000000	0.2700000
10988.0000000	0.2700000
10989.0000000	0.2750000
10990.0000000	0.2650000
10991.0000000	0.2650000
10992.0000000	0.2650000
10993.0000000	0.2600000
10994.0000000	0.2600000
10995.0000000	0.2600000
10996.0000000	0.2600000
10997.0000000	0.2550000
10998.0000000	0.2650000
10999.0000000	0.2600000
11000.0000000	0.2650000

11001.0000000	0.2550000
11002.0000000	0.2550000
11003.0000000	0.2550000
11004.0000000	0.2600000
11005.0000000	0.2600000
11006.0000000	0.2550000
11007.0000000	0.2600000
11008.0000000	0.2550000
11009.0000000	0.2600000
11010.0000000	0.2600000
11011.0000000	0.2550000
11012.0000000	0.2550000
11013.0000000	0.2650000
11014.0000000	0.2650000
11015.0000000	0.2600000
11016.0000000	0.2650000
11017.0000000	0.2550000
11018.0000000	0.2550000
11019.0000000	0.2500000
11020.0000000	0.2700000
11021.0000000	0.2600000
11022.0000000	0.2650000
11023.0000000	0.2500000
11024.0000000	0.2550000
11025.0000000	0.2550000
11026.0000000	0.2600000
11027.0000000	0.2500000
11028.0000000	0.2550000
11029.0000000	0.2450000
11030.0000000	0.2650000
11031.0000000	0.2550000
11032.0000000	0.2600000
11033.0000000	0.2500000
11034.0000000	0.2500000
11035.0000000	0.2550000
11036.0000000	0.2550000
11037.0000000	0.2600000
11038.0000000	0.2550000
11039.0000000	0.2550000
11040.0000000	0.2550000
11041.0000000	0.2450000
11042.0000000	0.2600000
11043.0000000	0.2450000
11044.0000000	0.2550000
11045.0000000	0.2500000
11046.0000000	0.2500000
11047.0000000	0.2500000
11048.0000000	0.2450000
11049.0000000	0.2400000
11050.0000000	0.2500000
11051.0000000	0.2600000
11052.0000000	0.2400000
11053.0000000	0.2650000
11054.0000000	0.2500000
11055.0000000	0.2450000
11056.0000000	0.2533333
11057.0000000	0.2500000

11058.0000000	0.2550000
11059.0000000	0.2500000
11060.0000000	0.2500000
11061.0000000	0.2550000
11062.0000000	0.2600000
11063.0000000	0.2550000
11064.0000000	0.2650000
11065.0000000	0.2550000
11066.0000000	0.2500000
11067.0000000	0.2550000
11068.0000000	0.2500000
11069.0000000	0.2550000
11070.0000000	0.2550000
11071.0000000	0.2600000
11072.0000000	0.2600000
11073.0000000	0.2550000
11074.0000000	0.2600000
11075.0000000	0.2500000
11076.0000000	0.2500000
11077.0000000	0.2600000
11078.0000000	0.2550000
11079.0000000	0.2550000
11080.0000000	0.2600000
11081.0000000	0.2600000
11082.0000000	0.2500000
11083.0000000	0.2500000
11084.0000000	0.2550000
11085.0000000	0.2550000
11086.0000000	0.2500000
11087.0000000	0.2533333
11088.0000000	0.2450000
11089.0000000	0.2400000
11090.0000000	0.2450000
11091.0000000	0.2550000
11092.0000000	0.2600000
11093.0000000	0.2500000
11094.0000000	0.2400000
11095.0000000	0.2500000
11096.0000000	0.2550000
11097.0000000	0.2550000
11098.0000000	0.2400000
11099.0000000	0.2450000
11100.0000000	0.2450000
11101.0000000	0.2500000
11102.0000000	0.2500000
11103.0000000	0.2550000
11104.0000000	0.2500000
11105.0000000	0.2500000
11106.0000000	0.2550000
11107.0000000	0.2500000
11108.0000000	0.2550000
11109.0000000	0.2450000
11110.0000000	0.2500000
11111.0000000	0.2450000
11112.0000000	0.2450000
11113.0000000	0.2400000
11114.0000000	0.2500000

11115.0000000	0.2500000
11116.0000000	0.2600000
11117.0000000	0.2433333
11118.0000000	0.2500000
11119.0000000	0.2550000
11120.0000000	0.2500000
11121.0000000	0.2450000
11122.0000000	0.2500000
11123.0000000	0.2500000
11124.0000000	0.2550000
11125.0000000	0.2450000
11126.0000000	0.2500000
11127.0000000	0.2500000
11128.0000000	0.2500000
11129.0000000	0.2650000
11130.0000000	0.2500000
11131.0000000	0.2550000
11132.0000000	0.2450000
11133.0000000	0.2550000
11134.0000000	0.2500000
11135.0000000	0.2650000
11136.0000000	0.2500000
11137.0000000	0.2600000
11138.0000000	0.2550000
11139.0000000	0.2550000
11140.0000000	0.2550000
11141.0000000	0.2550000
11142.0000000	0.2600000
11143.0000000	0.2500000
11144.0000000	0.2500000
11145.0000000	0.2500000
11146.0000000	0.2600000
11147.0000000	0.2500000
11148.0000000	0.2550000
11149.0000000	0.2600000
11150.0000000	0.2450000
11151.0000000	0.2500000
11152.0000000	0.2450000
11153.0000000	0.2600000
11154.0000000	0.2650000
11155.0000000	0.2550000
11156.0000000	0.2550000
11157.0000000	0.2500000
11158.0000000	0.2450000
11159.0000000	0.2550000
11160.0000000	0.2450000
11161.0000000	0.2450000
11162.0000000	0.2500000
11163.0000000	0.2450000
11164.0000000	0.2550000
11165.0000000	0.2450000
11166.0000000	0.2400000
11167.0000000	0.2350000
11168.0000000	0.2350000
11169.0000000	0.2400000
11170.0000000	0.2400000
11171.0000000	0.2350000

11172.0000000	0.2450000
11173.0000000	0.2500000
11174.0000000	0.2250000
11175.0000000	0.2350000
11176.0000000	0.2300000
11177.0000000	0.2400000
11178.0000000	0.2350000
11179.0000000	0.2350000
11180.0000000	0.2400000
11181.0000000	0.2350000
11182.0000000	0.2300000
11183.0000000	0.2300000
11184.0000000	0.2300000
11185.0000000	0.2400000
11186.0000000	0.2300000
11187.0000000	0.2350000
11188.0000000	0.2300000
11189.0000000	0.2350000
11190.0000000	0.2300000
11191.0000000	0.2400000
11192.0000000	0.2300000
11193.0000000	0.2250000
11194.0000000	0.2250000
11195.0000000	0.2250000
11196.0000000	0.2300000
11197.0000000	0.2250000
11198.0000000	0.2300000
11199.0000000	0.2200000
11200.0000000	0.2300000
11201.0000000	0.2250000
11202.0000000	0.2200000
11203.0000000	0.2250000
11204.0000000	0.2200000
11205.0000000	0.2350000
11206.0000000	0.2200000
11207.0000000	0.2300000
11208.0000000	0.2300000
11209.0000000	0.2200000
11210.0000000	0.2300000
11211.0000000	0.2150000
11212.0000000	0.2250000
11213.0000000	0.2200000
11214.0000000	0.2250000
11215.0000000	0.2300000
11216.0000000	0.2200000
11217.0000000	0.2200000
11218.0000000	0.2150000
11219.0000000	0.2150000
11220.0000000	0.2250000
11221.0000000	0.2200000
11222.0000000	0.2200000
11223.0000000	0.2250000
11224.0000000	0.2150000
11225.0000000	0.2200000
11226.0000000	0.2200000
11227.0000000	0.2200000
11228.0000000	0.2100000

11229.0000000	0.2150000
11230.0000000	0.2150000
11231.0000000	0.2150000
11232.0000000	0.2100000
11233.0000000	0.2150000
11234.0000000	0.2100000
11235.0000000	0.2150000
11236.0000000	0.2100000
11237.0000000	0.2200000
11238.0000000	0.2066667
11239.0000000	0.2100000
11240.0000000	0.2250000
11241.0000000	0.2100000
11242.0000000	0.2250000
11243.0000000	0.2100000
11244.0000000	0.2200000
11245.0000000	0.2100000
11246.0000000	0.2200000
11247.0000000	0.2150000
11248.0000000	0.2200000
11249.0000000	0.2050000
11250.0000000	0.2200000
11251.0000000	0.2100000
11252.0000000	0.2200000
11253.0000000	0.2150000
11254.0000000	0.2200000
11255.0000000	0.2150000
11256.0000000	0.2150000
11257.0000000	0.2250000
11258.0000000	0.2150000
11259.0000000	0.2200000
11260.0000000	0.2100000
11261.0000000	0.2250000
11262.0000000	0.2200000
11263.0000000	0.2200000
11264.0000000	0.2150000
11265.0000000	0.2150000
11266.0000000	0.2200000
11267.0000000	0.2200000
11268.0000000	0.2150000
11269.0000000	0.2150000
11270.0000000	0.2150000
11271.0000000	0.2100000
11272.0000000	0.2100000
11273.0000000	0.2150000
11274.0000000	0.2100000
11275.0000000	0.2150000
11276.0000000	0.2100000
11277.0000000	0.2000000
11278.0000000	0.2100000
11279.0000000	0.2050000
11280.0000000	0.2100000
11281.0000000	0.2050000
11282.0000000	0.2050000
11283.0000000	0.1950000
11284.0000000	0.1900000
11285.0000000	0.1950000

11286.0000000	0.1950000
11287.0000000	0.2000000
11288.0000000	0.1900000
11289.0000000	0.1900000
11290.0000000	0.2000000
11291.0000000	0.1900000
11292.0000000	0.1900000
11293.0000000	0.1800000
11294.0000000	0.1750000
11295.0000000	0.1900000
11296.0000000	0.1850000
11297.0000000	0.1900000
11298.0000000	0.1800000
11299.0000000	0.1800000
11300.0000000	0.1750000
11301.0000000	0.1700000
11302.0000000	0.1900000
11303.0000000	0.1750000
11304.0000000	0.1700000
11305.0000000	0.1700000
11306.0000000	0.1650000
11307.0000000	0.1700000
11308.0000000	0.1750000
11309.0000000	0.1700000
11310.0000000	0.1750000
11311.0000000	0.1700000
11312.0000000	0.1650000
11313.0000000	0.1800000
11314.0000000	0.1700000
11315.0000000	0.1700000
11316.0000000	0.1750000
11317.0000000	0.1600000
11318.0000000	0.1750000
11319.0000000	0.1700000
11320.0000000	0.1650000
11321.0000000	0.1700000
11322.0000000	0.1700000
11323.0000000	0.1750000
11324.0000000	0.1700000
11325.0000000	0.1650000
11326.0000000	0.1700000
11327.0000000	0.1650000
11328.0000000	0.1733333
11329.0000000	0.1700000
11330.0000000	0.1700000
11331.0000000	0.1700000
11332.0000000	0.1700000
11333.0000000	0.1750000
11334.0000000	0.1650000
11335.0000000	0.1700000
11336.0000000	0.1700000
11337.0000000	0.1650000
11338.0000000	0.1650000
11339.0000000	0.1700000
11340.0000000	0.1650000
11341.0000000	0.1650000
11342.0000000	0.1650000

11343.0000000	0.1650000
11344.0000000	0.1700000
11345.0000000	0.1700000
11346.0000000	0.1700000
11347.0000000	0.1600000
11348.0000000	0.1700000
11349.0000000	0.1600000
11350.0000000	0.1650000
11351.0000000	0.1600000
11352.0000000	0.1600000
11353.0000000	0.1600000
11354.0000000	0.1600000
11355.0000000	0.1600000
11356.0000000	0.1550000
11357.0000000	0.1600000
11358.0000000	0.1566667
11359.0000000	0.1450000
11360.0000000	0.1600000
11361.0000000	0.1500000
11362.0000000	0.1550000
11363.0000000	0.1550000
11364.0000000	0.1450000
11365.0000000	0.1550000
11366.0000000	0.1400000
11367.0000000	0.1400000
11368.0000000	0.1450000
11369.0000000	0.1500000
11370.0000000	0.1500000
11371.0000000	0.1500000
11372.0000000	0.1450000
11373.0000000	0.1500000
11374.0000000	0.1450000
11375.0000000	0.1500000
11376.0000000	0.1500000
11377.0000000	0.1450000
11378.0000000	0.1500000
11379.0000000	0.1450000
11380.0000000	0.1450000
11381.0000000	0.1450000
11382.0000000	0.1500000
11383.0000000	0.1500000
11384.0000000	0.1500000
11385.0000000	0.1500000
11386.0000000	0.1500000
11387.0000000	0.1400000
11388.0000000	0.1500000
11389.0000000	0.1500000
11390.0000000	0.1450000
11391.0000000	0.1550000
11392.0000000	0.1550000
11393.0000000	0.1500000
11394.0000000	0.1550000
11395.0000000	0.1500000
11396.0000000	0.1500000
11397.0000000	0.1550000
11398.0000000	0.1650000
11399.0000000	0.1550000

11400.0000000	0.1650000
11401.0000000	0.1550000
11402.0000000	0.1550000
11403.0000000	0.1450000
11404.0000000	0.1600000
11405.0000000	0.1450000
11406.0000000	0.1550000
11407.0000000	0.1650000
11408.0000000	0.1600000
11409.0000000	0.1600000
11410.0000000	0.1450000
11411.0000000	0.1550000
11412.0000000	0.1500000
11413.0000000	0.1500000
11414.0000000	0.1550000
11415.0000000	0.1600000
11416.0000000	0.1500000
11417.0000000	0.1500000
11418.0000000	0.1500000
11419.0000000	0.1533333
11420.0000000	0.1450000
11421.0000000	0.1400000
11422.0000000	0.1500000
11423.0000000	0.1350000
11424.0000000	0.1550000
11425.0000000	0.1450000
11426.0000000	0.1450000
11427.0000000	0.1300000
11428.0000000	0.1350000
11429.0000000	0.1350000
11430.0000000	0.1450000
11431.0000000	0.1400000
11432.0000000	0.1400000
11433.0000000	0.1350000
11434.0000000	0.1300000
11435.0000000	0.1350000
11436.0000000	0.1400000
11437.0000000	0.1350000
11438.0000000	0.1400000
11439.0000000	0.1400000
11440.0000000	0.1350000
11441.0000000	0.1400000
11442.0000000	0.1300000
11443.0000000	0.1300000
11444.0000000	0.1300000
11445.0000000	0.1400000
11446.0000000	0.1400000
11447.0000000	0.1400000
11448.0000000	0.1300000
11449.0000000	0.1366667
11450.0000000	0.1500000
11451.0000000	0.1300000
11452.0000000	0.1350000
11453.0000000	0.1450000
11454.0000000	0.1300000
11455.0000000	0.1350000
11456.0000000	0.1300000

11457.0000000	0.1300000
11458.0000000	0.1350000
11459.0000000	0.1350000
11460.0000000	0.1350000
11461.0000000	0.1300000
11462.0000000	0.1350000
11463.0000000	0.1350000
11464.0000000	0.1350000
11465.0000000	0.1350000
11466.0000000	0.1300000
11467.0000000	0.1350000
11468.0000000	0.1450000
11469.0000000	0.1300000
11470.0000000	0.1300000
11471.0000000	0.1300000
11472.0000000	0.1300000
11473.0000000	0.1250000
11474.0000000	0.1350000
11475.0000000	0.1350000
11476.0000000	0.1400000
11477.0000000	0.1300000
11478.0000000	0.1300000
11479.0000000	0.1400000
11480.0000000	0.1350000
11481.0000000	0.1350000
11482.0000000	0.1300000
11483.0000000	0.1350000
11484.0000000	0.1400000
11485.0000000	0.1350000
11486.0000000	0.1350000
11487.0000000	0.1400000
11488.0000000	0.1400000
11489.0000000	0.1450000
11490.0000000	0.1350000
11491.0000000	0.1450000
11492.0000000	0.1300000
11493.0000000	0.1500000
11494.0000000	0.1350000
11495.0000000	0.1500000
11496.0000000	0.1450000
11497.0000000	0.1450000
11498.0000000	0.1400000
11499.0000000	0.1400000
11500.0000000	0.1350000
11501.0000000	0.1400000
11502.0000000	0.1400000
11503.0000000	0.1450000
11504.0000000	0.1450000
11505.0000000	0.1400000
11506.0000000	0.1400000
11507.0000000	0.1400000
11508.0000000	0.1400000
11509.0000000	0.1466667
11510.0000000	0.1400000
11511.0000000	0.1400000
11512.0000000	0.1400000
11513.0000000	0.1400000

11514.0000000	0.1450000
11515.0000000	0.1400000
11516.0000000	0.1500000
11517.0000000	0.1350000
11518.0000000	0.1450000
11519.0000000	0.1400000
11520.0000000	0.1600000
11521.0000000	0.1450000
11522.0000000	0.1450000
11523.0000000	0.1350000
11524.0000000	0.1400000
11525.0000000	0.1400000
11526.0000000	0.1500000
11527.0000000	0.1400000
11528.0000000	0.1550000
11529.0000000	0.1400000
11530.0000000	0.1400000
11531.0000000	0.1450000
11532.0000000	0.1500000
11533.0000000	0.1550000
11534.0000000	0.1500000
11535.0000000	0.1500000
11536.0000000	0.1500000
11537.0000000	0.1450000
11538.0000000	0.1450000
11539.0000000	0.1500000
11540.0000000	0.1450000
11541.0000000	0.1500000
11542.0000000	0.1500000
11543.0000000	0.1550000
11544.0000000	0.1400000
11545.0000000	0.1500000
11546.0000000	0.1450000
11547.0000000	0.1500000
11548.0000000	0.1400000
11549.0000000	0.1600000
11550.0000000	0.1500000
11551.0000000	0.1550000
11552.0000000	0.1400000
11553.0000000	0.1500000
11554.0000000	0.1500000
11555.0000000	0.1550000
11556.0000000	0.1450000
11557.0000000	0.1500000
11558.0000000	0.1500000
11559.0000000	0.1450000
11560.0000000	0.1550000
11561.0000000	0.1550000
11562.0000000	0.1550000
11563.0000000	0.1500000
11564.0000000	0.1400000
11565.0000000	0.1500000
11566.0000000	0.1550000
11567.0000000	0.1550000
11568.0000000	0.1450000
11569.0000000	0.1600000
11570.0000000	0.1466667

11571.0000000	0.1550000
11572.0000000	0.1500000
11573.0000000	0.1550000
11574.0000000	0.1650000
11575.0000000	0.1500000
11576.0000000	0.1550000
11577.0000000	0.1450000
11578.0000000	0.1500000
11579.0000000	0.1550000
11580.0000000	0.1550000
11581.0000000	0.1500000
11582.0000000	0.1500000
11583.0000000	0.1550000
11584.0000000	0.1600000
11585.0000000	0.1500000
11586.0000000	0.1450000
11587.0000000	0.1450000
11588.0000000	0.1450000
11589.0000000	0.1600000
11590.0000000	0.1550000
11591.0000000	0.1450000
11592.0000000	0.1450000
11593.0000000	0.1600000
11594.0000000	0.1450000
11595.0000000	0.1400000
11596.0000000	0.1400000
11597.0000000	0.1450000
11598.0000000	0.1500000
11599.0000000	0.1550000
11600.0000000	0.1466667
11601.0000000	0.1550000
11602.0000000	0.1500000
11603.0000000	0.1500000
11604.0000000	0.1400000
11605.0000000	0.1450000
11606.0000000	0.1400000
11607.0000000	0.1450000
11608.0000000	0.1450000
11609.0000000	0.1500000
11610.0000000	0.1500000
11611.0000000	0.1500000
11612.0000000	0.1450000
11613.0000000	0.1450000
11614.0000000	0.1450000
11615.0000000	0.1450000
11616.0000000	0.1550000
11617.0000000	0.1350000
11618.0000000	0.1500000
11619.0000000	0.1350000
11620.0000000	0.1450000
11621.0000000	0.1400000
11622.0000000	0.1550000
11623.0000000	0.1400000
11624.0000000	0.1350000
11625.0000000	0.1400000
11626.0000000	0.1350000
11627.0000000	0.1500000

11628.0000000	0.1400000
11629.0000000	0.1350000
11630.0000000	0.1366667
11631.0000000	0.1300000
11632.0000000	0.1350000
11633.0000000	0.1350000
11634.0000000	0.1400000
11635.0000000	0.1350000
11636.0000000	0.1350000
11637.0000000	0.1450000
11638.0000000	0.1450000
11639.0000000	0.1450000
11640.0000000	0.1450000
11641.0000000	0.1350000
11642.0000000	0.1300000
11643.0000000	0.1400000
11644.0000000	0.1400000
11645.0000000	0.1400000
11646.0000000	0.1450000
11647.0000000	0.1400000
11648.0000000	0.1400000
11649.0000000	0.1350000
11650.0000000	0.1450000
11651.0000000	0.1400000
11652.0000000	0.1300000
11653.0000000	0.1450000
11654.0000000	0.1500000
11655.0000000	0.1400000
11656.0000000	0.1400000
11657.0000000	0.1300000
11658.0000000	0.1450000
11659.0000000	0.1350000
11660.0000000	0.1400000
11661.0000000	0.1450000
11662.0000000	0.1400000
11663.0000000	0.1400000
11664.0000000	0.1500000
11665.0000000	0.1400000
11666.0000000	0.1400000
11667.0000000	0.1400000
11668.0000000	0.1450000
11669.0000000	0.1450000
11670.0000000	0.1350000
11671.0000000	0.1300000
11672.0000000	0.1500000
11673.0000000	0.1400000
11674.0000000	0.1450000
11675.0000000	0.1350000
11676.0000000	0.1350000
11677.0000000	0.1400000
11678.0000000	0.1350000
11679.0000000	0.1300000
11680.0000000	0.1350000
11681.0000000	0.1300000
11682.0000000	0.1400000
11683.0000000	0.1400000
11684.0000000	0.1400000

11685.0000000	0.1350000
11686.0000000	0.1300000
11687.0000000	0.1400000
11688.0000000	0.1400000
11689.0000000	0.1400000
11690.0000000	0.1250000
11691.0000000	0.1266667
11692.0000000	0.1400000
11693.0000000	0.1400000
11694.0000000	0.1450000
11695.0000000	0.1250000
11696.0000000	0.1300000
11697.0000000	0.1300000
11698.0000000	0.1350000
11699.0000000	0.1350000
11700.0000000	0.1350000
11701.0000000	0.1300000
11702.0000000	0.1250000
11703.0000000	0.1300000
11704.0000000	0.1300000
11705.0000000	0.1350000
11706.0000000	0.1300000
11707.0000000	0.1250000
11708.0000000	0.1300000
11709.0000000	0.1300000
11710.0000000	0.1200000
11711.0000000	0.1300000
11712.0000000	0.1250000
11713.0000000	0.1250000
11714.0000000	0.1250000
11715.0000000	0.1250000
11716.0000000	0.1250000
11717.0000000	0.1300000
11718.0000000	0.1300000
11719.0000000	0.1250000
11720.0000000	0.1200000
11721.0000000	0.1300000
11722.0000000	0.1200000
11723.0000000	0.1250000
11724.0000000	0.1250000
11725.0000000	0.1150000
11726.0000000	0.1300000
11727.0000000	0.1300000
11728.0000000	0.1300000
11729.0000000	0.1200000
11730.0000000	0.1300000
11731.0000000	0.1200000
11732.0000000	0.1300000
11733.0000000	0.1200000
11734.0000000	0.1150000
11735.0000000	0.1250000
11736.0000000	0.1200000
11737.0000000	0.1250000
11738.0000000	0.1300000
11739.0000000	0.1250000
11740.0000000	0.1200000
11741.0000000	0.1200000

11742.0000000	0.1150000
11743.0000000	0.1250000
11744.0000000	0.1250000
11745.0000000	0.1150000
11746.0000000	0.1250000
11747.0000000	0.1350000
11748.0000000	0.1150000
11749.0000000	0.1200000
11750.0000000	0.1233333
11751.0000000	0.1150000
11752.0000000	0.1250000
11753.0000000	0.1250000
11754.0000000	0.1200000
11755.0000000	0.1200000
11756.0000000	0.1100000
11757.0000000	0.1200000
11758.0000000	0.1250000
11759.0000000	0.1100000
11760.0000000	0.1150000
11761.0000000	0.1200000
11762.0000000	0.1100000
11763.0000000	0.1250000
11764.0000000	0.1250000
11765.0000000	0.1150000
11766.0000000	0.1100000
11767.0000000	0.1150000
11768.0000000	0.1100000
11769.0000000	0.1000000
11770.0000000	0.1100000
11771.0000000	0.1200000
11772.0000000	0.1100000
11773.0000000	0.1150000
11774.0000000	0.1150000
11775.0000000	0.1050000
11776.0000000	0.1100000
11777.0000000	0.1100000
11778.0000000	0.1100000
11779.0000000	0.1000000
11780.0000000	0.1150000
11781.0000000	0.1100000
11782.0000000	0.1150000
11783.0000000	0.1200000
11784.0000000	0.1100000
11785.0000000	0.1000000
11786.0000000	0.1100000
11787.0000000	0.1100000
11788.0000000	0.1200000
11789.0000000	0.1150000
11790.0000000	0.1000000
11791.0000000	0.1100000
11792.0000000	0.1100000
11793.0000000	0.1100000
11794.0000000	0.1100000
11795.0000000	0.1100000
11796.0000000	0.1100000
11797.0000000	0.1050000
11798.0000000	0.1250000

11799.0000000	0.1100000
11800.0000000	0.1050000
11801.0000000	0.1100000
11802.0000000	0.1050000
11803.0000000	0.1100000
11804.0000000	0.1050000
11805.0000000	0.1100000
11806.0000000	0.1000000
11807.0000000	0.1050000
11808.0000000	0.1050000
11809.0000000	0.1050000
11810.0000000	0.1100000
11811.0000000	0.1066667
11812.0000000	0.1000000
11813.0000000	0.1050000
11814.0000000	0.1100000
11815.0000000	0.1000000
11816.0000000	0.1100000
11817.0000000	0.1150000
11818.0000000	0.1050000
11819.0000000	0.1100000
11820.0000000	0.1000000
11821.0000000	0.1100000
11822.0000000	0.1000000
11823.0000000	0.1000000
11824.0000000	0.1100000
11825.0000000	0.1000000
11826.0000000	0.1100000
11827.0000000	0.1150000
11828.0000000	0.1050000
11829.0000000	0.1100000
11830.0000000	0.1100000
11831.0000000	0.1150000
11832.0000000	0.1000000
11833.0000000	0.1000000
11834.0000000	0.1050000
11835.0000000	0.1000000
11836.0000000	0.1000000
11837.0000000	0.1050000
11838.0000000	0.1000000
11839.0000000	0.1000000
11840.0000000	0.1150000
11841.0000000	0.1000000
11842.0000000	0.1000000
11843.0000000	0.1100000
11844.0000000	0.1100000
11845.0000000	0.1050000
11846.0000000	0.1000000
11847.0000000	0.1050000
11848.0000000	0.1000000
11849.0000000	0.1100000
11850.0000000	0.1100000
11851.0000000	0.1050000
11852.0000000	0.1050000
11853.0000000	0.1050000
11854.0000000	0.1100000
11855.0000000	0.0950000

11856.0000000	0.1000000
11857.0000000	0.1000000
11858.0000000	0.1050000
11859.0000000	0.1050000
11860.0000000	0.1000000
11861.0000000	0.1000000
11862.0000000	0.0950000
11863.0000000	0.0950000
11864.0000000	0.0900000
11865.0000000	0.0900000
11866.0000000	0.0900000
11867.0000000	0.0900000
11868.0000000	0.0900000
11869.0000000	0.0900000
11870.0000000	0.0850000
11871.0000000	0.0866667
11872.0000000	0.0800000
11873.0000000	0.0800000
11874.0000000	0.0850000
11875.0000000	0.0900000
11876.0000000	0.0850000
11877.0000000	0.0900000
11878.0000000	0.0850000
11879.0000000	0.0900000
11880.0000000	0.0800000
11881.0000000	0.0900000
11882.0000000	0.0750000
11883.0000000	0.0800000
11884.0000000	0.0850000
11885.0000000	0.0800000
11886.0000000	0.0900000
11887.0000000	0.0800000
11888.0000000	0.0850000
11889.0000000	0.0850000
11890.0000000	0.0900000
11891.0000000	0.0900000
11892.0000000	0.0900000
11893.0000000	0.0900000
11894.0000000	0.0800000
11895.0000000	0.0900000
11896.0000000	0.0850000
11897.0000000	0.0900000
11898.0000000	0.0850000
11899.0000000	0.0850000
11900.0000000	0.0850000
11901.0000000	0.0950000
11902.0000000	0.0866667
11903.0000000	0.0850000
11904.0000000	0.0900000
11905.0000000	0.0800000
11906.0000000	0.0900000
11907.0000000	0.0850000
11908.0000000	0.0850000
11909.0000000	0.0900000
11910.0000000	0.0800000
11911.0000000	0.0800000
11912.0000000	0.0850000

11913.0000000	0.0900000
11914.0000000	0.0900000
11915.0000000	0.0900000
11916.0000000	0.0850000
11917.0000000	0.0900000
11918.0000000	0.0850000
11919.0000000	0.0900000
11920.0000000	0.0900000
11921.0000000	0.1050000
11922.0000000	0.0800000
11923.0000000	0.0950000
11924.0000000	0.0850000
11925.0000000	0.0900000
11926.0000000	0.0850000
11927.0000000	0.1000000
11928.0000000	0.0900000
11929.0000000	0.0850000
11930.0000000	0.0900000
11931.0000000	0.0900000
11932.0000000	0.0966667
11933.0000000	0.0900000
11934.0000000	0.1000000
11935.0000000	0.0950000
11936.0000000	0.0900000
11937.0000000	0.0850000
11938.0000000	0.0850000
11939.0000000	0.1050000
11940.0000000	0.0950000
11941.0000000	0.0950000
11942.0000000	0.0950000
11943.0000000	0.0850000
11944.0000000	0.0900000
11945.0000000	0.0950000
11946.0000000	0.0800000
11947.0000000	0.0850000
11948.0000000	0.1000000
11949.0000000	0.0850000
11950.0000000	0.0950000
11951.0000000	0.0850000
11952.0000000	0.0850000
11953.0000000	0.0900000
11954.0000000	0.0850000
11955.0000000	0.0850000
11956.0000000	0.0800000
11957.0000000	0.0900000
11958.0000000	0.0900000
11959.0000000	0.0850000
11960.0000000	0.0900000
11961.0000000	0.0750000
11962.0000000	0.0833333
11963.0000000	0.0700000
11964.0000000	0.0850000
11965.0000000	0.0850000
11966.0000000	0.0850000
11967.0000000	0.0800000
11968.0000000	0.0800000
11969.0000000	0.0750000

11970.0000000	0.0750000
11971.0000000	0.0800000
11972.0000000	0.0800000
11973.0000000	0.0850000
11974.0000000	0.0750000
11975.0000000	0.0800000
11976.0000000	0.0700000
11977.0000000	0.0800000
11978.0000000	0.0850000
11979.0000000	0.0900000
11980.0000000	0.0750000
11981.0000000	0.0700000
11982.0000000	0.0800000
11983.0000000	0.0750000
11984.0000000	0.0850000
11985.0000000	0.0850000
11986.0000000	0.0900000
11987.0000000	0.0700000
11988.0000000	0.0800000
11989.0000000	0.0750000
11990.0000000	0.0850000
11991.0000000	0.0850000
11992.0000000	0.0800000
11993.0000000	0.0800000
11994.0000000	0.0800000
11995.0000000	0.0800000
11996.0000000	0.0800000
11997.0000000	0.0750000
11998.0000000	0.0850000
11999.0000000	0.0750000
12000.0000000	0.0750000
12001.0000000	0.0800000
12002.0000000	0.0800000
12003.0000000	0.0800000
12004.0000000	0.0750000
12005.0000000	0.0850000
12006.0000000	0.0800000
12007.0000000	0.0750000
12008.0000000	0.0700000
12009.0000000	0.0900000
12010.0000000	0.0750000
12011.0000000	0.0800000
12012.0000000	0.0800000
12013.0000000	0.0800000
12014.0000000	0.0850000
12015.0000000	0.0850000
12016.0000000	0.0850000
12017.0000000	0.0700000
12018.0000000	0.0800000
12019.0000000	0.0800000
12020.0000000	0.0900000
12021.0000000	0.0750000
12022.0000000	0.0750000
12023.0000000	0.0766667
12024.0000000	0.0800000
12025.0000000	0.0850000
12026.0000000	0.0800000

12027.0000000	0.0800000
12028.0000000	0.0750000
12029.0000000	0.0800000
12030.0000000	0.0750000
12031.0000000	0.0750000
12032.0000000	0.0850000
12033.0000000	0.0750000
12034.0000000	0.0750000
12035.0000000	0.0800000
12036.0000000	0.0700000
12037.0000000	0.0750000
12038.0000000	0.0850000
12039.0000000	0.0650000
12040.0000000	0.0800000
12041.0000000	0.0700000
12042.0000000	0.0850000
12043.0000000	0.0800000
12044.0000000	0.0700000
12045.0000000	0.0700000
12046.0000000	0.0750000
12047.0000000	0.0850000
12048.0000000	0.0800000
12049.0000000	0.0850000
12050.0000000	0.0650000
12051.0000000	0.0700000
12052.0000000	0.0733333
12053.0000000	0.0850000
12054.0000000	0.0800000
12055.0000000	0.0750000
12056.0000000	0.0750000
12057.0000000	0.0850000
12058.0000000	0.0750000
12059.0000000	0.0750000
12060.0000000	0.0800000
12061.0000000	0.0750000
12062.0000000	0.0850000
12063.0000000	0.0800000
12064.0000000	0.0750000
12065.0000000	0.0750000
12066.0000000	0.0800000
12067.0000000	0.0800000
12068.0000000	0.0800000
12069.0000000	0.0750000
12070.0000000	0.0850000
12071.0000000	0.0750000
12072.0000000	0.0750000
12073.0000000	0.0750000
12074.0000000	0.0800000
12075.0000000	0.0850000
12076.0000000	0.0800000
12077.0000000	0.0750000
12078.0000000	0.0800000
12079.0000000	0.0750000
12080.0000000	0.0800000
12081.0000000	0.0800000
12082.0000000	0.0766667
12083.0000000	0.0800000

12084.0000000	0.0900000
12085.0000000	0.0900000
12086.0000000	0.0800000
12087.0000000	0.0750000
12088.0000000	0.0750000
12089.0000000	0.0900000
12090.0000000	0.0900000
12091.0000000	0.0850000
12092.0000000	0.0850000
12093.0000000	0.0800000
12094.0000000	0.0800000
12095.0000000	0.0850000
12096.0000000	0.0800000
12097.0000000	0.0800000
12098.0000000	0.0850000
12099.0000000	0.0850000
12100.0000000	0.0900000
12101.0000000	0.0850000
12102.0000000	0.0900000
12103.0000000	0.0850000
12104.0000000	0.0950000
12105.0000000	0.0850000
12106.0000000	0.1000000
12107.0000000	0.0900000
12108.0000000	0.0850000
12109.0000000	0.0900000
12110.0000000	0.0850000
12111.0000000	0.0900000
12112.0000000	0.1000000
12113.0000000	0.0866667
12114.0000000	0.0900000
12115.0000000	0.0900000
12116.0000000	0.1000000
12117.0000000	0.0900000
12118.0000000	0.0950000
12119.0000000	0.0900000
12120.0000000	0.0900000
12121.0000000	0.0900000
12122.0000000	0.0950000
12123.0000000	0.0950000
12124.0000000	0.0950000
12125.0000000	0.0950000
12126.0000000	0.0950000
12127.0000000	0.0950000
12128.0000000	0.0900000
12129.0000000	0.0950000
12130.0000000	0.0950000
12131.0000000	0.1000000
12132.0000000	0.1000000
12133.0000000	0.0950000
12134.0000000	0.0950000
12135.0000000	0.0900000
12136.0000000	0.1000000
12137.0000000	0.0950000
12138.0000000	0.0900000
12139.0000000	0.1000000
12140.0000000	0.1050000

12141.0000000	0.1050000
12142.0000000	0.1100000
12143.0000000	0.0966667
12144.0000000	0.1000000
12145.0000000	0.1000000
12146.0000000	0.1100000
12147.0000000	0.1000000
12148.0000000	0.0950000
12149.0000000	0.0950000
12150.0000000	0.1050000
12151.0000000	0.1100000
12152.0000000	0.1100000
12153.0000000	0.1100000
12154.0000000	0.1050000
12155.0000000	0.1050000
12156.0000000	0.1100000
12157.0000000	0.1050000
12158.0000000	0.1050000
12159.0000000	0.0950000
12160.0000000	0.1150000
12161.0000000	0.1100000
12162.0000000	0.1150000
12163.0000000	0.1000000
12164.0000000	0.1050000
12165.0000000	0.1000000
12166.0000000	0.1150000
12167.0000000	0.1150000
12168.0000000	0.1200000
12169.0000000	0.1000000
12170.0000000	0.1150000
12171.0000000	0.1000000
12172.0000000	0.1100000
12173.0000000	0.1133333
12174.0000000	0.1050000
12175.0000000	0.1100000
12176.0000000	0.1100000
12177.0000000	0.1200000
12178.0000000	0.1150000
12179.0000000	0.1200000
12180.0000000	0.1100000
12181.0000000	0.1100000
12182.0000000	0.1150000
12183.0000000	0.1200000
12184.0000000	0.1150000
12185.0000000	0.1150000
12186.0000000	0.1050000
12187.0000000	0.1100000
12188.0000000	0.1150000
12189.0000000	0.1100000
12190.0000000	0.1150000
12191.0000000	0.1150000
12192.0000000	0.1100000
12193.0000000	0.1150000
12194.0000000	0.1150000
12195.0000000	0.1150000
12196.0000000	0.1150000
12197.0000000	0.1150000

12198.0000000	0.1200000
12199.0000000	0.1200000
12200.0000000	0.1250000
12201.0000000	0.1100000
12202.0000000	0.1100000
12203.0000000	0.1166667
12204.0000000	0.1100000
12205.0000000	0.1200000
12206.0000000	0.1300000
12207.0000000	0.1250000
12208.0000000	0.1200000
12209.0000000	0.1200000
12210.0000000	0.1200000
12211.0000000	0.1250000
12212.0000000	0.1250000
12213.0000000	0.1300000
12214.0000000	0.1250000
12215.0000000	0.1250000
12216.0000000	0.1150000
12217.0000000	0.1250000
12218.0000000	0.1250000
12219.0000000	0.1300000
12220.0000000	0.1300000
12221.0000000	0.1300000
12222.0000000	0.1300000
12223.0000000	0.1300000
12224.0000000	0.1300000
12225.0000000	0.1300000
12226.0000000	0.1400000
12227.0000000	0.1300000
12228.0000000	0.1350000
12229.0000000	0.1250000
12230.0000000	0.1400000
12231.0000000	0.1300000
12232.0000000	0.1400000
12233.0000000	0.1300000
12234.0000000	0.1333333
12235.0000000	0.1500000
12236.0000000	0.1400000
12237.0000000	0.1450000
12238.0000000	0.1300000
12239.0000000	0.1350000
12240.0000000	0.1400000
12241.0000000	0.1500000
12242.0000000	0.1450000
12243.0000000	0.1500000
12244.0000000	0.1400000
12245.0000000	0.1450000
12246.0000000	0.1400000
12247.0000000	0.1600000
12248.0000000	0.1500000
12249.0000000	0.1600000
12250.0000000	0.1450000
12251.0000000	0.1500000
12252.0000000	0.1400000
12253.0000000	0.1550000
12254.0000000	0.1550000

12255.0000000	0.1550000
12256.0000000	0.1500000
12257.0000000	0.1450000
12258.0000000	0.1500000
12259.0000000	0.1450000
12260.0000000	0.1600000
12261.0000000	0.1600000
12262.0000000	0.1550000
12263.0000000	0.1550000
12264.0000000	0.1566667
12265.0000000	0.1500000
12266.0000000	0.1550000
12267.0000000	0.1650000
12268.0000000	0.1650000
12269.0000000	0.1700000
12270.0000000	0.1550000
12271.0000000	0.1600000
12272.0000000	0.1550000
12273.0000000	0.1700000
12274.0000000	0.1650000
12275.0000000	0.1650000
12276.0000000	0.1550000
12277.0000000	0.1600000
12278.0000000	0.1600000
12279.0000000	0.1650000
12280.0000000	0.1700000
12281.0000000	0.1650000
12282.0000000	0.1650000
12283.0000000	0.1550000
12284.0000000	0.1650000
12285.0000000	0.1550000
12286.0000000	0.1700000
12287.0000000	0.1600000
12288.0000000	0.1600000
12289.0000000	0.1600000
12290.0000000	0.1650000
12291.0000000	0.1700000
12292.0000000	0.1600000
12293.0000000	0.1700000
12294.0000000	0.1666667
12295.0000000	0.1700000
12296.0000000	0.1700000
12297.0000000	0.1750000
12298.0000000	0.1650000
12299.0000000	0.1700000
12300.0000000	0.1600000
12301.0000000	0.1650000
12302.0000000	0.1650000
12303.0000000	0.1700000
12304.0000000	0.1650000
12305.0000000	0.1600000
12306.0000000	0.1600000
12307.0000000	0.1600000
12308.0000000	0.1650000
12309.0000000	0.1650000
12310.0000000	0.1750000
12311.0000000	0.1650000

12312.0000000	0.1700000
12313.0000000	0.1650000
12314.0000000	0.1700000
12315.0000000	0.1700000
12316.0000000	0.1700000
12317.0000000	0.1700000
12318.0000000	0.1700000
12319.0000000	0.1600000
12320.0000000	0.1700000
12321.0000000	0.1650000
12322.0000000	0.1800000
12323.0000000	0.1650000
12324.0000000	0.1633333
12325.0000000	0.1700000
12326.0000000	0.1750000
12327.0000000	0.1700000
12328.0000000	0.1800000
12329.0000000	0.1600000
12330.0000000	0.1750000
12331.0000000	0.1700000
12332.0000000	0.1700000
12333.0000000	0.1700000
12334.0000000	0.1650000
12335.0000000	0.1800000
12336.0000000	0.1800000
12337.0000000	0.1750000
12338.0000000	0.1750000
12339.0000000	0.1800000
12340.0000000	0.1750000
12341.0000000	0.1800000
12342.0000000	0.1750000
12343.0000000	0.1800000
12344.0000000	0.1750000
12345.0000000	0.1800000
12346.0000000	0.1800000
12347.0000000	0.1900000
12348.0000000	0.1800000
12349.0000000	0.1800000
12350.0000000	0.1850000
12351.0000000	0.1850000
12352.0000000	0.1800000
12353.0000000	0.1900000
12354.0000000	0.1850000
12355.0000000	0.1833333
12356.0000000	0.1800000
12357.0000000	0.1900000
12358.0000000	0.1800000
12359.0000000	0.1950000
12360.0000000	0.1750000
12361.0000000	0.1850000
12362.0000000	0.1900000
12363.0000000	0.1900000
12364.0000000	0.1950000
12365.0000000	0.1950000
12366.0000000	0.1950000
12367.0000000	0.1850000
12368.0000000	0.1850000

12369.0000000	0.1900000
12370.0000000	0.2000000
12371.0000000	0.1900000
12372.0000000	0.1850000
12373.0000000	0.1950000
12374.0000000	0.1850000
12375.0000000	0.1950000
12376.0000000	0.1850000
12377.0000000	0.1900000
12378.0000000	0.1950000
12379.0000000	0.1900000
12380.0000000	0.2050000
12381.0000000	0.1950000
12382.0000000	0.1850000
12383.0000000	0.1900000
12384.0000000	0.1966667
12385.0000000	0.1950000
12386.0000000	0.2000000
12387.0000000	0.1950000
12388.0000000	0.1950000
12389.0000000	0.1850000
12390.0000000	0.1950000
12391.0000000	0.1900000
12392.0000000	0.2000000
12393.0000000	0.2000000
12394.0000000	0.2000000
12395.0000000	0.2000000
12396.0000000	0.1850000
12397.0000000	0.1950000
12398.0000000	0.1950000
12399.0000000	0.2000000
12400.0000000	0.2000000
12401.0000000	0.1950000
12402.0000000	0.2000000
12403.0000000	0.2000000
12404.0000000	0.1950000
12405.0000000	0.2050000
12406.0000000	0.2050000
12407.0000000	0.2100000
12408.0000000	0.1950000
12409.0000000	0.1950000
12410.0000000	0.1950000
12411.0000000	0.2000000
12412.0000000	0.2150000
12413.0000000	0.1950000
12414.0000000	0.2050000
12415.0000000	0.2000000
12416.0000000	0.2050000
12417.0000000	0.2050000
12418.0000000	0.2050000
12419.0000000	0.2000000
12420.0000000	0.2000000
12421.0000000	0.2100000
12422.0000000	0.2100000
12423.0000000	0.2100000
12424.0000000	0.2000000
12425.0000000	0.2000000

12426.0000000	0.2050000
12427.0000000	0.2050000
12428.0000000	0.2050000
12429.0000000	0.2050000
12430.0000000	0.2050000
12431.0000000	0.2100000
12432.0000000	0.2050000
12433.0000000	0.2150000
12434.0000000	0.2050000
12435.0000000	0.2000000
12436.0000000	0.2100000
12437.0000000	0.2000000
12438.0000000	0.2050000
12439.0000000	0.2050000
12440.0000000	0.2000000
12441.0000000	0.2050000
12442.0000000	0.2100000
12443.0000000	0.2100000
12444.0000000	0.2000000
12445.0000000	0.2000000
12446.0000000	0.2050000
12447.0000000	0.1950000
12448.0000000	0.1900000
12449.0000000	0.2000000
12450.0000000	0.2050000
12451.0000000	0.2050000
12452.0000000	0.2000000
12453.0000000	0.2000000
12454.0000000	0.2100000
12455.0000000	0.2100000
12456.0000000	0.2050000
12457.0000000	0.2050000
12458.0000000	0.2050000
12459.0000000	0.2000000
12460.0000000	0.2100000
12461.0000000	0.2000000
12462.0000000	0.2050000
12463.0000000	0.2050000
12464.0000000	0.2100000
12465.0000000	0.2000000
12466.0000000	0.2050000
12467.0000000	0.2000000
12468.0000000	0.2050000
12469.0000000	0.2050000
12470.0000000	0.2150000
12471.0000000	0.2050000
12472.0000000	0.2050000
12473.0000000	0.2000000
12474.0000000	0.2050000
12475.0000000	0.2100000
12476.0000000	0.2000000
12477.0000000	0.2100000
12478.0000000	0.2050000
12479.0000000	0.2100000
12480.0000000	0.2000000
12481.0000000	0.2050000
12482.0000000	0.2150000

12483.0000000	0.2050000
12484.0000000	0.2050000
12485.0000000	0.2050000
12486.0000000	0.2100000
12487.0000000	0.2150000
12488.0000000	0.2150000
12489.0000000	0.2150000
12490.0000000	0.2050000
12491.0000000	0.2100000
12492.0000000	0.2100000
12493.0000000	0.2100000
12494.0000000	0.2100000
12495.0000000	0.2150000
12496.0000000	0.2100000
12497.0000000	0.2100000
12498.0000000	0.2050000
12499.0000000	0.2050000
12500.0000000	0.2050000
12501.0000000	0.2100000
12502.0000000	0.2150000
12503.0000000	0.2100000
12504.0000000	0.2100000
12505.0000000	0.2066667
12506.0000000	0.2100000
12507.0000000	0.2150000
12508.0000000	0.2000000
12509.0000000	0.2100000
12510.0000000	0.2000000
12511.0000000	0.2100000
12512.0000000	0.2150000
12513.0000000	0.2150000
12514.0000000	0.2050000
12515.0000000	0.2050000
12516.0000000	0.2050000
12517.0000000	0.2000000
12518.0000000	0.2150000
12519.0000000	0.2150000
12520.0000000	0.2100000
12521.0000000	0.2100000
12522.0000000	0.2050000
12523.0000000	0.2100000
12524.0000000	0.2200000
12525.0000000	0.2150000
12526.0000000	0.2100000
12527.0000000	0.2100000
12528.0000000	0.2000000
12529.0000000	0.2050000
12530.0000000	0.2050000
12531.0000000	0.2200000
12532.0000000	0.2150000
12533.0000000	0.2100000
12534.0000000	0.2150000
12535.0000000	0.2000000
12536.0000000	0.2100000
12537.0000000	0.2050000
12538.0000000	0.2150000
12539.0000000	0.2150000

12540.0000000	0.2150000
12541.0000000	0.2100000
12542.0000000	0.2050000
12543.0000000	0.2100000
12544.0000000	0.2050000
12545.0000000	0.2100000
12546.0000000	0.2050000
12547.0000000	0.2100000
12548.0000000	0.2150000
12549.0000000	0.2100000
12550.0000000	0.2100000
12551.0000000	0.2050000
12552.0000000	0.2150000
12553.0000000	0.2100000
12554.0000000	0.2100000
12555.0000000	0.2100000
12556.0000000	0.2050000
12557.0000000	0.2050000
12558.0000000	0.2000000
12559.0000000	0.2000000
12560.0000000	0.2200000
12561.0000000	0.2100000
12562.0000000	0.2000000
12563.0000000	0.2200000
12564.0000000	0.2050000
12565.0000000	0.2100000
12566.0000000	0.2033333
12567.0000000	0.2100000
12568.0000000	0.2100000
12569.0000000	0.2050000
12570.0000000	0.2100000
12571.0000000	0.2200000
12572.0000000	0.1950000
12573.0000000	0.2150000
12574.0000000	0.2050000
12575.0000000	0.2050000
12576.0000000	0.2150000
12577.0000000	0.2150000
12578.0000000	0.2100000
12579.0000000	0.2150000
12580.0000000	0.2050000
12581.0000000	0.2100000
12582.0000000	0.2150000
12583.0000000	0.2100000
12584.0000000	0.2050000
12585.0000000	0.2150000
12586.0000000	0.2200000
12587.0000000	0.2150000
12588.0000000	0.2150000
12589.0000000	0.2050000
12590.0000000	0.2150000
12591.0000000	0.2150000
12592.0000000	0.2200000
12593.0000000	0.2250000
12594.0000000	0.2100000
12595.0000000	0.2200000
12596.0000000	0.2233333

12597.0000000	0.2150000
12598.0000000	0.2150000
12599.0000000	0.2200000
12600.0000000	0.2250000
12601.0000000	0.2200000
12602.0000000	0.2200000
12603.0000000	0.2150000
12604.0000000	0.2250000
12605.0000000	0.2200000
12606.0000000	0.2200000
12607.0000000	0.2200000
12608.0000000	0.2100000
12609.0000000	0.2200000
12610.0000000	0.2150000
12611.0000000	0.2250000
12612.0000000	0.2150000
12613.0000000	0.2250000
12614.0000000	0.2100000
12615.0000000	0.2200000
12616.0000000	0.2100000
12617.0000000	0.2200000
12618.0000000	0.2150000
12619.0000000	0.2250000
12620.0000000	0.2150000
12621.0000000	0.2100000
12622.0000000	0.2150000
12623.0000000	0.2200000
12624.0000000	0.2150000
12625.0000000	0.2150000
12626.0000000	0.2133333
12627.0000000	0.2050000
12628.0000000	0.2150000
12629.0000000	0.2100000
12630.0000000	0.2150000
12631.0000000	0.2150000
12632.0000000	0.2150000
12633.0000000	0.2100000
12634.0000000	0.2150000
12635.0000000	0.2150000
12636.0000000	0.2050000
12637.0000000	0.2100000
12638.0000000	0.2000000
12639.0000000	0.2050000
12640.0000000	0.2050000
12641.0000000	0.2050000
12642.0000000	0.2100000
12643.0000000	0.2000000
12644.0000000	0.2000000
12645.0000000	0.2000000
12646.0000000	0.1950000
12647.0000000	0.2000000
12648.0000000	0.1950000
12649.0000000	0.2000000
12650.0000000	0.2100000
12651.0000000	0.2050000
12652.0000000	0.1950000
12653.0000000	0.1950000

12654.0000000	0.2000000
12655.0000000	0.1900000
12656.0000000	0.2050000
12657.0000000	0.2000000
12658.0000000	0.1950000
12659.0000000	0.1950000
12660.0000000	0.1950000
12661.0000000	0.1950000
12662.0000000	0.1900000
12663.0000000	0.2000000
12664.0000000	0.2000000
12665.0000000	0.1900000
12666.0000000	0.1950000
12667.0000000	0.2000000
12668.0000000	0.1950000
12669.0000000	0.1950000
12670.0000000	0.1950000
12671.0000000	0.1900000
12672.0000000	0.1900000
12673.0000000	0.1900000
12674.0000000	0.1850000
12675.0000000	0.1900000
12676.0000000	0.2000000
12677.0000000	0.1900000
12678.0000000	0.1900000
12679.0000000	0.2000000
12680.0000000	0.1850000
12681.0000000	0.1900000
12682.0000000	0.1950000
12683.0000000	0.1900000
12684.0000000	0.1950000
12685.0000000	0.2000000
12686.0000000	0.1966667
12687.0000000	0.1950000
12688.0000000	0.2000000
12689.0000000	0.1950000
12690.0000000	0.2000000
12691.0000000	0.2000000
12692.0000000	0.1950000
12693.0000000	0.1900000
12694.0000000	0.1950000
12695.0000000	0.1900000
12696.0000000	0.1950000
12697.0000000	0.2050000
12698.0000000	0.1950000
12699.0000000	0.2050000
12700.0000000	0.2000000
12701.0000000	0.2000000
12702.0000000	0.1950000
12703.0000000	0.1950000
12704.0000000	0.2050000
12705.0000000	0.2100000
12706.0000000	0.2100000
12707.0000000	0.2050000
12708.0000000	0.2050000
12709.0000000	0.1950000
12710.0000000	0.2100000

12711.0000000	0.2000000
12712.0000000	0.2150000
12713.0000000	0.2050000
12714.0000000	0.2100000
12715.0000000	0.2000000
12716.0000000	0.2033333
12717.0000000	0.2050000
12718.0000000	0.1950000
12719.0000000	0.2000000
12720.0000000	0.1950000
12721.0000000	0.2050000
12722.0000000	0.2100000
12723.0000000	0.2000000
12724.0000000	0.2000000
12725.0000000	0.1950000
12726.0000000	0.2050000
12727.0000000	0.2050000
12728.0000000	0.2050000
12729.0000000	0.2000000
12730.0000000	0.2050000
12731.0000000	0.1900000
12732.0000000	0.2000000
12733.0000000	0.2100000
12734.0000000	0.2000000
12735.0000000	0.2100000
12736.0000000	0.2050000
12737.0000000	0.2050000
12738.0000000	0.2050000
12739.0000000	0.2050000
12740.0000000	0.2000000
12741.0000000	0.1900000
12742.0000000	0.2050000
12743.0000000	0.2100000
12744.0000000	0.2100000
12745.0000000	0.2150000
12746.0000000	0.2000000
12747.0000000	0.2033333
12748.0000000	0.2050000
12749.0000000	0.1950000
12750.0000000	0.2100000
12751.0000000	0.2150000
12752.0000000	0.2150000
12753.0000000	0.2000000
12754.0000000	0.2150000
12755.0000000	0.2100000
12756.0000000	0.2000000
12757.0000000	0.2050000
12758.0000000	0.2100000
12759.0000000	0.2000000
12760.0000000	0.2200000
12761.0000000	0.2150000
12762.0000000	0.2000000
12763.0000000	0.2200000
12764.0000000	0.2050000
12765.0000000	0.2100000
12766.0000000	0.2100000
12767.0000000	0.2000000

12768.0000000	0.2050000
12769.0000000	0.2150000
12770.0000000	0.2100000
12771.0000000	0.2100000
12772.0000000	0.2050000
12773.0000000	0.2100000
12774.0000000	0.2050000
12775.0000000	0.2100000
12776.0000000	0.2100000
12777.0000000	0.2100000
12778.0000000	0.2000000
12779.0000000	0.2050000
12780.0000000	0.2000000
12781.0000000	0.2000000
12782.0000000	0.2000000
12783.0000000	0.2150000
12784.0000000	0.2100000
12785.0000000	0.2100000
12786.0000000	0.1950000
12787.0000000	0.2100000
12788.0000000	0.2050000
12789.0000000	0.2100000
12790.0000000	0.2000000
12791.0000000	0.2050000
12792.0000000	0.2000000
12793.0000000	0.2000000
12794.0000000	0.2050000
12795.0000000	0.2050000
12796.0000000	0.2050000
12797.0000000	0.1950000
12798.0000000	0.2100000
12799.0000000	0.2050000
12800.0000000	0.2050000
12801.0000000	0.1950000
12802.0000000	0.2050000
12803.0000000	0.2000000
12804.0000000	0.2050000
12805.0000000	0.2000000
12806.0000000	0.2000000
12807.0000000	0.1933333
12808.0000000	0.2000000
12809.0000000	0.2000000
12810.0000000	0.2050000
12811.0000000	0.2000000
12812.0000000	0.2000000
12813.0000000	0.2000000
12814.0000000	0.1950000
12815.0000000	0.2000000
12816.0000000	0.1950000
12817.0000000	0.1950000
12818.0000000	0.2000000
12819.0000000	0.2000000
12820.0000000	0.1900000
12821.0000000	0.2000000
12822.0000000	0.1900000
12823.0000000	0.1950000
12824.0000000	0.1950000

12825.0000000	0.1950000
12826.0000000	0.2100000
12827.0000000	0.1900000
12828.0000000	0.1950000
12829.0000000	0.1900000
12830.0000000	0.2050000
12831.0000000	0.1950000
12832.0000000	0.2050000
12833.0000000	0.1900000
12834.0000000	0.2000000
12835.0000000	0.1900000
12836.0000000	0.2050000
12837.0000000	0.2000000
12838.0000000	0.2050000
12839.0000000	0.1950000
12840.0000000	0.1950000
12841.0000000	0.2000000
12842.0000000	0.2000000
12843.0000000	0.2050000
12844.0000000	0.1950000
12845.0000000	0.2000000
12846.0000000	0.1850000
12847.0000000	0.2050000
12848.0000000	0.1950000
12849.0000000	0.1900000
12850.0000000	0.1950000
12851.0000000	0.1950000
12852.0000000	0.1950000
12853.0000000	0.1950000
12854.0000000	0.2000000
12855.0000000	0.2000000
12856.0000000	0.1950000
12857.0000000	0.2100000
12858.0000000	0.2000000
12859.0000000	0.1900000
12860.0000000	0.1950000
12861.0000000	0.2000000
12862.0000000	0.1950000
12863.0000000	0.2000000
12864.0000000	0.1950000
12865.0000000	0.1950000
12866.0000000	0.1850000
12867.0000000	0.1950000
12868.0000000	0.2033333
12869.0000000	0.2000000
12870.0000000	0.1900000
12871.0000000	0.1950000
12872.0000000	0.1900000
12873.0000000	0.1950000
12874.0000000	0.2000000
12875.0000000	0.1950000
12876.0000000	0.2000000
12877.0000000	0.2050000
12878.0000000	0.2050000
12879.0000000	0.2000000
12880.0000000	0.1900000
12881.0000000	0.1950000

12882.0000000	0.1950000
12883.0000000	0.1900000
12884.0000000	0.1950000
12885.0000000	0.1950000
12886.0000000	0.1900000
12887.0000000	0.1900000
12888.0000000	0.1950000
12889.0000000	0.1900000
12890.0000000	0.1950000
12891.0000000	0.1950000
12892.0000000	0.1900000
12893.0000000	0.1900000
12894.0000000	0.2000000
12895.0000000	0.1850000
12896.0000000	0.1900000
12897.0000000	0.2050000
12898.0000000	0.1900000
12899.0000000	0.1850000
12900.0000000	0.1900000
12901.0000000	0.1950000
12902.0000000	0.1900000
12903.0000000	0.1950000
12904.0000000	0.1850000
12905.0000000	0.1850000
12906.0000000	0.1850000
12907.0000000	0.1850000
12908.0000000	0.1950000
12909.0000000	0.1950000
12910.0000000	0.1900000
12911.0000000	0.1800000
12912.0000000	0.1900000
12913.0000000	0.1900000
12914.0000000	0.1850000
12915.0000000	0.1900000
12916.0000000	0.1950000
12917.0000000	0.1950000
12918.0000000	0.1850000
12919.0000000	0.1950000
12920.0000000	0.1900000
12921.0000000	0.1900000
12922.0000000	0.1900000
12923.0000000	0.2000000
12924.0000000	0.1950000
12925.0000000	0.1950000
12926.0000000	0.1850000
12927.0000000	0.1900000
12928.0000000	0.1966667
12929.0000000	0.1900000
12930.0000000	0.1950000
12931.0000000	0.1900000
12932.0000000	0.1900000
12933.0000000	0.1950000
12934.0000000	0.2000000
12935.0000000	0.1900000
12936.0000000	0.1950000
12937.0000000	0.1800000
12938.0000000	0.1900000

12939.0000000	0.1850000
12940.0000000	0.1950000
12941.0000000	0.1850000
12942.0000000	0.1850000
12943.0000000	0.1750000
12944.0000000	0.1800000
12945.0000000	0.1800000
12946.0000000	0.1900000
12947.0000000	0.1850000
12948.0000000	0.1800000
12949.0000000	0.1700000
12950.0000000	0.1800000
12951.0000000	0.1850000
12952.0000000	0.1700000
12953.0000000	0.1700000
12954.0000000	0.1700000
12955.0000000	0.1750000
12956.0000000	0.1850000
12957.0000000	0.1800000
12958.0000000	0.1700000
12959.0000000	0.1750000
12960.0000000	0.1750000
12961.0000000	0.1750000
12962.0000000	0.1700000
12963.0000000	0.1700000
12964.0000000	0.1750000
12965.0000000	0.1800000
12966.0000000	0.1750000
12967.0000000	0.1750000
12968.0000000	0.1700000
12969.0000000	0.1600000
12970.0000000	0.1700000
12971.0000000	0.1700000
12972.0000000	0.1900000
12973.0000000	0.1700000
12974.0000000	0.1850000
12975.0000000	0.1650000
12976.0000000	0.1750000
12977.0000000	0.1700000
12978.0000000	0.1850000
12979.0000000	0.1700000
12980.0000000	0.1750000
12981.0000000	0.1700000
12982.0000000	0.1800000
12983.0000000	0.1700000
12984.0000000	0.1800000
12985.0000000	0.1850000
12986.0000000	0.1800000
12987.0000000	0.1800000
12988.0000000	0.1750000
12989.0000000	0.1833333
12990.0000000	0.1750000
12991.0000000	0.1700000
12992.0000000	0.1800000
12993.0000000	0.1750000
12994.0000000	0.1900000
12995.0000000	0.1900000

12996.0000000	0.1900000
12997.0000000	0.1850000
12998.0000000	0.1850000
12999.0000000	0.1850000
13000.0000000	0.1850000
13001.0000000	0.1900000
13002.0000000	0.1850000
13003.0000000	0.1850000
13004.0000000	0.1800000
13005.0000000	0.1850000
13006.0000000	0.1900000
13007.0000000	0.1950000
13008.0000000	0.1900000
13009.0000000	0.1900000
13010.0000000	0.1800000
13011.0000000	0.1900000
13012.0000000	0.1950000
13013.0000000	0.1950000
13014.0000000	0.1950000
13015.0000000	0.1900000
13016.0000000	0.1900000
13017.0000000	0.1900000
13018.0000000	0.1933333
13019.0000000	0.1950000
13020.0000000	0.1900000
13021.0000000	0.1900000
13022.0000000	0.1900000
13023.0000000	0.2000000
13024.0000000	0.2000000
13025.0000000	0.2000000
13026.0000000	0.1900000
13027.0000000	0.1950000
13028.0000000	0.2000000
13029.0000000	0.2000000
13030.0000000	0.1950000
13031.0000000	0.1950000
13032.0000000	0.2000000
13033.0000000	0.1900000
13034.0000000	0.1900000
13035.0000000	0.1950000
13036.0000000	0.2050000
13037.0000000	0.1950000
13038.0000000	0.1950000
13039.0000000	0.1950000
13040.0000000	0.2050000
13041.0000000	0.2000000
13042.0000000	0.1950000
13043.0000000	0.2050000
13044.0000000	0.1900000
13045.0000000	0.2000000
13046.0000000	0.1850000
13047.0000000	0.1950000
13048.0000000	0.1866667
13049.0000000	0.2100000
13050.0000000	0.2000000
13051.0000000	0.1950000
13052.0000000	0.1900000

13053.0000000	0.1950000
13054.0000000	0.1950000
13055.0000000	0.2000000
13056.0000000	0.2050000
13057.0000000	0.1900000
13058.0000000	0.2000000
13059.0000000	0.1950000
13060.0000000	0.1900000
13061.0000000	0.2000000
13062.0000000	0.1950000
13063.0000000	0.1950000
13064.0000000	0.1950000
13065.0000000	0.1950000
13066.0000000	0.2050000
13067.0000000	0.1950000
13068.0000000	0.1900000
13069.0000000	0.2000000
13070.0000000	0.1900000
13071.0000000	0.1950000
13072.0000000	0.2000000
13073.0000000	0.2000000
13074.0000000	0.2000000
13075.0000000	0.1950000
13076.0000000	0.1950000
13077.0000000	0.1900000
13078.0000000	0.1950000
13079.0000000	0.2000000
13080.0000000	0.1850000
13081.0000000	0.2000000
13082.0000000	0.1900000
13083.0000000	0.1950000
13084.0000000	0.1900000
13085.0000000	0.1900000
13086.0000000	0.1900000
13087.0000000	0.1900000
13088.0000000	0.2000000
13089.0000000	0.1850000
13090.0000000	0.2000000
13091.0000000	0.1800000
13092.0000000	0.1900000
13093.0000000	0.1850000
13094.0000000	0.2050000
13095.0000000	0.1850000
13096.0000000	0.2000000
13097.0000000	0.1850000
13098.0000000	0.2000000
13099.0000000	0.1900000
13100.0000000	0.1900000
13101.0000000	0.2000000
13102.0000000	0.1850000
13103.0000000	0.1950000
13104.0000000	0.1850000
13105.0000000	0.1900000
13106.0000000	0.2000000
13107.0000000	0.1900000
13108.0000000	0.1900000
13109.0000000	0.1900000

13110.0000000	0.1850000
13111.0000000	0.1950000
13112.0000000	0.1900000
13113.0000000	0.2000000
13114.0000000	0.1900000
13115.0000000	0.1950000
13116.0000000	0.1900000
13117.0000000	0.1850000
13118.0000000	0.1850000
13119.0000000	0.1850000
13120.0000000	0.1950000
13121.0000000	0.1900000
13122.0000000	0.2000000
13123.0000000	0.1800000
13124.0000000	0.1900000
13125.0000000	0.1900000
13126.0000000	0.1950000
13127.0000000	0.1950000
13128.0000000	0.1900000
13129.0000000	0.1850000
13130.0000000	0.1900000
13131.0000000	0.1950000
13132.0000000	0.1950000
13133.0000000	0.2000000
13134.0000000	0.1850000
13135.0000000	0.1850000
13136.0000000	0.1900000
13137.0000000	0.1900000
13138.0000000	0.1900000
13139.0000000	0.1866667
13140.0000000	0.1850000
13141.0000000	0.1900000
13142.0000000	0.1800000
13143.0000000	0.1800000
13144.0000000	0.1700000
13145.0000000	0.1800000
13146.0000000	0.1800000
13147.0000000	0.1700000
13148.0000000	0.1800000
13149.0000000	0.1650000
13150.0000000	0.1700000
13151.0000000	0.1700000
13152.0000000	0.1750000
13153.0000000	0.1650000
13154.0000000	0.1600000
13155.0000000	0.1600000
13156.0000000	0.1700000
13157.0000000	0.1700000
13158.0000000	0.1750000
13159.0000000	0.1700000
13160.0000000	0.1700000
13161.0000000	0.1550000
13162.0000000	0.1650000
13163.0000000	0.1650000
13164.0000000	0.1700000
13165.0000000	0.1600000
13166.0000000	0.1600000

13167.0000000	0.1700000
13168.0000000	0.1650000
13169.0000000	0.1666667
13170.0000000	0.1600000
13171.0000000	0.1600000
13172.0000000	0.1700000
13173.0000000	0.1550000
13174.0000000	0.1750000
13175.0000000	0.1650000
13176.0000000	0.1700000
13177.0000000	0.1650000
13178.0000000	0.1600000
13179.0000000	0.1700000
13180.0000000	0.1650000
13181.0000000	0.1700000
13182.0000000	0.1650000
13183.0000000	0.1600000
13184.0000000	0.1600000
13185.0000000	0.1600000
13186.0000000	0.1600000
13187.0000000	0.1600000
13188.0000000	0.1600000
13189.0000000	0.1700000
13190.0000000	0.1650000
13191.0000000	0.1650000
13192.0000000	0.1600000
13193.0000000	0.1650000
13194.0000000	0.1700000
13195.0000000	0.1650000
13196.0000000	0.1750000
13197.0000000	0.1650000
13198.0000000	0.1750000
13199.0000000	0.1550000
13200.0000000	0.1600000
13201.0000000	0.1650000
13202.0000000	0.1600000
13203.0000000	0.1700000
13204.0000000	0.1600000
13205.0000000	0.1650000
13206.0000000	0.1600000
13207.0000000	0.1650000
13208.0000000	0.1650000
13209.0000000	0.1600000
13210.0000000	0.1650000
13211.0000000	0.1650000
13212.0000000	0.1600000
13213.0000000	0.1600000
13214.0000000	0.1700000
13215.0000000	0.1650000
13216.0000000	0.1600000
13217.0000000	0.1650000
13218.0000000	0.1600000
13219.0000000	0.1750000
13220.0000000	0.1650000
13221.0000000	0.1600000
13222.0000000	0.1700000
13223.0000000	0.1650000

13224.0000000	0.1700000
13225.0000000	0.1650000
13226.0000000	0.1700000
13227.0000000	0.1750000
13228.0000000	0.1700000
13229.0000000	0.1650000
13230.0000000	0.1733333
13231.0000000	0.1650000
13232.0000000	0.1550000
13233.0000000	0.1750000
13234.0000000	0.1650000
13235.0000000	0.1750000
13236.0000000	0.1650000
13237.0000000	0.1650000
13238.0000000	0.1600000
13239.0000000	0.1650000
13240.0000000	0.1600000
13241.0000000	0.1650000
13242.0000000	0.1600000
13243.0000000	0.1600000
13244.0000000	0.1600000
13245.0000000	0.1600000
13246.0000000	0.1650000
13247.0000000	0.1650000
13248.0000000	0.1700000
13249.0000000	0.1600000
13250.0000000	0.1650000
13251.0000000	0.1550000
13252.0000000	0.1750000
13253.0000000	0.1650000
13254.0000000	0.1650000
13255.0000000	0.1600000
13256.0000000	0.1550000
13257.0000000	0.1600000
13258.0000000	0.1650000
13259.0000000	0.1700000
13260.0000000	0.1633333
13261.0000000	0.1700000
13262.0000000	0.1650000
13263.0000000	0.1650000
13264.0000000	0.1600000
13265.0000000	0.1600000
13266.0000000	0.1750000
13267.0000000	0.1600000
13268.0000000	0.1650000
13269.0000000	0.1600000
13270.0000000	0.1650000
13271.0000000	0.1650000
13272.0000000	0.1600000
13273.0000000	0.1550000
13274.0000000	0.1600000
13275.0000000	0.1700000
13276.0000000	0.1650000
13277.0000000	0.1650000
13278.0000000	0.1650000
13279.0000000	0.1550000
13280.0000000	0.1700000

13281.0000000	0.1650000
13282.0000000	0.1600000
13283.0000000	0.1600000
13284.0000000	0.1600000
13285.0000000	0.1600000
13286.0000000	0.1750000
13287.0000000	0.1550000
13288.0000000	0.1650000
13289.0000000	0.1600000
13290.0000000	0.1633333
13291.0000000	0.1550000
13292.0000000	0.1700000
13293.0000000	0.1700000
13294.0000000	0.1600000
13295.0000000	0.1550000
13296.0000000	0.1600000
13297.0000000	0.1650000
13298.0000000	0.1750000
13299.0000000	0.1650000
13300.0000000	0.1700000
13301.0000000	0.1650000
13302.0000000	0.1600000
13303.0000000	0.1650000
13304.0000000	0.1800000
13305.0000000	0.1750000
13306.0000000	0.1650000
13307.0000000	0.1700000
13308.0000000	0.1700000
13309.0000000	0.1700000
13310.0000000	0.1700000
13311.0000000	0.1750000
13312.0000000	0.1700000
13313.0000000	0.1700000
13314.0000000	0.1750000
13315.0000000	0.1650000
13316.0000000	0.1800000
13317.0000000	0.1600000
13318.0000000	0.1750000
13319.0000000	0.1700000
13320.0000000	0.1750000
13321.0000000	0.1600000
13322.0000000	0.1600000
13323.0000000	0.1600000
13324.0000000	0.1550000
13325.0000000	0.1650000
13326.0000000	0.1600000
13327.0000000	0.1700000
13328.0000000	0.1600000
13329.0000000	0.1600000
13330.0000000	0.1500000
13331.0000000	0.1600000
13332.0000000	0.1550000
13333.0000000	0.1650000
13334.0000000	0.1600000
13335.0000000	0.1650000
13336.0000000	0.1500000
13337.0000000	0.1500000

13338.0000000	0.1550000
13339.0000000	0.1500000
13340.0000000	0.1550000
13341.0000000	0.1500000
13342.0000000	0.1500000
13343.0000000	0.1450000
13344.0000000	0.1550000
13345.0000000	0.1500000
13346.0000000	0.1550000
13347.0000000	0.1500000
13348.0000000	0.1500000
13349.0000000	0.1450000
13350.0000000	0.1533333
13351.0000000	0.1450000
13352.0000000	0.1450000
13353.0000000	0.1500000
13354.0000000	0.1550000
13355.0000000	0.1500000
13356.0000000	0.1450000
13357.0000000	0.1500000
13358.0000000	0.1500000
13359.0000000	0.1550000
13360.0000000	0.1400000
13361.0000000	0.1500000
13362.0000000	0.1400000
13363.0000000	0.1450000
13364.0000000	0.1400000
13365.0000000	0.1550000
13366.0000000	0.1500000
13367.0000000	0.1500000
13368.0000000	0.1500000
13369.0000000	0.1400000
13370.0000000	0.1550000
13371.0000000	0.1550000
13372.0000000	0.1500000
13373.0000000	0.1500000
13374.0000000	0.1500000
13375.0000000	0.1500000
13376.0000000	0.1550000
13377.0000000	0.1450000
13378.0000000	0.1500000
13379.0000000	0.1400000
13380.0000000	0.1600000
13381.0000000	0.1450000
13382.0000000	0.1550000
13383.0000000	0.1500000
13384.0000000	0.1550000
13385.0000000	0.1500000
13386.0000000	0.1550000
13387.0000000	0.1500000
13388.0000000	0.1500000
13389.0000000	0.1500000
13390.0000000	0.1350000
13391.0000000	0.1500000
13392.0000000	0.1500000
13393.0000000	0.1500000
13394.0000000	0.1600000

13395.0000000	0.1450000
13396.0000000	0.1500000
13397.0000000	0.1400000
13398.0000000	0.1500000
13399.0000000	0.1500000
13400.0000000	0.1450000
13401.0000000	0.1550000
13402.0000000	0.1400000
13403.0000000	0.1450000
13404.0000000	0.1450000
13405.0000000	0.1500000
13406.0000000	0.1500000
13407.0000000	0.1500000
13408.0000000	0.1400000
13409.0000000	0.1600000
13410.0000000	0.1500000
13411.0000000	0.1500000
13412.0000000	0.1450000
13413.0000000	0.1450000
13414.0000000	0.1500000
13415.0000000	0.1500000
13416.0000000	0.1550000
13417.0000000	0.1500000
13418.0000000	0.1500000
13419.0000000	0.1500000
13420.0000000	0.1450000
13421.0000000	0.1550000
13422.0000000	0.1550000
13423.0000000	0.1600000
13424.0000000	0.1500000
13425.0000000	0.1500000
13426.0000000	0.1500000
13427.0000000	0.1500000
13428.0000000	0.1500000
13429.0000000	0.1600000
13430.0000000	0.1550000
13431.0000000	0.1400000
13432.0000000	0.1550000
13433.0000000	0.1450000
13434.0000000	0.1550000
13435.0000000	0.1600000
13436.0000000	0.1450000
13437.0000000	0.1550000
13438.0000000	0.1400000
13439.0000000	0.1500000
13440.0000000	0.1550000
13441.0000000	0.1533333
13442.0000000	0.1550000
13443.0000000	0.1450000
13444.0000000	0.1500000
13445.0000000	0.1550000
13446.0000000	0.1500000
13447.0000000	0.1500000
13448.0000000	0.1500000
13449.0000000	0.1500000
13450.0000000	0.1600000
13451.0000000	0.1500000

13452.0000000	0.1450000
13453.0000000	0.1550000
13454.0000000	0.1550000
13455.0000000	0.1500000
13456.0000000	0.1550000
13457.0000000	0.1600000
13458.0000000	0.1550000
13459.0000000	0.1450000
13460.0000000	0.1550000
13461.0000000	0.1500000
13462.0000000	0.1550000
13463.0000000	0.1500000
13464.0000000	0.1500000
13465.0000000	0.1600000
13466.0000000	0.1550000
13467.0000000	0.1550000
13468.0000000	0.1550000
13469.0000000	0.1500000
13470.0000000	0.1500000
13471.0000000	0.1633333
13472.0000000	0.1450000
13473.0000000	0.1500000
13474.0000000	0.1450000
13475.0000000	0.1600000
13476.0000000	0.1550000
13477.0000000	0.1550000
13478.0000000	0.1500000
13479.0000000	0.1600000
13480.0000000	0.1500000
13481.0000000	0.1550000
13482.0000000	0.1450000
13483.0000000	0.1600000
13484.0000000	0.1550000
13485.0000000	0.1500000
13486.0000000	0.1450000
13487.0000000	0.1500000
13488.0000000	0.1550000
13489.0000000	0.1650000
13490.0000000	0.1600000
13491.0000000	0.1500000
13492.0000000	0.1600000
13493.0000000	0.1550000
13494.0000000	0.1550000
13495.0000000	0.1600000
13496.0000000	0.1550000
13497.0000000	0.1500000
13498.0000000	0.1550000
13499.0000000	0.1550000
13500.0000000	0.1550000
13501.0000000	0.1666667
13502.0000000	0.1500000
13503.0000000	0.1550000
13504.0000000	0.1550000
13505.0000000	0.1600000
13506.0000000	0.1650000
13507.0000000	0.1600000
13508.0000000	0.1550000

13509.0000000	0.1500000
13510.0000000	0.1600000
13511.0000000	0.1550000
13512.0000000	0.1550000
13513.0000000	0.1500000
13514.0000000	0.1500000
13515.0000000	0.1500000
13516.0000000	0.1500000
13517.0000000	0.1500000
13518.0000000	0.1400000
13519.0000000	0.1450000
13520.0000000	0.1600000
13521.0000000	0.1500000
13522.0000000	0.1600000
13523.0000000	0.1450000
13524.0000000	0.1450000
13525.0000000	0.1400000
13526.0000000	0.1500000
13527.0000000	0.1500000
13528.0000000	0.1450000
13529.0000000	0.1450000
13530.0000000	0.1500000
13531.0000000	0.1500000
13532.0000000	0.1500000
13533.0000000	0.1500000
13534.0000000	0.1450000
13535.0000000	0.1450000
13536.0000000	0.1500000
13537.0000000	0.1450000
13538.0000000	0.1500000
13539.0000000	0.1450000
13540.0000000	0.1500000
13541.0000000	0.1450000
13542.0000000	0.1350000
13543.0000000	0.1500000
13544.0000000	0.1350000
13545.0000000	0.1500000
13546.0000000	0.1300000
13547.0000000	0.1500000
13548.0000000	0.1400000
13549.0000000	0.1350000
13550.0000000	0.1350000
13551.0000000	0.1450000
13552.0000000	0.1400000
13553.0000000	0.1450000
13554.0000000	0.1400000
13555.0000000	0.1400000
13556.0000000	0.1350000
13557.0000000	0.1500000
13558.0000000	0.1450000
13559.0000000	0.1500000
13560.0000000	0.1400000
13561.0000000	0.1500000
13562.0000000	0.1400000
13563.0000000	0.1450000
13564.0000000	0.1400000
13565.0000000	0.1500000

13566.0000000	0.1550000
13567.0000000	0.1500000
13568.0000000	0.1450000
13569.0000000	0.1450000
13570.0000000	0.1500000
13571.0000000	0.1500000
13572.0000000	0.1550000
13573.0000000	0.1450000
13574.0000000	0.1450000
13575.0000000	0.1450000
13576.0000000	0.1500000
13577.0000000	0.1450000
13578.0000000	0.1450000
13579.0000000	0.1500000
13580.0000000	0.1450000
13581.0000000	0.1600000
13582.0000000	0.1500000
13583.0000000	0.1550000
13584.0000000	0.1450000
13585.0000000	0.1450000
13586.0000000	0.1500000
13587.0000000	0.1500000
13588.0000000	0.1500000
13589.0000000	0.1500000
13590.0000000	0.1400000
13591.0000000	0.1500000
13592.0000000	0.1533334
13593.0000000	0.1500000
13594.0000000	0.1550000
13595.0000000	0.1600000
13596.0000000	0.1550000
13597.0000000	0.1600000
13598.0000000	0.1500000
13599.0000000	0.1600000
13600.0000000	0.1550000
13601.0000000	0.1600000
13602.0000000	0.1600000
13603.0000000	0.1550000
13604.0000000	0.1550000
13605.0000000	0.1650000
13606.0000000	0.1550000
13607.0000000	0.1700000
13608.0000000	0.1500000
13609.0000000	0.1550000
13610.0000000	0.1550000
13611.0000000	0.1550000
13612.0000000	0.1600000
13613.0000000	0.1700000
13614.0000000	0.1650000
13615.0000000	0.1650000
13616.0000000	0.1600000
13617.0000000	0.1650000
13618.0000000	0.1650000
13619.0000000	0.1600000
13620.0000000	0.1650000
13621.0000000	0.1700000
13622.0000000	0.1633333

13623.0000000	0.1700000
13624.0000000	0.1700000
13625.0000000	0.1650000
13626.0000000	0.1650000
13627.0000000	0.1650000
13628.0000000	0.1750000
13629.0000000	0.1750000
13630.0000000	0.1700000
13631.0000000	0.1750000
13632.0000000	0.1650000
13633.0000000	0.1700000
13634.0000000	0.1700000
13635.0000000	0.1750000
13636.0000000	0.1750000
13637.0000000	0.1850000
13638.0000000	0.1750000
13639.0000000	0.1700000
13640.0000000	0.1750000
13641.0000000	0.1850000
13642.0000000	0.1800000
13643.0000000	0.1850000
13644.0000000	0.1750000
13645.0000000	0.1800000
13646.0000000	0.1800000
13647.0000000	0.1900000
13648.0000000	0.1900000
13649.0000000	0.1850000
13650.0000000	0.1800000
13651.0000000	0.1800000
13652.0000000	0.1850000
13653.0000000	0.1900000
13654.0000000	0.1850000
13655.0000000	0.1900000
13656.0000000	0.1850000
13657.0000000	0.1900000
13658.0000000	0.1850000
13659.0000000	0.1900000
13660.0000000	0.1900000
13661.0000000	0.1850000
13662.0000000	0.1900000
13663.0000000	0.1850000
13664.0000000	0.1900000
13665.0000000	0.1850000
13666.0000000	0.1900000
13667.0000000	0.1900000
13668.0000000	0.1900000
13669.0000000	0.1900000
13670.0000000	0.1750000
13671.0000000	0.1900000
13672.0000000	0.1850000
13673.0000000	0.1850000
13674.0000000	0.1850000
13675.0000000	0.1750000
13676.0000000	0.1900000
13677.0000000	0.1850000
13678.0000000	0.1800000
13679.0000000	0.1900000

13680.0000000	0.1800000
13681.0000000	0.1850000
13682.0000000	0.1750000
13683.0000000	0.1866667
13684.0000000	0.1750000
13685.0000000	0.1800000
13686.0000000	0.1850000
13687.0000000	0.1800000
13688.0000000	0.1850000
13689.0000000	0.1800000
13690.0000000	0.1750000
13691.0000000	0.1800000
13692.0000000	0.1900000
13693.0000000	0.1850000
13694.0000000	0.1900000
13695.0000000	0.1850000
13696.0000000	0.1850000
13697.0000000	0.1750000
13698.0000000	0.1900000
13699.0000000	0.1800000
13700.0000000	0.1900000
13701.0000000	0.1850000
13702.0000000	0.1800000
13703.0000000	0.1800000
13704.0000000	0.1950000
13705.0000000	0.1850000
13706.0000000	0.1900000
13707.0000000	0.1800000
13708.0000000	0.1800000
13709.0000000	0.1800000
13710.0000000	0.1950000
13711.0000000	0.1800000
13712.0000000	0.1966667
13713.0000000	0.1900000
13714.0000000	0.1950000
13715.0000000	0.1950000
13716.0000000	0.1900000
13717.0000000	0.1950000
13718.0000000	0.1900000
13719.0000000	0.1900000
13720.0000000	0.1900000
13721.0000000	0.2000000
13722.0000000	0.1950000
13723.0000000	0.1900000
13724.0000000	0.1900000
13725.0000000	0.1800000
13726.0000000	0.1950000
13727.0000000	0.1950000
13728.0000000	0.2050000
13729.0000000	0.1950000
13730.0000000	0.1900000
13731.0000000	0.1950000
13732.0000000	0.1950000
13733.0000000	0.2050000
13734.0000000	0.2050000
13735.0000000	0.2050000
13736.0000000	0.2000000

13737.0000000	0.2000000
13738.0000000	0.2000000
13739.0000000	0.2050000
13740.0000000	0.1950000
13741.0000000	0.2100000
13742.0000000	0.1950000
13743.0000000	0.2033333
13744.0000000	0.2100000
13745.0000000	0.2000000
13746.0000000	0.2100000
13747.0000000	0.2000000
13748.0000000	0.2100000
13749.0000000	0.2050000
13750.0000000	0.2100000
13751.0000000	0.2050000
13752.0000000	0.2050000
13753.0000000	0.2250000
13754.0000000	0.2100000
13755.0000000	0.2100000
13756.0000000	0.2050000
13757.0000000	0.2050000
13758.0000000	0.2150000
13759.0000000	0.2150000
13760.0000000	0.2150000
13761.0000000	0.2100000
13762.0000000	0.2100000
13763.0000000	0.2100000
13764.0000000	0.2150000
13765.0000000	0.2150000
13766.0000000	0.2150000
13767.0000000	0.2150000
13768.0000000	0.2100000
13769.0000000	0.2100000
13770.0000000	0.2150000
13771.0000000	0.2150000
13772.0000000	0.2200000
13773.0000000	0.2166667
13774.0000000	0.2200000
13775.0000000	0.2200000
13776.0000000	0.2200000
13777.0000000	0.2200000
13778.0000000	0.2200000
13779.0000000	0.2200000
13780.0000000	0.2250000
13781.0000000	0.2250000
13782.0000000	0.2300000
13783.0000000	0.2300000
13784.0000000	0.2200000
13785.0000000	0.2300000
13786.0000000	0.2250000
13787.0000000	0.2250000
13788.0000000	0.2350000
13789.0000000	0.2300000
13790.0000000	0.2350000
13791.0000000	0.2350000
13792.0000000	0.2300000
13793.0000000	0.2350000

13794.0000000	0.2300000
13795.0000000	0.2300000
13796.0000000	0.2350000
13797.0000000	0.2350000
13798.0000000	0.2400000
13799.0000000	0.2350000
13800.0000000	0.2350000
13801.0000000	0.2400000
13802.0000000	0.2350000
13803.0000000	0.2400000
13804.0000000	0.2300000
13805.0000000	0.2250000
13806.0000000	0.2350000
13807.0000000	0.2350000
13808.0000000	0.2400000
13809.0000000	0.2400000
13810.0000000	0.2400000
13811.0000000	0.2400000
13812.0000000	0.2450000
13813.0000000	0.2350000
13814.0000000	0.2350000
13815.0000000	0.2350000
13816.0000000	0.2400000
13817.0000000	0.2450000
13818.0000000	0.2350000
13819.0000000	0.2450000
13820.0000000	0.2350000
13821.0000000	0.2350000
13822.0000000	0.2300000
13823.0000000	0.2500000
13824.0000000	0.2300000
13825.0000000	0.2400000
13826.0000000	0.2350000
13827.0000000	0.2250000
13828.0000000	0.2350000
13829.0000000	0.2350000
13830.0000000	0.2300000
13831.0000000	0.2350000
13832.0000000	0.2250000
13833.0000000	0.2366667
13834.0000000	0.2250000
13835.0000000	0.2350000
13836.0000000	0.2400000
13837.0000000	0.2400000
13838.0000000	0.2450000
13839.0000000	0.2300000
13840.0000000	0.2300000
13841.0000000	0.2400000
13842.0000000	0.2400000
13843.0000000	0.2350000
13844.0000000	0.2400000
13845.0000000	0.2350000
13846.0000000	0.2450000
13847.0000000	0.2400000
13848.0000000	0.2350000
13849.0000000	0.2300000
13850.0000000	0.2450000

13851.0000000	0.2400000
13852.0000000	0.2400000
13853.0000000	0.2450000
13854.0000000	0.2500000
13855.0000000	0.2450000
13856.0000000	0.2400000
13857.0000000	0.2500000
13858.0000000	0.2400000
13859.0000000	0.2350000
13860.0000000	0.2500000
13861.0000000	0.2450000
13862.0000000	0.2300000
13863.0000000	0.2500000
13864.0000000	0.2500000
13865.0000000	0.2500000
13866.0000000	0.2550000
13867.0000000	0.2500000
13868.0000000	0.2350000
13869.0000000	0.2450000
13870.0000000	0.2550000
13871.0000000	0.2450000
13872.0000000	0.2400000
13873.0000000	0.2450000
13874.0000000	0.2500000
13875.0000000	0.2450000
13876.0000000	0.2500000
13877.0000000	0.2450000
13878.0000000	0.2450000
13879.0000000	0.2500000
13880.0000000	0.2500000
13881.0000000	0.2400000
13882.0000000	0.2450000
13883.0000000	0.2400000
13884.0000000	0.2550000
13885.0000000	0.2500000
13886.0000000	0.2450000
13887.0000000	0.2450000
13888.0000000	0.2450000
13889.0000000	0.2550000
13890.0000000	0.2600000
13891.0000000	0.2550000
13892.0000000	0.2450000
13893.0000000	0.2450000
13894.0000000	0.2533333
13895.0000000	0.2550000
13896.0000000	0.2550000
13897.0000000	0.2500000
13898.0000000	0.2550000
13899.0000000	0.2550000
13900.0000000	0.2500000
13901.0000000	0.2550000
13902.0000000	0.2550000
13903.0000000	0.2550000
13904.0000000	0.2550000
13905.0000000	0.2550000
13906.0000000	0.2600000
13907.0000000	0.2600000

13908.0000000	0.2600000
13909.0000000	0.2500000
13910.0000000	0.2600000
13911.0000000	0.2550000
13912.0000000	0.2550000
13913.0000000	0.2650000
13914.0000000	0.2500000
13915.0000000	0.2550000
13916.0000000	0.2550000
13917.0000000	0.2600000
13918.0000000	0.2600000
13919.0000000	0.2500000
13920.0000000	0.2600000
13921.0000000	0.2600000
13922.0000000	0.2600000
13923.0000000	0.2550000
13924.0000000	0.2566667
13925.0000000	0.2500000
13926.0000000	0.2650000
13927.0000000	0.2700000
13928.0000000	0.2600000
13929.0000000	0.2600000
13930.0000000	0.2550000
13931.0000000	0.2600000
13932.0000000	0.2600000
13933.0000000	0.2650000
13934.0000000	0.2650000
13935.0000000	0.2600000
13936.0000000	0.2550000
13937.0000000	0.2550000
13938.0000000	0.2600000
13939.0000000	0.2550000
13940.0000000	0.2500000
13941.0000000	0.2600000
13942.0000000	0.2650000
13943.0000000	0.2600000
13944.0000000	0.2600000
13945.0000000	0.2600000
13946.0000000	0.2600000
13947.0000000	0.2550000
13948.0000000	0.2550000
13949.0000000	0.2550000
13950.0000000	0.2750000
13951.0000000	0.2600000
13952.0000000	0.2650000
13953.0000000	0.2550000
13954.0000000	0.2666667
13955.0000000	0.2650000
13956.0000000	0.2650000
13957.0000000	0.2700000
13958.0000000	0.2700000
13959.0000000	0.2700000
13960.0000000	0.2700000
13961.0000000	0.2650000
13962.0000000	0.2700000
13963.0000000	0.2700000
13964.0000000	0.2750000

13965.0000000	0.2700000
13966.0000000	0.2650000
13967.0000000	0.2650000
13968.0000000	0.2600000
13969.0000000	0.2750000
13970.0000000	0.2700000
13971.0000000	0.2800000
13972.0000000	0.2650000
13973.0000000	0.2700000
13974.0000000	0.2650000
13975.0000000	0.2750000
13976.0000000	0.2800000
13977.0000000	0.2750000
13978.0000000	0.2750000
13979.0000000	0.2650000
13980.0000000	0.2750000
13981.0000000	0.2750000
13982.0000000	0.2750000
13983.0000000	0.2700000
13984.0000000	0.2650000
13985.0000000	0.2800000
13986.0000000	0.2700000
13987.0000000	0.2750000
13988.0000000	0.2700000
13989.0000000	0.2850000
13990.0000000	0.2800000
13991.0000000	0.2850000
13992.0000000	0.2750000
13993.0000000	0.2750000
13994.0000000	0.2700000
13995.0000000	0.2850000
13996.0000000	0.2800000
13997.0000000	0.2850000
13998.0000000	0.2700000
13999.0000000	0.2800000
14000.0000000	0.2750000
14001.0000000	0.2900000
14002.0000000	0.2850000
14003.0000000	0.2950000
14004.0000000	0.2800000
14005.0000000	0.2800000
14006.0000000	0.2800000
14007.0000000	0.2800000
14008.0000000	0.2800000
14009.0000000	0.2900000
14010.0000000	0.2800000
14011.0000000	0.2800000
14012.0000000	0.2850000
14013.0000000	0.2850000
14014.0000000	0.2850000
14015.0000000	0.2833333
14016.0000000	0.2850000
14017.0000000	0.2900000
14018.0000000	0.2950000
14019.0000000	0.2900000
14020.0000000	0.2900000
14021.0000000	0.2850000

14022.0000000	0.2800000
14023.0000000	0.2900000
14024.0000000	0.3000000
14025.0000000	0.2950000
14026.0000000	0.2900000
14027.0000000	0.2900000
14028.0000000	0.2900000
14029.0000000	0.2900000
14030.0000000	0.2950000
14031.0000000	0.3000000
14032.0000000	0.3000000
14033.0000000	0.2900000
14034.0000000	0.2950000
14035.0000000	0.2900000
14036.0000000	0.3000000
14037.0000000	0.2950000
14038.0000000	0.3000000
14039.0000000	0.2900000
14040.0000000	0.3000000
14041.0000000	0.2900000
14042.0000000	0.3050000
14043.0000000	0.3000000
14044.0000000	0.2966667
14045.0000000	0.2950000
14046.0000000	0.3000000
14047.0000000	0.3000000
14048.0000000	0.3000000
14049.0000000	0.3000000
14050.0000000	0.3050000
14051.0000000	0.2900000
14052.0000000	0.3050000
14053.0000000	0.2950000
14054.0000000	0.3000000
14055.0000000	0.2900000
14056.0000000	0.3100000
14057.0000000	0.2950000
14058.0000000	0.3000000
14059.0000000	0.2900000
14060.0000000	0.3050000
14061.0000000	0.2950000
14062.0000000	0.3050000
14063.0000000	0.2950000
14064.0000000	0.3050000
14065.0000000	0.3050000
14066.0000000	0.3100000
14067.0000000	0.2950000
14068.0000000	0.3000000
14069.0000000	0.3050000
14070.0000000	0.3200000
14071.0000000	0.3000000
14072.0000000	0.3100000
14073.0000000	0.2950000
14074.0000000	0.3050000
14075.0000000	0.3100000
14076.0000000	0.3050000
14077.0000000	0.3050000
14078.0000000	0.3100000

14079.0000000	0.3100000
14080.0000000	0.3200000
14081.0000000	0.3050000
14082.0000000	0.3050000
14083.0000000	0.3150000
14084.0000000	0.3050000
14085.0000000	0.3200000
14086.0000000	0.3100000
14087.0000000	0.3150000
14088.0000000	0.3100000
14089.0000000	0.3150000
14090.0000000	0.3200000
14091.0000000	0.3250000
14092.0000000	0.3200000
14093.0000000	0.3150000
14094.0000000	0.3150000
14095.0000000	0.3000000
14096.0000000	0.3200000
14097.0000000	0.3250000
14098.0000000	0.3250000
14099.0000000	0.3200000
14100.0000000	0.3200000
14101.0000000	0.3250000
14102.0000000	0.3200000
14103.0000000	0.3250000
14104.0000000	0.3250000
14105.0000000	0.3250000
14106.0000000	0.3233334
14107.0000000	0.3200000
14108.0000000	0.3300000
14109.0000000	0.3200000
14110.0000000	0.3250000
14111.0000000	0.3350000
14112.0000000	0.3300000
14113.0000000	0.3300000
14114.0000000	0.3300000
14115.0000000	0.3300000
14116.0000000	0.3350000
14117.0000000	0.3400000
14118.0000000	0.3350000
14119.0000000	0.3300000
14120.0000000	0.3200000
14121.0000000	0.3300000
14122.0000000	0.3400000
14123.0000000	0.3300000
14124.0000000	0.3350000
14125.0000000	0.3350000
14126.0000000	0.3400000
14127.0000000	0.3400000
14128.0000000	0.3350000
14129.0000000	0.3300000
14130.0000000	0.3300000
14131.0000000	0.3350000
14132.0000000	0.3350000
14133.0000000	0.3300000
14134.0000000	0.3400000
14135.0000000	0.3433333

14136.0000000	0.3350000
14137.0000000	0.3300000
14138.0000000	0.3350000
14139.0000000	0.3350000
14140.0000000	0.3400000
14141.0000000	0.3400000
14142.0000000	0.3450000
14143.0000000	0.3300000
14144.0000000	0.3400000
14145.0000000	0.3350000
14146.0000000	0.3400000
14147.0000000	0.3350000
14148.0000000	0.3400000
14149.0000000	0.3350000
14150.0000000	0.3350000
14151.0000000	0.3400000
14152.0000000	0.3350000
14153.0000000	0.3350000
14154.0000000	0.3350000
14155.0000000	0.3350000
14156.0000000	0.3300000
14157.0000000	0.3500000
14158.0000000	0.3350000
14159.0000000	0.3450000
14160.0000000	0.3350000
14161.0000000	0.3500000
14162.0000000	0.3300000
14163.0000000	0.3450000
14164.0000000	0.3350000
14165.0000000	0.3400000
14166.0000000	0.3450000
14167.0000000	0.3450000
14168.0000000	0.3400000
14169.0000000	0.3450000
14170.0000000	0.3500000
14171.0000000	0.3500000
14172.0000000	0.3550000
14173.0000000	0.3500000
14174.0000000	0.3450000
14175.0000000	0.3500000
14176.0000000	0.3450000
14177.0000000	0.3550000
14178.0000000	0.3550000
14179.0000000	0.3500000
14180.0000000	0.3500000
14181.0000000	0.3450000
14182.0000000	0.3500000
14183.0000000	0.3500000
14184.0000000	0.3550000
14185.0000000	0.3500000
14186.0000000	0.3500000
14187.0000000	0.3500000
14188.0000000	0.3500000
14189.0000000	0.3550000
14190.0000000	0.3600000
14191.0000000	0.3550000
14192.0000000	0.3550000

14193.0000000	0.3500000
14194.0000000	0.3500000
14195.0000000	0.3450000
14196.0000000	0.3533334
14197.0000000	0.3600000
14198.0000000	0.3500000
14199.0000000	0.3550000
14200.0000000	0.3500000
14201.0000000	0.3550000
14202.0000000	0.3550000
14203.0000000	0.3550000
14204.0000000	0.3650000
14205.0000000	0.3600000
14206.0000000	0.3550000
14207.0000000	0.3500000
14208.0000000	0.3600000
14209.0000000	0.3500000
14210.0000000	0.3700000
14211.0000000	0.3650000
14212.0000000	0.3600000
14213.0000000	0.3500000
14214.0000000	0.3650000
14215.0000000	0.3600000
14216.0000000	0.3650000
14217.0000000	0.3550000
14218.0000000	0.3550000
14219.0000000	0.3600000
14220.0000000	0.3550000
14221.0000000	0.3650000
14222.0000000	0.3600000
14223.0000000	0.3600000
14224.0000000	0.3500000
14225.0000000	0.3550000
14226.0000000	0.3600000
14227.0000000	0.3550000
14228.0000000	0.3650000
14229.0000000	0.3700000
14230.0000000	0.3700000
14231.0000000	0.3600000
14232.0000000	0.3600000
14233.0000000	0.3650000
14234.0000000	0.3600000
14235.0000000	0.3750000
14236.0000000	0.3600000
14237.0000000	0.3650000
14238.0000000	0.3650000
14239.0000000	0.3650000
14240.0000000	0.3700000
14241.0000000	0.3650000
14242.0000000	0.3650000
14243.0000000	0.3700000
14244.0000000	0.3650000
14245.0000000	0.3850000
14246.0000000	0.3700000
14247.0000000	0.3650000
14248.0000000	0.3750000
14249.0000000	0.3700000

14250.0000000	0.3800000
14251.0000000	0.3800000
14252.0000000	0.3700000
14253.0000000	0.3750000
14254.0000000	0.3650000
14255.0000000	0.3700000
14256.0000000	0.3700000
14257.0000000	0.3650000
14258.0000000	0.3700000
14259.0000000	0.3700000
14260.0000000	0.3800000
14261.0000000	0.3650000
14262.0000000	0.3700000
14263.0000000	0.3650000
14264.0000000	0.3700000
14265.0000000	0.3650000
14266.0000000	0.3650000
14267.0000000	0.3700000
14268.0000000	0.3650000
14269.0000000	0.3750000
14270.0000000	0.3700000
14271.0000000	0.3700000
14272.0000000	0.3750000
14273.0000000	0.3650000
14274.0000000	0.3700000
14275.0000000	0.3650000
14276.0000000	0.3750000
14277.0000000	0.3700000
14278.0000000	0.3750000
14279.0000000	0.3600000
14280.0000000	0.3750000
14281.0000000	0.3600000
14282.0000000	0.3750000
14283.0000000	0.3750000
14284.0000000	0.3700000
14285.0000000	0.3750000
14286.0000000	0.3733333
14287.0000000	0.3750000
14288.0000000	0.3750000
14289.0000000	0.3750000
14290.0000000	0.3750000
14291.0000000	0.3750000
14292.0000000	0.3750000
14293.0000000	0.3650000
14294.0000000	0.3650000
14295.0000000	0.3700000
14296.0000000	0.3700000
14297.0000000	0.3800000
14298.0000000	0.3700000
14299.0000000	0.3700000
14300.0000000	0.3700000
14301.0000000	0.3700000
14302.0000000	0.3700000
14303.0000000	0.3750000
14304.0000000	0.3750000
14305.0000000	0.3800000
14306.0000000	0.3750000

14307.0000000	0.3750000
14308.0000000	0.3700000
14309.0000000	0.3700000
14310.0000000	0.3800000
14311.0000000	0.3700000
14312.0000000	0.3900000
14313.0000000	0.3750000
14314.0000000	0.3800000
14315.0000000	0.3800000
14316.0000000	0.3800000
14317.0000000	0.3866667
14318.0000000	0.3750000
14319.0000000	0.3800000
14320.0000000	0.3750000
14321.0000000	0.3850000
14322.0000000	0.3900000
14323.0000000	0.3900000
14324.0000000	0.3800000
14325.0000000	0.3750000
14326.0000000	0.3850000
14327.0000000	0.3850000
14328.0000000	0.3850000
14329.0000000	0.3850000
14330.0000000	0.3900000
14331.0000000	0.3800000
14332.0000000	0.3850000
14333.0000000	0.3900000
14334.0000000	0.3800000
14335.0000000	0.3850000
14336.0000000	0.3850000
14337.0000000	0.3850000
14338.0000000	0.3850000
14339.0000000	0.3850000
14340.0000000	0.3900000
14341.0000000	0.3800000
14342.0000000	0.3850000
14343.0000000	0.3750000
14344.0000000	0.3900000
14345.0000000	0.3800000
14346.0000000	0.3800000
14347.0000000	0.3866667
14348.0000000	0.3850000
14349.0000000	0.3900000
14350.0000000	0.3800000
14351.0000000	0.3800000
14352.0000000	0.3850000
14353.0000000	0.3800000
14354.0000000	0.3850000
14355.0000000	0.3850000
14356.0000000	0.3750000
14357.0000000	0.3750000
14358.0000000	0.3800000
14359.0000000	0.3800000
14360.0000000	0.3950000
14361.0000000	0.3700000
14362.0000000	0.3750000
14363.0000000	0.3850000

14364.0000000	0.3750000
14365.0000000	0.3800000
14366.0000000	0.3750000
14367.0000000	0.3750000
14368.0000000	0.3750000
14369.0000000	0.3750000
14370.0000000	0.3800000
14371.0000000	0.3700000
14372.0000000	0.3600000
14373.0000000	0.3650000
14374.0000000	0.3700000
14375.0000000	0.3750000
14376.0000000	0.3666667
14377.0000000	0.3700000
14378.0000000	0.3700000
14379.0000000	0.3650000
14380.0000000	0.3650000
14381.0000000	0.3550000
14382.0000000	0.3700000
14383.0000000	0.3600000
14384.0000000	0.3650000
14385.0000000	0.3600000
14386.0000000	0.3500000
14387.0000000	0.3650000
14388.0000000	0.3600000
14389.0000000	0.3550000
14390.0000000	0.3650000
14391.0000000	0.3600000
14392.0000000	0.3700000
14393.0000000	0.3600000
14394.0000000	0.3600000
14395.0000000	0.3450000
14396.0000000	0.3650000
14397.0000000	0.3600000
14398.0000000	0.3600000
14399.0000000	0.3600000
14400.0000000	0.3550000
14401.0000000	0.3550000
14402.0000000	0.3550000
14403.0000000	0.3550000
14404.0000000	0.3600000
14405.0000000	0.3550000
14406.0000000	0.3550000
14407.0000000	0.3433334
14408.0000000	0.3550000
14409.0000000	0.3500000
14410.0000000	0.3500000
14411.0000000	0.3400000
14412.0000000	0.3500000
14413.0000000	0.3450000
14414.0000000	0.3500000
14415.0000000	0.3450000
14416.0000000	0.3450000
14417.0000000	0.3350000
14418.0000000	0.3450000
14419.0000000	0.3450000
14420.0000000	0.3500000

14421.0000000	0.3550000
14422.0000000	0.3450000
14423.0000000	0.3500000
14424.0000000	0.3400000
14425.0000000	0.3450000
14426.0000000	0.3500000
14427.0000000	0.3450000
14428.0000000	0.3500000
14429.0000000	0.3500000
14430.0000000	0.3450000
14431.0000000	0.3550000
14432.0000000	0.3400000
14433.0000000	0.3400000
14434.0000000	0.3500000
14435.0000000	0.3400000
14436.0000000	0.3450000
14437.0000000	0.3400000
14438.0000000	0.3366667
14439.0000000	0.3300000
14440.0000000	0.3500000
14441.0000000	0.3350000
14442.0000000	0.3400000
14443.0000000	0.3400000
14444.0000000	0.3350000
14445.0000000	0.3300000
14446.0000000	0.3300000
14447.0000000	0.3300000
14448.0000000	0.3300000
14449.0000000	0.3350000
14450.0000000	0.3350000
14451.0000000	0.3200000
14452.0000000	0.3250000
14453.0000000	0.3200000
14454.0000000	0.3250000
14455.0000000	0.3300000
14456.0000000	0.3300000
14457.0000000	0.3300000
14458.0000000	0.3200000
14459.0000000	0.3200000
14460.0000000	0.3150000
14461.0000000	0.3300000
14462.0000000	0.3250000
14463.0000000	0.3300000
14464.0000000	0.3250000
14465.0000000	0.3250000
14466.0000000	0.3200000
14467.0000000	0.3300000
14468.0000000	0.3200000
14469.0000000	0.3150000
14470.0000000	0.3250000
14471.0000000	0.3250000
14472.0000000	0.3300000
14473.0000000	0.3150000
14474.0000000	0.3200000
14475.0000000	0.3150000
14476.0000000	0.3200000
14477.0000000	0.3200000

14478.0000000	0.3250000
14479.0000000	0.3250000
14480.0000000	0.3100000
14481.0000000	0.3150000
14482.0000000	0.3100000
14483.0000000	0.3300000
14484.0000000	0.3200000
14485.0000000	0.3250000
14486.0000000	0.3200000
14487.0000000	0.3200000
14488.0000000	0.3150000
14489.0000000	0.3250000
14490.0000000	0.3200000
14491.0000000	0.3250000
14492.0000000	0.3100000
14493.0000000	0.3200000
14494.0000000	0.3200000
14495.0000000	0.3200000
14496.0000000	0.3200000
14497.0000000	0.3166667
14498.0000000	0.3300000
14499.0000000	0.3050000
14500.0000000	0.3100000
14501.0000000	0.3150000
14502.0000000	0.3200000
14503.0000000	0.3250000
14504.0000000	0.3100000
14505.0000000	0.3250000
14506.0000000	0.3150000
14507.0000000	0.3200000
14508.0000000	0.3150000
14509.0000000	0.3100000
14510.0000000	0.3150000
14511.0000000	0.3200000
14512.0000000	0.3150000
14513.0000000	0.3150000
14514.0000000	0.3100000
14515.0000000	0.3100000
14516.0000000	0.3100000
14517.0000000	0.3100000
14518.0000000	0.3150000
14519.0000000	0.3100000
14520.0000000	0.3100000
14521.0000000	0.3100000
14522.0000000	0.3150000
14523.0000000	0.3100000
14524.0000000	0.3150000
14525.0000000	0.3100000
14526.0000000	0.3150000
14527.0000000	0.3050000
14528.0000000	0.3133333
14529.0000000	0.3150000
14530.0000000	0.3100000
14531.0000000	0.3000000
14532.0000000	0.3100000
14533.0000000	0.3100000
14534.0000000	0.3150000

14535.0000000	0.3150000
14536.0000000	0.3150000
14537.0000000	0.3050000
14538.0000000	0.3100000
14539.0000000	0.3050000
14540.0000000	0.3150000
14541.0000000	0.3050000
14542.0000000	0.3000000
14543.0000000	0.3150000
14544.0000000	0.3100000
14545.0000000	0.3150000
14546.0000000	0.3100000
14547.0000000	0.3050000
14548.0000000	0.3200000
14549.0000000	0.3100000
14550.0000000	0.3200000
14551.0000000	0.3100000
14552.0000000	0.3200000
14553.0000000	0.3150000
14554.0000000	0.3150000
14555.0000000	0.3100000
14556.0000000	0.3250000
14557.0000000	0.3150000
14558.0000000	0.3066666
14559.0000000	0.3200000
14560.0000000	0.3150000
14561.0000000	0.3200000
14562.0000000	0.3050000
14563.0000000	0.3050000
14564.0000000	0.3050000
14565.0000000	0.3150000
14566.0000000	0.3100000
14567.0000000	0.3000000
14568.0000000	0.3150000
14569.0000000	0.3000000
14570.0000000	0.3100000
14571.0000000	0.3100000
14572.0000000	0.2950000
14573.0000000	0.2950000
14574.0000000	0.3100000
14575.0000000	0.3050000
14576.0000000	0.3100000
14577.0000000	0.3000000
14578.0000000	0.2950000
14579.0000000	0.3100000
14580.0000000	0.3050000
14581.0000000	0.2950000
14582.0000000	0.3050000
14583.0000000	0.3000000
14584.0000000	0.3050000
14585.0000000	0.3050000
14586.0000000	0.3050000
14587.0000000	0.3000000
14588.0000000	0.3033333
14589.0000000	0.2950000
14590.0000000	0.3100000
14591.0000000	0.3050000

14592.0000000	0.3100000
14593.0000000	0.2950000
14594.0000000	0.2950000
14595.0000000	0.3050000
14596.0000000	0.3050000
14597.0000000	0.3150000
14598.0000000	0.3050000
14599.0000000	0.3050000
14600.0000000	0.3000000
14601.0000000	0.3150000
14602.0000000	0.2950000
14603.0000000	0.3050000
14604.0000000	0.2950000
14605.0000000	0.3050000
14606.0000000	0.3100000
14607.0000000	0.3150000
14608.0000000	0.3100000
14609.0000000	0.3050000
14610.0000000	0.3050000
14611.0000000	0.3100000
14612.0000000	0.3100000
14613.0000000	0.3200000
14614.0000000	0.3050000
14615.0000000	0.3100000
14616.0000000	0.3100000
14617.0000000	0.3150000
14618.0000000	0.3100000
14619.0000000	0.3150000
14620.0000000	0.3150000
14621.0000000	0.3200000
14622.0000000	0.3100000
14623.0000000	0.3100000
14624.0000000	0.3150000
14625.0000000	0.3150000
14626.0000000	0.3200000
14627.0000000	0.3250000
14628.0000000	0.3150000
14629.0000000	0.3200000
14630.0000000	0.3250000
14631.0000000	0.3250000
14632.0000000	0.3200000
14633.0000000	0.3150000
14634.0000000	0.3300000
14635.0000000	0.3200000
14636.0000000	0.3250000
14637.0000000	0.3200000
14638.0000000	0.3200000
14639.0000000	0.3300000
14640.0000000	0.3250000
14641.0000000	0.3200000
14642.0000000	0.3200000
14643.0000000	0.3250000
14644.0000000	0.3150000
14645.0000000	0.3300000
14646.0000000	0.3150000
14647.0000000	0.3250000
14648.0000000	0.3050000

14649.0000000	0.3133333
14650.0000000	0.3200000
14651.0000000	0.3100000
14652.0000000	0.3100000
14653.0000000	0.3050000
14654.0000000	0.3150000
14655.0000000	0.3100000
14656.0000000	0.3200000
14657.0000000	0.3100000
14658.0000000	0.3100000
14659.0000000	0.3000000
14660.0000000	0.3200000
14661.0000000	0.3050000
14662.0000000	0.3200000
14663.0000000	0.3000000
14664.0000000	0.3050000
14665.0000000	0.3050000
14666.0000000	0.3100000
14667.0000000	0.3100000
14668.0000000	0.3100000
14669.0000000	0.3050000
14670.0000000	0.3000000
14671.0000000	0.3000000
14672.0000000	0.3050000
14673.0000000	0.3050000
14674.0000000	0.3000000
14675.0000000	0.3000000
14676.0000000	0.3000000
14677.0000000	0.3000000
14678.0000000	0.3000000
14679.0000000	0.3066667
14680.0000000	0.3000000
14681.0000000	0.3000000
14682.0000000	0.3000000
14683.0000000	0.3050000
14684.0000000	0.2950000
14685.0000000	0.3050000
14686.0000000	0.3000000
14687.0000000	0.3000000
14688.0000000	0.3050000
14689.0000000	0.2950000
14690.0000000	0.2900000
14691.0000000	0.3000000
14692.0000000	0.3050000
14693.0000000	0.3000000
14694.0000000	0.3050000
14695.0000000	0.3000000
14696.0000000	0.3000000
14697.0000000	0.3000000
14698.0000000	0.3150000
14699.0000000	0.3000000
14700.0000000	0.3050000
14701.0000000	0.3050000
14702.0000000	0.3050000
14703.0000000	0.3050000
14704.0000000	0.3050000
14705.0000000	0.3150000

14706.0000000	0.3100000
14707.0000000	0.3100000
14708.0000000	0.3033333
14709.0000000	0.3050000
14710.0000000	0.3050000
14711.0000000	0.2950000
14712.0000000	0.3000000
14713.0000000	0.3000000
14714.0000000	0.3050000
14715.0000000	0.3000000
14716.0000000	0.3150000
14717.0000000	0.3000000
14718.0000000	0.2950000
14719.0000000	0.2900000
14720.0000000	0.3000000
14721.0000000	0.3000000
14722.0000000	0.2950000
14723.0000000	0.2900000
14724.0000000	0.2850000
14725.0000000	0.2900000
14726.0000000	0.2900000
14727.0000000	0.2900000
14728.0000000	0.2950000
14729.0000000	0.2900000
14730.0000000	0.2900000
14731.0000000	0.2900000
14732.0000000	0.2950000
14733.0000000	0.2850000
14734.0000000	0.2950000
14735.0000000	0.2950000
14736.0000000	0.2950000
14737.0000000	0.3000000
14738.0000000	0.2950000
14739.0000000	0.2933334
14740.0000000	0.3000000
14741.0000000	0.3000000
14742.0000000	0.2950000
14743.0000000	0.3050000
14744.0000000	0.3200000
14745.0000000	0.3000000
14746.0000000	0.3000000
14747.0000000	0.3000000
14748.0000000	0.2900000
14749.0000000	0.3150000
14750.0000000	0.3100000
14751.0000000	0.3000000
14752.0000000	0.3050000
14753.0000000	0.3050000
14754.0000000	0.3000000
14755.0000000	0.3050000
14756.0000000	0.2950000
14757.0000000	0.3100000
14758.0000000	0.3150000
14759.0000000	0.3150000
14760.0000000	0.3100000
14761.0000000	0.3100000
14762.0000000	0.3100000

14763.0000000	0.3050000
14764.0000000	0.3200000
14765.0000000	0.3000000
14766.0000000	0.3050000
14767.0000000	0.3150000
14768.0000000	0.3150000
14769.0000000	0.3100000
14770.0000000	0.3150000
14771.0000000	0.3050000
14772.0000000	0.3150000
14773.0000000	0.3050000
14774.0000000	0.3100000
14775.0000000	0.3100000
14776.0000000	0.3300000
14777.0000000	0.3150000
14778.0000000	0.3150000
14779.0000000	0.3050000
14780.0000000	0.3250000
14781.0000000	0.3100000
14782.0000000	0.3200000
14783.0000000	0.3100000
14784.0000000	0.3050000
14785.0000000	0.2950000
14786.0000000	0.3100000
14787.0000000	0.3050000
14788.0000000	0.3200000
14789.0000000	0.2950000
14790.0000000	0.3050000
14791.0000000	0.2950000
14792.0000000	0.3000000
14793.0000000	0.3050000
14794.0000000	0.3100000
14795.0000000	0.3000000
14796.0000000	0.3100000
14797.0000000	0.2950000
14798.0000000	0.3050000
14799.0000000	0.3100000
14800.0000000	0.3000000
14801.0000000	0.3050000
14802.0000000	0.2950000
14803.0000000	0.3150000
14804.0000000	0.3100000
14805.0000000	0.3150000
14806.0000000	0.3100000
14807.0000000	0.3000000
14808.0000000	0.3050000
14809.0000000	0.3100000
14810.0000000	0.3150000
14811.0000000	0.3250000
14812.0000000	0.3100000
14813.0000000	0.3050000
14814.0000000	0.3000000
14815.0000000	0.3000000
14816.0000000	0.3150000
14817.0000000	0.3100000
14818.0000000	0.3100000
14819.0000000	0.2950000

14820.0000000	0.3050000
14821.0000000	0.3050000
14822.0000000	0.3100000
14823.0000000	0.3100000
14824.0000000	0.3050000
14825.0000000	0.3000000
14826.0000000	0.3050000
14827.0000000	0.2950000
14828.0000000	0.3100000
14829.0000000	0.3033333
14830.0000000	0.2950000
14831.0000000	0.2950000
14832.0000000	0.3000000
14833.0000000	0.3000000
14834.0000000	0.2950000
14835.0000000	0.2950000
14836.0000000	0.2900000
14837.0000000	0.2950000
14838.0000000	0.3000000
14839.0000000	0.3000000
14840.0000000	0.2900000
14841.0000000	0.3000000
14842.0000000	0.2900000
14843.0000000	0.2900000
14844.0000000	0.2950000
14845.0000000	0.2900000
14846.0000000	0.2950000
14847.0000000	0.2950000
14848.0000000	0.2900000
14849.0000000	0.2900000
14850.0000000	0.2850000
14851.0000000	0.2900000
14852.0000000	0.2950000
14853.0000000	0.2950000
14854.0000000	0.2950000
14855.0000000	0.2950000
14856.0000000	0.3000000
14857.0000000	0.2900000
14858.0000000	0.2950000
14859.0000000	0.3000000
14860.0000000	0.3033333
14861.0000000	0.2950000
14862.0000000	0.2950000
14863.0000000	0.3000000
14864.0000000	0.3050000
14865.0000000	0.2950000
14866.0000000	0.3050000
14867.0000000	0.3000000
14868.0000000	0.3000000
14869.0000000	0.3050000
14870.0000000	0.2950000
14871.0000000	0.2950000
14872.0000000	0.3050000
14873.0000000	0.2950000
14874.0000000	0.3050000
14875.0000000	0.3050000
14876.0000000	0.3050000

14877.0000000	0.3000000
14878.0000000	0.2950000
14879.0000000	0.3050000
14880.0000000	0.3050000
14881.0000000	0.3000000
14882.0000000	0.3000000
14883.0000000	0.3000000
14884.0000000	0.3050000
14885.0000000	0.3000000
14886.0000000	0.2950000
14887.0000000	0.3000000
14888.0000000	0.3100000
14889.0000000	0.3000000
14890.0000000	0.2966667
14891.0000000	0.3050000
14892.0000000	0.3000000
14893.0000000	0.3050000
14894.0000000	0.3050000
14895.0000000	0.3000000
14896.0000000	0.3000000
14897.0000000	0.3100000
14898.0000000	0.3100000
14899.0000000	0.3050000
14900.0000000	0.3000000
14901.0000000	0.3100000
14902.0000000	0.3050000
14903.0000000	0.3050000
14904.0000000	0.3050000
14905.0000000	0.2950000
14906.0000000	0.3000000
14907.0000000	0.2950000
14908.0000000	0.3100000
14909.0000000	0.3050000
14910.0000000	0.3150000
14911.0000000	0.3000000
14912.0000000	0.3100000
14913.0000000	0.3050000
14914.0000000	0.3150000
14915.0000000	0.3050000
14916.0000000	0.3150000
14917.0000000	0.3050000
14918.0000000	0.3050000
14919.0000000	0.3050000
14920.0000000	0.3066667
14921.0000000	0.3050000
14922.0000000	0.3050000
14923.0000000	0.3150000
14924.0000000	0.3050000
14925.0000000	0.3000000
14926.0000000	0.2950000
14927.0000000	0.3000000
14928.0000000	0.3050000
14929.0000000	0.2900000
14930.0000000	0.3050000
14931.0000000	0.2950000
14932.0000000	0.2950000
14933.0000000	0.3000000

14934.0000000	0.2900000
14935.0000000	0.2950000
14936.0000000	0.3000000
14937.0000000	0.2950000
14938.0000000	0.3000000
14939.0000000	0.2950000
14940.0000000	0.2900000
14941.0000000	0.2900000
14942.0000000	0.3000000
14943.0000000	0.3000000
14944.0000000	0.3050000
14945.0000000	0.3000000
14946.0000000	0.2900000
14947.0000000	0.2950000
14948.0000000	0.2950000
14949.0000000	0.3100000
14950.0000000	0.2866667
14951.0000000	0.3100000
14952.0000000	0.2950000
14953.0000000	0.3000000
14954.0000000	0.2950000
14955.0000000	0.3000000
14956.0000000	0.3000000
14957.0000000	0.3000000
14958.0000000	0.2950000
14959.0000000	0.2900000
14960.0000000	0.3050000
14961.0000000	0.3000000
14962.0000000	0.2900000
14963.0000000	0.2800000
14964.0000000	0.2900000
14965.0000000	0.3000000
14966.0000000	0.2800000
14967.0000000	0.2900000
14968.0000000	0.2900000
14969.0000000	0.2950000
14970.0000000	0.2850000
14971.0000000	0.2900000
14972.0000000	0.2800000
14973.0000000	0.2750000
14974.0000000	0.2950000
14975.0000000	0.2800000
14976.0000000	0.2750000
14977.0000000	0.2900000
14978.0000000	0.2900000
14979.0000000	0.2850000
14980.0000000	0.2800000
14981.0000000	0.2866667
14982.0000000	0.2800000
14983.0000000	0.2800000
14984.0000000	0.2800000
14985.0000000	0.2850000
14986.0000000	0.2850000
14987.0000000	0.2800000
14988.0000000	0.2750000
14989.0000000	0.2800000
14990.0000000	0.2850000

14991.0000000	0.2850000
14992.0000000	0.2800000
14993.0000000	0.2850000
14994.0000000	0.2800000
14995.0000000	0.2900000
14996.0000000	0.2750000
14997.0000000	0.2850000
14998.0000000	0.2800000
14999.0000000	0.2800000
15000.0000000	0.2850000
15001.0000000	0.2950000
15002.0000000	0.2800000
15003.0000000	0.2900000
15004.0000000	0.2850000
15005.0000000	0.2850000
15006.0000000	0.2850000
15007.0000000	0.2850000
15008.0000000	0.2850000
15009.0000000	0.2900000
15010.0000000	0.2850000
15011.0000000	0.2866667
15012.0000000	0.2950000
15013.0000000	0.2900000
15014.0000000	0.2900000
15015.0000000	0.2900000
15016.0000000	0.2900000
15017.0000000	0.2950000
15018.0000000	0.3000000
15019.0000000	0.2950000
15020.0000000	0.2900000
15021.0000000	0.2900000
15022.0000000	0.2900000
15023.0000000	0.2900000
15024.0000000	0.2950000
15025.0000000	0.2900000
15026.0000000	0.2850000
15027.0000000	0.2950000
15028.0000000	0.2900000
15029.0000000	0.3000000
15030.0000000	0.2900000
15031.0000000	0.2950000
15032.0000000	0.2850000
15033.0000000	0.3000000
15034.0000000	0.2950000
15035.0000000	0.2950000
15036.0000000	0.2950000
15037.0000000	0.2900000
15038.0000000	0.2950000
15039.0000000	0.2900000
15040.0000000	0.2950000
15041.0000000	0.2900000
15042.0000000	0.2950000
15043.0000000	0.2950000
15044.0000000	0.3000000
15045.0000000	0.2800000
15046.0000000	0.2800000
15047.0000000	0.2900000

15048.0000000	0.2900000
15049.0000000	0.2950000
15050.0000000	0.2850000
15051.0000000	0.3000000
15052.0000000	0.2850000
15053.0000000	0.2950000
15054.0000000	0.2900000
15055.0000000	0.2900000
15056.0000000	0.2800000
15057.0000000	0.2850000
15058.0000000	0.2800000
15059.0000000	0.2900000
15060.0000000	0.2850000
15061.0000000	0.2900000
15062.0000000	0.2850000
15063.0000000	0.2750000
15064.0000000	0.2800000
15065.0000000	0.2800000
15066.0000000	0.2850000
15067.0000000	0.2750000
15068.0000000	0.2800000
15069.0000000	0.2800000
15070.0000000	0.2750000
15071.0000000	0.2766667
15072.0000000	0.2700000
15073.0000000	0.2750000
15074.0000000	0.2800000
15075.0000000	0.2850000
15076.0000000	0.2700000
15077.0000000	0.2700000
15078.0000000	0.2700000
15079.0000000	0.2750000
15080.0000000	0.2700000
15081.0000000	0.2700000
15082.0000000	0.2700000
15083.0000000	0.2800000
15084.0000000	0.2750000
15085.0000000	0.2750000
15086.0000000	0.2700000
15087.0000000	0.2650000
15088.0000000	0.2750000
15089.0000000	0.2700000
15090.0000000	0.2750000
15091.0000000	0.2750000
15092.0000000	0.2800000
15093.0000000	0.2800000
15094.0000000	0.2700000
15095.0000000	0.2800000
15096.0000000	0.2750000
15097.0000000	0.2850000
15098.0000000	0.2800000
15099.0000000	0.2850000
15100.0000000	0.2750000
15101.0000000	0.2866667
15102.0000000	0.2800000
15103.0000000	0.2700000
15104.0000000	0.2850000

15105.0000000	0.2800000
15106.0000000	0.2800000
15107.0000000	0.2700000
15108.0000000	0.2850000
15109.0000000	0.2800000
15110.0000000	0.2700000
15111.0000000	0.2750000
15112.0000000	0.2850000
15113.0000000	0.2700000
15114.0000000	0.2850000
15115.0000000	0.2750000
15116.0000000	0.2800000
15117.0000000	0.2750000
15118.0000000	0.2850000
15119.0000000	0.2650000
15120.0000000	0.2850000
15121.0000000	0.2700000
15122.0000000	0.2800000
15123.0000000	0.2750000
15124.0000000	0.2800000
15125.0000000	0.2750000
15126.0000000	0.2700000
15127.0000000	0.2750000
15128.0000000	0.2650000
15129.0000000	0.2750000
15130.0000000	0.2800000
15131.0000000	0.2766667
15132.0000000	0.2800000
15133.0000000	0.2750000
15134.0000000	0.2750000
15135.0000000	0.2650000
15136.0000000	0.2850000
15137.0000000	0.2750000
15138.0000000	0.2800000
15139.0000000	0.2750000
15140.0000000	0.2700000
15141.0000000	0.2750000
15142.0000000	0.2750000
15143.0000000	0.2800000
15144.0000000	0.2800000
15145.0000000	0.2700000
15146.0000000	0.2800000
15147.0000000	0.2850000
15148.0000000	0.2750000
15149.0000000	0.2850000
15150.0000000	0.2650000
15151.0000000	0.2650000
15152.0000000	0.2800000
15153.0000000	0.2800000
15154.0000000	0.2800000
15155.0000000	0.2650000
15156.0000000	0.2700000
15157.0000000	0.2700000
15158.0000000	0.2750000
15159.0000000	0.2700000
15160.0000000	0.2700000
15161.0000000	0.2733333

15162.0000000	0.2700000
15163.0000000	0.2700000
15164.0000000	0.2750000
15165.0000000	0.2650000
15166.0000000	0.2750000
15167.0000000	0.2600000
15168.0000000	0.2650000
15169.0000000	0.2750000
15170.0000000	0.2700000
15171.0000000	0.2650000
15172.0000000	0.2700000
15173.0000000	0.2800000
15174.0000000	0.2600000
15175.0000000	0.2700000
15176.0000000	0.2700000
15177.0000000	0.2750000
15178.0000000	0.2700000
15179.0000000	0.2750000
15180.0000000	0.2750000
15181.0000000	0.2650000
15182.0000000	0.2800000
15183.0000000	0.2700000
15184.0000000	0.2700000
15185.0000000	0.2700000
15186.0000000	0.2800000
15187.0000000	0.2750000
15188.0000000	0.2750000
15189.0000000	0.2700000
15190.0000000	0.2700000
15191.0000000	0.2900000
15192.0000000	0.2666667
15193.0000000	0.2850000
15194.0000000	0.2850000
15195.0000000	0.2750000
15196.0000000	0.2750000
15197.0000000	0.2800000
15198.0000000	0.2850000
15199.0000000	0.2850000
15200.0000000	0.2700000
15201.0000000	0.2800000
15202.0000000	0.2900000
15203.0000000	0.2850000
15204.0000000	0.2950000
15205.0000000	0.2800000
15206.0000000	0.2800000
15207.0000000	0.2900000
15208.0000000	0.2850000
15209.0000000	0.2850000
15210.0000000	0.2850000
15211.0000000	0.2850000
15212.0000000	0.2850000
15213.0000000	0.2950000
15214.0000000	0.2900000
15215.0000000	0.2850000
15216.0000000	0.2850000
15217.0000000	0.2800000
15218.0000000	0.2900000

15219.0000000	0.2850000
15220.0000000	0.2900000
15221.0000000	0.2900000
15222.0000000	0.2833333
15223.0000000	0.2900000
15224.0000000	0.2900000
15225.0000000	0.2900000
15226.0000000	0.2850000
15227.0000000	0.2850000
15228.0000000	0.2850000
15229.0000000	0.2900000
15230.0000000	0.2900000
15231.0000000	0.2800000
15232.0000000	0.2900000
15233.0000000	0.2750000
15234.0000000	0.2850000
15235.0000000	0.2850000
15236.0000000	0.2800000
15237.0000000	0.2900000
15238.0000000	0.2800000
15239.0000000	0.2800000
15240.0000000	0.2800000
15241.0000000	0.2800000
15242.0000000	0.2850000
15243.0000000	0.2800000
15244.0000000	0.2850000
15245.0000000	0.2800000
15246.0000000	0.2900000
15247.0000000	0.2850000
15248.0000000	0.2850000
15249.0000000	0.2800000
15250.0000000	0.2800000
15251.0000000	0.2850000
15252.0000000	0.2866667
15253.0000000	0.2900000
15254.0000000	0.2850000
15255.0000000	0.2950000
15256.0000000	0.2850000
15257.0000000	0.2850000
15258.0000000	0.2850000
15259.0000000	0.2800000
15260.0000000	0.2900000
15261.0000000	0.2750000
15262.0000000	0.2850000
15263.0000000	0.2850000
15264.0000000	0.2850000
15265.0000000	0.2850000
15266.0000000	0.2800000
15267.0000000	0.2850000
15268.0000000	0.2700000
15269.0000000	0.2850000
15270.0000000	0.2850000
15271.0000000	0.2800000
15272.0000000	0.2950000
15273.0000000	0.2850000
15274.0000000	0.2850000
15275.0000000	0.2850000

15276.0000000	0.2850000
15277.0000000	0.2900000
15278.0000000	0.2800000
15279.0000000	0.2950000
15280.0000000	0.2850000
15281.0000000	0.2900000
15282.0000000	0.2833333
15283.0000000	0.2850000
15284.0000000	0.2950000
15285.0000000	0.3000000
15286.0000000	0.2800000
15287.0000000	0.2900000
15288.0000000	0.2850000
15289.0000000	0.2900000
15290.0000000	0.2900000
15291.0000000	0.2900000
15292.0000000	0.2950000
15293.0000000	0.2800000
15294.0000000	0.3000000
15295.0000000	0.2850000
15296.0000000	0.3000000
15297.0000000	0.2950000
15298.0000000	0.3000000
15299.0000000	0.2800000
15300.0000000	0.3000000
15301.0000000	0.2900000
15302.0000000	0.2950000
15303.0000000	0.2900000
15304.0000000	0.2900000
15305.0000000	0.2850000
15306.0000000	0.2800000
15307.0000000	0.2900000
15308.0000000	0.2950000
15309.0000000	0.2950000
15310.0000000	0.2900000
15311.0000000	0.2900000
15312.0000000	0.2950000
15313.0000000	0.2933334
15314.0000000	0.2850000
15315.0000000	0.3000000
15316.0000000	0.3050000
15317.0000000	0.2950000
15318.0000000	0.2850000
15319.0000000	0.2950000
15320.0000000	0.2950000
15321.0000000	0.3000000
15322.0000000	0.3000000
15323.0000000	0.3000000
15324.0000000	0.2900000
15325.0000000	0.3100000
15326.0000000	0.2950000
15327.0000000	0.3000000
15328.0000000	0.2950000
15329.0000000	0.2950000
15330.0000000	0.3000000
15331.0000000	0.3050000
15332.0000000	0.2950000

15333.0000000	0.2950000
15334.0000000	0.3050000
15335.0000000	0.2900000
15336.0000000	0.3100000
15337.0000000	0.3000000
15338.0000000	0.2900000
15339.0000000	0.3100000
15340.0000000	0.3100000
15341.0000000	0.2900000
15342.0000000	0.3000000
15343.0000000	0.3033333
15344.0000000	0.2950000
15345.0000000	0.3000000
15346.0000000	0.3000000
15347.0000000	0.3000000
15348.0000000	0.3050000
15349.0000000	0.3050000
15350.0000000	0.3000000
15351.0000000	0.3150000
15352.0000000	0.3100000
15353.0000000	0.3000000
15354.0000000	0.3100000
15355.0000000	0.3050000
15356.0000000	0.3050000
15357.0000000	0.3100000
15358.0000000	0.3000000
15359.0000000	0.3050000
15360.0000000	0.3150000
15361.0000000	0.3000000
15362.0000000	0.3200000
15363.0000000	0.2900000
15364.0000000	0.3050000
15365.0000000	0.3100000
15366.0000000	0.3050000
15367.0000000	0.3000000
15368.0000000	0.3000000
15369.0000000	0.3000000
15370.0000000	0.3050000
15371.0000000	0.3000000
15372.0000000	0.3050000
15373.0000000	0.2933333
15374.0000000	0.3100000
15375.0000000	0.3050000
15376.0000000	0.2950000
15377.0000000	0.3050000
15378.0000000	0.2950000
15379.0000000	0.3100000
15380.0000000	0.3050000
15381.0000000	0.2950000
15382.0000000	0.3000000
15383.0000000	0.3050000
15384.0000000	0.3000000
15385.0000000	0.3050000
15386.0000000	0.3000000
15387.0000000	0.3000000
15388.0000000	0.3100000
15389.0000000	0.2950000

15390.0000000	0.3100000
15391.0000000	0.2900000
15392.0000000	0.3050000
15393.0000000	0.3000000
15394.0000000	0.3000000
15395.0000000	0.3050000
15396.0000000	0.3000000
15397.0000000	0.3000000
15398.0000000	0.2950000
15399.0000000	0.3100000
15400.0000000	0.3000000
15401.0000000	0.3100000
15402.0000000	0.2950000
15403.0000000	0.3033333
15404.0000000	0.3000000
15405.0000000	0.2950000
15406.0000000	0.3050000
15407.0000000	0.3000000
15408.0000000	0.3100000
15409.0000000	0.3000000
15410.0000000	0.3050000
15411.0000000	0.2950000
15412.0000000	0.3050000
15413.0000000	0.3000000
15414.0000000	0.3000000
15415.0000000	0.3000000
15416.0000000	0.3050000
15417.0000000	0.3100000
15418.0000000	0.3100000
15419.0000000	0.3050000
15420.0000000	0.3100000
15421.0000000	0.3100000
15422.0000000	0.3000000
15423.0000000	0.3000000
15424.0000000	0.3150000
15425.0000000	0.3100000
15426.0000000	0.3150000
15427.0000000	0.3100000
15428.0000000	0.3050000
15429.0000000	0.3100000
15430.0000000	0.3050000
15431.0000000	0.3050000
15432.0000000	0.3150000
15433.0000000	0.3100000
15434.0000000	0.3050000
15435.0000000	0.3050000
15436.0000000	0.3150000
15437.0000000	0.3100000
15438.0000000	0.3200000
15439.0000000	0.3100000
15440.0000000	0.3150000
15441.0000000	0.3100000
15442.0000000	0.3100000
15443.0000000	0.3150000
15444.0000000	0.3100000
15445.0000000	0.3100000
15446.0000000	0.3200000

15447.0000000	0.3150000
15448.0000000	0.3150000
15449.0000000	0.3050000
15450.0000000	0.3150000
15451.0000000	0.3150000
15452.0000000	0.3100000
15453.0000000	0.3250000
15454.0000000	0.3100000
15455.0000000	0.3200000
15456.0000000	0.3150000
15457.0000000	0.3100000
15458.0000000	0.3200000
15459.0000000	0.3100000
15460.0000000	0.3200000
15461.0000000	0.3200000
15462.0000000	0.3200000
15463.0000000	0.3200000
15464.0000000	0.3133333
15465.0000000	0.3150000
15466.0000000	0.3250000
15467.0000000	0.3250000
15468.0000000	0.3100000
15469.0000000	0.3250000
15470.0000000	0.3150000
15471.0000000	0.3250000
15472.0000000	0.3200000
15473.0000000	0.3200000
15474.0000000	0.3150000
15475.0000000	0.3250000
15476.0000000	0.3250000
15477.0000000	0.3200000
15478.0000000	0.3300000
15479.0000000	0.3200000
15480.0000000	0.3300000
15481.0000000	0.3250000
15482.0000000	0.3250000
15483.0000000	0.3250000
15484.0000000	0.3200000
15485.0000000	0.3250000
15486.0000000	0.3250000
15487.0000000	0.3150000
15488.0000000	0.3150000
15489.0000000	0.3150000
15490.0000000	0.3250000
15491.0000000	0.3200000
15492.0000000	0.3200000
15493.0000000	0.3133333
15494.0000000	0.3250000
15495.0000000	0.3300000
15496.0000000	0.3150000
15497.0000000	0.3150000
15498.0000000	0.3150000
15499.0000000	0.3250000
15500.0000000	0.3200000
15501.0000000	0.3300000
15502.0000000	0.3250000
15503.0000000	0.3300000

15504.0000000	0.3250000
15505.0000000	0.3200000
15506.0000000	0.3200000
15507.0000000	0.3200000
15508.0000000	0.3300000
15509.0000000	0.3150000
15510.0000000	0.3300000
15511.0000000	0.3200000
15512.0000000	0.3200000
15513.0000000	0.3250000
15514.0000000	0.3300000
15515.0000000	0.3250000
15516.0000000	0.3250000
15517.0000000	0.3200000
15518.0000000	0.3150000
15519.0000000	0.3200000
15520.0000000	0.3300000
15521.0000000	0.3250000
15522.0000000	0.3150000
15523.0000000	0.3200000
15524.0000000	0.3233334
15525.0000000	0.3250000
15526.0000000	0.3200000
15527.0000000	0.3250000
15528.0000000	0.3250000
15529.0000000	0.3250000
15530.0000000	0.3300000
15531.0000000	0.3250000
15532.0000000	0.3250000
15533.0000000	0.3250000
15534.0000000	0.3350000
15535.0000000	0.3200000
15536.0000000	0.3350000
15537.0000000	0.3200000
15538.0000000	0.3300000
15539.0000000	0.3300000
15540.0000000	0.3300000
15541.0000000	0.3350000
15542.0000000	0.3250000
15543.0000000	0.3300000
15544.0000000	0.3350000
15545.0000000	0.3400000
15546.0000000	0.3250000
15547.0000000	0.3250000
15548.0000000	0.3350000
15549.0000000	0.3350000
15550.0000000	0.3400000
15551.0000000	0.3250000
15552.0000000	0.3250000
15553.0000000	0.3300000
15554.0000000	0.3300000
15555.0000000	0.3400000
15556.0000000	0.3300000
15557.0000000	0.3400000
15558.0000000	0.3300000
15559.0000000	0.3250000
15560.0000000	0.3350000

15561.0000000	0.3350000
15562.0000000	0.3300000
15563.0000000	0.3350000
15564.0000000	0.3250000
15565.0000000	0.3300000
15566.0000000	0.3300000
15567.0000000	0.3250000
15568.0000000	0.3400000
15569.0000000	0.3250000
15570.0000000	0.3250000
15571.0000000	0.3300000
15572.0000000	0.3350000
15573.0000000	0.3400000
15574.0000000	0.3200000
15575.0000000	0.3250000
15576.0000000	0.3300000
15577.0000000	0.3350000
15578.0000000	0.3400000
15579.0000000	0.3350000
15580.0000000	0.3350000
15581.0000000	0.3250000
15582.0000000	0.3300000
15583.0000000	0.3300000
15584.0000000	0.3266667
15585.0000000	0.3250000
15586.0000000	0.3400000
15587.0000000	0.3250000
15588.0000000	0.3250000
15589.0000000	0.3200000
15590.0000000	0.3350000
15591.0000000	0.3300000
15592.0000000	0.3350000
15593.0000000	0.3250000
15594.0000000	0.3250000
15595.0000000	0.3200000
15596.0000000	0.3300000
15597.0000000	0.3300000
15598.0000000	0.3350000
15599.0000000	0.3300000
15600.0000000	0.3300000
15601.0000000	0.3200000
15602.0000000	0.3300000
15603.0000000	0.3250000
15604.0000000	0.3400000
15605.0000000	0.3300000
15606.0000000	0.3350000
15607.0000000	0.3200000
15608.0000000	0.3350000
15609.0000000	0.3250000
15610.0000000	0.3400000
15611.0000000	0.3300000
15612.0000000	0.3350000
15613.0000000	0.3250000
15614.0000000	0.3300000
15615.0000000	0.3300000
15616.0000000	0.3300000
15617.0000000	0.3200000

15618.0000000	0.3300000
15619.0000000	0.3250000
15620.0000000	0.3350000
15621.0000000	0.3300000
15622.0000000	0.3250000
15623.0000000	0.3300000
15624.0000000	0.3300000
15625.0000000	0.3300000
15626.0000000	0.3350000
15627.0000000	0.3300000
15628.0000000	0.3300000
15629.0000000	0.3300000
15630.0000000	0.3500000
15631.0000000	0.3250000
15632.0000000	0.3400000
15633.0000000	0.3300000
15634.0000000	0.3200000
15635.0000000	0.3450000
15636.0000000	0.3450000
15637.0000000	0.3350000
15638.0000000	0.3300000
15639.0000000	0.3250000
15640.0000000	0.3350000
15641.0000000	0.3400000
15642.0000000	0.3400000
15643.0000000	0.3400000
15644.0000000	0.3300000
15645.0000000	0.3433333
15646.0000000	0.3300000
15647.0000000	0.3400000
15648.0000000	0.3350000
15649.0000000	0.3450000
15650.0000000	0.3350000
15651.0000000	0.3350000
15652.0000000	0.3300000
15653.0000000	0.3350000
15654.0000000	0.3300000
15655.0000000	0.3400000
15656.0000000	0.3350000
15657.0000000	0.3450000
15658.0000000	0.3250000
15659.0000000	0.3300000
15660.0000000	0.3250000
15661.0000000	0.3400000
15662.0000000	0.3350000
15663.0000000	0.3350000
15664.0000000	0.3300000
15665.0000000	0.3300000
15666.0000000	0.3300000
15667.0000000	0.3350000
15668.0000000	0.3400000
15669.0000000	0.3350000
15670.0000000	0.3250000
15671.0000000	0.3250000
15672.0000000	0.3300000
15673.0000000	0.3300000
15674.0000000	0.3350000

15675.0000000	0.3333333
15676.0000000	0.3300000
15677.0000000	0.3350000
15678.0000000	0.3300000
15679.0000000	0.3400000
15680.0000000	0.3300000
15681.0000000	0.3300000
15682.0000000	0.3300000
15683.0000000	0.3150000
15684.0000000	0.3350000
15685.0000000	0.3350000
15686.0000000	0.3300000
15687.0000000	0.3200000
15688.0000000	0.3300000
15689.0000000	0.3300000
15690.0000000	0.3350000
15691.0000000	0.3400000
15692.0000000	0.3300000
15693.0000000	0.3350000
15694.0000000	0.3250000
15695.0000000	0.3350000
15696.0000000	0.3350000
15697.0000000	0.3400000
15698.0000000	0.3200000
15699.0000000	0.3300000
15700.0000000	0.3200000
15701.0000000	0.3250000
15702.0000000	0.3250000
15703.0000000	0.3300000
15704.0000000	0.3300000
15705.0000000	0.3266667
15706.0000000	0.3250000
15707.0000000	0.3250000
15708.0000000	0.3350000
15709.0000000	0.3200000
15710.0000000	0.3350000
15711.0000000	0.3150000
15712.0000000	0.3250000
15713.0000000	0.3250000
15714.0000000	0.3200000
15715.0000000	0.3250000
15716.0000000	0.3200000
15717.0000000	0.3300000
15718.0000000	0.3200000
15719.0000000	0.3200000
15720.0000000	0.3250000
15721.0000000	0.3200000
15722.0000000	0.3300000
15723.0000000	0.3350000
15724.0000000	0.3300000
15725.0000000	0.3250000
15726.0000000	0.3200000
15727.0000000	0.3150000
15728.0000000	0.3250000
15729.0000000	0.3250000
15730.0000000	0.3200000
15731.0000000	0.3200000

15732.0000000	0.3250000
15733.0000000	0.3200000
15734.0000000	0.3250000
15735.0000000	0.3300000
15736.0000000	0.3150000
15737.0000000	0.3200000
15738.0000000	0.3150000
15739.0000000	0.3250000
15740.0000000	0.3250000
15741.0000000	0.3250000
15742.0000000	0.3100000
15743.0000000	0.3200000
15744.0000000	0.3150000
15745.0000000	0.3200000
15746.0000000	0.3200000
15747.0000000	0.3150000
15748.0000000	0.3150000
15749.0000000	0.3100000
15750.0000000	0.3150000
15751.0000000	0.3100000
15752.0000000	0.3200000
15753.0000000	0.3200000
15754.0000000	0.3300000
15755.0000000	0.3150000
15756.0000000	0.3200000
15757.0000000	0.3150000
15758.0000000	0.3150000
15759.0000000	0.3250000
15760.0000000	0.3150000
15761.0000000	0.3250000
15762.0000000	0.3200000
15763.0000000	0.3300000
15764.0000000	0.3150000
15765.0000000	0.3166667
15766.0000000	0.3200000
15767.0000000	0.3150000
15768.0000000	0.3150000
15769.0000000	0.3150000
15770.0000000	0.3200000
15771.0000000	0.3200000
15772.0000000	0.3250000
15773.0000000	0.3150000
15774.0000000	0.3200000
15775.0000000	0.3150000
15776.0000000	0.3200000
15777.0000000	0.3200000
15778.0000000	0.3250000
15779.0000000	0.3150000
15780.0000000	0.3150000
15781.0000000	0.3100000
15782.0000000	0.3200000
15783.0000000	0.3150000
15784.0000000	0.3250000
15785.0000000	0.3100000
15786.0000000	0.3200000
15787.0000000	0.3200000
15788.0000000	0.3050000

15789.0000000	0.3200000
15790.0000000	0.3050000
15791.0000000	0.3200000
15792.0000000	0.3100000
15793.0000000	0.3150000
15794.0000000	0.3200000
15795.0000000	0.3150000
15796.0000000	0.3100000
15797.0000000	0.3150000
15798.0000000	0.3100000
15799.0000000	0.3250000
15800.0000000	0.3050000
15801.0000000	0.3250000
15802.0000000	0.3200000
15803.0000000	0.3050000
15804.0000000	0.3150000
15805.0000000	0.3150000
15806.0000000	0.3250000
15807.0000000	0.3300000
15808.0000000	0.3100000
15809.0000000	0.3200000
15810.0000000	0.3150000
15811.0000000	0.3150000
15812.0000000	0.3200000
15813.0000000	0.3100000
15814.0000000	0.3050000
15815.0000000	0.3200000
15816.0000000	0.3200000
15817.0000000	0.3250000
15818.0000000	0.3150000
15819.0000000	0.3100000
15820.0000000	0.3150000
15821.0000000	0.3100000
15822.0000000	0.3250000
15823.0000000	0.3150000
15824.0000000	0.3200000
15825.0000000	0.3200000
15826.0000000	0.3100000
15827.0000000	0.3050000
15828.0000000	0.3250000
15829.0000000	0.3250000
15830.0000000	0.3200000
15831.0000000	0.3250000
15832.0000000	0.3100000
15833.0000000	0.3200000
15834.0000000	0.3100000
15835.0000000	0.3200000
15836.0000000	0.3200000
15837.0000000	0.3150000
15838.0000000	0.3100000
15839.0000000	0.3100000
15840.0000000	0.3100000
15841.0000000	0.3150000
15842.0000000	0.3150000
15843.0000000	0.3150000
15844.0000000	0.3150000
15845.0000000	0.3050000

15846.0000000	0.3100000
15847.0000000	0.3100000
15848.0000000	0.3200000
15849.0000000	0.3200000
15850.0000000	0.3100000
15851.0000000	0.3100000
15852.0000000	0.3000000
15853.0000000	0.3150000
15854.0000000	0.3150000
15855.0000000	0.3150000
15856.0000000	0.3100000
15857.0000000	0.3100000
15858.0000000	0.3200000
15859.0000000	0.3250000
15860.0000000	0.3150000
15861.0000000	0.3150000
15862.0000000	0.3150000
15863.0000000	0.3200000
15864.0000000	0.3100000
15865.0000000	0.3200000
15866.0000000	0.3100000
15867.0000000	0.3200000
15868.0000000	0.3150000
15869.0000000	0.3150000
15870.0000000	0.3250000
15871.0000000	0.3100000
15872.0000000	0.3150000
15873.0000000	0.3100000
15874.0000000	0.3200000
15875.0000000	0.3250000
15876.0000000	0.3100000
15877.0000000	0.3100000
15878.0000000	0.3000000
15879.0000000	0.3150000
15880.0000000	0.3200000
15881.0000000	0.3100000
15882.0000000	0.3150000
15883.0000000	0.3100000
15884.0000000	0.3100000
15885.0000000	0.3100000
15886.0000000	0.3066667
15887.0000000	0.3200000
15888.0000000	0.3000000
15889.0000000	0.3150000
15890.0000000	0.3050000
15891.0000000	0.3150000
15892.0000000	0.3050000
15893.0000000	0.3150000
15894.0000000	0.3100000
15895.0000000	0.3150000
15896.0000000	0.3150000
15897.0000000	0.3150000
15898.0000000	0.3100000
15899.0000000	0.3100000
15900.0000000	0.3200000
15901.0000000	0.3050000
15902.0000000	0.3100000

15903.0000000	0.3100000
15904.0000000	0.3000000
15905.0000000	0.3100000
15906.0000000	0.3100000
15907.0000000	0.3100000
15908.0000000	0.3150000
15909.0000000	0.3100000
15910.0000000	0.3100000
15911.0000000	0.3200000
15912.0000000	0.3050000
15913.0000000	0.3100000
15914.0000000	0.3050000
15915.0000000	0.3150000
15916.0000000	0.3100000
15917.0000000	0.3100000
15918.0000000	0.3150000
15919.0000000	0.3150000
15920.0000000	0.3200000
15921.0000000	0.3150000
15922.0000000	0.3100000
15923.0000000	0.3100000
15924.0000000	0.3050000
15925.0000000	0.3150000
15926.0000000	0.3100000
15927.0000000	0.3150000
15928.0000000	0.3150000
15929.0000000	0.3000000
15930.0000000	0.3100000
15931.0000000	0.3100000
15932.0000000	0.3200000
15933.0000000	0.3050000
15934.0000000	0.3150000
15935.0000000	0.3050000
15936.0000000	0.3050000
15937.0000000	0.3200000
15938.0000000	0.3100000
15939.0000000	0.3050000
15940.0000000	0.3050000
15941.0000000	0.3100000
15942.0000000	0.3100000
15943.0000000	0.3150000
15944.0000000	0.3050000
15945.0000000	0.3050000
15946.0000000	0.3133334
15947.0000000	0.3100000
15948.0000000	0.3050000
15949.0000000	0.3150000
15950.0000000	0.3050000
15951.0000000	0.3100000
15952.0000000	0.3050000
15953.0000000	0.3000000
15954.0000000	0.3100000
15955.0000000	0.3100000
15956.0000000	0.3100000
15957.0000000	0.3150000
15958.0000000	0.3050000
15959.0000000	0.3100000

15960.0000000	0.3000000
15961.0000000	0.3050000
15962.0000000	0.3100000
15963.0000000	0.3050000
15964.0000000	0.3150000
15965.0000000	0.2950000
15966.0000000	0.3100000
15967.0000000	0.3050000
15968.0000000	0.3050000
15969.0000000	0.3050000
15970.0000000	0.3050000
15971.0000000	0.3100000
15972.0000000	0.3050000
15973.0000000	0.3050000
15974.0000000	0.3000000
15975.0000000	0.3050000
15976.0000000	0.3050000
15977.0000000	0.3100000
15978.0000000	0.3000000
15979.0000000	0.3100000
15980.0000000	0.3100000
15981.0000000	0.3150000
15982.0000000	0.3100000
15983.0000000	0.3150000
15984.0000000	0.3050000
15985.0000000	0.3100000
15986.0000000	0.3150000
15987.0000000	0.3250000
15988.0000000	0.3100000
15989.0000000	0.3150000
15990.0000000	0.3050000
15991.0000000	0.3100000
15992.0000000	0.3050000
15993.0000000	0.3200000
15994.0000000	0.3150000
15995.0000000	0.3150000
15996.0000000	0.3100000
15997.0000000	0.3150000
15998.0000000	0.3100000
15999.0000000	0.3250000
16000.0000000	0.3050000
16001.0000000	0.3050000
16002.0000000	0.3200000
16003.0000000	0.3100000
16004.0000000	0.3150000
16005.0000000	0.3150000
16006.0000000	0.3100000
16007.0000000	0.3100000
16008.0000000	0.3200000
16009.0000000	0.3150000
16010.0000000	0.3150000
16011.0000000	0.3100000
16012.0000000	0.3200000
16013.0000000	0.3100000
16014.0000000	0.3150000
16015.0000000	0.3100000
16016.0000000	0.3200000

16017.0000000	0.3000000
16018.0000000	0.3150000
16019.0000000	0.3050000
16020.0000000	0.3100000
16021.0000000	0.3150000
16022.0000000	0.3050000
16023.0000000	0.3100000
16024.0000000	0.3050000
16025.0000000	0.3150000
16026.0000000	0.3050000
16027.0000000	0.3050000
16028.0000000	0.3050000
16029.0000000	0.3000000
16030.0000000	0.3100000
16031.0000000	0.3050000
16032.0000000	0.3050000
16033.0000000	0.3050000
16034.0000000	0.3100000
16035.0000000	0.2900000
16036.0000000	0.3000000
16037.0000000	0.3000000
16038.0000000	0.2950000
16039.0000000	0.3000000
16040.0000000	0.2950000
16041.0000000	0.2950000
16042.0000000	0.3000000
16043.0000000	0.2950000
16044.0000000	0.3000000
16045.0000000	0.2900000
16046.0000000	0.2900000
16047.0000000	0.2950000
16048.0000000	0.2900000
16049.0000000	0.2950000
16050.0000000	0.2950000
16051.0000000	0.2800000
16052.0000000	0.2950000
16053.0000000	0.2850000
16054.0000000	0.2850000
16055.0000000	0.2900000
16056.0000000	0.2800000
16057.0000000	0.2900000
16058.0000000	0.2900000
16059.0000000	0.2850000
16060.0000000	0.2800000
16061.0000000	0.2850000
16062.0000000	0.2850000
16063.0000000	0.2750000
16064.0000000	0.2750000
16065.0000000	0.2750000
16066.0000000	0.2750000
16067.0000000	0.2766667
16068.0000000	0.2800000
16069.0000000	0.2700000
16070.0000000	0.2800000
16071.0000000	0.2750000
16072.0000000	0.2750000
16073.0000000	0.2750000

16074.0000000	0.2700000
16075.0000000	0.2750000
16076.0000000	0.2750000
16077.0000000	0.2800000
16078.0000000	0.2800000
16079.0000000	0.2800000
16080.0000000	0.2850000
16081.0000000	0.2700000
16082.0000000	0.2800000
16083.0000000	0.2700000
16084.0000000	0.2750000
16085.0000000	0.2750000
16086.0000000	0.2700000
16087.0000000	0.2800000
16088.0000000	0.2750000
16089.0000000	0.2850000
16090.0000000	0.2750000
16091.0000000	0.2800000
16092.0000000	0.2700000
16093.0000000	0.2850000
16094.0000000	0.2800000
16095.0000000	0.2850000
16096.0000000	0.2750000
16097.0000000	0.2850000
16098.0000000	0.2766667
16099.0000000	0.2800000
16100.0000000	0.2850000
16101.0000000	0.2850000
16102.0000000	0.2850000
16103.0000000	0.2850000
16104.0000000	0.2850000
16105.0000000	0.2850000
16106.0000000	0.2850000
16107.0000000	0.2850000
16108.0000000	0.2850000
16109.0000000	0.2750000
16110.0000000	0.2850000
16111.0000000	0.2900000
16112.0000000	0.2850000
16113.0000000	0.2800000
16114.0000000	0.2850000
16115.0000000	0.2850000
16116.0000000	0.2950000
16117.0000000	0.2900000
16118.0000000	0.2950000
16119.0000000	0.2900000
16120.0000000	0.2950000
16121.0000000	0.2850000
16122.0000000	0.2950000
16123.0000000	0.2900000
16124.0000000	0.2850000
16125.0000000	0.2850000
16126.0000000	0.2900000
16127.0000000	0.2950000
16128.0000000	0.2933333
16129.0000000	0.2900000
16130.0000000	0.3000000

16131.0000000	0.2900000
16132.0000000	0.2950000
16133.0000000	0.2900000
16134.0000000	0.3000000
16135.0000000	0.2950000
16136.0000000	0.3000000
16137.0000000	0.3000000
16138.0000000	0.3000000
16139.0000000	0.2950000
16140.0000000	0.3050000
16141.0000000	0.3000000
16142.0000000	0.3050000
16143.0000000	0.3000000
16144.0000000	0.2950000
16145.0000000	0.3000000
16146.0000000	0.3000000
16147.0000000	0.3050000
16148.0000000	0.3000000
16149.0000000	0.3100000
16150.0000000	0.3000000
16151.0000000	0.3000000
16152.0000000	0.2950000
16153.0000000	0.3000000
16154.0000000	0.3050000
16155.0000000	0.3100000
16156.0000000	0.2950000
16157.0000000	0.3100000
16158.0000000	0.3050000
16159.0000000	0.3100000
16160.0000000	0.3000000
16161.0000000	0.3050000
16162.0000000	0.3050000
16163.0000000	0.3050000
16164.0000000	0.3050000
16165.0000000	0.3050000
16166.0000000	0.3050000
16167.0000000	0.3150000
16168.0000000	0.3150000
16169.0000000	0.3150000
16170.0000000	0.3050000
16171.0000000	0.3100000
16172.0000000	0.3050000
16173.0000000	0.3050000
16174.0000000	0.3150000
16175.0000000	0.3050000
16176.0000000	0.3200000
16177.0000000	0.3100000
16178.0000000	0.3150000
16179.0000000	0.3150000
16180.0000000	0.3050000
16181.0000000	0.3100000
16182.0000000	0.3100000
16183.0000000	0.3150000
16184.0000000	0.3100000
16185.0000000	0.3150000
16186.0000000	0.3100000
16187.0000000	0.3050000

16188.0000000	0.3200000
16189.0000000	0.3100000
16190.0000000	0.3250000
16191.0000000	0.3050000
16192.0000000	0.3200000
16193.0000000	0.3150000
16194.0000000	0.2950000
16195.0000000	0.3150000
16196.0000000	0.3200000
16197.0000000	0.3200000
16198.0000000	0.3200000
16199.0000000	0.3050000
16200.0000000	0.3100000
16201.0000000	0.3200000
16202.0000000	0.3150000
16203.0000000	0.3100000
16204.0000000	0.3100000
16205.0000000	0.3100000
16206.0000000	0.3150000
16207.0000000	0.3200000
16208.0000000	0.3000000
16209.0000000	0.3150000
16210.0000000	0.3100000
16211.0000000	0.3250000
16212.0000000	0.3150000
16213.0000000	0.3150000
16214.0000000	0.3050000
16215.0000000	0.3050000
16216.0000000	0.3150000
16217.0000000	0.3100000
16218.0000000	0.3100000
16219.0000000	0.3100000
16220.0000000	0.3200000
16221.0000000	0.3100000
16222.0000000	0.3100000
16223.0000000	0.3100000
16224.0000000	0.3150000
16225.0000000	0.3200000
16226.0000000	0.3200000
16227.0000000	0.3200000
16228.0000000	0.3050000
16229.0000000	0.3150000
16230.0000000	0.3100000
16231.0000000	0.3200000
16232.0000000	0.3200000
16233.0000000	0.3200000
16234.0000000	0.3150000
16235.0000000	0.3150000
16236.0000000	0.3100000
16237.0000000	0.3150000
16238.0000000	0.3150000
16239.0000000	0.3200000
16240.0000000	0.3150000
16241.0000000	0.3100000
16242.0000000	0.3150000
16243.0000000	0.3150000
16244.0000000	0.3200000

16245.0000000	0.3200000
16246.0000000	0.3150000
16247.0000000	0.3200000
16248.0000000	0.3166667
16249.0000000	0.3150000
16250.0000000	0.3200000
16251.0000000	0.3200000
16252.0000000	0.3100000
16253.0000000	0.3200000
16254.0000000	0.3200000
16255.0000000	0.3250000
16256.0000000	0.3150000
16257.0000000	0.3150000
16258.0000000	0.3200000
16259.0000000	0.3200000
16260.0000000	0.3200000
16261.0000000	0.3150000
16262.0000000	0.3200000
16263.0000000	0.3200000
16264.0000000	0.3200000
16265.0000000	0.3250000
16266.0000000	0.3200000
16267.0000000	0.3250000
16268.0000000	0.3250000
16269.0000000	0.3250000
16270.0000000	0.3250000
16271.0000000	0.3200000
16272.0000000	0.3250000
16273.0000000	0.3200000
16274.0000000	0.3300000
16275.0000000	0.3250000
16276.0000000	0.3200000
16277.0000000	0.3250000
16278.0000000	0.3233334
16279.0000000	0.3250000
16280.0000000	0.3200000
16281.0000000	0.3150000
16282.0000000	0.3200000
16283.0000000	0.3250000
16284.0000000	0.3250000
16285.0000000	0.3200000
16286.0000000	0.3300000
16287.0000000	0.3200000
16288.0000000	0.3250000
16289.0000000	0.3250000
16290.0000000	0.3100000
16291.0000000	0.3250000
16292.0000000	0.3200000
16293.0000000	0.3200000
16294.0000000	0.3200000
16295.0000000	0.3250000
16296.0000000	0.3200000
16297.0000000	0.3200000
16298.0000000	0.3250000
16299.0000000	0.3150000
16300.0000000	0.3150000
16301.0000000	0.3150000

16302.0000000	0.3250000
16303.0000000	0.3200000
16304.0000000	0.3250000
16305.0000000	0.3200000
16306.0000000	0.3200000
16307.0000000	0.3250000
16308.0000000	0.3250000
16309.0000000	0.3233333
16310.0000000	0.3200000
16311.0000000	0.3300000
16312.0000000	0.3100000
16313.0000000	0.3200000
16314.0000000	0.3250000
16315.0000000	0.3250000
16316.0000000	0.3200000
16317.0000000	0.3250000
16318.0000000	0.3250000
16319.0000000	0.3300000
16320.0000000	0.3350000
16321.0000000	0.3200000
16322.0000000	0.3250000
16323.0000000	0.3150000
16324.0000000	0.3300000
16325.0000000	0.3200000
16326.0000000	0.3350000
16327.0000000	0.3150000
16328.0000000	0.3300000
16329.0000000	0.3200000
16330.0000000	0.3250000
16331.0000000	0.3200000
16332.0000000	0.3300000
16333.0000000	0.3150000
16334.0000000	0.3200000
16335.0000000	0.3200000
16336.0000000	0.3200000
16337.0000000	0.3200000
16338.0000000	0.3200000
16339.0000000	0.3233334
16340.0000000	0.3150000
16341.0000000	0.3150000
16342.0000000	0.3150000
16343.0000000	0.3200000
16344.0000000	0.3250000
16345.0000000	0.3150000
16346.0000000	0.3250000
16347.0000000	0.3100000
16348.0000000	0.3200000
16349.0000000	0.3150000
16350.0000000	0.3200000
16351.0000000	0.3200000
16352.0000000	0.3050000
16353.0000000	0.3250000
16354.0000000	0.3150000
16355.0000000	0.3150000
16356.0000000	0.3100000
16357.0000000	0.3100000
16358.0000000	0.3050000

16359.0000000	0.3150000
16360.0000000	0.3100000
16361.0000000	0.3200000
16362.0000000	0.3050000
16363.0000000	0.3100000
16364.0000000	0.3150000
16365.0000000	0.3100000
16366.0000000	0.3200000
16367.0000000	0.3100000
16368.0000000	0.3100000
16369.0000000	0.3100000
16370.0000000	0.3000000
16371.0000000	0.3050000
16372.0000000	0.3150000
16373.0000000	0.3150000
16374.0000000	0.3150000
16375.0000000	0.3000000
16376.0000000	0.3200000
16377.0000000	0.3100000
16378.0000000	0.3150000
16379.0000000	0.3050000
16380.0000000	0.3050000
16381.0000000	0.3100000
16382.0000000	0.3150000
16383.0000000	0.3100000
16384.0000000	0.3100000
16385.0000000	0.3100000
16386.0000000	0.3050000
16387.0000000	0.3150000
16388.0000000	0.3000000
16389.0000000	0.3050000
16390.0000000	0.3050000
16391.0000000	0.3050000
16392.0000000	0.3200000
16393.0000000	0.3150000
16394.0000000	0.3100000
16395.0000000	0.3050000
16396.0000000	0.3050000
16397.0000000	0.3100000
16398.0000000	0.3050000
16399.0000000	0.3100000
16400.0000000	0.3050000
16401.0000000	0.3050000
16402.0000000	0.3000000
16403.0000000	0.2900000
16404.0000000	0.3100000
16405.0000000	0.3150000
16406.0000000	0.3100000
16407.0000000	0.3050000
16408.0000000	0.2950000
16409.0000000	0.3050000
16410.0000000	0.3000000
16411.0000000	0.3050000
16412.0000000	0.3000000
16413.0000000	0.3000000
16414.0000000	0.3100000
16415.0000000	0.3000000

16416.0000000	0.3100000
16417.0000000	0.3050000
16418.0000000	0.3050000
16419.0000000	0.3000000
16420.0000000	0.3050000
16421.0000000	0.3050000
16422.0000000	0.3050000
16423.0000000	0.2950000
16424.0000000	0.3050000
16425.0000000	0.3000000
16426.0000000	0.3100000
16427.0000000	0.3050000
16428.0000000	0.3100000
16429.0000000	0.2966667
16430.0000000	0.3050000
16431.0000000	0.3000000
16432.0000000	0.2950000
16433.0000000	0.3000000
16434.0000000	0.2950000
16435.0000000	0.2850000
16436.0000000	0.2950000
16437.0000000	0.3100000
16438.0000000	0.3050000
16439.0000000	0.3100000
16440.0000000	0.2950000
16441.0000000	0.3050000
16442.0000000	0.2950000
16443.0000000	0.2900000
16444.0000000	0.2950000
16445.0000000	0.3000000
16446.0000000	0.2950000
16447.0000000	0.3000000
16448.0000000	0.2900000
16449.0000000	0.2950000
16450.0000000	0.2950000
16451.0000000	0.2950000
16452.0000000	0.3000000
16453.0000000	0.2950000
16454.0000000	0.2950000
16455.0000000	0.2950000
16456.0000000	0.2950000
16457.0000000	0.3050000
16458.0000000	0.2950000
16459.0000000	0.2950000
16460.0000000	0.3033333
16461.0000000	0.2900000
16462.0000000	0.2950000
16463.0000000	0.2900000
16464.0000000	0.2950000
16465.0000000	0.3050000
16466.0000000	0.3000000
16467.0000000	0.3000000
16468.0000000	0.2950000
16469.0000000	0.3000000
16470.0000000	0.2900000
16471.0000000	0.3050000
16472.0000000	0.3000000

16473.0000000	0.3050000
16474.0000000	0.3000000
16475.0000000	0.2900000
16476.0000000	0.2900000
16477.0000000	0.2950000
16478.0000000	0.3000000
16479.0000000	0.2950000
16480.0000000	0.3050000
16481.0000000	0.2950000
16482.0000000	0.3000000
16483.0000000	0.2950000
16484.0000000	0.2950000
16485.0000000	0.2900000
16486.0000000	0.2950000
16487.0000000	0.2850000
16488.0000000	0.2900000
16489.0000000	0.2933334
16490.0000000	0.2850000
16491.0000000	0.2900000
16492.0000000	0.2850000
16493.0000000	0.2900000
16494.0000000	0.2950000
16495.0000000	0.3000000
16496.0000000	0.2900000
16497.0000000	0.2900000
16498.0000000	0.2850000
16499.0000000	0.2850000
16500.0000000	0.2950000
16501.0000000	0.2900000
16502.0000000	0.2850000
16503.0000000	0.2850000
16504.0000000	0.2850000
16505.0000000	0.2800000
16506.0000000	0.2850000
16507.0000000	0.2900000
16508.0000000	0.2950000
16509.0000000	0.2900000
16510.0000000	0.2900000
16511.0000000	0.2900000
16512.0000000	0.2950000
16513.0000000	0.2850000
16514.0000000	0.2950000
16515.0000000	0.2900000
16516.0000000	0.2950000
16517.0000000	0.2900000
16518.0000000	0.2900000
16519.0000000	0.2900000
16520.0000000	0.2933333
16521.0000000	0.2950000
16522.0000000	0.2900000
16523.0000000	0.2850000
16524.0000000	0.2900000
16525.0000000	0.3050000
16526.0000000	0.2850000
16527.0000000	0.3050000
16528.0000000	0.3000000
16529.0000000	0.2950000

16530.0000000	0.3000000
16531.0000000	0.3000000
16532.0000000	0.3000000
16533.0000000	0.3000000
16534.0000000	0.3000000
16535.0000000	0.3000000
16536.0000000	0.3100000
16537.0000000	0.3000000
16538.0000000	0.2950000
16539.0000000	0.2950000
16540.0000000	0.3100000
16541.0000000	0.3050000
16542.0000000	0.3150000
16543.0000000	0.3150000
16544.0000000	0.3050000
16545.0000000	0.3000000
16546.0000000	0.3000000
16547.0000000	0.3000000
16548.0000000	0.3150000
16549.0000000	0.3100000
16550.0000000	0.3133333
16551.0000000	0.3150000
16552.0000000	0.3200000
16553.0000000	0.3150000
16554.0000000	0.3100000
16555.0000000	0.3150000
16556.0000000	0.3200000
16557.0000000	0.3150000
16558.0000000	0.3100000
16559.0000000	0.3100000
16560.0000000	0.3200000
16561.0000000	0.3200000
16562.0000000	0.3100000
16563.0000000	0.3150000
16564.0000000	0.3050000
16565.0000000	0.3100000
16566.0000000	0.3050000
16567.0000000	0.3100000
16568.0000000	0.3200000
16569.0000000	0.3150000
16570.0000000	0.3100000
16571.0000000	0.3050000
16572.0000000	0.3150000
16573.0000000	0.3050000
16574.0000000	0.3100000
16575.0000000	0.3050000
16576.0000000	0.3050000
16577.0000000	0.3100000
16578.0000000	0.3050000
16579.0000000	0.3050000
16580.0000000	0.3066667
16581.0000000	0.3100000
16582.0000000	0.3100000
16583.0000000	0.3100000
16584.0000000	0.3000000
16585.0000000	0.3000000
16586.0000000	0.3150000

16587.0000000	0.3100000
16588.0000000	0.3100000
16589.0000000	0.3100000
16590.0000000	0.3050000
16591.0000000	0.3150000
16592.0000000	0.3100000
16593.0000000	0.3050000
16594.0000000	0.3100000
16595.0000000	0.3100000
16596.0000000	0.3150000
16597.0000000	0.3200000
16598.0000000	0.3100000
16599.0000000	0.3200000
16600.0000000	0.3150000
16601.0000000	0.3100000
16602.0000000	0.3200000
16603.0000000	0.3050000
16604.0000000	0.3150000
16605.0000000	0.3200000
16606.0000000	0.3200000
16607.0000000	0.3250000
16608.0000000	0.3150000
16609.0000000	0.3050000
16610.0000000	0.3200000
16611.0000000	0.3200000
16612.0000000	0.3100000
16613.0000000	0.3150000
16614.0000000	0.3050000
16615.0000000	0.3150000
16616.0000000	0.3150000
16617.0000000	0.3100000
16618.0000000	0.3150000
16619.0000000	0.3050000
16620.0000000	0.3100000
16621.0000000	0.3100000
16622.0000000	0.3050000
16623.0000000	0.3100000
16624.0000000	0.3100000
16625.0000000	0.3100000
16626.0000000	0.3100000
16627.0000000	0.3150000
16628.0000000	0.3100000
16629.0000000	0.3050000
16630.0000000	0.2950000
16631.0000000	0.3100000
16632.0000000	0.3100000
16633.0000000	0.3100000
16634.0000000	0.3000000
16635.0000000	0.3000000
16636.0000000	0.3000000
16637.0000000	0.3000000
16638.0000000	0.3100000
16639.0000000	0.3000000
16640.0000000	0.2950000
16641.0000000	0.3066667
16642.0000000	0.2950000
16643.0000000	0.3050000

16644.0000000	0.2900000
16645.0000000	0.3100000
16646.0000000	0.3050000
16647.0000000	0.2950000
16648.0000000	0.3000000
16649.0000000	0.3100000
16650.0000000	0.3000000
16651.0000000	0.3050000
16652.0000000	0.3000000
16653.0000000	0.2900000
16654.0000000	0.3100000
16655.0000000	0.3100000
16656.0000000	0.3000000
16657.0000000	0.3050000
16658.0000000	0.3000000
16659.0000000	0.3050000
16660.0000000	0.3050000
16661.0000000	0.2950000
16662.0000000	0.3050000
16663.0000000	0.3100000
16664.0000000	0.3100000
16665.0000000	0.3050000
16666.0000000	0.3050000
16667.0000000	0.3000000
16668.0000000	0.2950000
16669.0000000	0.3050000
16670.0000000	0.3150000
16671.0000000	0.3100000
16672.0000000	0.3150000
16673.0000000	0.3150000
16674.0000000	0.3050000
16675.0000000	0.3050000
16676.0000000	0.3100000
16677.0000000	0.3150000
16678.0000000	0.3150000
16679.0000000	0.3150000
16680.0000000	0.3150000
16681.0000000	0.3050000
16682.0000000	0.3200000
16683.0000000	0.3050000
16684.0000000	0.3050000
16685.0000000	0.3200000
16686.0000000	0.3050000
16687.0000000	0.3250000
16688.0000000	0.3150000
16689.0000000	0.3150000
16690.0000000	0.3100000
16691.0000000	0.3000000
16692.0000000	0.3100000
16693.0000000	0.3150000
16694.0000000	0.3100000
16695.0000000	0.3150000
16696.0000000	0.3000000
16697.0000000	0.3100000
16698.0000000	0.3050000
16699.0000000	0.3100000
16700.0000000	0.3050000

16701.0000000	0.3100000
16702.0000000	0.3100000
16703.0000000	0.3050000
16704.0000000	0.3100000
16705.0000000	0.3050000
16706.0000000	0.3200000
16707.0000000	0.3100000
16708.0000000	0.3100000
16709.0000000	0.3100000
16710.0000000	0.3100000
16711.0000000	0.3050000
16712.0000000	0.3150000
16713.0000000	0.3150000
16714.0000000	0.3100000
16715.0000000	0.3150000
16716.0000000	0.3050000
16717.0000000	0.3150000
16718.0000000	0.3150000
16719.0000000	0.3150000
16720.0000000	0.3100000
16721.0000000	0.3050000
16722.0000000	0.3150000
16723.0000000	0.3100000
16724.0000000	0.3150000
16725.0000000	0.3000000
16726.0000000	0.3100000
16727.0000000	0.3000000
16728.0000000	0.3050000
16729.0000000	0.3100000
16730.0000000	0.3150000
16731.0000000	0.2966667
16732.0000000	0.3100000
16733.0000000	0.3100000
16734.0000000	0.3100000
16735.0000000	0.3000000
16736.0000000	0.3050000
16737.0000000	0.3000000
16738.0000000	0.3150000
16739.0000000	0.3050000
16740.0000000	0.3150000
16741.0000000	0.3000000
16742.0000000	0.3000000
16743.0000000	0.2950000
16744.0000000	0.3100000
16745.0000000	0.3050000
16746.0000000	0.3050000
16747.0000000	0.3000000
16748.0000000	0.2950000
16749.0000000	0.2950000
16750.0000000	0.3100000
16751.0000000	0.3000000
16752.0000000	0.3100000
16753.0000000	0.3050000
16754.0000000	0.3000000
16755.0000000	0.3000000
16756.0000000	0.3000000
16757.0000000	0.3000000

16758.0000000	0.3050000
16759.0000000	0.3050000
16760.0000000	0.2950000
16761.0000000	0.2966667
16762.0000000	0.2950000
16763.0000000	0.2950000
16764.0000000	0.2900000
16765.0000000	0.2950000
16766.0000000	0.2850000
16767.0000000	0.3000000
16768.0000000	0.3000000
16769.0000000	0.3000000
16770.0000000	0.3000000
16771.0000000	0.2900000
16772.0000000	0.2950000
16773.0000000	0.3000000
16774.0000000	0.3000000
16775.0000000	0.2900000
16776.0000000	0.3050000
16777.0000000	0.2950000
16778.0000000	0.3000000
16779.0000000	0.2950000
16780.0000000	0.2950000
16781.0000000	0.2950000
16782.0000000	0.3000000
16783.0000000	0.2900000
16784.0000000	0.2900000
16785.0000000	0.2900000
16786.0000000	0.2900000
16787.0000000	0.2850000
16788.0000000	0.2850000
16789.0000000	0.2950000
16790.0000000	0.2850000
16791.0000000	0.2850000
16792.0000000	0.2800000
16793.0000000	0.2800000
16794.0000000	0.2800000
16795.0000000	0.2750000
16796.0000000	0.2750000
16797.0000000	0.2650000
16798.0000000	0.2750000
16799.0000000	0.2800000
16800.0000000	0.2750000
16801.0000000	0.2800000
16802.0000000	0.2750000
16803.0000000	0.2700000
16804.0000000	0.2800000
16805.0000000	0.2600000
16806.0000000	0.2750000
16807.0000000	0.2550000
16808.0000000	0.2700000
16809.0000000	0.2700000
16810.0000000	0.2750000
16811.0000000	0.2700000
16812.0000000	0.2600000
16813.0000000	0.2650000
16814.0000000	0.2650000

16815.0000000	0.2700000
16816.0000000	0.2600000
16817.0000000	0.2700000
16818.0000000	0.2600000
16819.0000000	0.2550000
16820.0000000	0.2650000
16821.0000000	0.2633333
16822.0000000	0.2550000
16823.0000000	0.2600000
16824.0000000	0.2550000
16825.0000000	0.2650000
16826.0000000	0.2650000
16827.0000000	0.2650000
16828.0000000	0.2650000
16829.0000000	0.2550000
16830.0000000	0.2600000
16831.0000000	0.2500000
16832.0000000	0.2600000
16833.0000000	0.2550000
16834.0000000	0.2550000
16835.0000000	0.2500000
16836.0000000	0.2550000
16837.0000000	0.2550000
16838.0000000	0.2500000
16839.0000000	0.2550000
16840.0000000	0.2550000
16841.0000000	0.2550000
16842.0000000	0.2550000
16843.0000000	0.2500000
16844.0000000	0.2500000
16845.0000000	0.2500000
16846.0000000	0.2500000
16847.0000000	0.2550000
16848.0000000	0.2400000
16849.0000000	0.2600000
16850.0000000	0.2500000
16851.0000000	0.2550000
16852.0000000	0.2533333
16853.0000000	0.2500000
16854.0000000	0.2500000
16855.0000000	0.2600000
16856.0000000	0.2550000
16857.0000000	0.2550000
16858.0000000	0.2500000
16859.0000000	0.2550000
16860.0000000	0.2550000
16861.0000000	0.2650000
16862.0000000	0.2500000
16863.0000000	0.2450000
16864.0000000	0.2550000
16865.0000000	0.2550000
16866.0000000	0.2500000
16867.0000000	0.2450000
16868.0000000	0.2400000
16869.0000000	0.2500000
16870.0000000	0.2600000
16871.0000000	0.2450000

16872.0000000	0.2550000
16873.0000000	0.2450000
16874.0000000	0.2400000
16875.0000000	0.2500000
16876.0000000	0.2500000
16877.0000000	0.2500000
16878.0000000	0.2450000
16879.0000000	0.2500000
16880.0000000	0.2500000
16881.0000000	0.2450000
16882.0000000	0.2466667
16883.0000000	0.2350000
16884.0000000	0.2450000
16885.0000000	0.2500000
16886.0000000	0.2400000
16887.0000000	0.2450000
16888.0000000	0.2450000
16889.0000000	0.2500000
16890.0000000	0.2400000
16891.0000000	0.2450000
16892.0000000	0.2400000
16893.0000000	0.2400000
16894.0000000	0.2400000
16895.0000000	0.2550000
16896.0000000	0.2500000
16897.0000000	0.2450000
16898.0000000	0.2350000
16899.0000000	0.2450000
16900.0000000	0.2500000
16901.0000000	0.2450000
16902.0000000	0.2500000
16903.0000000	0.2400000
16904.0000000	0.2450000
16905.0000000	0.2400000
16906.0000000	0.2500000
16907.0000000	0.2400000
16908.0000000	0.2550000
16909.0000000	0.2450000
16910.0000000	0.2400000
16911.0000000	0.2500000
16912.0000000	0.2433333
16913.0000000	0.2450000
16914.0000000	0.2450000
16915.0000000	0.2550000
16916.0000000	0.2500000
16917.0000000	0.2500000
16918.0000000	0.2350000
16919.0000000	0.2500000
16920.0000000	0.2450000
16921.0000000	0.2500000
16922.0000000	0.2500000
16923.0000000	0.2450000
16924.0000000	0.2550000
16925.0000000	0.2500000
16926.0000000	0.2500000
16927.0000000	0.2500000
16928.0000000	0.2450000

16929.0000000	0.2550000
16930.0000000	0.2500000
16931.0000000	0.2650000
16932.0000000	0.2500000
16933.0000000	0.2450000
16934.0000000	0.2500000
16935.0000000	0.2400000
16936.0000000	0.2600000
16937.0000000	0.2550000
16938.0000000	0.2500000
16939.0000000	0.2550000
16940.0000000	0.2450000
16941.0000000	0.2500000
16942.0000000	0.2466667
16943.0000000	0.2500000
16944.0000000	0.2500000
16945.0000000	0.2600000
16946.0000000	0.2450000
16947.0000000	0.2450000
16948.0000000	0.2450000
16949.0000000	0.2450000
16950.0000000	0.2450000
16951.0000000	0.2600000
16952.0000000	0.2550000
16953.0000000	0.2450000
16954.0000000	0.2500000
16955.0000000	0.2500000
16956.0000000	0.2550000
16957.0000000	0.2500000
16958.0000000	0.2450000
16959.0000000	0.2500000
16960.0000000	0.2500000
16961.0000000	0.2450000
16962.0000000	0.2500000
16963.0000000	0.2400000
16964.0000000	0.2500000
16965.0000000	0.2450000
16966.0000000	0.2550000
16967.0000000	0.2550000
16968.0000000	0.2450000
16969.0000000	0.2450000
16970.0000000	0.2500000
16971.0000000	0.2400000
16972.0000000	0.2450000
16973.0000000	0.2466667
16974.0000000	0.2450000
16975.0000000	0.2500000
16976.0000000	0.2500000
16977.0000000	0.2550000
16978.0000000	0.2500000
16979.0000000	0.2550000
16980.0000000	0.2450000
16981.0000000	0.2600000
16982.0000000	0.2650000
16983.0000000	0.2550000
16984.0000000	0.2600000
16985.0000000	0.2550000

16986.0000000	0.2600000
16987.0000000	0.2600000
16988.0000000	0.2700000
16989.0000000	0.2450000
16990.0000000	0.2600000
16991.0000000	0.2750000
16992.0000000	0.2600000
16993.0000000	0.2600000
16994.0000000	0.2550000
16995.0000000	0.2600000
16996.0000000	0.2550000
16997.0000000	0.2650000
16998.0000000	0.2550000
16999.0000000	0.2500000
17000.0000000	0.2600000
17001.0000000	0.2700000
17002.0000000	0.2600000
17003.0000000	0.2633333
17004.0000000	0.2650000
17005.0000000	0.2600000
17006.0000000	0.2500000
17007.0000000	0.2650000
17008.0000000	0.2700000
17009.0000000	0.2650000
17010.0000000	0.2650000
17011.0000000	0.2550000
17012.0000000	0.2600000
17013.0000000	0.2650000
17014.0000000	0.2650000
17015.0000000	0.2500000
17016.0000000	0.2600000
17017.0000000	0.2500000
17018.0000000	0.2750000
17019.0000000	0.2650000
17020.0000000	0.2700000
17021.0000000	0.2550000
17022.0000000	0.2500000
17023.0000000	0.2600000
17024.0000000	0.2650000
17025.0000000	0.2700000
17026.0000000	0.2650000
17027.0000000	0.2500000
17028.0000000	0.2600000
17029.0000000	0.2550000
17030.0000000	0.2700000
17031.0000000	0.2550000
17032.0000000	0.2650000
17033.0000000	0.2566667
17034.0000000	0.2650000
17035.0000000	0.2650000
17036.0000000	0.2600000
17037.0000000	0.2600000
17038.0000000	0.2550000
17039.0000000	0.2500000
17040.0000000	0.2650000
17041.0000000	0.2550000
17042.0000000	0.2650000

17043.0000000	0.2500000
17044.0000000	0.2550000
17045.0000000	0.2600000
17046.0000000	0.2650000
17047.0000000	0.2600000
17048.0000000	0.2600000
17049.0000000	0.2550000
17050.0000000	0.2550000
17051.0000000	0.2550000
17052.0000000	0.2600000
17053.0000000	0.2600000
17054.0000000	0.2650000
17055.0000000	0.2500000
17056.0000000	0.2600000
17057.0000000	0.2550000
17058.0000000	0.2650000
17059.0000000	0.2600000
17060.0000000	0.2650000
17061.0000000	0.2500000
17062.0000000	0.2500000
17063.0000000	0.2600000
17064.0000000	0.2500000
17065.0000000	0.2550000
17066.0000000	0.2400000
17067.0000000	0.2500000
17068.0000000	0.2550000
17069.0000000	0.2450000
17070.0000000	0.2550000
17071.0000000	0.2500000
17072.0000000	0.2400000
17073.0000000	0.2450000
17074.0000000	0.2450000
17075.0000000	0.2500000
17076.0000000	0.2500000
17077.0000000	0.2550000
17078.0000000	0.2450000
17079.0000000	0.2350000
17080.0000000	0.2550000
17081.0000000	0.2450000
17082.0000000	0.2550000
17083.0000000	0.2400000
17084.0000000	0.2500000
17085.0000000	0.2450000
17086.0000000	0.2550000
17087.0000000	0.2500000
17088.0000000	0.2500000
17089.0000000	0.2450000
17090.0000000	0.2550000
17091.0000000	0.2400000
17092.0000000	0.2500000
17093.0000000	0.2400000
17094.0000000	0.2533333
17095.0000000	0.2400000
17096.0000000	0.2350000
17097.0000000	0.2550000
17098.0000000	0.2450000
17099.0000000	0.2450000

17100.0000000	0.2350000
17101.0000000	0.2400000
17102.0000000	0.2250000
17103.0000000	0.2500000
17104.0000000	0.2400000
17105.0000000	0.2450000
17106.0000000	0.2350000
17107.0000000	0.2350000
17108.0000000	0.2450000
17109.0000000	0.2400000
17110.0000000	0.2450000
17111.0000000	0.2450000
17112.0000000	0.2400000
17113.0000000	0.2450000
17114.0000000	0.2450000
17115.0000000	0.2350000
17116.0000000	0.2350000
17117.0000000	0.2350000
17118.0000000	0.2450000
17119.0000000	0.2400000
17120.0000000	0.2350000
17121.0000000	0.2400000
17122.0000000	0.2400000
17123.0000000	0.2450000
17124.0000000	0.2333333
17125.0000000	0.2450000
17126.0000000	0.2350000
17127.0000000	0.2500000
17128.0000000	0.2400000
17129.0000000	0.2350000
17130.0000000	0.2400000
17131.0000000	0.2300000
17132.0000000	0.2450000
17133.0000000	0.2450000
17134.0000000	0.2400000
17135.0000000	0.2400000
17136.0000000	0.2300000
17137.0000000	0.2400000
17138.0000000	0.2500000
17139.0000000	0.2350000
17140.0000000	0.2350000
17141.0000000	0.2400000
17142.0000000	0.2400000
17143.0000000	0.2400000
17144.0000000	0.2300000
17145.0000000	0.2450000
17146.0000000	0.2300000
17147.0000000	0.2450000
17148.0000000	0.2350000
17149.0000000	0.2450000
17150.0000000	0.2450000
17151.0000000	0.2400000
17152.0000000	0.2300000
17153.0000000	0.2400000
17154.0000000	0.2500000
17155.0000000	0.2300000
17156.0000000	0.2300000

17157.0000000	0.2300000
17158.0000000	0.2400000
17159.0000000	0.2400000
17160.0000000	0.2350000
17161.0000000	0.2400000
17162.0000000	0.2400000
17163.0000000	0.2400000
17164.0000000	0.2350000
17165.0000000	0.2350000
17166.0000000	0.2350000
17167.0000000	0.2350000
17168.0000000	0.2400000
17169.0000000	0.2350000
17170.0000000	0.2400000
17171.0000000	0.2300000
17172.0000000	0.2350000
17173.0000000	0.2450000
17174.0000000	0.2350000
17175.0000000	0.2400000
17176.0000000	0.2350000
17177.0000000	0.2400000
17178.0000000	0.2400000
17179.0000000	0.2350000
17180.0000000	0.2400000
17181.0000000	0.2450000
17182.0000000	0.2400000
17183.0000000	0.2300000
17184.0000000	0.2366667
17185.0000000	0.2400000
17186.0000000	0.2400000
17187.0000000	0.2350000
17188.0000000	0.2300000
17189.0000000	0.2350000
17190.0000000	0.2350000
17191.0000000	0.2250000
17192.0000000	0.2400000
17193.0000000	0.2400000
17194.0000000	0.2400000
17195.0000000	0.2450000
17196.0000000	0.2300000
17197.0000000	0.2400000
17198.0000000	0.2250000
17199.0000000	0.2450000
17200.0000000	0.2350000
17201.0000000	0.2450000
17202.0000000	0.2350000
17203.0000000	0.2300000
17204.0000000	0.2250000
17205.0000000	0.2450000
17206.0000000	0.2300000
17207.0000000	0.2450000
17208.0000000	0.2300000
17209.0000000	0.2400000
17210.0000000	0.2350000
17211.0000000	0.2400000
17212.0000000	0.2400000
17213.0000000	0.2400000

17214.0000000	0.2400000
17215.0000000	0.2350000
17216.0000000	0.2450000
17217.0000000	0.2400000
17218.0000000	0.2500000
17219.0000000	0.2350000
17220.0000000	0.2400000
17221.0000000	0.2300000
17222.0000000	0.2450000
17223.0000000	0.2400000
17224.0000000	0.2500000
17225.0000000	0.2400000
17226.0000000	0.2350000
17227.0000000	0.2350000
17228.0000000	0.2400000
17229.0000000	0.2400000
17230.0000000	0.2500000
17231.0000000	0.2250000
17232.0000000	0.2450000
17233.0000000	0.2350000
17234.0000000	0.2400000
17235.0000000	0.2350000
17236.0000000	0.2500000
17237.0000000	0.2400000
17238.0000000	0.2450000
17239.0000000	0.2400000
17240.0000000	0.2350000
17241.0000000	0.2400000
17242.0000000	0.2350000
17243.0000000	0.2450000
17244.0000000	0.2433333
17245.0000000	0.2350000
17246.0000000	0.2350000
17247.0000000	0.2350000
17248.0000000	0.2400000
17249.0000000	0.2350000
17250.0000000	0.2350000
17251.0000000	0.2350000
17252.0000000	0.2300000
17253.0000000	0.2350000
17254.0000000	0.2450000
17255.0000000	0.2400000
17256.0000000	0.2450000
17257.0000000	0.2300000
17258.0000000	0.2450000
17259.0000000	0.2350000
17260.0000000	0.2400000
17261.0000000	0.2400000
17262.0000000	0.2350000
17263.0000000	0.2400000
17264.0000000	0.2350000
17265.0000000	0.2400000
17266.0000000	0.2350000
17267.0000000	0.2300000
17268.0000000	0.2300000
17269.0000000	0.2300000
17270.0000000	0.2350000

17271.0000000	0.2400000
17272.0000000	0.2400000
17273.0000000	0.2400000
17274.0000000	0.2300000
17275.0000000	0.2300000
17276.0000000	0.2450000
17277.0000000	0.2300000
17278.0000000	0.2400000
17279.0000000	0.2250000
17280.0000000	0.2400000
17281.0000000	0.2450000
17282.0000000	0.2350000
17283.0000000	0.2400000
17284.0000000	0.2400000
17285.0000000	0.2450000
17286.0000000	0.2450000
17287.0000000	0.2400000
17288.0000000	0.2450000
17289.0000000	0.2400000
17290.0000000	0.2400000
17291.0000000	0.2400000
17292.0000000	0.2350000
17293.0000000	0.2450000
17294.0000000	0.2450000
17295.0000000	0.2450000
17296.0000000	0.2450000
17297.0000000	0.2350000
17298.0000000	0.2400000
17299.0000000	0.2350000
17300.0000000	0.2300000
17301.0000000	0.2400000
17302.0000000	0.2450000
17303.0000000	0.2500000
17304.0000000	0.2450000
17305.0000000	0.2400000
17306.0000000	0.2350000
17307.0000000	0.2450000
17308.0000000	0.2500000
17309.0000000	0.2350000
17310.0000000	0.2450000
17311.0000000	0.2400000
17312.0000000	0.2500000
17313.0000000	0.2500000
17314.0000000	0.2500000
17315.0000000	0.2450000
17316.0000000	0.2500000
17317.0000000	0.2550000
17318.0000000	0.2450000
17319.0000000	0.2500000
17320.0000000	0.2500000
17321.0000000	0.2500000
17322.0000000	0.2550000
17323.0000000	0.2550000
17324.0000000	0.2500000
17325.0000000	0.2550000
17326.0000000	0.2400000
17327.0000000	0.2400000

17328.0000000	0.2550000
17329.0000000	0.2600000
17330.0000000	0.2600000
17331.0000000	0.2500000
17332.0000000	0.2500000
17333.0000000	0.2500000
17334.0000000	0.2500000
17335.0000000	0.2533333
17336.0000000	0.2500000
17337.0000000	0.2450000
17338.0000000	0.2400000
17339.0000000	0.2450000
17340.0000000	0.2500000
17341.0000000	0.2500000
17342.0000000	0.2350000
17343.0000000	0.2400000
17344.0000000	0.2350000
17345.0000000	0.2450000
17346.0000000	0.2500000
17347.0000000	0.2500000
17348.0000000	0.2400000
17349.0000000	0.2350000
17350.0000000	0.2400000
17351.0000000	0.2400000
17352.0000000	0.2550000
17353.0000000	0.2400000
17354.0000000	0.2500000
17355.0000000	0.2400000
17356.0000000	0.2500000
17357.0000000	0.2500000
17358.0000000	0.2500000
17359.0000000	0.2550000
17360.0000000	0.2450000
17361.0000000	0.2500000
17362.0000000	0.2550000
17363.0000000	0.2500000
17364.0000000	0.2400000
17365.0000000	0.2466667
17366.0000000	0.2550000
17367.0000000	0.2400000
17368.0000000	0.2450000
17369.0000000	0.2450000
17370.0000000	0.2500000
17371.0000000	0.2450000
17372.0000000	0.2550000
17373.0000000	0.2500000
17374.0000000	0.2450000
17375.0000000	0.2450000
17376.0000000	0.2350000
17377.0000000	0.2500000
17378.0000000	0.2500000
17379.0000000	0.2500000
17380.0000000	0.2500000
17381.0000000	0.2450000
17382.0000000	0.2400000
17383.0000000	0.2450000
17384.0000000	0.2500000

17385.0000000	0.2400000
17386.0000000	0.2500000
17387.0000000	0.2450000
17388.0000000	0.2550000
17389.0000000	0.2450000
17390.0000000	0.2550000
17391.0000000	0.2450000
17392.0000000	0.2500000
17393.0000000	0.2500000
17394.0000000	0.2600000
17395.0000000	0.2533333
17396.0000000	0.2500000
17397.0000000	0.2550000
17398.0000000	0.2450000
17399.0000000	0.2500000
17400.0000000	0.2550000
17401.0000000	0.2500000
17402.0000000	0.2550000
17403.0000000	0.2500000
17404.0000000	0.2550000
17405.0000000	0.2450000
17406.0000000	0.2500000
17407.0000000	0.2450000
17408.0000000	0.2400000
17409.0000000	0.2500000
17410.0000000	0.2550000
17411.0000000	0.2600000
17412.0000000	0.2550000
17413.0000000	0.2450000
17414.0000000	0.2550000
17415.0000000	0.2500000
17416.0000000	0.2550000
17417.0000000	0.2400000
17418.0000000	0.2450000
17419.0000000	0.2600000
17420.0000000	0.2550000
17421.0000000	0.2550000
17422.0000000	0.2500000
17423.0000000	0.2500000
17424.0000000	0.2550000
17425.0000000	0.2450000
17426.0000000	0.2533333
17427.0000000	0.2450000
17428.0000000	0.2400000
17429.0000000	0.2500000
17430.0000000	0.2550000
17431.0000000	0.2500000
17432.0000000	0.2500000
17433.0000000	0.2500000
17434.0000000	0.2450000
17435.0000000	0.2500000
17436.0000000	0.2400000
17437.0000000	0.2400000
17438.0000000	0.2500000
17439.0000000	0.2450000
17440.0000000	0.2400000
17441.0000000	0.2450000

17442.0000000	0.2450000
17443.0000000	0.2450000
17444.0000000	0.2450000
17445.0000000	0.2450000
17446.0000000	0.2400000
17447.0000000	0.2450000
17448.0000000	0.2400000
17449.0000000	0.2400000
17450.0000000	0.2400000
17451.0000000	0.2400000
17452.0000000	0.2350000
17453.0000000	0.2500000
17454.0000000	0.2300000
17455.0000000	0.2450000
17456.0000000	0.2400000
17457.0000000	0.2400000
17458.0000000	0.2400000
17459.0000000	0.2350000
17460.0000000	0.2300000
17461.0000000	0.2400000
17462.0000000	0.2350000
17463.0000000	0.2350000
17464.0000000	0.2350000
17465.0000000	0.2300000
17466.0000000	0.2450000
17467.0000000	0.2350000
17468.0000000	0.2400000
17469.0000000	0.2250000
17470.0000000	0.2300000
17471.0000000	0.2350000
17472.0000000	0.2350000
17473.0000000	0.2300000
17474.0000000	0.2350000
17475.0000000	0.2400000
17476.0000000	0.2300000
17477.0000000	0.2300000
17478.0000000	0.2350000
17479.0000000	0.2250000
17480.0000000	0.2350000
17481.0000000	0.2350000
17482.0000000	0.2350000
17483.0000000	0.2300000
17484.0000000	0.2400000
17485.0000000	0.2400000
17486.0000000	0.2250000
17487.0000000	0.2400000
17488.0000000	0.2350000
17489.0000000	0.2400000
17490.0000000	0.2400000
17491.0000000	0.2350000
17492.0000000	0.2350000
17493.0000000	0.2400000
17494.0000000	0.2400000
17495.0000000	0.2450000
17496.0000000	0.2400000
17497.0000000	0.2450000
17498.0000000	0.2400000

17499.0000000	0.2450000
17500.0000000	0.2400000
17501.0000000	0.2350000
17502.0000000	0.2350000
17503.0000000	0.2350000
17504.0000000	0.2450000
17505.0000000	0.2450000
17506.0000000	0.2450000
17507.0000000	0.2400000
17508.0000000	0.2400000
17509.0000000	0.2400000
17510.0000000	0.2450000
17511.0000000	0.2500000
17512.0000000	0.2500000
17513.0000000	0.2500000
17514.0000000	0.2400000
17515.0000000	0.2500000
17516.0000000	0.2466667
17517.0000000	0.2400000
17518.0000000	0.2400000
17519.0000000	0.2350000
17520.0000000	0.2450000
17521.0000000	0.2400000
17522.0000000	0.2450000
17523.0000000	0.2450000
17524.0000000	0.2450000
17525.0000000	0.2450000
17526.0000000	0.2400000
17527.0000000	0.2400000
17528.0000000	0.2350000
17529.0000000	0.2350000
17530.0000000	0.2450000
17531.0000000	0.2400000
17532.0000000	0.2400000
17533.0000000	0.2300000
17534.0000000	0.2350000
17535.0000000	0.2300000
17536.0000000	0.2400000
17537.0000000	0.2400000
17538.0000000	0.2450000
17539.0000000	0.2350000
17540.0000000	0.2350000
17541.0000000	0.2400000
17542.0000000	0.2350000
17543.0000000	0.2450000
17544.0000000	0.2400000
17545.0000000	0.2450000
17546.0000000	0.2400000
17547.0000000	0.2350000
17548.0000000	0.2450000
17549.0000000	0.2450000
17550.0000000	0.2350000
17551.0000000	0.2450000
17552.0000000	0.2400000
17553.0000000	0.2500000
17554.0000000	0.2400000
17555.0000000	0.2400000

17556.0000000	0.2450000
17557.0000000	0.2450000
17558.0000000	0.2450000
17559.0000000	0.2400000
17560.0000000	0.2400000
17561.0000000	0.2350000
17562.0000000	0.2400000
17563.0000000	0.2500000
17564.0000000	0.2400000
17565.0000000	0.2450000
17566.0000000	0.2350000
17567.0000000	0.2400000
17568.0000000	0.2450000
17569.0000000	0.2500000
17570.0000000	0.2400000
17571.0000000	0.2450000
17572.0000000	0.2450000
17573.0000000	0.2500000
17574.0000000	0.2450000
17575.0000000	0.2450000
17576.0000000	0.2433333
17577.0000000	0.2300000
17578.0000000	0.2500000
17579.0000000	0.2450000
17580.0000000	0.2500000
17581.0000000	0.2400000
17582.0000000	0.2450000
17583.0000000	0.2400000
17584.0000000	0.2450000
17585.0000000	0.2450000
17586.0000000	0.2350000
17587.0000000	0.2450000
17588.0000000	0.2400000
17589.0000000	0.2500000
17590.0000000	0.2450000
17591.0000000	0.2500000
17592.0000000	0.2400000
17593.0000000	0.2400000
17594.0000000	0.2450000
17595.0000000	0.2550000
17596.0000000	0.2450000
17597.0000000	0.2550000
17598.0000000	0.2400000
17599.0000000	0.2450000
17600.0000000	0.2400000
17601.0000000	0.2550000
17602.0000000	0.2550000
17603.0000000	0.2500000
17604.0000000	0.2450000
17605.0000000	0.2450000
17606.0000000	0.2500000
17607.0000000	0.2400000
17608.0000000	0.2500000
17609.0000000	0.2550000
17610.0000000	0.2550000
17611.0000000	0.2500000
17612.0000000	0.2450000

17613.0000000	0.2450000
17614.0000000	0.2500000
17615.0000000	0.2450000
17616.0000000	0.2550000
17617.0000000	0.2450000
17618.0000000	0.2550000
17619.0000000	0.2450000
17620.0000000	0.2600000
17621.0000000	0.2550000
17622.0000000	0.2550000
17623.0000000	0.2500000
17624.0000000	0.2500000
17625.0000000	0.2500000
17626.0000000	0.2500000
17627.0000000	0.2500000
17628.0000000	0.2450000
17629.0000000	0.2450000
17630.0000000	0.2500000
17631.0000000	0.2550000
17632.0000000	0.2600000
17633.0000000	0.2400000
17634.0000000	0.2500000
17635.0000000	0.2450000
17636.0000000	0.2500000
17637.0000000	0.2566667
17638.0000000	0.2500000
17639.0000000	0.2450000
17640.0000000	0.2500000
17641.0000000	0.2550000
17642.0000000	0.2550000
17643.0000000	0.2550000
17644.0000000	0.2550000
17645.0000000	0.2500000
17646.0000000	0.2550000
17647.0000000	0.2500000
17648.0000000	0.2500000
17649.0000000	0.2550000
17650.0000000	0.2550000
17651.0000000	0.2600000
17652.0000000	0.2500000
17653.0000000	0.2500000
17654.0000000	0.2450000
17655.0000000	0.2600000
17656.0000000	0.2550000
17657.0000000	0.2600000
17658.0000000	0.2650000
17659.0000000	0.2500000
17660.0000000	0.2550000
17661.0000000	0.2450000
17662.0000000	0.2550000
17663.0000000	0.2600000
17664.0000000	0.2650000
17665.0000000	0.2500000
17666.0000000	0.2600000
17667.0000000	0.2566667
17668.0000000	0.2550000
17669.0000000	0.2550000

17670.0000000	0.2550000
17671.0000000	0.2500000
17672.0000000	0.2550000
17673.0000000	0.2550000
17674.0000000	0.2550000
17675.0000000	0.2650000
17676.0000000	0.2450000
17677.0000000	0.2550000
17678.0000000	0.2550000
17679.0000000	0.2600000
17680.0000000	0.2600000
17681.0000000	0.2700000
17682.0000000	0.2550000
17683.0000000	0.2600000
17684.0000000	0.2500000
17685.0000000	0.2650000
17686.0000000	0.2500000
17687.0000000	0.2650000
17688.0000000	0.2550000
17689.0000000	0.2600000
17690.0000000	0.2500000
17691.0000000	0.2650000
17692.0000000	0.2550000
17693.0000000	0.2700000
17694.0000000	0.2600000
17695.0000000	0.2600000
17696.0000000	0.2600000
17697.0000000	0.2566667
17698.0000000	0.2650000
17699.0000000	0.2600000
17700.0000000	0.2600000
17701.0000000	0.2600000
17702.0000000	0.2600000
17703.0000000	0.2650000
17704.0000000	0.2600000
17705.0000000	0.2550000
17706.0000000	0.2650000
17707.0000000	0.2650000
17708.0000000	0.2700000
17709.0000000	0.2550000
17710.0000000	0.2600000
17711.0000000	0.2700000
17712.0000000	0.2650000
17713.0000000	0.2650000
17714.0000000	0.2600000
17715.0000000	0.2600000
17716.0000000	0.2650000
17717.0000000	0.2650000
17718.0000000	0.2600000
17719.0000000	0.2650000
17720.0000000	0.2700000
17721.0000000	0.2700000
17722.0000000	0.2700000
17723.0000000	0.2650000
17724.0000000	0.2700000
17725.0000000	0.2700000
17726.0000000	0.2700000

17727.0000000	0.2633333
17728.0000000	0.2700000
17729.0000000	0.2600000
17730.0000000	0.2700000
17731.0000000	0.2650000
17732.0000000	0.2600000
17733.0000000	0.2650000
17734.0000000	0.2650000
17735.0000000	0.2700000
17736.0000000	0.2750000
17737.0000000	0.2700000
17738.0000000	0.2700000
17739.0000000	0.2650000
17740.0000000	0.2750000
17741.0000000	0.2700000
17742.0000000	0.2700000
17743.0000000	0.2650000
17744.0000000	0.2600000
17745.0000000	0.2700000
17746.0000000	0.2650000
17747.0000000	0.2750000
17748.0000000	0.2550000
17749.0000000	0.2550000
17750.0000000	0.2600000
17751.0000000	0.2650000
17752.0000000	0.2700000
17753.0000000	0.2600000
17754.0000000	0.2650000
17755.0000000	0.2650000
17756.0000000	0.2650000
17757.0000000	0.2600000
17758.0000000	0.2633333
17759.0000000	0.2600000
17760.0000000	0.2600000
17761.0000000	0.2650000
17762.0000000	0.2700000
17763.0000000	0.2700000
17764.0000000	0.2550000
17765.0000000	0.2550000
17766.0000000	0.2600000
17767.0000000	0.2600000
17768.0000000	0.2650000
17769.0000000	0.2600000
17770.0000000	0.2650000
17771.0000000	0.2550000
17772.0000000	0.2550000
17773.0000000	0.2650000
17774.0000000	0.2500000
17775.0000000	0.2500000
17776.0000000	0.2500000
17777.0000000	0.2550000
17778.0000000	0.2500000
17779.0000000	0.2450000
17780.0000000	0.2550000
17781.0000000	0.2500000
17782.0000000	0.2550000
17783.0000000	0.2450000

17784.0000000	0.2450000
17785.0000000	0.2550000
17786.0000000	0.2550000
17787.0000000	0.2500000
17788.0000000	0.2533333
17789.0000000	0.2400000
17790.0000000	0.2600000
17791.0000000	0.2400000
17792.0000000	0.2500000
17793.0000000	0.2500000
17794.0000000	0.2450000
17795.0000000	0.2500000
17796.0000000	0.2450000
17797.0000000	0.2500000
17798.0000000	0.2450000
17799.0000000	0.2450000
17800.0000000	0.2500000
17801.0000000	0.2400000
17802.0000000	0.2450000
17803.0000000	0.2550000
17804.0000000	0.2450000
17805.0000000	0.2450000
17806.0000000	0.2500000
17807.0000000	0.2500000
17808.0000000	0.2600000
17809.0000000	0.2450000
17810.0000000	0.2350000
17811.0000000	0.2500000
17812.0000000	0.2450000
17813.0000000	0.2450000
17814.0000000	0.2450000
17815.0000000	0.2350000
17816.0000000	0.2550000
17817.0000000	0.2466667
17818.0000000	0.2500000
17819.0000000	0.2500000
17820.0000000	0.2450000
17821.0000000	0.2450000
17822.0000000	0.2450000
17823.0000000	0.2400000
17824.0000000	0.2500000
17825.0000000	0.2400000
17826.0000000	0.2450000
17827.0000000	0.2450000
17828.0000000	0.2400000
17829.0000000	0.2400000
17830.0000000	0.2400000
17831.0000000	0.2400000
17832.0000000	0.2400000
17833.0000000	0.2300000
17834.0000000	0.2400000
17835.0000000	0.2400000
17836.0000000	0.2500000
17837.0000000	0.2450000
17838.0000000	0.2400000
17839.0000000	0.2350000
17840.0000000	0.2400000

17841.0000000	0.2450000
17842.0000000	0.2400000
17843.0000000	0.2500000
17844.0000000	0.2350000
17845.0000000	0.2450000
17846.0000000	0.2450000
17847.0000000	0.2400000
17848.0000000	0.2466667
17849.0000000	0.2450000
17850.0000000	0.2400000
17851.0000000	0.2400000
17852.0000000	0.2400000
17853.0000000	0.2450000
17854.0000000	0.2500000
17855.0000000	0.2450000
17856.0000000	0.2400000
17857.0000000	0.2450000
17858.0000000	0.2400000
17859.0000000	0.2400000
17860.0000000	0.2450000
17861.0000000	0.2400000
17862.0000000	0.2350000
17863.0000000	0.2400000
17864.0000000	0.2350000
17865.0000000	0.2400000
17866.0000000	0.2350000
17867.0000000	0.2400000
17868.0000000	0.2300000
17869.0000000	0.2450000
17870.0000000	0.2350000
17871.0000000	0.2350000
17872.0000000	0.2300000
17873.0000000	0.2350000
17874.0000000	0.2250000
17875.0000000	0.2400000
17876.0000000	0.2350000
17877.0000000	0.2350000
17878.0000000	0.2333333
17879.0000000	0.2300000
17880.0000000	0.2350000
17881.0000000	0.2300000
17882.0000000	0.2300000
17883.0000000	0.2350000
17884.0000000	0.2300000
17885.0000000	0.2400000
17886.0000000	0.2350000
17887.0000000	0.2350000
17888.0000000	0.2350000
17889.0000000	0.2350000
17890.0000000	0.2300000
17891.0000000	0.2450000
17892.0000000	0.2450000
17893.0000000	0.2400000
17894.0000000	0.2300000
17895.0000000	0.2350000
17896.0000000	0.2350000
17897.0000000	0.2400000

17898.0000000	0.2400000
17899.0000000	0.2450000
17900.0000000	0.2400000
17901.0000000	0.2400000
17902.0000000	0.2350000
17903.0000000	0.2500000
17904.0000000	0.2550000
17905.0000000	0.2500000
17906.0000000	0.2450000
17907.0000000	0.2400000
17908.0000000	0.2533333
17909.0000000	0.2400000
17910.0000000	0.2450000
17911.0000000	0.2400000
17912.0000000	0.2550000
17913.0000000	0.2500000
17914.0000000	0.2450000
17915.0000000	0.2400000
17916.0000000	0.2450000
17917.0000000	0.2550000
17918.0000000	0.2550000
17919.0000000	0.2500000
17920.0000000	0.2450000
17921.0000000	0.2450000
17922.0000000	0.2350000
17923.0000000	0.2500000
17924.0000000	0.2550000
17925.0000000	0.2500000
17926.0000000	0.2500000
17927.0000000	0.2400000
17928.0000000	0.2450000
17929.0000000	0.2500000
17930.0000000	0.2500000
17931.0000000	0.2550000
17932.0000000	0.2400000
17933.0000000	0.2550000
17934.0000000	0.2450000
17935.0000000	0.2500000
17936.0000000	0.2500000
17937.0000000	0.2450000
17938.0000000	0.2566667
17939.0000000	0.2550000
17940.0000000	0.2500000
17941.0000000	0.2500000
17942.0000000	0.2500000
17943.0000000	0.2500000
17944.0000000	0.2550000
17945.0000000	0.2400000
17946.0000000	0.2450000
17947.0000000	0.2600000
17948.0000000	0.2500000
17949.0000000	0.2500000
17950.0000000	0.2400000
17951.0000000	0.2450000
17952.0000000	0.2450000
17953.0000000	0.2600000
17954.0000000	0.2500000

17955.0000000	0.2500000
17956.0000000	0.2450000
17957.0000000	0.2450000
17958.0000000	0.2500000
17959.0000000	0.2500000
17960.0000000	0.2450000
17961.0000000	0.2550000
17962.0000000	0.2450000
17963.0000000	0.2500000
17964.0000000	0.2450000
17965.0000000	0.2500000
17966.0000000	0.2450000
17967.0000000	0.2600000
17968.0000000	0.2450000
17969.0000000	0.2500000
17970.0000000	0.2600000
17971.0000000	0.2500000
17972.0000000	0.2550000
17973.0000000	0.2500000
17974.0000000	0.2600000
17975.0000000	0.2500000
17976.0000000	0.2600000
17977.0000000	0.2450000
17978.0000000	0.2500000
17979.0000000	0.2450000
17980.0000000	0.2600000
17981.0000000	0.2450000
17982.0000000	0.2550000
17983.0000000	0.2550000
17984.0000000	0.2550000
17985.0000000	0.2500000
17986.0000000	0.2500000
17987.0000000	0.2500000
17988.0000000	0.2500000
17989.0000000	0.2600000
17990.0000000	0.2500000
17991.0000000	0.2450000
17992.0000000	0.2500000
17993.0000000	0.2500000
17994.0000000	0.2550000
17995.0000000	0.2450000
17996.0000000	0.2550000
17997.0000000	0.2550000
17998.0000000	0.2600000
17999.0000000	0.2500000
18000.0000000	0.2500000
18001.0000000	0.2500000
18002.0000000	0.2450000
18003.0000000	0.2550000
18004.0000000	0.2500000
18005.0000000	0.2600000
18006.0000000	0.2450000
18007.0000000	0.2550000
18008.0000000	0.2600000
18009.0000000	0.2550000
18010.0000000	0.2600000
18011.0000000	0.2500000

18012.0000000	0.2500000
18013.0000000	0.2500000
18014.0000000	0.2550000
18015.0000000	0.2550000
18016.0000000	0.2550000
18017.0000000	0.2550000
18018.0000000	0.2500000
18019.0000000	0.2450000
18020.0000000	0.2500000
18021.0000000	0.2450000
18022.0000000	0.2500000
18023.0000000	0.2500000
18024.0000000	0.2500000
18025.0000000	0.2550000
18026.0000000	0.2450000
18027.0000000	0.2550000
18028.0000000	0.2500000
18029.0000000	0.2466667
18030.0000000	0.2550000
18031.0000000	0.2400000
18032.0000000	0.2300000
18033.0000000	0.2450000
18034.0000000	0.2500000
18035.0000000	0.2600000
18036.0000000	0.2550000
18037.0000000	0.2450000
18038.0000000	0.2500000
18039.0000000	0.2450000
18040.0000000	0.2500000
18041.0000000	0.2450000
18042.0000000	0.2450000
18043.0000000	0.2550000
18044.0000000	0.2500000
18045.0000000	0.2500000
18046.0000000	0.2500000
18047.0000000	0.2500000
18048.0000000	0.2500000
18049.0000000	0.2600000
18050.0000000	0.2450000
18051.0000000	0.2500000
18052.0000000	0.2500000
18053.0000000	0.2400000
18054.0000000	0.2550000
18055.0000000	0.2450000
18056.0000000	0.2500000
18057.0000000	0.2500000
18058.0000000	0.2500000
18059.0000000	0.2433333
18060.0000000	0.2550000
18061.0000000	0.2450000
18062.0000000	0.2500000
18063.0000000	0.2500000
18064.0000000	0.2500000
18065.0000000	0.2400000
18066.0000000	0.2450000
18067.0000000	0.2500000
18068.0000000	0.2400000

18069.0000000	0.2500000
18070.0000000	0.2450000
18071.0000000	0.2450000
18072.0000000	0.2450000
18073.0000000	0.2450000
18074.0000000	0.2500000
18075.0000000	0.2450000
18076.0000000	0.2400000
18077.0000000	0.2450000
18078.0000000	0.2450000
18079.0000000	0.2400000
18080.0000000	0.2450000
18081.0000000	0.2400000
18082.0000000	0.2500000
18083.0000000	0.2400000
18084.0000000	0.2400000
18085.0000000	0.2400000
18086.0000000	0.2400000
18087.0000000	0.2400000
18088.0000000	0.2300000
18089.0000000	0.2400000
18090.0000000	0.2333333
18091.0000000	0.2300000
18092.0000000	0.2350000
18093.0000000	0.2350000
18094.0000000	0.2350000
18095.0000000	0.2350000
18096.0000000	0.2350000
18097.0000000	0.2350000
18098.0000000	0.2400000
18099.0000000	0.2350000
18100.0000000	0.2400000
18101.0000000	0.2300000
18102.0000000	0.2250000
18103.0000000	0.2350000
18104.0000000	0.2300000
18105.0000000	0.2350000
18106.0000000	0.2300000
18107.0000000	0.2400000
18108.0000000	0.2300000
18109.0000000	0.2350000
18110.0000000	0.2250000
18111.0000000	0.2350000
18112.0000000	0.2350000
18113.0000000	0.2400000
18114.0000000	0.2200000
18115.0000000	0.2250000
18116.0000000	0.2150000
18117.0000000	0.2300000
18118.0000000	0.2300000
18119.0000000	0.2300000
18120.0000000	0.2333333
18121.0000000	0.2300000
18122.0000000	0.2250000
18123.0000000	0.2300000
18124.0000000	0.2300000
18125.0000000	0.2350000

18126.0000000	0.2300000
18127.0000000	0.2300000
18128.0000000	0.2300000
18129.0000000	0.2350000
18130.0000000	0.2250000
18131.0000000	0.2350000
18132.0000000	0.2400000
18133.0000000	0.2300000
18134.0000000	0.2300000
18135.0000000	0.2300000
18136.0000000	0.2350000
18137.0000000	0.2250000
18138.0000000	0.2350000
18139.0000000	0.2300000
18140.0000000	0.2250000
18141.0000000	0.2350000
18142.0000000	0.2300000
18143.0000000	0.2400000
18144.0000000	0.2350000
18145.0000000	0.2300000
18146.0000000	0.2400000
18147.0000000	0.2300000
18148.0000000	0.2300000
18149.0000000	0.2333333
18150.0000000	0.2400000
18151.0000000	0.2400000
18152.0000000	0.2350000
18153.0000000	0.2300000
18154.0000000	0.2250000
18155.0000000	0.2300000
18156.0000000	0.2300000
18157.0000000	0.2300000
18158.0000000	0.2350000
18159.0000000	0.2300000
18160.0000000	0.2300000
18161.0000000	0.2450000
18162.0000000	0.2350000
18163.0000000	0.2400000
18164.0000000	0.2300000
18165.0000000	0.2300000
18166.0000000	0.2350000
18167.0000000	0.2350000
18168.0000000	0.2300000
18169.0000000	0.2300000
18170.0000000	0.2250000
18171.0000000	0.2350000
18172.0000000	0.2400000
18173.0000000	0.2350000
18174.0000000	0.2450000
18175.0000000	0.2250000
18176.0000000	0.2350000
18177.0000000	0.2350000
18178.0000000	0.2400000
18179.0000000	0.2250000
18180.0000000	0.2300000
18181.0000000	0.2350000
18182.0000000	0.2350000

18183.0000000	0.2300000
18184.0000000	0.2350000
18185.0000000	0.2250000
18186.0000000	0.2350000
18187.0000000	0.2350000
18188.0000000	0.2350000
18189.0000000	0.2300000
18190.0000000	0.2350000
18191.0000000	0.2250000
18192.0000000	0.2350000
18193.0000000	0.2300000
18194.0000000	0.2400000
18195.0000000	0.2300000
18196.0000000	0.2300000
18197.0000000	0.2250000
18198.0000000	0.2350000
18199.0000000	0.2350000
18200.0000000	0.2350000
18201.0000000	0.2300000
18202.0000000	0.2300000
18203.0000000	0.2250000
18204.0000000	0.2350000
18205.0000000	0.2400000
18206.0000000	0.2400000
18207.0000000	0.2350000
18208.0000000	0.2300000
18209.0000000	0.2250000
18210.0000000	0.2400000
18211.0000000	0.2366667
18212.0000000	0.2300000
18213.0000000	0.2350000
18214.0000000	0.2300000
18215.0000000	0.2350000
18216.0000000	0.2400000
18217.0000000	0.2400000
18218.0000000	0.2250000
18219.0000000	0.2300000
18220.0000000	0.2300000
18221.0000000	0.2300000
18222.0000000	0.2350000
18223.0000000	0.2300000
18224.0000000	0.2450000
18225.0000000	0.2300000
18226.0000000	0.2250000
18227.0000000	0.2350000
18228.0000000	0.2200000
18229.0000000	0.2250000
18230.0000000	0.2250000
18231.0000000	0.2300000
18232.0000000	0.2250000
18233.0000000	0.2350000
18234.0000000	0.2300000
18235.0000000	0.2400000
18236.0000000	0.2350000
18237.0000000	0.2350000
18238.0000000	0.2350000
18239.0000000	0.2250000

18240.0000000	0.2366667
18241.0000000	0.2350000
18242.0000000	0.2300000
18243.0000000	0.2350000
18244.0000000	0.2350000
18245.0000000	0.2350000
18246.0000000	0.2300000
18247.0000000	0.2350000
18248.0000000	0.2350000
18249.0000000	0.2300000
18250.0000000	0.2300000
18251.0000000	0.2300000
18252.0000000	0.2350000
18253.0000000	0.2300000
18254.0000000	0.2300000
18255.0000000	0.2300000
18256.0000000	0.2300000
18257.0000000	0.2350000
18258.0000000	0.2350000
18259.0000000	0.2350000
18260.0000000	0.2350000
18261.0000000	0.2400000
18262.0000000	0.2350000
18263.0000000	0.2300000
18264.0000000	0.2300000
18265.0000000	0.2350000
18266.0000000	0.2400000
18267.0000000	0.2300000
18268.0000000	0.2350000
18269.0000000	0.2400000
18270.0000000	0.2333333
18271.0000000	0.2450000
18272.0000000	0.2350000
18273.0000000	0.2350000
18274.0000000	0.2400000
18275.0000000	0.2350000
18276.0000000	0.2350000
18277.0000000	0.2350000
18278.0000000	0.2400000
18279.0000000	0.2350000
18280.0000000	0.2350000
18281.0000000	0.2300000
18282.0000000	0.2400000
18283.0000000	0.2450000
18284.0000000	0.2400000
18285.0000000	0.2400000
18286.0000000	0.2300000
18287.0000000	0.2250000
18288.0000000	0.2350000
18289.0000000	0.2350000
18290.0000000	0.2350000
18291.0000000	0.2350000
18292.0000000	0.2350000
18293.0000000	0.2400000
18294.0000000	0.2300000
18295.0000000	0.2400000
18296.0000000	0.2350000

18297.0000000	0.2300000
18298.0000000	0.2400000
18299.0000000	0.2350000
18300.0000000	0.2400000
18301.0000000	0.2366667
18302.0000000	0.2400000
18303.0000000	0.2400000
18304.0000000	0.2350000
18305.0000000	0.2400000
18306.0000000	0.2300000
18307.0000000	0.2350000
18308.0000000	0.2350000
18309.0000000	0.2500000
18310.0000000	0.2400000
18311.0000000	0.2400000
18312.0000000	0.2400000
18313.0000000	0.2300000
18314.0000000	0.2350000
18315.0000000	0.2400000
18316.0000000	0.2400000
18317.0000000	0.2400000
18318.0000000	0.2400000
18319.0000000	0.2300000
18320.0000000	0.2350000
18321.0000000	0.2350000
18322.0000000	0.2400000
18323.0000000	0.2350000
18324.0000000	0.2350000
18325.0000000	0.2300000
18326.0000000	0.2350000
18327.0000000	0.2300000
18328.0000000	0.2450000
18329.0000000	0.2350000
18330.0000000	0.2350000
18331.0000000	0.2300000
18332.0000000	0.2500000
18333.0000000	0.2350000
18334.0000000	0.2450000
18335.0000000	0.2350000
18336.0000000	0.2400000
18337.0000000	0.2450000
18338.0000000	0.2400000
18339.0000000	0.2350000
18340.0000000	0.2350000
18341.0000000	0.2450000
18342.0000000	0.2300000
18343.0000000	0.2450000
18344.0000000	0.2350000
18345.0000000	0.2450000
18346.0000000	0.2350000
18347.0000000	0.2400000
18348.0000000	0.2350000
18349.0000000	0.2400000
18350.0000000	0.2300000
18351.0000000	0.2400000
18352.0000000	0.2350000
18353.0000000	0.2400000

18354.0000000	0.2300000
18355.0000000	0.2250000
18356.0000000	0.2400000
18357.0000000	0.2400000
18358.0000000	0.2500000
18359.0000000	0.2300000
18360.0000000	0.2400000
18361.0000000	0.2366667
18362.0000000	0.2400000
18363.0000000	0.2400000
18364.0000000	0.2400000
18365.0000000	0.2350000
18366.0000000	0.2400000
18367.0000000	0.2450000
18368.0000000	0.2350000
18369.0000000	0.2500000
18370.0000000	0.2400000
18371.0000000	0.2400000
18372.0000000	0.2300000
18373.0000000	0.2500000
18374.0000000	0.2400000
18375.0000000	0.2500000
18376.0000000	0.2300000
18377.0000000	0.2450000
18378.0000000	0.2350000
18379.0000000	0.2450000
18380.0000000	0.2350000
18381.0000000	0.2450000
18382.0000000	0.2400000
18383.0000000	0.2400000
18384.0000000	0.2300000
18385.0000000	0.2500000
18386.0000000	0.2300000
18387.0000000	0.2500000
18388.0000000	0.2400000
18389.0000000	0.2500000
18390.0000000	0.2350000
18391.0000000	0.2433333
18392.0000000	0.2450000
18393.0000000	0.2350000
18394.0000000	0.2350000
18395.0000000	0.2350000
18396.0000000	0.2350000
18397.0000000	0.2450000
18398.0000000	0.2450000
18399.0000000	0.2350000
18400.0000000	0.2400000
18401.0000000	0.2350000
18402.0000000	0.2400000
18403.0000000	0.2350000
18404.0000000	0.2450000
18405.0000000	0.2400000
18406.0000000	0.2400000
18407.0000000	0.2500000
18408.0000000	0.2350000
18409.0000000	0.2350000
18410.0000000	0.2400000

18411.0000000	0.2300000
18412.0000000	0.2400000
18413.0000000	0.2450000
18414.0000000	0.2400000
18415.0000000	0.2400000
18416.0000000	0.2350000
18417.0000000	0.2400000
18418.0000000	0.2400000
18419.0000000	0.2350000
18420.0000000	0.2400000
18421.0000000	0.2400000
18422.0000000	0.2433333
18423.0000000	0.2350000
18424.0000000	0.2350000
18425.0000000	0.2300000
18426.0000000	0.2350000
18427.0000000	0.2400000
18428.0000000	0.2400000
18429.0000000	0.2350000
18430.0000000	0.2400000
18431.0000000	0.2350000
18432.0000000	0.2300000
18433.0000000	0.2350000
18434.0000000	0.2300000
18435.0000000	0.2350000
18436.0000000	0.2400000
18437.0000000	0.2300000
18438.0000000	0.2400000
18439.0000000	0.2350000
18440.0000000	0.2300000
18441.0000000	0.2450000
18442.0000000	0.2350000
18443.0000000	0.2400000
18444.0000000	0.2450000
18445.0000000	0.2400000
18446.0000000	0.2350000
18447.0000000	0.2350000
18448.0000000	0.2350000
18449.0000000	0.2200000
18450.0000000	0.2350000
18451.0000000	0.2350000
18452.0000000	0.2300000
18453.0000000	0.2300000
18454.0000000	0.2300000
18455.0000000	0.2400000
18456.0000000	0.2300000
18457.0000000	0.2300000
18458.0000000	0.2250000
18459.0000000	0.2250000
18460.0000000	0.2250000
18461.0000000	0.2250000
18462.0000000	0.2400000
18463.0000000	0.2250000
18464.0000000	0.2350000
18465.0000000	0.2250000
18466.0000000	0.2300000
18467.0000000	0.2300000

18468.0000000	0.2250000
18469.0000000	0.2300000
18470.0000000	0.2250000
18471.0000000	0.2250000
18472.0000000	0.2200000
18473.0000000	0.2200000
18474.0000000	0.2150000
18475.0000000	0.2200000
18476.0000000	0.2200000
18477.0000000	0.2250000
18478.0000000	0.2250000
18479.0000000	0.2200000
18480.0000000	0.2200000
18481.0000000	0.2200000
18482.0000000	0.2233333
18483.0000000	0.2200000
18484.0000000	0.2200000
18485.0000000	0.2200000
18486.0000000	0.2250000
18487.0000000	0.2250000
18488.0000000	0.2300000
18489.0000000	0.2200000
18490.0000000	0.2250000
18491.0000000	0.2150000
18492.0000000	0.2300000
18493.0000000	0.2100000
18494.0000000	0.2250000
18495.0000000	0.2150000
18496.0000000	0.2200000
18497.0000000	0.2150000
18498.0000000	0.2250000
18499.0000000	0.2200000
18500.0000000	0.2200000
18501.0000000	0.2200000
18502.0000000	0.2200000
18503.0000000	0.2250000
18504.0000000	0.2250000
18505.0000000	0.2200000
18506.0000000	0.2150000
18507.0000000	0.2100000
18508.0000000	0.2200000
18509.0000000	0.2300000
18510.0000000	0.2200000
18511.0000000	0.2300000
18512.0000000	0.2200000
18513.0000000	0.2200000
18514.0000000	0.2300000
18515.0000000	0.2200000
18516.0000000	0.2150000
18517.0000000	0.2150000
18518.0000000	0.2200000
18519.0000000	0.2200000
18520.0000000	0.2250000
18521.0000000	0.2150000
18522.0000000	0.2200000
18523.0000000	0.2100000
18524.0000000	0.2200000

18525.0000000	0.2150000
18526.0000000	0.2200000
18527.0000000	0.2150000
18528.0000000	0.2250000
18529.0000000	0.2100000
18530.0000000	0.2150000
18531.0000000	0.2100000
18532.0000000	0.2250000
18533.0000000	0.2200000
18534.0000000	0.2200000
18535.0000000	0.2050000
18536.0000000	0.2100000
18537.0000000	0.2100000
18538.0000000	0.2200000
18539.0000000	0.2150000
18540.0000000	0.2150000
18541.0000000	0.2000000
18542.0000000	0.2100000
18543.0000000	0.2100000
18544.0000000	0.2150000
18545.0000000	0.2050000
18546.0000000	0.2150000
18547.0000000	0.2100000
18548.0000000	0.2100000
18549.0000000	0.2100000
18550.0000000	0.2150000
18551.0000000	0.2050000
18552.0000000	0.2100000
18553.0000000	0.2050000
18554.0000000	0.2100000
18555.0000000	0.2050000
18556.0000000	0.2150000
18557.0000000	0.2000000
18558.0000000	0.2150000
18559.0000000	0.1900000
18560.0000000	0.2200000
18561.0000000	0.2050000
18562.0000000	0.2050000
18563.0000000	0.2050000
18564.0000000	0.2050000
18565.0000000	0.2000000
18566.0000000	0.2100000
18567.0000000	0.2050000
18568.0000000	0.2000000
18569.0000000	0.2100000
18570.0000000	0.2100000
18571.0000000	0.1950000
18572.0000000	0.2066667
18573.0000000	0.2050000
18574.0000000	0.2000000
18575.0000000	0.2150000
18576.0000000	0.2050000
18577.0000000	0.2050000
18578.0000000	0.2000000
18579.0000000	0.2050000
18580.0000000	0.2050000
18581.0000000	0.2050000

18582.0000000	0.2000000
18583.0000000	0.2050000
18584.0000000	0.1950000
18585.0000000	0.2000000
18586.0000000	0.2000000
18587.0000000	0.2100000
18588.0000000	0.2000000
18589.0000000	0.2100000
18590.0000000	0.1950000
18591.0000000	0.2050000
18592.0000000	0.2000000
18593.0000000	0.2100000
18594.0000000	0.1900000
18595.0000000	0.2050000
18596.0000000	0.2000000
18597.0000000	0.2150000
18598.0000000	0.2050000
18599.0000000	0.2050000
18600.0000000	0.2000000
18601.0000000	0.1950000
18602.0000000	0.2066667
18603.0000000	0.2050000
18604.0000000	0.2050000
18605.0000000	0.2100000
18606.0000000	0.2000000
18607.0000000	0.2100000
18608.0000000	0.2100000
18609.0000000	0.2050000
18610.0000000	0.2000000
18611.0000000	0.2000000
18612.0000000	0.2100000
18613.0000000	0.2150000
18614.0000000	0.2000000
18615.0000000	0.2150000
18616.0000000	0.2100000
18617.0000000	0.2100000
18618.0000000	0.2050000
18619.0000000	0.2050000
18620.0000000	0.2150000
18621.0000000	0.2100000
18622.0000000	0.2150000
18623.0000000	0.2150000
18624.0000000	0.2150000
18625.0000000	0.2050000
18626.0000000	0.2050000
18627.0000000	0.2100000
18628.0000000	0.2100000
18629.0000000	0.2100000
18630.0000000	0.2100000
18631.0000000	0.2150000
18632.0000000	0.2150000
18633.0000000	0.2066667
18634.0000000	0.2100000
18635.0000000	0.2200000
18636.0000000	0.2100000
18637.0000000	0.2100000
18638.0000000	0.2050000

18639.0000000	0.2150000
18640.0000000	0.2050000
18641.0000000	0.2150000
18642.0000000	0.2100000
18643.0000000	0.2150000
18644.0000000	0.2100000
18645.0000000	0.2100000
18646.0000000	0.2000000
18647.0000000	0.2050000
18648.0000000	0.2100000
18649.0000000	0.2100000
18650.0000000	0.2100000
18651.0000000	0.2000000
18652.0000000	0.2050000
18653.0000000	0.1950000
18654.0000000	0.2150000
18655.0000000	0.2050000
18656.0000000	0.2100000
18657.0000000	0.2050000
18658.0000000	0.2050000
18659.0000000	0.2000000
18660.0000000	0.2050000
18661.0000000	0.2050000
18662.0000000	0.2050000
18663.0000000	0.2066667
18664.0000000	0.2100000
18665.0000000	0.2050000
18666.0000000	0.2050000
18667.0000000	0.2100000
18668.0000000	0.2050000
18669.0000000	0.2050000
18670.0000000	0.2000000
18671.0000000	0.2000000
18672.0000000	0.2100000
18673.0000000	0.2000000
18674.0000000	0.2100000
18675.0000000	0.2050000
18676.0000000	0.2000000
18677.0000000	0.2050000
18678.0000000	0.2000000
18679.0000000	0.2050000
18680.0000000	0.1950000
18681.0000000	0.2050000
18682.0000000	0.2000000
18683.0000000	0.1950000
18684.0000000	0.1950000
18685.0000000	0.2050000
18686.0000000	0.2000000
18687.0000000	0.2050000
18688.0000000	0.2050000
18689.0000000	0.2000000
18690.0000000	0.2000000
18691.0000000	0.2000000
18692.0000000	0.2000000
18693.0000000	0.1933333
18694.0000000	0.2000000
18695.0000000	0.2050000

18696.0000000	0.1950000
18697.0000000	0.1950000
18698.0000000	0.1900000
18699.0000000	0.1950000
18700.0000000	0.1900000
18701.0000000	0.1900000
18702.0000000	0.1950000
18703.0000000	0.1850000
18704.0000000	0.2050000
18705.0000000	0.1950000
18706.0000000	0.1850000
18707.0000000	0.1900000
18708.0000000	0.1850000
18709.0000000	0.2050000
18710.0000000	0.1900000
18711.0000000	0.1950000
18712.0000000	0.1950000
18713.0000000	0.1850000
18714.0000000	0.1950000
18715.0000000	0.1900000
18716.0000000	0.1950000
18717.0000000	0.1850000
18718.0000000	0.1900000
18719.0000000	0.1900000
18720.0000000	0.2000000
18721.0000000	0.1950000
18722.0000000	0.1950000
18723.0000000	0.1900000
18724.0000000	0.1900000
18725.0000000	0.1950000
18726.0000000	0.1900000
18727.0000000	0.1900000
18728.0000000	0.1900000
18729.0000000	0.1900000
18730.0000000	0.1900000
18731.0000000	0.1900000
18732.0000000	0.1850000
18733.0000000	0.1900000
18734.0000000	0.1900000
18735.0000000	0.1900000
18736.0000000	0.1900000
18737.0000000	0.1900000
18738.0000000	0.1900000
18739.0000000	0.1900000
18740.0000000	0.1900000
18741.0000000	0.1850000
18742.0000000	0.1900000
18743.0000000	0.1900000
18744.0000000	0.1950000
18745.0000000	0.1900000
18746.0000000	0.1850000
18747.0000000	0.1800000
18748.0000000	0.1950000
18749.0000000	0.1950000
18750.0000000	0.1850000
18751.0000000	0.1900000
18752.0000000	0.1850000

18753.0000000	0.1750000
18754.0000000	0.1900000
18755.0000000	0.1850000
18756.0000000	0.1800000
18757.0000000	0.1900000
18758.0000000	0.1800000
18759.0000000	0.1850000
18760.0000000	0.1900000
18761.0000000	0.1850000
18762.0000000	0.1850000
18763.0000000	0.1850000
18764.0000000	0.1750000
18765.0000000	0.1800000
18766.0000000	0.1850000
18767.0000000	0.1950000
18768.0000000	0.1800000
18769.0000000	0.1850000
18770.0000000	0.1850000
18771.0000000	0.1900000
18772.0000000	0.1900000
18773.0000000	0.1900000
18774.0000000	0.1850000
18775.0000000	0.1800000
18776.0000000	0.1750000
18777.0000000	0.1850000
18778.0000000	0.1900000
18779.0000000	0.1850000
18780.0000000	0.1800000
18781.0000000	0.1800000
18782.0000000	0.1750000
18783.0000000	0.1800000
18784.0000000	0.1833333
18785.0000000	0.1850000
18786.0000000	0.1850000
18787.0000000	0.1850000
18788.0000000	0.1850000
18789.0000000	0.1850000
18790.0000000	0.1850000
18791.0000000	0.1850000
18792.0000000	0.1900000
18793.0000000	0.1800000
18794.0000000	0.1850000
18795.0000000	0.1750000
18796.0000000	0.1900000
18797.0000000	0.1800000
18798.0000000	0.1800000
18799.0000000	0.1850000
18800.0000000	0.1850000
18801.0000000	0.1900000
18802.0000000	0.1800000
18803.0000000	0.1850000
18804.0000000	0.1800000
18805.0000000	0.1800000
18806.0000000	0.1850000
18807.0000000	0.1950000
18808.0000000	0.1900000
18809.0000000	0.1850000

18810.0000000	0.1850000
18811.0000000	0.1750000
18812.0000000	0.1900000
18813.0000000	0.1850000
18814.0000000	0.1833333
18815.0000000	0.1850000
18816.0000000	0.1800000
18817.0000000	0.1900000
18818.0000000	0.1850000
18819.0000000	0.1900000
18820.0000000	0.1800000
18821.0000000	0.1750000
18822.0000000	0.1800000
18823.0000000	0.1850000
18824.0000000	0.1950000
18825.0000000	0.1800000
18826.0000000	0.1950000
18827.0000000	0.1750000
18828.0000000	0.1900000
18829.0000000	0.1800000
18830.0000000	0.1900000
18831.0000000	0.1900000
18832.0000000	0.1800000
18833.0000000	0.1900000
18834.0000000	0.1850000
18835.0000000	0.1950000
18836.0000000	0.1850000
18837.0000000	0.1800000
18838.0000000	0.1850000
18839.0000000	0.1850000
18840.0000000	0.1850000
18841.0000000	0.1850000
18842.0000000	0.1900000
18843.0000000	0.1950000
18844.0000000	0.1833333
18845.0000000	0.1850000
18846.0000000	0.1850000
18847.0000000	0.1850000
18848.0000000	0.1900000
18849.0000000	0.1850000
18850.0000000	0.1900000
18851.0000000	0.1900000
18852.0000000	0.1900000
18853.0000000	0.1900000
18854.0000000	0.1850000
18855.0000000	0.1800000
18856.0000000	0.1850000
18857.0000000	0.1850000
18858.0000000	0.1850000
18859.0000000	0.1950000
18860.0000000	0.1800000
18861.0000000	0.1950000
18862.0000000	0.1900000
18863.0000000	0.1850000
18864.0000000	0.1950000
18865.0000000	0.1800000
18866.0000000	0.1900000

18867.0000000	0.1850000
18868.0000000	0.1850000
18869.0000000	0.1850000
18870.0000000	0.1800000
18871.0000000	0.1850000
18872.0000000	0.1900000
18873.0000000	0.1900000
18874.0000000	0.1800000
18875.0000000	0.1850000
18876.0000000	0.1900000
18877.0000000	0.1800000
18878.0000000	0.1850000
18879.0000000	0.1900000
18880.0000000	0.1800000
18881.0000000	0.1850000
18882.0000000	0.1800000
18883.0000000	0.1900000
18884.0000000	0.1850000
18885.0000000	0.1800000
18886.0000000	0.1800000
18887.0000000	0.1850000
18888.0000000	0.1850000
18889.0000000	0.1900000
18890.0000000	0.1850000
18891.0000000	0.1750000
18892.0000000	0.1850000
18893.0000000	0.1850000
18894.0000000	0.1850000
18895.0000000	0.1800000
18896.0000000	0.1800000
18897.0000000	0.1850000
18898.0000000	0.1850000
18899.0000000	0.1850000
18900.0000000	0.1850000
18901.0000000	0.1800000
18902.0000000	0.1850000
18903.0000000	0.1800000
18904.0000000	0.1800000
18905.0000000	0.1900000
18906.0000000	0.1900000
18907.0000000	0.1850000
18908.0000000	0.1950000
18909.0000000	0.1850000
18910.0000000	0.1850000
18911.0000000	0.1750000
18912.0000000	0.1900000
18913.0000000	0.1800000
18914.0000000	0.1900000
18915.0000000	0.1850000
18916.0000000	0.1850000
18917.0000000	0.1850000
18918.0000000	0.1850000
18919.0000000	0.1850000
18920.0000000	0.1900000
18921.0000000	0.1900000
18922.0000000	0.1800000
18923.0000000	0.1900000

18924.0000000	0.1900000
18925.0000000	0.1900000
18926.0000000	0.1800000
18927.0000000	0.1850000
18928.0000000	0.1900000
18929.0000000	0.1850000
18930.0000000	0.1850000
18931.0000000	0.1800000
18932.0000000	0.1850000
18933.0000000	0.1800000
18934.0000000	0.1833333
18935.0000000	0.1850000
18936.0000000	0.1850000
18937.0000000	0.1850000
18938.0000000	0.1950000
18939.0000000	0.1850000
18940.0000000	0.1850000
18941.0000000	0.1850000
18942.0000000	0.1800000
18943.0000000	0.1850000
18944.0000000	0.1800000
18945.0000000	0.1800000
18946.0000000	0.1850000
18947.0000000	0.1800000
18948.0000000	0.1900000
18949.0000000	0.1750000
18950.0000000	0.1750000
18951.0000000	0.1850000
18952.0000000	0.1850000
18953.0000000	0.1900000
18954.0000000	0.1800000
18955.0000000	0.1800000
18956.0000000	0.1800000
18957.0000000	0.1800000
18958.0000000	0.1850000
18959.0000000	0.1750000
18960.0000000	0.1850000
18961.0000000	0.1800000
18962.0000000	0.1800000
18963.0000000	0.1800000
18964.0000000	0.1800000
18965.0000000	0.1733333
18966.0000000	0.1950000
18967.0000000	0.1800000
18968.0000000	0.1850000
18969.0000000	0.1850000
18970.0000000	0.1700000
18971.0000000	0.1850000
18972.0000000	0.1850000
18973.0000000	0.1950000
18974.0000000	0.1800000
18975.0000000	0.1750000
18976.0000000	0.1850000
18977.0000000	0.1800000
18978.0000000	0.1850000
18979.0000000	0.1800000
18980.0000000	0.1750000

18981.0000000	0.1750000
18982.0000000	0.1800000
18983.0000000	0.1850000
18984.0000000	0.1800000
18985.0000000	0.1850000
18986.0000000	0.1650000
18987.0000000	0.1850000
18988.0000000	0.1750000
18989.0000000	0.1900000
18990.0000000	0.1750000
18991.0000000	0.1800000
18992.0000000	0.1650000
18993.0000000	0.1650000
18994.0000000	0.1750000
18995.0000000	0.1800000
18996.0000000	0.1600000
18997.0000000	0.1750000
18998.0000000	0.1650000
18999.0000000	0.1800000
19000.0000000	0.1850000
19001.0000000	0.1750000
19002.0000000	0.1700000
19003.0000000	0.1700000
19004.0000000	0.1800000
19005.0000000	0.1700000
19006.0000000	0.1800000
19007.0000000	0.1750000
19008.0000000	0.1750000
19009.0000000	0.1750000
19010.0000000	0.1750000
19011.0000000	0.1700000
19012.0000000	0.1650000
19013.0000000	0.1700000
19014.0000000	0.1750000
19015.0000000	0.1750000
19016.0000000	0.1800000
19017.0000000	0.1700000
19018.0000000	0.1650000
19019.0000000	0.1700000
19020.0000000	0.1750000
19021.0000000	0.1800000
19022.0000000	0.1700000
19023.0000000	0.1750000
19024.0000000	0.1750000
19025.0000000	0.1633333
19026.0000000	0.1650000
19027.0000000	0.1700000
19028.0000000	0.1750000
19029.0000000	0.1750000
19030.0000000	0.1750000
19031.0000000	0.1700000
19032.0000000	0.1650000
19033.0000000	0.1650000
19034.0000000	0.1650000
19035.0000000	0.1650000
19036.0000000	0.1700000
19037.0000000	0.1650000

19038.0000000	0.1700000
19039.0000000	0.1700000
19040.0000000	0.1700000
19041.0000000	0.1750000
19042.0000000	0.1650000
19043.0000000	0.1700000
19044.0000000	0.1600000
19045.0000000	0.1600000
19046.0000000	0.1700000
19047.0000000	0.1700000
19048.0000000	0.1600000
19049.0000000	0.1700000
19050.0000000	0.1750000
19051.0000000	0.1700000
19052.0000000	0.1700000
19053.0000000	0.1600000
19054.0000000	0.1650000
19055.0000000	0.1733333
19056.0000000	0.1750000
19057.0000000	0.1700000
19058.0000000	0.1650000
19059.0000000	0.1700000
19060.0000000	0.1650000
19061.0000000	0.1750000
19062.0000000	0.1650000
19063.0000000	0.1700000
19064.0000000	0.1750000
19065.0000000	0.1700000
19066.0000000	0.1700000
19067.0000000	0.1700000
19068.0000000	0.1700000
19069.0000000	0.1700000
19070.0000000	0.1800000
19071.0000000	0.1650000
19072.0000000	0.1700000
19073.0000000	0.1800000
19074.0000000	0.1700000
19075.0000000	0.1800000
19076.0000000	0.1700000
19077.0000000	0.1750000
19078.0000000	0.1700000
19079.0000000	0.1800000
19080.0000000	0.1700000
19081.0000000	0.1750000
19082.0000000	0.1750000
19083.0000000	0.1800000
19084.0000000	0.1750000
19085.0000000	0.1750000
19086.0000000	0.1733333
19087.0000000	0.1750000
19088.0000000	0.1750000
19089.0000000	0.1800000
19090.0000000	0.1700000
19091.0000000	0.1650000
19092.0000000	0.1750000
19093.0000000	0.1750000
19094.0000000	0.1750000

19095.0000000	0.1750000
19096.0000000	0.1700000
19097.0000000	0.1800000
19098.0000000	0.1700000
19099.0000000	0.1800000
19100.0000000	0.1700000
19101.0000000	0.1700000
19102.0000000	0.1800000
19103.0000000	0.1750000
19104.0000000	0.1750000
19105.0000000	0.1700000
19106.0000000	0.1600000
19107.0000000	0.1650000
19108.0000000	0.1750000
19109.0000000	0.1650000
19110.0000000	0.1750000
19111.0000000	0.1700000
19112.0000000	0.1600000
19113.0000000	0.1650000
19114.0000000	0.1700000
19115.0000000	0.1750000
19116.0000000	0.1700000
19117.0000000	0.1650000
19118.0000000	0.1700000
19119.0000000	0.1700000
19120.0000000	0.1750000
19121.0000000	0.1650000
19122.0000000	0.1700000
19123.0000000	0.1650000
19124.0000000	0.1650000
19125.0000000	0.1700000
19126.0000000	0.1650000
19127.0000000	0.1650000
19128.0000000	0.1650000
19129.0000000	0.1650000
19130.0000000	0.1650000
19131.0000000	0.1600000
19132.0000000	0.1700000
19133.0000000	0.1650000
19134.0000000	0.1650000
19135.0000000	0.1550000
19136.0000000	0.1600000
19137.0000000	0.1650000
19138.0000000	0.1650000
19139.0000000	0.1700000
19140.0000000	0.1600000
19141.0000000	0.1650000
19142.0000000	0.1500000
19143.0000000	0.1600000
19144.0000000	0.1700000
19145.0000000	0.1650000
19146.0000000	0.1566667
19147.0000000	0.1600000
19148.0000000	0.1600000
19149.0000000	0.1600000
19150.0000000	0.1550000
19151.0000000	0.1650000

19152.0000000	0.1500000
19153.0000000	0.1650000
19154.0000000	0.1550000
19155.0000000	0.1500000
19156.0000000	0.1650000
19157.0000000	0.1600000
19158.0000000	0.1550000
19159.0000000	0.1600000
19160.0000000	0.1500000
19161.0000000	0.1600000
19162.0000000	0.1600000
19163.0000000	0.1550000
19164.0000000	0.1550000
19165.0000000	0.1500000
19166.0000000	0.1500000
19167.0000000	0.1550000
19168.0000000	0.1650000
19169.0000000	0.1450000
19170.0000000	0.1550000
19171.0000000	0.1500000
19172.0000000	0.1500000
19173.0000000	0.1550000
19174.0000000	0.1550000
19175.0000000	0.1550000
19176.0000000	0.1566667
19177.0000000	0.1500000
19178.0000000	0.1400000
19179.0000000	0.1500000
19180.0000000	0.1600000
19181.0000000	0.1500000
19182.0000000	0.1650000
19183.0000000	0.1500000
19184.0000000	0.1550000
19185.0000000	0.1500000
19186.0000000	0.1650000
19187.0000000	0.1600000
19188.0000000	0.1550000
19189.0000000	0.1600000
19190.0000000	0.1500000
19191.0000000	0.1500000
19192.0000000	0.1450000
19193.0000000	0.1550000
19194.0000000	0.1550000
19195.0000000	0.1450000
19196.0000000	0.1550000
19197.0000000	0.1450000
19198.0000000	0.1500000
19199.0000000	0.1500000
19200.0000000	0.1600000
19201.0000000	0.1450000
19202.0000000	0.1550000
19203.0000000	0.1450000
19204.0000000	0.1500000
19205.0000000	0.1400000
19206.0000000	0.1500000
19207.0000000	0.1466667
19208.0000000	0.1350000

19209.0000000	0.1550000
19210.0000000	0.1450000
19211.0000000	0.1500000
19212.0000000	0.1350000
19213.0000000	0.1450000
19214.0000000	0.1400000
19215.0000000	0.1400000
19216.0000000	0.1400000
19217.0000000	0.1400000
19218.0000000	0.1350000
19219.0000000	0.1350000
19220.0000000	0.1450000
19221.0000000	0.1450000
19222.0000000	0.1350000
19223.0000000	0.1300000
19224.0000000	0.1350000
19225.0000000	0.1350000
19226.0000000	0.1400000
19227.0000000	0.1350000
19228.0000000	0.1350000
19229.0000000	0.1350000
19230.0000000	0.1400000
19231.0000000	0.1300000
19232.0000000	0.1300000
19233.0000000	0.1300000
19234.0000000	0.1300000
19235.0000000	0.1200000
19236.0000000	0.1350000
19237.0000000	0.1366667
19238.0000000	0.1350000
19239.0000000	0.1300000
19240.0000000	0.1250000
19241.0000000	0.1250000
19242.0000000	0.1350000
19243.0000000	0.1300000
19244.0000000	0.1300000
19245.0000000	0.1250000
19246.0000000	0.1250000
19247.0000000	0.1250000
19248.0000000	0.1250000
19249.0000000	0.1300000
19250.0000000	0.1350000
19251.0000000	0.1300000
19252.0000000	0.1300000
19253.0000000	0.1300000
19254.0000000	0.1300000
19255.0000000	0.1250000
19256.0000000	0.1300000
19257.0000000	0.1250000
19258.0000000	0.1250000
19259.0000000	0.1300000
19260.0000000	0.1250000
19261.0000000	0.1250000
19262.0000000	0.1300000
19263.0000000	0.1200000
19264.0000000	0.1300000
19265.0000000	0.1200000

19266.0000000	0.1300000
19267.0000000	0.1250000
19268.0000000	0.1250000
19269.0000000	0.1250000
19270.0000000	0.1300000
19271.0000000	0.1300000
19272.0000000	0.1350000
19273.0000000	0.1250000
19274.0000000	0.1250000
19275.0000000	0.1300000
19276.0000000	0.1200000
19277.0000000	0.1250000
19278.0000000	0.1300000
19279.0000000	0.1250000
19280.0000000	0.1300000
19281.0000000	0.1200000
19282.0000000	0.1250000
19283.0000000	0.1250000
19284.0000000	0.1300000
19285.0000000	0.1250000
19286.0000000	0.1250000
19287.0000000	0.1350000
19288.0000000	0.1250000
19289.0000000	0.1300000
19290.0000000	0.1250000
19291.0000000	0.1200000
19292.0000000	0.1250000
19293.0000000	0.1250000
19294.0000000	0.1200000
19295.0000000	0.1300000
19296.0000000	0.1200000
19297.0000000	0.1300000
19298.0000000	0.1250000
19299.0000000	0.1150000
19300.0000000	0.1200000
19301.0000000	0.1250000
19302.0000000	0.1150000
19303.0000000	0.1200000
19304.0000000	0.1200000
19305.0000000	0.1200000
19306.0000000	0.1250000
19307.0000000	0.1250000
19308.0000000	0.1200000
19309.0000000	0.1250000
19310.0000000	0.1100000
19311.0000000	0.1300000
19312.0000000	0.1150000
19313.0000000	0.1200000
19314.0000000	0.1200000
19315.0000000	0.1150000
19316.0000000	0.1200000
19317.0000000	0.1150000
19318.0000000	0.1200000
19319.0000000	0.1150000
19320.0000000	0.1150000
19321.0000000	0.1150000
19322.0000000	0.1150000

19323.0000000	0.1150000
19324.0000000	0.1100000
19325.0000000	0.1150000
19326.0000000	0.1200000
19327.0000000	0.1100000
19328.0000000	0.1100000
19329.0000000	0.1200000
19330.0000000	0.1100000
19331.0000000	0.1150000
19332.0000000	0.1100000
19333.0000000	0.1050000
19334.0000000	0.1100000
19335.0000000	0.1100000
19336.0000000	0.1150000
19337.0000000	0.1100000
19338.0000000	0.1200000
19339.0000000	0.1050000
19340.0000000	0.1050000
19341.0000000	0.1100000
19342.0000000	0.1050000
19343.0000000	0.1100000
19344.0000000	0.1100000
19345.0000000	0.1050000
19346.0000000	0.1150000
19347.0000000	0.1100000
19348.0000000	0.1050000
19349.0000000	0.1100000
19350.0000000	0.1050000
19351.0000000	0.1150000
19352.0000000	0.1050000
19353.0000000	0.1050000
19354.0000000	0.1100000
19355.0000000	0.1100000
19356.0000000	0.1150000
19357.0000000	0.1033333
19358.0000000	0.1050000
19359.0000000	0.1050000
19360.0000000	0.1150000
19361.0000000	0.1050000
19362.0000000	0.0950000
19363.0000000	0.1100000
19364.0000000	0.1000000
19365.0000000	0.1050000
19366.0000000	0.1000000
19367.0000000	0.1050000
19368.0000000	0.1100000
19369.0000000	0.1000000
19370.0000000	0.1100000
19371.0000000	0.1050000
19372.0000000	0.1000000
19373.0000000	0.1000000
19374.0000000	0.1000000
19375.0000000	0.1050000
19376.0000000	0.1050000
19377.0000000	0.1000000
19378.0000000	0.1000000
19379.0000000	0.0900000

19380.0000000	0.1100000
19381.0000000	0.0950000
19382.0000000	0.0950000
19383.0000000	0.1000000
19384.0000000	0.1000000
19385.0000000	0.1050000
19386.0000000	0.1050000
19387.0000000	0.0966667
19388.0000000	0.1000000
19389.0000000	0.1000000
19390.0000000	0.1000000
19391.0000000	0.1050000
19392.0000000	0.0900000
19393.0000000	0.0950000
19394.0000000	0.1000000
19395.0000000	0.1050000
19396.0000000	0.1000000
19397.0000000	0.0950000
19398.0000000	0.0950000
19399.0000000	0.0950000
19400.0000000	0.0900000
19401.0000000	0.1050000
19402.0000000	0.1000000
19403.0000000	0.1000000
19404.0000000	0.0900000
19405.0000000	0.0900000
19406.0000000	0.0900000
19407.0000000	0.0950000
19408.0000000	0.1050000
19409.0000000	0.1000000
19410.0000000	0.0900000
19411.0000000	0.0850000
19412.0000000	0.0900000
19413.0000000	0.0900000
19414.0000000	0.1000000
19415.0000000	0.1000000
19416.0000000	0.1000000
19417.0000000	0.0900000
19418.0000000	0.0933333
19419.0000000	0.0900000
19420.0000000	0.0950000
19421.0000000	0.0850000
19422.0000000	0.0950000
19423.0000000	0.0900000
19424.0000000	0.1050000
19425.0000000	0.0800000
19426.0000000	0.1000000
19427.0000000	0.0950000
19428.0000000	0.1000000
19429.0000000	0.0850000
19430.0000000	0.1000000
19431.0000000	0.0950000
19432.0000000	0.0950000
19433.0000000	0.0800000
19434.0000000	0.0900000
19435.0000000	0.0850000
19436.0000000	0.0900000

19437.0000000	0.1000000
19438.0000000	0.0900000
19439.0000000	0.0950000
19440.0000000	0.0850000
19441.0000000	0.0950000
19442.0000000	0.0850000
19443.0000000	0.0950000
19444.0000000	0.0950000
19445.0000000	0.0900000
19446.0000000	0.0950000
19447.0000000	0.0900000
19448.0000000	0.0900000
19449.0000000	0.0950000
19450.0000000	0.0950000
19451.0000000	0.0850000
19452.0000000	0.0850000
19453.0000000	0.0900000
19454.0000000	0.0950000
19455.0000000	0.0900000
19456.0000000	0.0850000
19457.0000000	0.0900000
19458.0000000	0.0850000
19459.0000000	0.0900000
19460.0000000	0.0800000
19461.0000000	0.0850000
19462.0000000	0.0850000
19463.0000000	0.0900000
19464.0000000	0.0950000
19465.0000000	0.0850000
19466.0000000	0.0850000
19467.0000000	0.0950000
19468.0000000	0.0900000
19469.0000000	0.0850000
19470.0000000	0.0750000
19471.0000000	0.0850000
19472.0000000	0.0850000
19473.0000000	0.0850000
19474.0000000	0.0800000
19475.0000000	0.0800000
19476.0000000	0.0850000
19477.0000000	0.0850000
19478.0000000	0.0766667
19479.0000000	0.0800000
19480.0000000	0.0800000
19481.0000000	0.0800000
19482.0000000	0.0800000
19483.0000000	0.0700000
19484.0000000	0.0800000
19485.0000000	0.0800000
19486.0000000	0.0800000
19487.0000000	0.0800000
19488.0000000	0.0750000
19489.0000000	0.0800000
19490.0000000	0.0700000
19491.0000000	0.0800000
19492.0000000	0.0700000
19493.0000000	0.0750000

19494.0000000	0.0750000
19495.0000000	0.0750000
19496.0000000	0.0650000
19497.0000000	0.0700000
19498.0000000	0.0700000
19499.0000000	0.0750000
19500.0000000	0.0750000
19501.0000000	0.0700000
19502.0000000	0.0750000
19503.0000000	0.0700000
19504.0000000	0.0650000
19505.0000000	0.0650000
19506.0000000	0.0650000
19507.0000000	0.0700000
19508.0000000	0.0700000
19509.0000000	0.0800000
19510.0000000	0.0750000
19511.0000000	0.0700000
19512.0000000	0.0600000
19513.0000000	0.0700000
19514.0000000	0.0600000
19515.0000000	0.0700000
19516.0000000	0.0600000
19517.0000000	0.0600000
19518.0000000	0.0650000
19519.0000000	0.0550000
19520.0000000	0.0700000
19521.0000000	0.0450000
19522.0000000	0.0600000
19523.0000000	0.0550000
19524.0000000	0.0600000
19525.0000000	0.0500000
19526.0000000	0.0650000
19527.0000000	0.0600000
19528.0000000	0.0600000
19529.0000000	0.0550000
19530.0000000	0.0550000
19531.0000000	0.0550000
19532.0000000	0.0550000
19533.0000000	0.0550000
19534.0000000	0.0450000
19535.0000000	0.0600000
19536.0000000	0.0500000
19537.0000000	0.0500000
19538.0000000	0.0550000
19539.0000000	0.0566667
19540.0000000	0.0500000
19541.0000000	0.0650000
19542.0000000	0.0550000
19543.0000000	0.0600000
19544.0000000	0.0600000
19545.0000000	0.0550000
19546.0000000	0.0550000
19547.0000000	0.0450000
19548.0000000	0.0500000
19549.0000000	0.0500000
19550.0000000	0.0600000

19551.0000000	0.0550000
19552.0000000	0.0650000
19553.0000000	0.0500000
19554.0000000	0.0550000
19555.0000000	0.0550000
19556.0000000	0.0500000
19557.0000000	0.0650000
19558.0000000	0.0500000
19559.0000000	0.0550000
19560.0000000	0.0600000
19561.0000000	0.0600000
19562.0000000	0.0650000
19563.0000000	0.0550000
19564.0000000	0.0500000
19565.0000000	0.0600000
19566.0000000	0.0550000
19567.0000000	0.0700000
19568.0000000	0.0500000
19569.0000000	0.0533333
19570.0000000	0.0550000
19571.0000000	0.0550000
19572.0000000	0.0650000
19573.0000000	0.0500000
19574.0000000	0.0500000
19575.0000000	0.0500000
19576.0000000	0.0500000
19577.0000000	0.0500000
19578.0000000	0.0550000
19579.0000000	0.0500000
19580.0000000	0.0500000
19581.0000000	0.0500000
19582.0000000	0.0450000
19583.0000000	0.0500000
19584.0000000	0.0500000
19585.0000000	0.0450000
19586.0000000	0.0500000
19587.0000000	0.0550000
19588.0000000	0.0600000
19589.0000000	0.0500000
19590.0000000	0.0450000
19591.0000000	0.0500000
19592.0000000	0.0500000
19593.0000000	0.0600000
19594.0000000	0.0500000
19595.0000000	0.0500000
19596.0000000	0.0550000
19597.0000000	0.0500000
19598.0000000	0.0466667
19599.0000000	0.0550000
19600.0000000	0.0600000
19601.0000000	0.0500000
19602.0000000	0.0550000
19603.0000000	0.0550000
19604.0000000	0.0500000
19605.0000000	0.0550000
19606.0000000	0.0500000
19607.0000000	0.0500000

19608.0000000	0.0550000
19609.0000000	0.0550000
19610.0000000	0.0600000
19611.0000000	0.0500000
19612.0000000	0.0550000
19613.0000000	0.0500000
19614.0000000	0.0550000
19615.0000000	0.0550000
19616.0000000	0.0500000
19617.0000000	0.0550000
19618.0000000	0.0600000
19619.0000000	0.0600000
19620.0000000	0.0500000
19621.0000000	0.0550000
19622.0000000	0.0600000
19623.0000000	0.0600000
19624.0000000	0.0600000
19625.0000000	0.0550000
19626.0000000	0.0650000
19627.0000000	0.0550000
19628.0000000	0.0500000
19629.0000000	0.0566667
19630.0000000	0.0550000
19631.0000000	0.0550000
19632.0000000	0.0500000
19633.0000000	0.0550000
19634.0000000	0.0600000
19635.0000000	0.0550000
19636.0000000	0.0650000
19637.0000000	0.0550000
19638.0000000	0.0500000
19639.0000000	0.0600000
19640.0000000	0.0550000
19641.0000000	0.0500000
19642.0000000	0.0550000
19643.0000000	0.0600000
19644.0000000	0.0550000
19645.0000000	0.0600000
19646.0000000	0.0600000
19647.0000000	0.0600000
19648.0000000	0.0500000
19649.0000000	0.0650000
19650.0000000	0.0500000
19651.0000000	0.0650000
19652.0000000	0.0450000
19653.0000000	0.0650000
19654.0000000	0.0600000
19655.0000000	0.0550000
19656.0000000	0.0550000
19657.0000000	0.0550000
19658.0000000	0.0500000
19659.0000000	0.0566667
19660.0000000	0.0600000
19661.0000000	0.0550000
19662.0000000	0.0600000
19663.0000000	0.0550000
19664.0000000	0.0600000

19665.0000000	0.0600000
19666.0000000	0.0550000
19667.0000000	0.0550000
19668.0000000	0.0500000
19669.0000000	0.0600000
19670.0000000	0.0550000
19671.0000000	0.0650000
19672.0000000	0.0650000
19673.0000000	0.0550000
19674.0000000	0.0550000
19675.0000000	0.0500000
19676.0000000	0.0550000
19677.0000000	0.0600000
19678.0000000	0.0550000
19679.0000000	0.0550000
19680.0000000	0.0450000
19681.0000000	0.0550000
19682.0000000	0.0500000
19683.0000000	0.0500000
19684.0000000	0.0450000
19685.0000000	0.0500000
19686.0000000	0.0600000
19687.0000000	0.0550000
19688.0000000	0.0600000
19689.0000000	0.0533333
19690.0000000	0.0500000
19691.0000000	0.0600000
19692.0000000	0.0500000
19693.0000000	0.0500000
19694.0000000	0.0550000
19695.0000000	0.0500000
19696.0000000	0.0550000
19697.0000000	0.0650000
19698.0000000	0.0550000
19699.0000000	0.0500000
19700.0000000	0.0450000
19701.0000000	0.0500000
19702.0000000	0.0550000
19703.0000000	0.0550000
19704.0000000	0.0600000
19705.0000000	0.0550000
19706.0000000	0.0550000
19707.0000000	0.0550000
19708.0000000	0.0650000
19709.0000000	0.0600000
19710.0000000	0.0600000
19711.0000000	0.0600000
19712.0000000	0.0600000
19713.0000000	0.0550000
19714.0000000	0.0550000
19715.0000000	0.0600000
19716.0000000	0.0600000
19717.0000000	0.0600000
19718.0000000	0.0650000
19719.0000000	0.0533333
19720.0000000	0.0650000
19721.0000000	0.0600000

19722.0000000	0.0550000
19723.0000000	0.0600000
19724.0000000	0.0650000
19725.0000000	0.0600000
19726.0000000	0.0500000
19727.0000000	0.0600000
19728.0000000	0.0550000
19729.0000000	0.0550000
19730.0000000	0.0550000
19731.0000000	0.0550000
19732.0000000	0.0550000
19733.0000000	0.0500000
19734.0000000	0.0500000
19735.0000000	0.0650000
19736.0000000	0.0600000
19737.0000000	0.0600000
19738.0000000	0.0550000
19739.0000000	0.0550000
19740.0000000	0.0550000
19741.0000000	0.0650000
19742.0000000	0.0650000
19743.0000000	0.0600000
19744.0000000	0.0550000
19745.0000000	0.0550000
19746.0000000	0.0550000
19747.0000000	0.0550000
19748.0000000	0.0550000
19749.0000000	0.0550000
19750.0000000	0.0533333
19751.0000000	0.0650000
19752.0000000	0.0500000
19753.0000000	0.0550000
19754.0000000	0.0500000
19755.0000000	0.0650000
19756.0000000	0.0500000
19757.0000000	0.0700000
19758.0000000	0.0600000
19759.0000000	0.0700000
19760.0000000	0.0500000
19761.0000000	0.0600000
19762.0000000	0.0500000
19763.0000000	0.0650000
19764.0000000	0.0500000
19765.0000000	0.0600000
19766.0000000	0.0650000
19767.0000000	0.0500000
19768.0000000	0.0600000
19769.0000000	0.0500000
19770.0000000	0.0700000
19771.0000000	0.0650000
19772.0000000	0.0650000
19773.0000000	0.0600000
19774.0000000	0.0550000
19775.0000000	0.0600000
19776.0000000	0.0600000
19777.0000000	0.0550000
19778.0000000	0.0600000

19779.0000000	0.0650000
19780.0000000	0.0633333
19781.0000000	0.0650000
19782.0000000	0.0500000
19783.0000000	0.0700000
19784.0000000	0.0700000
19785.0000000	0.0700000
19786.0000000	0.0700000
19787.0000000	0.0600000
19788.0000000	0.0650000
19789.0000000	0.0600000
19790.0000000	0.0600000
19791.0000000	0.0700000
19792.0000000	0.0650000
19793.0000000	0.0600000
19794.0000000	0.0600000
19795.0000000	0.0650000
19796.0000000	0.0600000
19797.0000000	0.0700000
19798.0000000	0.0700000
19799.0000000	0.0650000
19800.0000000	0.0700000
19801.0000000	0.0600000
19802.0000000	0.0700000
19803.0000000	0.0650000
19804.0000000	0.0700000
19805.0000000	0.0600000
19806.0000000	0.0650000
19807.0000000	0.0650000
19808.0000000	0.0650000
19809.0000000	0.0650000
19810.0000000	0.0633333
19811.0000000	0.0700000
19812.0000000	0.0700000
19813.0000000	0.0600000
19814.0000000	0.0700000
19815.0000000	0.0700000
19816.0000000	0.0800000
19817.0000000	0.0650000
19818.0000000	0.0600000
19819.0000000	0.0700000
19820.0000000	0.0700000
19821.0000000	0.0650000
19822.0000000	0.0650000
19823.0000000	0.0700000
19824.0000000	0.0800000
19825.0000000	0.0650000
19826.0000000	0.0750000
19827.0000000	0.0750000
19828.0000000	0.0700000
19829.0000000	0.0650000
19830.0000000	0.0650000
19831.0000000	0.0750000
19832.0000000	0.0700000
19833.0000000	0.0800000
19834.0000000	0.0650000
19835.0000000	0.0700000

19836.0000000	0.0750000
19837.0000000	0.0700000
19838.0000000	0.0750000
19839.0000000	0.0700000
19840.0000000	0.0666667
19841.0000000	0.0700000
19842.0000000	0.0700000
19843.0000000	0.0800000
19844.0000000	0.0650000
19845.0000000	0.0750000
19846.0000000	0.0800000
19847.0000000	0.0750000
19848.0000000	0.0750000
19849.0000000	0.0700000
19850.0000000	0.0800000
19851.0000000	0.0800000
19852.0000000	0.0750000
19853.0000000	0.0800000
19854.0000000	0.0800000
19855.0000000	0.0700000
19856.0000000	0.0800000
19857.0000000	0.0800000
19858.0000000	0.0800000
19859.0000000	0.0750000
19860.0000000	0.0850000
19861.0000000	0.0850000
19862.0000000	0.0750000
19863.0000000	0.0800000
19864.0000000	0.0800000
19865.0000000	0.0800000
19866.0000000	0.0850000
19867.0000000	0.0750000
19868.0000000	0.0850000
19869.0000000	0.0850000
19870.0000000	0.0800000
19871.0000000	0.0800000
19872.0000000	0.0900000
19873.0000000	0.0700000
19874.0000000	0.0850000
19875.0000000	0.0750000
19876.0000000	0.0750000
19877.0000000	0.0850000
19878.0000000	0.0850000
19879.0000000	0.0850000
19880.0000000	0.0750000
19881.0000000	0.0850000
19882.0000000	0.0750000
19883.0000000	0.0850000
19884.0000000	0.0800000
19885.0000000	0.0900000
19886.0000000	0.0900000
19887.0000000	0.0850000
19888.0000000	0.0800000
19889.0000000	0.0750000
19890.0000000	0.0850000
19891.0000000	0.0800000
19892.0000000	0.0850000

19893.0000000	0.0850000
19894.0000000	0.0900000
19895.0000000	0.0800000
19896.0000000	0.0800000
19897.0000000	0.0900000
19898.0000000	0.0950000
19899.0000000	0.0850000
19900.0000000	0.0900000
19901.0000000	0.0900000
19902.0000000	0.0850000
19903.0000000	0.0850000
19904.0000000	0.0850000
19905.0000000	0.0950000
19906.0000000	0.0850000
19907.0000000	0.0950000
19908.0000000	0.0850000
19909.0000000	0.0900000
19910.0000000	0.0850000
19911.0000000	0.0950000
19912.0000000	0.0900000
19913.0000000	0.0900000
19914.0000000	0.0850000
19915.0000000	0.0950000
19916.0000000	0.0850000
19917.0000000	0.0900000
19918.0000000	0.0900000
19919.0000000	0.1000000
19920.0000000	0.0950000
19921.0000000	0.1000000
19922.0000000	0.0900000
19923.0000000	0.1000000
19924.0000000	0.0900000
19925.0000000	0.1100000
19926.0000000	0.0850000
19927.0000000	0.1000000
19928.0000000	0.0800000
19929.0000000	0.0950000
19930.0000000	0.1000000
19931.0000000	0.0900000
19932.0000000	0.1000000
19933.0000000	0.0950000
19934.0000000	0.1000000
19935.0000000	0.0950000
19936.0000000	0.0950000
19937.0000000	0.1050000
19938.0000000	0.0900000
19939.0000000	0.1050000
19940.0000000	0.1000000
19941.0000000	0.0850000
19942.0000000	0.1000000
19943.0000000	0.1000000
19944.0000000	0.1000000
19945.0000000	0.1000000
19946.0000000	0.1000000
19947.0000000	0.1000000
19948.0000000	0.1100000
19949.0000000	0.1050000

19950.0000000	0.0950000
19951.0000000	0.0950000
19952.0000000	0.1050000
19953.0000000	0.1050000
19954.0000000	0.1000000
19955.0000000	0.1050000
19956.0000000	0.1050000
19957.0000000	0.1050000
19958.0000000	0.1100000
19959.0000000	0.1000000
19960.0000000	0.1000000
19961.0000000	0.1033333
19962.0000000	0.0950000
19963.0000000	0.1000000
19964.0000000	0.1100000
19965.0000000	0.0950000
19966.0000000	0.0950000
19967.0000000	0.1150000
19968.0000000	0.1100000
19969.0000000	0.1050000
19970.0000000	0.1000000
19971.0000000	0.1100000
19972.0000000	0.1000000
19973.0000000	0.1000000
19974.0000000	0.1000000
19975.0000000	0.1050000
19976.0000000	0.1050000
19977.0000000	0.1100000
19978.0000000	0.1200000
19979.0000000	0.1050000
19980.0000000	0.1100000
19981.0000000	0.1100000
19982.0000000	0.1150000
19983.0000000	0.1050000
19984.0000000	0.1050000
19985.0000000	0.1050000
19986.0000000	0.1100000
19987.0000000	0.1150000
19988.0000000	0.1100000
19989.0000000	0.1050000
19990.0000000	0.1000000
19991.0000000	0.1100000
19992.0000000	0.1100000
19993.0000000	0.1100000
19994.0000000	0.1150000
19995.0000000	0.1050000
19996.0000000	0.1150000
19997.0000000	0.1000000
19998.0000000	0.1100000
19999.0000000	0.1050000
20000.0000000	0.1100000
20001.0000000	0.1200000
20002.0000000	0.1100000
20003.0000000	0.1100000
20004.0000000	0.1000000
20005.0000000	0.1150000
20006.0000000	0.1050000

20007.0000000	0.1200000
20008.0000000	0.1100000
20009.0000000	0.1100000
20010.0000000	0.1100000
20011.0000000	0.1100000
20012.0000000	0.1250000
20013.0000000	0.1200000
20014.0000000	0.1150000
20015.0000000	0.1100000
20016.0000000	0.1150000
20017.0000000	0.1050000
20018.0000000	0.1150000
20019.0000000	0.1150000
20020.0000000	0.1150000
20021.0000000	0.1133333
20022.0000000	0.1200000
20023.0000000	0.1150000
20024.0000000	0.1200000
20025.0000000	0.1150000
20026.0000000	0.1200000
20027.0000000	0.1300000
20028.0000000	0.1200000
20029.0000000	0.1250000
20030.0000000	0.1250000
20031.0000000	0.1150000
20032.0000000	0.1250000
20033.0000000	0.1200000
20034.0000000	0.1250000
20035.0000000	0.1200000
20036.0000000	0.1250000
20037.0000000	0.1250000
20038.0000000	0.1250000
20039.0000000	0.1250000
20040.0000000	0.1200000
20041.0000000	0.1200000
20042.0000000	0.1200000
20043.0000000	0.1350000
20044.0000000	0.1250000
20045.0000000	0.1150000
20046.0000000	0.1200000
20047.0000000	0.1250000
20048.0000000	0.1300000
20049.0000000	0.1200000
20050.0000000	0.1250000
20051.0000000	0.1300000
20052.0000000	0.1250000
20053.0000000	0.1200000
20054.0000000	0.1250000
20055.0000000	0.1350000
20056.0000000	0.1250000
20057.0000000	0.1250000
20058.0000000	0.1300000
20059.0000000	0.1200000
20060.0000000	0.1350000
20061.0000000	0.1250000
20062.0000000	0.1250000
20063.0000000	0.1250000

20064.0000000	0.1150000
20065.0000000	0.1250000
20066.0000000	0.1350000
20067.0000000	0.1200000
20068.0000000	0.1300000
20069.0000000	0.1350000
20070.0000000	0.1250000
20071.0000000	0.1250000
20072.0000000	0.1250000
20073.0000000	0.1250000
20074.0000000	0.1200000
20075.0000000	0.1250000
20076.0000000	0.1200000
20077.0000000	0.1300000
20078.0000000	0.1250000
20079.0000000	0.1250000
20080.0000000	0.1350000
20081.0000000	0.1250000
20082.0000000	0.1233333
20083.0000000	0.1200000
20084.0000000	0.1300000
20085.0000000	0.1350000
20086.0000000	0.1300000
20087.0000000	0.1250000
20088.0000000	0.1350000
20089.0000000	0.1250000
20090.0000000	0.1250000
20091.0000000	0.1300000
20092.0000000	0.1350000
20093.0000000	0.1200000
20094.0000000	0.1200000
20095.0000000	0.1350000
20096.0000000	0.1250000
20097.0000000	0.1250000
20098.0000000	0.1300000
20099.0000000	0.1300000
20100.0000000	0.1300000
20101.0000000	0.1300000
20102.0000000	0.1300000
20103.0000000	0.1250000
20104.0000000	0.1300000
20105.0000000	0.1250000
20106.0000000	0.1250000
20107.0000000	0.1250000
20108.0000000	0.1300000
20109.0000000	0.1300000
20110.0000000	0.1350000
20111.0000000	0.1250000
20112.0000000	0.1266667
20113.0000000	0.1300000
20114.0000000	0.1350000
20115.0000000	0.1300000
20116.0000000	0.1200000
20117.0000000	0.1350000
20118.0000000	0.1350000
20119.0000000	0.1300000
20120.0000000	0.1350000

20121.0000000	0.1300000
20122.0000000	0.1400000
20123.0000000	0.1400000
20124.0000000	0.1300000
20125.0000000	0.1300000
20126.0000000	0.1300000
20127.0000000	0.1350000
20128.0000000	0.1400000
20129.0000000	0.1250000
20130.0000000	0.1350000
20131.0000000	0.1250000
20132.0000000	0.1350000
20133.0000000	0.1350000
20134.0000000	0.1250000
20135.0000000	0.1300000
20136.0000000	0.1250000
20137.0000000	0.1400000
20138.0000000	0.1350000
20139.0000000	0.1400000
20140.0000000	0.1250000
20141.0000000	0.1350000
20142.0000000	0.1400000
20143.0000000	0.1400000
20144.0000000	0.1350000
20145.0000000	0.1350000
20146.0000000	0.1300000
20147.0000000	0.1450000
20148.0000000	0.1300000
20149.0000000	0.1350000
20150.0000000	0.1400000
20151.0000000	0.1400000
20152.0000000	0.1400000
20153.0000000	0.1300000
20154.0000000	0.1400000
20155.0000000	0.1350000
20156.0000000	0.1450000
20157.0000000	0.1350000
20158.0000000	0.1400000
20159.0000000	0.1350000
20160.0000000	0.1350000
20161.0000000	0.1350000
20162.0000000	0.1350000
20163.0000000	0.1400000
20164.0000000	0.1400000
20165.0000000	0.1350000
20166.0000000	0.1400000
20167.0000000	0.1300000
20168.0000000	0.1400000
20169.0000000	0.1350000
20170.0000000	0.1400000
20171.0000000	0.1450000
20172.0000000	0.1400000
20173.0000000	0.1400000
20174.0000000	0.1450000
20175.0000000	0.1400000
20176.0000000	0.1400000
20177.0000000	0.1450000

20178.0000000	0.1400000
20179.0000000	0.1400000
20180.0000000	0.1350000
20181.0000000	0.1300000
20182.0000000	0.1350000
20183.0000000	0.1300000
20184.0000000	0.1500000
20185.0000000	0.1350000
20186.0000000	0.1400000
20187.0000000	0.1400000
20188.0000000	0.1300000
20189.0000000	0.1350000
20190.0000000	0.1350000
20191.0000000	0.1400000
20192.0000000	0.1300000
20193.0000000	0.1400000
20194.0000000	0.1300000
20195.0000000	0.1450000
20196.0000000	0.1400000
20197.0000000	0.1500000
20198.0000000	0.1350000
20199.0000000	0.1350000
20200.0000000	0.1250000
20201.0000000	0.1350000
20202.0000000	0.1400000
20203.0000000	0.1366667
20204.0000000	0.1350000
20205.0000000	0.1350000
20206.0000000	0.1450000
20207.0000000	0.1400000
20208.0000000	0.1400000
20209.0000000	0.1400000
20210.0000000	0.1300000
20211.0000000	0.1350000
20212.0000000	0.1300000
20213.0000000	0.1400000
20214.0000000	0.1350000
20215.0000000	0.1400000
20216.0000000	0.1350000
20217.0000000	0.1300000
20218.0000000	0.1400000
20219.0000000	0.1300000
20220.0000000	0.1250000
20221.0000000	0.1300000
20222.0000000	0.1300000
20223.0000000	0.1350000
20224.0000000	0.1400000
20225.0000000	0.1300000
20226.0000000	0.1350000
20227.0000000	0.1400000
20228.0000000	0.1350000
20229.0000000	0.1300000
20230.0000000	0.1400000
20231.0000000	0.1250000
20232.0000000	0.1300000
20233.0000000	0.1333333
20234.0000000	0.1350000

20235.0000000	0.1300000
20236.0000000	0.1350000
20237.0000000	0.1350000
20238.0000000	0.1300000
20239.0000000	0.1300000
20240.0000000	0.1300000
20241.0000000	0.1350000
20242.0000000	0.1400000
20243.0000000	0.1250000
20244.0000000	0.1300000
20245.0000000	0.1350000
20246.0000000	0.1300000
20247.0000000	0.1400000
20248.0000000	0.1250000
20249.0000000	0.1400000
20250.0000000	0.1300000
20251.0000000	0.1400000
20252.0000000	0.1300000
20253.0000000	0.1400000
20254.0000000	0.1300000
20255.0000000	0.1400000
20256.0000000	0.1350000
20257.0000000	0.1300000
20258.0000000	0.1250000
20259.0000000	0.1400000
20260.0000000	0.1250000
20261.0000000	0.1400000
20262.0000000	0.1300000
20263.0000000	0.1300000
20264.0000000	0.1400000
20265.0000000	0.1300000
20266.0000000	0.1300000
20267.0000000	0.1300000
20268.0000000	0.1300000
20269.0000000	0.1300000
20270.0000000	0.1300000
20271.0000000	0.1350000
20272.0000000	0.1250000
20273.0000000	0.1250000
20274.0000000	0.1250000
20275.0000000	0.1300000
20276.0000000	0.1300000
20277.0000000	0.1250000
20278.0000000	0.1250000
20279.0000000	0.1250000
20280.0000000	0.1200000
20281.0000000	0.1250000
20282.0000000	0.1250000
20283.0000000	0.1250000
20284.0000000	0.1200000
20285.0000000	0.1250000
20286.0000000	0.1250000
20287.0000000	0.1300000
20288.0000000	0.1250000
20289.0000000	0.1300000
20290.0000000	0.1250000
20291.0000000	0.1200000

20292.0000000	0.1150000
20293.0000000	0.1266667
20294.0000000	0.1300000
20295.0000000	0.1150000
20296.0000000	0.1300000
20297.0000000	0.1200000
20298.0000000	0.1250000
20299.0000000	0.1200000
20300.0000000	0.1050000
20301.0000000	0.1300000
20302.0000000	0.1250000
20303.0000000	0.1400000
20304.0000000	0.1250000
20305.0000000	0.1200000
20306.0000000	0.1150000
20307.0000000	0.1250000
20308.0000000	0.1200000
20309.0000000	0.1250000
20310.0000000	0.1200000
20311.0000000	0.1250000
20312.0000000	0.1200000
20313.0000000	0.1200000
20314.0000000	0.1250000
20315.0000000	0.1250000
20316.0000000	0.1200000
20317.0000000	0.1200000
20318.0000000	0.1150000
20319.0000000	0.1250000
20320.0000000	0.1200000
20321.0000000	0.1200000
20322.0000000	0.1150000
20323.0000000	0.1233333
20324.0000000	0.1200000
20325.0000000	0.1250000
20326.0000000	0.1200000
20327.0000000	0.1250000
20328.0000000	0.1150000
20329.0000000	0.1150000
20330.0000000	0.1200000
20331.0000000	0.1150000
20332.0000000	0.1150000
20333.0000000	0.1200000
20334.0000000	0.1200000
20335.0000000	0.1150000
20336.0000000	0.1200000
20337.0000000	0.1200000
20338.0000000	0.1100000
20339.0000000	0.1150000
20340.0000000	0.1150000
20341.0000000	0.1200000
20342.0000000	0.1200000
20343.0000000	0.1150000
20344.0000000	0.1150000
20345.0000000	0.1100000
20346.0000000	0.1100000
20347.0000000	0.1150000
20348.0000000	0.1200000

20349.0000000	0.1150000
20350.0000000	0.1100000
20351.0000000	0.1100000
20352.0000000	0.1200000
20353.0000000	0.1166667
20354.0000000	0.1150000
20355.0000000	0.1150000
20356.0000000	0.1200000
20357.0000000	0.1150000
20358.0000000	0.1050000
20359.0000000	0.1200000
20360.0000000	0.1200000
20361.0000000	0.1150000
20362.0000000	0.1100000
20363.0000000	0.1200000
20364.0000000	0.1150000
20365.0000000	0.1200000
20366.0000000	0.1150000
20367.0000000	0.1150000
20368.0000000	0.1150000
20369.0000000	0.1150000
20370.0000000	0.1200000
20371.0000000	0.1200000
20372.0000000	0.1200000
20373.0000000	0.1100000
20374.0000000	0.1100000
20375.0000000	0.1100000
20376.0000000	0.1100000
20377.0000000	0.1250000
20378.0000000	0.1100000
20379.0000000	0.1150000
20380.0000000	0.1150000
20381.0000000	0.1100000
20382.0000000	0.1100000
20383.0000000	0.1200000
20384.0000000	0.1150000
20385.0000000	0.1050000
20386.0000000	0.1150000
20387.0000000	0.1150000
20388.0000000	0.1200000
20389.0000000	0.1100000
20390.0000000	0.1150000
20391.0000000	0.1100000
20392.0000000	0.1150000
20393.0000000	0.1150000
20394.0000000	0.1150000
20395.0000000	0.1200000
20396.0000000	0.1150000
20397.0000000	0.1250000
20398.0000000	0.1150000
20399.0000000	0.1150000
20400.0000000	0.1100000
20401.0000000	0.1200000
20402.0000000	0.1050000
20403.0000000	0.1150000
20404.0000000	0.1100000
20405.0000000	0.1200000

20406.0000000	0.1100000
20407.0000000	0.1150000
20408.0000000	0.1050000
20409.0000000	0.1200000
20410.0000000	0.1150000
20411.0000000	0.1200000
20412.0000000	0.1150000
20413.0000000	0.1250000
20414.0000000	0.1166667
20415.0000000	0.1150000
20416.0000000	0.1150000
20417.0000000	0.1200000
20418.0000000	0.1200000
20419.0000000	0.1200000
20420.0000000	0.1150000
20421.0000000	0.1250000
20422.0000000	0.1050000
20423.0000000	0.1200000
20424.0000000	0.1150000
20425.0000000	0.1250000
20426.0000000	0.1200000
20427.0000000	0.1300000
20428.0000000	0.1150000
20429.0000000	0.1300000
20430.0000000	0.1300000
20431.0000000	0.1250000
20432.0000000	0.1250000
20433.0000000	0.1150000
20434.0000000	0.1200000
20435.0000000	0.1200000
20436.0000000	0.1200000
20437.0000000	0.1250000
20438.0000000	0.1100000
20439.0000000	0.1250000
20440.0000000	0.1100000
20441.0000000	0.1250000
20442.0000000	0.1250000
20443.0000000	0.1250000
20444.0000000	0.1200000
20445.0000000	0.1250000
20446.0000000	0.1200000
20447.0000000	0.1200000
20448.0000000	0.1250000
20449.0000000	0.1300000
20450.0000000	0.1300000
20451.0000000	0.1300000
20452.0000000	0.1150000
20453.0000000	0.1200000
20454.0000000	0.1150000
20455.0000000	0.1250000
20456.0000000	0.1200000
20457.0000000	0.1250000
20458.0000000	0.1200000
20459.0000000	0.1150000
20460.0000000	0.1250000
20461.0000000	0.1150000
20462.0000000	0.1300000

20463.0000000	0.1200000
20464.0000000	0.1150000
20465.0000000	0.1100000
20466.0000000	0.1200000
20467.0000000	0.1150000
20468.0000000	0.1250000
20469.0000000	0.1250000
20470.0000000	0.1150000
20471.0000000	0.1250000
20472.0000000	0.1150000
20473.0000000	0.1200000
20474.0000000	0.1200000
20475.0000000	0.1250000
20476.0000000	0.1200000
20477.0000000	0.1250000
20478.0000000	0.1100000
20479.0000000	0.1200000
20480.0000000	0.1150000
20481.0000000	0.1300000
20482.0000000	0.1250000
20483.0000000	0.1250000
20484.0000000	0.1100000
20485.0000000	0.1150000
20486.0000000	0.1150000
20487.0000000	0.1300000
20488.0000000	0.1150000
20489.0000000	0.1200000
20490.0000000	0.1200000
20491.0000000	0.1150000
20492.0000000	0.1250000
20493.0000000	0.1200000
20494.0000000	0.1150000
20495.0000000	0.1150000
20496.0000000	0.1200000
20497.0000000	0.1150000
20498.0000000	0.1150000
20499.0000000	0.1200000
20500.0000000	0.1150000
20501.0000000	0.1100000
20502.0000000	0.1050000
20503.0000000	0.1050000
20504.0000000	0.1200000
20505.0000000	0.1150000
20506.0000000	0.1100000
20507.0000000	0.1150000
20508.0000000	0.1250000
20509.0000000	0.1150000
20510.0000000	0.1150000
20511.0000000	0.1150000
20512.0000000	0.1150000
20513.0000000	0.1100000
20514.0000000	0.1150000
20515.0000000	0.1100000
20516.0000000	0.1100000
20517.0000000	0.1150000
20518.0000000	0.1150000
20519.0000000	0.1100000

20520.0000000	0.1200000
20521.0000000	0.1050000
20522.0000000	0.1150000
20523.0000000	0.1100000
20524.0000000	0.1050000
20525.0000000	0.1150000
20526.0000000	0.1150000
20527.0000000	0.1150000
20528.0000000	0.1100000
20529.0000000	0.1100000
20530.0000000	0.1200000
20531.0000000	0.1150000
20532.0000000	0.1100000
20533.0000000	0.1150000
20534.0000000	0.1150000
20535.0000000	0.1133333
20536.0000000	0.1100000
20537.0000000	0.1100000
20538.0000000	0.1100000
20539.0000000	0.1050000
20540.0000000	0.1100000
20541.0000000	0.1150000
20542.0000000	0.1050000
20543.0000000	0.1100000
20544.0000000	0.1050000
20545.0000000	0.1050000
20546.0000000	0.1100000
20547.0000000	0.1150000
20548.0000000	0.1100000
20549.0000000	0.1100000
20550.0000000	0.1050000
20551.0000000	0.1050000
20552.0000000	0.1000000
20553.0000000	0.1100000
20554.0000000	0.1000000
20555.0000000	0.1100000
20556.0000000	0.1150000
20557.0000000	0.1100000
20558.0000000	0.1100000
20559.0000000	0.1050000
20560.0000000	0.1100000
20561.0000000	0.1100000
20562.0000000	0.1100000
20563.0000000	0.1100000
20564.0000000	0.1200000
20565.0000000	0.1066667
20566.0000000	0.1100000
20567.0000000	0.1050000
20568.0000000	0.1050000
20569.0000000	0.1100000
20570.0000000	0.1150000
20571.0000000	0.1150000
20572.0000000	0.1150000
20573.0000000	0.1100000
20574.0000000	0.1000000
20575.0000000	0.1100000
20576.0000000	0.1100000

20577.0000000	0.1200000
20578.0000000	0.1150000
20579.0000000	0.1000000
20580.0000000	0.1100000
20581.0000000	0.1050000
20582.0000000	0.1100000
20583.0000000	0.1150000
20584.0000000	0.1050000
20585.0000000	0.1000000
20586.0000000	0.1150000
20587.0000000	0.1100000
20588.0000000	0.1050000
20589.0000000	0.1050000
20590.0000000	0.1000000
20591.0000000	0.1050000
20592.0000000	0.1200000
20593.0000000	0.1100000
20594.0000000	0.1100000
20595.0000000	0.1200000
20596.0000000	0.1050000
20597.0000000	0.1100000
20598.0000000	0.1200000
20599.0000000	0.1100000
20600.0000000	0.1100000
20601.0000000	0.1100000
20602.0000000	0.1050000
20603.0000000	0.1150000
20604.0000000	0.1200000
20605.0000000	0.1100000
20606.0000000	0.1150000
20607.0000000	0.1050000
20608.0000000	0.1100000
20609.0000000	0.1200000
20610.0000000	0.1150000
20611.0000000	0.1100000
20612.0000000	0.1150000
20613.0000000	0.1100000
20614.0000000	0.1150000
20615.0000000	0.1100000
20616.0000000	0.1100000
20617.0000000	0.1250000
20618.0000000	0.1150000
20619.0000000	0.1250000
20620.0000000	0.1050000
20621.0000000	0.1100000
20622.0000000	0.1150000
20623.0000000	0.1100000
20624.0000000	0.1200000
20625.0000000	0.1100000
20626.0000000	0.1150000
20627.0000000	0.1150000
20628.0000000	0.1150000
20629.0000000	0.1050000
20630.0000000	0.1200000
20631.0000000	0.1150000
20632.0000000	0.1200000
20633.0000000	0.1100000

20634.0000000	0.1150000
20635.0000000	0.1150000
20636.0000000	0.1150000
20637.0000000	0.1200000
20638.0000000	0.1150000
20639.0000000	0.1100000
20640.0000000	0.1250000
20641.0000000	0.1200000
20642.0000000	0.1200000
20643.0000000	0.1150000
20644.0000000	0.1200000
20645.0000000	0.1200000
20646.0000000	0.1250000
20647.0000000	0.1100000
20648.0000000	0.1150000
20649.0000000	0.1150000
20650.0000000	0.1100000
20651.0000000	0.1250000
20652.0000000	0.1200000
20653.0000000	0.1200000
20654.0000000	0.1250000
20655.0000000	0.1150000
20656.0000000	0.1100000
20657.0000000	0.1100000
20658.0000000	0.1250000
20659.0000000	0.1200000
20660.0000000	0.1250000
20661.0000000	0.1150000
20662.0000000	0.1200000
20663.0000000	0.1250000
20664.0000000	0.1100000
20665.0000000	0.1200000
20666.0000000	0.1150000
20667.0000000	0.1100000
20668.0000000	0.1250000
20669.0000000	0.1200000
20670.0000000	0.1200000
20671.0000000	0.1200000
20672.0000000	0.1300000
20673.0000000	0.1100000
20674.0000000	0.1150000
20675.0000000	0.1150000
20676.0000000	0.1200000
20677.0000000	0.1200000
20678.0000000	0.1200000
20679.0000000	0.1250000
20680.0000000	0.1150000
20681.0000000	0.1150000
20682.0000000	0.1250000
20683.0000000	0.1300000
20684.0000000	0.1100000
20685.0000000	0.1200000
20686.0000000	0.1100000
20687.0000000	0.1150000
20688.0000000	0.1200000
20689.0000000	0.1100000
20690.0000000	0.1250000

20691.0000000	0.1100000
20692.0000000	0.1200000
20693.0000000	0.1150000
20694.0000000	0.1250000
20695.0000000	0.1200000
20696.0000000	0.1200000
20697.0000000	0.1200000
20698.0000000	0.1250000
20699.0000000	0.1150000
20700.0000000	0.1250000
20701.0000000	0.1100000
20702.0000000	0.1250000
20703.0000000	0.1200000
20704.0000000	0.1200000
20705.0000000	0.1150000
20706.0000000	0.1100000
20707.0000000	0.1250000
20708.0000000	0.1250000
20709.0000000	0.1100000
20710.0000000	0.1250000
20711.0000000	0.1100000
20712.0000000	0.1200000
20713.0000000	0.1100000
20714.0000000	0.1200000
20715.0000000	0.1133333
20716.0000000	0.1200000
20717.0000000	0.1150000
20718.0000000	0.1050000
20719.0000000	0.1200000
20720.0000000	0.1150000
20721.0000000	0.1300000
20722.0000000	0.1200000
20723.0000000	0.1050000
20724.0000000	0.1150000
20725.0000000	0.1100000
20726.0000000	0.1250000
20727.0000000	0.1150000
20728.0000000	0.1250000
20729.0000000	0.1150000
20730.0000000	0.1100000
20731.0000000	0.1100000
20732.0000000	0.1150000
20733.0000000	0.1200000
20734.0000000	0.1200000
20735.0000000	0.1150000
20736.0000000	0.1050000
20737.0000000	0.1150000
20738.0000000	0.1050000
20739.0000000	0.1200000
20740.0000000	0.1200000
20741.0000000	0.1200000
20742.0000000	0.1150000
20743.0000000	0.1150000
20744.0000000	0.1050000
20745.0000000	0.1150000
20746.0000000	0.1133333
20747.0000000	0.1100000

20748.0000000	0.1150000
20749.0000000	0.1100000
20750.0000000	0.1200000
20751.0000000	0.1200000
20752.0000000	0.1100000
20753.0000000	0.1200000
20754.0000000	0.1100000
20755.0000000	0.1250000
20756.0000000	0.1100000
20757.0000000	0.1250000
20758.0000000	0.1150000
20759.0000000	0.1150000
20760.0000000	0.1150000
20761.0000000	0.1150000
20762.0000000	0.1150000
20763.0000000	0.1100000
20764.0000000	0.1150000
20765.0000000	0.1150000
20766.0000000	0.1200000
20767.0000000	0.1150000
20768.0000000	0.1200000
20769.0000000	0.1100000
20770.0000000	0.1100000
20771.0000000	0.1100000
20772.0000000	0.1150000
20773.0000000	0.1200000
20774.0000000	0.1100000
20775.0000000	0.1150000
20776.0000000	0.1100000
20777.0000000	0.1200000
20778.0000000	0.1150000
20779.0000000	0.1100000
20780.0000000	0.1150000
20781.0000000	0.1000000
20782.0000000	0.1200000
20783.0000000	0.1150000
20784.0000000	0.1200000
20785.0000000	0.1200000
20786.0000000	0.1100000
20787.0000000	0.1100000
20788.0000000	0.1100000
20789.0000000	0.1150000
20790.0000000	0.1150000
20791.0000000	0.1100000
20792.0000000	0.1200000
20793.0000000	0.1200000
20794.0000000	0.1200000
20795.0000000	0.1100000
20796.0000000	0.1050000
20797.0000000	0.1100000
20798.0000000	0.1150000
20799.0000000	0.1100000
20800.0000000	0.1150000
20801.0000000	0.1150000
20802.0000000	0.1200000
20803.0000000	0.1100000
20804.0000000	0.1250000

20805.0000000	0.1150000
20806.0000000	0.1133333
20807.0000000	0.1050000
20808.0000000	0.1100000
20809.0000000	0.1150000
20810.0000000	0.1100000
20811.0000000	0.1200000
20812.0000000	0.1150000
20813.0000000	0.1100000
20814.0000000	0.1150000
20815.0000000	0.1100000
20816.0000000	0.1100000
20817.0000000	0.1150000
20818.0000000	0.1150000
20819.0000000	0.1200000
20820.0000000	0.1150000
20821.0000000	0.1100000
20822.0000000	0.1150000
20823.0000000	0.1100000
20824.0000000	0.1200000
20825.0000000	0.1000000
20826.0000000	0.1100000
20827.0000000	0.1100000
20828.0000000	0.1100000
20829.0000000	0.1250000
20830.0000000	0.1150000
20831.0000000	0.1200000
20832.0000000	0.1100000
20833.0000000	0.1100000
20834.0000000	0.1150000
20835.0000000	0.1100000
20836.0000000	0.1166667
20837.0000000	0.1200000
20838.0000000	0.1200000
20839.0000000	0.1100000
20840.0000000	0.1100000
20841.0000000	0.1100000
20842.0000000	0.1200000
20843.0000000	0.1250000
20844.0000000	0.1150000
20845.0000000	0.1100000
20846.0000000	0.1100000
20847.0000000	0.1150000
20848.0000000	0.1150000
20849.0000000	0.1100000
20850.0000000	0.1150000
20851.0000000	0.1100000
20852.0000000	0.1150000
20853.0000000	0.1150000
20854.0000000	0.1150000
20855.0000000	0.1100000
20856.0000000	0.1200000
20857.0000000	0.1250000
20858.0000000	0.1250000
20859.0000000	0.1100000
20860.0000000	0.1200000
20861.0000000	0.1100000

20862.0000000	0.1150000
20863.0000000	0.1100000
20864.0000000	0.1150000
20865.0000000	0.1150000
20866.0000000	0.1150000
20867.0000000	0.1133333
20868.0000000	0.1200000
20869.0000000	0.1150000
20870.0000000	0.1150000
20871.0000000	0.1200000
20872.0000000	0.1250000
20873.0000000	0.1150000
20874.0000000	0.1150000
20875.0000000	0.1150000
20876.0000000	0.1200000
20877.0000000	0.1250000
20878.0000000	0.1200000
20879.0000000	0.1200000
20880.0000000	0.1150000
20881.0000000	0.1100000
20882.0000000	0.1200000
20883.0000000	0.1100000
20884.0000000	0.1250000
20885.0000000	0.1150000
20886.0000000	0.1150000
20887.0000000	0.1250000
20888.0000000	0.1100000
20889.0000000	0.1200000
20890.0000000	0.1200000
20891.0000000	0.1100000
20892.0000000	0.1200000
20893.0000000	0.1200000
20894.0000000	0.1200000
20895.0000000	0.1250000
20896.0000000	0.1200000
20897.0000000	0.1166667
20898.0000000	0.1250000
20899.0000000	0.1150000
20900.0000000	0.1250000
20901.0000000	0.1100000
20902.0000000	0.1200000
20903.0000000	0.1200000
20904.0000000	0.1150000
20905.0000000	0.1200000
20906.0000000	0.1150000
20907.0000000	0.1250000
20908.0000000	0.1150000
20909.0000000	0.1300000
20910.0000000	0.1100000
20911.0000000	0.1100000
20912.0000000	0.1150000
20913.0000000	0.1300000
20914.0000000	0.1200000
20915.0000000	0.1300000
20916.0000000	0.1200000
20917.0000000	0.1200000
20918.0000000	0.1200000

20919.0000000	0.1250000
20920.0000000	0.1200000
20921.0000000	0.1300000
20922.0000000	0.1250000
20923.0000000	0.1200000
20924.0000000	0.1200000
20925.0000000	0.1200000
20926.0000000	0.1300000
20927.0000000	0.1233333
20928.0000000	0.1250000
20929.0000000	0.1200000
20930.0000000	0.1100000
20931.0000000	0.1150000
20932.0000000	0.1150000
20933.0000000	0.1250000
20934.0000000	0.1250000
20935.0000000	0.1250000
20936.0000000	0.1200000
20937.0000000	0.1100000
20938.0000000	0.1300000
20939.0000000	0.1150000
20940.0000000	0.1200000
20941.0000000	0.1250000
20942.0000000	0.1200000
20943.0000000	0.1200000
20944.0000000	0.1200000
20945.0000000	0.1200000
20946.0000000	0.1200000
20947.0000000	0.1100000
20948.0000000	0.1200000
20949.0000000	0.1200000
20950.0000000	0.1250000
20951.0000000	0.1100000
20952.0000000	0.1200000
20953.0000000	0.1150000
20954.0000000	0.1150000
20955.0000000	0.1200000
20956.0000000	0.1250000
20957.0000000	0.1166667
20958.0000000	0.1200000
20959.0000000	0.1100000
20960.0000000	0.1250000
20961.0000000	0.1200000
20962.0000000	0.1100000
20963.0000000	0.1100000
20964.0000000	0.1150000
20965.0000000	0.1200000
20966.0000000	0.1200000
20967.0000000	0.1200000
20968.0000000	0.1200000
20969.0000000	0.1100000
20970.0000000	0.1150000
20971.0000000	0.1200000
20972.0000000	0.1200000
20973.0000000	0.1250000
20974.0000000	0.1100000
20975.0000000	0.1200000

20976.0000000	0.1150000
20977.0000000	0.1100000
20978.0000000	0.1150000
20979.0000000	0.1150000
20980.0000000	0.1150000
20981.0000000	0.1200000
20982.0000000	0.1200000
20983.0000000	0.1250000
20984.0000000	0.1150000
20985.0000000	0.1100000
20986.0000000	0.1050000
20987.0000000	0.1200000
20988.0000000	0.1233333
20989.0000000	0.1050000
20990.0000000	0.1200000
20991.0000000	0.1100000
20992.0000000	0.1250000
20993.0000000	0.1200000
20994.0000000	0.1250000
20995.0000000	0.1050000
20996.0000000	0.1100000
20997.0000000	0.1100000
20998.0000000	0.1250000
20999.0000000	0.1300000
21000.0000000	0.1250000
21001.0000000	0.1200000
21002.0000000	0.1200000
21003.0000000	0.1200000
21004.0000000	0.1300000
21005.0000000	0.1200000
21006.0000000	0.1200000
21007.0000000	0.1150000
21008.0000000	0.1150000
21009.0000000	0.1200000
21010.0000000	0.1150000
21011.0000000	0.1250000
21012.0000000	0.1200000
21013.0000000	0.1150000
21014.0000000	0.1150000
21015.0000000	0.1150000
21016.0000000	0.1300000
21017.0000000	0.1166667
21018.0000000	0.1250000
21019.0000000	0.1250000
21020.0000000	0.1200000
21021.0000000	0.1250000
21022.0000000	0.1150000
21023.0000000	0.1250000
21024.0000000	0.1250000
21025.0000000	0.1150000
21026.0000000	0.1300000
21027.0000000	0.1200000
21028.0000000	0.1250000
21029.0000000	0.1250000
21030.0000000	0.1200000
21031.0000000	0.1200000
21032.0000000	0.1100000

21033.0000000	0.1200000
21034.0000000	0.1250000
21035.0000000	0.1250000
21036.0000000	0.1200000
21037.0000000	0.1200000
21038.0000000	0.1150000
21039.0000000	0.1300000
21040.0000000	0.1200000
21041.0000000	0.1150000
21042.0000000	0.1150000
21043.0000000	0.1150000
21044.0000000	0.1150000
21045.0000000	0.1150000
21046.0000000	0.1200000
21047.0000000	0.1100000
21048.0000000	0.1200000
21049.0000000	0.1150000
21050.0000000	0.1150000
21051.0000000	0.1100000
21052.0000000	0.1150000
21053.0000000	0.1200000
21054.0000000	0.1200000
21055.0000000	0.1300000
21056.0000000	0.1150000
21057.0000000	0.1200000
21058.0000000	0.1150000
21059.0000000	0.1200000
21060.0000000	0.1100000
21061.0000000	0.1200000
21062.0000000	0.1200000
21063.0000000	0.1200000
21064.0000000	0.1200000
21065.0000000	0.1200000
21066.0000000	0.1200000
21067.0000000	0.1150000
21068.0000000	0.1150000
21069.0000000	0.1250000
21070.0000000	0.1250000
21071.0000000	0.1250000
21072.0000000	0.1200000
21073.0000000	0.1200000
21074.0000000	0.1200000
21075.0000000	0.1200000
21076.0000000	0.1250000
21077.0000000	0.1250000
21078.0000000	0.1200000
21079.0000000	0.1200000
21080.0000000	0.1250000
21081.0000000	0.1200000
21082.0000000	0.1250000
21083.0000000	0.1200000
21084.0000000	0.1300000
21085.0000000	0.1250000
21086.0000000	0.1300000
21087.0000000	0.1250000
21088.0000000	0.1250000
21089.0000000	0.1250000

21090.0000000	0.1200000
21091.0000000	0.1350000
21092.0000000	0.1250000
21093.0000000	0.1300000
21094.0000000	0.1200000
21095.0000000	0.1350000
21096.0000000	0.1350000
21097.0000000	0.1350000
21098.0000000	0.1250000
21099.0000000	0.1300000
21100.0000000	0.1300000
21101.0000000	0.1200000
21102.0000000	0.1350000
21103.0000000	0.1350000
21104.0000000	0.1250000
21105.0000000	0.1300000
21106.0000000	0.1250000
21107.0000000	0.1300000
21108.0000000	0.1300000
21109.0000000	0.1350000
21110.0000000	0.1250000
21111.0000000	0.1350000
21112.0000000	0.1250000
21113.0000000	0.1300000
21114.0000000	0.1250000
21115.0000000	0.1350000
21116.0000000	0.1250000
21117.0000000	0.1450000
21118.0000000	0.1200000
21119.0000000	0.1350000
21120.0000000	0.1200000
21121.0000000	0.1350000
21122.0000000	0.1300000
21123.0000000	0.1400000
21124.0000000	0.1250000
21125.0000000	0.1350000
21126.0000000	0.1300000
21127.0000000	0.1300000
21128.0000000	0.1350000
21129.0000000	0.1350000
21130.0000000	0.1250000
21131.0000000	0.1400000
21132.0000000	0.1350000
21133.0000000	0.1300000
21134.0000000	0.1350000
21135.0000000	0.1300000
21136.0000000	0.1400000
21137.0000000	0.1350000
21138.0000000	0.1333333
21139.0000000	0.1350000
21140.0000000	0.1400000
21141.0000000	0.1350000
21142.0000000	0.1350000
21143.0000000	0.1350000
21144.0000000	0.1350000
21145.0000000	0.1400000
21146.0000000	0.1300000

21147.0000000	0.1400000
21148.0000000	0.1400000
21149.0000000	0.1350000
21150.0000000	0.1350000
21151.0000000	0.1350000
21152.0000000	0.1300000
21153.0000000	0.1350000
21154.0000000	0.1350000
21155.0000000	0.1400000
21156.0000000	0.1400000
21157.0000000	0.1400000
21158.0000000	0.1350000
21159.0000000	0.1250000
21160.0000000	0.1350000
21161.0000000	0.1450000
21162.0000000	0.1400000
21163.0000000	0.1450000
21164.0000000	0.1350000
21165.0000000	0.1250000
21166.0000000	0.1400000
21167.0000000	0.1400000
21168.0000000	0.1433333
21169.0000000	0.1350000
21170.0000000	0.1350000
21171.0000000	0.1400000
21172.0000000	0.1400000
21173.0000000	0.1400000
21174.0000000	0.1350000
21175.0000000	0.1350000
21176.0000000	0.1350000
21177.0000000	0.1350000
21178.0000000	0.1450000
21179.0000000	0.1400000
21180.0000000	0.1400000
21181.0000000	0.1400000
21182.0000000	0.1550000
21183.0000000	0.1350000
21184.0000000	0.1400000
21185.0000000	0.1350000
21186.0000000	0.1400000
21187.0000000	0.1350000
21188.0000000	0.1450000
21189.0000000	0.1450000
21190.0000000	0.1500000
21191.0000000	0.1400000
21192.0000000	0.1350000
21193.0000000	0.1400000
21194.0000000	0.1400000
21195.0000000	0.1500000
21196.0000000	0.1350000
21197.0000000	0.1450000
21198.0000000	0.1350000
21199.0000000	0.1466667
21200.0000000	0.1450000
21201.0000000	0.1450000
21202.0000000	0.1250000
21203.0000000	0.1450000

21204.0000000	0.1350000
21205.0000000	0.1450000
21206.0000000	0.1450000
21207.0000000	0.1400000
21208.0000000	0.1450000
21209.0000000	0.1400000
21210.0000000	0.1350000
21211.0000000	0.1400000
21212.0000000	0.1400000
21213.0000000	0.1350000
21214.0000000	0.1300000
21215.0000000	0.1400000
21216.0000000	0.1400000
21217.0000000	0.1500000
21218.0000000	0.1500000
21219.0000000	0.1450000
21220.0000000	0.1350000
21221.0000000	0.1400000
21222.0000000	0.1400000
21223.0000000	0.1450000
21224.0000000	0.1450000
21225.0000000	0.1450000
21226.0000000	0.1400000
21227.0000000	0.1400000
21228.0000000	0.1500000
21229.0000000	0.1366667
21230.0000000	0.1450000
21231.0000000	0.1500000
21232.0000000	0.1450000
21233.0000000	0.1350000
21234.0000000	0.1500000
21235.0000000	0.1450000
21236.0000000	0.1450000
21237.0000000	0.1500000
21238.0000000	0.1350000
21239.0000000	0.1350000
21240.0000000	0.1400000
21241.0000000	0.1400000
21242.0000000	0.1450000
21243.0000000	0.1350000
21244.0000000	0.1400000
21245.0000000	0.1400000
21246.0000000	0.1350000
21247.0000000	0.1500000
21248.0000000	0.1400000
21249.0000000	0.1400000
21250.0000000	0.1400000
21251.0000000	0.1350000
21252.0000000	0.1500000
21253.0000000	0.1400000
21254.0000000	0.1400000
21255.0000000	0.1350000
21256.0000000	0.1400000
21257.0000000	0.1400000
21258.0000000	0.1400000
21259.0000000	0.1366667
21260.0000000	0.1500000

21261.0000000	0.1450000
21262.0000000	0.1400000
21263.0000000	0.1350000
21264.0000000	0.1350000
21265.0000000	0.1350000
21266.0000000	0.1500000
21267.0000000	0.1450000
21268.0000000	0.1450000
21269.0000000	0.1400000
21270.0000000	0.1350000
21271.0000000	0.1400000
21272.0000000	0.1400000
21273.0000000	0.1450000
21274.0000000	0.1400000
21275.0000000	0.1450000
21276.0000000	0.1350000
21277.0000000	0.1450000
21278.0000000	0.1300000
21279.0000000	0.1450000
21280.0000000	0.1350000
21281.0000000	0.1400000
21282.0000000	0.1350000
21283.0000000	0.1400000
21284.0000000	0.1300000
21285.0000000	0.1400000
21286.0000000	0.1350000
21287.0000000	0.1450000
21288.0000000	0.1300000
21289.0000000	0.1366667
21290.0000000	0.1500000
21291.0000000	0.1400000
21292.0000000	0.1400000
21293.0000000	0.1300000
21294.0000000	0.1350000
21295.0000000	0.1400000
21296.0000000	0.1400000
21297.0000000	0.1400000
21298.0000000	0.1300000
21299.0000000	0.1350000
21300.0000000	0.1350000
21301.0000000	0.1300000
21302.0000000	0.1350000
21303.0000000	0.1350000
21304.0000000	0.1400000
21305.0000000	0.1250000
21306.0000000	0.1400000
21307.0000000	0.1350000
21308.0000000	0.1400000
21309.0000000	0.1300000
21310.0000000	0.1300000
21311.0000000	0.1250000
21312.0000000	0.1350000
21313.0000000	0.1350000
21314.0000000	0.1350000
21315.0000000	0.1300000
21316.0000000	0.1300000
21317.0000000	0.1300000

21318.0000000	0.1400000
21319.0000000	0.1400000
21320.0000000	0.1333333
21321.0000000	0.1350000
21322.0000000	0.1350000
21323.0000000	0.1300000
21324.0000000	0.1350000
21325.0000000	0.1250000
21326.0000000	0.1350000
21327.0000000	0.1300000
21328.0000000	0.1300000
21329.0000000	0.1300000
21330.0000000	0.1250000
21331.0000000	0.1350000
21332.0000000	0.1300000
21333.0000000	0.1450000
21334.0000000	0.1300000
21335.0000000	0.1200000
21336.0000000	0.1300000
21337.0000000	0.1300000
21338.0000000	0.1350000
21339.0000000	0.1300000
21340.0000000	0.1250000
21341.0000000	0.1300000
21342.0000000	0.1400000
21343.0000000	0.1200000
21344.0000000	0.1250000
21345.0000000	0.1200000
21346.0000000	0.1250000
21347.0000000	0.1350000
21348.0000000	0.1350000
21349.0000000	0.1233333
21350.0000000	0.1350000
21351.0000000	0.1200000
21352.0000000	0.1250000
21353.0000000	0.1200000
21354.0000000	0.1300000
21355.0000000	0.1350000
21356.0000000	0.1350000
21357.0000000	0.1300000
21358.0000000	0.1250000
21359.0000000	0.1200000
21360.0000000	0.1300000
21361.0000000	0.1350000
21362.0000000	0.1350000
21363.0000000	0.1300000
21364.0000000	0.1250000
21365.0000000	0.1250000
21366.0000000	0.1300000
21367.0000000	0.1250000
21368.0000000	0.1400000
21369.0000000	0.1200000
21370.0000000	0.1350000
21371.0000000	0.1250000
21372.0000000	0.1300000
21373.0000000	0.1300000
21374.0000000	0.1400000

21375.0000000	0.1250000
21376.0000000	0.1300000
21377.0000000	0.1250000
21378.0000000	0.1250000
21379.0000000	0.1333333
21380.0000000	0.1250000
21381.0000000	0.1250000
21382.0000000	0.1350000
21383.0000000	0.1300000
21384.0000000	0.1400000
21385.0000000	0.1200000
21386.0000000	0.1400000
21387.0000000	0.1300000
21388.0000000	0.1350000
21389.0000000	0.1300000
21390.0000000	0.1400000
21391.0000000	0.1250000
21392.0000000	0.1250000
21393.0000000	0.1250000
21394.0000000	0.1250000
21395.0000000	0.1250000
21396.0000000	0.1300000
21397.0000000	0.1300000
21398.0000000	0.1350000
21399.0000000	0.1350000
21400.0000000	0.1250000
21401.0000000	0.1250000
21402.0000000	0.1200000
21403.0000000	0.1350000
21404.0000000	0.1350000
21405.0000000	0.1350000
21406.0000000	0.1300000
21407.0000000	0.1300000
21408.0000000	0.1250000
21409.0000000	0.1350000
21410.0000000	0.1233333
21411.0000000	0.1250000
21412.0000000	0.1300000
21413.0000000	0.1300000
21414.0000000	0.1250000
21415.0000000	0.1300000
21416.0000000	0.1300000
21417.0000000	0.1300000
21418.0000000	0.1250000
21419.0000000	0.1350000
21420.0000000	0.1300000
21421.0000000	0.1300000
21422.0000000	0.1300000
21423.0000000	0.1300000
21424.0000000	0.1250000
21425.0000000	0.1300000
21426.0000000	0.1350000
21427.0000000	0.1350000
21428.0000000	0.1250000
21429.0000000	0.1300000
21430.0000000	0.1250000
21431.0000000	0.1300000

21432.0000000	0.1250000
21433.0000000	0.1300000
21434.0000000	0.1250000
21435.0000000	0.1300000
21436.0000000	0.1300000
21437.0000000	0.1300000
21438.0000000	0.1300000
21439.0000000	0.1400000
21440.0000000	0.1300000
21441.0000000	0.1300000
21442.0000000	0.1300000
21443.0000000	0.1250000
21444.0000000	0.1200000
21445.0000000	0.1300000
21446.0000000	0.1250000
21447.0000000	0.1300000
21448.0000000	0.1250000
21449.0000000	0.1200000
21450.0000000	0.1250000
21451.0000000	0.1250000
21452.0000000	0.1300000
21453.0000000	0.1350000
21454.0000000	0.1300000
21455.0000000	0.1250000
21456.0000000	0.1200000
21457.0000000	0.1250000
21458.0000000	0.1300000
21459.0000000	0.1350000
21460.0000000	0.1250000
21461.0000000	0.1250000
21462.0000000	0.1350000
21463.0000000	0.1250000
21464.0000000	0.1200000
21465.0000000	0.1200000
21466.0000000	0.1150000
21467.0000000	0.1200000
21468.0000000	0.1300000
21469.0000000	0.1300000
21470.0000000	0.1233333
21471.0000000	0.1250000
21472.0000000	0.1200000
21473.0000000	0.1150000
21474.0000000	0.1200000
21475.0000000	0.1300000
21476.0000000	0.1200000
21477.0000000	0.1300000
21478.0000000	0.1200000
21479.0000000	0.1250000
21480.0000000	0.1150000
21481.0000000	0.1250000
21482.0000000	0.1200000
21483.0000000	0.1300000
21484.0000000	0.1200000
21485.0000000	0.1250000
21486.0000000	0.1150000
21487.0000000	0.1200000
21488.0000000	0.1150000

21489.0000000	0.1250000
21490.0000000	0.1200000
21491.0000000	0.1200000
21492.0000000	0.1200000
21493.0000000	0.1150000
21494.0000000	0.1250000
21495.0000000	0.1200000
21496.0000000	0.1200000
21497.0000000	0.1250000
21498.0000000	0.1250000
21499.0000000	0.1200000
21500.0000000	0.1200000
21501.0000000	0.1200000
21502.0000000	0.1150000
21503.0000000	0.1200000
21504.0000000	0.1250000
21505.0000000	0.1250000
21506.0000000	0.1250000
21507.0000000	0.1300000
21508.0000000	0.1250000
21509.0000000	0.1200000
21510.0000000	0.1200000
21511.0000000	0.1200000
21512.0000000	0.1200000
21513.0000000	0.1250000
21514.0000000	0.1250000
21515.0000000	0.1300000
21516.0000000	0.1200000
21517.0000000	0.1300000
21518.0000000	0.1200000
21519.0000000	0.1250000
21520.0000000	0.1250000
21521.0000000	0.1250000
21522.0000000	0.1200000
21523.0000000	0.1200000
21524.0000000	0.1250000
21525.0000000	0.1200000
21526.0000000	0.1300000
21527.0000000	0.1200000
21528.0000000	0.1200000
21529.0000000	0.1200000
21530.0000000	0.1300000
21531.0000000	0.1233333
21532.0000000	0.1100000
21533.0000000	0.1300000
21534.0000000	0.1250000
21535.0000000	0.1300000
21536.0000000	0.1150000
21537.0000000	0.1150000
21538.0000000	0.1200000
21539.0000000	0.1200000
21540.0000000	0.1250000
21541.0000000	0.1250000
21542.0000000	0.1200000
21543.0000000	0.1200000
21544.0000000	0.1250000
21545.0000000	0.1200000

21546.0000000	0.1250000
21547.0000000	0.1300000
21548.0000000	0.1200000
21549.0000000	0.1250000
21550.0000000	0.1350000
21551.0000000	0.1150000
21552.0000000	0.1250000
21553.0000000	0.1200000
21554.0000000	0.1250000
21555.0000000	0.1200000
21556.0000000	0.1200000
21557.0000000	0.1300000
21558.0000000	0.1300000
21559.0000000	0.1200000
21560.0000000	0.1250000
21561.0000000	0.1233333
21562.0000000	0.1300000
21563.0000000	0.1200000
21564.0000000	0.1450000
21565.0000000	0.1250000
21566.0000000	0.1250000
21567.0000000	0.1250000
21568.0000000	0.1150000
21569.0000000	0.1300000
21570.0000000	0.1300000
21571.0000000	0.1400000
21572.0000000	0.1200000
21573.0000000	0.1250000
21574.0000000	0.1200000
21575.0000000	0.1250000
21576.0000000	0.1300000
21577.0000000	0.1300000
21578.0000000	0.1300000
21579.0000000	0.1250000
21580.0000000	0.1200000
21581.0000000	0.1300000
21582.0000000	0.1300000
21583.0000000	0.1300000
21584.0000000	0.1300000
21585.0000000	0.1300000
21586.0000000	0.1350000
21587.0000000	0.1250000
21588.0000000	0.1350000
21589.0000000	0.1300000
21590.0000000	0.1350000
21591.0000000	0.1233333
21592.0000000	0.1350000
21593.0000000	0.1250000
21594.0000000	0.1300000
21595.0000000	0.1300000
21596.0000000	0.1400000
21597.0000000	0.1300000
21598.0000000	0.1350000
21599.0000000	0.1250000
21600.0000000	0.1350000
21601.0000000	0.1250000
21602.0000000	0.1350000

21603.0000000	0.1400000
21604.0000000	0.1350000
21605.0000000	0.1300000
21606.0000000	0.1250000
21607.0000000	0.1350000
21608.0000000	0.1350000
21609.0000000	0.1300000
21610.0000000	0.1300000
21611.0000000	0.1300000
21612.0000000	0.1350000
21613.0000000	0.1350000
21614.0000000	0.1350000
21615.0000000	0.1400000
21616.0000000	0.1350000
21617.0000000	0.1350000
21618.0000000	0.1250000
21619.0000000	0.1350000
21620.0000000	0.1350000
21621.0000000	0.1366667
21622.0000000	0.1250000
21623.0000000	0.1400000
21624.0000000	0.1300000
21625.0000000	0.1450000
21626.0000000	0.1300000
21627.0000000	0.1300000
21628.0000000	0.1450000
21629.0000000	0.1300000
21630.0000000	0.1400000
21631.0000000	0.1350000
21632.0000000	0.1300000
21633.0000000	0.1400000
21634.0000000	0.1350000
21635.0000000	0.1500000
21636.0000000	0.1500000
21637.0000000	0.1300000
21638.0000000	0.1400000
21639.0000000	0.1350000
21640.0000000	0.1400000
21641.0000000	0.1450000
21642.0000000	0.1400000
21643.0000000	0.1450000
21644.0000000	0.1250000
21645.0000000	0.1400000
21646.0000000	0.1300000
21647.0000000	0.1450000
21648.0000000	0.1450000
21649.0000000	0.1400000
21650.0000000	0.1400000
21651.0000000	0.1350000
21652.0000000	0.1433333
21653.0000000	0.1350000
21654.0000000	0.1350000
21655.0000000	0.1350000
21656.0000000	0.1350000
21657.0000000	0.1450000
21658.0000000	0.1400000
21659.0000000	0.1400000

21660.0000000	0.1300000
21661.0000000	0.1400000
21662.0000000	0.1400000
21663.0000000	0.1300000
21664.0000000	0.1400000
21665.0000000	0.1350000
21666.0000000	0.1350000
21667.0000000	0.1450000
21668.0000000	0.1400000
21669.0000000	0.1300000
21670.0000000	0.1450000
21671.0000000	0.1350000
21672.0000000	0.1400000
21673.0000000	0.1400000
21674.0000000	0.1350000
21675.0000000	0.1350000
21676.0000000	0.1350000
21677.0000000	0.1350000
21678.0000000	0.1350000
21679.0000000	0.1400000
21680.0000000	0.1350000
21681.0000000	0.1400000
21682.0000000	0.1450000
21683.0000000	0.1350000
21684.0000000	0.1400000
21685.0000000	0.1400000
21686.0000000	0.1350000
21687.0000000	0.1350000
21688.0000000	0.1300000
21689.0000000	0.1400000
21690.0000000	0.1400000
21691.0000000	0.1400000
21692.0000000	0.1350000
21693.0000000	0.1350000
21694.0000000	0.1300000
21695.0000000	0.1350000
21696.0000000	0.1300000
21697.0000000	0.1350000
21698.0000000	0.1250000
21699.0000000	0.1400000
21700.0000000	0.1250000
21701.0000000	0.1400000
21702.0000000	0.1350000
21703.0000000	0.1300000
21704.0000000	0.1250000
21705.0000000	0.1350000
21706.0000000	0.1400000
21707.0000000	0.1350000
21708.0000000	0.1400000
21709.0000000	0.1250000
21710.0000000	0.1350000
21711.0000000	0.1300000
21712.0000000	0.1250000
21713.0000000	0.1300000
21714.0000000	0.1350000
21715.0000000	0.1300000
21716.0000000	0.1350000

21717.0000000	0.1300000
21718.0000000	0.1300000
21719.0000000	0.1250000
21720.0000000	0.1300000
21721.0000000	0.1250000
21722.0000000	0.1300000
21723.0000000	0.1300000
21724.0000000	0.1300000
21725.0000000	0.1300000
21726.0000000	0.1250000
21727.0000000	0.1300000
21728.0000000	0.1350000
21729.0000000	0.1300000
21730.0000000	0.1250000
21731.0000000	0.1300000
21732.0000000	0.1300000
21733.0000000	0.1350000
21734.0000000	0.1350000
21735.0000000	0.1150000
21736.0000000	0.1250000
21737.0000000	0.1250000
21738.0000000	0.1200000
21739.0000000	0.1400000
21740.0000000	0.1300000
21741.0000000	0.1300000
21742.0000000	0.1266667
21743.0000000	0.1150000
21744.0000000	0.1250000
21745.0000000	0.1300000
21746.0000000	0.1300000
21747.0000000	0.1350000
21748.0000000	0.1300000
21749.0000000	0.1250000
21750.0000000	0.1300000
21751.0000000	0.1250000
21752.0000000	0.1300000
21753.0000000	0.1100000
21754.0000000	0.1200000
21755.0000000	0.1250000
21756.0000000	0.1200000
21757.0000000	0.1300000
21758.0000000	0.1150000
21759.0000000	0.1200000
21760.0000000	0.1200000
21761.0000000	0.1350000
21762.0000000	0.1250000
21763.0000000	0.1300000
21764.0000000	0.1300000
21765.0000000	0.1200000
21766.0000000	0.1250000
21767.0000000	0.1150000
21768.0000000	0.1250000
21769.0000000	0.1250000
21770.0000000	0.1250000
21771.0000000	0.1200000
21772.0000000	0.1233333
21773.0000000	0.1250000

21774.0000000	0.1250000
21775.0000000	0.1200000
21776.0000000	0.1350000
21777.0000000	0.1200000
21778.0000000	0.1250000
21779.0000000	0.1250000
21780.0000000	0.1250000
21781.0000000	0.1200000
21782.0000000	0.1150000
21783.0000000	0.1200000
21784.0000000	0.1200000
21785.0000000	0.1300000
21786.0000000	0.1250000
21787.0000000	0.1300000
21788.0000000	0.1100000
21789.0000000	0.1200000
21790.0000000	0.1250000
21791.0000000	0.1250000
21792.0000000	0.1250000
21793.0000000	0.1200000
21794.0000000	0.1200000
21795.0000000	0.1100000
21796.0000000	0.1150000
21797.0000000	0.1100000
21798.0000000	0.1200000
21799.0000000	0.1150000
21800.0000000	0.1200000
21801.0000000	0.1100000
21802.0000000	0.1166667
21803.0000000	0.1300000
21804.0000000	0.1250000
21805.0000000	0.1150000
21806.0000000	0.1050000
21807.0000000	0.1150000
21808.0000000	0.1150000
21809.0000000	0.1150000
21810.0000000	0.1150000
21811.0000000	0.1150000
21812.0000000	0.1200000
21813.0000000	0.1200000
21814.0000000	0.1150000
21815.0000000	0.1150000
21816.0000000	0.1000000
21817.0000000	0.1100000
21818.0000000	0.1200000
21819.0000000	0.1050000
21820.0000000	0.1150000
21821.0000000	0.1050000
21822.0000000	0.1100000
21823.0000000	0.1100000
21824.0000000	0.1000000
21825.0000000	0.1100000
21826.0000000	0.1100000
21827.0000000	0.1050000
21828.0000000	0.1100000
21829.0000000	0.1150000
21830.0000000	0.1050000

21831.0000000	0.1200000
21832.0000000	0.1066667
21833.0000000	0.1000000
21834.0000000	0.1100000
21835.0000000	0.1150000
21836.0000000	0.1000000
21837.0000000	0.1100000
21838.0000000	0.1000000
21839.0000000	0.1000000
21840.0000000	0.1100000
21841.0000000	0.1050000
21842.0000000	0.1000000
21843.0000000	0.1050000
21844.0000000	0.1000000
21845.0000000	0.1100000
21846.0000000	0.1050000
21847.0000000	0.1050000
21848.0000000	0.1000000
21849.0000000	0.1000000
21850.0000000	0.1050000
21851.0000000	0.1050000
21852.0000000	0.1000000
21853.0000000	0.0900000
21854.0000000	0.1100000
21855.0000000	0.1000000
21856.0000000	0.0950000
21857.0000000	0.1050000
21858.0000000	0.1000000
21859.0000000	0.0950000
21860.0000000	0.1000000
21861.0000000	0.1000000
21862.0000000	0.0900000
21863.0000000	0.1033333
21864.0000000	0.1000000
21865.0000000	0.0950000
21866.0000000	0.0850000
21867.0000000	0.1000000
21868.0000000	0.0950000
21869.0000000	0.0950000
21870.0000000	0.0950000
21871.0000000	0.0950000
21872.0000000	0.0900000
21873.0000000	0.0900000
21874.0000000	0.0950000
21875.0000000	0.0900000
21876.0000000	0.0900000
21877.0000000	0.0950000
21878.0000000	0.0900000
21879.0000000	0.0950000
21880.0000000	0.0950000
21881.0000000	0.0950000
21882.0000000	0.0950000
21883.0000000	0.0850000
21884.0000000	0.0850000
21885.0000000	0.0850000
21886.0000000	0.0950000
21887.0000000	0.0900000

21888.0000000	0.0900000
21889.0000000	0.0850000
21890.0000000	0.0850000
21891.0000000	0.0800000
21892.0000000	0.0850000
21893.0000000	0.0933333
21894.0000000	0.0750000
21895.0000000	0.0850000
21896.0000000	0.0900000
21897.0000000	0.0900000
21898.0000000	0.0900000
21899.0000000	0.0750000
21900.0000000	0.0800000
21901.0000000	0.0800000
21902.0000000	0.0900000
21903.0000000	0.0850000
21904.0000000	0.0950000
21905.0000000	0.0800000
21906.0000000	0.0800000
21907.0000000	0.0700000
21908.0000000	0.0900000
21909.0000000	0.0800000
21910.0000000	0.0850000
21911.0000000	0.0700000
21912.0000000	0.0800000
21913.0000000	0.0700000
21914.0000000	0.0850000
21915.0000000	0.0750000
21916.0000000	0.0700000
21917.0000000	0.0650000
21918.0000000	0.0800000
21919.0000000	0.0800000
21920.0000000	0.0850000
21921.0000000	0.0750000
21922.0000000	0.0700000
21923.0000000	0.0800000
21924.0000000	0.0650000
21925.0000000	0.0800000
21926.0000000	0.0700000
21927.0000000	0.0750000
21928.0000000	0.0650000
21929.0000000	0.0700000
21930.0000000	0.0600000
21931.0000000	0.0750000
21932.0000000	0.0650000
21933.0000000	0.0700000
21934.0000000	0.0600000
21935.0000000	0.0750000
21936.0000000	0.0600000
21937.0000000	0.0600000
21938.0000000	0.0650000
21939.0000000	0.0700000
21940.0000000	0.0650000
21941.0000000	0.0650000
21942.0000000	0.0700000
21943.0000000	0.0500000
21944.0000000	0.0650000

21945.0000000	0.0600000
21946.0000000	0.0750000
21947.0000000	0.0650000
21948.0000000	0.0650000
21949.0000000	0.0600000
21950.0000000	0.0550000
21951.0000000	0.0600000
21952.0000000	0.0550000
21953.0000000	0.0566667
21954.0000000	0.0550000
21955.0000000	0.0600000
21956.0000000	0.0700000
21957.0000000	0.0550000
21958.0000000	0.0600000
21959.0000000	0.0500000
21960.0000000	0.0600000
21961.0000000	0.0550000
21962.0000000	0.0650000
21963.0000000	0.0650000
21964.0000000	0.0550000
21965.0000000	0.0500000
21966.0000000	0.0550000
21967.0000000	0.0550000
21968.0000000	0.0500000
21969.0000000	0.0600000
21970.0000000	0.0550000
21971.0000000	0.0550000
21972.0000000	0.0400000
21973.0000000	0.0550000
21974.0000000	0.0500000
21975.0000000	0.0650000
21976.0000000	0.0600000
21977.0000000	0.0500000
21978.0000000	0.0500000
21979.0000000	0.0550000
21980.0000000	0.0550000
21981.0000000	0.0550000
21982.0000000	0.0550000
21983.0000000	0.0550000
21984.0000000	0.0466667
21985.0000000	0.0500000
21986.0000000	0.0350000
21987.0000000	0.0450000
21988.0000000	0.0500000
21989.0000000	0.0450000
21990.0000000	0.0550000
21991.0000000	0.0400000
21992.0000000	0.0500000
21993.0000000	0.0450000
21994.0000000	0.0500000
21995.0000000	0.0500000
21996.0000000	0.0500000
21997.0000000	0.0550000
21998.0000000	0.0450000
21999.0000000	0.0500000
22000.0000000	0.0400000
22001.0000000	0.0550000

22002.0000000	0.0550000
22003.0000000	0.0450000
22004.0000000	0.0450000
22005.0000000	0.0350000
22006.0000000	0.0500000
22007.0000000	0.0400000
22008.0000000	0.0500000
22009.0000000	0.0350000
22010.0000000	0.0500000
22011.0000000	0.0400000
22012.0000000	0.0500000
22013.0000000	0.0466667
22014.0000000	0.0550000
22015.0000000	0.0400000
22016.0000000	0.0550000
22017.0000000	0.0550000
22018.0000000	0.0500000
22019.0000000	0.0400000
22020.0000000	0.0450000
22021.0000000	0.0500000
22022.0000000	0.0550000
22023.0000000	0.0500000
22024.0000000	0.0450000
22025.0000000	0.0450000
22026.0000000	0.0500000
22027.0000000	0.0400000
22028.0000000	0.0500000
22029.0000000	0.0400000
22030.0000000	0.0450000
22031.0000000	0.0450000
22032.0000000	0.0550000
22033.0000000	0.0450000
22034.0000000	0.0400000
22035.0000000	0.0450000
22036.0000000	0.0450000
22037.0000000	0.0550000
22038.0000000	0.0450000
22039.0000000	0.0500000
22040.0000000	0.0450000
22041.0000000	0.0300000
22042.0000000	0.0450000
22043.0000000	0.0466667
22044.0000000	0.0450000
22045.0000000	0.0450000
22046.0000000	0.0500000
22047.0000000	0.0400000
22048.0000000	0.0450000
22049.0000000	0.0350000
22050.0000000	0.0400000
22051.0000000	0.0500000
22052.0000000	0.0550000
22053.0000000	0.0450000
22054.0000000	0.0450000
22055.0000000	0.0500000
22056.0000000	0.0350000
22057.0000000	0.0450000
22058.0000000	0.0350000

22059.0000000	0.0400000
22060.0000000	0.0450000
22061.0000000	0.0550000
22062.0000000	0.0450000
22063.0000000	0.0450000
22064.0000000	0.0450000
22065.0000000	0.0400000
22066.0000000	0.0400000
22067.0000000	0.0350000
22068.0000000	0.0400000
22069.0000000	0.0450000
22070.0000000	0.0400000
22071.0000000	0.0400000
22072.0000000	0.0450000
22073.0000000	0.0450000
22074.0000000	0.0400000
22075.0000000	0.0400000
22076.0000000	0.0450000
22077.0000000	0.0400000
22078.0000000	0.0400000
22079.0000000	0.0450000
22080.0000000	0.0350000
22081.0000000	0.0450000
22082.0000000	0.0400000
22083.0000000	0.0350000
22084.0000000	0.0350000
22085.0000000	0.0450000
22086.0000000	0.0350000
22087.0000000	0.0450000
22088.0000000	0.0450000
22089.0000000	0.0400000
22090.0000000	0.0400000
22091.0000000	0.0300000
22092.0000000	0.0450000
22093.0000000	0.0400000
22094.0000000	0.0450000
22095.0000000	0.0450000
22096.0000000	0.0400000
22097.0000000	0.0450000
22098.0000000	0.0400000
22099.0000000	0.0400000
22100.0000000	0.0350000
22101.0000000	0.0450000
22102.0000000	0.0400000
22103.0000000	0.0500000
22104.0000000	0.0366667
22105.0000000	0.0400000
22106.0000000	0.0400000
22107.0000000	0.0350000
22108.0000000	0.0350000
22109.0000000	0.0350000
22110.0000000	0.0400000
22111.0000000	0.0450000
22112.0000000	0.0450000
22113.0000000	0.0400000
22114.0000000	0.0400000
22115.0000000	0.0400000

22116.0000000	0.0400000
22117.0000000	0.0450000
22118.0000000	0.0400000
22119.0000000	0.0450000
22120.0000000	0.0400000
22121.0000000	0.0400000
22122.0000000	0.0400000
22123.0000000	0.0450000
22124.0000000	0.0450000
22125.0000000	0.0450000
22126.0000000	0.0350000
22127.0000000	0.0450000
22128.0000000	0.0350000
22129.0000000	0.0400000
22130.0000000	0.0350000
22131.0000000	0.0400000
22132.0000000	0.0350000
22133.0000000	0.0350000
22134.0000000	0.0466667
22135.0000000	0.0350000
22136.0000000	0.0400000
22137.0000000	0.0400000
22138.0000000	0.0400000
22139.0000000	0.0500000
22140.0000000	0.0450000
22141.0000000	0.0400000
22142.0000000	0.0500000
22143.0000000	0.0350000
22144.0000000	0.0350000
22145.0000000	0.0350000
22146.0000000	0.0450000
22147.0000000	0.0400000
22148.0000000	0.0400000
22149.0000000	0.0350000
22150.0000000	0.0350000
22151.0000000	0.0400000
22152.0000000	0.0400000
22153.0000000	0.0450000
22154.0000000	0.0400000
22155.0000000	0.0350000
22156.0000000	0.0300000
22157.0000000	0.0450000
22158.0000000	0.0450000
22159.0000000	0.0350000
22160.0000000	0.0350000
22161.0000000	0.0400000
22162.0000000	0.0300000
22163.0000000	0.0450000
22164.0000000	0.0433333
22165.0000000	0.0300000
22166.0000000	0.0400000
22167.0000000	0.0450000
22168.0000000	0.0350000
22169.0000000	0.0350000
22170.0000000	0.0300000
22171.0000000	0.0400000
22172.0000000	0.0450000

22173.0000000	0.0450000
22174.0000000	0.0400000
22175.0000000	0.0400000
22176.0000000	0.0300000
22177.0000000	0.0400000
22178.0000000	0.0500000
22179.0000000	0.0450000
22180.0000000	0.0450000
22181.0000000	0.0350000
22182.0000000	0.0350000
22183.0000000	0.0400000
22184.0000000	0.0450000
22185.0000000	0.0550000
22186.0000000	0.0400000
22187.0000000	0.0400000
22188.0000000	0.0350000
22189.0000000	0.0400000
22190.0000000	0.0450000
22191.0000000	0.0450000
22192.0000000	0.0450000
22193.0000000	0.0400000
22194.0000000	0.0450000
22195.0000000	0.0466667
22196.0000000	0.0400000
22197.0000000	0.0400000
22198.0000000	0.0550000
22199.0000000	0.0450000
22200.0000000	0.0500000
22201.0000000	0.0400000
22202.0000000	0.0400000
22203.0000000	0.0400000
22204.0000000	0.0450000
22205.0000000	0.0400000
22206.0000000	0.0500000
22207.0000000	0.0400000
22208.0000000	0.0450000
22209.0000000	0.0400000
22210.0000000	0.0500000
22211.0000000	0.0500000
22212.0000000	0.0500000
22213.0000000	0.0450000
22214.0000000	0.0450000
22215.0000000	0.0400000
22216.0000000	0.0400000
22217.0000000	0.0400000
22218.0000000	0.0400000
22219.0000000	0.0450000
22220.0000000	0.0500000
22221.0000000	0.0450000
22222.0000000	0.0500000
22223.0000000	0.0400000
22224.0000000	0.0500000
22225.0000000	0.0500000
22226.0000000	0.0450000
22227.0000000	0.0500000
22228.0000000	0.0450000
22229.0000000	0.0450000

22230.0000000	0.0350000
22231.0000000	0.0400000
22232.0000000	0.0450000
22233.0000000	0.0450000
22234.0000000	0.0350000
22235.0000000	0.0450000
22236.0000000	0.0350000
22237.0000000	0.0550000
22238.0000000	0.0450000
22239.0000000	0.0550000
22240.0000000	0.0350000
22241.0000000	0.0450000
22242.0000000	0.0400000
22243.0000000	0.0500000
22244.0000000	0.0450000
22245.0000000	0.0500000
22246.0000000	0.0450000
22247.0000000	0.0500000
22248.0000000	0.0500000
22249.0000000	0.0500000
22250.0000000	0.0450000
22251.0000000	0.0500000
22252.0000000	0.0400000
22253.0000000	0.0400000
22254.0000000	0.0350000
22255.0000000	0.0466667
22256.0000000	0.0500000
22257.0000000	0.0400000
22258.0000000	0.0500000
22259.0000000	0.0400000
22260.0000000	0.0550000
22261.0000000	0.0500000
22262.0000000	0.0500000
22263.0000000	0.0450000
22264.0000000	0.0350000
22265.0000000	0.0500000
22266.0000000	0.0450000
22267.0000000	0.0500000
22268.0000000	0.0450000
22269.0000000	0.0500000
22270.0000000	0.0450000
22271.0000000	0.0400000
22272.0000000	0.0500000
22273.0000000	0.0450000
22274.0000000	0.0500000
22275.0000000	0.0550000
22276.0000000	0.0550000
22277.0000000	0.0400000
22278.0000000	0.0500000
22279.0000000	0.0400000
22280.0000000	0.0550000
22281.0000000	0.0500000
22282.0000000	0.0600000
22283.0000000	0.0550000
22284.0000000	0.0500000
22285.0000000	0.0500000
22286.0000000	0.0500000

22287.0000000	0.0550000
22288.0000000	0.0500000
22289.0000000	0.0450000
22290.0000000	0.0500000
22291.0000000	0.0500000
22292.0000000	0.0550000
22293.0000000	0.0600000
22294.0000000	0.0650000
22295.0000000	0.0500000
22296.0000000	0.0500000
22297.0000000	0.0450000
22298.0000000	0.0500000
22299.0000000	0.0500000
22300.0000000	0.0600000
22301.0000000	0.0500000
22302.0000000	0.0500000
22303.0000000	0.0400000
22304.0000000	0.0500000
22305.0000000	0.0500000
22306.0000000	0.0500000
22307.0000000	0.0550000
22308.0000000	0.0550000
22309.0000000	0.0450000
22310.0000000	0.0550000
22311.0000000	0.0500000
22312.0000000	0.0550000
22313.0000000	0.0550000
22314.0000000	0.0550000
22315.0000000	0.0450000
22316.0000000	0.0533333
22317.0000000	0.0500000
22318.0000000	0.0550000
22319.0000000	0.0450000
22320.0000000	0.0500000
22321.0000000	0.0550000
22322.0000000	0.0550000
22323.0000000	0.0600000
22324.0000000	0.0500000
22325.0000000	0.0450000
22326.0000000	0.0450000
22327.0000000	0.0500000
22328.0000000	0.0650000
22329.0000000	0.0550000
22330.0000000	0.0600000
22331.0000000	0.0500000
22332.0000000	0.0500000
22333.0000000	0.0500000
22334.0000000	0.0650000
22335.0000000	0.0550000
22336.0000000	0.0650000
22337.0000000	0.0500000
22338.0000000	0.0450000
22339.0000000	0.0550000
22340.0000000	0.0550000
22341.0000000	0.0700000
22342.0000000	0.0550000
22343.0000000	0.0700000

22344.0000000	0.0450000
22345.0000000	0.0550000
22346.0000000	0.0566667
22347.0000000	0.0600000
22348.0000000	0.0650000
22349.0000000	0.0550000
22350.0000000	0.0650000
22351.0000000	0.0500000
22352.0000000	0.0600000
22353.0000000	0.0550000
22354.0000000	0.0650000
22355.0000000	0.0550000
22356.0000000	0.0650000
22357.0000000	0.0500000
22358.0000000	0.0550000
22359.0000000	0.0500000
22360.0000000	0.0650000
22361.0000000	0.0600000
22362.0000000	0.0700000
22363.0000000	0.0500000
22364.0000000	0.0600000
22365.0000000	0.0550000
22366.0000000	0.0700000
22367.0000000	0.0650000
22368.0000000	0.0700000
22369.0000000	0.0700000
22370.0000000	0.0650000
22371.0000000	0.0600000
22372.0000000	0.0550000
22373.0000000	0.0650000
22374.0000000	0.0700000
22375.0000000	0.0666667
22376.0000000	0.0700000
22377.0000000	0.0650000
22378.0000000	0.0650000
22379.0000000	0.0650000
22380.0000000	0.0600000
22381.0000000	0.0650000
22382.0000000	0.0650000
22383.0000000	0.0650000
22384.0000000	0.0700000
22385.0000000	0.0600000
22386.0000000	0.0600000
22387.0000000	0.0600000
22388.0000000	0.0750000
22389.0000000	0.0650000
22390.0000000	0.0650000
22391.0000000	0.0600000
22392.0000000	0.0650000
22393.0000000	0.0700000
22394.0000000	0.0700000
22395.0000000	0.0650000
22396.0000000	0.0650000
22397.0000000	0.0600000
22398.0000000	0.0650000
22399.0000000	0.0600000
22400.0000000	0.0700000

22401.0000000	0.0700000
22402.0000000	0.0600000
22403.0000000	0.0800000
22404.0000000	0.0650000
22405.0000000	0.0750000
22406.0000000	0.0633333
22407.0000000	0.0750000
22408.0000000	0.0750000
22409.0000000	0.0650000
22410.0000000	0.0650000
22411.0000000	0.0800000
22412.0000000	0.0700000
22413.0000000	0.0800000
22414.0000000	0.0600000
22415.0000000	0.0700000
22416.0000000	0.0700000
22417.0000000	0.0750000
22418.0000000	0.0700000
22419.0000000	0.0750000
22420.0000000	0.0650000
22421.0000000	0.0700000
22422.0000000	0.0700000
22423.0000000	0.0700000
22424.0000000	0.0750000
22425.0000000	0.0700000
22426.0000000	0.0700000
22427.0000000	0.0700000
22428.0000000	0.0700000
22429.0000000	0.0700000
22430.0000000	0.0650000
22431.0000000	0.0700000
22432.0000000	0.0750000
22433.0000000	0.0800000
22434.0000000	0.0750000
22435.0000000	0.0700000
22436.0000000	0.0733333
22437.0000000	0.0700000
22438.0000000	0.0700000
22439.0000000	0.0750000
22440.0000000	0.0800000
22441.0000000	0.0750000
22442.0000000	0.0850000
22443.0000000	0.0700000
22444.0000000	0.0750000
22445.0000000	0.0800000
22446.0000000	0.0800000
22447.0000000	0.0750000
22448.0000000	0.0750000
22449.0000000	0.0750000
22450.0000000	0.0850000
22451.0000000	0.0750000
22452.0000000	0.0800000
22453.0000000	0.0750000
22454.0000000	0.0750000
22455.0000000	0.0800000
22456.0000000	0.0750000
22457.0000000	0.0850000

22458.0000000	0.0750000
22459.0000000	0.0750000
22460.0000000	0.0750000
22461.0000000	0.0900000
22462.0000000	0.0800000
22463.0000000	0.0850000
22464.0000000	0.0800000
22465.0000000	0.0850000
22466.0000000	0.0733333
22467.0000000	0.0850000
22468.0000000	0.0750000
22469.0000000	0.0800000
22470.0000000	0.0750000
22471.0000000	0.0800000
22472.0000000	0.0850000
22473.0000000	0.0850000
22474.0000000	0.0900000
22475.0000000	0.0700000
22476.0000000	0.0850000
22477.0000000	0.0750000
22478.0000000	0.0850000
22479.0000000	0.0850000
22480.0000000	0.0800000
22481.0000000	0.0750000
22482.0000000	0.0900000
22483.0000000	0.0850000
22484.0000000	0.0850000
22485.0000000	0.0750000
22486.0000000	0.0800000
22487.0000000	0.0900000
22488.0000000	0.0800000
22489.0000000	0.0950000
22490.0000000	0.0800000
22491.0000000	0.0900000
22492.0000000	0.0800000
22493.0000000	0.0850000
22494.0000000	0.0800000
22495.0000000	0.0800000
22496.0000000	0.0866667
22497.0000000	0.0800000
22498.0000000	0.0800000
22499.0000000	0.0900000
22500.0000000	0.0800000
22501.0000000	0.0900000
22502.0000000	0.0900000
22503.0000000	0.0850000
22504.0000000	0.0850000
22505.0000000	0.0900000
22506.0000000	0.0850000
22507.0000000	0.0900000
22508.0000000	0.0900000
22509.0000000	0.0850000
22510.0000000	0.0850000
22511.0000000	0.0850000
22512.0000000	0.0800000
22513.0000000	0.0800000
22514.0000000	0.0850000

22515.0000000	0.0900000
22516.0000000	0.0900000
22517.0000000	0.0850000
22518.0000000	0.0850000
22519.0000000	0.0900000
22520.0000000	0.0850000
22521.0000000	0.0850000
22522.0000000	0.0750000
22523.0000000	0.0900000
22524.0000000	0.0850000
22525.0000000	0.0950000
22526.0000000	0.0950000
22527.0000000	0.0833333
22528.0000000	0.0900000
22529.0000000	0.0800000
22530.0000000	0.0850000
22531.0000000	0.0900000
22532.0000000	0.0950000
22533.0000000	0.0850000
22534.0000000	0.0950000
22535.0000000	0.0900000
22536.0000000	0.0900000
22537.0000000	0.0850000
22538.0000000	0.0900000
22539.0000000	0.0900000
22540.0000000	0.0950000
22541.0000000	0.0900000
22542.0000000	0.0850000
22543.0000000	0.0900000
22544.0000000	0.0900000
22545.0000000	0.0900000
22546.0000000	0.0950000
22547.0000000	0.0850000
22548.0000000	0.0850000
22549.0000000	0.0900000
22550.0000000	0.0900000
22551.0000000	0.0850000
22552.0000000	0.0950000
22553.0000000	0.1000000
22554.0000000	0.1000000
22555.0000000	0.0950000
22556.0000000	0.0900000
22557.0000000	0.0966667
22558.0000000	0.0800000
22559.0000000	0.1000000
22560.0000000	0.0900000
22561.0000000	0.0950000
22562.0000000	0.1000000
22563.0000000	0.0900000
22564.0000000	0.0850000
22565.0000000	0.0900000
22566.0000000	0.1000000
22567.0000000	0.0950000
22568.0000000	0.1000000
22569.0000000	0.0950000
22570.0000000	0.0900000
22571.0000000	0.0950000

22572.0000000	0.0950000
22573.0000000	0.1100000
22574.0000000	0.0950000
22575.0000000	0.1000000
22576.0000000	0.0950000
22577.0000000	0.1000000
22578.0000000	0.0950000
22579.0000000	0.1050000
22580.0000000	0.1050000
22581.0000000	0.1000000
22582.0000000	0.0950000
22583.0000000	0.0950000
22584.0000000	0.1000000
22585.0000000	0.0950000
22586.0000000	0.1100000
22587.0000000	0.0966667
22588.0000000	0.1050000
22589.0000000	0.1000000
22590.0000000	0.0950000
22591.0000000	0.0950000
22592.0000000	0.0950000
22593.0000000	0.1000000
22594.0000000	0.1050000
22595.0000000	0.0950000
22596.0000000	0.1000000
22597.0000000	0.1000000
22598.0000000	0.1000000
22599.0000000	0.0950000
22600.0000000	0.1050000
22601.0000000	0.1000000
22602.0000000	0.1000000
22603.0000000	0.1050000
22604.0000000	0.1050000
22605.0000000	0.0950000
22606.0000000	0.1000000
22607.0000000	0.1050000
22608.0000000	0.1050000
22609.0000000	0.1050000
22610.0000000	0.1000000
22611.0000000	0.1050000
22612.0000000	0.0950000
22613.0000000	0.0950000
22614.0000000	0.1000000
22615.0000000	0.1050000
22616.0000000	0.1050000
22617.0000000	0.1000000
22618.0000000	0.1000000
22619.0000000	0.1050000
22620.0000000	0.0950000
22621.0000000	0.1000000
22622.0000000	0.1000000
22623.0000000	0.1050000
22624.0000000	0.1050000
22625.0000000	0.1000000
22626.0000000	0.0950000
22627.0000000	0.1000000
22628.0000000	0.1000000

22629.0000000	0.1050000
22630.0000000	0.1050000
22631.0000000	0.1050000
22632.0000000	0.1050000
22633.0000000	0.1050000
22634.0000000	0.1000000
22635.0000000	0.1050000
22636.0000000	0.1050000
22637.0000000	0.1050000
22638.0000000	0.1000000
22639.0000000	0.1100000
22640.0000000	0.1000000
22641.0000000	0.1050000
22642.0000000	0.1000000
22643.0000000	0.1050000
22644.0000000	0.1050000
22645.0000000	0.1100000
22646.0000000	0.1000000
22647.0000000	0.1100000
22648.0000000	0.1000000
22649.0000000	0.1100000
22650.0000000	0.1100000
22651.0000000	0.1100000
22652.0000000	0.1000000
22653.0000000	0.1000000
22654.0000000	0.1050000
22655.0000000	0.1050000
22656.0000000	0.1200000
22657.0000000	0.1100000
22658.0000000	0.1150000
22659.0000000	0.1050000
22660.0000000	0.1050000
22661.0000000	0.1050000
22662.0000000	0.1050000
22663.0000000	0.1050000
22664.0000000	0.1050000
22665.0000000	0.1150000
22666.0000000	0.1000000
22667.0000000	0.1100000
22668.0000000	0.1000000
22669.0000000	0.1000000
22670.0000000	0.1050000
22671.0000000	0.1100000
22672.0000000	0.1100000
22673.0000000	0.1050000
22674.0000000	0.1100000
22675.0000000	0.1100000
22676.0000000	0.1050000
22677.0000000	0.1133333
22678.0000000	0.1050000
22679.0000000	0.1050000
22680.0000000	0.1050000
22681.0000000	0.1050000
22682.0000000	0.1100000
22683.0000000	0.1050000
22684.0000000	0.1150000
22685.0000000	0.1050000

22686.0000000	0.1100000
22687.0000000	0.1050000
22688.0000000	0.1100000
22689.0000000	0.1000000
22690.0000000	0.1100000
22691.0000000	0.1100000
22692.0000000	0.1200000
22693.0000000	0.1100000
22694.0000000	0.1050000
22695.0000000	0.1050000
22696.0000000	0.1150000
22697.0000000	0.1150000
22698.0000000	0.1150000
22699.0000000	0.1150000
22700.0000000	0.1100000
22701.0000000	0.1200000
22702.0000000	0.1100000
22703.0000000	0.1150000
22704.0000000	0.1100000
22705.0000000	0.1100000
22706.0000000	0.1100000
22707.0000000	0.1133333
22708.0000000	0.1050000
22709.0000000	0.1050000
22710.0000000	0.1100000
22711.0000000	0.1150000
22712.0000000	0.1100000
22713.0000000	0.1150000
22714.0000000	0.1050000
22715.0000000	0.1100000
22716.0000000	0.1000000
22717.0000000	0.1150000
22718.0000000	0.1100000
22719.0000000	0.1200000
22720.0000000	0.1050000
22721.0000000	0.1050000
22722.0000000	0.1000000
22723.0000000	0.1050000
22724.0000000	0.1050000
22725.0000000	0.1150000
22726.0000000	0.1150000
22727.0000000	0.1000000
22728.0000000	0.1100000
22729.0000000	0.1000000
22730.0000000	0.1150000
22731.0000000	0.1100000
22732.0000000	0.1100000
22733.0000000	0.1000000
22734.0000000	0.1100000
22735.0000000	0.1100000
22736.0000000	0.1100000
22737.0000000	0.1150000
22738.0000000	0.1066667
22739.0000000	0.1150000
22740.0000000	0.1100000
22741.0000000	0.1100000
22742.0000000	0.1050000

22743.0000000	0.1100000
22744.0000000	0.1150000
22745.0000000	0.1100000
22746.0000000	0.1100000
22747.0000000	0.1000000
22748.0000000	0.1100000
22749.0000000	0.1100000
22750.0000000	0.1150000
22751.0000000	0.1100000
22752.0000000	0.1050000
22753.0000000	0.1100000
22754.0000000	0.1050000
22755.0000000	0.1100000
22756.0000000	0.1150000
22757.0000000	0.1100000
22758.0000000	0.1150000
22759.0000000	0.1000000
22760.0000000	0.1150000
22761.0000000	0.1000000
22762.0000000	0.1150000
22763.0000000	0.1050000
22764.0000000	0.1050000
22765.0000000	0.1100000
22766.0000000	0.1050000
22767.0000000	0.1200000
22768.0000000	0.1066667
22769.0000000	0.1100000
22770.0000000	0.1100000
22771.0000000	0.1100000
22772.0000000	0.1100000
22773.0000000	0.1100000
22774.0000000	0.1150000
22775.0000000	0.1050000
22776.0000000	0.1150000
22777.0000000	0.1000000
22778.0000000	0.1100000
22779.0000000	0.1100000
22780.0000000	0.1150000
22781.0000000	0.1050000
22782.0000000	0.1000000
22783.0000000	0.1100000
22784.0000000	0.1100000
22785.0000000	0.1100000
22786.0000000	0.1050000
22787.0000000	0.1150000
22788.0000000	0.1050000
22789.0000000	0.1050000
22790.0000000	0.1000000
22791.0000000	0.1100000
22792.0000000	0.1100000
22793.0000000	0.1100000
22794.0000000	0.1100000
22795.0000000	0.1050000
22796.0000000	0.1000000
22797.0000000	0.1000000
22798.0000000	0.1133333
22799.0000000	0.1100000

22800.0000000	0.1050000
22801.0000000	0.1050000
22802.0000000	0.1000000
22803.0000000	0.1150000
22804.0000000	0.1000000
22805.0000000	0.1050000
22806.0000000	0.1000000
22807.0000000	0.1050000
22808.0000000	0.1050000
22809.0000000	0.1100000
22810.0000000	0.1100000
22811.0000000	0.1100000
22812.0000000	0.1050000
22813.0000000	0.1050000
22814.0000000	0.1050000
22815.0000000	0.1200000
22816.0000000	0.1100000
22817.0000000	0.1150000
22818.0000000	0.1050000
22819.0000000	0.1100000
22820.0000000	0.1050000
22821.0000000	0.1100000
22822.0000000	0.1100000
22823.0000000	0.1100000
22824.0000000	0.0950000
22825.0000000	0.1050000
22826.0000000	0.1000000
22827.0000000	0.1150000
22828.0000000	0.1166667
22829.0000000	0.0950000
22830.0000000	0.1100000
22831.0000000	0.1100000
22832.0000000	0.1150000
22833.0000000	0.1050000
22834.0000000	0.1100000
22835.0000000	0.0950000
22836.0000000	0.0950000
22837.0000000	0.1050000
22838.0000000	0.1100000
22839.0000000	0.1100000
22840.0000000	0.1100000
22841.0000000	0.1100000
22842.0000000	0.1050000
22843.0000000	0.1100000
22844.0000000	0.1100000
22845.0000000	0.1150000
22846.0000000	0.1100000
22847.0000000	0.1100000
22848.0000000	0.1100000
22849.0000000	0.1100000
22850.0000000	0.1050000
22851.0000000	0.1100000
22852.0000000	0.1100000
22853.0000000	0.1000000
22854.0000000	0.1200000
22855.0000000	0.1200000
22856.0000000	0.1100000

22857.0000000	0.1050000
22858.0000000	0.1000000
22859.0000000	0.1100000
22860.0000000	0.1150000
22861.0000000	0.1100000
22862.0000000	0.1000000
22863.0000000	0.1100000
22864.0000000	0.1100000
22865.0000000	0.1050000
22866.0000000	0.1100000
22867.0000000	0.1050000
22868.0000000	0.1100000
22869.0000000	0.1150000
22870.0000000	0.1050000
22871.0000000	0.1150000
22872.0000000	0.1050000
22873.0000000	0.1100000
22874.0000000	0.1100000
22875.0000000	0.1100000
22876.0000000	0.1100000
22877.0000000	0.1050000
22878.0000000	0.1200000
22879.0000000	0.1050000
22880.0000000	0.1150000
22881.0000000	0.1100000
22882.0000000	0.1100000
22883.0000000	0.1100000
22884.0000000	0.1100000
22885.0000000	0.1200000
22886.0000000	0.1100000
22887.0000000	0.1050000
22888.0000000	0.1150000
22889.0000000	0.1166667
22890.0000000	0.1150000
22891.0000000	0.1050000
22892.0000000	0.1050000
22893.0000000	0.1250000
22894.0000000	0.1100000
22895.0000000	0.1200000
22896.0000000	0.1100000
22897.0000000	0.1100000
22898.0000000	0.1100000
22899.0000000	0.1150000
22900.0000000	0.1150000
22901.0000000	0.1200000
22902.0000000	0.1150000
22903.0000000	0.1200000
22904.0000000	0.1150000
22905.0000000	0.1100000
22906.0000000	0.1150000
22907.0000000	0.1200000
22908.0000000	0.1150000
22909.0000000	0.1200000
22910.0000000	0.1200000
22911.0000000	0.1100000
22912.0000000	0.1200000
22913.0000000	0.1050000

22914.0000000	0.1100000
22915.0000000	0.1150000
22916.0000000	0.1200000
22917.0000000	0.1200000
22918.0000000	0.1200000
22919.0000000	0.1066667
22920.0000000	0.1150000
22921.0000000	0.1200000
22922.0000000	0.1150000
22923.0000000	0.1200000
22924.0000000	0.1100000
22925.0000000	0.1100000
22926.0000000	0.1150000
22927.0000000	0.1200000
22928.0000000	0.1200000
22929.0000000	0.1150000
22930.0000000	0.1200000
22931.0000000	0.1200000
22932.0000000	0.1200000
22933.0000000	0.1150000
22934.0000000	0.1100000
22935.0000000	0.1150000
22936.0000000	0.1250000
22937.0000000	0.1100000
22938.0000000	0.1200000
22939.0000000	0.1150000
22940.0000000	0.1250000
22941.0000000	0.1200000
22942.0000000	0.1150000
22943.0000000	0.1150000
22944.0000000	0.1150000
22945.0000000	0.1250000
22946.0000000	0.1200000
22947.0000000	0.1200000
22948.0000000	0.1150000
22949.0000000	0.1200000
22950.0000000	0.1250000
22951.0000000	0.1200000
22952.0000000	0.1250000
22953.0000000	0.1200000
22954.0000000	0.1200000
22955.0000000	0.1250000
22956.0000000	0.1250000
22957.0000000	0.1250000
22958.0000000	0.1200000
22959.0000000	0.1250000
22960.0000000	0.1250000
22961.0000000	0.1250000
22962.0000000	0.1250000
22963.0000000	0.1150000
22964.0000000	0.1250000
22965.0000000	0.1200000
22966.0000000	0.1200000
22967.0000000	0.1250000
22968.0000000	0.1200000
22969.0000000	0.1200000
22970.0000000	0.1300000

22971.0000000	0.1200000
22972.0000000	0.1250000
22973.0000000	0.1250000
22974.0000000	0.1250000
22975.0000000	0.1250000
22976.0000000	0.1250000
22977.0000000	0.1250000
22978.0000000	0.1300000
22979.0000000	0.1250000
22980.0000000	0.1200000
22981.0000000	0.1250000
22982.0000000	0.1300000
22983.0000000	0.1200000
22984.0000000	0.1350000
22985.0000000	0.1200000
22986.0000000	0.1200000
22987.0000000	0.1300000
22988.0000000	0.1300000
22989.0000000	0.1300000
22990.0000000	0.1250000
22991.0000000	0.1250000
22992.0000000	0.1300000
22993.0000000	0.1300000
22994.0000000	0.1250000
22995.0000000	0.1200000
22996.0000000	0.1250000
22997.0000000	0.1250000
22998.0000000	0.1250000
22999.0000000	0.1200000
23000.0000000	0.1250000
23001.0000000	0.1250000
23002.0000000	0.1300000
23003.0000000	0.1300000
23004.0000000	0.1250000
23005.0000000	0.1300000
23006.0000000	0.1350000
23007.0000000	0.1250000
23008.0000000	0.1350000
23009.0000000	0.1200000
23010.0000000	0.1266667
23011.0000000	0.1350000
23012.0000000	0.1200000
23013.0000000	0.1300000
23014.0000000	0.1300000
23015.0000000	0.1300000
23016.0000000	0.1350000
23017.0000000	0.1250000
23018.0000000	0.1300000
23019.0000000	0.1250000
23020.0000000	0.1300000
23021.0000000	0.1300000
23022.0000000	0.1350000
23023.0000000	0.1300000
23024.0000000	0.1350000
23025.0000000	0.1250000
23026.0000000	0.1250000
23027.0000000	0.1300000

23028.0000000	0.1350000
23029.0000000	0.1300000
23030.0000000	0.1300000
23031.0000000	0.1250000
23032.0000000	0.1250000
23033.0000000	0.1250000
23034.0000000	0.1250000
23035.0000000	0.1300000
23036.0000000	0.1350000
23037.0000000	0.1250000
23038.0000000	0.1350000
23039.0000000	0.1300000
23040.0000000	0.1200000
23041.0000000	0.1350000
23042.0000000	0.1350000
23043.0000000	0.1350000
23044.0000000	0.1300000
23045.0000000	0.1300000
23046.0000000	0.1250000
23047.0000000	0.1250000
23048.0000000	0.1400000
23049.0000000	0.1350000
23050.0000000	0.1450000
23051.0000000	0.1250000
23052.0000000	0.1400000
23053.0000000	0.1250000
23054.0000000	0.1400000
23055.0000000	0.1300000
23056.0000000	0.1400000
23057.0000000	0.1300000
23058.0000000	0.1350000
23059.0000000	0.1300000
23060.0000000	0.1350000
23061.0000000	0.1300000
23062.0000000	0.1400000
23063.0000000	0.1350000
23064.0000000	0.1350000
23065.0000000	0.1350000
23066.0000000	0.1350000
23067.0000000	0.1350000
23068.0000000	0.1350000
23069.0000000	0.1350000
23070.0000000	0.1400000
23071.0000000	0.1350000
23072.0000000	0.1300000
23073.0000000	0.1350000
23074.0000000	0.1300000
23075.0000000	0.1400000
23076.0000000	0.1400000
23077.0000000	0.1350000
23078.0000000	0.1300000
23079.0000000	0.1250000
23080.0000000	0.1400000
23081.0000000	0.1350000
23082.0000000	0.1400000
23083.0000000	0.1350000
23084.0000000	0.1450000

23085.0000000	0.1350000
23086.0000000	0.1350000
23087.0000000	0.1350000
23088.0000000	0.1300000
23089.0000000	0.1350000
23090.0000000	0.1400000
23091.0000000	0.1400000
23092.0000000	0.1400000
23093.0000000	0.1350000
23094.0000000	0.1350000
23095.0000000	0.1350000
23096.0000000	0.1300000
23097.0000000	0.1300000
23098.0000000	0.1400000
23099.0000000	0.1300000
23100.0000000	0.1400000
23101.0000000	0.1400000
23102.0000000	0.1350000
23103.0000000	0.1300000
23104.0000000	0.1400000
23105.0000000	0.1450000
23106.0000000	0.1300000
23107.0000000	0.1400000
23108.0000000	0.1350000
23109.0000000	0.1350000
23110.0000000	0.1350000
23111.0000000	0.1350000
23112.0000000	0.1350000
23113.0000000	0.1350000
23114.0000000	0.1400000
23115.0000000	0.1400000
23116.0000000	0.1350000
23117.0000000	0.1350000
23118.0000000	0.1300000
23119.0000000	0.1300000
23120.0000000	0.1500000
23121.0000000	0.1250000
23122.0000000	0.1300000
23123.0000000	0.1450000
23124.0000000	0.1350000
23125.0000000	0.1400000
23126.0000000	0.1350000
23127.0000000	0.1350000
23128.0000000	0.1400000
23129.0000000	0.1350000
23130.0000000	0.1366667
23131.0000000	0.1350000
23132.0000000	0.1200000
23133.0000000	0.1400000
23134.0000000	0.1400000
23135.0000000	0.1350000
23136.0000000	0.1450000
23137.0000000	0.1350000
23138.0000000	0.1450000
23139.0000000	0.1350000
23140.0000000	0.1400000
23141.0000000	0.1400000

23142.0000000	0.1350000
23143.0000000	0.1450000
23144.0000000	0.1350000
23145.0000000	0.1450000
23146.0000000	0.1300000
23147.0000000	0.1400000
23148.0000000	0.1350000
23149.0000000	0.1450000
23150.0000000	0.1400000
23151.0000000	0.1450000
23152.0000000	0.1350000
23153.0000000	0.1400000
23154.0000000	0.1350000
23155.0000000	0.1450000
23156.0000000	0.1350000
23157.0000000	0.1450000
23158.0000000	0.1350000
23159.0000000	0.1400000
23160.0000000	0.1400000
23161.0000000	0.1400000
23162.0000000	0.1500000
23163.0000000	0.1400000
23164.0000000	0.1450000
23165.0000000	0.1400000
23166.0000000	0.1400000
23167.0000000	0.1450000
23168.0000000	0.1400000
23169.0000000	0.1400000
23170.0000000	0.1450000
23171.0000000	0.1500000
23172.0000000	0.1450000
23173.0000000	0.1400000
23174.0000000	0.1350000
23175.0000000	0.1450000
23176.0000000	0.1400000
23177.0000000	0.1450000
23178.0000000	0.1450000
23179.0000000	0.1450000
23180.0000000	0.1450000
23181.0000000	0.1450000
23182.0000000	0.1550000
23183.0000000	0.1400000
23184.0000000	0.1400000
23185.0000000	0.1500000
23186.0000000	0.1450000
23187.0000000	0.1500000
23188.0000000	0.1500000
23189.0000000	0.1500000
23190.0000000	0.1500000
23191.0000000	0.1433333
23192.0000000	0.1450000
23193.0000000	0.1550000
23194.0000000	0.1550000
23195.0000000	0.1450000
23196.0000000	0.1400000
23197.0000000	0.1500000
23198.0000000	0.1500000

23199.0000000	0.1500000
23200.0000000	0.1500000
23201.0000000	0.1500000
23202.0000000	0.1500000
23203.0000000	0.1450000
23204.0000000	0.1500000
23205.0000000	0.1450000
23206.0000000	0.1600000
23207.0000000	0.1550000
23208.0000000	0.1500000
23209.0000000	0.1500000
23210.0000000	0.1500000
23211.0000000	0.1500000
23212.0000000	0.1500000
23213.0000000	0.1450000
23214.0000000	0.1550000
23215.0000000	0.1450000
23216.0000000	0.1550000
23217.0000000	0.1500000
23218.0000000	0.1500000
23219.0000000	0.1450000
23220.0000000	0.1500000
23221.0000000	0.1566667
23222.0000000	0.1500000
23223.0000000	0.1450000
23224.0000000	0.1550000
23225.0000000	0.1500000
23226.0000000	0.1600000
23227.0000000	0.1550000
23228.0000000	0.1600000
23229.0000000	0.1450000
23230.0000000	0.1500000
23231.0000000	0.1500000
23232.0000000	0.1500000
23233.0000000	0.1600000
23234.0000000	0.1500000
23235.0000000	0.1550000
23236.0000000	0.1500000
23237.0000000	0.1500000
23238.0000000	0.1600000
23239.0000000	0.1450000
23240.0000000	0.1500000
23241.0000000	0.1500000
23242.0000000	0.1500000
23243.0000000	0.1600000
23244.0000000	0.1550000
23245.0000000	0.1550000
23246.0000000	0.1500000
23247.0000000	0.1550000
23248.0000000	0.1550000
23249.0000000	0.1450000
23250.0000000	0.1450000
23251.0000000	0.1533333
23252.0000000	0.1500000
23253.0000000	0.1500000
23254.0000000	0.1450000
23255.0000000	0.1550000

23256.0000000	0.1550000
23257.0000000	0.1500000
23258.0000000	0.1550000
23259.0000000	0.1600000
23260.0000000	0.1600000
23261.0000000	0.1550000
23262.0000000	0.1500000
23263.0000000	0.1550000
23264.0000000	0.1500000
23265.0000000	0.1500000
23266.0000000	0.1550000
23267.0000000	0.1550000
23268.0000000	0.1600000
23269.0000000	0.1550000
23270.0000000	0.1550000
23271.0000000	0.1550000
23272.0000000	0.1550000
23273.0000000	0.1650000
23274.0000000	0.1600000
23275.0000000	0.1550000
23276.0000000	0.1500000
23277.0000000	0.1600000
23278.0000000	0.1500000
23279.0000000	0.1550000
23280.0000000	0.1550000
23281.0000000	0.1533333
23282.0000000	0.1550000
23283.0000000	0.1550000
23284.0000000	0.1550000
23285.0000000	0.1600000
23286.0000000	0.1500000
23287.0000000	0.1550000
23288.0000000	0.1550000
23289.0000000	0.1600000
23290.0000000	0.1600000
23291.0000000	0.1500000
23292.0000000	0.1500000
23293.0000000	0.1550000
23294.0000000	0.1500000
23295.0000000	0.1550000
23296.0000000	0.1550000
23297.0000000	0.1500000
23298.0000000	0.1600000
23299.0000000	0.1550000
23300.0000000	0.1600000
23301.0000000	0.1500000
23302.0000000	0.1400000
23303.0000000	0.1500000
23304.0000000	0.1550000
23305.0000000	0.1550000
23306.0000000	0.1600000
23307.0000000	0.1550000
23308.0000000	0.1450000
23309.0000000	0.1500000
23310.0000000	0.1500000
23311.0000000	0.1500000
23312.0000000	0.1533333

23313.0000000	0.1550000
23314.0000000	0.1550000
23315.0000000	0.1600000
23316.0000000	0.1500000
23317.0000000	0.1550000
23318.0000000	0.1450000
23319.0000000	0.1450000
23320.0000000	0.1500000
23321.0000000	0.1600000
23322.0000000	0.1600000
23323.0000000	0.1550000
23324.0000000	0.1500000
23325.0000000	0.1400000
23326.0000000	0.1600000
23327.0000000	0.1500000
23328.0000000	0.1550000
23329.0000000	0.1600000
23330.0000000	0.1550000
23331.0000000	0.1550000
23332.0000000	0.1500000
23333.0000000	0.1550000
23334.0000000	0.1550000
23335.0000000	0.1550000
23336.0000000	0.1500000
23337.0000000	0.1450000
23338.0000000	0.1550000
23339.0000000	0.1500000
23340.0000000	0.1500000
23341.0000000	0.1500000
23342.0000000	0.1500000
23343.0000000	0.1500000
23344.0000000	0.1600000
23345.0000000	0.1400000
23346.0000000	0.1550000
23347.0000000	0.1550000
23348.0000000	0.1550000
23349.0000000	0.1550000
23350.0000000	0.1500000
23351.0000000	0.1500000
23352.0000000	0.1500000
23353.0000000	0.1550000
23354.0000000	0.1550000
23355.0000000	0.1550000
23356.0000000	0.1550000
23357.0000000	0.1500000
23358.0000000	0.1600000
23359.0000000	0.1500000
23360.0000000	0.1500000
23361.0000000	0.1450000
23362.0000000	0.1550000
23363.0000000	0.1450000
23364.0000000	0.1550000
23365.0000000	0.1450000
23366.0000000	0.1550000
23367.0000000	0.1500000
23368.0000000	0.1550000
23369.0000000	0.1450000

23370.0000000	0.1550000
23371.0000000	0.1533333
23372.0000000	0.1500000
23373.0000000	0.1450000
23374.0000000	0.1500000
23375.0000000	0.1550000
23376.0000000	0.1500000
23377.0000000	0.1450000
23378.0000000	0.1500000
23379.0000000	0.1500000
23380.0000000	0.1550000
23381.0000000	0.1550000
23382.0000000	0.1550000
23383.0000000	0.1500000
23384.0000000	0.1600000
23385.0000000	0.1450000
23386.0000000	0.1550000
23387.0000000	0.1550000
23388.0000000	0.1650000
23389.0000000	0.1500000
23390.0000000	0.1550000
23391.0000000	0.1450000
23392.0000000	0.1550000
23393.0000000	0.1500000
23394.0000000	0.1550000
23395.0000000	0.1500000
23396.0000000	0.1450000
23397.0000000	0.1600000
23398.0000000	0.1550000
23399.0000000	0.1550000
23400.0000000	0.1550000
23401.0000000	0.1550000
23402.0000000	0.1566667
23403.0000000	0.1500000
23404.0000000	0.1600000
23405.0000000	0.1550000
23406.0000000	0.1650000
23407.0000000	0.1600000
23408.0000000	0.1600000
23409.0000000	0.1500000
23410.0000000	0.1600000
23411.0000000	0.1500000
23412.0000000	0.1550000
23413.0000000	0.1500000
23414.0000000	0.1600000
23415.0000000	0.1550000
23416.0000000	0.1450000
23417.0000000	0.1500000
23418.0000000	0.1500000
23419.0000000	0.1650000
23420.0000000	0.1550000
23421.0000000	0.1650000
23422.0000000	0.1500000
23423.0000000	0.1550000
23424.0000000	0.1600000
23425.0000000	0.1550000
23426.0000000	0.1550000

23427.0000000	0.1500000
23428.0000000	0.1600000
23429.0000000	0.1550000
23430.0000000	0.1600000
23431.0000000	0.1500000
23432.0000000	0.1533333
23433.0000000	0.1500000
23434.0000000	0.1600000
23435.0000000	0.1550000
23436.0000000	0.1600000
23437.0000000	0.1550000
23438.0000000	0.1450000
23439.0000000	0.1650000
23440.0000000	0.1550000
23441.0000000	0.1600000
23442.0000000	0.1550000
23443.0000000	0.1500000
23444.0000000	0.1550000
23445.0000000	0.1600000
23446.0000000	0.1550000
23447.0000000	0.1500000
23448.0000000	0.1550000
23449.0000000	0.1600000
23450.0000000	0.1550000
23451.0000000	0.1550000
23452.0000000	0.1550000
23453.0000000	0.1500000
23454.0000000	0.1600000
23455.0000000	0.1550000
23456.0000000	0.1500000
23457.0000000	0.1550000
23458.0000000	0.1550000
23459.0000000	0.1500000
23460.0000000	0.1550000
23461.0000000	0.1550000
23462.0000000	0.1533333
23463.0000000	0.1550000
23464.0000000	0.1500000
23465.0000000	0.1500000
23466.0000000	0.1550000
23467.0000000	0.1550000
23468.0000000	0.1550000
23469.0000000	0.1550000
23470.0000000	0.1500000
23471.0000000	0.1550000
23472.0000000	0.1550000
23473.0000000	0.1500000
23474.0000000	0.1450000
23475.0000000	0.1550000
23476.0000000	0.1550000
23477.0000000	0.1550000
23478.0000000	0.1550000
23479.0000000	0.1450000
23480.0000000	0.1500000
23481.0000000	0.1550000
23482.0000000	0.1500000
23483.0000000	0.1450000

23484.0000000	0.1500000
23485.0000000	0.1600000
23486.0000000	0.1500000
23487.0000000	0.1600000
23488.0000000	0.1450000
23489.0000000	0.1550000
23490.0000000	0.1500000
23491.0000000	0.1550000
23492.0000000	0.1566667
23493.0000000	0.1500000
23494.0000000	0.1500000
23495.0000000	0.1500000
23496.0000000	0.1550000
23497.0000000	0.1600000
23498.0000000	0.1500000
23499.0000000	0.1500000
23500.0000000	0.1550000
23501.0000000	0.1550000
23502.0000000	0.1500000
23503.0000000	0.1500000
23504.0000000	0.1500000
23505.0000000	0.1550000
23506.0000000	0.1550000
23507.0000000	0.1550000
23508.0000000	0.1600000
23509.0000000	0.1450000
23510.0000000	0.1500000
23511.0000000	0.1500000
23512.0000000	0.1450000
23513.0000000	0.1600000
23514.0000000	0.1500000
23515.0000000	0.1500000
23516.0000000	0.1600000
23517.0000000	0.1500000
23518.0000000	0.1500000
23519.0000000	0.1400000
23520.0000000	0.1550000
23521.0000000	0.1500000
23522.0000000	0.1550000
23523.0000000	0.1566667
23524.0000000	0.1600000
23525.0000000	0.1450000
23526.0000000	0.1550000
23527.0000000	0.1500000
23528.0000000	0.1550000
23529.0000000	0.1550000
23530.0000000	0.1600000
23531.0000000	0.1500000
23532.0000000	0.1600000
23533.0000000	0.1550000
23534.0000000	0.1550000
23535.0000000	0.1600000
23536.0000000	0.1550000
23537.0000000	0.1500000
23538.0000000	0.1550000
23539.0000000	0.1650000
23540.0000000	0.1600000

23541.0000000	0.1500000
23542.0000000	0.1600000
23543.0000000	0.1600000
23544.0000000	0.1600000
23545.0000000	0.1600000
23546.0000000	0.1550000
23547.0000000	0.1550000
23548.0000000	0.1550000
23549.0000000	0.1650000
23550.0000000	0.1600000
23551.0000000	0.1650000
23552.0000000	0.1500000
23553.0000000	0.1600000
23554.0000000	0.1550000
23555.0000000	0.1600000
23556.0000000	0.1550000
23557.0000000	0.1600000
23558.0000000	0.1550000
23559.0000000	0.1600000
23560.0000000	0.1600000
23561.0000000	0.1650000
23562.0000000	0.1600000
23563.0000000	0.1650000
23564.0000000	0.1650000
23565.0000000	0.1600000
23566.0000000	0.1600000
23567.0000000	0.1550000
23568.0000000	0.1700000
23569.0000000	0.1650000
23570.0000000	0.1650000
23571.0000000	0.1650000
23572.0000000	0.1550000
23573.0000000	0.1650000
23574.0000000	0.1550000
23575.0000000	0.1650000
23576.0000000	0.1650000
23577.0000000	0.1650000
23578.0000000	0.1500000
23579.0000000	0.1650000
23580.0000000	0.1500000
23581.0000000	0.1700000
23582.0000000	0.1700000
23583.0000000	0.1600000
23584.0000000	0.1600000
23585.0000000	0.1600000
23586.0000000	0.1550000
23587.0000000	0.1550000
23588.0000000	0.1700000
23589.0000000	0.1600000
23590.0000000	0.1700000
23591.0000000	0.1600000
23592.0000000	0.1550000
23593.0000000	0.1600000
23594.0000000	0.1650000
23595.0000000	0.1700000
23596.0000000	0.1600000
23597.0000000	0.1650000

23598.0000000	0.1500000
23599.0000000	0.1650000
23600.0000000	0.1600000
23601.0000000	0.1650000
23602.0000000	0.1650000
23603.0000000	0.1600000
23604.0000000	0.1600000
23605.0000000	0.1650000
23606.0000000	0.1550000
23607.0000000	0.1650000
23608.0000000	0.1600000
23609.0000000	0.1600000
23610.0000000	0.1650000
23611.0000000	0.1600000
23612.0000000	0.1550000
23613.0000000	0.1633333
23614.0000000	0.1550000
23615.0000000	0.1500000
23616.0000000	0.1600000
23617.0000000	0.1600000
23618.0000000	0.1600000
23619.0000000	0.1650000
23620.0000000	0.1550000
23621.0000000	0.1650000
23622.0000000	0.1650000
23623.0000000	0.1700000
23624.0000000	0.1600000
23625.0000000	0.1600000
23626.0000000	0.1600000
23627.0000000	0.1650000
23628.0000000	0.1700000
23629.0000000	0.1600000
23630.0000000	0.1650000
23631.0000000	0.1650000
23632.0000000	0.1600000
23633.0000000	0.1650000
23634.0000000	0.1550000
23635.0000000	0.1550000
23636.0000000	0.1600000
23637.0000000	0.1650000
23638.0000000	0.1700000
23639.0000000	0.1650000
23640.0000000	0.1600000
23641.0000000	0.1650000
23642.0000000	0.1650000
23643.0000000	0.1600000
23644.0000000	0.1600000
23645.0000000	0.1600000
23646.0000000	0.1650000
23647.0000000	0.1550000
23648.0000000	0.1550000
23649.0000000	0.1600000
23650.0000000	0.1600000
23651.0000000	0.1600000
23652.0000000	0.1700000
23653.0000000	0.1600000
23654.0000000	0.1600000

23655.0000000	0.1600000
23656.0000000	0.1600000
23657.0000000	0.1700000
23658.0000000	0.1750000
23659.0000000	0.1650000
23660.0000000	0.1550000
23661.0000000	0.1600000
23662.0000000	0.1550000
23663.0000000	0.1600000
23664.0000000	0.1600000
23665.0000000	0.1650000
23666.0000000	0.1600000
23667.0000000	0.1650000
23668.0000000	0.1700000
23669.0000000	0.1600000
23670.0000000	0.1650000
23671.0000000	0.1700000
23672.0000000	0.1650000
23673.0000000	0.1600000
23674.0000000	0.1733333
23675.0000000	0.1650000
23676.0000000	0.1600000
23677.0000000	0.1650000
23678.0000000	0.1650000
23679.0000000	0.1600000
23680.0000000	0.1800000
23681.0000000	0.1700000
23682.0000000	0.1750000
23683.0000000	0.1650000
23684.0000000	0.1650000
23685.0000000	0.1650000
23686.0000000	0.1650000
23687.0000000	0.1750000
23688.0000000	0.1700000
23689.0000000	0.1650000
23690.0000000	0.1800000
23691.0000000	0.1750000
23692.0000000	0.1700000
23693.0000000	0.1650000
23694.0000000	0.1700000
23695.0000000	0.1700000
23696.0000000	0.1750000
23697.0000000	0.1750000
23698.0000000	0.1750000
23699.0000000	0.1750000
23700.0000000	0.1750000
23701.0000000	0.1700000
23702.0000000	0.1750000
23703.0000000	0.1700000
23704.0000000	0.1750000
23705.0000000	0.1750000
23706.0000000	0.1750000
23707.0000000	0.1700000
23708.0000000	0.1700000
23709.0000000	0.1700000
23710.0000000	0.1800000
23711.0000000	0.1750000

23712.0000000	0.1800000
23713.0000000	0.1700000
23714.0000000	0.1700000
23715.0000000	0.1800000
23716.0000000	0.1700000
23717.0000000	0.1800000
23718.0000000	0.1750000
23719.0000000	0.1750000
23720.0000000	0.1700000
23721.0000000	0.1750000
23722.0000000	0.1700000
23723.0000000	0.1750000
23724.0000000	0.1750000
23725.0000000	0.1750000
23726.0000000	0.1800000
23727.0000000	0.1700000
23728.0000000	0.1800000
23729.0000000	0.1750000
23730.0000000	0.1750000
23731.0000000	0.1750000
23732.0000000	0.1750000
23733.0000000	0.1700000
23734.0000000	0.1733333
23735.0000000	0.1750000
23736.0000000	0.1750000
23737.0000000	0.1700000
23738.0000000	0.1650000
23739.0000000	0.1850000
23740.0000000	0.1800000
23741.0000000	0.1700000
23742.0000000	0.1750000
23743.0000000	0.1700000
23744.0000000	0.1700000
23745.0000000	0.1800000
23746.0000000	0.1750000
23747.0000000	0.1750000
23748.0000000	0.1750000
23749.0000000	0.1700000
23750.0000000	0.1750000
23751.0000000	0.1750000
23752.0000000	0.1650000
23753.0000000	0.1750000
23754.0000000	0.1650000
23755.0000000	0.1750000
23756.0000000	0.1800000
23757.0000000	0.1700000
23758.0000000	0.1750000
23759.0000000	0.1750000
23760.0000000	0.1750000
23761.0000000	0.1850000
23762.0000000	0.1750000
23763.0000000	0.1700000
23764.0000000	0.1750000
23765.0000000	0.1800000
23766.0000000	0.1750000
23767.0000000	0.1700000
23768.0000000	0.1800000

23769.0000000	0.1700000
23770.0000000	0.1750000
23771.0000000	0.1750000
23772.0000000	0.1700000
23773.0000000	0.1800000
23774.0000000	0.1850000
23775.0000000	0.1750000
23776.0000000	0.1850000
23777.0000000	0.1750000
23778.0000000	0.1800000
23779.0000000	0.1850000
23780.0000000	0.1900000
23781.0000000	0.1800000
23782.0000000	0.1750000
23783.0000000	0.1900000
23784.0000000	0.1800000
23785.0000000	0.1850000
23786.0000000	0.1800000
23787.0000000	0.1750000
23788.0000000	0.1850000
23789.0000000	0.1900000
23790.0000000	0.1950000
23791.0000000	0.1800000
23792.0000000	0.1900000
23793.0000000	0.1850000
23794.0000000	0.1833333
23795.0000000	0.1800000
23796.0000000	0.1900000
23797.0000000	0.1850000
23798.0000000	0.1900000
23799.0000000	0.1950000
23800.0000000	0.1850000
23801.0000000	0.1800000
23802.0000000	0.1850000
23803.0000000	0.1900000
23804.0000000	0.1850000
23805.0000000	0.1900000
23806.0000000	0.1850000
23807.0000000	0.1950000
23808.0000000	0.1950000
23809.0000000	0.1800000
23810.0000000	0.1900000
23811.0000000	0.1800000
23812.0000000	0.1900000
23813.0000000	0.1850000
23814.0000000	0.1850000
23815.0000000	0.1850000
23816.0000000	0.1850000
23817.0000000	0.1900000
23818.0000000	0.1800000
23819.0000000	0.1850000
23820.0000000	0.1800000
23821.0000000	0.1750000
23822.0000000	0.1950000
23823.0000000	0.1850000
23824.0000000	0.1800000
23825.0000000	0.1750000

23826.0000000	0.1850000
23827.0000000	0.1750000
23828.0000000	0.1850000
23829.0000000	0.1800000
23830.0000000	0.1700000
23831.0000000	0.1800000
23832.0000000	0.1750000
23833.0000000	0.1800000
23834.0000000	0.1800000
23835.0000000	0.1750000
23836.0000000	0.1800000
23837.0000000	0.1900000
23838.0000000	0.1700000
23839.0000000	0.1750000
23840.0000000	0.1850000
23841.0000000	0.1800000
23842.0000000	0.1800000
23843.0000000	0.1850000
23844.0000000	0.1850000
23845.0000000	0.1900000
23846.0000000	0.1950000
23847.0000000	0.1750000
23848.0000000	0.1850000
23849.0000000	0.1800000
23850.0000000	0.1800000
23851.0000000	0.1800000
23852.0000000	0.1800000
23853.0000000	0.1850000
23854.0000000	0.1800000
23855.0000000	0.1833333
23856.0000000	0.1850000
23857.0000000	0.1800000
23858.0000000	0.1800000
23859.0000000	0.1850000
23860.0000000	0.1850000
23861.0000000	0.1800000
23862.0000000	0.1800000
23863.0000000	0.1850000
23864.0000000	0.1800000
23865.0000000	0.1800000
23866.0000000	0.1850000
23867.0000000	0.1800000
23868.0000000	0.1900000
23869.0000000	0.1800000
23870.0000000	0.1900000
23871.0000000	0.1850000
23872.0000000	0.1850000
23873.0000000	0.1900000
23874.0000000	0.1850000
23875.0000000	0.1950000
23876.0000000	0.1850000
23877.0000000	0.1800000
23878.0000000	0.1850000
23879.0000000	0.1800000
23880.0000000	0.1900000
23881.0000000	0.1900000
23882.0000000	0.1900000

23883.0000000	0.1900000
23884.0000000	0.1800000
23885.0000000	0.1800000
23886.0000000	0.1900000
23887.0000000	0.1850000
23888.0000000	0.1850000
23889.0000000	0.1950000
23890.0000000	0.1850000
23891.0000000	0.1800000
23892.0000000	0.1850000
23893.0000000	0.1900000
23894.0000000	0.1900000
23895.0000000	0.1800000
23896.0000000	0.1750000
23897.0000000	0.1900000
23898.0000000	0.1900000
23899.0000000	0.1800000
23900.0000000	0.1900000
23901.0000000	0.1800000
23902.0000000	0.1850000
23903.0000000	0.1900000
23904.0000000	0.1850000
23905.0000000	0.1850000
23906.0000000	0.1900000
23907.0000000	0.1900000
23908.0000000	0.1950000
23909.0000000	0.1900000
23910.0000000	0.1850000
23911.0000000	0.1900000
23912.0000000	0.1950000
23913.0000000	0.1850000
23914.0000000	0.1850000
23915.0000000	0.1900000
23916.0000000	0.1850000
23917.0000000	0.1950000
23918.0000000	0.1850000
23919.0000000	0.1850000
23920.0000000	0.1900000
23921.0000000	0.1900000
23922.0000000	0.1900000
23923.0000000	0.1850000
23924.0000000	0.1850000
23925.0000000	0.1950000
23926.0000000	0.1900000
23927.0000000	0.1900000
23928.0000000	0.1950000
23929.0000000	0.1850000
23930.0000000	0.1900000
23931.0000000	0.1900000
23932.0000000	0.1900000
23933.0000000	0.1850000
23934.0000000	0.1950000
23935.0000000	0.1950000
23936.0000000	0.1950000
23937.0000000	0.1950000
23938.0000000	0.1900000
23939.0000000	0.1950000

23940.0000000	0.1900000
23941.0000000	0.1850000
23942.0000000	0.1950000
23943.0000000	0.1900000
23944.0000000	0.2000000
23945.0000000	0.1933333
23946.0000000	0.1900000
23947.0000000	0.1900000
23948.0000000	0.1950000
23949.0000000	0.1950000
23950.0000000	0.1950000
23951.0000000	0.1950000
23952.0000000	0.1950000
23953.0000000	0.2000000
23954.0000000	0.1900000
23955.0000000	0.2000000
23956.0000000	0.1900000
23957.0000000	0.2000000
23958.0000000	0.1950000
23959.0000000	0.1900000
23960.0000000	0.1900000
23961.0000000	0.1850000
23962.0000000	0.1950000
23963.0000000	0.1900000
23964.0000000	0.2050000
23965.0000000	0.1900000
23966.0000000	0.2000000
23967.0000000	0.1900000
23968.0000000	0.1900000
23969.0000000	0.1950000
23970.0000000	0.1900000
23971.0000000	0.1950000
23972.0000000	0.1950000
23973.0000000	0.1950000
23974.0000000	0.1900000
23975.0000000	0.1950000
23976.0000000	0.1933333
23977.0000000	0.1950000
23978.0000000	0.1850000
23979.0000000	0.1900000
23980.0000000	0.1900000
23981.0000000	0.2000000
23982.0000000	0.1950000
23983.0000000	0.1950000
23984.0000000	0.1900000
23985.0000000	0.1850000
23986.0000000	0.1950000
23987.0000000	0.1950000
23988.0000000	0.1950000
23989.0000000	0.2000000
23990.0000000	0.1900000
23991.0000000	0.1950000
23992.0000000	0.1950000
23993.0000000	0.1950000
23994.0000000	0.2000000
23995.0000000	0.2000000
23996.0000000	0.1950000

23997.0000000	0.2000000
23998.0000000	0.1900000
23999.0000000	0.2000000
24000.0000000	0.2000000
24001.0000000	0.1950000
24002.0000000	0.1950000
24003.0000000	0.1950000
24004.0000000	0.2050000
24005.0000000	0.1900000
24006.0000000	0.1966667
24007.0000000	0.1950000
24008.0000000	0.1950000
24009.0000000	0.1950000
24010.0000000	0.1950000
24011.0000000	0.2000000
24012.0000000	0.2050000
24013.0000000	0.1950000
24014.0000000	0.2050000
24015.0000000	0.1900000
24016.0000000	0.2000000
24017.0000000	0.2050000
24018.0000000	0.2050000
24019.0000000	0.1950000
24020.0000000	0.2000000
24021.0000000	0.1950000
24022.0000000	0.1900000
24023.0000000	0.2050000
24024.0000000	0.2000000
24025.0000000	0.2050000
24026.0000000	0.1950000
24027.0000000	0.1950000
24028.0000000	0.1950000
24029.0000000	0.2000000
24030.0000000	0.2050000
24031.0000000	0.1950000
24032.0000000	0.2000000
24033.0000000	0.1900000
24034.0000000	0.2000000
24035.0000000	0.1933333
24036.0000000	0.2000000
24037.0000000	0.1950000
24038.0000000	0.2000000
24039.0000000	0.2000000
24040.0000000	0.1900000
24041.0000000	0.2000000
24042.0000000	0.1850000
24043.0000000	0.2000000
24044.0000000	0.2000000
24045.0000000	0.1900000
24046.0000000	0.1900000
24047.0000000	0.1900000
24048.0000000	0.1900000
24049.0000000	0.1900000
24050.0000000	0.2050000
24051.0000000	0.2000000
24052.0000000	0.2050000
24053.0000000	0.1800000

24054.0000000	0.1950000
24055.0000000	0.1900000
24056.0000000	0.2050000
24057.0000000	0.1900000
24058.0000000	0.1950000
24059.0000000	0.1900000
24060.0000000	0.1900000
24061.0000000	0.1950000
24062.0000000	0.1850000
24063.0000000	0.1900000
24064.0000000	0.1950000
24065.0000000	0.2000000
24066.0000000	0.1933333
24067.0000000	0.1950000
24068.0000000	0.1950000
24069.0000000	0.1950000
24070.0000000	0.1950000
24071.0000000	0.1900000
24072.0000000	0.2000000
24073.0000000	0.2000000
24074.0000000	0.1900000
24075.0000000	0.1950000
24076.0000000	0.1950000
24077.0000000	0.1900000
24078.0000000	0.2050000
24079.0000000	0.1950000
24080.0000000	0.2050000
24081.0000000	0.1950000
24082.0000000	0.1900000
24083.0000000	0.1950000
24084.0000000	0.2000000
24085.0000000	0.2000000
24086.0000000	0.2000000
24087.0000000	0.2050000
24088.0000000	0.2050000
24089.0000000	0.2050000
24090.0000000	0.2050000
24091.0000000	0.2150000
24092.0000000	0.1950000
24093.0000000	0.2000000
24094.0000000	0.1950000
24095.0000000	0.2050000
24096.0000000	0.2100000
24097.0000000	0.2000000
24098.0000000	0.2000000
24099.0000000	0.2050000
24100.0000000	0.2050000
24101.0000000	0.2000000
24102.0000000	0.2000000
24103.0000000	0.2050000
24104.0000000	0.2100000
24105.0000000	0.2050000
24106.0000000	0.2050000
24107.0000000	0.2000000
24108.0000000	0.2050000
24109.0000000	0.2050000
24110.0000000	0.2100000

24111.0000000	0.2100000
24112.0000000	0.2100000
24113.0000000	0.2050000
24114.0000000	0.2050000
24115.0000000	0.2050000
24116.0000000	0.2100000
24117.0000000	0.2050000
24118.0000000	0.2050000
24119.0000000	0.2050000
24120.0000000	0.2100000
24121.0000000	0.2050000
24122.0000000	0.2100000
24123.0000000	0.2050000
24124.0000000	0.2050000
24125.0000000	0.2100000
24126.0000000	0.2066667
24127.0000000	0.2100000
24128.0000000	0.2050000
24129.0000000	0.2100000
24130.0000000	0.2100000
24131.0000000	0.2100000
24132.0000000	0.2050000
24133.0000000	0.2050000
24134.0000000	0.2100000
24135.0000000	0.2100000
24136.0000000	0.2050000
24137.0000000	0.2050000
24138.0000000	0.2000000
24139.0000000	0.2050000
24140.0000000	0.2100000
24141.0000000	0.2100000
24142.0000000	0.2100000
24143.0000000	0.2050000
24144.0000000	0.2050000
24145.0000000	0.2050000
24146.0000000	0.2100000
24147.0000000	0.2050000
24148.0000000	0.1950000
24149.0000000	0.2050000
24150.0000000	0.2000000
24151.0000000	0.2100000
24152.0000000	0.2050000
24153.0000000	0.2050000
24154.0000000	0.2050000
24155.0000000	0.2150000
24156.0000000	0.1966667
24157.0000000	0.2050000
24158.0000000	0.2050000
24159.0000000	0.2050000
24160.0000000	0.2050000
24161.0000000	0.2100000
24162.0000000	0.1900000
24163.0000000	0.2050000
24164.0000000	0.2050000
24165.0000000	0.2050000
24166.0000000	0.2050000
24167.0000000	0.2000000

24168.0000000	0.2050000
24169.0000000	0.2050000
24170.0000000	0.2000000
24171.0000000	0.2050000
24172.0000000	0.2000000
24173.0000000	0.2000000
24174.0000000	0.2150000
24175.0000000	0.1950000
24176.0000000	0.2150000
24177.0000000	0.2050000
24178.0000000	0.2000000
24179.0000000	0.2050000
24180.0000000	0.2000000
24181.0000000	0.2100000
24182.0000000	0.2050000
24183.0000000	0.2050000
24184.0000000	0.2050000
24185.0000000	0.2100000
24186.0000000	0.2050000
24187.0000000	0.2066667
24188.0000000	0.2000000
24189.0000000	0.2100000
24190.0000000	0.1950000
24191.0000000	0.2100000
24192.0000000	0.2050000
24193.0000000	0.2200000
24194.0000000	0.2050000
24195.0000000	0.2150000
24196.0000000	0.2000000
24197.0000000	0.2100000
24198.0000000	0.2150000
24199.0000000	0.2200000
24200.0000000	0.2100000
24201.0000000	0.2050000
24202.0000000	0.2100000
24203.0000000	0.1950000
24204.0000000	0.2200000
24205.0000000	0.2150000
24206.0000000	0.2200000
24207.0000000	0.2100000
24208.0000000	0.2050000
24209.0000000	0.2100000
24210.0000000	0.2100000
24211.0000000	0.2200000
24212.0000000	0.2100000
24213.0000000	0.2100000
24214.0000000	0.2150000
24215.0000000	0.2100000
24216.0000000	0.2250000
24217.0000000	0.2133333
24218.0000000	0.2200000
24219.0000000	0.2100000
24220.0000000	0.2150000
24221.0000000	0.2100000
24222.0000000	0.2150000
24223.0000000	0.2200000
24224.0000000	0.2200000

24225.0000000	0.2150000
24226.0000000	0.2250000
24227.0000000	0.2150000
24228.0000000	0.2250000
24229.0000000	0.2200000
24230.0000000	0.2200000
24231.0000000	0.2150000
24232.0000000	0.2200000
24233.0000000	0.2200000
24234.0000000	0.2300000
24235.0000000	0.2300000
24236.0000000	0.2200000
24237.0000000	0.2200000
24238.0000000	0.2100000
24239.0000000	0.2250000
24240.0000000	0.2200000
24241.0000000	0.2200000
24242.0000000	0.2200000
24243.0000000	0.2300000
24244.0000000	0.2250000
24245.0000000	0.2350000
24246.0000000	0.2250000
24247.0000000	0.2233333
24248.0000000	0.2150000
24249.0000000	0.2200000
24250.0000000	0.2300000
24251.0000000	0.2300000
24252.0000000	0.2350000
24253.0000000	0.2250000
24254.0000000	0.2200000
24255.0000000	0.2150000
24256.0000000	0.2200000
24257.0000000	0.2350000
24258.0000000	0.2300000
24259.0000000	0.2400000
24260.0000000	0.2250000
24261.0000000	0.2250000
24262.0000000	0.2300000
24263.0000000	0.2300000
24264.0000000	0.2250000
24265.0000000	0.2350000
24266.0000000	0.2250000
24267.0000000	0.2350000
24268.0000000	0.2250000
24269.0000000	0.2250000
24270.0000000	0.2350000
24271.0000000	0.2350000
24272.0000000	0.2250000
24273.0000000	0.2300000
24274.0000000	0.2300000
24275.0000000	0.2250000
24276.0000000	0.2350000
24277.0000000	0.2333333
24278.0000000	0.2450000
24279.0000000	0.2350000
24280.0000000	0.2350000
24281.0000000	0.2300000

24282.0000000	0.2300000
24283.0000000	0.2300000
24284.0000000	0.2350000
24285.0000000	0.2350000
24286.0000000	0.2350000
24287.0000000	0.2350000
24288.0000000	0.2350000
24289.0000000	0.2350000
24290.0000000	0.2400000
24291.0000000	0.2400000
24292.0000000	0.2400000
24293.0000000	0.2400000
24294.0000000	0.2400000
24295.0000000	0.2350000
24296.0000000	0.2450000
24297.0000000	0.2350000
24298.0000000	0.2450000
24299.0000000	0.2400000
24300.0000000	0.2550000
24301.0000000	0.2450000
24302.0000000	0.2450000
24303.0000000	0.2400000
24304.0000000	0.2400000
24305.0000000	0.2400000
24306.0000000	0.2500000
24307.0000000	0.2450000
24308.0000000	0.2433333
24309.0000000	0.2450000
24310.0000000	0.2350000
24311.0000000	0.2500000
24312.0000000	0.2450000
24313.0000000	0.2450000
24314.0000000	0.2400000
24315.0000000	0.2450000
24316.0000000	0.2450000
24317.0000000	0.2550000
24318.0000000	0.2500000
24319.0000000	0.2500000
24320.0000000	0.2450000
24321.0000000	0.2450000
24322.0000000	0.2500000
24323.0000000	0.2450000
24324.0000000	0.2600000
24325.0000000	0.2500000
24326.0000000	0.2450000
24327.0000000	0.2550000
24328.0000000	0.2450000
24329.0000000	0.2500000
24330.0000000	0.2450000
24331.0000000	0.2500000
24332.0000000	0.2550000
24333.0000000	0.2500000
24334.0000000	0.2550000
24335.0000000	0.2500000
24336.0000000	0.2450000
24337.0000000	0.2450000
24338.0000000	0.2533333

24339.0000000	0.2650000
24340.0000000	0.2500000
24341.0000000	0.2450000
24342.0000000	0.2550000
24343.0000000	0.2550000
24344.0000000	0.2600000
24345.0000000	0.2550000
24346.0000000	0.2550000
24347.0000000	0.2600000
24348.0000000	0.2600000
24349.0000000	0.2550000
24350.0000000	0.2500000
24351.0000000	0.2550000
24352.0000000	0.2600000
24353.0000000	0.2600000
24354.0000000	0.2700000
24355.0000000	0.2500000
24356.0000000	0.2550000
24357.0000000	0.2500000
24358.0000000	0.2500000
24359.0000000	0.2550000
24360.0000000	0.2550000
24361.0000000	0.2500000
24362.0000000	0.2600000
24363.0000000	0.2650000
24364.0000000	0.2500000
24365.0000000	0.2650000
24366.0000000	0.2600000
24367.0000000	0.2600000
24368.0000000	0.2650000
24369.0000000	0.2600000
24370.0000000	0.2600000
24371.0000000	0.2600000
24372.0000000	0.2650000
24373.0000000	0.2500000
24374.0000000	0.2600000
24375.0000000	0.2650000
24376.0000000	0.2650000
24377.0000000	0.2650000
24378.0000000	0.2650000
24379.0000000	0.2650000
24380.0000000	0.2700000
24381.0000000	0.2650000
24382.0000000	0.2600000
24383.0000000	0.2600000
24384.0000000	0.2650000
24385.0000000	0.2650000
24386.0000000	0.2700000
24387.0000000	0.2550000
24388.0000000	0.2700000
24389.0000000	0.2550000
24390.0000000	0.2600000
24391.0000000	0.2650000
24392.0000000	0.2650000
24393.0000000	0.2600000
24394.0000000	0.2600000
24395.0000000	0.2750000

24396.0000000	0.2650000
24397.0000000	0.2700000
24398.0000000	0.2666667
24399.0000000	0.2650000
24400.0000000	0.2750000
24401.0000000	0.2600000
24402.0000000	0.2650000
24403.0000000	0.2600000
24404.0000000	0.2600000
24405.0000000	0.2700000
24406.0000000	0.2700000
24407.0000000	0.2750000
24408.0000000	0.2700000
24409.0000000	0.2650000
24410.0000000	0.2650000
24411.0000000	0.2700000
24412.0000000	0.2750000
24413.0000000	0.2650000
24414.0000000	0.2700000
24415.0000000	0.2700000
24416.0000000	0.2700000
24417.0000000	0.2700000
24418.0000000	0.2700000
24419.0000000	0.2700000
24420.0000000	0.2700000
24421.0000000	0.2700000
24422.0000000	0.2650000
24423.0000000	0.2750000
24424.0000000	0.2650000
24425.0000000	0.2700000
24426.0000000	0.2700000
24427.0000000	0.2750000
24428.0000000	0.2700000
24429.0000000	0.2733333
24430.0000000	0.2700000
24431.0000000	0.2750000
24432.0000000	0.2700000
24433.0000000	0.2600000
24434.0000000	0.2700000
24435.0000000	0.2750000
24436.0000000	0.2650000
24437.0000000	0.2800000
24438.0000000	0.2650000
24439.0000000	0.2700000
24440.0000000	0.2650000
24441.0000000	0.2800000
24442.0000000	0.2750000
24443.0000000	0.2750000
24444.0000000	0.2750000
24445.0000000	0.2700000
24446.0000000	0.2750000
24447.0000000	0.2700000
24448.0000000	0.2750000
24449.0000000	0.2750000
24450.0000000	0.2700000
24451.0000000	0.2750000
24452.0000000	0.2700000

24453.0000000	0.2700000
24454.0000000	0.2700000
24455.0000000	0.2800000
24456.0000000	0.2700000
24457.0000000	0.2750000
24458.0000000	0.2766666
24459.0000000	0.2750000
24460.0000000	0.2650000
24461.0000000	0.2800000
24462.0000000	0.2800000
24463.0000000	0.2800000
24464.0000000	0.2700000
24465.0000000	0.2850000
24466.0000000	0.2650000
24467.0000000	0.2800000
24468.0000000	0.2750000
24469.0000000	0.2850000
24470.0000000	0.2750000
24471.0000000	0.2750000
24472.0000000	0.2750000
24473.0000000	0.2850000
24474.0000000	0.2750000
24475.0000000	0.2800000
24476.0000000	0.2750000
24477.0000000	0.2800000
24478.0000000	0.2750000
24479.0000000	0.2850000
24480.0000000	0.2750000
24481.0000000	0.2700000
24482.0000000	0.2700000
24483.0000000	0.2850000
24484.0000000	0.2650000
24485.0000000	0.2850000
24486.0000000	0.2750000
24487.0000000	0.2700000
24488.0000000	0.2766666
24489.0000000	0.2700000
24490.0000000	0.2800000
24491.0000000	0.2600000
24492.0000000	0.2850000
24493.0000000	0.2650000
24494.0000000	0.2700000
24495.0000000	0.2700000
24496.0000000	0.2700000
24497.0000000	0.2700000
24498.0000000	0.2650000
24499.0000000	0.2750000
24500.0000000	0.2750000
24501.0000000	0.2850000
24502.0000000	0.2700000
24503.0000000	0.2750000
24504.0000000	0.2650000
24505.0000000	0.2700000
24506.0000000	0.2650000
24507.0000000	0.2700000
24508.0000000	0.2750000
24509.0000000	0.2750000

24510.0000000	0.2700000
24511.0000000	0.2650000
24512.0000000	0.2750000
24513.0000000	0.2700000
24514.0000000	0.2700000
24515.0000000	0.2700000
24516.0000000	0.2700000
24517.0000000	0.2750000
24518.0000000	0.2750000
24519.0000000	0.2733333
24520.0000000	0.2700000
24521.0000000	0.2650000
24522.0000000	0.2650000
24523.0000000	0.2750000
24524.0000000	0.2650000
24525.0000000	0.2600000
24526.0000000	0.2750000
24527.0000000	0.2650000
24528.0000000	0.2700000
24529.0000000	0.2750000
24530.0000000	0.2800000
24531.0000000	0.2650000
24532.0000000	0.2650000
24533.0000000	0.2650000
24534.0000000	0.2800000
24535.0000000	0.2700000
24536.0000000	0.2650000
24537.0000000	0.2650000
24538.0000000	0.2650000
24539.0000000	0.2800000
24540.0000000	0.2650000
24541.0000000	0.2800000
24542.0000000	0.2650000
24543.0000000	0.2750000
24544.0000000	0.2700000
24545.0000000	0.2650000
24546.0000000	0.2750000
24547.0000000	0.2650000
24548.0000000	0.2700000
24549.0000000	0.2733333
24550.0000000	0.2750000
24551.0000000	0.2700000
24552.0000000	0.2650000
24553.0000000	0.2650000
24554.0000000	0.2750000
24555.0000000	0.2800000
24556.0000000	0.2700000
24557.0000000	0.2750000
24558.0000000	0.2700000
24559.0000000	0.2750000
24560.0000000	0.2700000
24561.0000000	0.2650000
24562.0000000	0.2650000
24563.0000000	0.2700000
24564.0000000	0.2750000
24565.0000000	0.2750000
24566.0000000	0.2700000

24567.0000000	0.2650000
24568.0000000	0.2800000
24569.0000000	0.2700000
24570.0000000	0.2700000
24571.0000000	0.2650000
24572.0000000	0.2650000
24573.0000000	0.2750000
24574.0000000	0.2650000
24575.0000000	0.2700000
24576.0000000	0.2650000
24577.0000000	0.2700000
24578.0000000	0.2750000
24579.0000000	0.2733333
24580.0000000	0.2700000
24581.0000000	0.2650000
24582.0000000	0.2700000
24583.0000000	0.2650000
24584.0000000	0.2750000
24585.0000000	0.2650000
24586.0000000	0.2750000
24587.0000000	0.2700000
24588.0000000	0.2700000
24589.0000000	0.2650000
24590.0000000	0.2650000
24591.0000000	0.2700000
24592.0000000	0.2700000
24593.0000000	0.2750000
24594.0000000	0.2750000
24595.0000000	0.2700000
24596.0000000	0.2550000
24597.0000000	0.2650000
24598.0000000	0.2600000
24599.0000000	0.2700000
24600.0000000	0.2650000
24601.0000000	0.2700000
24602.0000000	0.2750000
24603.0000000	0.2650000
24604.0000000	0.2600000
24605.0000000	0.2700000
24606.0000000	0.2700000
24607.0000000	0.2700000
24608.0000000	0.2750000
24609.0000000	0.2600000
24610.0000000	0.2600000
24611.0000000	0.2550000
24612.0000000	0.2650000
24613.0000000	0.2650000
24614.0000000	0.2650000
24615.0000000	0.2650000
24616.0000000	0.2600000
24617.0000000	0.2550000
24618.0000000	0.2600000
24619.0000000	0.2600000
24620.0000000	0.2600000
24621.0000000	0.2800000
24622.0000000	0.2650000
24623.0000000	0.2550000

24624.0000000	0.2600000
24625.0000000	0.2600000
24626.0000000	0.2650000
24627.0000000	0.2650000
24628.0000000	0.2600000
24629.0000000	0.2700000
24630.0000000	0.2550000
24631.0000000	0.2600000
24632.0000000	0.2700000
24633.0000000	0.2600000
24634.0000000	0.2600000
24635.0000000	0.2600000
24636.0000000	0.2550000
24637.0000000	0.2600000
24638.0000000	0.2550000
24639.0000000	0.2600000
24640.0000000	0.2600000
24641.0000000	0.2650000
24642.0000000	0.2550000
24643.0000000	0.2600000
24644.0000000	0.2700000
24645.0000000	0.2550000
24646.0000000	0.2600000
24647.0000000	0.2550000
24648.0000000	0.2500000
24649.0000000	0.2550000
24650.0000000	0.2650000
24651.0000000	0.2550000
24652.0000000	0.2550000
24653.0000000	0.2500000
24654.0000000	0.2550000
24655.0000000	0.2550000
24656.0000000	0.2600000
24657.0000000	0.2600000
24658.0000000	0.2550000
24659.0000000	0.2600000
24660.0000000	0.2500000
24661.0000000	0.2500000
24662.0000000	0.2650000
24663.0000000	0.2550000
24664.0000000	0.2550000
24665.0000000	0.2450000
24666.0000000	0.2550000
24667.0000000	0.2500000
24668.0000000	0.2500000
24669.0000000	0.2450000
24670.0000000	0.2533333
24671.0000000	0.2550000
24672.0000000	0.2500000
24673.0000000	0.2550000
24674.0000000	0.2550000
24675.0000000	0.2600000
24676.0000000	0.2550000
24677.0000000	0.2500000
24678.0000000	0.2600000
24679.0000000	0.2450000
24680.0000000	0.2550000

24681.0000000	0.2500000
24682.0000000	0.2550000
24683.0000000	0.2500000
24684.0000000	0.2550000
24685.0000000	0.2450000
24686.0000000	0.2450000
24687.0000000	0.2450000
24688.0000000	0.2500000
24689.0000000	0.2500000
24690.0000000	0.2500000
24691.0000000	0.2450000
24692.0000000	0.2500000
24693.0000000	0.2550000
24694.0000000	0.2450000
24695.0000000	0.2450000
24696.0000000	0.2500000
24697.0000000	0.2500000
24698.0000000	0.2500000
24699.0000000	0.2500000
24700.0000000	0.2500000
24701.0000000	0.2500000
24702.0000000	0.2450000
24703.0000000	0.2550000
24704.0000000	0.2500000
24705.0000000	0.2450000
24706.0000000	0.2350000
24707.0000000	0.2500000
24708.0000000	0.2400000
24709.0000000	0.2500000
24710.0000000	0.2550000
24711.0000000	0.2500000
24712.0000000	0.2400000
24713.0000000	0.2400000
24714.0000000	0.2500000
24715.0000000	0.2500000
24716.0000000	0.2550000
24717.0000000	0.2500000
24718.0000000	0.2450000
24719.0000000	0.2450000
24720.0000000	0.2400000
24721.0000000	0.2550000
24722.0000000	0.2550000
24723.0000000	0.2550000
24724.0000000	0.2500000
24725.0000000	0.2500000
24726.0000000	0.2450000
24727.0000000	0.2500000
24728.0000000	0.2500000
24729.0000000	0.2450000
24730.0000000	0.2433333
24731.0000000	0.2400000
24732.0000000	0.2500000
24733.0000000	0.2450000
24734.0000000	0.2550000
24735.0000000	0.2400000
24736.0000000	0.2450000
24737.0000000	0.2350000

24738.0000000	0.2450000
24739.0000000	0.2500000
24740.0000000	0.2400000
24741.0000000	0.2450000
24742.0000000	0.2350000
24743.0000000	0.2400000
24744.0000000	0.2450000
24745.0000000	0.2500000
24746.0000000	0.2450000
24747.0000000	0.2500000
24748.0000000	0.2450000
24749.0000000	0.2450000
24750.0000000	0.2450000
24751.0000000	0.2500000
24752.0000000	0.2450000
24753.0000000	0.2500000
24754.0000000	0.2350000
24755.0000000	0.2500000
24756.0000000	0.2450000
24757.0000000	0.2450000
24758.0000000	0.2350000
24759.0000000	0.2450000
24760.0000000	0.2450000
24761.0000000	0.2466667
24762.0000000	0.2500000
24763.0000000	0.2350000
24764.0000000	0.2500000
24765.0000000	0.2500000
24766.0000000	0.2450000
24767.0000000	0.2400000
24768.0000000	0.2450000
24769.0000000	0.2350000
24770.0000000	0.2450000
24771.0000000	0.2400000
24772.0000000	0.2500000
24773.0000000	0.2450000
24774.0000000	0.2450000
24775.0000000	0.2350000
24776.0000000	0.2450000
24777.0000000	0.2300000
24778.0000000	0.2500000
24779.0000000	0.2400000
24780.0000000	0.2450000
24781.0000000	0.2350000
24782.0000000	0.2400000
24783.0000000	0.2400000
24784.0000000	0.2400000
24785.0000000	0.2500000
24786.0000000	0.2400000
24787.0000000	0.2450000
24788.0000000	0.2450000
24789.0000000	0.2450000
24790.0000000	0.2433333
24791.0000000	0.2500000
24792.0000000	0.2550000
24793.0000000	0.2450000
24794.0000000	0.2400000

24795.0000000	0.2350000
24796.0000000	0.2400000
24797.0000000	0.2500000
24798.0000000	0.2500000
24799.0000000	0.2450000
24800.0000000	0.2450000
24801.0000000	0.2500000
24802.0000000	0.2500000
24803.0000000	0.2400000
24804.0000000	0.2450000
24805.0000000	0.2350000
24806.0000000	0.2400000
24807.0000000	0.2450000
24808.0000000	0.2400000
24809.0000000	0.2500000
24810.0000000	0.2450000
24811.0000000	0.2400000
24812.0000000	0.2400000
24813.0000000	0.2450000
24814.0000000	0.2500000
24815.0000000	0.2500000
24816.0000000	0.2500000
24817.0000000	0.2350000
24818.0000000	0.2450000
24819.0000000	0.2450000
24820.0000000	0.2433333
24821.0000000	0.2500000
24822.0000000	0.2400000
24823.0000000	0.2450000
24824.0000000	0.2500000
24825.0000000	0.2450000
24826.0000000	0.2500000
24827.0000000	0.2400000
24828.0000000	0.2450000
24829.0000000	0.2450000
24830.0000000	0.2450000
24831.0000000	0.2450000
24832.0000000	0.2450000
24833.0000000	0.2400000
24834.0000000	0.2400000
24835.0000000	0.2350000
24836.0000000	0.2450000
24837.0000000	0.2500000
24838.0000000	0.2400000
24839.0000000	0.2450000
24840.0000000	0.2450000
24841.0000000	0.2450000
24842.0000000	0.2500000
24843.0000000	0.2400000
24844.0000000	0.2450000
24845.0000000	0.2500000
24846.0000000	0.2400000
24847.0000000	0.2400000
24848.0000000	0.2500000
24849.0000000	0.2400000
24850.0000000	0.2450000
24851.0000000	0.2400000

24852.0000000	0.2400000
24853.0000000	0.2450000
24854.0000000	0.2600000
24855.0000000	0.2500000
24856.0000000	0.2450000
24857.0000000	0.2500000
24858.0000000	0.2450000
24859.0000000	0.2500000
24860.0000000	0.2450000
24861.0000000	0.2500000
24862.0000000	0.2400000
24863.0000000	0.2450000
24864.0000000	0.2500000
24865.0000000	0.2450000
24866.0000000	0.2450000
24867.0000000	0.2500000
24868.0000000	0.2500000
24869.0000000	0.2400000
24870.0000000	0.2500000
24871.0000000	0.2450000
24872.0000000	0.2400000
24873.0000000	0.2400000
24874.0000000	0.2450000
24875.0000000	0.2500000
24876.0000000	0.2550000
24877.0000000	0.2550000
24878.0000000	0.2500000
24879.0000000	0.2500000
24880.0000000	0.2450000
24881.0000000	0.2500000
24882.0000000	0.2600000
24883.0000000	0.2400000
24884.0000000	0.2450000
24885.0000000	0.2450000
24886.0000000	0.2450000
24887.0000000	0.2500000
24888.0000000	0.2500000
24889.0000000	0.2500000
24890.0000000	0.2450000
24891.0000000	0.2500000
24892.0000000	0.2500000
24893.0000000	0.2450000
24894.0000000	0.2450000
24895.0000000	0.2500000
24896.0000000	0.2500000
24897.0000000	0.2550000
24898.0000000	0.2500000
24899.0000000	0.2550000
24900.0000000	0.2400000
24901.0000000	0.2500000
24902.0000000	0.2500000
24903.0000000	0.2600000
24904.0000000	0.2500000
24905.0000000	0.2500000
24906.0000000	0.2450000
24907.0000000	0.2450000
24908.0000000	0.2500000

24909.0000000	0.2550000
24910.0000000	0.2550000
24911.0000000	0.2566667
24912.0000000	0.2500000
24913.0000000	0.2550000
24914.0000000	0.2450000
24915.0000000	0.2550000
24916.0000000	0.2550000
24917.0000000	0.2550000
24918.0000000	0.2650000
24919.0000000	0.2500000
24920.0000000	0.2500000
24921.0000000	0.2500000
24922.0000000	0.2500000
24923.0000000	0.2600000
24924.0000000	0.2500000
24925.0000000	0.2550000
24926.0000000	0.2500000
24927.0000000	0.2500000
24928.0000000	0.2600000
24929.0000000	0.2650000
24930.0000000	0.2600000
24931.0000000	0.2550000
24932.0000000	0.2600000
24933.0000000	0.2550000
24934.0000000	0.2650000
24935.0000000	0.2550000
24936.0000000	0.2650000
24937.0000000	0.2650000
24938.0000000	0.2600000
24939.0000000	0.2550000
24940.0000000	0.2600000
24941.0000000	0.2566667
24942.0000000	0.2600000
24943.0000000	0.2650000
24944.0000000	0.2700000
24945.0000000	0.2650000
24946.0000000	0.2600000
24947.0000000	0.2600000
24948.0000000	0.2600000
24949.0000000	0.2650000
24950.0000000	0.2650000
24951.0000000	0.2650000
24952.0000000	0.2700000
24953.0000000	0.2650000
24954.0000000	0.2650000
24955.0000000	0.2650000
24956.0000000	0.2600000
24957.0000000	0.2650000
24958.0000000	0.2600000
24959.0000000	0.2700000
24960.0000000	0.2700000
24961.0000000	0.2800000
24962.0000000	0.2650000
24963.0000000	0.2700000
24964.0000000	0.2700000
24965.0000000	0.2700000

24966.0000000	0.2750000
24967.0000000	0.2600000
24968.0000000	0.2750000
24969.0000000	0.2750000
24970.0000000	0.2750000
24971.0000000	0.2750000
24972.0000000	0.2766666
24973.0000000	0.2750000
24974.0000000	0.2700000
24975.0000000	0.2750000
24976.0000000	0.2850000
24977.0000000	0.2750000
24978.0000000	0.2800000
24979.0000000	0.2800000
24980.0000000	0.2850000
24981.0000000	0.2750000
24982.0000000	0.2750000
24983.0000000	0.2700000
24984.0000000	0.2850000
24985.0000000	0.2800000
24986.0000000	0.2900000
24987.0000000	0.2850000
24988.0000000	0.2850000
24989.0000000	0.2900000
24990.0000000	0.2850000
24991.0000000	0.2900000
24992.0000000	0.2800000
24993.0000000	0.2850000
24994.0000000	0.2850000
24995.0000000	0.2950000
24996.0000000	0.2900000
24997.0000000	0.2950000
24998.0000000	0.2850000
24999.0000000	0.2950000
25000.0000000	0.3000000
25001.0000000	0.2900000
25002.0000000	0.2833333
25003.0000000	0.3000000
25004.0000000	0.3050000
25005.0000000	0.3000000
25006.0000000	0.2900000
25007.0000000	0.2900000
25008.0000000	0.2900000
25009.0000000	0.3000000
25010.0000000	0.2950000
25011.0000000	0.3000000
25012.0000000	0.3000000
25013.0000000	0.2950000
25014.0000000	0.3050000
25015.0000000	0.3000000
25016.0000000	0.2950000
25017.0000000	0.3000000
25018.0000000	0.3100000
25019.0000000	0.2950000
25020.0000000	0.2950000
25021.0000000	0.3050000
25022.0000000	0.3000000

25023.0000000	0.3050000
25024.0000000	0.3000000
25025.0000000	0.3000000
25026.0000000	0.3050000
25027.0000000	0.3100000
25028.0000000	0.3050000
25029.0000000	0.3000000
25030.0000000	0.3050000
25031.0000000	0.3150000
25032.0000000	0.3133333
25033.0000000	0.3100000
25034.0000000	0.3050000
25035.0000000	0.3100000
25036.0000000	0.3050000
25037.0000000	0.3050000
25038.0000000	0.3100000
25039.0000000	0.3100000
25040.0000000	0.3100000
25041.0000000	0.3100000
25042.0000000	0.2950000
25043.0000000	0.3150000
25044.0000000	0.3100000
25045.0000000	0.3050000
25046.0000000	0.3100000
25047.0000000	0.3000000
25048.0000000	0.3150000
25049.0000000	0.3100000
25050.0000000	0.3100000
25051.0000000	0.3150000
25052.0000000	0.3100000
25053.0000000	0.3150000
25054.0000000	0.3150000
25055.0000000	0.3150000
25056.0000000	0.3150000
25057.0000000	0.3150000
25058.0000000	0.3200000
25059.0000000	0.3100000
25060.0000000	0.3100000
25061.0000000	0.3200000
25062.0000000	0.3100000
25063.0000000	0.3200000
25064.0000000	0.3150000
25065.0000000	0.3100000
25066.0000000	0.3150000
25067.0000000	0.3200000
25068.0000000	0.3200000
25069.0000000	0.3300000
25070.0000000	0.3100000
25071.0000000	0.3300000
25072.0000000	0.3150000
25073.0000000	0.3250000
25074.0000000	0.3200000
25075.0000000	0.3300000
25076.0000000	0.3200000
25077.0000000	0.3300000
25078.0000000	0.3200000
25079.0000000	0.3300000

25080.0000000	0.3200000
25081.0000000	0.3300000
25082.0000000	0.3250000
25083.0000000	0.3250000
25084.0000000	0.3250000
25085.0000000	0.3100000
25086.0000000	0.3300000
25087.0000000	0.3300000
25088.0000000	0.3350000
25089.0000000	0.3350000
25090.0000000	0.3300000
25091.0000000	0.3250000
25092.0000000	0.3350000
25093.0000000	0.3400000
25094.0000000	0.3350000
25095.0000000	0.3300000
25096.0000000	0.3300000
25097.0000000	0.3400000
25098.0000000	0.3450000
25099.0000000	0.3400000
25100.0000000	0.3500000
25101.0000000	0.3350000
25102.0000000	0.3450000
25103.0000000	0.3450000
25104.0000000	0.3550000
25105.0000000	0.3500000
25106.0000000	0.3450000
25107.0000000	0.3500000
25108.0000000	0.3400000
25109.0000000	0.3450000
25110.0000000	0.3450000
25111.0000000	0.3500000
25112.0000000	0.3450000
25113.0000000	0.3550000
25114.0000000	0.3500000
25115.0000000	0.3500000
25116.0000000	0.3500000
25117.0000000	0.3550000
25118.0000000	0.3600000
25119.0000000	0.3500000
25120.0000000	0.3600000
25121.0000000	0.3550000
25122.0000000	0.3600000
25123.0000000	0.3550000
25124.0000000	0.3650000
25125.0000000	0.3600000
25126.0000000	0.3650000
25127.0000000	0.3600000
25128.0000000	0.3650000
25129.0000000	0.3650000
25130.0000000	0.3750000
25131.0000000	0.3650000
25132.0000000	0.3650000
25133.0000000	0.3700000
25134.0000000	0.3600000
25135.0000000	0.3650000
25136.0000000	0.3650000

25137.0000000	0.3650000
25138.0000000	0.3700000
25139.0000000	0.3600000
25140.0000000	0.3700000
25141.0000000	0.3650000
25142.0000000	0.3700000
25143.0000000	0.3750000
25144.0000000	0.3750000
25145.0000000	0.3650000
25146.0000000	0.3650000
25147.0000000	0.3800000
25148.0000000	0.3700000
25149.0000000	0.3800000
25150.0000000	0.3700000
25151.0000000	0.3800000
25152.0000000	0.3733333
25153.0000000	0.3800000
25154.0000000	0.3700000
25155.0000000	0.3800000
25156.0000000	0.3700000
25157.0000000	0.3850000
25158.0000000	0.3850000
25159.0000000	0.3900000
25160.0000000	0.3750000
25161.0000000	0.3850000
25162.0000000	0.3850000
25163.0000000	0.3900000
25164.0000000	0.3850000
25165.0000000	0.3850000
25166.0000000	0.3900000
25167.0000000	0.3800000
25168.0000000	0.3850000
25169.0000000	0.3850000
25170.0000000	0.3800000
25171.0000000	0.3850000
25172.0000000	0.3850000
25173.0000000	0.3900000
25174.0000000	0.3850000
25175.0000000	0.3850000
25176.0000000	0.3900000
25177.0000000	0.3850000
25178.0000000	0.3900000
25179.0000000	0.3900000
25180.0000000	0.3850000
25181.0000000	0.3900000
25182.0000000	0.3900000
25183.0000000	0.3866667
25184.0000000	0.3900000
25185.0000000	0.3900000
25186.0000000	0.3900000
25187.0000000	0.3950000
25188.0000000	0.3900000
25189.0000000	0.3850000
25190.0000000	0.3850000
25191.0000000	0.3900000
25192.0000000	0.3900000
25193.0000000	0.3950000

25194.0000000	0.3950000
25195.0000000	0.3900000
25196.0000000	0.4000000
25197.0000000	0.3950000
25198.0000000	0.3950000
25199.0000000	0.3900000
25200.0000000	0.3950000
25201.0000000	0.3950000
25202.0000000	0.3900000
25203.0000000	0.3900000
25204.0000000	0.3900000
25205.0000000	0.4000000
25206.0000000	0.3850000
25207.0000000	0.3900000
25208.0000000	0.3900000
25209.0000000	0.4050000
25210.0000000	0.3950000
25211.0000000	0.3900000
25212.0000000	0.4000000
25213.0000000	0.4000000
25214.0000000	0.3900000
25215.0000000	0.3950000
25216.0000000	0.3950000
25217.0000000	0.4000000
25218.0000000	0.4000000
25219.0000000	0.3950000
25220.0000000	0.4000000
25221.0000000	0.4000000
25222.0000000	0.3950000
25223.0000000	0.3950000
25224.0000000	0.4000000
25225.0000000	0.4000000
25226.0000000	0.3950000
25227.0000000	0.4000000
25228.0000000	0.4000000
25229.0000000	0.3950000
25230.0000000	0.4000000
25231.0000000	0.4050000
25232.0000000	0.4050000
25233.0000000	0.3950000
25234.0000000	0.4000000
25235.0000000	0.4000000
25236.0000000	0.3950000
25237.0000000	0.4000000
25238.0000000	0.4050000
25239.0000000	0.4000000
25240.0000000	0.4000000
25241.0000000	0.4000000
25242.0000000	0.4000000
25243.0000000	0.4066667
25244.0000000	0.3950000
25245.0000000	0.4000000
25246.0000000	0.4000000
25247.0000000	0.4050000
25248.0000000	0.4050000
25249.0000000	0.4000000
25250.0000000	0.4050000

25251.0000000	0.4000000
25252.0000000	0.3900000
25253.0000000	0.4000000
25254.0000000	0.3950000
25255.0000000	0.3950000
25256.0000000	0.4050000
25257.0000000	0.4050000
25258.0000000	0.3950000
25259.0000000	0.4100000
25260.0000000	0.4050000
25261.0000000	0.3950000
25262.0000000	0.3950000
25263.0000000	0.4000000
25264.0000000	0.4000000
25265.0000000	0.4000000
25266.0000000	0.3950000
25267.0000000	0.4000000
25268.0000000	0.3850000
25269.0000000	0.4000000
25270.0000000	0.3900000
25271.0000000	0.4100000
25272.0000000	0.4000000
25273.0000000	0.3933333
25274.0000000	0.4000000
25275.0000000	0.4000000
25276.0000000	0.3950000
25277.0000000	0.3900000
25278.0000000	0.3900000
25279.0000000	0.3850000
25280.0000000	0.3900000
25281.0000000	0.3900000
25282.0000000	0.3850000
25283.0000000	0.3900000
25284.0000000	0.3900000
25285.0000000	0.3850000
25286.0000000	0.3950000
25287.0000000	0.3800000
25288.0000000	0.3750000
25289.0000000	0.3800000
25290.0000000	0.3900000
25291.0000000	0.3750000
25292.0000000	0.3650000
25293.0000000	0.3750000
25294.0000000	0.3850000
25295.0000000	0.3850000
25296.0000000	0.3700000
25297.0000000	0.3700000
25298.0000000	0.3800000
25299.0000000	0.3850000
25300.0000000	0.3750000
25301.0000000	0.3700000
25302.0000000	0.3750000
25303.0000000	0.3750000
25304.0000000	0.3700000
25305.0000000	0.3700000
25306.0000000	0.3700000
25307.0000000	0.3700000

25308.0000000	0.3750000
25309.0000000	0.3750000
25310.0000000	0.3650000
25311.0000000	0.3700000
25312.0000000	0.3700000
25313.0000000	0.3750000
25314.0000000	0.3700000
25315.0000000	0.3700000
25316.0000000	0.3700000
25317.0000000	0.3550000
25318.0000000	0.3550000
25319.0000000	0.3700000
25320.0000000	0.3650000
25321.0000000	0.3650000
25322.0000000	0.3650000
25323.0000000	0.3700000
25324.0000000	0.3600000
25325.0000000	0.3600000
25326.0000000	0.3650000
25327.0000000	0.3750000
25328.0000000	0.3700000
25329.0000000	0.3600000
25330.0000000	0.3700000
25331.0000000	0.3700000
25332.0000000	0.3550000
25333.0000000	0.3550000
25334.0000000	0.3633333
25335.0000000	0.3650000
25336.0000000	0.3650000
25337.0000000	0.3600000
25338.0000000	0.3650000
25339.0000000	0.3700000
25340.0000000	0.3600000
25341.0000000	0.3600000
25342.0000000	0.3700000
25343.0000000	0.3600000
25344.0000000	0.3500000
25345.0000000	0.3650000
25346.0000000	0.3500000
25347.0000000	0.3400000
25348.0000000	0.3500000
25349.0000000	0.3500000
25350.0000000	0.3450000
25351.0000000	0.3500000
25352.0000000	0.3600000
25353.0000000	0.3600000
25354.0000000	0.3450000
25355.0000000	0.3550000
25356.0000000	0.3550000
25357.0000000	0.3400000
25358.0000000	0.3450000
25359.0000000	0.3400000
25360.0000000	0.3450000
25361.0000000	0.3450000
25362.0000000	0.3500000
25363.0000000	0.3550000
25364.0000000	0.3466667

25365.0000000	0.3550000
25366.0000000	0.3500000
25367.0000000	0.3500000
25368.0000000	0.3550000
25369.0000000	0.3500000
25370.0000000	0.3400000
25371.0000000	0.3450000
25372.0000000	0.3500000
25373.0000000	0.3450000
25374.0000000	0.3400000
25375.0000000	0.3400000
25376.0000000	0.3500000
25377.0000000	0.3500000
25378.0000000	0.3400000
25379.0000000	0.3350000
25380.0000000	0.3500000
25381.0000000	0.3450000
25382.0000000	0.3500000
25383.0000000	0.3450000
25384.0000000	0.3400000
25385.0000000	0.3400000
25386.0000000	0.3550000
25387.0000000	0.3450000
25388.0000000	0.3300000
25389.0000000	0.3450000
25390.0000000	0.3500000
25391.0000000	0.3450000
25392.0000000	0.3350000
25393.0000000	0.3450000
25394.0000000	0.3366667
25395.0000000	0.3350000
25396.0000000	0.3450000
25397.0000000	0.3450000
25398.0000000	0.3450000
25399.0000000	0.3400000
25400.0000000	0.3300000
25401.0000000	0.3350000
25402.0000000	0.3400000
25403.0000000	0.3400000
25404.0000000	0.3300000
25405.0000000	0.3400000
25406.0000000	0.3350000
25407.0000000	0.3400000
25408.0000000	0.3550000
25409.0000000	0.3300000
25410.0000000	0.3400000
25411.0000000	0.3400000
25412.0000000	0.3350000
25413.0000000	0.3400000
25414.0000000	0.3300000
25415.0000000	0.3400000
25416.0000000	0.3250000
25417.0000000	0.3400000
25418.0000000	0.3350000
25419.0000000	0.3350000
25420.0000000	0.3400000
25421.0000000	0.3350000

25422.0000000	0.3400000
25423.0000000	0.3400000
25424.0000000	0.3400000
25425.0000000	0.3366667
25426.0000000	0.3400000
25427.0000000	0.3450000
25428.0000000	0.3400000
25429.0000000	0.3350000
25430.0000000	0.3350000
25431.0000000	0.3400000
25432.0000000	0.3300000
25433.0000000	0.3400000
25434.0000000	0.3350000
25435.0000000	0.3300000
25436.0000000	0.3350000
25437.0000000	0.3350000
25438.0000000	0.3300000
25439.0000000	0.3350000
25440.0000000	0.3300000
25441.0000000	0.3250000
25442.0000000	0.3300000
25443.0000000	0.3350000
25444.0000000	0.3350000
25445.0000000	0.3200000
25446.0000000	0.3300000
25447.0000000	0.3250000
25448.0000000	0.3300000
25449.0000000	0.3350000
25450.0000000	0.3200000
25451.0000000	0.3300000
25452.0000000	0.3250000
25453.0000000	0.3250000
25454.0000000	0.3300000
25455.0000000	0.3200000
25456.0000000	0.3200000
25457.0000000	0.3300000
25458.0000000	0.3250000
25459.0000000	0.3150000
25460.0000000	0.3200000
25461.0000000	0.3150000
25462.0000000	0.3200000
25463.0000000	0.3250000
25464.0000000	0.3150000
25465.0000000	0.3300000
25466.0000000	0.3150000
25467.0000000	0.3200000
25468.0000000	0.3200000
25469.0000000	0.3200000
25470.0000000	0.3250000
25471.0000000	0.3150000
25472.0000000	0.3250000
25473.0000000	0.3200000
25474.0000000	0.3200000
25475.0000000	0.3100000
25476.0000000	0.3150000
25477.0000000	0.3150000
25478.0000000	0.3200000

25479.0000000	0.3200000
25480.0000000	0.3200000
25481.0000000	0.3150000
25482.0000000	0.3150000
25483.0000000	0.3150000
25484.0000000	0.3133333
25485.0000000	0.3150000
25486.0000000	0.3150000
25487.0000000	0.3200000
25488.0000000	0.3150000
25489.0000000	0.3200000
25490.0000000	0.3100000
25491.0000000	0.3200000
25492.0000000	0.3100000
25493.0000000	0.3200000
25494.0000000	0.3150000
25495.0000000	0.3050000
25496.0000000	0.3200000
25497.0000000	0.3150000
25498.0000000	0.3200000
25499.0000000	0.3150000
25500.0000000	0.3100000
25501.0000000	0.3050000
25502.0000000	0.3100000
25503.0000000	0.3150000
25504.0000000	0.3100000
25505.0000000	0.3000000
25506.0000000	0.3100000
25507.0000000	0.3000000
25508.0000000	0.3100000
25509.0000000	0.3050000
25510.0000000	0.3050000
25511.0000000	0.3000000
25512.0000000	0.3050000
25513.0000000	0.3050000
25514.0000000	0.3050000
25515.0000000	0.3100000
25516.0000000	0.3000000
25517.0000000	0.2950000
25518.0000000	0.3100000
25519.0000000	0.3000000
25520.0000000	0.3050000
25521.0000000	0.3000000
25522.0000000	0.3000000
25523.0000000	0.3000000
25524.0000000	0.2950000
25525.0000000	0.2950000
25526.0000000	0.2950000
25527.0000000	0.3000000
25528.0000000	0.2900000
25529.0000000	0.2950000
25530.0000000	0.2950000
25531.0000000	0.3000000
25532.0000000	0.2850000
25533.0000000	0.3000000
25534.0000000	0.2850000
25535.0000000	0.2900000

25536.0000000	0.2900000
25537.0000000	0.2950000
25538.0000000	0.3000000
25539.0000000	0.2950000
25540.0000000	0.2950000
25541.0000000	0.2950000
25542.0000000	0.2900000
25543.0000000	0.2900000
25544.0000000	0.2950000
25545.0000000	0.2933334
25546.0000000	0.2800000
25547.0000000	0.2950000
25548.0000000	0.2850000
25549.0000000	0.2900000
25550.0000000	0.2900000
25551.0000000	0.2850000
25552.0000000	0.2900000
25553.0000000	0.2850000
25554.0000000	0.2800000
25555.0000000	0.2750000
25556.0000000	0.2850000
25557.0000000	0.2850000
25558.0000000	0.2800000
25559.0000000	0.2800000
25560.0000000	0.2800000
25561.0000000	0.2800000
25562.0000000	0.2800000
25563.0000000	0.2800000
25564.0000000	0.2800000
25565.0000000	0.2750000
25566.0000000	0.2850000
25567.0000000	0.2800000
25568.0000000	0.2700000
25569.0000000	0.2800000
25570.0000000	0.2800000
25571.0000000	0.2650000
25572.0000000	0.2750000
25573.0000000	0.2650000
25574.0000000	0.2750000
25575.0000000	0.2766667
25576.0000000	0.2650000
25577.0000000	0.2700000
25578.0000000	0.2750000
25579.0000000	0.2700000
25580.0000000	0.2700000
25581.0000000	0.2700000
25582.0000000	0.2650000
25583.0000000	0.2700000
25584.0000000	0.2700000
25585.0000000	0.2600000
25586.0000000	0.2700000
25587.0000000	0.2650000
25588.0000000	0.2700000
25589.0000000	0.2750000
25590.0000000	0.2700000
25591.0000000	0.2700000
25592.0000000	0.2600000

25593.0000000	0.2650000
25594.0000000	0.2600000
25595.0000000	0.2600000
25596.0000000	0.2600000
25597.0000000	0.2600000
25598.0000000	0.2650000
25599.0000000	0.2500000
25600.0000000	0.2600000
25601.0000000	0.2450000
25602.0000000	0.2550000
25603.0000000	0.2500000
25604.0000000	0.2550000
25605.0000000	0.2566667
25606.0000000	0.2500000
25607.0000000	0.2500000
25608.0000000	0.2550000
25609.0000000	0.2500000
25610.0000000	0.2500000
25611.0000000	0.2450000
25612.0000000	0.2500000
25613.0000000	0.2450000
25614.0000000	0.2500000
25615.0000000	0.2450000
25616.0000000	0.2400000
25617.0000000	0.2450000
25618.0000000	0.2500000
25619.0000000	0.2400000
25620.0000000	0.2500000
25621.0000000	0.2400000
25622.0000000	0.2400000
25623.0000000	0.2450000
25624.0000000	0.2400000
25625.0000000	0.2350000
25626.0000000	0.2400000
25627.0000000	0.2350000
25628.0000000	0.2400000
25629.0000000	0.2450000
25630.0000000	0.2300000
25631.0000000	0.2350000
25632.0000000	0.2400000
25633.0000000	0.2300000
25634.0000000	0.2350000
25635.0000000	0.2300000
25636.0000000	0.2366667
25637.0000000	0.2250000
25638.0000000	0.2300000
25639.0000000	0.2300000
25640.0000000	0.2300000
25641.0000000	0.2350000
25642.0000000	0.2300000
25643.0000000	0.2300000
25644.0000000	0.2350000
25645.0000000	0.2300000
25646.0000000	0.2300000
25647.0000000	0.2250000
25648.0000000	0.2250000
25649.0000000	0.2250000

25650.0000000	0.2200000
25651.0000000	0.2200000
25652.0000000	0.2250000
25653.0000000	0.2250000
25654.0000000	0.2250000
25655.0000000	0.2350000
25656.0000000	0.2150000
25657.0000000	0.2250000
25658.0000000	0.2250000
25659.0000000	0.2250000
25660.0000000	0.2250000
25661.0000000	0.2200000
25662.0000000	0.2200000
25663.0000000	0.2250000
25664.0000000	0.2250000
25665.0000000	0.2200000
25666.0000000	0.2166667
25667.0000000	0.2150000
25668.0000000	0.2200000
25669.0000000	0.2300000
25670.0000000	0.2150000
25671.0000000	0.2200000
25672.0000000	0.2250000
25673.0000000	0.2200000
25674.0000000	0.2200000
25675.0000000	0.2150000
25676.0000000	0.2150000
25677.0000000	0.2250000
25678.0000000	0.2300000
25679.0000000	0.2150000
25680.0000000	0.2200000
25681.0000000	0.2150000
25682.0000000	0.2200000
25683.0000000	0.2250000
25684.0000000	0.2150000
25685.0000000	0.2150000
25686.0000000	0.2200000
25687.0000000	0.2150000
25688.0000000	0.2100000
25689.0000000	0.2250000
25690.0000000	0.2100000
25691.0000000	0.2200000
25692.0000000	0.2150000
25693.0000000	0.2050000
25694.0000000	0.2200000
25695.0000000	0.2100000
25696.0000000	0.2200000
25697.0000000	0.2150000
25698.0000000	0.2100000
25699.0000000	0.2100000
25700.0000000	0.2200000
25701.0000000	0.2150000
25702.0000000	0.2200000
25703.0000000	0.2150000
25704.0000000	0.2150000
25705.0000000	0.2200000
25706.0000000	0.2150000

25707.0000000	0.2150000
25708.0000000	0.2050000
25709.0000000	0.2150000
25710.0000000	0.2150000
25711.0000000	0.2100000
25712.0000000	0.2200000
25713.0000000	0.2050000
25714.0000000	0.2100000
25715.0000000	0.2100000
25716.0000000	0.2150000
25717.0000000	0.2150000
25718.0000000	0.2200000
25719.0000000	0.2150000
25720.0000000	0.2050000
25721.0000000	0.2150000
25722.0000000	0.2050000
25723.0000000	0.2150000
25724.0000000	0.2100000
25725.0000000	0.2200000
25726.0000000	0.2166667
25727.0000000	0.2150000
25728.0000000	0.2150000
25729.0000000	0.2150000
25730.0000000	0.2100000
25731.0000000	0.2100000
25732.0000000	0.2100000
25733.0000000	0.2100000
25734.0000000	0.2100000
25735.0000000	0.2100000
25736.0000000	0.2100000
25737.0000000	0.2100000
25738.0000000	0.2150000
25739.0000000	0.2100000
25740.0000000	0.2050000
25741.0000000	0.2100000
25742.0000000	0.2050000
25743.0000000	0.2150000
25744.0000000	0.2100000
25745.0000000	0.2150000
25746.0000000	0.2150000
25747.0000000	0.2100000
25748.0000000	0.2150000
25749.0000000	0.2100000
25750.0000000	0.2150000
25751.0000000	0.2100000
25752.0000000	0.2100000
25753.0000000	0.2100000
25754.0000000	0.2150000
25755.0000000	0.2150000
25756.0000000	0.2050000
25757.0000000	0.2133333
25758.0000000	0.2200000
25759.0000000	0.2100000
25760.0000000	0.2100000
25761.0000000	0.2050000
25762.0000000	0.2100000
25763.0000000	0.2200000

25764.0000000	0.2100000
25765.0000000	0.2150000
25766.0000000	0.2150000
25767.0000000	0.2100000
25768.0000000	0.2150000
25769.0000000	0.2050000
25770.0000000	0.2150000
25771.0000000	0.2000000
25772.0000000	0.2200000
25773.0000000	0.2150000
25774.0000000	0.2200000
25775.0000000	0.2150000
25776.0000000	0.2050000
25777.0000000	0.2100000
25778.0000000	0.2050000
25779.0000000	0.2150000
25780.0000000	0.2150000
25781.0000000	0.2150000
25782.0000000	0.2100000
25783.0000000	0.1950000
25784.0000000	0.2100000
25785.0000000	0.2100000
25786.0000000	0.2000000
25787.0000000	0.2033333
25788.0000000	0.2050000
25789.0000000	0.2000000
25790.0000000	0.2000000
25791.0000000	0.1950000
25792.0000000	0.2100000
25793.0000000	0.2100000
25794.0000000	0.2000000
25795.0000000	0.2100000
25796.0000000	0.1950000
25797.0000000	0.2000000
25798.0000000	0.2000000
25799.0000000	0.2000000
25800.0000000	0.2000000
25801.0000000	0.2000000
25802.0000000	0.2000000
25803.0000000	0.2000000
25804.0000000	0.2000000
25805.0000000	0.1950000
25806.0000000	0.1950000
25807.0000000	0.1950000
25808.0000000	0.2000000
25809.0000000	0.2050000
25810.0000000	0.2050000
25811.0000000	0.1950000
25812.0000000	0.1950000
25813.0000000	0.2000000
25814.0000000	0.1900000
25815.0000000	0.2000000
25816.0000000	0.2000000
25817.0000000	0.1900000
25818.0000000	0.1950000
25819.0000000	0.2000000
25820.0000000	0.2000000

25821.0000000	0.2050000
25822.0000000	0.2000000
25823.0000000	0.1900000
25824.0000000	0.1900000
25825.0000000	0.2050000
25826.0000000	0.1950000
25827.0000000	0.2000000
25828.0000000	0.2000000
25829.0000000	0.1950000
25830.0000000	0.1900000
25831.0000000	0.1950000
25832.0000000	0.1900000
25833.0000000	0.1950000
25834.0000000	0.1950000
25835.0000000	0.2000000
25836.0000000	0.1950000
25837.0000000	0.2000000
25838.0000000	0.1950000
25839.0000000	0.1950000
25840.0000000	0.1900000
25841.0000000	0.2050000
25842.0000000	0.2000000
25843.0000000	0.2050000
25844.0000000	0.1900000
25845.0000000	0.1950000
25846.0000000	0.1900000
25847.0000000	0.1966667
25848.0000000	0.1950000
25849.0000000	0.2000000
25850.0000000	0.1950000
25851.0000000	0.1950000
25852.0000000	0.1950000
25853.0000000	0.1900000
25854.0000000	0.1900000
25855.0000000	0.1950000
25856.0000000	0.2000000
25857.0000000	0.2000000
25858.0000000	0.1950000
25859.0000000	0.1900000
25860.0000000	0.1950000
25861.0000000	0.1950000
25862.0000000	0.1950000
25863.0000000	0.2000000
25864.0000000	0.1950000
25865.0000000	0.1950000
25866.0000000	0.1950000
25867.0000000	0.1950000
25868.0000000	0.1950000
25869.0000000	0.1950000
25870.0000000	0.2000000
25871.0000000	0.1950000
25872.0000000	0.1950000
25873.0000000	0.2000000
25874.0000000	0.1950000
25875.0000000	0.1900000
25876.0000000	0.1900000
25877.0000000	0.1950000

25878.0000000	0.1933333
25879.0000000	0.1950000
25880.0000000	0.1950000
25881.0000000	0.2000000
25882.0000000	0.1850000
25883.0000000	0.1950000
25884.0000000	0.1950000
25885.0000000	0.1950000
25886.0000000	0.1900000
25887.0000000	0.2000000
25888.0000000	0.2000000
25889.0000000	0.1950000
25890.0000000	0.1950000
25891.0000000	0.1900000
25892.0000000	0.1900000
25893.0000000	0.1900000
25894.0000000	0.1950000
25895.0000000	0.1950000
25896.0000000	0.2000000
25897.0000000	0.1950000
25898.0000000	0.1900000
25899.0000000	0.1900000
25900.0000000	0.1900000
25901.0000000	0.1950000
25902.0000000	0.1900000
25903.0000000	0.1950000
25904.0000000	0.1900000
25905.0000000	0.1950000
25906.0000000	0.1850000
25907.0000000	0.1866667
25908.0000000	0.1900000
25909.0000000	0.2000000
25910.0000000	0.1950000
25911.0000000	0.1900000
25912.0000000	0.1850000
25913.0000000	0.1900000
25914.0000000	0.1950000
25915.0000000	0.1900000
25916.0000000	0.1950000
25917.0000000	0.1950000
25918.0000000	0.1900000
25919.0000000	0.1900000
25920.0000000	0.1900000
25921.0000000	0.1850000
25922.0000000	0.1850000
25923.0000000	0.1850000
25924.0000000	0.1900000
25925.0000000	0.1950000
25926.0000000	0.1800000
25927.0000000	0.1900000
25928.0000000	0.1850000
25929.0000000	0.1900000
25930.0000000	0.1900000
25931.0000000	0.1800000
25932.0000000	0.1900000
25933.0000000	0.1900000
25934.0000000	0.1900000

25935.0000000	0.1850000
25936.0000000	0.1850000
25937.0000000	0.1766667
25938.0000000	0.1900000
25939.0000000	0.1800000
25940.0000000	0.1800000
25941.0000000	0.1800000
25942.0000000	0.1850000
25943.0000000	0.1800000
25944.0000000	0.1750000
25945.0000000	0.1800000
25946.0000000	0.1800000
25947.0000000	0.1800000
25948.0000000	0.1800000
25949.0000000	0.1750000
25950.0000000	0.1750000
25951.0000000	0.1900000
25952.0000000	0.1850000
25953.0000000	0.1800000
25954.0000000	0.1850000
25955.0000000	0.1850000
25956.0000000	0.1800000
25957.0000000	0.1850000
25958.0000000	0.1700000
25959.0000000	0.1850000
25960.0000000	0.1850000
25961.0000000	0.1850000
25962.0000000	0.1800000
25963.0000000	0.1750000
25964.0000000	0.1800000
25965.0000000	0.1700000
25966.0000000	0.1800000
25967.0000000	0.1800000
25968.0000000	0.1766667
25969.0000000	0.1700000
25970.0000000	0.1800000
25971.0000000	0.1800000
25972.0000000	0.1750000
25973.0000000	0.1800000
25974.0000000	0.1700000
25975.0000000	0.1700000
25976.0000000	0.1650000
25977.0000000	0.1800000
25978.0000000	0.1650000
25979.0000000	0.1800000
25980.0000000	0.1650000
25981.0000000	0.1700000
25982.0000000	0.1650000
25983.0000000	0.1700000
25984.0000000	0.1700000
25985.0000000	0.1700000
25986.0000000	0.1650000
25987.0000000	0.1650000
25988.0000000	0.1700000
25989.0000000	0.1600000
25990.0000000	0.1650000
25991.0000000	0.1650000

25992.0000000	0.1700000
25993.0000000	0.1650000
25994.0000000	0.1700000
25995.0000000	0.1700000
25996.0000000	0.1750000
25997.0000000	0.1650000
25998.0000000	0.1700000
25999.0000000	0.1700000
26000.0000000	0.1650000
26001.0000000	0.1700000
26002.0000000	0.1650000
26003.0000000	0.1700000
26004.0000000	0.1700000
26005.0000000	0.1650000
26006.0000000	0.1700000
26007.0000000	0.1700000
26008.0000000	0.1650000
26009.0000000	0.1650000
26010.0000000	0.1550000
26011.0000000	0.1650000
26012.0000000	0.1650000
26013.0000000	0.1600000
26014.0000000	0.1650000
26015.0000000	0.1550000
26016.0000000	0.1700000
26017.0000000	0.1600000
26018.0000000	0.1650000
26019.0000000	0.1600000
26020.0000000	0.1650000
26021.0000000	0.1550000
26022.0000000	0.1600000
26023.0000000	0.1550000
26024.0000000	0.1550000
26025.0000000	0.1650000
26026.0000000	0.1500000
26027.0000000	0.1550000
26028.0000000	0.1600000
26029.0000000	0.1700000
26030.0000000	0.1550000
26031.0000000	0.1600000
26032.0000000	0.1600000
26033.0000000	0.1600000
26034.0000000	0.1600000
26035.0000000	0.1700000
26036.0000000	0.1550000
26037.0000000	0.1600000
26038.0000000	0.1450000
26039.0000000	0.1600000
26040.0000000	0.1650000
26041.0000000	0.1600000
26042.0000000	0.1600000
26043.0000000	0.1600000
26044.0000000	0.1500000
26045.0000000	0.1600000
26046.0000000	0.1650000
26047.0000000	0.1650000
26048.0000000	0.1600000

26049.0000000	0.1550000
26050.0000000	0.1600000
26051.0000000	0.1500000
26052.0000000	0.1650000
26053.0000000	0.1550000
26054.0000000	0.1700000
26055.0000000	0.1550000
26056.0000000	0.1550000
26057.0000000	0.1600000
26058.0000000	0.1666667
26059.0000000	0.1550000
26060.0000000	0.1600000
26061.0000000	0.1650000
26062.0000000	0.1600000
26063.0000000	0.1550000
26064.0000000	0.1500000
26065.0000000	0.1550000
26066.0000000	0.1650000
26067.0000000	0.1550000
26068.0000000	0.1650000
26069.0000000	0.1550000
26070.0000000	0.1450000
26071.0000000	0.1550000
26072.0000000	0.1550000
26073.0000000	0.1600000
26074.0000000	0.1600000
26075.0000000	0.1500000
26076.0000000	0.1650000
26077.0000000	0.1500000
26078.0000000	0.1600000
26079.0000000	0.1550000
26080.0000000	0.1550000
26081.0000000	0.1550000
26082.0000000	0.1550000
26083.0000000	0.1600000
26084.0000000	0.1600000
26085.0000000	0.1600000
26086.0000000	0.1550000
26087.0000000	0.1600000
26088.0000000	0.1600000
26089.0000000	0.1566667
26090.0000000	0.1600000
26091.0000000	0.1550000
26092.0000000	0.1600000
26093.0000000	0.1600000
26094.0000000	0.1600000
26095.0000000	0.1550000
26096.0000000	0.1500000
26097.0000000	0.1550000
26098.0000000	0.1550000
26099.0000000	0.1650000
26100.0000000	0.1650000
26101.0000000	0.1550000
26102.0000000	0.1550000
26103.0000000	0.1400000
26104.0000000	0.1650000
26105.0000000	0.1500000

26106.0000000	0.1650000
26107.0000000	0.1550000
26108.0000000	0.1500000
26109.0000000	0.1550000
26110.0000000	0.1500000
26111.0000000	0.1600000
26112.0000000	0.1450000
26113.0000000	0.1500000
26114.0000000	0.1550000
26115.0000000	0.1550000
26116.0000000	0.1600000
26117.0000000	0.1500000
26118.0000000	0.1500000
26119.0000000	0.1500000
26120.0000000	0.1550000
26121.0000000	0.1550000
26122.0000000	0.1500000
26123.0000000	0.1550000
26124.0000000	0.1500000
26125.0000000	0.1550000
26126.0000000	0.1500000
26127.0000000	0.1550000
26128.0000000	0.1400000
26129.0000000	0.1450000
26130.0000000	0.1300000
26131.0000000	0.1500000
26132.0000000	0.1550000
26133.0000000	0.1450000
26134.0000000	0.1450000
26135.0000000	0.1450000
26136.0000000	0.1400000
26137.0000000	0.1350000
26138.0000000	0.1500000
26139.0000000	0.1450000
26140.0000000	0.1500000
26141.0000000	0.1400000
26142.0000000	0.1400000
26143.0000000	0.1350000
26144.0000000	0.1550000
26145.0000000	0.1450000
26146.0000000	0.1500000
26147.0000000	0.1300000
26148.0000000	0.1450000
26149.0000000	0.1433333
26150.0000000	0.1400000
26151.0000000	0.1400000
26152.0000000	0.1300000
26153.0000000	0.1350000
26154.0000000	0.1350000
26155.0000000	0.1450000
26156.0000000	0.1400000
26157.0000000	0.1450000
26158.0000000	0.1350000
26159.0000000	0.1300000
26160.0000000	0.1350000
26161.0000000	0.1400000
26162.0000000	0.1400000

26163.0000000	0.1350000
26164.0000000	0.1400000
26165.0000000	0.1350000
26166.0000000	0.1350000
26167.0000000	0.1400000
26168.0000000	0.1300000
26169.0000000	0.1400000
26170.0000000	0.1350000
26171.0000000	0.1400000
26172.0000000	0.1450000
26173.0000000	0.1250000
26174.0000000	0.1350000
26175.0000000	0.1250000
26176.0000000	0.1300000
26177.0000000	0.1350000
26178.0000000	0.1350000
26179.0000000	0.1333333
26180.0000000	0.1350000
26181.0000000	0.1400000
26182.0000000	0.1350000
26183.0000000	0.1400000
26184.0000000	0.1300000
26185.0000000	0.1300000
26186.0000000	0.1400000
26187.0000000	0.1350000
26188.0000000	0.1300000
26189.0000000	0.1300000
26190.0000000	0.1450000
26191.0000000	0.1350000
26192.0000000	0.1400000
26193.0000000	0.1350000
26194.0000000	0.1350000
26195.0000000	0.1300000
26196.0000000	0.1350000
26197.0000000	0.1400000
26198.0000000	0.1350000
26199.0000000	0.1400000
26200.0000000	0.1400000
26201.0000000	0.1400000
26202.0000000	0.1300000
26203.0000000	0.1400000
26204.0000000	0.1350000
26205.0000000	0.1350000
26206.0000000	0.1450000
26207.0000000	0.1400000
26208.0000000	0.1400000
26209.0000000	0.1366667
26210.0000000	0.1400000
26211.0000000	0.1400000
26212.0000000	0.1400000
26213.0000000	0.1400000
26214.0000000	0.1400000
26215.0000000	0.1400000
26216.0000000	0.1350000
26217.0000000	0.1400000
26218.0000000	0.1500000
26219.0000000	0.1400000

26220.0000000	0.1500000
26221.0000000	0.1350000
26222.0000000	0.1400000
26223.0000000	0.1400000
26224.0000000	0.1400000
26225.0000000	0.1450000
26226.0000000	0.1400000
26227.0000000	0.1450000
26228.0000000	0.1450000
26229.0000000	0.1400000
26230.0000000	0.1400000
26231.0000000	0.1400000
26232.0000000	0.1400000
26233.0000000	0.1450000
26234.0000000	0.1350000
26235.0000000	0.1500000
26236.0000000	0.1500000
26237.0000000	0.1450000
26238.0000000	0.1500000
26239.0000000	0.1400000
26240.0000000	0.1433333
26241.0000000	0.1400000
26242.0000000	0.1550000
26243.0000000	0.1450000
26244.0000000	0.1400000
26245.0000000	0.1450000
26246.0000000	0.1450000
26247.0000000	0.1450000
26248.0000000	0.1450000
26249.0000000	0.1400000
26250.0000000	0.1500000
26251.0000000	0.1500000
26252.0000000	0.1450000
26253.0000000	0.1400000
26254.0000000	0.1500000
26255.0000000	0.1450000
26256.0000000	0.1500000
26257.0000000	0.1500000
26258.0000000	0.1450000
26259.0000000	0.1500000
26260.0000000	0.1550000
26261.0000000	0.1450000
26262.0000000	0.1450000
26263.0000000	0.1550000
26264.0000000	0.1550000
26265.0000000	0.1500000
26266.0000000	0.1450000
26267.0000000	0.1450000
26268.0000000	0.1600000
26269.0000000	0.1600000
26270.0000000	0.1450000
26271.0000000	0.1500000
26272.0000000	0.1550000
26273.0000000	0.1550000
26274.0000000	0.1550000
26275.0000000	0.1600000
26276.0000000	0.1550000

26277.0000000	0.1550000
26278.0000000	0.1550000
26279.0000000	0.1550000
26280.0000000	0.1500000
26281.0000000	0.1500000
26282.0000000	0.1550000
26283.0000000	0.1600000
26284.0000000	0.1650000
26285.0000000	0.1550000
26286.0000000	0.1550000
26287.0000000	0.1650000
26288.0000000	0.1600000
26289.0000000	0.1600000
26290.0000000	0.1650000
26291.0000000	0.1700000
26292.0000000	0.1650000
26293.0000000	0.1700000
26294.0000000	0.1600000
26295.0000000	0.1600000
26296.0000000	0.1700000
26297.0000000	0.1700000
26298.0000000	0.1600000
26299.0000000	0.1600000
26300.0000000	0.1666667
26301.0000000	0.1750000
26302.0000000	0.1600000
26303.0000000	0.1700000
26304.0000000	0.1750000
26305.0000000	0.1650000
26306.0000000	0.1600000
26307.0000000	0.1650000
26308.0000000	0.1750000
26309.0000000	0.1650000
26310.0000000	0.1800000
26311.0000000	0.1700000
26312.0000000	0.1700000
26313.0000000	0.1800000
26314.0000000	0.1800000
26315.0000000	0.1700000
26316.0000000	0.1700000
26317.0000000	0.1700000
26318.0000000	0.1700000
26319.0000000	0.1850000
26320.0000000	0.1750000
26321.0000000	0.1800000
26322.0000000	0.1800000
26323.0000000	0.1800000
26324.0000000	0.1800000
26325.0000000	0.1800000
26326.0000000	0.1750000
26327.0000000	0.1800000
26328.0000000	0.1800000
26329.0000000	0.1850000
26330.0000000	0.1800000
26331.0000000	0.1750000
26332.0000000	0.1900000
26333.0000000	0.1850000

26334.0000000	0.2000000
26335.0000000	0.1850000
26336.0000000	0.1850000
26337.0000000	0.1950000
26338.0000000	0.1850000
26339.0000000	0.2000000
26340.0000000	0.1900000
26341.0000000	0.1950000
26342.0000000	0.1950000
26343.0000000	0.1950000
26344.0000000	0.1900000
26345.0000000	0.2050000
26346.0000000	0.1900000
26347.0000000	0.2000000
26348.0000000	0.1950000
26349.0000000	0.2050000
26350.0000000	0.1950000
26351.0000000	0.1950000
26352.0000000	0.1950000
26353.0000000	0.2000000
26354.0000000	0.2050000
26355.0000000	0.2050000
26356.0000000	0.2100000
26357.0000000	0.2000000
26358.0000000	0.2100000
26359.0000000	0.2050000
26360.0000000	0.2100000
26361.0000000	0.1950000
26362.0000000	0.2250000
26363.0000000	0.2100000
26364.0000000	0.2150000
26365.0000000	0.2100000
26366.0000000	0.2050000
26367.0000000	0.2200000
26368.0000000	0.2150000
26369.0000000	0.2250000
26370.0000000	0.2200000
26371.0000000	0.2150000
26372.0000000	0.2150000
26373.0000000	0.2200000
26374.0000000	0.2200000
26375.0000000	0.2250000
26376.0000000	0.2100000
26377.0000000	0.2200000
26378.0000000	0.2300000
26379.0000000	0.2250000
26380.0000000	0.2250000
26381.0000000	0.2200000
26382.0000000	0.2200000
26383.0000000	0.2300000
26384.0000000	0.2300000
26385.0000000	0.2300000
26386.0000000	0.2250000
26387.0000000	0.2200000
26388.0000000	0.2350000
26389.0000000	0.2350000
26390.0000000	0.2300000

26391.0000000	0.2300000
26392.0000000	0.2400000
26393.0000000	0.2400000
26394.0000000	0.2300000
26395.0000000	0.2350000
26396.0000000	0.2400000
26397.0000000	0.2350000
26398.0000000	0.2400000
26399.0000000	0.2350000
26400.0000000	0.2400000
26401.0000000	0.2400000
26402.0000000	0.2450000
26403.0000000	0.2400000
26404.0000000	0.2400000
26405.0000000	0.2400000
26406.0000000	0.2400000
26407.0000000	0.2400000
26408.0000000	0.2450000
26409.0000000	0.2450000
26410.0000000	0.2550000
26411.0000000	0.2400000
26412.0000000	0.2450000
26413.0000000	0.2450000
26414.0000000	0.2550000
26415.0000000	0.2600000
26416.0000000	0.2550000
26417.0000000	0.2500000
26418.0000000	0.2400000
26419.0000000	0.2500000
26420.0000000	0.2600000
26421.0000000	0.2600000
26422.0000000	0.2650000
26423.0000000	0.2650000
26424.0000000	0.2600000
26425.0000000	0.2600000
26426.0000000	0.2600000
26427.0000000	0.2600000
26428.0000000	0.2700000
26429.0000000	0.2600000
26430.0000000	0.2700000
26431.0000000	0.2700000
26432.0000000	0.2650000
26433.0000000	0.2650000
26434.0000000	0.2650000
26435.0000000	0.2650000
26436.0000000	0.2700000
26437.0000000	0.2700000
26438.0000000	0.2800000
26439.0000000	0.2750000
26440.0000000	0.2800000
26441.0000000	0.2800000
26442.0000000	0.2700000
26443.0000000	0.2750000
26444.0000000	0.2750000
26445.0000000	0.2850000
26446.0000000	0.2800000
26447.0000000	0.2850000

26448.0000000	0.2850000
26449.0000000	0.2800000
26450.0000000	0.2850000
26451.0000000	0.2833333
26452.0000000	0.2800000
26453.0000000	0.2900000
26454.0000000	0.2950000
26455.0000000	0.2850000
26456.0000000	0.2900000
26457.0000000	0.2800000
26458.0000000	0.2950000
26459.0000000	0.3000000
26460.0000000	0.2900000
26461.0000000	0.3000000
26462.0000000	0.2950000
26463.0000000	0.3000000
26464.0000000	0.3000000
26465.0000000	0.2950000
26466.0000000	0.3000000
26467.0000000	0.3000000
26468.0000000	0.3000000
26469.0000000	0.3000000
26470.0000000	0.3100000
26471.0000000	0.3050000
26472.0000000	0.3000000
26473.0000000	0.3050000
26474.0000000	0.3000000
26475.0000000	0.3000000
26476.0000000	0.3050000
26477.0000000	0.3050000
26478.0000000	0.3100000
26479.0000000	0.3100000
26480.0000000	0.3050000
26481.0000000	0.3066666
26482.0000000	0.3150000
26483.0000000	0.3100000
26484.0000000	0.3050000
26485.0000000	0.3200000
26486.0000000	0.3150000
26487.0000000	0.3150000
26488.0000000	0.3100000
26489.0000000	0.3050000
26490.0000000	0.3150000
26491.0000000	0.3200000
26492.0000000	0.3150000
26493.0000000	0.3200000
26494.0000000	0.3100000
26495.0000000	0.3200000
26496.0000000	0.3150000
26497.0000000	0.3200000
26498.0000000	0.3150000
26499.0000000	0.3200000
26500.0000000	0.3250000
26501.0000000	0.3200000
26502.0000000	0.3300000
26503.0000000	0.3250000
26504.0000000	0.3250000

26505.0000000	0.3200000
26506.0000000	0.3250000
26507.0000000	0.3300000
26508.0000000	0.3250000
26509.0000000	0.3300000
26510.0000000	0.3300000
26511.0000000	0.3266667
26512.0000000	0.3300000
26513.0000000	0.3300000
26514.0000000	0.3300000
26515.0000000	0.3400000
26516.0000000	0.3250000
26517.0000000	0.3250000
26518.0000000	0.3350000
26519.0000000	0.3300000
26520.0000000	0.3350000
26521.0000000	0.3350000
26522.0000000	0.3400000
26523.0000000	0.3450000
26524.0000000	0.3300000
26525.0000000	0.3400000
26526.0000000	0.3350000
26527.0000000	0.3350000
26528.0000000	0.3400000
26529.0000000	0.3500000
26530.0000000	0.3450000
26531.0000000	0.3500000
26532.0000000	0.3450000
26533.0000000	0.3450000
26534.0000000	0.3400000
26535.0000000	0.3450000
26536.0000000	0.3450000
26537.0000000	0.3500000
26538.0000000	0.3450000
26539.0000000	0.3500000
26540.0000000	0.3450000
26541.0000000	0.3433333
26542.0000000	0.3550000
26543.0000000	0.3500000
26544.0000000	0.3450000
26545.0000000	0.3400000
26546.0000000	0.3450000
26547.0000000	0.3450000
26548.0000000	0.3600000
26549.0000000	0.3450000
26550.0000000	0.3500000
26551.0000000	0.3450000
26552.0000000	0.3500000
26553.0000000	0.3500000
26554.0000000	0.3550000
26555.0000000	0.3450000
26556.0000000	0.3600000
26557.0000000	0.3450000
26558.0000000	0.3500000
26559.0000000	0.3500000
26560.0000000	0.3550000
26561.0000000	0.3450000

26562.0000000	0.3550000
26563.0000000	0.3550000
26564.0000000	0.3500000
26565.0000000	0.3500000
26566.0000000	0.3550000
26567.0000000	0.3550000
26568.0000000	0.3500000
26569.0000000	0.3500000
26570.0000000	0.3550000
26571.0000000	0.3500000
26572.0000000	0.3600000
26573.0000000	0.3550000
26574.0000000	0.3600000
26575.0000000	0.3550000
26576.0000000	0.3500000
26577.0000000	0.3550000
26578.0000000	0.3600000
26579.0000000	0.3550000
26580.0000000	0.3600000
26581.0000000	0.3550000
26582.0000000	0.3650000
26583.0000000	0.3500000
26584.0000000	0.3500000
26585.0000000	0.3550000
26586.0000000	0.3600000
26587.0000000	0.3600000
26588.0000000	0.3600000
26589.0000000	0.3600000
26590.0000000	0.3550000
26591.0000000	0.3550000
26592.0000000	0.3550000
26593.0000000	0.3650000
26594.0000000	0.3550000
26595.0000000	0.3550000
26596.0000000	0.3500000
26597.0000000	0.3550000
26598.0000000	0.3550000
26599.0000000	0.3600000
26600.0000000	0.3550000
26601.0000000	0.3533334
26602.0000000	0.3600000
26603.0000000	0.3550000
26604.0000000	0.3650000
26605.0000000	0.3550000
26606.0000000	0.3600000
26607.0000000	0.3500000
26608.0000000	0.3600000
26609.0000000	0.3650000
26610.0000000	0.3600000
26611.0000000	0.3500000
26612.0000000	0.3500000
26613.0000000	0.3600000
26614.0000000	0.3450000
26615.0000000	0.3550000
26616.0000000	0.3500000
26617.0000000	0.3550000
26618.0000000	0.3500000

26619.0000000	0.3500000
26620.0000000	0.3400000
26621.0000000	0.3650000
26622.0000000	0.3550000
26623.0000000	0.3600000
26624.0000000	0.3450000
26625.0000000	0.3550000
26626.0000000	0.3450000
26627.0000000	0.3550000
26628.0000000	0.3500000
26629.0000000	0.3600000
26630.0000000	0.3500000
26631.0000000	0.3500000
26632.0000000	0.3433334
26633.0000000	0.3550000
26634.0000000	0.3500000
26635.0000000	0.3400000
26636.0000000	0.3450000
26637.0000000	0.3500000
26638.0000000	0.3550000
26639.0000000	0.3500000
26640.0000000	0.3550000
26641.0000000	0.3500000
26642.0000000	0.3450000
26643.0000000	0.3500000
26644.0000000	0.3450000
26645.0000000	0.3600000
26646.0000000	0.3450000
26647.0000000	0.3450000
26648.0000000	0.3400000
26649.0000000	0.3450000
26650.0000000	0.3450000
26651.0000000	0.3500000
26652.0000000	0.3500000
26653.0000000	0.3600000
26654.0000000	0.3500000
26655.0000000	0.3450000
26656.0000000	0.3350000
26657.0000000	0.3500000
26658.0000000	0.3550000
26659.0000000	0.3500000
26660.0000000	0.3500000
26661.0000000	0.3400000
26662.0000000	0.3500000
26663.0000000	0.3350000
26664.0000000	0.3550000
26665.0000000	0.3450000
26666.0000000	0.3550000
26667.0000000	0.3450000
26668.0000000	0.3450000
26669.0000000	0.3400000
26670.0000000	0.3450000
26671.0000000	0.3450000
26672.0000000	0.3500000
26673.0000000	0.3450000
26674.0000000	0.3400000
26675.0000000	0.3350000

26676.0000000	0.3400000
26677.0000000	0.3500000
26678.0000000	0.3500000
26679.0000000	0.3400000
26680.0000000	0.3450000
26681.0000000	0.3350000
26682.0000000	0.3350000
26683.0000000	0.3350000
26684.0000000	0.3500000
26685.0000000	0.3450000
26686.0000000	0.3500000
26687.0000000	0.3300000
26688.0000000	0.3350000
26689.0000000	0.3350000
26690.0000000	0.3400000
26691.0000000	0.3450000
26692.0000000	0.3433334
26693.0000000	0.3300000
26694.0000000	0.3400000
26695.0000000	0.3350000
26696.0000000	0.3400000
26697.0000000	0.3400000
26698.0000000	0.3400000
26699.0000000	0.3500000
26700.0000000	0.3400000
26701.0000000	0.3350000
26702.0000000	0.3350000
26703.0000000	0.3400000
26704.0000000	0.3350000
26705.0000000	0.3400000
26706.0000000	0.3400000
26707.0000000	0.3350000
26708.0000000	0.3350000
26709.0000000	0.3350000
26710.0000000	0.3350000
26711.0000000	0.3250000
26712.0000000	0.3300000
26713.0000000	0.3350000
26714.0000000	0.3350000
26715.0000000	0.3350000
26716.0000000	0.3350000
26717.0000000	0.3300000
26718.0000000	0.3300000
26719.0000000	0.3250000
26720.0000000	0.3250000
26721.0000000	0.3300000
26722.0000000	0.3300000
26723.0000000	0.3250000
26724.0000000	0.3250000
26725.0000000	0.3300000
26726.0000000	0.3300000
26727.0000000	0.3250000
26728.0000000	0.3200000
26729.0000000	0.3250000
26730.0000000	0.3300000
26731.0000000	0.3250000
26732.0000000	0.3250000

26733.0000000	0.3200000
26734.0000000	0.3200000
26735.0000000	0.3150000
26736.0000000	0.3250000
26737.0000000	0.3250000
26738.0000000	0.3250000
26739.0000000	0.3300000
26740.0000000	0.3100000
26741.0000000	0.3150000
26742.0000000	0.3100000
26743.0000000	0.3200000
26744.0000000	0.3200000
26745.0000000	0.3200000
26746.0000000	0.3150000
26747.0000000	0.3100000
26748.0000000	0.3100000
26749.0000000	0.3100000
26750.0000000	0.3100000
26751.0000000	0.3050000
26752.0000000	0.3133333
26753.0000000	0.3100000
26754.0000000	0.3050000
26755.0000000	0.3100000
26756.0000000	0.3050000
26757.0000000	0.3150000
26758.0000000	0.3000000
26759.0000000	0.3050000
26760.0000000	0.3000000
26761.0000000	0.3000000
26762.0000000	0.2950000
26763.0000000	0.3050000
26764.0000000	0.3050000
26765.0000000	0.3000000
26766.0000000	0.3050000
26767.0000000	0.3000000
26768.0000000	0.3000000
26769.0000000	0.3050000
26770.0000000	0.3050000
26771.0000000	0.3050000
26772.0000000	0.3000000
26773.0000000	0.2900000
26774.0000000	0.2950000
26775.0000000	0.2900000
26776.0000000	0.2900000
26777.0000000	0.2950000
26778.0000000	0.3000000
26779.0000000	0.2900000
26780.0000000	0.2950000
26781.0000000	0.2850000
26782.0000000	0.2950000
26783.0000000	0.2966667
26784.0000000	0.2900000
26785.0000000	0.2800000
26786.0000000	0.2950000
26787.0000000	0.2950000
26788.0000000	0.2950000
26789.0000000	0.2900000

26790.0000000	0.2850000
26791.0000000	0.2850000
26792.0000000	0.2800000
26793.0000000	0.2750000
26794.0000000	0.2850000
26795.0000000	0.2900000
26796.0000000	0.2750000
26797.0000000	0.2850000
26798.0000000	0.2750000
26799.0000000	0.2700000
26800.0000000	0.2800000
26801.0000000	0.2850000
26802.0000000	0.2750000
26803.0000000	0.2750000
26804.0000000	0.2750000
26805.0000000	0.2700000
26806.0000000	0.2800000
26807.0000000	0.2750000
26808.0000000	0.2750000
26809.0000000	0.2800000
26810.0000000	0.2650000
26811.0000000	0.2800000
26812.0000000	0.2700000
26813.0000000	0.2800000
26814.0000000	0.2700000
26815.0000000	0.2650000
26816.0000000	0.2650000
26817.0000000	0.2700000
26818.0000000	0.2700000
26819.0000000	0.2700000
26820.0000000	0.2700000
26821.0000000	0.2700000
26822.0000000	0.2650000
26823.0000000	0.2700000
26824.0000000	0.2600000
26825.0000000	0.2700000
26826.0000000	0.2700000
26827.0000000	0.2700000
26828.0000000	0.2650000
26829.0000000	0.2700000
26830.0000000	0.2600000
26831.0000000	0.2600000
26832.0000000	0.2600000
26833.0000000	0.2700000
26834.0000000	0.2700000
26835.0000000	0.2700000
26836.0000000	0.2600000
26837.0000000	0.2650000
26838.0000000	0.2650000
26839.0000000	0.2700000
26840.0000000	0.2650000
26841.0000000	0.2650000
26842.0000000	0.2700000
26843.0000000	0.2633333
26844.0000000	0.2650000
26845.0000000	0.2700000
26846.0000000	0.2600000

26847.0000000	0.2700000
26848.0000000	0.2700000
26849.0000000	0.2700000
26850.0000000	0.2650000
26851.0000000	0.2600000
26852.0000000	0.2700000
26853.0000000	0.2650000
26854.0000000	0.2600000
26855.0000000	0.2650000
26856.0000000	0.2700000
26857.0000000	0.2600000
26858.0000000	0.2650000
26859.0000000	0.2700000
26860.0000000	0.2700000
26861.0000000	0.2700000
26862.0000000	0.2600000
26863.0000000	0.2700000
26864.0000000	0.2650000
26865.0000000	0.2700000
26866.0000000	0.2650000
26867.0000000	0.2650000
26868.0000000	0.2700000
26869.0000000	0.2600000
26870.0000000	0.2700000
26871.0000000	0.2600000
26872.0000000	0.2600000
26873.0000000	0.2666667
26874.0000000	0.2750000
26875.0000000	0.2650000
26876.0000000	0.2650000
26877.0000000	0.2650000
26878.0000000	0.2700000
26879.0000000	0.2750000
26880.0000000	0.2700000
26881.0000000	0.2700000
26882.0000000	0.2700000
26883.0000000	0.2700000
26884.0000000	0.2550000
26885.0000000	0.2650000
26886.0000000	0.2650000
26887.0000000	0.2600000
26888.0000000	0.2600000
26889.0000000	0.2650000
26890.0000000	0.2650000
26891.0000000	0.2700000
26892.0000000	0.2600000
26893.0000000	0.2650000
26894.0000000	0.2550000
26895.0000000	0.2600000
26896.0000000	0.2650000
26897.0000000	0.2550000
26898.0000000	0.2650000
26899.0000000	0.2600000
26900.0000000	0.2650000
26901.0000000	0.2650000
26902.0000000	0.2500000
26903.0000000	0.2600000

26904.0000000	0.2633333
26905.0000000	0.2600000
26906.0000000	0.2550000
26907.0000000	0.2600000
26908.0000000	0.2600000
26909.0000000	0.2550000
26910.0000000	0.2600000
26911.0000000	0.2500000
26912.0000000	0.2550000
26913.0000000	0.2600000
26914.0000000	0.2600000
26915.0000000	0.2500000
26916.0000000	0.2500000
26917.0000000	0.2500000
26918.0000000	0.2550000
26919.0000000	0.2500000
26920.0000000	0.2500000
26921.0000000	0.2550000
26922.0000000	0.2600000
26923.0000000	0.2600000
26924.0000000	0.2600000
26925.0000000	0.2550000
26926.0000000	0.2550000
26927.0000000	0.2650000
26928.0000000	0.2600000
26929.0000000	0.2550000
26930.0000000	0.2550000
26931.0000000	0.2500000
26932.0000000	0.2600000
26933.0000000	0.2550000
26934.0000000	0.2500000
26935.0000000	0.2550000
26936.0000000	0.2650000
26937.0000000	0.2600000
26938.0000000	0.2600000
26939.0000000	0.2600000
26940.0000000	0.2450000
26941.0000000	0.2550000
26942.0000000	0.2600000
26943.0000000	0.2500000
26944.0000000	0.2500000
26945.0000000	0.2550000
26946.0000000	0.2600000
26947.0000000	0.2550000
26948.0000000	0.2550000
26949.0000000	0.2500000
26950.0000000	0.2450000
26951.0000000	0.2550000
26952.0000000	0.2500000
26953.0000000	0.2500000
26954.0000000	0.2550000
26955.0000000	0.2500000
26956.0000000	0.2500000
26957.0000000	0.2500000
26958.0000000	0.2500000
26959.0000000	0.2450000
26960.0000000	0.2500000

26961.0000000	0.2500000
26962.0000000	0.2600000
26963.0000000	0.2466667
26964.0000000	0.2450000
26965.0000000	0.2550000
26966.0000000	0.2550000
26967.0000000	0.2550000
26968.0000000	0.2500000
26969.0000000	0.2450000
26970.0000000	0.2450000
26971.0000000	0.2450000
26972.0000000	0.2550000
26973.0000000	0.2500000
26974.0000000	0.2550000
26975.0000000	0.2450000
26976.0000000	0.2450000
26977.0000000	0.2350000
26978.0000000	0.2450000
26979.0000000	0.2450000
26980.0000000	0.2400000
26981.0000000	0.2500000
26982.0000000	0.2500000
26983.0000000	0.2500000
26984.0000000	0.2450000
26985.0000000	0.2450000
26986.0000000	0.2350000
26987.0000000	0.2500000
26988.0000000	0.2400000
26989.0000000	0.2450000
26990.0000000	0.2450000
26991.0000000	0.2450000
26992.0000000	0.2400000
26993.0000000	0.2450000
26994.0000000	0.2466667
26995.0000000	0.2350000
26996.0000000	0.2400000
26997.0000000	0.2400000
26998.0000000	0.2450000
26999.0000000	0.2450000
27000.0000000	0.2500000
27001.0000000	0.2350000
27002.0000000	0.2450000
27003.0000000	0.2300000
27004.0000000	0.2450000
27005.0000000	0.2350000
27006.0000000	0.2500000
27007.0000000	0.2350000
27008.0000000	0.2350000
27009.0000000	0.2300000
27010.0000000	0.2300000
27011.0000000	0.2450000
27012.0000000	0.2350000
27013.0000000	0.2450000
27014.0000000	0.2350000
27015.0000000	0.2450000
27016.0000000	0.2300000
27017.0000000	0.2450000

27018.0000000	0.2350000
27019.0000000	0.2350000
27020.0000000	0.2350000
27021.0000000	0.2400000
27022.0000000	0.2350000
27023.0000000	0.2350000
27024.0000000	0.2350000
27025.0000000	0.2333333
27026.0000000	0.2350000
27027.0000000	0.2350000
27028.0000000	0.2350000
27029.0000000	0.2400000
27030.0000000	0.2450000
27031.0000000	0.2400000
27032.0000000	0.2350000
27033.0000000	0.2350000
27034.0000000	0.2350000
27035.0000000	0.2400000
27036.0000000	0.2350000
27037.0000000	0.2350000
27038.0000000	0.2350000
27039.0000000	0.2400000
27040.0000000	0.2300000
27041.0000000	0.2350000
27042.0000000	0.2350000
27043.0000000	0.2400000
27044.0000000	0.2400000
27045.0000000	0.2450000
27046.0000000	0.2300000
27047.0000000	0.2400000
27048.0000000	0.2350000
27049.0000000	0.2400000
27050.0000000	0.2350000
27051.0000000	0.2450000
27052.0000000	0.2350000
27053.0000000	0.2400000
27054.0000000	0.2300000
27055.0000000	0.2350000
27056.0000000	0.2400000
27057.0000000	0.2350000
27058.0000000	0.2400000
27059.0000000	0.2350000
27060.0000000	0.2400000
27061.0000000	0.2350000
27062.0000000	0.2450000
27063.0000000	0.2400000
27064.0000000	0.2450000
27065.0000000	0.2350000
27066.0000000	0.2350000
27067.0000000	0.2350000
27068.0000000	0.2400000
27069.0000000	0.2350000
27070.0000000	0.2450000
27071.0000000	0.2350000
27072.0000000	0.2350000
27073.0000000	0.2300000
27074.0000000	0.2400000

27075.0000000	0.2400000
27076.0000000	0.2500000
27077.0000000	0.2350000
27078.0000000	0.2350000
27079.0000000	0.2300000
27080.0000000	0.2450000
27081.0000000	0.2400000
27082.0000000	0.2400000
27083.0000000	0.2400000
27084.0000000	0.2300000
27085.0000000	0.2400000
27086.0000000	0.2450000
27087.0000000	0.2400000
27088.0000000	0.2300000
27089.0000000	0.2350000
27090.0000000	0.2350000
27091.0000000	0.2350000
27092.0000000	0.2450000
27093.0000000	0.2400000
27094.0000000	0.2350000
27095.0000000	0.2300000
27096.0000000	0.2300000
27097.0000000	0.2400000
27098.0000000	0.2400000
27099.0000000	0.2400000
27100.0000000	0.2350000
27101.0000000	0.2400000
27102.0000000	0.2300000
27103.0000000	0.2350000
27104.0000000	0.2400000
27105.0000000	0.2300000
27106.0000000	0.2350000
27107.0000000	0.2250000
27108.0000000	0.2350000
27109.0000000	0.2350000
27110.0000000	0.2300000
27111.0000000	0.2300000
27112.0000000	0.2300000
27113.0000000	0.2250000
27114.0000000	0.2350000
27115.0000000	0.2300000
27116.0000000	0.2250000
27117.0000000	0.2300000
27118.0000000	0.2350000
27119.0000000	0.2200000
27120.0000000	0.2350000
27121.0000000	0.2250000
27122.0000000	0.2400000
27123.0000000	0.2350000
27124.0000000	0.2300000
27125.0000000	0.2300000
27126.0000000	0.2200000
27127.0000000	0.2250000
27128.0000000	0.2400000
27129.0000000	0.2250000
27130.0000000	0.2350000
27131.0000000	0.2400000

27132.0000000	0.2250000
27133.0000000	0.2350000
27134.0000000	0.2350000
27135.0000000	0.2300000
27136.0000000	0.2300000
27137.0000000	0.2300000
27138.0000000	0.2300000
27139.0000000	0.2400000
27140.0000000	0.2250000
27141.0000000	0.2300000
27142.0000000	0.2300000
27143.0000000	0.2300000
27144.0000000	0.2400000
27145.0000000	0.2300000
27146.0000000	0.2350000
27147.0000000	0.2350000
27148.0000000	0.2250000
27149.0000000	0.2300000
27150.0000000	0.2200000
27151.0000000	0.2250000
27152.0000000	0.2350000
27153.0000000	0.2300000
27154.0000000	0.2250000
27155.0000000	0.2250000
27156.0000000	0.2300000
27157.0000000	0.2350000
27158.0000000	0.2350000
27159.0000000	0.2300000
27160.0000000	0.2350000
27161.0000000	0.2300000
27162.0000000	0.2400000
27163.0000000	0.2300000
27164.0000000	0.2300000
27165.0000000	0.2300000
27166.0000000	0.2400000
27167.0000000	0.2300000
27168.0000000	0.2400000
27169.0000000	0.2250000
27170.0000000	0.2300000
27171.0000000	0.2350000
27172.0000000	0.2250000
27173.0000000	0.2450000
27174.0000000	0.2350000
27175.0000000	0.2366667
27176.0000000	0.2400000
27177.0000000	0.2300000
27178.0000000	0.2300000
27179.0000000	0.2350000
27180.0000000	0.2300000
27181.0000000	0.2350000
27182.0000000	0.2400000
27183.0000000	0.2300000
27184.0000000	0.2350000
27185.0000000	0.2300000
27186.0000000	0.2300000
27187.0000000	0.2350000
27188.0000000	0.2400000

27189.0000000	0.2400000
27190.0000000	0.2350000
27191.0000000	0.2350000
27192.0000000	0.2350000
27193.0000000	0.2400000
27194.0000000	0.2350000
27195.0000000	0.2450000
27196.0000000	0.2350000
27197.0000000	0.2250000
27198.0000000	0.2400000
27199.0000000	0.2350000
27200.0000000	0.2350000
27201.0000000	0.2400000
27202.0000000	0.2300000
27203.0000000	0.2350000
27204.0000000	0.2350000
27205.0000000	0.2400000
27206.0000000	0.2450000
27207.0000000	0.2350000
27208.0000000	0.2400000
27209.0000000	0.2400000
27210.0000000	0.2450000
27211.0000000	0.2400000
27212.0000000	0.2350000
27213.0000000	0.2350000
27214.0000000	0.2450000
27215.0000000	0.2400000
27216.0000000	0.2350000
27217.0000000	0.2400000
27218.0000000	0.2400000
27219.0000000	0.2450000
27220.0000000	0.2400000
27221.0000000	0.2400000
27222.0000000	0.2400000
27223.0000000	0.2400000
27224.0000000	0.2400000
27225.0000000	0.2450000
27226.0000000	0.2400000
27227.0000000	0.2500000
27228.0000000	0.2400000
27229.0000000	0.2450000
27230.0000000	0.2500000
27231.0000000	0.2400000
27232.0000000	0.2550000
27233.0000000	0.2450000
27234.0000000	0.2450000
27235.0000000	0.2450000
27236.0000000	0.2466667
27237.0000000	0.2450000
27238.0000000	0.2450000
27239.0000000	0.2500000
27240.0000000	0.2400000
27241.0000000	0.2500000
27242.0000000	0.2550000
27243.0000000	0.2450000
27244.0000000	0.2550000
27245.0000000	0.2550000

27246.0000000	0.2550000
27247.0000000	0.2550000
27248.0000000	0.2500000
27249.0000000	0.2550000
27250.0000000	0.2500000
27251.0000000	0.2550000
27252.0000000	0.2600000
27253.0000000	0.2600000
27254.0000000	0.2450000
27255.0000000	0.2650000
27256.0000000	0.2600000
27257.0000000	0.2600000
27258.0000000	0.2600000
27259.0000000	0.2600000
27260.0000000	0.2650000
27261.0000000	0.2700000
27262.0000000	0.2650000
27263.0000000	0.2650000
27264.0000000	0.2700000
27265.0000000	0.2700000
27266.0000000	0.2600000
27267.0000000	0.2700000
27268.0000000	0.2700000
27269.0000000	0.2700000
27270.0000000	0.2700000
27271.0000000	0.2650000
27272.0000000	0.2700000
27273.0000000	0.2700000
27274.0000000	0.2750000
27275.0000000	0.2800000
27276.0000000	0.2800000
27277.0000000	0.2700000
27278.0000000	0.2850000
27279.0000000	0.2800000
27280.0000000	0.2700000
27281.0000000	0.2750000
27282.0000000	0.2850000
27283.0000000	0.2850000
27284.0000000	0.2800000
27285.0000000	0.2850000
27286.0000000	0.2900000
27287.0000000	0.2900000
27288.0000000	0.2800000
27289.0000000	0.2900000
27290.0000000	0.2900000
27291.0000000	0.2900000
27292.0000000	0.2900000
27293.0000000	0.2900000
27294.0000000	0.2950000
27295.0000000	0.2900000
27296.0000000	0.2950000
27297.0000000	0.3050000
27298.0000000	0.2950000
27299.0000000	0.2950000
27300.0000000	0.3000000
27301.0000000	0.3000000
27302.0000000	0.3000000

27303.0000000	0.3000000
27304.0000000	0.3000000
27305.0000000	0.3000000
27306.0000000	0.3000000
27307.0000000	0.3050000
27308.0000000	0.2950000
27309.0000000	0.3050000
27310.0000000	0.3050000
27311.0000000	0.3050000
27312.0000000	0.3050000
27313.0000000	0.3050000
27314.0000000	0.3100000
27315.0000000	0.3050000
27316.0000000	0.3100000
27317.0000000	0.3150000
27318.0000000	0.3050000
27319.0000000	0.3100000
27320.0000000	0.3050000
27321.0000000	0.3250000
27322.0000000	0.3150000
27323.0000000	0.3100000
27324.0000000	0.3150000
27325.0000000	0.3200000
27326.0000000	0.3200000
27327.0000000	0.3150000
27328.0000000	0.3200000
27329.0000000	0.3100000
27330.0000000	0.3200000
27331.0000000	0.3250000
27332.0000000	0.3250000
27333.0000000	0.3250000
27334.0000000	0.3250000
27335.0000000	0.3200000
27336.0000000	0.3250000
27337.0000000	0.3200000
27338.0000000	0.3200000
27339.0000000	0.3200000
27340.0000000	0.3250000
27341.0000000	0.3250000
27342.0000000	0.3300000
27343.0000000	0.3300000
27344.0000000	0.3200000
27345.0000000	0.3250000
27346.0000000	0.3350000
27347.0000000	0.3250000
27348.0000000	0.3300000
27349.0000000	0.3200000
27350.0000000	0.3300000
27351.0000000	0.3300000
27352.0000000	0.3300000
27353.0000000	0.3300000
27354.0000000	0.3300000
27355.0000000	0.3350000
27356.0000000	0.3300000
27357.0000000	0.3433333
27358.0000000	0.3200000
27359.0000000	0.3450000

27360.0000000	0.3350000
27361.0000000	0.3400000
27362.0000000	0.3350000
27363.0000000	0.3300000
27364.0000000	0.3350000
27365.0000000	0.3350000
27366.0000000	0.3400000
27367.0000000	0.3350000
27368.0000000	0.3300000
27369.0000000	0.3400000
27370.0000000	0.3300000
27371.0000000	0.3500000
27372.0000000	0.3450000
27373.0000000	0.3350000
27374.0000000	0.3350000
27375.0000000	0.3400000
27376.0000000	0.3450000
27377.0000000	0.3400000
27378.0000000	0.3350000
27379.0000000	0.3400000
27380.0000000	0.3350000
27381.0000000	0.3400000
27382.0000000	0.3400000
27383.0000000	0.3400000
27384.0000000	0.3450000
27385.0000000	0.3350000
27386.0000000	0.3466667
27387.0000000	0.3350000
27388.0000000	0.3300000
27389.0000000	0.3400000
27390.0000000	0.3400000
27391.0000000	0.3400000
27392.0000000	0.3350000
27393.0000000	0.3300000
27394.0000000	0.3350000
27395.0000000	0.3300000
27396.0000000	0.3400000
27397.0000000	0.3400000
27398.0000000	0.3350000
27399.0000000	0.3450000
27400.0000000	0.3300000
27401.0000000	0.3350000
27402.0000000	0.3400000
27403.0000000	0.3400000
27404.0000000	0.3350000
27405.0000000	0.3350000
27406.0000000	0.3300000
27407.0000000	0.3300000
27408.0000000	0.3350000
27409.0000000	0.3300000
27410.0000000	0.3350000
27411.0000000	0.3300000
27412.0000000	0.3350000
27413.0000000	0.3400000
27414.0000000	0.3300000
27415.0000000	0.3250000
27416.0000000	0.3233334

27417.0000000	0.3200000
27418.0000000	0.3250000
27419.0000000	0.3250000
27420.0000000	0.3200000
27421.0000000	0.3200000
27422.0000000	0.3250000
27423.0000000	0.3250000
27424.0000000	0.3200000
27425.0000000	0.3250000
27426.0000000	0.3200000
27427.0000000	0.3200000
27428.0000000	0.3200000
27429.0000000	0.3150000
27430.0000000	0.3150000
27431.0000000	0.3200000
27432.0000000	0.3200000
27433.0000000	0.3250000
27434.0000000	0.3150000
27435.0000000	0.3100000
27436.0000000	0.3150000
27437.0000000	0.3100000
27438.0000000	0.3200000
27439.0000000	0.3200000
27440.0000000	0.3100000
27441.0000000	0.3100000
27442.0000000	0.3100000
27443.0000000	0.3100000
27444.0000000	0.3050000
27445.0000000	0.3100000
27446.0000000	0.3050000
27447.0000000	0.3000000
27448.0000000	0.3050000
27449.0000000	0.3000000
27450.0000000	0.3050000
27451.0000000	0.3000000
27452.0000000	0.3050000
27453.0000000	0.3000000
27454.0000000	0.3000000
27455.0000000	0.3050000
27456.0000000	0.2950000
27457.0000000	0.3050000
27458.0000000	0.3000000
27459.0000000	0.3000000
27460.0000000	0.2900000
27461.0000000	0.2850000
27462.0000000	0.2950000
27463.0000000	0.2950000
27464.0000000	0.2950000
27465.0000000	0.2900000
27466.0000000	0.2950000
27467.0000000	0.2850000
27468.0000000	0.2850000
27469.0000000	0.2900000
27470.0000000	0.2900000
27471.0000000	0.2850000
27472.0000000	0.2850000
27473.0000000	0.2800000

27474.0000000	0.2850000
27475.0000000	0.2750000
27476.0000000	0.2800000
27477.0000000	0.2800000
27478.0000000	0.2750000
27479.0000000	0.2750000
27480.0000000	0.2800000
27481.0000000	0.2850000
27482.0000000	0.2750000
27483.0000000	0.2750000
27484.0000000	0.2750000
27485.0000000	0.2750000
27486.0000000	0.2700000
27487.0000000	0.2650000
27488.0000000	0.2700000
27489.0000000	0.2700000
27490.0000000	0.2750000
27491.0000000	0.2700000
27492.0000000	0.2600000
27493.0000000	0.2600000
27494.0000000	0.2650000
27495.0000000	0.2550000
27496.0000000	0.2700000
27497.0000000	0.2650000
27498.0000000	0.2550000
27499.0000000	0.2600000
27500.0000000	0.2600000
27501.0000000	0.2600000
27502.0000000	0.2600000
27503.0000000	0.2650000
27504.0000000	0.2550000
27505.0000000	0.2600000
27506.0000000	0.2500000
27507.0000000	0.2500000
27508.0000000	0.2500000
27509.0000000	0.2400000
27510.0000000	0.2550000
27511.0000000	0.2450000
27512.0000000	0.2450000
27513.0000000	0.2400000
27514.0000000	0.2450000
27515.0000000	0.2450000
27516.0000000	0.2450000
27517.0000000	0.2450000
27518.0000000	0.2400000
27519.0000000	0.2450000
27520.0000000	0.2350000
27521.0000000	0.2400000
27522.0000000	0.2400000
27523.0000000	0.2400000
27524.0000000	0.2400000
27525.0000000	0.2350000
27526.0000000	0.2300000
27527.0000000	0.2400000
27528.0000000	0.2350000
27529.0000000	0.2300000
27530.0000000	0.2300000

27531.0000000	0.2350000
27532.0000000	0.2400000
27533.0000000	0.2350000
27534.0000000	0.2350000
27535.0000000	0.2300000
27536.0000000	0.2250000
27537.0000000	0.2366667
27538.0000000	0.2250000
27539.0000000	0.2350000
27540.0000000	0.2300000
27541.0000000	0.2250000
27542.0000000	0.2300000
27543.0000000	0.2350000
27544.0000000	0.2250000
27545.0000000	0.2250000
27546.0000000	0.2250000
27547.0000000	0.2250000
27548.0000000	0.2250000
27549.0000000	0.2350000
27550.0000000	0.2250000
27551.0000000	0.2300000
27552.0000000	0.2250000
27553.0000000	0.2300000
27554.0000000	0.2150000
27555.0000000	0.2250000
27556.0000000	0.2250000
27557.0000000	0.2250000
27558.0000000	0.2300000
27559.0000000	0.2250000
27560.0000000	0.2300000
27561.0000000	0.2200000
27562.0000000	0.2200000
27563.0000000	0.2150000
27564.0000000	0.2200000
27565.0000000	0.2200000
27566.0000000	0.2200000
27567.0000000	0.2200000
27568.0000000	0.2133333
27569.0000000	0.2250000
27570.0000000	0.2150000
27571.0000000	0.2250000
27572.0000000	0.2200000
27573.0000000	0.2200000
27574.0000000	0.2100000
27575.0000000	0.2100000
27576.0000000	0.2150000
27577.0000000	0.2250000
27578.0000000	0.2150000
27579.0000000	0.2200000
27580.0000000	0.2150000
27581.0000000	0.2200000
27582.0000000	0.2200000
27583.0000000	0.2250000
27584.0000000	0.2150000
27585.0000000	0.2200000
27586.0000000	0.2100000
27587.0000000	0.2150000

27588.0000000	0.2150000
27589.0000000	0.2250000
27590.0000000	0.2200000
27591.0000000	0.2200000
27592.0000000	0.2100000
27593.0000000	0.2150000
27594.0000000	0.2100000
27595.0000000	0.2250000
27596.0000000	0.2150000
27597.0000000	0.2150000
27598.0000000	0.2100000
27599.0000000	0.2200000
27600.0000000	0.2150000
27601.0000000	0.2250000
27602.0000000	0.2150000
27603.0000000	0.2150000
27604.0000000	0.2100000
27605.0000000	0.2150000
27606.0000000	0.2200000
27607.0000000	0.2200000
27608.0000000	0.2250000
27609.0000000	0.2050000
27610.0000000	0.2150000
27611.0000000	0.2100000
27612.0000000	0.2200000
27613.0000000	0.2200000
27614.0000000	0.2200000
27615.0000000	0.2150000
27616.0000000	0.2150000
27617.0000000	0.2200000
27618.0000000	0.2150000
27619.0000000	0.2200000
27620.0000000	0.2100000
27621.0000000	0.2150000
27622.0000000	0.2200000
27623.0000000	0.2200000
27624.0000000	0.2200000
27625.0000000	0.2150000
27626.0000000	0.2150000
27627.0000000	0.2200000
27628.0000000	0.2166667
27629.0000000	0.2150000
27630.0000000	0.2100000
27631.0000000	0.2150000
27632.0000000	0.2200000
27633.0000000	0.2150000
27634.0000000	0.2200000
27635.0000000	0.2150000
27636.0000000	0.2100000
27637.0000000	0.2200000
27638.0000000	0.2100000
27639.0000000	0.2150000
27640.0000000	0.2100000
27641.0000000	0.2150000
27642.0000000	0.2150000
27643.0000000	0.2200000
27644.0000000	0.2050000

27645.0000000	0.2200000
27646.0000000	0.2200000
27647.0000000	0.2200000
27648.0000000	0.2150000
27649.0000000	0.2150000
27650.0000000	0.2150000
27651.0000000	0.2150000
27652.0000000	0.2150000
27653.0000000	0.2100000
27654.0000000	0.2150000
27655.0000000	0.2100000
27656.0000000	0.2150000
27657.0000000	0.2150000
27658.0000000	0.2100000
27659.0000000	0.2200000
27660.0000000	0.2200000
27661.0000000	0.2250000
27662.0000000	0.2200000
27663.0000000	0.2100000
27664.0000000	0.2150000
27665.0000000	0.2150000
27666.0000000	0.2050000
27667.0000000	0.2150000
27668.0000000	0.2100000
27669.0000000	0.2200000
27670.0000000	0.2100000
27671.0000000	0.2150000
27672.0000000	0.2100000
27673.0000000	0.2150000
27674.0000000	0.2150000
27675.0000000	0.2050000
27676.0000000	0.2050000
27677.0000000	0.2100000
27678.0000000	0.2150000
27679.0000000	0.2200000
27680.0000000	0.2200000
27681.0000000	0.2100000
27682.0000000	0.2150000
27683.0000000	0.2100000
27684.0000000	0.2150000
27685.0000000	0.2150000
27686.0000000	0.2100000
27687.0000000	0.2150000
27688.0000000	0.2133333
27689.0000000	0.2100000
27690.0000000	0.2100000
27691.0000000	0.2100000
27692.0000000	0.2100000
27693.0000000	0.2150000
27694.0000000	0.2200000
27695.0000000	0.2100000
27696.0000000	0.2100000
27697.0000000	0.2150000
27698.0000000	0.2050000
27699.0000000	0.2100000
27700.0000000	0.2100000
27701.0000000	0.2100000

27702.0000000	0.2100000
27703.0000000	0.2150000
27704.0000000	0.2000000
27705.0000000	0.2100000
27706.0000000	0.2100000
27707.0000000	0.2050000
27708.0000000	0.2100000
27709.0000000	0.2100000
27710.0000000	0.1950000
27711.0000000	0.2100000
27712.0000000	0.2150000
27713.0000000	0.2050000
27714.0000000	0.2050000
27715.0000000	0.2200000
27716.0000000	0.2050000
27717.0000000	0.2050000
27718.0000000	0.2100000
27719.0000000	0.2033333
27720.0000000	0.2050000
27721.0000000	0.2050000
27722.0000000	0.2100000
27723.0000000	0.2050000
27724.0000000	0.2050000
27725.0000000	0.2050000
27726.0000000	0.2050000
27727.0000000	0.2000000
27728.0000000	0.2050000
27729.0000000	0.2100000
27730.0000000	0.2100000
27731.0000000	0.2050000
27732.0000000	0.2050000
27733.0000000	0.2000000
27734.0000000	0.2050000
27735.0000000	0.2050000
27736.0000000	0.2000000
27737.0000000	0.2000000
27738.0000000	0.2050000
27739.0000000	0.2000000
27740.0000000	0.2000000
27741.0000000	0.2050000
27742.0000000	0.1950000
27743.0000000	0.2000000
27744.0000000	0.1950000
27745.0000000	0.1950000
27746.0000000	0.2000000
27747.0000000	0.2000000
27748.0000000	0.2033333
27749.0000000	0.2000000
27750.0000000	0.1900000
27751.0000000	0.2000000
27752.0000000	0.2000000
27753.0000000	0.1950000
27754.0000000	0.2000000
27755.0000000	0.2050000
27756.0000000	0.2000000
27757.0000000	0.2000000
27758.0000000	0.2000000

27759.0000000	0.1950000
27760.0000000	0.2000000
27761.0000000	0.2000000
27762.0000000	0.2050000
27763.0000000	0.2000000
27764.0000000	0.2000000
27765.0000000	0.1900000
27766.0000000	0.2000000
27767.0000000	0.2000000
27768.0000000	0.2050000
27769.0000000	0.2050000
27770.0000000	0.2050000
27771.0000000	0.2000000
27772.0000000	0.1900000
27773.0000000	0.2050000
27774.0000000	0.2000000
27775.0000000	0.1950000
27776.0000000	0.2000000
27777.0000000	0.1900000
27778.0000000	0.1950000
27779.0000000	0.1900000
27780.0000000	0.2000000
27781.0000000	0.1950000
27782.0000000	0.2000000
27783.0000000	0.2000000
27784.0000000	0.1850000
27785.0000000	0.1950000
27786.0000000	0.2000000
27787.0000000	0.1800000
27788.0000000	0.1950000
27789.0000000	0.1900000
27790.0000000	0.1900000
27791.0000000	0.1950000
27792.0000000	0.1950000
27793.0000000	0.1900000
27794.0000000	0.1900000
27795.0000000	0.1800000
27796.0000000	0.1850000
27797.0000000	0.1950000
27798.0000000	0.1850000
27799.0000000	0.1850000
27800.0000000	0.1900000
27801.0000000	0.1850000
27802.0000000	0.2000000
27803.0000000	0.1950000
27804.0000000	0.1750000
27805.0000000	0.1850000
27806.0000000	0.1900000
27807.0000000	0.1800000
27808.0000000	0.1900000
27809.0000000	0.1833333
27810.0000000	0.1900000
27811.0000000	0.1950000
27812.0000000	0.1750000
27813.0000000	0.1850000
27814.0000000	0.1750000
27815.0000000	0.1850000

27816.0000000	0.1950000
27817.0000000	0.1800000
27818.0000000	0.1850000
27819.0000000	0.1800000
27820.0000000	0.1850000
27821.0000000	0.1800000
27822.0000000	0.1750000
27823.0000000	0.1850000
27824.0000000	0.1800000
27825.0000000	0.1850000
27826.0000000	0.1900000
27827.0000000	0.1850000
27828.0000000	0.1900000
27829.0000000	0.1750000
27830.0000000	0.1850000
27831.0000000	0.1850000
27832.0000000	0.1900000
27833.0000000	0.1850000
27834.0000000	0.1750000
27835.0000000	0.1700000
27836.0000000	0.1850000
27837.0000000	0.1900000
27838.0000000	0.1800000
27839.0000000	0.1833333
27840.0000000	0.1750000
27841.0000000	0.1800000
27842.0000000	0.1750000
27843.0000000	0.1800000
27844.0000000	0.1800000
27845.0000000	0.1750000
27846.0000000	0.1850000
27847.0000000	0.1800000
27848.0000000	0.1800000
27849.0000000	0.1750000
27850.0000000	0.1750000
27851.0000000	0.1750000
27852.0000000	0.1800000
27853.0000000	0.1800000
27854.0000000	0.1850000
27855.0000000	0.1800000
27856.0000000	0.1800000
27857.0000000	0.1800000
27858.0000000	0.1750000
27859.0000000	0.1800000
27860.0000000	0.1800000
27861.0000000	0.1850000
27862.0000000	0.1850000
27863.0000000	0.1800000
27864.0000000	0.1750000
27865.0000000	0.1650000
27866.0000000	0.1800000
27867.0000000	0.1750000
27868.0000000	0.1850000
27869.0000000	0.1766667
27870.0000000	0.1750000
27871.0000000	0.1800000
27872.0000000	0.1800000

27873.0000000	0.1800000
27874.0000000	0.1700000
27875.0000000	0.1750000
27876.0000000	0.1800000
27877.0000000	0.1750000
27878.0000000	0.1800000
27879.0000000	0.1750000
27880.0000000	0.1800000
27881.0000000	0.1700000
27882.0000000	0.1850000
27883.0000000	0.1750000
27884.0000000	0.1700000
27885.0000000	0.1750000
27886.0000000	0.1750000
27887.0000000	0.1850000
27888.0000000	0.1800000
27889.0000000	0.1800000
27890.0000000	0.1800000
27891.0000000	0.1700000
27892.0000000	0.1750000
27893.0000000	0.1750000
27894.0000000	0.1800000
27895.0000000	0.1750000
27896.0000000	0.1700000
27897.0000000	0.1800000
27898.0000000	0.1800000
27899.0000000	0.1800000
27900.0000000	0.1733333
27901.0000000	0.1800000
27902.0000000	0.1800000
27903.0000000	0.1850000
27904.0000000	0.1750000
27905.0000000	0.1850000
27906.0000000	0.1750000
27907.0000000	0.1800000
27908.0000000	0.1850000
27909.0000000	0.1750000
27910.0000000	0.1800000
27911.0000000	0.1800000
27912.0000000	0.1800000
27913.0000000	0.1750000
27914.0000000	0.1850000
27915.0000000	0.1650000
27916.0000000	0.1850000
27917.0000000	0.1750000
27918.0000000	0.1800000
27919.0000000	0.1750000
27920.0000000	0.1800000
27921.0000000	0.1850000
27922.0000000	0.1850000
27923.0000000	0.1800000
27924.0000000	0.1750000
27925.0000000	0.1900000
27926.0000000	0.1750000
27927.0000000	0.1850000
27928.0000000	0.1750000
27929.0000000	0.1800000

27930.0000000	0.1866667
27931.0000000	0.1800000
27932.0000000	0.1800000
27933.0000000	0.1850000
27934.0000000	0.1800000
27935.0000000	0.1800000
27936.0000000	0.1850000
27937.0000000	0.1800000
27938.0000000	0.1800000
27939.0000000	0.1750000
27940.0000000	0.1800000
27941.0000000	0.1900000
27942.0000000	0.1850000
27943.0000000	0.1800000
27944.0000000	0.1950000
27945.0000000	0.1850000
27946.0000000	0.1850000
27947.0000000	0.1850000
27948.0000000	0.1800000
27949.0000000	0.1900000
27950.0000000	0.1850000
27951.0000000	0.1850000
27952.0000000	0.1900000
27953.0000000	0.1850000
27954.0000000	0.1900000
27955.0000000	0.1800000
27956.0000000	0.1900000
27957.0000000	0.1800000
27958.0000000	0.1750000
27959.0000000	0.1900000
27960.0000000	0.1866667
27961.0000000	0.1800000
27962.0000000	0.1800000
27963.0000000	0.1850000
27964.0000000	0.1900000
27965.0000000	0.1850000
27966.0000000	0.1850000
27967.0000000	0.1900000
27968.0000000	0.1900000
27969.0000000	0.1850000
27970.0000000	0.1950000
27971.0000000	0.1900000
27972.0000000	0.1850000
27973.0000000	0.1950000
27974.0000000	0.1900000
27975.0000000	0.1950000
27976.0000000	0.1900000
27977.0000000	0.1950000
27978.0000000	0.1850000
27979.0000000	0.1900000
27980.0000000	0.1850000
27981.0000000	0.1850000
27982.0000000	0.1850000
27983.0000000	0.1900000
27984.0000000	0.1950000
27985.0000000	0.1800000
27986.0000000	0.1800000

27987.0000000	0.1800000
27988.0000000	0.1950000
27989.0000000	0.1850000
27990.0000000	0.1866667
27991.0000000	0.1900000
27992.0000000	0.1850000
27993.0000000	0.1900000
27994.0000000	0.1850000
27995.0000000	0.1850000
27996.0000000	0.1900000
27997.0000000	0.1800000
27998.0000000	0.1850000
27999.0000000	0.1900000
28000.0000000	0.1850000
28001.0000000	0.1850000
28002.0000000	0.1750000
28003.0000000	0.1850000
28004.0000000	0.1850000
28005.0000000	0.1800000
28006.0000000	0.1850000
28007.0000000	0.1750000
28008.0000000	0.1800000
28009.0000000	0.1800000
28010.0000000	0.1850000
28011.0000000	0.1800000
28012.0000000	0.1850000
28013.0000000	0.1800000
28014.0000000	0.1800000
28015.0000000	0.1750000
28016.0000000	0.1700000
28017.0000000	0.1700000
28018.0000000	0.1700000
28019.0000000	0.1850000
28020.0000000	0.1766667
28021.0000000	0.1700000
28022.0000000	0.1750000
28023.0000000	0.1750000
28024.0000000	0.1750000
28025.0000000	0.1750000
28026.0000000	0.1700000
28027.0000000	0.1750000
28028.0000000	0.1750000
28029.0000000	0.1750000
28030.0000000	0.1750000
28031.0000000	0.1650000
28032.0000000	0.1750000
28033.0000000	0.1800000
28034.0000000	0.1750000
28035.0000000	0.1800000
28036.0000000	0.1650000
28037.0000000	0.1750000
28038.0000000	0.1700000
28039.0000000	0.1750000
28040.0000000	0.1700000
28041.0000000	0.1750000
28042.0000000	0.1750000
28043.0000000	0.1750000

28044.0000000	0.1750000
28045.0000000	0.1650000
28046.0000000	0.1700000
28047.0000000	0.1650000
28048.0000000	0.1650000
28049.0000000	0.1800000
28050.0000000	0.1750000
28051.0000000	0.1733333
28052.0000000	0.1700000
28053.0000000	0.1750000
28054.0000000	0.1750000
28055.0000000	0.1750000
28056.0000000	0.1700000
28057.0000000	0.1750000
28058.0000000	0.1700000
28059.0000000	0.1750000
28060.0000000	0.1650000
28061.0000000	0.1650000
28062.0000000	0.1750000
28063.0000000	0.1700000
28064.0000000	0.1750000
28065.0000000	0.1700000
28066.0000000	0.1700000
28067.0000000	0.1650000
28068.0000000	0.1650000
28069.0000000	0.1700000
28070.0000000	0.1750000
28071.0000000	0.1600000
28072.0000000	0.1700000
28073.0000000	0.1650000
28074.0000000	0.1750000
28075.0000000	0.1650000
28076.0000000	0.1650000
28077.0000000	0.1750000
28078.0000000	0.1650000
28079.0000000	0.1700000
28080.0000000	0.1666667
28081.0000000	0.1650000
28082.0000000	0.1650000
28083.0000000	0.1700000
28084.0000000	0.1650000
28085.0000000	0.1700000
28086.0000000	0.1600000
28087.0000000	0.1700000
28088.0000000	0.1700000
28089.0000000	0.1650000
28090.0000000	0.1600000
28091.0000000	0.1550000
28092.0000000	0.1650000
28093.0000000	0.1750000
28094.0000000	0.1600000
28095.0000000	0.1700000
28096.0000000	0.1550000
28097.0000000	0.1600000
28098.0000000	0.1650000
28099.0000000	0.1600000
28100.0000000	0.1600000

28101.0000000	0.1600000
28102.0000000	0.1550000
28103.0000000	0.1550000
28104.0000000	0.1600000
28105.0000000	0.1550000
28106.0000000	0.1550000
28107.0000000	0.1600000
28108.0000000	0.1650000
28109.0000000	0.1550000
28110.0000000	0.1550000
28111.0000000	0.1533333
28112.0000000	0.1550000
28113.0000000	0.1500000
28114.0000000	0.1550000
28115.0000000	0.1500000
28116.0000000	0.1500000
28117.0000000	0.1550000
28118.0000000	0.1600000
28119.0000000	0.1550000
28120.0000000	0.1550000
28121.0000000	0.1450000
28122.0000000	0.1500000
28123.0000000	0.1600000
28124.0000000	0.1550000
28125.0000000	0.1550000
28126.0000000	0.1550000
28127.0000000	0.1500000
28128.0000000	0.1550000
28129.0000000	0.1500000
28130.0000000	0.1500000
28131.0000000	0.1500000
28132.0000000	0.1400000
28133.0000000	0.1450000
28134.0000000	0.1550000
28135.0000000	0.1500000
28136.0000000	0.1500000
28137.0000000	0.1500000
28138.0000000	0.1450000
28139.0000000	0.1450000
28140.0000000	0.1550000
28141.0000000	0.1500000
28142.0000000	0.1400000
28143.0000000	0.1500000
28144.0000000	0.1400000
28145.0000000	0.1450000
28146.0000000	0.1450000
28147.0000000	0.1400000
28148.0000000	0.1350000
28149.0000000	0.1450000
28150.0000000	0.1450000
28151.0000000	0.1400000
28152.0000000	0.1400000
28153.0000000	0.1400000
28154.0000000	0.1300000
28155.0000000	0.1400000
28156.0000000	0.1350000
28157.0000000	0.1300000

28158.0000000	0.1400000
28159.0000000	0.1350000
28160.0000000	0.1400000
28161.0000000	0.1350000
28162.0000000	0.1300000
28163.0000000	0.1300000
28164.0000000	0.1350000
28165.0000000	0.1350000
28166.0000000	0.1250000
28167.0000000	0.1400000
28168.0000000	0.1350000
28169.0000000	0.1300000
28170.0000000	0.1350000
28171.0000000	0.1300000
28172.0000000	0.1350000
28173.0000000	0.1350000
28174.0000000	0.1350000
28175.0000000	0.1350000
28176.0000000	0.1300000
28177.0000000	0.1300000
28178.0000000	0.1350000
28179.0000000	0.1300000
28180.0000000	0.1400000
28181.0000000	0.1300000
28182.0000000	0.1350000
28183.0000000	0.1300000
28184.0000000	0.1300000
28185.0000000	0.1350000
28186.0000000	0.1300000
28187.0000000	0.1350000
28188.0000000	0.1250000
28189.0000000	0.1250000
28190.0000000	0.1200000
28191.0000000	0.1300000
28192.0000000	0.1250000
28193.0000000	0.1250000
28194.0000000	0.1250000
28195.0000000	0.1350000
28196.0000000	0.1250000
28197.0000000	0.1300000
28198.0000000	0.1250000
28199.0000000	0.1200000
28200.0000000	0.1200000
28201.0000000	0.1233333
28202.0000000	0.1300000
28203.0000000	0.1200000
28204.0000000	0.1300000
28205.0000000	0.1200000
28206.0000000	0.1300000
28207.0000000	0.1250000
28208.0000000	0.1250000
28209.0000000	0.1200000
28210.0000000	0.1300000
28211.0000000	0.1200000
28212.0000000	0.1300000
28213.0000000	0.1200000
28214.0000000	0.1300000

28215.0000000	0.1150000
28216.0000000	0.1250000
28217.0000000	0.1200000
28218.0000000	0.1300000
28219.0000000	0.1200000
28220.0000000	0.1300000
28221.0000000	0.1150000
28222.0000000	0.1150000
28223.0000000	0.1250000
28224.0000000	0.1200000
28225.0000000	0.1250000
28226.0000000	0.1250000
28227.0000000	0.1250000
28228.0000000	0.1100000
28229.0000000	0.1100000
28230.0000000	0.1150000
28231.0000000	0.1200000
28232.0000000	0.1133333
28233.0000000	0.1250000
28234.0000000	0.1200000
28235.0000000	0.1200000
28236.0000000	0.1050000
28237.0000000	0.1100000
28238.0000000	0.1100000
28239.0000000	0.1200000
28240.0000000	0.1150000
28241.0000000	0.1150000
28242.0000000	0.1100000
28243.0000000	0.1100000
28244.0000000	0.1150000
28245.0000000	0.1100000
28246.0000000	0.1200000
28247.0000000	0.1200000
28248.0000000	0.1150000
28249.0000000	0.1200000
28250.0000000	0.1150000
28251.0000000	0.1150000
28252.0000000	0.1100000
28253.0000000	0.1250000
28254.0000000	0.1100000
28255.0000000	0.1200000
28256.0000000	0.1100000
28257.0000000	0.1100000
28258.0000000	0.1100000
28259.0000000	0.1150000
28260.0000000	0.1100000
28261.0000000	0.1200000
28262.0000000	0.1166667
28263.0000000	0.1150000
28264.0000000	0.1150000
28265.0000000	0.1100000
28266.0000000	0.1150000
28267.0000000	0.1150000
28268.0000000	0.1200000
28269.0000000	0.1050000
28270.0000000	0.1050000
28271.0000000	0.1150000

28272.0000000	0.1100000
28273.0000000	0.1150000
28274.0000000	0.1150000
28275.0000000	0.1150000
28276.0000000	0.1150000
28277.0000000	0.1100000
28278.0000000	0.1100000
28279.0000000	0.1050000
28280.0000000	0.1150000
28281.0000000	0.1100000
28282.0000000	0.1100000
28283.0000000	0.1100000
28284.0000000	0.1150000
28285.0000000	0.1050000
28286.0000000	0.1100000
28287.0000000	0.1100000
28288.0000000	0.1000000
28289.0000000	0.1100000
28290.0000000	0.1150000
28291.0000000	0.1100000
28292.0000000	0.1066667
28293.0000000	0.1150000
28294.0000000	0.1100000
28295.0000000	0.1050000
28296.0000000	0.1050000
28297.0000000	0.1000000
28298.0000000	0.1050000
28299.0000000	0.1100000
28300.0000000	0.1050000
28301.0000000	0.1200000
28302.0000000	0.1050000
28303.0000000	0.1100000
28304.0000000	0.1050000
28305.0000000	0.1050000
28306.0000000	0.1100000
28307.0000000	0.1000000
28308.0000000	0.1050000
28309.0000000	0.1050000
28310.0000000	0.1100000
28311.0000000	0.1050000
28312.0000000	0.1050000
28313.0000000	0.1100000
28314.0000000	0.1150000
28315.0000000	0.1000000
28316.0000000	0.1100000
28317.0000000	0.1100000
28318.0000000	0.1050000
28319.0000000	0.1100000
28320.0000000	0.1000000
28321.0000000	0.1100000
28322.0000000	0.1066667
28323.0000000	0.1050000
28324.0000000	0.1150000
28325.0000000	0.1100000
28326.0000000	0.1050000
28327.0000000	0.1150000
28328.0000000	0.1100000

28329.0000000	0.1050000
28330.0000000	0.1100000
28331.0000000	0.1150000
28332.0000000	0.1100000
28333.0000000	0.1200000
28334.0000000	0.1050000
28335.0000000	0.1100000
28336.0000000	0.1100000
28337.0000000	0.1100000
28338.0000000	0.1050000
28339.0000000	0.1100000
28340.0000000	0.1100000
28341.0000000	0.1050000
28342.0000000	0.1150000
28343.0000000	0.1100000
28344.0000000	0.1100000
28345.0000000	0.1050000
28346.0000000	0.1050000
28347.0000000	0.1150000
28348.0000000	0.1150000
28349.0000000	0.1000000
28350.0000000	0.1150000
28351.0000000	0.1150000
28352.0000000	0.1100000
28353.0000000	0.1050000
28354.0000000	0.1100000
28355.0000000	0.1050000
28356.0000000	0.1150000
28357.0000000	0.1050000
28358.0000000	0.1100000
28359.0000000	0.1100000
28360.0000000	0.1150000
28361.0000000	0.1050000
28362.0000000	0.1050000
28363.0000000	0.1100000
28364.0000000	0.1050000
28365.0000000	0.1100000
28366.0000000	0.1100000
28367.0000000	0.1150000
28368.0000000	0.1150000
28369.0000000	0.1100000
28370.0000000	0.1200000
28371.0000000	0.1050000
28372.0000000	0.0950000
28373.0000000	0.1150000
28374.0000000	0.1100000
28375.0000000	0.1100000
28376.0000000	0.1100000
28377.0000000	0.1050000
28378.0000000	0.1100000
28379.0000000	0.1150000
28380.0000000	0.1100000
28381.0000000	0.1150000
28382.0000000	0.1150000
28383.0000000	0.1033333
28384.0000000	0.1100000
28385.0000000	0.1100000

28386.0000000	0.1150000
28387.0000000	0.1100000
28388.0000000	0.1100000
28389.0000000	0.1050000
28390.0000000	0.1050000
28391.0000000	0.1100000
28392.0000000	0.1100000
28393.0000000	0.1100000
28394.0000000	0.1050000
28395.0000000	0.1050000
28396.0000000	0.1100000
28397.0000000	0.1100000
28398.0000000	0.1050000
28399.0000000	0.1050000
28400.0000000	0.1050000
28401.0000000	0.1100000
28402.0000000	0.1150000
28403.0000000	0.1000000
28404.0000000	0.1050000
28405.0000000	0.1100000
28406.0000000	0.1100000
28407.0000000	0.1100000
28408.0000000	0.1050000
28409.0000000	0.1000000
28410.0000000	0.1100000
28411.0000000	0.1050000
28412.0000000	0.1100000
28413.0000000	0.1050000
28414.0000000	0.1100000
28415.0000000	0.1100000
28416.0000000	0.1100000
28417.0000000	0.1100000
28418.0000000	0.1000000
28419.0000000	0.1000000
28420.0000000	0.1100000
28421.0000000	0.1050000
28422.0000000	0.1150000
28423.0000000	0.1050000
28424.0000000	0.1150000
28425.0000000	0.0950000
28426.0000000	0.0950000
28427.0000000	0.1050000
28428.0000000	0.1100000
28429.0000000	0.1050000
28430.0000000	0.1050000
28431.0000000	0.1000000
28432.0000000	0.0900000
28433.0000000	0.1050000
28434.0000000	0.0900000
28435.0000000	0.1000000
28436.0000000	0.0950000
28437.0000000	0.1050000
28438.0000000	0.0950000
28439.0000000	0.1000000
28440.0000000	0.0950000
28441.0000000	0.1000000
28442.0000000	0.1000000

28443.0000000	0.0966667
28444.0000000	0.1000000
28445.0000000	0.0850000
28446.0000000	0.0950000
28447.0000000	0.0950000
28448.0000000	0.0950000
28449.0000000	0.0950000
28450.0000000	0.0950000
28451.0000000	0.0950000
28452.0000000	0.0900000
28453.0000000	0.0900000
28454.0000000	0.0950000
28455.0000000	0.0900000
28456.0000000	0.0900000
28457.0000000	0.0900000
28458.0000000	0.0900000
28459.0000000	0.1000000
28460.0000000	0.0850000
28461.0000000	0.1000000
28462.0000000	0.0850000
28463.0000000	0.0950000
28464.0000000	0.0800000
28465.0000000	0.0900000
28466.0000000	0.0800000
28467.0000000	0.0900000
28468.0000000	0.0750000
28469.0000000	0.0950000
28470.0000000	0.0750000
28471.0000000	0.0800000
28472.0000000	0.0700000
28473.0000000	0.0800000
28474.0000000	0.0750000
28475.0000000	0.0750000
28476.0000000	0.0800000
28477.0000000	0.0850000
28478.0000000	0.0700000
28479.0000000	0.0750000
28480.0000000	0.0750000
28481.0000000	0.0750000
28482.0000000	0.0800000
28483.0000000	0.0700000
28484.0000000	0.0650000
28485.0000000	0.0650000
28486.0000000	0.0650000
28487.0000000	0.0700000
28488.0000000	0.0750000
28489.0000000	0.0650000
28490.0000000	0.0700000
28491.0000000	0.0650000
28492.0000000	0.0550000
28493.0000000	0.0650000
28494.0000000	0.0650000
28495.0000000	0.0650000
28496.0000000	0.0600000
28497.0000000	0.0600000
28498.0000000	0.0600000
28499.0000000	0.0600000

28500.0000000	0.0600000
28501.0000000	0.0500000
28502.0000000	0.0550000
28503.0000000	0.0566667
28504.0000000	0.0500000
28505.0000000	0.0600000
28506.0000000	0.0500000
28507.0000000	0.0550000
28508.0000000	0.0550000
28509.0000000	0.0550000
28510.0000000	0.0600000
28511.0000000	0.0450000
28512.0000000	0.0400000
28513.0000000	0.0450000
28514.0000000	0.0500000
28515.0000000	0.0350000
28516.0000000	0.0450000
28517.0000000	0.0400000
28518.0000000	0.0400000
28519.0000000	0.0450000
28520.0000000	0.0300000
28521.0000000	0.0400000
28522.0000000	0.0350000
28523.0000000	0.0400000
28524.0000000	0.0350000
28525.0000000	0.0350000
28526.0000000	0.0350000
28527.0000000	0.0300000
28528.0000000	0.0350000
28529.0000000	0.0300000
28530.0000000	0.0300000
28531.0000000	0.0300000
28532.0000000	0.0300000
28533.0000000	0.0300000
28534.0000000	0.0200000
28535.0000000	0.0250000
28536.0000000	0.0250000
28537.0000000	0.0200000
28538.0000000	0.0250000
28539.0000000	0.0200000
28540.0000000	0.0150000
28541.0000000	0.0200000
28542.0000000	0.0250000
28543.0000000	0.0300000
28544.0000000	0.0100000
28545.0000000	0.0200000
28546.0000000	0.0150000
28547.0000000	0.0100000
28548.0000000	0.0200000
28549.0000000	0.0050000
28550.0000000	0.0150000
28551.0000000	0.0100000
28552.0000000	0.0100000
28553.0000000	0.0100000
28554.0000000	0.0050000
28555.0000000	0.0050000
28556.0000000	0.0050000

28557.0000000	0.0100000
28558.0000000	0.0050000
28559.0000000	0.0100000
28560.0000000	-0.0050000
28561.0000000	0.0000000
28562.0000000	-0.0050000
28563.0000000	0.0050000
28564.0000000	-0.0033333
28565.0000000	-0.0050000
28566.0000000	-0.0100000
28567.0000000	0.0000000
28568.0000000	-0.0050000
28569.0000000	0.0000000
28570.0000000	-0.0150000
28571.0000000	-0.0100000
28572.0000000	-0.0200000
28573.0000000	-0.0100000
28574.0000000	-0.0100000
28575.0000000	-0.0100000
28576.0000000	-0.0200000
28577.0000000	-0.0150000
28578.0000000	-0.0200000
28579.0000000	-0.0100000
28580.0000000	-0.0150000
28581.0000000	-0.0100000
28582.0000000	-0.0150000
28583.0000000	-0.0300000
28584.0000000	-0.0200000
28585.0000000	-0.0200000
28586.0000000	-0.0200000
28587.0000000	-0.0250000
28588.0000000	-0.0250000
28589.0000000	-0.0350000
28590.0000000	-0.0350000
28591.0000000	-0.0250000
28592.0000000	-0.0300000
28593.0000000	-0.0300000
28594.0000000	-0.0433333
28595.0000000	-0.0200000
28596.0000000	-0.0350000
28597.0000000	-0.0300000
28598.0000000	-0.0450000
28599.0000000	-0.0400000
28600.0000000	-0.0500000
28601.0000000	-0.0350000
28602.0000000	-0.0400000
28603.0000000	-0.0400000
28604.0000000	-0.0450000
28605.0000000	-0.0400000
28606.0000000	-0.0400000
28607.0000000	-0.0350000
28608.0000000	-0.0450000
28609.0000000	-0.0450000
28610.0000000	-0.0550000
28611.0000000	-0.0450000
28612.0000000	-0.0600000
28613.0000000	-0.0450000

28614.0000000	-0.0500000
28615.0000000	-0.0550000
28616.0000000	-0.0500000
28617.0000000	-0.0600000
28618.0000000	-0.0500000
28619.0000000	-0.0600000
28620.0000000	-0.0500000
28621.0000000	-0.0650000
28622.0000000	-0.0550000
28623.0000000	-0.0550000
28624.0000000	-0.0600000
28625.0000000	-0.0550000
28626.0000000	-0.0700000
28627.0000000	-0.0600000
28628.0000000	-0.0650000
28629.0000000	-0.0650000
28630.0000000	-0.0700000
28631.0000000	-0.0600000
28632.0000000	-0.0700000
28633.0000000	-0.0650000
28634.0000000	-0.0650000
28635.0000000	-0.0650000
28636.0000000	-0.0650000
28637.0000000	-0.0750000
28638.0000000	-0.0700000
28639.0000000	-0.0700000
28640.0000000	-0.0650000
28641.0000000	-0.0750000
28642.0000000	-0.0700000
28643.0000000	-0.0800000
28644.0000000	-0.0750000
28645.0000000	-0.0750000
28646.0000000	-0.0800000
28647.0000000	-0.0800000
28648.0000000	-0.0800000
28649.0000000	-0.0800000
28650.0000000	-0.0800000
28651.0000000	-0.0750000
28652.0000000	-0.0800000
28653.0000000	-0.0850000
28654.0000000	-0.0800000
28655.0000000	-0.0850000
28656.0000000	-0.0850000
28657.0000000	-0.0800000
28658.0000000	-0.0850000
28659.0000000	-0.0850000
28660.0000000	-0.0850000
28661.0000000	-0.0850000
28662.0000000	-0.0800000
28663.0000000	-0.0900000
28664.0000000	-0.0950000
28665.0000000	-0.0900000
28666.0000000	-0.0950000
28667.0000000	-0.0900000
28668.0000000	-0.0900000
28669.0000000	-0.0900000
28670.0000000	-0.0900000

28671.0000000	-0.0950000
28672.0000000	-0.0900000
28673.0000000	-0.0950000
28674.0000000	-0.1000000
28675.0000000	-0.0950000
28676.0000000	-0.0950000
28677.0000000	-0.0850000
28678.0000000	-0.0950000
28679.0000000	-0.0950000
28680.0000000	-0.0900000
28681.0000000	-0.1000000
28682.0000000	-0.1000000
28683.0000000	-0.1100000
28684.0000000	-0.1000000
28685.0000000	-0.1000000
28686.0000000	-0.1000000
28687.0000000	-0.1050000
28688.0000000	-0.1000000
28689.0000000	-0.1100000
28690.0000000	-0.1100000
28691.0000000	-0.1000000
28692.0000000	-0.1050000
28693.0000000	-0.1000000
28694.0000000	-0.1050000
28695.0000000	-0.1100000
28696.0000000	-0.1100000
28697.0000000	-0.1100000
28698.0000000	-0.1100000
28699.0000000	-0.1150000
28700.0000000	-0.1050000
28701.0000000	-0.1150000
28702.0000000	-0.1050000
28703.0000000	-0.1150000
28704.0000000	-0.1150000
28705.0000000	-0.1100000
28706.0000000	-0.1100000
28707.0000000	-0.1150000
28708.0000000	-0.1100000
28709.0000000	-0.1150000
28710.0000000	-0.1200000
28711.0000000	-0.1150000
28712.0000000	-0.1150000
28713.0000000	-0.1150000
28714.0000000	-0.1150000
28715.0000000	-0.1166667
28716.0000000	-0.1100000
28717.0000000	-0.1200000
28718.0000000	-0.1200000
28719.0000000	-0.1150000
28720.0000000	-0.1150000
28721.0000000	-0.1200000
28722.0000000	-0.1200000
28723.0000000	-0.1200000
28724.0000000	-0.1250000
28725.0000000	-0.1200000
28726.0000000	-0.1150000
28727.0000000	-0.1150000

28728.0000000	-0.1200000
28729.0000000	-0.1250000
28730.0000000	-0.1250000
28731.0000000	-0.1250000
28732.0000000	-0.1250000
28733.0000000	-0.1150000
28734.0000000	-0.1250000
28735.0000000	-0.1250000
28736.0000000	-0.1350000
28737.0000000	-0.1250000
28738.0000000	-0.1250000
28739.0000000	-0.1200000
28740.0000000	-0.1200000
28741.0000000	-0.1250000
28742.0000000	-0.1200000
28743.0000000	-0.1350000
28744.0000000	-0.1250000
28745.0000000	-0.1333333
28746.0000000	-0.1250000
28747.0000000	-0.1300000
28748.0000000	-0.1200000
28749.0000000	-0.1450000
28750.0000000	-0.1350000
28751.0000000	-0.1350000
28752.0000000	-0.1300000
28753.0000000	-0.1300000
28754.0000000	-0.1250000
28755.0000000	-0.1300000
28756.0000000	-0.1300000
28757.0000000	-0.1350000
28758.0000000	-0.1300000
28759.0000000	-0.1300000
28760.0000000	-0.1300000
28761.0000000	-0.1300000
28762.0000000	-0.1350000
28763.0000000	-0.1400000
28764.0000000	-0.1300000
28765.0000000	-0.1400000
28766.0000000	-0.1250000
28767.0000000	-0.1350000
28768.0000000	-0.1250000
28769.0000000	-0.1400000
28770.0000000	-0.1350000
28771.0000000	-0.1350000
28772.0000000	-0.1350000
28773.0000000	-0.1400000
28774.0000000	-0.1350000
28775.0000000	-0.1466667
28776.0000000	-0.1350000
28777.0000000	-0.1400000
28778.0000000	-0.1450000
28779.0000000	-0.1450000
28780.0000000	-0.1350000
28781.0000000	-0.1400000
28782.0000000	-0.1350000
28783.0000000	-0.1500000
28784.0000000	-0.1350000

28785.0000000	-0.1450000
28786.0000000	-0.1400000
28787.0000000	-0.1500000
28788.0000000	-0.1350000
28789.0000000	-0.1400000
28790.0000000	-0.1400000
28791.0000000	-0.1450000
28792.0000000	-0.1450000
28793.0000000	-0.1500000
28794.0000000	-0.1500000
28795.0000000	-0.1450000
28796.0000000	-0.1400000
28797.0000000	-0.1350000
28798.0000000	-0.1500000
28799.0000000	-0.1400000
28800.0000000	-0.1500000
28801.0000000	-0.1500000
28802.0000000	-0.1500000
28803.0000000	-0.1500000
28804.0000000	-0.1400000
28805.0000000	-0.1433333
28806.0000000	-0.1450000
28807.0000000	-0.1500000
28808.0000000	-0.1450000
28809.0000000	-0.1500000
28810.0000000	-0.1400000
28811.0000000	-0.1500000
28812.0000000	-0.1550000
28813.0000000	-0.1500000
28814.0000000	-0.1550000
28815.0000000	-0.1450000
28816.0000000	-0.1500000
28817.0000000	-0.1500000
28818.0000000	-0.1500000
28819.0000000	-0.1500000
28820.0000000	-0.1500000
28821.0000000	-0.1500000
28822.0000000	-0.1550000
28823.0000000	-0.1550000
28824.0000000	-0.1500000
28825.0000000	-0.1550000
28826.0000000	-0.1550000
28827.0000000	-0.1500000
28828.0000000	-0.1500000
28829.0000000	-0.1500000
28830.0000000	-0.1450000
28831.0000000	-0.1500000
28832.0000000	-0.1500000
28833.0000000	-0.1550000
28834.0000000	-0.1550000
28835.0000000	-0.1550000
28836.0000000	-0.1533333
28837.0000000	-0.1500000
28838.0000000	-0.1600000
28839.0000000	-0.1600000
28840.0000000	-0.1600000
28841.0000000	-0.1550000

28842.0000000	-0.1550000
28843.0000000	-0.1550000
28844.0000000	-0.1550000
28845.0000000	-0.1550000
28846.0000000	-0.1550000
28847.0000000	-0.1600000
28848.0000000	-0.1550000
28849.0000000	-0.1650000
28850.0000000	-0.1650000
28851.0000000	-0.1550000
28852.0000000	-0.1600000
28853.0000000	-0.1650000
28854.0000000	-0.1600000
28855.0000000	-0.1550000
28856.0000000	-0.1600000
28857.0000000	-0.1650000
28858.0000000	-0.1650000
28859.0000000	-0.1600000
28860.0000000	-0.1650000
28861.0000000	-0.1550000
28862.0000000	-0.1650000
28863.0000000	-0.1600000
28864.0000000	-0.1650000
28865.0000000	-0.1700000
28866.0000000	-0.1600000
28867.0000000	-0.1650000
28868.0000000	-0.1650000
28869.0000000	-0.1650000
28870.0000000	-0.1650000
28871.0000000	-0.1700000
28872.0000000	-0.1700000
28873.0000000	-0.1750000
28874.0000000	-0.1650000
28875.0000000	-0.1600000
28876.0000000	-0.1600000
28877.0000000	-0.1650000
28878.0000000	-0.1650000
28879.0000000	-0.1750000
28880.0000000	-0.1700000
28881.0000000	-0.1650000
28882.0000000	-0.1750000
28883.0000000	-0.1600000
28884.0000000	-0.1700000
28885.0000000	-0.1650000
28886.0000000	-0.1700000
28887.0000000	-0.1700000
28888.0000000	-0.1700000
28889.0000000	-0.1750000
28890.0000000	-0.1800000
28891.0000000	-0.1650000
28892.0000000	-0.1750000
28893.0000000	-0.1800000
28894.0000000	-0.1700000
28895.0000000	-0.1700000
28896.0000000	-0.1750000
28897.0000000	-0.1650000
28898.0000000	-0.1850000

28899.0000000	-0.1650000
28900.0000000	-0.1800000
28901.0000000	-0.1750000
28902.0000000	-0.1750000
28903.0000000	-0.1800000
28904.0000000	-0.1750000
28905.0000000	-0.1750000
28906.0000000	-0.1750000
28907.0000000	-0.1800000
28908.0000000	-0.1800000
28909.0000000	-0.1850000
28910.0000000	-0.1800000
28911.0000000	-0.1750000
28912.0000000	-0.1800000
28913.0000000	-0.1800000
28914.0000000	-0.1800000
28915.0000000	-0.1800000
28916.0000000	-0.1750000
28917.0000000	-0.1850000
28918.0000000	-0.1850000
28919.0000000	-0.1900000
28920.0000000	-0.1900000
28921.0000000	-0.1850000
28922.0000000	-0.1900000
28923.0000000	-0.1800000
28924.0000000	-0.1900000
28925.0000000	-0.1850000
28926.0000000	-0.1866667
28927.0000000	-0.1900000
28928.0000000	-0.1900000
28929.0000000	-0.1900000
28930.0000000	-0.1850000
28931.0000000	-0.1900000
28932.0000000	-0.1850000
28933.0000000	-0.1900000
28934.0000000	-0.1850000
28935.0000000	-0.1950000
28936.0000000	-0.1900000
28937.0000000	-0.1950000
28938.0000000	-0.2000000
28939.0000000	-0.1950000
28940.0000000	-0.1900000
28941.0000000	-0.1900000
28942.0000000	-0.1850000
28943.0000000	-0.1900000
28944.0000000	-0.2000000
28945.0000000	-0.1950000
28946.0000000	-0.2000000
28947.0000000	-0.2000000
28948.0000000	-0.2050000
28949.0000000	-0.1900000
28950.0000000	-0.2050000
28951.0000000	-0.2050000
28952.0000000	-0.1950000
28953.0000000	-0.2150000
28954.0000000	-0.2000000
28955.0000000	-0.1950000

28956.0000000	-0.2033333
28957.0000000	-0.2000000
28958.0000000	-0.2050000
28959.0000000	-0.2100000
28960.0000000	-0.2150000
28961.0000000	-0.2100000
28962.0000000	-0.2000000
28963.0000000	-0.2000000
28964.0000000	-0.2000000
28965.0000000	-0.2100000
28966.0000000	-0.2150000
28967.0000000	-0.2000000
28968.0000000	-0.2050000
28969.0000000	-0.2050000
28970.0000000	-0.2050000
28971.0000000	-0.2150000
28972.0000000	-0.2050000
28973.0000000	-0.2050000
28974.0000000	-0.2050000
28975.0000000	-0.2100000
28976.0000000	-0.2150000
28977.0000000	-0.2050000
28978.0000000	-0.2100000
28979.0000000	-0.2100000
28980.0000000	-0.2100000
28981.0000000	-0.2150000
28982.0000000	-0.2150000
28983.0000000	-0.2200000
28984.0000000	-0.2050000
28985.0000000	-0.2100000
28986.0000000	-0.2133333
28987.0000000	-0.2100000
28988.0000000	-0.2150000
28989.0000000	-0.2150000
28990.0000000	-0.2150000
28991.0000000	-0.2200000
28992.0000000	-0.2050000
28993.0000000	-0.2200000
28994.0000000	-0.2200000
28995.0000000	-0.2200000
28996.0000000	-0.2200000
28997.0000000	-0.2200000
28998.0000000	-0.2250000
28999.0000000	-0.2150000
29000.0000000	-0.2250000
29001.0000000	-0.2200000
29002.0000000	-0.2200000
29003.0000000	-0.2200000
29004.0000000	-0.2200000
29005.0000000	-0.2250000
29006.0000000	-0.2300000
29007.0000000	-0.2150000
29008.0000000	-0.2200000
29009.0000000	-0.2200000
29010.0000000	-0.2300000
29011.0000000	-0.2300000
29012.0000000	-0.2350000

29013.0000000	-0.2250000
29014.0000000	-0.2200000
29015.0000000	-0.2250000
29016.0000000	-0.2233333
29017.0000000	-0.2300000
29018.0000000	-0.2250000
29019.0000000	-0.2250000
29020.0000000	-0.2300000
29021.0000000	-0.2300000
29022.0000000	-0.2450000
29023.0000000	-0.2300000
29024.0000000	-0.2350000
29025.0000000	-0.2350000
29026.0000000	-0.2300000
29027.0000000	-0.2350000
29028.0000000	-0.2300000
29029.0000000	-0.2450000
29030.0000000	-0.2300000
29031.0000000	-0.2400000
29032.0000000	-0.2350000
29033.0000000	-0.2450000
29034.0000000	-0.2350000
29035.0000000	-0.2450000
29036.0000000	-0.2350000
29037.0000000	-0.2350000
29038.0000000	-0.2500000
29039.0000000	-0.2450000
29040.0000000	-0.2400000
29041.0000000	-0.2350000
29042.0000000	-0.2400000
29043.0000000	-0.2350000
29044.0000000	-0.2450000
29045.0000000	-0.2400000
29046.0000000	-0.2450000
29047.0000000	-0.2400000
29048.0000000	-0.2500000
29049.0000000	-0.2500000
29050.0000000	-0.2450000
29051.0000000	-0.2400000
29052.0000000	-0.2550000
29053.0000000	-0.2400000
29054.0000000	-0.2500000
29055.0000000	-0.2450000
29056.0000000	-0.2500000
29057.0000000	-0.2400000
29058.0000000	-0.2450000
29059.0000000	-0.2450000
29060.0000000	-0.2450000
29061.0000000	-0.2550000
29062.0000000	-0.2500000
29063.0000000	-0.2500000
29064.0000000	-0.2500000
29065.0000000	-0.2450000
29066.0000000	-0.2550000
29067.0000000	-0.2500000
29068.0000000	-0.2600000
29069.0000000	-0.2550000

29070.0000000	-0.2500000
29071.0000000	-0.2450000
29072.0000000	-0.2550000
29073.0000000	-0.2500000
29074.0000000	-0.2600000
29075.0000000	-0.2550000
29076.0000000	-0.2550000
29077.0000000	-0.2533333
29078.0000000	-0.2550000
29079.0000000	-0.2650000
29080.0000000	-0.2600000
29081.0000000	-0.2700000
29082.0000000	-0.2500000
29083.0000000	-0.2600000
29084.0000000	-0.2600000
29085.0000000	-0.2650000
29086.0000000	-0.2650000
29087.0000000	-0.2550000
29088.0000000	-0.2550000
29089.0000000	-0.2600000
29090.0000000	-0.2600000
29091.0000000	-0.2600000
29092.0000000	-0.2700000
29093.0000000	-0.2600000
29094.0000000	-0.2700000
29095.0000000	-0.2650000
29096.0000000	-0.2650000
29097.0000000	-0.2600000
29098.0000000	-0.2550000
29099.0000000	-0.2650000
29100.0000000	-0.2600000
29101.0000000	-0.2700000
29102.0000000	-0.2650000
29103.0000000	-0.2650000
29104.0000000	-0.2600000
29105.0000000	-0.2550000
29106.0000000	-0.2700000
29107.0000000	-0.2700000
29108.0000000	-0.2700000
29109.0000000	-0.2600000
29110.0000000	-0.2700000
29111.0000000	-0.2700000
29112.0000000	-0.2700000
29113.0000000	-0.2650000
29114.0000000	-0.2650000
29115.0000000	-0.2600000
29116.0000000	-0.2650000
29117.0000000	-0.2650000
29118.0000000	-0.2700000
29119.0000000	-0.2650000
29120.0000000	-0.2700000
29121.0000000	-0.2650000
29122.0000000	-0.2700000
29123.0000000	-0.2650000
29124.0000000	-0.2700000
29125.0000000	-0.2700000
29126.0000000	-0.2700000

29127.0000000	-0.2700000
29128.0000000	-0.2700000
29129.0000000	-0.2700000
29130.0000000	-0.2650000
29131.0000000	-0.2750000
29132.0000000	-0.2600000
29133.0000000	-0.2750000
29134.0000000	-0.2700000
29135.0000000	-0.2700000
29136.0000000	-0.2700000
29137.0000000	-0.2633333
29138.0000000	-0.2700000
29139.0000000	-0.2700000
29140.0000000	-0.2700000
29141.0000000	-0.2700000
29142.0000000	-0.2700000
29143.0000000	-0.2700000
29144.0000000	-0.2700000
29145.0000000	-0.2700000
29146.0000000	-0.2650000
29147.0000000	-0.2750000
29148.0000000	-0.2700000
29149.0000000	-0.2800000
29150.0000000	-0.2750000
29151.0000000	-0.2750000
29152.0000000	-0.2600000
29153.0000000	-0.2750000
29154.0000000	-0.2700000
29155.0000000	-0.2750000
29156.0000000	-0.2700000
29157.0000000	-0.2750000
29158.0000000	-0.2650000
29159.0000000	-0.2800000
29160.0000000	-0.2700000
29161.0000000	-0.2700000
29162.0000000	-0.2700000
29163.0000000	-0.2750000
29164.0000000	-0.2750000
29165.0000000	-0.2800000
29166.0000000	-0.2700000
29167.0000000	-0.2750000
29168.0000000	-0.2766666
29169.0000000	-0.2750000
29170.0000000	-0.2650000
29171.0000000	-0.2750000
29172.0000000	-0.2800000
29173.0000000	-0.2800000
29174.0000000	-0.2800000
29175.0000000	-0.2800000
29176.0000000	-0.2750000
29177.0000000	-0.2700000
29178.0000000	-0.2800000
29179.0000000	-0.2700000
29180.0000000	-0.2800000
29181.0000000	-0.2650000
29182.0000000	-0.2800000
29183.0000000	-0.2800000

29184.0000000	-0.2700000
29185.0000000	-0.2700000
29186.0000000	-0.2650000
29187.0000000	-0.2800000
29188.0000000	-0.2750000
29189.0000000	-0.2750000
29190.0000000	-0.2750000
29191.0000000	-0.2650000
29192.0000000	-0.2750000
29193.0000000	-0.2700000
29194.0000000	-0.2800000
29195.0000000	-0.2700000
29196.0000000	-0.2750000
29197.0000000	-0.2766667
29198.0000000	-0.2700000
29199.0000000	-0.2800000
29200.0000000	-0.2750000
29201.0000000	-0.2600000
29202.0000000	-0.2750000
29203.0000000	-0.2750000
29204.0000000	-0.2800000
29205.0000000	-0.2700000
29206.0000000	-0.2650000
29207.0000000	-0.2800000
29208.0000000	-0.2700000
29209.0000000	-0.2750000
29210.0000000	-0.2750000
29211.0000000	-0.2750000
29212.0000000	-0.2800000
29213.0000000	-0.2800000
29214.0000000	-0.2800000
29215.0000000	-0.2800000
29216.0000000	-0.2850000
29217.0000000	-0.2800000
29218.0000000	-0.2850000
29219.0000000	-0.2800000
29220.0000000	-0.2700000
29221.0000000	-0.2850000
29222.0000000	-0.2800000
29223.0000000	-0.2800000
29224.0000000	-0.2800000
29225.0000000	-0.2900000
29226.0000000	-0.2750000
29227.0000000	-0.2800000
29228.0000000	-0.2850000
29229.0000000	-0.2850000
29230.0000000	-0.2800000
29231.0000000	-0.2850000
29232.0000000	-0.2800000
29233.0000000	-0.2800000
29234.0000000	-0.2850000
29235.0000000	-0.2850000
29236.0000000	-0.2850000
29237.0000000	-0.2900000
29238.0000000	-0.2900000
29239.0000000	-0.2950000
29240.0000000	-0.2900000

29241.0000000	-0.2800000
29242.0000000	-0.2950000
29243.0000000	-0.2850000
29244.0000000	-0.2850000
29245.0000000	-0.2850000
29246.0000000	-0.2950000
29247.0000000	-0.3000000
29248.0000000	-0.2900000
29249.0000000	-0.3050000
29250.0000000	-0.2950000
29251.0000000	-0.2950000
29252.0000000	-0.2900000
29253.0000000	-0.2950000
29254.0000000	-0.3000000
29255.0000000	-0.2900000
29256.0000000	-0.3000000
29257.0000000	-0.3000000
29258.0000000	-0.2966667
29259.0000000	-0.3050000
29260.0000000	-0.3000000
29261.0000000	-0.3000000
29262.0000000	-0.3050000
29263.0000000	-0.2950000
29264.0000000	-0.2950000
29265.0000000	-0.2950000
29266.0000000	-0.3050000
29267.0000000	-0.3050000
29268.0000000	-0.3000000
29269.0000000	-0.3100000
29270.0000000	-0.3000000
29271.0000000	-0.3050000
29272.0000000	-0.3050000
29273.0000000	-0.3000000
29274.0000000	-0.3050000
29275.0000000	-0.3000000
29276.0000000	-0.3000000
29277.0000000	-0.3100000
29278.0000000	-0.3100000
29279.0000000	-0.3150000
29280.0000000	-0.3000000
29281.0000000	-0.3050000
29282.0000000	-0.3050000
29283.0000000	-0.3050000
29284.0000000	-0.3150000
29285.0000000	-0.3100000
29286.0000000	-0.3100000
29287.0000000	-0.3050000
29288.0000000	-0.3100000
29289.0000000	-0.3150000
29290.0000000	-0.3050000
29291.0000000	-0.3050000
29292.0000000	-0.3100000
29293.0000000	-0.3050000
29294.0000000	-0.3100000
29295.0000000	-0.3050000
29296.0000000	-0.3050000
29297.0000000	-0.3200000

29298.0000000	-0.3100000
29299.0000000	-0.3200000
29300.0000000	-0.3050000
29301.0000000	-0.3150000
29302.0000000	-0.3200000
29303.0000000	-0.3100000
29304.0000000	-0.3200000
29305.0000000	-0.3150000
29306.0000000	-0.3150000
29307.0000000	-0.3200000
29308.0000000	-0.3200000
29309.0000000	-0.3150000
29310.0000000	-0.3050000
29311.0000000	-0.3200000
29312.0000000	-0.3050000
29313.0000000	-0.3250000
29314.0000000	-0.3150000
29315.0000000	-0.3150000
29316.0000000	-0.3250000
29317.0000000	-0.3150000
29318.0000000	-0.3166667
29319.0000000	-0.3200000
29320.0000000	-0.3200000
29321.0000000	-0.3200000
29322.0000000	-0.3200000
29323.0000000	-0.3150000
29324.0000000	-0.3150000
29325.0000000	-0.3200000
29326.0000000	-0.3250000
29327.0000000	-0.3250000
29328.0000000	-0.3150000
29329.0000000	-0.3250000
29330.0000000	-0.3150000
29331.0000000	-0.3250000
29332.0000000	-0.3200000
29333.0000000	-0.3200000
29334.0000000	-0.3250000
29335.0000000	-0.3200000
29336.0000000	-0.3200000
29337.0000000	-0.3300000
29338.0000000	-0.3250000
29339.0000000	-0.3250000
29340.0000000	-0.3250000
29341.0000000	-0.3250000
29342.0000000	-0.3200000
29343.0000000	-0.3350000
29344.0000000	-0.3350000
29345.0000000	-0.3350000
29346.0000000	-0.3300000
29347.0000000	-0.3250000
29348.0000000	-0.3300000
29349.0000000	-0.3250000
29350.0000000	-0.3300000
29351.0000000	-0.3300000
29352.0000000	-0.3300000
29353.0000000	-0.3250000
29354.0000000	-0.3350000

29355.0000000	-0.3350000
29356.0000000	-0.3250000
29357.0000000	-0.3250000
29358.0000000	-0.3250000
29359.0000000	-0.3300000
29360.0000000	-0.3350000
29361.0000000	-0.3400000
29362.0000000	-0.3350000
29363.0000000	-0.3400000
29364.0000000	-0.3300000
29365.0000000	-0.3350000
29366.0000000	-0.3350000
29367.0000000	-0.3350000
29368.0000000	-0.3400000
29369.0000000	-0.3350000
29370.0000000	-0.3400000
29371.0000000	-0.3350000
29372.0000000	-0.3350000
29373.0000000	-0.3350000
29374.0000000	-0.3400000
29375.0000000	-0.3400000
29376.0000000	-0.3400000
29377.0000000	-0.3400000
29378.0000000	-0.3350000
29379.0000000	-0.3466667
29380.0000000	-0.3350000
29381.0000000	-0.3350000
29382.0000000	-0.3350000
29383.0000000	-0.3450000
29384.0000000	-0.3350000
29385.0000000	-0.3500000
29386.0000000	-0.3350000
29387.0000000	-0.3400000
29388.0000000	-0.3300000
29389.0000000	-0.3450000
29390.0000000	-0.3300000
29391.0000000	-0.3500000
29392.0000000	-0.3350000
29393.0000000	-0.3350000
29394.0000000	-0.3350000
29395.0000000	-0.3400000
29396.0000000	-0.3400000
29397.0000000	-0.3300000
29398.0000000	-0.3400000
29399.0000000	-0.3350000
29400.0000000	-0.3450000
29401.0000000	-0.3400000
29402.0000000	-0.3400000
29403.0000000	-0.3400000
29404.0000000	-0.3350000
29405.0000000	-0.3450000
29406.0000000	-0.3300000
29407.0000000	-0.3450000
29408.0000000	-0.3300000
29409.0000000	-0.3366667
29410.0000000	-0.3400000
29411.0000000	-0.3400000

29412.0000000	-0.3350000
29413.0000000	-0.3350000
29414.0000000	-0.3300000
29415.0000000	-0.3300000
29416.0000000	-0.3450000
29417.0000000	-0.3400000
29418.0000000	-0.3400000
29419.0000000	-0.3350000
29420.0000000	-0.3400000
29421.0000000	-0.3300000
29422.0000000	-0.3400000
29423.0000000	-0.3350000
29424.0000000	-0.3350000
29425.0000000	-0.3400000
29426.0000000	-0.3350000
29427.0000000	-0.3400000
29428.0000000	-0.3350000
29429.0000000	-0.3350000
29430.0000000	-0.3200000
29431.0000000	-0.3300000
29432.0000000	-0.3300000
29433.0000000	-0.3350000
29434.0000000	-0.3350000
29435.0000000	-0.3300000
29436.0000000	-0.3200000
29437.0000000	-0.3200000
29438.0000000	-0.3300000
29439.0000000	-0.3266667
29440.0000000	-0.3200000
29441.0000000	-0.3250000
29442.0000000	-0.3250000
29443.0000000	-0.3200000
29444.0000000	-0.3200000
29445.0000000	-0.3300000
29446.0000000	-0.3200000
29447.0000000	-0.3150000
29448.0000000	-0.3250000
29449.0000000	-0.3150000
29450.0000000	-0.3200000
29451.0000000	-0.3150000
29452.0000000	-0.3200000
29453.0000000	-0.3200000
29454.0000000	-0.3200000
29455.0000000	-0.3250000
29456.0000000	-0.3100000
29457.0000000	-0.3100000
29458.0000000	-0.3200000
29459.0000000	-0.3150000
29460.0000000	-0.3250000
29461.0000000	-0.3200000
29462.0000000	-0.3200000
29463.0000000	-0.3150000
29464.0000000	-0.3200000
29465.0000000	-0.3200000
29466.0000000	-0.3200000
29467.0000000	-0.3050000
29468.0000000	-0.3200000

29469.0000000	-0.3166667
29470.0000000	-0.3100000
29471.0000000	-0.3150000
29472.0000000	-0.3100000
29473.0000000	-0.3150000
29474.0000000	-0.3100000
29475.0000000	-0.3150000
29476.0000000	-0.3100000
29477.0000000	-0.3100000
29478.0000000	-0.3050000
29479.0000000	-0.3050000
29480.0000000	-0.3100000
29481.0000000	-0.3050000
29482.0000000	-0.3150000
29483.0000000	-0.3100000
29484.0000000	-0.3100000
29485.0000000	-0.3050000
29486.0000000	-0.3050000
29487.0000000	-0.3000000
29488.0000000	-0.3050000
29489.0000000	-0.3100000
29490.0000000	-0.3050000
29491.0000000	-0.3050000
29492.0000000	-0.3050000
29493.0000000	-0.2950000
29494.0000000	-0.3050000
29495.0000000	-0.3000000
29496.0000000	-0.3100000
29497.0000000	-0.3000000
29498.0000000	-0.3050000
29499.0000000	-0.3050000
29500.0000000	-0.3000000
29501.0000000	-0.2950000
29502.0000000	-0.3000000
29503.0000000	-0.3050000
29504.0000000	-0.3050000
29505.0000000	-0.3000000
29506.0000000	-0.3000000
29507.0000000	-0.3000000
29508.0000000	-0.2900000
29509.0000000	-0.3000000
29510.0000000	-0.3000000
29511.0000000	-0.2950000
29512.0000000	-0.3000000
29513.0000000	-0.3000000
29514.0000000	-0.2950000
29515.0000000	-0.3000000
29516.0000000	-0.2950000
29517.0000000	-0.2950000
29518.0000000	-0.2950000
29519.0000000	-0.2950000
29520.0000000	-0.2900000
29521.0000000	-0.2950000
29522.0000000	-0.2900000
29523.0000000	-0.3000000
29524.0000000	-0.2950000
29525.0000000	-0.2900000

29526.0000000	-0.2950000
29527.0000000	-0.2850000
29528.0000000	-0.2900000
29529.0000000	-0.2900000
29530.0000000	-0.2900000
29531.0000000	-0.2950000
29532.0000000	-0.2900000
29533.0000000	-0.2850000
29534.0000000	-0.2850000
29535.0000000	-0.2850000
29536.0000000	-0.2950000
29537.0000000	-0.2900000
29538.0000000	-0.2850000
29539.0000000	-0.2850000
29540.0000000	-0.2850000
29541.0000000	-0.2900000
29542.0000000	-0.2800000
29543.0000000	-0.2850000
29544.0000000	-0.2800000
29545.0000000	-0.2800000
29546.0000000	-0.2800000
29547.0000000	-0.2800000
29548.0000000	-0.2900000
29549.0000000	-0.2850000
29550.0000000	-0.2850000
29551.0000000	-0.2800000
29552.0000000	-0.2750000
29553.0000000	-0.2750000
29554.0000000	-0.2850000
29555.0000000	-0.2800000
29556.0000000	-0.2900000
29557.0000000	-0.2850000
29558.0000000	-0.2800000
29559.0000000	-0.2766667
29560.0000000	-0.2850000
29561.0000000	-0.2800000
29562.0000000	-0.2800000
29563.0000000	-0.2800000
29564.0000000	-0.2750000
29565.0000000	-0.2800000
29566.0000000	-0.2800000
29567.0000000	-0.2850000
29568.0000000	-0.2850000
29569.0000000	-0.2700000
29570.0000000	-0.2750000
29571.0000000	-0.2700000
29572.0000000	-0.2850000
29573.0000000	-0.2700000
29574.0000000	-0.2900000
29575.0000000	-0.2750000
29576.0000000	-0.2900000
29577.0000000	-0.2700000
29578.0000000	-0.2800000
29579.0000000	-0.2750000
29580.0000000	-0.2850000
29581.0000000	-0.2750000
29582.0000000	-0.2800000

29583.0000000	-0.2650000
29584.0000000	-0.2750000
29585.0000000	-0.2800000
29586.0000000	-0.2850000
29587.0000000	-0.2800000
29588.0000000	-0.2800000
29589.0000000	-0.2800000
29590.0000000	-0.2766667
29591.0000000	-0.2850000
29592.0000000	-0.2800000
29593.0000000	-0.2700000
29594.0000000	-0.2750000
29595.0000000	-0.2700000
29596.0000000	-0.2850000
29597.0000000	-0.2750000
29598.0000000	-0.2800000
29599.0000000	-0.2850000
29600.0000000	-0.2800000
29601.0000000	-0.2800000
29602.0000000	-0.2750000
29603.0000000	-0.2800000
29604.0000000	-0.2750000
29605.0000000	-0.2750000
29606.0000000	-0.2800000
29607.0000000	-0.2900000
29608.0000000	-0.2750000
29609.0000000	-0.2850000
29610.0000000	-0.2750000
29611.0000000	-0.2800000
29612.0000000	-0.2800000
29613.0000000	-0.2750000
29614.0000000	-0.2850000
29615.0000000	-0.2800000
29616.0000000	-0.2800000
29617.0000000	-0.2850000
29618.0000000	-0.2850000
29619.0000000	-0.2750000
29620.0000000	-0.2750000
29621.0000000	-0.2833333
29622.0000000	-0.2750000
29623.0000000	-0.2800000
29624.0000000	-0.2750000
29625.0000000	-0.2750000
29626.0000000	-0.2800000
29627.0000000	-0.2850000
29628.0000000	-0.2800000
29629.0000000	-0.2750000
29630.0000000	-0.2750000
29631.0000000	-0.2800000
29632.0000000	-0.2750000
29633.0000000	-0.2800000
29634.0000000	-0.2800000
29635.0000000	-0.2800000
29636.0000000	-0.2800000
29637.0000000	-0.2800000
29638.0000000	-0.2800000
29639.0000000	-0.2700000

29640.0000000	-0.2800000
29641.0000000	-0.2800000
29642.0000000	-0.2800000
29643.0000000	-0.2850000
29644.0000000	-0.2800000
29645.0000000	-0.2800000
29646.0000000	-0.2850000
29647.0000000	-0.2800000
29648.0000000	-0.2800000
29649.0000000	-0.2800000
29650.0000000	-0.2833333
29651.0000000	-0.2850000
29652.0000000	-0.2750000
29653.0000000	-0.2700000
29654.0000000	-0.2900000
29655.0000000	-0.2800000
29656.0000000	-0.2900000
29657.0000000	-0.2800000
29658.0000000	-0.2800000
29659.0000000	-0.2800000
29660.0000000	-0.2850000
29661.0000000	-0.2800000
29662.0000000	-0.2800000
29663.0000000	-0.2800000
29664.0000000	-0.2800000
29665.0000000	-0.2850000
29666.0000000	-0.2850000
29667.0000000	-0.2850000
29668.0000000	-0.2800000
29669.0000000	-0.2800000
29670.0000000	-0.2700000
29671.0000000	-0.2800000
29672.0000000	-0.2800000
29673.0000000	-0.2800000
29674.0000000	-0.2800000
29675.0000000	-0.2700000
29676.0000000	-0.2750000
29677.0000000	-0.2750000
29678.0000000	-0.2850000
29679.0000000	-0.2800000
29680.0000000	-0.2733333
29681.0000000	-0.2800000
29682.0000000	-0.2800000
29683.0000000	-0.2800000
29684.0000000	-0.2700000
29685.0000000	-0.2700000
29686.0000000	-0.2700000
29687.0000000	-0.2750000
29688.0000000	-0.2650000
29689.0000000	-0.2800000
29690.0000000	-0.2650000
29691.0000000	-0.2650000
29692.0000000	-0.2650000
29693.0000000	-0.2700000
29694.0000000	-0.2750000
29695.0000000	-0.2750000
29696.0000000	-0.2700000

29697.0000000	-0.2650000
29698.0000000	-0.2700000
29699.0000000	-0.2700000
29700.0000000	-0.2600000
29701.0000000	-0.2650000
29702.0000000	-0.2650000
29703.0000000	-0.2650000
29704.0000000	-0.2650000
29705.0000000	-0.2700000
29706.0000000	-0.2550000
29707.0000000	-0.2650000
29708.0000000	-0.2650000
29709.0000000	-0.2750000
29710.0000000	-0.2600000
29711.0000000	-0.2600000
29712.0000000	-0.2650000
29713.0000000	-0.2600000
29714.0000000	-0.2550000
29715.0000000	-0.2600000
29716.0000000	-0.2650000
29717.0000000	-0.2550000
29718.0000000	-0.2550000
29719.0000000	-0.2600000
29720.0000000	-0.2650000
29721.0000000	-0.2600000
29722.0000000	-0.2600000
29723.0000000	-0.2500000
29724.0000000	-0.2500000
29725.0000000	-0.2550000
29726.0000000	-0.2550000
29727.0000000	-0.2550000
29728.0000000	-0.2600000
29729.0000000	-0.2450000
29730.0000000	-0.2500000
29731.0000000	-0.2500000
29732.0000000	-0.2550000
29733.0000000	-0.2550000
29734.0000000	-0.2450000
29735.0000000	-0.2550000
29736.0000000	-0.2500000
29737.0000000	-0.2600000
29738.0000000	-0.2400000
29739.0000000	-0.2500000
29740.0000000	-0.2400000
29741.0000000	-0.2433333
29742.0000000	-0.2500000
29743.0000000	-0.2450000
29744.0000000	-0.2500000
29745.0000000	-0.2400000
29746.0000000	-0.2500000
29747.0000000	-0.2350000
29748.0000000	-0.2500000
29749.0000000	-0.2400000
29750.0000000	-0.2400000
29751.0000000	-0.2450000
29752.0000000	-0.2400000
29753.0000000	-0.2500000

29754.0000000	-0.2450000
29755.0000000	-0.2450000
29756.0000000	-0.2350000
29757.0000000	-0.2400000
29758.0000000	-0.2400000
29759.0000000	-0.2400000
29760.0000000	-0.2350000
29761.0000000	-0.2350000
29762.0000000	-0.2350000
29763.0000000	-0.2400000
29764.0000000	-0.2300000
29765.0000000	-0.2400000
29766.0000000	-0.2300000
29767.0000000	-0.2400000
29768.0000000	-0.2450000
29769.0000000	-0.2450000
29770.0000000	-0.2350000
29771.0000000	-0.2300000
29772.0000000	-0.2400000
29773.0000000	-0.2350000
29774.0000000	-0.2400000
29775.0000000	-0.2350000
29776.0000000	-0.2350000
29777.0000000	-0.2300000
29778.0000000	-0.2200000
29779.0000000	-0.2400000
29780.0000000	-0.2300000
29781.0000000	-0.2300000
29782.0000000	-0.2200000
29783.0000000	-0.2300000
29784.0000000	-0.2200000
29785.0000000	-0.2300000
29786.0000000	-0.2250000
29787.0000000	-0.2250000
29788.0000000	-0.2300000
29789.0000000	-0.2250000
29790.0000000	-0.2300000
29791.0000000	-0.2250000
29792.0000000	-0.2300000
29793.0000000	-0.2150000
29794.0000000	-0.2100000
29795.0000000	-0.2200000
29796.0000000	-0.2200000
29797.0000000	-0.2300000
29798.0000000	-0.2250000
29799.0000000	-0.2200000
29800.0000000	-0.2250000
29801.0000000	-0.2166667
29802.0000000	-0.2250000
29803.0000000	-0.2150000
29804.0000000	-0.2050000
29805.0000000	-0.2150000
29806.0000000	-0.2100000
29807.0000000	-0.2150000
29808.0000000	-0.2050000
29809.0000000	-0.2200000
29810.0000000	-0.2100000

29811.0000000	-0.2250000
29812.0000000	-0.2100000
29813.0000000	-0.2050000
29814.0000000	-0.2050000
29815.0000000	-0.2050000
29816.0000000	-0.2100000
29817.0000000	-0.2000000
29818.0000000	-0.2100000
29819.0000000	-0.2000000
29820.0000000	-0.2050000
29821.0000000	-0.2000000
29822.0000000	-0.2100000
29823.0000000	-0.2150000
29824.0000000	-0.2000000
29825.0000000	-0.2000000
29826.0000000	-0.2000000
29827.0000000	-0.2000000
29828.0000000	-0.2000000
29829.0000000	-0.2000000
29830.0000000	-0.2050000
29831.0000000	-0.1950000
29832.0000000	-0.1966667
29833.0000000	-0.2000000
29834.0000000	-0.2000000
29835.0000000	-0.1850000
29836.0000000	-0.1950000
29837.0000000	-0.2000000
29838.0000000	-0.1900000
29839.0000000	-0.1900000
29840.0000000	-0.2000000
29841.0000000	-0.1900000
29842.0000000	-0.1950000
29843.0000000	-0.1900000
29844.0000000	-0.1850000
29845.0000000	-0.1850000
29846.0000000	-0.1850000
29847.0000000	-0.1850000
29848.0000000	-0.1900000
29849.0000000	-0.1900000
29850.0000000	-0.1900000
29851.0000000	-0.1850000
29852.0000000	-0.1850000
29853.0000000	-0.1850000
29854.0000000	-0.1850000
29855.0000000	-0.1800000
29856.0000000	-0.1850000
29857.0000000	-0.1850000
29858.0000000	-0.1900000
29859.0000000	-0.1850000
29860.0000000	-0.1900000
29861.0000000	-0.1800000
29862.0000000	-0.1766667
29863.0000000	-0.1900000
29864.0000000	-0.1750000
29865.0000000	-0.1850000
29866.0000000	-0.1800000
29867.0000000	-0.1750000

29868.0000000	-0.1850000
29869.0000000	-0.1800000
29870.0000000	-0.1850000
29871.0000000	-0.1850000
29872.0000000	-0.1800000
29873.0000000	-0.1800000
29874.0000000	-0.1750000
29875.0000000	-0.1700000
29876.0000000	-0.1800000
29877.0000000	-0.1800000
29878.0000000	-0.1850000
29879.0000000	-0.1800000
29880.0000000	-0.1750000
29881.0000000	-0.1800000
29882.0000000	-0.1800000
29883.0000000	-0.1750000
29884.0000000	-0.1750000
29885.0000000	-0.1750000
29886.0000000	-0.1750000
29887.0000000	-0.1800000
29888.0000000	-0.1850000
29889.0000000	-0.1750000
29890.0000000	-0.1750000
29891.0000000	-0.1833333
29892.0000000	-0.1750000
29893.0000000	-0.1700000
29894.0000000	-0.1750000
29895.0000000	-0.1700000
29896.0000000	-0.1850000
29897.0000000	-0.1750000
29898.0000000	-0.1750000
29899.0000000	-0.1700000
29900.0000000	-0.1800000
29901.0000000	-0.1750000
29902.0000000	-0.1650000
29903.0000000	-0.1750000
29904.0000000	-0.1700000
29905.0000000	-0.1750000
29906.0000000	-0.1700000
29907.0000000	-0.1700000
29908.0000000	-0.1700000
29909.0000000	-0.1750000
29910.0000000	-0.1750000
29911.0000000	-0.1700000
29912.0000000	-0.1700000
29913.0000000	-0.1750000
29914.0000000	-0.1750000
29915.0000000	-0.1700000
29916.0000000	-0.1700000
29917.0000000	-0.1700000
29918.0000000	-0.1650000
29919.0000000	-0.1650000
29920.0000000	-0.1700000
29921.0000000	-0.1700000
29922.0000000	-0.1733333
29923.0000000	-0.1600000
29924.0000000	-0.1650000

29925.0000000	-0.1650000
29926.0000000	-0.1750000
29927.0000000	-0.1700000
29928.0000000	-0.1650000
29929.0000000	-0.1650000
29930.0000000	-0.1550000
29931.0000000	-0.1650000
29932.0000000	-0.1600000
29933.0000000	-0.1700000
29934.0000000	-0.1700000
29935.0000000	-0.1700000
29936.0000000	-0.1600000
29937.0000000	-0.1550000
29938.0000000	-0.1600000
29939.0000000	-0.1600000
29940.0000000	-0.1600000
29941.0000000	-0.1550000
29942.0000000	-0.1600000
29943.0000000	-0.1650000
29944.0000000	-0.1600000
29945.0000000	-0.1600000
29946.0000000	-0.1500000
29947.0000000	-0.1550000
29948.0000000	-0.1550000
29949.0000000	-0.1550000
29950.0000000	-0.1550000
29951.0000000	-0.1600000
29952.0000000	-0.1450000
29953.0000000	-0.1466667
29954.0000000	-0.1400000
29955.0000000	-0.1450000
29956.0000000	-0.1450000
29957.0000000	-0.1450000
29958.0000000	-0.1450000
29959.0000000	-0.1500000
29960.0000000	-0.1450000
29961.0000000	-0.1450000
29962.0000000	-0.1450000
29963.0000000	-0.1450000
29964.0000000	-0.1400000
29965.0000000	-0.1450000
29966.0000000	-0.1450000
29967.0000000	-0.1500000
29968.0000000	-0.1400000
29969.0000000	-0.1400000
29970.0000000	-0.1350000
29971.0000000	-0.1450000
29972.0000000	-0.1450000
29973.0000000	-0.1450000
29974.0000000	-0.1350000
29975.0000000	-0.1400000
29976.0000000	-0.1450000
29977.0000000	-0.1400000
29978.0000000	-0.1450000
29979.0000000	-0.1400000
29980.0000000	-0.1500000
29981.0000000	-0.1350000

29982.0000000	-0.1433333
29983.0000000	-0.1400000
29984.0000000	-0.1350000
29985.0000000	-0.1450000
29986.0000000	-0.1350000
29987.0000000	-0.1400000
29988.0000000	-0.1400000
29989.0000000	-0.1400000
29990.0000000	-0.1350000
29991.0000000	-0.1450000
29992.0000000	-0.1300000
29993.0000000	-0.1450000
29994.0000000	-0.1300000
29995.0000000	-0.1400000
29996.0000000	-0.1400000
29997.0000000	-0.1450000
29998.0000000	-0.1350000
29999.0000000	-0.1350000
30000.0000000	-0.1400000
30001.0000000	-0.1350000
30002.0000000	-0.1450000
30003.0000000	-0.1350000
30004.0000000	-0.1400000
30005.0000000	-0.1350000
30006.0000000	-0.1350000
30007.0000000	-0.1400000
30008.0000000	-0.1350000
30009.0000000	-0.1350000
30010.0000000	-0.1300000
30011.0000000	-0.1400000
30012.0000000	-0.1400000
30013.0000000	-0.1350000
30014.0000000	-0.1400000
30015.0000000	-0.1300000
30016.0000000	-0.1350000
30017.0000000	-0.1400000
30018.0000000	-0.1300000
30019.0000000	-0.1400000
30020.0000000	-0.1350000
30021.0000000	-0.1300000
30022.0000000	-0.1350000
30023.0000000	-0.1250000
30024.0000000	-0.1350000
30025.0000000	-0.1250000
30026.0000000	-0.1300000
30027.0000000	-0.1350000
30028.0000000	-0.1300000
30029.0000000	-0.1250000
30030.0000000	-0.1200000
30031.0000000	-0.1250000
30032.0000000	-0.1250000
30033.0000000	-0.1300000
30034.0000000	-0.1300000
30035.0000000	-0.1200000
30036.0000000	-0.1150000
30037.0000000	-0.1300000
30038.0000000	-0.1200000

30039.0000000	-0.1350000
30040.0000000	-0.1200000
30041.0000000	-0.1250000
30042.0000000	-0.1350000
30043.0000000	-0.1233333
30044.0000000	-0.1150000
30045.0000000	-0.1250000
30046.0000000	-0.1250000
30047.0000000	-0.1250000
30048.0000000	-0.1300000
30049.0000000	-0.1250000
30050.0000000	-0.1150000
30051.0000000	-0.1200000
30052.0000000	-0.1150000
30053.0000000	-0.1200000
30054.0000000	-0.1200000
30055.0000000	-0.1100000
30056.0000000	-0.1250000
30057.0000000	-0.1150000
30058.0000000	-0.1200000
30059.0000000	-0.1200000
30060.0000000	-0.1150000
30061.0000000	-0.1200000
30062.0000000	-0.1200000
30063.0000000	-0.1200000
30064.0000000	-0.1150000
30065.0000000	-0.1200000
30066.0000000	-0.1200000
30067.0000000	-0.1200000
30068.0000000	-0.1100000
30069.0000000	-0.1100000
30070.0000000	-0.1100000
30071.0000000	-0.1100000
30072.0000000	-0.1200000
30073.0000000	-0.1133333
30074.0000000	-0.1100000
30075.0000000	-0.1100000
30076.0000000	-0.1200000
30077.0000000	-0.1150000
30078.0000000	-0.1200000
30079.0000000	-0.1100000
30080.0000000	-0.1100000
30081.0000000	-0.1050000
30082.0000000	-0.1200000
30083.0000000	-0.1150000
30084.0000000	-0.1200000
30085.0000000	-0.1200000
30086.0000000	-0.1200000
30087.0000000	-0.1250000
30088.0000000	-0.1150000
30089.0000000	-0.1200000
30090.0000000	-0.1100000
30091.0000000	-0.1100000
30092.0000000	-0.1200000
30093.0000000	-0.1200000
30094.0000000	-0.1200000
30095.0000000	-0.1150000

30096.0000000	-0.1150000
30097.0000000	-0.1250000
30098.0000000	-0.1150000
30099.0000000	-0.1250000
30100.0000000	-0.1150000
30101.0000000	-0.1150000
30102.0000000	-0.1250000
30103.0000000	-0.1200000
30104.0000000	-0.1150000
30105.0000000	-0.1150000
30106.0000000	-0.1200000
30107.0000000	-0.1200000
30108.0000000	-0.1150000
30109.0000000	-0.1200000
30110.0000000	-0.1100000
30111.0000000	-0.1200000
30112.0000000	-0.1150000
30113.0000000	-0.1150000
30114.0000000	-0.1200000
30115.0000000	-0.1150000
30116.0000000	-0.1200000
30117.0000000	-0.1200000
30118.0000000	-0.1150000
30119.0000000	-0.1200000
30120.0000000	-0.1150000
30121.0000000	-0.1200000
30122.0000000	-0.1200000
30123.0000000	-0.1150000
30124.0000000	-0.1200000
30125.0000000	-0.1100000
30126.0000000	-0.1150000
30127.0000000	-0.1150000
30128.0000000	-0.1150000
30129.0000000	-0.1100000
30130.0000000	-0.1050000
30131.0000000	-0.1050000
30132.0000000	-0.1150000
30133.0000000	-0.1166667
30134.0000000	-0.1100000
30135.0000000	-0.1100000
30136.0000000	-0.1100000
30137.0000000	-0.1050000
30138.0000000	-0.1150000
30139.0000000	-0.1150000
30140.0000000	-0.1200000
30141.0000000	-0.1050000
30142.0000000	-0.1200000
30143.0000000	-0.1100000
30144.0000000	-0.1100000
30145.0000000	-0.1200000
30146.0000000	-0.1150000
30147.0000000	-0.1200000
30148.0000000	-0.1100000
30149.0000000	-0.1150000
30150.0000000	-0.1050000
30151.0000000	-0.1150000
30152.0000000	-0.1200000

30153.0000000	-0.1150000
30154.0000000	-0.1150000
30155.0000000	-0.1150000
30156.0000000	-0.1100000
30157.0000000	-0.1200000
30158.0000000	-0.1200000
30159.0000000	-0.1100000
30160.0000000	-0.1200000
30161.0000000	-0.1150000
30162.0000000	-0.1200000
30163.0000000	-0.1100000
30164.0000000	-0.1166667
30165.0000000	-0.1200000
30166.0000000	-0.1100000
30167.0000000	-0.1150000
30168.0000000	-0.1150000
30169.0000000	-0.1150000
30170.0000000	-0.1200000
30171.0000000	-0.1150000
30172.0000000	-0.1250000
30173.0000000	-0.1100000
30174.0000000	-0.1150000
30175.0000000	-0.1050000
30176.0000000	-0.1200000
30177.0000000	-0.1100000
30178.0000000	-0.1200000
30179.0000000	-0.1150000
30180.0000000	-0.1200000
30181.0000000	-0.1100000
30182.0000000	-0.1200000
30183.0000000	-0.1100000
30184.0000000	-0.1150000
30185.0000000	-0.1050000
30186.0000000	-0.1200000
30187.0000000	-0.1100000
30188.0000000	-0.1150000
30189.0000000	-0.1050000
30190.0000000	-0.1150000
30191.0000000	-0.1100000
30192.0000000	-0.1200000
30193.0000000	-0.1050000
30194.0000000	-0.1133333
30195.0000000	-0.1150000
30196.0000000	-0.1000000
30197.0000000	-0.1150000
30198.0000000	-0.1050000
30199.0000000	-0.1150000
30200.0000000	-0.1100000
30201.0000000	-0.1150000
30202.0000000	-0.1100000
30203.0000000	-0.1150000
30204.0000000	-0.1100000
30205.0000000	-0.1100000
30206.0000000	-0.1150000
30207.0000000	-0.1000000
30208.0000000	-0.1050000
30209.0000000	-0.1100000

30210.0000000	-0.1100000
30211.0000000	-0.1150000
30212.0000000	-0.1050000
30213.0000000	-0.1150000
30214.0000000	-0.1150000
30215.0000000	-0.1100000
30216.0000000	-0.1150000
30217.0000000	-0.1100000
30218.0000000	-0.1100000
30219.0000000	-0.1100000
30220.0000000	-0.1150000
30221.0000000	-0.1200000
30222.0000000	-0.1050000
30223.0000000	-0.1100000
30224.0000000	-0.1066667
30225.0000000	-0.1050000
30226.0000000	-0.1200000
30227.0000000	-0.1050000
30228.0000000	-0.1050000
30229.0000000	-0.1100000
30230.0000000	-0.1100000
30231.0000000	-0.1050000
30232.0000000	-0.1100000
30233.0000000	-0.1100000
30234.0000000	-0.1150000
30235.0000000	-0.1100000
30236.0000000	-0.1050000
30237.0000000	-0.1100000
30238.0000000	-0.1000000
30239.0000000	-0.1050000
30240.0000000	-0.1050000
30241.0000000	-0.1050000
30242.0000000	-0.1000000
30243.0000000	-0.1050000
30244.0000000	-0.1100000
30245.0000000	-0.1000000
30246.0000000	-0.1100000
30247.0000000	-0.1100000
30248.0000000	-0.1050000
30249.0000000	-0.1100000
30250.0000000	-0.1050000
30251.0000000	-0.1100000
30252.0000000	-0.1050000
30253.0000000	-0.1100000
30254.0000000	-0.1066667
30255.0000000	-0.1050000
30256.0000000	-0.1100000
30257.0000000	-0.1050000
30258.0000000	-0.1050000
30259.0000000	-0.1100000
30260.0000000	-0.1100000
30261.0000000	-0.1050000
30262.0000000	-0.1050000
30263.0000000	-0.1150000
30264.0000000	-0.1000000
30265.0000000	-0.1050000
30266.0000000	-0.1050000

30267.0000000	-0.1050000
30268.0000000	-0.1050000
30269.0000000	-0.1050000
30270.0000000	-0.1100000
30271.0000000	-0.1100000
30272.0000000	-0.1050000
30273.0000000	-0.1050000
30274.0000000	-0.1050000
30275.0000000	-0.1100000
30276.0000000	-0.1050000
30277.0000000	-0.1050000
30278.0000000	-0.1100000
30279.0000000	-0.1100000
30280.0000000	-0.1150000
30281.0000000	-0.1100000
30282.0000000	-0.1200000
30283.0000000	-0.1100000
30284.0000000	-0.1100000
30285.0000000	-0.1150000
30286.0000000	-0.1000000
30287.0000000	-0.1150000
30288.0000000	-0.1050000
30289.0000000	-0.1100000
30290.0000000	-0.1100000
30291.0000000	-0.1050000
30292.0000000	-0.1150000
30293.0000000	-0.1000000
30294.0000000	-0.1100000
30295.0000000	-0.1050000
30296.0000000	-0.1150000
30297.0000000	-0.1100000
30298.0000000	-0.1100000
30299.0000000	-0.1150000
30300.0000000	-0.1150000
30301.0000000	-0.1200000
30302.0000000	-0.1100000
30303.0000000	-0.1100000
30304.0000000	-0.1050000
30305.0000000	-0.1100000
30306.0000000	-0.1150000
30307.0000000	-0.1100000
30308.0000000	-0.1150000
30309.0000000	-0.1100000
30310.0000000	-0.1100000
30311.0000000	-0.1050000
30312.0000000	-0.1050000
30313.0000000	-0.1100000
30314.0000000	-0.1100000
30315.0000000	-0.1100000
30316.0000000	-0.1150000
30317.0000000	-0.1100000
30318.0000000	-0.1150000
30319.0000000	-0.1100000
30320.0000000	-0.1200000
30321.0000000	-0.1050000
30322.0000000	-0.1150000
30323.0000000	-0.1100000

30324.0000000	-0.1150000
30325.0000000	-0.1050000
30326.0000000	-0.1150000
30327.0000000	-0.1150000
30328.0000000	-0.1150000
30329.0000000	-0.1050000
30330.0000000	-0.1050000
30331.0000000	-0.1150000
30332.0000000	-0.1100000
30333.0000000	-0.1200000
30334.0000000	-0.1150000
30335.0000000	-0.1200000
30336.0000000	-0.1050000
30337.0000000	-0.1150000
30338.0000000	-0.1050000
30339.0000000	-0.1200000
30340.0000000	-0.1050000
30341.0000000	-0.1150000
30342.0000000	-0.1100000
30343.0000000	-0.1150000
30344.0000000	-0.1133333
30345.0000000	-0.1150000
30346.0000000	-0.1200000
30347.0000000	-0.1100000
30348.0000000	-0.1100000
30349.0000000	-0.1100000
30350.0000000	-0.1200000
30351.0000000	-0.1150000
30352.0000000	-0.1250000
30353.0000000	-0.1100000
30354.0000000	-0.1200000
30355.0000000	-0.1100000
30356.0000000	-0.1200000
30357.0000000	-0.1050000
30358.0000000	-0.1200000
30359.0000000	-0.1050000
30360.0000000	-0.1150000
30361.0000000	-0.1000000
30362.0000000	-0.1150000
30363.0000000	-0.1150000
30364.0000000	-0.1150000
30365.0000000	-0.1100000
30366.0000000	-0.1150000
30367.0000000	-0.1150000
30368.0000000	-0.1150000
30369.0000000	-0.1150000
30370.0000000	-0.1100000
30371.0000000	-0.1200000
30372.0000000	-0.1100000
30373.0000000	-0.1100000
30374.0000000	-0.1100000
30375.0000000	-0.1100000
30376.0000000	-0.1150000
30377.0000000	-0.1100000
30378.0000000	-0.1000000
30379.0000000	-0.1200000
30380.0000000	-0.1100000

30381.0000000	-0.1200000
30382.0000000	-0.1050000
30383.0000000	-0.1100000
30384.0000000	-0.1100000
30385.0000000	-0.1050000
30386.0000000	-0.1150000
30387.0000000	-0.1150000
30388.0000000	-0.1150000
30389.0000000	-0.1100000
30390.0000000	-0.1100000
30391.0000000	-0.1250000
30392.0000000	-0.1150000
30393.0000000	-0.1150000
30394.0000000	-0.1100000
30395.0000000	-0.1150000
30396.0000000	-0.1150000
30397.0000000	-0.1150000
30398.0000000	-0.1150000
30399.0000000	-0.1150000
30400.0000000	-0.1100000
30401.0000000	-0.1100000
30402.0000000	-0.1150000
30403.0000000	-0.1150000
30404.0000000	-0.1150000
30405.0000000	-0.1133333
30406.0000000	-0.1050000
30407.0000000	-0.1250000
30408.0000000	-0.1200000
30409.0000000	-0.1150000
30410.0000000	-0.1150000
30411.0000000	-0.1100000
30412.0000000	-0.1100000
30413.0000000	-0.1150000
30414.0000000	-0.1200000
30415.0000000	-0.1100000
30416.0000000	-0.1150000
30417.0000000	-0.1150000
30418.0000000	-0.1150000
30419.0000000	-0.1150000
30420.0000000	-0.1050000
30421.0000000	-0.1050000
30422.0000000	-0.1200000
30423.0000000	-0.1100000
30424.0000000	-0.1150000
30425.0000000	-0.1100000
30426.0000000	-0.1200000
30427.0000000	-0.1050000
30428.0000000	-0.1050000
30429.0000000	-0.1150000
30430.0000000	-0.1100000
30431.0000000	-0.1150000
30432.0000000	-0.1050000
30433.0000000	-0.1150000
30434.0000000	-0.1250000
30435.0000000	-0.1066667
30436.0000000	-0.1100000
30437.0000000	-0.1150000

30438.0000000	-0.1150000
30439.0000000	-0.1150000
30440.0000000	-0.1150000
30441.0000000	-0.1100000
30442.0000000	-0.1200000
30443.0000000	-0.1150000
30444.0000000	-0.1050000
30445.0000000	-0.1100000
30446.0000000	-0.1150000
30447.0000000	-0.1150000
30448.0000000	-0.1050000
30449.0000000	-0.1100000
30450.0000000	-0.1150000
30451.0000000	-0.1100000
30452.0000000	-0.1200000
30453.0000000	-0.1050000
30454.0000000	-0.1150000
30455.0000000	-0.1150000
30456.0000000	-0.1100000
30457.0000000	-0.1050000
30458.0000000	-0.1050000
30459.0000000	-0.1050000
30460.0000000	-0.1050000
30461.0000000	-0.1150000
30462.0000000	-0.1050000
30463.0000000	-0.1100000
30464.0000000	-0.1100000
30465.0000000	-0.1066667
30466.0000000	-0.1100000
30467.0000000	-0.1150000
30468.0000000	-0.1150000
30469.0000000	-0.1100000
30470.0000000	-0.1050000
30471.0000000	-0.1050000
30472.0000000	-0.1100000
30473.0000000	-0.1200000
30474.0000000	-0.1050000
30475.0000000	-0.1150000
30476.0000000	-0.1150000
30477.0000000	-0.1150000
30478.0000000	-0.1100000
30479.0000000	-0.1050000
30480.0000000	-0.1050000
30481.0000000	-0.1000000
30482.0000000	-0.1100000
30483.0000000	-0.1150000
30484.0000000	-0.1100000
30485.0000000	-0.1150000
30486.0000000	-0.1050000
30487.0000000	-0.1050000
30488.0000000	-0.1150000
30489.0000000	-0.1000000
30490.0000000	-0.1000000
30491.0000000	-0.1100000
30492.0000000	-0.1050000
30493.0000000	-0.1150000
30494.0000000	-0.1050000

30495.0000000	-0.1033333
30496.0000000	-0.1050000
30497.0000000	-0.1000000
30498.0000000	-0.1100000
30499.0000000	-0.1050000
30500.0000000	-0.1050000
30501.0000000	-0.1000000
30502.0000000	-0.1050000
30503.0000000	-0.1050000
30504.0000000	-0.1050000
30505.0000000	-0.1100000
30506.0000000	-0.1100000
30507.0000000	-0.1100000
30508.0000000	-0.1100000
30509.0000000	-0.1100000
30510.0000000	-0.1100000
30511.0000000	-0.1050000
30512.0000000	-0.1100000
30513.0000000	-0.1100000
30514.0000000	-0.1050000
30515.0000000	-0.1050000
30516.0000000	-0.1100000
30517.0000000	-0.1100000
30518.0000000	-0.1100000
30519.0000000	-0.1100000
30520.0000000	-0.1050000
30521.0000000	-0.1100000
30522.0000000	-0.1050000
30523.0000000	-0.1100000
30524.0000000	-0.1100000
30525.0000000	-0.1100000
30526.0000000	-0.1100000
30527.0000000	-0.1000000
30528.0000000	-0.1150000
30529.0000000	-0.1050000
30530.0000000	-0.1050000
30531.0000000	-0.1000000
30532.0000000	-0.1000000
30533.0000000	-0.1150000
30534.0000000	-0.1100000
30535.0000000	-0.1050000
30536.0000000	-0.1050000
30537.0000000	-0.1100000
30538.0000000	-0.1050000
30539.0000000	-0.1050000
30540.0000000	-0.1000000
30541.0000000	-0.1050000
30542.0000000	-0.1000000
30543.0000000	-0.1050000
30544.0000000	-0.1050000
30545.0000000	-0.1000000
30546.0000000	-0.1000000
30547.0000000	-0.1050000
30548.0000000	-0.1000000
30549.0000000	-0.1050000
30550.0000000	-0.1050000
30551.0000000	-0.1000000

30552.0000000	-0.1000000
30553.0000000	-0.1000000
30554.0000000	-0.1050000
30555.0000000	-0.1050000
30556.0000000	-0.1000000
30557.0000000	-0.1000000
30558.0000000	-0.1050000
30559.0000000	-0.1050000
30560.0000000	-0.0950000
30561.0000000	-0.1000000
30562.0000000	-0.1000000
30563.0000000	-0.0950000
30564.0000000	-0.0950000
30565.0000000	-0.1000000
30566.0000000	-0.0950000
30567.0000000	-0.0950000
30568.0000000	-0.1000000
30569.0000000	-0.0950000
30570.0000000	-0.0900000
30571.0000000	-0.0950000
30572.0000000	-0.0950000
30573.0000000	-0.0900000
30574.0000000	-0.1000000
30575.0000000	-0.0950000
30576.0000000	-0.0950000
30577.0000000	-0.1000000
30578.0000000	-0.0950000
30579.0000000	-0.0950000
30580.0000000	-0.0900000
30581.0000000	-0.0900000
30582.0000000	-0.0900000
30583.0000000	-0.0950000
30584.0000000	-0.0900000
30585.0000000	-0.0900000
30586.0000000	-0.0900000
30587.0000000	-0.0900000
30588.0000000	-0.0900000
30589.0000000	-0.0800000
30590.0000000	-0.0900000
30591.0000000	-0.0800000
30592.0000000	-0.0900000
30593.0000000	-0.0800000
30594.0000000	-0.0850000
30595.0000000	-0.0850000
30596.0000000	-0.0800000
30597.0000000	-0.0800000
30598.0000000	-0.0800000
30599.0000000	-0.0850000
30600.0000000	-0.0800000
30601.0000000	-0.0900000
30602.0000000	-0.0800000
30603.0000000	-0.0750000
30604.0000000	-0.0700000
30605.0000000	-0.0800000
30606.0000000	-0.0800000
30607.0000000	-0.0850000
30608.0000000	-0.0800000

30609.0000000	-0.0750000
30610.0000000	-0.0700000
30611.0000000	-0.0800000
30612.0000000	-0.0750000
30613.0000000	-0.0850000
30614.0000000	-0.0800000
30615.0000000	-0.0850000
30616.0000000	-0.0766667
30617.0000000	-0.0750000
30618.0000000	-0.0750000
30619.0000000	-0.0800000
30620.0000000	-0.0750000
30621.0000000	-0.0800000
30622.0000000	-0.0800000
30623.0000000	-0.0800000
30624.0000000	-0.0750000
30625.0000000	-0.0850000
30626.0000000	-0.0700000
30627.0000000	-0.0850000
30628.0000000	-0.0750000
30629.0000000	-0.0750000
30630.0000000	-0.0750000
30631.0000000	-0.0750000
30632.0000000	-0.0750000
30633.0000000	-0.0700000
30634.0000000	-0.0850000
30635.0000000	-0.0750000
30636.0000000	-0.0800000
30637.0000000	-0.0750000
30638.0000000	-0.0750000
30639.0000000	-0.0700000
30640.0000000	-0.0700000
30641.0000000	-0.0750000
30642.0000000	-0.0700000
30643.0000000	-0.0700000
30644.0000000	-0.0650000
30645.0000000	-0.0800000
30646.0000000	-0.0750000
30647.0000000	-0.0733333
30648.0000000	-0.0600000
30649.0000000	-0.0700000
30650.0000000	-0.0750000
30651.0000000	-0.0750000
30652.0000000	-0.0700000
30653.0000000	-0.0600000
30654.0000000	-0.0650000
30655.0000000	-0.0700000
30656.0000000	-0.0700000
30657.0000000	-0.0750000
30658.0000000	-0.0700000
30659.0000000	-0.0700000
30660.0000000	-0.0600000
30661.0000000	-0.0600000
30662.0000000	-0.0700000
30663.0000000	-0.0700000
30664.0000000	-0.0700000
30665.0000000	-0.0650000

30666.0000000	-0.0650000
30667.0000000	-0.0600000
30668.0000000	-0.0700000
30669.0000000	-0.0600000
30670.0000000	-0.0700000
30671.0000000	-0.0750000
30672.0000000	-0.0650000
30673.0000000	-0.0550000
30674.0000000	-0.0650000
30675.0000000	-0.0600000
30676.0000000	-0.0666667
30677.0000000	-0.0650000
30678.0000000	-0.0600000
30679.0000000	-0.0600000
30680.0000000	-0.0600000
30681.0000000	-0.0700000
30682.0000000	-0.0650000
30683.0000000	-0.0700000
30684.0000000	-0.0600000
30685.0000000	-0.0650000
30686.0000000	-0.0600000
30687.0000000	-0.0700000
30688.0000000	-0.0600000
30689.0000000	-0.0650000
30690.0000000	-0.0550000
30691.0000000	-0.0600000
30692.0000000	-0.0600000
30693.0000000	-0.0650000
30694.0000000	-0.0600000
30695.0000000	-0.0600000
30696.0000000	-0.0650000
30697.0000000	-0.0600000
30698.0000000	-0.0600000
30699.0000000	-0.0650000
30700.0000000	-0.0600000
30701.0000000	-0.0600000
30702.0000000	-0.0550000
30703.0000000	-0.0600000
30704.0000000	-0.0650000
30705.0000000	-0.0550000
30706.0000000	-0.0600000
30707.0000000	-0.0533333
30708.0000000	-0.0600000
30709.0000000	-0.0650000
30710.0000000	-0.0600000
30711.0000000	-0.0600000
30712.0000000	-0.0550000
30713.0000000	-0.0550000
30714.0000000	-0.0600000
30715.0000000	-0.0500000
30716.0000000	-0.0550000
30717.0000000	-0.0650000
30718.0000000	-0.0600000
30719.0000000	-0.0450000
30720.0000000	-0.0600000
30721.0000000	-0.0500000
30722.0000000	-0.0550000

30723.0000000	-0.0550000
30724.0000000	-0.0500000
30725.0000000	-0.0500000
30726.0000000	-0.0550000
30727.0000000	-0.0500000
30728.0000000	-0.0450000
30729.0000000	-0.0550000
30730.0000000	-0.0500000
30731.0000000	-0.0450000
30732.0000000	-0.0500000
30733.0000000	-0.0450000
30734.0000000	-0.0500000
30735.0000000	-0.0500000
30736.0000000	-0.0500000
30737.0000000	-0.0500000
30738.0000000	-0.0450000
30739.0000000	-0.0550000
30740.0000000	-0.0450000
30741.0000000	-0.0450000
30742.0000000	-0.0500000
30743.0000000	-0.0450000
30744.0000000	-0.0500000
30745.0000000	-0.0400000
30746.0000000	-0.0500000
30747.0000000	-0.0450000
30748.0000000	-0.0550000
30749.0000000	-0.0500000
30750.0000000	-0.0350000
30751.0000000	-0.0450000
30752.0000000	-0.0400000
30753.0000000	-0.0450000
30754.0000000	-0.0400000
30755.0000000	-0.0450000
30756.0000000	-0.0450000
30757.0000000	-0.0550000
30758.0000000	-0.0400000
30759.0000000	-0.0450000
30760.0000000	-0.0500000
30761.0000000	-0.0400000
30762.0000000	-0.0450000
30763.0000000	-0.0350000
30764.0000000	-0.0400000
30765.0000000	-0.0350000
30766.0000000	-0.0400000
30767.0000000	-0.0366667
30768.0000000	-0.0350000
30769.0000000	-0.0450000
30770.0000000	-0.0400000
30771.0000000	-0.0300000
30772.0000000	-0.0400000
30773.0000000	-0.0350000
30774.0000000	-0.0350000
30775.0000000	-0.0350000
30776.0000000	-0.0350000
30777.0000000	-0.0300000
30778.0000000	-0.0350000
30779.0000000	-0.0350000

30780.0000000	-0.0350000
30781.0000000	-0.0350000
30782.0000000	-0.0300000
30783.0000000	-0.0250000
30784.0000000	-0.0250000
30785.0000000	-0.0300000
30786.0000000	-0.0300000
30787.0000000	-0.0300000
30788.0000000	-0.0300000
30789.0000000	-0.0250000
30790.0000000	-0.0250000
30791.0000000	-0.0300000
30792.0000000	-0.0300000
30793.0000000	-0.0300000
30794.0000000	-0.0300000
30795.0000000	-0.0300000
30796.0000000	-0.0200000
30797.0000000	-0.0233333
30798.0000000	-0.0250000
30799.0000000	-0.0200000
30800.0000000	-0.0200000
30801.0000000	-0.0150000
30802.0000000	-0.0300000
30803.0000000	-0.0200000
30804.0000000	-0.0200000
30805.0000000	-0.0200000
30806.0000000	-0.0200000
30807.0000000	-0.0250000
30808.0000000	-0.0300000
30809.0000000	-0.0150000
30810.0000000	-0.0200000
30811.0000000	-0.0250000
30812.0000000	-0.0200000
30813.0000000	-0.0200000
30814.0000000	-0.0250000
30815.0000000	-0.0150000
30816.0000000	-0.0250000
30817.0000000	-0.0200000
30818.0000000	-0.0200000
30819.0000000	-0.0150000
30820.0000000	-0.0150000
30821.0000000	-0.0250000
30822.0000000	-0.0150000
30823.0000000	-0.0150000
30824.0000000	-0.0100000
30825.0000000	-0.0200000
30826.0000000	-0.0200000
30827.0000000	-0.0166667
30828.0000000	-0.0100000
30829.0000000	-0.0200000
30830.0000000	-0.0200000
30831.0000000	-0.0150000
30832.0000000	-0.0200000
30833.0000000	-0.0150000
30834.0000000	-0.0100000
30835.0000000	-0.0200000
30836.0000000	-0.0200000

30837.0000000	-0.0100000
30838.0000000	-0.0100000
30839.0000000	-0.0050000
30840.0000000	-0.0100000
30841.0000000	-0.0200000
30842.0000000	-0.0100000
30843.0000000	-0.0150000
30844.0000000	-0.0050000
30845.0000000	-0.0150000
30846.0000000	0.0000000
30847.0000000	-0.0100000
30848.0000000	-0.0050000
30849.0000000	-0.0100000
30850.0000000	-0.0200000
30851.0000000	-0.0050000
30852.0000000	-0.0100000
30853.0000000	0.0000000
30854.0000000	-0.0050000
30855.0000000	-0.0050000
30856.0000000	-0.0100000
30857.0000000	-0.0050000
30858.0000000	-0.0033333
30859.0000000	0.0000000
30860.0000000	-0.0100000
30861.0000000	0.0050000
30862.0000000	-0.0150000
30863.0000000	-0.0050000
30864.0000000	-0.0100000
30865.0000000	0.0050000
30866.0000000	-0.0100000
30867.0000000	0.0050000
30868.0000000	-0.0100000
30869.0000000	0.0000000
30870.0000000	-0.0050000
30871.0000000	-0.0050000
30872.0000000	-0.0050000
30873.0000000	-0.0100000
30874.0000000	0.0000000
30875.0000000	-0.0100000
30876.0000000	0.0050000
30877.0000000	-0.0050000
30878.0000000	0.0000000
30879.0000000	0.0050000
30880.0000000	-0.0050000
30881.0000000	0.0100000
30882.0000000	-0.0100000
30883.0000000	0.0050000
30884.0000000	-0.0050000
30885.0000000	-0.0100000
30886.0000000	0.0050000
30887.0000000	0.0050000
30888.0000000	-0.0033333
30889.0000000	0.0050000
30890.0000000	0.0000000
30891.0000000	0.0100000
30892.0000000	0.0100000
30893.0000000	0.0000000

30894.0000000	0.0050000
30895.0000000	0.0000000
30896.0000000	0.0100000
30897.0000000	0.0100000
30898.0000000	0.0150000
30899.0000000	0.0150000
30900.0000000	0.0150000
30901.0000000	0.0100000
30902.0000000	0.0100000
30903.0000000	0.0150000
30904.0000000	0.0100000
30905.0000000	0.0150000
30906.0000000	0.0150000
30907.0000000	0.0200000
30908.0000000	0.0150000
30909.0000000	0.0150000
30910.0000000	0.0200000
30911.0000000	0.0100000
30912.0000000	0.0200000
30913.0000000	0.0200000
30914.0000000	0.0150000
30915.0000000	0.0150000
30916.0000000	0.0200000
30917.0000000	0.0100000
30918.0000000	0.0200000
30919.0000000	0.0200000
30920.0000000	0.0200000
30921.0000000	0.0100000
30922.0000000	0.0200000
30923.0000000	0.0150000
30924.0000000	0.0250000
30925.0000000	0.0300000
30926.0000000	0.0200000
30927.0000000	0.0200000
30928.0000000	0.0200000
30929.0000000	0.0250000
30930.0000000	0.0300000
30931.0000000	0.0150000
30932.0000000	0.0300000
30933.0000000	0.0200000
30934.0000000	0.0250000
30935.0000000	0.0250000
30936.0000000	0.0200000
30937.0000000	0.0350000
30938.0000000	0.0250000
30939.0000000	0.0300000
30940.0000000	0.0300000
30941.0000000	0.0350000
30942.0000000	0.0300000
30943.0000000	0.0400000
30944.0000000	0.0300000
30945.0000000	0.0400000
30946.0000000	0.0250000
30947.0000000	0.0400000
30948.0000000	0.0366667
30949.0000000	0.0300000
30950.0000000	0.0400000

30951.0000000	0.0350000
30952.0000000	0.0500000
30953.0000000	0.0400000
30954.0000000	0.0450000
30955.0000000	0.0400000
30956.0000000	0.0350000
30957.0000000	0.0350000
30958.0000000	0.0500000
30959.0000000	0.0500000
30960.0000000	0.0550000
30961.0000000	0.0450000
30962.0000000	0.0500000
30963.0000000	0.0400000
30964.0000000	0.0500000
30965.0000000	0.0450000
30966.0000000	0.0500000
30967.0000000	0.0450000
30968.0000000	0.0550000
30969.0000000	0.0500000
30970.0000000	0.0550000
30971.0000000	0.0500000
30972.0000000	0.0500000
30973.0000000	0.0550000
30974.0000000	0.0600000
30975.0000000	0.0500000
30976.0000000	0.0550000
30977.0000000	0.0400000
30978.0000000	0.0500000
30979.0000000	0.0600000
30980.0000000	0.0600000
30981.0000000	0.0600000
30982.0000000	0.0600000
30983.0000000	0.0550000
30984.0000000	0.0550000
30985.0000000	0.0500000
30986.0000000	0.0500000
30987.0000000	0.0550000
30988.0000000	0.0550000
30989.0000000	0.0550000
30990.0000000	0.0550000
30991.0000000	0.0600000
30992.0000000	0.0600000
30993.0000000	0.0550000
30994.0000000	0.0600000
30995.0000000	0.0600000
30996.0000000	0.0600000
30997.0000000	0.0650000
30998.0000000	0.0550000
30999.0000000	0.0700000
31000.0000000	0.0550000
31001.0000000	0.0600000
31002.0000000	0.0550000
31003.0000000	0.0650000
31004.0000000	0.0500000
31005.0000000	0.0700000
31006.0000000	0.0600000
31007.0000000	0.0600000

31008.0000000	0.0566667
31009.0000000	0.0550000
31010.0000000	0.0600000
31011.0000000	0.0600000
31012.0000000	0.0700000
31013.0000000	0.0600000
31014.0000000	0.0650000
31015.0000000	0.0550000
31016.0000000	0.0600000
31017.0000000	0.0550000
31018.0000000	0.0700000
31019.0000000	0.0550000
31020.0000000	0.0650000
31021.0000000	0.0550000
31022.0000000	0.0700000
31023.0000000	0.0600000
31024.0000000	0.0600000
31025.0000000	0.0550000
31026.0000000	0.0650000
31027.0000000	0.0650000
31028.0000000	0.0550000
31029.0000000	0.0700000
31030.0000000	0.0600000
31031.0000000	0.0650000
31032.0000000	0.0550000
31033.0000000	0.0650000
31034.0000000	0.0600000
31035.0000000	0.0600000
31036.0000000	0.0650000
31037.0000000	0.0650000
31038.0000000	0.0700000
31039.0000000	0.0600000
31040.0000000	0.0600000
31041.0000000	0.0600000
31042.0000000	0.0700000
31043.0000000	0.0600000
31044.0000000	0.0650000
31045.0000000	0.0700000
31046.0000000	0.0550000
31047.0000000	0.0700000
31048.0000000	0.0700000
31049.0000000	0.0700000
31050.0000000	0.0650000
31051.0000000	0.0700000
31052.0000000	0.0650000
31053.0000000	0.0750000
31054.0000000	0.0650000
31055.0000000	0.0650000
31056.0000000	0.0800000
31057.0000000	0.0650000
31058.0000000	0.0650000
31059.0000000	0.0700000
31060.0000000	0.0700000
31061.0000000	0.0700000
31062.0000000	0.0750000
31063.0000000	0.0700000
31064.0000000	0.0750000

31065.0000000	0.0800000
31066.0000000	0.0750000
31067.0000000	0.0650000
31068.0000000	0.0750000
31069.0000000	0.0766667
31070.0000000	0.0700000
31071.0000000	0.0750000
31072.0000000	0.0700000
31073.0000000	0.0700000
31074.0000000	0.0750000
31075.0000000	0.0700000
31076.0000000	0.0650000
31077.0000000	0.0700000
31078.0000000	0.0800000
31079.0000000	0.0800000
31080.0000000	0.0750000
31081.0000000	0.0750000
31082.0000000	0.0750000
31083.0000000	0.0800000
31084.0000000	0.0750000
31085.0000000	0.0800000
31086.0000000	0.0700000
31087.0000000	0.0750000
31088.0000000	0.0750000
31089.0000000	0.0750000
31090.0000000	0.0800000
31091.0000000	0.0800000
31092.0000000	0.0850000
31093.0000000	0.0800000
31094.0000000	0.0750000
31095.0000000	0.0750000
31096.0000000	0.0800000
31097.0000000	0.0800000
31098.0000000	0.0750000
31099.0000000	0.0800000
31100.0000000	0.0800000
31101.0000000	0.0800000
31102.0000000	0.0800000
31103.0000000	0.0850000
31104.0000000	0.0850000
31105.0000000	0.0900000
31106.0000000	0.0850000
31107.0000000	0.0800000
31108.0000000	0.0800000
31109.0000000	0.0850000
31110.0000000	0.0800000
31111.0000000	0.0850000
31112.0000000	0.1000000
31113.0000000	0.0800000
31114.0000000	0.0850000
31115.0000000	0.0750000
31116.0000000	0.0850000
31117.0000000	0.0800000
31118.0000000	0.0950000
31119.0000000	0.0850000
31120.0000000	0.0950000
31121.0000000	0.0850000

31122.0000000	0.0900000
31123.0000000	0.0850000
31124.0000000	0.0900000
31125.0000000	0.0900000
31126.0000000	0.1000000
31127.0000000	0.0900000
31128.0000000	0.0900000
31129.0000000	0.0900000
31130.0000000	0.0950000
31131.0000000	0.0900000
31132.0000000	0.0900000
31133.0000000	0.0950000
31134.0000000	0.0850000
31135.0000000	0.0950000
31136.0000000	0.0900000
31137.0000000	0.0900000
31138.0000000	0.0900000
31139.0000000	0.0900000
31140.0000000	0.1000000
31141.0000000	0.0900000
31142.0000000	0.1050000
31143.0000000	0.0900000
31144.0000000	0.0900000
31145.0000000	0.1000000
31146.0000000	0.0900000
31147.0000000	0.1000000
31148.0000000	0.0850000
31149.0000000	0.0950000
31150.0000000	0.0900000
31151.0000000	0.0950000
31152.0000000	0.1050000
31153.0000000	0.0900000
31154.0000000	0.1050000
31155.0000000	0.0850000
31156.0000000	0.1000000
31157.0000000	0.0900000
31158.0000000	0.1050000
31159.0000000	0.0966667
31160.0000000	0.1050000
31161.0000000	0.1000000
31162.0000000	0.1000000
31163.0000000	0.0950000
31164.0000000	0.1000000
31165.0000000	0.0950000
31166.0000000	0.1000000
31167.0000000	0.1000000
31168.0000000	0.1000000
31169.0000000	0.1050000
31170.0000000	0.0950000
31171.0000000	0.0950000
31172.0000000	0.1000000
31173.0000000	0.1050000
31174.0000000	0.1100000
31175.0000000	0.1050000
31176.0000000	0.1000000
31177.0000000	0.1000000
31178.0000000	0.1000000

31179.0000000	0.0950000
31180.0000000	0.1000000
31181.0000000	0.1050000
31182.0000000	0.1050000
31183.0000000	0.1050000
31184.0000000	0.1000000
31185.0000000	0.1000000
31186.0000000	0.1050000
31187.0000000	0.1050000
31188.0000000	0.1050000
31189.0000000	0.1000000
31190.0000000	0.1066667
31191.0000000	0.1100000
31192.0000000	0.1100000
31193.0000000	0.1150000
31194.0000000	0.1050000
31195.0000000	0.1100000
31196.0000000	0.1050000
31197.0000000	0.1100000
31198.0000000	0.1100000
31199.0000000	0.1000000
31200.0000000	0.1100000
31201.0000000	0.1100000
31202.0000000	0.1150000
31203.0000000	0.1100000
31204.0000000	0.1100000
31205.0000000	0.1000000
31206.0000000	0.1150000
31207.0000000	0.1050000
31208.0000000	0.1100000
31209.0000000	0.1200000
31210.0000000	0.1050000
31211.0000000	0.1100000
31212.0000000	0.1100000
31213.0000000	0.1150000
31214.0000000	0.1100000
31215.0000000	0.1150000
31216.0000000	0.1150000
31217.0000000	0.1150000
31218.0000000	0.1150000
31219.0000000	0.1200000
31220.0000000	0.1200000
31221.0000000	0.1150000
31222.0000000	0.1100000
31223.0000000	0.1100000
31224.0000000	0.1200000
31225.0000000	0.1200000
31226.0000000	0.1150000
31227.0000000	0.1150000
31228.0000000	0.1150000
31229.0000000	0.1150000
31230.0000000	0.1150000
31231.0000000	0.1200000
31232.0000000	0.1150000
31233.0000000	0.1250000
31234.0000000	0.1200000
31235.0000000	0.1300000

31236.0000000	0.1150000
31237.0000000	0.1250000
31238.0000000	0.1200000
31239.0000000	0.1250000
31240.0000000	0.1250000
31241.0000000	0.1300000
31242.0000000	0.1350000
31243.0000000	0.1200000
31244.0000000	0.1250000
31245.0000000	0.1200000
31246.0000000	0.1250000
31247.0000000	0.1300000
31248.0000000	0.1300000
31249.0000000	0.1300000
31250.0000000	0.1300000
31251.0000000	0.1250000
31252.0000000	0.1250000
31253.0000000	0.1300000
31254.0000000	0.1300000
31255.0000000	0.1350000
31256.0000000	0.1300000
31257.0000000	0.1300000
31258.0000000	0.1300000
31259.0000000	0.1350000
31260.0000000	0.1300000
31261.0000000	0.1300000
31262.0000000	0.1300000
31263.0000000	0.1300000
31264.0000000	0.1350000
31265.0000000	0.1300000
31266.0000000	0.1350000
31267.0000000	0.1400000
31268.0000000	0.1350000
31269.0000000	0.1400000
31270.0000000	0.1400000
31271.0000000	0.1400000
31272.0000000	0.1350000
31273.0000000	0.1400000
31274.0000000	0.1400000
31275.0000000	0.1350000
31276.0000000	0.1400000
31277.0000000	0.1450000
31278.0000000	0.1400000
31279.0000000	0.1450000
31280.0000000	0.1400000
31281.0000000	0.1450000
31282.0000000	0.1350000
31283.0000000	0.1450000
31284.0000000	0.1450000
31285.0000000	0.1400000
31286.0000000	0.1450000
31287.0000000	0.1500000
31288.0000000	0.1400000
31289.0000000	0.1400000
31290.0000000	0.1450000
31291.0000000	0.1450000
31292.0000000	0.1450000

31293.0000000	0.1450000
31294.0000000	0.1400000
31295.0000000	0.1500000
31296.0000000	0.1450000
31297.0000000	0.1450000
31298.0000000	0.1450000
31299.0000000	0.1550000
31300.0000000	0.1500000
31301.0000000	0.1450000
31302.0000000	0.1450000
31303.0000000	0.1550000
31304.0000000	0.1550000
31305.0000000	0.1450000
31306.0000000	0.1550000
31307.0000000	0.1600000
31308.0000000	0.1550000
31309.0000000	0.1550000
31310.0000000	0.1500000
31311.0000000	0.1500000
31312.0000000	0.1600000
31313.0000000	0.1500000
31314.0000000	0.1650000
31315.0000000	0.1600000
31316.0000000	0.1600000
31317.0000000	0.1600000
31318.0000000	0.1550000
31319.0000000	0.1500000
31320.0000000	0.1600000
31321.0000000	0.1650000
31322.0000000	0.1550000
31323.0000000	0.1650000
31324.0000000	0.1600000
31325.0000000	0.1650000
31326.0000000	0.1700000
31327.0000000	0.1600000
31328.0000000	0.1650000
31329.0000000	0.1700000
31330.0000000	0.1600000
31331.0000000	0.1650000
31332.0000000	0.1650000
31333.0000000	0.1700000
31334.0000000	0.1750000
31335.0000000	0.1750000
31336.0000000	0.1750000
31337.0000000	0.1750000
31338.0000000	0.1650000
31339.0000000	0.1700000
31340.0000000	0.1700000
31341.0000000	0.1650000
31342.0000000	0.1650000
31343.0000000	0.1750000
31344.0000000	0.1750000
31345.0000000	0.1600000
31346.0000000	0.1700000
31347.0000000	0.1700000
31348.0000000	0.1750000
31349.0000000	0.1750000

31350.0000000	0.1700000
31351.0000000	0.1750000
31352.0000000	0.1750000
31353.0000000	0.1750000
31354.0000000	0.1800000
31355.0000000	0.1700000
31356.0000000	0.1800000
31357.0000000	0.1750000
31358.0000000	0.1800000
31359.0000000	0.1800000
31360.0000000	0.1750000
31361.0000000	0.1800000
31362.0000000	0.1800000
31363.0000000	0.1850000
31364.0000000	0.1750000
31365.0000000	0.1850000
31366.0000000	0.1800000
31367.0000000	0.1800000
31368.0000000	0.1850000
31369.0000000	0.1850000
31370.0000000	0.1800000
31371.0000000	0.1933333
31372.0000000	0.1800000
31373.0000000	0.1850000
31374.0000000	0.1850000
31375.0000000	0.1950000
31376.0000000	0.1850000
31377.0000000	0.1850000
31378.0000000	0.1850000
31379.0000000	0.1800000
31380.0000000	0.1950000
31381.0000000	0.1850000
31382.0000000	0.2000000
31383.0000000	0.1900000
31384.0000000	0.1850000
31385.0000000	0.1850000
31386.0000000	0.1900000
31387.0000000	0.1900000
31388.0000000	0.1900000
31389.0000000	0.2000000
31390.0000000	0.1950000
31391.0000000	0.1950000
31392.0000000	0.1900000
31393.0000000	0.2000000
31394.0000000	0.1950000
31395.0000000	0.2000000
31396.0000000	0.1900000
31397.0000000	0.2000000
31398.0000000	0.1950000
31399.0000000	0.2000000
31400.0000000	0.1950000
31401.0000000	0.2000000
31402.0000000	0.2000000
31403.0000000	0.2000000
31404.0000000	0.2000000
31405.0000000	0.2050000
31406.0000000	0.1950000

31407.0000000	0.2050000
31408.0000000	0.1950000
31409.0000000	0.2100000
31410.0000000	0.2050000
31411.0000000	0.2100000
31412.0000000	0.2000000
31413.0000000	0.2100000
31414.0000000	0.2050000
31415.0000000	0.2000000
31416.0000000	0.2050000
31417.0000000	0.2050000
31418.0000000	0.2100000
31419.0000000	0.2050000
31420.0000000	0.2150000
31421.0000000	0.2050000
31422.0000000	0.2000000
31423.0000000	0.2050000
31424.0000000	0.2100000
31425.0000000	0.2100000
31426.0000000	0.2050000
31427.0000000	0.2100000
31428.0000000	0.2100000
31429.0000000	0.2100000
31430.0000000	0.2050000
31431.0000000	0.2100000
31432.0000000	0.2100000
31433.0000000	0.2150000
31434.0000000	0.2150000
31435.0000000	0.2100000
31436.0000000	0.2200000
31437.0000000	0.2100000
31438.0000000	0.2150000
31439.0000000	0.2200000
31440.0000000	0.2150000
31441.0000000	0.2150000
31442.0000000	0.2150000
31443.0000000	0.2150000
31444.0000000	0.2200000
31445.0000000	0.2200000
31446.0000000	0.2150000
31447.0000000	0.2150000
31448.0000000	0.2200000
31449.0000000	0.2150000
31450.0000000	0.2150000
31451.0000000	0.2200000
31452.0000000	0.2150000
31453.0000000	0.2300000
31454.0000000	0.2250000
31455.0000000	0.2250000
31456.0000000	0.2200000
31457.0000000	0.2150000
31458.0000000	0.2200000
31459.0000000	0.2200000
31460.0000000	0.2250000
31461.0000000	0.2233333
31462.0000000	0.2100000
31463.0000000	0.2200000

31464.0000000	0.2200000
31465.0000000	0.2300000
31466.0000000	0.2250000
31467.0000000	0.2150000
31468.0000000	0.2250000
31469.0000000	0.2150000
31470.0000000	0.2200000
31471.0000000	0.2150000
31472.0000000	0.2250000
31473.0000000	0.2150000
31474.0000000	0.2300000
31475.0000000	0.2250000
31476.0000000	0.2300000
31477.0000000	0.2200000
31478.0000000	0.2250000
31479.0000000	0.2250000
31480.0000000	0.2300000
31481.0000000	0.2300000
31482.0000000	0.2250000
31483.0000000	0.2250000
31484.0000000	0.2250000
31485.0000000	0.2200000
31486.0000000	0.2200000
31487.0000000	0.2300000
31488.0000000	0.2250000
31489.0000000	0.2250000
31490.0000000	0.2250000
31491.0000000	0.2233333
31492.0000000	0.2250000
31493.0000000	0.2350000
31494.0000000	0.2250000
31495.0000000	0.2200000
31496.0000000	0.2250000
31497.0000000	0.2300000
31498.0000000	0.2350000
31499.0000000	0.2150000
31500.0000000	0.2300000
31501.0000000	0.2400000
31502.0000000	0.2250000
31503.0000000	0.2350000
31504.0000000	0.2200000
31505.0000000	0.2250000
31506.0000000	0.2250000
31507.0000000	0.2300000
31508.0000000	0.2250000
31509.0000000	0.2300000
31510.0000000	0.2250000
31511.0000000	0.2300000
31512.0000000	0.2300000
31513.0000000	0.2300000
31514.0000000	0.2200000
31515.0000000	0.2250000
31516.0000000	0.2350000
31517.0000000	0.2250000
31518.0000000	0.2250000
31519.0000000	0.2350000
31520.0000000	0.2200000

31521.0000000	0.2350000
31522.0000000	0.2266666
31523.0000000	0.2350000
31524.0000000	0.2300000
31525.0000000	0.2300000
31526.0000000	0.2300000
31527.0000000	0.2300000
31528.0000000	0.2250000
31529.0000000	0.2350000
31530.0000000	0.2250000
31531.0000000	0.2400000
31532.0000000	0.2400000
31533.0000000	0.2400000
31534.0000000	0.2300000
31535.0000000	0.2250000
31536.0000000	0.2300000
31537.0000000	0.2300000
31538.0000000	0.2300000
31539.0000000	0.2300000
31540.0000000	0.2350000
31541.0000000	0.2400000
31542.0000000	0.2450000
31543.0000000	0.2350000
31544.0000000	0.2400000
31545.0000000	0.2300000
31546.0000000	0.2350000
31547.0000000	0.2350000
31548.0000000	0.2350000
31549.0000000	0.2450000
31550.0000000	0.2300000
31551.0000000	0.2400000
31552.0000000	0.2366667
31553.0000000	0.2400000
31554.0000000	0.2400000
31555.0000000	0.2400000
31556.0000000	0.2350000
31557.0000000	0.2400000
31558.0000000	0.2450000
31559.0000000	0.2400000
31560.0000000	0.2400000
31561.0000000	0.2450000
31562.0000000	0.2500000
31563.0000000	0.2450000
31564.0000000	0.2350000
31565.0000000	0.2450000
31566.0000000	0.2400000
31567.0000000	0.2500000
31568.0000000	0.2500000
31569.0000000	0.2400000
31570.0000000	0.2500000
31571.0000000	0.2500000
31572.0000000	0.2450000
31573.0000000	0.2500000
31574.0000000	0.2450000
31575.0000000	0.2400000
31576.0000000	0.2500000
31577.0000000	0.2450000

31578.0000000	0.2550000
31579.0000000	0.2500000
31580.0000000	0.2500000
31581.0000000	0.2550000
31582.0000000	0.2466667
31583.0000000	0.2500000
31584.0000000	0.2450000
31585.0000000	0.2600000
31586.0000000	0.2500000
31587.0000000	0.2450000
31588.0000000	0.2500000
31589.0000000	0.2450000
31590.0000000	0.2550000
31591.0000000	0.2500000
31592.0000000	0.2500000
31593.0000000	0.2450000
31594.0000000	0.2550000
31595.0000000	0.2550000
31596.0000000	0.2600000
31597.0000000	0.2500000
31598.0000000	0.2500000
31599.0000000	0.2450000
31600.0000000	0.2500000
31601.0000000	0.2600000
31602.0000000	0.2400000
31603.0000000	0.2550000
31604.0000000	0.2600000
31605.0000000	0.2500000
31606.0000000	0.2550000
31607.0000000	0.2500000
31608.0000000	0.2550000
31609.0000000	0.2500000
31610.0000000	0.2550000
31611.0000000	0.2600000
31612.0000000	0.2566667
31613.0000000	0.2450000
31614.0000000	0.2600000
31615.0000000	0.2650000
31616.0000000	0.2550000
31617.0000000	0.2650000
31618.0000000	0.2600000
31619.0000000	0.2500000
31620.0000000	0.2600000
31621.0000000	0.2500000
31622.0000000	0.2450000
31623.0000000	0.2550000
31624.0000000	0.2550000
31625.0000000	0.2600000
31626.0000000	0.2600000
31627.0000000	0.2600000
31628.0000000	0.2600000
31629.0000000	0.2650000
31630.0000000	0.2550000
31631.0000000	0.2600000
31632.0000000	0.2600000
31633.0000000	0.2550000
31634.0000000	0.2500000

31635.0000000	0.2550000
31636.0000000	0.2550000
31637.0000000	0.2550000
31638.0000000	0.2550000
31639.0000000	0.2500000
31640.0000000	0.2550000
31641.0000000	0.2550000
31642.0000000	0.2550000
31643.0000000	0.2566667
31644.0000000	0.2600000
31645.0000000	0.2550000
31646.0000000	0.2600000
31647.0000000	0.2600000
31648.0000000	0.2550000
31649.0000000	0.2500000
31650.0000000	0.2600000
31651.0000000	0.2600000
31652.0000000	0.2550000
31653.0000000	0.2600000
31654.0000000	0.2550000
31655.0000000	0.2550000
31656.0000000	0.2550000
31657.0000000	0.2550000
31658.0000000	0.2550000
31659.0000000	0.2500000
31660.0000000	0.2500000
31661.0000000	0.2550000
31662.0000000	0.2600000
31663.0000000	0.2600000
31664.0000000	0.2600000
31665.0000000	0.2550000
31666.0000000	0.2550000
31667.0000000	0.2500000
31668.0000000	0.2650000
31669.0000000	0.2550000
31670.0000000	0.2500000
31671.0000000	0.2500000
31672.0000000	0.2600000
31673.0000000	0.2550000
31674.0000000	0.2550000
31675.0000000	0.2500000
31676.0000000	0.2500000
31677.0000000	0.2600000
31678.0000000	0.2600000
31679.0000000	0.2500000
31680.0000000	0.2600000
31681.0000000	0.2550000
31682.0000000	0.2650000
31683.0000000	0.2600000
31684.0000000	0.2600000
31685.0000000	0.2550000
31686.0000000	0.2550000
31687.0000000	0.2600000
31688.0000000	0.2500000
31689.0000000	0.2600000
31690.0000000	0.2550000
31691.0000000	0.2600000

31692.0000000	0.2600000
31693.0000000	0.2550000
31694.0000000	0.2500000
31695.0000000	0.2550000
31696.0000000	0.2500000
31697.0000000	0.2550000
31698.0000000	0.2600000
31699.0000000	0.2550000
31700.0000000	0.2650000
31701.0000000	0.2600000
31702.0000000	0.2600000
31703.0000000	0.2533333
31704.0000000	0.2600000
31705.0000000	0.2550000
31706.0000000	0.2600000
31707.0000000	0.2550000
31708.0000000	0.2600000
31709.0000000	0.2600000
31710.0000000	0.2550000
31711.0000000	0.2550000
31712.0000000	0.2450000
31713.0000000	0.2600000
31714.0000000	0.2650000
31715.0000000	0.2550000
31716.0000000	0.2600000
31717.0000000	0.2550000
31718.0000000	0.2650000
31719.0000000	0.2550000
31720.0000000	0.2600000
31721.0000000	0.2650000
31722.0000000	0.2600000
31723.0000000	0.2650000
31724.0000000	0.2550000
31725.0000000	0.2450000
31726.0000000	0.2550000
31727.0000000	0.2600000
31728.0000000	0.2500000
31729.0000000	0.2600000
31730.0000000	0.2600000
31731.0000000	0.2600000
31732.0000000	0.2600000
31733.0000000	0.2566667
31734.0000000	0.2650000
31735.0000000	0.2550000
31736.0000000	0.2450000
31737.0000000	0.2600000
31738.0000000	0.2600000
31739.0000000	0.2550000
31740.0000000	0.2550000
31741.0000000	0.2500000
31742.0000000	0.2700000
31743.0000000	0.2600000
31744.0000000	0.2550000
31745.0000000	0.2600000
31746.0000000	0.2600000
31747.0000000	0.2550000
31748.0000000	0.2650000

31749.0000000	0.2600000
31750.0000000	0.2450000
31751.0000000	0.2600000
31752.0000000	0.2550000
31753.0000000	0.2650000
31754.0000000	0.2600000
31755.0000000	0.2500000
31756.0000000	0.2650000
31757.0000000	0.2600000
31758.0000000	0.2700000
31759.0000000	0.2600000
31760.0000000	0.2650000
31761.0000000	0.2550000
31762.0000000	0.2550000
31763.0000000	0.2666667
31764.0000000	0.2550000
31765.0000000	0.2550000
31766.0000000	0.2650000
31767.0000000	0.2600000
31768.0000000	0.2600000
31769.0000000	0.2600000
31770.0000000	0.2550000
31771.0000000	0.2650000
31772.0000000	0.2600000
31773.0000000	0.2600000
31774.0000000	0.2550000
31775.0000000	0.2650000
31776.0000000	0.2600000
31777.0000000	0.2600000
31778.0000000	0.2650000
31779.0000000	0.2600000
31780.0000000	0.2650000
31781.0000000	0.2550000
31782.0000000	0.2600000
31783.0000000	0.2700000
31784.0000000	0.2550000
31785.0000000	0.2700000
31786.0000000	0.2550000
31787.0000000	0.2700000
31788.0000000	0.2650000
31789.0000000	0.2650000
31790.0000000	0.2700000
31791.0000000	0.2600000
31792.0000000	0.2700000
31793.0000000	0.2766666
31794.0000000	0.2600000
31795.0000000	0.2650000
31796.0000000	0.2600000
31797.0000000	0.2700000
31798.0000000	0.2700000
31799.0000000	0.2650000
31800.0000000	0.2700000
31801.0000000	0.2650000
31802.0000000	0.2700000
31803.0000000	0.2650000
31804.0000000	0.2700000
31805.0000000	0.2700000

31806.0000000	0.2700000
31807.0000000	0.2600000
31808.0000000	0.2800000
31809.0000000	0.2700000
31810.0000000	0.2650000
31811.0000000	0.2650000
31812.0000000	0.2700000
31813.0000000	0.2650000
31814.0000000	0.2750000
31815.0000000	0.2800000
31816.0000000	0.2700000
31817.0000000	0.2700000
31818.0000000	0.2600000
31819.0000000	0.2700000
31820.0000000	0.2750000
31821.0000000	0.2750000
31822.0000000	0.2700000
31823.0000000	0.2733333
31824.0000000	0.2750000
31825.0000000	0.2700000
31826.0000000	0.2700000
31827.0000000	0.2750000
31828.0000000	0.2700000
31829.0000000	0.2700000
31830.0000000	0.2700000
31831.0000000	0.2650000
31832.0000000	0.2750000
31833.0000000	0.2700000
31834.0000000	0.2750000
31835.0000000	0.2750000
31836.0000000	0.2650000
31837.0000000	0.2700000
31838.0000000	0.2700000
31839.0000000	0.2650000
31840.0000000	0.2700000
31841.0000000	0.2700000
31842.0000000	0.2700000
31843.0000000	0.2700000
31844.0000000	0.2750000
31845.0000000	0.2700000
31846.0000000	0.2750000
31847.0000000	0.2600000
31848.0000000	0.2750000
31849.0000000	0.2650000
31850.0000000	0.2800000
31851.0000000	0.2750000
31852.0000000	0.2800000
31853.0000000	0.2700000
31854.0000000	0.2700000
31855.0000000	0.2750000
31856.0000000	0.2750000
31857.0000000	0.2750000
31858.0000000	0.2700000
31859.0000000	0.2750000
31860.0000000	0.2800000
31861.0000000	0.2800000
31862.0000000	0.2900000

31863.0000000	0.2750000
31864.0000000	0.2750000
31865.0000000	0.2800000
31866.0000000	0.2750000
31867.0000000	0.2800000
31868.0000000	0.2750000
31869.0000000	0.2750000
31870.0000000	0.2800000
31871.0000000	0.2800000
31872.0000000	0.2850000
31873.0000000	0.2650000
31874.0000000	0.2800000
31875.0000000	0.2750000
31876.0000000	0.2850000
31877.0000000	0.2750000
31878.0000000	0.2850000
31879.0000000	0.2850000
31880.0000000	0.2800000
31881.0000000	0.2900000
31882.0000000	0.2800000
31883.0000000	0.2850000
31884.0000000	0.2866667
31885.0000000	0.2800000
31886.0000000	0.2850000
31887.0000000	0.2900000
31888.0000000	0.2750000
31889.0000000	0.2900000
31890.0000000	0.2850000
31891.0000000	0.2900000
31892.0000000	0.2850000
31893.0000000	0.2850000
31894.0000000	0.2900000
31895.0000000	0.2850000
31896.0000000	0.2850000
31897.0000000	0.2850000
31898.0000000	0.2900000
31899.0000000	0.2850000
31900.0000000	0.2900000
31901.0000000	0.2850000
31902.0000000	0.2850000
31903.0000000	0.2850000
31904.0000000	0.2900000
31905.0000000	0.2850000
31906.0000000	0.2900000
31907.0000000	0.2850000
31908.0000000	0.2900000
31909.0000000	0.2850000
31910.0000000	0.2950000
31911.0000000	0.2750000
31912.0000000	0.2950000
31913.0000000	0.2850000
31914.0000000	0.2833333
31915.0000000	0.2950000
31916.0000000	0.2800000
31917.0000000	0.2900000
31918.0000000	0.2850000
31919.0000000	0.2750000

31920.0000000	0.2800000
31921.0000000	0.2800000
31922.0000000	0.2800000
31923.0000000	0.2850000
31924.0000000	0.2850000
31925.0000000	0.2950000
31926.0000000	0.2850000
31927.0000000	0.2850000
31928.0000000	0.2800000
31929.0000000	0.2900000
31930.0000000	0.2900000
31931.0000000	0.2850000
31932.0000000	0.2850000
31933.0000000	0.2750000
31934.0000000	0.2850000
31935.0000000	0.2850000
31936.0000000	0.2900000
31937.0000000	0.2850000
31938.0000000	0.2850000
31939.0000000	0.2850000
31940.0000000	0.2900000
31941.0000000	0.2900000
31942.0000000	0.2850000
31943.0000000	0.2800000
31944.0000000	0.2833333
31945.0000000	0.2850000
31946.0000000	0.2850000
31947.0000000	0.2850000
31948.0000000	0.2800000
31949.0000000	0.2850000
31950.0000000	0.2850000
31951.0000000	0.2850000
31952.0000000	0.2900000
31953.0000000	0.2800000
31954.0000000	0.2750000
31955.0000000	0.2900000
31956.0000000	0.2850000
31957.0000000	0.2850000
31958.0000000	0.2850000
31959.0000000	0.2900000
31960.0000000	0.2800000
31961.0000000	0.2850000
31962.0000000	0.2850000
31963.0000000	0.2850000
31964.0000000	0.2850000
31965.0000000	0.2850000
31966.0000000	0.2850000
31967.0000000	0.2850000
31968.0000000	0.2800000
31969.0000000	0.2850000
31970.0000000	0.2800000
31971.0000000	0.2850000
31972.0000000	0.2900000
31973.0000000	0.2900000
31974.0000000	0.2800000
31975.0000000	0.2833333
31976.0000000	0.2900000

31977.0000000	0.2850000
31978.0000000	0.2750000
31979.0000000	0.2850000
31980.0000000	0.2850000
31981.0000000	0.2900000
31982.0000000	0.2850000
31983.0000000	0.2850000
31984.0000000	0.2900000
31985.0000000	0.2900000
31986.0000000	0.2850000
31987.0000000	0.2800000
31988.0000000	0.2800000
31989.0000000	0.2850000
31990.0000000	0.2850000
31991.0000000	0.2750000
31992.0000000	0.2900000
31993.0000000	0.2850000
31994.0000000	0.2850000
31995.0000000	0.2750000
31996.0000000	0.2900000
31997.0000000	0.2850000
31998.0000000	0.2900000
31999.0000000	0.2800000
32000.0000000	0.2950000
32001.0000000	0.2800000
32002.0000000	0.2900000
32003.0000000	0.2750000
32004.0000000	0.2866667
32005.0000000	0.2850000
32006.0000000	0.2850000
32007.0000000	0.2850000
32008.0000000	0.2800000
32009.0000000	0.2850000
32010.0000000	0.2800000
32011.0000000	0.2800000
32012.0000000	0.2800000
32013.0000000	0.2850000
32014.0000000	0.2850000
32015.0000000	0.2850000
32016.0000000	0.2850000
32017.0000000	0.2850000
32018.0000000	0.2800000
32019.0000000	0.2800000
32020.0000000	0.2800000
32021.0000000	0.2850000
32022.0000000	0.2900000
32023.0000000	0.2850000
32024.0000000	0.2800000
32025.0000000	0.2800000
32026.0000000	0.2800000
32027.0000000	0.2850000
32028.0000000	0.2800000
32029.0000000	0.2900000
32030.0000000	0.2850000
32031.0000000	0.2850000
32032.0000000	0.2850000
32033.0000000	0.2800000

32034.0000000	0.2800000
32035.0000000	0.2866667
32036.0000000	0.2850000
32037.0000000	0.2800000
32038.0000000	0.2800000
32039.0000000	0.2850000
32040.0000000	0.2900000
32041.0000000	0.2900000
32042.0000000	0.2850000
32043.0000000	0.2900000
32044.0000000	0.2800000
32045.0000000	0.2800000
32046.0000000	0.2850000
32047.0000000	0.2900000
32048.0000000	0.2850000
32049.0000000	0.2950000
32050.0000000	0.2850000
32051.0000000	0.2950000
32052.0000000	0.2850000
32053.0000000	0.2850000
32054.0000000	0.2850000
32055.0000000	0.2800000
32056.0000000	0.2850000
32057.0000000	0.2800000
32058.0000000	0.2950000
32059.0000000	0.2800000
32060.0000000	0.2900000
32061.0000000	0.2800000
32062.0000000	0.2900000
32063.0000000	0.2800000
32064.0000000	0.2900000
32065.0000000	0.2900000
32066.0000000	0.2850000
32067.0000000	0.2900000
32068.0000000	0.2850000
32069.0000000	0.2900000
32070.0000000	0.2800000
32071.0000000	0.2800000
32072.0000000	0.2750000
32073.0000000	0.2900000
32074.0000000	0.2850000
32075.0000000	0.2900000
32076.0000000	0.2800000
32077.0000000	0.2900000
32078.0000000	0.2750000
32079.0000000	0.2850000
32080.0000000	0.2850000
32081.0000000	0.2950000
32082.0000000	0.2850000
32083.0000000	0.2850000
32084.0000000	0.2900000
32085.0000000	0.2800000
32086.0000000	0.2900000
32087.0000000	0.2850000
32088.0000000	0.2950000
32089.0000000	0.2850000
32090.0000000	0.2850000

32091.0000000	0.2800000
32092.0000000	0.2850000
32093.0000000	0.2900000
32094.0000000	0.2900000
32095.0000000	0.2866667
32096.0000000	0.2850000
32097.0000000	0.2850000
32098.0000000	0.2900000
32099.0000000	0.2850000
32100.0000000	0.2850000
32101.0000000	0.2900000
32102.0000000	0.2800000
32103.0000000	0.2850000
32104.0000000	0.2800000
32105.0000000	0.2900000
32106.0000000	0.2850000
32107.0000000	0.2850000
32108.0000000	0.2900000
32109.0000000	0.2850000
32110.0000000	0.2800000
32111.0000000	0.2900000
32112.0000000	0.2850000
32113.0000000	0.2900000
32114.0000000	0.2850000
32115.0000000	0.2850000
32116.0000000	0.2900000
32117.0000000	0.2900000
32118.0000000	0.2900000
32119.0000000	0.2850000
32120.0000000	0.2900000
32121.0000000	0.2850000
32122.0000000	0.2900000
32123.0000000	0.2850000
32124.0000000	0.2950000
32125.0000000	0.2833333
32126.0000000	0.2900000
32127.0000000	0.2900000
32128.0000000	0.2900000
32129.0000000	0.2900000
32130.0000000	0.2950000
32131.0000000	0.2900000
32132.0000000	0.2850000
32133.0000000	0.2900000
32134.0000000	0.2850000
32135.0000000	0.2850000
32136.0000000	0.2850000
32137.0000000	0.2900000
32138.0000000	0.2900000
32139.0000000	0.2900000
32140.0000000	0.2950000
32141.0000000	0.2850000
32142.0000000	0.2850000
32143.0000000	0.2900000
32144.0000000	0.2900000
32145.0000000	0.2950000
32146.0000000	0.2900000
32147.0000000	0.2950000

32148.0000000	0.2950000
32149.0000000	0.2900000
32150.0000000	0.2850000
32151.0000000	0.2800000
32152.0000000	0.2900000
32153.0000000	0.2900000
32154.0000000	0.2950000
32155.0000000	0.2966667
32156.0000000	0.2850000
32157.0000000	0.2900000
32158.0000000	0.2900000
32159.0000000	0.2900000
32160.0000000	0.2900000
32161.0000000	0.2850000
32162.0000000	0.2900000
32163.0000000	0.2800000
32164.0000000	0.2900000
32165.0000000	0.2850000
32166.0000000	0.2850000
32167.0000000	0.2950000
32168.0000000	0.2900000
32169.0000000	0.2850000
32170.0000000	0.2950000
32171.0000000	0.2850000
32172.0000000	0.2850000
32173.0000000	0.2900000
32174.0000000	0.2750000
32175.0000000	0.2850000
32176.0000000	0.2850000
32177.0000000	0.2850000
32178.0000000	0.2900000
32179.0000000	0.2900000
32180.0000000	0.2850000
32181.0000000	0.2850000
32182.0000000	0.2900000
32183.0000000	0.2850000
32184.0000000	0.2800000
32185.0000000	0.2850000
32186.0000000	0.2900000
32187.0000000	0.2900000
32188.0000000	0.2800000
32189.0000000	0.2800000
32190.0000000	0.2850000
32191.0000000	0.2850000
32192.0000000	0.2900000
32193.0000000	0.2800000
32194.0000000	0.2900000
32195.0000000	0.2900000
32196.0000000	0.2900000
32197.0000000	0.2850000
32198.0000000	0.2800000
32199.0000000	0.2850000
32200.0000000	0.2850000
32201.0000000	0.2900000
32202.0000000	0.2850000
32203.0000000	0.2900000
32204.0000000	0.2850000

32205.0000000	0.2850000
32206.0000000	0.2850000
32207.0000000	0.2800000
32208.0000000	0.2850000
32209.0000000	0.2850000
32210.0000000	0.2850000
32211.0000000	0.2800000
32212.0000000	0.2900000
32213.0000000	0.2850000
32214.0000000	0.2800000
32215.0000000	0.2850000
32216.0000000	0.2833333
32217.0000000	0.2850000
32218.0000000	0.2900000
32219.0000000	0.2800000
32220.0000000	0.2800000
32221.0000000	0.2900000
32222.0000000	0.2750000
32223.0000000	0.2900000
32224.0000000	0.2850000
32225.0000000	0.2850000
32226.0000000	0.2800000
32227.0000000	0.2900000
32228.0000000	0.2800000
32229.0000000	0.2900000
32230.0000000	0.2800000
32231.0000000	0.2800000
32232.0000000	0.2850000
32233.0000000	0.2750000
32234.0000000	0.2900000
32235.0000000	0.2850000
32236.0000000	0.2900000
32237.0000000	0.2800000
32238.0000000	0.2850000
32239.0000000	0.2800000
32240.0000000	0.2850000
32241.0000000	0.2850000
32242.0000000	0.2900000
32243.0000000	0.2850000
32244.0000000	0.2800000
32245.0000000	0.2850000
32246.0000000	0.2833333
32247.0000000	0.2850000
32248.0000000	0.2800000
32249.0000000	0.2800000
32250.0000000	0.2850000
32251.0000000	0.2800000
32252.0000000	0.2900000
32253.0000000	0.2750000
32254.0000000	0.2900000
32255.0000000	0.2750000
32256.0000000	0.2850000
32257.0000000	0.2800000
32258.0000000	0.2800000
32259.0000000	0.2900000
32260.0000000	0.2850000
32261.0000000	0.2850000

32262.0000000	0.2850000
32263.0000000	0.2900000
32264.0000000	0.2800000
32265.0000000	0.2800000
32266.0000000	0.2800000
32267.0000000	0.2850000
32268.0000000	0.2850000
32269.0000000	0.2900000
32270.0000000	0.2850000
32271.0000000	0.2850000
32272.0000000	0.2850000
32273.0000000	0.2800000
32274.0000000	0.2900000
32275.0000000	0.2750000
32276.0000000	0.2800000
32277.0000000	0.2850000
32278.0000000	0.2850000
32279.0000000	0.2750000
32280.0000000	0.2800000
32281.0000000	0.2750000
32282.0000000	0.2800000
32283.0000000	0.2900000
32284.0000000	0.2800000
32285.0000000	0.2800000
32286.0000000	0.2900000
32287.0000000	0.2850000
32288.0000000	0.2750000
32289.0000000	0.2800000
32290.0000000	0.2800000
32291.0000000	0.2750000
32292.0000000	0.2850000
32293.0000000	0.2700000
32294.0000000	0.2850000
32295.0000000	0.2800000
32296.0000000	0.2800000
32297.0000000	0.2900000
32298.0000000	0.2750000
32299.0000000	0.2850000
32300.0000000	0.2800000
32301.0000000	0.2800000
32302.0000000	0.2850000
32303.0000000	0.2850000
32304.0000000	0.2800000
32305.0000000	0.2800000
32306.0000000	0.2850000
32307.0000000	0.2833333
32308.0000000	0.2800000
32309.0000000	0.2800000
32310.0000000	0.2750000
32311.0000000	0.2800000
32312.0000000	0.2850000
32313.0000000	0.2800000
32314.0000000	0.2800000
32315.0000000	0.2800000
32316.0000000	0.2750000
32317.0000000	0.2850000
32318.0000000	0.2850000

32319.0000000	0.2800000
32320.0000000	0.2800000
32321.0000000	0.2800000
32322.0000000	0.2850000
32323.0000000	0.2800000
32324.0000000	0.2800000
32325.0000000	0.2800000
32326.0000000	0.2900000
32327.0000000	0.2850000
32328.0000000	0.2850000
32329.0000000	0.2700000
32330.0000000	0.2900000
32331.0000000	0.2800000
32332.0000000	0.2950000
32333.0000000	0.2850000
32334.0000000	0.2850000
32335.0000000	0.2850000
32336.0000000	0.2800000
32337.0000000	0.2900000
32338.0000000	0.2900000
32339.0000000	0.2800000
32340.0000000	0.2900000
32341.0000000	0.2850000
32342.0000000	0.2950000
32343.0000000	0.2950000
32344.0000000	0.2800000
32345.0000000	0.2900000
32346.0000000	0.2850000
32347.0000000	0.2900000
32348.0000000	0.2900000
32349.0000000	0.2950000
32350.0000000	0.2850000
32351.0000000	0.2900000
32352.0000000	0.2850000
32353.0000000	0.2850000
32354.0000000	0.2900000
32355.0000000	0.2900000
32356.0000000	0.2950000
32357.0000000	0.2850000
32358.0000000	0.2900000
32359.0000000	0.2800000
32360.0000000	0.2900000
32361.0000000	0.3000000
32362.0000000	0.2950000
32363.0000000	0.2850000
32364.0000000	0.2850000
32365.0000000	0.2900000
32366.0000000	0.2800000
32367.0000000	0.2933334
32368.0000000	0.2850000
32369.0000000	0.2800000
32370.0000000	0.2900000
32371.0000000	0.2850000
32372.0000000	0.2950000
32373.0000000	0.2850000
32374.0000000	0.2950000
32375.0000000	0.2800000

32376.0000000	0.2850000
32377.0000000	0.2850000
32378.0000000	0.2900000
32379.0000000	0.2850000
32380.0000000	0.2950000
32381.0000000	0.2900000
32382.0000000	0.2900000
32383.0000000	0.2900000
32384.0000000	0.2850000
32385.0000000	0.2900000
32386.0000000	0.2850000
32387.0000000	0.2900000
32388.0000000	0.2850000
32389.0000000	0.2950000
32390.0000000	0.2850000
32391.0000000	0.2850000
32392.0000000	0.2900000
32393.0000000	0.2900000
32394.0000000	0.2950000
32395.0000000	0.2900000
32396.0000000	0.3000000
32397.0000000	0.2900000
32398.0000000	0.2950000
32399.0000000	0.2900000
32400.0000000	0.2900000
32401.0000000	0.2850000
32402.0000000	0.2900000
32403.0000000	0.3000000
32404.0000000	0.2950000
32405.0000000	0.2850000
32406.0000000	0.2900000
32407.0000000	0.2800000
32408.0000000	0.2900000
32409.0000000	0.2900000
32410.0000000	0.3000000
32411.0000000	0.2950000
32412.0000000	0.2900000
32413.0000000	0.2950000
32414.0000000	0.2900000
32415.0000000	0.2900000
32416.0000000	0.2850000
32417.0000000	0.2900000
32418.0000000	0.2900000
32419.0000000	0.2900000
32420.0000000	0.2900000
32421.0000000	0.2850000
32422.0000000	0.2900000
32423.0000000	0.2850000
32424.0000000	0.2850000
32425.0000000	0.2850000
32426.0000000	0.2950000
32427.0000000	0.2950000
32428.0000000	0.2866667
32429.0000000	0.2900000
32430.0000000	0.2800000
32431.0000000	0.2950000
32432.0000000	0.2900000

32433.0000000	0.2900000
32434.0000000	0.2850000
32435.0000000	0.2850000
32436.0000000	0.2900000
32437.0000000	0.2900000
32438.0000000	0.2800000
32439.0000000	0.2800000
32440.0000000	0.2800000
32441.0000000	0.2900000
32442.0000000	0.2950000
32443.0000000	0.2900000
32444.0000000	0.2900000
32445.0000000	0.2850000
32446.0000000	0.2850000
32447.0000000	0.2950000
32448.0000000	0.2800000
32449.0000000	0.2950000
32450.0000000	0.2850000
32451.0000000	0.2850000
32452.0000000	0.2950000
32453.0000000	0.2850000
32454.0000000	0.2900000
32455.0000000	0.2850000
32456.0000000	0.2850000
32457.0000000	0.2900000
32458.0000000	0.2900000
32459.0000000	0.2900000
32460.0000000	0.2900000
32461.0000000	0.2900000
32462.0000000	0.2900000
32463.0000000	0.2850000
32464.0000000	0.2850000
32465.0000000	0.2900000
32466.0000000	0.2950000
32467.0000000	0.2850000
32468.0000000	0.2950000
32469.0000000	0.2900000
32470.0000000	0.2950000
32471.0000000	0.2900000
32472.0000000	0.2850000
32473.0000000	0.2850000
32474.0000000	0.2900000
32475.0000000	0.2850000
32476.0000000	0.2900000
32477.0000000	0.2950000
32478.0000000	0.2850000
32479.0000000	0.2900000
32480.0000000	0.2900000
32481.0000000	0.2900000
32482.0000000	0.2900000
32483.0000000	0.2950000
32484.0000000	0.2950000
32485.0000000	0.2900000
32486.0000000	0.2850000
32487.0000000	0.2900000
32488.0000000	0.2950000
32489.0000000	0.2950000

32490.0000000	0.2950000
32491.0000000	0.2900000
32492.0000000	0.2900000
32493.0000000	0.2900000
32494.0000000	0.2900000
32495.0000000	0.3000000
32496.0000000	0.2950000
32497.0000000	0.2950000
32498.0000000	0.2900000
32499.0000000	0.3000000
32500.0000000	0.2950000
32501.0000000	0.2900000
32502.0000000	0.2950000
32503.0000000	0.2950000
32504.0000000	0.3000000
32505.0000000	0.2900000
32506.0000000	0.2950000
32507.0000000	0.2950000
32508.0000000	0.2950000
32509.0000000	0.3050000
32510.0000000	0.2950000
32511.0000000	0.3000000
32512.0000000	0.2950000
32513.0000000	0.2950000
32514.0000000	0.2950000
32515.0000000	0.3000000
32516.0000000	0.2900000
32517.0000000	0.2950000
32518.0000000	0.2933333
32519.0000000	0.2900000
32520.0000000	0.3000000
32521.0000000	0.2900000
32522.0000000	0.3000000
32523.0000000	0.2950000
32524.0000000	0.3000000
32525.0000000	0.3000000
32526.0000000	0.2900000
32527.0000000	0.3000000
32528.0000000	0.2950000
32529.0000000	0.2950000
32530.0000000	0.3100000
32531.0000000	0.3000000
32532.0000000	0.2950000
32533.0000000	0.3050000
32534.0000000	0.2950000
32535.0000000	0.2950000
32536.0000000	0.2950000
32537.0000000	0.2900000
32538.0000000	0.3000000
32539.0000000	0.2950000
32540.0000000	0.2950000
32541.0000000	0.3000000
32542.0000000	0.2950000
32543.0000000	0.3000000
32544.0000000	0.3000000
32545.0000000	0.2900000
32546.0000000	0.2900000

32547.0000000	0.3000000
32548.0000000	0.3000000
32549.0000000	0.2900000
32550.0000000	0.3000000
32551.0000000	0.2950000
32552.0000000	0.2950000
32553.0000000	0.2950000
32554.0000000	0.2950000
32555.0000000	0.3000000
32556.0000000	0.3000000
32557.0000000	0.2950000
32558.0000000	0.2900000
32559.0000000	0.2950000
32560.0000000	0.2950000
32561.0000000	0.2950000
32562.0000000	0.2900000
32563.0000000	0.3050000
32564.0000000	0.2950000
32565.0000000	0.3000000
32566.0000000	0.2950000
32567.0000000	0.3000000
32568.0000000	0.2900000
32569.0000000	0.2950000
32570.0000000	0.3050000
32571.0000000	0.2950000
32572.0000000	0.3000000
32573.0000000	0.2950000
32574.0000000	0.2950000
32575.0000000	0.2900000
32576.0000000	0.2950000
32577.0000000	0.2850000
32578.0000000	0.2966667
32579.0000000	0.2950000
32580.0000000	0.2850000
32581.0000000	0.2900000
32582.0000000	0.2950000
32583.0000000	0.2950000
32584.0000000	0.2950000
32585.0000000	0.2900000
32586.0000000	0.2950000
32587.0000000	0.2900000
32588.0000000	0.2900000
32589.0000000	0.3000000
32590.0000000	0.2850000
32591.0000000	0.2900000
32592.0000000	0.2850000
32593.0000000	0.2950000
32594.0000000	0.2950000
32595.0000000	0.2850000
32596.0000000	0.2950000
32597.0000000	0.2900000
32598.0000000	0.2950000
32599.0000000	0.2850000
32600.0000000	0.2900000
32601.0000000	0.2900000
32602.0000000	0.2850000
32603.0000000	0.2950000

32604.0000000	0.2850000
32605.0000000	0.2900000
32606.0000000	0.2800000
32607.0000000	0.2900000
32608.0000000	0.2833333
32609.0000000	0.2850000
32610.0000000	0.2800000
32611.0000000	0.2850000
32612.0000000	0.2850000
32613.0000000	0.2800000
32614.0000000	0.2850000
32615.0000000	0.2750000
32616.0000000	0.2800000
32617.0000000	0.2750000
32618.0000000	0.2800000
32619.0000000	0.2750000
32620.0000000	0.2900000
32621.0000000	0.2750000
32622.0000000	0.2800000
32623.0000000	0.2750000
32624.0000000	0.2800000
32625.0000000	0.2700000
32626.0000000	0.2850000
32627.0000000	0.2750000
32628.0000000	0.2800000
32629.0000000	0.2750000
32630.0000000	0.2800000
32631.0000000	0.2800000
32632.0000000	0.2800000
32633.0000000	0.2800000
32634.0000000	0.2750000
32635.0000000	0.2800000
32636.0000000	0.2800000
32637.0000000	0.2800000
32638.0000000	0.2800000
32639.0000000	0.2733333
32640.0000000	0.2750000
32641.0000000	0.2700000
32642.0000000	0.2750000
32643.0000000	0.2750000
32644.0000000	0.2700000
32645.0000000	0.2750000
32646.0000000	0.2700000
32647.0000000	0.2850000
32648.0000000	0.2800000
32649.0000000	0.2700000
32650.0000000	0.2700000
32651.0000000	0.2700000
32652.0000000	0.2750000
32653.0000000	0.2800000
32654.0000000	0.2700000
32655.0000000	0.2700000
32656.0000000	0.2700000
32657.0000000	0.2800000
32658.0000000	0.2650000
32659.0000000	0.2800000
32660.0000000	0.2700000

32661.0000000	0.2750000
32662.0000000	0.2650000
32663.0000000	0.2800000
32664.0000000	0.2750000
32665.0000000	0.2750000
32666.0000000	0.2700000
32667.0000000	0.2650000
32668.0000000	0.2700000
32669.0000000	0.2800000
32670.0000000	0.2700000
32671.0000000	0.2650000
32672.0000000	0.2700000
32673.0000000	0.2650000
32674.0000000	0.2750000
32675.0000000	0.2750000
32676.0000000	0.2750000
32677.0000000	0.2650000
32678.0000000	0.2700000
32679.0000000	0.2700000
32680.0000000	0.2800000
32681.0000000	0.2750000
32682.0000000	0.2750000
32683.0000000	0.2650000
32684.0000000	0.2650000
32685.0000000	0.2700000
32686.0000000	0.2700000
32687.0000000	0.2700000
32688.0000000	0.2700000
32689.0000000	0.2700000
32690.0000000	0.2700000
32691.0000000	0.2750000
32692.0000000	0.2650000
32693.0000000	0.2750000
32694.0000000	0.2650000
32695.0000000	0.2700000
32696.0000000	0.2750000
32697.0000000	0.2700000
32698.0000000	0.2700000
32699.0000000	0.2700000
32700.0000000	0.2650000
32701.0000000	0.2800000
32702.0000000	0.2700000
32703.0000000	0.2650000
32704.0000000	0.2600000
32705.0000000	0.2700000
32706.0000000	0.2700000
32707.0000000	0.2750000
32708.0000000	0.2750000
32709.0000000	0.2650000
32710.0000000	0.2650000
32711.0000000	0.2600000
32712.0000000	0.2650000
32713.0000000	0.2650000
32714.0000000	0.2800000
32715.0000000	0.2700000
32716.0000000	0.2700000
32717.0000000	0.2750000

32718.0000000	0.2650000
32719.0000000	0.2700000
32720.0000000	0.2650000
32721.0000000	0.2700000
32722.0000000	0.2750000
32723.0000000	0.2700000
32724.0000000	0.2750000
32725.0000000	0.2700000
32726.0000000	0.2650000
32727.0000000	0.2800000
32728.0000000	0.2750000
32729.0000000	0.2700000
32730.0000000	0.2750000
32731.0000000	0.2750000
32732.0000000	0.2700000
32733.0000000	0.2700000
32734.0000000	0.2700000
32735.0000000	0.2600000
32736.0000000	0.2700000
32737.0000000	0.2750000
32738.0000000	0.2700000
32739.0000000	0.2650000
32740.0000000	0.2750000
32741.0000000	0.2700000
32742.0000000	0.2700000
32743.0000000	0.2650000
32744.0000000	0.2750000
32745.0000000	0.2700000
32746.0000000	0.2650000
32747.0000000	0.2750000
32748.0000000	0.2650000
32749.0000000	0.2700000
32750.0000000	0.2750000
32751.0000000	0.2750000
32752.0000000	0.2700000
32753.0000000	0.2700000
32754.0000000	0.2750000
32755.0000000	0.2650000
32756.0000000	0.2650000
32757.0000000	0.2700000
32758.0000000	0.2700000
32759.0000000	0.2700000
32760.0000000	0.2700000
32761.0000000	0.2700000
32762.0000000	0.2700000
32763.0000000	0.2700000
32764.0000000	0.2700000
32765.0000000	0.2800000
32766.0000000	0.2700000
32767.0000000	0.2750000
32768.0000000	0.2700000
32769.0000000	0.2650000
32770.0000000	0.2700000
32771.0000000	0.2650000
32772.0000000	0.2700000
32773.0000000	0.2650000
32774.0000000	0.2750000

32775.0000000	0.2700000
32776.0000000	0.2700000
32777.0000000	0.2700000
32778.0000000	0.2650000
32779.0000000	0.2700000
32780.0000000	0.2700000
32781.0000000	0.2650000
32782.0000000	0.2650000
32783.0000000	0.2650000
32784.0000000	0.2750000
32785.0000000	0.2700000
32786.0000000	0.2650000
32787.0000000	0.2750000
32788.0000000	0.2600000
32789.0000000	0.2766666
32790.0000000	0.2750000
32791.0000000	0.2650000
32792.0000000	0.2750000
32793.0000000	0.2700000
32794.0000000	0.2700000
32795.0000000	0.2800000
32796.0000000	0.2700000
32797.0000000	0.2700000
32798.0000000	0.2700000
32799.0000000	0.2750000
32800.0000000	0.2650000
32801.0000000	0.2650000
32802.0000000	0.2650000
32803.0000000	0.2650000
32804.0000000	0.2700000
32805.0000000	0.2700000
32806.0000000	0.2750000
32807.0000000	0.2650000
32808.0000000	0.2650000
32809.0000000	0.2600000
32810.0000000	0.2750000
32811.0000000	0.2700000
32812.0000000	0.2750000
32813.0000000	0.2650000
32814.0000000	0.2700000
32815.0000000	0.2650000
32816.0000000	0.2700000
32817.0000000	0.2700000
32818.0000000	0.2650000
32819.0000000	0.2700000
32820.0000000	0.2600000
32821.0000000	0.2700000
32822.0000000	0.2750000
32823.0000000	0.2700000
32824.0000000	0.2800000
32825.0000000	0.2650000
32826.0000000	0.2700000
32827.0000000	0.2650000
32828.0000000	0.2700000
32829.0000000	0.2550000
32830.0000000	0.2700000
32831.0000000	0.2700000

32832.0000000	0.2700000
32833.0000000	0.2650000
32834.0000000	0.2600000
32835.0000000	0.2600000
32836.0000000	0.2600000
32837.0000000	0.2750000
32838.0000000	0.2650000
32839.0000000	0.2750000
32840.0000000	0.2600000
32841.0000000	0.2600000
32842.0000000	0.2650000
32843.0000000	0.2650000
32844.0000000	0.2750000
32845.0000000	0.2650000
32846.0000000	0.2650000
32847.0000000	0.2700000
32848.0000000	0.2700000
32849.0000000	0.2650000
32850.0000000	0.2633333
32851.0000000	0.2650000
32852.0000000	0.2650000
32853.0000000	0.2600000
32854.0000000	0.2600000
32855.0000000	0.2600000
32856.0000000	0.2650000
32857.0000000	0.2600000
32858.0000000	0.2650000
32859.0000000	0.2750000
32860.0000000	0.2650000
32861.0000000	0.2750000
32862.0000000	0.2700000
32863.0000000	0.2650000
32864.0000000	0.2650000
32865.0000000	0.2550000
32866.0000000	0.2600000
32867.0000000	0.2650000
32868.0000000	0.2650000
32869.0000000	0.2650000
32870.0000000	0.2650000
32871.0000000	0.2700000
32872.0000000	0.2650000
32873.0000000	0.2650000
32874.0000000	0.2650000
32875.0000000	0.2650000
32876.0000000	0.2650000
32877.0000000	0.2700000
32878.0000000	0.2650000
32879.0000000	0.2650000
32880.0000000	0.2600000
32881.0000000	0.2650000
32882.0000000	0.2700000
32883.0000000	0.2650000
32884.0000000	0.2700000
32885.0000000	0.2550000
32886.0000000	0.2750000
32887.0000000	0.2650000
32888.0000000	0.2650000

32889.0000000	0.2600000
32890.0000000	0.2600000
32891.0000000	0.2700000
32892.0000000	0.2600000
32893.0000000	0.2650000
32894.0000000	0.2650000
32895.0000000	0.2650000
32896.0000000	0.2650000
32897.0000000	0.2600000
32898.0000000	0.2650000
32899.0000000	0.2650000
32900.0000000	0.2650000
32901.0000000	0.2650000
32902.0000000	0.2750000
32903.0000000	0.2600000
32904.0000000	0.2650000
32905.0000000	0.2550000
32906.0000000	0.2550000
32907.0000000	0.2650000
32908.0000000	0.2650000
32909.0000000	0.2700000
32910.0000000	0.2600000
32911.0000000	0.2550000
32912.0000000	0.2650000
32913.0000000	0.2700000
32914.0000000	0.2650000
32915.0000000	0.2650000
32916.0000000	0.2600000
32917.0000000	0.2700000
32918.0000000	0.2650000
32919.0000000	0.2550000
32920.0000000	0.2650000
32921.0000000	0.2600000
32922.0000000	0.2700000
32923.0000000	0.2650000
32924.0000000	0.2650000
32925.0000000	0.2600000
32926.0000000	0.2550000
32927.0000000	0.2650000
32928.0000000	0.2600000
32929.0000000	0.2650000
32930.0000000	0.2650000
32931.0000000	0.2650000
32932.0000000	0.2600000
32933.0000000	0.2700000
32934.0000000	0.2600000
32935.0000000	0.2650000
32936.0000000	0.2650000
32937.0000000	0.2650000
32938.0000000	0.2650000
32939.0000000	0.2650000
32940.0000000	0.2600000
32941.0000000	0.2650000
32942.0000000	0.2600000
32943.0000000	0.2600000
32944.0000000	0.2600000
32945.0000000	0.2550000

32946.0000000	0.2700000
32947.0000000	0.2600000
32948.0000000	0.2600000
32949.0000000	0.2600000
32950.0000000	0.2600000
32951.0000000	0.2650000
32952.0000000	0.2550000
32953.0000000	0.2700000
32954.0000000	0.2600000
32955.0000000	0.2600000
32956.0000000	0.2500000
32957.0000000	0.2600000
32958.0000000	0.2550000
32959.0000000	0.2650000
32960.0000000	0.2550000
32961.0000000	0.2600000
32962.0000000	0.2550000
32963.0000000	0.2550000
32964.0000000	0.2550000
32965.0000000	0.2650000
32966.0000000	0.2600000
32967.0000000	0.2600000
32968.0000000	0.2500000
32969.0000000	0.2550000
32970.0000000	0.2500000
32971.0000000	0.2566667
32972.0000000	0.2600000
32973.0000000	0.2450000
32974.0000000	0.2550000
32975.0000000	0.2550000
32976.0000000	0.2600000
32977.0000000	0.2550000
32978.0000000	0.2500000
32979.0000000	0.2500000
32980.0000000	0.2550000
32981.0000000	0.2450000
32982.0000000	0.2550000
32983.0000000	0.2450000
32984.0000000	0.2450000
32985.0000000	0.2550000
32986.0000000	0.2500000
32987.0000000	0.2550000
32988.0000000	0.2500000
32989.0000000	0.2500000
32990.0000000	0.2500000
32991.0000000	0.2550000
32992.0000000	0.2500000
32993.0000000	0.2450000
32994.0000000	0.2400000
32995.0000000	0.2500000
32996.0000000	0.2450000
32997.0000000	0.2550000
32998.0000000	0.2500000
32999.0000000	0.2450000
33000.0000000	0.2450000
33001.0000000	0.2433333
33002.0000000	0.2450000

33003.0000000	0.2500000
33004.0000000	0.2450000
33005.0000000	0.2450000
33006.0000000	0.2450000
33007.0000000	0.2450000
33008.0000000	0.2450000
33009.0000000	0.2400000
33010.0000000	0.2450000
33011.0000000	0.2400000
33012.0000000	0.2500000
33013.0000000	0.2500000
33014.0000000	0.2350000
33015.0000000	0.2450000
33016.0000000	0.2400000
33017.0000000	0.2400000
33018.0000000	0.2400000
33019.0000000	0.2350000
33020.0000000	0.2350000
33021.0000000	0.2500000
33022.0000000	0.2300000
33023.0000000	0.2400000
33024.0000000	0.2400000
33025.0000000	0.2350000
33026.0000000	0.2400000
33027.0000000	0.2300000
33028.0000000	0.2400000
33029.0000000	0.2300000
33030.0000000	0.2400000
33031.0000000	0.2266666
33032.0000000	0.2300000
33033.0000000	0.2350000
33034.0000000	0.2350000
33035.0000000	0.2350000
33036.0000000	0.2350000
33037.0000000	0.2300000
33038.0000000	0.2300000
33039.0000000	0.2250000
33040.0000000	0.2250000
33041.0000000	0.2300000
33042.0000000	0.2300000
33043.0000000	0.2300000
33044.0000000	0.2300000
33045.0000000	0.2300000
33046.0000000	0.2250000
33047.0000000	0.2200000
33048.0000000	0.2250000
33049.0000000	0.2250000
33050.0000000	0.2300000
33051.0000000	0.2350000
33052.0000000	0.2200000
33053.0000000	0.2250000
33054.0000000	0.2300000
33055.0000000	0.2250000
33056.0000000	0.2250000
33057.0000000	0.2100000
33058.0000000	0.2200000
33059.0000000	0.2200000

33060.0000000	0.2200000
33061.0000000	0.2233333
33062.0000000	0.2150000
33063.0000000	0.2200000
33064.0000000	0.2200000
33065.0000000	0.2150000
33066.0000000	0.2200000
33067.0000000	0.2250000
33068.0000000	0.2200000
33069.0000000	0.2200000
33070.0000000	0.2200000
33071.0000000	0.2200000
33072.0000000	0.2250000
33073.0000000	0.2150000
33074.0000000	0.2150000
33075.0000000	0.2200000
33076.0000000	0.2100000
33077.0000000	0.2200000
33078.0000000	0.2150000
33079.0000000	0.2150000
33080.0000000	0.2150000
33081.0000000	0.2100000
33082.0000000	0.2150000
33083.0000000	0.2150000
33084.0000000	0.2250000
33085.0000000	0.2100000
33086.0000000	0.2200000
33087.0000000	0.2050000
33088.0000000	0.2100000
33089.0000000	0.2100000
33090.0000000	0.2250000
33091.0000000	0.2150000
33092.0000000	0.2066667
33093.0000000	0.2150000
33094.0000000	0.2050000
33095.0000000	0.2200000
33096.0000000	0.2050000
33097.0000000	0.2000000
33098.0000000	0.2050000
33099.0000000	0.2000000
33100.0000000	0.2050000
33101.0000000	0.2050000
33102.0000000	0.2100000
33103.0000000	0.2050000
33104.0000000	0.2050000
33105.0000000	0.2050000
33106.0000000	0.2050000
33107.0000000	0.2000000
33108.0000000	0.2050000
33109.0000000	0.2100000
33110.0000000	0.2100000
33111.0000000	0.2050000
33112.0000000	0.2000000
33113.0000000	0.2000000
33114.0000000	0.2000000
33115.0000000	0.2000000
33116.0000000	0.2100000

33117.0000000	0.2050000
33118.0000000	0.2050000
33119.0000000	0.1950000
33120.0000000	0.2100000
33121.0000000	0.2050000
33122.0000000	0.2033333
33123.0000000	0.2100000
33124.0000000	0.2050000
33125.0000000	0.2000000
33126.0000000	0.2050000
33127.0000000	0.2000000
33128.0000000	0.2000000
33129.0000000	0.2050000
33130.0000000	0.2050000
33131.0000000	0.2050000
33132.0000000	0.2050000
33133.0000000	0.2100000
33134.0000000	0.2000000
33135.0000000	0.2000000
33136.0000000	0.2000000
33137.0000000	0.2000000
33138.0000000	0.2050000
33139.0000000	0.2000000
33140.0000000	0.2050000
33141.0000000	0.2000000
33142.0000000	0.2000000
33143.0000000	0.1950000
33144.0000000	0.2000000
33145.0000000	0.2000000
33146.0000000	0.2100000
33147.0000000	0.2000000
33148.0000000	0.2050000
33149.0000000	0.1900000
33150.0000000	0.2000000
33151.0000000	0.1933333
33152.0000000	0.2000000
33153.0000000	0.1950000
33154.0000000	0.1950000
33155.0000000	0.2000000
33156.0000000	0.2050000
33157.0000000	0.2000000
33158.0000000	0.2050000
33159.0000000	0.2000000
33160.0000000	0.2000000
33161.0000000	0.1950000
33162.0000000	0.2000000
33163.0000000	0.2000000
33164.0000000	0.2000000
33165.0000000	0.2000000
33166.0000000	0.1950000
33167.0000000	0.2000000
33168.0000000	0.1900000
33169.0000000	0.2050000
33170.0000000	0.1950000
33171.0000000	0.2000000
33172.0000000	0.1950000
33173.0000000	0.1950000

33174.0000000	0.2050000
33175.0000000	0.2000000
33176.0000000	0.2000000
33177.0000000	0.1950000
33178.0000000	0.1950000
33179.0000000	0.2100000
33180.0000000	0.1950000
33181.0000000	0.2050000
33182.0000000	0.1933333
33183.0000000	0.2000000
33184.0000000	0.1950000
33185.0000000	0.2000000
33186.0000000	0.1950000
33187.0000000	0.2000000
33188.0000000	0.1900000
33189.0000000	0.2000000
33190.0000000	0.1950000
33191.0000000	0.2000000
33192.0000000	0.1950000
33193.0000000	0.1950000
33194.0000000	0.1900000
33195.0000000	0.1950000
33196.0000000	0.1950000
33197.0000000	0.2000000
33198.0000000	0.2050000
33199.0000000	0.1900000
33200.0000000	0.1950000
33201.0000000	0.1850000
33202.0000000	0.2000000
33203.0000000	0.1900000
33204.0000000	0.1950000
33205.0000000	0.1950000
33206.0000000	0.1900000
33207.0000000	0.2000000
33208.0000000	0.2000000
33209.0000000	0.1950000
33210.0000000	0.1800000
33211.0000000	0.1950000
33212.0000000	0.1966667
33213.0000000	0.1950000
33214.0000000	0.1950000
33215.0000000	0.1950000
33216.0000000	0.1900000
33217.0000000	0.1950000
33218.0000000	0.2050000
33219.0000000	0.1900000
33220.0000000	0.1950000
33221.0000000	0.1850000
33222.0000000	0.1900000
33223.0000000	0.1950000
33224.0000000	0.2000000
33225.0000000	0.1900000
33226.0000000	0.1900000
33227.0000000	0.1900000
33228.0000000	0.1950000
33229.0000000	0.1950000
33230.0000000	0.1950000

33231.0000000	0.2000000
33232.0000000	0.1900000
33233.0000000	0.1950000
33234.0000000	0.1900000
33235.0000000	0.1950000
33236.0000000	0.1850000
33237.0000000	0.2000000
33238.0000000	0.1900000
33239.0000000	0.1900000
33240.0000000	0.1950000
33241.0000000	0.2000000
33242.0000000	0.1966667
33243.0000000	0.1950000
33244.0000000	0.1950000
33245.0000000	0.2000000
33246.0000000	0.1950000
33247.0000000	0.1950000
33248.0000000	0.1850000
33249.0000000	0.1950000
33250.0000000	0.1850000
33251.0000000	0.1950000
33252.0000000	0.1950000
33253.0000000	0.1900000
33254.0000000	0.1950000
33255.0000000	0.1850000
33256.0000000	0.2000000
33257.0000000	0.1900000
33258.0000000	0.2050000
33259.0000000	0.1900000
33260.0000000	0.2000000
33261.0000000	0.1900000
33262.0000000	0.1900000
33263.0000000	0.1850000
33264.0000000	0.1900000
33265.0000000	0.1850000
33266.0000000	0.1850000
33267.0000000	0.1900000
33268.0000000	0.1950000
33269.0000000	0.1950000
33270.0000000	0.1800000
33271.0000000	0.1900000
33272.0000000	0.1900000
33273.0000000	0.1850000
33274.0000000	0.1900000
33275.0000000	0.1850000
33276.0000000	0.1900000
33277.0000000	0.1850000
33278.0000000	0.1950000
33279.0000000	0.1850000
33280.0000000	0.1850000
33281.0000000	0.1900000
33282.0000000	0.1850000
33283.0000000	0.1850000
33284.0000000	0.1900000
33285.0000000	0.1800000
33286.0000000	0.1900000
33287.0000000	0.1900000

33288.0000000	0.1850000
33289.0000000	0.1850000
33290.0000000	0.1950000
33291.0000000	0.1800000
33292.0000000	0.1850000
33293.0000000	0.1850000
33294.0000000	0.1850000
33295.0000000	0.1900000
33296.0000000	0.1850000
33297.0000000	0.1800000
33298.0000000	0.1800000
33299.0000000	0.1750000
33300.0000000	0.1800000
33301.0000000	0.1850000
33302.0000000	0.1850000
33303.0000000	0.1800000
33304.0000000	0.1750000
33305.0000000	0.1850000
33306.0000000	0.1900000
33307.0000000	0.1850000
33308.0000000	0.1850000
33309.0000000	0.1750000
33310.0000000	0.1850000
33311.0000000	0.1850000
33312.0000000	0.1800000
33313.0000000	0.1800000
33314.0000000	0.1800000
33315.0000000	0.1850000
33316.0000000	0.1750000
33317.0000000	0.1800000
33318.0000000	0.1850000
33319.0000000	0.1800000
33320.0000000	0.1800000
33321.0000000	0.1900000
33322.0000000	0.1800000
33323.0000000	0.1900000
33324.0000000	0.1750000
33325.0000000	0.1850000
33326.0000000	0.1800000
33327.0000000	0.1800000
33328.0000000	0.1800000
33329.0000000	0.1850000
33330.0000000	0.1800000
33331.0000000	0.1850000
33332.0000000	0.1800000
33333.0000000	0.1733333
33334.0000000	0.1800000
33335.0000000	0.1800000
33336.0000000	0.1800000
33337.0000000	0.1800000
33338.0000000	0.1750000
33339.0000000	0.1800000
33340.0000000	0.1850000
33341.0000000	0.1800000
33342.0000000	0.1800000
33343.0000000	0.1850000
33344.0000000	0.1750000

33345.0000000	0.1750000
33346.0000000	0.1750000
33347.0000000	0.1800000
33348.0000000	0.1850000
33349.0000000	0.1850000
33350.0000000	0.1750000
33351.0000000	0.1800000
33352.0000000	0.1750000
33353.0000000	0.1750000
33354.0000000	0.1800000
33355.0000000	0.1750000
33356.0000000	0.1700000
33357.0000000	0.1750000
33358.0000000	0.1800000
33359.0000000	0.1750000
33360.0000000	0.1750000
33361.0000000	0.1700000
33362.0000000	0.1750000
33363.0000000	0.1800000
33364.0000000	0.1800000
33365.0000000	0.1750000
33366.0000000	0.1750000
33367.0000000	0.1750000
33368.0000000	0.1800000
33369.0000000	0.1700000
33370.0000000	0.1800000
33371.0000000	0.1700000
33372.0000000	0.1700000
33373.0000000	0.1600000
33374.0000000	0.1700000
33375.0000000	0.1750000
33376.0000000	0.1700000
33377.0000000	0.1700000
33378.0000000	0.1700000
33379.0000000	0.1550000
33380.0000000	0.1650000
33381.0000000	0.1700000
33382.0000000	0.1750000
33383.0000000	0.1700000
33384.0000000	0.1700000
33385.0000000	0.1650000
33386.0000000	0.1700000
33387.0000000	0.1700000
33388.0000000	0.1650000
33389.0000000	0.1700000
33390.0000000	0.1700000
33391.0000000	0.1750000
33392.0000000	0.1600000
33393.0000000	0.1733333
33394.0000000	0.1800000
33395.0000000	0.1650000
33396.0000000	0.1700000
33397.0000000	0.1800000
33398.0000000	0.1750000
33399.0000000	0.1800000
33400.0000000	0.1750000
33401.0000000	0.1800000

33402.0000000	0.1700000
33403.0000000	0.1750000
33404.0000000	0.1700000
33405.0000000	0.1800000
33406.0000000	0.1850000
33407.0000000	0.1800000
33408.0000000	0.1750000
33409.0000000	0.1750000
33410.0000000	0.1800000
33411.0000000	0.1900000
33412.0000000	0.1800000
33413.0000000	0.1850000
33414.0000000	0.1750000
33415.0000000	0.1750000
33416.0000000	0.1800000
33417.0000000	0.1750000
33418.0000000	0.1900000
33419.0000000	0.1750000
33420.0000000	0.1850000
33421.0000000	0.1750000
33422.0000000	0.1800000
33423.0000000	0.1850000
33424.0000000	0.1733333
33425.0000000	0.1800000
33426.0000000	0.1850000
33427.0000000	0.1800000
33428.0000000	0.1800000
33429.0000000	0.1750000
33430.0000000	0.1700000
33431.0000000	0.1750000
33432.0000000	0.1850000
33433.0000000	0.1750000
33434.0000000	0.1800000
33435.0000000	0.1750000
33436.0000000	0.1850000
33437.0000000	0.1800000
33438.0000000	0.1750000
33439.0000000	0.1700000
33440.0000000	0.1750000
33441.0000000	0.1850000
33442.0000000	0.1750000
33443.0000000	0.1850000
33444.0000000	0.1750000
33445.0000000	0.1800000
33446.0000000	0.1800000
33447.0000000	0.1800000
33448.0000000	0.1800000
33449.0000000	0.1700000
33450.0000000	0.1700000
33451.0000000	0.1800000
33452.0000000	0.1800000
33453.0000000	0.1800000
33454.0000000	0.1766667
33455.0000000	0.1850000
33456.0000000	0.1700000
33457.0000000	0.1800000
33458.0000000	0.1800000

33459.0000000	0.1800000
33460.0000000	0.1750000
33461.0000000	0.1850000
33462.0000000	0.1750000
33463.0000000	0.1750000
33464.0000000	0.1800000
33465.0000000	0.1800000
33466.0000000	0.1800000
33467.0000000	0.1800000
33468.0000000	0.1700000
33469.0000000	0.1750000
33470.0000000	0.1750000
33471.0000000	0.1750000
33472.0000000	0.1850000
33473.0000000	0.1750000
33474.0000000	0.1850000
33475.0000000	0.1750000
33476.0000000	0.1800000
33477.0000000	0.1800000
33478.0000000	0.1750000
33479.0000000	0.1750000
33480.0000000	0.1800000
33481.0000000	0.1750000
33482.0000000	0.1750000
33483.0000000	0.1766667
33484.0000000	0.1900000
33485.0000000	0.1800000
33486.0000000	0.1850000
33487.0000000	0.1800000
33488.0000000	0.1850000
33489.0000000	0.1900000
33490.0000000	0.1750000
33491.0000000	0.1800000
33492.0000000	0.1750000
33493.0000000	0.1700000
33494.0000000	0.1750000
33495.0000000	0.1850000
33496.0000000	0.1700000
33497.0000000	0.1850000
33498.0000000	0.1800000
33499.0000000	0.1850000
33500.0000000	0.1850000
33501.0000000	0.1800000
33502.0000000	0.1750000
33503.0000000	0.1800000
33504.0000000	0.1800000
33505.0000000	0.1750000
33506.0000000	0.1750000
33507.0000000	0.1750000
33508.0000000	0.1750000
33509.0000000	0.1800000
33510.0000000	0.1800000
33511.0000000	0.1800000
33512.0000000	0.1850000
33513.0000000	0.1800000
33514.0000000	0.1766667
33515.0000000	0.1800000

33516.0000000	0.1800000
33517.0000000	0.1800000
33518.0000000	0.1800000
33519.0000000	0.1850000
33520.0000000	0.1700000
33521.0000000	0.1850000
33522.0000000	0.1800000
33523.0000000	0.1850000
33524.0000000	0.1800000
33525.0000000	0.1850000
33526.0000000	0.1900000
33527.0000000	0.1750000
33528.0000000	0.1800000
33529.0000000	0.1750000
33530.0000000	0.1850000
33531.0000000	0.1800000
33532.0000000	0.1850000
33533.0000000	0.1850000
33534.0000000	0.1850000
33535.0000000	0.1750000
33536.0000000	0.1800000
33537.0000000	0.1850000
33538.0000000	0.1900000
33539.0000000	0.1850000
33540.0000000	0.1900000
33541.0000000	0.1800000
33542.0000000	0.1850000
33543.0000000	0.1800000
33544.0000000	0.1900000
33545.0000000	0.1850000
33546.0000000	0.1900000
33547.0000000	0.1750000
33548.0000000	0.1950000
33549.0000000	0.1750000
33550.0000000	0.1800000
33551.0000000	0.1900000
33552.0000000	0.1900000
33553.0000000	0.1950000
33554.0000000	0.1900000
33555.0000000	0.1800000
33556.0000000	0.1850000
33557.0000000	0.1800000
33558.0000000	0.1900000
33559.0000000	0.1900000
33560.0000000	0.1950000
33561.0000000	0.1850000
33562.0000000	0.1800000
33563.0000000	0.1900000
33564.0000000	0.1900000
33565.0000000	0.1900000
33566.0000000	0.1950000
33567.0000000	0.1950000
33568.0000000	0.1850000
33569.0000000	0.1850000
33570.0000000	0.1950000
33571.0000000	0.1900000
33572.0000000	0.1950000

33573.0000000	0.1950000
33574.0000000	0.1966667
33575.0000000	0.1850000
33576.0000000	0.1900000
33577.0000000	0.1900000
33578.0000000	0.1950000
33579.0000000	0.1950000
33580.0000000	0.2000000
33581.0000000	0.1950000
33582.0000000	0.1950000
33583.0000000	0.1950000
33584.0000000	0.1950000
33585.0000000	0.1900000
33586.0000000	0.1950000
33587.0000000	0.1950000
33588.0000000	0.1950000
33589.0000000	0.2000000
33590.0000000	0.1950000
33591.0000000	0.1950000
33592.0000000	0.1900000
33593.0000000	0.1850000
33594.0000000	0.1950000
33595.0000000	0.1900000
33596.0000000	0.1950000
33597.0000000	0.2000000
33598.0000000	0.1950000
33599.0000000	0.1950000
33600.0000000	0.1950000
33601.0000000	0.1950000
33602.0000000	0.2000000
33603.0000000	0.1950000
33604.0000000	0.1966667
33605.0000000	0.1900000
33606.0000000	0.1950000
33607.0000000	0.1900000
33608.0000000	0.1900000
33609.0000000	0.2000000
33610.0000000	0.2000000
33611.0000000	0.1900000
33612.0000000	0.2000000
33613.0000000	0.1950000
33614.0000000	0.1950000
33615.0000000	0.1950000
33616.0000000	0.2000000
33617.0000000	0.2000000
33618.0000000	0.1900000
33619.0000000	0.1950000
33620.0000000	0.2000000
33621.0000000	0.2000000
33622.0000000	0.1900000
33623.0000000	0.1900000
33624.0000000	0.2000000
33625.0000000	0.1900000
33626.0000000	0.1950000
33627.0000000	0.1950000
33628.0000000	0.2000000
33629.0000000	0.1950000

33630.0000000	0.1900000
33631.0000000	0.2000000
33632.0000000	0.1900000
33633.0000000	0.1950000
33634.0000000	0.2000000
33635.0000000	0.1966667
33636.0000000	0.1900000
33637.0000000	0.2000000
33638.0000000	0.2050000
33639.0000000	0.2000000
33640.0000000	0.1950000
33641.0000000	0.1950000
33642.0000000	0.1950000
33643.0000000	0.2000000
33644.0000000	0.1950000
33645.0000000	0.2000000
33646.0000000	0.1950000
33647.0000000	0.1950000
33648.0000000	0.2000000
33649.0000000	0.1900000
33650.0000000	0.2000000
33651.0000000	0.1950000
33652.0000000	0.1900000
33653.0000000	0.1950000
33654.0000000	0.1900000
33655.0000000	0.2050000
33656.0000000	0.1950000
33657.0000000	0.1950000
33658.0000000	0.1900000
33659.0000000	0.1900000
33660.0000000	0.1950000
33661.0000000	0.1900000
33662.0000000	0.1950000
33663.0000000	0.2000000
33664.0000000	0.1950000
33665.0000000	0.1966667
33666.0000000	0.1850000
33667.0000000	0.2000000
33668.0000000	0.2000000
33669.0000000	0.1950000
33670.0000000	0.2000000
33671.0000000	0.1900000
33672.0000000	0.2000000
33673.0000000	0.2000000
33674.0000000	0.2000000
33675.0000000	0.1950000
33676.0000000	0.1900000
33677.0000000	0.1950000
33678.0000000	0.2000000
33679.0000000	0.1900000
33680.0000000	0.1900000
33681.0000000	0.2050000
33682.0000000	0.1950000
33683.0000000	0.2000000
33684.0000000	0.2000000
33685.0000000	0.2000000
33686.0000000	0.1950000

33687.0000000	0.2050000
33688.0000000	0.1900000
33689.0000000	0.1950000
33690.0000000	0.2000000
33691.0000000	0.2000000
33692.0000000	0.1950000
33693.0000000	0.1950000
33694.0000000	0.2000000
33695.0000000	0.1900000
33696.0000000	0.2050000
33697.0000000	0.1900000
33698.0000000	0.1950000
33699.0000000	0.2000000
33700.0000000	0.1900000
33701.0000000	0.1950000
33702.0000000	0.2000000
33703.0000000	0.2050000
33704.0000000	0.2000000
33705.0000000	0.2100000
33706.0000000	0.1950000
33707.0000000	0.2000000
33708.0000000	0.1950000
33709.0000000	0.2050000
33710.0000000	0.2050000
33711.0000000	0.2000000
33712.0000000	0.1950000
33713.0000000	0.1950000
33714.0000000	0.2050000
33715.0000000	0.1950000
33716.0000000	0.1950000
33717.0000000	0.2000000
33718.0000000	0.2050000
33719.0000000	0.2100000
33720.0000000	0.2000000
33721.0000000	0.2100000
33722.0000000	0.2000000
33723.0000000	0.1900000
33724.0000000	0.2000000
33725.0000000	0.2066667
33726.0000000	0.1950000
33727.0000000	0.2000000
33728.0000000	0.2050000
33729.0000000	0.2000000
33730.0000000	0.2050000
33731.0000000	0.2000000
33732.0000000	0.1950000
33733.0000000	0.2100000
33734.0000000	0.2100000
33735.0000000	0.2000000
33736.0000000	0.2050000
33737.0000000	0.2000000
33738.0000000	0.2050000
33739.0000000	0.2050000
33740.0000000	0.2000000
33741.0000000	0.2100000
33742.0000000	0.2050000
33743.0000000	0.2050000

33744.0000000	0.2050000
33745.0000000	0.2050000
33746.0000000	0.2050000
33747.0000000	0.2050000
33748.0000000	0.2000000
33749.0000000	0.2000000
33750.0000000	0.2000000
33751.0000000	0.2050000
33752.0000000	0.2000000
33753.0000000	0.2050000
33754.0000000	0.2100000
33755.0000000	0.2066667
33756.0000000	0.2050000
33757.0000000	0.2000000
33758.0000000	0.2100000
33759.0000000	0.2050000
33760.0000000	0.2050000
33761.0000000	0.2050000
33762.0000000	0.2050000
33763.0000000	0.2100000
33764.0000000	0.2100000
33765.0000000	0.2050000
33766.0000000	0.2050000
33767.0000000	0.2050000
33768.0000000	0.2050000
33769.0000000	0.2100000
33770.0000000	0.2050000
33771.0000000	0.2150000
33772.0000000	0.2050000
33773.0000000	0.2100000
33774.0000000	0.2100000
33775.0000000	0.2050000
33776.0000000	0.2050000
33777.0000000	0.2000000
33778.0000000	0.2050000
33779.0000000	0.2000000
33780.0000000	0.2200000
33781.0000000	0.2000000
33782.0000000	0.2100000
33783.0000000	0.1950000
33784.0000000	0.1950000
33785.0000000	0.2000000
33786.0000000	0.2066667
33787.0000000	0.2000000
33788.0000000	0.2000000
33789.0000000	0.1900000
33790.0000000	0.2050000
33791.0000000	0.2050000
33792.0000000	0.2000000
33793.0000000	0.2000000
33794.0000000	0.2000000
33795.0000000	0.1900000
33796.0000000	0.2050000
33797.0000000	0.2000000
33798.0000000	0.2050000
33799.0000000	0.2000000
33800.0000000	0.2000000

33801.0000000	0.2000000
33802.0000000	0.2050000
33803.0000000	0.2000000
33804.0000000	0.2050000
33805.0000000	0.2050000
33806.0000000	0.2000000
33807.0000000	0.2050000
33808.0000000	0.1950000
33809.0000000	0.2000000
33810.0000000	0.1950000
33811.0000000	0.2100000
33812.0000000	0.2050000
33813.0000000	0.2050000
33814.0000000	0.1950000
33815.0000000	0.2000000
33816.0000000	0.2000000
33817.0000000	0.2100000
33818.0000000	0.2000000
33819.0000000	0.1950000
33820.0000000	0.2050000
33821.0000000	0.2100000
33822.0000000	0.2050000
33823.0000000	0.2000000
33824.0000000	0.2000000
33825.0000000	0.2000000
33826.0000000	0.2000000
33827.0000000	0.2000000
33828.0000000	0.2050000
33829.0000000	0.2000000
33830.0000000	0.2100000
33831.0000000	0.1950000
33832.0000000	0.1900000
33833.0000000	0.1950000
33834.0000000	0.2050000
33835.0000000	0.2050000
33836.0000000	0.2050000
33837.0000000	0.2050000
33838.0000000	0.1950000
33839.0000000	0.2050000
33840.0000000	0.2000000
33841.0000000	0.2000000
33842.0000000	0.2000000
33843.0000000	0.2000000
33844.0000000	0.2050000
33845.0000000	0.2000000
33846.0000000	0.2066667
33847.0000000	0.2050000
33848.0000000	0.2050000
33849.0000000	0.2050000
33850.0000000	0.2050000
33851.0000000	0.2000000
33852.0000000	0.2000000
33853.0000000	0.2100000
33854.0000000	0.2100000
33855.0000000	0.1950000
33856.0000000	0.2050000
33857.0000000	0.2050000

33858.0000000	0.2050000
33859.0000000	0.2050000
33860.0000000	0.2100000
33861.0000000	0.1950000
33862.0000000	0.2100000
33863.0000000	0.2050000
33864.0000000	0.2050000
33865.0000000	0.2050000
33866.0000000	0.2100000
33867.0000000	0.2050000
33868.0000000	0.2050000
33869.0000000	0.2000000
33870.0000000	0.2050000
33871.0000000	0.2100000
33872.0000000	0.2050000
33873.0000000	0.2050000
33874.0000000	0.2100000
33875.0000000	0.2050000
33876.0000000	0.2100000
33877.0000000	0.2000000
33878.0000000	0.2000000
33879.0000000	0.2000000
33880.0000000	0.2050000
33881.0000000	0.2050000
33882.0000000	0.2100000
33883.0000000	0.2050000
33884.0000000	0.2050000
33885.0000000	0.2000000
33886.0000000	0.2050000
33887.0000000	0.2050000
33888.0000000	0.2000000
33889.0000000	0.2050000
33890.0000000	0.2000000
33891.0000000	0.2000000
33892.0000000	0.2000000
33893.0000000	0.2000000
33894.0000000	0.2050000
33895.0000000	0.2050000
33896.0000000	0.2100000
33897.0000000	0.1900000
33898.0000000	0.2000000
33899.0000000	0.2000000
33900.0000000	0.2000000
33901.0000000	0.1950000
33902.0000000	0.2000000
33903.0000000	0.2000000
33904.0000000	0.2000000
33905.0000000	0.1950000
33906.0000000	0.2000000
33907.0000000	0.2000000
33908.0000000	0.1950000
33909.0000000	0.1950000
33910.0000000	0.2000000
33911.0000000	0.2050000
33912.0000000	0.2000000
33913.0000000	0.2000000
33914.0000000	0.2050000

33915.0000000	0.1950000
33916.0000000	0.2000000
33917.0000000	0.2050000
33918.0000000	0.1950000
33919.0000000	0.2000000
33920.0000000	0.2000000
33921.0000000	0.1950000
33922.0000000	0.2000000
33923.0000000	0.1950000
33924.0000000	0.1900000
33925.0000000	0.2050000
33926.0000000	0.1950000
33927.0000000	0.2000000
33928.0000000	0.1950000
33929.0000000	0.1900000
33930.0000000	0.2000000
33931.0000000	0.2000000
33932.0000000	0.1850000
33933.0000000	0.1950000
33934.0000000	0.1950000
33935.0000000	0.1900000
33936.0000000	0.1966667
33937.0000000	0.1900000
33938.0000000	0.1850000
33939.0000000	0.1950000
33940.0000000	0.1900000
33941.0000000	0.1950000
33942.0000000	0.1950000
33943.0000000	0.1850000
33944.0000000	0.1950000
33945.0000000	0.1900000
33946.0000000	0.1900000
33947.0000000	0.1850000
33948.0000000	0.1900000
33949.0000000	0.1900000
33950.0000000	0.2000000
33951.0000000	0.1850000
33952.0000000	0.1900000
33953.0000000	0.1800000
33954.0000000	0.1800000
33955.0000000	0.1900000
33956.0000000	0.1850000
33957.0000000	0.1850000
33958.0000000	0.1850000
33959.0000000	0.1900000
33960.0000000	0.1850000
33961.0000000	0.1900000
33962.0000000	0.1850000
33963.0000000	0.1800000
33964.0000000	0.1850000
33965.0000000	0.1850000
33966.0000000	0.1850000
33967.0000000	0.1833333
33968.0000000	0.1800000
33969.0000000	0.1900000
33970.0000000	0.1900000
33971.0000000	0.1900000

33972.0000000	0.1850000
33973.0000000	0.1850000
33974.0000000	0.1800000
33975.0000000	0.1900000
33976.0000000	0.1800000
33977.0000000	0.1850000
33978.0000000	0.1850000
33979.0000000	0.1900000
33980.0000000	0.1800000
33981.0000000	0.1950000
33982.0000000	0.1900000
33983.0000000	0.1900000
33984.0000000	0.1900000
33985.0000000	0.1900000
33986.0000000	0.1900000
33987.0000000	0.1900000
33988.0000000	0.1950000
33989.0000000	0.1850000
33990.0000000	0.1800000
33991.0000000	0.1950000
33992.0000000	0.1850000
33993.0000000	0.1900000
33994.0000000	0.1850000
33995.0000000	0.1900000
33996.0000000	0.1950000
33997.0000000	0.1866667
33998.0000000	0.1900000
33999.0000000	0.1900000
34000.0000000	0.1850000
34001.0000000	0.1900000
34002.0000000	0.1850000
34003.0000000	0.1950000
34004.0000000	0.1850000
34005.0000000	0.1850000
34006.0000000	0.1950000
34007.0000000	0.1900000
34008.0000000	0.1950000
34009.0000000	0.1900000
34010.0000000	0.1900000
34011.0000000	0.1850000
34012.0000000	0.1900000
34013.0000000	0.1950000
34014.0000000	0.1850000
34015.0000000	0.1950000
34016.0000000	0.1850000
34017.0000000	0.1850000
34018.0000000	0.1900000
34019.0000000	0.1800000
34020.0000000	0.1900000
34021.0000000	0.1800000
34022.0000000	0.1900000
34023.0000000	0.1900000
34024.0000000	0.1850000
34025.0000000	0.1900000
34026.0000000	0.1850000
34027.0000000	0.1866667
34028.0000000	0.1950000

34029.0000000	0.1900000
34030.0000000	0.1900000
34031.0000000	0.1850000
34032.0000000	0.1900000
34033.0000000	0.1950000
34034.0000000	0.1850000
34035.0000000	0.1800000
34036.0000000	0.1850000
34037.0000000	0.1950000
34038.0000000	0.1950000
34039.0000000	0.1900000
34040.0000000	0.1850000
34041.0000000	0.1800000
34042.0000000	0.1800000
34043.0000000	0.1900000
34044.0000000	0.1950000
34045.0000000	0.1850000
34046.0000000	0.1950000
34047.0000000	0.1900000
34048.0000000	0.1900000
34049.0000000	0.1950000
34050.0000000	0.1900000
34051.0000000	0.1900000
34052.0000000	0.1900000
34053.0000000	0.1900000
34054.0000000	0.1900000
34055.0000000	0.1900000
34056.0000000	0.1900000
34057.0000000	0.1900000
34058.0000000	0.2000000
34059.0000000	0.1850000
34060.0000000	0.1900000
34061.0000000	0.1900000
34062.0000000	0.1950000
34063.0000000	0.1950000
34064.0000000	0.1800000
34065.0000000	0.1900000
34066.0000000	0.1900000
34067.0000000	0.1900000
34068.0000000	0.1850000
34069.0000000	0.1900000
34070.0000000	0.1900000
34071.0000000	0.1950000
34072.0000000	0.1950000
34073.0000000	0.1950000
34074.0000000	0.1850000
34075.0000000	0.1900000
34076.0000000	0.2000000
34077.0000000	0.1900000
34078.0000000	0.2000000
34079.0000000	0.1850000
34080.0000000	0.1950000
34081.0000000	0.1900000
34082.0000000	0.1950000
34083.0000000	0.1900000
34084.0000000	0.2050000
34085.0000000	0.1850000

34086.0000000	0.2000000
34087.0000000	0.1866667
34088.0000000	0.1950000
34089.0000000	0.1900000
34090.0000000	0.1950000
34091.0000000	0.1900000
34092.0000000	0.1900000
34093.0000000	0.1850000
34094.0000000	0.1900000
34095.0000000	0.1900000
34096.0000000	0.1900000
34097.0000000	0.1950000
34098.0000000	0.1950000
34099.0000000	0.1900000
34100.0000000	0.1900000
34101.0000000	0.1950000
34102.0000000	0.1900000
34103.0000000	0.1900000
34104.0000000	0.1950000
34105.0000000	0.1950000
34106.0000000	0.2000000
34107.0000000	0.1950000
34108.0000000	0.1950000
34109.0000000	0.1900000
34110.0000000	0.1950000
34111.0000000	0.1950000
34112.0000000	0.2000000
34113.0000000	0.1950000
34114.0000000	0.2000000
34115.0000000	0.1900000
34116.0000000	0.1950000
34117.0000000	0.1950000
34118.0000000	0.2000000
34119.0000000	0.1950000
34120.0000000	0.1950000
34121.0000000	0.2000000
34122.0000000	0.1950000
34123.0000000	0.2050000
34124.0000000	0.2050000
34125.0000000	0.1950000
34126.0000000	0.2000000
34127.0000000	0.2000000
34128.0000000	0.1950000
34129.0000000	0.1950000
34130.0000000	0.1950000
34131.0000000	0.1950000
34132.0000000	0.2000000
34133.0000000	0.1950000
34134.0000000	0.1950000
34135.0000000	0.2000000
34136.0000000	0.1900000
34137.0000000	0.1900000
34138.0000000	0.1950000
34139.0000000	0.2000000
34140.0000000	0.1950000
34141.0000000	0.2000000
34142.0000000	0.1900000

34143.0000000	0.1950000
34144.0000000	0.1950000
34145.0000000	0.1900000
34146.0000000	0.2000000
34147.0000000	0.1950000
34148.0000000	0.1900000
34149.0000000	0.1850000
34150.0000000	0.1950000
34151.0000000	0.1900000
34152.0000000	0.1950000
34153.0000000	0.1850000
34154.0000000	0.1900000
34155.0000000	0.1900000
34156.0000000	0.1900000
34157.0000000	0.1950000
34158.0000000	0.1900000
34159.0000000	0.1900000
34160.0000000	0.1850000
34161.0000000	0.1850000
34162.0000000	0.1900000
34163.0000000	0.1950000
34164.0000000	0.1850000
34165.0000000	0.1900000
34166.0000000	0.1900000
34167.0000000	0.1900000
34168.0000000	0.1850000
34169.0000000	0.1900000
34170.0000000	0.1850000
34171.0000000	0.1850000
34172.0000000	0.1800000
34173.0000000	0.1900000
34174.0000000	0.1900000
34175.0000000	0.1800000
34176.0000000	0.1850000
34177.0000000	0.1800000
34178.0000000	0.1900000
34179.0000000	0.1850000
34180.0000000	0.1800000
34181.0000000	0.1850000
34182.0000000	0.1800000
34183.0000000	0.1900000
34184.0000000	0.1850000
34185.0000000	0.1900000
34186.0000000	0.1800000
34187.0000000	0.1850000
34188.0000000	0.1850000
34189.0000000	0.1900000
34190.0000000	0.1900000
34191.0000000	0.1850000
34192.0000000	0.1900000
34193.0000000	0.1900000
34194.0000000	0.1950000
34195.0000000	0.1850000
34196.0000000	0.1850000
34197.0000000	0.1900000
34198.0000000	0.1900000
34199.0000000	0.1900000

34200.0000000	0.1900000
34201.0000000	0.1900000
34202.0000000	0.1900000
34203.0000000	0.1850000
34204.0000000	0.1850000
34205.0000000	0.1850000
34206.0000000	0.1900000
34207.0000000	0.1800000
34208.0000000	0.1866667
34209.0000000	0.1850000
34210.0000000	0.1900000
34211.0000000	0.1850000
34212.0000000	0.1900000
34213.0000000	0.1850000
34214.0000000	0.1900000
34215.0000000	0.1900000
34216.0000000	0.1900000
34217.0000000	0.1800000
34218.0000000	0.1800000
34219.0000000	0.1850000
34220.0000000	0.1900000
34221.0000000	0.1750000
34222.0000000	0.1900000
34223.0000000	0.1850000
34224.0000000	0.1950000
34225.0000000	0.1900000
34226.0000000	0.1750000
34227.0000000	0.1850000
34228.0000000	0.1750000
34229.0000000	0.1850000
34230.0000000	0.1850000
34231.0000000	0.1850000
34232.0000000	0.1900000
34233.0000000	0.1850000
34234.0000000	0.1900000
34235.0000000	0.1750000
34236.0000000	0.1850000
34237.0000000	0.1800000
34238.0000000	0.1800000
34239.0000000	0.1900000
34240.0000000	0.1850000
34241.0000000	0.1750000
34242.0000000	0.1800000
34243.0000000	0.1900000
34244.0000000	0.1950000
34245.0000000	0.1900000
34246.0000000	0.1850000
34247.0000000	0.1900000
34248.0000000	0.1750000
34249.0000000	0.1900000
34250.0000000	0.1800000
34251.0000000	0.1900000
34252.0000000	0.1800000
34253.0000000	0.1900000
34254.0000000	0.1900000
34255.0000000	0.1900000
34256.0000000	0.1900000

34257.0000000	0.1850000
34258.0000000	0.1900000
34259.0000000	0.1800000
34260.0000000	0.2000000
34261.0000000	0.1800000
34262.0000000	0.1950000
34263.0000000	0.1900000
34264.0000000	0.1800000
34265.0000000	0.1900000
34266.0000000	0.1850000
34267.0000000	0.1900000
34268.0000000	0.1866667
34269.0000000	0.1900000
34270.0000000	0.1900000
34271.0000000	0.1850000
34272.0000000	0.1900000
34273.0000000	0.1950000
34274.0000000	0.1950000
34275.0000000	0.1850000
34276.0000000	0.1900000
34277.0000000	0.1900000
34278.0000000	0.1900000
34279.0000000	0.1850000
34280.0000000	0.1900000
34281.0000000	0.1900000
34282.0000000	0.1900000
34283.0000000	0.1900000
34284.0000000	0.1900000
34285.0000000	0.1950000
34286.0000000	0.1850000
34287.0000000	0.1950000
34288.0000000	0.1850000
34289.0000000	0.1900000
34290.0000000	0.1950000
34291.0000000	0.1900000
34292.0000000	0.1900000
34293.0000000	0.1900000
34294.0000000	0.1850000
34295.0000000	0.1950000
34296.0000000	0.1900000
34297.0000000	0.2000000
34298.0000000	0.1866667
34299.0000000	0.1850000
34300.0000000	0.1950000
34301.0000000	0.1900000
34302.0000000	0.1850000
34303.0000000	0.1900000
34304.0000000	0.1850000
34305.0000000	0.2000000
34306.0000000	0.1950000
34307.0000000	0.1900000
34308.0000000	0.1900000
34309.0000000	0.1850000
34310.0000000	0.2000000
34311.0000000	0.1950000
34312.0000000	0.1850000
34313.0000000	0.1900000

34314.0000000	0.1850000
34315.0000000	0.1900000
34316.0000000	0.1900000
34317.0000000	0.1950000
34318.0000000	0.2000000
34319.0000000	0.1950000
34320.0000000	0.1850000
34321.0000000	0.1950000
34322.0000000	0.1850000
34323.0000000	0.1850000
34324.0000000	0.1950000
34325.0000000	0.1950000
34326.0000000	0.1900000
34327.0000000	0.1900000
34328.0000000	0.1900000
34329.0000000	0.1833333
34330.0000000	0.2000000
34331.0000000	0.1900000
34332.0000000	0.1950000
34333.0000000	0.1900000
34334.0000000	0.1900000
34335.0000000	0.1900000
34336.0000000	0.1800000
34337.0000000	0.1900000
34338.0000000	0.1950000
34339.0000000	0.1950000
34340.0000000	0.1950000
34341.0000000	0.1950000
34342.0000000	0.1900000
34343.0000000	0.1900000
34344.0000000	0.1950000
34345.0000000	0.1950000
34346.0000000	0.1950000
34347.0000000	0.1900000
34348.0000000	0.1850000
34349.0000000	0.1900000
34350.0000000	0.1950000
34351.0000000	0.1950000
34352.0000000	0.1900000
34353.0000000	0.1850000
34354.0000000	0.2000000
34355.0000000	0.1900000
34356.0000000	0.1900000
34357.0000000	0.1900000
34358.0000000	0.1850000
34359.0000000	0.1900000
34360.0000000	0.1900000
34361.0000000	0.1950000
34362.0000000	0.1900000
34363.0000000	0.1850000
34364.0000000	0.1950000
34365.0000000	0.1900000
34366.0000000	0.1900000
34367.0000000	0.1850000
34368.0000000	0.1900000
34369.0000000	0.1850000
34370.0000000	0.1900000

34371.0000000	0.1850000
34372.0000000	0.1800000
34373.0000000	0.1850000
34374.0000000	0.1850000
34375.0000000	0.1850000
34376.0000000	0.1900000
34377.0000000	0.1900000
34378.0000000	0.1800000
34379.0000000	0.1900000
34380.0000000	0.1850000
34381.0000000	0.1800000
34382.0000000	0.1900000
34383.0000000	0.1850000
34384.0000000	0.1900000
34385.0000000	0.1850000
34386.0000000	0.1900000
34387.0000000	0.1850000
34388.0000000	0.1900000
34389.0000000	0.1833333
34390.0000000	0.1800000
34391.0000000	0.1800000
34392.0000000	0.1850000
34393.0000000	0.1800000
34394.0000000	0.1900000
34395.0000000	0.1800000
34396.0000000	0.1900000
34397.0000000	0.1850000
34398.0000000	0.1850000
34399.0000000	0.1850000
34400.0000000	0.1850000
34401.0000000	0.1950000
34402.0000000	0.1900000
34403.0000000	0.1850000
34404.0000000	0.1800000
34405.0000000	0.1900000
34406.0000000	0.1850000
34407.0000000	0.1950000
34408.0000000	0.1850000
34409.0000000	0.1900000
34410.0000000	0.1950000
34411.0000000	0.1950000
34412.0000000	0.1900000
34413.0000000	0.1800000
34414.0000000	0.1900000
34415.0000000	0.1900000
34416.0000000	0.1950000
34417.0000000	0.1850000
34418.0000000	0.1850000
34419.0000000	0.2000000
34420.0000000	0.1900000
34421.0000000	0.1850000
34422.0000000	0.1900000
34423.0000000	0.1850000
34424.0000000	0.2000000
34425.0000000	0.1950000
34426.0000000	0.1900000
34427.0000000	0.1950000

34428.0000000	0.1800000
34429.0000000	0.1950000
34430.0000000	0.1850000
34431.0000000	0.1900000
34432.0000000	0.1950000
34433.0000000	0.1900000
34434.0000000	0.2000000
34435.0000000	0.1950000
34436.0000000	0.1950000
34437.0000000	0.1850000
34438.0000000	0.1950000
34439.0000000	0.1850000
34440.0000000	0.1950000
34441.0000000	0.2000000
34442.0000000	0.1900000
34443.0000000	0.1950000
34444.0000000	0.1950000
34445.0000000	0.2000000
34446.0000000	0.1900000
34447.0000000	0.1950000
34448.0000000	0.1900000
34449.0000000	0.1950000
34450.0000000	0.1966667
34451.0000000	0.1900000
34452.0000000	0.2050000
34453.0000000	0.1950000
34454.0000000	0.1950000
34455.0000000	0.1900000
34456.0000000	0.2050000
34457.0000000	0.1900000
34458.0000000	0.1950000
34459.0000000	0.1900000
34460.0000000	0.1850000
34461.0000000	0.2000000
34462.0000000	0.1950000
34463.0000000	0.2050000
34464.0000000	0.1900000
34465.0000000	0.1950000
34466.0000000	0.1900000
34467.0000000	0.2000000
34468.0000000	0.1950000
34469.0000000	0.1900000
34470.0000000	0.1950000
34471.0000000	0.1950000
34472.0000000	0.1950000
34473.0000000	0.1950000
34474.0000000	0.1950000
34475.0000000	0.1850000
34476.0000000	0.1900000
34477.0000000	0.1900000
34478.0000000	0.1950000
34479.0000000	0.2000000
34480.0000000	0.2000000
34481.0000000	0.1950000
34482.0000000	0.1900000
34483.0000000	0.1950000
34484.0000000	0.1900000

34485.0000000	0.1900000
34486.0000000	0.1900000
34487.0000000	0.1950000
34488.0000000	0.1900000
34489.0000000	0.1900000
34490.0000000	0.1950000
34491.0000000	0.1900000
34492.0000000	0.2000000
34493.0000000	0.1850000
34494.0000000	0.1900000
34495.0000000	0.1950000
34496.0000000	0.1950000
34497.0000000	0.1950000
34498.0000000	0.1900000
34499.0000000	0.1900000
34500.0000000	0.1900000
34501.0000000	0.1850000
34502.0000000	0.1850000
34503.0000000	0.1900000
34504.0000000	0.1900000
34505.0000000	0.1850000
34506.0000000	0.1900000
34507.0000000	0.1900000
34508.0000000	0.1950000
34509.0000000	0.1900000
34510.0000000	0.1900000
34511.0000000	0.1900000
34512.0000000	0.1950000
34513.0000000	0.1950000
34514.0000000	0.1950000
34515.0000000	0.1900000
34516.0000000	0.1950000
34517.0000000	0.1950000
34518.0000000	0.1850000
34519.0000000	0.1950000
34520.0000000	0.1850000
34521.0000000	0.1900000
34522.0000000	0.1900000
34523.0000000	0.1850000
34524.0000000	0.1950000
34525.0000000	0.1950000
34526.0000000	0.1900000
34527.0000000	0.2000000
34528.0000000	0.1900000
34529.0000000	0.1950000
34530.0000000	0.1950000
34531.0000000	0.1950000
34532.0000000	0.1950000
34533.0000000	0.1900000
34534.0000000	0.1950000
34535.0000000	0.1900000
34536.0000000	0.1900000
34537.0000000	0.1900000
34538.0000000	0.1900000
34539.0000000	0.1900000
34540.0000000	0.1900000
34541.0000000	0.1900000

34542.0000000	0.1950000
34543.0000000	0.1950000
34544.0000000	0.1900000
34545.0000000	0.2050000
34546.0000000	0.1900000
34547.0000000	0.2000000
34548.0000000	0.1950000
34549.0000000	0.1950000
34550.0000000	0.1900000
34551.0000000	0.1900000
34552.0000000	0.1900000
34553.0000000	0.1900000
34554.0000000	0.2000000
34555.0000000	0.1900000
34556.0000000	0.1900000
34557.0000000	0.1950000
34558.0000000	0.1900000
34559.0000000	0.1900000
34560.0000000	0.2000000
34561.0000000	0.1900000
34562.0000000	0.1950000
34563.0000000	0.1900000
34564.0000000	0.2000000
34565.0000000	0.1950000
34566.0000000	0.1850000
34567.0000000	0.1900000
34568.0000000	0.1900000
34569.0000000	0.1900000
34570.0000000	0.2000000
34571.0000000	0.1966667
34572.0000000	0.1900000
34573.0000000	0.1950000
34574.0000000	0.1900000
34575.0000000	0.1900000
34576.0000000	0.1950000
34577.0000000	0.1900000
34578.0000000	0.1950000
34579.0000000	0.1900000
34580.0000000	0.1900000
34581.0000000	0.1900000
34582.0000000	0.1900000
34583.0000000	0.1850000
34584.0000000	0.2000000
34585.0000000	0.1900000
34586.0000000	0.2000000
34587.0000000	0.1900000
34588.0000000	0.1950000
34589.0000000	0.1950000
34590.0000000	0.1850000
34591.0000000	0.1900000
34592.0000000	0.1900000
34593.0000000	0.1950000
34594.0000000	0.1900000
34595.0000000	0.1900000
34596.0000000	0.1950000
34597.0000000	0.1950000
34598.0000000	0.1850000

34599.0000000	0.1900000
34600.0000000	0.1900000
34601.0000000	0.1933333
34602.0000000	0.1950000
34603.0000000	0.1900000
34604.0000000	0.1900000
34605.0000000	0.2050000
34606.0000000	0.1900000
34607.0000000	0.2000000
34608.0000000	0.1900000
34609.0000000	0.1950000
34610.0000000	0.1900000
34611.0000000	0.1900000
34612.0000000	0.1950000
34613.0000000	0.1950000
34614.0000000	0.2000000
34615.0000000	0.1900000
34616.0000000	0.1950000
34617.0000000	0.1950000
34618.0000000	0.1900000
34619.0000000	0.1950000
34620.0000000	0.1950000
34621.0000000	0.1950000
34622.0000000	0.2000000
34623.0000000	0.1850000
34624.0000000	0.2000000
34625.0000000	0.1900000
34626.0000000	0.1900000
34627.0000000	0.1950000
34628.0000000	0.1900000
34629.0000000	0.1900000
34630.0000000	0.1966667
34631.0000000	0.1950000
34632.0000000	0.1950000
34633.0000000	0.1950000
34634.0000000	0.1950000
34635.0000000	0.1850000
34636.0000000	0.1900000
34637.0000000	0.2000000
34638.0000000	0.1950000
34639.0000000	0.2000000
34640.0000000	0.2000000
34641.0000000	0.2000000
34642.0000000	0.1950000
34643.0000000	0.2000000
34644.0000000	0.2000000
34645.0000000	0.1950000
34646.0000000	0.2000000
34647.0000000	0.1900000
34648.0000000	0.2000000
34649.0000000	0.2000000
34650.0000000	0.1950000
34651.0000000	0.2000000
34652.0000000	0.2000000
34653.0000000	0.2000000
34654.0000000	0.2000000
34655.0000000	0.1950000

34656.0000000	0.1950000
34657.0000000	0.2000000
34658.0000000	0.2000000
34659.0000000	0.1950000
34660.0000000	0.1950000
34661.0000000	0.2033333
34662.0000000	0.2000000
34663.0000000	0.1950000
34664.0000000	0.1950000
34665.0000000	0.2000000
34666.0000000	0.2000000
34667.0000000	0.1950000
34668.0000000	0.2000000
34669.0000000	0.2000000
34670.0000000	0.2000000
34671.0000000	0.2000000
34672.0000000	0.2000000
34673.0000000	0.1900000
34674.0000000	0.1950000
34675.0000000	0.2000000
34676.0000000	0.2000000
34677.0000000	0.1950000
34678.0000000	0.1950000
34679.0000000	0.2050000
34680.0000000	0.2000000
34681.0000000	0.2000000
34682.0000000	0.1950000
34683.0000000	0.1950000
34684.0000000	0.2000000
34685.0000000	0.2050000
34686.0000000	0.1950000
34687.0000000	0.2050000
34688.0000000	0.2000000
34689.0000000	0.2050000
34690.0000000	0.2000000
34691.0000000	0.1933333
34692.0000000	0.1950000
34693.0000000	0.2000000
34694.0000000	0.1950000
34695.0000000	0.2000000
34696.0000000	0.2000000
34697.0000000	0.2050000
34698.0000000	0.2050000
34699.0000000	0.1950000
34700.0000000	0.2050000
34701.0000000	0.2050000
34702.0000000	0.2000000
34703.0000000	0.2000000
34704.0000000	0.2050000
34705.0000000	0.1950000
34706.0000000	0.1950000
34707.0000000	0.1950000
34708.0000000	0.1950000
34709.0000000	0.2050000
34710.0000000	0.2050000
34711.0000000	0.2050000
34712.0000000	0.2050000

34713.0000000	0.2000000
34714.0000000	0.2000000
34715.0000000	0.2050000
34716.0000000	0.2000000
34717.0000000	0.2050000
34718.0000000	0.2000000
34719.0000000	0.2000000
34720.0000000	0.2050000
34721.0000000	0.2000000
34722.0000000	0.2050000
34723.0000000	0.2100000
34724.0000000	0.2000000
34725.0000000	0.2000000
34726.0000000	0.2050000
34727.0000000	0.1950000
34728.0000000	0.2000000
34729.0000000	0.2000000
34730.0000000	0.2000000
34731.0000000	0.2100000
34732.0000000	0.2050000
34733.0000000	0.1950000
34734.0000000	0.2050000
34735.0000000	0.2000000
34736.0000000	0.2000000
34737.0000000	0.2050000
34738.0000000	0.2000000
34739.0000000	0.2050000
34740.0000000	0.2050000
34741.0000000	0.2050000
34742.0000000	0.2100000
34743.0000000	0.2000000
34744.0000000	0.2000000
34745.0000000	0.1950000
34746.0000000	0.2000000
34747.0000000	0.2050000
34748.0000000	0.2000000
34749.0000000	0.2050000
34750.0000000	0.2100000
34751.0000000	0.2066667
34752.0000000	0.2000000
34753.0000000	0.2050000
34754.0000000	0.2050000
34755.0000000	0.2100000
34756.0000000	0.2150000
34757.0000000	0.2050000
34758.0000000	0.2100000
34759.0000000	0.2100000
34760.0000000	0.2100000
34761.0000000	0.2100000
34762.0000000	0.2050000
34763.0000000	0.2100000
34764.0000000	0.2100000
34765.0000000	0.2150000
34766.0000000	0.2100000
34767.0000000	0.2150000
34768.0000000	0.2100000
34769.0000000	0.2050000

34770.0000000	0.2150000
34771.0000000	0.2100000
34772.0000000	0.2150000
34773.0000000	0.2150000
34774.0000000	0.2150000
34775.0000000	0.2150000
34776.0000000	0.2100000
34777.0000000	0.2100000
34778.0000000	0.2100000
34779.0000000	0.2100000
34780.0000000	0.2100000
34781.0000000	0.2150000
34782.0000000	0.2100000
34783.0000000	0.2150000
34784.0000000	0.2150000
34785.0000000	0.2150000
34786.0000000	0.2100000
34787.0000000	0.2150000
34788.0000000	0.2100000
34789.0000000	0.2100000
34790.0000000	0.2150000
34791.0000000	0.2200000
34792.0000000	0.2100000
34793.0000000	0.2150000
34794.0000000	0.2200000
34795.0000000	0.2100000
34796.0000000	0.2150000
34797.0000000	0.2100000
34798.0000000	0.2200000
34799.0000000	0.2200000
34800.0000000	0.2250000
34801.0000000	0.2150000
34802.0000000	0.2150000
34803.0000000	0.2200000
34804.0000000	0.2100000
34805.0000000	0.2150000
34806.0000000	0.2100000
34807.0000000	0.2200000
34808.0000000	0.2200000
34809.0000000	0.2250000
34810.0000000	0.2150000
34811.0000000	0.2200000
34812.0000000	0.2166667
34813.0000000	0.2150000
34814.0000000	0.2150000
34815.0000000	0.2150000
34816.0000000	0.2100000
34817.0000000	0.2150000
34818.0000000	0.2100000
34819.0000000	0.2200000
34820.0000000	0.2250000
34821.0000000	0.2150000
34822.0000000	0.2250000
34823.0000000	0.2150000
34824.0000000	0.2200000
34825.0000000	0.2150000
34826.0000000	0.2150000

34827.0000000	0.2200000
34828.0000000	0.2200000
34829.0000000	0.2250000
34830.0000000	0.2200000
34831.0000000	0.2250000
34832.0000000	0.2150000
34833.0000000	0.2200000
34834.0000000	0.2150000
34835.0000000	0.2200000
34836.0000000	0.2150000
34837.0000000	0.2300000
34838.0000000	0.2100000
34839.0000000	0.2200000
34840.0000000	0.2100000
34841.0000000	0.2200000
34842.0000000	0.2266667
34843.0000000	0.2150000
34844.0000000	0.2200000
34845.0000000	0.2150000
34846.0000000	0.2250000
34847.0000000	0.2250000
34848.0000000	0.2250000
34849.0000000	0.2200000
34850.0000000	0.2150000
34851.0000000	0.2200000
34852.0000000	0.2200000
34853.0000000	0.2200000
34854.0000000	0.2200000
34855.0000000	0.2300000
34856.0000000	0.2150000
34857.0000000	0.2200000
34858.0000000	0.2150000
34859.0000000	0.2150000
34860.0000000	0.2300000
34861.0000000	0.2150000
34862.0000000	0.2250000
34863.0000000	0.2250000
34864.0000000	0.2200000
34865.0000000	0.2300000
34866.0000000	0.2150000
34867.0000000	0.2200000
34868.0000000	0.2250000
34869.0000000	0.2200000
34870.0000000	0.2150000
34871.0000000	0.2200000
34872.0000000	0.2266667
34873.0000000	0.2250000
34874.0000000	0.2150000
34875.0000000	0.2200000
34876.0000000	0.2250000
34877.0000000	0.2250000
34878.0000000	0.2250000
34879.0000000	0.2250000
34880.0000000	0.2200000
34881.0000000	0.2350000
34882.0000000	0.2250000
34883.0000000	0.2250000

34884.0000000	0.2250000
34885.0000000	0.2300000
34886.0000000	0.2250000
34887.0000000	0.2250000
34888.0000000	0.2200000
34889.0000000	0.2250000
34890.0000000	0.2250000
34891.0000000	0.2300000
34892.0000000	0.2300000
34893.0000000	0.2300000
34894.0000000	0.2300000
34895.0000000	0.2300000
34896.0000000	0.2300000
34897.0000000	0.2350000
34898.0000000	0.2300000
34899.0000000	0.2350000
34900.0000000	0.2250000
34901.0000000	0.2250000
34902.0000000	0.2350000
34903.0000000	0.2266666
34904.0000000	0.2350000
34905.0000000	0.2350000
34906.0000000	0.2350000
34907.0000000	0.2350000
34908.0000000	0.2300000
34909.0000000	0.2300000
34910.0000000	0.2350000
34911.0000000	0.2300000
34912.0000000	0.2350000
34913.0000000	0.2300000
34914.0000000	0.2300000
34915.0000000	0.2300000
34916.0000000	0.2300000
34917.0000000	0.2350000
34918.0000000	0.2300000
34919.0000000	0.2300000
34920.0000000	0.2300000
34921.0000000	0.2300000
34922.0000000	0.2350000
34923.0000000	0.2300000
34924.0000000	0.2250000
34925.0000000	0.2300000
34926.0000000	0.2300000
34927.0000000	0.2300000
34928.0000000	0.2300000
34929.0000000	0.2200000
34930.0000000	0.2250000
34931.0000000	0.2300000
34932.0000000	0.2300000
34933.0000000	0.2300000
34934.0000000	0.2250000
34935.0000000	0.2250000
34936.0000000	0.2300000
34937.0000000	0.2250000
34938.0000000	0.2400000
34939.0000000	0.2250000
34940.0000000	0.2350000

34941.0000000	0.2350000
34942.0000000	0.2250000
34943.0000000	0.2200000
34944.0000000	0.2300000
34945.0000000	0.2350000
34946.0000000	0.2350000
34947.0000000	0.2350000
34948.0000000	0.2300000
34949.0000000	0.2300000
34950.0000000	0.2250000
34951.0000000	0.2350000
34952.0000000	0.2300000
34953.0000000	0.2350000
34954.0000000	0.2200000
34955.0000000	0.2400000
34956.0000000	0.2250000
34957.0000000	0.2250000
34958.0000000	0.2200000
34959.0000000	0.2300000
34960.0000000	0.2350000
34961.0000000	0.2300000
34962.0000000	0.2266666
34963.0000000	0.2350000
34964.0000000	0.2350000
34965.0000000	0.2250000
34966.0000000	0.2250000
34967.0000000	0.2300000
34968.0000000	0.2350000
34969.0000000	0.2350000
34970.0000000	0.2400000
34971.0000000	0.2300000
34972.0000000	0.2300000
34973.0000000	0.2350000
34974.0000000	0.2300000
34975.0000000	0.2350000
34976.0000000	0.2400000
34977.0000000	0.2400000
34978.0000000	0.2300000
34979.0000000	0.2350000
34980.0000000	0.2400000
34981.0000000	0.2400000
34982.0000000	0.2350000
34983.0000000	0.2400000
34984.0000000	0.2250000
34985.0000000	0.2250000
34986.0000000	0.2350000
34987.0000000	0.2350000
34988.0000000	0.2450000
34989.0000000	0.2300000
34990.0000000	0.2400000
34991.0000000	0.2350000
34992.0000000	0.2400000
34993.0000000	0.2366667
34994.0000000	0.2400000
34995.0000000	0.2300000
34996.0000000	0.2500000
34997.0000000	0.2350000

34998.0000000	0.2400000
34999.0000000	0.2300000
35000.0000000	0.2350000
35001.0000000	0.2350000
35002.0000000	0.2400000
35003.0000000	0.2350000
35004.0000000	0.2450000
35005.0000000	0.2350000
35006.0000000	0.2400000
35007.0000000	0.2350000
35008.0000000	0.2350000
35009.0000000	0.2250000
35010.0000000	0.2350000
35011.0000000	0.2400000
35012.0000000	0.2400000
35013.0000000	0.2450000
35014.0000000	0.2300000
35015.0000000	0.2400000
35016.0000000	0.2300000
35017.0000000	0.2400000
35018.0000000	0.2350000
35019.0000000	0.2450000
35020.0000000	0.2350000
35021.0000000	0.2400000
35022.0000000	0.2400000
35023.0000000	0.2350000
35024.0000000	0.2400000
35025.0000000	0.2400000
35026.0000000	0.2400000
35027.0000000	0.2400000
35028.0000000	0.2350000
35029.0000000	0.2400000
35030.0000000	0.2350000
35031.0000000	0.2350000
35032.0000000	0.2350000
35033.0000000	0.2400000
35034.0000000	0.2400000
35035.0000000	0.2350000
35036.0000000	0.2350000
35037.0000000	0.2400000
35038.0000000	0.2400000
35039.0000000	0.2350000
35040.0000000	0.2350000
35041.0000000	0.2400000
35042.0000000	0.2400000
35043.0000000	0.2300000
35044.0000000	0.2400000
35045.0000000	0.2400000
35046.0000000	0.2350000
35047.0000000	0.2400000
35048.0000000	0.2400000
35049.0000000	0.2450000
35050.0000000	0.2450000
35051.0000000	0.2350000
35052.0000000	0.2400000
35053.0000000	0.2333333
35054.0000000	0.2400000

35055.0000000	0.2450000
35056.0000000	0.2350000
35057.0000000	0.2400000
35058.0000000	0.2400000
35059.0000000	0.2300000
35060.0000000	0.2400000
35061.0000000	0.2400000
35062.0000000	0.2350000
35063.0000000	0.2350000
35064.0000000	0.2400000
35065.0000000	0.2300000
35066.0000000	0.2400000
35067.0000000	0.2400000
35068.0000000	0.2400000
35069.0000000	0.2400000
35070.0000000	0.2350000
35071.0000000	0.2300000
35072.0000000	0.2400000
35073.0000000	0.2350000
35074.0000000	0.2300000
35075.0000000	0.2300000
35076.0000000	0.2300000
35077.0000000	0.2350000
35078.0000000	0.2300000
35079.0000000	0.2350000
35080.0000000	0.2400000
35081.0000000	0.2350000
35082.0000000	0.2400000
35083.0000000	0.2400000
35084.0000000	0.2300000
35085.0000000	0.2350000
35086.0000000	0.2350000
35087.0000000	0.2350000
35088.0000000	0.2300000
35089.0000000	0.2400000
35090.0000000	0.2350000
35091.0000000	0.2400000
35092.0000000	0.2400000
35093.0000000	0.2350000
35094.0000000	0.2400000
35095.0000000	0.2350000
35096.0000000	0.2300000
35097.0000000	0.2300000
35098.0000000	0.2350000
35099.0000000	0.2300000
35100.0000000	0.2350000
35101.0000000	0.2400000
35102.0000000	0.2300000
35103.0000000	0.2450000
35104.0000000	0.2300000
35105.0000000	0.2400000
35106.0000000	0.2300000
35107.0000000	0.2300000
35108.0000000	0.2400000
35109.0000000	0.2300000
35110.0000000	0.2350000
35111.0000000	0.2400000

35112.0000000	0.2300000
35113.0000000	0.2300000
35114.0000000	0.2366667
35115.0000000	0.2300000
35116.0000000	0.2400000
35117.0000000	0.2300000
35118.0000000	0.2300000
35119.0000000	0.2300000
35120.0000000	0.2300000
35121.0000000	0.2400000
35122.0000000	0.2300000
35123.0000000	0.2350000
35124.0000000	0.2300000
35125.0000000	0.2250000
35126.0000000	0.2250000
35127.0000000	0.2350000
35128.0000000	0.2250000
35129.0000000	0.2300000
35130.0000000	0.2200000
35131.0000000	0.2350000
35132.0000000	0.2300000
35133.0000000	0.2300000
35134.0000000	0.2250000
35135.0000000	0.2200000
35136.0000000	0.2200000
35137.0000000	0.2300000
35138.0000000	0.2300000
35139.0000000	0.2350000
35140.0000000	0.2150000
35141.0000000	0.2200000
35142.0000000	0.2200000
35143.0000000	0.2200000
35144.0000000	0.2233333
35145.0000000	0.2100000
35146.0000000	0.2200000
35147.0000000	0.2150000
35148.0000000	0.2250000
35149.0000000	0.2250000
35150.0000000	0.2250000
35151.0000000	0.2150000
35152.0000000	0.2200000
35153.0000000	0.2150000
35154.0000000	0.2150000
35155.0000000	0.2300000
35156.0000000	0.2150000
35157.0000000	0.2250000
35158.0000000	0.2200000
35159.0000000	0.2200000
35160.0000000	0.2150000
35161.0000000	0.2200000
35162.0000000	0.2200000
35163.0000000	0.2200000
35164.0000000	0.2250000
35165.0000000	0.2200000
35166.0000000	0.2150000
35167.0000000	0.2150000
35168.0000000	0.2250000

35169.0000000	0.2150000
35170.0000000	0.2150000
35171.0000000	0.2200000
35172.0000000	0.2200000
35173.0000000	0.2100000
35174.0000000	0.2166667
35175.0000000	0.2150000
35176.0000000	0.2150000
35177.0000000	0.2150000
35178.0000000	0.2150000
35179.0000000	0.2200000
35180.0000000	0.2200000
35181.0000000	0.2150000
35182.0000000	0.2100000
35183.0000000	0.2100000
35184.0000000	0.2150000
35185.0000000	0.2150000
35186.0000000	0.2250000
35187.0000000	0.2150000
35188.0000000	0.2150000
35189.0000000	0.2150000
35190.0000000	0.2150000
35191.0000000	0.2150000
35192.0000000	0.2100000
35193.0000000	0.2150000
35194.0000000	0.2150000
35195.0000000	0.2200000
35196.0000000	0.2100000
35197.0000000	0.2100000
35198.0000000	0.2100000
35199.0000000	0.2100000
35200.0000000	0.2150000
35201.0000000	0.2100000
35202.0000000	0.2200000
35203.0000000	0.2100000
35204.0000000	0.2066667
35205.0000000	0.2200000
35206.0000000	0.2150000
35207.0000000	0.2150000
35208.0000000	0.2050000
35209.0000000	0.2050000
35210.0000000	0.2050000
35211.0000000	0.2050000
35212.0000000	0.2100000
35213.0000000	0.2050000
35214.0000000	0.2100000
35215.0000000	0.2150000
35216.0000000	0.2100000
35217.0000000	0.2150000
35218.0000000	0.2000000
35219.0000000	0.2100000
35220.0000000	0.2100000
35221.0000000	0.2150000
35222.0000000	0.2050000
35223.0000000	0.2150000
35224.0000000	0.2050000
35225.0000000	0.2100000

35226.0000000	0.2100000
35227.0000000	0.2050000
35228.0000000	0.2100000
35229.0000000	0.2150000
35230.0000000	0.2100000
35231.0000000	0.2050000
35232.0000000	0.2200000
35233.0000000	0.2100000
35234.0000000	0.2250000
35235.0000000	0.2100000
35236.0000000	0.2100000
35237.0000000	0.2200000
35238.0000000	0.2150000
35239.0000000	0.2100000
35240.0000000	0.2150000
35241.0000000	0.2100000
35242.0000000	0.2150000
35243.0000000	0.2200000
35244.0000000	0.2200000
35245.0000000	0.2150000
35246.0000000	0.2250000
35247.0000000	0.2250000
35248.0000000	0.2200000
35249.0000000	0.2150000
35250.0000000	0.2150000
35251.0000000	0.2200000
35252.0000000	0.2250000
35253.0000000	0.2200000
35254.0000000	0.2250000
35255.0000000	0.2250000
35256.0000000	0.2250000
35257.0000000	0.2300000
35258.0000000	0.2250000
35259.0000000	0.2250000
35260.0000000	0.2250000
35261.0000000	0.2200000
35262.0000000	0.2300000
35263.0000000	0.2200000
35264.0000000	0.2250000
35265.0000000	0.2233333
35266.0000000	0.2200000
35267.0000000	0.2250000
35268.0000000	0.2250000
35269.0000000	0.2200000
35270.0000000	0.2250000
35271.0000000	0.2300000
35272.0000000	0.2200000
35273.0000000	0.2250000
35274.0000000	0.2300000
35275.0000000	0.2250000
35276.0000000	0.2250000
35277.0000000	0.2200000
35278.0000000	0.2250000
35279.0000000	0.2150000
35280.0000000	0.2200000
35281.0000000	0.2150000
35282.0000000	0.2250000

35283.0000000	0.2250000
35284.0000000	0.2300000
35285.0000000	0.2200000
35286.0000000	0.2150000
35287.0000000	0.2250000
35288.0000000	0.2250000
35289.0000000	0.2150000
35290.0000000	0.2200000
35291.0000000	0.2250000
35292.0000000	0.2250000
35293.0000000	0.2200000
35294.0000000	0.2266667
35295.0000000	0.2250000
35296.0000000	0.2250000
35297.0000000	0.2200000
35298.0000000	0.2150000
35299.0000000	0.2200000
35300.0000000	0.2250000
35301.0000000	0.2250000
35302.0000000	0.2200000
35303.0000000	0.2300000
35304.0000000	0.2200000
35305.0000000	0.2300000
35306.0000000	0.2300000
35307.0000000	0.2300000
35308.0000000	0.2350000
35309.0000000	0.2300000
35310.0000000	0.2250000
35311.0000000	0.2300000
35312.0000000	0.2350000
35313.0000000	0.2300000
35314.0000000	0.2400000
35315.0000000	0.2350000
35316.0000000	0.2350000
35317.0000000	0.2350000
35318.0000000	0.2350000
35319.0000000	0.2350000
35320.0000000	0.2300000
35321.0000000	0.2350000
35322.0000000	0.2350000
35323.0000000	0.2250000
35324.0000000	0.2350000
35325.0000000	0.2366667
35326.0000000	0.2300000
35327.0000000	0.2350000
35328.0000000	0.2300000
35329.0000000	0.2400000
35330.0000000	0.2350000
35331.0000000	0.2350000
35332.0000000	0.2300000
35333.0000000	0.2350000
35334.0000000	0.2350000
35335.0000000	0.2400000
35336.0000000	0.2350000
35337.0000000	0.2300000
35338.0000000	0.2300000
35339.0000000	0.2400000

35340.0000000	0.2350000
35341.0000000	0.2350000
35342.0000000	0.2400000
35343.0000000	0.2400000
35344.0000000	0.2400000
35345.0000000	0.2450000
35346.0000000	0.2300000
35347.0000000	0.2350000
35348.0000000	0.2400000
35349.0000000	0.2300000
35350.0000000	0.2400000
35351.0000000	0.2400000
35352.0000000	0.2450000
35353.0000000	0.2400000
35354.0000000	0.2450000
35355.0000000	0.2350000
35356.0000000	0.2400000
35357.0000000	0.2450000
35358.0000000	0.2450000
35359.0000000	0.2400000
35360.0000000	0.2400000
35361.0000000	0.2450000
35362.0000000	0.2400000
35363.0000000	0.2450000
35364.0000000	0.2400000
35365.0000000	0.2450000
35366.0000000	0.2300000
35367.0000000	0.2500000
35368.0000000	0.2450000
35369.0000000	0.2450000
35370.0000000	0.2400000
35371.0000000	0.2450000
35372.0000000	0.2450000
35373.0000000	0.2350000
35374.0000000	0.2450000
35375.0000000	0.2350000
35376.0000000	0.2450000
35377.0000000	0.2300000
35378.0000000	0.2450000
35379.0000000	0.2400000
35380.0000000	0.2400000
35381.0000000	0.2400000
35382.0000000	0.2450000
35383.0000000	0.2500000
35384.0000000	0.2400000
35385.0000000	0.2433333
35386.0000000	0.2400000
35387.0000000	0.2450000
35388.0000000	0.2500000
35389.0000000	0.2500000
35390.0000000	0.2500000
35391.0000000	0.2400000
35392.0000000	0.2450000
35393.0000000	0.2400000
35394.0000000	0.2500000
35395.0000000	0.2550000
35396.0000000	0.2500000

35397.0000000	0.2500000
35398.0000000	0.2400000
35399.0000000	0.2450000
35400.0000000	0.2450000
35401.0000000	0.2500000
35402.0000000	0.2450000
35403.0000000	0.2500000
35404.0000000	0.2450000
35405.0000000	0.2500000
35406.0000000	0.2600000
35407.0000000	0.2450000
35408.0000000	0.2500000
35409.0000000	0.2450000
35410.0000000	0.2400000
35411.0000000	0.2450000
35412.0000000	0.2550000
35413.0000000	0.2500000
35414.0000000	0.2500000
35415.0000000	0.2466667
35416.0000000	0.2600000
35417.0000000	0.2500000
35418.0000000	0.2450000
35419.0000000	0.2450000
35420.0000000	0.2550000
35421.0000000	0.2500000
35422.0000000	0.2550000
35423.0000000	0.2500000
35424.0000000	0.2500000
35425.0000000	0.2500000
35426.0000000	0.2500000
35427.0000000	0.2500000
35428.0000000	0.2450000
35429.0000000	0.2450000
35430.0000000	0.2450000
35431.0000000	0.2500000
35432.0000000	0.2550000
35433.0000000	0.2550000
35434.0000000	0.2450000
35435.0000000	0.2600000
35436.0000000	0.2550000
35437.0000000	0.2500000
35438.0000000	0.2500000
35439.0000000	0.2500000
35440.0000000	0.2500000
35441.0000000	0.2500000
35442.0000000	0.2550000
35443.0000000	0.2500000
35444.0000000	0.2550000
35445.0000000	0.2550000
35446.0000000	0.2500000
35447.0000000	0.2550000
35448.0000000	0.2500000
35449.0000000	0.2450000
35450.0000000	0.2500000
35451.0000000	0.2550000
35452.0000000	0.2400000
35453.0000000	0.2500000

35454.0000000	0.2500000
35455.0000000	0.2600000
35456.0000000	0.2550000
35457.0000000	0.2450000
35458.0000000	0.2550000
35459.0000000	0.2400000
35460.0000000	0.2550000
35461.0000000	0.2600000
35462.0000000	0.2500000
35463.0000000	0.2500000
35464.0000000	0.2550000
35465.0000000	0.2600000
35466.0000000	0.2650000
35467.0000000	0.2550000
35468.0000000	0.2600000
35469.0000000	0.2550000
35470.0000000	0.2550000
35471.0000000	0.2650000
35472.0000000	0.2550000
35473.0000000	0.2600000
35474.0000000	0.2550000
35475.0000000	0.2650000
35476.0000000	0.2566667
35477.0000000	0.2600000
35478.0000000	0.2600000
35479.0000000	0.2650000
35480.0000000	0.2550000
35481.0000000	0.2650000
35482.0000000	0.2600000
35483.0000000	0.2550000
35484.0000000	0.2550000
35485.0000000	0.2600000
35486.0000000	0.2600000
35487.0000000	0.2600000
35488.0000000	0.2650000
35489.0000000	0.2700000
35490.0000000	0.2650000
35491.0000000	0.2600000
35492.0000000	0.2550000
35493.0000000	0.2650000
35494.0000000	0.2550000
35495.0000000	0.2700000
35496.0000000	0.2650000
35497.0000000	0.2650000
35498.0000000	0.2600000
35499.0000000	0.2550000
35500.0000000	0.2600000
35501.0000000	0.2650000
35502.0000000	0.2650000
35503.0000000	0.2700000
35504.0000000	0.2650000
35505.0000000	0.2650000
35506.0000000	0.2600000
35507.0000000	0.2650000
35508.0000000	0.2700000
35509.0000000	0.2600000
35510.0000000	0.2600000

35511.0000000	0.2550000
35512.0000000	0.2600000
35513.0000000	0.2600000
35514.0000000	0.2700000
35515.0000000	0.2600000
35516.0000000	0.2600000
35517.0000000	0.2500000
35518.0000000	0.2600000
35519.0000000	0.2550000
35520.0000000	0.2550000
35521.0000000	0.2600000
35522.0000000	0.2650000
35523.0000000	0.2550000
35524.0000000	0.2600000
35525.0000000	0.2500000
35526.0000000	0.2500000
35527.0000000	0.2600000
35528.0000000	0.2650000
35529.0000000	0.2650000
35530.0000000	0.2650000
35531.0000000	0.2650000
35532.0000000	0.2600000
35533.0000000	0.2550000
35534.0000000	0.2500000
35535.0000000	0.2600000
35536.0000000	0.2566667
35537.0000000	0.2550000
35538.0000000	0.2600000
35539.0000000	0.2600000
35540.0000000	0.2600000
35541.0000000	0.2650000
35542.0000000	0.2550000
35543.0000000	0.2600000
35544.0000000	0.2550000
35545.0000000	0.2550000
35546.0000000	0.2600000
35547.0000000	0.2650000
35548.0000000	0.2550000
35549.0000000	0.2600000
35550.0000000	0.2500000
35551.0000000	0.2550000
35552.0000000	0.2550000
35553.0000000	0.2650000
35554.0000000	0.2600000
35555.0000000	0.2700000
35556.0000000	0.2500000
35557.0000000	0.2600000
35558.0000000	0.2550000
35559.0000000	0.2650000
35560.0000000	0.2550000
35561.0000000	0.2650000
35562.0000000	0.2550000
35563.0000000	0.2600000
35564.0000000	0.2550000
35565.0000000	0.2600000
35566.0000000	0.2650000
35567.0000000	0.2633333

35568.0000000	0.2650000
35569.0000000	0.2550000
35570.0000000	0.2700000
35571.0000000	0.2600000
35572.0000000	0.2700000
35573.0000000	0.2550000
35574.0000000	0.2600000
35575.0000000	0.2550000
35576.0000000	0.2650000
35577.0000000	0.2600000
35578.0000000	0.2650000
35579.0000000	0.2650000
35580.0000000	0.2650000
35581.0000000	0.2700000
35582.0000000	0.2600000
35583.0000000	0.2600000
35584.0000000	0.2600000
35585.0000000	0.2550000
35586.0000000	0.2700000
35587.0000000	0.2650000
35588.0000000	0.2600000
35589.0000000	0.2650000
35590.0000000	0.2700000
35591.0000000	0.2600000
35592.0000000	0.2700000
35593.0000000	0.2600000
35594.0000000	0.2600000
35595.0000000	0.2700000
35596.0000000	0.2650000
35597.0000000	0.2633333
35598.0000000	0.2600000
35599.0000000	0.2600000
35600.0000000	0.2600000
35601.0000000	0.2600000
35602.0000000	0.2650000
35603.0000000	0.2600000
35604.0000000	0.2600000
35605.0000000	0.2600000
35606.0000000	0.2550000
35607.0000000	0.2550000
35608.0000000	0.2650000
35609.0000000	0.2700000
35610.0000000	0.2550000
35611.0000000	0.2600000
35612.0000000	0.2650000
35613.0000000	0.2600000
35614.0000000	0.2600000
35615.0000000	0.2550000
35616.0000000	0.2650000
35617.0000000	0.2650000
35618.0000000	0.2550000
35619.0000000	0.2650000
35620.0000000	0.2650000
35621.0000000	0.2650000
35622.0000000	0.2650000
35623.0000000	0.2650000
35624.0000000	0.2600000

35625.0000000	0.2650000
35626.0000000	0.2566667
35627.0000000	0.2600000
35628.0000000	0.2600000
35629.0000000	0.2650000
35630.0000000	0.2600000
35631.0000000	0.2600000
35632.0000000	0.2650000
35633.0000000	0.2600000
35634.0000000	0.2600000
35635.0000000	0.2600000
35636.0000000	0.2600000
35637.0000000	0.2650000
35638.0000000	0.2550000
35639.0000000	0.2550000
35640.0000000	0.2650000
35641.0000000	0.2600000
35642.0000000	0.2550000
35643.0000000	0.2550000
35644.0000000	0.2600000
35645.0000000	0.2650000
35646.0000000	0.2600000
35647.0000000	0.2650000
35648.0000000	0.2650000
35649.0000000	0.2700000
35650.0000000	0.2600000
35651.0000000	0.2600000
35652.0000000	0.2600000
35653.0000000	0.2700000
35654.0000000	0.2600000
35655.0000000	0.2550000
35656.0000000	0.2650000
35657.0000000	0.2566667
35658.0000000	0.2600000
35659.0000000	0.2600000
35660.0000000	0.2650000
35661.0000000	0.2650000
35662.0000000	0.2650000
35663.0000000	0.2600000
35664.0000000	0.2650000
35665.0000000	0.2600000
35666.0000000	0.2500000
35667.0000000	0.2550000
35668.0000000	0.2600000
35669.0000000	0.2650000
35670.0000000	0.2650000
35671.0000000	0.2650000
35672.0000000	0.2600000
35673.0000000	0.2700000
35674.0000000	0.2600000
35675.0000000	0.2500000
35676.0000000	0.2600000
35677.0000000	0.2550000
35678.0000000	0.2550000
35679.0000000	0.2550000
35680.0000000	0.2650000
35681.0000000	0.2550000

35682.0000000	0.2600000
35683.0000000	0.2650000
35684.0000000	0.2600000
35685.0000000	0.2550000
35686.0000000	0.2550000
35687.0000000	0.2500000
35688.0000000	0.2666667
35689.0000000	0.2550000
35690.0000000	0.2550000
35691.0000000	0.2500000
35692.0000000	0.2550000
35693.0000000	0.2600000
35694.0000000	0.2500000
35695.0000000	0.2550000
35696.0000000	0.2500000
35697.0000000	0.2550000
35698.0000000	0.2550000
35699.0000000	0.2500000
35700.0000000	0.2500000
35701.0000000	0.2550000
35702.0000000	0.2500000
35703.0000000	0.2500000
35704.0000000	0.2500000
35705.0000000	0.2500000
35706.0000000	0.2500000
35707.0000000	0.2500000
35708.0000000	0.2600000
35709.0000000	0.2550000
35710.0000000	0.2500000
35711.0000000	0.2600000
35712.0000000	0.2500000
35713.0000000	0.2500000
35714.0000000	0.2550000
35715.0000000	0.2450000
35716.0000000	0.2450000
35717.0000000	0.2500000
35718.0000000	0.2450000
35719.0000000	0.2450000
35720.0000000	0.2500000
35721.0000000	0.2450000
35722.0000000	0.2400000
35723.0000000	0.2600000
35724.0000000	0.2400000
35725.0000000	0.2450000
35726.0000000	0.2450000
35727.0000000	0.2450000
35728.0000000	0.2500000
35729.0000000	0.2500000
35730.0000000	0.2450000
35731.0000000	0.2400000
35732.0000000	0.2450000
35733.0000000	0.2450000
35734.0000000	0.2450000
35735.0000000	0.2450000
35736.0000000	0.2400000
35737.0000000	0.2400000
35738.0000000	0.2400000

35739.0000000	0.2400000
35740.0000000	0.2400000
35741.0000000	0.2350000
35742.0000000	0.2450000
35743.0000000	0.2300000
35744.0000000	0.2450000
35745.0000000	0.2400000
35746.0000000	0.2500000
35747.0000000	0.2433333
35748.0000000	0.2350000
35749.0000000	0.2450000
35750.0000000	0.2450000
35751.0000000	0.2350000
35752.0000000	0.2400000
35753.0000000	0.2400000
35754.0000000	0.2400000
35755.0000000	0.2450000
35756.0000000	0.2500000
35757.0000000	0.2500000
35758.0000000	0.2500000
35759.0000000	0.2400000
35760.0000000	0.2450000
35761.0000000	0.2350000
35762.0000000	0.2450000
35763.0000000	0.2450000
35764.0000000	0.2400000
35765.0000000	0.2550000
35766.0000000	0.2450000
35767.0000000	0.2450000
35768.0000000	0.2400000
35769.0000000	0.2400000
35770.0000000	0.2450000
35771.0000000	0.2400000
35772.0000000	0.2500000
35773.0000000	0.2450000
35774.0000000	0.2500000
35775.0000000	0.2400000
35776.0000000	0.2550000
35777.0000000	0.2400000
35778.0000000	0.2400000
35779.0000000	0.2500000
35780.0000000	0.2400000
35781.0000000	0.2450000
35782.0000000	0.2350000
35783.0000000	0.2500000
35784.0000000	0.2400000
35785.0000000	0.2500000
35786.0000000	0.2400000
35787.0000000	0.2400000
35788.0000000	0.2400000
35789.0000000	0.2450000
35790.0000000	0.2400000
35791.0000000	0.2450000
35792.0000000	0.2500000
35793.0000000	0.2400000
35794.0000000	0.2400000
35795.0000000	0.2300000

35796.0000000	0.2450000
35797.0000000	0.2300000
35798.0000000	0.2400000
35799.0000000	0.2400000
35800.0000000	0.2400000
35801.0000000	0.2350000
35802.0000000	0.2350000
35803.0000000	0.2350000
35804.0000000	0.2450000
35805.0000000	0.2400000
35806.0000000	0.2400000
35807.0000000	0.2350000
35808.0000000	0.2333333
35809.0000000	0.2500000
35810.0000000	0.2400000
35811.0000000	0.2400000
35812.0000000	0.2300000
35813.0000000	0.2400000
35814.0000000	0.2400000
35815.0000000	0.2400000
35816.0000000	0.2450000
35817.0000000	0.2350000
35818.0000000	0.2400000
35819.0000000	0.2300000
35820.0000000	0.2300000
35821.0000000	0.2400000
35822.0000000	0.2400000
35823.0000000	0.2400000
35824.0000000	0.2400000
35825.0000000	0.2350000
35826.0000000	0.2350000
35827.0000000	0.2300000
35828.0000000	0.2450000
35829.0000000	0.2300000
35830.0000000	0.2400000
35831.0000000	0.2350000
35832.0000000	0.2450000
35833.0000000	0.2450000
35834.0000000	0.2350000
35835.0000000	0.2450000
35836.0000000	0.2450000
35837.0000000	0.2350000
35838.0000000	0.2400000
35839.0000000	0.2400000
35840.0000000	0.2350000
35841.0000000	0.2400000
35842.0000000	0.2450000
35843.0000000	0.2400000
35844.0000000	0.2300000
35845.0000000	0.2400000
35846.0000000	0.2350000
35847.0000000	0.2350000
35848.0000000	0.2350000
35849.0000000	0.2400000
35850.0000000	0.2400000
35851.0000000	0.2250000
35852.0000000	0.2400000

35853.0000000	0.2350000
35854.0000000	0.2350000
35855.0000000	0.2350000
35856.0000000	0.2300000
35857.0000000	0.2300000
35858.0000000	0.2300000
35859.0000000	0.2400000
35860.0000000	0.2400000
35861.0000000	0.2300000
35862.0000000	0.2350000
35863.0000000	0.2400000
35864.0000000	0.2350000
35865.0000000	0.2300000
35866.0000000	0.2350000
35867.0000000	0.2350000
35868.0000000	0.2300000
35869.0000000	0.2300000
35870.0000000	0.2250000
35871.0000000	0.2250000
35872.0000000	0.2300000
35873.0000000	0.2250000
35874.0000000	0.2250000
35875.0000000	0.2300000
35876.0000000	0.2300000
35877.0000000	0.2250000
35878.0000000	0.2250000
35879.0000000	0.2200000
35880.0000000	0.2150000
35881.0000000	0.2250000
35882.0000000	0.2300000
35883.0000000	0.2250000
35884.0000000	0.2200000
35885.0000000	0.2250000
35886.0000000	0.2250000
35887.0000000	0.2250000
35888.0000000	0.2300000
35889.0000000	0.2300000
35890.0000000	0.2150000
35891.0000000	0.2150000
35892.0000000	0.2200000
35893.0000000	0.2200000
35894.0000000	0.2250000
35895.0000000	0.2200000
35896.0000000	0.2250000
35897.0000000	0.2200000
35898.0000000	0.2200000
35899.0000000	0.2200000
35900.0000000	0.2250000
35901.0000000	0.2250000
35902.0000000	0.2200000
35903.0000000	0.2250000
35904.0000000	0.2300000
35905.0000000	0.2200000
35906.0000000	0.2200000
35907.0000000	0.2250000
35908.0000000	0.2150000
35909.0000000	0.2250000

35910.0000000	0.2250000
35911.0000000	0.2200000
35912.0000000	0.2300000
35913.0000000	0.2200000
35914.0000000	0.2250000
35915.0000000	0.2150000
35916.0000000	0.2250000
35917.0000000	0.2250000
35918.0000000	0.2250000
35919.0000000	0.2250000
35920.0000000	0.2250000
35921.0000000	0.2250000
35922.0000000	0.2200000
35923.0000000	0.2200000
35924.0000000	0.2150000
35925.0000000	0.2250000
35926.0000000	0.2200000
35927.0000000	0.2300000
35928.0000000	0.2200000
35929.0000000	0.2200000
35930.0000000	0.2250000
35931.0000000	0.2250000
35932.0000000	0.2300000
35933.0000000	0.2200000
35934.0000000	0.2300000
35935.0000000	0.2200000
35936.0000000	0.2300000
35937.0000000	0.2300000
35938.0000000	0.2350000
35939.0000000	0.2300000
35940.0000000	0.2300000
35941.0000000	0.2200000
35942.0000000	0.2200000
35943.0000000	0.2250000
35944.0000000	0.2400000
35945.0000000	0.2300000
35946.0000000	0.2300000
35947.0000000	0.2200000
35948.0000000	0.2200000
35949.0000000	0.2250000
35950.0000000	0.2350000
35951.0000000	0.2400000
35952.0000000	0.2250000
35953.0000000	0.2300000
35954.0000000	0.2250000
35955.0000000	0.2250000
35956.0000000	0.2350000
35957.0000000	0.2300000
35958.0000000	0.2300000
35959.0000000	0.2233333
35960.0000000	0.2300000
35961.0000000	0.2350000
35962.0000000	0.2200000
35963.0000000	0.2300000
35964.0000000	0.2250000
35965.0000000	0.2400000
35966.0000000	0.2300000

35967.0000000	0.2350000
35968.0000000	0.2250000
35969.0000000	0.2350000
35970.0000000	0.2300000
35971.0000000	0.2350000
35972.0000000	0.2400000
35973.0000000	0.2300000
35974.0000000	0.2300000
35975.0000000	0.2250000
35976.0000000	0.2350000
35977.0000000	0.2350000
35978.0000000	0.2400000
35979.0000000	0.2350000
35980.0000000	0.2300000
35981.0000000	0.2400000
35982.0000000	0.2250000
35983.0000000	0.2350000
35984.0000000	0.2250000
35985.0000000	0.2400000
35986.0000000	0.2300000
35987.0000000	0.2300000
35988.0000000	0.2300000
35989.0000000	0.2266666
35990.0000000	0.2450000
35991.0000000	0.2300000
35992.0000000	0.2300000
35993.0000000	0.2300000
35994.0000000	0.2250000
35995.0000000	0.2350000
35996.0000000	0.2200000
35997.0000000	0.2250000
35998.0000000	0.2350000
35999.0000000	0.2300000
36000.0000000	0.2400000
36001.0000000	0.2350000
36002.0000000	0.2350000
36003.0000000	0.2350000
36004.0000000	0.2300000
36005.0000000	0.2350000
36006.0000000	0.2350000
36007.0000000	0.2350000
36008.0000000	0.2300000
36009.0000000	0.2350000
36010.0000000	0.2300000
36011.0000000	0.2300000
36012.0000000	0.2400000
36013.0000000	0.2350000
36014.0000000	0.2400000
36015.0000000	0.2350000
36016.0000000	0.2350000
36017.0000000	0.2300000
36018.0000000	0.2350000
36019.0000000	0.2350000
36020.0000000	0.2400000
36021.0000000	0.2400000
36022.0000000	0.2350000
36023.0000000	0.2400000

36024.0000000	0.2350000
36025.0000000	0.2400000
36026.0000000	0.2350000
36027.0000000	0.2400000
36028.0000000	0.2400000
36029.0000000	0.2350000
36030.0000000	0.2450000
36031.0000000	0.2350000
36032.0000000	0.2450000
36033.0000000	0.2350000
36034.0000000	0.2400000
36035.0000000	0.2350000
36036.0000000	0.2400000
36037.0000000	0.2400000
36038.0000000	0.2400000
36039.0000000	0.2400000
36040.0000000	0.2400000
36041.0000000	0.2350000
36042.0000000	0.2400000
36043.0000000	0.2350000
36044.0000000	0.2250000
36045.0000000	0.2350000
36046.0000000	0.2500000
36047.0000000	0.2300000
36048.0000000	0.2350000
36049.0000000	0.2400000
36050.0000000	0.2333333
36051.0000000	0.2350000
36052.0000000	0.2350000
36053.0000000	0.2400000
36054.0000000	0.2350000
36055.0000000	0.2400000
36056.0000000	0.2350000
36057.0000000	0.2350000
36058.0000000	0.2450000
36059.0000000	0.2350000
36060.0000000	0.2300000
36061.0000000	0.2350000
36062.0000000	0.2400000
36063.0000000	0.2350000
36064.0000000	0.2400000
36065.0000000	0.2400000
36066.0000000	0.2400000
36067.0000000	0.2400000
36068.0000000	0.2400000
36069.0000000	0.2350000
36070.0000000	0.2400000
36071.0000000	0.2350000
36072.0000000	0.2350000
36073.0000000	0.2400000
36074.0000000	0.2350000
36075.0000000	0.2300000
36076.0000000	0.2400000
36077.0000000	0.2500000
36078.0000000	0.2300000
36079.0000000	0.2366667
36080.0000000	0.2350000

36081.0000000	0.2400000
36082.0000000	0.2350000
36083.0000000	0.2450000
36084.0000000	0.2350000
36085.0000000	0.2400000
36086.0000000	0.2400000
36087.0000000	0.2450000
36088.0000000	0.2350000
36089.0000000	0.2400000
36090.0000000	0.2400000
36091.0000000	0.2400000
36092.0000000	0.2400000
36093.0000000	0.2400000
36094.0000000	0.2350000
36095.0000000	0.2400000
36096.0000000	0.2400000
36097.0000000	0.2400000
36098.0000000	0.2350000
36099.0000000	0.2400000
36100.0000000	0.2350000
36101.0000000	0.2350000
36102.0000000	0.2400000
36103.0000000	0.2350000
36104.0000000	0.2400000
36105.0000000	0.2350000
36106.0000000	0.2350000
36107.0000000	0.2400000
36108.0000000	0.2400000
36109.0000000	0.2350000
36110.0000000	0.2333333
36111.0000000	0.2350000
36112.0000000	0.2350000
36113.0000000	0.2400000
36114.0000000	0.2350000
36115.0000000	0.2350000
36116.0000000	0.2300000
36117.0000000	0.2350000
36118.0000000	0.2300000
36119.0000000	0.2300000
36120.0000000	0.2300000
36121.0000000	0.2300000
36122.0000000	0.2250000
36123.0000000	0.2200000
36124.0000000	0.2300000
36125.0000000	0.2300000
36126.0000000	0.2250000
36127.0000000	0.2300000
36128.0000000	0.2250000
36129.0000000	0.2200000
36130.0000000	0.2300000
36131.0000000	0.2150000
36132.0000000	0.2250000
36133.0000000	0.2150000
36134.0000000	0.2150000
36135.0000000	0.2100000
36136.0000000	0.2150000
36137.0000000	0.2100000

36138.0000000	0.2200000
36139.0000000	0.2250000
36140.0000000	0.2100000
36141.0000000	0.2100000
36142.0000000	0.2200000
36143.0000000	0.2100000
36144.0000000	0.2150000
36145.0000000	0.2150000
36146.0000000	0.2200000
36147.0000000	0.2100000
36148.0000000	0.2150000
36149.0000000	0.2100000
36150.0000000	0.2050000
36151.0000000	0.2150000
36152.0000000	0.2100000
36153.0000000	0.2200000
36154.0000000	0.2050000
36155.0000000	0.2100000
36156.0000000	0.2000000
36157.0000000	0.2000000
36158.0000000	0.2000000
36159.0000000	0.2100000
36160.0000000	0.1950000
36161.0000000	0.2100000
36162.0000000	0.2000000
36163.0000000	0.2100000
36164.0000000	0.2050000
36165.0000000	0.2150000
36166.0000000	0.1950000
36167.0000000	0.2100000
36168.0000000	0.2000000
36169.0000000	0.2100000
36170.0000000	0.2000000
36171.0000000	0.2150000
36172.0000000	0.2050000
36173.0000000	0.2000000
36174.0000000	0.2100000
36175.0000000	0.2050000
36176.0000000	0.2000000
36177.0000000	0.2050000
36178.0000000	0.2050000
36179.0000000	0.2150000
36180.0000000	0.2100000
36181.0000000	0.2100000
36182.0000000	0.2100000
36183.0000000	0.2050000
36184.0000000	0.2050000
36185.0000000	0.2000000
36186.0000000	0.2100000
36187.0000000	0.2100000
36188.0000000	0.2100000
36189.0000000	0.2100000
36190.0000000	0.2000000
36191.0000000	0.2050000
36192.0000000	0.2050000
36193.0000000	0.2150000
36194.0000000	0.2000000

36195.0000000	0.2050000
36196.0000000	0.2000000
36197.0000000	0.2000000
36198.0000000	0.1950000
36199.0000000	0.1900000
36200.0000000	0.2000000
36201.0000000	0.2000000
36202.0000000	0.1950000
36203.0000000	0.2000000
36204.0000000	0.1950000
36205.0000000	0.1950000
36206.0000000	0.2050000
36207.0000000	0.2000000
36208.0000000	0.1950000
36209.0000000	0.2000000
36210.0000000	0.1950000
36211.0000000	0.2000000
36212.0000000	0.2000000
36213.0000000	0.1900000
36214.0000000	0.1900000
36215.0000000	0.2000000
36216.0000000	0.1900000
36217.0000000	0.1900000
36218.0000000	0.1950000
36219.0000000	0.2000000
36220.0000000	0.1950000
36221.0000000	0.2000000
36222.0000000	0.2000000
36223.0000000	0.1950000
36224.0000000	0.2000000
36225.0000000	0.2000000
36226.0000000	0.2000000
36227.0000000	0.1950000
36228.0000000	0.1950000
36229.0000000	0.2000000
36230.0000000	0.1950000
36231.0000000	0.2000000
36232.0000000	0.2000000
36233.0000000	0.1950000
36234.0000000	0.1950000
36235.0000000	0.1900000
36236.0000000	0.2050000
36237.0000000	0.2000000
36238.0000000	0.1950000
36239.0000000	0.1950000
36240.0000000	0.2050000
36241.0000000	0.2050000
36242.0000000	0.2000000
36243.0000000	0.2000000
36244.0000000	0.1950000
36245.0000000	0.2000000
36246.0000000	0.2050000
36247.0000000	0.1950000
36248.0000000	0.2000000
36249.0000000	0.2000000
36250.0000000	0.2000000
36251.0000000	0.2000000

36252.0000000	0.2000000
36253.0000000	0.2000000
36254.0000000	0.2000000
36255.0000000	0.2100000
36256.0000000	0.2100000
36257.0000000	0.1950000
36258.0000000	0.2100000
36259.0000000	0.2000000
36260.0000000	0.2050000
36261.0000000	0.2033333
36262.0000000	0.2000000
36263.0000000	0.2050000
36264.0000000	0.2000000
36265.0000000	0.2000000
36266.0000000	0.2050000
36267.0000000	0.2100000
36268.0000000	0.2000000
36269.0000000	0.2000000
36270.0000000	0.2000000
36271.0000000	0.2050000
36272.0000000	0.2000000
36273.0000000	0.1900000
36274.0000000	0.2050000
36275.0000000	0.2100000
36276.0000000	0.2000000
36277.0000000	0.2000000
36278.0000000	0.2100000
36279.0000000	0.2100000
36280.0000000	0.2000000
36281.0000000	0.2000000
36282.0000000	0.2000000
36283.0000000	0.2050000
36284.0000000	0.2050000
36285.0000000	0.2000000
36286.0000000	0.2050000
36287.0000000	0.2000000
36288.0000000	0.2050000
36289.0000000	0.1950000
36290.0000000	0.2100000
36291.0000000	0.2133333
36292.0000000	0.2050000
36293.0000000	0.2000000
36294.0000000	0.2050000
36295.0000000	0.2050000
36296.0000000	0.2050000
36297.0000000	0.2050000
36298.0000000	0.2100000
36299.0000000	0.2100000
36300.0000000	0.2000000
36301.0000000	0.2000000
36302.0000000	0.2100000
36303.0000000	0.2050000
36304.0000000	0.2050000
36305.0000000	0.2100000
36306.0000000	0.2000000
36307.0000000	0.2100000
36308.0000000	0.2000000

36309.0000000	0.2000000
36310.0000000	0.2050000
36311.0000000	0.2050000
36312.0000000	0.2000000
36313.0000000	0.2050000
36314.0000000	0.2050000
36315.0000000	0.2000000
36316.0000000	0.1950000
36317.0000000	0.1950000
36318.0000000	0.2000000
36319.0000000	0.1900000
36320.0000000	0.1900000
36321.0000000	0.2000000
36322.0000000	0.1900000
36323.0000000	0.1950000
36324.0000000	0.1900000
36325.0000000	0.1900000
36326.0000000	0.2000000
36327.0000000	0.1950000
36328.0000000	0.2000000
36329.0000000	0.1950000
36330.0000000	0.1850000
36331.0000000	0.1950000
36332.0000000	0.1900000
36333.0000000	0.1950000
36334.0000000	0.1900000
36335.0000000	0.1900000
36336.0000000	0.1950000
36337.0000000	0.1900000
36338.0000000	0.1900000
36339.0000000	0.1850000
36340.0000000	0.1900000
36341.0000000	0.1900000
36342.0000000	0.1850000
36343.0000000	0.1950000
36344.0000000	0.1900000
36345.0000000	0.1900000
36346.0000000	0.1900000
36347.0000000	0.1900000
36348.0000000	0.1900000
36349.0000000	0.1900000
36350.0000000	0.1800000
36351.0000000	0.1933333
36352.0000000	0.1950000
36353.0000000	0.1800000
36354.0000000	0.1850000
36355.0000000	0.1850000
36356.0000000	0.1800000
36357.0000000	0.1900000
36358.0000000	0.1900000
36359.0000000	0.1900000
36360.0000000	0.1950000
36361.0000000	0.1900000
36362.0000000	0.1900000
36363.0000000	0.1850000
36364.0000000	0.1900000
36365.0000000	0.1900000

36366.0000000	0.1850000
36367.0000000	0.1900000
36368.0000000	0.1900000
36369.0000000	0.1950000
36370.0000000	0.1900000
36371.0000000	0.1900000
36372.0000000	0.1800000
36373.0000000	0.1900000
36374.0000000	0.1800000
36375.0000000	0.1900000
36376.0000000	0.1900000
36377.0000000	0.1900000
36378.0000000	0.1850000
36379.0000000	0.1850000
36380.0000000	0.1950000
36381.0000000	0.1950000
36382.0000000	0.1866667
36383.0000000	0.1900000
36384.0000000	0.2000000
36385.0000000	0.1900000
36386.0000000	0.1850000
36387.0000000	0.1900000
36388.0000000	0.1900000
36389.0000000	0.1900000
36390.0000000	0.1900000
36391.0000000	0.1900000
36392.0000000	0.1900000
36393.0000000	0.1950000
36394.0000000	0.1950000
36395.0000000	0.2000000
36396.0000000	0.1900000
36397.0000000	0.1850000
36398.0000000	0.1900000
36399.0000000	0.1900000
36400.0000000	0.1950000
36401.0000000	0.1900000
36402.0000000	0.1850000
36403.0000000	0.1950000
36404.0000000	0.1950000
36405.0000000	0.1900000
36406.0000000	0.1950000
36407.0000000	0.1900000
36408.0000000	0.1950000
36409.0000000	0.1900000
36410.0000000	0.1900000
36411.0000000	0.1900000
36412.0000000	0.1950000
36413.0000000	0.1950000
36414.0000000	0.1950000
36415.0000000	0.1900000
36416.0000000	0.1950000
36417.0000000	0.1900000
36418.0000000	0.1850000
36419.0000000	0.1950000
36420.0000000	0.1850000
36421.0000000	0.1950000
36422.0000000	0.1900000

36423.0000000	0.1900000
36424.0000000	0.1900000
36425.0000000	0.1900000
36426.0000000	0.1850000
36427.0000000	0.1850000
36428.0000000	0.1900000
36429.0000000	0.1900000
36430.0000000	0.1900000
36431.0000000	0.1950000
36432.0000000	0.2000000
36433.0000000	0.1900000
36434.0000000	0.1900000
36435.0000000	0.1900000
36436.0000000	0.1900000
36437.0000000	0.1850000
36438.0000000	0.1850000
36439.0000000	0.1900000
36440.0000000	0.1850000
36441.0000000	0.1950000
36442.0000000	0.1866667
36443.0000000	0.1900000
36444.0000000	0.1950000
36445.0000000	0.1850000
36446.0000000	0.1900000
36447.0000000	0.1850000
36448.0000000	0.1900000
36449.0000000	0.1900000
36450.0000000	0.1850000
36451.0000000	0.1850000
36452.0000000	0.1800000
36453.0000000	0.1850000
36454.0000000	0.1900000
36455.0000000	0.1850000
36456.0000000	0.1900000
36457.0000000	0.1800000
36458.0000000	0.1950000
36459.0000000	0.1950000
36460.0000000	0.1900000
36461.0000000	0.1850000
36462.0000000	0.1800000
36463.0000000	0.1900000
36464.0000000	0.1800000
36465.0000000	0.1950000
36466.0000000	0.1800000
36467.0000000	0.1900000
36468.0000000	0.1900000
36469.0000000	0.1950000
36470.0000000	0.1850000
36471.0000000	0.1850000
36472.0000000	0.1900000
36473.0000000	0.1850000
36474.0000000	0.1900000
36475.0000000	0.1800000
36476.0000000	0.1850000
36477.0000000	0.1850000
36478.0000000	0.1900000
36479.0000000	0.1900000

36480.0000000	0.1850000
36481.0000000	0.1950000
36482.0000000	0.1850000
36483.0000000	0.1850000
36484.0000000	0.1750000
36485.0000000	0.1900000
36486.0000000	0.1850000
36487.0000000	0.1900000
36488.0000000	0.1800000
36489.0000000	0.1950000
36490.0000000	0.1850000
36491.0000000	0.1800000
36492.0000000	0.1850000
36493.0000000	0.1850000
36494.0000000	0.1900000
36495.0000000	0.1900000
36496.0000000	0.1950000
36497.0000000	0.1850000
36498.0000000	0.1900000
36499.0000000	0.1850000
36500.0000000	0.1800000
36501.0000000	0.1900000
36502.0000000	0.1900000
36503.0000000	0.1850000
36504.0000000	0.1900000
36505.0000000	0.1850000
36506.0000000	0.1950000
36507.0000000	0.1850000
36508.0000000	0.1850000
36509.0000000	0.1750000
36510.0000000	0.1900000
36511.0000000	0.1750000
36512.0000000	0.1900000
36513.0000000	0.1750000
36514.0000000	0.1900000
36515.0000000	0.1800000
36516.0000000	0.1850000
36517.0000000	0.1700000
36518.0000000	0.1850000
36519.0000000	0.1900000
36520.0000000	0.1900000
36521.0000000	0.1850000
36522.0000000	0.1850000
36523.0000000	0.1800000
36524.0000000	0.1800000
36525.0000000	0.1850000
36526.0000000	0.1900000
36527.0000000	0.1850000
36528.0000000	0.1950000
36529.0000000	0.1750000
36530.0000000	0.1850000
36531.0000000	0.1700000
36532.0000000	0.1866667
36533.0000000	0.1750000
36534.0000000	0.1800000
36535.0000000	0.1800000
36536.0000000	0.1700000

36537.0000000	0.1850000
36538.0000000	0.1800000
36539.0000000	0.1700000
36540.0000000	0.1850000
36541.0000000	0.1750000
36542.0000000	0.1850000
36543.0000000	0.1750000
36544.0000000	0.1750000
36545.0000000	0.1850000
36546.0000000	0.1800000
36547.0000000	0.1850000
36548.0000000	0.1800000
36549.0000000	0.1850000
36550.0000000	0.1750000
36551.0000000	0.1800000
36552.0000000	0.1800000
36553.0000000	0.1800000
36554.0000000	0.1850000
36555.0000000	0.1800000
36556.0000000	0.1700000
36557.0000000	0.1750000
36558.0000000	0.1850000
36559.0000000	0.1850000
36560.0000000	0.1750000
36561.0000000	0.1700000
36562.0000000	0.1800000
36563.0000000	0.1700000
36564.0000000	0.1900000
36565.0000000	0.1700000
36566.0000000	0.1750000
36567.0000000	0.1800000
36568.0000000	0.1700000
36569.0000000	0.1800000
36570.0000000	0.1750000
36571.0000000	0.1800000
36572.0000000	0.1850000
36573.0000000	0.1700000
36574.0000000	0.1800000
36575.0000000	0.1750000
36576.0000000	0.1750000
36577.0000000	0.1800000
36578.0000000	0.1750000
36579.0000000	0.1750000
36580.0000000	0.1800000
36581.0000000	0.1800000
36582.0000000	0.1800000
36583.0000000	0.1800000
36584.0000000	0.1750000
36585.0000000	0.1750000
36586.0000000	0.1850000
36587.0000000	0.1800000
36588.0000000	0.1700000
36589.0000000	0.1800000
36590.0000000	0.1750000
36591.0000000	0.1800000
36592.0000000	0.1800000
36593.0000000	0.1733333

36594.0000000	0.1750000
36595.0000000	0.1750000
36596.0000000	0.1750000
36597.0000000	0.1750000
36598.0000000	0.1750000
36599.0000000	0.1750000
36600.0000000	0.1750000
36601.0000000	0.1750000
36602.0000000	0.1800000
36603.0000000	0.1750000
36604.0000000	0.1800000
36605.0000000	0.1700000
36606.0000000	0.1750000
36607.0000000	0.1700000
36608.0000000	0.1750000
36609.0000000	0.1800000
36610.0000000	0.1800000
36611.0000000	0.1750000
36612.0000000	0.1800000
36613.0000000	0.1750000
36614.0000000	0.1800000
36615.0000000	0.1700000
36616.0000000	0.1700000
36617.0000000	0.1700000
36618.0000000	0.1750000
36619.0000000	0.1750000
36620.0000000	0.1750000
36621.0000000	0.1700000
36622.0000000	0.1700000
36623.0000000	0.1766667
36624.0000000	0.1750000
36625.0000000	0.1750000
36626.0000000	0.1650000
36627.0000000	0.1800000
36628.0000000	0.1800000
36629.0000000	0.1700000
36630.0000000	0.1700000
36631.0000000	0.1800000
36632.0000000	0.1850000
36633.0000000	0.1750000
36634.0000000	0.1700000
36635.0000000	0.1850000
36636.0000000	0.1800000
36637.0000000	0.1750000
36638.0000000	0.1800000
36639.0000000	0.1800000
36640.0000000	0.1850000
36641.0000000	0.1750000
36642.0000000	0.1800000
36643.0000000	0.1850000
36644.0000000	0.1800000
36645.0000000	0.1800000
36646.0000000	0.1750000
36647.0000000	0.1850000
36648.0000000	0.1750000
36649.0000000	0.1850000
36650.0000000	0.1850000

36651.0000000	0.1750000
36652.0000000	0.1800000
36653.0000000	0.1733333
36654.0000000	0.1700000
36655.0000000	0.1850000
36656.0000000	0.1800000
36657.0000000	0.1800000
36658.0000000	0.1800000
36659.0000000	0.1700000
36660.0000000	0.1650000
36661.0000000	0.1750000
36662.0000000	0.1700000
36663.0000000	0.1700000
36664.0000000	0.1650000
36665.0000000	0.1700000
36666.0000000	0.1800000
36667.0000000	0.1700000
36668.0000000	0.1750000
36669.0000000	0.1650000
36670.0000000	0.1750000
36671.0000000	0.1650000
36672.0000000	0.1700000
36673.0000000	0.1700000
36674.0000000	0.1700000
36675.0000000	0.1750000
36676.0000000	0.1750000
36677.0000000	0.1750000
36678.0000000	0.1800000
36679.0000000	0.1650000
36680.0000000	0.1700000
36681.0000000	0.1700000
36682.0000000	0.1650000
36683.0000000	0.1700000
36684.0000000	0.1750000
36685.0000000	0.1700000
36686.0000000	0.1700000
36687.0000000	0.1700000
36688.0000000	0.1700000
36689.0000000	0.1650000
36690.0000000	0.1800000
36691.0000000	0.1750000
36692.0000000	0.1650000
36693.0000000	0.1750000
36694.0000000	0.1750000
36695.0000000	0.1700000
36696.0000000	0.1800000
36697.0000000	0.1650000
36698.0000000	0.1700000
36699.0000000	0.1750000
36700.0000000	0.1650000
36701.0000000	0.1700000
36702.0000000	0.1700000
36703.0000000	0.1700000
36704.0000000	0.1650000
36705.0000000	0.1600000
36706.0000000	0.1650000
36707.0000000	0.1650000

36708.0000000	0.1650000
36709.0000000	0.1700000
36710.0000000	0.1650000
36711.0000000	0.1700000
36712.0000000	0.1650000
36713.0000000	0.1650000
36714.0000000	0.1666667
36715.0000000	0.1700000
36716.0000000	0.1700000
36717.0000000	0.1650000
36718.0000000	0.1750000
36719.0000000	0.1750000
36720.0000000	0.1650000
36721.0000000	0.1700000
36722.0000000	0.1750000
36723.0000000	0.1700000
36724.0000000	0.1700000
36725.0000000	0.1650000
36726.0000000	0.1700000
36727.0000000	0.1700000
36728.0000000	0.1750000
36729.0000000	0.1700000
36730.0000000	0.1800000
36731.0000000	0.1750000
36732.0000000	0.1700000
36733.0000000	0.1700000
36734.0000000	0.1700000
36735.0000000	0.1750000
36736.0000000	0.1750000
36737.0000000	0.1800000
36738.0000000	0.1700000
36739.0000000	0.1750000
36740.0000000	0.1850000
36741.0000000	0.1800000
36742.0000000	0.1750000
36743.0000000	0.1800000
36744.0000000	0.1733333
36745.0000000	0.1700000
36746.0000000	0.1800000
36747.0000000	0.1800000
36748.0000000	0.1750000
36749.0000000	0.1850000
36750.0000000	0.1800000
36751.0000000	0.1750000
36752.0000000	0.1800000
36753.0000000	0.1800000
36754.0000000	0.1750000
36755.0000000	0.1800000
36756.0000000	0.1800000
36757.0000000	0.1750000
36758.0000000	0.1800000
36759.0000000	0.1800000
36760.0000000	0.1750000
36761.0000000	0.1800000
36762.0000000	0.1750000
36763.0000000	0.1850000
36764.0000000	0.1750000

36765.0000000	0.1700000
36766.0000000	0.1800000
36767.0000000	0.1750000
36768.0000000	0.1800000
36769.0000000	0.1800000
36770.0000000	0.1700000
36771.0000000	0.1750000
36772.0000000	0.1750000
36773.0000000	0.1700000
36774.0000000	0.1700000
36775.0000000	0.1650000
36776.0000000	0.1750000
36777.0000000	0.1700000
36778.0000000	0.1700000
36779.0000000	0.1700000
36780.0000000	0.1650000
36781.0000000	0.1700000
36782.0000000	0.1750000
36783.0000000	0.1650000
36784.0000000	0.1700000
36785.0000000	0.1750000
36786.0000000	0.1700000
36787.0000000	0.1700000
36788.0000000	0.1700000
36789.0000000	0.1700000
36790.0000000	0.1750000
36791.0000000	0.1650000
36792.0000000	0.1650000
36793.0000000	0.1700000
36794.0000000	0.1700000
36795.0000000	0.1650000
36796.0000000	0.1700000
36797.0000000	0.1650000
36798.0000000	0.1650000
36799.0000000	0.1700000
36800.0000000	0.1650000
36801.0000000	0.1700000
36802.0000000	0.1550000
36803.0000000	0.1650000
36804.0000000	0.1666667
36805.0000000	0.1700000
36806.0000000	0.1700000
36807.0000000	0.1600000
36808.0000000	0.1600000
36809.0000000	0.1600000
36810.0000000	0.1650000
36811.0000000	0.1650000
36812.0000000	0.1650000
36813.0000000	0.1700000
36814.0000000	0.1650000
36815.0000000	0.1750000
36816.0000000	0.1650000
36817.0000000	0.1650000
36818.0000000	0.1650000
36819.0000000	0.1650000
36820.0000000	0.1650000
36821.0000000	0.1600000

36822.0000000	0.1700000
36823.0000000	0.1650000
36824.0000000	0.1700000
36825.0000000	0.1650000
36826.0000000	0.1750000
36827.0000000	0.1750000
36828.0000000	0.1700000
36829.0000000	0.1750000
36830.0000000	0.1650000
36831.0000000	0.1700000
36832.0000000	0.1650000
36833.0000000	0.1700000
36834.0000000	0.1700000
36835.0000000	0.1666667
36836.0000000	0.1700000
36837.0000000	0.1700000
36838.0000000	0.1700000
36839.0000000	0.1700000
36840.0000000	0.1650000
36841.0000000	0.1650000
36842.0000000	0.1600000
36843.0000000	0.1650000
36844.0000000	0.1800000
36845.0000000	0.1650000
36846.0000000	0.1700000
36847.0000000	0.1650000
36848.0000000	0.1700000
36849.0000000	0.1650000
36850.0000000	0.1700000
36851.0000000	0.1700000
36852.0000000	0.1650000
36853.0000000	0.1700000
36854.0000000	0.1650000
36855.0000000	0.1750000
36856.0000000	0.1650000
36857.0000000	0.1700000
36858.0000000	0.1650000
36859.0000000	0.1650000
36860.0000000	0.1700000
36861.0000000	0.1650000
36862.0000000	0.1700000
36863.0000000	0.1650000
36864.0000000	0.1733333
36865.0000000	0.1700000
36866.0000000	0.1650000
36867.0000000	0.1650000
36868.0000000	0.1700000
36869.0000000	0.1700000
36870.0000000	0.1650000
36871.0000000	0.1700000
36872.0000000	0.1600000
36873.0000000	0.1700000
36874.0000000	0.1650000
36875.0000000	0.1750000
36876.0000000	0.1650000
36877.0000000	0.1700000
36878.0000000	0.1550000

36879.0000000	0.1650000
36880.0000000	0.1550000
36881.0000000	0.1750000
36882.0000000	0.1650000
36883.0000000	0.1700000
36884.0000000	0.1700000
36885.0000000	0.1600000
36886.0000000	0.1600000
36887.0000000	0.1600000
36888.0000000	0.1600000
36889.0000000	0.1700000
36890.0000000	0.1600000
36891.0000000	0.1700000
36892.0000000	0.1650000
36893.0000000	0.1650000
36894.0000000	0.1666667
36895.0000000	0.1650000
36896.0000000	0.1600000
36897.0000000	0.1700000
36898.0000000	0.1700000
36899.0000000	0.1650000
36900.0000000	0.1650000
36901.0000000	0.1600000
36902.0000000	0.1700000
36903.0000000	0.1700000
36904.0000000	0.1650000
36905.0000000	0.1700000
36906.0000000	0.1700000
36907.0000000	0.1650000
36908.0000000	0.1700000
36909.0000000	0.1700000
36910.0000000	0.1650000
36911.0000000	0.1650000
36912.0000000	0.1650000
36913.0000000	0.1650000
36914.0000000	0.1600000
36915.0000000	0.1650000
36916.0000000	0.1700000
36917.0000000	0.1700000
36918.0000000	0.1700000
36919.0000000	0.1700000
36920.0000000	0.1700000
36921.0000000	0.1750000
36922.0000000	0.1650000
36923.0000000	0.1600000
36924.0000000	0.1700000
36925.0000000	0.1733333
36926.0000000	0.1600000
36927.0000000	0.1700000
36928.0000000	0.1650000
36929.0000000	0.1600000
36930.0000000	0.1700000
36931.0000000	0.1650000
36932.0000000	0.1650000
36933.0000000	0.1700000
36934.0000000	0.1700000
36935.0000000	0.1650000

36936.0000000	0.1750000
36937.0000000	0.1650000
36938.0000000	0.1650000
36939.0000000	0.1700000
36940.0000000	0.1700000
36941.0000000	0.1650000
36942.0000000	0.1700000
36943.0000000	0.1750000
36944.0000000	0.1700000
36945.0000000	0.1650000
36946.0000000	0.1700000
36947.0000000	0.1750000
36948.0000000	0.1700000
36949.0000000	0.1750000
36950.0000000	0.1700000
36951.0000000	0.1750000
36952.0000000	0.1700000
36953.0000000	0.1800000
36954.0000000	0.1800000
36955.0000000	0.1766667
36956.0000000	0.1800000
36957.0000000	0.1650000
36958.0000000	0.1800000
36959.0000000	0.1850000
36960.0000000	0.1700000
36961.0000000	0.1700000
36962.0000000	0.1750000
36963.0000000	0.1700000
36964.0000000	0.1750000
36965.0000000	0.1700000
36966.0000000	0.1700000
36967.0000000	0.1700000
36968.0000000	0.1800000
36969.0000000	0.1700000
36970.0000000	0.1750000
36971.0000000	0.1800000
36972.0000000	0.1750000
36973.0000000	0.1700000
36974.0000000	0.1700000
36975.0000000	0.1700000
36976.0000000	0.1750000
36977.0000000	0.1850000
36978.0000000	0.1750000
36979.0000000	0.1850000
36980.0000000	0.1850000
36981.0000000	0.1800000
36982.0000000	0.1800000
36983.0000000	0.1750000
36984.0000000	0.1800000
36985.0000000	0.1800000
36986.0000000	0.1850000
36987.0000000	0.1850000
36988.0000000	0.1850000
36989.0000000	0.1850000
36990.0000000	0.1900000
36991.0000000	0.1850000
36992.0000000	0.1850000

36993.0000000	0.1800000
36994.0000000	0.1850000
36995.0000000	0.1800000
36996.0000000	0.1800000
36997.0000000	0.1850000
36998.0000000	0.1800000
36999.0000000	0.1850000
37000.0000000	0.1850000
37001.0000000	0.1900000
37002.0000000	0.1900000
37003.0000000	0.1850000
37004.0000000	0.1900000
37005.0000000	0.1850000
37006.0000000	0.1800000
37007.0000000	0.1800000
37008.0000000	0.1800000
37009.0000000	0.1800000
37010.0000000	0.1850000
37011.0000000	0.1800000
37012.0000000	0.1900000
37013.0000000	0.1850000
37014.0000000	0.1800000
37015.0000000	0.1800000
37016.0000000	0.1850000
37017.0000000	0.1850000
37018.0000000	0.1800000
37019.0000000	0.1850000
37020.0000000	0.1800000
37021.0000000	0.1800000
37022.0000000	0.1800000
37023.0000000	0.1800000
37024.0000000	0.1750000
37025.0000000	0.1900000
37026.0000000	0.1750000
37027.0000000	0.1800000
37028.0000000	0.1900000
37029.0000000	0.1900000
37030.0000000	0.1850000
37031.0000000	0.1900000
37032.0000000	0.1750000
37033.0000000	0.1750000
37034.0000000	0.1900000
37035.0000000	0.1850000
37036.0000000	0.1900000
37037.0000000	0.1850000
37038.0000000	0.1850000
37039.0000000	0.1750000
37040.0000000	0.1850000
37041.0000000	0.1750000
37042.0000000	0.1800000
37043.0000000	0.1850000
37044.0000000	0.1800000
37045.0000000	0.1700000
37046.0000000	0.1800000
37047.0000000	0.1800000
37048.0000000	0.1750000
37049.0000000	0.1800000

37050.0000000	0.1850000
37051.0000000	0.1800000
37052.0000000	0.1750000
37053.0000000	0.1750000
37054.0000000	0.1800000
37055.0000000	0.1800000
37056.0000000	0.1850000
37057.0000000	0.1800000
37058.0000000	0.1750000
37059.0000000	0.1800000
37060.0000000	0.1750000
37061.0000000	0.1800000
37062.0000000	0.1750000
37063.0000000	0.1700000
37064.0000000	0.1800000
37065.0000000	0.1700000
37066.0000000	0.1750000
37067.0000000	0.1750000
37068.0000000	0.1750000
37069.0000000	0.1800000
37070.0000000	0.1800000
37071.0000000	0.1800000
37072.0000000	0.1850000
37073.0000000	0.1900000
37074.0000000	0.1850000
37075.0000000	0.1850000
37076.0000000	0.1800000
37077.0000000	0.1800000
37078.0000000	0.1850000
37079.0000000	0.1800000
37080.0000000	0.1800000
37081.0000000	0.1850000
37082.0000000	0.1850000
37083.0000000	0.1800000
37084.0000000	0.2000000
37085.0000000	0.1800000
37086.0000000	0.1850000
37087.0000000	0.1900000
37088.0000000	0.1900000
37089.0000000	0.1900000
37090.0000000	0.1800000
37091.0000000	0.1900000
37092.0000000	0.1850000
37093.0000000	0.1900000
37094.0000000	0.1900000
37095.0000000	0.1850000
37096.0000000	0.1950000
37097.0000000	0.1900000
37098.0000000	0.1900000
37099.0000000	0.1900000
37100.0000000	0.1850000
37101.0000000	0.1850000
37102.0000000	0.1900000
37103.0000000	0.1950000
37104.0000000	0.2050000
37105.0000000	0.1950000
37106.0000000	0.1900000

37107.0000000	0.2000000
37108.0000000	0.1950000
37109.0000000	0.2000000
37110.0000000	0.1950000
37111.0000000	0.1900000
37112.0000000	0.1900000
37113.0000000	0.1900000
37114.0000000	0.1900000
37115.0000000	0.1850000
37116.0000000	0.1900000
37117.0000000	0.1850000
37118.0000000	0.2000000
37119.0000000	0.1950000
37120.0000000	0.1950000
37121.0000000	0.1950000
37122.0000000	0.1900000
37123.0000000	0.1950000
37124.0000000	0.1900000
37125.0000000	0.2050000
37126.0000000	0.1900000
37127.0000000	0.2000000
37128.0000000	0.1950000
37129.0000000	0.2050000
37130.0000000	0.1950000
37131.0000000	0.2050000
37132.0000000	0.2000000
37133.0000000	0.1950000
37134.0000000	0.2000000
37135.0000000	0.2050000
37136.0000000	0.1966667
37137.0000000	0.2050000
37138.0000000	0.2050000
37139.0000000	0.2050000
37140.0000000	0.2050000
37141.0000000	0.2000000
37142.0000000	0.2000000
37143.0000000	0.2050000
37144.0000000	0.2050000
37145.0000000	0.2100000
37146.0000000	0.2100000
37147.0000000	0.2050000
37148.0000000	0.2050000
37149.0000000	0.2100000
37150.0000000	0.1950000
37151.0000000	0.2000000
37152.0000000	0.2050000
37153.0000000	0.2000000
37154.0000000	0.2100000
37155.0000000	0.2050000
37156.0000000	0.2050000
37157.0000000	0.2000000
37158.0000000	0.2050000
37159.0000000	0.2000000
37160.0000000	0.2100000
37161.0000000	0.2050000
37162.0000000	0.2100000
37163.0000000	0.2050000

37164.0000000	0.2050000
37165.0000000	0.2100000
37166.0000000	0.2050000
37167.0000000	0.2033333
37168.0000000	0.2100000
37169.0000000	0.2000000
37170.0000000	0.2050000
37171.0000000	0.2050000
37172.0000000	0.2050000
37173.0000000	0.2100000
37174.0000000	0.2100000
37175.0000000	0.2050000
37176.0000000	0.2000000
37177.0000000	0.2150000
37178.0000000	0.2000000
37179.0000000	0.2100000
37180.0000000	0.2050000
37181.0000000	0.2050000
37182.0000000	0.2100000
37183.0000000	0.2100000
37184.0000000	0.2150000
37185.0000000	0.2150000
37186.0000000	0.2050000
37187.0000000	0.2200000
37188.0000000	0.2100000
37189.0000000	0.2050000
37190.0000000	0.2150000
37191.0000000	0.2050000
37192.0000000	0.2050000
37193.0000000	0.2100000
37194.0000000	0.2050000
37195.0000000	0.2150000
37196.0000000	0.2100000
37197.0000000	0.2133333
37198.0000000	0.2050000
37199.0000000	0.2100000
37200.0000000	0.2100000
37201.0000000	0.2150000
37202.0000000	0.2100000
37203.0000000	0.2150000
37204.0000000	0.2150000
37205.0000000	0.2100000
37206.0000000	0.2050000
37207.0000000	0.2200000
37208.0000000	0.2100000
37209.0000000	0.2100000
37210.0000000	0.2150000
37211.0000000	0.2150000
37212.0000000	0.2200000
37213.0000000	0.2100000
37214.0000000	0.2150000
37215.0000000	0.2150000
37216.0000000	0.2100000
37217.0000000	0.2100000
37218.0000000	0.2100000
37219.0000000	0.2100000
37220.0000000	0.2100000

37221.0000000	0.2050000
37222.0000000	0.2100000
37223.0000000	0.2100000
37224.0000000	0.2000000
37225.0000000	0.2150000
37226.0000000	0.2066667
37227.0000000	0.2150000
37228.0000000	0.2100000
37229.0000000	0.2200000
37230.0000000	0.2150000
37231.0000000	0.2100000
37232.0000000	0.2150000
37233.0000000	0.2150000
37234.0000000	0.2150000
37235.0000000	0.2100000
37236.0000000	0.2100000
37237.0000000	0.2100000
37238.0000000	0.2150000
37239.0000000	0.2200000
37240.0000000	0.2100000
37241.0000000	0.2150000
37242.0000000	0.2150000
37243.0000000	0.2150000
37244.0000000	0.2100000
37245.0000000	0.2100000
37246.0000000	0.2150000
37247.0000000	0.2100000
37248.0000000	0.2150000
37249.0000000	0.2100000
37250.0000000	0.2150000
37251.0000000	0.2100000
37252.0000000	0.2100000
37253.0000000	0.2150000
37254.0000000	0.2100000
37255.0000000	0.2150000
37256.0000000	0.2100000
37257.0000000	0.2033333
37258.0000000	0.2100000
37259.0000000	0.2150000
37260.0000000	0.2100000
37261.0000000	0.2150000
37262.0000000	0.2100000
37263.0000000	0.2100000
37264.0000000	0.2100000
37265.0000000	0.2050000
37266.0000000	0.2150000
37267.0000000	0.2050000
37268.0000000	0.2150000
37269.0000000	0.2150000
37270.0000000	0.2150000
37271.0000000	0.2050000
37272.0000000	0.2150000
37273.0000000	0.2150000
37274.0000000	0.2150000
37275.0000000	0.2150000
37276.0000000	0.2200000
37277.0000000	0.2150000

37278.0000000	0.2150000
37279.0000000	0.2200000
37280.0000000	0.2150000
37281.0000000	0.2100000
37282.0000000	0.2150000
37283.0000000	0.2100000
37284.0000000	0.2150000
37285.0000000	0.2150000
37286.0000000	0.2150000
37287.0000000	0.2100000
37288.0000000	0.2200000
37289.0000000	0.2150000
37290.0000000	0.2200000
37291.0000000	0.2200000
37292.0000000	0.2250000
37293.0000000	0.2200000
37294.0000000	0.2200000
37295.0000000	0.2200000
37296.0000000	0.2200000
37297.0000000	0.2300000
37298.0000000	0.2250000
37299.0000000	0.2300000
37300.0000000	0.2250000
37301.0000000	0.2150000
37302.0000000	0.2250000
37303.0000000	0.2250000
37304.0000000	0.2250000
37305.0000000	0.2200000
37306.0000000	0.2250000
37307.0000000	0.2300000
37308.0000000	0.2250000
37309.0000000	0.2300000
37310.0000000	0.2300000
37311.0000000	0.2250000
37312.0000000	0.2300000
37313.0000000	0.2150000
37314.0000000	0.2250000
37315.0000000	0.2250000
37316.0000000	0.2300000
37317.0000000	0.2233333
37318.0000000	0.2200000
37319.0000000	0.2150000
37320.0000000	0.2250000
37321.0000000	0.2200000
37322.0000000	0.2250000
37323.0000000	0.2250000
37324.0000000	0.2200000
37325.0000000	0.2250000
37326.0000000	0.2250000
37327.0000000	0.2200000
37328.0000000	0.2200000
37329.0000000	0.2150000
37330.0000000	0.2250000
37331.0000000	0.2150000
37332.0000000	0.2200000
37333.0000000	0.2200000
37334.0000000	0.2200000

37335.0000000	0.2250000
37336.0000000	0.2200000
37337.0000000	0.2200000
37338.0000000	0.2250000
37339.0000000	0.2250000
37340.0000000	0.2300000
37341.0000000	0.2350000
37342.0000000	0.2300000
37343.0000000	0.2300000
37344.0000000	0.2300000
37345.0000000	0.2250000
37346.0000000	0.2200000
37347.0000000	0.2266667
37348.0000000	0.2250000
37349.0000000	0.2150000
37350.0000000	0.2300000
37351.0000000	0.2150000
37352.0000000	0.2300000
37353.0000000	0.2200000
37354.0000000	0.2300000
37355.0000000	0.2200000
37356.0000000	0.2250000
37357.0000000	0.2200000
37358.0000000	0.2300000
37359.0000000	0.2200000
37360.0000000	0.2250000
37361.0000000	0.2250000
37362.0000000	0.2100000
37363.0000000	0.2200000
37364.0000000	0.2150000
37365.0000000	0.2100000
37366.0000000	0.2100000
37367.0000000	0.2150000
37368.0000000	0.2200000
37369.0000000	0.2150000
37370.0000000	0.2100000
37371.0000000	0.2050000
37372.0000000	0.2100000
37373.0000000	0.2000000
37374.0000000	0.2150000
37375.0000000	0.2200000
37376.0000000	0.2200000
37377.0000000	0.2100000
37378.0000000	0.2000000
37379.0000000	0.2100000
37380.0000000	0.2100000
37381.0000000	0.2050000
37382.0000000	0.2150000
37383.0000000	0.2050000
37384.0000000	0.2150000
37385.0000000	0.2050000
37386.0000000	0.2100000
37387.0000000	0.2000000
37388.0000000	0.2050000
37389.0000000	0.2150000
37390.0000000	0.2100000
37391.0000000	0.2100000

37392.0000000	0.2050000
37393.0000000	0.2050000
37394.0000000	0.2000000
37395.0000000	0.2100000
37396.0000000	0.2050000
37397.0000000	0.2100000
37398.0000000	0.2100000
37399.0000000	0.2050000
37400.0000000	0.2100000
37401.0000000	0.2000000
37402.0000000	0.2100000
37403.0000000	0.2050000
37404.0000000	0.2100000
37405.0000000	0.2150000
37406.0000000	0.2000000
37407.0000000	0.1950000
37408.0000000	0.2100000
37409.0000000	0.2100000
37410.0000000	0.2100000
37411.0000000	0.2050000
37412.0000000	0.2000000
37413.0000000	0.2050000
37414.0000000	0.2050000
37415.0000000	0.2000000
37416.0000000	0.2050000
37417.0000000	0.1950000
37418.0000000	0.2050000
37419.0000000	0.2050000
37420.0000000	0.2000000
37421.0000000	0.2100000
37422.0000000	0.2000000
37423.0000000	0.2000000
37424.0000000	0.2050000
37425.0000000	0.2000000
37426.0000000	0.1950000
37427.0000000	0.2000000
37428.0000000	0.2000000
37429.0000000	0.2050000
37430.0000000	0.2050000
37431.0000000	0.2050000
37432.0000000	0.2100000
37433.0000000	0.2050000
37434.0000000	0.2050000
37435.0000000	0.2000000
37436.0000000	0.2050000
37437.0000000	0.2050000
37438.0000000	0.1966667
37439.0000000	0.2050000
37440.0000000	0.2050000
37441.0000000	0.2050000
37442.0000000	0.2000000
37443.0000000	0.2050000
37444.0000000	0.2000000
37445.0000000	0.2050000
37446.0000000	0.2000000
37447.0000000	0.2000000
37448.0000000	0.2000000

37449.0000000	0.2050000
37450.0000000	0.2050000
37451.0000000	0.2000000
37452.0000000	0.1950000
37453.0000000	0.1950000
37454.0000000	0.2050000
37455.0000000	0.2000000
37456.0000000	0.2050000
37457.0000000	0.1950000
37458.0000000	0.2000000
37459.0000000	0.1950000
37460.0000000	0.1950000
37461.0000000	0.2000000
37462.0000000	0.1950000
37463.0000000	0.1950000
37464.0000000	0.2000000
37465.0000000	0.1950000
37466.0000000	0.1950000
37467.0000000	0.1950000
37468.0000000	0.1966667
37469.0000000	0.1950000
37470.0000000	0.1950000
37471.0000000	0.1950000
37472.0000000	0.1950000
37473.0000000	0.2000000
37474.0000000	0.1900000
37475.0000000	0.1900000
37476.0000000	0.2000000
37477.0000000	0.1900000
37478.0000000	0.1950000
37479.0000000	0.1950000
37480.0000000	0.1900000
37481.0000000	0.1800000
37482.0000000	0.1900000
37483.0000000	0.1950000
37484.0000000	0.1900000
37485.0000000	0.1900000
37486.0000000	0.1800000
37487.0000000	0.1950000
37488.0000000	0.1950000
37489.0000000	0.1800000
37490.0000000	0.1850000
37491.0000000	0.1850000
37492.0000000	0.1850000
37493.0000000	0.1900000
37494.0000000	0.1900000
37495.0000000	0.1900000
37496.0000000	0.1900000
37497.0000000	0.1900000
37498.0000000	0.1933333
37499.0000000	0.1800000
37500.0000000	0.1850000
37501.0000000	0.1900000
37502.0000000	0.1850000
37503.0000000	0.1800000
37504.0000000	0.1900000
37505.0000000	0.1900000

37506.0000000	0.1900000
37507.0000000	0.1900000
37508.0000000	0.1900000
37509.0000000	0.1800000
37510.0000000	0.1900000
37511.0000000	0.1900000
37512.0000000	0.1900000
37513.0000000	0.1900000
37514.0000000	0.1800000
37515.0000000	0.1800000
37516.0000000	0.1850000
37517.0000000	0.1900000
37518.0000000	0.1800000
37519.0000000	0.1850000
37520.0000000	0.1850000
37521.0000000	0.1800000
37522.0000000	0.1800000
37523.0000000	0.1850000
37524.0000000	0.1800000
37525.0000000	0.1850000
37526.0000000	0.1750000
37527.0000000	0.1800000
37528.0000000	0.1850000
37529.0000000	0.1766667
37530.0000000	0.1800000
37531.0000000	0.1750000
37532.0000000	0.1850000
37533.0000000	0.1800000
37534.0000000	0.1750000
37535.0000000	0.1850000
37536.0000000	0.1750000
37537.0000000	0.1750000
37538.0000000	0.1700000
37539.0000000	0.1800000
37540.0000000	0.1750000
37541.0000000	0.1800000
37542.0000000	0.1850000
37543.0000000	0.1750000
37544.0000000	0.1850000
37545.0000000	0.1750000
37546.0000000	0.1850000
37547.0000000	0.1750000
37548.0000000	0.1900000
37549.0000000	0.1800000
37550.0000000	0.1850000
37551.0000000	0.1700000
37552.0000000	0.1800000
37553.0000000	0.1700000
37554.0000000	0.1800000
37555.0000000	0.1800000
37556.0000000	0.1900000
37557.0000000	0.1850000
37558.0000000	0.1800000
37559.0000000	0.1766667
37560.0000000	0.1850000
37561.0000000	0.1850000
37562.0000000	0.1950000

37563.0000000	0.1750000
37564.0000000	0.1900000
37565.0000000	0.1900000
37566.0000000	0.1950000
37567.0000000	0.1900000
37568.0000000	0.1850000
37569.0000000	0.1800000
37570.0000000	0.1800000
37571.0000000	0.1850000
37572.0000000	0.1950000
37573.0000000	0.1900000
37574.0000000	0.1900000
37575.0000000	0.1800000
37576.0000000	0.1950000
37577.0000000	0.1950000
37578.0000000	0.1950000
37579.0000000	0.1900000
37580.0000000	0.1750000
37581.0000000	0.1900000
37582.0000000	0.1900000
37583.0000000	0.1900000
37584.0000000	0.1900000
37585.0000000	0.1850000
37586.0000000	0.1950000
37587.0000000	0.1800000
37588.0000000	0.1900000
37589.0000000	0.1900000
37590.0000000	0.1900000
37591.0000000	0.1950000
37592.0000000	0.1900000
37593.0000000	0.1850000
37594.0000000	0.1850000
37595.0000000	0.1900000
37596.0000000	0.1900000
37597.0000000	0.1900000
37598.0000000	0.2000000
37599.0000000	0.1900000
37600.0000000	0.1900000
37601.0000000	0.1800000
37602.0000000	0.1850000
37603.0000000	0.1950000
37604.0000000	0.1950000
37605.0000000	0.2050000
37606.0000000	0.1800000
37607.0000000	0.1900000
37608.0000000	0.1900000
37609.0000000	0.1900000
37610.0000000	0.2000000
37611.0000000	0.1900000
37612.0000000	0.2000000
37613.0000000	0.1850000
37614.0000000	0.1900000
37615.0000000	0.1850000
37616.0000000	0.1900000
37617.0000000	0.1950000
37618.0000000	0.1950000
37619.0000000	0.1866667

37620.0000000	0.1950000
37621.0000000	0.2000000
37622.0000000	0.1900000
37623.0000000	0.1950000
37624.0000000	0.1850000
37625.0000000	0.2000000
37626.0000000	0.1900000
37627.0000000	0.1950000
37628.0000000	0.1950000
37629.0000000	0.2000000
37630.0000000	0.1900000
37631.0000000	0.1900000
37632.0000000	0.1850000
37633.0000000	0.1950000
37634.0000000	0.1900000
37635.0000000	0.1950000
37636.0000000	0.1950000
37637.0000000	0.1900000
37638.0000000	0.1950000
37639.0000000	0.1900000
37640.0000000	0.1900000
37641.0000000	0.1800000
37642.0000000	0.1900000
37643.0000000	0.1900000
37644.0000000	0.1950000
37645.0000000	0.2000000
37646.0000000	0.1900000
37647.0000000	0.1950000
37648.0000000	0.1900000
37649.0000000	0.1900000
37650.0000000	0.1900000
37651.0000000	0.2000000
37652.0000000	0.1900000
37653.0000000	0.1900000
37654.0000000	0.1950000
37655.0000000	0.1900000
37656.0000000	0.1950000
37657.0000000	0.1850000
37658.0000000	0.1900000
37659.0000000	0.1900000
37660.0000000	0.1900000
37661.0000000	0.1950000
37662.0000000	0.1950000
37663.0000000	0.1950000
37664.0000000	0.1850000
37665.0000000	0.1900000
37666.0000000	0.1900000
37667.0000000	0.1900000
37668.0000000	0.1850000
37669.0000000	0.1900000
37670.0000000	0.1950000
37671.0000000	0.1900000
37672.0000000	0.1850000
37673.0000000	0.1850000
37674.0000000	0.1900000
37675.0000000	0.1800000
37676.0000000	0.1850000

37677.0000000	0.1850000
37678.0000000	0.1900000
37679.0000000	0.1866667
37680.0000000	0.1850000
37681.0000000	0.1800000
37682.0000000	0.1900000
37683.0000000	0.1900000
37684.0000000	0.1850000
37685.0000000	0.1850000
37686.0000000	0.1850000
37687.0000000	0.1800000
37688.0000000	0.1850000
37689.0000000	0.1900000
37690.0000000	0.1950000
37691.0000000	0.1900000
37692.0000000	0.1850000
37693.0000000	0.1900000
37694.0000000	0.1900000
37695.0000000	0.1850000
37696.0000000	0.1900000
37697.0000000	0.1900000
37698.0000000	0.1900000
37699.0000000	0.1900000
37700.0000000	0.1850000
37701.0000000	0.1850000
37702.0000000	0.1900000
37703.0000000	0.1950000
37704.0000000	0.1900000
37705.0000000	0.1900000
37706.0000000	0.1900000
37707.0000000	0.1900000
37708.0000000	0.1950000
37709.0000000	0.1800000
37710.0000000	0.1950000
37711.0000000	0.1950000
37712.0000000	0.1900000
37713.0000000	0.1850000
37714.0000000	0.1900000
37715.0000000	0.1900000
37716.0000000	0.1900000
37717.0000000	0.1900000
37718.0000000	0.1900000
37719.0000000	0.1900000
37720.0000000	0.1850000
37721.0000000	0.1900000
37722.0000000	0.1950000
37723.0000000	0.1900000
37724.0000000	0.1900000
37725.0000000	0.1950000
37726.0000000	0.1950000
37727.0000000	0.2000000
37728.0000000	0.1900000
37729.0000000	0.1950000
37730.0000000	0.1950000
37731.0000000	0.2050000
37732.0000000	0.1900000
37733.0000000	0.1950000

37734.0000000	0.2000000
37735.0000000	0.2000000
37736.0000000	0.1950000
37737.0000000	0.1900000
37738.0000000	0.1950000
37739.0000000	0.1900000
37740.0000000	0.1966667
37741.0000000	0.2000000
37742.0000000	0.2000000
37743.0000000	0.1950000
37744.0000000	0.1950000
37745.0000000	0.2000000
37746.0000000	0.2100000
37747.0000000	0.1950000
37748.0000000	0.2000000
37749.0000000	0.2000000
37750.0000000	0.1950000
37751.0000000	0.2050000
37752.0000000	0.2000000
37753.0000000	0.2050000
37754.0000000	0.2050000
37755.0000000	0.2000000
37756.0000000	0.2000000
37757.0000000	0.2000000
37758.0000000	0.2050000
37759.0000000	0.2050000
37760.0000000	0.1950000
37761.0000000	0.2050000
37762.0000000	0.1950000
37763.0000000	0.2050000
37764.0000000	0.2050000
37765.0000000	0.2000000
37766.0000000	0.1950000
37767.0000000	0.2050000
37768.0000000	0.2050000
37769.0000000	0.2050000
37770.0000000	0.2033333
37771.0000000	0.2050000
37772.0000000	0.2000000
37773.0000000	0.2100000
37774.0000000	0.2100000
37775.0000000	0.2100000
37776.0000000	0.2150000
37777.0000000	0.2150000
37778.0000000	0.2150000
37779.0000000	0.2050000
37780.0000000	0.2100000
37781.0000000	0.2050000
37782.0000000	0.2100000
37783.0000000	0.2050000
37784.0000000	0.2100000
37785.0000000	0.2100000
37786.0000000	0.2100000
37787.0000000	0.2200000
37788.0000000	0.2100000
37789.0000000	0.2150000
37790.0000000	0.2200000

37791.0000000	0.2100000
37792.0000000	0.2200000
37793.0000000	0.2200000
37794.0000000	0.2100000
37795.0000000	0.2200000
37796.0000000	0.2300000
37797.0000000	0.2150000
37798.0000000	0.2200000
37799.0000000	0.2100000
37800.0000000	0.2166667
37801.0000000	0.2250000
37802.0000000	0.2200000
37803.0000000	0.2100000
37804.0000000	0.2250000
37805.0000000	0.2300000
37806.0000000	0.2150000
37807.0000000	0.2250000
37808.0000000	0.2250000
37809.0000000	0.2150000
37810.0000000	0.2200000
37811.0000000	0.2150000
37812.0000000	0.2200000
37813.0000000	0.2150000
37814.0000000	0.2250000
37815.0000000	0.2150000
37816.0000000	0.2250000
37817.0000000	0.2300000
37818.0000000	0.2200000
37819.0000000	0.2250000
37820.0000000	0.2200000
37821.0000000	0.2150000
37822.0000000	0.2200000
37823.0000000	0.2200000
37824.0000000	0.2200000
37825.0000000	0.2200000
37826.0000000	0.2250000
37827.0000000	0.2250000
37828.0000000	0.2250000
37829.0000000	0.2300000
37830.0000000	0.2266667
37831.0000000	0.2250000
37832.0000000	0.2250000
37833.0000000	0.2200000
37834.0000000	0.2250000
37835.0000000	0.2350000
37836.0000000	0.2250000
37837.0000000	0.2300000
37838.0000000	0.2250000
37839.0000000	0.2250000
37840.0000000	0.2350000
37841.0000000	0.2350000
37842.0000000	0.2300000
37843.0000000	0.2350000
37844.0000000	0.2200000
37845.0000000	0.2350000
37846.0000000	0.2300000
37847.0000000	0.2250000

37848.0000000	0.2250000
37849.0000000	0.2300000
37850.0000000	0.2300000
37851.0000000	0.2300000
37852.0000000	0.2350000
37853.0000000	0.2250000
37854.0000000	0.2300000
37855.0000000	0.2400000
37856.0000000	0.2300000
37857.0000000	0.2300000
37858.0000000	0.2300000
37859.0000000	0.2350000
37860.0000000	0.2350000
37861.0000000	0.2300000
37862.0000000	0.2350000
37863.0000000	0.2300000
37864.0000000	0.2450000
37865.0000000	0.2300000
37866.0000000	0.2350000
37867.0000000	0.2400000
37868.0000000	0.2500000
37869.0000000	0.2400000
37870.0000000	0.2400000
37871.0000000	0.2450000
37872.0000000	0.2450000
37873.0000000	0.2450000
37874.0000000	0.2500000
37875.0000000	0.2450000
37876.0000000	0.2500000
37877.0000000	0.2500000
37878.0000000	0.2450000
37879.0000000	0.2500000
37880.0000000	0.2500000
37881.0000000	0.2500000
37882.0000000	0.2550000
37883.0000000	0.2450000
37884.0000000	0.2500000
37885.0000000	0.2550000
37886.0000000	0.2550000
37887.0000000	0.2550000
37888.0000000	0.2550000
37889.0000000	0.2600000
37890.0000000	0.2600000
37891.0000000	0.2533333
37892.0000000	0.2550000
37893.0000000	0.2550000
37894.0000000	0.2550000
37895.0000000	0.2550000
37896.0000000	0.2650000
37897.0000000	0.2550000
37898.0000000	0.2500000
37899.0000000	0.2550000
37900.0000000	0.2600000
37901.0000000	0.2600000
37902.0000000	0.2600000
37903.0000000	0.2650000
37904.0000000	0.2650000

37905.0000000	0.2600000
37906.0000000	0.2600000
37907.0000000	0.2550000
37908.0000000	0.2600000
37909.0000000	0.2600000
37910.0000000	0.2600000
37911.0000000	0.2600000
37912.0000000	0.2550000
37913.0000000	0.2600000
37914.0000000	0.2550000
37915.0000000	0.2550000
37916.0000000	0.2550000
37917.0000000	0.2550000
37918.0000000	0.2650000
37919.0000000	0.2600000
37920.0000000	0.2633333
37921.0000000	0.2600000
37922.0000000	0.2650000
37923.0000000	0.2600000
37924.0000000	0.2650000
37925.0000000	0.2650000
37926.0000000	0.2650000
37927.0000000	0.2550000
37928.0000000	0.2650000
37929.0000000	0.2600000
37930.0000000	0.2600000
37931.0000000	0.2650000
37932.0000000	0.2550000
37933.0000000	0.2600000
37934.0000000	0.2650000
37935.0000000	0.2650000
37936.0000000	0.2650000
37937.0000000	0.2650000
37938.0000000	0.2750000
37939.0000000	0.2700000
37940.0000000	0.2650000
37941.0000000	0.2650000
37942.0000000	0.2700000
37943.0000000	0.2650000
37944.0000000	0.2750000
37945.0000000	0.2750000
37946.0000000	0.2650000
37947.0000000	0.2700000
37948.0000000	0.2800000
37949.0000000	0.2700000
37950.0000000	0.2700000
37951.0000000	0.2766667
37952.0000000	0.2750000
37953.0000000	0.2700000
37954.0000000	0.2800000
37955.0000000	0.2700000
37956.0000000	0.2750000
37957.0000000	0.2750000
37958.0000000	0.2700000
37959.0000000	0.2750000
37960.0000000	0.2650000
37961.0000000	0.2750000

37962.0000000	0.2750000
37963.0000000	0.2750000
37964.0000000	0.2800000
37965.0000000	0.2750000
37966.0000000	0.2700000
37967.0000000	0.2800000
37968.0000000	0.2700000
37969.0000000	0.2750000
37970.0000000	0.2750000
37971.0000000	0.2750000
37972.0000000	0.2750000
37973.0000000	0.2700000
37974.0000000	0.2750000
37975.0000000	0.2800000
37976.0000000	0.2750000
37977.0000000	0.2750000
37978.0000000	0.2700000
37979.0000000	0.2650000
37980.0000000	0.2750000
37981.0000000	0.2750000
37982.0000000	0.2700000
37983.0000000	0.2750000
37984.0000000	0.2700000
37985.0000000	0.2750000
37986.0000000	0.2800000
37987.0000000	0.2750000
37988.0000000	0.2750000
37989.0000000	0.2750000
37990.0000000	0.2800000
37991.0000000	0.2850000
37992.0000000	0.2750000
37993.0000000	0.2750000
37994.0000000	0.2750000
37995.0000000	0.2750000
37996.0000000	0.2900000
37997.0000000	0.2750000
37998.0000000	0.2850000
37999.0000000	0.2850000
38000.0000000	0.2800000
38001.0000000	0.2800000
38002.0000000	0.2800000
38003.0000000	0.2850000
38004.0000000	0.2850000
38005.0000000	0.2750000
38006.0000000	0.2800000
38007.0000000	0.2850000
38008.0000000	0.2850000
38009.0000000	0.2900000
38010.0000000	0.2800000
38011.0000000	0.2833333
38012.0000000	0.2850000
38013.0000000	0.2850000
38014.0000000	0.2750000
38015.0000000	0.2700000
38016.0000000	0.2750000
38017.0000000	0.2750000
38018.0000000	0.2800000

38019.0000000	0.2750000
38020.0000000	0.2800000
38021.0000000	0.2850000
38022.0000000	0.2700000
38023.0000000	0.2700000
38024.0000000	0.2700000
38025.0000000	0.2750000
38026.0000000	0.2800000
38027.0000000	0.2700000
38028.0000000	0.2750000
38029.0000000	0.2750000
38030.0000000	0.2850000
38031.0000000	0.2750000
38032.0000000	0.2800000
38033.0000000	0.2800000
38034.0000000	0.2750000
38035.0000000	0.2750000
38036.0000000	0.2750000
38037.0000000	0.2800000
38038.0000000	0.2800000
38039.0000000	0.2750000
38040.0000000	0.2800000
38041.0000000	0.2866667
38042.0000000	0.2850000
38043.0000000	0.2850000
38044.0000000	0.2800000
38045.0000000	0.2850000
38046.0000000	0.2850000
38047.0000000	0.2800000
38048.0000000	0.2800000
38049.0000000	0.2850000
38050.0000000	0.2850000
38051.0000000	0.2850000
38052.0000000	0.2850000
38053.0000000	0.2800000
38054.0000000	0.2750000
38055.0000000	0.2750000
38056.0000000	0.2750000
38057.0000000	0.2700000
38058.0000000	0.2800000
38059.0000000	0.2800000
38060.0000000	0.2650000
38061.0000000	0.2800000
38062.0000000	0.2650000
38063.0000000	0.2650000
38064.0000000	0.2800000
38065.0000000	0.2800000
38066.0000000	0.2750000
38067.0000000	0.2750000
38068.0000000	0.2700000
38069.0000000	0.2800000
38070.0000000	0.2700000
38071.0000000	0.2650000
38072.0000000	0.2766667
38073.0000000	0.2700000
38074.0000000	0.2750000
38075.0000000	0.2700000

38076.0000000	0.2800000
38077.0000000	0.2800000
38078.0000000	0.2700000
38079.0000000	0.2750000
38080.0000000	0.2750000
38081.0000000	0.2750000
38082.0000000	0.2800000
38083.0000000	0.2850000
38084.0000000	0.2700000
38085.0000000	0.2750000
38086.0000000	0.2800000
38087.0000000	0.2700000
38088.0000000	0.2700000
38089.0000000	0.2700000
38090.0000000	0.2700000
38091.0000000	0.2650000
38092.0000000	0.2700000
38093.0000000	0.2700000
38094.0000000	0.2700000
38095.0000000	0.2700000
38096.0000000	0.2750000
38097.0000000	0.2700000
38098.0000000	0.2750000
38099.0000000	0.2650000
38100.0000000	0.2650000
38101.0000000	0.2650000
38102.0000000	0.2733333
38103.0000000	0.2750000
38104.0000000	0.2750000
38105.0000000	0.2700000
38106.0000000	0.2700000
38107.0000000	0.2800000
38108.0000000	0.2750000
38109.0000000	0.2700000
38110.0000000	0.2700000
38111.0000000	0.2800000
38112.0000000	0.2750000
38113.0000000	0.2800000
38114.0000000	0.2750000
38115.0000000	0.2750000
38116.0000000	0.2800000
38117.0000000	0.2750000
38118.0000000	0.2800000
38119.0000000	0.2750000
38120.0000000	0.2800000
38121.0000000	0.2900000
38122.0000000	0.2800000
38123.0000000	0.2850000
38124.0000000	0.2800000
38125.0000000	0.2850000
38126.0000000	0.2800000
38127.0000000	0.2750000
38128.0000000	0.2800000
38129.0000000	0.2800000
38130.0000000	0.2800000
38131.0000000	0.2800000
38132.0000000	0.2800000

38133.0000000	0.2800000
38134.0000000	0.2850000
38135.0000000	0.2800000
38136.0000000	0.2900000
38137.0000000	0.2800000
38138.0000000	0.2850000
38139.0000000	0.2750000
38140.0000000	0.2750000
38141.0000000	0.2850000
38142.0000000	0.2850000
38143.0000000	0.2850000
38144.0000000	0.2850000
38145.0000000	0.2800000
38146.0000000	0.2850000
38147.0000000	0.2800000
38148.0000000	0.2900000
38149.0000000	0.2750000
38150.0000000	0.2800000
38151.0000000	0.2850000
38152.0000000	0.2750000
38153.0000000	0.2850000
38154.0000000	0.2850000
38155.0000000	0.2850000
38156.0000000	0.2750000
38157.0000000	0.2800000
38158.0000000	0.2850000
38159.0000000	0.2700000
38160.0000000	0.2800000
38161.0000000	0.2750000
38162.0000000	0.2866667
38163.0000000	0.2750000
38164.0000000	0.2750000
38165.0000000	0.2850000
38166.0000000	0.2850000
38167.0000000	0.2800000
38168.0000000	0.2800000
38169.0000000	0.2750000
38170.0000000	0.2750000
38171.0000000	0.2750000
38172.0000000	0.2850000
38173.0000000	0.2850000
38174.0000000	0.2800000
38175.0000000	0.2750000
38176.0000000	0.2850000
38177.0000000	0.2750000
38178.0000000	0.2750000
38179.0000000	0.2750000
38180.0000000	0.2800000
38181.0000000	0.2800000
38182.0000000	0.2850000
38183.0000000	0.2900000
38184.0000000	0.2850000
38185.0000000	0.2750000
38186.0000000	0.2800000
38187.0000000	0.2800000
38188.0000000	0.2800000
38189.0000000	0.2750000

38190.0000000	0.2750000
38191.0000000	0.2850000
38192.0000000	0.2750000
38193.0000000	0.2833333
38194.0000000	0.2800000
38195.0000000	0.2850000
38196.0000000	0.2850000
38197.0000000	0.2700000
38198.0000000	0.2750000
38199.0000000	0.2700000
38200.0000000	0.2800000
38201.0000000	0.2750000
38202.0000000	0.2750000
38203.0000000	0.2800000
38204.0000000	0.2750000
38205.0000000	0.2800000
38206.0000000	0.2700000
38207.0000000	0.2750000
38208.0000000	0.2700000
38209.0000000	0.2650000
38210.0000000	0.2800000
38211.0000000	0.2800000
38212.0000000	0.2700000
38213.0000000	0.2800000
38214.0000000	0.2750000
38215.0000000	0.2750000
38216.0000000	0.2800000
38217.0000000	0.2700000
38218.0000000	0.2800000
38219.0000000	0.2800000
38220.0000000	0.2750000
38221.0000000	0.2850000
38222.0000000	0.2750000
38223.0000000	0.2766666
38224.0000000	0.2800000
38225.0000000	0.2850000
38226.0000000	0.2750000
38227.0000000	0.2850000
38228.0000000	0.2850000
38229.0000000	0.2850000
38230.0000000	0.2800000
38231.0000000	0.2800000
38232.0000000	0.2800000
38233.0000000	0.2800000
38234.0000000	0.2900000
38235.0000000	0.2750000
38236.0000000	0.2800000
38237.0000000	0.2800000
38238.0000000	0.2800000
38239.0000000	0.2900000
38240.0000000	0.2750000
38241.0000000	0.2850000
38242.0000000	0.2800000
38243.0000000	0.2800000
38244.0000000	0.2800000
38245.0000000	0.2700000
38246.0000000	0.2750000

38247.0000000	0.2750000
38248.0000000	0.2800000
38249.0000000	0.2750000
38250.0000000	0.2800000
38251.0000000	0.2800000
38252.0000000	0.2750000
38253.0000000	0.2766667
38254.0000000	0.2750000
38255.0000000	0.2750000
38256.0000000	0.2700000
38257.0000000	0.2650000
38258.0000000	0.2700000
38259.0000000	0.2700000
38260.0000000	0.2750000
38261.0000000	0.2750000
38262.0000000	0.2650000
38263.0000000	0.2700000
38264.0000000	0.2600000
38265.0000000	0.2650000
38266.0000000	0.2550000
38267.0000000	0.2750000
38268.0000000	0.2650000
38269.0000000	0.2750000
38270.0000000	0.2600000
38271.0000000	0.2600000
38272.0000000	0.2600000
38273.0000000	0.2700000
38274.0000000	0.2650000
38275.0000000	0.2750000
38276.0000000	0.2650000
38277.0000000	0.2750000
38278.0000000	0.2650000
38279.0000000	0.2700000
38280.0000000	0.2700000
38281.0000000	0.2750000
38282.0000000	0.2800000
38283.0000000	0.2700000
38284.0000000	0.2750000
38285.0000000	0.2700000
38286.0000000	0.2800000
38287.0000000	0.2850000
38288.0000000	0.2800000
38289.0000000	0.2800000
38290.0000000	0.2800000
38291.0000000	0.2750000
38292.0000000	0.2850000
38293.0000000	0.2900000
38294.0000000	0.2700000
38295.0000000	0.2900000
38296.0000000	0.2800000
38297.0000000	0.2900000
38298.0000000	0.2850000
38299.0000000	0.2900000
38300.0000000	0.2900000
38301.0000000	0.2800000
38302.0000000	0.2900000
38303.0000000	0.2800000

38304.0000000	0.2800000
38305.0000000	0.2850000
38306.0000000	0.2800000
38307.0000000	0.2900000
38308.0000000	0.2850000
38309.0000000	0.2900000
38310.0000000	0.2800000
38311.0000000	0.2800000
38312.0000000	0.2800000
38313.0000000	0.2850000
38314.0000000	0.2800000
38315.0000000	0.2800000
38316.0000000	0.2750000
38317.0000000	0.2850000
38318.0000000	0.2800000
38319.0000000	0.2800000
38320.0000000	0.2800000
38321.0000000	0.2750000
38322.0000000	0.2750000
38323.0000000	0.2750000
38324.0000000	0.2850000
38325.0000000	0.2750000
38326.0000000	0.2900000
38327.0000000	0.2850000
38328.0000000	0.2800000
38329.0000000	0.2850000
38330.0000000	0.2800000
38331.0000000	0.2700000
38332.0000000	0.2850000
38333.0000000	0.2800000
38334.0000000	0.2800000
38335.0000000	0.2850000
38336.0000000	0.2900000
38337.0000000	0.2750000
38338.0000000	0.2750000
38339.0000000	0.2700000
38340.0000000	0.2800000
38341.0000000	0.2900000
38342.0000000	0.2850000
38343.0000000	0.2900000
38344.0000000	0.2733333
38345.0000000	0.2800000
38346.0000000	0.2800000
38347.0000000	0.2850000
38348.0000000	0.2850000
38349.0000000	0.2750000
38350.0000000	0.2900000
38351.0000000	0.2750000
38352.0000000	0.2850000
38353.0000000	0.2750000
38354.0000000	0.2800000
38355.0000000	0.2800000
38356.0000000	0.2750000
38357.0000000	0.2800000
38358.0000000	0.2700000
38359.0000000	0.2700000
38360.0000000	0.2700000

38361.0000000	0.2800000
38362.0000000	0.2750000
38363.0000000	0.2750000
38364.0000000	0.2750000
38365.0000000	0.2700000
38366.0000000	0.2700000
38367.0000000	0.2650000
38368.0000000	0.2700000
38369.0000000	0.2650000
38370.0000000	0.2700000
38371.0000000	0.2700000
38372.0000000	0.2600000
38373.0000000	0.2666667
38374.0000000	0.2700000
38375.0000000	0.2700000
38376.0000000	0.2750000
38377.0000000	0.2600000
38378.0000000	0.2650000
38379.0000000	0.2700000
38380.0000000	0.2650000
38381.0000000	0.2700000
38382.0000000	0.2650000
38383.0000000	0.2750000
38384.0000000	0.2750000
38385.0000000	0.2650000
38386.0000000	0.2650000
38387.0000000	0.2650000
38388.0000000	0.2700000
38389.0000000	0.2650000
38390.0000000	0.2650000
38391.0000000	0.2700000
38392.0000000	0.2750000
38393.0000000	0.2700000
38394.0000000	0.2700000
38395.0000000	0.2700000
38396.0000000	0.2650000
38397.0000000	0.2650000
38398.0000000	0.2700000
38399.0000000	0.2750000
38400.0000000	0.2650000
38401.0000000	0.2600000
38402.0000000	0.2700000
38403.0000000	0.2750000
38404.0000000	0.2633333
38405.0000000	0.2700000
38406.0000000	0.2750000
38407.0000000	0.2750000
38408.0000000	0.2750000
38409.0000000	0.2800000
38410.0000000	0.2750000
38411.0000000	0.2800000
38412.0000000	0.2850000
38413.0000000	0.2750000
38414.0000000	0.2800000
38415.0000000	0.2750000
38416.0000000	0.2750000
38417.0000000	0.2800000

38418.0000000	0.2850000
38419.0000000	0.2800000
38420.0000000	0.2800000
38421.0000000	0.2750000
38422.0000000	0.2800000
38423.0000000	0.2800000
38424.0000000	0.2850000
38425.0000000	0.2800000
38426.0000000	0.2800000
38427.0000000	0.2850000
38428.0000000	0.2700000
38429.0000000	0.2800000
38430.0000000	0.2800000
38431.0000000	0.2750000
38432.0000000	0.2750000
38433.0000000	0.2850000
38434.0000000	0.2800000
38435.0000000	0.2850000
38436.0000000	0.2800000
38437.0000000	0.2850000
38438.0000000	0.2800000
38439.0000000	0.2800000
38440.0000000	0.2700000
38441.0000000	0.2850000
38442.0000000	0.2900000
38443.0000000	0.2850000
38444.0000000	0.2800000
38445.0000000	0.2900000
38446.0000000	0.2850000
38447.0000000	0.2900000
38448.0000000	0.2750000
38449.0000000	0.2900000
38450.0000000	0.2900000
38451.0000000	0.2850000
38452.0000000	0.2850000
38453.0000000	0.2900000
38454.0000000	0.2850000
38455.0000000	0.2800000
38456.0000000	0.2900000
38457.0000000	0.2950000
38458.0000000	0.2900000
38459.0000000	0.2900000
38460.0000000	0.2900000
38461.0000000	0.2900000
38462.0000000	0.2950000
38463.0000000	0.3050000
38464.0000000	0.2933333
38465.0000000	0.2950000
38466.0000000	0.2950000
38467.0000000	0.3050000
38468.0000000	0.2900000
38469.0000000	0.2950000
38470.0000000	0.3050000
38471.0000000	0.3000000
38472.0000000	0.2950000
38473.0000000	0.3050000
38474.0000000	0.3050000

38475.0000000	0.2950000
38476.0000000	0.3000000
38477.0000000	0.3050000
38478.0000000	0.3050000
38479.0000000	0.3000000
38480.0000000	0.2950000
38481.0000000	0.3000000
38482.0000000	0.3050000
38483.0000000	0.3050000
38484.0000000	0.3050000
38485.0000000	0.3100000
38486.0000000	0.3100000
38487.0000000	0.3100000
38488.0000000	0.3050000
38489.0000000	0.3100000
38490.0000000	0.3050000
38491.0000000	0.3100000
38492.0000000	0.3100000
38493.0000000	0.3100000
38494.0000000	0.3100000
38495.0000000	0.3050000
38496.0000000	0.3150000
38497.0000000	0.3050000
38498.0000000	0.3100000
38499.0000000	0.3050000
38500.0000000	0.3100000
38501.0000000	0.3150000
38502.0000000	0.3050000
38503.0000000	0.3100000
38504.0000000	0.3000000
38505.0000000	0.3150000
38506.0000000	0.3150000
38507.0000000	0.3150000
38508.0000000	0.3050000
38509.0000000	0.3150000
38510.0000000	0.3150000
38511.0000000	0.3050000
38512.0000000	0.3050000
38513.0000000	0.3050000
38514.0000000	0.3200000
38515.0000000	0.3150000
38516.0000000	0.3200000
38517.0000000	0.3050000
38518.0000000	0.3150000
38519.0000000	0.3200000
38520.0000000	0.3100000
38521.0000000	0.3150000
38522.0000000	0.3100000
38523.0000000	0.3100000
38524.0000000	0.3100000
38525.0000000	0.3133333
38526.0000000	0.3100000
38527.0000000	0.3000000
38528.0000000	0.3000000
38529.0000000	0.3000000
38530.0000000	0.3050000
38531.0000000	0.3050000

38532.0000000	0.3000000
38533.0000000	0.3000000
38534.0000000	0.3050000
38535.0000000	0.3000000
38536.0000000	0.3050000
38537.0000000	0.2900000
38538.0000000	0.2950000
38539.0000000	0.2900000
38540.0000000	0.3000000
38541.0000000	0.3000000
38542.0000000	0.2950000
38543.0000000	0.2950000
38544.0000000	0.2850000
38545.0000000	0.2950000
38546.0000000	0.2900000
38547.0000000	0.3000000
38548.0000000	0.2850000
38549.0000000	0.2950000
38550.0000000	0.2950000
38551.0000000	0.2950000
38552.0000000	0.3000000
38553.0000000	0.2950000
38554.0000000	0.2900000
38555.0000000	0.2900000
38556.0000000	0.2950000
38557.0000000	0.2950000
38558.0000000	0.2900000
38559.0000000	0.2850000
38560.0000000	0.2950000
38561.0000000	0.2850000
38562.0000000	0.2950000
38563.0000000	0.2950000
38564.0000000	0.2950000
38565.0000000	0.2950000
38566.0000000	0.3000000
38567.0000000	0.2900000
38568.0000000	0.2950000
38569.0000000	0.2900000
38570.0000000	0.2950000
38571.0000000	0.3000000
38572.0000000	0.2950000
38573.0000000	0.2900000
38574.0000000	0.2950000
38575.0000000	0.2900000
38576.0000000	0.3000000
38577.0000000	0.2850000
38578.0000000	0.2950000
38579.0000000	0.2850000
38580.0000000	0.3000000
38581.0000000	0.2950000
38582.0000000	0.2950000
38583.0000000	0.2950000
38584.0000000	0.2950000
38585.0000000	0.2933333
38586.0000000	0.2950000
38587.0000000	0.2900000
38588.0000000	0.2950000

38589.0000000	0.2850000
38590.0000000	0.2900000
38591.0000000	0.2850000
38592.0000000	0.3000000
38593.0000000	0.2900000
38594.0000000	0.3000000
38595.0000000	0.2950000
38596.0000000	0.2900000
38597.0000000	0.2900000
38598.0000000	0.2950000
38599.0000000	0.3000000
38600.0000000	0.2900000
38601.0000000	0.3000000
38602.0000000	0.2850000
38603.0000000	0.2900000
38604.0000000	0.2900000
38605.0000000	0.3000000
38606.0000000	0.2950000
38607.0000000	0.2950000
38608.0000000	0.3000000
38609.0000000	0.2850000
38610.0000000	0.2950000
38611.0000000	0.2900000
38612.0000000	0.3050000
38613.0000000	0.3000000
38614.0000000	0.3050000
38615.0000000	0.2900000
38616.0000000	0.3100000
38617.0000000	0.2900000
38618.0000000	0.3050000
38619.0000000	0.3000000
38620.0000000	0.3050000
38621.0000000	0.2950000
38622.0000000	0.3050000
38623.0000000	0.2950000
38624.0000000	0.3050000
38625.0000000	0.2900000
38626.0000000	0.2950000
38627.0000000	0.2950000
38628.0000000	0.2950000
38629.0000000	0.3050000
38630.0000000	0.2950000
38631.0000000	0.3100000
38632.0000000	0.2900000
38633.0000000	0.3000000
38634.0000000	0.2850000
38635.0000000	0.3000000
38636.0000000	0.3000000
38637.0000000	0.2950000
38638.0000000	0.2950000
38639.0000000	0.2950000
38640.0000000	0.3000000
38641.0000000	0.3050000
38642.0000000	0.2950000
38643.0000000	0.2900000
38644.0000000	0.2850000
38645.0000000	0.2900000

38646.0000000	0.2833333
38647.0000000	0.2900000
38648.0000000	0.2900000
38649.0000000	0.2900000
38650.0000000	0.2900000
38651.0000000	0.2850000
38652.0000000	0.2900000
38653.0000000	0.2950000
38654.0000000	0.2850000
38655.0000000	0.2900000
38656.0000000	0.2850000
38657.0000000	0.2850000
38658.0000000	0.2850000
38659.0000000	0.2900000
38660.0000000	0.2900000
38661.0000000	0.2850000
38662.0000000	0.2800000
38663.0000000	0.2900000
38664.0000000	0.2800000
38665.0000000	0.2850000
38666.0000000	0.2850000
38667.0000000	0.2850000
38668.0000000	0.2850000
38669.0000000	0.2850000
38670.0000000	0.2850000
38671.0000000	0.2850000
38672.0000000	0.2850000
38673.0000000	0.2850000
38674.0000000	0.2800000
38675.0000000	0.2750000
38676.0000000	0.2800000
38677.0000000	0.2850000
38678.0000000	0.2800000
38679.0000000	0.2800000
38680.0000000	0.2850000
38681.0000000	0.2850000
38682.0000000	0.2750000
38683.0000000	0.2800000
38684.0000000	0.2850000
38685.0000000	0.2900000
38686.0000000	0.2750000
38687.0000000	0.2850000
38688.0000000	0.2850000
38689.0000000	0.2800000
38690.0000000	0.2850000
38691.0000000	0.2800000
38692.0000000	0.2800000
38693.0000000	0.2850000
38694.0000000	0.2850000
38695.0000000	0.2850000
38696.0000000	0.2750000
38697.0000000	0.2850000
38698.0000000	0.2700000
38699.0000000	0.2750000
38700.0000000	0.2750000
38701.0000000	0.2750000
38702.0000000	0.2800000

38703.0000000	0.2750000
38704.0000000	0.2800000
38705.0000000	0.2733333
38706.0000000	0.2850000
38707.0000000	0.2850000
38708.0000000	0.2800000
38709.0000000	0.2750000
38710.0000000	0.2750000
38711.0000000	0.2800000
38712.0000000	0.2700000
38713.0000000	0.2850000
38714.0000000	0.2650000
38715.0000000	0.2800000
38716.0000000	0.2750000
38717.0000000	0.2800000
38718.0000000	0.2750000
38719.0000000	0.2750000
38720.0000000	0.2800000
38721.0000000	0.2700000
38722.0000000	0.2800000
38723.0000000	0.2700000
38724.0000000	0.2900000
38725.0000000	0.2700000
38726.0000000	0.2800000
38727.0000000	0.2700000
38728.0000000	0.2850000
38729.0000000	0.2700000
38730.0000000	0.2800000
38731.0000000	0.2700000
38732.0000000	0.2750000
38733.0000000	0.2650000
38734.0000000	0.2750000
38735.0000000	0.2700000
38736.0000000	0.2700000
38737.0000000	0.2800000
38738.0000000	0.2750000
38739.0000000	0.2800000
38740.0000000	0.2750000
38741.0000000	0.2950000
38742.0000000	0.2750000
38743.0000000	0.2800000
38744.0000000	0.2750000
38745.0000000	0.2700000
38746.0000000	0.2700000
38747.0000000	0.2800000
38748.0000000	0.2750000
38749.0000000	0.2750000
38750.0000000	0.2750000
38751.0000000	0.2750000
38752.0000000	0.2650000
38753.0000000	0.2800000
38754.0000000	0.2700000
38755.0000000	0.2700000
38756.0000000	0.2750000
38757.0000000	0.2750000
38758.0000000	0.2750000
38759.0000000	0.2750000

38760.0000000	0.2650000
38761.0000000	0.2700000
38762.0000000	0.2700000
38763.0000000	0.2750000
38764.0000000	0.2800000
38765.0000000	0.2700000
38766.0000000	0.2666667
38767.0000000	0.2700000
38768.0000000	0.2700000
38769.0000000	0.2800000
38770.0000000	0.2650000
38771.0000000	0.2700000
38772.0000000	0.2700000
38773.0000000	0.2700000
38774.0000000	0.2750000
38775.0000000	0.2700000
38776.0000000	0.2700000
38777.0000000	0.2750000
38778.0000000	0.2850000
38779.0000000	0.2750000
38780.0000000	0.2800000
38781.0000000	0.2800000
38782.0000000	0.2750000
38783.0000000	0.2800000
38784.0000000	0.2750000
38785.0000000	0.2750000
38786.0000000	0.2800000
38787.0000000	0.2750000
38788.0000000	0.2750000
38789.0000000	0.2800000
38790.0000000	0.2850000
38791.0000000	0.2800000
38792.0000000	0.2700000
38793.0000000	0.2800000
38794.0000000	0.2800000
38795.0000000	0.2850000
38796.0000000	0.2866667
38797.0000000	0.2850000
38798.0000000	0.2800000
38799.0000000	0.2800000
38800.0000000	0.2800000
38801.0000000	0.2850000
38802.0000000	0.2850000
38803.0000000	0.2850000
38804.0000000	0.2750000
38805.0000000	0.2750000
38806.0000000	0.2900000
38807.0000000	0.2800000
38808.0000000	0.2800000
38809.0000000	0.2850000
38810.0000000	0.2800000
38811.0000000	0.2850000
38812.0000000	0.2850000
38813.0000000	0.2850000
38814.0000000	0.2800000
38815.0000000	0.2800000
38816.0000000	0.2850000

38817.0000000	0.2800000
38818.0000000	0.2800000
38819.0000000	0.2750000
38820.0000000	0.2800000
38821.0000000	0.2800000
38822.0000000	0.2750000
38823.0000000	0.2850000
38824.0000000	0.2750000
38825.0000000	0.2700000
38826.0000000	0.2766667
38827.0000000	0.2850000
38828.0000000	0.2850000
38829.0000000	0.2700000
38830.0000000	0.2800000
38831.0000000	0.2750000
38832.0000000	0.2750000
38833.0000000	0.2800000
38834.0000000	0.2750000
38835.0000000	0.2800000
38836.0000000	0.2750000
38837.0000000	0.2800000
38838.0000000	0.2850000
38839.0000000	0.2750000
38840.0000000	0.2800000
38841.0000000	0.2800000
38842.0000000	0.2750000
38843.0000000	0.2750000
38844.0000000	0.2750000
38845.0000000	0.2700000
38846.0000000	0.2800000
38847.0000000	0.2750000
38848.0000000	0.2750000
38849.0000000	0.2700000
38850.0000000	0.2650000
38851.0000000	0.2750000
38852.0000000	0.2800000
38853.0000000	0.2800000
38854.0000000	0.2700000
38855.0000000	0.2750000
38856.0000000	0.2750000
38857.0000000	0.2800000
38858.0000000	0.2750000
38859.0000000	0.2750000
38860.0000000	0.2750000
38861.0000000	0.2800000
38862.0000000	0.2800000
38863.0000000	0.2700000
38864.0000000	0.2900000
38865.0000000	0.2850000
38866.0000000	0.2800000
38867.0000000	0.2800000
38868.0000000	0.2800000
38869.0000000	0.2800000
38870.0000000	0.2800000
38871.0000000	0.2700000
38872.0000000	0.2800000
38873.0000000	0.2800000

38874.0000000	0.2750000
38875.0000000	0.2800000
38876.0000000	0.2900000
38877.0000000	0.2800000
38878.0000000	0.2900000
38879.0000000	0.2850000
38880.0000000	0.2700000
38881.0000000	0.2850000
38882.0000000	0.2750000
38883.0000000	0.2800000
38884.0000000	0.2750000
38885.0000000	0.2800000
38886.0000000	0.2700000
38887.0000000	0.2733333
38888.0000000	0.2850000
38889.0000000	0.2800000
38890.0000000	0.2800000
38891.0000000	0.2700000
38892.0000000	0.2700000
38893.0000000	0.2800000
38894.0000000	0.2700000
38895.0000000	0.2800000
38896.0000000	0.2700000
38897.0000000	0.2750000
38898.0000000	0.2750000
38899.0000000	0.2800000
38900.0000000	0.2800000
38901.0000000	0.2700000
38902.0000000	0.2700000
38903.0000000	0.2700000
38904.0000000	0.2800000
38905.0000000	0.2800000
38906.0000000	0.2800000
38907.0000000	0.2750000
38908.0000000	0.2750000
38909.0000000	0.2750000
38910.0000000	0.2850000
38911.0000000	0.2800000
38912.0000000	0.2800000
38913.0000000	0.2800000
38914.0000000	0.2800000
38915.0000000	0.2800000
38916.0000000	0.2850000
38917.0000000	0.2866667
38918.0000000	0.2800000
38919.0000000	0.2800000
38920.0000000	0.2750000
38921.0000000	0.2850000
38922.0000000	0.2850000
38923.0000000	0.2850000
38924.0000000	0.2800000
38925.0000000	0.2850000
38926.0000000	0.2900000
38927.0000000	0.2850000
38928.0000000	0.2950000
38929.0000000	0.2850000
38930.0000000	0.2900000

38931.0000000	0.2750000
38932.0000000	0.2850000
38933.0000000	0.2950000
38934.0000000	0.2800000
38935.0000000	0.2900000
38936.0000000	0.2850000
38937.0000000	0.2850000
38938.0000000	0.2850000
38939.0000000	0.2850000
38940.0000000	0.2800000
38941.0000000	0.2900000
38942.0000000	0.2900000
38943.0000000	0.2850000
38944.0000000	0.2800000
38945.0000000	0.2800000
38946.0000000	0.2850000
38947.0000000	0.2800000
38948.0000000	0.2700000
38949.0000000	0.2800000
38950.0000000	0.2800000
38951.0000000	0.2700000
38952.0000000	0.2650000
38953.0000000	0.2800000
38954.0000000	0.2700000
38955.0000000	0.2650000
38956.0000000	0.2700000
38957.0000000	0.2700000
38958.0000000	0.2700000
38959.0000000	0.2600000
38960.0000000	0.2700000
38961.0000000	0.2650000
38962.0000000	0.2600000
38963.0000000	0.2700000
38964.0000000	0.2550000
38965.0000000	0.2600000
38966.0000000	0.2650000
38967.0000000	0.2600000
38968.0000000	0.2700000
38969.0000000	0.2600000
38970.0000000	0.2700000
38971.0000000	0.2650000
38972.0000000	0.2700000
38973.0000000	0.2600000
38974.0000000	0.2600000
38975.0000000	0.2700000
38976.0000000	0.2700000
38977.0000000	0.2600000
38978.0000000	0.2666667
38979.0000000	0.2650000
38980.0000000	0.2700000
38981.0000000	0.2700000
38982.0000000	0.2650000
38983.0000000	0.2700000
38984.0000000	0.2700000
38985.0000000	0.2650000
38986.0000000	0.2600000
38987.0000000	0.2650000

38988.0000000	0.2600000
38989.0000000	0.2550000
38990.0000000	0.2550000
38991.0000000	0.2650000
38992.0000000	0.2700000
38993.0000000	0.2650000
38994.0000000	0.2650000
38995.0000000	0.2700000
38996.0000000	0.2600000
38997.0000000	0.2700000
38998.0000000	0.2700000
38999.0000000	0.2750000
39000.0000000	0.2700000
39001.0000000	0.2650000
39002.0000000	0.2650000
39003.0000000	0.2650000
39004.0000000	0.2650000
39005.0000000	0.2600000
39006.0000000	0.2650000
39007.0000000	0.2650000
39008.0000000	0.2666667
39009.0000000	0.2650000
39010.0000000	0.2650000
39011.0000000	0.2650000
39012.0000000	0.2650000
39013.0000000	0.2700000
39014.0000000	0.2700000
39015.0000000	0.2650000
39016.0000000	0.2650000
39017.0000000	0.2650000
39018.0000000	0.2700000
39019.0000000	0.2650000
39020.0000000	0.2650000
39021.0000000	0.2700000
39022.0000000	0.2650000
39023.0000000	0.2700000
39024.0000000	0.2650000
39025.0000000	0.2750000
39026.0000000	0.2750000
39027.0000000	0.2650000
39028.0000000	0.2750000
39029.0000000	0.2700000
39030.0000000	0.2700000
39031.0000000	0.2650000
39032.0000000	0.2650000
39033.0000000	0.2700000
39034.0000000	0.2600000
39035.0000000	0.2700000
39036.0000000	0.2650000
39037.0000000	0.2733333
39038.0000000	0.2650000
39039.0000000	0.2700000
39040.0000000	0.2700000
39041.0000000	0.2650000
39042.0000000	0.2700000
39043.0000000	0.2700000
39044.0000000	0.2700000

39045.0000000	0.2750000
39046.0000000	0.2600000
39047.0000000	0.2700000
39048.0000000	0.2750000
39049.0000000	0.2650000
39050.0000000	0.2650000
39051.0000000	0.2700000
39052.0000000	0.2650000
39053.0000000	0.2750000
39054.0000000	0.2700000
39055.0000000	0.2650000
39056.0000000	0.2650000
39057.0000000	0.2700000
39058.0000000	0.2650000
39059.0000000	0.2750000
39060.0000000	0.2650000
39061.0000000	0.2750000
39062.0000000	0.2700000
39063.0000000	0.2650000
39064.0000000	0.2750000
39065.0000000	0.2700000
39066.0000000	0.2700000
39067.0000000	0.2650000
39068.0000000	0.2666667
39069.0000000	0.2650000
39070.0000000	0.2700000
39071.0000000	0.2700000
39072.0000000	0.2650000
39073.0000000	0.2700000
39074.0000000	0.2650000
39075.0000000	0.2600000
39076.0000000	0.2700000
39077.0000000	0.2650000
39078.0000000	0.2700000
39079.0000000	0.2650000
39080.0000000	0.2650000
39081.0000000	0.2650000
39082.0000000	0.2600000
39083.0000000	0.2650000
39084.0000000	0.2600000
39085.0000000	0.2750000
39086.0000000	0.2600000
39087.0000000	0.2700000
39088.0000000	0.2600000
39089.0000000	0.2650000
39090.0000000	0.2600000
39091.0000000	0.2700000
39092.0000000	0.2650000
39093.0000000	0.2600000
39094.0000000	0.2600000
39095.0000000	0.2550000
39096.0000000	0.2550000
39097.0000000	0.2600000
39098.0000000	0.2600000
39099.0000000	0.2500000
39100.0000000	0.2450000
39101.0000000	0.2550000

39102.0000000	0.2550000
39103.0000000	0.2550000
39104.0000000	0.2550000
39105.0000000	0.2550000
39106.0000000	0.2500000
39107.0000000	0.2500000
39108.0000000	0.2600000
39109.0000000	0.2500000
39110.0000000	0.2650000
39111.0000000	0.2500000
39112.0000000	0.2650000
39113.0000000	0.2500000
39114.0000000	0.2550000
39115.0000000	0.2550000
39116.0000000	0.2650000
39117.0000000	0.2550000
39118.0000000	0.2500000
39119.0000000	0.2600000
39120.0000000	0.2450000
39121.0000000	0.2500000
39122.0000000	0.2500000
39123.0000000	0.2450000
39124.0000000	0.2550000
39125.0000000	0.2550000
39126.0000000	0.2450000
39127.0000000	0.2450000
39128.0000000	0.2400000
39129.0000000	0.2400000
39130.0000000	0.2500000
39131.0000000	0.2450000
39132.0000000	0.2400000
39133.0000000	0.2500000
39134.0000000	0.2600000
39135.0000000	0.2450000
39136.0000000	0.2450000
39137.0000000	0.2500000
39138.0000000	0.2450000
39139.0000000	0.2500000
39140.0000000	0.2500000
39141.0000000	0.2400000
39142.0000000	0.2500000
39143.0000000	0.2450000
39144.0000000	0.2450000
39145.0000000	0.2400000
39146.0000000	0.2450000
39147.0000000	0.2450000
39148.0000000	0.2500000
39149.0000000	0.2450000
39150.0000000	0.2500000
39151.0000000	0.2500000
39152.0000000	0.2550000
39153.0000000	0.2450000
39154.0000000	0.2450000
39155.0000000	0.2450000
39156.0000000	0.2500000
39157.0000000	0.2450000
39158.0000000	0.2566667

39159.0000000	0.2500000
39160.0000000	0.2500000
39161.0000000	0.2400000
39162.0000000	0.2400000
39163.0000000	0.2450000
39164.0000000	0.2500000
39165.0000000	0.2500000
39166.0000000	0.2500000
39167.0000000	0.2450000
39168.0000000	0.2500000
39169.0000000	0.2500000
39170.0000000	0.2450000
39171.0000000	0.2500000
39172.0000000	0.2450000
39173.0000000	0.2450000
39174.0000000	0.2500000
39175.0000000	0.2500000
39176.0000000	0.2550000
39177.0000000	0.2450000
39178.0000000	0.2450000
39179.0000000	0.2550000
39180.0000000	0.2450000
39181.0000000	0.2400000
39182.0000000	0.2450000
39183.0000000	0.2450000
39184.0000000	0.2550000
39185.0000000	0.2600000
39186.0000000	0.2500000
39187.0000000	0.2550000
39188.0000000	0.2533333
39189.0000000	0.2500000
39190.0000000	0.2550000
39191.0000000	0.2500000
39192.0000000	0.2650000
39193.0000000	0.2500000
39194.0000000	0.2500000
39195.0000000	0.2600000
39196.0000000	0.2600000
39197.0000000	0.2500000
39198.0000000	0.2500000
39199.0000000	0.2500000
39200.0000000	0.2550000
39201.0000000	0.2500000
39202.0000000	0.2500000
39203.0000000	0.2400000
39204.0000000	0.2450000
39205.0000000	0.2450000
39206.0000000	0.2400000
39207.0000000	0.2350000
39208.0000000	0.2450000
39209.0000000	0.2550000
39210.0000000	0.2450000
39211.0000000	0.2450000
39212.0000000	0.2500000
39213.0000000	0.2500000
39214.0000000	0.2450000
39215.0000000	0.2350000

39216.0000000	0.2400000
39217.0000000	0.2400000
39218.0000000	0.2500000
39219.0000000	0.2466667
39220.0000000	0.2400000
39221.0000000	0.2400000
39222.0000000	0.2450000
39223.0000000	0.2350000
39224.0000000	0.2400000
39225.0000000	0.2400000
39226.0000000	0.2450000
39227.0000000	0.2350000
39228.0000000	0.2450000
39229.0000000	0.2400000
39230.0000000	0.2450000
39231.0000000	0.2400000
39232.0000000	0.2350000
39233.0000000	0.2350000
39234.0000000	0.2350000
39235.0000000	0.2300000
39236.0000000	0.2400000
39237.0000000	0.2350000
39238.0000000	0.2300000
39239.0000000	0.2350000
39240.0000000	0.2350000
39241.0000000	0.2250000
39242.0000000	0.2300000
39243.0000000	0.2300000
39244.0000000	0.2250000
39245.0000000	0.2300000
39246.0000000	0.2150000
39247.0000000	0.2250000
39248.0000000	0.2250000
39249.0000000	0.2300000
39250.0000000	0.2250000
39251.0000000	0.2200000
39252.0000000	0.2200000
39253.0000000	0.2200000
39254.0000000	0.2300000
39255.0000000	0.2200000
39256.0000000	0.2250000
39257.0000000	0.2300000
39258.0000000	0.2300000
39259.0000000	0.2300000
39260.0000000	0.2250000
39261.0000000	0.2200000
39262.0000000	0.2250000
39263.0000000	0.2250000
39264.0000000	0.2150000
39265.0000000	0.2250000
39266.0000000	0.2200000
39267.0000000	0.2300000
39268.0000000	0.2250000
39269.0000000	0.2250000
39270.0000000	0.2300000
39271.0000000	0.2250000
39272.0000000	0.2250000

39273.0000000	0.2250000
39274.0000000	0.2250000
39275.0000000	0.2250000
39276.0000000	0.2300000
39277.0000000	0.2300000
39278.0000000	0.2350000
39279.0000000	0.2233333
39280.0000000	0.2300000
39281.0000000	0.2350000
39282.0000000	0.2350000
39283.0000000	0.2250000
39284.0000000	0.2350000
39285.0000000	0.2300000
39286.0000000	0.2350000
39287.0000000	0.2300000
39288.0000000	0.2250000
39289.0000000	0.2200000
39290.0000000	0.2250000
39291.0000000	0.2350000
39292.0000000	0.2300000
39293.0000000	0.2300000
39294.0000000	0.2250000
39295.0000000	0.2250000
39296.0000000	0.2250000
39297.0000000	0.2200000
39298.0000000	0.2200000
39299.0000000	0.2200000
39300.0000000	0.2250000
39301.0000000	0.2200000
39302.0000000	0.2300000
39303.0000000	0.2200000
39304.0000000	0.2200000
39305.0000000	0.2200000
39306.0000000	0.2150000
39307.0000000	0.2300000
39308.0000000	0.2250000
39309.0000000	0.2266667
39310.0000000	0.2250000
39311.0000000	0.2200000
39312.0000000	0.2300000
39313.0000000	0.2300000
39314.0000000	0.2200000
39315.0000000	0.2200000
39316.0000000	0.2100000
39317.0000000	0.2200000
39318.0000000	0.2200000
39319.0000000	0.2250000
39320.0000000	0.2100000
39321.0000000	0.2250000
39322.0000000	0.2150000
39323.0000000	0.2200000
39324.0000000	0.2150000
39325.0000000	0.2100000
39326.0000000	0.2150000
39327.0000000	0.2050000
39328.0000000	0.2100000
39329.0000000	0.2050000

39330.0000000	0.2100000
39331.0000000	0.2100000
39332.0000000	0.2100000
39333.0000000	0.2000000
39334.0000000	0.2000000
39335.0000000	0.2050000
39336.0000000	0.2000000
39337.0000000	0.2000000
39338.0000000	0.2000000
39339.0000000	0.2100000
39340.0000000	0.2000000
39341.0000000	0.2000000
39342.0000000	0.2050000
39343.0000000	0.2050000
39344.0000000	0.2050000
39345.0000000	0.2000000
39346.0000000	0.1950000
39347.0000000	0.2000000
39348.0000000	0.1900000
39349.0000000	0.1950000
39350.0000000	0.2050000
39351.0000000	0.1950000
39352.0000000	0.2050000
39353.0000000	0.2000000
39354.0000000	0.2000000
39355.0000000	0.2000000
39356.0000000	0.1950000
39357.0000000	0.1900000
39358.0000000	0.2000000
39359.0000000	0.1900000
39360.0000000	0.1950000
39361.0000000	0.1950000
39362.0000000	0.1900000
39363.0000000	0.1950000
39364.0000000	0.2000000
39365.0000000	0.2000000
39366.0000000	0.1900000
39367.0000000	0.1950000
39368.0000000	0.1950000
39369.0000000	0.1900000
39370.0000000	0.2000000
39371.0000000	0.1900000
39372.0000000	0.1900000
39373.0000000	0.1950000
39374.0000000	0.1950000
39375.0000000	0.2000000
39376.0000000	0.1900000
39377.0000000	0.1950000
39378.0000000	0.1850000
39379.0000000	0.1900000
39380.0000000	0.1800000
39381.0000000	0.1800000
39382.0000000	0.1800000
39383.0000000	0.1850000
39384.0000000	0.1900000
39385.0000000	0.1850000
39386.0000000	0.1850000

39387.0000000	0.1800000
39388.0000000	0.1850000
39389.0000000	0.1750000
39390.0000000	0.1800000
39391.0000000	0.1750000
39392.0000000	0.1800000
39393.0000000	0.1750000
39394.0000000	0.1800000
39395.0000000	0.1750000
39396.0000000	0.1700000
39397.0000000	0.1800000
39398.0000000	0.1700000
39399.0000000	0.1766667
39400.0000000	0.1750000
39401.0000000	0.1650000
39402.0000000	0.1750000
39403.0000000	0.1700000
39404.0000000	0.1700000
39405.0000000	0.1700000
39406.0000000	0.1800000
39407.0000000	0.1800000
39408.0000000	0.1650000
39409.0000000	0.1700000
39410.0000000	0.1650000
39411.0000000	0.1750000
39412.0000000	0.1700000
39413.0000000	0.1800000
39414.0000000	0.1700000
39415.0000000	0.1700000
39416.0000000	0.1750000
39417.0000000	0.1750000
39418.0000000	0.1650000
39419.0000000	0.1700000
39420.0000000	0.1700000
39421.0000000	0.1750000
39422.0000000	0.1700000
39423.0000000	0.1700000
39424.0000000	0.1650000
39425.0000000	0.1700000
39426.0000000	0.1750000
39427.0000000	0.1750000
39428.0000000	0.1700000
39429.0000000	0.1600000
39430.0000000	0.1733333
39431.0000000	0.1700000
39432.0000000	0.1650000
39433.0000000	0.1650000
39434.0000000	0.1700000
39435.0000000	0.1700000
39436.0000000	0.1650000
39437.0000000	0.1650000
39438.0000000	0.1750000
39439.0000000	0.1650000
39440.0000000	0.1700000
39441.0000000	0.1600000
39442.0000000	0.1650000
39443.0000000	0.1650000

39444.0000000	0.1650000
39445.0000000	0.1650000
39446.0000000	0.1600000
39447.0000000	0.1600000
39448.0000000	0.1700000
39449.0000000	0.1700000
39450.0000000	0.1650000
39451.0000000	0.1700000
39452.0000000	0.1700000
39453.0000000	0.1650000
39454.0000000	0.1650000
39455.0000000	0.1700000
39456.0000000	0.1600000
39457.0000000	0.1650000
39458.0000000	0.1650000
39459.0000000	0.1600000
39460.0000000	0.1600000
39461.0000000	0.1566667
39462.0000000	0.1550000
39463.0000000	0.1650000
39464.0000000	0.1600000
39465.0000000	0.1650000
39466.0000000	0.1600000
39467.0000000	0.1550000
39468.0000000	0.1600000
39469.0000000	0.1650000
39470.0000000	0.1500000
39471.0000000	0.1550000
39472.0000000	0.1550000
39473.0000000	0.1550000
39474.0000000	0.1500000
39475.0000000	0.1450000
39476.0000000	0.1600000
39477.0000000	0.1550000
39478.0000000	0.1550000
39479.0000000	0.1550000
39480.0000000	0.1550000
39481.0000000	0.1500000
39482.0000000	0.1550000
39483.0000000	0.1500000
39484.0000000	0.1500000
39485.0000000	0.1450000
39486.0000000	0.1450000
39487.0000000	0.1400000
39488.0000000	0.1500000
39489.0000000	0.1550000
39490.0000000	0.1533333
39491.0000000	0.1450000
39492.0000000	0.1400000
39493.0000000	0.1450000
39494.0000000	0.1500000
39495.0000000	0.1450000
39496.0000000	0.1600000
39497.0000000	0.1450000
39498.0000000	0.1550000
39499.0000000	0.1550000
39500.0000000	0.1500000

39501.0000000	0.1500000
39502.0000000	0.1500000
39503.0000000	0.1500000
39504.0000000	0.1550000
39505.0000000	0.1450000
39506.0000000	0.1550000
39507.0000000	0.1550000
39508.0000000	0.1600000
39509.0000000	0.1550000
39510.0000000	0.1550000
39511.0000000	0.1550000
39512.0000000	0.1500000
39513.0000000	0.1500000
39514.0000000	0.1450000
39515.0000000	0.1500000
39516.0000000	0.1550000
39517.0000000	0.1550000
39518.0000000	0.1550000
39519.0000000	0.1550000
39520.0000000	0.1500000
39521.0000000	0.1500000
39522.0000000	0.1550000
39523.0000000	0.1600000
39524.0000000	0.1450000
39525.0000000	0.1550000
39526.0000000	0.1550000
39527.0000000	0.1500000
39528.0000000	0.1500000
39529.0000000	0.1550000
39530.0000000	0.1450000
39531.0000000	0.1500000
39532.0000000	0.1550000
39533.0000000	0.1450000
39534.0000000	0.1500000
39535.0000000	0.1450000
39536.0000000	0.1500000
39537.0000000	0.1500000
39538.0000000	0.1550000
39539.0000000	0.1550000
39540.0000000	0.1550000
39541.0000000	0.1500000
39542.0000000	0.1450000
39543.0000000	0.1450000
39544.0000000	0.1450000
39545.0000000	0.1450000
39546.0000000	0.1450000
39547.0000000	0.1500000
39548.0000000	0.1500000
39549.0000000	0.1500000
39550.0000000	0.1550000
39551.0000000	0.1533333
39552.0000000	0.1450000
39553.0000000	0.1500000
39554.0000000	0.1400000
39555.0000000	0.1500000
39556.0000000	0.1500000
39557.0000000	0.1550000

39558.0000000	0.1400000
39559.0000000	0.1350000
39560.0000000	0.1450000
39561.0000000	0.1500000
39562.0000000	0.1500000
39563.0000000	0.1500000
39564.0000000	0.1500000
39565.0000000	0.1500000
39566.0000000	0.1450000
39567.0000000	0.1550000
39568.0000000	0.1500000
39569.0000000	0.1500000
39570.0000000	0.1550000
39571.0000000	0.1550000
39572.0000000	0.1500000
39573.0000000	0.1500000
39574.0000000	0.1500000
39575.0000000	0.1550000
39576.0000000	0.1500000
39577.0000000	0.1550000
39578.0000000	0.1500000
39579.0000000	0.1500000
39580.0000000	0.1500000
39581.0000000	0.1500000
39582.0000000	0.1550000
39583.0000000	0.1500000
39584.0000000	0.1400000
39585.0000000	0.1500000
39586.0000000	0.1450000
39587.0000000	0.1550000
39588.0000000	0.1500000
39589.0000000	0.1500000
39590.0000000	0.1500000
39591.0000000	0.1550000
39592.0000000	0.1450000
39593.0000000	0.1400000
39594.0000000	0.1500000
39595.0000000	0.1500000
39596.0000000	0.1550000
39597.0000000	0.1550000
39598.0000000	0.1500000
39599.0000000	0.1500000
39600.0000000	0.1500000
39601.0000000	0.1500000
39602.0000000	0.1450000
39603.0000000	0.1450000
39604.0000000	0.1500000
39605.0000000	0.1450000
39606.0000000	0.1450000
39607.0000000	0.1550000
39608.0000000	0.1450000
39609.0000000	0.1500000
39610.0000000	0.1450000
39611.0000000	0.1466667
39612.0000000	0.1450000
39613.0000000	0.1500000
39614.0000000	0.1500000

39615.0000000	0.1450000
39616.0000000	0.1450000
39617.0000000	0.1450000
39618.0000000	0.1350000
39619.0000000	0.1400000
39620.0000000	0.1400000
39621.0000000	0.1400000
39622.0000000	0.1350000
39623.0000000	0.1400000
39624.0000000	0.1500000
39625.0000000	0.1400000
39626.0000000	0.1500000
39627.0000000	0.1500000
39628.0000000	0.1400000
39629.0000000	0.1450000
39630.0000000	0.1350000
39631.0000000	0.1400000
39632.0000000	0.1450000
39633.0000000	0.1400000
39634.0000000	0.1450000
39635.0000000	0.1500000
39636.0000000	0.1400000
39637.0000000	0.1500000
39638.0000000	0.1350000
39639.0000000	0.1350000
39640.0000000	0.1500000
39641.0000000	0.1433333
39642.0000000	0.1400000
39643.0000000	0.1500000
39644.0000000	0.1400000
39645.0000000	0.1450000
39646.0000000	0.1400000
39647.0000000	0.1350000
39648.0000000	0.1450000
39649.0000000	0.1550000
39650.0000000	0.1400000
39651.0000000	0.1450000
39652.0000000	0.1450000
39653.0000000	0.1400000
39654.0000000	0.1450000
39655.0000000	0.1350000
39656.0000000	0.1300000
39657.0000000	0.1400000
39658.0000000	0.1400000
39659.0000000	0.1350000
39660.0000000	0.1350000
39661.0000000	0.1250000
39662.0000000	0.1300000
39663.0000000	0.1400000
39664.0000000	0.1350000
39665.0000000	0.1300000
39666.0000000	0.1400000
39667.0000000	0.1300000
39668.0000000	0.1250000
39669.0000000	0.1350000
39670.0000000	0.1300000
39671.0000000	0.1300000

39672.0000000	0.1366667
39673.0000000	0.1350000
39674.0000000	0.1300000
39675.0000000	0.1350000
39676.0000000	0.1350000
39677.0000000	0.1350000
39678.0000000	0.1400000
39679.0000000	0.1300000
39680.0000000	0.1350000
39681.0000000	0.1300000
39682.0000000	0.1350000
39683.0000000	0.1250000
39684.0000000	0.1250000
39685.0000000	0.1400000
39686.0000000	0.1400000
39687.0000000	0.1350000
39688.0000000	0.1350000
39689.0000000	0.1400000
39690.0000000	0.1300000
39691.0000000	0.1400000
39692.0000000	0.1400000
39693.0000000	0.1400000
39694.0000000	0.1450000
39695.0000000	0.1400000
39696.0000000	0.1500000
39697.0000000	0.1450000
39698.0000000	0.1400000
39699.0000000	0.1400000
39700.0000000	0.1300000
39701.0000000	0.1450000
39702.0000000	0.1400000
39703.0000000	0.1350000
39704.0000000	0.1400000
39705.0000000	0.1350000
39706.0000000	0.1450000
39707.0000000	0.1450000
39708.0000000	0.1400000
39709.0000000	0.1450000
39710.0000000	0.1400000
39711.0000000	0.1450000
39712.0000000	0.1450000
39713.0000000	0.1450000
39714.0000000	0.1450000
39715.0000000	0.1500000
39716.0000000	0.1500000
39717.0000000	0.1500000
39718.0000000	0.1450000
39719.0000000	0.1450000
39720.0000000	0.1500000
39721.0000000	0.1450000
39722.0000000	0.1450000
39723.0000000	0.1450000
39724.0000000	0.1450000
39725.0000000	0.1450000
39726.0000000	0.1450000
39727.0000000	0.1450000
39728.0000000	0.1450000

39729.0000000	0.1450000
39730.0000000	0.1500000
39731.0000000	0.1500000
39732.0000000	0.1500000
39733.0000000	0.1500000
39734.0000000	0.1400000
39735.0000000	0.1550000
39736.0000000	0.1500000
39737.0000000	0.1550000
39738.0000000	0.1500000
39739.0000000	0.1350000
39740.0000000	0.1600000
39741.0000000	0.1500000
39742.0000000	0.1450000
39743.0000000	0.1500000
39744.0000000	0.1500000
39745.0000000	0.1500000
39746.0000000	0.1500000
39747.0000000	0.1450000
39748.0000000	0.1500000
39749.0000000	0.1550000
39750.0000000	0.1500000
39751.0000000	0.1450000
39752.0000000	0.1600000
39753.0000000	0.1500000
39754.0000000	0.1600000
39755.0000000	0.1450000
39756.0000000	0.1600000
39757.0000000	0.1500000
39758.0000000	0.1600000
39759.0000000	0.1450000
39760.0000000	0.1500000
39761.0000000	0.1400000
39762.0000000	0.1566667
39763.0000000	0.1450000
39764.0000000	0.1450000
39765.0000000	0.1450000
39766.0000000	0.1500000
39767.0000000	0.1450000
39768.0000000	0.1450000
39769.0000000	0.1400000
39770.0000000	0.1350000
39771.0000000	0.1400000
39772.0000000	0.1400000
39773.0000000	0.1550000
39774.0000000	0.1450000
39775.0000000	0.1450000
39776.0000000	0.1350000
39777.0000000	0.1400000
39778.0000000	0.1450000
39779.0000000	0.1500000
39780.0000000	0.1450000
39781.0000000	0.1450000
39782.0000000	0.1500000
39783.0000000	0.1400000
39784.0000000	0.1400000
39785.0000000	0.1450000

39786.0000000	0.1550000
39787.0000000	0.1500000
39788.0000000	0.1450000
39789.0000000	0.1450000
39790.0000000	0.1450000
39791.0000000	0.1450000
39792.0000000	0.1450000
39793.0000000	0.1466667
39794.0000000	0.1500000
39795.0000000	0.1450000
39796.0000000	0.1450000
39797.0000000	0.1450000
39798.0000000	0.1500000
39799.0000000	0.1450000
39800.0000000	0.1450000
39801.0000000	0.1400000
39802.0000000	0.1550000
39803.0000000	0.1450000
39804.0000000	0.1500000
39805.0000000	0.1350000
39806.0000000	0.1450000
39807.0000000	0.1400000
39808.0000000	0.1400000
39809.0000000	0.1500000
39810.0000000	0.1400000
39811.0000000	0.1450000
39812.0000000	0.1400000
39813.0000000	0.1300000
39814.0000000	0.1350000
39815.0000000	0.1400000
39816.0000000	0.1450000
39817.0000000	0.1450000
39818.0000000	0.1400000
39819.0000000	0.1400000
39820.0000000	0.1300000
39821.0000000	0.1300000
39822.0000000	0.1300000
39823.0000000	0.1366667
39824.0000000	0.1300000
39825.0000000	0.1300000
39826.0000000	0.1300000
39827.0000000	0.1250000
39828.0000000	0.1400000
39829.0000000	0.1350000
39830.0000000	0.1400000
39831.0000000	0.1300000
39832.0000000	0.1200000
39833.0000000	0.1350000
39834.0000000	0.1300000
39835.0000000	0.1400000
39836.0000000	0.1300000
39837.0000000	0.1400000
39838.0000000	0.1300000
39839.0000000	0.1300000
39840.0000000	0.1300000
39841.0000000	0.1250000
39842.0000000	0.1250000

39843.0000000	0.1300000
39844.0000000	0.1400000
39845.0000000	0.1300000
39846.0000000	0.1350000
39847.0000000	0.1350000
39848.0000000	0.1300000
39849.0000000	0.1400000
39850.0000000	0.1350000
39851.0000000	0.1250000
39852.0000000	0.1333333
39853.0000000	0.1300000
39854.0000000	0.1350000
39855.0000000	0.1300000
39856.0000000	0.1350000
39857.0000000	0.1400000
39858.0000000	0.1250000
39859.0000000	0.1250000
39860.0000000	0.1350000
39861.0000000	0.1350000
39862.0000000	0.1250000
39863.0000000	0.1350000
39864.0000000	0.1300000
39865.0000000	0.1350000
39866.0000000	0.1400000
39867.0000000	0.1400000
39868.0000000	0.1300000
39869.0000000	0.1400000
39870.0000000	0.1450000
39871.0000000	0.1400000
39872.0000000	0.1400000
39873.0000000	0.1450000
39874.0000000	0.1400000
39875.0000000	0.1300000
39876.0000000	0.1300000
39877.0000000	0.1400000
39878.0000000	0.1450000
39879.0000000	0.1400000
39880.0000000	0.1400000
39881.0000000	0.1450000
39882.0000000	0.1400000
39883.0000000	0.1400000
39884.0000000	0.1400000
39885.0000000	0.1400000
39886.0000000	0.1400000
39887.0000000	0.1300000
39888.0000000	0.1350000
39889.0000000	0.1450000
39890.0000000	0.1450000
39891.0000000	0.1350000
39892.0000000	0.1350000
39893.0000000	0.1400000
39894.0000000	0.1400000
39895.0000000	0.1350000
39896.0000000	0.1350000
39897.0000000	0.1350000
39898.0000000	0.1300000
39899.0000000	0.1200000

39900.0000000	0.1200000
39901.0000000	0.1250000
39902.0000000	0.1250000
39903.0000000	0.1300000
39904.0000000	0.1250000
39905.0000000	0.1250000
39906.0000000	0.1300000
39907.0000000	0.1300000
39908.0000000	0.1250000
39909.0000000	0.1200000
39910.0000000	0.1200000
39911.0000000	0.1350000
39912.0000000	0.1200000
39913.0000000	0.1200000
39914.0000000	0.1266667
39915.0000000	0.1350000
39916.0000000	0.1200000
39917.0000000	0.1300000
39918.0000000	0.1250000
39919.0000000	0.1200000
39920.0000000	0.1250000
39921.0000000	0.1200000
39922.0000000	0.1150000
39923.0000000	0.1150000
39924.0000000	0.1250000
39925.0000000	0.1200000
39926.0000000	0.1200000
39927.0000000	0.1200000
39928.0000000	0.1200000
39929.0000000	0.1200000
39930.0000000	0.1150000
39931.0000000	0.1200000
39932.0000000	0.1250000
39933.0000000	0.1200000
39934.0000000	0.1250000
39935.0000000	0.1200000
39936.0000000	0.1200000
39937.0000000	0.1300000
39938.0000000	0.1250000
39939.0000000	0.1150000
39940.0000000	0.1200000
39941.0000000	0.1100000
39942.0000000	0.1150000
39943.0000000	0.1266667
39944.0000000	0.1200000
39945.0000000	0.1150000
39946.0000000	0.1200000
39947.0000000	0.1150000
39948.0000000	0.1150000
39949.0000000	0.1150000
39950.0000000	0.1150000
39951.0000000	0.1200000
39952.0000000	0.1150000
39953.0000000	0.1200000
39954.0000000	0.1150000
39955.0000000	0.1100000
39956.0000000	0.1150000

39957.0000000	0.1150000
39958.0000000	0.1200000
39959.0000000	0.1100000
39960.0000000	0.1150000
39961.0000000	0.1200000
39962.0000000	0.1200000
39963.0000000	0.1200000
39964.0000000	0.1200000
39965.0000000	0.1250000
39966.0000000	0.1100000
39967.0000000	0.1150000
39968.0000000	0.1100000
39969.0000000	0.1100000
39970.0000000	0.1150000
39971.0000000	0.1150000
39972.0000000	0.1100000
39973.0000000	0.1166667
39974.0000000	0.1200000
39975.0000000	0.1150000
39976.0000000	0.1150000
39977.0000000	0.1150000
39978.0000000	0.1150000
39979.0000000	0.1200000
39980.0000000	0.1200000
39981.0000000	0.1250000
39982.0000000	0.1150000
39983.0000000	0.1200000
39984.0000000	0.1250000
39985.0000000	0.1200000
39986.0000000	0.1150000
39987.0000000	0.1150000
39988.0000000	0.1300000
39989.0000000	0.1250000
39990.0000000	0.1150000
39991.0000000	0.1300000
39992.0000000	0.1200000
39993.0000000	0.1200000
39994.0000000	0.1100000
39995.0000000	0.1200000
39996.0000000	0.1250000
39997.0000000	0.1250000
39998.0000000	0.1250000
39999.0000000	0.1250000
40000.0000000	0.1300000
40001.0000000	0.1200000
40002.0000000	0.1100000
40003.0000000	0.1200000
40004.0000000	0.1200000
40005.0000000	0.1200000
40006.0000000	0.1200000
40007.0000000	0.1250000
40008.0000000	0.1200000
40009.0000000	0.1250000
40010.0000000	0.1150000
40011.0000000	0.1200000
40012.0000000	0.1250000
40013.0000000	0.1200000

40014.0000000	0.1250000
40015.0000000	0.1250000
40016.0000000	0.1350000
40017.0000000	0.1200000
40018.0000000	0.1150000
40019.0000000	0.1350000
40020.0000000	0.1250000
40021.0000000	0.1200000
40022.0000000	0.1200000
40023.0000000	0.1250000
40024.0000000	0.1250000
40025.0000000	0.1200000
40026.0000000	0.1200000
40027.0000000	0.1200000
40028.0000000	0.1250000
40029.0000000	0.1250000
40030.0000000	0.1150000
40031.0000000	0.1250000
40032.0000000	0.1200000
40033.0000000	0.1250000
40034.0000000	0.1200000
40035.0000000	0.1250000
40036.0000000	0.1200000
40037.0000000	0.1250000
40038.0000000	0.1250000
40039.0000000	0.1250000
40040.0000000	0.1250000
40041.0000000	0.1200000
40042.0000000	0.1300000
40043.0000000	0.1250000
40044.0000000	0.1250000
40045.0000000	0.1250000
40046.0000000	0.1250000
40047.0000000	0.1350000
40048.0000000	0.1250000
40049.0000000	0.1250000
40050.0000000	0.1300000
40051.0000000	0.1300000
40052.0000000	0.1250000
40053.0000000	0.1250000
40054.0000000	0.1250000
40055.0000000	0.1150000
40056.0000000	0.1350000
40057.0000000	0.1250000
40058.0000000	0.1350000
40059.0000000	0.1250000
40060.0000000	0.1300000
40061.0000000	0.1250000
40062.0000000	0.1300000
40063.0000000	0.1300000
40064.0000000	0.1266667
40065.0000000	0.1300000
40066.0000000	0.1350000
40067.0000000	0.1300000
40068.0000000	0.1350000
40069.0000000	0.1300000
40070.0000000	0.1250000

40071.0000000	0.1250000
40072.0000000	0.1300000
40073.0000000	0.1350000
40074.0000000	0.1300000
40075.0000000	0.1300000
40076.0000000	0.1350000
40077.0000000	0.1300000
40078.0000000	0.1300000
40079.0000000	0.1300000
40080.0000000	0.1300000
40081.0000000	0.1250000
40082.0000000	0.1250000
40083.0000000	0.1300000
40084.0000000	0.1200000
40085.0000000	0.1300000
40086.0000000	0.1200000
40087.0000000	0.1250000
40088.0000000	0.1300000
40089.0000000	0.1250000
40090.0000000	0.1300000
40091.0000000	0.1250000
40092.0000000	0.1300000
40093.0000000	0.1200000
40094.0000000	0.1233333
40095.0000000	0.1250000
40096.0000000	0.1200000
40097.0000000	0.1300000
40098.0000000	0.1300000
40099.0000000	0.1250000
40100.0000000	0.1200000
40101.0000000	0.1300000
40102.0000000	0.1300000
40103.0000000	0.1300000
40104.0000000	0.1200000
40105.0000000	0.1250000
40106.0000000	0.1250000
40107.0000000	0.1300000
40108.0000000	0.1250000
40109.0000000	0.1350000
40110.0000000	0.1200000
40111.0000000	0.1350000
40112.0000000	0.1250000
40113.0000000	0.1300000
40114.0000000	0.1250000
40115.0000000	0.1250000
40116.0000000	0.1250000
40117.0000000	0.1300000
40118.0000000	0.1200000
40119.0000000	0.1350000
40120.0000000	0.1300000
40121.0000000	0.1250000
40122.0000000	0.1200000
40123.0000000	0.1200000
40124.0000000	0.1250000
40125.0000000	0.1333333
40126.0000000	0.1250000
40127.0000000	0.1200000

40128.0000000	0.1200000
40129.0000000	0.1200000
40130.0000000	0.1250000
40131.0000000	0.1300000
40132.0000000	0.1250000
40133.0000000	0.1250000
40134.0000000	0.1300000
40135.0000000	0.1250000
40136.0000000	0.1300000
40137.0000000	0.1200000
40138.0000000	0.1200000
40139.0000000	0.1250000
40140.0000000	0.1300000
40141.0000000	0.1300000
40142.0000000	0.1250000
40143.0000000	0.1300000
40144.0000000	0.1300000
40145.0000000	0.1250000
40146.0000000	0.1350000
40147.0000000	0.1250000
40148.0000000	0.1300000
40149.0000000	0.1300000
40150.0000000	0.1300000
40151.0000000	0.1350000
40152.0000000	0.1350000
40153.0000000	0.1350000
40154.0000000	0.1300000
40155.0000000	0.1366667
40156.0000000	0.1400000
40157.0000000	0.1350000
40158.0000000	0.1350000
40159.0000000	0.1400000
40160.0000000	0.1400000
40161.0000000	0.1400000
40162.0000000	0.1400000
40163.0000000	0.1350000
40164.0000000	0.1450000
40165.0000000	0.1400000
40166.0000000	0.1400000
40167.0000000	0.1400000
40168.0000000	0.1300000
40169.0000000	0.1400000
40170.0000000	0.1350000
40171.0000000	0.1350000
40172.0000000	0.1350000
40173.0000000	0.1350000
40174.0000000	0.1450000
40175.0000000	0.1300000
40176.0000000	0.1350000
40177.0000000	0.1350000
40178.0000000	0.1350000
40179.0000000	0.1350000
40180.0000000	0.1350000
40181.0000000	0.1450000
40182.0000000	0.1350000
40183.0000000	0.1300000
40184.0000000	0.1433333

40185.0000000	0.1350000
40186.0000000	0.1400000
40187.0000000	0.1400000
40188.0000000	0.1450000
40189.0000000	0.1400000
40190.0000000	0.1450000
40191.0000000	0.1450000
40192.0000000	0.1450000
40193.0000000	0.1400000
40194.0000000	0.1400000
40195.0000000	0.1400000
40196.0000000	0.1500000
40197.0000000	0.1400000
40198.0000000	0.1400000
40199.0000000	0.1400000
40200.0000000	0.1450000
40201.0000000	0.1450000
40202.0000000	0.1500000
40203.0000000	0.1500000
40204.0000000	0.1450000
40205.0000000	0.1450000
40206.0000000	0.1350000
40207.0000000	0.1450000
40208.0000000	0.1500000
40209.0000000	0.1450000
40210.0000000	0.1500000
40211.0000000	0.1450000
40212.0000000	0.1550000
40213.0000000	0.1550000
40214.0000000	0.1500000
40215.0000000	0.1466667
40216.0000000	0.1450000
40217.0000000	0.1450000
40218.0000000	0.1450000
40219.0000000	0.1400000
40220.0000000	0.1450000
40221.0000000	0.1400000
40222.0000000	0.1500000
40223.0000000	0.1500000
40224.0000000	0.1450000
40225.0000000	0.1500000
40226.0000000	0.1500000
40227.0000000	0.1450000
40228.0000000	0.1500000
40229.0000000	0.1450000
40230.0000000	0.1450000
40231.0000000	0.1500000
40232.0000000	0.1450000
40233.0000000	0.1550000
40234.0000000	0.1500000
40235.0000000	0.1500000
40236.0000000	0.1600000
40237.0000000	0.1500000
40238.0000000	0.1550000
40239.0000000	0.1550000
40240.0000000	0.1500000
40241.0000000	0.1600000

40242.0000000	0.1500000
40243.0000000	0.1500000
40244.0000000	0.1450000
40245.0000000	0.1550000
40246.0000000	0.1533333
40247.0000000	0.1500000
40248.0000000	0.1500000
40249.0000000	0.1550000
40250.0000000	0.1550000
40251.0000000	0.1550000
40252.0000000	0.1550000
40253.0000000	0.1450000
40254.0000000	0.1450000
40255.0000000	0.1450000
40256.0000000	0.1450000
40257.0000000	0.1500000
40258.0000000	0.1450000
40259.0000000	0.1450000
40260.0000000	0.1350000
40261.0000000	0.1450000
40262.0000000	0.1400000
40263.0000000	0.1450000
40264.0000000	0.1300000
40265.0000000	0.1450000
40266.0000000	0.1400000
40267.0000000	0.1350000
40268.0000000	0.1300000
40269.0000000	0.1400000
40270.0000000	0.1350000
40271.0000000	0.1300000
40272.0000000	0.1400000
40273.0000000	0.1350000
40274.0000000	0.1300000
40275.0000000	0.1400000
40276.0000000	0.1400000
40277.0000000	0.1350000
40278.0000000	0.1350000
40279.0000000	0.1400000
40280.0000000	0.1350000
40281.0000000	0.1400000
40282.0000000	0.1450000
40283.0000000	0.1350000
40284.0000000	0.1450000
40285.0000000	0.1500000
40286.0000000	0.1400000
40287.0000000	0.1450000
40288.0000000	0.1450000
40289.0000000	0.1450000
40290.0000000	0.1400000
40291.0000000	0.1450000
40292.0000000	0.1450000
40293.0000000	0.1350000
40294.0000000	0.1450000
40295.0000000	0.1400000
40296.0000000	0.1300000
40297.0000000	0.1400000
40298.0000000	0.1350000

40299.0000000	0.1400000
40300.0000000	0.1400000
40301.0000000	0.1350000
40302.0000000	0.1350000
40303.0000000	0.1400000
40304.0000000	0.1350000
40305.0000000	0.1400000
40306.0000000	0.1350000
40307.0000000	0.1450000
40308.0000000	0.1400000
40309.0000000	0.1350000
40310.0000000	0.1350000
40311.0000000	0.1350000
40312.0000000	0.1350000
40313.0000000	0.1400000
40314.0000000	0.1400000
40315.0000000	0.1400000
40316.0000000	0.1350000
40317.0000000	0.1300000
40318.0000000	0.1450000
40319.0000000	0.1400000
40320.0000000	0.1350000
40321.0000000	0.1300000
40322.0000000	0.1350000
40323.0000000	0.1350000
40324.0000000	0.1300000
40325.0000000	0.1300000
40326.0000000	0.1300000
40327.0000000	0.1400000
40328.0000000	0.1300000
40329.0000000	0.1300000
40330.0000000	0.1250000
40331.0000000	0.1400000
40332.0000000	0.1350000
40333.0000000	0.1300000
40334.0000000	0.1350000
40335.0000000	0.1250000
40336.0000000	0.1266667
40337.0000000	0.1400000
40338.0000000	0.1300000
40339.0000000	0.1350000
40340.0000000	0.1400000
40341.0000000	0.1350000
40342.0000000	0.1350000
40343.0000000	0.1350000
40344.0000000	0.1450000
40345.0000000	0.1350000
40346.0000000	0.1400000
40347.0000000	0.1400000
40348.0000000	0.1350000
40349.0000000	0.1400000
40350.0000000	0.1350000
40351.0000000	0.1400000
40352.0000000	0.1350000
40353.0000000	0.1450000
40354.0000000	0.1400000
40355.0000000	0.1300000

40356.0000000	0.1350000
40357.0000000	0.1350000
40358.0000000	0.1400000
40359.0000000	0.1350000
40360.0000000	0.1350000
40361.0000000	0.1300000
40362.0000000	0.1300000
40363.0000000	0.1400000
40364.0000000	0.1300000
40365.0000000	0.1350000
40366.0000000	0.1300000
40367.0000000	0.1350000
40368.0000000	0.1400000
40369.0000000	0.1350000
40370.0000000	0.1350000
40371.0000000	0.1350000
40372.0000000	0.1350000
40373.0000000	0.1450000
40374.0000000	0.1350000
40375.0000000	0.1400000
40376.0000000	0.1450000
40377.0000000	0.1300000
40378.0000000	0.1400000
40379.0000000	0.1400000
40380.0000000	0.1450000
40381.0000000	0.1450000
40382.0000000	0.1350000
40383.0000000	0.1500000
40384.0000000	0.1350000
40385.0000000	0.1400000
40386.0000000	0.1350000
40387.0000000	0.1300000
40388.0000000	0.1400000
40389.0000000	0.1500000
40390.0000000	0.1400000
40391.0000000	0.1350000
40392.0000000	0.1400000
40393.0000000	0.1400000
40394.0000000	0.1500000
40395.0000000	0.1400000
40396.0000000	0.1433333
40397.0000000	0.1400000
40398.0000000	0.1400000
40399.0000000	0.1400000
40400.0000000	0.1500000
40401.0000000	0.1450000
40402.0000000	0.1500000
40403.0000000	0.1450000
40404.0000000	0.1450000
40405.0000000	0.1450000
40406.0000000	0.1400000
40407.0000000	0.1500000
40408.0000000	0.1450000
40409.0000000	0.1500000
40410.0000000	0.1500000
40411.0000000	0.1500000
40412.0000000	0.1500000

40413.0000000	0.1400000
40414.0000000	0.1400000
40415.0000000	0.1500000
40416.0000000	0.1400000
40417.0000000	0.1500000
40418.0000000	0.1500000
40419.0000000	0.1450000
40420.0000000	0.1450000
40421.0000000	0.1500000
40422.0000000	0.1500000
40423.0000000	0.1500000
40424.0000000	0.1450000
40425.0000000	0.1500000
40426.0000000	0.1533333
40427.0000000	0.1500000
40428.0000000	0.1450000
40429.0000000	0.1500000
40430.0000000	0.1450000
40431.0000000	0.1450000
40432.0000000	0.1500000
40433.0000000	0.1500000
40434.0000000	0.1450000
40435.0000000	0.1550000
40436.0000000	0.1500000
40437.0000000	0.1600000
40438.0000000	0.1500000
40439.0000000	0.1450000
40440.0000000	0.1450000
40441.0000000	0.1450000
40442.0000000	0.1500000
40443.0000000	0.1500000
40444.0000000	0.1450000
40445.0000000	0.1500000
40446.0000000	0.1500000
40447.0000000	0.1600000
40448.0000000	0.1450000
40449.0000000	0.1500000
40450.0000000	0.1450000
40451.0000000	0.1450000
40452.0000000	0.1500000
40453.0000000	0.1400000
40454.0000000	0.1550000
40455.0000000	0.1450000
40456.0000000	0.1500000
40457.0000000	0.1400000
40458.0000000	0.1400000
40459.0000000	0.1450000
40460.0000000	0.1500000
40461.0000000	0.1500000
40462.0000000	0.1400000
40463.0000000	0.1400000
40464.0000000	0.1400000
40465.0000000	0.1400000
40466.0000000	0.1500000
40467.0000000	0.1400000
40468.0000000	0.1500000
40469.0000000	0.1500000

40470.0000000	0.1450000
40471.0000000	0.1500000
40472.0000000	0.1450000
40473.0000000	0.1550000
40474.0000000	0.1500000
40475.0000000	0.1550000
40476.0000000	0.1550000
40477.0000000	0.1550000
40478.0000000	0.1500000
40479.0000000	0.1550000
40480.0000000	0.1500000
40481.0000000	0.1500000
40482.0000000	0.1400000
40483.0000000	0.1500000
40484.0000000	0.1500000
40485.0000000	0.1550000
40486.0000000	0.1450000
40487.0000000	0.1533333
40488.0000000	0.1500000
40489.0000000	0.1550000
40490.0000000	0.1550000
40491.0000000	0.1550000
40492.0000000	0.1500000
40493.0000000	0.1550000
40494.0000000	0.1450000
40495.0000000	0.1600000
40496.0000000	0.1500000
40497.0000000	0.1500000
40498.0000000	0.1600000
40499.0000000	0.1550000
40500.0000000	0.1550000
40501.0000000	0.1500000
40502.0000000	0.1550000
40503.0000000	0.1550000
40504.0000000	0.1600000
40505.0000000	0.1600000
40506.0000000	0.1550000
40507.0000000	0.1600000
40508.0000000	0.1550000
40509.0000000	0.1600000
40510.0000000	0.1550000
40511.0000000	0.1550000
40512.0000000	0.1550000
40513.0000000	0.1550000
40514.0000000	0.1650000
40515.0000000	0.1650000
40516.0000000	0.1600000
40517.0000000	0.1566667
40518.0000000	0.1500000
40519.0000000	0.1700000
40520.0000000	0.1650000
40521.0000000	0.1700000
40522.0000000	0.1600000
40523.0000000	0.1550000
40524.0000000	0.1500000
40525.0000000	0.1650000
40526.0000000	0.1700000

40527.0000000	0.1550000
40528.0000000	0.1650000
40529.0000000	0.1650000
40530.0000000	0.1700000
40531.0000000	0.1550000
40532.0000000	0.1650000
40533.0000000	0.1600000
40534.0000000	0.1650000
40535.0000000	0.1600000
40536.0000000	0.1700000
40537.0000000	0.1650000
40538.0000000	0.1550000
40539.0000000	0.1600000
40540.0000000	0.1650000
40541.0000000	0.1700000
40542.0000000	0.1650000
40543.0000000	0.1650000
40544.0000000	0.1550000
40545.0000000	0.1650000
40546.0000000	0.1550000
40547.0000000	0.1600000
40548.0000000	0.1700000
40549.0000000	0.1600000
40550.0000000	0.1600000
40551.0000000	0.1650000
40552.0000000	0.1550000
40553.0000000	0.1600000
40554.0000000	0.1550000
40555.0000000	0.1600000
40556.0000000	0.1600000
40557.0000000	0.1650000
40558.0000000	0.1500000
40559.0000000	0.1550000
40560.0000000	0.1650000
40561.0000000	0.1600000
40562.0000000	0.1650000
40563.0000000	0.1500000
40564.0000000	0.1500000
40565.0000000	0.1600000
40566.0000000	0.1550000
40567.0000000	0.1600000
40568.0000000	0.1600000
40569.0000000	0.1550000
40570.0000000	0.1600000
40571.0000000	0.1600000
40572.0000000	0.1500000
40573.0000000	0.1550000
40574.0000000	0.1450000
40575.0000000	0.1550000
40576.0000000	0.1500000
40577.0000000	0.1600000
40578.0000000	0.1500000
40579.0000000	0.1500000
40580.0000000	0.1500000
40581.0000000	0.1550000
40582.0000000	0.1500000
40583.0000000	0.1550000

40584.0000000	0.1550000
40585.0000000	0.1550000
40586.0000000	0.1550000
40587.0000000	0.1500000
40588.0000000	0.1550000
40589.0000000	0.1500000
40590.0000000	0.1550000
40591.0000000	0.1550000
40592.0000000	0.1500000
40593.0000000	0.1650000
40594.0000000	0.1550000
40595.0000000	0.1600000
40596.0000000	0.1550000
40597.0000000	0.1500000
40598.0000000	0.1600000
40599.0000000	0.1550000
40600.0000000	0.1500000
40601.0000000	0.1550000
40602.0000000	0.1600000
40603.0000000	0.1500000
40604.0000000	0.1600000
40605.0000000	0.1500000
40606.0000000	0.1600000
40607.0000000	0.1600000
40608.0000000	0.1600000
40609.0000000	0.1500000
40610.0000000	0.1550000
40611.0000000	0.1550000
40612.0000000	0.1600000
40613.0000000	0.1600000
40614.0000000	0.1550000
40615.0000000	0.1600000
40616.0000000	0.1550000
40617.0000000	0.1550000
40618.0000000	0.1500000
40619.0000000	0.1600000
40620.0000000	0.1550000
40621.0000000	0.1500000
40622.0000000	0.1600000
40623.0000000	0.1550000
40624.0000000	0.1600000
40625.0000000	0.1500000
40626.0000000	0.1550000
40627.0000000	0.1550000
40628.0000000	0.1500000
40629.0000000	0.1500000
40630.0000000	0.1500000
40631.0000000	0.1600000
40632.0000000	0.1600000
40633.0000000	0.1550000
40634.0000000	0.1500000
40635.0000000	0.1500000
40636.0000000	0.1550000
40637.0000000	0.1500000
40638.0000000	0.1550000
40639.0000000	0.1500000
40640.0000000	0.1500000

40641.0000000	0.1500000
40642.0000000	0.1500000
40643.0000000	0.1500000
40644.0000000	0.1650000
40645.0000000	0.1600000
40646.0000000	0.1600000
40647.0000000	0.1550000
40648.0000000	0.1550000
40649.0000000	0.1600000
40650.0000000	0.1550000
40651.0000000	0.1600000
40652.0000000	0.1600000
40653.0000000	0.1700000
40654.0000000	0.1650000
40655.0000000	0.1650000
40656.0000000	0.1600000
40657.0000000	0.1600000
40658.0000000	0.1650000
40659.0000000	0.1550000
40660.0000000	0.1700000
40661.0000000	0.1650000
40662.0000000	0.1550000
40663.0000000	0.1700000
40664.0000000	0.1750000
40665.0000000	0.1600000
40666.0000000	0.1700000
40667.0000000	0.1600000
40668.0000000	0.1633333
40669.0000000	0.1650000
40670.0000000	0.1650000
40671.0000000	0.1600000
40672.0000000	0.1650000
40673.0000000	0.1550000
40674.0000000	0.1500000
40675.0000000	0.1550000
40676.0000000	0.1550000
40677.0000000	0.1550000
40678.0000000	0.1650000
40679.0000000	0.1550000
40680.0000000	0.1600000
40681.0000000	0.1600000
40682.0000000	0.1600000
40683.0000000	0.1550000
40684.0000000	0.1550000
40685.0000000	0.1550000
40686.0000000	0.1500000
40687.0000000	0.1600000
40688.0000000	0.1550000
40689.0000000	0.1550000
40690.0000000	0.1600000
40691.0000000	0.1500000
40692.0000000	0.1600000
40693.0000000	0.1500000
40694.0000000	0.1550000
40695.0000000	0.1550000
40696.0000000	0.1400000
40697.0000000	0.1550000

40698.0000000	0.1566667
40699.0000000	0.1500000
40700.0000000	0.1500000
40701.0000000	0.1650000
40702.0000000	0.1550000
40703.0000000	0.1500000
40704.0000000	0.1550000
40705.0000000	0.1600000
40706.0000000	0.1600000
40707.0000000	0.1650000
40708.0000000	0.1650000
40709.0000000	0.1600000
40710.0000000	0.1550000
40711.0000000	0.1550000
40712.0000000	0.1600000
40713.0000000	0.1600000
40714.0000000	0.1500000
40715.0000000	0.1650000
40716.0000000	0.1550000
40717.0000000	0.1500000
40718.0000000	0.1500000
40719.0000000	0.1550000
40720.0000000	0.1550000
40721.0000000	0.1500000
40722.0000000	0.1550000
40723.0000000	0.1600000
40724.0000000	0.1500000
40725.0000000	0.1500000
40726.0000000	0.1550000
40727.0000000	0.1500000
40728.0000000	0.1500000
40729.0000000	0.1550000
40730.0000000	0.1500000
40731.0000000	0.1550000
40732.0000000	0.1500000
40733.0000000	0.1400000
40734.0000000	0.1550000
40735.0000000	0.1600000
40736.0000000	0.1500000
40737.0000000	0.1450000
40738.0000000	0.1500000
40739.0000000	0.1500000
40740.0000000	0.1450000
40741.0000000	0.1500000
40742.0000000	0.1600000
40743.0000000	0.1600000
40744.0000000	0.1500000
40745.0000000	0.1600000
40746.0000000	0.1500000
40747.0000000	0.1550000
40748.0000000	0.1550000
40749.0000000	0.1550000
40750.0000000	0.1550000
40751.0000000	0.1600000
40752.0000000	0.1550000
40753.0000000	0.1550000
40754.0000000	0.1650000

40755.0000000	0.1550000
40756.0000000	0.1600000
40757.0000000	0.1550000
40758.0000000	0.1566667
40759.0000000	0.1550000
40760.0000000	0.1650000
40761.0000000	0.1550000
40762.0000000	0.1600000
40763.0000000	0.1550000
40764.0000000	0.1600000
40765.0000000	0.1650000
40766.0000000	0.1550000
40767.0000000	0.1550000
40768.0000000	0.1500000
40769.0000000	0.1600000
40770.0000000	0.1600000
40771.0000000	0.1600000
40772.0000000	0.1600000
40773.0000000	0.1600000
40774.0000000	0.1500000
40775.0000000	0.1550000
40776.0000000	0.1600000
40777.0000000	0.1600000
40778.0000000	0.1600000
40779.0000000	0.1600000
40780.0000000	0.1600000
40781.0000000	0.1550000
40782.0000000	0.1600000
40783.0000000	0.1600000
40784.0000000	0.1600000
40785.0000000	0.1600000
40786.0000000	0.1650000
40787.0000000	0.1650000
40788.0000000	0.1666667
40789.0000000	0.1700000
40790.0000000	0.1700000
40791.0000000	0.1600000
40792.0000000	0.1600000
40793.0000000	0.1700000
40794.0000000	0.1650000
40795.0000000	0.1650000
40796.0000000	0.1600000
40797.0000000	0.1700000
40798.0000000	0.1650000
40799.0000000	0.1650000
40800.0000000	0.1650000
40801.0000000	0.1650000
40802.0000000	0.1750000
40803.0000000	0.1700000
40804.0000000	0.1650000
40805.0000000	0.1700000
40806.0000000	0.1700000
40807.0000000	0.1650000
40808.0000000	0.1650000
40809.0000000	0.1650000
40810.0000000	0.1700000
40811.0000000	0.1600000

40812.0000000	0.1750000
40813.0000000	0.1650000
40814.0000000	0.1700000
40815.0000000	0.1650000
40816.0000000	0.1650000
40817.0000000	0.1700000
40818.0000000	0.1650000
40819.0000000	0.1666667
40820.0000000	0.1700000
40821.0000000	0.1600000
40822.0000000	0.1700000
40823.0000000	0.1750000
40824.0000000	0.1700000
40825.0000000	0.1650000
40826.0000000	0.1650000
40827.0000000	0.1700000
40828.0000000	0.1600000
40829.0000000	0.1650000
40830.0000000	0.1650000
40831.0000000	0.1600000
40832.0000000	0.1600000
40833.0000000	0.1650000
40834.0000000	0.1650000
40835.0000000	0.1700000
40836.0000000	0.1650000
40837.0000000	0.1700000
40838.0000000	0.1650000
40839.0000000	0.1550000
40840.0000000	0.1700000
40841.0000000	0.1600000
40842.0000000	0.1650000
40843.0000000	0.1650000
40844.0000000	0.1650000
40845.0000000	0.1650000
40846.0000000	0.1650000
40847.0000000	0.1700000
40848.0000000	0.1650000
40849.0000000	0.1666667
40850.0000000	0.1700000
40851.0000000	0.1600000
40852.0000000	0.1700000
40853.0000000	0.1600000
40854.0000000	0.1750000
40855.0000000	0.1700000
40856.0000000	0.1750000
40857.0000000	0.1700000
40858.0000000	0.1800000
40859.0000000	0.1700000
40860.0000000	0.1750000
40861.0000000	0.1650000
40862.0000000	0.1750000
40863.0000000	0.1750000
40864.0000000	0.1850000
40865.0000000	0.1700000
40866.0000000	0.1800000
40867.0000000	0.1700000
40868.0000000	0.1750000

40869.0000000	0.1700000
40870.0000000	0.1800000
40871.0000000	0.1700000
40872.0000000	0.1700000
40873.0000000	0.1700000
40874.0000000	0.1700000
40875.0000000	0.1700000
40876.0000000	0.1750000
40877.0000000	0.1750000
40878.0000000	0.1700000
40879.0000000	0.1733333
40880.0000000	0.1750000
40881.0000000	0.1850000
40882.0000000	0.1650000
40883.0000000	0.1750000
40884.0000000	0.1800000
40885.0000000	0.1750000
40886.0000000	0.1900000
40887.0000000	0.1750000
40888.0000000	0.1850000
40889.0000000	0.1850000
40890.0000000	0.1850000
40891.0000000	0.1750000
40892.0000000	0.1750000
40893.0000000	0.1650000
40894.0000000	0.1800000
40895.0000000	0.1800000
40896.0000000	0.1800000
40897.0000000	0.1850000
40898.0000000	0.1800000
40899.0000000	0.1800000
40900.0000000	0.1800000
40901.0000000	0.1800000
40902.0000000	0.1700000
40903.0000000	0.1750000
40904.0000000	0.1800000
40905.0000000	0.1700000
40906.0000000	0.1850000
40907.0000000	0.1750000
40908.0000000	0.1700000
40909.0000000	0.1733333
40910.0000000	0.1750000
40911.0000000	0.1800000
40912.0000000	0.1700000
40913.0000000	0.1700000
40914.0000000	0.1750000
40915.0000000	0.1750000
40916.0000000	0.1750000
40917.0000000	0.1750000
40918.0000000	0.1700000
40919.0000000	0.1750000
40920.0000000	0.1850000
40921.0000000	0.1750000
40922.0000000	0.1800000
40923.0000000	0.1750000
40924.0000000	0.1700000
40925.0000000	0.1750000

40926.0000000	0.1750000
40927.0000000	0.1800000
40928.0000000	0.1800000
40929.0000000	0.1800000
40930.0000000	0.1700000
40931.0000000	0.1800000
40932.0000000	0.1700000
40933.0000000	0.1850000
40934.0000000	0.1850000
40935.0000000	0.1750000
40936.0000000	0.1850000
40937.0000000	0.1800000
40938.0000000	0.1800000
40939.0000000	0.1850000
40940.0000000	0.1866667
40941.0000000	0.1850000
40942.0000000	0.1850000
40943.0000000	0.1900000
40944.0000000	0.1850000
40945.0000000	0.1850000
40946.0000000	0.1900000
40947.0000000	0.1900000
40948.0000000	0.1850000
40949.0000000	0.1950000
40950.0000000	0.1900000
40951.0000000	0.1950000
40952.0000000	0.1900000
40953.0000000	0.1950000
40954.0000000	0.1850000
40955.0000000	0.1950000
40956.0000000	0.1850000
40957.0000000	0.1850000
40958.0000000	0.1900000
40959.0000000	0.1850000
40960.0000000	0.2000000
40961.0000000	0.1900000
40962.0000000	0.1950000
40963.0000000	0.1900000
40964.0000000	0.1900000
40965.0000000	0.1900000
40966.0000000	0.1900000
40967.0000000	0.2000000
40968.0000000	0.1900000
40969.0000000	0.1933333
40970.0000000	0.1900000
40971.0000000	0.2000000
40972.0000000	0.2000000
40973.0000000	0.1950000
40974.0000000	0.1950000
40975.0000000	0.1950000
40976.0000000	0.1950000
40977.0000000	0.2000000
40978.0000000	0.1950000
40979.0000000	0.1950000
40980.0000000	0.1900000
40981.0000000	0.2000000
40982.0000000	0.2000000

40983.0000000	0.1950000
40984.0000000	0.2000000
40985.0000000	0.1900000
40986.0000000	0.2050000
40987.0000000	0.2000000
40988.0000000	0.1950000
40989.0000000	0.1900000
40990.0000000	0.1900000
40991.0000000	0.1950000
40992.0000000	0.1950000
40993.0000000	0.1950000
40994.0000000	0.2000000
40995.0000000	0.2050000
40996.0000000	0.2000000
40997.0000000	0.1900000
40998.0000000	0.2000000
40999.0000000	0.1966667
41000.0000000	0.2000000
41001.0000000	0.2000000
41002.0000000	0.1900000
41003.0000000	0.1950000
41004.0000000	0.1950000
41005.0000000	0.1900000
41006.0000000	0.2000000
41007.0000000	0.1900000
41008.0000000	0.1850000
41009.0000000	0.2000000
41010.0000000	0.1950000
41011.0000000	0.1900000
41012.0000000	0.1900000
41013.0000000	0.1900000
41014.0000000	0.1900000
41015.0000000	0.1850000
41016.0000000	0.1950000
41017.0000000	0.1850000
41018.0000000	0.1950000
41019.0000000	0.1950000
41020.0000000	0.2000000
41021.0000000	0.1950000
41022.0000000	0.1900000
41023.0000000	0.1950000
41024.0000000	0.1900000
41025.0000000	0.1950000
41026.0000000	0.1900000
41027.0000000	0.2000000
41028.0000000	0.1950000
41029.0000000	0.1950000
41030.0000000	0.1933333
41031.0000000	0.1950000
41032.0000000	0.2050000
41033.0000000	0.1950000
41034.0000000	0.1950000
41035.0000000	0.2000000
41036.0000000	0.2000000
41037.0000000	0.1950000
41038.0000000	0.2000000
41039.0000000	0.2000000

41040.0000000	0.2000000
41041.0000000	0.2000000
41042.0000000	0.1900000
41043.0000000	0.2000000
41044.0000000	0.2000000
41045.0000000	0.1950000
41046.0000000	0.1950000
41047.0000000	0.1950000
41048.0000000	0.2050000
41049.0000000	0.2050000
41050.0000000	0.1950000
41051.0000000	0.1950000
41052.0000000	0.2000000
41053.0000000	0.2050000
41054.0000000	0.2050000
41055.0000000	0.2050000
41056.0000000	0.2000000
41057.0000000	0.2000000
41058.0000000	0.2000000
41059.0000000	0.2000000
41060.0000000	0.2033333
41061.0000000	0.2050000
41062.0000000	0.2100000
41063.0000000	0.2000000
41064.0000000	0.2050000
41065.0000000	0.2050000
41066.0000000	0.1950000
41067.0000000	0.2050000
41068.0000000	0.2000000
41069.0000000	0.2050000
41070.0000000	0.2050000
41071.0000000	0.2000000
41072.0000000	0.2050000
41073.0000000	0.2000000
41074.0000000	0.2000000
41075.0000000	0.1950000
41076.0000000	0.2050000
41077.0000000	0.2000000
41078.0000000	0.2050000
41079.0000000	0.1950000
41080.0000000	0.2000000
41081.0000000	0.2050000
41082.0000000	0.1950000
41083.0000000	0.2050000
41084.0000000	0.2000000
41085.0000000	0.2000000
41086.0000000	0.2000000
41087.0000000	0.1950000
41088.0000000	0.1950000
41089.0000000	0.1950000
41090.0000000	0.2066667
41091.0000000	0.1950000
41092.0000000	0.2000000
41093.0000000	0.1950000
41094.0000000	0.2000000
41095.0000000	0.2000000
41096.0000000	0.2000000

41097.0000000	0.2050000
41098.0000000	0.2000000
41099.0000000	0.2000000
41100.0000000	0.2050000
41101.0000000	0.2000000
41102.0000000	0.1950000
41103.0000000	0.2050000
41104.0000000	0.2000000
41105.0000000	0.2050000
41106.0000000	0.2100000
41107.0000000	0.2050000
41108.0000000	0.2000000
41109.0000000	0.2100000
41110.0000000	0.2050000
41111.0000000	0.2050000
41112.0000000	0.2050000
41113.0000000	0.2100000
41114.0000000	0.2050000
41115.0000000	0.2050000
41116.0000000	0.2000000
41117.0000000	0.2100000
41118.0000000	0.2100000
41119.0000000	0.2100000
41120.0000000	0.2100000
41121.0000000	0.2100000
41122.0000000	0.2100000
41123.0000000	0.2050000
41124.0000000	0.2050000
41125.0000000	0.2100000
41126.0000000	0.2150000
41127.0000000	0.2150000
41128.0000000	0.2050000
41129.0000000	0.2100000
41130.0000000	0.2100000
41131.0000000	0.2100000
41132.0000000	0.2150000
41133.0000000	0.2100000
41134.0000000	0.2100000
41135.0000000	0.2100000
41136.0000000	0.2100000
41137.0000000	0.2050000
41138.0000000	0.2050000
41139.0000000	0.2100000
41140.0000000	0.2150000
41141.0000000	0.2100000
41142.0000000	0.2200000
41143.0000000	0.2100000
41144.0000000	0.2150000
41145.0000000	0.2150000
41146.0000000	0.2050000
41147.0000000	0.2100000
41148.0000000	0.2200000
41149.0000000	0.2150000
41150.0000000	0.2100000
41151.0000000	0.2200000
41152.0000000	0.2250000
41153.0000000	0.2200000

41154.0000000	0.2200000
41155.0000000	0.2200000
41156.0000000	0.2200000
41157.0000000	0.2200000
41158.0000000	0.2200000
41159.0000000	0.2150000
41160.0000000	0.2250000
41161.0000000	0.2200000
41162.0000000	0.2200000
41163.0000000	0.2250000
41164.0000000	0.2150000
41165.0000000	0.2150000
41166.0000000	0.2200000
41167.0000000	0.2200000
41168.0000000	0.2150000
41169.0000000	0.2200000
41170.0000000	0.2150000
41171.0000000	0.2250000
41172.0000000	0.2200000
41173.0000000	0.2150000
41174.0000000	0.2200000
41175.0000000	0.2150000
41176.0000000	0.2150000
41177.0000000	0.2200000
41178.0000000	0.2150000
41179.0000000	0.2100000
41180.0000000	0.2200000
41181.0000000	0.2200000
41182.0000000	0.2150000
41183.0000000	0.2200000
41184.0000000	0.2150000
41185.0000000	0.2200000
41186.0000000	0.2150000
41187.0000000	0.2200000
41188.0000000	0.2200000
41189.0000000	0.2200000
41190.0000000	0.2200000
41191.0000000	0.2150000
41192.0000000	0.2200000
41193.0000000	0.2100000
41194.0000000	0.2150000
41195.0000000	0.2200000
41196.0000000	0.2100000
41197.0000000	0.2150000
41198.0000000	0.2150000
41199.0000000	0.2200000
41200.0000000	0.2200000
41201.0000000	0.2150000
41202.0000000	0.2200000
41203.0000000	0.2250000
41204.0000000	0.2150000
41205.0000000	0.2300000
41206.0000000	0.2150000
41207.0000000	0.2150000
41208.0000000	0.2250000
41209.0000000	0.2200000
41210.0000000	0.2250000

41211.0000000	0.2166667
41212.0000000	0.2200000
41213.0000000	0.2300000
41214.0000000	0.2200000
41215.0000000	0.2250000
41216.0000000	0.2200000
41217.0000000	0.2250000
41218.0000000	0.2300000
41219.0000000	0.2250000
41220.0000000	0.2250000
41221.0000000	0.2250000
41222.0000000	0.2150000
41223.0000000	0.2250000
41224.0000000	0.2350000
41225.0000000	0.2250000
41226.0000000	0.2250000
41227.0000000	0.2300000
41228.0000000	0.2150000
41229.0000000	0.2250000
41230.0000000	0.2300000
41231.0000000	0.2200000
41232.0000000	0.2250000
41233.0000000	0.2250000
41234.0000000	0.2200000
41235.0000000	0.2250000
41236.0000000	0.2300000
41237.0000000	0.2250000
41238.0000000	0.2250000
41239.0000000	0.2200000
41240.0000000	0.2200000
41241.0000000	0.2233333
41242.0000000	0.2200000
41243.0000000	0.2300000
41244.0000000	0.2200000
41245.0000000	0.2250000
41246.0000000	0.2250000
41247.0000000	0.2250000
41248.0000000	0.2250000
41249.0000000	0.2300000
41250.0000000	0.2250000
41251.0000000	0.2250000
41252.0000000	0.2150000
41253.0000000	0.2250000
41254.0000000	0.2200000
41255.0000000	0.2250000
41256.0000000	0.2200000
41257.0000000	0.2250000
41258.0000000	0.2250000
41259.0000000	0.2150000
41260.0000000	0.2250000
41261.0000000	0.2200000
41262.0000000	0.2200000
41263.0000000	0.2300000
41264.0000000	0.2200000
41265.0000000	0.2200000
41266.0000000	0.2200000
41267.0000000	0.2250000

41268.0000000	0.2250000
41269.0000000	0.2250000
41270.0000000	0.2300000
41271.0000000	0.2300000
41272.0000000	0.2300000
41273.0000000	0.2200000
41274.0000000	0.2300000
41275.0000000	0.2300000
41276.0000000	0.2250000
41277.0000000	0.2300000
41278.0000000	0.2350000
41279.0000000	0.2300000
41280.0000000	0.2300000
41281.0000000	0.2250000
41282.0000000	0.2250000
41283.0000000	0.2300000
41284.0000000	0.2250000
41285.0000000	0.2250000
41286.0000000	0.2300000
41287.0000000	0.2350000
41288.0000000	0.2300000
41289.0000000	0.2300000
41290.0000000	0.2400000
41291.0000000	0.2300000
41292.0000000	0.2400000
41293.0000000	0.2400000
41294.0000000	0.2300000
41295.0000000	0.2350000
41296.0000000	0.2400000
41297.0000000	0.2350000
41298.0000000	0.2450000
41299.0000000	0.2300000
41300.0000000	0.2250000
41301.0000000	0.2350000
41302.0000000	0.2400000
41303.0000000	0.2250000
41304.0000000	0.2250000
41305.0000000	0.2250000
41306.0000000	0.2250000
41307.0000000	0.2200000
41308.0000000	0.2250000
41309.0000000	0.2200000
41310.0000000	0.2200000
41311.0000000	0.2250000
41312.0000000	0.2200000
41313.0000000	0.2150000
41314.0000000	0.2200000
41315.0000000	0.2150000
41316.0000000	0.2150000
41317.0000000	0.2150000
41318.0000000	0.2150000
41319.0000000	0.2100000
41320.0000000	0.2050000
41321.0000000	0.2100000
41322.0000000	0.2100000
41323.0000000	0.2200000
41324.0000000	0.2150000

41325.0000000	0.2100000
41326.0000000	0.2150000
41327.0000000	0.2150000
41328.0000000	0.2150000
41329.0000000	0.2200000
41330.0000000	0.2100000
41331.0000000	0.2166667
41332.0000000	0.2100000
41333.0000000	0.2200000
41334.0000000	0.2100000
41335.0000000	0.2100000
41336.0000000	0.2150000
41337.0000000	0.2100000
41338.0000000	0.2100000
41339.0000000	0.2150000
41340.0000000	0.2200000
41341.0000000	0.2100000
41342.0000000	0.2200000
41343.0000000	0.2200000
41344.0000000	0.2150000
41345.0000000	0.2200000
41346.0000000	0.2150000
41347.0000000	0.2150000
41348.0000000	0.2200000
41349.0000000	0.2250000
41350.0000000	0.2200000
41351.0000000	0.2100000
41352.0000000	0.2150000
41353.0000000	0.2150000
41354.0000000	0.2150000
41355.0000000	0.2150000
41356.0000000	0.2200000
41357.0000000	0.2150000
41358.0000000	0.2200000
41359.0000000	0.2250000
41360.0000000	0.2200000
41361.0000000	0.2100000
41362.0000000	0.2166667
41363.0000000	0.2200000
41364.0000000	0.2200000
41365.0000000	0.2150000
41366.0000000	0.2200000
41367.0000000	0.2200000
41368.0000000	0.2200000
41369.0000000	0.2150000
41370.0000000	0.2200000
41371.0000000	0.2150000
41372.0000000	0.2150000
41373.0000000	0.2100000
41374.0000000	0.2200000
41375.0000000	0.2150000
41376.0000000	0.2200000
41377.0000000	0.2200000
41378.0000000	0.2150000
41379.0000000	0.2200000
41380.0000000	0.2200000
41381.0000000	0.2100000

41382.0000000	0.2200000
41383.0000000	0.2100000
41384.0000000	0.2150000
41385.0000000	0.2150000
41386.0000000	0.2050000
41387.0000000	0.2150000
41388.0000000	0.2250000
41389.0000000	0.2150000
41390.0000000	0.2200000
41391.0000000	0.2200000
41392.0000000	0.2250000
41393.0000000	0.2200000
41394.0000000	0.2150000
41395.0000000	0.2200000
41396.0000000	0.2200000
41397.0000000	0.2200000
41398.0000000	0.2200000
41399.0000000	0.2150000
41400.0000000	0.2250000
41401.0000000	0.2250000
41402.0000000	0.2200000
41403.0000000	0.2150000
41404.0000000	0.2150000
41405.0000000	0.2200000
41406.0000000	0.2200000
41407.0000000	0.2300000
41408.0000000	0.2250000
41409.0000000	0.2250000
41410.0000000	0.2150000
41411.0000000	0.2250000
41412.0000000	0.2200000
41413.0000000	0.2150000
41414.0000000	0.2150000
41415.0000000	0.2200000
41416.0000000	0.2250000
41417.0000000	0.2250000
41418.0000000	0.2200000
41419.0000000	0.2200000
41420.0000000	0.2250000
41421.0000000	0.2200000
41422.0000000	0.2233333
41423.0000000	0.2300000
41424.0000000	0.2250000
41425.0000000	0.2200000
41426.0000000	0.2250000
41427.0000000	0.2150000
41428.0000000	0.2250000
41429.0000000	0.2250000
41430.0000000	0.2200000
41431.0000000	0.2250000
41432.0000000	0.2200000
41433.0000000	0.2250000
41434.0000000	0.2250000
41435.0000000	0.2250000
41436.0000000	0.2250000
41437.0000000	0.2250000
41438.0000000	0.2200000

41439.0000000	0.2250000
41440.0000000	0.2250000
41441.0000000	0.2200000
41442.0000000	0.2250000
41443.0000000	0.2300000
41444.0000000	0.2250000
41445.0000000	0.2300000
41446.0000000	0.2350000
41447.0000000	0.2300000
41448.0000000	0.2250000
41449.0000000	0.2350000
41450.0000000	0.2250000
41451.0000000	0.2200000
41452.0000000	0.2300000
41453.0000000	0.2200000
41454.0000000	0.2200000
41455.0000000	0.2250000
41456.0000000	0.2300000
41457.0000000	0.2200000
41458.0000000	0.2200000
41459.0000000	0.2200000
41460.0000000	0.2150000
41461.0000000	0.2150000
41462.0000000	0.2150000
41463.0000000	0.2200000
41464.0000000	0.2150000
41465.0000000	0.2150000
41466.0000000	0.2200000
41467.0000000	0.2200000
41468.0000000	0.2200000
41469.0000000	0.2200000
41470.0000000	0.2250000
41471.0000000	0.2250000
41472.0000000	0.2300000
41473.0000000	0.2150000
41474.0000000	0.2250000
41475.0000000	0.2200000
41476.0000000	0.2150000
41477.0000000	0.2200000
41478.0000000	0.2150000
41479.0000000	0.2200000
41480.0000000	0.2250000
41481.0000000	0.2250000
41482.0000000	0.2250000
41483.0000000	0.2200000
41484.0000000	0.2250000
41485.0000000	0.2150000
41486.0000000	0.2300000
41487.0000000	0.2200000
41488.0000000	0.2200000
41489.0000000	0.2300000
41490.0000000	0.2250000
41491.0000000	0.2250000
41492.0000000	0.2200000
41493.0000000	0.2150000
41494.0000000	0.2200000
41495.0000000	0.2300000

41496.0000000	0.2250000
41497.0000000	0.2300000
41498.0000000	0.2250000
41499.0000000	0.2300000
41500.0000000	0.2300000
41501.0000000	0.2300000
41502.0000000	0.2250000
41503.0000000	0.2250000
41504.0000000	0.2200000
41505.0000000	0.2250000
41506.0000000	0.2250000
41507.0000000	0.2200000
41508.0000000	0.2350000
41509.0000000	0.2250000
41510.0000000	0.2200000
41511.0000000	0.2250000
41512.0000000	0.2200000
41513.0000000	0.2233333
41514.0000000	0.2250000
41515.0000000	0.2250000
41516.0000000	0.2200000
41517.0000000	0.2150000
41518.0000000	0.2150000
41519.0000000	0.2200000
41520.0000000	0.2250000
41521.0000000	0.2150000
41522.0000000	0.2150000
41523.0000000	0.2200000
41524.0000000	0.2200000
41525.0000000	0.2200000
41526.0000000	0.2200000
41527.0000000	0.2150000
41528.0000000	0.2200000
41529.0000000	0.2300000
41530.0000000	0.2200000
41531.0000000	0.2200000
41532.0000000	0.2200000
41533.0000000	0.2250000
41534.0000000	0.2250000
41535.0000000	0.2150000
41536.0000000	0.2250000
41537.0000000	0.2200000
41538.0000000	0.2250000
41539.0000000	0.2250000
41540.0000000	0.2350000
41541.0000000	0.2350000
41542.0000000	0.2200000
41543.0000000	0.2166667
41544.0000000	0.2300000
41545.0000000	0.2250000
41546.0000000	0.2250000
41547.0000000	0.2150000
41548.0000000	0.2200000
41549.0000000	0.2150000
41550.0000000	0.2250000
41551.0000000	0.2300000
41552.0000000	0.2300000

41553.0000000	0.2250000
41554.0000000	0.2150000
41555.0000000	0.2200000
41556.0000000	0.2200000
41557.0000000	0.2250000
41558.0000000	0.2100000
41559.0000000	0.2250000
41560.0000000	0.2150000
41561.0000000	0.2250000
41562.0000000	0.2150000
41563.0000000	0.2250000
41564.0000000	0.2250000
41565.0000000	0.2150000
41566.0000000	0.2200000
41567.0000000	0.2200000
41568.0000000	0.2300000
41569.0000000	0.2150000
41570.0000000	0.2300000
41571.0000000	0.2200000
41572.0000000	0.2300000
41573.0000000	0.2266667
41574.0000000	0.2300000
41575.0000000	0.2200000
41576.0000000	0.2350000
41577.0000000	0.2200000
41578.0000000	0.2250000
41579.0000000	0.2300000
41580.0000000	0.2250000
41581.0000000	0.2200000
41582.0000000	0.2300000
41583.0000000	0.2200000
41584.0000000	0.2200000
41585.0000000	0.2300000
41586.0000000	0.2300000
41587.0000000	0.2250000
41588.0000000	0.2200000
41589.0000000	0.2300000
41590.0000000	0.2300000
41591.0000000	0.2250000
41592.0000000	0.2250000
41593.0000000	0.2150000
41594.0000000	0.2200000
41595.0000000	0.2150000
41596.0000000	0.2200000
41597.0000000	0.2200000
41598.0000000	0.2200000
41599.0000000	0.2300000
41600.0000000	0.2200000
41601.0000000	0.2150000
41602.0000000	0.2100000
41603.0000000	0.2100000
41604.0000000	0.2133333
41605.0000000	0.2100000
41606.0000000	0.2100000
41607.0000000	0.2050000
41608.0000000	0.2150000
41609.0000000	0.2000000

41610.0000000	0.2050000
41611.0000000	0.2150000
41612.0000000	0.2100000
41613.0000000	0.2100000
41614.0000000	0.2100000
41615.0000000	0.2200000
41616.0000000	0.2050000
41617.0000000	0.2050000
41618.0000000	0.2050000
41619.0000000	0.2150000
41620.0000000	0.2150000
41621.0000000	0.2100000
41622.0000000	0.2200000
41623.0000000	0.2150000
41624.0000000	0.2150000
41625.0000000	0.2150000
41626.0000000	0.2100000
41627.0000000	0.2150000
41628.0000000	0.2100000
41629.0000000	0.2200000
41630.0000000	0.2250000
41631.0000000	0.2150000
41632.0000000	0.2250000
41633.0000000	0.2200000
41634.0000000	0.2166667
41635.0000000	0.2150000
41636.0000000	0.2200000
41637.0000000	0.2150000
41638.0000000	0.2200000
41639.0000000	0.2150000
41640.0000000	0.2150000
41641.0000000	0.2150000
41642.0000000	0.2050000
41643.0000000	0.2150000
41644.0000000	0.2150000
41645.0000000	0.2200000
41646.0000000	0.2150000
41647.0000000	0.2200000
41648.0000000	0.2200000
41649.0000000	0.2200000
41650.0000000	0.2150000
41651.0000000	0.2200000
41652.0000000	0.2200000
41653.0000000	0.2150000
41654.0000000	0.2250000
41655.0000000	0.2300000
41656.0000000	0.2250000
41657.0000000	0.2200000
41658.0000000	0.2250000
41659.0000000	0.2150000
41660.0000000	0.2200000
41661.0000000	0.2300000
41662.0000000	0.2250000
41663.0000000	0.2200000
41664.0000000	0.2233333
41665.0000000	0.2200000
41666.0000000	0.2250000

41667.0000000	0.2200000
41668.0000000	0.2150000
41669.0000000	0.2300000
41670.0000000	0.2250000
41671.0000000	0.2250000
41672.0000000	0.2250000
41673.0000000	0.2250000
41674.0000000	0.2250000
41675.0000000	0.2200000
41676.0000000	0.2200000
41677.0000000	0.2200000
41678.0000000	0.2150000
41679.0000000	0.2250000
41680.0000000	0.2300000
41681.0000000	0.2150000
41682.0000000	0.2300000
41683.0000000	0.2200000
41684.0000000	0.2250000
41685.0000000	0.2250000
41686.0000000	0.2200000
41687.0000000	0.2250000
41688.0000000	0.2250000
41689.0000000	0.2300000
41690.0000000	0.2250000
41691.0000000	0.2250000
41692.0000000	0.2300000
41693.0000000	0.2350000
41694.0000000	0.2200000
41695.0000000	0.2200000
41696.0000000	0.2300000
41697.0000000	0.2300000
41698.0000000	0.2250000
41699.0000000	0.2300000
41700.0000000	0.2250000
41701.0000000	0.2250000
41702.0000000	0.2250000
41703.0000000	0.2250000
41704.0000000	0.2250000
41705.0000000	0.2250000
41706.0000000	0.2200000
41707.0000000	0.2300000
41708.0000000	0.2300000
41709.0000000	0.2300000
41710.0000000	0.2350000
41711.0000000	0.2250000
41712.0000000	0.2300000
41713.0000000	0.2350000
41714.0000000	0.2200000
41715.0000000	0.2300000
41716.0000000	0.2300000
41717.0000000	0.2300000
41718.0000000	0.2300000
41719.0000000	0.2200000
41720.0000000	0.2200000
41721.0000000	0.2150000
41722.0000000	0.2150000
41723.0000000	0.2250000

41724.0000000	0.2300000
41725.0000000	0.2200000
41726.0000000	0.2200000
41727.0000000	0.2200000
41728.0000000	0.2150000
41729.0000000	0.2200000
41730.0000000	0.2200000
41731.0000000	0.2250000
41732.0000000	0.2250000
41733.0000000	0.2200000
41734.0000000	0.2300000
41735.0000000	0.2200000
41736.0000000	0.2250000
41737.0000000	0.2150000
41738.0000000	0.2200000
41739.0000000	0.2250000
41740.0000000	0.2250000
41741.0000000	0.2350000
41742.0000000	0.2400000
41743.0000000	0.2200000
41744.0000000	0.2300000
41745.0000000	0.2250000
41746.0000000	0.2300000
41747.0000000	0.2250000
41748.0000000	0.2300000
41749.0000000	0.2250000
41750.0000000	0.2250000
41751.0000000	0.2350000
41752.0000000	0.2300000
41753.0000000	0.2350000
41754.0000000	0.2350000
41755.0000000	0.2300000
41756.0000000	0.2400000
41757.0000000	0.2300000
41758.0000000	0.2350000
41759.0000000	0.2350000
41760.0000000	0.2350000
41761.0000000	0.2350000
41762.0000000	0.2350000
41763.0000000	0.2300000
41764.0000000	0.2350000
41765.0000000	0.2300000
41766.0000000	0.2400000
41767.0000000	0.2300000
41768.0000000	0.2350000
41769.0000000	0.2300000
41770.0000000	0.2250000
41771.0000000	0.2300000
41772.0000000	0.2350000
41773.0000000	0.2450000
41774.0000000	0.2350000
41775.0000000	0.2350000
41776.0000000	0.2300000
41777.0000000	0.2350000
41778.0000000	0.2350000
41779.0000000	0.2400000
41780.0000000	0.2400000

41781.0000000	0.2400000
41782.0000000	0.2400000
41783.0000000	0.2300000
41784.0000000	0.2366667
41785.0000000	0.2400000
41786.0000000	0.2300000
41787.0000000	0.2250000
41788.0000000	0.2200000
41789.0000000	0.2300000
41790.0000000	0.2400000
41791.0000000	0.2350000
41792.0000000	0.2350000
41793.0000000	0.2200000
41794.0000000	0.2250000
41795.0000000	0.2200000
41796.0000000	0.2350000
41797.0000000	0.2300000
41798.0000000	0.2300000
41799.0000000	0.2200000
41800.0000000	0.2350000
41801.0000000	0.2200000
41802.0000000	0.2250000
41803.0000000	0.2250000
41804.0000000	0.2300000
41805.0000000	0.2300000
41806.0000000	0.2300000
41807.0000000	0.2350000
41808.0000000	0.2300000
41809.0000000	0.2300000
41810.0000000	0.2250000
41811.0000000	0.2300000
41812.0000000	0.2300000
41813.0000000	0.2300000
41814.0000000	0.2300000
41815.0000000	0.2300000
41816.0000000	0.2250000
41817.0000000	0.2250000
41818.0000000	0.2400000
41819.0000000	0.2250000
41820.0000000	0.2250000
41821.0000000	0.2400000
41822.0000000	0.2350000
41823.0000000	0.2350000
41824.0000000	0.2300000
41825.0000000	0.2300000
41826.0000000	0.2300000
41827.0000000	0.2350000
41828.0000000	0.2200000
41829.0000000	0.2300000
41830.0000000	0.2250000
41831.0000000	0.2300000
41832.0000000	0.2350000
41833.0000000	0.2300000
41834.0000000	0.2250000
41835.0000000	0.2350000
41836.0000000	0.2350000
41837.0000000	0.2300000

41838.0000000	0.2350000
41839.0000000	0.2300000
41840.0000000	0.2350000
41841.0000000	0.2400000
41842.0000000	0.2350000
41843.0000000	0.2300000
41844.0000000	0.2250000
41845.0000000	0.2433333
41846.0000000	0.2300000
41847.0000000	0.2300000
41848.0000000	0.2300000
41849.0000000	0.2400000
41850.0000000	0.2300000
41851.0000000	0.2300000
41852.0000000	0.2400000
41853.0000000	0.2300000
41854.0000000	0.2300000
41855.0000000	0.2300000
41856.0000000	0.2250000
41857.0000000	0.2350000
41858.0000000	0.2250000
41859.0000000	0.2350000
41860.0000000	0.2350000
41861.0000000	0.2400000
41862.0000000	0.2300000
41863.0000000	0.2400000
41864.0000000	0.2250000
41865.0000000	0.2300000
41866.0000000	0.2400000
41867.0000000	0.2300000
41868.0000000	0.2350000
41869.0000000	0.2350000
41870.0000000	0.2300000
41871.0000000	0.2350000
41872.0000000	0.2400000
41873.0000000	0.2350000
41874.0000000	0.2300000
41875.0000000	0.2400000
41876.0000000	0.2400000
41877.0000000	0.2300000
41878.0000000	0.2350000
41879.0000000	0.2450000
41880.0000000	0.2400000
41881.0000000	0.2350000
41882.0000000	0.2400000
41883.0000000	0.2400000
41884.0000000	0.2450000
41885.0000000	0.2450000
41886.0000000	0.2400000
41887.0000000	0.2450000
41888.0000000	0.2350000
41889.0000000	0.2400000
41890.0000000	0.2400000
41891.0000000	0.2350000
41892.0000000	0.2400000
41893.0000000	0.2400000
41894.0000000	0.2400000

41895.0000000	0.2400000
41896.0000000	0.2350000
41897.0000000	0.2350000
41898.0000000	0.2300000
41899.0000000	0.2400000
41900.0000000	0.2400000
41901.0000000	0.2350000
41902.0000000	0.2400000
41903.0000000	0.2450000
41904.0000000	0.2450000
41905.0000000	0.2366667
41906.0000000	0.2350000
41907.0000000	0.2350000
41908.0000000	0.2450000
41909.0000000	0.2450000
41910.0000000	0.2400000
41911.0000000	0.2300000
41912.0000000	0.2350000
41913.0000000	0.2400000
41914.0000000	0.2350000
41915.0000000	0.2350000
41916.0000000	0.2400000
41917.0000000	0.2350000
41918.0000000	0.2350000
41919.0000000	0.2450000
41920.0000000	0.2400000
41921.0000000	0.2400000
41922.0000000	0.2450000
41923.0000000	0.2450000
41924.0000000	0.2400000
41925.0000000	0.2450000
41926.0000000	0.2450000
41927.0000000	0.2400000
41928.0000000	0.2400000
41929.0000000	0.2450000
41930.0000000	0.2400000
41931.0000000	0.2500000
41932.0000000	0.2500000
41933.0000000	0.2550000
41934.0000000	0.2400000
41935.0000000	0.2466667
41936.0000000	0.2450000
41937.0000000	0.2500000
41938.0000000	0.2450000
41939.0000000	0.2400000
41940.0000000	0.2450000
41941.0000000	0.2350000
41942.0000000	0.2400000
41943.0000000	0.2350000
41944.0000000	0.2450000
41945.0000000	0.2250000
41946.0000000	0.2450000
41947.0000000	0.2350000
41948.0000000	0.2400000
41949.0000000	0.2250000
41950.0000000	0.2350000
41951.0000000	0.2200000

41952.0000000	0.2300000
41953.0000000	0.2350000
41954.0000000	0.2300000
41955.0000000	0.2300000
41956.0000000	0.2300000
41957.0000000	0.2300000
41958.0000000	0.2350000
41959.0000000	0.2300000
41960.0000000	0.2300000
41961.0000000	0.2350000
41962.0000000	0.2400000
41963.0000000	0.2350000
41964.0000000	0.2400000
41965.0000000	0.2350000
41966.0000000	0.2300000
41967.0000000	0.2250000
41968.0000000	0.2350000
41969.0000000	0.2350000
41970.0000000	0.2350000
41971.0000000	0.2300000
41972.0000000	0.2300000
41973.0000000	0.2350000
41974.0000000	0.2400000
41975.0000000	0.2400000
41976.0000000	0.2350000
41977.0000000	0.2350000
41978.0000000	0.2400000
41979.0000000	0.2400000
41980.0000000	0.2350000
41981.0000000	0.2350000
41982.0000000	0.2350000
41983.0000000	0.2350000
41984.0000000	0.2350000
41985.0000000	0.2350000
41986.0000000	0.2450000
41987.0000000	0.2400000
41988.0000000	0.2400000
41989.0000000	0.2400000
41990.0000000	0.2400000
41991.0000000	0.2350000
41992.0000000	0.2350000
41993.0000000	0.2400000
41994.0000000	0.2400000
41995.0000000	0.2450000
41996.0000000	0.2333333
41997.0000000	0.2350000
41998.0000000	0.2450000
41999.0000000	0.2350000
42000.0000000	0.2400000
42001.0000000	0.2250000
42002.0000000	0.2300000
42003.0000000	0.2300000
42004.0000000	0.2400000
42005.0000000	0.2350000
42006.0000000	0.2450000
42007.0000000	0.2250000
42008.0000000	0.2300000

42009.0000000	0.2200000
42010.0000000	0.2300000
42011.0000000	0.2300000
42012.0000000	0.2400000
42013.0000000	0.2200000
42014.0000000	0.2400000
42015.0000000	0.2300000
42016.0000000	0.2350000
42017.0000000	0.2250000
42018.0000000	0.2250000
42019.0000000	0.2350000
42020.0000000	0.2250000
42021.0000000	0.2200000
42022.0000000	0.2250000
42023.0000000	0.2300000
42024.0000000	0.2300000
42025.0000000	0.2300000
42026.0000000	0.2266666
42027.0000000	0.2300000
42028.0000000	0.2250000
42029.0000000	0.2350000
42030.0000000	0.2300000
42031.0000000	0.2350000
42032.0000000	0.2250000
42033.0000000	0.2350000
42034.0000000	0.2200000
42035.0000000	0.2350000
42036.0000000	0.2300000
42037.0000000	0.2450000
42038.0000000	0.2250000
42039.0000000	0.2300000
42040.0000000	0.2250000
42041.0000000	0.2250000
42042.0000000	0.2250000
42043.0000000	0.2400000
42044.0000000	0.2350000
42045.0000000	0.2400000
42046.0000000	0.2400000
42047.0000000	0.2300000
42048.0000000	0.2250000
42049.0000000	0.2350000
42050.0000000	0.2350000
42051.0000000	0.2300000
42052.0000000	0.2400000
42053.0000000	0.2300000
42054.0000000	0.2350000
42055.0000000	0.2300000
42056.0000000	0.2300000
42057.0000000	0.2400000
42058.0000000	0.2300000
42059.0000000	0.2350000
42060.0000000	0.2300000
42061.0000000	0.2450000
42062.0000000	0.2300000
42063.0000000	0.2350000
42064.0000000	0.2350000
42065.0000000	0.2350000

42066.0000000	0.2400000
42067.0000000	0.2350000
42068.0000000	0.2350000
42069.0000000	0.2400000
42070.0000000	0.2300000
42071.0000000	0.2400000
42072.0000000	0.2350000
42073.0000000	0.2250000
42074.0000000	0.2250000
42075.0000000	0.2350000
42076.0000000	0.2250000
42077.0000000	0.2300000
42078.0000000	0.2250000
42079.0000000	0.2300000
42080.0000000	0.2350000
42081.0000000	0.2300000
42082.0000000	0.2300000
42083.0000000	0.2350000
42084.0000000	0.2350000
42085.0000000	0.2350000
42086.0000000	0.2300000
42087.0000000	0.2300000
42088.0000000	0.2400000
42089.0000000	0.2400000
42090.0000000	0.2350000
42091.0000000	0.2400000
42092.0000000	0.2400000
42093.0000000	0.2350000
42094.0000000	0.2400000
42095.0000000	0.2350000
42096.0000000	0.2300000
42097.0000000	0.2300000
42098.0000000	0.2350000
42099.0000000	0.2300000
42100.0000000	0.2250000
42101.0000000	0.2250000
42102.0000000	0.2300000
42103.0000000	0.2200000
42104.0000000	0.2250000
42105.0000000	0.2250000
42106.0000000	0.2300000
42107.0000000	0.2200000
42108.0000000	0.2250000
42109.0000000	0.2200000
42110.0000000	0.2250000
42111.0000000	0.2200000
42112.0000000	0.2200000
42113.0000000	0.2250000
42114.0000000	0.2300000
42115.0000000	0.2200000
42116.0000000	0.2300000
42117.0000000	0.2250000
42118.0000000	0.2300000
42119.0000000	0.2300000
42120.0000000	0.2200000
42121.0000000	0.2300000
42122.0000000	0.2250000

42123.0000000	0.2300000
42124.0000000	0.2350000
42125.0000000	0.2300000
42126.0000000	0.2250000
42127.0000000	0.2250000
42128.0000000	0.2300000
42129.0000000	0.2350000
42130.0000000	0.2250000
42131.0000000	0.2300000
42132.0000000	0.2300000
42133.0000000	0.2350000
42134.0000000	0.2350000
42135.0000000	0.2350000
42136.0000000	0.2350000
42137.0000000	0.2350000
42138.0000000	0.2400000
42139.0000000	0.2400000
42140.0000000	0.2450000
42141.0000000	0.2450000
42142.0000000	0.2450000
42143.0000000	0.2450000
42144.0000000	0.2400000
42145.0000000	0.2450000
42146.0000000	0.2400000
42147.0000000	0.2450000
42148.0000000	0.2450000
42149.0000000	0.2450000
42150.0000000	0.2400000
42151.0000000	0.2350000
42152.0000000	0.2500000
42153.0000000	0.2500000
42154.0000000	0.2450000
42155.0000000	0.2400000
42156.0000000	0.2450000
42157.0000000	0.2500000
42158.0000000	0.2500000
42159.0000000	0.2500000
42160.0000000	0.2450000
42161.0000000	0.2500000
42162.0000000	0.2500000
42163.0000000	0.2450000
42164.0000000	0.2400000
42165.0000000	0.2400000
42166.0000000	0.2400000
42167.0000000	0.2400000
42168.0000000	0.2350000
42169.0000000	0.2450000
42170.0000000	0.2450000
42171.0000000	0.2350000
42172.0000000	0.2450000
42173.0000000	0.2450000
42174.0000000	0.2400000
42175.0000000	0.2400000
42176.0000000	0.2400000
42177.0000000	0.2433333
42178.0000000	0.2400000
42179.0000000	0.2350000

42180.0000000	0.2450000
42181.0000000	0.2450000
42182.0000000	0.2450000
42183.0000000	0.2450000
42184.0000000	0.2500000
42185.0000000	0.2450000
42186.0000000	0.2500000
42187.0000000	0.2450000
42188.0000000	0.2400000
42189.0000000	0.2500000
42190.0000000	0.2400000
42191.0000000	0.2650000
42192.0000000	0.2550000
42193.0000000	0.2450000
42194.0000000	0.2450000
42195.0000000	0.2450000
42196.0000000	0.2550000
42197.0000000	0.2550000
42198.0000000	0.2600000
42199.0000000	0.2500000
42200.0000000	0.2550000
42201.0000000	0.2500000
42202.0000000	0.2600000
42203.0000000	0.2550000
42204.0000000	0.2650000
42205.0000000	0.2600000
42206.0000000	0.2600000
42207.0000000	0.2666667
42208.0000000	0.2550000
42209.0000000	0.2600000
42210.0000000	0.2550000
42211.0000000	0.2600000
42212.0000000	0.2450000
42213.0000000	0.2600000
42214.0000000	0.2450000
42215.0000000	0.2500000
42216.0000000	0.2500000
42217.0000000	0.2550000
42218.0000000	0.2500000
42219.0000000	0.2600000
42220.0000000	0.2550000
42221.0000000	0.2600000
42222.0000000	0.2450000
42223.0000000	0.2550000
42224.0000000	0.2550000
42225.0000000	0.2500000
42226.0000000	0.2600000
42227.0000000	0.2500000
42228.0000000	0.2600000
42229.0000000	0.2500000
42230.0000000	0.2550000
42231.0000000	0.2450000
42232.0000000	0.2550000
42233.0000000	0.2550000
42234.0000000	0.2550000
42235.0000000	0.2500000
42236.0000000	0.2350000

42237.0000000	0.2466667
42238.0000000	0.2450000
42239.0000000	0.2350000
42240.0000000	0.2400000
42241.0000000	0.2400000
42242.0000000	0.2400000
42243.0000000	0.2450000
42244.0000000	0.2450000
42245.0000000	0.2350000
42246.0000000	0.2350000
42247.0000000	0.2400000
42248.0000000	0.2300000
42249.0000000	0.2300000
42250.0000000	0.2350000
42251.0000000	0.2400000
42252.0000000	0.2350000
42253.0000000	0.2400000
42254.0000000	0.2350000
42255.0000000	0.2400000
42256.0000000	0.2400000
42257.0000000	0.2450000
42258.0000000	0.2400000
42259.0000000	0.2400000
42260.0000000	0.2300000
42261.0000000	0.2400000
42262.0000000	0.2350000
42263.0000000	0.2400000
42264.0000000	0.2450000
42265.0000000	0.2350000
42266.0000000	0.2350000
42267.0000000	0.2300000
42268.0000000	0.2400000
42269.0000000	0.2350000
42270.0000000	0.2350000
42271.0000000	0.2300000
42272.0000000	0.2250000
42273.0000000	0.2400000
42274.0000000	0.2250000
42275.0000000	0.2350000
42276.0000000	0.2350000
42277.0000000	0.2300000
42278.0000000	0.2450000
42279.0000000	0.2400000
42280.0000000	0.2450000
42281.0000000	0.2300000
42282.0000000	0.2400000
42283.0000000	0.2400000
42284.0000000	0.2400000
42285.0000000	0.2400000
42286.0000000	0.2350000
42287.0000000	0.2400000
42288.0000000	0.2450000
42289.0000000	0.2350000
42290.0000000	0.2500000
42291.0000000	0.2400000
42292.0000000	0.2350000
42293.0000000	0.2450000

42294.0000000	0.2500000
42295.0000000	0.2350000
42296.0000000	0.2450000
42297.0000000	0.2400000
42298.0000000	0.2433333
42299.0000000	0.2550000
42300.0000000	0.2400000
42301.0000000	0.2400000
42302.0000000	0.2550000
42303.0000000	0.2500000
42304.0000000	0.2450000
42305.0000000	0.2500000
42306.0000000	0.2500000
42307.0000000	0.2500000
42308.0000000	0.2500000
42309.0000000	0.2400000
42310.0000000	0.2400000
42311.0000000	0.2500000
42312.0000000	0.2450000
42313.0000000	0.2550000
42314.0000000	0.2450000
42315.0000000	0.2450000
42316.0000000	0.2450000
42317.0000000	0.2450000
42318.0000000	0.2450000
42319.0000000	0.2400000
42320.0000000	0.2400000
42321.0000000	0.2400000
42322.0000000	0.2450000
42323.0000000	0.2450000
42324.0000000	0.2400000
42325.0000000	0.2400000
42326.0000000	0.2350000
42327.0000000	0.2400000
42328.0000000	0.2366667
42329.0000000	0.2400000
42330.0000000	0.2300000
42331.0000000	0.2350000
42332.0000000	0.2350000
42333.0000000	0.2300000
42334.0000000	0.2250000
42335.0000000	0.2300000
42336.0000000	0.2350000
42337.0000000	0.2300000
42338.0000000	0.2300000
42339.0000000	0.2350000
42340.0000000	0.2350000
42341.0000000	0.2400000
42342.0000000	0.2300000
42343.0000000	0.2350000
42344.0000000	0.2350000
42345.0000000	0.2250000
42346.0000000	0.2300000
42347.0000000	0.2300000
42348.0000000	0.2250000
42349.0000000	0.2350000
42350.0000000	0.2300000

42351.0000000	0.2300000
42352.0000000	0.2300000
42353.0000000	0.2350000
42354.0000000	0.2350000
42355.0000000	0.2300000
42356.0000000	0.2250000
42357.0000000	0.2300000
42358.0000000	0.2266666
42359.0000000	0.2300000
42360.0000000	0.2300000
42361.0000000	0.2300000
42362.0000000	0.2350000
42363.0000000	0.2250000
42364.0000000	0.2300000
42365.0000000	0.2350000
42366.0000000	0.2200000
42367.0000000	0.2400000
42368.0000000	0.2250000
42369.0000000	0.2200000
42370.0000000	0.2400000
42371.0000000	0.2300000
42372.0000000	0.2350000
42373.0000000	0.2300000
42374.0000000	0.2200000
42375.0000000	0.2350000
42376.0000000	0.2250000
42377.0000000	0.2250000
42378.0000000	0.2200000
42379.0000000	0.2300000
42380.0000000	0.2300000
42381.0000000	0.2250000
42382.0000000	0.2350000
42383.0000000	0.2350000
42384.0000000	0.2250000
42385.0000000	0.2350000
42386.0000000	0.2300000
42387.0000000	0.2400000
42388.0000000	0.2333333
42389.0000000	0.2300000
42390.0000000	0.2300000
42391.0000000	0.2350000
42392.0000000	0.2300000
42393.0000000	0.2350000
42394.0000000	0.2350000
42395.0000000	0.2400000
42396.0000000	0.2450000
42397.0000000	0.2400000
42398.0000000	0.2400000
42399.0000000	0.2400000
42400.0000000	0.2500000
42401.0000000	0.2500000
42402.0000000	0.2350000
42403.0000000	0.2400000
42404.0000000	0.2400000
42405.0000000	0.2450000
42406.0000000	0.2500000
42407.0000000	0.2450000

42408.0000000	0.2500000
42409.0000000	0.2400000
42410.0000000	0.2500000
42411.0000000	0.2450000
42412.0000000	0.2400000
42413.0000000	0.2450000
42414.0000000	0.2500000
42415.0000000	0.2450000
42416.0000000	0.2500000
42417.0000000	0.2450000
42418.0000000	0.2400000
42419.0000000	0.2466667
42420.0000000	0.2500000
42421.0000000	0.2550000
42422.0000000	0.2450000
42423.0000000	0.2550000
42424.0000000	0.2500000
42425.0000000	0.2400000
42426.0000000	0.2500000
42427.0000000	0.2400000
42428.0000000	0.2400000
42429.0000000	0.2500000
42430.0000000	0.2450000
42431.0000000	0.2500000
42432.0000000	0.2400000
42433.0000000	0.2450000
42434.0000000	0.2350000
42435.0000000	0.2500000
42436.0000000	0.2400000
42437.0000000	0.2450000
42438.0000000	0.2350000
42439.0000000	0.2500000
42440.0000000	0.2400000
42441.0000000	0.2450000
42442.0000000	0.2350000
42443.0000000	0.2450000
42444.0000000	0.2400000
42445.0000000	0.2450000
42446.0000000	0.2450000
42447.0000000	0.2500000
42448.0000000	0.2400000
42449.0000000	0.2433333
42450.0000000	0.2500000
42451.0000000	0.2500000
42452.0000000	0.2400000
42453.0000000	0.2400000
42454.0000000	0.2300000
42455.0000000	0.2400000
42456.0000000	0.2350000
42457.0000000	0.2400000
42458.0000000	0.2350000
42459.0000000	0.2350000
42460.0000000	0.2350000
42461.0000000	0.2400000
42462.0000000	0.2450000
42463.0000000	0.2350000
42464.0000000	0.2400000

42465.0000000	0.2350000
42466.0000000	0.2450000
42467.0000000	0.2400000
42468.0000000	0.2400000
42469.0000000	0.2450000
42470.0000000	0.2300000
42471.0000000	0.2250000
42472.0000000	0.2400000
42473.0000000	0.2350000
42474.0000000	0.2500000
42475.0000000	0.2450000
42476.0000000	0.2350000
42477.0000000	0.2300000
42478.0000000	0.2300000
42479.0000000	0.2250000
42480.0000000	0.2250000
42481.0000000	0.2300000
42482.0000000	0.2300000
42483.0000000	0.2250000
42484.0000000	0.2300000
42485.0000000	0.2300000
42486.0000000	0.2300000
42487.0000000	0.2300000
42488.0000000	0.2300000
42489.0000000	0.2300000
42490.0000000	0.2300000
42491.0000000	0.2250000
42492.0000000	0.2250000
42493.0000000	0.2300000
42494.0000000	0.2200000
42495.0000000	0.2250000
42496.0000000	0.2150000
42497.0000000	0.2200000
42498.0000000	0.2200000
42499.0000000	0.2250000
42500.0000000	0.2250000
42501.0000000	0.2250000
42502.0000000	0.2200000
42503.0000000	0.2150000
42504.0000000	0.2200000
42505.0000000	0.2200000
42506.0000000	0.2250000
42507.0000000	0.2200000
42508.0000000	0.2250000
42509.0000000	0.2166667
42510.0000000	0.2250000
42511.0000000	0.2200000
42512.0000000	0.2250000
42513.0000000	0.2200000
42514.0000000	0.2200000
42515.0000000	0.2200000
42516.0000000	0.2200000
42517.0000000	0.2200000
42518.0000000	0.2200000
42519.0000000	0.2250000
42520.0000000	0.2200000
42521.0000000	0.2200000

42522.0000000	0.2250000
42523.0000000	0.2250000
42524.0000000	0.2300000
42525.0000000	0.2300000
42526.0000000	0.2300000
42527.0000000	0.2200000
42528.0000000	0.2250000
42529.0000000	0.2250000
42530.0000000	0.2150000
42531.0000000	0.2200000
42532.0000000	0.2200000
42533.0000000	0.2350000
42534.0000000	0.2300000
42535.0000000	0.2250000
42536.0000000	0.2300000
42537.0000000	0.2250000
42538.0000000	0.2250000
42539.0000000	0.2300000
42540.0000000	0.2200000
42541.0000000	0.2350000
42542.0000000	0.2300000
42543.0000000	0.2300000
42544.0000000	0.2350000
42545.0000000	0.2350000
42546.0000000	0.2300000
42547.0000000	0.2300000
42548.0000000	0.2350000
42549.0000000	0.2350000
42550.0000000	0.2300000
42551.0000000	0.2350000
42552.0000000	0.2350000
42553.0000000	0.2300000
42554.0000000	0.2300000
42555.0000000	0.2350000
42556.0000000	0.2300000
42557.0000000	0.2300000
42558.0000000	0.2300000
42559.0000000	0.2300000
42560.0000000	0.2250000
42561.0000000	0.2400000
42562.0000000	0.2350000
42563.0000000	0.2350000
42564.0000000	0.2350000
42565.0000000	0.2300000
42566.0000000	0.2350000
42567.0000000	0.2250000
42568.0000000	0.2350000
42569.0000000	0.2366667
42570.0000000	0.2400000
42571.0000000	0.2350000
42572.0000000	0.2350000
42573.0000000	0.2300000
42574.0000000	0.2350000
42575.0000000	0.2400000
42576.0000000	0.2350000
42577.0000000	0.2400000
42578.0000000	0.2300000

42579.0000000	0.2350000
42580.0000000	0.2350000
42581.0000000	0.2350000
42582.0000000	0.2250000
42583.0000000	0.2350000
42584.0000000	0.2350000
42585.0000000	0.2300000
42586.0000000	0.2300000
42587.0000000	0.2250000
42588.0000000	0.2250000
42589.0000000	0.2300000
42590.0000000	0.2250000
42591.0000000	0.2200000
42592.0000000	0.2150000
42593.0000000	0.2250000
42594.0000000	0.2250000
42595.0000000	0.2150000
42596.0000000	0.2150000
42597.0000000	0.2200000
42598.0000000	0.2100000
42599.0000000	0.2233333
42600.0000000	0.2150000
42601.0000000	0.2200000
42602.0000000	0.2150000
42603.0000000	0.2050000
42604.0000000	0.2100000
42605.0000000	0.2050000
42606.0000000	0.2050000
42607.0000000	0.2050000
42608.0000000	0.2050000
42609.0000000	0.2000000
42610.0000000	0.2050000
42611.0000000	0.2100000
42612.0000000	0.2050000
42613.0000000	0.2050000
42614.0000000	0.2050000
42615.0000000	0.2050000
42616.0000000	0.2100000
42617.0000000	0.2000000
42618.0000000	0.2050000
42619.0000000	0.2050000
42620.0000000	0.2050000
42621.0000000	0.2050000
42622.0000000	0.2050000
42623.0000000	0.2000000
42624.0000000	0.2000000
42625.0000000	0.2000000
42626.0000000	0.2050000
42627.0000000	0.2000000
42628.0000000	0.1950000
42629.0000000	0.2000000
42630.0000000	0.2000000
42631.0000000	0.1900000
42632.0000000	0.1950000
42633.0000000	0.1900000
42634.0000000	0.1900000
42635.0000000	0.1900000

42636.0000000	0.1850000
42637.0000000	0.1900000
42638.0000000	0.1900000
42639.0000000	0.1950000
42640.0000000	0.1800000
42641.0000000	0.1750000
42642.0000000	0.1900000
42643.0000000	0.1850000
42644.0000000	0.1750000
42645.0000000	0.1850000
42646.0000000	0.1850000
42647.0000000	0.1850000
42648.0000000	0.1900000
42649.0000000	0.1800000
42650.0000000	0.1850000
42651.0000000	0.1950000
42652.0000000	0.1750000
42653.0000000	0.1850000
42654.0000000	0.1850000
42655.0000000	0.1800000
42656.0000000	0.1850000
42657.0000000	0.1950000
42658.0000000	0.1850000
42659.0000000	0.1850000
42660.0000000	0.1966667
42661.0000000	0.1950000
42662.0000000	0.1850000
42663.0000000	0.1850000
42664.0000000	0.1900000
42665.0000000	0.1900000
42666.0000000	0.1850000
42667.0000000	0.1900000
42668.0000000	0.1850000
42669.0000000	0.1950000
42670.0000000	0.1850000
42671.0000000	0.1950000
42672.0000000	0.1900000
42673.0000000	0.1850000
42674.0000000	0.1950000
42675.0000000	0.1950000
42676.0000000	0.1900000
42677.0000000	0.1950000
42678.0000000	0.1950000
42679.0000000	0.1950000
42680.0000000	0.1950000
42681.0000000	0.1900000
42682.0000000	0.1850000
42683.0000000	0.1950000
42684.0000000	0.1900000
42685.0000000	0.1900000
42686.0000000	0.1950000
42687.0000000	0.1900000
42688.0000000	0.1900000
42689.0000000	0.1800000
42690.0000000	0.1866667
42691.0000000	0.1850000
42692.0000000	0.1850000

42693.0000000	0.1900000
42694.0000000	0.1850000
42695.0000000	0.1850000
42696.0000000	0.1700000
42697.0000000	0.1750000
42698.0000000	0.1800000
42699.0000000	0.1650000
42700.0000000	0.1700000
42701.0000000	0.1700000
42702.0000000	0.1700000
42703.0000000	0.1750000
42704.0000000	0.1750000
42705.0000000	0.1750000
42706.0000000	0.1650000
42707.0000000	0.1700000
42708.0000000	0.1750000
42709.0000000	0.1700000
42710.0000000	0.1650000
42711.0000000	0.1600000
42712.0000000	0.1700000
42713.0000000	0.1550000
42714.0000000	0.1700000
42715.0000000	0.1650000
42716.0000000	0.1700000
42717.0000000	0.1650000
42718.0000000	0.1650000
42719.0000000	0.1800000
42720.0000000	0.1700000
42721.0000000	0.1650000
42722.0000000	0.1650000
42723.0000000	0.1700000
42724.0000000	0.1700000
42725.0000000	0.1650000
42726.0000000	0.1650000
42727.0000000	0.1700000
42728.0000000	0.1700000
42729.0000000	0.1600000
42730.0000000	0.1650000
42731.0000000	0.1550000
42732.0000000	0.1650000
42733.0000000	0.1600000
42734.0000000	0.1550000
42735.0000000	0.1650000
42736.0000000	0.1650000
42737.0000000	0.1550000
42738.0000000	0.1650000
42739.0000000	0.1600000
42740.0000000	0.1650000
42741.0000000	0.1550000
42742.0000000	0.1600000
42743.0000000	0.1550000
42744.0000000	0.1650000
42745.0000000	0.1600000
42746.0000000	0.1650000
42747.0000000	0.1650000
42748.0000000	0.1550000
42749.0000000	0.1700000

42750.0000000	0.1600000
42751.0000000	0.1633333
42752.0000000	0.1750000
42753.0000000	0.1700000
42754.0000000	0.1650000
42755.0000000	0.1700000
42756.0000000	0.1650000
42757.0000000	0.1650000
42758.0000000	0.1700000
42759.0000000	0.1650000
42760.0000000	0.1650000
42761.0000000	0.1700000
42762.0000000	0.1700000
42763.0000000	0.1750000
42764.0000000	0.1750000
42765.0000000	0.1700000
42766.0000000	0.1700000
42767.0000000	0.1700000
42768.0000000	0.1750000
42769.0000000	0.1700000
42770.0000000	0.1650000
42771.0000000	0.1750000
42772.0000000	0.1650000
42773.0000000	0.1600000
42774.0000000	0.1750000
42775.0000000	0.1600000
42776.0000000	0.1700000
42777.0000000	0.1700000
42778.0000000	0.1600000
42779.0000000	0.1650000
42780.0000000	0.1650000
42781.0000000	0.1766667
42782.0000000	0.1700000
42783.0000000	0.1600000
42784.0000000	0.1600000
42785.0000000	0.1650000
42786.0000000	0.1650000
42787.0000000	0.1600000
42788.0000000	0.1650000
42789.0000000	0.1700000
42790.0000000	0.1650000
42791.0000000	0.1750000
42792.0000000	0.1650000
42793.0000000	0.1600000
42794.0000000	0.1700000
42795.0000000	0.1650000
42796.0000000	0.1550000
42797.0000000	0.1650000
42798.0000000	0.1650000
42799.0000000	0.1650000
42800.0000000	0.1700000
42801.0000000	0.1600000
42802.0000000	0.1650000
42803.0000000	0.1700000
42804.0000000	0.1700000
42805.0000000	0.1650000
42806.0000000	0.1700000

42807.0000000	0.1650000
42808.0000000	0.1700000
42809.0000000	0.1800000
42810.0000000	0.1650000
42811.0000000	0.1733333
42812.0000000	0.1750000
42813.0000000	0.1700000
42814.0000000	0.1600000
42815.0000000	0.1700000
42816.0000000	0.1750000
42817.0000000	0.1750000
42818.0000000	0.1750000
42819.0000000	0.1750000
42820.0000000	0.1700000
42821.0000000	0.1800000
42822.0000000	0.1800000
42823.0000000	0.1800000
42824.0000000	0.1800000
42825.0000000	0.1750000
42826.0000000	0.1850000
42827.0000000	0.1650000
42828.0000000	0.1850000
42829.0000000	0.1800000
42830.0000000	0.1850000
42831.0000000	0.1900000
42832.0000000	0.1800000
42833.0000000	0.1850000
42834.0000000	0.1750000
42835.0000000	0.1850000
42836.0000000	0.1750000
42837.0000000	0.1800000
42838.0000000	0.1800000
42839.0000000	0.1800000
42840.0000000	0.1850000
42841.0000000	0.1733333
42842.0000000	0.1800000
42843.0000000	0.1850000
42844.0000000	0.1750000
42845.0000000	0.1800000
42846.0000000	0.1800000
42847.0000000	0.1750000
42848.0000000	0.1750000
42849.0000000	0.1800000
42850.0000000	0.1750000
42851.0000000	0.1700000
42852.0000000	0.1850000
42853.0000000	0.1900000
42854.0000000	0.1800000
42855.0000000	0.1750000
42856.0000000	0.1750000
42857.0000000	0.1800000
42858.0000000	0.1800000
42859.0000000	0.1800000
42860.0000000	0.1850000
42861.0000000	0.1750000
42862.0000000	0.1900000
42863.0000000	0.1800000

42864.0000000	0.1750000
42865.0000000	0.1750000
42866.0000000	0.1800000
42867.0000000	0.1800000
42868.0000000	0.1750000
42869.0000000	0.1700000
42870.0000000	0.1750000
42871.0000000	0.1750000
42872.0000000	0.1833333
42873.0000000	0.1850000
42874.0000000	0.1800000
42875.0000000	0.1850000
42876.0000000	0.1800000
42877.0000000	0.1800000
42878.0000000	0.1900000
42879.0000000	0.1750000
42880.0000000	0.1800000
42881.0000000	0.1900000
42882.0000000	0.1850000
42883.0000000	0.1850000
42884.0000000	0.1900000
42885.0000000	0.1900000
42886.0000000	0.1850000
42887.0000000	0.1950000
42888.0000000	0.1900000
42889.0000000	0.1900000
42890.0000000	0.1800000
42891.0000000	0.1800000
42892.0000000	0.1800000
42893.0000000	0.1700000
42894.0000000	0.1800000
42895.0000000	0.1800000
42896.0000000	0.1800000
42897.0000000	0.1850000
42898.0000000	0.1850000
42899.0000000	0.1700000
42900.0000000	0.1750000
42901.0000000	0.1766667
42902.0000000	0.1750000
42903.0000000	0.1800000
42904.0000000	0.1800000
42905.0000000	0.1650000
42906.0000000	0.1750000
42907.0000000	0.1800000
42908.0000000	0.1650000
42909.0000000	0.1700000
42910.0000000	0.1650000
42911.0000000	0.1650000
42912.0000000	0.1700000
42913.0000000	0.1750000
42914.0000000	0.1750000
42915.0000000	0.1650000
42916.0000000	0.1700000
42917.0000000	0.1750000
42918.0000000	0.1650000
42919.0000000	0.1650000
42920.0000000	0.1750000

42921.0000000	0.1750000
42922.0000000	0.1650000
42923.0000000	0.1800000
42924.0000000	0.1700000
42925.0000000	0.1700000
42926.0000000	0.1750000
42927.0000000	0.1650000
42928.0000000	0.1700000
42929.0000000	0.1750000
42930.0000000	0.1850000
42931.0000000	0.1833333
42932.0000000	0.1700000
42933.0000000	0.1650000
42934.0000000	0.1800000
42935.0000000	0.1700000
42936.0000000	0.1750000
42937.0000000	0.1800000
42938.0000000	0.1750000
42939.0000000	0.1800000
42940.0000000	0.1800000
42941.0000000	0.1700000
42942.0000000	0.1800000
42943.0000000	0.1850000
42944.0000000	0.1800000
42945.0000000	0.1750000
42946.0000000	0.1800000
42947.0000000	0.1800000
42948.0000000	0.1850000
42949.0000000	0.1750000
42950.0000000	0.1800000
42951.0000000	0.1800000
42952.0000000	0.1800000
42953.0000000	0.1900000
42954.0000000	0.1850000
42955.0000000	0.1800000
42956.0000000	0.1850000
42957.0000000	0.1850000
42958.0000000	0.1850000
42959.0000000	0.1850000
42960.0000000	0.1900000
42961.0000000	0.1900000
42962.0000000	0.1866667
42963.0000000	0.1850000
42964.0000000	0.1850000
42965.0000000	0.1850000
42966.0000000	0.1950000
42967.0000000	0.1800000
42968.0000000	0.1900000
42969.0000000	0.2000000
42970.0000000	0.1900000
42971.0000000	0.1900000
42972.0000000	0.1950000
42973.0000000	0.1900000
42974.0000000	0.1900000
42975.0000000	0.1950000
42976.0000000	0.1850000
42977.0000000	0.1900000

42978.0000000	0.1900000
42979.0000000	0.1950000
42980.0000000	0.1950000
42981.0000000	0.1800000
42982.0000000	0.1900000
42983.0000000	0.1850000
42984.0000000	0.1900000
42985.0000000	0.1900000
42986.0000000	0.1900000
42987.0000000	0.1900000
42988.0000000	0.2000000
42989.0000000	0.1800000
42990.0000000	0.1900000
42991.0000000	0.1900000
42992.0000000	0.1900000
42993.0000000	0.1950000
42994.0000000	0.1850000
42995.0000000	0.1900000
42996.0000000	0.1850000
42997.0000000	0.1950000
42998.0000000	0.1950000
42999.0000000	0.2000000
43000.0000000	0.1900000
43001.0000000	0.1800000
43002.0000000	0.1950000
43003.0000000	0.1850000
43004.0000000	0.1950000
43005.0000000	0.1950000
43006.0000000	0.1950000
43007.0000000	0.1950000
43008.0000000	0.1900000
43009.0000000	0.1950000
43010.0000000	0.1900000
43011.0000000	0.1950000
43012.0000000	0.2000000
43013.0000000	0.1900000
43014.0000000	0.1900000
43015.0000000	0.1950000
43016.0000000	0.2000000
43017.0000000	0.1900000
43018.0000000	0.1950000
43019.0000000	0.1950000
43020.0000000	0.1950000
43021.0000000	0.1950000
43022.0000000	0.1966667
43023.0000000	0.1900000
43024.0000000	0.1900000
43025.0000000	0.1950000
43026.0000000	0.1900000
43027.0000000	0.1950000
43028.0000000	0.2000000
43029.0000000	0.1950000
43030.0000000	0.1950000
43031.0000000	0.1900000
43032.0000000	0.1900000
43033.0000000	0.1900000
43034.0000000	0.1850000

43035.0000000	0.1850000
43036.0000000	0.1950000
43037.0000000	0.1850000
43038.0000000	0.1800000
43039.0000000	0.1850000
43040.0000000	0.1900000
43041.0000000	0.1850000
43042.0000000	0.1850000
43043.0000000	0.1850000
43044.0000000	0.1900000
43045.0000000	0.1850000
43046.0000000	0.1800000
43047.0000000	0.1850000
43048.0000000	0.1850000
43049.0000000	0.1900000
43050.0000000	0.1800000
43051.0000000	0.1900000
43052.0000000	0.1833333
43053.0000000	0.1800000
43054.0000000	0.1850000
43055.0000000	0.1900000
43056.0000000	0.1850000
43057.0000000	0.1850000
43058.0000000	0.1850000
43059.0000000	0.1900000
43060.0000000	0.1750000
43061.0000000	0.1900000
43062.0000000	0.1900000
43063.0000000	0.1850000
43064.0000000	0.1900000
43065.0000000	0.1850000
43066.0000000	0.1900000
43067.0000000	0.1900000
43068.0000000	0.1900000
43069.0000000	0.1800000
43070.0000000	0.1900000
43071.0000000	0.2000000
43072.0000000	0.1900000
43073.0000000	0.1900000
43074.0000000	0.1850000
43075.0000000	0.1900000
43076.0000000	0.1950000
43077.0000000	0.1900000
43078.0000000	0.1900000
43079.0000000	0.1850000
43080.0000000	0.1950000
43081.0000000	0.1900000
43082.0000000	0.1850000
43083.0000000	0.1933333
43084.0000000	0.1900000
43085.0000000	0.1950000
43086.0000000	0.1850000
43087.0000000	0.1800000
43088.0000000	0.1900000
43089.0000000	0.1800000
43090.0000000	0.1900000
43091.0000000	0.1850000

43092.0000000	0.1800000
43093.0000000	0.1800000
43094.0000000	0.1750000
43095.0000000	0.1750000
43096.0000000	0.1800000
43097.0000000	0.1800000
43098.0000000	0.1800000
43099.0000000	0.1850000
43100.0000000	0.1750000
43101.0000000	0.1850000
43102.0000000	0.1750000
43103.0000000	0.1800000
43104.0000000	0.1850000
43105.0000000	0.1750000
43106.0000000	0.1750000
43107.0000000	0.1750000
43108.0000000	0.1750000
43109.0000000	0.1700000
43110.0000000	0.1700000
43111.0000000	0.1700000
43112.0000000	0.1750000
43113.0000000	0.1733333
43114.0000000	0.1650000
43115.0000000	0.1750000
43116.0000000	0.1700000
43117.0000000	0.1700000
43118.0000000	0.1650000
43119.0000000	0.1800000
43120.0000000	0.1600000
43121.0000000	0.1750000
43122.0000000	0.1700000
43123.0000000	0.1600000
43124.0000000	0.1650000
43125.0000000	0.1550000
43126.0000000	0.1650000
43127.0000000	0.1550000
43128.0000000	0.1550000
43129.0000000	0.1500000
43130.0000000	0.1550000
43131.0000000	0.1550000
43132.0000000	0.1550000
43133.0000000	0.1550000
43134.0000000	0.1600000
43135.0000000	0.1500000
43136.0000000	0.1550000
43137.0000000	0.1500000
43138.0000000	0.1550000
43139.0000000	0.1450000
43140.0000000	0.1550000
43141.0000000	0.1500000
43142.0000000	0.1550000
43143.0000000	0.1500000
43144.0000000	0.1400000
43145.0000000	0.1550000
43146.0000000	0.1450000
43147.0000000	0.1500000
43148.0000000	0.1450000

43149.0000000	0.1550000
43150.0000000	0.1450000
43151.0000000	0.1500000
43152.0000000	0.1450000
43153.0000000	0.1550000
43154.0000000	0.1400000
43155.0000000	0.1450000
43156.0000000	0.1300000
43157.0000000	0.1450000
43158.0000000	0.1350000
43159.0000000	0.1400000
43160.0000000	0.1350000
43161.0000000	0.1400000
43162.0000000	0.1350000
43163.0000000	0.1300000
43164.0000000	0.1350000
43165.0000000	0.1400000
43166.0000000	0.1400000
43167.0000000	0.1400000
43168.0000000	0.1350000
43169.0000000	0.1300000
43170.0000000	0.1200000
43171.0000000	0.1300000
43172.0000000	0.1350000
43173.0000000	0.1366667
43174.0000000	0.1250000
43175.0000000	0.1400000
43176.0000000	0.1350000
43177.0000000	0.1350000
43178.0000000	0.1350000
43179.0000000	0.1350000
43180.0000000	0.1250000
43181.0000000	0.1300000
43182.0000000	0.1300000
43183.0000000	0.1400000
43184.0000000	0.1300000
43185.0000000	0.1400000
43186.0000000	0.1350000
43187.0000000	0.1300000
43188.0000000	0.1250000
43189.0000000	0.1300000
43190.0000000	0.1250000
43191.0000000	0.1350000
43192.0000000	0.1200000
43193.0000000	0.1250000
43194.0000000	0.1250000
43195.0000000	0.1150000
43196.0000000	0.1300000
43197.0000000	0.1200000
43198.0000000	0.1150000
43199.0000000	0.1150000
43200.0000000	0.1150000
43201.0000000	0.1150000
43202.0000000	0.1150000
43203.0000000	0.1100000
43204.0000000	0.1200000
43205.0000000	0.1150000

43206.0000000	0.1150000
43207.0000000	0.1200000
43208.0000000	0.1250000
43209.0000000	0.1150000
43210.0000000	0.1200000
43211.0000000	0.1050000
43212.0000000	0.1150000
43213.0000000	0.1150000
43214.0000000	0.1200000
43215.0000000	0.1200000
43216.0000000	0.1150000
43217.0000000	0.1250000
43218.0000000	0.1200000
43219.0000000	0.1250000
43220.0000000	0.1150000
43221.0000000	0.1200000
43222.0000000	0.1150000
43223.0000000	0.1100000
43224.0000000	0.1100000
43225.0000000	0.1050000
43226.0000000	0.1100000
43227.0000000	0.1100000
43228.0000000	0.1150000
43229.0000000	0.1100000
43230.0000000	0.1100000
43231.0000000	0.1050000
43232.0000000	0.1100000
43233.0000000	0.1000000
43234.0000000	0.1000000
43235.0000000	0.1050000
43236.0000000	0.1050000
43237.0000000	0.0950000
43238.0000000	0.1000000
43239.0000000	0.0900000
43240.0000000	0.0950000
43241.0000000	0.0900000
43242.0000000	0.0850000
43243.0000000	0.0950000
43244.0000000	0.0900000
43245.0000000	0.0950000
43246.0000000	0.0950000
43247.0000000	0.0950000
43248.0000000	0.0900000
43249.0000000	0.0850000
43250.0000000	0.0850000
43251.0000000	0.0900000
43252.0000000	0.0850000
43253.0000000	0.1000000
43254.0000000	0.0850000
43255.0000000	0.0950000
43256.0000000	0.0950000
43257.0000000	0.0950000
43258.0000000	0.0950000
43259.0000000	0.0800000
43260.0000000	0.0850000
43261.0000000	0.0800000
43262.0000000	0.0900000

43263.0000000	0.0933333
43264.0000000	0.0800000
43265.0000000	0.0800000
43266.0000000	0.0850000
43267.0000000	0.0850000
43268.0000000	0.0850000
43269.0000000	0.0850000
43270.0000000	0.0900000
43271.0000000	0.0750000
43272.0000000	0.0900000
43273.0000000	0.0800000
43274.0000000	0.0850000
43275.0000000	0.0850000
43276.0000000	0.0850000
43277.0000000	0.0850000
43278.0000000	0.0900000
43279.0000000	0.0850000
43280.0000000	0.0850000
43281.0000000	0.0850000
43282.0000000	0.0800000
43283.0000000	0.0950000
43284.0000000	0.0900000
43285.0000000	0.1000000
43286.0000000	0.0900000
43287.0000000	0.0900000
43288.0000000	0.0850000
43289.0000000	0.0900000
43290.0000000	0.0950000
43291.0000000	0.0800000
43292.0000000	0.0900000
43293.0000000	0.0800000
43294.0000000	0.0933333
43295.0000000	0.0950000
43296.0000000	0.0900000
43297.0000000	0.0950000
43298.0000000	0.0900000
43299.0000000	0.0850000
43300.0000000	0.0950000
43301.0000000	0.0850000
43302.0000000	0.0950000
43303.0000000	0.0850000
43304.0000000	0.0900000
43305.0000000	0.0900000
43306.0000000	0.0850000
43307.0000000	0.0950000
43308.0000000	0.0900000
43309.0000000	0.0950000
43310.0000000	0.0850000
43311.0000000	0.0900000
43312.0000000	0.0850000
43313.0000000	0.0750000
43314.0000000	0.0850000
43315.0000000	0.0800000
43316.0000000	0.0800000
43317.0000000	0.0850000
43318.0000000	0.0800000
43319.0000000	0.0800000

43320.0000000	0.0900000
43321.0000000	0.0700000
43322.0000000	0.0800000
43323.0000000	0.0700000
43324.0000000	0.0733333
43325.0000000	0.0800000
43326.0000000	0.0700000
43327.0000000	0.0700000
43328.0000000	0.0700000
43329.0000000	0.0750000
43330.0000000	0.0650000
43331.0000000	0.0650000
43332.0000000	0.0650000
43333.0000000	0.0650000
43334.0000000	0.0650000
43335.0000000	0.0550000
43336.0000000	0.0600000
43337.0000000	0.0500000
43338.0000000	0.0550000
43339.0000000	0.0500000
43340.0000000	0.0550000
43341.0000000	0.0500000
43342.0000000	0.0550000
43343.0000000	0.0400000
43344.0000000	0.0450000
43345.0000000	0.0350000
43346.0000000	0.0450000
43347.0000000	0.0400000
43348.0000000	0.0500000
43349.0000000	0.0350000
43350.0000000	0.0450000
43351.0000000	0.0300000
43352.0000000	0.0350000
43353.0000000	0.0350000
43354.0000000	0.0366667
43355.0000000	0.0300000
43356.0000000	0.0350000
43357.0000000	0.0350000
43358.0000000	0.0350000
43359.0000000	0.0400000
43360.0000000	0.0350000
43361.0000000	0.0300000
43362.0000000	0.0350000
43363.0000000	0.0300000
43364.0000000	0.0350000
43365.0000000	0.0400000
43366.0000000	0.0300000
43367.0000000	0.0300000
43368.0000000	0.0250000
43369.0000000	0.0300000
43370.0000000	0.0300000
43371.0000000	0.0350000
43372.0000000	0.0250000
43373.0000000	0.0300000
43374.0000000	0.0200000
43375.0000000	0.0300000
43376.0000000	0.0150000

43377.0000000	0.0300000
43378.0000000	0.0250000
43379.0000000	0.0300000
43380.0000000	0.0250000
43381.0000000	0.0250000
43382.0000000	0.0250000
43383.0000000	0.0200000
43384.0000000	0.0233333
43385.0000000	0.0250000
43386.0000000	0.0200000
43387.0000000	0.0250000
43388.0000000	0.0250000
43389.0000000	0.0250000
43390.0000000	0.0200000
43391.0000000	0.0250000
43392.0000000	0.0150000
43393.0000000	0.0150000
43394.0000000	0.0200000
43395.0000000	0.0200000
43396.0000000	0.0300000
43397.0000000	0.0200000
43398.0000000	0.0250000
43399.0000000	0.0300000
43400.0000000	0.0250000
43401.0000000	0.0250000
43402.0000000	0.0150000
43403.0000000	0.0250000
43404.0000000	0.0250000
43405.0000000	0.0250000
43406.0000000	0.0150000
43407.0000000	0.0150000
43408.0000000	0.0150000
43409.0000000	0.0150000
43410.0000000	0.0100000
43411.0000000	0.0100000
43412.0000000	0.0150000
43413.0000000	0.0100000
43414.0000000	0.0100000
43415.0000000	0.0150000
43416.0000000	0.0100000
43417.0000000	0.0100000
43418.0000000	0.0150000
43419.0000000	0.0100000
43420.0000000	0.0100000
43421.0000000	0.0100000
43422.0000000	0.0050000
43423.0000000	0.0100000
43424.0000000	0.0050000
43425.0000000	0.0000000
43426.0000000	0.0100000
43427.0000000	0.0050000
43428.0000000	0.0100000
43429.0000000	0.0100000
43430.0000000	0.0000000
43431.0000000	-0.0050000
43432.0000000	0.0100000
43433.0000000	0.0000000

43434.0000000	-0.0050000
43435.0000000	0.0000000
43436.0000000	0.0000000
43437.0000000	-0.0050000
43438.0000000	-0.0050000
43439.0000000	-0.0100000
43440.0000000	0.0000000
43441.0000000	-0.0050000
43442.0000000	-0.0050000
43443.0000000	-0.0100000
43444.0000000	-0.0150000
43445.0000000	-0.0066667
43446.0000000	-0.0050000
43447.0000000	-0.0100000
43448.0000000	-0.0100000
43449.0000000	-0.0150000
43450.0000000	-0.0150000
43451.0000000	-0.0150000
43452.0000000	-0.0150000
43453.0000000	-0.0150000
43454.0000000	-0.0200000
43455.0000000	-0.0150000
43456.0000000	-0.0200000
43457.0000000	-0.0150000
43458.0000000	-0.0200000
43459.0000000	-0.0150000
43460.0000000	-0.0150000
43461.0000000	-0.0250000
43462.0000000	-0.0200000
43463.0000000	-0.0200000
43464.0000000	-0.0150000
43465.0000000	-0.0250000
43466.0000000	-0.0200000
43467.0000000	-0.0100000
43468.0000000	-0.0250000
43469.0000000	-0.0150000
43470.0000000	-0.0150000
43471.0000000	-0.0200000
43472.0000000	-0.0200000
43473.0000000	-0.0200000
43474.0000000	-0.0100000
43475.0000000	-0.0200000
43476.0000000	-0.0200000
43477.0000000	-0.0200000
43478.0000000	-0.0200000
43479.0000000	-0.0150000
43480.0000000	-0.0250000
43481.0000000	-0.0200000
43482.0000000	-0.0200000
43483.0000000	-0.0250000
43484.0000000	-0.0400000
43485.0000000	-0.0250000
43486.0000000	-0.0200000
43487.0000000	-0.0250000
43488.0000000	-0.0250000
43489.0000000	-0.0300000
43490.0000000	-0.0300000

43491.0000000	-0.0300000
43492.0000000	-0.0350000
43493.0000000	-0.0350000
43494.0000000	-0.0350000
43495.0000000	-0.0400000
43496.0000000	-0.0350000
43497.0000000	-0.0350000
43498.0000000	-0.0350000
43499.0000000	-0.0350000
43500.0000000	-0.0400000
43501.0000000	-0.0350000
43502.0000000	-0.0300000
43503.0000000	-0.0350000
43504.0000000	-0.0350000
43505.0000000	-0.0333333
43506.0000000	-0.0350000
43507.0000000	-0.0400000
43508.0000000	-0.0400000
43509.0000000	-0.0500000
43510.0000000	-0.0450000
43511.0000000	-0.0400000
43512.0000000	-0.0400000
43513.0000000	-0.0450000
43514.0000000	-0.0400000
43515.0000000	-0.0350000
43516.0000000	-0.0400000
43517.0000000	-0.0400000
43518.0000000	-0.0400000
43519.0000000	-0.0400000
43520.0000000	-0.0400000
43521.0000000	-0.0450000
43522.0000000	-0.0400000
43523.0000000	-0.0300000
43524.0000000	-0.0450000
43525.0000000	-0.0500000
43526.0000000	-0.0400000
43527.0000000	-0.0400000
43528.0000000	-0.0400000
43529.0000000	-0.0450000
43530.0000000	-0.0400000
43531.0000000	-0.0400000
43532.0000000	-0.0350000
43533.0000000	-0.0350000
43534.0000000	-0.0400000
43535.0000000	-0.0400000
43536.0000000	-0.0300000
43537.0000000	-0.0350000
43538.0000000	-0.0400000
43539.0000000	-0.0400000
43540.0000000	-0.0400000
43541.0000000	-0.0350000
43542.0000000	-0.0450000
43543.0000000	-0.0550000
43544.0000000	-0.0350000
43545.0000000	-0.0300000
43546.0000000	-0.0400000
43547.0000000	-0.0400000

43548.0000000	-0.0450000
43549.0000000	-0.0400000
43550.0000000	-0.0350000
43551.0000000	-0.0400000
43552.0000000	-0.0350000
43553.0000000	-0.0350000
43554.0000000	-0.0400000
43555.0000000	-0.0450000
43556.0000000	-0.0450000
43557.0000000	-0.0450000
43558.0000000	-0.0450000
43559.0000000	-0.0500000
43560.0000000	-0.0450000
43561.0000000	-0.0500000
43562.0000000	-0.0450000
43563.0000000	-0.0500000
43564.0000000	-0.0500000
43565.0000000	-0.0500000
43566.0000000	-0.0633333
43567.0000000	-0.0500000
43568.0000000	-0.0500000
43569.0000000	-0.0550000
43570.0000000	-0.0650000
43571.0000000	-0.0500000
43572.0000000	-0.0550000
43573.0000000	-0.0500000
43574.0000000	-0.0500000
43575.0000000	-0.0600000
43576.0000000	-0.0550000
43577.0000000	-0.0550000
43578.0000000	-0.0550000
43579.0000000	-0.0600000
43580.0000000	-0.0550000
43581.0000000	-0.0550000
43582.0000000	-0.0600000
43583.0000000	-0.0550000
43584.0000000	-0.0550000
43585.0000000	-0.0600000
43586.0000000	-0.0600000
43587.0000000	-0.0650000
43588.0000000	-0.0600000
43589.0000000	-0.0600000
43590.0000000	-0.0550000
43591.0000000	-0.0500000
43592.0000000	-0.0550000
43593.0000000	-0.0600000
43594.0000000	-0.0600000
43595.0000000	-0.0600000
43596.0000000	-0.0533333
43597.0000000	-0.0600000
43598.0000000	-0.0700000
43599.0000000	-0.0550000
43600.0000000	-0.0550000
43601.0000000	-0.0650000
43602.0000000	-0.0600000
43603.0000000	-0.0650000
43604.0000000	-0.0550000

43605.0000000	-0.0600000
43606.0000000	-0.0550000
43607.0000000	-0.0600000
43608.0000000	-0.0550000
43609.0000000	-0.0600000
43610.0000000	-0.0550000
43611.0000000	-0.0600000
43612.0000000	-0.0600000
43613.0000000	-0.0600000
43614.0000000	-0.0600000
43615.0000000	-0.0600000
43616.0000000	-0.0650000
43617.0000000	-0.0600000
43618.0000000	-0.0650000
43619.0000000	-0.0650000
43620.0000000	-0.0600000
43621.0000000	-0.0550000
43622.0000000	-0.0650000
43623.0000000	-0.0550000
43624.0000000	-0.0700000
43625.0000000	-0.0633333
43626.0000000	-0.0550000
43627.0000000	-0.0600000
43628.0000000	-0.0600000
43629.0000000	-0.0650000
43630.0000000	-0.0600000
43631.0000000	-0.0700000
43632.0000000	-0.0550000
43633.0000000	-0.0650000
43634.0000000	-0.0550000
43635.0000000	-0.0700000
43636.0000000	-0.0650000
43637.0000000	-0.0650000
43638.0000000	-0.0600000
43639.0000000	-0.0600000
43640.0000000	-0.0700000
43641.0000000	-0.0650000
43642.0000000	-0.0650000
43643.0000000	-0.0600000
43644.0000000	-0.0650000
43645.0000000	-0.0650000
43646.0000000	-0.0700000
43647.0000000	-0.0650000
43648.0000000	-0.0650000
43649.0000000	-0.0650000
43650.0000000	-0.0700000
43651.0000000	-0.0700000
43652.0000000	-0.0650000
43653.0000000	-0.0800000
43654.0000000	-0.0750000
43655.0000000	-0.0700000
43656.0000000	-0.0666667
43657.0000000	-0.0700000
43658.0000000	-0.0700000
43659.0000000	-0.0650000
43660.0000000	-0.0650000
43661.0000000	-0.0700000

43662.0000000	-0.0650000
43663.0000000	-0.0750000
43664.0000000	-0.0650000
43665.0000000	-0.0700000
43666.0000000	-0.0700000
43667.0000000	-0.0700000
43668.0000000	-0.0750000
43669.0000000	-0.0700000
43670.0000000	-0.0700000
43671.0000000	-0.0650000
43672.0000000	-0.0650000
43673.0000000	-0.0700000
43674.0000000	-0.0750000
43675.0000000	-0.0700000
43676.0000000	-0.0700000
43677.0000000	-0.0700000
43678.0000000	-0.0700000
43679.0000000	-0.0700000
43680.0000000	-0.0650000
43681.0000000	-0.0650000
43682.0000000	-0.0700000
43683.0000000	-0.0750000
43684.0000000	-0.0600000
43685.0000000	-0.0650000
43686.0000000	-0.0700000
43687.0000000	-0.0700000
43688.0000000	-0.0700000
43689.0000000	-0.0550000
43690.0000000	-0.0750000
43691.0000000	-0.0700000
43692.0000000	-0.0700000
43693.0000000	-0.0700000
43694.0000000	-0.0650000
43695.0000000	-0.0700000
43696.0000000	-0.0700000
43697.0000000	-0.0650000
43698.0000000	-0.0700000
43699.0000000	-0.0700000
43700.0000000	-0.0700000
43701.0000000	-0.0650000
43702.0000000	-0.0650000
43703.0000000	-0.0700000
43704.0000000	-0.0700000
43705.0000000	-0.0650000
43706.0000000	-0.0700000
43707.0000000	-0.0650000
43708.0000000	-0.0700000
43709.0000000	-0.0700000
43710.0000000	-0.0650000
43711.0000000	-0.0750000
43712.0000000	-0.0650000
43713.0000000	-0.0600000
43714.0000000	-0.0700000
43715.0000000	-0.0700000
43716.0000000	-0.0600000
43717.0000000	-0.0650000
43718.0000000	-0.0650000

43719.0000000	-0.0700000
43720.0000000	-0.0750000
43721.0000000	-0.0650000
43722.0000000	-0.0700000
43723.0000000	-0.0600000
43724.0000000	-0.0650000
43725.0000000	-0.0700000
43726.0000000	-0.0600000
43727.0000000	-0.0650000
43728.0000000	-0.0650000
43729.0000000	-0.0600000
43730.0000000	-0.0550000
43731.0000000	-0.0650000
43732.0000000	-0.0750000
43733.0000000	-0.0650000
43734.0000000	-0.0650000
43735.0000000	-0.0700000
43736.0000000	-0.0650000
43737.0000000	-0.0600000
43738.0000000	-0.0650000
43739.0000000	-0.0700000
43740.0000000	-0.0650000
43741.0000000	-0.0700000
43742.0000000	-0.0650000
43743.0000000	-0.0600000
43744.0000000	-0.0650000
43745.0000000	-0.0700000
43746.0000000	-0.0566667
43747.0000000	-0.0700000
43748.0000000	-0.0650000
43749.0000000	-0.0650000
43750.0000000	-0.0650000
43751.0000000	-0.0650000
43752.0000000	-0.0700000
43753.0000000	-0.0650000
43754.0000000	-0.0600000
43755.0000000	-0.0600000
43756.0000000	-0.0700000
43757.0000000	-0.0650000
43758.0000000	-0.0650000
43759.0000000	-0.0650000
43760.0000000	-0.0650000
43761.0000000	-0.0700000
43762.0000000	-0.0700000
43763.0000000	-0.0650000
43764.0000000	-0.0650000
43765.0000000	-0.0650000
43766.0000000	-0.0650000
43767.0000000	-0.0650000
43768.0000000	-0.0600000
43769.0000000	-0.0650000
43770.0000000	-0.0650000
43771.0000000	-0.0650000
43772.0000000	-0.0700000
43773.0000000	-0.0650000
43774.0000000	-0.0650000
43775.0000000	-0.0650000

43776.0000000	-0.0600000
43777.0000000	-0.0633333
43778.0000000	-0.0650000
43779.0000000	-0.0650000
43780.0000000	-0.0600000
43781.0000000	-0.0550000
43782.0000000	-0.0600000
43783.0000000	-0.0650000
43784.0000000	-0.0500000
43785.0000000	-0.0650000
43786.0000000	-0.0600000
43787.0000000	-0.0750000
43788.0000000	-0.0600000
43789.0000000	-0.0600000
43790.0000000	-0.0650000
43791.0000000	-0.0600000
43792.0000000	-0.0600000
43793.0000000	-0.0700000
43794.0000000	-0.0600000
43795.0000000	-0.0700000
43796.0000000	-0.0700000
43797.0000000	-0.0650000
43798.0000000	-0.0550000
43799.0000000	-0.0650000
43800.0000000	-0.0600000
43801.0000000	-0.0700000
43802.0000000	-0.0650000
43803.0000000	-0.0650000
43804.0000000	-0.0600000
43805.0000000	-0.0650000
43806.0000000	-0.0650000
43807.0000000	-0.0700000
43808.0000000	-0.0500000
43809.0000000	-0.0650000
43810.0000000	-0.0650000
43811.0000000	-0.0700000
43812.0000000	-0.0700000
43813.0000000	-0.0700000
43814.0000000	-0.0550000
43815.0000000	-0.0650000
43816.0000000	-0.0600000
43817.0000000	-0.0600000
43818.0000000	-0.0750000
43819.0000000	-0.0650000
43820.0000000	-0.0600000
43821.0000000	-0.0650000
43822.0000000	-0.0600000
43823.0000000	-0.0750000
43824.0000000	-0.0700000
43825.0000000	-0.0650000
43826.0000000	-0.0650000
43827.0000000	-0.0500000
43828.0000000	-0.0650000
43829.0000000	-0.0650000
43830.0000000	-0.0700000
43831.0000000	-0.0700000
43832.0000000	-0.0650000

43833.0000000	-0.0650000
43834.0000000	-0.0650000
43835.0000000	-0.0700000
43836.0000000	-0.0700000
43837.0000000	-0.0600000
43838.0000000	-0.0650000
43839.0000000	-0.0650000
43840.0000000	-0.0600000
43841.0000000	-0.0550000
43842.0000000	-0.0650000
43843.0000000	-0.0650000
43844.0000000	-0.0600000
43845.0000000	-0.0600000
43846.0000000	-0.0650000
43847.0000000	-0.0650000
43848.0000000	-0.0600000
43849.0000000	-0.0600000
43850.0000000	-0.0600000
43851.0000000	-0.0650000
43852.0000000	-0.0700000
43853.0000000	-0.0600000
43854.0000000	-0.0650000
43855.0000000	-0.0550000
43856.0000000	-0.0650000
43857.0000000	-0.0600000
43858.0000000	-0.0650000
43859.0000000	-0.0600000
43860.0000000	-0.0600000
43861.0000000	-0.0650000
43862.0000000	-0.0650000
43863.0000000	-0.0750000
43864.0000000	-0.0650000
43865.0000000	-0.0650000
43866.0000000	-0.0650000
43867.0000000	-0.0600000
43868.0000000	-0.0700000
43869.0000000	-0.0750000
43870.0000000	-0.0700000
43871.0000000	-0.0650000
43872.0000000	-0.0700000
43873.0000000	-0.0600000
43874.0000000	-0.0650000
43875.0000000	-0.0650000
43876.0000000	-0.0650000
43877.0000000	-0.0700000
43878.0000000	-0.0650000
43879.0000000	-0.0600000
43880.0000000	-0.0750000
43881.0000000	-0.0700000
43882.0000000	-0.0600000
43883.0000000	-0.0700000
43884.0000000	-0.0600000
43885.0000000	-0.0750000
43886.0000000	-0.0600000
43887.0000000	-0.0650000
43888.0000000	-0.0650000
43889.0000000	-0.0600000

43890.0000000	-0.0750000
43891.0000000	-0.0700000
43892.0000000	-0.0650000
43893.0000000	-0.0550000
43894.0000000	-0.0600000
43895.0000000	-0.0600000
43896.0000000	-0.0650000
43897.0000000	-0.0600000
43898.0000000	-0.0633333
43899.0000000	-0.0650000
43900.0000000	-0.0600000
43901.0000000	-0.0650000
43902.0000000	-0.0650000
43903.0000000	-0.0650000
43904.0000000	-0.0600000
43905.0000000	-0.0600000
43906.0000000	-0.0600000
43907.0000000	-0.0600000
43908.0000000	-0.0650000
43909.0000000	-0.0550000
43910.0000000	-0.0600000
43911.0000000	-0.0700000
43912.0000000	-0.0700000
43913.0000000	-0.0650000
43914.0000000	-0.0650000
43915.0000000	-0.0550000
43916.0000000	-0.0650000
43917.0000000	-0.0650000
43918.0000000	-0.0600000
43919.0000000	-0.0550000
43920.0000000	-0.0600000
43921.0000000	-0.0650000
43922.0000000	-0.0650000
43923.0000000	-0.0600000
43924.0000000	-0.0650000
43925.0000000	-0.0600000
43926.0000000	-0.0600000
43927.0000000	-0.0600000
43928.0000000	-0.0633333
43929.0000000	-0.0600000
43930.0000000	-0.0600000
43931.0000000	-0.0550000
43932.0000000	-0.0650000
43933.0000000	-0.0650000
43934.0000000	-0.0600000
43935.0000000	-0.0550000
43936.0000000	-0.0600000
43937.0000000	-0.0600000
43938.0000000	-0.0600000
43939.0000000	-0.0600000
43940.0000000	-0.0650000
43941.0000000	-0.0550000
43942.0000000	-0.0600000
43943.0000000	-0.0550000
43944.0000000	-0.0650000
43945.0000000	-0.0600000
43946.0000000	-0.0600000

43947.0000000	-0.0650000
43948.0000000	-0.0600000
43949.0000000	-0.0600000
43950.0000000	-0.0700000
43951.0000000	-0.0600000
43952.0000000	-0.0550000
43953.0000000	-0.0600000
43954.0000000	-0.0650000
43955.0000000	-0.0600000
43956.0000000	-0.0550000
43957.0000000	-0.0650000
43958.0000000	-0.0533333
43959.0000000	-0.0600000
43960.0000000	-0.0550000
43961.0000000	-0.0600000
43962.0000000	-0.0600000
43963.0000000	-0.0500000
43964.0000000	-0.0550000
43965.0000000	-0.0550000
43966.0000000	-0.0600000
43967.0000000	-0.0550000
43968.0000000	-0.0550000
43969.0000000	-0.0600000
43970.0000000	-0.0550000
43971.0000000	-0.0600000
43972.0000000	-0.0600000
43973.0000000	-0.0550000
43974.0000000	-0.0600000
43975.0000000	-0.0600000
43976.0000000	-0.0600000
43977.0000000	-0.0550000
43978.0000000	-0.0500000
43979.0000000	-0.0550000
43980.0000000	-0.0650000
43981.0000000	-0.0550000
43982.0000000	-0.0600000
43983.0000000	-0.0600000
43984.0000000	-0.0600000
43985.0000000	-0.0650000
43986.0000000	-0.0600000
43987.0000000	-0.0600000
43988.0000000	-0.0633333
43989.0000000	-0.0650000
43990.0000000	-0.0600000
43991.0000000	-0.0650000
43992.0000000	-0.0650000
43993.0000000	-0.0650000
43994.0000000	-0.0650000
43995.0000000	-0.0600000
43996.0000000	-0.0600000
43997.0000000	-0.0650000
43998.0000000	-0.0600000
43999.0000000	-0.0700000
44000.0000000	-0.0650000
44001.0000000	-0.0600000
44002.0000000	-0.0700000
44003.0000000	-0.0650000

44004.0000000	-0.0650000
44005.0000000	-0.0600000
44006.0000000	-0.0600000
44007.0000000	-0.0700000
44008.0000000	-0.0700000
44009.0000000	-0.0650000
44010.0000000	-0.0600000
44011.0000000	-0.0650000
44012.0000000	-0.0650000
44013.0000000	-0.0600000
44014.0000000	-0.0650000
44015.0000000	-0.0600000
44016.0000000	-0.0550000
44017.0000000	-0.0700000
44018.0000000	-0.0633333
44019.0000000	-0.0700000
44020.0000000	-0.0650000
44021.0000000	-0.0600000
44022.0000000	-0.0600000
44023.0000000	-0.0650000
44024.0000000	-0.0650000
44025.0000000	-0.0600000
44026.0000000	-0.0650000
44027.0000000	-0.0550000
44028.0000000	-0.0700000
44029.0000000	-0.0600000
44030.0000000	-0.0600000
44031.0000000	-0.0650000
44032.0000000	-0.0600000
44033.0000000	-0.0650000
44034.0000000	-0.0700000
44035.0000000	-0.0700000
44036.0000000	-0.0650000
44037.0000000	-0.0600000
44038.0000000	-0.0600000
44039.0000000	-0.0650000
44040.0000000	-0.0600000
44041.0000000	-0.0650000
44042.0000000	-0.0550000
44043.0000000	-0.0700000
44044.0000000	-0.0600000
44045.0000000	-0.0600000
44046.0000000	-0.0600000
44047.0000000	-0.0650000
44048.0000000	-0.0566667
44049.0000000	-0.0650000
44050.0000000	-0.0600000
44051.0000000	-0.0700000
44052.0000000	-0.0650000
44053.0000000	-0.0700000
44054.0000000	-0.0650000
44055.0000000	-0.0650000
44056.0000000	-0.0600000
44057.0000000	-0.0600000
44058.0000000	-0.0600000
44059.0000000	-0.0650000
44060.0000000	-0.0600000

44061.0000000	-0.0700000
44062.0000000	-0.0700000
44063.0000000	-0.0650000
44064.0000000	-0.0700000
44065.0000000	-0.0700000
44066.0000000	-0.0700000
44067.0000000	-0.0600000
44068.0000000	-0.0700000
44069.0000000	-0.0700000
44070.0000000	-0.0700000
44071.0000000	-0.0650000
44072.0000000	-0.0700000
44073.0000000	-0.0650000
44074.0000000	-0.0700000
44075.0000000	-0.0650000
44076.0000000	-0.0700000
44077.0000000	-0.0600000
44078.0000000	-0.0733333
44079.0000000	-0.0700000
44080.0000000	-0.0700000
44081.0000000	-0.0650000
44082.0000000	-0.0650000
44083.0000000	-0.0700000
44084.0000000	-0.0700000
44085.0000000	-0.0650000
44086.0000000	-0.0700000
44087.0000000	-0.0750000
44088.0000000	-0.0700000
44089.0000000	-0.0800000
44090.0000000	-0.0750000
44091.0000000	-0.0700000
44092.0000000	-0.0700000
44093.0000000	-0.0700000
44094.0000000	-0.0700000
44095.0000000	-0.0800000
44096.0000000	-0.0700000
44097.0000000	-0.0750000
44098.0000000	-0.0700000
44099.0000000	-0.0700000
44100.0000000	-0.0700000
44101.0000000	-0.0750000
44102.0000000	-0.0700000
44103.0000000	-0.0750000
44104.0000000	-0.0750000
44105.0000000	-0.0700000
44106.0000000	-0.0650000
44107.0000000	-0.0750000
44108.0000000	-0.0750000
44109.0000000	-0.0733333
44110.0000000	-0.0700000
44111.0000000	-0.0750000
44112.0000000	-0.0750000
44113.0000000	-0.0800000
44114.0000000	-0.0700000
44115.0000000	-0.0750000
44116.0000000	-0.0800000
44117.0000000	-0.0750000

44118.0000000	-0.0650000
44119.0000000	-0.0750000
44120.0000000	-0.0800000
44121.0000000	-0.0650000
44122.0000000	-0.0700000
44123.0000000	-0.0750000
44124.0000000	-0.0850000
44125.0000000	-0.0750000
44126.0000000	-0.0850000
44127.0000000	-0.0850000
44128.0000000	-0.0800000
44129.0000000	-0.0700000
44130.0000000	-0.0800000
44131.0000000	-0.0800000
44132.0000000	-0.0900000
44133.0000000	-0.0750000
44134.0000000	-0.0800000
44135.0000000	-0.0850000
44136.0000000	-0.0800000
44137.0000000	-0.0800000
44138.0000000	-0.0800000
44139.0000000	-0.0833333
44140.0000000	-0.0850000
44141.0000000	-0.0800000
44142.0000000	-0.0800000
44143.0000000	-0.0850000
44144.0000000	-0.0800000
44145.0000000	-0.0800000
44146.0000000	-0.0800000
44147.0000000	-0.0850000
44148.0000000	-0.0800000
44149.0000000	-0.0800000
44150.0000000	-0.0850000
44151.0000000	-0.0850000
44152.0000000	-0.0850000
44153.0000000	-0.0850000
44154.0000000	-0.0900000
44155.0000000	-0.0900000
44156.0000000	-0.0950000
44157.0000000	-0.0850000
44158.0000000	-0.0900000
44159.0000000	-0.0900000
44160.0000000	-0.1000000
44161.0000000	-0.0900000
44162.0000000	-0.0900000
44163.0000000	-0.1000000
44164.0000000	-0.0900000
44165.0000000	-0.0900000
44166.0000000	-0.1000000
44167.0000000	-0.0900000
44168.0000000	-0.1000000
44169.0000000	-0.0966667
44170.0000000	-0.0900000
44171.0000000	-0.0950000
44172.0000000	-0.1000000
44173.0000000	-0.1050000
44174.0000000	-0.1000000

44175.0000000	-0.0950000
44176.0000000	-0.0950000
44177.0000000	-0.1050000
44178.0000000	-0.0950000
44179.0000000	-0.0950000
44180.0000000	-0.1050000
44181.0000000	-0.1050000
44182.0000000	-0.0950000
44183.0000000	-0.1100000
44184.0000000	-0.1000000
44185.0000000	-0.1100000
44186.0000000	-0.1000000
44187.0000000	-0.1000000
44188.0000000	-0.1050000
44189.0000000	-0.1050000
44190.0000000	-0.1150000
44191.0000000	-0.1100000
44192.0000000	-0.1150000
44193.0000000	-0.1000000
44194.0000000	-0.1100000
44195.0000000	-0.1050000
44196.0000000	-0.1100000
44197.0000000	-0.1050000
44198.0000000	-0.1100000
44199.0000000	-0.1100000
44200.0000000	-0.1050000
44201.0000000	-0.1100000
44202.0000000	-0.1100000
44203.0000000	-0.1050000
44204.0000000	-0.1200000
44205.0000000	-0.1050000
44206.0000000	-0.1200000
44207.0000000	-0.1100000
44208.0000000	-0.1100000
44209.0000000	-0.1050000
44210.0000000	-0.1200000
44211.0000000	-0.1150000
44212.0000000	-0.1200000
44213.0000000	-0.1100000
44214.0000000	-0.1150000
44215.0000000	-0.1000000
44216.0000000	-0.1200000
44217.0000000	-0.1150000
44218.0000000	-0.1250000
44219.0000000	-0.1100000
44220.0000000	-0.1200000
44221.0000000	-0.1050000
44222.0000000	-0.1200000
44223.0000000	-0.1200000
44224.0000000	-0.1200000
44225.0000000	-0.1250000
44226.0000000	-0.1200000
44227.0000000	-0.1150000
44228.0000000	-0.1200000
44229.0000000	-0.1150000
44230.0000000	-0.1266667
44231.0000000	-0.1100000

44232.0000000	-0.1250000
44233.0000000	-0.1150000
44234.0000000	-0.1250000
44235.0000000	-0.1250000
44236.0000000	-0.1300000
44237.0000000	-0.1200000
44238.0000000	-0.1250000
44239.0000000	-0.1150000
44240.0000000	-0.1250000
44241.0000000	-0.1250000
44242.0000000	-0.1250000
44243.0000000	-0.1250000
44244.0000000	-0.1300000
44245.0000000	-0.1200000
44246.0000000	-0.1200000
44247.0000000	-0.1300000
44248.0000000	-0.1300000
44249.0000000	-0.1300000
44250.0000000	-0.1250000
44251.0000000	-0.1300000
44252.0000000	-0.1150000
44253.0000000	-0.1300000
44254.0000000	-0.1300000
44255.0000000	-0.1300000
44256.0000000	-0.1300000
44257.0000000	-0.1300000
44258.0000000	-0.1300000
44259.0000000	-0.1300000
44260.0000000	-0.1300000
44261.0000000	-0.1300000
44262.0000000	-0.1300000
44263.0000000	-0.1400000
44264.0000000	-0.1300000
44265.0000000	-0.1400000
44266.0000000	-0.1300000
44267.0000000	-0.1450000
44268.0000000	-0.1250000
44269.0000000	-0.1300000
44270.0000000	-0.1250000
44271.0000000	-0.1350000
44272.0000000	-0.1400000
44273.0000000	-0.1450000
44274.0000000	-0.1450000
44275.0000000	-0.1350000
44276.0000000	-0.1350000
44277.0000000	-0.1350000
44278.0000000	-0.1400000
44279.0000000	-0.1300000
44280.0000000	-0.1400000
44281.0000000	-0.1400000
44282.0000000	-0.1450000
44283.0000000	-0.1450000
44284.0000000	-0.1500000
44285.0000000	-0.1500000
44286.0000000	-0.1300000
44287.0000000	-0.1400000
44288.0000000	-0.1400000

44289.0000000	-0.1500000
44290.0000000	-0.1500000
44291.0000000	-0.1450000
44292.0000000	-0.1400000
44293.0000000	-0.1450000
44294.0000000	-0.1450000
44295.0000000	-0.1450000
44296.0000000	-0.1450000
44297.0000000	-0.1500000
44298.0000000	-0.1500000
44299.0000000	-0.1400000
44300.0000000	-0.1500000
44301.0000000	-0.1600000
44302.0000000	-0.1500000
44303.0000000	-0.1500000
44304.0000000	-0.1500000
44305.0000000	-0.1500000
44306.0000000	-0.1500000
44307.0000000	-0.1450000
44308.0000000	-0.1550000
44309.0000000	-0.1550000
44310.0000000	-0.1500000
44311.0000000	-0.1550000
44312.0000000	-0.1550000
44313.0000000	-0.1500000
44314.0000000	-0.1550000
44315.0000000	-0.1650000
44316.0000000	-0.1500000
44317.0000000	-0.1500000
44318.0000000	-0.1550000
44319.0000000	-0.1550000
44320.0000000	-0.1566667
44321.0000000	-0.1600000
44322.0000000	-0.1600000
44323.0000000	-0.1700000
44324.0000000	-0.1550000
44325.0000000	-0.1650000
44326.0000000	-0.1600000
44327.0000000	-0.1600000
44328.0000000	-0.1600000
44329.0000000	-0.1650000
44330.0000000	-0.1600000
44331.0000000	-0.1600000
44332.0000000	-0.1600000
44333.0000000	-0.1600000
44334.0000000	-0.1600000
44335.0000000	-0.1600000
44336.0000000	-0.1600000
44337.0000000	-0.1600000
44338.0000000	-0.1650000
44339.0000000	-0.1700000
44340.0000000	-0.1600000
44341.0000000	-0.1600000
44342.0000000	-0.1650000
44343.0000000	-0.1650000
44344.0000000	-0.1650000
44345.0000000	-0.1650000

44346.0000000	-0.1600000
44347.0000000	-0.1650000
44348.0000000	-0.1700000
44349.0000000	-0.1600000
44350.0000000	-0.1666667
44351.0000000	-0.1650000
44352.0000000	-0.1600000
44353.0000000	-0.1700000
44354.0000000	-0.1700000
44355.0000000	-0.1650000
44356.0000000	-0.1650000
44357.0000000	-0.1700000
44358.0000000	-0.1700000
44359.0000000	-0.1650000
44360.0000000	-0.1650000
44361.0000000	-0.1700000
44362.0000000	-0.1750000
44363.0000000	-0.1700000
44364.0000000	-0.1650000
44365.0000000	-0.1650000
44366.0000000	-0.1700000
44367.0000000	-0.1700000
44368.0000000	-0.1700000
44369.0000000	-0.1700000
44370.0000000	-0.1700000
44371.0000000	-0.1700000
44372.0000000	-0.1700000
44373.0000000	-0.1750000
44374.0000000	-0.1800000
44375.0000000	-0.1750000
44376.0000000	-0.1750000
44377.0000000	-0.1800000
44378.0000000	-0.1750000
44379.0000000	-0.1750000
44380.0000000	-0.1750000
44381.0000000	-0.1733333
44382.0000000	-0.1800000
44383.0000000	-0.1800000
44384.0000000	-0.1750000
44385.0000000	-0.1750000
44386.0000000	-0.1800000
44387.0000000	-0.1800000
44388.0000000	-0.1800000
44389.0000000	-0.1800000
44390.0000000	-0.1750000
44391.0000000	-0.1700000
44392.0000000	-0.1800000
44393.0000000	-0.1700000
44394.0000000	-0.1800000
44395.0000000	-0.1850000
44396.0000000	-0.1800000
44397.0000000	-0.1800000
44398.0000000	-0.1700000
44399.0000000	-0.1750000
44400.0000000	-0.1800000
44401.0000000	-0.1900000
44402.0000000	-0.1800000

44403.0000000	-0.1750000
44404.0000000	-0.1900000
44405.0000000	-0.1800000
44406.0000000	-0.1800000
44407.0000000	-0.1900000
44408.0000000	-0.1750000
44409.0000000	-0.1800000
44410.0000000	-0.1766667
44411.0000000	-0.1800000
44412.0000000	-0.1900000
44413.0000000	-0.1850000
44414.0000000	-0.1900000
44415.0000000	-0.1850000
44416.0000000	-0.1800000
44417.0000000	-0.1900000
44418.0000000	-0.1800000
44419.0000000	-0.1750000
44420.0000000	-0.1800000
44421.0000000	-0.1750000
44422.0000000	-0.1850000
44423.0000000	-0.1850000
44424.0000000	-0.1850000
44425.0000000	-0.1850000
44426.0000000	-0.1850000
44427.0000000	-0.1900000
44428.0000000	-0.1850000
44429.0000000	-0.1850000
44430.0000000	-0.1750000
44431.0000000	-0.1850000
44432.0000000	-0.1750000
44433.0000000	-0.1900000
44434.0000000	-0.1800000
44435.0000000	-0.1800000
44436.0000000	-0.1850000
44437.0000000	-0.1850000
44438.0000000	-0.1750000
44439.0000000	-0.1850000
44440.0000000	-0.1900000
44441.0000000	-0.1833333
44442.0000000	-0.1900000
44443.0000000	-0.1800000
44444.0000000	-0.1900000
44445.0000000	-0.1850000
44446.0000000	-0.1850000
44447.0000000	-0.1950000
44448.0000000	-0.1850000
44449.0000000	-0.1900000
44450.0000000	-0.1850000
44451.0000000	-0.1900000
44452.0000000	-0.1900000
44453.0000000	-0.1900000
44454.0000000	-0.1900000
44455.0000000	-0.1950000
44456.0000000	-0.1900000
44457.0000000	-0.1900000
44458.0000000	-0.1950000
44459.0000000	-0.2000000

44460.0000000	-0.1900000
44461.0000000	-0.1900000
44462.0000000	-0.2000000
44463.0000000	-0.2000000
44464.0000000	-0.1900000
44465.0000000	-0.1800000
44466.0000000	-0.1950000
44467.0000000	-0.1950000
44468.0000000	-0.1900000
44469.0000000	-0.1900000
44470.0000000	-0.2000000
44471.0000000	-0.2000000
44472.0000000	-0.1900000
44473.0000000	-0.1950000
44474.0000000	-0.2000000
44475.0000000	-0.1950000
44476.0000000	-0.1950000
44477.0000000	-0.1950000
44478.0000000	-0.2000000
44479.0000000	-0.1950000
44480.0000000	-0.1950000
44481.0000000	-0.1950000
44482.0000000	-0.1950000
44483.0000000	-0.1950000
44484.0000000	-0.2000000
44485.0000000	-0.1900000
44486.0000000	-0.2000000
44487.0000000	-0.2000000
44488.0000000	-0.2000000
44489.0000000	-0.1950000
44490.0000000	-0.2050000
44491.0000000	-0.1950000
44492.0000000	-0.1950000
44493.0000000	-0.1900000
44494.0000000	-0.1950000
44495.0000000	-0.2050000
44496.0000000	-0.2000000
44497.0000000	-0.1900000
44498.0000000	-0.2000000
44499.0000000	-0.2050000
44500.0000000	-0.2000000
44501.0000000	-0.1933333
44502.0000000	-0.2000000
44503.0000000	-0.2000000
44504.0000000	-0.1950000
44505.0000000	-0.2000000
44506.0000000	-0.1950000
44507.0000000	-0.1950000
44508.0000000	-0.1950000
44509.0000000	-0.1950000
44510.0000000	-0.2000000
44511.0000000	-0.1950000
44512.0000000	-0.2000000
44513.0000000	-0.1950000
44514.0000000	-0.2000000
44515.0000000	-0.2000000
44516.0000000	-0.1950000

44517.0000000	-0.2000000
44518.0000000	-0.2050000
44519.0000000	-0.1950000
44520.0000000	-0.1950000
44521.0000000	-0.1950000
44522.0000000	-0.1950000
44523.0000000	-0.1950000
44524.0000000	-0.1900000
44525.0000000	-0.1900000
44526.0000000	-0.1950000
44527.0000000	-0.2050000
44528.0000000	-0.1950000
44529.0000000	-0.1950000
44530.0000000	-0.1900000
44531.0000000	-0.1933333
44532.0000000	-0.2000000
44533.0000000	-0.2000000
44534.0000000	-0.1900000
44535.0000000	-0.2000000
44536.0000000	-0.1950000
44537.0000000	-0.1950000
44538.0000000	-0.1950000
44539.0000000	-0.1900000
44540.0000000	-0.2000000
44541.0000000	-0.1900000
44542.0000000	-0.2000000
44543.0000000	-0.1950000
44544.0000000	-0.1950000
44545.0000000	-0.1950000
44546.0000000	-0.1900000
44547.0000000	-0.1950000
44548.0000000	-0.1950000
44549.0000000	-0.1900000
44550.0000000	-0.1900000
44551.0000000	-0.1950000
44552.0000000	-0.1950000
44553.0000000	-0.2050000
44554.0000000	-0.1950000
44555.0000000	-0.1950000
44556.0000000	-0.1900000
44557.0000000	-0.1950000
44558.0000000	-0.1950000
44559.0000000	-0.1900000
44560.0000000	-0.1950000
44561.0000000	-0.1933333
44562.0000000	-0.2000000
44563.0000000	-0.1900000
44564.0000000	-0.1900000
44565.0000000	-0.1900000
44566.0000000	-0.2000000
44567.0000000	-0.1900000
44568.0000000	-0.1950000
44569.0000000	-0.2050000
44570.0000000	-0.1950000
44571.0000000	-0.1950000
44572.0000000	-0.1950000
44573.0000000	-0.1900000

44574.0000000	-0.1900000
44575.0000000	-0.2000000
44576.0000000	-0.1950000
44577.0000000	-0.1950000
44578.0000000	-0.1900000
44579.0000000	-0.2000000
44580.0000000	-0.1900000
44581.0000000	-0.1950000
44582.0000000	-0.2000000
44583.0000000	-0.1900000
44584.0000000	-0.2000000
44585.0000000	-0.1950000
44586.0000000	-0.1900000
44587.0000000	-0.2000000
44588.0000000	-0.1900000
44589.0000000	-0.1950000
44590.0000000	-0.1950000
44591.0000000	-0.1950000
44592.0000000	-0.1900000
44593.0000000	-0.2000000
44594.0000000	-0.1900000
44595.0000000	-0.1900000
44596.0000000	-0.2000000
44597.0000000	-0.1850000
44598.0000000	-0.1950000
44599.0000000	-0.1800000
44600.0000000	-0.1950000
44601.0000000	-0.1850000
44602.0000000	-0.1900000
44603.0000000	-0.1950000
44604.0000000	-0.1900000
44605.0000000	-0.1900000
44606.0000000	-0.1950000
44607.0000000	-0.1950000
44608.0000000	-0.1850000
44609.0000000	-0.1950000
44610.0000000	-0.1900000
44611.0000000	-0.1900000
44612.0000000	-0.1950000
44613.0000000	-0.1900000
44614.0000000	-0.1850000
44615.0000000	-0.1900000
44616.0000000	-0.1800000
44617.0000000	-0.1850000
44618.0000000	-0.1900000
44619.0000000	-0.1950000
44620.0000000	-0.1900000
44621.0000000	-0.1950000
44622.0000000	-0.1833333
44623.0000000	-0.1950000
44624.0000000	-0.1950000
44625.0000000	-0.1800000
44626.0000000	-0.1900000
44627.0000000	-0.1850000
44628.0000000	-0.1900000
44629.0000000	-0.1900000
44630.0000000	-0.1800000

44631.0000000	-0.1950000
44632.0000000	-0.1900000
44633.0000000	-0.1900000
44634.0000000	-0.1950000
44635.0000000	-0.1900000
44636.0000000	-0.1900000
44637.0000000	-0.1950000
44638.0000000	-0.1850000
44639.0000000	-0.1900000
44640.0000000	-0.1900000
44641.0000000	-0.1900000
44642.0000000	-0.1850000
44643.0000000	-0.1900000
44644.0000000	-0.1900000
44645.0000000	-0.1950000
44646.0000000	-0.1950000
44647.0000000	-0.1900000
44648.0000000	-0.1850000
44649.0000000	-0.1900000
44650.0000000	-0.1950000
44651.0000000	-0.1900000
44652.0000000	-0.1866667
44653.0000000	-0.1900000
44654.0000000	-0.1950000
44655.0000000	-0.1900000
44656.0000000	-0.1900000
44657.0000000	-0.1900000
44658.0000000	-0.1850000
44659.0000000	-0.1850000
44660.0000000	-0.1900000
44661.0000000	-0.1850000
44662.0000000	-0.1800000
44663.0000000	-0.1900000
44664.0000000	-0.1900000
44665.0000000	-0.1900000
44666.0000000	-0.1950000
44667.0000000	-0.1900000
44668.0000000	-0.1850000
44669.0000000	-0.2000000
44670.0000000	-0.1900000
44671.0000000	-0.1900000
44672.0000000	-0.1900000
44673.0000000	-0.2000000
44674.0000000	-0.1850000
44675.0000000	-0.1800000
44676.0000000	-0.1950000
44677.0000000	-0.1850000
44678.0000000	-0.1850000
44679.0000000	-0.1850000
44680.0000000	-0.1900000
44681.0000000	-0.1950000
44682.0000000	-0.1866667
44683.0000000	-0.1850000
44684.0000000	-0.1950000
44685.0000000	-0.1900000
44686.0000000	-0.1850000
44687.0000000	-0.1900000

44688.0000000	-0.1900000
44689.0000000	-0.1850000
44690.0000000	-0.1900000
44691.0000000	-0.1850000
44692.0000000	-0.1950000
44693.0000000	-0.1950000
44694.0000000	-0.1900000
44695.0000000	-0.1900000
44696.0000000	-0.1900000
44697.0000000	-0.1900000
44698.0000000	-0.1950000
44699.0000000	-0.1900000
44700.0000000	-0.1900000
44701.0000000	-0.1900000
44702.0000000	-0.1950000
44703.0000000	-0.1850000
44704.0000000	-0.1900000
44705.0000000	-0.1950000
44706.0000000	-0.1800000
44707.0000000	-0.1950000
44708.0000000	-0.1900000
44709.0000000	-0.1950000
44710.0000000	-0.1800000
44711.0000000	-0.1900000
44712.0000000	-0.1850000
44713.0000000	-0.1833333
44714.0000000	-0.1900000
44715.0000000	-0.1900000
44716.0000000	-0.1850000
44717.0000000	-0.1850000
44718.0000000	-0.1850000
44719.0000000	-0.1900000
44720.0000000	-0.1800000
44721.0000000	-0.1950000
44722.0000000	-0.1950000
44723.0000000	-0.1800000
44724.0000000	-0.1900000
44725.0000000	-0.1800000
44726.0000000	-0.1950000
44727.0000000	-0.1900000
44728.0000000	-0.1850000
44729.0000000	-0.1900000
44730.0000000	-0.1900000
44731.0000000	-0.1900000
44732.0000000	-0.1900000
44733.0000000	-0.1850000
44734.0000000	-0.1850000
44735.0000000	-0.1900000
44736.0000000	-0.1900000
44737.0000000	-0.1850000
44738.0000000	-0.1800000
44739.0000000	-0.1850000
44740.0000000	-0.1900000
44741.0000000	-0.1950000
44742.0000000	-0.1900000
44743.0000000	-0.1966667
44744.0000000	-0.1800000

44745.0000000	-0.1850000
44746.0000000	-0.1850000
44747.0000000	-0.1850000
44748.0000000	-0.1800000
44749.0000000	-0.1850000
44750.0000000	-0.1850000
44751.0000000	-0.1950000
44752.0000000	-0.1900000
44753.0000000	-0.1850000
44754.0000000	-0.1800000
44755.0000000	-0.1800000
44756.0000000	-0.1850000
44757.0000000	-0.1850000
44758.0000000	-0.1850000
44759.0000000	-0.1850000
44760.0000000	-0.1850000
44761.0000000	-0.1900000
44762.0000000	-0.1850000
44763.0000000	-0.1850000
44764.0000000	-0.1850000
44765.0000000	-0.1900000
44766.0000000	-0.1800000
44767.0000000	-0.1850000
44768.0000000	-0.1850000
44769.0000000	-0.1900000
44770.0000000	-0.1900000
44771.0000000	-0.1950000
44772.0000000	-0.1866667
44773.0000000	-0.1800000
44774.0000000	-0.1950000
44775.0000000	-0.1900000
44776.0000000	-0.1850000
44777.0000000	-0.1900000
44778.0000000	-0.1850000
44779.0000000	-0.1850000
44780.0000000	-0.1850000
44781.0000000	-0.1850000
44782.0000000	-0.1900000
44783.0000000	-0.1900000
44784.0000000	-0.1950000
44785.0000000	-0.1850000
44786.0000000	-0.1850000
44787.0000000	-0.1900000
44788.0000000	-0.1850000
44789.0000000	-0.1800000
44790.0000000	-0.1800000
44791.0000000	-0.1950000
44792.0000000	-0.1850000
44793.0000000	-0.1800000
44794.0000000	-0.1800000
44795.0000000	-0.1850000
44796.0000000	-0.1900000
44797.0000000	-0.1850000
44798.0000000	-0.1800000
44799.0000000	-0.1850000
44800.0000000	-0.1850000
44801.0000000	-0.1900000

44802.0000000	-0.1900000
44803.0000000	-0.1866667
44804.0000000	-0.1850000
44805.0000000	-0.1900000
44806.0000000	-0.1900000
44807.0000000	-0.1800000
44808.0000000	-0.1850000
44809.0000000	-0.1900000
44810.0000000	-0.1900000
44811.0000000	-0.1950000
44812.0000000	-0.1900000
44813.0000000	-0.1850000
44814.0000000	-0.1850000
44815.0000000	-0.1850000
44816.0000000	-0.1850000
44817.0000000	-0.1850000
44818.0000000	-0.1850000
44819.0000000	-0.1850000
44820.0000000	-0.1950000
44821.0000000	-0.1900000
44822.0000000	-0.1850000
44823.0000000	-0.1900000
44824.0000000	-0.1900000
44825.0000000	-0.1850000
44826.0000000	-0.1850000
44827.0000000	-0.1950000
44828.0000000	-0.1900000
44829.0000000	-0.1900000
44830.0000000	-0.1950000
44831.0000000	-0.1900000
44832.0000000	-0.1950000
44833.0000000	-0.1900000
44834.0000000	-0.1900000
44835.0000000	-0.1850000
44836.0000000	-0.1850000
44837.0000000	-0.1900000
44838.0000000	-0.1950000
44839.0000000	-0.1900000
44840.0000000	-0.1950000
44841.0000000	-0.1900000
44842.0000000	-0.1850000
44843.0000000	-0.1900000
44844.0000000	-0.1900000
44845.0000000	-0.1900000
44846.0000000	-0.1850000
44847.0000000	-0.1850000
44848.0000000	-0.1900000
44849.0000000	-0.1950000
44850.0000000	-0.1900000
44851.0000000	-0.1900000
44852.0000000	-0.1900000
44853.0000000	-0.1850000
44854.0000000	-0.1950000
44855.0000000	-0.1950000
44856.0000000	-0.1900000
44857.0000000	-0.1850000
44858.0000000	-0.1800000

44859.0000000	-0.1800000
44860.0000000	-0.1850000
44861.0000000	-0.1800000
44862.0000000	-0.1850000
44863.0000000	-0.1966667
44864.0000000	-0.1900000
44865.0000000	-0.1900000
44866.0000000	-0.1800000
44867.0000000	-0.1850000
44868.0000000	-0.1900000
44869.0000000	-0.1900000
44870.0000000	-0.1900000
44871.0000000	-0.1950000
44872.0000000	-0.1900000
44873.0000000	-0.1900000
44874.0000000	-0.1850000
44875.0000000	-0.1850000
44876.0000000	-0.1850000
44877.0000000	-0.1900000
44878.0000000	-0.1900000
44879.0000000	-0.1850000
44880.0000000	-0.1800000
44881.0000000	-0.1850000
44882.0000000	-0.1900000
44883.0000000	-0.1900000
44884.0000000	-0.1850000
44885.0000000	-0.1950000
44886.0000000	-0.1900000
44887.0000000	-0.1850000
44888.0000000	-0.1850000
44889.0000000	-0.1850000
44890.0000000	-0.1800000
44891.0000000	-0.1900000
44892.0000000	-0.1800000
44893.0000000	-0.1966667
44894.0000000	-0.1900000
44895.0000000	-0.1850000
44896.0000000	-0.1950000
44897.0000000	-0.1850000
44898.0000000	-0.1900000
44899.0000000	-0.1900000
44900.0000000	-0.1900000
44901.0000000	-0.1900000
44902.0000000	-0.1900000
44903.0000000	-0.1900000
44904.0000000	-0.1800000
44905.0000000	-0.1950000
44906.0000000	-0.1850000
44907.0000000	-0.1900000
44908.0000000	-0.1850000
44909.0000000	-0.1950000
44910.0000000	-0.1850000
44911.0000000	-0.1900000
44912.0000000	-0.1850000
44913.0000000	-0.1950000
44914.0000000	-0.1950000
44915.0000000	-0.1950000

44916.0000000	-0.1900000
44917.0000000	-0.2050000
44918.0000000	-0.1900000
44919.0000000	-0.2000000
44920.0000000	-0.1850000
44921.0000000	-0.2000000
44922.0000000	-0.1850000
44923.0000000	-0.2000000
44924.0000000	-0.1933333
44925.0000000	-0.1900000
44926.0000000	-0.1900000
44927.0000000	-0.1950000
44928.0000000	-0.1950000
44929.0000000	-0.1950000
44930.0000000	-0.1950000
44931.0000000	-0.2000000
44932.0000000	-0.2000000
44933.0000000	-0.1950000
44934.0000000	-0.2000000
44935.0000000	-0.1950000
44936.0000000	-0.1950000
44937.0000000	-0.1950000
44938.0000000	-0.1950000
44939.0000000	-0.1950000
44940.0000000	-0.2000000
44941.0000000	-0.1950000
44942.0000000	-0.2000000
44943.0000000	-0.1950000
44944.0000000	-0.1950000
44945.0000000	-0.1950000
44946.0000000	-0.2000000
44947.0000000	-0.1950000
44948.0000000	-0.2050000
44949.0000000	-0.1950000
44950.0000000	-0.2000000
44951.0000000	-0.1950000
44952.0000000	-0.1950000
44953.0000000	-0.2050000
44954.0000000	-0.2000000
44955.0000000	-0.1950000
44956.0000000	-0.1950000
44957.0000000	-0.1950000
44958.0000000	-0.1950000
44959.0000000	-0.2050000
44960.0000000	-0.2050000
44961.0000000	-0.2000000
44962.0000000	-0.2050000
44963.0000000	-0.1950000
44964.0000000	-0.2050000
44965.0000000	-0.2000000
44966.0000000	-0.1950000
44967.0000000	-0.2050000
44968.0000000	-0.2000000
44969.0000000	-0.2050000
44970.0000000	-0.2000000
44971.0000000	-0.2100000
44972.0000000	-0.2000000

44973.0000000	-0.2000000
44974.0000000	-0.1950000
44975.0000000	-0.2100000
44976.0000000	-0.1950000
44977.0000000	-0.2050000
44978.0000000	-0.2100000
44979.0000000	-0.2100000
44980.0000000	-0.2000000
44981.0000000	-0.2100000
44982.0000000	-0.2050000
44983.0000000	-0.2150000
44984.0000000	-0.2033333
44985.0000000	-0.2000000
44986.0000000	-0.2050000
44987.0000000	-0.2100000
44988.0000000	-0.2050000
44989.0000000	-0.2250000
44990.0000000	-0.2050000
44991.0000000	-0.2000000
44992.0000000	-0.2050000
44993.0000000	-0.2100000
44994.0000000	-0.2100000
44995.0000000	-0.2100000
44996.0000000	-0.2100000
44997.0000000	-0.2100000
44998.0000000	-0.2050000
44999.0000000	-0.2150000
45000.0000000	-0.2000000
45001.0000000	-0.2150000
45002.0000000	-0.2100000
45003.0000000	-0.2200000
45004.0000000	-0.2100000
45005.0000000	-0.2150000
45006.0000000	-0.2150000
45007.0000000	-0.2150000
45008.0000000	-0.2100000
45009.0000000	-0.2100000
45010.0000000	-0.2150000
45011.0000000	-0.2200000
45012.0000000	-0.2200000
45013.0000000	-0.2150000
45014.0000000	-0.2033333
45015.0000000	-0.2250000
45016.0000000	-0.2150000
45017.0000000	-0.2250000
45018.0000000	-0.2200000
45019.0000000	-0.2150000
45020.0000000	-0.2200000
45021.0000000	-0.2100000
45022.0000000	-0.2250000
45023.0000000	-0.2200000
45024.0000000	-0.2150000
45025.0000000	-0.2200000
45026.0000000	-0.2200000
45027.0000000	-0.2200000
45028.0000000	-0.2300000
45029.0000000	-0.2200000

45030.0000000	-0.2250000
45031.0000000	-0.2150000
45032.0000000	-0.2200000
45033.0000000	-0.2150000
45034.0000000	-0.2200000
45035.0000000	-0.2200000
45036.0000000	-0.2250000
45037.0000000	-0.2300000
45038.0000000	-0.2200000
45039.0000000	-0.2150000
45040.0000000	-0.2150000
45041.0000000	-0.2200000
45042.0000000	-0.2200000
45043.0000000	-0.2250000
45044.0000000	-0.2200000
45045.0000000	-0.2166667
45046.0000000	-0.2250000
45047.0000000	-0.2250000
45048.0000000	-0.2250000
45049.0000000	-0.2200000
45050.0000000	-0.2200000
45051.0000000	-0.2250000
45052.0000000	-0.2200000
45053.0000000	-0.2250000
45054.0000000	-0.2250000
45055.0000000	-0.2250000
45056.0000000	-0.2150000
45057.0000000	-0.2250000
45058.0000000	-0.2200000
45059.0000000	-0.2200000
45060.0000000	-0.2150000
45061.0000000	-0.2250000
45062.0000000	-0.2150000
45063.0000000	-0.2250000
45064.0000000	-0.2200000
45065.0000000	-0.2300000
45066.0000000	-0.2250000
45067.0000000	-0.2200000
45068.0000000	-0.2300000
45069.0000000	-0.2200000
45070.0000000	-0.2250000
45071.0000000	-0.2150000
45072.0000000	-0.2250000
45073.0000000	-0.2200000
45074.0000000	-0.2300000
45075.0000000	-0.2266667
45076.0000000	-0.2200000
45077.0000000	-0.2200000
45078.0000000	-0.2250000
45079.0000000	-0.2250000
45080.0000000	-0.2350000
45081.0000000	-0.2150000
45082.0000000	-0.2250000
45083.0000000	-0.2200000
45084.0000000	-0.2250000
45085.0000000	-0.2250000
45086.0000000	-0.2200000

45087.0000000	-0.2250000
45088.0000000	-0.2250000
45089.0000000	-0.2300000
45090.0000000	-0.2200000
45091.0000000	-0.2200000
45092.0000000	-0.2200000
45093.0000000	-0.2250000
45094.0000000	-0.2200000
45095.0000000	-0.2250000
45096.0000000	-0.2300000
45097.0000000	-0.2200000
45098.0000000	-0.2300000
45099.0000000	-0.2200000
45100.0000000	-0.2300000
45101.0000000	-0.2250000
45102.0000000	-0.2200000
45103.0000000	-0.2300000
45104.0000000	-0.2200000
45105.0000000	-0.2200000
45106.0000000	-0.2300000
45107.0000000	-0.2200000
45108.0000000	-0.2200000
45109.0000000	-0.2250000
45110.0000000	-0.2250000
45111.0000000	-0.2200000
45112.0000000	-0.2250000
45113.0000000	-0.2300000
45114.0000000	-0.2250000
45115.0000000	-0.2200000
45116.0000000	-0.2250000
45117.0000000	-0.2250000
45118.0000000	-0.2200000
45119.0000000	-0.2300000
45120.0000000	-0.2150000
45121.0000000	-0.2250000
45122.0000000	-0.2200000
45123.0000000	-0.2200000
45124.0000000	-0.2250000
45125.0000000	-0.2250000
45126.0000000	-0.2250000
45127.0000000	-0.2300000
45128.0000000	-0.2250000
45129.0000000	-0.2250000
45130.0000000	-0.2250000
45131.0000000	-0.2250000
45132.0000000	-0.2350000
45133.0000000	-0.2250000
45134.0000000	-0.2300000
45135.0000000	-0.2366667
45136.0000000	-0.2350000
45137.0000000	-0.2300000
45138.0000000	-0.2300000
45139.0000000	-0.2300000
45140.0000000	-0.2250000
45141.0000000	-0.2300000
45142.0000000	-0.2200000
45143.0000000	-0.2300000

45144.0000000	-0.2300000
45145.0000000	-0.2300000
45146.0000000	-0.2350000
45147.0000000	-0.2250000
45148.0000000	-0.2300000
45149.0000000	-0.2250000
45150.0000000	-0.2300000
45151.0000000	-0.2300000
45152.0000000	-0.2350000
45153.0000000	-0.2300000
45154.0000000	-0.2350000
45155.0000000	-0.2300000
45156.0000000	-0.2300000
45157.0000000	-0.2350000
45158.0000000	-0.2300000
45159.0000000	-0.2250000
45160.0000000	-0.2400000
45161.0000000	-0.2300000
45162.0000000	-0.2350000
45163.0000000	-0.2400000
45164.0000000	-0.2300000
45165.0000000	-0.2300000
45166.0000000	-0.2366667
45167.0000000	-0.2400000
45168.0000000	-0.2300000
45169.0000000	-0.2350000
45170.0000000	-0.2250000
45171.0000000	-0.2400000
45172.0000000	-0.2300000
45173.0000000	-0.2400000
45174.0000000	-0.2350000
45175.0000000	-0.2350000
45176.0000000	-0.2300000
45177.0000000	-0.2350000
45178.0000000	-0.2400000
45179.0000000	-0.2350000
45180.0000000	-0.2300000
45181.0000000	-0.2350000
45182.0000000	-0.2300000
45183.0000000	-0.2400000
45184.0000000	-0.2350000
45185.0000000	-0.2300000
45186.0000000	-0.2400000
45187.0000000	-0.2300000
45188.0000000	-0.2400000
45189.0000000	-0.2350000
45190.0000000	-0.2400000
45191.0000000	-0.2400000
45192.0000000	-0.2400000
45193.0000000	-0.2450000
45194.0000000	-0.2400000
45195.0000000	-0.2400000
45196.0000000	-0.2400000
45197.0000000	-0.2450000
45198.0000000	-0.2550000
45199.0000000	-0.2350000
45200.0000000	-0.2400000

45201.0000000	-0.2400000
45202.0000000	-0.2450000
45203.0000000	-0.2450000
45204.0000000	-0.2400000
45205.0000000	-0.2400000
45206.0000000	-0.2450000
45207.0000000	-0.2450000
45208.0000000	-0.2400000
45209.0000000	-0.2500000
45210.0000000	-0.2300000
45211.0000000	-0.2450000
45212.0000000	-0.2450000
45213.0000000	-0.2450000
45214.0000000	-0.2500000
45215.0000000	-0.2550000
45216.0000000	-0.2400000
45217.0000000	-0.2500000
45218.0000000	-0.2400000
45219.0000000	-0.2450000
45220.0000000	-0.2450000
45221.0000000	-0.2500000
45222.0000000	-0.2450000
45223.0000000	-0.2500000
45224.0000000	-0.2450000
45225.0000000	-0.2533333
45226.0000000	-0.2400000
45227.0000000	-0.2500000
45228.0000000	-0.2500000
45229.0000000	-0.2500000
45230.0000000	-0.2500000
45231.0000000	-0.2500000
45232.0000000	-0.2450000
45233.0000000	-0.2550000
45234.0000000	-0.2550000
45235.0000000	-0.2550000
45236.0000000	-0.2550000
45237.0000000	-0.2450000
45238.0000000	-0.2550000
45239.0000000	-0.2500000
45240.0000000	-0.2500000
45241.0000000	-0.2550000
45242.0000000	-0.2550000
45243.0000000	-0.2500000
45244.0000000	-0.2600000
45245.0000000	-0.2500000
45246.0000000	-0.2550000
45247.0000000	-0.2600000
45248.0000000	-0.2500000
45249.0000000	-0.2550000
45250.0000000	-0.2500000
45251.0000000	-0.2550000
45252.0000000	-0.2500000
45253.0000000	-0.2500000
45254.0000000	-0.2650000
45255.0000000	-0.2500000
45256.0000000	-0.2466667
45257.0000000	-0.2550000

45258.0000000	-0.2550000
45259.0000000	-0.2550000
45260.0000000	-0.2500000
45261.0000000	-0.2500000
45262.0000000	-0.2600000
45263.0000000	-0.2550000
45264.0000000	-0.2450000
45265.0000000	-0.2550000
45266.0000000	-0.2500000
45267.0000000	-0.2550000
45268.0000000	-0.2600000
45269.0000000	-0.2550000
45270.0000000	-0.2600000
45271.0000000	-0.2550000
45272.0000000	-0.2600000
45273.0000000	-0.2600000
45274.0000000	-0.2550000
45275.0000000	-0.2500000
45276.0000000	-0.2600000
45277.0000000	-0.2600000
45278.0000000	-0.2600000
45279.0000000	-0.2550000
45280.0000000	-0.2550000
45281.0000000	-0.2550000
45282.0000000	-0.2550000
45283.0000000	-0.2550000
45284.0000000	-0.2550000
45285.0000000	-0.2600000
45286.0000000	-0.2600000
45287.0000000	-0.2600000
45288.0000000	-0.2600000
45289.0000000	-0.2650000
45290.0000000	-0.2550000
45291.0000000	-0.2650000
45292.0000000	-0.2700000
45293.0000000	-0.2600000
45294.0000000	-0.2700000
45295.0000000	-0.2600000
45296.0000000	-0.2650000
45297.0000000	-0.2700000
45298.0000000	-0.2650000
45299.0000000	-0.2600000
45300.0000000	-0.2700000
45301.0000000	-0.2550000
45302.0000000	-0.2650000
45303.0000000	-0.2600000
45304.0000000	-0.2650000
45305.0000000	-0.2700000
45306.0000000	-0.2650000
45307.0000000	-0.2700000
45308.0000000	-0.2700000
45309.0000000	-0.2600000
45310.0000000	-0.2650000
45311.0000000	-0.2650000
45312.0000000	-0.2600000
45313.0000000	-0.2550000
45314.0000000	-0.2650000

45315.0000000	-0.2550000
45316.0000000	-0.2666667
45317.0000000	-0.2650000
45318.0000000	-0.2550000
45319.0000000	-0.2600000
45320.0000000	-0.2600000
45321.0000000	-0.2600000
45322.0000000	-0.2650000
45323.0000000	-0.2650000
45324.0000000	-0.2500000
45325.0000000	-0.2600000
45326.0000000	-0.2550000
45327.0000000	-0.2600000
45328.0000000	-0.2550000
45329.0000000	-0.2550000
45330.0000000	-0.2650000
45331.0000000	-0.2650000
45332.0000000	-0.2600000
45333.0000000	-0.2550000
45334.0000000	-0.2550000
45335.0000000	-0.2600000
45336.0000000	-0.2700000
45337.0000000	-0.2650000
45338.0000000	-0.2600000
45339.0000000	-0.2550000
45340.0000000	-0.2650000
45341.0000000	-0.2550000
45342.0000000	-0.2550000
45343.0000000	-0.2600000
45344.0000000	-0.2700000
45345.0000000	-0.2650000
45346.0000000	-0.2566667
45347.0000000	-0.2600000
45348.0000000	-0.2600000
45349.0000000	-0.2600000
45350.0000000	-0.2600000
45351.0000000	-0.2600000
45352.0000000	-0.2650000
45353.0000000	-0.2700000
45354.0000000	-0.2550000
45355.0000000	-0.2650000
45356.0000000	-0.2600000
45357.0000000	-0.2550000
45358.0000000	-0.2650000
45359.0000000	-0.2650000
45360.0000000	-0.2650000
45361.0000000	-0.2650000
45362.0000000	-0.2650000
45363.0000000	-0.2650000
45364.0000000	-0.2650000
45365.0000000	-0.2600000
45366.0000000	-0.2550000
45367.0000000	-0.2600000
45368.0000000	-0.2650000
45369.0000000	-0.2750000
45370.0000000	-0.2650000
45371.0000000	-0.2650000

45372.0000000	-0.2650000
45373.0000000	-0.2600000
45374.0000000	-0.2700000
45375.0000000	-0.2600000
45376.0000000	-0.2550000
45377.0000000	-0.2633333
45378.0000000	-0.2750000
45379.0000000	-0.2650000
45380.0000000	-0.2550000
45381.0000000	-0.2700000
45382.0000000	-0.2600000
45383.0000000	-0.2600000
45384.0000000	-0.2600000
45385.0000000	-0.2550000
45386.0000000	-0.2650000
45387.0000000	-0.2650000
45388.0000000	-0.2700000
45389.0000000	-0.2650000
45390.0000000	-0.2650000
45391.0000000	-0.2550000
45392.0000000	-0.2550000
45393.0000000	-0.2650000
45394.0000000	-0.2600000
45395.0000000	-0.2650000
45396.0000000	-0.2600000
45397.0000000	-0.2700000
45398.0000000	-0.2650000
45399.0000000	-0.2700000
45400.0000000	-0.2550000
45401.0000000	-0.2600000
45402.0000000	-0.2550000
45403.0000000	-0.2700000
45404.0000000	-0.2600000
45405.0000000	-0.2650000
45406.0000000	-0.2550000
45407.0000000	-0.2633333
45408.0000000	-0.2650000
45409.0000000	-0.2650000
45410.0000000	-0.2650000
45411.0000000	-0.2650000
45412.0000000	-0.2600000
45413.0000000	-0.2700000
45414.0000000	-0.2600000
45415.0000000	-0.2700000
45416.0000000	-0.2650000
45417.0000000	-0.2700000
45418.0000000	-0.2700000
45419.0000000	-0.2600000
45420.0000000	-0.2650000
45421.0000000	-0.2650000
45422.0000000	-0.2650000
45423.0000000	-0.2700000
45424.0000000	-0.2800000
45425.0000000	-0.2650000
45426.0000000	-0.2650000
45427.0000000	-0.2700000
45428.0000000	-0.2600000

45429.0000000	-0.2600000
45430.0000000	-0.2500000
45431.0000000	-0.2700000
45432.0000000	-0.2650000
45433.0000000	-0.2700000
45434.0000000	-0.2550000
45435.0000000	-0.2650000
45436.0000000	-0.2650000
45437.0000000	-0.2566667
45438.0000000	-0.2650000
45439.0000000	-0.2650000
45440.0000000	-0.2650000
45441.0000000	-0.2700000
45442.0000000	-0.2600000
45443.0000000	-0.2650000
45444.0000000	-0.2650000
45445.0000000	-0.2600000
45446.0000000	-0.2650000
45447.0000000	-0.2650000
45448.0000000	-0.2650000
45449.0000000	-0.2650000
45450.0000000	-0.2700000
45451.0000000	-0.2550000
45452.0000000	-0.2650000
45453.0000000	-0.2650000
45454.0000000	-0.2600000
45455.0000000	-0.2600000
45456.0000000	-0.2550000
45457.0000000	-0.2600000
45458.0000000	-0.2600000
45459.0000000	-0.2650000
45460.0000000	-0.2600000
45461.0000000	-0.2550000
45462.0000000	-0.2650000
45463.0000000	-0.2600000
45464.0000000	-0.2500000
45465.0000000	-0.2650000
45466.0000000	-0.2600000
45467.0000000	-0.2633333
45468.0000000	-0.2550000
45469.0000000	-0.2500000
45470.0000000	-0.2650000
45471.0000000	-0.2550000
45472.0000000	-0.2550000
45473.0000000	-0.2600000
45474.0000000	-0.2600000
45475.0000000	-0.2600000
45476.0000000	-0.2650000
45477.0000000	-0.2550000
45478.0000000	-0.2500000
45479.0000000	-0.2650000
45480.0000000	-0.2600000
45481.0000000	-0.2500000
45482.0000000	-0.2550000
45483.0000000	-0.2550000
45484.0000000	-0.2650000
45485.0000000	-0.2600000

45486.0000000	-0.2550000
45487.0000000	-0.2500000
45488.0000000	-0.2600000
45489.0000000	-0.2550000
45490.0000000	-0.2550000
45491.0000000	-0.2600000
45492.0000000	-0.2550000
45493.0000000	-0.2600000
45494.0000000	-0.2550000
45495.0000000	-0.2600000
45496.0000000	-0.2650000
45497.0000000	-0.2533333
45498.0000000	-0.2650000
45499.0000000	-0.2600000
45500.0000000	-0.2650000
45501.0000000	-0.2600000
45502.0000000	-0.2550000
45503.0000000	-0.2600000
45504.0000000	-0.2650000
45505.0000000	-0.2600000
45506.0000000	-0.2550000
45507.0000000	-0.2600000
45508.0000000	-0.2600000
45509.0000000	-0.2500000
45510.0000000	-0.2550000
45511.0000000	-0.2600000
45512.0000000	-0.2600000
45513.0000000	-0.2600000
45514.0000000	-0.2550000
45515.0000000	-0.2500000
45516.0000000	-0.2650000
45517.0000000	-0.2650000
45518.0000000	-0.2600000
45519.0000000	-0.2550000
45520.0000000	-0.2650000
45521.0000000	-0.2600000
45522.0000000	-0.2550000
45523.0000000	-0.2500000
45524.0000000	-0.2600000
45525.0000000	-0.2550000
45526.0000000	-0.2600000
45527.0000000	-0.2500000
45528.0000000	-0.2633333
45529.0000000	-0.2600000
45530.0000000	-0.2550000
45531.0000000	-0.2550000
45532.0000000	-0.2550000
45533.0000000	-0.2500000
45534.0000000	-0.2600000
45535.0000000	-0.2600000
45536.0000000	-0.2550000
45537.0000000	-0.2600000
45538.0000000	-0.2550000
45539.0000000	-0.2550000
45540.0000000	-0.2550000
45541.0000000	-0.2600000
45542.0000000	-0.2500000

45543.0000000	-0.2550000
45544.0000000	-0.2650000
45545.0000000	-0.2550000
45546.0000000	-0.2600000
45547.0000000	-0.2550000
45548.0000000	-0.2450000
45549.0000000	-0.2600000
45550.0000000	-0.2550000
45551.0000000	-0.2500000
45552.0000000	-0.2550000
45553.0000000	-0.2550000
45554.0000000	-0.2600000
45555.0000000	-0.2550000
45556.0000000	-0.2600000
45557.0000000	-0.2533333
45558.0000000	-0.2650000
45559.0000000	-0.2650000
45560.0000000	-0.2600000
45561.0000000	-0.2650000
45562.0000000	-0.2550000
45563.0000000	-0.2650000
45564.0000000	-0.2600000
45565.0000000	-0.2650000
45566.0000000	-0.2600000
45567.0000000	-0.2550000
45568.0000000	-0.2600000
45569.0000000	-0.2600000
45570.0000000	-0.2650000
45571.0000000	-0.2600000
45572.0000000	-0.2600000
45573.0000000	-0.2650000
45574.0000000	-0.2650000
45575.0000000	-0.2600000
45576.0000000	-0.2600000
45577.0000000	-0.2600000
45578.0000000	-0.2550000
45579.0000000	-0.2600000
45580.0000000	-0.2600000
45581.0000000	-0.2600000
45582.0000000	-0.2600000
45583.0000000	-0.2600000
45584.0000000	-0.2650000
45585.0000000	-0.2600000
45586.0000000	-0.2650000
45587.0000000	-0.2600000
45588.0000000	-0.2666667
45589.0000000	-0.2600000
45590.0000000	-0.2600000
45591.0000000	-0.2600000
45592.0000000	-0.2650000
45593.0000000	-0.2650000
45594.0000000	-0.2650000
45595.0000000	-0.2550000
45596.0000000	-0.2550000
45597.0000000	-0.2700000
45598.0000000	-0.2650000
45599.0000000	-0.2650000

45600.0000000	-0.2650000
45601.0000000	-0.2650000
45602.0000000	-0.2750000
45603.0000000	-0.2650000
45604.0000000	-0.2650000
45605.0000000	-0.2650000
45606.0000000	-0.2600000
45607.0000000	-0.2600000
45608.0000000	-0.2650000
45609.0000000	-0.2600000
45610.0000000	-0.2600000
45611.0000000	-0.2650000
45612.0000000	-0.2650000
45613.0000000	-0.2600000
45614.0000000	-0.2650000
45615.0000000	-0.2650000
45616.0000000	-0.2600000
45617.0000000	-0.2650000
45618.0000000	-0.2533333
45619.0000000	-0.2650000
45620.0000000	-0.2600000
45621.0000000	-0.2700000
45622.0000000	-0.2650000
45623.0000000	-0.2650000
45624.0000000	-0.2700000
45625.0000000	-0.2650000
45626.0000000	-0.2700000
45627.0000000	-0.2600000
45628.0000000	-0.2600000
45629.0000000	-0.2700000
45630.0000000	-0.2600000
45631.0000000	-0.2650000
45632.0000000	-0.2650000
45633.0000000	-0.2750000
45634.0000000	-0.2650000
45635.0000000	-0.2650000
45636.0000000	-0.2750000
45637.0000000	-0.2700000
45638.0000000	-0.2700000
45639.0000000	-0.2650000
45640.0000000	-0.2600000
45641.0000000	-0.2750000
45642.0000000	-0.2750000
45643.0000000	-0.2600000
45644.0000000	-0.2700000
45645.0000000	-0.2700000
45646.0000000	-0.2750000
45647.0000000	-0.2650000
45648.0000000	-0.2733333
45649.0000000	-0.2700000
45650.0000000	-0.2700000
45651.0000000	-0.2700000
45652.0000000	-0.2750000
45653.0000000	-0.2800000
45654.0000000	-0.2800000
45655.0000000	-0.2650000
45656.0000000	-0.2700000

45657.0000000	-0.2800000
45658.0000000	-0.2850000
45659.0000000	-0.2750000
45660.0000000	-0.2800000
45661.0000000	-0.2850000
45662.0000000	-0.2800000
45663.0000000	-0.2750000
45664.0000000	-0.2750000
45665.0000000	-0.2800000
45666.0000000	-0.2800000
45667.0000000	-0.2800000
45668.0000000	-0.2750000
45669.0000000	-0.2800000
45670.0000000	-0.2750000
45671.0000000	-0.2750000
45672.0000000	-0.2800000
45673.0000000	-0.2750000
45674.0000000	-0.2900000
45675.0000000	-0.2750000
45676.0000000	-0.2750000
45677.0000000	-0.2800000
45678.0000000	-0.2833333
45679.0000000	-0.2750000
45680.0000000	-0.2850000
45681.0000000	-0.2850000
45682.0000000	-0.2750000
45683.0000000	-0.2800000
45684.0000000	-0.2800000
45685.0000000	-0.2800000
45686.0000000	-0.2850000
45687.0000000	-0.2850000
45688.0000000	-0.2800000
45689.0000000	-0.2850000
45690.0000000	-0.2850000
45691.0000000	-0.2800000
45692.0000000	-0.2900000
45693.0000000	-0.2800000
45694.0000000	-0.2850000
45695.0000000	-0.2850000
45696.0000000	-0.2850000
45697.0000000	-0.2850000
45698.0000000	-0.2800000
45699.0000000	-0.2900000
45700.0000000	-0.2900000
45701.0000000	-0.2850000
45702.0000000	-0.2900000
45703.0000000	-0.2750000
45704.0000000	-0.2800000
45705.0000000	-0.2850000
45706.0000000	-0.2850000
45707.0000000	-0.2800000
45708.0000000	-0.2800000
45709.0000000	-0.2850000
45710.0000000	-0.2850000
45711.0000000	-0.2950000
45712.0000000	-0.2850000
45713.0000000	-0.2800000

45714.0000000	-0.2900000
45715.0000000	-0.2850000
45716.0000000	-0.2900000
45717.0000000	-0.2900000
45718.0000000	-0.2850000
45719.0000000	-0.2900000
45720.0000000	-0.2800000
45721.0000000	-0.2900000
45722.0000000	-0.2800000
45723.0000000	-0.2850000
45724.0000000	-0.2950000
45725.0000000	-0.2850000
45726.0000000	-0.2900000
45727.0000000	-0.2900000
45728.0000000	-0.2850000
45729.0000000	-0.2850000
45730.0000000	-0.2900000
45731.0000000	-0.2900000
45732.0000000	-0.2850000
45733.0000000	-0.2800000
45734.0000000	-0.2750000
45735.0000000	-0.2800000
45736.0000000	-0.2850000
45737.0000000	-0.2850000
45738.0000000	-0.2900000
45739.0000000	-0.2866667
45740.0000000	-0.2750000
45741.0000000	-0.2800000
45742.0000000	-0.2850000
45743.0000000	-0.2900000
45744.0000000	-0.2800000
45745.0000000	-0.2850000
45746.0000000	-0.2850000
45747.0000000	-0.2900000
45748.0000000	-0.2850000
45749.0000000	-0.2850000
45750.0000000	-0.2850000
45751.0000000	-0.2800000
45752.0000000	-0.2800000
45753.0000000	-0.2850000
45754.0000000	-0.2850000
45755.0000000	-0.2850000
45756.0000000	-0.2900000
45757.0000000	-0.2950000
45758.0000000	-0.2750000
45759.0000000	-0.2900000
45760.0000000	-0.2850000
45761.0000000	-0.2950000
45762.0000000	-0.2950000
45763.0000000	-0.2850000
45764.0000000	-0.2900000
45765.0000000	-0.2900000
45766.0000000	-0.2900000
45767.0000000	-0.2950000
45768.0000000	-0.2950000
45769.0000000	-0.2900000
45770.0000000	-0.2950000

45771.0000000	-0.2950000
45772.0000000	-0.2950000
45773.0000000	-0.2950000
45774.0000000	-0.3000000
45775.0000000	-0.3000000
45776.0000000	-0.2950000
45777.0000000	-0.2950000
45778.0000000	-0.2900000
45779.0000000	-0.2950000
45780.0000000	-0.2950000
45781.0000000	-0.2950000
45782.0000000	-0.2900000
45783.0000000	-0.2950000
45784.0000000	-0.2950000
45785.0000000	-0.2950000
45786.0000000	-0.2900000
45787.0000000	-0.2950000
45788.0000000	-0.3000000
45789.0000000	-0.3050000
45790.0000000	-0.2950000
45791.0000000	-0.3000000
45792.0000000	-0.3050000
45793.0000000	-0.2950000
45794.0000000	-0.3000000
45795.0000000	-0.3000000
45796.0000000	-0.2900000
45797.0000000	-0.3100000
45798.0000000	-0.3000000
45799.0000000	-0.3033333
45800.0000000	-0.2950000
45801.0000000	-0.2950000
45802.0000000	-0.3050000
45803.0000000	-0.3050000
45804.0000000	-0.3000000
45805.0000000	-0.2950000
45806.0000000	-0.2950000
45807.0000000	-0.3000000
45808.0000000	-0.3050000
45809.0000000	-0.3000000
45810.0000000	-0.3000000
45811.0000000	-0.2950000
45812.0000000	-0.2950000
45813.0000000	-0.3050000
45814.0000000	-0.2950000
45815.0000000	-0.3000000
45816.0000000	-0.3050000
45817.0000000	-0.3050000
45818.0000000	-0.3100000
45819.0000000	-0.2900000
45820.0000000	-0.3050000
45821.0000000	-0.2950000
45822.0000000	-0.3000000
45823.0000000	-0.3050000
45824.0000000	-0.3000000
45825.0000000	-0.3050000
45826.0000000	-0.2950000
45827.0000000	-0.3050000

45828.0000000	-0.3000000
45829.0000000	-0.2966667
45830.0000000	-0.2950000
45831.0000000	-0.3100000
45832.0000000	-0.2950000
45833.0000000	-0.3000000
45834.0000000	-0.2950000
45835.0000000	-0.2900000
45836.0000000	-0.2950000
45837.0000000	-0.2950000
45838.0000000	-0.2950000
45839.0000000	-0.2900000
45840.0000000	-0.3050000
45841.0000000	-0.3000000
45842.0000000	-0.3000000
45843.0000000	-0.3000000
45844.0000000	-0.2950000
45845.0000000	-0.2900000
45846.0000000	-0.2950000
45847.0000000	-0.2900000
45848.0000000	-0.3050000
45849.0000000	-0.3000000
45850.0000000	-0.2900000
45851.0000000	-0.2950000
45852.0000000	-0.2850000
45853.0000000	-0.3000000
45854.0000000	-0.2950000
45855.0000000	-0.3000000
45856.0000000	-0.2850000
45857.0000000	-0.3000000
45858.0000000	-0.3000000
45859.0000000	-0.2900000
45860.0000000	-0.2933333
45861.0000000	-0.2950000
45862.0000000	-0.3000000
45863.0000000	-0.3000000
45864.0000000	-0.3050000
45865.0000000	-0.2950000
45866.0000000	-0.3000000
45867.0000000	-0.2950000
45868.0000000	-0.2950000
45869.0000000	-0.3000000
45870.0000000	-0.3000000
45871.0000000	-0.3000000
45872.0000000	-0.2950000
45873.0000000	-0.3000000
45874.0000000	-0.3000000
45875.0000000	-0.2950000
45876.0000000	-0.3000000
45877.0000000	-0.3050000
45878.0000000	-0.3000000
45879.0000000	-0.3000000
45880.0000000	-0.2900000
45881.0000000	-0.2950000
45882.0000000	-0.3000000
45883.0000000	-0.3050000
45884.0000000	-0.3000000

45885.0000000	-0.2950000
45886.0000000	-0.3000000
45887.0000000	-0.2950000
45888.0000000	-0.3000000
45889.0000000	-0.3000000
45890.0000000	-0.3000000
45891.0000000	-0.3000000
45892.0000000	-0.3000000
45893.0000000	-0.2950000
45894.0000000	-0.2950000
45895.0000000	-0.3050000
45896.0000000	-0.2950000
45897.0000000	-0.3000000
45898.0000000	-0.3050000
45899.0000000	-0.3000000
45900.0000000	-0.3050000
45901.0000000	-0.3000000
45902.0000000	-0.2950000
45903.0000000	-0.2850000
45904.0000000	-0.3000000
45905.0000000	-0.2950000
45906.0000000	-0.2950000
45907.0000000	-0.3000000
45908.0000000	-0.2950000
45909.0000000	-0.3000000
45910.0000000	-0.3000000
45911.0000000	-0.2900000
45912.0000000	-0.3000000
45913.0000000	-0.2900000
45914.0000000	-0.3000000
45915.0000000	-0.3050000
45916.0000000	-0.2950000
45917.0000000	-0.2950000
45918.0000000	-0.3000000
45919.0000000	-0.2966667
45920.0000000	-0.2950000
45921.0000000	-0.2900000
45922.0000000	-0.2950000
45923.0000000	-0.2950000
45924.0000000	-0.2950000
45925.0000000	-0.3000000
45926.0000000	-0.2950000
45927.0000000	-0.3000000
45928.0000000	-0.2900000
45929.0000000	-0.2950000
45930.0000000	-0.2950000
45931.0000000	-0.3000000
45932.0000000	-0.2900000
45933.0000000	-0.2950000
45934.0000000	-0.2850000
45935.0000000	-0.2900000
45936.0000000	-0.2850000
45937.0000000	-0.2950000
45938.0000000	-0.2950000
45939.0000000	-0.2950000
45940.0000000	-0.2950000
45941.0000000	-0.2900000

45942.0000000	-0.2900000
45943.0000000	-0.2900000
45944.0000000	-0.2950000
45945.0000000	-0.2950000
45946.0000000	-0.2950000
45947.0000000	-0.2950000
45948.0000000	-0.2900000
45949.0000000	-0.2950000
45950.0000000	-0.2966667
45951.0000000	-0.2950000
45952.0000000	-0.2950000
45953.0000000	-0.2900000
45954.0000000	-0.3000000
45955.0000000	-0.2950000
45956.0000000	-0.3000000
45957.0000000	-0.3000000
45958.0000000	-0.2950000
45959.0000000	-0.2950000
45960.0000000	-0.2950000
45961.0000000	-0.3050000
45962.0000000	-0.3000000
45963.0000000	-0.2950000
45964.0000000	-0.3000000
45965.0000000	-0.2950000
45966.0000000	-0.2900000
45967.0000000	-0.2900000
45968.0000000	-0.3000000
45969.0000000	-0.2950000
45970.0000000	-0.2950000
45971.0000000	-0.2950000
45972.0000000	-0.3000000
45973.0000000	-0.3100000
45974.0000000	-0.2950000
45975.0000000	-0.2850000
45976.0000000	-0.3000000
45977.0000000	-0.2950000
45978.0000000	-0.3000000
45979.0000000	-0.2900000
45980.0000000	-0.2900000
45981.0000000	-0.2966667
45982.0000000	-0.2950000
45983.0000000	-0.2900000
45984.0000000	-0.2850000
45985.0000000	-0.2900000
45986.0000000	-0.2950000
45987.0000000	-0.3000000
45988.0000000	-0.2900000
45989.0000000	-0.2950000
45990.0000000	-0.3000000
45991.0000000	-0.2950000
45992.0000000	-0.2850000
45993.0000000	-0.2950000
45994.0000000	-0.2850000
45995.0000000	-0.2900000
45996.0000000	-0.2850000
45997.0000000	-0.2950000
45998.0000000	-0.2900000

45999.0000000	-0.2900000
46000.0000000	-0.2950000
46001.0000000	-0.2950000
46002.0000000	-0.2950000
46003.0000000	-0.2850000
46004.0000000	-0.2850000
46005.0000000	-0.2900000
46006.0000000	-0.2950000
46007.0000000	-0.2950000
46008.0000000	-0.2900000
46009.0000000	-0.2900000
46010.0000000	-0.2866667
46011.0000000	-0.2900000
46012.0000000	-0.2900000
46013.0000000	-0.2900000
46014.0000000	-0.2850000
46015.0000000	-0.2800000
46016.0000000	-0.2850000
46017.0000000	-0.2850000
46018.0000000	-0.2850000
46019.0000000	-0.2800000
46020.0000000	-0.2850000
46021.0000000	-0.2750000
46022.0000000	-0.2900000
46023.0000000	-0.2850000
46024.0000000	-0.2850000
46025.0000000	-0.2800000
46026.0000000	-0.2700000
46027.0000000	-0.2800000
46028.0000000	-0.2850000
46029.0000000	-0.2850000
46030.0000000	-0.2750000
46031.0000000	-0.2750000
46032.0000000	-0.2850000
46033.0000000	-0.2750000
46034.0000000	-0.2700000
46035.0000000	-0.2750000
46036.0000000	-0.2700000
46037.0000000	-0.2750000
46038.0000000	-0.2750000
46039.0000000	-0.2800000
46040.0000000	-0.2733333
46041.0000000	-0.2700000
46042.0000000	-0.2800000
46043.0000000	-0.2700000
46044.0000000	-0.2750000
46045.0000000	-0.2700000
46046.0000000	-0.2650000
46047.0000000	-0.2750000
46048.0000000	-0.2700000
46049.0000000	-0.2750000
46050.0000000	-0.2650000
46051.0000000	-0.2700000
46052.0000000	-0.2650000
46053.0000000	-0.2650000
46054.0000000	-0.2550000
46055.0000000	-0.2700000

46056.0000000	-0.2700000
46057.0000000	-0.2600000
46058.0000000	-0.2650000
46059.0000000	-0.2650000
46060.0000000	-0.2600000
46061.0000000	-0.2700000
46062.0000000	-0.2650000
46063.0000000	-0.2550000
46064.0000000	-0.2550000
46065.0000000	-0.2600000
46066.0000000	-0.2550000
46067.0000000	-0.2650000
46068.0000000	-0.2500000
46069.0000000	-0.2550000
46070.0000000	-0.2550000
46071.0000000	-0.2533333
46072.0000000	-0.2500000
46073.0000000	-0.2500000
46074.0000000	-0.2500000
46075.0000000	-0.2600000
46076.0000000	-0.2500000
46077.0000000	-0.2500000
46078.0000000	-0.2450000
46079.0000000	-0.2450000
46080.0000000	-0.2500000
46081.0000000	-0.2450000
46082.0000000	-0.2500000
46083.0000000	-0.2500000
46084.0000000	-0.2400000
46085.0000000	-0.2500000
46086.0000000	-0.2450000
46087.0000000	-0.2450000
46088.0000000	-0.2450000
46089.0000000	-0.2500000
46090.0000000	-0.2350000
46091.0000000	-0.2450000
46092.0000000	-0.2350000
46093.0000000	-0.2450000
46094.0000000	-0.2400000
46095.0000000	-0.2350000
46096.0000000	-0.2400000
46097.0000000	-0.2400000
46098.0000000	-0.2300000
46099.0000000	-0.2450000
46100.0000000	-0.2400000
46101.0000000	-0.2266666
46102.0000000	-0.2350000
46103.0000000	-0.2400000
46104.0000000	-0.2350000
46105.0000000	-0.2350000
46106.0000000	-0.2350000
46107.0000000	-0.2300000
46108.0000000	-0.2400000
46109.0000000	-0.2300000
46110.0000000	-0.2350000
46111.0000000	-0.2300000
46112.0000000	-0.2200000

46113.0000000	-0.2350000
46114.0000000	-0.2350000
46115.0000000	-0.2300000
46116.0000000	-0.2300000
46117.0000000	-0.2250000
46118.0000000	-0.2300000
46119.0000000	-0.2300000
46120.0000000	-0.2200000
46121.0000000	-0.2300000
46122.0000000	-0.2200000
46123.0000000	-0.2250000
46124.0000000	-0.2300000
46125.0000000	-0.2250000
46126.0000000	-0.2250000
46127.0000000	-0.2200000
46128.0000000	-0.2250000
46129.0000000	-0.2150000
46130.0000000	-0.2200000
46131.0000000	-0.2233333
46132.0000000	-0.2150000
46133.0000000	-0.2200000
46134.0000000	-0.2200000
46135.0000000	-0.2250000
46136.0000000	-0.2200000
46137.0000000	-0.2200000
46138.0000000	-0.2150000
46139.0000000	-0.2250000
46140.0000000	-0.2100000
46141.0000000	-0.2200000
46142.0000000	-0.2100000
46143.0000000	-0.2100000
46144.0000000	-0.2100000
46145.0000000	-0.2150000
46146.0000000	-0.2200000
46147.0000000	-0.2150000
46148.0000000	-0.2150000
46149.0000000	-0.2100000
46150.0000000	-0.2150000
46151.0000000	-0.2150000
46152.0000000	-0.2150000
46153.0000000	-0.2050000
46154.0000000	-0.2050000
46155.0000000	-0.2150000
46156.0000000	-0.2100000
46157.0000000	-0.2200000
46158.0000000	-0.2100000
46159.0000000	-0.2000000
46160.0000000	-0.2150000
46161.0000000	-0.2066667
46162.0000000	-0.2050000
46163.0000000	-0.2050000
46164.0000000	-0.2150000
46165.0000000	-0.2150000
46166.0000000	-0.2050000
46167.0000000	-0.2100000
46168.0000000	-0.2150000
46169.0000000	-0.2100000

46170.0000000	-0.2050000
46171.0000000	-0.2000000
46172.0000000	-0.2050000
46173.0000000	-0.2050000
46174.0000000	-0.2100000
46175.0000000	-0.2000000
46176.0000000	-0.2050000
46177.0000000	-0.2000000
46178.0000000	-0.2000000
46179.0000000	-0.2050000
46180.0000000	-0.2000000
46181.0000000	-0.2000000
46182.0000000	-0.2000000
46183.0000000	-0.2000000
46184.0000000	-0.1950000
46185.0000000	-0.2000000
46186.0000000	-0.2000000
46187.0000000	-0.2000000
46188.0000000	-0.2050000
46189.0000000	-0.2000000
46190.0000000	-0.2000000
46191.0000000	-0.1950000
46192.0000000	-0.1966667
46193.0000000	-0.1900000
46194.0000000	-0.1950000
46195.0000000	-0.1900000
46196.0000000	-0.1900000
46197.0000000	-0.1850000
46198.0000000	-0.1900000
46199.0000000	-0.1850000
46200.0000000	-0.1900000
46201.0000000	-0.1900000
46202.0000000	-0.1900000
46203.0000000	-0.1900000
46204.0000000	-0.1850000
46205.0000000	-0.1800000
46206.0000000	-0.1800000
46207.0000000	-0.1800000
46208.0000000	-0.1800000
46209.0000000	-0.1700000
46210.0000000	-0.1800000
46211.0000000	-0.1800000
46212.0000000	-0.1750000
46213.0000000	-0.1800000
46214.0000000	-0.1750000
46215.0000000	-0.1850000
46216.0000000	-0.1750000
46217.0000000	-0.1700000
46218.0000000	-0.1750000
46219.0000000	-0.1800000
46220.0000000	-0.1650000
46221.0000000	-0.1750000
46222.0000000	-0.1766667
46223.0000000	-0.1700000
46224.0000000	-0.1700000
46225.0000000	-0.1650000
46226.0000000	-0.1750000

46227.0000000	-0.1700000
46228.0000000	-0.1600000
46229.0000000	-0.1650000
46230.0000000	-0.1650000
46231.0000000	-0.1650000
46232.0000000	-0.1600000
46233.0000000	-0.1650000
46234.0000000	-0.1600000
46235.0000000	-0.1650000
46236.0000000	-0.1650000
46237.0000000	-0.1600000
46238.0000000	-0.1550000
46239.0000000	-0.1650000
46240.0000000	-0.1600000
46241.0000000	-0.1600000
46242.0000000	-0.1500000
46243.0000000	-0.1550000
46244.0000000	-0.1500000
46245.0000000	-0.1550000
46246.0000000	-0.1500000
46247.0000000	-0.1500000
46248.0000000	-0.1600000
46249.0000000	-0.1550000
46250.0000000	-0.1500000
46251.0000000	-0.1500000
46252.0000000	-0.1500000
46253.0000000	-0.1450000
46254.0000000	-0.1500000
46255.0000000	-0.1550000
46256.0000000	-0.1450000
46257.0000000	-0.1500000
46258.0000000	-0.1450000
46259.0000000	-0.1400000
46260.0000000	-0.1500000
46261.0000000	-0.1350000
46262.0000000	-0.1400000
46263.0000000	-0.1400000
46264.0000000	-0.1350000
46265.0000000	-0.1400000
46266.0000000	-0.1450000
46267.0000000	-0.1300000
46268.0000000	-0.1350000
46269.0000000	-0.1350000
46270.0000000	-0.1400000
46271.0000000	-0.1350000
46272.0000000	-0.1400000
46273.0000000	-0.1350000
46274.0000000	-0.1300000
46275.0000000	-0.1350000
46276.0000000	-0.1350000
46277.0000000	-0.1200000
46278.0000000	-0.1300000
46279.0000000	-0.1300000
46280.0000000	-0.1350000
46281.0000000	-0.1300000
46282.0000000	-0.1266667
46283.0000000	-0.1250000

46284.0000000	-0.1200000
46285.0000000	-0.1200000
46286.0000000	-0.1300000
46287.0000000	-0.1200000
46288.0000000	-0.1250000
46289.0000000	-0.1300000
46290.0000000	-0.1200000
46291.0000000	-0.1200000
46292.0000000	-0.1100000
46293.0000000	-0.1150000
46294.0000000	-0.1200000
46295.0000000	-0.1150000
46296.0000000	-0.1200000
46297.0000000	-0.1150000
46298.0000000	-0.1100000
46299.0000000	-0.1150000
46300.0000000	-0.1150000
46301.0000000	-0.1100000
46302.0000000	-0.1100000
46303.0000000	-0.1150000
46304.0000000	-0.1050000
46305.0000000	-0.1100000
46306.0000000	-0.1050000
46307.0000000	-0.1000000
46308.0000000	-0.1100000
46309.0000000	-0.1050000
46310.0000000	-0.1000000
46311.0000000	-0.1000000
46312.0000000	-0.1100000
46313.0000000	-0.1033333
46314.0000000	-0.0900000
46315.0000000	-0.0950000
46316.0000000	-0.1000000
46317.0000000	-0.0950000
46318.0000000	-0.0950000
46319.0000000	-0.1000000
46320.0000000	-0.0950000
46321.0000000	-0.0950000
46322.0000000	-0.1000000
46323.0000000	-0.0950000
46324.0000000	-0.0900000
46325.0000000	-0.0950000
46326.0000000	-0.0950000
46327.0000000	-0.0900000
46328.0000000	-0.0850000
46329.0000000	-0.0950000
46330.0000000	-0.0900000
46331.0000000	-0.0900000
46332.0000000	-0.0950000
46333.0000000	-0.0850000
46334.0000000	-0.0900000
46335.0000000	-0.0850000
46336.0000000	-0.0800000
46337.0000000	-0.0850000
46338.0000000	-0.0850000
46339.0000000	-0.0800000
46340.0000000	-0.0850000

46341.0000000	-0.0850000
46342.0000000	-0.0800000
46343.0000000	-0.0800000
46344.0000000	-0.0800000
46345.0000000	-0.0750000
46346.0000000	-0.0800000
46347.0000000	-0.0800000
46348.0000000	-0.0750000
46349.0000000	-0.0750000
46350.0000000	-0.0650000
46351.0000000	-0.0800000
46352.0000000	-0.0700000
46353.0000000	-0.0700000
46354.0000000	-0.0750000
46355.0000000	-0.0700000
46356.0000000	-0.0700000
46357.0000000	-0.0700000
46358.0000000	-0.0700000
46359.0000000	-0.0700000
46360.0000000	-0.0650000
46361.0000000	-0.0700000
46362.0000000	-0.0700000
46363.0000000	-0.0600000
46364.0000000	-0.0600000
46365.0000000	-0.0600000
46366.0000000	-0.0650000
46367.0000000	-0.0600000
46368.0000000	-0.0650000
46369.0000000	-0.0650000
46370.0000000	-0.0600000
46371.0000000	-0.0700000
46372.0000000	-0.0600000
46373.0000000	-0.0600000
46374.0000000	-0.0650000
46375.0000000	-0.0550000
46376.0000000	-0.0600000
46377.0000000	-0.0500000
46378.0000000	-0.0600000
46379.0000000	-0.0550000
46380.0000000	-0.0550000
46381.0000000	-0.0550000
46382.0000000	-0.0600000
46383.0000000	-0.0600000
46384.0000000	-0.0600000
46385.0000000	-0.0550000
46386.0000000	-0.0450000
46387.0000000	-0.0650000
46388.0000000	-0.0550000
46389.0000000	-0.0550000
46390.0000000	-0.0500000
46391.0000000	-0.0500000
46392.0000000	-0.0600000
46393.0000000	-0.0550000
46394.0000000	-0.0650000
46395.0000000	-0.0500000
46396.0000000	-0.0500000
46397.0000000	-0.0500000

46398.0000000	-0.0550000
46399.0000000	-0.0400000
46400.0000000	-0.0500000
46401.0000000	-0.0550000
46402.0000000	-0.0450000
46403.0000000	-0.0500000
46404.0000000	-0.0500000
46405.0000000	-0.0500000
46406.0000000	-0.0500000
46407.0000000	-0.0500000
46408.0000000	-0.0450000
46409.0000000	-0.0450000
46410.0000000	-0.0500000
46411.0000000	-0.0550000
46412.0000000	-0.0500000
46413.0000000	-0.0500000
46414.0000000	-0.0500000
46415.0000000	-0.0450000
46416.0000000	-0.0500000
46417.0000000	-0.0450000
46418.0000000	-0.0450000
46419.0000000	-0.0500000
46420.0000000	-0.0450000
46421.0000000	-0.0450000
46422.0000000	-0.0500000
46423.0000000	-0.0350000
46424.0000000	-0.0500000
46425.0000000	-0.0450000
46426.0000000	-0.0450000
46427.0000000	-0.0450000
46428.0000000	-0.0450000
46429.0000000	-0.0450000
46430.0000000	-0.0450000
46431.0000000	-0.0500000
46432.0000000	-0.0400000
46433.0000000	-0.0400000
46434.0000000	-0.0450000
46435.0000000	-0.0450000
46436.0000000	-0.0450000
46437.0000000	-0.0450000
46438.0000000	-0.0500000
46439.0000000	-0.0450000
46440.0000000	-0.0500000
46441.0000000	-0.0450000
46442.0000000	-0.0500000
46443.0000000	-0.0500000
46444.0000000	-0.0450000
46445.0000000	-0.0500000
46446.0000000	-0.0450000
46447.0000000	-0.0450000
46448.0000000	-0.0500000
46449.0000000	-0.0500000
46450.0000000	-0.0500000
46451.0000000	-0.0450000
46452.0000000	-0.0450000
46453.0000000	-0.0500000
46454.0000000	-0.0500000

46455.0000000	-0.0500000
46456.0000000	-0.0450000
46457.0000000	-0.0600000
46458.0000000	-0.0450000
46459.0000000	-0.0500000
46460.0000000	-0.0450000
46461.0000000	-0.0500000
46462.0000000	-0.0550000
46463.0000000	-0.0466667
46464.0000000	-0.0550000
46465.0000000	-0.0500000
46466.0000000	-0.0550000
46467.0000000	-0.0550000
46468.0000000	-0.0450000
46469.0000000	-0.0550000
46470.0000000	-0.0500000
46471.0000000	-0.0550000
46472.0000000	-0.0500000
46473.0000000	-0.0600000
46474.0000000	-0.0500000
46475.0000000	-0.0550000
46476.0000000	-0.0500000
46477.0000000	-0.0550000
46478.0000000	-0.0550000
46479.0000000	-0.0500000
46480.0000000	-0.0550000
46481.0000000	-0.0500000
46482.0000000	-0.0550000
46483.0000000	-0.0500000
46484.0000000	-0.0500000
46485.0000000	-0.0550000
46486.0000000	-0.0550000
46487.0000000	-0.0550000
46488.0000000	-0.0550000
46489.0000000	-0.0550000
46490.0000000	-0.0550000
46491.0000000	-0.0550000
46492.0000000	-0.0550000
46493.0000000	-0.0600000
46494.0000000	-0.0550000
46495.0000000	-0.0600000
46496.0000000	-0.0550000
46497.0000000	-0.0500000
46498.0000000	-0.0600000
46499.0000000	-0.0600000
46500.0000000	-0.0550000
46501.0000000	-0.0550000
46502.0000000	-0.0550000
46503.0000000	-0.0600000
46504.0000000	-0.0600000
46505.0000000	-0.0550000
46506.0000000	-0.0600000
46507.0000000	-0.0600000
46508.0000000	-0.0550000
46509.0000000	-0.0600000
46510.0000000	-0.0600000
46511.0000000	-0.0600000

46512.0000000	-0.0600000
46513.0000000	-0.0650000
46514.0000000	-0.0600000
46515.0000000	-0.0600000
46516.0000000	-0.0600000
46517.0000000	-0.0600000
46518.0000000	-0.0550000
46519.0000000	-0.0550000
46520.0000000	-0.0650000
46521.0000000	-0.0600000
46522.0000000	-0.0650000
46523.0000000	-0.0650000
46524.0000000	-0.0633333
46525.0000000	-0.0600000
46526.0000000	-0.0650000
46527.0000000	-0.0600000
46528.0000000	-0.0550000
46529.0000000	-0.0650000
46530.0000000	-0.0650000
46531.0000000	-0.0550000
46532.0000000	-0.0600000
46533.0000000	-0.0700000
46534.0000000	-0.0700000
46535.0000000	-0.0550000
46536.0000000	-0.0700000
46537.0000000	-0.0650000
46538.0000000	-0.0600000
46539.0000000	-0.0650000
46540.0000000	-0.0600000
46541.0000000	-0.0550000
46542.0000000	-0.0650000
46543.0000000	-0.0600000
46544.0000000	-0.0650000
46545.0000000	-0.0600000
46546.0000000	-0.0600000
46547.0000000	-0.0700000
46548.0000000	-0.0650000
46549.0000000	-0.0650000
46550.0000000	-0.0650000
46551.0000000	-0.0600000
46552.0000000	-0.0650000
46553.0000000	-0.0600000
46554.0000000	-0.0633333
46555.0000000	-0.0550000
46556.0000000	-0.0650000
46557.0000000	-0.0650000
46558.0000000	-0.0550000
46559.0000000	-0.0600000
46560.0000000	-0.0550000
46561.0000000	-0.0650000
46562.0000000	-0.0600000
46563.0000000	-0.0500000
46564.0000000	-0.0600000
46565.0000000	-0.0650000
46566.0000000	-0.0600000
46567.0000000	-0.0700000
46568.0000000	-0.0600000

46569.0000000	-0.0550000
46570.0000000	-0.0650000
46571.0000000	-0.0650000
46572.0000000	-0.0600000
46573.0000000	-0.0600000
46574.0000000	-0.0600000
46575.0000000	-0.0600000
46576.0000000	-0.0550000
46577.0000000	-0.0600000
46578.0000000	-0.0600000
46579.0000000	-0.0600000
46580.0000000	-0.0600000
46581.0000000	-0.0550000
46582.0000000	-0.0500000
46583.0000000	-0.0600000
46584.0000000	-0.0566667
46585.0000000	-0.0550000
46586.0000000	-0.0550000
46587.0000000	-0.0600000
46588.0000000	-0.0600000
46589.0000000	-0.0700000
46590.0000000	-0.0500000
46591.0000000	-0.0600000
46592.0000000	-0.0550000
46593.0000000	-0.0550000
46594.0000000	-0.0600000
46595.0000000	-0.0550000
46596.0000000	-0.0550000
46597.0000000	-0.0600000
46598.0000000	-0.0600000
46599.0000000	-0.0500000
46600.0000000	-0.0550000
46601.0000000	-0.0650000
46602.0000000	-0.0600000
46603.0000000	-0.0650000
46604.0000000	-0.0550000
46605.0000000	-0.0550000
46606.0000000	-0.0600000
46607.0000000	-0.0550000
46608.0000000	-0.0500000
46609.0000000	-0.0600000
46610.0000000	-0.0550000
46611.0000000	-0.0600000
46612.0000000	-0.0600000
46613.0000000	-0.0550000
46614.0000000	-0.0600000
46615.0000000	-0.0600000
46616.0000000	-0.0600000
46617.0000000	-0.0550000
46618.0000000	-0.0550000
46619.0000000	-0.0550000
46620.0000000	-0.0550000
46621.0000000	-0.0650000
46622.0000000	-0.0550000
46623.0000000	-0.0600000
46624.0000000	-0.0450000
46625.0000000	-0.0600000

46626.0000000	-0.0500000
46627.0000000	-0.0550000
46628.0000000	-0.0600000
46629.0000000	-0.0450000
46630.0000000	-0.0550000
46631.0000000	-0.0550000
46632.0000000	-0.0550000
46633.0000000	-0.0600000
46634.0000000	-0.0500000
46635.0000000	-0.0600000
46636.0000000	-0.0550000
46637.0000000	-0.0500000
46638.0000000	-0.0450000
46639.0000000	-0.0550000
46640.0000000	-0.0500000
46641.0000000	-0.0500000
46642.0000000	-0.0600000
46643.0000000	-0.0500000
46644.0000000	-0.0466667
46645.0000000	-0.0500000
46646.0000000	-0.0500000
46647.0000000	-0.0400000
46648.0000000	-0.0550000
46649.0000000	-0.0450000
46650.0000000	-0.0500000
46651.0000000	-0.0450000
46652.0000000	-0.0450000
46653.0000000	-0.0500000
46654.0000000	-0.0400000
46655.0000000	-0.0500000
46656.0000000	-0.0450000
46657.0000000	-0.0400000
46658.0000000	-0.0550000
46659.0000000	-0.0450000
46660.0000000	-0.0450000
46661.0000000	-0.0450000
46662.0000000	-0.0450000
46663.0000000	-0.0400000
46664.0000000	-0.0550000
46665.0000000	-0.0450000
46666.0000000	-0.0450000
46667.0000000	-0.0450000
46668.0000000	-0.0500000
46669.0000000	-0.0400000
46670.0000000	-0.0500000
46671.0000000	-0.0450000
46672.0000000	-0.0400000
46673.0000000	-0.0450000
46674.0000000	-0.0400000
46675.0000000	-0.0466667
46676.0000000	-0.0450000
46677.0000000	-0.0400000
46678.0000000	-0.0450000
46679.0000000	-0.0400000
46680.0000000	-0.0400000
46681.0000000	-0.0400000
46682.0000000	-0.0350000

46683.0000000	-0.0450000
46684.0000000	-0.0400000
46685.0000000	-0.0450000
46686.0000000	-0.0350000
46687.0000000	-0.0500000
46688.0000000	-0.0350000
46689.0000000	-0.0350000
46690.0000000	-0.0350000
46691.0000000	-0.0350000
46692.0000000	-0.0450000
46693.0000000	-0.0400000
46694.0000000	-0.0450000
46695.0000000	-0.0450000
46696.0000000	-0.0450000
46697.0000000	-0.0450000
46698.0000000	-0.0450000
46699.0000000	-0.0350000
46700.0000000	-0.0400000
46701.0000000	-0.0400000
46702.0000000	-0.0500000
46703.0000000	-0.0500000
46704.0000000	-0.0433333
46705.0000000	-0.0500000
46706.0000000	-0.0400000
46707.0000000	-0.0500000
46708.0000000	-0.0500000
46709.0000000	-0.0500000
46710.0000000	-0.0400000
46711.0000000	-0.0450000
46712.0000000	-0.0500000
46713.0000000	-0.0500000
46714.0000000	-0.0500000
46715.0000000	-0.0500000
46716.0000000	-0.0500000
46717.0000000	-0.0550000
46718.0000000	-0.0600000
46719.0000000	-0.0550000
46720.0000000	-0.0450000
46721.0000000	-0.0400000
46722.0000000	-0.0400000
46723.0000000	-0.0450000
46724.0000000	-0.0500000
46725.0000000	-0.0550000
46726.0000000	-0.0500000
46727.0000000	-0.0500000
46728.0000000	-0.0550000
46729.0000000	-0.0500000
46730.0000000	-0.0500000
46731.0000000	-0.0450000
46732.0000000	-0.0450000
46733.0000000	-0.0450000
46734.0000000	-0.0450000
46735.0000000	-0.0500000
46736.0000000	-0.0500000
46737.0000000	-0.0450000
46738.0000000	-0.0450000
46739.0000000	-0.0500000

46740.0000000	-0.0500000
46741.0000000	-0.0400000
46742.0000000	-0.0500000
46743.0000000	-0.0450000
46744.0000000	-0.0500000
46745.0000000	-0.0450000
46746.0000000	-0.0450000
46747.0000000	-0.0400000
46748.0000000	-0.0450000
46749.0000000	-0.0500000
46750.0000000	-0.0400000
46751.0000000	-0.0500000
46752.0000000	-0.0400000
46753.0000000	-0.0450000
46754.0000000	-0.0400000
46755.0000000	-0.0400000
46756.0000000	-0.0400000
46757.0000000	-0.0450000
46758.0000000	-0.0350000
46759.0000000	-0.0400000
46760.0000000	-0.0300000
46761.0000000	-0.0450000
46762.0000000	-0.0350000
46763.0000000	-0.0350000
46764.0000000	-0.0350000
46765.0000000	-0.0433333
46766.0000000	-0.0250000
46767.0000000	-0.0450000
46768.0000000	-0.0400000
46769.0000000	-0.0450000
46770.0000000	-0.0400000
46771.0000000	-0.0350000
46772.0000000	-0.0350000
46773.0000000	-0.0300000
46774.0000000	-0.0450000
46775.0000000	-0.0350000
46776.0000000	-0.0450000
46777.0000000	-0.0350000
46778.0000000	-0.0350000
46779.0000000	-0.0300000
46780.0000000	-0.0350000
46781.0000000	-0.0350000
46782.0000000	-0.0400000
46783.0000000	-0.0400000
46784.0000000	-0.0300000
46785.0000000	-0.0350000
46786.0000000	-0.0300000
46787.0000000	-0.0350000
46788.0000000	-0.0250000
46789.0000000	-0.0400000
46790.0000000	-0.0350000
46791.0000000	-0.0350000
46792.0000000	-0.0300000
46793.0000000	-0.0400000
46794.0000000	-0.0300000
46795.0000000	-0.0333333
46796.0000000	-0.0400000

46797.0000000	-0.0300000
46798.0000000	-0.0350000
46799.0000000	-0.0300000
46800.0000000	-0.0400000
46801.0000000	-0.0300000
46802.0000000	-0.0350000
46803.0000000	-0.0300000
46804.0000000	-0.0300000
46805.0000000	-0.0350000
46806.0000000	-0.0300000
46807.0000000	-0.0300000
46808.0000000	-0.0250000
46809.0000000	-0.0300000
46810.0000000	-0.0350000
46811.0000000	-0.0350000
46812.0000000	-0.0250000
46813.0000000	-0.0300000
46814.0000000	-0.0350000
46815.0000000	-0.0350000
46816.0000000	-0.0300000
46817.0000000	-0.0300000
46818.0000000	-0.0350000
46819.0000000	-0.0400000
46820.0000000	-0.0350000
46821.0000000	-0.0350000
46822.0000000	-0.0250000
46823.0000000	-0.0250000
46824.0000000	-0.0400000
46825.0000000	-0.0300000
46826.0000000	-0.0300000
46827.0000000	-0.0350000
46828.0000000	-0.0400000
46829.0000000	-0.0400000
46830.0000000	-0.0400000
46831.0000000	-0.0350000
46832.0000000	-0.0400000
46833.0000000	-0.0400000
46834.0000000	-0.0300000
46835.0000000	-0.0400000
46836.0000000	-0.0300000
46837.0000000	-0.0300000
46838.0000000	-0.0400000
46839.0000000	-0.0300000
46840.0000000	-0.0350000
46841.0000000	-0.0300000
46842.0000000	-0.0350000
46843.0000000	-0.0400000
46844.0000000	-0.0350000
46845.0000000	-0.0350000
46846.0000000	-0.0300000
46847.0000000	-0.0300000
46848.0000000	-0.0350000
46849.0000000	-0.0300000
46850.0000000	-0.0300000
46851.0000000	-0.0250000
46852.0000000	-0.0350000
46853.0000000	-0.0350000

46854.0000000	-0.0350000
46855.0000000	-0.0333333
46856.0000000	-0.0250000
46857.0000000	-0.0400000
46858.0000000	-0.0300000
46859.0000000	-0.0250000
46860.0000000	-0.0350000
46861.0000000	-0.0400000
46862.0000000	-0.0300000
46863.0000000	-0.0250000
46864.0000000	-0.0350000
46865.0000000	-0.0350000
46866.0000000	-0.0300000
46867.0000000	-0.0300000
46868.0000000	-0.0250000
46869.0000000	-0.0350000
46870.0000000	-0.0300000
46871.0000000	-0.0350000
46872.0000000	-0.0350000
46873.0000000	-0.0300000
46874.0000000	-0.0300000
46875.0000000	-0.0300000
46876.0000000	-0.0350000
46877.0000000	-0.0300000
46878.0000000	-0.0250000
46879.0000000	-0.0300000
46880.0000000	-0.0300000
46881.0000000	-0.0250000
46882.0000000	-0.0250000
46883.0000000	-0.0300000
46884.0000000	-0.0300000
46885.0000000	-0.0250000
46886.0000000	-0.0266667
46887.0000000	-0.0300000
46888.0000000	-0.0300000
46889.0000000	-0.0300000
46890.0000000	-0.0250000
46891.0000000	-0.0350000
46892.0000000	-0.0250000
46893.0000000	-0.0250000
46894.0000000	-0.0250000
46895.0000000	-0.0250000
46896.0000000	-0.0350000
46897.0000000	-0.0300000
46898.0000000	-0.0200000
46899.0000000	-0.0300000
46900.0000000	-0.0250000
46901.0000000	-0.0300000
46902.0000000	-0.0250000
46903.0000000	-0.0300000
46904.0000000	-0.0250000
46905.0000000	-0.0250000
46906.0000000	-0.0250000
46907.0000000	-0.0300000
46908.0000000	-0.0300000
46909.0000000	-0.0300000
46910.0000000	-0.0250000

46911.0000000	-0.0300000
46912.0000000	-0.0300000
46913.0000000	-0.0300000
46914.0000000	-0.0250000
46915.0000000	-0.0200000
46916.0000000	-0.0300000
46917.0000000	-0.0200000
46918.0000000	-0.0300000
46919.0000000	-0.0300000
46920.0000000	-0.0250000
46921.0000000	-0.0300000
46922.0000000	-0.0200000
46923.0000000	-0.0250000
46924.0000000	-0.0250000
46925.0000000	-0.0250000
46926.0000000	-0.0200000
46927.0000000	-0.0200000
46928.0000000	-0.0200000
46929.0000000	-0.0250000
46930.0000000	-0.0250000
46931.0000000	-0.0250000
46932.0000000	-0.0300000
46933.0000000	-0.0250000
46934.0000000	-0.0200000
46935.0000000	-0.0300000
46936.0000000	-0.0250000
46937.0000000	-0.0200000
46938.0000000	-0.0250000
46939.0000000	-0.0250000
46940.0000000	-0.0250000
46941.0000000	-0.0250000
46942.0000000	-0.0250000
46943.0000000	-0.0300000
46944.0000000	-0.0250000
46945.0000000	-0.0200000
46946.0000000	-0.0333333
46947.0000000	-0.0300000
46948.0000000	-0.0300000
46949.0000000	-0.0250000
46950.0000000	-0.0200000
46951.0000000	-0.0200000
46952.0000000	-0.0250000
46953.0000000	-0.0300000
46954.0000000	-0.0250000
46955.0000000	-0.0250000
46956.0000000	-0.0250000
46957.0000000	-0.0250000
46958.0000000	-0.0250000
46959.0000000	-0.0200000
46960.0000000	-0.0200000
46961.0000000	-0.0250000
46962.0000000	-0.0250000
46963.0000000	-0.0200000
46964.0000000	-0.0200000
46965.0000000	-0.0150000
46966.0000000	-0.0150000
46967.0000000	-0.0150000

46968.0000000	-0.0200000
46969.0000000	-0.0150000
46970.0000000	-0.0200000
46971.0000000	-0.0200000
46972.0000000	-0.0200000
46973.0000000	-0.0200000
46974.0000000	-0.0200000
46975.0000000	-0.0150000
46976.0000000	-0.0133333
46977.0000000	-0.0100000
46978.0000000	-0.0150000
46979.0000000	-0.0150000
46980.0000000	-0.0150000
46981.0000000	-0.0200000
46982.0000000	-0.0100000
46983.0000000	-0.0150000
46984.0000000	-0.0100000
46985.0000000	-0.0150000
46986.0000000	-0.0200000
46987.0000000	-0.0150000
46988.0000000	-0.0150000
46989.0000000	-0.0200000
46990.0000000	-0.0150000
46991.0000000	-0.0150000
46992.0000000	-0.0150000
46993.0000000	-0.0150000
46994.0000000	-0.0250000
46995.0000000	-0.0150000
46996.0000000	-0.0200000
46997.0000000	-0.0150000
46998.0000000	-0.0200000
46999.0000000	-0.0300000
47000.0000000	-0.0150000
47001.0000000	-0.0200000
47002.0000000	-0.0250000
47003.0000000	-0.0250000
47004.0000000	-0.0350000
47005.0000000	-0.0200000
47006.0000000	-0.0250000
47007.0000000	-0.0266667
47008.0000000	-0.0250000
47009.0000000	-0.0250000
47010.0000000	-0.0200000
47011.0000000	-0.0200000
47012.0000000	-0.0200000
47013.0000000	-0.0300000
47014.0000000	-0.0400000
47015.0000000	-0.0200000
47016.0000000	-0.0350000
47017.0000000	-0.0250000
47018.0000000	-0.0350000
47019.0000000	-0.0250000
47020.0000000	-0.0300000
47021.0000000	-0.0200000
47022.0000000	-0.0250000
47023.0000000	-0.0350000
47024.0000000	-0.0300000

47025.0000000	-0.0350000
47026.0000000	-0.0350000
47027.0000000	-0.0300000
47028.0000000	-0.0300000
47029.0000000	-0.0300000
47030.0000000	-0.0350000
47031.0000000	-0.0200000
47032.0000000	-0.0200000
47033.0000000	-0.0300000
47034.0000000	-0.0300000
47035.0000000	-0.0350000
47036.0000000	-0.0350000
47037.0000000	-0.0266667
47038.0000000	-0.0350000
47039.0000000	-0.0250000
47040.0000000	-0.0350000
47041.0000000	-0.0300000
47042.0000000	-0.0250000
47043.0000000	-0.0250000
47044.0000000	-0.0350000
47045.0000000	-0.0250000
47046.0000000	-0.0300000
47047.0000000	-0.0250000
47048.0000000	-0.0350000
47049.0000000	-0.0300000
47050.0000000	-0.0300000
47051.0000000	-0.0250000
47052.0000000	-0.0300000
47053.0000000	-0.0250000
47054.0000000	-0.0250000
47055.0000000	-0.0300000
47056.0000000	-0.0250000
47057.0000000	-0.0250000
47058.0000000	-0.0300000
47059.0000000	-0.0250000
47060.0000000	-0.0250000
47061.0000000	-0.0350000
47062.0000000	-0.0350000
47063.0000000	-0.0350000
47064.0000000	-0.0250000
47065.0000000	-0.0300000
47066.0000000	-0.0333333
47067.0000000	-0.0250000
47068.0000000	-0.0350000
47069.0000000	-0.0250000
47070.0000000	-0.0300000
47071.0000000	-0.0300000
47072.0000000	-0.0250000
47073.0000000	-0.0250000
47074.0000000	-0.0350000
47075.0000000	-0.0250000
47076.0000000	-0.0250000
47077.0000000	-0.0300000
47078.0000000	-0.0250000
47079.0000000	-0.0250000
47080.0000000	-0.0300000
47081.0000000	-0.0350000

47082.0000000	-0.0250000
47083.0000000	-0.0300000
47084.0000000	-0.0300000
47085.0000000	-0.0250000
47086.0000000	-0.0250000
47087.0000000	-0.0300000
47088.0000000	-0.0300000
47089.0000000	-0.0250000
47090.0000000	-0.0250000
47091.0000000	-0.0250000
47092.0000000	-0.0300000
47093.0000000	-0.0300000
47094.0000000	-0.0350000
47095.0000000	-0.0350000
47096.0000000	-0.0300000
47097.0000000	-0.0333333
47098.0000000	-0.0250000
47099.0000000	-0.0250000
47100.0000000	-0.0200000
47101.0000000	-0.0300000
47102.0000000	-0.0250000
47103.0000000	-0.0250000
47104.0000000	-0.0250000
47105.0000000	-0.0250000
47106.0000000	-0.0250000
47107.0000000	-0.0250000
47108.0000000	-0.0300000
47109.0000000	-0.0300000
47110.0000000	-0.0250000
47111.0000000	-0.0200000
47112.0000000	-0.0300000
47113.0000000	-0.0350000
47114.0000000	-0.0300000
47115.0000000	-0.0300000
47116.0000000	-0.0300000
47117.0000000	-0.0300000
47118.0000000	-0.0300000
47119.0000000	-0.0300000
47120.0000000	-0.0250000
47121.0000000	-0.0300000
47122.0000000	-0.0300000
47123.0000000	-0.0350000
47124.0000000	-0.0350000
47125.0000000	-0.0300000
47126.0000000	-0.0400000
47127.0000000	-0.0350000
47128.0000000	-0.0333333
47129.0000000	-0.0250000
47130.0000000	-0.0350000
47131.0000000	-0.0300000
47132.0000000	-0.0300000
47133.0000000	-0.0300000
47134.0000000	-0.0300000
47135.0000000	-0.0350000
47136.0000000	-0.0350000
47137.0000000	-0.0300000
47138.0000000	-0.0350000

47139.0000000	-0.0300000
47140.0000000	-0.0300000
47141.0000000	-0.0350000
47142.0000000	-0.0300000
47143.0000000	-0.0300000
47144.0000000	-0.0300000
47145.0000000	-0.0250000
47146.0000000	-0.0300000
47147.0000000	-0.0350000
47148.0000000	-0.0300000
47149.0000000	-0.0400000
47150.0000000	-0.0400000
47151.0000000	-0.0350000
47152.0000000	-0.0300000
47153.0000000	-0.0300000
47154.0000000	-0.0400000
47155.0000000	-0.0400000
47156.0000000	-0.0350000
47157.0000000	-0.0333333
47158.0000000	-0.0400000
47159.0000000	-0.0400000
47160.0000000	-0.0400000
47161.0000000	-0.0400000
47162.0000000	-0.0350000
47163.0000000	-0.0450000
47164.0000000	-0.0350000
47165.0000000	-0.0500000
47166.0000000	-0.0400000
47167.0000000	-0.0450000
47168.0000000	-0.0400000
47169.0000000	-0.0350000
47170.0000000	-0.0400000
47171.0000000	-0.0450000
47172.0000000	-0.0400000
47173.0000000	-0.0400000
47174.0000000	-0.0450000
47175.0000000	-0.0400000
47176.0000000	-0.0500000
47177.0000000	-0.0450000
47178.0000000	-0.0400000
47179.0000000	-0.0450000
47180.0000000	-0.0350000
47181.0000000	-0.0500000
47182.0000000	-0.0400000
47183.0000000	-0.0450000
47184.0000000	-0.0500000
47185.0000000	-0.0450000
47186.0000000	-0.0500000
47187.0000000	-0.0466667
47188.0000000	-0.0400000
47189.0000000	-0.0500000
47190.0000000	-0.0550000
47191.0000000	-0.0500000
47192.0000000	-0.0550000
47193.0000000	-0.0450000
47194.0000000	-0.0400000
47195.0000000	-0.0450000

47196.0000000	-0.0450000
47197.0000000	-0.0400000
47198.0000000	-0.0400000
47199.0000000	-0.0400000
47200.0000000	-0.0500000
47201.0000000	-0.0500000
47202.0000000	-0.0450000
47203.0000000	-0.0500000
47204.0000000	-0.0500000
47205.0000000	-0.0500000
47206.0000000	-0.0500000
47207.0000000	-0.0500000
47208.0000000	-0.0450000
47209.0000000	-0.0500000
47210.0000000	-0.0450000
47211.0000000	-0.0450000
47212.0000000	-0.0550000
47213.0000000	-0.0500000
47214.0000000	-0.0500000
47215.0000000	-0.0550000
47216.0000000	-0.0500000
47217.0000000	-0.0450000
47218.0000000	-0.0566667
47219.0000000	-0.0550000
47220.0000000	-0.0550000
47221.0000000	-0.0500000
47222.0000000	-0.0500000
47223.0000000	-0.0500000
47224.0000000	-0.0550000
47225.0000000	-0.0550000
47226.0000000	-0.0550000
47227.0000000	-0.0600000
47228.0000000	-0.0550000
47229.0000000	-0.0600000
47230.0000000	-0.0500000
47231.0000000	-0.0650000
47232.0000000	-0.0600000
47233.0000000	-0.0650000
47234.0000000	-0.0600000
47235.0000000	-0.0550000
47236.0000000	-0.0550000
47237.0000000	-0.0600000
47238.0000000	-0.0550000
47239.0000000	-0.0600000
47240.0000000	-0.0500000
47241.0000000	-0.0550000
47242.0000000	-0.0550000
47243.0000000	-0.0600000
47244.0000000	-0.0650000
47245.0000000	-0.0550000
47246.0000000	-0.0600000
47247.0000000	-0.0550000
47248.0000000	-0.0600000
47249.0000000	-0.0550000
47250.0000000	-0.0650000
47251.0000000	-0.0600000
47252.0000000	-0.0600000

47253.0000000	-0.0600000
47254.0000000	-0.0600000
47255.0000000	-0.0650000
47256.0000000	-0.0550000
47257.0000000	-0.0600000
47258.0000000	-0.0700000
47259.0000000	-0.0650000
47260.0000000	-0.0600000
47261.0000000	-0.0600000
47262.0000000	-0.0600000
47263.0000000	-0.0650000
47264.0000000	-0.0650000
47265.0000000	-0.0600000
47266.0000000	-0.0650000
47267.0000000	-0.0650000
47268.0000000	-0.0600000
47269.0000000	-0.0700000
47270.0000000	-0.0700000
47271.0000000	-0.0600000
47272.0000000	-0.0600000
47273.0000000	-0.0650000
47274.0000000	-0.0650000
47275.0000000	-0.0650000
47276.0000000	-0.0700000
47277.0000000	-0.0700000
47278.0000000	-0.0666667
47279.0000000	-0.0650000
47280.0000000	-0.0600000
47281.0000000	-0.0700000
47282.0000000	-0.0650000
47283.0000000	-0.0700000
47284.0000000	-0.0700000
47285.0000000	-0.0650000
47286.0000000	-0.0700000
47287.0000000	-0.0650000
47288.0000000	-0.0650000
47289.0000000	-0.0650000
47290.0000000	-0.0700000
47291.0000000	-0.0700000
47292.0000000	-0.0600000
47293.0000000	-0.0650000
47294.0000000	-0.0700000
47295.0000000	-0.0700000
47296.0000000	-0.0650000
47297.0000000	-0.0700000
47298.0000000	-0.0650000
47299.0000000	-0.0700000
47300.0000000	-0.0700000
47301.0000000	-0.0700000
47302.0000000	-0.0700000
47303.0000000	-0.0700000
47304.0000000	-0.0700000
47305.0000000	-0.0650000
47306.0000000	-0.0700000
47307.0000000	-0.0700000
47308.0000000	-0.0733333
47309.0000000	-0.0750000

47310.0000000	-0.0750000
47311.0000000	-0.0700000
47312.0000000	-0.0650000
47313.0000000	-0.0750000
47314.0000000	-0.0750000
47315.0000000	-0.0750000
47316.0000000	-0.0700000
47317.0000000	-0.0750000
47318.0000000	-0.0750000
47319.0000000	-0.0750000
47320.0000000	-0.0700000
47321.0000000	-0.0750000
47322.0000000	-0.0750000
47323.0000000	-0.0700000
47324.0000000	-0.0700000
47325.0000000	-0.0750000
47326.0000000	-0.0750000
47327.0000000	-0.0750000
47328.0000000	-0.0750000
47329.0000000	-0.0800000
47330.0000000	-0.0800000
47331.0000000	-0.0700000
47332.0000000	-0.0700000
47333.0000000	-0.0650000
47334.0000000	-0.0700000
47335.0000000	-0.0650000
47336.0000000	-0.0700000
47337.0000000	-0.0700000
47338.0000000	-0.0650000
47339.0000000	-0.0700000
47340.0000000	-0.0650000
47341.0000000	-0.0800000
47342.0000000	-0.0750000
47343.0000000	-0.0650000
47344.0000000	-0.0750000
47345.0000000	-0.0750000
47346.0000000	-0.0700000
47347.0000000	-0.0650000
47348.0000000	-0.0650000
47349.0000000	-0.0700000
47350.0000000	-0.0700000
47351.0000000	-0.0700000
47352.0000000	-0.0700000
47353.0000000	-0.0700000
47354.0000000	-0.0600000
47355.0000000	-0.0650000
47356.0000000	-0.0700000
47357.0000000	-0.0650000
47358.0000000	-0.0700000
47359.0000000	-0.0650000
47360.0000000	-0.0600000
47361.0000000	-0.0650000
47362.0000000	-0.0700000
47363.0000000	-0.0700000
47364.0000000	-0.0650000
47365.0000000	-0.0550000
47366.0000000	-0.0700000

47367.0000000	-0.0650000
47368.0000000	-0.0550000
47369.0000000	-0.0600000
47370.0000000	-0.0650000
47371.0000000	-0.0650000
47372.0000000	-0.0650000
47373.0000000	-0.0600000
47374.0000000	-0.0600000
47375.0000000	-0.0650000
47376.0000000	-0.0550000
47377.0000000	-0.0650000
47378.0000000	-0.0650000
47379.0000000	-0.0600000
47380.0000000	-0.0550000
47381.0000000	-0.0600000
47382.0000000	-0.0550000
47383.0000000	-0.0650000
47384.0000000	-0.0600000
47385.0000000	-0.0600000
47386.0000000	-0.0600000
47387.0000000	-0.0550000
47388.0000000	-0.0550000
47389.0000000	-0.0600000
47390.0000000	-0.0600000
47391.0000000	-0.0600000
47392.0000000	-0.0600000
47393.0000000	-0.0650000
47394.0000000	-0.0500000
47395.0000000	-0.0550000
47396.0000000	-0.0550000
47397.0000000	-0.0550000
47398.0000000	-0.0550000
47399.0000000	-0.0566667
47400.0000000	-0.0500000
47401.0000000	-0.0500000
47402.0000000	-0.0550000
47403.0000000	-0.0500000
47404.0000000	-0.0500000
47405.0000000	-0.0550000
47406.0000000	-0.0550000
47407.0000000	-0.0600000
47408.0000000	-0.0500000
47409.0000000	-0.0500000
47410.0000000	-0.0550000
47411.0000000	-0.0500000
47412.0000000	-0.0550000
47413.0000000	-0.0500000
47414.0000000	-0.0500000
47415.0000000	-0.0500000
47416.0000000	-0.0550000
47417.0000000	-0.0500000
47418.0000000	-0.0450000
47419.0000000	-0.0550000
47420.0000000	-0.0450000
47421.0000000	-0.0550000
47422.0000000	-0.0450000
47423.0000000	-0.0450000

47424.0000000	-0.0450000
47425.0000000	-0.0500000
47426.0000000	-0.0550000
47427.0000000	-0.0500000
47428.0000000	-0.0450000
47429.0000000	-0.0466667
47430.0000000	-0.0400000
47431.0000000	-0.0400000
47432.0000000	-0.0500000
47433.0000000	-0.0400000
47434.0000000	-0.0400000
47435.0000000	-0.0550000
47436.0000000	-0.0450000
47437.0000000	-0.0400000
47438.0000000	-0.0450000
47439.0000000	-0.0350000
47440.0000000	-0.0400000
47441.0000000	-0.0400000
47442.0000000	-0.0400000
47443.0000000	-0.0400000
47444.0000000	-0.0350000
47445.0000000	-0.0400000
47446.0000000	-0.0450000
47447.0000000	-0.0350000
47448.0000000	-0.0400000
47449.0000000	-0.0350000
47450.0000000	-0.0350000
47451.0000000	-0.0400000
47452.0000000	-0.0400000
47453.0000000	-0.0300000
47454.0000000	-0.0300000
47455.0000000	-0.0350000
47456.0000000	-0.0250000
47457.0000000	-0.0300000
47458.0000000	-0.0300000
47459.0000000	-0.0333333
47460.0000000	-0.0300000
47461.0000000	-0.0250000
47462.0000000	-0.0250000
47463.0000000	-0.0200000
47464.0000000	-0.0200000
47465.0000000	-0.0250000
47466.0000000	-0.0250000
47467.0000000	-0.0250000
47468.0000000	-0.0250000
47469.0000000	-0.0150000
47470.0000000	-0.0250000
47471.0000000	-0.0300000
47472.0000000	-0.0200000
47473.0000000	-0.0300000
47474.0000000	-0.0200000
47475.0000000	-0.0200000
47476.0000000	-0.0200000
47477.0000000	-0.0200000
47478.0000000	-0.0200000
47479.0000000	-0.0100000
47480.0000000	-0.0150000

47481.0000000	-0.0200000
47482.0000000	-0.0100000
47483.0000000	-0.0150000
47484.0000000	-0.0150000
47485.0000000	-0.0150000
47486.0000000	-0.0100000
47487.0000000	-0.0100000
47488.0000000	-0.0100000
47489.0000000	-0.0100000
47490.0000000	-0.0066667
47491.0000000	-0.0050000
47492.0000000	-0.0150000
47493.0000000	0.0000000
47494.0000000	0.0000000
47495.0000000	0.0000000
47496.0000000	-0.0100000
47497.0000000	-0.0050000
47498.0000000	0.0000000
47499.0000000	-0.0050000
47500.0000000	0.0000000
47501.0000000	0.0000000
47502.0000000	0.0000000
47503.0000000	0.0000000
47504.0000000	0.0050000
47505.0000000	0.0050000
47506.0000000	0.0000000
47507.0000000	0.0000000
47508.0000000	0.0000000
47509.0000000	0.0100000
47510.0000000	0.0100000
47511.0000000	0.0100000
47512.0000000	0.0050000
47513.0000000	0.0050000
47514.0000000	0.0050000
47515.0000000	0.0100000
47516.0000000	0.0100000
47517.0000000	0.0050000
47518.0000000	0.0150000
47519.0000000	0.0133333
47520.0000000	0.0150000
47521.0000000	0.0150000
47522.0000000	0.0150000
47523.0000000	0.0200000
47524.0000000	0.0200000
47525.0000000	0.0250000
47526.0000000	0.0150000
47527.0000000	0.0200000
47528.0000000	0.0200000
47529.0000000	0.0200000
47530.0000000	0.0250000
47531.0000000	0.0200000
47532.0000000	0.0250000
47533.0000000	0.0250000
47534.0000000	0.0200000
47535.0000000	0.0250000
47536.0000000	0.0300000
47537.0000000	0.0300000

47538.0000000	0.0300000
47539.0000000	0.0250000
47540.0000000	0.0300000
47541.0000000	0.0300000
47542.0000000	0.0300000
47543.0000000	0.0300000
47544.0000000	0.0300000
47545.0000000	0.0300000
47546.0000000	0.0300000
47547.0000000	0.0300000
47548.0000000	0.0450000
47549.0000000	0.0300000
47550.0000000	0.0266667
47551.0000000	0.0350000
47552.0000000	0.0350000
47553.0000000	0.0400000
47554.0000000	0.0400000
47555.0000000	0.0450000
47556.0000000	0.0350000
47557.0000000	0.0450000
47558.0000000	0.0400000
47559.0000000	0.0400000
47560.0000000	0.0400000
47561.0000000	0.0350000
47562.0000000	0.0400000
47563.0000000	0.0400000
47564.0000000	0.0450000
47565.0000000	0.0400000
47566.0000000	0.0450000
47567.0000000	0.0450000
47568.0000000	0.0450000
47569.0000000	0.0450000
47570.0000000	0.0550000
47571.0000000	0.0450000
47572.0000000	0.0500000
47573.0000000	0.0450000
47574.0000000	0.0500000
47575.0000000	0.0650000
47576.0000000	0.0500000
47577.0000000	0.0500000
47578.0000000	0.0550000
47579.0000000	0.0500000
47580.0000000	0.0566667
47581.0000000	0.0550000
47582.0000000	0.0500000
47583.0000000	0.0550000
47584.0000000	0.0550000
47585.0000000	0.0500000
47586.0000000	0.0600000
47587.0000000	0.0600000
47588.0000000	0.0600000
47589.0000000	0.0600000
47590.0000000	0.0550000
47591.0000000	0.0550000
47592.0000000	0.0600000
47593.0000000	0.0550000
47594.0000000	0.0550000

47595.0000000	0.0550000
47596.0000000	0.0500000
47597.0000000	0.0600000
47598.0000000	0.0550000
47599.0000000	0.0550000
47600.0000000	0.0650000
47601.0000000	0.0600000
47602.0000000	0.0650000
47603.0000000	0.0650000
47604.0000000	0.0650000
47605.0000000	0.0600000
47606.0000000	0.0700000
47607.0000000	0.0700000
47608.0000000	0.0600000
47609.0000000	0.0600000
47610.0000000	0.0666667
47611.0000000	0.0700000
47612.0000000	0.0650000
47613.0000000	0.0700000
47614.0000000	0.0750000
47615.0000000	0.0700000
47616.0000000	0.0700000
47617.0000000	0.0700000
47618.0000000	0.0650000
47619.0000000	0.0800000
47620.0000000	0.0750000
47621.0000000	0.0750000
47622.0000000	0.0700000
47623.0000000	0.0800000
47624.0000000	0.0750000
47625.0000000	0.0750000
47626.0000000	0.0800000
47627.0000000	0.0700000
47628.0000000	0.0800000
47629.0000000	0.0750000
47630.0000000	0.0750000
47631.0000000	0.0800000
47632.0000000	0.0750000
47633.0000000	0.0750000
47634.0000000	0.0800000
47635.0000000	0.0700000
47636.0000000	0.0700000
47637.0000000	0.0800000
47638.0000000	0.0800000
47639.0000000	0.0850000
47640.0000000	0.0900000
47641.0000000	0.0800000
47642.0000000	0.0850000
47643.0000000	0.0850000
47644.0000000	0.0900000
47645.0000000	0.0800000
47646.0000000	0.0800000
47647.0000000	0.0900000
47648.0000000	0.0850000
47649.0000000	0.0900000
47650.0000000	0.0900000
47651.0000000	0.0800000

47652.0000000	0.0950000
47653.0000000	0.0850000
47654.0000000	0.0850000
47655.0000000	0.0850000
47656.0000000	0.0850000
47657.0000000	0.0850000
47658.0000000	0.0900000
47659.0000000	0.0900000
47660.0000000	0.0950000
47661.0000000	0.0900000
47662.0000000	0.0850000
47663.0000000	0.0950000
47664.0000000	0.0950000
47665.0000000	0.0900000
47666.0000000	0.0950000
47667.0000000	0.0900000
47668.0000000	0.0900000
47669.0000000	0.0950000
47670.0000000	0.0966667
47671.0000000	0.0900000
47672.0000000	0.1000000
47673.0000000	0.1000000
47674.0000000	0.0950000
47675.0000000	0.0950000
47676.0000000	0.0950000
47677.0000000	0.0900000
47678.0000000	0.0950000
47679.0000000	0.1000000
47680.0000000	0.0950000
47681.0000000	0.0950000
47682.0000000	0.0950000
47683.0000000	0.1000000
47684.0000000	0.0950000
47685.0000000	0.0950000
47686.0000000	0.1050000
47687.0000000	0.1000000
47688.0000000	0.0950000
47689.0000000	0.1000000
47690.0000000	0.1000000
47691.0000000	0.1050000
47692.0000000	0.1000000
47693.0000000	0.1100000
47694.0000000	0.1000000
47695.0000000	0.1000000
47696.0000000	0.1050000
47697.0000000	0.1000000
47698.0000000	0.1000000
47699.0000000	0.1100000
47700.0000000	0.1000000
47701.0000000	0.0966667
47702.0000000	0.1050000
47703.0000000	0.1000000
47704.0000000	0.1100000
47705.0000000	0.1100000
47706.0000000	0.1050000
47707.0000000	0.1000000
47708.0000000	0.1100000

47709.0000000	0.1050000
47710.0000000	0.1050000
47711.0000000	0.1050000
47712.0000000	0.1100000
47713.0000000	0.1050000
47714.0000000	0.1050000
47715.0000000	0.1050000
47716.0000000	0.1100000
47717.0000000	0.1050000
47718.0000000	0.1000000
47719.0000000	0.1050000
47720.0000000	0.1050000
47721.0000000	0.1050000
47722.0000000	0.1050000
47723.0000000	0.1050000
47724.0000000	0.1050000
47725.0000000	0.1050000
47726.0000000	0.1050000
47727.0000000	0.1050000
47728.0000000	0.1100000
47729.0000000	0.1100000
47730.0000000	0.1150000
47731.0000000	0.1133333
47732.0000000	0.1100000
47733.0000000	0.1100000
47734.0000000	0.1100000
47735.0000000	0.1100000
47736.0000000	0.1150000
47737.0000000	0.1050000
47738.0000000	0.1100000
47739.0000000	0.1100000
47740.0000000	0.1150000
47741.0000000	0.1100000
47742.0000000	0.1150000
47743.0000000	0.1100000
47744.0000000	0.1150000
47745.0000000	0.1150000
47746.0000000	0.1150000
47747.0000000	0.1200000
47748.0000000	0.1100000
47749.0000000	0.1150000
47750.0000000	0.1250000
47751.0000000	0.1150000
47752.0000000	0.1100000
47753.0000000	0.1200000
47754.0000000	0.1150000
47755.0000000	0.1150000
47756.0000000	0.1100000
47757.0000000	0.1150000
47758.0000000	0.1150000
47759.0000000	0.1150000
47760.0000000	0.1150000
47761.0000000	0.1133333
47762.0000000	0.1100000
47763.0000000	0.1200000
47764.0000000	0.1200000
47765.0000000	0.1150000

47766.0000000	0.1100000
47767.0000000	0.1200000
47768.0000000	0.1200000
47769.0000000	0.1250000
47770.0000000	0.1200000
47771.0000000	0.1150000
47772.0000000	0.1200000
47773.0000000	0.1200000
47774.0000000	0.1200000
47775.0000000	0.1250000
47776.0000000	0.1200000
47777.0000000	0.1200000
47778.0000000	0.1150000
47779.0000000	0.1200000
47780.0000000	0.1200000
47781.0000000	0.1200000
47782.0000000	0.1200000
47783.0000000	0.1200000
47784.0000000	0.1100000
47785.0000000	0.1200000
47786.0000000	0.1250000
47787.0000000	0.1200000
47788.0000000	0.1300000
47789.0000000	0.1200000
47790.0000000	0.1200000
47791.0000000	0.1300000
47792.0000000	0.1300000
47793.0000000	0.1250000
47794.0000000	0.1250000
47795.0000000	0.1250000
47796.0000000	0.1250000
47797.0000000	0.1300000
47798.0000000	0.1250000
47799.0000000	0.1300000
47800.0000000	0.1300000
47801.0000000	0.1300000
47802.0000000	0.1400000
47803.0000000	0.1350000
47804.0000000	0.1350000
47805.0000000	0.1300000
47806.0000000	0.1300000
47807.0000000	0.1350000
47808.0000000	0.1300000
47809.0000000	0.1350000
47810.0000000	0.1350000
47811.0000000	0.1350000
47812.0000000	0.1300000
47813.0000000	0.1300000
47814.0000000	0.1400000
47815.0000000	0.1300000
47816.0000000	0.1350000
47817.0000000	0.1350000
47818.0000000	0.1350000
47819.0000000	0.1350000
47820.0000000	0.1400000
47821.0000000	0.1300000
47822.0000000	0.1333333

47823.0000000	0.1300000
47824.0000000	0.1300000
47825.0000000	0.1300000
47826.0000000	0.1300000
47827.0000000	0.1300000
47828.0000000	0.1350000
47829.0000000	0.1350000
47830.0000000	0.1350000
47831.0000000	0.1350000
47832.0000000	0.1400000
47833.0000000	0.1300000
47834.0000000	0.1400000
47835.0000000	0.1300000
47836.0000000	0.1350000
47837.0000000	0.1350000
47838.0000000	0.1350000
47839.0000000	0.1350000
47840.0000000	0.1400000
47841.0000000	0.1350000
47842.0000000	0.1350000
47843.0000000	0.1350000
47844.0000000	0.1300000
47845.0000000	0.1400000
47846.0000000	0.1400000
47847.0000000	0.1350000
47848.0000000	0.1400000
47849.0000000	0.1400000
47850.0000000	0.1400000
47851.0000000	0.1350000
47852.0000000	0.1366667
47853.0000000	0.1450000
47854.0000000	0.1450000
47855.0000000	0.1400000
47856.0000000	0.1450000
47857.0000000	0.1450000
47858.0000000	0.1450000
47859.0000000	0.1450000
47860.0000000	0.1400000
47861.0000000	0.1450000
47862.0000000	0.1450000
47863.0000000	0.1400000
47864.0000000	0.1400000
47865.0000000	0.1350000
47866.0000000	0.1350000
47867.0000000	0.1400000
47868.0000000	0.1450000
47869.0000000	0.1400000
47870.0000000	0.1400000
47871.0000000	0.1450000
47872.0000000	0.1400000
47873.0000000	0.1400000
47874.0000000	0.1350000
47875.0000000	0.1450000
47876.0000000	0.1400000
47877.0000000	0.1400000
47878.0000000	0.1400000
47879.0000000	0.1450000

47880.0000000	0.1500000
47881.0000000	0.1433333
47882.0000000	0.1400000
47883.0000000	0.1400000
47884.0000000	0.1450000
47885.0000000	0.1450000
47886.0000000	0.1400000
47887.0000000	0.1400000
47888.0000000	0.1450000
47889.0000000	0.1450000
47890.0000000	0.1400000
47891.0000000	0.1350000
47892.0000000	0.1400000
47893.0000000	0.1400000
47894.0000000	0.1450000
47895.0000000	0.1450000
47896.0000000	0.1400000
47897.0000000	0.1350000
47898.0000000	0.1400000
47899.0000000	0.1450000
47900.0000000	0.1450000
47901.0000000	0.1450000
47902.0000000	0.1450000
47903.0000000	0.1400000
47904.0000000	0.1450000
47905.0000000	0.1450000
47906.0000000	0.1500000
47907.0000000	0.1450000
47908.0000000	0.1450000
47909.0000000	0.1500000
47910.0000000	0.1500000
47911.0000000	0.1500000
47912.0000000	0.1433333
47913.0000000	0.1450000
47914.0000000	0.1500000
47915.0000000	0.1450000
47916.0000000	0.1500000
47917.0000000	0.1550000
47918.0000000	0.1450000
47919.0000000	0.1500000
47920.0000000	0.1450000
47921.0000000	0.1500000
47922.0000000	0.1500000
47923.0000000	0.1450000
47924.0000000	0.1500000
47925.0000000	0.1550000
47926.0000000	0.1400000
47927.0000000	0.1450000
47928.0000000	0.1550000
47929.0000000	0.1450000
47930.0000000	0.1500000
47931.0000000	0.1450000
47932.0000000	0.1550000
47933.0000000	0.1500000
47934.0000000	0.1450000
47935.0000000	0.1450000
47936.0000000	0.1500000

47937.0000000	0.1500000
47938.0000000	0.1450000
47939.0000000	0.1450000
47940.0000000	0.1400000
47941.0000000	0.1400000
47942.0000000	0.1450000
47943.0000000	0.1466667
47944.0000000	0.1450000
47945.0000000	0.1400000
47946.0000000	0.1400000
47947.0000000	0.1400000
47948.0000000	0.1450000
47949.0000000	0.1400000
47950.0000000	0.1400000
47951.0000000	0.1350000
47952.0000000	0.1350000
47953.0000000	0.1400000
47954.0000000	0.1400000
47955.0000000	0.1400000
47956.0000000	0.1350000
47957.0000000	0.1400000
47958.0000000	0.1350000
47959.0000000	0.1400000
47960.0000000	0.1400000
47961.0000000	0.1350000
47962.0000000	0.1350000
47963.0000000	0.1350000
47964.0000000	0.1350000
47965.0000000	0.1350000
47966.0000000	0.1350000
47967.0000000	0.1300000
47968.0000000	0.1300000
47969.0000000	0.1350000
47970.0000000	0.1350000
47971.0000000	0.1300000
47972.0000000	0.1333333
47973.0000000	0.1350000
47974.0000000	0.1350000
47975.0000000	0.1300000
47976.0000000	0.1300000
47977.0000000	0.1350000
47978.0000000	0.1250000
47979.0000000	0.1350000
47980.0000000	0.1300000
47981.0000000	0.1300000
47982.0000000	0.1400000
47983.0000000	0.1300000
47984.0000000	0.1250000
47985.0000000	0.1300000
47986.0000000	0.1300000
47987.0000000	0.1300000
47988.0000000	0.1300000
47989.0000000	0.1300000
47990.0000000	0.1300000
47991.0000000	0.1350000
47992.0000000	0.1300000
47993.0000000	0.1300000

47994.0000000	0.1350000
47995.0000000	0.1300000
47996.0000000	0.1300000
47997.0000000	0.1250000
47998.0000000	0.1300000
47999.0000000	0.1250000
48000.0000000	0.1300000
48001.0000000	0.1400000
48002.0000000	0.1333333
48003.0000000	0.1350000
48004.0000000	0.1350000
48005.0000000	0.1350000
48006.0000000	0.1250000
48007.0000000	0.1350000
48008.0000000	0.1350000
48009.0000000	0.1300000
48010.0000000	0.1250000
48011.0000000	0.1300000
48012.0000000	0.1350000
48013.0000000	0.1300000
48014.0000000	0.1300000
48015.0000000	0.1300000
48016.0000000	0.1350000
48017.0000000	0.1300000
48018.0000000	0.1350000
48019.0000000	0.1350000
48020.0000000	0.1300000
48021.0000000	0.1300000
48022.0000000	0.1300000
48023.0000000	0.1300000
48024.0000000	0.1350000
48025.0000000	0.1300000
48026.0000000	0.1250000
48027.0000000	0.1250000
48028.0000000	0.1200000
48029.0000000	0.1300000
48030.0000000	0.1300000
48031.0000000	0.1250000
48032.0000000	0.1350000
48033.0000000	0.1400000
48034.0000000	0.1300000
48035.0000000	0.1200000
48036.0000000	0.1350000
48037.0000000	0.1250000
48038.0000000	0.1300000
48039.0000000	0.1200000
48040.0000000	0.1250000
48041.0000000	0.1300000
48042.0000000	0.1300000
48043.0000000	0.1300000
48044.0000000	0.1250000
48045.0000000	0.1300000
48046.0000000	0.1300000
48047.0000000	0.1250000
48048.0000000	0.1200000
48049.0000000	0.1200000
48050.0000000	0.1300000

48051.0000000	0.1250000
48052.0000000	0.1250000
48053.0000000	0.1200000
48054.0000000	0.1150000
48055.0000000	0.1200000
48056.0000000	0.1200000
48057.0000000	0.1200000
48058.0000000	0.1150000
48059.0000000	0.1250000
48060.0000000	0.1200000
48061.0000000	0.1200000
48062.0000000	0.1200000
48063.0000000	0.1200000
48064.0000000	0.1150000
48065.0000000	0.1200000
48066.0000000	0.1200000
48067.0000000	0.1150000
48068.0000000	0.1200000
48069.0000000	0.1250000
48070.0000000	0.1200000
48071.0000000	0.1100000
48072.0000000	0.1200000
48073.0000000	0.1200000
48074.0000000	0.1150000
48075.0000000	0.1150000
48076.0000000	0.1150000
48077.0000000	0.1100000
48078.0000000	0.1150000
48079.0000000	0.1100000
48080.0000000	0.1200000
48081.0000000	0.1150000
48082.0000000	0.1100000
48083.0000000	0.1050000
48084.0000000	0.1150000
48085.0000000	0.1150000
48086.0000000	0.1100000
48087.0000000	0.1150000
48088.0000000	0.1100000
48089.0000000	0.1200000
48090.0000000	0.1050000
48091.0000000	0.1100000
48092.0000000	0.1100000
48093.0000000	0.1133333
48094.0000000	0.1100000
48095.0000000	0.1100000
48096.0000000	0.1100000
48097.0000000	0.1100000
48098.0000000	0.1050000
48099.0000000	0.1100000
48100.0000000	0.1100000
48101.0000000	0.1050000
48102.0000000	0.1050000
48103.0000000	0.1050000
48104.0000000	0.1100000
48105.0000000	0.1000000
48106.0000000	0.1000000
48107.0000000	0.1100000

48108.0000000	0.1100000
48109.0000000	0.1000000
48110.0000000	0.1000000
48111.0000000	0.1100000
48112.0000000	0.1100000
48113.0000000	0.1050000
48114.0000000	0.1000000
48115.0000000	0.0950000
48116.0000000	0.1000000
48117.0000000	0.1050000
48118.0000000	0.0950000
48119.0000000	0.0950000
48120.0000000	0.1050000
48121.0000000	0.1000000
48122.0000000	0.0950000
48123.0000000	0.0966667
48124.0000000	0.1000000
48125.0000000	0.1050000
48126.0000000	0.0950000
48127.0000000	0.1000000
48128.0000000	0.0950000
48129.0000000	0.0900000
48130.0000000	0.0950000
48131.0000000	0.0950000
48132.0000000	0.0950000
48133.0000000	0.0950000
48134.0000000	0.0950000
48135.0000000	0.0850000
48136.0000000	0.0950000
48137.0000000	0.0950000
48138.0000000	0.0900000
48139.0000000	0.0950000
48140.0000000	0.0900000
48141.0000000	0.0900000
48142.0000000	0.0900000
48143.0000000	0.0950000
48144.0000000	0.0900000
48145.0000000	0.0850000
48146.0000000	0.0850000
48147.0000000	0.0900000
48148.0000000	0.0900000
48149.0000000	0.0900000
48150.0000000	0.0850000
48151.0000000	0.0850000
48152.0000000	0.0850000
48153.0000000	0.0800000
48154.0000000	0.0766667
48155.0000000	0.0850000
48156.0000000	0.0850000
48157.0000000	0.0850000
48158.0000000	0.0900000
48159.0000000	0.0850000
48160.0000000	0.0850000
48161.0000000	0.0750000
48162.0000000	0.0850000
48163.0000000	0.0750000
48164.0000000	0.0750000

48165.0000000	0.0800000
48166.0000000	0.0800000
48167.0000000	0.0750000
48168.0000000	0.0800000
48169.0000000	0.0800000
48170.0000000	0.0800000
48171.0000000	0.0700000
48172.0000000	0.0750000
48173.0000000	0.0750000
48174.0000000	0.0750000
48175.0000000	0.0700000
48176.0000000	0.0700000
48177.0000000	0.0700000
48178.0000000	0.0750000
48179.0000000	0.0700000
48180.0000000	0.0700000
48181.0000000	0.0700000
48182.0000000	0.0700000
48183.0000000	0.0600000
48184.0000000	0.0666667
48185.0000000	0.0650000
48186.0000000	0.0700000
48187.0000000	0.0650000
48188.0000000	0.0650000
48189.0000000	0.0600000
48190.0000000	0.0650000
48191.0000000	0.0650000
48192.0000000	0.0600000
48193.0000000	0.0650000
48194.0000000	0.0700000
48195.0000000	0.0650000
48196.0000000	0.0700000
48197.0000000	0.0600000
48198.0000000	0.0700000
48199.0000000	0.0650000
48200.0000000	0.0600000
48201.0000000	0.0650000
48202.0000000	0.0750000
48203.0000000	0.0700000
48204.0000000	0.0700000
48205.0000000	0.0700000
48206.0000000	0.0700000
48207.0000000	0.0700000
48208.0000000	0.0750000
48209.0000000	0.0700000
48210.0000000	0.0700000
48211.0000000	0.0650000
48212.0000000	0.0750000
48213.0000000	0.0750000
48214.0000000	0.0666667
48215.0000000	0.0650000
48216.0000000	0.0650000
48217.0000000	0.0650000
48218.0000000	0.0650000
48219.0000000	0.0650000
48220.0000000	0.0650000
48221.0000000	0.0600000

48222.0000000	0.0600000
48223.0000000	0.0650000
48224.0000000	0.0600000
48225.0000000	0.0650000
48226.0000000	0.0600000
48227.0000000	0.0550000
48228.0000000	0.0550000
48229.0000000	0.0550000
48230.0000000	0.0550000
48231.0000000	0.0550000
48232.0000000	0.0550000
48233.0000000	0.0550000
48234.0000000	0.0500000
48235.0000000	0.0400000
48236.0000000	0.0500000
48237.0000000	0.0450000
48238.0000000	0.0400000
48239.0000000	0.0400000
48240.0000000	0.0400000
48241.0000000	0.0400000
48242.0000000	0.0450000
48243.0000000	0.0400000
48244.0000000	0.0433333
48245.0000000	0.0500000
48246.0000000	0.0400000
48247.0000000	0.0500000
48248.0000000	0.0450000
48249.0000000	0.0500000
48250.0000000	0.0500000
48251.0000000	0.0550000
48252.0000000	0.0500000
48253.0000000	0.0550000
48254.0000000	0.0450000
48255.0000000	0.0550000
48256.0000000	0.0500000
48257.0000000	0.0550000
48258.0000000	0.0550000
48259.0000000	0.0450000
48260.0000000	0.0500000
48261.0000000	0.0500000
48262.0000000	0.0550000
48263.0000000	0.0450000
48264.0000000	0.0500000
48265.0000000	0.0500000
48266.0000000	0.0500000
48267.0000000	0.0400000
48268.0000000	0.0450000
48269.0000000	0.0450000
48270.0000000	0.0450000
48271.0000000	0.0400000
48272.0000000	0.0400000
48273.0000000	0.0350000
48274.0000000	0.0350000
48275.0000000	0.0366667
48276.0000000	0.0350000
48277.0000000	0.0300000
48278.0000000	0.0350000

48279.0000000	0.0300000
48280.0000000	0.0300000
48281.0000000	0.0250000
48282.0000000	0.0300000
48283.0000000	0.0300000
48284.0000000	0.0350000
48285.0000000	0.0300000
48286.0000000	0.0300000
48287.0000000	0.0300000
48288.0000000	0.0350000
48289.0000000	0.0400000
48290.0000000	0.0300000
48291.0000000	0.0350000
48292.0000000	0.0400000
48293.0000000	0.0350000
48294.0000000	0.0300000
48295.0000000	0.0300000
48296.0000000	0.0350000
48297.0000000	0.0300000
48298.0000000	0.0300000
48299.0000000	0.0300000
48300.0000000	0.0350000
48301.0000000	0.0300000
48302.0000000	0.0300000
48303.0000000	0.0300000
48304.0000000	0.0300000
48305.0000000	0.0300000
48306.0000000	0.0250000
48307.0000000	0.0250000
48308.0000000	0.0250000
48309.0000000	0.0200000
48310.0000000	0.0200000
48311.0000000	0.0300000
48312.0000000	0.0250000
48313.0000000	0.0250000
48314.0000000	0.0250000
48315.0000000	0.0250000
48316.0000000	0.0200000
48317.0000000	0.0200000
48318.0000000	0.0150000
48319.0000000	0.0200000
48320.0000000	0.0150000
48321.0000000	0.0200000
48322.0000000	0.0150000
48323.0000000	0.0150000
48324.0000000	0.0100000
48325.0000000	0.0150000
48326.0000000	0.0150000
48327.0000000	0.0100000
48328.0000000	0.0100000
48329.0000000	0.0050000
48330.0000000	0.0050000
48331.0000000	0.0000000
48332.0000000	0.0000000
48333.0000000	0.0000000
48334.0000000	0.0033333
48335.0000000	-0.0050000

48336.0000000	-0.0050000
48337.0000000	-0.0050000
48338.0000000	-0.0050000
48339.0000000	-0.0100000
48340.0000000	-0.0050000
48341.0000000	-0.0150000
48342.0000000	-0.0100000
48343.0000000	-0.0100000
48344.0000000	-0.0100000
48345.0000000	-0.0050000
48346.0000000	-0.0050000
48347.0000000	-0.0150000
48348.0000000	-0.0150000
48349.0000000	-0.0150000
48350.0000000	-0.0050000
48351.0000000	-0.0100000
48352.0000000	-0.0150000
48353.0000000	-0.0250000
48354.0000000	-0.0200000
48355.0000000	-0.0200000
48356.0000000	-0.0150000
48357.0000000	-0.0150000
48358.0000000	-0.0200000
48359.0000000	-0.0150000
48360.0000000	-0.0150000
48361.0000000	-0.0250000
48362.0000000	-0.0250000
48363.0000000	-0.0150000
48364.0000000	-0.0250000
48365.0000000	-0.0300000
48366.0000000	-0.0250000
48367.0000000	-0.0250000
48368.0000000	-0.0300000
48369.0000000	-0.0300000
48370.0000000	-0.0300000
48371.0000000	-0.0350000
48372.0000000	-0.0400000
48373.0000000	-0.0350000
48374.0000000	-0.0350000
48375.0000000	-0.0450000
48376.0000000	-0.0450000
48377.0000000	-0.0450000
48378.0000000	-0.0400000
48379.0000000	-0.0450000
48380.0000000	-0.0550000
48381.0000000	-0.0450000
48382.0000000	-0.0500000
48383.0000000	-0.0550000
48384.0000000	-0.0500000
48385.0000000	-0.0500000
48386.0000000	-0.0550000
48387.0000000	-0.0550000
48388.0000000	-0.0550000
48389.0000000	-0.0550000
48390.0000000	-0.0650000
48391.0000000	-0.0650000
48392.0000000	-0.0600000

48393.0000000	-0.0650000
48394.0000000	-0.0650000
48395.0000000	-0.0633333
48396.0000000	-0.0700000
48397.0000000	-0.0700000
48398.0000000	-0.0750000
48399.0000000	-0.0750000
48400.0000000	-0.0800000
48401.0000000	-0.0800000
48402.0000000	-0.0800000
48403.0000000	-0.0750000
48404.0000000	-0.0750000
48405.0000000	-0.0900000
48406.0000000	-0.0850000
48407.0000000	-0.0800000
48408.0000000	-0.0850000
48409.0000000	-0.0950000
48410.0000000	-0.0900000
48411.0000000	-0.0900000
48412.0000000	-0.0900000
48413.0000000	-0.0900000
48414.0000000	-0.0900000
48415.0000000	-0.0900000
48416.0000000	-0.0950000
48417.0000000	-0.0950000
48418.0000000	-0.1050000
48419.0000000	-0.1000000
48420.0000000	-0.0900000
48421.0000000	-0.0900000
48422.0000000	-0.1050000
48423.0000000	-0.1000000
48424.0000000	-0.1100000
48425.0000000	-0.1000000
48426.0000000	-0.1000000
48427.0000000	-0.1050000
48428.0000000	-0.1050000
48429.0000000	-0.1050000
48430.0000000	-0.1050000
48431.0000000	-0.1050000
48432.0000000	-0.1100000
48433.0000000	-0.1100000
48434.0000000	-0.1000000
48435.0000000	-0.1100000
48436.0000000	-0.1050000
48437.0000000	-0.1000000
48438.0000000	-0.1050000
48439.0000000	-0.1000000
48440.0000000	-0.1050000
48441.0000000	-0.1100000
48442.0000000	-0.1050000
48443.0000000	-0.1000000
48444.0000000	-0.1000000
48445.0000000	-0.0900000
48446.0000000	-0.1000000
48447.0000000	-0.0950000
48448.0000000	-0.0900000
48449.0000000	-0.0900000

48450.0000000	-0.0900000
48451.0000000	-0.0900000
48452.0000000	-0.0950000
48453.0000000	-0.0850000
48454.0000000	-0.0950000
48455.0000000	-0.0966667
48456.0000000	-0.0900000
48457.0000000	-0.0850000
48458.0000000	-0.0850000
48459.0000000	-0.0850000
48460.0000000	-0.0900000
48461.0000000	-0.0900000
48462.0000000	-0.0800000
48463.0000000	-0.0850000
48464.0000000	-0.0900000
48465.0000000	-0.0900000
48466.0000000	-0.0900000
48467.0000000	-0.0850000
48468.0000000	-0.0900000
48469.0000000	-0.0900000
48470.0000000	-0.0950000
48471.0000000	-0.0900000
48472.0000000	-0.1000000
48473.0000000	-0.0900000
48474.0000000	-0.0850000
48475.0000000	-0.0900000
48476.0000000	-0.0950000
48477.0000000	-0.0900000
48478.0000000	-0.0900000
48479.0000000	-0.0900000
48480.0000000	-0.0900000
48481.0000000	-0.0850000
48482.0000000	-0.0900000
48483.0000000	-0.0900000
48484.0000000	-0.0900000
48485.0000000	-0.0866667
48486.0000000	-0.0900000
48487.0000000	-0.0900000
48488.0000000	-0.0850000
48489.0000000	-0.0850000
48490.0000000	-0.0850000
48491.0000000	-0.0950000
48492.0000000	-0.0900000
48493.0000000	-0.0850000
48494.0000000	-0.0850000
48495.0000000	-0.0850000
48496.0000000	-0.0800000
48497.0000000	-0.0750000
48498.0000000	-0.0800000
48499.0000000	-0.0850000
48500.0000000	-0.0850000
48501.0000000	-0.0750000
48502.0000000	-0.0750000
48503.0000000	-0.0700000
48504.0000000	-0.0800000
48505.0000000	-0.0800000
48506.0000000	-0.0750000

48507.0000000	-0.0750000
48508.0000000	-0.0750000
48509.0000000	-0.0750000
48510.0000000	-0.0700000
48511.0000000	-0.0750000
48512.0000000	-0.0750000
48513.0000000	-0.0700000
48514.0000000	-0.0750000
48515.0000000	-0.0800000
48516.0000000	-0.0766667
48517.0000000	-0.0750000
48518.0000000	-0.0800000
48519.0000000	-0.0700000
48520.0000000	-0.0750000
48521.0000000	-0.0850000
48522.0000000	-0.0850000
48523.0000000	-0.0800000
48524.0000000	-0.0850000
48525.0000000	-0.0900000
48526.0000000	-0.0850000
48527.0000000	-0.0850000
48528.0000000	-0.0850000
48529.0000000	-0.0900000
48530.0000000	-0.0850000
48531.0000000	-0.0850000
48532.0000000	-0.0950000
48533.0000000	-0.0850000
48534.0000000	-0.0800000
48535.0000000	-0.0850000
48536.0000000	-0.0900000
48537.0000000	-0.0850000
48538.0000000	-0.0850000
48539.0000000	-0.0850000
48540.0000000	-0.0800000
48541.0000000	-0.0850000
48542.0000000	-0.0800000
48543.0000000	-0.0800000
48544.0000000	-0.0900000
48545.0000000	-0.0900000
48546.0000000	-0.0766667
48547.0000000	-0.0850000
48548.0000000	-0.0850000
48549.0000000	-0.0750000
48550.0000000	-0.0800000
48551.0000000	-0.0850000
48552.0000000	-0.0750000
48553.0000000	-0.0750000
48554.0000000	-0.0800000
48555.0000000	-0.0750000
48556.0000000	-0.0700000
48557.0000000	-0.0750000
48558.0000000	-0.0750000
48559.0000000	-0.0700000
48560.0000000	-0.0750000
48561.0000000	-0.0750000
48562.0000000	-0.0750000
48563.0000000	-0.0800000

48564.0000000	-0.0750000
48565.0000000	-0.0700000
48566.0000000	-0.0750000
48567.0000000	-0.0800000
48568.0000000	-0.0750000
48569.0000000	-0.0700000
48570.0000000	-0.0800000
48571.0000000	-0.0800000
48572.0000000	-0.0750000
48573.0000000	-0.0800000
48574.0000000	-0.0750000
48575.0000000	-0.0750000
48576.0000000	-0.0766667
48577.0000000	-0.0750000
48578.0000000	-0.0650000
48579.0000000	-0.0750000
48580.0000000	-0.0750000
48581.0000000	-0.0700000
48582.0000000	-0.0750000
48583.0000000	-0.0700000
48584.0000000	-0.0800000
48585.0000000	-0.0750000
48586.0000000	-0.0750000
48587.0000000	-0.0700000
48588.0000000	-0.0750000
48589.0000000	-0.0750000
48590.0000000	-0.0800000
48591.0000000	-0.0750000
48592.0000000	-0.0800000
48593.0000000	-0.0800000
48594.0000000	-0.0850000
48595.0000000	-0.0850000
48596.0000000	-0.0850000
48597.0000000	-0.0850000
48598.0000000	-0.0800000
48599.0000000	-0.0750000
48600.0000000	-0.0850000
48601.0000000	-0.0900000
48602.0000000	-0.0850000
48603.0000000	-0.0800000
48604.0000000	-0.0900000
48605.0000000	-0.0900000
48606.0000000	-0.0833333
48607.0000000	-0.0800000
48608.0000000	-0.0950000
48609.0000000	-0.0850000
48610.0000000	-0.0800000
48611.0000000	-0.0800000
48612.0000000	-0.0850000
48613.0000000	-0.0800000
48614.0000000	-0.0850000
48615.0000000	-0.0850000
48616.0000000	-0.0800000
48617.0000000	-0.0850000
48618.0000000	-0.0800000
48619.0000000	-0.0800000
48620.0000000	-0.0750000

48621.0000000	-0.0750000
48622.0000000	-0.0800000
48623.0000000	-0.0750000
48624.0000000	-0.0700000
48625.0000000	-0.0700000
48626.0000000	-0.0800000
48627.0000000	-0.0750000
48628.0000000	-0.0800000
48629.0000000	-0.0750000
48630.0000000	-0.0750000
48631.0000000	-0.0800000
48632.0000000	-0.0750000
48633.0000000	-0.0700000
48634.0000000	-0.0700000
48635.0000000	-0.0650000
48636.0000000	-0.0700000
48637.0000000	-0.0733333
48638.0000000	-0.0650000
48639.0000000	-0.0700000
48640.0000000	-0.0700000
48641.0000000	-0.0700000
48642.0000000	-0.0750000
48643.0000000	-0.0650000
48644.0000000	-0.0700000
48645.0000000	-0.0700000
48646.0000000	-0.0700000
48647.0000000	-0.0750000
48648.0000000	-0.0700000
48649.0000000	-0.0700000
48650.0000000	-0.0750000
48651.0000000	-0.0750000
48652.0000000	-0.0700000
48653.0000000	-0.0700000
48654.0000000	-0.0650000
48655.0000000	-0.0750000
48656.0000000	-0.0700000
48657.0000000	-0.0750000
48658.0000000	-0.0700000
48659.0000000	-0.0750000
48660.0000000	-0.0750000
48661.0000000	-0.0700000
48662.0000000	-0.0800000
48663.0000000	-0.0750000
48664.0000000	-0.0800000
48665.0000000	-0.0800000
48666.0000000	-0.0800000
48667.0000000	-0.0733333
48668.0000000	-0.0750000
48669.0000000	-0.0800000
48670.0000000	-0.0750000
48671.0000000	-0.0750000
48672.0000000	-0.0800000
48673.0000000	-0.0850000
48674.0000000	-0.0800000
48675.0000000	-0.0800000
48676.0000000	-0.0800000
48677.0000000	-0.0850000

48678.0000000	-0.0850000
48679.0000000	-0.0850000
48680.0000000	-0.0850000
48681.0000000	-0.0750000
48682.0000000	-0.0800000
48683.0000000	-0.0850000
48684.0000000	-0.0800000
48685.0000000	-0.0800000
48686.0000000	-0.0850000
48687.0000000	-0.0850000
48688.0000000	-0.0850000
48689.0000000	-0.0850000
48690.0000000	-0.0900000
48691.0000000	-0.0850000
48692.0000000	-0.0800000
48693.0000000	-0.0900000
48694.0000000	-0.0900000
48695.0000000	-0.0850000
48696.0000000	-0.0866667
48697.0000000	-0.0900000
48698.0000000	-0.0850000
48699.0000000	-0.0900000
48700.0000000	-0.0800000
48701.0000000	-0.0900000
48702.0000000	-0.0850000
48703.0000000	-0.0850000
48704.0000000	-0.0850000
48705.0000000	-0.0800000
48706.0000000	-0.0850000
48707.0000000	-0.0800000
48708.0000000	-0.0750000
48709.0000000	-0.0800000
48710.0000000	-0.0800000
48711.0000000	-0.0750000
48712.0000000	-0.0850000
48713.0000000	-0.0750000
48714.0000000	-0.0750000
48715.0000000	-0.0750000
48716.0000000	-0.0700000
48717.0000000	-0.0750000
48718.0000000	-0.0700000
48719.0000000	-0.0650000
48720.0000000	-0.0700000
48721.0000000	-0.0700000
48722.0000000	-0.0650000
48723.0000000	-0.0650000
48724.0000000	-0.0650000
48725.0000000	-0.0700000
48726.0000000	-0.0650000
48727.0000000	-0.0666667
48728.0000000	-0.0650000
48729.0000000	-0.0650000
48730.0000000	-0.0650000
48731.0000000	-0.0650000
48732.0000000	-0.0650000
48733.0000000	-0.0550000
48734.0000000	-0.0650000

48735.0000000	-0.0650000
48736.0000000	-0.0600000
48737.0000000	-0.0600000
48738.0000000	-0.0600000
48739.0000000	-0.0650000
48740.0000000	-0.0600000
48741.0000000	-0.0600000
48742.0000000	-0.0600000
48743.0000000	-0.0650000
48744.0000000	-0.0600000
48745.0000000	-0.0600000
48746.0000000	-0.0550000
48747.0000000	-0.0600000
48748.0000000	-0.0600000
48749.0000000	-0.0650000
48750.0000000	-0.0550000
48751.0000000	-0.0600000
48752.0000000	-0.0600000
48753.0000000	-0.0650000
48754.0000000	-0.0550000
48755.0000000	-0.0550000
48756.0000000	-0.0650000
48757.0000000	-0.0600000
48758.0000000	-0.0600000
48759.0000000	-0.0550000
48760.0000000	-0.0650000
48761.0000000	-0.0600000
48762.0000000	-0.0550000
48763.0000000	-0.0650000
48764.0000000	-0.0650000
48765.0000000	-0.0650000
48766.0000000	-0.0650000
48767.0000000	-0.0600000
48768.0000000	-0.0650000
48769.0000000	-0.0700000
48770.0000000	-0.0650000
48771.0000000	-0.0700000
48772.0000000	-0.0650000
48773.0000000	-0.0600000
48774.0000000	-0.0700000
48775.0000000	-0.0650000
48776.0000000	-0.0750000
48777.0000000	-0.0700000
48778.0000000	-0.0700000
48779.0000000	-0.0800000
48780.0000000	-0.0800000
48781.0000000	-0.0800000
48782.0000000	-0.0800000
48783.0000000	-0.0750000
48784.0000000	-0.0800000
48785.0000000	-0.0750000
48786.0000000	-0.0800000
48787.0000000	-0.0733333
48788.0000000	-0.0850000
48789.0000000	-0.0800000
48790.0000000	-0.0850000
48791.0000000	-0.0800000

48792.0000000	-0.0800000
48793.0000000	-0.0900000
48794.0000000	-0.0850000
48795.0000000	-0.0850000
48796.0000000	-0.0850000
48797.0000000	-0.0800000
48798.0000000	-0.0900000
48799.0000000	-0.0850000
48800.0000000	-0.0800000
48801.0000000	-0.0950000
48802.0000000	-0.0850000
48803.0000000	-0.0900000
48804.0000000	-0.0900000
48805.0000000	-0.0900000
48806.0000000	-0.0950000
48807.0000000	-0.0900000
48808.0000000	-0.0900000
48809.0000000	-0.0900000
48810.0000000	-0.0900000
48811.0000000	-0.0950000
48812.0000000	-0.0900000
48813.0000000	-0.0900000
48814.0000000	-0.0900000
48815.0000000	-0.0800000
48816.0000000	-0.0900000
48817.0000000	-0.0933333
48818.0000000	-0.0850000
48819.0000000	-0.0800000
48820.0000000	-0.0900000
48821.0000000	-0.0800000
48822.0000000	-0.0800000
48823.0000000	-0.0750000
48824.0000000	-0.0750000
48825.0000000	-0.0750000
48826.0000000	-0.0800000
48827.0000000	-0.0800000
48828.0000000	-0.0700000
48829.0000000	-0.0800000
48830.0000000	-0.0800000
48831.0000000	-0.0800000
48832.0000000	-0.0750000
48833.0000000	-0.0750000
48834.0000000	-0.0700000
48835.0000000	-0.0800000
48836.0000000	-0.0750000
48837.0000000	-0.0800000
48838.0000000	-0.0800000
48839.0000000	-0.0800000
48840.0000000	-0.0850000
48841.0000000	-0.0800000
48842.0000000	-0.0900000
48843.0000000	-0.0800000
48844.0000000	-0.0800000
48845.0000000	-0.0850000
48846.0000000	-0.0850000
48847.0000000	-0.0900000
48848.0000000	-0.0900000

48849.0000000	-0.0850000
48850.0000000	-0.1050000
48851.0000000	-0.1100000
48852.0000000	-0.1050000
48853.0000000	-0.1050000
48854.0000000	-0.1050000
48855.0000000	-0.1100000
48856.0000000	-0.1100000
48857.0000000	-0.1100000
48858.0000000	-0.1100000
48859.0000000	-0.1050000
48860.0000000	-0.1050000
48861.0000000	-0.1100000
48862.0000000	-0.1100000
48863.0000000	-0.1100000
48864.0000000	-0.1100000
48865.0000000	-0.1100000
48866.0000000	-0.1100000
48867.0000000	-0.1050000
48868.0000000	-0.1000000
48869.0000000	-0.1050000
48870.0000000	-0.1050000
48871.0000000	-0.1050000
48872.0000000	-0.0950000
48873.0000000	-0.1050000
48874.0000000	-0.0950000
48875.0000000	-0.1000000
48876.0000000	-0.1050000
48877.0000000	-0.1000000
48878.0000000	-0.0933333
48879.0000000	-0.1000000
48880.0000000	-0.1000000
48881.0000000	-0.0950000
48882.0000000	-0.0950000
48883.0000000	-0.0950000
48884.0000000	-0.0950000
48885.0000000	-0.0900000
48886.0000000	-0.0950000
48887.0000000	-0.0900000
48888.0000000	-0.0950000
48889.0000000	-0.0900000
48890.0000000	-0.1000000
48891.0000000	-0.0900000
48892.0000000	-0.0850000
48893.0000000	-0.0800000
48894.0000000	-0.0900000
48895.0000000	-0.0900000
48896.0000000	-0.0850000
48897.0000000	-0.0850000
48898.0000000	-0.0900000
48899.0000000	-0.0900000
48900.0000000	-0.0850000
48901.0000000	-0.0800000
48902.0000000	-0.0800000
48903.0000000	-0.0800000
48904.0000000	-0.0800000
48905.0000000	-0.0750000

48906.0000000	-0.0750000
48907.0000000	-0.0750000
48908.0000000	-0.0766667
48909.0000000	-0.0800000
48910.0000000	-0.0850000
48911.0000000	-0.0750000
48912.0000000	-0.0800000
48913.0000000	-0.0900000
48914.0000000	-0.0850000
48915.0000000	-0.0850000
48916.0000000	-0.0850000
48917.0000000	-0.0950000
48918.0000000	-0.0950000
48919.0000000	-0.0850000
48920.0000000	-0.0900000
48921.0000000	-0.0950000
48922.0000000	-0.1000000
48923.0000000	-0.0850000
48924.0000000	-0.0950000
48925.0000000	-0.0900000
48926.0000000	-0.0900000
48927.0000000	-0.0950000
48928.0000000	-0.0900000
48929.0000000	-0.1000000
48930.0000000	-0.0950000
48931.0000000	-0.0950000
48932.0000000	-0.1000000
48933.0000000	-0.1000000
48934.0000000	-0.1000000
48935.0000000	-0.1000000
48936.0000000	-0.0950000
48937.0000000	-0.1000000
48938.0000000	-0.0966667
48939.0000000	-0.1000000
48940.0000000	-0.1000000
48941.0000000	-0.1050000
48942.0000000	-0.0900000
48943.0000000	-0.1000000
48944.0000000	-0.1050000
48945.0000000	-0.1050000
48946.0000000	-0.0950000
48947.0000000	-0.1000000
48948.0000000	-0.1050000
48949.0000000	-0.1000000
48950.0000000	-0.0950000
48951.0000000	-0.0950000
48952.0000000	-0.0950000
48953.0000000	-0.1000000
48954.0000000	-0.1000000
48955.0000000	-0.1000000
48956.0000000	-0.0950000
48957.0000000	-0.0950000
48958.0000000	-0.0950000
48959.0000000	-0.0950000
48960.0000000	-0.0950000
48961.0000000	-0.1000000
48962.0000000	-0.1000000

48963.0000000	-0.1000000
48964.0000000	-0.0900000
48965.0000000	-0.1000000
48966.0000000	-0.0950000
48967.0000000	-0.1050000
48968.0000000	-0.1000000
48969.0000000	-0.1000000
48970.0000000	-0.1050000
48971.0000000	-0.1100000
48972.0000000	-0.0950000
48973.0000000	-0.1050000
48974.0000000	-0.1000000
48975.0000000	-0.1100000
48976.0000000	-0.1100000
48977.0000000	-0.1100000
48978.0000000	-0.1100000
48979.0000000	-0.1100000
48980.0000000	-0.1200000
48981.0000000	-0.1150000
48982.0000000	-0.1250000
48983.0000000	-0.1200000
48984.0000000	-0.1200000
48985.0000000	-0.1200000
48986.0000000	-0.1250000
48987.0000000	-0.1250000
48988.0000000	-0.1250000
48989.0000000	-0.1300000
48990.0000000	-0.1200000
48991.0000000	-0.1250000
48992.0000000	-0.1300000
48993.0000000	-0.1300000
48994.0000000	-0.1250000
48995.0000000	-0.1300000
48996.0000000	-0.1250000
48997.0000000	-0.1250000
48998.0000000	-0.1300000
48999.0000000	-0.1233333
49000.0000000	-0.1250000
49001.0000000	-0.1300000
49002.0000000	-0.1300000
49003.0000000	-0.1250000
49004.0000000	-0.1250000
49005.0000000	-0.1300000
49006.0000000	-0.1250000
49007.0000000	-0.1300000
49008.0000000	-0.1200000
49009.0000000	-0.1200000
49010.0000000	-0.1250000
49011.0000000	-0.1300000
49012.0000000	-0.1300000
49013.0000000	-0.1250000
49014.0000000	-0.1300000
49015.0000000	-0.1400000
49016.0000000	-0.1200000
49017.0000000	-0.1250000
49018.0000000	-0.1350000
49019.0000000	-0.1350000

49020.0000000	-0.1300000
49021.0000000	-0.1300000
49022.0000000	-0.1300000
49023.0000000	-0.1350000
49024.0000000	-0.1300000
49025.0000000	-0.1350000
49026.0000000	-0.1400000
49027.0000000	-0.1350000
49028.0000000	-0.1350000
49029.0000000	-0.1333333
49030.0000000	-0.1300000
49031.0000000	-0.1400000
49032.0000000	-0.1350000
49033.0000000	-0.1250000
49034.0000000	-0.1300000
49035.0000000	-0.1300000
49036.0000000	-0.1250000
49037.0000000	-0.1300000
49038.0000000	-0.1300000
49039.0000000	-0.1300000
49040.0000000	-0.1250000
49041.0000000	-0.1350000
49042.0000000	-0.1250000
49043.0000000	-0.1300000
49044.0000000	-0.1250000
49045.0000000	-0.1250000
49046.0000000	-0.1300000
49047.0000000	-0.1250000
49048.0000000	-0.1300000
49049.0000000	-0.1300000
49050.0000000	-0.1300000
49051.0000000	-0.1200000
49052.0000000	-0.1300000
49053.0000000	-0.1300000
49054.0000000	-0.1300000
49055.0000000	-0.1400000
49056.0000000	-0.1200000
49057.0000000	-0.1250000
49058.0000000	-0.1300000
49059.0000000	-0.1266667
49060.0000000	-0.1250000
49061.0000000	-0.1250000
49062.0000000	-0.1350000
49063.0000000	-0.1300000
49064.0000000	-0.1350000
49065.0000000	-0.1300000
49066.0000000	-0.1200000
49067.0000000	-0.1250000
49068.0000000	-0.1300000
49069.0000000	-0.1200000
49070.0000000	-0.1250000
49071.0000000	-0.1300000
49072.0000000	-0.1300000
49073.0000000	-0.1250000
49074.0000000	-0.1200000
49075.0000000	-0.1300000
49076.0000000	-0.1250000

49077.0000000	-0.1200000
49078.0000000	-0.1200000
49079.0000000	-0.1300000
49080.0000000	-0.1200000
49081.0000000	-0.1200000
49082.0000000	-0.1250000
49083.0000000	-0.1300000
49084.0000000	-0.1250000
49085.0000000	-0.1200000
49086.0000000	-0.1300000
49087.0000000	-0.1300000
49088.0000000	-0.1300000
49089.0000000	-0.1333333
49090.0000000	-0.1250000
49091.0000000	-0.1300000
49092.0000000	-0.1250000
49093.0000000	-0.1200000
49094.0000000	-0.1350000
49095.0000000	-0.1300000
49096.0000000	-0.1300000
49097.0000000	-0.1350000
49098.0000000	-0.1300000
49099.0000000	-0.1400000
49100.0000000	-0.1350000
49101.0000000	-0.1350000
49102.0000000	-0.1400000
49103.0000000	-0.1350000
49104.0000000	-0.1300000
49105.0000000	-0.1350000
49106.0000000	-0.1300000
49107.0000000	-0.1350000
49108.0000000	-0.1300000
49109.0000000	-0.1350000
49110.0000000	-0.1300000
49111.0000000	-0.1350000
49112.0000000	-0.1350000
49113.0000000	-0.1300000
49114.0000000	-0.1350000
49115.0000000	-0.1400000
49116.0000000	-0.1350000
49117.0000000	-0.1350000
49118.0000000	-0.1300000
49119.0000000	-0.1350000
49120.0000000	-0.1400000
49121.0000000	-0.1400000
49122.0000000	-0.1350000
49123.0000000	-0.1350000
49124.0000000	-0.1400000
49125.0000000	-0.1400000
49126.0000000	-0.1350000
49127.0000000	-0.1400000
49128.0000000	-0.1350000
49129.0000000	-0.1400000
49130.0000000	-0.1400000
49131.0000000	-0.1350000
49132.0000000	-0.1400000
49133.0000000	-0.1350000

49134.0000000	-0.1400000
49135.0000000	-0.1400000
49136.0000000	-0.1400000
49137.0000000	-0.1400000
49138.0000000	-0.1400000
49139.0000000	-0.1350000
49140.0000000	-0.1400000
49141.0000000	-0.1400000
49142.0000000	-0.1450000
49143.0000000	-0.1400000
49144.0000000	-0.1350000
49145.0000000	-0.1350000
49146.0000000	-0.1400000
49147.0000000	-0.1450000
49148.0000000	-0.1400000
49149.0000000	-0.1466667
49150.0000000	-0.1500000
49151.0000000	-0.1450000
49152.0000000	-0.1500000
49153.0000000	-0.1500000
49154.0000000	-0.1500000
49155.0000000	-0.1500000
49156.0000000	-0.1500000
49157.0000000	-0.1500000
49158.0000000	-0.1600000
49159.0000000	-0.1650000
49160.0000000	-0.1600000
49161.0000000	-0.1500000
49162.0000000	-0.1700000
49163.0000000	-0.1550000
49164.0000000	-0.1650000
49165.0000000	-0.1650000
49166.0000000	-0.1600000
49167.0000000	-0.1600000
49168.0000000	-0.1650000
49169.0000000	-0.1600000
49170.0000000	-0.1600000
49171.0000000	-0.1550000
49172.0000000	-0.1600000
49173.0000000	-0.1550000
49174.0000000	-0.1600000
49175.0000000	-0.1600000
49176.0000000	-0.1550000
49177.0000000	-0.1500000
49178.0000000	-0.1550000
49179.0000000	-0.1600000
49180.0000000	-0.1533333
49181.0000000	-0.1500000
49182.0000000	-0.1500000
49183.0000000	-0.1550000
49184.0000000	-0.1500000
49185.0000000	-0.1450000
49186.0000000	-0.1400000
49187.0000000	-0.1400000
49188.0000000	-0.1400000
49189.0000000	-0.1300000
49190.0000000	-0.1250000

49191.0000000	-0.1250000
49192.0000000	-0.1250000
49193.0000000	-0.1150000
49194.0000000	-0.1300000
49195.0000000	-0.1250000
49196.0000000	-0.1250000
49197.0000000	-0.1200000
49198.0000000	-0.1150000
49199.0000000	-0.1200000
49200.0000000	-0.1150000
49201.0000000	-0.1150000
49202.0000000	-0.1100000
49203.0000000	-0.1150000
49204.0000000	-0.1200000
49205.0000000	-0.1150000
49206.0000000	-0.1150000
49207.0000000	-0.1150000
49208.0000000	-0.1250000
49209.0000000	-0.1150000
49210.0000000	-0.1133333
49211.0000000	-0.1200000
49212.0000000	-0.1150000
49213.0000000	-0.1100000
49214.0000000	-0.1150000
49215.0000000	-0.1100000
49216.0000000	-0.1100000
49217.0000000	-0.1100000
49218.0000000	-0.1100000
49219.0000000	-0.1100000
49220.0000000	-0.1050000
49221.0000000	-0.1100000
49222.0000000	-0.1100000
49223.0000000	-0.1050000
49224.0000000	-0.1050000
49225.0000000	-0.1050000
49226.0000000	-0.1050000
49227.0000000	-0.1050000
49228.0000000	-0.1100000
49229.0000000	-0.1050000
49230.0000000	-0.0950000
49231.0000000	-0.1050000
49232.0000000	-0.1000000
49233.0000000	-0.1050000
49234.0000000	-0.1050000
49235.0000000	-0.1100000
49236.0000000	-0.1050000
49237.0000000	-0.1000000
49238.0000000	-0.1050000
49239.0000000	-0.1050000
49240.0000000	-0.1033333
49241.0000000	-0.1050000
49242.0000000	-0.1050000
49243.0000000	-0.1050000
49244.0000000	-0.1050000
49245.0000000	-0.1100000
49246.0000000	-0.1100000
49247.0000000	-0.1050000

49248.0000000	-0.1050000
49249.0000000	-0.1100000
49250.0000000	-0.1100000
49251.0000000	-0.1050000
49252.0000000	-0.1150000
49253.0000000	-0.1150000
49254.0000000	-0.1200000
49255.0000000	-0.1150000
49256.0000000	-0.1150000
49257.0000000	-0.1150000
49258.0000000	-0.1200000
49259.0000000	-0.1250000
49260.0000000	-0.1200000
49261.0000000	-0.1200000
49262.0000000	-0.1300000
49263.0000000	-0.1200000
49264.0000000	-0.1200000
49265.0000000	-0.1250000
49266.0000000	-0.1300000
49267.0000000	-0.1250000
49268.0000000	-0.1300000
49269.0000000	-0.1300000
49270.0000000	-0.1400000
49271.0000000	-0.1350000
49272.0000000	-0.1350000
49273.0000000	-0.1400000
49274.0000000	-0.1350000
49275.0000000	-0.1400000
49276.0000000	-0.1400000
49277.0000000	-0.1450000
49278.0000000	-0.1400000
49279.0000000	-0.1350000
49280.0000000	-0.1400000
49281.0000000	-0.1400000
49282.0000000	-0.1400000
49283.0000000	-0.1400000
49284.0000000	-0.1400000
49285.0000000	-0.1350000
49286.0000000	-0.1450000
49287.0000000	-0.1400000
49288.0000000	-0.1400000
49289.0000000	-0.1400000
49290.0000000	-0.1450000
49291.0000000	-0.1500000
49292.0000000	-0.1450000
49293.0000000	-0.1450000
49294.0000000	-0.1450000
49295.0000000	-0.1500000
49296.0000000	-0.1450000
49297.0000000	-0.1500000
49298.0000000	-0.1500000
49299.0000000	-0.1550000
49300.0000000	-0.1533333
49301.0000000	-0.1600000
49302.0000000	-0.1550000
49303.0000000	-0.1550000
49304.0000000	-0.1600000

49305.0000000	-0.1550000
49306.0000000	-0.1550000
49307.0000000	-0.1550000
49308.0000000	-0.1550000
49309.0000000	-0.1500000
49310.0000000	-0.1550000
49311.0000000	-0.1550000
49312.0000000	-0.1500000
49313.0000000	-0.1550000
49314.0000000	-0.1500000
49315.0000000	-0.1550000
49316.0000000	-0.1500000
49317.0000000	-0.1500000
49318.0000000	-0.1550000
49319.0000000	-0.1550000
49320.0000000	-0.1550000
49321.0000000	-0.1550000
49322.0000000	-0.1550000
49323.0000000	-0.1550000
49324.0000000	-0.1550000
49325.0000000	-0.1600000
49326.0000000	-0.1600000
49327.0000000	-0.1600000
49328.0000000	-0.1600000
49329.0000000	-0.1550000
49330.0000000	-0.1650000
49331.0000000	-0.1633333
49332.0000000	-0.1650000
49333.0000000	-0.1600000
49334.0000000	-0.1600000
49335.0000000	-0.1650000
49336.0000000	-0.1650000
49337.0000000	-0.1600000
49338.0000000	-0.1650000
49339.0000000	-0.1650000
49340.0000000	-0.1700000
49341.0000000	-0.1700000
49342.0000000	-0.1700000
49343.0000000	-0.1700000
49344.0000000	-0.1700000
49345.0000000	-0.1650000
49346.0000000	-0.1700000
49347.0000000	-0.1700000
49348.0000000	-0.1650000
49349.0000000	-0.1650000
49350.0000000	-0.1700000
49351.0000000	-0.1600000
49352.0000000	-0.1650000
49353.0000000	-0.1600000
49354.0000000	-0.1600000
49355.0000000	-0.1550000
49356.0000000	-0.1600000
49357.0000000	-0.1650000
49358.0000000	-0.1600000
49359.0000000	-0.1550000
49360.0000000	-0.1500000
49361.0000000	-0.1533333

49362.0000000	-0.1550000
49363.0000000	-0.1500000
49364.0000000	-0.1500000
49365.0000000	-0.1600000
49366.0000000	-0.1500000
49367.0000000	-0.1450000
49368.0000000	-0.1550000
49369.0000000	-0.1550000
49370.0000000	-0.1550000
49371.0000000	-0.1500000
49372.0000000	-0.1500000
49373.0000000	-0.1500000
49374.0000000	-0.1500000
49375.0000000	-0.1550000
49376.0000000	-0.1550000
49377.0000000	-0.1550000
49378.0000000	-0.1550000
49379.0000000	-0.1600000
49380.0000000	-0.1550000
49381.0000000	-0.1450000
49382.0000000	-0.1500000
49383.0000000	-0.1450000
49384.0000000	-0.1500000
49385.0000000	-0.1500000
49386.0000000	-0.1500000
49387.0000000	-0.1400000
49388.0000000	-0.1450000
49389.0000000	-0.1450000
49390.0000000	-0.1500000
49391.0000000	-0.1500000
49392.0000000	-0.1550000
49393.0000000	-0.1500000
49394.0000000	-0.1500000
49395.0000000	-0.1550000
49396.0000000	-0.1550000
49397.0000000	-0.1500000
49398.0000000	-0.1550000
49399.0000000	-0.1550000
49400.0000000	-0.1550000
49401.0000000	-0.1600000
49402.0000000	-0.1550000
49403.0000000	-0.1550000
49404.0000000	-0.1550000
49405.0000000	-0.1500000
49406.0000000	-0.1550000
49407.0000000	-0.1500000
49408.0000000	-0.1450000
49409.0000000	-0.1450000
49410.0000000	-0.1500000
49411.0000000	-0.1550000
49412.0000000	-0.1450000
49413.0000000	-0.1400000
49414.0000000	-0.1500000
49415.0000000	-0.1450000
49416.0000000	-0.1450000
49417.0000000	-0.1450000
49418.0000000	-0.1500000

49419.0000000	-0.1500000
49420.0000000	-0.1550000
49421.0000000	-0.1500000
49422.0000000	-0.1600000
49423.0000000	-0.1550000
49424.0000000	-0.1600000
49425.0000000	-0.1600000
49426.0000000	-0.1600000
49427.0000000	-0.1650000
49428.0000000	-0.1600000
49429.0000000	-0.1700000
49430.0000000	-0.1700000
49431.0000000	-0.1700000
49432.0000000	-0.1750000
49433.0000000	-0.1750000
49434.0000000	-0.1700000
49435.0000000	-0.1750000
49436.0000000	-0.1800000
49437.0000000	-0.1850000
49438.0000000	-0.1850000
49439.0000000	-0.1850000
49440.0000000	-0.1850000
49441.0000000	-0.1800000
49442.0000000	-0.1850000
49443.0000000	-0.1850000
49444.0000000	-0.1900000
49445.0000000	-0.1900000
49446.0000000	-0.1900000
49447.0000000	-0.1950000
49448.0000000	-0.1950000
49449.0000000	-0.1950000
49450.0000000	-0.1950000
49451.0000000	-0.1950000
49452.0000000	-0.1933333
49453.0000000	-0.1900000
49454.0000000	-0.1950000
49455.0000000	-0.1950000
49456.0000000	-0.1950000
49457.0000000	-0.2000000
49458.0000000	-0.1950000
49459.0000000	-0.1850000
49460.0000000	-0.1950000
49461.0000000	-0.1950000
49462.0000000	-0.1950000
49463.0000000	-0.1950000
49464.0000000	-0.1950000
49465.0000000	-0.2000000
49466.0000000	-0.2000000
49467.0000000	-0.1850000
49468.0000000	-0.2000000
49469.0000000	-0.2000000
49470.0000000	-0.2000000
49471.0000000	-0.1850000
49472.0000000	-0.2000000
49473.0000000	-0.2000000
49474.0000000	-0.1950000
49475.0000000	-0.1950000

49476.0000000	-0.2000000
49477.0000000	-0.2000000
49478.0000000	-0.1950000
49479.0000000	-0.1950000
49480.0000000	-0.2000000
49481.0000000	-0.2050000
49482.0000000	-0.1966667
49483.0000000	-0.1900000
49484.0000000	-0.2000000
49485.0000000	-0.2000000
49486.0000000	-0.1950000
49487.0000000	-0.2000000
49488.0000000	-0.2000000
49489.0000000	-0.2000000
49490.0000000	-0.1900000
49491.0000000	-0.1950000
49492.0000000	-0.2000000
49493.0000000	-0.1900000
49494.0000000	-0.1950000
49495.0000000	-0.1900000
49496.0000000	-0.1950000
49497.0000000	-0.1850000
49498.0000000	-0.1950000
49499.0000000	-0.1950000
49500.0000000	-0.1850000
49501.0000000	-0.1850000
49502.0000000	-0.1900000
49503.0000000	-0.1900000
49504.0000000	-0.1900000
49505.0000000	-0.1850000
49506.0000000	-0.1900000
49507.0000000	-0.1950000
49508.0000000	-0.1900000
49509.0000000	-0.1950000
49510.0000000	-0.1900000
49511.0000000	-0.1900000
49512.0000000	-0.1850000
49513.0000000	-0.1900000
49514.0000000	-0.1850000
49515.0000000	-0.1850000
49516.0000000	-0.1900000
49517.0000000	-0.1850000
49518.0000000	-0.1800000
49519.0000000	-0.1900000
49520.0000000	-0.1850000
49521.0000000	-0.1800000
49522.0000000	-0.1800000
49523.0000000	-0.1850000
49524.0000000	-0.1800000
49525.0000000	-0.1800000
49526.0000000	-0.1850000
49527.0000000	-0.1800000
49528.0000000	-0.1800000
49529.0000000	-0.1750000
49530.0000000	-0.1750000
49531.0000000	-0.1800000
49532.0000000	-0.1750000

49533.0000000	-0.1700000
49534.0000000	-0.1700000
49535.0000000	-0.1850000
49536.0000000	-0.1750000
49537.0000000	-0.1650000
49538.0000000	-0.1700000
49539.0000000	-0.1750000
49540.0000000	-0.1800000
49541.0000000	-0.1700000
49542.0000000	-0.1700000
49543.0000000	-0.1750000
49544.0000000	-0.1700000
49545.0000000	-0.1700000
49546.0000000	-0.1700000
49547.0000000	-0.1650000
49548.0000000	-0.1650000
49549.0000000	-0.1650000
49550.0000000	-0.1650000
49551.0000000	-0.1650000
49552.0000000	-0.1700000
49553.0000000	-0.1650000
49554.0000000	-0.1650000
49555.0000000	-0.1650000
49556.0000000	-0.1650000
49557.0000000	-0.1650000
49558.0000000	-0.1650000
49559.0000000	-0.1650000
49560.0000000	-0.1650000
49561.0000000	-0.1700000
49562.0000000	-0.1600000
49563.0000000	-0.1650000
49564.0000000	-0.1650000
49565.0000000	-0.1700000
49566.0000000	-0.1650000
49567.0000000	-0.1650000
49568.0000000	-0.1700000
49569.0000000	-0.1700000
49570.0000000	-0.1600000
49571.0000000	-0.1600000
49572.0000000	-0.1600000
49573.0000000	-0.1650000
49574.0000000	-0.1550000
49575.0000000	-0.1650000
49576.0000000	-0.1650000
49577.0000000	-0.1650000
49578.0000000	-0.1650000
49579.0000000	-0.1650000
49580.0000000	-0.1650000
49581.0000000	-0.1650000
49582.0000000	-0.1700000
49583.0000000	-0.1700000
49584.0000000	-0.1650000
49585.0000000	-0.1650000
49586.0000000	-0.1700000
49587.0000000	-0.1650000
49588.0000000	-0.1700000
49589.0000000	-0.1700000

49590.0000000	-0.1650000
49591.0000000	-0.1700000
49592.0000000	-0.1750000
49593.0000000	-0.1700000
49594.0000000	-0.1750000
49595.0000000	-0.1700000
49596.0000000	-0.1650000
49597.0000000	-0.1700000
49598.0000000	-0.1700000
49599.0000000	-0.1650000
49600.0000000	-0.1750000
49601.0000000	-0.1700000
49602.0000000	-0.1633333
49603.0000000	-0.1600000
49604.0000000	-0.1700000
49605.0000000	-0.1700000
49606.0000000	-0.1650000
49607.0000000	-0.1700000
49608.0000000	-0.1700000
49609.0000000	-0.1700000
49610.0000000	-0.1650000
49611.0000000	-0.1700000
49612.0000000	-0.1700000
49613.0000000	-0.1750000
49614.0000000	-0.1700000
49615.0000000	-0.1700000
49616.0000000	-0.1700000
49617.0000000	-0.1650000
49618.0000000	-0.1750000
49619.0000000	-0.1700000
49620.0000000	-0.1700000
49621.0000000	-0.1750000
49622.0000000	-0.1700000
49623.0000000	-0.1750000
49624.0000000	-0.1700000
49625.0000000	-0.1700000
49626.0000000	-0.1700000
49627.0000000	-0.1700000
49628.0000000	-0.1700000
49629.0000000	-0.1700000
49630.0000000	-0.1750000
49631.0000000	-0.1750000
49632.0000000	-0.1733333
49633.0000000	-0.1750000
49634.0000000	-0.1700000
49635.0000000	-0.1750000
49636.0000000	-0.1700000
49637.0000000	-0.1750000
49638.0000000	-0.1750000
49639.0000000	-0.1700000
49640.0000000	-0.1750000
49641.0000000	-0.1700000
49642.0000000	-0.1750000
49643.0000000	-0.1750000
49644.0000000	-0.1750000
49645.0000000	-0.1800000
49646.0000000	-0.1750000

49647.0000000	-0.1700000
49648.0000000	-0.1750000
49649.0000000	-0.1750000
49650.0000000	-0.1700000
49651.0000000	-0.1750000
49652.0000000	-0.1750000
49653.0000000	-0.1700000
49654.0000000	-0.1700000
49655.0000000	-0.1700000
49656.0000000	-0.1700000
49657.0000000	-0.1700000
49658.0000000	-0.1650000
49659.0000000	-0.1600000
49660.0000000	-0.1650000
49661.0000000	-0.1650000
49662.0000000	-0.1600000
49663.0000000	-0.1666667
49664.0000000	-0.1600000
49665.0000000	-0.1600000
49666.0000000	-0.1550000
49667.0000000	-0.1600000
49668.0000000	-0.1550000
49669.0000000	-0.1550000
49670.0000000	-0.1550000
49671.0000000	-0.1600000
49672.0000000	-0.1600000
49673.0000000	-0.1550000
49674.0000000	-0.1550000
49675.0000000	-0.1600000
49676.0000000	-0.1600000
49677.0000000	-0.1600000
49678.0000000	-0.1600000
49679.0000000	-0.1600000
49680.0000000	-0.1600000
49681.0000000	-0.1600000
49682.0000000	-0.1600000
49683.0000000	-0.1550000
49684.0000000	-0.1600000
49685.0000000	-0.1650000
49686.0000000	-0.1650000
49687.0000000	-0.1650000
49688.0000000	-0.1650000
49689.0000000	-0.1650000
49690.0000000	-0.1650000
49691.0000000	-0.1700000
49692.0000000	-0.1700000
49693.0000000	-0.1733333
49694.0000000	-0.1750000
49695.0000000	-0.1750000
49696.0000000	-0.1800000
49697.0000000	-0.1700000
49698.0000000	-0.1750000
49699.0000000	-0.1850000
49700.0000000	-0.1800000
49701.0000000	-0.1750000
49702.0000000	-0.1800000
49703.0000000	-0.1800000

49704.0000000	-0.1750000
49705.0000000	-0.1800000
49706.0000000	-0.1800000
49707.0000000	-0.1850000
49708.0000000	-0.1750000
49709.0000000	-0.1800000
49710.0000000	-0.1800000
49711.0000000	-0.1850000
49712.0000000	-0.1850000
49713.0000000	-0.1850000
49714.0000000	-0.1800000
49715.0000000	-0.1850000
49716.0000000	-0.1850000
49717.0000000	-0.1800000
49718.0000000	-0.1850000
49719.0000000	-0.1900000
49720.0000000	-0.1850000
49721.0000000	-0.1850000
49722.0000000	-0.1850000
49723.0000000	-0.1866667
49724.0000000	-0.1850000
49725.0000000	-0.1850000
49726.0000000	-0.1900000
49727.0000000	-0.1850000
49728.0000000	-0.1850000
49729.0000000	-0.1900000
49730.0000000	-0.1900000
49731.0000000	-0.1850000
49732.0000000	-0.1800000
49733.0000000	-0.1850000
49734.0000000	-0.1850000
49735.0000000	-0.1850000
49736.0000000	-0.1800000
49737.0000000	-0.1850000
49738.0000000	-0.1850000
49739.0000000	-0.1850000
49740.0000000	-0.1850000
49741.0000000	-0.1850000
49742.0000000	-0.1850000
49743.0000000	-0.1900000
49744.0000000	-0.1850000
49745.0000000	-0.1900000
49746.0000000	-0.1850000
49747.0000000	-0.1850000
49748.0000000	-0.1850000
49749.0000000	-0.1850000
49750.0000000	-0.1800000
49751.0000000	-0.1850000
49752.0000000	-0.1850000
49753.0000000	-0.1866667
49754.0000000	-0.1800000
49755.0000000	-0.1900000
49756.0000000	-0.1850000
49757.0000000	-0.1800000
49758.0000000	-0.1800000
49759.0000000	-0.1850000
49760.0000000	-0.1850000

49761.0000000	-0.1850000
49762.0000000	-0.1800000
49763.0000000	-0.1800000
49764.0000000	-0.1800000
49765.0000000	-0.1800000
49766.0000000	-0.1800000
49767.0000000	-0.1850000
49768.0000000	-0.1800000
49769.0000000	-0.1750000
49770.0000000	-0.1800000
49771.0000000	-0.1750000
49772.0000000	-0.1750000
49773.0000000	-0.1800000
49774.0000000	-0.1750000
49775.0000000	-0.1750000
49776.0000000	-0.1800000
49777.0000000	-0.1750000
49778.0000000	-0.1750000
49779.0000000	-0.1800000
49780.0000000	-0.1700000
49781.0000000	-0.1750000
49782.0000000	-0.1800000
49783.0000000	-0.1766667
49784.0000000	-0.1750000
49785.0000000	-0.1800000
49786.0000000	-0.1750000
49787.0000000	-0.1750000
49788.0000000	-0.1800000
49789.0000000	-0.1800000
49790.0000000	-0.1750000
49791.0000000	-0.1800000
49792.0000000	-0.1750000
49793.0000000	-0.1800000
49794.0000000	-0.1750000
49795.0000000	-0.1800000
49796.0000000	-0.1800000
49797.0000000	-0.1750000
49798.0000000	-0.1800000
49799.0000000	-0.1850000
49800.0000000	-0.1800000
49801.0000000	-0.1800000
49802.0000000	-0.1800000
49803.0000000	-0.1800000
49804.0000000	-0.1850000
49805.0000000	-0.1850000
49806.0000000	-0.1800000
49807.0000000	-0.1850000
49808.0000000	-0.1850000
49809.0000000	-0.1800000
49810.0000000	-0.1850000
49811.0000000	-0.1850000
49812.0000000	-0.1900000
49813.0000000	-0.1950000
49814.0000000	-0.1933333
49815.0000000	-0.1950000
49816.0000000	-0.1900000
49817.0000000	-0.1950000

49818.0000000	-0.2000000
49819.0000000	-0.2000000
49820.0000000	-0.1950000
49821.0000000	-0.2050000
49822.0000000	-0.2050000
49823.0000000	-0.2100000
49824.0000000	-0.2050000
49825.0000000	-0.2100000
49826.0000000	-0.2100000
49827.0000000	-0.2100000
49828.0000000	-0.2050000
49829.0000000	-0.2100000
49830.0000000	-0.2100000
49831.0000000	-0.2100000
49832.0000000	-0.2050000
49833.0000000	-0.2150000
49834.0000000	-0.2150000
49835.0000000	-0.2150000
49836.0000000	-0.2150000
49837.0000000	-0.2150000
49838.0000000	-0.2150000
49839.0000000	-0.2150000
49840.0000000	-0.2200000
49841.0000000	-0.2150000
49842.0000000	-0.2150000
49843.0000000	-0.2200000
49844.0000000	-0.2133333
49845.0000000	-0.2150000
49846.0000000	-0.2150000
49847.0000000	-0.2150000
49848.0000000	-0.2200000
49849.0000000	-0.2200000
49850.0000000	-0.2150000
49851.0000000	-0.2100000
49852.0000000	-0.2150000
49853.0000000	-0.2150000
49854.0000000	-0.2100000
49855.0000000	-0.2150000
49856.0000000	-0.2200000
49857.0000000	-0.2150000
49858.0000000	-0.2150000
49859.0000000	-0.2100000
49860.0000000	-0.2150000
49861.0000000	-0.2100000
49862.0000000	-0.2100000
49863.0000000	-0.2050000
49864.0000000	-0.2100000
49865.0000000	-0.2100000
49866.0000000	-0.2000000
49867.0000000	-0.2050000
49868.0000000	-0.2050000
49869.0000000	-0.2050000
49870.0000000	-0.2000000
49871.0000000	-0.2050000
49872.0000000	-0.2000000
49873.0000000	-0.2000000
49874.0000000	-0.2000000

49875.0000000	-0.2000000
49876.0000000	-0.1850000
49877.0000000	-0.1950000
49878.0000000	-0.1900000
49879.0000000	-0.1850000
49880.0000000	-0.1950000
49881.0000000	-0.1900000
49882.0000000	-0.1950000
49883.0000000	-0.1900000
49884.0000000	-0.1900000
49885.0000000	-0.1900000
49886.0000000	-0.1950000
49887.0000000	-0.1950000
49888.0000000	-0.1950000
49889.0000000	-0.1950000
49890.0000000	-0.1850000
49891.0000000	-0.1950000
49892.0000000	-0.2000000
49893.0000000	-0.1950000
49894.0000000	-0.1950000
49895.0000000	-0.1950000
49896.0000000	-0.1900000
49897.0000000	-0.1900000
49898.0000000	-0.1850000
49899.0000000	-0.1950000
49900.0000000	-0.1950000
49901.0000000	-0.1900000
49902.0000000	-0.1950000
49903.0000000	-0.2000000
49904.0000000	-0.1933333
49905.0000000	-0.1950000
49906.0000000	-0.1900000
49907.0000000	-0.2000000
49908.0000000	-0.2000000
49909.0000000	-0.1950000
49910.0000000	-0.1950000
49911.0000000	-0.1950000
49912.0000000	-0.2050000
49913.0000000	-0.2000000
49914.0000000	-0.2000000
49915.0000000	-0.1950000
49916.0000000	-0.2000000
49917.0000000	-0.1950000
49918.0000000	-0.2000000
49919.0000000	-0.2000000
49920.0000000	-0.2000000
49921.0000000	-0.2050000
49922.0000000	-0.2050000
49923.0000000	-0.2050000
49924.0000000	-0.2100000
49925.0000000	-0.2050000
49926.0000000	-0.2050000
49927.0000000	-0.2100000
49928.0000000	-0.2100000
49929.0000000	-0.2100000
49930.0000000	-0.2150000
49931.0000000	-0.2100000

49932.0000000	-0.2100000
49933.0000000	-0.2100000
49934.0000000	-0.2200000
49935.0000000	-0.2166667
49936.0000000	-0.2150000
49937.0000000	-0.2200000
49938.0000000	-0.2150000
49939.0000000	-0.2200000
49940.0000000	-0.2200000
49941.0000000	-0.2200000
49942.0000000	-0.2200000
49943.0000000	-0.2250000
49944.0000000	-0.2250000
49945.0000000	-0.2200000
49946.0000000	-0.2250000
49947.0000000	-0.2300000
49948.0000000	-0.2300000
49949.0000000	-0.2200000
49950.0000000	-0.2250000
49951.0000000	-0.2250000
49952.0000000	-0.2250000
49953.0000000	-0.2200000
49954.0000000	-0.2150000
49955.0000000	-0.2200000
49956.0000000	-0.2200000
49957.0000000	-0.2150000
49958.0000000	-0.2150000
49959.0000000	-0.2150000
49960.0000000	-0.2250000
49961.0000000	-0.2200000
49962.0000000	-0.2150000
49963.0000000	-0.2200000
49964.0000000	-0.2200000
49965.0000000	-0.2200000
49966.0000000	-0.2150000
49967.0000000	-0.2200000
49968.0000000	-0.2200000
49969.0000000	-0.2150000
49970.0000000	-0.2150000
49971.0000000	-0.2150000
49972.0000000	-0.2100000
49973.0000000	-0.2200000
49974.0000000	-0.2150000
49975.0000000	-0.2150000
49976.0000000	-0.2150000
49977.0000000	-0.2100000
49978.0000000	-0.2100000
49979.0000000	-0.2100000
49980.0000000	-0.2100000
49981.0000000	-0.2050000
49982.0000000	-0.2100000
49983.0000000	-0.2100000
49984.0000000	-0.2100000
49985.0000000	-0.2100000
49986.0000000	-0.2150000
49987.0000000	-0.2100000
49988.0000000	-0.2100000

49989.0000000	-0.2150000
49990.0000000	-0.2150000
49991.0000000	-0.2150000
49992.0000000	-0.2150000
49993.0000000	-0.2150000
49994.0000000	-0.2133333
49995.0000000	-0.2150000
49996.0000000	-0.2150000
49997.0000000	-0.2200000
49998.0000000	-0.2150000
49999.0000000	-0.2150000
50000.0000000	-0.2250000
50001.0000000	-0.2200000
50002.0000000	-0.2150000
50003.0000000	-0.2200000
50004.0000000	-0.2200000
50005.0000000	-0.2250000
50006.0000000	-0.2300000
50007.0000000	-0.2200000
50008.0000000	-0.2250000
50009.0000000	-0.2300000
50010.0000000	-0.2300000
50011.0000000	-0.2350000
50012.0000000	-0.2300000
50013.0000000	-0.2300000
50014.0000000	-0.2300000
50015.0000000	-0.2300000
50016.0000000	-0.2400000
50017.0000000	-0.2400000
50018.0000000	-0.2400000
50019.0000000	-0.2400000
50020.0000000	-0.2500000
50021.0000000	-0.2400000
50022.0000000	-0.2400000
50023.0000000	-0.2400000
50024.0000000	-0.2450000
50025.0000000	-0.2433333
50026.0000000	-0.2400000
50027.0000000	-0.2450000
50028.0000000	-0.2500000
50029.0000000	-0.2450000
50030.0000000	-0.2450000
50031.0000000	-0.2500000
50032.0000000	-0.2500000
50033.0000000	-0.2450000
50034.0000000	-0.2500000
50035.0000000	-0.2450000
50036.0000000	-0.2550000
50037.0000000	-0.2450000
50038.0000000	-0.2500000
50039.0000000	-0.2500000
50040.0000000	-0.2500000
50041.0000000	-0.2450000
50042.0000000	-0.2450000
50043.0000000	-0.2500000
50044.0000000	-0.2600000
50045.0000000	-0.2450000

50046.0000000	-0.2500000
50047.0000000	-0.2500000
50048.0000000	-0.2450000
50049.0000000	-0.2500000
50050.0000000	-0.2550000
50051.0000000	-0.2450000
50052.0000000	-0.2500000
50053.0000000	-0.2450000
50054.0000000	-0.2450000
50055.0000000	-0.2500000
50056.0000000	-0.2500000
50057.0000000	-0.2450000
50058.0000000	-0.2500000
50059.0000000	-0.2500000
50060.0000000	-0.2500000
50061.0000000	-0.2500000
50062.0000000	-0.2500000
50063.0000000	-0.2500000
50064.0000000	-0.2450000
50065.0000000	-0.2500000
50066.0000000	-0.2500000
50067.0000000	-0.2500000
50068.0000000	-0.2400000
50069.0000000	-0.2450000
50070.0000000	-0.2450000
50071.0000000	-0.2500000
50072.0000000	-0.2450000
50073.0000000	-0.2500000
50074.0000000	-0.2500000
50075.0000000	-0.2500000
50076.0000000	-0.2500000
50077.0000000	-0.2450000
50078.0000000	-0.2500000
50079.0000000	-0.2500000
50080.0000000	-0.2400000
50081.0000000	-0.2450000
50082.0000000	-0.2450000
50083.0000000	-0.2450000
50084.0000000	-0.2450000
50085.0000000	-0.2433333
50086.0000000	-0.2450000
50087.0000000	-0.2500000
50088.0000000	-0.2500000
50089.0000000	-0.2450000
50090.0000000	-0.2450000
50091.0000000	-0.2450000
50092.0000000	-0.2450000
50093.0000000	-0.2400000
50094.0000000	-0.2400000
50095.0000000	-0.2450000
50096.0000000	-0.2400000
50097.0000000	-0.2350000
50098.0000000	-0.2450000
50099.0000000	-0.2400000
50100.0000000	-0.2350000
50101.0000000	-0.2400000
50102.0000000	-0.2350000

50103.0000000	-0.2400000
50104.0000000	-0.2400000
50105.0000000	-0.2400000
50106.0000000	-0.2350000
50107.0000000	-0.2400000
50108.0000000	-0.2400000
50109.0000000	-0.2400000
50110.0000000	-0.2350000
50111.0000000	-0.2450000
50112.0000000	-0.2400000
50113.0000000	-0.2400000
50114.0000000	-0.2400000
50115.0000000	-0.2400000
50116.0000000	-0.2400000
50117.0000000	-0.2400000
50118.0000000	-0.2350000
50119.0000000	-0.2400000
50120.0000000	-0.2400000
50121.0000000	-0.2350000
50122.0000000	-0.2350000
50123.0000000	-0.2400000
50124.0000000	-0.2450000
50125.0000000	-0.2350000
50126.0000000	-0.2350000
50127.0000000	-0.2300000
50128.0000000	-0.2350000
50129.0000000	-0.2350000
50130.0000000	-0.2350000
50131.0000000	-0.2350000
50132.0000000	-0.2400000
50133.0000000	-0.2350000
50134.0000000	-0.2350000
50135.0000000	-0.2400000
50136.0000000	-0.2400000
50137.0000000	-0.2350000
50138.0000000	-0.2350000
50139.0000000	-0.2350000
50140.0000000	-0.2400000
50141.0000000	-0.2400000
50142.0000000	-0.2350000
50143.0000000	-0.2400000
50144.0000000	-0.2400000
50145.0000000	-0.2400000
50146.0000000	-0.2366667
50147.0000000	-0.2350000
50148.0000000	-0.2350000
50149.0000000	-0.2350000
50150.0000000	-0.2400000
50151.0000000	-0.2400000
50152.0000000	-0.2400000
50153.0000000	-0.2400000
50154.0000000	-0.2400000
50155.0000000	-0.2300000
50156.0000000	-0.2350000
50157.0000000	-0.2400000
50158.0000000	-0.2350000
50159.0000000	-0.2400000

50160.0000000	-0.2400000
50161.0000000	-0.2350000
50162.0000000	-0.2400000
50163.0000000	-0.2450000
50164.0000000	-0.2450000
50165.0000000	-0.2400000
50166.0000000	-0.2400000
50167.0000000	-0.2450000
50168.0000000	-0.2450000
50169.0000000	-0.2400000
50170.0000000	-0.2400000
50171.0000000	-0.2400000
50172.0000000	-0.2400000
50173.0000000	-0.2400000
50174.0000000	-0.2450000
50175.0000000	-0.2450000
50176.0000000	-0.2433333
50177.0000000	-0.2450000
50178.0000000	-0.2450000
50179.0000000	-0.2400000
50180.0000000	-0.2500000
50181.0000000	-0.2400000
50182.0000000	-0.2400000
50183.0000000	-0.2450000
50184.0000000	-0.2450000
50185.0000000	-0.2400000
50186.0000000	-0.2400000
50187.0000000	-0.2400000
50188.0000000	-0.2400000
50189.0000000	-0.2400000
50190.0000000	-0.2350000
50191.0000000	-0.2350000
50192.0000000	-0.2350000
50193.0000000	-0.2350000
50194.0000000	-0.2350000
50195.0000000	-0.2250000
50196.0000000	-0.2300000
50197.0000000	-0.2300000
50198.0000000	-0.2300000
50199.0000000	-0.2200000
50200.0000000	-0.2250000
50201.0000000	-0.2250000
50202.0000000	-0.2200000
50203.0000000	-0.2250000
50204.0000000	-0.2150000
50205.0000000	-0.2200000
50206.0000000	-0.2200000
50207.0000000	-0.2200000
50208.0000000	-0.2150000
50209.0000000	-0.2200000
50210.0000000	-0.2100000
50211.0000000	-0.2150000
50212.0000000	-0.2050000
50213.0000000	-0.2150000
50214.0000000	-0.2150000
50215.0000000	-0.2100000
50216.0000000	-0.2100000

50217.0000000	-0.2150000
50218.0000000	-0.2100000
50219.0000000	-0.2100000
50220.0000000	-0.2050000
50221.0000000	-0.2150000
50222.0000000	-0.2100000
50223.0000000	-0.2100000
50224.0000000	-0.2100000
50225.0000000	-0.2050000
50226.0000000	-0.2150000
50227.0000000	-0.2100000
50228.0000000	-0.2100000
50229.0000000	-0.2100000
50230.0000000	-0.2050000
50231.0000000	-0.2100000
50232.0000000	-0.2100000
50233.0000000	-0.2150000
50234.0000000	-0.2050000
50235.0000000	-0.2100000
50236.0000000	-0.2100000
50237.0000000	-0.2100000
50238.0000000	-0.2050000
50239.0000000	-0.2050000
50240.0000000	-0.2050000
50241.0000000	-0.2100000
50242.0000000	-0.2100000
50243.0000000	-0.2100000
50244.0000000	-0.2050000
50245.0000000	-0.2050000
50246.0000000	-0.2050000
50247.0000000	-0.2050000
50248.0000000	-0.2050000
50249.0000000	-0.2050000
50250.0000000	-0.2100000
50251.0000000	-0.2100000
50252.0000000	-0.2000000
50253.0000000	-0.2050000
50254.0000000	-0.2050000
50255.0000000	-0.2050000
50256.0000000	-0.2050000
50257.0000000	-0.2000000
50258.0000000	-0.2000000
50259.0000000	-0.2050000
50260.0000000	-0.2000000
50261.0000000	-0.2050000
50262.0000000	-0.2000000
50263.0000000	-0.1950000
50264.0000000	-0.2050000
50265.0000000	-0.1950000
50266.0000000	-0.2000000
50267.0000000	-0.1966667
50268.0000000	-0.1950000
50269.0000000	-0.1950000
50270.0000000	-0.1900000
50271.0000000	-0.1850000
50272.0000000	-0.1850000
50273.0000000	-0.1900000

50274.0000000	-0.1850000
50275.0000000	-0.1800000
50276.0000000	-0.1800000
50277.0000000	-0.1800000
50278.0000000	-0.1800000
50279.0000000	-0.1700000
50280.0000000	-0.1700000
50281.0000000	-0.1750000
50282.0000000	-0.1750000
50283.0000000	-0.1650000
50284.0000000	-0.1750000
50285.0000000	-0.1700000
50286.0000000	-0.1650000
50287.0000000	-0.1650000
50288.0000000	-0.1700000
50289.0000000	-0.1650000
50290.0000000	-0.1600000
50291.0000000	-0.1600000
50292.0000000	-0.1650000
50293.0000000	-0.1550000
50294.0000000	-0.1500000
50295.0000000	-0.1550000
50296.0000000	-0.1500000
50297.0000000	-0.1600000
50298.0000000	-0.1550000
50299.0000000	-0.1550000
50300.0000000	-0.1550000
50301.0000000	-0.1600000
50302.0000000	-0.1500000
50303.0000000	-0.1550000
50304.0000000	-0.1550000
50305.0000000	-0.1500000
50306.0000000	-0.1500000
50307.0000000	-0.1500000
50308.0000000	-0.1550000
50309.0000000	-0.1500000
50310.0000000	-0.1500000
50311.0000000	-0.1500000
50312.0000000	-0.1550000
50313.0000000	-0.1450000
50314.0000000	-0.1500000
50315.0000000	-0.1500000
50316.0000000	-0.1450000
50317.0000000	-0.1500000
50318.0000000	-0.1500000
50319.0000000	-0.1500000
50320.0000000	-0.1400000
50321.0000000	-0.1500000
50322.0000000	-0.1500000
50323.0000000	-0.1450000
50324.0000000	-0.1450000
50325.0000000	-0.1500000
50326.0000000	-0.1500000
50327.0000000	-0.1433333
50328.0000000	-0.1450000
50329.0000000	-0.1500000
50330.0000000	-0.1450000

50331.0000000	-0.1400000
50332.0000000	-0.1500000
50333.0000000	-0.1500000
50334.0000000	-0.1450000
50335.0000000	-0.1450000
50336.0000000	-0.1450000
50337.0000000	-0.1450000
50338.0000000	-0.1450000
50339.0000000	-0.1450000
50340.0000000	-0.1450000
50341.0000000	-0.1350000
50342.0000000	-0.1500000
50343.0000000	-0.1450000
50344.0000000	-0.1400000
50345.0000000	-0.1450000
50346.0000000	-0.1400000
50347.0000000	-0.1450000
50348.0000000	-0.1450000
50349.0000000	-0.1450000
50350.0000000	-0.1400000
50351.0000000	-0.1450000
50352.0000000	-0.1400000
50353.0000000	-0.1350000
50354.0000000	-0.1400000
50355.0000000	-0.1400000
50356.0000000	-0.1400000
50357.0000000	-0.1433333
50358.0000000	-0.1350000
50359.0000000	-0.1350000
50360.0000000	-0.1400000
50361.0000000	-0.1350000
50362.0000000	-0.1300000
50363.0000000	-0.1350000
50364.0000000	-0.1350000
50365.0000000	-0.1300000
50366.0000000	-0.1350000
50367.0000000	-0.1300000
50368.0000000	-0.1300000
50369.0000000	-0.1300000
50370.0000000	-0.1300000
50371.0000000	-0.1250000
50372.0000000	-0.1250000
50373.0000000	-0.1300000
50374.0000000	-0.1250000
50375.0000000	-0.1250000
50376.0000000	-0.1300000
50377.0000000	-0.1300000
50378.0000000	-0.1250000
50379.0000000	-0.1300000
50380.0000000	-0.1250000
50381.0000000	-0.1250000
50382.0000000	-0.1300000
50383.0000000	-0.1250000
50384.0000000	-0.1250000
50385.0000000	-0.1300000
50386.0000000	-0.1250000
50387.0000000	-0.1233333

50388.0000000	-0.1250000
50389.0000000	-0.1300000
50390.0000000	-0.1250000
50391.0000000	-0.1200000
50392.0000000	-0.1300000
50393.0000000	-0.1250000
50394.0000000	-0.1200000
50395.0000000	-0.1250000
50396.0000000	-0.1200000
50397.0000000	-0.1250000
50398.0000000	-0.1250000
50399.0000000	-0.1200000
50400.0000000	-0.1200000
50401.0000000	-0.1250000
50402.0000000	-0.1250000
50403.0000000	-0.1250000
50404.0000000	-0.1200000
50405.0000000	-0.1250000
50406.0000000	-0.1200000
50407.0000000	-0.1200000
50408.0000000	-0.1200000
50409.0000000	-0.1200000
50410.0000000	-0.1150000
50411.0000000	-0.1250000
50412.0000000	-0.1150000
50413.0000000	-0.1200000
50414.0000000	-0.1200000
50415.0000000	-0.1150000
50416.0000000	-0.1200000
50417.0000000	-0.1150000
50418.0000000	-0.1166667
50419.0000000	-0.1200000
50420.0000000	-0.1150000
50421.0000000	-0.1150000
50422.0000000	-0.1100000
50423.0000000	-0.1100000
50424.0000000	-0.1050000
50425.0000000	-0.1100000
50426.0000000	-0.1050000
50427.0000000	-0.1050000
50428.0000000	-0.1050000
50429.0000000	-0.1050000
50430.0000000	-0.1050000
50431.0000000	-0.1050000
50432.0000000	-0.1050000
50433.0000000	-0.1050000
50434.0000000	-0.0950000
50435.0000000	-0.0950000
50436.0000000	-0.1000000
50437.0000000	-0.1000000
50438.0000000	-0.1000000
50439.0000000	-0.0950000
50440.0000000	-0.0950000
50441.0000000	-0.0850000
50442.0000000	-0.0900000
50443.0000000	-0.0850000
50444.0000000	-0.0900000

50445.0000000	-0.0850000
50446.0000000	-0.0850000
50447.0000000	-0.0866667
50448.0000000	-0.0900000
50449.0000000	-0.0850000
50450.0000000	-0.0850000
50451.0000000	-0.0800000
50452.0000000	-0.0850000
50453.0000000	-0.0800000
50454.0000000	-0.0800000
50455.0000000	-0.0800000
50456.0000000	-0.0800000
50457.0000000	-0.0850000
50458.0000000	-0.0750000
50459.0000000	-0.0800000
50460.0000000	-0.0800000
50461.0000000	-0.0850000
50462.0000000	-0.0750000
50463.0000000	-0.0800000
50464.0000000	-0.0700000
50465.0000000	-0.0850000
50466.0000000	-0.0750000
50467.0000000	-0.0750000
50468.0000000	-0.0800000
50469.0000000	-0.0750000
50470.0000000	-0.0750000
50471.0000000	-0.0700000
50472.0000000	-0.0800000
50473.0000000	-0.0750000
50474.0000000	-0.0750000
50475.0000000	-0.0800000
50476.0000000	-0.0750000
50477.0000000	-0.0766667
50478.0000000	-0.0700000
50479.0000000	-0.0750000
50480.0000000	-0.0750000
50481.0000000	-0.0800000
50482.0000000	-0.0750000
50483.0000000	-0.0800000
50484.0000000	-0.0750000
50485.0000000	-0.0700000
50486.0000000	-0.0700000
50487.0000000	-0.0750000
50488.0000000	-0.0750000
50489.0000000	-0.0700000
50490.0000000	-0.0750000
50491.0000000	-0.0700000
50492.0000000	-0.0750000
50493.0000000	-0.0700000
50494.0000000	-0.0750000
50495.0000000	-0.0750000
50496.0000000	-0.0700000
50497.0000000	-0.0800000
50498.0000000	-0.0750000
50499.0000000	-0.0750000
50500.0000000	-0.0750000
50501.0000000	-0.0700000

50502.0000000	-0.0800000
50503.0000000	-0.0750000
50504.0000000	-0.0750000
50505.0000000	-0.0700000
50506.0000000	-0.0750000
50507.0000000	-0.0800000
50508.0000000	-0.0800000
50509.0000000	-0.0750000
50510.0000000	-0.0800000
50511.0000000	-0.0750000
50512.0000000	-0.0700000
50513.0000000	-0.0800000
50514.0000000	-0.0800000
50515.0000000	-0.0800000
50516.0000000	-0.0800000
50517.0000000	-0.0850000
50518.0000000	-0.0850000
50519.0000000	-0.0800000
50520.0000000	-0.0750000
50521.0000000	-0.0850000
50522.0000000	-0.0800000
50523.0000000	-0.0850000
50524.0000000	-0.0850000
50525.0000000	-0.0800000
50526.0000000	-0.0900000
50527.0000000	-0.0850000
50528.0000000	-0.0800000
50529.0000000	-0.0800000
50530.0000000	-0.0800000
50531.0000000	-0.0800000
50532.0000000	-0.0800000
50533.0000000	-0.0850000
50534.0000000	-0.0800000
50535.0000000	-0.0800000
50536.0000000	-0.0800000
50537.0000000	-0.0850000
50538.0000000	-0.0866667
50539.0000000	-0.0800000
50540.0000000	-0.0900000
50541.0000000	-0.0900000
50542.0000000	-0.0800000
50543.0000000	-0.0850000
50544.0000000	-0.0900000
50545.0000000	-0.0850000
50546.0000000	-0.0900000
50547.0000000	-0.0850000
50548.0000000	-0.0850000
50549.0000000	-0.0850000
50550.0000000	-0.0900000
50551.0000000	-0.0900000
50552.0000000	-0.0850000
50553.0000000	-0.0900000
50554.0000000	-0.0900000
50555.0000000	-0.0850000
50556.0000000	-0.0900000
50557.0000000	-0.0900000
50558.0000000	-0.0900000

50559.0000000	-0.0850000
50560.0000000	-0.0900000
50561.0000000	-0.0900000
50562.0000000	-0.0850000
50563.0000000	-0.0900000
50564.0000000	-0.0900000
50565.0000000	-0.0900000
50566.0000000	-0.0850000
50567.0000000	-0.0850000
50568.0000000	-0.0800000
50569.0000000	-0.0850000
50570.0000000	-0.0900000
50571.0000000	-0.0900000
50572.0000000	-0.0850000
50573.0000000	-0.0750000
50574.0000000	-0.0850000
50575.0000000	-0.0850000
50576.0000000	-0.0800000
50577.0000000	-0.0800000
50578.0000000	-0.0850000
50579.0000000	-0.0850000
50580.0000000	-0.0850000
50581.0000000	-0.0800000
50582.0000000	-0.0850000
50583.0000000	-0.0800000
50584.0000000	-0.0800000
50585.0000000	-0.0800000
50586.0000000	-0.0750000
50587.0000000	-0.0800000
50588.0000000	-0.0750000
50589.0000000	-0.0750000
50590.0000000	-0.0700000
50591.0000000	-0.0700000
50592.0000000	-0.0700000
50593.0000000	-0.0650000
50594.0000000	-0.0700000
50595.0000000	-0.0650000
50596.0000000	-0.0600000
50597.0000000	-0.0650000
50598.0000000	-0.0633333
50599.0000000	-0.0650000
50600.0000000	-0.0550000
50601.0000000	-0.0600000
50602.0000000	-0.0550000
50603.0000000	-0.0600000
50604.0000000	-0.0650000
50605.0000000	-0.0600000
50606.0000000	-0.0550000
50607.0000000	-0.0550000
50608.0000000	-0.0550000
50609.0000000	-0.0600000
50610.0000000	-0.0600000
50611.0000000	-0.0600000
50612.0000000	-0.0500000
50613.0000000	-0.0550000
50614.0000000	-0.0600000
50615.0000000	-0.0550000

50616.0000000	-0.0550000
50617.0000000	-0.0550000
50618.0000000	-0.0550000
50619.0000000	-0.0600000
50620.0000000	-0.0600000
50621.0000000	-0.0550000
50622.0000000	-0.0550000
50623.0000000	-0.0600000
50624.0000000	-0.0550000
50625.0000000	-0.0600000
50626.0000000	-0.0600000
50627.0000000	-0.0600000
50628.0000000	-0.0550000
50629.0000000	-0.0600000
50630.0000000	-0.0550000
50631.0000000	-0.0550000
50632.0000000	-0.0550000
50633.0000000	-0.0600000
50634.0000000	-0.0600000
50635.0000000	-0.0550000
50636.0000000	-0.0550000
50637.0000000	-0.0550000
50638.0000000	-0.0550000
50639.0000000	-0.0550000
50640.0000000	-0.0550000
50641.0000000	-0.0600000
50642.0000000	-0.0550000
50643.0000000	-0.0550000
50644.0000000	-0.0550000
50645.0000000	-0.0550000
50646.0000000	-0.0550000
50647.0000000	-0.0550000
50648.0000000	-0.0500000
50649.0000000	-0.0550000
50650.0000000	-0.0550000
50651.0000000	-0.0550000
50652.0000000	-0.0550000
50653.0000000	-0.0500000
50654.0000000	-0.0500000
50655.0000000	-0.0500000
50656.0000000	-0.0550000
50657.0000000	-0.0500000
50658.0000000	-0.0500000
50659.0000000	-0.0500000
50660.0000000	-0.0550000
50661.0000000	-0.0550000
50662.0000000	-0.0550000
50663.0000000	-0.0500000
50664.0000000	-0.0500000
50665.0000000	-0.0500000
50666.0000000	-0.0500000
50667.0000000	-0.0500000
50668.0000000	-0.0500000
50669.0000000	-0.0450000
50670.0000000	-0.0450000
50671.0000000	-0.0500000
50672.0000000	-0.0450000

50673.0000000	-0.0400000
50674.0000000	-0.0450000
50675.0000000	-0.0500000
50676.0000000	-0.0450000
50677.0000000	-0.0450000
50678.0000000	-0.0450000
50679.0000000	-0.0500000
50680.0000000	-0.0500000
50681.0000000	-0.0450000
50682.0000000	-0.0450000
50683.0000000	-0.0450000
50684.0000000	-0.0450000
50685.0000000	-0.0500000
50686.0000000	-0.0550000
50687.0000000	-0.0500000
50688.0000000	-0.0500000
50689.0000000	-0.0533333
50690.0000000	-0.0500000
50691.0000000	-0.0500000
50692.0000000	-0.0550000
50693.0000000	-0.0500000
50694.0000000	-0.0450000
50695.0000000	-0.0500000
50696.0000000	-0.0500000
50697.0000000	-0.0550000
50698.0000000	-0.0550000
50699.0000000	-0.0550000
50700.0000000	-0.0500000
50701.0000000	-0.0500000
50702.0000000	-0.0500000
50703.0000000	-0.0550000
50704.0000000	-0.0550000
50705.0000000	-0.0500000
50706.0000000	-0.0550000
50707.0000000	-0.0550000
50708.0000000	-0.0600000
50709.0000000	-0.0550000
50710.0000000	-0.0500000
50711.0000000	-0.0550000
50712.0000000	-0.0550000
50713.0000000	-0.0550000
50714.0000000	-0.0550000
50715.0000000	-0.0550000
50716.0000000	-0.0500000
50717.0000000	-0.0550000
50718.0000000	-0.0550000
50719.0000000	-0.0533333
50720.0000000	-0.0500000
50721.0000000	-0.0550000
50722.0000000	-0.0550000
50723.0000000	-0.0500000
50724.0000000	-0.0550000
50725.0000000	-0.0500000
50726.0000000	-0.0450000
50727.0000000	-0.0500000
50728.0000000	-0.0500000
50729.0000000	-0.0450000

50730.0000000	-0.0450000
50731.0000000	-0.0500000
50732.0000000	-0.0500000
50733.0000000	-0.0450000
50734.0000000	-0.0450000
50735.0000000	-0.0450000
50736.0000000	-0.0450000
50737.0000000	-0.0450000
50738.0000000	-0.0400000
50739.0000000	-0.0400000
50740.0000000	-0.0400000
50741.0000000	-0.0400000
50742.0000000	-0.0350000
50743.0000000	-0.0450000
50744.0000000	-0.0400000
50745.0000000	-0.0350000
50746.0000000	-0.0350000
50747.0000000	-0.0350000
50748.0000000	-0.0400000
50749.0000000	-0.0366667
50750.0000000	-0.0350000
50751.0000000	-0.0400000
50752.0000000	-0.0350000
50753.0000000	-0.0300000
50754.0000000	-0.0350000
50755.0000000	-0.0300000
50756.0000000	-0.0300000
50757.0000000	-0.0350000
50758.0000000	-0.0200000
50759.0000000	-0.0250000
50760.0000000	-0.0300000
50761.0000000	-0.0250000
50762.0000000	-0.0200000
50763.0000000	-0.0250000
50764.0000000	-0.0200000
50765.0000000	-0.0250000
50766.0000000	-0.0200000
50767.0000000	-0.0250000
50768.0000000	-0.0150000
50769.0000000	-0.0200000
50770.0000000	-0.0200000
50771.0000000	-0.0200000
50772.0000000	-0.0150000
50773.0000000	-0.0100000
50774.0000000	-0.0150000
50775.0000000	-0.0150000
50776.0000000	-0.0150000
50777.0000000	-0.0150000
50778.0000000	-0.0100000
50779.0000000	-0.0100000
50780.0000000	-0.0100000
50781.0000000	-0.0100000
50782.0000000	-0.0100000
50783.0000000	-0.0150000
50784.0000000	-0.0100000
50785.0000000	-0.0100000
50786.0000000	-0.0050000

50787.0000000	-0.0050000
50788.0000000	-0.0050000
50789.0000000	-0.0100000
50790.0000000	0.0000000
50791.0000000	-0.0050000
50792.0000000	0.0000000
50793.0000000	0.0000000
50794.0000000	-0.0050000
50795.0000000	0.0000000
50796.0000000	0.0050000
50797.0000000	0.0000000
50798.0000000	-0.0050000
50799.0000000	0.0000000
50800.0000000	0.0050000
50801.0000000	0.0050000
50802.0000000	0.0000000
50803.0000000	0.0050000
50804.0000000	0.0050000
50805.0000000	0.0050000
50806.0000000	0.0050000
50807.0000000	0.0000000
50808.0000000	0.0050000
50809.0000000	0.0066667
50810.0000000	0.0100000
50811.0000000	0.0100000
50812.0000000	0.0100000
50813.0000000	0.0100000
50814.0000000	0.0100000
50815.0000000	0.0100000
50816.0000000	0.0100000
50817.0000000	0.0050000
50818.0000000	0.0100000
50819.0000000	0.0100000
50820.0000000	0.0100000
50821.0000000	0.0100000
50822.0000000	0.0150000
50823.0000000	0.0100000
50824.0000000	0.0050000
50825.0000000	0.0100000
50826.0000000	0.0100000
50827.0000000	0.0150000
50828.0000000	0.0150000
50829.0000000	0.0200000
50830.0000000	0.0200000
50831.0000000	0.0150000
50832.0000000	0.0150000
50833.0000000	0.0200000
50834.0000000	0.0150000
50835.0000000	0.0200000
50836.0000000	0.0150000
50837.0000000	0.0150000
50838.0000000	0.0150000
50839.0000000	0.0200000
50840.0000000	0.0200000
50841.0000000	0.0150000
50842.0000000	0.0150000
50843.0000000	0.0200000

50844.0000000	0.0200000
50845.0000000	0.0150000
50846.0000000	0.0150000
50847.0000000	0.0150000
50848.0000000	0.0150000
50849.0000000	0.0100000
50850.0000000	0.0100000
50851.0000000	0.0200000
50852.0000000	0.0100000
50853.0000000	0.0100000
50854.0000000	0.0050000
50855.0000000	0.0100000
50856.0000000	0.0050000
50857.0000000	0.0100000
50858.0000000	0.0050000
50859.0000000	0.0050000
50860.0000000	0.0050000
50861.0000000	0.0050000
50862.0000000	0.0050000
50863.0000000	0.0000000
50864.0000000	0.0050000
50865.0000000	0.0000000
50866.0000000	0.0050000
50867.0000000	0.0050000
50868.0000000	0.0050000
50869.0000000	0.0050000
50870.0000000	0.0033333
50871.0000000	0.0000000
50872.0000000	0.0000000
50873.0000000	0.0050000
50874.0000000	0.0050000
50875.0000000	0.0050000
50876.0000000	0.0000000
50877.0000000	0.0000000
50878.0000000	0.0050000
50879.0000000	0.0000000
50880.0000000	0.0100000
50881.0000000	0.0000000
50882.0000000	0.0050000
50883.0000000	0.0050000
50884.0000000	0.0050000
50885.0000000	0.0050000
50886.0000000	0.0050000
50887.0000000	0.0000000
50888.0000000	0.0050000
50889.0000000	0.0100000
50890.0000000	0.0050000
50891.0000000	0.0050000
50892.0000000	0.0050000
50893.0000000	0.0050000
50894.0000000	0.0050000
50895.0000000	0.0100000
50896.0000000	0.0100000
50897.0000000	0.0050000
50898.0000000	0.0000000
50899.0000000	0.0050000
50900.0000000	0.0133333

50901.0000000	0.0050000
50902.0000000	0.0050000
50903.0000000	0.0000000
50904.0000000	0.0050000
50905.0000000	0.0050000
50906.0000000	0.0050000
50907.0000000	0.0050000
50908.0000000	-0.0050000
50909.0000000	0.0000000
50910.0000000	0.0000000
50911.0000000	-0.0100000
50912.0000000	0.0000000
50913.0000000	-0.0050000
50914.0000000	-0.0050000
50915.0000000	-0.0050000
50916.0000000	-0.0050000
50917.0000000	-0.0050000
50918.0000000	-0.0050000
50919.0000000	-0.0050000
50920.0000000	-0.0050000
50921.0000000	-0.0100000
50922.0000000	-0.0050000
50923.0000000	-0.0050000
50924.0000000	-0.0100000
50925.0000000	-0.0150000
50926.0000000	-0.0100000
50927.0000000	-0.0100000
50928.0000000	-0.0100000
50929.0000000	-0.0100000
50930.0000000	-0.0133333
50931.0000000	-0.0100000
50932.0000000	-0.0100000
50933.0000000	-0.0150000
50934.0000000	-0.0150000
50935.0000000	-0.0100000
50936.0000000	-0.0100000
50937.0000000	-0.0100000
50938.0000000	-0.0150000
50939.0000000	-0.0150000
50940.0000000	-0.0150000
50941.0000000	-0.0150000
50942.0000000	-0.0150000
50943.0000000	-0.0150000
50944.0000000	-0.0150000
50945.0000000	-0.0150000
50946.0000000	-0.0100000
50947.0000000	-0.0100000
50948.0000000	-0.0150000
50949.0000000	-0.0150000
50950.0000000	-0.0150000
50951.0000000	-0.0100000
50952.0000000	-0.0200000
50953.0000000	-0.0250000
50954.0000000	-0.0200000
50955.0000000	-0.0150000
50956.0000000	-0.0200000
50957.0000000	-0.0200000

50958.0000000	-0.0150000
50959.0000000	-0.0200000
50960.0000000	-0.0233333
50961.0000000	-0.0200000
50962.0000000	-0.0200000
50963.0000000	-0.0150000
50964.0000000	-0.0200000
50965.0000000	-0.0200000
50966.0000000	-0.0200000
50967.0000000	-0.0200000
50968.0000000	-0.0150000
50969.0000000	-0.0250000
50970.0000000	-0.0200000
50971.0000000	-0.0150000
50972.0000000	-0.0200000
50973.0000000	-0.0200000
50974.0000000	-0.0200000
50975.0000000	-0.0150000
50976.0000000	-0.0200000
50977.0000000	-0.0250000
50978.0000000	-0.0200000
50979.0000000	-0.0200000
50980.0000000	-0.0200000
50981.0000000	-0.0200000
50982.0000000	-0.0150000
50983.0000000	-0.0150000
50984.0000000	-0.0200000
50985.0000000	-0.0200000
50986.0000000	-0.0200000
50987.0000000	-0.0150000
50988.0000000	-0.0200000
50989.0000000	-0.0150000
50990.0000000	-0.0150000
50991.0000000	-0.0133333
50992.0000000	-0.0150000
50993.0000000	-0.0150000
50994.0000000	-0.0100000
50995.0000000	-0.0100000
50996.0000000	-0.0150000
50997.0000000	-0.0100000
50998.0000000	-0.0100000
50999.0000000	-0.0150000
51000.0000000	-0.0100000
51001.0000000	-0.0200000
51002.0000000	-0.0150000
51003.0000000	-0.0100000
51004.0000000	-0.0150000
51005.0000000	-0.0150000
51006.0000000	-0.0150000
51007.0000000	-0.0100000
51008.0000000	-0.0100000
51009.0000000	-0.0100000
51010.0000000	-0.0100000
51011.0000000	-0.0050000
51012.0000000	-0.0100000
51013.0000000	-0.0100000
51014.0000000	-0.0100000

51015.0000000	-0.0100000
51016.0000000	-0.0050000
51017.0000000	-0.0050000
51018.0000000	-0.0100000
51019.0000000	-0.0050000
51020.0000000	-0.0100000
51021.0000000	-0.0066667
51022.0000000	-0.0050000
51023.0000000	0.0000000
51024.0000000	0.0000000
51025.0000000	-0.0100000
51026.0000000	-0.0100000
51027.0000000	0.0000000
51028.0000000	-0.0050000
51029.0000000	-0.0050000
51030.0000000	0.0000000
51031.0000000	0.0000000
51032.0000000	0.0000000
51033.0000000	0.0050000
51034.0000000	0.0050000
51035.0000000	0.0050000
51036.0000000	0.0050000
51037.0000000	0.0050000
51038.0000000	0.0050000
51039.0000000	0.0050000
51040.0000000	0.0050000
51041.0000000	0.0100000
51042.0000000	0.0150000
51043.0000000	0.0150000
51044.0000000	0.0150000
51045.0000000	0.0150000
51046.0000000	0.0200000
51047.0000000	0.0150000
51048.0000000	0.0200000
51049.0000000	0.0200000
51050.0000000	0.0250000
51051.0000000	0.0200000
51052.0000000	0.0200000
51053.0000000	0.0250000
51054.0000000	0.0250000
51055.0000000	0.0250000
51056.0000000	0.0250000
51057.0000000	0.0300000
51058.0000000	0.0250000
51059.0000000	0.0250000
51060.0000000	0.0300000
51061.0000000	0.0250000
51062.0000000	0.0250000
51063.0000000	0.0250000
51064.0000000	0.0300000
51065.0000000	0.0300000
51066.0000000	0.0250000
51067.0000000	0.0300000
51068.0000000	0.0300000
51069.0000000	0.0350000
51070.0000000	0.0300000
51071.0000000	0.0300000

51072.0000000	0.0350000
51073.0000000	0.0350000
51074.0000000	0.0300000
51075.0000000	0.0350000
51076.0000000	0.0250000
51077.0000000	0.0250000
51078.0000000	0.0300000
51079.0000000	0.0300000
51080.0000000	0.0250000
51081.0000000	0.0300000
51082.0000000	0.0300000
51083.0000000	0.0300000
51084.0000000	0.0300000
51085.0000000	0.0300000
51086.0000000	0.0350000
51087.0000000	0.0300000
51088.0000000	0.0350000
51089.0000000	0.0350000
51090.0000000	0.0300000
51091.0000000	0.0300000
51092.0000000	0.0350000
51093.0000000	0.0300000
51094.0000000	0.0350000
51095.0000000	0.0300000
51096.0000000	0.0350000
51097.0000000	0.0300000
51098.0000000	0.0300000
51099.0000000	0.0300000
51100.0000000	0.0300000
51101.0000000	0.0300000
51102.0000000	0.0350000
51103.0000000	0.0350000
51104.0000000	0.0300000
51105.0000000	0.0350000
51106.0000000	0.0300000
51107.0000000	0.0350000
51108.0000000	0.0300000
51109.0000000	0.0250000
51110.0000000	0.0300000
51111.0000000	0.0300000
51112.0000000	0.0333333
51113.0000000	0.0300000
51114.0000000	0.0300000
51115.0000000	0.0300000
51116.0000000	0.0300000
51117.0000000	0.0300000
51118.0000000	0.0250000
51119.0000000	0.0250000
51120.0000000	0.0250000
51121.0000000	0.0250000
51122.0000000	0.0250000
51123.0000000	0.0250000
51124.0000000	0.0250000
51125.0000000	0.0250000
51126.0000000	0.0200000
51127.0000000	0.0250000
51128.0000000	0.0250000

51129.0000000	0.0250000
51130.0000000	0.0250000
51131.0000000	0.0250000
51132.0000000	0.0250000
51133.0000000	0.0300000
51134.0000000	0.0250000
51135.0000000	0.0300000
51136.0000000	0.0300000
51137.0000000	0.0300000
51138.0000000	0.0250000
51139.0000000	0.0300000
51140.0000000	0.0300000
51141.0000000	0.0300000
51142.0000000	0.0266667
51143.0000000	0.0300000
51144.0000000	0.0250000
51145.0000000	0.0250000
51146.0000000	0.0300000
51147.0000000	0.0300000
51148.0000000	0.0300000
51149.0000000	0.0300000
51150.0000000	0.0300000
51151.0000000	0.0250000
51152.0000000	0.0300000
51153.0000000	0.0250000
51154.0000000	0.0300000
51155.0000000	0.0300000
51156.0000000	0.0300000
51157.0000000	0.0300000
51158.0000000	0.0300000
51159.0000000	0.0300000
51160.0000000	0.0350000
51161.0000000	0.0350000
51162.0000000	0.0350000
51163.0000000	0.0300000
51164.0000000	0.0400000
51165.0000000	0.0350000
51166.0000000	0.0300000
51167.0000000	0.0350000
51168.0000000	0.0350000
51169.0000000	0.0350000
51170.0000000	0.0350000
51171.0000000	0.0366667
51172.0000000	0.0350000
51173.0000000	0.0400000
51174.0000000	0.0350000
51175.0000000	0.0350000
51176.0000000	0.0350000
51177.0000000	0.0350000
51178.0000000	0.0350000
51179.0000000	0.0350000
51180.0000000	0.0400000
51181.0000000	0.0400000
51182.0000000	0.0400000
51183.0000000	0.0400000
51184.0000000	0.0400000
51185.0000000	0.0350000

51186.0000000	0.0350000
51187.0000000	0.0400000
51188.0000000	0.0350000
51189.0000000	0.0350000
51190.0000000	0.0400000
51191.0000000	0.0350000
51192.0000000	0.0350000
51193.0000000	0.0400000
51194.0000000	0.0350000
51195.0000000	0.0400000
51196.0000000	0.0400000
51197.0000000	0.0400000
51198.0000000	0.0400000
51199.0000000	0.0400000
51200.0000000	0.0350000
51201.0000000	0.0350000
51202.0000000	0.0400000
51203.0000000	0.0400000
51204.0000000	0.0400000
51205.0000000	0.0350000
51206.0000000	0.0350000
51207.0000000	0.0450000
51208.0000000	0.0350000
51209.0000000	0.0350000
51210.0000000	0.0350000
51211.0000000	0.0400000
51212.0000000	0.0400000
51213.0000000	0.0400000
51214.0000000	0.0350000
51215.0000000	0.0400000
51216.0000000	0.0350000
51217.0000000	0.0350000
51218.0000000	0.0350000
51219.0000000	0.0350000
51220.0000000	0.0350000
51221.0000000	0.0350000
51222.0000000	0.0300000
51223.0000000	0.0300000
51224.0000000	0.0300000
51225.0000000	0.0250000
51226.0000000	0.0250000
51227.0000000	0.0300000
51228.0000000	0.0300000
51229.0000000	0.0300000
51230.0000000	0.0350000
51231.0000000	0.0250000
51232.0000000	0.0300000
51233.0000000	0.0266667
51234.0000000	0.0250000
51235.0000000	0.0300000
51236.0000000	0.0250000
51237.0000000	0.0250000
51238.0000000	0.0250000
51239.0000000	0.0250000
51240.0000000	0.0200000
51241.0000000	0.0300000
51242.0000000	0.0250000

51243.0000000	0.0250000
51244.0000000	0.0250000
51245.0000000	0.0250000
51246.0000000	0.0250000
51247.0000000	0.0250000
51248.0000000	0.0300000
51249.0000000	0.0250000
51250.0000000	0.0250000
51251.0000000	0.0250000
51252.0000000	0.0200000
51253.0000000	0.0250000
51254.0000000	0.0250000
51255.0000000	0.0250000
51256.0000000	0.0200000
51257.0000000	0.0250000
51258.0000000	0.0250000
51259.0000000	0.0250000
51260.0000000	0.0300000
51261.0000000	0.0250000
51262.0000000	0.0233333
51263.0000000	0.0300000
51264.0000000	0.0250000
51265.0000000	0.0200000
51266.0000000	0.0250000
51267.0000000	0.0250000
51268.0000000	0.0250000
51269.0000000	0.0300000
51270.0000000	0.0300000
51271.0000000	0.0300000
51272.0000000	0.0300000
51273.0000000	0.0300000
51274.0000000	0.0300000
51275.0000000	0.0300000
51276.0000000	0.0350000
51277.0000000	0.0350000
51278.0000000	0.0300000
51279.0000000	0.0400000
51280.0000000	0.0400000
51281.0000000	0.0400000
51282.0000000	0.0400000
51283.0000000	0.0400000
51284.0000000	0.0400000
51285.0000000	0.0400000
51286.0000000	0.0450000
51287.0000000	0.0450000
51288.0000000	0.0450000
51289.0000000	0.0450000
51290.0000000	0.0450000
51291.0000000	0.0450000
51292.0000000	0.0400000
51293.0000000	0.0450000
51294.0000000	0.0450000
51295.0000000	0.0450000
51296.0000000	0.0500000
51297.0000000	0.0400000
51298.0000000	0.0450000
51299.0000000	0.0450000

51300.0000000	0.0400000
51301.0000000	0.0450000
51302.0000000	0.0450000
51303.0000000	0.0450000
51304.0000000	0.0450000
51305.0000000	0.0450000
51306.0000000	0.0450000
51307.0000000	0.0450000
51308.0000000	0.0450000
51309.0000000	0.0450000
51310.0000000	0.0350000
51311.0000000	0.0450000
51312.0000000	0.0500000
51313.0000000	0.0400000
51314.0000000	0.0400000
51315.0000000	0.0500000
51316.0000000	0.0500000
51317.0000000	0.0450000
51318.0000000	0.0450000
51319.0000000	0.0450000
51320.0000000	0.0450000
51321.0000000	0.0400000
51322.0000000	0.0400000
51323.0000000	0.0500000
51324.0000000	0.0450000
51325.0000000	0.0450000
51326.0000000	0.0450000
51327.0000000	0.0500000
51328.0000000	0.0450000
51329.0000000	0.0500000
51330.0000000	0.0500000
51331.0000000	0.0500000
51332.0000000	0.0500000
51333.0000000	0.0450000
51334.0000000	0.0550000
51335.0000000	0.0550000
51336.0000000	0.0500000
51337.0000000	0.0550000
51338.0000000	0.0550000
51339.0000000	0.0550000
51340.0000000	0.0550000
51341.0000000	0.0550000
51342.0000000	0.0550000
51343.0000000	0.0550000
51344.0000000	0.0550000
51345.0000000	0.0500000
51346.0000000	0.0500000
51347.0000000	0.0500000
51348.0000000	0.0500000
51349.0000000	0.0500000
51350.0000000	0.0550000
51351.0000000	0.0600000
51352.0000000	0.0550000
51353.0000000	0.0533333
51354.0000000	0.0600000
51355.0000000	0.0550000
51356.0000000	0.0550000

51357.0000000	0.0550000
51358.0000000	0.0550000
51359.0000000	0.0550000
51360.0000000	0.0600000
51361.0000000	0.0650000
51362.0000000	0.0600000
51363.0000000	0.0600000
51364.0000000	0.0600000
51365.0000000	0.0600000
51366.0000000	0.0600000
51367.0000000	0.0600000
51368.0000000	0.0600000
51369.0000000	0.0650000
51370.0000000	0.0650000
51371.0000000	0.0650000
51372.0000000	0.0650000
51373.0000000	0.0700000
51374.0000000	0.0700000
51375.0000000	0.0700000
51376.0000000	0.0650000
51377.0000000	0.0700000
51378.0000000	0.0700000
51379.0000000	0.0700000
51380.0000000	0.0700000
51381.0000000	0.0750000
51382.0000000	0.0700000
51383.0000000	0.0766667
51384.0000000	0.0750000
51385.0000000	0.0750000
51386.0000000	0.0800000
51387.0000000	0.0800000
51388.0000000	0.0800000
51389.0000000	0.0800000
51390.0000000	0.0800000
51391.0000000	0.0800000
51392.0000000	0.0800000
51393.0000000	0.0800000
51394.0000000	0.0850000
51395.0000000	0.0850000
51396.0000000	0.0900000
51397.0000000	0.0900000
51398.0000000	0.0900000
51399.0000000	0.0950000
51400.0000000	0.0900000
51401.0000000	0.0950000
51402.0000000	0.0950000
51403.0000000	0.1000000
51404.0000000	0.1000000
51405.0000000	0.1000000
51406.0000000	0.1000000
51407.0000000	0.1050000
51408.0000000	0.0950000
51409.0000000	0.1000000
51410.0000000	0.1100000
51411.0000000	0.1050000
51412.0000000	0.1000000
51413.0000000	0.1000000

51414.0000000	0.1050000
51415.0000000	0.1050000
51416.0000000	0.1050000
51417.0000000	0.1050000
51418.0000000	0.1000000
51419.0000000	0.1050000
51420.0000000	0.1000000
51421.0000000	0.1050000
51422.0000000	0.1050000
51423.0000000	0.1050000
51424.0000000	0.1050000
51425.0000000	0.1050000
51426.0000000	0.1050000
51427.0000000	0.1050000
51428.0000000	0.1000000
51429.0000000	0.1000000
51430.0000000	0.1050000
51431.0000000	0.1050000
51432.0000000	0.1050000
51433.0000000	0.1050000
51434.0000000	0.1050000
51435.0000000	0.1050000
51436.0000000	0.1050000
51437.0000000	0.1000000
51438.0000000	0.1050000
51439.0000000	0.1050000
51440.0000000	0.1000000
51441.0000000	0.1000000
51442.0000000	0.1000000
51443.0000000	0.1033333
51444.0000000	0.0950000
51445.0000000	0.1000000
51446.0000000	0.1000000
51447.0000000	0.1000000
51448.0000000	0.1000000
51449.0000000	0.1000000
51450.0000000	0.1000000
51451.0000000	0.1000000
51452.0000000	0.1050000
51453.0000000	0.1000000
51454.0000000	0.1000000
51455.0000000	0.1000000
51456.0000000	0.1050000
51457.0000000	0.1000000
51458.0000000	0.1000000
51459.0000000	0.1000000
51460.0000000	0.1000000
51461.0000000	0.1050000
51462.0000000	0.1000000
51463.0000000	0.1000000
51464.0000000	0.1000000
51465.0000000	0.1050000
51466.0000000	0.1000000
51467.0000000	0.1000000
51468.0000000	0.1000000
51469.0000000	0.1050000
51470.0000000	0.1000000

51471.0000000	0.1000000
51472.0000000	0.1050000
51473.0000000	0.1000000
51474.0000000	0.1033333
51475.0000000	0.0950000
51476.0000000	0.1000000
51477.0000000	0.1000000
51478.0000000	0.1000000
51479.0000000	0.0950000
51480.0000000	0.1000000
51481.0000000	0.1000000
51482.0000000	0.1000000
51483.0000000	0.1050000
51484.0000000	0.1050000
51485.0000000	0.1050000
51486.0000000	0.1050000
51487.0000000	0.1050000
51488.0000000	0.1000000
51489.0000000	0.1000000
51490.0000000	0.1000000
51491.0000000	0.1050000
51492.0000000	0.1050000
51493.0000000	0.1050000
51494.0000000	0.1000000
51495.0000000	0.1050000
51496.0000000	0.1050000
51497.0000000	0.1100000
51498.0000000	0.1100000
51499.0000000	0.1100000
51500.0000000	0.1150000
51501.0000000	0.1150000
51502.0000000	0.1100000
51503.0000000	0.1100000
51504.0000000	0.1100000
51505.0000000	0.1150000
51506.0000000	0.1150000
51507.0000000	0.1200000
51508.0000000	0.1150000
51509.0000000	0.1150000
51510.0000000	0.1200000
51511.0000000	0.1150000
51512.0000000	0.1200000
51513.0000000	0.1250000
51514.0000000	0.1300000
51515.0000000	0.1200000
51516.0000000	0.1200000
51517.0000000	0.1250000
51518.0000000	0.1250000
51519.0000000	0.1200000
51520.0000000	0.1250000
51521.0000000	0.1250000
51522.0000000	0.1250000
51523.0000000	0.1250000
51524.0000000	0.1300000
51525.0000000	0.1250000
51526.0000000	0.1250000
51527.0000000	0.1250000

51528.0000000	0.1200000
51529.0000000	0.1200000
51530.0000000	0.1200000
51531.0000000	0.1200000
51532.0000000	0.1300000
51533.0000000	0.1150000
51534.0000000	0.1266667
51535.0000000	0.1250000
51536.0000000	0.1300000
51537.0000000	0.1300000
51538.0000000	0.1200000
51539.0000000	0.1250000
51540.0000000	0.1250000
51541.0000000	0.1250000
51542.0000000	0.1250000
51543.0000000	0.1300000
51544.0000000	0.1300000
51545.0000000	0.1250000
51546.0000000	0.1200000
51547.0000000	0.1250000
51548.0000000	0.1300000
51549.0000000	0.1250000
51550.0000000	0.1250000
51551.0000000	0.1300000
51552.0000000	0.1250000
51553.0000000	0.1250000
51554.0000000	0.1300000
51555.0000000	0.1250000
51556.0000000	0.1200000
51557.0000000	0.1200000
51558.0000000	0.1250000
51559.0000000	0.1250000
51560.0000000	0.1200000
51561.0000000	0.1200000
51562.0000000	0.1250000
51563.0000000	0.1200000
51564.0000000	0.1233333
51565.0000000	0.1250000
51566.0000000	0.1200000
51567.0000000	0.1200000
51568.0000000	0.1150000
51569.0000000	0.1200000
51570.0000000	0.1150000
51571.0000000	0.1200000
51572.0000000	0.1150000
51573.0000000	0.1150000
51574.0000000	0.1150000
51575.0000000	0.1100000
51576.0000000	0.1150000
51577.0000000	0.1150000
51578.0000000	0.1100000
51579.0000000	0.1150000
51580.0000000	0.1100000
51581.0000000	0.1100000
51582.0000000	0.1150000
51583.0000000	0.1100000
51584.0000000	0.1100000

51585.0000000	0.1100000
51586.0000000	0.1100000
51587.0000000	0.1050000
51588.0000000	0.1150000
51589.0000000	0.1050000
51590.0000000	0.1050000
51591.0000000	0.1050000
51592.0000000	0.1050000
51593.0000000	0.1050000
51594.0000000	0.1000000
51595.0000000	0.1033333
51596.0000000	0.1000000
51597.0000000	0.1000000
51598.0000000	0.1000000
51599.0000000	0.1000000
51600.0000000	0.1000000
51601.0000000	0.1000000
51602.0000000	0.1050000
51603.0000000	0.0950000
51604.0000000	0.0950000
51605.0000000	0.0950000
51606.0000000	0.0950000
51607.0000000	0.1000000
51608.0000000	0.1000000
51609.0000000	0.1000000
51610.0000000	0.1000000
51611.0000000	0.0950000
51612.0000000	0.1000000
51613.0000000	0.1000000
51614.0000000	0.1000000
51615.0000000	0.1000000
51616.0000000	0.1000000
51617.0000000	0.1000000
51618.0000000	0.1000000
51619.0000000	0.1100000
51620.0000000	0.1050000
51621.0000000	0.1050000
51622.0000000	0.1050000
51623.0000000	0.1100000
51624.0000000	0.1100000
51625.0000000	0.1133333
51626.0000000	0.1100000
51627.0000000	0.1150000
51628.0000000	0.1150000
51629.0000000	0.1150000
51630.0000000	0.1100000
51631.0000000	0.1100000
51632.0000000	0.1100000
51633.0000000	0.1150000
51634.0000000	0.1100000
51635.0000000	0.1100000
51636.0000000	0.1100000
51637.0000000	0.1050000
51638.0000000	0.1050000
51639.0000000	0.1100000
51640.0000000	0.1100000
51641.0000000	0.1100000

51642.0000000	0.1050000
51643.0000000	0.1100000
51644.0000000	0.1050000
51645.0000000	0.1050000
51646.0000000	0.1000000
51647.0000000	0.1050000
51648.0000000	0.1000000
51649.0000000	0.1050000
51650.0000000	0.1000000
51651.0000000	0.1000000
51652.0000000	0.0950000
51653.0000000	0.1000000
51654.0000000	0.0966667
51655.0000000	0.0950000
51656.0000000	0.0950000
51657.0000000	0.0950000
51658.0000000	0.0950000
51659.0000000	0.1000000
51660.0000000	0.0950000
51661.0000000	0.1000000
51662.0000000	0.1000000
51663.0000000	0.1000000
51664.0000000	0.1000000
51665.0000000	0.1000000
51666.0000000	0.1000000
51667.0000000	0.0950000
51668.0000000	0.0950000
51669.0000000	0.1050000
51670.0000000	0.1050000
51671.0000000	0.1000000
51672.0000000	0.1050000
51673.0000000	0.1050000
51674.0000000	0.1050000
51675.0000000	0.1050000
51676.0000000	0.1150000
51677.0000000	0.1050000
51678.0000000	0.1100000
51679.0000000	0.1100000
51680.0000000	0.1050000
51681.0000000	0.1050000
51682.0000000	0.1100000
51683.0000000	0.1100000
51684.0000000	0.1050000
51685.0000000	0.1066667
51686.0000000	0.1100000
51687.0000000	0.1100000
51688.0000000	0.1050000
51689.0000000	0.1100000
51690.0000000	0.1100000
51691.0000000	0.1150000
51692.0000000	0.1100000
51693.0000000	0.1150000
51694.0000000	0.1150000
51695.0000000	0.1050000
51696.0000000	0.1150000
51697.0000000	0.1200000
51698.0000000	0.1150000

51699.0000000	0.1200000
51700.0000000	0.1250000
51701.0000000	0.1150000
51702.0000000	0.1250000
51703.0000000	0.1250000
51704.0000000	0.1150000
51705.0000000	0.1200000
51706.0000000	0.1200000
51707.0000000	0.1150000
51708.0000000	0.1100000
51709.0000000	0.1150000
51710.0000000	0.1100000
51711.0000000	0.1150000
51712.0000000	0.1150000
51713.0000000	0.1150000
51714.0000000	0.1100000
51715.0000000	0.1100000
51716.0000000	0.1100000
51717.0000000	0.1050000
51718.0000000	0.1050000
51719.0000000	0.1050000
51720.0000000	0.1100000
51721.0000000	0.1050000
51722.0000000	0.1100000
51723.0000000	0.1050000
51724.0000000	0.1050000
51725.0000000	0.1050000
51726.0000000	0.1050000
51727.0000000	0.1050000
51728.0000000	0.1050000
51729.0000000	0.1050000
51730.0000000	0.1050000
51731.0000000	0.1000000
51732.0000000	0.1000000
51733.0000000	0.1050000
51734.0000000	0.1000000
51735.0000000	0.1000000
51736.0000000	0.1000000
51737.0000000	0.1000000
51738.0000000	0.1000000
51739.0000000	0.1000000
51740.0000000	0.0950000
51741.0000000	0.1000000
51742.0000000	0.0950000
51743.0000000	0.0950000
51744.0000000	0.1000000
51745.0000000	0.1033333
51746.0000000	0.0950000
51747.0000000	0.1000000
51748.0000000	0.1000000
51749.0000000	0.1000000
51750.0000000	0.1000000
51751.0000000	0.0950000
51752.0000000	0.1000000
51753.0000000	0.1000000
51754.0000000	0.1000000
51755.0000000	0.1000000

51756.0000000	0.1000000
51757.0000000	0.0950000
51758.0000000	0.1000000
51759.0000000	0.0950000
51760.0000000	0.1000000
51761.0000000	0.1000000
51762.0000000	0.1000000
51763.0000000	0.0950000
51764.0000000	0.0950000
51765.0000000	0.0950000
51766.0000000	0.1000000
51767.0000000	0.0950000
51768.0000000	0.0950000
51769.0000000	0.1000000
51770.0000000	0.0900000
51771.0000000	0.0950000
51772.0000000	0.0950000
51773.0000000	0.0950000
51774.0000000	0.0900000
51775.0000000	0.0933333
51776.0000000	0.0950000
51777.0000000	0.0950000
51778.0000000	0.0900000
51779.0000000	0.0850000
51780.0000000	0.0850000
51781.0000000	0.0900000
51782.0000000	0.0900000
51783.0000000	0.0900000
51784.0000000	0.0900000
51785.0000000	0.0900000
51786.0000000	0.0900000
51787.0000000	0.0800000
51788.0000000	0.0800000
51789.0000000	0.0850000
51790.0000000	0.0800000
51791.0000000	0.0800000
51792.0000000	0.0800000
51793.0000000	0.0800000
51794.0000000	0.0750000
51795.0000000	0.0800000
51796.0000000	0.0800000
51797.0000000	0.0800000
51798.0000000	0.0800000
51799.0000000	0.0800000
51800.0000000	0.0800000
51801.0000000	0.0800000
51802.0000000	0.0850000
51803.0000000	0.0800000
51804.0000000	0.0800000
51805.0000000	0.0800000
51806.0000000	0.0833333
51807.0000000	0.0850000
51808.0000000	0.0800000
51809.0000000	0.0850000
51810.0000000	0.0850000
51811.0000000	0.0850000
51812.0000000	0.0900000

51813.0000000	0.0900000
51814.0000000	0.0850000
51815.0000000	0.0850000
51816.0000000	0.0850000
51817.0000000	0.0850000
51818.0000000	0.0850000
51819.0000000	0.0900000
51820.0000000	0.0900000
51821.0000000	0.0850000
51822.0000000	0.0850000
51823.0000000	0.0850000
51824.0000000	0.0850000
51825.0000000	0.0850000
51826.0000000	0.0850000
51827.0000000	0.0900000
51828.0000000	0.0850000
51829.0000000	0.0800000
51830.0000000	0.0850000
51831.0000000	0.0850000
51832.0000000	0.0850000
51833.0000000	0.0850000
51834.0000000	0.0850000
51835.0000000	0.0800000
51836.0000000	0.0800000
51837.0000000	0.0850000
51838.0000000	0.0800000
51839.0000000	0.0800000
51840.0000000	0.0800000
51841.0000000	0.0800000
51842.0000000	0.0800000
51843.0000000	0.0750000
51844.0000000	0.0800000
51845.0000000	0.0750000
51846.0000000	0.0750000
51847.0000000	0.0750000
51848.0000000	0.0750000
51849.0000000	0.0700000
51850.0000000	0.0700000
51851.0000000	0.0700000
51852.0000000	0.0700000
51853.0000000	0.0650000
51854.0000000	0.0700000
51855.0000000	0.0650000
51856.0000000	0.0650000
51857.0000000	0.0650000
51858.0000000	0.0650000
51859.0000000	0.0650000
51860.0000000	0.0650000
51861.0000000	0.0700000
51862.0000000	0.0650000
51863.0000000	0.0650000
51864.0000000	0.0650000
51865.0000000	0.0650000
51866.0000000	0.0666667
51867.0000000	0.0700000
51868.0000000	0.0700000
51869.0000000	0.0650000

51870.0000000	0.0700000
51871.0000000	0.0750000
51872.0000000	0.0700000
51873.0000000	0.0700000
51874.0000000	0.0750000
51875.0000000	0.0700000
51876.0000000	0.0750000
51877.0000000	0.0750000
51878.0000000	0.0750000
51879.0000000	0.0750000
51880.0000000	0.0750000
51881.0000000	0.0750000
51882.0000000	0.0750000
51883.0000000	0.0750000
51884.0000000	0.0750000
51885.0000000	0.0750000
51886.0000000	0.0800000
51887.0000000	0.0750000
51888.0000000	0.0750000
51889.0000000	0.0750000
51890.0000000	0.0800000
51891.0000000	0.0700000
51892.0000000	0.0750000
51893.0000000	0.0750000
51894.0000000	0.0800000
51895.0000000	0.0800000
51896.0000000	0.0866667
51897.0000000	0.0750000
51898.0000000	0.0850000
51899.0000000	0.0800000
51900.0000000	0.0850000
51901.0000000	0.0800000
51902.0000000	0.0900000
51903.0000000	0.0900000
51904.0000000	0.0800000
51905.0000000	0.0850000
51906.0000000	0.0900000
51907.0000000	0.0900000
51908.0000000	0.0850000
51909.0000000	0.0900000
51910.0000000	0.0850000
51911.0000000	0.0900000
51912.0000000	0.0850000
51913.0000000	0.0800000
51914.0000000	0.0850000
51915.0000000	0.0850000
51916.0000000	0.0850000
51917.0000000	0.0800000
51918.0000000	0.0800000
51919.0000000	0.0800000
51920.0000000	0.0750000
51921.0000000	0.0850000
51922.0000000	0.0850000
51923.0000000	0.0800000
51924.0000000	0.0800000
51925.0000000	0.0800000
51926.0000000	0.0800000

51927.0000000	0.0833333
51928.0000000	0.0800000
51929.0000000	0.0850000
51930.0000000	0.0850000
51931.0000000	0.0800000
51932.0000000	0.0800000
51933.0000000	0.0800000
51934.0000000	0.0850000
51935.0000000	0.0800000
51936.0000000	0.0800000
51937.0000000	0.0800000
51938.0000000	0.0850000
51939.0000000	0.0750000
51940.0000000	0.0850000
51941.0000000	0.0850000
51942.0000000	0.0800000
51943.0000000	0.0850000
51944.0000000	0.0850000
51945.0000000	0.0800000
51946.0000000	0.0800000
51947.0000000	0.0800000
51948.0000000	0.0800000
51949.0000000	0.0800000
51950.0000000	0.0800000
51951.0000000	0.0800000
51952.0000000	0.0850000
51953.0000000	0.0750000
51954.0000000	0.0800000
51955.0000000	0.0800000
51956.0000000	0.0800000
51957.0000000	0.0766667
51958.0000000	0.0750000
51959.0000000	0.0850000
51960.0000000	0.0800000
51961.0000000	0.0800000
51962.0000000	0.0800000
51963.0000000	0.0800000
51964.0000000	0.0850000
51965.0000000	0.0850000
51966.0000000	0.0800000
51967.0000000	0.0900000
51968.0000000	0.0850000
51969.0000000	0.0850000
51970.0000000	0.0900000
51971.0000000	0.0900000
51972.0000000	0.0950000
51973.0000000	0.0850000
51974.0000000	0.0950000
51975.0000000	0.1000000
51976.0000000	0.1000000
51977.0000000	0.1000000
51978.0000000	0.1000000
51979.0000000	0.1000000
51980.0000000	0.1000000
51981.0000000	0.1050000
51982.0000000	0.1050000
51983.0000000	0.1050000

51984.0000000	0.1050000
51985.0000000	0.1050000
51986.0000000	0.1050000
51987.0000000	0.1100000
51988.0000000	0.1100000
51989.0000000	0.1050000
51990.0000000	0.1050000
51991.0000000	0.1000000
51992.0000000	0.1050000
51993.0000000	0.1050000
51994.0000000	0.1050000
51995.0000000	0.1000000
51996.0000000	0.1000000
51997.0000000	0.1000000
51998.0000000	0.0950000
51999.0000000	0.1000000
52000.0000000	0.0950000
52001.0000000	0.0950000
52002.0000000	0.0950000
52003.0000000	0.0950000
52004.0000000	0.0950000
52005.0000000	0.0900000
52006.0000000	0.0900000
52007.0000000	0.0900000
52008.0000000	0.0850000
52009.0000000	0.0900000
52010.0000000	0.0900000
52011.0000000	0.0850000
52012.0000000	0.0900000
52013.0000000	0.0850000
52014.0000000	0.0900000
52015.0000000	0.0850000
52016.0000000	0.0900000
52017.0000000	0.0866667
52018.0000000	0.0850000
52019.0000000	0.0900000
52020.0000000	0.0850000
52021.0000000	0.0850000
52022.0000000	0.0900000
52023.0000000	0.0850000
52024.0000000	0.0850000
52025.0000000	0.0850000
52026.0000000	0.0850000
52027.0000000	0.0850000
52028.0000000	0.0850000
52029.0000000	0.0850000
52030.0000000	0.0850000
52031.0000000	0.0850000
52032.0000000	0.0850000
52033.0000000	0.0850000
52034.0000000	0.0850000
52035.0000000	0.0850000
52036.0000000	0.0850000
52037.0000000	0.0850000
52038.0000000	0.0850000
52039.0000000	0.0850000
52040.0000000	0.0850000

52041.0000000	0.0850000
52042.0000000	0.0850000
52043.0000000	0.0800000
52044.0000000	0.0800000
52045.0000000	0.0850000
52046.0000000	0.0850000
52047.0000000	0.0900000
52048.0000000	0.0850000
52049.0000000	0.0800000
52050.0000000	0.0850000
52051.0000000	0.0900000
52052.0000000	0.0850000
52053.0000000	0.0850000
52054.0000000	0.0850000
52055.0000000	0.0850000
52056.0000000	0.0900000
52057.0000000	0.0850000
52058.0000000	0.0900000
52059.0000000	0.0950000
52060.0000000	0.0900000
52061.0000000	0.0900000
52062.0000000	0.0950000
52063.0000000	0.0950000
52064.0000000	0.0950000
52065.0000000	0.0900000
52066.0000000	0.0950000
52067.0000000	0.0950000
52068.0000000	0.0950000
52069.0000000	0.1000000
52070.0000000	0.1000000
52071.0000000	0.1000000
52072.0000000	0.0950000
52073.0000000	0.1000000
52074.0000000	0.0950000
52075.0000000	0.1000000
52076.0000000	0.1000000
52077.0000000	0.1000000
52078.0000000	0.1000000
52079.0000000	0.1000000
52080.0000000	0.1000000
52081.0000000	0.1000000
52082.0000000	0.1000000
52083.0000000	0.1000000
52084.0000000	0.1000000
52085.0000000	0.1000000
52086.0000000	0.1050000
52087.0000000	0.1050000
52088.0000000	0.1050000
52089.0000000	0.1050000
52090.0000000	0.1050000
52091.0000000	0.1050000
52092.0000000	0.1100000
52093.0000000	0.1050000
52094.0000000	0.1050000
52095.0000000	0.1000000
52096.0000000	0.1050000
52097.0000000	0.1050000

52098.0000000	0.1050000
52099.0000000	0.1000000
52100.0000000	0.1000000
52101.0000000	0.1050000
52102.0000000	0.1050000
52103.0000000	0.1050000
52104.0000000	0.1000000
52105.0000000	0.1000000
52106.0000000	0.1050000
52107.0000000	0.1066667
52108.0000000	0.1050000
52109.0000000	0.1050000
52110.0000000	0.1050000
52111.0000000	0.1050000
52112.0000000	0.1050000
52113.0000000	0.1000000
52114.0000000	0.1050000
52115.0000000	0.1000000
52116.0000000	0.1050000
52117.0000000	0.1050000
52118.0000000	0.1050000
52119.0000000	0.1000000
52120.0000000	0.1100000
52121.0000000	0.1050000
52122.0000000	0.1100000
52123.0000000	0.1050000
52124.0000000	0.1100000
52125.0000000	0.1050000
52126.0000000	0.1100000
52127.0000000	0.1100000
52128.0000000	0.1100000
52129.0000000	0.1100000
52130.0000000	0.1150000
52131.0000000	0.1150000
52132.0000000	0.1100000
52133.0000000	0.1150000
52134.0000000	0.1200000
52135.0000000	0.1150000
52136.0000000	0.1250000
52137.0000000	0.1200000
52138.0000000	0.1250000
52139.0000000	0.1200000
52140.0000000	0.1200000
52141.0000000	0.1300000
52142.0000000	0.1250000
52143.0000000	0.1250000
52144.0000000	0.1250000
52145.0000000	0.1250000
52146.0000000	0.1200000
52147.0000000	0.1250000
52148.0000000	0.1250000
52149.0000000	0.1250000
52150.0000000	0.1200000
52151.0000000	0.1200000
52152.0000000	0.1150000
52153.0000000	0.1200000
52154.0000000	0.1200000

52155.0000000	0.1200000
52156.0000000	0.1150000
52157.0000000	0.1200000
52158.0000000	0.1150000
52159.0000000	0.1200000
52160.0000000	0.1150000
52161.0000000	0.1150000
52162.0000000	0.1150000
52163.0000000	0.1150000
52164.0000000	0.1150000
52165.0000000	0.1150000
52166.0000000	0.1050000
52167.0000000	0.1100000
52168.0000000	0.1100000
52169.0000000	0.1100000
52170.0000000	0.1100000
52171.0000000	0.1050000
52172.0000000	0.1050000
52173.0000000	0.1050000
52174.0000000	0.1000000
52175.0000000	0.1000000
52176.0000000	0.1050000
52177.0000000	0.1000000
52178.0000000	0.1000000
52179.0000000	0.1000000
52180.0000000	0.1000000
52181.0000000	0.0950000
52182.0000000	0.0950000
52183.0000000	0.0950000
52184.0000000	0.0900000
52185.0000000	0.0950000
52186.0000000	0.0950000
52187.0000000	0.0850000
52188.0000000	0.0950000
52189.0000000	0.0900000
52190.0000000	0.0850000
52191.0000000	0.0900000
52192.0000000	0.0900000
52193.0000000	0.0900000
52194.0000000	0.0850000
52195.0000000	0.0950000
52196.0000000	0.0900000
52197.0000000	0.0900000
52198.0000000	0.0950000
52199.0000000	0.0933333
52200.0000000	0.0950000
52201.0000000	0.0900000
52202.0000000	0.0950000
52203.0000000	0.0950000
52204.0000000	0.0950000
52205.0000000	0.0950000
52206.0000000	0.0950000
52207.0000000	0.0950000
52208.0000000	0.0900000
52209.0000000	0.0950000
52210.0000000	0.0950000
52211.0000000	0.0900000

52212.0000000	0.0900000
52213.0000000	0.1000000
52214.0000000	0.0950000
52215.0000000	0.1000000
52216.0000000	0.0950000
52217.0000000	0.1000000
52218.0000000	0.0950000
52219.0000000	0.1000000
52220.0000000	0.1000000
52221.0000000	0.0950000
52222.0000000	0.1000000
52223.0000000	0.1050000
52224.0000000	0.1050000
52225.0000000	0.1050000
52226.0000000	0.1050000
52227.0000000	0.1100000
52228.0000000	0.1066667
52229.0000000	0.1100000
52230.0000000	0.1100000
52231.0000000	0.1150000
52232.0000000	0.1150000
52233.0000000	0.1200000
52234.0000000	0.1150000
52235.0000000	0.1150000
52236.0000000	0.1200000
52237.0000000	0.1200000
52238.0000000	0.1250000
52239.0000000	0.1250000
52240.0000000	0.1200000
52241.0000000	0.1250000
52242.0000000	0.1200000
52243.0000000	0.1300000
52244.0000000	0.1250000
52245.0000000	0.1200000
52246.0000000	0.1250000
52247.0000000	0.1250000
52248.0000000	0.1250000
52249.0000000	0.1250000
52250.0000000	0.1300000
52251.0000000	0.1300000
52252.0000000	0.1300000
52253.0000000	0.1250000
52254.0000000	0.1250000
52255.0000000	0.1250000
52256.0000000	0.1250000
52257.0000000	0.1250000
52258.0000000	0.1266667
52259.0000000	0.1250000
52260.0000000	0.1300000
52261.0000000	0.1250000
52262.0000000	0.1300000
52263.0000000	0.1250000
52264.0000000	0.1250000
52265.0000000	0.1200000
52266.0000000	0.1250000
52267.0000000	0.1250000
52268.0000000	0.1250000

52269.0000000	0.1250000
52270.0000000	0.1250000
52271.0000000	0.1250000
52272.0000000	0.1250000
52273.0000000	0.1300000
52274.0000000	0.1250000
52275.0000000	0.1300000
52276.0000000	0.1250000
52277.0000000	0.1250000
52278.0000000	0.1250000
52279.0000000	0.1250000
52280.0000000	0.1250000
52281.0000000	0.1250000
52282.0000000	0.1250000
52283.0000000	0.1250000
52284.0000000	0.1250000
52285.0000000	0.1250000
52286.0000000	0.1250000
52287.0000000	0.1300000
52288.0000000	0.1300000
52289.0000000	0.1333333
52290.0000000	0.1300000
52291.0000000	0.1300000
52292.0000000	0.1250000
52293.0000000	0.1250000
52294.0000000	0.1300000
52295.0000000	0.1300000
52296.0000000	0.1250000
52297.0000000	0.1250000
52298.0000000	0.1250000
52299.0000000	0.1200000
52300.0000000	0.1200000
52301.0000000	0.1200000
52302.0000000	0.1200000
52303.0000000	0.1200000
52304.0000000	0.1200000
52305.0000000	0.1150000
52306.0000000	0.1100000
52307.0000000	0.1100000
52308.0000000	0.1150000
52309.0000000	0.1150000
52310.0000000	0.1150000
52311.0000000	0.1150000
52312.0000000	0.1100000
52313.0000000	0.1100000
52314.0000000	0.1100000
52315.0000000	0.1100000
52316.0000000	0.1100000
52317.0000000	0.1100000
52318.0000000	0.1150000
52319.0000000	0.1133333
52320.0000000	0.1150000
52321.0000000	0.1150000
52322.0000000	0.1150000
52323.0000000	0.1150000
52324.0000000	0.1100000
52325.0000000	0.1150000

52326.0000000	0.1150000
52327.0000000	0.1100000
52328.0000000	0.1100000
52329.0000000	0.1200000
52330.0000000	0.1150000
52331.0000000	0.1200000
52332.0000000	0.1150000
52333.0000000	0.1200000
52334.0000000	0.1150000
52335.0000000	0.1150000
52336.0000000	0.1150000
52337.0000000	0.1200000
52338.0000000	0.1200000
52339.0000000	0.1250000
52340.0000000	0.1200000
52341.0000000	0.1250000
52342.0000000	0.1250000
52343.0000000	0.1200000
52344.0000000	0.1250000
52345.0000000	0.1300000
52346.0000000	0.1250000
52347.0000000	0.1300000
52348.0000000	0.1300000
52349.0000000	0.1300000
52350.0000000	0.1300000
52351.0000000	0.1300000
52352.0000000	0.1350000
52353.0000000	0.1300000
52354.0000000	0.1400000
52355.0000000	0.1400000
52356.0000000	0.1350000
52357.0000000	0.1350000
52358.0000000	0.1400000
52359.0000000	0.1350000
52360.0000000	0.1400000
52361.0000000	0.1400000
52362.0000000	0.1400000
52363.0000000	0.1400000
52364.0000000	0.1350000
52365.0000000	0.1400000
52366.0000000	0.1450000
52367.0000000	0.1400000
52368.0000000	0.1450000
52369.0000000	0.1450000
52370.0000000	0.1400000
52371.0000000	0.1450000
52372.0000000	0.1400000
52373.0000000	0.1400000
52374.0000000	0.1450000
52375.0000000	0.1400000
52376.0000000	0.1450000
52377.0000000	0.1450000
52378.0000000	0.1400000
52379.0000000	0.1466667
52380.0000000	0.1450000
52381.0000000	0.1550000
52382.0000000	0.1450000

52383.0000000	0.1550000
52384.0000000	0.1550000
52385.0000000	0.1550000
52386.0000000	0.1550000
52387.0000000	0.1550000
52388.0000000	0.1550000
52389.0000000	0.1550000
52390.0000000	0.1550000
52391.0000000	0.1600000
52392.0000000	0.1600000
52393.0000000	0.1600000
52394.0000000	0.1650000
52395.0000000	0.1600000
52396.0000000	0.1650000
52397.0000000	0.1600000
52398.0000000	0.1700000
52399.0000000	0.1650000
52400.0000000	0.1700000
52401.0000000	0.1650000
52402.0000000	0.1700000
52403.0000000	0.1750000
52404.0000000	0.1700000
52405.0000000	0.1750000
52406.0000000	0.1750000
52407.0000000	0.1750000
52408.0000000	0.1800000
52409.0000000	0.1800000
52410.0000000	0.1800000
52411.0000000	0.1750000
52412.0000000	0.1750000
52413.0000000	0.1800000
52414.0000000	0.1750000
52415.0000000	0.1750000
52416.0000000	0.1800000
52417.0000000	0.1800000
52418.0000000	0.1800000
52419.0000000	0.1800000
52420.0000000	0.1850000
52421.0000000	0.1850000
52422.0000000	0.1800000
52423.0000000	0.1800000
52424.0000000	0.1850000
52425.0000000	0.1850000
52426.0000000	0.1850000
52427.0000000	0.1800000
52428.0000000	0.1850000
52429.0000000	0.1850000
52430.0000000	0.1750000
52431.0000000	0.1800000
52432.0000000	0.1900000
52433.0000000	0.1850000
52434.0000000	0.1850000
52435.0000000	0.1850000
52436.0000000	0.1850000
52437.0000000	0.1850000
52438.0000000	0.1850000
52439.0000000	0.1850000

52440.0000000	0.1866667
52441.0000000	0.1850000
52442.0000000	0.1850000
52443.0000000	0.1850000
52444.0000000	0.1900000
52445.0000000	0.1800000
52446.0000000	0.1900000
52447.0000000	0.1900000
52448.0000000	0.1850000
52449.0000000	0.1850000
52450.0000000	0.1900000
52451.0000000	0.1900000
52452.0000000	0.1900000
52453.0000000	0.1800000
52454.0000000	0.1900000
52455.0000000	0.1900000
52456.0000000	0.1850000
52457.0000000	0.1900000
52458.0000000	0.1900000
52459.0000000	0.1900000
52460.0000000	0.1850000
52461.0000000	0.1900000
52462.0000000	0.1900000
52463.0000000	0.1850000
52464.0000000	0.1900000
52465.0000000	0.1900000
52466.0000000	0.1950000
52467.0000000	0.1950000
52468.0000000	0.1900000
52469.0000000	0.1950000
52470.0000000	0.1966667
52471.0000000	0.1950000
52472.0000000	0.1950000
52473.0000000	0.1950000
52474.0000000	0.1950000
52475.0000000	0.1900000
52476.0000000	0.1900000
52477.0000000	0.1950000
52478.0000000	0.1950000
52479.0000000	0.1950000
52480.0000000	0.1950000
52481.0000000	0.1950000
52482.0000000	0.1950000
52483.0000000	0.1950000
52484.0000000	0.2000000
52485.0000000	0.1950000
52486.0000000	0.1950000
52487.0000000	0.2000000
52488.0000000	0.1950000
52489.0000000	0.2000000
52490.0000000	0.1950000
52491.0000000	0.2000000
52492.0000000	0.2000000
52493.0000000	0.2000000
52494.0000000	0.2000000
52495.0000000	0.2050000
52496.0000000	0.2000000

52497.0000000	0.2000000
52498.0000000	0.1950000
52499.0000000	0.1950000
52500.0000000	0.2000000
52501.0000000	0.2000000
52502.0000000	0.2050000
52503.0000000	0.2050000
52504.0000000	0.2000000
52505.0000000	0.2050000
52506.0000000	0.2000000
52507.0000000	0.2050000
52508.0000000	0.2050000
52509.0000000	0.2050000
52510.0000000	0.2050000
52511.0000000	0.2050000
52512.0000000	0.2050000
52513.0000000	0.2100000
52514.0000000	0.2000000
52515.0000000	0.2050000
52516.0000000	0.2050000
52517.0000000	0.2000000
52518.0000000	0.2050000
52519.0000000	0.2050000
52520.0000000	0.2050000
52521.0000000	0.2050000
52522.0000000	0.2050000
52523.0000000	0.2050000
52524.0000000	0.2050000
52525.0000000	0.2000000
52526.0000000	0.2050000
52527.0000000	0.2100000
52528.0000000	0.2050000
52529.0000000	0.2050000
52530.0000000	0.2066667
52531.0000000	0.2100000
52532.0000000	0.2050000
52533.0000000	0.2100000
52534.0000000	0.2100000
52535.0000000	0.2100000
52536.0000000	0.2100000
52537.0000000	0.2100000
52538.0000000	0.2100000
52539.0000000	0.2150000
52540.0000000	0.2150000
52541.0000000	0.2100000
52542.0000000	0.2100000
52543.0000000	0.2100000
52544.0000000	0.2100000
52545.0000000	0.2100000
52546.0000000	0.2150000
52547.0000000	0.2150000
52548.0000000	0.2100000
52549.0000000	0.2100000
52550.0000000	0.2100000
52551.0000000	0.2100000
52552.0000000	0.2100000
52553.0000000	0.2150000

52554.0000000	0.2100000
52555.0000000	0.2100000
52556.0000000	0.2050000
52557.0000000	0.2100000
52558.0000000	0.2150000
52559.0000000	0.2100000
52560.0000000	0.2100000
52561.0000000	0.2100000
52562.0000000	0.2100000
52563.0000000	0.2100000
52564.0000000	0.2100000
52565.0000000	0.2150000
52566.0000000	0.2050000
52567.0000000	0.2100000
52568.0000000	0.2150000
52569.0000000	0.2100000
52570.0000000	0.2100000
52571.0000000	0.2050000
52572.0000000	0.2100000
52573.0000000	0.2150000
52574.0000000	0.2100000
52575.0000000	0.2100000
52576.0000000	0.2150000
52577.0000000	0.2100000
52578.0000000	0.2150000
52579.0000000	0.2150000
52580.0000000	0.2100000
52581.0000000	0.2100000
52582.0000000	0.2150000
52583.0000000	0.2150000
52584.0000000	0.2100000
52585.0000000	0.2100000
52586.0000000	0.2150000
52587.0000000	0.2150000
52588.0000000	0.2150000
52589.0000000	0.2100000
52590.0000000	0.2100000
52591.0000000	0.2150000
52592.0000000	0.2150000
52593.0000000	0.2100000
52594.0000000	0.2100000
52595.0000000	0.2150000
52596.0000000	0.2150000
52597.0000000	0.2150000
52598.0000000	0.2150000
52599.0000000	0.2150000
52600.0000000	0.2150000
52601.0000000	0.2100000
52602.0000000	0.2100000
52603.0000000	0.2100000
52604.0000000	0.2150000
52605.0000000	0.2100000
52606.0000000	0.2150000
52607.0000000	0.2200000
52608.0000000	0.2200000
52609.0000000	0.2150000
52610.0000000	0.2200000

52611.0000000	0.2200000
52612.0000000	0.2200000
52613.0000000	0.2200000
52614.0000000	0.2200000
52615.0000000	0.2150000
52616.0000000	0.2250000
52617.0000000	0.2250000
52618.0000000	0.2250000
52619.0000000	0.2250000
52620.0000000	0.2233333
52621.0000000	0.2250000
52622.0000000	0.2250000
52623.0000000	0.2300000
52624.0000000	0.2350000
52625.0000000	0.2250000
52626.0000000	0.2300000
52627.0000000	0.2300000
52628.0000000	0.2300000
52629.0000000	0.2250000
52630.0000000	0.2300000
52631.0000000	0.2300000
52632.0000000	0.2250000
52633.0000000	0.2250000
52634.0000000	0.2300000
52635.0000000	0.2300000
52636.0000000	0.2250000
52637.0000000	0.2250000
52638.0000000	0.2250000
52639.0000000	0.2300000
52640.0000000	0.2250000
52641.0000000	0.2250000
52642.0000000	0.2300000
52643.0000000	0.2250000
52644.0000000	0.2200000
52645.0000000	0.2300000
52646.0000000	0.2250000
52647.0000000	0.2200000
52648.0000000	0.2250000
52649.0000000	0.2250000
52650.0000000	0.2200000
52651.0000000	0.2266667
52652.0000000	0.2300000
52653.0000000	0.2250000
52654.0000000	0.2250000
52655.0000000	0.2250000
52656.0000000	0.2200000
52657.0000000	0.2250000
52658.0000000	0.2300000
52659.0000000	0.2200000
52660.0000000	0.2250000
52661.0000000	0.2250000
52662.0000000	0.2200000
52663.0000000	0.2250000
52664.0000000	0.2200000
52665.0000000	0.2250000
52666.0000000	0.2250000
52667.0000000	0.2200000

52668.0000000	0.2250000
52669.0000000	0.2250000
52670.0000000	0.2250000
52671.0000000	0.2250000
52672.0000000	0.2300000
52673.0000000	0.2250000
52674.0000000	0.2250000
52675.0000000	0.2250000
52676.0000000	0.2250000
52677.0000000	0.2250000
52678.0000000	0.2250000
52679.0000000	0.2300000
52680.0000000	0.2300000
52681.0000000	0.2300000
52682.0000000	0.2300000
52683.0000000	0.2300000
52684.0000000	0.2300000
52685.0000000	0.2350000
52686.0000000	0.2350000
52687.0000000	0.2400000
52688.0000000	0.2400000
52689.0000000	0.2350000
52690.0000000	0.2350000
52691.0000000	0.2400000
52692.0000000	0.2400000
52693.0000000	0.2400000
52694.0000000	0.2400000
52695.0000000	0.2400000
52696.0000000	0.2400000
52697.0000000	0.2400000
52698.0000000	0.2450000
52699.0000000	0.2450000
52700.0000000	0.2450000
52701.0000000	0.2450000
52702.0000000	0.2450000
52703.0000000	0.2450000
52704.0000000	0.2500000
52705.0000000	0.2550000
52706.0000000	0.2450000
52707.0000000	0.2500000
52708.0000000	0.2500000
52709.0000000	0.2500000
52710.0000000	0.2500000
52711.0000000	0.2533333
52712.0000000	0.2500000
52713.0000000	0.2500000
52714.0000000	0.2450000
52715.0000000	0.2500000
52716.0000000	0.2500000
52717.0000000	0.2500000
52718.0000000	0.2500000
52719.0000000	0.2450000
52720.0000000	0.2450000
52721.0000000	0.2450000
52722.0000000	0.2450000
52723.0000000	0.2450000
52724.0000000	0.2400000

52725.0000000	0.2450000
52726.0000000	0.2450000
52727.0000000	0.2450000
52728.0000000	0.2400000
52729.0000000	0.2450000
52730.0000000	0.2400000
52731.0000000	0.2500000
52732.0000000	0.2400000
52733.0000000	0.2350000
52734.0000000	0.2450000
52735.0000000	0.2450000
52736.0000000	0.2450000
52737.0000000	0.2400000
52738.0000000	0.2450000
52739.0000000	0.2450000
52740.0000000	0.2400000
52741.0000000	0.2433333
52742.0000000	0.2450000
52743.0000000	0.2500000
52744.0000000	0.2450000
52745.0000000	0.2450000
52746.0000000	0.2500000
52747.0000000	0.2500000
52748.0000000	0.2500000
52749.0000000	0.2500000
52750.0000000	0.2550000
52751.0000000	0.2500000
52752.0000000	0.2550000
52753.0000000	0.2600000
52754.0000000	0.2500000
52755.0000000	0.2600000
52756.0000000	0.2600000
52757.0000000	0.2600000
52758.0000000	0.2550000
52759.0000000	0.2600000
52760.0000000	0.2600000
52761.0000000	0.2650000
52762.0000000	0.2600000
52763.0000000	0.2600000
52764.0000000	0.2600000
52765.0000000	0.2650000
52766.0000000	0.2600000
52767.0000000	0.2550000
52768.0000000	0.2650000
52769.0000000	0.2650000
52770.0000000	0.2600000
52771.0000000	0.2600000
52772.0000000	0.2633333
52773.0000000	0.2600000
52774.0000000	0.2650000
52775.0000000	0.2650000
52776.0000000	0.2600000
52777.0000000	0.2550000
52778.0000000	0.2600000
52779.0000000	0.2650000
52780.0000000	0.2600000
52781.0000000	0.2600000

52782.0000000	0.2550000
52783.0000000	0.2600000
52784.0000000	0.2600000
52785.0000000	0.2500000
52786.0000000	0.2550000
52787.0000000	0.2600000
52788.0000000	0.2550000
52789.0000000	0.2600000
52790.0000000	0.2550000
52791.0000000	0.2550000
52792.0000000	0.2550000
52793.0000000	0.2500000
52794.0000000	0.2500000
52795.0000000	0.2550000
52796.0000000	0.2550000
52797.0000000	0.2500000
52798.0000000	0.2500000
52799.0000000	0.2500000
52800.0000000	0.2550000
52801.0000000	0.2550000
52802.0000000	0.2533333
52803.0000000	0.2500000
52804.0000000	0.2550000
52805.0000000	0.2500000
52806.0000000	0.2500000
52807.0000000	0.2500000
52808.0000000	0.2500000
52809.0000000	0.2550000
52810.0000000	0.2550000
52811.0000000	0.2500000
52812.0000000	0.2550000
52813.0000000	0.2500000
52814.0000000	0.2450000
52815.0000000	0.2450000
52816.0000000	0.2450000
52817.0000000	0.2450000
52818.0000000	0.2500000
52819.0000000	0.2400000
52820.0000000	0.2450000
52821.0000000	0.2450000
52822.0000000	0.2400000
52823.0000000	0.2400000
52824.0000000	0.2450000
52825.0000000	0.2450000
52826.0000000	0.2400000
52827.0000000	0.2400000
52828.0000000	0.2450000
52829.0000000	0.2450000
52830.0000000	0.2400000
52831.0000000	0.2450000
52832.0000000	0.2466667
52833.0000000	0.2400000
52834.0000000	0.2400000
52835.0000000	0.2400000
52836.0000000	0.2400000
52837.0000000	0.2400000
52838.0000000	0.2400000

52839.0000000	0.2400000
52840.0000000	0.2350000
52841.0000000	0.2350000
52842.0000000	0.2400000
52843.0000000	0.2350000
52844.0000000	0.2350000
52845.0000000	0.2300000
52846.0000000	0.2300000
52847.0000000	0.2400000
52848.0000000	0.2350000
52849.0000000	0.2350000
52850.0000000	0.2350000
52851.0000000	0.2400000
52852.0000000	0.2400000
52853.0000000	0.2400000
52854.0000000	0.2350000
52855.0000000	0.2400000
52856.0000000	0.2350000
52857.0000000	0.2400000
52858.0000000	0.2400000
52859.0000000	0.2450000
52860.0000000	0.2400000
52861.0000000	0.2400000
52862.0000000	0.2400000
52863.0000000	0.2400000
52864.0000000	0.2400000
52865.0000000	0.2400000
52866.0000000	0.2350000
52867.0000000	0.2400000
52868.0000000	0.2350000
52869.0000000	0.2350000
52870.0000000	0.2300000
52871.0000000	0.2350000
52872.0000000	0.2250000
52873.0000000	0.2250000
52874.0000000	0.2300000
52875.0000000	0.2250000
52876.0000000	0.2250000
52877.0000000	0.2250000
52878.0000000	0.2200000
52879.0000000	0.2200000
52880.0000000	0.2200000
52881.0000000	0.2200000
52882.0000000	0.2150000
52883.0000000	0.2200000
52884.0000000	0.2200000
52885.0000000	0.2150000
52886.0000000	0.2150000
52887.0000000	0.2150000
52888.0000000	0.2150000
52889.0000000	0.2150000
52890.0000000	0.2150000
52891.0000000	0.2100000
52892.0000000	0.2100000
52893.0000000	0.2166667
52894.0000000	0.2100000
52895.0000000	0.2050000

52896.0000000	0.2050000
52897.0000000	0.2100000
52898.0000000	0.2050000
52899.0000000	0.2050000
52900.0000000	0.2050000
52901.0000000	0.2000000
52902.0000000	0.2000000
52903.0000000	0.2050000
52904.0000000	0.2000000
52905.0000000	0.2000000
52906.0000000	0.2050000
52907.0000000	0.2000000
52908.0000000	0.1950000
52909.0000000	0.1950000
52910.0000000	0.1950000
52911.0000000	0.1950000
52912.0000000	0.1950000
52913.0000000	0.1900000
52914.0000000	0.1900000
52915.0000000	0.1900000
52916.0000000	0.1900000
52917.0000000	0.1850000
52918.0000000	0.1800000
52919.0000000	0.1850000
52920.0000000	0.1850000
52921.0000000	0.1800000
52922.0000000	0.1800000
52923.0000000	0.1833333
52924.0000000	0.1750000
52925.0000000	0.1850000
52926.0000000	0.1800000
52927.0000000	0.1800000
52928.0000000	0.1800000
52929.0000000	0.1850000
52930.0000000	0.1900000
52931.0000000	0.1800000
52932.0000000	0.1850000
52933.0000000	0.1850000
52934.0000000	0.1850000
52935.0000000	0.1800000
52936.0000000	0.1850000
52937.0000000	0.1900000
52938.0000000	0.1800000
52939.0000000	0.1850000
52940.0000000	0.1850000
52941.0000000	0.1900000
52942.0000000	0.1850000
52943.0000000	0.1850000
52944.0000000	0.1900000
52945.0000000	0.1850000
52946.0000000	0.1850000
52947.0000000	0.1900000
52948.0000000	0.1900000
52949.0000000	0.1900000
52950.0000000	0.1900000
52951.0000000	0.1900000
52952.0000000	0.1900000

52953.0000000	0.1850000
52954.0000000	0.2000000
52955.0000000	0.1950000
52956.0000000	0.1900000
52957.0000000	0.1900000
52958.0000000	0.1900000
52959.0000000	0.1900000
52960.0000000	0.1900000
52961.0000000	0.1900000
52962.0000000	0.1900000
52963.0000000	0.1900000
52964.0000000	0.1900000
52965.0000000	0.1900000
52966.0000000	0.1900000
52967.0000000	0.1850000
52968.0000000	0.1950000
52969.0000000	0.1950000
52970.0000000	0.1900000
52971.0000000	0.1850000
52972.0000000	0.1950000
52973.0000000	0.1900000
52974.0000000	0.1900000
52975.0000000	0.1900000
52976.0000000	0.1950000
52977.0000000	0.1850000
52978.0000000	0.1850000
52979.0000000	0.1900000
52980.0000000	0.1900000
52981.0000000	0.1850000
52982.0000000	0.1900000
52983.0000000	0.1866667
52984.0000000	0.1850000
52985.0000000	0.1850000
52986.0000000	0.1800000
52987.0000000	0.1800000
52988.0000000	0.1850000
52989.0000000	0.1850000
52990.0000000	0.1800000
52991.0000000	0.1900000
52992.0000000	0.1850000
52993.0000000	0.1800000
52994.0000000	0.1800000
52995.0000000	0.1850000
52996.0000000	0.1850000
52997.0000000	0.1800000
52998.0000000	0.1850000
52999.0000000	0.1850000
53000.0000000	0.1850000
53001.0000000	0.1800000
53002.0000000	0.1850000
53003.0000000	0.1800000
53004.0000000	0.1800000
53005.0000000	0.1800000
53006.0000000	0.1850000
53007.0000000	0.1850000
53008.0000000	0.1850000
53009.0000000	0.1800000

53010.0000000	0.1850000
53011.0000000	0.1900000
53012.0000000	0.1900000
53013.0000000	0.1866667
53014.0000000	0.1950000
53015.0000000	0.1900000
53016.0000000	0.1900000
53017.0000000	0.1850000
53018.0000000	0.1950000
53019.0000000	0.2000000
53020.0000000	0.1900000
53021.0000000	0.1950000
53022.0000000	0.2000000
53023.0000000	0.2000000
53024.0000000	0.2000000
53025.0000000	0.2000000
53026.0000000	0.2050000
53027.0000000	0.2100000
53028.0000000	0.2100000
53029.0000000	0.2100000
53030.0000000	0.2100000
53031.0000000	0.2100000
53032.0000000	0.2100000
53033.0000000	0.2150000
53034.0000000	0.2150000
53035.0000000	0.2150000
53036.0000000	0.2150000
53037.0000000	0.2200000
53038.0000000	0.2250000
53039.0000000	0.2250000
53040.0000000	0.2200000
53041.0000000	0.2250000
53042.0000000	0.2250000
53043.0000000	0.2233333
53044.0000000	0.2300000
53045.0000000	0.2300000
53046.0000000	0.2250000
53047.0000000	0.2250000
53048.0000000	0.2200000
53049.0000000	0.2200000
53050.0000000	0.2250000
53051.0000000	0.2250000
53052.0000000	0.2250000
53053.0000000	0.2250000
53054.0000000	0.2250000
53055.0000000	0.2200000
53056.0000000	0.2250000
53057.0000000	0.2250000
53058.0000000	0.2250000
53059.0000000	0.2250000
53060.0000000	0.2200000
53061.0000000	0.2200000
53062.0000000	0.2200000
53063.0000000	0.2250000
53064.0000000	0.2250000
53065.0000000	0.2200000
53066.0000000	0.2250000

53067.0000000	0.2250000
53068.0000000	0.2250000
53069.0000000	0.2300000
53070.0000000	0.2250000
53071.0000000	0.2300000
53072.0000000	0.2250000
53073.0000000	0.2233333
53074.0000000	0.2250000
53075.0000000	0.2250000
53076.0000000	0.2250000
53077.0000000	0.2350000
53078.0000000	0.2250000
53079.0000000	0.2300000
53080.0000000	0.2250000
53081.0000000	0.2300000
53082.0000000	0.2250000
53083.0000000	0.2350000
53084.0000000	0.2300000
53085.0000000	0.2300000
53086.0000000	0.2300000
53087.0000000	0.2350000
53088.0000000	0.2300000
53089.0000000	0.2300000
53090.0000000	0.2350000
53091.0000000	0.2300000
53092.0000000	0.2350000
53093.0000000	0.2300000
53094.0000000	0.2350000
53095.0000000	0.2300000
53096.0000000	0.2300000
53097.0000000	0.2350000
53098.0000000	0.2350000
53099.0000000	0.2350000
53100.0000000	0.2350000
53101.0000000	0.2350000
53102.0000000	0.2350000
53103.0000000	0.2333333
53104.0000000	0.2350000
53105.0000000	0.2300000
53106.0000000	0.2300000
53107.0000000	0.2350000
53108.0000000	0.2350000
53109.0000000	0.2350000
53110.0000000	0.2350000
53111.0000000	0.2350000
53112.0000000	0.2350000
53113.0000000	0.2300000
53114.0000000	0.2300000
53115.0000000	0.2350000
53116.0000000	0.2350000
53117.0000000	0.2300000
53118.0000000	0.2300000
53119.0000000	0.2300000
53120.0000000	0.2300000
53121.0000000	0.2300000
53122.0000000	0.2250000
53123.0000000	0.2250000

53124.0000000	0.2300000
53125.0000000	0.2250000
53126.0000000	0.2250000
53127.0000000	0.2250000
53128.0000000	0.2250000
53129.0000000	0.2200000
53130.0000000	0.2200000
53131.0000000	0.2200000
53132.0000000	0.2200000
53133.0000000	0.2200000
53134.0000000	0.2266667
53135.0000000	0.2200000
53136.0000000	0.2200000
53137.0000000	0.2200000
53138.0000000	0.2200000
53139.0000000	0.2200000
53140.0000000	0.2200000
53141.0000000	0.2200000
53142.0000000	0.2200000
53143.0000000	0.2200000
53144.0000000	0.2150000
53145.0000000	0.2150000
53146.0000000	0.2200000
53147.0000000	0.2150000
53148.0000000	0.2150000
53149.0000000	0.2200000
53150.0000000	0.2150000
53151.0000000	0.2150000
53152.0000000	0.2150000
53153.0000000	0.2200000
53154.0000000	0.2200000
53155.0000000	0.2200000
53156.0000000	0.2150000
53157.0000000	0.2150000
53158.0000000	0.2200000
53159.0000000	0.2200000
53160.0000000	0.2250000
53161.0000000	0.2250000
53162.0000000	0.2250000
53163.0000000	0.2250000
53164.0000000	0.2266667
53165.0000000	0.2250000
53166.0000000	0.2250000
53167.0000000	0.2250000
53168.0000000	0.2250000
53169.0000000	0.2200000
53170.0000000	0.2250000
53171.0000000	0.2300000
53172.0000000	0.2250000
53173.0000000	0.2300000
53174.0000000	0.2300000
53175.0000000	0.2300000
53176.0000000	0.2300000
53177.0000000	0.2300000
53178.0000000	0.2350000
53179.0000000	0.2300000
53180.0000000	0.2300000

53181.0000000	0.2300000
53182.0000000	0.2300000
53183.0000000	0.2250000
53184.0000000	0.2300000
53185.0000000	0.2300000
53186.0000000	0.2300000
53187.0000000	0.2300000
53188.0000000	0.2250000
53189.0000000	0.2250000
53190.0000000	0.2300000
53191.0000000	0.2250000
53192.0000000	0.2300000
53193.0000000	0.2300000
53194.0000000	0.2266667
53195.0000000	0.2200000
53196.0000000	0.2250000
53197.0000000	0.2250000
53198.0000000	0.2250000
53199.0000000	0.2250000
53200.0000000	0.2250000
53201.0000000	0.2200000
53202.0000000	0.2200000
53203.0000000	0.2200000
53204.0000000	0.2200000
53205.0000000	0.2200000
53206.0000000	0.2200000
53207.0000000	0.2150000
53208.0000000	0.2200000
53209.0000000	0.2200000
53210.0000000	0.2200000
53211.0000000	0.2150000
53212.0000000	0.2150000
53213.0000000	0.2100000
53214.0000000	0.2100000
53215.0000000	0.2150000
53216.0000000	0.2050000
53217.0000000	0.2050000
53218.0000000	0.2100000
53219.0000000	0.2100000
53220.0000000	0.2050000
53221.0000000	0.2000000
53222.0000000	0.2050000
53223.0000000	0.2000000
53224.0000000	0.2000000
53225.0000000	0.2000000
53226.0000000	0.2050000
53227.0000000	0.1950000
53228.0000000	0.2000000
53229.0000000	0.2050000
53230.0000000	0.1950000
53231.0000000	0.1950000
53232.0000000	0.1900000
53233.0000000	0.1950000
53234.0000000	0.1950000
53235.0000000	0.1950000
53236.0000000	0.1950000
53237.0000000	0.1950000

53238.0000000	0.2000000
53239.0000000	0.1950000
53240.0000000	0.1900000
53241.0000000	0.1950000
53242.0000000	0.1950000
53243.0000000	0.1950000
53244.0000000	0.1950000
53245.0000000	0.1950000
53246.0000000	0.1950000
53247.0000000	0.1900000
53248.0000000	0.1900000
53249.0000000	0.1950000
53250.0000000	0.1950000
53251.0000000	0.1950000
53252.0000000	0.1950000
53253.0000000	0.1900000
53254.0000000	0.1950000
53255.0000000	0.1966667
53256.0000000	0.1950000
53257.0000000	0.1950000
53258.0000000	0.1950000
53259.0000000	0.1950000
53260.0000000	0.1950000
53261.0000000	0.1950000
53262.0000000	0.1950000
53263.0000000	0.2000000
53264.0000000	0.1900000
53265.0000000	0.1950000
53266.0000000	0.1950000
53267.0000000	0.1950000
53268.0000000	0.1950000
53269.0000000	0.1900000
53270.0000000	0.1950000
53271.0000000	0.1950000
53272.0000000	0.1950000
53273.0000000	0.2000000
53274.0000000	0.1950000
53275.0000000	0.2000000
53276.0000000	0.1950000
53277.0000000	0.1950000
53278.0000000	0.1950000
53279.0000000	0.2000000
53280.0000000	0.2000000
53281.0000000	0.2000000
53282.0000000	0.2000000
53283.0000000	0.2000000
53284.0000000	0.2000000
53285.0000000	0.1966667
53286.0000000	0.2000000
53287.0000000	0.2000000
53288.0000000	0.2000000
53289.0000000	0.2000000
53290.0000000	0.2000000
53291.0000000	0.2000000
53292.0000000	0.2050000
53293.0000000	0.2050000
53294.0000000	0.2050000

53295.0000000	0.2050000
53296.0000000	0.2050000
53297.0000000	0.2050000
53298.0000000	0.2050000
53299.0000000	0.2050000
53300.0000000	0.2050000
53301.0000000	0.2050000
53302.0000000	0.2050000
53303.0000000	0.2050000
53304.0000000	0.2100000
53305.0000000	0.2050000
53306.0000000	0.2050000
53307.0000000	0.2050000
53308.0000000	0.2100000
53309.0000000	0.2150000
53310.0000000	0.2100000
53311.0000000	0.2100000
53312.0000000	0.2150000
53313.0000000	0.2150000
53314.0000000	0.2100000
53315.0000000	0.2166667
53316.0000000	0.2100000
53317.0000000	0.2150000
53318.0000000	0.2200000
53319.0000000	0.2150000
53320.0000000	0.2150000
53321.0000000	0.2200000
53322.0000000	0.2150000
53323.0000000	0.2200000
53324.0000000	0.2200000
53325.0000000	0.2200000
53326.0000000	0.2200000
53327.0000000	0.2200000
53328.0000000	0.2250000
53329.0000000	0.2250000
53330.0000000	0.2150000
53331.0000000	0.2250000
53332.0000000	0.2250000
53333.0000000	0.2200000
53334.0000000	0.2250000
53335.0000000	0.2250000
53336.0000000	0.2200000
53337.0000000	0.2200000
53338.0000000	0.2200000
53339.0000000	0.2200000
53340.0000000	0.2200000
53341.0000000	0.2150000
53342.0000000	0.2200000
53343.0000000	0.2200000
53344.0000000	0.2150000
53345.0000000	0.2133333
53346.0000000	0.2100000
53347.0000000	0.2150000
53348.0000000	0.2100000
53349.0000000	0.2100000
53350.0000000	0.2150000
53351.0000000	0.2100000

53352.0000000	0.2100000
53353.0000000	0.2050000
53354.0000000	0.2100000
53355.0000000	0.2100000
53356.0000000	0.2050000
53357.0000000	0.2100000
53358.0000000	0.2050000
53359.0000000	0.2000000
53360.0000000	0.2050000
53361.0000000	0.2000000
53362.0000000	0.2000000
53363.0000000	0.2000000
53364.0000000	0.2050000
53365.0000000	0.2050000
53366.0000000	0.2050000
53367.0000000	0.1950000
53368.0000000	0.2000000
53369.0000000	0.1950000
53370.0000000	0.2000000
53371.0000000	0.2000000
53372.0000000	0.2000000
53373.0000000	0.2000000
53374.0000000	0.2000000
53375.0000000	0.2000000
53376.0000000	0.2000000
53377.0000000	0.1950000
53378.0000000	0.1950000
53379.0000000	0.1950000
53380.0000000	0.1950000
53381.0000000	0.1950000
53382.0000000	0.1900000
53383.0000000	0.1900000
53384.0000000	0.1900000
53385.0000000	0.1900000
53386.0000000	0.1950000
53387.0000000	0.1850000
53388.0000000	0.1900000
53389.0000000	0.1950000
53390.0000000	0.1850000
53391.0000000	0.1900000
53392.0000000	0.1900000
53393.0000000	0.1850000
53394.0000000	0.1900000
53395.0000000	0.1850000
53396.0000000	0.1900000
53397.0000000	0.1900000
53398.0000000	0.1850000
53399.0000000	0.1900000
53400.0000000	0.1800000
53401.0000000	0.1850000
53402.0000000	0.1900000
53403.0000000	0.1850000
53404.0000000	0.1900000
53405.0000000	0.1850000
53406.0000000	0.1900000
53407.0000000	0.1900000
53408.0000000	0.1800000

53409.0000000	0.1850000
53410.0000000	0.1850000
53411.0000000	0.1800000
53412.0000000	0.1800000
53413.0000000	0.1850000
53414.0000000	0.1750000
53415.0000000	0.1800000
53416.0000000	0.1800000
53417.0000000	0.1750000
53418.0000000	0.1800000
53419.0000000	0.1750000
53420.0000000	0.1800000
53421.0000000	0.1750000
53422.0000000	0.1750000
53423.0000000	0.1750000
53424.0000000	0.1750000
53425.0000000	0.1750000
53426.0000000	0.1700000
53427.0000000	0.1750000
53428.0000000	0.1800000
53429.0000000	0.1750000
53430.0000000	0.1750000
53431.0000000	0.1800000
53432.0000000	0.1800000
53433.0000000	0.1800000
53434.0000000	0.1850000
53435.0000000	0.1800000
53436.0000000	0.1800000
53437.0000000	0.1850000
53438.0000000	0.1850000
53439.0000000	0.1850000
53440.0000000	0.1800000
53441.0000000	0.1850000
53442.0000000	0.1800000
53443.0000000	0.1850000
53444.0000000	0.1850000
53445.0000000	0.1900000
53446.0000000	0.1900000
53447.0000000	0.1900000
53448.0000000	0.1900000
53449.0000000	0.1900000
53450.0000000	0.1900000
53451.0000000	0.1850000
53452.0000000	0.1900000
53453.0000000	0.1800000
53454.0000000	0.1900000
53455.0000000	0.1850000
53456.0000000	0.1900000
53457.0000000	0.1850000
53458.0000000	0.1950000
53459.0000000	0.1850000
53460.0000000	0.1900000
53461.0000000	0.1850000
53462.0000000	0.1850000
53463.0000000	0.1750000
53464.0000000	0.1900000
53465.0000000	0.1800000

53466.0000000	0.1900000
53467.0000000	0.1800000
53468.0000000	0.1800000
53469.0000000	0.1750000
53470.0000000	0.1800000
53471.0000000	0.1750000
53472.0000000	0.1800000
53473.0000000	0.1750000
53474.0000000	0.1800000
53475.0000000	0.1750000
53476.0000000	0.1800000
53477.0000000	0.1700000
53478.0000000	0.1800000
53479.0000000	0.1750000
53480.0000000	0.1800000
53481.0000000	0.1700000
53482.0000000	0.1750000
53483.0000000	0.1750000
53484.0000000	0.1750000
53485.0000000	0.1750000
53486.0000000	0.1800000
53487.0000000	0.1650000
53488.0000000	0.1750000
53489.0000000	0.1700000
53490.0000000	0.1750000
53491.0000000	0.1700000
53492.0000000	0.1700000
53493.0000000	0.1700000
53494.0000000	0.1700000
53495.0000000	0.1700000
53496.0000000	0.1733333
53497.0000000	0.1700000
53498.0000000	0.1650000
53499.0000000	0.1600000
53500.0000000	0.1650000
53501.0000000	0.1600000
53502.0000000	0.1650000
53503.0000000	0.1600000
53504.0000000	0.1600000
53505.0000000	0.1550000
53506.0000000	0.1600000
53507.0000000	0.1600000
53508.0000000	0.1600000
53509.0000000	0.1600000
53510.0000000	0.1550000
53511.0000000	0.1600000
53512.0000000	0.1500000
53513.0000000	0.1550000
53514.0000000	0.1500000
53515.0000000	0.1450000
53516.0000000	0.1500000
53517.0000000	0.1450000
53518.0000000	0.1450000
53519.0000000	0.1450000
53520.0000000	0.1400000
53521.0000000	0.1500000
53522.0000000	0.1400000

53523.0000000	0.1450000
53524.0000000	0.1400000
53525.0000000	0.1350000
53526.0000000	0.1400000
53527.0000000	0.1400000
53528.0000000	0.1350000
53529.0000000	0.1400000
53530.0000000	0.1350000
53531.0000000	0.1400000
53532.0000000	0.1400000
53533.0000000	0.1350000
53534.0000000	0.1450000
53535.0000000	0.1400000
53536.0000000	0.1450000
53537.0000000	0.1450000
53538.0000000	0.1450000
53539.0000000	0.1450000
53540.0000000	0.1400000
53541.0000000	0.1450000
53542.0000000	0.1450000
53543.0000000	0.1350000
53544.0000000	0.1400000
53545.0000000	0.1450000
53546.0000000	0.1450000
53547.0000000	0.1400000
53548.0000000	0.1350000
53549.0000000	0.1450000
53550.0000000	0.1450000
53551.0000000	0.1450000
53552.0000000	0.1500000
53553.0000000	0.1450000
53554.0000000	0.1450000
53555.0000000	0.1400000
53556.0000000	0.1433333
53557.0000000	0.1500000
53558.0000000	0.1500000
53559.0000000	0.1500000
53560.0000000	0.1450000
53561.0000000	0.1450000
53562.0000000	0.1450000
53563.0000000	0.1400000
53564.0000000	0.1450000
53565.0000000	0.1450000
53566.0000000	0.1400000
53567.0000000	0.1400000
53568.0000000	0.1450000
53569.0000000	0.1400000
53570.0000000	0.1450000
53571.0000000	0.1450000
53572.0000000	0.1400000
53573.0000000	0.1350000
53574.0000000	0.1400000
53575.0000000	0.1450000
53576.0000000	0.1400000
53577.0000000	0.1450000
53578.0000000	0.1450000
53579.0000000	0.1400000

53580.0000000	0.1400000
53581.0000000	0.1400000
53582.0000000	0.1400000
53583.0000000	0.1400000
53584.0000000	0.1400000
53585.0000000	0.1400000
53586.0000000	0.1433333
53587.0000000	0.1400000
53588.0000000	0.1400000
53589.0000000	0.1450000
53590.0000000	0.1400000
53591.0000000	0.1400000
53592.0000000	0.1400000
53593.0000000	0.1400000
53594.0000000	0.1400000
53595.0000000	0.1350000
53596.0000000	0.1400000
53597.0000000	0.1450000
53598.0000000	0.1400000
53599.0000000	0.1400000
53600.0000000	0.1450000
53601.0000000	0.1400000
53602.0000000	0.1400000
53603.0000000	0.1400000
53604.0000000	0.1350000
53605.0000000	0.1350000
53606.0000000	0.1350000
53607.0000000	0.1400000
53608.0000000	0.1350000
53609.0000000	0.1400000
53610.0000000	0.1400000
53611.0000000	0.1350000
53612.0000000	0.1300000
53613.0000000	0.1300000
53614.0000000	0.1300000
53615.0000000	0.1300000
53616.0000000	0.1300000
53617.0000000	0.1233333
53618.0000000	0.1300000
53619.0000000	0.1200000
53620.0000000	0.1300000
53621.0000000	0.1250000
53622.0000000	0.1200000
53623.0000000	0.1250000
53624.0000000	0.1250000
53625.0000000	0.1200000
53626.0000000	0.1250000
53627.0000000	0.1200000
53628.0000000	0.1200000
53629.0000000	0.1150000
53630.0000000	0.1100000
53631.0000000	0.1100000
53632.0000000	0.1100000
53633.0000000	0.1100000
53634.0000000	0.1100000
53635.0000000	0.1100000
53636.0000000	0.1100000

53637.0000000	0.1050000
53638.0000000	0.1050000
53639.0000000	0.1000000
53640.0000000	0.1000000
53641.0000000	0.1050000
53642.0000000	0.1050000
53643.0000000	0.1000000
53644.0000000	0.1000000
53645.0000000	0.1000000
53646.0000000	0.0950000
53647.0000000	0.0933333
53648.0000000	0.0900000
53649.0000000	0.0950000
53650.0000000	0.0900000
53651.0000000	0.0900000
53652.0000000	0.0900000
53653.0000000	0.0850000
53654.0000000	0.0850000
53655.0000000	0.0900000
53656.0000000	0.0850000
53657.0000000	0.0850000
53658.0000000	0.0850000
53659.0000000	0.0850000
53660.0000000	0.0850000
53661.0000000	0.0800000
53662.0000000	0.0850000
53663.0000000	0.0900000
53664.0000000	0.0850000
53665.0000000	0.0800000
53666.0000000	0.0850000
53667.0000000	0.0850000
53668.0000000	0.0800000
53669.0000000	0.0850000
53670.0000000	0.0850000
53671.0000000	0.0800000
53672.0000000	0.0800000
53673.0000000	0.0850000
53674.0000000	0.0850000
53675.0000000	0.0850000
53676.0000000	0.0850000
53677.0000000	0.0866667
53678.0000000	0.0800000
53679.0000000	0.0800000
53680.0000000	0.0850000
53681.0000000	0.0850000
53682.0000000	0.0800000
53683.0000000	0.0800000
53684.0000000	0.0750000
53685.0000000	0.0850000
53686.0000000	0.0800000
53687.0000000	0.0800000
53688.0000000	0.0800000
53689.0000000	0.0850000
53690.0000000	0.0800000
53691.0000000	0.0850000
53692.0000000	0.0850000
53693.0000000	0.0750000

53694.0000000	0.0800000
53695.0000000	0.0800000
53696.0000000	0.0800000
53697.0000000	0.0800000
53698.0000000	0.0850000
53699.0000000	0.0800000
53700.0000000	0.0850000
53701.0000000	0.0850000
53702.0000000	0.0800000
53703.0000000	0.0800000
53704.0000000	0.0800000
53705.0000000	0.0800000
53706.0000000	0.0800000
53707.0000000	0.0866667
53708.0000000	0.0800000
53709.0000000	0.0850000
53710.0000000	0.0800000
53711.0000000	0.0800000
53712.0000000	0.0800000
53713.0000000	0.0800000
53714.0000000	0.0750000
53715.0000000	0.0800000
53716.0000000	0.0800000
53717.0000000	0.0800000
53718.0000000	0.0750000
53719.0000000	0.0800000
53720.0000000	0.0750000
53721.0000000	0.0750000
53722.0000000	0.0750000
53723.0000000	0.0800000
53724.0000000	0.0750000
53725.0000000	0.0750000
53726.0000000	0.0750000
53727.0000000	0.0750000
53728.0000000	0.0750000
53729.0000000	0.0750000
53730.0000000	0.0750000
53731.0000000	0.0700000
53732.0000000	0.0700000
53733.0000000	0.0700000
53734.0000000	0.0750000
53735.0000000	0.0700000
53736.0000000	0.0700000
53737.0000000	0.0700000
53738.0000000	0.0700000
53739.0000000	0.0700000
53740.0000000	0.0650000
53741.0000000	0.0750000
53742.0000000	0.0700000
53743.0000000	0.0700000
53744.0000000	0.0650000
53745.0000000	0.0700000
53746.0000000	0.0700000
53747.0000000	0.0700000
53748.0000000	0.0700000
53749.0000000	0.0650000
53750.0000000	0.0700000

53751.0000000	0.0700000
53752.0000000	0.0700000
53753.0000000	0.0700000
53754.0000000	0.0700000
53755.0000000	0.0650000
53756.0000000	0.0700000
53757.0000000	0.0700000
53758.0000000	0.0650000
53759.0000000	0.0700000
53760.0000000	0.0650000
53761.0000000	0.0700000
53762.0000000	0.0700000
53763.0000000	0.0700000
53764.0000000	0.0650000
53765.0000000	0.0650000
53766.0000000	0.0650000
53767.0000000	0.0650000
53768.0000000	0.0666667
53769.0000000	0.0650000
53770.0000000	0.0700000
53771.0000000	0.0650000
53772.0000000	0.0650000
53773.0000000	0.0650000
53774.0000000	0.0650000
53775.0000000	0.0600000
53776.0000000	0.0650000
53777.0000000	0.0650000
53778.0000000	0.0600000
53779.0000000	0.0650000
53780.0000000	0.0650000
53781.0000000	0.0650000
53782.0000000	0.0600000
53783.0000000	0.0600000
53784.0000000	0.0600000
53785.0000000	0.0550000
53786.0000000	0.0600000
53787.0000000	0.0550000
53788.0000000	0.0600000
53789.0000000	0.0600000
53790.0000000	0.0550000
53791.0000000	0.0550000
53792.0000000	0.0500000
53793.0000000	0.0550000
53794.0000000	0.0550000
53795.0000000	0.0550000
53796.0000000	0.0500000
53797.0000000	0.0550000
53798.0000000	0.0500000
53799.0000000	0.0500000
53800.0000000	0.0500000
53801.0000000	0.0500000
53802.0000000	0.0550000
53803.0000000	0.0450000
53804.0000000	0.0450000
53805.0000000	0.0450000
53806.0000000	0.0500000
53807.0000000	0.0450000

53808.0000000	0.0450000
53809.0000000	0.0450000
53810.0000000	0.0450000
53811.0000000	0.0450000
53812.0000000	0.0450000
53813.0000000	0.0500000
53814.0000000	0.0400000
53815.0000000	0.0450000
53816.0000000	0.0350000
53817.0000000	0.0450000
53818.0000000	0.0400000
53819.0000000	0.0400000
53820.0000000	0.0400000
53821.0000000	0.0450000
53822.0000000	0.0400000
53823.0000000	0.0450000
53824.0000000	0.0450000
53825.0000000	0.0450000
53826.0000000	0.0450000
53827.0000000	0.0450000
53828.0000000	0.0433333
53829.0000000	0.0450000
53830.0000000	0.0500000
53831.0000000	0.0500000
53832.0000000	0.0500000
53833.0000000	0.0500000
53834.0000000	0.0550000
53835.0000000	0.0550000
53836.0000000	0.0550000
53837.0000000	0.0550000
53838.0000000	0.0600000
53839.0000000	0.0550000
53840.0000000	0.0550000
53841.0000000	0.0550000
53842.0000000	0.0600000
53843.0000000	0.0550000
53844.0000000	0.0550000
53845.0000000	0.0550000
53846.0000000	0.0600000
53847.0000000	0.0550000
53848.0000000	0.0550000
53849.0000000	0.0550000
53850.0000000	0.0500000
53851.0000000	0.0500000
53852.0000000	0.0550000
53853.0000000	0.0500000
53854.0000000	0.0500000
53855.0000000	0.0500000
53856.0000000	0.0450000
53857.0000000	0.0450000
53858.0000000	0.0500000
53859.0000000	0.0466667
53860.0000000	0.0450000
53861.0000000	0.0500000
53862.0000000	0.0500000
53863.0000000	0.0400000
53864.0000000	0.0450000

53865.0000000	0.0450000
53866.0000000	0.0450000
53867.0000000	0.0400000
53868.0000000	0.0400000
53869.0000000	0.0350000
53870.0000000	0.0400000
53871.0000000	0.0400000
53872.0000000	0.0350000
53873.0000000	0.0350000
53874.0000000	0.0350000
53875.0000000	0.0350000
53876.0000000	0.0300000
53877.0000000	0.0250000
53878.0000000	0.0300000
53879.0000000	0.0250000
53880.0000000	0.0250000
53881.0000000	0.0300000
53882.0000000	0.0250000
53883.0000000	0.0250000
53884.0000000	0.0200000
53885.0000000	0.0200000
53886.0000000	0.0200000
53887.0000000	0.0200000
53888.0000000	0.0200000
53889.0000000	0.0233333
53890.0000000	0.0150000
53891.0000000	0.0200000
53892.0000000	0.0150000
53893.0000000	0.0150000
53894.0000000	0.0100000
53895.0000000	0.0100000
53896.0000000	0.0100000
53897.0000000	0.0050000
53898.0000000	0.0000000
53899.0000000	0.0050000
53900.0000000	0.0050000
53901.0000000	0.0050000
53902.0000000	0.0000000
53903.0000000	0.0050000
53904.0000000	0.0050000
53905.0000000	0.0000000
53906.0000000	0.0000000
53907.0000000	-0.0050000
53908.0000000	0.0000000
53909.0000000	0.0000000
53910.0000000	-0.0100000
53911.0000000	-0.0050000
53912.0000000	-0.0050000
53913.0000000	-0.0100000
53914.0000000	-0.0100000
53915.0000000	-0.0100000
53916.0000000	-0.0150000
53917.0000000	-0.0150000
53918.0000000	-0.0100000
53919.0000000	-0.0200000
53920.0000000	-0.0150000
53921.0000000	-0.0200000

53922.0000000	-0.0200000
53923.0000000	-0.0200000
53924.0000000	-0.0250000
53925.0000000	-0.0250000
53926.0000000	-0.0250000
53927.0000000	-0.0250000
53928.0000000	-0.0250000
53929.0000000	-0.0250000
53930.0000000	-0.0300000
53931.0000000	-0.0300000
53932.0000000	-0.0350000
53933.0000000	-0.0350000
53934.0000000	-0.0300000
53935.0000000	-0.0400000
53936.0000000	-0.0350000
53937.0000000	-0.0350000
53938.0000000	-0.0400000
53939.0000000	-0.0350000
53940.0000000	-0.0350000
53941.0000000	-0.0350000
53942.0000000	-0.0400000
53943.0000000	-0.0400000
53944.0000000	-0.0400000
53945.0000000	-0.0400000
53946.0000000	-0.0350000
53947.0000000	-0.0400000
53948.0000000	-0.0400000
53949.0000000	-0.0366667
53950.0000000	-0.0400000
53951.0000000	-0.0400000
53952.0000000	-0.0450000
53953.0000000	-0.0400000
53954.0000000	-0.0400000
53955.0000000	-0.0400000
53956.0000000	-0.0400000
53957.0000000	-0.0400000
53958.0000000	-0.0450000
53959.0000000	-0.0450000
53960.0000000	-0.0400000
53961.0000000	-0.0400000
53962.0000000	-0.0450000
53963.0000000	-0.0450000
53964.0000000	-0.0450000
53965.0000000	-0.0550000
53966.0000000	-0.0500000
53967.0000000	-0.0500000
53968.0000000	-0.0550000
53969.0000000	-0.0550000
53970.0000000	-0.0550000
53971.0000000	-0.0600000
53972.0000000	-0.0550000
53973.0000000	-0.0600000
53974.0000000	-0.0600000
53975.0000000	-0.0600000
53976.0000000	-0.0600000
53977.0000000	-0.0650000
53978.0000000	-0.0600000

53979.0000000	-0.0700000
53980.0000000	-0.0650000
53981.0000000	-0.0650000
53982.0000000	-0.0650000
53983.0000000	-0.0600000
53984.0000000	-0.0650000
53985.0000000	-0.0650000
53986.0000000	-0.0650000
53987.0000000	-0.0650000
53988.0000000	-0.0700000
53989.0000000	-0.0650000
53990.0000000	-0.0650000
53991.0000000	-0.0650000
53992.0000000	-0.0650000
53993.0000000	-0.0650000
53994.0000000	-0.0700000
53995.0000000	-0.0650000
53996.0000000	-0.0650000
53997.0000000	-0.0650000
53998.0000000	-0.0650000
53999.0000000	-0.0650000
54000.0000000	-0.0650000
54001.0000000	-0.0650000
54002.0000000	-0.0650000
54003.0000000	-0.0650000
54004.0000000	-0.0650000
54005.0000000	-0.0650000
54006.0000000	-0.0600000
54007.0000000	-0.0600000
54008.0000000	-0.0600000
54009.0000000	-0.0566667
54010.0000000	-0.0650000
54011.0000000	-0.0600000
54012.0000000	-0.0550000
54013.0000000	-0.0550000
54014.0000000	-0.0550000
54015.0000000	-0.0550000
54016.0000000	-0.0550000
54017.0000000	-0.0550000
54018.0000000	-0.0500000
54019.0000000	-0.0550000
54020.0000000	-0.0500000
54021.0000000	-0.0550000
54022.0000000	-0.0500000
54023.0000000	-0.0500000
54024.0000000	-0.0500000
54025.0000000	-0.0500000
54026.0000000	-0.0450000
54027.0000000	-0.0450000
54028.0000000	-0.0450000
54029.0000000	-0.0450000
54030.0000000	-0.0450000
54031.0000000	-0.0450000
54032.0000000	-0.0450000
54033.0000000	-0.0500000
54034.0000000	-0.0450000
54035.0000000	-0.0450000

54036.0000000	-0.0450000
54037.0000000	-0.0450000
54038.0000000	-0.0400000
54039.0000000	-0.0466667
54040.0000000	-0.0450000
54041.0000000	-0.0450000
54042.0000000	-0.0450000
54043.0000000	-0.0500000
54044.0000000	-0.0450000
54045.0000000	-0.0450000
54046.0000000	-0.0500000
54047.0000000	-0.0500000
54048.0000000	-0.0500000
54049.0000000	-0.0500000
54050.0000000	-0.0500000
54051.0000000	-0.0500000
54052.0000000	-0.0600000
54053.0000000	-0.0550000
54054.0000000	-0.0500000
54055.0000000	-0.0550000
54056.0000000	-0.0550000
54057.0000000	-0.0500000
54058.0000000	-0.0550000
54059.0000000	-0.0550000
54060.0000000	-0.0600000
54061.0000000	-0.0550000
54062.0000000	-0.0550000
54063.0000000	-0.0550000
54064.0000000	-0.0500000
54065.0000000	-0.0500000
54066.0000000	-0.0450000
54067.0000000	-0.0500000
54068.0000000	-0.0500000
54069.0000000	-0.0466667
54070.0000000	-0.0450000
54071.0000000	-0.0500000
54072.0000000	-0.0450000
54073.0000000	-0.0500000
54074.0000000	-0.0500000
54075.0000000	-0.0500000
54076.0000000	-0.0500000
54077.0000000	-0.0450000
54078.0000000	-0.0500000
54079.0000000	-0.0500000
54080.0000000	-0.0500000
54081.0000000	-0.0550000
54082.0000000	-0.0500000
54083.0000000	-0.0500000
54084.0000000	-0.0500000
54085.0000000	-0.0450000
54086.0000000	-0.0500000
54087.0000000	-0.0500000
54088.0000000	-0.0500000
54089.0000000	-0.0450000
54090.0000000	-0.0500000
54091.0000000	-0.0500000
54092.0000000	-0.0450000

54093.0000000	-0.0450000
54094.0000000	-0.0500000
54095.0000000	-0.0450000
54096.0000000	-0.0450000
54097.0000000	-0.0450000
54098.0000000	-0.0450000
54099.0000000	-0.0500000
54100.0000000	-0.0500000
54101.0000000	-0.0450000
54102.0000000	-0.0500000
54103.0000000	-0.0550000
54104.0000000	-0.0450000
54105.0000000	-0.0500000
54106.0000000	-0.0550000
54107.0000000	-0.0500000
54108.0000000	-0.0550000
54109.0000000	-0.0500000
54110.0000000	-0.0500000
54111.0000000	-0.0500000
54112.0000000	-0.0500000
54113.0000000	-0.0550000
54114.0000000	-0.0500000
54115.0000000	-0.0450000
54116.0000000	-0.0550000
54117.0000000	-0.0500000
54118.0000000	-0.0550000
54119.0000000	-0.0550000
54120.0000000	-0.0550000
54121.0000000	-0.0600000
54122.0000000	-0.0550000
54123.0000000	-0.0600000
54124.0000000	-0.0550000
54125.0000000	-0.0600000
54126.0000000	-0.0550000
54127.0000000	-0.0550000
54128.0000000	-0.0550000
54129.0000000	-0.0550000
54130.0000000	-0.0633333
54131.0000000	-0.0550000
54132.0000000	-0.0550000
54133.0000000	-0.0550000
54134.0000000	-0.0600000
54135.0000000	-0.0550000
54136.0000000	-0.0550000
54137.0000000	-0.0550000
54138.0000000	-0.0600000
54139.0000000	-0.0550000
54140.0000000	-0.0550000
54141.0000000	-0.0600000
54142.0000000	-0.0600000
54143.0000000	-0.0550000
54144.0000000	-0.0550000
54145.0000000	-0.0600000
54146.0000000	-0.0500000
54147.0000000	-0.0550000
54148.0000000	-0.0550000
54149.0000000	-0.0600000

54150.0000000	-0.0600000
54151.0000000	-0.0550000
54152.0000000	-0.0550000
54153.0000000	-0.0550000
54154.0000000	-0.0600000
54155.0000000	-0.0550000
54156.0000000	-0.0550000
54157.0000000	-0.0600000
54158.0000000	-0.0600000
54159.0000000	-0.0550000
54160.0000000	-0.0566667
54161.0000000	-0.0600000
54162.0000000	-0.0600000
54163.0000000	-0.0600000
54164.0000000	-0.0650000
54165.0000000	-0.0600000
54166.0000000	-0.0650000
54167.0000000	-0.0650000
54168.0000000	-0.0650000
54169.0000000	-0.0700000
54170.0000000	-0.0650000
54171.0000000	-0.0650000
54172.0000000	-0.0700000
54173.0000000	-0.0700000
54174.0000000	-0.0700000
54175.0000000	-0.0800000
54176.0000000	-0.0750000
54177.0000000	-0.0800000
54178.0000000	-0.0800000
54179.0000000	-0.0800000
54180.0000000	-0.0750000
54181.0000000	-0.0800000
54182.0000000	-0.0800000
54183.0000000	-0.0800000
54184.0000000	-0.0800000
54185.0000000	-0.0850000
54186.0000000	-0.0850000
54187.0000000	-0.0900000
54188.0000000	-0.0850000
54189.0000000	-0.0950000
54190.0000000	-0.0933333
54191.0000000	-0.0950000
54192.0000000	-0.0950000
54193.0000000	-0.0950000
54194.0000000	-0.1050000
54195.0000000	-0.1000000
54196.0000000	-0.1050000
54197.0000000	-0.1050000
54198.0000000	-0.1050000
54199.0000000	-0.1050000
54200.0000000	-0.1100000
54201.0000000	-0.1100000
54202.0000000	-0.1150000
54203.0000000	-0.1150000
54204.0000000	-0.1150000
54205.0000000	-0.1150000
54206.0000000	-0.1200000

54207.0000000	-0.1150000
54208.0000000	-0.1150000
54209.0000000	-0.1250000
54210.0000000	-0.1250000
54211.0000000	-0.1200000
54212.0000000	-0.1250000
54213.0000000	-0.1250000
54214.0000000	-0.1250000
54215.0000000	-0.1200000
54216.0000000	-0.1250000
54217.0000000	-0.1250000
54218.0000000	-0.1200000
54219.0000000	-0.1200000
54220.0000000	-0.1250000
54221.0000000	-0.1200000
54222.0000000	-0.1150000
54223.0000000	-0.1200000
54224.0000000	-0.1200000
54225.0000000	-0.1150000
54226.0000000	-0.1150000
54227.0000000	-0.1100000
54228.0000000	-0.1100000
54229.0000000	-0.1100000
54230.0000000	-0.1100000
54231.0000000	-0.1050000
54232.0000000	-0.1050000
54233.0000000	-0.1050000
54234.0000000	-0.1000000
54235.0000000	-0.1000000
54236.0000000	-0.1000000
54237.0000000	-0.0950000
54238.0000000	-0.0950000
54239.0000000	-0.0950000
54240.0000000	-0.0950000
54241.0000000	-0.0900000
54242.0000000	-0.0900000
54243.0000000	-0.0900000
54244.0000000	-0.0900000
54245.0000000	-0.0900000
54246.0000000	-0.0900000
54247.0000000	-0.0850000
54248.0000000	-0.0850000
54249.0000000	-0.0850000
54250.0000000	-0.0900000
54251.0000000	-0.0833333
54252.0000000	-0.0900000
54253.0000000	-0.0850000
54254.0000000	-0.0900000
54255.0000000	-0.0850000
54256.0000000	-0.0850000
54257.0000000	-0.0850000
54258.0000000	-0.0850000
54259.0000000	-0.0850000
54260.0000000	-0.0850000
54261.0000000	-0.0800000
54262.0000000	-0.0800000
54263.0000000	-0.0800000

54264.0000000	-0.0800000
54265.0000000	-0.0800000
54266.0000000	-0.0750000
54267.0000000	-0.0750000
54268.0000000	-0.0700000
54269.0000000	-0.0700000
54270.0000000	-0.0650000
54271.0000000	-0.0700000
54272.0000000	-0.0650000
54273.0000000	-0.0600000
54274.0000000	-0.0650000
54275.0000000	-0.0650000
54276.0000000	-0.0650000
54277.0000000	-0.0600000
54278.0000000	-0.0600000
54279.0000000	-0.0650000
54280.0000000	-0.0566667
54281.0000000	-0.0600000
54282.0000000	-0.0550000
54283.0000000	-0.0600000
54284.0000000	-0.0600000
54285.0000000	-0.0600000
54286.0000000	-0.0550000
54287.0000000	-0.0550000
54288.0000000	-0.0600000
54289.0000000	-0.0500000
54290.0000000	-0.0550000
54291.0000000	-0.0550000
54292.0000000	-0.0550000
54293.0000000	-0.0600000
54294.0000000	-0.0550000
54295.0000000	-0.0600000
54296.0000000	-0.0550000
54297.0000000	-0.0600000
54298.0000000	-0.0600000
54299.0000000	-0.0600000
54300.0000000	-0.0550000
54301.0000000	-0.0600000
54302.0000000	-0.0600000
54303.0000000	-0.0550000
54304.0000000	-0.0600000
54305.0000000	-0.0600000
54306.0000000	-0.0600000
54307.0000000	-0.0600000
54308.0000000	-0.0600000
54309.0000000	-0.0600000
54310.0000000	-0.0600000
54311.0000000	-0.0566667
54312.0000000	-0.0550000
54313.0000000	-0.0600000
54314.0000000	-0.0600000
54315.0000000	-0.0550000
54316.0000000	-0.0550000
54317.0000000	-0.0600000
54318.0000000	-0.0550000
54319.0000000	-0.0550000
54320.0000000	-0.0550000

54321.0000000	-0.0550000
54322.0000000	-0.0500000
54323.0000000	-0.0600000
54324.0000000	-0.0550000
54325.0000000	-0.0550000
54326.0000000	-0.0450000
54327.0000000	-0.0450000
54328.0000000	-0.0450000
54329.0000000	-0.0450000
54330.0000000	-0.0500000
54331.0000000	-0.0450000
54332.0000000	-0.0450000
54333.0000000	-0.0400000
54334.0000000	-0.0450000
54335.0000000	-0.0400000
54336.0000000	-0.0450000
54337.0000000	-0.0400000
54338.0000000	-0.0450000
54339.0000000	-0.0400000
54340.0000000	-0.0350000
54341.0000000	-0.0400000
54342.0000000	-0.0366667
54343.0000000	-0.0400000
54344.0000000	-0.0350000
54345.0000000	-0.0350000
54346.0000000	-0.0350000
54347.0000000	-0.0400000
54348.0000000	-0.0300000
54349.0000000	-0.0400000
54350.0000000	-0.0350000
54351.0000000	-0.0350000
54352.0000000	-0.0350000
54353.0000000	-0.0350000
54354.0000000	-0.0350000
54355.0000000	-0.0300000
54356.0000000	-0.0300000
54357.0000000	-0.0350000
54358.0000000	-0.0300000
54359.0000000	-0.0350000
54360.0000000	-0.0300000
54361.0000000	-0.0350000
54362.0000000	-0.0400000
54363.0000000	-0.0350000
54364.0000000	-0.0350000
54365.0000000	-0.0350000
54366.0000000	-0.0350000
54367.0000000	-0.0400000
54368.0000000	-0.0350000
54369.0000000	-0.0400000
54370.0000000	-0.0450000
54371.0000000	-0.0433333
54372.0000000	-0.0400000
54373.0000000	-0.0400000
54374.0000000	-0.0400000
54375.0000000	-0.0450000
54376.0000000	-0.0500000
54377.0000000	-0.0450000

54378.0000000	-0.0450000
54379.0000000	-0.0450000
54380.0000000	-0.0450000
54381.0000000	-0.0450000
54382.0000000	-0.0500000
54383.0000000	-0.0500000
54384.0000000	-0.0450000
54385.0000000	-0.0500000
54386.0000000	-0.0500000
54387.0000000	-0.0550000
54388.0000000	-0.0550000
54389.0000000	-0.0600000
54390.0000000	-0.0600000
54391.0000000	-0.0550000
54392.0000000	-0.0550000
54393.0000000	-0.0600000
54394.0000000	-0.0550000
54395.0000000	-0.0550000
54396.0000000	-0.0550000
54397.0000000	-0.0600000
54398.0000000	-0.0600000
54399.0000000	-0.0650000
54400.0000000	-0.0600000
54401.0000000	-0.0666667
54402.0000000	-0.0650000
54403.0000000	-0.0700000
54404.0000000	-0.0750000
54405.0000000	-0.0700000
54406.0000000	-0.0750000
54407.0000000	-0.0800000
54408.0000000	-0.0700000
54409.0000000	-0.0750000
54410.0000000	-0.0750000
54411.0000000	-0.0850000
54412.0000000	-0.0800000
54413.0000000	-0.0800000
54414.0000000	-0.0750000
54415.0000000	-0.0800000
54416.0000000	-0.0800000
54417.0000000	-0.0800000
54418.0000000	-0.0800000
54419.0000000	-0.0850000
54420.0000000	-0.0850000
54421.0000000	-0.0850000
54422.0000000	-0.0850000
54423.0000000	-0.0850000
54424.0000000	-0.0850000
54425.0000000	-0.0850000
54426.0000000	-0.0850000
54427.0000000	-0.0900000
54428.0000000	-0.0850000
54429.0000000	-0.0850000
54430.0000000	-0.0850000
54431.0000000	-0.0900000
54432.0000000	-0.0866667
54433.0000000	-0.0850000
54434.0000000	-0.0850000

54435.0000000	-0.0800000
54436.0000000	-0.0850000
54437.0000000	-0.0850000
54438.0000000	-0.0800000
54439.0000000	-0.0800000
54440.0000000	-0.0750000
54441.0000000	-0.0800000
54442.0000000	-0.0700000
54443.0000000	-0.0750000
54444.0000000	-0.0700000
54445.0000000	-0.0750000
54446.0000000	-0.0700000
54447.0000000	-0.0650000
54448.0000000	-0.0650000
54449.0000000	-0.0650000
54450.0000000	-0.0600000
54451.0000000	-0.0650000
54452.0000000	-0.0600000
54453.0000000	-0.0650000
54454.0000000	-0.0550000
54455.0000000	-0.0550000
54456.0000000	-0.0550000
54457.0000000	-0.0550000
54458.0000000	-0.0550000
54459.0000000	-0.0550000
54460.0000000	-0.0550000
54461.0000000	-0.0600000
54462.0000000	-0.0600000
54463.0000000	-0.0500000
54464.0000000	-0.0600000
54465.0000000	-0.0600000
54466.0000000	-0.0550000
54467.0000000	-0.0550000
54468.0000000	-0.0600000
54469.0000000	-0.0600000
54470.0000000	-0.0600000
54471.0000000	-0.0550000
54472.0000000	-0.0600000
54473.0000000	-0.0600000
54474.0000000	-0.0600000
54475.0000000	-0.0600000
54476.0000000	-0.0600000
54477.0000000	-0.0550000
54478.0000000	-0.0600000
54479.0000000	-0.0650000
54480.0000000	-0.0600000
54481.0000000	-0.0600000
54482.0000000	-0.0600000
54483.0000000	-0.0550000
54484.0000000	-0.0550000
54485.0000000	-0.0600000
54486.0000000	-0.0600000
54487.0000000	-0.0600000
54488.0000000	-0.0600000
54489.0000000	-0.0600000
54490.0000000	-0.0600000
54491.0000000	-0.0600000

54492.0000000	-0.0600000
54493.0000000	-0.0600000
54494.0000000	-0.0600000
54495.0000000	-0.0600000
54496.0000000	-0.0600000
54497.0000000	-0.0600000
54498.0000000	-0.0600000
54499.0000000	-0.0650000
54500.0000000	-0.0650000
54501.0000000	-0.0600000
54502.0000000	-0.0650000
54503.0000000	-0.0650000
54504.0000000	-0.0650000
54505.0000000	-0.0700000
54506.0000000	-0.0750000
54507.0000000	-0.0700000
54508.0000000	-0.0800000
54509.0000000	-0.0800000
54510.0000000	-0.0800000
54511.0000000	-0.0850000
54512.0000000	-0.0800000
54513.0000000	-0.0850000
54514.0000000	-0.0850000
54515.0000000	-0.0850000
54516.0000000	-0.0850000
54517.0000000	-0.0850000
54518.0000000	-0.0900000
54519.0000000	-0.0950000
54520.0000000	-0.0950000
54521.0000000	-0.0950000
54522.0000000	-0.0933333
54523.0000000	-0.0950000
54524.0000000	-0.1000000
54525.0000000	-0.1000000
54526.0000000	-0.1000000
54527.0000000	-0.1050000
54528.0000000	-0.1000000
54529.0000000	-0.1100000
54530.0000000	-0.1050000
54531.0000000	-0.1050000
54532.0000000	-0.1100000
54533.0000000	-0.1100000
54534.0000000	-0.1100000
54535.0000000	-0.1150000
54536.0000000	-0.1150000
54537.0000000	-0.1150000
54538.0000000	-0.1150000
54539.0000000	-0.1150000
54540.0000000	-0.1200000
54541.0000000	-0.1150000
54542.0000000	-0.1150000
54543.0000000	-0.1150000
54544.0000000	-0.1100000
54545.0000000	-0.1150000
54546.0000000	-0.1150000
54547.0000000	-0.1100000
54548.0000000	-0.1100000

54549.0000000	-0.1150000
54550.0000000	-0.1150000
54551.0000000	-0.1150000
54552.0000000	-0.1133333
54553.0000000	-0.1200000
54554.0000000	-0.1200000
54555.0000000	-0.1200000
54556.0000000	-0.1250000
54557.0000000	-0.1200000
54558.0000000	-0.1200000
54559.0000000	-0.1300000
54560.0000000	-0.1250000
54561.0000000	-0.1300000
54562.0000000	-0.1300000
54563.0000000	-0.1350000
54564.0000000	-0.1400000
54565.0000000	-0.1350000
54566.0000000	-0.1450000
54567.0000000	-0.1450000
54568.0000000	-0.1450000
54569.0000000	-0.1450000
54570.0000000	-0.1450000
54571.0000000	-0.1500000
54572.0000000	-0.1500000
54573.0000000	-0.1550000
54574.0000000	-0.1550000
54575.0000000	-0.1550000
54576.0000000	-0.1600000
54577.0000000	-0.1600000
54578.0000000	-0.1600000
54579.0000000	-0.1600000
54580.0000000	-0.1650000
54581.0000000	-0.1700000
54582.0000000	-0.1700000
54583.0000000	-0.1700000
54584.0000000	-0.1700000
54585.0000000	-0.1700000
54586.0000000	-0.1750000
54587.0000000	-0.1750000
54588.0000000	-0.1700000
54589.0000000	-0.1750000
54590.0000000	-0.1750000
54591.0000000	-0.1700000
54592.0000000	-0.1700000
54593.0000000	-0.1700000
54594.0000000	-0.1750000
54595.0000000	-0.1750000
54596.0000000	-0.1750000
54597.0000000	-0.1750000
54598.0000000	-0.1800000
54599.0000000	-0.1800000
54600.0000000	-0.1800000
54601.0000000	-0.1800000
54602.0000000	-0.1800000
54603.0000000	-0.1900000
54604.0000000	-0.1850000
54605.0000000	-0.1900000

54606.0000000	-0.1850000
54607.0000000	-0.1900000
54608.0000000	-0.1850000
54609.0000000	-0.1900000
54610.0000000	-0.1950000
54611.0000000	-0.1900000
54612.0000000	-0.1900000
54613.0000000	-0.1900000
54614.0000000	-0.1950000
54615.0000000	-0.2000000
54616.0000000	-0.1950000
54617.0000000	-0.2000000
54618.0000000	-0.2000000
54619.0000000	-0.2000000
54620.0000000	-0.1950000
54621.0000000	-0.2000000
54622.0000000	-0.1950000
54623.0000000	-0.1950000
54624.0000000	-0.2000000
54625.0000000	-0.2000000
54626.0000000	-0.1950000
54627.0000000	-0.1950000
54628.0000000	-0.2000000
54629.0000000	-0.1950000
54630.0000000	-0.1950000
54631.0000000	-0.1950000
54632.0000000	-0.1950000
54633.0000000	-0.1950000
54634.0000000	-0.1950000
54635.0000000	-0.2000000
54636.0000000	-0.2000000
54637.0000000	-0.2000000
54638.0000000	-0.2000000
54639.0000000	-0.2050000
54640.0000000	-0.2050000
54641.0000000	-0.2050000
54642.0000000	-0.2100000
54643.0000000	-0.2033333
54644.0000000	-0.2100000
54645.0000000	-0.2100000
54646.0000000	-0.2100000
54647.0000000	-0.2150000
54648.0000000	-0.2150000
54649.0000000	-0.2150000
54650.0000000	-0.2200000
54651.0000000	-0.2150000
54652.0000000	-0.2150000
54653.0000000	-0.2150000
54654.0000000	-0.2150000
54655.0000000	-0.2150000
54656.0000000	-0.2150000
54657.0000000	-0.2150000
54658.0000000	-0.2200000
54659.0000000	-0.2150000
54660.0000000	-0.2150000
54661.0000000	-0.2150000
54662.0000000	-0.2100000

54663.0000000	-0.2150000
54664.0000000	-0.2100000
54665.0000000	-0.2100000
54666.0000000	-0.2100000
54667.0000000	-0.2050000
54668.0000000	-0.2050000
54669.0000000	-0.2050000
54670.0000000	-0.2000000
54671.0000000	-0.2050000
54672.0000000	-0.2000000
54673.0000000	-0.1933333
54674.0000000	-0.1950000
54675.0000000	-0.1950000
54676.0000000	-0.2000000
54677.0000000	-0.2000000
54678.0000000	-0.1950000
54679.0000000	-0.1900000
54680.0000000	-0.1900000
54681.0000000	-0.1850000
54682.0000000	-0.1850000
54683.0000000	-0.1850000
54684.0000000	-0.1850000
54685.0000000	-0.1850000
54686.0000000	-0.1750000
54687.0000000	-0.1750000
54688.0000000	-0.1700000
54689.0000000	-0.1700000
54690.0000000	-0.1750000
54691.0000000	-0.1750000
54692.0000000	-0.1700000
54693.0000000	-0.1700000
54694.0000000	-0.1650000
54695.0000000	-0.1650000
54696.0000000	-0.1650000
54697.0000000	-0.1650000
54698.0000000	-0.1650000
54699.0000000	-0.1650000
54700.0000000	-0.1600000
54701.0000000	-0.1600000
54702.0000000	-0.1550000
54703.0000000	-0.1600000
54704.0000000	-0.1600000
54705.0000000	-0.1550000
54706.0000000	-0.1550000
54707.0000000	-0.1550000
54708.0000000	-0.1500000
54709.0000000	-0.1550000
54710.0000000	-0.1500000
54711.0000000	-0.1500000
54712.0000000	-0.1450000
54713.0000000	-0.1500000
54714.0000000	-0.1500000
54715.0000000	-0.1450000
54716.0000000	-0.1450000
54717.0000000	-0.1400000
54718.0000000	-0.1400000
54719.0000000	-0.1350000

54720.0000000	-0.1350000
54721.0000000	-0.1350000
54722.0000000	-0.1350000
54723.0000000	-0.1350000
54724.0000000	-0.1250000
54725.0000000	-0.1250000
54726.0000000	-0.1250000
54727.0000000	-0.1250000
54728.0000000	-0.1150000
54729.0000000	-0.1200000
54730.0000000	-0.1100000
54731.0000000	-0.1100000
54732.0000000	-0.1100000
54733.0000000	-0.1100000
54734.0000000	-0.1066667
54735.0000000	-0.1000000
54736.0000000	-0.0950000
54737.0000000	-0.0950000
54738.0000000	-0.0900000
54739.0000000	-0.0850000
54740.0000000	-0.0800000
54741.0000000	-0.0850000
54742.0000000	-0.0800000
54743.0000000	-0.0800000
54744.0000000	-0.0850000
54745.0000000	-0.0750000
54746.0000000	-0.0750000
54747.0000000	-0.0750000
54748.0000000	-0.0750000
54749.0000000	-0.0750000
54750.0000000	-0.0750000
54751.0000000	-0.0750000
54752.0000000	-0.0750000
54753.0000000	-0.0750000
54754.0000000	-0.0700000
54755.0000000	-0.0750000
54756.0000000	-0.0750000
54757.0000000	-0.0800000
54758.0000000	-0.0750000
54759.0000000	-0.0700000
54760.0000000	-0.0750000
54761.0000000	-0.0750000
54762.0000000	-0.0700000
54763.0000000	-0.0633333
54764.0000000	-0.0650000
54765.0000000	-0.0600000
54766.0000000	-0.0600000
54767.0000000	-0.0500000
54768.0000000	-0.0500000
54769.0000000	-0.0400000
54770.0000000	-0.0400000
54771.0000000	-0.0350000
54772.0000000	-0.0300000
54773.0000000	-0.0250000
54774.0000000	-0.0250000
54775.0000000	-0.0250000
54776.0000000	-0.0250000

54777.0000000	-0.0200000
54778.0000000	-0.0200000
54779.0000000	-0.0150000
54780.0000000	-0.0150000
54781.0000000	-0.0150000
54782.0000000	-0.0150000
54783.0000000	-0.0050000
54784.0000000	-0.0100000
54785.0000000	-0.0100000
54786.0000000	-0.0050000
54787.0000000	-0.0050000
54788.0000000	-0.0050000
54789.0000000	-0.0100000
54790.0000000	-0.0050000
54791.0000000	-0.0050000
54792.0000000	-0.0050000
54793.0000000	0.0000000
54794.0000000	-0.0066667
54795.0000000	0.0000000
54796.0000000	0.0050000
54797.0000000	0.0000000
54798.0000000	0.0000000
54799.0000000	0.0000000
54800.0000000	-0.0050000
54801.0000000	-0.0050000
54802.0000000	-0.0050000
54803.0000000	-0.0050000
54804.0000000	-0.0050000
54805.0000000	-0.0100000
54806.0000000	-0.0100000
54807.0000000	-0.0150000
54808.0000000	-0.0100000
54809.0000000	-0.0050000
54810.0000000	-0.0100000
54811.0000000	-0.0050000
54812.0000000	-0.0050000
54813.0000000	-0.0050000
54814.0000000	-0.0050000
54815.0000000	-0.0100000
54816.0000000	-0.0050000
54817.0000000	0.0000000
54818.0000000	-0.0050000
54819.0000000	-0.0050000
54820.0000000	0.0000000
54821.0000000	0.0000000
54822.0000000	0.0000000
54823.0000000	0.0000000
54824.0000000	0.0000000
54825.0000000	0.0000000
54826.0000000	0.0000000
54827.0000000	0.0050000
54828.0000000	0.0000000
54829.0000000	0.0000000
54830.0000000	0.0050000
54831.0000000	0.0050000
54832.0000000	0.0050000
54833.0000000	0.0050000

54834.0000000	0.0050000
54835.0000000	0.0100000
54836.0000000	0.0100000
54837.0000000	0.0050000
54838.0000000	0.0100000
54839.0000000	0.0150000
54840.0000000	0.0150000
54841.0000000	0.0100000
54842.0000000	0.0150000
54843.0000000	0.0150000
54844.0000000	0.0100000
54845.0000000	0.0150000
54846.0000000	0.0150000
54847.0000000	0.0200000
54848.0000000	0.0150000
54849.0000000	0.0100000
54850.0000000	0.0150000
54851.0000000	0.0150000
54852.0000000	0.0200000
54853.0000000	0.0250000
54854.0000000	0.0300000
54855.0000000	0.0250000
54856.0000000	0.0250000
54857.0000000	0.0300000
54858.0000000	0.0300000
54859.0000000	0.0300000
54860.0000000	0.0300000
54861.0000000	0.0350000
54862.0000000	0.0350000
54863.0000000	0.0350000
54864.0000000	0.0350000
54865.0000000	0.0300000
54866.0000000	0.0350000
54867.0000000	0.0300000
54868.0000000	0.0350000
54869.0000000	0.0300000
54870.0000000	0.0350000
54871.0000000	0.0350000
54872.0000000	0.0350000
54873.0000000	0.0300000
54874.0000000	0.0250000
54875.0000000	0.0300000
54876.0000000	0.0250000
54877.0000000	0.0250000
54878.0000000	0.0300000
54879.0000000	0.0300000
54880.0000000	0.0250000
54881.0000000	0.0200000
54882.0000000	0.0250000
54883.0000000	0.0200000
54884.0000000	0.0266667
54885.0000000	0.0200000
54886.0000000	0.0250000
54887.0000000	0.0250000
54888.0000000	0.0250000
54889.0000000	0.0200000
54890.0000000	0.0250000

54891.0000000	0.0250000
54892.0000000	0.0250000
54893.0000000	0.0300000
54894.0000000	0.0300000
54895.0000000	0.0300000
54896.0000000	0.0300000
54897.0000000	0.0300000
54898.0000000	0.0300000
54899.0000000	0.0250000
54900.0000000	0.0300000
54901.0000000	0.0250000
54902.0000000	0.0250000
54903.0000000	0.0250000
54904.0000000	0.0300000
54905.0000000	0.0250000
54906.0000000	0.0250000
54907.0000000	0.0250000
54908.0000000	0.0250000
54909.0000000	0.0200000
54910.0000000	0.0200000
54911.0000000	0.0200000
54912.0000000	0.0250000
54913.0000000	0.0250000
54914.0000000	0.0200000
54915.0000000	0.0233333
54916.0000000	0.0250000
54917.0000000	0.0200000
54918.0000000	0.0250000
54919.0000000	0.0200000
54920.0000000	0.0250000
54921.0000000	0.0300000
54922.0000000	0.0250000
54923.0000000	0.0250000
54924.0000000	0.0200000
54925.0000000	0.0300000
54926.0000000	0.0300000
54927.0000000	0.0350000
54928.0000000	0.0300000
54929.0000000	0.0350000
54930.0000000	0.0300000
54931.0000000	0.0350000
54932.0000000	0.0400000
54933.0000000	0.0350000
54934.0000000	0.0350000
54935.0000000	0.0400000
54936.0000000	0.0400000
54937.0000000	0.0400000
54938.0000000	0.0400000
54939.0000000	0.0450000
54940.0000000	0.0400000
54941.0000000	0.0450000
54942.0000000	0.0500000
54943.0000000	0.0500000
54944.0000000	0.0500000
54945.0000000	0.0433333
54946.0000000	0.0500000
54947.0000000	0.0500000

54948.0000000	0.0550000
54949.0000000	0.0550000
54950.0000000	0.0550000
54951.0000000	0.0550000
54952.0000000	0.0600000
54953.0000000	0.0600000
54954.0000000	0.0700000
54955.0000000	0.0700000
54956.0000000	0.0750000
54957.0000000	0.0750000
54958.0000000	0.0750000
54959.0000000	0.0800000
54960.0000000	0.0800000
54961.0000000	0.0800000
54962.0000000	0.0850000
54963.0000000	0.0850000
54964.0000000	0.0950000
54965.0000000	0.0950000
54966.0000000	0.1000000
54967.0000000	0.1000000
54968.0000000	0.1050000
54969.0000000	0.1100000
54970.0000000	0.1100000
54971.0000000	0.1150000
54972.0000000	0.1150000
54973.0000000	0.1150000
54974.0000000	0.1100000
54975.0000000	0.1166667
54976.0000000	0.1150000
54977.0000000	0.1150000
54978.0000000	0.1150000
54979.0000000	0.1100000
54980.0000000	0.1200000
54981.0000000	0.1200000
54982.0000000	0.1150000
54983.0000000	0.1150000
54984.0000000	0.1200000
54985.0000000	0.1150000
54986.0000000	0.1200000
54987.0000000	0.1250000
54988.0000000	0.1200000
54989.0000000	0.1250000
54990.0000000	0.1300000
54991.0000000	0.1350000
54992.0000000	0.1350000
54993.0000000	0.1350000
54994.0000000	0.1400000
54995.0000000	0.1450000
54996.0000000	0.1500000
54997.0000000	0.1550000
54998.0000000	0.1500000
54999.0000000	0.1550000
55000.0000000	0.1550000
55001.0000000	0.1600000
55002.0000000	0.1650000
55003.0000000	0.1700000
55004.0000000	0.1700000

55005.0000000	0.1700000
55006.0000000	0.1750000
55007.0000000	0.1750000
55008.0000000	0.1850000
55009.0000000	0.1850000
55010.0000000	0.1800000
55011.0000000	0.1850000
55012.0000000	0.1900000
55013.0000000	0.1950000
55014.0000000	0.1950000
55015.0000000	0.1950000
55016.0000000	0.1950000
55017.0000000	0.1900000
55018.0000000	0.2100000
55019.0000000	0.2100000
55020.0000000	0.2100000
55021.0000000	0.2100000
55022.0000000	0.2100000
55023.0000000	0.2200000
55024.0000000	0.2200000
55025.0000000	0.2250000
55026.0000000	0.2300000
55027.0000000	0.2300000
55028.0000000	0.2250000
55029.0000000	0.2350000
55030.0000000	0.2300000
55031.0000000	0.2350000
55032.0000000	0.2350000
55033.0000000	0.2350000
55034.0000000	0.2350000
55035.0000000	0.2333333
55036.0000000	0.2400000
55037.0000000	0.2450000
55038.0000000	0.2450000
55039.0000000	0.2500000
55040.0000000	0.2450000
55041.0000000	0.2450000
55042.0000000	0.2500000
55043.0000000	0.2550000
55044.0000000	0.2500000
55045.0000000	0.2500000
55046.0000000	0.2500000
55047.0000000	0.2450000
55048.0000000	0.2550000
55049.0000000	0.2550000
55050.0000000	0.2550000
55051.0000000	0.2650000
55052.0000000	0.2650000
55053.0000000	0.2650000
55054.0000000	0.2650000
55055.0000000	0.2650000
55056.0000000	0.2650000
55057.0000000	0.2700000
55058.0000000	0.2650000
55059.0000000	0.2650000
55060.0000000	0.2600000
55061.0000000	0.2600000

55062.0000000	0.2600000
55063.0000000	0.2600000
55064.0000000	0.2600000
55065.0000000	0.2650000
55066.0000000	0.2700000
55067.0000000	0.2650000
55068.0000000	0.2650000
55069.0000000	0.2700000
55070.0000000	0.2700000
55071.0000000	0.2750000
55072.0000000	0.2800000
55073.0000000	0.2900000
55074.0000000	0.2850000
55075.0000000	0.2900000
55076.0000000	0.2900000
55077.0000000	0.2900000
55078.0000000	0.3000000
55079.0000000	0.3000000
55080.0000000	0.3000000
55081.0000000	0.3000000
55082.0000000	0.3100000
55083.0000000	0.3200000
55084.0000000	0.3200000
55085.0000000	0.3200000
55086.0000000	0.3250000
55087.0000000	0.3200000
55088.0000000	0.3200000
55089.0000000	0.3250000
55090.0000000	0.3250000
55091.0000000	0.3300000
55092.0000000	0.3300000
55093.0000000	0.3300000
55094.0000000	0.3400000
55095.0000000	0.3400000
55096.0000000	0.3400000
55097.0000000	0.3450000
55098.0000000	0.3550000
55099.0000000	0.3600000
55100.0000000	0.3650000
55101.0000000	0.3700000
55102.0000000	0.3650000
55103.0000000	0.3700000
55104.0000000	0.3750000
55105.0000000	0.3750000
55106.0000000	0.3800000
55107.0000000	0.3800000
55108.0000000	0.3850000
55109.0000000	0.3900000
55110.0000000	0.3950000
55111.0000000	0.3950000
55112.0000000	0.4000000
55113.0000000	0.4000000
55114.0000000	0.4050000
55115.0000000	0.4000000
55116.0000000	0.4050000
55117.0000000	0.4150000
55118.0000000	0.4200000

55119.0000000	0.4250000
55120.0000000	0.4300000
55121.0000000	0.4350000
55122.0000000	0.4350000
55123.0000000	0.4400000
55124.0000000	0.4450000
55125.0000000	0.4533333
55126.0000000	0.4550000
55127.0000000	0.4600000
55128.0000000	0.4650000
55129.0000000	0.4700000
55130.0000000	0.4700000
55131.0000000	0.4750000
55132.0000000	0.4850000
55133.0000000	0.4800000
55134.0000000	0.4850000
55135.0000000	0.4950000
55136.0000000	0.4950000
55137.0000000	0.5000000
55138.0000000	0.5000000
55139.0000000	0.5050000
55140.0000000	0.5100000
55141.0000000	0.5050000
55142.0000000	0.5000000
55143.0000000	0.5150000
55144.0000000	0.5100000
55145.0000000	0.5200000
55146.0000000	0.5200000
55147.0000000	0.5200000
55148.0000000	0.5200000
55149.0000000	0.5350000
55150.0000000	0.5350000
55151.0000000	0.5400000
55152.0000000	0.5350000
55153.0000000	0.5450000
55154.0000000	0.5450000
55155.0000000	0.5550000
55156.0000000	0.5600000
55157.0000000	0.5550000
55158.0000000	0.5600000
55159.0000000	0.5600000
55160.0000000	0.5650000
55161.0000000	0.5700000
55162.0000000	0.5750000
55163.0000000	0.5700000
55164.0000000	0.5800000
55165.0000000	0.5800000
55166.0000000	0.5850000
55167.0000000	0.5850000
55168.0000000	0.5900000
55169.0000000	0.5950000
55170.0000000	0.6000000
55171.0000000	0.6000000
55172.0000000	0.6150000
55173.0000000	0.6200000
55174.0000000	0.6300000
55175.0000000	0.6300000

55176.0000000	0.6400000
55177.0000000	0.6500000
55178.0000000	0.6600000
55179.0000000	0.6700000
55180.0000000	0.6750000
55181.0000000	0.6800000
55182.0000000	0.6850000
55183.0000000	0.6950000
55184.0000000	0.7000000
55185.0000000	0.7000000
55186.0000000	0.7100000
55187.0000000	0.7166667
55188.0000000	0.7200000
55189.0000000	0.7200000
55190.0000000	0.7250000
55191.0000000	0.7350000
55192.0000000	0.7300000
55193.0000000	0.7350000
55194.0000000	0.7350000
55195.0000000	0.7350000
55196.0000000	0.7350000
55197.0000000	0.7350000
55198.0000000	0.7350000
55199.0000000	0.7350000
55200.0000000	0.7350000
55201.0000000	0.7350000
55202.0000000	0.7400000
55203.0000000	0.7400000
55204.0000000	0.7450000
55205.0000000	0.7500000
55206.0000000	0.7500000
55207.0000000	0.7500000
55208.0000000	0.7600000
55209.0000000	0.7650000
55210.0000000	0.7600000
55211.0000000	0.7650000
55212.0000000	0.7700000
55213.0000000	0.7750000
55214.0000000	0.7750000
55215.0000000	0.7700000
55216.0000000	0.7800000
55217.0000000	0.7833333
55218.0000000	0.7800000
55219.0000000	0.7900000
55220.0000000	0.7850000
55221.0000000	0.7900000
55222.0000000	0.7900000
55223.0000000	0.7900000
55224.0000000	0.7950000
55225.0000000	0.7950000
55226.0000000	0.7900000
55227.0000000	0.7950000
55228.0000000	0.7950000
55229.0000000	0.8000000
55230.0000000	0.8000000
55231.0000000	0.8000000
55232.0000000	0.8050000

55233.0000000	0.8050000
55234.0000000	0.8050000
55235.0000000	0.8050000
55236.0000000	0.8150000
55237.0000000	0.8100000
55238.0000000	0.8150000
55239.0000000	0.8150000
55240.0000000	0.8200000
55241.0000000	0.8200000
55242.0000000	0.8200000
55243.0000000	0.8250000
55244.0000000	0.8250000
55245.0000000	0.8300000
55246.0000000	0.8333333
55247.0000000	0.8350000
55248.0000000	0.8300000
55249.0000000	0.8300000
55250.0000000	0.8400000
55251.0000000	0.8450000
55252.0000000	0.8450000
55253.0000000	0.8500000
55254.0000000	0.8550000
55255.0000000	0.8550000
55256.0000000	0.8550000
55257.0000000	0.8600000
55258.0000000	0.8600000
55259.0000000	0.8650000
55260.0000000	0.8650000
55261.0000000	0.8700000
55262.0000000	0.8750000
55263.0000000	0.8800000
55264.0000000	0.8800000
55265.0000000	0.8850000
55266.0000000	0.8900000
55267.0000000	0.8850000
55268.0000000	0.8900000
55269.0000000	0.8900000
55270.0000000	0.8950000
55271.0000000	0.9050000
55272.0000000	0.9100000
55273.0000000	0.9050000
55274.0000000	0.9150000
55275.0000000	0.9100000
55276.0000000	0.9200000
55277.0000000	0.9233333
55278.0000000	0.9250000
55279.0000000	0.9300000
55280.0000000	0.9350000
55281.0000000	0.9450000
55282.0000000	0.9500000
55283.0000000	0.9450000
55284.0000000	0.9500000
55285.0000000	0.9550000
55286.0000000	0.9600000
55287.0000000	0.9700000
55288.0000000	0.9700000
55289.0000000	0.9700000

55290.0000000	0.9800000
55291.0000000	0.9800000
55292.0000000	0.9800000
55293.0000000	0.9900000
55294.0000000	0.9950000
55295.0000000	0.9950000
55296.0000000	0.9950000
55297.0000000	1.0000000
55298.0000000	1.0100000
55299.0000000	1.0050000
55300.0000000	1.0050000
55301.0000000	1.0150000
55302.0000000	1.0100000
55303.0000000	1.0100000
55304.0000000	1.0150000
55305.0000000	1.0200000
55306.0000000	1.0250000
55307.0000000	1.0266666
55308.0000000	1.0300000
55309.0000000	1.0350000
55310.0000000	1.0350000
55311.0000000	1.0300000
55312.0000000	1.0350000
55313.0000000	1.0350000
55314.0000000	1.0350000
55315.0000000	1.0450000
55316.0000000	1.0400000
55317.0000000	1.0400000
55318.0000000	1.0400000
55319.0000000	1.0450000
55320.0000000	1.0450000
55321.0000000	1.0450000
55322.0000000	1.0450000
55323.0000000	1.0450000
55324.0000000	1.0500000
55325.0000000	1.0500000
55326.0000000	1.0500000
55327.0000000	1.0450000
55328.0000000	1.0549999
55329.0000000	1.0500000
55330.0000000	1.0500000
55331.0000000	1.0549999
55332.0000000	1.0599999
55333.0000000	1.0549999
55334.0000000	1.0599999
55335.0000000	1.0549999
55336.0000000	1.0500000
55337.0000000	1.0599999
55338.0000000	1.0599999
55339.0000000	1.0650001
55340.0000000	1.0650001
55341.0000000	1.0599999
55342.0000000	1.0650001
55343.0000000	1.0650001
55344.0000000	1.0700001
55345.0000000	1.0650001
55346.0000000	1.0599999

55347.0000000	1.0599999
55348.0000000	1.0599999
55349.0000000	1.0650001
55350.0000000	1.0650001
55351.0000000	1.0549999
55352.0000000	1.0599999
55353.0000000	1.0500000
55354.0000000	1.0549999
55355.0000000	1.0599999
55356.0000000	1.0599999
55357.0000000	1.0500000
55358.0000000	1.0549999
55359.0000000	1.0500000
55360.0000000	1.0500000
55361.0000000	1.0450000
55362.0000000	1.0450000
55363.0000000	1.0450000
55364.0000000	1.0450000
55365.0000000	1.0450000
55366.0000000	1.0500000
55367.0000000	1.0533333
55368.0000000	1.0550001
55369.0000000	1.0500000
55370.0000000	1.0500000
55371.0000000	1.0549999
55372.0000000	1.0549999
55373.0000000	1.0549999
55374.0000000	1.0549999
55375.0000000	1.0549999
55376.0000000	1.0549999
55377.0000000	1.0549999
55378.0000000	1.0599999
55379.0000000	1.0549999
55380.0000000	1.0549999
55381.0000000	1.0599999
55382.0000000	1.0599999
55383.0000000	1.0599999
55384.0000000	1.0650001
55385.0000000	1.0599999
55386.0000000	1.0599999
55387.0000000	1.0599999
55388.0000000	1.0599999
55389.0000000	1.0549999
55390.0000000	1.0599999
55391.0000000	1.0650001
55392.0000000	1.0599999
55393.0000000	1.0599999
55394.0000000	1.0650001
55395.0000000	1.0599999
55396.0000000	1.0650001
55397.0000000	1.0650001
55398.0000000	1.0599999
55399.0000000	1.0700001
55400.0000000	1.0700001
55401.0000000	1.0650001
55402.0000000	1.0750000
55403.0000000	1.0700001

55404.0000000	1.0800000
55405.0000000	1.0800000
55406.0000000	1.0800000
55407.0000000	1.0800000
55408.0000000	1.0800000
55409.0000000	1.0850000
55410.0000000	1.0800000
55411.0000000	1.0800000
55412.0000000	1.0850000
55413.0000000	1.0850000
55414.0000000	1.0900000
55415.0000000	1.0850000
55416.0000000	1.0900000
55417.0000000	1.0850000
55418.0000000	1.0900000
55419.0000000	1.0850000
55420.0000000	1.0900000
55421.0000000	1.0950000
55422.0000000	1.0950000
55423.0000000	1.0950000
55424.0000000	1.0950000
55425.0000000	1.0950000
55426.0000000	1.0950000
55427.0000000	1.0950000
55428.0000000	1.1033334
55429.0000000	1.0950000
55430.0000000	1.1050000
55431.0000000	1.1050000
55432.0000000	1.1000000
55433.0000000	1.1050000
55434.0000000	1.1050000
55435.0000000	1.1100000
55436.0000000	1.1100000
55437.0000000	1.1150000
55438.0000000	1.1150000
55439.0000000	1.1150000
55440.0000000	1.1250000
55441.0000000	1.1250000
55442.0000000	1.1250000
55443.0000000	1.1250000
55444.0000000	1.1300000
55445.0000000	1.1350000
55446.0000000	1.1300000
55447.0000000	1.1300000
55448.0000000	1.1250000
55449.0000000	1.1350000
55450.0000000	1.1400000
55451.0000000	1.1450000
55452.0000000	1.1450000
55453.0000000	1.1550000
55454.0000000	1.1600000
55455.0000000	1.1500000
55456.0000000	1.1600000
55457.0000000	1.1600000
55458.0000000	1.1600000
55459.0000000	1.1650000
55460.0000000	1.1700000

55461.0000000	1.1700000
55462.0000000	1.1700000
55463.0000000	1.1800001
55464.0000000	1.1800001
55465.0000000	1.1849999
55466.0000000	1.1900001
55467.0000000	1.1950001
55468.0000000	1.1950001
55469.0000000	1.2000000
55470.0000000	1.2000000
55471.0000000	1.2000000
55472.0000000	1.2150000
55473.0000000	1.2050000
55474.0000000	1.2200000
55475.0000000	1.2250000
55476.0000000	1.2250000
55477.0000000	1.2400000
55478.0000000	1.2350000
55479.0000000	1.2350000
55480.0000000	1.2400000
55481.0000000	1.2400000
55482.0000000	1.2450000
55483.0000000	1.2450000
55484.0000000	1.2500000
55485.0000000	1.2550000
55486.0000000	1.2550000
55487.0000000	1.2550000
55488.0000000	1.2566667
55489.0000000	1.2600000
55490.0000000	1.2650000
55491.0000000	1.2700000
55492.0000000	1.2700000
55493.0000000	1.2750000
55494.0000000	1.2750000
55495.0000000	1.2900000
55496.0000000	1.2800000
55497.0000000	1.2900000
55498.0000000	1.2900000
55499.0000000	1.2950000
55500.0000000	1.2950000
55501.0000000	1.3000000
55502.0000000	1.3049999
55503.0000000	1.3099999
55504.0000000	1.3150001
55505.0000000	1.3199999
55506.0000000	1.3250000
55507.0000000	1.3150001
55508.0000000	1.3300000
55509.0000000	1.3400000
55510.0000000	1.3400000
55511.0000000	1.3400000
55512.0000000	1.3450000
55513.0000000	1.3500000
55514.0000000	1.3600000
55515.0000000	1.3600000
55516.0000000	1.3600000
55517.0000000	1.3700000

55518.0000000	1.3700000
55519.0000000	1.3750000
55520.0000000	1.3800000
55521.0000000	1.3850000
55522.0000000	1.3900000
55523.0000000	1.3850000
55524.0000000	1.4000000
55525.0000000	1.4050000
55526.0000000	1.4100000
55527.0000000	1.4150000
55528.0000000	1.4200000
55529.0000000	1.4200000
55530.0000000	1.4200000
55531.0000000	1.4250000
55532.0000000	1.4250000
55533.0000000	1.4299999
55534.0000000	1.4400001
55535.0000000	1.4450001
55536.0000000	1.4500000
55537.0000000	1.4550000
55538.0000000	1.4600000
55539.0000000	1.4650000
55540.0000000	1.4750000
55541.0000000	1.4650000
55542.0000000	1.4850000
55543.0000000	1.4850000
55544.0000000	1.4900000
55545.0000000	1.4950000
55546.0000000	1.4950000
55547.0000000	1.5000000
55548.0000000	1.5050000
55549.0000000	1.5066667
55550.0000000	1.5150000
55551.0000000	1.5200000
55552.0000000	1.5250000
55553.0000000	1.5300000
55554.0000000	1.5300000
55555.0000000	1.5350000
55556.0000000	1.5450000
55557.0000000	1.5450000
55558.0000000	1.5549999
55559.0000000	1.5549999
55560.0000000	1.5599999
55561.0000000	1.5650001
55562.0000000	1.5700001
55563.0000000	1.5800000
55564.0000000	1.5800000
55565.0000000	1.5900000
55566.0000000	1.6000000
55567.0000000	1.6050000
55568.0000000	1.6100000
55569.0000000	1.6200000
55570.0000000	1.6250000
55571.0000000	1.6250000
55572.0000000	1.6350000
55573.0000000	1.6350000
55574.0000000	1.6350000

55575.0000000	1.6400000
55576.0000000	1.6400000
55577.0000000	1.6500000
55578.0000000	1.6500000
55579.0000000	1.6566666
55580.0000000	1.6650000
55581.0000000	1.6750000
55582.0000000	1.6750000
55583.0000000	1.6800001
55584.0000000	1.6900001
55585.0000000	1.6849999
55586.0000000	1.7050000
55587.0000000	1.7100000
55588.0000000	1.7100000
55589.0000000	1.7200000
55590.0000000	1.7250000
55591.0000000	1.7300000
55592.0000000	1.7350000
55593.0000000	1.7400000
55594.0000000	1.7450000
55595.0000000	1.7500000
55596.0000000	1.7600000
55597.0000000	1.7700000
55598.0000000	1.7650000
55599.0000000	1.7800000
55600.0000000	1.7850000
55601.0000000	1.7900000
55602.0000000	1.7950000
55603.0000000	1.8049999
55604.0000000	1.8049999
55605.0000000	1.8200001
55606.0000000	1.8300000
55607.0000000	1.8300000
55608.0000000	1.8433334
55609.0000000	1.8500000
55610.0000000	1.8600000
55611.0000000	1.8600000
55612.0000000	1.8750000
55613.0000000	1.8850000
55614.0000000	1.8900000
55615.0000000	1.9000000
55616.0000000	1.9100000
55617.0000000	1.9100000
55618.0000000	1.9200000
55619.0000000	1.9150000
55620.0000000	1.9349999
55621.0000000	1.9450001
55622.0000000	1.9500000
55623.0000000	1.9600000
55624.0000000	1.9700000
55625.0000000	1.9900000
55626.0000000	1.9900000
55627.0000000	2.0000000
55628.0000000	2.0100000
55629.0000000	2.0200000
55630.0000000	2.0250001
55631.0000000	2.0400000

55632.0000000	2.0450001
55633.0000000	2.0549998
55634.0000000	2.0599999
55635.0000000	2.0650001
55636.0000000	2.0749998
55637.0000000	2.0850000
55638.0000000	2.0899999
55639.0000000	2.1033332
55640.0000000	2.1099999
55641.0000000	2.1199999
55642.0000000	2.1250000
55643.0000000	2.1300001
55644.0000000	2.1350002
55645.0000000	2.1400001
55646.0000000	2.1500001
55647.0000000	2.1550002
55648.0000000	2.1600001
55649.0000000	2.1550002
55650.0000000	2.1650000
55651.0000000	2.1750002
55652.0000000	2.1750002
55653.0000000	2.1849999
55654.0000000	2.1950002
55655.0000000	2.1950002
55656.0000000	2.2000000
55657.0000000	2.2049999
55658.0000000	2.1950002
55659.0000000	2.2100000
55660.0000000	2.2150002
55661.0000000	2.2150002
55662.0000000	2.2249999
55663.0000000	2.2300000
55664.0000000	2.2300000
55665.0000000	2.2350001
55666.0000000	2.2400000
55667.0000000	2.2400000
55668.0000000	2.2449999
55669.0000000	2.2500000
55670.0000000	2.2533333
55671.0000000	2.2500000
55672.0000000	2.2550001
55673.0000000	2.2600000
55674.0000000	2.2649999
55675.0000000	2.2700000
55676.0000000	2.2750001
55677.0000000	2.2800000
55678.0000000	2.2849998
55679.0000000	2.2950001
55680.0000000	2.3000000
55681.0000000	2.3049998
55682.0000000	2.3049998
55683.0000000	2.3099999
55684.0000000	2.3150001
55685.0000000	2.3150001
55686.0000000	2.3249998
55687.0000000	2.3249998
55688.0000000	2.3299999

55689.0000000	2.3299999
55690.0000000	2.3350000
55691.0000000	2.3350000
55692.0000000	2.3199999
55693.0000000	2.3350000
55694.0000000	2.3350000
55695.0000000	2.3399999
55696.0000000	2.3399999
55697.0000000	2.3399999
55698.0000000	2.3449998
55699.0000000	2.3466666
55700.0000000	2.3399999
55701.0000000	2.3399999
55702.0000000	2.3399999
55703.0000000	2.3399999
55704.0000000	2.3499999
55705.0000000	2.3449998
55706.0000000	2.3449998
55707.0000000	2.3399999
55708.0000000	2.3350000
55709.0000000	2.3399999
55710.0000000	2.3350000
55711.0000000	2.3350000
55712.0000000	2.3449998
55713.0000000	2.3499999
55714.0000000	2.3599999
55715.0000000	2.3499999
55716.0000000	2.3649998
55717.0000000	2.3699999
55718.0000000	2.3800001
55719.0000000	2.3900001
55720.0000000	2.4050002
55721.0000000	2.4100001
55722.0000000	2.4150000
55723.0000000	2.4250002
55724.0000000	2.4300001
55725.0000000	2.4400001
55726.0000000	2.4549999
55727.0000000	2.4600000
55728.0000000	2.4650002
55729.0000000	2.4866667
55730.0000000	2.5100000
55731.0000000	2.5100000
55732.0000000	2.5349998
55733.0000000	2.5549998
55734.0000000	2.5699999
55735.0000000	2.5899999
55736.0000000	2.6099999
55737.0000000	2.6350002
55738.0000000	2.6500001
55739.0000000	2.6700001
55740.0000000	2.6849999
55741.0000000	2.7049999
55742.0000000	2.7200000
55743.0000000	2.7449999
55744.0000000	2.7600000
55745.0000000	2.7750001

55746.0000000	2.7849998
55747.0000000	2.8150001
55748.0000000	2.8299999
55749.0000000	2.8449998
55750.0000000	2.8599999
55751.0000000	2.8750000
55752.0000000	2.8750000
55753.0000000	2.8850002
55754.0000000	2.9000001
55755.0000000	2.9050002
55756.0000000	2.9200001
55757.0000000	2.9250002
55758.0000000	2.9400001
55759.0000000	2.9500000
55760.0000000	2.9633334
55761.0000000	2.9749999
55762.0000000	2.9900000
55763.0000000	3.0000000
55764.0000000	2.9900000
55765.0000000	3.0300000
55766.0000000	3.0500000
55767.0000000	3.0599999
55768.0000000	3.0699999
55769.0000000	3.0799999
55770.0000000	3.0799999
55771.0000000	3.0999999
55772.0000000	3.1149998
55773.0000000	3.1300001
55774.0000000	3.1450000
55775.0000000	3.1600001
55776.0000000	3.1800001
55777.0000000	3.2000000
55778.0000000	3.2150002
55779.0000000	3.2249999
55780.0000000	3.2449999
55781.0000000	3.2500000
55782.0000000	3.2800000
55783.0000000	3.2900000
55784.0000000	3.3099999
55785.0000000	3.3350000
55786.0000000	3.3449998
55787.0000000	3.3649998
55788.0000000	3.3900001
55789.0000000	3.4000001
55790.0000000	3.4300003
55791.0000000	3.4500000
55792.0000000	3.4700000
55793.0000000	3.4900000
55794.0000000	3.5050001
55795.0000000	3.5300000
55796.0000000	3.5349998
55797.0000000	3.5699999
55798.0000000	3.5899999
55799.0000000	3.5999999
55800.0000000	3.6149998
55801.0000000	3.6300001
55802.0000000	3.6400001

55803.0000000	3.6700001
55804.0000000	3.6900001
55805.0000000	3.7000000
55806.0000000	3.7350001
55807.0000000	3.7550001
55808.0000000	3.7750001
55809.0000000	3.7950001
55810.0000000	3.8150001
55811.0000000	3.8249998
55812.0000000	3.8350000
55813.0000000	3.8550000
55814.0000000	3.8750000
55815.0000000	3.8950000
55816.0000000	3.9200001
55817.0000000	3.9450002
55818.0000000	3.9600000
55819.0000000	3.9949999
55820.0000000	4.0100002
55821.0000000	4.0299997
55822.0000000	4.0400000
55823.0000000	4.0200000
55824.0000000	4.0550003
55825.0000000	4.0749998
55826.0000000	4.1149998
55827.0000000	4.1350002
55828.0000000	4.1599998
55829.0000000	4.1800003
55830.0000000	4.2100000
55831.0000000	4.2349997
55832.0000000	4.2600002
55833.0000000	4.2800002
55834.0000000	4.3000002
55835.0000000	4.3150001
55836.0000000	4.3200002
55837.0000000	4.3500004
55838.0000000	4.3500004
55839.0000000	4.3599997
55840.0000000	4.3649998
55841.0000000	4.3800001
55842.0000000	4.3850002
55843.0000000	4.3850002
55844.0000000	4.4000001
55845.0000000	4.4049997
55846.0000000	4.3950000
55847.0000000	4.4150000
55848.0000000	4.4449997
55849.0000000	4.4699998
55850.0000000	4.5033336
55851.0000000	4.5050001
55852.0000000	4.5200005
55853.0000000	4.5250001
55854.0000000	4.5250001
55855.0000000	4.5650001
55856.0000000	4.5850000
55857.0000000	4.6149998
55858.0000000	4.6199999
55859.0000000	4.6500001

55860.0000000	4.6599998
55861.0000000	4.6750002
55862.0000000	4.6999998
55863.0000000	4.7249999
55864.0000000	4.7300000
55865.0000000	4.7750001
55866.0000000	4.8050003
55867.0000000	4.8400002
55868.0000000	4.8550000
55869.0000000	4.8850002
55870.0000000	4.8900003
55871.0000000	4.9200001
55872.0000000	4.9349999
55873.0000000	4.9549999
55874.0000000	4.9749999
55875.0000000	4.9849997
55876.0000000	5.0100002
55877.0000000	5.0400000
55878.0000000	5.0699997
55879.0000000	5.0950003
55880.0000000	5.1199999
55881.0000000	5.1399999
55882.0000000	5.1650000
55883.0000000	5.1750002
55884.0000000	5.1549997
55885.0000000	5.2049999
55886.0000000	5.1899996
55887.0000000	5.2100000
55888.0000000	5.2500000
55889.0000000	5.2799997
55890.0000000	5.2900000
55891.0000000	5.3200002
55892.0000000	5.3499999
55893.0000000	5.3649998
55894.0000000	5.3999996
55895.0000000	5.4049997
55896.0000000	5.4300003
55897.0000000	5.4300003
55898.0000000	5.4499998
55899.0000000	5.4650002
55900.0000000	5.5000000
55901.0000000	5.4949999
55902.0000000	5.5100002
55903.0000000	5.5250001
55904.0000000	5.5299997
55905.0000000	5.5300002
55906.0000000	5.5650001
55907.0000000	5.5950003
55908.0000000	5.6050000
55909.0000000	5.5850000
55910.0000000	5.6149998
55911.0000000	5.5966668
55912.0000000	5.5450001
55913.0000000	5.5450001
55914.0000000	5.5100002
55915.0000000	5.5200000
55916.0000000	5.4800000

55917.0000000	5.5349998
55918.0000000	5.5749998
55919.0000000	5.5900002
55920.0000000	5.5999999
55921.0000000	5.6100001
55922.0000000	5.6199999
55923.0000000	5.6399999
55924.0000000	5.6700001
55925.0000000	5.6849999
55926.0000000	5.7150002
55927.0000000	5.7249999
55928.0000000	5.7449999
55929.0000000	5.7349997
55930.0000000	5.7799997
55931.0000000	5.7800002
55932.0000000	5.7449999
55933.0000000	5.7449999
55934.0000000	5.7399998
55935.0000000	5.7349997
55936.0000000	5.7550001
55937.0000000	5.8150001
55938.0000000	5.8500004
55939.0000000	5.8800001
55940.0000000	5.9049997
55941.0000000	5.9433331
55942.0000000	5.9749999
55943.0000000	5.9849997
55944.0000000	5.9849997
55945.0000000	5.9600000
55946.0000000	5.9600000
55947.0000000	5.9549999
55948.0000000	5.9449997
55949.0000000	5.9349999
55950.0000000	5.9250002
55951.0000000	5.9200001
55952.0000000	5.9150000
55953.0000000	5.9049997
55954.0000000	5.9150000
55955.0000000	5.9099998
55956.0000000	5.9049997
55957.0000000	5.8950000
55958.0000000	5.9000001
55959.0000000	5.9099998
55960.0000000	5.9150000
55961.0000000	5.9099998
55962.0000000	5.9049997
55963.0000000	5.8950000
55964.0000000	5.9150000
55965.0000000	5.9150000
55966.0000000	5.9000001
55967.0000000	5.9399996
55968.0000000	5.9949999
55969.0000000	6.0600004
55970.0000000	6.0950003
55971.0000000	6.1233330
55972.0000000	6.1549997
55973.0000000	6.1700001

55974.0000000	6.1750002
55975.0000000	6.2049999
55976.0000000	6.2300000
55977.0000000	6.2550001
55978.0000000	6.2700000
55979.0000000	6.2800002
55980.0000000	6.3000002
55981.0000000	6.3200002
55982.0000000	6.3400002
55983.0000000	6.3649998
55984.0000000	6.3699999
55985.0000000	6.3600001
55986.0000000	6.3800001
55987.0000000	6.3750000
55988.0000000	6.3900003
55989.0000000	6.4099998
55990.0000000	6.4200001
55991.0000000	6.4399996
55992.0000000	6.4549999
55993.0000000	6.4700003
55994.0000000	6.4849997
55995.0000000	6.4949999
55996.0000000	6.5050001
55997.0000000	6.5150003
55998.0000000	6.5150003
55999.0000000	6.5250001
56000.0000000	6.5400000
56001.0000000	6.5366669
56002.0000000	6.5450001
56003.0000000	6.5500002
56004.0000000	6.5500002
56005.0000000	6.5450001
56006.0000000	6.5450001
56007.0000000	6.5550003
56008.0000000	6.5600004
56009.0000000	6.5749998
56010.0000000	6.5799999
56011.0000000	6.6000004
56012.0000000	6.6199999
56013.0000000	6.6300001
56014.0000000	6.6500001
56015.0000000	6.6650000
56016.0000000	6.6750002
56017.0000000	6.6999998
56018.0000000	6.7199998
56019.0000000	6.7349997
56020.0000000	6.7550001
56021.0000000	6.7550001
56022.0000000	6.7900000
56023.0000000	6.7900000
56024.0000000	6.7849998
56025.0000000	6.8150001
56026.0000000	6.8249998
56027.0000000	6.8249998
56028.0000000	6.8200002
56029.0000000	6.8050003
56030.0000000	6.8000002

56031.0000000	6.7900000
56032.0000000	6.7633338
56033.0000000	6.7299995
56034.0000000	6.7100000
56035.0000000	6.6849999
56036.0000000	6.6499996
56037.0000000	6.6100001
56038.0000000	6.5799999
56039.0000000	6.5500002
56040.0000000	6.5450001
56041.0000000	6.5349998
56042.0000000	6.5550003
56043.0000000	6.5850000
56044.0000000	6.6050000
56045.0000000	6.6350002
56046.0000000	6.6500001
56047.0000000	6.6599998
56048.0000000	6.6750002
56049.0000000	6.6750002
56050.0000000	6.6599998
56051.0000000	6.6300001
56052.0000000	6.6350002
56053.0000000	6.6399999
56054.0000000	6.6450000
56055.0000000	6.6399999
56056.0000000	6.6650000
56057.0000000	6.6849999
56058.0000000	6.7150002
56059.0000000	6.7349997
56060.0000000	6.7349997
56061.0000000	6.7650003
56062.0000000	6.7800002
56063.0000000	6.7849998
56064.0000000	6.8050003
56065.0000000	6.8100004
56066.0000000	6.8200002
56067.0000000	6.8249998
56068.0000000	6.8200002
56069.0000000	6.8350000
56070.0000000	6.8350000
56071.0000000	6.8350000
56072.0000000	6.8550000
56073.0000000	6.8649998
56074.0000000	6.8800001
56075.0000000	6.8900003
56076.0000000	6.8850002
56077.0000000	6.9099998
56078.0000000	6.9200001
56079.0000000	6.9349999
56080.0000000	6.9400001
56081.0000000	6.9449997
56082.0000000	6.9549999
56083.0000000	6.9600000
56084.0000000	6.9600000
56085.0000000	6.9650002
56086.0000000	6.9650002
56087.0000000	6.9600000

56088.0000000	6.9700003
56089.0000000	6.9200001
56090.0000000	6.9849997
56091.0000000	6.9766669
56092.0000000	6.9700003
56093.0000000	6.9549999
56094.0000000	6.9349999
56095.0000000	6.9150000
56096.0000000	6.8800001
56097.0000000	6.8550000
56098.0000000	6.7950001
56099.0000000	6.7200003
56100.0000000	6.6599998
56101.0000000	6.5650001
56102.0000000	6.4899998
56103.0000000	6.4150000
56104.0000000	6.3450003
56105.0000000	6.2800002
56106.0000000	6.2200003
56107.0000000	6.1599998
56108.0000000	6.1050000
56109.0000000	6.0450001
56110.0000000	5.9849997
56111.0000000	5.9349999
56112.0000000	5.9399996
56113.0000000	5.9949999
56114.0000000	6.0500002
56115.0000000	6.0699997
56116.0000000	6.0799999
56117.0000000	6.0550003
56118.0000000	6.0149999
56119.0000000	6.0450001
56120.0000000	6.0999999
56121.0000000	6.1999998
56122.0000000	6.3033333
56123.0000000	6.5050001
56124.0000000	6.6450000
56125.0000000	6.7350001
56126.0000000	6.8000002
56127.0000000	6.8400002
56128.0000000	6.8450003
56129.0000000	6.8850002
56130.0000000	6.8899999
56131.0000000	6.8950000
56132.0000000	6.8900003
56133.0000000	6.8750000
56134.0000000	6.8699999
56135.0000000	6.8450003
56136.0000000	6.8550000
56137.0000000	6.8450003
56138.0000000	6.8400002
56139.0000000	6.8400002
56140.0000000	6.8450003
56141.0000000	6.8500004
56142.0000000	6.8699999
56143.0000000	6.8899999
56144.0000000	6.9200001

56145.0000000	6.9499998
56146.0000000	6.9749999
56147.0000000	7.0150003
56148.0000000	7.0650001
56149.0000000	7.1000004
56150.0000000	7.1300001
56151.0000000	7.1400003
56152.0000000	7.1949997
56153.0000000	7.2433333
56154.0000000	7.2750001
56155.0000000	7.3050003
56156.0000000	7.3150001
56157.0000000	7.3299999
56158.0000000	7.3350000
56159.0000000	7.3350000
56160.0000000	7.3400002
56161.0000000	7.3450003
56162.0000000	7.3299999
56163.0000000	7.2900000
56164.0000000	7.2799997
56165.0000000	7.2399998
56166.0000000	7.2100000
56167.0000000	7.1650000
56168.0000000	7.1199999
56169.0000000	7.0700002
56170.0000000	7.0100002
56171.0000000	6.9600000
56172.0000000	6.9099998
56173.0000000	6.9000001
56174.0000000	6.9049997
56175.0000000	6.9150000
56176.0000000	6.9600000
56177.0000000	7.0150003
56178.0000000	7.0550003
56179.0000000	7.0850000
56180.0000000	7.0900002
56181.0000000	7.0749998
56182.0000000	7.0166664
56183.0000000	6.9549999
56184.0000000	6.9099998
56185.0000000	6.8999996
56186.0000000	6.9449997
56187.0000000	6.9949999
56188.0000000	7.0500002
56189.0000000	7.0999999
56190.0000000	7.1300001
56191.0000000	7.1250000
56192.0000000	7.1100001
56193.0000000	7.0850000
56194.0000000	7.0600004
56195.0000000	7.0250001
56196.0000000	6.9600000
56197.0000000	6.9200001
56198.0000000	6.9049997
56199.0000000	6.8950000
56200.0000000	6.8899999
56201.0000000	6.8899999

56202.0000000	6.9200001
56203.0000000	6.9600000
56204.0000000	6.9899998
56205.0000000	7.0150003
56206.0000000	7.0150003
56207.0000000	6.9800000
56208.0000000	6.9349999
56209.0000000	6.9300003
56210.0000000	6.8850002
56211.0000000	6.7849998
56212.0000000	6.6399999
56213.0000000	6.4700003
56214.0000000	6.3400002
56215.0000000	6.2500000
56216.0000000	6.0500002
56217.0000000	5.7300000
56218.0000000	5.9850001
56219.0000000	6.1700001
56220.0000000	6.3000002
56221.0000000	6.3299999
56222.0000000	6.3750000
56223.0000000	6.4449997
56224.0000000	6.4600000
56225.0000000	6.4699998
56226.0000000	6.4549999
56227.0000000	6.4449997
56228.0000000	6.5050001
56229.0000000	6.5749998
56230.0000000	6.5799999
56231.0000000	6.5250001
56232.0000000	6.5250001
56233.0000000	6.5999999
56234.0000000	6.7700000
56235.0000000	6.8500004
56236.0000000	6.8649998
56237.0000000	6.8400002
56238.0000000	6.8050003
56239.0000000	6.8100004
56240.0000000	6.8199997
56241.0000000	6.8249998
56242.0000000	6.8649998
56243.0000000	6.8433337
56244.0000000	6.8099999
56245.0000000	6.8200002
56246.0000000	6.8450003
56247.0000000	6.8649998
56248.0000000	6.8550000
56249.0000000	6.8800001
56250.0000000	6.8550000
56251.0000000	6.8249998
56252.0000000	6.8000002
56253.0000000	6.7750001
56254.0000000	6.7700000
56255.0000000	6.7750001
56256.0000000	6.7550001
56257.0000000	6.8150001
56258.0000000	6.8099999

56259.0000000	6.7950001
56260.0000000	6.7600002
56261.0000000	6.7100000
56262.0000000	6.7049999
56263.0000000	6.6849999
56264.0000000	6.6549997
56265.0000000	6.6849999
56266.0000000	6.7550001
56267.0000000	6.8350000
56268.0000000	6.9150000
56269.0000000	6.9749999
56270.0000000	7.0400000
56271.0000000	7.0799999
56272.0000000	7.1100001
56273.0000000	7.1666665
56274.0000000	7.2199998
56275.0000000	7.2750001
56276.0000000	7.3000002
56277.0000000	7.2900000
56278.0000000	7.2399998
56279.0000000	7.1650000
56280.0000000	7.0600004
56281.0000000	7.0249996
56282.0000000	6.9449997
56283.0000000	6.9850001
56284.0000000	7.0000000
56285.0000000	6.9650002
56286.0000000	6.9000001
56287.0000000	6.7500000
56288.0000000	6.6000004
56289.0000000	6.4499998
56290.0000000	6.3750000
56291.0000000	6.3500004
56292.0000000	6.3699999
56293.0000000	6.5050001
56294.0000000	6.6700001
56295.0000000	6.7950001
56296.0000000	6.8699999
56297.0000000	6.9000001
56298.0000000	6.9099998
56299.0000000	6.9049997
56300.0000000	6.8350000
56301.0000000	6.8950000
56302.0000000	6.9600000
56303.0000000	6.9766669
56304.0000000	6.9549999
56305.0000000	6.9499998
56306.0000000	6.9499998
56307.0000000	6.9399996
56308.0000000	6.8850002
56309.0000000	6.8350000
56310.0000000	6.7600002
56311.0000000	6.7950001
56312.0000000	6.8350000
56313.0000000	6.8099999
56314.0000000	6.8249998
56315.0000000	6.8150001

56316.0000000	6.7700005
56317.0000000	6.6949997
56318.0000000	6.6250000
56319.0000000	6.6499996
56320.0000000	6.6050000
56321.0000000	6.4600000
56322.0000000	6.3500004
56323.0000000	6.2449999
56324.0000000	6.1900001
56325.0000000	6.1700001
56326.0000000	6.2500000
56327.0000000	6.2800002
56328.0000000	6.2449999
56329.0000000	6.1800003
56330.0000000	6.1849999
56331.0000000	6.2200003
56332.0000000	6.2600002
56333.0000000	6.1866670
56334.0000000	5.9800000
56335.0000000	5.7049999
56336.0000000	5.5500002
56337.0000000	5.5950003
56338.0000000	5.7500000
56339.0000000	5.9049997
56340.0000000	6.3000002
56341.0000000	6.7550001
56342.0000000	7.0299997
56343.0000000	7.1999998
56344.0000000	7.2950001
56345.0000000	7.3400002
56346.0000000	7.3699999
56347.0000000	7.3600001
56348.0000000	7.3699999
56349.0000000	7.3150001
56350.0000000	7.2350001
56351.0000000	7.1399999
56352.0000000	7.0400000
56353.0000000	6.9049997
56354.0000000	6.7399998
56355.0000000	6.5400000
56356.0000000	6.3750000
56357.0000000	6.3249998
56358.0000000	6.4099998
56359.0000000	6.5200000
56360.0000000	6.5650001
56361.0000000	6.6100001
56362.0000000	6.6549997
56363.0000000	6.6933331
56364.0000000	6.6499996
56365.0000000	6.5900002
56366.0000000	6.5850000
56367.0000000	6.7150002
56368.0000000	6.7800002
56369.0000000	6.8299999
56370.0000000	6.8649998
56371.0000000	6.9000001
56372.0000000	7.0100002

56373.0000000	7.0699997
56374.0000000	7.0799999
56375.0000000	7.0799999
56376.0000000	7.0250001
56377.0000000	6.9399996
56378.0000000	6.8750000
56379.0000000	6.8649998
56380.0000000	6.9349999
56381.0000000	7.0250001
56382.0000000	7.1300001
56383.0000000	7.1849999
56384.0000000	7.2200003
56385.0000000	7.4099998
56386.0000000	7.5400000
56387.0000000	7.6199999
56388.0000000	7.6650000
56389.0000000	7.6949997
56390.0000000	7.7249999
56391.0000000	7.7550001
56392.0000000	7.7849998
56393.0000000	7.8150001
56394.0000000	7.8566666
56395.0000000	7.9000001
56396.0000000	7.9299998
56397.0000000	7.9650002
56398.0000000	7.9949999
56399.0000000	8.0349998
56400.0000000	8.0699997
56401.0000000	8.1000004
56402.0000000	8.1049995
56403.0000000	8.1250000
56404.0000000	8.1200008
56405.0000000	8.1049995
56406.0000000	8.0750008
56407.0000000	8.0249996
56408.0000000	7.9650002
56409.0000000	7.9150000
56410.0000000	7.8099999
56411.0000000	7.7700005
56412.0000000	7.7049999
56413.0000000	7.6350002
56414.0000000	7.5600004
56415.0000000	7.4800000
56416.0000000	7.4049997
56417.0000000	7.3550000
56418.0000000	7.3299999
56419.0000000	7.3050003
56420.0000000	7.2600002
56421.0000000	7.2100000
56422.0000000	7.1599998
56423.0000000	7.1050000
56424.0000000	7.0599999
56425.0000000	7.0349998
56426.0000000	7.0300002
56427.0000000	7.0450001
56428.0000000	7.0500002
56429.0000000	7.0699997

56430.0000000	7.0850000
56431.0000000	7.0400000
56432.0000000	7.0850000
56433.0000000	7.0450001
56434.0000000	6.9899998
56435.0000000	6.8950000
56436.0000000	6.6850004
56437.0000000	6.3699999
56438.0000000	6.1400003
56439.0000000	6.3400002
56440.0000000	6.6849999
56441.0000000	6.8050003
56442.0000000	6.8400002
56443.0000000	7.2600002
56444.0000000	7.4600000
56445.0000000	7.4949999
56446.0000000	7.5150003
56447.0000000	7.5850000
56448.0000000	7.6549997
56449.0000000	7.6350002
56450.0000000	7.5950003
56451.0000000	7.5500002
56452.0000000	7.5150003
56453.0000000	7.4849997
56454.0000000	7.4333329
56455.0000000	7.4250002
56456.0000000	7.4699998
56457.0000000	7.5900002
56458.0000000	7.7150002
56459.0000000	7.8150001
56460.0000000	7.8999996
56461.0000000	7.9699998
56462.0000000	8.0200005
56463.0000000	8.0799999
56464.0000000	8.1300001
56465.0000000	8.1849995
56466.0000000	8.2399998
56467.0000000	8.2849998
56468.0000000	8.3400002
56469.0000000	8.3850002
56470.0000000	8.4200001
56471.0000000	8.4449997
56472.0000000	8.4600000
56473.0000000	8.4700003
56474.0000000	8.4949999
56475.0000000	8.5249996
56476.0000000	8.5600004
56477.0000000	8.5500002
56478.0000000	8.5650005
56479.0000000	8.5799999
56480.0000000	8.5900002
56481.0000000	8.6000004
56482.0000000	8.6300001
56483.0000000	8.6300001
56484.0000000	8.6800003
56485.0000000	8.7349997
56486.0000000	8.7800007

56487.0000000	8.8249998
56488.0000000	8.8500004
56489.0000000	8.8750000
56490.0000000	8.8950005
56491.0000000	8.9150000
56492.0000000	8.9300003
56493.0000000	8.9300003
56494.0000000	8.9400005
56495.0000000	8.9349995
56496.0000000	8.9349995
56497.0000000	8.9349995
56498.0000000	8.9300003
56499.0000000	8.9249992
56500.0000000	8.9349995
56501.0000000	8.9300003
56502.0000000	8.9349995
56503.0000000	8.9349995
56504.0000000	8.9300003
56505.0000000	8.9300003
56506.0000000	8.9200001
56507.0000000	8.9099998
56508.0000000	8.8999996
56509.0000000	8.8649998
56510.0000000	8.8249998
56511.0000000	8.7799997
56512.0000000	8.7049999
56513.0000000	8.6450005
56514.0000000	8.5850000
56515.0000000	8.5666666
56516.0000000	8.5550003
56517.0000000	8.5500002
56518.0000000	8.5550003
56519.0000000	8.5599995
56520.0000000	8.5699997
56521.0000000	8.5749998
56522.0000000	8.5600004
56523.0000000	8.5349998
56524.0000000	8.5000000
56525.0000000	8.4249992
56526.0000000	8.3400002
56527.0000000	8.2549992
56528.0000000	8.1650000
56529.0000000	8.0799999
56530.0000000	8.0000000
56531.0000000	7.9449997
56532.0000000	7.9049997
56533.0000000	7.9049997
56534.0000000	7.9300003
56535.0000000	7.9399996
56536.0000000	7.9699998
56537.0000000	7.9749999
56538.0000000	7.9250002
56539.0000000	7.8699999
56540.0000000	7.7849998
56541.0000000	7.6750002
56542.0000000	7.5900002
56543.0000000	7.5000000

56544.0000000	7.4349999
56545.0000000	7.3466668
56546.0000000	7.2650003
56547.0000000	7.1799998
56548.0000000	7.1000004
56549.0000000	7.0999999
56550.0000000	7.1599998
56551.0000000	7.2199998
56552.0000000	7.2449999
56553.0000000	7.2349997
56554.0000000	7.2049999
56555.0000000	7.1999998
56556.0000000	7.2100000
56557.0000000	7.2249999
56558.0000000	7.2349997
56559.0000000	7.2750001
56560.0000000	7.3649998
56561.0000000	7.4800000
56562.0000000	7.6199999
56563.0000000	7.7550001
56564.0000000	7.8249998
56565.0000000	7.8550000
56566.0000000	7.8199997
56567.0000000	7.7399998
56568.0000000	7.6450000
56569.0000000	7.5050001
56570.0000000	7.3450003
56571.0000000	7.2350001
56572.0000000	7.1650000
56573.0000000	7.1599998
56574.0000000	7.1966667
56575.0000000	7.2349997
56576.0000000	7.2800002
56577.0000000	7.3150001
56578.0000000	7.3350000
56579.0000000	7.3850002
56580.0000000	7.4499998
56581.0000000	7.5450001
56582.0000000	7.6700001
56583.0000000	7.7700005
56584.0000000	7.8100004
56585.0000000	7.7800002
56586.0000000	7.7449999
56587.0000000	7.6700001
56588.0000000	7.5650001
56589.0000000	7.5249996
56590.0000000	7.4600000
56591.0000000	7.4449997
56592.0000000	7.4549999
56593.0000000	7.5100002
56594.0000000	7.5900002
56595.0000000	7.6999998
56596.0000000	7.8000002
56597.0000000	7.8649998
56598.0000000	7.9200001
56599.0000000	7.9449997
56600.0000000	7.9449997

56601.0000000	7.9400001
56602.0000000	7.9250002
56603.0000000	7.8850002
56604.0000000	7.8649998
56605.0000000	7.8400002
56606.0000000	7.8400002
56607.0000000	7.8550000
56608.0000000	7.8699999
56609.0000000	7.8800001
56610.0000000	7.8850002
56611.0000000	7.8800001
56612.0000000	7.8850002
56613.0000000	7.8800001
56614.0000000	7.8800001
56615.0000000	7.8699999
56616.0000000	7.8499999
56617.0000000	7.8450003
56618.0000000	7.8450003
56619.0000000	7.8899999
56620.0000000	7.9449997
56621.0000000	7.9650002
56622.0000000	7.9700003
56623.0000000	7.9200001
56624.0000000	7.8400002
56625.0000000	7.7550001
56626.0000000	7.6599998
56627.0000000	7.5850000
56628.0000000	7.5650001
56629.0000000	7.5450001
56630.0000000	7.5500002
56631.0000000	7.5450001
56632.0000000	7.5349998
56633.0000000	7.5450001
56634.0000000	7.5600004
56635.0000000	7.5850000
56636.0000000	7.6366668
56637.0000000	7.6400003
56638.0000000	7.6700001
56639.0000000	7.6599998
56640.0000000	7.6450000
56641.0000000	7.6350002
56642.0000000	7.6350002
56643.0000000	7.6450000
56644.0000000	7.6650000
56645.0000000	7.7249999
56646.0000000	7.8100004
56647.0000000	7.9150000
56648.0000000	8.0200005
56649.0000000	8.1700001
56650.0000000	8.2449999
56651.0000000	8.3199997
56652.0000000	8.3649998
56653.0000000	8.3950005
56654.0000000	8.4150000
56655.0000000	8.4099998
56656.0000000	8.3750000
56657.0000000	8.3800001

56658.0000000	8.3500004
56659.0000000	8.3199997
56660.0000000	8.3150005
56661.0000000	8.3199997
56662.0000000	8.3500004
56663.0000000	8.3850002
56664.0000000	8.4099998
56665.0000000	8.4566660
56666.0000000	8.4799995
56667.0000000	8.4899998
56668.0000000	8.4949999
56669.0000000	8.5249996
56670.0000000	8.5550003
56671.0000000	8.5799999
56672.0000000	8.6000004
56673.0000000	8.6499996
56674.0000000	8.6650000
56675.0000000	8.6599998
56676.0000000	8.6549997
56677.0000000	8.6549997
56678.0000000	8.6199999
56679.0000000	8.5850000
56680.0000000	8.5450001
56681.0000000	8.4949999
56682.0000000	8.4349995
56683.0000000	8.3750000
56684.0000000	8.3199997
56685.0000000	8.2700005
56686.0000000	8.2150002
56687.0000000	8.1650000
56688.0000000	8.1299992
56689.0000000	8.1499996
56690.0000000	8.1800003
56691.0000000	8.2199993
56692.0000000	8.2350006
56693.0000000	8.2700005
56694.0000000	8.2700005
56695.0000000	8.2566671
56696.0000000	8.2299995
56697.0000000	8.1650000
56698.0000000	8.1149998
56699.0000000	8.0400009
56700.0000000	7.9749999
56701.0000000	7.9000001
56702.0000000	7.8249998
56703.0000000	7.7650003
56704.0000000	7.7100000
56705.0000000	7.6849999
56706.0000000	7.6800003
56707.0000000	7.6999998
56708.0000000	7.7500000
56709.0000000	7.8150001
56710.0000000	7.8699999
56711.0000000	7.9250002
56712.0000000	7.9700003
56713.0000000	8.0000000
56714.0000000	7.9899998

56715.0000000	8.0699997
56716.0000000	8.1049995
56717.0000000	8.1250000
56718.0000000	8.1350002
56719.0000000	8.1650000
56720.0000000	8.1750002
56721.0000000	8.1800003
56722.0000000	8.1849995
56723.0000000	8.1599998
56724.0000000	8.1199999
56725.0000000	8.0799999
56726.0000000	8.0200005
56727.0000000	7.9700003
56728.0000000	7.9449997
56729.0000000	7.9449997
56730.0000000	7.9650002
56731.0000000	8.0000000
56732.0000000	8.0550003
56733.0000000	8.1049995
56734.0000000	8.1800003
56735.0000000	8.2399998
56736.0000000	8.2700005
56737.0000000	8.3199997
56738.0000000	8.3299999
56739.0000000	8.3150005
56740.0000000	8.2849998
56741.0000000	8.2900000
56742.0000000	8.2749996
56743.0000000	8.2550001
56744.0000000	8.2299995
56745.0000000	8.2200003
56746.0000000	8.2150002
56747.0000000	8.2100000
56748.0000000	8.2200003
56749.0000000	8.2349997
56750.0000000	8.2550001
56751.0000000	8.2500000
56752.0000000	8.3000002
56753.0000000	8.3250008
56754.0000000	8.3299999
56755.0000000	8.3850002
56756.0000000	8.4299994
56757.0000000	8.4650002
56758.0000000	8.5149994
56759.0000000	8.5500002
56760.0000000	8.5850000
56761.0000000	8.6250000
56762.0000000	8.6499996
56763.0000000	8.6750002
56764.0000000	8.6999998
56765.0000000	8.6949997
56766.0000000	8.6949997
56767.0000000	8.6700001
56768.0000000	8.6450005
56769.0000000	8.6049995
56770.0000000	8.5450001
56771.0000000	8.5050001

56772.0000000	8.4449997
56773.0000000	8.3850002
56774.0000000	8.3600006
56775.0000000	8.3249998
56776.0000000	8.3000002
56777.0000000	8.2849998
56778.0000000	8.2700005
56779.0000000	8.2749996
56780.0000000	8.2749996
56781.0000000	8.2950001
56782.0000000	8.3199997
56783.0000000	8.3299999
56784.0000000	8.3549995
56785.0000000	8.3750000
56786.0000000	8.3866663
56787.0000000	8.3950005
56788.0000000	8.3899994
56789.0000000	8.3800001
56790.0000000	8.3249998
56791.0000000	8.3299999
56792.0000000	8.2950001
56793.0000000	8.2500000
56794.0000000	8.1949997
56795.0000000	8.1200008
56796.0000000	8.0400009
56797.0000000	7.9749999
56798.0000000	7.8950000
56799.0000000	7.8150001
56800.0000000	7.7350001
56801.0000000	7.6599998
56802.0000000	7.5950003
56803.0000000	7.5500002
56804.0000000	7.5000000
56805.0000000	7.4849997
56806.0000000	7.4700003
56807.0000000	7.4749999
56808.0000000	7.5100002
56809.0000000	7.5550003
56810.0000000	7.6099997
56811.0000000	7.6700001
56812.0000000	7.7150002
56813.0000000	7.8099999
56814.0000000	7.8599997
56815.0000000	7.9499998
56816.0000000	8.0500002
56817.0000000	8.1450005
56818.0000000	8.2049999
56819.0000000	8.2950001
56820.0000000	8.3700008
56821.0000000	8.4150000
56822.0000000	8.4599991
56823.0000000	8.4850006
56824.0000000	8.4849997
56825.0000000	8.4799995
56826.0000000	8.4499998
56827.0000000	8.4050007
56828.0000000	8.3350000

56829.0000000	8.2900000
56830.0000000	8.2199993
56831.0000000	8.1599998
56832.0000000	8.0949993
56833.0000000	8.0450001
56834.0000000	7.9949999
56835.0000000	7.9449997
56836.0000000	7.8999996
56837.0000000	7.8450003
56838.0000000	7.7950001
56839.0000000	7.7399998
56840.0000000	7.6549997
56841.0000000	7.6099997
56842.0000000	7.5500002
56843.0000000	7.4899998
56844.0000000	7.4349999
56845.0000000	7.3750000
56846.0000000	7.3233337
56847.0000000	7.2500000
56848.0000000	7.1849999
56849.0000000	7.1899996
56850.0000000	7.2150002
56851.0000000	7.2800002
56852.0000000	7.3800001
56853.0000000	7.4800000
56854.0000000	7.5799999
56855.0000000	7.6650000
56856.0000000	7.7249999
56857.0000000	7.7750001
56858.0000000	7.8000002
56859.0000000	7.8249998
56860.0000000	7.8350000
56861.0000000	7.8299999
56862.0000000	7.8350000
56863.0000000	7.8499999
56864.0000000	7.8499999
56865.0000000	7.8600001
56866.0000000	7.8550000
56867.0000000	7.8450003
56868.0000000	7.8299999
56869.0000000	7.7950001
56870.0000000	7.7449999
56871.0000000	7.7299995
56872.0000000	7.6949997
56873.0000000	7.6800003
56874.0000000	7.6549997
56875.0000000	7.6700001
56876.0000000	7.6650000
56877.0000000	7.6833329
56878.0000000	7.7349997
56879.0000000	7.7600002
56880.0000000	7.7900000
56881.0000000	7.8150001
56882.0000000	7.8249998
56883.0000000	7.8199997
56884.0000000	7.8150001
56885.0000000	7.8000002

56886.0000000	7.7900000
56887.0000000	7.7849998
56888.0000000	7.7750001
56889.0000000	7.7700000
56890.0000000	7.7750001
56891.0000000	7.7950001
56892.0000000	7.8200002
56893.0000000	7.8299999
56894.0000000	7.8649998
56895.0000000	7.8950000
56896.0000000	7.9250002
56897.0000000	7.9399996
56898.0000000	7.9449997
56899.0000000	7.9499998
56900.0000000	7.9549999
56901.0000000	7.9650002
56902.0000000	7.9600000
56903.0000000	7.9600000
56904.0000000	7.9549999
56905.0000000	7.9150000
56906.0000000	7.8999996
56907.0000000	7.8799996
56908.0000000	7.8350000
56909.0000000	7.7950001
56910.0000000	7.7449999
56911.0000000	7.7100000
56912.0000000	7.6700001
56913.0000000	7.6149998
56914.0000000	7.5799999
56915.0000000	7.5300002
56916.0000000	7.4899998
56917.0000000	7.4700003
56918.0000000	7.4399996
56919.0000000	7.4300003
56920.0000000	7.3850002
56921.0000000	7.4349999
56922.0000000	7.4400001
56923.0000000	7.4449997
56924.0000000	7.4600000
56925.0000000	7.4799995
56926.0000000	7.4949999
56927.0000000	7.5250001
56928.0000000	7.5599999
56929.0000000	7.5950003
56930.0000000	7.6350002
56931.0000000	7.6750002
56932.0000000	7.7299995
56933.0000000	7.7799997
56934.0000000	7.8249998
56935.0000000	7.8699999
56936.0000000	7.9150000
56937.0000000	7.9466667
56938.0000000	7.9749999
56939.0000000	7.9799995
56940.0000000	7.9899998
56941.0000000	7.9849997
56942.0000000	7.9849997

56943.0000000	7.9800000
56944.0000000	7.9800000
56945.0000000	7.9849997
56946.0000000	7.9949999
56947.0000000	8.0000000
56948.0000000	8.0200005
56949.0000000	8.0400000
56950.0000000	8.0600004
56951.0000000	8.0900002
56952.0000000	8.1199999
56953.0000000	8.1499996
56954.0000000	8.1700001
56955.0000000	8.1849995
56956.0000000	8.2049999
56957.0000000	8.2199993
56958.0000000	8.2449999
56959.0000000	8.2600002
56960.0000000	8.2650003
56961.0000000	8.2649994
56962.0000000	8.2399998
56963.0000000	8.2250004
56964.0000000	8.2150002
56965.0000000	8.1749992
56966.0000000	8.1450005
56967.0000000	8.1133337
56968.0000000	8.1000004
56969.0000000	8.1000004
56970.0000000	8.1000004
56971.0000000	8.1049995
56972.0000000	8.0900002
56973.0000000	8.0850000
56974.0000000	8.0699997
56975.0000000	8.0400000
56976.0000000	8.0100002
56977.0000000	7.9800000
56978.0000000	7.9250002
56979.0000000	7.9049997
56980.0000000	7.8649998
56981.0000000	7.8450003
56982.0000000	7.8299999
56983.0000000	7.8299999
56984.0000000	7.8400002
56985.0000000	7.8550000
56986.0000000	7.8649998
56987.0000000	7.9099998
56988.0000000	7.9399996
56989.0000000	8.0000000
56990.0000000	8.0699997
56991.0000000	8.1200008
56992.0000000	8.1849995
56993.0000000	8.2449999
56994.0000000	8.2849998
56995.0000000	8.3350000
56996.0000000	8.3299999
56997.0000000	8.4099998
56998.0000000	8.4533339
56999.0000000	8.4600000

57000.0000000	8.4799995
57001.0000000	8.4750004
57002.0000000	8.4650002
57003.0000000	8.4600000
57004.0000000	8.4300003
57005.0000000	8.4250002
57006.0000000	8.4150000
57007.0000000	8.4150000
57008.0000000	8.4250002
57009.0000000	8.4300003
57010.0000000	8.4449997
57011.0000000	8.4700003
57012.0000000	8.4799995
57013.0000000	8.5000000
57014.0000000	8.5200005
57015.0000000	8.5400009
57016.0000000	8.5400009
57017.0000000	8.6149998
57018.0000000	8.6450005
57019.0000000	8.6700001
57020.0000000	8.6700001
57021.0000000	8.6999998
57022.0000000	8.6949997
57023.0000000	8.7150002
57024.0000000	8.7200003
57025.0000000	8.7550001
57026.0000000	8.7950001
57027.0000000	8.8249998
57028.0000000	8.8633337
57029.0000000	8.9050007
57030.0000000	8.9150000
57031.0000000	8.9300003
57032.0000000	8.9399996
57033.0000000	8.9449997
57034.0000000	8.9549999
57035.0000000	8.9600000
57036.0000000	8.9799995
57037.0000000	8.9799995
57038.0000000	8.9949999
57039.0000000	9.0000000
57040.0000000	9.0100002
57041.0000000	9.0249996
57042.0000000	9.0300007
57043.0000000	9.0349998
57044.0000000	9.0349998
57045.0000000	9.0249996
57046.0000000	9.0299997
57047.0000000	9.0200005
57048.0000000	9.0150003
57049.0000000	9.0100002
57050.0000000	9.0050001
57051.0000000	8.9849997
57052.0000000	8.9949999
57053.0000000	8.9899998
57054.0000000	9.0050001
57055.0000000	9.0200005
57056.0000000	9.0200005

57057.0000000	9.0500002
57058.0000000	9.0650005
57059.0000000	9.0850000
57060.0000000	9.1049995
57061.0000000	9.1049995
57062.0000000	9.1149998
57063.0000000	9.1100006
57064.0000000	9.1000004
57065.0000000	9.0799999
57066.0000000	9.0450001
57067.0000000	9.0200005
57068.0000000	8.9750004
57069.0000000	8.9449997
57070.0000000	8.9050007
57071.0000000	8.8649998
57072.0000000	8.8400002
57073.0000000	8.8500004
57074.0000000	8.8649998
57075.0000000	8.8999996
57076.0000000	8.9150000
57077.0000000	8.8999996
57078.0000000	8.9600000
57079.0000000	8.9699993
57080.0000000	8.9650002
57081.0000000	8.9600000
57082.0000000	8.9349995
57083.0000000	8.9099998
57084.0000000	8.8800001
57085.0000000	8.8750000
57086.0000000	8.8900003
57087.0000000	8.8850002
57088.0000000	8.8966665
57089.0000000	8.9099998
57090.0000000	8.9200001
57091.0000000	8.9250002
57092.0000000	8.8549995
57093.0000000	8.9099998
57094.0000000	8.8899994
57095.0000000	8.8549995
57096.0000000	8.8250008
57097.0000000	8.7800007
57098.0000000	8.7600002
57099.0000000	8.7600002
57100.0000000	8.7449999
57101.0000000	8.7900000
57102.0000000	8.8150005
57103.0000000	8.8400002
57104.0000000	8.8649998
57105.0000000	8.8850002
57106.0000000	8.8950005
57107.0000000	8.9250002
57108.0000000	8.9250002
57109.0000000	8.9349995
57110.0000000	8.9349995
57111.0000000	8.9300003
57112.0000000	8.9250002
57113.0000000	8.8999996

57114.0000000	8.8700008
57115.0000000	8.8999996
57116.0000000	8.8850002
57117.0000000	8.8899994
57118.0000000	8.8800001
57119.0000000	8.8966665
57120.0000000	8.9099998
57121.0000000	8.9250002
57122.0000000	8.9499998
57123.0000000	8.9549999
57124.0000000	8.9650002
57125.0000000	8.9750004
57126.0000000	8.9449997
57127.0000000	8.9349995
57128.0000000	8.9249992
57129.0000000	8.9250002
57130.0000000	8.9150000
57131.0000000	8.8850002
57132.0000000	8.8950005
57133.0000000	8.8999996
57134.0000000	8.8999996
57135.0000000	8.9049997
57136.0000000	8.8850002
57137.0000000	8.8850002
57138.0000000	8.8800001
57139.0000000	8.8600006
57140.0000000	8.8350000
57141.0000000	8.8050003
57142.0000000	8.7749996
57143.0000000	8.7550001
57144.0000000	8.7349997
57145.0000000	8.7250004
57146.0000000	8.7250004
57147.0000000	8.7150002
57148.0000000	8.7133331
57149.0000000	8.7049999
57150.0000000	8.6999998
57151.0000000	8.6949997
57152.0000000	8.6900005
57153.0000000	8.6899996
57154.0000000	8.6750002
57155.0000000	8.6599998
57156.0000000	8.6949997
57157.0000000	8.6899996
57158.0000000	8.6849995
57159.0000000	8.6949997
57160.0000000	8.6749992
57161.0000000	8.6800003
57162.0000000	8.6949997
57163.0000000	8.6999998
57164.0000000	8.7299995
57165.0000000	8.7600002
57166.0000000	8.7749996
57167.0000000	8.8100004
57168.0000000	8.8199997
57169.0000000	8.8549995
57170.0000000	8.8699999

57171.0000000	8.8699999
57172.0000000	8.8750000
57173.0000000	8.8500004
57174.0000000	8.8249998
57175.0000000	8.7399998
57176.0000000	8.7299995
57177.0000000	8.7049999
57178.0000000	8.6500006
57179.0000000	8.6549997
57180.0000000	8.6450005
57181.0000000	8.6400003
57182.0000000	8.6450005
57183.0000000	8.6450005
57184.0000000	8.6650000
57185.0000000	8.6800003
57186.0000000	8.6750002
57187.0000000	8.6899996
57188.0000000	8.6700001
57189.0000000	8.6499996
57190.0000000	8.6400003
57191.0000000	8.6199999
57192.0000000	8.6000004
57193.0000000	8.5900002
57194.0000000	8.5799999
57195.0000000	8.6000004
57196.0000000	8.6000004
57197.0000000	8.5850000
57198.0000000	8.5699997
57199.0000000	8.5500002
57200.0000000	8.5349998
57201.0000000	8.5050001
57202.0000000	8.4750004
57203.0000000	8.4499998
57204.0000000	8.4150000
57205.0000000	8.3950005
57206.0000000	8.3750000
57207.0000000	8.3950005
57208.0000000	8.4099998
57209.0000000	8.4200001
57210.0000000	8.4449997
57211.0000000	8.4600000
57212.0000000	8.4699993
57213.0000000	8.4949999
57214.0000000	8.5050001
57215.0000000	8.5000000
57216.0000000	8.5050001
57217.0000000	8.4899998
57218.0000000	8.4650002
57219.0000000	8.4300003
57220.0000000	8.4099998
57221.0000000	8.3850002
57222.0000000	8.3350000
57223.0000000	8.2800007
57224.0000000	8.2100000
57225.0000000	8.0950003
57226.0000000	7.9600000
57227.0000000	7.8100004

57228.0000000	7.7049999
57229.0000000	7.6500001
57230.0000000	7.6350002
57231.0000000	7.6599998
57232.0000000	7.7200003
57233.0000000	7.7750001
57234.0000000	7.8500004
57235.0000000	7.9150000
57236.0000000	7.9700003
57237.0000000	8.0300007
57238.0000000	8.0500002
57239.0000000	8.0799999
57240.0000000	8.0850000
57241.0000000	8.0650005
57242.0000000	8.0550003
57243.0000000	8.0200005
57244.0000000	7.9899998
57245.0000000	7.9549999
57246.0000000	7.8999996
57247.0000000	7.8699999
57248.0000000	7.8100004
57249.0000000	7.7700000
57250.0000000	7.7399998
57251.0000000	7.7199998
57252.0000000	7.6999998
57253.0000000	7.6949997
57254.0000000	7.7150002
57255.0000000	7.7299995
57256.0000000	7.7349997
57257.0000000	7.6900001
57258.0000000	7.7199998
57259.0000000	7.6650000
57260.0000000	7.6350002
57261.0000000	7.5850000
57262.0000000	7.5400000
57263.0000000	7.5300002
57264.0000000	7.5250001
57265.0000000	7.5150003
57266.0000000	7.5650001
57267.0000000	7.5749998
57268.0000000	7.5999999
57269.0000000	7.6566663
57270.0000000	7.7300000
57271.0000000	7.8200002
57272.0000000	7.9200001
57273.0000000	8.0550003
57274.0000000	8.1499996
57275.0000000	8.2299995
57276.0000000	8.2999992
57277.0000000	8.3350000
57278.0000000	8.3699999
57279.0000000	8.3999996
57280.0000000	8.3999996
57281.0000000	8.4049997
57282.0000000	8.4049997
57283.0000000	8.3950005
57284.0000000	8.4049997

57285.0000000	8.3599997
57286.0000000	8.3450003
57287.0000000	8.3150005
57288.0000000	8.2550001
57289.0000000	8.1499996
57290.0000000	8.1550007
57291.0000000	8.1100006
57292.0000000	7.9750004
57293.0000000	7.9549999
57294.0000000	7.8699999
57295.0000000	7.7950001
57296.0000000	7.7249999
57297.0000000	7.6499996
57298.0000000	7.5550003
57299.0000000	7.4533334
57300.0000000	7.3600001
57301.0000000	7.3249998
57302.0000000	7.3450003
57303.0000000	7.3500004
57304.0000000	7.3550000
57305.0000000	7.3649998
57306.0000000	7.3699999
57307.0000000	7.4049997
57308.0000000	7.4099998
57309.0000000	7.3950000
57310.0000000	7.3750000
57311.0000000	7.3350000
57312.0000000	7.2849998
57313.0000000	7.2349997
57314.0000000	7.1599998
57315.0000000	7.0999999
57316.0000000	7.0450001
57317.0000000	7.0250001
57318.0000000	7.0400000
57319.0000000	7.0650001
57320.0000000	7.1250000
57321.0000000	7.1849999
57322.0000000	7.2500000
57323.0000000	7.3200002
57324.0000000	7.3599997
57325.0000000	7.4150000
57326.0000000	7.4549999
57327.0000000	7.4699998
57328.0000000	7.4899998
57329.0000000	7.4899998
57330.0000000	7.4499998
57331.0000000	7.4700003
57332.0000000	7.4549999
57333.0000000	7.4299998
57334.0000000	7.4000001
57335.0000000	7.3699999
57336.0000000	7.3350000
57337.0000000	7.2849998
57338.0000000	7.2650003
57339.0000000	7.2349997
57340.0000000	7.2100000
57341.0000000	7.2049999

57342.0000000	7.1949997
57343.0000000	7.1949997
57344.0000000	7.1999998
57345.0000000	7.1799998
57346.0000000	7.1849999
57347.0000000	7.1750002
57348.0000000	7.1700001
57349.0000000	7.1499996
57350.0000000	7.1250000
57351.0000000	7.0999999
57352.0000000	7.0650001
57353.0000000	7.0200000
57354.0000000	6.9799995
57355.0000000	6.9549999
57356.0000000	6.9299998
57357.0000000	6.9300003
57358.0000000	6.9250002
57359.0000000	6.9449997
57360.0000000	6.9633331
57361.0000000	7.0250001
57362.0000000	7.0400000
57363.0000000	7.0450001
57364.0000000	7.0400000
57365.0000000	7.0300002
57366.0000000	7.0100002
57367.0000000	6.9899998
57368.0000000	6.9549999
57369.0000000	6.9250002
57370.0000000	6.8800001
57371.0000000	6.8500004
57372.0000000	6.8299999
57373.0000000	6.7849998
57374.0000000	6.7650003
57375.0000000	6.7500000
57376.0000000	6.7449999
57377.0000000	6.7449999
57378.0000000	6.7500000
57379.0000000	6.7300000
57380.0000000	6.7249999
57381.0000000	6.7150002
57382.0000000	6.6849999
57383.0000000	6.6650000
57384.0000000	6.6250000
57385.0000000	6.6050000
57386.0000000	6.5950003
57387.0000000	6.5650001
57388.0000000	6.5349998
57389.0000000	6.5200005
57390.0000000	6.4799995
57391.0000000	6.4399996
57392.0000000	6.4049997
57393.0000000	6.4000001
57394.0000000	6.3950000
57395.0000000	6.3950000
57396.0000000	6.4099998
57397.0000000	6.4200001
57398.0000000	6.4400001

57399.0000000	6.4499998
57400.0000000	6.4499998
57401.0000000	6.4449997
57402.0000000	6.4150000
57403.0000000	6.4449997
57404.0000000	6.4299998
57405.0000000	6.4700003
57406.0000000	6.4849997
57407.0000000	6.5150003
57408.0000000	6.5450001
57409.0000000	6.5699997
57410.0000000	6.6300001
57411.0000000	6.6750002
57412.0000000	6.7299995
57413.0000000	6.7750001
57414.0000000	6.8249998
57415.0000000	6.8699999
57416.0000000	6.9099998
57417.0000000	6.9499998
57418.0000000	6.9749999
57419.0000000	7.0050001
57420.0000000	7.0200005
57421.0000000	7.0349998
57422.0000000	7.0550003
57423.0000000	7.0450001
57424.0000000	7.0500002
57425.0000000	7.0450001
57426.0000000	7.0450001
57427.0000000	7.0299997
57428.0000000	7.0100002
57429.0000000	6.9899998
57430.0000000	6.9549999
57431.0000000	6.9000001
57432.0000000	6.8500004
57433.0000000	6.7900000
57434.0000000	6.7199998
57435.0000000	6.6499996
57436.0000000	6.5500002
57437.0000000	6.5000000
57438.0000000	6.4099998
57439.0000000	6.3050003
57440.0000000	6.1949997
57441.0000000	6.1000004
57442.0000000	6.0450001
57443.0000000	5.9899998
57444.0000000	5.9099998
57445.0000000	5.8649998
57446.0000000	5.8200002
57447.0000000	5.7700000
57448.0000000	5.7500000
57449.0000000	5.7700000
57450.0000000	5.8233337
57451.0000000	5.9349999
57452.0000000	6.0500002
57453.0000000	6.1650000
57454.0000000	6.2649999
57455.0000000	6.3600001

57456.0000000	6.4349999
57457.0000000	6.4899998
57458.0000000	6.5400000
57459.0000000	6.5600004
57460.0000000	6.5900002
57461.0000000	6.6100001
57462.0000000	6.6350002
57463.0000000	6.6650000
57464.0000000	6.6800003
57465.0000000	6.6899996
57466.0000000	6.7049999
57467.0000000	6.7100000
57468.0000000	6.7100000
57469.0000000	6.7100000
57470.0000000	6.6999998
57471.0000000	6.7049999
57472.0000000	6.6949997
57473.0000000	6.6949997
57474.0000000	6.6949997
57475.0000000	6.6849999
57476.0000000	6.6750002
57477.0000000	6.6750002
57478.0000000	6.6549997
57479.0000000	6.6549997
57480.0000000	6.6549997
57481.0000000	6.6533332
57482.0000000	6.6450000
57483.0000000	6.6350002
57484.0000000	6.6300001
57485.0000000	6.6050000
57486.0000000	6.5850000
57487.0000000	6.5400000
57488.0000000	6.5000000
57489.0000000	6.4549999
57490.0000000	6.4200001
57491.0000000	6.3750000
57492.0000000	6.3499999
57493.0000000	6.3000002
57494.0000000	6.2550001
57495.0000000	6.2249999
57496.0000000	6.1849999
57497.0000000	6.1650000
57498.0000000	6.1450000
57499.0000000	6.1250000
57500.0000000	6.1199999
57501.0000000	6.1100001
57502.0000000	6.1149998
57503.0000000	6.1099997
57504.0000000	6.0950003
57505.0000000	6.0900002
57506.0000000	6.0600004
57507.0000000	6.0749998
57508.0000000	6.0749998
57509.0000000	6.0550003
57510.0000000	6.0450001
57511.0000000	6.0233331
57512.0000000	6.0100002

57513.0000000	6.0200000
57514.0000000	6.0299997
57515.0000000	6.0550003
57516.0000000	6.0950003
57517.0000000	6.1350002
57518.0000000	6.1799998
57519.0000000	6.2150002
57520.0000000	6.2650003
57521.0000000	6.3050003
57522.0000000	6.3050003
57523.0000000	6.3550000
57524.0000000	6.3600001
57525.0000000	6.3500004
57526.0000000	6.3400002
57527.0000000	6.3099999
57528.0000000	6.2800002
57529.0000000	6.2299995
57530.0000000	6.1599998
57531.0000000	6.0850000
57532.0000000	5.9700003
57533.0000000	5.8199997
57534.0000000	5.6350002
57535.0000000	5.3800001
57536.0000000	5.1199999
57537.0000000	4.9299998
57538.0000000	4.7250004
57539.0000000	4.5500002
57540.0000000	4.6333332
57541.0000000	5.0400000
57542.0000000	5.3750000
57543.0000000	5.5999999
57544.0000000	5.7200003
57545.0000000	5.7799997
57546.0000000	5.8050003
57547.0000000	5.8100004
57548.0000000	5.7750001
57549.0000000	5.7500000
57550.0000000	5.6900001
57551.0000000	5.6149998
57552.0000000	5.5299997
57553.0000000	5.4899998
57554.0000000	5.4399996
57555.0000000	5.4250002
57556.0000000	5.4000001
57557.0000000	5.4099998
57558.0000000	5.4200001
57559.0000000	5.4049997
57560.0000000	5.4000001
57561.0000000	5.3600001
57562.0000000	5.3200002
57563.0000000	5.2700005
57564.0000000	5.2049999
57565.0000000	5.1549997
57566.0000000	5.1100001
57567.0000000	5.0850000
57568.0000000	5.0450001
57569.0000000	4.9849997

57570.0000000	4.9299998
57571.0000000	4.9299998
57572.0000000	4.9899998
57573.0000000	5.0100002
57574.0000000	5.0150003
57575.0000000	5.0500002
57576.0000000	5.0950003
57577.0000000	5.1199999
57578.0000000	5.1250000
57579.0000000	5.1149998
57580.0000000	5.1149998
57581.0000000	5.1100001
57582.0000000	5.0999999
57583.0000000	5.1300001
57584.0000000	5.1400003
57585.0000000	5.1549997
57586.0000000	5.1750002
57587.0000000	5.1700001
57588.0000000	5.1599998
57589.0000000	5.1549997
57590.0000000	5.1599998
57591.0000000	5.1650000
57592.0000000	5.1500001
57593.0000000	5.0950003
57594.0000000	5.0799999
57595.0000000	5.0600004
57596.0000000	5.0900002
57597.0000000	5.1050000
57598.0000000	5.1350002
57599.0000000	5.1650000
57600.0000000	5.1750002
57601.0000000	5.2066665
57602.0000000	5.2249999
57603.0000000	5.2249999
57604.0000000	5.2550001
57605.0000000	5.2650003
57606.0000000	5.2650003
57607.0000000	5.2849998
57608.0000000	5.2950001
57609.0000000	5.3100004
57610.0000000	5.3150001
57611.0000000	5.3350000
57612.0000000	5.3450003
57613.0000000	5.3450003
57614.0000000	5.3550000
57615.0000000	5.3750000
57616.0000000	5.3999996
57617.0000000	5.4150000
57618.0000000	5.4400001
57619.0000000	5.4499998
57620.0000000	5.4600000
57621.0000000	5.4650002
57622.0000000	5.4650002
57623.0000000	5.4549999
57624.0000000	5.4549999
57625.0000000	5.4600000
57626.0000000	5.4449997

57627.0000000	5.4349999
57628.0000000	5.4300003
57629.0000000	5.4049997
57630.0000000	5.4099998
57631.0000000	5.3866668
57632.0000000	5.3800001
57633.0000000	5.3699999
57634.0000000	5.3550000
57635.0000000	5.3450003
57636.0000000	5.3299999
57637.0000000	5.3150001
57638.0000000	5.3050003
57639.0000000	5.2900000
57640.0000000	5.2750001
57641.0000000	5.2650003
57642.0000000	5.2349997
57643.0000000	5.2249999
57644.0000000	5.2100000
57645.0000000	5.1899996
57646.0000000	5.1700001
57647.0000000	5.1500001
57648.0000000	5.1300001
57649.0000000	5.1099997
57650.0000000	5.0799999
57651.0000000	5.0650001
57652.0000000	5.0450001
57653.0000000	5.0250001
57654.0000000	5.0000000
57655.0000000	4.9899998
57656.0000000	4.9749999
57657.0000000	4.9499998
57658.0000000	4.9400001
57659.0000000	4.9200001
57660.0000000	4.8950000
57661.0000000	4.8800001
57662.0000000	4.8599997
57663.0000000	4.8500004
57664.0000000	4.8350000
57665.0000000	4.8249998
57666.0000000	4.8249998
57667.0000000	4.8150001
57668.0000000	4.8150001
57669.0000000	4.8150001
57670.0000000	4.8150001
57671.0000000	4.8249998
57672.0000000	4.8249998
57673.0000000	4.8350000
57674.0000000	4.8499999
57675.0000000	4.8500004
57676.0000000	4.8599997
57677.0000000	4.8649998
57678.0000000	4.8699999
57679.0000000	4.8800001
57680.0000000	4.8850002
57681.0000000	4.8850002
57682.0000000	4.8950000
57683.0000000	4.8999996

57684.0000000	4.9049997
57685.0000000	4.9049997
57686.0000000	4.9099998
57687.0000000	4.9099998
57688.0000000	4.9099998
57689.0000000	4.9049997
57690.0000000	4.9049997
57691.0000000	4.9000001
57692.0000000	4.8950000
57693.0000000	4.8899999
57694.0000000	4.8750000
57695.0000000	4.8649998
57696.0000000	4.8450003
57697.0000000	4.8400002
57698.0000000	4.8299999
57699.0000000	4.8050003
57700.0000000	4.7900000
57701.0000000	4.7750001
57702.0000000	4.7550001
57703.0000000	4.7349997
57704.0000000	4.6999998
57705.0000000	4.6899996
57706.0000000	4.6750002
57707.0000000	4.6450000
57708.0000000	4.6250000
57709.0000000	4.6099997
57710.0000000	4.5850000
57711.0000000	4.5699997
57712.0000000	4.5799999
57713.0000000	4.5900002
57714.0000000	4.5950003
57715.0000000	4.5900002
57716.0000000	4.5900002
57717.0000000	4.5700002
57718.0000000	4.5500002
57719.0000000	4.5349998
57720.0000000	4.5000000
57721.0000000	4.4749999
57722.0000000	4.4366665
57723.0000000	4.3900003
57724.0000000	4.3649998
57725.0000000	4.3350000
57726.0000000	4.3150001
57727.0000000	4.3000002
57728.0000000	4.2799997
57729.0000000	4.2799997
57730.0000000	4.2700005
57731.0000000	4.2650003
57732.0000000	4.2500000
57733.0000000	4.2500000
57734.0000000	4.2550001
57735.0000000	4.2449999
57736.0000000	4.2299995
57737.0000000	4.2349997
57738.0000000	4.2199998
57739.0000000	4.2150002
57740.0000000	4.2100000

57741.0000000	4.2100000
57742.0000000	4.2100000
57743.0000000	4.1999998
57744.0000000	4.1799998
57745.0000000	4.1700001
57746.0000000	4.1400003
57747.0000000	4.1199999
57748.0000000	4.0950003
57749.0000000	4.0550003
57750.0000000	4.0500002
57751.0000000	4.0200005
57752.0000000	3.9933331
57753.0000000	3.9600000
57754.0000000	3.9349999
57755.0000000	3.9100001
57756.0000000	3.8850002
57757.0000000	3.8599999
57758.0000000	3.8299999
57759.0000000	3.8000000
57760.0000000	3.7700000
57761.0000000	3.7350001
57762.0000000	3.7100000
57763.0000000	3.6750002
57764.0000000	3.6450000
57765.0000000	3.5999999
57766.0000000	3.5749998
57767.0000000	3.5400000
57768.0000000	3.5050001
57769.0000000	3.4650002
57770.0000000	3.4300001
57771.0000000	3.3950000
57772.0000000	3.3600001
57773.0000000	3.3299999
57774.0000000	3.2950001
57775.0000000	3.2600000
57776.0000000	3.2200000
57777.0000000	3.1849999
57778.0000000	3.1550002
57779.0000000	3.1199999
57780.0000000	3.0850000
57781.0000000	3.0500000
57782.0000000	3.0000000
57783.0000000	2.9549999
57784.0000000	2.9150000
57785.0000000	2.8800001
57786.0000000	2.8449998
57787.0000000	2.8000000
57788.0000000	2.7649999
57789.0000000	2.7300000
57790.0000000	2.6900001
57791.0000000	2.6500001
57792.0000000	2.6050000
57793.0000000	2.5799999
57794.0000000	2.5400000
57795.0000000	2.5050001
57796.0000000	2.4700000
57797.0000000	2.4400001

57798.0000000	2.4000001
57799.0000000	2.3699999
57800.0000000	2.3350000
57801.0000000	2.3049998
57802.0000000	2.2800000
57803.0000000	2.2449999
57804.0000000	2.2200000
57805.0000000	2.1849999
57806.0000000	2.1600001
57807.0000000	2.1300001
57808.0000000	2.1050000
57809.0000000	2.0799999
57810.0000000	2.0599999
57811.0000000	2.0250001
57812.0000000	2.0066667
57813.0000000	1.9700000
57814.0000000	1.9600000
57815.0000000	1.9400001
57816.0000000	1.9200000
57817.0000000	1.9000000
57818.0000000	1.8900000
57819.0000000	1.8650000
57820.0000000	1.8550000
57821.0000000	1.8350000
57822.0000000	1.8150001
57823.0000000	1.7950000
57824.0000000	1.7850000
57825.0000000	1.7650000
57826.0000000	1.7500000
57827.0000000	1.7350000
57828.0000000	1.7200000
57829.0000000	1.6950001
57830.0000000	1.6900001
57831.0000000	1.6750000
57832.0000000	1.6500000
57833.0000000	1.6300000
57834.0000000	1.6200000
57835.0000000	1.6000000
57836.0000000	1.5800000
57837.0000000	1.5599999
57838.0000000	1.5400000
57839.0000000	1.5200000
57840.0000000	1.5000000
57841.0000000	1.4750000
57842.0000000	1.4500000
57843.0000000	1.4266666
57844.0000000	1.4150000
57845.0000000	1.3900000
57846.0000000	1.3750000
57847.0000000	1.3500000
57848.0000000	1.3350000
57849.0000000	1.3250000
57850.0000000	1.3099999
57851.0000000	1.2950000
57852.0000000	1.2750000
57853.0000000	1.2600000
57854.0000000	1.2500000

57855.0000000	1.2400000
57856.0000000	1.2350000
57857.0000000	1.2200000
57858.0000000	1.2100000
57859.0000000	1.2000000
57860.0000000	1.1900001
57861.0000000	1.1750000
57862.0000000	1.1700000
57863.0000000	1.1600000
57864.0000000	1.1550000
57865.0000000	1.1450000
57866.0000000	1.1350000
57867.0000000	1.1300000
57868.0000000	1.1250000
57869.0000000	1.1150000
57870.0000000	1.1050000
57871.0000000	1.0900000
57872.0000000	1.0900000
57873.0000000	1.0700001
57874.0000000	1.0650001
57875.0000000	1.0549999
57876.0000000	1.0450000
57877.0000000	1.0400000
57878.0000000	1.0300000
57879.0000000	1.0150000
57880.0000000	1.0000000
57881.0000000	0.9850000
57882.0000000	0.9750000
57883.0000000	0.9600000
57884.0000000	0.9450000
57885.0000000	0.9350000
57886.0000000	0.9250000
57887.0000000	0.9100000
57888.0000000	0.9000000
57889.0000000	0.8850000
57890.0000000	0.8700000
57891.0000000	0.8500000
57892.0000000	0.8450000
57893.0000000	0.8300000
57894.0000000	0.8100000
57895.0000000	0.7950000
57896.0000000	0.7900000
57897.0000000	0.7750000
57898.0000000	0.7650000
57899.0000000	0.7550000
57900.0000000	0.7400000
57901.0000000	0.7300000
57902.0000000	0.7150000
57903.0000000	0.7033333
57904.0000000	0.6950000
57905.0000000	0.6850000
57906.0000000	0.6700000
57907.0000000	0.6600000
57908.0000000	0.6550000
57909.0000000	0.6450000
57910.0000000	0.6300000
57911.0000000	0.6250000

57912.0000000	0.6100000
57913.0000000	0.6050000
57914.0000000	0.6050000
57915.0000000	0.5950000
57916.0000000	0.5850000
57917.0000000	0.5800000
57918.0000000	0.5650000
57919.0000000	0.5700000
57920.0000000	0.5550000
57921.0000000	0.5550000
57922.0000000	0.5500000
57923.0000000	0.5400000
57924.0000000	0.5300000
57925.0000000	0.5300000
57926.0000000	0.5250000
57927.0000000	0.5200000
57928.0000000	0.5150000
57929.0000000	0.5100000
57930.0000000	0.5150000
57931.0000000	0.5100000
57932.0000000	0.5000000
57933.0000000	0.4933333
57934.0000000	0.4900000
57935.0000000	0.4850000
57936.0000000	0.4800000
57937.0000000	0.4700000
57938.0000000	0.4600000
57939.0000000	0.4550000
57940.0000000	0.4500000
57941.0000000	0.4450000
57942.0000000	0.4350000
57943.0000000	0.4300000
57944.0000000	0.4250000
57945.0000000	0.4150000
57946.0000000	0.4100000
57947.0000000	0.3950000
57948.0000000	0.3950000
57949.0000000	0.3850000
57950.0000000	0.3750000
57951.0000000	0.3600000
57952.0000000	0.3500000
57953.0000000	0.3400000
57954.0000000	0.3250000
57955.0000000	0.3150000
57956.0000000	0.3050000
57957.0000000	0.2950000
57958.0000000	0.2850000
57959.0000000	0.2900000
57960.0000000	0.2800000
57961.0000000	0.2750000
57962.0000000	0.2700000
57963.0000000	0.2650000
57964.0000000	0.2533333
57965.0000000	0.2450000
57966.0000000	0.2400000
57967.0000000	0.2300000
57968.0000000	0.2250000

57969.0000000	0.2200000
57970.0000000	0.2150000
57971.0000000	0.2150000
57972.0000000	0.2100000
57973.0000000	0.2050000
57974.0000000	0.1950000
57975.0000000	0.1900000
57976.0000000	0.1900000
57977.0000000	0.1800000
57978.0000000	0.1750000
57979.0000000	0.1650000
57980.0000000	0.1650000
57981.0000000	0.1550000
57982.0000000	0.1450000
57983.0000000	0.1400000
57984.0000000	0.1350000
57985.0000000	0.1200000
57986.0000000	0.1100000
57987.0000000	0.1050000
57988.0000000	0.0900000
57989.0000000	0.0800000
57990.0000000	0.0700000
57991.0000000	0.0600000
57992.0000000	0.0550000
57993.0000000	0.0450000
57994.0000000	0.0300000
57995.0000000	0.0200000
57996.0000000	0.0050000
57997.0000000	-0.0050000
57998.0000000	-0.0200000
57999.0000000	-0.0300000
58000.0000000	-0.0450000
58001.0000000	-0.0550000
58002.0000000	-0.0700000
58003.0000000	-0.0850000
58004.0000000	-0.0950000
58005.0000000	-0.1050000
58006.0000000	-0.1200000
58007.0000000	-0.1350000
58008.0000000	-0.1500000
58009.0000000	-0.1600000
58010.0000000	-0.1750000
58011.0000000	-0.1900000
58012.0000000	-0.2000000
58013.0000000	-0.2100000
58014.0000000	-0.2250000
58015.0000000	-0.2350000
58016.0000000	-0.2500000
58017.0000000	-0.2550000
58018.0000000	-0.2700000
58019.0000000	-0.2850000
58020.0000000	-0.3050000
58021.0000000	-0.3100000
58022.0000000	-0.3250000
58023.0000000	-0.3400000
58024.0000000	-0.3550000
58025.0000000	-0.3650000

58026.0000000	-0.3800000
58027.0000000	-0.3900000
58028.0000000	-0.4050000
58029.0000000	-0.4200000
58030.0000000	-0.4250000
58031.0000000	-0.4400000
58032.0000000	-0.4500000
58033.0000000	-0.4550000
58034.0000000	-0.4750000
58035.0000000	-0.4900000
58036.0000000	-0.4950000
58037.0000000	-0.5100000
58038.0000000	-0.5200000
58039.0000000	-0.5250000
58040.0000000	-0.5400000
58041.0000000	-0.5500000
58042.0000000	-0.5600000
58043.0000000	-0.5700000
58044.0000000	-0.5850000
58045.0000000	-0.5900000
58046.0000000	-0.6000000
58047.0000000	-0.6050000
58048.0000000	-0.6150000
58049.0000000	-0.6200000
58050.0000000	-0.6300000
58051.0000000	-0.6350000
58052.0000000	-0.6400000
58053.0000000	-0.6450000
58054.0000000	-0.6500000
58055.0000000	-0.6650000
58056.0000000	-0.6600000
58057.0000000	-0.6600000
58058.0000000	-0.6650000
58059.0000000	-0.6650000
58060.0000000	-0.6650000
58061.0000000	-0.6700000
58062.0000000	-0.6700000
58063.0000000	-0.6700000
58064.0000000	-0.6700000
58065.0000000	-0.6700000
58066.0000000	-0.6700000
58067.0000000	-0.6700000
58068.0000000	-0.6650000
58069.0000000	-0.6650000
58070.0000000	-0.6700000
58071.0000000	-0.6650000
58072.0000000	-0.6700000
58073.0000000	-0.6650000
58074.0000000	-0.6700000
58075.0000000	-0.6750000
58076.0000000	-0.6750000
58077.0000000	-0.6850000
58078.0000000	-0.6800000
58079.0000000	-0.6850000
58080.0000000	-0.6900000
58081.0000000	-0.6900000
58082.0000000	-0.6950000

58083.0000000	-0.7000000
58084.0000000	-0.7000000
58085.0000000	-0.7050000
58086.0000000	-0.7050000
58087.0000000	-0.7150000
58088.0000000	-0.7150000
58089.0000000	-0.7200000
58090.0000000	-0.7150000
58091.0000000	-0.7200000
58092.0000000	-0.7200000
58093.0000000	-0.7250000
58094.0000000	-0.7250000
58095.0000000	-0.7400000
58096.0000000	-0.7550000
58097.0000000	-0.7550000
58098.0000000	-0.7600000
58099.0000000	-0.7550000
58100.0000000	-0.7500000
58101.0000000	-0.7600000
58102.0000000	-0.7600000
58103.0000000	-0.7650000
58104.0000000	-0.7700000
58105.0000000	-0.7700000
58106.0000000	-0.7800000
58107.0000000	-0.7900000
58108.0000000	-0.7950000
58109.0000000	-0.7900000
58110.0000000	-0.7950000
58111.0000000	-0.8050000
58112.0000000	-0.8000000
58113.0000000	-0.8000000
58114.0000000	-0.8033333
58115.0000000	-0.8000000
58116.0000000	-0.8000000
58117.0000000	-0.8000000
58118.0000000	-0.7950000
58119.0000000	-0.8000000
58120.0000000	-0.7950000
58121.0000000	-0.8000000
58122.0000000	-0.7850000
58123.0000000	-0.7850000
58124.0000000	-0.7800000
58125.0000000	-0.7800000
58126.0000000	-0.7800000
58127.0000000	-0.7850000
58128.0000000	-0.8000000
58129.0000000	-0.8000000
58130.0000000	-0.8050000
58131.0000000	-0.8000000
58132.0000000	-0.8050000
58133.0000000	-0.8200000
58134.0000000	-0.8250000
58135.0000000	-0.8200000
58136.0000000	-0.8250000
58137.0000000	-0.8400000
58138.0000000	-0.8350000
58139.0000000	-0.8450000

58140.0000000	-0.8500000
58141.0000000	-0.8550000
58142.0000000	-0.8700000
58143.0000000	-0.8800000
58144.0000000	-0.8966667
58145.0000000	-0.9100000
58146.0000000	-0.9200000
58147.0000000	-0.9350000
58148.0000000	-0.9500000
58149.0000000	-0.9750000
58150.0000000	-0.9850000
58151.0000000	-0.9950000
58152.0000000	-1.0050000
58153.0000000	-1.0150000
58154.0000000	-1.0250000
58155.0000000	-1.0350000
58156.0000000	-1.0450000
58157.0000000	-1.0549999
58158.0000000	-1.0549999
58159.0000000	-1.0650001
58160.0000000	-1.0699999
58161.0000000	-1.0750000
58162.0000000	-1.0850000
58163.0000000	-1.0950000
58164.0000000	-1.1050000
58165.0000000	-1.1200000
58166.0000000	-1.1250000
58167.0000000	-1.1250000
58168.0000000	-1.1250000
58169.0000000	-1.1300000
58170.0000000	-1.1250000
58171.0000000	-1.1400000
58172.0000000	-1.1500000
58173.0000000	-1.1550000
58174.0000000	-1.1633333
58175.0000000	-1.1700000
58176.0000000	-1.1799999
58177.0000000	-1.1950001
58178.0000000	-1.2050000
58179.0000000	-1.2150000
58180.0000000	-1.2200000
58181.0000000	-1.2300000
58182.0000000	-1.2250000
58183.0000000	-1.2250000
58184.0000000	-1.2400000
58185.0000000	-1.2500000
58186.0000000	-1.2600000
58187.0000000	-1.2750000
58188.0000000	-1.2850000
58189.0000000	-1.3049999
58190.0000000	-1.3300000
58191.0000000	-1.3400000
58192.0000000	-1.3500000
58193.0000000	-1.3650000
58194.0000000	-1.3550000
58195.0000000	-1.3800000
58196.0000000	-1.3900000

58197.0000000	-1.3900000
58198.0000000	-1.4100000
58199.0000000	-1.4349999
58200.0000000	-1.4450001
58201.0000000	-1.4550000
58202.0000000	-1.4750000
58203.0000000	-1.4850000
58204.0000000	-1.5050000
58205.0000000	-1.5266666
58206.0000000	-1.5400000
58207.0000000	-1.5500000
58208.0000000	-1.5549999
58209.0000000	-1.5699999
58210.0000000	-1.5850000
58211.0000000	-1.5850000
58212.0000000	-1.6100000
58213.0000000	-1.6250000
58214.0000000	-1.6450000
58215.0000000	-1.6550000
58216.0000000	-1.6650000
58217.0000000	-1.6700000
58218.0000000	-1.6799999
58219.0000000	-1.6950001
58220.0000000	-1.7050000
58221.0000000	-1.7200000
58222.0000000	-1.7200000
58223.0000000	-1.7400000
58224.0000000	-1.7500000
58225.0000000	-1.7600000
58226.0000000	-1.7650000
58227.0000000	-1.7800000
58228.0000000	-1.7900000
58229.0000000	-1.7950000
58230.0000000	-1.8099999
58231.0000000	-1.8200001
58232.0000000	-1.8250000
58233.0000000	-1.8400000
58234.0000000	-1.8500000
58235.0000000	-1.8566667
58236.0000000	-1.8700000
58237.0000000	-1.8800000
58238.0000000	-1.8900000
58239.0000000	-1.8950000
58240.0000000	-1.9050000
58241.0000000	-1.9150000
58242.0000000	-1.9200000
58243.0000000	-1.9200000
58244.0000000	-1.9200000
58245.0000000	-1.9150000
58246.0000000	-1.9300001
58247.0000000	-1.9300001
58248.0000000	-1.9300001
58249.0000000	-1.9250000
58250.0000000	-1.9400001
58251.0000000	-1.9400001
58252.0000000	-1.9500000
58253.0000000	-1.9500000

58254.0000000	-1.9550000
58255.0000000	-1.9600000
58256.0000000	-1.9750000
58257.0000000	-1.9850000
58258.0000000	-1.9950000
58259.0000000	-2.0050001
58260.0000000	-2.0200000
58261.0000000	-2.0250001
58262.0000000	-2.0250001
58263.0000000	-2.0300000
58264.0000000	-2.0349998
58265.0000000	-2.0366666
58266.0000000	-2.0450001
58267.0000000	-2.0500000
58268.0000000	-2.0500000
58269.0000000	-2.0450001
58270.0000000	-2.0500000
58271.0000000	-2.0400000
58272.0000000	-2.0450001
58273.0000000	-2.0500000
58274.0000000	-2.0349998
58275.0000000	-2.0400000
58276.0000000	-2.0400000
58277.0000000	-2.0349998
58278.0000000	-2.0400000
58279.0000000	-2.0400000
58280.0000000	-2.0349998
58281.0000000	-2.0349998
58282.0000000	-2.0349998
58283.0000000	-2.0400000
58284.0000000	-2.0500000
58285.0000000	-2.0450001
58286.0000000	-2.0500000
58287.0000000	-2.0549998
58288.0000000	-2.0549998
58289.0000000	-2.0400000
58290.0000000	-2.0500000
58291.0000000	-2.0500000
58292.0000000	-2.0500000
58293.0000000	-2.0450001
58294.0000000	-2.0650001
58295.0000000	-2.0633333
58296.0000000	-2.0699999
58297.0000000	-2.0699999
58298.0000000	-2.0749998
58299.0000000	-2.0749998
58300.0000000	-2.0850000
58301.0000000	-2.0899999
58302.0000000	-2.0850000
58303.0000000	-2.0899999
58304.0000000	-2.0949998
58305.0000000	-2.1050000
58306.0000000	-2.1149998
58307.0000000	-2.1099999
58308.0000000	-2.1149998
58309.0000000	-2.1199999
58310.0000000	-2.1250000

58311.0000000	-2.1300001
58312.0000000	-2.1400001
58313.0000000	-2.1450000
58314.0000000	-2.1550002
58315.0000000	-2.1750002
58316.0000000	-2.1800001
58317.0000000	-2.1900001
58318.0000000	-2.1950002
58319.0000000	-2.2100000
58320.0000000	-2.2150002
58321.0000000	-2.2200000
58322.0000000	-2.2300000
58323.0000000	-2.2400000
58324.0000000	-2.2500000
58325.0000000	-2.2600000
58326.0000000	-2.2766666
58327.0000000	-2.2900000
58328.0000000	-2.2950001
58329.0000000	-2.3049998
58330.0000000	-2.3150001
58331.0000000	-2.3399999
58332.0000000	-2.3499999
58333.0000000	-2.3550000
58334.0000000	-2.3699999
58335.0000000	-2.3699999
58336.0000000	-2.3900001
58337.0000000	-2.4050002
58338.0000000	-2.4300001
58339.0000000	-2.4400001
58340.0000000	-2.4450002
58341.0000000	-2.4549999
58342.0000000	-2.4650002
58343.0000000	-2.4800000
58344.0000000	-2.4949999
58345.0000000	-2.5000000
58346.0000000	-2.5149999
58347.0000000	-2.5250001
58348.0000000	-2.5300000
58349.0000000	-2.5300000
58350.0000000	-2.5400000
58351.0000000	-2.5500000
58352.0000000	-2.5650001
58353.0000000	-2.5850000
58354.0000000	-2.5949998
58355.0000000	-2.5999999
58356.0000000	-2.6033332
58357.0000000	-2.6099999
58358.0000000	-2.6199999
58359.0000000	-2.6350002
58360.0000000	-2.6300001
58361.0000000	-2.6500001
58362.0000000	-2.6650000
58363.0000000	-2.6700001
58364.0000000	-2.6750002
58365.0000000	-2.6800001
58366.0000000	-2.7049999
58367.0000000	-2.7150002

58368.0000000	-2.7150002
58369.0000000	-2.7249999
58370.0000000	-2.7249999
58371.0000000	-2.7249999
58372.0000000	-2.7249999
58373.0000000	-2.7400000
58374.0000000	-2.7500000
58375.0000000	-2.7500000
58376.0000000	-2.7600000
58377.0000000	-2.7700000
58378.0000000	-2.7649999
58379.0000000	-2.7750001
58380.0000000	-2.7849998
58381.0000000	-2.7800000
58382.0000000	-2.7900000
58383.0000000	-2.8000000
58384.0000000	-2.8000000
58385.0000000	-2.8049998
58386.0000000	-2.8199999
58387.0000000	-2.8399999
58388.0000000	-2.8550000
58389.0000000	-2.8649998
58390.0000000	-2.8750000
58391.0000000	-2.8800001
58392.0000000	-2.8950000
58393.0000000	-2.9050002
58394.0000000	-2.9150000
58395.0000000	-2.9250002
58396.0000000	-2.9349999
58397.0000000	-2.9349999
58398.0000000	-2.9500000
58399.0000000	-2.9650002
58400.0000000	-2.9749999
58401.0000000	-2.9800000
58402.0000000	-2.9850001
58403.0000000	-2.9949999
58404.0000000	-3.0050001
58405.0000000	-3.0200000
58406.0000000	-3.0349998
58407.0000000	-3.0400000
58408.0000000	-3.0500000
58409.0000000	-3.0599999
58410.0000000	-3.0699999
58411.0000000	-3.0799999
58412.0000000	-3.0899999
58413.0000000	-3.0899999
58414.0000000	-3.0699999
58415.0000000	-3.1099999
58416.0000000	-3.1233332
58417.0000000	-3.1250000
58418.0000000	-3.1350002
58419.0000000	-3.1350002
58420.0000000	-3.1350002
58421.0000000	-3.1350002
58422.0000000	-3.1300001
58423.0000000	-3.1300001
58424.0000000	-3.1400001

58425.0000000	-3.1300001
58426.0000000	-3.1300001
58427.0000000	-3.1350002
58428.0000000	-3.1300001
58429.0000000	-3.1350002
58430.0000000	-3.1350002
58431.0000000	-3.1250000
58432.0000000	-3.1250000
58433.0000000	-3.0949998
58434.0000000	-3.1199999
58435.0000000	-3.1050000
58436.0000000	-3.1149998
58437.0000000	-3.1149998
58438.0000000	-3.1250000
58439.0000000	-3.1300001
58440.0000000	-3.1400001
58441.0000000	-3.1450000
58442.0000000	-3.1500001
58443.0000000	-3.1650000
58444.0000000	-3.1900001
58445.0000000	-3.2000000
58446.0000000	-3.2100000
58447.0000000	-3.2000000
58448.0000000	-3.2249999
58449.0000000	-3.2350001
58450.0000000	-3.2350001
58451.0000000	-3.2449999
58452.0000000	-3.2550001
58453.0000000	-3.2550001
58454.0000000	-3.2600000
58455.0000000	-3.2750001
58456.0000000	-3.2800000
58457.0000000	-3.2950001
58458.0000000	-3.3000000
58459.0000000	-3.3099999
58460.0000000	-3.3249998
58461.0000000	-3.3249998
58462.0000000	-3.3350000
58463.0000000	-3.3399999
58464.0000000	-3.3399999
58465.0000000	-3.3449998
58466.0000000	-3.3399999
58467.0000000	-3.3550000
58468.0000000	-3.3649998
58469.0000000	-3.3699999
58470.0000000	-3.3800001
58471.0000000	-3.3850002
58472.0000000	-3.3900001
58473.0000000	-3.4000001
58474.0000000	-3.4000001
58475.0000000	-3.4100001
58476.0000000	-3.4150000
58477.0000000	-3.4333334
58478.0000000	-3.4400001
58479.0000000	-3.4349999
58480.0000000	-3.4400001
58481.0000000	-3.4450002

58482.0000000	-3.4500000
58483.0000000	-3.4500000
58484.0000000	-3.4549999
58485.0000000	-3.4549999
58486.0000000	-3.4700000
58487.0000000	-3.4749999
58488.0000000	-3.4800000
58489.0000000	-3.4850001
58490.0000000	-3.4949999
58491.0000000	-3.4949999
58492.0000000	-3.5050001
58493.0000000	-3.5100000
58494.0000000	-3.5200000
58495.0000000	-3.5149999
58496.0000000	-3.5200000
58497.0000000	-3.5300000
58498.0000000	-3.5200000
58499.0000000	-3.5450001
58500.0000000	-3.5500000
58501.0000000	-3.5549998
58502.0000000	-3.5549998
58503.0000000	-3.5650001
58504.0000000	-3.5699999
58505.0000000	-3.5699999
58506.0000000	-3.5766666
58507.0000000	-3.5949998
58508.0000000	-3.6099999
58509.0000000	-3.6149998
58510.0000000	-3.6250000
58511.0000000	-3.6300001
58512.0000000	-3.6350002
58513.0000000	-3.6350002
58514.0000000	-3.6400001
58515.0000000	-3.6400001
58516.0000000	-3.6450000
58517.0000000	-3.6500001
58518.0000000	-3.6500001
58519.0000000	-3.6550002
58520.0000000	-3.6700001
58521.0000000	-3.6800001
58522.0000000	-3.6800001
58523.0000000	-3.6800001
58524.0000000	-3.6800001
58525.0000000	-3.6900001
58526.0000000	-3.6900001
58527.0000000	-3.6950002
58528.0000000	-3.6849999
58529.0000000	-3.6800001
58530.0000000	-3.6900001
58531.0000000	-3.7000000
58532.0000000	-3.7049999
58533.0000000	-3.7100000
58534.0000000	-3.7000000
58535.0000000	-3.7100000
58536.0000000	-3.7100000
58537.0000000	-3.7133334
58538.0000000	-3.7249999

58539.0000000	-3.7300000
58540.0000000	-3.7400000
58541.0000000	-3.7550001
58542.0000000	-3.7600000
58543.0000000	-3.7700000
58544.0000000	-3.7800000
58545.0000000	-3.7849998
58546.0000000	-3.7950001
58547.0000000	-3.7950001
58548.0000000	-3.8099999
58549.0000000	-3.8199999
58550.0000000	-3.8199999
58551.0000000	-3.8299999
58552.0000000	-3.8249998
58553.0000000	-3.8499999
58554.0000000	-3.8550000
58555.0000000	-3.8649998
58556.0000000	-3.8750000
58557.0000000	-3.8750000
58558.0000000	-3.8900001
58559.0000000	-3.8900001
58560.0000000	-3.8900001
58561.0000000	-3.8900001
58562.0000000	-3.8850002
58563.0000000	-3.8800001
58564.0000000	-3.8800001
58565.0000000	-3.8699999
58566.0000000	-3.8800001
58567.0000000	-3.8800001
58568.0000000	-3.8800001
58569.0000000	-3.8850000
58570.0000000	-3.8699999
58571.0000000	-3.8800001
58572.0000000	-3.8800001
58573.0000000	-3.8950000
58574.0000000	-3.8900001
58575.0000000	-3.8900001
58576.0000000	-3.9000001
58577.0000000	-3.9150000
58578.0000000	-3.9250002
58579.0000000	-3.9300001
58580.0000000	-3.9300001
58581.0000000	-3.9400001
58582.0000000	-3.9400001
58583.0000000	-3.9300001
58584.0000000	-3.9400001
58585.0000000	-3.9600000
58586.0000000	-3.9600000
58587.0000000	-3.9600000
58588.0000000	-3.9749999
58589.0000000	-3.9749999
58590.0000000	-3.9850001
58591.0000000	-3.9900000
58592.0000000	-4.0000000
58593.0000000	-4.0000000
58594.0000000	-4.0100002
58595.0000000	-4.0100002

58596.0000000	-4.0150003
58597.0000000	-4.0200005
58598.0000000	-4.0299997
58599.0000000	-4.0450001
58600.0000000	-4.0500002
58601.0000000	-4.0699997
58602.0000000	-4.0799999
58603.0000000	-4.0850000
58604.0000000	-4.0850000
58605.0000000	-4.0999999
58606.0000000	-4.0999999
58607.0000000	-4.1149998
58608.0000000	-4.1149998
58609.0000000	-4.1199999
58610.0000000	-4.1199999
58611.0000000	-4.0950003
58612.0000000	-4.1099997
58613.0000000	-4.1149998
58614.0000000	-4.1250000
58615.0000000	-4.1149998
58616.0000000	-4.1350002
58617.0000000	-4.1400003
58618.0000000	-4.1399999
58619.0000000	-4.1450000
58620.0000000	-4.1400003
58621.0000000	-4.1549997
58622.0000000	-4.1650000
58623.0000000	-4.1650000
58624.0000000	-4.1700001
58625.0000000	-4.1750002
58626.0000000	-4.1849999
58627.0000000	-4.1766667
58628.0000000	-4.1750002
58629.0000000	-4.1700001
58630.0000000	-4.1799998
58631.0000000	-4.1899996
58632.0000000	-4.1949997
58633.0000000	-4.1999998
58634.0000000	-4.2150002
58635.0000000	-4.2199998
58636.0000000	-4.2300000
58637.0000000	-4.2349997
58638.0000000	-4.2399998
58639.0000000	-4.2449999
58640.0000000	-4.2500000
58641.0000000	-4.2500000
58642.0000000	-4.2550001
58643.0000000	-4.2300000
58644.0000000	-4.2600002
58645.0000000	-4.2600002
58646.0000000	-4.2650003
58647.0000000	-4.2550001
58648.0000000	-4.2750001
58649.0000000	-4.2799997
58650.0000000	-4.2799997
58651.0000000	-4.2849998
58652.0000000	-4.3050003

58653.0000000	-4.3000002
58654.0000000	-4.3150001
58655.0000000	-4.3200002
58656.0000000	-4.3150001
58657.0000000	-4.3266664
58658.0000000	-4.3350000
58659.0000000	-4.3350000
58660.0000000	-4.3500004
58661.0000000	-4.3550000
58662.0000000	-4.3600001
58663.0000000	-4.3649998
58664.0000000	-4.3550000
58665.0000000	-4.3450003
58666.0000000	-4.3299999
58667.0000000	-4.3450003
58668.0000000	-4.3450003
58669.0000000	-4.3450003
58670.0000000	-4.3649998
58671.0000000	-4.3699999
58672.0000000	-4.3800001
58673.0000000	-4.3850002
58674.0000000	-4.3850002
58675.0000000	-4.4000001
58676.0000000	-4.4049997
58677.0000000	-4.4150000
58678.0000000	-4.4250002
58679.0000000	-4.4300003
58680.0000000	-4.4349999
58681.0000000	-4.4449997
58682.0000000	-4.4549999
58683.0000000	-4.4650002
58684.0000000	-4.4749999
58685.0000000	-4.4699998
58686.0000000	-4.4849997
58687.0000000	-4.4899998
58688.0000000	-4.4866662
58689.0000000	-4.4849997
58690.0000000	-4.5000000
58691.0000000	-4.5050001
58692.0000000	-4.5100002
58693.0000000	-4.5299997
58694.0000000	-4.5349998
58695.0000000	-4.5450001
58696.0000000	-4.5500002
58697.0000000	-4.5599999
58698.0000000	-4.5650001
58699.0000000	-4.5900002
58700.0000000	-4.5950003
58701.0000000	-4.6050000
58702.0000000	-4.6050000
58703.0000000	-4.6050000
58704.0000000	-4.6099997
58705.0000000	-4.6050000
58706.0000000	-4.6300001
58707.0000000	-4.6300001
58708.0000000	-4.6350002
58709.0000000	-4.6250000

58710.0000000	-4.6450000
58711.0000000	-4.6450000
58712.0000000	-4.6599998
58713.0000000	-4.6599998
58714.0000000	-4.6700001
58715.0000000	-4.6750002
58716.0000000	-4.6750002
58717.0000000	-4.6800003
58718.0000000	-4.6966662
58719.0000000	-4.7049999
58720.0000000	-4.6949997
58721.0000000	-4.7049999
58722.0000000	-4.7150002
58723.0000000	-4.7200003
58724.0000000	-4.7249999
58725.0000000	-4.7299995
58726.0000000	-4.7349997
58727.0000000	-4.7449999
58728.0000000	-4.7500000
58729.0000000	-4.7600002
58730.0000000	-4.7550001
58731.0000000	-4.7449999
58732.0000000	-4.7500000
58733.0000000	-4.7449999
58734.0000000	-4.7449999
58735.0000000	-4.7349997
58736.0000000	-4.7600002
58737.0000000	-4.7550001
58738.0000000	-4.7550001
58739.0000000	-4.7600002
58740.0000000	-4.7700005
58741.0000000	-4.7600002
58742.0000000	-4.7750001
58743.0000000	-4.7750001
58744.0000000	-4.7700000
58745.0000000	-4.7750001
58746.0000000	-4.7900000
58747.0000000	-4.7950001
58748.0000000	-4.8099999
58749.0000000	-4.8249998
58750.0000000	-4.8299999
58751.0000000	-4.8400002
58752.0000000	-4.8450003
58753.0000000	-4.8550000
58754.0000000	-4.8550000
58755.0000000	-4.8550000
58756.0000000	-4.8649998
58757.0000000	-4.8649998
58758.0000000	-4.8850002
58759.0000000	-4.8850002
58760.0000000	-4.8899999
58761.0000000	-4.8950000
58762.0000000	-4.8899999
58763.0000000	-4.8899999
58764.0000000	-4.8950000
58765.0000000	-4.8950000
58766.0000000	-4.8999996

58767.0000000	-4.8999996
58768.0000000	-4.8999996
58769.0000000	-4.8999996
58770.0000000	-4.9049997
58771.0000000	-4.9000001
58772.0000000	-4.9049997
58773.0000000	-4.8899999
58774.0000000	-4.8999996
58775.0000000	-4.8999996
58776.0000000	-4.9049997
58777.0000000	-4.9049997
58778.0000000	-4.8933334
58779.0000000	-4.8950000
58780.0000000	-4.8900003
58781.0000000	-4.8899999
58782.0000000	-4.8899999
58783.0000000	-4.8950000
58784.0000000	-4.8950000
58785.0000000	-4.8950000
58786.0000000	-4.9049997
58787.0000000	-4.9099998
58788.0000000	-4.9150000
58789.0000000	-4.9200001
58790.0000000	-4.9200001
58791.0000000	-4.9150000
58792.0000000	-4.9200001
58793.0000000	-4.9200001
58794.0000000	-4.9200001
58795.0000000	-4.9099998
58796.0000000	-4.9200001
58797.0000000	-4.9200001
58798.0000000	-4.9250002
58799.0000000	-4.9250002
58800.0000000	-4.9300003
58801.0000000	-4.9300003
58802.0000000	-4.9349999
58803.0000000	-4.9299998
58804.0000000	-4.9150000
58805.0000000	-4.9300003
58806.0000000	-4.9349999
58807.0000000	-4.9399996
58808.0000000	-4.9399996
58809.0000000	-4.9300003
58810.0000000	-4.9400001
58811.0000000	-4.8999996
58812.0000000	-4.9300003
58813.0000000	-4.9300003
58814.0000000	-4.9300003
58815.0000000	-4.9250002
58816.0000000	-4.9250002
58817.0000000	-4.9250002
58818.0000000	-4.9250002
58819.0000000	-4.9200001
58820.0000000	-4.9200001
58821.0000000	-4.9200001
58822.0000000	-4.9150000
58823.0000000	-4.9200001

58824.0000000	-4.9150000
58825.0000000	-4.9099998
58826.0000000	-4.9049997
58827.0000000	-4.8950000
58828.0000000	-4.8950000
58829.0000000	-4.8850002
58830.0000000	-4.8850002
58831.0000000	-4.8850002
58832.0000000	-4.8800001
58833.0000000	-4.8750000
58834.0000000	-4.8750000
58835.0000000	-4.8750000
58836.0000000	-4.8750000
58837.0000000	-4.8699999
58838.0000000	-4.8599997
58839.0000000	-4.8633332
58840.0000000	-4.8599997
58841.0000000	-4.8649998
58842.0000000	-4.8550000
58843.0000000	-4.8649998
58844.0000000	-4.8699999
58845.0000000	-4.8649998
58846.0000000	-4.8800001
58847.0000000	-4.8750000
58848.0000000	-4.8800001
58849.0000000	-4.8800001
58850.0000000	-4.8800001
58851.0000000	-4.8699999
58852.0000000	-4.8900003
58853.0000000	-4.8900003
58854.0000000	-4.9000001
58855.0000000	-4.8999996
58856.0000000	-4.8950000
58857.0000000	-4.8950000
58858.0000000	-4.8950000
58859.0000000	-4.8950000
58860.0000000	-4.8999996
58861.0000000	-4.9049997
58862.0000000	-4.9000001
58863.0000000	-4.8950000
58864.0000000	-4.8900003
58865.0000000	-4.8899999
58866.0000000	-4.8800001
58867.0000000	-4.8950000
58868.0000000	-4.8799996
58869.0000000	-4.9000001
58870.0000000	-4.9000001
58871.0000000	-4.8950000
58872.0000000	-4.9000001
58873.0000000	-4.8899999
58874.0000000	-4.8950000
58875.0000000	-4.8950000
58876.0000000	-4.8900003
58877.0000000	-4.9000001
58878.0000000	-4.8999996
58879.0000000	-4.8999996
58880.0000000	-4.8950000

58881.0000000	-4.8900003
58882.0000000	-4.8850002
58883.0000000	-4.8850002
58884.0000000	-4.8750000
58885.0000000	-4.8800001
58886.0000000	-4.8699999
58887.0000000	-4.8599997
58888.0000000	-4.8649998
58889.0000000	-4.8599997
58890.0000000	-4.8649998
58891.0000000	-4.8599997
58892.0000000	-4.8500004
58893.0000000	-4.8499999
58894.0000000	-4.8400002
58895.0000000	-4.8350000
58896.0000000	-4.8299999
58897.0000000	-4.8099999
58898.0000000	-4.8099999
58899.0000000	-4.7966666
58900.0000000	-4.7900000
58901.0000000	-4.7750001
58902.0000000	-4.7650003
58903.0000000	-4.7550001
58904.0000000	-4.7449999
58905.0000000	-4.7349997
58906.0000000	-4.7199998
58907.0000000	-4.7100000
58908.0000000	-4.7049999
58909.0000000	-4.6899996
58910.0000000	-4.6700001
58911.0000000	-4.6750002
58912.0000000	-4.6549997
58913.0000000	-4.6450000
58914.0000000	-4.6300001
58915.0000000	-4.6250000
58916.0000000	-4.6149998
58917.0000000	-4.5950003
58918.0000000	-4.5799999
58919.0000000	-4.5650001
58920.0000000	-4.5450001
58921.0000000	-4.5299997
58922.0000000	-4.5100002
58923.0000000	-4.4899998
58924.0000000	-4.4699998
58925.0000000	-4.4499998
58926.0000000	-4.4250002
58927.0000000	-4.4099998
58928.0000000	-4.3750000
58929.0000000	-4.3566666
58930.0000000	-4.3249998
58931.0000000	-4.3100004
58932.0000000	-4.2900000
58933.0000000	-4.2849998
58934.0000000	-4.2750001
58935.0000000	-4.2799997
58936.0000000	-4.2750001
58937.0000000	-4.2700005

58938.0000000	-4.2650003
58939.0000000	-4.2600002
58940.0000000	-4.2650003
58941.0000000	-4.2650003
58942.0000000	-4.2600002
58943.0000000	-4.2500000
58944.0000000	-4.2449999
58945.0000000	-4.2500000
58946.0000000	-4.2550001
58947.0000000	-4.2600002
58948.0000000	-4.2600002
58949.0000000	-4.2600002
58950.0000000	-4.2800002
58951.0000000	-4.2900000
58952.0000000	-4.2849998
58953.0000000	-4.3000002
58954.0000000	-4.3050003
58955.0000000	-4.3150001
58956.0000000	-4.3100004
58957.0000000	-4.2950001
58958.0000000	-4.3000002
58959.0000000	-4.3050003
58960.0000000	-4.3066669
58961.0000000	-4.3050003
58962.0000000	-4.3100004
58963.0000000	-4.3100004
58964.0000000	-4.3100004
58965.0000000	-4.3100004
58966.0000000	-4.3150001
58967.0000000	-4.3150001
58968.0000000	-4.3100004
58969.0000000	-4.3249998
58970.0000000	-4.3249998
58971.0000000	-4.3299999
58972.0000000	-4.3350000
58973.0000000	-4.3499999
58974.0000000	-4.3450003
58975.0000000	-4.3550000
58976.0000000	-4.3550000
58977.0000000	-4.3649998
58978.0000000	-4.3699999
58979.0000000	-4.3699999
58980.0000000	-4.3699999
58981.0000000	-4.3750000
58982.0000000	-4.3699999
58983.0000000	-4.3800001
58984.0000000	-4.3800001
58985.0000000	-4.3750000
58986.0000000	-4.3750000
58987.0000000	-4.3750000
58988.0000000	-4.3649998
58989.0000000	-4.3699999
58990.0000000	-4.3649998
58991.0000000	-4.3599997
58992.0000000	-4.3550000
58993.0000000	-4.3649998
58994.0000000	-4.3600001

58995.0000000	-4.3499999
58996.0000000	-4.3450003
58997.0000000	-4.3350000
58998.0000000	-4.3199997
58999.0000000	-4.3150001
59000.0000000	-4.3050003
59001.0000000	-4.2800002
59002.0000000	-4.2750001
59003.0000000	-4.2500000
59004.0000000	-4.2399998
59005.0000000	-4.2049999
59006.0000000	-4.1999998
59007.0000000	-4.1799998
59008.0000000	-4.1599998
59009.0000000	-4.1300001
59010.0000000	-4.1199999
59011.0000000	-4.0900002
59012.0000000	-4.0799999
59013.0000000	-4.0550003
59014.0000000	-4.0400000
59015.0000000	-4.0250001
59016.0000000	-4.0000000
59017.0000000	-3.9749999
59018.0000000	-3.9549999
59019.0000000	-3.9349999
59020.0000000	-3.9033334
59021.0000000	-3.8850002
59022.0000000	-3.8699999
59023.0000000	-3.8900001
59024.0000000	-3.8900001
59025.0000000	-3.8900001
59026.0000000	-3.8850002
59027.0000000	-3.8850002
59028.0000000	-3.8800001
59029.0000000	-3.8750000
59030.0000000	-3.8649998
59031.0000000	-3.8599999
59032.0000000	-3.8499999
59033.0000000	-3.8350000
59034.0000000	-3.8099999
59035.0000000	-3.7900000
59036.0000000	-3.7750001
59037.0000000	-3.7750001
59038.0000000	-3.7750001
59039.0000000	-3.7600000
59040.0000000	-3.7449999
59041.0000000	-3.7200000
59042.0000000	-3.7150002
59043.0000000	-3.7000000
59044.0000000	-3.6849999
59045.0000000	-3.6900001
59046.0000000	-3.6900001
59047.0000000	-3.6950002
59048.0000000	-3.7000000
59049.0000000	-3.7000000
59050.0000000	-3.7033336
59051.0000000	-3.7049999

59052.0000000	-3.7000000
59053.0000000	-3.6950002
59054.0000000	-3.6849999
59055.0000000	-3.6849999
59056.0000000	-3.6750002
59057.0000000	-3.6849999
59058.0000000	-3.6800001
59059.0000000	-3.6750002
59060.0000000	-3.6800001
59061.0000000	-3.6700001
59062.0000000	-3.6700001
59063.0000000	-3.6550002
59064.0000000	-3.6550002
59065.0000000	-3.6450000
59066.0000000	-3.6400001
59067.0000000	-3.6300001
59068.0000000	-3.6250000
59069.0000000	-3.6199999
59070.0000000	-3.6149998
59071.0000000	-3.5899999
59072.0000000	-3.5850000
59073.0000000	-3.5349998
59074.0000000	-3.5599999
59075.0000000	-3.5549998
59076.0000000	-3.5650001
59077.0000000	-3.5650001
59078.0000000	-3.5650001
59079.0000000	-3.5549998
59080.0000000	-3.5600002
59081.0000000	-3.5549998
59082.0000000	-3.5450001
59083.0000000	-3.5500000
59084.0000000	-3.5500000
59085.0000000	-3.5450001
59086.0000000	-3.5500000
59087.0000000	-3.5599999
59088.0000000	-3.5549998
59089.0000000	-3.5500000
59090.0000000	-3.5549998
59091.0000000	-3.5500000
59092.0000000	-3.5450001
59093.0000000	-3.5349998
59094.0000000	-3.5200000
59095.0000000	-3.5250001
59096.0000000	-3.5200000
59097.0000000	-3.5149999
59098.0000000	-3.5149999
59099.0000000	-3.5200000
59100.0000000	-3.5100000
59101.0000000	-3.5000000
59102.0000000	-3.5050001
59103.0000000	-3.5050001
59104.0000000	-3.5050001
59105.0000000	-3.5000000
59106.0000000	-3.5050001
59107.0000000	-3.5000000
59108.0000000	-3.4900000

59109.0000000	-3.4749999
59110.0000000	-3.4700000
59111.0000000	-3.4500000
59112.0000000	-3.4500000
59113.0000000	-3.4200001
59114.0000000	-3.4150000
59115.0000000	-3.3950000
59116.0000000	-3.3800001
59117.0000000	-3.3650000
59118.0000000	-3.3599999
59119.0000000	-3.3399999
59120.0000000	-3.3150001
59121.0000000	-3.3000000
59122.0000000	-3.2900000
59123.0000000	-3.2700000
59124.0000000	-3.2649999
59125.0000000	-3.2449999
59126.0000000	-3.2200000
59127.0000000	-3.2100000
59128.0000000	-3.1950002
59129.0000000	-3.1700001
59130.0000000	-3.1700001
59131.0000000	-3.1650000
59132.0000000	-3.1550002
59133.0000000	-3.1400001
59134.0000000	-3.1350002
59135.0000000	-3.1250000
59136.0000000	-3.1149998
59137.0000000	-3.1099999
59138.0000000	-3.1050000
59139.0000000	-3.0999999
59140.0000000	-3.0899999
59141.0000000	-3.0850000
59142.0000000	-3.0799999
59143.0000000	-3.0699999
59144.0000000	-3.0650001
59145.0000000	-3.0500000
59146.0000000	-3.0450001
59147.0000000	-3.0400000
59148.0000000	-3.0300000
59149.0000000	-3.0250001
59150.0000000	-3.0149999
59151.0000000	-3.0000000
59152.0000000	-2.9900000
59153.0000000	-2.9850001
59154.0000000	-2.9800000
59155.0000000	-2.9700000
59156.0000000	-2.9450002
59157.0000000	-2.9600000
59158.0000000	-2.9549999
59159.0000000	-2.9400001
59160.0000000	-2.9349999
59161.0000000	-2.9200001
59162.0000000	-2.9250002
59163.0000000	-2.9200001
59164.0000000	-2.9100001
59165.0000000	-2.9050002

59166.0000000	-2.9100001
59167.0000000	-2.9000001
59168.0000000	-2.8900001
59169.0000000	-2.8900001
59170.0000000	-2.8900001
59171.0000000	-2.8866665
59172.0000000	-2.8800001
59173.0000000	-2.8750000
59174.0000000	-2.8699999
59175.0000000	-2.8649998
59176.0000000	-2.8649998
59177.0000000	-2.8599999
59178.0000000	-2.8599999
59179.0000000	-2.8599999
59180.0000000	-2.8499999
59181.0000000	-2.8550000
59182.0000000	-2.8449998
59183.0000000	-2.8449998
59184.0000000	-2.8449998
59185.0000000	-2.8399999
59186.0000000	-2.8350000
59187.0000000	-2.8449998
59188.0000000	-2.8350000
59189.0000000	-2.8399999
59190.0000000	-2.8299999
59191.0000000	-2.8249998
59192.0000000	-2.8150001
59193.0000000	-2.8099999
59194.0000000	-2.8000000
59195.0000000	-2.7950001
59196.0000000	-2.7849998
59197.0000000	-2.7750001
59198.0000000	-2.7800000
59199.0000000	-2.7700000
59200.0000000	-2.7649999
59201.0000000	-2.7600000
59202.0000000	-2.7449999
59203.0000000	-2.7350001
59204.0000000	-2.7350001
59205.0000000	-2.7350001
59206.0000000	-2.7249999
59207.0000000	-2.7100000
59208.0000000	-2.7100000
59209.0000000	-2.7049999
59210.0000000	-2.6950002
59211.0000000	-2.6900001
59212.0000000	-2.6849999
59213.0000000	-2.6750002
59214.0000000	-2.6650000
59215.0000000	-2.6550002
59216.0000000	-2.6350000
59217.0000000	-2.6300001
59218.0000000	-2.6149998
59219.0000000	-2.6099999
59220.0000000	-2.6050000
59221.0000000	-2.5949998
59222.0000000	-2.5850000

59223.0000000	-2.5799999
59224.0000000	-2.5749998
59225.0000000	-2.5749998
59226.0000000	-2.5599999
59227.0000000	-2.5699999
59228.0000000	-2.5650001
59229.0000000	-2.5650001
59230.0000000	-2.5699999
59231.0000000	-2.5699999
59232.0000000	-2.5599999
59233.0000000	-2.5749998
59234.0000000	-2.5749998
59235.0000000	-2.5749998
59236.0000000	-2.5799999
59237.0000000	-2.5799999
59238.0000000	-2.5749998
59239.0000000	-2.5749998
59240.0000000	-2.5749998
59241.0000000	-2.5799999
59242.0000000	-2.5799999
59243.0000000	-2.5799999
59244.0000000	-2.5850000
59245.0000000	-2.5850000
59246.0000000	-2.5850000
59247.0000000	-2.5799999
59248.0000000	-2.5799999
59249.0000000	-2.5799999
59250.0000000	-2.5799999
59251.0000000	-2.5799999
59252.0000000	-2.5850000
59253.0000000	-2.5850000
59254.0000000	-2.5850000
59255.0000000	-2.5850000
59256.0000000	-2.5850000
59257.0000000	-2.5749998
59258.0000000	-2.5850000
59259.0000000	-2.5850000
59260.0000000	-2.5899999
59261.0000000	-2.5899999
59262.0000000	-2.5749998
59263.0000000	-2.5799999
59264.0000000	-2.5749998
59265.0000000	-2.5699999
59266.0000000	-2.5699999
59267.0000000	-2.5650001
59268.0000000	-2.5599999
59269.0000000	-2.5500000
59270.0000000	-2.5450001
59271.0000000	-2.5250001
59272.0000000	-2.5250001
59273.0000000	-2.5149999
59274.0000000	-2.5100000
59275.0000000	-2.5050001
59276.0000000	-2.5050001
59277.0000000	-2.5000000
59278.0000000	-2.5000000
59279.0000000	-2.4949999

59280.0000000	-2.4900000
59281.0000000	-2.4949999
59282.0000000	-2.4900000
59283.0000000	-2.4850001
59284.0000000	-2.4800000
59285.0000000	-2.4600000
59286.0000000	-2.4600000
59287.0000000	-2.4549999
59288.0000000	-2.4450002
59289.0000000	-2.4349999
59290.0000000	-2.4300001
59291.0000000	-2.4250002
59292.0000000	-2.4200001
59293.0000000	-2.4200001
59294.0000000	-2.4250002
59295.0000000	-2.4200001
59296.0000000	-2.4200001
59297.0000000	-2.4250002
59298.0000000	-2.4200001
59299.0000000	-2.4250002
59300.0000000	-2.4300001
59301.0000000	-2.4250002
59302.0000000	-2.4349999
59303.0000000	-2.4349999
59304.0000000	-2.4349999
59305.0000000	-2.4300001
59306.0000000	-2.4349999
59307.0000000	-2.4349999
59308.0000000	-2.4400001
59309.0000000	-2.4349999
59310.0000000	-2.4400001
59311.0000000	-2.4349999
59312.0000000	-2.4349999
59313.0000000	-2.4349999
59314.0000000	-2.4300001
59315.0000000	-2.4400001
59316.0000000	-2.4349999
59317.0000000	-2.4150000
59318.0000000	-2.4300001
59319.0000000	-2.4250002
59320.0000000	-2.4300001
59321.0000000	-2.4250002
59322.0000000	-2.4166667
59323.0000000	-2.4250002
59324.0000000	-2.4250002
59325.0000000	-2.4150000
59326.0000000	-2.4200001
59327.0000000	-2.4300001
59328.0000000	-2.4349999
59329.0000000	-2.4349999
59330.0000000	-2.4450002
59331.0000000	-2.4349999
59332.0000000	-2.4349999
59333.0000000	-2.4349999
59334.0000000	-2.4400001
59335.0000000	-2.4400001
59336.0000000	-2.4400001

59337.0000000	-2.4450002
59338.0000000	-2.4450002
59339.0000000	-2.4349999
59340.0000000	-2.4400001
59341.0000000	-2.4450002
59342.0000000	-2.4450002
59343.0000000	-2.4450002
59344.0000000	-2.4349999
59345.0000000	-2.4349999
59346.0000000	-2.4400001
59347.0000000	-2.4300001
59348.0000000	-2.4300001
59349.0000000	-2.4349999
59350.0000000	-2.4300001
59351.0000000	-2.4300001
59352.0000000	-2.4233334
59353.0000000	-2.4300001
59354.0000000	-2.4250002
59355.0000000	-2.4349999
59356.0000000	-2.4349999
59357.0000000	-2.4349999
59358.0000000	-2.4349999
59359.0000000	-2.4349999
59360.0000000	-2.4450002
59361.0000000	-2.4450002
59362.0000000	-2.4500000
59363.0000000	-2.4500000
59364.0000000	-2.4450002
59365.0000000	-2.4500000
59366.0000000	-2.4500000
59367.0000000	-2.4500000
59368.0000000	-2.4500000
59369.0000000	-2.4500000
59370.0000000	-2.4500000
59371.0000000	-2.4450002
59372.0000000	-2.4400001
59373.0000000	-2.4349999
59374.0000000	-2.4300001
59375.0000000	-2.4349999
59376.0000000	-2.4250002
59377.0000000	-2.4250002
59378.0000000	-2.4250002
59379.0000000	-2.4300001
59380.0000000	-2.4349999
59381.0000000	-2.4349999
59382.0000000	-2.4400001
59383.0000000	-2.4349999
59384.0000000	-2.4450002
59385.0000000	-2.4400001
59386.0000000	-2.4349999
59387.0000000	-2.4400001
59388.0000000	-2.4400001
59389.0000000	-2.4349999
59390.0000000	-2.4300001
59391.0000000	-2.4250002
59392.0000000	-2.4200001
59393.0000000	-2.4100001

59394.0000000	-2.4000001
59395.0000000	-2.4100001
59396.0000000	-2.4100001
59397.0000000	-2.4050002
59398.0000000	-2.4150000
59399.0000000	-2.4150000
59400.0000000	-2.4150000
59401.0000000	-2.4100001
59402.0000000	-2.4050002
59403.0000000	-2.4100001
59404.0000000	-2.4050002
59405.0000000	-2.4150000
59406.0000000	-2.4150000
59407.0000000	-2.4100001
59408.0000000	-2.4100001
59409.0000000	-2.4150000
59410.0000000	-2.4100001
59411.0000000	-2.4150000
59412.0000000	-2.4166667
59413.0000000	-2.4150000
59414.0000000	-2.4150000
59415.0000000	-2.4150000
59416.0000000	-2.4150000
59417.0000000	-2.4200001
59418.0000000	-2.4150000
59419.0000000	-2.3900001
59420.0000000	-2.4100001
59421.0000000	-2.4100001
59422.0000000	-2.4050002
59423.0000000	-2.4150000
59424.0000000	-2.4150000
59425.0000000	-2.4100001
59426.0000000	-2.4150000
59427.0000000	-2.4200001
59428.0000000	-2.4200001
59429.0000000	-2.4200001
59430.0000000	-2.4150000
59431.0000000	-2.4200001
59432.0000000	-2.4100001
59433.0000000	-2.4100001
59434.0000000	-2.4100001
59435.0000000	-2.4050002
59436.0000000	-2.4000001
59437.0000000	-2.4000001
59438.0000000	-2.4150000
59439.0000000	-2.4150000
59440.0000000	-2.4100001
59441.0000000	-2.4050002
59442.0000000	-2.4050002
59443.0000000	-2.4033334
59444.0000000	-2.4050002
59445.0000000	-2.4050002
59446.0000000	-2.4000001
59447.0000000	-2.3950000
59448.0000000	-2.3950000
59449.0000000	-2.4050002
59450.0000000	-2.4050002

59451.0000000	-2.4100001
59452.0000000	-2.4250002
59453.0000000	-2.4250002
59454.0000000	-2.4300001
59455.0000000	-2.4349999
59456.0000000	-2.4300001
59457.0000000	-2.4300001
59458.0000000	-2.4250002
59459.0000000	-2.4349999
59460.0000000	-2.4250002
59461.0000000	-2.4150000
59462.0000000	-2.4050002
59463.0000000	-2.4100001
59464.0000000	-2.4100001
59465.0000000	-2.4150000
59466.0000000	-2.4100001
59467.0000000	-2.4050002
59468.0000000	-2.4050002
59469.0000000	-2.4000001
59470.0000000	-2.3950000
59471.0000000	-2.3950000
59472.0000000	-2.3933334
59473.0000000	-2.3900001
59474.0000000	-2.3850002
59475.0000000	-2.3800001
59476.0000000	-2.3800001
59477.0000000	-2.3599999
59478.0000000	-2.3699999
59479.0000000	-2.3499999
59480.0000000	-2.3699999
59481.0000000	-2.3649998
59482.0000000	-2.3649998
59483.0000000	-2.3599999
59484.0000000	-2.3599999
59485.0000000	-2.3599999
59486.0000000	-2.3550000
59487.0000000	-2.3499999
59488.0000000	-2.3399999
59489.0000000	-2.3350000
59490.0000000	-2.3299999
59491.0000000	-2.3249998
59492.0000000	-2.3150001
59493.0000000	-2.3199999
59494.0000000	-2.3150001
59495.0000000	-2.3000000
59496.0000000	-2.3199999
59497.0000000	-2.3249998
59498.0000000	-2.3199999
59499.0000000	-2.3199999
59500.0000000	-2.3150001
59501.0000000	-2.3199999
59502.0000000	-2.3166666
59503.0000000	-2.3049998
59504.0000000	-2.3049998
59505.0000000	-2.3000000
59506.0000000	-2.3049998
59507.0000000	-2.3000000

59508.0000000	-2.2900000
59509.0000000	-2.2849998
59510.0000000	-2.2750001
59511.0000000	-2.2750001
59512.0000000	-2.2700000
59513.0000000	-2.2550001
59514.0000000	-2.2600000
59515.0000000	-2.2600000
59516.0000000	-2.2600000
59517.0000000	-2.2600000
59518.0000000	-2.2550001
59519.0000000	-2.2550001
59520.0000000	-2.2600000
59521.0000000	-2.2550001
59522.0000000	-2.2550001
59523.0000000	-2.2500000
59524.0000000	-2.2500000
59525.0000000	-2.2400000
59526.0000000	-2.2350001
59527.0000000	-2.2300000
59528.0000000	-2.2300000
59529.0000000	-2.2350001
59530.0000000	-2.2350001
59531.0000000	-2.2249999
59532.0000000	-2.2150002
59533.0000000	-2.2000000
59534.0000000	-2.2049999
59535.0000000	-2.2049999
59536.0000000	-2.2049999
59537.0000000	-2.2150002
59538.0000000	-2.2100000
59539.0000000	-2.2150002
59540.0000000	-2.2100000
59541.0000000	-2.2100000
59542.0000000	-2.2100000
59543.0000000	-2.2049999
59544.0000000	-2.2000000
59545.0000000	-2.1950002
59546.0000000	-2.1900001
59547.0000000	-2.1900001
59548.0000000	-2.1950002
59549.0000000	-2.1950002
59550.0000000	-2.1900001
59551.0000000	-2.1849999
59552.0000000	-2.1800001
59553.0000000	-2.1800001
59554.0000000	-2.1650000
59555.0000000	-2.1650000
59556.0000000	-2.1550002
59557.0000000	-2.1400001
59558.0000000	-2.1199999
59559.0000000	-2.1099999
59560.0000000	-2.1050000
59561.0000000	-2.0999999
59562.0000000	-2.0999999
59563.0000000	-2.0966666
59564.0000000	-2.0799999

59565.0000000	-2.0699999
59566.0000000	-2.0650001
59567.0000000	-2.0599999
59568.0000000	-2.0500000
59569.0000000	-2.0400000
59570.0000000	-2.0300000
59571.0000000	-2.0200000
59572.0000000	-2.0050001
59573.0000000	-1.9900000
59574.0000000	-1.9800000
59575.0000000	-1.9700000
59576.0000000	-1.9650000
59577.0000000	-1.9650000
59578.0000000	-1.9550000
59579.0000000	-1.9450001
59580.0000000	-1.9450001
59581.0000000	-1.9400001
59582.0000000	-1.9300001
59583.0000000	-1.9250000
59584.0000000	-1.9100000
59585.0000000	-1.9100000
59586.0000000	-1.9000000
59587.0000000	-1.8900000
59588.0000000	-1.8900000
59589.0000000	-1.8900000
59590.0000000	-1.8700000
59591.0000000	-1.8800000
59592.0000000	-1.8700000
59593.0000000	-1.8666667
59594.0000000	-1.8600000
59595.0000000	-1.8500000
59596.0000000	-1.8500000
59597.0000000	-1.8450000
59598.0000000	-1.8400000
59599.0000000	-1.8400000
59600.0000000	-1.8350000
59601.0000000	-1.8300000
59602.0000000	-1.8250000
59603.0000000	-1.8200001
59604.0000000	-1.8250000
59605.0000000	-1.8199999
59606.0000000	-1.8300000
59607.0000000	-1.8300000
59608.0000000	-1.8199999
59609.0000000	-1.8150001
59610.0000000	-1.8150001
59611.0000000	-1.8150001
59612.0000000	-1.8199999
59613.0000000	-1.8199999
59614.0000000	-1.8199999
59615.0000000	-1.8250000
59616.0000000	-1.8250000
59617.0000000	-1.8300000
59618.0000000	-1.8199999
59619.0000000	-1.8199999
59620.0000000	-1.8199999
59621.0000000	-1.8250000

59622.0000000	-1.8199999
59623.0000000	-1.8099999
59624.0000000	-1.8000000
59625.0000000	-1.7900000
59626.0000000	-1.7900000
59627.0000000	-1.7850000
59628.0000000	-1.7750000
59629.0000000	-1.7600000
59630.0000000	-1.7550000
59631.0000000	-1.7500000
59632.0000000	-1.7500000
59633.0000000	-1.7450000
59634.0000000	-1.7500000
59635.0000000	-1.7500000
59636.0000000	-1.7500000
59637.0000000	-1.7450000
59638.0000000	-1.7350000
59639.0000000	-1.7350000
59640.0000000	-1.7400000
59641.0000000	-1.7400000
59642.0000000	-1.7350000
59643.0000000	-1.7250000
59644.0000000	-1.7250000
59645.0000000	-1.7200000
59646.0000000	-1.7150000
59647.0000000	-1.7150000
59648.0000000	-1.7200000
59649.0000000	-1.7200000
59650.0000000	-1.7150000
59651.0000000	-1.7150000
59652.0000000	-1.7100000
59653.0000000	-1.7000000
59654.0000000	-1.7000002
59655.0000000	-1.6950001
59656.0000000	-1.7000000
59657.0000000	-1.7000000
59658.0000000	-1.7000000
59659.0000000	-1.7000000
59660.0000000	-1.6950001
59661.0000000	-1.6900001
59662.0000000	-1.6900001
59663.0000000	-1.6950001
59664.0000000	-1.6949999
59665.0000000	-1.6849999
59666.0000000	-1.6750000
59667.0000000	-1.6700000
59668.0000000	-1.6600000
59669.0000000	-1.6600000
59670.0000000	-1.6450000
59671.0000000	-1.6450000
59672.0000000	-1.6550000
59673.0000000	-1.6600000
59674.0000000	-1.6650000
59675.0000000	-1.6700000
59676.0000000	-1.6600000
59677.0000000	-1.6400000
59678.0000000	-1.6450000

59679.0000000	-1.6450000
59680.0000000	-1.6600000
59681.0000000	-1.6500000
59682.0000000	-1.6350000
59683.0000000	-1.6200000
59684.0000000	-1.6133333
59685.0000000	-1.6100000
59686.0000000	-1.6100000
59687.0000000	-1.6100000
59688.0000000	-1.6050000
59689.0000000	-1.6100000
59690.0000000	-1.6100000
59691.0000000	-1.6100000
59692.0000000	-1.6050000
59693.0000000	-1.5950000
59694.0000000	-1.5900000
59695.0000000	-1.5800000
59696.0000000	-1.5800000
59697.0000000	-1.5850000
59698.0000000	-1.5850000
59699.0000000	-1.5850000
59700.0000000	-1.5800000
59701.0000000	-1.5700001
59702.0000000	-1.5650001
59703.0000000	-1.5650001
59704.0000000	-1.5550001
59705.0000000	-1.5599999
59706.0000000	-1.5500000
59707.0000000	-1.5450000
59708.0000000	-1.5400000
59709.0000000	-1.5400000
59710.0000000	-1.5450000
59711.0000000	-1.5400000
59712.0000000	-1.5350000
59713.0000000	-1.5350000
59714.0000000	-1.5300001
59715.0000000	-1.5200000
59716.0000000	-1.5200000
59717.0000000	-1.5150000
59718.0000000	-1.5300000
59719.0000000	-1.5200000
59720.0000000	-1.5300000
59721.0000000	-1.5250000
59722.0000000	-1.5250000
59723.0000000	-1.5200000
59724.0000000	-1.5200000
59725.0000000	-1.5200000
59726.0000000	-1.5100000
59727.0000000	-1.5100000
59728.0000000	-1.5000000
59729.0000000	-1.5000000
59730.0000000	-1.4950000
59731.0000000	-1.4850000
59732.0000000	-1.4850000
59733.0000000	-1.4800000
59734.0000000	-1.4800000
59735.0000000	-1.4750000

59736.0000000	-1.4650000
59737.0000000	-1.4600000
59738.0000000	-1.4550000
59739.0000000	-1.4550000
59740.0000000	-1.4500000
59741.0000000	-1.4550000
59742.0000000	-1.4500000
59743.0000000	-1.4500000
59744.0000000	-1.4433333
59745.0000000	-1.4450001
59746.0000000	-1.4500000
59747.0000000	-1.4450001
59748.0000000	-1.4400001
59749.0000000	-1.4349999
59750.0000000	-1.4349999
59751.0000000	-1.4450001
59752.0000000	-1.4400001
59753.0000000	-1.4400001
59754.0000000	-1.4349999
59755.0000000	-1.4349999
59756.0000000	-1.4299999
59757.0000000	-1.4300001
59758.0000000	-1.4300001
59759.0000000	-1.4250000
59760.0000000	-1.4250000
59761.0000000	-1.4200000
59762.0000000	-1.4150000
59763.0000000	-1.4150000
59764.0000000	-1.4150000
59765.0000000	-1.4200000
59766.0000000	-1.4200000
59767.0000000	-1.4100000
59768.0000000	-1.4050000
59769.0000000	-1.4000000
59770.0000000	-1.3950000
59771.0000000	-1.3950000
59772.0000000	-1.3950000
59773.0000000	-1.4000000
59774.0000000	-1.4033333
59775.0000000	-1.3950000
59776.0000000	-1.3900000
59777.0000000	-1.3800000
59778.0000000	-1.3800000
59779.0000000	-1.3750000
59780.0000000	-1.3700000
59781.0000000	-1.3650000
59782.0000000	-1.3550000
59783.0000000	-1.3550000
59784.0000000	-1.3500000
59785.0000000	-1.3350000
59786.0000000	-1.3400000
59787.0000000	-1.3350000
59788.0000000	-1.3400000
59789.0000000	-1.3400000
59790.0000000	-1.3400000
59791.0000000	-1.3350000
59792.0000000	-1.3400000

59793.0000000	-1.3300000
59794.0000000	-1.3250000
59795.0000000	-1.3200001
59796.0000000	-1.3150001
59797.0000000	-1.3150001
59798.0000000	-1.3099999
59799.0000000	-1.3049999
59800.0000000	-1.3099999
59801.0000000	-1.3099999
59802.0000000	-1.3099999
59803.0000000	-1.3049999
59804.0000000	-1.3150001
59805.0000000	-1.3200001
59806.0000000	-1.3099999
59807.0000000	-1.3150001
59808.0000000	-1.3199999
59809.0000000	-1.3199999
59810.0000000	-1.3250000
59811.0000000	-1.3250000
59812.0000000	-1.3350000
59813.0000000	-1.3300000
59814.0000000	-1.3250000
59815.0000000	-1.3250000
59816.0000000	-1.3250000
59817.0000000	-1.3300000
59818.0000000	-1.3300000
59819.0000000	-1.3350000
59820.0000000	-1.3350000
59821.0000000	-1.3300000
59822.0000000	-1.3300000
59823.0000000	-1.3350000
59824.0000000	-1.3250000
59825.0000000	-1.3199999
59826.0000000	-1.3250000
59827.0000000	-1.3150001
59828.0000000	-1.3150001
59829.0000000	-1.3150001
59830.0000000	-1.3150001
59831.0000000	-1.3099999
59832.0000000	-1.3150001
59833.0000000	-1.3150001
59834.0000000	-1.3099999
59835.0000000	-1.3066666
59836.0000000	-1.3000000
59837.0000000	-1.2950000
59838.0000000	-1.3000000
59839.0000000	-1.2950000
59840.0000000	-1.2900000
59841.0000000	-1.2950000
59842.0000000	-1.2950000
59843.0000000	-1.3000000
59844.0000000	-1.3000000
59845.0000000	-1.2900000
59846.0000000	-1.3000000
59847.0000000	-1.3049999
59848.0000000	-1.3099999
59849.0000000	-1.3099999

59850.0000000	-1.3150001
59851.0000000	-1.3099999
59852.0000000	-1.3150001
59853.0000000	-1.3099999
59854.0000000	-1.3099999
59855.0000000	-1.3099999
59856.0000000	-1.3049999
59857.0000000	-1.2950000
59858.0000000	-1.2950000
59859.0000000	-1.2950000
59860.0000000	-1.2950000
59861.0000000	-1.2900000
59862.0000000	-1.2950000
59863.0000000	-1.2900000
59864.0000000	-1.2900000
59865.0000000	-1.2850000
59866.0000000	-1.2850000
59867.0000000	-1.2850000
59868.0000000	-1.2800000
59869.0000000	-1.2800000
59870.0000000	-1.2750000
59871.0000000	-1.2800000
59872.0000000	-1.2800000
59873.0000000	-1.2800000
59874.0000000	-1.2750000
59875.0000000	-1.2800000
59876.0000000	-1.2750000
59877.0000000	-1.2700000
59878.0000000	-1.2700000
59879.0000000	-1.2700000
59880.0000000	-1.2750000
59881.0000000	-1.2750000
59882.0000000	-1.2750000
59883.0000000	-1.2700000
59884.0000000	-1.2650000
59885.0000000	-1.2600000
59886.0000000	-1.2650000
59887.0000000	-1.2600000
59888.0000000	-1.2600000
59889.0000000	-1.2550000
59890.0000000	-1.2550000
59891.0000000	-1.2500000
59892.0000000	-1.2500000
59893.0000000	-1.2400000
59894.0000000	-1.2400000
59895.0000000	-1.2400000
59896.0000000	-1.2350000
59897.0000000	-1.2250000
59898.0000000	-1.2300000
59899.0000000	-1.2250000
59900.0000000	-1.2200000
59901.0000000	-1.2200000
59902.0000000	-1.2150000
59903.0000000	-1.2200000
59904.0000000	-1.2150000
59905.0000000	-1.2150000
59906.0000000	-1.2150000

59907.0000000	-1.2150000
59908.0000000	-1.2100000
59909.0000000	-1.2050000
59910.0000000	-1.1949999
59911.0000000	-1.2050000
59912.0000000	-1.2000000
59913.0000000	-1.2000000
59914.0000000	-1.2000000
59915.0000000	-1.2000000
59916.0000000	-1.2000000
59917.0000000	-1.1950001
59918.0000000	-1.2000000
59919.0000000	-1.2000000
59920.0000000	-1.1900001
59921.0000000	-1.1950001
59922.0000000	-1.1900001
59923.0000000	-1.1849999
59924.0000000	-1.1700000
59925.0000000	-1.1700000
59926.0000000	-1.1666666
59927.0000000	-1.1650000
59928.0000000	-1.1550000
59929.0000000	-1.1450000
59930.0000000	-1.1450000
59931.0000000	-1.1400000
59932.0000000	-1.1300000
59933.0000000	-1.1350000
59934.0000000	-1.1400000
59935.0000000	-1.1350000
59936.0000000	-1.1400000
59937.0000000	-1.1350000
59938.0000000	-1.1350000
59939.0000000	-1.1300000
59940.0000000	-1.1300000
59941.0000000	-1.1150000
59942.0000000	-1.1250000
59943.0000000	-1.1250000
59944.0000000	-1.1300000
59945.0000000	-1.1200000
59946.0000000	-1.1200000
59947.0000000	-1.1200000
59948.0000000	-1.1150000
59949.0000000	-1.1000000
59950.0000000	-1.1000000
59951.0000000	-1.0950000
59952.0000000	-1.0850000
59953.0000000	-1.0900000
59954.0000000	-1.0900000
59955.0000000	-1.0766667
59956.0000000	-1.0699999
59957.0000000	-1.0650001
59958.0000000	-1.0599999
59959.0000000	-1.0650001
59960.0000000	-1.0549999
59961.0000000	-1.0549999
59962.0000000	-1.0500000
59963.0000000	-1.0450000

59964.0000000	-1.0400000
59965.0000000	-1.0300000
59966.0000000	-1.0300000
59967.0000000	-1.0250000
59968.0000000	-1.0200000
59969.0000000	-1.0150000
59970.0000000	-1.0050000
59971.0000000	-0.9950000
59972.0000000	-0.9900000
59973.0000000	-0.9800000
59974.0000000	-0.9700000
59975.0000000	-0.9600000
59976.0000000	-0.9600000
59977.0000000	-0.9450000
59978.0000000	-0.9350000
59979.0000000	-0.9250000
59980.0000000	-0.9200000
59981.0000000	-0.9100000
59982.0000000	-0.9100000
59983.0000000	-0.8950000
59984.0000000	-0.8950000
59985.0000000	-0.8900000
59986.0000000	-0.8800000
59987.0000000	-0.8800000
59988.0000000	-0.8800000
59989.0000000	-0.8800000
59990.0000000	-0.8750000
59991.0000000	-0.8750000
59992.0000000	-0.8650000
59993.0000000	-0.8650000
59994.0000000	-0.8650000
59995.0000000	-0.8650000
59996.0000000	-0.8550000
59997.0000000	-0.8450000
59998.0000000	-0.8400000
59999.0000000	-0.8400000
60000.0000000	-0.8350000
60001.0000000	-0.8300000
60002.0000000	-0.8300000
60003.0000000	-0.8300000
60004.0000000	-0.8250000
60005.0000000	-0.8250000
60006.0000000	-0.8250000
60007.0000000	-0.8250000
60008.0000000	-0.8250000
60009.0000000	-0.8250000
60010.0000000	-0.8150000
60011.0000000	-0.8150000
60012.0000000	-0.8150000
60013.0000000	-0.8100000
60014.0000000	-0.8000000
60015.0000000	-0.8000000
60016.0000000	-0.7966667
60017.0000000	-0.8050000
60018.0000000	-0.7950000
60019.0000000	-0.7950000
60020.0000000	-0.7950000

60021.0000000	-0.7900000
60022.0000000	-0.8000000
60023.0000000	-0.8000000
60024.0000000	-0.8050000
60025.0000000	-0.8000000
60026.0000000	-0.8000000
60027.0000000	-0.8000000
60028.0000000	-0.8000000
60029.0000000	-0.8000000
60030.0000000	-0.7950000
60031.0000000	-0.7900000
60032.0000000	-0.7850000
60033.0000000	-0.7800000
60034.0000000	-0.7850000
60035.0000000	-0.7900000
60036.0000000	-0.7900000
60037.0000000	-0.7950000
60038.0000000	-0.7950000
60039.0000000	-0.7900000
60040.0000000	-0.7950000
60041.0000000	-0.7900000
60042.0000000	-0.8000000
60043.0000000	-0.7950000
60044.0000000	-0.7950000
60045.0000000	-0.7900000
60046.0000000	-0.7966667
60047.0000000	-0.7950000
60048.0000000	-0.7950000
60049.0000000	-0.7900000
60050.0000000	-0.7900000
60051.0000000	-0.7850000
60052.0000000	-0.7850000
60053.0000000	-0.7800000
60054.0000000	-0.7750000
60055.0000000	-0.7700000
60056.0000000	-0.7650000
60057.0000000	-0.7600000
60058.0000000	-0.7700000
60059.0000000	-0.7650000
60060.0000000	-0.7700000
60061.0000000	-0.7700000
60062.0000000	-0.7700000
60063.0000000	-0.7650000
60064.0000000	-0.7650000
60065.0000000	-0.7550000
60066.0000000	-0.7600000
60067.0000000	-0.7500000
60068.0000000	-0.7450000
60069.0000000	-0.7450000
60070.0000000	-0.7450000
60071.0000000	-0.7400000
60072.0000000	-0.7350000
60073.0000000	-0.7400000
60074.0000000	-0.7300000
60075.0000000	-0.7300000
60076.0000000	-0.7266667
60077.0000000	-0.7250000

60078.0000000	-0.7300000
60079.0000000	-0.7250000
60080.0000000	-0.7250000
60081.0000000	-0.7300000
60082.0000000	-0.7200000
60083.0000000	-0.7250000
60084.0000000	-0.7250000
60085.0000000	-0.7200000
60086.0000000	-0.7200000
60087.0000000	-0.7250000
60088.0000000	-0.7150000
60089.0000000	-0.7200000
60090.0000000	-0.7150000
60091.0000000	-0.7200000
60092.0000000	-0.7200000
60093.0000000	-0.7150000
60094.0000000	-0.7100000
60095.0000000	-0.7100000
60096.0000000	-0.7100000
60097.0000000	-0.7100000
60098.0000000	-0.7000000
60099.0000000	-0.7050000
60100.0000000	-0.7100000
60101.0000000	-0.7100000
60102.0000000	-0.7050000
60103.0000000	-0.6950000
60104.0000000	-0.7000000
60105.0000000	-0.6850000
60106.0000000	-0.6933333
60107.0000000	-0.6900000
60108.0000000	-0.6850000
60109.0000000	-0.6950000
60110.0000000	-0.6950000
60111.0000000	-0.6900000
60112.0000000	-0.6850000
60113.0000000	-0.6850000
60114.0000000	-0.6800000
60115.0000000	-0.6800000
60116.0000000	-0.6800000
60117.0000000	-0.6750000
60118.0000000	-0.6750000
60119.0000000	-0.6750000
60120.0000000	-0.6700000
60121.0000000	-0.6650000
60122.0000000	-0.6650000
60123.0000000	-0.6600000
60124.0000000	-0.6500000
60125.0000000	-0.6450000
60126.0000000	-0.6400000
60127.0000000	-0.6350000
60128.0000000	-0.6300000
60129.0000000	-0.6300000
60130.0000000	-0.6200000
60131.0000000	-0.6250000
60132.0000000	-0.6250000
60133.0000000	-0.6250000
60134.0000000	-0.6200000

60135.0000000	-0.6000000
60136.0000000	-0.5966666
60137.0000000	-0.5850000
60138.0000000	-0.5850000
60139.0000000	-0.5750000
60140.0000000	-0.5850000
60141.0000000	-0.5800000
60142.0000000	-0.5800000
60143.0000000	-0.5800000
60144.0000000	-0.5800000
60145.0000000	-0.5850000
60146.0000000	-0.5850000
60147.0000000	-0.5800000
60148.0000000	-0.5750000
60149.0000000	-0.5750000
60150.0000000	-0.5650000
60151.0000000	-0.5550000
60152.0000000	-0.5450000
60153.0000000	-0.5450000
60154.0000000	-0.5400000
60155.0000000	-0.5400000
60156.0000000	-0.5350000
60157.0000000	-0.5300000
60158.0000000	-0.5300000
60159.0000000	-0.5300000
60160.0000000	-0.5300000
60161.0000000	-0.5250000
60162.0000000	-0.5300000
60163.0000000	-0.5300000
60164.0000000	-0.5300000
60165.0000000	-0.5300000
60166.0000000	-0.5250000
60167.0000000	-0.5233333
60168.0000000	-0.5200000
60169.0000000	-0.5200000
60170.0000000	-0.5150000
60171.0000000	-0.5200000
60172.0000000	-0.5200000
60173.0000000	-0.5200000
60174.0000000	-0.5200000
60175.0000000	-0.5150000
60176.0000000	-0.5100000
60177.0000000	-0.5100000
60178.0000000	-0.5000000
60179.0000000	-0.5050000
60180.0000000	-0.5050000
60181.0000000	-0.5000000
60182.0000000	-0.4950000
60183.0000000	-0.4850000
60184.0000000	-0.4850000
60185.0000000	-0.4750000
60186.0000000	-0.4600000
60187.0000000	-0.4500000
60188.0000000	-0.4450000
60189.0000000	-0.4250000
60190.0000000	-0.4150000
60191.0000000	-0.4150000

60192.0000000	-0.4000000
60193.0000000	-0.3900000
60194.0000000	-0.3900000
60195.0000000	-0.3850000
60196.0000000	-0.3850000
60197.0000000	-0.3833333
60198.0000000	-0.3750000
60199.0000000	-0.3800000
60200.0000000	-0.3750000
60201.0000000	-0.3700000
60202.0000000	-0.3600000
60203.0000000	-0.3550000
60204.0000000	-0.3450000
60205.0000000	-0.3400000
60206.0000000	-0.3450000
60207.0000000	-0.3400000
60208.0000000	-0.3450000
60209.0000000	-0.3350000
60210.0000000	-0.3350000
60211.0000000	-0.3300000
60212.0000000	-0.3300000
60213.0000000	-0.3250000
60214.0000000	-0.3200000
60215.0000000	-0.3250000
60216.0000000	-0.3300000
60217.0000000	-0.3200000
60218.0000000	-0.3200000
60219.0000000	-0.3250000
60220.0000000	-0.3250000
60221.0000000	-0.3200000
60222.0000000	-0.3200000
60223.0000000	-0.3250000
60224.0000000	-0.3250000
60225.0000000	-0.3200000
60226.0000000	-0.3050000
60227.0000000	-0.3100000
60228.0000000	-0.3050000
60229.0000000	-0.2950000
60230.0000000	-0.2950000
60231.0000000	-0.2900000
60232.0000000	-0.2850000
60233.0000000	-0.2850000
60234.0000000	-0.2850000
60235.0000000	-0.2800000
60236.0000000	-0.2700000
60237.0000000	-0.2700000
60238.0000000	-0.2650000
60239.0000000	-0.2600000
60240.0000000	-0.2550000
60241.0000000	-0.2500000
60242.0000000	-0.2400000
60243.0000000	-0.2400000
60244.0000000	-0.2450000
60245.0000000	-0.2400000
60246.0000000	-0.2300000
60247.0000000	-0.2250000
60248.0000000	-0.2200000

60249.0000000	-0.2100000
60250.0000000	-0.2000000
60251.0000000	-0.1900000
60252.0000000	-0.1750000
60253.0000000	-0.1700000
60254.0000000	-0.1650000
60255.0000000	-0.1500000
60256.0000000	-0.1450000
60257.0000000	-0.1366667
60258.0000000	-0.1400000
60259.0000000	-0.1350000
60260.0000000	-0.1300000
60261.0000000	-0.1250000
60262.0000000	-0.1200000
60263.0000000	-0.1200000
60264.0000000	-0.1150000
60265.0000000	-0.1100000
60266.0000000	-0.1100000
60267.0000000	-0.1000000
60268.0000000	-0.1000000
60269.0000000	-0.0950000
60270.0000000	-0.0900000
60271.0000000	-0.0850000
60272.0000000	-0.0850000
60273.0000000	-0.0800000
60274.0000000	-0.0850000
60275.0000000	-0.0800000
60276.0000000	-0.0800000
60277.0000000	-0.0750000
60278.0000000	-0.0650000
60279.0000000	-0.0650000
60280.0000000	-0.0550000
60281.0000000	-0.0500000
60282.0000000	-0.0500000
60283.0000000	-0.0500000
60284.0000000	-0.0500000
60285.0000000	-0.0500000
60286.0000000	-0.0450000
60287.0000000	-0.0450000
60288.0000000	-0.0533333
60289.0000000	-0.0450000
60290.0000000	-0.0450000
60291.0000000	-0.0500000
60292.0000000	-0.0550000
60293.0000000	-0.0550000
60294.0000000	-0.0600000
60295.0000000	-0.0450000
60296.0000000	-0.0400000
60297.0000000	-0.0400000
60298.0000000	-0.0350000
60299.0000000	-0.0350000
60300.0000000	-0.0350000
60301.0000000	-0.0300000
60302.0000000	-0.0350000
60303.0000000	-0.0350000
60304.0000000	-0.0400000
60305.0000000	-0.0450000

60306.0000000	-0.0400000
60307.0000000	-0.0350000
60308.0000000	-0.0350000
60309.0000000	-0.0350000
60310.0000000	-0.0300000
60311.0000000	-0.0250000
60312.0000000	-0.0200000
60313.0000000	-0.0150000
60314.0000000	-0.0100000
60315.0000000	-0.0050000
60316.0000000	0.0000000
60317.0000000	0.0050000
60318.0000000	0.0066667
60319.0000000	0.0100000
60320.0000000	0.0150000
60321.0000000	0.0200000
60322.0000000	0.0250000
60323.0000000	0.0250000
60324.0000000	0.0250000
60325.0000000	0.0300000
60326.0000000	0.0350000
60327.0000000	0.0350000
60328.0000000	0.0450000
60329.0000000	0.0500000
60330.0000000	0.0500000
60331.0000000	0.0600000
60332.0000000	0.0600000
60333.0000000	0.0650000
60334.0000000	0.0650000
60335.0000000	0.0650000
60336.0000000	0.0750000
60337.0000000	0.0800000
60338.0000000	0.0900000
60339.0000000	0.1000000
60340.0000000	0.1000000
60341.0000000	0.1150000
60342.0000000	0.1250000
60343.0000000	0.1300000
60344.0000000	0.1350000
60345.0000000	0.1350000
60346.0000000	0.1400000
60347.0000000	0.1400000
60348.0000000	0.1500000
60349.0000000	0.1500000
60350.0000000	0.1600000
60351.0000000	0.1600000
60352.0000000	0.1700000
60353.0000000	0.1750000
60354.0000000	0.1850000
60355.0000000	0.1850000
60356.0000000	0.1850000
60357.0000000	0.1950000
60358.0000000	0.1950000
60359.0000000	0.1950000
60360.0000000	0.1950000
60361.0000000	0.2050000
60362.0000000	0.2050000

60363.0000000	0.2050000
60364.0000000	0.2100000
60365.0000000	0.2000000
60366.0000000	0.2050000
60367.0000000	0.2100000
60368.0000000	0.2050000
60369.0000000	0.2000000
60370.0000000	0.1900000
60371.0000000	0.1950000
60372.0000000	0.1950000
60373.0000000	0.1950000
60374.0000000	0.1950000
60375.0000000	0.1900000
60376.0000000	0.1900000
60377.0000000	0.2000000
60378.0000000	0.2100000
60379.0000000	0.2000000
60380.0000000	0.2100000
60381.0000000	0.2100000
60382.0000000	0.2250000
60383.0000000	0.2300000
60384.0000000	0.2350000
60385.0000000	0.2450000
60386.0000000	0.2500000
60387.0000000	0.2550000
60388.0000000	0.2600000
60389.0000000	0.2650000
60390.0000000	0.2700000
60391.0000000	0.2800000
60392.0000000	0.2750000
60393.0000000	0.2800000
60394.0000000	0.2750000
60395.0000000	0.2800000
60396.0000000	0.2800000
60397.0000000	0.2800000
60398.0000000	0.2900000
60399.0000000	0.2900000
60400.0000000	0.3000000
60401.0000000	0.3000000
60402.0000000	0.3000000
60403.0000000	0.3050000
60404.0000000	0.3000000
60405.0000000	0.3050000
60406.0000000	0.3050000
60407.0000000	0.3050000
60408.0000000	0.3066667
60409.0000000	0.3100000
60410.0000000	0.3150000
60411.0000000	0.3200000
60412.0000000	0.3150000
60413.0000000	0.3250000
60414.0000000	0.3250000
60415.0000000	0.3250000
60416.0000000	0.3250000
60417.0000000	0.3250000
60418.0000000	0.3300000
60419.0000000	0.3300000

60420.0000000	0.3300000
60421.0000000	0.3350000
60422.0000000	0.3400000
60423.0000000	0.3500000
60424.0000000	0.3500000
60425.0000000	0.3550000
60426.0000000	0.3600000
60427.0000000	0.3650000
60428.0000000	0.3700000
60429.0000000	0.3750000
60430.0000000	0.3800000
60431.0000000	0.3850000
60432.0000000	0.3850000
60433.0000000	0.3850000
60434.0000000	0.3900000
60435.0000000	0.3900000
60436.0000000	0.3950000
60437.0000000	0.3900000
60438.0000000	0.3950000
60439.0000000	0.3966666
60440.0000000	0.3950000
60441.0000000	0.3950000
60442.0000000	0.4000000
60443.0000000	0.3950000
60444.0000000	0.4000000
60445.0000000	0.4000000
60446.0000000	0.4000000
60447.0000000	0.4000000
60448.0000000	0.4050000
60449.0000000	0.4000000
60450.0000000	0.4000000
60451.0000000	0.3950000
60452.0000000	0.4000000
60453.0000000	0.4000000
60454.0000000	0.3950000
60455.0000000	0.4000000
60456.0000000	0.4000000
60457.0000000	0.3900000
60458.0000000	0.3950000
60459.0000000	0.3950000
60460.0000000	0.3950000
60461.0000000	0.3950000
60462.0000000	0.3900000
60463.0000000	0.3900000
60464.0000000	0.3900000
60465.0000000	0.3950000
60466.0000000	0.3950000
60467.0000000	0.4000000
60468.0000000	0.4000000
60469.0000000	0.3950000
60470.0000000	0.4000000
60471.0000000	0.4050000
60472.0000000	0.4100000
60473.0000000	0.4000000
60474.0000000	0.4050000
60475.0000000	0.4000000
60476.0000000	0.4000000

60477.0000000	0.3950000
60478.0000000	0.3950000
60479.0000000	0.3950000
60480.0000000	0.3950000
60481.0000000	0.3950000
60482.0000000	0.3950000
60483.0000000	0.3950000
60484.0000000	0.3950000
60485.0000000	0.3950000
60486.0000000	0.3950000
60487.0000000	0.3850000
60488.0000000	0.3950000
60489.0000000	0.3950000
60490.0000000	0.3950000
60491.0000000	0.3950000
60492.0000000	0.3900000
60493.0000000	0.3900000
60494.0000000	0.3850000
60495.0000000	0.3950000
60496.0000000	0.3900000
60497.0000000	0.3850000
60498.0000000	0.3966666
60499.0000000	0.3950000
60500.0000000	0.3950000
60501.0000000	0.3950000
60502.0000000	0.4000000
60503.0000000	0.4050000
60504.0000000	0.4050000
60505.0000000	0.4100000
60506.0000000	0.4050000
60507.0000000	0.4100000
60508.0000000	0.4150000
60509.0000000	0.4100000
60510.0000000	0.4100000
60511.0000000	0.4050000
60512.0000000	0.4100000
60513.0000000	0.4150000
60514.0000000	0.4100000
60515.0000000	0.4100000
60516.0000000	0.4150000
60517.0000000	0.4100000
60518.0000000	0.4100000
60519.0000000	0.4050000
60520.0000000	0.3950000
60521.0000000	0.3950000
60522.0000000	0.3900000
60523.0000000	0.3900000
60524.0000000	0.3950000
60525.0000000	0.3950000
60526.0000000	0.4050000
60527.0000000	0.4050000
60528.0000000	0.4100000
60529.0000000	0.4100000
60530.0000000	0.4200000
60531.0000000	0.4200000
60532.0000000	0.4300000
60533.0000000	0.4250000

60534.0000000	0.4250000
60535.0000000	0.4250000
60536.0000000	0.4250000
60537.0000000	0.4300000
60538.0000000	0.4250000
60539.0000000	0.4300000
60540.0000000	0.4200000
60541.0000000	0.4150000
60542.0000000	0.4250000
60543.0000000	0.4200000
60544.0000000	0.4200000
60545.0000000	0.4200000
60546.0000000	0.4250000
60547.0000000	0.4200000
60548.0000000	0.4300000
60549.0000000	0.4350000
60550.0000000	0.4350000
60551.0000000	0.4350000
60552.0000000	0.4400000
60553.0000000	0.4450000
60554.0000000	0.4500000
60555.0000000	0.4500000
60556.0000000	0.4550000
60557.0000000	0.4600000
60558.0000000	0.4650000
60559.0000000	0.4633333
60560.0000000	0.4700000
60561.0000000	0.4750000
60562.0000000	0.4800000
60563.0000000	0.4800000
60564.0000000	0.4800000
60565.0000000	0.4850000
60566.0000000	0.4950000
60567.0000000	0.5000000
60568.0000000	0.5050000
60569.0000000	0.5100000
60570.0000000	0.5150000
60571.0000000	0.5200000
60572.0000000	0.5150000
60573.0000000	0.5100000
60574.0000000	0.5150000
60575.0000000	0.5200000
60576.0000000	0.5200000
60577.0000000	0.5250000
60578.0000000	0.5250000
60579.0000000	0.5250000
60580.0000000	0.5250000
60581.0000000	0.5200000
60582.0000000	0.5200000
60583.0000000	0.5200000
60584.0000000	0.5250000
60585.0000000	0.5250000
60586.0000000	0.5200000
60587.0000000	0.5250000
60588.0000000	0.5250000
60589.0000000	0.5233333
60590.0000000	0.5250000

60591.0000000	0.5200000
60592.0000000	0.5300000
60593.0000000	0.5250000
60594.0000000	0.5300000
60595.0000000	0.5350000
60596.0000000	0.5300000
60597.0000000	0.5350000
60598.0000000	0.5400000
60599.0000000	0.5400000
60600.0000000	0.5450000
60601.0000000	0.5450000
60602.0000000	0.5350000
60603.0000000	0.5450000
60604.0000000	0.5400000
60605.0000000	0.5500000
60606.0000000	0.5500000
60607.0000000	0.5550000
60608.0000000	0.5550000
60609.0000000	0.5550000
60610.0000000	0.5550000
60611.0000000	0.5600000
60612.0000000	0.5650000
60613.0000000	0.5650000
60614.0000000	0.5700000
60615.0000000	0.5650000
60616.0000000	0.5700000
60617.0000000	0.5700000
60618.0000000	0.5700000
60619.0000000	0.5833333
60620.0000000	0.5800000
60621.0000000	0.5850000
60622.0000000	0.5800000
60623.0000000	0.5900000
60624.0000000	0.5950000
60625.0000000	0.5950000
60626.0000000	0.5950000
60627.0000000	0.5950000
60628.0000000	0.6000000
60629.0000000	0.6000000
60630.0000000	0.6050000
60631.0000000	0.6050000
60632.0000000	0.6100000
60633.0000000	0.6100000
60634.0000000	0.6100000
60635.0000000	0.6100000
60636.0000000	0.6100000
60637.0000000	0.6250000
60638.0000000	0.6300000
60639.0000000	0.6300000
60640.0000000	0.6250000
60641.0000000	0.6250000
60642.0000000	0.6350000
60643.0000000	0.6300000
60644.0000000	0.6350000
60645.0000000	0.6300000
60646.0000000	0.6300000
60647.0000000	0.6350000

60648.0000000	0.6300000
60649.0000000	0.6300000
60650.0000000	0.6266667
60651.0000000	0.6300000
60652.0000000	0.6250000
60653.0000000	0.6300000
60654.0000000	0.6300000
60655.0000000	0.6250000
60656.0000000	0.6250000
60657.0000000	0.6300000
60658.0000000	0.6250000
60659.0000000	0.6250000
60660.0000000	0.6250000
60661.0000000	0.6250000
60662.0000000	0.6300000
60663.0000000	0.6350000
60664.0000000	0.6450000
60665.0000000	0.6500000
60666.0000000	0.6450000
60667.0000000	0.6450000
60668.0000000	0.6400000
60669.0000000	0.6500000
60670.0000000	0.6500000
60671.0000000	0.6500000
60672.0000000	0.6500000
60673.0000000	0.6450000
60674.0000000	0.6450000
60675.0000000	0.6350000
60676.0000000	0.6350000
60677.0000000	0.6400000
60678.0000000	0.6500000
60679.0000000	0.6500000
60680.0000000	0.6566666
60681.0000000	0.6550000
60682.0000000	0.6550000
60683.0000000	0.6550000
60684.0000000	0.6450000
60685.0000000	0.6500000
60686.0000000	0.6350000
60687.0000000	0.6450000
60688.0000000	0.6450000
60689.0000000	0.6450000
60690.0000000	0.6450000
60691.0000000	0.6550000
60692.0000000	0.6550000
60693.0000000	0.6500000
60694.0000000	0.6500000
60695.0000000	0.6500000
60696.0000000	0.6500000
60697.0000000	0.6500000
60698.0000000	0.6500000
60699.0000000	0.6600000
60700.0000000	0.6650000
60701.0000000	0.6650000
60702.0000000	0.6650000
60703.0000000	0.6650000
60704.0000000	0.6600000

60705.0000000	0.6650000
60706.0000000	0.6650000
60707.0000000	0.6650000
60708.0000000	0.6650000
60709.0000000	0.6700000
60710.0000000	0.6700000
60711.0000000	0.6700000
60712.0000000	0.6650000
60713.0000000	0.6650000
60714.0000000	0.6700000
60715.0000000	0.6750000
60716.0000000	0.6800000
60717.0000000	0.6850000
60718.0000000	0.6900000
60719.0000000	0.6900000
60720.0000000	0.6900000
60721.0000000	0.6900000
60722.0000000	0.6900000
60723.0000000	0.6950000
60724.0000000	0.6950000
60725.0000000	0.6950000
60726.0000000	0.6950000
60727.0000000	0.6900000
60728.0000000	0.6950000
60729.0000000	0.6950000
60730.0000000	0.6950000
60731.0000000	0.6950000
60732.0000000	0.6850000
60733.0000000	0.6850000
60734.0000000	0.6800000
60735.0000000	0.6800000
60736.0000000	0.6800000
60737.0000000	0.6700000
60738.0000000	0.6650000
60739.0000000	0.6650000
60740.0000000	0.6566667
60741.0000000	0.6550000
60742.0000000	0.6550000
60743.0000000	0.6500000
60744.0000000	0.6500000
60745.0000000	0.6500000
60746.0000000	0.6400000
60747.0000000	0.6450000
60748.0000000	0.6400000
60749.0000000	0.6400000
60750.0000000	0.6300000
60751.0000000	0.6350000
60752.0000000	0.6250000
60753.0000000	0.6300000
60754.0000000	0.6250000
60755.0000000	0.6250000
60756.0000000	0.6250000
60757.0000000	0.6250000
60758.0000000	0.6250000
60759.0000000	0.6250000
60760.0000000	0.6200000
60761.0000000	0.6200000

60762.0000000	0.6200000
60763.0000000	0.6150000
60764.0000000	0.6250000
60765.0000000	0.6250000
60766.0000000	0.6200000
60767.0000000	0.6250000
60768.0000000	0.6200000
60769.0000000	0.6200000
60770.0000000	0.6266667
60771.0000000	0.6250000
60772.0000000	0.6250000
60773.0000000	0.6200000
60774.0000000	0.6200000
60775.0000000	0.6150000
60776.0000000	0.6150000
60777.0000000	0.6250000
60778.0000000	0.6250000
60779.0000000	0.6200000
60780.0000000	0.6200000
60781.0000000	0.6200000
60782.0000000	0.6300000
60783.0000000	0.6250000
60784.0000000	0.6250000
60785.0000000	0.6250000
60786.0000000	0.6150000
60787.0000000	0.6200000
60788.0000000	0.6200000
60789.0000000	0.6200000
60790.0000000	0.6250000
60791.0000000	0.6150000
60792.0000000	0.6150000
60793.0000000	0.6200000
60794.0000000	0.6150000
60795.0000000	0.6250000
60796.0000000	0.6250000
60797.0000000	0.6350000
60798.0000000	0.6300000
60799.0000000	0.6350000
60800.0000000	0.6350000
60801.0000000	0.6400000
60802.0000000	0.6450000
60803.0000000	0.6450000
60804.0000000	0.6350000
60805.0000000	0.6350000
60806.0000000	0.6300000
60807.0000000	0.6350000
60808.0000000	0.6400000
60809.0000000	0.6250000
60810.0000000	0.6300000
60811.0000000	0.6300000
60812.0000000	0.6250000
60813.0000000	0.6250000
60814.0000000	0.6300000
60815.0000000	0.6250000
60816.0000000	0.6250000
60817.0000000	0.6200000
60818.0000000	0.6200000

60819.0000000	0.6150000
60820.0000000	0.6250000
60821.0000000	0.6250000
60822.0000000	0.6200000
60823.0000000	0.6250000
60824.0000000	0.6200000
60825.0000000	0.6250000
60826.0000000	0.6200000
60827.0000000	0.6200000
60828.0000000	0.6250000
60829.0000000	0.6250000
60830.0000000	0.6250000
60831.0000000	0.6166667
60832.0000000	0.6050000
60833.0000000	0.6150000
60834.0000000	0.6150000
60835.0000000	0.6150000
60836.0000000	0.6200000
60837.0000000	0.6200000
60838.0000000	0.6150000
60839.0000000	0.6150000
60840.0000000	0.6100000
60841.0000000	0.6100000
60842.0000000	0.6150000
60843.0000000	0.6200000
60844.0000000	0.6200000
60845.0000000	0.6250000
60846.0000000	0.6250000
60847.0000000	0.6200000
60848.0000000	0.6150000
60849.0000000	0.6150000
60850.0000000	0.6150000
60851.0000000	0.6150000
60852.0000000	0.6150000
60853.0000000	0.6200000
60854.0000000	0.6150000
60855.0000000	0.6150000
60856.0000000	0.6150000
60857.0000000	0.6150000
60858.0000000	0.6150000
60859.0000000	0.6200000
60860.0000000	0.6200000
60861.0000000	0.6200000
60862.0000000	0.6200000
60863.0000000	0.6100000
60864.0000000	0.6150000
60865.0000000	0.6100000
60866.0000000	0.6100000
60867.0000000	0.6150000
60868.0000000	0.6150000
60869.0000000	0.6150000
60870.0000000	0.6200000
60871.0000000	0.6200000
60872.0000000	0.6200000
60873.0000000	0.6100000
60874.0000000	0.6150000
60875.0000000	0.6150000

60876.0000000	0.6150000
60877.0000000	0.6150000
60878.0000000	0.6150000
60879.0000000	0.6200000
60880.0000000	0.6250000
60881.0000000	0.6250000
60882.0000000	0.6200000
60883.0000000	0.6250000
60884.0000000	0.6300000
60885.0000000	0.6300000
60886.0000000	0.6350000
60887.0000000	0.6300000
60888.0000000	0.6250000
60889.0000000	0.6300000
60890.0000000	0.6400000
60891.0000000	0.6400000
60892.0000000	0.6400000
60893.0000000	0.6350000
60894.0000000	0.6400000
60895.0000000	0.6400000
60896.0000000	0.6350000
60897.0000000	0.6350000
60898.0000000	0.6250000
60899.0000000	0.6250000
60900.0000000	0.6300000
60901.0000000	0.6300000
60902.0000000	0.6350000
60903.0000000	0.6350000
60904.0000000	0.6300000
60905.0000000	0.6300000
60906.0000000	0.6300000
60907.0000000	0.6300000
60908.0000000	0.6300000
60909.0000000	0.6300000
60910.0000000	0.6250000
60911.0000000	0.6150000
60912.0000000	0.6150000
60913.0000000	0.6200000
60914.0000000	0.6100000
60915.0000000	0.6150000
60916.0000000	0.6200000
60917.0000000	0.6150000
60918.0000000	0.6100000
60919.0000000	0.6150000
60920.0000000	0.6150000
60921.0000000	0.6150000
60922.0000000	0.6100000
60923.0000000	0.6100000
60924.0000000	0.6150000
60925.0000000	0.6100000
60926.0000000	0.6050000
60927.0000000	0.6050000
60928.0000000	0.6000000
60929.0000000	0.6050000
60930.0000000	0.6050000
60931.0000000	0.6100000
60932.0000000	0.6050000

60933.0000000	0.6000000
60934.0000000	0.6000000
60935.0000000	0.6000000
60936.0000000	0.6000000
60937.0000000	0.6000000
60938.0000000	0.6000000
60939.0000000	0.5900000
60940.0000000	0.5850000
60941.0000000	0.5900000
60942.0000000	0.5900000
60943.0000000	0.5900000
60944.0000000	0.5850000
60945.0000000	0.5800000
60946.0000000	0.5850000
60947.0000000	0.5850000
60948.0000000	0.5800000
60949.0000000	0.5800000
60950.0000000	0.5750000
60951.0000000	0.5733333
60952.0000000	0.5650000
60953.0000000	0.5650000
60954.0000000	0.5650000
60955.0000000	0.5550000
60956.0000000	0.5650000
60957.0000000	0.5500000
60958.0000000	0.5450000
60959.0000000	0.5400000
60960.0000000	0.5350000
60961.0000000	0.5450000
60962.0000000	0.5500000
60963.0000000	0.5350000
60964.0000000	0.5350000
60965.0000000	0.5250000
60966.0000000	0.5200000
60967.0000000	0.5250000
60968.0000000	0.5250000
60969.0000000	0.5200000
60970.0000000	0.5200000
60971.0000000	0.5150000
60972.0000000	0.5200000
60973.0000000	0.5150000
60974.0000000	0.5200000
60975.0000000	0.5150000
60976.0000000	0.5150000
60977.0000000	0.5150000
60978.0000000	0.5050000
60979.0000000	0.5150000
60980.0000000	0.5150000
60981.0000000	0.5100000
60982.0000000	0.5050000
60983.0000000	0.5000000
60984.0000000	0.5000000
60985.0000000	0.4900000
60986.0000000	0.4850000
60987.0000000	0.4850000
60988.0000000	0.4800000
60989.0000000	0.4750000

60990.0000000	0.4750000
60991.0000000	0.4750000
60992.0000000	0.4650000
60993.0000000	0.4600000
60994.0000000	0.4500000
60995.0000000	0.4500000
60996.0000000	0.4450000
60997.0000000	0.4500000
60998.0000000	0.4400000
60999.0000000	0.4350000
61000.0000000	0.4300000
61001.0000000	0.4250000
61002.0000000	0.4200000
61003.0000000	0.4150000
61004.0000000	0.4150000
61005.0000000	0.4100000
61006.0000000	0.4050000
61007.0000000	0.3950000
61008.0000000	0.4000000
61009.0000000	0.3900000
61010.0000000	0.3900000
61011.0000000	0.3850000
61012.0000000	0.3833333
61013.0000000	0.3700000
61014.0000000	0.3550000
61015.0000000	0.3500000
61016.0000000	0.3600000
61017.0000000	0.3550000
61018.0000000	0.3450000
61019.0000000	0.3350000
61020.0000000	0.3350000
61021.0000000	0.3300000
61022.0000000	0.3250000
61023.0000000	0.3250000
61024.0000000	0.3250000
61025.0000000	0.3250000
61026.0000000	0.3150000
61027.0000000	0.3200000
61028.0000000	0.3150000
61029.0000000	0.3050000
61030.0000000	0.3050000
61031.0000000	0.3000000
61032.0000000	0.3000000
61033.0000000	0.2950000
61034.0000000	0.2850000
61035.0000000	0.2850000
61036.0000000	0.2750000
61037.0000000	0.2750000
61038.0000000	0.2750000
61039.0000000	0.2650000
61040.0000000	0.2750000
61041.0000000	0.2750000
61042.0000000	0.2800000
61043.0000000	0.2800000
61044.0000000	0.2850000
61045.0000000	0.2850000
61046.0000000	0.2900000

61047.0000000	0.2850000
61048.0000000	0.2850000
61049.0000000	0.2950000
61050.0000000	0.3100000
61051.0000000	0.3250000
61052.0000000	0.3400000
61053.0000000	0.3550000
61054.0000000	0.3600000
61055.0000000	0.3600000
61056.0000000	0.3600000
61057.0000000	0.3600000
61058.0000000	0.3600000
61059.0000000	0.3600000
61060.0000000	0.3650000
61061.0000000	0.3650000
61062.0000000	0.3650000
61063.0000000	0.3750000
61064.0000000	0.3750000
61065.0000000	0.3700000
61066.0000000	0.3700000
61067.0000000	0.3650000
61068.0000000	0.3650000
61069.0000000	0.3700000
61070.0000000	0.3650000
61071.0000000	0.3650000
61072.0000000	0.3666667
61073.0000000	0.3600000
61074.0000000	0.3600000
61075.0000000	0.3550000
61076.0000000	0.3700000
61077.0000000	0.3700000
61078.0000000	0.3700000
61079.0000000	0.3650000
61080.0000000	0.3600000
61081.0000000	0.3550000
61082.0000000	0.3500000
61083.0000000	0.3500000
61084.0000000	0.3600000
61085.0000000	0.3650000
61086.0000000	0.3700000
61087.0000000	0.3750000
61088.0000000	0.3750000
61089.0000000	0.3800000
61090.0000000	0.3800000
61091.0000000	0.3850000
61092.0000000	0.3900000
61093.0000000	0.3850000
61094.0000000	0.3900000
61095.0000000	0.3950000
61096.0000000	0.3950000
61097.0000000	0.4000000
61098.0000000	0.3950000
61099.0000000	0.3850000
61100.0000000	0.3750000
61101.0000000	0.3550000
61102.0000000	0.3566667
61103.0000000	0.3600000

61104.0000000	0.3550000
61105.0000000	0.3550000
61106.0000000	0.3600000
61107.0000000	0.3700000
61108.0000000	0.3650000
61109.0000000	0.3650000
61110.0000000	0.3650000
61111.0000000	0.3550000
61112.0000000	0.3650000
61113.0000000	0.3550000
61114.0000000	0.3600000
61115.0000000	0.3600000
61116.0000000	0.3600000
61117.0000000	0.3600000
61118.0000000	0.3650000
61119.0000000	0.3650000
61120.0000000	0.3650000
61121.0000000	0.3700000
61122.0000000	0.3650000
61123.0000000	0.3700000
61124.0000000	0.3700000
61125.0000000	0.3800000
61126.0000000	0.3900000
61127.0000000	0.4000000
61128.0000000	0.4050000
61129.0000000	0.4000000
61130.0000000	0.4000000
61131.0000000	0.4000000
61132.0000000	0.3866667
61133.0000000	0.3900000
61134.0000000	0.3900000
61135.0000000	0.3850000
61136.0000000	0.3800000
61137.0000000	0.3800000
61138.0000000	0.3800000
61139.0000000	0.3750000
61140.0000000	0.3800000
61141.0000000	0.3800000
61142.0000000	0.3750000
61143.0000000	0.3750000
61144.0000000	0.3700000
61145.0000000	0.3750000
61146.0000000	0.3700000
61147.0000000	0.3750000
61148.0000000	0.3700000
61149.0000000	0.3750000
61150.0000000	0.3800000
61151.0000000	0.3800000
61152.0000000	0.3850000
61153.0000000	0.3950000
61154.0000000	0.3850000
61155.0000000	0.3850000
61156.0000000	0.3850000
61157.0000000	0.3800000
61158.0000000	0.3850000
61159.0000000	0.3850000
61160.0000000	0.3750000

61161.0000000	0.3800000
61162.0000000	0.3900000
61163.0000000	0.3933333
61164.0000000	0.3900000
61165.0000000	0.3900000
61166.0000000	0.3950000
61167.0000000	0.4000000
61168.0000000	0.4000000
61169.0000000	0.3950000
61170.0000000	0.4000000
61171.0000000	0.4000000
61172.0000000	0.4050000
61173.0000000	0.4100000
61174.0000000	0.4100000
61175.0000000	0.4050000
61176.0000000	0.4100000
61177.0000000	0.4050000
61178.0000000	0.3950000
61179.0000000	0.3950000
61180.0000000	0.4000000
61181.0000000	0.3950000
61182.0000000	0.4000000
61183.0000000	0.4050000
61184.0000000	0.4100000
61185.0000000	0.4050000
61186.0000000	0.4050000
61187.0000000	0.4150000
61188.0000000	0.4200000
61189.0000000	0.4250000
61190.0000000	0.4200000
61191.0000000	0.4250000
61192.0000000	0.4250000
61193.0000000	0.4266666
61194.0000000	0.4250000
61195.0000000	0.4250000
61196.0000000	0.4200000
61197.0000000	0.4350000
61198.0000000	0.4250000
61199.0000000	0.4200000
61200.0000000	0.4150000
61201.0000000	0.4150000
61202.0000000	0.4100000
61203.0000000	0.4100000
61204.0000000	0.4050000
61205.0000000	0.4100000
61206.0000000	0.4100000
61207.0000000	0.4150000
61208.0000000	0.4150000
61209.0000000	0.4100000
61210.0000000	0.4100000
61211.0000000	0.4050000
61212.0000000	0.4000000
61213.0000000	0.4000000
61214.0000000	0.4000000
61215.0000000	0.4050000
61216.0000000	0.3950000
61217.0000000	0.4000000

61218.0000000	0.3950000
61219.0000000	0.3850000
61220.0000000	0.3900000
61221.0000000	0.3800000
61222.0000000	0.3800000
61223.0000000	0.3700000
61224.0000000	0.3700000
61225.0000000	0.3700000
61226.0000000	0.3700000
61227.0000000	0.3600000
61228.0000000	0.3600000
61229.0000000	0.3600000
61230.0000000	0.3600000
61231.0000000	0.3600000
61232.0000000	0.3600000
61233.0000000	0.3600000
61234.0000000	0.3600000
61235.0000000	0.3600000
61236.0000000	0.3650000
61237.0000000	0.3650000
61238.0000000	0.3650000
61239.0000000	0.3700000
61240.0000000	0.3750000
61241.0000000	0.3750000
61242.0000000	0.3650000
61243.0000000	0.3700000
61244.0000000	0.3650000
61245.0000000	0.3550000
61246.0000000	0.3550000
61247.0000000	0.3650000
61248.0000000	0.3750000
61249.0000000	0.3700000
61250.0000000	0.3700000
61251.0000000	0.3700000
61252.0000000	0.3700000
61253.0000000	0.3700000
61254.0000000	0.3650000
61255.0000000	0.3600000
61256.0000000	0.3600000
61257.0000000	0.3650000
61258.0000000	0.3650000
61259.0000000	0.3600000
61260.0000000	0.3650000
61261.0000000	0.3650000
61262.0000000	0.3650000
61263.0000000	0.3650000
61264.0000000	0.3650000
61265.0000000	0.3650000
61266.0000000	0.3700000
61267.0000000	0.3700000
61268.0000000	0.3750000
61269.0000000	0.3800000
61270.0000000	0.3800000
61271.0000000	0.3900000
61272.0000000	0.3900000
61273.0000000	0.4000000
61274.0000000	0.4000000

61275.0000000	0.4050000
61276.0000000	0.4100000
61277.0000000	0.4150000
61278.0000000	0.4150000
61279.0000000	0.4250000
61280.0000000	0.4250000
61281.0000000	0.4300000
61282.0000000	0.4250000
61283.0000000	0.4300000
61284.0000000	0.4333333
61285.0000000	0.4300000
61286.0000000	0.4400000
61287.0000000	0.4400000
61288.0000000	0.4450000
61289.0000000	0.4450000
61290.0000000	0.4400000
61291.0000000	0.4400000
61292.0000000	0.4350000
61293.0000000	0.4400000
61294.0000000	0.4400000
61295.0000000	0.4350000
61296.0000000	0.4450000
61297.0000000	0.4350000
61298.0000000	0.4300000
61299.0000000	0.4250000
61300.0000000	0.4150000
61301.0000000	0.4150000
61302.0000000	0.4100000
61303.0000000	0.4100000
61304.0000000	0.4050000
61305.0000000	0.3950000
61306.0000000	0.3900000
61307.0000000	0.3900000
61308.0000000	0.3900000
61309.0000000	0.3800000
61310.0000000	0.3800000
61311.0000000	0.3750000
61312.0000000	0.3600000
61313.0000000	0.3500000
61314.0000000	0.3533334
61315.0000000	0.3600000
61316.0000000	0.3450000
61317.0000000	0.3450000
61318.0000000	0.3450000
61319.0000000	0.3450000
61320.0000000	0.3350000
61321.0000000	0.3400000
61322.0000000	0.3400000
61323.0000000	0.3350000
61324.0000000	0.3400000
61325.0000000	0.3350000
61326.0000000	0.3300000
61327.0000000	0.3350000
61328.0000000	0.3300000
61329.0000000	0.3300000
61330.0000000	0.3400000
61331.0000000	0.3400000

61332.0000000	0.3450000
61333.0000000	0.3450000
61334.0000000	0.3500000
61335.0000000	0.3450000
61336.0000000	0.3500000
61337.0000000	0.3400000
61338.0000000	0.3500000
61339.0000000	0.3450000
61340.0000000	0.3500000
61341.0000000	0.3450000
61342.0000000	0.3450000
61343.0000000	0.3400000
61344.0000000	0.3366667
61345.0000000	0.3300000
61346.0000000	0.3250000
61347.0000000	0.3200000
61348.0000000	0.3250000
61349.0000000	0.3300000
61350.0000000	0.3300000
61351.0000000	0.3300000
61352.0000000	0.3300000
61353.0000000	0.3350000
61354.0000000	0.3400000
61355.0000000	0.3350000
61356.0000000	0.3400000
61357.0000000	0.3400000
61358.0000000	0.3400000
61359.0000000	0.3400000
61360.0000000	0.3450000
61361.0000000	0.3400000
61362.0000000	0.3450000
61363.0000000	0.3400000
61364.0000000	0.3400000
61365.0000000	0.3400000
61366.0000000	0.3350000
61367.0000000	0.3350000
61368.0000000	0.3350000
61369.0000000	0.3250000
61370.0000000	0.3200000
61371.0000000	0.3200000
61372.0000000	0.3250000
61373.0000000	0.3200000
61374.0000000	0.3066667
61375.0000000	0.3100000
61376.0000000	0.3050000
61377.0000000	0.3050000
61378.0000000	0.3000000
61379.0000000	0.2900000
61380.0000000	0.2900000
61381.0000000	0.2900000
61382.0000000	0.2900000
61383.0000000	0.2900000
61384.0000000	0.2900000
61385.0000000	0.2850000
61386.0000000	0.2800000
61387.0000000	0.2750000
61388.0000000	0.2750000

61389.0000000	0.2700000
61390.0000000	0.2700000
61391.0000000	0.2650000
61392.0000000	0.2600000
61393.0000000	0.2600000
61394.0000000	0.2550000
61395.0000000	0.2550000
61396.0000000	0.2550000
61397.0000000	0.2550000
61398.0000000	0.2500000
61399.0000000	0.2450000
61400.0000000	0.2500000
61401.0000000	0.2500000
61402.0000000	0.2550000
61403.0000000	0.2550000
61404.0000000	0.2500000
61405.0000000	0.2600000
61406.0000000	0.2550000
61407.0000000	0.2600000
61408.0000000	0.2600000
61409.0000000	0.2600000
61410.0000000	0.2650000
61411.0000000	0.2650000
61412.0000000	0.2700000
61413.0000000	0.2650000
61414.0000000	0.2650000
61415.0000000	0.2650000
61416.0000000	0.2700000
61417.0000000	0.2700000
61418.0000000	0.2700000
61419.0000000	0.2800000
61420.0000000	0.2700000
61421.0000000	0.2750000
61422.0000000	0.2700000
61423.0000000	0.2750000
61424.0000000	0.2800000
61425.0000000	0.2750000
61426.0000000	0.2750000
61427.0000000	0.2750000
61428.0000000	0.2650000
61429.0000000	0.2700000
61430.0000000	0.2600000
61431.0000000	0.2500000
61432.0000000	0.2550000
61433.0000000	0.2550000
61434.0000000	0.2600000
61435.0000000	0.2500000
61436.0000000	0.2450000
61437.0000000	0.2450000
61438.0000000	0.2400000
61439.0000000	0.2450000
61440.0000000	0.2300000
61441.0000000	0.2350000
61442.0000000	0.2250000
61443.0000000	0.2250000
61444.0000000	0.2250000
61445.0000000	0.2250000

61446.0000000	0.2200000
61447.0000000	0.2250000
61448.0000000	0.2150000
61449.0000000	0.2150000
61450.0000000	0.2050000
61451.0000000	0.2000000
61452.0000000	0.2000000
61453.0000000	0.2100000
61454.0000000	0.2150000
61455.0000000	0.2100000
61456.0000000	0.2050000
61457.0000000	0.2100000
61458.0000000	0.2100000
61459.0000000	0.2000000
61460.0000000	0.1950000
61461.0000000	0.2000000
61462.0000000	0.2050000
61463.0000000	0.2100000
61464.0000000	0.2100000
61465.0000000	0.2100000
61466.0000000	0.2100000
61467.0000000	0.2250000
61468.0000000	0.2200000
61469.0000000	0.2250000
61470.0000000	0.2250000
61471.0000000	0.2200000
61472.0000000	0.2300000
61473.0000000	0.2300000
61474.0000000	0.2350000
61475.0000000	0.2350000
61476.0000000	0.2400000
61477.0000000	0.2400000
61478.0000000	0.2450000
61479.0000000	0.2450000
61480.0000000	0.2550000
61481.0000000	0.2550000
61482.0000000	0.2500000
61483.0000000	0.2550000
61484.0000000	0.2550000
61485.0000000	0.2550000
61486.0000000	0.2600000
61487.0000000	0.2550000
61488.0000000	0.2600000
61489.0000000	0.2550000
61490.0000000	0.2500000
61491.0000000	0.2450000
61492.0000000	0.2450000
61493.0000000	0.2500000
61494.0000000	0.2466667
61495.0000000	0.2400000
61496.0000000	0.2300000
61497.0000000	0.2200000
61498.0000000	0.2150000
61499.0000000	0.2150000
61500.0000000	0.2250000
61501.0000000	0.2250000
61502.0000000	0.2250000

61503.0000000	0.2250000
61504.0000000	0.2300000
61505.0000000	0.2250000
61506.0000000	0.2300000
61507.0000000	0.2250000
61508.0000000	0.2300000
61509.0000000	0.2400000
61510.0000000	0.2450000
61511.0000000	0.2500000
61512.0000000	0.2600000
61513.0000000	0.2650000
61514.0000000	0.2700000
61515.0000000	0.2700000
61516.0000000	0.2700000
61517.0000000	0.2700000
61518.0000000	0.2700000
61519.0000000	0.2800000
61520.0000000	0.2950000
61521.0000000	0.2900000
61522.0000000	0.3000000
61523.0000000	0.3050000
61524.0000000	0.3100000
61525.0000000	0.3166667
61526.0000000	0.3250000
61527.0000000	0.3300000
61528.0000000	0.3450000
61529.0000000	0.3500000
61530.0000000	0.3550000
61531.0000000	0.3550000
61532.0000000	0.3550000
61533.0000000	0.3600000
61534.0000000	0.3650000
61535.0000000	0.3700000
61536.0000000	0.3700000
61537.0000000	0.3700000
61538.0000000	0.3700000
61539.0000000	0.3700000
61540.0000000	0.3700000
61541.0000000	0.3750000
61542.0000000	0.3850000
61543.0000000	0.3950000
61544.0000000	0.3900000
61545.0000000	0.3900000
61546.0000000	0.3900000
61547.0000000	0.3850000
61548.0000000	0.3850000
61549.0000000	0.3900000
61550.0000000	0.3850000
61551.0000000	0.3850000
61552.0000000	0.3900000
61553.0000000	0.3900000
61554.0000000	0.3900000
61555.0000000	0.3800000
61556.0000000	0.3850000
61557.0000000	0.3850000
61558.0000000	0.3850000
61559.0000000	0.3850000

61560.0000000	0.3800000
61561.0000000	0.3700000
61562.0000000	0.3600000
61563.0000000	0.3550000
61564.0000000	0.3500000
61565.0000000	0.3500000
61566.0000000	0.3500000
61567.0000000	0.3550000
61568.0000000	0.3450000
61569.0000000	0.3450000
61570.0000000	0.3400000
61571.0000000	0.3400000
61572.0000000	0.3400000
61573.0000000	0.3350000
61574.0000000	0.3400000
61575.0000000	0.3350000
61576.0000000	0.3250000
61577.0000000	0.3150000
61578.0000000	0.3050000
61579.0000000	0.3050000
61580.0000000	0.3000000
61581.0000000	0.2950000
61582.0000000	0.2900000
61583.0000000	0.2800000
61584.0000000	0.2700000
61585.0000000	0.2566667
61586.0000000	0.2650000
61587.0000000	0.2550000
61588.0000000	0.2500000
61589.0000000	0.2500000
61590.0000000	0.2500000
61591.0000000	0.2350000
61592.0000000	0.2300000
61593.0000000	0.2300000
61594.0000000	0.2300000
61595.0000000	0.2250000
61596.0000000	0.2200000
61597.0000000	0.2050000
61598.0000000	0.1900000
61599.0000000	0.1800000
61600.0000000	0.1700000
61601.0000000	0.1550000
61602.0000000	0.1500000
61603.0000000	0.1500000
61604.0000000	0.1400000
61605.0000000	0.1400000
61606.0000000	0.1300000
61607.0000000	0.1300000
61608.0000000	0.1150000
61609.0000000	0.1100000
61610.0000000	0.1100000
61611.0000000	0.1000000
61612.0000000	0.0950000
61613.0000000	0.1000000
61614.0000000	0.0950000
61615.0000000	0.0933333
61616.0000000	0.0800000

61617.0000000	0.0750000
61618.0000000	0.0750000
61619.0000000	0.0700000
61620.0000000	0.0650000
61621.0000000	0.0650000
61622.0000000	0.0500000
61623.0000000	0.0500000
61624.0000000	0.0450000
61625.0000000	0.0400000
61626.0000000	0.0300000
61627.0000000	0.0200000
61628.0000000	0.0150000
61629.0000000	0.0000000
61630.0000000	-0.0050000
61631.0000000	-0.0150000
61632.0000000	-0.0300000
61633.0000000	-0.0400000
61634.0000000	-0.0450000
61635.0000000	-0.0550000
61636.0000000	-0.0650000
61637.0000000	-0.0700000
61638.0000000	-0.0700000
61639.0000000	-0.0750000
61640.0000000	-0.0850000
61641.0000000	-0.0800000
61642.0000000	-0.0850000
61643.0000000	-0.0850000
61644.0000000	-0.0950000
61645.0000000	-0.1000000
61646.0000000	-0.1033333
61647.0000000	-0.1100000
61648.0000000	-0.1200000
61649.0000000	-0.1200000
61650.0000000	-0.1200000
61651.0000000	-0.1250000
61652.0000000	-0.1250000
61653.0000000	-0.1350000
61654.0000000	-0.1400000
61655.0000000	-0.1450000
61656.0000000	-0.1500000
61657.0000000	-0.1500000
61658.0000000	-0.1550000
61659.0000000	-0.1600000
61660.0000000	-0.1750000
61661.0000000	-0.1850000
61662.0000000	-0.1900000
61663.0000000	-0.1950000
61664.0000000	-0.2050000
61665.0000000	-0.2150000
61666.0000000	-0.2250000
61667.0000000	-0.2300000
61668.0000000	-0.2450000
61669.0000000	-0.2450000
61670.0000000	-0.2500000
61671.0000000	-0.2550000
61672.0000000	-0.2650000
61673.0000000	-0.2750000

61674.0000000	-0.2800000
61675.0000000	-0.2850000
61676.0000000	-0.2933333
61677.0000000	-0.3000000
61678.0000000	-0.3050000
61679.0000000	-0.3100000
61680.0000000	-0.3200000
61681.0000000	-0.3200000
61682.0000000	-0.3250000
61683.0000000	-0.3300000
61684.0000000	-0.3350000
61685.0000000	-0.3400000
61686.0000000	-0.3500000
61687.0000000	-0.3500000
61688.0000000	-0.3600000
61689.0000000	-0.3650000
61690.0000000	-0.3750000
61691.0000000	-0.3850000
61692.0000000	-0.3950000
61693.0000000	-0.4150000
61694.0000000	-0.4250000
61695.0000000	-0.4150000
61696.0000000	-0.4200000
61697.0000000	-0.4200000
61698.0000000	-0.4250000
61699.0000000	-0.4300000
61700.0000000	-0.4400000
61701.0000000	-0.4450000
61702.0000000	-0.4600000
61703.0000000	-0.4700000
61704.0000000	-0.4800000
61705.0000000	-0.4850000
61706.0000000	-0.4866667
61707.0000000	-0.4750000
61708.0000000	-0.4800000
61709.0000000	-0.4750000
61710.0000000	-0.4850000
61711.0000000	-0.4950000
61712.0000000	-0.5000000
61713.0000000	-0.5100000
61714.0000000	-0.5200000
61715.0000000	-0.5250000
61716.0000000	-0.5300000
61717.0000000	-0.5450000
61718.0000000	-0.5500000
61719.0000000	-0.5550000
61720.0000000	-0.5750000
61721.0000000	-0.5800000
61722.0000000	-0.5850000
61723.0000000	-0.5900000
61724.0000000	-0.5950000
61725.0000000	-0.6000000
61726.0000000	-0.6150000
61727.0000000	-0.6200000
61728.0000000	-0.6200000
61729.0000000	-0.6300000
61730.0000000	-0.6300000

61731.0000000	-0.6350000
61732.0000000	-0.6450000
61733.0000000	-0.6500000
61734.0000000	-0.6550000
61735.0000000	-0.6650000
61736.0000000	-0.6733333
61737.0000000	-0.6800000
61738.0000000	-0.6850000
61739.0000000	-0.6900000
61740.0000000	-0.6950000
61741.0000000	-0.7050000
61742.0000000	-0.7100000
61743.0000000	-0.7300000
61744.0000000	-0.7300000
61745.0000000	-0.7250000
61746.0000000	-0.7300000
61747.0000000	-0.7400000
61748.0000000	-0.7400000
61749.0000000	-0.7500000
61750.0000000	-0.7600000
61751.0000000	-0.7650000
61752.0000000	-0.7800000
61753.0000000	-0.7800000
61754.0000000	-0.7750000
61755.0000000	-0.7750000
61756.0000000	-0.7850000
61757.0000000	-0.7850000
61758.0000000	-0.7900000
61759.0000000	-0.7950000
61760.0000000	-0.8000000
61761.0000000	-0.8050000
61762.0000000	-0.8100000
61763.0000000	-0.8150000
61764.0000000	-0.8200000
61765.0000000	-0.8300000
61766.0000000	-0.8400000
61767.0000000	-0.8550000
61768.0000000	-0.8550000
61769.0000000	-0.8600000
61770.0000000	-0.8600000
61771.0000000	-0.8600000
61772.0000000	-0.8600000
61773.0000000	-0.8600000
61774.0000000	-0.8600000
61775.0000000	-0.8650000
61776.0000000	-0.8700000
61777.0000000	-0.8750000
61778.0000000	-0.8750000
61779.0000000	-0.8850000
61780.0000000	-0.8850000
61781.0000000	-0.8800000
61782.0000000	-0.8800000
61783.0000000	-0.8900000
61784.0000000	-0.8950000
61785.0000000	-0.8900000
61786.0000000	-0.8800000
61787.0000000	-0.8800000

61788.0000000	-0.8800000
61789.0000000	-0.8850000
61790.0000000	-0.8900000
61791.0000000	-0.8950000
61792.0000000	-0.9000000
61793.0000000	-0.9050000
61794.0000000	-0.9150000
61795.0000000	-0.9100000
61796.0000000	-0.9050000
61797.0000000	-0.9100000
61798.0000000	-0.9150000
61799.0000000	-0.9350000
61800.0000000	-0.9300000
61801.0000000	-0.9250000
61802.0000000	-0.9250000
61803.0000000	-0.9400000
61804.0000000	-0.9550000
61805.0000000	-0.9500000
61806.0000000	-0.9450000
61807.0000000	-0.9450000
61808.0000000	-0.9400000
61809.0000000	-0.9400000
61810.0000000	-0.9400000
61811.0000000	-0.9400000
61812.0000000	-0.9300000
61813.0000000	-0.9400000
61814.0000000	-0.9400000
61815.0000000	-0.9400000
61816.0000000	-0.9350000
61817.0000000	-0.9400000
61818.0000000	-0.9350000
61819.0000000	-0.9350000
61820.0000000	-0.9350000
61821.0000000	-0.9400000
61822.0000000	-0.9300000
61823.0000000	-0.9300000
61824.0000000	-0.9300000
61825.0000000	-0.9300000
61826.0000000	-0.9250000
61827.0000000	-0.9266667
61828.0000000	-0.9250000
61829.0000000	-0.9200000
61830.0000000	-0.9050000
61831.0000000	-0.9000000
61832.0000000	-0.9000000
61833.0000000	-0.9000000
61834.0000000	-0.9050000
61835.0000000	-0.8950000
61836.0000000	-0.8900000
61837.0000000	-0.8850000
61838.0000000	-0.8800000
61839.0000000	-0.8750000
61840.0000000	-0.8700000
61841.0000000	-0.8700000
61842.0000000	-0.8700000
61843.0000000	-0.8650000
61844.0000000	-0.8600000

61845.0000000	-0.8550000
61846.0000000	-0.8500000
61847.0000000	-0.8600000
61848.0000000	-0.8500000
61849.0000000	-0.8550000
61850.0000000	-0.8600000
61851.0000000	-0.8600000
61852.0000000	-0.8600000
61853.0000000	-0.8750000
61854.0000000	-0.8700000
61855.0000000	-0.8650000
61856.0000000	-0.8600000
61857.0000000	-0.8566667
61858.0000000	-0.8500000
61859.0000000	-0.8500000
61860.0000000	-0.8550000
61861.0000000	-0.8400000
61862.0000000	-0.8400000
61863.0000000	-0.8300000
61864.0000000	-0.8250000
61865.0000000	-0.8200000
61866.0000000	-0.8050000
61867.0000000	-0.7900000
61868.0000000	-0.7900000
61869.0000000	-0.7950000
61870.0000000	-0.8000000
61871.0000000	-0.7950000
61872.0000000	-0.7950000
61873.0000000	-0.8000000
61874.0000000	-0.8050000
61875.0000000	-0.8200000
61876.0000000	-0.8350000
61877.0000000	-0.8250000
61878.0000000	-0.8200000
61879.0000000	-0.8150000
61880.0000000	-0.8200000
61881.0000000	-0.8200000
61882.0000000	-0.8200000
61883.0000000	-0.8250000
61884.0000000	-0.8250000
61885.0000000	-0.8250000
61886.0000000	-0.8300000
61887.0000000	-0.8233333
61888.0000000	-0.8200000
61889.0000000	-0.8200000
61890.0000000	-0.8150000
61891.0000000	-0.8100000
61892.0000000	-0.8150000
61893.0000000	-0.8100000
61894.0000000	-0.8100000
61895.0000000	-0.8150000
61896.0000000	-0.8150000
61897.0000000	-0.8100000
61898.0000000	-0.8050000
61899.0000000	-0.8000000
61900.0000000	-0.8050000
61901.0000000	-0.8050000

61902.0000000	-0.8000000
61903.0000000	-0.8050000
61904.0000000	-0.8100000
61905.0000000	-0.8150000
61906.0000000	-0.8100000
61907.0000000	-0.8100000
61908.0000000	-0.8000000
61909.0000000	-0.8000000
61910.0000000	-0.8050000
61911.0000000	-0.8100000
61912.0000000	-0.8100000
61913.0000000	-0.8150000
61914.0000000	-0.8200000
61915.0000000	-0.8150000
61916.0000000	-0.8100000
61917.0000000	-0.8100000
61918.0000000	-0.8100000
61919.0000000	-0.8150000
61920.0000000	-0.8100000
61921.0000000	-0.8150000
61922.0000000	-0.8150000
61923.0000000	-0.8150000
61924.0000000	-0.8150000
61925.0000000	-0.8150000
61926.0000000	-0.8200000
61927.0000000	-0.8250000
61928.0000000	-0.8250000
61929.0000000	-0.8300000
61930.0000000	-0.8300000
61931.0000000	-0.8300000
61932.0000000	-0.8300000
61933.0000000	-0.8350000
61934.0000000	-0.8350000
61935.0000000	-0.8300000
61936.0000000	-0.8400000
61937.0000000	-0.8400000
61938.0000000	-0.8350000
61939.0000000	-0.8350000
61940.0000000	-0.8350000
61941.0000000	-0.8350000
61942.0000000	-0.8350000
61943.0000000	-0.8350000
61944.0000000	-0.8350000
61945.0000000	-0.8400000
61946.0000000	-0.8400000
61947.0000000	-0.8400000
61948.0000000	-0.8333333
61949.0000000	-0.8350000
61950.0000000	-0.8300000
61951.0000000	-0.8250000
61952.0000000	-0.8200000
61953.0000000	-0.8100000
61954.0000000	-0.8100000
61955.0000000	-0.8050000
61956.0000000	-0.8050000
61957.0000000	-0.8100000
61958.0000000	-0.8100000

61959.0000000	-0.8100000
61960.0000000	-0.8050000
61961.0000000	-0.8050000
61962.0000000	-0.8000000
61963.0000000	-0.7950000
61964.0000000	-0.8000000
61965.0000000	-0.8050000
61966.0000000	-0.8050000
61967.0000000	-0.8100000
61968.0000000	-0.8100000
61969.0000000	-0.8200000
61970.0000000	-0.8050000
61971.0000000	-0.8050000
61972.0000000	-0.8050000
61973.0000000	-0.8100000
61974.0000000	-0.8100000
61975.0000000	-0.8100000
61976.0000000	-0.8150000
61977.0000000	-0.8100000
61978.0000000	-0.8150000
61979.0000000	-0.8100000
61980.0000000	-0.8100000
61981.0000000	-0.8150000
61982.0000000	-0.8100000
61983.0000000	-0.8150000
61984.0000000	-0.8200000
61985.0000000	-0.8200000
61986.0000000	-0.8250000
61987.0000000	-0.8200000
61988.0000000	-0.8250000
61989.0000000	-0.8150000
61990.0000000	-0.8250000
61991.0000000	-0.8200000
61992.0000000	-0.8200000
61993.0000000	-0.8200000
61994.0000000	-0.8250000
61995.0000000	-0.8250000
61996.0000000	-0.8200000
61997.0000000	-0.8200000
61998.0000000	-0.8250000
61999.0000000	-0.8200000
62000.0000000	-0.8150000
62001.0000000	-0.8150000
62002.0000000	-0.8100000
62003.0000000	-0.8050000
62004.0000000	-0.8150000
62005.0000000	-0.8100000
62006.0000000	-0.8100000
62007.0000000	-0.8150000
62008.0000000	-0.8200000
62009.0000000	-0.8200000
62010.0000000	-0.8300000
62011.0000000	-0.8300000
62012.0000000	-0.8350000
62013.0000000	-0.8350000
62014.0000000	-0.8250000
62015.0000000	-0.8250000

62016.0000000	-0.8300000
62017.0000000	-0.8350000
62018.0000000	-0.8400000
62019.0000000	-0.8400000
62020.0000000	-0.8350000
62021.0000000	-0.8300000
62022.0000000	-0.8300000
62023.0000000	-0.8350000
62024.0000000	-0.8450000
62025.0000000	-0.8400000
62026.0000000	-0.8400000
62027.0000000	-0.8350000
62028.0000000	-0.8400000
62029.0000000	-0.8300000
62030.0000000	-0.8300000
62031.0000000	-0.8300000
62032.0000000	-0.8250000
62033.0000000	-0.8300000
62034.0000000	-0.8250000
62035.0000000	-0.8200000
62036.0000000	-0.8150000
62037.0000000	-0.8200000
62038.0000000	-0.8150000
62039.0000000	-0.8133333
62040.0000000	-0.8150000
62041.0000000	-0.8100000
62042.0000000	-0.8050000
62043.0000000	-0.8050000
62044.0000000	-0.8000000
62045.0000000	-0.8000000
62046.0000000	-0.8050000
62047.0000000	-0.8000000
62048.0000000	-0.8050000
62049.0000000	-0.8100000
62050.0000000	-0.8050000
62051.0000000	-0.8100000
62052.0000000	-0.8100000
62053.0000000	-0.8200000
62054.0000000	-0.8200000
62055.0000000	-0.8250000
62056.0000000	-0.8200000
62057.0000000	-0.8200000
62058.0000000	-0.8200000
62059.0000000	-0.8250000
62060.0000000	-0.8250000
62061.0000000	-0.8300000
62062.0000000	-0.8400000
62063.0000000	-0.8350000
62064.0000000	-0.8400000
62065.0000000	-0.8450000
62066.0000000	-0.8450000
62067.0000000	-0.8550000
62068.0000000	-0.8566667
62069.0000000	-0.8600000
62070.0000000	-0.8650000
62071.0000000	-0.8750000
62072.0000000	-0.8800000

62073.0000000	-0.8850000
62074.0000000	-0.8850000
62075.0000000	-0.8850000
62076.0000000	-0.8850000
62077.0000000	-0.8900000
62078.0000000	-0.8950000
62079.0000000	-0.9000000
62080.0000000	-0.9050000
62081.0000000	-0.9000000
62082.0000000	-0.9000000
62083.0000000	-0.9000000
62084.0000000	-0.9050000
62085.0000000	-0.9000000
62086.0000000	-0.9050000
62087.0000000	-0.9050000
62088.0000000	-0.9050000
62089.0000000	-0.9000000
62090.0000000	-0.9000000
62091.0000000	-0.9100000
62092.0000000	-0.9100000
62093.0000000	-0.9000000
62094.0000000	-0.9050000
62095.0000000	-0.9050000
62096.0000000	-0.9000000
62097.0000000	-0.9000000
62098.0000000	-0.8999999
62099.0000000	-0.8850000
62100.0000000	-0.8900000
62101.0000000	-0.8850000
62102.0000000	-0.8850000
62103.0000000	-0.8850000
62104.0000000	-0.8850000
62105.0000000	-0.8850000
62106.0000000	-0.8850000
62107.0000000	-0.8850000
62108.0000000	-0.8850000
62109.0000000	-0.8900000
62110.0000000	-0.8950000
62111.0000000	-0.8900000
62112.0000000	-0.9000000
62113.0000000	-0.9000000
62114.0000000	-0.9100000
62115.0000000	-0.9100000
62116.0000000	-0.9150000
62117.0000000	-0.9200000
62118.0000000	-0.9250000
62119.0000000	-0.9300000
62120.0000000	-0.9300000
62121.0000000	-0.9300000
62122.0000000	-0.9350000
62123.0000000	-0.9350000
62124.0000000	-0.9400000
62125.0000000	-0.9450000
62126.0000000	-0.9450000
62127.0000000	-0.9500000
62128.0000000	-0.9450000
62129.0000000	-0.9500000

62130.0000000	-0.9550000
62131.0000000	-0.9550000
62132.0000000	-0.9600000
62133.0000000	-0.9600000
62134.0000000	-0.9600000
62135.0000000	-0.9600000
62136.0000000	-0.9550000
62137.0000000	-0.9550000
62138.0000000	-0.9550000
62139.0000000	-0.9550000
62140.0000000	-0.9450000
62141.0000000	-0.9500000
62142.0000000	-0.9500000
62143.0000000	-0.9550000
62144.0000000	-0.9600000
62145.0000000	-0.9650000
62146.0000000	-0.9700000
62147.0000000	-0.9800000
62148.0000000	-0.9750000
62149.0000000	-0.9800000
62150.0000000	-0.9800000
62151.0000000	-0.9800000
62152.0000000	-0.9850000
62153.0000000	-0.9950000
62154.0000000	-0.9900000
62155.0000000	-0.9850000
62156.0000000	-0.9800000
62157.0000000	-0.9800000
62158.0000000	-0.9750000
62159.0000000	-0.9766667
62160.0000000	-0.9700000
62161.0000000	-0.9700000
62162.0000000	-0.9700000
62163.0000000	-0.9700000
62164.0000000	-0.9700000
62165.0000000	-0.9700000
62166.0000000	-0.9800000
62167.0000000	-0.9750000
62168.0000000	-0.9800000
62169.0000000	-0.9900000
62170.0000000	-0.9950000
62171.0000000	-0.9950000
62172.0000000	-1.0000000
62173.0000000	-1.0000000
62174.0000000	-1.0100000
62175.0000000	-1.0150000
62176.0000000	-1.0150000
62177.0000000	-1.0250000
62178.0000000	-1.0300000
62179.0000000	-1.0300000
62180.0000000	-1.0300000
62181.0000000	-1.0350000
62182.0000000	-1.0400000
62183.0000000	-1.0400000
62184.0000000	-1.0350000
62185.0000000	-1.0450000
62186.0000000	-1.0400000

62187.0000000	-1.0350000
62188.0000000	-1.0450000
62189.0000000	-1.0466666
62190.0000000	-1.0500000
62191.0000000	-1.0550001
62192.0000000	-1.0500000
62193.0000000	-1.0549999
62194.0000000	-1.0500000
62195.0000000	-1.0549999
62196.0000000	-1.0500000
62197.0000000	-1.0450000
62198.0000000	-1.0550001
62199.0000000	-1.0549999
62200.0000000	-1.0549999
62201.0000000	-1.0549999
62202.0000000	-1.0500000
62203.0000000	-1.0549999
62204.0000000	-1.0599999
62205.0000000	-1.0699999
62206.0000000	-1.0750000
62207.0000000	-1.0750000
62208.0000000	-1.0800000
62209.0000000	-1.0750000
62210.0000000	-1.0850000
62211.0000000	-1.0950000
62212.0000000	-1.1050000
62213.0000000	-1.1200000
62214.0000000	-1.1150000
62215.0000000	-1.1100000
62216.0000000	-1.1150000
62217.0000000	-1.1150000
62218.0000000	-1.1150000
62219.0000000	-1.1200000
62220.0000000	-1.1250000
62221.0000000	-1.1350000
62222.0000000	-1.1400000
62223.0000000	-1.1400000
62224.0000000	-1.1450000
62225.0000000	-1.1500000
62226.0000000	-1.1650000
62227.0000000	-1.1700000
62228.0000000	-1.1700000
62229.0000000	-1.1750000
62230.0000000	-1.1750000
62231.0000000	-1.1849999
62232.0000000	-1.1900001
62233.0000000	-1.1950001
62234.0000000	-1.1950001
62235.0000000	-1.2100000
62236.0000000	-1.2150000
62237.0000000	-1.2250000
62238.0000000	-1.2200000
62239.0000000	-1.2300000
62240.0000000	-1.2400000
62241.0000000	-1.2450000
62242.0000000	-1.2600000
62243.0000000	-1.2550000

62244.0000000	-1.2700000
62245.0000000	-1.2800000
62246.0000000	-1.2900000
62247.0000000	-1.2950000
62248.0000000	-1.2950000
62249.0000000	-1.2966666
62250.0000000	-1.2900000
62251.0000000	-1.2900000
62252.0000000	-1.3000000
62253.0000000	-1.3049999
62254.0000000	-1.3000000
62255.0000000	-1.3099999
62256.0000000	-1.3199999
62257.0000000	-1.3150001
62258.0000000	-1.3300000
62259.0000000	-1.3300000
62260.0000000	-1.3350000
62261.0000000	-1.3450000
62262.0000000	-1.3450000
62263.0000000	-1.3500000
62264.0000000	-1.3600000
62265.0000000	-1.3700000
62266.0000000	-1.3650000
62267.0000000	-1.3750000
62268.0000000	-1.3700000
62269.0000000	-1.3650000
62270.0000000	-1.3750000
62271.0000000	-1.3850000
62272.0000000	-1.3900000
62273.0000000	-1.3950000
62274.0000000	-1.3950000
62275.0000000	-1.3950000
62276.0000000	-1.3900000
62277.0000000	-1.3900000
62278.0000000	-1.3900000
62279.0000000	-1.4050000
62280.0000000	-1.3966666
62281.0000000	-1.4000000
62282.0000000	-1.4050000
62283.0000000	-1.4150000
62284.0000000	-1.4200000
62285.0000000	-1.4300001
62286.0000000	-1.4400001
62287.0000000	-1.4349999
62288.0000000	-1.4500000
62289.0000000	-1.4550000
62290.0000000	-1.4650000
62291.0000000	-1.4750000
62292.0000000	-1.4800000
62293.0000000	-1.4800000
62294.0000000	-1.4800000
62295.0000000	-1.4900000
62296.0000000	-1.4850000
62297.0000000	-1.4850000
62298.0000000	-1.4900000
62299.0000000	-1.4850000
62300.0000000	-1.4750000

62301.0000000	-1.4650000
62302.0000000	-1.4700000
62303.0000000	-1.4750000
62304.0000000	-1.4950000
62305.0000000	-1.4900000
62306.0000000	-1.4900000
62307.0000000	-1.4850000
62308.0000000	-1.4800000
62309.0000000	-1.4750000
62310.0000000	-1.4699999
62311.0000000	-1.4700000
62312.0000000	-1.4800000
62313.0000000	-1.4750000
62314.0000000	-1.4800000
62315.0000000	-1.4800000
62316.0000000	-1.4850000
62317.0000000	-1.4900000
62318.0000000	-1.4900000
62319.0000000	-1.4900000
62320.0000000	-1.4900000
62321.0000000	-1.4900000
62322.0000000	-1.5050000
62323.0000000	-1.5050000
62324.0000000	-1.5000000
62325.0000000	-1.5000000
62326.0000000	-1.4900000
62327.0000000	-1.4900000
62328.0000000	-1.4900000
62329.0000000	-1.4850000
62330.0000000	-1.4850000
62331.0000000	-1.4900000
62332.0000000	-1.4850000
62333.0000000	-1.4750000
62334.0000000	-1.4750000
62335.0000000	-1.4700000
62336.0000000	-1.4550000
62337.0000000	-1.4400001
62338.0000000	-1.4550000
62339.0000000	-1.4500002
62340.0000000	-1.4550000
62341.0000000	-1.4600000
62342.0000000	-1.4600000
62343.0000000	-1.4650000
62344.0000000	-1.4650000
62345.0000000	-1.4750000
62346.0000000	-1.4850000
62347.0000000	-1.4900000
62348.0000000	-1.4950000
62349.0000000	-1.4950000
62350.0000000	-1.4900000
62351.0000000	-1.5050000
62352.0000000	-1.5050000
62353.0000000	-1.4950000
62354.0000000	-1.4850000
62355.0000000	-1.4800000
62356.0000000	-1.4750000
62357.0000000	-1.4800000

62358.0000000	-1.4900000
62359.0000000	-1.4850000
62360.0000000	-1.4750000
62361.0000000	-1.4800000
62362.0000000	-1.4900000
62363.0000000	-1.4950000
62364.0000000	-1.5000000
62365.0000000	-1.5000000
62366.0000000	-1.5000000
62367.0000000	-1.5000000
62368.0000000	-1.5000000
62369.0000000	-1.5050000
62370.0000000	-1.5066667
62371.0000000	-1.5100000
62372.0000000	-1.5150000
62373.0000000	-1.5150000
62374.0000000	-1.5100000
62375.0000000	-1.5050000
62376.0000000	-1.5100000
62377.0000000	-1.5100000
62378.0000000	-1.5150000
62379.0000000	-1.5150000
62380.0000000	-1.5150000
62381.0000000	-1.5150000
62382.0000000	-1.5150000
62383.0000000	-1.5250000
62384.0000000	-1.5300000
62385.0000000	-1.5250000
62386.0000000	-1.5200000
62387.0000000	-1.5250000
62388.0000000	-1.5300000
62389.0000000	-1.5300000
62390.0000000	-1.5250000
62391.0000000	-1.5150000
62392.0000000	-1.5150000
62393.0000000	-1.5100000
62394.0000000	-1.5200000
62395.0000000	-1.5250000
62396.0000000	-1.5200000
62397.0000000	-1.5050000
62398.0000000	-1.5150000
62399.0000000	-1.5200000
62400.0000000	-1.5250000
62401.0000000	-1.5200000
62402.0000000	-1.5300000
62403.0000000	-1.5300000
62404.0000000	-1.5300000
62405.0000000	-1.5300000
62406.0000000	-1.5350000
62407.0000000	-1.5350000
62408.0000000	-1.5350000
62409.0000000	-1.5400000
62410.0000000	-1.5400000
62411.0000000	-1.5450000
62412.0000000	-1.5450000
62413.0000000	-1.5450000
62414.0000000	-1.5450000

62415.0000000	-1.5549999
62416.0000000	-1.5549999
62417.0000000	-1.5599999
62418.0000000	-1.5549999
62419.0000000	-1.5549999
62420.0000000	-1.5450000
62421.0000000	-1.5450000
62422.0000000	-1.5450000
62423.0000000	-1.5500000
62424.0000000	-1.5450000
62425.0000000	-1.5400000
62426.0000000	-1.5300000
62427.0000000	-1.5350000
62428.0000000	-1.5400000
62429.0000000	-1.5400000
62430.0000000	-1.5400000
62431.0000000	-1.5466666
62432.0000000	-1.5450000
62433.0000000	-1.5500000
62434.0000000	-1.5549999
62435.0000000	-1.5500000
62436.0000000	-1.5549999
62437.0000000	-1.5599999
62438.0000000	-1.5599999
62439.0000000	-1.5650001
62440.0000000	-1.5650001
62441.0000000	-1.5750000
62442.0000000	-1.5699999
62443.0000000	-1.5750000
62444.0000000	-1.5850000
62445.0000000	-1.5900000
62446.0000000	-1.5850000
62447.0000000	-1.5950000
62448.0000000	-1.6000000
62449.0000000	-1.6050000
62450.0000000	-1.6050000
62451.0000000	-1.6100000
62452.0000000	-1.6050000
62453.0000000	-1.6050000
62454.0000000	-1.6050000
62455.0000000	-1.6000000
62456.0000000	-1.6050000
62457.0000000	-1.6000000
62458.0000000	-1.5950000
62459.0000000	-1.5950000
62460.0000000	-1.5900000
62461.0000000	-1.5900000
62462.0000000	-1.5850000
62463.0000000	-1.5850000
62464.0000000	-1.5900000
62465.0000000	-1.5900000
62466.0000000	-1.5950000
62467.0000000	-1.6000000
62468.0000000	-1.5950000
62469.0000000	-1.5950000
62470.0000000	-1.6000000
62471.0000000	-1.5900000

62472.0000000	-1.5900000
62473.0000000	-1.5900000
62474.0000000	-1.5850000
62475.0000000	-1.5800000
62476.0000000	-1.5700001
62477.0000000	-1.5750000
62478.0000000	-1.5750000
62479.0000000	-1.5699999
62480.0000000	-1.5650001
62481.0000000	-1.5750000
62482.0000000	-1.5699999
62483.0000000	-1.5650001
62484.0000000	-1.5800000
62485.0000000	-1.5800000
62486.0000000	-1.5750000
62487.0000000	-1.5700001
62488.0000000	-1.5700001
62489.0000000	-1.5800000
62490.0000000	-1.5850000
62491.0000000	-1.5900000
62492.0000000	-1.6050000
62493.0000000	-1.6100000
62494.0000000	-1.6250000
62495.0000000	-1.6350000
62496.0000000	-1.6350000
62497.0000000	-1.6350000
62498.0000000	-1.6350000
62499.0000000	-1.6450000
62500.0000000	-1.6450000
62501.0000000	-1.6550000
62502.0000000	-1.6500000
62503.0000000	-1.6450000
62504.0000000	-1.6400000
62505.0000000	-1.6350000
62506.0000000	-1.6300000
62507.0000000	-1.6250000
62508.0000000	-1.6250000
62509.0000000	-1.6350000
62510.0000000	-1.6400000
62511.0000000	-1.6450000
62512.0000000	-1.6550000
62513.0000000	-1.6650000
62514.0000000	-1.6700000
62515.0000000	-1.6700000
62516.0000000	-1.6700001
62517.0000000	-1.6600000
62518.0000000	-1.6600000
62519.0000000	-1.6550000
62520.0000000	-1.6650000
62521.0000000	-1.6533333
62522.0000000	-1.6500000
62523.0000000	-1.6600000
62524.0000000	-1.6700000
62525.0000000	-1.6849999
62526.0000000	-1.6900001
62527.0000000	-1.6900001
62528.0000000	-1.6849999

62529.0000000	-1.6600000
62530.0000000	-1.6700000
62531.0000000	-1.6550000
62532.0000000	-1.6500000
62533.0000000	-1.6550000
62534.0000000	-1.6500000
62535.0000000	-1.6600000
62536.0000000	-1.6650000
62537.0000000	-1.6750000
62538.0000000	-1.6799999
62539.0000000	-1.6799999
62540.0000000	-1.6900001
62541.0000000	-1.6900001
62542.0000000	-1.6950001
62543.0000000	-1.6900001
62544.0000000	-1.6849999
62545.0000000	-1.6849999
62546.0000000	-1.6950001
62547.0000000	-1.7000000
62548.0000000	-1.7050000
62549.0000000	-1.7150000
62550.0000000	-1.7200000
62551.0000000	-1.7233334
62552.0000000	-1.7400000
62553.0000000	-1.7400000
62554.0000000	-1.7400000
62555.0000000	-1.7400000
62556.0000000	-1.7450000
62557.0000000	-1.7600000
62558.0000000	-1.7650000
62559.0000000	-1.7650000
62560.0000000	-1.7700000
62561.0000000	-1.7700000
62562.0000000	-1.7700000
62563.0000000	-1.7700000
62564.0000000	-1.7700000
62565.0000000	-1.7650000
62566.0000000	-1.7700000
62567.0000000	-1.7600000
62568.0000000	-1.7600000
62569.0000000	-1.7500000
62570.0000000	-1.7550000
62571.0000000	-1.7500000
62572.0000000	-1.7650000
62573.0000000	-1.7550000
62574.0000000	-1.7550000
62575.0000000	-1.7550000
62576.0000000	-1.7550000
62577.0000000	-1.7600000
62578.0000000	-1.7700000
62579.0000000	-1.7800000
62580.0000000	-1.7900000
62581.0000000	-1.8099999
62582.0000000	-1.8199999
62583.0000000	-1.8400000
62584.0000000	-1.8600000
62585.0000000	-1.8800000

62586.0000000	-1.8900000
62587.0000000	-1.8900000
62588.0000000	-1.8800000
62589.0000000	-1.8750000
62590.0000000	-1.8750000
62591.0000000	-1.8750000
62592.0000000	-1.8750000
62593.0000000	-1.8650000
62594.0000000	-1.8600000
62595.0000000	-1.8750000
62596.0000000	-1.8800000
62597.0000000	-1.8750000
62598.0000000	-1.8900000
62599.0000000	-1.8850000
62600.0000000	-1.8750000
62601.0000000	-1.8900000
62602.0000000	-1.8850000
62603.0000000	-1.9050000
62604.0000000	-1.9100000
62605.0000000	-1.9050000
62606.0000000	-1.9100000
62607.0000000	-1.9150000
62608.0000000	-1.9349999
62609.0000000	-1.9349999
62610.0000000	-1.9400001
62611.0000000	-1.9533334
62612.0000000	-1.9650000
62613.0000000	-1.9650000
62614.0000000	-1.9750000
62615.0000000	-1.9850000
62616.0000000	-1.9900000
62617.0000000	-1.9950000
62618.0000000	-2.0100000
62619.0000000	-2.0100000
62620.0000000	-2.0100000
62621.0000000	-2.0149999
62622.0000000	-2.0300000
62623.0000000	-2.0349998
62624.0000000	-2.0400000
62625.0000000	-2.0599999
62626.0000000	-2.0650001
62627.0000000	-2.0599999
62628.0000000	-2.0500000
62629.0000000	-2.0650001
62630.0000000	-2.0650001
62631.0000000	-2.0650001
62632.0000000	-2.0599999
62633.0000000	-2.0650001
62634.0000000	-2.0549998
62635.0000000	-2.0450001
62636.0000000	-2.0450001
62637.0000000	-2.0300000
62638.0000000	-2.0400000
62639.0000000	-2.0300000
62640.0000000	-2.0100000
62641.0000000	-2.0200000
62642.0000000	-2.0033333

62643.0000000	-2.0149999
62644.0000000	-2.0200000
62645.0000000	-2.0200000
62646.0000000	-2.0149999
62647.0000000	-2.0149999
62648.0000000	-2.0250001
62649.0000000	-2.0200000
62650.0000000	-2.0250001
62651.0000000	-2.0300000
62652.0000000	-2.0450001
62653.0000000	-2.0500000
62654.0000000	-2.0650001
62655.0000000	-2.0599999
62656.0000000	-2.0699999
62657.0000000	-2.0749998
62658.0000000	-2.0650001
62659.0000000	-2.0799999
62660.0000000	-2.0850000
62661.0000000	-2.0850000
62662.0000000	-2.0850000
62663.0000000	-2.0899999
62664.0000000	-2.0949998
62665.0000000	-2.1050000
62666.0000000	-2.1099999
62667.0000000	-2.1149998
62668.0000000	-2.1199999
62669.0000000	-2.1250000
62670.0000000	-2.1250000
62671.0000000	-2.1199999
62672.0000000	-2.1266668
62673.0000000	-2.1350002
62674.0000000	-2.1500001
62675.0000000	-2.1500001
62676.0000000	-2.1550002
62677.0000000	-2.1550002
62678.0000000	-2.1500001
62679.0000000	-2.1450000
62680.0000000	-2.1600001
62681.0000000	-2.1550002
62682.0000000	-2.1450000
62683.0000000	-2.1400001
62684.0000000	-2.1350002
62685.0000000	-2.1350002
62686.0000000	-2.1250000
62687.0000000	-2.1199999
62688.0000000	-2.1199999
62689.0000000	-2.1050000
62690.0000000	-2.0949998
62691.0000000	-2.0949998
62692.0000000	-2.0949998
62693.0000000	-2.1050000
62694.0000000	-2.1149998
62695.0000000	-2.1250000
62696.0000000	-2.1400001
62697.0000000	-2.1400001
62698.0000000	-2.1450000
62699.0000000	-2.1500001

62700.0000000	-2.1550002
62701.0000000	-2.1550002
62702.0000000	-2.1466668
62703.0000000	-2.1400001
62704.0000000	-2.1350002
62705.0000000	-2.1250000
62706.0000000	-2.1350002
62707.0000000	-2.1400001
62708.0000000	-2.1400001
62709.0000000	-2.1500001
62710.0000000	-2.1500001
62711.0000000	-2.1650000
62712.0000000	-2.1700001
62713.0000000	-2.1700001
62714.0000000	-2.1750002
62715.0000000	-2.1800001
62716.0000000	-2.1700001
62717.0000000	-2.1900001
62718.0000000	-2.2000000
62719.0000000	-2.2049999
62720.0000000	-2.2150002
62721.0000000	-2.2200000
62722.0000000	-2.2300000
62723.0000000	-2.2449999
62724.0000000	-2.2500000
62725.0000000	-2.2600000
62726.0000000	-2.2700000
62727.0000000	-2.2700000
62728.0000000	-2.2750001
62729.0000000	-2.2800000
62730.0000000	-2.2800000
62731.0000000	-2.2849998
62732.0000000	-2.2833331
62733.0000000	-2.2849998
62734.0000000	-2.2849998
62735.0000000	-2.2950001
62736.0000000	-2.2849998
62737.0000000	-2.2700000
62738.0000000	-2.2750001
62739.0000000	-2.2849998
62740.0000000	-2.2849998
62741.0000000	-2.2800000
62742.0000000	-2.2750001
62743.0000000	-2.2750001
62744.0000000	-2.2700000
62745.0000000	-2.2600000
62746.0000000	-2.2600000
62747.0000000	-2.2649999
62748.0000000	-2.2550001
62749.0000000	-2.2500000
62750.0000000	-2.2400000
62751.0000000	-2.2200000
62752.0000000	-2.1900001
62753.0000000	-2.1750002
62754.0000000	-2.1700001
62755.0000000	-2.1650000
62756.0000000	-2.1600001

62757.0000000	-2.1600001
62758.0000000	-2.1700001
62759.0000000	-2.1800001
62760.0000000	-2.1849999
62761.0000000	-2.1849999
62762.0000000	-2.1900001
62763.0000000	-2.1933334
62764.0000000	-2.2100000
62765.0000000	-2.2049999
62766.0000000	-2.1950002
62767.0000000	-2.2000000
62768.0000000	-2.1849999
62769.0000000	-2.1849999
62770.0000000	-2.1750002
62771.0000000	-2.1600001
62772.0000000	-2.1550002
62773.0000000	-2.1500001
62774.0000000	-2.1700001
62775.0000000	-2.1750002
62776.0000000	-2.1750002
62777.0000000	-2.1700001
62778.0000000	-2.1700001
62779.0000000	-2.1700001
62780.0000000	-2.1849999
62781.0000000	-2.1950002
62782.0000000	-2.2200000
62783.0000000	-2.2200000
62784.0000000	-2.2300000
62785.0000000	-2.2350001
62786.0000000	-2.2449999
62787.0000000	-2.2449999
62788.0000000	-2.2500000
62789.0000000	-2.2550001
62790.0000000	-2.2600000
62791.0000000	-2.2500000
62792.0000000	-2.2400000
62793.0000000	-2.2433331
62794.0000000	-2.2400000
62795.0000000	-2.2249999
62796.0000000	-2.2249999
62797.0000000	-2.2150002
62798.0000000	-2.2100000
62799.0000000	-2.2200000
62800.0000000	-2.2049999
62801.0000000	-2.1849999
62802.0000000	-2.1849999
62803.0000000	-2.1650000
62804.0000000	-2.1650000
62805.0000000	-2.1350002
62806.0000000	-2.1300001
62807.0000000	-2.1250000
62808.0000000	-2.1250000
62809.0000000	-2.1149998
62810.0000000	-2.1050000
62811.0000000	-2.0899999
62812.0000000	-2.1050000
62813.0000000	-2.0999999

62814.0000000	-2.0999999
62815.0000000	-2.1050000
62816.0000000	-2.0949998
62817.0000000	-2.0749998
62818.0000000	-2.0899999
62819.0000000	-2.0899999
62820.0000000	-2.0949998
62821.0000000	-2.0999999
62822.0000000	-2.0949998
62823.0000000	-2.1099999
62824.0000000	-2.0999999
62825.0000000	-2.0999999
62826.0000000	-2.0999999
62827.0000000	-2.0899999
62828.0000000	-2.0749998
62829.0000000	-2.0699999
62830.0000000	-2.0749998
62831.0000000	-2.0699999
62832.0000000	-2.0650001
62833.0000000	-2.0599999
62834.0000000	-2.0549998
62835.0000000	-2.0450001
62836.0000000	-2.0250001
62837.0000000	-2.0300000
62838.0000000	-2.0149999
62839.0000000	-1.9850000
62840.0000000	-1.9650000
62841.0000000	-1.9449999
62842.0000000	-1.9349999
62843.0000000	-1.9250000
62844.0000000	-1.9050000
62845.0000000	-1.9050000
62846.0000000	-1.9150000
62847.0000000	-1.9100000
62848.0000000	-1.9100000
62849.0000000	-1.8950000
62850.0000000	-1.9150000
62851.0000000	-1.9150000
62852.0000000	-1.9200000
62853.0000000	-1.9299999
62854.0000000	-1.9400001
62855.0000000	-1.9450001
62856.0000000	-1.9250000
62857.0000000	-1.9349999
62858.0000000	-1.9450001
62859.0000000	-1.9400001
62860.0000000	-1.9349999
62861.0000000	-1.9349999
62862.0000000	-1.9349999
62863.0000000	-1.9349999
62864.0000000	-1.9300001
62865.0000000	-1.9250000
62866.0000000	-1.9250000
62867.0000000	-1.9300001
62868.0000000	-1.9250000
62869.0000000	-1.9300001
62870.0000000	-1.9300001

62871.0000000	-1.9400001
62872.0000000	-1.9450001
62873.0000000	-1.9450001
62874.0000000	-1.9349999
62875.0000000	-1.9250000
62876.0000000	-1.9100000
62877.0000000	-1.9000000
62878.0000000	-1.8900000
62879.0000000	-1.8850000
62880.0000000	-1.8850000
62881.0000000	-1.9000000
62882.0000000	-1.8800000
62883.0000000	-1.8733333
62884.0000000	-1.8650000
62885.0000000	-1.8550000
62886.0000000	-1.8450000
62887.0000000	-1.8450000
62888.0000000	-1.8450000
62889.0000000	-1.8350000
62890.0000000	-1.8450000
62891.0000000	-1.8400000
62892.0000000	-1.8350000
62893.0000000	-1.8400000
62894.0000000	-1.8350000
62895.0000000	-1.8250000
62896.0000000	-1.8150001
62897.0000000	-1.8000000
62898.0000000	-1.7900000
62899.0000000	-1.7750000
62900.0000000	-1.7600000
62901.0000000	-1.7400000
62902.0000000	-1.7350000
62903.0000000	-1.7350000
62904.0000000	-1.7400000
62905.0000000	-1.7350000
62906.0000000	-1.7350000
62907.0000000	-1.7350000
62908.0000000	-1.7400000
62909.0000000	-1.7450000
62910.0000000	-1.7450000
62911.0000000	-1.7400000
62912.0000000	-1.7400000
62913.0000000	-1.7550000
62914.0000000	-1.7766666
62915.0000000	-1.7750000
62916.0000000	-1.7650000
62917.0000000	-1.7550000
62918.0000000	-1.7350000
62919.0000000	-1.7350000
62920.0000000	-1.7300000
62921.0000000	-1.7300000
62922.0000000	-1.7400000
62923.0000000	-1.7500000
62924.0000000	-1.7550000
62925.0000000	-1.7400000
62926.0000000	-1.7550000
62927.0000000	-1.7400000

62928.0000000	-1.7400000
62929.0000000	-1.7350000
62930.0000000	-1.7300000
62931.0000000	-1.7300000
62932.0000000	-1.7250000
62933.0000000	-1.7200000
62934.0000000	-1.7200000
62935.0000000	-1.7200000
62936.0000000	-1.7150000
62937.0000000	-1.7100000
62938.0000000	-1.7150000
62939.0000000	-1.7100000
62940.0000000	-1.7100000
62941.0000000	-1.7050000
62942.0000000	-1.7050000
62943.0000000	-1.7050000
62944.0000000	-1.6933333
62945.0000000	-1.6849999
62946.0000000	-1.6849999
62947.0000000	-1.6700000
62948.0000000	-1.6600000
62949.0000000	-1.6600000
62950.0000000	-1.6500000
62951.0000000	-1.6500000
62952.0000000	-1.6500000
62953.0000000	-1.6500000
62954.0000000	-1.6300000
62955.0000000	-1.6200000
62956.0000000	-1.6250000
62957.0000000	-1.6300000
62958.0000000	-1.6300000
62959.0000000	-1.6150000
62960.0000000	-1.6050000
62961.0000000	-1.5950000
62962.0000000	-1.5900000
62963.0000000	-1.5850000
62964.0000000	-1.5800000
62965.0000000	-1.5800000
62966.0000000	-1.5850000
62967.0000000	-1.5750000
62968.0000000	-1.5750000
62969.0000000	-1.5650001
62970.0000000	-1.5750000
62971.0000000	-1.5850000
62972.0000000	-1.5850000
62973.0000000	-1.5766667
62974.0000000	-1.5750000
62975.0000000	-1.5500000
62976.0000000	-1.5300000
62977.0000000	-1.5250000
62978.0000000	-1.5250000
62979.0000000	-1.5300000
62980.0000000	-1.5350000
62981.0000000	-1.5350000
62982.0000000	-1.5350000
62983.0000000	-1.5400000
62984.0000000	-1.5400000

62985.0000000	-1.5400000
62986.0000000	-1.5500000
62987.0000000	-1.5500000
62988.0000000	-1.5500000
62989.0000000	-1.5400000
62990.0000000	-1.5250000
62991.0000000	-1.5200000
62992.0000000	-1.5200000
62993.0000000	-1.5050000
62994.0000000	-1.5050000
62995.0000000	-1.5150000
62996.0000000	-1.5100000
62997.0000000	-1.5100000
62998.0000000	-1.4950000
62999.0000000	-1.4900000
63000.0000000	-1.4850000
63001.0000000	-1.4950000
63002.0000000	-1.4950000
63003.0000000	-1.4850000
63004.0000000	-1.4833332
63005.0000000	-1.5050000
63006.0000000	-1.4950000
63007.0000000	-1.4900000
63008.0000000	-1.4950000
63009.0000000	-1.5050000
63010.0000000	-1.5050000
63011.0000000	-1.4950000
63012.0000000	-1.4850000
63013.0000000	-1.5000000
63014.0000000	-1.5000000
63015.0000000	-1.4900000
63016.0000000	-1.4900000
63017.0000000	-1.4900000
63018.0000000	-1.4950000
63019.0000000	-1.4850000
63020.0000000	-1.4700000
63021.0000000	-1.4650000
63022.0000000	-1.4600000
63023.0000000	-1.4650000
63024.0000000	-1.4600000
63025.0000000	-1.4600000
63026.0000000	-1.4500000
63027.0000000	-1.4400001
63028.0000000	-1.4349999
63029.0000000	-1.4349999
63030.0000000	-1.4300001
63031.0000000	-1.4250000
63032.0000000	-1.4200000
63033.0000000	-1.4150000
63034.0000000	-1.4150000
63035.0000000	-1.4166666
63036.0000000	-1.4100000
63037.0000000	-1.4100000
63038.0000000	-1.4150000
63039.0000000	-1.4250000
63040.0000000	-1.4200000
63041.0000000	-1.4150000

63042.0000000	-1.4200000
63043.0000000	-1.4150000
63044.0000000	-1.4200000
63045.0000000	-1.4200000
63046.0000000	-1.4150000
63047.0000000	-1.4100000
63048.0000000	-1.4200000
63049.0000000	-1.4200000
63050.0000000	-1.4150000
63051.0000000	-1.4200000
63052.0000000	-1.4200000
63053.0000000	-1.4250000
63054.0000000	-1.4200000
63055.0000000	-1.4100000
63056.0000000	-1.4200000
63057.0000000	-1.4200000
63058.0000000	-1.4200000
63059.0000000	-1.4250000
63060.0000000	-1.4150000
63061.0000000	-1.4150000
63062.0000000	-1.4050000
63063.0000000	-1.4100000
63064.0000000	-1.4000000
63065.0000000	-1.4000000
63066.0000000	-1.4000000
63067.0000000	-1.3900000
63068.0000000	-1.3800000
63069.0000000	-1.3750000
63070.0000000	-1.3750000
63071.0000000	-1.3700000
63072.0000000	-1.3600000
63073.0000000	-1.3450000
63074.0000000	-1.3450000
63075.0000000	-1.3400000
63076.0000000	-1.3350000
63077.0000000	-1.3300000
63078.0000000	-1.3199999
63079.0000000	-1.3250000
63080.0000000	-1.3350000
63081.0000000	-1.3500000
63082.0000000	-1.3450000
63083.0000000	-1.3450000
63084.0000000	-1.3500000
63085.0000000	-1.3450000
63086.0000000	-1.3400000
63087.0000000	-1.3600000
63088.0000000	-1.3650000
63089.0000000	-1.3600000
63090.0000000	-1.3500000
63091.0000000	-1.3500000
63092.0000000	-1.3350000
63093.0000000	-1.3400000
63094.0000000	-1.3433334
63095.0000000	-1.3450000
63096.0000000	-1.3450000
63097.0000000	-1.3450000
63098.0000000	-1.3450000

63099.0000000	-1.3550000
63100.0000000	-1.3500000
63101.0000000	-1.3550000
63102.0000000	-1.3600000
63103.0000000	-1.3600000
63104.0000000	-1.3650000
63105.0000000	-1.3750000
63106.0000000	-1.3750000
63107.0000000	-1.3750000
63108.0000000	-1.3750000
63109.0000000	-1.3800000
63110.0000000	-1.3800000
63111.0000000	-1.3850000
63112.0000000	-1.3950000
63113.0000000	-1.3850000
63114.0000000	-1.3900000
63115.0000000	-1.3900000
63116.0000000	-1.3900000
63117.0000000	-1.3900000
63118.0000000	-1.3850000
63119.0000000	-1.3850000
63120.0000000	-1.3850000
63121.0000000	-1.3900000
63122.0000000	-1.3950000
63123.0000000	-1.3950000
63124.0000000	-1.3950000
63125.0000000	-1.3933333
63126.0000000	-1.4000000
63127.0000000	-1.4000000
63128.0000000	-1.3900000
63129.0000000	-1.4050000
63130.0000000	-1.4000000
63131.0000000	-1.4050000
63132.0000000	-1.4050000
63133.0000000	-1.4050000
63134.0000000	-1.4050000
63135.0000000	-1.4000000
63136.0000000	-1.4050000
63137.0000000	-1.4000000
63138.0000000	-1.4050000
63139.0000000	-1.4050000
63140.0000000	-1.4050000
63141.0000000	-1.4000000
63142.0000000	-1.4000000
63143.0000000	-1.4000000
63144.0000000	-1.4000000
63145.0000000	-1.4050000
63146.0000000	-1.4100000
63147.0000000	-1.4050000
63148.0000000	-1.4000000
63149.0000000	-1.4000000
63150.0000000	-1.3950000
63151.0000000	-1.3950000
63152.0000000	-1.3950000
63153.0000000	-1.3850000
63154.0000000	-1.3950000
63155.0000000	-1.3933333

63156.0000000	-1.3800000
63157.0000000	-1.3750000
63158.0000000	-1.3650000
63159.0000000	-1.3650000
63160.0000000	-1.3550000
63161.0000000	-1.3400000
63162.0000000	-1.3350000
63163.0000000	-1.3350000
63164.0000000	-1.3250000
63165.0000000	-1.3150001
63166.0000000	-1.3000000
63167.0000000	-1.2950000
63168.0000000	-1.2950000
63169.0000000	-1.3049999
63170.0000000	-1.3099999
63171.0000000	-1.3099999
63172.0000000	-1.3150001
63173.0000000	-1.3200001
63174.0000000	-1.3150001
63175.0000000	-1.3200001
63176.0000000	-1.3199999
63177.0000000	-1.3200001
63178.0000000	-1.3200001
63179.0000000	-1.3250000
63180.0000000	-1.3199999
63181.0000000	-1.3250000
63182.0000000	-1.3199999
63183.0000000	-1.3250000
63184.0000000	-1.3250000
63185.0000000	-1.3200001
63186.0000000	-1.3049999
63187.0000000	-1.3099999
63188.0000000	-1.3099999
63189.0000000	-1.3099999
63190.0000000	-1.3099999
63191.0000000	-1.3049999
63192.0000000	-1.3049999
63193.0000000	-1.3099999
63194.0000000	-1.3099999
63195.0000000	-1.3099999
63196.0000000	-1.3099999
63197.0000000	-1.3099999
63198.0000000	-1.3150001
63199.0000000	-1.3200001
63200.0000000	-1.3250000
63201.0000000	-1.3199999
63202.0000000	-1.3200001
63203.0000000	-1.3200001
63204.0000000	-1.3300000
63205.0000000	-1.3300000
63206.0000000	-1.3250000
63207.0000000	-1.3099999
63208.0000000	-1.3000000
63209.0000000	-1.2950000
63210.0000000	-1.2850000
63211.0000000	-1.2900000
63212.0000000	-1.2800000

63213.0000000	-1.2650000
63214.0000000	-1.2650000
63215.0000000	-1.2600000
63216.0000000	-1.2450000
63217.0000000	-1.2400000
63218.0000000	-1.2400000
63219.0000000	-1.2250000
63220.0000000	-1.2100000
63221.0000000	-1.2000000
63222.0000000	-1.2050000
63223.0000000	-1.2150000
63224.0000000	-1.2150000
63225.0000000	-1.2150000
63226.0000000	-1.2250000
63227.0000000	-1.2200000
63228.0000000	-1.2200000
63229.0000000	-1.2200000
63230.0000000	-1.2300000
63231.0000000	-1.2350000
63232.0000000	-1.2450000
63233.0000000	-1.2450000
63234.0000000	-1.2500000
63235.0000000	-1.2550000
63236.0000000	-1.2600000
63237.0000000	-1.2650000
63238.0000000	-1.2650000
63239.0000000	-1.2650000
63240.0000000	-1.2700000
63241.0000000	-1.2700000
63242.0000000	-1.2700000
63243.0000000	-1.2600000
63244.0000000	-1.2650000
63245.0000000	-1.2633333
63246.0000000	-1.2650000
63247.0000000	-1.2600000
63248.0000000	-1.2550000
63249.0000000	-1.2550000
63250.0000000	-1.2600000
63251.0000000	-1.2600000
63252.0000000	-1.2650000
63253.0000000	-1.2650000
63254.0000000	-1.2650000
63255.0000000	-1.2650000
63256.0000000	-1.2650000
63257.0000000	-1.2550000
63258.0000000	-1.2550000
63259.0000000	-1.2500000
63260.0000000	-1.2500000
63261.0000000	-1.2600000
63262.0000000	-1.2650000
63263.0000000	-1.2650000
63264.0000000	-1.2700000
63265.0000000	-1.2750000
63266.0000000	-1.2800000
63267.0000000	-1.2800000
63268.0000000	-1.2700000
63269.0000000	-1.2700000

63270.0000000	-1.2700000
63271.0000000	-1.2750000
63272.0000000	-1.2700000
63273.0000000	-1.2550000
63274.0000000	-1.2400000
63275.0000000	-1.2500000
63276.0000000	-1.2533333
63277.0000000	-1.2650000
63278.0000000	-1.2800000
63279.0000000	-1.2850000
63280.0000000	-1.2750000
63281.0000000	-1.2850000
63282.0000000	-1.2950000
63283.0000000	-1.3000000
63284.0000000	-1.3049999
63285.0000000	-1.3000000
63286.0000000	-1.3000000
63287.0000000	-1.3049999
63288.0000000	-1.3099999
63289.0000000	-1.3150001
63290.0000000	-1.3150001
63291.0000000	-1.3150001
63292.0000000	-1.3150001
63293.0000000	-1.3200001
63294.0000000	-1.3150001
63295.0000000	-1.3049999
63296.0000000	-1.2950000
63297.0000000	-1.2900000
63298.0000000	-1.2950000
63299.0000000	-1.2900000
63300.0000000	-1.2750000
63301.0000000	-1.2700000
63302.0000000	-1.2600000
63303.0000000	-1.2450000
63304.0000000	-1.2400000
63305.0000000	-1.2400000
63306.0000000	-1.2433333
63307.0000000	-1.2350000
63308.0000000	-1.2350000
63309.0000000	-1.2250000
63310.0000000	-1.2200000
63311.0000000	-1.2150000
63312.0000000	-1.2100000
63313.0000000	-1.2000000
63314.0000000	-1.1950001
63315.0000000	-1.1849999
63316.0000000	-1.1750000
63317.0000000	-1.1800001
63318.0000000	-1.1799999
63319.0000000	-1.1799999
63320.0000000	-1.1700000
63321.0000000	-1.1650000
63322.0000000	-1.1700000
63323.0000000	-1.1700000
63324.0000000	-1.1700000
63325.0000000	-1.1600000
63326.0000000	-1.1700000

63327.0000000	-1.1700000
63328.0000000	-1.1600000
63329.0000000	-1.1450000
63330.0000000	-1.1350000
63331.0000000	-1.1350000
63332.0000000	-1.1150000
63333.0000000	-1.1050000
63334.0000000	-1.1050000
63335.0000000	-1.1050000
63336.0000000	-1.1100000
63337.0000000	-1.1000000
63338.0000000	-1.1100000
63339.0000000	-1.1200000
63340.0000000	-1.1350000
63341.0000000	-1.1350000
63342.0000000	-1.1300000
63343.0000000	-1.1150000
63344.0000000	-1.1150000
63345.0000000	-1.1250000
63346.0000000	-1.1350000
63347.0000000	-1.1300000
63348.0000000	-1.1350000
63349.0000000	-1.1250000
63350.0000000	-1.1300000
63351.0000000	-1.1250000
63352.0000000	-1.1150000
63353.0000000	-1.1000000
63354.0000000	-1.0900000
63355.0000000	-1.0900000
63356.0000000	-1.0950000
63357.0000000	-1.0900000
63358.0000000	-1.0850000
63359.0000000	-1.0800000
63360.0000000	-1.0750000
63361.0000000	-1.0700001
63362.0000000	-1.0599999
63363.0000000	-1.0500000
63364.0000000	-1.0450000
63365.0000000	-1.0400000
63366.0000000	-1.0366666
63367.0000000	-1.0250000
63368.0000000	-1.0200000
63369.0000000	-1.0200000
63370.0000000	-1.0150000
63371.0000000	-1.0150000
63372.0000000	-1.0100000
63373.0000000	-1.0100000
63374.0000000	-1.0150000
63375.0000000	-1.0050000
63376.0000000	-0.9950000
63377.0000000	-0.9950000
63378.0000000	-0.9950000
63379.0000000	-0.9850000
63380.0000000	-0.9800000
63381.0000000	-0.9700000
63382.0000000	-0.9700000
63383.0000000	-0.9550000

63384.0000000	-0.9450000
63385.0000000	-0.9500000
63386.0000000	-0.9450000
63387.0000000	-0.9450000
63388.0000000	-0.9400000
63389.0000000	-0.9350000
63390.0000000	-0.9300000
63391.0000000	-0.9300000
63392.0000000	-0.9300000
63393.0000000	-0.9300000
63394.0000000	-0.9350000
63395.0000000	-0.9300000
63396.0000000	-0.9300000
63397.0000000	-0.9266667
63398.0000000	-0.9350000
63399.0000000	-0.9300000
63400.0000000	-0.9200000
63401.0000000	-0.9250000
63402.0000000	-0.9050000
63403.0000000	-0.8950000
63404.0000000	-0.8900000
63405.0000000	-0.8900000
63406.0000000	-0.9000000
63407.0000000	-0.9100000
63408.0000000	-0.9150000
63409.0000000	-0.9100000
63410.0000000	-0.9200000
63411.0000000	-0.9300000
63412.0000000	-0.9300000
63413.0000000	-0.9350000
63414.0000000	-0.9350000
63415.0000000	-0.9350000
63416.0000000	-0.9300000
63417.0000000	-0.9100000
63418.0000000	-0.9000000
63419.0000000	-0.9000000
63420.0000000	-0.8950000
63421.0000000	-0.8950000
63422.0000000	-0.8800000
63423.0000000	-0.8700000
63424.0000000	-0.8700000
63425.0000000	-0.8700000
63426.0000000	-0.8700000
63427.0000000	-0.8700001
63428.0000000	-0.8500000
63429.0000000	-0.8400000
63430.0000000	-0.8450000
63431.0000000	-0.8400000
63432.0000000	-0.8400000
63433.0000000	-0.8250000
63434.0000000	-0.8100000
63435.0000000	-0.8050000
63436.0000000	-0.7950000
63437.0000000	-0.7850000
63438.0000000	-0.7850000
63439.0000000	-0.7800000
63440.0000000	-0.7650000

63441.0000000	-0.7550000
63442.0000000	-0.7450000
63443.0000000	-0.7300000
63444.0000000	-0.7300000
63445.0000000	-0.7250000
63446.0000000	-0.7200000
63447.0000000	-0.7150000
63448.0000000	-0.7150000
63449.0000000	-0.7150000
63450.0000000	-0.7050000
63451.0000000	-0.6950000
63452.0000000	-0.7000000
63453.0000000	-0.7000000
63454.0000000	-0.7000000
63455.0000000	-0.7050000
63456.0000000	-0.7033333
63457.0000000	-0.7000000
63458.0000000	-0.7100000
63459.0000000	-0.7100000
63460.0000000	-0.7100000
63461.0000000	-0.7100000
63462.0000000	-0.7300000
63463.0000000	-0.7300000
63464.0000000	-0.7250000
63465.0000000	-0.7350000
63466.0000000	-0.7200000
63467.0000000	-0.7050000
63468.0000000	-0.7000000
63469.0000000	-0.6900000
63470.0000000	-0.6850000
63471.0000000	-0.6800000
63472.0000000	-0.6850000
63473.0000000	-0.6800000
63474.0000000	-0.6700000
63475.0000000	-0.6650000
63476.0000000	-0.6600000
63477.0000000	-0.6550000
63478.0000000	-0.6650000
63479.0000000	-0.6650000
63480.0000000	-0.6600000
63481.0000000	-0.6550000
63482.0000000	-0.6500000
63483.0000000	-0.6450000
63484.0000000	-0.6550000
63485.0000000	-0.6550000
63486.0000000	-0.6400000
63487.0000000	-0.6300000
63488.0000000	-0.6100000
63489.0000000	-0.6000000
63490.0000000	-0.6000000
63491.0000000	-0.5850000
63492.0000000	-0.5700000
63493.0000000	-0.5550000
63494.0000000	-0.5350000
63495.0000000	-0.5350000
63496.0000000	-0.5350000
63497.0000000	-0.5400000

63498.0000000	-0.5300000
63499.0000000	-0.5250000
63500.0000000	-0.5300000
63501.0000000	-0.5400000
63502.0000000	-0.5450000
63503.0000000	-0.5250000
63504.0000000	-0.5350000
63505.0000000	-0.5450000
63506.0000000	-0.5400000
63507.0000000	-0.5500000
63508.0000000	-0.5500000
63509.0000000	-0.5550000
63510.0000000	-0.5550000
63511.0000000	-0.5650000
63512.0000000	-0.5750000
63513.0000000	-0.5750000
63514.0000000	-0.5700000
63515.0000000	-0.5700000
63516.0000000	-0.5700000
63517.0000000	-0.5600000
63518.0000000	-0.5550000
63519.0000000	-0.5400000
63520.0000000	-0.5200000
63521.0000000	-0.5150000
63522.0000000	-0.5200000
63523.0000000	-0.5250000
63524.0000000	-0.5150000
63525.0000000	-0.5000000
63526.0000000	-0.5000000
63527.0000000	-0.4900000
63528.0000000	-0.4950000
63529.0000000	-0.5100000
63530.0000000	-0.5300000
63531.0000000	-0.5450000
63532.0000000	-0.5600000
63533.0000000	-0.5600000
63534.0000000	-0.5600000
63535.0000000	-0.5650000
63536.0000000	-0.5650000
63537.0000000	-0.5650000
63538.0000000	-0.5650000
63539.0000000	-0.5550000
63540.0000000	-0.5550000
63541.0000000	-0.5550000
63542.0000000	-0.5550000
63543.0000000	-0.5550000
63544.0000000	-0.5500000
63545.0000000	-0.5500000
63546.0000000	-0.5650000
63547.0000000	-0.5833333
63548.0000000	-0.6000000
63549.0000000	-0.6000000
63550.0000000	-0.6050000
63551.0000000	-0.5900000
63552.0000000	-0.5850000
63553.0000000	-0.5850000
63554.0000000	-0.5850000

63555.0000000	-0.5950000
63556.0000000	-0.6000000
63557.0000000	-0.6000000
63558.0000000	-0.6100000
63559.0000000	-0.6250000
63560.0000000	-0.6300000
63561.0000000	-0.6300000
63562.0000000	-0.6200000
63563.0000000	-0.6300000
63564.0000000	-0.6300000
63565.0000000	-0.6250000
63566.0000000	-0.6100000
63567.0000000	-0.6150000
63568.0000000	-0.6100000
63569.0000000	-0.6050000
63570.0000000	-0.6000000
63571.0000000	-0.5950000
63572.0000000	-0.5800000
63573.0000000	-0.5750000
63574.0000000	-0.5650000
63575.0000000	-0.5600000
63576.0000000	-0.5350000
63577.0000000	-0.5233333
63578.0000000	-0.5100000
63579.0000000	-0.5100000
63580.0000000	-0.5100000
63581.0000000	-0.5150000
63582.0000000	-0.5250000
63583.0000000	-0.5350000
63584.0000000	-0.5350000
63585.0000000	-0.5550000
63586.0000000	-0.5650000
63587.0000000	-0.5700000
63588.0000000	-0.5800000
63589.0000000	-0.5750000
63590.0000000	-0.5900000
63591.0000000	-0.6000000
63592.0000000	-0.6100000
63593.0000000	-0.6200000
63594.0000000	-0.6250000
63595.0000000	-0.6300000
63596.0000000	-0.6350000
63597.0000000	-0.6500000
63598.0000000	-0.6550000
63599.0000000	-0.6650000
63600.0000000	-0.6800000
63601.0000000	-0.6750000
63602.0000000	-0.6800000
63603.0000000	-0.6900000
63604.0000000	-0.7000000
63605.0000000	-0.7100000
63606.0000000	-0.7050000
63607.0000000	-0.7100000
63608.0000000	-0.7100000
63609.0000000	-0.7100000
63610.0000000	-0.7150000
63611.0000000	-0.7150000

63612.0000000	-0.7200000
63613.0000000	-0.7200000
63614.0000000	-0.7150000
63615.0000000	-0.7200000
63616.0000000	-0.7200000
63617.0000000	-0.7200000
63618.0000000	-0.7100000
63619.0000000	-0.6950000
63620.0000000	-0.6900000
63621.0000000	-0.6900000
63622.0000000	-0.7000000
63623.0000000	-0.6900000
63624.0000000	-0.6900000
63625.0000000	-0.6900000
63626.0000000	-0.6900000
63627.0000000	-0.6900000
63628.0000000	-0.6800000
63629.0000000	-0.6700000
63630.0000000	-0.6600000
63631.0000000	-0.6600000
63632.0000000	-0.6550000
63633.0000000	-0.6550000
63634.0000000	-0.6650000
63635.0000000	-0.6700000
63636.0000000	-0.6750000
63637.0000000	-0.6850000
63638.0000000	-0.6966667
63639.0000000	-0.7050000
63640.0000000	-0.7200000
63641.0000000	-0.7350000
63642.0000000	-0.7500000
63643.0000000	-0.7500000
63644.0000000	-0.7600000
63645.0000000	-0.7750000
63646.0000000	-0.7700000
63647.0000000	-0.7650000
63648.0000000	-0.7700000
63649.0000000	-0.7650000
63650.0000000	-0.7700000
63651.0000000	-0.7750000
63652.0000000	-0.7750000
63653.0000000	-0.7650000
63654.0000000	-0.7650000
63655.0000000	-0.7700000
63656.0000000	-0.7650000
63657.0000000	-0.7600000
63658.0000000	-0.7550000
63659.0000000	-0.7600000
63660.0000000	-0.7550000
63661.0000000	-0.7650000
63662.0000000	-0.7700000
63663.0000000	-0.7700000
63664.0000000	-0.7700000
63665.0000000	-0.7700000
63666.0000000	-0.7800000
63667.0000000	-0.7750000
63668.0000000	-0.7733333

63669.0000000	-0.7850000
63670.0000000	-0.7900000
63671.0000000	-0.8050000
63672.0000000	-0.8150000
63673.0000000	-0.8150000
63674.0000000	-0.8250000
63675.0000000	-0.8400000
63676.0000000	-0.8500000
63677.0000000	-0.8600000
63678.0000000	-0.8700000
63679.0000000	-0.8750000
63680.0000000	-0.8900000
63681.0000000	-0.9050000
63682.0000000	-0.9200000
63683.0000000	-0.9300000
63684.0000000	-0.9500000
63685.0000000	-0.9550000
63686.0000000	-0.9600000
63687.0000000	-0.9500000
63688.0000000	-0.9550000
63689.0000000	-0.9650000
63690.0000000	-0.9750000
63691.0000000	-0.9850000
63692.0000000	-0.9900000
63693.0000000	-1.0000000
63694.0000000	-1.0050000
63695.0000000	-0.9950000
63696.0000000	-0.9950000
63697.0000000	-0.9900000
63698.0000000	-0.9866667
63699.0000000	-0.9750000
63700.0000000	-0.9800000
63701.0000000	-0.9800000
63702.0000000	-0.9850000
63703.0000000	-0.9850000
63704.0000000	-0.9750000
63705.0000000	-0.9750000
63706.0000000	-0.9800000
63707.0000000	-0.9800000
63708.0000000	-0.9850000
63709.0000000	-0.9800000
63710.0000000	-0.9750000
63711.0000000	-0.9800000
63712.0000000	-0.9650000
63713.0000000	-0.9500000
63714.0000000	-0.9350000
63715.0000000	-0.9200000
63716.0000000	-0.8850000
63717.0000000	-0.8800000
63718.0000000	-0.8550000
63719.0000000	-0.8550000
63720.0000000	-0.8550000
63721.0000000	-0.8550000
63722.0000000	-0.8500000
63723.0000000	-0.8500000
63724.0000000	-0.8550000
63725.0000000	-0.8550000

63726.0000000	-0.8450000
63727.0000000	-0.8550000
63728.0000000	-0.8500000
63729.0000000	-0.8600000
63730.0000000	-0.8650000
63731.0000000	-0.8650000
63732.0000000	-0.8700000
63733.0000000	-0.8700000
63734.0000000	-0.8750000
63735.0000000	-0.8800000
63736.0000000	-0.8900000
63737.0000000	-0.9000000
63738.0000000	-0.9100000
63739.0000000	-0.9250000
63740.0000000	-0.9250000
63741.0000000	-0.9300000
63742.0000000	-0.9500000
63743.0000000	-0.9550000
63744.0000000	-0.9500000
63745.0000000	-0.9550000
63746.0000000	-0.9500000
63747.0000000	-0.9450000
63748.0000000	-0.9250000
63749.0000000	-0.9050000
63750.0000000	-0.9000000
63751.0000000	-0.8950000
63752.0000000	-0.8900000
63753.0000000	-0.8950000
63754.0000000	-0.8850000
63755.0000000	-0.9000000
63756.0000000	-0.9050000
63757.0000000	-0.9050000
63758.0000000	-0.9000000
63759.0000000	-0.8966667
63760.0000000	-0.8950000
63761.0000000	-0.8850000
63762.0000000	-0.8800000
63763.0000000	-0.8700000
63764.0000000	-0.8600000
63765.0000000	-0.8600000
63766.0000000	-0.8600000
63767.0000000	-0.8550000
63768.0000000	-0.8600000
63769.0000000	-0.8600000
63770.0000000	-0.8700000
63771.0000000	-0.8650000
63772.0000000	-0.8600000
63773.0000000	-0.8650000
63774.0000000	-0.8600000
63775.0000000	-0.8650000
63776.0000000	-0.8650000
63777.0000000	-0.8400000
63778.0000000	-0.8350000
63779.0000000	-0.8350000
63780.0000000	-0.8350000
63781.0000000	-0.8500000
63782.0000000	-0.8500000

63783.0000000	-0.8500000
63784.0000000	-0.8550000
63785.0000000	-0.8650000
63786.0000000	-0.8650000
63787.0000000	-0.8600000
63788.0000000	-0.8500000
63789.0000000	-0.8566667
63790.0000000	-0.8600000
63791.0000000	-0.8650000
63792.0000000	-0.8550000
63793.0000000	-0.8550000
63794.0000000	-0.8450000
63795.0000000	-0.8550000
63796.0000000	-0.8550000
63797.0000000	-0.8550000
63798.0000000	-0.8600000
63799.0000000	-0.8600000
63800.0000000	-0.8600000
63801.0000000	-0.8600000
63802.0000000	-0.8500000
63803.0000000	-0.8450000
63804.0000000	-0.8450000
63805.0000000	-0.8350000
63806.0000000	-0.8500000
63807.0000000	-0.8550000
63808.0000000	-0.8650000
63809.0000000	-0.8650000
63810.0000000	-0.8600000
63811.0000000	-0.8600000
63812.0000000	-0.8600000
63813.0000000	-0.8650000
63814.0000000	-0.8650000
63815.0000000	-0.8650000
63816.0000000	-0.8750000
63817.0000000	-0.8800000
63818.0000000	-0.8750000
63819.0000000	-0.8600000
63820.0000000	-0.8550000
63821.0000000	-0.8500000
63822.0000000	-0.8350000
63823.0000000	-0.8350000
63824.0000000	-0.8350000
63825.0000000	-0.8250000
63826.0000000	-0.8150000
63827.0000000	-0.8050000
63828.0000000	-0.8000000
63829.0000000	-0.7800000
63830.0000000	-0.7700000
63831.0000000	-0.7700000
63832.0000000	-0.7600000
63833.0000000	-0.7600000
63834.0000000	-0.7550000
63835.0000000	-0.7500000
63836.0000000	-0.7550000
63837.0000000	-0.7550000
63838.0000000	-0.7550000
63839.0000000	-0.7500000

63840.0000000	-0.7550000
63841.0000000	-0.7550000
63842.0000000	-0.7500000
63843.0000000	-0.7550000
63844.0000000	-0.7600000
63845.0000000	-0.7600000
63846.0000000	-0.7700000
63847.0000000	-0.7650000
63848.0000000	-0.7450000
63849.0000000	-0.7366667
63850.0000000	-0.7400000
63851.0000000	-0.7550000
63852.0000000	-0.7650000
63853.0000000	-0.7800000
63854.0000000	-0.7950000
63855.0000000	-0.8050000
63856.0000000	-0.7950000
63857.0000000	-0.8000000
63858.0000000	-0.8000000
63859.0000000	-0.8000000
63860.0000000	-0.8000000
63861.0000000	-0.8000000
63862.0000000	-0.8000000
63863.0000000	-0.8000000
63864.0000000	-0.8000000
63865.0000000	-0.8000000
63866.0000000	-0.8000000
63867.0000000	-0.7950000
63868.0000000	-0.7950000
63869.0000000	-0.7900000
63870.0000000	-0.7750000
63871.0000000	-0.7650000
63872.0000000	-0.7400000
63873.0000000	-0.7350000
63874.0000000	-0.7300000
63875.0000000	-0.7250000
63876.0000000	-0.7200000
63877.0000000	-0.7000000
63878.0000000	-0.7100000
63879.0000000	-0.7033334
63880.0000000	-0.6950000
63881.0000000	-0.7050000
63882.0000000	-0.7050000
63883.0000000	-0.7050000
63884.0000000	-0.7000000
63885.0000000	-0.7050000
63886.0000000	-0.7100000
63887.0000000	-0.7150000
63888.0000000	-0.7250000
63889.0000000	-0.7300000
63890.0000000	-0.7300000
63891.0000000	-0.7450000
63892.0000000	-0.7500000
63893.0000000	-0.7550000
63894.0000000	-0.7600000
63895.0000000	-0.7650000
63896.0000000	-0.7650000

63897.0000000	-0.7650000
63898.0000000	-0.7750000
63899.0000000	-0.7750000
63900.0000000	-0.7750000
63901.0000000	-0.7800000
63902.0000000	-0.7800000
63903.0000000	-0.7800000
63904.0000000	-0.7850000
63905.0000000	-0.7800000
63906.0000000	-0.7800000
63907.0000000	-0.7700000
63908.0000000	-0.7750000
63909.0000000	-0.7750000
63910.0000000	-0.7633333
63911.0000000	-0.7650000
63912.0000000	-0.7500000
63913.0000000	-0.7500000
63914.0000000	-0.7450000
63915.0000000	-0.7500000
63916.0000000	-0.7550000
63917.0000000	-0.7600000
63918.0000000	-0.7700000
63919.0000000	-0.7650000
63920.0000000	-0.7750000
63921.0000000	-0.7950000
63922.0000000	-0.8000000
63923.0000000	-0.8000000
63924.0000000	-0.8050000
63925.0000000	-0.8150000
63926.0000000	-0.8150000
63927.0000000	-0.8150000
63928.0000000	-0.8150000
63929.0000000	-0.8150000
63930.0000000	-0.8200000
63931.0000000	-0.8200000
63932.0000000	-0.8200000
63933.0000000	-0.8100000
63934.0000000	-0.7900000
63935.0000000	-0.7750000
63936.0000000	-0.7650000
63937.0000000	-0.7650000
63938.0000000	-0.7550000
63939.0000000	-0.7500000
63940.0000000	-0.7100000
63941.0000000	-0.6900000
63942.0000000	-0.6800000
63943.0000000	-0.6650000
63944.0000000	-0.6650000
63945.0000000	-0.6550000
63946.0000000	-0.6600000
63947.0000000	-0.6600000
63948.0000000	-0.6600000
63949.0000000	-0.6650000
63950.0000000	-0.6700000
63951.0000000	-0.6550000
63952.0000000	-0.6500000
63953.0000000	-0.6450000

63954.0000000	-0.6500000
63955.0000000	-0.6550000
63956.0000000	-0.6550000
63957.0000000	-0.6500000
63958.0000000	-0.6500000
63959.0000000	-0.6450000
63960.0000000	-0.6400000
63961.0000000	-0.6450000
63962.0000000	-0.6500000
63963.0000000	-0.6400000
63964.0000000	-0.6250000
63965.0000000	-0.6050000
63966.0000000	-0.5900000
63967.0000000	-0.5850000
63968.0000000	-0.5850000
63969.0000000	-0.5866666
63970.0000000	-0.5750000
63971.0000000	-0.5850000
63972.0000000	-0.5800000
63973.0000000	-0.5800000
63974.0000000	-0.5850000
63975.0000000	-0.5750000
63976.0000000	-0.5850000
63977.0000000	-0.5900000
63978.0000000	-0.5950000
63979.0000000	-0.6000000
63980.0000000	-0.6150000
63981.0000000	-0.6250000
63982.0000000	-0.6300000
63983.0000000	-0.6400000
63984.0000000	-0.6350000
63985.0000000	-0.6350000
63986.0000000	-0.6350000
63987.0000000	-0.6200000
63988.0000000	-0.6250000
63989.0000000	-0.6350000
63990.0000000	-0.6400000
63991.0000000	-0.6600000
63992.0000000	-0.6700000
63993.0000000	-0.6800000
63994.0000000	-0.6800000
63995.0000000	-0.6700000
63996.0000000	-0.6800000
63997.0000000	-0.6800000
63998.0000000	-0.6850000
63999.0000000	-0.6850000
64000.0000000	-0.6900000
64001.0000000	-0.6800000
64002.0000000	-0.6750000
64003.0000000	-0.6800000
64004.0000000	-0.6900000
64005.0000000	-0.6900000
64006.0000000	-0.6900000
64007.0000000	-0.7000000
64008.0000000	-0.6900000
64009.0000000	-0.6850000
64010.0000000	-0.6950000

64011.0000000	-0.7050000
64012.0000000	-0.7100000
64013.0000000	-0.7250000
64014.0000000	-0.7100000
64015.0000000	-0.7050000
64016.0000000	-0.7150000
64017.0000000	-0.7100000
64018.0000000	-0.7200000
64019.0000000	-0.7250000
64020.0000000	-0.7300000
64021.0000000	-0.7350000
64022.0000000	-0.7450000
64023.0000000	-0.7450000
64024.0000000	-0.7600000
64025.0000000	-0.7600000
64026.0000000	-0.7600000
64027.0000000	-0.7650000
64028.0000000	-0.7800000
64029.0000000	-0.7900000
64030.0000000	-0.7950000
64031.0000000	-0.8066667
64032.0000000	-0.8200000
64033.0000000	-0.8250000
64034.0000000	-0.8400000
64035.0000000	-0.8450000
64036.0000000	-0.8500000
64037.0000000	-0.8550000
64038.0000000	-0.8600000
64039.0000000	-0.8700000
64040.0000000	-0.8850000
64041.0000000	-0.8950000
64042.0000000	-0.9100000
64043.0000000	-0.9250000
64044.0000000	-0.9300000
64045.0000000	-0.9250000
64046.0000000	-0.9300000
64047.0000000	-0.9350000
64048.0000000	-0.9500000
64049.0000000	-0.9600000
64050.0000000	-0.9600000
64051.0000000	-0.9650000
64052.0000000	-0.9700000
64053.0000000	-0.9850000
64054.0000000	-0.9950000
64055.0000000	-1.0050000
64056.0000000	-1.0000000
64057.0000000	-1.0000000
64058.0000000	-0.9950000
64059.0000000	-1.0050000
64060.0000000	-1.0166667
64061.0000000	-1.0200000
64062.0000000	-1.0250000
64063.0000000	-1.0400000
64064.0000000	-1.0549999
64065.0000000	-1.0549999
64066.0000000	-1.0400000
64067.0000000	-1.0300000

64068.0000000	-1.0350000
64069.0000000	-1.0450000
64070.0000000	-1.0450000
64071.0000000	-1.0400000
64072.0000000	-1.0500000
64073.0000000	-1.0450000
64074.0000000	-1.0500000
64075.0000000	-1.0599999
64076.0000000	-1.0650001
64077.0000000	-1.0549999
64078.0000000	-1.0599999
64079.0000000	-1.0650001
64080.0000000	-1.0550001
64081.0000000	-1.0650001
64082.0000000	-1.0650001
64083.0000000	-1.0750000
64084.0000000	-1.0800000
64085.0000000	-1.0850000
64086.0000000	-1.0800000
64087.0000000	-1.0850000
64088.0000000	-1.0850000
64089.0000000	-1.0850000
64090.0000000	-1.0866667
64091.0000000	-1.0950000
64092.0000000	-1.0950000
64093.0000000	-1.0950000
64094.0000000	-1.0800000
64095.0000000	-1.0800000
64096.0000000	-1.0850000
64097.0000000	-1.0900000
64098.0000000	-1.0850000
64099.0000000	-1.0800000
64100.0000000	-1.0850000
64101.0000000	-1.0850000
64102.0000000	-1.0750000
64103.0000000	-1.0699999
64104.0000000	-1.0700001
64105.0000000	-1.0750000
64106.0000000	-1.0800000
64107.0000000	-1.0800000
64108.0000000	-1.0850000
64109.0000000	-1.0900000
64110.0000000	-1.1000000
64111.0000000	-1.1150000
64112.0000000	-1.1250000
64113.0000000	-1.1300000
64114.0000000	-1.1350000
64115.0000000	-1.1550000
64116.0000000	-1.1750000
64117.0000000	-1.1800001
64118.0000000	-1.1900001
64119.0000000	-1.2150000
64120.0000000	-1.2300000
64121.0000000	-1.2433333
64122.0000000	-1.2500000
64123.0000000	-1.2550000
64124.0000000	-1.2600000

64125.0000000	-1.2750000
64126.0000000	-1.2850000
64127.0000000	-1.2850000
64128.0000000	-1.2850000
64129.0000000	-1.2850000
64130.0000000	-1.2850000
64131.0000000	-1.2850000
64132.0000000	-1.2950000
64133.0000000	-1.2850000
64134.0000000	-1.2900000
64135.0000000	-1.2950000
64136.0000000	-1.3049999
64137.0000000	-1.3150001
64138.0000000	-1.3099999
64139.0000000	-1.3200001
64140.0000000	-1.3350000
64141.0000000	-1.3500000
64142.0000000	-1.3350000
64143.0000000	-1.3300000
64144.0000000	-1.3300000
64145.0000000	-1.3350000
64146.0000000	-1.3350000
64147.0000000	-1.3450000
64148.0000000	-1.3500000
64149.0000000	-1.3500000
64150.0000000	-1.3550000
64151.0000000	-1.3666667
64152.0000000	-1.3800000
64153.0000000	-1.3800000
64154.0000000	-1.3800000
64155.0000000	-1.3850000
64156.0000000	-1.3850000
64157.0000000	-1.3900000
64158.0000000	-1.3950000
64159.0000000	-1.3950000
64160.0000000	-1.3900000
64161.0000000	-1.3900000
64162.0000000	-1.3900000
64163.0000000	-1.4000000
64164.0000000	-1.3950000
64165.0000000	-1.3900000
64166.0000000	-1.3800000
64167.0000000	-1.3700000
64168.0000000	-1.3700000
64169.0000000	-1.3650000
64170.0000000	-1.3550000
64171.0000000	-1.3400000
64172.0000000	-1.3250000
64173.0000000	-1.3199999
64174.0000000	-1.3150001
64175.0000000	-1.3049999
64176.0000000	-1.2950000
64177.0000000	-1.2850000
64178.0000000	-1.2850000
64179.0000000	-1.2950000
64180.0000000	-1.3050001
64181.0000000	-1.3066667

64182.0000000	-1.3099999
64183.0000000	-1.3049999
64184.0000000	-1.3050001
64185.0000000	-1.3200001
64186.0000000	-1.3199999
64187.0000000	-1.3150001
64188.0000000	-1.3099999
64189.0000000	-1.3250000
64190.0000000	-1.3200001
64191.0000000	-1.3200001
64192.0000000	-1.3200001
64193.0000000	-1.3300000
64194.0000000	-1.3300000
64195.0000000	-1.3300000
64196.0000000	-1.3350000
64197.0000000	-1.3199999
64198.0000000	-1.3150001
64199.0000000	-1.3099999
64200.0000000	-1.3000000
64201.0000000	-1.3049999
64202.0000000	-1.2950000
64203.0000000	-1.2900000
64204.0000000	-1.3000000
64205.0000000	-1.2950000
64206.0000000	-1.3000000
64207.0000000	-1.3049999
64208.0000000	-1.3199999
64209.0000000	-1.3200001
64210.0000000	-1.3199999
64211.0000000	-1.3266667
64212.0000000	-1.3300000
64213.0000000	-1.3400000
64214.0000000	-1.3500000
64215.0000000	-1.3600000
64216.0000000	-1.3600000
64217.0000000	-1.3550000
64218.0000000	-1.3350000
64219.0000000	-1.3550000
64220.0000000	-1.3550000
64221.0000000	-1.3750000
64222.0000000	-1.3800000
64223.0000000	-1.3750000
64224.0000000	-1.3850000
64225.0000000	-1.3950000
64226.0000000	-1.4050000
64227.0000000	-1.4299999
64228.0000000	-1.4300001
64229.0000000	-1.4250000
64230.0000000	-1.4250000
64231.0000000	-1.4500000
64232.0000000	-1.4550000
64233.0000000	-1.4600000
64234.0000000	-1.4600000
64235.0000000	-1.4550000
64236.0000000	-1.4650000
64237.0000000	-1.4550000
64238.0000000	-1.4449999

64239.0000000	-1.4550000
64240.0000000	-1.4550000
64241.0000000	-1.4633335
64242.0000000	-1.4750000
64243.0000000	-1.4700000
64244.0000000	-1.4600000
64245.0000000	-1.4700000
64246.0000000	-1.4800000
64247.0000000	-1.4800000
64248.0000000	-1.4850000
64249.0000000	-1.4850000
64250.0000000	-1.4650000
64251.0000000	-1.4800000
64252.0000000	-1.4950000
64253.0000000	-1.5000000
64254.0000000	-1.5050000
64255.0000000	-1.5100000
64256.0000000	-1.5150000
64257.0000000	-1.5050000
64258.0000000	-1.5200000
64259.0000000	-1.5200000
64260.0000000	-1.5100000
64261.0000000	-1.5100000
64262.0000000	-1.5100000
64263.0000000	-1.5200000
64264.0000000	-1.5350000
64265.0000000	-1.5450000
64266.0000000	-1.5599999
64267.0000000	-1.5650001
64268.0000000	-1.5650001
64269.0000000	-1.5800000
64270.0000000	-1.5850000
64271.0000000	-1.5900000
64272.0000000	-1.6100000
64273.0000000	-1.6150000
64274.0000000	-1.6350000
64275.0000000	-1.6550000
64276.0000000	-1.6700000
64277.0000000	-1.6750000
64278.0000000	-1.6849999
64279.0000000	-1.6849999
64280.0000000	-1.6950001
64281.0000000	-1.6950001
64282.0000000	-1.6900001
64283.0000000	-1.7000000
64284.0000000	-1.7050000
64285.0000000	-1.7050000
64286.0000000	-1.7050000
64287.0000000	-1.7100000
64288.0000000	-1.7150000
64289.0000000	-1.7200000
64290.0000000	-1.7200000
64291.0000000	-1.7400000
64292.0000000	-1.7500000
64293.0000000	-1.7500000
64294.0000000	-1.7600000
64295.0000000	-1.7700000

64296.0000000	-1.7800000
64297.0000000	-1.7700000
64298.0000000	-1.7650000
64299.0000000	-1.7550000
64300.0000000	-1.7700000
64301.0000000	-1.7650000
64302.0000000	-1.7566667
64303.0000000	-1.7600000
64304.0000000	-1.7500000
64305.0000000	-1.7550000
64306.0000000	-1.7650000
64307.0000000	-1.7700000
64308.0000000	-1.7800000
64309.0000000	-1.7950000
64310.0000000	-1.8000000
64311.0000000	-1.7950000
64312.0000000	-1.8099999
64313.0000000	-1.8300000
64314.0000000	-1.8250000
64315.0000000	-1.8300000
64316.0000000	-1.8400000
64317.0000000	-1.8450000
64318.0000000	-1.8450000
64319.0000000	-1.8450000
64320.0000000	-1.8550000
64321.0000000	-1.8550000
64322.0000000	-1.8400000
64323.0000000	-1.8400000
64324.0000000	-1.8300000
64325.0000000	-1.8199999
64326.0000000	-1.8200001
64327.0000000	-1.8050001
64328.0000000	-1.8099999
64329.0000000	-1.8099999
64330.0000000	-1.8300000
64331.0000000	-1.8350000
64332.0000000	-1.8466667
64333.0000000	-1.8700000
64334.0000000	-1.8800000
64335.0000000	-1.8800000
64336.0000000	-1.8750000
64337.0000000	-1.8650000
64338.0000000	-1.8700000
64339.0000000	-1.8750000
64340.0000000	-1.8700000
64341.0000000	-1.8800000
64342.0000000	-1.8950000
64343.0000000	-1.9000000
64344.0000000	-1.9100000
64345.0000000	-1.9200000
64346.0000000	-1.9349999
64347.0000000	-1.9500000
64348.0000000	-1.9550000
64349.0000000	-1.9700000
64350.0000000	-1.9800000
64351.0000000	-1.9950000
64352.0000000	-2.0050001

64353.0000000	-2.0149999
64354.0000000	-2.0149999
64355.0000000	-2.0149999
64356.0000000	-2.0349998
64357.0000000	-2.0400000
64358.0000000	-2.0599999
64359.0000000	-2.0650001
64360.0000000	-2.0850000
64361.0000000	-2.1050000
64362.0000000	-2.1266668
64363.0000000	-2.1450000
64364.0000000	-2.1550002
64365.0000000	-2.1450000
64366.0000000	-2.1400001
64367.0000000	-2.1450000
64368.0000000	-2.1550002
64369.0000000	-2.1550002
64370.0000000	-2.1600001
64371.0000000	-2.1750002
64372.0000000	-2.1800001
64373.0000000	-2.2000000
64374.0000000	-2.2100000
64375.0000000	-2.2200000
64376.0000000	-2.2350001
64377.0000000	-2.2600000
64378.0000000	-2.2649999
64379.0000000	-2.2750001
64380.0000000	-2.3049998
64381.0000000	-2.3150001
64382.0000000	-2.3299999
64383.0000000	-2.3249998
64384.0000000	-2.3399999
64385.0000000	-2.3550000
64386.0000000	-2.3550000
64387.0000000	-2.3699999
64388.0000000	-2.3750000
64389.0000000	-2.3850002
64390.0000000	-2.3950000
64391.0000000	-2.3950000
64392.0000000	-2.4100001
64393.0000000	-2.4233334
64394.0000000	-2.4349999
64395.0000000	-2.4400001
64396.0000000	-2.4500000
64397.0000000	-2.4600000
64398.0000000	-2.4700000
64399.0000000	-2.4800000
64400.0000000	-2.4800000
64401.0000000	-2.4900000
64402.0000000	-2.5050001
64403.0000000	-2.5050001
64404.0000000	-2.5100000
64405.0000000	-2.5250001
64406.0000000	-2.5349998
64407.0000000	-2.5450001
64408.0000000	-2.5549998
64409.0000000	-2.5599999

64410.0000000	-2.5749998
64411.0000000	-2.5850000
64412.0000000	-2.5999999
64413.0000000	-2.6099999
64414.0000000	-2.6149998
64415.0000000	-2.6300001
64416.0000000	-2.6350002
64417.0000000	-2.6450000
64418.0000000	-2.6550002
64419.0000000	-2.6750002
64420.0000000	-2.6849999
64421.0000000	-2.7000000
64422.0000000	-2.7049999
64423.0000000	-2.7266667
64424.0000000	-2.7300000
64425.0000000	-2.7400000
64426.0000000	-2.7500000
64427.0000000	-2.7449999
64428.0000000	-2.7600000
64429.0000000	-2.7649999
64430.0000000	-2.7800000
64431.0000000	-2.7849998
64432.0000000	-2.7900000
64433.0000000	-2.7900000
64434.0000000	-2.7950001
64435.0000000	-2.8099999
64436.0000000	-2.8150001
64437.0000000	-2.8099999
64438.0000000	-2.8000000
64439.0000000	-2.7900000
64440.0000000	-2.7750001
64441.0000000	-2.7550001
64442.0000000	-2.7700000
64443.0000000	-2.7750001
64444.0000000	-2.7750001
64445.0000000	-2.7849998
64446.0000000	-2.7950001
64447.0000000	-2.8150001
64448.0000000	-2.8350000
64449.0000000	-2.8550000
64450.0000000	-2.8699999
64451.0000000	-2.8900001
64452.0000000	-2.9100001
64453.0000000	-2.9399998
64454.0000000	-2.9600000
64455.0000000	-2.9749999
64456.0000000	-2.9900000
64457.0000000	-3.0050001
64458.0000000	-2.9949999
64459.0000000	-3.0100000
64460.0000000	-3.0250001
64461.0000000	-3.0450001
64462.0000000	-3.0650001
64463.0000000	-3.0749998
64464.0000000	-3.0899999
64465.0000000	-3.0850000
64466.0000000	-3.0949998

64467.0000000	-3.1050000
64468.0000000	-3.1050000
64469.0000000	-3.1099999
64470.0000000	-3.1149998
64471.0000000	-3.1149998
64472.0000000	-3.1199999
64473.0000000	-3.1250000
64474.0000000	-3.1350002
64475.0000000	-3.1450000
64476.0000000	-3.1550002
64477.0000000	-3.1550002
64478.0000000	-3.1600001
64479.0000000	-3.1550002
64480.0000000	-3.1450000
64481.0000000	-3.1450000
64482.0000000	-3.1400001
64483.0000000	-3.1366665
64484.0000000	-3.1600001
64485.0000000	-3.1600001
64486.0000000	-3.1650000
64487.0000000	-3.1700001
64488.0000000	-3.1750002
64489.0000000	-3.1750002
64490.0000000	-3.1700001
64491.0000000	-3.1650000
64492.0000000	-3.1650000
64493.0000000	-3.1500001
64494.0000000	-3.1350000
64495.0000000	-3.0899999
64496.0000000	-3.0850000
64497.0000000	-3.0999999
64498.0000000	-3.1050000
64499.0000000	-3.0949998
64500.0000000	-3.1050000
64501.0000000	-3.1149998
64502.0000000	-3.1350002
64503.0000000	-3.1500001
64504.0000000	-3.1750002
64505.0000000	-3.1900001
64506.0000000	-3.2000000
64507.0000000	-3.2150002
64508.0000000	-3.2100000
64509.0000000	-3.2200000
64510.0000000	-3.2200000
64511.0000000	-3.2150002
64512.0000000	-3.2249999
64513.0000000	-3.2366667
64514.0000000	-3.2350001
64515.0000000	-3.2249999
64516.0000000	-3.2249999
64517.0000000	-3.2350001
64518.0000000	-3.2449999
64519.0000000	-3.2550001
64520.0000000	-3.2600000
64521.0000000	-3.2649999
64522.0000000	-3.2750001
64523.0000000	-3.2800000

64524.0000000	-3.2649999
64525.0000000	-3.2550001
64526.0000000	-3.2750001
64527.0000000	-3.2849998
64528.0000000	-3.2950001
64529.0000000	-3.3000000
64530.0000000	-3.3099999
64531.0000000	-3.3099999
64532.0000000	-3.3199999
64533.0000000	-3.3350000
64534.0000000	-3.3399999
64535.0000000	-3.3249998
64536.0000000	-3.3299999
64537.0000000	-3.3299999
64538.0000000	-3.3299999
64539.0000000	-3.3350000
64540.0000000	-3.3299999
64541.0000000	-3.3350000
64542.0000000	-3.3399999
64543.0000000	-3.3649998
64544.0000000	-3.3900001
64545.0000000	-3.4000001
64546.0000000	-3.4250002
64547.0000000	-3.4300001
64548.0000000	-3.4400001
64549.0000000	-3.4600000
64550.0000000	-3.4800000
64551.0000000	-3.4900000
64552.0000000	-3.4949999
64553.0000000	-3.4949999
64554.0000000	-3.5050001
64555.0000000	-3.5200000
64556.0000000	-3.5300000
64557.0000000	-3.5500000
64558.0000000	-3.5599999
64559.0000000	-3.5699999
64560.0000000	-3.5899999
64561.0000000	-3.5999999
64562.0000000	-3.6099999
64563.0000000	-3.6199999
64564.0000000	-3.6350002
64565.0000000	-3.6450000
64566.0000000	-3.6550002
64567.0000000	-3.6800001
64568.0000000	-3.6849999
64569.0000000	-3.6950002
64570.0000000	-3.7000000
64571.0000000	-3.7049999
64572.0000000	-3.7100000
64573.0000000	-3.7133334
64574.0000000	-3.7249999
64575.0000000	-3.7000000
64576.0000000	-3.7249999
64577.0000000	-3.7049999
64578.0000000	-3.6700001
64579.0000000	-3.6950002
64580.0000000	-3.7000000

64581.0000000	-3.7000000
64582.0000000	-3.6900001
64583.0000000	-3.6900001
64584.0000000	-3.6650000
64585.0000000	-3.6199999
64586.0000000	-3.6050000
64587.0000000	-3.5850000
64588.0000000	-3.5799999
64589.0000000	-3.5850000
64590.0000000	-3.5850000
64591.0000000	-3.5749998
64592.0000000	-3.5999999
64593.0000000	-3.6149998
64594.0000000	-3.6199999
64595.0000000	-3.6300001
64596.0000000	-3.6550002
64597.0000000	-3.6800001
64598.0000000	-3.6950002
64599.0000000	-3.7300000
64600.0000000	-3.7400000
64601.0000000	-3.7449999
64602.0000000	-3.7550001
64603.0000000	-3.7466667
64604.0000000	-3.7500000
64605.0000000	-3.7449999
64606.0000000	-3.7500000
64607.0000000	-3.7449999
64608.0000000	-3.7200000
64609.0000000	-3.7249999
64610.0000000	-3.7000000
64611.0000000	-3.6800001
64612.0000000	-3.6700001
64613.0000000	-3.6700001
64614.0000000	-3.6650000
64615.0000000	-3.6650000
64616.0000000	-3.6750002
64617.0000000	-3.6700001
64618.0000000	-3.6650000
64619.0000000	-3.6650000
64620.0000000	-3.6550002
64621.0000000	-3.6650000
64622.0000000	-3.6650000
64623.0000000	-3.6650000
64624.0000000	-3.6800001
64625.0000000	-3.7000000
64626.0000000	-3.7000000
64627.0000000	-3.7000000
64628.0000000	-3.7000000
64629.0000000	-3.7150002
64630.0000000	-3.7350001
64631.0000000	-3.7449999
64632.0000000	-3.7600000
64633.0000000	-3.7700000
64634.0000000	-3.7766666
64635.0000000	-3.7600000
64636.0000000	-3.7700000
64637.0000000	-3.7649999

64638.0000000	-3.7900000
64639.0000000	-3.8099999
64640.0000000	-3.8199999
64641.0000000	-3.8199999
64642.0000000	-3.8199999
64643.0000000	-3.8000000
64644.0000000	-3.8199999
64645.0000000	-3.8299999
64646.0000000	-3.8249998
64647.0000000	-3.8150001
64648.0000000	-3.8150001
64649.0000000	-3.8049998
64650.0000000	-3.7950001
64651.0000000	-3.8000000
64652.0000000	-3.8000000
64653.0000000	-3.8049998
64654.0000000	-3.8199999
64655.0000000	-3.8350000
64656.0000000	-3.8399999
64657.0000000	-3.8449998
64658.0000000	-3.8550000
64659.0000000	-3.8499999
64660.0000000	-3.8499999
64661.0000000	-3.8399999
64662.0000000	-3.8399999
64663.0000000	-3.8550000
64664.0000000	-3.8733332
64665.0000000	-3.8800001
64666.0000000	-3.8800001
64667.0000000	-3.8900001
64668.0000000	-3.8800001
64669.0000000	-3.8699999
64670.0000000	-3.8800001
64671.0000000	-3.8900001
64672.0000000	-3.8800001
64673.0000000	-3.8950000
64674.0000000	-3.8900001
64675.0000000	-3.8900001
64676.0000000	-3.8950000
64677.0000000	-3.8699999
64678.0000000	-3.8800001
64679.0000000	-3.8950000
64680.0000000	-3.9050002
64681.0000000	-3.9100001
64682.0000000	-3.9100001
64683.0000000	-3.9150000
64684.0000000	-3.9250002
64685.0000000	-3.9250002
64686.0000000	-3.9150000
64687.0000000	-3.9349999
64688.0000000	-3.9349999
64689.0000000	-3.9400001
64690.0000000	-3.9500000
64691.0000000	-3.9450002
64692.0000000	-3.9349999
64693.0000000	-3.9300001
64694.0000000	-3.9633334

64695.0000000	-3.9850001
64696.0000000	-3.9749999
64697.0000000	-3.9850001
64698.0000000	-3.9949999
64699.0000000	-3.9900000
64700.0000000	-3.9800000
64701.0000000	-3.9800000
64702.0000000	-3.9700000
64703.0000000	-3.9700000
64704.0000000	-3.9749999
64705.0000000	-3.9900000
64706.0000000	-3.9949999
64707.0000000	-4.0050001
64708.0000000	-4.0000000
64709.0000000	-4.0000000
64710.0000000	-4.0050001
64711.0000000	-3.9850001
64712.0000000	-3.9949999
64713.0000000	-3.9900000
64714.0000000	-3.9749999
64715.0000000	-3.9749999
64716.0000000	-3.9700000
64717.0000000	-3.9650002
64718.0000000	-3.9600000
64719.0000000	-3.9549999
64720.0000000	-3.9400001
64721.0000000	-3.9450002
64722.0000000	-3.9349999
64723.0000000	-3.9349999
64724.0000000	-3.9233334
64725.0000000	-3.8800001
64726.0000000	-3.8800001
64727.0000000	-3.8800001
64728.0000000	-3.8650000
64729.0000000	-3.8699999
64730.0000000	-3.8599999
64731.0000000	-3.8599999
64732.0000000	-3.8699999
64733.0000000	-3.8750000
64734.0000000	-3.8850002
64735.0000000	-3.8950000
64736.0000000	-3.8950000
64737.0000000	-3.8850002
64738.0000000	-3.8750000
64739.0000000	-3.8649998
64740.0000000	-3.8599999
64741.0000000	-3.8499999
64742.0000000	-3.8499999
64743.0000000	-3.8350000
64744.0000000	-3.8049998
64745.0000000	-3.8099999
64746.0000000	-3.7900000
64747.0000000	-3.7750001
64748.0000000	-3.7600000
64749.0000000	-3.7500000
64750.0000000	-3.7550001
64751.0000000	-3.7449999

64752.0000000	-3.7249999
64753.0000000	-3.7249999
64754.0000000	-3.7200000
64755.0000000	-3.6833334
64756.0000000	-3.6849999
64757.0000000	-3.6849999
64758.0000000	-3.6700001
64759.0000000	-3.6700001
64760.0000000	-3.6650000
64761.0000000	-3.6500001
64762.0000000	-3.6800001
64763.0000000	-3.6800001
64764.0000000	-3.6750002
64765.0000000	-3.6750002
64766.0000000	-3.6600001
64767.0000000	-3.6550002
64768.0000000	-3.6300001
64769.0000000	-3.6250000
64770.0000000	-3.6300001
64771.0000000	-3.6150000
64772.0000000	-3.6199999
64773.0000000	-3.6149998
64774.0000000	-3.6149998
64775.0000000	-3.6050000
64776.0000000	-3.6050000
64777.0000000	-3.5949998
64778.0000000	-3.5850000
64779.0000000	-3.5699999
64780.0000000	-3.5650001
64781.0000000	-3.5400000
64782.0000000	-3.5050001
64783.0000000	-3.4800000
64784.0000000	-3.4650002
64785.0000000	-3.4533336
64786.0000000	-3.4549999
64787.0000000	-3.4500000
64788.0000000	-3.4600000
64789.0000000	-3.4650002
64790.0000000	-3.4600000
64791.0000000	-3.4400001
64792.0000000	-3.4450002
64793.0000000	-3.4650002
64794.0000000	-3.4800000
64795.0000000	-3.4650002
64796.0000000	-3.4250002
64797.0000000	-3.4050002
64798.0000000	-3.3950000
64799.0000000	-3.4200001
64800.0000000	-3.4200001
64801.0000000	-3.4000001
64802.0000000	-3.3900001
64803.0000000	-3.3800001
64804.0000000	-3.3800001
64805.0000000	-3.3850002
64806.0000000	-3.3850002
64807.0000000	-3.3900001
64808.0000000	-3.3800001

64809.0000000	-3.3850002
64810.0000000	-3.3800001
64811.0000000	-3.3800001
64812.0000000	-3.3499999
64813.0000000	-3.3449998
64814.0000000	-3.3399999
64815.0000000	-3.3333333
64816.0000000	-3.3249998
64817.0000000	-3.3399999
64818.0000000	-3.3449998
64819.0000000	-3.3449998
64820.0000000	-3.3350000
64821.0000000	-3.3449998
64822.0000000	-3.3550000
64823.0000000	-3.3550000
64824.0000000	-3.3449998
64825.0000000	-3.3649998
64826.0000000	-3.3850002
64827.0000000	-3.3699999
64828.0000000	-3.3599999
64829.0000000	-3.3699999
64830.0000000	-3.3900001
64831.0000000	-3.3950000
64832.0000000	-3.3950000
64833.0000000	-3.4050002
64834.0000000	-3.4150000
64835.0000000	-3.4050002
64836.0000000	-3.3950000
64837.0000000	-3.3750000
64838.0000000	-3.3550000
64839.0000000	-3.3299999
64840.0000000	-3.3199999
64841.0000000	-3.2550001
64842.0000000	-3.2750001
64843.0000000	-3.2600000
64844.0000000	-3.2500000
64845.0000000	-3.2400000
64846.0000000	-3.2550001
64847.0000000	-3.2500000
64848.0000000	-3.2400000
64849.0000000	-3.2449999
64850.0000000	-3.2350001
64851.0000000	-3.2300000
64852.0000000	-3.2350001
64853.0000000	-3.2400000
64854.0000000	-3.2249999
64855.0000000	-3.2049999
64856.0000000	-3.1700001
64857.0000000	-3.1849999
64858.0000000	-3.1600001
64859.0000000	-3.1350002
64860.0000000	-3.0949998
64861.0000000	-3.0850000
64862.0000000	-3.0699999
64863.0000000	-3.0400000
64864.0000000	-3.0450001
64865.0000000	-3.0250001

64866.0000000	-2.9949999
64867.0000000	-2.9850001
64868.0000000	-2.9749999
64869.0000000	-2.9600000
64870.0000000	-2.9600000
64871.0000000	-2.9700000
64872.0000000	-2.9500000
64873.0000000	-2.9500000
64874.0000000	-2.9349999
64875.0000000	-2.9233334
64876.0000000	-2.9100001
64877.0000000	-2.8750000
64878.0000000	-2.8599999
64879.0000000	-2.8499999
64880.0000000	-2.8399999
64881.0000000	-2.8249998
64882.0000000	-2.8299999
64883.0000000	-2.8099999
64884.0000000	-2.7700000
64885.0000000	-2.7600000
64886.0000000	-2.7400000
64887.0000000	-2.7300000
64888.0000000	-2.7500000
64889.0000000	-2.7500000
64890.0000000	-2.7550001
64891.0000000	-2.7500000
64892.0000000	-2.7300000
64893.0000000	-2.7449999
64894.0000000	-2.7350001
64895.0000000	-2.7100000
64896.0000000	-2.7000000
64897.0000000	-2.6849999
64898.0000000	-2.6700001
64899.0000000	-2.6600001
64900.0000000	-2.6199999
64901.0000000	-2.6100001
64902.0000000	-2.6250000
64903.0000000	-2.6300001
64904.0000000	-2.6149998
64905.0000000	-2.6099999
64906.0000000	-2.6166666
64907.0000000	-2.6149998
64908.0000000	-2.6149998
64909.0000000	-2.5949998
64910.0000000	-2.5949998
64911.0000000	-2.5699999
64912.0000000	-2.5599999
64913.0000000	-2.5650001
64914.0000000	-2.5699999
64915.0000000	-2.5749998
64916.0000000	-2.5749998
64917.0000000	-2.5749998
64918.0000000	-2.5799999
64919.0000000	-2.5650001
64920.0000000	-2.5400000
64921.0000000	-2.5300000
64922.0000000	-2.5349998

64923.0000000	-2.5349998
64924.0000000	-2.5349998
64925.0000000	-2.5349998
64926.0000000	-2.5300000
64927.0000000	-2.5149999
64928.0000000	-2.5149999
64929.0000000	-2.5149999
64930.0000000	-2.5149999
64931.0000000	-2.5200000
64932.0000000	-2.5250001
64933.0000000	-2.5250001
64934.0000000	-2.5250001
64935.0000000	-2.5200000
64936.0000000	-2.5300000
64937.0000000	-2.5300000
64938.0000000	-2.5300000
64939.0000000	-2.5300000
64940.0000000	-2.5349998
64941.0000000	-2.5200000
64942.0000000	-2.5149999
64943.0000000	-2.5000000
64944.0000000	-2.4900000
64945.0000000	-2.4850001
64946.0000000	-2.4900000
64947.0000000	-2.4800000
64948.0000000	-2.4800000
64949.0000000	-2.4850001
64950.0000000	-2.4949999
64951.0000000	-2.5000000
64952.0000000	-2.4900000
64953.0000000	-2.4800000
64954.0000000	-2.4700000
64955.0000000	-2.4549999
64956.0000000	-2.4700000
64957.0000000	-2.4700000
64958.0000000	-2.4600000
64959.0000000	-2.4450002
64960.0000000	-2.4349999
64961.0000000	-2.4349999
64962.0000000	-2.4349999
64963.0000000	-2.4300001
64964.0000000	-2.4150000
64965.0000000	-2.4200001
64966.0000000	-2.4200001
64967.0000000	-2.4200001
64968.0000000	-2.4300001
64969.0000000	-2.4250002
64970.0000000	-2.4150000
64971.0000000	-2.4100001
64972.0000000	-2.4150000
64973.0000000	-2.4200001
64974.0000000	-2.4200001
64975.0000000	-2.4100001
64976.0000000	-2.4200001
64977.0000000	-2.4150000
64978.0000000	-2.4150000
64979.0000000	-2.4000001

64980.0000000	-2.4000001
64981.0000000	-2.4000001
64982.0000000	-2.4100001
64983.0000000	-2.4100001
64984.0000000	-2.4100001
64985.0000000	-2.4300001
64986.0000000	-2.4349999
64987.0000000	-2.4450002
64988.0000000	-2.4500000
64989.0000000	-2.4400001
64990.0000000	-2.4400001
64991.0000000	-2.4150000
64992.0000000	-2.4200001
64993.0000000	-2.4150000
64994.0000000	-2.4450002
64995.0000000	-2.4450002
64996.0000000	-2.4466667
64997.0000000	-2.4500000
64998.0000000	-2.4549999
64999.0000000	-2.4600000
65000.0000000	-2.4800000
65001.0000000	-2.4749999
65002.0000000	-2.4850001
65003.0000000	-2.4949999
65004.0000000	-2.5000000
65005.0000000	-2.5050001
65006.0000000	-2.4949999
65007.0000000	-2.5000000
65008.0000000	-2.4949999
65009.0000000	-2.4949999
65010.0000000	-2.4800000
65011.0000000	-2.4900000
65012.0000000	-2.5000000
65013.0000000	-2.5100000
65014.0000000	-2.5050001
65015.0000000	-2.5100000
65016.0000000	-2.5250001
65017.0000000	-2.5250001
65018.0000000	-2.5250001
65019.0000000	-2.5250001
65020.0000000	-2.5300000
65021.0000000	-2.5400000
65022.0000000	-2.5349998
65023.0000000	-2.5300000
65024.0000000	-2.5050001
65025.0000000	-2.5400000
65026.0000000	-2.5400000
65027.0000000	-2.5450001
65028.0000000	-2.5500000
65029.0000000	-2.5450001
65030.0000000	-2.5500000
65031.0000000	-2.5500000
65032.0000000	-2.5400000
65033.0000000	-2.5200000
65034.0000000	-2.5149999
65035.0000000	-2.5400000
65036.0000000	-2.5500000

65037.0000000	-2.5650001
65038.0000000	-2.5699999
65039.0000000	-2.5749998
65040.0000000	-2.5799999
65041.0000000	-2.5899999
65042.0000000	-2.5949998
65043.0000000	-2.5949998
65044.0000000	-2.5999999
65045.0000000	-2.6050000
65046.0000000	-2.6099999
65047.0000000	-2.5999999
65048.0000000	-2.6250000
65049.0000000	-2.6400001
65050.0000000	-2.6450000
65051.0000000	-2.6550002
65052.0000000	-2.6500001
65053.0000000	-2.6600001
65054.0000000	-2.6700001
65055.0000000	-2.6849999
65056.0000000	-2.6950002
65057.0000000	-2.6966667
65058.0000000	-2.7100000
65059.0000000	-2.7200000
65060.0000000	-2.7300000
65061.0000000	-2.7350001
65062.0000000	-2.7400000
65063.0000000	-2.7449999
65064.0000000	-2.7500000
65065.0000000	-2.7350001
65066.0000000	-2.7400000
65067.0000000	-2.7400000
65068.0000000	-2.7449999
65069.0000000	-2.7300000
65070.0000000	-2.7150002
65071.0000000	-2.7150002
65072.0000000	-2.6950002
65073.0000000	-2.6849999
65074.0000000	-2.6750002
65075.0000000	-2.6849999
65076.0000000	-2.6849999
65077.0000000	-2.6750002
65078.0000000	-2.6750002
65079.0000000	-2.6650000
65080.0000000	-2.6450000
65081.0000000	-2.6099999
65082.0000000	-2.5949998
65083.0000000	-2.5949998
65084.0000000	-2.5749998
65085.0000000	-2.5599999
65086.0000000	-2.5599997
65087.0000000	-2.5650001
65088.0000000	-2.5599999
65089.0000000	-2.5699999
65090.0000000	-2.5699999
65091.0000000	-2.5549998
65092.0000000	-2.5749998
65093.0000000	-2.5850000

65094.0000000	-2.5799999
65095.0000000	-2.5650001
65096.0000000	-2.5749998
65097.0000000	-2.5650001
65098.0000000	-2.5549998
65099.0000000	-2.5699999
65100.0000000	-2.5699999
65101.0000000	-2.5799999
65102.0000000	-2.5799999
65103.0000000	-2.5799999
65104.0000000	-2.5749998
65105.0000000	-2.5850000
65106.0000000	-2.5949998
65107.0000000	-2.5999999
65108.0000000	-2.5949998
65109.0000000	-2.6050000
65110.0000000	-2.6199999
65111.0000000	-2.6300001
65112.0000000	-2.6250000
65113.0000000	-2.6350002
65114.0000000	-2.6450000
65115.0000000	-2.6500001
65116.0000000	-2.6500003
65117.0000000	-2.6500001
65118.0000000	-2.6550002
65119.0000000	-2.6500001
65120.0000000	-2.6450000
65121.0000000	-2.6350000
65122.0000000	-2.6500001
65123.0000000	-2.6500001
65124.0000000	-2.6350002
65125.0000000	-2.6350002
65126.0000000	-2.6350002
65127.0000000	-2.6350002
65128.0000000	-2.6450000
65129.0000000	-2.6400001
65130.0000000	-2.6450000
65131.0000000	-2.6500001
65132.0000000	-2.6550002
65133.0000000	-2.6550002
65134.0000000	-2.6550002
65135.0000000	-2.6600001
65136.0000000	-2.6550002
65137.0000000	-2.6500001
65138.0000000	-2.6500001
65139.0000000	-2.6350002
65140.0000000	-2.6300001
65141.0000000	-2.6250000
65142.0000000	-2.6300001
65143.0000000	-2.6350002
65144.0000000	-2.6350002
65145.0000000	-2.6400001
65146.0000000	-2.6150000
65147.0000000	-2.6433334
65148.0000000	-2.6500001
65149.0000000	-2.6550002
65150.0000000	-2.6750002

65151.0000000	-2.6750002
65152.0000000	-2.6750002
65153.0000000	-2.6750002
65154.0000000	-2.6849999
65155.0000000	-2.6849999
65156.0000000	-2.6750002
65157.0000000	-2.6700001
65158.0000000	-2.6800001
65159.0000000	-2.6800001
65160.0000000	-2.6800001
65161.0000000	-2.6800001
65162.0000000	-2.6849999
65163.0000000	-2.6849999
65164.0000000	-2.6650000
65165.0000000	-2.6750002
65166.0000000	-2.6800001
65167.0000000	-2.6800001
65168.0000000	-2.6950002
65169.0000000	-2.7049999
65170.0000000	-2.7049999
65171.0000000	-2.7049999
65172.0000000	-2.7150002
65173.0000000	-2.7300000
65174.0000000	-2.7300000
65175.0000000	-2.7350001
65176.0000000	-2.7300000
65177.0000000	-2.7350001
65178.0000000	-2.7300003
65179.0000000	-2.7449999
65180.0000000	-2.7449999
65181.0000000	-2.7550001
65182.0000000	-2.7700000
65183.0000000	-2.7750001
65184.0000000	-2.7600000
65185.0000000	-2.7500000
65186.0000000	-2.7550001
65187.0000000	-2.7600000
65188.0000000	-2.7750001
65189.0000000	-2.7849998
65190.0000000	-2.7849998
65191.0000000	-2.7950001
65192.0000000	-2.8099999
65193.0000000	-2.8199999
65194.0000000	-2.8150001
65195.0000000	-2.8150001
65196.0000000	-2.8150001
65197.0000000	-2.8099999
65198.0000000	-2.8049998
65199.0000000	-2.8099999
65200.0000000	-2.7950001
65201.0000000	-2.7950001
65202.0000000	-2.7950001
65203.0000000	-2.8000000
65204.0000000	-2.7950001
65205.0000000	-2.7800000
65206.0000000	-2.8150001
65207.0000000	-2.8099997

65208.0000000	-2.8049998
65209.0000000	-2.8150001
65210.0000000	-2.8249998
65211.0000000	-2.8299999
65212.0000000	-2.8350000
65213.0000000	-2.8350000
65214.0000000	-2.8249998
65215.0000000	-2.8299999
65216.0000000	-2.8449998
65217.0000000	-2.8449998
65218.0000000	-2.8350000
65219.0000000	-2.8399999
65220.0000000	-2.8299999
65221.0000000	-2.8350000
65222.0000000	-2.8449998
65223.0000000	-2.8649998
65224.0000000	-2.8550000
65225.0000000	-2.8900001
65226.0000000	-2.8900001
65227.0000000	-2.9000001
65228.0000000	-2.9150000
65229.0000000	-2.9349999
65230.0000000	-2.9250002
65231.0000000	-2.9300001
65232.0000000	-2.9549999
65233.0000000	-2.9749999
65234.0000000	-2.9900000
65235.0000000	-2.9949999
65236.0000000	-3.0050001
65237.0000000	-3.0266666
65238.0000000	-3.0349998
65239.0000000	-3.0450001
65240.0000000	-3.0549998
65241.0000000	-3.0699999
65242.0000000	-3.0599999
65243.0000000	-3.0599999
65244.0000000	-3.0699999
65245.0000000	-3.0850000
65246.0000000	-3.0949998
65247.0000000	-3.0999999
65248.0000000	-3.1099999
65249.0000000	-3.1099999
65250.0000000	-3.1199999
65251.0000000	-3.1350002
65252.0000000	-3.1350002
65253.0000000	-3.1450000
65254.0000000	-3.1450000
65255.0000000	-3.1450000
65256.0000000	-3.1450000
65257.0000000	-3.1600001
65258.0000000	-3.1700001
65259.0000000	-3.1700001
65260.0000000	-3.1650000
65261.0000000	-3.1650000
65262.0000000	-3.1500001
65263.0000000	-3.1550002
65264.0000000	-3.1500001

65265.0000000	-3.1650000
65266.0000000	-3.1800001
65267.0000000	-3.1950002
65268.0000000	-3.2066667
65269.0000000	-3.2249999
65270.0000000	-3.2350001
65271.0000000	-3.2500000
65272.0000000	-3.2600000
65273.0000000	-3.2849998
65274.0000000	-3.3049998
65275.0000000	-3.3150001
65276.0000000	-3.3150001
65277.0000000	-3.3199999
65278.0000000	-3.3099999
65279.0000000	-3.2950001
65280.0000000	-3.3000000
65281.0000000	-3.3049998
65282.0000000	-3.3000000
65283.0000000	-3.3000000
65284.0000000	-3.3049998
65285.0000000	-3.3049998
65286.0000000	-3.3150001
65287.0000000	-3.3249998
65288.0000000	-3.3199999
65289.0000000	-3.3199999
65290.0000000	-3.3099999
65291.0000000	-3.3199999
65292.0000000	-3.3249998
65293.0000000	-3.3350000
65294.0000000	-3.3399999
65295.0000000	-3.3449998
65296.0000000	-3.3550000
65297.0000000	-3.3599999
65298.0000000	-3.3666666
65299.0000000	-3.3750000
65300.0000000	-3.3950000
65301.0000000	-3.3950000
65302.0000000	-3.4050002
65303.0000000	-3.4100001
65304.0000000	-3.4150000
65305.0000000	-3.4100001
65306.0000000	-3.4000001
65307.0000000	-3.4300001
65308.0000000	-3.4349999
65309.0000000	-3.4349999
65310.0000000	-3.4450002
65311.0000000	-3.4400001
65312.0000000	-3.4549999
65313.0000000	-3.4650002
65314.0000000	-3.4700000
65315.0000000	-3.4700000
65316.0000000	-3.4800000
65317.0000000	-3.4749999
65318.0000000	-3.4749999
65319.0000000	-3.4800000
65320.0000000	-3.4800000
65321.0000000	-3.4700000

65322.0000000	-3.4500000
65323.0000000	-3.4349999
65324.0000000	-3.4200001
65325.0000000	-3.3950000
65326.0000000	-3.3649998
65327.0000000	-3.3599999
65328.0000000	-3.3366668
65329.0000000	-3.3299999
65330.0000000	-3.3299999
65331.0000000	-3.3399999
65332.0000000	-3.3150001
65333.0000000	-3.3249998
65334.0000000	-3.3299999
65335.0000000	-3.3649998
65336.0000000	-3.3550000
65337.0000000	-3.3699999
65338.0000000	-3.3950000
65339.0000000	-3.4150000
65340.0000000	-3.4300001
65341.0000000	-3.4300001
65342.0000000	-3.4549999
65343.0000000	-3.4650002
65344.0000000	-3.4700000
65345.0000000	-3.4700000
65346.0000000	-3.4749999
65347.0000000	-3.4800000
65348.0000000	-3.4749999
65349.0000000	-3.4800000
65350.0000000	-3.4850001
65351.0000000	-3.4850001
65352.0000000	-3.4949999
65353.0000000	-3.4800000
65354.0000000	-3.4650002
65355.0000000	-3.4749999
65356.0000000	-3.4700000
65357.0000000	-3.4500000
65358.0000000	-3.4366665
65359.0000000	-3.4300001
65360.0000000	-3.4400001
65361.0000000	-3.4400001
65362.0000000	-3.4300001
65363.0000000	-3.4250002
65364.0000000	-3.4300001
65365.0000000	-3.4250002
65366.0000000	-3.4300001
65367.0000000	-3.4250002
65368.0000000	-3.4300001
65369.0000000	-3.4349999
65370.0000000	-3.4250002
65371.0000000	-3.4100001
65372.0000000	-3.4100001
65373.0000000	-3.4150000
65374.0000000	-3.4200001
65375.0000000	-3.4150000
65376.0000000	-3.4250002
65377.0000000	-3.4349999
65378.0000000	-3.4300001

65379.0000000	-3.4250002
65380.0000000	-3.4250002
65381.0000000	-3.4200001
65382.0000000	-3.4100001
65383.0000000	-3.3800001
65384.0000000	-3.3800001
65385.0000000	-3.3750000
65386.0000000	-3.3449998
65387.0000000	-3.3299999
65388.0000000	-3.3099997
65389.0000000	-3.3150001
65390.0000000	-3.3150001
65391.0000000	-3.3000000
65392.0000000	-3.3199999
65393.0000000	-3.3249998
65394.0000000	-3.3350000
65395.0000000	-3.3499999
65396.0000000	-3.3550000
65397.0000000	-3.3550000
65398.0000000	-3.3449998
65399.0000000	-3.3649998
65400.0000000	-3.3750000
65401.0000000	-3.3699999
65402.0000000	-3.3950000
65403.0000000	-3.4100001
65404.0000000	-3.4100001
65405.0000000	-3.4050002
65406.0000000	-3.4100001
65407.0000000	-3.4050002
65408.0000000	-3.4000001
65409.0000000	-3.4000001
65410.0000000	-3.3900001
65411.0000000	-3.3800001
65412.0000000	-3.3850002
65413.0000000	-3.3800001
65414.0000000	-3.3649998
65415.0000000	-3.3550000
65416.0000000	-3.3399999
65417.0000000	-3.3249998
65418.0000000	-3.3150001
65419.0000000	-3.2866666
65420.0000000	-3.2649999
65421.0000000	-3.2400000
65422.0000000	-3.2300000
65423.0000000	-3.2200000
65424.0000000	-3.1900001
65425.0000000	-3.1700001
65426.0000000	-3.1600001
65427.0000000	-3.1350002
65428.0000000	-3.1450000
65429.0000000	-3.1450000
65430.0000000	-3.1550002
65431.0000000	-3.1450000
65432.0000000	-3.1500001
65433.0000000	-3.1550002
65434.0000000	-3.1550002
65435.0000000	-3.1500001

65436.0000000	-3.1550002
65437.0000000	-3.1300001
65438.0000000	-3.1350002
65439.0000000	-3.1400001
65440.0000000	-3.1199999
65441.0000000	-3.1149998
65442.0000000	-3.1149998
65443.0000000	-3.1350002
65444.0000000	-3.1250000
65445.0000000	-3.1400001
65446.0000000	-3.1300001
65447.0000000	-3.1250000
65448.0000000	-3.1199999
65449.0000000	-3.1300001
65450.0000000	-3.1250000
65451.0000000	-3.1099999
65452.0000000	-3.1050000
65453.0000000	-3.1250000
65454.0000000	-3.1149998
65455.0000000	-3.1300001
65456.0000000	-3.1350002
65457.0000000	-3.1400001
65458.0000000	-3.1500001
65459.0000000	-3.1400001
65460.0000000	-3.1450000
65461.0000000	-3.1550002
65462.0000000	-3.1550002
65463.0000000	-3.1450000
65464.0000000	-3.1400001
65465.0000000	-3.1400001
65466.0000000	-3.1099999
65467.0000000	-3.1250000
65468.0000000	-3.1300001
65469.0000000	-3.1250000
65470.0000000	-3.1149998
65471.0000000	-3.1149998
65472.0000000	-3.1050000
65473.0000000	-3.1050000
65474.0000000	-3.1050000
65475.0000000	-3.0999999
65476.0000000	-3.0850000
65477.0000000	-3.0999999
65478.0000000	-3.0949998
65479.0000000	-3.0799999
65480.0000000	-3.0749998
65481.0000000	-3.0699999
65482.0000000	-3.0699999
65483.0000000	-3.0650001
65484.0000000	-3.0650001
65485.0000000	-3.0650001
65486.0000000	-3.0749998
65487.0000000	-3.0699999
65488.0000000	-3.0599999
65489.0000000	-3.0650001
65490.0000000	-3.0699999
65491.0000000	-3.0799999
65492.0000000	-3.0749998

65493.0000000	-3.0749998
65494.0000000	-3.0699999
65495.0000000	-3.0749998
65496.0000000	-3.0749998
65497.0000000	-3.0699999
65498.0000000	-3.0699999
65499.0000000	-3.0699999
65500.0000000	-3.0699999
65501.0000000	-3.0699999
65502.0000000	-3.0699999
65503.0000000	-3.0599999
65504.0000000	-3.0500000
65505.0000000	-3.0450001
65506.0000000	-3.0250001
65507.0000000	-3.0149999
65508.0000000	-3.0100000
65509.0000000	-2.9933331
65510.0000000	-2.9800000
65511.0000000	-2.9700000
65512.0000000	-2.9600000
65513.0000000	-2.9500000
65514.0000000	-2.9450002
65515.0000000	-2.9349999
65516.0000000	-2.9100001
65517.0000000	-2.9250002
65518.0000000	-2.9200001
65519.0000000	-2.9200001
65520.0000000	-2.9250002
65521.0000000	-2.9300001
65522.0000000	-2.9400001
65523.0000000	-2.9450002
65524.0000000	-2.9549999
65525.0000000	-2.9549999
65526.0000000	-2.9549999
65527.0000000	-2.9549999
65528.0000000	-2.9549999
65529.0000000	-2.9549999
65530.0000000	-2.9700000
65531.0000000	-2.9900000
65532.0000000	-2.9900000
65533.0000000	-3.0000000
65534.0000000	-3.0000000
65535.0000000	-3.0149999
65536.0000000	-3.0100000
65537.0000000	-3.0500000
65538.0000000	-3.0549998
65539.0000000	-3.0650001
65540.0000000	-3.0666666
65541.0000000	-3.0599999
65542.0000000	-3.0549998
65543.0000000	-3.0500000
65544.0000000	-3.0450001
65545.0000000	-3.0450001
65546.0000000	-3.0349998
65547.0000000	-3.0200000
65548.0000000	-3.0050001
65549.0000000	-2.9700000

65550.0000000	-2.9250002
65551.0000000	-2.8950000
65552.0000000	-2.8499999
65553.0000000	-2.8199999
65554.0000000	-2.8049998
65555.0000000	-2.7900000
65556.0000000	-2.7900000
65557.0000000	-2.7800000
65558.0000000	-2.7600000
65559.0000000	-2.7649999
65560.0000000	-2.7649999
65561.0000000	-2.7700000
65562.0000000	-2.7750001
65563.0000000	-2.7750001
65564.0000000	-2.7700000
65565.0000000	-2.7800000
65566.0000000	-2.7900000
65567.0000000	-2.7900000
65568.0000000	-2.7800000
65569.0000000	-2.7750001
65570.0000000	-2.7633333
65571.0000000	-2.7649999
65572.0000000	-2.7600000
65573.0000000	-2.7649999
65574.0000000	-2.7500000
65575.0000000	-2.7649999
65576.0000000	-2.7649999
65577.0000000	-2.7400000
65578.0000000	-2.7700000
65579.0000000	-2.7649999
65580.0000000	-2.7700000
65581.0000000	-2.7649999
65582.0000000	-2.7600000
65583.0000000	-2.7600000
65584.0000000	-2.7500000
65585.0000000	-2.7449999
65586.0000000	-2.7249999
65587.0000000	-2.7100000
65588.0000000	-2.7049999
65589.0000000	-2.6900001
65590.0000000	-2.6550002
65591.0000000	-2.6400001
65592.0000000	-2.6350002
65593.0000000	-2.6149998
65594.0000000	-2.6099999
65595.0000000	-2.5850000
65596.0000000	-2.5850000
65597.0000000	-2.5599999
65598.0000000	-2.5699999
65599.0000000	-2.5633333
65600.0000000	-2.5650001
65601.0000000	-2.5650001
65602.0000000	-2.5500000
65603.0000000	-2.5450001
65604.0000000	-2.5349998
65605.0000000	-2.5349998
65606.0000000	-2.5349998

65607.0000000	-2.5250001
65608.0000000	-2.5100000
65609.0000000	-2.4900000
65610.0000000	-2.4800000
65611.0000000	-2.4549999
65612.0000000	-2.4400001
65613.0000000	-2.4250002
65614.0000000	-2.4250002
65615.0000000	-2.4300001
65616.0000000	-2.4150000
65617.0000000	-2.4100001
65618.0000000	-2.4100001
65619.0000000	-2.4100001
65620.0000000	-2.3950000
65621.0000000	-2.3800001
65622.0000000	-2.3649998
65623.0000000	-2.3599999
65624.0000000	-2.3550000
65625.0000000	-2.3449998
65626.0000000	-2.3399999
65627.0000000	-2.3550000
65628.0000000	-2.3550000
65629.0000000	-2.3650000
65630.0000000	-2.3866668
65631.0000000	-2.3950000
65632.0000000	-2.3900001
65633.0000000	-2.3850000
65634.0000000	-2.3699999
65635.0000000	-2.3449998
65636.0000000	-2.3350000
65637.0000000	-2.3499999
65638.0000000	-2.3399999
65639.0000000	-2.3350000
65640.0000000	-2.3350000
65641.0000000	-2.3299999
65642.0000000	-2.3449998
65643.0000000	-2.3299999
65644.0000000	-2.3449998
65645.0000000	-2.3399999
65646.0000000	-2.3449998
65647.0000000	-2.3499999
65648.0000000	-2.3449998
65649.0000000	-2.3350000
65650.0000000	-2.3299999
65651.0000000	-2.3249998
65652.0000000	-2.3199999
65653.0000000	-2.3049998
65654.0000000	-2.3099999
65655.0000000	-2.3000000
65656.0000000	-2.2900000
65657.0000000	-2.2900000
65658.0000000	-2.2900000
65659.0000000	-2.2900000
65660.0000000	-2.2833331
65661.0000000	-2.2800000
65662.0000000	-2.2700000
65663.0000000	-2.2750001

65664.0000000	-2.2849998
65665.0000000	-2.2800000
65666.0000000	-2.2800000
65667.0000000	-2.2800000
65668.0000000	-2.2849998
65669.0000000	-2.2800000
65670.0000000	-2.2700000
65671.0000000	-2.2649999
65672.0000000	-2.2700000
65673.0000000	-2.2700000
65674.0000000	-2.2600000
65675.0000000	-2.2550001
65676.0000000	-2.2600000
65677.0000000	-2.2649999
65678.0000000	-2.2500000
65679.0000000	-2.2500000
65680.0000000	-2.2500000
65681.0000000	-2.2550001
65682.0000000	-2.2449999
65683.0000000	-2.2449999
65684.0000000	-2.2449999
65685.0000000	-2.2550001
65686.0000000	-2.2600000
65687.0000000	-2.2649999
65688.0000000	-2.2600000
65689.0000000	-2.2649999
65690.0000000	-2.2600000
65691.0000000	-2.2633333
65692.0000000	-2.2550001
65693.0000000	-2.2600000
65694.0000000	-2.2500000
65695.0000000	-2.2600000
65696.0000000	-2.2550001
65697.0000000	-2.2550001
65698.0000000	-2.2449999
65699.0000000	-2.2449999
65700.0000000	-2.2449999
65701.0000000	-2.2350001
65702.0000000	-2.2150002
65703.0000000	-2.2000000
65704.0000000	-2.1900001
65705.0000000	-2.1750002
65706.0000000	-2.1600001
65707.0000000	-2.1600001
65708.0000000	-2.1650000
65709.0000000	-2.1650000
65710.0000000	-2.1600001
65711.0000000	-2.1650000
65712.0000000	-2.1800001
65713.0000000	-2.1849999
65714.0000000	-2.1849999
65715.0000000	-2.1800001
65716.0000000	-2.1849999
65717.0000000	-2.1900001
65718.0000000	-2.1849999
65719.0000000	-2.1800001
65720.0000000	-2.1700001

65721.0000000	-2.1650000
65722.0000000	-2.1650000
65723.0000000	-2.1700001
65724.0000000	-2.1750002
65725.0000000	-2.1750002
65726.0000000	-2.1750002
65727.0000000	-2.1900001
65728.0000000	-2.1900001
65729.0000000	-2.1849999
65730.0000000	-2.1800001
65731.0000000	-2.1800001
65732.0000000	-2.1849999
65733.0000000	-2.1849999
65734.0000000	-2.1750002
65735.0000000	-2.1750002
65736.0000000	-2.1700001
65737.0000000	-2.1700001
65738.0000000	-2.1600001
65739.0000000	-2.1550002
65740.0000000	-2.1550002
65741.0000000	-2.1500001
65742.0000000	-2.1450000
65743.0000000	-2.1300001
65744.0000000	-2.1300001
65745.0000000	-2.1300001
65746.0000000	-2.1199999
65747.0000000	-2.0999999
65748.0000000	-2.0949998
65749.0000000	-2.0949998
65750.0000000	-2.0733333
65751.0000000	-2.0699999
65752.0000000	-2.0650001
65753.0000000	-2.0450001
65754.0000000	-2.0349998
65755.0000000	-2.0400000
65756.0000000	-2.0300000
65757.0000000	-2.0300000
65758.0000000	-2.0300000
65759.0000000	-2.0300000
65760.0000000	-2.0300000
65761.0000000	-2.0300000
65762.0000000	-2.0300000
65763.0000000	-2.0250001
65764.0000000	-2.0300000
65765.0000000	-2.0300000
65766.0000000	-2.0200000
65767.0000000	-2.0149999
65768.0000000	-2.0200000
65769.0000000	-2.0300000
65770.0000000	-2.0300000
65771.0000000	-2.0250001
65772.0000000	-2.0349998
65773.0000000	-2.0400000
65774.0000000	-2.0400000
65775.0000000	-2.0450001
65776.0000000	-2.0400000
65777.0000000	-2.0400000

65778.0000000	-2.0400000
65779.0000000	-2.0300000
65780.0000000	-2.0250001
65781.0000000	-2.0233333
65782.0000000	-2.0149999
65783.0000000	-2.0149999
65784.0000000	-2.0100000
65785.0000000	-2.0000000
65786.0000000	-1.9950000
65787.0000000	-1.9950000
65788.0000000	-1.9950000
65789.0000000	-1.9850000
65790.0000000	-1.9750000
65791.0000000	-1.9700000
65792.0000000	-1.9550000
65793.0000000	-1.9349999
65794.0000000	-1.9250000
65795.0000000	-1.9300001
65796.0000000	-1.9400001
65797.0000000	-1.9400001
65798.0000000	-1.9349999
65799.0000000	-1.9349999
65800.0000000	-1.9400001
65801.0000000	-1.9500000
65802.0000000	-1.9400001
65803.0000000	-1.9450001
65804.0000000	-1.9449999
65805.0000000	-1.9450001
65806.0000000	-1.9450001
65807.0000000	-1.9400001
65808.0000000	-1.9450001
65809.0000000	-1.9400001
65810.0000000	-1.9400001
65811.0000000	-1.9333333
65812.0000000	-1.9349999
65813.0000000	-1.9300001
65814.0000000	-1.9299999
65815.0000000	-1.9300001
65816.0000000	-1.9250000
65817.0000000	-1.9300001
65818.0000000	-1.9200000
65819.0000000	-1.9300001
65820.0000000	-1.9299999
65821.0000000	-1.9200000
65822.0000000	-1.9200000
65823.0000000	-1.9250000
65824.0000000	-1.9200000
65825.0000000	-1.9150000
65826.0000000	-1.9150000
65827.0000000	-1.9100000
65828.0000000	-1.9100000
65829.0000000	-1.9100000
65830.0000000	-1.9000000
65831.0000000	-1.8900000
65832.0000000	-1.8900000
65833.0000000	-1.8850000
65834.0000000	-1.8750000

65835.0000000	-1.8600000
[truncated: 607,370 more chars]
