# Supplementary material for: Periodic oscillations of atmospheric electric field during snowfall in the Tokyo metropolitan area
Source: Sci Rep. 2021 Jan 26;11:2280. doi: 10.1038/s41598-021-82091-w (PMC7838252; doi:10.1038/s41598-021-82091-w)
Supplement: Supplementary file 2 — Supplementary Information 2. [file 41598_2021_82091_MOESM2_ESM.pdf]

0.0000000	0.3416000	-36.6878815
0.0000000	0.3904000	-38.9682541
0.0000000	0.4392000	-48.8525200
0.0000000	0.4880000	-38.1078720
0.0000000	0.5368000	-39.6725121
0.0000000	0.5856000	-56.0023422
0.0000000	0.6344000	-9.5442772
0.0000000	0.6832000	-154.4703217
0.0000000	0.7320000	-30.9840393
0.0000000	0.7808000	-96.5936279
0.0000000	0.8296000	-45.7269287
0.0000000	0.8784000	-35.7620201
0.0000000	0.9272000	-33.5323830
0.0000000	0.9760000	-30.4338264
0.0000000	1.0247999	-28.7891998
0.0000000	1.0735999	-29.0709724
0.0000000	1.1224000	-29.0808430
0.0000000	1.1711999	-29.1791000
0.0000000	1.2200000	-30.5091496
0.0000000	1.2687999	-31.0353241
0.0000000	1.3176000	-30.8465710
0.0000000	1.3664000	-29.8186741
0.0000000	1.4152000	-30.1414642
0.0000000	1.4640000	-29.7896862
0.0000000	1.5128000	-49.2873459
0.0000000	1.5616000	-37.8980713
0.0000000	1.6104001	-1816.5826416
0.0000000	1.6592000	-103.2392578
0.0000000	1.7079999	-27.2455254
0.0000000	1.7567999	-24.7109890
0.0000000	1.8055999	-24.3221798
0.0000000	1.8544000	-26.1321201
0.0000000	1.9031999	-26.5512962
0.0000000	1.9520000	-25.3542042
0.0000000	2.0007999	-30.9828243
0.0000000	2.0495999	-10.3691444
0.0000000	2.0983999	-298.5036011
0.0000000	2.1471999	25.5866280
0.0000000	2.1960001	-25.8658028
0.0000000	2.2448001	-27.6547108
0.0000000	2.2935998	-33.7620316
0.0000000	2.3423998	-19.5205803
0.0000000	2.3912001	95.2782059
0.0000000	2.4400001	-22.5838432
0.0000000	2.4888000	-23.4822502
0.0000000	2.5375998	-10.5213995
0.0000000	2.5863998	-8.3712749
0.0000000	2.6352000	-6.1380310
0.0000000	2.6840000	-14.1884575
0.0000000	2.7328000	160.4391327
0.0000000	2.7815998	188.5453796
0.0000000	2.8304000	-23.9281578
0.0000000	2.8792000	-20.2845516
0.0000000	2.9280000	-22.6301708
0.0000000	2.9768000	-15.9305267
0.0000000	3.0256000	-20.2076588
0.0000000	3.0743999	-30.6853104
0.0000000	3.1231999	-24.6096992

0.0000000	3.1719999	-30.4568138
0.0000000	3.2208002	-528.6297607
0.0000000	3.2695999	-1035.9666748
0.0000000	3.3183999	-20.4694042
0.0000000	3.3671999	-3545.7485352
0.0000000	3.4159999	1273.1462402
0.0000000	3.4648001	-6.5582819
0.0000000	3.5135999	-27.3421898
0.0000000	3.5623999	-335.1669617
0.0000000	3.6111999	-2110.5068359
0.0000000	3.6600001	-477.1573486
0.0000000	3.7088001	-630.3519287
0.0000000	3.7575998	-987.4083862
0.0000000	3.8063998	-1792.4201660
0.0000000	3.8552001	-2602.7106934
0.0000000	3.9040000	-2551.7666016
0.0000000	3.9528000	1168.0538330
0.0000000	4.0015998	-254.2689056
0.0000000	4.0503998	715.4755249
0.0000000	4.0991998	91.8264923
0.0000000	4.1479998	-679.8249512
0.0000000	4.1967998	3.3983917
0.0000000	4.2456002	-257.5597534
0.0000000	4.2943997	-506.4634705
0.0000000	4.3431997	-13.1306381
0.0000000	4.3920002	-257.1728821
0.0000000	4.4407997	-22.6240120
0.0000000	4.4896002	-15.3929930
0.0000000	4.5383997	-679.0933838
0.0000000	4.5871997	-12.6531448
0.0000000	4.6360002	-21.7629681
0.0000000	4.6847997	-28.6433830
0.0000000	4.7336001	-19.6288528
0.0000000	4.7824001	-16.4705734
0.0000000	4.8311996	-1523.1695557
0.0000000	4.8800001	236.4813843
0.0000000	4.9287996	-207.6296692
0.0000000	4.9776001	1095.6180420
0.0000000	5.0264001	89.6206589
0.0000000	5.0751996	-310.6539001
0.0000000	5.1240001	-12.2237625
0.0000000	5.1727996	569.0734863
0.0000000	5.2216001	32.9122734
0.0000000	5.2704000	-161.5428009
0.0000000	5.3191996	-39.6240005
0.0000000	5.3680000	-3603.0876465
0.0000000	5.4168000	537.5005493
0.0000000	5.4656000	-204.2545319
0.0000000	5.5144000	39.1025543
0.0000000	5.5631995	497.9049683
0.0000000	5.6120000	400.9147339
0.0000000	5.6608000	-37.4821777
0.0000000	5.7096000	64.0305710
0.0000000	5.7584000	135.8534546
0.0000000	5.8071995	-11.4356222
0.0000000	5.8559999	30.6784534
0.0000000	5.9047999	-16.6248798
0.0000000	5.9535999	-40.2377815

0.0000000	6.0023999	-1602.6459961
0.0000000	6.0511999	-14.5475760
0.0000000	6.0999999	-19.4905815
0.0000000	6.1487999	-13.2281113
0.0000000	6.1975999	-23.3928318
0.0000000	6.2463999	-1825.8367920
0.0000000	6.2951999	-19.1500206
0.0000000	6.3439999	-348.9850769
0.0000000	6.3927999	-15.0499229
0.0000000	6.4416003	-12.3283291
0.0000000	6.4903998	-11.1972275
0.0000000	6.5391998	-11.7391586
0.0000000	6.5879998	453.1596069
0.0000000	6.6367998	-12.0299397
0.0000000	6.6856003	-13.7980843
0.0000000	6.7343998	-13.4342890
0.0000000	6.7831998	-14.4138041
0.0000000	6.8319998	60.3386612
0.0000000	6.8807998	-14.0632124
0.0000000	6.9296002	-525.3755493
0.0000000	6.9783998	-3.8050013
0.0000000	7.0271997	-14.2400475
0.0000000	7.0760002	15.4097548
0.0000000	7.1247997	-2527.2314453
0.0000000	7.1736002	-2026.5137939
0.0000000	7.2223997	-1152.2697754
0.0000000	7.2711997	-246.5575562
0.0000000	7.3200002	-20.0877647
0.0000000	7.3687997	225.5754089
0.0000000	7.4176002	467.9898987
0.0000000	7.4663997	368.0511475
0.0000000	7.5151997	-1127.9826660
0.0000000	7.5640001	-1246.1699219
0.0000000	7.6127996	-2940.0795898
0.0000000	7.6616001	-942.7943726
0.0000000	7.7104001	-483.2994995
0.0000000	7.7591996	53.4228668
0.0000000	7.8080001	486.3283997
0.0000000	7.8567996	639.9927368
0.0000000	7.9056001	-1320.5405273
0.0000000	7.9544001	2102.8813477
0.0000000	8.0031996	2713.2917480
0.0000000	8.0520000	2708.2023926
0.0000000	8.1007996	2865.7614746
0.0000000	8.1496000	3056.1228027
0.0000000	8.1983995	1901.4589844
0.0000000	8.2472000	-4349.2954102
0.0000000	8.2959995	-1541.4616699
0.0000000	8.3448000	-1228.0290527
0.0000000	8.3935995	-1125.4218750
0.0000000	8.4423990	-484.4241943
0.0000000	8.4912004	-1131.5966797
0.0000000	8.5400000	-3053.3974609
0.0000000	8.5887995	-1479.5439453
0.0000000	8.6375999	289.8039246
0.0000000	8.6863995	2613.0617676
0.0000000	8.7351999	-970.5446167
0.0000000	8.7840004	1110.5856934

0.0000000	8.8327999	61.8698196
0.0000000	8.8815994	-11.4787340
0.0000000	8.9303989	-24.3231392
0.0000000	8.9792004	-11.5707369
0.0000000	9.0279999	-5.6570816
0.0000000	9.0767994	7.6209965
0.0000000	9.1255999	-17.4164886
0.0000000	9.1743994	-10.0326509
0.0000000	9.2231998	38.6141205
0.0000000	9.2720003	-8.3246965
0.0000000	9.3207998	-9.7431278
0.0000000	9.3695993	-10.7406855
0.0000000	9.4183998	-11.7665806
0.0000000	9.4672003	-11.1571302
0.0000000	9.5159998	-14.2550926
0.0000000	9.5648003	-0.0490646
0.0000000	9.6135998	-13.4766607
0.0000000	9.6623993	-10.1932793
0.0000000	9.7111998	-10.1300049
0.0000000	9.7600002	-10.8533220
0.0000000	9.8087997	-10.9939671
0.0000000	9.8575993	3.6409855
0.0000000	9.9063997	-7.8758488
0.0000000	9.9552002	-10.9061623
0.0000000	10.0039997	-10.0494690
10.0000000	0.3416000	-36.9113998
10.0000000	0.3904000	-39.6928520
10.0000000	0.4392000	-48.6869965
10.0000000	0.4880000	-38.7310905
10.0000000	0.5368000	-39.6772232
10.0000000	0.5856000	-55.6141891
10.0000000	0.6344000	-10.2143364
10.0000000	0.6832000	-151.2127991
10.0000000	0.7320000	-31.1832924
10.0000000	0.7808000	-94.5099792
10.0000000	0.8296000	-45.3353157
10.0000000	0.8784000	-35.1850433
10.0000000	0.9272000	-33.3416481
10.0000000	0.9760000	-30.7720432
10.0000000	1.0247999	-28.9417114
10.0000000	1.0735999	-28.3867207
10.0000000	1.1224000	-29.1112080
10.0000000	1.1711999	-30.0159988
10.0000000	1.2200000	-30.5418472
10.0000000	1.2687999	-30.6584110
10.0000000	1.3176000	-30.1623898
10.0000000	1.3664000	-30.0850086
10.0000000	1.4152000	-30.1416683
10.0000000	1.4640000	-29.7879772
10.0000000	1.5128000	-49.1901703
10.0000000	1.5616000	-37.8562317
10.0000000	1.6104001	-1814.0397949
10.0000000	1.6592000	-102.9453278
10.0000000	1.7079999	-26.9238892
10.0000000	1.7567999	-24.4926414
10.0000000	1.8055999	-24.7814903
10.0000000	1.8544000	-26.7717209
10.0000000	1.9031999	-26.3976669

10.0000000	1.9520000	-25.7703304
10.0000000	2.0007999	-30.9202423
10.0000000	2.0495999	-10.5557613
10.0000000	2.0983999	-297.0278320
10.0000000	2.1471999	25.3193588
10.0000000	2.1960001	-25.8617115
10.0000000	2.2448001	-27.6367989
10.0000000	2.2935998	-33.7024078
10.0000000	2.3423998	-19.5349846
10.0000000	2.3912001	94.6566620
10.0000000	2.4400001	-22.5863304
10.0000000	2.4888000	-23.4820328
10.0000000	2.5375998	-10.5385065
10.0000000	2.5863998	-8.3910341
10.0000000	2.6352000	-6.1607761
10.0000000	2.6840000	-14.2004004
10.0000000	2.7328000	160.1731110
10.0000000	2.7815998	188.2389526
10.0000000	2.8304000	-23.9268532
10.0000000	2.8792000	-20.2878151
10.0000000	2.9280000	-22.6296005
10.0000000	2.9768000	-15.9388962
10.0000000	3.0256000	-20.2102432
10.0000000	3.0743999	-30.6739235
10.0000000	3.1231999	-24.6062431
10.0000000	3.1719999	-30.4485207
10.0000000	3.2208002	-528.3920288
10.0000000	3.2695999	-1035.4916992
10.0000000	3.3183999	-20.4696007
10.0000000	3.3671999	-3544.2868652
10.0000000	3.4159999	1272.6109619
10.0000000	3.4648001	-6.5639896
10.0000000	3.5135999	-27.3391991
10.0000000	3.5623999	-335.0373840
10.0000000	3.6111999	-2109.6472168
10.0000000	3.6600001	-476.9692383
10.0000000	3.7088001	-630.1005859
10.0000000	3.7575998	-987.0098267
10.0000000	3.8063998	-1791.6903076
10.0000000	3.8552001	-2601.6472168
10.0000000	3.9040000	-2550.7241211
10.0000000	3.9528000	1167.5654297
10.0000000	4.0015998	-254.1717987
10.0000000	4.0503998	715.1729126
10.0000000	4.0991998	91.7808151
10.0000000	4.1479998	-679.5527344
10.0000000	4.1967998	3.3891354
10.0000000	4.2456002	-257.4614563
10.0000000	4.2943997	-506.2629089
10.0000000	4.3431997	-13.1328192
10.0000000	4.3920002	-257.0747681
10.0000000	4.4407997	-22.6222363
10.0000000	4.4896002	-15.3938751
10.0000000	4.5383997	-678.8322754
10.0000000	4.5871997	-12.6551619
10.0000000	4.6360002	-21.7614250
10.0000000	4.6847997	-28.6394577
10.0000000	4.7336001	-19.6281300

10.0000000	4.7824001	-16.4710274
10.0000000	4.8311996	-1522.6383057
10.0000000	4.8800001	236.3918304
10.0000000	4.9287996	-207.5624084
10.0000000	4.9776001	1095.2254639
10.0000000	5.0264001	89.5831375
10.0000000	5.0751996	-310.5502930
10.0000000	5.1240001	-12.2246838
10.0000000	5.1727996	568.9575806
10.0000000	5.2216001	32.9028320
10.0000000	5.2704000	-161.5146790
10.0000000	5.3191996	-39.6194496
10.0000000	5.3680000	-3602.3952637
10.0000000	5.4168000	537.3937378
10.0000000	5.4656000	-204.2183533
10.0000000	5.5144000	39.0919037
10.0000000	5.5631995	497.8063965
10.0000000	5.6120000	400.8347473
10.0000000	5.6608000	-37.4780922
10.0000000	5.7096000	64.0151443
10.0000000	5.7584000	135.8245087
10.0000000	5.8071995	-11.4364929
10.0000000	5.8559999	30.6696033
10.0000000	5.9047999	-16.6246815
10.0000000	5.9535999	-40.2330437
10.0000000	6.0023999	-1602.3416748
10.0000000	6.0511999	-14.5477867
10.0000000	6.0999999	-19.4897480
10.0000000	6.1487999	-13.2285748
10.0000000	6.1975999	-23.3910675
10.0000000	6.2463999	-1825.4893799
10.0000000	6.2951999	-19.1492062
10.0000000	6.3439999	-348.9210510
10.0000000	6.3927999	-15.0499048
10.0000000	6.4416003	-12.3289156
10.0000000	6.4903998	-11.1979036
10.0000000	6.5391998	-11.7398396
10.0000000	6.5879998	452.4689331
10.0000000	6.6367998	-12.0304890
10.0000000	6.6856003	-13.7982540
10.0000000	6.7343998	-13.4345045
10.0000000	6.7831998	-14.4133892
10.0000000	6.8319998	60.3195419
10.0000000	6.8807998	-14.0630741
10.0000000	6.9296002	-524.6140747
10.0000000	6.9783998	-3.8178244
10.0000000	7.0271997	-14.2390203
10.0000000	7.0760002	15.3675680
10.0000000	7.1247997	-2523.2854004
10.0000000	7.1736002	-2023.3509521
10.0000000	7.2223997	-1150.4765625
10.0000000	7.2711997	-246.1897736
10.0000000	7.3200002	-20.0757751
10.0000000	7.3687997	225.2008820
10.0000000	7.4176002	467.2340698
10.0000000	7.4663997	367.4534912
10.0000000	7.5151997	-1126.2216797
10.0000000	7.5640001	-1244.2225342

10.0000000	7.6127996	-2935.4619141
10.0000000	7.6616001	-941.3229980
10.0000000	7.7104001	-482.5532227
10.0000000	7.7591996	53.3218117
10.0000000	7.8080001	485.5430298
10.0000000	7.8567996	638.9646606
10.0000000	7.9056001	-1318.4675293
10.0000000	7.9544001	2099.5715332
10.0000000	8.0031996	2709.0222168
10.0000000	8.0520000	2703.9404297
10.0000000	8.1007996	2861.2519531
10.0000000	8.1496000	3051.3146973
10.0000000	8.1983995	1898.4664307
10.0000000	8.2472000	-4342.4609375
10.0000000	8.2959995	-1539.0415039
10.0000000	8.3448000	-1226.0998535
10.0000000	8.3935995	-1123.6541748
10.0000000	8.4423990	-483.6661987
10.0000000	8.4912004	-1129.8204346
10.0000000	8.5400000	-3048.5976563
10.0000000	8.5887995	-1477.2224121
10.0000000	8.6375999	289.3425903
10.0000000	8.6863995	2608.9436035
10.0000000	8.7351999	-969.0203857
10.0000000	8.7840004	1108.8288574
10.0000000	8.8327999	61.7582092
10.0000000	8.8815994	-11.4789076
10.0000000	8.9303989	-24.3194199
10.0000000	8.9792004	-11.5708189
10.0000000	9.0279999	-5.6588497
10.0000000	9.0767994	7.6152782
10.0000000	9.1255999	-17.4147739
10.0000000	9.1743994	-10.0330534
10.0000000	9.2231998	38.5983887
10.0000000	9.2720003	-8.3256330
10.0000000	9.3207998	-9.7436676
10.0000000	9.3695993	-10.7409115
10.0000000	9.4183998	-11.7664566
10.0000000	9.4672003	-11.1571712
10.0000000	9.5159998	-14.2542019
10.0000000	9.5648003	-0.0524378
10.0000000	9.6135998	-13.4758701
10.0000000	9.6623993	-10.1935539
10.0000000	9.7111998	-10.1302767
10.0000000	9.7600002	-10.8533640
10.0000000	9.8087997	-10.9939747
10.0000000	9.8575993	3.6365643
10.0000000	9.9063997	-7.8767624
10.0000000	9.9552002	-10.9061661
10.0000000	10.0039997	-10.0497169
20.0000000	0.3416000	-36.1300125
20.0000000	0.3904000	-38.4631271
20.0000000	0.4392000	-48.5214729
20.0000000	0.4880000	-39.3543091
20.0000000	0.5368000	-39.6819305
20.0000000	0.5856000	-55.2260361
20.0000000	0.6344000	-10.8843956
20.0000000	0.6832000	-147.9552917

20.0000000	0.7320000	-31.3825455
20.0000000	0.7808000	-92.4263306
20.0000000	0.8296000	-44.9437027
20.0000000	0.8784000	-34.6080666
20.0000000	0.9272000	-32.6063042
20.0000000	0.9760000	-30.7913055
20.0000000	1.0247999	-29.2786140
20.0000000	1.0735999	-28.7081833
20.0000000	1.1224000	-28.7264690
20.0000000	1.1711999	-29.1730785
20.0000000	1.2200000	-29.7952595
20.0000000	1.2687999	-30.2873306
20.0000000	1.3176000	-30.6267757
20.0000000	1.3664000	-30.3513432
20.0000000	1.4152000	-30.1418724
20.0000000	1.4640000	-29.7862663
20.0000000	1.5128000	-49.0929909
20.0000000	1.5616000	-37.8143921
20.0000000	1.6104001	-1811.4970703
20.0000000	1.6592000	-102.6513901
20.0000000	1.7079999	-26.9274807
20.0000000	1.7567999	-24.9034195
20.0000000	1.8055999	-25.1931820
20.0000000	1.8544000	-25.7986126
20.0000000	1.9031999	-26.2440376
20.0000000	1.9520000	-26.1864567
20.0000000	2.0007999	-30.8576603
20.0000000	2.0495999	-10.7423782
20.0000000	2.0983999	-295.5520325
20.0000000	2.1471999	25.0520897
20.0000000	2.1960001	-25.8576202
20.0000000	2.2448001	-27.6188889
20.0000000	2.2935998	-33.6427841
20.0000000	2.3423998	-19.5493908
20.0000000	2.3912001	94.0351181
20.0000000	2.4400001	-22.5888176
20.0000000	2.4888000	-23.4818153
20.0000000	2.5375998	-10.5556145
20.0000000	2.5863998	-8.4107924
20.0000000	2.6352000	-6.1835194
20.0000000	2.6840000	-14.2123423
20.0000000	2.7328000	159.9070892
20.0000000	2.7815998	187.9325562
20.0000000	2.8304000	-23.9255486
20.0000000	2.8792000	-20.2910786
20.0000000	2.9280000	-22.6290321
20.0000000	2.9768000	-15.9472675
20.0000000	3.0256000	-20.2128277
20.0000000	3.0743999	-30.6625366
20.0000000	3.1231999	-24.6027870
20.0000000	3.1719999	-30.4402275
20.0000000	3.2208002	-528.1542969
20.0000000	3.2695999	-1035.0167236
20.0000000	3.3183999	-20.4697990
20.0000000	3.3671999	-3542.8251953
20.0000000	3.4159999	1272.0756836
20.0000000	3.4648001	-6.5696964
20.0000000	3.5135999	-27.3362083

20.0000000	3.5623999	-334.9077759
20.0000000	3.6111999	-2108.7873535
20.0000000	3.6600001	-476.7810669
20.0000000	3.7088001	-629.8491821
20.0000000	3.7575998	-986.6112671
20.0000000	3.8063998	-1790.9605713
20.0000000	3.8552001	-2600.5837402
20.0000000	3.9040000	-2549.6816406
20.0000000	3.9528000	1167.0771484
20.0000000	4.0015998	-254.0746918
20.0000000	4.0503998	714.8703613
20.0000000	4.0991998	91.7351303
20.0000000	4.1479998	-679.2804565
20.0000000	4.1967998	3.3798790
20.0000000	4.2456002	-257.3631592
20.0000000	4.2943997	-506.0623169
20.0000000	4.3431997	-13.1349993
20.0000000	4.3920002	-256.9766541
20.0000000	4.4407997	-22.6204605
20.0000000	4.4896002	-15.3947582
20.0000000	4.5383997	-678.5712280
20.0000000	4.5871997	-12.6571779
20.0000000	4.6360002	-21.7598839
20.0000000	4.6847997	-28.6355324
20.0000000	4.7336001	-19.6274071
20.0000000	4.7824001	-16.4714832
20.0000000	4.8311996	-1522.1070557
20.0000000	4.8800001	236.3022766
20.0000000	4.9287996	-207.4951477
20.0000000	4.9776001	1094.8330078
20.0000000	5.0264001	89.5456161
20.0000000	5.0751996	-310.4466553
20.0000000	5.1240001	-12.2256050
20.0000000	5.1727996	568.8417358
20.0000000	5.2216001	32.8933868
20.0000000	5.2704000	-161.4865875
20.0000000	5.3191996	-39.6149025
20.0000000	5.3680000	-3601.7028809
20.0000000	5.4168000	537.2869873
20.0000000	5.4656000	-204.1821594
20.0000000	5.5144000	39.0812454
20.0000000	5.5631995	497.7078247
20.0000000	5.6120000	400.7547913
20.0000000	5.6608000	-37.4740028
20.0000000	5.7096000	63.9997177
20.0000000	5.7584000	135.7955780
20.0000000	5.8071995	-11.4373636
20.0000000	5.8559999	30.6607533
20.0000000	5.9047999	-16.6244831
20.0000000	5.9535999	-40.2283020
20.0000000	6.0023999	-1602.0371094
20.0000000	6.0511999	-14.5479984
20.0000000	6.0999999	-19.4889164
20.0000000	6.1487999	-13.2290373
20.0000000	6.1975999	-23.3893013
20.0000000	6.2463999	-1825.1420898
20.0000000	6.2951999	-19.1483917
20.0000000	6.3439999	-348.8569946

20.0000000	6.3927999	-15.0498867
20.0000000	6.4416003	-12.3295031
20.0000000	6.4903998	-11.1985788
20.0000000	6.5391998	-11.7405205
20.0000000	6.5879998	451.7782288
20.0000000	6.6367998	-12.0310383
20.0000000	6.6856003	-13.7984247
20.0000000	6.7343998	-13.4347200
20.0000000	6.7831998	-14.4129734
20.0000000	6.8319998	60.3004227
20.0000000	6.8807998	-14.0629349
20.0000000	6.9296002	-523.8526611
20.0000000	6.9783998	-3.8306484
20.0000000	7.0271997	-14.2379923
20.0000000	7.0760002	15.3253832
20.0000000	7.1247997	-2519.3395996
20.0000000	7.1736002	-2020.1881104
20.0000000	7.2223997	-1148.6834717
20.0000000	7.2711997	-245.8219910
20.0000000	7.3200002	-20.0637875
20.0000000	7.3687997	224.8263550
20.0000000	7.4176002	466.4782410
20.0000000	7.4663997	366.8558350
20.0000000	7.5151997	-1124.4605713
20.0000000	7.5640001	-1242.2751465
20.0000000	7.6127996	-2930.8442383
20.0000000	7.6616001	-939.8516846
20.0000000	7.7104001	-481.8069458
20.0000000	7.7591996	53.2207642
20.0000000	7.8080001	484.7576599
20.0000000	7.8567996	637.9365845
20.0000000	7.9056001	-1316.3946533
20.0000000	7.9544001	2096.2617188
20.0000000	8.0031996	2704.7526855
20.0000000	8.0520000	2699.6784668
20.0000000	8.1007996	2856.7424316
20.0000000	8.1496000	3046.5068359
20.0000000	8.1983995	1895.4739990
20.0000000	8.2472000	-4335.6259766
20.0000000	8.2959995	-1536.6213379
20.0000000	8.3448000	-1224.1706543
20.0000000	8.3935995	-1121.8864746
20.0000000	8.4423990	-482.9081726
20.0000000	8.4912004	-1128.0441895
20.0000000	8.5400000	-3043.7978516
20.0000000	8.5887995	-1474.9008789
20.0000000	8.6375999	288.8812561
20.0000000	8.6863995	2604.8251953
20.0000000	8.7351999	-967.4960938
20.0000000	8.7840004	1107.0721436
20.0000000	8.8327999	61.6466064
20.0000000	8.8815994	-11.4790821
20.0000000	8.9303989	-24.3157005
20.0000000	8.9792004	-11.5709009
20.0000000	9.0279999	-5.6606178
20.0000000	9.0767994	7.6095619
20.0000000	9.1255999	-17.4130611
20.0000000	9.1743994	-10.0334549

20.0000000	9.2231998	38.5826569
20.0000000	9.2720003	-8.3265696
20.0000000	9.3207998	-9.7442064
20.0000000	9.3695993	-10.7411385
20.0000000	9.4183998	-11.7663317
20.0000000	9.4672003	-11.1572123
20.0000000	9.5159998	-14.2533112
20.0000000	9.5648003	-0.0558109
20.0000000	9.6135998	-13.4750786
20.0000000	9.6623993	-10.1938295
20.0000000	9.7111998	-10.1305485
20.0000000	9.7600002	-10.8534050
20.0000000	9.8087997	-10.9939823
20.0000000	9.8575993	3.6321421
20.0000000	9.9063997	-7.8776765
20.0000000	9.9552002	-10.9061708
20.0000000	10.0039997	-10.0499649
30.0000000	0.3416000	-36.2285042
30.0000000	0.3904000	-38.8761139
30.0000000	0.4392000	-48.3559494
30.0000000	0.4880000	-39.9775276
30.0000000	0.5368000	-39.6866379
30.0000000	0.5856000	-54.8378830
30.0000000	0.6344000	-11.5544548
30.0000000	0.6832000	-144.6977692
30.0000000	0.7320000	-31.5817986
30.0000000	0.7808000	-90.3426819
30.0000000	0.8296000	-44.5520897
30.0000000	0.8784000	-35.2300301
30.0000000	0.9272000	-33.1800232
30.0000000	0.9760000	-30.9844398
30.0000000	1.0247999	-29.5585899
30.0000000	1.0735999	-29.3056240
30.0000000	1.1224000	-29.6835442
30.0000000	1.1711999	-30.0722504
30.0000000	1.2200000	-30.1186829
30.0000000	1.2687999	-30.2735615
30.0000000	1.3176000	-30.2178574
30.0000000	1.3664000	-30.6176777
30.0000000	1.4152000	-30.1420784
30.0000000	1.4640000	-29.7845573
30.0000000	1.5128000	-48.9958115
30.0000000	1.5616000	-37.7725487
30.0000000	1.6104001	-1808.9542236
30.0000000	1.6592000	-102.3574600
30.0000000	1.7079999	-27.1082115
30.0000000	1.7567999	-23.8585415
30.0000000	1.8055999	-23.8786488
30.0000000	1.8544000	-25.3720646
30.0000000	1.9031999	-26.0904083
30.0000000	1.9520000	-26.6025829
30.0000000	2.0007999	-30.7950783
30.0000000	2.0495999	-10.9289942
30.0000000	2.0983999	-294.0762634
30.0000000	2.1471999	24.7848244
30.0000000	2.1960001	-25.8535290
30.0000000	2.2448001	-27.6009769
30.0000000	2.2935998	-33.5831604

30.0000000	2.3423998	-19.5637951
30.0000000	2.3912001	93.4135666
30.0000000	2.4400001	-22.5913029
30.0000000	2.4888000	-23.4815979
30.0000000	2.5375998	-10.5727224
30.0000000	2.5863998	-8.4305515
30.0000000	2.6352000	-6.2062645
30.0000000	2.6840000	-14.2242842
30.0000000	2.7328000	159.6410828
30.0000000	2.7815998	187.6261292
30.0000000	2.8304000	-23.9242458
30.0000000	2.8792000	-20.2943420
30.0000000	2.9280000	-22.6284637
30.0000000	2.9768000	-15.9556379
30.0000000	3.0256000	-20.2154121
30.0000000	3.0743999	-30.6511497
30.0000000	3.1231999	-24.5993328
30.0000000	3.1719999	-30.4319344
30.0000000	3.2208002	-527.9165649
30.0000000	3.2695999	-1034.5417480
30.0000000	3.3183999	-20.4699974
30.0000000	3.3671999	-3541.3635254
30.0000000	3.4159999	1271.5402832
30.0000000	3.4648001	-6.5754051
30.0000000	3.5135999	-27.3332176
30.0000000	3.5623999	-334.7781982
30.0000000	3.6111999	-2107.9277344
30.0000000	3.6600001	-476.5929565
30.0000000	3.7088001	-629.5977783
30.0000000	3.7575998	-986.2127686
30.0000000	3.8063998	-1790.2307129
30.0000000	3.8552001	-2599.5202637
30.0000000	3.9040000	-2548.6391602
30.0000000	3.9528000	1166.5888672
30.0000000	4.0015998	-253.9775696
30.0000000	4.0503998	714.5678101
30.0000000	4.0991998	91.6894455
30.0000000	4.1479998	-679.0082397
30.0000000	4.1967998	3.3706226
30.0000000	4.2456002	-257.2648315
30.0000000	4.2943997	-505.8617554
30.0000000	4.3431997	-13.1371803
30.0000000	4.3920002	-256.8785400
30.0000000	4.4407997	-22.6186867
30.0000000	4.4896002	-15.3956413
30.0000000	4.5383997	-678.3101196
30.0000000	4.5871997	-12.6591949
30.0000000	4.6360002	-21.7583408
30.0000000	4.6847997	-28.6316071
30.0000000	4.7336001	-19.6266861
30.0000000	4.7824001	-16.4719372
30.0000000	4.8311996	-1521.5758057
30.0000000	4.8800001	236.2127228
30.0000000	4.9287996	-207.4278870
30.0000000	4.9776001	1094.4405518
30.0000000	5.0264001	89.5080948
30.0000000	5.0751996	-310.3430176
30.0000000	5.1240001	-12.2265263

30.0000000	5.1727996	568.7258911
30.0000000	5.2216001	32.8839417
30.0000000	5.2704000	−161.4584656
30.0000000	5.3191996	−39.6103516
30.0000000	5.3680000	−3601.0104980
30.0000000	5.4168000	537.1802368
30.0000000	5.4656000	−204.1459808
30.0000000	5.5144000	39.0705872
30.0000000	5.5631995	497.6092529
30.0000000	5.6120000	400.6748047
30.0000000	5.6608000	−37.4699173
30.0000000	5.7096000	63.9842834
30.0000000	5.7584000	135.7666473
30.0000000	5.8071995	−11.4382343
30.0000000	5.8559999	30.6519032
30.0000000	5.9047999	−16.6242867
30.0000000	5.9535999	−40.2235641
30.0000000	6.0023999	−1601.7327881
30.0000000	6.0511999	−14.5482101
30.0000000	6.0999999	−19.4880829
30.0000000	6.1487999	−13.2294998
30.0000000	6.1975999	−23.3875370
30.0000000	6.2463999	−1824.7946777
30.0000000	6.2951999	−19.1475773
30.0000000	6.3439999	−348.7929382
30.0000000	6.3927999	−15.0498686
30.0000000	6.4416003	−12.3300896
30.0000000	6.4903998	−11.1992550
30.0000000	6.5391998	−11.7412024
30.0000000	6.5879998	451.0875244
30.0000000	6.6367998	−12.0315876
30.0000000	6.6856003	−13.7985945
30.0000000	6.7343998	−13.4349356
30.0000000	6.7831998	−14.4125576
30.0000000	6.8319998	60.2813110
30.0000000	6.8807998	−14.0627966
30.0000000	6.9296002	−523.0911865
30.0000000	6.9783998	−3.8434725
30.0000000	7.0271997	−14.2369642
30.0000000	7.0760002	15.2831984
30.0000000	7.1247997	−2515.3937988
30.0000000	7.1736002	−2017.0251465
30.0000000	7.2223997	−1146.8902588
30.0000000	7.2711997	−245.4542084
30.0000000	7.3200002	−20.0517998
30.0000000	7.3687997	224.4518280
30.0000000	7.4176002	465.7224121
30.0000000	7.4663997	366.2581787
30.0000000	7.5151997	−1122.6995850
30.0000000	7.5640001	−1240.3277588
30.0000000	7.6127996	−2926.2265625
30.0000000	7.6616001	−938.3803101
30.0000000	7.7104001	−481.0606689
30.0000000	7.7591996	53.1197166
30.0000000	7.8080001	483.9723206
30.0000000	7.8567996	636.9084473
30.0000000	7.9056001	−1314.3217773
30.0000000	7.9544001	2092.9516602

30.0000000	8.0031996	2700.4831543
30.0000000	8.0520000	2695.4162598
30.0000000	8.1007996	2852.2329102
30.0000000	8.1496000	3041.6987305
30.0000000	8.1983995	1892.4814453
30.0000000	8.2472000	-4328.7910156
30.0000000	8.2959995	-1534.2011719
30.0000000	8.3448000	-1222.2414551
30.0000000	8.3935995	-1120.1188965
30.0000000	8.4423990	-482.1501770
30.0000000	8.4912004	-1126.2679443
30.0000000	8.5400000	-3038.9980469
30.0000000	8.5887995	-1472.5793457
30.0000000	8.6375999	288.4199524
30.0000000	8.6863995	2600.7067871
30.0000000	8.7351999	-965.9718628
30.0000000	8.7840004	1105.3154297
30.0000000	8.8327999	61.5349960
30.0000000	8.8815994	-11.4792566
30.0000000	8.9303989	-24.3119793
30.0000000	8.9792004	-11.5709839
30.0000000	9.0279999	-5.6623864
30.0000000	9.0767994	7.6038437
30.0000000	9.1255999	-17.4113464
30.0000000	9.1743994	-10.0338573
30.0000000	9.2231998	38.5669250
30.0000000	9.2720003	-8.3275061
30.0000000	9.3207998	-9.7447453
30.0000000	9.3695993	-10.7413645
30.0000000	9.4183998	-11.7662067
30.0000000	9.4672003	-11.1572533
30.0000000	9.5159998	-14.2524204
30.0000000	9.5648003	-0.0591831
30.0000000	9.6135998	-13.4742880
30.0000000	9.6623993	-10.1941042
30.0000000	9.7111998	-10.1308203
30.0000000	9.7600002	-10.8534470
30.0000000	9.8087997	-10.9939909
30.0000000	9.8575993	3.6277180
30.0000000	9.9063997	-7.8785901
30.0000000	9.9552002	-10.9061747
30.0000000	10.0039997	-10.0502119
40.0000000	0.3416000	-36.3901329
40.0000000	0.3904000	-39.2594109
40.0000000	0.4392000	-48.1904297
40.0000000	0.4880000	-39.7538223
40.0000000	0.5368000	-39.6913490
40.0000000	0.5856000	-54.4497261
40.0000000	0.6344000	-12.2245140
40.0000000	0.6832000	-141.4402618
40.0000000	0.7320000	-31.7810516
40.0000000	0.7808000	-88.2590332
40.0000000	0.8296000	-44.1604767
40.0000000	0.8784000	-35.2688293
40.0000000	0.9272000	-33.6537323
40.0000000	0.9760000	-31.6114044
40.0000000	1.0247999	-30.3343277
40.0000000	1.0735999	-29.9992104

40.0000000	1.1224000	-29.7233715
40.0000000	1.1711999	-30.3055210
40.0000000	1.2200000	-30.8832531
40.0000000	1.2687999	-30.5306168
40.0000000	1.3176000	-30.8473034
40.0000000	1.3664000	-30.5142307
40.0000000	1.4152000	-30.1422825
40.0000000	1.4640000	-29.7828484
40.0000000	1.5128000	-48.8986359
40.0000000	1.5616000	-37.7307091
40.0000000	1.6104001	-1806.4114990
40.0000000	1.6592000	-102.0635300
40.0000000	1.7079999	-27.6265907
40.0000000	1.7567999	-24.6469040
40.0000000	1.8055999	-24.8899632
40.0000000	1.8544000	-25.5943260
40.0000000	1.9031999	-26.2429638
40.0000000	1.9520000	-26.6446838
40.0000000	2.0007999	-30.7324963
40.0000000	2.0495999	-11.1156101
40.0000000	2.0983999	-292.6004944
40.0000000	2.1471999	24.5175591
40.0000000	2.1960001	-25.8494377
40.0000000	2.2448001	-27.5830650
40.0000000	2.2935998	-33.5235405
40.0000000	2.3423998	-19.5781994
40.0000000	2.3912001	92.7920227
40.0000000	2.4400001	-22.5937901
40.0000000	2.4888000	-23.4813805
40.0000000	2.5375998	-10.5898314
40.0000000	2.5863998	-8.4503107
40.0000000	2.6352000	-6.2290077
40.0000000	2.6840000	-14.2362251
40.0000000	2.7328000	159.3750610
40.0000000	2.7815998	187.3197021
40.0000000	2.8304000	-23.9229412
40.0000000	2.8792000	-20.2976055
40.0000000	2.9280000	-22.6278934
40.0000000	2.9768000	-15.9640083
40.0000000	3.0256000	-20.2179966
40.0000000	3.0743999	-30.6397629
40.0000000	3.1231999	-24.5958767
40.0000000	3.1719999	-30.4236412
40.0000000	3.2208002	-527.6788330
40.0000000	3.2695999	-1034.0667725
40.0000000	3.3183999	-20.4701958
40.0000000	3.3671999	-3539.9018555
40.0000000	3.4159999	1271.0050049
40.0000000	3.4648001	-6.5811138
40.0000000	3.5135999	-27.3302269
40.0000000	3.5623999	-334.6485901
40.0000000	3.6111999	-2107.0678711
40.0000000	3.6600001	-476.4048462
40.0000000	3.7088001	-629.3464355
40.0000000	3.7575998	-985.8142090
40.0000000	3.8063998	-1789.5008545
40.0000000	3.8552001	-2598.4567871
40.0000000	3.9040000	-2547.5966797

40.0000000	3.9528000	1166.1005859
40.0000000	4.0015998	-253.8804626
40.0000000	4.0503998	714.2652588
40.0000000	4.0991998	91.6437607
40.0000000	4.1479998	-678.7360229
40.0000000	4.1967998	3.3613663
40.0000000	4.2456002	-257.1665344
40.0000000	4.2943997	-505.6611938
40.0000000	4.3431997	-13.1393604
40.0000000	4.3920002	-256.7804260
40.0000000	4.4407997	-22.6169109
40.0000000	4.4896002	-15.3965235
40.0000000	4.5383997	-678.0490723
40.0000000	4.5871997	-12.6612101
40.0000000	4.6360002	-21.7567978
40.0000000	4.6847997	-28.6276817
40.0000000	4.7336001	-19.6259632
40.0000000	4.7824001	-16.4723911
40.0000000	4.8311996	-1521.0445557
40.0000000	4.8800001	236.1231689
40.0000000	4.9287996	-207.3606262
40.0000000	4.9776001	1094.0479736
40.0000000	5.0264001	89.4705734
40.0000000	5.0751996	-310.2394104
40.0000000	5.1240001	-12.2274475
40.0000000	5.1727996	568.6099854
40.0000000	5.2216001	32.8745003
40.0000000	5.2704000	-161.4303741
40.0000000	5.3191996	-39.6058044
40.0000000	5.3680000	-3600.3181152
40.0000000	5.4168000	537.0734253
40.0000000	5.4656000	-204.1098022
40.0000000	5.5144000	39.0599289
40.0000000	5.5631995	497.5106812
40.0000000	5.6120000	400.5948181
40.0000000	5.6608000	-37.4658279
40.0000000	5.7096000	63.9688492
40.0000000	5.7584000	135.7377167
40.0000000	5.8071995	-11.4391041
40.0000000	5.8559999	30.6430531
40.0000000	5.9047999	-16.6240883
40.0000000	5.9535999	-40.2188263
40.0000000	6.0023999	-1601.4282227
40.0000000	6.0511999	-14.5484209
40.0000000	6.0999999	-19.4872513
40.0000000	6.1487999	-13.2299623
40.0000000	6.1975999	-23.3857727
40.0000000	6.2463999	-1824.4472656
40.0000000	6.2951999	-19.1467628
40.0000000	6.3439999	-348.7288818
40.0000000	6.3927999	-15.0498495
40.0000000	6.4416003	-12.3306770
40.0000000	6.4903998	-11.1999311
40.0000000	6.5391998	-11.7418833
40.0000000	6.5879998	450.3968201
40.0000000	6.6367998	-12.0321369
40.0000000	6.6856003	-13.7987652
40.0000000	6.7343998	-13.4351521

40.0000000	6.7831998	-14.4121418
40.0000000	6.8319998	60.2621994
40.0000000	6.8807998	-14.0626574
40.0000000	6.9296002	-522.3297119
40.0000000	6.9783998	-3.8562956
40.0000000	7.0271997	-14.2359362
40.0000000	7.0760002	15.2410116
40.0000000	7.1247997	-2511.4479980
40.0000000	7.1736002	-2013.8623047
40.0000000	7.2223997	-1145.0971680
40.0000000	7.2711997	-245.0864258
40.0000000	7.3200002	-20.0398102
40.0000000	7.3687997	224.0773010
40.0000000	7.4176002	464.9665833
40.0000000	7.4663997	365.6605530
40.0000000	7.5151997	-1120.9384766
40.0000000	7.5640001	-1238.3803711
40.0000000	7.6127996	-2921.6091309
40.0000000	7.6616001	-936.9089355
40.0000000	7.7104001	-480.3144531
40.0000000	7.7591996	53.0186653
40.0000000	7.8080001	483.1869507
40.0000000	7.8567996	635.8803711
40.0000000	7.9056001	-1312.2489014
40.0000000	7.9544001	2089.6418457
40.0000000	8.0031996	2696.2133789
40.0000000	8.0520000	2691.1542969
40.0000000	8.1007996	2847.7233887
40.0000000	8.1496000	3036.8908691
40.0000000	8.1983995	1889.4888916
40.0000000	8.2472000	-4321.9565430
40.0000000	8.2959995	-1531.7810059
40.0000000	8.3448000	-1220.3123779
40.0000000	8.3935995	-1118.3511963
40.0000000	8.4423990	-481.3921509
40.0000000	8.4912004	-1124.4918213
40.0000000	8.5400000	-3034.1982422
40.0000000	8.5887995	-1470.2578125
40.0000000	8.6375999	287.9586182
40.0000000	8.6863995	2596.5883789
40.0000000	8.7351999	-964.4475708
40.0000000	8.7840004	1103.5587158
40.0000000	8.8327999	61.4233856
40.0000000	8.8815994	-11.4794312
40.0000000	8.9303989	-24.3082581
40.0000000	8.9792004	-11.5710659
40.0000000	9.0279999	-5.6641550
40.0000000	9.0767994	7.5981255
40.0000000	9.1255999	-17.4096317
40.0000000	9.1743994	-10.0342598
40.0000000	9.2231998	38.5511932
40.0000000	9.2720003	-8.3284416
40.0000000	9.3207998	-9.7452850
40.0000000	9.3695993	-10.7415905
40.0000000	9.4183998	-11.7660828
40.0000000	9.4672003	-11.1572943
40.0000000	9.5159998	-14.2515287
40.0000000	9.5648003	-0.0625553

40.0000000	9.6135998	-13.4734974
40.0000000	9.6623993	-10.1943789
40.0000000	9.7111998	-10.1310911
40.0000000	9.7600002	-10.8534889
40.0000000	9.8087997	-10.9939985
40.0000000	9.8575993	3.6232967
40.0000000	9.9063997	-7.8795042
40.0000000	9.9552002	-10.9061794
40.0000000	10.0039997	-10.0504599
50.0000000	0.3416000	-36.6196861
50.0000000	0.3904000	-39.1627159
50.0000000	0.4392000	-48.0249062
50.0000000	0.4880000	-39.5301170
50.0000000	0.5368000	-39.6960564
50.0000000	0.5856000	-54.0615730
50.0000000	0.6344000	-12.8945732
50.0000000	0.6832000	-138.1827393
50.0000000	0.7320000	-31.9803047
50.0000000	0.7808000	-86.1753845
50.0000000	0.8296000	-43.7688637
50.0000000	0.8784000	-35.3076324
50.0000000	0.9272000	-33.6716537
50.0000000	0.9760000	-31.3799114
50.0000000	1.0247999	-30.0385933
50.0000000	1.0735999	-29.6668320
50.0000000	1.1224000	-29.6179295
50.0000000	1.1711999	-30.5719414
50.0000000	1.2200000	-31.3284187
50.0000000	1.2687999	-31.3403568
50.0000000	1.3176000	-30.7816620
50.0000000	1.3664000	-30.4107838
50.0000000	1.4152000	-30.1424866
50.0000000	1.4640000	-29.7811394
50.0000000	1.5128000	-48.8014603
50.0000000	1.5616000	-37.6888657
50.0000000	1.6104001	-1803.8686523
50.0000000	1.6592000	-101.7695923
50.0000000	1.7079999	-26.1761112
50.0000000	1.7567999	-23.4803448
50.0000000	1.8055999	-23.7876816
50.0000000	1.8544000	-25.7987442
50.0000000	1.9031999	-26.3809891
50.0000000	1.9520000	-26.6867867
50.0000000	2.0007999	-30.6699142
50.0000000	2.0495999	-11.3022270
50.0000000	2.0983999	-291.1246948
50.0000000	2.1471999	24.2502899
50.0000000	2.1960001	-25.8453465
50.0000000	2.2448001	-27.5651550
50.0000000	2.2935998	-33.4639130
50.0000000	2.3423998	-19.5926037
50.0000000	2.3912001	92.1704788
50.0000000	2.4400001	-22.5962753
50.0000000	2.4888000	-23.4811630
50.0000000	2.5375998	-10.6069393
50.0000000	2.5863998	-8.4700699
50.0000000	2.6352000	-6.2517529
50.0000000	2.6840000	-14.2481670

50.0000000	2.7328000	159.1090546
50.0000000	2.7815998	187.0132751
50.0000000	2.8304000	-23.9216366
50.0000000	2.8792000	-20.3008690
50.0000000	2.9280000	-22.6273251
50.0000000	2.9768000	-15.9723787
50.0000000	3.0256000	-20.2205811
50.0000000	3.0743999	-30.6283760
50.0000000	3.1231999	-24.5924225
50.0000000	3.1719999	-30.4153481
50.0000000	3.2208002	-527.4410400
50.0000000	3.2695999	-1033.5917969
50.0000000	3.3183999	-20.4703941
50.0000000	3.3671999	-3538.4401855
50.0000000	3.4159999	1270.4697266
50.0000000	3.4648001	-6.5868216
50.0000000	3.5135999	-27.3272362
50.0000000	3.5623999	-334.5190125
50.0000000	3.6111999	-2106.2082520
50.0000000	3.6600001	-476.2166748
50.0000000	3.7088001	-629.0950317
50.0000000	3.7575998	-985.4157104
50.0000000	3.8063998	-1788.7709961
50.0000000	3.8552001	-2597.3935547
50.0000000	3.9040000	-2546.5541992
50.0000000	3.9528000	1165.6123047
50.0000000	4.0015998	-253.7833557
50.0000000	4.0503998	713.9627075
50.0000000	4.0991998	91.5980759
50.0000000	4.1479998	-678.4638062
50.0000000	4.1967998	3.3521099
50.0000000	4.2456002	-257.0682373
50.0000000	4.2943997	-505.4606323
50.0000000	4.3431997	-13.1415415
50.0000000	4.3920002	-256.6823120
50.0000000	4.4407997	-22.6151352
50.0000000	4.4896002	-15.3974056
50.0000000	4.5383997	-677.7880249
50.0000000	4.5871997	-12.6632271
50.0000000	4.6360002	-21.7552567
50.0000000	4.6847997	-28.6237564
50.0000000	4.7336001	-19.6252403
50.0000000	4.7824001	-16.4728451
50.0000000	4.8311996	-1520.5131836
50.0000000	4.8800001	236.0336304
50.0000000	4.9287996	-207.2933655
50.0000000	4.9776001	1093.6555176
50.0000000	5.0264001	89.4330521
50.0000000	5.0751996	-310.1357727
50.0000000	5.1240001	-12.2283688
50.0000000	5.1727996	568.4941406
50.0000000	5.2216001	32.8650551
50.0000000	5.2704000	-161.4022522
50.0000000	5.3191996	-39.6012535
50.0000000	5.3680000	-3599.6257324
50.0000000	5.4168000	536.9666138
50.0000000	5.4656000	-204.0736084
50.0000000	5.5144000	39.0492783

50.0000000	5.5631995	497.4121094
50.0000000	5.6120000	400.5148621
50.0000000	5.6608000	-37.4617386
50.0000000	5.7096000	63.9534225
50.0000000	5.7584000	135.7087708
50.0000000	5.8071995	-11.4399757
50.0000000	5.8559999	30.6341991
50.0000000	5.9047999	-16.6238899
50.0000000	5.9535999	-40.2140846
50.0000000	6.0023999	-1601.1239014
50.0000000	6.0511999	-14.5486326
50.0000000	6.0999999	-19.4864197
50.0000000	6.1487999	-13.2304249
50.0000000	6.1975999	-23.3840065
50.0000000	6.2463999	-1824.0998535
50.0000000	6.2951999	-19.1459484
50.0000000	6.3439999	-348.6648560
50.0000000	6.3927999	-15.0498314
50.0000000	6.4416003	-12.3312635
50.0000000	6.4903998	-11.2006073
50.0000000	6.5391998	-11.7425642
50.0000000	6.5879998	449.7061157
50.0000000	6.6367998	-12.0326862
50.0000000	6.6856003	-13.7989349
50.0000000	6.7343998	-13.4353676
50.0000000	6.7831998	-14.4117260
50.0000000	6.8319998	60.2430801
50.0000000	6.8807998	-14.0625191
50.0000000	6.9296002	-521.5682983
50.0000000	6.9783998	-3.8691187
50.0000000	7.0271997	-14.2349081
50.0000000	7.0760002	15.1988268
50.0000000	7.1247997	-2507.5019531
50.0000000	7.1736002	-2010.6994629
50.0000000	7.2223997	-1143.3039551
50.0000000	7.2711997	-244.7186584
50.0000000	7.3200002	-20.0278206
50.0000000	7.3687997	223.7027588
50.0000000	7.4176002	464.2107544
50.0000000	7.4663997	365.0628967
50.0000000	7.5151997	-1119.1774902
50.0000000	7.5640001	-1236.4329834
50.0000000	7.6127996	-2916.9914551
50.0000000	7.6616001	-935.4376221
50.0000000	7.7104001	-479.5681763
50.0000000	7.7591996	52.9176178
50.0000000	7.8080001	482.4015808
50.0000000	7.8567996	634.8522949
50.0000000	7.9056001	-1310.1759033
50.0000000	7.9544001	2086.3317871
50.0000000	8.0031996	2691.9438477
50.0000000	8.0520000	2686.8923340
50.0000000	8.1007996	2843.2138672
50.0000000	8.1496000	3032.0827637
50.0000000	8.1983995	1886.4964600
50.0000000	8.2472000	-4315.1215820
50.0000000	8.2959995	-1529.3608398
50.0000000	8.3448000	-1218.3831787

50.0000000	8.3935995	-1116.5834961
50.0000000	8.4423990	-480.6341553
50.0000000	8.4912004	-1122.7155762
50.0000000	8.5400000	-3029.3984375
50.0000000	8.5887995	-1467.9362793
50.0000000	8.6375999	287.4973145
50.0000000	8.6863995	2592.4699707
50.0000000	8.7351999	-962.9233398
50.0000000	8.7840004	1101.8018799
50.0000000	8.8327999	61.3117828
50.0000000	8.8815994	-11.4796057
50.0000000	8.9303989	-24.3045387
50.0000000	8.9792004	-11.5711479
50.0000000	9.0279999	-5.6659231
50.0000000	9.0767994	7.5924072
50.0000000	9.1255999	-17.4079189
50.0000000	9.1743994	-10.0346613
50.0000000	9.2231998	38.5354614
50.0000000	9.2720003	-8.3293781
50.0000000	9.3207998	-9.7458248
50.0000000	9.3695993	-10.7418175
50.0000000	9.4183998	-11.7659578
50.0000000	9.4672003	-11.1573362
50.0000000	9.5159998	-14.2506380
50.0000000	9.5648003	-0.0659285
50.0000000	9.6135998	-13.4727068
50.0000000	9.6623993	-10.1946545
50.0000000	9.7111998	-10.1313629
50.0000000	9.7600002	-10.8535309
50.0000000	9.8087997	-10.9940062
50.0000000	9.8575993	3.6188745
50.0000000	9.9063997	-7.8804178
50.0000000	9.9552002	-10.9061832
50.0000000	10.0039997	-10.0507069
60.0000000	0.3416000	-36.6253090
60.0000000	0.3904000	-39.1240921
60.0000000	0.4392000	-47.8593826
60.0000000	0.4880000	-39.4372406
60.0000000	0.5368000	-39.7007675
60.0000000	0.5856000	-53.6734200
60.0000000	0.6344000	-13.5646324
60.0000000	0.6832000	-134.9252319
60.0000000	0.7320000	-32.1795578
60.0000000	0.7808000	-84.0917358
60.0000000	0.8296000	-43.3772507
60.0000000	0.8784000	-35.2472229
60.0000000	0.9272000	-33.0638542
60.0000000	0.9760000	-30.9334183
60.0000000	1.0247999	-29.9045506
60.0000000	1.0735999	-29.8826771
60.0000000	1.1224000	-30.1977501
60.0000000	1.1711999	-30.2616959
60.0000000	1.2200000	-30.5512466
60.0000000	1.2687999	-30.7856064
60.0000000	1.3176000	-30.1865940
60.0000000	1.3664000	-30.3073368
60.0000000	1.4152000	-30.1426907
60.0000000	1.4640000	-29.7794285

60.0000000	1.5128000	-48.7042809
60.0000000	1.5616000	-37.6470261
60.0000000	1.6104001	-1801.3258057
60.0000000	1.6592000	-101.4756622
60.0000000	1.7079999	-26.9237804
60.0000000	1.7567999	-24.1346207
60.0000000	1.8055999	-24.9191132
60.0000000	1.8544000	-27.2115841
60.0000000	1.9031999	-26.5190163
60.0000000	1.9520000	-26.7288895
60.0000000	2.0007999	-30.6073322
60.0000000	2.0495999	-11.4888439
60.0000000	2.0983999	-289.6489258
60.0000000	2.1471999	23.9830208
60.0000000	2.1960001	-25.8412552
60.0000000	2.2448001	-27.5472431
60.0000000	2.2935998	-33.4042931
60.0000000	2.3423998	-19.6070080
60.0000000	2.3912001	91.5489273
60.0000000	2.4400001	-22.5987625
60.0000000	2.4888000	-23.4809475
60.0000000	2.5375998	-10.6240473
60.0000000	2.5863998	-8.4898291
60.0000000	2.6352000	-6.2744961
60.0000000	2.6840000	-14.2601089
60.0000000	2.7328000	158.8430328
60.0000000	2.7815998	186.7068787
60.0000000	2.8304000	-23.9203320
60.0000000	2.8792000	-20.3041325
60.0000000	2.9280000	-22.6267567
60.0000000	2.9768000	-15.9807491
60.0000000	3.0256000	-20.2231655
60.0000000	3.0743999	-30.6169891
60.0000000	3.1231999	-24.5889664
60.0000000	3.1719999	-30.4070549
60.0000000	3.2208002	-527.2033081
60.0000000	3.2695999	-1033.1168213
60.0000000	3.3183999	-20.4705906
60.0000000	3.3671999	-3536.9785156
60.0000000	3.4159999	1269.9344482
60.0000000	3.4648001	-6.5925303
60.0000000	3.5135999	-27.3242455
60.0000000	3.5623999	-334.3894043
60.0000000	3.6111999	-2105.3483887
60.0000000	3.6600001	-476.0285645
60.0000000	3.7088001	-628.8436279
60.0000000	3.7575998	-985.0171509
60.0000000	3.8063998	-1788.0411377
60.0000000	3.8552001	-2596.3300781
60.0000000	3.9040000	-2545.5117188
60.0000000	3.9528000	1165.1240234
60.0000000	4.0015998	-253.6862488
60.0000000	4.0503998	713.6601563
60.0000000	4.0991998	91.5523911
60.0000000	4.1479998	-678.1915894
60.0000000	4.1967998	3.3428535
60.0000000	4.2456002	-256.9699402
60.0000000	4.2943997	-505.2600403

60.0000000	4.3431997	-13.1437225
60.0000000	4.3920002	-256.5841980
60.0000000	4.4407997	-22.6133595
60.0000000	4.4896002	-15.3982887
60.0000000	4.5383997	-677.5269165
60.0000000	4.5871997	-12.6652431
60.0000000	4.6360002	-21.7537136
60.0000000	4.6847997	-28.6198311
60.0000000	4.7336001	-19.6245193
60.0000000	4.7824001	-16.4733009
60.0000000	4.8311996	-1519.9819336
60.0000000	4.8800001	235.9440765
60.0000000	4.9287996	-207.2261047
60.0000000	4.9776001	1093.2630615
60.0000000	5.0264001	89.3955307
60.0000000	5.0751996	-310.0321350
60.0000000	5.1240001	-12.2292900
60.0000000	5.1727996	568.3782959
60.0000000	5.2216001	32.8556099
60.0000000	5.2704000	-161.3741608
60.0000000	5.3191996	-39.5967026
60.0000000	5.3680000	-3598.9333496
60.0000000	5.4168000	536.8598633
60.0000000	5.4656000	-204.0374298
60.0000000	5.5144000	39.0386200
60.0000000	5.5631995	497.3135376
60.0000000	5.6120000	400.4348755
60.0000000	5.6608000	-37.4576492
60.0000000	5.7096000	63.9379883
60.0000000	5.7584000	135.6798401
60.0000000	5.8071995	-11.4408455
60.0000000	5.8559999	30.6253490
60.0000000	5.9047999	-16.6236935
60.0000000	5.9535999	-40.2093506
60.0000000	6.0023999	-1600.8194580
60.0000000	6.0511999	-14.5488443
60.0000000	6.0999999	-19.4855862
60.0000000	6.1487999	-13.2308884
60.0000000	6.1975999	-23.3822403
60.0000000	6.2463999	-1823.7525635
60.0000000	6.2951999	-19.1451340
60.0000000	6.3439999	-348.6007690
60.0000000	6.3927999	-15.0498133
60.0000000	6.4416003	-12.3318501
60.0000000	6.4903998	-11.2012825
60.0000000	6.5391998	-11.7432451
60.0000000	6.5879998	449.0154419
60.0000000	6.6367998	-12.0332346
60.0000000	6.6856003	-13.7991056
60.0000000	6.7343998	-13.4355831
60.0000000	6.7831998	-14.4113102
60.0000000	6.8319998	60.2239685
60.0000000	6.8807998	-14.0623808
60.0000000	6.9296002	-520.8068237
60.0000000	6.9783998	-3.8819427
60.0000000	7.0271997	-14.2338810
60.0000000	7.0760002	15.1566420
60.0000000	7.1247997	-2503.5561523

60.0000000	7.1736002	-2007.5366211
60.0000000	7.2223997	-1141.5107422
60.0000000	7.2711997	-244.3508759
60.0000000	7.3200002	-20.0158329
60.0000000	7.3687997	223.3282318
60.0000000	7.4176002	463.4549255
60.0000000	7.4663997	364.4652405
60.0000000	7.5151997	-1117.4163818
60.0000000	7.5640001	-1234.4855957
60.0000000	7.6127996	-2912.3737793
60.0000000	7.6616001	-933.9663086
60.0000000	7.7104001	-478.8218994
60.0000000	7.7591996	52.8165703
60.0000000	7.8080001	481.6162415
60.0000000	7.8567996	633.8242188
60.0000000	7.9056001	-1308.1030273
60.0000000	7.9544001	2083.0219727
60.0000000	8.0031996	2687.6743164
60.0000000	8.0520000	2682.6303711
60.0000000	8.1007996	2838.7043457
60.0000000	8.1496000	3027.2746582
60.0000000	8.1983995	1883.5039063
60.0000000	8.2472000	-4308.2866211
60.0000000	8.2959995	-1526.9406738
60.0000000	8.3448000	-1216.4539795
60.0000000	8.3935995	-1114.8157959
60.0000000	8.4423990	-479.8761292
60.0000000	8.4912004	-1120.9393311
60.0000000	8.5400000	-3024.5986328
60.0000000	8.5887995	-1465.6147461
60.0000000	8.6375999	287.0359802
60.0000000	8.6863995	2588.3518066
60.0000000	8.7351999	-961.3991089
60.0000000	8.7840004	1100.0451660
60.0000000	8.8327999	61.2001724
60.0000000	8.8815994	-11.4797792
60.0000000	8.9303989	-24.3008194
60.0000000	8.9792004	-11.5712309
60.0000000	9.0279999	-5.6676912
60.0000000	9.0767994	7.5866909
60.0000000	9.1255999	-17.4062042
60.0000000	9.1743994	-10.0350637
60.0000000	9.2231998	38.5197220
60.0000000	9.2720003	-8.3303146
60.0000000	9.3207998	-9.7463636
60.0000000	9.3695993	-10.7420435
60.0000000	9.4183998	-11.7658339
60.0000000	9.4672003	-11.1573772
60.0000000	9.5159998	-14.2497473
60.0000000	9.5648003	-0.0693016
60.0000000	9.6135998	-13.4719162
60.0000000	9.6623993	-10.1949291
60.0000000	9.7111998	-10.1316347
60.0000000	9.7600002	-10.8535719
60.0000000	9.8087997	-10.9940147
60.0000000	9.8575993	3.6144505
60.0000000	9.9063997	-7.8813314
60.0000000	9.9552002	-10.9061880

60.0000000	10.0039997	-10.0509548
70.0000000	0.3416000	-35.7535019
70.0000000	0.3904000	-38.3465958
70.0000000	0.4392000	-47.6938591
70.0000000	0.4880000	-39.3443680
70.0000000	0.5368000	-39.7054749
70.0000000	0.5856000	-53.2852631
70.0000000	0.6344000	-14.2346916
70.0000000	0.6832000	-131.6677094
70.0000000	0.7320000	-32.3788109
70.0000000	0.7808000	-82.0080872
70.0000000	0.8296000	-42.9856377
70.0000000	0.8784000	-35.1868134
70.0000000	0.9272000	-33.5983467
70.0000000	0.9760000	-31.4839535
70.0000000	1.0247999	-30.2505798
70.0000000	1.0735999	-29.6971149
70.0000000	1.1224000	-29.4075375
70.0000000	1.1711999	-29.5981369
70.0000000	1.2200000	-30.5692139
70.0000000	1.2687999	-30.7412453
70.0000000	1.3176000	-30.2410183
70.0000000	1.3664000	-30.5297985
70.0000000	1.4152000	-30.1428947
70.0000000	1.4640000	-29.7777195
70.0000000	1.5128000	-48.6071014
70.0000000	1.5616000	-37.6051865
70.0000000	1.6104001	-1798.7830811
70.0000000	1.6592000	-101.1817322
70.0000000	1.7079999	-26.1232433
70.0000000	1.7567999	-23.4164829
70.0000000	1.8055999	-24.0448227
70.0000000	1.8544000	-25.8709259
70.0000000	1.9031999	-26.6570415
70.0000000	1.9520000	-26.7709904
70.0000000	2.0007999	-30.5447502
70.0000000	2.0495999	-11.6754599
70.0000000	2.0983999	-288.1731567
70.0000000	2.1471999	23.7157555
70.0000000	2.1960001	-25.8371639
70.0000000	2.2448001	-27.5293312
70.0000000	2.2935998	-33.3446693
70.0000000	2.3423998	-19.6214123
70.0000000	2.3912001	90.9273834
70.0000000	2.4400001	-22.6012497
70.0000000	2.4888000	-23.4807301
70.0000000	2.5375998	-10.6411552
70.0000000	2.5863998	-8.5095873
70.0000000	2.6352000	-6.2972412
70.0000000	2.6840000	-14.2720509
70.0000000	2.7328000	158.5770111
70.0000000	2.7815998	186.4004517
70.0000000	2.8304000	-23.9190292
70.0000000	2.8792000	-20.3073959
70.0000000	2.9280000	-22.6261864
70.0000000	2.9768000	-15.9891205
70.0000000	3.0256000	-20.2257500
70.0000000	3.0743999	-30.6056023

70.0000000	3.1231999	-24.5855103
70.0000000	3.1719999	-30.3987617
70.0000000	3.2208002	-526.9655762
70.0000000	3.2695999	-1032.6418457
70.0000000	3.3183999	-20.4707890
70.0000000	3.3671999	-3535.5168457
70.0000000	3.4159999	1269.3991699
70.0000000	3.4648001	-6.5982389
70.0000000	3.5135999	-27.3212547
70.0000000	3.5623999	-334.2598267
70.0000000	3.6111999	-2104.4885254
70.0000000	3.6600001	-475.8404541
70.0000000	3.7088001	-628.5922852
70.0000000	3.7575998	-984.6185913
70.0000000	3.8063998	-1787.3112793
70.0000000	3.8552001	-2595.2666016
70.0000000	3.9040000	-2544.4692383
70.0000000	3.9528000	1164.6356201
70.0000000	4.0015998	-253.5891418
70.0000000	4.0503998	713.3576050
70.0000000	4.0991998	91.5067062
70.0000000	4.1479998	-677.9193115
70.0000000	4.1967998	3.3335972
70.0000000	4.2456002	-256.8716431
70.0000000	4.2943997	-505.0594788
70.0000000	4.3431997	-13.1459026
70.0000000	4.3920002	-256.4860840
70.0000000	4.4407997	-22.6115837
70.0000000	4.4896002	-15.3991718
70.0000000	4.5383997	-677.2658691
70.0000000	4.5871997	-12.6672592
70.0000000	4.6360002	-21.7521706
70.0000000	4.6847997	-28.6159058
70.0000000	4.7336001	-19.6237965
70.0000000	4.7824001	-16.4737549
70.0000000	4.8311996	-1519.4506836
70.0000000	4.8800001	235.8545227
70.0000000	4.9287996	-207.1588287
70.0000000	4.9776001	1092.8704834
70.0000000	5.0264001	89.3580170
70.0000000	5.0751996	-309.9285278
70.0000000	5.1240001	-12.2302113
70.0000000	5.1727996	568.2623901
70.0000000	5.2216001	32.8461685
70.0000000	5.2704000	-161.3460388
70.0000000	5.3191996	-39.5921555
70.0000000	5.3680000	-3598.2409668
70.0000000	5.4168000	536.7531128
70.0000000	5.4656000	-204.0012512
70.0000000	5.5144000	39.0279617
70.0000000	5.5631995	497.2149658
70.0000000	5.6120000	400.3548889
70.0000000	5.6608000	-37.4535637
70.0000000	5.7096000	63.9225540
70.0000000	5.7584000	135.6509094
70.0000000	5.8071995	-11.4417171
70.0000000	5.8559999	30.6164951
70.0000000	5.9047999	-16.6234951

70.0000000	5.9535999	-40.2046089
70.0000000	6.0023999	-1600.5148926
70.0000000	6.0511999	-14.5490551
70.0000000	6.0999999	-19.4847546
70.0000000	6.1487999	-13.2313509
70.0000000	6.1975999	-23.3804760
70.0000000	6.2463999	-1823.4051514
70.0000000	6.2951999	-19.1443195
70.0000000	6.3439999	-348.5367432
70.0000000	6.3927999	-15.0497942
70.0000000	6.4416003	-12.3324375
70.0000000	6.4903998	-11.2019587
70.0000000	6.5391998	-11.7439260
70.0000000	6.5879998	448.3247375
70.0000000	6.6367998	-12.0337839
70.0000000	6.6856003	-13.7992754
70.0000000	6.7343998	-13.4357996
70.0000000	6.7831998	-14.4108944
70.0000000	6.8319998	60.2048569
70.0000000	6.8807998	-14.0622416
70.0000000	6.9296002	-520.0454102
70.0000000	6.9783998	-3.8947659
70.0000000	7.0271997	-14.2328529
70.0000000	7.0760002	15.1144552
70.0000000	7.1247997	-2499.6103516
70.0000000	7.1736002	-2004.3737793
70.0000000	7.2223997	-1139.7176514
70.0000000	7.2711997	-243.9830933
70.0000000	7.3200002	-20.0038452
70.0000000	7.3687997	222.9537048
70.0000000	7.4176002	462.6990967
70.0000000	7.4663997	363.8675842
70.0000000	7.5151997	-1115.6553955
70.0000000	7.5640001	-1232.5380859
70.0000000	7.6127996	-2907.7561035
70.0000000	7.6616001	-932.4949951
70.0000000	7.7104001	-478.0756226
70.0000000	7.7591996	52.7155190
70.0000000	7.8080001	480.8308716
70.0000000	7.8567996	632.7961426
70.0000000	7.9056001	-1306.0301514
70.0000000	7.9544001	2079.7119141
70.0000000	8.0031996	2683.4045410
70.0000000	8.0520000	2678.3684082
70.0000000	8.1007996	2834.1948242
70.0000000	8.1496000	3022.4667969
70.0000000	8.1983995	1880.5113525
70.0000000	8.2472000	-4301.4521484
70.0000000	8.2959995	-1524.5205078
70.0000000	8.3448000	-1214.5247803
70.0000000	8.3935995	-1113.0480957
70.0000000	8.4423990	-479.1181335
70.0000000	8.4912004	-1119.1630859
70.0000000	8.5400000	-3019.7988281
70.0000000	8.5887995	-1463.2932129
70.0000000	8.6375999	286.5746765
70.0000000	8.6863995	2584.2333984
70.0000000	8.7351999	-959.8748169

70.0000000	8.7840004	1098.2884521
70.0000000	8.8327999	61.0885696
70.0000000	8.8815994	-11.4799538
70.0000000	8.9303989	-24.2971001
70.0000000	8.9792004	-11.5713129
70.0000000	9.0279999	-5.6694593
70.0000000	9.0767994	7.5809727
70.0000000	9.1255999	-17.4044914
70.0000000	9.1743994	-10.0354652
70.0000000	9.2231998	38.5039902
70.0000000	9.2720003	-8.3312511
70.0000000	9.3207998	-9.7469034
70.0000000	9.3695993	-10.7422705
70.0000000	9.4183998	-11.7657089
70.0000000	9.4672003	-11.1574183
70.0000000	9.5159998	-14.2488556
70.0000000	9.5648003	-0.0726738
70.0000000	9.6135998	-13.4711256
70.0000000	9.6623993	-10.1952047
70.0000000	9.7111998	-10.1319065
70.0000000	9.7600002	-10.8536139
70.0000000	9.8087997	-10.9940224
70.0000000	9.8575993	3.6100292
70.0000000	9.9063997	-7.8822460
70.0000000	9.9552002	-10.9061918
70.0000000	10.0039997	-10.0512018
80.0000000	0.3416000	-36.7178764
80.0000000	0.3904000	-38.9254837
80.0000000	0.4392000	-47.5283356
80.0000000	0.4880000	-39.2766533
80.0000000	0.5368000	-39.7628250
80.0000000	0.5856000	-52.8971100
80.0000000	0.6344000	-14.9047508
80.0000000	0.6832000	-128.4102020
80.0000000	0.7320000	-32.5780640
80.0000000	0.7808000	-79.9244385
80.0000000	0.8296000	-42.5940247
80.0000000	0.8784000	-35.1264038
80.0000000	0.9272000	-33.6012383
80.0000000	0.9760000	-31.8922596
80.0000000	1.0247999	-31.1090736
80.0000000	1.0735999	-30.6037731
80.0000000	1.1224000	-31.0122032
80.0000000	1.1711999	-30.6336155
80.0000000	1.2200000	-31.0140953
80.0000000	1.2687999	-30.8404598
80.0000000	1.3176000	-29.8575268
80.0000000	1.3664000	-30.0805740
80.0000000	1.4152000	-30.1430988
80.0000000	1.4640000	-29.7760105
80.0000000	1.5128000	-48.5099258
80.0000000	1.5616000	-37.5633430
80.0000000	1.6104001	-1796.2402344
80.0000000	1.6592000	-100.8878021
80.0000000	1.7079999	-26.6303329
80.0000000	1.7567999	-24.0719070
80.0000000	1.8055999	-24.5795937
80.0000000	1.8544000	-26.6227837

80.0000000	1.9031999	-26.5660362
80.0000000	1.9520000	-26.7193069
80.0000000	2.0007999	-30.4821682
80.0000000	2.0495999	-11.8620758
80.0000000	2.0983999	-286.6973572
80.0000000	2.1471999	23.4484901
80.0000000	2.1960001	-25.8330727
80.0000000	2.2448001	-27.5114212
80.0000000	2.2935998	-33.2850456
80.0000000	2.3423998	-19.6358185
80.0000000	2.3912001	90.3058395
80.0000000	2.4400001	-22.6037350
80.0000000	2.4888000	-23.4805126
80.0000000	2.5375998	-10.6582632
80.0000000	2.5863998	-8.5293465
80.0000000	2.6352000	-6.3199844
80.0000000	2.6840000	-14.2839928
80.0000000	2.7328000	158.3110046
80.0000000	2.7815998	186.0940247
80.0000000	2.8304000	-23.9177246
80.0000000	2.8792000	-20.3106594
80.0000000	2.9280000	-22.6256180
80.0000000	2.9768000	-15.9974899
80.0000000	3.0256000	-20.2283344
80.0000000	3.0743999	-30.5942154
80.0000000	3.1231999	-24.5820560
80.0000000	3.1719999	-30.3904686
80.0000000	3.2208002	-526.7278442
80.0000000	3.2695999	-1032.1668701
80.0000000	3.3183999	-20.4709873
80.0000000	3.3671999	-3534.0551758
80.0000000	3.4159999	1268.8638916
80.0000000	3.4648001	-6.6039467
80.0000000	3.5135999	-27.3182640
80.0000000	3.5623999	-334.1302185
80.0000000	3.6111999	-2103.6289063
80.0000000	3.6600001	-475.6522827
80.0000000	3.7088001	-628.3408813
80.0000000	3.7575998	-984.2200928
80.0000000	3.8063998	-1786.5814209
80.0000000	3.8552001	-2594.2031250
80.0000000	3.9040000	-2543.4267578
80.0000000	3.9528000	1164.1473389
80.0000000	4.0015998	-253.4920349
80.0000000	4.0503998	713.0549927
80.0000000	4.0991998	91.4610291
80.0000000	4.1479998	-677.6470947
80.0000000	4.1967998	3.3243408
80.0000000	4.2456002	-256.7733459
80.0000000	4.2943997	-504.8589172
80.0000000	4.3431997	-13.1480827
80.0000000	4.3920002	-256.3879700
80.0000000	4.4407997	-22.6098080
80.0000000	4.4896002	-15.4000540
80.0000000	4.5383997	-677.0048218
80.0000000	4.5871997	-12.6692753
80.0000000	4.6360002	-21.7506294
80.0000000	4.6847997	-28.6119804

80.0000000	4.7336001	-19.6230736
80.0000000	4.7824001	-16.4742088
80.0000000	4.8311996	-1518.9194336
80.0000000	4.8800001	235.7649841
80.0000000	4.9287996	-207.0915680
80.0000000	4.9776001	1092.4780273
80.0000000	5.0264001	89.3204956
80.0000000	5.0751996	-309.8248901
80.0000000	5.1240001	-12.2311325
80.0000000	5.1727996	568.1465454
80.0000000	5.2216001	32.8367195
80.0000000	5.2704000	-161.3179474
80.0000000	5.3191996	-39.5876083
80.0000000	5.3680000	-3597.5485840
80.0000000	5.4168000	536.6463013
80.0000000	5.4656000	-203.9650574
80.0000000	5.5144000	39.0173035
80.0000000	5.5631995	497.1163940
80.0000000	5.6120000	400.2749329
80.0000000	5.6608000	-37.4494743
80.0000000	5.7096000	63.9071274
80.0000000	5.7584000	135.6219788
80.0000000	5.8071995	-11.4425869
80.0000000	5.8559999	30.6076450
80.0000000	5.9047999	-16.6232967
80.0000000	5.9535999	-40.1998711
80.0000000	6.0023999	-1600.2105713
80.0000000	6.0511999	-14.5492668
80.0000000	6.0999999	-19.4839211
80.0000000	6.1487999	-13.2318134
80.0000000	6.1975999	-23.3787117
80.0000000	6.2463999	-1823.0577393
80.0000000	6.2951999	-19.1435051
80.0000000	6.3439999	-348.4726868
80.0000000	6.3927999	-15.0497761
80.0000000	6.4416003	-12.3330240
80.0000000	6.4903998	-11.2026348
80.0000000	6.5391998	-11.7446079
80.0000000	6.5879998	447.6340332
80.0000000	6.6367998	-12.0343332
80.0000000	6.6856003	-13.7994461
80.0000000	6.7343998	-13.4360151
80.0000000	6.7831998	-14.4104786
80.0000000	6.8319998	60.1857376
80.0000000	6.8807998	-14.0621033
80.0000000	6.9296002	-519.2839355
80.0000000	6.9783998	-3.9075899
80.0000000	7.0271997	-14.2318249
80.0000000	7.0760002	15.0722704
80.0000000	7.1247997	-2495.6645508
80.0000000	7.1736002	-2001.2108154
80.0000000	7.2223997	-1137.9244385
80.0000000	7.2711997	-243.6153259
80.0000000	7.3200002	-19.9918556
80.0000000	7.3687997	222.5791626
80.0000000	7.4176002	461.9432678
80.0000000	7.4663997	363.2699280
80.0000000	7.5151997	-1113.8944092

80.0000000	7.5640001	-1230.5906982
80.0000000	7.6127996	-2903.1386719
80.0000000	7.6616001	-931.0236206
80.0000000	7.7104001	-477.3293457
80.0000000	7.7591996	52.6144714
80.0000000	7.8080001	480.0455017
80.0000000	7.8567996	631.7680664
80.0000000	7.9056001	-1303.9572754
80.0000000	7.9544001	2076.4020996
80.0000000	8.0031996	2679.1350098
80.0000000	8.0520000	2674.1064453
80.0000000	8.1007996	2829.6853027
80.0000000	8.1496000	3017.6586914
80.0000000	8.1983995	1877.5189209
80.0000000	8.2472000	-4294.6171875
80.0000000	8.2959995	-1522.1003418
80.0000000	8.3448000	-1212.5955811
80.0000000	8.3935995	-1111.2805176
80.0000000	8.4423990	-478.3601074
80.0000000	8.4912004	-1117.3868408
80.0000000	8.5400000	-3014.9990234
80.0000000	8.5887995	-1460.9716797
80.0000000	8.6375999	286.1133423
80.0000000	8.6863995	2580.1149902
80.0000000	8.7351999	-958.3505859
80.0000000	8.7840004	1096.5317383
80.0000000	8.8327999	60.9769592
80.0000000	8.8815994	-11.4801283
80.0000000	8.9303989	-24.2933807
80.0000000	8.9792004	-11.5713949
80.0000000	9.0279999	-5.6712279
80.0000000	9.0767994	7.5752544
80.0000000	9.1255999	-17.4027767
80.0000000	9.1743994	-10.0358677
80.0000000	9.2231998	38.4882584
80.0000000	9.2720003	-8.3321877
80.0000000	9.3207998	-9.7474422
80.0000000	9.3695993	-10.7424965
80.0000000	9.4183998	-11.7655849
80.0000000	9.4672003	-11.1574593
80.0000000	9.5159998	-14.2479649
80.0000000	9.5648003	-0.0760460
80.0000000	9.6135998	-13.4703350
80.0000000	9.6623993	-10.1954794
80.0000000	9.7111998	-10.1321774
80.0000000	9.7600002	-10.8536558
80.0000000	9.8087997	-10.9940310
80.0000000	9.8575993	3.6056070
80.0000000	9.9063997	-7.8831596
80.0000000	9.9552002	-10.9061966
80.0000000	10.0039997	-10.0514498
90.0000000	0.3416000	-36.6012077
90.0000000	0.3904000	-39.5043755
90.0000000	0.4392000	-47.3628159
90.0000000	0.4880000	-39.2089386
90.0000000	0.5368000	-39.8201790
90.0000000	0.5856000	-52.5089569
90.0000000	0.6344000	-15.5748100

90.0000000	0.6832000	-125.1526794
90.0000000	0.7320000	-32.7773170
90.0000000	0.7808000	-77.8407898
90.0000000	0.8296000	-42.2024117
90.0000000	0.8784000	-35.0659943
90.0000000	0.9272000	-33.6041260
90.0000000	0.9760000	-32.3005638
90.0000000	1.0247999	-31.9675674
90.0000000	1.0735999	-31.5104294
90.0000000	1.1224000	-32.6168671
90.0000000	1.1711999	-31.6690941
90.0000000	1.2200000	-31.4589748
90.0000000	1.2687999	-30.9396744
90.0000000	1.3176000	-29.4740353
90.0000000	1.3664000	-29.6313515
90.0000000	1.4152000	-30.1433048
90.0000000	1.4640000	-29.7743015
90.0000000	1.5128000	-48.4127464
90.0000000	1.5616000	-37.5215034
90.0000000	1.6104001	-1793.6973877
90.0000000	1.6592000	-100.5938721
90.0000000	1.7079999	-26.4704552
90.0000000	1.7567999	-23.5884743
90.0000000	1.8055999	-23.9877300
90.0000000	1.8544000	-25.7995548
90.0000000	1.9031999	-26.4750309
90.0000000	1.9520000	-26.6676235
90.0000000	2.0007999	-30.4195862
90.0000000	2.0495999	-12.0486927
90.0000000	2.0983999	-285.2215881
90.0000000	2.1471999	23.1812210
90.0000000	2.1960001	-25.8289814
90.0000000	2.2448001	-27.4935093
90.0000000	2.2935998	-33.2254219
90.0000000	2.3423998	-19.6502228
90.0000000	2.3912001	89.6842957
90.0000000	2.4400001	-22.6062222
90.0000000	2.4888000	-23.4802952
90.0000000	2.5375998	-10.6753712
90.0000000	2.5863998	-8.5491047
90.0000000	2.6352000	-6.3427296
90.0000000	2.6840000	-14.2959347
90.0000000	2.7328000	158.0449829
90.0000000	2.7815998	185.7876282
90.0000000	2.8304000	-23.9164200
90.0000000	2.8792000	-20.3139229
90.0000000	2.9280000	-22.6250496
90.0000000	2.9768000	-16.0058613
90.0000000	3.0256000	-20.2309189
90.0000000	3.0743999	-30.5828285
90.0000000	3.1231999	-24.5785999
90.0000000	3.1719999	-30.3821754
90.0000000	3.2208002	-526.4901123
90.0000000	3.2695999	-1031.6918945
90.0000000	3.3183999	-20.4711857
90.0000000	3.3671999	-3532.5935059
90.0000000	3.4159999	1268.3286133
90.0000000	3.4648001	-6.6096535

90.0000000	3.5135999	-27.3152733
90.0000000	3.5623999	-334.0006409
90.0000000	3.6111999	-2102.7690430
90.0000000	3.6600001	-475.4641724
90.0000000	3.7088001	-628.0894775
90.0000000	3.7575998	-983.8215332
90.0000000	3.8063998	-1785.8516846
90.0000000	3.8552001	-2593.1398926
90.0000000	3.9040000	-2542.3842773
90.0000000	3.9528000	1163.6590576
90.0000000	4.0015998	-253.3949280
90.0000000	4.0503998	712.7524414
90.0000000	4.0991998	91.4153442
90.0000000	4.1479998	-677.3748779
90.0000000	4.1967998	3.3150845
90.0000000	4.2456002	-256.6750183
90.0000000	4.2943997	-504.6583557
90.0000000	4.3431997	-13.1502638
90.0000000	4.3920002	-256.2898560
90.0000000	4.4407997	-22.6080322
90.0000000	4.4896002	-15.4009371
90.0000000	4.5383997	-676.7437134
90.0000000	4.5871997	-12.6712914
90.0000000	4.6360002	-21.7490864
90.0000000	4.6847997	-28.6080551
90.0000000	4.7336001	-19.6223526
90.0000000	4.7824001	-16.4746647
90.0000000	4.8311996	-1518.3881836
90.0000000	4.8800001	235.6754303
90.0000000	4.9287996	-207.0243073
90.0000000	4.9776001	1092.0855713
90.0000000	5.0264001	89.2829742
90.0000000	5.0751996	-309.7212524
90.0000000	5.1240001	-12.2320538
90.0000000	5.1727996	568.0306396
90.0000000	5.2216001	32.8272781
90.0000000	5.2704000	-161.2898254
90.0000000	5.3191996	-39.5830536
90.0000000	5.3680000	-3596.8562012
90.0000000	5.4168000	536.5394897
90.0000000	5.4656000	-203.9288788
90.0000000	5.5144000	39.0066452
90.0000000	5.5631995	497.0178223
90.0000000	5.6120000	400.1949463
90.0000000	5.6608000	-37.4453850
90.0000000	5.7096000	63.8917007
90.0000000	5.7584000	135.5930481
90.0000000	5.8071995	-11.4434586
90.0000000	5.8559999	30.5987949
90.0000000	5.9047999	-16.6231003
90.0000000	5.9535999	-40.1951332
90.0000000	6.0023999	-1599.9061279
90.0000000	6.0511999	-14.5494785
90.0000000	6.0999999	-19.4830894
90.0000000	6.1487999	-13.2322769
90.0000000	6.1975999	-23.3769455
90.0000000	6.2463999	-1822.7104492
90.0000000	6.2951999	-19.1426907

90.0000000	6.3439999	-348.4086304
90.0000000	6.3927999	-15.0497580
90.0000000	6.4416003	-12.3336115
90.0000000	6.4903998	-11.2033110
90.0000000	6.5391998	-11.7452888
90.0000000	6.5879998	446.9433594
90.0000000	6.6367998	-12.0348825
90.0000000	6.6856003	-13.7996159
90.0000000	6.7343998	-13.4362307
90.0000000	6.7831998	-14.4100628
90.0000000	6.8319998	60.1666183
90.0000000	6.8807998	-14.0619640
90.0000000	6.9296002	-518.5224609
90.0000000	6.9783998	-3.9204140
90.0000000	7.0271997	-14.2307968
90.0000000	7.0760002	15.0300856
90.0000000	7.1247997	-2491.7185059
90.0000000	7.1736002	-1998.0479736
90.0000000	7.2223997	-1136.1313477
90.0000000	7.2711997	-243.2475433
90.0000000	7.3200002	-19.9798660
90.0000000	7.3687997	222.2046356
90.0000000	7.4176002	461.1874390
90.0000000	7.4663997	362.6722717
90.0000000	7.5151997	-1112.1333008
90.0000000	7.5640001	-1228.6433105
90.0000000	7.6127996	-2898.5209961
90.0000000	7.6616001	-929.5522461
90.0000000	7.7104001	-476.5831299
90.0000000	7.7591996	52.5134239
90.0000000	7.8080001	479.2601624
90.0000000	7.8567996	630.7399292
90.0000000	7.9056001	-1301.8842773
90.0000000	7.9544001	2073.0920410
90.0000000	8.0031996	2674.8654785
90.0000000	8.0520000	2669.8444824
90.0000000	8.1007996	2825.1757813
90.0000000	8.1496000	3012.8508301
90.0000000	8.1983995	1874.5263672
90.0000000	8.2472000	-4287.7822266
90.0000000	8.2959995	-1519.6801758
90.0000000	8.3448000	-1210.6663818
90.0000000	8.3935995	-1109.5128174
90.0000000	8.4423990	-477.6021118
90.0000000	8.4912004	-1115.6107178
90.0000000	8.5400000	-3010.1992188
90.0000000	8.5887995	-1458.6501465
90.0000000	8.6375999	285.6520081
90.0000000	8.6863995	2575.9965820
90.0000000	8.7351999	-956.8262939
90.0000000	8.7840004	1094.7749023
90.0000000	8.8327999	60.8653488
90.0000000	8.8815994	-11.4803028
90.0000000	8.9303989	-24.2896595
90.0000000	8.9792004	-11.5714779
90.0000000	9.0279999	-5.6729960
90.0000000	9.0767994	7.5695362
90.0000000	9.1255999	-17.4010620

90.0000000	9.1743994	-10.0362701
90.0000000	9.2231998	38.4725266
90.0000000	9.2720003	-8.3331242
90.0000000	9.3207998	-9.7479820
90.0000000	9.3695993	-10.7427235
90.0000000	9.4183998	-11.7654600
90.0000000	9.4672003	-11.1575003
90.0000000	9.5159998	-14.2470741
90.0000000	9.5648003	-0.0794191
90.0000000	9.6135998	-13.4695444
90.0000000	9.6623993	-10.1957541
90.0000000	9.7111998	-10.1324492
90.0000000	9.7600002	-10.8536978
90.0000000	9.8087997	-10.9940386
90.0000000	9.8575993	3.6011848
90.0000000	9.9063997	-7.8840733
90.0000000	9.9552002	-10.9062004
90.0000000	10.0039997	-10.0516977
100.0000000	0.3416000	-36.5466805
100.0000000	0.3904000	-39.7184830
100.0000000	0.4392000	-47.1972923
100.0000000	0.4880000	-39.2334671
100.0000000	0.5368000	-39.8387451
100.0000000	0.5856000	-52.1208000
100.0000000	0.6344000	-16.2448692
100.0000000	0.6832000	-121.8951721
100.0000000	0.7320000	-32.9765701
100.0000000	0.7808000	-75.7571411
100.0000000	0.8296000	-41.8107986
100.0000000	0.8784000	-35.0055809
100.0000000	0.9272000	-33.6070175
100.0000000	0.9760000	-32.7088699
100.0000000	1.0247999	-32.8260612
100.0000000	1.0735999	-32.4170876
100.0000000	1.1224000	-32.6858788
100.0000000	1.1711999	-31.7784386
100.0000000	1.2200000	-31.4395542
100.0000000	1.2687999	-31.1899834
100.0000000	1.3176000	-30.1946793
100.0000000	1.3664000	-30.6068077
100.0000000	1.4152000	-30.1435089
100.0000000	1.4640000	-29.7725906
100.0000000	1.5128000	-48.3155708
100.0000000	1.5616000	-37.4796600
100.0000000	1.6104001	-1791.1546631
100.0000000	1.6592000	-100.2999344
100.0000000	1.7079999	-26.9886074
100.0000000	1.7567999	-24.2109814
100.0000000	1.8055999	-24.4812393
100.0000000	1.8544000	-25.7772923
100.0000000	1.9031999	-26.2361488
100.0000000	1.9520000	-26.7638626
100.0000000	2.0007999	-30.3570042
100.0000000	2.0495999	-12.2353096
100.0000000	2.0983999	-283.7458191
100.0000000	2.1471999	22.9139519
100.0000000	2.1960001	-25.8248901
100.0000000	2.2448001	-27.4755974

100.0000000	2.2935998	-33.1657982
100.0000000	2.3423998	-19.6646271
100.0000000	2.3912001	89.0627518
100.0000000	2.4400001	-22.6087093
100.0000000	2.4888000	-23.4800777
100.0000000	2.5375998	-10.6924791
100.0000000	2.5863998	-8.5688639
100.0000000	2.6352000	-6.3654728
100.0000000	2.6840000	-14.3078766
100.0000000	2.7328000	157.7789612
100.0000000	2.7815998	185.4812012
100.0000000	2.8304000	-23.9151154
100.0000000	2.8792000	-20.3171864
100.0000000	2.9280000	-22.6244793
100.0000000	2.9768000	-16.0142326
100.0000000	3.0256000	-20.2335033
100.0000000	3.0743999	-30.5714417
100.0000000	3.1231999	-24.5751457
100.0000000	3.1719999	-30.3738823
100.0000000	3.2208002	-526.2523804
100.0000000	3.2695999	-1031.2169189
100.0000000	3.3183999	-20.4713840
100.0000000	3.3671999	-3531.1320801
100.0000000	3.4159999	1267.7933350
100.0000000	3.4648001	-6.6153622
100.0000000	3.5135999	-27.3122826
100.0000000	3.5623999	-333.8710632
100.0000000	3.6111999	-2101.9094238
100.0000000	3.6600001	-475.2760620
100.0000000	3.7088001	-627.8381348
100.0000000	3.7575998	-983.4230347
100.0000000	3.8063998	-1785.1218262
100.0000000	3.8552001	-2592.0764160
100.0000000	3.9040000	-2541.3417969
100.0000000	3.9528000	1163.1707764
100.0000000	4.0015998	-253.2978058
100.0000000	4.0503998	712.4498901
100.0000000	4.0991998	91.3696594
100.0000000	4.1479998	-677.1026611
100.0000000	4.1967998	3.3058281
100.0000000	4.2456002	-256.5767212
100.0000000	4.2943997	-504.4577942
100.0000000	4.3431997	-13.1524448
100.0000000	4.3920002	-256.1917419
100.0000000	4.4407997	-22.6062565
100.0000000	4.4896002	-15.4018192
100.0000000	4.5383997	-676.4826660
100.0000000	4.5871997	-12.6733074
100.0000000	4.6360002	-21.7475433
100.0000000	4.6847997	-28.6041298
100.0000000	4.7336001	-19.6216297
100.0000000	4.7824001	-16.4751186
100.0000000	4.8311996	-1517.8569336
100.0000000	4.8800001	235.5858765
100.0000000	4.9287996	-206.9570465
100.0000000	4.9776001	1091.6929932
100.0000000	5.0264001	89.2454529
100.0000000	5.0751996	-309.6176453

100.0000000	5.1240001	-12.2329750
100.0000000	5.1727996	567.9147949
100.0000000	5.2216001	32.8178368
100.0000000	5.2704000	-161.2617340
100.0000000	5.3191996	-39.5785065
100.0000000	5.3680000	-3596.1638184
100.0000000	5.4168000	536.4327393
100.0000000	5.4656000	-203.8927002
100.0000000	5.5144000	38.9959946
100.0000000	5.5631995	496.9192505
100.0000000	5.6120000	400.1149597
100.0000000	5.6608000	-37.4412994
100.0000000	5.7096000	63.8762665
100.0000000	5.7584000	135.5641174
100.0000000	5.8071995	-11.4443283
100.0000000	5.8559999	30.5899410
100.0000000	5.9047999	-16.6229019
100.0000000	5.9535999	-40.1903954
100.0000000	6.0023999	-1599.6018066
100.0000000	6.0511999	-14.5496893
100.0000000	6.0999999	-19.4822578
100.0000000	6.1487999	-13.2327394
100.0000000	6.1975999	-23.3751793
100.0000000	6.2463999	-1822.3630371
100.0000000	6.2951999	-19.1418762
100.0000000	6.3439999	-348.3445740
100.0000000	6.3927999	-15.0497398
100.0000000	6.4416003	-12.3341980
100.0000000	6.4903998	-11.2039862
100.0000000	6.5391998	-11.7459698
100.0000000	6.5879998	446.2526550
100.0000000	6.6367998	-12.0354319
100.0000000	6.6856003	-13.7997866
100.0000000	6.7343998	-13.4364462
100.0000000	6.7831998	-14.4096470
100.0000000	6.8319998	60.1475067
100.0000000	6.8807998	-14.0618258
100.0000000	6.9296002	-517.7610474
100.0000000	6.9783998	-3.9332371
100.0000000	7.0271997	-14.2297688
100.0000000	7.0760002	14.9878988
100.0000000	7.1247997	-2487.7727051
100.0000000	7.1736002	-1994.8851318
100.0000000	7.2223997	-1134.3381348
100.0000000	7.2711997	-242.8797607
100.0000000	7.3200002	-19.9678783
100.0000000	7.3687997	221.8301086
100.0000000	7.4176002	460.4316101
100.0000000	7.4663997	362.0746155
100.0000000	7.5151997	-1110.3723145
100.0000000	7.5640001	-1226.6959229
100.0000000	7.6127996	-2893.9033203
100.0000000	7.6616001	-928.0809326
100.0000000	7.7104001	-475.8368530
100.0000000	7.7591996	52.4123726
100.0000000	7.8080001	478.4747925
100.0000000	7.8567996	629.7118530
100.0000000	7.9056001	-1299.8114014

100.0000000	7.9544001	2069.7822266
100.0000000	8.0031996	2670.5959473
100.0000000	8.0520000	2665.5822754
100.0000000	8.1007996	2820.6662598
100.0000000	8.1496000	3008.0427246
100.0000000	8.1983995	1871.5338135
100.0000000	8.2472000	-4280.9477539
100.0000000	8.2959995	-1517.2600098
100.0000000	8.3448000	-1208.7373047
100.0000000	8.3935995	-1107.7451172
100.0000000	8.4423990	-476.8440857
100.0000000	8.4912004	-1113.8344727
100.0000000	8.5400000	-3005.3994141
100.0000000	8.5887995	-1456.3286133
100.0000000	8.6375999	285.1907043
100.0000000	8.6863995	2571.8784180
100.0000000	8.7351999	-955.3020630
100.0000000	8.7840004	1093.0181885
100.0000000	8.8327999	60.7537384
100.0000000	8.8815994	-11.4804764
100.0000000	8.9303989	-24.2859383
100.0000000	8.9792004	-11.5715599
100.0000000	9.0279999	-5.6747646
100.0000000	9.0767994	7.5638199
100.0000000	9.1255999	-17.3993492
100.0000000	9.1743994	-10.0366716
100.0000000	9.2231998	38.4567947
100.0000000	9.2720003	-8.3340597
100.0000000	9.3207998	-9.7485209
100.0000000	9.3695993	-10.7429495
100.0000000	9.4183998	-11.7653351
100.0000000	9.4672003	-11.1575422
100.0000000	9.5159998	-14.2461824
100.0000000	9.5648003	-0.0827923
100.0000000	9.6135998	-13.4687538
100.0000000	9.6623993	-10.1960297
100.0000000	9.7111998	-10.1327209
100.0000000	9.7600002	-10.8537388
100.0000000	9.8087997	-10.9940462
100.0000000	9.8575993	3.5967627
100.0000000	9.9063997	-7.8849874
100.0000000	9.9552002	-10.9062052
100.0000000	10.0039997	-10.0519447
110.0000000	0.3416000	-36.1800461
110.0000000	0.3904000	-39.0817223
110.0000000	0.4392000	-47.0317688
110.0000000	0.4880000	-39.2579994
110.0000000	0.5368000	-39.8573074
110.0000000	0.5856000	-51.7326469
110.0000000	0.6344000	-16.9149284
110.0000000	0.6832000	-118.6376495
110.0000000	0.7320000	-33.1758232
110.0000000	0.7808000	-73.6734924
110.0000000	0.8296000	-41.4191856
110.0000000	0.8784000	-34.9451714
110.0000000	0.9272000	-33.6099052
110.0000000	0.9760000	-33.1171761
110.0000000	1.0247999	-33.6845551

110.0000000	1.0735999	-33.3237457
110.0000000	1.1224000	-32.7548866
110.0000000	1.1711999	-31.7375278
110.0000000	1.2200000	-31.4201317
110.0000000	1.2687999	-31.4402943
110.0000000	1.3176000	-29.5011730
110.0000000	1.3664000	-28.9125862
110.0000000	1.4152000	-30.1437130
110.0000000	1.4640000	-29.7708817
110.0000000	1.5128000	-48.2183914
110.0000000	1.5616000	-37.4378204
110.0000000	1.6104001	-1788.6118164
110.0000000	1.6592000	-100.0060043
110.0000000	1.7079999	-26.0521336
110.0000000	1.7567999	-23.8490810
110.0000000	1.8055999	-23.9503441
110.0000000	1.8544000	-24.5200806
110.0000000	1.9031999	-25.9972687
110.0000000	1.9520000	-26.8601036
110.0000000	2.0007999	-30.2944221
110.0000000	2.0495999	-12.4219255
110.0000000	2.0983999	-282.2700195
110.0000000	2.1471999	22.6466866
110.0000000	2.1960001	-25.8207989
110.0000000	2.2448001	-27.4576874
110.0000000	2.2935998	-33.1061745
110.0000000	2.3423998	-19.6790314
110.0000000	2.3912001	88.4412003
110.0000000	2.4400001	-22.6111946
110.0000000	2.4888000	-23.4798603
110.0000000	2.5375998	-10.7095871
110.0000000	2.5863998	-8.5886230
110.0000000	2.6352000	-6.3882179
110.0000000	2.6840000	-14.3198185
110.0000000	2.7328000	157.5129547
110.0000000	2.7815998	185.1747742
110.0000000	2.8304000	-23.9138126
110.0000000	2.8792000	-20.3204498
110.0000000	2.9280000	-22.6239109
110.0000000	2.9768000	-16.0226021
110.0000000	3.0256000	-20.2360878
110.0000000	3.0743999	-30.5600548
110.0000000	3.1231999	-24.5716896
110.0000000	3.1719999	-30.3655891
110.0000000	3.2208002	-526.0146484
110.0000000	3.2695999	-1030.7419434
110.0000000	3.3183999	-20.4715805
110.0000000	3.3671999	-3529.6704102
110.0000000	3.4159999	1267.2580566
110.0000000	3.4648001	-6.6210709
110.0000000	3.5135999	-27.3092918
110.0000000	3.5623999	-333.7414551
110.0000000	3.6111999	-2101.0495605
110.0000000	3.6600001	-475.0878906
110.0000000	3.7088001	-627.5867310
110.0000000	3.7575998	-983.0244751
110.0000000	3.8063998	-1784.3919678
110.0000000	3.8552001	-2591.0129395

110.0000000	3.9040000	-2540.2993164
110.0000000	3.9528000	1162.6824951
110.0000000	4.0015998	-253.2006989
110.0000000	4.0503998	712.1473389
110.0000000	4.0991998	91.3239746
110.0000000	4.1479998	-676.8304443
110.0000000	4.1967998	3.2965717
110.0000000	4.2456002	-256.4784241
110.0000000	4.2943997	-504.2571716
110.0000000	4.3431997	-13.1546249
110.0000000	4.3920002	-256.0936279
110.0000000	4.4407997	-22.6044807
110.0000000	4.4896002	-15.4027023
110.0000000	4.5383997	-676.2216187
110.0000000	4.5871997	-12.6753235
110.0000000	4.6360002	-21.7460022
110.0000000	4.6847997	-28.6002045
110.0000000	4.7336001	-19.6209068
110.0000000	4.7824001	-16.4755726
110.0000000	4.8311996	-1517.3255615
110.0000000	4.8800001	235.4963226
110.0000000	4.9287996	-206.8897858
110.0000000	4.9776001	1091.3005371
110.0000000	5.0264001	89.2079315
110.0000000	5.0751996	-309.5140076
110.0000000	5.1240001	-12.2338963
110.0000000	5.1727996	567.7989502
110.0000000	5.2216001	32.8083878
110.0000000	5.2704000	-161.2336121
110.0000000	5.3191996	-39.5739594
110.0000000	5.3680000	-3595.4714355
110.0000000	5.4168000	536.3259888
110.0000000	5.4656000	-203.8565063
110.0000000	5.5144000	38.9853363
110.0000000	5.5631995	496.8206787
110.0000000	5.6120000	400.0350037
110.0000000	5.6608000	-37.4372101
110.0000000	5.7096000	63.8608322
110.0000000	5.7584000	135.5351868
110.0000000	5.8071995	-11.4451990
110.0000000	5.8559999	30.5810909
110.0000000	5.9047999	-16.6227036
110.0000000	5.9535999	-40.1856537
110.0000000	6.0023999	-1599.2972412
110.0000000	6.0511999	-14.5499010
110.0000000	6.0999999	-19.4814243
110.0000000	6.1487999	-13.2332020
110.0000000	6.1975999	-23.3734150
110.0000000	6.2463999	-1822.0156250
110.0000000	6.2951999	-19.1410637
110.0000000	6.3439999	-348.2805481
110.0000000	6.3927999	-15.0497208
110.0000000	6.4416003	-12.3347855
110.0000000	6.4903998	-11.2046623
110.0000000	6.5391998	-11.7466507
110.0000000	6.5879998	445.5619507
110.0000000	6.6367998	-12.0359812
110.0000000	6.6856003	-13.7999563

110.0000000	6.7343998	-13.4366627
110.0000000	6.7831998	-14.4092312
110.0000000	6.8319998	60.1283951
110.0000000	6.8807998	-14.0616875
110.0000000	6.9296002	-516.9995728
110.0000000	6.9783998	-3.9460611
110.0000000	7.0271997	-14.2287416
110.0000000	7.0760002	14.9457140
110.0000000	7.1247997	-2483.8269043
110.0000000	7.1736002	-1991.7221680
110.0000000	7.2223997	-1132.5449219
110.0000000	7.2711997	-242.5119781
110.0000000	7.3200002	-19.9558907
110.0000000	7.3687997	221.4555817
110.0000000	7.4176002	459.6757813
110.0000000	7.4663997	361.4769897
110.0000000	7.5151997	-1108.6112061
110.0000000	7.5640001	-1224.7485352
110.0000000	7.6127996	-2889.2858887
110.0000000	7.6616001	-926.6095581
110.0000000	7.7104001	-475.0905762
110.0000000	7.7591996	52.3113251
110.0000000	7.8080001	477.6894226
110.0000000	7.8567996	628.6837769
110.0000000	7.9056001	-1297.7385254
110.0000000	7.9544001	2066.4721680
110.0000000	8.0031996	2666.3261719
110.0000000	8.0520000	2661.3203125
110.0000000	8.1007996	2816.1567383
110.0000000	8.1496000	3003.2346191
110.0000000	8.1983995	1868.5413818
110.0000000	8.2472000	-4274.1127930
110.0000000	8.2959995	-1514.8398438
110.0000000	8.3448000	-1206.8081055
110.0000000	8.3935995	-1105.9774170
110.0000000	8.4423990	-476.0860901
110.0000000	8.4912004	-1112.0582275
110.0000000	8.5400000	-3000.5996094
110.0000000	8.5887995	-1454.0070801
110.0000000	8.6375999	284.7293701
110.0000000	8.6863995	2567.7600098
110.0000000	8.7351999	-953.7778320
110.0000000	8.7840004	1091.2614746
110.0000000	8.8327999	60.6421356
110.0000000	8.8815994	-11.4806509
110.0000000	8.9303989	-24.2822189
110.0000000	8.9792004	-11.5716419
110.0000000	9.0279999	-5.6765327
110.0000000	9.0767994	7.5581017
110.0000000	9.1255999	-17.3976345
110.0000000	9.1743994	-10.0370741
110.0000000	9.2231998	38.4410629
110.0000000	9.2720003	-8.3349962
110.0000000	9.3207998	-9.7490606
110.0000000	9.3695993	-10.7431755
110.0000000	9.4183998	-11.7652111
110.0000000	9.4672003	-11.1575832
110.0000000	9.5159998	-14.2452917

110.0000000	9.5648003	-0.0861645
110.0000000	9.6135998	-13.4679623
110.0000000	9.6623993	-10.1963043
110.0000000	9.7111998	-10.1329927
110.0000000	9.7600002	-10.8537807
110.0000000	9.8087997	-10.9940548
110.0000000	9.8575993	3.5923395
110.0000000	9.9063997	-7.8859015
110.0000000	9.9552002	-10.9062090
110.0000000	10.0039997	-10.0521927
120.0000000	0.3416000	-36.8257828
120.0000000	0.3904000	-39.9339027
120.0000000	0.4392000	-46.8662453
120.0000000	0.4880000	-39.4623146
120.0000000	0.5368000	-39.8401299
120.0000000	0.5856000	-51.3444939
120.0000000	0.6344000	-17.5849876
120.0000000	0.6832000	-115.3801422
120.0000000	0.7320000	-33.3750763
120.0000000	0.7808000	-71.5898438
120.0000000	0.8296000	-41.0275726
120.0000000	0.8784000	-34.8847618
120.0000000	0.9272000	-33.6127968
120.0000000	0.9760000	-33.5254784
120.0000000	1.0247999	-33.7075081
120.0000000	1.0735999	-33.2561417
120.0000000	1.1224000	-32.8238983
120.0000000	1.1711999	-31.6966152
120.0000000	1.2200000	-31.4007111
120.0000000	1.2687999	-31.4749355
120.0000000	1.3176000	-29.7609749
120.0000000	1.3664000	-29.8734455
120.0000000	1.4152000	-30.1439171
120.0000000	1.4640000	-29.7691727
120.0000000	1.5128000	-48.1212158
120.0000000	1.5616000	-37.3959808
120.0000000	1.6104001	-1786.0689697
120.0000000	1.6592000	-99.7120743
120.0000000	1.7079999	-27.4510269
120.0000000	1.7567999	-24.5572948
120.0000000	1.8055999	-24.3955650
120.0000000	1.8544000	-25.2532215
120.0000000	1.9031999	-26.8805294
120.0000000	1.9520000	-26.8354836
120.0000000	2.0007999	-30.2318401
120.0000000	2.0495999	-12.6085415
120.0000000	2.0983999	-280.7942505
120.0000000	2.1471999	22.3794212
120.0000000	2.1960001	-25.8167076
120.0000000	2.2448001	-27.4397755
120.0000000	2.2935998	-33.0465508
120.0000000	2.3423998	-19.6934357
120.0000000	2.3912001	87.8196564
120.0000000	2.4400001	-22.6136818
120.0000000	2.4888000	-23.4796429
120.0000000	2.5375998	-10.7266941
120.0000000	2.5863998	-8.6083813
120.0000000	2.6352000	-6.4109621

120.0000000	2.6840000	-14.3317604
120.0000000	2.7328000	157.2469330
120.0000000	2.7815998	184.8683777
120.0000000	2.8304000	-23.9125080
120.0000000	2.8792000	-20.3237133
120.0000000	2.9280000	-22.6233406
120.0000000	2.9768000	-16.0309715
120.0000000	3.0256000	-20.2386723
120.0000000	3.0743999	-30.5486660
120.0000000	3.1231999	-24.5682335
120.0000000	3.1719999	-30.3572960
120.0000000	3.2208002	-525.7769165
120.0000000	3.2695999	-1030.2669678
120.0000000	3.3183999	-20.4717789
120.0000000	3.3671999	-3528.2087402
120.0000000	3.4159999	1266.7227783
120.0000000	3.4648001	-6.6267786
120.0000000	3.5135999	-27.3063011
120.0000000	3.5623999	-333.6118774
120.0000000	3.6111999	-2100.1896973
120.0000000	3.6600001	-474.8997803
120.0000000	3.7088001	-627.3353882
120.0000000	3.7575998	-982.6259155
120.0000000	3.8063998	-1783.6621094
120.0000000	3.8552001	-2589.9494629
120.0000000	3.9040000	-2539.2568359
120.0000000	3.9528000	1162.1942139
120.0000000	4.0015998	-253.1035919
120.0000000	4.0503998	711.8447876
120.0000000	4.0991998	91.2782898
120.0000000	4.1479998	-676.5582275
120.0000000	4.1967998	3.2873154
120.0000000	4.2456002	-256.3801270
120.0000000	4.2943997	-504.0566101
120.0000000	4.3431997	-13.1568050
120.0000000	4.3920002	-255.9954987
120.0000000	4.4407997	-22.6027050
120.0000000	4.4896002	-15.4035845
120.0000000	4.5383997	-675.9605103
120.0000000	4.5871997	-12.6773396
120.0000000	4.6360002	-21.7444592
120.0000000	4.6847997	-28.5962791
120.0000000	4.7336001	-19.6201839
120.0000000	4.7824001	-16.4760265
120.0000000	4.8311996	-1516.7943115
120.0000000	4.8800001	235.4067688
120.0000000	4.9287996	-206.8225250
120.0000000	4.9776001	1090.9080811
120.0000000	5.0264001	89.1704102
120.0000000	5.0751996	-309.4103699
120.0000000	5.1240001	-12.2348175
120.0000000	5.1727996	567.6830444
120.0000000	5.2216001	32.7989464
120.0000000	5.2704000	-161.2055206
120.0000000	5.3191996	-39.5694122
120.0000000	5.3680000	-3594.7790527
120.0000000	5.4168000	536.2191772
120.0000000	5.4656000	-203.8203278

120.0000000	5.5144000	38.9746780
120.0000000	5.5631995	496.7221069
120.0000000	5.6120000	399.9550171
120.0000000	5.6608000	-37.4331207
120.0000000	5.7096000	63.8454056
120.0000000	5.7584000	135.5062408
120.0000000	5.8071995	-11.4460697
120.0000000	5.8559999	30.5722370
120.0000000	5.9047999	-16.6225052
120.0000000	5.9535999	-40.1809158
120.0000000	6.0023999	-1598.9929199
120.0000000	6.0511999	-14.5501118
120.0000000	6.0999999	-19.4805927
120.0000000	6.1487999	-13.2336655
120.0000000	6.1975999	-23.3716507
120.0000000	6.2463999	-1821.6683350
120.0000000	6.2951999	-19.1402493
120.0000000	6.3439999	-348.2164917
120.0000000	6.3927999	-15.0497026
120.0000000	6.4416003	-12.3353720
120.0000000	6.4903998	-11.2053385
120.0000000	6.5391998	-11.7473316
120.0000000	6.5879998	444.8712463
120.0000000	6.6367998	-12.0365295
120.0000000	6.6856003	-13.8001270
120.0000000	6.7343998	-13.4368782
120.0000000	6.7831998	-14.4088154
120.0000000	6.8319998	60.1092758
120.0000000	6.8807998	-14.0615482
120.0000000	6.9296002	-516.2381592
120.0000000	6.9783998	-3.9588842
120.0000000	7.0271997	-14.2277136
120.0000000	7.0760002	14.9035292
120.0000000	7.1247997	-2479.8808594
120.0000000	7.1736002	-1988.5593262
120.0000000	7.2223997	-1130.7518311
120.0000000	7.2711997	-242.1441956
120.0000000	7.3200002	-19.9439011
120.0000000	7.3687997	221.0810547
120.0000000	7.4176002	458.9199524
120.0000000	7.4663997	360.8793335
120.0000000	7.5151997	-1106.8502197
120.0000000	7.5640001	-1222.8011475
120.0000000	7.6127996	-2884.6682129
120.0000000	7.6616001	-925.1381836
120.0000000	7.7104001	-474.3442993
120.0000000	7.7591996	52.2102776
120.0000000	7.8080001	476.9040833
120.0000000	7.8567996	627.6556396
120.0000000	7.9056001	-1295.6656494
120.0000000	7.9544001	2063.1623535
120.0000000	8.0031996	2662.0566406
120.0000000	8.0520000	2657.0583496
120.0000000	8.1007996	2811.6472168
120.0000000	8.1496000	2998.4267578
120.0000000	8.1983995	1865.5488281
120.0000000	8.2472000	-4267.2778320
120.0000000	8.2959995	-1512.4196777

120.0000000	8.3448000	-1204.8789063
120.0000000	8.3935995	-1104.2098389
120.0000000	8.4423990	-475.3280640
120.0000000	8.4912004	-1110.2819824
120.0000000	8.5400000	-2995.7995605
120.0000000	8.5887995	-1451.6855469
120.0000000	8.6375999	284.2680664
120.0000000	8.6863995	2563.6416016
120.0000000	8.7351999	-952.2535400
120.0000000	8.7840004	1089.5047607
120.0000000	8.8327999	60.5305328
120.0000000	8.8815994	-11.4808254
120.0000000	8.9303989	-24.2784996
120.0000000	8.9792004	-11.5717239
120.0000000	9.0279999	-5.6783009
120.0000000	9.0767994	7.5523834
120.0000000	9.1255999	-17.3959198
120.0000000	9.1743994	-10.0374756
120.0000000	9.2231998	38.4253311
120.0000000	9.2720003	-8.3359327
120.0000000	9.3207998	-9.7495995
120.0000000	9.3695993	-10.7434025
120.0000000	9.4183998	-11.7650862
120.0000000	9.4672003	-11.1576242
120.0000000	9.5159998	-14.2444010
120.0000000	9.5648003	-0.0895367
120.0000000	9.6135998	-13.4671726
120.0000000	9.6623993	-10.1965799
120.0000000	9.7111998	-10.1332636
120.0000000	9.7600002	-10.8538227
120.0000000	9.8087997	-10.9940624
120.0000000	9.8575993	3.5879173
120.0000000	9.9063997	-7.8868151
120.0000000	9.9552002	-10.9062138
120.0000000	10.0039997	-10.0524397
130.0000000	0.3416000	-36.5177650
130.0000000	0.3904000	-39.6869698
130.0000000	0.4392000	-46.7007217
130.0000000	0.4880000	-39.6666298
130.0000000	0.5368000	-39.8229523
130.0000000	0.5856000	-50.9563370
130.0000000	0.6344000	-18.2550468
130.0000000	0.6832000	-112.1226196
130.0000000	0.7320000	-33.5743294
130.0000000	0.7808000	-69.5061951
130.0000000	0.8296000	-40.6359596
130.0000000	0.8784000	-34.8243523
130.0000000	0.9272000	-33.6156883
130.0000000	0.9760000	-33.9337845
130.0000000	1.0247999	-33.7304573
130.0000000	1.0735999	-33.1885376
130.0000000	1.1224000	-32.4081993
130.0000000	1.1711999	-31.6557045
130.0000000	1.2200000	-31.3812904
130.0000000	1.2687999	-31.1473541
130.0000000	1.3176000	-29.7870846
130.0000000	1.3664000	-29.8811951
130.0000000	1.4152000	-30.1441212

130.0000000	1.4640000	-29.7674618
130.0000000	1.5128000	-48.0240364
130.0000000	1.5616000	-37.3541374
130.0000000	1.6104001	-1783.5262451
130.0000000	1.6592000	-99.4181366
130.0000000	1.7079999	-26.6636829
130.0000000	1.7567999	-24.2130795
130.0000000	1.8055999	-24.4392548
130.0000000	1.8544000	-25.8683262
130.0000000	1.9031999	-26.8934765
130.0000000	1.9520000	-26.8108635
130.0000000	2.0007999	-30.1692581
130.0000000	2.0495999	-12.7951584
130.0000000	2.0983999	-279.3184814
130.0000000	2.1471999	22.1121521
130.0000000	2.1960001	-25.8126163
130.0000000	2.2448001	-27.4218636
130.0000000	2.2935998	-32.9869270
130.0000000	2.3423998	-19.7078400
130.0000000	2.3912001	87.1981125
130.0000000	2.4400001	-22.6161690
130.0000000	2.4888000	-23.4794254
130.0000000	2.5375998	-10.7438030
130.0000000	2.5863998	-8.6281404
130.0000000	2.6352000	-6.4337072
130.0000000	2.6840000	-14.3437023
130.0000000	2.7328000	156.9809113
130.0000000	2.7815998	184.5619507
130.0000000	2.8304000	-23.9112034
130.0000000	2.8792000	-20.3269768
130.0000000	2.9280000	-22.6227722
130.0000000	2.9768000	-16.0393429
130.0000000	3.0256000	-20.2412567
130.0000000	3.0743999	-30.5372791
130.0000000	3.1231999	-24.5647793
130.0000000	3.1719999	-30.3490028
130.0000000	3.2208002	-525.5391846
130.0000000	3.2695999	-1029.7921143
130.0000000	3.3183999	-20.4719772
130.0000000	3.3671999	-3526.7470703
130.0000000	3.4159999	1266.1875000
130.0000000	3.4648001	-6.6324873
130.0000000	3.5135999	-27.3033104
130.0000000	3.5623999	-333.4822693
130.0000000	3.6111999	-2099.3300781
130.0000000	3.6600001	-474.7116699
130.0000000	3.7088001	-627.0839844
130.0000000	3.7575998	-982.2274170
130.0000000	3.8063998	-1782.9322510
130.0000000	3.8552001	-2588.8859863
130.0000000	3.9040000	-2538.2143555
130.0000000	3.9528000	1161.7058105
130.0000000	4.0015998	-253.0064850
130.0000000	4.0503998	711.5422363
130.0000000	4.0991998	91.2326050
130.0000000	4.1479998	-676.2860107
130.0000000	4.1967998	3.2780590
130.0000000	4.2456002	-256.2818298

130.0000000	4.2943997	-503.8560486
130.0000000	4.3431997	-13.1589851
130.0000000	4.3920002	-255.8973846
130.0000000	4.4407997	-22.6009293
130.0000000	4.4896002	-15.4044676
130.0000000	4.5383997	-675.6994629
130.0000000	4.5871997	-12.6793566
130.0000000	4.6360002	-21.7429161
130.0000000	4.6847997	-28.5923538
130.0000000	4.7336001	-19.6194630
130.0000000	4.7824001	-16.4764824
130.0000000	4.8311996	-1516.2630615
130.0000000	4.8800001	235.3172302
130.0000000	4.9287996	-206.7552643
130.0000000	4.9776001	1090.5156250
130.0000000	5.0264001	89.1328888
130.0000000	5.0751996	-309.3067322
130.0000000	5.1240001	-12.2357388
130.0000000	5.1727996	567.5671997
130.0000000	5.2216001	32.7895050
130.0000000	5.2704000	-161.1773987
130.0000000	5.3191996	-39.5648575
130.0000000	5.3680000	-3594.0866699
130.0000000	5.4168000	536.1123657
130.0000000	5.4656000	-203.7841492
130.0000000	5.5144000	38.9640274
130.0000000	5.5631995	496.6235352
130.0000000	5.6120000	399.8750610
130.0000000	5.6608000	-37.4290314
130.0000000	5.7096000	63.8299713
130.0000000	5.7584000	135.4773102
130.0000000	5.8071995	-11.4469404
130.0000000	5.8559999	30.5633869
130.0000000	5.9047999	-16.6223087
130.0000000	5.9535999	-40.1761780
130.0000000	6.0023999	-1598.6883545
130.0000000	6.0511999	-14.5503235
130.0000000	6.0999999	-19.4797592
130.0000000	6.1487999	-13.2341280
130.0000000	6.1975999	-23.3698845
130.0000000	6.2463999	-1821.3209229
130.0000000	6.2951999	-19.1394348
130.0000000	6.3439999	-348.1524353
130.0000000	6.3927999	-15.0496845
130.0000000	6.4416003	-12.3359585
130.0000000	6.4903998	-11.2060146
130.0000000	6.5391998	-11.7480135
130.0000000	6.5879998	444.1805420
130.0000000	6.6367998	-12.0370789
130.0000000	6.6856003	-13.8002968
130.0000000	6.7343998	-13.4370937
130.0000000	6.7831998	-14.4083996
130.0000000	6.8319998	60.0901642
130.0000000	6.8807998	-14.0614100
130.0000000	6.9296002	-515.4766846
130.0000000	6.9783998	-3.9717083
130.0000000	7.0271997	-14.2266855
130.0000000	7.0760002	14.8613443

130.0000000	7.1247997	-2475.9350586
130.0000000	7.1736002	-1985.3964844
130.0000000	7.2223997	-1128.9586182
130.0000000	7.2711997	-241.7764282
130.0000000	7.3200002	-19.9319115
130.0000000	7.3687997	220.7065125
130.0000000	7.4176002	458.1641235
130.0000000	7.4663997	360.2816772
130.0000000	7.5151997	-1105.0891113
130.0000000	7.5640001	-1220.8537598
130.0000000	7.6127996	-2880.0505371
130.0000000	7.6616001	-923.6669312
130.0000000	7.7104001	-473.5980225
130.0000000	7.7591996	52.1092262
130.0000000	7.8080001	476.1187134
130.0000000	7.8567996	626.6275635
130.0000000	7.9056001	-1293.5926514
130.0000000	7.9544001	2059.8522949
130.0000000	8.0031996	2657.7871094
130.0000000	8.0520000	2652.7963867
130.0000000	8.1007996	2807.1376953
130.0000000	8.1496000	2993.6186523
130.0000000	8.1983995	1862.5562744
130.0000000	8.2472000	-4260.4433594
130.0000000	8.2959995	-1509.9995117
130.0000000	8.3448000	-1202.9497070
130.0000000	8.3935995	-1102.4421387
130.0000000	8.4423990	-474.5700684
130.0000000	8.4912004	-1108.5057373
130.0000000	8.5400000	-2990.9997559
130.0000000	8.5887995	-1449.3638916
130.0000000	8.6375999	283.8067322
130.0000000	8.6863995	2559.5231934
130.0000000	8.7351999	-950.7293091
130.0000000	8.7840004	1087.7479248
130.0000000	8.8327999	60.4189224
130.0000000	8.8815994	-11.4809999
130.0000000	8.9303989	-24.2747803
130.0000000	8.9792004	-11.5718069
130.0000000	9.0279999	-5.6800694
130.0000000	9.0767994	7.5466652
130.0000000	9.1255999	-17.3942070
130.0000000	9.1743994	-10.0378780
130.0000000	9.2231998	38.4095993
130.0000000	9.2720003	-8.3368683
130.0000000	9.3207998	-9.7501392
130.0000000	9.3695993	-10.7436285
130.0000000	9.4183998	-11.7649622
130.0000000	9.4672003	-11.1576653
130.0000000	9.5159998	-14.2435093
130.0000000	9.5648003	-0.0929098
130.0000000	9.6135998	-13.4663811
130.0000000	9.6623993	-10.1968546
130.0000000	9.7111998	-10.1335354
130.0000000	9.7600002	-10.8538637
130.0000000	9.8087997	-10.9940701
130.0000000	9.8575993	3.5834951
130.0000000	9.9063997	-7.8877287

130.0000000	9.9552002	-10.9062185
130.0000000	10.0039997	-10.0526876
140.0000000	0.3416000	-36.9295311
140.0000000	0.3904000	-39.6182251
140.0000000	0.4392000	-46.5352020
140.0000000	0.4880000	-39.8709450
140.0000000	0.5368000	-39.8057747
140.0000000	0.5856000	-50.5681839
140.0000000	0.6344000	-18.9251060
140.0000000	0.6832000	-108.8651123
140.0000000	0.7320000	-33.7735825
140.0000000	0.7808000	-67.4225464
140.0000000	0.8296000	-40.2443466
140.0000000	0.8784000	-34.7639427
140.0000000	0.9272000	-33.6185760
140.0000000	0.9760000	-34.3420906
140.0000000	1.0247999	-33.7534103
140.0000000	1.0735999	-33.1209373
140.0000000	1.1224000	-32.4645691
140.0000000	1.1711999	-31.2277756
140.0000000	1.2200000	-30.6239262
140.0000000	1.2687999	-31.0532265
140.0000000	1.3176000	-29.8131924
140.0000000	1.3664000	-29.8889446
140.0000000	1.4152000	-30.1443272
140.0000000	1.4640000	-29.7657528
140.0000000	1.5128000	-47.9268570
140.0000000	1.5616000	-37.3122978
140.0000000	1.6104001	-1780.9833984
140.0000000	1.6592000	-99.1242065
140.0000000	1.7079999	-26.0799809
140.0000000	1.7567999	-24.3507977
140.0000000	1.8055999	-24.9480629
140.0000000	1.8544000	-25.8187981
140.0000000	1.9031999	-26.9064236
140.0000000	1.9520000	-26.7862434
140.0000000	2.0007999	-30.1066761
140.0000000	2.0495999	-12.9817753
140.0000000	2.0983999	-277.8426819
140.0000000	2.1471999	21.8448830
140.0000000	2.1960001	-25.8085270
140.0000000	2.2448001	-27.4039536
140.0000000	2.2935998	-32.9273033
140.0000000	2.3423998	-19.7222443
140.0000000	2.3912001	86.5765610
140.0000000	2.4400001	-22.6186543
140.0000000	2.4888000	-23.4792080
140.0000000	2.5375998	-10.7609110
140.0000000	2.5863998	-8.6478996
140.0000000	2.6352000	-6.4564505
140.0000000	2.6840000	-14.3556442
140.0000000	2.7328000	156.7149048
140.0000000	2.7815998	184.2555237
140.0000000	2.8304000	-23.9098988
140.0000000	2.8792000	-20.3302402
140.0000000	2.9280000	-22.6222038
140.0000000	2.9768000	-16.0477142
140.0000000	3.0256000	-20.2438412

140.0000000	3.0743999	-30.5258923
140.0000000	3.1231999	-24.5613232
140.0000000	3.1719999	-30.3407097
140.0000000	3.2208002	-525.3014526
140.0000000	3.2695999	-1029.3170166
140.0000000	3.3183999	-20.4721756
140.0000000	3.3671999	-3525.2854004
140.0000000	3.4159999	1265.6522217
140.0000000	3.4648001	-6.6381960
140.0000000	3.5135999	-27.3003197
140.0000000	3.5623999	-333.3526917
140.0000000	3.6111999	-2098.4702148
140.0000000	3.6600001	-474.5234985
140.0000000	3.7088001	-626.8325806
140.0000000	3.7575998	-981.8288574
140.0000000	3.8063998	-1782.2023926
140.0000000	3.8552001	-2587.8227539
140.0000000	3.9040000	-2537.1718750
140.0000000	3.9528000	1161.2175293
140.0000000	4.0015998	-252.9093781
140.0000000	4.0503998	711.2396851
140.0000000	4.0991998	91.1869202
140.0000000	4.1479998	-676.0137939
140.0000000	4.1967998	3.2688026
140.0000000	4.2456002	-256.1835022
140.0000000	4.2943997	-503.6554871
140.0000000	4.3431997	-13.1611671
140.0000000	4.3920002	-255.7992706
140.0000000	4.4407997	-22.5991554
140.0000000	4.4896002	-15.4053497
140.0000000	4.5383997	-675.4384155
140.0000000	4.5871997	-12.6813726
140.0000000	4.6360002	-21.7413750
140.0000000	4.6847997	-28.5884285
140.0000000	4.7336001	-19.6187401
140.0000000	4.7824001	-16.4769363
140.0000000	4.8311996	-1515.7318115
140.0000000	4.8800001	235.2276764
140.0000000	4.9287996	-206.6879883
140.0000000	4.9776001	1090.1230469
140.0000000	5.0264001	89.0953751
140.0000000	5.0751996	-309.2031250
140.0000000	5.1240001	-12.2366600
140.0000000	5.1727996	567.4513550
140.0000000	5.2216001	32.7800560
140.0000000	5.2704000	-161.1493073
140.0000000	5.3191996	-39.5603104
140.0000000	5.3680000	-3593.3942871
140.0000000	5.4168000	536.0056152
140.0000000	5.4656000	-203.7479553
140.0000000	5.5144000	38.9533691
140.0000000	5.5631995	496.5249634
140.0000000	5.6120000	399.7950745
140.0000000	5.6608000	-37.4249420
140.0000000	5.7096000	63.8145447
140.0000000	5.7584000	135.4483795
140.0000000	5.8071995	-11.4478111
140.0000000	5.8559999	30.5545368

140.0000000	5.9047999	-16.6221104
140.0000000	5.9535999	-40.1714401
140.0000000	6.0023999	-1598.3840332
140.0000000	6.0511999	-14.5505352
140.0000000	6.0999999	-19.4789276
140.0000000	6.1487999	-13.2345905
140.0000000	6.1975999	-23.3681183
140.0000000	6.2463999	-1820.9735107
140.0000000	6.2951999	-19.1386204
140.0000000	6.3439999	-348.0883789
140.0000000	6.3927999	-15.0496664
140.0000000	6.4416003	-12.3365459
140.0000000	6.4903998	-11.2066898
140.0000000	6.5391998	-11.7486944
140.0000000	6.5879998	443.4898682
140.0000000	6.6367998	-12.0376282
140.0000000	6.6856003	-13.8004675
140.0000000	6.7343998	-13.4373093
140.0000000	6.7831998	-14.4079838
140.0000000	6.8319998	60.0710526
140.0000000	6.8807998	-14.0612707
140.0000000	6.9296002	-514.7152100
140.0000000	6.9783998	-3.9845314
140.0000000	7.0271997	-14.2256575
140.0000000	7.0760002	14.8191576
140.0000000	7.1247997	-2471.9892578
140.0000000	7.1736002	-1982.2336426
140.0000000	7.2223997	-1127.1655273
140.0000000	7.2711997	-241.4086456
140.0000000	7.3200002	-19.9199238
140.0000000	7.3687997	220.3319855
140.0000000	7.4176002	457.4082947
140.0000000	7.4663997	359.6840210
140.0000000	7.5151997	-1103.3281250
140.0000000	7.5640001	-1218.9063721
140.0000000	7.6127996	-2875.4328613
140.0000000	7.6616001	-922.1955566
140.0000000	7.7104001	-472.8518066
140.0000000	7.7591996	52.0081787
140.0000000	7.8080001	475.3333435
140.0000000	7.8567996	625.5994873
140.0000000	7.9056001	-1291.5197754
140.0000000	7.9544001	2056.5424805
140.0000000	8.0031996	2653.5173340
140.0000000	8.0520000	2648.5344238
140.0000000	8.1007996	2802.6281738
140.0000000	8.1496000	2988.8107910
140.0000000	8.1983995	1859.5638428
140.0000000	8.2472000	-4253.6083984
140.0000000	8.2959995	-1507.5792236
140.0000000	8.3448000	-1201.0205078
140.0000000	8.3935995	-1100.6744385
140.0000000	8.4423990	-473.8120422
140.0000000	8.4912004	-1106.7296143
140.0000000	8.5400000	-2986.1999512
140.0000000	8.5887995	-1447.0423584
140.0000000	8.6375999	283.3453979
140.0000000	8.6863995	2555.4050293

140.0000000	8.7351999	-949.2050171
140.0000000	8.7840004	1085.9912109
140.0000000	8.8327999	60.3073120
140.0000000	8.8815994	-11.4811735
140.0000000	8.9303989	-24.2710590
140.0000000	8.9792004	-11.5718889
140.0000000	9.0279999	-5.6818376
140.0000000	9.0767994	7.5409489
140.0000000	9.1255999	-17.3924923
140.0000000	9.1743994	-10.0382795
140.0000000	9.2231998	38.3938599
140.0000000	9.2720003	-8.3378057
140.0000000	9.3207998	-9.7506781
140.0000000	9.3695993	-10.7438555
140.0000000	9.4183998	-11.7648373
140.0000000	9.4672003	-11.1577063
140.0000000	9.5159998	-14.2426186
140.0000000	9.5648003	-0.0962830
140.0000000	9.6135998	-13.4655905
140.0000000	9.6623993	-10.1971292
140.0000000	9.7111998	-10.1338072
140.0000000	9.7600002	-10.8539057
140.0000000	9.8087997	-10.9940786
140.0000000	9.8575993	3.5790720
140.0000000	9.9063997	-7.8886428
140.0000000	9.9552002	-10.9062223
140.0000000	10.0039997	-10.0529346
150.0000000	0.3416000	-36.4272232
150.0000000	0.3904000	-39.5494804
150.0000000	0.4392000	-46.3696785
150.0000000	0.4880000	-39.5201988
150.0000000	0.5368000	-39.7885971
150.0000000	0.5856000	-50.1800308
150.0000000	0.6344000	-19.5951653
150.0000000	0.6832000	-105.6075897
150.0000000	0.7320000	-33.9728355
150.0000000	0.7808000	-65.3388977
150.0000000	0.8296000	-39.8527336
150.0000000	0.8784000	-34.7035332
150.0000000	0.9272000	-33.6214676
150.0000000	0.9760000	-34.2347412
150.0000000	1.0247999	-33.4003754
150.0000000	1.0735999	-33.0533333
150.0000000	1.1224000	-32.4587326
150.0000000	1.1711999	-30.7602386
150.0000000	1.2200000	-30.3030643
150.0000000	1.2687999	-30.8576927
150.0000000	1.3176000	-29.8393021
150.0000000	1.3664000	-29.8966942
150.0000000	1.4152000	-30.1445313
150.0000000	1.4640000	-29.7640438
150.0000000	1.5128000	-47.8296814
150.0000000	1.5616000	-37.2704544
150.0000000	1.6104001	-1778.4406738
150.0000000	1.6592000	-98.8302765
150.0000000	1.7079999	-25.3650837
150.0000000	1.7567999	-24.0397224
150.0000000	1.8055999	-24.6240215

150.0000000	1.8544000	-25.3959637
150.0000000	1.9031999	-26.2887135
150.0000000	1.9520000	-26.7616234
150.0000000	2.0007999	-30.0440941
150.0000000	2.0495999	-13.1683912
150.0000000	2.0983999	-276.3669128
150.0000000	2.1471999	21.5776176
150.0000000	2.1960001	-25.8044357
150.0000000	2.2448001	-27.3860416
150.0000000	2.2935998	-32.8676796
150.0000000	2.3423998	-19.7366505
150.0000000	2.3912001	85.9550171
150.0000000	2.4400001	-22.6211414
150.0000000	2.4888000	-23.4789906
150.0000000	2.5375998	-10.7780190
150.0000000	2.5863998	-8.6676588
150.0000000	2.6352000	-6.4791956
150.0000000	2.6840000	-14.3675852
150.0000000	2.7328000	156.4488831
150.0000000	2.7815998	183.9491272
150.0000000	2.8304000	-23.9085941
150.0000000	2.8792000	-20.3335037
150.0000000	2.9280000	-22.6216335
150.0000000	2.9768000	-16.0560837
150.0000000	3.0256000	-20.2464256
150.0000000	3.0743999	-30.5145054
150.0000000	3.1231999	-24.5578671
150.0000000	3.1719999	-30.3324165
150.0000000	3.2208002	-525.0637207
150.0000000	3.2695999	-1028.8421631
150.0000000	3.3183999	-20.4723740
150.0000000	3.3671999	-3523.8237305
150.0000000	3.4159999	1265.1169434
150.0000000	3.4648001	-6.6439047
150.0000000	3.5135999	-27.2973289
150.0000000	3.5623999	-333.2230835
150.0000000	3.6111999	-2097.6105957
150.0000000	3.6600001	-474.3353882
150.0000000	3.7088001	-626.5812378
150.0000000	3.7575998	-981.4303589
150.0000000	3.8063998	-1781.4725342
150.0000000	3.8552001	-2586.7592773
150.0000000	3.9040000	-2536.1293945
150.0000000	3.9528000	1160.7292480
150.0000000	4.0015998	-252.8122711
150.0000000	4.0503998	710.9371338
150.0000000	4.0991998	91.1412354
150.0000000	4.1479998	-675.7415161
150.0000000	4.1967998	3.2595463
150.0000000	4.2456002	-256.0852051
150.0000000	4.2943997	-503.4548950
150.0000000	4.3431997	-13.1633472
150.0000000	4.3920002	-255.7011566
150.0000000	4.4407997	-22.5973797
150.0000000	4.4896002	-15.4062328
150.0000000	4.5383997	-675.1773071
150.0000000	4.5871997	-12.6833887
150.0000000	4.6360002	-21.7398319

150.0000000	4.6847997	-28.5845032
150.0000000	4.7336001	-19.6180172
150.0000000	4.7824001	-16.4773903
150.0000000	4.8311996	-1515.2005615
150.0000000	4.8800001	235.1381226
150.0000000	4.9287996	-206.6207275
150.0000000	4.9776001	1089.7305908
150.0000000	5.0264001	89.0578537
150.0000000	5.0751996	-309.0994873
150.0000000	5.1240001	-12.2375813
150.0000000	5.1727996	567.3354492
150.0000000	5.2216001	32.7706146
150.0000000	5.2704000	-161.1212006
150.0000000	5.3191996	-39.5557632
150.0000000	5.3680000	-3592.7019043
150.0000000	5.4168000	535.8988647
150.0000000	5.4656000	-203.7117767
150.0000000	5.5144000	38.9427109
150.0000000	5.5631995	496.4263916
150.0000000	5.6120000	399.7150879
150.0000000	5.6608000	-37.4208565
150.0000000	5.7096000	63.7991104
150.0000000	5.7584000	135.4194489
150.0000000	5.8071995	-11.4486809
150.0000000	5.8559999	30.5456867
150.0000000	5.9047999	-16.6219120
150.0000000	5.9535999	-40.1666985
150.0000000	6.0023999	-1598.0795898
150.0000000	6.0511999	-14.5507460
150.0000000	6.0999999	-19.4780960
150.0000000	6.1487999	-13.2350540
150.0000000	6.1975999	-23.3663540
150.0000000	6.2463999	-1820.6262207
150.0000000	6.2951999	-19.1378059
150.0000000	6.3439999	-348.0243530
150.0000000	6.3927999	-15.0496473
150.0000000	6.4416003	-12.3371325
150.0000000	6.4903998	-11.2073660
150.0000000	6.5391998	-11.7493753
150.0000000	6.5879998	442.7991638
150.0000000	6.6367998	-12.0381775
150.0000000	6.6856003	-13.8006372
150.0000000	6.7343998	-13.4375257
150.0000000	6.7831998	-14.4075680
150.0000000	6.8319998	60.0519333
150.0000000	6.8807998	-14.0611324
150.0000000	6.9296002	-513.9537964
150.0000000	6.9783998	-3.9973545
150.0000000	7.0271997	-14.2246294
150.0000000	7.0760002	14.7769728
150.0000000	7.1247997	-2468.0434570
150.0000000	7.1736002	-1979.0708008
150.0000000	7.2223997	-1125.3723145
150.0000000	7.2711997	-241.0408630
150.0000000	7.3200002	-19.9079361
150.0000000	7.3687997	219.9574585
150.0000000	7.4176002	456.6524658
150.0000000	7.4663997	359.0863647

150.0000000	7.5151997	-1101.5671387
150.0000000	7.5640001	-1216.9589844
150.0000000	7.6127996	-2870.8154297
150.0000000	7.6616001	-920.7242432
150.0000000	7.7104001	-472.1055298
150.0000000	7.7591996	51.9071312
150.0000000	7.8080001	474.5480042
150.0000000	7.8567996	624.5714111
150.0000000	7.9056001	-1289.4468994
150.0000000	7.9544001	2053.2324219
150.0000000	8.0031996	2649.2478027
150.0000000	8.0520000	2644.2724609
150.0000000	8.1007996	2798.1186523
150.0000000	8.1496000	2984.0026855
150.0000000	8.1983995	1856.5712891
150.0000000	8.2472000	-4246.7734375
150.0000000	8.2959995	-1505.1590576
150.0000000	8.3448000	-1199.0913086
150.0000000	8.3935995	-1098.9067383
150.0000000	8.4423990	-473.0540466
150.0000000	8.4912004	-1104.9533691
150.0000000	8.5400000	-2981.4001465
150.0000000	8.5887995	-1444.7208252
150.0000000	8.6375999	282.8840942
150.0000000	8.6863995	2551.2866211
150.0000000	8.7351999	-947.6807861
150.0000000	8.7840004	1084.2344971
150.0000000	8.8327999	60.1957016
150.0000000	8.8815994	-11.4813480
150.0000000	8.9303989	-24.2673378
150.0000000	8.9792004	-11.5719709
150.0000000	9.0279999	-5.6836061
150.0000000	9.0767994	7.5352306
150.0000000	9.1255999	-17.3907776
150.0000000	9.1743994	-10.0386820
150.0000000	9.2231998	38.3781281
150.0000000	9.2720003	-8.3387413
150.0000000	9.3207998	-9.7512178
150.0000000	9.3695993	-10.7440815
150.0000000	9.4183998	-11.7647123
150.0000000	9.4672003	-11.1577482
150.0000000	9.5159998	-14.2417278
150.0000000	9.5648003	-0.0996552
150.0000000	9.6135998	-13.4647999
150.0000000	9.6623993	-10.1974049
150.0000000	9.7111998	-10.1340790
150.0000000	9.7600002	-10.8539476
150.0000000	9.8087997	-10.9940863
150.0000000	9.8575993	3.5746498
150.0000000	9.9063997	-7.8895569
150.0000000	9.9552002	-10.9062271
150.0000000	10.0039997	-10.0531826
160.0000000	0.3416000	-36.9704666
160.0000000	0.3904000	-39.7042694
160.0000000	0.4392000	-46.2041550
160.0000000	0.4880000	-39.5768852
160.0000000	0.5368000	-39.7714195
160.0000000	0.5856000	-49.7918739

160.0000000	0.6344000	-20.2652245
160.0000000	0.6832000	-102.3500824
160.0000000	0.7320000	-34.1720886
160.0000000	0.7808000	-63.2552490
160.0000000	0.8296000	-39.4611206
160.0000000	0.8784000	-34.6431236
160.0000000	0.9272000	-33.6243553
160.0000000	0.9760000	-34.1273918
160.0000000	1.0247999	-33.0473366
160.0000000	1.0735999	-32.9857292
160.0000000	1.1224000	-31.1310978
160.0000000	1.1711999	-30.7015476
160.0000000	1.2200000	-30.9957581
160.0000000	1.2687999	-31.0472679
160.0000000	1.3176000	-29.8654099
160.0000000	1.3664000	-29.9044437
160.0000000	1.4152000	-30.1447353
160.0000000	1.4640000	-29.7623348
160.0000000	1.5128000	-47.7325020
160.0000000	1.5616000	-37.2286148
160.0000000	1.6104001	-1775.8978271
160.0000000	1.6592000	-98.5363464
160.0000000	1.7079999	-27.1197128
160.0000000	1.7567999	-24.5838356
160.0000000	1.8055999	-24.8208218
160.0000000	1.8544000	-25.5547943
160.0000000	1.9031999	-26.2602139
160.0000000	1.9520000	-26.7370033
160.0000000	2.0007999	-29.9815121
160.0000000	2.0495999	-13.3550072
160.0000000	2.0983999	-274.8911438
160.0000000	2.1471999	21.3103523
160.0000000	2.1960001	-25.8003445
160.0000000	2.2448001	-27.3681297
160.0000000	2.2935998	-32.8080559
160.0000000	2.3423998	-19.7510548
160.0000000	2.3912001	85.3334732
160.0000000	2.4400001	-22.6236267
160.0000000	2.4888000	-23.4787750
160.0000000	2.5375998	-10.7951269
160.0000000	2.5863998	-8.6874180
160.0000000	2.6352000	-6.5019388
160.0000000	2.6840000	-14.3795271
160.0000000	2.7328000	156.1828766
160.0000000	2.7815998	183.6427002
160.0000000	2.8304000	-23.9072914
160.0000000	2.8792000	-20.3367672
160.0000000	2.9280000	-22.6210651
160.0000000	2.9768000	-16.0644550
160.0000000	3.0256000	-20.2490101
160.0000000	3.0743999	-30.5031185
160.0000000	3.1231999	-24.5544128
160.0000000	3.1719999	-30.3241234
160.0000000	3.2208002	-524.8259888
160.0000000	3.2695999	-1028.3670654
160.0000000	3.3183999	-20.4725704
160.0000000	3.3671999	-3522.3620605
160.0000000	3.4159999	1264.5816650

160.0000000	3.4648001	-6.6496124
160.0000000	3.5135999	-27.2943382
160.0000000	3.5623999	-333.0935059
160.0000000	3.6111999	-2096.7507324
160.0000000	3.6600001	-474.1472778
160.0000000	3.7088001	-626.3298340
160.0000000	3.7575998	-981.0317993
160.0000000	3.8063998	-1780.7426758
160.0000000	3.8552001	-2585.6958008
160.0000000	3.9040000	-2535.0869141
160.0000000	3.9528000	1160.2409668
160.0000000	4.0015998	-252.7151642
160.0000000	4.0503998	710.6345825
160.0000000	4.0991998	91.0955505
160.0000000	4.1479998	-675.4692993
160.0000000	4.1967998	3.2502899
160.0000000	4.2456002	-255.9869080
160.0000000	4.2943997	-503.2543335
160.0000000	4.3431997	-13.1655273
160.0000000	4.3920002	-255.6030426
160.0000000	4.4407997	-22.5956039
160.0000000	4.4896002	-15.4071150
160.0000000	4.5383997	-674.9162598
160.0000000	4.5871997	-12.6854057
160.0000000	4.6360002	-21.7382889
160.0000000	4.6847997	-28.5805779
160.0000000	4.7336001	-19.6172962
160.0000000	4.7824001	-16.4778442
160.0000000	4.8311996	-1514.6691895
160.0000000	4.8800001	235.0485840
160.0000000	4.9287996	-206.5534668
160.0000000	4.9776001	1089.3381348
160.0000000	5.0264001	89.0203323
160.0000000	5.0751996	-308.9958496
160.0000000	5.1240001	-12.2385025
160.0000000	5.1727996	567.2196045
160.0000000	5.2216001	32.7611732
160.0000000	5.2704000	-161.0930939
160.0000000	5.3191996	-39.5512123
160.0000000	5.3680000	-3592.0095215
160.0000000	5.4168000	535.7920532
160.0000000	5.4656000	-203.6755981
160.0000000	5.5144000	38.9320602
160.0000000	5.5631995	496.3278198
160.0000000	5.6120000	399.6351318
160.0000000	5.6608000	-37.4167671
160.0000000	5.7096000	63.7836838
160.0000000	5.7584000	135.3905029
160.0000000	5.8071995	-11.4495525
160.0000000	5.8559999	30.5368366
160.0000000	5.9047999	-16.6217155
160.0000000	5.9535999	-40.1619606
160.0000000	6.0023999	-1597.7752686
160.0000000	6.0511999	-14.5509577
160.0000000	6.0999999	-19.4772625
160.0000000	6.1487999	-13.2355165
160.0000000	6.1975999	-23.3645897
160.0000000	6.2463999	-1820.2788086

160.0000000	6.2951999	-19.1369915
160.0000000	6.3439999	-347.9602661
160.0000000	6.3927999	-15.0496292
160.0000000	6.4416003	-12.3377199
160.0000000	6.4903998	-11.2080421
160.0000000	6.5391998	-11.7500563
160.0000000	6.5879998	442.1084595
160.0000000	6.6367998	-12.0387268
160.0000000	6.6856003	-13.8008080
160.0000000	6.7343998	-13.4377413
160.0000000	6.7831998	-14.4071531
160.0000000	6.8319998	60.0328140
160.0000000	6.8807998	-14.0609941
160.0000000	6.9296002	-513.1923218
160.0000000	6.9783998	-4.0101786
160.0000000	7.0271997	-14.2236023
160.0000000	7.0760002	14.7347879
160.0000000	7.1247997	-2464.0974121
160.0000000	7.1736002	-1975.9078369
160.0000000	7.2223997	-1123.5792236
160.0000000	7.2711997	-240.6730957
160.0000000	7.3200002	-19.8959465
160.0000000	7.3687997	219.5829163
160.0000000	7.4176002	455.8966370
160.0000000	7.4663997	358.4887085
160.0000000	7.5151997	-1099.8060303
160.0000000	7.5640001	-1215.0114746
160.0000000	7.6127996	-2866.1977539
160.0000000	7.6616001	-919.2528687
160.0000000	7.7104001	-471.3592529
160.0000000	7.7591996	51.8060760
160.0000000	7.8080001	473.7626343
160.0000000	7.8567996	623.5433350
160.0000000	7.9056001	-1287.3740234
160.0000000	7.9544001	2049.9226074
160.0000000	8.0031996	2644.9782715
160.0000000	8.0520000	2640.0104980
160.0000000	8.1007996	2793.6091309
160.0000000	8.1496000	2979.1945801
160.0000000	8.1983995	1853.5787354
160.0000000	8.2472000	-4239.9389648
160.0000000	8.2959995	-1502.7388916
160.0000000	8.3448000	-1197.1622314
160.0000000	8.3935995	-1097.1391602
160.0000000	8.4423990	-472.2960205
160.0000000	8.4912004	-1103.1771240
160.0000000	8.5400000	-2976.6003418
160.0000000	8.5887995	-1442.3992920
160.0000000	8.6375999	282.4227600
160.0000000	8.6863995	2547.1682129
160.0000000	8.7351999	-946.1565552
160.0000000	8.7840004	1082.4777832
160.0000000	8.8327999	60.0840988
160.0000000	8.8815994	-11.4815226
160.0000000	8.9303989	-24.2636185
160.0000000	8.9792004	-11.5720539
160.0000000	9.0279999	-5.6853738
160.0000000	9.0767994	7.5295124

160.0000000	9.1255999	-17.3890648
160.0000000	9.1743994	-10.0390835
160.0000000	9.2231998	38.3623962
160.0000000	9.2720003	-8.3396778
160.0000000	9.3207998	-9.7517567
160.0000000	9.3695993	-10.7443085
160.0000000	9.4183998	-11.7645884
160.0000000	9.4672003	-11.1577892
160.0000000	9.5159998	-14.2408371
160.0000000	9.5648003	-0.1030273
160.0000000	9.6135998	-13.4640093
160.0000000	9.6623993	-10.1976795
160.0000000	9.7111998	-10.1343508
160.0000000	9.7600002	-10.8539896
160.0000000	9.8087997	-10.9940939
160.0000000	9.8575993	3.5702276
160.0000000	9.9063997	-7.8904705
160.0000000	9.9552002	-10.9062309
160.0000000	10.0039997	-10.0534306
170.0000000	0.3416000	-36.7660599
170.0000000	0.3904000	-39.8590584
170.0000000	0.4392000	-46.0386314
170.0000000	0.4880000	-39.6335716
170.0000000	0.5368000	-39.7542419
170.0000000	0.5856000	-49.4037209
170.0000000	0.6344000	-20.9352837
170.0000000	0.6832000	-99.0925598
170.0000000	0.7320000	-34.3713417
170.0000000	0.7808000	-61.1716003
170.0000000	0.8296000	-39.0695076
170.0000000	0.8784000	-34.5827141
170.0000000	0.9272000	-33.6272469
170.0000000	0.9760000	-34.0200424
170.0000000	1.0247999	-32.6943016
170.0000000	1.0735999	-30.7224712
170.0000000	1.1224000	-29.7143688
170.0000000	1.1711999	-29.6481972
170.0000000	1.2200000	-30.1952019
170.0000000	1.2687999	-31.2368450
170.0000000	1.3176000	-29.8915195
170.0000000	1.3664000	-29.9121933
170.0000000	1.4152000	-30.1449394
170.0000000	1.4640000	-29.7606239
170.0000000	1.5128000	-47.6353264
170.0000000	1.5616000	-37.1867752
170.0000000	1.6104001	-1773.3549805
170.0000000	1.6592000	-98.2424164
170.0000000	1.7079999	-26.4195061
170.0000000	1.7567999	-24.0553608
170.0000000	1.8055999	-24.2053127
170.0000000	1.8544000	-25.2205467
170.0000000	1.9031999	-25.8687687
170.0000000	1.9520000	-26.3899708
170.0000000	2.0007999	-29.9189301
170.0000000	2.0495999	-13.5416241
170.0000000	2.0983999	-273.4153442
170.0000000	2.1471999	21.0430832
170.0000000	2.1960001	-25.7962532

170.0000000	2.2448001	-27.3502197
170.0000000	2.2935998	-32.7484322
170.0000000	2.3423998	-19.7654591
170.0000000	2.3912001	84.7119293
170.0000000	2.4400001	-22.6261139
170.0000000	2.4888000	-23.4785576
170.0000000	2.5375998	-10.8122349
170.0000000	2.5863998	-8.7071772
170.0000000	2.6352000	-6.5246820
170.0000000	2.6840000	-14.3914690
170.0000000	2.7328000	155.9168549
170.0000000	2.7815998	183.3362732
170.0000000	2.8304000	-23.9059868
170.0000000	2.8792000	-20.3400307
170.0000000	2.9280000	-22.6204967
170.0000000	2.9768000	-16.0728264
170.0000000	3.0256000	-20.2515945
170.0000000	3.0743999	-30.4917316
170.0000000	3.1231999	-24.5509567
170.0000000	3.1719999	-30.3158302
170.0000000	3.2208002	-524.5882568
170.0000000	3.2695999	-1027.8922119
170.0000000	3.3183999	-20.4727688
170.0000000	3.3671999	-3520.9003906
170.0000000	3.4159999	1264.0463867
170.0000000	3.4648001	-6.6553211
170.0000000	3.5135999	-27.2913475
170.0000000	3.5623999	-332.9639282
170.0000000	3.6111999	-2095.8911133
170.0000000	3.6600001	-473.9591675
170.0000000	3.7088001	-626.0784302
170.0000000	3.7575998	-980.6333008
170.0000000	3.8063998	-1780.0128174
170.0000000	3.8552001	-2584.6323242
170.0000000	3.9040000	-2534.0444336
170.0000000	3.9528000	1159.7526855
170.0000000	4.0015998	-252.6180573
170.0000000	4.0503998	710.3319702
170.0000000	4.0991998	91.0498734
170.0000000	4.1479998	-675.1970825
170.0000000	4.1967998	3.2410336
170.0000000	4.2456002	-255.8885956
170.0000000	4.2943997	-503.0537720
170.0000000	4.3431997	-13.1677074
170.0000000	4.3920002	-255.5049286
170.0000000	4.4407997	-22.5938282
170.0000000	4.4896002	-15.4079981
170.0000000	4.5383997	-674.6551514
170.0000000	4.5871997	-12.6874218
170.0000000	4.6360002	-21.7367458
170.0000000	4.6847997	-28.5766525
170.0000000	4.7336001	-19.6165733
170.0000000	4.7824001	-16.4783001
170.0000000	4.8311996	-1514.1379395
170.0000000	4.8800001	234.9590302
170.0000000	4.9287996	-206.4862061
170.0000000	4.9776001	1088.9455566
170.0000000	5.0264001	88.9828110

170.0000000	5.0751996	-308.8922424
170.0000000	5.1240001	-12.2394238
170.0000000	5.1727996	567.1037598
170.0000000	5.2216001	32.7517242
170.0000000	5.2704000	-161.0649872
170.0000000	5.3191996	-39.5466614
170.0000000	5.3680000	-3591.3171387
170.0000000	5.4168000	535.6852417
170.0000000	5.4656000	-203.6394043
170.0000000	5.5144000	38.9214020
170.0000000	5.5631995	496.2292480
170.0000000	5.6120000	399.5551453
170.0000000	5.6608000	-37.4126816
170.0000000	5.7096000	63.7682495
170.0000000	5.7584000	135.3615723
170.0000000	5.8071995	-11.4504223
170.0000000	5.8559999	30.5279865
170.0000000	5.9047999	-16.6215172
170.0000000	5.9535999	-40.1572227
170.0000000	6.0023999	-1597.4707031
170.0000000	6.0511999	-14.5511694
170.0000000	6.0999999	-19.4764309
170.0000000	6.1487999	-13.2359791
170.0000000	6.1975999	-23.3628235
170.0000000	6.2463999	-1819.9313965
170.0000000	6.2951999	-19.1361771
170.0000000	6.3439999	-347.8962402
170.0000000	6.3927999	-15.0496111
170.0000000	6.4416003	-12.3383064
170.0000000	6.4903998	-11.2087183
170.0000000	6.5391998	-11.7507372
170.0000000	6.5879998	441.4177856
170.0000000	6.6367998	-12.0392752
170.0000000	6.6856003	-13.8009777
170.0000000	6.7343998	-13.4379568
170.0000000	6.7831998	-14.4067373
170.0000000	6.8319998	60.0137024
170.0000000	6.8807998	-14.0608549
170.0000000	6.9296002	-512.4309082
170.0000000	6.9783998	-4.0230026
170.0000000	7.0271997	-14.2225742
170.0000000	7.0760002	14.6926012
170.0000000	7.1247997	-2460.1516113
170.0000000	7.1736002	-1972.7449951
170.0000000	7.2223997	-1121.7860107
170.0000000	7.2711997	-240.3053131
170.0000000	7.3200002	-19.8839569
170.0000000	7.3687997	219.2083893
170.0000000	7.4176002	455.1408081
170.0000000	7.4663997	357.8910828
170.0000000	7.5151997	-1098.0450439
170.0000000	7.5640001	-1213.0640869
170.0000000	7.6127996	-2861.5800781
170.0000000	7.6616001	-917.7814941
170.0000000	7.7104001	-470.6130371
170.0000000	7.7591996	51.7050285
170.0000000	7.8080001	472.9772644
170.0000000	7.8567996	622.5152588

170.0000000	7.9056001	-1285.3010254
170.0000000	7.9544001	2046.6125488
170.0000000	8.0031996	2640.7087402
170.0000000	8.0520000	2635.7482910
170.0000000	8.1007996	2789.0996094
170.0000000	8.1496000	2974.3867188
170.0000000	8.1983995	1850.5863037
170.0000000	8.2472000	-4233.1040039
170.0000000	8.2959995	-1500.3187256
170.0000000	8.3448000	-1195.2330322
170.0000000	8.3935995	-1095.3714600
170.0000000	8.4423990	-471.5379944
170.0000000	8.4912004	-1101.4008789
170.0000000	8.5400000	-2971.8005371
170.0000000	8.5887995	-1440.0777588
170.0000000	8.6375999	281.9614563
170.0000000	8.6863995	2543.0498047
170.0000000	8.7351999	-944.6322632
170.0000000	8.7840004	1080.7209473
170.0000000	8.8327999	59.9724884
170.0000000	8.8815994	-11.4816971
170.0000000	8.9303989	-24.2598991
170.0000000	8.9792004	-11.5721359
170.0000000	9.0279999	-5.6871424
170.0000000	9.0767994	7.5237942
170.0000000	9.1255999	-17.3873501
170.0000000	9.1743994	-10.0394859
170.0000000	9.2231998	38.3466644
170.0000000	9.2720003	-8.3406143
170.0000000	9.3207998	-9.7522964
170.0000000	9.3695993	-10.7445345
170.0000000	9.4183998	-11.7644634
170.0000000	9.4672003	-11.1578302
170.0000000	9.5159998	-14.2399454
170.0000000	9.5648003	-0.1064005
170.0000000	9.6135998	-13.4632187
170.0000000	9.6623993	-10.1979551
170.0000000	9.7111998	-10.1346216
170.0000000	9.7600002	-10.8540306
170.0000000	9.8087997	-10.9941025
170.0000000	9.8575993	3.5658054
170.0000000	9.9063997	-7.8913846
170.0000000	9.9552002	-10.9062357
170.0000000	10.0039997	-10.0536776
180.0000000	0.3416000	-36.7673302
180.0000000	0.3904000	-39.7727089
180.0000000	0.4392000	-45.8731079
180.0000000	0.4880000	-39.6732407
180.0000000	0.5368000	-39.7370644
180.0000000	0.5856000	-49.0155678
180.0000000	0.6344000	-21.6053429
180.0000000	0.6832000	-95.8350525
180.0000000	0.7320000	-34.5705948
180.0000000	0.7808000	-59.0879517
180.0000000	0.8296000	-38.6778946
180.0000000	0.8784000	-34.5223045
180.0000000	0.9272000	-33.6301384
180.0000000	0.9760000	-33.9126930

180.0000000	1.0247999	-31.9986572
180.0000000	1.0735999	-29.7129250
180.0000000	1.1224000	-28.1077404
180.0000000	1.1711999	-28.2074184
180.0000000	1.2200000	-29.9577789
180.0000000	1.2687999	-31.2039089
180.0000000	1.3176000	-29.9176292
180.0000000	1.3664000	-29.9199429
180.0000000	1.4152000	-30.1451435
180.0000000	1.4640000	-29.7589149
180.0000000	1.5128000	-47.5381470
180.0000000	1.5616000	-37.1449318
180.0000000	1.6104001	-1770.8122559
180.0000000	1.6592000	-97.9484787
180.0000000	1.7079999	-27.2432175
180.0000000	1.7567999	-24.5465813
180.0000000	1.8055999	-24.0489407
180.0000000	1.8544000	-24.6267567
180.0000000	1.9031999	-25.4222679
180.0000000	1.9520000	-26.1187286
180.0000000	2.0007999	-29.8563480
180.0000000	2.0495999	-13.7282410
180.0000000	2.0983999	-271.9395752
180.0000000	2.1471999	20.7758141
180.0000000	2.1960001	-25.7921619
180.0000000	2.2448001	-27.3323078
180.0000000	2.2935998	-32.6888084
180.0000000	2.3423998	-19.7798634
180.0000000	2.3912001	84.0903854
180.0000000	2.4400001	-22.6286011
180.0000000	2.4888000	-23.4783401
180.0000000	2.5375998	-10.8293428
180.0000000	2.5863998	-8.7269354
180.0000000	2.6352000	-6.5474272
180.0000000	2.6840000	-14.4034109
180.0000000	2.7328000	155.6508331
180.0000000	2.7815998	183.0298767
180.0000000	2.8304000	-23.9046822
180.0000000	2.8792000	-20.3432941
180.0000000	2.9280000	-22.6199265
180.0000000	2.9768000	-16.0811958
180.0000000	3.0256000	-20.2541790
180.0000000	3.0743999	-30.4803448
180.0000000	3.1231999	-24.5475006
180.0000000	3.1719999	-30.3075371
180.0000000	3.2208002	-524.3504639
180.0000000	3.2695999	-1027.4171143
180.0000000	3.3183999	-20.4729671
180.0000000	3.3671999	-3519.4387207
180.0000000	3.4159999	1263.5111084
180.0000000	3.4648001	-6.6610279
180.0000000	3.5135999	-27.2883568
180.0000000	3.5623999	-332.8343201
180.0000000	3.6111999	-2095.0312500
180.0000000	3.6600001	-473.7709961
180.0000000	3.7088001	-625.8270874
180.0000000	3.7575998	-980.2347412
180.0000000	3.8063998	-1779.2830811

180.0000000	3.8552001	-2583.5688477
180.0000000	3.9040000	-2533.0019531
180.0000000	3.9528000	1159.2644043
180.0000000	4.0015998	-252.5209503
180.0000000	4.0503998	710.0294189
180.0000000	4.0991998	91.0041885
180.0000000	4.1479998	-674.9248047
180.0000000	4.1967998	3.2317772
180.0000000	4.2456002	-255.7902985
180.0000000	4.2943997	-502.8532104
180.0000000	4.3431997	-13.1698895
180.0000000	4.3920002	-255.4068146
180.0000000	4.4407997	-22.5920525
180.0000000	4.4896002	-15.4088802
180.0000000	4.5383997	-674.3941040
180.0000000	4.5871997	-12.6894379
180.0000000	4.6360002	-21.7352047
180.0000000	4.6847997	-28.5727272
180.0000000	4.7336001	-19.6158504
180.0000000	4.7824001	-16.4787540
180.0000000	4.8311996	-1513.6066895
180.0000000	4.8800001	234.8694763
180.0000000	4.9287996	-206.4189453
180.0000000	4.9776001	1088.5531006
180.0000000	5.0264001	88.9452896
180.0000000	5.0751996	-308.7886047
180.0000000	5.1240001	-12.2403450
180.0000000	5.1727996	566.9878540
180.0000000	5.2216001	32.7422829
180.0000000	5.2704000	-161.0368805
180.0000000	5.3191996	-39.5421143
180.0000000	5.3680000	-3590.6247559
180.0000000	5.4168000	535.5784912
180.0000000	5.4656000	-203.6032257
180.0000000	5.5144000	38.9107437
180.0000000	5.5631995	496.1306763
180.0000000	5.6120000	399.4751892
180.0000000	5.6608000	-37.4085922
180.0000000	5.7096000	63.7528152
180.0000000	5.7584000	135.3326416
180.0000000	5.8071995	-11.4512939
180.0000000	5.8559999	30.5191326
180.0000000	5.9047999	-16.6213188
180.0000000	5.9535999	-40.1524849
180.0000000	6.0023999	-1597.1663818
180.0000000	6.0511999	-14.5513802
180.0000000	6.0999999	-19.4755974
180.0000000	6.1487999	-13.2364416
180.0000000	6.1975999	-23.3610592
180.0000000	6.2463999	-1819.5841064
180.0000000	6.2951999	-19.1353626
180.0000000	6.3439999	-347.8321838
180.0000000	6.3927999	-15.0495930
180.0000000	6.4416003	-12.3388929
180.0000000	6.4903998	-11.2093935
180.0000000	6.5391998	-11.7514191
180.0000000	6.5879998	440.7270813
180.0000000	6.6367998	-12.0398245

180.0000000	6.6856003	-13.8011484
180.0000000	6.7343998	-13.4381733
180.0000000	6.7831998	-14.4063215
180.0000000	6.8319998	59.9945908
180.0000000	6.8807998	-14.0607166
180.0000000	6.9296002	-511.6694641
180.0000000	6.9783998	-4.0358257
180.0000000	7.0271997	-14.2215462
180.0000000	7.0760002	14.6504183
180.0000000	7.1247997	-2456.2058105
180.0000000	7.1736002	-1969.5821533
180.0000000	7.2223997	-1119.9927979
180.0000000	7.2711997	-239.9375305
180.0000000	7.3200002	-19.8719692
180.0000000	7.3687997	218.8338623
180.0000000	7.4176002	454.3849792
180.0000000	7.4663997	357.2934265
180.0000000	7.5151997	-1096.2839355
180.0000000	7.5640001	-1211.1166992
180.0000000	7.6127996	-2856.9624023
180.0000000	7.6616001	-916.3101807
180.0000000	7.7104001	-469.8666992
180.0000000	7.7591996	51.6039772
180.0000000	7.8080001	472.1919250
180.0000000	7.8567996	621.4871216
180.0000000	7.9056001	-1283.2281494
180.0000000	7.9544001	2043.3027344
180.0000000	8.0031996	2636.4389648
180.0000000	8.0520000	2631.4863281
180.0000000	8.1007996	2784.5900879
180.0000000	8.1496000	2969.5786133
180.0000000	8.1983995	1847.5937500
180.0000000	8.2472000	-4226.2690430
180.0000000	8.2959995	-1497.8985596
180.0000000	8.3448000	-1193.3038330
180.0000000	8.3935995	-1093.6037598
180.0000000	8.4423990	-470.7799988
180.0000000	8.4912004	-1099.6246338
180.0000000	8.5400000	-2967.0007324
180.0000000	8.5887995	-1437.7562256
180.0000000	8.6375999	281.5001221
180.0000000	8.6863995	2538.9313965
180.0000000	8.7351999	-943.1080322
180.0000000	8.7840004	1078.9642334
180.0000000	8.8327999	59.8608856
180.0000000	8.8815994	-11.4818716
180.0000000	8.9303989	-24.2561798
180.0000000	8.9792004	-11.5722179
180.0000000	9.0279999	-5.6889110
180.0000000	9.0767994	7.5180779
180.0000000	9.1255999	-17.3856373
180.0000000	9.1743994	-10.0398884
180.0000000	9.2231998	38.3309326
180.0000000	9.2720003	-8.3415508
180.0000000	9.3207998	-9.7528353
180.0000000	9.3695993	-10.7447605
180.0000000	9.4183998	-11.7643394
180.0000000	9.4672003	-11.1578712

180.0000000	9.5159998	-14.2390547
180.0000000	9.5648003	-0.1097736
180.0000000	9.6135998	-13.4624281
180.0000000	9.6623993	-10.1982298
180.0000000	9.7111998	-10.1348934
180.0000000	9.7600002	-10.8540726
180.0000000	9.8087997	-10.9941101
180.0000000	9.8575993	3.5613842
180.0000000	9.9063997	-7.8922987
180.0000000	9.9552002	-10.9062395
180.0000000	10.0039997	-10.0539255
190.0000000	0.3416000	-36.9763451
190.0000000	0.3904000	-39.6863556
190.0000000	0.4392000	-45.7075882
190.0000000	0.4880000	-39.7129135
190.0000000	0.5368000	-39.7198868
190.0000000	0.5856000	-48.6274109
190.0000000	0.6344000	-22.2754021
190.0000000	0.6832000	-92.5775299
190.0000000	0.7320000	-34.7698479
190.0000000	0.7808000	-57.0043030
190.0000000	0.8296000	-38.2862816
190.0000000	0.8784000	-34.4618912
190.0000000	0.9272000	-33.6330261
190.0000000	0.9760000	-33.0514145
190.0000000	1.0247999	-30.9228630
190.0000000	1.0735999	-29.5433922
190.0000000	1.1224000	-28.8643475
190.0000000	1.1711999	-29.0423069
190.0000000	1.2200000	-30.5054226
190.0000000	1.2687999	-31.1709728
190.0000000	1.3176000	-29.9437370
190.0000000	1.3664000	-29.9276924
190.0000000	1.4152000	-30.1453476
190.0000000	1.4640000	-29.7572060
190.0000000	1.5128000	-47.4409714
190.0000000	1.5616000	-37.1030922
190.0000000	1.6104001	-1768.2694092
190.0000000	1.6592000	-97.6545486
190.0000000	1.7079999	-27.3790092
190.0000000	1.7567999	-24.3271904
190.0000000	1.8055999	-23.8352070
190.0000000	1.8544000	-24.8904552
190.0000000	1.9031999	-25.6339474
190.0000000	1.9520000	-25.8667030
190.0000000	2.0007999	-29.7937660
190.0000000	2.0495999	-13.9148569
190.0000000	2.0983999	-270.4638062
190.0000000	2.1471999	20.5085487
190.0000000	2.1960001	-25.7880707
190.0000000	2.2448001	-27.3143959
190.0000000	2.2935998	-32.6291847
190.0000000	2.3423998	-19.7942677
190.0000000	2.3912001	83.4688339
190.0000000	2.4400001	-22.6310863
190.0000000	2.4888000	-23.4781227
190.0000000	2.5375998	-10.8464508
190.0000000	2.5863998	-8.7466946

190.0000000	2.6352000	-6.5701714
190.0000000	2.6840000	-14.4153528
190.0000000	2.7328000	155.3848267
190.0000000	2.7815998	182.7234497
190.0000000	2.8304000	-23.9033775
190.0000000	2.8792000	-20.3465576
190.0000000	2.9280000	-22.6193581
190.0000000	2.9768000	-16.0895653
190.0000000	3.0256000	-20.2567635
190.0000000	3.0743999	-30.4689560
190.0000000	3.1231999	-24.5440464
190.0000000	3.1719999	-30.2992439
190.0000000	3.2208002	-524.1127319
190.0000000	3.2695999	-1026.9422607
190.0000000	3.3183999	-20.4731655
190.0000000	3.3671999	-3517.9770508
190.0000000	3.4159999	1262.9758301
190.0000000	3.4648001	-6.6667366
190.0000000	3.5135999	-27.2853661
190.0000000	3.5623999	-332.7047424
190.0000000	3.6111999	-2094.1713867
190.0000000	3.6600001	-473.5828857
190.0000000	3.7088001	-625.5756836
190.0000000	3.7575998	-979.8361816
190.0000000	3.8063998	-1778.5532227
190.0000000	3.8552001	-2582.5056152
190.0000000	3.9040000	-2531.9594727
190.0000000	3.9528000	1158.7760010
190.0000000	4.0015998	-252.4238281
190.0000000	4.0503998	709.7268677
190.0000000	4.0991998	90.9585037
190.0000000	4.1479998	-674.6525879
190.0000000	4.1967998	3.2225208
190.0000000	4.2456002	-255.6920013
190.0000000	4.2943997	-502.6526489
190.0000000	4.3431997	-13.1720695
190.0000000	4.3920002	-255.3087006
190.0000000	4.4407997	-22.5902767
190.0000000	4.4896002	-15.4097633
190.0000000	4.5383997	-674.1330566
190.0000000	4.5871997	-12.6914539
190.0000000	4.6360002	-21.7336617
190.0000000	4.6847997	-28.5688019
190.0000000	4.7336001	-19.6151295
190.0000000	4.7824001	-16.4792080
190.0000000	4.8311996	-1513.0754395
190.0000000	4.8800001	234.7799225
190.0000000	4.9287996	-206.3516846
190.0000000	4.9776001	1088.1606445
190.0000000	5.0264001	88.9077682
190.0000000	5.0751996	-308.6849670
190.0000000	5.1240001	-12.2412663
190.0000000	5.1727996	566.8720093
190.0000000	5.2216001	32.7328377
190.0000000	5.2704000	-161.0087738
190.0000000	5.3191996	-39.5375633
190.0000000	5.3680000	-3589.9323730
190.0000000	5.4168000	535.4717407

190.0000000	5.4656000	-203.5670319
190.0000000	5.5144000	38.9000854
190.0000000	5.5631995	496.0321045
190.0000000	5.6120000	399.3952026
190.0000000	5.6608000	-37.4045029
190.0000000	5.7096000	63.7373886
190.0000000	5.7584000	135.3037109
190.0000000	5.8071995	-11.4521637
190.0000000	5.8559999	30.5102825
190.0000000	5.9047999	-16.6211224
190.0000000	5.9535999	-40.1477432
190.0000000	6.0023999	-1596.8618164
190.0000000	6.0511999	-14.5515919
190.0000000	6.0999999	-19.4747658
190.0000000	6.1487999	-13.2369051
190.0000000	6.1975999	-23.3592949
190.0000000	6.2463999	-1819.2366943
190.0000000	6.2951999	-19.1345482
190.0000000	6.3439999	-347.7681274
190.0000000	6.3927999	-15.0495739
190.0000000	6.4416003	-12.3394804
190.0000000	6.4903998	-11.2100697
190.0000000	6.5391998	-11.7521000
190.0000000	6.5879998	440.0363770
190.0000000	6.6367998	-12.0403738
190.0000000	6.6856003	-13.8013182
190.0000000	6.7343998	-13.4383888
190.0000000	6.7831998	-14.4059057
190.0000000	6.8319998	59.9754715
190.0000000	6.8807998	-14.0605774
190.0000000	6.9296002	-510.9079895
190.0000000	6.9783998	-4.0486498
190.0000000	7.0271997	-14.2205181
190.0000000	7.0760002	14.6082315
190.0000000	7.1247997	-2452.2600098
190.0000000	7.1736002	-1966.4191895
190.0000000	7.2223997	-1118.1997070
190.0000000	7.2711997	-239.5697479
190.0000000	7.3200002	-19.8599815
190.0000000	7.3687997	218.4593353
190.0000000	7.4176002	453.6291504
190.0000000	7.4663997	356.6957703
190.0000000	7.5151997	-1094.5229492
190.0000000	7.5640001	-1209.1693115
190.0000000	7.6127996	-2852.3449707
190.0000000	7.6616001	-914.8388062
190.0000000	7.7104001	-469.1204834
190.0000000	7.7591996	51.5029297
190.0000000	7.8080001	471.4065552
190.0000000	7.8567996	620.4590454
190.0000000	7.9056001	-1281.1552734
190.0000000	7.9544001	2039.9927979
190.0000000	8.0031996	2632.1694336
190.0000000	8.0520000	2627.2243652
190.0000000	8.1007996	2780.0805664
190.0000000	8.1496000	2964.7707520
190.0000000	8.1983995	1844.6011963
190.0000000	8.2472000	-4219.4340820

190.0000000	8.2959995	-1495.4783936
190.0000000	8.3448000	-1191.3746338
190.0000000	8.3935995	-1091.8360596
190.0000000	8.4423990	-470.0219727
190.0000000	8.4912004	-1097.8485107
190.0000000	8.5400000	-2962.2009277
190.0000000	8.5887995	-1435.4346924
190.0000000	8.6375999	281.0388184
190.0000000	8.6863995	2534.8132324
190.0000000	8.7351999	-941.5837402
190.0000000	8.7840004	1077.2075195
190.0000000	8.8327999	59.7492752
190.0000000	8.8815994	-11.4820452
190.0000000	8.9303989	-24.2524605
190.0000000	8.9792004	-11.5723009
190.0000000	9.0279999	-5.6906791
190.0000000	9.0767994	7.5123596
190.0000000	9.1255999	-17.3839226
190.0000000	9.1743994	-10.0402899
190.0000000	9.2231998	38.3152008
190.0000000	9.2720003	-8.3424873
190.0000000	9.3207998	-9.7533751
190.0000000	9.3695993	-10.7449875
190.0000000	9.4183998	-11.7642145
190.0000000	9.4672003	-11.1579123
190.0000000	9.5159998	-14.2381639
190.0000000	9.5648003	-0.1131458
190.0000000	9.6135998	-13.4616375
190.0000000	9.6623993	-10.1985044
190.0000000	9.7111998	-10.1351652
190.0000000	9.7600002	-10.8541145
190.0000000	9.8087997	-10.9941177
190.0000000	9.8575993	3.5569601
190.0000000	9.9063997	-7.8932123
190.0000000	9.9552002	-10.9062443
190.0000000	10.0039997	-10.0541725
200.0000000	0.3416000	-36.3343811
200.0000000	0.3904000	-39.6000061
200.0000000	0.4392000	-45.5420647
200.0000000	0.4880000	-39.7525826
200.0000000	0.5368000	-39.7027092
200.0000000	0.5856000	-48.2392578
200.0000000	0.6344000	-22.9454613
200.0000000	0.6832000	-89.3200226
200.0000000	0.7320000	-34.9691010
200.0000000	0.7808000	-54.9206543
200.0000000	0.8296000	-37.8946686
200.0000000	0.8784000	-34.4014816
200.0000000	0.9272000	-33.6359177
200.0000000	0.9760000	-32.5968513
200.0000000	1.0247999	-30.3096790
200.0000000	1.0735999	-28.5931187
200.0000000	1.1224000	-27.9363308
200.0000000	1.1711999	-28.4034424
200.0000000	1.2200000	-30.0014648
200.0000000	1.2687999	-31.1380386
200.0000000	1.3176000	-29.9698467
200.0000000	1.3664000	-29.9354420

200.0000000	1.4152000	-30.1455536
200.0000000	1.4640000	-29.7554970
200.0000000	1.5128000	-47.3437920
200.0000000	1.5616000	-37.0612488
200.0000000	1.6104001	-1765.7266846
200.0000000	1.6592000	-97.3606186
200.0000000	1.7079999	-27.8216076
200.0000000	1.7567999	-24.0708809
200.0000000	1.8055999	-23.3391762
200.0000000	1.8544000	-24.0157318
200.0000000	1.9031999	-25.3105392
200.0000000	1.9520000	-25.9160404
200.0000000	2.0007999	-29.7311840
200.0000000	2.0495999	-14.1014729
200.0000000	2.0983999	-268.9880066
200.0000000	2.1471999	20.2412834
200.0000000	2.1960001	-25.7839794
200.0000000	2.2448001	-27.2964859
200.0000000	2.2935998	-32.5695648
200.0000000	2.3423998	-19.8086720
200.0000000	2.3912001	82.8472900
200.0000000	2.4400001	-22.6335735
200.0000000	2.4888000	-23.4779053
200.0000000	2.5375998	-10.8635588
200.0000000	2.5863998	-8.7664537
200.0000000	2.6352000	-6.5929155
200.0000000	2.6840000	-14.4272938
200.0000000	2.7328000	155.1188049
200.0000000	2.7815998	182.4170227
200.0000000	2.8304000	-23.9020748
200.0000000	2.8792000	-20.3498211
200.0000000	2.9280000	-22.6187878
200.0000000	2.9768000	-16.0979366
200.0000000	3.0256000	-20.2593479
200.0000000	3.0743999	-30.4575691
200.0000000	3.1231999	-24.5405903
200.0000000	3.1719999	-30.2909508
200.0000000	3.2208002	-523.8750000
200.0000000	3.2695999	-1026.4671631
200.0000000	3.3183999	-20.4733639
200.0000000	3.3671999	-3516.5153809
200.0000000	3.4159999	1262.4405518
200.0000000	3.4648001	-6.6724443
200.0000000	3.5135999	-27.2823753
200.0000000	3.5623999	-332.5751343
200.0000000	3.6111999	-2093.3117676
200.0000000	3.6600001	-473.3947754
200.0000000	3.7088001	-625.3242798
200.0000000	3.7575998	-979.4376831
200.0000000	3.8063998	-1777.8233643
200.0000000	3.8552001	-2581.4421387
200.0000000	3.9040000	-2530.9169922
200.0000000	3.9528000	1158.2877197
200.0000000	4.0015998	-252.3267212
200.0000000	4.0503998	709.4243164
200.0000000	4.0991998	90.9128189
200.0000000	4.1479998	-674.3803711
200.0000000	4.1967998	3.2132645

200.0000000	4.2456002	-255.5936890
200.0000000	4.2943997	-502.4520569
200.0000000	4.3431997	-13.1742496
200.0000000	4.3920002	-255.2105865
200.0000000	4.4407997	-22.5885010
200.0000000	4.4896002	-15.4106455
200.0000000	4.5383997	-673.8719482
200.0000000	4.5871997	-12.6934700
200.0000000	4.6360002	-21.7321186
200.0000000	4.6847997	-28.5648766
200.0000000	4.7336001	-19.6144066
200.0000000	4.7824001	-16.4796638
200.0000000	4.8311996	-1512.5441895
200.0000000	4.8800001	234.6903687
200.0000000	4.9287996	-206.2844238
200.0000000	4.9776001	1087.7680664
200.0000000	5.0264001	88.8702469
200.0000000	5.0751996	-308.5813599
200.0000000	5.1240001	-12.2421875
200.0000000	5.1727996	566.7561035
200.0000000	5.2216001	32.7233925
200.0000000	5.2704000	-160.9806671
200.0000000	5.3191996	-39.5330162
200.0000000	5.3680000	-3589.2399902
200.0000000	5.4168000	535.3649292
200.0000000	5.4656000	-203.5308533
200.0000000	5.5144000	38.8894348
200.0000000	5.5631995	495.9335632
200.0000000	5.6120000	399.3152161
200.0000000	5.6608000	-37.4004135
200.0000000	5.7096000	63.7219543
200.0000000	5.7584000	135.2747803
200.0000000	5.8071995	-11.4530354
200.0000000	5.8559999	30.5014324
200.0000000	5.9047999	-16.6209240
200.0000000	5.9535999	-40.1430054
200.0000000	6.0023999	-1596.5574951
200.0000000	6.0511999	-14.5518036
200.0000000	6.0999999	-19.4739342
200.0000000	6.1487999	-13.2373676
200.0000000	6.1975999	-23.3575287
200.0000000	6.2463999	-1818.8892822
200.0000000	6.2951999	-19.1337337
200.0000000	6.3439999	-347.7040710
200.0000000	6.3927999	-15.0495558
200.0000000	6.4416003	-12.3400669
200.0000000	6.4903998	-11.2107458
200.0000000	6.5391998	-11.7527809
200.0000000	6.5879998	439.3456726
200.0000000	6.6367998	-12.0409231
200.0000000	6.6856003	-13.8014889
200.0000000	6.7343998	-13.4386044
200.0000000	6.7831998	-14.4054899
200.0000000	6.8319998	59.9563599
200.0000000	6.8807998	-14.0604391
200.0000000	6.9296002	-510.1465454
200.0000000	6.9783998	-4.0614729
200.0000000	7.0271997	-14.2194901

200.0000000	7.0760002	14.5660448
200.0000000	7.1247997	-2448.3139648
200.0000000	7.1736002	-1963.2563477
200.0000000	7.2223997	-1116.4064941
200.0000000	7.2711997	-239.2019653
200.0000000	7.3200002	-19.8479919
200.0000000	7.3687997	218.0848083
200.0000000	7.4176002	452.8733215
200.0000000	7.4663997	356.0981140
200.0000000	7.5151997	-1092.7618408
200.0000000	7.5640001	-1207.2219238
200.0000000	7.6127996	-2847.7272949
200.0000000	7.6616001	-913.3675537
200.0000000	7.7104001	-468.3742065
200.0000000	7.7591996	51.4018822
200.0000000	7.8080001	470.6211853
200.0000000	7.8567996	619.4309692
200.0000000	7.9056001	-1279.0823975
200.0000000	7.9544001	2036.6828613
200.0000000	8.0031996	2627.8999023
200.0000000	8.0520000	2622.9624023
200.0000000	8.1007996	2775.5712891
200.0000000	8.1496000	2959.9626465
200.0000000	8.1983995	1841.6087646
200.0000000	8.2472000	-4212.5996094
200.0000000	8.2959995	-1493.0582275
200.0000000	8.3448000	-1189.4454346
200.0000000	8.3935995	-1090.0683594
200.0000000	8.4423990	-469.2639771
200.0000000	8.4912004	-1096.0722656
200.0000000	8.5400000	-2957.4011230
200.0000000	8.5887995	-1433.1131592
200.0000000	8.6375999	280.5774841
200.0000000	8.6863995	2530.6948242
200.0000000	8.7351999	-940.0595093
200.0000000	8.7840004	1075.4508057
200.0000000	8.8327999	59.6376648
200.0000000	8.8815994	-11.4822197
200.0000000	8.9303989	-24.2487392
200.0000000	8.9792004	-11.5723829
200.0000000	9.0279999	-5.6924477
200.0000000	9.0767994	7.5066414
200.0000000	9.1255999	-17.3822079
200.0000000	9.1743994	-10.0406923
200.0000000	9.2231998	38.2994690
200.0000000	9.2720003	-8.3434229
200.0000000	9.3207998	-9.7539148
200.0000000	9.3695993	-10.7452135
200.0000000	9.4183998	-11.7640896
200.0000000	9.4672003	-11.1579533
200.0000000	9.5159998	-14.2372723
200.0000000	9.5648003	-0.1165180
200.0000000	9.6135998	-13.4608469
200.0000000	9.6623993	-10.1987801
200.0000000	9.7111998	-10.1354370
200.0000000	9.7600002	-10.8541565
200.0000000	9.8087997	-10.9941263
200.0000000	9.8575993	3.5525379

200.0000000	9.9063997	-7.8941259
200.0000000	9.9552002	-10.9062481
200.0000000	10.0039997	-10.0544205
210.0000000	0.3416000	-36.8220711
210.0000000	0.3904000	-39.6014061
210.0000000	0.4392000	-45.3765411
210.0000000	0.4880000	-39.7251358
210.0000000	0.5368000	-39.6855316
210.0000000	0.5856000	-47.8511047
210.0000000	0.6344000	-23.6155205
210.0000000	0.6832000	-86.0625000
210.0000000	0.7320000	-35.1683540
210.0000000	0.7808000	-52.8370056
210.0000000	0.8296000	-37.5030556
210.0000000	0.8784000	-34.3410721
210.0000000	0.9272000	-33.6388054
210.0000000	0.9760000	-31.6533947
210.0000000	1.0247999	-28.7971783
210.0000000	1.0735999	-27.9473972
210.0000000	1.1224000	-28.3394566
210.0000000	1.1711999	-29.0128136
210.0000000	1.2200000	-29.6188335
210.0000000	1.2687999	-31.1051025
210.0000000	1.3176000	-29.9959545
210.0000000	1.3664000	-29.9431915
210.0000000	1.4152000	-30.1457577
210.0000000	1.4640000	-29.7537861
210.0000000	1.5128000	-47.2466164
210.0000000	1.5616000	-37.0194092
210.0000000	1.6104001	-1763.1838379
210.0000000	1.6592000	-97.0666809
210.0000000	1.7079999	-27.8322029
210.0000000	1.7567999	-24.9226761
210.0000000	1.8055999	-23.4950199
210.0000000	1.8544000	-23.7216949
210.0000000	1.9031999	-24.6398487
210.0000000	1.9520000	-26.2542095
210.0000000	2.0007999	-29.6686020
210.0000000	2.0495999	-14.2880898
210.0000000	2.0983999	-267.5122375
210.0000000	2.1471999	19.9740143
210.0000000	2.1960001	-25.7798882
210.0000000	2.2448001	-27.2785740
210.0000000	2.2935998	-32.5099411
210.0000000	2.3423998	-19.8230782
210.0000000	2.3912001	82.2257462
210.0000000	2.4400001	-22.6360607
210.0000000	2.4888000	-23.4776878
210.0000000	2.5375998	-10.8806667
210.0000000	2.5863998	-8.7862129
210.0000000	2.6352000	-6.6156597
210.0000000	2.6840000	-14.4392357
210.0000000	2.7328000	154.8527985
210.0000000	2.7815998	182.1105957
210.0000000	2.8304000	-23.9007702
210.0000000	2.8792000	-20.3530846
210.0000000	2.9280000	-22.6182194
210.0000000	2.9768000	-16.1063080

210.0000000	3.0256000	-20.2619324
210.0000000	3.0743999	-30.4461823
210.0000000	3.1231999	-24.5371361
210.0000000	3.1719999	-30.2826576
210.0000000	3.2208002	-523.6372681
210.0000000	3.2695999	-1025.9923096
210.0000000	3.3183999	-20.4735603
210.0000000	3.3671999	-3515.0539551
210.0000000	3.4159999	1261.9052734
210.0000000	3.4648001	-6.6781530
210.0000000	3.5135999	-27.2793846
210.0000000	3.5623999	-332.4455566
210.0000000	3.6111999	-2092.4519043
210.0000000	3.6600001	-473.2066040
210.0000000	3.7088001	-625.0729370
210.0000000	3.7575998	-979.0391235
210.0000000	3.8063998	-1777.0935059
210.0000000	3.8552001	-2580.3786621
210.0000000	3.9040000	-2529.8745117
210.0000000	3.9528000	1157.7994385
210.0000000	4.0015998	-252.2296143
210.0000000	4.0503998	709.1217651
210.0000000	4.0991998	90.8671341
210.0000000	4.1479998	-674.1081543
210.0000000	4.1967998	3.2040081
210.0000000	4.2456002	-255.4953918
210.0000000	4.2943997	-502.2514954
210.0000000	4.3431997	-13.1764307
210.0000000	4.3920002	-255.1124725
210.0000000	4.4407997	-22.5867252
210.0000000	4.4896002	-15.4115286
210.0000000	4.5383997	-673.6109009
210.0000000	4.5871997	-12.6954861
210.0000000	4.6360002	-21.7305775
210.0000000	4.6847997	-28.5609512
210.0000000	4.7336001	-19.6136837
210.0000000	4.7824001	-16.4801178
210.0000000	4.8311996	-1512.0128174
210.0000000	4.8800001	234.6008301
210.0000000	4.9287996	-206.2171631
210.0000000	4.9776001	1087.3756104
210.0000000	5.0264001	88.8327255
210.0000000	5.0751996	-308.4777222
210.0000000	5.1240001	-12.2431087
210.0000000	5.1727996	566.6402588
210.0000000	5.2216001	32.7139511
210.0000000	5.2704000	-160.9525604
210.0000000	5.3191996	-39.5284653
210.0000000	5.3680000	-3588.5476074
210.0000000	5.4168000	535.2581177
210.0000000	5.4656000	-203.4946747
210.0000000	5.5144000	38.8787766
210.0000000	5.5631995	495.8349915
210.0000000	5.6120000	399.2352600
210.0000000	5.6608000	-37.3963242
210.0000000	5.7096000	63.7065277
210.0000000	5.7584000	135.2458496
210.0000000	5.8071995	-11.4539051

210.0000000	5.8559999	30.4925785
210.0000000	5.9047999	-16.6207256
210.0000000	5.9535999	-40.1382675
210.0000000	6.0023999	-1596.2530518
210.0000000	6.0511999	-14.5520144
210.0000000	6.0999999	-19.4731007
210.0000000	6.1487999	-13.2378302
210.0000000	6.1975999	-23.3557625
210.0000000	6.2463999	-1818.5419922
210.0000000	6.2951999	-19.1329193
210.0000000	6.3439999	-347.6400452
210.0000000	6.3927999	-15.0495377
210.0000000	6.4416003	-12.3406544
210.0000000	6.4903998	-11.2114220
210.0000000	6.5391998	-11.7534618
210.0000000	6.5879998	438.6549683
210.0000000	6.6367998	-12.0414724
210.0000000	6.6856003	-13.8016586
210.0000000	6.7343998	-13.4388199
210.0000000	6.7831998	-14.4050741
210.0000000	6.8319998	59.9372406
210.0000000	6.8807998	-14.0602999
210.0000000	6.9296002	-509.3851013
210.0000000	6.9783998	-4.0742970
210.0000000	7.0271997	-14.2184629
210.0000000	7.0760002	14.5238619
210.0000000	7.1247997	-2444.3681641
210.0000000	7.1736002	-1960.0935059
210.0000000	7.2223997	-1114.6134033
210.0000000	7.2711997	-238.8341980
210.0000000	7.3200002	-19.8360023
210.0000000	7.3687997	217.7102661
210.0000000	7.4176002	452.1174927
210.0000000	7.4663997	355.5004578
210.0000000	7.5151997	-1091.0008545
210.0000000	7.5640001	-1205.2745361
210.0000000	7.6127996	-2843.1096191
210.0000000	7.6616001	-911.8961792
210.0000000	7.7104001	-467.6279297
210.0000000	7.7591996	51.3008308
210.0000000	7.8080001	469.8358459
210.0000000	7.8567996	618.4028320
210.0000000	7.9056001	-1277.0093994
210.0000000	7.9544001	2033.3729248
210.0000000	8.0031996	2623.6301270
210.0000000	8.0520000	2618.7004395
210.0000000	8.1007996	2771.0617676
210.0000000	8.1496000	2955.1547852
210.0000000	8.1983995	1838.6162109
210.0000000	8.2472000	-4205.7646484
210.0000000	8.2959995	-1490.6380615
210.0000000	8.3448000	-1187.5162354
210.0000000	8.3935995	-1088.3007813
210.0000000	8.4423990	-468.5059509
210.0000000	8.4912004	-1094.2960205
210.0000000	8.5400000	-2952.6013184
210.0000000	8.5887995	-1430.7916260
210.0000000	8.6375999	280.1161499

210.0000000	8.6863995	2526.5764160
210.0000000	8.7351999	-938.5352783
210.0000000	8.7840004	1073.6939697
210.0000000	8.8327999	59.5260620
210.0000000	8.8815994	-11.4823942
210.0000000	8.9303989	-24.2450180
210.0000000	8.9792004	-11.5724649
210.0000000	9.0279999	-5.6942153
210.0000000	9.0767994	7.5009251
210.0000000	9.1255999	-17.3804951
210.0000000	9.1743994	-10.0410938
210.0000000	9.2231998	38.2837372
210.0000000	9.2720003	-8.3443594
210.0000000	9.3207998	-9.7544537
210.0000000	9.3695993	-10.7454405
210.0000000	9.4183998	-11.7639656
210.0000000	9.4672003	-11.1579952
210.0000000	9.5159998	-14.2363815
210.0000000	9.5648003	-0.1198912
210.0000000	9.6135998	-13.4600563
210.0000000	9.6623993	-10.1990547
210.0000000	9.7111998	-10.1357079
210.0000000	9.7600002	-10.8541975
210.0000000	9.8087997	-10.9941339
210.0000000	9.8575993	3.5481167
210.0000000	9.9063997	-7.8950400
210.0000000	9.9552002	-10.9062529
210.0000000	10.0039997	-10.0546675
220.0000000	0.3416000	-36.4698296
220.0000000	0.3904000	-39.6028023
220.0000000	0.4392000	-45.2110176
220.0000000	0.4880000	-39.6976891
220.0000000	0.5368000	-39.6683540
220.0000000	0.5856000	-47.4629478
220.0000000	0.6344000	-24.2855797
220.0000000	0.6832000	-82.8049927
220.0000000	0.7320000	-35.3676071
220.0000000	0.7808000	-50.7533569
220.0000000	0.8296000	-37.1114426
220.0000000	0.8784000	-34.2806625
220.0000000	0.9272000	-33.6416969
220.0000000	0.9760000	-29.7424488
220.0000000	1.0247999	-26.8866329
220.0000000	1.0735999	-26.4357700
220.0000000	1.1224000	-27.6495857
220.0000000	1.1711999	-29.7414722
220.0000000	1.2200000	-30.8932362
220.0000000	1.2687999	-31.0721664
220.0000000	1.3176000	-30.0220642
220.0000000	1.3664000	-29.9509411
220.0000000	1.4152000	-30.1459618
220.0000000	1.4640000	-29.7520771
220.0000000	1.5128000	-47.1494370
220.0000000	1.5616000	-36.9775696
220.0000000	1.6104001	-1760.6409912
220.0000000	1.6592000	-96.7727509
220.0000000	1.7079999	-27.8427963
220.0000000	1.7567999	-25.0618591

220.0000000	1.8055999	-23.9524231
220.0000000	1.8544000	-24.5549202
220.0000000	1.9031999	-25.8031902
220.0000000	1.9520000	-26.2970428
220.0000000	2.0007999	-29.6060200
220.0000000	2.0495999	-14.4747066
220.0000000	2.0983999	-266.0364685
220.0000000	2.1471999	19.7067451
220.0000000	2.1960001	-25.7757969
220.0000000	2.2448001	-27.2606621
220.0000000	2.2935998	-32.4503174
220.0000000	2.3423998	-19.8374825
220.0000000	2.3912001	81.6041946
220.0000000	2.4400001	-22.6385460
220.0000000	2.4888000	-23.4774704
220.0000000	2.5375998	-10.8977747
220.0000000	2.5863998	-8.8059721
220.0000000	2.6352000	-6.6384039
220.0000000	2.6840000	-14.4511786
220.0000000	2.7328000	154.5867767
220.0000000	2.7815998	181.8041992
220.0000000	2.8304000	-23.8994656
220.0000000	2.8792000	-20.3563480
220.0000000	2.9280000	-22.6176510
220.0000000	2.9768000	-16.1146774
220.0000000	3.0256000	-20.2645168
220.0000000	3.0743999	-30.4347954
220.0000000	3.1231999	-24.5336800
220.0000000	3.1719999	-30.2743645
220.0000000	3.2208002	-523.3995361
220.0000000	3.2695999	-1025.5172119
220.0000000	3.3183999	-20.4737587
220.0000000	3.3671999	-3513.5922852
220.0000000	3.4159999	1261.3699951
220.0000000	3.4648001	-6.6838617
220.0000000	3.5135999	-27.2763939
220.0000000	3.5623999	-332.3159485
220.0000000	3.6111999	-2091.5922852
220.0000000	3.6600001	-473.0184937
220.0000000	3.7088001	-624.8215332
220.0000000	3.7575998	-978.6406250
220.0000000	3.8063998	-1776.3636475
220.0000000	3.8552001	-2579.3151855
220.0000000	3.9040000	-2528.8320313
220.0000000	3.9528000	1157.3111572
220.0000000	4.0015998	-252.1325073
220.0000000	4.0503998	708.8192139
220.0000000	4.0991998	90.8214493
220.0000000	4.1479998	-673.8359375
220.0000000	4.1967998	3.1947517
220.0000000	4.2456002	-255.3970947
220.0000000	4.2943997	-502.0509338
220.0000000	4.3431997	-13.1786108
220.0000000	4.3920002	-255.0143585
220.0000000	4.4407997	-22.5849495
220.0000000	4.4896002	-15.4124107
220.0000000	4.5383997	-673.3498535
220.0000000	4.5871997	-12.6975021

220.0000000	4.6360002	-21.7290344
220.0000000	4.6847997	-28.5570259
220.0000000	4.7336001	-19.6129608
220.0000000	4.7824001	-16.4805717
220.0000000	4.8311996	-1511.4815674
220.0000000	4.8800001	234.5112762
220.0000000	4.9287996	-206.1499023
220.0000000	4.9776001	1086.9831543
220.0000000	5.0264001	88.7952042
220.0000000	5.0751996	-308.3740845
220.0000000	5.1240001	-12.2440300
220.0000000	5.1727996	566.5244141
220.0000000	5.2216001	32.7045059
220.0000000	5.2704000	-160.9244537
220.0000000	5.3191996	-39.5239182
220.0000000	5.3680000	-3587.8552246
220.0000000	5.4168000	535.1513672
220.0000000	5.4656000	-203.4584808
220.0000000	5.5144000	38.8681183
220.0000000	5.5631995	495.7364197
220.0000000	5.6120000	399.1552734
220.0000000	5.6608000	-37.3922386
220.0000000	5.7096000	63.6910934
220.0000000	5.7584000	135.2169189
220.0000000	5.8071995	-11.4547758
220.0000000	5.8559999	30.4837284
220.0000000	5.9047999	-16.6205292
220.0000000	5.9535999	-40.1335297
220.0000000	6.0023999	-1595.9484863
220.0000000	6.0511999	-14.5522261
220.0000000	6.0999999	-19.4722691
220.0000000	6.1487999	-13.2382927
220.0000000	6.1975999	-23.3539963
220.0000000	6.2463999	-1818.1945801
220.0000000	6.2951999	-19.1321068
220.0000000	6.3439999	-347.5759888
220.0000000	6.3927999	-15.0495195
220.0000000	6.4416003	-12.3412409
220.0000000	6.4903998	-11.2120972
220.0000000	6.5391998	-11.7541428
220.0000000	6.5879998	437.9642944
220.0000000	6.6367998	-12.0420218
220.0000000	6.6856003	-13.8018293
220.0000000	6.7343998	-13.4390364
220.0000000	6.7831998	-14.4046583
220.0000000	6.8319998	59.9181290
220.0000000	6.8807998	-14.0601616
220.0000000	6.9296002	-508.6236572
220.0000000	6.9783998	-4.0871210
220.0000000	7.0271997	-14.2174349
220.0000000	7.0760002	14.4816751
220.0000000	7.1247997	-2440.4223633
220.0000000	7.1736002	-1956.9306641
220.0000000	7.2223997	-1112.8201904
220.0000000	7.2711997	-238.4664154
220.0000000	7.3200002	-19.8240147
220.0000000	7.3687997	217.3357391
220.0000000	7.4176002	451.3616638

220.0000000	7.4663997	354.9028015
220.0000000	7.5151997	-1089.2398682
220.0000000	7.5640001	-1203.3271484
220.0000000	7.6127996	-2838.4919434
220.0000000	7.6616001	-910.4248047
220.0000000	7.7104001	-466.8817139
220.0000000	7.7591996	51.1997833
220.0000000	7.8080001	469.0504761
220.0000000	7.8567996	617.3747559
220.0000000	7.9056001	-1274.9365234
220.0000000	7.9544001	2030.0629883
220.0000000	8.0031996	2619.3605957
220.0000000	8.0520000	2614.4384766
220.0000000	8.1007996	2766.5522461
220.0000000	8.1496000	2950.3466797
220.0000000	8.1983995	1835.6237793
220.0000000	8.2472000	-4198.9296875
220.0000000	8.2959995	-1488.2178955
220.0000000	8.3448000	-1185.5871582
220.0000000	8.3935995	-1086.5330811
220.0000000	8.4423990	-467.7479553
220.0000000	8.4912004	-1092.5197754
220.0000000	8.5400000	-2947.8015137
220.0000000	8.5887995	-1428.4700928
220.0000000	8.6375999	279.6548462
220.0000000	8.6863995	2522.4580078
220.0000000	8.7351999	-937.0109863
220.0000000	8.7840004	1071.9372559
220.0000000	8.8327999	59.4144516
220.0000000	8.8815994	-11.4825687
220.0000000	8.9303989	-24.2412987
220.0000000	8.9792004	-11.5725479
220.0000000	9.0279999	-5.6959839
220.0000000	9.0767994	7.4952068
220.0000000	9.1255999	-17.3787804
220.0000000	9.1743994	-10.0414963
220.0000000	9.2231998	38.2679977
220.0000000	9.2720003	-8.3452959
220.0000000	9.3207998	-9.7549934
220.0000000	9.3695993	-10.7456665
220.0000000	9.4183998	-11.7638407
220.0000000	9.4672003	-11.1580362
220.0000000	9.5159998	-14.2354908
220.0000000	9.5648003	-0.1232643
220.0000000	9.6135998	-13.4592648
220.0000000	9.6623993	-10.1993303
220.0000000	9.7111998	-10.1359797
220.0000000	9.7600002	-10.8542395
220.0000000	9.8087997	-10.9941416
220.0000000	9.8575993	3.5436926
220.0000000	9.9063997	-7.8959537
220.0000000	9.9552002	-10.9062567
220.0000000	10.0039997	-10.0549154
230.0000000	0.3416000	-36.7222977
230.0000000	0.3904000	-39.6042023
230.0000000	0.4392000	-45.0454941
230.0000000	0.4880000	-39.6702423
230.0000000	0.5368000	-39.6511765

230.0000000	0.5856000	-47.0747948
230.0000000	0.6344000	-24.9556389
230.0000000	0.6832000	-79.5474701
230.0000000	0.7320000	-35.5668602
230.0000000	0.7808000	-48.6697083
230.0000000	0.8296000	-36.7198296
230.0000000	0.8784000	-34.2202530
230.0000000	0.9272000	-29.8056507
230.0000000	0.9760000	-26.8968925
230.0000000	1.0247999	-26.9201126
230.0000000	1.0735999	-29.2859745
230.0000000	1.1224000	-29.9852104
230.0000000	1.1711999	-30.4047070
230.0000000	1.2200000	-30.4352913
230.0000000	1.2687999	-31.1186390
230.0000000	1.3176000	-30.0481739
230.0000000	1.3664000	-29.9586887
230.0000000	1.4152000	-30.1461658
230.0000000	1.4640000	-29.7503681
230.0000000	1.5128000	-47.0522614
230.0000000	1.5616000	-36.9357262
230.0000000	1.6104001	-1758.0982666
230.0000000	1.6592000	-96.4788208
230.0000000	1.7079999	-27.8533916
230.0000000	1.7567999	-25.1449375
230.0000000	1.8055999	-23.9813690
230.0000000	1.8544000	-24.2451077
230.0000000	1.9031999	-24.9753475
230.0000000	1.9520000	-26.3398743
230.0000000	2.0007999	-29.5434380
230.0000000	2.0495999	-14.6613226
230.0000000	2.0983999	-264.5606689
230.0000000	2.1471999	19.4394798
230.0000000	2.1960001	-25.7717056
230.0000000	2.2448001	-27.2427521
230.0000000	2.2935998	-32.3906937
230.0000000	2.3423998	-19.8518867
230.0000000	2.3912001	80.9826508
230.0000000	2.4400001	-22.6410332
230.0000000	2.4888000	-23.4772530
230.0000000	2.5375998	-10.9148827
230.0000000	2.5863998	-8.8257303
230.0000000	2.6352000	-6.6611481
230.0000000	2.6840000	-14.4631205
230.0000000	2.7328000	154.3207550
230.0000000	2.7815998	181.4977722
230.0000000	2.8304000	-23.8981609
230.0000000	2.8792000	-20.3596115
230.0000000	2.9280000	-22.6170807
230.0000000	2.9768000	-16.1230488
230.0000000	3.0256000	-20.2671013
230.0000000	3.0743999	-30.4234085
230.0000000	3.1231999	-24.5302238
230.0000000	3.1719999	-30.2660713
230.0000000	3.2208002	-523.1618042
230.0000000	3.2695999	-1025.0423584
230.0000000	3.3183999	-20.4739571
230.0000000	3.3671999	-3512.1306152

230.0000000	3.4159999	1260.8347168
230.0000000	3.4648001	-6.6895695
230.0000000	3.5135999	-27.2734032
230.0000000	3.5623999	-332.1863708
230.0000000	3.6111999	-2090.7324219
230.0000000	3.6600001	-472.8303833
230.0000000	3.7088001	-624.5701294
230.0000000	3.7575998	-978.2420654
230.0000000	3.8063998	-1775.6337891
230.0000000	3.8552001	-2578.2517090
230.0000000	3.9040000	-2527.7895508
230.0000000	3.9528000	1156.8228760
230.0000000	4.0015998	-252.0354004
230.0000000	4.0503998	708.5166626
230.0000000	4.0991998	90.7757645
230.0000000	4.1479998	-673.5637207
230.0000000	4.1967998	3.1854954
230.0000000	4.2456002	-255.2987823
230.0000000	4.2943997	-501.8503723
230.0000000	4.3431997	-13.1807919
230.0000000	4.3920002	-254.9162445
230.0000000	4.4407997	-22.5831757
230.0000000	4.4896002	-15.4132938
230.0000000	4.5383997	-673.0887451
230.0000000	4.5871997	-12.6995182
230.0000000	4.6360002	-21.7274914
230.0000000	4.6847997	-28.5531006
230.0000000	4.7336001	-19.6122398
230.0000000	4.7824001	-16.4810257
230.0000000	4.8311996	-1510.9503174
230.0000000	4.8800001	234.4217224
230.0000000	4.9287996	-206.0826263
230.0000000	4.9776001	1086.5905762
230.0000000	5.0264001	88.7576904
230.0000000	5.0751996	-308.2704773
230.0000000	5.1240001	-12.2449512
230.0000000	5.1727996	566.4085083
230.0000000	5.2216001	32.6950607
230.0000000	5.2704000	-160.8963470
230.0000000	5.3191996	-39.5193672
230.0000000	5.3680000	-3587.1628418
230.0000000	5.4168000	535.0446167
230.0000000	5.4656000	-203.4223022
230.0000000	5.5144000	38.8574600
230.0000000	5.5631995	495.6378479
230.0000000	5.6120000	399.0752869
230.0000000	5.6608000	-37.3881493
230.0000000	5.7096000	63.6756668
230.0000000	5.7584000	135.1879730
230.0000000	5.8071995	-11.4556465
230.0000000	5.8559999	30.4748745
230.0000000	5.9047999	-16.6203308
230.0000000	5.9535999	-40.1287880
230.0000000	6.0023999	-1595.6441650
230.0000000	6.0511999	-14.5524378
230.0000000	6.0999999	-19.4714375
230.0000000	6.1487999	-13.2387562
230.0000000	6.1975999	-23.3522320

230.0000000	6.2463999	-1817.8471680
230.0000000	6.2951999	-19.1312923
230.0000000	6.3439999	-347.5119324
230.0000000	6.3927999	-15.0495005
230.0000000	6.4416003	-12.3418283
230.0000000	6.4903998	-11.2127733
230.0000000	6.5391998	-11.7548237
230.0000000	6.5879998	437.2735901
230.0000000	6.6367998	-12.0425701
230.0000000	6.6856003	-13.8020000
230.0000000	6.7343998	-13.4392519
230.0000000	6.7831998	-14.4042425
230.0000000	6.8319998	59.8990097
230.0000000	6.8807998	-14.0600233
230.0000000	6.9296002	-507.8622131
230.0000000	6.9783998	-4.0999432
230.0000000	7.0271997	-14.2164068
230.0000000	7.0760002	14.4394884
230.0000000	7.1247997	-2436.4763184
230.0000000	7.1736002	-1953.7678223
230.0000000	7.2223997	-1111.0269775
230.0000000	7.2711997	-238.0986328
230.0000000	7.3200002	-19.8120270
230.0000000	7.3687997	216.9612122
230.0000000	7.4176002	450.6058350
230.0000000	7.4663997	354.3051758
230.0000000	7.5151997	-1087.4787598
230.0000000	7.5640001	-1201.3797607
230.0000000	7.6127996	-2833.8745117
230.0000000	7.6616001	-908.9534912
230.0000000	7.7104001	-466.1353760
230.0000000	7.7591996	51.0987358
230.0000000	7.8080001	468.2651062
230.0000000	7.8567996	616.3466797
230.0000000	7.9056001	-1272.8636475
230.0000000	7.9544001	2026.7530518
230.0000000	8.0031996	2615.0910645
230.0000000	8.0520000	2610.1765137
230.0000000	8.1007996	2762.0427246
230.0000000	8.1496000	2945.5385742
230.0000000	8.1983995	1832.6312256
230.0000000	8.2472000	-4192.0952148
230.0000000	8.2959995	-1485.7977295
230.0000000	8.3448000	-1183.6579590
230.0000000	8.3935995	-1084.7653809
230.0000000	8.4423990	-466.9899292
230.0000000	8.4912004	-1090.7436523
230.0000000	8.5400000	-2943.0017090
230.0000000	8.5887995	-1426.1485596
230.0000000	8.6375999	279.1935120
230.0000000	8.6863995	2518.3398438
230.0000000	8.7351999	-935.4867554
230.0000000	8.7840004	1070.1805420
230.0000000	8.8327999	59.3028412
230.0000000	8.8815994	-11.4827423
230.0000000	8.9303989	-24.2375793
230.0000000	8.9792004	-11.5726299
230.0000000	9.0279999	-5.6977525

230.0000000	9.0767994	7.4894886
230.0000000	9.1255999	-17.3770676
230.0000000	9.1743994	-10.0418978
230.0000000	9.2231998	38.2522659
230.0000000	9.2720003	-8.3462324
230.0000000	9.3207998	-9.7555323
230.0000000	9.3695993	-10.7458935
230.0000000	9.4183998	-11.7637167
230.0000000	9.4672003	-11.1580772
230.0000000	9.5159998	-14.2345991
230.0000000	9.5648003	-0.1266365
230.0000000	9.6135998	-13.4584742
230.0000000	9.6623993	-10.1996050
230.0000000	9.7111998	-10.1362514
230.0000000	9.7600002	-10.8542814
230.0000000	9.8087997	-10.9941502
230.0000000	9.8575993	3.5392704
230.0000000	9.9063997	-7.8968678
230.0000000	9.9552002	-10.9062614
230.0000000	10.0039997	-10.0551634
240.0000000	0.3416000	-36.8752022
240.0000000	0.3904000	-39.6056023
240.0000000	0.4392000	-44.8799744
240.0000000	0.4880000	-39.6427994
240.0000000	0.5368000	-39.6339989
240.0000000	0.5856000	-46.6866417
240.0000000	0.6344000	-25.6256981
240.0000000	0.6832000	-76.2899628
240.0000000	0.7320000	-35.7661133
240.0000000	0.7808000	-46.5860596
240.0000000	0.8296000	-36.3282166
240.0000000	0.8784000	-33.1875725
240.0000000	0.9272000	-30.0935879
240.0000000	0.9760000	-27.1254616
240.0000000	1.0247999	-27.0274372
240.0000000	1.0735999	-28.2810783
240.0000000	1.1224000	-29.2837696
240.0000000	1.1711999	-30.0968342
240.0000000	1.2200000	-30.8056736
240.0000000	1.2687999	-31.1278725
240.0000000	1.3176000	-30.0742817
240.0000000	1.3664000	-29.9664383
240.0000000	1.4152000	-30.1463699
240.0000000	1.4640000	-29.7486591
240.0000000	1.5128000	-46.9550819
240.0000000	1.5616000	-36.8938866
240.0000000	1.6104001	-1755.5554199
240.0000000	1.6592000	-96.1848907
240.0000000	1.7079999	-27.8639851
240.0000000	1.7567999	-25.1259403
240.0000000	1.8055999	-23.8576221
240.0000000	1.8544000	-24.4813290
240.0000000	1.9031999	-24.8643112
240.0000000	1.9520000	-26.5322323
240.0000000	2.0007999	-29.4808559
240.0000000	2.0495999	-14.8479385
240.0000000	2.0983999	-263.0848999
240.0000000	2.1471999	19.1722145

240.0000000	2.1960001	-25.7676144
240.0000000	2.2448001	-27.2248402
240.0000000	2.2935998	-32.3310699
240.0000000	2.3423998	-19.8662910
240.0000000	2.3912001	80.3611069
240.0000000	2.4400001	-22.6435184
240.0000000	2.4888000	-23.4770355
240.0000000	2.5375998	-10.9319906
240.0000000	2.5863998	-8.8454895
240.0000000	2.6352000	-6.6838923
240.0000000	2.6840000	-14.4750624
240.0000000	2.7328000	154.0547485
240.0000000	2.7815998	181.1913452
240.0000000	2.8304000	-23.8968582
240.0000000	2.8792000	-20.3628750
240.0000000	2.9280000	-22.6165123
240.0000000	2.9768000	-16.1314201
240.0000000	3.0256000	-20.2696857
240.0000000	3.0743999	-30.4120216
240.0000000	3.1231999	-24.5267696
240.0000000	3.1719999	-30.2577782
240.0000000	3.2208002	-522.9240723
240.0000000	3.2695999	-1024.5673828
240.0000000	3.3183999	-20.4741554
240.0000000	3.3671999	-3510.6689453
240.0000000	3.4159999	1260.2994385
240.0000000	3.4648001	-6.6952782
240.0000000	3.5135999	-27.2704124
240.0000000	3.5623999	-332.0567932
240.0000000	3.6111999	-2089.8728027
240.0000000	3.6600001	-472.6422119
240.0000000	3.7088001	-624.3187866
240.0000000	3.7575998	-977.8435059
240.0000000	3.8063998	-1774.9039307
240.0000000	3.8552001	-2577.1884766
240.0000000	3.9040000	-2526.7470703
240.0000000	3.9528000	1156.3345947
240.0000000	4.0015998	-251.9382935
240.0000000	4.0503998	708.2140503
240.0000000	4.0991998	90.7300873
240.0000000	4.1479998	-673.2915039
240.0000000	4.1967998	3.1762390
240.0000000	4.2456002	-255.2004852
240.0000000	4.2943997	-501.6498108
240.0000000	4.3431997	-13.1829720
240.0000000	4.3920002	-254.8181305
240.0000000	4.4407997	-22.5813980
240.0000000	4.4896002	-15.4141760
240.0000000	4.5383997	-672.8276978
240.0000000	4.5871997	-12.7015343
240.0000000	4.6360002	-21.7259502
240.0000000	4.6847997	-28.5491753
240.0000000	4.7336001	-19.6115170
240.0000000	4.7824001	-16.4814816
240.0000000	4.8311996	-1510.4190674
240.0000000	4.8800001	234.3321838
240.0000000	4.9287996	-206.0153656
240.0000000	4.9776001	1086.1981201

240.0000000	5.0264001	88.7201691
240.0000000	5.0751996	-308.1668396
240.0000000	5.1240001	-12.2458725
240.0000000	5.1727996	566.2926636
240.0000000	5.2216001	32.6856194
240.0000000	5.2704000	-160.8682404
240.0000000	5.3191996	-39.5148163
240.0000000	5.3680000	-3586.4704590
240.0000000	5.4168000	534.9378052
240.0000000	5.4656000	-203.3861237
240.0000000	5.5144000	38.8468018
240.0000000	5.5631995	495.5392761
240.0000000	5.6120000	398.9953308
240.0000000	5.6608000	-37.3840599
240.0000000	5.7096000	63.6602325
240.0000000	5.7584000	135.1590424
240.0000000	5.8071995	-11.4565172
240.0000000	5.8559999	30.4660244
240.0000000	5.9047999	-16.6201324
240.0000000	5.9535999	-40.1240501
240.0000000	6.0023999	-1595.3397217
240.0000000	6.0511999	-14.5526485
240.0000000	6.0999999	-19.4706039
240.0000000	6.1487999	-13.2392187
240.0000000	6.1975999	-23.3504677
240.0000000	6.2463999	-1817.4997559
240.0000000	6.2951999	-19.1304779
240.0000000	6.3439999	-347.4478760
240.0000000	6.3927999	-15.0494823
240.0000000	6.4416003	-12.3424149
240.0000000	6.4903998	-11.2134495
240.0000000	6.5391998	-11.7555056
240.0000000	6.5879998	436.5828857
240.0000000	6.6367998	-12.0431194
240.0000000	6.6856003	-13.8021698
240.0000000	6.7343998	-13.4394674
240.0000000	6.7831998	-14.4038267
240.0000000	6.8319998	59.8798981
240.0000000	6.8807998	-14.0598841
240.0000000	6.9296002	-507.1007690
240.0000000	6.9783998	-4.1127672
240.0000000	7.0271997	-14.2153788
240.0000000	7.0760002	14.3973055
240.0000000	7.1247997	-2432.5305176
240.0000000	7.1736002	-1950.6048584
240.0000000	7.2223997	-1109.2338867
240.0000000	7.2711997	-237.7308655
240.0000000	7.3200002	-19.8000374
240.0000000	7.3687997	216.5866699
240.0000000	7.4176002	449.8500061
240.0000000	7.4663997	353.7075195
240.0000000	7.5151997	-1085.7177734
240.0000000	7.5640001	-1199.4323730
240.0000000	7.6127996	-2829.2568359
240.0000000	7.6616001	-907.4821167
240.0000000	7.7104001	-465.3891602
240.0000000	7.7591996	50.9976845
240.0000000	7.8080001	467.4797668

240.0000000	7.8567996	615.3186035
240.0000000	7.9056001	-1270.7907715
240.0000000	7.9544001	2023.4431152
240.0000000	8.0031996	2610.8215332
240.0000000	8.0520000	2605.9143066
240.0000000	8.1007996	2757.5332031
240.0000000	8.1496000	2940.7307129
240.0000000	8.1983995	1829.6386719
240.0000000	8.2472000	-4185.2602539
240.0000000	8.2959995	-1483.3775635
240.0000000	8.3448000	-1181.7287598
240.0000000	8.3935995	-1082.9976807
240.0000000	8.4423990	-466.2319336
240.0000000	8.4912004	-1088.9674072
240.0000000	8.5400000	-2938.2019043
240.0000000	8.5887995	-1423.8270264
240.0000000	8.6375999	278.7322083
240.0000000	8.6863995	2514.2214355
240.0000000	8.7351999	-933.9624634
240.0000000	8.7840004	1068.4238281
240.0000000	8.8327999	59.1912384
240.0000000	8.8815994	-11.4829168
240.0000000	8.9303989	-24.2338600
240.0000000	8.9792004	-11.5727119
240.0000000	9.0279999	-5.6995206
240.0000000	9.0767994	7.4837704
240.0000000	9.1255999	-17.3753529
240.0000000	9.1743994	-10.0423002
240.0000000	9.2231998	38.2365341
240.0000000	9.2720003	-8.3471680
240.0000000	9.3207998	-9.7560720
240.0000000	9.3695993	-10.7461195
240.0000000	9.4183998	-11.7635918
240.0000000	9.4672003	-11.1581182
240.0000000	9.5159998	-14.2337084
240.0000000	9.5648003	-0.1300087
240.0000000	9.6135998	-13.4576836
240.0000000	9.6623993	-10.1998796
240.0000000	9.7111998	-10.1365232
240.0000000	9.7600002	-10.8543224
240.0000000	9.8087997	-10.9941578
240.0000000	9.8575993	3.5348492
240.0000000	9.9063997	-7.8977814
240.0000000	9.9552002	-10.9062653
240.0000000	10.0039997	-10.0554104
250.0000000	0.3416000	-36.7948990
250.0000000	0.3904000	-39.6070023
250.0000000	0.4392000	-44.7144508
250.0000000	0.4880000	-39.6153526
250.0000000	0.5368000	-39.6168213
250.0000000	0.5856000	-46.2984848
250.0000000	0.6344000	-26.2957573
250.0000000	0.6832000	-73.0324402
250.0000000	0.7320000	-35.9653664
250.0000000	0.7808000	-44.5024109
250.0000000	0.8296000	-35.9366035
250.0000000	0.8784000	-32.1548920
250.0000000	0.9272000	-29.5339851

250.0000000	0.9760000	-28.1501255
250.0000000	1.0247999	-27.7350807
250.0000000	1.0735999	-28.7157211
250.0000000	1.1224000	-29.6034641
250.0000000	1.1711999	-30.7748966
250.0000000	1.2200000	-31.1288738
250.0000000	1.2687999	-31.1371059
250.0000000	1.3176000	-30.1003914
250.0000000	1.3664000	-29.9741879
250.0000000	1.4152000	-30.1465740
250.0000000	1.4640000	-29.7469482
250.0000000	1.5128000	-46.8579025
250.0000000	1.5616000	-36.8520432
250.0000000	1.6104001	-1753.0125732
250.0000000	1.6592000	-95.8909607
250.0000000	1.7079999	-27.8745804
250.0000000	1.7567999	-25.4332581
250.0000000	1.8055999	-23.6413403
250.0000000	1.8544000	-24.4239464
250.0000000	1.9031999	-25.5540829
250.0000000	1.9520000	-26.5326881
250.0000000	2.0007999	-29.4182739
250.0000000	2.0495999	-15.0345554
250.0000000	2.0983999	-261.6091309
250.0000000	2.1471999	18.9049454
250.0000000	2.1960001	-25.7635231
250.0000000	2.2448001	-27.2069283
250.0000000	2.2935998	-32.2714462
250.0000000	2.3423998	-19.8806953
250.0000000	2.3912001	79.7395630
250.0000000	2.4400001	-22.6460056
250.0000000	2.4888000	-23.4768181
250.0000000	2.5375998	-10.9490986
250.0000000	2.5863998	-8.8652487
250.0000000	2.6352000	-6.7066374
250.0000000	2.6840000	-14.4870033
250.0000000	2.7328000	153.7887268
250.0000000	2.7815998	180.8849487
250.0000000	2.8304000	-23.8955536
250.0000000	2.8792000	-20.3661385
250.0000000	2.9280000	-22.6159439
250.0000000	2.9768000	-16.1397896
250.0000000	3.0256000	-20.2722702
250.0000000	3.0743999	-30.4006348
250.0000000	3.1231999	-24.5233135
250.0000000	3.1719999	-30.2494850
250.0000000	3.2208002	-522.6863403
250.0000000	3.2695999	-1024.0924072
250.0000000	3.3183999	-20.4743538
250.0000000	3.3671999	-3509.2072754
250.0000000	3.4159999	1259.7641602
250.0000000	3.4648001	-6.7009850
250.0000000	3.5135999	-27.2674217
250.0000000	3.5623999	-331.9271851
250.0000000	3.6111999	-2089.0129395
250.0000000	3.6600001	-472.4541016
250.0000000	3.7088001	-624.0673828
250.0000000	3.7575998	-977.4450073

250.0000000	3.8063998	-1774.1741943
250.0000000	3.8552001	-2576.1250000
250.0000000	3.9040000	-2525.7045898
250.0000000	3.9528000	1155.8461914
250.0000000	4.0015998	-251.8411865
250.0000000	4.0503998	707.9114990
250.0000000	4.0991998	90.6844025
250.0000000	4.1479998	-673.0192871
250.0000000	4.1967998	3.1669827
250.0000000	4.2456002	-255.1021881
250.0000000	4.2943997	-501.4491882
250.0000000	4.3431997	-13.1851530
250.0000000	4.3920002	-254.7200165
250.0000000	4.4407997	-22.5796242
250.0000000	4.4896002	-15.4150591
250.0000000	4.5383997	-672.5666504
250.0000000	4.5871997	-12.7035503
250.0000000	4.6360002	-21.7244072
250.0000000	4.6847997	-28.5452499
250.0000000	4.7336001	-19.6107941
250.0000000	4.7824001	-16.4819355
250.0000000	4.8311996	-1509.8878174
250.0000000	4.8800001	234.2426300
250.0000000	4.9287996	-205.9481049
250.0000000	4.9776001	1085.8056641
250.0000000	5.0264001	88.6826477
250.0000000	5.0751996	-308.0632019
250.0000000	5.1240001	-12.2467937
250.0000000	5.1727996	566.1768188
250.0000000	5.2216001	32.6761742
250.0000000	5.2704000	-160.8401337
250.0000000	5.3191996	-39.5102692
250.0000000	5.3680000	-3585.7780762
250.0000000	5.4168000	534.8309937
250.0000000	5.4656000	-203.3499298
250.0000000	5.5144000	38.8361511
250.0000000	5.5631995	495.4407349
250.0000000	5.6120000	398.9153442
250.0000000	5.6608000	-37.3799744
250.0000000	5.7096000	63.6447983
250.0000000	5.7584000	135.1301117
250.0000000	5.8071995	-11.4573879
250.0000000	5.8559999	30.4571743
250.0000000	5.9047999	-16.6199341
250.0000000	5.9535999	-40.1193123
250.0000000	6.0023999	-1595.0354004
250.0000000	6.0511999	-14.5528603
250.0000000	6.0999999	-19.4697723
250.0000000	6.1487999	-13.2396812
250.0000000	6.1975999	-23.3487015
250.0000000	6.2463999	-1817.1524658
250.0000000	6.2951999	-19.1296635
250.0000000	6.3439999	-347.3838501
250.0000000	6.3927999	-15.0494642
250.0000000	6.4416003	-12.3430014
250.0000000	6.4903998	-11.2141256
250.0000000	6.5391998	-11.7561865
250.0000000	6.5879998	435.8922119

250.0000000	6.6367998	-12.0436687
250.0000000	6.6856003	-13.8023405
250.0000000	6.7343998	-13.4396830
250.0000000	6.7831998	-14.4034109
250.0000000	6.8319998	59.8607864
250.0000000	6.8807998	-14.0597458
250.0000000	6.9296002	-506.3392944
250.0000000	6.9783998	-4.1255913
250.0000000	7.0271997	-14.2143507
250.0000000	7.0760002	14.3551188
250.0000000	7.1247997	-2428.5847168
250.0000000	7.1736002	-1947.4420166
250.0000000	7.2223997	-1107.4406738
250.0000000	7.2711997	-237.3630829
250.0000000	7.3200002	-19.7880478
250.0000000	7.3687997	216.2121429
250.0000000	7.4176002	449.0941772
250.0000000	7.4663997	353.1098633
250.0000000	7.5151997	-1083.9566650
250.0000000	7.5640001	-1197.4848633
250.0000000	7.6127996	-2824.6391602
250.0000000	7.6616001	-906.0107422
250.0000000	7.7104001	-464.6428833
250.0000000	7.7591996	50.8966370
250.0000000	7.8080001	466.6943665
250.0000000	7.8567996	614.2905273
250.0000000	7.9056001	-1268.7177734
250.0000000	7.9544001	2020.1331787
250.0000000	8.0031996	2606.5517578
250.0000000	8.0520000	2601.6523438
250.0000000	8.1007996	2753.0236816
250.0000000	8.1496000	2935.9226074
250.0000000	8.1983995	1826.6462402
250.0000000	8.2472000	-4178.4252930
250.0000000	8.2959995	-1480.9573975
250.0000000	8.3448000	-1179.7995605
250.0000000	8.3935995	-1081.2301025
250.0000000	8.4423990	-465.4739075
250.0000000	8.4912004	-1087.1911621
250.0000000	8.5400000	-2933.4020996
250.0000000	8.5887995	-1421.5054932
250.0000000	8.6375999	278.2708740
250.0000000	8.6863995	2510.1030273
250.0000000	8.7351999	-932.4382324
250.0000000	8.7840004	1066.6669922
250.0000000	8.8327999	59.0796356
250.0000000	8.8815994	-11.4830914
250.0000000	8.9303989	-24.2301407
250.0000000	8.9792004	-11.5727949
250.0000000	9.0279999	-5.7012887
250.0000000	9.0767994	7.4780540
250.0000000	9.1255999	-17.3736382
250.0000000	9.1743994	-10.0427017
250.0000000	9.2231998	38.2208023
250.0000000	9.2720003	-8.3481045
250.0000000	9.3207998	-9.7566109
250.0000000	9.3695993	-10.7463455
250.0000000	9.4183998	-11.7634668

250.0000000	9.4672003	-11.1581593
250.0000000	9.5159998	-14.2328176
250.0000000	9.5648003	-0.1333818
250.0000000	9.6135998	-13.4568930
250.0000000	9.6623993	-10.2001553
250.0000000	9.7111998	-10.1367941
250.0000000	9.7600002	-10.8543644
250.0000000	9.8087997	-10.9941654
250.0000000	9.8575993	3.5304270
250.0000000	9.9063997	-7.8986955
250.0000000	9.9552002	-10.9062700
250.0000000	10.0039997	-10.0556583
260.0000000	0.3416000	-37.1619530
260.0000000	0.3904000	-39.6083984
260.0000000	0.4392000	-44.5489273
260.0000000	0.4880000	-39.5879059
260.0000000	0.5368000	-39.5996437
260.0000000	0.5856000	-45.9103317
260.0000000	0.6344000	-26.9658165
260.0000000	0.6832000	-69.7749329
260.0000000	0.7320000	-36.1646194
260.0000000	0.7808000	-42.4187622
260.0000000	0.8296000	-35.5449905
260.0000000	0.8784000	-31.8266296
260.0000000	0.9272000	-28.7768364
260.0000000	0.9760000	-27.5716171
260.0000000	1.0247999	-27.6488914
260.0000000	1.0735999	-28.1337147
260.0000000	1.1224000	-28.9587975
260.0000000	1.1711999	-30.5861645
260.0000000	1.2200000	-31.4520741
260.0000000	1.2687999	-31.1463375
260.0000000	1.3176000	-30.1264992
260.0000000	1.3664000	-29.9819374
260.0000000	1.4152000	-30.1467800
260.0000000	1.4640000	-29.7452393
260.0000000	1.5128000	-46.7607269
260.0000000	1.5616000	-36.8102036
260.0000000	1.6104001	-1750.4698486
260.0000000	1.6592000	-95.5970230
260.0000000	1.7079999	-27.8851738
260.0000000	1.7567999	-27.3575439
260.0000000	1.8055999	-24.9000511
260.0000000	1.8544000	-24.3117676
260.0000000	1.9031999	-25.2612038
260.0000000	1.9520000	-26.5331421
260.0000000	2.0007999	-29.3556919
260.0000000	2.0495999	-15.2211723
260.0000000	2.0983999	-260.1333313
260.0000000	2.1471999	18.6376762
260.0000000	2.1960001	-25.7594318
260.0000000	2.2448001	-27.1890182
260.0000000	2.2935998	-32.2118225
260.0000000	2.3423998	-19.8950996
260.0000000	2.3912001	79.1180191
260.0000000	2.4400001	-22.6484928
260.0000000	2.4888000	-23.4766026
260.0000000	2.5375998	-10.9662066

260.0000000	2.5863998	-8.8850069
260.0000000	2.6352000	-6.7293816
260.0000000	2.6840000	-14.4989452
260.0000000	2.7328000	153.5227051
260.0000000	2.7815998	180.5785217
260.0000000	2.8304000	-23.8942490
260.0000000	2.8792000	-20.3694019
260.0000000	2.9280000	-22.6153736
260.0000000	2.9768000	-16.1481590
260.0000000	3.0256000	-20.2748547
260.0000000	3.0743999	-30.3892479
260.0000000	3.1231999	-24.5198593
260.0000000	3.1719999	-30.2411919
260.0000000	3.2208002	-522.4486084
260.0000000	3.2695999	-1023.6174316
260.0000000	3.3183999	-20.4745502
260.0000000	3.3671999	-3507.7456055
260.0000000	3.4159999	1259.2288818
260.0000000	3.4648001	-6.7066936
260.0000000	3.5135999	-27.2644310
260.0000000	3.5623999	-331.7976074
260.0000000	3.6111999	-2088.1530762
260.0000000	3.6600001	-472.2659912
260.0000000	3.7088001	-623.8159790
260.0000000	3.7575998	-977.0464478
260.0000000	3.8063998	-1773.4443359
260.0000000	3.8552001	-2575.0615234
260.0000000	3.9040000	-2524.6621094
260.0000000	3.9528000	1155.3579102
260.0000000	4.0015998	-251.7440643
260.0000000	4.0503998	707.6089478
260.0000000	4.0991998	90.6387177
260.0000000	4.1479998	-672.7470093
260.0000000	4.1967998	3.1577263
260.0000000	4.2456002	-255.0038757
260.0000000	4.2943997	-501.2486267
260.0000000	4.3431997	-13.1873341
260.0000000	4.3920002	-254.6219025
260.0000000	4.4407997	-22.5778484
260.0000000	4.4896002	-15.4159412
260.0000000	4.5383997	-672.3055420
260.0000000	4.5871997	-12.7055674
260.0000000	4.6360002	-21.7228642
260.0000000	4.6847997	-28.5413246
260.0000000	4.7336001	-19.6100731
260.0000000	4.7824001	-16.4823895
260.0000000	4.8311996	-1509.3564453
260.0000000	4.8800001	234.1530762
260.0000000	4.9287996	-205.8808441
260.0000000	4.9776001	1085.4132080
260.0000000	5.0264001	88.6451263
260.0000000	5.0751996	-307.9595642
260.0000000	5.1240001	-12.2477150
260.0000000	5.1727996	566.0609131
260.0000000	5.2216001	32.6667328
260.0000000	5.2704000	-160.8120270
260.0000000	5.3191996	-39.5057220
260.0000000	5.3680000	-3585.0856934

260.0000000	5.4168000	534.7242432
260.0000000	5.4656000	-203.3137512
260.0000000	5.5144000	38.8254929
260.0000000	5.5631995	495.3421631
260.0000000	5.6120000	398.8353577
260.0000000	5.6608000	-37.3758850
260.0000000	5.7096000	63.6293716
260.0000000	5.7584000	135.1011810
260.0000000	5.8071995	-11.4582586
260.0000000	5.8559999	30.4483204
260.0000000	5.9047999	-16.6197376
260.0000000	5.9535999	-40.1145744
260.0000000	6.0023999	-1594.7308350
260.0000000	6.0511999	-14.5530710
260.0000000	6.0999999	-19.4689388
260.0000000	6.1487999	-13.2401447
260.0000000	6.1975999	-23.3469372
260.0000000	6.2463999	-1816.8050537
260.0000000	6.2951999	-19.1288490
260.0000000	6.3439999	-347.3197632
260.0000000	6.3927999	-15.0494452
260.0000000	6.4416003	-12.3435888
260.0000000	6.4903998	-11.2148008
260.0000000	6.5391998	-11.7568674
260.0000000	6.5879998	435.2015076
260.0000000	6.6367998	-12.0442181
260.0000000	6.6856003	-13.8025103
260.0000000	6.7343998	-13.4398994
260.0000000	6.7831998	-14.4029951
260.0000000	6.8319998	59.8416672
260.0000000	6.8807998	-14.0596066
260.0000000	6.9296002	-505.5778503
260.0000000	6.9783998	-4.1384153
260.0000000	7.0271997	-14.2133236
260.0000000	7.0760002	14.3129320
260.0000000	7.1247997	-2424.6389160
260.0000000	7.1736002	-1944.2791748
260.0000000	7.2223997	-1105.6475830
260.0000000	7.2711997	-236.9953003
260.0000000	7.3200002	-19.7760601
260.0000000	7.3687997	215.8376160
260.0000000	7.4176002	448.3383484
260.0000000	7.4663997	352.5122070
260.0000000	7.5151997	-1082.1956787
260.0000000	7.5640001	-1195.5374756
260.0000000	7.6127996	-2820.0214844
260.0000000	7.6616001	-904.5394287
260.0000000	7.7104001	-463.8966064
260.0000000	7.7591996	50.7955894
260.0000000	7.8080001	465.9089966
260.0000000	7.8567996	613.2624512
260.0000000	7.9056001	-1266.6448975
260.0000000	7.9544001	2016.8232422
260.0000000	8.0031996	2602.2822266
260.0000000	8.0520000	2597.3903809
260.0000000	8.1007996	2748.5141602
260.0000000	8.1496000	2931.1147461
260.0000000	8.1983995	1823.6536865

260.0000000	8.2472000	-4171.5908203
260.0000000	8.2959995	-1478.5372314
260.0000000	8.3448000	-1177.8703613
260.0000000	8.3935995	-1079.4624023
260.0000000	8.4423990	-464.7159119
260.0000000	8.4912004	-1085.4149170
260.0000000	8.5400000	-2928.6022949
260.0000000	8.5887995	-1419.1839600
260.0000000	8.6375999	277.8095398
260.0000000	8.6863995	2505.9846191
260.0000000	8.7351999	-930.9140015
260.0000000	8.7840004	1064.9102783
260.0000000	8.8327999	58.9680252
260.0000000	8.8815994	-11.4832659
260.0000000	8.9303989	-24.2264194
260.0000000	8.9792004	-11.5728769
260.0000000	9.0279999	-5.7030568
260.0000000	9.0767994	7.4723358
260.0000000	9.1255999	-17.3719254
260.0000000	9.1743994	-10.0431042
260.0000000	9.2231998	38.2050705
260.0000000	9.2720003	-8.3490410
260.0000000	9.3207998	-9.7571507
260.0000000	9.3695993	-10.7465725
260.0000000	9.4183998	-11.7633429
260.0000000	9.4672003	-11.1582012
260.0000000	9.5159998	-14.2319260
260.0000000	9.5648003	-0.1367550
260.0000000	9.6135998	-13.4561024
260.0000000	9.6623993	-10.2004299
260.0000000	9.7111998	-10.1370659
260.0000000	9.7600002	-10.8544064
260.0000000	9.8087997	-10.9941740
260.0000000	9.8575993	3.5260048
260.0000000	9.9063997	-7.8996096
260.0000000	9.9552002	-10.9062738
260.0000000	10.0039997	-10.0559053
270.0000000	0.3416000	-35.6101952
270.0000000	0.3904000	-39.6097984
270.0000000	0.4392000	-44.3834038
270.0000000	0.4880000	-39.5604591
270.0000000	0.5368000	-39.5824661
270.0000000	0.5856000	-45.5221786
270.0000000	0.6344000	-27.6358757
270.0000000	0.6832000	-66.5174103
270.0000000	0.7320000	-36.3638725
270.0000000	0.7808000	-40.3351135
270.0000000	0.8296000	-32.8423653
270.0000000	0.8784000	-29.1703587
270.0000000	0.9272000	-27.7113991
270.0000000	0.9760000	-27.2967205
270.0000000	1.0247999	-27.2331963
270.0000000	1.0735999	-27.3111839
270.0000000	1.1224000	-28.1812325
270.0000000	1.1711999	-29.9347706
270.0000000	1.2200000	-31.7752743
270.0000000	1.2687999	-31.1555710
270.0000000	1.3176000	-30.1526089

270.0000000	1.3664000	-29.9896870
270.0000000	1.4152000	-30.1469841
270.0000000	1.4640000	-29.7435303
270.0000000	1.5128000	-46.6635475
270.0000000	1.5616000	-36.7683640
270.0000000	1.6104001	-1747.9270020
270.0000000	1.6592000	-95.3030930
270.0000000	1.7079999	-27.8957691
270.0000000	1.7567999	-26.8624706
270.0000000	1.8055999	-24.0732975
270.0000000	1.8544000	-24.4670048
270.0000000	1.9031999	-26.6520405
270.0000000	1.9520000	-26.5335979
270.0000000	2.0007999	-29.2931099
270.0000000	2.0495999	-15.4077883
270.0000000	2.0983999	-258.6575623
270.0000000	2.1471999	18.3704109
270.0000000	2.1960001	-25.7553406
270.0000000	2.2448001	-27.1711063
270.0000000	2.2935998	-32.1521988
270.0000000	2.3423998	-19.9095058
270.0000000	2.3912001	78.4964676
270.0000000	2.4400001	-22.6509781
270.0000000	2.4888000	-23.4763851
270.0000000	2.5375998	-10.9833145
270.0000000	2.5863998	-8.9047661
270.0000000	2.6352000	-6.7521257
270.0000000	2.6840000	-14.5108871
270.0000000	2.7328000	153.2566986
270.0000000	2.7815998	180.2720947
270.0000000	2.8304000	-23.8929443
270.0000000	2.8792000	-20.3726654
270.0000000	2.9280000	-22.6148052
270.0000000	2.9768000	-16.1565304
270.0000000	3.0256000	-20.2774391
270.0000000	3.0743999	-30.3778610
270.0000000	3.1231999	-24.5164032
270.0000000	3.1719999	-30.2328987
270.0000000	3.2208002	-522.2108765
270.0000000	3.2695999	-1023.1424561
270.0000000	3.3183999	-20.4747486
270.0000000	3.3671999	-3506.2839355
270.0000000	3.4159999	1258.6936035
270.0000000	3.4648001	-6.7124014
270.0000000	3.5135999	-27.2614403
270.0000000	3.5623999	-331.6679993
270.0000000	3.6111999	-2087.2934570
270.0000000	3.6600001	-472.0778198
270.0000000	3.7088001	-623.5646362
270.0000000	3.7575998	-976.6479492
270.0000000	3.8063998	-1772.7144775
270.0000000	3.8552001	-2573.9980469
270.0000000	3.9040000	-2523.6196289
270.0000000	3.9528000	1154.8696289
270.0000000	4.0015998	-251.6469574
270.0000000	4.0503998	707.3063965
270.0000000	4.0991998	90.5930328
270.0000000	4.1479998	-672.4747925

270.0000000	4.1967998	3.1484699
270.0000000	4.2456002	-254.9055786
270.0000000	4.2943997	-501.0480652
270.0000000	4.3431997	-13.1895142
270.0000000	4.3920002	-254.5237885
270.0000000	4.4407997	-22.5760727
270.0000000	4.4896002	-15.4168243
270.0000000	4.5383997	-672.0444946
270.0000000	4.5871997	-12.7075834
270.0000000	4.6360002	-21.7213211
270.0000000	4.6847997	-28.5373993
270.0000000	4.7336001	-19.6093502
270.0000000	4.7824001	-16.4828434
270.0000000	4.8311996	-1508.8251953
270.0000000	4.8800001	234.0635223
270.0000000	4.9287996	-205.8135834
270.0000000	4.9776001	1085.0206299
270.0000000	5.0264001	88.6076050
270.0000000	5.0751996	-307.8559570
270.0000000	5.1240001	-12.2486362
270.0000000	5.1727996	565.9450684
270.0000000	5.2216001	32.6572876
270.0000000	5.2704000	-160.7839203
270.0000000	5.3191996	-39.5011711
270.0000000	5.3680000	-3584.3933105
270.0000000	5.4168000	534.6174927
270.0000000	5.4656000	-203.2775726
270.0000000	5.5144000	38.8148346
270.0000000	5.5631995	495.2435913
270.0000000	5.6120000	398.7554016
270.0000000	5.6608000	-37.3717957
270.0000000	5.7096000	63.6139374
270.0000000	5.7584000	135.0722351
270.0000000	5.8071995	-11.4591293
270.0000000	5.8559999	30.4394703
270.0000000	5.9047999	-16.6195393
270.0000000	5.9535999	-40.1098328
270.0000000	6.0023999	-1594.4265137
270.0000000	6.0511999	-14.5532827
270.0000000	6.0999999	-19.4681072
270.0000000	6.1487999	-13.2406073
270.0000000	6.1975999	-23.3451729
270.0000000	6.2463999	-1816.4576416
270.0000000	6.2951999	-19.1280346
270.0000000	6.3439999	-347.2557373
270.0000000	6.3927999	-15.0494270
270.0000000	6.4416003	-12.3441753
270.0000000	6.4903998	-11.2154770
270.0000000	6.5391998	-11.7575483
270.0000000	6.5879998	434.5108032
270.0000000	6.6367998	-12.0447674
270.0000000	6.6856003	-13.8026810
270.0000000	6.7343998	-13.4401150
270.0000000	6.7831998	-14.4025793
270.0000000	6.8319998	59.8225555
270.0000000	6.8807998	-14.0594683
270.0000000	6.9296002	-504.8164063
270.0000000	6.9783998	-4.1512384

270.0000000	7.0271997	-14.2122955
270.0000000	7.0760002	14.2707491
270.0000000	7.1247997	-2420.6928711
270.0000000	7.1736002	-1941.1162109
270.0000000	7.2223997	-1103.8543701
270.0000000	7.2711997	-236.6275177
270.0000000	7.3200002	-19.7640724
270.0000000	7.3687997	215.4630890
270.0000000	7.4176002	447.5825195
270.0000000	7.4663997	351.9145508
270.0000000	7.5151997	-1080.4345703
270.0000000	7.5640001	-1193.5900879
270.0000000	7.6127996	-2815.4040527
270.0000000	7.6616001	-903.0681152
270.0000000	7.7104001	-463.1503906
270.0000000	7.7591996	50.6945381
270.0000000	7.8080001	465.1236267
270.0000000	7.8567996	612.2343750
270.0000000	7.9056001	-1264.5720215
270.0000000	7.9544001	2013.5133057
270.0000000	8.0031996	2598.0126953
270.0000000	8.0520000	2593.1284180
270.0000000	8.1007996	2744.0046387
270.0000000	8.1496000	2926.3066406
270.0000000	8.1983995	1820.6611328
270.0000000	8.2472000	-4164.7558594
270.0000000	8.2959995	-1476.1170654
270.0000000	8.3448000	-1175.9411621
270.0000000	8.3935995	-1077.6947021
270.0000000	8.4423990	-463.9578857
270.0000000	8.4912004	-1083.6386719
270.0000000	8.5400000	-2923.8024902
270.0000000	8.5887995	-1416.8624268
270.0000000	8.6375999	277.3482361
270.0000000	8.6863995	2501.8662109
270.0000000	8.7351999	-929.3897095
270.0000000	8.7840004	1063.1535645
270.0000000	8.8327999	58.8564148
270.0000000	8.8815994	-11.4834394
270.0000000	8.9303989	-24.2226982
270.0000000	8.9792004	-11.5729589
270.0000000	9.0279999	-5.7048254
270.0000000	9.0767994	7.4666176
270.0000000	9.1255999	-17.3702106
270.0000000	9.1743994	-10.0435066
270.0000000	9.2231998	38.1893387
270.0000000	9.2720003	-8.3499775
270.0000000	9.3207998	-9.7576895
270.0000000	9.3695993	-10.7467985
270.0000000	9.4183998	-11.7632179
270.0000000	9.4672003	-11.1582422
270.0000000	9.5159998	-14.2310352
270.0000000	9.5648003	-0.1401272
270.0000000	9.6135998	-13.4553118
270.0000000	9.6623993	-10.2007055
270.0000000	9.7111998	-10.1373377
270.0000000	9.7600002	-10.8544483
270.0000000	9.8087997	-10.9941816

270.0000000	9.8575993	3.5215816
270.0000000	9.9063997	-7.9005232
270.0000000	9.9552002	-10.9062786
270.0000000	10.0039997	-10.0561533
280.0000000	0.3416000	-37.0960770
280.0000000	0.3904000	-39.6587067
280.0000000	0.4392000	-44.2178802
280.0000000	0.4880000	-39.5330124
280.0000000	0.5368000	-39.5652847
280.0000000	0.5856000	-45.1340218
280.0000000	0.6344000	-28.3059349
280.0000000	0.6832000	-63.2598991
280.0000000	0.7320000	-36.5631256
280.0000000	0.7808000	-38.2514648
280.0000000	0.8296000	-33.4743805
280.0000000	0.8784000	-30.3034840
280.0000000	0.9272000	-28.4914150
280.0000000	0.9760000	-26.8155975
280.0000000	1.0247999	-26.3049507
280.0000000	1.0735999	-27.5285053
280.0000000	1.1224000	-29.0360317
280.0000000	1.1711999	-30.7907200
280.0000000	1.2200000	-31.9297142
280.0000000	1.2687999	-31.1648045
280.0000000	1.3176000	-30.1787186
280.0000000	1.3664000	-29.9974365
280.0000000	1.4152000	-30.1471882
280.0000000	1.4640000	-29.7418213
280.0000000	1.5128000	-46.5663719
280.0000000	1.5616000	-36.7265205
280.0000000	1.6104001	-1745.3841553
280.0000000	1.6592000	-95.0091629
280.0000000	1.7079999	-27.9063625
280.0000000	1.7567999	-27.8011837
280.0000000	1.8055999	-25.5325851
280.0000000	1.8544000	-25.4094410
280.0000000	1.9031999	-26.1420326
280.0000000	1.9520000	-26.5340538
280.0000000	2.0007999	-29.2305279
280.0000000	2.0495999	-15.5944042
280.0000000	2.0983999	-257.1817932
280.0000000	2.1471999	18.1031456
280.0000000	2.1960001	-25.7512493
280.0000000	2.2448001	-27.1531944
280.0000000	2.2935998	-32.0925751
280.0000000	2.3423998	-19.9239101
280.0000000	2.3912001	77.8749237
280.0000000	2.4400001	-22.6534653
280.0000000	2.4888000	-23.4761677
280.0000000	2.5375998	-11.0004225
280.0000000	2.5863998	-8.9245243
280.0000000	2.6352000	-6.7748699
280.0000000	2.6840000	-14.5228291
280.0000000	2.7328000	152.9906769
280.0000000	2.7815998	179.9656982
280.0000000	2.8304000	-23.8916397
280.0000000	2.8792000	-20.3759289
280.0000000	2.9280000	-22.6142368

280.0000000	2.9768000	-16.1649017
280.0000000	3.0256000	-20.2800236
280.0000000	3.0743999	-30.3664742
280.0000000	3.1231999	-24.5129471
280.0000000	3.1719999	-30.2246056
280.0000000	3.2208002	-521.9731445
280.0000000	3.2695999	-1022.6674805
280.0000000	3.3183999	-20.4749470
280.0000000	3.3671999	-3504.8222656
280.0000000	3.4159999	1258.1583252
280.0000000	3.4648001	-6.7181101
280.0000000	3.5135999	-27.2584496
280.0000000	3.5623999	-331.5384216
280.0000000	3.6111999	-2086.4335938
280.0000000	3.6600001	-471.8897095
280.0000000	3.7088001	-623.3132324
280.0000000	3.7575998	-976.2493896
280.0000000	3.8063998	-1771.9846191
280.0000000	3.8552001	-2572.9348145
280.0000000	3.9040000	-2522.5771484
280.0000000	3.9528000	1154.3813477
280.0000000	4.0015998	-251.5498505
280.0000000	4.0503998	707.0038452
280.0000000	4.0991998	90.5473480
280.0000000	4.1479998	-672.2025757
280.0000000	4.1967998	3.1392136
280.0000000	4.2456002	-254.8072815
280.0000000	4.2943997	-500.8475037
280.0000000	4.3431997	-13.1916943
280.0000000	4.3920002	-254.4256744
280.0000000	4.4407997	-22.5742970
280.0000000	4.4896002	-15.4177074
280.0000000	4.5383997	-671.7834473
280.0000000	4.5871997	-12.7096004
280.0000000	4.6360002	-21.7197800
280.0000000	4.6847997	-28.5334740
280.0000000	4.7336001	-19.6086273
280.0000000	4.7824001	-16.4832993
280.0000000	4.8311996	-1508.2939453
280.0000000	4.8800001	233.9739685
280.0000000	4.9287996	-205.7463226
280.0000000	4.9776001	1084.6281738
280.0000000	5.0264001	88.5700836
280.0000000	5.0751996	-307.7523193
280.0000000	5.1240001	-12.2495575
280.0000000	5.1727996	565.8291626
280.0000000	5.2216001	32.6478424
280.0000000	5.2704000	-160.7558136
280.0000000	5.3191996	-39.4966202
280.0000000	5.3680000	-3583.7009277
280.0000000	5.4168000	534.5106812
280.0000000	5.4656000	-203.2413788
280.0000000	5.5144000	38.8041840
280.0000000	5.5631995	495.1450195
280.0000000	5.6120000	398.6754150
280.0000000	5.6608000	-37.3677063
280.0000000	5.7096000	63.5985107
280.0000000	5.7584000	135.0433044

280.0000000	5.8071995	-11.4599991
280.0000000	5.8559999	30.4306202
280.0000000	5.9047999	-16.6193409
280.0000000	5.9535999	-40.1050949
280.0000000	6.0023999	-1594.1219482
280.0000000	6.0511999	-14.5534945
280.0000000	6.0999999	-19.4672737
280.0000000	6.1487999	-13.2410698
280.0000000	6.1975999	-23.3434067
280.0000000	6.2463999	-1816.1103516
280.0000000	6.2951999	-19.1272202
280.0000000	6.3439999	-347.1916809
280.0000000	6.3927999	-15.0494089
280.0000000	6.4416003	-12.3447628
280.0000000	6.4903998	-11.2161531
280.0000000	6.5391998	-11.7582293
280.0000000	6.5879998	433.8200989
280.0000000	6.6367998	-12.0453167
280.0000000	6.6856003	-13.8028507
280.0000000	6.7343998	-13.4403305
280.0000000	6.7831998	-14.4021635
280.0000000	6.8319998	59.8034363
280.0000000	6.8807998	-14.0593300
280.0000000	6.9296002	-504.0549622
280.0000000	6.9783998	-4.1640615
280.0000000	7.0271997	-14.2112675
280.0000000	7.0760002	14.2285624
280.0000000	7.1247997	-2416.7470703
280.0000000	7.1736002	-1937.9533691
280.0000000	7.2223997	-1102.0611572
280.0000000	7.2711997	-236.2597351
280.0000000	7.3200002	-19.7520828
280.0000000	7.3687997	215.0885620
280.0000000	7.4176002	446.8266907
280.0000000	7.4663997	351.3168945
280.0000000	7.5151997	-1078.6735840
280.0000000	7.5640001	-1191.6427002
280.0000000	7.6127996	-2810.7863770
280.0000000	7.6616001	-901.5968018
280.0000000	7.7104001	-462.4041138
280.0000000	7.7591996	50.5934906
280.0000000	7.8080001	464.3382874
280.0000000	7.8567996	611.2062378
280.0000000	7.9056001	-1262.4991455
280.0000000	7.9544001	2010.2033691
280.0000000	8.0031996	2593.7429199
280.0000000	8.0520000	2588.8664551
280.0000000	8.1007996	2739.4951172
280.0000000	8.1496000	2921.4985352
280.0000000	8.1983995	1817.6687012
280.0000000	8.2472000	-4157.9208984
280.0000000	8.2959995	-1473.6967773
280.0000000	8.3448000	-1174.0120850
280.0000000	8.3935995	-1075.9270020
280.0000000	8.4423990	-463.1998901
280.0000000	8.4912004	-1081.8625488
280.0000000	8.5400000	-2919.0026855
280.0000000	8.5887995	-1414.5408936

280.0000000	8.6375999	276.8869019
280.0000000	8.6863995	2497.7480469
280.0000000	8.7351999	-927.8654785
280.0000000	8.7840004	1061.3967285
280.0000000	8.8327999	58.7448044
280.0000000	8.8815994	-11.4836140
280.0000000	8.9303989	-24.2189789
280.0000000	8.9792004	-11.5730419
280.0000000	9.0279999	-5.7065935
280.0000000	9.0767994	7.4608994
280.0000000	9.1255999	-17.3684959
280.0000000	9.1743994	-10.0439081
280.0000000	9.2231998	38.1736069
280.0000000	9.2720003	-8.3509140
280.0000000	9.3207998	-9.7582293
280.0000000	9.3695993	-10.7470255
280.0000000	9.4183998	-11.7630939
280.0000000	9.4672003	-11.1582832
280.0000000	9.5159998	-14.2301445
280.0000000	9.5648003	-0.1434994
280.0000000	9.6135998	-13.4545212
280.0000000	9.6623993	-10.2009802
280.0000000	9.7111998	-10.1376095
280.0000000	9.7600002	-10.8544893
280.0000000	9.8087997	-10.9941893
280.0000000	9.8575993	3.5171595
280.0000000	9.9063997	-7.9014373
280.0000000	9.9552002	-10.9062824
280.0000000	10.0039997	-10.0564003
290.0000000	0.3416000	-36.8299446
290.0000000	0.3904000	-39.7076111
290.0000000	0.4392000	-44.0523605
290.0000000	0.4880000	-39.5055695
290.0000000	0.5368000	-39.5481071
290.0000000	0.5856000	-44.7458687
290.0000000	0.6344000	-28.9759941
290.0000000	0.6832000	-60.0023842
290.0000000	0.7320000	-36.7623787
290.0000000	0.7808000	-36.1678162
290.0000000	0.8296000	-31.9765816
290.0000000	0.8784000	-28.1216259
290.0000000	0.9272000	-26.3410320
290.0000000	0.9760000	-25.6241093
290.0000000	1.0247999	-26.0095749
290.0000000	1.0735999	-27.3893661
290.0000000	1.1224000	-29.4523335
290.0000000	1.1711999	-31.6362514
290.0000000	1.2200000	-31.9295712
290.0000000	1.2687999	-31.1740379
290.0000000	1.3176000	-30.2048264
290.0000000	1.3664000	-30.0051861
290.0000000	1.4152000	-30.1473923
290.0000000	1.4640000	-29.7401104
290.0000000	1.5128000	-46.4691925
290.0000000	1.5616000	-36.6846809
290.0000000	1.6104001	-1742.8414307
290.0000000	1.6592000	-94.7152252
290.0000000	1.7079999	-27.9169579

290.0000000	1.7567999	-27.7860222
290.0000000	1.8055999	-25.4656887
290.0000000	1.8544000	-25.4883976
290.0000000	1.9031999	-26.2029324
290.0000000	1.9520000	-26.5345078
290.0000000	2.0007999	-29.1679459
290.0000000	2.0495999	-15.7810211
290.0000000	2.0983999	-255.7060242
290.0000000	2.1471999	17.8358765
290.0000000	2.1960001	-25.7471581
290.0000000	2.2448001	-27.1352844
290.0000000	2.2935998	-32.0329514
290.0000000	2.3423998	-19.9383144
290.0000000	2.3912001	77.2533798
290.0000000	2.4400001	-22.6559525
290.0000000	2.4888000	-23.4759502
290.0000000	2.5375998	-11.0175304
290.0000000	2.5863998	-8.9442835
290.0000000	2.6352000	-6.7976141
290.0000000	2.6840000	-14.5347710
290.0000000	2.7328000	152.7246552
290.0000000	2.7815998	179.6592712
290.0000000	2.8304000	-23.8903370
290.0000000	2.8792000	-20.3791924
290.0000000	2.9280000	-22.6136665
290.0000000	2.9768000	-16.1732712
290.0000000	3.0256000	-20.2826080
290.0000000	3.0743999	-30.3550873
290.0000000	3.1231999	-24.5094929
290.0000000	3.1719999	-30.2163124
290.0000000	3.2208002	-521.7354126
290.0000000	3.2695999	-1022.1925049
290.0000000	3.3183999	-20.4751453
290.0000000	3.3671999	-3503.3605957
290.0000000	3.4159999	1257.6230469
290.0000000	3.4648001	-6.7238188
290.0000000	3.5135999	-27.2554588
290.0000000	3.5623999	-331.4088135
290.0000000	3.6111999	-2085.5739746
290.0000000	3.6600001	-471.7015991
290.0000000	3.7088001	-623.0618286
290.0000000	3.7575998	-975.8508301
290.0000000	3.8063998	-1771.2547607
290.0000000	3.8552001	-2571.8713379
290.0000000	3.9040000	-2521.5346680
290.0000000	3.9528000	1153.8930664
290.0000000	4.0015998	-251.4527435
290.0000000	4.0503998	706.7012939
290.0000000	4.0991998	90.5016632
290.0000000	4.1479998	-671.9303589
290.0000000	4.1967998	3.1299572
290.0000000	4.2456002	-254.7089691
290.0000000	4.2943997	-500.6469116
290.0000000	4.3431997	-13.1938753
290.0000000	4.3920002	-254.3275604
290.0000000	4.4407997	-22.5725212
290.0000000	4.4896002	-15.4185896
290.0000000	4.5383997	-671.5223389

290.0000000	4.5871997	-12.7116165
290.0000000	4.6360002	-21.7182369
290.0000000	4.6847997	-28.5295486
290.0000000	4.7336001	-19.6079063
290.0000000	4.7824001	-16.4837532
290.0000000	4.8311996	-1507.7626953
290.0000000	4.8800001	233.8844299
290.0000000	4.9287996	-205.6790619
290.0000000	4.9776001	1084.2357178
290.0000000	5.0264001	88.5325623
290.0000000	5.0751996	-307.6486816
290.0000000	5.1240001	-12.2504787
290.0000000	5.1727996	565.7133179
290.0000000	5.2216001	32.6384010
290.0000000	5.2704000	-160.7277069
290.0000000	5.3191996	-39.4920731
290.0000000	5.3680000	-3583.0085449
290.0000000	5.4168000	534.4038696
290.0000000	5.4656000	-203.2052002
290.0000000	5.5144000	38.7935257
290.0000000	5.5631995	495.0464478
290.0000000	5.6120000	398.5954590
290.0000000	5.6608000	-37.3636169
290.0000000	5.7096000	63.5830765
290.0000000	5.7584000	135.0143738
290.0000000	5.8071995	-11.4608707
290.0000000	5.8559999	30.4217701
290.0000000	5.9047999	-16.6191444
290.0000000	5.9535999	-40.1003571
290.0000000	6.0023999	-1593.8176270
290.0000000	6.0511999	-14.5537052
290.0000000	6.0999999	-19.4664421
290.0000000	6.1487999	-13.2415333
290.0000000	6.1975999	-23.3416405
290.0000000	6.2463999	-1815.7629395
290.0000000	6.2951999	-19.1264057
290.0000000	6.3439999	-347.1276245
290.0000000	6.3927999	-15.0493908
290.0000000	6.4416003	-12.3453493
290.0000000	6.4903998	-11.2168293
290.0000000	6.5391998	-11.7589111
290.0000000	6.5879998	433.1293945
290.0000000	6.6367998	-12.0458651
290.0000000	6.6856003	-13.8030214
290.0000000	6.7343998	-13.4405460
290.0000000	6.7831998	-14.4017487
290.0000000	6.8319998	59.7843246
290.0000000	6.8807998	-14.0591908
290.0000000	6.9296002	-503.2935181
290.0000000	6.9783998	-4.1768856
290.0000000	7.0271997	-14.2102394
290.0000000	7.0760002	14.1863775
290.0000000	7.1247997	-2412.8012695
290.0000000	7.1736002	-1934.7905273
290.0000000	7.2223997	-1100.2680664
290.0000000	7.2711997	-235.8919678
290.0000000	7.3200002	-19.7400932
290.0000000	7.3687997	214.7140198

290.0000000	7.4176002	446.0708618
290.0000000	7.4663997	350.7192383
290.0000000	7.5151997	-1076.9125977
290.0000000	7.5640001	-1189.6953125
290.0000000	7.6127996	-2806.1687012
290.0000000	7.6616001	-900.1254272
290.0000000	7.7104001	-461.6578369
290.0000000	7.7591996	50.4924431
290.0000000	7.8080001	463.5529175
290.0000000	7.8567996	610.1781616
290.0000000	7.9056001	-1260.4261475
290.0000000	7.9544001	2006.8934326
290.0000000	8.0031996	2589.4733887
290.0000000	8.0520000	2584.6044922
290.0000000	8.1007996	2734.9855957
290.0000000	8.1496000	2916.6906738
290.0000000	8.1983995	1814.6761475
290.0000000	8.2472000	-4151.0864258
290.0000000	8.2959995	-1471.2766113
290.0000000	8.3448000	-1172.0828857
290.0000000	8.3935995	-1074.1594238
290.0000000	8.4423990	-462.4418640
290.0000000	8.4912004	-1080.0863037
290.0000000	8.5400000	-2914.2028809
290.0000000	8.5887995	-1412.2193604
290.0000000	8.6375999	276.4255981
290.0000000	8.6863995	2493.6296387
290.0000000	8.7351999	-926.3411865
290.0000000	8.7840004	1059.6400146
290.0000000	8.8327999	58.6331940
290.0000000	8.8815994	-11.4837885
290.0000000	8.9303989	-24.2152596
290.0000000	8.9792004	-11.5731239
290.0000000	9.0279999	-5.7083621
290.0000000	9.0767994	7.4551830
290.0000000	9.1255999	-17.3667831
290.0000000	9.1743994	-10.0443106
290.0000000	9.2231998	38.1578751
290.0000000	9.2720003	-8.3518505
290.0000000	9.3207998	-9.7587681
290.0000000	9.3695993	-10.7472515
290.0000000	9.4183998	-11.7629690
290.0000000	9.4672003	-11.1583242
290.0000000	9.5159998	-14.2292538
290.0000000	9.5648003	-0.1468725
290.0000000	9.6135998	-13.4537306
290.0000000	9.6623993	-10.2012558
290.0000000	9.7111998	-10.1378813
290.0000000	9.7600002	-10.8545313
290.0000000	9.8087997	-10.9941978
290.0000000	9.8575993	3.5127373
290.0000000	9.9063997	-7.9023509
290.0000000	9.9552002	-10.9062872
290.0000000	10.0039997	-10.0566483
300.0000000	0.3416000	-37.3666191
300.0000000	0.3904000	-39.7565155
300.0000000	0.4392000	-43.8868370
300.0000000	0.4880000	-39.4781227

300.0000000	0.5368000	-39.5309296
300.0000000	0.5856000	-44.3577156
300.0000000	0.6344000	-29.6460533
300.0000000	0.6832000	-56.7448692
300.0000000	0.7320000	-36.9616318
300.0000000	0.7808000	-34.0841675
300.0000000	0.8296000	-30.8031693
300.0000000	0.8784000	-27.5811977
300.0000000	0.9272000	-26.1052780
300.0000000	0.9760000	-25.9090233
300.0000000	1.0247999	-27.1526337
300.0000000	1.0735999	-29.7277164
300.0000000	1.1224000	-31.1891689
300.0000000	1.1711999	-32.0378609
300.0000000	1.2200000	-31.9294300
300.0000000	1.2687999	-31.1832714
300.0000000	1.3176000	-30.2309361
300.0000000	1.3664000	-30.0129356
300.0000000	1.4152000	-30.1475964
300.0000000	1.4640000	-29.7384014
300.0000000	1.5128000	-46.3720169
300.0000000	1.5616000	-36.6428375
300.0000000	1.6104001	-1740.2985840
300.0000000	1.6592000	-94.4212952
300.0000000	1.7079999	-27.9275513
300.0000000	1.7567999	-27.4605675
300.0000000	1.8055999	-25.1143875
300.0000000	1.8544000	-24.0488529
300.0000000	1.9031999	-24.2699432
300.0000000	1.9520000	-26.5349636
300.0000000	2.0007999	-29.1053638
300.0000000	2.0495999	-15.9676380
300.0000000	2.0983999	-254.2302246
300.0000000	2.1471999	17.5686073
300.0000000	2.1960001	-25.7430668
300.0000000	2.2448001	-27.1173725
300.0000000	2.2935998	-31.9733276
300.0000000	2.3423998	-19.9527187
300.0000000	2.3912001	76.6318283
300.0000000	2.4400001	-22.6584377
300.0000000	2.4888000	-23.4757328
300.0000000	2.5375998	-11.0346384
300.0000000	2.5863998	-8.9640427
300.0000000	2.6352000	-6.8203583
300.0000000	2.6840000	-14.5467129
300.0000000	2.7328000	152.4586487
300.0000000	2.7815998	179.3528442
300.0000000	2.8304000	-23.8890324
300.0000000	2.8792000	-20.3824558
300.0000000	2.9280000	-22.6130981
300.0000000	2.9768000	-16.1816425
300.0000000	3.0256000	-20.2851925
300.0000000	3.0743999	-30.3437004
300.0000000	3.1231999	-24.5060368
300.0000000	3.1719999	-30.2080193
300.0000000	3.2208002	-521.4976807
300.0000000	3.2695999	-1021.7175293
300.0000000	3.3183999	-20.4753437

300.0000000	3.3671999	-3501.8989258
300.0000000	3.4159999	1257.0877686
300.0000000	3.4648001	-6.7295265
300.0000000	3.5135999	-27.2524681
300.0000000	3.5623999	-331.2792358
300.0000000	3.6111999	-2084.7141113
300.0000000	3.6600001	-471.5134277
300.0000000	3.7088001	-622.8104858
300.0000000	3.7575998	-975.4523315
300.0000000	3.8063998	-1770.5249023
300.0000000	3.8552001	-2570.8078613
300.0000000	3.9040000	-2520.4919434
300.0000000	3.9528000	1153.4046631
300.0000000	4.0015998	-251.3556366
300.0000000	4.0503998	706.3987427
300.0000000	4.0991998	90.4559784
300.0000000	4.1479998	-671.6581421
300.0000000	4.1967998	3.1207008
300.0000000	4.2456002	-254.6106720
300.0000000	4.2943997	-500.4463501
300.0000000	4.3431997	-13.1960564
300.0000000	4.3920002	-254.2294464
300.0000000	4.4407997	-22.5707455
300.0000000	4.4896002	-15.4194717
300.0000000	4.5383997	-671.2612915
300.0000000	4.5871997	-12.7136326
300.0000000	4.6360002	-21.7166939
300.0000000	4.6847997	-28.5256233
300.0000000	4.7336001	-19.6071835
300.0000000	4.7824001	-16.4842072
300.0000000	4.8311996	-1507.2314453
300.0000000	4.8800001	233.7948761
300.0000000	4.9287996	-205.6117859
300.0000000	4.9776001	1083.8431396
300.0000000	5.0264001	88.4950485
300.0000000	5.0751996	-307.5450745
300.0000000	5.1240001	-12.2514000
300.0000000	5.1727996	565.5974731
300.0000000	5.2216001	32.6289558
300.0000000	5.2704000	-160.6996002
300.0000000	5.3191996	-39.4875259
300.0000000	5.3680000	-3582.3161621
300.0000000	5.4168000	534.2971191
300.0000000	5.4656000	-203.1690216
300.0000000	5.5144000	38.7828751
300.0000000	5.5631995	494.9478760
300.0000000	5.6120000	398.5154724
300.0000000	5.6608000	-37.3595314
300.0000000	5.7096000	63.5676498
300.0000000	5.7584000	134.9854431
300.0000000	5.8071995	-11.4617405
300.0000000	5.8559999	30.4129200
300.0000000	5.9047999	-16.6189461
300.0000000	5.9535999	-40.0956192
300.0000000	6.0023999	-1593.5131836
300.0000000	6.0511999	-14.5539169
300.0000000	6.0999999	-19.4656105
300.0000000	6.1487999	-13.2419958

300.0000000	6.1975999	-23.3398762
300.0000000	6.2463999	-1815.4155273
300.0000000	6.2951999	-19.1255913
300.0000000	6.3439999	-347.0635681
300.0000000	6.3927999	-15.0493717
300.0000000	6.4416003	-12.3459358
300.0000000	6.4903998	-11.2175045
300.0000000	6.5391998	-11.7595921
300.0000000	6.5879998	432.4387207
300.0000000	6.6367998	-12.0464144
300.0000000	6.6856003	-13.8031912
300.0000000	6.7343998	-13.4407625
300.0000000	6.7831998	-14.4013329
300.0000000	6.8319998	59.7652054
300.0000000	6.8807998	-14.0590525
300.0000000	6.9296002	-502.5320435
300.0000000	6.9783998	-4.1897097
300.0000000	7.0271997	-14.2092113
300.0000000	7.0760002	14.1441927
300.0000000	7.1247997	-2408.8554688
300.0000000	7.1736002	-1931.6276855
300.0000000	7.2223997	-1098.4748535
300.0000000	7.2711997	-235.5241852
300.0000000	7.3200002	-19.7281055
300.0000000	7.3687997	214.3394928
300.0000000	7.4176002	445.3150330
300.0000000	7.4663997	350.1216125
300.0000000	7.5151997	-1075.1514893
300.0000000	7.5640001	-1187.7479248
300.0000000	7.6127996	-2801.5512695
300.0000000	7.6616001	-898.6540527
300.0000000	7.7104001	-460.9115601
300.0000000	7.7591996	50.3913918
300.0000000	7.8080001	462.7675476
300.0000000	7.8567996	609.1500854
300.0000000	7.9056001	-1258.3532715
300.0000000	7.9544001	2003.5834961
300.0000000	8.0031996	2585.2038574
300.0000000	8.0520000	2580.3425293
300.0000000	8.1007996	2730.4760742
300.0000000	8.1496000	2911.8825684
300.0000000	8.1983995	1811.6835938
300.0000000	8.2472000	-4144.2514648
300.0000000	8.2959995	-1468.8564453
300.0000000	8.3448000	-1170.1536865
300.0000000	8.3935995	-1072.3917236
300.0000000	8.4423990	-461.6838684
300.0000000	8.4912004	-1078.3100586
300.0000000	8.5400000	-2909.4030762
300.0000000	8.5887995	-1409.8977051
300.0000000	8.6375999	275.9642639
300.0000000	8.6863995	2489.5112305
300.0000000	8.7351999	-924.8169556
300.0000000	8.7840004	1057.8833008
300.0000000	8.8327999	58.5215912
300.0000000	8.8815994	-11.4839630
300.0000000	8.9303989	-24.2115402
300.0000000	8.9792004	-11.5732059

300.0000000	9.0279999	-5.7101297
300.0000000	9.0767994	7.4494648
300.0000000	9.1255999	-17.3650703
300.0000000	9.1743994	-10.0447121
300.0000000	9.2231998	38.1421356
300.0000000	9.2720003	-8.3527870
300.0000000	9.3207998	-9.7593079
300.0000000	9.3695993	-10.7474785
300.0000000	9.4183998	-11.7628441
300.0000000	9.4672003	-11.1583652
300.0000000	9.5159998	-14.2283621
300.0000000	9.5648003	-0.1502457
300.0000000	9.6135998	-13.4529400
300.0000000	9.6623993	-10.2015305
300.0000000	9.7111998	-10.1381521
300.0000000	9.7600002	-10.8545732
300.0000000	9.8087997	-10.9942055
300.0000000	9.8575993	3.5083141
300.0000000	9.9063997	-7.9032650
300.0000000	9.9552002	-10.9062910
300.0000000	10.0039997	-10.0568962
310.0000000	0.3416000	-37.0738487
310.0000000	0.3904000	-39.8054237
310.0000000	0.4392000	-43.7213135
310.0000000	0.4880000	-39.4506760
310.0000000	0.5368000	-39.5137520
310.0000000	0.5856000	-43.9695587
310.0000000	0.6344000	-30.3161125
310.0000000	0.6832000	-53.4873543
310.0000000	0.7320000	-37.1608849
310.0000000	0.7808000	-33.6021156
310.0000000	0.8296000	-29.2618046
310.0000000	0.8784000	-26.5467949
310.0000000	0.9272000	-26.1880131
310.0000000	0.9760000	-27.9538136
310.0000000	1.0247999	-29.7723770
310.0000000	1.0735999	-31.7510281
310.0000000	1.1224000	-32.5373039
310.0000000	1.1711999	-32.0230789
310.0000000	1.2200000	-31.9292870
310.0000000	1.2687999	-31.1925049
310.0000000	1.3176000	-30.2570438
310.0000000	1.3664000	-30.0206852
310.0000000	1.4152000	-30.1478004
310.0000000	1.4640000	-29.7366924
310.0000000	1.5128000	-46.2748375
310.0000000	1.5616000	-36.6009979
310.0000000	1.6104001	-1737.7558594
310.0000000	1.6592000	-94.1273651
310.0000000	1.7079999	-27.9381466
310.0000000	1.7567999	-27.7479515
310.0000000	1.8055999	-25.0257225
310.0000000	1.8544000	-24.0403748
310.0000000	1.9031999	-25.2636337
310.0000000	1.9520000	-26.5354176
310.0000000	2.0007999	-29.0427818
310.0000000	2.0495999	-16.1542549
310.0000000	2.0983999	-252.7544556

310.0000000	2.1471999	17.3013420
310.0000000	2.1960001	-25.7389755
310.0000000	2.2448001	-27.0994606
310.0000000	2.2935998	-31.9137039
310.0000000	2.3423998	-19.9671230
310.0000000	2.3912001	76.0102844
310.0000000	2.4400001	-22.6609249
310.0000000	2.4888000	-23.4755154
310.0000000	2.5375998	-11.0517464
310.0000000	2.5863998	-8.9838018
310.0000000	2.6352000	-6.8431025
310.0000000	2.6840000	-14.5586548
310.0000000	2.7328000	152.1926270
310.0000000	2.7815998	179.0464478
310.0000000	2.8304000	-23.8877277
310.0000000	2.8792000	-20.3857193
310.0000000	2.9280000	-22.6125278
310.0000000	2.9768000	-16.1900139
310.0000000	3.0256000	-20.2877769
310.0000000	3.0743999	-30.3323135
310.0000000	3.1231999	-24.5025826
310.0000000	3.1719999	-30.1997261
310.0000000	3.2208002	-521.2598877
310.0000000	3.2695999	-1021.2425537
310.0000000	3.3183999	-20.4755402
310.0000000	3.3671999	-3500.4372559
310.0000000	3.4159999	1256.5524902
310.0000000	3.4648001	-6.7352352
310.0000000	3.5135999	-27.2494774
310.0000000	3.5623999	-331.1496277
310.0000000	3.6111999	-2083.8544922
310.0000000	3.6600001	-471.3253174
310.0000000	3.7088001	-622.5590820
310.0000000	3.7575998	-975.0537720
310.0000000	3.8063998	-1769.7950439
310.0000000	3.8552001	-2569.7443848
310.0000000	3.9040000	-2519.4494629
310.0000000	3.9528000	1152.9163818
310.0000000	4.0015998	-251.2585297
310.0000000	4.0503998	706.0961914
310.0000000	4.0991998	90.4102936
310.0000000	4.1479998	-671.3858643
310.0000000	4.1967998	3.1114445
310.0000000	4.2456002	-254.5123749
310.0000000	4.2943997	-500.2457886
310.0000000	4.3431997	-13.1982365
310.0000000	4.3920002	-254.1313324
310.0000000	4.4407997	-22.5689697
310.0000000	4.4896002	-15.4203548
310.0000000	4.5383997	-671.0001831
310.0000000	4.5871997	-12.7156487
310.0000000	4.6360002	-21.7151527
310.0000000	4.6847997	-28.5216980
310.0000000	4.7336001	-19.6064606
310.0000000	4.7824001	-16.4846630
310.0000000	4.8311996	-1506.7001953
310.0000000	4.8800001	233.7053223
310.0000000	4.9287996	-205.5445251

310.0000000	4.9776001	1083.4506836
310.0000000	5.0264001	88.4575272
310.0000000	5.0751996	-307.4414368
310.0000000	5.1240001	-12.2523212
310.0000000	5.1727996	565.4815674
310.0000000	5.2216001	32.6195107
310.0000000	5.2704000	-160.6714935
310.0000000	5.3191996	-39.4829712
310.0000000	5.3680000	-3581.6237793
310.0000000	5.4168000	534.1903687
310.0000000	5.4656000	-203.1328278
310.0000000	5.5144000	38.7722168
310.0000000	5.5631995	494.8493042
310.0000000	5.6120000	398.4354858
310.0000000	5.6608000	-37.3554459
310.0000000	5.7096000	63.5522156
310.0000000	5.7584000	134.9565125
310.0000000	5.8071995	-11.4626122
310.0000000	5.8559999	30.4040661
310.0000000	5.9047999	-16.6187477
310.0000000	5.9535999	-40.0908813
310.0000000	6.0023999	-1593.2088623
310.0000000	6.0511999	-14.5541286
310.0000000	6.0999999	-19.4647770
310.0000000	6.1487999	-13.2424583
310.0000000	6.1975999	-23.3381119
310.0000000	6.2463999	-1815.0682373
310.0000000	6.2951999	-19.1247768
310.0000000	6.3439999	-346.9995422
310.0000000	6.3927999	-15.0493536
310.0000000	6.4416003	-12.3465233
310.0000000	6.4903998	-11.2181807
310.0000000	6.5391998	-11.7602730
310.0000000	6.5879998	431.7480164
310.0000000	6.6367998	-12.0469637
310.0000000	6.6856003	-13.8033619
310.0000000	6.7343998	-13.4409781
310.0000000	6.7831998	-14.4009171
310.0000000	6.8319998	59.7460938
310.0000000	6.8807998	-14.0589132
310.0000000	6.9296002	-501.7705994
310.0000000	6.9783998	-4.2025328
310.0000000	7.0271997	-14.2081842
310.0000000	7.0760002	14.1020060
310.0000000	7.1247997	-2404.9094238
310.0000000	7.1736002	-1928.4648438
310.0000000	7.2223997	-1096.6817627
310.0000000	7.2711997	-235.1564026
310.0000000	7.3200002	-19.7161179
310.0000000	7.3687997	213.9649658
310.0000000	7.4176002	444.5592041
310.0000000	7.4663997	349.5239563
310.0000000	7.5151997	-1073.3905029
310.0000000	7.5640001	-1185.8005371
310.0000000	7.6127996	-2796.9335938
310.0000000	7.6616001	-897.1827393
310.0000000	7.7104001	-460.1652832
310.0000000	7.7591996	50.2903442

310.0000000	7.8080001	461.9822083
310.0000000	7.8567996	608.1219482
310.0000000	7.9056001	-1256.2803955
310.0000000	7.9544001	2000.2735596
310.0000000	8.0031996	2580.9343262
310.0000000	8.0520000	2576.0803223
310.0000000	8.1007996	2725.9665527
310.0000000	8.1496000	2907.0747070
310.0000000	8.1983995	1808.6911621
310.0000000	8.2472000	-4137.4165039
310.0000000	8.2959995	-1466.4362793
310.0000000	8.3448000	-1168.2244873
310.0000000	8.3935995	-1070.6240234
310.0000000	8.4423990	-460.9258423
310.0000000	8.4912004	-1076.5338135
310.0000000	8.5400000	-2904.6032715
310.0000000	8.5887995	-1407.5761719
310.0000000	8.6375999	275.5029602
310.0000000	8.6863995	2485.3928223
310.0000000	8.7351999	-923.2927246
310.0000000	8.7840004	1056.1265869
310.0000000	8.8327999	58.4099884
310.0000000	8.8815994	-11.4841375
310.0000000	8.9303989	-24.2078190
310.0000000	8.9792004	-11.5732889
310.0000000	9.0279999	-5.7118983
310.0000000	9.0767994	7.4437466
310.0000000	9.1255999	-17.3633556
310.0000000	9.1743994	-10.0451145
310.0000000	9.2231998	38.1264038
310.0000000	9.2720003	-8.3537226
310.0000000	9.3207998	-9.7598467
310.0000000	9.3695993	-10.7477045
310.0000000	9.4183998	-11.7627201
310.0000000	9.4672003	-11.1584072
310.0000000	9.5159998	-14.2274714
310.0000000	9.5648003	-0.1536179
310.0000000	9.6135998	-13.4521484
310.0000000	9.6623993	-10.2018051
310.0000000	9.7111998	-10.1384239
310.0000000	9.7600002	-10.8546152
310.0000000	9.8087997	-10.9942131
310.0000000	9.8575993	3.5038919
310.0000000	9.9063997	-7.9041786
310.0000000	9.9552002	-10.9062958
310.0000000	10.0039997	-10.0571432
320.0000000	0.3416000	-37.2117538
320.0000000	0.3904000	-39.8543320
320.0000000	0.4392000	-43.5557899
320.0000000	0.4880000	-39.4232292
320.0000000	0.5368000	-39.4965744
320.0000000	0.5856000	-43.5814056
320.0000000	0.6344000	-30.9861717
320.0000000	0.6832000	-50.2298393
320.0000000	0.7320000	-36.2252235
320.0000000	0.7808000	-30.8580284
320.0000000	0.8296000	-27.7978344
320.0000000	0.8784000	-26.3695831

320.0000000	0.9272000	-26.3623695
320.0000000	0.9760000	-27.7511520
320.0000000	1.0247999	-29.5249176
320.0000000	1.0735999	-31.3236866
320.0000000	1.1224000	-31.9654408
320.0000000	1.1711999	-32.0083008
320.0000000	1.2200000	-31.9291439
320.0000000	1.2687999	-31.2017365
320.0000000	1.3176000	-30.2831535
320.0000000	1.3664000	-30.0284348
320.0000000	1.4152000	-30.1480064
320.0000000	1.4640000	-29.7349834
320.0000000	1.5128000	-46.1776581
320.0000000	1.5616000	-36.5591583
320.0000000	1.6104001	-1735.2130127
320.0000000	1.6592000	-93.8334351
320.0000000	1.7079999	-27.9487400
320.0000000	1.7567999	-27.8754120
320.0000000	1.8055999	-25.3207150
320.0000000	1.8544000	-24.6326084
320.0000000	1.9031999	-25.5632839
320.0000000	1.9520000	-26.5358734
320.0000000	2.0007999	-28.9801998
320.0000000	2.0495999	-16.3408699
320.0000000	2.0983999	-251.2786865
320.0000000	2.1471999	17.0340767
320.0000000	2.1960001	-25.7348843
320.0000000	2.2448001	-27.0815506
320.0000000	2.2935998	-31.8540802
320.0000000	2.3423998	-19.9815273
320.0000000	2.3912001	75.3887405
320.0000000	2.4400001	-22.6634102
320.0000000	2.4888000	-23.4752979
320.0000000	2.5375998	-11.0688553
320.0000000	2.5863998	-9.0035610
320.0000000	2.6352000	-6.8658466
320.0000000	2.6840000	-14.5705967
320.0000000	2.7328000	151.9266205
320.0000000	2.7815998	178.7400208
320.0000000	2.8304000	-23.8864231
320.0000000	2.8792000	-20.3889828
320.0000000	2.9280000	-22.6119595
320.0000000	2.9768000	-16.1983833
320.0000000	3.0256000	-20.2903614
320.0000000	3.0743999	-30.3209267
320.0000000	3.1231999	-24.4991264
320.0000000	3.1719999	-30.1914330
320.0000000	3.2208002	-521.0221558
320.0000000	3.2695999	-1020.7675781
320.0000000	3.3183999	-20.4757385
320.0000000	3.3671999	-3498.9755859
320.0000000	3.4159999	1256.0172119
320.0000000	3.4648001	-6.7409439
320.0000000	3.5135999	-27.2464867
320.0000000	3.5623999	-331.0200500
320.0000000	3.6111999	-2082.9946289
320.0000000	3.6600001	-471.1372070
320.0000000	3.7088001	-622.3076782

320.0000000	3.7575998	-974.6552734
320.0000000	3.8063998	-1769.0651855
320.0000000	3.8552001	-2568.6809082
320.0000000	3.9040000	-2518.4069824
320.0000000	3.9528000	1152.4281006
320.0000000	4.0015998	-251.1614227
320.0000000	4.0503998	705.7936401
320.0000000	4.0991998	90.3646088
320.0000000	4.1479998	-671.1136475
320.0000000	4.1967998	3.1021881
320.0000000	4.2456002	-254.4140625
320.0000000	4.2943997	-500.0452271
320.0000000	4.3431997	-13.2004175
320.0000000	4.3920002	-254.0332184
320.0000000	4.4407997	-22.5671959
320.0000000	4.4896002	-15.4212370
320.0000000	4.5383997	-670.7391357
320.0000000	4.5871997	-12.7176647
320.0000000	4.6360002	-21.7136097
320.0000000	4.6847997	-28.5177727
320.0000000	4.7336001	-19.6057377
320.0000000	4.7824001	-16.4851170
320.0000000	4.8311996	-1506.1688232
320.0000000	4.8800001	233.6157837
320.0000000	4.9287996	-205.4772644
320.0000000	4.9776001	1083.0582275
320.0000000	5.0264001	88.4200058
320.0000000	5.0751996	-307.3377991
320.0000000	5.1240001	-12.2532425
320.0000000	5.1727996	565.3657227
320.0000000	5.2216001	32.6100693
320.0000000	5.2704000	-160.6433868
320.0000000	5.3191996	-39.4784241
320.0000000	5.3680000	-3580.9313965
320.0000000	5.4168000	534.0835571
320.0000000	5.4656000	-203.0966492
320.0000000	5.5144000	38.7615585
320.0000000	5.5631995	494.7507324
320.0000000	5.6120000	398.3555298
320.0000000	5.6608000	-37.3513565
320.0000000	5.7096000	63.5367813
320.0000000	5.7584000	134.9275818
320.0000000	5.8071995	-11.4634819
320.0000000	5.8559999	30.3952160
320.0000000	5.9047999	-16.6185513
320.0000000	5.9535999	-40.0861397
320.0000000	6.0023999	-1592.9042969
320.0000000	6.0511999	-14.5543394
320.0000000	6.0999999	-19.4639454
320.0000000	6.1487999	-13.2429218
320.0000000	6.1975999	-23.3363457
320.0000000	6.2463999	-1814.7208252
320.0000000	6.2951999	-19.1239624
320.0000000	6.3439999	-346.9354858
320.0000000	6.3927999	-15.0493355
320.0000000	6.4416003	-12.3471098
320.0000000	6.4903998	-11.2188568
320.0000000	6.5391998	-11.7609539

320.0000000	6.5879998	431.0573120
320.0000000	6.6367998	-12.0475130
320.0000000	6.6856003	-13.8035316
320.0000000	6.7343998	-13.4411936
320.0000000	6.7831998	-14.4005013
320.0000000	6.8319998	59.7269821
320.0000000	6.8807998	-14.0587749
320.0000000	6.9296002	-501.0091553
320.0000000	6.9783998	-4.2153559
320.0000000	7.0271997	-14.2071562
320.0000000	7.0760002	14.0598211
320.0000000	7.1247997	-2400.9636230
320.0000000	7.1736002	-1925.3018799
320.0000000	7.2223997	-1094.8885498
320.0000000	7.2711997	-234.7886353
320.0000000	7.3200002	-19.7041283
320.0000000	7.3687997	213.5904236
320.0000000	7.4176002	443.8033752
320.0000000	7.4663997	348.9263000
320.0000000	7.5151997	-1071.6293945
320.0000000	7.5640001	-1183.8531494
320.0000000	7.6127996	-2792.3159180
320.0000000	7.6616001	-895.7113647
320.0000000	7.7104001	-459.4190674
320.0000000	7.7591996	50.1892967
320.0000000	7.8080001	461.1968384
320.0000000	7.8567996	607.0938721
320.0000000	7.9056001	-1254.2075195
320.0000000	7.9544001	1996.9637451
320.0000000	8.0031996	2576.6645508
320.0000000	8.0520000	2571.8183594
320.0000000	8.1007996	2721.4570313
320.0000000	8.1496000	2902.2666016
320.0000000	8.1983995	1805.6986084
320.0000000	8.2472000	-4130.5820313
320.0000000	8.2959995	-1464.0161133
320.0000000	8.3448000	-1166.2952881
320.0000000	8.3935995	-1068.8563232
320.0000000	8.4423990	-460.1678467
320.0000000	8.4912004	-1074.7575684
320.0000000	8.5400000	-2899.8034668
320.0000000	8.5887995	-1405.2546387
320.0000000	8.6375999	275.0416260
320.0000000	8.6863995	2481.2746582
320.0000000	8.7351999	-921.7684326
320.0000000	8.7840004	1054.3697510
320.0000000	8.8327999	58.2983780
320.0000000	8.8815994	-11.4843111
320.0000000	8.9303989	-24.2040997
320.0000000	8.9792004	-11.5733709
320.0000000	9.0279999	-5.7136669
320.0000000	9.0767994	7.4380283
320.0000000	9.1255999	-17.3616409
320.0000000	9.1743994	-10.0455170
320.0000000	9.2231998	38.1106720
320.0000000	9.2720003	-8.3546591
320.0000000	9.3207998	-9.7603865
320.0000000	9.3695993	-10.7479305

320.0000000	9.4183998	-11.7625952
320.0000000	9.4672003	-11.1584482
320.0000000	9.5159998	-14.2265797
320.0000000	9.5648003	-0.1569901
320.0000000	9.6135998	-13.4513588
320.0000000	9.6623993	-10.2020807
320.0000000	9.7111998	-10.1386957
320.0000000	9.7600002	-10.8546562
320.0000000	9.8087997	-10.9942217
320.0000000	9.8575993	3.4994698
320.0000000	9.9063997	-7.9050927
320.0000000	9.9552002	-10.9062996
320.0000000	10.0039997	-10.0573912
330.0000000	0.3416000	-36.3610802
330.0000000	0.3904000	-39.9032364
330.0000000	0.4392000	-43.3902664
330.0000000	0.4880000	-39.3957825
330.0000000	0.5368000	-39.4793968
330.0000000	0.5856000	-43.1932526
330.0000000	0.6344000	-31.6562309
330.0000000	0.6832000	-46.9723244
330.0000000	0.7320000	-36.1907043
330.0000000	0.7808000	-31.7765999
330.0000000	0.8296000	-27.9257088
330.0000000	0.8784000	-25.8237724
330.0000000	0.9272000	-25.6455688
330.0000000	0.9760000	-27.6817570
330.0000000	1.0247999	-30.8133526
330.0000000	1.0735999	-32.8008995
330.0000000	1.1224000	-31.9470139
330.0000000	1.1711999	-31.9935188
330.0000000	1.2200000	-31.9290028
330.0000000	1.2687999	-31.2109699
330.0000000	1.3176000	-30.3092632
330.0000000	1.3664000	-30.0361843
330.0000000	1.4152000	-30.1482105
330.0000000	1.4640000	-29.7332726
330.0000000	1.5128000	-46.0804825
330.0000000	1.5616000	-36.5173149
330.0000000	1.6104001	-1732.6701660
330.0000000	1.6592000	-93.5395050
330.0000000	1.7079999	-27.9593353
330.0000000	1.7567999	-27.8169365
330.0000000	1.8055999	-26.0747547
330.0000000	1.8544000	-24.8873405
330.0000000	1.9031999	-25.6955338
330.0000000	1.9520000	-26.5363293
330.0000000	2.0007999	-28.9176178
330.0000000	2.0495999	-16.5274868
330.0000000	2.0983999	-249.8028870
330.0000000	2.1471999	16.7668076
330.0000000	2.1960001	-25.7307930
330.0000000	2.2448001	-27.0636387
330.0000000	2.2935998	-31.7944565
330.0000000	2.3423998	-19.9959335
330.0000000	2.3912001	74.7671967
330.0000000	2.4400001	-22.6658974
330.0000000	2.4888000	-23.4750805

330.0000000	2.5375998	-11.0859632
330.0000000	2.5863998	-9.0233192
330.0000000	2.6352000	-6.8885899
330.0000000	2.6840000	-14.5825386
330.0000000	2.7328000	151.6605988
330.0000000	2.7815998	178.4335938
330.0000000	2.8304000	-23.8851204
330.0000000	2.8792000	-20.3922462
330.0000000	2.9280000	-22.6113911
330.0000000	2.9768000	-16.2067528
330.0000000	3.0256000	-20.2929459
330.0000000	3.0743999	-30.3095398
330.0000000	3.1231999	-24.4956703
330.0000000	3.1719999	-30.1831398
330.0000000	3.2208002	-520.7844238
330.0000000	3.2695999	-1020.2926025
330.0000000	3.3183999	-20.4759369
330.0000000	3.3671999	-3497.5141602
330.0000000	3.4159999	1255.4819336
330.0000000	3.4648001	-6.7466516
330.0000000	3.5135999	-27.2434959
330.0000000	3.5623999	-330.8904724
330.0000000	3.6111999	-2082.1347656
330.0000000	3.6600001	-470.9490967
330.0000000	3.7088001	-622.0563354
330.0000000	3.7575998	-974.2567139
330.0000000	3.8063998	-1768.3353271
330.0000000	3.8552001	-2567.6176758
330.0000000	3.9040000	-2517.3645020
330.0000000	3.9528000	1151.9398193
330.0000000	4.0015998	-251.0643158
330.0000000	4.0503998	705.4910278
330.0000000	4.0991998	90.3189316
330.0000000	4.1479998	-670.8414307
330.0000000	4.1967998	3.0929317
330.0000000	4.2456002	-254.3157654
330.0000000	4.2943997	-499.8446655
330.0000000	4.3431997	-13.2025976
330.0000000	4.3920002	-253.9351044
330.0000000	4.4407997	-22.5654182
330.0000000	4.4896002	-15.4221201
330.0000000	4.5383997	-670.4780884
330.0000000	4.5871997	-12.7196808
330.0000000	4.6360002	-21.7120667
330.0000000	4.6847997	-28.5138474
330.0000000	4.7336001	-19.6050167
330.0000000	4.7824001	-16.4855709
330.0000000	4.8311996	-1505.6375732
330.0000000	4.8800001	233.5262299
330.0000000	4.9287996	-205.4100037
330.0000000	4.9776001	1082.6656494
330.0000000	5.0264001	88.3824844
330.0000000	5.0751996	-307.2341919
330.0000000	5.1240001	-12.2541637
330.0000000	5.1727996	565.2498779
330.0000000	5.2216001	32.6006241
330.0000000	5.2704000	-160.6152802
330.0000000	5.3191996	-39.4738770

330.0000000	5.3680000	-3580.2390137
330.0000000	5.4168000	533.9767456
330.0000000	5.4656000	-203.0604706
330.0000000	5.5144000	38.7509003
330.0000000	5.5631995	494.6521606
330.0000000	5.6120000	398.2755432
330.0000000	5.6608000	-37.3472672
330.0000000	5.7096000	63.5213547
330.0000000	5.7584000	134.8986511
330.0000000	5.8071995	-11.4643526
330.0000000	5.8559999	30.3863659
330.0000000	5.9047999	-16.6183529
330.0000000	5.9535999	-40.0814018
330.0000000	6.0023999	-1592.5999756
330.0000000	6.0511999	-14.5545511
330.0000000	6.0999999	-19.4631119
330.0000000	6.1487999	-13.2433844
330.0000000	6.1975999	-23.3345795
330.0000000	6.2463999	-1814.3734131
330.0000000	6.2951999	-19.1231480
330.0000000	6.3439999	-346.8714294
330.0000000	6.3927999	-15.0493174
330.0000000	6.4416003	-12.3476973
330.0000000	6.4903998	-11.2195330
330.0000000	6.5391998	-11.7616348
330.0000000	6.5879998	430.3666382
330.0000000	6.6367998	-12.0480623
330.0000000	6.6856003	-13.8037024
330.0000000	6.7343998	-13.4414101
330.0000000	6.7831998	-14.4000854
330.0000000	6.8319998	59.7078629
330.0000000	6.8807998	-14.0586357
330.0000000	6.9296002	-500.2477112
330.0000000	6.9783998	-4.2281799
330.0000000	7.0271997	-14.2061281
330.0000000	7.0760002	14.0176363
330.0000000	7.1247997	-2397.0178223
330.0000000	7.1736002	-1922.1390381
330.0000000	7.2223997	-1093.0953369
330.0000000	7.2711997	-234.4208527
330.0000000	7.3200002	-19.6921387
330.0000000	7.3687997	213.2158966
330.0000000	7.4176002	443.0475464
330.0000000	7.4663997	348.3286438
330.0000000	7.5151997	-1069.8684082
330.0000000	7.5640001	-1181.9057617
330.0000000	7.6127996	-2787.6982422
330.0000000	7.6616001	-894.2399902
330.0000000	7.7104001	-458.6727905
330.0000000	7.7591996	50.0882416
330.0000000	7.8080001	460.4114685
330.0000000	7.8567996	606.0657959
330.0000000	7.9056001	-1252.1345215
330.0000000	7.9544001	1993.6538086
330.0000000	8.0031996	2572.3950195
330.0000000	8.0520000	2567.5563965
330.0000000	8.1007996	2716.9475098
330.0000000	8.1496000	2897.4584961

330.0000000	8.1983995	1802.7060547
330.0000000	8.2472000	-4123.7470703
330.0000000	8.2959995	-1461.5959473
330.0000000	8.3448000	-1164.3660889
330.0000000	8.3935995	-1067.0886230
330.0000000	8.4423990	-459.4098206
330.0000000	8.4912004	-1072.9814453
330.0000000	8.5400000	-2895.0036621
330.0000000	8.5887995	-1402.9331055
330.0000000	8.6375999	274.5802917
330.0000000	8.6863995	2477.1562500
330.0000000	8.7351999	-920.2442017
330.0000000	8.7840004	1052.6130371
330.0000000	8.8327999	58.1867676
330.0000000	8.8815994	-11.4844856
330.0000000	8.9303989	-24.2003784
330.0000000	8.9792004	-11.5734529
330.0000000	9.0279999	-5.7154350
330.0000000	9.0767994	7.4323120
330.0000000	9.1255999	-17.3599281
330.0000000	9.1743994	-10.0459185
330.0000000	9.2231998	38.0949402
330.0000000	9.2720003	-8.3555956
330.0000000	9.3207998	-9.7609253
330.0000000	9.3695993	-10.7481575
330.0000000	9.4183998	-11.7624712
330.0000000	9.4672003	-11.1584892
330.0000000	9.5159998	-14.2256889
330.0000000	9.5648003	-0.1603632
330.0000000	9.6135998	-13.4505672
330.0000000	9.6623993	-10.2023554
330.0000000	9.7111998	-10.1389675
330.0000000	9.7600002	-10.8546982
330.0000000	9.8087997	-10.9942293
330.0000000	9.8575993	3.4950476
330.0000000	9.9063997	-7.9060068
330.0000000	9.9552002	-10.9063044
330.0000000	10.0039997	-10.0576382
340.0000000	0.3416000	-36.6139183
340.0000000	0.3904000	-39.9192810
340.0000000	0.4392000	-43.2247467
340.0000000	0.4880000	-39.3683357
340.0000000	0.5368000	-39.4622192
340.0000000	0.5856000	-42.8050957
340.0000000	0.6344000	-32.3262901
340.0000000	0.6832000	-43.7148094
340.0000000	0.7320000	-36.3132477
340.0000000	0.7808000	-32.8421211
340.0000000	0.8296000	-29.2692909
340.0000000	0.8784000	-26.8062649
340.0000000	0.9272000	-26.4255409
340.0000000	0.9760000	-28.5799694
340.0000000	1.0247999	-31.8095112
340.0000000	1.0735999	-32.7387848
340.0000000	1.1224000	-31.9285851
340.0000000	1.1711999	-31.9787369
340.0000000	1.2200000	-31.9288597
340.0000000	1.2687999	-31.2202034

340.0000000	1.3176000	-30.3353710
340.0000000	1.3664000	-30.0439339
340.0000000	1.4152000	-30.1484146
340.0000000	1.4640000	-29.7315636
340.0000000	1.5128000	-45.9833069
340.0000000	1.5616000	-36.4754753
340.0000000	1.6104001	-1730.1274414
340.0000000	1.6592000	-93.2455673
340.0000000	1.7079999	-27.9699306
340.0000000	1.7567999	-27.7584610
340.0000000	1.8055999	-25.3014297
340.0000000	1.8544000	-24.4590492
340.0000000	1.9031999	-25.9571571
340.0000000	1.9520000	-26.5367832
340.0000000	2.0007999	-28.8550358
340.0000000	2.0495999	-16.7141037
340.0000000	2.0983999	-248.3271179
340.0000000	2.1471999	16.4995384
340.0000000	2.1960001	-25.7267036
340.0000000	2.2448001	-27.0457268
340.0000000	2.2935998	-31.7348328
340.0000000	2.3423998	-20.0103378
340.0000000	2.3912001	74.1456528
340.0000000	2.4400001	-22.6683846
340.0000000	2.4888000	-23.4748631
340.0000000	2.5375998	-11.1030703
340.0000000	2.5863998	-9.0430784
340.0000000	2.6352000	-6.9113340
340.0000000	2.6840000	-14.5944805
340.0000000	2.7328000	151.3945770
340.0000000	2.7815998	178.1271973
340.0000000	2.8304000	-23.8838158
340.0000000	2.8792000	-20.3955097
340.0000000	2.9280000	-22.6108208
340.0000000	2.9768000	-16.2151241
340.0000000	3.0256000	-20.2955303
340.0000000	3.0743999	-30.2981529
340.0000000	3.1231999	-24.4922161
340.0000000	3.1719999	-30.1748466
340.0000000	3.2208002	-520.5466919
340.0000000	3.2695999	-1019.8176270
340.0000000	3.3183999	-20.4761353
340.0000000	3.3671999	-3496.0524902
340.0000000	3.4159999	1254.9466553
340.0000000	3.4648001	-6.7523584
340.0000000	3.5135999	-27.2405052
340.0000000	3.5623999	-330.7608643
340.0000000	3.6111999	-2081.2751465
340.0000000	3.6600001	-470.7609253
340.0000000	3.7088001	-621.8049316
340.0000000	3.7575998	-973.8581543
340.0000000	3.8063998	-1767.6055908
340.0000000	3.8552001	-2566.5541992
340.0000000	3.9040000	-2516.3220215
340.0000000	3.9528000	1151.4515381
340.0000000	4.0015998	-250.9672089
340.0000000	4.0503998	705.1884766
340.0000000	4.0991998	90.2732468

340.0000000	4.1479998	-670.5692139
340.0000000	4.1967998	3.0836754
340.0000000	4.2456002	-254.2174683
340.0000000	4.2943997	-499.6440735
340.0000000	4.3431997	-13.2047787
340.0000000	4.3920002	-253.8369904
340.0000000	4.4407997	-22.5636444
340.0000000	4.4896002	-15.4230022
340.0000000	4.5383997	-670.2169800
340.0000000	4.5871997	-12.7216969
340.0000000	4.6360002	-21.7105255
340.0000000	4.6847997	-28.5099220
340.0000000	4.7336001	-19.6042938
340.0000000	4.7824001	-16.4860249
340.0000000	4.8311996	-1505.1063232
340.0000000	4.8800001	233.4366760
340.0000000	4.9287996	-205.3427429
340.0000000	4.9776001	1082.2731934
340.0000000	5.0264001	88.3449631
340.0000000	5.0751996	-307.1305542
340.0000000	5.1240001	-12.2550850
340.0000000	5.1727996	565.1339722
340.0000000	5.2216001	32.5911789
340.0000000	5.2704000	-160.5871735
340.0000000	5.3191996	-39.4693260
340.0000000	5.3680000	-3579.5466309
340.0000000	5.4168000	533.8699951
340.0000000	5.4656000	-203.0242767
340.0000000	5.5144000	38.7402420
340.0000000	5.5631995	494.5535889
340.0000000	5.6120000	398.1955872
340.0000000	5.6608000	-37.3431778
340.0000000	5.7096000	63.5059204
340.0000000	5.7584000	134.8697052
340.0000000	5.8071995	-11.4652233
340.0000000	5.8559999	30.3775120
340.0000000	5.9047999	-16.6181545
340.0000000	5.9535999	-40.0766640
340.0000000	6.0023999	-1592.2954102
340.0000000	6.0511999	-14.5547628
340.0000000	6.0999999	-19.4622803
340.0000000	6.1487999	-13.2438469
340.0000000	6.1975999	-23.3328152
340.0000000	6.2463999	-1814.0261230
340.0000000	6.2951999	-19.1223335
340.0000000	6.3439999	-346.8073730
340.0000000	6.3927999	-15.0492983
340.0000000	6.4416003	-12.3482838
340.0000000	6.4903998	-11.2202082
340.0000000	6.5391998	-11.7623167
340.0000000	6.5879998	429.6759338
340.0000000	6.6367998	-12.0486116
340.0000000	6.6856003	-13.8038721
340.0000000	6.7343998	-13.4416256
340.0000000	6.7831998	-14.3996696
340.0000000	6.8319998	59.6887436
340.0000000	6.8807998	-14.0584974
340.0000000	6.9296002	-499.4862671

340.0000000	6.9783998	-4.2410040
340.0000000	7.0271997	-14.2051001
340.0000000	7.0760002	13.9754515
340.0000000	7.1247997	-2393.0720215
340.0000000	7.1736002	-1918.9761963
340.0000000	7.2223997	-1091.3022461
340.0000000	7.2711997	-234.0530701
340.0000000	7.3200002	-19.6801510
340.0000000	7.3687997	212.8413696
340.0000000	7.4176002	442.2917175
340.0000000	7.4663997	347.7309875
340.0000000	7.5151997	-1068.1072998
340.0000000	7.5640001	-1179.9583740
340.0000000	7.6127996	-2783.0808105
340.0000000	7.6616001	-892.7687378
340.0000000	7.7104001	-457.9265137
340.0000000	7.7591996	49.9871941
340.0000000	7.8080001	459.6261292
340.0000000	7.8567996	605.0377197
340.0000000	7.9056001	-1250.0616455
340.0000000	7.9544001	1990.3438721
340.0000000	8.0031996	2568.1254883
340.0000000	8.0520000	2563.2944336
340.0000000	8.1007996	2712.4379883
340.0000000	8.1496000	2892.6506348
340.0000000	8.1983995	1799.7136230
340.0000000	8.2472000	-4116.9121094
340.0000000	8.2959995	-1459.1757813
340.0000000	8.3448000	-1162.4370117
340.0000000	8.3935995	-1065.3210449
340.0000000	8.4423990	-458.6518250
340.0000000	8.4912004	-1071.2052002
340.0000000	8.5400000	-2890.2038574
340.0000000	8.5887995	-1400.6115723
340.0000000	8.6375999	274.1189880
340.0000000	8.6863995	2473.0378418
340.0000000	8.7351999	-918.7199097
340.0000000	8.7840004	1050.8563232
340.0000000	8.8327999	58.0751648
340.0000000	8.8815994	-11.4846601
340.0000000	8.9303989	-24.1966591
340.0000000	8.9792004	-11.5735359
340.0000000	9.0279999	-5.7172036
340.0000000	9.0767994	7.4265938
340.0000000	9.1255999	-17.3582134
340.0000000	9.1743994	-10.0463209
340.0000000	9.2231998	38.0792084
340.0000000	9.2720003	-8.3565311
340.0000000	9.3207998	-9.7614651
340.0000000	9.3695993	-10.7483835
340.0000000	9.4183998	-11.7623463
340.0000000	9.4672003	-11.1585302
340.0000000	9.5159998	-14.2247982
340.0000000	9.5648003	-0.1637363
340.0000000	9.6135998	-13.4497766
340.0000000	9.6623993	-10.2026310
340.0000000	9.7111998	-10.1392384
340.0000000	9.7600002	-10.8547401

340.0000000	9.8087997	-10.9942369
340.0000000	9.8575993	3.4906254
340.0000000	9.9063997	-7.9069204
340.0000000	9.9552002	-10.9063082
340.0000000	10.0039997	-10.0578861
350.0000000	0.3416000	-37.3584442
350.0000000	0.3904000	-39.9353256
350.0000000	0.4392000	-43.0592232
350.0000000	0.4880000	-39.3408890
350.0000000	0.5368000	-39.4450417
350.0000000	0.5856000	-42.4169426
350.0000000	0.6344000	-32.9963493
350.0000000	0.6832000	-40.4572945
350.0000000	0.7320000	-35.9245110
350.0000000	0.7808000	-32.8580818
350.0000000	0.8296000	-29.3552055
350.0000000	0.8784000	-26.9572086
350.0000000	0.9272000	-27.2929001
350.0000000	0.9760000	-29.4586735
350.0000000	1.0247999	-31.4865837
350.0000000	1.0735999	-32.6766663
350.0000000	1.1224000	-31.9101582
350.0000000	1.1711999	-31.9639568
350.0000000	1.2200000	-31.9287186
350.0000000	1.2687999	-31.2294369
350.0000000	1.3176000	-30.3614807
350.0000000	1.3664000	-30.0516834
350.0000000	1.4152000	-30.1486187
350.0000000	1.4640000	-29.7298546
350.0000000	1.5128000	-45.8861275
350.0000000	1.5616000	-36.4336319
350.0000000	1.6104001	-1727.5845947
350.0000000	1.6592000	-92.9516373
350.0000000	1.7079999	-27.9805241
350.0000000	1.7567999	-27.6999855
350.0000000	1.8055999	-24.6663685
350.0000000	1.8544000	-24.4090366
350.0000000	1.9031999	-26.4030972
350.0000000	1.9520000	-26.5372391
350.0000000	2.0007999	-28.7924538
350.0000000	2.0495999	-16.9007187
350.0000000	2.0983999	-246.8513489
350.0000000	2.1471999	16.2322731
350.0000000	2.1960001	-25.7226124
350.0000000	2.2448001	-27.0278168
350.0000000	2.2935998	-31.6752090
350.0000000	2.3423998	-20.0247421
350.0000000	2.3912001	73.5241013
350.0000000	2.4400001	-22.6708698
350.0000000	2.4888000	-23.4746456
350.0000000	2.5375998	-11.1201782
350.0000000	2.5863998	-9.0628376
350.0000000	2.6352000	-6.9340782
350.0000000	2.6840000	-14.6064224
350.0000000	2.7328000	151.1285706
350.0000000	2.7815998	177.8207703
350.0000000	2.8304000	-23.8825111
350.0000000	2.8792000	-20.3987732

350.0000000	2.9280000	-22.6102524
350.0000000	2.9768000	-16.2234955
350.0000000	3.0256000	-20.2981148
350.0000000	3.0743999	-30.2867661
350.0000000	3.1231999	-24.4887600
350.0000000	3.1719999	-30.1665516
350.0000000	3.2208002	-520.3089600
350.0000000	3.2695999	-1019.3426514
350.0000000	3.3183999	-20.4763336
350.0000000	3.3671999	-3494.5908203
350.0000000	3.4159999	1254.4113770
350.0000000	3.4648001	-6.7580671
350.0000000	3.5135999	-27.2375145
350.0000000	3.5623999	-330.6312866
350.0000000	3.6111999	-2080.4152832
350.0000000	3.6600001	-470.5728149
350.0000000	3.7088001	-621.5535278
350.0000000	3.7575998	-973.4596558
350.0000000	3.8063998	-1766.8757324
350.0000000	3.8552001	-2565.4907227
350.0000000	3.9040000	-2515.2795410
350.0000000	3.9528000	1150.9632568
350.0000000	4.0015998	-250.8700867
350.0000000	4.0503998	704.8859253
350.0000000	4.0991998	90.2275620
350.0000000	4.1479998	-670.2969971
350.0000000	4.1967998	3.0744190
350.0000000	4.2456002	-254.1191559
350.0000000	4.2943997	-499.4435120
350.0000000	4.3431997	-13.2069588
350.0000000	4.3920002	-253.7388763
350.0000000	4.4407997	-22.5618687
350.0000000	4.4896002	-15.4238853
350.0000000	4.5383997	-669.9559326
350.0000000	4.5871997	-12.7237129
350.0000000	4.6360002	-21.7089825
350.0000000	4.6847997	-28.5059967
350.0000000	4.7336001	-19.6035709
350.0000000	4.7824001	-16.4864807
350.0000000	4.8311996	-1504.5750732
350.0000000	4.8800001	233.3471222
350.0000000	4.9287996	-205.2754822
350.0000000	4.9776001	1081.8807373
350.0000000	5.0264001	88.3074417
350.0000000	5.0751996	-307.0269165
350.0000000	5.1240001	-12.2560062
350.0000000	5.1727996	565.0181274
350.0000000	5.2216001	32.5817375
350.0000000	5.2704000	-160.5590668
350.0000000	5.3191996	-39.4647751
350.0000000	5.3680000	-3578.8542480
350.0000000	5.4168000	533.7632446
350.0000000	5.4656000	-202.9880981
350.0000000	5.5144000	38.7295914
350.0000000	5.5631995	494.4550171
350.0000000	5.6120000	398.1156006
350.0000000	5.6608000	-37.3390884
350.0000000	5.7096000	63.4904938

350.0000000	5.7584000	134.8407745
350.0000000	5.8071995	-11.4660940
350.0000000	5.8559999	30.3686619
350.0000000	5.9047999	-16.6179581
350.0000000	5.9535999	-40.0719261
350.0000000	6.0023999	-1591.9910889
350.0000000	6.0511999	-14.5549736
350.0000000	6.0999999	-19.4614487
350.0000000	6.1487999	-13.2443094
350.0000000	6.1975999	-23.3310509
350.0000000	6.2463999	-1813.6787109
350.0000000	6.2951999	-19.1215210
350.0000000	6.3439999	-346.7433472
350.0000000	6.3927999	-15.0492802
350.0000000	6.4416003	-12.3488712
350.0000000	6.4903998	-11.2208843
350.0000000	6.5391998	-11.7629976
350.0000000	6.5879998	428.9852295
350.0000000	6.6367998	-12.0491600
350.0000000	6.6856003	-13.8040428
350.0000000	6.7343998	-13.4418411
350.0000000	6.7831998	-14.3992538
350.0000000	6.8319998	59.6696320
350.0000000	6.8807998	-14.0583591
350.0000000	6.9296002	-498.7247925
350.0000000	6.9783998	-4.2538280
350.0000000	7.0271997	-14.2040720
350.0000000	7.0760002	13.9332647
350.0000000	7.1247997	-2389.1259766
350.0000000	7.1736002	-1915.8132324
350.0000000	7.2223997	-1089.5090332
350.0000000	7.2711997	-233.6852875
350.0000000	7.3200002	-19.6681633
350.0000000	7.3687997	212.4668427
350.0000000	7.4176002	441.5358887
350.0000000	7.4663997	347.1333313
350.0000000	7.5151997	-1066.3463135
350.0000000	7.5640001	-1178.0108643
350.0000000	7.6127996	-2778.4631348
350.0000000	7.6616001	-891.2973633
350.0000000	7.7104001	-457.1802368
350.0000000	7.7591996	49.8861427
350.0000000	7.8080001	458.8407593
350.0000000	7.8567996	604.0096436
350.0000000	7.9056001	-1247.9887695
350.0000000	7.9544001	1987.0339355
350.0000000	8.0031996	2563.8557129
350.0000000	8.0520000	2559.0324707
350.0000000	8.1007996	2707.9284668
350.0000000	8.1496000	2887.8425293
350.0000000	8.1983995	1796.7210693
350.0000000	8.2472000	-4110.0776367
350.0000000	8.2959995	-1456.7556152
350.0000000	8.3448000	-1160.5078125
350.0000000	8.3935995	-1063.5533447
350.0000000	8.4423990	-457.8937988
350.0000000	8.4912004	-1069.4289551
350.0000000	8.5400000	-2885.4040527

350.0000000	8.5887995	-1398.2900391
350.0000000	8.6375999	273.6576538
350.0000000	8.6863995	2468.9194336
350.0000000	8.7351999	-917.1956787
350.0000000	8.7840004	1049.0996094
350.0000000	8.8327999	57.9635544
350.0000000	8.8815994	-11.4848347
350.0000000	8.9303989	-24.1929398
350.0000000	8.9792004	-11.5736179
350.0000000	9.0279999	-5.7189713
350.0000000	9.0767994	7.4208775
350.0000000	9.1255999	-17.3564987
350.0000000	9.1743994	-10.0467224
350.0000000	9.2231998	38.0634766
350.0000000	9.2720003	-8.3574677
350.0000000	9.3207998	-9.7620039
350.0000000	9.3695993	-10.7486105
350.0000000	9.4183998	-11.7622213
350.0000000	9.4672003	-11.1585712
350.0000000	9.5159998	-14.2239075
350.0000000	9.5648003	-0.1671085
350.0000000	9.6135998	-13.4489861
350.0000000	9.6623993	-10.2029057
350.0000000	9.7111998	-10.1395102
350.0000000	9.7600002	-10.8547812
350.0000000	9.8087997	-10.9942455
350.0000000	9.8575993	3.4862022
350.0000000	9.9063997	-7.9078341
350.0000000	9.9552002	-10.9063129
350.0000000	10.0039997	-10.0581331
360.0000000	0.3416000	-37.3670654
360.0000000	0.3904000	-39.9513702
360.0000000	0.4392000	-42.8936996
360.0000000	0.4880000	-39.3134460
360.0000000	0.5368000	-39.4278641
360.0000000	0.5856000	-42.0287895
360.0000000	0.6344000	-33.6664085
360.0000000	0.6832000	-37.1997795
360.0000000	0.7320000	-31.3722172
360.0000000	0.7808000	-27.7441082
360.0000000	0.8296000	-26.3514614
360.0000000	0.8784000	-25.8291550
360.0000000	0.9272000	-27.0126953
360.0000000	0.9760000	-29.3207607
360.0000000	1.0247999	-30.9785995
360.0000000	1.0735999	-31.6859512
360.0000000	1.1224000	-31.8917294
360.0000000	1.1711999	-31.9491749
360.0000000	1.2200000	-31.9285755
360.0000000	1.2687999	-31.2386703
360.0000000	1.3176000	-30.3875885
360.0000000	1.3664000	-30.0594330
360.0000000	1.4152000	-30.1488228
360.0000000	1.4640000	-29.7281437
360.0000000	1.5128000	-45.7889481
360.0000000	1.5616000	-36.3917923
360.0000000	1.6104001	-1725.0418701
360.0000000	1.6592000	-92.6577072

360.0000000	1.7079999	-27.9911194
360.0000000	1.7567999	-27.6415100
360.0000000	1.8055999	-26.2147007
360.0000000	1.8544000	-25.2500000
360.0000000	1.9031999	-25.8178329
360.0000000	1.9520000	-26.5376930
360.0000000	2.0007999	-28.7298717
360.0000000	2.0495999	-17.0873356
360.0000000	2.0983999	-245.3755493
360.0000000	2.1471999	15.9650078
360.0000000	2.1960001	-25.7185211
360.0000000	2.2448001	-27.0099049
360.0000000	2.2935998	-31.6155872
360.0000000	2.3423998	-20.0391464
360.0000000	2.3912001	72.9025574
360.0000000	2.4400001	-22.6733570
360.0000000	2.4888000	-23.4744282
360.0000000	2.5375998	-11.1372862
360.0000000	2.5863998	-9.0825968
360.0000000	2.6352000	-6.9568224
360.0000000	2.6840000	-14.6183643
360.0000000	2.7328000	150.8625488
360.0000000	2.7815998	177.5143433
360.0000000	2.8304000	-23.8812065
360.0000000	2.8792000	-20.4020367
360.0000000	2.9280000	-22.6096840
360.0000000	2.9768000	-16.2318649
360.0000000	3.0256000	-20.3006992
360.0000000	3.0743999	-30.2753792
360.0000000	3.1231999	-24.4853039
360.0000000	3.1719999	-30.1582584
360.0000000	3.2208002	-520.0712280
360.0000000	3.2695999	-1018.8676758
360.0000000	3.3183999	-20.4765301
360.0000000	3.3671999	-3493.1291504
360.0000000	3.4159999	1253.8760986
360.0000000	3.4648001	-6.7637758
360.0000000	3.5135999	-27.2345238
360.0000000	3.5623999	-330.5016785
360.0000000	3.6111999	-2079.5556641
360.0000000	3.6600001	-470.3847046
360.0000000	3.7088001	-621.3021851
360.0000000	3.7575998	-973.0610962
360.0000000	3.8063998	-1766.1458740
360.0000000	3.8552001	-2564.4272461
360.0000000	3.9040000	-2514.2370605
360.0000000	3.9528000	1150.4748535
360.0000000	4.0015998	-250.7729797
360.0000000	4.0503998	704.5833740
360.0000000	4.0991998	90.1818771
360.0000000	4.1479998	-670.0247803
360.0000000	4.1967998	3.0651627
360.0000000	4.2456002	-254.0208588
360.0000000	4.2943997	-499.2429504
360.0000000	4.3431997	-13.2091389
360.0000000	4.3920002	-253.6407623
360.0000000	4.4407997	-22.5600929
360.0000000	4.4896002	-15.4247684

360.0000000	4.5383997	-669.6948853
360.0000000	4.5871997	-12.7257290
360.0000000	4.6360002	-21.7074394
360.0000000	4.6847997	-28.5020714
360.0000000	4.7336001	-19.6028500
360.0000000	4.7824001	-16.4869347
360.0000000	4.8311996	-1504.0438232
360.0000000	4.8800001	233.2575684
360.0000000	4.9287996	-205.2082214
360.0000000	4.9776001	1081.4881592
360.0000000	5.0264001	88.2699203
360.0000000	5.0751996	-306.9233093
360.0000000	5.1240001	-12.2569275
360.0000000	5.1727996	564.9022827
360.0000000	5.2216001	32.5722923
360.0000000	5.2704000	-160.5309601
360.0000000	5.3191996	-39.4602280
360.0000000	5.3680000	-3578.1618652
360.0000000	5.4168000	533.6564331
360.0000000	5.4656000	-202.9519196
360.0000000	5.5144000	38.7189331
360.0000000	5.5631995	494.3564758
360.0000000	5.6120000	398.0356140
360.0000000	5.6608000	-37.3350029
360.0000000	5.7096000	63.4750595
360.0000000	5.7584000	134.8118439
360.0000000	5.8071995	-11.4669647
360.0000000	5.8559999	30.3598118
360.0000000	5.9047999	-16.6177597
360.0000000	5.9535999	-40.0671844
360.0000000	6.0023999	-1591.6866455
360.0000000	6.0511999	-14.5551853
360.0000000	6.0999999	-19.4606152
360.0000000	6.1487999	-13.2447720
360.0000000	6.1975999	-23.3292847
360.0000000	6.2463999	-1813.3312988
360.0000000	6.2951999	-19.1207066
360.0000000	6.3439999	-346.6792603
360.0000000	6.3927999	-15.0492620
360.0000000	6.4416003	-12.3494577
360.0000000	6.4903998	-11.2215605
360.0000000	6.5391998	-11.7636786
360.0000000	6.5879998	428.2945251
360.0000000	6.6367998	-12.0497093
360.0000000	6.6856003	-13.8042126
360.0000000	6.7343998	-13.4420567
360.0000000	6.7831998	-14.3988380
360.0000000	6.8319998	59.6505203
360.0000000	6.8807998	-14.0582199
360.0000000	6.9296002	-497.9633484
360.0000000	6.9783998	-4.2666512
360.0000000	7.0271997	-14.2030449
360.0000000	7.0760002	13.8910799
360.0000000	7.1247997	-2385.1801758
360.0000000	7.1736002	-1912.6503906
360.0000000	7.2223997	-1087.7159424
360.0000000	7.2711997	-233.3175049
360.0000000	7.3200002	-19.6561737

360.0000000	7.3687997	212.0923157
360.0000000	7.4176002	440.7800598
360.0000000	7.4663997	346.5357056
360.0000000	7.5151997	-1064.5853271
360.0000000	7.5640001	-1176.0634766
360.0000000	7.6127996	-2773.8454590
360.0000000	7.6616001	-889.8260498
360.0000000	7.7104001	-456.4339600
360.0000000	7.7591996	49.7850952
360.0000000	7.8080001	458.0553894
360.0000000	7.8567996	602.9815674
360.0000000	7.9056001	-1245.9158936
360.0000000	7.9544001	1983.7239990
360.0000000	8.0031996	2559.5861816
360.0000000	8.0520000	2554.7705078
360.0000000	8.1007996	2703.4189453
360.0000000	8.1496000	2883.0346680
360.0000000	8.1983995	1793.7285156
360.0000000	8.2472000	-4103.2426758
360.0000000	8.2959995	-1454.3354492
360.0000000	8.3448000	-1158.5786133
360.0000000	8.3935995	-1061.7856445
360.0000000	8.4423990	-457.1358032
360.0000000	8.4912004	-1067.6527100
360.0000000	8.5400000	-2880.6042480
360.0000000	8.5887995	-1395.9685059
360.0000000	8.6375999	273.1963501
360.0000000	8.6863995	2464.8010254
360.0000000	8.7351999	-915.6714478
360.0000000	8.7840004	1047.3427734
360.0000000	8.8327999	57.8519440
360.0000000	8.8815994	-11.4850082
360.0000000	8.9303989	-24.1892204
360.0000000	8.9792004	-11.5737000
360.0000000	9.0279999	-5.7207398
360.0000000	9.0767994	7.4151592
360.0000000	9.1255999	-17.3547859
360.0000000	9.1743994	-10.0471249
360.0000000	9.2231998	38.0477448
360.0000000	9.2720003	-8.3584042
360.0000000	9.3207998	-9.7625437
360.0000000	9.3695993	-10.7488365
360.0000000	9.4183998	-11.7620974
360.0000000	9.4672003	-11.1586132
360.0000000	9.5159998	-14.2230167
360.0000000	9.5648003	-0.1704807
360.0000000	9.6135998	-13.4481955
360.0000000	9.6623993	-10.2031803
360.0000000	9.7111998	-10.1397820
360.0000000	9.7600002	-10.8548231
360.0000000	9.8087997	-10.9942532
360.0000000	9.8575993	3.4817801
360.0000000	9.9063997	-7.9087481
360.0000000	9.9552002	-10.9063168
360.0000000	10.0039997	-10.0583811
370.0000000	0.3416000	-36.8558731
370.0000000	0.3904000	-39.9674187
370.0000000	0.4392000	-42.7281761

370.0000000	0.4880000	-39.2859993
370.0000000	0.5368000	-39.4106865
370.0000000	0.5856000	-41.6406326
370.0000000	0.6344000	-34.3364677
370.0000000	0.6832000	-33.9422646
370.0000000	0.7320000	-30.4344616
370.0000000	0.7808000	-29.0619869
370.0000000	0.8296000	-28.4209366
370.0000000	0.8784000	-29.1163387
370.0000000	0.9272000	-30.2540665
370.0000000	0.9760000	-31.1229820
370.0000000	1.0247999	-32.0183449
370.0000000	1.0735999	-32.8467369
370.0000000	1.1224000	-31.8733025
370.0000000	1.1711999	-31.9343948
370.0000000	1.2200000	-31.9284325
370.0000000	1.2687999	-31.2479038
370.0000000	1.3176000	-30.4136982
370.0000000	1.3664000	-30.0671825
370.0000000	1.4152000	-30.1490288
370.0000000	1.4640000	-29.7264347
370.0000000	1.5128000	-45.6917725
370.0000000	1.5616000	-36.3499527
370.0000000	1.6104001	-1722.4990234
370.0000000	1.6592000	-92.3637695
370.0000000	1.7079999	-28.0017128
370.0000000	1.7567999	-27.5830345
370.0000000	1.8055999	-26.3711243
370.0000000	1.8544000	-25.4477062
370.0000000	1.9031999	-26.9985275
370.0000000	1.9520000	-26.5381489
370.0000000	2.0007999	-28.6672897
370.0000000	2.0495999	-17.2739525
370.0000000	2.0983999	-243.8997803
370.0000000	2.1471999	15.6977386
370.0000000	2.1960001	-25.7144299
370.0000000	2.2448001	-26.9919930
370.0000000	2.2935998	-31.5559635
370.0000000	2.3423998	-20.0535507
370.0000000	2.3912001	72.2810135
370.0000000	2.4400001	-22.6758442
370.0000000	2.4888000	-23.4742126
370.0000000	2.5375998	-11.1543941
370.0000000	2.5863998	-9.1023560
370.0000000	2.6352000	-6.9795666
370.0000000	2.6840000	-14.6303062
370.0000000	2.7328000	150.5965424
370.0000000	2.7815998	177.2079163
370.0000000	2.8304000	-23.8799038
370.0000000	2.8792000	-20.4053001
370.0000000	2.9280000	-22.6091137
370.0000000	2.9768000	-16.2402363
370.0000000	3.0256000	-20.3032837
370.0000000	3.0743999	-30.2639923
370.0000000	3.1231999	-24.4818497
370.0000000	3.1719999	-30.1499653
370.0000000	3.2208002	-519.8334961
370.0000000	3.2695999	-1018.3927002

370.0000000	3.3183999	-20.4767284
370.0000000	3.3671999	-3491.6674805
370.0000000	3.4159999	1253.3408203
370.0000000	3.4648001	-6.7694845
370.0000000	3.5135999	-27.2315331
370.0000000	3.5623999	-330.3721008
370.0000000	3.6111999	-2078.6958008
370.0000000	3.6600001	-470.1965332
370.0000000	3.7088001	-621.0507813
370.0000000	3.7575998	-972.6625977
370.0000000	3.8063998	-1765.4160156
370.0000000	3.8552001	-2563.3637695
370.0000000	3.9040000	-2513.1945801
370.0000000	3.9528000	1149.9865723
370.0000000	4.0015998	-250.6758728
370.0000000	4.0503998	704.2808228
370.0000000	4.0991998	90.1361923
370.0000000	4.1479998	-669.7525635
370.0000000	4.1967998	3.0559063
370.0000000	4.2456002	-253.9225616
370.0000000	4.2943997	-499.0423889
370.0000000	4.3431997	-13.2113209
370.0000000	4.3920002	-253.5426483
370.0000000	4.4407997	-22.5583172
370.0000000	4.4896002	-15.4256506
370.0000000	4.5383997	-669.4337769
370.0000000	4.5871997	-12.7277451
370.0000000	4.6360002	-21.7058983
370.0000000	4.6847997	-28.4981461
370.0000000	4.7336001	-19.6021271
370.0000000	4.7824001	-16.4873886
370.0000000	4.8311996	-1503.5124512
370.0000000	4.8800001	233.1680298
370.0000000	4.9287996	-205.1409607
370.0000000	4.9776001	1081.0957031
370.0000000	5.0264001	88.2323990
370.0000000	5.0751996	-306.8196716
370.0000000	5.1240001	-12.2578487
370.0000000	5.1727996	564.7863770
370.0000000	5.2216001	32.5628471
370.0000000	5.2704000	-160.5028534
370.0000000	5.3191996	-39.4556808
370.0000000	5.3680000	-3577.4694824
370.0000000	5.4168000	533.5496216
370.0000000	5.4656000	-202.9157257
370.0000000	5.5144000	38.7082748
370.0000000	5.5631995	494.2579041
370.0000000	5.6120000	397.9556580
370.0000000	5.6608000	-37.3309135
370.0000000	5.7096000	63.4596329
370.0000000	5.7584000	134.7829132
370.0000000	5.8071995	-11.4678354
370.0000000	5.8559999	30.3509579
370.0000000	5.9047999	-16.6175613
370.0000000	5.9535999	-40.0624466
370.0000000	6.0023999	-1591.3820801
370.0000000	6.0511999	-14.5553970
370.0000000	6.0999999	-19.4597836

370.0000000	6.1487999	-13.2452354
370.0000000	6.1975999	-23.3275185
370.0000000	6.2463999	-1812.9838867
370.0000000	6.2951999	-19.1198921
370.0000000	6.3439999	-346.6152344
370.0000000	6.3927999	-15.0492439
370.0000000	6.4416003	-12.3500443
370.0000000	6.4903998	-11.2222366
370.0000000	6.5391998	-11.7643595
370.0000000	6.5879998	427.6038208
370.0000000	6.6367998	-12.0502586
370.0000000	6.6856003	-13.8043833
370.0000000	6.7343998	-13.4422731
370.0000000	6.7831998	-14.3984222
370.0000000	6.8319998	59.6314011
370.0000000	6.8807998	-14.0580816
370.0000000	6.9296002	-497.2019043
370.0000000	6.9783998	-4.2794743
370.0000000	7.0271997	-14.2020168
370.0000000	7.0760002	13.8488951
370.0000000	7.1247997	-2381.2343750
370.0000000	7.1736002	-1909.4875488
370.0000000	7.2223997	-1085.9227295
370.0000000	7.2711997	-232.9497375
370.0000000	7.3200002	-19.6441841
370.0000000	7.3687997	211.7177734
370.0000000	7.4176002	440.0242310
370.0000000	7.4663997	345.9380493
370.0000000	7.5151997	-1062.8242188
370.0000000	7.5640001	-1174.1160889
370.0000000	7.6127996	-2769.2277832
370.0000000	7.6616001	-888.3546753
370.0000000	7.7104001	-455.6877441
370.0000000	7.7591996	49.6840477
370.0000000	7.8080001	457.2700500
370.0000000	7.8567996	601.9534302
370.0000000	7.9056001	-1243.8428955
370.0000000	7.9544001	1980.4140625
370.0000000	8.0031996	2555.3166504
370.0000000	8.0520000	2550.5085449
370.0000000	8.1007996	2698.9094238
370.0000000	8.1496000	2878.2265625
370.0000000	8.1983995	1790.7360840
370.0000000	8.2472000	-4096.4077148
370.0000000	8.2959995	-1451.9152832
370.0000000	8.3448000	-1156.6494141
370.0000000	8.3935995	-1060.0179443
370.0000000	8.4423990	-456.3777771
370.0000000	8.4912004	-1065.8764648
370.0000000	8.5400000	-2875.8044434
370.0000000	8.5887995	-1393.6469727
370.0000000	8.6375999	272.7350159
370.0000000	8.6863995	2460.6828613
370.0000000	8.7351999	-914.1471558
370.0000000	8.7840004	1045.5860596
370.0000000	8.8327999	57.7403412
370.0000000	8.8815994	-11.4851828
370.0000000	8.9303989	-24.1854992

370.0000000	8.9792004	-11.5737829
370.0000000	9.0279999	-5.7225084
370.0000000	9.0767994	7.4094410
370.0000000	9.1255999	-17.3530712
370.0000000	9.1743994	-10.0475264
370.0000000	9.2231998	38.0320129
370.0000000	9.2720003	-8.3593407
370.0000000	9.3207998	-9.7630835
370.0000000	9.3695993	-10.7490635
370.0000000	9.4183998	-11.7619724
370.0000000	9.4672003	-11.1586542
370.0000000	9.5159998	-14.2221251
370.0000000	9.5648003	-0.1738539
370.0000000	9.6135998	-13.4474049
370.0000000	9.6623993	-10.2034559
370.0000000	9.7111998	-10.1400537
370.0000000	9.7600002	-10.8548651
370.0000000	9.8087997	-10.9942608
370.0000000	9.8575993	3.4773579
370.0000000	9.9063997	-7.9096622
370.0000000	9.9552002	-10.9063215
370.0000000	10.0039997	-10.0586290
380.0000000	0.3416000	-37.0339165
380.0000000	0.3904000	-39.9834633
380.0000000	0.4392000	-42.5626526
380.0000000	0.4880000	-39.2585526
380.0000000	0.5368000	-39.3935089
380.0000000	0.5856000	-41.2524796
380.0000000	0.6344000	-35.0065269
380.0000000	0.6832000	-33.4381104
380.0000000	0.7320000	-28.4608402
380.0000000	0.7808000	-27.9826641
380.0000000	0.8296000	-29.8643169
380.0000000	0.8784000	-31.4637642
380.0000000	0.9272000	-32.5798874
380.0000000	0.9760000	-32.7146988
380.0000000	1.0247999	-32.9070625
380.0000000	1.0735999	-33.2265320
380.0000000	1.1224000	-31.8548737
380.0000000	1.1711999	-31.9196148
380.0000000	1.2200000	-31.9282913
380.0000000	1.2687999	-31.2571354
380.0000000	1.3176000	-30.4398079
380.0000000	1.3664000	-30.0749321
380.0000000	1.4152000	-30.1492329
380.0000000	1.4640000	-29.7247257
380.0000000	1.5128000	-45.5945930
380.0000000	1.5616000	-36.3081093
380.0000000	1.6104001	-1719.9561768
380.0000000	1.6592000	-92.0698395
380.0000000	1.7079999	-28.0123081
380.0000000	1.7567999	-27.5245571
380.0000000	1.8055999	-26.5634575
380.0000000	1.8544000	-26.3904572
380.0000000	1.9031999	-26.9704494
380.0000000	1.9520000	-26.5386047
380.0000000	2.0007999	-28.6047077
380.0000000	2.0495999	-17.4605694

380.0000000	2.0983999	-242.4240112
380.0000000	2.1471999	15.4304695
380.0000000	2.1960001	-25.7103386
380.0000000	2.2448001	-26.9740829
380.0000000	2.2935998	-31.4963398
380.0000000	2.3423998	-20.0679550
380.0000000	2.3912001	71.6594620
380.0000000	2.4400001	-22.6783295
380.0000000	2.4888000	-23.4739952
380.0000000	2.5375998	-11.1715021
380.0000000	2.5863998	-9.1221142
380.0000000	2.6352000	-7.0023117
380.0000000	2.6840000	-14.6422482
380.0000000	2.7328000	150.3305206
380.0000000	2.7815998	176.9015198
380.0000000	2.8304000	-23.8785992
380.0000000	2.8792000	-20.4085636
380.0000000	2.9280000	-22.6085453
380.0000000	2.9768000	-16.2486076
380.0000000	3.0256000	-20.3058681
380.0000000	3.0743999	-30.2526054
380.0000000	3.1231999	-24.4783936
380.0000000	3.1719999	-30.1416721
380.0000000	3.2208002	-519.5957642
380.0000000	3.2695999	-1017.9177246
380.0000000	3.3183999	-20.4769268
380.0000000	3.3671999	-3490.2058105
380.0000000	3.4159999	1252.8055420
380.0000000	3.4648001	-6.7751923
380.0000000	3.5135999	-27.2285423
380.0000000	3.5623999	-330.2424927
380.0000000	3.6111999	-2077.8359375
380.0000000	3.6600001	-470.0084229
380.0000000	3.7088001	-620.7993774
380.0000000	3.7575998	-972.2640381
380.0000000	3.8063998	-1764.6861572
380.0000000	3.8552001	-2562.3005371
380.0000000	3.9040000	-2512.1520996
380.0000000	3.9528000	1149.4982910
380.0000000	4.0015998	-250.5787659
380.0000000	4.0503998	703.9782715
380.0000000	4.0991998	90.0905075
380.0000000	4.1479998	-669.4803467
380.0000000	4.1967998	3.0466499
380.0000000	4.2456002	-253.8242493
380.0000000	4.2943997	-498.8417664
380.0000000	4.3431997	-13.2135010
380.0000000	4.3920002	-253.4445343
380.0000000	4.4407997	-22.5565414
380.0000000	4.4896002	-15.4265327
380.0000000	4.5383997	-669.1727295
380.0000000	4.5871997	-12.7297621
380.0000000	4.6360002	-21.7043552
380.0000000	4.6847997	-28.4942207
380.0000000	4.7336001	-19.6014042
380.0000000	4.7824001	-16.4878426
380.0000000	4.8311996	-1502.9812012
380.0000000	4.8800001	233.0784760

380.0000000	4.9287996	-205.0737000
380.0000000	4.9776001	1080.7032471
380.0000000	5.0264001	88.1948776
380.0000000	5.0751996	-306.7160339
380.0000000	5.1240001	-12.2587700
380.0000000	5.1727996	564.6705322
380.0000000	5.2216001	32.5534058
380.0000000	5.2704000	-160.4747467
380.0000000	5.3191996	-39.4511299
380.0000000	5.3680000	-3576.7770996
380.0000000	5.4168000	533.4428711
380.0000000	5.4656000	-202.8795471
380.0000000	5.5144000	38.6976166
380.0000000	5.5631995	494.1593323
380.0000000	5.6120000	397.8756714
380.0000000	5.6608000	-37.3268242
380.0000000	5.7096000	63.4441986
380.0000000	5.7584000	134.7539673
380.0000000	5.8071995	-11.4687061
380.0000000	5.8559999	30.3421078
380.0000000	5.9047999	-16.6173630
380.0000000	5.9535999	-40.0577087
380.0000000	6.0023999	-1591.0777588
380.0000000	6.0511999	-14.5556078
380.0000000	6.0999999	-19.4589500
380.0000000	6.1487999	-13.2456980
380.0000000	6.1975999	-23.3257561
380.0000000	6.2463999	-1812.6365967
380.0000000	6.2951999	-19.1190777
380.0000000	6.3439999	-346.5511780
380.0000000	6.3927999	-15.0492249
380.0000000	6.4416003	-12.3506317
380.0000000	6.4903998	-11.2229118
380.0000000	6.5391998	-11.7650404
380.0000000	6.5879998	426.9131470
380.0000000	6.6367998	-12.0508080
380.0000000	6.6856003	-13.8045530
380.0000000	6.7343998	-13.4424887
380.0000000	6.7831998	-14.3980064
380.0000000	6.8319998	59.6122894
380.0000000	6.8807998	-14.0579424
380.0000000	6.9296002	-496.4404602
380.0000000	6.9783998	-4.2922983
380.0000000	7.0271997	-14.2009888
380.0000000	7.0760002	13.8067083
380.0000000	7.1247997	-2377.2883301
380.0000000	7.1736002	-1906.3247070
380.0000000	7.2223997	-1084.1295166
380.0000000	7.2711997	-232.5819550
380.0000000	7.3200002	-19.6321964
380.0000000	7.3687997	211.3432465
380.0000000	7.4176002	439.2684021
380.0000000	7.4663997	345.3403931
380.0000000	7.5151997	-1061.0632324
380.0000000	7.5640001	-1172.1687012
380.0000000	7.6127996	-2764.6103516
380.0000000	7.6616001	-886.8833008
380.0000000	7.7104001	-454.9414673

380.0000000	7.7591996	49.5829964
380.0000000	7.8080001	456.4846802
380.0000000	7.8567996	600.9253540
380.0000000	7.9056001	-1241.7700195
380.0000000	7.9544001	1977.1041260
380.0000000	8.0031996	2551.0471191
380.0000000	8.0520000	2546.2463379
380.0000000	8.1007996	2694.3999023
380.0000000	8.1496000	2873.4187012
380.0000000	8.1983995	1787.7435303
380.0000000	8.2472000	-4089.5729980
380.0000000	8.2959995	-1449.4951172
380.0000000	8.3448000	-1154.7202148
380.0000000	8.3935995	-1058.2503662
380.0000000	8.4423990	-455.6197510
380.0000000	8.4912004	-1064.1003418
380.0000000	8.5400000	-2871.0046387
380.0000000	8.5887995	-1391.3254395
380.0000000	8.6375999	272.2736816
380.0000000	8.6863995	2456.5644531
380.0000000	8.7351999	-912.6229248
380.0000000	8.7840004	1043.8293457
380.0000000	8.8327999	57.6287308
380.0000000	8.8815994	-11.4853573
380.0000000	8.9303989	-24.1817780
380.0000000	8.9792004	-11.5738649
380.0000000	9.0279999	-5.7242765
380.0000000	9.0767994	7.4037247
380.0000000	9.1255999	-17.3513565
380.0000000	9.1743994	-10.0479288
380.0000000	9.2231998	38.0162735
380.0000000	9.2720003	-8.3602772
380.0000000	9.3207998	-9.7636223
380.0000000	9.3695993	-10.7492895
380.0000000	9.4183998	-11.7618484
380.0000000	9.4672003	-11.1586952
380.0000000	9.5159998	-14.2212343
380.0000000	9.5648003	-0.1772270
380.0000000	9.6135998	-13.4466143
380.0000000	9.6623993	-10.2037306
380.0000000	9.7111998	-10.1403246
380.0000000	9.7600002	-10.8549070
380.0000000	9.8087997	-10.9942694
380.0000000	9.8575993	3.4729347
380.0000000	9.9063997	-7.9105759
380.0000000	9.9552002	-10.9063253
380.0000000	10.0039997	-10.0588760
390.0000000	0.3416000	-36.8954620
390.0000000	0.3904000	-39.9995079
390.0000000	0.4392000	-42.3971329
390.0000000	0.4880000	-39.2311058
390.0000000	0.5368000	-39.3763313
390.0000000	0.5856000	-40.8643265
390.0000000	0.6344000	-35.6765862
390.0000000	0.6832000	-35.3173676
390.0000000	0.7320000	-30.4519901
390.0000000	0.7808000	-28.6838493
390.0000000	0.8296000	-29.8532124

390.0000000	0.8784000	-32.0636024
390.0000000	0.9272000	-33.3253326
390.0000000	0.9760000	-32.8695984
390.0000000	1.0247999	-32.4192581
390.0000000	1.0735999	-33.0674019
390.0000000	1.1224000	-31.8364468
390.0000000	1.1711999	-31.9048328
390.0000000	1.2200000	-31.9281483
390.0000000	1.2687999	-31.2663689
390.0000000	1.3176000	-30.4659157
390.0000000	1.3664000	-30.0826817
390.0000000	1.4152000	-30.1494370
390.0000000	1.4640000	-29.7230167
390.0000000	1.5128000	-45.4974174
390.0000000	1.5616000	-36.2662697
390.0000000	1.6104001	-1717.4134521
390.0000000	1.6592000	-91.7759094
390.0000000	1.7079999	-28.0229015
390.0000000	1.7567999	-27.4660816
390.0000000	1.8055999	-26.1421947
390.0000000	1.8544000	-25.4087982
390.0000000	1.9031999	-26.2099762
390.0000000	1.9520000	-26.5390587
390.0000000	2.0007999	-28.5421257
390.0000000	2.0495999	-17.6471863
390.0000000	2.0983999	-240.9482117
390.0000000	2.1471999	15.1632042
390.0000000	2.1960001	-25.7062473
390.0000000	2.2448001	-26.9561710
390.0000000	2.2935998	-31.4367161
390.0000000	2.3423998	-20.0823593
390.0000000	2.3912001	71.0379181
390.0000000	2.4400001	-22.6808167
390.0000000	2.4888000	-23.4737778
390.0000000	2.5375998	-11.1886101
390.0000000	2.5863998	-9.1418734
390.0000000	2.6352000	-7.0250559
390.0000000	2.6840000	-14.6541901
390.0000000	2.7328000	150.0644989
390.0000000	2.7815998	176.5950928
390.0000000	2.8304000	-23.8772945
390.0000000	2.8792000	-20.4118271
390.0000000	2.9280000	-22.6079750
390.0000000	2.9768000	-16.2569771
390.0000000	3.0256000	-20.3084526
390.0000000	3.0743999	-30.2412186
390.0000000	3.1231999	-24.4749393
390.0000000	3.1719999	-30.1333790
390.0000000	3.2208002	-519.3580322
390.0000000	3.2695999	-1017.4427490
390.0000000	3.3183999	-20.4771252
390.0000000	3.3671999	-3488.7441406
390.0000000	3.4159999	1252.2702637
390.0000000	3.4648001	-6.7809010
390.0000000	3.5135999	-27.2255516
390.0000000	3.5623999	-330.1129150
390.0000000	3.6111999	-2076.9763184
390.0000000	3.6600001	-469.8203125

390.0000000	3.7088001	-620.5480347
390.0000000	3.7575998	-971.8654785
390.0000000	3.8063998	-1763.9562988
390.0000000	3.8552001	-2561.2370605
390.0000000	3.9040000	-2511.1096191
390.0000000	3.9528000	1149.0100098
390.0000000	4.0015998	-250.4816589
390.0000000	4.0503998	703.6757202
390.0000000	4.0991998	90.0448227
390.0000000	4.1479998	-669.2080688
390.0000000	4.1967998	3.0373936
390.0000000	4.2456002	-253.7259521
390.0000000	4.2943997	-498.6412048
390.0000000	4.3431997	-13.2156811
390.0000000	4.3920002	-253.3464203
390.0000000	4.4407997	-22.5547657
390.0000000	4.4896002	-15.4274158
390.0000000	4.5383997	-668.9116821
390.0000000	4.5871997	-12.7317781
390.0000000	4.6360002	-21.7028122
390.0000000	4.6847997	-28.4902954
390.0000000	4.7336001	-19.6006813
390.0000000	4.7824001	-16.4882984
390.0000000	4.8311996	-1502.4499512
390.0000000	4.8800001	232.9889221
390.0000000	4.9287996	-205.0064240
390.0000000	4.9776001	1080.3106689
390.0000000	5.0264001	88.1573639
390.0000000	5.0751996	-306.6124268
390.0000000	5.1240001	-12.2596912
390.0000000	5.1727996	564.5546265
390.0000000	5.2216001	32.5439606
390.0000000	5.2704000	-160.4466400
390.0000000	5.3191996	-39.4465790
390.0000000	5.3680000	-3576.0849609
390.0000000	5.4168000	533.3361206
390.0000000	5.4656000	-202.8433685
390.0000000	5.5144000	38.6869659
390.0000000	5.5631995	494.0607605
390.0000000	5.6120000	397.7956848
390.0000000	5.6608000	-37.3227386
390.0000000	5.7096000	63.4287643
390.0000000	5.7584000	134.7250366
390.0000000	5.8071995	-11.4695759
390.0000000	5.8559999	30.3332539
390.0000000	5.9047999	-16.6171665
390.0000000	5.9535999	-40.0529709
390.0000000	6.0023999	-1590.7733154
390.0000000	6.0511999	-14.5558195
390.0000000	6.0999999	-19.4581184
390.0000000	6.1487999	-13.2461605
390.0000000	6.1975999	-23.3239899
390.0000000	6.2463999	-1812.2891846
390.0000000	6.2951999	-19.1182632
390.0000000	6.3439999	-346.4871216
390.0000000	6.3927999	-15.0492067
390.0000000	6.4416003	-12.3512182
390.0000000	6.4903998	-11.2235880

390.0000000	6.5391998	-11.7657223
390.0000000	6.5879998	426.2224426
390.0000000	6.6367998	-12.0513573
390.0000000	6.6856003	-13.8047237
390.0000000	6.7343998	-13.4427042
390.0000000	6.7831998	-14.3975906
390.0000000	6.8319998	59.5931778
390.0000000	6.8807998	-14.0578041
390.0000000	6.9296002	-495.6790161
390.0000000	6.9783998	-4.3051214
390.0000000	7.0271997	-14.1999607
390.0000000	7.0760002	13.7645235
390.0000000	7.1247997	-2373.3425293
390.0000000	7.1736002	-1903.1618652
390.0000000	7.2223997	-1082.3364258
390.0000000	7.2711997	-232.2141724
390.0000000	7.3200002	-19.6202087
390.0000000	7.3687997	210.9687195
390.0000000	7.4176002	438.5125732
390.0000000	7.4663997	344.7427368
390.0000000	7.5151997	-1059.3021240
390.0000000	7.5640001	-1170.2213135
390.0000000	7.6127996	-2759.9926758
390.0000000	7.6616001	-885.4119873
390.0000000	7.7104001	-454.1951904
390.0000000	7.7591996	49.4819489
390.0000000	7.8080001	455.6993103
390.0000000	7.8567996	599.8972778
390.0000000	7.9056001	-1239.6971436
390.0000000	7.9544001	1973.7941895
390.0000000	8.0031996	2546.7773438
390.0000000	8.0520000	2541.9843750
390.0000000	8.1007996	2689.8903809
390.0000000	8.1496000	2868.6105957
390.0000000	8.1983995	1784.7509766
390.0000000	8.2472000	-4082.7382813
390.0000000	8.2959995	-1447.0749512
390.0000000	8.3448000	-1152.7910156
390.0000000	8.3935995	-1056.4826660
390.0000000	8.4423990	-454.8617554
390.0000000	8.4912004	-1062.3240967
390.0000000	8.5400000	-2866.2048340
390.0000000	8.5887995	-1389.0039063
390.0000000	8.6375999	271.8123779
390.0000000	8.6863995	2452.4460449
390.0000000	8.7351999	-911.0986328
390.0000000	8.7840004	1042.0726318
390.0000000	8.8327999	57.5171280
390.0000000	8.8815994	-11.4855318
390.0000000	8.9303989	-24.1780586
390.0000000	8.9792004	-11.5739470
390.0000000	9.0279999	-5.7260451
390.0000000	9.0767994	7.3980064
390.0000000	9.1255999	-17.3496437
390.0000000	9.1743994	-10.0483303
390.0000000	9.2231998	38.0005417
390.0000000	9.2720003	-8.3612137
390.0000000	9.3207998	-9.7641621

390.0000000	9.3695993	-10.7495165
390.0000000	9.4183998	-11.7617235
390.0000000	9.4672003	-11.1587362
390.0000000	9.5159998	-14.2203426
390.0000000	9.5648003	-0.1805992
390.0000000	9.6135998	-13.4458237
390.0000000	9.6623993	-10.2040062
390.0000000	9.7111998	-10.1405964
390.0000000	9.7600002	-10.8549480
390.0000000	9.8087997	-10.9942770
390.0000000	9.8575993	3.4685125
390.0000000	9.9063997	-7.9114900
390.0000000	9.9552002	-10.9063301
390.0000000	10.0039997	-10.0591240
400.0000000	0.3416000	-37.2096939
400.0000000	0.3904000	-39.9355164
400.0000000	0.4392000	-42.2316093
400.0000000	0.4880000	-39.2036591
400.0000000	0.5368000	-39.3591537
400.0000000	0.5856000	-40.4761696
400.0000000	0.6344000	-36.3466454
400.0000000	0.6832000	-35.6302948
400.0000000	0.7320000	-30.2069645
400.0000000	0.7808000	-28.5589428
400.0000000	0.8296000	-29.8554935
400.0000000	0.8784000	-32.3364296
400.0000000	0.9272000	-32.6070099
400.0000000	0.9760000	-31.4870110
400.0000000	1.0247999	-31.8846951
400.0000000	1.0735999	-32.8231735
400.0000000	1.1224000	-31.8180199
400.0000000	1.1711999	-31.8900528
400.0000000	1.2200000	-31.9280052
400.0000000	1.2687999	-31.2756023
400.0000000	1.3176000	-30.4920254
400.0000000	1.3664000	-30.0904312
400.0000000	1.4152000	-30.1496410
400.0000000	1.4640000	-29.7213058
400.0000000	1.5128000	-45.4002380
400.0000000	1.5616000	-36.2244263
400.0000000	1.6104001	-1714.8706055
400.0000000	1.6592000	-91.4819794
400.0000000	1.7079999	-28.0334969
400.0000000	1.7567999	-27.4076061
400.0000000	1.8055999	-26.0548000
400.0000000	1.8544000	-25.6206837
400.0000000	1.9031999	-26.5437832
400.0000000	1.9520000	-26.5395145
400.0000000	2.0007999	-28.4795437
400.0000000	2.0495999	-17.8338013
400.0000000	2.0983999	-239.4724426
400.0000000	2.1471999	14.8959389
400.0000000	2.1960001	-25.7021561
400.0000000	2.2448001	-26.9382591
400.0000000	2.2935998	-31.3770924
400.0000000	2.3423998	-20.0967655
400.0000000	2.3912001	70.4163742
400.0000000	2.4400001	-22.6833038

400.0000000	2.4888000	-23.4735603
400.0000000	2.5375998	-11.2057180
400.0000000	2.5863998	-9.1616325
400.0000000	2.6352000	-7.0478001
400.0000000	2.6840000	-14.6661320
400.0000000	2.7328000	149.7984924
400.0000000	2.7815998	176.2886658
400.0000000	2.8304000	-23.8759899
400.0000000	2.8792000	-20.4150906
400.0000000	2.9280000	-22.6074066
400.0000000	2.9768000	-16.2653465
400.0000000	3.0256000	-20.3110371
400.0000000	3.0743999	-30.2298317
400.0000000	3.1231999	-24.4714832
400.0000000	3.1719999	-30.1250858
400.0000000	3.2208002	-519.1203003
400.0000000	3.2695999	-1016.9677734
400.0000000	3.3183999	-20.4773235
400.0000000	3.3671999	-3487.2824707
400.0000000	3.4159999	1251.7349854
400.0000000	3.4648001	-6.7866096
400.0000000	3.5135999	-27.2225609
400.0000000	3.5623999	-329.9833069
400.0000000	3.6111999	-2076.1164551
400.0000000	3.6600001	-469.6321411
400.0000000	3.7088001	-620.2966309
400.0000000	3.7575998	-971.4669800
400.0000000	3.8063998	-1763.2264404
400.0000000	3.8552001	-2560.1735840
400.0000000	3.9040000	-2510.0671387
400.0000000	3.9528000	1148.5217285
400.0000000	4.0015998	-250.3845520
400.0000000	4.0503998	703.3731079
400.0000000	4.0991998	89.9991455
400.0000000	4.1479998	-668.9358521
400.0000000	4.1967998	3.0281372
400.0000000	4.2456002	-253.6276550
400.0000000	4.2943997	-498.4406433
400.0000000	4.3431997	-13.2178612
400.0000000	4.3920002	-253.2483063
400.0000000	4.4407997	-22.5529900
400.0000000	4.4896002	-15.4282990
400.0000000	4.5383997	-668.6505737
400.0000000	4.5871997	-12.7337942
400.0000000	4.6360002	-21.7012711
400.0000000	4.6847997	-28.4863701
400.0000000	4.7336001	-19.5999603
400.0000000	4.7824001	-16.4887524
400.0000000	4.8311996	-1501.9187012
400.0000000	4.8800001	232.8993835
400.0000000	4.9287996	-204.9391632
400.0000000	4.9776001	1079.9182129
400.0000000	5.0264001	88.1198425
400.0000000	5.0751996	-306.5087891
400.0000000	5.1240001	-12.2606125
400.0000000	5.1727996	564.4387817
400.0000000	5.2216001	32.5345154
400.0000000	5.2704000	-160.4185333

400.0000000	5.3191996	-39.4420319
400.0000000	5.3680000	-3575.3925781
400.0000000	5.4168000	533.2293091
400.0000000	5.4656000	-202.8071747
400.0000000	5.5144000	38.6763077
400.0000000	5.5631995	493.9621887
400.0000000	5.6120000	397.7157288
400.0000000	5.6608000	-37.3186493
400.0000000	5.7096000	63.4133377
400.0000000	5.7584000	134.6961060
400.0000000	5.8071995	-11.4704475
400.0000000	5.8559999	30.3244038
400.0000000	5.9047999	-16.6169682
400.0000000	5.9535999	-40.0482292
400.0000000	6.0023999	-1590.4689941
400.0000000	6.0511999	-14.5560303
400.0000000	6.0999999	-19.4572868
400.0000000	6.1487999	-13.2466240
400.0000000	6.1975999	-23.3222237
400.0000000	6.2463999	-1811.9417725
400.0000000	6.2951999	-19.1174488
400.0000000	6.3439999	-346.4230652
400.0000000	6.3927999	-15.0491886
400.0000000	6.4416003	-12.3518057
400.0000000	6.4903998	-11.2242641
400.0000000	6.5391998	-11.7664032
400.0000000	6.5879998	425.5317383
400.0000000	6.6367998	-12.0519066
400.0000000	6.6856003	-13.8048935
400.0000000	6.7343998	-13.4429197
400.0000000	6.7831998	-14.3971748
400.0000000	6.8319998	59.5740585
400.0000000	6.8807998	-14.0576658
400.0000000	6.9296002	-494.9175415
400.0000000	6.9783998	-4.3179455
400.0000000	7.0271997	-14.1989326
400.0000000	7.0760002	13.7223387
400.0000000	7.1247997	-2369.3967285
400.0000000	7.1736002	-1899.9989014
400.0000000	7.2223997	-1080.5432129
400.0000000	7.2711997	-231.8464050
400.0000000	7.3200002	-19.6082191
400.0000000	7.3687997	210.5941772
400.0000000	7.4176002	437.7567444
400.0000000	7.4663997	344.1450806
400.0000000	7.5151997	-1057.5411377
400.0000000	7.5640001	-1168.2739258
400.0000000	7.6127996	-2755.3750000
400.0000000	7.6616001	-883.9406128
400.0000000	7.7104001	-453.4489136
400.0000000	7.7591996	49.3809013
400.0000000	7.8080001	454.9139709
400.0000000	7.8567996	598.8691406
400.0000000	7.9056001	-1237.6242676
400.0000000	7.9544001	1970.4842529
400.0000000	8.0031996	2542.5078125
400.0000000	8.0520000	2537.7224121
400.0000000	8.1007996	2685.3808594

400.0000000	8.1496000	2863.8024902
400.0000000	8.1983995	1781.7585449
400.0000000	8.2472000	-4075.9035645
400.0000000	8.2959995	-1444.6547852
400.0000000	8.3448000	-1150.8619385
400.0000000	8.3935995	-1054.7149658
400.0000000	8.4423990	-454.1037292
400.0000000	8.4912004	-1060.5478516
400.0000000	8.5400000	-2861.4050293
400.0000000	8.5887995	-1386.6823730
400.0000000	8.6375999	271.3510437
400.0000000	8.6863995	2448.3276367
400.0000000	8.7351999	-909.5744019
400.0000000	8.7840004	1040.3157959
400.0000000	8.8327999	57.4055176
400.0000000	8.8815994	-11.4857063
400.0000000	8.9303989	-24.1743393
400.0000000	8.9792004	-11.5740299
400.0000000	9.0279999	-5.7278128
400.0000000	9.0767994	7.3922882
400.0000000	9.1255999	-17.3479290
400.0000000	9.1743994	-10.0487328
400.0000000	9.2231998	37.9848099
400.0000000	9.2720003	-8.3621492
400.0000000	9.3207998	-9.7647009
400.0000000	9.3695993	-10.7497425
400.0000000	9.4183998	-11.7615986
400.0000000	9.4672003	-11.1587772
400.0000000	9.5159998	-14.2194519
400.0000000	9.5648003	-0.1839714
400.0000000	9.6135998	-13.4450321
400.0000000	9.6623993	-10.2042809
400.0000000	9.7111998	-10.1408682
400.0000000	9.7600002	-10.8549900
400.0000000	9.8087997	-10.9942846
400.0000000	9.8575993	3.4640903
400.0000000	9.9063997	-7.9124036
400.0000000	9.9552002	-10.9063349
400.0000000	10.0039997	-10.0593710
410.0000000	0.3416000	-36.8932648
410.0000000	0.3904000	-39.8715248
410.0000000	0.4392000	-42.0660858
410.0000000	0.4880000	-39.1762161
410.0000000	0.5368000	-39.3419762
410.0000000	0.5856000	-40.0880165
410.0000000	0.6344000	-37.0167046
410.0000000	0.6832000	-34.4440765
410.0000000	0.7320000	-31.0776196
410.0000000	0.7808000	-30.8351822
410.0000000	0.8296000	-31.9719715
410.0000000	0.8784000	-33.3282814
410.0000000	0.9272000	-32.7473755
410.0000000	0.9760000	-32.2658310
410.0000000	1.0247999	-32.3206406
410.0000000	1.0735999	-32.7862129
410.0000000	1.1224000	-31.7995911
410.0000000	1.1711999	-31.8752708
410.0000000	1.2200000	-31.9278641

410.0000000	1.2687999	-31.2848358
410.0000000	1.3176000	-30.5181351
410.0000000	1.3664000	-30.0981808
410.0000000	1.4152000	-30.1498451
410.0000000	1.4640000	-29.7195969
410.0000000	1.5128000	-45.3030624
410.0000000	1.5616000	-36.1825867
410.0000000	1.6104001	-1712.3277588
410.0000000	1.6592000	-91.1880493
410.0000000	1.7079999	-28.0440903
410.0000000	1.7567999	-27.3491306
410.0000000	1.8055999	-25.6420078
410.0000000	1.8544000	-25.1137428
410.0000000	1.9031999	-26.5581379
410.0000000	1.9520000	-26.5399704
410.0000000	2.0007999	-28.4169617
410.0000000	2.0495999	-18.0204182
410.0000000	2.0983999	-237.9966736
410.0000000	2.1471999	14.6286697
410.0000000	2.1960001	-25.6980648
410.0000000	2.2448001	-26.9203491
410.0000000	2.2935998	-31.3174686
410.0000000	2.3423998	-20.1111698
410.0000000	2.3912001	69.7948303
410.0000000	2.4400001	-22.6857891
410.0000000	2.4888000	-23.4733429
410.0000000	2.5375998	-11.2228270
410.0000000	2.5863998	-9.1813908
410.0000000	2.6352000	-7.0705442
410.0000000	2.6840000	-14.6780739
410.0000000	2.7328000	149.5324707
410.0000000	2.7815998	175.9822693
410.0000000	2.8304000	-23.8746872
410.0000000	2.8792000	-20.4183540
410.0000000	2.9280000	-22.6068382
410.0000000	2.9768000	-16.2737179
410.0000000	3.0256000	-20.3136215
410.0000000	3.0743999	-30.2184448
410.0000000	3.1231999	-24.4680271
410.0000000	3.1719999	-30.1167927
410.0000000	3.2208002	-518.8825684
410.0000000	3.2695999	-1016.4927979
410.0000000	3.3183999	-20.4775200
410.0000000	3.3671999	-3485.8208008
410.0000000	3.4159999	1251.1997070
410.0000000	3.4648001	-6.7923164
410.0000000	3.5135999	-27.2195702
410.0000000	3.5623999	-329.8537292
410.0000000	3.6111999	-2075.2568359
410.0000000	3.6600001	-469.4440308
410.0000000	3.7088001	-620.0452271
410.0000000	3.7575998	-971.0684204
410.0000000	3.8063998	-1762.4967041
410.0000000	3.8552001	-2559.1101074
410.0000000	3.9040000	-2509.0246582
410.0000000	3.9528000	1148.0334473
410.0000000	4.0015998	-250.2874451
410.0000000	4.0503998	703.0705566

410.0000000	4.0991998	89.9534607
410.0000000	4.1479998	-668.6636353
410.0000000	4.1967998	3.0188808
410.0000000	4.2456002	-253.5293427
410.0000000	4.2943997	-498.2400818
410.0000000	4.3431997	-13.2200422
410.0000000	4.3920002	-253.1501923
410.0000000	4.4407997	-22.5512142
410.0000000	4.4896002	-15.4291811
410.0000000	4.5383997	-668.3895264
410.0000000	4.5871997	-12.7358112
410.0000000	4.6360002	-21.6997280
410.0000000	4.6847997	-28.4824448
410.0000000	4.7336001	-19.5992374
410.0000000	4.7824001	-16.4892063
410.0000000	4.8311996	-1501.3874512
410.0000000	4.8800001	232.8098297
410.0000000	4.9287996	-204.8719025
410.0000000	4.9776001	1079.5257568
410.0000000	5.0264001	88.0823212
410.0000000	5.0751996	-306.4051514
410.0000000	5.1240001	-12.2615337
410.0000000	5.1727996	564.3229370
410.0000000	5.2216001	32.5250740
410.0000000	5.2704000	-160.3904266
410.0000000	5.3191996	-39.4374809
410.0000000	5.3680000	-3574.7001953
410.0000000	5.4168000	533.1224976
410.0000000	5.4656000	-202.7709961
410.0000000	5.5144000	38.6656494
410.0000000	5.5631995	493.8636169
410.0000000	5.6120000	397.6357422
410.0000000	5.6608000	-37.3145599
410.0000000	5.7096000	63.3979111
410.0000000	5.7584000	134.6671753
410.0000000	5.8071995	-11.4713173
410.0000000	5.8559999	30.3155537
410.0000000	5.9047999	-16.6167698
410.0000000	5.9535999	-40.0434914
410.0000000	6.0023999	-1590.1644287
410.0000000	6.0511999	-14.5562420
410.0000000	6.0999999	-19.4564533
410.0000000	6.1487999	-13.2470865
410.0000000	6.1975999	-23.3204594
410.0000000	6.2463999	-1811.5944824
410.0000000	6.2951999	-19.1166344
410.0000000	6.3439999	-346.3590393
410.0000000	6.3927999	-15.0491705
410.0000000	6.4416003	-12.3523922
410.0000000	6.4903998	-11.2249403
410.0000000	6.5391998	-11.7670841
410.0000000	6.5879998	424.8410645
410.0000000	6.6367998	-12.0524549
410.0000000	6.6856003	-13.8050642
410.0000000	6.7343998	-13.4431362
410.0000000	6.7831998	-14.3967590
410.0000000	6.8319998	59.5549393
410.0000000	6.8807998	-14.0575266

410.0000000	6.9296002	-494.1560974
410.0000000	6.9783998	-4.3307686
410.0000000	7.0271997	-14.1979055
410.0000000	7.0760002	13.6801519
410.0000000	7.1247997	-2365.4509277
410.0000000	7.1736002	-1896.8360596
410.0000000	7.2223997	-1078.7501221
410.0000000	7.2711997	-231.4786224
410.0000000	7.3200002	-19.5962296
410.0000000	7.3687997	210.2196503
410.0000000	7.4176002	437.0009155
410.0000000	7.4663997	343.5474243
410.0000000	7.5151997	-1055.7800293
410.0000000	7.5640001	-1166.3265381
410.0000000	7.6127996	-2750.7573242
410.0000000	7.6616001	-882.4693604
410.0000000	7.7104001	-452.7026367
410.0000000	7.7591996	49.2798500
410.0000000	7.8080001	454.1286011
410.0000000	7.8567996	597.8410645
410.0000000	7.9056001	-1235.5512695
410.0000000	7.9544001	1967.1743164
410.0000000	8.0031996	2538.2382813
410.0000000	8.0520000	2533.4604492
410.0000000	8.1007996	2680.8713379
410.0000000	8.1496000	2858.9946289
410.0000000	8.1983995	1778.7659912
410.0000000	8.2472000	-4069.0686035
410.0000000	8.2959995	-1442.2346191
410.0000000	8.3448000	-1148.9327393
410.0000000	8.3935995	-1052.9472656
410.0000000	8.4423990	-453.3457336
410.0000000	8.4912004	-1058.7716064
410.0000000	8.5400000	-2856.6052246
410.0000000	8.5887995	-1384.3608398
410.0000000	8.6375999	270.8897400
410.0000000	8.6863995	2444.2094727
410.0000000	8.7351999	-908.0501709
410.0000000	8.7840004	1038.5590820
410.0000000	8.8327999	57.2939072
410.0000000	8.8815994	-11.4858799
410.0000000	8.9303989	-24.1706200
410.0000000	8.9792004	-11.5741119
410.0000000	9.0279999	-5.7295814
410.0000000	9.0767994	7.3865700
410.0000000	9.1255999	-17.3462143
410.0000000	9.1743994	-10.0491352
410.0000000	9.2231998	37.9690781
410.0000000	9.2720003	-8.3630857
410.0000000	9.3207998	-9.7652407
410.0000000	9.3695993	-10.7499685
410.0000000	9.4183998	-11.7614746
410.0000000	9.4672003	-11.1588192
410.0000000	9.5159998	-14.2185612
410.0000000	9.5648003	-0.1873446
410.0000000	9.6135998	-13.4442425
410.0000000	9.6623993	-10.2045555
410.0000000	9.7111998	-10.1411400

410.0000000	9.7600002	-10.8550320
410.0000000	9.8087997	-10.9942932
410.0000000	9.8575993	3.4596691
410.0000000	9.9063997	-7.9133177
410.0000000	9.9552002	-10.9063387
410.0000000	10.0039997	-10.0596189
420.0000000	0.3416000	-36.3953667
420.0000000	0.3904000	-39.8075333
420.0000000	0.4392000	-41.9005623
420.0000000	0.4880000	-39.1487694
420.0000000	0.5368000	-39.3247986
420.0000000	0.5856000	-39.6998634
420.0000000	0.6344000	-37.6867638
420.0000000	0.6832000	-32.4331436
420.0000000	0.7320000	-29.8429470
420.0000000	0.7808000	-30.0212269
420.0000000	0.8296000	-31.0424652
420.0000000	0.8784000	-30.4926434
420.0000000	0.9272000	-30.6700592
420.0000000	0.9760000	-31.4769611
420.0000000	1.0247999	-32.6650200
420.0000000	1.0735999	-32.7350998
420.0000000	1.1224000	-31.7811642
420.0000000	1.1711999	-31.8604908
420.0000000	1.2200000	-31.9277210
420.0000000	1.2687999	-31.2940693
420.0000000	1.3176000	-30.5442429
420.0000000	1.3664000	-30.1059303
420.0000000	1.4152000	-30.1500492
420.0000000	1.4640000	-29.7178879
420.0000000	1.5128000	-45.2058830
420.0000000	1.5616000	-36.1407471
420.0000000	1.6104001	-1709.7850342
420.0000000	1.6592000	-90.8941116
420.0000000	1.7079999	-28.0546856
420.0000000	1.7567999	-27.2906551
420.0000000	1.8055999	-25.1810379
420.0000000	1.8544000	-25.0939503
420.0000000	1.9031999	-26.5724907
420.0000000	1.9520000	-26.5404243
420.0000000	2.0007999	-28.3543797
420.0000000	2.0495999	-18.2070351
420.0000000	2.0983999	-236.5208740
420.0000000	2.1471999	14.3614006
420.0000000	2.1960001	-25.6939735
420.0000000	2.2448001	-26.9024372
420.0000000	2.2935998	-31.2578449
420.0000000	2.3423998	-20.1255741
420.0000000	2.3912001	69.1732864
420.0000000	2.4400001	-22.6882763
420.0000000	2.4888000	-23.4731255
420.0000000	2.5375998	-11.2399349
420.0000000	2.5863998	-9.2011499
420.0000000	2.6352000	-7.0932884
420.0000000	2.6840000	-14.6900148
420.0000000	2.7328000	149.2664490
420.0000000	2.7815998	175.6758423
420.0000000	2.8304000	-23.8733826

420.0000000	2.8792000	-20.4216175
420.0000000	2.9280000	-22.6062679
420.0000000	2.9768000	-16.2820892
420.0000000	3.0256000	-20.3162060
420.0000000	3.0743999	-30.2070580
420.0000000	3.1231999	-24.4645729
420.0000000	3.1719999	-30.1084995
420.0000000	3.2208002	-518.6448364
420.0000000	3.2695999	-1016.0178223
420.0000000	3.3183999	-20.4777184
420.0000000	3.3671999	-3484.3591309
420.0000000	3.4159999	1250.6644287
420.0000000	3.4648001	-6.7980242
420.0000000	3.5135999	-27.2165794
420.0000000	3.5623999	-329.7241516
420.0000000	3.6111999	-2074.3969727
420.0000000	3.6600001	-469.2559204
420.0000000	3.7088001	-619.7938843
420.0000000	3.7575998	-970.6699219
420.0000000	3.8063998	-1761.7668457
420.0000000	3.8552001	-2558.0466309
420.0000000	3.9040000	-2507.9821777
420.0000000	3.9528000	1147.5450439
420.0000000	4.0015998	-250.1903229
420.0000000	4.0503998	702.7680054
420.0000000	4.0991998	89.9077759
420.0000000	4.1479998	-668.3913574
420.0000000	4.1967998	3.0096245
420.0000000	4.2456002	-253.4310455
420.0000000	4.2943997	-498.0395203
420.0000000	4.3431997	-13.2222233
420.0000000	4.3920002	-253.0520782
420.0000000	4.4407997	-22.5494385
420.0000000	4.4896002	-15.4300632
420.0000000	4.5383997	-668.1284180
420.0000000	4.5871997	-12.7378273
420.0000000	4.6360002	-21.6981850
420.0000000	4.6847997	-28.4785194
420.0000000	4.7336001	-19.5985146
420.0000000	4.7824001	-16.4896622
420.0000000	4.8311996	-1500.8562012
420.0000000	4.8800001	232.7202759
420.0000000	4.9287996	-204.8046417
420.0000000	4.9776001	1079.1331787
420.0000000	5.0264001	88.0447998
420.0000000	5.0751996	-306.3015442
420.0000000	5.1240001	-12.2624550
420.0000000	5.1727996	564.2070313
420.0000000	5.2216001	32.5156288
420.0000000	5.2704000	-160.3623199
420.0000000	5.3191996	-39.4329338
420.0000000	5.3680000	-3574.0078125
420.0000000	5.4168000	533.0157471
420.0000000	5.4656000	-202.7348175
420.0000000	5.5144000	38.6549911
420.0000000	5.5631995	493.7650452
420.0000000	5.6120000	397.5557556
420.0000000	5.6608000	-37.3104706

420.0000000	5.7096000	63.3824768
420.0000000	5.7584000	134.6382446
420.0000000	5.8071995	-11.4721889
420.0000000	5.8559999	30.3067036
420.0000000	5.9047999	-16.6165733
420.0000000	5.9535999	-40.0387535
420.0000000	6.0023999	-1589.8601074
420.0000000	6.0511999	-14.5564537
420.0000000	6.0999999	-19.4556217
420.0000000	6.1487999	-13.2475491
420.0000000	6.1975999	-23.3186951
420.0000000	6.2463999	-1811.2470703
420.0000000	6.2951999	-19.1158199
420.0000000	6.3439999	-346.2949829
420.0000000	6.3927999	-15.0491514
420.0000000	6.4416003	-12.3529797
420.0000000	6.4903998	-11.2256155
420.0000000	6.5391998	-11.7677650
420.0000000	6.5879998	424.1503601
420.0000000	6.6367998	-12.0530043
420.0000000	6.6856003	-13.8052340
420.0000000	6.7343998	-13.4433517
420.0000000	6.7831998	-14.3963432
420.0000000	6.8319998	59.5358276
420.0000000	6.8807998	-14.0573883
420.0000000	6.9296002	-493.3946533
420.0000000	6.9783998	-4.3435926
420.0000000	7.0271997	-14.1968775
420.0000000	7.0760002	13.6379671
420.0000000	7.1247997	-2361.5048828
420.0000000	7.1736002	-1893.6732178
420.0000000	7.2223997	-1076.9569092
420.0000000	7.2711997	-231.1108398
420.0000000	7.3200002	-19.5842419
420.0000000	7.3687997	209.8451233
420.0000000	7.4176002	436.2450867
420.0000000	7.4663997	342.9497986
420.0000000	7.5151997	-1054.0190430
420.0000000	7.5640001	-1164.3791504
420.0000000	7.6127996	-2746.1398926
420.0000000	7.6616001	-880.9979858
420.0000000	7.7104001	-451.9564209
420.0000000	7.7591996	49.1788025
420.0000000	7.8080001	453.3432312
420.0000000	7.8567996	596.8129883
420.0000000	7.9056001	-1233.4783936
420.0000000	7.9544001	1963.8643799
420.0000000	8.0031996	2533.9685059
420.0000000	8.0520000	2529.1984863
420.0000000	8.1007996	2676.3618164
420.0000000	8.1496000	2854.1865234
420.0000000	8.1983995	1775.7734375
420.0000000	8.2472000	-4062.2338867
420.0000000	8.2959995	-1439.8144531
420.0000000	8.3448000	-1147.0035400
420.0000000	8.3935995	-1051.1795654
420.0000000	8.4423990	-452.5877075
420.0000000	8.4912004	-1056.9953613

420.0000000	8.5400000	-2851.8054199
420.0000000	8.5887995	-1382.0393066
420.0000000	8.6375999	270.4284058
420.0000000	8.6863995	2440.0910645
420.0000000	8.7351999	-906.5258789
420.0000000	8.7840004	1036.8023682
420.0000000	8.8327999	57.1822968
420.0000000	8.8815994	-11.4860544
420.0000000	8.9303989	-24.1669006
420.0000000	8.9792004	-11.5741940
420.0000000	9.0279999	-5.7313495
420.0000000	9.0767994	7.3808537
420.0000000	9.1255999	-17.3445015
420.0000000	9.1743994	-10.0495367
420.0000000	9.2231998	37.9533463
420.0000000	9.2720003	-8.3640223
420.0000000	9.3207998	-9.7657795
420.0000000	9.3695993	-10.7501955
420.0000000	9.4183998	-11.7613497
420.0000000	9.4672003	-11.1588602
420.0000000	9.5159998	-14.2176704
420.0000000	9.5648003	-0.1907177
420.0000000	9.6135998	-13.4434509
420.0000000	9.6623993	-10.2048311
420.0000000	9.7111998	-10.1414108
420.0000000	9.7600002	-10.8550739
420.0000000	9.8087997	-10.9943008
420.0000000	9.8575993	3.4552469
420.0000000	9.9063997	-7.9142313
420.0000000	9.9552002	-10.9063435
420.0000000	10.0039997	-10.0598660
430.0000000	0.3416000	-36.6156769
430.0000000	0.3904000	-39.5826874
430.0000000	0.4392000	-41.7350388
430.0000000	0.4880000	-39.1213226
430.0000000	0.5368000	-39.3076210
430.0000000	0.5856000	-39.3117065
430.0000000	0.6344000	-38.3568230
430.0000000	0.6832000	-33.5798035
430.0000000	0.7320000	-31.3004780
430.0000000	0.7808000	-31.3995323
430.0000000	0.8296000	-31.9156952
430.0000000	0.8784000	-32.1316147
430.0000000	0.9272000	-31.7722511
430.0000000	0.9760000	-31.8159676
430.0000000	1.0247999	-33.2787323
430.0000000	1.0735999	-32.6839867
430.0000000	1.1224000	-31.7627354
430.0000000	1.1711999	-31.8457108
430.0000000	1.2200000	-31.9275780
430.0000000	1.2687999	-31.3033028
430.0000000	1.3176000	-30.5703526
430.0000000	1.3664000	-30.1136799
430.0000000	1.4152000	-30.1502552
430.0000000	1.4640000	-29.7161789
430.0000000	1.5128000	-45.1087036
430.0000000	1.5616000	-36.0989037
430.0000000	1.6104001	-1707.2421875

430.0000000	1.6592000	-90.6001816
430.0000000	1.7079999	-28.0652790
430.0000000	1.7567999	-26.3919964
430.0000000	1.8055999	-25.6010761
430.0000000	1.8544000	-27.1272202
430.0000000	1.9031999	-26.5868454
430.0000000	1.9520000	-26.5408802
430.0000000	2.0007999	-28.2917976
430.0000000	2.0495999	-18.3936501
430.0000000	2.0983999	-235.0451050
430.0000000	2.1471999	14.0941353
430.0000000	2.1960001	-25.6898823
430.0000000	2.2448001	-26.8845253
430.0000000	2.2935998	-31.1982231
430.0000000	2.3423998	-20.1399784
430.0000000	2.3912001	68.5517349
430.0000000	2.4400001	-22.6907616
430.0000000	2.4888000	-23.4729080
430.0000000	2.5375998	-11.2570429
430.0000000	2.5863998	-9.2209091
430.0000000	2.6352000	-7.1160326
430.0000000	2.6840000	-14.7019567
430.0000000	2.7328000	149.0004425
430.0000000	2.7815998	175.3694153
430.0000000	2.8304000	-23.8720779
430.0000000	2.8792000	-20.4248810
430.0000000	2.9280000	-22.6056995
430.0000000	2.9768000	-16.2904587
430.0000000	3.0256000	-20.3187904
430.0000000	3.0743999	-30.1956711
430.0000000	3.1231999	-24.4611168
430.0000000	3.1719999	-30.1002064
430.0000000	3.2208002	-518.4071045
430.0000000	3.2695999	-1015.5428467
430.0000000	3.3183999	-20.4779167
430.0000000	3.3671999	-3482.8974609
430.0000000	3.4159999	1250.1291504
430.0000000	3.4648001	-6.8037329
430.0000000	3.5135999	-27.2135887
430.0000000	3.5623999	-329.5945435
430.0000000	3.6111999	-2073.5373535
430.0000000	3.6600001	-469.0677490
430.0000000	3.7088001	-619.5424805
430.0000000	3.7575998	-970.2713623
430.0000000	3.8063998	-1761.0369873
430.0000000	3.8552001	-2556.9833984
430.0000000	3.9040000	-2506.9396973
430.0000000	3.9528000	1147.0567627
430.0000000	4.0015998	-250.0932159
430.0000000	4.0503998	702.4654541
430.0000000	4.0991998	89.8620911
430.0000000	4.1479998	-668.1191406
430.0000000	4.1967998	3.0003681
430.0000000	4.2456002	-253.3327484
430.0000000	4.2943997	-497.8389282
430.0000000	4.3431997	-13.2244034
430.0000000	4.3920002	-252.9539642
430.0000000	4.4407997	-22.5476646

430.0000000	4.4896002	-15.4309464
430.0000000	4.5383997	-667.8673706
430.0000000	4.5871997	-12.7398434
430.0000000	4.6360002	-21.6966419
430.0000000	4.6847997	-28.4745941
430.0000000	4.7336001	-19.5977936
430.0000000	4.7824001	-16.4901161
430.0000000	4.8311996	-1500.3248291
430.0000000	4.8800001	232.6307220
430.0000000	4.9287996	-204.7373810
430.0000000	4.9776001	1078.7407227
430.0000000	5.0264001	88.0072784
430.0000000	5.0751996	-306.1979065
430.0000000	5.1240001	-12.2633762
430.0000000	5.1727996	564.0911865
430.0000000	5.2216001	32.5061836
430.0000000	5.2704000	-160.3342133
430.0000000	5.3191996	-39.4283829
430.0000000	5.3680000	-3573.3154297
430.0000000	5.4168000	532.9089966
430.0000000	5.4656000	-202.6986237
430.0000000	5.5144000	38.6443405
430.0000000	5.5631995	493.6664734
430.0000000	5.6120000	397.4757996
430.0000000	5.6608000	-37.3063812
430.0000000	5.7096000	63.3670425
430.0000000	5.7584000	134.6093140
430.0000000	5.8071995	-11.4730587
430.0000000	5.8559999	30.2978535
430.0000000	5.9047999	-16.6163750
430.0000000	5.9535999	-40.0340157
430.0000000	6.0023999	-1589.5555420
430.0000000	6.0511999	-14.5566645
430.0000000	6.0999999	-19.4547882
430.0000000	6.1487999	-13.2480125
430.0000000	6.1975999	-23.3169289
430.0000000	6.2463999	-1810.8996582
430.0000000	6.2951999	-19.1150055
430.0000000	6.3439999	-346.2309265
430.0000000	6.3927999	-15.0491333
430.0000000	6.4416003	-12.3535662
430.0000000	6.4903998	-11.2262917
430.0000000	6.5391998	-11.7684460
430.0000000	6.5879998	423.4596558
430.0000000	6.6367998	-12.0535536
430.0000000	6.6856003	-13.8054047
430.0000000	6.7343998	-13.4435673
430.0000000	6.7831998	-14.3959274
430.0000000	6.8319998	59.5167160
430.0000000	6.8807998	-14.0572491
430.0000000	6.9296002	-492.6332092
430.0000000	6.9783998	-4.3564167
430.0000000	7.0271997	-14.1958494
430.0000000	7.0760002	13.5957823
430.0000000	7.1247997	-2357.5590820
430.0000000	7.1736002	-1890.5102539
430.0000000	7.2223997	-1075.1638184
430.0000000	7.2711997	-230.7430573

430.0000000	7.3200002	-19.5722542
430.0000000	7.3687997	209.4705963
430.0000000	7.4176002	435.4892578
430.0000000	7.4663997	342.3521423
430.0000000	7.5151997	-1052.2580566
430.0000000	7.5640001	-1162.4317627
430.0000000	7.6127996	-2741.5222168
430.0000000	7.6616001	-879.5266113
430.0000000	7.7104001	-451.2101440
430.0000000	7.7591996	49.0777550
430.0000000	7.8080001	452.5578918
430.0000000	7.8567996	595.7849121
430.0000000	7.9056001	-1231.4055176
430.0000000	7.9544001	1960.5544434
430.0000000	8.0031996	2529.6989746
430.0000000	8.0520000	2524.9365234
430.0000000	8.1007996	2671.8525391
430.0000000	8.1496000	2849.3786621
430.0000000	8.1983995	1772.7810059
430.0000000	8.2472000	-4055.3989258
430.0000000	8.2959995	-1437.3941650
430.0000000	8.3448000	-1145.0743408
430.0000000	8.3935995	-1049.4119873
430.0000000	8.4423990	-451.8297119
430.0000000	8.4912004	-1055.2192383
430.0000000	8.5400000	-2847.0053711
430.0000000	8.5887995	-1379.7177734
430.0000000	8.6375999	269.9671021
430.0000000	8.6863995	2435.9726563
430.0000000	8.7351999	-905.0016479
430.0000000	8.7840004	1035.0456543
430.0000000	8.8327999	57.0706940
430.0000000	8.8815994	-11.4862289
430.0000000	8.9303989	-24.1631794
430.0000000	8.9792004	-11.5742769
430.0000000	9.0279999	-5.7331181
430.0000000	9.0767994	7.3751354
430.0000000	9.1255999	-17.3427868
430.0000000	9.1743994	-10.0499392
430.0000000	9.2231998	37.9376144
430.0000000	9.2720003	-8.3649588
430.0000000	9.3207998	-9.7663193
430.0000000	9.3695993	-10.7504215
430.0000000	9.4183998	-11.7612257
430.0000000	9.4672003	-11.1589012
430.0000000	9.5159998	-14.2167788
430.0000000	9.5648003	-0.1940899
430.0000000	9.6135998	-13.4426603
430.0000000	9.6623993	-10.2051058
430.0000000	9.7111998	-10.1416826
430.0000000	9.7600002	-10.8551149
430.0000000	9.8087997	-10.9943085
430.0000000	9.8575993	3.4508228
430.0000000	9.9063997	-7.9151449
430.0000000	9.9552002	-10.9063473
430.0000000	10.0039997	-10.0601139
440.0000000	0.3416000	-36.4226341
440.0000000	0.3904000	-39.1519318

440.0000000	0.4392000	-41.5695190
440.0000000	0.4880000	-39.0938759
440.0000000	0.5368000	-39.2904434
440.0000000	0.5856000	-38.9235535
440.0000000	0.6344000	-35.6965790
440.0000000	0.6832000	-32.3275948
440.0000000	0.7320000	-30.8629551
440.0000000	0.7808000	-31.6843262
440.0000000	0.8296000	-33.5602036
440.0000000	0.8784000	-33.3831482
440.0000000	0.9272000	-32.9180260
440.0000000	0.9760000	-32.5711594
440.0000000	1.0247999	-33.5893784
440.0000000	1.0735999	-32.6328735
440.0000000	1.1224000	-31.7443085
440.0000000	1.1711999	-31.8309288
440.0000000	1.2200000	-31.9274368
440.0000000	1.2687999	-31.3125343
440.0000000	1.3176000	-30.5964603
440.0000000	1.3664000	-30.1214275
440.0000000	1.4152000	-30.1504593
440.0000000	1.4640000	-29.7144680
440.0000000	1.5128000	-45.0115280
440.0000000	1.5616000	-36.0570641
440.0000000	1.6104001	-1704.6993408
440.0000000	1.6592000	-90.3062515
440.0000000	1.7079999	-28.0758743
440.0000000	1.7567999	-25.5791168
440.0000000	1.8055999	-24.7237339
440.0000000	1.8544000	-26.2417908
440.0000000	1.9031999	-26.6011982
440.0000000	1.9520000	-26.5413361
440.0000000	2.0007999	-28.2292156
440.0000000	2.0495999	-18.5802670
440.0000000	2.0983999	-233.5693359
440.0000000	2.1471999	13.8268700
440.0000000	2.1960001	-25.6857910
440.0000000	2.2448001	-26.8666153
440.0000000	2.2935998	-31.1385994
440.0000000	2.3423998	-20.1543827
440.0000000	2.3912001	67.9301910
440.0000000	2.4400001	-22.6932487
440.0000000	2.4888000	-23.4726906
440.0000000	2.5375998	-11.2741499
440.0000000	2.5863998	-9.2406673
440.0000000	2.6352000	-7.1387768
440.0000000	2.6840000	-14.7138987
440.0000000	2.7328000	148.7344208
440.0000000	2.7815998	175.0630188
440.0000000	2.8304000	-23.8707733
440.0000000	2.8792000	-20.4281445
440.0000000	2.9280000	-22.6051311
440.0000000	2.9768000	-16.2988300
440.0000000	3.0256000	-20.3213749
440.0000000	3.0743999	-30.1842842
440.0000000	3.1231999	-24.4576607
440.0000000	3.1719999	-30.0919113
440.0000000	3.2208002	-518.1693115

440.0000000	3.2695999	-1015.0678711
440.0000000	3.3183999	-20.4781151
440.0000000	3.3671999	-3481.4357910
440.0000000	3.4159999	1249.5938721
440.0000000	3.4648001	-6.8094416
440.0000000	3.5135999	-27.2105980
440.0000000	3.5623999	-329.4649658
440.0000000	3.6111999	-2072.6774902
440.0000000	3.6600001	-468.8796387
440.0000000	3.7088001	-619.2910767
440.0000000	3.7575998	-969.8728638
440.0000000	3.8063998	-1760.3071289
440.0000000	3.8552001	-2555.9199219
440.0000000	3.9040000	-2505.8972168
440.0000000	3.9528000	1146.5684814
440.0000000	4.0015998	-249.9961090
440.0000000	4.0503998	702.1629028
440.0000000	4.0991998	89.8164063
440.0000000	4.1479998	-667.8469238
440.0000000	4.1967998	2.9911118
440.0000000	4.2456002	-253.2344360
440.0000000	4.2943997	-497.6383667
440.0000000	4.3431997	-13.2265835
440.0000000	4.3920002	-252.8558502
440.0000000	4.4407997	-22.5458870
440.0000000	4.4896002	-15.4318295
440.0000000	4.5383997	-667.6063232
440.0000000	4.5871997	-12.7418594
440.0000000	4.6360002	-21.6951008
440.0000000	4.6847997	-28.4706688
440.0000000	4.7336001	-19.5970707
440.0000000	4.7824001	-16.4905701
440.0000000	4.8311996	-1499.7935791
440.0000000	4.8800001	232.5411682
440.0000000	4.9287996	-204.6701202
440.0000000	4.9776001	1078.3482666
440.0000000	5.0264001	87.9697571
440.0000000	5.0751996	-306.0942688
440.0000000	5.1240001	-12.2642975
440.0000000	5.1727996	563.9753418
440.0000000	5.2216001	32.4967422
440.0000000	5.2704000	-160.3061066
440.0000000	5.3191996	-39.4238358
440.0000000	5.3680000	-3572.6230469
440.0000000	5.4168000	532.8021851
440.0000000	5.4656000	-202.6624451
440.0000000	5.5144000	38.6336823
440.0000000	5.5631995	493.5679016
440.0000000	5.6120000	397.3958130
440.0000000	5.6608000	-37.3022957
440.0000000	5.7096000	63.3516159
440.0000000	5.7584000	134.5803833
440.0000000	5.8071995	-11.4739304
440.0000000	5.8559999	30.2889996
440.0000000	5.9047999	-16.6161766
440.0000000	5.9535999	-40.0292740
440.0000000	6.0023999	-1589.2512207
440.0000000	6.0511999	-14.5568762

440.0000000	6.0999999	-19.4539566
440.0000000	6.1487999	-13.2484751
440.0000000	6.1975999	-23.3151627
440.0000000	6.2463999	-1810.5523682
440.0000000	6.2951999	-19.1141911
440.0000000	6.3439999	-346.1668701
440.0000000	6.3927999	-15.0491152
440.0000000	6.4416003	-12.3541527
440.0000000	6.4903998	-11.2269678
440.0000000	6.5391998	-11.7691278
440.0000000	6.5879998	422.7689514
440.0000000	6.6367998	-12.0541029
440.0000000	6.6856003	-13.8055744
440.0000000	6.7343998	-13.4437838
440.0000000	6.7831998	-14.3955126
440.0000000	6.8319998	59.4975967
440.0000000	6.8807998	-14.0571108
440.0000000	6.9296002	-491.8717651
440.0000000	6.9783998	-4.3692398
440.0000000	7.0271997	-14.1948214
440.0000000	7.0760002	13.5535955
440.0000000	7.1247997	-2353.6132813
440.0000000	7.1736002	-1887.3474121
440.0000000	7.2223997	-1073.3706055
440.0000000	7.2711997	-230.3752747
440.0000000	7.3200002	-19.5602646
440.0000000	7.3687997	209.0960693
440.0000000	7.4176002	434.7334290
440.0000000	7.4663997	341.7544861
440.0000000	7.5151997	-1050.4969482
440.0000000	7.5640001	-1160.4842529
440.0000000	7.6127996	-2736.9045410
440.0000000	7.6616001	-878.0552979
440.0000000	7.7104001	-450.4638672
440.0000000	7.7591996	48.9767036
440.0000000	7.8080001	451.7725220
440.0000000	7.8567996	594.7568359
440.0000000	7.9056001	-1229.3326416
440.0000000	7.9544001	1957.2445068
440.0000000	8.0031996	2525.4294434
440.0000000	8.0520000	2520.6745605
440.0000000	8.1007996	2667.3430176
440.0000000	8.1496000	2844.5705566
440.0000000	8.1983995	1769.7884521
440.0000000	8.2472000	-4048.5642090
440.0000000	8.2959995	-1434.9739990
440.0000000	8.3448000	-1143.1451416
440.0000000	8.3935995	-1047.6442871
440.0000000	8.4423990	-451.0716858
440.0000000	8.4912004	-1053.4429932
440.0000000	8.5400000	-2842.2055664
440.0000000	8.5887995	-1377.3962402
440.0000000	8.6375999	269.5057678
440.0000000	8.6863995	2431.8542480
440.0000000	8.7351999	-903.4773560
440.0000000	8.7840004	1033.2888184
440.0000000	8.8327999	56.9590912
440.0000000	8.8815994	-11.4864035

440.0000000	8.9303989	-24.1594582
440.0000000	8.9792004	-11.5743589
440.0000000	9.0279999	-5.7348866
440.0000000	9.0767994	7.3694172
440.0000000	9.1255999	-17.3410721
440.0000000	9.1743994	-10.0503407
440.0000000	9.2231998	37.9218826
440.0000000	9.2720003	-8.3658953
440.0000000	9.3207998	-9.7668581
440.0000000	9.3695993	-10.7506485
440.0000000	9.4183998	-11.7611008
440.0000000	9.4672003	-11.1589422
440.0000000	9.5159998	-14.2158880
440.0000000	9.5648003	-0.1974621
440.0000000	9.6135998	-13.4418697
440.0000000	9.6623993	-10.2053814
440.0000000	9.7111998	-10.1419544
440.0000000	9.7600002	-10.8551569
440.0000000	9.8087997	-10.9943171
440.0000000	9.8575993	3.4464016
440.0000000	9.9063997	-7.9160595
440.0000000	9.9552002	-10.9063520
440.0000000	10.0039997	-10.0603619
450.0000000	0.3416000	-36.5100288
450.0000000	0.3904000	-39.7274132
450.0000000	0.4392000	-41.4039955
450.0000000	0.4880000	-39.1758575
450.0000000	0.5368000	-39.3106728
450.0000000	0.5856000	-38.5354004
450.0000000	0.6344000	-35.6018219
450.0000000	0.6832000	-32.7574883
450.0000000	0.7320000	-32.9006310
450.0000000	0.7808000	-32.7067032
450.0000000	0.8296000	-32.3464432
450.0000000	0.8784000	-33.1922035
450.0000000	0.9272000	-32.7892189
450.0000000	0.9760000	-32.5993919
450.0000000	1.0247999	-33.5327263
450.0000000	1.0735999	-32.5817642
450.0000000	1.1224000	-31.7258816
450.0000000	1.1711999	-31.8161488
450.0000000	1.2200000	-31.9272938
450.0000000	1.2687999	-31.3217678
450.0000000	1.3176000	-30.6225700
450.0000000	1.3664000	-30.1291771
450.0000000	1.4152000	-30.1506634
450.0000000	1.4640000	-29.7127590
450.0000000	1.5128000	-44.9143524
450.0000000	1.5616000	-36.0152206
450.0000000	1.6104001	-1702.1566162
450.0000000	1.6592000	-90.0123215
450.0000000	1.7079999	-28.0864677
450.0000000	1.7567999	-25.9516354
450.0000000	1.8055999	-25.6204491
450.0000000	1.8544000	-27.1364307
450.0000000	1.9031999	-26.6155529
450.0000000	1.9520000	-26.5417900
450.0000000	2.0007999	-28.1666336

450.0000000	2.0495999	-18.7668839
450.0000000	2.0983999	-232.0935669
450.0000000	2.1471999	13.5596008
450.0000000	2.1960001	-25.6816998
450.0000000	2.2448001	-26.8487034
450.0000000	2.2935998	-31.0789757
450.0000000	2.3423998	-20.1687889
450.0000000	2.3912001	67.3086472
450.0000000	2.4400001	-22.6957359
450.0000000	2.4888000	-23.4724731
450.0000000	2.5375998	-11.2912579
450.0000000	2.5863998	-9.2604265
450.0000000	2.6352000	-7.1615210
450.0000000	2.6840000	-14.7258406
450.0000000	2.7328000	148.4683990
450.0000000	2.7815998	174.7565918
450.0000000	2.8304000	-23.8694687
450.0000000	2.8792000	-20.4314079
450.0000000	2.9280000	-22.6045609
450.0000000	2.9768000	-16.3072014
450.0000000	3.0256000	-20.3239594
450.0000000	3.0743999	-30.1728973
450.0000000	3.1231999	-24.4542065
450.0000000	3.1719999	-30.0836182
450.0000000	3.2208002	-517.9315796
450.0000000	3.2695999	-1014.5928955
450.0000000	3.3183999	-20.4783134
450.0000000	3.3671999	-3479.9743652
450.0000000	3.4159999	1249.0585938
450.0000000	3.4648001	-6.8151493
450.0000000	3.5135999	-27.2076073
450.0000000	3.5623999	-329.3353577
450.0000000	3.6111999	-2071.8176270
450.0000000	3.6600001	-468.6915283
450.0000000	3.7088001	-619.0397339
450.0000000	3.7575998	-969.4743042
450.0000000	3.8063998	-1759.5772705
450.0000000	3.8552001	-2554.8564453
450.0000000	3.9040000	-2504.8547363
450.0000000	3.9528000	1146.0802002
450.0000000	4.0015998	-249.8990021
450.0000000	4.0503998	701.8603516
450.0000000	4.0991998	89.7707214
450.0000000	4.1479998	-667.5747070
450.0000000	4.1967998	2.9818554
450.0000000	4.2456002	-253.1361389
450.0000000	4.2943997	-497.4378052
450.0000000	4.3431997	-13.2287645
450.0000000	4.3920002	-252.7577362
450.0000000	4.4407997	-22.5441132
450.0000000	4.4896002	-15.4327116
450.0000000	4.5383997	-667.3452148
450.0000000	4.5871997	-12.7438755
450.0000000	4.6360002	-21.6935577
450.0000000	4.6847997	-28.4667435
450.0000000	4.7336001	-19.5963478
450.0000000	4.7824001	-16.4910240
450.0000000	4.8311996	-1499.2623291

450.0000000	4.8800001	232.4516296
450.0000000	4.9287996	-204.6028595
450.0000000	4.9776001	1077.9558105
450.0000000	5.0264001	87.9322357
450.0000000	5.0751996	-305.9906311
450.0000000	5.1240001	-12.2652187
450.0000000	5.1727996	563.8594360
450.0000000	5.2216001	32.4872932
450.0000000	5.2704000	-160.2779999
450.0000000	5.3191996	-39.4192848
450.0000000	5.3680000	-3571.9306641
450.0000000	5.4168000	532.6953735
450.0000000	5.4656000	-202.6262665
450.0000000	5.5144000	38.6230316
450.0000000	5.5631995	493.4693298
450.0000000	5.6120000	397.3158569
450.0000000	5.6608000	-37.2982063
450.0000000	5.7096000	63.3361816
450.0000000	5.7584000	134.5514374
450.0000000	5.8071995	-11.4748001
450.0000000	5.8559999	30.2801495
450.0000000	5.9047999	-16.6159801
450.0000000	5.9535999	-40.0245361
450.0000000	6.0023999	-1588.9467773
450.0000000	6.0511999	-14.5570879
450.0000000	6.0999999	-19.4531250
450.0000000	6.1487999	-13.2489376
450.0000000	6.1975999	-23.3133984
450.0000000	6.2463999	-1810.2049561
450.0000000	6.2951999	-19.1133766
450.0000000	6.3439999	-346.1028442
450.0000000	6.3927999	-15.0490961
450.0000000	6.4416003	-12.3547401
450.0000000	6.4903998	-11.2276440
450.0000000	6.5391998	-11.7698088
450.0000000	6.5879998	422.0782471
450.0000000	6.6367998	-12.0546522
450.0000000	6.6856003	-13.8057451
450.0000000	6.7343998	-13.4439993
450.0000000	6.7831998	-14.3950968
450.0000000	6.8319998	59.4784851
450.0000000	6.8807998	-14.0569725
450.0000000	6.9296002	-491.1103210
450.0000000	6.9783998	-4.3820629
450.0000000	7.0271997	-14.1937933
450.0000000	7.0760002	13.5114126
450.0000000	7.1247997	-2349.6674805
450.0000000	7.1736002	-1884.1845703
450.0000000	7.2223997	-1071.5773926
450.0000000	7.2711997	-230.0075073
450.0000000	7.3200002	-19.5482750
450.0000000	7.3687997	208.7215271
450.0000000	7.4176002	433.9776001
450.0000000	7.4663997	341.1568298
450.0000000	7.5151997	-1048.7359619
450.0000000	7.5640001	-1158.5368652
450.0000000	7.6127996	-2732.2871094
450.0000000	7.6616001	-876.5839233

450.0000000	7.7104001	-449.7175903
450.0000000	7.7591996	48.8756561
450.0000000	7.8080001	450.9871521
450.0000000	7.8567996	593.7287598
450.0000000	7.9056001	-1227.2596436
450.0000000	7.9544001	1953.9345703
450.0000000	8.0031996	2521.1599121
450.0000000	8.0520000	2516.4123535
450.0000000	8.1007996	2662.8334961
450.0000000	8.1496000	2839.7624512
450.0000000	8.1983995	1766.7960205
450.0000000	8.2472000	-4041.7294922
450.0000000	8.2959995	-1432.5538330
450.0000000	8.3448000	-1141.2159424
450.0000000	8.3935995	-1045.8765869
450.0000000	8.4423990	-450.3136902
450.0000000	8.4912004	-1051.6667480
450.0000000	8.5400000	-2837.4057617
450.0000000	8.5887995	-1375.0747070
450.0000000	8.6375999	269.0444336
450.0000000	8.6863995	2427.7358398
450.0000000	8.7351999	-901.9531250
450.0000000	8.7840004	1031.5321045
450.0000000	8.8327999	56.8474808
450.0000000	8.8815994	-11.4865770
450.0000000	8.9303989	-24.1557388
450.0000000	8.9792004	-11.5744410
450.0000000	9.0279999	-5.7366543
450.0000000	9.0767994	7.3636990
450.0000000	9.1255999	-17.3393593
450.0000000	9.1743994	-10.0507431
450.0000000	9.2231998	37.9061508
450.0000000	9.2720003	-8.3668308
450.0000000	9.3207998	-9.7673979
450.0000000	9.3695993	-10.7508745
450.0000000	9.4183998	-11.7609758
450.0000000	9.4672003	-11.1589832
450.0000000	9.5159998	-14.2149973
450.0000000	9.5648003	-0.2008352
450.0000000	9.6135998	-13.4410791
450.0000000	9.6623993	-10.2056561
450.0000000	9.7111998	-10.1422262
450.0000000	9.7600002	-10.8551989
450.0000000	9.8087997	-10.9943247
450.0000000	9.8575993	3.4419794
450.0000000	9.9063997	-7.9169731
450.0000000	9.9552002	-10.9063559
450.0000000	10.0039997	-10.0606089
460.0000000	0.3416000	-36.5135574
460.0000000	0.3904000	-39.9851837
460.0000000	0.4392000	-41.2384720
460.0000000	0.4880000	-39.2578354
460.0000000	0.5368000	-39.3308983
460.0000000	0.5856000	-38.1472435
460.0000000	0.6344000	-37.6830063
460.0000000	0.6832000	-33.3354301
460.0000000	0.7320000	-32.5720139
460.0000000	0.7808000	-32.5686684

460.0000000	0.8296000	-31.1577187
460.0000000	0.8784000	-32.1358337
460.0000000	0.9272000	-34.2983055
460.0000000	0.9760000	-33.2935944
460.0000000	1.0247999	-33.4760742
460.0000000	1.0735999	-32.5306511
460.0000000	1.1224000	-31.7074528
460.0000000	1.1711999	-31.8013668
460.0000000	1.2200000	-31.9271507
460.0000000	1.2687999	-31.3310013
460.0000000	1.3176000	-30.6486797
460.0000000	1.3664000	-30.1369267
460.0000000	1.4152000	-30.1508675
460.0000000	1.4640000	-29.7110500
460.0000000	1.5128000	-44.8171730
460.0000000	1.5616000	-35.9733810
460.0000000	1.6104001	-1699.6137695
460.0000000	1.6592000	-89.7183838
460.0000000	1.7079999	-28.0970631
460.0000000	1.7567999	-25.7073956
460.0000000	1.8055999	-25.7165661
460.0000000	1.8544000	-27.1603584
460.0000000	1.9031999	-26.6299057
460.0000000	1.9520000	-26.5422459
460.0000000	2.0007999	-28.1040516
460.0000000	2.0495999	-18.9535007
460.0000000	2.0983999	-230.6177673
460.0000000	2.1471999	13.2923317
460.0000000	2.1960001	-25.6776085
460.0000000	2.2448001	-26.8307915
460.0000000	2.2935998	-31.0193520
460.0000000	2.3423998	-20.1831932
460.0000000	2.3912001	66.6870956
460.0000000	2.4400001	-22.6982212
460.0000000	2.4888000	-23.4722557
460.0000000	2.5375998	-11.3083658
460.0000000	2.5863998	-9.2801857
460.0000000	2.6352000	-7.1842651
460.0000000	2.6840000	-14.7377825
460.0000000	2.7328000	148.2023926
460.0000000	2.7815998	174.4501648
460.0000000	2.8304000	-23.8681660
460.0000000	2.8792000	-20.4346714
460.0000000	2.9280000	-22.6039925
460.0000000	2.9768000	-16.3155708
460.0000000	3.0256000	-20.3265438
460.0000000	3.0743999	-30.1615105
460.0000000	3.1231999	-24.4507504
460.0000000	3.1719999	-30.0753250
460.0000000	3.2208002	-517.6938477
460.0000000	3.2695999	-1014.1179199
460.0000000	3.3183999	-20.4785099
460.0000000	3.3671999	-3478.5126953
460.0000000	3.4159999	1248.5231934
460.0000000	3.4648001	-6.8208580
460.0000000	3.5135999	-27.2046165
460.0000000	3.5623999	-329.2057800
460.0000000	3.6111999	-2070.9580078

460.0000000	3.6600001	-468.5033569
460.0000000	3.7088001	-618.7883301
460.0000000	3.7575998	-969.0757446
460.0000000	3.8063998	-1758.8474121
460.0000000	3.8552001	-2553.7929688
460.0000000	3.9040000	-2503.8122559
460.0000000	3.9528000	1145.5919189
460.0000000	4.0015998	-249.8018951
460.0000000	4.0503998	701.5578003
460.0000000	4.0991998	89.7250366
460.0000000	4.1479998	-667.3024902
460.0000000	4.1967998	2.9725990
460.0000000	4.2456002	-253.0378418
460.0000000	4.2943997	-497.2372437
460.0000000	4.3431997	-13.2309456
460.0000000	4.3920002	-252.6596222
460.0000000	4.4407997	-22.5423374
460.0000000	4.4896002	-15.4335938
460.0000000	4.5383997	-667.0841675
460.0000000	4.5871997	-12.7458916
460.0000000	4.6360002	-21.6920147
460.0000000	4.6847997	-28.4628181
460.0000000	4.7336001	-19.5956268
460.0000000	4.7824001	-16.4914799
460.0000000	4.8311996	-1498.7310791
460.0000000	4.8800001	232.3620758
460.0000000	4.9287996	-204.5355835
460.0000000	4.9776001	1077.5632324
460.0000000	5.0264001	87.8947220
460.0000000	5.0751996	-305.8870239
460.0000000	5.1240001	-12.2661400
460.0000000	5.1727996	563.7435913
460.0000000	5.2216001	32.4778519
460.0000000	5.2704000	-160.2498932
460.0000000	5.3191996	-39.4147339
460.0000000	5.3680000	-3571.2382813
460.0000000	5.4168000	532.5886230
460.0000000	5.4656000	-202.5900726
460.0000000	5.5144000	38.6123734
460.0000000	5.5631995	493.3707886
460.0000000	5.6120000	397.2358704
460.0000000	5.6608000	-37.2941208
460.0000000	5.7096000	63.3207474
460.0000000	5.7584000	134.5225067
460.0000000	5.8071995	-11.4756708
460.0000000	5.8559999	30.2712994
460.0000000	5.9047999	-16.6157818
460.0000000	5.9535999	-40.0197983
460.0000000	6.0023999	-1588.6424561
460.0000000	6.0511999	-14.5572987
460.0000000	6.0999999	-19.4522915
460.0000000	6.1487999	-13.2494011
460.0000000	6.1975999	-23.3116341
460.0000000	6.2463999	-1809.8575439
460.0000000	6.2951999	-19.1125641
460.0000000	6.3439999	-346.0387573
460.0000000	6.3927999	-15.0490780
460.0000000	6.4416003	-12.3553267

460.0000000	6.4903998	-11.2283192
460.0000000	6.5391998	-11.7704897
460.0000000	6.5879998	421.3875732
460.0000000	6.6367998	-12.0552015
460.0000000	6.6856003	-13.8059149
460.0000000	6.7343998	-13.4442148
460.0000000	6.7831998	-14.3946810
460.0000000	6.8319998	59.4593735
460.0000000	6.8807998	-14.0568333
460.0000000	6.9296002	-490.3488464
460.0000000	6.9783998	-4.3948870
460.0000000	7.0271997	-14.1927662
460.0000000	7.0760002	13.4692259
460.0000000	7.1247997	-2345.7214355
460.0000000	7.1736002	-1881.0217285
460.0000000	7.2223997	-1069.7843018
460.0000000	7.2711997	-229.6397247
460.0000000	7.3200002	-19.5362873
460.0000000	7.3687997	208.3470001
460.0000000	7.4176002	433.2217712
460.0000000	7.4663997	340.5591736
460.0000000	7.5151997	-1046.9748535
460.0000000	7.5640001	-1156.5894775
460.0000000	7.6127996	-2727.6694336
460.0000000	7.6616001	-875.1125488
460.0000000	7.7104001	-448.9713135
460.0000000	7.7591996	48.7746086
460.0000000	7.8080001	450.2018127
460.0000000	7.8567996	592.7006226
460.0000000	7.9056001	-1225.1867676
460.0000000	7.9544001	1950.6246338
460.0000000	8.0031996	2516.8901367
460.0000000	8.0520000	2512.1503906
460.0000000	8.1007996	2658.3239746
460.0000000	8.1496000	2834.9545898
460.0000000	8.1983995	1763.8034668
460.0000000	8.2472000	-4034.8945313
460.0000000	8.2959995	-1430.1336670
460.0000000	8.3448000	-1139.2868652
460.0000000	8.3935995	-1044.1088867
460.0000000	8.4423990	-449.5556641
460.0000000	8.4912004	-1049.8905029
460.0000000	8.5400000	-2832.6059570
460.0000000	8.5887995	-1372.7531738
460.0000000	8.6375999	268.5831299
460.0000000	8.6863995	2423.6176758
460.0000000	8.7351999	-900.4288940
460.0000000	8.7840004	1029.7753906
460.0000000	8.8327999	56.7358704
460.0000000	8.8815994	-11.4867516
460.0000000	8.9303989	-24.1520195
460.0000000	8.9792004	-11.5745239
460.0000000	9.0279999	-5.7384229
460.0000000	9.0767994	7.3579826
460.0000000	9.1255999	-17.3376465
460.0000000	9.1743994	-10.0511456
460.0000000	9.2231998	37.8904114
460.0000000	9.2720003	-8.3677673

460.0000000	9.3207998	-9.7679367
460.0000000	9.3695993	-10.7511015
460.0000000	9.4183998	-11.7608519
460.0000000	9.4672003	-11.1590242
460.0000000	9.5159998	-14.2141056
460.0000000	9.5648003	-0.2042084
460.0000000	9.6135998	-13.4402885
460.0000000	9.6623993	-10.2059307
460.0000000	9.7111998	-10.1424980
460.0000000	9.7600002	-10.8552399
460.0000000	9.8087997	-10.9943333
460.0000000	9.8575993	3.4375553
460.0000000	9.9063997	-7.9178867
460.0000000	9.9552002	-10.9063606
460.0000000	10.0039997	-10.0608568
470.0000000	0.3416000	-36.5941086
470.0000000	0.3904000	-39.4755592
470.0000000	0.4392000	-41.0729485
470.0000000	0.4880000	-39.3398170
470.0000000	0.5368000	-39.3511276
470.0000000	0.5856000	-37.7590904
470.0000000	0.6344000	-38.1198158
470.0000000	0.6832000	-34.3063889
470.0000000	0.7320000	-32.7262917
470.0000000	0.7808000	-31.8519135
470.0000000	0.8296000	-31.5739059
470.0000000	0.8784000	-32.5944595
470.0000000	0.9272000	-33.4249420
470.0000000	0.9760000	-33.9877968
470.0000000	1.0247999	-33.4194183
470.0000000	1.0735999	-32.4795380
470.0000000	1.1224000	-31.6890259
470.0000000	1.1711999	-31.7865868
470.0000000	1.2200000	-31.9270096
470.0000000	1.2687999	-31.3402348
470.0000000	1.3176000	-30.6747875
470.0000000	1.3664000	-30.1446762
470.0000000	1.4152000	-30.1510715
470.0000000	1.4640000	-29.7093410
470.0000000	1.5128000	-44.7199936
470.0000000	1.5616000	-35.9315414
470.0000000	1.6104001	-1697.0710449
470.0000000	1.6592000	-89.4244537
470.0000000	1.7079999	-27.8152905
470.0000000	1.7567999	-25.8732586
470.0000000	1.8055999	-25.9654903
470.0000000	1.8544000	-27.1842861
470.0000000	1.9031999	-26.6442604
470.0000000	1.9520000	-26.5426998
470.0000000	2.0007999	-28.0414696
470.0000000	2.0495999	-19.1401176
470.0000000	2.0983999	-229.1419983
470.0000000	2.1471999	13.0250664
470.0000000	2.1960001	-25.6735172
470.0000000	2.2448001	-26.8128815
470.0000000	2.2935998	-30.9597282
470.0000000	2.3423998	-20.1975975
470.0000000	2.3912001	66.0655518

470.0000000	2.4400001	-22.7007084
470.0000000	2.4888000	-23.4720402
470.0000000	2.5375998	-11.3254738
470.0000000	2.5863998	-9.2999449
470.0000000	2.6352000	-7.2070093
470.0000000	2.6840000	-14.7497234
470.0000000	2.7328000	147.9363708
470.0000000	2.7815998	174.1437683
470.0000000	2.8304000	-23.8668613
470.0000000	2.8792000	-20.4379349
470.0000000	2.9280000	-22.6034241
470.0000000	2.9768000	-16.3239403
470.0000000	3.0256000	-20.3291283
470.0000000	3.0743999	-30.1501236
470.0000000	3.1231999	-24.4472961
470.0000000	3.1719999	-30.0670319
470.0000000	3.2208002	-517.4561157
470.0000000	3.2695999	-1013.6429443
470.0000000	3.3183999	-20.4787083
470.0000000	3.3671999	-3477.0510254
470.0000000	3.4159999	1247.9879150
470.0000000	3.4648001	-6.8265667
470.0000000	3.5135999	-27.2016258
470.0000000	3.5623999	-329.0761719
470.0000000	3.6111999	-2070.0981445
470.0000000	3.6600001	-468.3152466
470.0000000	3.7088001	-618.5369873
470.0000000	3.7575998	-968.6772461
470.0000000	3.8063998	-1758.1175537
470.0000000	3.8552001	-2552.7297363
470.0000000	3.9040000	-2502.7697754
470.0000000	3.9528000	1145.1036377
470.0000000	4.0015998	-249.7047882
470.0000000	4.0503998	701.2552490
470.0000000	4.0991998	89.6793518
470.0000000	4.1479998	-667.0302734
470.0000000	4.1967998	2.9633427
470.0000000	4.2456002	-252.9395294
470.0000000	4.2943997	-497.0366821
470.0000000	4.3431997	-13.2331257
470.0000000	4.3920002	-252.5615082
470.0000000	4.4407997	-22.5405617
470.0000000	4.4896002	-15.4344769
470.0000000	4.5383997	-666.8231201
470.0000000	4.5871997	-12.7479076
470.0000000	4.6360002	-21.6904736
470.0000000	4.6847997	-28.4588928
470.0000000	4.7336001	-19.5949039
470.0000000	4.7824001	-16.4919338
470.0000000	4.8311996	-1498.1998291
470.0000000	4.8800001	232.2725220
470.0000000	4.9287996	-204.4683228
470.0000000	4.9776001	1077.1707764
470.0000000	5.0264001	87.8572006
470.0000000	5.0751996	-305.7833862
470.0000000	5.1240001	-12.2670612
470.0000000	5.1727996	563.6277466
470.0000000	5.2216001	32.4684105

470.0000000	5.2704000	-160.2217865
470.0000000	5.3191996	-39.4101868
470.0000000	5.3680000	-3570.5458984
470.0000000	5.4168000	532.4818726
470.0000000	5.4656000	-202.5538940
470.0000000	5.5144000	38.6017151
470.0000000	5.5631995	493.2722168
470.0000000	5.6120000	397.1558838
470.0000000	5.6608000	-37.2900314
470.0000000	5.7096000	63.3053207
470.0000000	5.7584000	134.4935760
470.0000000	5.8071995	-11.4765415
470.0000000	5.8559999	30.2624454
470.0000000	5.9047999	-16.6155834
470.0000000	5.9535999	-40.0150604
470.0000000	6.0023999	-1588.3378906
470.0000000	6.0511999	-14.5575104
470.0000000	6.0999999	-19.4514599
470.0000000	6.1487999	-13.2498636
470.0000000	6.1975999	-23.3098679
470.0000000	6.2463999	-1809.5102539
470.0000000	6.2951999	-19.1117496
470.0000000	6.3439999	-345.9747314
470.0000000	6.3927999	-15.0490599
470.0000000	6.4416003	-12.3559141
470.0000000	6.4903998	-11.2289953
470.0000000	6.5391998	-11.7711706
470.0000000	6.5879998	420.6968689
470.0000000	6.6367998	-12.0557499
470.0000000	6.6856003	-13.8060856
470.0000000	6.7343998	-13.4444304
470.0000000	6.7831998	-14.3942652
470.0000000	6.8319998	59.4402542
470.0000000	6.8807998	-14.0566950
470.0000000	6.9296002	-489.5874023
470.0000000	6.9783998	-4.4077101
470.0000000	7.0271997	-14.1917381
470.0000000	7.0760002	13.4270391
470.0000000	7.1247997	-2341.7756348
470.0000000	7.1736002	-1877.8588867
470.0000000	7.2223997	-1067.9910889
470.0000000	7.2711997	-229.2719421
470.0000000	7.3200002	-19.5242996
470.0000000	7.3687997	207.9724731
470.0000000	7.4176002	432.4659424
470.0000000	7.4663997	339.9615173
470.0000000	7.5151997	-1045.2138672
470.0000000	7.5640001	-1154.6420898
470.0000000	7.6127996	-2723.0517578
470.0000000	7.6616001	-873.6412354
470.0000000	7.7104001	-448.2250977
470.0000000	7.7591996	48.6735573
470.0000000	7.8080001	449.4164429
470.0000000	7.8567996	591.6725464
470.0000000	7.9056001	-1223.1138916
470.0000000	7.9544001	1947.3146973
470.0000000	8.0031996	2512.6206055
470.0000000	8.0520000	2507.8884277

470.0000000	8.1007996	2653.8144531
470.0000000	8.1496000	2830.1464844
470.0000000	8.1983995	1760.8109131
470.0000000	8.2472000	-4028.0598145
470.0000000	8.2959995	-1427.7135010
470.0000000	8.3448000	-1137.3576660
470.0000000	8.3935995	-1042.3413086
470.0000000	8.4423990	-448.7976685
470.0000000	8.4912004	-1048.1143799
470.0000000	8.5400000	-2827.8061523
470.0000000	8.5887995	-1370.4315186
470.0000000	8.6375999	268.1217957
470.0000000	8.6863995	2419.4992676
470.0000000	8.7351999	-898.9046021
470.0000000	8.7840004	1028.0186768
470.0000000	8.8327999	56.6242599
470.0000000	8.8815994	-11.4869261
470.0000000	8.9303989	-24.1483002
470.0000000	8.9792004	-11.5746059
470.0000000	9.0279999	-5.7401910
470.0000000	9.0767994	7.3522644
470.0000000	9.1255999	-17.3359318
470.0000000	9.1743994	-10.0515471
470.0000000	9.2231998	37.8746796
470.0000000	9.2720003	-8.3687038
470.0000000	9.3207998	-9.7684765
470.0000000	9.3695993	-10.7513275
470.0000000	9.4183998	-11.7607269
470.0000000	9.4672003	-11.1590662
470.0000000	9.5159998	-14.2132149
470.0000000	9.5648003	-0.2075806
470.0000000	9.6135998	-13.4394979
470.0000000	9.6623993	-10.2062063
470.0000000	9.7111998	-10.1427689
470.0000000	9.7600002	-10.8552818
470.0000000	9.8087997	-10.9943409
470.0000000	9.8575993	3.4331341
470.0000000	9.9063997	-7.9188008
470.0000000	9.9552002	-10.9063644
470.0000000	10.0039997	-10.0611038
480.0000000	0.3416000	-36.8417397
480.0000000	0.3904000	-39.6408997
480.0000000	0.4392000	-40.9074249
480.0000000	0.4880000	-39.3646240
480.0000000	0.5368000	-39.3713570
480.0000000	0.5856000	-37.3709373
480.0000000	0.6344000	-37.7701874
480.0000000	0.6832000	-34.6063919
480.0000000	0.7320000	-32.4971352
480.0000000	0.7808000	-31.9327126
480.0000000	0.8296000	-32.7267609
480.0000000	0.8784000	-33.6974297
480.0000000	0.9272000	-33.6782761
480.0000000	0.9760000	-34.1688156
480.0000000	1.0247999	-33.3627663
480.0000000	1.0735999	-32.4284248
480.0000000	1.1224000	-31.6705971
480.0000000	1.1711999	-31.7718048

480.0000000	1.2200000	-31.9268665
480.0000000	1.2687999	-31.3494682
480.0000000	1.3176000	-30.7008972
480.0000000	1.3664000	-30.1524258
480.0000000	1.4152000	-30.1512756
480.0000000	1.4640000	-29.7076302
480.0000000	1.5128000	-44.6228180
480.0000000	1.5616000	-35.8896980
480.0000000	1.6104001	-1694.5281982
480.0000000	1.6592000	-89.1305237
480.0000000	1.7079999	-27.5335159
480.0000000	1.7567999	-26.6556511
480.0000000	1.8055999	-26.6354866
480.0000000	1.8544000	-27.2082138
480.0000000	1.9031999	-26.6586132
480.0000000	1.9520000	-26.5431557
480.0000000	2.0007999	-27.9788876
480.0000000	2.0495999	-19.3267326
480.0000000	2.0983999	-227.6662292
480.0000000	2.1471999	12.7578011
480.0000000	2.1960001	-25.6694260
480.0000000	2.2448001	-26.7949696
480.0000000	2.2935998	-30.9001045
480.0000000	2.3423998	-20.2120018
480.0000000	2.3912001	65.4440079
480.0000000	2.4400001	-22.7031956
480.0000000	2.4888000	-23.4718227
480.0000000	2.5375998	-11.3425817
480.0000000	2.5863998	-9.3197041
480.0000000	2.6352000	-7.2297535
480.0000000	2.6840000	-14.7616653
480.0000000	2.7328000	147.6703644
480.0000000	2.7815998	173.8373413
480.0000000	2.8304000	-23.8655567
480.0000000	2.8792000	-20.4411983
480.0000000	2.9280000	-22.6028538
480.0000000	2.9768000	-16.3323116
480.0000000	3.0256000	-20.3317127
480.0000000	3.0743999	-30.1387367
480.0000000	3.1231999	-24.4438400
480.0000000	3.1719999	-30.0587387
480.0000000	3.2208002	-517.2183838
480.0000000	3.2695999	-1013.1679688
480.0000000	3.3183999	-20.4789066
480.0000000	3.3671999	-3475.5893555
480.0000000	3.4159999	1247.4526367
480.0000000	3.4648001	-6.8322744
480.0000000	3.5135999	-27.1986351
480.0000000	3.5623999	-328.9465942
480.0000000	3.6111999	-2069.2385254
480.0000000	3.6600001	-468.1271362
480.0000000	3.7088001	-618.2855835
480.0000000	3.7575998	-968.2786865
480.0000000	3.8063998	-1757.3876953
480.0000000	3.8552001	-2551.6662598
480.0000000	3.9040000	-2501.7272949
480.0000000	3.9528000	1144.6152344
480.0000000	4.0015998	-249.6076813

480.0000000	4.0503998	700.9526978
480.0000000	4.0991998	89.6336670
480.0000000	4.1479998	-666.7580566
480.0000000	4.1967998	2.9540863
480.0000000	4.2456002	-252.8412323
480.0000000	4.2943997	-496.8360901
480.0000000	4.3431997	-13.2353058
480.0000000	4.3920002	-252.4633942
480.0000000	4.4407997	-22.5387859
480.0000000	4.4896002	-15.4353600
480.0000000	4.5383997	-666.5620117
480.0000000	4.5871997	-12.7499237
480.0000000	4.6360002	-21.6889305
480.0000000	4.6847997	-28.4549675
480.0000000	4.7336001	-19.5941811
480.0000000	4.7824001	-16.4923878
480.0000000	4.8311996	-1497.6684570
480.0000000	4.8800001	232.1829834
480.0000000	4.9287996	-204.4010620
480.0000000	4.9776001	1076.7783203
480.0000000	5.0264001	87.8196793
480.0000000	5.0751996	-305.6797485
480.0000000	5.1240001	-12.2679825
480.0000000	5.1727996	563.5118408
480.0000000	5.2216001	32.4589615
480.0000000	5.2704000	-160.1936798
480.0000000	5.3191996	-39.4056396
480.0000000	5.3680000	-3569.8535156
480.0000000	5.4168000	532.3750610
480.0000000	5.4656000	-202.5177155
480.0000000	5.5144000	38.5910568
480.0000000	5.5631995	493.1736450
480.0000000	5.6120000	397.0759277
480.0000000	5.6608000	-37.2859421
480.0000000	5.7096000	63.2898941
480.0000000	5.7584000	134.4646454
480.0000000	5.8071995	-11.4774122
480.0000000	5.8559999	30.2535954
480.0000000	5.9047999	-16.6153870
480.0000000	5.9535999	-40.0103188
480.0000000	6.0023999	-1588.0335693
480.0000000	6.0511999	-14.5577221
480.0000000	6.0999999	-19.4506283
480.0000000	6.1487999	-13.2503262
480.0000000	6.1975999	-23.3081017
480.0000000	6.2463999	-1809.1628418
480.0000000	6.2951999	-19.1109352
480.0000000	6.3439999	-345.9106750
480.0000000	6.3927999	-15.0490417
480.0000000	6.4416003	-12.3565006
480.0000000	6.4903998	-11.2296715
480.0000000	6.5391998	-11.7718515
480.0000000	6.5879998	420.0061646
480.0000000	6.6367998	-12.0562992
480.0000000	6.6856003	-13.8062553
480.0000000	6.7343998	-13.4446468
480.0000000	6.7831998	-14.3938494
480.0000000	6.8319998	59.4211349

480.0000000	6.8807998	-14.0565557
480.0000000	6.9296002	-488.8259583
480.0000000	6.9783998	-4.4205341
480.0000000	7.0271997	-14.1907101
480.0000000	7.0760002	13.3848562
480.0000000	7.1247997	-2337.8298340
480.0000000	7.1736002	-1874.6959229
480.0000000	7.2223997	-1066.1979980
480.0000000	7.2711997	-228.9041748
480.0000000	7.3200002	-19.5123100
480.0000000	7.3687997	207.5979309
480.0000000	7.4176002	431.7101135
480.0000000	7.4663997	339.3638611
480.0000000	7.5151997	-1043.4527588
480.0000000	7.5640001	-1152.6947021
480.0000000	7.6127996	-2718.4340820
480.0000000	7.6616001	-872.1699219
480.0000000	7.7104001	-447.4788208
480.0000000	7.7591996	48.5725060
480.0000000	7.8080001	448.6310730
480.0000000	7.8567996	590.6444702
480.0000000	7.9056001	-1221.0410156
480.0000000	7.9544001	1944.0047607
480.0000000	8.0031996	2508.3510742
480.0000000	8.0520000	2503.6264648
480.0000000	8.1007996	2649.3049316
480.0000000	8.1496000	2825.3386230
480.0000000	8.1983995	1757.8184814
480.0000000	8.2472000	-4021.2250977
480.0000000	8.2959995	-1425.2933350
480.0000000	8.3448000	-1135.4284668
480.0000000	8.3935995	-1040.5736084
480.0000000	8.4423990	-448.0396423
480.0000000	8.4912004	-1046.3381348
480.0000000	8.5400000	-2823.0063477
480.0000000	8.5887995	-1368.1099854
480.0000000	8.6375999	267.6604919
480.0000000	8.6863995	2415.3808594
480.0000000	8.7351999	-897.3803711
480.0000000	8.7840004	1026.2618408
480.0000000	8.8327999	56.5126572
480.0000000	8.8815994	-11.4871006
480.0000000	8.9303989	-24.1445808
480.0000000	8.9792004	-11.5746880
480.0000000	9.0279999	-5.7419596
480.0000000	9.0767994	7.3465462
480.0000000	9.1255999	-17.3342171
480.0000000	9.1743994	-10.0519495
480.0000000	9.2231998	37.8589478
480.0000000	9.2720003	-8.3696404
480.0000000	9.3207998	-9.7690153
480.0000000	9.3695993	-10.7515535
480.0000000	9.4183998	-11.7606030
480.0000000	9.4672003	-11.1591072
480.0000000	9.5159998	-14.2123241
480.0000000	9.5648003	-0.2109528
480.0000000	9.6135998	-13.4387074
480.0000000	9.6623993	-10.2064810

480.0000000	9.7111998	-10.1430407
480.0000000	9.7600002	-10.8553238
480.0000000	9.8087997	-10.9943485
480.0000000	9.8575993	3.4287119
480.0000000	9.9063997	-7.9197149
480.0000000	9.9552002	-10.9063692
480.0000000	10.0039997	-10.0613518
490.0000000	0.3416000	-36.7556877
490.0000000	0.3904000	-39.8062401
490.0000000	0.4392000	-40.7419052
490.0000000	0.4880000	-39.3894310
490.0000000	0.5368000	-39.3915825
490.0000000	0.5856000	-36.9827805
490.0000000	0.6344000	-37.4205589
490.0000000	0.6832000	-32.9969749
490.0000000	0.7320000	-31.3213005
490.0000000	0.7808000	-31.3408527
490.0000000	0.8296000	-31.9374390
490.0000000	0.8784000	-32.9989166
490.0000000	0.9272000	-34.6151085
490.0000000	0.9760000	-34.0326843
490.0000000	1.0247999	-33.3061142
490.0000000	1.0735999	-32.3773117
490.0000000	1.1224000	-31.6521702
490.0000000	1.1711999	-31.7570248
490.0000000	1.2200000	-31.9267235
490.0000000	1.2687999	-31.3587017
490.0000000	1.3176000	-30.7270050
490.0000000	1.3664000	-30.1601753
490.0000000	1.4152000	-30.1514816
490.0000000	1.4640000	-29.7059212
490.0000000	1.5128000	-44.5256386
490.0000000	1.5616000	-35.8478584
490.0000000	1.6104001	-1691.9853516
490.0000000	1.6592000	-88.8365936
490.0000000	1.7079999	-27.2517433
490.0000000	1.7567999	-26.4131317
490.0000000	1.8055999	-27.3054848
490.0000000	1.8544000	-27.2321396
490.0000000	1.9031999	-26.6729679
490.0000000	1.9520000	-26.5436115
490.0000000	2.0007999	-27.9163055
490.0000000	2.0495999	-19.5133495
490.0000000	2.0983999	-226.1904297
490.0000000	2.1471999	12.4905319
490.0000000	2.1960001	-25.6653347
490.0000000	2.2448001	-26.7770576
490.0000000	2.2935998	-30.8404808
490.0000000	2.3423998	-20.2264061
490.0000000	2.3912001	64.8224640
490.0000000	2.4400001	-22.7056808
490.0000000	2.4888000	-23.4716053
490.0000000	2.5375998	-11.3596897
490.0000000	2.5863998	-9.3394623
490.0000000	2.6352000	-7.2524977
490.0000000	2.6840000	-14.7736073
490.0000000	2.7328000	147.4043427
490.0000000	2.7815998	173.5309143

490.0000000	2.8304000	-23.8642521
490.0000000	2.8792000	-20.4444618
490.0000000	2.9280000	-22.6022854
490.0000000	2.9768000	-16.3406830
490.0000000	3.0256000	-20.3342972
490.0000000	3.0743999	-30.1273499
490.0000000	3.1231999	-24.4403839
490.0000000	3.1719999	-30.0504456
490.0000000	3.2208002	-516.9806519
490.0000000	3.2695999	-1012.6929932
490.0000000	3.3183999	-20.4791050
490.0000000	3.3671999	-3474.1276855
490.0000000	3.4159999	1246.9173584
490.0000000	3.4648001	-6.8379831
490.0000000	3.5135999	-27.1956444
490.0000000	3.5623999	-328.8170166
490.0000000	3.6111999	-2068.3786621
490.0000000	3.6600001	-467.9390259
490.0000000	3.7088001	-618.0341797
490.0000000	3.7575998	-967.8801880
490.0000000	3.8063998	-1756.6578369
490.0000000	3.8552001	-2550.6027832
490.0000000	3.9040000	-2500.6848145
490.0000000	3.9528000	1144.1269531
490.0000000	4.0015998	-249.5105743
490.0000000	4.0503998	700.6500854
490.0000000	4.0991998	89.5879898
490.0000000	4.1479998	-666.4858398
490.0000000	4.1967998	2.9448299
490.0000000	4.2456002	-252.7429199
490.0000000	4.2943997	-496.6355286
490.0000000	4.3431997	-13.2374878
490.0000000	4.3920002	-252.3652802
490.0000000	4.4407997	-22.5370102
490.0000000	4.4896002	-15.4362421
490.0000000	4.5383997	-666.3009644
490.0000000	4.5871997	-12.7519398
490.0000000	4.6360002	-21.6873875
490.0000000	4.6847997	-28.4510422
490.0000000	4.7336001	-19.5934582
490.0000000	4.7824001	-16.4928417
490.0000000	4.8311996	-1497.1372070
490.0000000	4.8800001	232.0934296
490.0000000	4.9287996	-204.3338013
490.0000000	4.9776001	1076.3857422
490.0000000	5.0264001	87.7821579
490.0000000	5.0751996	-305.5761414
490.0000000	5.1240001	-12.2689037
490.0000000	5.1727996	563.3959961
490.0000000	5.2216001	32.4495201
490.0000000	5.2704000	-160.1655884
490.0000000	5.3191996	-39.4010849
490.0000000	5.3680000	-3569.1611328
490.0000000	5.4168000	532.2682495
490.0000000	5.4656000	-202.4815216
490.0000000	5.5144000	38.5804062
490.0000000	5.5631995	493.0750732
490.0000000	5.6120000	396.9959412

490.0000000	5.6608000	-37.2818527
490.0000000	5.7096000	63.2744598
490.0000000	5.7584000	134.4356995
490.0000000	5.8071995	-11.4782829
490.0000000	5.8559999	30.2447453
490.0000000	5.9047999	-16.6151886
490.0000000	5.9535999	-40.0055847
490.0000000	6.0023999	-1587.7290039
490.0000000	6.0511999	-14.5579329
490.0000000	6.0999999	-19.4497948
490.0000000	6.1487999	-13.2507887
490.0000000	6.1975999	-23.3063374
490.0000000	6.2463999	-1808.8154297
490.0000000	6.2951999	-19.1101208
490.0000000	6.3439999	-345.8466187
490.0000000	6.3927999	-15.0490227
490.0000000	6.4416003	-12.3570871
490.0000000	6.4903998	-11.2303467
490.0000000	6.5391998	-11.7725334
490.0000000	6.5879998	419.3154907
490.0000000	6.6367998	-12.0568485
490.0000000	6.6856003	-13.8064260
490.0000000	6.7343998	-13.4448624
490.0000000	6.7831998	-14.3934336
490.0000000	6.8319998	59.4020233
490.0000000	6.8807998	-14.0564175
490.0000000	6.9296002	-488.0645142
490.0000000	6.9783998	-4.4333582
490.0000000	7.0271997	-14.1896820
490.0000000	7.0760002	13.3426695
490.0000000	7.1247997	-2333.8837891
490.0000000	7.1736002	-1871.5330811
490.0000000	7.2223997	-1064.4047852
490.0000000	7.2711997	-228.5363922
490.0000000	7.3200002	-19.5003204
490.0000000	7.3687997	207.2234039
490.0000000	7.4176002	430.9542847
490.0000000	7.4663997	338.7662354
490.0000000	7.5151997	-1041.6917725
490.0000000	7.5640001	-1150.7473145
490.0000000	7.6127996	-2713.8166504
490.0000000	7.6616001	-870.6986084
490.0000000	7.7104001	-446.7325439
490.0000000	7.7591996	48.4714546
490.0000000	7.8080001	447.8457336
490.0000000	7.8567996	589.6163330
490.0000000	7.9056001	-1218.9680176
490.0000000	7.9544001	1940.6948242
490.0000000	8.0031996	2504.0812988
490.0000000	8.0520000	2499.3645020
490.0000000	8.1007996	2644.7954102
490.0000000	8.1496000	2820.5305176
490.0000000	8.1983995	1754.8259277
490.0000000	8.2472000	-4014.3901367
490.0000000	8.2959995	-1422.8731689
490.0000000	8.3448000	-1133.4992676
490.0000000	8.3935995	-1038.8059082
490.0000000	8.4423990	-447.2816467

490.0000000	8.4912004	-1044.5618896
490.0000000	8.5400000	-2818.2065430
490.0000000	8.5887995	-1365.7884521
490.0000000	8.6375999	267.1991577
490.0000000	8.6863995	2411.2624512
490.0000000	8.7351999	-895.8560791
490.0000000	8.7840004	1024.5051270
490.0000000	8.8327999	56.4010468
490.0000000	8.8815994	-11.4872742
490.0000000	8.9303989	-24.1408596
490.0000000	8.9792004	-11.5747700
490.0000000	9.0279999	-5.7437277
490.0000000	9.0767994	7.3408298
490.0000000	9.1255999	-17.3325043
490.0000000	9.1743994	-10.0523510
490.0000000	9.2231998	37.8432159
490.0000000	9.2720003	-8.3705769
490.0000000	9.3207998	-9.7695551
490.0000000	9.3695993	-10.7517805
490.0000000	9.4183998	-11.7604780
490.0000000	9.4672003	-11.1591482
490.0000000	9.5159998	-14.2114334
490.0000000	9.5648003	-0.2143259
490.0000000	9.6135998	-13.4379168
490.0000000	9.6623993	-10.2067566
490.0000000	9.7111998	-10.1433125
490.0000000	9.7600002	-10.8553658
490.0000000	9.8087997	-10.9943571
490.0000000	9.8575993	3.4242897
490.0000000	9.9063997	-7.9206285
490.0000000	9.9552002	-10.9063730
490.0000000	10.0039997	-10.0615988
500.0000000	0.3416000	-35.9693489
500.0000000	0.3904000	-38.7989731
500.0000000	0.4392000	-40.5763817
500.0000000	0.4880000	-39.4142380
500.0000000	0.5368000	-39.4118118
500.0000000	0.5856000	-36.5946274
500.0000000	0.6344000	-32.3926392
500.0000000	0.6832000	-30.2212372
500.0000000	0.7320000	-30.6920643
500.0000000	0.7808000	-31.8325253
500.0000000	0.8296000	-31.9292984
500.0000000	0.8784000	-32.8474922
500.0000000	0.9272000	-33.5171394
500.0000000	0.9760000	-33.8965530
500.0000000	1.0247999	-33.2494583
500.0000000	1.0735999	-32.3261986
500.0000000	1.1224000	-31.6337433
500.0000000	1.1711999	-31.7422447
500.0000000	1.2200000	-31.9265823
500.0000000	1.2687999	-31.3679333
500.0000000	1.3176000	-30.7531147
500.0000000	1.3664000	-30.1679249
500.0000000	1.4152000	-30.1516857
500.0000000	1.4640000	-29.7042122
500.0000000	1.5128000	-44.4284630
500.0000000	1.5616000	-35.8060150

500.0000000	1.6104001	-1689.4426270
500.0000000	1.6592000	-88.5426559
500.0000000	1.7079999	-26.9699707
500.0000000	1.7567999	-26.1023178
500.0000000	1.8055999	-27.5955048
500.0000000	1.8544000	-27.2560673
500.0000000	1.9031999	-26.6873207
500.0000000	1.9520000	-26.5440655
500.0000000	2.0007999	-27.8537235
500.0000000	2.0495999	-19.6999664
500.0000000	2.0983999	-224.7146606
500.0000000	2.1471999	12.2232628
500.0000000	2.1960001	-25.6612434
500.0000000	2.2448001	-26.7591476
500.0000000	2.2935998	-30.7808571
500.0000000	2.3423998	-20.2408104
500.0000000	2.3912001	64.2009201
500.0000000	2.4400001	-22.7081680
500.0000000	2.4888000	-23.4713879
500.0000000	2.5375998	-11.3767986
500.0000000	2.5863998	-9.3592215
500.0000000	2.6352000	-7.2752419
500.0000000	2.6840000	-14.7855492
500.0000000	2.7328000	147.1383209
500.0000000	2.7815998	173.2245178
500.0000000	2.8304000	-23.8629494
500.0000000	2.8792000	-20.4477253
500.0000000	2.9280000	-22.6017151
500.0000000	2.9768000	-16.3490524
500.0000000	3.0256000	-20.3368816
500.0000000	3.0743999	-30.1159630
500.0000000	3.1231999	-24.4369297
500.0000000	3.1719999	-30.0421524
500.0000000	3.2208002	-516.7429199
500.0000000	3.2695999	-1012.2181396
500.0000000	3.3183999	-20.4793015
500.0000000	3.3671999	-3472.6660156
500.0000000	3.4159999	1246.3820801
500.0000000	3.4648001	-6.8436899
500.0000000	3.5135999	-27.1926537
500.0000000	3.5623999	-328.6874084
500.0000000	3.6111999	-2067.5187988
500.0000000	3.6600001	-467.7508545
500.0000000	3.7088001	-617.7828369
500.0000000	3.7575998	-967.4816284
500.0000000	3.8063998	-1755.9281006
500.0000000	3.8552001	-2549.5393066
500.0000000	3.9040000	-2499.6423340
500.0000000	3.9528000	1143.6386719
500.0000000	4.0015998	-249.4134674
500.0000000	4.0503998	700.3475342
500.0000000	4.0991998	89.5423050
500.0000000	4.1479998	-666.2135620
500.0000000	4.1967998	2.9355736
500.0000000	4.2456002	-252.6446228
500.0000000	4.2943997	-496.4349670
500.0000000	4.3431997	-13.2396679
500.0000000	4.3920002	-252.2671661

500.0000000	4.4407997	-22.5352345
500.0000000	4.4896002	-15.4371252
500.0000000	4.5383997	-666.0399170
500.0000000	4.5871997	-12.7539558
500.0000000	4.6360002	-21.6858463
500.0000000	4.6847997	-28.4471169
500.0000000	4.7336001	-19.5927372
500.0000000	4.7824001	-16.4932976
500.0000000	4.8311996	-1496.6059570
500.0000000	4.8800001	232.0038757
500.0000000	4.9287996	-204.2665405
500.0000000	4.9776001	1075.9932861
500.0000000	5.0264001	87.7446365
500.0000000	5.0751996	-305.4725037
500.0000000	5.1240001	-12.2698250
500.0000000	5.1727996	563.2800903
500.0000000	5.2216001	32.4400787
500.0000000	5.2704000	-160.1374664
500.0000000	5.3191996	-39.3965378
500.0000000	5.3680000	-3568.4687500
500.0000000	5.4168000	532.1614990
500.0000000	5.4656000	-202.4453430
500.0000000	5.5144000	38.5697479
500.0000000	5.5631995	492.9765015
500.0000000	5.6120000	396.9159851
500.0000000	5.6608000	-37.2777634
500.0000000	5.7096000	63.2590256
500.0000000	5.7584000	134.4067688
500.0000000	5.8071995	-11.4791527
500.0000000	5.8559999	30.2358913
500.0000000	5.9047999	-16.6149902
500.0000000	5.9535999	-40.0008430
500.0000000	6.0023999	-1587.4245605
500.0000000	6.0511999	-14.5581446
500.0000000	6.0999999	-19.4489632
500.0000000	6.1487999	-13.2512522
500.0000000	6.1975999	-23.3045731
500.0000000	6.2463999	-1808.4681396
500.0000000	6.2951999	-19.1093063
500.0000000	6.3439999	-345.7825623
500.0000000	6.3927999	-15.0490046
500.0000000	6.4416003	-12.3576746
500.0000000	6.4903998	-11.2310228
500.0000000	6.5391998	-11.7732143
500.0000000	6.5879998	418.6247864
500.0000000	6.6367998	-12.0573978
500.0000000	6.6856003	-13.8065958
500.0000000	6.7343998	-13.4450779
500.0000000	6.7831998	-14.3930178
500.0000000	6.8319998	59.3829117
500.0000000	6.8807998	-14.0562782
500.0000000	6.9296002	-487.3030701
500.0000000	6.9783998	-4.4461818
500.0000000	7.0271997	-14.1886539
500.0000000	7.0760002	13.3004866
500.0000000	7.1247997	-2329.9379883
500.0000000	7.1736002	-1868.3702393
500.0000000	7.2223997	-1062.6115723

500.0000000	7.2711997	-228.1686096
500.0000000	7.3200002	-19.4883327
500.0000000	7.3687997	206.8488770
500.0000000	7.4176002	430.1984558
500.0000000	7.4663997	338.1685791
500.0000000	7.5151997	-1039.9307861
500.0000000	7.5640001	-1148.7999268
500.0000000	7.6127996	-2709.1989746
500.0000000	7.6616001	-869.2272339
500.0000000	7.7104001	-445.9863281
500.0000000	7.7591996	48.3704071
500.0000000	7.8080001	447.0603638
500.0000000	7.8567996	588.5882568
500.0000000	7.9056001	-1216.8951416
500.0000000	7.9544001	1937.3848877
500.0000000	8.0031996	2499.8117676
500.0000000	8.0520000	2495.1025391
500.0000000	8.1007996	2640.2858887
500.0000000	8.1496000	2815.7226563
500.0000000	8.1983995	1751.8333740
500.0000000	8.2472000	-4007.5554199
500.0000000	8.2959995	-1420.4530029
500.0000000	8.3448000	-1131.5700684
500.0000000	8.3935995	-1037.0382080
500.0000000	8.4423990	-446.5236206
500.0000000	8.4912004	-1042.7856445
500.0000000	8.5400000	-2813.4067383
500.0000000	8.5887995	-1363.4669189
500.0000000	8.6375999	266.7378235
500.0000000	8.6863995	2407.1442871
500.0000000	8.7351999	-894.3318481
500.0000000	8.7840004	1022.7483521
500.0000000	8.8327999	56.2894440
500.0000000	8.8815994	-11.4874487
500.0000000	8.9303989	-24.1371384
500.0000000	8.9792004	-11.5748529
500.0000000	9.0279999	-5.7454958
500.0000000	9.0767994	7.3351116
500.0000000	9.1255999	-17.3307896
500.0000000	9.1743994	-10.0527534
500.0000000	9.2231998	37.8274841
500.0000000	9.2720003	-8.3715134
500.0000000	9.3207998	-9.7700939
500.0000000	9.3695993	-10.7520065
500.0000000	9.4183998	-11.7603531
500.0000000	9.4672003	-11.1591892
500.0000000	9.5159998	-14.2105417
500.0000000	9.5648003	-0.2176991
500.0000000	9.6135998	-13.4371262
500.0000000	9.6623993	-10.2070313
500.0000000	9.7111998	-10.1435843
500.0000000	9.7600002	-10.8554068
500.0000000	9.8087997	-10.9943647
500.0000000	9.8575993	3.4198675
500.0000000	9.9063997	-7.9215422
500.0000000	9.9552002	-10.9063778
500.0000000	10.0039997	-10.0618467
510.0000000	0.3416000	-36.4992676

510.0000000	0.3904000	-39.5576591
510.0000000	0.4392000	-40.4108582
510.0000000	0.4880000	-38.6079788
510.0000000	0.5368000	-36.7014313
510.0000000	0.5856000	-33.4556541
510.0000000	0.6344000	-32.3153687
510.0000000	0.6832000	-33.4874191
510.0000000	0.7320000	-34.8204803
510.0000000	0.7808000	-33.9337730
510.0000000	0.8296000	-33.8443756
510.0000000	0.8784000	-34.0346451
510.0000000	0.9272000	-34.6155052
510.0000000	0.9760000	-33.9226685
510.0000000	1.0247999	-33.1928062
510.0000000	1.0735999	-32.2750854
510.0000000	1.1224000	-31.6153145
510.0000000	1.1711999	-31.7274628
510.0000000	1.2200000	-31.9264393
510.0000000	1.2687999	-31.3771667
510.0000000	1.3176000	-30.7792244
510.0000000	1.3664000	-30.1756744
510.0000000	1.4152000	-30.1518898
510.0000000	1.4640000	-29.7025032
510.0000000	1.5128000	-44.3312836
510.0000000	1.5616000	-35.7641754
510.0000000	1.6104001	-1686.8997803
510.0000000	1.6592000	-88.2487259
510.0000000	1.7079999	-27.0664310
510.0000000	1.7567999	-25.7011814
510.0000000	1.8055999	-26.3799744
510.0000000	1.8544000	-27.2799950
510.0000000	1.9031999	-26.7016754
510.0000000	1.9520000	-26.5445213
510.0000000	2.0007999	-27.7911415
510.0000000	2.0495999	-19.8865814
510.0000000	2.0983999	-223.2388916
510.0000000	2.1471999	11.9559975
510.0000000	2.1960001	-25.6571522
510.0000000	2.2448001	-26.7412357
510.0000000	2.2935998	-30.7212334
510.0000000	2.3423998	-20.2552147
510.0000000	2.3912001	63.5793686
510.0000000	2.4400001	-22.7106533
510.0000000	2.4888000	-23.4711704
510.0000000	2.5375998	-11.3939066
510.0000000	2.5863998	-9.3789806
510.0000000	2.6352000	-7.2979860
510.0000000	2.6840000	-14.7974920
510.0000000	2.7328000	146.8723145
510.0000000	2.7815998	172.9180908
510.0000000	2.8304000	-23.8616447
510.0000000	2.8792000	-20.4509888
510.0000000	2.9280000	-22.6011467
510.0000000	2.9768000	-16.3574238
510.0000000	3.0256000	-20.3394661
510.0000000	3.0743999	-30.1045761
510.0000000	3.1231999	-24.4334736
510.0000000	3.1719999	-30.0338593

510.0000000	3.2208002	-516.5051880
510.0000000	3.2695999	-1011.7430420
510.0000000	3.3183999	-20.4794998
510.0000000	3.3671999	-3471.2043457
510.0000000	3.4159999	1245.8468018
510.0000000	3.4648001	-6.8493986
510.0000000	3.5135999	-27.1896629
510.0000000	3.5623999	-328.5578308
510.0000000	3.6111999	-2066.6591797
510.0000000	3.6600001	-467.5627441
510.0000000	3.7088001	-617.5314331
510.0000000	3.7575998	-967.0830688
510.0000000	3.8063998	-1755.1982422
510.0000000	3.8552001	-2548.4758301
510.0000000	3.9040000	-2498.5998535
510.0000000	3.9528000	1143.1503906
510.0000000	4.0015998	-249.3163452
510.0000000	4.0503998	700.0449829
510.0000000	4.0991998	89.4966202
510.0000000	4.1479998	-665.9413452
510.0000000	4.1967998	2.9263172
510.0000000	4.2456002	-252.5463257
510.0000000	4.2943997	-496.2344055
510.0000000	4.3431997	-13.2418480
510.0000000	4.3920002	-252.1690521
510.0000000	4.4407997	-22.5334587
510.0000000	4.4896002	-15.4380074
510.0000000	4.5383997	-665.7788086
510.0000000	4.5871997	-12.7559729
510.0000000	4.6360002	-21.6843033
510.0000000	4.6847997	-28.4431915
510.0000000	4.7336001	-19.5920143
510.0000000	4.7824001	-16.4937515
510.0000000	4.8311996	-1496.0747070
510.0000000	4.8800001	231.9143219
510.0000000	4.9287996	-204.1992798
510.0000000	4.9776001	1075.6008301
510.0000000	5.0264001	87.7071152
510.0000000	5.0751996	-305.3688660
510.0000000	5.1240001	-12.2707462
510.0000000	5.1727996	563.1642456
510.0000000	5.2216001	32.4306335
510.0000000	5.2704000	-160.1093750
510.0000000	5.3191996	-39.3919907
510.0000000	5.3680000	-3567.7763672
510.0000000	5.4168000	532.0547485
510.0000000	5.4656000	-202.4091644
510.0000000	5.5144000	38.5590897
510.0000000	5.5631995	492.8779297
510.0000000	5.6120000	396.8359985
510.0000000	5.6608000	-37.2736778
510.0000000	5.7096000	63.2435989
510.0000000	5.7584000	134.3778381
510.0000000	5.8071995	-11.4800243
510.0000000	5.8559999	30.2270412
510.0000000	5.9047999	-16.6147938
510.0000000	5.9535999	-39.9961052
510.0000000	6.0023999	-1587.1202393

510.0000000	6.0511999	-14.5583553
510.0000000	6.0999999	-19.4481297
510.0000000	6.1487999	-13.2517147
510.0000000	6.1975999	-23.3028069
510.0000000	6.2463999	-1808.1207275
510.0000000	6.2951999	-19.1084919
510.0000000	6.3439999	-345.7185364
510.0000000	6.3927999	-15.0489864
510.0000000	6.4416003	-12.3582611
510.0000000	6.4903998	-11.2316990
510.0000000	6.5391998	-11.7738953
510.0000000	6.5879998	417.9340820
510.0000000	6.6367998	-12.0579472
510.0000000	6.6856003	-13.8067665
510.0000000	6.7343998	-13.4452934
510.0000000	6.7831998	-14.3926020
510.0000000	6.8319998	59.3637924
510.0000000	6.8807998	-14.0561399
510.0000000	6.9296002	-486.5415955
510.0000000	6.9783998	-4.4590054
510.0000000	7.0271997	-14.1876268
510.0000000	7.0760002	13.2582998
510.0000000	7.1247997	-2325.9921875
510.0000000	7.1736002	-1865.2072754
510.0000000	7.2223997	-1060.8184814
510.0000000	7.2711997	-227.8008270
510.0000000	7.3200002	-19.4763451
510.0000000	7.3687997	206.4743500
510.0000000	7.4176002	429.4426270
510.0000000	7.4663997	337.5709229
510.0000000	7.5151997	-1038.1696777
510.0000000	7.5640001	-1146.8525391
510.0000000	7.6127996	-2704.5812988
510.0000000	7.6616001	-867.7558594
510.0000000	7.7104001	-445.2399902
510.0000000	7.7591996	48.2693596
510.0000000	7.8080001	446.2749939
510.0000000	7.8567996	587.5601807
510.0000000	7.9056001	-1214.8222656
510.0000000	7.9544001	1934.0749512
510.0000000	8.0031996	2495.5422363
510.0000000	8.0520000	2490.8405762
510.0000000	8.1007996	2635.7763672
510.0000000	8.1496000	2810.9145508
510.0000000	8.1983995	1748.8409424
510.0000000	8.2472000	-4000.7207031
510.0000000	8.2959995	-1418.0328369
510.0000000	8.3448000	-1129.6408691
510.0000000	8.3935995	-1035.2706299
510.0000000	8.4423990	-445.7656250
510.0000000	8.4912004	-1041.0093994
510.0000000	8.5400000	-2808.6069336
510.0000000	8.5887995	-1361.1453857
510.0000000	8.6375999	266.2765198
510.0000000	8.6863995	2403.0258789
510.0000000	8.7351999	-892.8076172
510.0000000	8.7840004	1020.9916382
510.0000000	8.8327999	56.1778336

510.0000000	8.8815994	-11.4876232
510.0000000	8.9303989	-24.1334190
510.0000000	8.9792004	-11.5749350
510.0000000	9.0279999	-5.7472644
510.0000000	9.0767994	7.3293934
510.0000000	9.1255999	-17.3290749
510.0000000	9.1743994	-10.0531549
510.0000000	9.2231998	37.8117523
510.0000000	9.2720003	-8.3724489
510.0000000	9.3207998	-9.7706337
510.0000000	9.3695993	-10.7522335
510.0000000	9.4183998	-11.7602291
510.0000000	9.4672003	-11.1592302
510.0000000	9.5159998	-14.2096510
510.0000000	9.5648003	-0.2210712
510.0000000	9.6135998	-13.4363346
510.0000000	9.6623993	-10.2073059
510.0000000	9.7111998	-10.1438551
510.0000000	9.7600002	-10.8554487
510.0000000	9.8087997	-10.9943724
510.0000000	9.8575993	3.4154444
510.0000000	9.9063997	-7.9224563
510.0000000	9.9552002	-10.9063816
510.0000000	10.0039997	-10.0620947
520.0000000	0.3416000	-36.6958885
520.0000000	0.3904000	-39.8862343
520.0000000	0.4392000	-40.2453346
520.0000000	0.4880000	-39.4268417
520.0000000	0.5368000	-37.0867195
520.0000000	0.5856000	-33.7711487
520.0000000	0.6344000	-33.3258095
520.0000000	0.6832000	-34.2143593
520.0000000	0.7320000	-33.9571037
520.0000000	0.7808000	-34.0839043
520.0000000	0.8296000	-34.3197365
520.0000000	0.8784000	-34.7244606
520.0000000	0.9272000	-34.8762016
520.0000000	0.9760000	-33.9487839
520.0000000	1.0247999	-33.1361542
520.0000000	1.0735999	-32.2239723
520.0000000	1.1224000	-31.5968876
520.0000000	1.1711999	-31.7126827
520.0000000	1.2200000	-31.9262981
520.0000000	1.2687999	-31.3864002
520.0000000	1.3176000	-30.8053322
520.0000000	1.3664000	-30.1834240
520.0000000	1.4152000	-30.1520939
520.0000000	1.4640000	-29.7007923
520.0000000	1.5128000	-44.2341080
520.0000000	1.5616000	-35.7223358
520.0000000	1.6104001	-1684.3570557
520.0000000	1.6592000	-87.9547958
520.0000000	1.7079999	-27.0701790
520.0000000	1.7567999	-26.6524181
520.0000000	1.8055999	-27.1178493
520.0000000	1.8544000	-27.3039227
520.0000000	1.9031999	-26.7160282
520.0000000	1.9520000	-26.5449753

520.0000000	2.0007999	-27.7285595
520.0000000	2.0495999	-20.0731983
520.0000000	2.0983999	-221.7630920
520.0000000	2.1471999	11.6887321
520.0000000	2.1960001	-25.6530609
520.0000000	2.2448001	-26.7233238
520.0000000	2.2935998	-30.6616096
520.0000000	2.3423998	-20.2696190
520.0000000	2.3912001	62.9578247
520.0000000	2.4400001	-22.7131405
520.0000000	2.4888000	-23.4709530
520.0000000	2.5375998	-11.4110146
520.0000000	2.5863998	-9.3987398
520.0000000	2.6352000	-7.3207312
520.0000000	2.6840000	-14.8094330
520.0000000	2.7328000	146.6062927
520.0000000	2.7815998	172.6116638
520.0000000	2.8304000	-23.8603401
520.0000000	2.8792000	-20.4542522
520.0000000	2.9280000	-22.6005783
520.0000000	2.9768000	-16.3657951
520.0000000	3.0256000	-20.3420506
520.0000000	3.0743999	-30.0931892
520.0000000	3.1231999	-24.4300194
520.0000000	3.1719999	-30.0255661
520.0000000	3.2208002	-516.2674561
520.0000000	3.2695999	-1011.2681885
520.0000000	3.3183999	-20.4796982
520.0000000	3.3671999	-3469.7426758
520.0000000	3.4159999	1245.3115234
520.0000000	3.4648001	-6.8551064
520.0000000	3.5135999	-27.1866722
520.0000000	3.5623999	-328.4282227
520.0000000	3.6111999	-2065.7993164
520.0000000	3.6600001	-467.3746338
520.0000000	3.7088001	-617.2800293
520.0000000	3.7575998	-966.6845703
520.0000000	3.8063998	-1754.4683838
520.0000000	3.8552001	-2547.4125977
520.0000000	3.9040000	-2497.5573730
520.0000000	3.9528000	1142.6621094
520.0000000	4.0015998	-249.2192383
520.0000000	4.0503998	699.7424316
520.0000000	4.0991998	89.4509354
520.0000000	4.1479998	-665.6691284
520.0000000	4.1967998	2.9170609
520.0000000	4.2456002	-252.4480133
520.0000000	4.2943997	-496.0337830
520.0000000	4.3431997	-13.2440281
520.0000000	4.3920002	-252.0709381
520.0000000	4.4407997	-22.5316849
520.0000000	4.4896002	-15.4388905
520.0000000	4.5383997	-665.5177612
520.0000000	4.5871997	-12.7579889
520.0000000	4.6360002	-21.6827602
520.0000000	4.6847997	-28.4392662
520.0000000	4.7336001	-19.5912914
520.0000000	4.7824001	-16.4942055

520.0000000	4.8311996	-1495.5434570
520.0000000	4.8800001	231.8247681
520.0000000	4.9287996	-204.1320190
520.0000000	4.9776001	1075.2082520
520.0000000	5.0264001	87.6695938
520.0000000	5.0751996	-305.2652588
520.0000000	5.1240001	-12.2716675
520.0000000	5.1727996	563.0484009
520.0000000	5.2216001	32.4211884
520.0000000	5.2704000	-160.0812531
520.0000000	5.3191996	-39.3874435
520.0000000	5.3680000	-3567.0839844
520.0000000	5.4168000	531.9479370
520.0000000	5.4656000	-202.3729706
520.0000000	5.5144000	38.5484314
520.0000000	5.5631995	492.7793579
520.0000000	5.6120000	396.7560120
520.0000000	5.6608000	-37.2695885
520.0000000	5.7096000	63.2281647
520.0000000	5.7584000	134.3489075
520.0000000	5.8071995	-11.4808941
520.0000000	5.8559999	30.2181911
520.0000000	5.9047999	-16.6145954
520.0000000	5.9535999	-39.9913635
520.0000000	6.0023999	-1586.8156738
520.0000000	6.0511999	-14.5585670
520.0000000	6.0999999	-19.4472980
520.0000000	6.1487999	-13.2521772
520.0000000	6.1975999	-23.3010406
520.0000000	6.2463999	-1807.7733154
520.0000000	6.2951999	-19.1076775
520.0000000	6.3439999	-345.6544800
520.0000000	6.3927999	-15.0489683
520.0000000	6.4416003	-12.3588486
520.0000000	6.4903998	-11.2323751
520.0000000	6.5391998	-11.7745762
520.0000000	6.5879998	417.2433777
520.0000000	6.6367998	-12.0584965
520.0000000	6.6856003	-13.8069363
520.0000000	6.7343998	-13.4455099
520.0000000	6.7831998	-14.3921862
520.0000000	6.8319998	59.3446808
520.0000000	6.8807998	-14.0560017
520.0000000	6.9296002	-485.7801514
520.0000000	6.9783998	-4.4718285
520.0000000	7.0271997	-14.1865988
520.0000000	7.0760002	13.2161131
520.0000000	7.1247997	-2322.0463867
520.0000000	7.1736002	-1862.0444336
520.0000000	7.2223997	-1059.0252686
520.0000000	7.2711997	-227.4330444
520.0000000	7.3200002	-19.4643555
520.0000000	7.3687997	206.0998230
520.0000000	7.4176002	428.6867981
520.0000000	7.4663997	336.9732666
520.0000000	7.5151997	-1036.4086914
520.0000000	7.5640001	-1144.9051514
520.0000000	7.6127996	-2699.9636230

520.0000000	7.6616001	-866.2845459
520.0000000	7.7104001	-444.4937744
520.0000000	7.7591996	48.1683083
520.0000000	7.8080001	445.4896545
520.0000000	7.8567996	586.5321045
520.0000000	7.9056001	-1212.7493896
520.0000000	7.9544001	1930.7650146
520.0000000	8.0031996	2491.2727051
520.0000000	8.0520000	2486.5783691
520.0000000	8.1007996	2631.2668457
520.0000000	8.1496000	2806.1064453
520.0000000	8.1983995	1745.8483887
520.0000000	8.2472000	-3993.8857422
520.0000000	8.2959995	-1415.6126709
520.0000000	8.3448000	-1127.7117920
520.0000000	8.3935995	-1033.5029297
520.0000000	8.4423990	-445.0075989
520.0000000	8.4912004	-1039.2332764
520.0000000	8.5400000	-2803.8071289
520.0000000	8.5887995	-1358.8238525
520.0000000	8.6375999	265.8151855
520.0000000	8.6863995	2398.9074707
520.0000000	8.7351999	-891.2833252
520.0000000	8.7840004	1019.2348022
520.0000000	8.8327999	56.0662231
520.0000000	8.8815994	-11.4877977
520.0000000	8.9303989	-24.1296997
520.0000000	8.9792004	-11.5750170
520.0000000	9.0279999	-5.7490325
520.0000000	9.0767994	7.3236752
520.0000000	9.1255999	-17.3273621
520.0000000	9.1743994	-10.0535574
520.0000000	9.2231998	37.7960205
520.0000000	9.2720003	-8.3733854
520.0000000	9.3207998	-9.7711735
520.0000000	9.3695993	-10.7524595
520.0000000	9.4183998	-11.7601042
520.0000000	9.4672003	-11.1592722
520.0000000	9.5159998	-14.2087593
520.0000000	9.5648003	-0.2244434
520.0000000	9.6135998	-13.4355440
520.0000000	9.6623993	-10.2075815
520.0000000	9.7111998	-10.1441269
520.0000000	9.7600002	-10.8554907
520.0000000	9.8087997	-10.9943810
520.0000000	9.8575993	3.4110222
520.0000000	9.9063997	-7.9233704
520.0000000	9.9552002	-10.9063864
520.0000000	10.0039997	-10.0623417
530.0000000	0.3416000	-36.4711418
530.0000000	0.3904000	-39.6768456
530.0000000	0.4392000	-40.0798111
530.0000000	0.4880000	-38.7715683
530.0000000	0.5368000	-37.0078049
530.0000000	0.5856000	-33.3556404
530.0000000	0.6344000	-32.5332146
530.0000000	0.6832000	-34.3990135
530.0000000	0.7320000	-34.3813210

530.0000000	0.7808000	-34.3514557
530.0000000	0.8296000	-34.8783340
530.0000000	0.8784000	-34.8872833
530.0000000	0.9272000	-34.6662331
530.0000000	0.9760000	-33.9748993
530.0000000	1.0247999	-33.0795021
530.0000000	1.0735999	-32.1728630
530.0000000	1.1224000	-31.5784588
530.0000000	1.1711999	-31.6979008
530.0000000	1.2200000	-31.9261551
530.0000000	1.2687999	-31.3956337
530.0000000	1.3176000	-30.8314419
530.0000000	1.3664000	-30.1911736
530.0000000	1.4152000	-30.1522980
530.0000000	1.4640000	-29.6990833
530.0000000	1.5128000	-44.1369286
530.0000000	1.5616000	-35.6804924
530.0000000	1.6104001	-1681.8142090
530.0000000	1.6592000	-87.6608658
530.0000000	1.7079999	-27.0739250
530.0000000	1.7567999	-27.6019382
530.0000000	1.8055999	-27.1903419
530.0000000	1.8544000	-27.3278503
530.0000000	1.9031999	-26.7303829
530.0000000	1.9520000	-26.5454311
530.0000000	2.0007999	-27.6659775
530.0000000	2.0495999	-20.2598152
530.0000000	2.0983999	-220.2873230
530.0000000	2.1471999	11.4214630
530.0000000	2.1960001	-25.6489697
530.0000000	2.2448001	-26.7054138
530.0000000	2.2935998	-30.6019859
530.0000000	2.3423998	-20.2840252
530.0000000	2.3912001	62.3362808
530.0000000	2.4400001	-22.7156277
530.0000000	2.4888000	-23.4707355
530.0000000	2.5375998	-11.4281225
530.0000000	2.5863998	-9.4184990
530.0000000	2.6352000	-7.3434753
530.0000000	2.6840000	-14.8213749
530.0000000	2.7328000	146.3402863
530.0000000	2.7815998	172.3052368
530.0000000	2.8304000	-23.8590355
530.0000000	2.8792000	-20.4575157
530.0000000	2.9280000	-22.6000080
530.0000000	2.9768000	-16.3741646
530.0000000	3.0256000	-20.3446350
530.0000000	3.0743999	-30.0818024
530.0000000	3.1231999	-24.4265633
530.0000000	3.1719999	-30.0172729
530.0000000	3.2208002	-516.0297241
530.0000000	3.2695999	-1010.7930908
530.0000000	3.3183999	-20.4798965
530.0000000	3.3671999	-3468.2810059
530.0000000	3.4159999	1244.7762451
530.0000000	3.4648001	-6.8608150
530.0000000	3.5135999	-27.1836815
530.0000000	3.5623999	-328.2986450

530.0000000	3.6111999	-2064.9396973
530.0000000	3.6600001	-467.1864624
530.0000000	3.7088001	-617.0286865
530.0000000	3.7575998	-966.2860107
530.0000000	3.8063998	-1753.7385254
530.0000000	3.8552001	-2546.3491211
530.0000000	3.9040000	-2496.5148926
530.0000000	3.9528000	1142.1738281
530.0000000	4.0015998	-249.1221313
530.0000000	4.0503998	699.4398804
530.0000000	4.0991998	89.4052505
530.0000000	4.1479998	-665.3969116
530.0000000	4.1967998	2.9078045
530.0000000	4.2456002	-252.3497162
530.0000000	4.2943997	-495.8332214
530.0000000	4.3431997	-13.2462091
530.0000000	4.3920002	-251.9728241
530.0000000	4.4407997	-22.5299072
530.0000000	4.4896002	-15.4397726
530.0000000	4.5383997	-665.2567139
530.0000000	4.5871997	-12.7600060
530.0000000	4.6360002	-21.6812172
530.0000000	4.6847997	-28.4353409
530.0000000	4.7336001	-19.5905704
530.0000000	4.7824001	-16.4946613
530.0000000	4.8311996	-1495.0120850
530.0000000	4.8800001	231.7352295
530.0000000	4.9287996	-204.0647583
530.0000000	4.9776001	1074.8157959
530.0000000	5.0264001	87.6320724
530.0000000	5.0751996	-305.1616211
530.0000000	5.1240001	-12.2725887
530.0000000	5.1727996	562.9324951
530.0000000	5.2216001	32.4117470
530.0000000	5.2704000	-160.0531616
530.0000000	5.3191996	-39.3828888
530.0000000	5.3680000	-3566.3916016
530.0000000	5.4168000	531.8411255
530.0000000	5.4656000	-202.3367920
530.0000000	5.5144000	38.5377731
530.0000000	5.5631995	492.6807861
530.0000000	5.6120000	396.6760559
530.0000000	5.6608000	-37.2654991
530.0000000	5.7096000	63.2127304
530.0000000	5.7584000	134.3199768
530.0000000	5.8071995	-11.4817657
530.0000000	5.8559999	30.2093372
530.0000000	5.9047999	-16.6143970
530.0000000	5.9535999	-39.9866257
530.0000000	6.0023999	-1586.5113525
530.0000000	6.0511999	-14.5587788
530.0000000	6.0999999	-19.4464645
530.0000000	6.1487999	-13.2526398
530.0000000	6.1975999	-23.2992764
530.0000000	6.2463999	-1807.4260254
530.0000000	6.2951999	-19.1068630
530.0000000	6.3439999	-345.5904236
530.0000000	6.3927999	-15.0489492

530.0000000	6.4416003	-12.3594351
530.0000000	6.4903998	-11.2330503
530.0000000	6.5391998	-11.7752571
530.0000000	6.5879998	416.5526733
530.0000000	6.6367998	-12.0590448
530.0000000	6.6856003	-13.8071070
530.0000000	6.7343998	-13.4457254
530.0000000	6.7831998	-14.3917704
530.0000000	6.8319998	59.3255615
530.0000000	6.8807998	-14.0558624
530.0000000	6.9296002	-485.0187073
530.0000000	6.9783998	-4.4846520
530.0000000	7.0271997	-14.1855707
530.0000000	7.0760002	13.1739302
530.0000000	7.1247997	-2318.1003418
530.0000000	7.1736002	-1858.8815918
530.0000000	7.2223997	-1057.2321777
530.0000000	7.2711997	-227.0652771
530.0000000	7.3200002	-19.4523659
530.0000000	7.3687997	205.7252808
530.0000000	7.4176002	427.9309692
530.0000000	7.4663997	336.3756104
530.0000000	7.5151997	-1034.6475830
530.0000000	7.5640001	-1142.9576416
530.0000000	7.6127996	-2695.3461914
530.0000000	7.6616001	-864.8131714
530.0000000	7.7104001	-443.7474976
530.0000000	7.7591996	48.0672607
530.0000000	7.8080001	444.7042847
530.0000000	7.8567996	585.5040283
530.0000000	7.9056001	-1210.6763916
530.0000000	7.9544001	1927.4552002
530.0000000	8.0031996	2487.0029297
530.0000000	8.0520000	2482.3164063
530.0000000	8.1007996	2626.7573242
530.0000000	8.1496000	2801.2985840
530.0000000	8.1983995	1742.8558350
530.0000000	8.2472000	-3987.0510254
530.0000000	8.2959995	-1413.1925049
530.0000000	8.3448000	-1125.7825928
530.0000000	8.3935995	-1031.7352295
530.0000000	8.4423990	-444.2496033
530.0000000	8.4912004	-1037.4570313
530.0000000	8.5400000	-2799.0073242
530.0000000	8.5887995	-1356.5023193
530.0000000	8.6375999	265.3538818
530.0000000	8.6863995	2394.7890625
530.0000000	8.7351999	-889.7590942
530.0000000	8.7840004	1017.4780884
530.0000000	8.8327999	55.9546204
530.0000000	8.8815994	-11.4879723
530.0000000	8.9303989	-24.1259804
530.0000000	8.9792004	-11.5750999
530.0000000	9.0279999	-5.7508011
530.0000000	9.0767994	7.3179588
530.0000000	9.1255999	-17.3256474
530.0000000	9.1743994	-10.0539589
530.0000000	9.2231998	37.7802887

530.0000000	9.2720003	-8.3743219
530.0000000	9.3207998	-9.7717123
530.0000000	9.3695993	-10.7526865
530.0000000	9.4183998	-11.7599802
530.0000000	9.4672003	-11.1593132
530.0000000	9.5159998	-14.2078686
530.0000000	9.5648003	-0.2278166
530.0000000	9.6135998	-13.4347534
530.0000000	9.6623993	-10.2078562
530.0000000	9.7111998	-10.1443987
530.0000000	9.7600002	-10.8555326
530.0000000	9.8087997	-10.9943886
530.0000000	9.8575993	3.4066000
530.0000000	9.9063997	-7.9242840
530.0000000	9.9552002	-10.9063902
530.0000000	10.0039997	-10.0625896
540.0000000	0.3416000	-36.7561226
540.0000000	0.3904000	-39.7287064
540.0000000	0.4392000	-39.9142914
540.0000000	0.4880000	-38.4458542
540.0000000	0.5368000	-35.2831726
540.0000000	0.5856000	-33.9245567
540.0000000	0.6344000	-34.5534172
540.0000000	0.6832000	-33.1205826
540.0000000	0.7320000	-32.6130753
540.0000000	0.7808000	-33.7891998
540.0000000	0.8296000	-34.6330833
540.0000000	0.8784000	-35.0501099
540.0000000	0.9272000	-34.4562607
540.0000000	0.9760000	-34.0010109
540.0000000	1.0247999	-33.0228500
540.0000000	1.0735999	-32.1217499
540.0000000	1.1224000	-31.5600319
540.0000000	1.1711999	-31.6831207
540.0000000	1.2200000	-31.9260120
540.0000000	1.2687999	-31.4048672
540.0000000	1.3176000	-30.8575497
540.0000000	1.3664000	-30.1989231
540.0000000	1.4152000	-30.1525021
540.0000000	1.4640000	-29.6973743
540.0000000	1.5128000	-44.0397491
540.0000000	1.5616000	-35.6386528
540.0000000	1.6104001	-1679.2713623
540.0000000	1.6592000	-87.3669281
540.0000000	1.7079999	-27.0776730
540.0000000	1.7567999	-26.8815212
540.0000000	1.8055999	-26.6179466
540.0000000	1.8544000	-27.2652588
540.0000000	1.9031999	-26.7447357
540.0000000	1.9520000	-26.5458870
540.0000000	2.0007999	-27.6033955
540.0000000	2.0495999	-20.4464321
540.0000000	2.0983999	-218.8115540
540.0000000	2.1471999	11.1541939
540.0000000	2.1960001	-25.6448784
540.0000000	2.2448001	-26.6875019
540.0000000	2.2935998	-30.5423622
540.0000000	2.3423998	-20.2984295

540.0000000	2.3912001	61.7147293
540.0000000	2.4400001	-22.7181129
540.0000000	2.4888000	-23.4705181
540.0000000	2.5375998	-11.4452305
540.0000000	2.5863998	-9.4382572
540.0000000	2.6352000	-7.3662195
540.0000000	2.6840000	-14.8333168
540.0000000	2.7328000	146.0742645
540.0000000	2.7815998	171.9988403
540.0000000	2.8304000	-23.8577328
540.0000000	2.8792000	-20.4607792
540.0000000	2.9280000	-22.5994396
540.0000000	2.9768000	-16.3825340
540.0000000	3.0256000	-20.3472195
540.0000000	3.0743999	-30.0704155
540.0000000	3.1231999	-24.4231071
540.0000000	3.1719999	-30.0089798
540.0000000	3.2208002	-515.7919922
540.0000000	3.2695999	-1010.3182373
540.0000000	3.3183999	-20.4800949
540.0000000	3.3671999	-3466.8193359
540.0000000	3.4159999	1244.2409668
540.0000000	3.4648001	-6.8665237
540.0000000	3.5135999	-27.1806908
540.0000000	3.5623999	-328.1690369
540.0000000	3.6111999	-2064.0798340
540.0000000	3.6600001	-466.9983521
540.0000000	3.7088001	-616.7772827
540.0000000	3.7575998	-965.8875122
540.0000000	3.8063998	-1753.0086670
540.0000000	3.8552001	-2545.2856445
540.0000000	3.9040000	-2495.4724121
540.0000000	3.9528000	1141.6854248
540.0000000	4.0015998	-249.0250244
540.0000000	4.0503998	699.1373291
540.0000000	4.0991998	89.3595657
540.0000000	4.1479998	-665.1246948
540.0000000	4.1967998	2.8985481
540.0000000	4.2456002	-252.2514191
540.0000000	4.2943997	-495.6326599
540.0000000	4.3431997	-13.2483902
540.0000000	4.3920002	-251.8747101
540.0000000	4.4407997	-22.5281334
540.0000000	4.4896002	-15.4406548
540.0000000	4.5383997	-664.9956055
540.0000000	4.5871997	-12.7620220
540.0000000	4.6360002	-21.6796761
540.0000000	4.6847997	-28.4314156
540.0000000	4.7336001	-19.5898476
540.0000000	4.7824001	-16.4951153
540.0000000	4.8311996	-1494.4808350
540.0000000	4.8800001	231.6456757
540.0000000	4.9287996	-203.9974976
540.0000000	4.9776001	1074.4233398
540.0000000	5.0264001	87.5945511
540.0000000	5.0751996	-305.0579834
540.0000000	5.1240001	-12.2735100
540.0000000	5.1727996	562.8166504

540.0000000	5.2216001	32.4023018
540.0000000	5.2704000	-160.0250397
540.0000000	5.3191996	-39.3783417
540.0000000	5.3680000	-3565.6992188
540.0000000	5.4168000	531.7343750
540.0000000	5.4656000	-202.3005981
540.0000000	5.5144000	38.5271225
540.0000000	5.5631995	492.5822144
540.0000000	5.6120000	396.5960693
540.0000000	5.6608000	-37.2614136
540.0000000	5.7096000	63.1973038
540.0000000	5.7584000	134.2910461
540.0000000	5.8071995	-11.4826355
540.0000000	5.8559999	30.2004871
540.0000000	5.9047999	-16.6141987
540.0000000	5.9535999	-39.9818878
540.0000000	6.0023999	-1586.2069092
540.0000000	6.0511999	-14.5589895
540.0000000	6.0999999	-19.4456329
540.0000000	6.1487999	-13.2531033
540.0000000	6.1975999	-23.2975121
540.0000000	6.2463999	-1807.0786133
540.0000000	6.2951999	-19.1060486
540.0000000	6.3439999	-345.5263672
540.0000000	6.3927999	-15.0489311
540.0000000	6.4416003	-12.3600216
540.0000000	6.4903998	-11.2337265
540.0000000	6.5391998	-11.7759380
540.0000000	6.5879998	415.8619995
540.0000000	6.6367998	-12.0595942
540.0000000	6.6856003	-13.8072767
540.0000000	6.7343998	-13.4459410
540.0000000	6.7831998	-14.3913546
540.0000000	6.8319998	59.3064499
540.0000000	6.8807998	-14.0557241
540.0000000	6.9296002	-484.2572632
540.0000000	6.9783998	-4.4974761
540.0000000	7.0271997	-14.1845427
540.0000000	7.0760002	13.1317434
540.0000000	7.1247997	-2314.1545410
540.0000000	7.1736002	-1855.7187500
540.0000000	7.2223997	-1055.4389648
540.0000000	7.2711997	-226.6974945
540.0000000	7.3200002	-19.4403782
540.0000000	7.3687997	205.3507538
540.0000000	7.4176002	427.1751404
540.0000000	7.4663997	335.7779541
540.0000000	7.5151997	-1032.8865967
540.0000000	7.5640001	-1141.0102539
540.0000000	7.6127996	-2690.7285156
540.0000000	7.6616001	-863.3417969
540.0000000	7.7104001	-443.0012207
540.0000000	7.7591996	47.9662132
540.0000000	7.8080001	443.9189148
540.0000000	7.8567996	584.4759521
540.0000000	7.9056001	-1208.6035156
540.0000000	7.9544001	1924.1452637
540.0000000	8.0031996	2482.7333984

540.0000000	8.0520000	2478.0544434
540.0000000	8.1007996	2622.2478027
540.0000000	8.1496000	2796.4904785
540.0000000	8.1983995	1739.8634033
540.0000000	8.2472000	-3980.2163086
540.0000000	8.2959995	-1410.7723389
540.0000000	8.3448000	-1123.8533936
540.0000000	8.3935995	-1029.9675293
540.0000000	8.4423990	-443.4915771
540.0000000	8.4912004	-1035.6807861
540.0000000	8.5400000	-2794.2075195
540.0000000	8.5887995	-1354.1807861
540.0000000	8.6375999	264.8925476
540.0000000	8.6863995	2390.6708984
540.0000000	8.7351999	-888.2348022
540.0000000	8.7840004	1015.7213745
540.0000000	8.8327999	55.8430099
540.0000000	8.8815994	-11.4881458
540.0000000	8.9303989	-24.1222591
540.0000000	8.9792004	-11.5751820
540.0000000	9.0279999	-5.7525692
540.0000000	9.0767994	7.3122406
540.0000000	9.1255999	-17.3239346
540.0000000	9.1743994	-10.0543613
540.0000000	9.2231998	37.7645493
540.0000000	9.2720003	-8.3752584
540.0000000	9.3207998	-9.7722511
540.0000000	9.3695993	-10.7529125
540.0000000	9.4183998	-11.7598553
540.0000000	9.4672003	-11.1593542
540.0000000	9.5159998	-14.2069778
540.0000000	9.5648003	-0.2311897
540.0000000	9.6135998	-13.4339628
540.0000000	9.6623993	-10.2081318
540.0000000	9.7111998	-10.1446705
540.0000000	9.7600002	-10.8555737
540.0000000	9.8087997	-10.9943962
540.0000000	9.8575993	3.4021769
540.0000000	9.9063997	-7.9251976
540.0000000	9.9552002	-10.9063950
540.0000000	10.0039997	-10.0628366
550.0000000	0.3416000	-36.6116333
550.0000000	0.3904000	-39.1743393
550.0000000	0.4392000	-39.7487679
550.0000000	0.4880000	-39.1883736
550.0000000	0.5368000	-36.2699852
550.0000000	0.5856000	-34.9325180
550.0000000	0.6344000	-35.5909958
550.0000000	0.6832000	-35.2953300
550.0000000	0.7320000	-33.9555168
550.0000000	0.7808000	-33.9458275
550.0000000	0.8296000	-35.7062263
550.0000000	0.8784000	-35.2129326
550.0000000	0.9272000	-34.2462921
550.0000000	0.9760000	-34.0271263
550.0000000	1.0247999	-32.9661942
550.0000000	1.0735999	-32.0706367
550.0000000	1.1224000	-31.5416031

550.0000000	1.1711999	-31.6683388
550.0000000	1.2200000	-31.9258709
550.0000000	1.2687999	-31.4141006
550.0000000	1.3176000	-30.8836594
550.0000000	1.3664000	-30.2066727
550.0000000	1.4152000	-30.1527081
550.0000000	1.4640000	-29.6956654
550.0000000	1.5128000	-43.9425735
550.0000000	1.5616000	-35.5968094
550.0000000	1.6104001	-1676.7286377
550.0000000	1.6592000	-87.0729980
550.0000000	1.7079999	-27.0814190
550.0000000	1.7567999	-27.7724533
550.0000000	1.8055999	-26.6364021
550.0000000	1.8544000	-27.3625469
550.0000000	1.9031999	-26.7590904
550.0000000	1.9520000	-26.5463409
550.0000000	2.0007999	-27.5408134
550.0000000	2.0495999	-20.6330490
550.0000000	2.0983999	-217.3357544
550.0000000	2.1471999	10.8869286
550.0000000	2.1960001	-25.6407890
550.0000000	2.2448001	-26.6695900
550.0000000	2.2935998	-30.4827404
550.0000000	2.3423998	-20.3128338
550.0000000	2.3912001	61.0931854
550.0000000	2.4400001	-22.7206001
550.0000000	2.4888000	-23.4703007
550.0000000	2.5375998	-11.4623375
550.0000000	2.5863998	-9.4580164
550.0000000	2.6352000	-7.3889637
550.0000000	2.6840000	-14.8452587
550.0000000	2.7328000	145.8082428
550.0000000	2.7815998	171.6924133
550.0000000	2.8304000	-23.8564281
550.0000000	2.8792000	-20.4640427
550.0000000	2.9280000	-22.5988712
550.0000000	2.9768000	-16.3909054
550.0000000	3.0256000	-20.3498039
550.0000000	3.0743999	-30.0590286
550.0000000	3.1231999	-24.4196529
550.0000000	3.1719999	-30.0006866
550.0000000	3.2208002	-515.5542603
550.0000000	3.2695999	-1009.8431396
550.0000000	3.3183999	-20.4802914
550.0000000	3.3671999	-3465.3576660
550.0000000	3.4159999	1243.7056885
550.0000000	3.4648001	-6.8722315
550.0000000	3.5135999	-27.1777000
550.0000000	3.5623999	-328.0394592
550.0000000	3.6111999	-2063.2199707
550.0000000	3.6600001	-466.8102417
550.0000000	3.7088001	-616.5258789
550.0000000	3.7575998	-965.4889526
550.0000000	3.8063998	-1752.2788086
550.0000000	3.8552001	-2544.2221680
550.0000000	3.9040000	-2494.4299316
550.0000000	3.9528000	1141.1971436

550.0000000	4.0015998	-248.9279175
550.0000000	4.0503998	698.8347778
550.0000000	4.0991998	89.3138809
550.0000000	4.1479998	-664.8524170
550.0000000	4.1967998	2.8892918
550.0000000	4.2456002	-252.1531067
550.0000000	4.2943997	-495.4320984
550.0000000	4.3431997	-13.2505703
550.0000000	4.3920002	-251.7765961
550.0000000	4.4407997	-22.5263577
550.0000000	4.4896002	-15.4415379
550.0000000	4.5383997	-664.7345581
550.0000000	4.5871997	-12.7640381
550.0000000	4.6360002	-21.6781330
550.0000000	4.6847997	-28.4274902
550.0000000	4.7336001	-19.5891247
550.0000000	4.7824001	-16.4955692
550.0000000	4.8311996	-1493.9495850
550.0000000	4.8800001	231.5561218
550.0000000	4.9287996	-203.9302216
550.0000000	4.9776001	1074.0307617
550.0000000	5.0264001	87.5570374
550.0000000	5.0751996	-304.9543762
550.0000000	5.1240001	-12.2744312
550.0000000	5.1727996	562.7008057
550.0000000	5.2216001	32.3928566
550.0000000	5.2704000	-159.9969482
550.0000000	5.3191996	-39.3737946
550.0000000	5.3680000	-3565.0068359
550.0000000	5.4168000	531.6276245
550.0000000	5.4656000	-202.2644196
550.0000000	5.5144000	38.5164642
550.0000000	5.5631995	492.4836426
550.0000000	5.6120000	396.5160828
550.0000000	5.6608000	-37.2573242
550.0000000	5.7096000	63.1818771
550.0000000	5.7584000	134.2621155
550.0000000	5.8071995	-11.4835072
550.0000000	5.8559999	30.1916370
550.0000000	5.9047999	-16.6140022
550.0000000	5.9535999	-39.9771500
550.0000000	6.0023999	-1585.9025879
550.0000000	6.0511999	-14.5592012
550.0000000	6.0999999	-19.4448013
550.0000000	6.1487999	-13.2535658
550.0000000	6.1975999	-23.2957458
550.0000000	6.2463999	-1806.7312012
550.0000000	6.2951999	-19.1052341
550.0000000	6.3439999	-345.4623413
550.0000000	6.3927999	-15.0489130
550.0000000	6.4416003	-12.3606091
550.0000000	6.4903998	-11.2344027
550.0000000	6.5391998	-11.7766199
550.0000000	6.5879998	415.1712952
550.0000000	6.6367998	-12.0601435
550.0000000	6.6856003	-13.8074474
550.0000000	6.7343998	-13.4461575
550.0000000	6.7831998	-14.3909388

550.0000000	6.8319998	59.2873306
550.0000000	6.8807998	-14.0555849
550.0000000	6.9296002	-483.4958191
550.0000000	6.9783998	-4.5102992
550.0000000	7.0271997	-14.1835146
550.0000000	7.0760002	13.0895567
550.0000000	7.1247997	-2310.2087402
550.0000000	7.1736002	-1852.5559082
550.0000000	7.2223997	-1053.6457520
550.0000000	7.2711997	-226.3297119
550.0000000	7.3200002	-19.4283905
550.0000000	7.3687997	204.9762268
550.0000000	7.4176002	426.4193115
550.0000000	7.4663997	335.1803284
550.0000000	7.5151997	-1031.1256104
550.0000000	7.5640001	-1139.0628662
550.0000000	7.6127996	-2686.1108398
550.0000000	7.6616001	-861.8705444
550.0000000	7.7104001	-442.2550049
550.0000000	7.7591996	47.8651619
550.0000000	7.8080001	443.1335754
550.0000000	7.8567996	583.4478149
550.0000000	7.9056001	-1206.5306396
550.0000000	7.9544001	1920.8353271
550.0000000	8.0031996	2478.4638672
550.0000000	8.0520000	2473.7924805
550.0000000	8.1007996	2617.7382813
550.0000000	8.1496000	2791.6826172
550.0000000	8.1983995	1736.8708496
550.0000000	8.2472000	-3973.3813477
550.0000000	8.2959995	-1408.3521729
550.0000000	8.3448000	-1121.9241943
550.0000000	8.3935995	-1028.1998291
550.0000000	8.4423990	-442.7335815
550.0000000	8.4912004	-1033.9045410
550.0000000	8.5400000	-2789.4077148
550.0000000	8.5887995	-1351.8592529
550.0000000	8.6375999	264.4312439
550.0000000	8.6863995	2386.5524902
550.0000000	8.7351999	-886.7105713
550.0000000	8.7840004	1013.9645996
550.0000000	8.8327999	55.7313995
550.0000000	8.8815994	-11.4883204
550.0000000	8.9303989	-24.1185398
550.0000000	8.9792004	-11.5752640
550.0000000	9.0279999	-5.7543373
550.0000000	9.0767994	7.3065224
550.0000000	9.1255999	-17.3222198
550.0000000	9.1743994	-10.0547638
550.0000000	9.2231998	37.7488174
550.0000000	9.2720003	-8.3761940
550.0000000	9.3207998	-9.7727909
550.0000000	9.3695993	-10.7531385
550.0000000	9.4183998	-11.7597303
550.0000000	9.4672003	-11.1593952
550.0000000	9.5159998	-14.2060871
550.0000000	9.5648003	-0.2345619
550.0000000	9.6135998	-13.4331722

550.0000000	9.6623993	-10.2084064
550.0000000	9.7111998	-10.1449413
550.0000000	9.7600002	-10.8556156
550.0000000	9.8087997	-10.9944048
550.0000000	9.8575993	3.3977547
550.0000000	9.9063997	-7.9261122
550.0000000	9.9552002	-10.9063988
550.0000000	10.0039997	-10.0630846
560.0000000	0.3416000	-36.2482071
560.0000000	0.3904000	-38.9085426
560.0000000	0.4392000	-39.5832443
560.0000000	0.4880000	-35.6767426
560.0000000	0.5368000	-33.3256416
560.0000000	0.5856000	-33.6737213
560.0000000	0.6344000	-35.3687744
560.0000000	0.6832000	-35.4628258
560.0000000	0.7320000	-34.5105019
560.0000000	0.7808000	-34.2399979
560.0000000	0.8296000	-34.6301994
560.0000000	0.8784000	-35.3757553
560.0000000	0.9272000	-34.0363197
560.0000000	0.9760000	-34.0532417
560.0000000	1.0247999	-32.9095421
560.0000000	1.0735999	-32.0195236
560.0000000	1.1224000	-31.5231762
560.0000000	1.1711999	-31.6535587
560.0000000	1.2200000	-31.9257278
560.0000000	1.2687999	-31.4233322
560.0000000	1.3176000	-30.9097691
560.0000000	1.3664000	-30.2144222
560.0000000	1.4152000	-30.1529121
560.0000000	1.4640000	-29.6939545
560.0000000	1.5128000	-43.8453979
560.0000000	1.5616000	-35.5549698
560.0000000	1.6104001	-1674.1857910
560.0000000	1.6592000	-86.7790680
560.0000000	1.7079999	-27.0851669
560.0000000	1.7567999	-26.7793255
560.0000000	1.8055999	-26.0738182
560.0000000	1.8544000	-27.2412319
560.0000000	1.9031999	-26.7734432
560.0000000	1.9520000	-26.5467968
560.0000000	2.0007999	-27.4782314
560.0000000	2.0495999	-20.8196640
560.0000000	2.0983999	-215.8599854
560.0000000	2.1471999	10.6196632
560.0000000	2.1960001	-25.6366978
560.0000000	2.2448001	-26.6516800
560.0000000	2.2935998	-30.4231167
560.0000000	2.3423998	-20.3272381
560.0000000	2.3912001	60.4716415
560.0000000	2.4400001	-22.7230873
560.0000000	2.4888000	-23.4700832
560.0000000	2.5375998	-11.4794455
560.0000000	2.5863998	-9.4777756
560.0000000	2.6352000	-7.4117079
560.0000000	2.6840000	-14.8572006
560.0000000	2.7328000	145.5422363

560.0000000	2.7815998	171.3859863
560.0000000	2.8304000	-23.8551235
560.0000000	2.8792000	-20.4673061
560.0000000	2.9280000	-22.5983009
560.0000000	2.9768000	-16.3992767
560.0000000	3.0256000	-20.3523884
560.0000000	3.0743999	-30.0476418
560.0000000	3.1231999	-24.4161968
560.0000000	3.1719999	-29.9923935
560.0000000	3.2208002	-515.3165283
560.0000000	3.2695999	-1009.3682861
560.0000000	3.3183999	-20.4804897
560.0000000	3.3671999	-3463.8962402
560.0000000	3.4159999	1243.1704102
560.0000000	3.4648001	-6.8779392
560.0000000	3.5135999	-27.1747093
560.0000000	3.5623999	-327.9098816
560.0000000	3.6111999	-2062.3603516
560.0000000	3.6600001	-466.6220703
560.0000000	3.7088001	-616.2745361
560.0000000	3.7575998	-965.0903931
560.0000000	3.8063998	-1751.5489502
560.0000000	3.8552001	-2543.1586914
560.0000000	3.9040000	-2493.3874512
560.0000000	3.9528000	1140.7088623
560.0000000	4.0015998	-248.8308105
560.0000000	4.0503998	698.5321655
560.0000000	4.0991998	89.2682037
560.0000000	4.1479998	-664.5802002
560.0000000	4.1967998	2.8800354
560.0000000	4.2456002	-252.0548096
560.0000000	4.2943997	-495.2315369
560.0000000	4.3431997	-13.2527514
560.0000000	4.3920002	-251.6784821
560.0000000	4.4407997	-22.5245819
560.0000000	4.4896002	-15.4424210
560.0000000	4.5383997	-664.4734497
560.0000000	4.5871997	-12.7660542
560.0000000	4.6360002	-21.6765900
560.0000000	4.6847997	-28.4235649
560.0000000	4.7336001	-19.5884037
560.0000000	4.7824001	-16.4960232
560.0000000	4.8311996	-1493.4183350
560.0000000	4.8800001	231.4665833
560.0000000	4.9287996	-203.8629608
560.0000000	4.9776001	1073.6383057
560.0000000	5.0264001	87.5195160
560.0000000	5.0751996	-304.8507385
560.0000000	5.1240001	-12.2753525
560.0000000	5.1727996	562.5848999
560.0000000	5.2216001	32.3834114
560.0000000	5.2704000	-159.9688263
560.0000000	5.3191996	-39.3692436
560.0000000	5.3680000	-3564.3144531
560.0000000	5.4168000	531.5208130
560.0000000	5.4656000	-202.2282410
560.0000000	5.5144000	38.5058060
560.0000000	5.5631995	492.3850708

560.0000000	5.6120000	396.4361267
560.0000000	5.6608000	-37.2532349
560.0000000	5.7096000	63.1664429
560.0000000	5.7584000	134.2331696
560.0000000	5.8071995	-11.4843769
560.0000000	5.8559999	30.1827869
560.0000000	5.9047999	-16.6138039
560.0000000	5.9535999	-39.9724121
560.0000000	6.0023999	-1585.5980225
560.0000000	6.0511999	-14.5594130
560.0000000	6.0999999	-19.4439678
560.0000000	6.1487999	-13.2540283
560.0000000	6.1975999	-23.2939796
560.0000000	6.2463999	-1806.3837891
560.0000000	6.2951999	-19.1044197
560.0000000	6.3439999	-345.3982544
560.0000000	6.3927999	-15.0488949
560.0000000	6.4416003	-12.3611956
560.0000000	6.4903998	-11.2350788
560.0000000	6.5391998	-11.7773008
560.0000000	6.5879998	414.4805908
560.0000000	6.6367998	-12.0606928
560.0000000	6.6856003	-13.8076172
560.0000000	6.7343998	-13.4463730
560.0000000	6.7831998	-14.3905230
560.0000000	6.8319998	59.2682190
560.0000000	6.8807998	-14.0554466
560.0000000	6.9296002	-482.7343750
560.0000000	6.9783998	-4.5231228
560.0000000	7.0271997	-14.1824875
560.0000000	7.0760002	13.0473738
560.0000000	7.1247997	-2306.2629395
560.0000000	7.1736002	-1849.3929443
560.0000000	7.2223997	-1051.8526611
560.0000000	7.2711997	-225.9619446
560.0000000	7.3200002	-19.4164009
560.0000000	7.3687997	204.6016846
560.0000000	7.4176002	425.6634827
560.0000000	7.4663997	334.5826721
560.0000000	7.5151997	-1029.3645020
560.0000000	7.5640001	-1137.1154785
560.0000000	7.6127996	-2681.4931641
560.0000000	7.6616001	-860.3991699
560.0000000	7.7104001	-441.5087280
560.0000000	7.7591996	47.7641144
560.0000000	7.8080001	442.3482056
560.0000000	7.8567996	582.4197388
560.0000000	7.9056001	-1204.4577637
560.0000000	7.9544001	1917.5253906
560.0000000	8.0031996	2474.1940918
560.0000000	8.0520000	2469.5305176
560.0000000	8.1007996	2613.2287598
560.0000000	8.1496000	2786.8745117
560.0000000	8.1983995	1733.8782959
560.0000000	8.2472000	-3966.5466309
560.0000000	8.2959995	-1405.9320068
560.0000000	8.3448000	-1119.9949951
560.0000000	8.3935995	-1026.4322510

560.0000000	8.4423990	-441.9755554
560.0000000	8.4912004	-1032.1282959
560.0000000	8.5400000	-2784.6079102
560.0000000	8.5887995	-1349.5377197
560.0000000	8.6375999	263.9699097
560.0000000	8.6863995	2382.4340820
560.0000000	8.7351999	-885.1863403
560.0000000	8.7840004	1012.2078857
560.0000000	8.8327999	55.6197968
560.0000000	8.8815994	-11.4884949
560.0000000	8.9303989	-24.1148186
560.0000000	8.9792004	-11.5753469
560.0000000	9.0279999	-5.7561054
560.0000000	9.0767994	7.3008041
560.0000000	9.1255999	-17.3205051
560.0000000	9.1743994	-10.0551653
560.0000000	9.2231998	37.7330856
560.0000000	9.2720003	-8.3771305
560.0000000	9.3207998	-9.7733307
560.0000000	9.3695993	-10.7533655
560.0000000	9.4183998	-11.7596064
560.0000000	9.4672003	-11.1594362
560.0000000	9.5159998	-14.2051964
560.0000000	9.5648003	-0.2379341
560.0000000	9.6135998	-13.4323816
560.0000000	9.6623993	-10.2086811
560.0000000	9.7111998	-10.1452131
560.0000000	9.7600002	-10.8556576
560.0000000	9.8087997	-10.9944124
560.0000000	9.8575993	3.3933325
560.0000000	9.9063997	-7.9270258
560.0000000	9.9552002	-10.9064035
560.0000000	10.0039997	-10.0633316
570.0000000	0.3416000	-36.9312897
570.0000000	0.3904000	-39.1005554
570.0000000	0.4392000	-39.4177208
570.0000000	0.4880000	-38.6365700
570.0000000	0.5368000	-35.8503036
570.0000000	0.5856000	-34.6227303
570.0000000	0.6344000	-35.1313553
570.0000000	0.6832000	-35.2261162
570.0000000	0.7320000	-34.8506737
570.0000000	0.7808000	-35.1113396
570.0000000	0.8296000	-35.7992592
570.0000000	0.8784000	-34.8886147
570.0000000	0.9272000	-33.8263512
570.0000000	0.9760000	-31.1716557
570.0000000	1.0247999	-28.4428234
570.0000000	1.0735999	-28.1688538
570.0000000	1.1224000	-29.1433010
570.0000000	1.1711999	-29.8741188
570.0000000	1.2200000	-30.3327999
570.0000000	1.2687999	-31.4325657
570.0000000	1.3176000	-30.9358768
570.0000000	1.3664000	-30.2221718
570.0000000	1.4152000	-30.1531162
570.0000000	1.4640000	-29.6922455
570.0000000	1.5128000	-43.7482185

570.0000000	1.5616000	-35.5131302
570.0000000	1.6104001	-1671.6429443
570.0000000	1.6592000	-86.4851379
570.0000000	1.7079999	-27.0889149
570.0000000	1.7567999	-27.1388321
570.0000000	1.8055999	-27.1376553
570.0000000	1.8544000	-26.9472713
570.0000000	1.9031999	-26.7877979
570.0000000	1.9520000	-26.5472527
570.0000000	2.0007999	-27.4156494
570.0000000	2.0495999	-21.0062809
570.0000000	2.0983999	-214.3842163
570.0000000	2.1471999	10.3523941
570.0000000	2.1960001	-25.6326065
570.0000000	2.2448001	-26.6337681
570.0000000	2.2935998	-30.3634930
570.0000000	2.3423998	-20.3416424
570.0000000	2.3912001	59.8500977
570.0000000	2.4400001	-22.7255726
570.0000000	2.4888000	-23.4698677
570.0000000	2.5375998	-11.4965534
570.0000000	2.5863998	-9.4975348
570.0000000	2.6352000	-7.4344521
570.0000000	2.6840000	-14.8691425
570.0000000	2.7328000	145.2762146
570.0000000	2.7815998	171.0795898
570.0000000	2.8304000	-23.8538189
570.0000000	2.8792000	-20.4705696
570.0000000	2.9280000	-22.5977325
570.0000000	2.9768000	-16.4076462
570.0000000	3.0256000	-20.3549728
570.0000000	3.0743999	-30.0362530
570.0000000	3.1231999	-24.4127426
570.0000000	3.1719999	-29.9841003
570.0000000	3.2208002	-515.0787354
570.0000000	3.2695999	-1008.8931885
570.0000000	3.3183999	-20.4806881
570.0000000	3.3671999	-3462.4345703
570.0000000	3.4159999	1242.6351318
570.0000000	3.4648001	-6.8836479
570.0000000	3.5135999	-27.1717186
570.0000000	3.5623999	-327.7802734
570.0000000	3.6111999	-2061.5004883
570.0000000	3.6600001	-466.4339600
570.0000000	3.7088001	-616.0231323
570.0000000	3.7575998	-964.6918945
570.0000000	3.8063998	-1750.8192139
570.0000000	3.8552001	-2542.0954590
570.0000000	3.9040000	-2492.3449707
570.0000000	3.9528000	1140.2205811
570.0000000	4.0015998	-248.7337036
570.0000000	4.0503998	698.2296143
570.0000000	4.0991998	89.2225189
570.0000000	4.1479998	-664.3079834
570.0000000	4.1967998	2.8707790
570.0000000	4.2456002	-251.9565125
570.0000000	4.2943997	-495.0309448
570.0000000	4.3431997	-13.2549314

570.0000000	4.3920002	-251.5803680
570.0000000	4.4407997	-22.5228062
570.0000000	4.4896002	-15.4433031
570.0000000	4.5383997	-664.2124023
570.0000000	4.5871997	-12.7680702
570.0000000	4.6360002	-21.6750488
570.0000000	4.6847997	-28.4196396
570.0000000	4.7336001	-19.5876808
570.0000000	4.7824001	-16.4964790
570.0000000	4.8311996	-1492.8870850
570.0000000	4.8800001	231.3770294
570.0000000	4.9287996	-203.7957001
570.0000000	4.9776001	1073.2458496
570.0000000	5.0264001	87.4819946
570.0000000	5.0751996	-304.7471008
570.0000000	5.1240001	-12.2762737
570.0000000	5.1727996	562.4690552
570.0000000	5.2216001	32.3739700
570.0000000	5.2704000	-159.9407349
570.0000000	5.3191996	-39.3646927
570.0000000	5.3680000	-3563.6220703
570.0000000	5.4168000	531.4140015
570.0000000	5.4656000	-202.1920471
570.0000000	5.5144000	38.4951553
570.0000000	5.5631995	492.2865295
570.0000000	5.6120000	396.3561401
570.0000000	5.6608000	-37.2491455
570.0000000	5.7096000	63.1510086
570.0000000	5.7584000	134.2042389
570.0000000	5.8071995	-11.4852476
570.0000000	5.8559999	30.1739368
570.0000000	5.9047999	-16.6136055
570.0000000	5.9535999	-39.9676743
570.0000000	6.0023999	-1585.2937012
570.0000000	6.0511999	-14.5596237
570.0000000	6.0999999	-19.4431362
570.0000000	6.1487999	-13.2544918
570.0000000	6.1975999	-23.2922153
570.0000000	6.2463999	-1806.0364990
570.0000000	6.2951999	-19.1036053
570.0000000	6.3439999	-345.3342285
570.0000000	6.3927999	-15.0488758
570.0000000	6.4416003	-12.3617830
570.0000000	6.4903998	-11.2357540
570.0000000	6.5391998	-11.7779818
570.0000000	6.5879998	413.7899170
570.0000000	6.6367998	-12.0612421
570.0000000	6.6856003	-13.8077879
570.0000000	6.7343998	-13.4465885
570.0000000	6.7831998	-14.3901072
570.0000000	6.8319998	59.2491074
570.0000000	6.8807998	-14.0553083
570.0000000	6.9296002	-481.9729004
570.0000000	6.9783998	-4.5359468
570.0000000	7.0271997	-14.1814594
570.0000000	7.0760002	13.0051870
570.0000000	7.1247997	-2302.3168945
570.0000000	7.1736002	-1846.2301025

570.0000000	7.2223997	-1050.0594482
570.0000000	7.2711997	-225.5941620
570.0000000	7.3200002	-19.4044113
570.0000000	7.3687997	204.2271576
570.0000000	7.4176002	424.9076538
570.0000000	7.4663997	333.9850159
570.0000000	7.5151997	-1027.6035156
570.0000000	7.5640001	-1135.1680908
570.0000000	7.6127996	-2676.8757324
570.0000000	7.6616001	-858.9278564
570.0000000	7.7104001	-440.7624512
570.0000000	7.7591996	47.6630669
570.0000000	7.8080001	441.5628357
570.0000000	7.8567996	581.3916626
570.0000000	7.9056001	-1202.3848877
570.0000000	7.9544001	1914.2154541
570.0000000	8.0031996	2469.9245605
570.0000000	8.0520000	2465.2685547
570.0000000	8.1007996	2608.7192383
570.0000000	8.1496000	2782.0664063
570.0000000	8.1983995	1730.8858643
570.0000000	8.2472000	-3959.7116699
570.0000000	8.2959995	-1403.5118408
570.0000000	8.3448000	-1118.0657959
570.0000000	8.3935995	-1024.6645508
570.0000000	8.4423990	-441.2175598
570.0000000	8.4912004	-1030.3521729
570.0000000	8.5400000	-2779.8081055
570.0000000	8.5887995	-1347.2161865
570.0000000	8.6375999	263.5085754
570.0000000	8.6863995	2378.3156738
570.0000000	8.7351999	-883.6620483
570.0000000	8.7840004	1010.4511108
570.0000000	8.8327999	55.5081940
570.0000000	8.8815994	-11.4886694
570.0000000	8.9303989	-24.1110992
570.0000000	8.9792004	-11.5754290
570.0000000	9.0279999	-5.7578740
570.0000000	9.0767994	7.2950878
570.0000000	9.1255999	-17.3187923
570.0000000	9.1743994	-10.0555677
570.0000000	9.2231998	37.7173538
570.0000000	9.2720003	-8.3780670
570.0000000	9.3207998	-9.7738695
570.0000000	9.3695993	-10.7535915
570.0000000	9.4183998	-11.7594814
570.0000000	9.4672003	-11.1594782
570.0000000	9.5159998	-14.2043047
570.0000000	9.5648003	-0.2413073
570.0000000	9.6135998	-13.4315910
570.0000000	9.6623993	-10.2089567
570.0000000	9.7111998	-10.1454849
570.0000000	9.7600002	-10.8556995
570.0000000	9.8087997	-10.9944201
570.0000000	9.8575993	3.3889103
570.0000000	9.9063997	-7.9279394
570.0000000	9.9552002	-10.9064074
570.0000000	10.0039997	-10.0635796

580.0000000	0.3416000	-36.6560364
580.0000000	0.3904000	-38.9129448
580.0000000	0.4392000	-39.2521973
580.0000000	0.4880000	-38.8682022
580.0000000	0.5368000	-36.3837891
580.0000000	0.5856000	-36.3944321
580.0000000	0.6344000	-37.2890472
580.0000000	0.6832000	-35.7118340
580.0000000	0.7320000	-35.0651970
580.0000000	0.7808000	-35.2758789
580.0000000	0.8296000	-35.3162575
580.0000000	0.8784000	-34.6444969
580.0000000	0.9272000	-32.9282379
580.0000000	0.9760000	-30.3213444
580.0000000	1.0247999	-29.0041409
580.0000000	1.0735999	-28.0759048
580.0000000	1.1224000	-28.3930798
580.0000000	1.1711999	-30.0374680
580.0000000	1.2200000	-30.8830986
580.0000000	1.2687999	-31.4417992
580.0000000	1.3176000	-30.9619865
580.0000000	1.3664000	-30.2299213
580.0000000	1.4152000	-30.1533203
580.0000000	1.4640000	-29.6905365
580.0000000	1.5128000	-43.6510391
580.0000000	1.5616000	-35.4712868
580.0000000	1.6104001	-1669.1002197
580.0000000	1.6592000	-86.1912003
580.0000000	1.7079999	-27.0926609
580.0000000	1.7567999	-27.4983406
580.0000000	1.8055999	-27.5237579
580.0000000	1.8544000	-26.8342743
580.0000000	1.9031999	-26.8021507
580.0000000	1.9520000	-26.5477066
580.0000000	2.0007999	-27.3530674
580.0000000	2.0495999	-21.1928978
580.0000000	2.0983999	-212.9084167
580.0000000	2.1471999	10.0851250
580.0000000	2.1960001	-25.6285152
580.0000000	2.2448001	-26.6158562
580.0000000	2.2935998	-30.3038692
580.0000000	2.3423998	-20.3560486
580.0000000	2.3912001	59.2285538
580.0000000	2.4400001	-22.7280598
580.0000000	2.4888000	-23.4696503
580.0000000	2.5375998	-11.5136614
580.0000000	2.5863998	-9.5172930
580.0000000	2.6352000	-7.4571962
580.0000000	2.6840000	-14.8810844
580.0000000	2.7328000	145.0101929
580.0000000	2.7815998	170.7731628
580.0000000	2.8304000	-23.8525143
580.0000000	2.8792000	-20.4738331
580.0000000	2.9280000	-22.5971622
580.0000000	2.9768000	-16.4160175
580.0000000	3.0256000	-20.3575573
580.0000000	3.0743999	-30.0248661
580.0000000	3.1231999	-24.4092865

580.0000000	3.1719999	-29.9758072
580.0000000	3.2208002	-514.8410034
580.0000000	3.2695999	-1008.4183350
580.0000000	3.3183999	-20.4808865
580.0000000	3.3671999	-3460.9729004
580.0000000	3.4159999	1242.0998535
580.0000000	3.4648001	-6.8893566
580.0000000	3.5135999	-27.1687279
580.0000000	3.5623999	-327.6506958
580.0000000	3.6111999	-2060.6408691
580.0000000	3.6600001	-466.2458496
580.0000000	3.7088001	-615.7717285
580.0000000	3.7575998	-964.2933350
580.0000000	3.8063998	-1750.0893555
580.0000000	3.8552001	-2541.0319824
580.0000000	3.9040000	-2491.3024902
580.0000000	3.9528000	1139.7322998
580.0000000	4.0015998	-248.6365814
580.0000000	4.0503998	697.9270630
580.0000000	4.0991998	89.1768341
580.0000000	4.1479998	-664.0357666
580.0000000	4.1967998	2.8615227
580.0000000	4.2456002	-251.8582001
580.0000000	4.2943997	-494.8303833
580.0000000	4.3431997	-13.2571125
580.0000000	4.3920002	-251.4822540
580.0000000	4.4407997	-22.5210304
580.0000000	4.4896002	-15.4441862
580.0000000	4.5383997	-663.9513550
580.0000000	4.5871997	-12.7700863
580.0000000	4.6360002	-21.6735058
580.0000000	4.6847997	-28.4157143
580.0000000	4.7336001	-19.5869579
580.0000000	4.7824001	-16.4969330
580.0000000	4.8311996	-1492.3557129
580.0000000	4.8800001	231.2874756
580.0000000	4.9287996	-203.7284393
580.0000000	4.9776001	1072.8533936
580.0000000	5.0264001	87.4444733
580.0000000	5.0751996	-304.6434631
580.0000000	5.1240001	-12.2771950
580.0000000	5.1727996	562.3531494
580.0000000	5.2216001	32.3645248
580.0000000	5.2704000	-159.9126129
580.0000000	5.3191996	-39.3601456
580.0000000	5.3680000	-3562.9296875
580.0000000	5.4168000	531.3072510
580.0000000	5.4656000	-202.1558685
580.0000000	5.5144000	38.4844971
580.0000000	5.5631995	492.1879578
580.0000000	5.6120000	396.2761536
580.0000000	5.6608000	-37.2450600
580.0000000	5.7096000	63.1355820
580.0000000	5.7584000	134.1753082
580.0000000	5.8071995	-11.4861183
580.0000000	5.8559999	30.1650829
580.0000000	5.9047999	-16.6134090
580.0000000	5.9535999	-39.9629326

580.0000000	6.0023999	-1584.9891357
580.0000000	6.0511999	-14.5598354
580.0000000	6.0999999	-19.4423046
580.0000000	6.1487999	-13.2549543
580.0000000	6.1975999	-23.2904510
580.0000000	6.2463999	-1805.6890869
580.0000000	6.2951999	-19.1027908
580.0000000	6.3439999	-345.2701721
580.0000000	6.3927999	-15.0488577
580.0000000	6.4416003	-12.3623695
580.0000000	6.4903998	-11.2364302
580.0000000	6.5391998	-11.7786627
580.0000000	6.5879998	413.0992126
580.0000000	6.6367998	-12.0617914
580.0000000	6.6856003	-13.8079576
580.0000000	6.7343998	-13.4468040
580.0000000	6.7831998	-14.3896923
580.0000000	6.8319998	59.2299881
580.0000000	6.8807998	-14.0551691
580.0000000	6.9296002	-481.2114563
580.0000000	6.9783998	-4.5487704
580.0000000	7.0271997	-14.1804314
580.0000000	7.0760002	12.9630003
580.0000000	7.1247997	-2298.3710938
580.0000000	7.1736002	-1843.0672607
580.0000000	7.2223997	-1048.2663574
580.0000000	7.2711997	-225.2263794
580.0000000	7.3200002	-19.3924236
580.0000000	7.3687997	203.8526306
580.0000000	7.4176002	424.1518250
580.0000000	7.4663997	333.3873596
580.0000000	7.5151997	-1025.8424072
580.0000000	7.5640001	-1133.2207031
580.0000000	7.6127996	-2672.2580566
580.0000000	7.6616001	-857.4564819
580.0000000	7.7104001	-440.0161743
580.0000000	7.7591996	47.5620155
580.0000000	7.8080001	440.7774963
580.0000000	7.8567996	580.3635254
580.0000000	7.9056001	-1200.3118896
580.0000000	7.9544001	1910.9055176
580.0000000	8.0031996	2465.6550293
580.0000000	8.0520000	2461.0065918
580.0000000	8.1007996	2604.2097168
580.0000000	8.1496000	2777.2585449
580.0000000	8.1983995	1727.8933105
580.0000000	8.2472000	-3952.8769531
580.0000000	8.2959995	-1401.0915527
580.0000000	8.3448000	-1116.1367188
580.0000000	8.3935995	-1022.8968506
580.0000000	8.4423990	-440.4595337
580.0000000	8.4912004	-1028.5759277
580.0000000	8.5400000	-2775.0083008
580.0000000	8.5887995	-1344.8946533
580.0000000	8.6375999	263.0472717
580.0000000	8.6863995	2374.1972656
580.0000000	8.7351999	-882.1378174
580.0000000	8.7840004	1008.6943970

580.0000000	8.8327999	55.3965836
580.0000000	8.8815994	-11.4888430
580.0000000	8.9303989	-24.1073799
580.0000000	8.9792004	-11.5755110
580.0000000	9.0279999	-5.7596426
580.0000000	9.0767994	7.2893696
580.0000000	9.1255999	-17.3170776
580.0000000	9.1743994	-10.0559692
580.0000000	9.2231998	37.7016220
580.0000000	9.2720003	-8.3790035
580.0000000	9.3207998	-9.7744093
580.0000000	9.3695993	-10.7538185
580.0000000	9.4183998	-11.7593575
580.0000000	9.4672003	-11.1595192
580.0000000	9.5159998	-14.2034140
580.0000000	9.5648003	-0.2446804
580.0000000	9.6135998	-13.4307995
580.0000000	9.6623993	-10.2092314
580.0000000	9.7111998	-10.1457567
580.0000000	9.7600002	-10.8557405
580.0000000	9.8087997	-10.9944286
580.0000000	9.8575993	3.3844891
580.0000000	9.9063997	-7.9288535
580.0000000	9.9552002	-10.9064121
580.0000000	10.0039997	-10.0638275
590.0000000	0.3416000	-36.4502945
590.0000000	0.3904000	-38.9898720
590.0000000	0.4392000	-39.0866776
590.0000000	0.4880000	-37.7617378
590.0000000	0.5368000	-35.8638268
590.0000000	0.5856000	-35.5633926
590.0000000	0.6344000	-36.1794281
590.0000000	0.6832000	-36.8718300
590.0000000	0.7320000	-36.8108902
590.0000000	0.7808000	-36.5231285
590.0000000	0.8296000	-36.0239220
590.0000000	0.8784000	-34.8971596
590.0000000	0.9272000	-32.5834045
590.0000000	0.9760000	-29.8885765
590.0000000	1.0247999	-28.6099987
590.0000000	1.0735999	-28.0180569
590.0000000	1.1224000	-28.2305336
590.0000000	1.1711999	-29.4709377
590.0000000	1.2200000	-29.8149834
590.0000000	1.2687999	-30.6847115
590.0000000	1.3176000	-30.9880943
590.0000000	1.3664000	-30.2376709
590.0000000	1.4152000	-30.1535244
590.0000000	1.4640000	-29.6888256
590.0000000	1.5128000	-43.5538635
590.0000000	1.5616000	-35.4294472
590.0000000	1.6104001	-1666.5573730
590.0000000	1.6592000	-85.8972702
590.0000000	1.7079999	-27.0964088
590.0000000	1.7567999	-27.8578472
590.0000000	1.8055999	-27.4562359
590.0000000	1.8544000	-26.7212753
590.0000000	1.9031999	-26.8165054

590.0000000	1.9520000	-26.5481625
590.0000000	2.0007999	-27.2904854
590.0000000	2.0495999	-21.3795128
590.0000000	2.0983999	-211.4326477
590.0000000	2.1471999	9.8178596
590.0000000	2.1960001	-25.6244240
590.0000000	2.2448001	-26.5979462
590.0000000	2.2935998	-30.2442455
590.0000000	2.3423998	-20.3704529
590.0000000	2.3912001	58.6070023
590.0000000	2.4400001	-22.7305470
590.0000000	2.4888000	-23.4694328
590.0000000	2.5375998	-11.5307703
590.0000000	2.5863998	-9.5370512
590.0000000	2.6352000	-7.4799404
590.0000000	2.6840000	-14.8930264
590.0000000	2.7328000	144.7441864
590.0000000	2.7815998	170.4667358
590.0000000	2.8304000	-23.8512115
590.0000000	2.8792000	-20.4770966
590.0000000	2.9280000	-22.5965939
590.0000000	2.9768000	-16.4243889
590.0000000	3.0256000	-20.3601418
590.0000000	3.0743999	-30.0134792
590.0000000	3.1231999	-24.4058304
590.0000000	3.1719999	-29.9675140
590.0000000	3.2208002	-514.6032715
590.0000000	3.2695999	-1007.9432373
590.0000000	3.3183999	-20.4810848
590.0000000	3.3671999	-3459.5112305
590.0000000	3.4159999	1241.5645752
590.0000000	3.4648001	-6.8950644
590.0000000	3.5135999	-27.1657372
590.0000000	3.5623999	-327.5210876
590.0000000	3.6111999	-2059.7810059
590.0000000	3.6600001	-466.0576782
590.0000000	3.7088001	-615.5203857
590.0000000	3.7575998	-963.8948364
590.0000000	3.8063998	-1749.3594971
590.0000000	3.8552001	-2539.9685059
590.0000000	3.9040000	-2490.2600098
590.0000000	3.9528000	1139.2440186
590.0000000	4.0015998	-248.5394745
590.0000000	4.0503998	697.6245117
590.0000000	4.0991998	89.1311493
590.0000000	4.1479998	-663.7635498
590.0000000	4.1967998	2.8522663
590.0000000	4.2456002	-251.7599030
590.0000000	4.2943997	-494.6298218
590.0000000	4.3431997	-13.2592926
590.0000000	4.3920002	-251.3841400
590.0000000	4.4407997	-22.5192547
590.0000000	4.4896002	-15.4450684
590.0000000	4.5383997	-663.6902466
590.0000000	4.5871997	-12.7721024
590.0000000	4.6360002	-21.6719627
590.0000000	4.6847997	-28.4117889
590.0000000	4.7336001	-19.5862350

590.0000000	4.7824001	-16.4973869
590.0000000	4.8311996	-1491.8244629
590.0000000	4.8800001	231.1979218
590.0000000	4.9287996	-203.6611786
590.0000000	4.9776001	1072.4608154
590.0000000	5.0264001	87.4069519
590.0000000	5.0751996	-304.5398560
590.0000000	5.1240001	-12.2781162
590.0000000	5.1727996	562.2373047
590.0000000	5.2216001	32.3550797
590.0000000	5.2704000	-159.8845215
590.0000000	5.3191996	-39.3555946
590.0000000	5.3680000	-3562.2373047
590.0000000	5.4168000	531.2005005
590.0000000	5.4656000	-202.1196899
590.0000000	5.5144000	38.4738388
590.0000000	5.5631995	492.0893860
590.0000000	5.6120000	396.1961975
590.0000000	5.6608000	-37.2409706
590.0000000	5.7096000	63.1201477
590.0000000	5.7584000	134.1463776
590.0000000	5.8071995	-11.4869890
590.0000000	5.8559999	30.1562328
590.0000000	5.9047999	-16.6132107
590.0000000	5.9535999	-39.9581947
590.0000000	6.0023999	-1584.6848145
590.0000000	6.0511999	-14.5600471
590.0000000	6.0999999	-19.4414711
590.0000000	6.1487999	-13.2554169
590.0000000	6.1975999	-23.2886848
590.0000000	6.2463999	-1805.3416748
590.0000000	6.2951999	-19.1019783
590.0000000	6.3439999	-345.2061157
590.0000000	6.3927999	-15.0488396
590.0000000	6.4416003	-12.3629570
590.0000000	6.4903998	-11.2371063
590.0000000	6.5391998	-11.7793436
590.0000000	6.5879998	412.4085083
590.0000000	6.6367998	-12.0623398
590.0000000	6.6856003	-13.8081284
590.0000000	6.7343998	-13.4470205
590.0000000	6.7831998	-14.3892765
590.0000000	6.8319998	59.2108765
590.0000000	6.8807998	-14.0550308
590.0000000	6.9296002	-480.4500122
590.0000000	6.9783998	-4.5615945
590.0000000	7.0271997	-14.1794033
590.0000000	7.0760002	12.9208174
590.0000000	7.1247997	-2294.4252930
590.0000000	7.1736002	-1839.9042969
590.0000000	7.2223997	-1046.4731445
590.0000000	7.2711997	-224.8585968
590.0000000	7.3200002	-19.3804359
590.0000000	7.3687997	203.4781036
590.0000000	7.4176002	423.3959961
590.0000000	7.4663997	332.7897034
590.0000000	7.5151997	-1024.0814209
590.0000000	7.5640001	-1131.2733154

590.0000000	7.6127996	-2667.6403809
590.0000000	7.6616001	-855.9851074
590.0000000	7.7104001	-439.2698975
590.0000000	7.7591996	47.4609680
590.0000000	7.8080001	439.9921265
590.0000000	7.8567996	579.3354492
590.0000000	7.9056001	-1198.2390137
590.0000000	7.9544001	1907.5955811
590.0000000	8.0031996	2461.3854980
590.0000000	8.0520000	2456.7443848
590.0000000	8.1007996	2599.7001953
590.0000000	8.1496000	2772.4504395
590.0000000	8.1983995	1724.9007568
590.0000000	8.2472000	-3946.0422363
590.0000000	8.2959995	-1398.6713867
590.0000000	8.3448000	-1114.2075195
590.0000000	8.3935995	-1021.1292114
590.0000000	8.4423990	-439.7015381
590.0000000	8.4912004	-1026.7996826
590.0000000	8.5400000	-2770.2084961
590.0000000	8.5887995	-1342.5731201
590.0000000	8.6375999	262.5859375
590.0000000	8.6863995	2370.0791016
590.0000000	8.7351999	-880.6135864
590.0000000	8.7840004	1006.9376221
590.0000000	8.8327999	55.2849731
590.0000000	8.8815994	-11.4890175
590.0000000	8.9303989	-24.1036606
590.0000000	8.9792004	-11.5755939
590.0000000	9.0279999	-5.7614107
590.0000000	9.0767994	7.2836514
590.0000000	9.1255999	-17.3153648
590.0000000	9.1743994	-10.0563717
590.0000000	9.2231998	37.6858902
590.0000000	9.2720003	-8.3799400
590.0000000	9.3207998	-9.7749481
590.0000000	9.3695993	-10.7540445
590.0000000	9.4183998	-11.7592325
590.0000000	9.4672003	-11.1595602
590.0000000	9.5159998	-14.2025223
590.0000000	9.5648003	-0.2480526
590.0000000	9.6135998	-13.4300098
590.0000000	9.6623993	-10.2095070
590.0000000	9.7111998	-10.1460285
590.0000000	9.7600002	-10.8557825
590.0000000	9.8087997	-10.9944363
590.0000000	9.8575993	3.3800650
590.0000000	9.9063997	-7.9297671
590.0000000	9.9552002	-10.9064159
590.0000000	10.0039997	-10.0640745
600.0000000	0.3416000	-36.7404594
600.0000000	0.3904000	-38.9010162
600.0000000	0.4392000	-38.9211540
600.0000000	0.4880000	-38.1276665
600.0000000	0.5368000	-37.5429840
600.0000000	0.5856000	-38.6753616
600.0000000	0.6344000	-37.3429298
600.0000000	0.6832000	-37.2042732

600.0000000	0.7320000	-36.8943329
600.0000000	0.7808000	-36.5232964
600.0000000	0.8296000	-35.9842377
600.0000000	0.8784000	-34.7711639
600.0000000	0.9272000	-33.4176598
600.0000000	0.9760000	-30.6901608
600.0000000	1.0247999	-27.8582401
600.0000000	1.0735999	-27.6897621
600.0000000	1.1224000	-28.7590008
600.0000000	1.1711999	-29.2470608
600.0000000	1.2200000	-29.8642731
600.0000000	1.2687999	-31.2926292
600.0000000	1.3176000	-31.0142040
600.0000000	1.3664000	-30.2454205
600.0000000	1.4152000	-30.1537304
600.0000000	1.4640000	-29.6871166
600.0000000	1.5128000	-43.4566841
600.0000000	1.5616000	-35.3876038
600.0000000	1.6104001	-1664.0145264
600.0000000	1.6592000	-85.6033401
600.0000000	1.7079999	-27.1001549
600.0000000	1.7567999	-27.8030529
600.0000000	1.8055999	-27.4669342
600.0000000	1.8544000	-26.6082783
600.0000000	1.9031999	-26.8308582
600.0000000	1.9520000	-26.5486183
600.0000000	2.0007999	-27.2279034
600.0000000	2.0495999	-21.5661297
600.0000000	2.0983999	-209.9568787
600.0000000	2.1471999	9.5505943
600.0000000	2.1960001	-25.6203327
600.0000000	2.2448001	-26.5800343
600.0000000	2.2935998	-30.1846218
600.0000000	2.3423998	-20.3848572
600.0000000	2.3912001	57.9854584
600.0000000	2.4400001	-22.7330322
600.0000000	2.4888000	-23.4692154
600.0000000	2.5375998	-11.5478783
600.0000000	2.5863998	-9.5568104
600.0000000	2.6352000	-7.5026846
600.0000000	2.6840000	-14.9049683
600.0000000	2.7328000	144.4781647
600.0000000	2.7815998	170.1603394
600.0000000	2.8304000	-23.8499069
600.0000000	2.8792000	-20.4803600
600.0000000	2.9280000	-22.5960255
600.0000000	2.9768000	-16.4327583
600.0000000	3.0256000	-20.3627262
600.0000000	3.0743999	-30.0020924
600.0000000	3.1231999	-24.4023762
600.0000000	3.1719999	-29.9592209
600.0000000	3.2208002	-514.3655396
600.0000000	3.2695999	-1007.4683838
600.0000000	3.3183999	-20.4812813
600.0000000	3.3671999	-3458.0495605
600.0000000	3.4159999	1241.0292969
600.0000000	3.4648001	-6.9007721
600.0000000	3.5135999	-27.1627464

600.0000000	3.5623999	-327.3915100
600.0000000	3.6111999	-2058.9211426
600.0000000	3.6600001	-465.8695679
600.0000000	3.7088001	-615.2689819
600.0000000	3.7575998	-963.4962769
600.0000000	3.8063998	-1748.6296387
600.0000000	3.8552001	-2538.9050293
600.0000000	3.9040000	-2489.2175293
600.0000000	3.9528000	1138.7556152
600.0000000	4.0015998	-248.4423676
600.0000000	4.0503998	697.3219604
600.0000000	4.0991998	89.0854645
600.0000000	4.1479998	-663.4913330
600.0000000	4.1967998	2.8430099
600.0000000	4.2456002	-251.6616058
600.0000000	4.2943997	-494.4292603
600.0000000	4.3431997	-13.2614727
600.0000000	4.3920002	-251.2860260
600.0000000	4.4407997	-22.5174789
600.0000000	4.4896002	-15.4459515
600.0000000	4.5383997	-663.4291992
600.0000000	4.5871997	-12.7741184
600.0000000	4.6360002	-21.6704216
600.0000000	4.6847997	-28.4078636
600.0000000	4.7336001	-19.5855141
600.0000000	4.7824001	-16.4978409
600.0000000	4.8311996	-1491.2932129
600.0000000	4.8800001	231.1083679
600.0000000	4.9287996	-203.5939178
600.0000000	4.9776001	1072.0683594
600.0000000	5.0264001	87.3694305
600.0000000	5.0751996	-304.4362183
600.0000000	5.1240001	-12.2790375
600.0000000	5.1727996	562.1214600
600.0000000	5.2216001	32.3456383
600.0000000	5.2704000	-159.8563995
600.0000000	5.3191996	-39.3510475
600.0000000	5.3680000	-3561.5449219
600.0000000	5.4168000	531.0936890
600.0000000	5.4656000	-202.0834961
600.0000000	5.5144000	38.4631882
600.0000000	5.5631995	491.9908142
600.0000000	5.6120000	396.1162109
600.0000000	5.6608000	-37.2368851
600.0000000	5.7096000	63.1047134
600.0000000	5.7584000	134.1174316
600.0000000	5.8071995	-11.4878597
600.0000000	5.8559999	30.1473789
600.0000000	5.9047999	-16.6130123
600.0000000	5.9535999	-39.9534569
600.0000000	6.0023999	-1584.3803711
600.0000000	6.0511999	-14.5602579
600.0000000	6.0999999	-19.4406395
600.0000000	6.1487999	-13.2558804
600.0000000	6.1975999	-23.2869205
600.0000000	6.2463999	-1804.9943848
600.0000000	6.2951999	-19.1011639
600.0000000	6.3439999	-345.1420593

600.0000000	6.3927999	-15.0488214
600.0000000	6.4416003	-12.3635435
600.0000000	6.4903998	-11.2377825
600.0000000	6.5391998	-11.7800255
600.0000000	6.5879998	411.7178040
600.0000000	6.6367998	-12.0628891
600.0000000	6.6856003	-13.8082981
600.0000000	6.7343998	-13.4472361
600.0000000	6.7831998	-14.3888607
600.0000000	6.8319998	59.1917572
600.0000000	6.8807998	-14.0548916
600.0000000	6.9296002	-479.6885681
600.0000000	6.9783998	-4.5744171
600.0000000	7.0271997	-14.1783752
600.0000000	7.0760002	12.8786306
600.0000000	7.1247997	-2290.4794922
600.0000000	7.1736002	-1836.7414551
600.0000000	7.2223997	-1044.6799316
600.0000000	7.2711997	-224.4908142
600.0000000	7.3200002	-19.3684464
600.0000000	7.3687997	203.1035767
600.0000000	7.4176002	422.6401672
600.0000000	7.4663997	332.1920471
600.0000000	7.5151997	-1022.3203125
600.0000000	7.5640001	-1129.3259277
600.0000000	7.6127996	-2663.0229492
600.0000000	7.6616001	-854.5137939
600.0000000	7.7104001	-438.5236816
600.0000000	7.7591996	47.3599205
600.0000000	7.8080001	439.2067566
600.0000000	7.8567996	578.3073730
600.0000000	7.9056001	-1196.1661377
600.0000000	7.9544001	1904.2856445
600.0000000	8.0031996	2457.1157227
600.0000000	8.0520000	2452.4824219
600.0000000	8.1007996	2595.1906738
600.0000000	8.1496000	2767.6425781
600.0000000	8.1983995	1721.9083252
600.0000000	8.2472000	-3939.2072754
600.0000000	8.2959995	-1396.2512207
600.0000000	8.3448000	-1112.2783203
600.0000000	8.3935995	-1019.3615112
600.0000000	8.4423990	-438.9435120
600.0000000	8.4912004	-1025.0235596
600.0000000	8.5400000	-2765.4086914
600.0000000	8.5887995	-1340.2515869
600.0000000	8.6375999	262.1246338
600.0000000	8.6863995	2365.9606934
600.0000000	8.7351999	-879.0892944
600.0000000	8.7840004	1005.1808472
600.0000000	8.8327999	55.1733627
600.0000000	8.8815994	-11.4891920
600.0000000	8.9303989	-24.0999393
600.0000000	8.9792004	-11.5756760
600.0000000	9.0279999	-5.7631788
600.0000000	9.0767994	7.2779331
600.0000000	9.1255999	-17.3136501
600.0000000	9.1743994	-10.0567741

600.0000000	9.2231998	37.6701584
600.0000000	9.2720003	-8.3808765
600.0000000	9.3207998	-9.7754879
600.0000000	9.3695993	-10.7542715
600.0000000	9.4183998	-11.7591076
600.0000000	9.4672003	-11.1596012
600.0000000	9.5159998	-14.2016315
600.0000000	9.5648003	-0.2514248
600.0000000	9.6135998	-13.4292183
600.0000000	9.6623993	-10.2097816
600.0000000	9.7111998	-10.1462994
600.0000000	9.7600002	-10.8558245
600.0000000	9.8087997	-10.9944439
600.0000000	9.8575993	3.3756428
600.0000000	9.9063997	-7.9306812
600.0000000	9.9552002	-10.9064207
600.0000000	10.0039997	-10.0643225
610.0000000	0.3416000	-36.9954414
610.0000000	0.3904000	-39.6728745
610.0000000	0.4392000	-38.7556305
610.0000000	0.4880000	-38.4935951
610.0000000	0.5368000	-39.2221413
610.0000000	0.5856000	-37.5957108
610.0000000	0.6344000	-37.1584358
610.0000000	0.6832000	-36.6888733
610.0000000	0.7320000	-36.6286240
610.0000000	0.7808000	-36.5234680
610.0000000	0.8296000	-35.9445534
610.0000000	0.8784000	-34.5839272
610.0000000	0.9272000	-32.9521637
610.0000000	0.9760000	-30.9864445
610.0000000	1.0247999	-29.4184761
610.0000000	1.0735999	-28.5134716
610.0000000	1.1224000	-28.7230129
610.0000000	1.1711999	-29.6926479
610.0000000	1.2200000	-30.7804451
610.0000000	1.2687999	-31.1836071
610.0000000	1.3176000	-31.0860310
610.0000000	1.3664000	-30.2531700
610.0000000	1.4152000	-30.1539345
610.0000000	1.4640000	-29.6854076
610.0000000	1.5128000	-43.3595085
610.0000000	1.5616000	-35.3457642
610.0000000	1.6104001	-1661.4718018
610.0000000	1.6592000	-85.3094101
610.0000000	1.7079999	-27.1039028
610.0000000	1.7567999	-27.5609550
610.0000000	1.8055999	-26.1958771
610.0000000	1.8544000	-26.4952812
610.0000000	1.9031999	-26.8452129
610.0000000	1.9520000	-26.5490723
610.0000000	2.0007999	-27.1653214
610.0000000	2.0495999	-21.7527466
610.0000000	2.0983999	-208.4811096
610.0000000	2.1471999	9.2833252
610.0000000	2.1960001	-25.6162415
610.0000000	2.2448001	-26.5621223
610.0000000	2.2935998	-30.1250000

610.0000000	2.3423998	-20.3992615
610.0000000	2.3912001	57.3639145
610.0000000	2.4400001	-22.7355194
610.0000000	2.4888000	-23.4689980
610.0000000	2.5375998	-11.5649862
610.0000000	2.5863998	-9.5765696
610.0000000	2.6352000	-7.5254288
610.0000000	2.6840000	-14.9169102
610.0000000	2.7328000	144.2121429
610.0000000	2.7815998	169.8539124
610.0000000	2.8304000	-23.8486023
610.0000000	2.8792000	-20.4836235
610.0000000	2.9280000	-22.5954552
610.0000000	2.9768000	-16.4411278
610.0000000	3.0256000	-20.3653107
610.0000000	3.0743999	-29.9907055
610.0000000	3.1231999	-24.3989201
610.0000000	3.1719999	-29.9509277
610.0000000	3.2208002	-514.1278076
610.0000000	3.2695999	-1006.9932861
610.0000000	3.3183999	-20.4814796
610.0000000	3.3671999	-3456.5878906
610.0000000	3.4159999	1240.4940186
610.0000000	3.4648001	-6.9064808
610.0000000	3.5135999	-27.1597557
610.0000000	3.5623999	-327.2619019
610.0000000	3.6111999	-2058.0615234
610.0000000	3.6600001	-465.6814575
610.0000000	3.7088001	-615.0175781
610.0000000	3.7575998	-963.0977173
610.0000000	3.8063998	-1747.8997803
610.0000000	3.8552001	-2537.8415527
610.0000000	3.9040000	-2488.1750488
610.0000000	3.9528000	1138.2673340
610.0000000	4.0015998	-248.3452606
610.0000000	4.0503998	697.0194092
610.0000000	4.0991998	89.0397797
610.0000000	4.1479998	-663.2191162
610.0000000	4.1967998	2.8337536
610.0000000	4.2456002	-251.5632935
610.0000000	4.2943997	-494.2286682
610.0000000	4.3431997	-13.2636547
610.0000000	4.3920002	-251.1879120
610.0000000	4.4407997	-22.5157032
610.0000000	4.4896002	-15.4468336
610.0000000	4.5383997	-663.1681519
610.0000000	4.5871997	-12.7761345
610.0000000	4.6360002	-21.6688786
610.0000000	4.6847997	-28.4039383
610.0000000	4.7336001	-19.5847912
610.0000000	4.7824001	-16.4982967
610.0000000	4.8311996	-1490.7619629
610.0000000	4.8800001	231.0188293
610.0000000	4.9287996	-203.5266571
610.0000000	4.9776001	1071.6759033
610.0000000	5.0264001	87.3319092
610.0000000	5.0751996	-304.3325806
610.0000000	5.1240001	-12.2799587

610.0000000	5.1727996	562.0055542
610.0000000	5.2216001	32.3361931
610.0000000	5.2704000	-159.8283081
610.0000000	5.3191996	-39.3464966
610.0000000	5.3680000	-3560.8525391
610.0000000	5.4168000	530.9868774
610.0000000	5.4656000	-202.0473175
610.0000000	5.5144000	38.4525299
610.0000000	5.5631995	491.8922424
610.0000000	5.6120000	396.0362549
610.0000000	5.6608000	-37.2327957
610.0000000	5.7096000	63.0892868
610.0000000	5.7584000	134.0885010
610.0000000	5.8071995	-11.4887295
610.0000000	5.8559999	30.1385288
610.0000000	5.9047999	-16.6128159
610.0000000	5.9535999	-39.9487152
610.0000000	6.0023999	-1584.0760498
610.0000000	6.0511999	-14.5604696
610.0000000	6.0999999	-19.4398060
610.0000000	6.1487999	-13.2563429
610.0000000	6.1975999	-23.2851563
610.0000000	6.2463999	-1804.6469727
610.0000000	6.2951999	-19.1003494
610.0000000	6.3439999	-345.0780334
610.0000000	6.3927999	-15.0488024
610.0000000	6.4416003	-12.3641300
610.0000000	6.4903998	-11.2384577
610.0000000	6.5391998	-11.7807064
610.0000000	6.5879998	411.0270996
610.0000000	6.6367998	-12.0634384
610.0000000	6.6856003	-13.8084688
610.0000000	6.7343998	-13.4474516
610.0000000	6.7831998	-14.3884449
610.0000000	6.8319998	59.1726456
610.0000000	6.8807998	-14.0547533
610.0000000	6.9296002	-478.9271240
610.0000000	6.9783998	-4.5872412
610.0000000	7.0271997	-14.1773481
610.0000000	7.0760002	12.8364458
610.0000000	7.1247997	-2286.5334473
610.0000000	7.1736002	-1833.5786133
610.0000000	7.2223997	-1042.8868408
610.0000000	7.2711997	-224.1230469
610.0000000	7.3200002	-19.3564568
610.0000000	7.3687997	202.7290344
610.0000000	7.4176002	421.8843384
610.0000000	7.4663997	331.5943909
610.0000000	7.5151997	-1020.5593262
610.0000000	7.5640001	-1127.3785400
610.0000000	7.6127996	-2658.4052734
610.0000000	7.6616001	-853.0424194
610.0000000	7.7104001	-437.7774048
610.0000000	7.7591996	47.2588692
610.0000000	7.8080001	438.4214172
610.0000000	7.8567996	577.2792969
610.0000000	7.9056001	-1194.0932617
610.0000000	7.9544001	1900.9757080

610.0000000	8.0031996	2452.8461914
610.0000000	8.0520000	2448.2204590
610.0000000	8.1007996	2590.6811523
610.0000000	8.1496000	2762.8344727
610.0000000	8.1983995	1718.9157715
610.0000000	8.2472000	-3932.3725586
610.0000000	8.2959995	-1393.8310547
610.0000000	8.3448000	-1110.3491211
610.0000000	8.3935995	-1017.5938721
610.0000000	8.4423990	-438.1854858
610.0000000	8.4912004	-1023.2473145
610.0000000	8.5400000	-2760.6088867
610.0000000	8.5887995	-1337.9300537
610.0000000	8.6375999	261.6632996
610.0000000	8.6863995	2361.8422852
610.0000000	8.7351999	-877.5650635
610.0000000	8.7840004	1003.4241333
610.0000000	8.8327999	55.0617523
610.0000000	8.8815994	-11.4893665
610.0000000	8.9303989	-24.0962181
610.0000000	8.9792004	-11.5757580
610.0000000	9.0279999	-5.7649469
610.0000000	9.0767994	7.2722168
610.0000000	9.1255999	-17.3119354
610.0000000	9.1743994	-10.0571756
610.0000000	9.2231998	37.6544266
610.0000000	9.2720003	-8.3818121
610.0000000	9.3207998	-9.7760267
610.0000000	9.3695993	-10.7544975
610.0000000	9.4183998	-11.7589836
610.0000000	9.4672003	-11.1596422
610.0000000	9.5159998	-14.2007408
610.0000000	9.5648003	-0.2547979
610.0000000	9.6135998	-13.4284286
610.0000000	9.6623993	-10.2100563
610.0000000	9.7111998	-10.1465712
610.0000000	9.7600002	-10.8558655
610.0000000	9.8087997	-10.9944525
610.0000000	9.8575993	3.3712215
610.0000000	9.9063997	-7.9315948
610.0000000	9.9552002	-10.9064245
610.0000000	10.0039997	-10.0645695
620.0000000	0.3416000	-36.4322166
620.0000000	0.3904000	-38.9557419
620.0000000	0.4392000	-38.5901070
620.0000000	0.4880000	-38.8595238
620.0000000	0.5368000	-36.4220810
620.0000000	0.5856000	-36.5537682
620.0000000	0.6344000	-36.9680061
620.0000000	0.6832000	-36.5662422
620.0000000	0.7320000	-36.9209747
620.0000000	0.7808000	-36.5236359
620.0000000	0.8296000	-35.9452820
620.0000000	0.8784000	-34.9503021
620.0000000	0.9272000	-33.1416321
620.0000000	0.9760000	-31.4858322
620.0000000	1.0247999	-30.0089130
620.0000000	1.0735999	-29.5849781

620.0000000	1.1224000	-29.8941441
620.0000000	1.1711999	-30.5607033
620.0000000	1.2200000	-30.9814186
620.0000000	1.2687999	-31.4106293
620.0000000	1.3176000	-31.0817604
620.0000000	1.3664000	-30.2609196
620.0000000	1.4152000	-30.1541386
620.0000000	1.4640000	-29.6836987
620.0000000	1.5128000	-43.2623291
620.0000000	1.5616000	-35.3039246
620.0000000	1.6104001	-1658.9289551
620.0000000	1.6592000	-85.0154724
620.0000000	1.7079999	-27.1076508
620.0000000	1.7567999	-27.3519115
620.0000000	1.8055999	-26.6287727
620.0000000	1.8544000	-26.6800003
620.0000000	1.9031999	-26.8595657
620.0000000	1.9520000	-26.5495281
620.0000000	2.0007999	-27.1027393
620.0000000	2.0495999	-21.9393635
620.0000000	2.0983999	-207.0053101
620.0000000	2.1471999	9.0160561
620.0000000	2.1960001	-25.6121502
620.0000000	2.2448001	-26.5442123
620.0000000	2.2935998	-30.0653763
620.0000000	2.3423998	-20.4136658
620.0000000	2.3912001	56.7423630
620.0000000	2.4400001	-22.7380047
620.0000000	2.4888000	-23.4687805
620.0000000	2.5375998	-11.5820942
620.0000000	2.5863998	-9.5963287
620.0000000	2.6352000	-7.5481730
620.0000000	2.6840000	-14.9288521
620.0000000	2.7328000	143.9461365
620.0000000	2.7815998	169.5474854
620.0000000	2.8304000	-23.8472977
620.0000000	2.8792000	-20.4868870
620.0000000	2.9280000	-22.5948868
620.0000000	2.9768000	-16.4494991
620.0000000	3.0256000	-20.3678951
620.0000000	3.0743999	-29.9793186
620.0000000	3.1231999	-24.3954639
620.0000000	3.1719999	-29.9426346
620.0000000	3.2208002	-513.8900757
620.0000000	3.2695999	-1006.5184326
620.0000000	3.3183999	-20.4816780
620.0000000	3.3671999	-3455.1262207
620.0000000	3.4159999	1239.9587402
620.0000000	3.4648001	-6.9121895
620.0000000	3.5135999	-27.1567650
620.0000000	3.5623999	-327.1323242
620.0000000	3.6111999	-2057.2016602
620.0000000	3.6600001	-465.4932861
620.0000000	3.7088001	-614.7662354
620.0000000	3.7575998	-962.6992188
620.0000000	3.8063998	-1747.1699219
620.0000000	3.8552001	-2536.7783203
620.0000000	3.9040000	-2487.1325684

620.0000000	3.9528000	1137.7790527
620.0000000	4.0015998	-248.2481537
620.0000000	4.0503998	696.7168579
620.0000000	4.0991998	88.9940948
620.0000000	4.1479998	-662.9468994
620.0000000	4.1967998	2.8244972
620.0000000	4.2456002	-251.4649963
620.0000000	4.2943997	-494.0281067
620.0000000	4.3431997	-13.2658348
620.0000000	4.3920002	-251.0897980
620.0000000	4.4407997	-22.5139275
620.0000000	4.4896002	-15.4477167
620.0000000	4.5383997	-662.9070435
620.0000000	4.5871997	-12.7781506
620.0000000	4.6360002	-21.6673355
620.0000000	4.6847997	-28.4000130
620.0000000	4.7336001	-19.5840683
620.0000000	4.7824001	-16.4987507
620.0000000	4.8311996	-1490.2307129
620.0000000	4.8800001	230.9292755
620.0000000	4.9287996	-203.4593811
620.0000000	4.9776001	1071.2833252
620.0000000	5.0264001	87.2943954
620.0000000	5.0751996	-304.2289734
620.0000000	5.1240001	-12.2808800
620.0000000	5.1727996	561.8897095
620.0000000	5.2216001	32.3267479
620.0000000	5.2704000	-159.8001862
620.0000000	5.3191996	-39.3419495
620.0000000	5.3680000	-3560.1601563
620.0000000	5.4168000	530.8801270
620.0000000	5.4656000	-202.0111389
620.0000000	5.5144000	38.4418716
620.0000000	5.5631995	491.7936707
620.0000000	5.6120000	395.9562683
620.0000000	5.6608000	-37.2287064
620.0000000	5.7096000	63.0738602
620.0000000	5.7584000	134.0595703
620.0000000	5.8071995	-11.4896011
620.0000000	5.8559999	30.1296787
620.0000000	5.9047999	-16.6126175
620.0000000	5.9535999	-39.9439774
620.0000000	6.0023999	-1583.7714844
620.0000000	6.0511999	-14.5606804
620.0000000	6.0999999	-19.4389744
620.0000000	6.1487999	-13.2568054
620.0000000	6.1975999	-23.2833900
620.0000000	6.2463999	-1804.2995605
620.0000000	6.2951999	-19.0995350
620.0000000	6.3439999	-345.0139771
620.0000000	6.3927999	-15.0487843
620.0000000	6.4416003	-12.3647175
620.0000000	6.4903998	-11.2391338
620.0000000	6.5391998	-11.7813873
620.0000000	6.5879998	410.3364258
620.0000000	6.6367998	-12.0639877
620.0000000	6.6856003	-13.8086386
620.0000000	6.7343998	-13.4476671

620.0000000	6.7831998	-14.3880291
620.0000000	6.8319998	59.1535263
620.0000000	6.8807998	-14.0546141
620.0000000	6.9296002	-478.1656494
620.0000000	6.9783998	-4.6000648
620.0000000	7.0271997	-14.1763201
620.0000000	7.0760002	12.7942610
620.0000000	7.1247997	-2282.5876465
620.0000000	7.1736002	-1830.4157715
620.0000000	7.2223997	-1041.0936279
620.0000000	7.2711997	-223.7552643
620.0000000	7.3200002	-19.3444691
620.0000000	7.3687997	202.3545074
620.0000000	7.4176002	421.1285095
620.0000000	7.4663997	330.9967651
620.0000000	7.5151997	-1018.7982788
620.0000000	7.5640001	-1125.4310303
620.0000000	7.6127996	-2653.7875977
620.0000000	7.6616001	-851.5711670
620.0000000	7.7104001	-437.0311279
620.0000000	7.7591996	47.1578217
620.0000000	7.8080001	437.6360474
620.0000000	7.8567996	576.2512207
620.0000000	7.9056001	-1192.0202637
620.0000000	7.9544001	1897.6657715
620.0000000	8.0031996	2448.5766602
620.0000000	8.0520000	2443.9584961
620.0000000	8.1007996	2586.1716309
620.0000000	8.1496000	2758.0263672
620.0000000	8.1983995	1715.9232178
620.0000000	8.2472000	-3925.5378418
620.0000000	8.2959995	-1391.4108887
620.0000000	8.3448000	-1108.4199219
620.0000000	8.3935995	-1015.8261719
620.0000000	8.4423990	-437.4274902
620.0000000	8.4912004	-1021.4710693
620.0000000	8.5400000	-2755.8090820
620.0000000	8.5887995	-1335.6085205
620.0000000	8.6375999	261.2019958
620.0000000	8.6863995	2357.7238770
620.0000000	8.7351999	-876.0407715
620.0000000	8.7840004	1001.6673584
620.0000000	8.8327999	54.9501534
620.0000000	8.8815994	-11.4895401
620.0000000	8.9303989	-24.0924988
620.0000000	8.9792004	-11.5758410
620.0000000	9.0279999	-5.7667155
620.0000000	9.0767994	7.2664986
620.0000000	9.1255999	-17.3102226
620.0000000	9.1743994	-10.0575781
620.0000000	9.2231998	37.6386871
620.0000000	9.2720003	-8.3827486
620.0000000	9.3207998	-9.7765665
620.0000000	9.3695993	-10.7547235
620.0000000	9.4183998	-11.7588587
620.0000000	9.4672003	-11.1596842
620.0000000	9.5159998	-14.1998491
620.0000000	9.5648003	-0.2581711

620.0000000	9.6135998	-13.4276371
620.0000000	9.6623993	-10.2103319
620.0000000	9.7111998	-10.1468430
620.0000000	9.7600002	-10.8559074
620.0000000	9.8087997	-10.9944601
620.0000000	9.8575993	3.3667974
620.0000000	9.9063997	-7.9325089
620.0000000	9.9552002	-10.9064293
620.0000000	10.0039997	-10.0648174
630.0000000	0.3416000	-36.4863396
630.0000000	0.3904000	-38.7605667
630.0000000	0.4392000	-38.4245834
630.0000000	0.4880000	-38.6741486
630.0000000	0.5368000	-35.9439049
630.0000000	0.5856000	-35.4115715
630.0000000	0.6344000	-36.3335800
630.0000000	0.6832000	-36.0790977
630.0000000	0.7320000	-35.9215775
630.0000000	0.7808000	-36.5238037
630.0000000	0.8296000	-35.9460106
630.0000000	0.8784000	-35.0130692
630.0000000	0.9272000	-33.7103386
630.0000000	0.9760000	-31.5250225
630.0000000	1.0247999	-29.9848824
630.0000000	1.0735999	-29.8864708
630.0000000	1.1224000	-30.1759186
630.0000000	1.1711999	-30.3901272
630.0000000	1.2200000	-31.0265923
630.0000000	1.2687999	-31.4231911
630.0000000	1.3176000	-31.0774879
630.0000000	1.3664000	-30.2686691
630.0000000	1.4152000	-30.1543427
630.0000000	1.4640000	-29.6819878
630.0000000	1.5128000	-43.1651535
630.0000000	1.5616000	-35.2620811
630.0000000	1.6104001	-1656.3862305
630.0000000	1.6592000	-84.7215424
630.0000000	1.7079999	-27.1113968
630.0000000	1.7567999	-27.6079540
630.0000000	1.8055999	-27.5238991
630.0000000	1.8544000	-26.8647175
630.0000000	1.9031999	-26.8739204
630.0000000	1.9520000	-26.5499821
630.0000000	2.0007999	-27.0401573
630.0000000	2.0495999	-22.1259804
630.0000000	2.0983999	-205.5295410
630.0000000	2.1471999	8.7487907
630.0000000	2.1960001	-25.6080589
630.0000000	2.2448001	-26.5263004
630.0000000	2.2935998	-30.0057526
630.0000000	2.3423998	-20.4280701
630.0000000	2.3912001	56.1208191
630.0000000	2.4400001	-22.7404919
630.0000000	2.4888000	-23.4685631
630.0000000	2.5375998	-11.5992022
630.0000000	2.5863998	-9.6160879
630.0000000	2.6352000	-7.5709171
630.0000000	2.6840000	-14.9407930

630.0000000	2.7328000	143.6801147
630.0000000	2.7815998	169.2410889
630.0000000	2.8304000	-23.8459949
630.0000000	2.8792000	-20.4901505
630.0000000	2.9280000	-22.5943184
630.0000000	2.9768000	-16.4578705
630.0000000	3.0256000	-20.3704796
630.0000000	3.0743999	-29.9679317
630.0000000	3.1231999	-24.3920097
630.0000000	3.1719999	-29.9343414
630.0000000	3.2208002	-513.6523438
630.0000000	3.2695999	-1006.0434570
630.0000000	3.3183999	-20.4818764
630.0000000	3.3671999	-3453.6645508
630.0000000	3.4159999	1239.4234619
630.0000000	3.4648001	-6.9178972
630.0000000	3.5135999	-27.1537743
630.0000000	3.5623999	-327.0027161
630.0000000	3.6111999	-2056.3420410
630.0000000	3.6600001	-465.3051758
630.0000000	3.7088001	-614.5148315
630.0000000	3.7575998	-962.3006592
630.0000000	3.8063998	-1746.4400635
630.0000000	3.8552001	-2535.7148438
630.0000000	3.9040000	-2486.0900879
630.0000000	3.9528000	1137.2907715
630.0000000	4.0015998	-248.1510468
630.0000000	4.0503998	696.4143066
630.0000000	4.0991998	88.9484100
630.0000000	4.1479998	-662.6746216
630.0000000	4.1967998	2.8152409
630.0000000	4.2456002	-251.3666992
630.0000000	4.2943997	-493.8275452
630.0000000	4.3431997	-13.2680149
630.0000000	4.3920002	-250.9916840
630.0000000	4.4407997	-22.5121536
630.0000000	4.4896002	-15.4485989
630.0000000	4.5383997	-662.6459961
630.0000000	4.5871997	-12.7801676
630.0000000	4.6360002	-21.6657944
630.0000000	4.6847997	-28.3960876
630.0000000	4.7336001	-19.5833473
630.0000000	4.7824001	-16.4992046
630.0000000	4.8311996	-1489.6994629
630.0000000	4.8800001	230.8397217
630.0000000	4.9287996	-203.3921204
630.0000000	4.9776001	1070.8908691
630.0000000	5.0264001	87.2568741
630.0000000	5.0751996	-304.1253357
630.0000000	5.1240001	-12.2818012
630.0000000	5.1727996	561.7738647
630.0000000	5.2216001	32.3173065
630.0000000	5.2704000	-159.7720947
630.0000000	5.3191996	-39.3373985
630.0000000	5.3680000	-3559.4677734
630.0000000	5.4168000	530.7733154
630.0000000	5.4656000	-201.9749451
630.0000000	5.5144000	38.4312134

630.0000000	5.5631995	491.6950989
630.0000000	5.6120000	395.8762817
630.0000000	5.6608000	-37.2246170
630.0000000	5.7096000	63.0584259
630.0000000	5.7584000	134.0306396
630.0000000	5.8071995	-11.4904709
630.0000000	5.8559999	30.1208248
630.0000000	5.9047999	-16.6124191
630.0000000	5.9535999	-39.9392395
630.0000000	6.0023999	-1583.4671631
630.0000000	6.0511999	-14.5608921
630.0000000	6.0999999	-19.4381428
630.0000000	6.1487999	-13.2572680
630.0000000	6.1975999	-23.2816238
630.0000000	6.2463999	-1803.9522705
630.0000000	6.2951999	-19.0987206
630.0000000	6.3439999	-344.9499207
630.0000000	6.3927999	-15.0487661
630.0000000	6.4416003	-12.3653040
630.0000000	6.4903998	-11.2398100
630.0000000	6.5391998	-11.7820683
630.0000000	6.5879998	409.6457214
630.0000000	6.6367998	-12.0645370
630.0000000	6.6856003	-13.8088093
630.0000000	6.7343998	-13.4478836
630.0000000	6.7831998	-14.3876133
630.0000000	6.8319998	59.1344147
630.0000000	6.8807998	-14.0544758
630.0000000	6.9296002	-477.4042053
630.0000000	6.9783998	-4.6128879
630.0000000	7.0271997	-14.1752920
630.0000000	7.0760002	12.7520742
630.0000000	7.1247997	-2278.6418457
630.0000000	7.1736002	-1827.2529297
630.0000000	7.2223997	-1039.3005371
630.0000000	7.2711997	-223.3874817
630.0000000	7.3200002	-19.3324814
630.0000000	7.3687997	201.9799805
630.0000000	7.4176002	420.3726807
630.0000000	7.4663997	330.3991089
630.0000000	7.5151997	-1017.0372314
630.0000000	7.5640001	-1123.4836426
630.0000000	7.6127996	-2649.1699219
630.0000000	7.6616001	-850.0997925
630.0000000	7.7104001	-436.2848511
630.0000000	7.7591996	47.0567741
630.0000000	7.8080001	436.8506775
630.0000000	7.8567996	575.2231445
630.0000000	7.9056001	-1189.9473877
630.0000000	7.9544001	1894.3558350
630.0000000	8.0031996	2444.3068848
630.0000000	8.0520000	2439.6965332
630.0000000	8.1007996	2581.6621094
630.0000000	8.1496000	2753.2185059
630.0000000	8.1983995	1712.9307861
630.0000000	8.2472000	-3918.7028809
630.0000000	8.2959995	-1388.9907227
630.0000000	8.3448000	-1106.4907227

630.0000000	8.3935995	-1014.0585327
630.0000000	8.4423990	-436.6694641
630.0000000	8.4912004	-1019.6948853
630.0000000	8.5400000	-2751.0092773
630.0000000	8.5887995	-1333.2869873
630.0000000	8.6375999	260.7406616
630.0000000	8.6863995	2353.6057129
630.0000000	8.7351999	-874.5165405
630.0000000	8.7840004	999.9106445
630.0000000	8.8327999	54.8385429
630.0000000	8.8815994	-11.4897146
630.0000000	8.9303989	-24.0887794
630.0000000	8.9792004	-11.5759230
630.0000000	9.0279999	-5.7684841
630.0000000	9.0767994	7.2607803
630.0000000	9.1255999	-17.3085079
630.0000000	9.1743994	-10.0579796
630.0000000	9.2231998	37.6229553
630.0000000	9.2720003	-8.3836851
630.0000000	9.3207998	-9.7771053
630.0000000	9.3695993	-10.7549505
630.0000000	9.4183998	-11.7587347
630.0000000	9.4672003	-11.1597252
630.0000000	9.5159998	-14.1989584
630.0000000	9.5648003	-0.2615433
630.0000000	9.6135998	-13.4268465
630.0000000	9.6623993	-10.2106066
630.0000000	9.7111998	-10.1471148
630.0000000	9.7600002	-10.8559494
630.0000000	9.8087997	-10.9944677
630.0000000	9.8575993	3.3623753
630.0000000	9.9063997	-7.9334230
630.0000000	9.9552002	-10.9064331
630.0000000	10.0039997	-10.0650644
640.0000000	0.3416000	-36.5495110
640.0000000	0.3904000	-38.9477463
640.0000000	0.4392000	-38.2590637
640.0000000	0.4880000	-38.5219841
640.0000000	0.5368000	-37.6485634
640.0000000	0.5856000	-37.8357964
640.0000000	0.6344000	-38.4556503
640.0000000	0.6832000	-36.0813217
640.0000000	0.7320000	-35.9236298
640.0000000	0.7808000	-36.5239754
640.0000000	0.8296000	-35.9467392
640.0000000	0.8784000	-35.0758362
640.0000000	0.9272000	-34.1906624
640.0000000	0.9760000	-31.9386101
640.0000000	1.0247999	-30.1273384
640.0000000	1.0735999	-29.5037289
640.0000000	1.1224000	-30.3319530
640.0000000	1.1711999	-31.2806854
640.0000000	1.2200000	-31.2516365
640.0000000	1.2687999	-31.4357548
640.0000000	1.3176000	-31.0732174
640.0000000	1.3664000	-30.2764187
640.0000000	1.4152000	-30.1545467
640.0000000	1.4640000	-29.6802788

640.0000000	1.5128000	-43.0679741
640.0000000	1.5616000	-35.2202415
640.0000000	1.6104001	-1653.8433838
640.0000000	1.6592000	-84.4276123
640.0000000	1.7079999	-27.1151447
640.0000000	1.7567999	-27.8639965
640.0000000	1.8055999	-27.1340504
640.0000000	1.8544000	-27.0494366
640.0000000	1.9031999	-26.8882732
640.0000000	1.9520000	-26.5504379
640.0000000	2.0007999	-26.9775753
640.0000000	2.0495999	-22.3125954
640.0000000	2.0983999	-204.0537720
640.0000000	2.1471999	8.4815254
640.0000000	2.1960001	-25.6039677
640.0000000	2.2448001	-26.5083885
640.0000000	2.2935998	-29.9461288
640.0000000	2.3423998	-20.4424744
640.0000000	2.3912001	55.4992752
640.0000000	2.4400001	-22.7429790
640.0000000	2.4888000	-23.4683456
640.0000000	2.5375998	-11.6163101
640.0000000	2.5863998	-9.6358471
640.0000000	2.6352000	-7.5936613
640.0000000	2.6840000	-14.9527349
640.0000000	2.7328000	143.4141083
640.0000000	2.7815998	168.9346619
640.0000000	2.8304000	-23.8446903
640.0000000	2.8792000	-20.4934139
640.0000000	2.9280000	-22.5937481
640.0000000	2.9768000	-16.4662399
640.0000000	3.0256000	-20.3730640
640.0000000	3.0743999	-29.9565449
640.0000000	3.1231999	-24.3885536
640.0000000	3.1719999	-29.9260483
640.0000000	3.2208002	-513.4146118
640.0000000	3.2695999	-1005.5684814
640.0000000	3.3183999	-20.4820747
640.0000000	3.3671999	-3452.2028809
640.0000000	3.4159999	1238.8881836
640.0000000	3.4648001	-6.9236050
640.0000000	3.5135999	-27.1507835
640.0000000	3.5623999	-326.8731384
640.0000000	3.6111999	-2055.4821777
640.0000000	3.6600001	-465.1170654
640.0000000	3.7088001	-614.2634277
640.0000000	3.7575998	-961.9021606
640.0000000	3.8063998	-1745.7102051
640.0000000	3.8552001	-2534.6513672
640.0000000	3.9040000	-2485.0476074
640.0000000	3.9528000	1136.8024902
640.0000000	4.0015998	-248.0539398
640.0000000	4.0503998	696.1117554
640.0000000	4.0991998	88.9027252
640.0000000	4.1479998	-662.4024048
640.0000000	4.1967998	2.8059845
640.0000000	4.2456002	-251.2683868
640.0000000	4.2943997	-493.6269836

640.0000000	4.3431997	-13.2701960
640.0000000	4.3920002	-250.8935699
640.0000000	4.4407997	-22.5103760
640.0000000	4.4896002	-15.4494820
640.0000000	4.5383997	-662.3849487
640.0000000	4.5871997	-12.7821836
640.0000000	4.6360002	-21.6642513
640.0000000	4.6847997	-28.3921623
640.0000000	4.7336001	-19.5826244
640.0000000	4.7824001	-16.4996605
640.0000000	4.8311996	-1489.1680908
640.0000000	4.8800001	230.7501831
640.0000000	4.9287996	-203.3248596
640.0000000	4.9776001	1070.4984131
640.0000000	5.0264001	87.2193527
640.0000000	5.0751996	-304.0216980
640.0000000	5.1240001	-12.2827225
640.0000000	5.1727996	561.6579590
640.0000000	5.2216001	32.3078613
640.0000000	5.2704000	-159.7439880
640.0000000	5.3191996	-39.3328476
640.0000000	5.3680000	-3558.7753906
640.0000000	5.4168000	530.6665649
640.0000000	5.4656000	-201.9387665
640.0000000	5.5144000	38.4205627
640.0000000	5.5631995	491.5965271
640.0000000	5.6120000	395.7963257
640.0000000	5.6608000	-37.2205276
640.0000000	5.7096000	63.0429916
640.0000000	5.7584000	134.0017090
640.0000000	5.8071995	-11.4913425
640.0000000	5.8559999	30.1119747
640.0000000	5.9047999	-16.6122227
640.0000000	5.9535999	-39.9345016
640.0000000	6.0023999	-1583.1625977
640.0000000	6.0511999	-14.5611038
640.0000000	6.0999999	-19.4373093
640.0000000	6.1487999	-13.2577314
640.0000000	6.1975999	-23.2798576
640.0000000	6.2463999	-1803.6048584
640.0000000	6.2951999	-19.0979061
640.0000000	6.3439999	-344.8858643
640.0000000	6.3927999	-15.0487471
640.0000000	6.4416003	-12.3658915
640.0000000	6.4903998	-11.2404861
640.0000000	6.5391998	-11.7827492
640.0000000	6.5879998	408.9550171
640.0000000	6.6367998	-12.0650864
640.0000000	6.6856003	-13.8089790
640.0000000	6.7343998	-13.4480991
640.0000000	6.7831998	-14.3871975
640.0000000	6.8319998	59.1153030
640.0000000	6.8807998	-14.0543375
640.0000000	6.9296002	-476.6427612
640.0000000	6.9783998	-4.6257119
640.0000000	7.0271997	-14.1742640
640.0000000	7.0760002	12.7098894
640.0000000	7.1247997	-2274.6958008

640.0000000	7.1736002	-1824.0899658
640.0000000	7.2223997	-1037.5073242
640.0000000	7.2711997	-223.0197144
640.0000000	7.3200002	-19.3204918
640.0000000	7.3687997	201.6054382
640.0000000	7.4176002	419.6168518
640.0000000	7.4663997	329.8014526
640.0000000	7.5151997	-1015.2761841
640.0000000	7.5640001	-1121.5362549
640.0000000	7.6127996	-2644.5524902
640.0000000	7.6616001	-848.6284180
640.0000000	7.7104001	-435.5385742
640.0000000	7.7591996	46.9557228
640.0000000	7.8080001	436.0653381
640.0000000	7.8567996	574.1950073
640.0000000	7.9056001	-1187.8745117
640.0000000	7.9544001	1891.0458984
640.0000000	8.0031996	2440.0373535
640.0000000	8.0520000	2435.4345703
640.0000000	8.1007996	2577.1525879
640.0000000	8.1496000	2748.4104004
640.0000000	8.1983995	1709.9382324
640.0000000	8.2472000	-3911.8681641
640.0000000	8.2959995	-1386.5705566
640.0000000	8.3448000	-1104.5616455
640.0000000	8.3935995	-1012.2908325
640.0000000	8.4423990	-435.9114685
640.0000000	8.4912004	-1017.9186401
640.0000000	8.5400000	-2746.2094727
640.0000000	8.5887995	-1330.9653320
640.0000000	8.6375999	260.2793274
640.0000000	8.6863995	2349.4873047
640.0000000	8.7351999	-872.9923096
640.0000000	8.7840004	998.1538696
640.0000000	8.8327999	54.7269363
640.0000000	8.8815994	-11.4898891
640.0000000	8.9303989	-24.0850601
640.0000000	8.9792004	-11.5760050
640.0000000	9.0279999	-5.7702522
640.0000000	9.0767994	7.2550621
640.0000000	9.1255999	-17.3067932
640.0000000	9.1743994	-10.0583820
640.0000000	9.2231998	37.6072235
640.0000000	9.2720003	-8.3846216
640.0000000	9.3207998	-9.7776451
640.0000000	9.3695993	-10.7551765
640.0000000	9.4183998	-11.7586098
640.0000000	9.4672003	-11.1597662
640.0000000	9.5159998	-14.1980677
640.0000000	9.5648003	-0.2649155
640.0000000	9.6135998	-13.4260559
640.0000000	9.6623993	-10.2108822
640.0000000	9.7111998	-10.1473856
640.0000000	9.7600002	-10.8559914
640.0000000	9.8087997	-10.9944763
640.0000000	9.8575993	3.3579540
640.0000000	9.9063997	-7.9343367
640.0000000	9.9552002	-10.9064379

640.0000000	10.0039997	-10.0653124
650.0000000	0.3416000	-36.3351440
650.0000000	0.3904000	-38.5735130
650.0000000	0.4392000	-38.0935402
650.0000000	0.4880000	-38.8365440
650.0000000	0.5368000	-37.5636749
650.0000000	0.5856000	-37.3208694
650.0000000	0.6344000	-38.2139626
650.0000000	0.6832000	-36.0835495
650.0000000	0.7320000	-35.9256821
650.0000000	0.7808000	-36.5241432
650.0000000	0.8296000	-35.9474716
650.0000000	0.8784000	-35.1386032
650.0000000	0.9272000	-34.0085831
650.0000000	0.9760000	-31.7154198
650.0000000	1.0247999	-30.3225098
650.0000000	1.0735999	-30.0983791
650.0000000	1.1224000	-30.3613148
650.0000000	1.1711999	-30.8127651
650.0000000	1.2200000	-31.3608074
650.0000000	1.2687999	-31.5316181
650.0000000	1.3176000	-31.0689449
650.0000000	1.3664000	-30.2841663
650.0000000	1.4152000	-30.1547508
650.0000000	1.4640000	-29.6785698
650.0000000	1.5128000	-42.9707947
650.0000000	1.5616000	-35.1783981
650.0000000	1.6104001	-1651.3005371
650.0000000	1.6592000	-84.1336823
650.0000000	1.7079999	-27.1188908
650.0000000	1.7567999	-27.9040070
650.0000000	1.8055999	-27.2946949
650.0000000	1.8544000	-27.2341537
650.0000000	1.9031999	-26.9026279
650.0000000	1.9520000	-26.5508938
650.0000000	2.0007999	-26.9149933
650.0000000	2.0495999	-22.4992123
650.0000000	2.0983999	-202.5779724
650.0000000	2.1471999	8.2142563
650.0000000	2.1960001	-25.5998764
650.0000000	2.2448001	-26.4904785
650.0000000	2.2935998	-29.8865051
650.0000000	2.3423998	-20.4568806
650.0000000	2.3912001	54.8777313
650.0000000	2.4400001	-22.7454643
650.0000000	2.4888000	-23.4681282
650.0000000	2.5375998	-11.6334171
650.0000000	2.5863998	-9.6556053
650.0000000	2.6352000	-7.6164064
650.0000000	2.6840000	-14.9646778
650.0000000	2.7328000	143.1480865
650.0000000	2.7815998	168.6282349
650.0000000	2.8304000	-23.8433857
650.0000000	2.8792000	-20.4966774
650.0000000	2.9280000	-22.5931797
650.0000000	2.9768000	-16.4746113
650.0000000	3.0256000	-20.3756485
650.0000000	3.0743999	-29.9451580

650.0000000	3.1231999	-24.3850975
650.0000000	3.1719999	-29.9177551
650.0000000	3.2208002	-513.1768799
650.0000000	3.2695999	-1005.0935059
650.0000000	3.3183999	-20.4822712
650.0000000	3.3671999	-3450.7412109
650.0000000	3.4159999	1238.3529053
650.0000000	3.4648001	-6.9293137
650.0000000	3.5135999	-27.1477928
650.0000000	3.5623999	-326.7435608
650.0000000	3.6111999	-2054.6225586
650.0000000	3.6600001	-464.9289551
650.0000000	3.7088001	-614.0120850
650.0000000	3.7575998	-961.5036011
650.0000000	3.8063998	-1744.9803467
650.0000000	3.8552001	-2533.5878906
650.0000000	3.9040000	-2484.0051270
650.0000000	3.9528000	1136.3140869
650.0000000	4.0015998	-247.9568329
650.0000000	4.0503998	695.8091431
650.0000000	4.0991998	88.8570480
650.0000000	4.1479998	-662.1301880
650.0000000	4.1967998	2.7967281
650.0000000	4.2456002	-251.1700897
650.0000000	4.2943997	-493.4264221
650.0000000	4.3431997	-13.2723770
650.0000000	4.3920002	-250.7954559
650.0000000	4.4407997	-22.5086021
650.0000000	4.4896002	-15.4503641
650.0000000	4.5383997	-662.1238403
650.0000000	4.5871997	-12.7841997
650.0000000	4.6360002	-21.6627083
650.0000000	4.6847997	-28.3882370
650.0000000	4.7336001	-19.5819016
650.0000000	4.7824001	-16.5001144
650.0000000	4.8311996	-1488.6368408
650.0000000	4.8800001	230.6606293
650.0000000	4.9287996	-203.2575989
650.0000000	4.9776001	1070.1058350
650.0000000	5.0264001	87.1818314
650.0000000	5.0751996	-303.9180908
650.0000000	5.1240001	-12.2836437
650.0000000	5.1727996	561.5421143
650.0000000	5.2216001	32.2984161
650.0000000	5.2704000	-159.7158813
650.0000000	5.3191996	-39.3283005
650.0000000	5.3680000	-3558.0830078
650.0000000	5.4168000	530.5597534
650.0000000	5.4656000	-201.9025879
650.0000000	5.5144000	38.4099045
650.0000000	5.5631995	491.4979553
650.0000000	5.6120000	395.7163391
650.0000000	5.6608000	-37.2164421
650.0000000	5.7096000	63.0275650
650.0000000	5.7584000	133.9727783
650.0000000	5.8071995	-11.4922123
650.0000000	5.8559999	30.1031246
650.0000000	5.9047999	-16.6120243

650.0000000	5.9535999	-39.9297638
650.0000000	6.0023999	-1582.8581543
650.0000000	6.0511999	-14.5613146
650.0000000	6.0999999	-19.4364777
650.0000000	6.1487999	-13.2581940
650.0000000	6.1975999	-23.2780952
650.0000000	6.2463999	-1803.2574463
650.0000000	6.2951999	-19.0970917
650.0000000	6.3439999	-344.8218384
650.0000000	6.3927999	-15.0487289
650.0000000	6.4416003	-12.3664780
650.0000000	6.4903998	-11.2411613
650.0000000	6.5391998	-11.7834311
650.0000000	6.5879998	408.2643433
650.0000000	6.6367998	-12.0656347
650.0000000	6.6856003	-13.8091497
650.0000000	6.7343998	-13.4483147
650.0000000	6.7831998	-14.3867817
650.0000000	6.8319998	59.0961838
650.0000000	6.8807998	-14.0541983
650.0000000	6.9296002	-475.8813171
650.0000000	6.9783998	-4.6385355
650.0000000	7.0271997	-14.1732359
650.0000000	7.0760002	12.6677046
650.0000000	7.1247997	-2270.7500000
650.0000000	7.1736002	-1820.9271240
650.0000000	7.2223997	-1035.7142334
650.0000000	7.2711997	-222.6519318
650.0000000	7.3200002	-19.3085022
650.0000000	7.3687997	201.2309113
650.0000000	7.4176002	418.8610229
650.0000000	7.4663997	329.2037964
650.0000000	7.5151997	-1013.5151367
650.0000000	7.5640001	-1119.5888672
650.0000000	7.6127996	-2639.9348145
650.0000000	7.6616001	-847.1571045
650.0000000	7.7104001	-434.7923584
650.0000000	7.7591996	46.8546715
650.0000000	7.8080001	435.2799683
650.0000000	7.8567996	573.1669312
650.0000000	7.9056001	-1185.8016357
650.0000000	7.9544001	1887.7359619
650.0000000	8.0031996	2435.7678223
650.0000000	8.0520000	2431.1726074
650.0000000	8.1007996	2572.6430664
650.0000000	8.1496000	2743.6025391
650.0000000	8.1983995	1706.9456787
650.0000000	8.2472000	-3905.0334473
650.0000000	8.2959995	-1384.1503906
650.0000000	8.3448000	-1102.6324463
650.0000000	8.3935995	-1010.5231323
650.0000000	8.4423990	-435.1534424
650.0000000	8.4912004	-1016.1424561
650.0000000	8.5400000	-2741.4096680
650.0000000	8.5887995	-1328.6437988
650.0000000	8.6375999	259.8180237
650.0000000	8.6863995	2345.3688965
650.0000000	8.7351999	-871.4680176

650.0000000	8.7840004	996.3971558
650.0000000	8.8327999	54.6153259
650.0000000	8.8815994	-11.4900637
650.0000000	8.9303989	-24.0813408
650.0000000	8.9792004	-11.5760880
650.0000000	9.0279999	-5.7720203
650.0000000	9.0767994	7.2493458
650.0000000	9.1255999	-17.3050804
650.0000000	9.1743994	-10.0587835
650.0000000	9.2231998	37.5914917
650.0000000	9.2720003	-8.3855581
650.0000000	9.3207998	-9.7781839
650.0000000	9.3695993	-10.7554035
650.0000000	9.4183998	-11.7584848
650.0000000	9.4672003	-11.1598072
650.0000000	9.5159998	-14.1971760
650.0000000	9.5648003	-0.2682886
650.0000000	9.6135998	-13.4252653
650.0000000	9.6623993	-10.2111568
650.0000000	9.7111998	-10.1476574
650.0000000	9.7600002	-10.8560324
650.0000000	9.8087997	-10.9944839
650.0000000	9.8575993	3.3535318
650.0000000	9.9063997	-7.9352508
650.0000000	9.9552002	-10.9064426
650.0000000	10.0039997	-10.0655603
660.0000000	0.3416000	-36.4268265
660.0000000	0.3904000	-37.7609444
660.0000000	0.4392000	-37.9280167
660.0000000	0.4880000	-36.9143143
660.0000000	0.5368000	-37.7687035
660.0000000	0.5856000	-39.1445618
660.0000000	0.6344000	-38.2142715
660.0000000	0.6832000	-36.0857735
660.0000000	0.7320000	-35.9277344
660.0000000	0.7808000	-36.5243149
660.0000000	0.8296000	-35.9482002
660.0000000	0.8784000	-35.2013702
660.0000000	0.9272000	-33.1842575
660.0000000	0.9760000	-31.3581066
660.0000000	1.0247999	-30.3544865
660.0000000	1.0735999	-30.0824261
660.0000000	1.1224000	-30.6309376
660.0000000	1.1711999	-31.1460571
660.0000000	1.2200000	-31.2115250
660.0000000	1.2687999	-31.0063457
660.0000000	1.3176000	-31.0646744
660.0000000	1.3664000	-30.2919159
660.0000000	1.4152000	-30.1549568
660.0000000	1.4640000	-29.6768608
660.0000000	1.5128000	-42.8736191
660.0000000	1.5616000	-35.1365585
660.0000000	1.6104001	-1648.7578125
660.0000000	1.6592000	-83.8397446
660.0000000	1.7079999	-27.1226387
660.0000000	1.7567999	-27.9440193
660.0000000	1.8055999	-27.4105644
660.0000000	1.8544000	-27.4188728

660.0000000	1.9031999	-26.9169807
660.0000000	1.9520000	-26.5513477
660.0000000	2.0007999	-26.8524113
660.0000000	2.0495999	-22.6858292
660.0000000	2.0983999	-201.1022034
660.0000000	2.1471999	7.9469872
660.0000000	2.1960001	-25.5957851
660.0000000	2.2448001	-26.4725666
660.0000000	2.2935998	-29.8268814
660.0000000	2.3423998	-20.4712849
660.0000000	2.3912001	54.2561874
660.0000000	2.4400001	-22.7479515
660.0000000	2.4888000	-23.4679108
660.0000000	2.5375998	-11.6505251
660.0000000	2.5863998	-9.6753645
660.0000000	2.6352000	-7.6391497
660.0000000	2.6840000	-14.9766197
660.0000000	2.7328000	142.8820648
660.0000000	2.7815998	168.3218384
660.0000000	2.8304000	-23.8420811
660.0000000	2.8792000	-20.4999409
660.0000000	2.9280000	-22.5926094
660.0000000	2.9768000	-16.4829826
660.0000000	3.0256000	-20.3782330
660.0000000	3.0743999	-29.9337711
660.0000000	3.1231999	-24.3816433
660.0000000	3.1719999	-29.9094620
660.0000000	3.2208002	-512.9391479
660.0000000	3.2695999	-1004.6185303
660.0000000	3.3183999	-20.4824696
660.0000000	3.3671999	-3449.2795410
660.0000000	3.4159999	1237.8176270
660.0000000	3.4648001	-6.9350214
660.0000000	3.5135999	-27.1448021
660.0000000	3.5623999	-326.6139526
660.0000000	3.6111999	-2053.7626953
660.0000000	3.6600001	-464.7407837
660.0000000	3.7088001	-613.7606812
660.0000000	3.7575998	-961.1050415
660.0000000	3.8063998	-1744.2506104
660.0000000	3.8552001	-2532.5246582
660.0000000	3.9040000	-2482.9626465
660.0000000	3.9528000	1135.8258057
660.0000000	4.0015998	-247.8597260
660.0000000	4.0503998	695.5065918
660.0000000	4.0991998	88.8113632
660.0000000	4.1479998	-661.8579102
660.0000000	4.1967998	2.7874718
660.0000000	4.2456002	-251.0717926
660.0000000	4.2943997	-493.2257996
660.0000000	4.3431997	-13.2745571
660.0000000	4.3920002	-250.6973419
660.0000000	4.4407997	-22.5068264
660.0000000	4.4896002	-15.4512472
660.0000000	4.5383997	-661.8627930
660.0000000	4.5871997	-12.7862167
660.0000000	4.6360002	-21.6611671
660.0000000	4.6847997	-28.3843117

660.0000000	4.7336001	-19.5811806
660.0000000	4.7824001	-16.5005684
660.0000000	4.8311996	-1488.1055908
660.0000000	4.8800001	230.5710754
660.0000000	4.9287996	-203.1903381
660.0000000	4.9776001	1069.7133789
660.0000000	5.0264001	87.1443100
660.0000000	5.0751996	-303.8144531
660.0000000	5.1240001	-12.2845650
660.0000000	5.1727996	561.4262695
660.0000000	5.2216001	32.2889748
660.0000000	5.2704000	-159.6877747
660.0000000	5.3191996	-39.3237534
660.0000000	5.3680000	-3557.3906250
660.0000000	5.4168000	530.4530029
660.0000000	5.4656000	-201.8663940
660.0000000	5.5144000	38.3992462
660.0000000	5.5631995	491.3993835
660.0000000	5.6120000	395.6363831
660.0000000	5.6608000	-37.2123528
660.0000000	5.7096000	63.0121307
660.0000000	5.7584000	133.9438477
660.0000000	5.8071995	-11.4930840
660.0000000	5.8559999	30.0942707
660.0000000	5.9047999	-16.6118259
660.0000000	5.9535999	-39.9250221
660.0000000	6.0023999	-1582.5538330
660.0000000	6.0511999	-14.5615263
660.0000000	6.0999999	-19.4356441
660.0000000	6.1487999	-13.2586565
660.0000000	6.1975999	-23.2763290
660.0000000	6.2463999	-1802.9101563
660.0000000	6.2951999	-19.0962772
660.0000000	6.3439999	-344.7577515
660.0000000	6.3927999	-15.0487108
660.0000000	6.4416003	-12.3670654
660.0000000	6.4903998	-11.2418375
660.0000000	6.5391998	-11.7841120
660.0000000	6.5879998	407.5736389
660.0000000	6.6367998	-12.0661840
660.0000000	6.6856003	-13.8093195
660.0000000	6.7343998	-13.4485302
660.0000000	6.7831998	-14.3863659
660.0000000	6.8319998	59.0770645
660.0000000	6.8807998	-14.0540600
660.0000000	6.9296002	-475.1198730
660.0000000	6.9783998	-4.6513591
660.0000000	7.0271997	-14.1722088
660.0000000	7.0760002	12.6255198
660.0000000	7.1247997	-2266.8041992
660.0000000	7.1736002	-1817.7642822
660.0000000	7.2223997	-1033.9210205
660.0000000	7.2711997	-222.2841492
660.0000000	7.3200002	-19.2965145
660.0000000	7.3687997	200.8563843
660.0000000	7.4176002	418.1051941
660.0000000	7.4663997	328.6061401
660.0000000	7.5151997	-1011.7540894

660.0000000	7.5640001	-1117.6414795
660.0000000	7.6127996	-2635.3171387
660.0000000	7.6616001	-845.6857300
660.0000000	7.7104001	-434.0460815
660.0000000	7.7591996	46.7536201
660.0000000	7.8080001	434.4945984
660.0000000	7.8567996	572.1388550
660.0000000	7.9056001	-1183.7286377
660.0000000	7.9544001	1884.4260254
660.0000000	8.0031996	2431.4982910
660.0000000	8.0520000	2426.9104004
660.0000000	8.1007996	2568.1337891
660.0000000	8.1496000	2738.7944336
660.0000000	8.1983995	1703.9532471
660.0000000	8.2472000	-3898.1984863
660.0000000	8.2959995	-1381.7302246
660.0000000	8.3448000	-1100.7032471
660.0000000	8.3935995	-1008.7554932
660.0000000	8.4423990	-434.3954468
660.0000000	8.4912004	-1014.3662109
660.0000000	8.5400000	-2736.6098633
660.0000000	8.5887995	-1326.3222656
660.0000000	8.6375999	259.3566895
660.0000000	8.6863995	2341.2504883
660.0000000	8.7351999	-869.9437866
660.0000000	8.7840004	994.6403809
660.0000000	8.8327999	54.5037231
660.0000000	8.8815994	-11.4902382
660.0000000	8.9303989	-24.0776196
660.0000000	8.9792004	-11.5761700
660.0000000	9.0279999	-5.7737885
660.0000000	9.0767994	7.2436275
660.0000000	9.1255999	-17.3033657
660.0000000	9.1743994	-10.0591860
660.0000000	9.2231998	37.5757599
660.0000000	9.2720003	-8.3864937
660.0000000	9.3207998	-9.7787237
660.0000000	9.3695993	-10.7556295
660.0000000	9.4183998	-11.7583609
660.0000000	9.4672003	-11.1598482
660.0000000	9.5159998	-14.1962852
660.0000000	9.5648003	-0.2716618
660.0000000	9.6135998	-13.4244747
660.0000000	9.6623993	-10.2114315
660.0000000	9.7111998	-10.1479292
660.0000000	9.7600002	-10.8560743
660.0000000	9.8087997	-10.9944916
660.0000000	9.8575993	3.3491096
660.0000000	9.9063997	-7.9361644
660.0000000	9.9552002	-10.9064465
660.0000000	10.0039997	-10.0658073
670.0000000	0.3416000	-35.9607086
670.0000000	0.3904000	-37.0685654
670.0000000	0.4392000	-38.6577568
670.0000000	0.4880000	-38.7521477
670.0000000	0.5368000	-39.1791267
670.0000000	0.5856000	-39.0937805
670.0000000	0.6344000	-38.2145844

670.0000000	0.6832000	-36.0880013
670.0000000	0.7320000	-35.9297867
670.0000000	0.7808000	-36.5244827
670.0000000	0.8296000	-35.9489288
670.0000000	0.8784000	-34.9378090
670.0000000	0.9272000	-32.7714272
670.0000000	0.9760000	-30.8672905
670.0000000	1.0247999	-29.3781071
670.0000000	1.0735999	-28.8688068
670.0000000	1.1224000	-29.2703171
670.0000000	1.1711999	-30.0411053
670.0000000	1.2200000	-30.5945950
670.0000000	1.2687999	-30.8636150
670.0000000	1.3176000	-31.0604019
670.0000000	1.3664000	-30.2996655
670.0000000	1.4152000	-30.1551609
670.0000000	1.4640000	-29.6751499
670.0000000	1.5128000	-42.7764397
670.0000000	1.5616000	-35.0947189
670.0000000	1.6104001	-1646.2149658
670.0000000	1.6592000	-83.5458145
670.0000000	1.7079999	-27.1263866
670.0000000	1.7567999	-27.9840298
670.0000000	1.8055999	-26.5867329
670.0000000	1.8544000	-26.2978325
670.0000000	1.9031999	-26.9313354
670.0000000	1.9520000	-26.5518036
670.0000000	2.0007999	-26.7898293
670.0000000	2.0495999	-22.8724442
670.0000000	2.0983999	-199.6264343
670.0000000	2.1471999	7.6797218
670.0000000	2.1960001	-25.5916939
670.0000000	2.2448001	-26.4546547
670.0000000	2.2935998	-29.7672577
670.0000000	2.3423998	-20.4856892
670.0000000	2.3912001	53.6346359
670.0000000	2.4400001	-22.7504387
670.0000000	2.4888000	-23.4676952
670.0000000	2.5375998	-11.6676331
670.0000000	2.5863998	-9.6951237
670.0000000	2.6352000	-7.6618938
670.0000000	2.6840000	-14.9885616
670.0000000	2.7328000	142.6160583
670.0000000	2.7815998	168.0154114
670.0000000	2.8304000	-23.8407784
670.0000000	2.8792000	-20.5032043
670.0000000	2.9280000	-22.5920410
670.0000000	2.9768000	-16.4913521
670.0000000	3.0256000	-20.3808174
670.0000000	3.0743999	-29.9223843
670.0000000	3.1231999	-24.3781872
670.0000000	3.1719999	-29.9011688
670.0000000	3.2208002	-512.7014160
670.0000000	3.2695999	-1004.1435547
670.0000000	3.3183999	-20.4826679
670.0000000	3.3671999	-3447.8178711
670.0000000	3.4159999	1237.2823486
670.0000000	3.4648001	-6.9407291

670.0000000	3.5135999	-27.1418114
670.0000000	3.5623999	-326.4843750
670.0000000	3.6111999	-2052.9028320
670.0000000	3.6600001	-464.5526733
670.0000000	3.7088001	-613.5092773
670.0000000	3.7575998	-960.7065430
670.0000000	3.8063998	-1743.5207520
670.0000000	3.8552001	-2531.4611816
670.0000000	3.9040000	-2481.9201660
670.0000000	3.9528000	1135.3375244
670.0000000	4.0015998	-247.7626038
670.0000000	4.0503998	695.2040405
670.0000000	4.0991998	88.7656784
670.0000000	4.1479998	-661.5856934
670.0000000	4.1967998	2.7782154
670.0000000	4.2456002	-250.9734802
670.0000000	4.2943997	-493.0252380
670.0000000	4.3431997	-13.2767372
670.0000000	4.3920002	-250.5992279
670.0000000	4.4407997	-22.5050507
670.0000000	4.4896002	-15.4521294
670.0000000	4.5383997	-661.6017456
670.0000000	4.5871997	-12.7882328
670.0000000	4.6360002	-21.6596241
670.0000000	4.6847997	-28.3803864
670.0000000	4.7336001	-19.5804577
670.0000000	4.7824001	-16.5010223
670.0000000	4.8311996	-1487.5743408
670.0000000	4.8800001	230.4815216
670.0000000	4.9287996	-203.1230774
670.0000000	4.9776001	1069.3209229
670.0000000	5.0264001	87.1067886
670.0000000	5.0751996	-303.7108154
670.0000000	5.1240001	-12.2854862
670.0000000	5.1727996	561.3103638
670.0000000	5.2216001	32.2795296
670.0000000	5.2704000	-159.6596680
670.0000000	5.3191996	-39.3192024
670.0000000	5.3680000	-3556.6982422
670.0000000	5.4168000	530.3461914
670.0000000	5.4656000	-201.8302155
670.0000000	5.5144000	38.3885880
670.0000000	5.5631995	491.3008118
670.0000000	5.6120000	395.5563965
670.0000000	5.6608000	-37.2082634
670.0000000	5.7096000	62.9966965
670.0000000	5.7584000	133.9149017
670.0000000	5.8071995	-11.4939537
670.0000000	5.8559999	30.0854206
670.0000000	5.9047999	-16.6116276
670.0000000	5.9535999	-39.9202843
670.0000000	6.0023999	-1582.2492676
670.0000000	6.0511999	-14.5617380
670.0000000	6.0999999	-19.4348125
670.0000000	6.1487999	-13.2591190
670.0000000	6.1975999	-23.2745628
670.0000000	6.2463999	-1802.5627441
670.0000000	6.2951999	-19.0954628

670.0000000	6.3439999	-344.6937256
670.0000000	6.3927999	-15.0486927
670.0000000	6.4416003	-12.3676519
670.0000000	6.4903998	-11.2425137
670.0000000	6.5391998	-11.7847929
670.0000000	6.5879998	406.8829346
670.0000000	6.6367998	-12.0667334
670.0000000	6.6856003	-13.8094902
670.0000000	6.7343998	-13.4487467
670.0000000	6.7831998	-14.3859501
670.0000000	6.8319998	59.0579529
670.0000000	6.8807998	-14.0539207
670.0000000	6.9296002	-474.3583984
670.0000000	6.9783998	-4.6641831
670.0000000	7.0271997	-14.1711807
670.0000000	7.0760002	12.5833330
670.0000000	7.1247997	-2262.8583984
670.0000000	7.1736002	-1814.6013184
670.0000000	7.2223997	-1032.1278076
670.0000000	7.2711997	-221.9163666
670.0000000	7.3200002	-19.2845268
670.0000000	7.3687997	200.4818573
670.0000000	7.4176002	417.3493652
670.0000000	7.4663997	328.0084839
670.0000000	7.5151997	-1009.9930420
670.0000000	7.5640001	-1115.6940918
670.0000000	7.6127996	-2630.6994629
670.0000000	7.6616001	-844.2143555
670.0000000	7.7104001	-433.2998047
670.0000000	7.7591996	46.6525726
670.0000000	7.8080001	433.7092590
670.0000000	7.8567996	571.1107178
670.0000000	7.9056001	-1181.6557617
670.0000000	7.9544001	1881.1160889
670.0000000	8.0031996	2427.2285156
670.0000000	8.0520000	2422.6484375
670.0000000	8.1007996	2563.6242676
670.0000000	8.1496000	2733.9865723
670.0000000	8.1983995	1700.9606934
670.0000000	8.2472000	-3891.3637695
670.0000000	8.2959995	-1379.3100586
670.0000000	8.3448000	-1098.7740479
670.0000000	8.3935995	-1006.9877930
670.0000000	8.4423990	-433.6374207
670.0000000	8.4912004	-1012.5899658
670.0000000	8.5400000	-2731.8100586
670.0000000	8.5887995	-1324.0007324
670.0000000	8.6375999	258.8953857
670.0000000	8.6863995	2337.1320801
670.0000000	8.7351999	-868.4194946
670.0000000	8.7840004	992.8836670
670.0000000	8.8327999	54.3921127
670.0000000	8.8815994	-11.4904118
670.0000000	8.9303989	-24.0738983
670.0000000	8.9792004	-11.5762520
670.0000000	9.0279999	-5.7755570
670.0000000	9.0767994	7.2379093
670.0000000	9.1255999	-17.3016510

670.0000000	9.1743994	-10.0595875
670.0000000	9.2231998	37.5600281
670.0000000	9.2720003	-8.3874302
670.0000000	9.3207998	-9.7792625
670.0000000	9.3695993	-10.7558565
670.0000000	9.4183998	-11.7582359
670.0000000	9.4672003	-11.1598892
670.0000000	9.5159998	-14.1953945
670.0000000	9.5648003	-0.2750340
670.0000000	9.6135998	-13.4236841
670.0000000	9.6623993	-10.2117071
670.0000000	9.7111998	-10.1482010
670.0000000	9.7600002	-10.8561163
670.0000000	9.8087997	-10.9945002
670.0000000	9.8575993	3.3446865
670.0000000	9.9063997	-7.9370785
670.0000000	9.9552002	-10.9064512
670.0000000	10.0039997	-10.0660553
680.0000000	0.3416000	-35.6825104
680.0000000	0.3904000	-37.6498833
680.0000000	0.4392000	-39.6035957
680.0000000	0.4880000	-39.3472290
680.0000000	0.5368000	-39.1199417
680.0000000	0.5856000	-39.0554771
680.0000000	0.6344000	-38.2148933
680.0000000	0.6832000	-36.0902252
680.0000000	0.7320000	-35.9318390
680.0000000	0.7808000	-36.5246506
680.0000000	0.8296000	-35.9496574
680.0000000	0.8784000	-34.9451828
680.0000000	0.9272000	-32.7742043
680.0000000	0.9760000	-30.8598385
680.0000000	1.0247999	-29.3870964
680.0000000	1.0735999	-28.8832417
680.0000000	1.1224000	-29.2673168
680.0000000	1.1711999	-30.0337276
680.0000000	1.2200000	-30.6025105
680.0000000	1.2687999	-30.8646221
680.0000000	1.3176000	-31.0561314
680.0000000	1.3664000	-30.3074150
680.0000000	1.4152000	-30.1553650
680.0000000	1.4640000	-29.6734409
680.0000000	1.5128000	-42.6792641
680.0000000	1.5616000	-35.0528755
680.0000000	1.6104001	-1643.6722412
680.0000000	1.6592000	-83.2518845
680.0000000	1.7079999	-27.1301327
680.0000000	1.7567999	-27.6184158
680.0000000	1.8055999	-26.5184689
680.0000000	1.8544000	-26.5753307
680.0000000	1.9031999	-26.7603607
680.0000000	1.9520000	-26.5522575
680.0000000	2.0007999	-26.7272472
680.0000000	2.0495999	-23.0590611
680.0000000	2.0983999	-198.1506348
680.0000000	2.1471999	7.4124546
680.0000000	2.1960001	-25.5876026
680.0000000	2.2448001	-26.4367447

680.0000000	2.2935998	-29.7076340
680.0000000	2.3423998	-20.5000935
680.0000000	2.3912001	53.0130920
680.0000000	2.4400001	-22.7529240
680.0000000	2.4888000	-23.4674778
680.0000000	2.5375998	-11.6847420
680.0000000	2.5863998	-9.7148829
680.0000000	2.6352000	-7.6846380
680.0000000	2.6840000	-15.0005016
680.0000000	2.7328000	142.3500366
680.0000000	2.7815998	167.7089844
680.0000000	2.8304000	-23.8394737
680.0000000	2.8792000	-20.5064678
680.0000000	2.9280000	-22.5914726
680.0000000	2.9768000	-16.4997215
680.0000000	3.0256000	-20.3834019
680.0000000	3.0743999	-29.9109974
680.0000000	3.1231999	-24.3747330
680.0000000	3.1719999	-29.8928757
680.0000000	3.2208002	-512.4636841
680.0000000	3.2695999	-1003.6685791
680.0000000	3.3183999	-20.4828663
680.0000000	3.3671999	-3446.3564453
680.0000000	3.4159999	1236.7470703
680.0000000	3.4648001	-6.9464378
680.0000000	3.5135999	-27.1388206
680.0000000	3.5623999	-326.3547668
680.0000000	3.6111999	-2052.0432129
680.0000000	3.6600001	-464.3645630
680.0000000	3.7088001	-613.2579346
680.0000000	3.7575998	-960.3079834
680.0000000	3.8063998	-1742.7908936
680.0000000	3.8552001	-2530.3977051
680.0000000	3.9040000	-2480.8776855
680.0000000	3.9528000	1134.8492432
680.0000000	4.0015998	-247.6654968
680.0000000	4.0503998	694.9014893
680.0000000	4.0991998	88.7199936
680.0000000	4.1479998	-661.3134766
680.0000000	4.1967998	2.7689590
680.0000000	4.2456002	-250.8751831
680.0000000	4.2943997	-492.8246765
680.0000000	4.3431997	-13.2789183
680.0000000	4.3920002	-250.5011139
680.0000000	4.4407997	-22.5032749
680.0000000	4.4896002	-15.4530125
680.0000000	4.5383997	-661.3406372
680.0000000	4.5871997	-12.7902489
680.0000000	4.6360002	-21.6580811
680.0000000	4.6847997	-28.3764610
680.0000000	4.7336001	-19.5797348
680.0000000	4.7824001	-16.5014782
680.0000000	4.8311996	-1487.0430908
680.0000000	4.8800001	230.3919678
680.0000000	4.9287996	-203.0558167
680.0000000	4.9776001	1068.9283447
680.0000000	5.0264001	87.0692673
680.0000000	5.0751996	-303.6072083

680.0000000	5.1240001	-12.2864075
680.0000000	5.1727996	561.1945190
680.0000000	5.2216001	32.2700844
680.0000000	5.2704000	-159.6315613
680.0000000	5.3191996	-39.3146515
680.0000000	5.3680000	-3556.0058594
680.0000000	5.4168000	530.2394409
680.0000000	5.4656000	-201.7940369
680.0000000	5.5144000	38.3779297
680.0000000	5.5631995	491.2022705
680.0000000	5.6120000	395.4764099
680.0000000	5.6608000	-37.2041779
680.0000000	5.7096000	62.9812698
680.0000000	5.7584000	133.8859711
680.0000000	5.8071995	-11.4948244
680.0000000	5.8559999	30.0765705
680.0000000	5.9047999	-16.6114311
680.0000000	5.9535999	-39.9155464
680.0000000	6.0023999	-1581.9449463
680.0000000	6.0511999	-14.5619488
680.0000000	6.0999999	-19.4339790
680.0000000	6.1487999	-13.2595825
680.0000000	6.1975999	-23.2727985
680.0000000	6.2463999	-1802.2153320
680.0000000	6.2951999	-19.0946484
680.0000000	6.3439999	-344.6296692
680.0000000	6.3927999	-15.0486736
680.0000000	6.4416003	-12.3682384
680.0000000	6.4903998	-11.2431898
680.0000000	6.5391998	-11.7854738
680.0000000	6.5879998	406.1922302
680.0000000	6.6367998	-12.0672827
680.0000000	6.6856003	-13.8096600
680.0000000	6.7343998	-13.4489622
680.0000000	6.7831998	-14.3855343
680.0000000	6.8319998	59.0388412
680.0000000	6.8807998	-14.0537825
680.0000000	6.9296002	-473.5969543
680.0000000	6.9783998	-4.6770062
680.0000000	7.0271997	-14.1701527
680.0000000	7.0760002	12.5411482
680.0000000	7.1247997	-2258.9123535
680.0000000	7.1736002	-1811.4384766
680.0000000	7.2223997	-1030.3347168
680.0000000	7.2711997	-221.5485840
680.0000000	7.3200002	-19.2725372
680.0000000	7.3687997	200.1073303
680.0000000	7.4176002	416.5935364
680.0000000	7.4663997	327.4108582
680.0000000	7.5151997	-1008.2320557
680.0000000	7.5640001	-1113.7467041
680.0000000	7.6127996	-2626.0820313
680.0000000	7.6616001	-842.7430420
680.0000000	7.7104001	-432.5535278
680.0000000	7.7591996	46.5515251
680.0000000	7.8080001	432.9238892
680.0000000	7.8567996	570.0826416
680.0000000	7.9056001	-1179.5828857

680.0000000	7.9544001	1877.8061523
680.0000000	8.0031996	2422.9589844
680.0000000	8.0520000	2418.3864746
680.0000000	8.1007996	2559.1147461
680.0000000	8.1496000	2729.1784668
680.0000000	8.1983995	1697.9682617
680.0000000	8.2472000	-3884.5290527
680.0000000	8.2959995	-1376.8898926
680.0000000	8.3448000	-1096.8448486
680.0000000	8.3935995	-1005.2201538
680.0000000	8.4423990	-432.8794250
680.0000000	8.4912004	-1010.8137817
680.0000000	8.5400000	-2727.0102539
680.0000000	8.5887995	-1321.6791992
680.0000000	8.6375999	258.4340515
680.0000000	8.6863995	2333.0139160
680.0000000	8.7351999	-866.8952637
680.0000000	8.7840004	991.1268921
680.0000000	8.8327999	54.2805061
680.0000000	8.8815994	-11.4905863
680.0000000	8.9303989	-24.0701790
680.0000000	8.9792004	-11.5763350
680.0000000	9.0279999	-5.7773252
680.0000000	9.0767994	7.2321930
680.0000000	9.1255999	-17.2999382
680.0000000	9.1743994	-10.0599899
680.0000000	9.2231998	37.5442963
680.0000000	9.2720003	-8.3883667
680.0000000	9.3207998	-9.7798023
680.0000000	9.3695993	-10.7560825
680.0000000	9.4183998	-11.7581120
680.0000000	9.4672003	-11.1599312
680.0000000	9.5159998	-14.1945038
680.0000000	9.5648003	-0.2784061
680.0000000	9.6135998	-13.4228935
680.0000000	9.6623993	-10.2119818
680.0000000	9.7111998	-10.1484718
680.0000000	9.7600002	-10.8561583
680.0000000	9.8087997	-10.9945078
680.0000000	9.8575993	3.3402643
680.0000000	9.9063997	-7.9379921
680.0000000	9.9552002	-10.9064550
680.0000000	10.0039997	-10.0663023
690.0000000	0.3416000	-36.4108162
690.0000000	0.3904000	-38.9825249
690.0000000	0.4392000	-39.9104729
690.0000000	0.4880000	-38.3707733
690.0000000	0.5368000	-39.0607567
690.0000000	0.5856000	-39.0171738
690.0000000	0.6344000	-38.2152023
690.0000000	0.6832000	-36.0924492
690.0000000	0.7320000	-35.9338913
690.0000000	0.7808000	-36.5248222
690.0000000	0.8296000	-35.9503860
690.0000000	0.8784000	-34.9525604
690.0000000	0.9272000	-32.7769814
690.0000000	0.9760000	-30.8523846
690.0000000	1.0247999	-29.3960857

690.0000000	1.0735999	-28.8976765
690.0000000	1.1224000	-29.2643185
690.0000000	1.1711999	-30.0263500
690.0000000	1.2200000	-30.6104259
690.0000000	1.2687999	-30.8656311
690.0000000	1.3176000	-31.0518589
690.0000000	1.3664000	-30.3151646
690.0000000	1.4152000	-30.1555691
690.0000000	1.4640000	-29.6717319
690.0000000	1.5128000	-42.5820847
690.0000000	1.5616000	-35.0110359
690.0000000	1.6104001	-1641.1293945
690.0000000	1.6592000	-82.9579544
690.0000000	1.7079999	-27.1338806
690.0000000	1.7567999	-27.4622211
690.0000000	1.8055999	-26.4859867
690.0000000	1.8544000	-26.0631733
690.0000000	1.9031999	-26.5893860
690.0000000	1.9520000	-26.5527134
690.0000000	2.0007999	-26.6646652
690.0000000	2.0495999	-23.2456779
690.0000000	2.0983999	-196.6748657
690.0000000	2.1471999	7.1451874
690.0000000	2.1960001	-25.5835114
690.0000000	2.2448001	-26.4188328
690.0000000	2.2935998	-29.6480103
690.0000000	2.3423998	-20.5144978
690.0000000	2.3912001	52.3915482
690.0000000	2.4400001	-22.7554111
690.0000000	2.4888000	-23.4672604
690.0000000	2.5375998	-11.7018499
690.0000000	2.5863998	-9.7346420
690.0000000	2.6352000	-7.7073822
690.0000000	2.6840000	-15.0124435
690.0000000	2.7328000	142.0840302
690.0000000	2.7815998	167.4025574
690.0000000	2.8304000	-23.8381691
690.0000000	2.8792000	-20.5097313
690.0000000	2.9280000	-22.5909023
690.0000000	2.9768000	-16.5080929
690.0000000	3.0256000	-20.3859863
690.0000000	3.0743999	-29.8996105
690.0000000	3.1231999	-24.3712769
690.0000000	3.1719999	-29.8845825
690.0000000	3.2208002	-512.2258911
690.0000000	3.2695999	-1003.1936035
690.0000000	3.3183999	-20.4830647
690.0000000	3.3671999	-3444.8947754
690.0000000	3.4159999	1236.2117920
690.0000000	3.4648001	-6.9521465
690.0000000	3.5135999	-27.1358299
690.0000000	3.5623999	-326.2251892
690.0000000	3.6111999	-2051.1833496
690.0000000	3.6600001	-464.1763916
690.0000000	3.7088001	-613.0065308
690.0000000	3.7575998	-959.9094849
690.0000000	3.8063998	-1742.0610352
690.0000000	3.8552001	-2529.3342285

690.0000000	3.9040000	-2479.8352051
690.0000000	3.9528000	1134.3609619
690.0000000	4.0015998	-247.5683899
690.0000000	4.0503998	694.5989380
690.0000000	4.0991998	88.6743088
690.0000000	4.1479998	-661.0412598
690.0000000	4.1967998	2.7597027
690.0000000	4.2456002	-250.7768860
690.0000000	4.2943997	-492.6241150
690.0000000	4.3431997	-13.2810993
690.0000000	4.3920002	-250.4029999
690.0000000	4.4407997	-22.5014992
690.0000000	4.4896002	-15.4538946
690.0000000	4.5383997	-661.0795898
690.0000000	4.5871997	-12.7922649
690.0000000	4.6360002	-21.6565399
690.0000000	4.6847997	-28.3725357
690.0000000	4.7336001	-19.5790138
690.0000000	4.7824001	-16.5019321
690.0000000	4.8311996	-1486.5117188
690.0000000	4.8800001	230.3024292
690.0000000	4.9287996	-202.9885559
690.0000000	4.9776001	1068.5358887
690.0000000	5.0264001	87.0317459
690.0000000	5.0751996	-303.5035706
690.0000000	5.1240001	-12.2873287
690.0000000	5.1727996	561.0786133
690.0000000	5.2216001	32.2606430
690.0000000	5.2704000	-159.6034546
690.0000000	5.3191996	-39.3101044
690.0000000	5.3680000	-3555.3134766
690.0000000	5.4168000	530.1326294
690.0000000	5.4656000	-201.7578430
690.0000000	5.5144000	38.3672791
690.0000000	5.5631995	491.1036987
690.0000000	5.6120000	395.3964539
690.0000000	5.6608000	-37.2000885
690.0000000	5.7096000	62.9658432
690.0000000	5.7584000	133.8570404
690.0000000	5.8071995	-11.4956951
690.0000000	5.8559999	30.0677204
690.0000000	5.9047999	-16.6112328
690.0000000	5.9535999	-39.9108047
690.0000000	6.0023999	-1581.6405029
690.0000000	6.0511999	-14.5621605
690.0000000	6.0999999	-19.4331474
690.0000000	6.1487999	-13.2600451
690.0000000	6.1975999	-23.2710342
690.0000000	6.2463999	-1801.8679199
690.0000000	6.2951999	-19.0938339
690.0000000	6.3439999	-344.5656128
690.0000000	6.3927999	-15.0486555
690.0000000	6.4416003	-12.3688259
690.0000000	6.4903998	-11.2438650
690.0000000	6.5391998	-11.7861547
690.0000000	6.5879998	405.5015259
690.0000000	6.6367998	-12.0678320
690.0000000	6.6856003	-13.8098307

690.0000000	6.7343998	-13.4491777
690.0000000	6.7831998	-14.3851185
690.0000000	6.8319998	59.0197220
690.0000000	6.8807998	-14.0536442
690.0000000	6.9296002	-472.8355103
690.0000000	6.9783998	-4.6898298
690.0000000	7.0271997	-14.1691246
690.0000000	7.0760002	12.4989634
690.0000000	7.1247997	-2254.9665527
690.0000000	7.1736002	-1808.2756348
690.0000000	7.2223997	-1028.5415039
690.0000000	7.2711997	-221.1808167
690.0000000	7.3200002	-19.2605476
690.0000000	7.3687997	199.7327881
690.0000000	7.4176002	415.8377075
690.0000000	7.4663997	326.8132019
690.0000000	7.5151997	-1006.4710083
690.0000000	7.5640001	-1111.7993164
690.0000000	7.6127996	-2621.4643555
690.0000000	7.6616001	-841.2717285
690.0000000	7.7104001	-431.8072510
690.0000000	7.7591996	46.4504738
690.0000000	7.8080001	432.1385193
690.0000000	7.8567996	569.0545654
690.0000000	7.9056001	-1177.5100098
690.0000000	7.9544001	1874.4962158
690.0000000	8.0031996	2418.6894531
690.0000000	8.0520000	2414.1245117
690.0000000	8.1007996	2554.6052246
690.0000000	8.1496000	2724.3703613
690.0000000	8.1983995	1694.9757080
690.0000000	8.2472000	-3877.6940918
690.0000000	8.2959995	-1374.4697266
690.0000000	8.3448000	-1094.9156494
690.0000000	8.3935995	-1003.4524536
690.0000000	8.4423990	-432.1213989
690.0000000	8.4912004	-1009.0375366
690.0000000	8.5400000	-2722.2104492
690.0000000	8.5887995	-1319.3576660
690.0000000	8.6375999	257.9727173
690.0000000	8.6863995	2328.8955078
690.0000000	8.7351999	-865.3710327
690.0000000	8.7840004	989.3701782
690.0000000	8.8327999	54.1688957
690.0000000	8.8815994	-11.4907608
690.0000000	8.9303989	-24.0664597
690.0000000	8.9792004	-11.5764170
690.0000000	9.0279999	-5.7790937
690.0000000	9.0767994	7.2264748
690.0000000	9.1255999	-17.2982235
690.0000000	9.1743994	-10.0603924
690.0000000	9.2231998	37.5285645
690.0000000	9.2720003	-8.3893032
690.0000000	9.3207998	-9.7803421
690.0000000	9.3695993	-10.7563086
690.0000000	9.4183998	-11.7579870
690.0000000	9.4672003	-11.1599722
690.0000000	9.5159998	-14.1936121

690.0000000	9.5648003	-0.2817793
690.0000000	9.6135998	-13.4221020
690.0000000	9.6623993	-10.2122574
690.0000000	9.7111998	-10.1487436
690.0000000	9.7600002	-10.8561993
690.0000000	9.8087997	-10.9945154
690.0000000	9.8575993	3.3358421
690.0000000	9.9063997	-7.9389062
690.0000000	9.9552002	-10.9064598
690.0000000	10.0039997	-10.0665503
700.0000000	0.3416000	-36.0587158
700.0000000	0.3904000	-39.2363472
700.0000000	0.4392000	-39.9086342
700.0000000	0.4880000	-39.2232742
700.0000000	0.5368000	-39.8263817
700.0000000	0.5856000	-39.0559120
700.0000000	0.6344000	-38.2155151
700.0000000	0.6832000	-36.0946770
700.0000000	0.7320000	-35.9359436
700.0000000	0.7808000	-36.5249901
700.0000000	0.8296000	-35.9511147
700.0000000	0.8784000	-34.9599342
700.0000000	0.9272000	-32.7797585
700.0000000	0.9760000	-30.8449326
700.0000000	1.0247999	-29.4050751
700.0000000	1.0735999	-28.9121113
700.0000000	1.1224000	-29.2613182
700.0000000	1.1711999	-30.0189743
700.0000000	1.2200000	-30.6183395
700.0000000	1.2687999	-30.8666382
700.0000000	1.3176000	-31.0475883
700.0000000	1.3664000	-30.3229141
700.0000000	1.4152000	-30.1557732
700.0000000	1.4640000	-29.6700230
700.0000000	1.5128000	-42.4849091
700.0000000	1.5616000	-34.9691925
700.0000000	1.6104001	-1638.5865479
700.0000000	1.6592000	-82.6640167
700.0000000	1.7079999	-27.1376266
700.0000000	1.7567999	-27.1857471
700.0000000	1.8055999	-25.7599163
700.0000000	1.8544000	-26.0648746
700.0000000	1.9031999	-26.8775940
700.0000000	1.9520000	-26.5531693
700.0000000	2.0007999	-26.6020832
700.0000000	2.0495999	-23.4322948
700.0000000	2.0983999	-195.1990967
700.0000000	2.1471999	6.8779202
700.0000000	2.1960001	-25.5794201
700.0000000	2.2448001	-26.4009209
700.0000000	2.2935998	-29.5883865
700.0000000	2.3423998	-20.5289021
700.0000000	2.3912001	51.7699966
700.0000000	2.4400001	-22.7578964
700.0000000	2.4888000	-23.4670429
700.0000000	2.5375998	-11.7189579
700.0000000	2.5863998	-9.7544003
700.0000000	2.6352000	-7.7301264

700.0000000	2.6840000	-15.0243864
700.0000000	2.7328000	141.8180084
700.0000000	2.7815998	167.0961609
700.0000000	2.8304000	-23.8368645
700.0000000	2.8792000	-20.5129948
700.0000000	2.9280000	-22.5903339
700.0000000	2.9768000	-16.5164642
700.0000000	3.0256000	-20.3885708
700.0000000	3.0743999	-29.8882236
700.0000000	3.1231999	-24.3678207
700.0000000	3.1719999	-29.8762894
700.0000000	3.2208002	-511.9881897
700.0000000	3.2695999	-1002.7186279
700.0000000	3.3183999	-20.4832611
700.0000000	3.3671999	-3443.4331055
700.0000000	3.4159999	1235.6765137
700.0000000	3.4648001	-6.9578543
700.0000000	3.5135999	-27.1328392
700.0000000	3.5623999	-326.0955811
700.0000000	3.6111999	-2050.3237305
700.0000000	3.6600001	-463.9882813
700.0000000	3.7088001	-612.7551270
700.0000000	3.7575998	-959.5109253
700.0000000	3.8063998	-1741.3311768
700.0000000	3.8552001	-2528.2707520
700.0000000	3.9040000	-2478.7927246
700.0000000	3.9528000	1133.8726807
700.0000000	4.0015998	-247.4712830
700.0000000	4.0503998	694.2963867
700.0000000	4.0991998	88.6286240
700.0000000	4.1479998	-660.7690430
700.0000000	4.1967998	2.7504463
700.0000000	4.2456002	-250.6785736
700.0000000	4.2943997	-492.4235229
700.0000000	4.3431997	-13.2832794
700.0000000	4.3920002	-250.3048859
700.0000000	4.4407997	-22.4997234
700.0000000	4.4896002	-15.4547777
700.0000000	4.5383997	-660.8184814
700.0000000	4.5871997	-12.7942810
700.0000000	4.6360002	-21.6549969
700.0000000	4.6847997	-28.3686104
700.0000000	4.7336001	-19.5782909
700.0000000	4.7824001	-16.5023861
700.0000000	4.8311996	-1485.9804688
700.0000000	4.8800001	230.2128754
700.0000000	4.9287996	-202.9212952
700.0000000	4.9776001	1068.1434326
700.0000000	5.0264001	86.9942245
700.0000000	5.0751996	-303.3999329
700.0000000	5.1240001	-12.2882500
700.0000000	5.1727996	560.9627686
700.0000000	5.2216001	32.2511978
700.0000000	5.2704000	-159.5753479
700.0000000	5.3191996	-39.3055573
700.0000000	5.3680000	-3554.6210938
700.0000000	5.4168000	530.0258789
700.0000000	5.4656000	-201.7216644

700.0000000	5.5144000	38.3566208
700.0000000	5.5631995	491.0051270
700.0000000	5.6120000	395.3164673
700.0000000	5.6608000	-37.1959991
700.0000000	5.7096000	62.9504089
700.0000000	5.7584000	133.8281097
700.0000000	5.8071995	-11.4965658
700.0000000	5.8559999	30.0588703
700.0000000	5.9047999	-16.6110344
700.0000000	5.9535999	-39.9060669
700.0000000	6.0023999	-1581.3361816
700.0000000	6.0511999	-14.5623722
700.0000000	6.0999999	-19.4323158
700.0000000	6.1487999	-13.2605076
700.0000000	6.1975999	-23.2692680
700.0000000	6.2463999	-1801.5206299
700.0000000	6.2951999	-19.0930214
700.0000000	6.3439999	-344.5015564
700.0000000	6.3927999	-15.0486374
700.0000000	6.4416003	-12.3694124
700.0000000	6.4903998	-11.2445412
700.0000000	6.5391998	-11.7868366
700.0000000	6.5879998	404.8108521
700.0000000	6.6367998	-12.0683804
700.0000000	6.6856003	-13.8100004
700.0000000	6.7343998	-13.4493942
700.0000000	6.7831998	-14.3847027
700.0000000	6.8319998	59.0006104
700.0000000	6.8807998	-14.0535049
700.0000000	6.9296002	-472.0740662
700.0000000	6.9783998	-4.7026539
700.0000000	7.0271997	-14.1680965
700.0000000	7.0760002	12.4567766
700.0000000	7.1247997	-2251.0207520
700.0000000	7.1736002	-1805.1127930
700.0000000	7.2223997	-1026.7484131
700.0000000	7.2711997	-220.8130341
700.0000000	7.3200002	-19.2485600
700.0000000	7.3687997	199.3582611
700.0000000	7.4176002	415.0818787
700.0000000	7.4663997	326.2155457
700.0000000	7.5151997	-1004.7099609
700.0000000	7.5640001	-1109.8519287
700.0000000	7.6127996	-2616.8466797
700.0000000	7.6616001	-839.8004150
700.0000000	7.7104001	-431.0610352
700.0000000	7.7591996	46.3494263
700.0000000	7.8080001	431.3531799
700.0000000	7.8567996	568.0264893
700.0000000	7.9056001	-1175.4370117
700.0000000	7.9544001	1871.1862793
700.0000000	8.0031996	2414.4196777
700.0000000	8.0520000	2409.8625488
700.0000000	8.1007996	2550.0957031
700.0000000	8.1496000	2719.5625000
700.0000000	8.1983995	1691.9831543
700.0000000	8.2472000	-3870.8593750
700.0000000	8.2959995	-1372.0495605

700.0000000	8.3448000	-1092.9865723
700.0000000	8.3935995	-1001.6848145
700.0000000	8.4423990	-431.3634033
700.0000000	8.4912004	-1007.2613525
700.0000000	8.5400000	-2717.4106445
700.0000000	8.5887995	-1317.0361328
700.0000000	8.6375999	257.5114136
700.0000000	8.6863995	2324.7770996
700.0000000	8.7351999	-863.8467407
700.0000000	8.7840004	987.6134033
700.0000000	8.8327999	54.0572891
700.0000000	8.8815994	-11.4909353
700.0000000	8.9303989	-24.0627403
700.0000000	8.9792004	-11.5764990
700.0000000	9.0279999	-5.7808614
700.0000000	9.0767994	7.2207565
700.0000000	9.1255999	-17.2965107
700.0000000	9.1743994	-10.0607939
700.0000000	9.2231998	37.5128250
700.0000000	9.2720003	-8.3902397
700.0000000	9.3207998	-9.7808809
700.0000000	9.3695993	-10.7565355
700.0000000	9.4183998	-11.7578621
700.0000000	9.4672003	-11.1600132
700.0000000	9.5159998	-14.1927214
700.0000000	9.5648003	-0.2851524
700.0000000	9.6135998	-13.4213114
700.0000000	9.6623993	-10.2125320
700.0000000	9.7111998	-10.1490154
700.0000000	9.7600002	-10.8562412
700.0000000	9.8087997	-10.9945240
700.0000000	9.8575993	3.3314190
700.0000000	9.9063997	-7.9398198
700.0000000	9.9552002	-10.9064636
700.0000000	10.0039997	-10.0667973
710.0000000	0.3416000	-36.4264984
710.0000000	0.3904000	-38.9666214
710.0000000	0.4392000	-39.9067917
710.0000000	0.4880000	-39.0513229
710.0000000	0.5368000	-39.9596939
710.0000000	0.5856000	-39.0946465
710.0000000	0.6344000	-38.2158241
710.0000000	0.6832000	-36.0969009
710.0000000	0.7320000	-35.9379997
710.0000000	0.7808000	-36.5251579
710.0000000	0.8296000	-35.9518433
710.0000000	0.8784000	-34.9673080
710.0000000	0.9272000	-32.7825356
710.0000000	0.9760000	-30.8374786
710.0000000	1.0247999	-29.4140644
710.0000000	1.0735999	-28.9265480
710.0000000	1.1224000	-29.2583199
710.0000000	1.1711999	-30.0115967
710.0000000	1.2200000	-30.6262550
710.0000000	1.2687999	-30.8676453
710.0000000	1.3176000	-31.0433159
710.0000000	1.3664000	-30.3306637
710.0000000	1.4152000	-30.1559772

710.0000000	1.4640000	-29.6683121
710.0000000	1.5128000	-42.3877296
710.0000000	1.5616000	-34.9273529
710.0000000	1.6104001	-1636.0438232
710.0000000	1.6592000	-82.3700867
710.0000000	1.7079999	-27.1413746
710.0000000	1.7567999	-27.5086670
710.0000000	1.8055999	-26.2574081
710.0000000	1.8544000	-26.2018909
710.0000000	1.9031999	-26.1927605
710.0000000	1.9520000	-26.5536232
710.0000000	2.0007999	-26.5395012
710.0000000	2.0495999	-23.6189117
710.0000000	2.0983999	-193.7232971
710.0000000	2.1471999	6.6106529
710.0000000	2.1960001	-25.5753288
710.0000000	2.2448001	-26.3830109
710.0000000	2.2935998	-29.5287628
710.0000000	2.3423998	-20.5433083
710.0000000	2.3912001	51.1484528
710.0000000	2.4400001	-22.7603836
710.0000000	2.4888000	-23.4668255
710.0000000	2.5375998	-11.7360659
710.0000000	2.5863998	-9.7741594
710.0000000	2.6352000	-7.7528706
710.0000000	2.6840000	-15.0363283
710.0000000	2.7328000	141.5519867
710.0000000	2.7815998	166.7897339
710.0000000	2.8304000	-23.8355598
710.0000000	2.8792000	-20.5162582
710.0000000	2.9280000	-22.5897655
710.0000000	2.9768000	-16.5248337
710.0000000	3.0256000	-20.3911552
710.0000000	3.0743999	-29.8768368
710.0000000	3.1231999	-24.3643665
710.0000000	3.1719999	-29.8679962
710.0000000	3.2208002	-511.7504578
710.0000000	3.2695999	-1002.2436523
710.0000000	3.3183999	-20.4834595
710.0000000	3.3671999	-3441.9714355
710.0000000	3.4159999	1235.1412354
710.0000000	3.4648001	-6.9635630
710.0000000	3.5135999	-27.1298485
710.0000000	3.5623999	-325.9660034
710.0000000	3.6111999	-2049.4638672
710.0000000	3.6600001	-463.8001709
710.0000000	3.7088001	-612.5037842
710.0000000	3.7575998	-959.1123657
710.0000000	3.8063998	-1740.6013184
710.0000000	3.8552001	-2527.2075195
710.0000000	3.9040000	-2477.7502441
710.0000000	3.9528000	1133.3842773
710.0000000	4.0015998	-247.3741760
710.0000000	4.0503998	693.9938354
710.0000000	4.0991998	88.5829391
710.0000000	4.1479998	-660.4968262
710.0000000	4.1967998	2.7411900
710.0000000	4.2456002	-250.5802765

710.0000000	4.2943997	-492.2229614
710.0000000	4.3431997	-13.2854595
710.0000000	4.3920002	-250.2067719
710.0000000	4.4407997	-22.4979477
710.0000000	4.4896002	-15.4556599
710.0000000	4.5383997	-660.5574341
710.0000000	4.5871997	-12.7962971
710.0000000	4.6360002	-21.6534538
710.0000000	4.6847997	-28.3646851
710.0000000	4.7336001	-19.5775681
710.0000000	4.7824001	-16.5028419
710.0000000	4.8311996	-1485.4492188
710.0000000	4.8800001	230.1233215
710.0000000	4.9287996	-202.8540192
710.0000000	4.9776001	1067.7508545
710.0000000	5.0264001	86.9567108
710.0000000	5.0751996	-303.2963257
710.0000000	5.1240001	-12.2891712
710.0000000	5.1727996	560.8469238
710.0000000	5.2216001	32.2417526
710.0000000	5.2704000	-159.5472412
710.0000000	5.3191996	-39.3010025
710.0000000	5.3680000	-3553.9287109
710.0000000	5.4168000	529.9190674
710.0000000	5.4656000	-201.6854858
710.0000000	5.5144000	38.3459625
710.0000000	5.5631995	490.9065552
710.0000000	5.6120000	395.2364807
710.0000000	5.6608000	-37.1919098
710.0000000	5.7096000	62.9349747
710.0000000	5.7584000	133.7991638
710.0000000	5.8071995	-11.4974365
710.0000000	5.8559999	30.0500164
710.0000000	5.9047999	-16.6108379
710.0000000	5.9535999	-39.9013290
710.0000000	6.0023999	-1581.0316162
710.0000000	6.0511999	-14.5625830
710.0000000	6.0999999	-19.4314823
710.0000000	6.1487999	-13.2609711
710.0000000	6.1975999	-23.2675018
710.0000000	6.2463999	-1801.1732178
710.0000000	6.2951999	-19.0922070
710.0000000	6.3439999	-344.4375305
710.0000000	6.3927999	-15.0486193
710.0000000	6.4416003	-12.3699999
710.0000000	6.4903998	-11.2452173
710.0000000	6.5391998	-11.7875175
710.0000000	6.5879998	404.1201477
710.0000000	6.6367998	-12.0689297
710.0000000	6.6856003	-13.8101711
710.0000000	6.7343998	-13.4496098
710.0000000	6.7831998	-14.3842869
710.0000000	6.8319998	58.9814987
710.0000000	6.8807998	-14.0533667
710.0000000	6.9296002	-471.3126221
710.0000000	6.9783998	-4.7154765
710.0000000	7.0271997	-14.1670694
710.0000000	7.0760002	12.4145918

710.0000000	7.1247997	-2247.0749512
710.0000000	7.1736002	-1801.9499512
710.0000000	7.2223997	-1024.9552002
710.0000000	7.2711997	-220.4452515
710.0000000	7.3200002	-19.2365723
710.0000000	7.3687997	198.9837341
710.0000000	7.4176002	414.3260498
710.0000000	7.4663997	325.6178894
710.0000000	7.5151997	-1002.9489136
710.0000000	7.5640001	-1107.9044189
710.0000000	7.6127996	-2612.2290039
710.0000000	7.6616001	-838.3290405
710.0000000	7.7104001	-430.3147583
710.0000000	7.7591996	46.2483788
710.0000000	7.8080001	430.5678101
710.0000000	7.8567996	566.9984131
710.0000000	7.9056001	-1173.3641357
710.0000000	7.9544001	1867.8763428
710.0000000	8.0031996	2410.1501465
710.0000000	8.0520000	2405.6005859
710.0000000	8.1007996	2545.5861816
710.0000000	8.1496000	2714.7543945
710.0000000	8.1983995	1688.9907227
710.0000000	8.2472000	-3864.0244141
710.0000000	8.2959995	-1369.6293945
710.0000000	8.3448000	-1091.0573730
710.0000000	8.3935995	-999.9171143
710.0000000	8.4423990	-430.6053772
710.0000000	8.4912004	-1005.4851074
710.0000000	8.5400000	-2712.6108398
710.0000000	8.5887995	-1314.7145996
710.0000000	8.6375999	257.0500793
710.0000000	8.6863995	2320.6586914
710.0000000	8.7351999	-862.3225098
710.0000000	8.7840004	985.8566895
710.0000000	8.8327999	53.9456863
710.0000000	8.8815994	-11.4911089
710.0000000	8.9303989	-24.0590210
710.0000000	8.9792004	-11.5765820
710.0000000	9.0279999	-5.7826300
710.0000000	9.0767994	7.2150383
710.0000000	9.1255999	-17.2947960
710.0000000	9.1743994	-10.0611963
710.0000000	9.2231998	37.4970932
710.0000000	9.2720003	-8.3911762
710.0000000	9.3207998	-9.7814198
710.0000000	9.3695993	-10.7567616
710.0000000	9.4183998	-11.7577381
710.0000000	9.4672003	-11.1600542
710.0000000	9.5159998	-14.1918297
710.0000000	9.5648003	-0.2885246
710.0000000	9.6135998	-13.4205208
710.0000000	9.6623993	-10.2128067
710.0000000	9.7111998	-10.1492872
710.0000000	9.7600002	-10.8562832
710.0000000	9.8087997	-10.9945316
710.0000000	9.8575993	3.3269968
710.0000000	9.9063997	-7.9407339

710.0000000	9.9552002	-10.9064684
710.0000000	10.0039997	-10.0670452
720.0000000	0.3416000	-36.5131111
720.0000000	0.3904000	-39.3775330
720.0000000	0.4392000	-39.9049530
720.0000000	0.4880000	-39.4874229
720.0000000	0.5368000	-39.8932419
720.0000000	0.5856000	-39.1333847
720.0000000	0.6344000	-38.2161369
720.0000000	0.6832000	-36.0991287
720.0000000	0.7320000	-35.9400520
720.0000000	0.7808000	-36.5253296
720.0000000	0.8296000	-35.9525757
720.0000000	0.8784000	-34.9746857
720.0000000	0.9272000	-32.7853127
720.0000000	0.9760000	-30.8300266
720.0000000	1.0247999	-29.4230537
720.0000000	1.0735999	-28.9409828
720.0000000	1.1224000	-29.2553196
720.0000000	1.1711999	-30.0042191
720.0000000	1.2200000	-30.6341705
720.0000000	1.2687999	-30.8686543
720.0000000	1.3176000	-31.0390453
720.0000000	1.3664000	-30.3384132
720.0000000	1.4152000	-30.1561832
720.0000000	1.4640000	-29.6666031
720.0000000	1.5128000	-42.2905540
720.0000000	1.5616000	-34.8855133
720.0000000	1.6104001	-1633.5009766
720.0000000	1.6592000	-82.0761566
720.0000000	1.7079999	-27.1451225
720.0000000	1.7567999	-27.8315849
720.0000000	1.8055999	-26.0967636
720.0000000	1.8544000	-25.7052517
720.0000000	1.9031999	-26.0147724
720.0000000	1.9520000	-26.5540791
720.0000000	2.0007999	-26.4769192
720.0000000	2.0495999	-23.8055267
720.0000000	2.0983999	-192.2475281
720.0000000	2.1471999	6.3433857
720.0000000	2.1960001	-25.5712376
720.0000000	2.2448001	-26.3650990
720.0000000	2.2935998	-29.4691410
720.0000000	2.3423998	-20.5577126
720.0000000	2.3912001	50.5269089
720.0000000	2.4400001	-22.7628708
720.0000000	2.4888000	-23.4666080
720.0000000	2.5375998	-11.7531738
720.0000000	2.5863998	-9.7939186
720.0000000	2.6352000	-7.7756147
720.0000000	2.6840000	-15.0482702
720.0000000	2.7328000	141.2859802
720.0000000	2.7815998	166.4833069
720.0000000	2.8304000	-23.8342571
720.0000000	2.8792000	-20.5195217
720.0000000	2.9280000	-22.5891953
720.0000000	2.9768000	-16.5332050
720.0000000	3.0256000	-20.3937397

720.0000000	3.0743999	-29.8654499
720.0000000	3.1231999	-24.3609104
720.0000000	3.1719999	-29.8597031
720.0000000	3.2208002	-511.5127258
720.0000000	3.2695999	-1001.7686768
720.0000000	3.3183999	-20.4836578
720.0000000	3.3671999	-3440.5097656
720.0000000	3.4159999	1234.6059570
720.0000000	3.4648001	-6.9692707
720.0000000	3.5135999	-27.1268578
720.0000000	3.5623999	-325.8363953
720.0000000	3.6111999	-2048.6042480
720.0000000	3.6600001	-463.6119995
720.0000000	3.7088001	-612.2523804
720.0000000	3.7575998	-958.7138672
720.0000000	3.8063998	-1739.8714600
720.0000000	3.8552001	-2526.1440430
720.0000000	3.9040000	-2476.7077637
720.0000000	3.9528000	1132.8959961
720.0000000	4.0015998	-247.2770691
720.0000000	4.0503998	693.6912231
720.0000000	4.0991998	88.5372620
720.0000000	4.1479998	-660.2246094
720.0000000	4.1967998	2.7319336
720.0000000	4.2456002	-250.4819794
720.0000000	4.2943997	-492.0223999
720.0000000	4.3431997	-13.2876406
720.0000000	4.3920002	-250.1086578
720.0000000	4.4407997	-22.4961739
720.0000000	4.4896002	-15.4565430
720.0000000	4.5383997	-660.2963867
720.0000000	4.5871997	-12.7983131
720.0000000	4.6360002	-21.6519108
720.0000000	4.6847997	-28.3607597
720.0000000	4.7336001	-19.5768452
720.0000000	4.7824001	-16.5032959
720.0000000	4.8311996	-1484.9179688
720.0000000	4.8800001	230.0337830
720.0000000	4.9287996	-202.7867584
720.0000000	4.9776001	1067.3583984
720.0000000	5.0264001	86.9191895
720.0000000	5.0751996	-303.1926880
720.0000000	5.1240001	-12.2900925
720.0000000	5.1727996	560.7310181
720.0000000	5.2216001	32.2323112
720.0000000	5.2704000	-159.5191345
720.0000000	5.3191996	-39.2964554
720.0000000	5.3680000	-3553.2363281
720.0000000	5.4168000	529.8123169
720.0000000	5.4656000	-201.6492920
720.0000000	5.5144000	38.3353119
720.0000000	5.5631995	490.8079834
720.0000000	5.6120000	395.1565247
720.0000000	5.6608000	-37.1878204
720.0000000	5.7096000	62.9195480
720.0000000	5.7584000	133.7702332
720.0000000	5.8071995	-11.4983063
720.0000000	5.8559999	30.0411663

720.0000000	5.9047999	-16.6106396
720.0000000	5.9535999	-39.8965912
720.0000000	6.0023999	-1580.7272949
720.0000000	6.0511999	-14.5627947
720.0000000	6.0999999	-19.4306507
720.0000000	6.1487999	-13.2614336
720.0000000	6.1975999	-23.2657394
720.0000000	6.2463999	-1800.8258057
720.0000000	6.2951999	-19.0913925
720.0000000	6.3439999	-344.3734741
720.0000000	6.3927999	-15.0486002
720.0000000	6.4416003	-12.3705864
720.0000000	6.4903998	-11.2458935
720.0000000	6.5391998	-11.7881985
720.0000000	6.5879998	403.4294434
720.0000000	6.6367998	-12.0694790
720.0000000	6.6856003	-13.8103409
720.0000000	6.7343998	-13.4498253
720.0000000	6.7831998	-14.3838720
720.0000000	6.8319998	58.9623795
720.0000000	6.8807998	-14.0532274
720.0000000	6.9296002	-470.5511475
720.0000000	6.9783998	-4.7283006
720.0000000	7.0271997	-14.1660414
720.0000000	7.0760002	12.3724070
720.0000000	7.1247997	-2243.1289063
720.0000000	7.1736002	-1798.7869873
720.0000000	7.2223997	-1023.1620483
720.0000000	7.2711997	-220.0774841
720.0000000	7.3200002	-19.2245827
720.0000000	7.3687997	198.6091919
720.0000000	7.4176002	413.5702209
720.0000000	7.4663997	325.0202332
720.0000000	7.5151997	-1001.1878662
720.0000000	7.5640001	-1105.9570313
720.0000000	7.6127996	-2607.6115723
720.0000000	7.6616001	-836.8576660
720.0000000	7.7104001	-429.5684814
720.0000000	7.7591996	46.1473274
720.0000000	7.8080001	429.7824402
720.0000000	7.8567996	565.9703369
720.0000000	7.9056001	-1171.2912598
720.0000000	7.9544001	1864.5664063
720.0000000	8.0031996	2405.8806152
720.0000000	8.0520000	2401.3386230
720.0000000	8.1007996	2541.0766602
720.0000000	8.1496000	2709.9465332
720.0000000	8.1983995	1685.9981689
720.0000000	8.2472000	-3857.1896973
720.0000000	8.2959995	-1367.2091064
720.0000000	8.3448000	-1089.1281738
720.0000000	8.3935995	-998.1494751
720.0000000	8.4423990	-429.8473816
720.0000000	8.4912004	-1003.7089233
720.0000000	8.5400000	-2707.8110352
720.0000000	8.5887995	-1312.3930664
720.0000000	8.6375999	256.5887756
720.0000000	8.6863995	2316.5405273

720.0000000	8.7351999	-860.7982178
720.0000000	8.7840004	984.0999146
720.0000000	8.8327999	53.8340759
720.0000000	8.8815994	-11.4912834
720.0000000	8.9303989	-24.0552998
720.0000000	8.9792004	-11.5766640
720.0000000	9.0279999	-5.7843986
720.0000000	9.0767994	7.2093220
720.0000000	9.1255999	-17.2930813
720.0000000	9.1743994	-10.0615978
720.0000000	9.2231998	37.4813614
720.0000000	9.2720003	-8.3921118
720.0000000	9.3207998	-9.7819595
720.0000000	9.3695993	-10.7569885
720.0000000	9.4183998	-11.7576132
720.0000000	9.4672003	-11.1600952
720.0000000	9.5159998	-14.1909389
720.0000000	9.5648003	-0.2918968
720.0000000	9.6135998	-13.4197302
720.0000000	9.6623993	-10.2130823
720.0000000	9.7111998	-10.1495590
720.0000000	9.7600002	-10.8563242
720.0000000	9.8087997	-10.9945393
720.0000000	9.8575993	3.3225746
720.0000000	9.9063997	-7.9416475
720.0000000	9.9552002	-10.9064722
720.0000000	10.0039997	-10.0672932
730.0000000	0.3416000	-36.3787842
730.0000000	0.3904000	-39.3888168
730.0000000	0.4392000	-39.9031143
730.0000000	0.4880000	-39.0384483
730.0000000	0.5368000	-39.8267937
730.0000000	0.5856000	-39.1721230
730.0000000	0.6344000	-38.2164459
730.0000000	0.6832000	-36.1013527
730.0000000	0.7320000	-35.9421043
730.0000000	0.7808000	-36.5254974
730.0000000	0.8296000	-35.9533043
730.0000000	0.8784000	-34.9820595
730.0000000	0.9272000	-32.7880898
730.0000000	0.9760000	-30.8225746
730.0000000	1.0247999	-29.4320431
730.0000000	1.0735999	-28.9554176
730.0000000	1.1224000	-29.2523193
730.0000000	1.1711999	-29.9968414
730.0000000	1.2200000	-30.6420860
730.0000000	1.2687999	-30.8696613
730.0000000	1.3176000	-31.0347748
730.0000000	1.3664000	-30.3461628
730.0000000	1.4152000	-30.1563873
730.0000000	1.4640000	-29.6648941
730.0000000	1.5128000	-42.1933746
730.0000000	1.5616000	-34.8436699
730.0000000	1.6104001	-1630.9581299
730.0000000	1.6592000	-81.7822266
730.0000000	1.7079999	-27.1488686
730.0000000	1.7567999	-27.7615891
730.0000000	1.8055999	-26.3605785

730.0000000	1.8544000	-25.6445084
730.0000000	1.9031999	-25.7590065
730.0000000	1.9520000	-26.1488094
730.0000000	2.0007999	-26.4143372
730.0000000	2.0495999	-23.9921436
730.0000000	2.0983999	-190.7717590
730.0000000	2.1471999	6.0761185
730.0000000	2.1960001	-25.5671463
730.0000000	2.2448001	-26.3471870
730.0000000	2.2935998	-29.4095173
730.0000000	2.3423998	-20.5721169
730.0000000	2.3912001	49.9053650
730.0000000	2.4400001	-22.7653561
730.0000000	2.4888000	-23.4663906
730.0000000	2.5375998	-11.7702818
730.0000000	2.5863998	-9.8136768
730.0000000	2.6352000	-7.7983589
730.0000000	2.6840000	-15.0602121
730.0000000	2.7328000	141.0199585
730.0000000	2.7815998	166.1769104
730.0000000	2.8304000	-23.8329525
730.0000000	2.8792000	-20.5227852
730.0000000	2.9280000	-22.5886269
730.0000000	2.9768000	-16.5415764
730.0000000	3.0256000	-20.3963242
730.0000000	3.0743999	-29.8540630
730.0000000	3.1231999	-24.3574562
730.0000000	3.1719999	-29.8514099
730.0000000	3.2208002	-511.2749939
730.0000000	3.2695999	-1001.2937012
730.0000000	3.3183999	-20.4838562
730.0000000	3.3671999	-3439.0480957
730.0000000	3.4159999	1234.0706787
730.0000000	3.4648001	-6.9749794
730.0000000	3.5135999	-27.1238670
730.0000000	3.5623999	-325.7068176
730.0000000	3.6111999	-2047.7442627
730.0000000	3.6600001	-463.4238892
730.0000000	3.7088001	-612.0009766
730.0000000	3.7575998	-958.3153076
730.0000000	3.8063998	-1739.1417236
730.0000000	3.8552001	-2525.0805664
730.0000000	3.9040000	-2475.6652832
730.0000000	3.9528000	1132.4077148
730.0000000	4.0015998	-247.1799622
730.0000000	4.0503998	693.3886719
730.0000000	4.0991998	88.4915771
730.0000000	4.1479998	-659.9523926
730.0000000	4.1967998	2.7226772
730.0000000	4.2456002	-250.3836670
730.0000000	4.2943997	-491.8218384
730.0000000	4.3431997	-13.2898216
730.0000000	4.3920002	-250.0105438
730.0000000	4.4407997	-22.4943962
730.0000000	4.4896002	-15.4574261
730.0000000	4.5383997	-660.0352783
730.0000000	4.5871997	-12.8003292
730.0000000	4.6360002	-21.6503696

730.0000000	4.6847997	-28.3568344
730.0000000	4.7336001	-19.5761242
730.0000000	4.7824001	-16.5037498
730.0000000	4.8311996	-1484.3867188
730.0000000	4.8800001	229.9442291
730.0000000	4.9287996	-202.7194977
730.0000000	4.9776001	1066.9659424
730.0000000	5.0264001	86.8816681
730.0000000	5.0751996	-303.0890503
730.0000000	5.1240001	-12.2910137
730.0000000	5.1727996	560.6151733
730.0000000	5.2216001	32.2228661
730.0000000	5.2704000	-159.4910278
730.0000000	5.3191996	-39.2919083
730.0000000	5.3680000	-3552.5439453
730.0000000	5.4168000	529.7055054
730.0000000	5.4656000	-201.6131134
730.0000000	5.5144000	38.3246536
730.0000000	5.5631995	490.7094116
730.0000000	5.6120000	395.0765381
730.0000000	5.6608000	-37.1837349
730.0000000	5.7096000	62.9041138
730.0000000	5.7584000	133.7413025
730.0000000	5.8071995	-11.4991779
730.0000000	5.8559999	30.0323162
730.0000000	5.9047999	-16.6104412
730.0000000	5.9535999	-39.8918533
730.0000000	6.0023999	-1580.4227295
730.0000000	6.0511999	-14.5630054
730.0000000	6.0999999	-19.4298191
730.0000000	6.1487999	-13.2618961
730.0000000	6.1975999	-23.2639732
730.0000000	6.2463999	-1800.4785156
730.0000000	6.2951999	-19.0905781
730.0000000	6.3439999	-344.3094177
730.0000000	6.3927999	-15.0485821
730.0000000	6.4416003	-12.3711729
730.0000000	6.4903998	-11.2465687
730.0000000	6.5391998	-11.7888794
730.0000000	6.5879998	402.7387695
730.0000000	6.6367998	-12.0700283
730.0000000	6.6856003	-13.8105116
730.0000000	6.7343998	-13.4500408
730.0000000	6.7831998	-14.3834562
730.0000000	6.8319998	58.9432602
730.0000000	6.8807998	-14.0530891
730.0000000	6.9296002	-469.7897034
730.0000000	6.9783998	-4.7411242
730.0000000	7.0271997	-14.1650133
730.0000000	7.0760002	12.3302202
730.0000000	7.1247997	-2239.1831055
730.0000000	7.1736002	-1795.6241455
730.0000000	7.2223997	-1021.3688354
730.0000000	7.2711997	-219.7097015
730.0000000	7.3200002	-19.2125931
730.0000000	7.3687997	198.2346649
730.0000000	7.4176002	412.8143921
730.0000000	7.4663997	324.4225769

730.0000000	7.5151997	-999.4268188
730.0000000	7.5640001	-1104.0096436
730.0000000	7.6127996	-2602.9938965
730.0000000	7.6616001	-835.3863525
730.0000000	7.7104001	-428.8222046
730.0000000	7.7591996	46.0462799
730.0000000	7.8080001	428.9971008
730.0000000	7.8567996	564.9421997
730.0000000	7.9056001	-1169.2183838
730.0000000	7.9544001	1861.2565918
730.0000000	8.0031996	2401.6110840
730.0000000	8.0520000	2397.0764160
730.0000000	8.1007996	2536.5671387
730.0000000	8.1496000	2705.1384277
730.0000000	8.1983995	1683.0056152
730.0000000	8.2472000	-3850.3549805
730.0000000	8.2959995	-1364.7889404
730.0000000	8.3448000	-1087.1989746
730.0000000	8.3935995	-996.3817749
730.0000000	8.4423990	-429.0893555
730.0000000	8.4912004	-1001.9326782
730.0000000	8.5400000	-2703.0112305
730.0000000	8.5887995	-1310.0715332
730.0000000	8.6375999	256.1274414
730.0000000	8.6863995	2312.4221191
730.0000000	8.7351999	-859.2739868
730.0000000	8.7840004	982.3432007
730.0000000	8.8327999	53.7224655
730.0000000	8.8815994	-11.4914579
730.0000000	8.9303989	-24.0515785
730.0000000	8.9792004	-11.5767460
730.0000000	9.0279999	-5.7861667
730.0000000	9.0767994	7.2036037
730.0000000	9.1255999	-17.2913685
730.0000000	9.1743994	-10.0620003
730.0000000	9.2231998	37.4656296
730.0000000	9.2720003	-8.3930483
730.0000000	9.3207998	-9.7824993
730.0000000	9.3695993	-10.7572145
730.0000000	9.4183998	-11.7574892
730.0000000	9.4672003	-11.1601372
730.0000000	9.5159998	-14.1900482
730.0000000	9.5648003	-0.2952700
730.0000000	9.6135998	-13.4189396
730.0000000	9.6623993	-10.2133570
730.0000000	9.7111998	-10.1498299
730.0000000	9.7600002	-10.8563662
730.0000000	9.8087997	-10.9945478
730.0000000	9.8575993	3.3181524
730.0000000	9.9063997	-7.9425611
730.0000000	9.9552002	-10.9064770
730.0000000	10.0039997	-10.0675402
740.0000000	0.3416000	-36.5479088
740.0000000	0.3904000	-39.0932732
740.0000000	0.4392000	-39.9012718
740.0000000	0.4880000	-38.6388054
740.0000000	0.5368000	-39.9518127
740.0000000	0.5856000	-39.2108574

740.0000000	0.6344000	-38.2167549
740.0000000	0.6832000	-36.1035767
740.0000000	0.7320000	-35.9441566
740.0000000	0.7808000	-36.5256691
740.0000000	0.8296000	-35.9540329
740.0000000	0.8784000	-34.9894333
740.0000000	0.9272000	-32.7908669
740.0000000	0.9760000	-30.8151207
740.0000000	1.0247999	-29.4410324
740.0000000	1.0735999	-28.9698524
740.0000000	1.1224000	-29.2493210
740.0000000	1.1711999	-29.9894638
740.0000000	1.2200000	-30.6500015
740.0000000	1.2687999	-30.8706684
740.0000000	1.3176000	-31.0305023
740.0000000	1.3664000	-30.3539124
740.0000000	1.4152000	-30.1565914
740.0000000	1.4640000	-29.6631851
740.0000000	1.5128000	-42.0961990
740.0000000	1.5616000	-34.8018303
740.0000000	1.6104001	-1628.4154053
740.0000000	1.6592000	-81.4882889
740.0000000	1.7079999	-27.1526165
740.0000000	1.7567999	-27.7616081
740.0000000	1.8055999	-26.4665260
740.0000000	1.8544000	-25.7096443
740.0000000	1.9031999	-26.3456669
740.0000000	1.9520000	-26.4309120
740.0000000	2.0007999	-26.3517551
740.0000000	2.0495999	-24.1787605
740.0000000	2.0983999	-189.2959595
740.0000000	2.1471999	5.8088512
740.0000000	2.1960001	-25.5630550
740.0000000	2.2448001	-26.3292770
740.0000000	2.2935998	-29.3498936
740.0000000	2.3423998	-20.5865211
740.0000000	2.3912001	49.2838211
740.0000000	2.4400001	-22.7678432
740.0000000	2.4888000	-23.4661732
740.0000000	2.5375998	-11.7873898
740.0000000	2.5863998	-9.8334360
740.0000000	2.6352000	-7.8211031
740.0000000	2.6840000	-15.0721531
740.0000000	2.7328000	140.7539368
740.0000000	2.7815998	165.8704834
740.0000000	2.8304000	-23.8316479
740.0000000	2.8792000	-20.5260487
740.0000000	2.9280000	-22.5880585
740.0000000	2.9768000	-16.5499458
740.0000000	3.0256000	-20.3989086
740.0000000	3.0743999	-29.8426762
740.0000000	3.1231999	-24.3540001
740.0000000	3.1719999	-29.8431168
740.0000000	3.2208002	-511.0372620
740.0000000	3.2695999	-1000.8187256
740.0000000	3.3183999	-20.4840546
740.0000000	3.3671999	-3437.5864258
740.0000000	3.4159999	1233.5354004

740.0000000	3.4648001	-6.9806871
740.0000000	3.5135999	-27.1208763
740.0000000	3.5623999	-325.5772400
740.0000000	3.6111999	-2046.8845215
740.0000000	3.6600001	-463.2357788
740.0000000	3.7088001	-611.7496338
740.0000000	3.7575998	-957.9168091
740.0000000	3.8063998	-1738.4118652
740.0000000	3.8552001	-2524.0170898
740.0000000	3.9040000	-2474.6228027
740.0000000	3.9528000	1131.9194336
740.0000000	4.0015998	-247.0828400
740.0000000	4.0503998	693.0861206
740.0000000	4.0991998	88.4458923
740.0000000	4.1479998	-659.6801147
740.0000000	4.1967998	2.7134209
740.0000000	4.2456002	-250.2853699
740.0000000	4.2943997	-491.6212769
740.0000000	4.3431997	-13.2920017
740.0000000	4.3920002	-249.9124298
740.0000000	4.4407997	-22.4926224
740.0000000	4.4896002	-15.4583082
740.0000000	4.5383997	-659.7742310
740.0000000	4.5871997	-12.8023453
740.0000000	4.6360002	-21.6488266
740.0000000	4.6847997	-28.3529091
740.0000000	4.7336001	-19.5754013
740.0000000	4.7824001	-16.5042038
740.0000000	4.8311996	-1483.8554688
740.0000000	4.8800001	229.8546753
740.0000000	4.9287996	-202.6522369
740.0000000	4.9776001	1066.5733643
740.0000000	5.0264001	86.8441467
740.0000000	5.0751996	-302.9854431
740.0000000	5.1240001	-12.2919350
740.0000000	5.1727996	560.4993286
740.0000000	5.2216001	32.2134209
740.0000000	5.2704000	-159.4629211
740.0000000	5.3191996	-39.2873573
740.0000000	5.3680000	-3551.8515625
740.0000000	5.4168000	529.5987549
740.0000000	5.4656000	-201.5769348
740.0000000	5.5144000	38.3140030
740.0000000	5.5631995	490.6108398
740.0000000	5.6120000	394.9965515
740.0000000	5.6608000	-37.1796494
740.0000000	5.7096000	62.8886795
740.0000000	5.7584000	133.7123718
740.0000000	5.8071995	-11.5000477
740.0000000	5.8559999	30.0234623
740.0000000	5.9047999	-16.6102448
740.0000000	5.9535999	-39.8871155
740.0000000	6.0023999	-1580.1184082
740.0000000	6.0511999	-14.5632172
740.0000000	6.0999999	-19.4289856
740.0000000	6.1487999	-13.2623596
740.0000000	6.1975999	-23.2622070
740.0000000	6.2463999	-1800.1311035

740.0000000	6.2951999	-19.0897636
740.0000000	6.3439999	-344.2453613
740.0000000	6.3927999	-15.0485640
740.0000000	6.4416003	-12.3717604
740.0000000	6.4903998	-11.2472448
740.0000000	6.5391998	-11.7895603
740.0000000	6.5879998	402.0480652
740.0000000	6.6367998	-12.0705776
740.0000000	6.6856003	-13.8106813
740.0000000	6.7343998	-13.4502573
740.0000000	6.7831998	-14.3830404
740.0000000	6.8319998	58.9241486
740.0000000	6.8807998	-14.0529509
740.0000000	6.9296002	-469.0282593
740.0000000	6.9783998	-4.7539482
740.0000000	7.0271997	-14.1639853
740.0000000	7.0760002	12.2880354
740.0000000	7.1247997	-2235.2373047
740.0000000	7.1736002	-1792.4613037
740.0000000	7.2223997	-1019.5756836
740.0000000	7.2711997	-219.3419189
740.0000000	7.3200002	-19.2006054
740.0000000	7.3687997	197.8601379
740.0000000	7.4176002	412.0585632
740.0000000	7.4663997	323.8249207
740.0000000	7.5151997	-997.6657715
740.0000000	7.5640001	-1102.0622559
740.0000000	7.6127996	-2598.3762207
740.0000000	7.6616001	-833.9149780
740.0000000	7.7104001	-428.0759277
740.0000000	7.7591996	45.9452324
740.0000000	7.8080001	428.2117310
740.0000000	7.8567996	563.9141235
740.0000000	7.9056001	-1167.1453857
740.0000000	7.9544001	1857.9466553
740.0000000	8.0031996	2397.3413086
740.0000000	8.0520000	2392.8144531
740.0000000	8.1007996	2532.0576172
740.0000000	8.1496000	2700.3303223
740.0000000	8.1983995	1680.0131836
740.0000000	8.2472000	-3843.5200195
740.0000000	8.2959995	-1362.3687744
740.0000000	8.3448000	-1085.2697754
740.0000000	8.3935995	-994.6141357
740.0000000	8.4423990	-428.3313599
740.0000000	8.4912004	-1000.1564331
740.0000000	8.5400000	-2698.2114258
740.0000000	8.5887995	-1307.7500000
740.0000000	8.6375999	255.6661224
740.0000000	8.6863995	2308.3037109
740.0000000	8.7351999	-857.7497559
740.0000000	8.7840004	980.5864258
740.0000000	8.8327999	53.6108589
740.0000000	8.8815994	-11.4916325
740.0000000	8.9303989	-24.0478592
740.0000000	8.9792004	-11.5768290
740.0000000	9.0279999	-5.7879353
740.0000000	9.0767994	7.1978855

740.0000000	9.1255999	-17.2896538
740.0000000	9.1743994	-10.0624027
740.0000000	9.2231998	37.4498978
740.0000000	9.2720003	-8.3939848
740.0000000	9.3207998	-9.7830381
740.0000000	9.3695993	-10.7574415
740.0000000	9.4183998	-11.7573643
740.0000000	9.4672003	-11.1601782
740.0000000	9.5159998	-14.1891575
740.0000000	9.5648003	-0.2986431
740.0000000	9.6135998	-13.4181490
740.0000000	9.6623993	-10.2136326
740.0000000	9.7111998	-10.1501017
740.0000000	9.7600002	-10.8564081
740.0000000	9.8087997	-10.9945555
740.0000000	9.8575993	3.3137302
740.0000000	9.9063997	-7.9434757
740.0000000	9.9552002	-10.9064808
740.0000000	10.0039997	-10.0677881
750.0000000	0.3416000	-35.9389877
750.0000000	0.3904000	-39.0488739
750.0000000	0.4392000	-39.8994331
750.0000000	0.4880000	-39.0287819
750.0000000	0.5368000	-39.7306290
750.0000000	0.5856000	-39.2495956
750.0000000	0.6344000	-38.2170677
750.0000000	0.6832000	-36.1058044
750.0000000	0.7320000	-35.9462090
750.0000000	0.7808000	-36.5258369
750.0000000	0.8296000	-35.9547615
750.0000000	0.8784000	-34.9968109
750.0000000	0.9272000	-32.7936440
750.0000000	0.9760000	-30.8076687
750.0000000	1.0247999	-29.4500217
750.0000000	1.0735999	-28.9842873
750.0000000	1.1224000	-29.2463207
750.0000000	1.1711999	-29.9820862
750.0000000	1.2200000	-30.6579170
750.0000000	1.2687999	-30.8716774
750.0000000	1.3176000	-31.0262318
750.0000000	1.3664000	-30.3616619
750.0000000	1.4152000	-30.1567955
750.0000000	1.4640000	-29.6614742
750.0000000	1.5128000	-41.9990196
750.0000000	1.5616000	-34.7599869
750.0000000	1.6104001	-1625.8725586
750.0000000	1.6592000	-81.1943588
750.0000000	1.7079999	-27.1563625
750.0000000	1.7567999	-27.7616272
750.0000000	1.8055999	-26.1169243
750.0000000	1.8544000	-24.8140812
750.0000000	1.9031999	-24.5459766
750.0000000	1.9520000	-25.3804245
750.0000000	2.0007999	-26.2891731
750.0000000	2.0495999	-24.3653755
750.0000000	2.0983999	-187.8201904
750.0000000	2.1471999	5.5415840
750.0000000	2.1960001	-25.5589657

750.0000000	2.2448001	-26.3113651
750.0000000	2.2935998	-29.2902699
750.0000000	2.3423998	-20.6009254
750.0000000	2.3912001	48.6622696
750.0000000	2.4400001	-22.7703304
750.0000000	2.4888000	-23.4659557
750.0000000	2.5375998	-11.8044977
750.0000000	2.5863998	-9.8531942
750.0000000	2.6352000	-7.8438473
750.0000000	2.6840000	-15.0840950
750.0000000	2.7328000	140.4879303
750.0000000	2.7815998	165.5640564
750.0000000	2.8304000	-23.8303432
750.0000000	2.8792000	-20.5293121
750.0000000	2.9280000	-22.5874882
750.0000000	2.9768000	-16.5583153
750.0000000	3.0256000	-20.4014931
750.0000000	3.0743999	-29.8312893
750.0000000	3.1231999	-24.3505440
750.0000000	3.1719999	-29.8348236
750.0000000	3.2208002	-510.7995300
750.0000000	3.2695999	-1000.3437500
750.0000000	3.3183999	-20.4842510
750.0000000	3.3671999	-3436.1247559
750.0000000	3.4159999	1233.0001221
750.0000000	3.4648001	-6.9863949
750.0000000	3.5135999	-27.1178856
750.0000000	3.5623999	-325.4476318
750.0000000	3.6111999	-2046.0247803
750.0000000	3.6600001	-463.0476074
750.0000000	3.7088001	-611.4982300
750.0000000	3.7575998	-957.5182495
750.0000000	3.8063998	-1737.6820068
750.0000000	3.8552001	-2522.9536133
750.0000000	3.9040000	-2473.5803223
750.0000000	3.9528000	1131.4311523
750.0000000	4.0015998	-246.9857330
750.0000000	4.0503998	692.7835693
750.0000000	4.0991998	88.4002075
750.0000000	4.1479998	-659.4078979
750.0000000	4.1967998	2.7041645
750.0000000	4.2456002	-250.1870728
750.0000000	4.2943997	-491.4206848
750.0000000	4.3431997	-13.2941818
750.0000000	4.3920002	-249.8143158
750.0000000	4.4407997	-22.4908466
750.0000000	4.4896002	-15.4591904
750.0000000	4.5383997	-659.5131836
750.0000000	4.5871997	-12.8043613
750.0000000	4.6360002	-21.6472836
750.0000000	4.6847997	-28.3489838
750.0000000	4.7336001	-19.5746784
750.0000000	4.7824001	-16.5046597
750.0000000	4.8311996	-1483.3240967
750.0000000	4.8800001	229.7651215
750.0000000	4.9287996	-202.5849762
750.0000000	4.9776001	1066.1809082
750.0000000	5.0264001	86.8066254

750.0000000	5.0751996	-302.8818054
750.0000000	5.1240001	-12.2928562
750.0000000	5.1727996	560.3834229
750.0000000	5.2216001	32.2039795
750.0000000	5.2704000	-159.4348145
750.0000000	5.3191996	-39.2828064
750.0000000	5.3680000	-3551.1591797
750.0000000	5.4168000	529.4919434
750.0000000	5.4656000	-201.5407410
750.0000000	5.5144000	38.3033447
750.0000000	5.5631995	490.5122681
750.0000000	5.6120000	394.9165955
750.0000000	5.6608000	-37.1755600
750.0000000	5.7096000	62.8732529
750.0000000	5.7584000	133.6834412
750.0000000	5.8071995	-11.5009193
750.0000000	5.8559999	30.0146122
750.0000000	5.9047999	-16.6100464
750.0000000	5.9535999	-39.8823738
750.0000000	6.0023999	-1579.8139648
750.0000000	6.0511999	-14.5634289
750.0000000	6.0999999	-19.4281540
750.0000000	6.1487999	-13.2628222
750.0000000	6.1975999	-23.2604408
750.0000000	6.2463999	-1799.7836914
750.0000000	6.2951999	-19.0889492
750.0000000	6.3439999	-344.1813354
750.0000000	6.3927999	-15.0485458
750.0000000	6.4416003	-12.3723469
750.0000000	6.4903998	-11.2479210
750.0000000	6.5391998	-11.7902422
750.0000000	6.5879998	401.3573608
750.0000000	6.6367998	-12.0711269
750.0000000	6.6856003	-13.8108521
750.0000000	6.7343998	-13.4504728
750.0000000	6.7831998	-14.3826246
750.0000000	6.8319998	58.9050369
750.0000000	6.8807998	-14.0528116
750.0000000	6.9296002	-468.2668152
750.0000000	6.9783998	-4.7667718
750.0000000	7.0271997	-14.1629572
750.0000000	7.0760002	12.2458506
750.0000000	7.1247997	-2231.2912598
750.0000000	7.1736002	-1789.2983398
750.0000000	7.2223997	-1017.7825317
750.0000000	7.2711997	-218.9741364
750.0000000	7.3200002	-19.1886177
750.0000000	7.3687997	197.4856110
750.0000000	7.4176002	411.3027344
750.0000000	7.4663997	323.2272949
750.0000000	7.5151997	-995.9047852
750.0000000	7.5640001	-1100.1148682
750.0000000	7.6127996	-2593.7587891
750.0000000	7.6616001	-832.4436035
750.0000000	7.7104001	-427.3297119
750.0000000	7.7591996	45.8441811
750.0000000	7.8080001	427.4263611
750.0000000	7.8567996	562.8860474

750.0000000	7.9056001	-1165.0725098
750.0000000	7.9544001	1854.6367188
750.0000000	8.0031996	2393.0717773
750.0000000	8.0520000	2388.5524902
750.0000000	8.1007996	2527.5480957
750.0000000	8.1496000	2695.5224609
750.0000000	8.1983995	1677.0206299
750.0000000	8.2472000	-3836.6853027
750.0000000	8.2959995	-1359.9486084
750.0000000	8.3448000	-1083.3405762
750.0000000	8.3935995	-992.8464355
750.0000000	8.4423990	-427.5733337
750.0000000	8.4912004	-998.3802490
750.0000000	8.5400000	-2693.4116211
750.0000000	8.5887995	-1305.4284668
750.0000000	8.6375999	255.2047882
750.0000000	8.6863995	2304.1853027
750.0000000	8.7351999	-856.2254639
750.0000000	8.7840004	978.8297119
750.0000000	8.8327999	53.4992561
750.0000000	8.8815994	-11.4918060
750.0000000	8.9303989	-24.0441399
750.0000000	8.9792004	-11.5769110
750.0000000	9.0279999	-5.7897029
750.0000000	9.0767994	7.1921673
750.0000000	9.1255999	-17.2879410
750.0000000	9.1743994	-10.0628042
750.0000000	9.2231998	37.4341660
750.0000000	9.2720003	-8.3949203
750.0000000	9.3207998	-9.7835779
750.0000000	9.3695993	-10.7576675
750.0000000	9.4183998	-11.7572393
750.0000000	9.4672003	-11.1602192
750.0000000	9.5159998	-14.1882668
750.0000000	9.5648003	-0.3020153
750.0000000	9.6135998	-13.4173584
750.0000000	9.6623993	-10.2139072
750.0000000	9.7111998	-10.1503735
750.0000000	9.7600002	-10.8564501
750.0000000	9.8087997	-10.9945631
750.0000000	9.8575993	3.3093071
750.0000000	9.9063997	-7.9443893
750.0000000	9.9552002	-10.9064856
750.0000000	10.0039997	-10.0680351
760.0000000	0.3416000	-36.4733849
760.0000000	0.3904000	-39.4396095
760.0000000	0.4392000	-39.8975945
760.0000000	0.4880000	-38.5297356
760.0000000	0.5368000	-38.9406281
760.0000000	0.5856000	-39.2883339
760.0000000	0.6344000	-38.2173767
760.0000000	0.6832000	-36.1080284
760.0000000	0.7320000	-35.9482613
760.0000000	0.7808000	-36.5260048
760.0000000	0.8296000	-35.9554901
760.0000000	0.8784000	-35.0041847
760.0000000	0.9272000	-32.7964211
760.0000000	0.9760000	-30.8002167

760.0000000	1.0247999	-29.4590111
760.0000000	1.0735999	-28.9987221
760.0000000	1.1224000	-29.2433224
760.0000000	1.1711999	-29.9747105
760.0000000	1.2200000	-30.6658306
760.0000000	1.2687999	-30.8726845
760.0000000	1.3176000	-31.0219593
760.0000000	1.3664000	-30.3694115
760.0000000	1.4152000	-30.1569996
760.0000000	1.4640000	-29.6597652
760.0000000	1.5128000	-41.9018402
760.0000000	1.5616000	-34.7181473
760.0000000	1.6104001	-1623.3297119
760.0000000	1.6592000	-80.9004288
760.0000000	1.7079999	-27.1601105
760.0000000	1.7567999	-27.7616463
760.0000000	1.8055999	-27.1809177
760.0000000	1.8544000	-25.5289383
760.0000000	1.9031999	-25.4182777
760.0000000	1.9520000	-26.4631348
760.0000000	2.0007999	-26.2265911
760.0000000	2.0495999	-24.5519924
760.0000000	2.0983999	-186.3444214
760.0000000	2.1471999	5.2743168
760.0000000	2.1960001	-25.5548744
760.0000000	2.2448001	-26.2934532
760.0000000	2.2935998	-29.2306461
760.0000000	2.3423998	-20.6153297
760.0000000	2.3912001	48.0407257
760.0000000	2.4400001	-22.7728157
760.0000000	2.4888000	-23.4657383
760.0000000	2.5375998	-11.8216047
760.0000000	2.5863998	-9.8729534
760.0000000	2.6352000	-7.8665915
760.0000000	2.6840000	-15.0960369
760.0000000	2.7328000	140.2219086
760.0000000	2.7815998	165.2576599
760.0000000	2.8304000	-23.8290405
760.0000000	2.8792000	-20.5325756
760.0000000	2.9280000	-22.5869198
760.0000000	2.9768000	-16.5666866
760.0000000	3.0256000	-20.4040775
760.0000000	3.0743999	-29.8199005
760.0000000	3.1231999	-24.3470898
760.0000000	3.1719999	-29.8265305
760.0000000	3.2208002	-510.5617676
760.0000000	3.2695999	-999.8687744
760.0000000	3.3183999	-20.4844494
760.0000000	3.3671999	-3434.6630859
760.0000000	3.4159999	1232.4648438
760.0000000	3.4648001	-6.9921036
760.0000000	3.5135999	-27.1148949
760.0000000	3.5623999	-325.3180542
760.0000000	3.6111999	-2045.1650391
760.0000000	3.6600001	-462.8594971
760.0000000	3.7088001	-611.2468872
760.0000000	3.7575998	-957.1196899
760.0000000	3.8063998	-1736.9521484

760.0000000	3.8552001	-2521.8903809
760.0000000	3.9040000	-2472.5378418
760.0000000	3.9528000	1130.9428711
760.0000000	4.0015998	-246.8886261
760.0000000	4.0503998	692.4810181
760.0000000	4.0991998	88.3545227
760.0000000	4.1479998	-659.1356812
760.0000000	4.1967998	2.6949081
760.0000000	4.2456002	-250.0887604
760.0000000	4.2943997	-491.2201233
760.0000000	4.3431997	-13.2963629
760.0000000	4.3920002	-249.7162018
760.0000000	4.4407997	-22.4890709
760.0000000	4.4896002	-15.4600735
760.0000000	4.5383997	-659.2520752
760.0000000	4.5871997	-12.8063784
760.0000000	4.6360002	-21.6457424
760.0000000	4.6847997	-28.3450584
760.0000000	4.7336001	-19.5739574
760.0000000	4.7824001	-16.5051136
760.0000000	4.8311996	-1482.7928467
760.0000000	4.8800001	229.6755676
760.0000000	4.9287996	-202.5177155
760.0000000	4.9776001	1065.7884521
760.0000000	5.0264001	86.7691040
760.0000000	5.0751996	-302.7781677
760.0000000	5.1240001	-12.2937775
760.0000000	5.1727996	560.2675781
760.0000000	5.2216001	32.1945343
760.0000000	5.2704000	-159.4067078
760.0000000	5.3191996	-39.2782593
760.0000000	5.3680000	-3550.4667969
760.0000000	5.4168000	529.3851929
760.0000000	5.4656000	-201.5045624
760.0000000	5.5144000	38.2926865
760.0000000	5.5631995	490.4136963
760.0000000	5.6120000	394.8366089
760.0000000	5.6608000	-37.1714706
760.0000000	5.7096000	62.8578262
760.0000000	5.7584000	133.6545105
760.0000000	5.8071995	-11.5017891
760.0000000	5.8559999	30.0057583
760.0000000	5.9047999	-16.6098480
760.0000000	5.9535999	-39.8776360
760.0000000	6.0023999	-1579.5096436
760.0000000	6.0511999	-14.5636396
760.0000000	6.0999999	-19.4273205
760.0000000	6.1487999	-13.2632847
760.0000000	6.1975999	-23.2586765
760.0000000	6.2463999	-1799.4364014
760.0000000	6.2951999	-19.0881348
760.0000000	6.3439999	-344.1172485
760.0000000	6.3927999	-15.0485268
760.0000000	6.4416003	-12.3729343
760.0000000	6.4903998	-11.2485971
760.0000000	6.5391998	-11.7909231
760.0000000	6.5879998	400.6666565
760.0000000	6.6367998	-12.0716763

760.0000000	6.6856003	-13.8110218
760.0000000	6.7343998	-13.4506884
760.0000000	6.7831998	-14.3822088
760.0000000	6.8319998	58.8859177
760.0000000	6.8807998	-14.0526733
760.0000000	6.9296002	-467.5053711
760.0000000	6.9783998	-4.7795949
760.0000000	7.0271997	-14.1619301
760.0000000	7.0760002	12.2036638
760.0000000	7.1247997	-2227.3454590
760.0000000	7.1736002	-1786.1354980
760.0000000	7.2223997	-1015.9893799
760.0000000	7.2711997	-218.6063538
760.0000000	7.3200002	-19.1766281
760.0000000	7.3687997	197.1110840
760.0000000	7.4176002	410.5469055
760.0000000	7.4663997	322.6296387
760.0000000	7.5151997	-994.1437378
760.0000000	7.5640001	-1098.1674805
760.0000000	7.6127996	-2589.1411133
760.0000000	7.6616001	-830.9723511
760.0000000	7.7104001	-426.5834351
760.0000000	7.7591996	45.7431335
760.0000000	7.8080001	426.6410217
760.0000000	7.8567996	561.8579102
760.0000000	7.9056001	-1162.9996338
760.0000000	7.9544001	1851.3267822
760.0000000	8.0031996	2388.8022461
760.0000000	8.0520000	2384.2905273
760.0000000	8.1007996	2523.0385742
760.0000000	8.1496000	2690.7143555
760.0000000	8.1983995	1674.0280762
760.0000000	8.2472000	-3829.8505859
760.0000000	8.2959995	-1357.5284424
760.0000000	8.3448000	-1081.4114990
760.0000000	8.3935995	-991.0787964
760.0000000	8.4423990	-426.8153381
760.0000000	8.4912004	-996.6040039
760.0000000	8.5400000	-2688.6115723
760.0000000	8.5887995	-1303.1069336
760.0000000	8.6375999	254.7434540
760.0000000	8.6863995	2300.0668945
760.0000000	8.7351999	-854.7012329
760.0000000	8.7840004	977.0729370
760.0000000	8.8327999	53.3876457
760.0000000	8.8815994	-11.4919806
760.0000000	8.9303989	-24.0404205
760.0000000	8.9792004	-11.5769930
760.0000000	9.0279999	-5.7914715
760.0000000	9.0767994	7.1864510
760.0000000	9.1255999	-17.2862263
760.0000000	9.1743994	-10.0632067
760.0000000	9.2231998	37.4184341
760.0000000	9.2720003	-8.3958569
760.0000000	9.3207998	-9.7841167
760.0000000	9.3695993	-10.7578945
760.0000000	9.4183998	-11.7571154
760.0000000	9.4672003	-11.1602602

760.0000000	9.5159998	-14.1873751
760.0000000	9.5648003	-0.3053875
760.0000000	9.6135998	-13.4165678
760.0000000	9.6623993	-10.2141829
760.0000000	9.7111998	-10.1506453
760.0000000	9.7600002	-10.8564911
760.0000000	9.8087997	-10.9945717
760.0000000	9.8575993	3.3048849
760.0000000	9.9063997	-7.9453034
760.0000000	9.9552002	-10.9064894
760.0000000	10.0039997	-10.0682831
770.0000000	0.3416000	-36.1719666
770.0000000	0.3904000	-38.9459229
770.0000000	0.4392000	-39.8957520
770.0000000	0.4880000	-39.3889122
770.0000000	0.5368000	-39.7149429
770.0000000	0.5856000	-39.3270683
770.0000000	0.6344000	-38.2176857
770.0000000	0.6832000	-36.1102562
770.0000000	0.7320000	-35.9503136
770.0000000	0.7808000	-36.5261765
770.0000000	0.8296000	-35.9562187
770.0000000	0.8784000	-35.0115585
770.0000000	0.9272000	-32.7991982
770.0000000	0.9760000	-30.7927628
770.0000000	1.0247999	-29.4680004
770.0000000	1.0735999	-29.0131569
770.0000000	1.1224000	-29.2403221
770.0000000	1.1711999	-29.9673328
770.0000000	1.2200000	-30.6737461
770.0000000	1.2687999	-30.8736916
770.0000000	1.3176000	-31.0176888
770.0000000	1.3664000	-30.3771610
770.0000000	1.4152000	-30.1572037
770.0000000	1.4640000	-29.6580563
770.0000000	1.5128000	-41.8046646
770.0000000	1.5616000	-34.6763077
770.0000000	1.6104001	-1620.7869873
770.0000000	1.6592000	-80.6064987
770.0000000	1.7079999	-27.1638584
770.0000000	1.7567999	-27.7616653
770.0000000	1.8055999	-27.3695087
770.0000000	1.8544000	-25.9962921
770.0000000	1.9031999	-25.2798042
770.0000000	1.9520000	-25.9426727
770.0000000	2.0007999	-26.1640091
770.0000000	2.0495999	-24.7386093
770.0000000	2.0983999	-184.8686523
770.0000000	2.1471999	5.0070496
770.0000000	2.1960001	-25.5507832
770.0000000	2.2448001	-26.2755432
770.0000000	2.2935998	-29.1710224
770.0000000	2.3423998	-20.6297359
770.0000000	2.3912001	47.4191818
770.0000000	2.4400001	-22.7753029
770.0000000	2.4888000	-23.4655228
770.0000000	2.5375998	-11.8387136
770.0000000	2.5863998	-9.8927126

770.0000000	2.6352000	-7.8893356
770.0000000	2.6840000	-15.1079788
770.0000000	2.7328000	139.9558868
770.0000000	2.7815998	164.9512329
770.0000000	2.8304000	-23.8277359
770.0000000	2.8792000	-20.5358391
770.0000000	2.9280000	-22.5863495
770.0000000	2.9768000	-16.5750580
770.0000000	3.0256000	-20.4066620
770.0000000	3.0743999	-29.8085136
770.0000000	3.1231999	-24.3436337
770.0000000	3.1719999	-29.8182373
770.0000000	3.2208002	-510.3240356
770.0000000	3.2695999	-999.3937988
770.0000000	3.3183999	-20.4846478
770.0000000	3.3671999	-3433.2014160
770.0000000	3.4159999	1231.9295654
770.0000000	3.4648001	-6.9978113
770.0000000	3.5135999	-27.1119041
770.0000000	3.5623999	-325.1884460
770.0000000	3.6111999	-2044.3052979
770.0000000	3.6600001	-462.6713867
770.0000000	3.7088001	-610.9954834
770.0000000	3.7575998	-956.7211914
770.0000000	3.8063998	-1736.2222900
770.0000000	3.8552001	-2520.8269043
770.0000000	3.9040000	-2471.4953613
770.0000000	3.9528000	1130.4544678
770.0000000	4.0015998	-246.7915192
770.0000000	4.0503998	692.1784668
770.0000000	4.0991998	88.3088379
770.0000000	4.1479998	-658.8634644
770.0000000	4.1967998	2.6856518
770.0000000	4.2456002	-249.9904633
770.0000000	4.2943997	-491.0195618
770.0000000	4.3431997	-13.2985439
770.0000000	4.3920002	-249.6180878
770.0000000	4.4407997	-22.4872952
770.0000000	4.4896002	-15.4609566
770.0000000	4.5383997	-658.9910278
770.0000000	4.5871997	-12.8083944
770.0000000	4.6360002	-21.6441994
770.0000000	4.6847997	-28.3411331
770.0000000	4.7336001	-19.5732346
770.0000000	4.7824001	-16.5055676
770.0000000	4.8311996	-1482.2615967
770.0000000	4.8800001	229.5860291
770.0000000	4.9287996	-202.4504547
770.0000000	4.9776001	1065.3959961
770.0000000	5.0264001	86.7315826
770.0000000	5.0751996	-302.6745300
770.0000000	5.1240001	-12.2946987
770.0000000	5.1727996	560.1516724
770.0000000	5.2216001	32.1850891
770.0000000	5.2704000	-159.3786011
770.0000000	5.3191996	-39.2737122
770.0000000	5.3680000	-3549.7744141
770.0000000	5.4168000	529.2783813

770.0000000	5.4656000	-201.4683838
770.0000000	5.5144000	38.2820282
770.0000000	5.5631995	490.3151245
770.0000000	5.6120000	394.7566528
770.0000000	5.6608000	-37.1673813
770.0000000	5.7096000	62.8423920
770.0000000	5.7584000	133.6255798
770.0000000	5.8071995	-11.5026608
770.0000000	5.8559999	29.9969082
770.0000000	5.9047999	-16.6096516
770.0000000	5.9535999	-39.8728943
770.0000000	6.0023999	-1579.2050781
770.0000000	6.0511999	-14.5638514
770.0000000	6.0999999	-19.4264889
770.0000000	6.1487999	-13.2637482
770.0000000	6.1975999	-23.2569122
770.0000000	6.2463999	-1799.0889893
770.0000000	6.2951999	-19.0873203
770.0000000	6.3439999	-344.0532227
770.0000000	6.3927999	-15.0485086
770.0000000	6.4416003	-12.3735209
770.0000000	6.4903998	-11.2492723
770.0000000	6.5391998	-11.7916040
770.0000000	6.5879998	399.9759521
770.0000000	6.6367998	-12.0722246
770.0000000	6.6856003	-13.8111925
770.0000000	6.7343998	-13.4509039
770.0000000	6.7831998	-14.3817930
770.0000000	6.8319998	58.8668060
770.0000000	6.8807998	-14.0525341
770.0000000	6.9296002	-466.7439270
770.0000000	6.9783998	-4.7924190
770.0000000	7.0271997	-14.1609020
770.0000000	7.0760002	12.1614809
770.0000000	7.1247997	-2223.3996582
770.0000000	7.1736002	-1782.9726563
770.0000000	7.2223997	-1014.1962280
770.0000000	7.2711997	-218.2385864
770.0000000	7.3200002	-19.1646385
770.0000000	7.3687997	196.7365417
770.0000000	7.4176002	409.7910767
770.0000000	7.4663997	322.0319824
770.0000000	7.5151997	-992.3826904
770.0000000	7.5640001	-1096.2200928
770.0000000	7.6127996	-2584.5234375
770.0000000	7.6616001	-829.5009766
770.0000000	7.7104001	-425.8371582
770.0000000	7.7591996	45.6420860
770.0000000	7.8080001	425.8556519
770.0000000	7.8567996	560.8298340
770.0000000	7.9056001	-1160.9267578
770.0000000	7.9544001	1848.0168457
770.0000000	8.0031996	2384.5324707
770.0000000	8.0520000	2380.0285645
770.0000000	8.1007996	2518.5290527
770.0000000	8.1496000	2685.9064941
770.0000000	8.1983995	1671.0356445
770.0000000	8.2472000	-3823.0156250

770.0000000	8.2959995	-1355.1082764
770.0000000	8.3448000	-1079.4822998
770.0000000	8.3935995	-989.3110962
770.0000000	8.4423990	-426.0573120
770.0000000	8.4912004	-994.8278198
770.0000000	8.5400000	-2683.8117676
770.0000000	8.5887995	-1300.7854004
770.0000000	8.6375999	254.2821503
770.0000000	8.6863995	2295.9487305
770.0000000	8.7351999	-853.1769409
770.0000000	8.7840004	975.3162231
770.0000000	8.8327999	53.2760391
770.0000000	8.8815994	-11.4921551
770.0000000	8.9303989	-24.0366993
770.0000000	8.9792004	-11.5770760
770.0000000	9.0279999	-5.7932401
770.0000000	9.0767994	7.1807327
770.0000000	9.1255999	-17.2845116
770.0000000	9.1743994	-10.0636082
770.0000000	9.2231998	37.4027023
770.0000000	9.2720003	-8.3967934
770.0000000	9.3207998	-9.7846565
770.0000000	9.3695993	-10.7581205
770.0000000	9.4183998	-11.7569904
770.0000000	9.4672003	-11.1603012
770.0000000	9.5159998	-14.1864843
770.0000000	9.5648003	-0.3087606
770.0000000	9.6135998	-13.4157772
770.0000000	9.6623993	-10.2144575
770.0000000	9.7111998	-10.1509161
770.0000000	9.7600002	-10.8565331
770.0000000	9.8087997	-10.9945793
770.0000000	9.8575993	3.3004627
770.0000000	9.9063997	-7.9462171
770.0000000	9.9552002	-10.9064941
770.0000000	10.0039997	-10.0685301
780.0000000	0.3416000	-36.1171570
780.0000000	0.3904000	-38.8171387
780.0000000	0.4392000	-39.8939133
780.0000000	0.4880000	-37.7979317
780.0000000	0.5368000	-37.8536530
780.0000000	0.5856000	-39.3658066
780.0000000	0.6344000	-38.2179985
780.0000000	0.6832000	-36.1124802
780.0000000	0.7320000	-35.9523659
780.0000000	0.7808000	-36.5263443
780.0000000	0.8296000	-35.9569473
780.0000000	0.8784000	-35.0189362
780.0000000	0.9272000	-32.8019753
780.0000000	0.9760000	-30.7853107
780.0000000	1.0247999	-29.4769897
780.0000000	1.0735999	-29.0275917
780.0000000	1.1224000	-29.2373238
780.0000000	1.1711999	-29.9599552
780.0000000	1.2200000	-30.6816616
780.0000000	1.2687999	-30.8746986
780.0000000	1.3176000	-31.0134163
780.0000000	1.3664000	-30.3849106

780.0000000	1.4152000	-30.1574097
780.0000000	1.4640000	-29.6563473
780.0000000	1.5128000	-41.7074852
780.0000000	1.5616000	-34.6344643
780.0000000	1.6104001	-1618.2441406
780.0000000	1.6592000	-80.3125610
780.0000000	1.7079999	-27.1676044
780.0000000	1.7567999	-27.7616844
780.0000000	1.8055999	-27.5580997
780.0000000	1.8544000	-25.7729759
780.0000000	1.9031999	-25.5540466
780.0000000	1.9520000	-25.8877563
780.0000000	2.0007999	-26.2517262
780.0000000	2.0495999	-24.9252262
780.0000000	2.0983999	-183.3928528
780.0000000	2.1471999	4.7397823
780.0000000	2.1960001	-25.5466919
780.0000000	2.2448001	-26.2576313
780.0000000	2.2935998	-29.1113987
780.0000000	2.3423998	-20.6441402
780.0000000	2.3912001	46.7976303
780.0000000	2.4400001	-22.7777882
780.0000000	2.4888000	-23.4653053
780.0000000	2.5375998	-11.8558216
780.0000000	2.5863998	-9.9124718
780.0000000	2.6352000	-7.9120808
780.0000000	2.6840000	-15.1199207
780.0000000	2.7328000	139.6898804
780.0000000	2.7815998	164.6448059
780.0000000	2.8304000	-23.8264313
780.0000000	2.8792000	-20.5391026
780.0000000	2.9280000	-22.5857811
780.0000000	2.9768000	-16.5834274
780.0000000	3.0256000	-20.4092464
780.0000000	3.0743999	-29.7971268
780.0000000	3.1231999	-24.3401794
780.0000000	3.1719999	-29.8099442
780.0000000	3.2208002	-510.0863037
780.0000000	3.2695999	-998.9188232
780.0000000	3.3183999	-20.4848461
780.0000000	3.3671999	-3431.7397461
780.0000000	3.4159999	1231.3942871
780.0000000	3.4648001	-7.0035200
780.0000000	3.5135999	-27.1089134
780.0000000	3.5623999	-325.0588684
780.0000000	3.6111999	-2043.4455566
780.0000000	3.6600001	-462.4832153
780.0000000	3.7088001	-610.7440796
780.0000000	3.7575998	-956.3226318
780.0000000	3.8063998	-1735.4924316
780.0000000	3.8552001	-2519.7634277
780.0000000	3.9040000	-2470.4528809
780.0000000	3.9528000	1129.9661865
780.0000000	4.0015998	-246.6944122
780.0000000	4.0503998	691.8759155
780.0000000	4.0991998	88.2631531
780.0000000	4.1479998	-658.5912476
780.0000000	4.1967998	2.6763954

780.0000000	4.2456002	-249.8921509
780.0000000	4.2943997	-490.8190002
780.0000000	4.3431997	-13.3007240
780.0000000	4.3920002	-249.5199738
780.0000000	4.4407997	-22.4855194
780.0000000	4.4896002	-15.4618387
780.0000000	4.5383997	-658.7299805
780.0000000	4.5871997	-12.8104105
780.0000000	4.6360002	-21.6426563
780.0000000	4.6847997	-28.3372078
780.0000000	4.7336001	-19.5725117
780.0000000	4.7824001	-16.5060215
780.0000000	4.8311996	-1481.7303467
780.0000000	4.8800001	229.4964752
780.0000000	4.9287996	-202.3831787
780.0000000	4.9776001	1065.0034180
780.0000000	5.0264001	86.6940689
780.0000000	5.0751996	-302.5709229
780.0000000	5.1240001	-12.2956200
780.0000000	5.1727996	560.0358276
780.0000000	5.2216001	32.1756477
780.0000000	5.2704000	-159.3504944
780.0000000	5.3191996	-39.2691612
780.0000000	5.3680000	-3549.0820313
780.0000000	5.4168000	529.1716309
780.0000000	5.4656000	-201.4321899
780.0000000	5.5144000	38.2713699
780.0000000	5.5631995	490.2165527
780.0000000	5.6120000	394.6766663
780.0000000	5.6608000	-37.1632919
780.0000000	5.7096000	62.8269577
780.0000000	5.7584000	133.5966339
780.0000000	5.8071995	-11.5035305
780.0000000	5.8559999	29.9880581
780.0000000	5.9047999	-16.6094532
780.0000000	5.9535999	-39.8681602
780.0000000	6.0023999	-1578.9007568
780.0000000	6.0511999	-14.5640631
780.0000000	6.0999999	-19.4256554
780.0000000	6.1487999	-13.2642107
780.0000000	6.1975999	-23.2551460
780.0000000	6.2463999	-1798.7415771
780.0000000	6.2951999	-19.0865059
780.0000000	6.3439999	-343.9891663
780.0000000	6.3927999	-15.0484905
780.0000000	6.4416003	-12.3741083
780.0000000	6.4903998	-11.2499485
780.0000000	6.5391998	-11.7922850
780.0000000	6.5879998	399.2852783
780.0000000	6.6367998	-12.0727739
780.0000000	6.6856003	-13.8113623
780.0000000	6.7343998	-13.4511204
780.0000000	6.7831998	-14.3813772
780.0000000	6.8319998	58.8476944
780.0000000	6.8807998	-14.0523958
780.0000000	6.9296002	-465.9824524
780.0000000	6.9783998	-4.8052425
780.0000000	7.0271997	-14.1598740

780.0000000	7.0760002	12.1192942
780.0000000	7.1247997	-2219.4538574
780.0000000	7.1736002	-1779.8098145
780.0000000	7.2223997	-1012.4030151
780.0000000	7.2711997	-217.8708038
780.0000000	7.3200002	-19.1526508
780.0000000	7.3687997	196.3620148
780.0000000	7.4176002	409.0352478
780.0000000	7.4663997	321.4343262
780.0000000	7.5151997	-990.6216431
780.0000000	7.5640001	-1094.2727051
780.0000000	7.6127996	-2579.9057617
780.0000000	7.6616001	-828.0296631
780.0000000	7.7104001	-425.0909424
780.0000000	7.7591996	45.5410347
780.0000000	7.8080001	425.0702820
780.0000000	7.8567996	559.8017578
780.0000000	7.9056001	-1158.8537598
780.0000000	7.9544001	1844.7069092
780.0000000	8.0031996	2380.2629395
780.0000000	8.0520000	2375.7666016
780.0000000	8.1007996	2514.0195313
780.0000000	8.1496000	2681.0983887
780.0000000	8.1983995	1668.0430908
780.0000000	8.2472000	-3816.1809082
780.0000000	8.2959995	-1352.6881104
780.0000000	8.3448000	-1077.5531006
780.0000000	8.3935995	-987.5433960
780.0000000	8.4423990	-425.2993164
780.0000000	8.4912004	-993.0515747
780.0000000	8.5400000	-2679.0119629
780.0000000	8.5887995	-1298.4638672
780.0000000	8.6375999	253.8208160
780.0000000	8.6863995	2291.8303223
780.0000000	8.7351999	-851.6527100
780.0000000	8.7840004	973.5594482
780.0000000	8.8327999	53.1644287
780.0000000	8.8815994	-11.4923296
780.0000000	8.9303989	-24.0329781
780.0000000	8.9792004	-11.5771580
780.0000000	9.0279999	-5.7950082
780.0000000	9.0767994	7.1750145
780.0000000	9.1255999	-17.2827988
780.0000000	9.1743994	-10.0640106
780.0000000	9.2231998	37.3869629
780.0000000	9.2720003	-8.3977299
780.0000000	9.3207998	-9.7851954
780.0000000	9.3695993	-10.7583466
780.0000000	9.4183998	-11.7568665
780.0000000	9.4672003	-11.1603432
780.0000000	9.5159998	-14.1855927
780.0000000	9.5648003	-0.3121338
780.0000000	9.6135998	-13.4149857
780.0000000	9.6623993	-10.2147322
780.0000000	9.7111998	-10.1511879
780.0000000	9.7600002	-10.8565750
780.0000000	9.8087997	-10.9945869
780.0000000	9.8575993	3.2960396

780.0000000	9.9063997	-7.9471312
780.0000000	9.9552002	-10.9064980
780.0000000	10.0039997	-10.0687780
790.0000000	0.3416000	-36.5191689
790.0000000	0.3904000	-39.6120529
790.0000000	0.4392000	-39.8875694
790.0000000	0.4880000	-39.2816887
790.0000000	0.5368000	-39.5867653
790.0000000	0.5856000	-39.3644257
790.0000000	0.6344000	-38.2183075
790.0000000	0.6832000	-36.1147041
790.0000000	0.7320000	-35.9544182
790.0000000	0.7808000	-36.5265121
790.0000000	0.8296000	-35.9576797
790.0000000	0.8784000	-35.0263100
790.0000000	0.9272000	-32.8047523
790.0000000	0.9760000	-30.7778587
790.0000000	1.0247999	-29.4859791
790.0000000	1.0735999	-29.0420265
790.0000000	1.1224000	-29.2343235
790.0000000	1.1711999	-29.9525776
790.0000000	1.2200000	-30.6895771
790.0000000	1.2687999	-30.8757076
790.0000000	1.3176000	-31.0091457
790.0000000	1.3664000	-30.3926601
790.0000000	1.4152000	-30.1576138
790.0000000	1.4640000	-29.6546364
790.0000000	1.5128000	-41.6103096
790.0000000	1.5616000	-34.5926247
790.0000000	1.6104001	-1615.7014160
790.0000000	1.6592000	-80.0186310
790.0000000	1.7079999	-27.1713524
790.0000000	1.7567999	-27.7617035
790.0000000	1.8055999	-27.5619869
790.0000000	1.8544000	-26.4769802
790.0000000	1.9031999	-25.4544144
790.0000000	1.9520000	-26.5492287
790.0000000	2.0007999	-26.0624104
790.0000000	2.0495999	-25.1118431
790.0000000	2.0983999	-181.9170837
790.0000000	2.1471999	4.4725151
790.0000000	2.1960001	-25.5426006
790.0000000	2.2448001	-26.2397194
790.0000000	2.2935998	-29.0517769
790.0000000	2.3423998	-20.6585445
790.0000000	2.3912001	46.1760864
790.0000000	2.4400001	-22.7802753
790.0000000	2.4888000	-23.4650879
790.0000000	2.5375998	-11.8729296
790.0000000	2.5863998	-9.9322309
790.0000000	2.6352000	-7.9348249
790.0000000	2.6840000	-15.1318626
790.0000000	2.7328000	139.4238586
790.0000000	2.7815998	164.3384094
790.0000000	2.8304000	-23.8251266
790.0000000	2.8792000	-20.5423660
790.0000000	2.9280000	-22.5852127
790.0000000	2.9768000	-16.5917988

790.0000000	3.0256000	-20.4118309
790.0000000	3.0743999	-29.7857399
790.0000000	3.1231999	-24.3367233
790.0000000	3.1719999	-29.8016510
790.0000000	3.2208002	-509.8485718
790.0000000	3.2695999	-998.4438477
790.0000000	3.3183999	-20.4850445
790.0000000	3.3671999	-3430.2783203
790.0000000	3.4159999	1230.8590088
790.0000000	3.4648001	-7.0092287
790.0000000	3.5135999	-27.1059227
790.0000000	3.5623999	-324.9292603
790.0000000	3.6111999	-2042.5858154
790.0000000	3.6600001	-462.2951050
790.0000000	3.7088001	-610.4927368
790.0000000	3.7575998	-955.9241333
790.0000000	3.8063998	-1734.7625732
790.0000000	3.8552001	-2518.6999512
790.0000000	3.9040000	-2469.4104004
790.0000000	3.9528000	1129.4779053
790.0000000	4.0015998	-246.5973053
790.0000000	4.0503998	691.5733643
790.0000000	4.0991998	88.2174683
790.0000000	4.1479998	-658.3189697
790.0000000	4.1967998	2.6671391
790.0000000	4.2456002	-249.7938538
790.0000000	4.2943997	-490.6183777
790.0000000	4.3431997	-13.3029041
790.0000000	4.3920002	-249.4218597
790.0000000	4.4407997	-22.4837437
790.0000000	4.4896002	-15.4627209
790.0000000	4.5383997	-658.4688721
790.0000000	4.5871997	-12.8124275
790.0000000	4.6360002	-21.6411152
790.0000000	4.6847997	-28.3332825
790.0000000	4.7336001	-19.5717888
790.0000000	4.7824001	-16.5064774
790.0000000	4.8311996	-1481.1990967
790.0000000	4.8800001	229.4069214
790.0000000	4.9287996	-202.3159180
790.0000000	4.9776001	1064.6109619
790.0000000	5.0264001	86.6565475
790.0000000	5.0751996	-302.4672852
790.0000000	5.1240001	-12.2965412
790.0000000	5.1727996	559.9199829
790.0000000	5.2216001	32.1662025
790.0000000	5.2704000	-159.3224030
790.0000000	5.3191996	-39.2646103
790.0000000	5.3680000	-3548.3896484
790.0000000	5.4168000	529.0648193
790.0000000	5.4656000	-201.3960114
790.0000000	5.5144000	38.2607193
790.0000000	5.5631995	490.1180115
790.0000000	5.6120000	394.5966797
790.0000000	5.6608000	-37.1592064
790.0000000	5.7096000	62.8115311
790.0000000	5.7584000	133.5677032
790.0000000	5.8071995	-11.5044012

790.0000000	5.8559999	29.9792042
790.0000000	5.9047999	-16.6092548
790.0000000	5.9535999	-39.8634186
790.0000000	6.0023999	-1578.5961914
790.0000000	6.0511999	-14.5642738
790.0000000	6.0999999	-19.4248238
790.0000000	6.1487999	-13.2646732
790.0000000	6.1975999	-23.2533817
790.0000000	6.2463999	-1798.3942871
790.0000000	6.2951999	-19.0856915
790.0000000	6.3439999	-343.9251099
790.0000000	6.3927999	-15.0484724
790.0000000	6.4416003	-12.3746948
790.0000000	6.4903998	-11.2506247
790.0000000	6.5391998	-11.7929659
790.0000000	6.5879998	398.5945740
790.0000000	6.6367998	-12.0733232
790.0000000	6.6856003	-13.8115330
790.0000000	6.7343998	-13.4513359
790.0000000	6.7831998	-14.3809614
790.0000000	6.8319998	58.8285751
790.0000000	6.8807998	-14.0522566
790.0000000	6.9296002	-465.2210083
790.0000000	6.9783998	-4.8180656
790.0000000	7.0271997	-14.1588459
790.0000000	7.0760002	12.0771074
790.0000000	7.1247997	-2215.5078125
790.0000000	7.1736002	-1776.6469727
790.0000000	7.2223997	-1010.6098633
790.0000000	7.2711997	-217.5030212
790.0000000	7.3200002	-19.1406631
790.0000000	7.3687997	195.9874878
790.0000000	7.4176002	408.2794189
790.0000000	7.4663997	320.8366699
790.0000000	7.5151997	-988.8605957
790.0000000	7.5640001	-1092.3253174
790.0000000	7.6127996	-2575.2883301
790.0000000	7.6616001	-826.5582886
790.0000000	7.7104001	-424.3446045
790.0000000	7.7591996	45.4399872
790.0000000	7.8080001	424.2849426
790.0000000	7.8567996	558.7736816
790.0000000	7.9056001	-1156.7808838
790.0000000	7.9544001	1841.3969727
790.0000000	8.0031996	2375.9934082
790.0000000	8.0520000	2371.5046387
790.0000000	8.1007996	2509.5100098
790.0000000	8.1496000	2676.2902832
790.0000000	8.1983995	1665.0505371
790.0000000	8.2472000	-3809.3461914
790.0000000	8.2959995	-1350.2679443
790.0000000	8.3448000	-1075.6239014
790.0000000	8.3935995	-985.7757568
790.0000000	8.4423990	-424.5412903
790.0000000	8.4912004	-991.2753906
790.0000000	8.5400000	-2674.2121582
790.0000000	8.5887995	-1296.1423340
790.0000000	8.6375999	253.3595123

790.0000000	8.6863995	2287.7119141
790.0000000	8.7351999	-850.1284790
790.0000000	8.7840004	971.8027344
790.0000000	8.8327999	53.0528183
790.0000000	8.8815994	-11.4925041
790.0000000	8.9303989	-24.0292587
790.0000000	8.9792004	-11.5772400
790.0000000	9.0279999	-5.7967768
790.0000000	9.0767994	7.1692963
790.0000000	9.1255999	-17.2810841
790.0000000	9.1743994	-10.0644121
790.0000000	9.2231998	37.3712311
790.0000000	9.2720003	-8.3986664
790.0000000	9.3207998	-9.7857351
790.0000000	9.3695993	-10.7585735
790.0000000	9.4183998	-11.7567415
790.0000000	9.4672003	-11.1603842
790.0000000	9.5159998	-14.1847019
790.0000000	9.5648003	-0.3155060
790.0000000	9.6135998	-13.4141951
790.0000000	9.6623993	-10.2150078
790.0000000	9.7111998	-10.1514597
790.0000000	9.7600002	-10.8566170
790.0000000	9.8087997	-10.9945955
790.0000000	9.8575993	3.2916174
790.0000000	9.9063997	-7.9480448
790.0000000	9.9552002	-10.9065027
790.0000000	10.0039997	-10.0690260
800.0000000	0.3416000	-36.0727425
800.0000000	0.3904000	-38.8952942
800.0000000	0.4392000	-39.8812256
800.0000000	0.4880000	-39.6178551
800.0000000	0.5368000	-39.5879402
800.0000000	0.5856000	-39.3630447
800.0000000	0.6344000	-38.2186203
800.0000000	0.6832000	-36.1169319
800.0000000	0.7320000	-35.9564705
800.0000000	0.7808000	-36.5266838
800.0000000	0.8296000	-35.9584084
800.0000000	0.8784000	-35.0336876
800.0000000	0.9272000	-32.8075294
800.0000000	0.9760000	-30.7704048
800.0000000	1.0247999	-29.4949684
800.0000000	1.0735999	-29.0564632
800.0000000	1.1224000	-29.2313232
800.0000000	1.1711999	-29.9452000
800.0000000	1.2200000	-30.6974926
800.0000000	1.2687999	-30.8767147
800.0000000	1.3176000	-31.0048733
800.0000000	1.3664000	-30.4004097
800.0000000	1.4152000	-30.1578178
800.0000000	1.4640000	-29.6529274
800.0000000	1.5128000	-41.5131302
800.0000000	1.5616000	-34.5507813
800.0000000	1.6104001	-1613.1585693
800.0000000	1.6592000	-79.7247009
800.0000000	1.7079999	-27.1750984
800.0000000	1.7567999	-27.7617226

800.0000000	1.8055999	-27.5658760
800.0000000	1.8544000	-26.3198433
800.0000000	1.9031999	-25.1189671
800.0000000	1.9520000	-25.5821800
800.0000000	2.0007999	-25.8730946
800.0000000	2.0495999	-25.2984581
800.0000000	2.0983999	-180.4413147
800.0000000	2.1471999	4.2052479
800.0000000	2.1960001	-25.5385094
800.0000000	2.2448001	-26.2218094
800.0000000	2.2935998	-28.9921532
800.0000000	2.3423998	-20.6729488
800.0000000	2.3912001	45.5545425
800.0000000	2.4400001	-22.7827625
800.0000000	2.4888000	-23.4648705
800.0000000	2.5375998	-11.8900375
800.0000000	2.5863998	-9.9519892
800.0000000	2.6352000	-7.9575691
800.0000000	2.6840000	-15.1438046
800.0000000	2.7328000	139.1578522
800.0000000	2.7815998	164.0319824
800.0000000	2.8304000	-23.8238239
800.0000000	2.8792000	-20.5456295
800.0000000	2.9280000	-22.5846424
800.0000000	2.9768000	-16.6001701
800.0000000	3.0256000	-20.4144154
800.0000000	3.0743999	-29.7743530
800.0000000	3.1231999	-24.3332672
800.0000000	3.1719999	-29.7933578
800.0000000	3.2208002	-509.6108398
800.0000000	3.2695999	-997.9688721
800.0000000	3.3183999	-20.4852409
800.0000000	3.3671999	-3428.8166504
800.0000000	3.4159999	1230.3237305
800.0000000	3.4648001	-7.0149364
800.0000000	3.5135999	-27.1029320
800.0000000	3.5623999	-324.7996826
800.0000000	3.6111999	-2041.7259521
800.0000000	3.6600001	-462.1069946
800.0000000	3.7088001	-610.2413330
800.0000000	3.7575998	-955.5255737
800.0000000	3.8063998	-1734.0327148
800.0000000	3.8552001	-2517.6367188
800.0000000	3.9040000	-2468.3679199
800.0000000	3.9528000	1128.9896240
800.0000000	4.0015998	-246.5001984
800.0000000	4.0503998	691.2708130
800.0000000	4.0991998	88.1717834
800.0000000	4.1479998	-658.0467529
800.0000000	4.1967998	2.6578827
800.0000000	4.2456002	-249.6955566
800.0000000	4.2943997	-490.4178162
800.0000000	4.3431997	-13.3050852
800.0000000	4.3920002	-249.3237457
800.0000000	4.4407997	-22.4819679
800.0000000	4.4896002	-15.4636040
800.0000000	4.5383997	-658.2078247
800.0000000	4.5871997	-12.8144436

800.0000000	4.6360002	-21.6395721
800.0000000	4.6847997	-28.3293571
800.0000000	4.7336001	-19.5710678
800.0000000	4.7824001	-16.5069313
800.0000000	4.8311996	-1480.6677246
800.0000000	4.8800001	229.3173828
800.0000000	4.9287996	-202.2486572
800.0000000	4.9776001	1064.2185059
800.0000000	5.0264001	86.6190262
800.0000000	5.0751996	-302.3636475
800.0000000	5.1240001	-12.2974625
800.0000000	5.1727996	559.8040771
800.0000000	5.2216001	32.1567574
800.0000000	5.2704000	-159.2942810
800.0000000	5.3191996	-39.2600632
800.0000000	5.3680000	-3547.6972656
800.0000000	5.4168000	528.9580688
800.0000000	5.4656000	-201.3598328
800.0000000	5.5144000	38.2500610
800.0000000	5.5631995	490.0194397
800.0000000	5.6120000	394.5167236
800.0000000	5.6608000	-37.1551170
800.0000000	5.7096000	62.7960968
800.0000000	5.7584000	133.5387726
800.0000000	5.8071995	-11.5052719
800.0000000	5.8559999	29.9703541
800.0000000	5.9047999	-16.6090565
800.0000000	5.9535999	-39.8586807
800.0000000	6.0023999	-1578.2917480
800.0000000	6.0511999	-14.5644855
800.0000000	6.0999999	-19.4239922
800.0000000	6.1487999	-13.2651358
800.0000000	6.1975999	-23.2516174
800.0000000	6.2463999	-1798.0468750
800.0000000	6.2951999	-19.0848770
800.0000000	6.3439999	-343.8610535
800.0000000	6.3927999	-15.0484533
800.0000000	6.4416003	-12.3752813
800.0000000	6.4903998	-11.2513008
800.0000000	6.5391998	-11.7936478
800.0000000	6.5879998	397.9038696
800.0000000	6.6367998	-12.0738726
800.0000000	6.6856003	-13.8117027
800.0000000	6.7343998	-13.4515514
800.0000000	6.7831998	-14.3805456
800.0000000	6.8319998	58.8094559
800.0000000	6.8807998	-14.0521183
800.0000000	6.9296002	-464.4595642
800.0000000	6.9783998	-4.8308892
800.0000000	7.0271997	-14.1578178
800.0000000	7.0760002	12.0349245
800.0000000	7.1247997	-2211.5620117
800.0000000	7.1736002	-1773.4840088
800.0000000	7.2223997	-1008.8167114
800.0000000	7.2711997	-217.1352539
800.0000000	7.3200002	-19.1286736
800.0000000	7.3687997	195.6129456
800.0000000	7.4176002	407.5235901

800.0000000	7.4663997	320.2390137
800.0000000	7.5151997	-987.0995483
800.0000000	7.5640001	-1090.3778076
800.0000000	7.6127996	-2570.6706543
800.0000000	7.6616001	-825.0869141
800.0000000	7.7104001	-423.5983887
800.0000000	7.7591996	45.3389320
800.0000000	7.8080001	423.4995728
800.0000000	7.8567996	557.7456055
800.0000000	7.9056001	-1154.7080078
800.0000000	7.9544001	1838.0870361
800.0000000	8.0031996	2371.7238770
800.0000000	8.0520000	2367.2424316
800.0000000	8.1007996	2505.0004883
800.0000000	8.1496000	2671.4824219
800.0000000	8.1983995	1662.0581055
800.0000000	8.2472000	-3802.5112305
800.0000000	8.2959995	-1347.8477783
800.0000000	8.3448000	-1073.6947021
800.0000000	8.3935995	-984.0080566
800.0000000	8.4423990	-423.7832947
800.0000000	8.4912004	-989.4991455
800.0000000	8.5400000	-2669.4123535
800.0000000	8.5887995	-1293.8208008
800.0000000	8.6375999	252.8981781
800.0000000	8.6863995	2283.5935059
800.0000000	8.7351999	-848.6041870
800.0000000	8.7840004	970.0459595
800.0000000	8.8327999	52.9412155
800.0000000	8.8815994	-11.4926777
800.0000000	8.9303989	-24.0255394
800.0000000	8.9792004	-11.5773230
800.0000000	9.0279999	-5.7985444
800.0000000	9.0767994	7.1635799
800.0000000	9.1255999	-17.2793694
800.0000000	9.1743994	-10.0648146
800.0000000	9.2231998	37.3554993
800.0000000	9.2720003	-8.3996029
800.0000000	9.3207998	-9.7862740
800.0000000	9.3695993	-10.7587996
800.0000000	9.4183998	-11.7566166
800.0000000	9.4672003	-11.1604252
800.0000000	9.5159998	-14.1838112
800.0000000	9.5648003	-0.3188782
800.0000000	9.6135998	-13.4134045
800.0000000	9.6623993	-10.2152824
800.0000000	9.7111998	-10.1517315
800.0000000	9.7600002	-10.8566580
800.0000000	9.8087997	-10.9946032
800.0000000	9.8575993	3.2871952
800.0000000	9.9063997	-7.9489584
800.0000000	9.9552002	-10.9065065
800.0000000	10.0039997	-10.0692730
810.0000000	0.3416000	-36.1448860
810.0000000	0.3904000	-38.7826233
810.0000000	0.4392000	-39.8748817
810.0000000	0.4880000	-39.6166458
810.0000000	0.5368000	-39.5891113

810.0000000	0.5856000	-39.3616638
810.0000000	0.6344000	-38.2189293
810.0000000	0.6832000	-36.1191559
810.0000000	0.7320000	-35.9585228
810.0000000	0.7808000	-36.5268517
810.0000000	0.8296000	-35.9591370
810.0000000	0.8784000	-35.0410614
810.0000000	0.9272000	-32.8103065
810.0000000	0.9760000	-30.7629528
810.0000000	1.0247999	-29.5039577
810.0000000	1.0735999	-29.0708981
810.0000000	1.1224000	-29.2283249
810.0000000	1.1711999	-29.9378242
810.0000000	1.2200000	-30.7054062
810.0000000	1.2687999	-30.8777218
810.0000000	1.3176000	-31.0006027
810.0000000	1.3664000	-30.4081593
810.0000000	1.4152000	-30.1580219
810.0000000	1.4640000	-29.6512184
810.0000000	1.5128000	-41.4159546
810.0000000	1.5616000	-34.5089417
810.0000000	1.6104001	-1610.6157227
810.0000000	1.6592000	-79.4307709
810.0000000	1.7079999	-27.1788464
810.0000000	1.7567999	-27.7617416
810.0000000	1.8055999	-27.5697632
810.0000000	1.8544000	-26.5942554
810.0000000	1.9031999	-25.7443428
810.0000000	1.9520000	-25.9642105
810.0000000	2.0007999	-25.6837788
810.0000000	2.0495999	-25.4850750
810.0000000	2.0983999	-178.9655151
810.0000000	2.1471999	3.9379807
810.0000000	2.1960001	-25.5344181
810.0000000	2.2448001	-26.2038975
810.0000000	2.2935998	-28.9325294
810.0000000	2.3423998	-20.6873531
810.0000000	2.3912001	44.9329987
810.0000000	2.4400001	-22.7852478
810.0000000	2.4888000	-23.4646530
810.0000000	2.5375998	-11.9071455
810.0000000	2.5863998	-9.9717484
810.0000000	2.6352000	-7.9803133
810.0000000	2.6840000	-15.1557465
810.0000000	2.7328000	138.8918304
810.0000000	2.7815998	163.7255554
810.0000000	2.8304000	-23.8225193
810.0000000	2.8792000	-20.5488930
810.0000000	2.9280000	-22.5840740
810.0000000	2.9768000	-16.6085396
810.0000000	3.0256000	-20.4169998
810.0000000	3.0743999	-29.7629662
810.0000000	3.1231999	-24.3298130
810.0000000	3.1719999	-29.7850647
810.0000000	3.2208002	-509.3731079
810.0000000	3.2695999	-997.4938965
810.0000000	3.3183999	-20.4854393
810.0000000	3.3671999	-3427.3549805

810.0000000	3.4159999	1229.7884521
810.0000000	3.4648001	-7.0206442
810.0000000	3.5135999	-27.0999413
810.0000000	3.5623999	-324.6701050
810.0000000	3.6111999	-2040.8662109
810.0000000	3.6600001	-461.9188843
810.0000000	3.7088001	-609.9899292
810.0000000	3.7575998	-955.1270752
810.0000000	3.8063998	-1733.3028564
810.0000000	3.8552001	-2516.5732422
810.0000000	3.9040000	-2467.3254395
810.0000000	3.9528000	1128.5013428
810.0000000	4.0015998	-246.4030914
810.0000000	4.0503998	690.9682007
810.0000000	4.0991998	88.1261063
810.0000000	4.1479998	-657.7745361
810.0000000	4.1967998	2.6486263
810.0000000	4.2456002	-249.5972443
810.0000000	4.2943997	-490.2172546
810.0000000	4.3431997	-13.3072662
810.0000000	4.3920002	-249.2256317
810.0000000	4.4407997	-22.4801922
810.0000000	4.4896002	-15.4644871
810.0000000	4.5383997	-657.9467163
810.0000000	4.5871997	-12.8164597
810.0000000	4.6360002	-21.6380291
810.0000000	4.6847997	-28.3254318
810.0000000	4.7336001	-19.5703449
810.0000000	4.7824001	-16.5073853
810.0000000	4.8311996	-1480.1364746
810.0000000	4.8800001	229.2278290
810.0000000	4.9287996	-202.1813965
810.0000000	4.9776001	1063.8259277
810.0000000	5.0264001	86.5815048
810.0000000	5.0751996	-302.2600403
810.0000000	5.1240001	-12.2983837
810.0000000	5.1727996	559.6882324
810.0000000	5.2216001	32.1473160
810.0000000	5.2704000	-159.2661896
810.0000000	5.3191996	-39.2555122
810.0000000	5.3680000	-3547.0048828
810.0000000	5.4168000	528.8512573
810.0000000	5.4656000	-201.3236389
810.0000000	5.5144000	38.2394028
810.0000000	5.5631995	489.9208679
810.0000000	5.6120000	394.4367371
810.0000000	5.6608000	-37.1510277
810.0000000	5.7096000	62.7806625
810.0000000	5.7584000	133.5098419
810.0000000	5.8071995	-11.5061426
810.0000000	5.8559999	29.9615040
810.0000000	5.9047999	-16.6088600
810.0000000	5.9535999	-39.8539429
810.0000000	6.0023999	-1577.9874268
810.0000000	6.0511999	-14.5646973
810.0000000	6.0999999	-19.4231586
810.0000000	6.1487999	-13.2655983
810.0000000	6.1975999	-23.2498512

810.0000000	6.2463999	-1797.6994629
810.0000000	6.2951999	-19.0840626
810.0000000	6.3439999	-343.7970276
810.0000000	6.3927999	-15.0484352
810.0000000	6.4416003	-12.3758688
810.0000000	6.4903998	-11.2519760
810.0000000	6.5391998	-11.7943287
810.0000000	6.5879998	397.2131958
810.0000000	6.6367998	-12.0744219
810.0000000	6.6856003	-13.8118734
810.0000000	6.7343998	-13.4517679
810.0000000	6.7831998	-14.3801298
810.0000000	6.8319998	58.7903442
810.0000000	6.8807998	-14.0519800
810.0000000	6.9296002	-463.6981201
810.0000000	6.9783998	-4.8437133
810.0000000	7.0271997	-14.1567907
810.0000000	7.0760002	11.9927378
810.0000000	7.1247997	-2207.6162109
810.0000000	7.1736002	-1770.3211670
810.0000000	7.2223997	-1007.0235596
810.0000000	7.2711997	-216.7674713
810.0000000	7.3200002	-19.1166840
810.0000000	7.3687997	195.2384186
810.0000000	7.4176002	406.7677612
810.0000000	7.4663997	319.6413879
810.0000000	7.5151997	-985.3385010
810.0000000	7.5640001	-1088.4304199
810.0000000	7.6127996	-2566.0529785
810.0000000	7.6616001	-823.6156006
810.0000000	7.7104001	-422.8521118
810.0000000	7.7591996	45.2378845
810.0000000	7.8080001	422.7142029
810.0000000	7.8567996	556.7175293
810.0000000	7.9056001	-1152.6351318
810.0000000	7.9544001	1834.7770996
810.0000000	8.0031996	2367.4541016
810.0000000	8.0520000	2362.9804688
810.0000000	8.1007996	2500.4909668
810.0000000	8.1496000	2666.6743164
810.0000000	8.1983995	1659.0655518
810.0000000	8.2472000	-3795.6765137
810.0000000	8.2959995	-1345.4276123
810.0000000	8.3448000	-1071.7655029
810.0000000	8.3935995	-982.2404175
810.0000000	8.4423990	-423.0252686
810.0000000	8.4912004	-987.7229004
810.0000000	8.5400000	-2664.6125488
810.0000000	8.5887995	-1291.4991455
810.0000000	8.6375999	252.4368591
810.0000000	8.6863995	2279.4753418
810.0000000	8.7351999	-847.0799561
810.0000000	8.7840004	968.2892456
810.0000000	8.8327999	52.8296089
810.0000000	8.8815994	-11.4928522
810.0000000	8.9303989	-24.0218201
810.0000000	8.9792004	-11.5774050
810.0000000	9.0279999	-5.8003130

810.0000000	9.0767994	7.1578617
810.0000000	9.1255999	-17.2776566
810.0000000	9.1743994	-10.0652161
810.0000000	9.2231998	37.3397675
810.0000000	9.2720003	-8.4005394
810.0000000	9.3207998	-9.7868137
810.0000000	9.3695993	-10.7590265
810.0000000	9.4183998	-11.7564926
810.0000000	9.4672003	-11.1604662
810.0000000	9.5159998	-14.1829205
810.0000000	9.5648003	-0.3222513
810.0000000	9.6135998	-13.4126139
810.0000000	9.6623993	-10.2155581
810.0000000	9.7111998	-10.1520023
810.0000000	9.7600002	-10.8566999
810.0000000	9.8087997	-10.9946108
810.0000000	9.8575993	3.2827740
810.0000000	9.9063997	-7.9498730
810.0000000	9.9552002	-10.9065113
810.0000000	10.0039997	-10.0695210
820.0000000	0.3416000	-36.5548019
820.0000000	0.3904000	-39.6922569
820.0000000	0.4392000	-39.8685417
820.0000000	0.4880000	-39.6154366
820.0000000	0.5368000	-39.5902863
820.0000000	0.5856000	-39.3602791
820.0000000	0.6344000	-38.2192383
820.0000000	0.6832000	-36.1213837
820.0000000	0.7320000	-35.9605751
820.0000000	0.7808000	-36.5270233
820.0000000	0.8296000	-35.9598656
820.0000000	0.8784000	-35.0484352
820.0000000	0.9272000	-32.8130836
820.0000000	0.9760000	-30.7554989
820.0000000	1.0247999	-29.5129471
820.0000000	1.0735999	-29.0853329
820.0000000	1.1224000	-29.2253246
820.0000000	1.1711999	-29.9304466
820.0000000	1.2200000	-30.7133217
820.0000000	1.2687999	-30.8787308
820.0000000	1.3176000	-30.9963303
820.0000000	1.3664000	-30.4159088
820.0000000	1.4152000	-30.1582260
820.0000000	1.4640000	-29.6495075
820.0000000	1.5128000	-41.3187752
820.0000000	1.5616000	-34.4671021
820.0000000	1.6104001	-1608.0729980
820.0000000	1.6592000	-79.1368332
820.0000000	1.7079999	-27.1825943
820.0000000	1.7567999	-27.7617607
820.0000000	1.8055999	-27.5736523
820.0000000	1.8544000	-26.6374397
820.0000000	1.9031999	-26.0559082
820.0000000	1.9520000	-26.0712185
820.0000000	2.0007999	-25.7263355
820.0000000	2.0495999	-25.6716919
820.0000000	2.0983999	-177.4897461
820.0000000	2.1471999	3.6707134

820.0000000	2.1960001	-25.5303268
820.0000000	2.2448001	-26.1859856
820.0000000	2.2935998	-28.8729057
820.0000000	2.3423998	-20.7017574
820.0000000	2.3912001	44.3114548
820.0000000	2.4400001	-22.7877350
820.0000000	2.4888000	-23.4644356
820.0000000	2.5375998	-11.9242535
820.0000000	2.5863998	-9.9915075
820.0000000	2.6352000	-8.0030575
820.0000000	2.6840000	-15.1676884
820.0000000	2.7328000	138.6258087
820.0000000	2.7815998	163.4191589
820.0000000	2.8304000	-23.8212147
820.0000000	2.8792000	-20.5521564
820.0000000	2.9280000	-22.5835056
820.0000000	2.9768000	-16.6169090
820.0000000	3.0256000	-20.4195843
820.0000000	3.0743999	-29.7515793
820.0000000	3.1231999	-24.3263569
820.0000000	3.1719999	-29.7767715
820.0000000	3.2208002	-509.1353455
820.0000000	3.2695999	-997.0189209
820.0000000	3.3183999	-20.4856377
820.0000000	3.3671999	-3425.8933105
820.0000000	3.4159999	1229.2531738
820.0000000	3.4648001	-7.0263529
820.0000000	3.5135999	-27.0969505
820.0000000	3.5623999	-324.5404968
820.0000000	3.6111999	-2040.0064697
820.0000000	3.6600001	-461.7307129
820.0000000	3.7088001	-609.7385864
820.0000000	3.7575998	-954.7285156
820.0000000	3.8063998	-1732.5731201
820.0000000	3.8552001	-2515.5097656
820.0000000	3.9040000	-2466.2829590
820.0000000	3.9528000	1128.0130615
820.0000000	4.0015998	-246.3059845
820.0000000	4.0503998	690.6656494
820.0000000	4.0991998	88.0804214
820.0000000	4.1479998	-657.5023193
820.0000000	4.1967998	2.6393700
820.0000000	4.2456002	-249.4989471
820.0000000	4.2943997	-490.0166931
820.0000000	4.3431997	-13.3094463
820.0000000	4.3920002	-249.1275177
820.0000000	4.4407997	-22.4784164
820.0000000	4.4896002	-15.4653692
820.0000000	4.5383997	-657.6856689
820.0000000	4.5871997	-12.8184757
820.0000000	4.6360002	-21.6364861
820.0000000	4.6847997	-28.3215065
820.0000000	4.7336001	-19.5696220
820.0000000	4.7824001	-16.5078392
820.0000000	4.8311996	-1479.6052246
820.0000000	4.8800001	229.1382751
820.0000000	4.9287996	-202.1141357
820.0000000	4.9776001	1063.4334717

820.0000000	5.0264001	86.5439835
820.0000000	5.0751996	-302.1564026
820.0000000	5.1240001	-12.2993050
820.0000000	5.1727996	559.5723877
820.0000000	5.2216001	32.1378708
820.0000000	5.2704000	-159.2380676
820.0000000	5.3191996	-39.2509651
820.0000000	5.3680000	-3546.3127441
820.0000000	5.4168000	528.7445068
820.0000000	5.4656000	-201.2874603
820.0000000	5.5144000	38.2287445
820.0000000	5.5631995	489.8222961
820.0000000	5.6120000	394.3567810
820.0000000	5.6608000	-37.1469383
820.0000000	5.7096000	62.7652359
820.0000000	5.7584000	133.4808960
820.0000000	5.8071995	-11.5070133
820.0000000	5.8559999	29.9526539
820.0000000	5.9047999	-16.6086617
820.0000000	5.9535999	-39.8492050
820.0000000	6.0023999	-1577.6828613
820.0000000	6.0511999	-14.5649080
820.0000000	6.0999999	-19.4223270
820.0000000	6.1487999	-13.2660618
820.0000000	6.1975999	-23.2480850
820.0000000	6.2463999	-1797.3521729
820.0000000	6.2951999	-19.0832481
820.0000000	6.3439999	-343.7329712
820.0000000	6.3927999	-15.0484171
820.0000000	6.4416003	-12.3764553
820.0000000	6.4903998	-11.2526522
820.0000000	6.5391998	-11.7950096
820.0000000	6.5879998	396.5224915
820.0000000	6.6367998	-12.0749702
820.0000000	6.6856003	-13.8120432
820.0000000	6.7343998	-13.4519835
820.0000000	6.7831998	-14.3797140
820.0000000	6.8319998	58.7712326
820.0000000	6.8807998	-14.0518408
820.0000000	6.9296002	-462.9366760
820.0000000	6.9783998	-4.8565369
820.0000000	7.0271997	-14.1557627
820.0000000	7.0760002	11.9505529
820.0000000	7.1247997	-2203.6704102
820.0000000	7.1736002	-1767.1583252
820.0000000	7.2223997	-1005.2304077
820.0000000	7.2711997	-216.3996887
820.0000000	7.3200002	-19.1046963
820.0000000	7.3687997	194.8638916
820.0000000	7.4176002	406.0119324
820.0000000	7.4663997	319.0437317
820.0000000	7.5151997	-983.5775146
820.0000000	7.5640001	-1086.4830322
820.0000000	7.6127996	-2561.4353027
820.0000000	7.6616001	-822.1442871
820.0000000	7.7104001	-422.1058350
820.0000000	7.7591996	45.1368370
820.0000000	7.8080001	421.9288635

820.0000000	7.8567996	555.6893921
820.0000000	7.9056001	-1150.5621338
820.0000000	7.9544001	1831.4671631
820.0000000	8.0031996	2363.1845703
820.0000000	8.0520000	2358.7185059
820.0000000	8.1007996	2495.9814453
820.0000000	8.1496000	2661.8664551
820.0000000	8.1983995	1656.0729980
820.0000000	8.2472000	-3788.8417969
820.0000000	8.2959995	-1343.0074463
820.0000000	8.3448000	-1069.8364258
820.0000000	8.3935995	-980.4727173
820.0000000	8.4423990	-422.2672424
820.0000000	8.4912004	-985.9467163
820.0000000	8.5400000	-2659.8127441
820.0000000	8.5887995	-1289.1776123
820.0000000	8.6375999	251.9755402
820.0000000	8.6863995	2275.3569336
820.0000000	8.7351999	-845.5556641
820.0000000	8.7840004	966.5324707
820.0000000	8.8327999	52.7179985
820.0000000	8.8815994	-11.4930267
820.0000000	8.9303989	-24.0181007
820.0000000	8.9792004	-11.5774870
820.0000000	9.0279999	-5.8020811
820.0000000	9.0767994	7.1521435
820.0000000	9.1255999	-17.2759438
820.0000000	9.1743994	-10.0656185
820.0000000	9.2231998	37.3240356
820.0000000	9.2720003	-8.4014750
820.0000000	9.3207998	-9.7873526
820.0000000	9.3695993	-10.7592525
820.0000000	9.4183998	-11.7563677
820.0000000	9.4672003	-11.1605072
820.0000000	9.5159998	-14.1820288
820.0000000	9.5648003	-0.3256245
820.0000000	9.6135998	-13.4118233
820.0000000	9.6623993	-10.2158327
820.0000000	9.7111998	-10.1522741
820.0000000	9.7600002	-10.8567419
820.0000000	9.8087997	-10.9946194
820.0000000	9.8575993	3.2783518
820.0000000	9.9063997	-7.9507866
820.0000000	9.9552002	-10.9065151
820.0000000	10.0039997	-10.0697680
830.0000000	0.3416000	-36.5550690
830.0000000	0.3904000	-39.6752586
830.0000000	0.4392000	-39.8621979
830.0000000	0.4880000	-39.6142273
830.0000000	0.5368000	-39.5914612
830.0000000	0.5856000	-39.3588982
830.0000000	0.6344000	-38.2195511
830.0000000	0.6832000	-36.1236076
830.0000000	0.7320000	-35.9626274
830.0000000	0.7808000	-36.5271912
830.0000000	0.8296000	-35.9605942
830.0000000	0.8784000	-35.0558128
830.0000000	0.9272000	-32.8158607

830.0000000	0.9760000	-30.7480469
830.0000000	1.0247999	-29.5219364
830.0000000	1.0735999	-29.0997677
830.0000000	1.1224000	-29.2223263
830.0000000	1.1711999	-29.9230690
830.0000000	1.2200000	-30.7212372
830.0000000	1.2687999	-30.8797379
830.0000000	1.3176000	-30.9920597
830.0000000	1.3664000	-30.4236584
830.0000000	1.4152000	-30.1584320
830.0000000	1.4640000	-29.6477985
830.0000000	1.5128000	-41.2215958
830.0000000	1.5616000	-34.4252586
830.0000000	1.6104001	-1605.5301514
830.0000000	1.6592000	-78.8429031
830.0000000	1.7079999	-27.1863403
830.0000000	1.7567999	-27.7617798
830.0000000	1.8055999	-27.5775394
830.0000000	1.8544000	-26.6806259
830.0000000	1.9031999	-26.0166473
830.0000000	1.9520000	-26.8863544
830.0000000	2.0007999	-25.7688904
830.0000000	2.0495999	-25.8583069
830.0000000	2.0983999	-176.0139771
830.0000000	2.1471999	3.4034462
830.0000000	2.1960001	-25.5262356
830.0000000	2.2448001	-26.1680756
830.0000000	2.2935998	-28.8132820
830.0000000	2.3423998	-20.7161636
830.0000000	2.3912001	43.6899033
830.0000000	2.4400001	-22.7902222
830.0000000	2.4888000	-23.4642181
830.0000000	2.5375998	-11.9413614
830.0000000	2.5863998	-10.0112667
830.0000000	2.6352000	-8.0258017
830.0000000	2.6840000	-15.1796303
830.0000000	2.7328000	138.3598022
830.0000000	2.7815998	163.1127319
830.0000000	2.8304000	-23.8199100
830.0000000	2.8792000	-20.5554199
830.0000000	2.9280000	-22.5829353
830.0000000	2.9768000	-16.6252804
830.0000000	3.0256000	-20.4221687
830.0000000	3.0743999	-29.7401924
830.0000000	3.1231999	-24.3229027
830.0000000	3.1719999	-29.7684765
830.0000000	3.2208002	-508.8976135
830.0000000	3.2695999	-996.5439453
830.0000000	3.3183999	-20.4858360
830.0000000	3.3671999	-3424.4316406
830.0000000	3.4159999	1228.7178955
830.0000000	3.4648001	-7.0320606
830.0000000	3.5135999	-27.0939598
830.0000000	3.5623999	-324.4109192
830.0000000	3.6111999	-2039.1467285
830.0000000	3.6600001	-461.5426025
830.0000000	3.7088001	-609.4871826
830.0000000	3.7575998	-954.3299561

830.0000000	3.8063998	-1731.8432617
830.0000000	3.8552001	-2514.4462891
830.0000000	3.9040000	-2465.2404785
830.0000000	3.9528000	1127.5246582
830.0000000	4.0015998	-246.2088623
830.0000000	4.0503998	690.3630981
830.0000000	4.0991998	88.0347366
830.0000000	4.1479998	-657.2301025
830.0000000	4.1967998	2.6301136
830.0000000	4.2456002	-249.4006500
830.0000000	4.2943997	-489.8161316
830.0000000	4.3431997	-13.3116264
830.0000000	4.3920002	-249.0294037
830.0000000	4.4407997	-22.4766426
830.0000000	4.4896002	-15.4662514
830.0000000	4.5383997	-657.4246216
830.0000000	4.5871997	-12.8204918
830.0000000	4.6360002	-21.6349449
830.0000000	4.6847997	-28.3175812
830.0000000	4.7336001	-19.5689011
830.0000000	4.7824001	-16.5082951
830.0000000	4.8311996	-1479.0739746
830.0000000	4.8800001	229.0487213
830.0000000	4.9287996	-202.0468750
830.0000000	4.9776001	1063.0410156
830.0000000	5.0264001	86.5064621
830.0000000	5.0751996	-302.0527649
830.0000000	5.1240001	-12.3002262
830.0000000	5.1727996	559.4564819
830.0000000	5.2216001	32.1284256
830.0000000	5.2704000	-159.2099762
830.0000000	5.3191996	-39.2464142
830.0000000	5.3680000	-3545.6203613
830.0000000	5.4168000	528.6376953
830.0000000	5.4656000	-201.2512665
830.0000000	5.5144000	38.2180862
830.0000000	5.5631995	489.7237244
830.0000000	5.6120000	394.2767944
830.0000000	5.6608000	-37.1428528
830.0000000	5.7096000	62.7498093
830.0000000	5.7584000	133.4519653
830.0000000	5.8071995	-11.5078840
830.0000000	5.8559999	29.9438038
830.0000000	5.9047999	-16.6084633
830.0000000	5.9535999	-39.8444633
830.0000000	6.0023999	-1577.3785400
830.0000000	6.0511999	-14.5651197
830.0000000	6.0999999	-19.4214954
830.0000000	6.1487999	-13.2665243
830.0000000	6.1975999	-23.2463207
830.0000000	6.2463999	-1797.0047607
830.0000000	6.2951999	-19.0824356
830.0000000	6.3439999	-343.6689148
830.0000000	6.3927999	-15.0483980
830.0000000	6.4416003	-12.3770428
830.0000000	6.4903998	-11.2533283
830.0000000	6.5391998	-11.7956905
830.0000000	6.5879998	395.8317871

830.0000000	6.6367998	-12.0755196
830.0000000	6.6856003	-13.8122139
830.0000000	6.7343998	-13.4521990
830.0000000	6.7831998	-14.3792982
830.0000000	6.8319998	58.7521133
830.0000000	6.8807998	-14.0517025
830.0000000	6.9296002	-462.1752014
830.0000000	6.9783998	-4.8693609
830.0000000	7.0271997	-14.1547346
830.0000000	7.0760002	11.9083681
830.0000000	7.1247997	-2199.7243652
830.0000000	7.1736002	-1763.9953613
830.0000000	7.2223997	-1003.4372559
830.0000000	7.2711997	-216.0319061
830.0000000	7.3200002	-19.0927086
830.0000000	7.3687997	194.4893646
830.0000000	7.4176002	405.2561035
830.0000000	7.4663997	318.4460754
830.0000000	7.5151997	-981.8164673
830.0000000	7.5640001	-1084.5356445
830.0000000	7.6127996	-2556.8178711
830.0000000	7.6616001	-820.6729736
830.0000000	7.7104001	-421.3596191
830.0000000	7.7591996	45.0357857
830.0000000	7.8080001	421.1434937
830.0000000	7.8567996	554.6613159
830.0000000	7.9056001	-1148.4892578
830.0000000	7.9544001	1828.1572266
830.0000000	8.0031996	2358.9150391
830.0000000	8.0520000	2354.4565430
830.0000000	8.1007996	2491.4719238
830.0000000	8.1496000	2657.0583496
830.0000000	8.1983995	1653.0805664
830.0000000	8.2472000	-3782.0068359
830.0000000	8.2959995	-1340.5872803
830.0000000	8.3448000	-1067.9072266
830.0000000	8.3935995	-978.7050781
830.0000000	8.4423990	-421.5092468
830.0000000	8.4912004	-984.1704712
830.0000000	8.5400000	-2655.0129395
830.0000000	8.5887995	-1286.8560791
830.0000000	8.6375999	251.5142212
830.0000000	8.6863995	2271.2385254
830.0000000	8.7351999	-844.0314331
830.0000000	8.7840004	964.7757568
830.0000000	8.8327999	52.6063919
830.0000000	8.8815994	-11.4932013
830.0000000	8.9303989	-24.0143795
830.0000000	8.9792004	-11.5775700
830.0000000	9.0279999	-5.8038497
830.0000000	9.0767994	7.1464272
830.0000000	9.1255999	-17.2742290
830.0000000	9.1743994	-10.0660210
830.0000000	9.2231998	37.3083038
830.0000000	9.2720003	-8.4024115
830.0000000	9.3207998	-9.7878923
830.0000000	9.3695993	-10.7594795
830.0000000	9.4183998	-11.7562437

830.0000000	9.4672003	-11.1605492
830.0000000	9.5159998	-14.1811380
830.0000000	9.5648003	-0.3289967
830.0000000	9.6135998	-13.4110327
830.0000000	9.6623993	-10.2161074
830.0000000	9.7111998	-10.1525459
830.0000000	9.7600002	-10.8567829
830.0000000	9.8087997	-10.9946270
830.0000000	9.8575993	3.2739277
830.0000000	9.9063997	-7.9517002
830.0000000	9.9552002	-10.9065199
830.0000000	10.0039997	-10.0700159
840.0000000	0.3416000	-36.9205856
840.0000000	0.3904000	-39.6582603
840.0000000	0.4392000	-39.8558540
840.0000000	0.4880000	-39.6130180
840.0000000	0.5368000	-39.5926323
840.0000000	0.5856000	-39.3575172
840.0000000	0.6344000	-38.2198601
840.0000000	0.6832000	-36.1258316
840.0000000	0.7320000	-35.9646797
840.0000000	0.7808000	-36.5273590
840.0000000	0.8296000	-35.9613228
840.0000000	0.8784000	-35.0631866
840.0000000	0.9272000	-32.8186378
840.0000000	0.9760000	-30.7405949
840.0000000	1.0247999	-29.5309258
840.0000000	1.0735999	-29.1142025
840.0000000	1.1224000	-29.2193260
840.0000000	1.1711999	-29.9156914
840.0000000	1.2200000	-30.7291527
840.0000000	1.2687999	-30.8807449
840.0000000	1.3176000	-30.9877892
840.0000000	1.3664000	-30.4314079
840.0000000	1.4152000	-30.1586361
840.0000000	1.4640000	-29.6460896
840.0000000	1.5128000	-41.1244202
840.0000000	1.5616000	-34.3834190
840.0000000	1.6104001	-1602.9874268
840.0000000	1.6592000	-78.5489731
840.0000000	1.7079999	-27.1900883
840.0000000	1.7567999	-27.7617989
840.0000000	1.8055999	-27.5814285
840.0000000	1.8544000	-27.0199261
840.0000000	1.9031999	-26.6268044
840.0000000	1.9520000	-26.0478153
840.0000000	2.0007999	-25.8114471
840.0000000	2.0495999	-26.0449238
840.0000000	2.0983999	-174.5381775
840.0000000	2.1471999	3.1361790
840.0000000	2.1960001	-25.5221443
840.0000000	2.2448001	-26.1501637
840.0000000	2.2935998	-28.7536583
840.0000000	2.3423998	-20.7305679
840.0000000	2.3912001	43.0683594
840.0000000	2.4400001	-22.7927074
840.0000000	2.4888000	-23.4640007
840.0000000	2.5375998	-11.9584694

840.0000000	2.5863998	-10.0310259
840.0000000	2.6352000	-8.0485458
840.0000000	2.6840000	-15.1915722
840.0000000	2.7328000	138.0937805
840.0000000	2.7815998	162.8063049
840.0000000	2.8304000	-23.8186054
840.0000000	2.8792000	-20.5586834
840.0000000	2.9280000	-22.5823669
840.0000000	2.9768000	-16.6336517
840.0000000	3.0256000	-20.4247532
840.0000000	3.0743999	-29.7288055
840.0000000	3.1231999	-24.3194466
840.0000000	3.1719999	-29.7601833
840.0000000	3.2208002	-508.6598816
840.0000000	3.2695999	-996.0689697
840.0000000	3.3183999	-20.4860344
840.0000000	3.3671999	-3422.9699707
840.0000000	3.4159999	1228.1826172
840.0000000	3.4648001	-7.0377693
840.0000000	3.5135999	-27.0909691
840.0000000	3.5623999	-324.2813110
840.0000000	3.6111999	-2038.2869873
840.0000000	3.6600001	-461.3544922
840.0000000	3.7088001	-609.2357788
840.0000000	3.7575998	-953.9314575
840.0000000	3.8063998	-1731.1134033
840.0000000	3.8552001	-2513.3828125
840.0000000	3.9040000	-2464.1979980
840.0000000	3.9528000	1127.0363770
840.0000000	4.0015998	-246.1117554
840.0000000	4.0503998	690.0605469
840.0000000	4.0991998	87.9890518
840.0000000	4.1479998	-656.9578857
840.0000000	4.1967998	2.6208572
840.0000000	4.2456002	-249.3023376
840.0000000	4.2943997	-489.6155396
840.0000000	4.3431997	-13.3138075
840.0000000	4.3920002	-248.9312897
840.0000000	4.4407997	-22.4748650
840.0000000	4.4896002	-15.4671345
840.0000000	4.5383997	-657.1635132
840.0000000	4.5871997	-12.8225079
840.0000000	4.6360002	-21.6334019
840.0000000	4.6847997	-28.3136559
840.0000000	4.7336001	-19.5681782
840.0000000	4.7824001	-16.5087490
840.0000000	4.8311996	-1478.5427246
840.0000000	4.8800001	228.9591675
840.0000000	4.9287996	-201.9796143
840.0000000	4.9776001	1062.6484375
840.0000000	5.0264001	86.4689407
840.0000000	5.0751996	-301.9491577
840.0000000	5.1240001	-12.3011475
840.0000000	5.1727996	559.3406372
840.0000000	5.2216001	32.1189842
840.0000000	5.2704000	-159.1818542
840.0000000	5.3191996	-39.2418633
840.0000000	5.3680000	-3544.9279785

840.0000000	5.4168000	528.5309448
840.0000000	5.4656000	-201.2150879
840.0000000	5.5144000	38.2074356
840.0000000	5.5631995	489.6251526
840.0000000	5.6120000	394.1968079
840.0000000	5.6608000	-37.1387634
840.0000000	5.7096000	62.7343750
840.0000000	5.7584000	133.4230347
840.0000000	5.8071995	-11.5087547
840.0000000	5.8559999	29.9349537
840.0000000	5.9047999	-16.6082668
840.0000000	5.9535999	-39.8397255
840.0000000	6.0023999	-1577.0740967
840.0000000	6.0511999	-14.5653315
840.0000000	6.0999999	-19.4206619
840.0000000	6.1487999	-13.2669868
840.0000000	6.1975999	-23.2445564
840.0000000	6.2463999	-1796.6573486
840.0000000	6.2951999	-19.0816212
840.0000000	6.3439999	-343.6048584
840.0000000	6.3927999	-15.0483799
840.0000000	6.4416003	-12.3776293
840.0000000	6.4903998	-11.2540045
840.0000000	6.5391998	-11.7963715
840.0000000	6.5879998	395.1410828
840.0000000	6.6367998	-12.0760689
840.0000000	6.6856003	-13.8123837
840.0000000	6.7343998	-13.4524145
840.0000000	6.7831998	-14.3788824
840.0000000	6.8319998	58.7330017
840.0000000	6.8807998	-14.0515633
840.0000000	6.9296002	-461.4137573
840.0000000	6.9783998	-4.8821840
840.0000000	7.0271997	-14.1537066
840.0000000	7.0760002	11.8661814
840.0000000	7.1247997	-2195.7785645
840.0000000	7.1736002	-1760.8325195
840.0000000	7.2223997	-1001.6440430
840.0000000	7.2711997	-215.6641235
840.0000000	7.3200002	-19.0807190
840.0000000	7.3687997	194.1148376
840.0000000	7.4176002	404.5002747
840.0000000	7.4663997	317.8484192
840.0000000	7.5151997	-980.0554199
840.0000000	7.5640001	-1082.5882568
840.0000000	7.6127996	-2552.2001953
840.0000000	7.6616001	-819.2015991
840.0000000	7.7104001	-420.6132813
840.0000000	7.7591996	44.9347382
840.0000000	7.8080001	420.3581238
840.0000000	7.8567996	553.6332397
840.0000000	7.9056001	-1146.4163818
840.0000000	7.9544001	1824.8472900
840.0000000	8.0031996	2354.6452637
840.0000000	8.0520000	2350.1945801
840.0000000	8.1007996	2486.9624023
840.0000000	8.1496000	2652.2504883
840.0000000	8.1983995	1650.0880127

840.0000000	8.2472000	-3775.1721191
840.0000000	8.2959995	-1338.1671143
840.0000000	8.3448000	-1065.9780273
840.0000000	8.3935995	-976.9373779
840.0000000	8.4423990	-420.7512207
840.0000000	8.4912004	-982.3942871
840.0000000	8.5400000	-2650.2131348
840.0000000	8.5887995	-1284.5345459
840.0000000	8.6375999	251.0528870
840.0000000	8.6863995	2267.1201172
840.0000000	8.7351999	-842.5072021
840.0000000	8.7840004	963.0189819
840.0000000	8.8327999	52.4947815
840.0000000	8.8815994	-11.4933748
840.0000000	8.9303989	-24.0106583
840.0000000	8.9792004	-11.5776520
840.0000000	9.0279999	-5.8056183
840.0000000	9.0767994	7.1407089
840.0000000	9.1255999	-17.2725143
840.0000000	9.1743994	-10.0664225
840.0000000	9.2231998	37.2925720
840.0000000	9.2720003	-8.4033480
840.0000000	9.3207998	-9.7884312
840.0000000	9.3695993	-10.7597055
840.0000000	9.4183998	-11.7561188
840.0000000	9.4672003	-11.1605902
840.0000000	9.5159998	-14.1802464
840.0000000	9.5648003	-0.3323689
840.0000000	9.6135998	-13.4102421
840.0000000	9.6623993	-10.2163830
840.0000000	9.7111998	-10.1528177
840.0000000	9.7600002	-10.8568249
840.0000000	9.8087997	-10.9946356
840.0000000	9.8575993	3.2695065
840.0000000	9.9063997	-7.9526143
840.0000000	9.9552002	-10.9065237
840.0000000	10.0039997	-10.0702629
850.0000000	0.3416000	-36.4462242
850.0000000	0.3904000	-39.6412621
850.0000000	0.4392000	-39.8495102
850.0000000	0.4880000	-39.6118088
850.0000000	0.5368000	-39.5938072
850.0000000	0.5856000	-39.3561363
850.0000000	0.6344000	-38.2201691
850.0000000	0.6832000	-36.1280594
850.0000000	0.7320000	-35.9667358
850.0000000	0.7808000	-36.5275307
850.0000000	0.8296000	-35.9620514
850.0000000	0.8784000	-35.0705605
850.0000000	0.9272000	-32.8214149
850.0000000	0.9760000	-30.7331409
850.0000000	1.0247999	-29.5399151
850.0000000	1.0735999	-29.1286373
850.0000000	1.1224000	-29.2163277
850.0000000	1.1711999	-29.9083138
850.0000000	1.2200000	-30.7370682
850.0000000	1.2687999	-30.8817539
850.0000000	1.3176000	-30.9835167

850.0000000	1.3664000	-30.4391575
850.0000000	1.4152000	-30.1588402
850.0000000	1.4640000	-29.6443806
850.0000000	1.5128000	-41.0272446
850.0000000	1.5616000	-34.3415756
850.0000000	1.6104001	-1600.4445801
850.0000000	1.6592000	-78.2550430
850.0000000	1.7079999	-27.1938343
850.0000000	1.7567999	-27.7618179
850.0000000	1.8055999	-27.5853157
850.0000000	1.8544000	-27.3592262
850.0000000	1.9031999	-26.2582378
850.0000000	1.9520000	-26.3200283
850.0000000	2.0007999	-25.6574783
850.0000000	2.0495999	-26.2315407
850.0000000	2.0983999	-173.0624084
850.0000000	2.1471999	2.8689117
850.0000000	2.1960001	-25.5180531
850.0000000	2.2448001	-26.1322517
850.0000000	2.2935998	-28.6940346
850.0000000	2.3423998	-20.7449722
850.0000000	2.3912001	42.4468155
850.0000000	2.4400001	-22.7951946
850.0000000	2.4888000	-23.4637833
850.0000000	2.5375998	-11.9755774
850.0000000	2.5863998	-10.0507841
850.0000000	2.6352000	-8.0712900
850.0000000	2.6840000	-15.2035141
850.0000000	2.7328000	137.8277740
850.0000000	2.7815998	162.4998779
850.0000000	2.8304000	-23.8173027
850.0000000	2.8792000	-20.5619469
850.0000000	2.9280000	-22.5817966
850.0000000	2.9768000	-16.6420212
850.0000000	3.0256000	-20.4273376
850.0000000	3.0743999	-29.7174187
850.0000000	3.1231999	-24.3159904
850.0000000	3.1719999	-29.7518902
850.0000000	3.2208002	-508.4221497
850.0000000	3.2695999	-995.5939941
850.0000000	3.3183999	-20.4862309
850.0000000	3.3671999	-3421.5083008
850.0000000	3.4159999	1227.6473389
850.0000000	3.4648001	-7.0434771
850.0000000	3.5135999	-27.0879784
850.0000000	3.5623999	-324.1517334
850.0000000	3.6111999	-2037.4272461
850.0000000	3.6600001	-461.1663208
850.0000000	3.7088001	-608.9844360
850.0000000	3.7575998	-953.5328979
850.0000000	3.8063998	-1730.3835449
850.0000000	3.8552001	-2512.3195801
850.0000000	3.9040000	-2463.1555176
850.0000000	3.9528000	1126.5480957
850.0000000	4.0015998	-246.0146484
850.0000000	4.0503998	689.7579956
850.0000000	4.0991998	87.9433670
850.0000000	4.1479998	-656.6856689

850.0000000	4.1967998	2.6116009
850.0000000	4.2456002	-249.2040405
850.0000000	4.2943997	-489.4149780
850.0000000	4.3431997	-13.3159885
850.0000000	4.3920002	-248.8331757
850.0000000	4.4407997	-22.4730911
850.0000000	4.4896002	-15.4680176
850.0000000	4.5383997	-656.9024658
850.0000000	4.5871997	-12.8245239
850.0000000	4.6360002	-21.6318588
850.0000000	4.6847997	-28.3097305
850.0000000	4.7336001	-19.5674553
850.0000000	4.7824001	-16.5092030
850.0000000	4.8311996	-1478.0113525
850.0000000	4.8800001	228.8696289
850.0000000	4.9287996	-201.9123535
850.0000000	4.9776001	1062.2559814
850.0000000	5.0264001	86.4314194
850.0000000	5.0751996	-301.8455200
850.0000000	5.1240001	-12.3020687
850.0000000	5.1727996	559.2247925
850.0000000	5.2216001	32.1095390
850.0000000	5.2704000	-159.1537628
850.0000000	5.3191996	-39.2373161
850.0000000	5.3680000	-3544.2355957
850.0000000	5.4168000	528.4241333
850.0000000	5.4656000	-201.1789093
850.0000000	5.5144000	38.1967773
850.0000000	5.5631995	489.5265808
850.0000000	5.6120000	394.1168518
850.0000000	5.6608000	-37.1346741
850.0000000	5.7096000	62.7189407
850.0000000	5.7584000	133.3941040
850.0000000	5.8071995	-11.5096245
850.0000000	5.8559999	29.9260998
850.0000000	5.9047999	-16.6080685
850.0000000	5.9535999	-39.8349876
850.0000000	6.0023999	-1576.7697754
850.0000000	6.0511999	-14.5655422
850.0000000	6.0999999	-19.4198303
850.0000000	6.1487999	-13.2674503
850.0000000	6.1975999	-23.2427902
850.0000000	6.2463999	-1796.3100586
850.0000000	6.2951999	-19.0808067
850.0000000	6.3439999	-343.5408325
850.0000000	6.3927999	-15.0483618
850.0000000	6.4416003	-12.3782167
850.0000000	6.4903998	-11.2546797
850.0000000	6.5391998	-11.7970524
850.0000000	6.5879998	394.4503784
850.0000000	6.6367998	-12.0766182
850.0000000	6.6856003	-13.8125544
850.0000000	6.7343998	-13.4526310
850.0000000	6.7831998	-14.3784666
850.0000000	6.8319998	58.7138824
850.0000000	6.8807998	-14.0514250
850.0000000	6.9296002	-460.6523132
850.0000000	6.9783998	-4.8950076

850.0000000	7.0271997	-14.1526785
850.0000000	7.0760002	11.8239965
850.0000000	7.1247997	-2191.8327637
850.0000000	7.1736002	-1757.6696777
850.0000000	7.2223997	-999.8508911
850.0000000	7.2711997	-215.2963562
850.0000000	7.3200002	-19.0687294
850.0000000	7.3687997	193.7402954
850.0000000	7.4176002	403.7444458
850.0000000	7.4663997	317.2507629
850.0000000	7.5151997	-978.2943726
850.0000000	7.5640001	-1080.6408691
850.0000000	7.6127996	-2547.5825195
850.0000000	7.6616001	-817.7302246
850.0000000	7.7104001	-419.8670654
850.0000000	7.7591996	44.8336906
850.0000000	7.8080001	419.5727844
850.0000000	7.8567996	552.6051025
850.0000000	7.9056001	-1144.3435059
850.0000000	7.9544001	1821.5373535
850.0000000	8.0031996	2350.3757324
850.0000000	8.0520000	2345.9326172
850.0000000	8.1007996	2482.4528809
850.0000000	8.1496000	2647.4423828
850.0000000	8.1983995	1647.0954590
850.0000000	8.2472000	-3768.3374023
850.0000000	8.2959995	-1335.7469482
850.0000000	8.3448000	-1064.0488281
850.0000000	8.3935995	-975.1697388
850.0000000	8.4423990	-419.9932251
850.0000000	8.4912004	-980.6180420
850.0000000	8.5400000	-2645.4133301
850.0000000	8.5887995	-1282.2130127
850.0000000	8.6375999	250.5915680
850.0000000	8.6863995	2263.0017090
850.0000000	8.7351999	-840.9829102
850.0000000	8.7840004	961.2622681
850.0000000	8.8327999	52.3831787
850.0000000	8.8815994	-11.4935493
850.0000000	8.9303989	-24.0069389
850.0000000	8.9792004	-11.5777340
850.0000000	9.0279999	-5.8073859
850.0000000	9.0767994	7.1349907
850.0000000	9.1255999	-17.2708015
850.0000000	9.1743994	-10.0668249
850.0000000	9.2231998	37.2768402
850.0000000	9.2720003	-8.4042845
850.0000000	9.3207998	-9.7889709
850.0000000	9.3695993	-10.7599316
850.0000000	9.4183998	-11.7559938
850.0000000	9.4672003	-11.1606312
850.0000000	9.5159998	-14.1793556
850.0000000	9.5648003	-0.3357420
850.0000000	9.6135998	-13.4094515
850.0000000	9.6623993	-10.2166576
850.0000000	9.7111998	-10.1530895
850.0000000	9.7600002	-10.8568668
850.0000000	9.8087997	-10.9946432

850.0000000	9.8575993	3.2650843
850.0000000	9.9063997	-7.9535284
850.0000000	9.9552002	-10.9065285
850.0000000	10.0039997	-10.0705109
860.0000000	0.3416000	-36.4722404
860.0000000	0.3904000	-39.8194580
860.0000000	0.4392000	-39.8431664
860.0000000	0.4880000	-39.6105995
860.0000000	0.5368000	-39.5949821
860.0000000	0.5856000	-39.3547554
860.0000000	0.6344000	-38.2204819
860.0000000	0.6832000	-36.1302834
860.0000000	0.7320000	-35.9687881
860.0000000	0.7808000	-36.5276985
860.0000000	0.8296000	-35.9627838
860.0000000	0.8784000	-35.0779381
860.0000000	0.9272000	-32.8241920
860.0000000	0.9760000	-30.7256889
860.0000000	1.0247999	-29.5489044
860.0000000	1.0735999	-29.1430721
860.0000000	1.1224000	-29.2133274
860.0000000	1.1711999	-29.9009380
860.0000000	1.2200000	-30.7449818
860.0000000	1.2687999	-30.8827610
860.0000000	1.3176000	-30.9792461
860.0000000	1.3664000	-30.4469070
860.0000000	1.4152000	-30.1590443
860.0000000	1.4640000	-29.6426697
860.0000000	1.5128000	-40.9300652
860.0000000	1.5616000	-34.2997360
860.0000000	1.6104001	-1597.9017334
860.0000000	1.6592000	-77.9611053
860.0000000	1.7079999	-27.1975822
860.0000000	1.7567999	-27.7618370
860.0000000	1.8055999	-27.5892048
860.0000000	1.8544000	-26.1099358
860.0000000	1.9031999	-26.1818333
860.0000000	1.9520000	-26.3720398
860.0000000	2.0007999	-26.5691414
860.0000000	2.0495999	-26.2213650
860.0000000	2.0983999	-171.5866394
860.0000000	2.1471999	2.6016445
860.0000000	2.1960001	-25.5139618
860.0000000	2.2448001	-26.1143417
860.0000000	2.2935998	-28.6344109
860.0000000	2.3423998	-20.7593765
860.0000000	2.3912001	41.8252640
860.0000000	2.4400001	-22.7976818
860.0000000	2.4888000	-23.4635658
860.0000000	2.5375998	-11.9926853
860.0000000	2.5863998	-10.0705433
860.0000000	2.6352000	-8.0940342
860.0000000	2.6840000	-15.2154560
860.0000000	2.7328000	137.5617523
860.0000000	2.7815998	162.1934814
860.0000000	2.8304000	-23.8159981
860.0000000	2.8792000	-20.5652103
860.0000000	2.9280000	-22.5812283

860.0000000	2.9768000	-16.6503925
860.0000000	3.0256000	-20.4299221
860.0000000	3.0743999	-29.7060318
860.0000000	3.1231999	-24.3125362
860.0000000	3.1719999	-29.7435970
860.0000000	3.2208002	-508.1844177
860.0000000	3.2695999	-995.1190186
860.0000000	3.3183999	-20.4864292
860.0000000	3.3671999	-3420.0466309
860.0000000	3.4159999	1227.1120605
860.0000000	3.4648001	-7.0491858
860.0000000	3.5135999	-27.0849876
860.0000000	3.5623999	-324.0221252
860.0000000	3.6111999	-2036.5673828
860.0000000	3.6600001	-460.9782104
860.0000000	3.7088001	-608.7330322
860.0000000	3.7575998	-953.1343994
860.0000000	3.8063998	-1729.6536865
860.0000000	3.8552001	-2511.2561035
860.0000000	3.9040000	-2462.1130371
860.0000000	3.9528000	1126.0598145
860.0000000	4.0015998	-245.9175415
860.0000000	4.0503998	689.4554443
860.0000000	4.0991998	87.8976822
860.0000000	4.1479998	-656.4134521
860.0000000	4.1967998	2.6023445
860.0000000	4.2456002	-249.1057434
860.0000000	4.2943997	-489.2144165
860.0000000	4.3431997	-13.3181686
860.0000000	4.3920002	-248.7350616
860.0000000	4.4407997	-22.4713154
860.0000000	4.4896002	-15.4688997
860.0000000	4.5383997	-656.6414185
860.0000000	4.5871997	-12.8265400
860.0000000	4.6360002	-21.6303177
860.0000000	4.6847997	-28.3058052
860.0000000	4.7336001	-19.5667343
860.0000000	4.7824001	-16.5096588
860.0000000	4.8311996	-1477.4801025
860.0000000	4.8800001	228.7800751
860.0000000	4.9287996	-201.8450928
860.0000000	4.9776001	1061.8635254
860.0000000	5.0264001	86.3938980
860.0000000	5.0751996	-301.7418823
860.0000000	5.1240001	-12.3029900
860.0000000	5.1727996	559.1088867
860.0000000	5.2216001	32.1000938
860.0000000	5.2704000	-159.1256409
860.0000000	5.3191996	-39.2327652
860.0000000	5.3680000	-3543.5432129
860.0000000	5.4168000	528.3173828
860.0000000	5.4656000	-201.1427155
860.0000000	5.5144000	38.1861191
860.0000000	5.5631995	489.4280090
860.0000000	5.6120000	394.0368652
860.0000000	5.6608000	-37.1305847
860.0000000	5.7096000	62.7035141
860.0000000	5.7584000	133.3651733

860.0000000	5.8071995	-11.5104961
860.0000000	5.8559999	29.9172497
860.0000000	5.9047999	-16.6078701
860.0000000	5.9535999	-39.8302498
860.0000000	6.0023999	-1576.4652100
860.0000000	6.0511999	-14.5657539
860.0000000	6.0999999	-19.4189987
860.0000000	6.1487999	-13.2679129
860.0000000	6.1975999	-23.2410240
860.0000000	6.2463999	-1795.9626465
860.0000000	6.2951999	-19.0799923
860.0000000	6.3439999	-343.4767456
860.0000000	6.3927999	-15.0483437
860.0000000	6.4416003	-12.3788033
860.0000000	6.4903998	-11.2553558
860.0000000	6.5391998	-11.7977343
860.0000000	6.5879998	393.7597046
860.0000000	6.6367998	-12.0771675
860.0000000	6.6856003	-13.8127241
860.0000000	6.7343998	-13.4528465
860.0000000	6.7831998	-14.3780518
860.0000000	6.8319998	58.6947708
860.0000000	6.8807998	-14.0512867
860.0000000	6.9296002	-459.8908691
860.0000000	6.9783998	-4.9078317
860.0000000	7.0271997	-14.1516514
860.0000000	7.0760002	11.7818117
860.0000000	7.1247997	-2187.8869629
860.0000000	7.1736002	-1754.5068359
860.0000000	7.2223997	-998.0577393
860.0000000	7.2711997	-214.9285736
860.0000000	7.3200002	-19.0567417
860.0000000	7.3687997	193.3657684
860.0000000	7.4176002	402.9886169
860.0000000	7.4663997	316.6531067
860.0000000	7.5151997	-976.5333252
860.0000000	7.5640001	-1078.6934814
860.0000000	7.6127996	-2542.9648438
860.0000000	7.6616001	-816.2589111
860.0000000	7.7104001	-419.1207886
860.0000000	7.7591996	44.7326393
860.0000000	7.8080001	418.7874146
860.0000000	7.8567996	551.5770264
860.0000000	7.9056001	-1142.2705078
860.0000000	7.9544001	1818.2274170
860.0000000	8.0031996	2346.1062012
860.0000000	8.0520000	2341.6704102
860.0000000	8.1007996	2477.9433594
860.0000000	8.1496000	2642.6342773
860.0000000	8.1983995	1644.1030273
860.0000000	8.2472000	-3761.5024414
860.0000000	8.2959995	-1333.3267822
860.0000000	8.3448000	-1062.1196289
860.0000000	8.3935995	-973.4020386
860.0000000	8.4423990	-419.2351990
860.0000000	8.4912004	-978.8417969
860.0000000	8.5400000	-2640.6135254
860.0000000	8.5887995	-1279.8914795

860.0000000	8.6375999	250.1302490
860.0000000	8.6863995	2258.8835449
860.0000000	8.7351999	-839.4586792
860.0000000	8.7840004	959.5054932
860.0000000	8.8327999	52.2715683
860.0000000	8.8815994	-11.4937239
860.0000000	8.9303989	-24.0032196
860.0000000	8.9792004	-11.5778160
860.0000000	9.0279999	-5.8091545
860.0000000	9.0767994	7.1292725
860.0000000	9.1255999	-17.2690868
860.0000000	9.1743994	-10.0672264
860.0000000	9.2231998	37.2611008
860.0000000	9.2720003	-8.4052200
860.0000000	9.3207998	-9.7895098
860.0000000	9.3695993	-10.7601585
860.0000000	9.4183998	-11.7558699
860.0000000	9.4672003	-11.1606722
860.0000000	9.5159998	-14.1784649
860.0000000	9.5648003	-0.3391151
860.0000000	9.6135998	-13.4086609
860.0000000	9.6623993	-10.2169333
860.0000000	9.7111998	-10.1533604
860.0000000	9.7600002	-10.8569088
860.0000000	9.8087997	-10.9946508
860.0000000	9.8575993	3.2606602
860.0000000	9.9063997	-7.9544420
860.0000000	9.9552002	-10.9065323
860.0000000	10.0039997	-10.0707588
870.0000000	0.3416000	-36.8278732
870.0000000	0.3904000	-39.3651428
870.0000000	0.4392000	-39.8368225
870.0000000	0.4880000	-39.6093903
870.0000000	0.5368000	-39.5961533
870.0000000	0.5856000	-39.3533745
870.0000000	0.6344000	-38.2207909
870.0000000	0.6832000	-36.1325111
870.0000000	0.7320000	-35.9708405
870.0000000	0.7808000	-36.5278664
870.0000000	0.8296000	-35.9635124
870.0000000	0.8784000	-35.0853119
870.0000000	0.9272000	-32.8269691
870.0000000	0.9760000	-30.7182350
870.0000000	1.0247999	-29.5578938
870.0000000	1.0735999	-29.1575089
870.0000000	1.1224000	-29.2103271
870.0000000	1.1711999	-29.8935604
870.0000000	1.2200000	-30.7528973
870.0000000	1.2687999	-30.8837681
870.0000000	1.3176000	-30.9749737
870.0000000	1.3664000	-30.4546547
870.0000000	1.4152000	-30.1592484
870.0000000	1.4640000	-29.6409607
870.0000000	1.5128000	-40.8328857
870.0000000	1.5616000	-34.2578964
870.0000000	1.6104001	-1595.3590088
870.0000000	1.6592000	-77.6671753
870.0000000	1.7079999	-27.2013302

870.0000000	1.7567999	-27.7618561
870.0000000	1.8055999	-27.5930920
870.0000000	1.8544000	-26.0388660
870.0000000	1.9031999	-25.5193806
870.0000000	1.9520000	-26.4240532
870.0000000	2.0007999	-26.5621986
870.0000000	2.0495999	-26.2111893
870.0000000	2.0983999	-170.1108398
870.0000000	2.1471999	2.3343773
870.0000000	2.1960001	-25.5098705
870.0000000	2.2448001	-26.0964298
870.0000000	2.2935998	-28.5747871
870.0000000	2.3423998	-20.7737808
870.0000000	2.3912001	41.2037201
870.0000000	2.4400001	-22.8001671
870.0000000	2.4888000	-23.4633503
870.0000000	2.5375998	-12.0097933
870.0000000	2.5863998	-10.0903025
870.0000000	2.6352000	-8.1167784
870.0000000	2.6840000	-15.2273979
870.0000000	2.7328000	137.2957306
870.0000000	2.7815998	161.8870544
870.0000000	2.8304000	-23.8146935
870.0000000	2.8792000	-20.5684738
870.0000000	2.9280000	-22.5806599
870.0000000	2.9768000	-16.6587639
870.0000000	3.0256000	-20.4325066
870.0000000	3.0743999	-29.6946449
870.0000000	3.1231999	-24.3090801
870.0000000	3.1719999	-29.7353039
870.0000000	3.2208002	-507.9466858
870.0000000	3.2695999	-994.6440430
870.0000000	3.3183999	-20.4866276
870.0000000	3.3671999	-3418.5849609
870.0000000	3.4159999	1226.5767822
870.0000000	3.4648001	-7.0548944
870.0000000	3.5135999	-27.0819969
870.0000000	3.5623999	-323.8925476
870.0000000	3.6111999	-2035.7076416
870.0000000	3.6600001	-460.7901001
870.0000000	3.7088001	-608.4816284
870.0000000	3.7575998	-952.7358398
870.0000000	3.8063998	-1728.9238281
870.0000000	3.8552001	-2510.1926270
870.0000000	3.9040000	-2461.0705566
870.0000000	3.9528000	1125.5715332
870.0000000	4.0015998	-245.8204346
870.0000000	4.0503998	689.1528931
870.0000000	4.0991998	87.8519974
870.0000000	4.1479998	-656.1411743
870.0000000	4.1967998	2.5930882
870.0000000	4.2456002	-249.0074310
870.0000000	4.2943997	-489.0138550
870.0000000	4.3431997	-13.3203487
870.0000000	4.3920002	-248.6369476
870.0000000	4.4407997	-22.4695396
870.0000000	4.4896002	-15.4697819
870.0000000	4.5383997	-656.3803101

870.0000000	4.5871997	-12.8285561
870.0000000	4.6360002	-21.6287746
870.0000000	4.6847997	-28.3018799
870.0000000	4.7336001	-19.5660114
870.0000000	4.7824001	-16.5101128
870.0000000	4.8311996	-1476.9488525
870.0000000	4.8800001	228.6905212
870.0000000	4.9287996	-201.7778168
870.0000000	4.9776001	1061.4709473
870.0000000	5.0264001	86.3563843
870.0000000	5.0751996	-301.6382751
870.0000000	5.1240001	-12.3039112
870.0000000	5.1727996	558.9930420
870.0000000	5.2216001	32.0906525
870.0000000	5.2704000	-159.0975494
870.0000000	5.3191996	-39.2282181
870.0000000	5.3680000	-3542.8508301
870.0000000	5.4168000	528.2105713
870.0000000	5.4656000	-201.1065369
870.0000000	5.5144000	38.1754684
870.0000000	5.5631995	489.3294373
870.0000000	5.6120000	393.9568787
870.0000000	5.6608000	-37.1264992
870.0000000	5.7096000	62.6880798
870.0000000	5.7584000	133.3362427
870.0000000	5.8071995	-11.5113659
870.0000000	5.8559999	29.9083958
870.0000000	5.9047999	-16.6076736
870.0000000	5.9535999	-39.8255081
870.0000000	6.0023999	-1576.1608887
870.0000000	6.0511999	-14.5659657
870.0000000	6.0999999	-19.4181652
870.0000000	6.1487999	-13.2683754
870.0000000	6.1975999	-23.2392597
870.0000000	6.2463999	-1795.6152344
870.0000000	6.2951999	-19.0791779
870.0000000	6.3439999	-343.4127197
870.0000000	6.3927999	-15.0483246
870.0000000	6.4416003	-12.3793898
870.0000000	6.4903998	-11.2560320
870.0000000	6.5391998	-11.7984152
870.0000000	6.5879998	393.0690002
870.0000000	6.6367998	-12.0777168
870.0000000	6.6856003	-13.8128948
870.0000000	6.7343998	-13.4530621
870.0000000	6.7831998	-14.3776360
870.0000000	6.8319998	58.6756516
870.0000000	6.8807998	-14.0511475
870.0000000	6.9296002	-459.1294250
870.0000000	6.9783998	-4.9206543
870.0000000	7.0271997	-14.1506233
870.0000000	7.0760002	11.7396250
870.0000000	7.1247997	-2183.9409180
870.0000000	7.1736002	-1751.3439941
870.0000000	7.2223997	-996.2645874
870.0000000	7.2711997	-214.5607910
870.0000000	7.3200002	-19.0447540
870.0000000	7.3687997	192.9912415

870.0000000	7.4176002	402.2327881
870.0000000	7.4663997	316.0554810
870.0000000	7.5151997	-974.7722778
870.0000000	7.5640001	-1076.7460938
870.0000000	7.6127996	-2538.3474121
870.0000000	7.6616001	-814.7875366
870.0000000	7.7104001	-418.3745117
870.0000000	7.7591996	44.6315918
870.0000000	7.8080001	418.0020447
870.0000000	7.8567996	550.5489502
870.0000000	7.9056001	-1140.1976318
870.0000000	7.9544001	1814.9174805
870.0000000	8.0031996	2341.8366699
870.0000000	8.0520000	2337.4084473
870.0000000	8.1007996	2473.4338379
870.0000000	8.1496000	2637.8264160
870.0000000	8.1983995	1641.1104736
870.0000000	8.2472000	-3754.6677246
870.0000000	8.2959995	-1330.9064941
870.0000000	8.3448000	-1060.1904297
870.0000000	8.3935995	-971.6343994
870.0000000	8.4423990	-418.4772034
870.0000000	8.4912004	-977.0656128
870.0000000	8.5400000	-2635.8137207
870.0000000	8.5887995	-1277.5699463
870.0000000	8.6375999	249.6689301
870.0000000	8.6863995	2254.7651367
870.0000000	8.7351999	-837.9343872
870.0000000	8.7840004	957.7487793
870.0000000	8.8327999	52.1599617
870.0000000	8.8815994	-11.4938984
870.0000000	8.9303989	-23.9995003
870.0000000	8.9792004	-11.5778990
870.0000000	9.0279999	-5.8109226
870.0000000	9.0767994	7.1235561
870.0000000	9.1255999	-17.2673721
870.0000000	9.1743994	-10.0676289
870.0000000	9.2231998	37.2453690
870.0000000	9.2720003	-8.4061565
870.0000000	9.3207998	-9.7900496
870.0000000	9.3695993	-10.7603846
870.0000000	9.4183998	-11.7557449
870.0000000	9.4672003	-11.1607132
870.0000000	9.5159998	-14.1775742
870.0000000	9.5648003	-0.3424873
870.0000000	9.6135998	-13.4078693
870.0000000	9.6623993	-10.2172079
870.0000000	9.7111998	-10.1536322
870.0000000	9.7600002	-10.8569498
870.0000000	9.8087997	-10.9946594
870.0000000	9.8575993	3.2562389
870.0000000	9.9063997	-7.9553556
870.0000000	9.9552002	-10.9065371
870.0000000	10.0039997	-10.0710058
880.0000000	0.3416000	-36.7843361
880.0000000	0.3904000	-39.5210304
880.0000000	0.4392000	-39.8304787
880.0000000	0.4880000	-39.6081810

880.0000000	0.5368000	-39.5973282
880.0000000	0.5856000	-39.3519936
880.0000000	0.6344000	-38.2211037
880.0000000	0.6832000	-36.1347351
880.0000000	0.7320000	-35.9728928
880.0000000	0.7808000	-36.5280380
880.0000000	0.8296000	-35.9642410
880.0000000	0.8784000	-35.0926857
880.0000000	0.9272000	-32.8297462
880.0000000	0.9760000	-30.7107830
880.0000000	1.0247999	-29.5668831
880.0000000	1.0735999	-29.1719437
880.0000000	1.1224000	-29.2073288
880.0000000	1.1711999	-29.8861828
880.0000000	1.2200000	-30.7608128
880.0000000	1.2687999	-30.8847771
880.0000000	1.3176000	-30.9707031
880.0000000	1.3664000	-30.4624043
880.0000000	1.4152000	-30.1594524
880.0000000	1.4640000	-29.6392517
880.0000000	1.5128000	-40.7357101
880.0000000	1.5616000	-34.2160530
880.0000000	1.6104001	-1592.8161621
880.0000000	1.6592000	-77.3732452
880.0000000	1.7079999	-27.2050762
880.0000000	1.7567999	-27.7618752
880.0000000	1.8055999	-27.5969810
880.0000000	1.8544000	-26.3625793
880.0000000	1.9031999	-26.0423565
880.0000000	1.9520000	-26.5908890
880.0000000	2.0007999	-26.5552559
880.0000000	2.0495999	-26.2010117
880.0000000	2.0983999	-168.6350708
880.0000000	2.1471999	2.0671101
880.0000000	2.1960001	-25.5057793
880.0000000	2.2448001	-26.0785179
880.0000000	2.2935998	-28.5151634
880.0000000	2.3423998	-20.7881851
880.0000000	2.3912001	40.5821762
880.0000000	2.4400001	-22.8026543
880.0000000	2.4888000	-23.4631329
880.0000000	2.5375998	-12.0269012
880.0000000	2.5863998	-10.1100616
880.0000000	2.6352000	-8.1395226
880.0000000	2.6840000	-15.2393398
880.0000000	2.7328000	137.0297241
880.0000000	2.7815998	161.5806274
880.0000000	2.8304000	-23.8133888
880.0000000	2.8792000	-20.5717373
880.0000000	2.9280000	-22.5800896
880.0000000	2.9768000	-16.6671333
880.0000000	3.0256000	-20.4350910
880.0000000	3.0743999	-29.6832581
880.0000000	3.1231999	-24.3056240
880.0000000	3.1719999	-29.7270107
880.0000000	3.2208002	-507.7089539
880.0000000	3.2695999	-994.1690674
880.0000000	3.3183999	-20.4868259

880.0000000	3.3671999	-3417.1232910
880.0000000	3.4159999	1226.0413818
880.0000000	3.4648001	-7.0606012
880.0000000	3.5135999	-27.0790062
880.0000000	3.5623999	-323.7629700
880.0000000	3.6111999	-2034.8479004
880.0000000	3.6600001	-460.6019287
880.0000000	3.7088001	-608.2302856
880.0000000	3.7575998	-952.3372803
880.0000000	3.8063998	-1728.1939697
880.0000000	3.8552001	-2509.1291504
880.0000000	3.9040000	-2460.0280762
880.0000000	3.9528000	1125.0832520
880.0000000	4.0015998	-245.7233276
880.0000000	4.0503998	688.8502808
880.0000000	4.0991998	87.8063202
880.0000000	4.1479998	-655.8689575
880.0000000	4.1967998	2.5838318
880.0000000	4.2456002	-248.9091339
880.0000000	4.2943997	-488.8132935
880.0000000	4.3431997	-13.3225298
880.0000000	4.3920002	-248.5388336
880.0000000	4.4407997	-22.4677639
880.0000000	4.4896002	-15.4706650
880.0000000	4.5383997	-656.1192627
880.0000000	4.5871997	-12.8305731
880.0000000	4.6360002	-21.6272316
880.0000000	4.6847997	-28.2979546
880.0000000	4.7336001	-19.5652885
880.0000000	4.7824001	-16.5105667
880.0000000	4.8311996	-1476.4176025
880.0000000	4.8800001	228.6009827
880.0000000	4.9287996	-201.7105560
880.0000000	4.9776001	1061.0784912
880.0000000	5.0264001	86.3188629
880.0000000	5.0751996	-301.5346375
880.0000000	5.1240001	-12.3048325
880.0000000	5.1727996	558.8771362
880.0000000	5.2216001	32.0812073
880.0000000	5.2704000	-159.0694275
880.0000000	5.3191996	-39.2236710
880.0000000	5.3680000	-3542.1584473
880.0000000	5.4168000	528.1038208
880.0000000	5.4656000	-201.0703583
880.0000000	5.5144000	38.1648102
880.0000000	5.5631995	489.2308655
880.0000000	5.6120000	393.8769226
880.0000000	5.6608000	-37.1224098
880.0000000	5.7096000	62.6726456
880.0000000	5.7584000	133.3073120
880.0000000	5.8071995	-11.5122375
880.0000000	5.8559999	29.8995457
880.0000000	5.9047999	-16.6074753
880.0000000	5.9535999	-39.8207703
880.0000000	6.0023999	-1575.8563232
880.0000000	6.0511999	-14.5661764
880.0000000	6.0999999	-19.4173336
880.0000000	6.1487999	-13.2688389

880.0000000	6.1975999	-23.2374954
880.0000000	6.2463999	-1795.2678223
880.0000000	6.2951999	-19.0783634
880.0000000	6.3439999	-343.3486633
880.0000000	6.3927999	-15.0483065
880.0000000	6.4416003	-12.3799772
880.0000000	6.4903998	-11.2567081
880.0000000	6.5391998	-11.7990961
880.0000000	6.5879998	392.3782959
880.0000000	6.6367998	-12.0782652
880.0000000	6.6856003	-13.8130646
880.0000000	6.7343998	-13.4532776
880.0000000	6.7831998	-14.3772202
880.0000000	6.8319998	58.6565399
880.0000000	6.8807998	-14.0510092
880.0000000	6.9296002	-458.3679810
880.0000000	6.9783998	-4.9334784
880.0000000	7.0271997	-14.1495953
880.0000000	7.0760002	11.6974401
880.0000000	7.1247997	-2179.9951172
880.0000000	7.1736002	-1748.1810303
880.0000000	7.2223997	-994.4714355
880.0000000	7.2711997	-214.1930237
880.0000000	7.3200002	-19.0327644
880.0000000	7.3687997	192.6166992
880.0000000	7.4176002	401.4769592
880.0000000	7.4663997	315.4578247
880.0000000	7.5151997	-973.0112305
880.0000000	7.5640001	-1074.7987061
880.0000000	7.6127996	-2533.7297363
880.0000000	7.6616001	-813.3161621
880.0000000	7.7104001	-417.6282959
880.0000000	7.7591996	44.5305443
880.0000000	7.8080001	417.2166443
880.0000000	7.8567996	549.5208740
880.0000000	7.9056001	-1138.1247559
880.0000000	7.9544001	1811.6075439
880.0000000	8.0031996	2337.5668945
880.0000000	8.0520000	2333.1464844
880.0000000	8.1007996	2468.9243164
880.0000000	8.1496000	2633.0183105
880.0000000	8.1983995	1638.1179199
880.0000000	8.2472000	-3747.8327637
880.0000000	8.2959995	-1328.4863281
880.0000000	8.3448000	-1058.2613525
880.0000000	8.3935995	-969.8666992
880.0000000	8.4423990	-417.7191772
880.0000000	8.4912004	-975.2893677
880.0000000	8.5400000	-2631.0139160
880.0000000	8.5887995	-1275.2484131
880.0000000	8.6375999	249.2076111
880.0000000	8.6863995	2250.6467285
880.0000000	8.7351999	-836.4101563
880.0000000	8.7840004	955.9920044
880.0000000	8.8327999	52.0483513
880.0000000	8.8815994	-11.4940720
880.0000000	8.9303989	-23.9957809
880.0000000	8.9792004	-11.5779810

880.0000000	9.0279999	-5.8126912
880.0000000	9.0767994	7.1178379
880.0000000	9.1255999	-17.2656593
880.0000000	9.1743994	-10.0680304
880.0000000	9.2231998	37.2296371
880.0000000	9.2720003	-8.4070930
880.0000000	9.3207998	-9.7905884
880.0000000	9.3695993	-10.7606115
880.0000000	9.4183998	-11.7556210
880.0000000	9.4672003	-11.1607552
880.0000000	9.5159998	-14.1766834
880.0000000	9.5648003	-0.3458595
880.0000000	9.6135998	-13.4070797
880.0000000	9.6623993	-10.2174826
880.0000000	9.7111998	-10.1539040
880.0000000	9.7600002	-10.8569918
880.0000000	9.8087997	-10.9946671
880.0000000	9.8575993	3.2518167
880.0000000	9.9063997	-7.9562697
880.0000000	9.9552002	-10.9065409
880.0000000	10.0039997	-10.0712538
890.0000000	0.3416000	-36.6034584
890.0000000	0.3904000	-39.7795525
890.0000000	0.4392000	-39.8241348
890.0000000	0.4880000	-39.6069717
890.0000000	0.5368000	-39.5985031
890.0000000	0.5856000	-39.3506088
890.0000000	0.6344000	-38.2214127
890.0000000	0.6832000	-36.1369591
890.0000000	0.7320000	-35.9749451
890.0000000	0.7808000	-36.5282059
890.0000000	0.8296000	-35.9649696
890.0000000	0.8784000	-35.1000633
890.0000000	0.9272000	-32.8325233
890.0000000	0.9760000	-30.7033310
890.0000000	1.0247999	-29.5758724
890.0000000	1.0735999	-29.1863785
890.0000000	1.1224000	-29.2043285
890.0000000	1.1711999	-29.8788052
890.0000000	1.2200000	-30.7687283
890.0000000	1.2687999	-30.8857841
890.0000000	1.3176000	-30.9664307
890.0000000	1.3664000	-30.4701538
890.0000000	1.4152000	-30.1596584
890.0000000	1.4640000	-29.6375427
890.0000000	1.5128000	-40.6385307
890.0000000	1.5616000	-34.1742134
890.0000000	1.6104001	-1590.2733154
890.0000000	1.6592000	-77.0793152
890.0000000	1.7079999	-27.2088242
890.0000000	1.7567999	-27.7618942
890.0000000	1.8055999	-27.6008682
890.0000000	1.8544000	-26.4853344
890.0000000	1.9031999	-25.7024975
890.0000000	1.9520000	-26.3445168
890.0000000	2.0007999	-26.5483131
890.0000000	2.0495999	-26.1908360
890.0000000	2.0983999	-167.1593018

890.0000000	2.1471999	1.7998428
890.0000000	2.1960001	-25.5016880
890.0000000	2.2448001	-26.0606079
890.0000000	2.2935998	-28.4555416
890.0000000	2.3423998	-20.8025894
890.0000000	2.3912001	39.9606323
890.0000000	2.4400001	-22.8051395
890.0000000	2.4888000	-23.4629154
890.0000000	2.5375998	-12.0440092
890.0000000	2.5863998	-10.1298208
890.0000000	2.6352000	-8.1622667
890.0000000	2.6840000	-15.2512808
890.0000000	2.7328000	136.7637024
890.0000000	2.7815998	161.2742310
890.0000000	2.8304000	-23.8120861
890.0000000	2.8792000	-20.5750008
890.0000000	2.9280000	-22.5795212
890.0000000	2.9768000	-16.6755028
890.0000000	3.0256000	-20.4376755
890.0000000	3.0743999	-29.6718712
890.0000000	3.1231999	-24.3021698
890.0000000	3.1719999	-29.7187176
890.0000000	3.2208002	-507.4711914
890.0000000	3.2695999	-993.6942139
890.0000000	3.3183999	-20.4870243
890.0000000	3.3671999	-3415.6616211
890.0000000	3.4159999	1225.5061035
890.0000000	3.4648001	-7.0663099
890.0000000	3.5135999	-27.0760155
890.0000000	3.5623999	-323.6333618
890.0000000	3.6111999	-2033.9881592
890.0000000	3.6600001	-460.4138184
890.0000000	3.7088001	-607.9788818
890.0000000	3.7575998	-951.9387817
890.0000000	3.8063998	-1727.4642334
890.0000000	3.8552001	-2508.0656738
890.0000000	3.9040000	-2458.9855957
890.0000000	3.9528000	1124.5948486
890.0000000	4.0015998	-245.6262207
890.0000000	4.0503998	688.5477295
890.0000000	4.0991998	87.7606354
890.0000000	4.1479998	-655.5967407
890.0000000	4.1967998	2.5745754
890.0000000	4.2456002	-248.8108368
890.0000000	4.2943997	-488.6127014
890.0000000	4.3431997	-13.3247108
890.0000000	4.3920002	-248.4407196
890.0000000	4.4407997	-22.4659882
890.0000000	4.4896002	-15.4715481
890.0000000	4.5383997	-655.8582153
890.0000000	4.5871997	-12.8325891
890.0000000	4.6360002	-21.6256905
890.0000000	4.6847997	-28.2940292
890.0000000	4.7336001	-19.5645676
890.0000000	4.7824001	-16.5110207
890.0000000	4.8311996	-1475.8863525
890.0000000	4.8800001	228.5114288
890.0000000	4.9287996	-201.6432953

890.0000000	4.9776001	1060.6860352
890.0000000	5.0264001	86.2813416
890.0000000	5.0751996	-301.4309998
890.0000000	5.1240001	-12.3057537
890.0000000	5.1727996	558.7612915
890.0000000	5.2216001	32.0717621
890.0000000	5.2704000	-159.0413361
890.0000000	5.3191996	-39.2191162
890.0000000	5.3680000	-3541.4660645
890.0000000	5.4168000	527.9970093
890.0000000	5.4656000	-201.0341644
890.0000000	5.5144000	38.1541595
890.0000000	5.5631995	489.1323242
890.0000000	5.6120000	393.7969360
890.0000000	5.6608000	-37.1183243
890.0000000	5.7096000	62.6572189
890.0000000	5.7584000	133.2783661
890.0000000	5.8071995	-11.5131073
890.0000000	5.8559999	29.8906956
890.0000000	5.9047999	-16.6072769
890.0000000	5.9535999	-39.8160324
890.0000000	6.0023999	-1575.5520020
890.0000000	6.0511999	-14.5663881
890.0000000	6.0999999	-19.4165001
890.0000000	6.1487999	-13.2693014
890.0000000	6.1975999	-23.2357292
890.0000000	6.2463999	-1794.9205322
890.0000000	6.2951999	-19.0775490
890.0000000	6.3439999	-343.2846069
890.0000000	6.3927999	-15.0482883
890.0000000	6.4416003	-12.3805637
890.0000000	6.4903998	-11.2573833
890.0000000	6.5391998	-11.7997770
890.0000000	6.5879998	391.6876221
890.0000000	6.6367998	-12.0788145
890.0000000	6.6856003	-13.8132353
890.0000000	6.7343998	-13.4534941
890.0000000	6.7831998	-14.3768044
890.0000000	6.8319998	58.6374283
890.0000000	6.8807998	-14.0508699
890.0000000	6.9296002	-457.6065063
890.0000000	6.9783998	-4.9463019
890.0000000	7.0271997	-14.1485672
890.0000000	7.0760002	11.6552553
890.0000000	7.1247997	-2176.0493164
890.0000000	7.1736002	-1745.0181885
890.0000000	7.2223997	-992.6782227
890.0000000	7.2711997	-213.8252411
890.0000000	7.3200002	-19.0207748
890.0000000	7.3687997	192.2421722
890.0000000	7.4176002	400.7211304
890.0000000	7.4663997	314.8601685
890.0000000	7.5151997	-971.2502441
890.0000000	7.5640001	-1072.8513184
890.0000000	7.6127996	-2529.1120605
890.0000000	7.6616001	-811.8449097
890.0000000	7.7104001	-416.8820190
890.0000000	7.7591996	44.4294930

890.0000000	7.8080001	416.4313049
890.0000000	7.8567996	548.4927979
890.0000000	7.9056001	-1136.0518799
890.0000000	7.9544001	1808.2976074
890.0000000	8.0031996	2333.2973633
890.0000000	8.0520000	2328.8845215
890.0000000	8.1007996	2464.4147949
890.0000000	8.1496000	2628.2104492
890.0000000	8.1983995	1635.1254883
890.0000000	8.2472000	-3740.9980469
890.0000000	8.2959995	-1326.0661621
890.0000000	8.3448000	-1056.3321533
890.0000000	8.3935995	-968.0989990
890.0000000	8.4423990	-416.9611816
890.0000000	8.4912004	-973.5131836
890.0000000	8.5400000	-2626.2141113
890.0000000	8.5887995	-1272.9268799
890.0000000	8.6375999	248.7462921
890.0000000	8.6863995	2246.5283203
890.0000000	8.7351999	-834.8859253
890.0000000	8.7840004	954.2352905
890.0000000	8.8327999	51.9367485
890.0000000	8.8815994	-11.4942465
890.0000000	8.9303989	-23.9920597
890.0000000	8.9792004	-11.5780630
890.0000000	9.0279999	-5.8144598
890.0000000	9.0767994	7.1121197
890.0000000	9.1255999	-17.2639446
890.0000000	9.1743994	-10.0684328
890.0000000	9.2231998	37.2139053
890.0000000	9.2720003	-8.4080296
890.0000000	9.3207998	-9.7911282
890.0000000	9.3695993	-10.7608376
890.0000000	9.4183998	-11.7554960
890.0000000	9.4672003	-11.1607962
890.0000000	9.5159998	-14.1757917
890.0000000	9.5648003	-0.3492327
890.0000000	9.6135998	-13.4062881
890.0000000	9.6623993	-10.2177582
890.0000000	9.7111998	-10.1541758
890.0000000	9.7600002	-10.8570337
890.0000000	9.8087997	-10.9946747
890.0000000	9.8575993	3.2473946
890.0000000	9.9063997	-7.9571834
890.0000000	9.9552002	-10.9065456
890.0000000	10.0039997	-10.0715008
900.0000000	0.3416000	-36.9195671
900.0000000	0.3904000	-39.7943687
900.0000000	0.4392000	-39.8177948
900.0000000	0.4880000	-39.6057625
900.0000000	0.5368000	-39.5996742
900.0000000	0.5856000	-39.3492279
900.0000000	0.6344000	-38.2217216
900.0000000	0.6832000	-36.1391869
900.0000000	0.7320000	-35.9769974
900.0000000	0.7808000	-36.5283775
900.0000000	0.8296000	-35.9656982
900.0000000	0.8784000	-35.1074371

900.0000000	0.9272000	-32.8353004
900.0000000	0.9760000	-30.6958771
900.0000000	1.0247999	-29.5848618
900.0000000	1.0735999	-29.2008133
900.0000000	1.1224000	-29.2013302
900.0000000	1.1711999	-29.8714275
900.0000000	1.2200000	-30.7766438
900.0000000	1.2687999	-30.8867912
900.0000000	1.3176000	-30.9621601
900.0000000	1.3664000	-30.4779034
900.0000000	1.4152000	-30.1598625
900.0000000	1.4640000	-29.6358318
900.0000000	1.5128000	-40.5413551
900.0000000	1.5616000	-34.1323700
900.0000000	1.6104001	-1587.7305908
900.0000000	1.6592000	-76.7853775
900.0000000	1.7079999	-27.2125702
900.0000000	1.7567999	-27.7619133
900.0000000	1.8055999	-27.6047554
900.0000000	1.8544000	-26.5206203
900.0000000	1.9031999	-26.2648888
900.0000000	1.9520000	-26.3675117
900.0000000	2.0007999	-26.5413723
900.0000000	2.0495999	-26.1806602
900.0000000	2.0983999	-165.6835022
900.0000000	2.1471999	1.5325756
900.0000000	2.1960001	-25.4975967
900.0000000	2.2448001	-26.0426960
900.0000000	2.2935998	-28.3959179
900.0000000	2.3423998	-20.8169956
900.0000000	2.3912001	39.3390808
900.0000000	2.4400001	-22.8076267
900.0000000	2.4888000	-23.4626980
900.0000000	2.5375998	-12.0611172
900.0000000	2.5863998	-10.1495790
900.0000000	2.6352000	-8.1850109
900.0000000	2.6840000	-15.2632227
900.0000000	2.7328000	136.4976807
900.0000000	2.7815998	160.9678040
900.0000000	2.8304000	-23.8107815
900.0000000	2.8792000	-20.5782642
900.0000000	2.9280000	-22.5789528
900.0000000	2.9768000	-16.6838741
900.0000000	3.0256000	-20.4402599
900.0000000	3.0743999	-29.6604843
900.0000000	3.1231999	-24.2987137
900.0000000	3.1719999	-29.7104244
900.0000000	3.2208002	-507.2334595
900.0000000	3.2695999	-993.2191162
900.0000000	3.3183999	-20.4872208
900.0000000	3.3671999	-3414.1999512
900.0000000	3.4159999	1224.9708252
900.0000000	3.4648001	-7.0720186
900.0000000	3.5135999	-27.0730247
900.0000000	3.5623999	-323.5037842
900.0000000	3.6111999	-2033.1284180
900.0000000	3.6600001	-460.2257080
900.0000000	3.7088001	-607.7274780

900.0000000	3.7575998	-951.5402222
900.0000000	3.8063998	-1726.7343750
900.0000000	3.8552001	-2507.0024414
900.0000000	3.9040000	-2457.9431152
900.0000000	3.9528000	1124.1065674
900.0000000	4.0015998	-245.5290985
900.0000000	4.0503998	688.2451782
900.0000000	4.0991998	87.7149506
900.0000000	4.1479998	-655.3244629
900.0000000	4.1967998	2.5653191
900.0000000	4.2456002	-248.7125244
900.0000000	4.2943997	-488.4121399
900.0000000	4.3431997	-13.3268909
900.0000000	4.3920002	-248.3426056
900.0000000	4.4407997	-22.4642124
900.0000000	4.4896002	-15.4724302
900.0000000	4.5383997	-655.5971069
900.0000000	4.5871997	-12.8346052
900.0000000	4.6360002	-21.6241474
900.0000000	4.6847997	-28.2901039
900.0000000	4.7336001	-19.5638447
900.0000000	4.7824001	-16.5114765
900.0000000	4.8311996	-1475.3549805
900.0000000	4.8800001	228.4218750
900.0000000	4.9287996	-201.5760345
900.0000000	4.9776001	1060.2935791
900.0000000	5.0264001	86.2438202
900.0000000	5.0751996	-301.3273621
900.0000000	5.1240001	-12.3066750
900.0000000	5.1727996	558.6454468
900.0000000	5.2216001	32.0623207
900.0000000	5.2704000	-159.0132141
900.0000000	5.3191996	-39.2145691
900.0000000	5.3680000	-3540.7736816
900.0000000	5.4168000	527.8902588
900.0000000	5.4656000	-200.9979858
900.0000000	5.5144000	38.1435013
900.0000000	5.5631995	489.0337524
900.0000000	5.6120000	393.7169495
900.0000000	5.6608000	-37.1142349
900.0000000	5.7096000	62.6417923
900.0000000	5.7584000	133.2494354
900.0000000	5.8071995	-11.5139790
900.0000000	5.8559999	29.8818417
900.0000000	5.9047999	-16.6070805
900.0000000	5.9535999	-39.8112946
900.0000000	6.0023999	-1575.2475586
900.0000000	6.0511999	-14.5665989
900.0000000	6.0999999	-19.4156685
900.0000000	6.1487999	-13.2697639
900.0000000	6.1975999	-23.2339630
900.0000000	6.2463999	-1794.5731201
900.0000000	6.2951999	-19.0767345
900.0000000	6.3439999	-343.2205505
900.0000000	6.3927999	-15.0482702
900.0000000	6.4416003	-12.3811512
900.0000000	6.4903998	-11.2580595
900.0000000	6.5391998	-11.8004580

900.0000000	6.5879998	390.9969177
900.0000000	6.6367998	-12.0793638
900.0000000	6.6856003	-13.8134050
900.0000000	6.7343998	-13.4537096
900.0000000	6.7831998	-14.3763885
900.0000000	6.8319998	58.6183090
900.0000000	6.8807998	-14.0507317
900.0000000	6.9296002	-456.8450623
900.0000000	6.9783998	-4.9591260
900.0000000	7.0271997	-14.1475391
900.0000000	7.0760002	11.6130686
900.0000000	7.1247997	-2172.1032715
900.0000000	7.1736002	-1741.8553467
900.0000000	7.2223997	-990.8850708
900.0000000	7.2711997	-213.4574585
900.0000000	7.3200002	-19.0087872
900.0000000	7.3687997	191.8676453
900.0000000	7.4176002	399.9653015
900.0000000	7.4663997	314.2625122
900.0000000	7.5151997	-969.4891968
900.0000000	7.5640001	-1070.9038086
900.0000000	7.6127996	-2524.4946289
900.0000000	7.6616001	-810.3735352
900.0000000	7.7104001	-416.1357422
900.0000000	7.7591996	44.3284454
900.0000000	7.8080001	415.6459351
900.0000000	7.8567996	547.4647217
900.0000000	7.9056001	-1133.9788818
900.0000000	7.9544001	1804.9876709
900.0000000	8.0031996	2329.0278320
900.0000000	8.0520000	2324.6225586
900.0000000	8.1007996	2459.9055176
900.0000000	8.1496000	2623.4023438
900.0000000	8.1983995	1632.1329346
900.0000000	8.2472000	-3734.1633301
900.0000000	8.2959995	-1323.6459961
900.0000000	8.3448000	-1054.4029541
900.0000000	8.3935995	-966.3313599
900.0000000	8.4423990	-416.2031555
900.0000000	8.4912004	-971.7369385
900.0000000	8.5400000	-2621.4143066
900.0000000	8.5887995	-1270.6053467
900.0000000	8.6375999	248.2849579
900.0000000	8.6863995	2242.4101563
900.0000000	8.7351999	-833.3616333
900.0000000	8.7840004	952.4785156
900.0000000	8.8327999	51.8251419
900.0000000	8.8815994	-11.4944210
900.0000000	8.9303989	-23.9883385
900.0000000	8.9792004	-11.5781460
900.0000000	9.0279999	-5.8162274
900.0000000	9.0767994	7.1064014
900.0000000	9.1255999	-17.2622299
900.0000000	9.1743994	-10.0688343
900.0000000	9.2231998	37.1981735
900.0000000	9.2720003	-8.4089661
900.0000000	9.3207998	-9.7916679
900.0000000	9.3695993	-10.7610636

900.0000000	9.4183998	-11.7553711
900.0000000	9.4672003	-11.1608372
900.0000000	9.5159998	-14.1749010
900.0000000	9.5648003	-0.3526058
900.0000000	9.6135998	-13.4054976
900.0000000	9.6623993	-10.2180328
900.0000000	9.7111998	-10.1544466
900.0000000	9.7600002	-10.8570757
900.0000000	9.8087997	-10.9946833
900.0000000	9.8575993	3.2429724
900.0000000	9.9063997	-7.9580975
900.0000000	9.9552002	-10.9065504
900.0000000	10.0039997	-10.0717487
910.0000000	0.3416000	-36.8532066
910.0000000	0.3904000	-39.8091812
910.0000000	0.4392000	-39.8114510
910.0000000	0.4880000	-39.6045532
910.0000000	0.5368000	-39.6008492
910.0000000	0.5856000	-39.3478470
910.0000000	0.6344000	-38.2220345
910.0000000	0.6832000	-36.1414108
910.0000000	0.7320000	-35.9790497
910.0000000	0.7808000	-36.5285454
910.0000000	0.8296000	-35.9664268
910.0000000	0.8784000	-35.1148109
910.0000000	0.9272000	-32.8380737
910.0000000	0.9760000	-30.6884251
910.0000000	1.0247999	-29.5938492
910.0000000	1.0735999	-29.2152481
910.0000000	1.1224000	-29.1983299
910.0000000	1.1711999	-29.8640518
910.0000000	1.2200000	-30.7845573
910.0000000	1.2687999	-30.8877983
910.0000000	1.3176000	-30.9578876
910.0000000	1.3664000	-30.4856529
910.0000000	1.4152000	-30.1600666
910.0000000	1.4640000	-29.6341228
910.0000000	1.5128000	-40.4441757
910.0000000	1.5616000	-34.0905304
910.0000000	1.6104001	-1585.1877441
910.0000000	1.6592000	-76.4914474
910.0000000	1.7079999	-27.2163181
910.0000000	1.7567999	-27.7619343
910.0000000	1.8055999	-27.6086445
910.0000000	1.8544000	-26.2440395
910.0000000	1.9031999	-25.8019295
910.0000000	1.9520000	-26.3905067
910.0000000	2.0007999	-26.5344296
910.0000000	2.0495999	-26.1704826
910.0000000	2.0983999	-164.2077332
910.0000000	2.1471999	1.2653084
910.0000000	2.1960001	-25.4935055
910.0000000	2.2448001	-26.0247841
910.0000000	2.2935998	-28.3362942
910.0000000	2.3423998	-20.8313999
910.0000000	2.3912001	38.7175369
910.0000000	2.4400001	-22.8101139
910.0000000	2.4888000	-23.4624805

910.0000000	2.5375998	-12.0782251
910.0000000	2.5863998	-10.1693373
910.0000000	2.6352000	-8.2077560
910.0000000	2.6840000	-15.2751646
910.0000000	2.7328000	136.2316742
910.0000000	2.7815998	160.6613770
910.0000000	2.8304000	-23.8094769
910.0000000	2.8792000	-20.5815277
910.0000000	2.9280000	-22.5783825
910.0000000	2.9768000	-16.6922455
910.0000000	3.0256000	-20.4428444
910.0000000	3.0743999	-29.6490974
910.0000000	3.1231999	-24.2952576
910.0000000	3.1719999	-29.7021313
910.0000000	3.2208002	-506.9957275
910.0000000	3.2695999	-992.7442627
910.0000000	3.3183999	-20.4874191
910.0000000	3.3671999	-3412.7385254
910.0000000	3.4159999	1224.4355469
910.0000000	3.4648001	-7.0777264
910.0000000	3.5135999	-27.0700340
910.0000000	3.5623999	-323.3741760
910.0000000	3.6111999	-2032.2686768
910.0000000	3.6600001	-460.0375366
910.0000000	3.7088001	-607.4761353
910.0000000	3.7575998	-951.1417236
910.0000000	3.8063998	-1726.0045166
910.0000000	3.8552001	-2505.9389648
910.0000000	3.9040000	-2456.9006348
910.0000000	3.9528000	1123.6182861
910.0000000	4.0015998	-245.4319916
910.0000000	4.0503998	687.9426270
910.0000000	4.0991998	87.6692657
910.0000000	4.1479998	-655.0522461
910.0000000	4.1967998	2.5560627
910.0000000	4.2456002	-248.6142273
910.0000000	4.2943997	-488.2115784
910.0000000	4.3431997	-13.3290710
910.0000000	4.3920002	-248.2444916
910.0000000	4.4407997	-22.4624367
910.0000000	4.4896002	-15.4733124
910.0000000	4.5383997	-655.3360596
910.0000000	4.5871997	-12.8366222
910.0000000	4.6360002	-21.6226044
910.0000000	4.6847997	-28.2861786
910.0000000	4.7336001	-19.5631218
910.0000000	4.7824001	-16.5119305
910.0000000	4.8311996	-1474.8237305
910.0000000	4.8800001	228.3323212
910.0000000	4.9287996	-201.5087738
910.0000000	4.9776001	1059.9010010
910.0000000	5.0264001	86.2062988
910.0000000	5.0751996	-301.2237549
910.0000000	5.1240001	-12.3075962
910.0000000	5.1727996	558.5295410
910.0000000	5.2216001	32.0528755
910.0000000	5.2704000	-158.9851227
910.0000000	5.3191996	-39.2100220

910.0000000	5.3680000	-3540.0812988
910.0000000	5.4168000	527.7834473
910.0000000	5.4656000	-200.9618073
910.0000000	5.5144000	38.1328430
910.0000000	5.5631995	488.9351807
910.0000000	5.6120000	393.6369934
910.0000000	5.6608000	-37.1101456
910.0000000	5.7096000	62.6263580
910.0000000	5.7584000	133.2205048
910.0000000	5.8071995	-11.5148487
910.0000000	5.8559999	29.8729916
910.0000000	5.9047999	-16.6068821
910.0000000	5.9535999	-39.8065529
910.0000000	6.0023999	-1574.9432373
910.0000000	6.0511999	-14.5668106
910.0000000	6.0999999	-19.4148350
910.0000000	6.1487999	-13.2702274
910.0000000	6.1975999	-23.2321987
910.0000000	6.2463999	-1794.2257080
910.0000000	6.2951999	-19.0759201
910.0000000	6.3439999	-343.1565247
910.0000000	6.3927999	-15.0482512
910.0000000	6.4416003	-12.3817377
910.0000000	6.4903998	-11.2587357
910.0000000	6.5391998	-11.8011398
910.0000000	6.5879998	390.3062134
910.0000000	6.6367998	-12.0799131
910.0000000	6.6856003	-13.8135757
910.0000000	6.7343998	-13.4539251
910.0000000	6.7831998	-14.3759727
910.0000000	6.8319998	58.5991974
910.0000000	6.8807998	-14.0505924
910.0000000	6.9296002	-456.0836182
910.0000000	6.9783998	-4.9719496
910.0000000	7.0271997	-14.1465120
910.0000000	7.0760002	11.5708838
910.0000000	7.1247997	-2168.1574707
910.0000000	7.1736002	-1738.6923828
910.0000000	7.2223997	-989.0919189
910.0000000	7.2711997	-213.0896759
910.0000000	7.3200002	-18.9967995
910.0000000	7.3687997	191.4931183
910.0000000	7.4176002	399.2094727
910.0000000	7.4663997	313.6648560
910.0000000	7.5151997	-967.7281494
910.0000000	7.5640001	-1068.9564209
910.0000000	7.6127996	-2519.8769531
910.0000000	7.6616001	-808.9022217
910.0000000	7.7104001	-415.3894653
910.0000000	7.7591996	44.2273979
910.0000000	7.8080001	414.8605652
910.0000000	7.8567996	546.4366455
910.0000000	7.9056001	-1131.9060059
910.0000000	7.9544001	1801.6777344
910.0000000	8.0031996	2324.7580566
910.0000000	8.0520000	2320.3605957
910.0000000	8.1007996	2455.3959961
910.0000000	8.1496000	2618.5942383

910.0000000	8.1983995	1629.1405029
910.0000000	8.2472000	-3727.3283691
910.0000000	8.2959995	-1321.2258301
910.0000000	8.3448000	-1052.4737549
910.0000000	8.3935995	-964.5636597
910.0000000	8.4423990	-415.4451599
910.0000000	8.4912004	-969.9607544
910.0000000	8.5400000	-2616.6145020
910.0000000	8.5887995	-1268.2838135
910.0000000	8.6375999	247.8236389
910.0000000	8.6863995	2238.2917480
910.0000000	8.7351999	-831.8374023
910.0000000	8.7840004	950.7217407
910.0000000	8.8327999	51.7135315
910.0000000	8.8815994	-11.4945955
910.0000000	8.9303989	-23.9846191
910.0000000	8.9792004	-11.5782280
910.0000000	9.0279999	-5.8179960
910.0000000	9.0767994	7.1006851
910.0000000	9.1255999	-17.2605171
910.0000000	9.1743994	-10.0692368
910.0000000	9.2231998	37.1824417
910.0000000	9.2720003	-8.4099016
910.0000000	9.3207998	-9.7922068
910.0000000	9.3695993	-10.7612906
910.0000000	9.4183998	-11.7552471
910.0000000	9.4672003	-11.1608782
910.0000000	9.5159998	-14.1740093
910.0000000	9.5648003	-0.3559780
910.0000000	9.6135998	-13.4047070
910.0000000	9.6623993	-10.2183084
910.0000000	9.7111998	-10.1547184
910.0000000	9.7600002	-10.8571167
910.0000000	9.8087997	-10.9946909
910.0000000	9.8575993	3.2385492
910.0000000	9.9063997	-7.9590111
910.0000000	9.9552002	-10.9065542
910.0000000	10.0039997	-10.0719957
920.0000000	0.3416000	-37.0008926
920.0000000	0.3904000	-39.8239975
920.0000000	0.4392000	-39.8051071
920.0000000	0.4880000	-39.6033478
920.0000000	0.5368000	-39.6020241
920.0000000	0.5856000	-39.3464661
920.0000000	0.6344000	-38.2223434
920.0000000	0.6832000	-36.1436386
920.0000000	0.7320000	-35.9811020
920.0000000	0.7808000	-36.5287132
920.0000000	0.8296000	-35.9671555
920.0000000	0.8784000	-35.1221886
920.0000000	0.9272000	-32.8408508
920.0000000	0.9760000	-30.6809731
920.0000000	1.0247999	-29.6028385
920.0000000	1.0735999	-29.2296829
920.0000000	1.1224000	-29.1953297
920.0000000	1.1711999	-29.8566742
920.0000000	1.2200000	-30.7924728
920.0000000	1.2687999	-30.8888073

920.0000000	1.3176000	-30.9536171
920.0000000	1.3664000	-30.4934025
920.0000000	1.4152000	-30.1602707
920.0000000	1.4640000	-29.6324139
920.0000000	1.5128000	-40.3470001
920.0000000	1.5616000	-34.0486908
920.0000000	1.6104001	-1582.6448975
920.0000000	1.6592000	-76.1975174
920.0000000	1.7079999	-27.2200661
920.0000000	1.7567999	-27.7619534
920.0000000	1.8055999	-27.6125317
920.0000000	1.8544000	-26.1843510
920.0000000	1.9031999	-25.8203125
920.0000000	1.9520000	-26.4135036
920.0000000	2.0007999	-26.5274868
920.0000000	2.0495999	-26.1603069
920.0000000	2.0983999	-162.7319641
920.0000000	2.1471999	0.9980412
920.0000000	2.1960001	-25.4894142
920.0000000	2.2448001	-26.0068741
920.0000000	2.2935998	-28.2766705
920.0000000	2.3423998	-20.8458042
920.0000000	2.3912001	38.0959930
920.0000000	2.4400001	-22.8125992
920.0000000	2.4888000	-23.4622631
920.0000000	2.5375998	-12.0953331
920.0000000	2.5863998	-10.1890965
920.0000000	2.6352000	-8.2305002
920.0000000	2.6840000	-15.2871065
920.0000000	2.7328000	135.9656525
920.0000000	2.7815998	160.3549805
920.0000000	2.8304000	-23.8081722
920.0000000	2.8792000	-20.5847912
920.0000000	2.9280000	-22.5778141
920.0000000	2.9768000	-16.7006149
920.0000000	3.0256000	-20.4454288
920.0000000	3.0743999	-29.6377106
920.0000000	3.1231999	-24.2918034
920.0000000	3.1719999	-29.6938362
920.0000000	3.2208002	-506.7579956
920.0000000	3.2695999	-992.2691650
920.0000000	3.3183999	-20.4876175
920.0000000	3.3671999	-3411.2768555
920.0000000	3.4159999	1223.9002686
920.0000000	3.4648001	-7.0834341
920.0000000	3.5135999	-27.0670433
920.0000000	3.5623999	-323.2445984
920.0000000	3.6111999	-2031.4088135
920.0000000	3.6600001	-459.8494263
920.0000000	3.7088001	-607.2247314
920.0000000	3.7575998	-950.7431641
920.0000000	3.8063998	-1725.2746582
920.0000000	3.8552001	-2504.8754883
920.0000000	3.9040000	-2455.8581543
920.0000000	3.9528000	1123.1300049
920.0000000	4.0015998	-245.3348846
920.0000000	4.0503998	687.6400757
920.0000000	4.0991998	87.6235809

920.0000000	4.1479998	-654.7800293
920.0000000	4.1967998	2.5468063
920.0000000	4.2456002	-248.5159302
920.0000000	4.2943997	-488.0110168
920.0000000	4.3431997	-13.3312521
920.0000000	4.3920002	-248.1463776
920.0000000	4.4407997	-22.4606609
920.0000000	4.4896002	-15.4741955
920.0000000	4.5383997	-655.0750122
920.0000000	4.5871997	-12.8386383
920.0000000	4.6360002	-21.6210632
920.0000000	4.6847997	-28.2822533
920.0000000	4.7336001	-19.5623989
920.0000000	4.7824001	-16.5123844
920.0000000	4.8311996	-1474.2924805
920.0000000	4.8800001	228.2427673
920.0000000	4.9287996	-201.4415131
920.0000000	4.9776001	1059.5085449
920.0000000	5.0264001	86.1687775
920.0000000	5.0751996	-301.1201172
920.0000000	5.1240001	-12.3085175
920.0000000	5.1727996	558.4136963
920.0000000	5.2216001	32.0434303
920.0000000	5.2704000	-158.9570007
920.0000000	5.3191996	-39.2054749
920.0000000	5.3680000	-3539.3889160
920.0000000	5.4168000	527.6766968
920.0000000	5.4656000	-200.9256134
920.0000000	5.5144000	38.1221848
920.0000000	5.5631995	488.8366089
920.0000000	5.6120000	393.5570068
920.0000000	5.6608000	-37.1060562
920.0000000	5.7096000	62.6109238
920.0000000	5.7584000	133.1915741
920.0000000	5.8071995	-11.5157194
920.0000000	5.8559999	29.8641376
920.0000000	5.9047999	-16.6066837
920.0000000	5.9535999	-39.8018150
920.0000000	6.0023999	-1574.6386719
920.0000000	6.0511999	-14.5670223
920.0000000	6.0999999	-19.4140034
920.0000000	6.1487999	-13.2706900
920.0000000	6.1975999	-23.2304344
920.0000000	6.2463999	-1793.8784180
920.0000000	6.2951999	-19.0751057
920.0000000	6.3439999	-343.0924683
920.0000000	6.3927999	-15.0482330
920.0000000	6.4416003	-12.3823242
920.0000000	6.4903998	-11.2594118
920.0000000	6.5391998	-11.8018208
920.0000000	6.5879998	389.6155090
920.0000000	6.6367998	-12.0804625
920.0000000	6.6856003	-13.8137455
920.0000000	6.7343998	-13.4541416
920.0000000	6.7831998	-14.3755569
920.0000000	6.8319998	58.5800781
920.0000000	6.8807998	-14.0504541
920.0000000	6.9296002	-455.3221741

920.0000000	6.9783998	-4.9847727
920.0000000	7.0271997	-14.1454840
920.0000000	7.0760002	11.5286989
920.0000000	7.1247997	-2164.2116699
920.0000000	7.1736002	-1735.5295410
920.0000000	7.2223997	-987.2987671
920.0000000	7.2711997	-212.7218933
920.0000000	7.3200002	-18.9848099
920.0000000	7.3687997	191.1185913
920.0000000	7.4176002	398.4536438
920.0000000	7.4663997	313.0671997
920.0000000	7.5151997	-965.9671021
920.0000000	7.5640001	-1067.0090332
920.0000000	7.6127996	-2515.2592773
920.0000000	7.6616001	-807.4308472
920.0000000	7.7104001	-414.6431885
920.0000000	7.7591996	44.1263466
920.0000000	7.8080001	414.0752258
920.0000000	7.8567996	545.4085083
920.0000000	7.9056001	-1129.8331299
920.0000000	7.9544001	1798.3677979
920.0000000	8.0031996	2320.4885254
920.0000000	8.0520000	2316.0986328
920.0000000	8.1007996	2450.8864746
920.0000000	8.1496000	2613.7863770
920.0000000	8.1983995	1626.1479492
920.0000000	8.2472000	-3720.4936523
920.0000000	8.2959995	-1318.8056641
920.0000000	8.3448000	-1050.5445557
920.0000000	8.3935995	-962.7960205
920.0000000	8.4423990	-414.6871338
920.0000000	8.4912004	-968.1845093
920.0000000	8.5400000	-2611.8146973
920.0000000	8.5887995	-1265.9622803
920.0000000	8.6375999	247.3623199
920.0000000	8.6863995	2234.1733398
920.0000000	8.7351999	-830.3131104
920.0000000	8.7840004	948.9650269
920.0000000	8.8327999	51.6019211
920.0000000	8.8815994	-11.4947701
920.0000000	8.9303989	-23.9808998
920.0000000	8.9792004	-11.5783100
920.0000000	9.0279999	-5.8197641
920.0000000	9.0767994	7.0949669
920.0000000	9.1255999	-17.2588024
920.0000000	9.1743994	-10.0696392
920.0000000	9.2231998	37.1667099
920.0000000	9.2720003	-8.4108391
920.0000000	9.3207998	-9.7927465
920.0000000	9.3695993	-10.7615166
920.0000000	9.4183998	-11.7551222
920.0000000	9.4672003	-11.1609192
920.0000000	9.5159998	-14.1731186
920.0000000	9.5648003	-0.3593502
920.0000000	9.6135998	-13.4039164
920.0000000	9.6623993	-10.2185831
920.0000000	9.7111998	-10.1549902
920.0000000	9.7600002	-10.8571587

920.0000000	9.8087997	-10.9946985
920.0000000	9.8575993	3.2341270
920.0000000	9.9063997	-7.9599257
920.0000000	9.9552002	-10.9065590
920.0000000	10.0039997	-10.0722437
930.0000000	0.3416000	-36.7828484
930.0000000	0.3904000	-39.8388138
930.0000000	0.4392000	-39.7987633
930.0000000	0.4880000	-39.6021385
930.0000000	0.5368000	-39.6031952
930.0000000	0.5856000	-39.3450851
930.0000000	0.6344000	-38.2226524
930.0000000	0.6832000	-36.1458626
930.0000000	0.7320000	-35.9831543
930.0000000	0.7808000	-36.5288849
930.0000000	0.8296000	-35.9678879
930.0000000	0.8784000	-35.1295624
930.0000000	0.9272000	-32.8436279
930.0000000	0.9760000	-30.6735191
930.0000000	1.0247999	-29.6118279
930.0000000	1.0735999	-29.2441177
930.0000000	1.1224000	-29.1923313
930.0000000	1.1711999	-29.8492966
930.0000000	1.2200000	-30.8003883
930.0000000	1.2687999	-30.8898144
930.0000000	1.3176000	-30.9493446
930.0000000	1.3664000	-30.5011520
930.0000000	1.4152000	-30.1604748
930.0000000	1.4640000	-29.6307049
930.0000000	1.5128000	-40.2498207
930.0000000	1.5616000	-34.0068474
930.0000000	1.6104001	-1580.1021729
930.0000000	1.6592000	-75.9035873
930.0000000	1.7079999	-27.2238121
930.0000000	1.7567999	-27.7619724
930.0000000	1.8055999	-27.6164207
930.0000000	1.8544000	-26.0797195
930.0000000	1.9031999	-25.3865871
930.0000000	1.9520000	-26.4364986
930.0000000	2.0007999	-26.5205441
930.0000000	2.0495999	-26.1501312
930.0000000	2.0983999	-161.2561951
930.0000000	2.1471999	0.7307739
930.0000000	2.1960001	-25.4853230
930.0000000	2.2448001	-25.9889622
930.0000000	2.2935998	-28.2170467
930.0000000	2.3423998	-20.8602085
930.0000000	2.3912001	37.4744492
930.0000000	2.4400001	-22.8150864
930.0000000	2.4888000	-23.4620457
930.0000000	2.5375998	-12.1124411
930.0000000	2.5863998	-10.2088556
930.0000000	2.6352000	-8.2532444
930.0000000	2.6840000	-15.2990484
930.0000000	2.7328000	135.6996307
930.0000000	2.7815998	160.0485535
930.0000000	2.8304000	-23.8068695
930.0000000	2.8792000	-20.5880547

930.0000000	2.9280000	-22.5772457
930.0000000	2.9768000	-16.7089863
930.0000000	3.0256000	-20.4480133
930.0000000	3.0743999	-29.6263237
930.0000000	3.1231999	-24.2883472
930.0000000	3.1719999	-29.6855431
930.0000000	3.2208002	-506.5202637
930.0000000	3.2695999	-991.7943115
930.0000000	3.3183999	-20.4878159
930.0000000	3.3671999	-3409.8151855
930.0000000	3.4159999	1223.3649902
930.0000000	3.4648001	-7.0891428
930.0000000	3.5135999	-27.0640526
930.0000000	3.5623999	-323.1149902
930.0000000	3.6111999	-2030.5490723
930.0000000	3.6600001	-459.6613159
930.0000000	3.7088001	-606.9733276
930.0000000	3.7575998	-950.3446045
930.0000000	3.8063998	-1724.5447998
930.0000000	3.8552001	-2503.8120117
930.0000000	3.9040000	-2454.8154297
930.0000000	3.9528000	1122.6417236
930.0000000	4.0015998	-245.2377777
930.0000000	4.0503998	687.3375244
930.0000000	4.0991998	87.5778961
930.0000000	4.1479998	-654.5078125
930.0000000	4.1967998	2.5375500
930.0000000	4.2456002	-248.4176178
930.0000000	4.2943997	-487.8103943
930.0000000	4.3431997	-13.3334332
930.0000000	4.3920002	-248.0482635
930.0000000	4.4407997	-22.4588852
930.0000000	4.4896002	-15.4750786
930.0000000	4.5383997	-654.8139038
930.0000000	4.5871997	-12.8406544
930.0000000	4.6360002	-21.6195202
930.0000000	4.6847997	-28.2783279
930.0000000	4.7336001	-19.5616779
930.0000000	4.7824001	-16.5128384
930.0000000	4.8311996	-1473.7612305
930.0000000	4.8800001	228.1532288
930.0000000	4.9287996	-201.3742523
930.0000000	4.9776001	1059.1160889
930.0000000	5.0264001	86.1312561
930.0000000	5.0751996	-301.0164795
930.0000000	5.1240001	-12.3094387
930.0000000	5.1727996	558.2978516
930.0000000	5.2216001	32.0339851
930.0000000	5.2704000	-158.9289093
930.0000000	5.3191996	-39.2009201
930.0000000	5.3680000	-3538.6965332
930.0000000	5.4168000	527.5698853
930.0000000	5.4656000	-200.8894348
930.0000000	5.5144000	38.1115265
930.0000000	5.5631995	488.7380371
930.0000000	5.6120000	393.4770508
930.0000000	5.6608000	-37.1019669
930.0000000	5.7096000	62.5954971

930.0000000	5.7584000	133.1626282
930.0000000	5.8071995	-11.5165901
930.0000000	5.8559999	29.8552876
930.0000000	5.9047999	-16.6064873
930.0000000	5.9535999	-39.7970772
930.0000000	6.0023999	-1574.3343506
930.0000000	6.0511999	-14.5672331
930.0000000	6.0999999	-19.4131718
930.0000000	6.1487999	-13.2711525
930.0000000	6.1975999	-23.2286682
930.0000000	6.2463999	-1793.5310059
930.0000000	6.2951999	-19.0742912
930.0000000	6.3439999	-343.0284119
930.0000000	6.3927999	-15.0482149
930.0000000	6.4416003	-12.3829117
930.0000000	6.4903998	-11.2600870
930.0000000	6.5391998	-11.8025017
930.0000000	6.5879998	388.9248047
930.0000000	6.6367998	-12.0810118
930.0000000	6.6856003	-13.8139162
930.0000000	6.7343998	-13.4543571
930.0000000	6.7831998	-14.3751411
930.0000000	6.8319998	58.5609665
930.0000000	6.8807998	-14.0503159
930.0000000	6.9296002	-454.5607300
930.0000000	6.9783998	-4.9975963
930.0000000	7.0271997	-14.1444559
930.0000000	7.0760002	11.4865141
930.0000000	7.1247997	-2160.2658691
930.0000000	7.1736002	-1732.3666992
930.0000000	7.2223997	-985.5056152
930.0000000	7.2711997	-212.3541260
930.0000000	7.3200002	-18.9728203
930.0000000	7.3687997	190.7440491
930.0000000	7.4176002	397.6978149
930.0000000	7.4663997	312.4695435
930.0000000	7.5151997	-964.2060547
930.0000000	7.5640001	-1065.0616455
930.0000000	7.6127996	-2510.6416016
930.0000000	7.6616001	-805.9594727
930.0000000	7.7104001	-413.8969727
930.0000000	7.7591996	44.0252991
930.0000000	7.8080001	413.2898560
930.0000000	7.8567996	544.3804321
930.0000000	7.9056001	-1127.7602539
930.0000000	7.9544001	1795.0578613
930.0000000	8.0031996	2316.2189941
930.0000000	8.0520000	2311.8364258
930.0000000	8.1007996	2446.3769531
930.0000000	8.1496000	2608.9782715
930.0000000	8.1983995	1623.1553955
930.0000000	8.2472000	-3713.6589355
930.0000000	8.2959995	-1316.3854980
930.0000000	8.3448000	-1048.6153564
930.0000000	8.3935995	-961.0283203
930.0000000	8.4423990	-413.9291382
930.0000000	8.4912004	-966.4082642
930.0000000	8.5400000	-2607.0148926

930.0000000	8.5887995	-1263.6407471
930.0000000	8.6375999	246.9010010
930.0000000	8.6863995	2230.0549316
930.0000000	8.7351999	-828.7888794
930.0000000	8.7840004	947.2082520
930.0000000	8.8327999	51.4903145
930.0000000	8.8815994	-11.4949436
930.0000000	8.9303989	-23.9771805
930.0000000	8.9792004	-11.5783930
930.0000000	9.0279999	-5.8215327
930.0000000	9.0767994	7.0892487
930.0000000	9.1255999	-17.2570877
930.0000000	9.1743994	-10.0700407
930.0000000	9.2231998	37.1509781
930.0000000	9.2720003	-8.4117746
930.0000000	9.3207998	-9.7932854
930.0000000	9.3695993	-10.7617435
930.0000000	9.4183998	-11.7549982
930.0000000	9.4672003	-11.1609602
930.0000000	9.5159998	-14.1722279
930.0000000	9.5648003	-0.3627234
930.0000000	9.6135998	-13.4031258
930.0000000	9.6623993	-10.2188578
930.0000000	9.7111998	-10.1552620
930.0000000	9.7600002	-10.8572006
930.0000000	9.8087997	-10.9947071
930.0000000	9.8575993	3.2297049
930.0000000	9.9063997	-7.9608393
930.0000000	9.9552002	-10.9065628
930.0000000	10.0039997	-10.0724916
940.0000000	0.3416000	-36.6366806
940.0000000	0.3904000	-39.8536263
940.0000000	0.4392000	-39.7924194
940.0000000	0.4880000	-39.6009293
940.0000000	0.5368000	-39.6043701
940.0000000	0.5856000	-39.3437042
940.0000000	0.6344000	-38.2229652
940.0000000	0.6832000	-36.1480865
940.0000000	0.7320000	-35.9852066
940.0000000	0.7808000	-36.5290527
940.0000000	0.8296000	-35.9686165
940.0000000	0.8784000	-35.1369362
940.0000000	0.9272000	-32.8464050
940.0000000	0.9760000	-30.6660671
940.0000000	1.0247999	-29.6208172
940.0000000	1.0735999	-29.2585526
940.0000000	1.1224000	-29.1893311
940.0000000	1.1711999	-29.8419189
940.0000000	1.2200000	-30.8083038
940.0000000	1.2687999	-30.8908215
940.0000000	1.3176000	-30.9450741
940.0000000	1.3664000	-30.5089016
940.0000000	1.4152000	-30.1606789
940.0000000	1.4640000	-29.6289940
940.0000000	1.5128000	-40.1526413
940.0000000	1.5616000	-33.9650078
940.0000000	1.6104001	-1577.5593262
940.0000000	1.6592000	-75.6096497

940.0000000	1.7079999	-27.2275600
940.0000000	1.7567999	-27.7619915
940.0000000	1.8055999	-27.6203079
940.0000000	1.8544000	-26.6388912
940.0000000	1.9031999	-26.2109337
940.0000000	1.9520000	-26.4329262
940.0000000	2.0007999	-26.5136013
940.0000000	2.0495999	-26.1521492
940.0000000	2.0983999	-159.7803955
940.0000000	2.1471999	0.4635067
940.0000000	2.1960001	-25.4812317
940.0000000	2.2448001	-25.9710503
940.0000000	2.2935998	-28.1574230
940.0000000	2.3423998	-20.8746128
940.0000000	2.3912001	36.8528976
940.0000000	2.4400001	-22.8175735
940.0000000	2.4888000	-23.4618282
940.0000000	2.5375998	-12.1295490
940.0000000	2.5863998	-10.2286148
940.0000000	2.6352000	-8.2759886
940.0000000	2.6840000	-15.3109913
940.0000000	2.7328000	135.4336243
940.0000000	2.7815998	159.7421265
940.0000000	2.8304000	-23.8055649
940.0000000	2.8792000	-20.5913181
940.0000000	2.9280000	-22.5766754
940.0000000	2.9768000	-16.7173576
940.0000000	3.0256000	-20.4505978
940.0000000	3.0743999	-29.6149368
940.0000000	3.1231999	-24.2848930
940.0000000	3.1719999	-29.6772499
940.0000000	3.2208002	-506.2825317
940.0000000	3.2695999	-991.3192139
940.0000000	3.3183999	-20.4880142
940.0000000	3.3671999	-3408.3535156
940.0000000	3.4159999	1222.8297119
940.0000000	3.4648001	-7.0948515
940.0000000	3.5135999	-27.0610619
940.0000000	3.5623999	-322.9854126
940.0000000	3.6111999	-2029.6893311
940.0000000	3.6600001	-459.4731445
940.0000000	3.7088001	-606.7219849
940.0000000	3.7575998	-949.9461060
940.0000000	3.8063998	-1723.8149414
940.0000000	3.8552001	-2502.7485352
940.0000000	3.9040000	-2453.7729492
940.0000000	3.9528000	1122.1534424
940.0000000	4.0015998	-245.1406708
940.0000000	4.0503998	687.0349731
940.0000000	4.0991998	87.5322113
940.0000000	4.1479998	-654.2355957
940.0000000	4.1967998	2.5282936
940.0000000	4.2456002	-248.3193207
940.0000000	4.2943997	-487.6098328
940.0000000	4.3431997	-13.3356133
940.0000000	4.3920002	-247.9501495
940.0000000	4.4407997	-22.4571114
940.0000000	4.4896002	-15.4759607

940.0000000	4.5383997	-654.5528564
940.0000000	4.5871997	-12.8426704
940.0000000	4.6360002	-21.6179771
940.0000000	4.6847997	-28.2744026
940.0000000	4.7336001	-19.5609550
940.0000000	4.7824001	-16.5132942
940.0000000	4.8311996	-1473.2299805
940.0000000	4.8800001	228.0636749
940.0000000	4.9287996	-201.3069763
940.0000000	4.9776001	1058.7235107
940.0000000	5.0264001	86.0937424
940.0000000	5.0751996	-300.9128723
940.0000000	5.1240001	-12.3103600
940.0000000	5.1727996	558.1819458
940.0000000	5.2216001	32.0245438
940.0000000	5.2704000	-158.9007874
940.0000000	5.3191996	-39.1963730
940.0000000	5.3680000	-3538.0041504
940.0000000	5.4168000	527.4631348
940.0000000	5.4656000	-200.8532562
940.0000000	5.5144000	38.1008759
940.0000000	5.5631995	488.6394653
940.0000000	5.6120000	393.3970642
940.0000000	5.6608000	-37.0978813
940.0000000	5.7096000	62.5800629
940.0000000	5.7584000	133.1336975
940.0000000	5.8071995	-11.5174608
940.0000000	5.8559999	29.8464375
940.0000000	5.9047999	-16.6062889
940.0000000	5.9535999	-39.7923393
940.0000000	6.0023999	-1574.0297852
940.0000000	6.0511999	-14.5674448
940.0000000	6.0999999	-19.4123383
940.0000000	6.1487999	-13.2716150
940.0000000	6.1975999	-23.2269039
940.0000000	6.2463999	-1793.1835938
940.0000000	6.2951999	-19.0734787
940.0000000	6.3439999	-342.9643555
940.0000000	6.3927999	-15.0481968
940.0000000	6.4416003	-12.3834982
940.0000000	6.4903998	-11.2607632
940.0000000	6.5391998	-11.8031826
940.0000000	6.5879998	388.2341309
940.0000000	6.6367998	-12.0815601
940.0000000	6.6856003	-13.8140860
940.0000000	6.7343998	-13.4545727
940.0000000	6.7831998	-14.3747253
940.0000000	6.8319998	58.5418472
940.0000000	6.8807998	-14.0501766
940.0000000	6.9296002	-453.7992554
940.0000000	6.9783998	-5.0104203
940.0000000	7.0271997	-14.1434278
940.0000000	7.0760002	11.4443274
940.0000000	7.1247997	-2156.3198242
940.0000000	7.1736002	-1729.2038574
940.0000000	7.2223997	-983.7124023
940.0000000	7.2711997	-211.9863434
940.0000000	7.3200002	-18.9608326

940.0000000	7.3687997	190.3695221
940.0000000	7.4176002	396.9419861
940.0000000	7.4663997	311.8719177
940.0000000	7.5151997	-962.4450073
940.0000000	7.5640001	-1063.1142578
940.0000000	7.6127996	-2506.0241699
940.0000000	7.6616001	-804.4881592
940.0000000	7.7104001	-413.1506958
940.0000000	7.7591996	43.9242516
940.0000000	7.8080001	412.5044861
940.0000000	7.8567996	543.3523560
940.0000000	7.9056001	-1125.6872559
940.0000000	7.9544001	1791.7480469
940.0000000	8.0031996	2311.9494629
940.0000000	8.0520000	2307.5744629
940.0000000	8.1007996	2441.8674316
940.0000000	8.1496000	2604.1704102
940.0000000	8.1983995	1620.1629639
940.0000000	8.2472000	-3706.8239746
940.0000000	8.2959995	-1313.9653320
940.0000000	8.3448000	-1046.6862793
940.0000000	8.3935995	-959.2606812
940.0000000	8.4423990	-413.1711121
940.0000000	8.4912004	-964.6320801
940.0000000	8.5400000	-2602.2150879
940.0000000	8.5887995	-1261.3192139
940.0000000	8.6375999	246.4396820
940.0000000	8.6863995	2225.9365234
940.0000000	8.7351999	-827.2646484
940.0000000	8.7840004	945.4515381
940.0000000	8.8327999	51.3787117
940.0000000	8.8815994	-11.4951181
940.0000000	8.9303989	-23.9734592
940.0000000	8.9792004	-11.5784750
940.0000000	9.0279999	-5.8233004
940.0000000	9.0767994	7.0835304
940.0000000	9.1255999	-17.2553749
940.0000000	9.1743994	-10.0704432
940.0000000	9.2231998	37.1352386
940.0000000	9.2720003	-8.4127111
940.0000000	9.3207998	-9.7938251
940.0000000	9.3695993	-10.7619696
940.0000000	9.4183998	-11.7548733
940.0000000	9.4672003	-11.1610022
940.0000000	9.5159998	-14.1713371
940.0000000	9.5648003	-0.3660965
940.0000000	9.6135998	-13.4023352
940.0000000	9.6623993	-10.2191334
940.0000000	9.7111998	-10.1555328
940.0000000	9.7600002	-10.8572426
940.0000000	9.8087997	-10.9947147
940.0000000	9.8575993	3.2252817
940.0000000	9.9063997	-7.9617529
940.0000000	9.9552002	-10.9065676
940.0000000	10.0039997	-10.0727386
950.0000000	0.3416000	-37.1072502
950.0000000	0.3904000	-39.8684425
950.0000000	0.4392000	-39.7860756

950.0000000	0.4880000	-39.5997200
950.0000000	0.5368000	-39.6055450
950.0000000	0.5856000	-39.3423233
950.0000000	0.6344000	-38.2232742
950.0000000	0.6832000	-36.1503143
950.0000000	0.7320000	-35.9872589
950.0000000	0.7808000	-36.5292206
950.0000000	0.8296000	-35.9693451
950.0000000	0.8784000	-35.1443138
950.0000000	0.9272000	-32.8491821
950.0000000	0.9760000	-30.6586151
950.0000000	1.0247999	-29.6298065
950.0000000	1.0735999	-29.2729874
950.0000000	1.1224000	-29.1863327
950.0000000	1.1711999	-29.8345413
950.0000000	1.2200000	-30.8162193
950.0000000	1.2687999	-30.8918304
950.0000000	1.3176000	-30.9408035
950.0000000	1.3664000	-30.5166512
950.0000000	1.4152000	-30.1608849
950.0000000	1.4640000	-29.6272850
950.0000000	1.5128000	-40.0554657
950.0000000	1.5616000	-33.9231644
950.0000000	1.6104001	-1575.0166016
950.0000000	1.6592000	-75.3157196
950.0000000	1.7079999	-27.2313061
950.0000000	1.7567999	-27.7620106
950.0000000	1.8055999	-27.6241970
950.0000000	1.8544000	-26.9392891
950.0000000	1.9031999	-27.0352821
950.0000000	1.9520000	-26.4293556
950.0000000	2.0007999	-26.5066586
950.0000000	2.0495999	-26.1541691
950.0000000	2.0983999	-158.3046265
950.0000000	2.1471999	0.1962395
950.0000000	2.1960001	-25.4771404
950.0000000	2.2448001	-25.9531403
950.0000000	2.2935998	-28.0977993
950.0000000	2.3423998	-20.8890171
950.0000000	2.3912001	36.2313538
950.0000000	2.4400001	-22.8200588
950.0000000	2.4888000	-23.4616108
950.0000000	2.5375998	-12.1466570
950.0000000	2.5863998	-10.2483740
950.0000000	2.6352000	-8.2987328
950.0000000	2.6840000	-15.3229313
950.0000000	2.7328000	135.1676025
950.0000000	2.7815998	159.4357300
950.0000000	2.8304000	-23.8042603
950.0000000	2.8792000	-20.5945816
950.0000000	2.9280000	-22.5761070
950.0000000	2.9768000	-16.7257271
950.0000000	3.0256000	-20.4531822
950.0000000	3.0743999	-29.6035500
950.0000000	3.1231999	-24.2814369
950.0000000	3.1719999	-29.6689568
950.0000000	3.2208002	-506.0447693
950.0000000	3.2695999	-990.8443604

950.0000000	3.3183999	-20.4882107
950.0000000	3.3671999	-3406.8918457
950.0000000	3.4159999	1222.2944336
950.0000000	3.4648001	-7.1005592
950.0000000	3.5135999	-27.0580711
950.0000000	3.5623999	-322.8558044
950.0000000	3.6111999	-2028.8295898
950.0000000	3.6600001	-459.2850342
950.0000000	3.7088001	-606.4705811
950.0000000	3.7575998	-949.5475464
950.0000000	3.8063998	-1723.0850830
950.0000000	3.8552001	-2501.6853027
950.0000000	3.9040000	-2452.7304688
950.0000000	3.9528000	1121.6650391
950.0000000	4.0015998	-245.0435638
950.0000000	4.0503998	686.7324219
950.0000000	4.0991998	87.4865265
950.0000000	4.1479998	-653.9633789
950.0000000	4.1967998	2.5190372
950.0000000	4.2456002	-248.2210236
950.0000000	4.2943997	-487.4092712
950.0000000	4.3431997	-13.3377934
950.0000000	4.3920002	-247.8520355
950.0000000	4.4407997	-22.4553337
950.0000000	4.4896002	-15.4768438
950.0000000	4.5383997	-654.2917480
950.0000000	4.5871997	-12.8446865
950.0000000	4.6360002	-21.6164360
950.0000000	4.6847997	-28.2704773
950.0000000	4.7336001	-19.5602322
950.0000000	4.7824001	-16.5137482
950.0000000	4.8311996	-1472.6987305
950.0000000	4.8800001	227.9741211
950.0000000	4.9287996	-201.2397156
950.0000000	4.9776001	1058.3310547
950.0000000	5.0264001	86.0562210
950.0000000	5.0751996	-300.8092346
950.0000000	5.1240001	-12.3112812
950.0000000	5.1727996	558.0661011
950.0000000	5.2216001	32.0150986
950.0000000	5.2704000	-158.8726959
950.0000000	5.3191996	-39.1918259
950.0000000	5.3680000	-3537.3117676
950.0000000	5.4168000	527.3563232
950.0000000	5.4656000	-200.8170624
950.0000000	5.5144000	38.0902176
950.0000000	5.5631995	488.5408936
950.0000000	5.6120000	393.3170776
950.0000000	5.6608000	-37.0937920
950.0000000	5.7096000	62.5646362
950.0000000	5.7584000	133.1047668
950.0000000	5.8071995	-11.5183315
950.0000000	5.8559999	29.8375874
950.0000000	5.9047999	-16.6060905
950.0000000	5.9535999	-39.7875977
950.0000000	6.0023999	-1573.7253418
950.0000000	6.0511999	-14.5676565
950.0000000	6.0999999	-19.4115067

950.0000000	6.1487999	-13.2720785
950.0000000	6.1975999	-23.2251377
950.0000000	6.2463999	-1792.8363037
950.0000000	6.2951999	-19.0726643
950.0000000	6.3439999	-342.9002991
950.0000000	6.3927999	-15.0481777
950.0000000	6.4416003	-12.3840857
950.0000000	6.4903998	-11.2614393
950.0000000	6.5391998	-11.8038635
950.0000000	6.5879998	387.5434265
950.0000000	6.6367998	-12.0821095
950.0000000	6.6856003	-13.8142567
950.0000000	6.7343998	-13.4547882
950.0000000	6.7831998	-14.3743095
950.0000000	6.8319998	58.5227356
950.0000000	6.8807998	-14.0500383
950.0000000	6.9296002	-453.0378113
950.0000000	6.9783998	-5.0232434
950.0000000	7.0271997	-14.1423998
950.0000000	7.0760002	11.4021425
950.0000000	7.1247997	-2152.3740234
950.0000000	7.1736002	-1726.0410156
950.0000000	7.2223997	-981.9192505
950.0000000	7.2711997	-211.6185608
950.0000000	7.3200002	-18.9488449
950.0000000	7.3687997	189.9949951
950.0000000	7.4176002	396.1861572
950.0000000	7.4663997	311.2742615
950.0000000	7.5151997	-960.6839600
950.0000000	7.5640001	-1061.1668701
950.0000000	7.6127996	-2501.4064941
950.0000000	7.6616001	-803.0167847
950.0000000	7.7104001	-412.4044189
950.0000000	7.7591996	43.8232002
950.0000000	7.8080001	411.7191467
950.0000000	7.8567996	542.3242188
950.0000000	7.9056001	-1123.6143799
950.0000000	7.9544001	1788.4381104
950.0000000	8.0031996	2307.6796875
950.0000000	8.0520000	2303.3125000
950.0000000	8.1007996	2437.3579102
950.0000000	8.1496000	2599.3623047
950.0000000	8.1983995	1617.1704102
950.0000000	8.2472000	-3699.9892578
950.0000000	8.2959995	-1311.5451660
950.0000000	8.3448000	-1044.7570801
950.0000000	8.3935995	-957.4929810
950.0000000	8.4423990	-412.4131165
950.0000000	8.4912004	-962.8558350
950.0000000	8.5400000	-2597.4152832
950.0000000	8.5887995	-1258.9976807
950.0000000	8.6375999	245.9783630
950.0000000	8.6863995	2221.8183594
950.0000000	8.7351999	-825.7403564
950.0000000	8.7840004	943.6947632
950.0000000	8.8327999	51.2671013
950.0000000	8.8815994	-11.4952927
950.0000000	8.9303989	-23.9697380

950.0000000	8.9792004	-11.5785570
950.0000000	9.0279999	-5.8250690
950.0000000	9.0767994	7.0778141
950.0000000	9.1255999	-17.2536602
950.0000000	9.1743994	-10.0708447
950.0000000	9.2231998	37.1195068
950.0000000	9.2720003	-8.4136477
950.0000000	9.3207998	-9.7943640
950.0000000	9.3695993	-10.7621965
950.0000000	9.4183998	-11.7547483
950.0000000	9.4672003	-11.1610432
950.0000000	9.5159998	-14.1704464
950.0000000	9.5648003	-0.3694687
950.0000000	9.6135998	-13.4015446
950.0000000	9.6623993	-10.2194080
950.0000000	9.7111998	-10.1558046
950.0000000	9.7600002	-10.8572836
950.0000000	9.8087997	-10.9947224
950.0000000	9.8575993	3.2208595
950.0000000	9.9063997	-7.9626670
950.0000000	9.9552002	-10.9065714
950.0000000	10.0039997	-10.0729866
960.0000000	0.3416000	-36.8917809
960.0000000	0.3904000	-39.8832550
960.0000000	0.4392000	-39.7797318
960.0000000	0.4880000	-39.5985107
960.0000000	0.5368000	-39.6067162
960.0000000	0.5856000	-39.3409386
960.0000000	0.6344000	-38.2235870
960.0000000	0.6832000	-36.1525383
960.0000000	0.7320000	-35.9893112
960.0000000	0.7808000	-36.5293922
960.0000000	0.8296000	-35.9700737
960.0000000	0.8784000	-35.1516876
960.0000000	0.9272000	-32.8519592
960.0000000	0.9760000	-30.6511612
960.0000000	1.0247999	-29.6387959
960.0000000	1.0735999	-29.2874241
960.0000000	1.1224000	-29.1833324
960.0000000	1.1711999	-29.8271637
960.0000000	1.2200000	-30.8241348
960.0000000	1.2687999	-30.8928375
960.0000000	1.3176000	-30.9365311
960.0000000	1.3664000	-30.5244007
960.0000000	1.4152000	-30.1610889
960.0000000	1.4640000	-29.6255760
960.0000000	1.5128000	-39.9582901
960.0000000	1.5616000	-33.8813248
960.0000000	1.6104001	-1572.4737549
960.0000000	1.6592000	-75.0217896
960.0000000	1.7079999	-27.2350540
960.0000000	1.7567999	-27.7620296
960.0000000	1.8055999	-27.6280842
960.0000000	1.8544000	-26.4727421
960.0000000	1.9031999	-26.8590603
960.0000000	1.9520000	-26.4257832
960.0000000	2.0007999	-26.4997158
960.0000000	2.0495999	-26.1561871

960.0000000	2.0983999	-156.8288574
960.0000000	2.1471999	-0.0710278
960.0000000	2.1960001	-25.4730511
960.0000000	2.2448001	-25.9352283
960.0000000	2.2935998	-28.0381756
960.0000000	2.3423998	-20.9034233
960.0000000	2.3912001	35.6098099
960.0000000	2.4400001	-22.8225460
960.0000000	2.4888000	-23.4613934
960.0000000	2.5375998	-12.1637659
960.0000000	2.5863998	-10.2681322
960.0000000	2.6352000	-8.3214769
960.0000000	2.6840000	-15.3348732
960.0000000	2.7328000	134.9015961
960.0000000	2.7815998	159.1293030
960.0000000	2.8304000	-23.8029556
960.0000000	2.8792000	-20.5978451
960.0000000	2.9280000	-22.5755367
960.0000000	2.9768000	-16.7340965
960.0000000	3.0256000	-20.4557667
960.0000000	3.0743999	-29.5921631
960.0000000	3.1231999	-24.2779808
960.0000000	3.1719999	-29.6606636
960.0000000	3.2208002	-505.8070374
960.0000000	3.2695999	-990.3692627
960.0000000	3.3183999	-20.4884090
960.0000000	3.3671999	-3405.4301758
960.0000000	3.4159999	1221.7591553
960.0000000	3.4648001	-7.1062670
960.0000000	3.5135999	-27.0550804
960.0000000	3.5623999	-322.7262268
960.0000000	3.6111999	-2027.9698486
960.0000000	3.6600001	-459.0969238
960.0000000	3.7088001	-606.2191772
960.0000000	3.7575998	-949.1490479
960.0000000	3.8063998	-1722.3552246
960.0000000	3.8552001	-2500.6218262
960.0000000	3.9040000	-2451.6879883
960.0000000	3.9528000	1121.1767578
960.0000000	4.0015998	-244.9464569
960.0000000	4.0503998	686.4298706
960.0000000	4.0991998	87.4408417
960.0000000	4.1479998	-653.6911621
960.0000000	4.1967998	2.5097809
960.0000000	4.2456002	-248.1227112
960.0000000	4.2943997	-487.2087097
960.0000000	4.3431997	-13.3399744
960.0000000	4.3920002	-247.7539215
960.0000000	4.4407997	-22.4535599
960.0000000	4.4896002	-15.4777260
960.0000000	4.5383997	-654.0307007
960.0000000	4.5871997	-12.8467026
960.0000000	4.6360002	-21.6148930
960.0000000	4.6847997	-28.2665520
960.0000000	4.7336001	-19.5595112
960.0000000	4.7824001	-16.5142021
960.0000000	4.8311996	-1472.1673584
960.0000000	4.8800001	227.8845825

960.0000000	4.9287996	-201.1724548
960.0000000	4.9776001	1057.9385986
960.0000000	5.0264001	86.0186996
960.0000000	5.0751996	-300.7055969
960.0000000	5.1240001	-12.3122025
960.0000000	5.1727996	557.9502563
960.0000000	5.2216001	32.0056534
960.0000000	5.2704000	-158.8445892
960.0000000	5.3191996	-39.1872711
960.0000000	5.3680000	-3536.6193848
960.0000000	5.4168000	527.2495728
960.0000000	5.4656000	-200.7808838
960.0000000	5.5144000	38.0795593
960.0000000	5.5631995	488.4423218
960.0000000	5.6120000	393.2371216
960.0000000	5.6608000	-37.0897026
960.0000000	5.7096000	62.5492020
960.0000000	5.7584000	133.0758362
960.0000000	5.8071995	-11.5192013
960.0000000	5.8559999	29.8287373
960.0000000	5.9047999	-16.6058922
960.0000000	5.9535999	-39.7828598
960.0000000	6.0023999	-1573.4210205
960.0000000	6.0511999	-14.5678673
960.0000000	6.0999999	-19.4106750
960.0000000	6.1487999	-13.2725410
960.0000000	6.1975999	-23.2233734
960.0000000	6.2463999	-1792.4888916
960.0000000	6.2951999	-19.0718498
960.0000000	6.3439999	-342.8362427
960.0000000	6.3927999	-15.0481596
960.0000000	6.4416003	-12.3846722
960.0000000	6.4903998	-11.2621155
960.0000000	6.5391998	-11.8045454
960.0000000	6.5879998	386.8527222
960.0000000	6.6367998	-12.0826588
960.0000000	6.6856003	-13.8144264
960.0000000	6.7343998	-13.4550047
960.0000000	6.7831998	-14.3738937
960.0000000	6.8319998	58.5036240
960.0000000	6.8807998	-14.0498991
960.0000000	6.9296002	-452.2763672
960.0000000	6.9783998	-5.0360670
960.0000000	7.0271997	-14.1413727
960.0000000	7.0760002	11.3599577
960.0000000	7.1247997	-2148.4282227
960.0000000	7.1736002	-1722.8780518
960.0000000	7.2223997	-980.1260986
960.0000000	7.2711997	-211.2507935
960.0000000	7.3200002	-18.9368553
960.0000000	7.3687997	189.6204529
960.0000000	7.4176002	395.4303284
960.0000000	7.4663997	310.6766052
960.0000000	7.5151997	-958.9229126
960.0000000	7.5640001	-1059.2194824
960.0000000	7.6127996	-2496.7888184
960.0000000	7.6616001	-801.5455322
960.0000000	7.7104001	-411.6581421

960.0000000	7.7591996	43.7221527
960.0000000	7.8080001	410.9337769
960.0000000	7.8567996	541.2961426
960.0000000	7.9056001	-1121.5415039
960.0000000	7.9544001	1785.1281738
960.0000000	8.0031996	2303.4101563
960.0000000	8.0520000	2299.0505371
960.0000000	8.1007996	2432.8483887
960.0000000	8.1496000	2594.5541992
960.0000000	8.1983995	1614.1778564
960.0000000	8.2472000	-3693.1545410
960.0000000	8.2959995	-1309.1250000
960.0000000	8.3448000	-1042.8278809
960.0000000	8.3935995	-955.7253418
960.0000000	8.4423990	-411.6550903
960.0000000	8.4912004	-961.0796509
960.0000000	8.5400000	-2592.6154785
960.0000000	8.5887995	-1256.6761475
960.0000000	8.6375999	245.5170288
960.0000000	8.6863995	2217.6999512
960.0000000	8.7351999	-824.2161255
960.0000000	8.7840004	941.9380493
960.0000000	8.8327999	51.1554947
960.0000000	8.8815994	-11.4954672
960.0000000	8.9303989	-23.9660187
960.0000000	8.9792004	-11.5786400
960.0000000	9.0279999	-5.8268371
960.0000000	9.0767994	7.0720959
960.0000000	9.1255999	-17.2519455
960.0000000	9.1743994	-10.0712471
960.0000000	9.2231998	37.1037750
960.0000000	9.2720003	-8.4145832
960.0000000	9.3207998	-9.7949038
960.0000000	9.3695993	-10.7624226
960.0000000	9.4183998	-11.7546244
960.0000000	9.4672003	-11.1610842
960.0000000	9.5159998	-14.1695547
960.0000000	9.5648003	-0.3728409
960.0000000	9.6135998	-13.4007540
960.0000000	9.6623993	-10.2196836
960.0000000	9.7111998	-10.1560764
960.0000000	9.7600002	-10.8573256
960.0000000	9.8087997	-10.9947309
960.0000000	9.8575993	3.2164373
960.0000000	9.9063997	-7.9635806
960.0000000	9.9552002	-10.9065762
960.0000000	10.0039997	-10.0732336
970.0000000	0.3416000	-36.8864403
970.0000000	0.3904000	-39.8980713
970.0000000	0.4392000	-39.7733879
970.0000000	0.4880000	-39.5973015
970.0000000	0.5368000	-39.6078911
970.0000000	0.5856000	-39.3395576
970.0000000	0.6344000	-38.2238960
970.0000000	0.6832000	-36.1547661
970.0000000	0.7320000	-35.9913635
970.0000000	0.7808000	-36.5295601
970.0000000	0.8296000	-35.9708023

970.0000000	0.8784000	-35.1590614
970.0000000	0.9272000	-32.8547363
970.0000000	0.9760000	-30.6437092
970.0000000	1.0247999	-29.6477852
970.0000000	1.0735999	-29.3018589
970.0000000	1.1224000	-29.1803322
970.0000000	1.1711999	-29.8197880
970.0000000	1.2200000	-30.8320484
970.0000000	1.2687999	-30.8938446
970.0000000	1.3176000	-30.9322605
970.0000000	1.3664000	-30.5321503
970.0000000	1.4152000	-30.1612930
970.0000000	1.4640000	-29.6238670
970.0000000	1.5128000	-39.8611107
970.0000000	1.5616000	-33.8394852
970.0000000	1.6104001	-1569.9309082
970.0000000	1.6592000	-74.7278595
970.0000000	1.7079999	-27.2388020
970.0000000	1.7567999	-27.7620487
970.0000000	1.8055999	-27.6319733
970.0000000	1.8544000	-26.6620750
970.0000000	1.9031999	-27.1277256
970.0000000	1.9520000	-26.4222126
970.0000000	2.0007999	-26.4927750
970.0000000	2.0495999	-26.1582069
970.0000000	2.0983999	-155.3530579
970.0000000	2.1471999	-0.3382950
970.0000000	2.1960001	-25.4689598
970.0000000	2.2448001	-25.9173164
970.0000000	2.2935998	-27.9785538
970.0000000	2.3423998	-20.9178276
970.0000000	2.3912001	34.9882660
970.0000000	2.4400001	-22.8250313
970.0000000	2.4888000	-23.4611778
970.0000000	2.5375998	-12.1808739
970.0000000	2.5863998	-10.2878914
970.0000000	2.6352000	-8.3442202
970.0000000	2.6840000	-15.3468151
970.0000000	2.7328000	134.6355743
970.0000000	2.7815998	158.8228760
970.0000000	2.8304000	-23.8016510
970.0000000	2.8792000	-20.6011086
970.0000000	2.9280000	-22.5749683
970.0000000	2.9768000	-16.7424679
970.0000000	3.0256000	-20.4583511
970.0000000	3.0743999	-29.5807762
970.0000000	3.1231999	-24.2745266
970.0000000	3.1719999	-29.6523705
970.0000000	3.2208002	-505.5693054
970.0000000	3.2695999	-989.8944092
970.0000000	3.3183999	-20.4886074
970.0000000	3.3671999	-3403.9685059
970.0000000	3.4159999	1221.2238770
970.0000000	3.4648001	-7.1119757
970.0000000	3.5135999	-27.0520897
970.0000000	3.5623999	-322.5966492
970.0000000	3.6111999	-2027.1101074
970.0000000	3.6600001	-458.9088135

970.0000000	3.7088001	-605.9678345
970.0000000	3.7575998	-948.7504883
970.0000000	3.8063998	-1721.6253662
970.0000000	3.8552001	-2499.5583496
970.0000000	3.9040000	-2450.6455078
970.0000000	3.9528000	1120.6884766
970.0000000	4.0015998	-244.8493500
970.0000000	4.0503998	686.1272583
970.0000000	4.0991998	87.3951645
970.0000000	4.1479998	-653.4189453
970.0000000	4.1967998	2.5005245
970.0000000	4.2456002	-248.0244141
970.0000000	4.2943997	-487.0081482
970.0000000	4.3431997	-13.3421555
970.0000000	4.3920002	-247.6558075
970.0000000	4.4407997	-22.4517841
970.0000000	4.4896002	-15.4786091
970.0000000	4.5383997	-653.7696533
970.0000000	4.5871997	-12.8487186
970.0000000	4.6360002	-21.6133499
970.0000000	4.6847997	-28.2626266
970.0000000	4.7336001	-19.5587883
970.0000000	4.7824001	-16.5146580
970.0000000	4.8311996	-1471.6361084
970.0000000	4.8800001	227.7950287
970.0000000	4.9287996	-201.1051941
970.0000000	4.9776001	1057.5460205
970.0000000	5.0264001	85.9811783
970.0000000	5.0751996	-300.6019897
970.0000000	5.1240001	-12.3131237
970.0000000	5.1727996	557.8343506
970.0000000	5.2216001	31.9962120
970.0000000	5.2704000	-158.8164825
970.0000000	5.3191996	-39.1827240
970.0000000	5.3680000	-3535.9270020
970.0000000	5.4168000	527.1427612
970.0000000	5.4656000	-200.7447052
970.0000000	5.5144000	38.0689011
970.0000000	5.5631995	488.3437500
970.0000000	5.6120000	393.1571350
970.0000000	5.6608000	-37.0856171
970.0000000	5.7096000	62.5337753
970.0000000	5.7584000	133.0469055
970.0000000	5.8071995	-11.5200729
970.0000000	5.8559999	29.8198872
970.0000000	5.9047999	-16.6056957
970.0000000	5.9535999	-39.7781219
970.0000000	6.0023999	-1573.1164551
970.0000000	6.0511999	-14.5680790
970.0000000	6.0999999	-19.4098415
970.0000000	6.1487999	-13.2730036
970.0000000	6.1975999	-23.2216072
970.0000000	6.2463999	-1792.1414795
970.0000000	6.2951999	-19.0710354
970.0000000	6.3439999	-342.7721863
970.0000000	6.3927999	-15.0481415
970.0000000	6.4416003	-12.3852596
970.0000000	6.4903998	-11.2627907

970.0000000	6.5391998	-11.8052263
970.0000000	6.5879998	386.1620483
970.0000000	6.6367998	-12.0832081
970.0000000	6.6856003	-13.8145971
970.0000000	6.7343998	-13.4552202
970.0000000	6.7831998	-14.3734779
970.0000000	6.8319998	58.4845047
970.0000000	6.8807998	-14.0497608
970.0000000	6.9296002	-451.5149231
970.0000000	6.9783998	-5.0488911
970.0000000	7.0271997	-14.1403446
970.0000000	7.0760002	11.3177710
970.0000000	7.1247997	-2144.4824219
970.0000000	7.1736002	-1719.7152100
970.0000000	7.2223997	-978.3329468
970.0000000	7.2711997	-210.8830109
970.0000000	7.3200002	-18.9248657
970.0000000	7.3687997	189.2459259
970.0000000	7.4176002	394.6744995
970.0000000	7.4663997	310.0789490
970.0000000	7.5151997	-957.1619263
970.0000000	7.5640001	-1057.2720947
970.0000000	7.6127996	-2492.1711426
970.0000000	7.6616001	-800.0741577
970.0000000	7.7104001	-410.9118652
970.0000000	7.7591996	43.6210976
970.0000000	7.8080001	410.1484070
970.0000000	7.8567996	540.2680664
970.0000000	7.9056001	-1119.4686279
970.0000000	7.9544001	1781.8182373
970.0000000	8.0031996	2299.1406250
970.0000000	8.0520000	2294.7885742
970.0000000	8.1007996	2428.3388672
970.0000000	8.1496000	2589.7463379
970.0000000	8.1983995	1611.1854248
970.0000000	8.2472000	-3686.3195801
970.0000000	8.2959995	-1306.7048340
970.0000000	8.3448000	-1040.8986816
970.0000000	8.3935995	-953.9576416
970.0000000	8.4423990	-410.8970947
970.0000000	8.4912004	-959.3034058
970.0000000	8.5400000	-2587.8156738
970.0000000	8.5887995	-1254.3546143
970.0000000	8.6375999	245.0557098
970.0000000	8.6863995	2213.5815430
970.0000000	8.7351999	-822.6918335
970.0000000	8.7840004	940.1812744
970.0000000	8.8327999	51.0438843
970.0000000	8.8815994	-11.4956408
970.0000000	8.9303989	-23.9622993
970.0000000	8.9792004	-11.5787220
970.0000000	9.0279999	-5.8286057
970.0000000	9.0767994	7.0663776
970.0000000	9.1255999	-17.2502327
970.0000000	9.1743994	-10.0716496
970.0000000	9.2231998	37.0880432
970.0000000	9.2720003	-8.4155197
970.0000000	9.3207998	-9.7954426

970.0000000	9.3695993	-10.7626486
970.0000000	9.4183998	-11.7544994
970.0000000	9.4672003	-11.1611252
970.0000000	9.5159998	-14.1686640
970.0000000	9.5648003	-0.3762140
970.0000000	9.6135998	-13.3999634
970.0000000	9.6623993	-10.2199583
970.0000000	9.7111998	-10.1563482
970.0000000	9.7600002	-10.8573675
970.0000000	9.8087997	-10.9947386
970.0000000	9.8575993	3.2120152
970.0000000	9.9063997	-7.9644947
970.0000000	9.9552002	-10.9065800
970.0000000	10.0039997	-10.0734816
980.0000000	0.3416000	-36.5473175
980.0000000	0.3904000	-39.9887466
980.0000000	0.4392000	-39.7670479
980.0000000	0.4880000	-39.5960922
980.0000000	0.5368000	-39.6090660
980.0000000	0.5856000	-39.3381767
980.0000000	0.6344000	-38.2242050
980.0000000	0.6832000	-36.1569901
980.0000000	0.7320000	-35.9934158
980.0000000	0.7808000	-36.5297318
980.0000000	0.8296000	-35.9715309
980.0000000	0.8784000	-35.1664391
980.0000000	0.9272000	-32.8575134
980.0000000	0.9760000	-30.6362553
980.0000000	1.0247999	-29.6567745
980.0000000	1.0735999	-29.3162937
980.0000000	1.1224000	-29.1773338
980.0000000	1.1711999	-29.8124104
980.0000000	1.2200000	-30.8399639
980.0000000	1.2687999	-30.8948536
980.0000000	1.3176000	-30.9279881
980.0000000	1.3664000	-30.5398998
980.0000000	1.4152000	-30.1614971
980.0000000	1.4640000	-29.6221561
980.0000000	1.5128000	-39.7639313
980.0000000	1.5616000	-33.7976418
980.0000000	1.6104001	-1567.3881836
980.0000000	1.6592000	-74.4339218
980.0000000	1.7079999	-27.2425480
980.0000000	1.7567999	-27.7620678
980.0000000	1.8055999	-27.6358604
980.0000000	1.8544000	-26.6720028
980.0000000	1.9031999	-26.7112255
980.0000000	1.9520000	-26.4186401
980.0000000	2.0007999	-26.4858322
980.0000000	2.0495999	-26.1602249
980.0000000	2.0983999	-153.8772888
980.0000000	2.1471999	-0.6055622
980.0000000	2.1960001	-25.4648685
980.0000000	2.2448001	-25.8994064
980.0000000	2.2935998	-27.9189301
980.0000000	2.3423998	-20.9322319
980.0000000	2.3912001	34.3667145
980.0000000	2.4400001	-22.8275185

980.0000000	2.4888000	-23.4609604
980.0000000	2.5375998	-12.1979809
980.0000000	2.5863998	-10.3076506
980.0000000	2.6352000	-8.3669643
980.0000000	2.6840000	-15.3587570
980.0000000	2.7328000	134.3695526
980.0000000	2.7815998	158.5164795
980.0000000	2.8304000	-23.8003483
980.0000000	2.8792000	-20.6043720
980.0000000	2.9280000	-22.5743999
980.0000000	2.9768000	-16.7508392
980.0000000	3.0256000	-20.4609356
980.0000000	3.0743999	-29.5693893
980.0000000	3.1231999	-24.2710705
980.0000000	3.1719999	-29.6440773
980.0000000	3.2208002	-505.3315735
980.0000000	3.2695999	-989.4193115
980.0000000	3.3183999	-20.4888058
980.0000000	3.3671999	-3402.5068359
980.0000000	3.4159999	1220.6885986
980.0000000	3.4648001	-7.1176844
980.0000000	3.5135999	-27.0490990
980.0000000	3.5623999	-322.4670410
980.0000000	3.6111999	-2026.2503662
980.0000000	3.6600001	-458.7206421
980.0000000	3.7088001	-605.7164307
980.0000000	3.7575998	-948.3519287
980.0000000	3.8063998	-1720.8956299
980.0000000	3.8552001	-2498.4948730
980.0000000	3.9040000	-2449.6030273
980.0000000	3.9528000	1120.2001953
980.0000000	4.0015998	-244.7522430
980.0000000	4.0503998	685.8247070
980.0000000	4.0991998	87.3494797
980.0000000	4.1479998	-653.1466675
980.0000000	4.1967998	2.4912682
980.0000000	4.2456002	-247.9261169
980.0000000	4.2943997	-486.8075562
980.0000000	4.3431997	-13.3443356
980.0000000	4.3920002	-247.5576935
980.0000000	4.4407997	-22.4500084
980.0000000	4.4896002	-15.4794912
980.0000000	4.5383997	-653.5085449
980.0000000	4.5871997	-12.8507347
980.0000000	4.6360002	-21.6118069
980.0000000	4.6847997	-28.2587013
980.0000000	4.7336001	-19.5580654
980.0000000	4.7824001	-16.5151119
980.0000000	4.8311996	-1471.1048584
980.0000000	4.8800001	227.7054749
980.0000000	4.9287996	-201.0379333
980.0000000	4.9776001	1057.1535645
980.0000000	5.0264001	85.9436569
980.0000000	5.0751996	-300.4983521
980.0000000	5.1240001	-12.3140450
980.0000000	5.1727996	557.7185059
980.0000000	5.2216001	31.9867668
980.0000000	5.2704000	-158.7883759

980.0000000	5.3191996	-39.1781769
980.0000000	5.3680000	-3535.2346191
980.0000000	5.4168000	527.0360107
980.0000000	5.4656000	-200.7085114
980.0000000	5.5144000	38.0582428
980.0000000	5.5631995	488.2451782
980.0000000	5.6120000	393.0771790
980.0000000	5.6608000	-37.0815277
980.0000000	5.7096000	62.5183411
980.0000000	5.7584000	133.0179749
980.0000000	5.8071995	-11.5209427
980.0000000	5.8559999	29.8110332
980.0000000	5.9047999	-16.6054974
980.0000000	5.9535999	-39.7733841
980.0000000	6.0023999	-1572.8121338
980.0000000	6.0511999	-14.5682907
980.0000000	6.0999999	-19.4090099
980.0000000	6.1487999	-13.2734661
980.0000000	6.1975999	-23.2198410
980.0000000	6.2463999	-1791.7941895
980.0000000	6.2951999	-19.0702209
980.0000000	6.3439999	-342.7081604
980.0000000	6.3927999	-15.0481224
980.0000000	6.4416003	-12.3858461
980.0000000	6.4903998	-11.2634668
980.0000000	6.5391998	-11.8059072
980.0000000	6.5879998	385.4713440
980.0000000	6.6367998	-12.0837574
980.0000000	6.6856003	-13.8147678
980.0000000	6.7343998	-13.4554358
980.0000000	6.7831998	-14.3730621
980.0000000	6.8319998	58.4653854
980.0000000	6.8807998	-14.0496225
980.0000000	6.9296002	-450.7534790
980.0000000	6.9783998	-5.0617146
980.0000000	7.0271997	-14.1393166
980.0000000	7.0760002	11.2755880
980.0000000	7.1247997	-2140.5363770
980.0000000	7.1736002	-1716.5523682
980.0000000	7.2223997	-976.5397949
980.0000000	7.2711997	-210.5152283
980.0000000	7.3200002	-18.9128780
980.0000000	7.3687997	188.8713989
980.0000000	7.4176002	393.9186707
980.0000000	7.4663997	309.4812927
980.0000000	7.5151997	-955.4008789
980.0000000	7.5640001	-1055.3247070
980.0000000	7.6127996	-2487.5537109
980.0000000	7.6616001	-798.6027832
980.0000000	7.7104001	-410.1656494
980.0000000	7.7591996	43.5200500
980.0000000	7.8080001	409.3630676
980.0000000	7.8567996	539.2399902
980.0000000	7.9056001	-1117.3956299
980.0000000	7.9544001	1778.5083008
980.0000000	8.0031996	2294.8708496
980.0000000	8.0520000	2290.5266113
980.0000000	8.1007996	2423.8293457

980.0000000	8.1496000	2584.9382324
980.0000000	8.1983995	1608.1928711
980.0000000	8.2472000	-3679.4848633
980.0000000	8.2959995	-1304.2846680
980.0000000	8.3448000	-1038.9694824
980.0000000	8.3935995	-952.1900024
980.0000000	8.4423990	-410.1390686
980.0000000	8.4912004	-957.5272217
980.0000000	8.5400000	-2583.0158691
980.0000000	8.5887995	-1252.0329590
980.0000000	8.6375999	244.5943909
980.0000000	8.6863995	2209.4631348
980.0000000	8.7351999	-821.1676025
980.0000000	8.7840004	938.4245605
980.0000000	8.8327999	50.9322815
980.0000000	8.8815994	-11.4958153
980.0000000	8.9303989	-23.9585800
980.0000000	8.9792004	-11.5788040
980.0000000	9.0279999	-5.8303742
980.0000000	9.0767994	7.0606613
980.0000000	9.1255999	-17.2485199
980.0000000	9.1743994	-10.0720510
980.0000000	9.2231998	37.0723114
980.0000000	9.2720003	-8.4164562
980.0000000	9.3207998	-9.7959824
980.0000000	9.3695993	-10.7628756
980.0000000	9.4183998	-11.7543755
980.0000000	9.4672003	-11.1611662
980.0000000	9.5159998	-14.1677723
980.0000000	9.5648003	-0.3795872
980.0000000	9.6135998	-13.3991718
980.0000000	9.6623993	-10.2202330
980.0000000	9.7111998	-10.1566200
980.0000000	9.7600002	-10.8574085
980.0000000	9.8087997	-10.9947462
980.0000000	9.8575993	3.2075939
980.0000000	9.9063997	-7.9654083
980.0000000	9.9552002	-10.9065847
980.0000000	10.0039997	-10.0737286
990.0000000	0.3416000	-37.1530876
990.0000000	0.3904000	-39.9536667
990.0000000	0.4392000	-39.7607040
990.0000000	0.4880000	-39.5948830
990.0000000	0.5368000	-39.6102371
990.0000000	0.5856000	-39.3367958
990.0000000	0.6344000	-38.2245178
990.0000000	0.6832000	-36.1592140
990.0000000	0.7320000	-35.9954681
990.0000000	0.7808000	-36.5298996
990.0000000	0.8296000	-35.9722595
990.0000000	0.8784000	-35.1738129
990.0000000	0.9272000	-32.8602905
990.0000000	0.9760000	-30.6288033
990.0000000	1.0247999	-29.6657639
990.0000000	1.0735999	-29.3307285
990.0000000	1.1224000	-29.1743336
990.0000000	1.1711999	-29.8050327
990.0000000	1.2200000	-30.8478794

990.0000000	1.2687999	-30.8958607
990.0000000	1.3176000	-30.9237175
990.0000000	1.3664000	-30.5476494
990.0000000	1.4152000	-30.1617012
990.0000000	1.4640000	-29.6204472
990.0000000	1.5128000	-39.6667557
990.0000000	1.5616000	-33.7558022
990.0000000	1.6104001	-1564.8453369
990.0000000	1.6592000	-74.1399918
990.0000000	1.7079999	-27.2462959
990.0000000	1.7567999	-27.7620869
990.0000000	1.8055999	-27.6397495
990.0000000	1.8544000	-26.6819305
990.0000000	1.9031999	-26.7084312
990.0000000	1.9520000	-26.4150677
990.0000000	2.0007999	-26.4788895
990.0000000	2.0495999	-26.1622448
990.0000000	2.0983999	-152.4015198
990.0000000	2.1471999	-0.8728294
990.0000000	2.1960001	-25.4607773
990.0000000	2.2448001	-25.8814945
990.0000000	2.2935998	-27.8593063
990.0000000	2.3423998	-20.9466362
990.0000000	2.3912001	33.7451706
990.0000000	2.4400001	-22.8300056
990.0000000	2.4888000	-23.4607430
990.0000000	2.5375998	-12.2150888
990.0000000	2.5863998	-10.3274097
990.0000000	2.6352000	-8.3897085
990.0000000	2.6840000	-15.3706999
990.0000000	2.7328000	134.1035461
990.0000000	2.7815998	158.2100525
990.0000000	2.8304000	-23.7990437
990.0000000	2.8792000	-20.6076355
990.0000000	2.9280000	-22.5738297
990.0000000	2.9768000	-16.7592087
990.0000000	3.0256000	-20.4635201
990.0000000	3.0743999	-29.5580006
990.0000000	3.1231999	-24.2676163
990.0000000	3.1719999	-29.6357841
990.0000000	3.2208002	-505.0938416
990.0000000	3.2695999	-988.9444580
990.0000000	3.3183999	-20.4890041
990.0000000	3.3671999	-3401.0451660
990.0000000	3.4159999	1220.1533203
990.0000000	3.4648001	-7.1233912
990.0000000	3.5135999	-27.0461082
990.0000000	3.5623999	-322.3374634
990.0000000	3.6111999	-2025.3905029
990.0000000	3.6600001	-458.5325317
990.0000000	3.7088001	-605.4650269
990.0000000	3.7575998	-947.9534302
990.0000000	3.8063998	-1720.1657715
990.0000000	3.8552001	-2497.4316406
990.0000000	3.9040000	-2448.5605469
990.0000000	3.9528000	1119.7119141
990.0000000	4.0015998	-244.6551208
990.0000000	4.0503998	685.5221558

990.0000000	4.0991998	87.3037949
990.0000000	4.1479998	-652.8744507
990.0000000	4.1967998	2.4820118
990.0000000	4.2456002	-247.8278046
990.0000000	4.2943997	-486.6069946
990.0000000	4.3431997	-13.3465157
990.0000000	4.3920002	-247.4595795
990.0000000	4.4407997	-22.4482327
990.0000000	4.4896002	-15.4803743
990.0000000	4.5383997	-653.2474976
990.0000000	4.5871997	-12.8527508
990.0000000	4.6360002	-21.6102657
990.0000000	4.6847997	-28.2547760
990.0000000	4.7336001	-19.5573425
990.0000000	4.7824001	-16.5155659
990.0000000	4.8311996	-1470.5736084
990.0000000	4.8800001	227.6159210
990.0000000	4.9287996	-200.9706726
990.0000000	4.9776001	1056.7611084
990.0000000	5.0264001	85.9061356
990.0000000	5.0751996	-300.3947144
990.0000000	5.1240001	-12.3149662
990.0000000	5.1727996	557.6026001
990.0000000	5.2216001	31.9773216
990.0000000	5.2704000	-158.7602692
990.0000000	5.3191996	-39.1736259
990.0000000	5.3680000	-3534.5422363
990.0000000	5.4168000	526.9291992
990.0000000	5.4656000	-200.6723328
990.0000000	5.5144000	38.0475922
990.0000000	5.5631995	488.1466064
990.0000000	5.6120000	392.9971924
990.0000000	5.6608000	-37.0774384
990.0000000	5.7096000	62.5029068
990.0000000	5.7584000	132.9890442
990.0000000	5.8071995	-11.5218143
990.0000000	5.8559999	29.8021832
990.0000000	5.9047999	-16.6052990
990.0000000	5.9535999	-39.7686462
990.0000000	6.0023999	-1572.5076904
990.0000000	6.0511999	-14.5685015
990.0000000	6.0999999	-19.4081764
990.0000000	6.1487999	-13.2739296
990.0000000	6.1975999	-23.2180767
990.0000000	6.2463999	-1791.4467773
990.0000000	6.2951999	-19.0694065
990.0000000	6.3439999	-342.6441040
990.0000000	6.3927999	-15.0481043
990.0000000	6.4416003	-12.3864326
990.0000000	6.4903998	-11.2641430
990.0000000	6.5391998	-11.8065882
990.0000000	6.5879998	384.7806396
990.0000000	6.6367998	-12.0843067
990.0000000	6.6856003	-13.8149376
990.0000000	6.7343998	-13.4556513
990.0000000	6.7831998	-14.3726463
990.0000000	6.8319998	58.4462738
990.0000000	6.8807998	-14.0494833

990.0000000	6.9296002	-449.9920044
990.0000000	6.9783998	-5.0745387
990.0000000	7.0271997	-14.1382885
990.0000000	7.0760002	11.2334013
990.0000000	7.1247997	-2136.5905762
990.0000000	7.1736002	-1713.3894043
990.0000000	7.2223997	-974.7465820
990.0000000	7.2711997	-210.1474457
990.0000000	7.3200002	-18.9008904
990.0000000	7.3687997	188.4968719
990.0000000	7.4176002	393.1628418
990.0000000	7.4663997	308.8836365
990.0000000	7.5151997	-953.6398315
990.0000000	7.5640001	-1053.3771973
990.0000000	7.6127996	-2482.9360352
990.0000000	7.6616001	-797.1314697
990.0000000	7.7104001	-409.4193726
990.0000000	7.7591996	43.4190025
990.0000000	7.8080001	408.5776978
990.0000000	7.8567996	538.2119141
990.0000000	7.9056001	-1115.3227539
990.0000000	7.9544001	1775.1983643
990.0000000	8.0031996	2290.6013184
990.0000000	8.0520000	2286.2646484
990.0000000	8.1007996	2419.3198242
990.0000000	8.1496000	2580.1303711
990.0000000	8.1983995	1605.2003174
990.0000000	8.2472000	-3672.6501465
990.0000000	8.2959995	-1301.8645020
990.0000000	8.3448000	-1037.0402832
990.0000000	8.3935995	-950.4223022
990.0000000	8.4423990	-409.3810730
990.0000000	8.4912004	-955.7509766
990.0000000	8.5400000	-2578.2160645
990.0000000	8.5887995	-1249.7114258
990.0000000	8.6375999	244.1330719
990.0000000	8.6863995	2205.3449707
990.0000000	8.7351999	-819.6433716
990.0000000	8.7840004	936.6677856
990.0000000	8.8327999	50.8206711
990.0000000	8.8815994	-11.4959898
990.0000000	8.9303989	-23.9548607
990.0000000	8.9792004	-11.5788870
990.0000000	9.0279999	-5.8321419
990.0000000	9.0767994	7.0549431
990.0000000	9.1255999	-17.2468052
990.0000000	9.1743994	-10.0724535
990.0000000	9.2231998	37.0565796
990.0000000	9.2720003	-8.4173927
990.0000000	9.3207998	-9.7965212
990.0000000	9.3695993	-10.7631016
990.0000000	9.4183998	-11.7542505
990.0000000	9.4672003	-11.1612082
990.0000000	9.5159998	-14.1668816
990.0000000	9.5648003	-0.3829594
990.0000000	9.6135998	-13.3983812
990.0000000	9.6623993	-10.2205086
990.0000000	9.7111998	-10.1568909

990.0000000	9.7600002	-10.8574505
990.0000000	9.8087997	-10.9947548
990.0000000	9.8575993	3.2031698
990.0000000	9.9063997	-7.9663224
990.0000000	9.9552002	-10.9065886
990.0000000	10.0039997	-10.0739765
1000.0000000	0.3416000	-37.1873512
1000.0000000	0.3904000	-39.9185829
1000.0000000	0.4392000	-39.7543602
1000.0000000	0.4880000	-39.5936737
1000.0000000	0.5368000	-39.6114120
1000.0000000	0.5856000	-39.3354149
1000.0000000	0.6344000	-38.2248268
1000.0000000	0.6832000	-36.1614418
1000.0000000	0.7320000	-35.9975243
1000.0000000	0.7808000	-36.5300674
1000.0000000	0.8296000	-35.9729919
1000.0000000	0.8784000	-35.1811867
1000.0000000	0.9272000	-32.8630676
1000.0000000	0.9760000	-30.6213512
1000.0000000	1.0247999	-29.6747532
1000.0000000	1.0735999	-29.3451633
1000.0000000	1.1224000	-29.1713352
1000.0000000	1.1711999	-29.7976551
1000.0000000	1.2200000	-30.8557949
1000.0000000	1.2687999	-30.8968678
1000.0000000	1.3176000	-30.9194450
1000.0000000	1.3664000	-30.5553989
1000.0000000	1.4152000	-30.1619053
1000.0000000	1.4640000	-29.6187382
1000.0000000	1.5128000	-39.5695763
1000.0000000	1.5616000	-33.7139587
1000.0000000	1.6104001	-1562.3026123
1000.0000000	1.6592000	-73.8460617
1000.0000000	1.7079999	-27.2500420
1000.0000000	1.7567999	-27.7621059
1000.0000000	1.8055999	-27.6436367
1000.0000000	1.8544000	-26.6918583
1000.0000000	1.9031999	-26.7056370
1000.0000000	1.9520000	-26.4114971
1000.0000000	2.0007999	-26.4719467
1000.0000000	2.0495999	-26.1642628
1000.0000000	2.0983999	-150.9257202
1000.0000000	2.1471999	-1.1400967
1000.0000000	2.1960001	-25.4566860
1000.0000000	2.2448001	-25.8635826
1000.0000000	2.2935998	-27.7996826
1000.0000000	2.3423998	-20.9610405
1000.0000000	2.3912001	33.1236267
1000.0000000	2.4400001	-22.8324909
1000.0000000	2.4888000	-23.4605255
1000.0000000	2.5375998	-12.2321968
1000.0000000	2.5863998	-10.3471689
1000.0000000	2.6352000	-8.4124527
1000.0000000	2.6840000	-15.3826408
1000.0000000	2.7328000	133.8375244
1000.0000000	2.7815998	157.9036255
1000.0000000	2.8304000	-23.7977390

1000.0000000	2.8792000	-20.6108990
1000.0000000	2.9280000	-22.5732613
1000.0000000	2.9768000	-16.7675800
1000.0000000	3.0256000	-20.4661045
1000.0000000	3.0743999	-29.5466137
1000.0000000	3.1231999	-24.2641602
1000.0000000	3.1719999	-29.6274910
1000.0000000	3.2208002	-504.8561096
1000.0000000	3.2695999	-988.4693604
1000.0000000	3.3183999	-20.4892006
1000.0000000	3.3671999	-3399.5834961
1000.0000000	3.4159999	1219.6180420
1000.0000000	3.4648001	-7.1290998
1000.0000000	3.5135999	-27.0431175
1000.0000000	3.5623999	-322.2078552
1000.0000000	3.6111999	-2024.5307617
1000.0000000	3.6600001	-458.3444214
1000.0000000	3.7088001	-605.2136841
1000.0000000	3.7575998	-947.5548706
1000.0000000	3.8063998	-1719.4359131
1000.0000000	3.8552001	-2496.3681641
1000.0000000	3.9040000	-2447.5180664
1000.0000000	3.9528000	1119.2235107
1000.0000000	4.0015998	-244.5580139
1000.0000000	4.0503998	685.2196045
1000.0000000	4.0991998	87.2581100
1000.0000000	4.1479998	-652.6022339
1000.0000000	4.1967998	2.4727554
1000.0000000	4.2456002	-247.7295074
1000.0000000	4.2943997	-486.4064331
1000.0000000	4.3431997	-13.3486967
1000.0000000	4.3920002	-247.3614655
1000.0000000	4.4407997	-22.4464569
1000.0000000	4.4896002	-15.4812565
1000.0000000	4.5383997	-652.9864502
1000.0000000	4.5871997	-12.8547668
1000.0000000	4.6360002	-21.6087227
1000.0000000	4.6847997	-28.2508507
1000.0000000	4.7336001	-19.5566216
1000.0000000	4.7824001	-16.5160198
1000.0000000	4.8311996	-1470.0423584
1000.0000000	4.8800001	227.5263672
1000.0000000	4.9287996	-200.9034119
1000.0000000	4.9776001	1056.3685303
1000.0000000	5.0264001	85.8686142
1000.0000000	5.0751996	-300.2911072
1000.0000000	5.1240001	-12.3158875
1000.0000000	5.1727996	557.4867554
1000.0000000	5.2216001	31.9678802
1000.0000000	5.2704000	-158.7321625
1000.0000000	5.3191996	-39.1690750
1000.0000000	5.3680000	-3533.8498535
1000.0000000	5.4168000	526.8224487
1000.0000000	5.4656000	-200.6361542
1000.0000000	5.5144000	38.0369339
1000.0000000	5.5631995	488.0480652
1000.0000000	5.6120000	392.9172058
1000.0000000	5.6608000	-37.0733490

1000.0000000	5.7096000	62.4874802
1000.0000000	5.7584000	132.9600983
1000.0000000	5.8071995	-11.5226841
1000.0000000	5.8559999	29.7933331
1000.0000000	5.9047999	-16.6051025
1000.0000000	5.9535999	-39.7639046
1000.0000000	6.0023999	-1572.2033691
1000.0000000	6.0511999	-14.5687132
1000.0000000	6.0999999	-19.4073448
1000.0000000	6.1487999	-13.2743921
1000.0000000	6.1975999	-23.2163124
1000.0000000	6.2463999	-1791.0993652
1000.0000000	6.2951999	-19.0685921
1000.0000000	6.3439999	-342.5800476
1000.0000000	6.3927999	-15.0480862
1000.0000000	6.4416003	-12.3870201
1000.0000000	6.4903998	-11.2648182
1000.0000000	6.5391998	-11.8072691
1000.0000000	6.5879998	384.0899353
1000.0000000	6.6367998	-12.0848551
1000.0000000	6.6856003	-13.8151083
1000.0000000	6.7343998	-13.4558678
1000.0000000	6.7831998	-14.3722315
1000.0000000	6.8319998	58.4271622
1000.0000000	6.8807998	-14.0493450
1000.0000000	6.9296002	-449.2305603
1000.0000000	6.9783998	-5.0873613
1000.0000000	7.0271997	-14.1372604
1000.0000000	7.0760002	11.1912146
1000.0000000	7.1247997	-2132.6447754
1000.0000000	7.1736002	-1710.2265625
1000.0000000	7.2223997	-972.9534302
1000.0000000	7.2711997	-209.7796631
1000.0000000	7.3200002	-18.8889008
1000.0000000	7.3687997	188.1223450
1000.0000000	7.4176002	392.4070129
1000.0000000	7.4663997	308.2860107
1000.0000000	7.5151997	-951.8787842
1000.0000000	7.5640001	-1051.4298096
1000.0000000	7.6127996	-2478.3183594
1000.0000000	7.6616001	-795.6600952
1000.0000000	7.7104001	-408.6730957
1000.0000000	7.7591996	43.3179512
1000.0000000	7.8080001	407.7923279
1000.0000000	7.8567996	537.1838379
1000.0000000	7.9056001	-1113.2498779
1000.0000000	7.9544001	1771.8884277
1000.0000000	8.0031996	2286.3317871
1000.0000000	8.0520000	2282.0024414
1000.0000000	8.1007996	2414.8103027
1000.0000000	8.1496000	2575.3222656
1000.0000000	8.1983995	1602.2078857
1000.0000000	8.2472000	-3665.8151855
1000.0000000	8.2959995	-1299.4443359
1000.0000000	8.3448000	-1035.1112061
1000.0000000	8.3935995	-948.6546021
1000.0000000	8.4423990	-408.6230469
1000.0000000	8.4912004	-953.9747314

1000.0000000	8.5400000	-2573.4162598
1000.0000000	8.5887995	-1247.3898926
1000.0000000	8.6375999	243.6717529
1000.0000000	8.6863995	2201.2265625
1000.0000000	8.7351999	-818.1190796
1000.0000000	8.7840004	934.9110718
1000.0000000	8.8327999	50.7090645
1000.0000000	8.8815994	-11.4961643
1000.0000000	8.9303989	-23.9511395
1000.0000000	8.9792004	-11.5789690
1000.0000000	9.0279999	-5.8339105
1000.0000000	9.0767994	7.0492268
1000.0000000	9.1255999	-17.2450905
1000.0000000	9.1743994	-10.0728550
1000.0000000	9.2231998	37.0408478
1000.0000000	9.2720003	-8.4183292
1000.0000000	9.3207998	-9.7970610
1000.0000000	9.3695993	-10.7633286
1000.0000000	9.4183998	-11.7541256
1000.0000000	9.4672003	-11.1612492
1000.0000000	9.5159998	-14.1659908
1000.0000000	9.5648003	-0.3863316
1000.0000000	9.6135998	-13.3975906
1000.0000000	9.6623993	-10.2207832
1000.0000000	9.7111998	-10.1571627
1000.0000000	9.7600002	-10.8574924
1000.0000000	9.8087997	-10.9947624
1000.0000000	9.8575993	3.1987476
1000.0000000	9.9063997	-7.9672365
1000.0000000	9.9552002	-10.9065933
1000.0000000	10.0039997	-10.0742245
1010.0000000	0.3416000	-37.3550072
1010.0000000	0.3904000	-39.8835030
1010.0000000	0.4392000	-39.7480164
1010.0000000	0.4880000	-39.5924644
1010.0000000	0.5368000	-39.6125870
1010.0000000	0.5856000	-39.3340340
1010.0000000	0.6344000	-38.2251358
1010.0000000	0.6832000	-36.1636658
1010.0000000	0.7320000	-35.9995766
1010.0000000	0.7808000	-36.5302391
1010.0000000	0.8296000	-35.9737206
1010.0000000	0.8784000	-35.1885643
1010.0000000	0.9272000	-32.8658447
1010.0000000	0.9760000	-30.6138973
1010.0000000	1.0247999	-29.6837425
1010.0000000	1.0735999	-29.3595982
1010.0000000	1.1224000	-29.1683350
1010.0000000	1.1711999	-29.7902775
1010.0000000	1.2200000	-30.8637104
1010.0000000	1.2687999	-30.8978767
1010.0000000	1.3176000	-30.9151745
1010.0000000	1.3664000	-30.5631485
1010.0000000	1.4152000	-30.1621113
1010.0000000	1.4640000	-29.6170273
1010.0000000	1.5128000	-39.4723969
1010.0000000	1.5616000	-33.6721191
1010.0000000	1.6104001	-1559.7597656

1010.0000000	1.6592000	-73.5521317
1010.0000000	1.7079999	-27.2537899
1010.0000000	1.7567999	-27.7621250
1010.0000000	1.8055999	-27.6475258
1010.0000000	1.8544000	-26.7017860
1010.0000000	1.9031999	-26.7028427
1010.0000000	1.9520000	-26.4079247
1010.0000000	2.0007999	-26.4650040
1010.0000000	2.0495999	-26.1662827
1010.0000000	2.0983999	-149.4499512
1010.0000000	2.1471999	-1.4073639
1010.0000000	2.1960001	-25.4525948
1010.0000000	2.2448001	-25.8456726
1010.0000000	2.2935998	-27.7400589
1010.0000000	2.3423998	-20.9754448
1010.0000000	2.3912001	32.5020828
1010.0000000	2.4400001	-22.8349781
1010.0000000	2.4888000	-23.4603081
1010.0000000	2.5375998	-12.2493048
1010.0000000	2.5863998	-10.3669271
1010.0000000	2.6352000	-8.4351969
1010.0000000	2.6840000	-15.3945827
1010.0000000	2.7328000	133.5715179
1010.0000000	2.7815998	157.5971985
1010.0000000	2.8304000	-23.7964344
1010.0000000	2.8792000	-20.6141624
1010.0000000	2.9280000	-22.5726929
1010.0000000	2.9768000	-16.7759514
1010.0000000	3.0256000	-20.4686890
1010.0000000	3.0743999	-29.5352268
1010.0000000	3.1231999	-24.2607040
1010.0000000	3.1719999	-29.6191978
1010.0000000	3.2208002	-504.6183777
1010.0000000	3.2695999	-987.9945068
1010.0000000	3.3183999	-20.4893990
1010.0000000	3.3671999	-3398.1218262
1010.0000000	3.4159999	1219.0827637
1010.0000000	3.4648001	-7.1348085
1010.0000000	3.5135999	-27.0401268
1010.0000000	3.5623999	-322.0782776
1010.0000000	3.6111999	-2023.6710205
1010.0000000	3.6600001	-458.1562500
1010.0000000	3.7088001	-604.9622803
1010.0000000	3.7575998	-947.1563721
1010.0000000	3.8063998	-1718.7060547
1010.0000000	3.8552001	-2495.3046875
1010.0000000	3.9040000	-2446.4755859
1010.0000000	3.9528000	1118.7352295
1010.0000000	4.0015998	-244.4609070
1010.0000000	4.0503998	684.9170532
1010.0000000	4.0991998	87.2124252
1010.0000000	4.1479998	-652.3300171
1010.0000000	4.1967998	2.4634991
1010.0000000	4.2456002	-247.6312103
1010.0000000	4.2943997	-486.2058716
1010.0000000	4.3431997	-13.3508778
1010.0000000	4.3920002	-247.2633514
1010.0000000	4.4407997	-22.4446812

1010.0000000	4.4896002	-15.4821396
1010.0000000	4.5383997	-652.7253418
1010.0000000	4.5871997	-12.8567839
1010.0000000	4.6360002	-21.6071796
1010.0000000	4.6847997	-28.2469254
1010.0000000	4.7336001	-19.5558987
1010.0000000	4.7824001	-16.5164757
1010.0000000	4.8311996	-1469.5109863
1010.0000000	4.8800001	227.4368286
1010.0000000	4.9287996	-200.8361511
1010.0000000	4.9776001	1055.9760742
1010.0000000	5.0264001	85.8310928
1010.0000000	5.0751996	-300.1874695
1010.0000000	5.1240001	-12.3168087
1010.0000000	5.1727996	557.3709106
1010.0000000	5.2216001	31.9584351
1010.0000000	5.2704000	-158.7040558
1010.0000000	5.3191996	-39.1645279
1010.0000000	5.3680000	-3533.1574707
1010.0000000	5.4168000	526.7156372
1010.0000000	5.4656000	-200.5999603
1010.0000000	5.5144000	38.0262833
1010.0000000	5.5631995	487.9494934
1010.0000000	5.6120000	392.8372498
1010.0000000	5.6608000	-37.0692596
1010.0000000	5.7096000	62.4720459
1010.0000000	5.7584000	132.9311676
1010.0000000	5.8071995	-11.5235558
1010.0000000	5.8559999	29.7844791
1010.0000000	5.9047999	-16.6049042
1010.0000000	5.9535999	-39.7591667
1010.0000000	6.0023999	-1571.8988037
1010.0000000	6.0511999	-14.5689240
1010.0000000	6.0999999	-19.4065113
1010.0000000	6.1487999	-13.2748547
1010.0000000	6.1975999	-23.2145462
1010.0000000	6.2463999	-1790.7519531
1010.0000000	6.2951999	-19.0677776
1010.0000000	6.3439999	-342.5159912
1010.0000000	6.3927999	-15.0480680
1010.0000000	6.4416003	-12.3876066
1010.0000000	6.4903998	-11.2654943
1010.0000000	6.5391998	-11.8079510
1010.0000000	6.5879998	383.3992310
1010.0000000	6.6367998	-12.0854044
1010.0000000	6.6856003	-13.8152781
1010.0000000	6.7343998	-13.4560833
1010.0000000	6.7831998	-14.3718157
1010.0000000	6.8319998	58.4080429
1010.0000000	6.8807998	-14.0492058
1010.0000000	6.9296002	-448.4691162
1010.0000000	6.9783998	-5.1001854
1010.0000000	7.0271997	-14.1362333
1010.0000000	7.0760002	11.1490316
1010.0000000	7.1247997	-2128.6987305
1010.0000000	7.1736002	-1707.0637207
1010.0000000	7.2223997	-971.1602783
1010.0000000	7.2711997	-209.4118958

1010.0000000	7.3200002	-18.8769112
1010.0000000	7.3687997	187.7478027
1010.0000000	7.4176002	391.6511841
1010.0000000	7.4663997	307.6883545
1010.0000000	7.5151997	-950.1177368
1010.0000000	7.5640001	-1049.4824219
1010.0000000	7.6127996	-2473.7006836
1010.0000000	7.6616001	-794.1887207
1010.0000000	7.7104001	-407.9268188
1010.0000000	7.7591996	43.2169037
1010.0000000	7.8080001	407.0069885
1010.0000000	7.8567996	536.1557007
1010.0000000	7.9056001	-1111.1770020
1010.0000000	7.9544001	1768.5784912
1010.0000000	8.0031996	2282.0622559
1010.0000000	8.0520000	2277.7404785
1010.0000000	8.1007996	2410.3007813
1010.0000000	8.1496000	2570.5144043
1010.0000000	8.1983995	1599.2153320
1010.0000000	8.2472000	-3658.9804688
1010.0000000	8.2959995	-1297.0241699
1010.0000000	8.3448000	-1033.1820068
1010.0000000	8.3935995	-946.8869629
1010.0000000	8.4423990	-407.8650513
1010.0000000	8.4912004	-952.1985474
1010.0000000	8.5400000	-2568.6164551
1010.0000000	8.5887995	-1245.0683594
1010.0000000	8.6375999	243.2104340
1010.0000000	8.6863995	2197.1081543
1010.0000000	8.7351999	-816.5948486
1010.0000000	8.7840004	933.1542969
1010.0000000	8.8327999	50.5974541
1010.0000000	8.8815994	-11.4963379
1010.0000000	8.9303989	-23.9474182
1010.0000000	8.9792004	-11.5790510
1010.0000000	9.0279999	-5.8356786
1010.0000000	9.0767994	7.0435085
1010.0000000	9.1255999	-17.2433777
1010.0000000	9.1743994	-10.0732574
1010.0000000	9.2231998	37.0251160
1010.0000000	9.2720003	-8.4192657
1010.0000000	9.3207998	-9.7975998
1010.0000000	9.3695993	-10.7635546
1010.0000000	9.4183998	-11.7540016
1010.0000000	9.4672003	-11.1612902
1010.0000000	9.5159998	-14.1650991
1010.0000000	9.5648003	-0.3897047
1010.0000000	9.6135998	-13.3968000
1010.0000000	9.6623993	-10.2210588
1010.0000000	9.7111998	-10.1574345
1010.0000000	9.7600002	-10.8575344
1010.0000000	9.8087997	-10.9947701
1010.0000000	9.8575993	3.1943264
1010.0000000	9.9063997	-7.9681501
1010.0000000	9.9552002	-10.9065971
1010.0000000	10.0039997	-10.0744715
1020.0000000	0.3416000	-37.2310333
1020.0000000	0.3904000	-39.8484230

1020.0000000	0.4392000	-39.7416725
1020.0000000	0.4880000	-39.5912552
1020.0000000	0.5368000	-39.6137581
1020.0000000	0.5856000	-39.3326530
1020.0000000	0.6344000	-38.2254486
1020.0000000	0.6832000	-36.1658936
1020.0000000	0.7320000	-36.0016289
1020.0000000	0.7808000	-36.5304070
1020.0000000	0.8296000	-35.9744492
1020.0000000	0.8784000	-35.1959381
1020.0000000	0.9272000	-32.8686218
1020.0000000	0.9760000	-30.6064453
1020.0000000	1.0247999	-29.6927319
1020.0000000	1.0735999	-29.3740330
1020.0000000	1.1224000	-29.1653366
1020.0000000	1.1711999	-29.7829018
1020.0000000	1.2200000	-30.8716240
1020.0000000	1.2687999	-30.8988838
1020.0000000	1.3176000	-30.9109020
1020.0000000	1.3664000	-30.5708981
1020.0000000	1.4152000	-30.1623154
1020.0000000	1.4640000	-29.6153183
1020.0000000	1.5128000	-39.3752213
1020.0000000	1.5616000	-33.6302795
1020.0000000	1.6104001	-1557.2169189
1020.0000000	1.6592000	-73.2581940
1020.0000000	1.7079999	-27.2575378
1020.0000000	1.7567999	-27.7621441
1020.0000000	1.8055999	-27.6514130
1020.0000000	1.8544000	-26.7117138
1020.0000000	1.9031999	-26.7000484
1020.0000000	1.9520000	-26.4043541
1020.0000000	2.0007999	-26.4580612
1020.0000000	2.0495999	-26.1683006
1020.0000000	2.0983999	-147.9741821
1020.0000000	2.1471999	-1.6746311
1020.0000000	2.1960001	-25.4485035
1020.0000000	2.2448001	-25.8277607
1020.0000000	2.2935998	-27.6804352
1020.0000000	2.3423998	-20.9898510
1020.0000000	2.3912001	31.8805332
1020.0000000	2.4400001	-22.8374653
1020.0000000	2.4888000	-23.4600906
1020.0000000	2.5375998	-12.2664127
1020.0000000	2.5863998	-10.3866863
1020.0000000	2.6352000	-8.4579411
1020.0000000	2.6840000	-15.4065247
1020.0000000	2.7328000	133.3054962
1020.0000000	2.7815998	157.2908020
1020.0000000	2.8304000	-23.7951317
1020.0000000	2.8792000	-20.6174259
1020.0000000	2.9280000	-22.5721226
1020.0000000	2.9768000	-16.7843208
1020.0000000	3.0256000	-20.4712734
1020.0000000	3.0743999	-29.5238400
1020.0000000	3.1231999	-24.2572498
1020.0000000	3.1719999	-29.6109047
1020.0000000	3.2208002	-504.3806152

1020.0000000	3.2695999	-987.5195313
1020.0000000	3.3183999	-20.4895973
1020.0000000	3.3671999	-3396.6601563
1020.0000000	3.4159999	1218.5474854
1020.0000000	3.4648001	-7.1405172
1020.0000000	3.5135999	-27.0371361
1020.0000000	3.5623999	-321.9486694
1020.0000000	3.6111999	-2022.8112793
1020.0000000	3.6600001	-457.9681396
1020.0000000	3.7088001	-604.7108765
1020.0000000	3.7575998	-946.7578125
1020.0000000	3.8063998	-1717.9761963
1020.0000000	3.8552001	-2494.2412109
1020.0000000	3.9040000	-2445.4331055
1020.0000000	3.9528000	1118.2469482
1020.0000000	4.0015998	-244.3638000
1020.0000000	4.0503998	684.6145020
1020.0000000	4.0991998	87.1667404
1020.0000000	4.1479998	-652.0578003
1020.0000000	4.1967998	2.4542427
1020.0000000	4.2456002	-247.5328979
1020.0000000	4.2943997	-486.0052795
1020.0000000	4.3431997	-13.3530579
1020.0000000	4.3920002	-247.1652374
1020.0000000	4.4407997	-22.4429054
1020.0000000	4.4896002	-15.4830217
1020.0000000	4.5383997	-652.4642944
1020.0000000	4.5871997	-12.8587999
1020.0000000	4.6360002	-21.6056385
1020.0000000	4.6847997	-28.2430000
1020.0000000	4.7336001	-19.5551758
1020.0000000	4.7824001	-16.5169296
1020.0000000	4.8311996	-1468.9797363
1020.0000000	4.8800001	227.3472748
1020.0000000	4.9287996	-200.7688904
1020.0000000	4.9776001	1055.5836182
1020.0000000	5.0264001	85.7935715
1020.0000000	5.0751996	-300.0838318
1020.0000000	5.1240001	-12.3177299
1020.0000000	5.1727996	557.2550049
1020.0000000	5.2216001	31.9489899
1020.0000000	5.2704000	-158.6759491
1020.0000000	5.3191996	-39.1599808
1020.0000000	5.3680000	-3532.4650879
1020.0000000	5.4168000	526.6088867
1020.0000000	5.4656000	-200.5637817
1020.0000000	5.5144000	38.0156250
1020.0000000	5.5631995	487.8509216
1020.0000000	5.6120000	392.7572632
1020.0000000	5.6608000	-37.0651741
1020.0000000	5.7096000	62.4566193
1020.0000000	5.7584000	132.9022369
1020.0000000	5.8071995	-11.5244255
1020.0000000	5.8559999	29.7756290
1020.0000000	5.9047999	-16.6047058
1020.0000000	5.9535999	-39.7544289
1020.0000000	6.0023999	-1571.5944824
1020.0000000	6.0511999	-14.5691357

1020.0000000	6.0999999	-19.4056797
1020.0000000	6.1487999	-13.2753181
1020.0000000	6.1975999	-23.2127819
1020.0000000	6.2463999	-1790.4046631
1020.0000000	6.2951999	-19.0669632
1020.0000000	6.3439999	-342.4519653
1020.0000000	6.3927999	-15.0480490
1020.0000000	6.4416003	-12.3881941
1020.0000000	6.4903998	-11.2661705
1020.0000000	6.5391998	-11.8086319
1020.0000000	6.5879998	382.7085571
1020.0000000	6.6367998	-12.0859537
1020.0000000	6.6856003	-13.8154488
1020.0000000	6.7343998	-13.4562988
1020.0000000	6.7831998	-14.3713999
1020.0000000	6.8319998	58.3889313
1020.0000000	6.8807998	-14.0490675
1020.0000000	6.9296002	-447.7076721
1020.0000000	6.9783998	-5.1130090
1020.0000000	7.0271997	-14.1352053
1020.0000000	7.0760002	11.1068449
1020.0000000	7.1247997	-2124.7529297
1020.0000000	7.1736002	-1703.9008789
1020.0000000	7.2223997	-969.3671265
1020.0000000	7.2711997	-209.0441132
1020.0000000	7.3200002	-18.8649235
1020.0000000	7.3687997	187.3732758
1020.0000000	7.4176002	390.8953552
1020.0000000	7.4663997	307.0906982
1020.0000000	7.5151997	-948.3566895
1020.0000000	7.5640001	-1047.5350342
1020.0000000	7.6127996	-2469.0832520
1020.0000000	7.6616001	-792.7174072
1020.0000000	7.7104001	-407.1805420
1020.0000000	7.7591996	43.1158562
1020.0000000	7.8080001	406.2216187
1020.0000000	7.8567996	535.1276245
1020.0000000	7.9056001	-1109.1040039
1020.0000000	7.9544001	1765.2685547
1020.0000000	8.0031996	2277.7924805
1020.0000000	8.0520000	2273.4785156
1020.0000000	8.1007996	2405.7912598
1020.0000000	8.1496000	2565.7062988
1020.0000000	8.1983995	1596.2227783
1020.0000000	8.2472000	-3652.1455078
1020.0000000	8.2959995	-1294.6038818
1020.0000000	8.3448000	-1031.2528076
1020.0000000	8.3935995	-945.1192627
1020.0000000	8.4423990	-407.1070251
1020.0000000	8.4912004	-950.4223022
1020.0000000	8.5400000	-2563.8166504
1020.0000000	8.5887995	-1242.7468262
1020.0000000	8.6375999	242.7490997
1020.0000000	8.6863995	2192.9897461
1020.0000000	8.7351999	-815.0705566
1020.0000000	8.7840004	931.3975830
1020.0000000	8.8327999	50.4858475
1020.0000000	8.8815994	-11.4965124

1020.0000000	8.9303989	-23.9436989
1020.0000000	8.9792004	-11.5791340
1020.0000000	9.0279999	-5.8374472
1020.0000000	9.0767994	7.0377903
1020.0000000	9.1255999	-17.2416630
1020.0000000	9.1743994	-10.0736599
1020.0000000	9.2231998	37.0093765
1020.0000000	9.2720003	-8.4202013
1020.0000000	9.3207998	-9.7981396
1020.0000000	9.3695993	-10.7637815
1020.0000000	9.4183998	-11.7538767
1020.0000000	9.4672003	-11.1613312
1020.0000000	9.5159998	-14.1642084
1020.0000000	9.5648003	-0.3930779
1020.0000000	9.6135998	-13.3960094
1020.0000000	9.6623993	-10.2213335
1020.0000000	9.7111998	-10.1577053
1020.0000000	9.7600002	-10.8575754
1020.0000000	9.8087997	-10.9947786
1020.0000000	9.8575993	3.1899023
1020.0000000	9.9063997	-7.9690638
1020.0000000	9.9552002	-10.9066019
1020.0000000	10.0039997	-10.0747194
1030.0000000	0.3416000	-37.2406883
1030.0000000	0.3904000	-39.8133392
1030.0000000	0.4392000	-39.7353287
1030.0000000	0.4880000	-39.5900459
1030.0000000	0.5368000	-39.6149330
1030.0000000	0.5856000	-39.3312683
1030.0000000	0.6344000	-38.2257576
1030.0000000	0.6832000	-36.1681175
1030.0000000	0.7320000	-36.0036812
1030.0000000	0.7808000	-36.5305748
1030.0000000	0.8296000	-35.9751778
1030.0000000	0.8784000	-35.2033119
1030.0000000	0.9272000	-32.8713989
1030.0000000	0.9760000	-30.5989914
1030.0000000	1.0247999	-29.7017212
1030.0000000	1.0735999	-29.3884697
1030.0000000	1.1224000	-29.1623363
1030.0000000	1.1711999	-29.7755241
1030.0000000	1.2200000	-30.8795395
1030.0000000	1.2687999	-30.8998909
1030.0000000	1.3176000	-30.9066315
1030.0000000	1.3664000	-30.5786476
1030.0000000	1.4152000	-30.1625195
1030.0000000	1.4640000	-29.6136093
1030.0000000	1.5128000	-39.2780457
1030.0000000	1.5616000	-33.5884361
1030.0000000	1.6104001	-1554.6741943
1030.0000000	1.6592000	-72.9642639
1030.0000000	1.7079999	-27.2612839
1030.0000000	1.7567999	-27.7621632
1030.0000000	1.8055999	-27.6553001
1030.0000000	1.8544000	-26.7216415
1030.0000000	1.9031999	-26.6972542
1030.0000000	1.9520000	-26.4007816
1030.0000000	2.0007999	-26.4511185

1030.0000000	2.0495999	-26.1703205
1030.0000000	2.0983999	-146.4983826
1030.0000000	2.1471999	-1.9418983
1030.0000000	2.1960001	-25.4444122
1030.0000000	2.2448001	-25.8098488
1030.0000000	2.2935998	-27.6208115
1030.0000000	2.3423998	-21.0042553
1030.0000000	2.3912001	31.2589855
1030.0000000	2.4400001	-22.8399506
1030.0000000	2.4888000	-23.4598732
1030.0000000	2.5375998	-12.2835207
1030.0000000	2.5863998	-10.4064455
1030.0000000	2.6352000	-8.4806852
1030.0000000	2.6840000	-15.4184666
1030.0000000	2.7328000	133.0394745
1030.0000000	2.7815998	156.9843750
1030.0000000	2.8304000	-23.7938271
1030.0000000	2.8792000	-20.6206894
1030.0000000	2.9280000	-22.5715542
1030.0000000	2.9768000	-16.7926903
1030.0000000	3.0256000	-20.4738579
1030.0000000	3.0743999	-29.5124531
1030.0000000	3.1231999	-24.2537937
1030.0000000	3.1719999	-29.6026115
1030.0000000	3.2208002	-504.1428833
1030.0000000	3.2695999	-987.0445557
1030.0000000	3.3183999	-20.4897957
1030.0000000	3.3671999	-3395.1987305
1030.0000000	3.4159999	1218.0122070
1030.0000000	3.4648001	-7.1462250
1030.0000000	3.5135999	-27.0341454
1030.0000000	3.5623999	-321.8190918
1030.0000000	3.6111999	-2021.9515381
1030.0000000	3.6600001	-457.7800293
1030.0000000	3.7088001	-604.4595337
1030.0000000	3.7575998	-946.3592529
1030.0000000	3.8063998	-1717.2463379
1030.0000000	3.8552001	-2493.1777344
1030.0000000	3.9040000	-2444.3906250
1030.0000000	3.9528000	1117.7586670
1030.0000000	4.0015998	-244.2666931
1030.0000000	4.0503998	684.3119507
1030.0000000	4.0991998	87.1210556
1030.0000000	4.1479998	-651.7855225
1030.0000000	4.1967998	2.4449863
1030.0000000	4.2456002	-247.4346008
1030.0000000	4.2943997	-485.8047180
1030.0000000	4.3431997	-13.3552380
1030.0000000	4.3920002	-247.0671234
1030.0000000	4.4407997	-22.4411316
1030.0000000	4.4896002	-15.4839048
1030.0000000	4.5383997	-652.2032471
1030.0000000	4.5871997	-12.8608160
1030.0000000	4.6360002	-21.6040955
1030.0000000	4.6847997	-28.2390747
1030.0000000	4.7336001	-19.5544548
1030.0000000	4.7824001	-16.5173836
1030.0000000	4.8311996	-1468.4484863

1030.0000000	4.8800001	227.2577209
1030.0000000	4.9287996	-200.7016144
1030.0000000	4.9776001	1055.1910400
1030.0000000	5.0264001	85.7560577
1030.0000000	5.0751996	-299.9802246
1030.0000000	5.1240001	-12.3186512
1030.0000000	5.1727996	557.1391602
1030.0000000	5.2216001	31.9395485
1030.0000000	5.2704000	-158.6478424
1030.0000000	5.3191996	-39.1554298
1030.0000000	5.3680000	-3531.7727051
1030.0000000	5.4168000	526.5020752
1030.0000000	5.4656000	-200.5276031
1030.0000000	5.5144000	38.0049667
1030.0000000	5.5631995	487.7523499
1030.0000000	5.6120000	392.6772766
1030.0000000	5.6608000	-37.0610886
1030.0000000	5.7096000	62.4411850
1030.0000000	5.7584000	132.8733063
1030.0000000	5.8071995	-11.5252962
1030.0000000	5.8559999	29.7667751
1030.0000000	5.9047999	-16.6045094
1030.0000000	5.9535999	-39.7496910
1030.0000000	6.0023999	-1571.2899170
1030.0000000	6.0511999	-14.5693474
1030.0000000	6.0999999	-19.4048481
1030.0000000	6.1487999	-13.2757807
1030.0000000	6.1975999	-23.2110157
1030.0000000	6.2463999	-1790.0572510
1030.0000000	6.2951999	-19.0661488
1030.0000000	6.3439999	-342.3878784
1030.0000000	6.3927999	-15.0480309
1030.0000000	6.4416003	-12.3887806
1030.0000000	6.4903998	-11.2668467
1030.0000000	6.5391998	-11.8093128
1030.0000000	6.5879998	382.0178528
1030.0000000	6.6367998	-12.0865030
1030.0000000	6.6856003	-13.8156185
1030.0000000	6.7343998	-13.4565144
1030.0000000	6.7831998	-14.3709841
1030.0000000	6.8319998	58.3698196
1030.0000000	6.8807998	-14.0489292
1030.0000000	6.9296002	-446.9462280
1030.0000000	6.9783998	-5.1258321
1030.0000000	7.0271997	-14.1341772
1030.0000000	7.0760002	11.0646582
1030.0000000	7.1247997	-2120.8071289
1030.0000000	7.1736002	-1700.7380371
1030.0000000	7.2223997	-967.5739746
1030.0000000	7.2711997	-208.6763306
1030.0000000	7.3200002	-18.8529358
1030.0000000	7.3687997	186.9987488
1030.0000000	7.4176002	390.1395264
1030.0000000	7.4663997	306.4930420
1030.0000000	7.5151997	-946.5956421
1030.0000000	7.5640001	-1045.5876465
1030.0000000	7.6127996	-2464.4655762
1030.0000000	7.6616001	-791.2460938

1030.0000000	7.7104001	-406.4343262
1030.0000000	7.7591996	43.0148048
1030.0000000	7.8080001	405.4362488
1030.0000000	7.8567996	534.0995483
1030.0000000	7.9056001	-1107.0311279
1030.0000000	7.9544001	1761.9586182
1030.0000000	8.0031996	2273.5229492
1030.0000000	8.0520000	2269.2165527
1030.0000000	8.1007996	2401.2817383
1030.0000000	8.1496000	2560.8981934
1030.0000000	8.1983995	1593.2303467
1030.0000000	8.2472000	-3645.3107910
1030.0000000	8.2959995	-1292.1837158
1030.0000000	8.3448000	-1029.3236084
1030.0000000	8.3935995	-943.3516235
1030.0000000	8.4423990	-406.3489990
1030.0000000	8.4912004	-948.6461182
1030.0000000	8.5400000	-2559.0168457
1030.0000000	8.5887995	-1240.4252930
1030.0000000	8.6375999	242.2877808
1030.0000000	8.6863995	2188.8715820
1030.0000000	8.7351999	-813.5463257
1030.0000000	8.7840004	929.6408081
1030.0000000	8.8327999	50.3742447
1030.0000000	8.8815994	-11.4966869
1030.0000000	8.9303989	-23.9399796
1030.0000000	8.9792004	-11.5792160
1030.0000000	9.0279999	-5.8392158
1030.0000000	9.0767994	7.0320721
1030.0000000	9.1255999	-17.2399483
1030.0000000	9.1743994	-10.0740614
1030.0000000	9.2231998	36.9936447
1030.0000000	9.2720003	-8.4211378
1030.0000000	9.3207998	-9.7986784
1030.0000000	9.3695993	-10.7640076
1030.0000000	9.4183998	-11.7537527
1030.0000000	9.4672003	-11.1613722
1030.0000000	9.5159998	-14.1633177
1030.0000000	9.5648003	-0.3964500
1030.0000000	9.6135998	-13.3952188
1030.0000000	9.6623993	-10.2216082
1030.0000000	9.7111998	-10.1579771
1030.0000000	9.7600002	-10.8576174
1030.0000000	9.8087997	-10.9947863
1030.0000000	9.8575993	3.1854801
1030.0000000	9.9063997	-7.9699779
1030.0000000	9.9552002	-10.9066057
1030.0000000	10.0039997	-10.0749664
1040.0000000	0.3416000	-37.3329468
1040.0000000	0.3904000	-39.7782593
1040.0000000	0.4392000	-39.7289848
1040.0000000	0.4880000	-39.5888367
1040.0000000	0.5368000	-39.6161079
1040.0000000	0.5856000	-39.3298874
1040.0000000	0.6344000	-38.2260704
1040.0000000	0.6832000	-36.1703415
1040.0000000	0.7320000	-36.0057335
1040.0000000	0.7808000	-36.5307465

1040.0000000	0.8296000	-35.9759064
1040.0000000	0.8784000	-35.2106895
1040.0000000	0.9272000	-32.8741760
1040.0000000	0.9760000	-30.5915394
1040.0000000	1.0247999	-29.7107105
1040.0000000	1.0735999	-29.4029045
1040.0000000	1.1224000	-29.1593361
1040.0000000	1.1711999	-29.7681465
1040.0000000	1.2200000	-30.8874550
1040.0000000	1.2687999	-30.9008999
1040.0000000	1.3176000	-30.9023590
1040.0000000	1.3664000	-30.5863972
1040.0000000	1.4152000	-30.1627235
1040.0000000	1.4640000	-29.6119003
1040.0000000	1.5128000	-39.1808662
1040.0000000	1.5616000	-33.5465965
1040.0000000	1.6104001	-1552.1313477
1040.0000000	1.6592000	-72.6703339
1040.0000000	1.7079999	-27.2650318
1040.0000000	1.7567999	-27.7621822
1040.0000000	1.8055999	-27.6591892
1040.0000000	1.8544000	-26.7315693
1040.0000000	1.9031999	-26.6944599
1040.0000000	1.9520000	-26.3972092
1040.0000000	2.0007999	-26.4441757
1040.0000000	2.0495999	-26.1723385
1040.0000000	2.0983999	-145.0226135
1040.0000000	2.1471999	-2.2091656
1040.0000000	2.1960001	-25.4403210
1040.0000000	2.2448001	-25.7919388
1040.0000000	2.2935998	-27.5611877
1040.0000000	2.3423998	-21.0186596
1040.0000000	2.3912001	30.6374416
1040.0000000	2.4400001	-22.8424377
1040.0000000	2.4888000	-23.4596558
1040.0000000	2.5375998	-12.3006287
1040.0000000	2.5863998	-10.4262047
1040.0000000	2.6352000	-8.5034304
1040.0000000	2.6840000	-15.4304085
1040.0000000	2.7328000	132.7734680
1040.0000000	2.7815998	156.6779480
1040.0000000	2.8304000	-23.7925224
1040.0000000	2.8792000	-20.6239529
1040.0000000	2.9280000	-22.5709839
1040.0000000	2.9768000	-16.8010616
1040.0000000	3.0256000	-20.4764423
1040.0000000	3.0743999	-29.5010662
1040.0000000	3.1231999	-24.2503395
1040.0000000	3.1719999	-29.5943184
1040.0000000	3.2208002	-503.9051514
1040.0000000	3.2695999	-986.5695801
1040.0000000	3.3183999	-20.4899940
1040.0000000	3.3671999	-3393.7370605
1040.0000000	3.4159999	1217.4769287
1040.0000000	3.4648001	-7.1519327
1040.0000000	3.5135999	-27.0311546
1040.0000000	3.5623999	-321.6894836
1040.0000000	3.6111999	-2021.0917969

1040.0000000	3.6600001	-457.5918579
1040.0000000	3.7088001	-604.2081299
1040.0000000	3.7575998	-945.9607544
1040.0000000	3.8063998	-1716.5164795
1040.0000000	3.8552001	-2492.1145020
1040.0000000	3.9040000	-2443.3481445
1040.0000000	3.9528000	1117.2703857
1040.0000000	4.0015998	-244.1695862
1040.0000000	4.0503998	684.0093384
1040.0000000	4.0991998	87.0753784
1040.0000000	4.1479998	-651.5133057
1040.0000000	4.1967998	2.4357300
1040.0000000	4.2456002	-247.3363037
1040.0000000	4.2943997	-485.6041565
1040.0000000	4.3431997	-13.3574200
1040.0000000	4.3920002	-246.9690094
1040.0000000	4.4407997	-22.4393539
1040.0000000	4.4896002	-15.4847870
1040.0000000	4.5383997	-651.9421387
1040.0000000	4.5871997	-12.8628330
1040.0000000	4.6360002	-21.6025524
1040.0000000	4.6847997	-28.2351494
1040.0000000	4.7336001	-19.5537319
1040.0000000	4.7824001	-16.5178375
1040.0000000	4.8311996	-1467.9172363
1040.0000000	4.8800001	227.1681824
1040.0000000	4.9287996	-200.6343536
1040.0000000	4.9776001	1054.7985840
1040.0000000	5.0264001	85.7185364
1040.0000000	5.0751996	-299.8765869
1040.0000000	5.1240001	-12.3195724
1040.0000000	5.1727996	557.0233154
1040.0000000	5.2216001	31.9301033
1040.0000000	5.2704000	-158.6197357
1040.0000000	5.3191996	-39.1508789
1040.0000000	5.3680000	-3531.0803223
1040.0000000	5.4168000	526.3953247
1040.0000000	5.4656000	-200.4914093
1040.0000000	5.5144000	37.9943161
1040.0000000	5.5631995	487.6537781
1040.0000000	5.6120000	392.5973206
1040.0000000	5.6608000	-37.0569992
1040.0000000	5.7096000	62.4257584
1040.0000000	5.7584000	132.8443604
1040.0000000	5.8071995	-11.5261669
1040.0000000	5.8559999	29.7579250
1040.0000000	5.9047999	-16.6043110
1040.0000000	5.9535999	-39.7449493
1040.0000000	6.0023999	-1570.9855957
1040.0000000	6.0511999	-14.5695581
1040.0000000	6.0999999	-19.4040146
1040.0000000	6.1487999	-13.2762432
1040.0000000	6.1975999	-23.2092514
1040.0000000	6.2463999	-1789.7098389
1040.0000000	6.2951999	-19.0653343
1040.0000000	6.3439999	-342.3238525
1040.0000000	6.3927999	-15.0480127
1040.0000000	6.4416003	-12.3893671

1040.0000000	6.4903998	-11.2675228
1040.0000000	6.5391998	-11.8099937
1040.0000000	6.5879998	381.3271484
1040.0000000	6.6367998	-12.0870523
1040.0000000	6.6856003	-13.8157892
1040.0000000	6.7343998	-13.4567308
1040.0000000	6.7831998	-14.3705683
1040.0000000	6.8319998	58.3507004
1040.0000000	6.8807998	-14.0487900
1040.0000000	6.9296002	-446.1847534
1040.0000000	6.9783998	-5.1386561
1040.0000000	7.0271997	-14.1331491
1040.0000000	7.0760002	11.0224752
1040.0000000	7.1247997	-2116.8613281
1040.0000000	7.1736002	-1697.5750732
1040.0000000	7.2223997	-965.7808228
1040.0000000	7.2711997	-208.3085632
1040.0000000	7.3200002	-18.8409462
1040.0000000	7.3687997	186.6242065
1040.0000000	7.4176002	389.3836975
1040.0000000	7.4663997	305.8953857
1040.0000000	7.5151997	-944.8346558
1040.0000000	7.5640001	-1043.6402588
1040.0000000	7.6127996	-2459.8479004
1040.0000000	7.6616001	-789.7747803
1040.0000000	7.7104001	-405.6880493
1040.0000000	7.7591996	42.9137573
1040.0000000	7.8080001	404.6509094
1040.0000000	7.8567996	533.0714111
1040.0000000	7.9056001	-1104.9582520
1040.0000000	7.9544001	1758.6486816
1040.0000000	8.0031996	2269.2534180
1040.0000000	8.0520000	2264.9545898
1040.0000000	8.1007996	2396.7722168
1040.0000000	8.1496000	2556.0903320
1040.0000000	8.1983995	1590.2377930
1040.0000000	8.2472000	-3638.4760742
1040.0000000	8.2959995	-1289.7635498
1040.0000000	8.3448000	-1027.3944092
1040.0000000	8.3935995	-941.5839233
1040.0000000	8.4423990	-405.5910034
1040.0000000	8.4912004	-946.8698730
1040.0000000	8.5400000	-2554.2170410
1040.0000000	8.5887995	-1238.1037598
1040.0000000	8.6375999	241.8264618
1040.0000000	8.6863995	2184.7531738
1040.0000000	8.7351999	-812.0220947
1040.0000000	8.7840004	927.8840942
1040.0000000	8.8327999	50.2626343
1040.0000000	8.8815994	-11.4968615
1040.0000000	8.9303989	-23.9362602
1040.0000000	8.9792004	-11.5792980
1040.0000000	9.0279999	-5.8409834
1040.0000000	9.0767994	7.0263557
1040.0000000	9.1255999	-17.2382355
1040.0000000	9.1743994	-10.0744638
1040.0000000	9.2231998	36.9779129
1040.0000000	9.2720003	-8.4220743

1040.0000000	9.3207998	-9.7992182
1040.0000000	9.3695993	-10.7642345
1040.0000000	9.4183998	-11.7536278
1040.0000000	9.4672003	-11.1614141
1040.0000000	9.5159998	-14.1624260
1040.0000000	9.5648003	-0.3998222
1040.0000000	9.6135998	-13.3944283
1040.0000000	9.6623993	-10.2218838
1040.0000000	9.7111998	-10.1582489
1040.0000000	9.7600002	-10.8576593
1040.0000000	9.8087997	-10.9947939
1040.0000000	9.8575993	3.1810589
1040.0000000	9.9063997	-7.9708920
1040.0000000	9.9552002	-10.9066105
1040.0000000	10.0039997	-10.0752144
1050.0000000	0.3416000	-37.2869797
1050.0000000	0.3904000	-39.7431793
1050.0000000	0.4392000	-39.7226410
1050.0000000	0.4880000	-39.5876274
1050.0000000	0.5368000	-39.6172791
1050.0000000	0.5856000	-39.3285065
1050.0000000	0.6344000	-38.2263794
1050.0000000	0.6832000	-36.1725693
1050.0000000	0.7320000	-36.0077858
1050.0000000	0.7808000	-36.5309143
1050.0000000	0.8296000	-35.9766350
1050.0000000	0.8784000	-35.2180634
1050.0000000	0.9272000	-32.8769531
1050.0000000	0.9760000	-30.5840874
1050.0000000	1.0247999	-29.7196999
1050.0000000	1.0735999	-29.4173393
1050.0000000	1.1224000	-29.1563377
1050.0000000	1.1711999	-29.7607689
1050.0000000	1.2200000	-30.8953705
1050.0000000	1.2687999	-30.9019070
1050.0000000	1.3176000	-30.8980885
1050.0000000	1.3664000	-30.5941467
1050.0000000	1.4152000	-30.1629276
1050.0000000	1.4640000	-29.6101894
1050.0000000	1.5128000	-39.0836868
1050.0000000	1.5616000	-33.5047531
1050.0000000	1.6104001	-1549.5885010
1050.0000000	1.6592000	-72.3764038
1050.0000000	1.7079999	-27.2687778
1050.0000000	1.7567999	-27.7622013
1050.0000000	1.8055999	-27.6630764
1050.0000000	1.8544000	-26.7414970
1050.0000000	1.9031999	-26.6916656
1050.0000000	1.9520000	-26.3936386
1050.0000000	2.0007999	-26.4372349
1050.0000000	2.0495999	-26.1743584
1050.0000000	2.0983999	-143.5468445
1050.0000000	2.1471999	-2.4764328
1050.0000000	2.1960001	-25.4362297
1050.0000000	2.2448001	-25.7740269
1050.0000000	2.2935998	-27.5015640
1050.0000000	2.3423998	-21.0330639
1050.0000000	2.3912001	30.0158978

1050.0000000	2.4400001	-22.8449249
1050.0000000	2.4888000	-23.4594383
1050.0000000	2.5375998	-12.3177376
1050.0000000	2.5863998	-10.4459629
1050.0000000	2.6352000	-8.5261745
1050.0000000	2.6840000	-15.4423504
1050.0000000	2.7328000	132.5074463
1050.0000000	2.7815998	156.3715515
1050.0000000	2.8304000	-23.7912178
1050.0000000	2.8792000	-20.6272163
1050.0000000	2.9280000	-22.5704155
1050.0000000	2.9768000	-16.8094330
1050.0000000	3.0256000	-20.4790268
1050.0000000	3.0743999	-29.4896793
1050.0000000	3.1231999	-24.2468834
1050.0000000	3.1719999	-29.5860252
1050.0000000	3.2208002	-503.6674194
1050.0000000	3.2695999	-986.0946045
1050.0000000	3.3183999	-20.4901905
1050.0000000	3.3671999	-3392.2753906
1050.0000000	3.4159999	1216.9416504
1050.0000000	3.4648001	-7.1576414
1050.0000000	3.5135999	-27.0281639
1050.0000000	3.5623999	-321.5599060
1050.0000000	3.6111999	-2020.2319336
1050.0000000	3.6600001	-457.4037476
1050.0000000	3.7088001	-603.9567261
1050.0000000	3.7575998	-945.5621948
1050.0000000	3.8063998	-1715.7867432
1050.0000000	3.8552001	-2491.0510254
1050.0000000	3.9040000	-2442.3056641
1050.0000000	3.9528000	1116.7821045
1050.0000000	4.0015998	-244.0724792
1050.0000000	4.0503998	683.7067871
1050.0000000	4.0991998	87.0296936
1050.0000000	4.1479998	-651.2410889
1050.0000000	4.1967998	2.4264736
1050.0000000	4.2456002	-247.2379913
1050.0000000	4.2943997	-485.4035950
1050.0000000	4.3431997	-13.3596001
1050.0000000	4.3920002	-246.8708954
1050.0000000	4.4407997	-22.4375801
1050.0000000	4.4896002	-15.4856701
1050.0000000	4.5383997	-651.6810913
1050.0000000	4.5871997	-12.8648491
1050.0000000	4.6360002	-21.6010113
1050.0000000	4.6847997	-28.2312241
1050.0000000	4.7336001	-19.5530090
1050.0000000	4.7824001	-16.5182934
1050.0000000	4.8311996	-1467.3859863
1050.0000000	4.8800001	227.0786285
1050.0000000	4.9287996	-200.5670929
1050.0000000	4.9776001	1054.4061279
1050.0000000	5.0264001	85.6810150
1050.0000000	5.0751996	-299.7729492
1050.0000000	5.1240001	-12.3204937
1050.0000000	5.1727996	556.9074097
1050.0000000	5.2216001	31.9206581

1050.0000000	5.2704000	-158.5916290
1050.0000000	5.3191996	-39.1463318
1050.0000000	5.3680000	-3530.3879395
1050.0000000	5.4168000	526.2885132
1050.0000000	5.4656000	-200.4552307
1050.0000000	5.5144000	37.9836578
1050.0000000	5.5631995	487.5552063
1050.0000000	5.6120000	392.5173340
1050.0000000	5.6608000	-37.0529099
1050.0000000	5.7096000	62.4103241
1050.0000000	5.7584000	132.8154297
1050.0000000	5.8071995	-11.5270376
1050.0000000	5.8559999	29.7490749
1050.0000000	5.9047999	-16.6041126
1050.0000000	5.9535999	-39.7402115
1050.0000000	6.0023999	-1570.6811523
1050.0000000	6.0511999	-14.5697699
1050.0000000	6.0999999	-19.4031830
1050.0000000	6.1487999	-13.2767067
1050.0000000	6.1975999	-23.2074852
1050.0000000	6.2463999	-1789.3625488
1050.0000000	6.2951999	-19.0645218
1050.0000000	6.3439999	-342.2597961
1050.0000000	6.3927999	-15.0479946
1050.0000000	6.4416003	-12.3899546
1050.0000000	6.4903998	-11.2681980
1050.0000000	6.5391998	-11.8106747
1050.0000000	6.5879998	380.6364746
1050.0000000	6.6367998	-12.0876017
1050.0000000	6.6856003	-13.8159590
1050.0000000	6.7343998	-13.4569464
1050.0000000	6.7831998	-14.3701525
1050.0000000	6.8319998	58.3315811
1050.0000000	6.8807998	-14.0486517
1050.0000000	6.9296002	-445.4233093
1050.0000000	6.9783998	-5.1514797
1050.0000000	7.0271997	-14.1321211
1050.0000000	7.0760002	10.9802885
1050.0000000	7.1247997	-2112.9152832
1050.0000000	7.1736002	-1694.4122314
1050.0000000	7.2223997	-963.9876099
1050.0000000	7.2711997	-207.9407806
1050.0000000	7.3200002	-18.8289566
1050.0000000	7.3687997	186.2496796
1050.0000000	7.4176002	388.6278687
1050.0000000	7.4663997	305.2977295
1050.0000000	7.5151997	-943.0736084
1050.0000000	7.5640001	-1041.6928711
1050.0000000	7.6127996	-2455.2302246
1050.0000000	7.6616001	-788.3034058
1050.0000000	7.7104001	-404.9417725
1050.0000000	7.7591996	42.8127098
1050.0000000	7.8080001	403.8655396
1050.0000000	7.8567996	532.0433350
1050.0000000	7.9056001	-1102.8853760
1050.0000000	7.9544001	1755.3387451
1050.0000000	8.0031996	2264.9836426
1050.0000000	8.0520000	2260.6926270

1050.0000000	8.1007996	2392.2626953
1050.0000000	8.1496000	2551.2822266
1050.0000000	8.1983995	1587.2452393
1050.0000000	8.2472000	-3631.6411133
1050.0000000	8.2959995	-1287.3433838
1050.0000000	8.3448000	-1025.4652100
1050.0000000	8.3935995	-939.8162842
1050.0000000	8.4423990	-404.8329773
1050.0000000	8.4912004	-945.0936279
1050.0000000	8.5400000	-2549.4172363
1050.0000000	8.5887995	-1235.7822266
1050.0000000	8.6375999	241.3651428
1050.0000000	8.6863995	2180.6347656
1050.0000000	8.7351999	-810.4978027
1050.0000000	8.7840004	926.1273193
1050.0000000	8.8327999	50.1510239
1050.0000000	8.8815994	-11.4970360
1050.0000000	8.9303989	-23.9325409
1050.0000000	8.9792004	-11.5793810
1050.0000000	9.0279999	-5.8427520
1050.0000000	9.0767994	7.0206375
1050.0000000	9.1255999	-17.2365208
1050.0000000	9.1743994	-10.0748653
1050.0000000	9.2231998	36.9621811
1050.0000000	9.2720003	-8.4230108
1050.0000000	9.3207998	-9.7997570
1050.0000000	9.3695993	-10.7644606
1050.0000000	9.4183998	-11.7535028
1050.0000000	9.4672003	-11.1614552
1050.0000000	9.5159998	-14.1615353
1050.0000000	9.5648003	-0.4031954
1050.0000000	9.6135998	-13.3936377
1050.0000000	9.6623993	-10.2221584
1050.0000000	9.7111998	-10.1585207
1050.0000000	9.7600002	-10.8577013
1050.0000000	9.8087997	-10.9948025
1050.0000000	9.8575993	3.1766367
1050.0000000	9.9063997	-7.9718056
1050.0000000	9.9552002	-10.9066143
1050.0000000	10.0039997	-10.0754614
1060.0000000	0.3416000	-36.8788490
1060.0000000	0.3904000	-39.7080956
1060.0000000	0.4392000	-39.7163010
1060.0000000	0.4880000	-39.5864182
1060.0000000	0.5368000	-39.6184540
1060.0000000	0.5856000	-39.3271255
1060.0000000	0.6344000	-38.2266884
1060.0000000	0.6832000	-36.1747932
1060.0000000	0.7320000	-36.0098381
1060.0000000	0.7808000	-36.5310860
1060.0000000	0.8296000	-35.9773636
1060.0000000	0.8784000	-35.2254410
1060.0000000	0.9272000	-32.8797302
1060.0000000	0.9760000	-30.5766335
1060.0000000	1.0247999	-29.7286892
1060.0000000	1.0735999	-29.4317741
1060.0000000	1.1224000	-29.1533375
1060.0000000	1.1711999	-29.7533913

1060.0000000	1.2200000	-30.9032860
1060.0000000	1.2687999	-30.9029140
1060.0000000	1.3176000	-30.8938179
1060.0000000	1.3664000	-30.6018963
1060.0000000	1.4152000	-30.1631336
1060.0000000	1.4640000	-29.6084805
1060.0000000	1.5128000	-38.9865112
1060.0000000	1.5616000	-33.4629135
1060.0000000	1.6104001	-1547.0457764
1060.0000000	1.6592000	-72.0824661
1060.0000000	1.7079999	-27.2725258
1060.0000000	1.7567999	-27.7622204
1060.0000000	1.8055999	-27.6669655
1060.0000000	1.8544000	-26.7514248
1060.0000000	1.9031999	-26.6888714
1060.0000000	1.9520000	-26.3900661
1060.0000000	2.0007999	-26.4302921
1060.0000000	2.0495999	-26.1763763
1060.0000000	2.0983999	-142.0710602
1060.0000000	2.1471999	-2.7437000
1060.0000000	2.1960001	-25.4321384
1060.0000000	2.2448001	-25.7561150
1060.0000000	2.2935998	-27.4419403
1060.0000000	2.3423998	-21.0474682
1060.0000000	2.3912001	29.3943501
1060.0000000	2.4400001	-22.8474102
1060.0000000	2.4888000	-23.4592209
1060.0000000	2.5375998	-12.3348455
1060.0000000	2.5863998	-10.4657221
1060.0000000	2.6352000	-8.5489187
1060.0000000	2.6840000	-15.4542923
1060.0000000	2.7328000	132.2414246
1060.0000000	2.7815998	156.0651245
1060.0000000	2.8304000	-23.7899151
1060.0000000	2.8792000	-20.6304798
1060.0000000	2.9280000	-22.5698471
1060.0000000	2.9768000	-16.8178024
1060.0000000	3.0256000	-20.4816113
1060.0000000	3.0743999	-29.4782925
1060.0000000	3.1231999	-24.2434273
1060.0000000	3.1719999	-29.5777321
1060.0000000	3.2208002	-503.4296875
1060.0000000	3.2695999	-985.6196289
1060.0000000	3.3183999	-20.4903889
1060.0000000	3.3671999	-3390.8137207
1060.0000000	3.4159999	1216.4063721
1060.0000000	3.4648001	-7.1633492
1060.0000000	3.5135999	-27.0251732
1060.0000000	3.5623999	-321.4303284
1060.0000000	3.6111999	-2019.3721924
1060.0000000	3.6600001	-457.2156372
1060.0000000	3.7088001	-603.7053833
1060.0000000	3.7575998	-945.1636963
1060.0000000	3.8063998	-1715.0568848
1060.0000000	3.8552001	-2489.9875488
1060.0000000	3.9040000	-2441.2631836
1060.0000000	3.9528000	1116.2937012
1060.0000000	4.0015998	-243.9753571

1060.0000000	4.0503998	683.4042358
1060.0000000	4.0991998	86.9840088
1060.0000000	4.1479998	-650.9688721
1060.0000000	4.1967998	2.4172173
1060.0000000	4.2456002	-247.1396942
1060.0000000	4.2943997	-485.2030334
1060.0000000	4.3431997	-13.3617802
1060.0000000	4.3920002	-246.7727814
1060.0000000	4.4407997	-22.4358044
1060.0000000	4.4896002	-15.4865522
1060.0000000	4.5383997	-651.4199829
1060.0000000	4.5871997	-12.8668652
1060.0000000	4.6360002	-21.5994682
1060.0000000	4.6847997	-28.2272987
1060.0000000	4.7336001	-19.5522881
1060.0000000	4.7824001	-16.5187473
1060.0000000	4.8311996	-1466.8547363
1060.0000000	4.8800001	226.9890747
1060.0000000	4.9287996	-200.4998322
1060.0000000	4.9776001	1054.0135498
1060.0000000	5.0264001	85.6434937
1060.0000000	5.0751996	-299.6693420
1060.0000000	5.1240001	-12.3214149
1060.0000000	5.1727996	556.7915649
1060.0000000	5.2216001	31.9112167
1060.0000000	5.2704000	-158.5635223
1060.0000000	5.3191996	-39.1417809
1060.0000000	5.3680000	-3529.6955566
1060.0000000	5.4168000	526.1817627
1060.0000000	5.4656000	-200.4190521
1060.0000000	5.5144000	37.9729996
1060.0000000	5.5631995	487.4566345
1060.0000000	5.6120000	392.4373474
1060.0000000	5.6608000	-37.0488205
1060.0000000	5.7096000	62.3948898
1060.0000000	5.7584000	132.7864990
1060.0000000	5.8071995	-11.5279083
1060.0000000	5.8559999	29.7402210
1060.0000000	5.9047999	-16.6039162
1060.0000000	5.9535999	-39.7354736
1060.0000000	6.0023999	-1570.3768311
1060.0000000	6.0511999	-14.5699816
1060.0000000	6.0999999	-19.4023514
1060.0000000	6.1487999	-13.2771692
1060.0000000	6.1975999	-23.2057209
1060.0000000	6.2463999	-1789.0151367
1060.0000000	6.2951999	-19.0637054
1060.0000000	6.3439999	-342.1957397
1060.0000000	6.3927999	-15.0479755
1060.0000000	6.4416003	-12.3905411
1060.0000000	6.4903998	-11.2688742
1060.0000000	6.5391998	-11.8113565
1060.0000000	6.5879998	379.9457703
1060.0000000	6.6367998	-12.0881500
1060.0000000	6.6856003	-13.8161297
1060.0000000	6.7343998	-13.4571619
1060.0000000	6.7831998	-14.3697367
1060.0000000	6.8319998	58.3124695

1060.0000000	6.8807998	-14.0485125
1060.0000000	6.9296002	-444.6618652
1060.0000000	6.9783998	-5.1643038
1060.0000000	7.0271997	-14.1310940
1060.0000000	7.0760002	10.9381018
1060.0000000	7.1247997	-2108.9694824
1060.0000000	7.1736002	-1691.2493896
1060.0000000	7.2223997	-962.1944580
1060.0000000	7.2711997	-207.5729980
1060.0000000	7.3200002	-18.8169689
1060.0000000	7.3687997	185.8751526
1060.0000000	7.4176002	387.8720398
1060.0000000	7.4663997	304.7001038
1060.0000000	7.5151997	-941.3125610
1060.0000000	7.5640001	-1039.7454834
1060.0000000	7.6127996	-2450.6127930
1060.0000000	7.6616001	-786.8320313
1060.0000000	7.7104001	-404.1955566
1060.0000000	7.7591996	42.7116585
1060.0000000	7.8080001	403.0801697
1060.0000000	7.8567996	531.0152588
1060.0000000	7.9056001	-1100.8123779
1060.0000000	7.9544001	1752.0288086
1060.0000000	8.0031996	2260.7141113
1060.0000000	8.0520000	2256.4306641
1060.0000000	8.1007996	2387.7531738
1060.0000000	8.1496000	2546.4743652
1060.0000000	8.1983995	1584.2528076
1060.0000000	8.2472000	-3624.8063965
1060.0000000	8.2959995	-1284.9232178
1060.0000000	8.3448000	-1023.5360107
1060.0000000	8.3935995	-938.0485840
1060.0000000	8.4423990	-404.0749817
1060.0000000	8.4912004	-943.3174438
1060.0000000	8.5400000	-2544.6174316
1060.0000000	8.5887995	-1233.4606934
1060.0000000	8.6375999	240.9038239
1060.0000000	8.6863995	2176.5163574
1060.0000000	8.7351999	-808.9735718
1060.0000000	8.7840004	924.3706055
1060.0000000	8.8327999	50.0394173
1060.0000000	8.8815994	-11.4972095
1060.0000000	8.9303989	-23.9288197
1060.0000000	8.9792004	-11.5794630
1060.0000000	9.0279999	-5.8445201
1060.0000000	9.0767994	7.0149193
1060.0000000	9.1255999	-17.2348080
1060.0000000	9.1743994	-10.0752678
1060.0000000	9.2231998	36.9464493
1060.0000000	9.2720003	-8.4239464
1060.0000000	9.3207998	-9.8002968
1060.0000000	9.3695993	-10.7646866
1060.0000000	9.4183998	-11.7533789
1060.0000000	9.4672003	-11.1614962
1060.0000000	9.5159998	-14.1606445
1060.0000000	9.5648003	-0.4065685
1060.0000000	9.6135998	-13.3928471
1060.0000000	9.6623993	-10.2224340

1060.0000000	9.7111998	-10.1587925
1060.0000000	9.7600002	-10.8577423
1060.0000000	9.8087997	-10.9948101
1060.0000000	9.8575993	3.1722145
1060.0000000	9.9063997	-7.9727197
1060.0000000	9.9552002	-10.9066191
1060.0000000	10.0039997	-10.0757093
1070.0000000	0.3416000	-37.2031708
1070.0000000	0.3904000	-39.6730156
1070.0000000	0.4392000	-39.7099571
1070.0000000	0.4880000	-39.5852089
1070.0000000	0.5368000	-39.6196289
1070.0000000	0.5856000	-39.3257446
1070.0000000	0.6344000	-38.2270012
1070.0000000	0.6832000	-36.1770210
1070.0000000	0.7320000	-36.0118904
1070.0000000	0.7808000	-36.5312538
1070.0000000	0.8296000	-35.9780960
1070.0000000	0.8784000	-35.2328148
1070.0000000	0.9272000	-32.8825073
1070.0000000	0.9760000	-30.5691814
1070.0000000	1.0247999	-29.7376785
1070.0000000	1.0735999	-29.4462090
1070.0000000	1.1224000	-29.1503391
1070.0000000	1.1711999	-29.7460136
1070.0000000	1.2200000	-30.9112015
1070.0000000	1.2687999	-30.9039230
1070.0000000	1.3176000	-30.8895454
1070.0000000	1.3664000	-30.6096458
1070.0000000	1.4152000	-30.1633377
1070.0000000	1.4640000	-29.6067715
1070.0000000	1.5128000	-38.8893356
1070.0000000	1.5616000	-33.4210739
1070.0000000	1.6104001	-1544.5029297
1070.0000000	1.6592000	-71.7885361
1070.0000000	1.7079999	-27.2762737
1070.0000000	1.7567999	-27.7622395
1070.0000000	1.8055999	-27.6708527
1070.0000000	1.8544000	-26.7613525
1070.0000000	1.9031999	-26.6860771
1070.0000000	1.9520000	-26.3864956
1070.0000000	2.0007999	-26.4233494
1070.0000000	2.0495999	-26.1783962
1070.0000000	2.0983999	-140.5952759
1070.0000000	2.1471999	-3.0109673
1070.0000000	2.1960001	-25.4280472
1070.0000000	2.2448001	-25.7382050
1070.0000000	2.2935998	-27.3823166
1070.0000000	2.3423998	-21.0618725
1070.0000000	2.3912001	28.7728024
1070.0000000	2.4400001	-22.8498974
1070.0000000	2.4888000	-23.4590054
1070.0000000	2.5375998	-12.3519535
1070.0000000	2.5863998	-10.4854803
1070.0000000	2.6352000	-8.5716629
1070.0000000	2.6840000	-15.4662342
1070.0000000	2.7328000	131.9754181
1070.0000000	2.7815998	155.7586975

1070.0000000	2.8304000	-23.7886105
1070.0000000	2.8792000	-20.6337433
1070.0000000	2.9280000	-22.5692768
1070.0000000	2.9768000	-16.8261738
1070.0000000	3.0256000	-20.4841957
1070.0000000	3.0743999	-29.4669056
1070.0000000	3.1231999	-24.2399731
1070.0000000	3.1719999	-29.5694389
1070.0000000	3.2208002	-503.1919556
1070.0000000	3.2695999	-985.1446533
1070.0000000	3.3183999	-20.4905872
1070.0000000	3.3671999	-3389.3520508
1070.0000000	3.4159999	1215.8710938
1070.0000000	3.4648001	-7.1690569
1070.0000000	3.5135999	-27.0221825
1070.0000000	3.5623999	-321.3007202
1070.0000000	3.6111999	-2018.5124512
1070.0000000	3.6600001	-457.0274658
1070.0000000	3.7088001	-603.4539795
1070.0000000	3.7575998	-944.7651367
1070.0000000	3.8063998	-1714.3270264
1070.0000000	3.8552001	-2488.9240723
1070.0000000	3.9040000	-2440.2207031
1070.0000000	3.9528000	1115.8054199
1070.0000000	4.0015998	-243.8782501
1070.0000000	4.0503998	683.1016846
1070.0000000	4.0991998	86.9383240
1070.0000000	4.1479998	-650.6966553
1070.0000000	4.1967998	2.4079609
1070.0000000	4.2456002	-247.0413971
1070.0000000	4.2943997	-485.0024109
1070.0000000	4.3431997	-13.3639612
1070.0000000	4.3920002	-246.6746674
1070.0000000	4.4407997	-22.4340286
1070.0000000	4.4896002	-15.4874353
1070.0000000	4.5383997	-651.1589355
1070.0000000	4.5871997	-12.8688812
1070.0000000	4.6360002	-21.5979252
1070.0000000	4.6847997	-28.2233734
1070.0000000	4.7336001	-19.5515652
1070.0000000	4.7824001	-16.5192013
1070.0000000	4.8311996	-1466.3233643
1070.0000000	4.8800001	226.8995209
1070.0000000	4.9287996	-200.4325714
1070.0000000	4.9776001	1053.6210938
1070.0000000	5.0264001	85.6059723
1070.0000000	5.0751996	-299.5657043
1070.0000000	5.1240001	-12.3223362
1070.0000000	5.1727996	556.6756592
1070.0000000	5.2216001	31.9017715
1070.0000000	5.2704000	-158.5354156
1070.0000000	5.3191996	-39.1372337
1070.0000000	5.3680000	-3529.0031738
1070.0000000	5.4168000	526.0749512
1070.0000000	5.4656000	-200.3828583
1070.0000000	5.5144000	37.9623413
1070.0000000	5.5631995	487.3580627
1070.0000000	5.6120000	392.3573914

1070.0000000	5.6608000	-37.0447311
1070.0000000	5.7096000	62.3794632
1070.0000000	5.7584000	132.7575684
1070.0000000	5.8071995	-11.5287781
1070.0000000	5.8559999	29.7313709
1070.0000000	5.9047999	-16.6037178
1070.0000000	5.9535999	-39.7307358
1070.0000000	6.0023999	-1570.0722656
1070.0000000	6.0511999	-14.5701923
1070.0000000	6.0999999	-19.4015179
1070.0000000	6.1487999	-13.2776318
1070.0000000	6.1975999	-23.2039566
1070.0000000	6.2463999	-1788.6677246
1070.0000000	6.2951999	-19.0628929
1070.0000000	6.3439999	-342.1316833
1070.0000000	6.3927999	-15.0479574
1070.0000000	6.4416003	-12.3911285
1070.0000000	6.4903998	-11.2695503
1070.0000000	6.5391998	-11.8120375
1070.0000000	6.5879998	379.2550659
1070.0000000	6.6367998	-12.0886993
1070.0000000	6.6856003	-13.8162994
1070.0000000	6.7343998	-13.4573784
1070.0000000	6.7831998	-14.3693209
1070.0000000	6.8319998	58.2933578
1070.0000000	6.8807998	-14.0483742
1070.0000000	6.9296002	-443.9004211
1070.0000000	6.9783998	-5.1771274
1070.0000000	7.0271997	-14.1300659
1070.0000000	7.0760002	10.8959188
1070.0000000	7.1247997	-2105.0236816
1070.0000000	7.1736002	-1688.0864258
1070.0000000	7.2223997	-960.4013062
1070.0000000	7.2711997	-207.2052155
1070.0000000	7.3200002	-18.8049812
1070.0000000	7.3687997	185.5006256
1070.0000000	7.4176002	387.1162109
1070.0000000	7.4663997	304.1024475
1070.0000000	7.5151997	-939.5515137
1070.0000000	7.5640001	-1037.7980957
1070.0000000	7.6127996	-2445.9951172
1070.0000000	7.6616001	-785.3607178
1070.0000000	7.7104001	-403.4492188
1070.0000000	7.7591996	42.6106110
1070.0000000	7.8080001	402.2948303
1070.0000000	7.8567996	529.9871826
1070.0000000	7.9056001	-1098.7395020
1070.0000000	7.9544001	1748.7188721
1070.0000000	8.0031996	2256.4445801
1070.0000000	8.0520000	2252.1684570
1070.0000000	8.1007996	2383.2436523
1070.0000000	8.1496000	2541.6662598
1070.0000000	8.1983995	1581.2602539
1070.0000000	8.2472000	-3617.9716797
1070.0000000	8.2959995	-1282.5030518
1070.0000000	8.3448000	-1021.6068726
1070.0000000	8.3935995	-936.2809448
1070.0000000	8.4423990	-403.3169556

1070.0000000	8.4912004	-941.5411987
1070.0000000	8.5400000	-2539.8173828
1070.0000000	8.5887995	-1231.1391602
1070.0000000	8.6375999	240.4425049
1070.0000000	8.6863995	2172.3979492
1070.0000000	8.7351999	-807.4492798
1070.0000000	8.7840004	922.6138306
1070.0000000	8.8327999	49.9278069
1070.0000000	8.8815994	-11.4973841
1070.0000000	8.9303989	-23.9250984
1070.0000000	8.9792004	-11.5795450
1070.0000000	9.0279999	-5.8462887
1070.0000000	9.0767994	7.0092010
1070.0000000	9.1255999	-17.2330933
1070.0000000	9.1743994	-10.0756693
1070.0000000	9.2231998	36.9307175
1070.0000000	9.2720003	-8.4248829
1070.0000000	9.3207998	-9.8008366
1070.0000000	9.3695993	-10.7649136
1070.0000000	9.4183998	-11.7532539
1070.0000000	9.4672003	-11.1615372
1070.0000000	9.5159998	-14.1597538
1070.0000000	9.5648003	-0.4099407
1070.0000000	9.6135998	-13.3920555
1070.0000000	9.6623993	-10.2227087
1070.0000000	9.7111998	-10.1590633
1070.0000000	9.7600002	-10.8577843
1070.0000000	9.8087997	-10.9948177
1070.0000000	9.8575993	3.1677914
1070.0000000	9.9063997	-7.9736333
1070.0000000	9.9552002	-10.9066229
1070.0000000	10.0039997	-10.0759573
1080.0000000	0.3416000	-36.8979149
1080.0000000	0.3904000	-39.6379318
1080.0000000	0.4392000	-39.7036133
1080.0000000	0.4880000	-39.5839996
1080.0000000	0.5368000	-39.6208000
1080.0000000	0.5856000	-39.3243637
1080.0000000	0.6344000	-38.2273102
1080.0000000	0.6832000	-36.1792450
1080.0000000	0.7320000	-36.0139427
1080.0000000	0.7808000	-36.5314217
1080.0000000	0.8296000	-35.9788246
1080.0000000	0.8784000	-35.2401886
1080.0000000	0.9272000	-32.8852844
1080.0000000	0.9760000	-30.5617294
1080.0000000	1.0247999	-29.7466679
1080.0000000	1.0735999	-29.4606438
1080.0000000	1.1224000	-29.1473389
1080.0000000	1.1711999	-29.7386379
1080.0000000	1.2200000	-30.9191151
1080.0000000	1.2687999	-30.9049301
1080.0000000	1.3176000	-30.8852749
1080.0000000	1.3664000	-30.6173935
1080.0000000	1.4152000	-30.1635418
1080.0000000	1.4640000	-29.6050625
1080.0000000	1.5128000	-38.7921562
1080.0000000	1.5616000	-33.3792305

1080.0000000	1.6104001	-1541.9600830
1080.0000000	1.6592000	-71.4946060
1080.0000000	1.7079999	-27.2800198
1080.0000000	1.7567999	-27.7622585
1080.0000000	1.8055999	-27.6747417
1080.0000000	1.8544000	-26.7712803
1080.0000000	1.9031999	-26.6832829
1080.0000000	1.9520000	-26.3829231
1080.0000000	2.0007999	-26.4164066
1080.0000000	2.0495999	-26.1804142
1080.0000000	2.0983999	-139.1195068
1080.0000000	2.1471999	-3.2782345
1080.0000000	2.1960001	-25.4239559
1080.0000000	2.2448001	-25.7202930
1080.0000000	2.2935998	-27.3226948
1080.0000000	2.3423998	-21.0762787
1080.0000000	2.3912001	28.1512585
1080.0000000	2.4400001	-22.8523827
1080.0000000	2.4888000	-23.4587879
1080.0000000	2.5375998	-12.3690605
1080.0000000	2.5863998	-10.5052395
1080.0000000	2.6352000	-8.5944071
1080.0000000	2.6840000	-15.4781761
1080.0000000	2.7328000	131.7093964
1080.0000000	2.7815998	155.4523010
1080.0000000	2.8304000	-23.7873058
1080.0000000	2.8792000	-20.6370068
1080.0000000	2.9280000	-22.5687084
1080.0000000	2.9768000	-16.8345451
1080.0000000	3.0256000	-20.4867802
1080.0000000	3.0743999	-29.4555187
1080.0000000	3.1231999	-24.2365170
1080.0000000	3.1719999	-29.5611458
1080.0000000	3.2208002	-502.9541931
1080.0000000	3.2695999	-984.6696777
1080.0000000	3.3183999	-20.4907856
1080.0000000	3.3671999	-3387.8903809
1080.0000000	3.4159999	1215.3358154
1080.0000000	3.4648001	-7.1747656
1080.0000000	3.5135999	-27.0191917
1080.0000000	3.5623999	-321.1711426
1080.0000000	3.6111999	-2017.6527100
1080.0000000	3.6600001	-456.8393555
1080.0000000	3.7088001	-603.2025757
1080.0000000	3.7575998	-944.3666382
1080.0000000	3.8063998	-1713.5971680
1080.0000000	3.8552001	-2487.8605957
1080.0000000	3.9040000	-2439.1782227
1080.0000000	3.9528000	1115.3171387
1080.0000000	4.0015998	-243.7811432
1080.0000000	4.0503998	682.7991333
1080.0000000	4.0991998	86.8926392
1080.0000000	4.1479998	-650.4244385
1080.0000000	4.1967998	2.3987045
1080.0000000	4.2456002	-246.9430847
1080.0000000	4.2943997	-484.8018494
1080.0000000	4.3431997	-13.3661413
1080.0000000	4.3920002	-246.5765533

1080.0000000	4.4407997	-22.4322529
1080.0000000	4.4896002	-15.4883175
1080.0000000	4.5383997	-650.8978882
1080.0000000	4.5871997	-12.8708973
1080.0000000	4.6360002	-21.5963821
1080.0000000	4.6847997	-28.2194481
1080.0000000	4.7336001	-19.5508423
1080.0000000	4.7824001	-16.5196571
1080.0000000	4.8311996	-1465.7921143
1080.0000000	4.8800001	226.8099670
1080.0000000	4.9287996	-200.3653107
1080.0000000	4.9776001	1053.2286377
1080.0000000	5.0264001	85.5684509
1080.0000000	5.0751996	-299.4620667
1080.0000000	5.1240001	-12.3232574
1080.0000000	5.1727996	556.5598145
1080.0000000	5.2216001	31.8923264
1080.0000000	5.2704000	-158.5073090
1080.0000000	5.3191996	-39.1326828
1080.0000000	5.3680000	-3528.3107910
1080.0000000	5.4168000	525.9682007
1080.0000000	5.4656000	-200.3466797
1080.0000000	5.5144000	37.9516907
1080.0000000	5.5631995	487.2594910
1080.0000000	5.6120000	392.2774048
1080.0000000	5.6608000	-37.0406456
1080.0000000	5.7096000	62.3640289
1080.0000000	5.7584000	132.7286377
1080.0000000	5.8071995	-11.5296497
1080.0000000	5.8559999	29.7225208
1080.0000000	5.9047999	-16.6035194
1080.0000000	5.9535999	-39.7259941
1080.0000000	6.0023999	-1569.7678223
1080.0000000	6.0511999	-14.5704041
1080.0000000	6.0999999	-19.4006863
1080.0000000	6.1487999	-13.2780943
1080.0000000	6.1975999	-23.2021904
1080.0000000	6.2463999	-1788.3204346
1080.0000000	6.2951999	-19.0620785
1080.0000000	6.3439999	-342.0676575
1080.0000000	6.3927999	-15.0479393
1080.0000000	6.4416003	-12.3917150
1080.0000000	6.4903998	-11.2702255
1080.0000000	6.5391998	-11.8127184
1080.0000000	6.5879998	378.5643616
1080.0000000	6.6367998	-12.0892487
1080.0000000	6.6856003	-13.8164701
1080.0000000	6.7343998	-13.4575939
1080.0000000	6.7831998	-14.3689051
1080.0000000	6.8319998	58.2742386
1080.0000000	6.8807998	-14.0482349
1080.0000000	6.9296002	-443.1389771
1080.0000000	6.9783998	-5.1899505
1080.0000000	7.0271997	-14.1290379
1080.0000000	7.0760002	10.8537321
1080.0000000	7.1247997	-2101.0778809
1080.0000000	7.1736002	-1684.9235840
1080.0000000	7.2223997	-958.6081543

1080.0000000	7.2711997	-206.8374329
1080.0000000	7.3200002	-18.7929916
1080.0000000	7.3687997	185.1260986
1080.0000000	7.4176002	386.3603821
1080.0000000	7.4663997	303.5047913
1080.0000000	7.5151997	-937.7904663
1080.0000000	7.5640001	-1035.8507080
1080.0000000	7.6127996	-2441.3774414
1080.0000000	7.6616001	-783.8893433
1080.0000000	7.7104001	-402.7030029
1080.0000000	7.7591996	42.5095634
1080.0000000	7.8080001	401.5094604
1080.0000000	7.8567996	528.9591064
1080.0000000	7.9056001	-1096.6666260
1080.0000000	7.9544001	1745.4089355
1080.0000000	8.0031996	2252.1750488
1080.0000000	8.0520000	2247.9064941
1080.0000000	8.1007996	2378.7341309
1080.0000000	8.1496000	2536.8581543
1080.0000000	8.1983995	1578.2677002
1080.0000000	8.2472000	-3611.1367188
1080.0000000	8.2959995	-1280.0828857
1080.0000000	8.3448000	-1019.6776733
1080.0000000	8.3935995	-934.5132446
1080.0000000	8.4423990	-402.5589600
1080.0000000	8.4912004	-939.7650146
1080.0000000	8.5400000	-2535.0175781
1080.0000000	8.5887995	-1228.8176270
1080.0000000	8.6375999	239.9811707
1080.0000000	8.6863995	2168.2797852
1080.0000000	8.7351999	-805.9250488
1080.0000000	8.7840004	920.8571167
1080.0000000	8.8327999	49.8162041
1080.0000000	8.8815994	-11.4975586
1080.0000000	8.9303989	-23.9213791
1080.0000000	8.9792004	-11.5796280
1080.0000000	9.0279999	-5.8480568
1080.0000000	9.0767994	7.0034847
1080.0000000	9.1255999	-17.2313786
1080.0000000	9.1743994	-10.0760717
1080.0000000	9.2231998	36.9149857
1080.0000000	9.2720003	-8.4258194
1080.0000000	9.3207998	-9.8013754
1080.0000000	9.3695993	-10.7651396
1080.0000000	9.4183998	-11.7531300
1080.0000000	9.4672003	-11.1615782
1080.0000000	9.5159998	-14.1588621
1080.0000000	9.5648003	-0.4133129
1080.0000000	9.6135998	-13.3912649
1080.0000000	9.6623993	-10.2229843
1080.0000000	9.7111998	-10.1593351
1080.0000000	9.7600002	-10.8578262
1080.0000000	9.8087997	-10.9948263
1080.0000000	9.8575993	3.1633692
1080.0000000	9.9063997	-7.9745474
1080.0000000	9.9552002	-10.9066277
1080.0000000	10.0039997	-10.0762043
1090.0000000	0.3416000	-36.8092308

1090.0000000	0.3904000	-39.6028519
1090.0000000	0.4392000	-39.6972694
1090.0000000	0.4880000	-39.5827904
1090.0000000	0.5368000	-39.6219749
1090.0000000	0.5856000	-39.3229828
1090.0000000	0.6344000	-38.2276192
1090.0000000	0.6832000	-36.1814728
1090.0000000	0.7320000	-36.0159950
1090.0000000	0.7808000	-36.5315933
1090.0000000	0.8296000	-35.9795532
1090.0000000	0.8784000	-35.2475662
1090.0000000	0.9272000	-32.8880615
1090.0000000	0.9760000	-30.5542755
1090.0000000	1.0247999	-29.7556572
1090.0000000	1.0735999	-29.4750786
1090.0000000	1.1224000	-29.1443405
1090.0000000	1.1711999	-29.7312603
1090.0000000	1.2200000	-30.9270306
1090.0000000	1.2687999	-30.9059372
1090.0000000	1.3176000	-30.8810024
1090.0000000	1.3664000	-30.6251431
1090.0000000	1.4152000	-30.1637459
1090.0000000	1.4640000	-29.6033516
1090.0000000	1.5128000	-38.6949768
1090.0000000	1.5616000	-33.3373909
1090.0000000	1.6104001	-1539.4173584
1090.0000000	1.6592000	-71.2006760
1090.0000000	1.7079999	-27.2837677
1090.0000000	1.7567999	-27.7622776
1090.0000000	1.8055999	-27.6786289
1090.0000000	1.8544000	-26.7812080
1090.0000000	1.9031999	-26.6804886
1090.0000000	1.9520000	-26.3793507
1090.0000000	2.0007999	-26.4094639
1090.0000000	2.0495999	-26.1824341
1090.0000000	2.0983999	-137.6437225
1090.0000000	2.1471999	-3.5455017
1090.0000000	2.1960001	-25.4198647
1090.0000000	2.2448001	-25.7023811
1090.0000000	2.2935998	-27.2630711
1090.0000000	2.3423998	-21.0906830
1090.0000000	2.3912001	27.5297146
1090.0000000	2.4400001	-22.8548698
1090.0000000	2.4888000	-23.4585705
1090.0000000	2.5375998	-12.3861685
1090.0000000	2.5863998	-10.5249987
1090.0000000	2.6352000	-8.6171513
1090.0000000	2.6840000	-15.4901180
1090.0000000	2.7328000	131.4433746
1090.0000000	2.7815998	155.1458740
1090.0000000	2.8304000	-23.7860012
1090.0000000	2.8792000	-20.6402702
1090.0000000	2.9280000	-22.5681400
1090.0000000	2.9768000	-16.8429146
1090.0000000	3.0256000	-20.4893646
1090.0000000	3.0743999	-29.4441319
1090.0000000	3.1231999	-24.2330627
1090.0000000	3.1719999	-29.5528526

1090.0000000	3.2208002	-502.7164612
1090.0000000	3.2695999	-984.1947021
1090.0000000	3.3183999	-20.4909840
1090.0000000	3.3671999	-3386.4287109
1090.0000000	3.4159999	1214.8005371
1090.0000000	3.4648001	-7.1804743
1090.0000000	3.5135999	-27.0162010
1090.0000000	3.5623999	-321.0415344
1090.0000000	3.6111999	-2016.7929688
1090.0000000	3.6600001	-456.6512451
1090.0000000	3.7088001	-602.9512329
1090.0000000	3.7575998	-943.9680786
1090.0000000	3.8063998	-1712.8673096
1090.0000000	3.8552001	-2486.7973633
1090.0000000	3.9040000	-2438.1357422
1090.0000000	3.9528000	1114.8288574
1090.0000000	4.0015998	-243.6840363
1090.0000000	4.0503998	682.4965820
1090.0000000	4.0991998	86.8469543
1090.0000000	4.1479998	-650.1522217
1090.0000000	4.1967998	2.3894482
1090.0000000	4.2456002	-246.8447876
1090.0000000	4.2943997	-484.6012878
1090.0000000	4.3431997	-13.3683224
1090.0000000	4.3920002	-246.4784393
1090.0000000	4.4407997	-22.4304771
1090.0000000	4.4896002	-15.4892006
1090.0000000	4.5383997	-650.6367798
1090.0000000	4.5871997	-12.8729134
1090.0000000	4.6360002	-21.5948410
1090.0000000	4.6847997	-28.2155228
1090.0000000	4.7336001	-19.5501213
1090.0000000	4.7824001	-16.5201111
1090.0000000	4.8311996	-1465.2608643
1090.0000000	4.8800001	226.7204285
1090.0000000	4.9287996	-200.2980499
1090.0000000	4.9776001	1052.8361816
1090.0000000	5.0264001	85.5309296
1090.0000000	5.0751996	-299.3584290
1090.0000000	5.1240001	-12.3241787
1090.0000000	5.1727996	556.4439697
1090.0000000	5.2216001	31.8828850
1090.0000000	5.2704000	-158.4792023
1090.0000000	5.3191996	-39.1281357
1090.0000000	5.3680000	-3527.6184082
1090.0000000	5.4168000	525.8613892
1090.0000000	5.4656000	-200.3105011
1090.0000000	5.5144000	37.9410324
1090.0000000	5.5631995	487.1609192
1090.0000000	5.6120000	392.1974487
1090.0000000	5.6608000	-37.0365562
1090.0000000	5.7096000	62.3486023
1090.0000000	5.7584000	132.6997070
1090.0000000	5.8071995	-11.5305195
1090.0000000	5.8559999	29.7136707
1090.0000000	5.9047999	-16.6033211
1090.0000000	5.9535999	-39.7212563
1090.0000000	6.0023999	-1569.4633789

1090.0000000	6.0511999	-14.5706158
1090.0000000	6.0999999	-19.3998528
1090.0000000	6.1487999	-13.2785578
1090.0000000	6.1975999	-23.2004242
1090.0000000	6.2463999	-1787.9730225
1090.0000000	6.2951999	-19.0612640
1090.0000000	6.3439999	-342.0036011
1090.0000000	6.3927999	-15.0479212
1090.0000000	6.4416003	-12.3923025
1090.0000000	6.4903998	-11.2709017
1090.0000000	6.5391998	-11.8133993
1090.0000000	6.5879998	377.8736572
1090.0000000	6.6367998	-12.0897980
1090.0000000	6.6856003	-13.8166399
1090.0000000	6.7343998	-13.4578094
1090.0000000	6.7831998	-14.3684893
1090.0000000	6.8319998	58.2551270
1090.0000000	6.8807998	-14.0480967
1090.0000000	6.9296002	-442.3775330
1090.0000000	6.9783998	-5.2027740
1090.0000000	7.0271997	-14.1280098
1090.0000000	7.0760002	10.8115492
1090.0000000	7.1247997	-2097.1318359
1090.0000000	7.1736002	-1681.7607422
1090.0000000	7.2223997	-956.8150024
1090.0000000	7.2711997	-206.4696655
1090.0000000	7.3200002	-18.7810020
1090.0000000	7.3687997	184.7515564
1090.0000000	7.4176002	385.6045532
1090.0000000	7.4663997	302.9071350
1090.0000000	7.5151997	-936.0294189
1090.0000000	7.5640001	-1033.9033203
1090.0000000	7.6127996	-2436.7600098
1090.0000000	7.6616001	-782.4179688
1090.0000000	7.7104001	-401.9567261
1090.0000000	7.7591996	42.4085121
1090.0000000	7.8080001	400.7240906
1090.0000000	7.8567996	527.9310303
1090.0000000	7.9056001	-1094.5937500
1090.0000000	7.9544001	1742.0989990
1090.0000000	8.0031996	2247.9052734
1090.0000000	8.0520000	2243.6445313
1090.0000000	8.1007996	2374.2246094
1090.0000000	8.1496000	2532.0502930
1090.0000000	8.1983995	1575.2752686
1090.0000000	8.2472000	-3604.3020020
1090.0000000	8.2959995	-1277.6627197
1090.0000000	8.3448000	-1017.7484741
1090.0000000	8.3935995	-932.7456055
1090.0000000	8.4423990	-401.8009338
1090.0000000	8.4912004	-937.9887695
1090.0000000	8.5400000	-2530.2177734
1090.0000000	8.5887995	-1226.4960938
1090.0000000	8.6375999	239.5198517
1090.0000000	8.6863995	2164.1613770
1090.0000000	8.7351999	-804.4008179
1090.0000000	8.7840004	919.1003418
1090.0000000	8.8327999	49.7045975

1090.0000000	8.8815994	-11.4977331
1090.0000000	8.9303989	-23.9176598
1090.0000000	8.9792004	-11.5797100
1090.0000000	9.0279999	-5.8498249
1090.0000000	9.0767994	6.9977665
1090.0000000	9.1255999	-17.2296658
1090.0000000	9.1743994	-10.0764732
1090.0000000	9.2231998	36.8992538
1090.0000000	9.2720003	-8.4267559
1090.0000000	9.3207998	-9.8019142
1090.0000000	9.3695993	-10.7653666
1090.0000000	9.4183998	-11.7530050
1090.0000000	9.4672003	-11.1616192
1090.0000000	9.5159998	-14.1579714
1090.0000000	9.5648003	-0.4166861
1090.0000000	9.6135998	-13.3904743
1090.0000000	9.6623993	-10.2232590
1090.0000000	9.7111998	-10.1596069
1090.0000000	9.7600002	-10.8578672
1090.0000000	9.8087997	-10.9948339
1090.0000000	9.8575993	3.1589470
1090.0000000	9.9063997	-7.9754610
1090.0000000	9.9552002	-10.9066315
1090.0000000	10.0039997	-10.0764523
1100.0000000	0.3416000	-37.5325241
1100.0000000	0.3904000	-39.5677719
1100.0000000	0.4392000	-39.6909256
1100.0000000	0.4880000	-39.5815811
1100.0000000	0.5368000	-39.6231499
1100.0000000	0.5856000	-39.3215981
1100.0000000	0.6344000	-38.2279320
1100.0000000	0.6832000	-36.1836967
1100.0000000	0.7320000	-36.0180473
1100.0000000	0.7808000	-36.5317612
1100.0000000	0.8296000	-35.9802818
1100.0000000	0.8784000	-35.2549400
1100.0000000	0.9272000	-32.8908386
1100.0000000	0.9760000	-30.5468235
1100.0000000	1.0247999	-29.7646465
1100.0000000	1.0735999	-29.4895134
1100.0000000	1.1224000	-29.1413403
1100.0000000	1.1711999	-29.7238827
1100.0000000	1.2200000	-30.9349461
1100.0000000	1.2687999	-30.9069443
1100.0000000	1.3176000	-30.8767319
1100.0000000	1.3664000	-30.6328926
1100.0000000	1.4152000	-30.1639500
1100.0000000	1.4640000	-29.6016426
1100.0000000	1.5128000	-38.5978012
1100.0000000	1.5616000	-33.2955475
1100.0000000	1.6104001	-1536.8745117
1100.0000000	1.6592000	-70.9067383
1100.0000000	1.7079999	-27.2875137
1100.0000000	1.7567999	-27.7622967
1100.0000000	1.8055999	-27.6825180
1100.0000000	1.8544000	-26.7911358
1100.0000000	1.9031999	-26.6776943
1100.0000000	1.9520000	-26.3757801

1100.0000000	2.0007999	-26.4025211
1100.0000000	2.0495999	-26.1844521
1100.0000000	2.0983999	-136.1679382
1100.0000000	2.1471999	-3.8127689
1100.0000000	2.1960001	-25.4157734
1100.0000000	2.2448001	-25.6844711
1100.0000000	2.2935998	-27.2034473
1100.0000000	2.3423998	-21.1050873
1100.0000000	2.3912001	26.9081669
1100.0000000	2.4400001	-22.8573570
1100.0000000	2.4888000	-23.4583530
1100.0000000	2.5375998	-12.4032764
1100.0000000	2.5863998	-10.5447578
1100.0000000	2.6352000	-8.6398954
1100.0000000	2.6840000	-15.5020599
1100.0000000	2.7328000	131.1773682
1100.0000000	2.7815998	154.8394470
1100.0000000	2.8304000	-23.7846985
1100.0000000	2.8792000	-20.6435337
1100.0000000	2.9280000	-22.5675697
1100.0000000	2.9768000	-16.8512840
1100.0000000	3.0256000	-20.4919491
1100.0000000	3.0743999	-29.4327450
1100.0000000	3.1231999	-24.2296066
1100.0000000	3.1719999	-29.5445595
1100.0000000	3.2208002	-502.4787292
1100.0000000	3.2695999	-983.7197266
1100.0000000	3.3183999	-20.4911804
1100.0000000	3.3671999	-3384.9670410
1100.0000000	3.4159999	1214.2652588
1100.0000000	3.4648001	-7.1861820
1100.0000000	3.5135999	-27.0132103
1100.0000000	3.5623999	-320.9119568
1100.0000000	3.6111999	-2015.9332275
1100.0000000	3.6600001	-456.4630737
1100.0000000	3.7088001	-602.6998291
1100.0000000	3.7575998	-943.5695190
1100.0000000	3.8063998	-1712.1374512
1100.0000000	3.8552001	-2485.7338867
1100.0000000	3.9040000	-2437.0932617
1100.0000000	3.9528000	1114.3405762
1100.0000000	4.0015998	-243.5869293
1100.0000000	4.0503998	682.1940308
1100.0000000	4.0991998	86.8012695
1100.0000000	4.1479998	-649.8800049
1100.0000000	4.1967998	2.3801918
1100.0000000	4.2456002	-246.7464905
1100.0000000	4.2943997	-484.4007263
1100.0000000	4.3431997	-13.3705025
1100.0000000	4.3920002	-246.3803253
1100.0000000	4.4407997	-22.4287014
1100.0000000	4.4896002	-15.4900827
1100.0000000	4.5383997	-650.3757324
1100.0000000	4.5871997	-12.8749294
1100.0000000	4.6360002	-21.5932980
1100.0000000	4.6847997	-28.2115974
1100.0000000	4.7336001	-19.5493984
1100.0000000	4.7824001	-16.5205650

1100.0000000	4.8311996	-1464.7296143
1100.0000000	4.8800001	226.6308746
1100.0000000	4.9287996	-200.2307739
1100.0000000	4.9776001	1052.4436035
1100.0000000	5.0264001	85.4934158
1100.0000000	5.0751996	-299.2548218
1100.0000000	5.1240001	-12.3250999
1100.0000000	5.1727996	556.3280640
1100.0000000	5.2216001	31.8734398
1100.0000000	5.2704000	-158.4510956
1100.0000000	5.3191996	-39.1235847
1100.0000000	5.3680000	-3526.9260254
1100.0000000	5.4168000	525.7546387
1100.0000000	5.4656000	-200.2743073
1100.0000000	5.5144000	37.9303741
1100.0000000	5.5631995	487.0623779
1100.0000000	5.6120000	392.1174622
1100.0000000	5.6608000	-37.0324669
1100.0000000	5.7096000	62.3331680
1100.0000000	5.7584000	132.6707764
1100.0000000	5.8071995	-11.5313911
1100.0000000	5.8559999	29.7048206
1100.0000000	5.9047999	-16.6031246
1100.0000000	5.9535999	-39.7165184
1100.0000000	6.0023999	-1569.1589355
1100.0000000	6.0511999	-14.5708265
1100.0000000	6.0999999	-19.3990211
1100.0000000	6.1487999	-13.2790203
1100.0000000	6.1975999	-23.1986599
1100.0000000	6.2463999	-1787.6256104
1100.0000000	6.2951999	-19.0604496
1100.0000000	6.3439999	-341.9395447
1100.0000000	6.3927999	-15.0479021
1100.0000000	6.4416003	-12.3928890
1100.0000000	6.4903998	-11.2715778
1100.0000000	6.5391998	-11.8140802
1100.0000000	6.5879998	377.1829834
1100.0000000	6.6367998	-12.0903473
1100.0000000	6.6856003	-13.8168106
1100.0000000	6.7343998	-13.4580250
1100.0000000	6.7831998	-14.3680735
1100.0000000	6.8319998	58.2360153
1100.0000000	6.8807998	-14.0479584
1100.0000000	6.9296002	-441.6160583
1100.0000000	6.9783998	-5.2155981
1100.0000000	7.0271997	-14.1269817
1100.0000000	7.0760002	10.7693624
1100.0000000	7.1247997	-2093.1860352
1100.0000000	7.1736002	-1678.5979004
1100.0000000	7.2223997	-955.0217896
1100.0000000	7.2711997	-206.1018829
1100.0000000	7.3200002	-18.7690144
1100.0000000	7.3687997	184.3770294
1100.0000000	7.4176002	384.8487244
1100.0000000	7.4663997	302.3094788
1100.0000000	7.5151997	-934.2683716
1100.0000000	7.5640001	-1031.9558105
1100.0000000	7.6127996	-2432.1423340

1100.0000000	7.6616001	-780.9467163
1100.0000000	7.7104001	-401.2104492
1100.0000000	7.7591996	42.3074646
1100.0000000	7.8080001	399.9387512
1100.0000000	7.8567996	526.9028931
1100.0000000	7.9056001	-1092.5207520
1100.0000000	7.9544001	1738.7890625
1100.0000000	8.0031996	2243.6357422
1100.0000000	8.0520000	2239.3825684
1100.0000000	8.1007996	2369.7150879
1100.0000000	8.1496000	2527.2421875
1100.0000000	8.1983995	1572.2827148
1100.0000000	8.2472000	-3597.4672852
1100.0000000	8.2959995	-1275.2425537
1100.0000000	8.3448000	-1015.8193359
1100.0000000	8.3935995	-930.9779053
1100.0000000	8.4423990	-401.0429382
1100.0000000	8.4912004	-936.2125854
1100.0000000	8.5400000	-2525.4179688
1100.0000000	8.5887995	-1224.1745605
1100.0000000	8.6375999	239.0585327
1100.0000000	8.6863995	2160.0429688
1100.0000000	8.7351999	-802.8765259
1100.0000000	8.7840004	917.3436279
1100.0000000	8.8327999	49.5929871
1100.0000000	8.8815994	-11.4979067
1100.0000000	8.9303989	-23.9139404
1100.0000000	8.9792004	-11.5797920
1100.0000000	9.0279999	-5.8515930
1100.0000000	9.0767994	6.9920483
1100.0000000	9.1255999	-17.2279510
1100.0000000	9.1743994	-10.0768757
1100.0000000	9.2231998	36.8835144
1100.0000000	9.2720003	-8.4276924
1100.0000000	9.3207998	-9.8024540
1100.0000000	9.3695993	-10.7655926
1100.0000000	9.4183998	-11.7528801
1100.0000000	9.4672003	-11.1616611
1100.0000000	9.5159998	-14.1570807
1100.0000000	9.5648003	-0.4200592
1100.0000000	9.6135998	-13.3896837
1100.0000000	9.6623993	-10.2235336
1100.0000000	9.7111998	-10.1598787
1100.0000000	9.7600002	-10.8579092
1100.0000000	9.8087997	-10.9948416
1100.0000000	9.8575993	3.1545238
1100.0000000	9.9063997	-7.9763746
1100.0000000	9.9552002	-10.9066362
1100.0000000	10.0039997	-10.0766993
1110.0000000	0.3416000	-36.9423561
1110.0000000	0.3904000	-39.5326881
1110.0000000	0.4392000	-39.6845818
1110.0000000	0.4880000	-39.5803719
1110.0000000	0.5368000	-39.6243210
1110.0000000	0.5856000	-39.3202171
1110.0000000	0.6344000	-38.2282410
1110.0000000	0.6832000	-36.1859207
1110.0000000	0.7320000	-36.0200996

1110.0000000	0.7808000	-36.5319290
1110.0000000	0.8296000	-35.9810104
1110.0000000	0.8784000	-35.2623138
1110.0000000	0.9272000	-32.8936157
1110.0000000	0.9760000	-30.5393715
1110.0000000	1.0247999	-29.7736359
1110.0000000	1.0735999	-29.5039482
1110.0000000	1.1224000	-29.1383400
1110.0000000	1.1711999	-29.7165051
1110.0000000	1.2200000	-30.9428616
1110.0000000	1.2687999	-30.9079533
1110.0000000	1.3176000	-30.8724594
1110.0000000	1.3664000	-30.6406422
1110.0000000	1.4152000	-30.1641541
1110.0000000	1.4640000	-29.5999336
1110.0000000	1.5128000	-38.5006218
1110.0000000	1.5616000	-33.2537079
1110.0000000	1.6104001	-1534.3317871
1110.0000000	1.6592000	-70.6128082
1110.0000000	1.7079999	-27.2912617
1110.0000000	1.7567999	-27.7623158
1110.0000000	1.8055999	-27.6864052
1110.0000000	1.8544000	-26.8010635
1110.0000000	1.9031999	-26.6749001
1110.0000000	1.9520000	-26.3722076
1110.0000000	2.0007999	-26.3955784
1110.0000000	2.0495999	-26.1864719
1110.0000000	2.0983999	-134.6921692
1110.0000000	2.1471999	-4.0800362
1110.0000000	2.1960001	-25.4116821
1110.0000000	2.2448001	-25.6665592
1110.0000000	2.2935998	-27.1438236
1110.0000000	2.3423998	-21.1194916
1110.0000000	2.3912001	26.2866192
1110.0000000	2.4400001	-22.8598423
1110.0000000	2.4888000	-23.4581356
1110.0000000	2.5375998	-12.4203844
1110.0000000	2.5863998	-10.5645170
1110.0000000	2.6352000	-8.6626396
1110.0000000	2.6840000	-15.5140018
1110.0000000	2.7328000	130.9113464
1110.0000000	2.7815998	154.5330505
1110.0000000	2.8304000	-23.7833939
1110.0000000	2.8792000	-20.6467972
1110.0000000	2.9280000	-22.5670013
1110.0000000	2.9768000	-16.8596554
1110.0000000	3.0256000	-20.4945335
1110.0000000	3.0743999	-29.4213581
1110.0000000	3.1231999	-24.2261505
1110.0000000	3.1719999	-29.5362663
1110.0000000	3.2208002	-502.2409973
1110.0000000	3.2695999	-983.2447510
1110.0000000	3.3183999	-20.4913788
1110.0000000	3.3671999	-3383.5053711
1110.0000000	3.4159999	1213.7299805
1110.0000000	3.4648001	-7.1918907
1110.0000000	3.5135999	-27.0102196
1110.0000000	3.5623999	-320.7823486

1110.0000000	3.6111999	-2015.0733643
1110.0000000	3.6600001	-456.2749634
1110.0000000	3.7088001	-602.4484863
1110.0000000	3.7575998	-943.1710205
1110.0000000	3.8063998	-1711.4075928
1110.0000000	3.8552001	-2484.6704102
1110.0000000	3.9040000	-2436.0507813
1110.0000000	3.9528000	1113.8522949
1110.0000000	4.0015998	-243.4898224
1110.0000000	4.0503998	681.8914795
1110.0000000	4.0991998	86.7555847
1110.0000000	4.1479998	-649.6077271
1110.0000000	4.1967998	2.3709354
1110.0000000	4.2456002	-246.6481781
1110.0000000	4.2943997	-484.2001648
1110.0000000	4.3431997	-13.3726835
1110.0000000	4.3920002	-246.2822113
1110.0000000	4.4407997	-22.4269257
1110.0000000	4.4896002	-15.4909658
1110.0000000	4.5383997	-650.1146851
1110.0000000	4.5871997	-12.8769455
1110.0000000	4.6360002	-21.5917549
1110.0000000	4.6847997	-28.2076721
1110.0000000	4.7336001	-19.5486755
1110.0000000	4.7824001	-16.5210190
1110.0000000	4.8311996	-1464.1983643
1110.0000000	4.8800001	226.5413208
1110.0000000	4.9287996	-200.1635132
1110.0000000	4.9776001	1052.0511475
1110.0000000	5.0264001	85.4558945
1110.0000000	5.0751996	-299.1511841
1110.0000000	5.1240001	-12.3260212
1110.0000000	5.1727996	556.2122192
1110.0000000	5.2216001	31.8639946
1110.0000000	5.2704000	-158.4229889
1110.0000000	5.3191996	-39.1190338
1110.0000000	5.3680000	-3526.2336426
1110.0000000	5.4168000	525.6478271
1110.0000000	5.4656000	-200.2381287
1110.0000000	5.5144000	37.9197159
1110.0000000	5.5631995	486.9638062
1110.0000000	5.6120000	392.0374756
1110.0000000	5.6608000	-37.0283813
1110.0000000	5.7096000	62.3177414
1110.0000000	5.7584000	132.6418304
1110.0000000	5.8071995	-11.5322609
1110.0000000	5.8559999	29.6959667
1110.0000000	5.9047999	-16.6029263
1110.0000000	5.9535999	-39.7117805
1110.0000000	6.0023999	-1568.8546143
1110.0000000	6.0511999	-14.5710382
1110.0000000	6.0999999	-19.3981895
1110.0000000	6.1487999	-13.2794828
1110.0000000	6.1975999	-23.1968956
1110.0000000	6.2463999	-1787.2783203
1110.0000000	6.2951999	-19.0596352
1110.0000000	6.3439999	-341.8754883
1110.0000000	6.3927999	-15.0478840

1110.0000000	6.4416003	-12.3934755
1110.0000000	6.4903998	-11.2722540
1110.0000000	6.5391998	-11.8147621
1110.0000000	6.5879998	376.4922791
1110.0000000	6.6367998	-12.0908966
1110.0000000	6.6856003	-13.8169804
1110.0000000	6.7343998	-13.4582415
1110.0000000	6.7831998	-14.3676577
1110.0000000	6.8319998	58.2168961
1110.0000000	6.8807998	-14.0478191
1110.0000000	6.9296002	-440.8546143
1110.0000000	6.9783998	-5.2284212
1110.0000000	7.0271997	-14.1259546
1110.0000000	7.0760002	10.7271757
1110.0000000	7.1247997	-2089.2402344
1110.0000000	7.1736002	-1675.4350586
1110.0000000	7.2223997	-953.2286377
1110.0000000	7.2711997	-205.7341003
1110.0000000	7.3200002	-18.7570267
1110.0000000	7.3687997	184.0025024
1110.0000000	7.4176002	384.0928955
1110.0000000	7.4663997	301.7118225
1110.0000000	7.5151997	-932.5073853
1110.0000000	7.5640001	-1030.0084229
1110.0000000	7.6127996	-2427.5246582
1110.0000000	7.6616001	-779.4753418
1110.0000000	7.7104001	-400.4642334
1110.0000000	7.7591996	42.2064171
1110.0000000	7.8080001	399.1533813
1110.0000000	7.8567996	525.8748169
1110.0000000	7.9056001	-1090.4478760
1110.0000000	7.9544001	1735.4791260
1110.0000000	8.0031996	2239.3662109
1110.0000000	8.0520000	2235.1206055
1110.0000000	8.1007996	2365.2055664
1110.0000000	8.1496000	2522.4343262
1110.0000000	8.1983995	1569.2901611
1110.0000000	8.2472000	-3590.6323242
1110.0000000	8.2959995	-1272.8223877
1110.0000000	8.3448000	-1013.8901367
1110.0000000	8.3935995	-929.2102661
1110.0000000	8.4423990	-400.2849121
1110.0000000	8.4912004	-934.4363403
1110.0000000	8.5400000	-2520.6181641
1110.0000000	8.5887995	-1221.8530273
1110.0000000	8.6375999	238.5972137
1110.0000000	8.6863995	2155.9245605
1110.0000000	8.7351999	-801.3522949
1110.0000000	8.7840004	915.5868530
1110.0000000	8.8327999	49.4813766
1110.0000000	8.8815994	-11.4980812
1110.0000000	8.9303989	-23.9102192
1110.0000000	8.9792004	-11.5798750
1110.0000000	9.0279999	-5.8533616
1110.0000000	9.0767994	6.9863319
1110.0000000	9.1255999	-17.2262383
1110.0000000	9.1743994	-10.0772781
1110.0000000	9.2231998	36.8677826

1110.0000000	9.2720003	-8.4286289
1110.0000000	9.3207998	-9.8029938
1110.0000000	9.3695993	-10.7658195
1110.0000000	9.4183998	-11.7527561
1110.0000000	9.4672003	-11.1617022
1110.0000000	9.5159998	-14.1561890
1110.0000000	9.5648003	-0.4234314
1110.0000000	9.6135998	-13.3888931
1110.0000000	9.6623993	-10.2238092
1110.0000000	9.7111998	-10.1601496
1110.0000000	9.7600002	-10.8579512
1110.0000000	9.8087997	-10.9948502
1110.0000000	9.8575993	3.1501017
1110.0000000	9.9063997	-7.9772892
1110.0000000	9.9552002	-10.9066401
1110.0000000	10.0039997	-10.0769472
1120.0000000	0.3416000	-36.6149063
1120.0000000	0.3904000	-39.4976082
1120.0000000	0.4392000	-39.6782379
1120.0000000	0.4880000	-39.5791626
1120.0000000	0.5368000	-39.6254959
1120.0000000	0.5856000	-39.3188362
1120.0000000	0.6344000	-38.2285538
1120.0000000	0.6832000	-36.1881485
1120.0000000	0.7320000	-36.0221519
1120.0000000	0.7808000	-36.5321007
1120.0000000	0.8296000	-35.9817390
1120.0000000	0.8784000	-35.2696915
1120.0000000	0.9272000	-32.8963928
1120.0000000	0.9760000	-30.5319176
1120.0000000	1.0247999	-29.7826252
1120.0000000	1.0735999	-29.5183849
1120.0000000	1.1224000	-29.1353416
1120.0000000	1.1711999	-29.7091274
1120.0000000	1.2200000	-30.9507771
1120.0000000	1.2687999	-30.9089603
1120.0000000	1.3176000	-30.8681889
1120.0000000	1.3664000	-30.6483917
1120.0000000	1.4152000	-30.1643600
1120.0000000	1.4640000	-29.5982246
1120.0000000	1.5128000	-38.4034424
1120.0000000	1.5616000	-33.2118683
1120.0000000	1.6104001	-1531.7889404
1120.0000000	1.6592000	-70.3188782
1120.0000000	1.7079999	-27.2950096
1120.0000000	1.7567999	-27.7623348
1120.0000000	1.8055999	-27.6902943
1120.0000000	1.8544000	-26.8109913
1120.0000000	1.9031999	-26.6721058
1120.0000000	1.9520000	-26.3686371
1120.0000000	2.0007999	-26.3886375
1120.0000000	2.0495999	-26.1884899
1120.0000000	2.0983999	-133.2163849
1120.0000000	2.1471999	-4.3473034
1120.0000000	2.1960001	-25.4075909
1120.0000000	2.2448001	-25.6486473
1120.0000000	2.2935998	-27.0841999
1120.0000000	2.3423998	-21.1338959

1120.0000000	2.3912001	25.6650753
1120.0000000	2.4400001	-22.8623295
1120.0000000	2.4888000	-23.4579182
1120.0000000	2.5375998	-12.4374924
1120.0000000	2.5863998	-10.5842752
1120.0000000	2.6352000	-8.6853838
1120.0000000	2.6840000	-15.5259438
1120.0000000	2.7328000	130.6453400
1120.0000000	2.7815998	154.2266235
1120.0000000	2.8304000	-23.7820892
1120.0000000	2.8792000	-20.6500607
1120.0000000	2.9280000	-22.5664330
1120.0000000	2.9768000	-16.8680267
1120.0000000	3.0256000	-20.4971180
1120.0000000	3.0743999	-29.4099712
1120.0000000	3.1231999	-24.2226963
1120.0000000	3.1719999	-29.5279732
1120.0000000	3.2208002	-502.0032654
1120.0000000	3.2695999	-982.7697754
1120.0000000	3.3183999	-20.4915771
1120.0000000	3.3671999	-3382.0437012
1120.0000000	3.4159999	1213.1947021
1120.0000000	3.4648001	-7.1975985
1120.0000000	3.5135999	-27.0072289
1120.0000000	3.5623999	-320.6527710
1120.0000000	3.6111999	-2014.2136230
1120.0000000	3.6600001	-456.0868530
1120.0000000	3.7088001	-602.1970825
1120.0000000	3.7575998	-942.7724609
1120.0000000	3.8063998	-1710.6777344
1120.0000000	3.8552001	-2483.6069336
1120.0000000	3.9040000	-2435.0083008
1120.0000000	3.9528000	1113.3638916
1120.0000000	4.0015998	-243.3927155
1120.0000000	4.0503998	681.5889282
1120.0000000	4.0991998	86.7098999
1120.0000000	4.1479998	-649.3355103
1120.0000000	4.1967998	2.3616791
1120.0000000	4.2456002	-246.5498810
1120.0000000	4.2943997	-483.9995728
1120.0000000	4.3431997	-13.3748646
1120.0000000	4.3920002	-246.1840973
1120.0000000	4.4407997	-22.4251499
1120.0000000	4.4896002	-15.4918480
1120.0000000	4.5383997	-649.8535767
1120.0000000	4.5871997	-12.8789616
1120.0000000	4.6360002	-21.5902138
1120.0000000	4.6847997	-28.2037468
1120.0000000	4.7336001	-19.5479527
1120.0000000	4.7824001	-16.5214748
1120.0000000	4.8311996	-1463.6669922
1120.0000000	4.8800001	226.4517822
1120.0000000	4.9287996	-200.0962524
1120.0000000	4.9776001	1051.6586914
1120.0000000	5.0264001	85.4183731
1120.0000000	5.0751996	-299.0475464
1120.0000000	5.1240001	-12.3269424
1120.0000000	5.1727996	556.0963745

1120.0000000	5.2216001	31.8545532
1120.0000000	5.2704000	-158.3948822
1120.0000000	5.3191996	-39.1144867
1120.0000000	5.3680000	-3525.5412598
1120.0000000	5.4168000	525.5410767
1120.0000000	5.4656000	-200.2019501
1120.0000000	5.5144000	37.9090576
1120.0000000	5.5631995	486.8652344
1120.0000000	5.6120000	391.9575195
1120.0000000	5.6608000	-37.0242920
1120.0000000	5.7096000	62.3023071
1120.0000000	5.7584000	132.6128998
1120.0000000	5.8071995	-11.5331326
1120.0000000	5.8559999	29.6871166
1120.0000000	5.9047999	-16.6027279
1120.0000000	5.9535999	-39.7070389
1120.0000000	6.0023999	-1568.5500488
1120.0000000	6.0511999	-14.5712490
1120.0000000	6.0999999	-19.3973560
1120.0000000	6.1487999	-13.2799454
1120.0000000	6.1975999	-23.1951294
1120.0000000	6.2463999	-1786.9309082
1120.0000000	6.2951999	-19.0588207
1120.0000000	6.3439999	-341.8114624
1120.0000000	6.3927999	-15.0478659
1120.0000000	6.4416003	-12.3940630
1120.0000000	6.4903998	-11.2729292
1120.0000000	6.5391998	-11.8154430
1120.0000000	6.5879998	375.8015747
1120.0000000	6.6367998	-12.0914450
1120.0000000	6.6856003	-13.8171511
1120.0000000	6.7343998	-13.4584570
1120.0000000	6.7831998	-14.3672419
1120.0000000	6.8319998	58.1977768
1120.0000000	6.8807998	-14.0476809
1120.0000000	6.9296002	-440.0931702
1120.0000000	6.9783998	-5.2412448
1120.0000000	7.0271997	-14.1249266
1120.0000000	7.0760002	10.6849928
1120.0000000	7.1247997	-2085.2944336
1120.0000000	7.1736002	-1672.2720947
1120.0000000	7.2223997	-951.4354858
1120.0000000	7.2711997	-205.3663330
1120.0000000	7.3200002	-18.7450371
1120.0000000	7.3687997	183.6279602
1120.0000000	7.4176002	383.3370667
1120.0000000	7.4663997	301.1141663
1120.0000000	7.5151997	-930.7463379
1120.0000000	7.5640001	-1028.0610352
1120.0000000	7.6127996	-2422.9069824
1120.0000000	7.6616001	-778.0040283
1120.0000000	7.7104001	-399.7178955
1120.0000000	7.7591996	42.1053619
1120.0000000	7.8080001	398.3680115
1120.0000000	7.8567996	524.8467407
1120.0000000	7.9056001	-1088.3750000
1120.0000000	7.9544001	1732.1691895
1120.0000000	8.0031996	2235.0964355

1120.0000000	8.0520000	2230.8586426
1120.0000000	8.1007996	2360.6960449
1120.0000000	8.1496000	2517.6262207
1120.0000000	8.1983995	1566.2977295
1120.0000000	8.2472000	-3583.7976074
1120.0000000	8.2959995	-1270.4022217
1120.0000000	8.3448000	-1011.9609375
1120.0000000	8.3935995	-927.4425659
1120.0000000	8.4423990	-399.5269165
1120.0000000	8.4912004	-932.6600952
1120.0000000	8.5400000	-2515.8183594
1120.0000000	8.5887995	-1219.5314941
1120.0000000	8.6375999	238.1358948
1120.0000000	8.6863995	2151.8063965
1120.0000000	8.7351999	-799.8280029
1120.0000000	8.7840004	913.8301392
1120.0000000	8.8327999	49.3697739
1120.0000000	8.8815994	-11.4982557
1120.0000000	8.9303989	-23.9064999
1120.0000000	8.9792004	-11.5799570
1120.0000000	9.0279999	-5.8551302
1120.0000000	9.0767994	6.9806137
1120.0000000	9.1255999	-17.2245235
1120.0000000	9.1743994	-10.0776796
1120.0000000	9.2231998	36.8520508
1120.0000000	9.2720003	-8.4295645
1120.0000000	9.3207998	-9.8035326
1120.0000000	9.3695993	-10.7660456
1120.0000000	9.4183998	-11.7526312
1120.0000000	9.4672003	-11.1617432
1120.0000000	9.5159998	-14.1552982
1120.0000000	9.5648003	-0.4268036
1120.0000000	9.6135998	-13.3881025
1120.0000000	9.6623993	-10.2240839
1120.0000000	9.7111998	-10.1604214
1120.0000000	9.7600002	-10.8579931
1120.0000000	9.8087997	-10.9948578
1120.0000000	9.8575993	3.1456795
1120.0000000	9.9063997	-7.9782028
1120.0000000	9.9552002	-10.9066448
1120.0000000	10.0039997	-10.0771942
1130.0000000	0.3416000	-36.9640503
1130.0000000	0.3904000	-39.4625282
1130.0000000	0.4392000	-39.6718979
1130.0000000	0.4880000	-39.5779533
1130.0000000	0.5368000	-39.6266708
1130.0000000	0.5856000	-39.3174553
1130.0000000	0.6344000	-38.2288628
1130.0000000	0.6832000	-36.1903725
1130.0000000	0.7320000	-36.0242043
1130.0000000	0.7808000	-36.5322685
1130.0000000	0.8296000	-35.9824677
1130.0000000	0.8784000	-35.2770653
1130.0000000	0.9272000	-32.8991699
1130.0000000	0.9760000	-30.5244656
1130.0000000	1.0247999	-29.7916145
1130.0000000	1.0735999	-29.5328197
1130.0000000	1.1224000	-29.1323414

1130.0000000	1.1711999	-29.7017517
1130.0000000	1.2200000	-30.9586906
1130.0000000	1.2687999	-30.9099674
1130.0000000	1.3176000	-30.8639164
1130.0000000	1.3664000	-30.6561413
1130.0000000	1.4152000	-30.1645641
1130.0000000	1.4640000	-29.5965137
1130.0000000	1.5128000	-38.3062668
1130.0000000	1.5616000	-33.1700249
1130.0000000	1.6104001	-1529.2460938
1130.0000000	1.6592000	-70.0249481
1130.0000000	1.7079999	-27.2987556
1130.0000000	1.7567999	-27.7623539
1130.0000000	1.8055999	-27.6941814
1130.0000000	1.8544000	-26.8209190
1130.0000000	1.9031999	-26.6693115
1130.0000000	1.9520000	-26.3650646
1130.0000000	2.0007999	-26.3816948
1130.0000000	2.0495999	-26.1905098
1130.0000000	2.0983999	-131.7406006
1130.0000000	2.1471999	-4.6145706
1130.0000000	2.1960001	-25.4034996
1130.0000000	2.2448001	-25.6307373
1130.0000000	2.2935998	-27.0245762
1130.0000000	2.3423998	-21.1483002
1130.0000000	2.3912001	25.0435314
1130.0000000	2.4400001	-22.8648167
1130.0000000	2.4888000	-23.4577007
1130.0000000	2.5375998	-12.4546003
1130.0000000	2.5863998	-10.6040344
1130.0000000	2.6352000	-8.7081280
1130.0000000	2.6840000	-15.5378857
1130.0000000	2.7328000	130.3793182
1130.0000000	2.7815998	153.9201965
1130.0000000	2.8304000	-23.7807846
1130.0000000	2.8792000	-20.6533241
1130.0000000	2.9280000	-22.5658627
1130.0000000	2.9768000	-16.8763962
1130.0000000	3.0256000	-20.4997025
1130.0000000	3.0743999	-29.3985844
1130.0000000	3.1231999	-24.2192402
1130.0000000	3.1719999	-29.5196800
1130.0000000	3.2208002	-501.7655334
1130.0000000	3.2695999	-982.2947998
1130.0000000	3.3183999	-20.4917755
1130.0000000	3.3671999	-3380.5820313
1130.0000000	3.4159999	1212.6594238
1130.0000000	3.4648001	-7.2033072
1130.0000000	3.5135999	-27.0042381
1130.0000000	3.5623999	-320.5231934
1130.0000000	3.6111999	-2013.3538818
1130.0000000	3.6600001	-455.8987427
1130.0000000	3.7088001	-601.9456787
1130.0000000	3.7575998	-942.3739624
1130.0000000	3.8063998	-1709.9478760
1130.0000000	3.8552001	-2482.5434570
1130.0000000	3.9040000	-2433.9658203
1130.0000000	3.9528000	1112.8756104

1130.0000000	4.0015998	-243.2956085
1130.0000000	4.0503998	681.2863159
1130.0000000	4.0991998	86.6642227
1130.0000000	4.1479998	-649.0632935
1130.0000000	4.1967998	2.3524227
1130.0000000	4.2456002	-246.4515686
1130.0000000	4.2943997	-483.7990112
1130.0000000	4.3431997	-13.3770447
1130.0000000	4.3920002	-246.0859833
1130.0000000	4.4407997	-22.4233742
1130.0000000	4.4896002	-15.4927311
1130.0000000	4.5383997	-649.5925293
1130.0000000	4.5871997	-12.8809786
1130.0000000	4.6360002	-21.5886707
1130.0000000	4.6847997	-28.1998215
1130.0000000	4.7336001	-19.5472317
1130.0000000	4.7824001	-16.5219288
1130.0000000	4.8311996	-1463.1357422
1130.0000000	4.8800001	226.3622284
1130.0000000	4.9287996	-200.0289917
1130.0000000	4.9776001	1051.2661133
1130.0000000	5.0264001	85.3808517
1130.0000000	5.0751996	-298.9439392
1130.0000000	5.1240001	-12.3278637
1130.0000000	5.1727996	555.9804688
1130.0000000	5.2216001	31.8451080
1130.0000000	5.2704000	-158.3667755
1130.0000000	5.3191996	-39.1099396
1130.0000000	5.3680000	-3524.8488770
1130.0000000	5.4168000	525.4342651
1130.0000000	5.4656000	-200.1657562
1130.0000000	5.5144000	37.8984070
1130.0000000	5.5631995	486.7666626
1130.0000000	5.6120000	391.8775330
1130.0000000	5.6608000	-37.0202026
1130.0000000	5.7096000	62.2868729
1130.0000000	5.7584000	132.5839691
1130.0000000	5.8071995	-11.5340023
1130.0000000	5.8559999	29.6782665
1130.0000000	5.9047999	-16.6025314
1130.0000000	5.9535999	-39.7023010
1130.0000000	6.0023999	-1568.2457275
1130.0000000	6.0511999	-14.5714607
1130.0000000	6.0999999	-19.3965244
1130.0000000	6.1487999	-13.2804089
1130.0000000	6.1975999	-23.1933632
1130.0000000	6.2463999	-1786.5834961
1130.0000000	6.2951999	-19.0580063
1130.0000000	6.3439999	-341.7473755
1130.0000000	6.3927999	-15.0478477
1130.0000000	6.4416003	-12.3946495
1130.0000000	6.4903998	-11.2736053
1130.0000000	6.5391998	-11.8161240
1130.0000000	6.5879998	375.1109009
1130.0000000	6.6367998	-12.0919943
1130.0000000	6.6856003	-13.8173208
1130.0000000	6.7343998	-13.4586725
1130.0000000	6.7831998	-14.3668261

1130.0000000	6.8319998	58.1786652
1130.0000000	6.8807998	-14.0475416
1130.0000000	6.9296002	-439.3317261
1130.0000000	6.9783998	-5.2540689
1130.0000000	7.0271997	-14.1238985
1130.0000000	7.0760002	10.6428061
1130.0000000	7.1247997	-2081.3483887
1130.0000000	7.1736002	-1669.1092529
1130.0000000	7.2223997	-949.6423340
1130.0000000	7.2711997	-204.9985504
1130.0000000	7.3200002	-18.7330475
1130.0000000	7.3687997	183.2534332
1130.0000000	7.4176002	382.5812378
1130.0000000	7.4663997	300.5165405
1130.0000000	7.5151997	-928.9852905
1130.0000000	7.5640001	-1026.1136475
1130.0000000	7.6127996	-2418.2895508
1130.0000000	7.6616001	-776.5326538
1130.0000000	7.7104001	-398.9716797
1130.0000000	7.7591996	42.0043144
1130.0000000	7.8080001	397.5826721
1130.0000000	7.8567996	523.8186035
1130.0000000	7.9056001	-1086.3021240
1130.0000000	7.9544001	1728.8592529
1130.0000000	8.0031996	2230.8269043
1130.0000000	8.0520000	2226.5966797
1130.0000000	8.1007996	2356.1867676
1130.0000000	8.1496000	2512.8181152
1130.0000000	8.1983995	1563.3051758
1130.0000000	8.2472000	-3576.9628906
1130.0000000	8.2959995	-1267.9820557
1130.0000000	8.3448000	-1010.0317993
1130.0000000	8.3935995	-925.6748657
1130.0000000	8.4423990	-398.7688904
1130.0000000	8.4912004	-930.8839111
1130.0000000	8.5400000	-2511.0185547
1130.0000000	8.5887995	-1217.2099609
1130.0000000	8.6375999	237.6745758
1130.0000000	8.6863995	2147.6879883
1130.0000000	8.7351999	-798.3037720
1130.0000000	8.7840004	912.0733643
1130.0000000	8.8327999	49.2581673
1130.0000000	8.8815994	-11.4984303
1130.0000000	8.9303989	-23.9027786
1130.0000000	8.9792004	-11.5800390
1130.0000000	9.0279999	-5.8568983
1130.0000000	9.0767994	6.9748955
1130.0000000	9.1255999	-17.2228088
1130.0000000	9.1743994	-10.0780821
1130.0000000	9.2231998	36.8363190
1130.0000000	9.2720003	-8.4305010
1130.0000000	9.3207998	-9.8040724
1130.0000000	9.3695993	-10.7662716
1130.0000000	9.4183998	-11.7525072
1130.0000000	9.4672003	-11.1617842
1130.0000000	9.5159998	-14.1544075
1130.0000000	9.5648003	-0.4301767
1130.0000000	9.6135998	-13.3873119

1130.0000000	9.6623993	-10.2243595
1130.0000000	9.7111998	-10.1606932
1130.0000000	9.7600002	-10.8580341
1130.0000000	9.8087997	-10.9948654
1130.0000000	9.8575993	3.1412573
1130.0000000	9.9063997	-7.9791169
1130.0000000	9.9552002	-10.9066486
1130.0000000	10.0039997	-10.0774422
1140.0000000	0.3416000	-37.3347054
1140.0000000	0.3904000	-39.4274445
1140.0000000	0.4392000	-39.6655540
1140.0000000	0.4880000	-39.5767441
1140.0000000	0.5368000	-39.6278419
1140.0000000	0.5856000	-39.3160744
1140.0000000	0.6344000	-38.2291718
1140.0000000	0.6832000	-36.1926003
1140.0000000	0.7320000	-36.0262566
1140.0000000	0.7808000	-36.5324402
1140.0000000	0.8296000	-35.9832001
1140.0000000	0.8784000	-35.2844391
1140.0000000	0.9272000	-32.9019470
1140.0000000	0.9760000	-30.5170116
1140.0000000	1.0247999	-29.8006039
1140.0000000	1.0735999	-29.5472546
1140.0000000	1.1224000	-29.1293430
1140.0000000	1.1711999	-29.6943741
1140.0000000	1.2200000	-30.9666061
1140.0000000	1.2687999	-30.9109764
1140.0000000	1.3176000	-30.8596458
1140.0000000	1.3664000	-30.6638908
1140.0000000	1.4152000	-30.1647682
1140.0000000	1.4640000	-29.5948048
1140.0000000	1.5128000	-38.2090912
1140.0000000	1.5616000	-33.1281853
1140.0000000	1.6104001	-1526.7033691
1140.0000000	1.6592000	-69.7310104
1140.0000000	1.7079999	-27.3025036
1140.0000000	1.7567999	-27.7623730
1140.0000000	1.8055999	-27.5316391
1140.0000000	1.8544000	-26.9420013
1140.0000000	1.9031999	-26.6665173
1140.0000000	1.9520000	-26.3614922
1140.0000000	2.0007999	-26.3747520
1140.0000000	2.0495999	-26.1925278
1140.0000000	2.0983999	-130.2648315
1140.0000000	2.1471999	-4.8818378
1140.0000000	2.1960001	-25.3994083
1140.0000000	2.2448001	-25.6128254
1140.0000000	2.2935998	-26.9649525
1140.0000000	2.3423998	-21.1627045
1140.0000000	2.3912001	24.4219837
1140.0000000	2.4400001	-22.8673019
1140.0000000	2.4888000	-23.4574833
1140.0000000	2.5375998	-12.4717093
1140.0000000	2.5863998	-10.6237936
1140.0000000	2.6352000	-8.7308722
1140.0000000	2.6840000	-15.5498276
1140.0000000	2.7328000	130.1132965

1140.0000000	2.7815998	153.6138000
1140.0000000	2.8304000	-23.7794800
1140.0000000	2.8792000	-20.6565876
1140.0000000	2.9280000	-22.5652943
1140.0000000	2.9768000	-16.8847675
1140.0000000	3.0256000	-20.5022869
1140.0000000	3.0743999	-29.3871975
1140.0000000	3.1231999	-24.2157841
1140.0000000	3.1719999	-29.5113869
1140.0000000	3.2208002	-501.5277710
1140.0000000	3.2695999	-981.8198242
1140.0000000	3.3183999	-20.4919739
1140.0000000	3.3671999	-3379.1206055
1140.0000000	3.4159999	1212.1241455
1140.0000000	3.4648001	-7.2090149
1140.0000000	3.5135999	-27.0012474
1140.0000000	3.5623999	-320.3935852
1140.0000000	3.6111999	-2012.4941406
1140.0000000	3.6600001	-455.7105713
1140.0000000	3.7088001	-601.6943359
1140.0000000	3.7575998	-941.9754028
1140.0000000	3.8063998	-1709.2181396
1140.0000000	3.8552001	-2481.4802246
1140.0000000	3.9040000	-2432.9233398
1140.0000000	3.9528000	1112.3873291
1140.0000000	4.0015998	-243.1985016
1140.0000000	4.0503998	680.9837646
1140.0000000	4.0991998	86.6185379
1140.0000000	4.1479998	-648.7910156
1140.0000000	4.1967998	2.3431664
1140.0000000	4.2456002	-246.3532715
1140.0000000	4.2943997	-483.5984497
1140.0000000	4.3431997	-13.3792248
1140.0000000	4.3920002	-245.9878693
1140.0000000	4.4407997	-22.4216003
1140.0000000	4.4896002	-15.4936142
1140.0000000	4.5383997	-649.3314819
1140.0000000	4.5871997	-12.8829947
1140.0000000	4.6360002	-21.5871277
1140.0000000	4.6847997	-28.1958961
1140.0000000	4.7336001	-19.5465088
1140.0000000	4.7824001	-16.5223827
1140.0000000	4.8311996	-1462.6044922
1140.0000000	4.8800001	226.2726746
1140.0000000	4.9287996	-199.9617310
1140.0000000	4.9776001	1050.8736572
1140.0000000	5.0264001	85.3433304
1140.0000000	5.0751996	-298.8403015
1140.0000000	5.1240001	-12.3287849
1140.0000000	5.1727996	555.8646240
1140.0000000	5.2216001	31.8356628
1140.0000000	5.2704000	-158.3386688
1140.0000000	5.3191996	-39.1053848
1140.0000000	5.3680000	-3524.1564941
1140.0000000	5.4168000	525.3275146
1140.0000000	5.4656000	-200.1295776
1140.0000000	5.5144000	37.8877487
1140.0000000	5.5631995	486.6680908

1140.0000000	5.6120000	391.7975769
1140.0000000	5.6608000	-37.0161133
1140.0000000	5.7096000	62.2714462
1140.0000000	5.7584000	132.5550385
1140.0000000	5.8071995	-11.5348730
1140.0000000	5.8559999	29.6694126
1140.0000000	5.9047999	-16.6023331
1140.0000000	5.9535999	-39.6975632
1140.0000000	6.0023999	-1567.9412842
1140.0000000	6.0511999	-14.5716724
1140.0000000	6.0999999	-19.3956909
1140.0000000	6.1487999	-13.2808714
1140.0000000	6.1975999	-23.1915989
1140.0000000	6.2463999	-1786.2362061
1140.0000000	6.2951999	-19.0571918
1140.0000000	6.3439999	-341.6833496
1140.0000000	6.3927999	-15.0478287
1140.0000000	6.4416003	-12.3952370
1140.0000000	6.4903998	-11.2742815
1140.0000000	6.5391998	-11.8168049
1140.0000000	6.5879998	374.4201965
1140.0000000	6.6367998	-12.0925436
1140.0000000	6.6856003	-13.8174915
1140.0000000	6.7343998	-13.4588881
1140.0000000	6.7831998	-14.3664103
1140.0000000	6.8319998	58.1595535
1140.0000000	6.8807998	-14.0474033
1140.0000000	6.9296002	-438.5702820
1140.0000000	6.9783998	-5.2668924
1140.0000000	7.0271997	-14.1228704
1140.0000000	7.0760002	10.6006212
1140.0000000	7.1247997	-2077.4025879
1140.0000000	7.1736002	-1665.9464111
1140.0000000	7.2223997	-947.8491821
1140.0000000	7.2711997	-204.6307678
1140.0000000	7.3200002	-18.7210598
1140.0000000	7.3687997	182.8789063
1140.0000000	7.4176002	381.8254089
1140.0000000	7.4663997	299.9188843
1140.0000000	7.5151997	-927.2242432
1140.0000000	7.5640001	-1024.1662598
1140.0000000	7.6127996	-2413.6718750
1140.0000000	7.6616001	-775.0612793
1140.0000000	7.7104001	-398.2254028
1140.0000000	7.7591996	41.9032631
1140.0000000	7.8080001	396.7973022
1140.0000000	7.8567996	522.7905273
1140.0000000	7.9056001	-1084.2291260
1140.0000000	7.9544001	1725.5494385
1140.0000000	8.0031996	2226.5573730
1140.0000000	8.0520000	2222.3344727
1140.0000000	8.1007996	2351.6772461
1140.0000000	8.1496000	2508.0102539
1140.0000000	8.1983995	1560.3127441
1140.0000000	8.2472000	-3570.1279297
1140.0000000	8.2959995	-1265.5618896
1140.0000000	8.3448000	-1008.1026001
1140.0000000	8.3935995	-923.9072266

1140.0000000	8.4423990	-398.0108948
1140.0000000	8.4912004	-929.1076660
1140.0000000	8.5400000	-2506.2187500
1140.0000000	8.5887995	-1214.8884277
1140.0000000	8.6375999	237.2132416
1140.0000000	8.6863995	2143.5695801
1140.0000000	8.7351999	-796.7795410
1140.0000000	8.7840004	910.3166504
1140.0000000	8.8327999	49.1465569
1140.0000000	8.8815994	-11.4986038
1140.0000000	8.9303989	-23.8990593
1140.0000000	8.9792004	-11.5801220
1140.0000000	9.0279999	-5.8586664
1140.0000000	9.0767994	6.9691772
1140.0000000	9.1255999	-17.2210960
1140.0000000	9.1743994	-10.0784836
1140.0000000	9.2231998	36.8205872
1140.0000000	9.2720003	-8.4314375
1140.0000000	9.3207998	-9.8046112
1140.0000000	9.3695993	-10.7664986
1140.0000000	9.4183998	-11.7523823
1140.0000000	9.4672003	-11.1618252
1140.0000000	9.5159998	-14.1535168
1140.0000000	9.5648003	-0.4335499
1140.0000000	9.6135998	-13.3865204
1140.0000000	9.6623993	-10.2246342
1140.0000000	9.7111998	-10.1609650
1140.0000000	9.7600002	-10.8580761
1140.0000000	9.8087997	-10.9948740
1140.0000000	9.8575993	3.1368351
1140.0000000	9.9063997	-7.9800305
1140.0000000	9.9552002	-10.9066534
1140.0000000	10.0039997	-10.0776901
1150.0000000	0.3416000	-36.8572655
1150.0000000	0.3904000	-39.3923645
1150.0000000	0.4392000	-39.6592102
1150.0000000	0.4880000	-39.5755386
1150.0000000	0.5368000	-39.6290169
1150.0000000	0.5856000	-39.3146935
1150.0000000	0.6344000	-38.2294846
1150.0000000	0.6832000	-36.1948242
1150.0000000	0.7320000	-36.0283127
1150.0000000	0.7808000	-36.5326080
1150.0000000	0.8296000	-35.9839287
1150.0000000	0.8784000	-35.2918167
1150.0000000	0.9272000	-32.9047241
1150.0000000	0.9760000	-30.5095596
1150.0000000	1.0247999	-29.8095932
1150.0000000	1.0735999	-29.5616894
1150.0000000	1.1224000	-29.1263428
1150.0000000	1.1711999	-29.6869965
1150.0000000	1.2200000	-30.9745216
1150.0000000	1.2687999	-30.9119835
1150.0000000	1.3176000	-30.8553734
1150.0000000	1.3664000	-30.6716404
1150.0000000	1.4152000	-30.1649723
1150.0000000	1.4640000	-29.5930958
1150.0000000	1.5128000	-38.1119118

1150.0000000	1.5616000	-33.0863419
1150.0000000	1.6104001	-1524.1605225
1150.0000000	1.6592000	-69.4370804
1150.0000000	1.7079999	-27.3062496
1150.0000000	1.7567999	-27.7623920
1150.0000000	1.8055999	-27.3690948
1150.0000000	1.8544000	-27.0630836
1150.0000000	1.9031999	-26.6637230
1150.0000000	1.9520000	-26.3579216
1150.0000000	2.0007999	-26.3678093
1150.0000000	2.0495999	-26.1945477
1150.0000000	2.0983999	-128.7890625
1150.0000000	2.1471999	-5.1491051
1150.0000000	2.1960001	-25.3953171
1150.0000000	2.2448001	-25.5949135
1150.0000000	2.2935998	-26.9053288
1150.0000000	2.3423998	-21.1771107
1150.0000000	2.3912001	23.8004360
1150.0000000	2.4400001	-22.8697891
1150.0000000	2.4888000	-23.4572659
1150.0000000	2.5375998	-12.4888172
1150.0000000	2.5863998	-10.6435528
1150.0000000	2.6352000	-8.7536163
1150.0000000	2.6840000	-15.5617685
1150.0000000	2.7328000	129.8472900
1150.0000000	2.7815998	153.3073730
1150.0000000	2.8304000	-23.7781773
1150.0000000	2.8792000	-20.6598511
1150.0000000	2.9280000	-22.5647240
1150.0000000	2.9768000	-16.8931389
1150.0000000	3.0256000	-20.5048714
1150.0000000	3.0743999	-29.3758106
1150.0000000	3.1231999	-24.2123299
1150.0000000	3.1719999	-29.5030937
1150.0000000	3.2208002	-501.2900391
1150.0000000	3.2695999	-981.3448486
1150.0000000	3.3183999	-20.4921703
1150.0000000	3.3671999	-3377.6589355
1150.0000000	3.4159999	1211.5888672
1150.0000000	3.4648001	-7.2147226
1150.0000000	3.5135999	-26.9982567
1150.0000000	3.5623999	-320.2640076
1150.0000000	3.6111999	-2011.6343994
1150.0000000	3.6600001	-455.5224609
1150.0000000	3.7088001	-601.4429321
1150.0000000	3.7575998	-941.5768433
1150.0000000	3.8063998	-1708.4882813
1150.0000000	3.8552001	-2480.4167480
1150.0000000	3.9040000	-2431.8808594
1150.0000000	3.9528000	1111.8990479
1150.0000000	4.0015998	-243.1013794
1150.0000000	4.0503998	680.6812134
1150.0000000	4.0991998	86.5728531
1150.0000000	4.1479998	-648.5187988
1150.0000000	4.1967998	2.3339100
1150.0000000	4.2456002	-246.2549744
1150.0000000	4.2943997	-483.3978882
1150.0000000	4.3431997	-13.3814049

1150.0000000	4.3920002	-245.8897552
1150.0000000	4.4407997	-22.4198227
1150.0000000	4.4896002	-15.4944963
1150.0000000	4.5383997	-649.0703735
1150.0000000	4.5871997	-12.8850107
1150.0000000	4.6360002	-21.5855865
1150.0000000	4.6847997	-28.1919708
1150.0000000	4.7336001	-19.5457859
1150.0000000	4.7824001	-16.5228386
1150.0000000	4.8311996	-1462.0732422
1150.0000000	4.8800001	226.1831207
1150.0000000	4.9287996	-199.8944702
1150.0000000	4.9776001	1050.4812012
1150.0000000	5.0264001	85.3058090
1150.0000000	5.0751996	-298.7366638
1150.0000000	5.1240001	-12.3297062
1150.0000000	5.1727996	555.7487793
1150.0000000	5.2216001	31.8262215
1150.0000000	5.2704000	-158.3105621
1150.0000000	5.3191996	-39.1008377
1150.0000000	5.3680000	-3523.4641113
1150.0000000	5.4168000	525.2207031
1150.0000000	5.4656000	-200.0933990
1150.0000000	5.5144000	37.8770905
1150.0000000	5.5631995	486.5695190
1150.0000000	5.6120000	391.7175903
1150.0000000	5.6608000	-37.0120239
1150.0000000	5.7096000	62.2560196
1150.0000000	5.7584000	132.5260925
1150.0000000	5.8071995	-11.5357437
1150.0000000	5.8559999	29.6605625
1150.0000000	5.9047999	-16.6021347
1150.0000000	5.9535999	-39.6928253
1150.0000000	6.0023999	-1567.6369629
1150.0000000	6.0511999	-14.5718832
1150.0000000	6.0999999	-19.3948593
1150.0000000	6.1487999	-13.2813339
1150.0000000	6.1975999	-23.1898346
1150.0000000	6.2463999	-1785.8887939
1150.0000000	6.2951999	-19.0563774
1150.0000000	6.3439999	-341.6192932
1150.0000000	6.3927999	-15.0478106
1150.0000000	6.4416003	-12.3958235
1150.0000000	6.4903998	-11.2749577
1150.0000000	6.5391998	-11.8174858
1150.0000000	6.5879998	373.7294922
1150.0000000	6.6367998	-12.0930929
1150.0000000	6.6856003	-13.8176613
1150.0000000	6.7343998	-13.4591045
1150.0000000	6.7831998	-14.3659954
1150.0000000	6.8319998	58.1404343
1150.0000000	6.8807998	-14.0472651
1150.0000000	6.9296002	-437.8088074
1150.0000000	6.9783998	-5.2797160
1150.0000000	7.0271997	-14.1218424
1150.0000000	7.0760002	10.5584364
1150.0000000	7.1247997	-2073.4567871
1150.0000000	7.1736002	-1662.7834473

1150.0000000	7.2223997	-946.0560303
1150.0000000	7.2711997	-204.2629852
1150.0000000	7.3200002	-18.7090721
1150.0000000	7.3687997	182.5043793
1150.0000000	7.4176002	381.0695801
1150.0000000	7.4663997	299.3212280
1150.0000000	7.5151997	-925.4631958
1150.0000000	7.5640001	-1022.2188110
1150.0000000	7.6127996	-2409.0541992
1150.0000000	7.6616001	-773.5899658
1150.0000000	7.7104001	-397.4791260
1150.0000000	7.7591996	41.8022156
1150.0000000	7.8080001	396.0119324
1150.0000000	7.8567996	521.7624512
1150.0000000	7.9056001	-1082.1562500
1150.0000000	7.9544001	1722.2395020
1150.0000000	8.0031996	2222.2878418
1150.0000000	8.0520000	2218.0725098
1150.0000000	8.1007996	2347.1677246
1150.0000000	8.1496000	2503.2021484
1150.0000000	8.1983995	1557.3201904
1150.0000000	8.2472000	-3563.2932129
1150.0000000	8.2959995	-1263.1417236
1150.0000000	8.3448000	-1006.1734009
1150.0000000	8.3935995	-922.1395264
1150.0000000	8.4423990	-397.2528687
1150.0000000	8.4912004	-927.3314819
1150.0000000	8.5400000	-2501.4189453
1150.0000000	8.5887995	-1212.5667725
1150.0000000	8.6375999	236.7519226
1150.0000000	8.6863995	2139.4511719
1150.0000000	8.7351999	-795.2552490
1150.0000000	8.7840004	908.5598755
1150.0000000	8.8327999	49.0349503
1150.0000000	8.8815994	-11.4987783
1150.0000000	8.9303989	-23.8953400
1150.0000000	8.9792004	-11.5802040
1150.0000000	9.0279999	-5.8604345
1150.0000000	9.0767994	6.9634609
1150.0000000	9.1255999	-17.2193813
1150.0000000	9.1743994	-10.0788860
1150.0000000	9.2231998	36.8048553
1150.0000000	9.2720003	-8.4323740
1150.0000000	9.3207998	-9.8051510
1150.0000000	9.3695993	-10.7667246
1150.0000000	9.4183998	-11.7522573
1150.0000000	9.4672003	-11.1618671
1150.0000000	9.5159998	-14.1526251
1150.0000000	9.5648003	-0.4369221
1150.0000000	9.6135998	-13.3857307
1150.0000000	9.6623993	-10.2249088
1150.0000000	9.7111998	-10.1612368
1150.0000000	9.7600002	-10.8581181
1150.0000000	9.8087997	-10.9948816
1150.0000000	9.8575993	3.1324120
1150.0000000	9.9063997	-7.9809446
1150.0000000	9.9552002	-10.9066582
1150.0000000	10.0039997	-10.0779371

1160.0000000	0.3416000	-36.5489082
1160.0000000	0.3904000	-38.2926216
1160.0000000	0.4392000	-39.6528664
1160.0000000	0.4880000	-39.5743294
1160.0000000	0.5368000	-39.6301918
1160.0000000	0.5856000	-39.3133125
1160.0000000	0.6344000	-38.2297935
1160.0000000	0.6832000	-36.1970482
1160.0000000	0.7320000	-36.0303650
1160.0000000	0.7808000	-36.5327759
1160.0000000	0.8296000	-35.9846573
1160.0000000	0.8784000	-35.2991905
1160.0000000	0.9272000	-32.6905899
1160.0000000	0.9760000	-29.9651871
1160.0000000	1.0247999	-27.9246464
1160.0000000	1.0735999	-27.4815578
1160.0000000	1.1224000	-28.2599621
1160.0000000	1.1711999	-29.7721825
1160.0000000	1.2200000	-30.5943031
1160.0000000	1.2687999	-30.2161255
1160.0000000	1.3176000	-30.8511028
1160.0000000	1.3664000	-30.6793900
1160.0000000	1.4152000	-30.1651764
1160.0000000	1.4640000	-29.5913868
1160.0000000	1.5128000	-38.0147324
1160.0000000	1.5616000	-33.0445023
1160.0000000	1.6104001	-1521.6177979
1160.0000000	1.6592000	-69.1431503
1160.0000000	1.7079999	-27.3099976
1160.0000000	1.7567999	-27.7624111
1160.0000000	1.8055999	-27.2065525
1160.0000000	1.8544000	-26.7477150
1160.0000000	1.9031999	-26.7745667
1160.0000000	1.9520000	-26.3543491
1160.0000000	2.0007999	-26.3441601
1160.0000000	2.0495999	-26.1965656
1160.0000000	2.0983999	-127.3132706
1160.0000000	2.1471999	-5.4163723
1160.0000000	2.1960001	-25.3912277
1160.0000000	2.2448001	-25.5770035
1160.0000000	2.2935998	-26.8457069
1160.0000000	2.3423998	-21.1915150
1160.0000000	2.3912001	23.1788921
1160.0000000	2.4400001	-22.8722744
1160.0000000	2.4888000	-23.4570484
1160.0000000	2.5375998	-12.5059252
1160.0000000	2.5863998	-10.6633120
1160.0000000	2.6352000	-8.7763605
1160.0000000	2.6840000	-15.5737104
1160.0000000	2.7328000	129.5812683
1160.0000000	2.7815998	153.0009460
1160.0000000	2.8304000	-23.7768726
1160.0000000	2.8792000	-20.6631145
1160.0000000	2.9280000	-22.5641556
1160.0000000	2.9768000	-16.9015083
1160.0000000	3.0256000	-20.5074558
1160.0000000	3.0743999	-29.3644238
1160.0000000	3.1231999	-24.2088737

1160.0000000	3.1719999	-29.4948006
1160.0000000	3.2208002	-501.0523071
1160.0000000	3.2695999	-980.8698730
1160.0000000	3.3183999	-20.4923687
1160.0000000	3.3671999	-3376.1972656
1160.0000000	3.4159999	1211.0535889
1160.0000000	3.4648001	-7.2204313
1160.0000000	3.5135999	-26.9952660
1160.0000000	3.5623999	-320.1343994
1160.0000000	3.6111999	-2010.7746582
1160.0000000	3.6600001	-455.3343506
1160.0000000	3.7088001	-601.1915283
1160.0000000	3.7575998	-941.1783447
1160.0000000	3.8063998	-1707.7584229
1160.0000000	3.8552001	-2479.3532715
1160.0000000	3.9040000	-2430.8383789
1160.0000000	3.9528000	1111.4107666
1160.0000000	4.0015998	-243.0042725
1160.0000000	4.0503998	680.3786621
1160.0000000	4.0991998	86.5271683
1160.0000000	4.1479998	-648.2465820
1160.0000000	4.1967998	2.3246536
1160.0000000	4.2456002	-246.1566620
1160.0000000	4.2943997	-483.1972961
1160.0000000	4.3431997	-13.3835869
1160.0000000	4.3920002	-245.7916412
1160.0000000	4.4407997	-22.4180489
1160.0000000	4.4896002	-15.4953785
1160.0000000	4.5383997	-648.8093262
1160.0000000	4.5871997	-12.8870277
1160.0000000	4.6360002	-21.5840435
1160.0000000	4.6847997	-28.1880455
1160.0000000	4.7336001	-19.5450649
1160.0000000	4.7824001	-16.5232925
1160.0000000	4.8311996	-1461.5419922
1160.0000000	4.8800001	226.0935669
1160.0000000	4.9287996	-199.8272095
1160.0000000	4.9776001	1050.0886230
1160.0000000	5.0264001	85.2682877
1160.0000000	5.0751996	-298.6330566
1160.0000000	5.1240001	-12.3306274
1160.0000000	5.1727996	555.6328735
1160.0000000	5.2216001	31.8167763
1160.0000000	5.2704000	-158.2824554
1160.0000000	5.3191996	-39.0962906
1160.0000000	5.3680000	-3522.7717285
1160.0000000	5.4168000	525.1139526
1160.0000000	5.4656000	-200.0572052
1160.0000000	5.5144000	37.8664398
1160.0000000	5.5631995	486.4709473
1160.0000000	5.6120000	391.6376038
1160.0000000	5.6608000	-37.0079384
1160.0000000	5.7096000	62.2405853
1160.0000000	5.7584000	132.4971619
1160.0000000	5.8071995	-11.5366144
1160.0000000	5.8559999	29.6517124
1160.0000000	5.9047999	-16.6019382
1160.0000000	5.9535999	-39.6880836

1160.0000000	6.0023999	-1567.3323975
1160.0000000	6.0511999	-14.5720949
1160.0000000	6.0999999	-19.3940258
1160.0000000	6.1487999	-13.2817974
1160.0000000	6.1975999	-23.1880684
1160.0000000	6.2463999	-1785.5413818
1160.0000000	6.2951999	-19.0555630
1160.0000000	6.3439999	-341.5552368
1160.0000000	6.3927999	-15.0477924
1160.0000000	6.4416003	-12.3964100
1160.0000000	6.4903998	-11.2756329
1160.0000000	6.5391998	-11.8181667
1160.0000000	6.5879998	373.0387878
1160.0000000	6.6367998	-12.0936422
1160.0000000	6.6856003	-13.8178320
1160.0000000	6.7343998	-13.4593201
1160.0000000	6.7831998	-14.3655796
1160.0000000	6.8319998	58.1213226
1160.0000000	6.8807998	-14.0471258
1160.0000000	6.9296002	-437.0473633
1160.0000000	6.9783998	-5.2925391
1160.0000000	7.0271997	-14.1208153
1160.0000000	7.0760002	10.5162497
1160.0000000	7.1247997	-2069.5107422
1160.0000000	7.1736002	-1659.6206055
1160.0000000	7.2223997	-944.2628174
1160.0000000	7.2711997	-203.8952026
1160.0000000	7.3200002	-18.6970825
1160.0000000	7.3687997	182.1298523
1160.0000000	7.4176002	380.3137512
1160.0000000	7.4663997	298.7235718
1160.0000000	7.5151997	-923.7021484
1160.0000000	7.5640001	-1020.2714233
1160.0000000	7.6127996	-2404.4365234
1160.0000000	7.6616001	-772.1185913
1160.0000000	7.7104001	-396.7329102
1160.0000000	7.7591996	41.7011681
1160.0000000	7.8080001	395.2265930
1160.0000000	7.8567996	520.7343750
1160.0000000	7.9056001	-1080.0833740
1160.0000000	7.9544001	1718.9295654
1160.0000000	8.0031996	2218.0180664
1160.0000000	8.0520000	2213.8105469
1160.0000000	8.1007996	2342.6582031
1160.0000000	8.1496000	2498.3942871
1160.0000000	8.1983995	1554.3276367
1160.0000000	8.2472000	-3556.4582520
1160.0000000	8.2959995	-1260.7214355
1160.0000000	8.3448000	-1004.2442627
1160.0000000	8.3935995	-920.3718872
1160.0000000	8.4423990	-396.4948730
1160.0000000	8.4912004	-925.5552368
1160.0000000	8.5400000	-2496.6191406
1160.0000000	8.5887995	-1210.2452393
1160.0000000	8.6375999	236.2906036
1160.0000000	8.6863995	2135.3327637
1160.0000000	8.7351999	-793.7310181
1160.0000000	8.7840004	906.8031616

1160.0000000	8.8327999	48.9233398
1160.0000000	8.8815994	-11.4989529
1160.0000000	8.9303989	-23.8916206
1160.0000000	8.9792004	-11.5802860
1160.0000000	9.0279999	-5.8622031
1160.0000000	9.0767994	6.9577427
1160.0000000	9.1255999	-17.2176666
1160.0000000	9.1743994	-10.0792875
1160.0000000	9.2231998	36.7891235
1160.0000000	9.2720003	-8.4333105
1160.0000000	9.3207998	-9.8056898
1160.0000000	9.3695993	-10.7669516
1160.0000000	9.4183998	-11.7521334
1160.0000000	9.4672003	-11.1619081
1160.0000000	9.5159998	-14.1517344
1160.0000000	9.5648003	-0.4402943
1160.0000000	9.6135998	-13.3849392
1160.0000000	9.6623993	-10.2251844
1160.0000000	9.7111998	-10.1615076
1160.0000000	9.7600002	-10.8581600
1160.0000000	9.8087997	-10.9948893
1160.0000000	9.8575993	3.1279898
1160.0000000	9.9063997	-7.9818583
1160.0000000	9.9552002	-10.9066620
1160.0000000	10.0039997	-10.0781851
1170.0000000	0.3416000	-36.2651711
1170.0000000	0.3904000	-38.5821075
1170.0000000	0.4392000	-39.6465225
1170.0000000	0.4880000	-39.5731201
1170.0000000	0.5368000	-39.6313629
1170.0000000	0.5856000	-39.3119278
1170.0000000	0.6344000	-38.2301025
1170.0000000	0.6832000	-36.1992760
1170.0000000	0.7320000	-36.0324173
1170.0000000	0.7808000	-36.5329475
1170.0000000	0.8296000	-35.9853859
1170.0000000	0.8784000	-34.1810188
1170.0000000	0.9272000	-32.2623405
1170.0000000	0.9760000	-29.9584141
1170.0000000	1.0247999	-27.8493671
1170.0000000	1.0735999	-27.6988125
1170.0000000	1.1224000	-28.6244068
1170.0000000	1.1711999	-28.9053001
1170.0000000	1.2200000	-29.3361683
1170.0000000	1.2687999	-30.8119736
1170.0000000	1.3176000	-30.9735661
1170.0000000	1.3664000	-30.6871395
1170.0000000	1.4152000	-30.1653805
1170.0000000	1.4640000	-29.5896759
1170.0000000	1.5128000	-37.9175568
1170.0000000	1.5616000	-33.0026627
1170.0000000	1.6104001	-1519.0749512
1170.0000000	1.6592000	-68.8492203
1170.0000000	1.7079999	-27.3137455
1170.0000000	1.7567999	-27.7624302
1170.0000000	1.8055999	-27.3687878
1170.0000000	1.8544000	-27.1224060
1170.0000000	1.9031999	-26.8854084

1170.0000000	1.9520000	-26.3507786
1170.0000000	2.0007999	-26.3205090
1170.0000000	2.0495999	-26.1985855
1170.0000000	2.0983999	-125.8374939
1170.0000000	2.1471999	-5.6836395
1170.0000000	2.1960001	-25.3871365
1170.0000000	2.2448001	-25.5590916
1170.0000000	2.2935998	-26.7860832
1170.0000000	2.3423998	-21.2059193
1170.0000000	2.3912001	22.5573483
1170.0000000	2.4400001	-22.8747616
1170.0000000	2.4888000	-23.4568310
1170.0000000	2.5375998	-12.5230331
1170.0000000	2.5863998	-10.6830702
1170.0000000	2.6352000	-8.7991056
1170.0000000	2.6840000	-15.5856524
1170.0000000	2.7328000	129.3152618
1170.0000000	2.7815998	152.6945190
1170.0000000	2.8304000	-23.7755680
1170.0000000	2.8792000	-20.6663780
1170.0000000	2.9280000	-22.5635872
1170.0000000	2.9768000	-16.9098778
1170.0000000	3.0256000	-20.5100403
1170.0000000	3.0743999	-29.3530369
1170.0000000	3.1231999	-24.2054176
1170.0000000	3.1719999	-29.4865074
1170.0000000	3.2208002	-500.8145752
1170.0000000	3.2695999	-980.3948975
1170.0000000	3.3183999	-20.4925671
1170.0000000	3.3671999	-3374.7355957
1170.0000000	3.4159999	1210.5183105
1170.0000000	3.4648001	-7.2261391
1170.0000000	3.5135999	-26.9922752
1170.0000000	3.5623999	-320.0048218
1170.0000000	3.6111999	-2009.9149170
1170.0000000	3.6600001	-455.1461792
1170.0000000	3.7088001	-600.9401855
1170.0000000	3.7575998	-940.7797852
1170.0000000	3.8063998	-1707.0285645
1170.0000000	3.8552001	-2478.2897949
1170.0000000	3.9040000	-2429.7958984
1170.0000000	3.9528000	1110.9224854
1170.0000000	4.0015998	-242.9071655
1170.0000000	4.0503998	680.0761108
1170.0000000	4.0991998	86.4814835
1170.0000000	4.1479998	-647.9743652
1170.0000000	4.1967998	2.3153973
1170.0000000	4.2456002	-246.0583649
1170.0000000	4.2943997	-482.9967346
1170.0000000	4.3431997	-13.3857670
1170.0000000	4.3920002	-245.6935272
1170.0000000	4.4407997	-22.4162731
1170.0000000	4.4896002	-15.4962616
1170.0000000	4.5383997	-648.5482788
1170.0000000	4.5871997	-12.8890438
1170.0000000	4.6360002	-21.5825005
1170.0000000	4.6847997	-28.1841202
1170.0000000	4.7336001	-19.5443420

1170.0000000	4.7824001	-16.5237465
1170.0000000	4.8311996	-1461.0106201
1170.0000000	4.8800001	226.0040283
1170.0000000	4.9287996	-199.7599487
1170.0000000	4.9776001	1049.6961670
1170.0000000	5.0264001	85.2307663
1170.0000000	5.0751996	-298.5294189
1170.0000000	5.1240001	-12.3315487
1170.0000000	5.1727996	555.5170288
1170.0000000	5.2216001	31.8073311
1170.0000000	5.2704000	-158.2543488
1170.0000000	5.3191996	-39.0917435
1170.0000000	5.3680000	-3522.0793457
1170.0000000	5.4168000	525.0071411
1170.0000000	5.4656000	-200.0210266
1170.0000000	5.5144000	37.8557816
1170.0000000	5.5631995	486.3723755
1170.0000000	5.6120000	391.5576477
1170.0000000	5.6608000	-37.0038490
1170.0000000	5.7096000	62.2251511
1170.0000000	5.7584000	132.4682312
1170.0000000	5.8071995	-11.5374851
1170.0000000	5.8559999	29.6428585
1170.0000000	5.9047999	-16.6017399
1170.0000000	5.9535999	-39.6833458
1170.0000000	6.0023999	-1567.0280762
1170.0000000	6.0511999	-14.5723066
1170.0000000	6.0999999	-19.3931942
1170.0000000	6.1487999	-13.2822599
1170.0000000	6.1975999	-23.1863041
1170.0000000	6.2463999	-1785.1940918
1170.0000000	6.2951999	-19.0547485
1170.0000000	6.3439999	-341.4911804
1170.0000000	6.3927999	-15.0477734
1170.0000000	6.4416003	-12.3969975
1170.0000000	6.4903998	-11.2763090
1170.0000000	6.5391998	-11.8188486
1170.0000000	6.5879998	372.3480835
1170.0000000	6.6367998	-12.0941916
1170.0000000	6.6856003	-13.8180017
1170.0000000	6.7343998	-13.4595356
1170.0000000	6.7831998	-14.3651638
1170.0000000	6.8319998	58.1022034
1170.0000000	6.8807998	-14.0469875
1170.0000000	6.9296002	-436.2859192
1170.0000000	6.9783998	-5.3053632
1170.0000000	7.0271997	-14.1197872
1170.0000000	7.0760002	10.4740648
1170.0000000	7.1247997	-2065.5649414
1170.0000000	7.1736002	-1656.4577637
1170.0000000	7.2223997	-942.4696655
1170.0000000	7.2711997	-203.5274353
1170.0000000	7.3200002	-18.6850929
1170.0000000	7.3687997	181.7553101
1170.0000000	7.4176002	379.5579224
1170.0000000	7.4663997	298.1259155
1170.0000000	7.5151997	-921.9411011
1170.0000000	7.5640001	-1018.3240356

1170.0000000	7.6127996	-2399.8190918
1170.0000000	7.6616001	-770.6473389
1170.0000000	7.7104001	-395.9866333
1170.0000000	7.7591996	41.6001167
1170.0000000	7.8080001	394.4412231
1170.0000000	7.8567996	519.7062988
1170.0000000	7.9056001	-1078.0104980
1170.0000000	7.9544001	1715.6196289
1170.0000000	8.0031996	2213.7485352
1170.0000000	8.0520000	2209.5485840
1170.0000000	8.1007996	2338.1486816
1170.0000000	8.1496000	2493.5861816
1170.0000000	8.1983995	1551.3352051
1170.0000000	8.2472000	-3549.6235352
1170.0000000	8.2959995	-1258.3012695
1170.0000000	8.3448000	-1002.3150635
1170.0000000	8.3935995	-918.6041870
1170.0000000	8.4423990	-395.7368469
1170.0000000	8.4912004	-923.7789917
1170.0000000	8.5400000	-2491.8193359
1170.0000000	8.5887995	-1207.9237061
1170.0000000	8.6375999	235.8292847
1170.0000000	8.6863995	2131.2145996
1170.0000000	8.7351999	-792.2067261
1170.0000000	8.7840004	905.0463867
1170.0000000	8.8327999	48.8117371
1170.0000000	8.8815994	-11.4991274
1170.0000000	8.9303989	-23.8878994
1170.0000000	8.9792004	-11.5803690
1170.0000000	9.0279999	-5.8639717
1170.0000000	9.0767994	6.9520245
1170.0000000	9.1255999	-17.2159538
1170.0000000	9.1743994	-10.0796900
1170.0000000	9.2231998	36.7733917
1170.0000000	9.2720003	-8.4342461
1170.0000000	9.3207998	-9.8062296
1170.0000000	9.3695993	-10.7671776
1170.0000000	9.4183998	-11.7520084
1170.0000000	9.4672003	-11.1619492
1170.0000000	9.5159998	-14.1508436
1170.0000000	9.5648003	-0.4436674
1170.0000000	9.6135998	-13.3841496
1170.0000000	9.6623993	-10.2254591
1170.0000000	9.7111998	-10.1617794
1170.0000000	9.7600002	-10.8582010
1170.0000000	9.8087997	-10.9948978
1170.0000000	9.8575993	3.1235676
1170.0000000	9.9063997	-7.9827719
1170.0000000	9.9552002	-10.9066668
1170.0000000	10.0039997	-10.0784321
1180.0000000	0.3416000	-36.5890770
1180.0000000	0.3904000	-38.2559929
1180.0000000	0.4392000	-39.6401787
1180.0000000	0.4880000	-39.5719109
1180.0000000	0.5368000	-39.6325378
1180.0000000	0.5856000	-39.3105469
1180.0000000	0.6344000	-38.2304153
1180.0000000	0.6832000	-36.2014999

1180.0000000	0.7320000	-36.0344696
1180.0000000	0.7808000	-36.5331154
1180.0000000	0.8296000	-35.9835396
1180.0000000	0.8784000	-35.1883583
1180.0000000	0.9272000	-32.3783379
1180.0000000	0.9760000	-29.9758778
1180.0000000	1.0247999	-28.8884773
1180.0000000	1.0735999	-27.9462814
1180.0000000	1.1224000	-28.3821354
1180.0000000	1.1711999	-29.9498405
1180.0000000	1.2200000	-30.0035419
1180.0000000	1.2687999	-30.8449287
1180.0000000	1.3176000	-31.0960293
1180.0000000	1.3664000	-30.6948891
1180.0000000	1.4152000	-30.1655865
1180.0000000	1.4640000	-29.5879669
1180.0000000	1.5128000	-37.8203773
1180.0000000	1.5616000	-32.9608192
1180.0000000	1.6104001	-1516.5321045
1180.0000000	1.6592000	-68.5552826
1180.0000000	1.7079999	-27.3174915
1180.0000000	1.7567999	-27.7624493
1180.0000000	1.8055999	-27.5310211
1180.0000000	1.8544000	-27.4418621
1180.0000000	1.9031999	-26.8001232
1180.0000000	1.9520000	-26.3472061
1180.0000000	2.0007999	-26.2968597
1180.0000000	2.0495999	-26.2006035
1180.0000000	2.0983999	-124.3617172
1180.0000000	2.1471999	-5.9509068
1180.0000000	2.1960001	-25.3830452
1180.0000000	2.2448001	-25.5411797
1180.0000000	2.2935998	-26.7264595
1180.0000000	2.3423998	-21.2203236
1180.0000000	2.3912001	21.9358006
1180.0000000	2.4400001	-22.8772488
1180.0000000	2.4888000	-23.4566154
1180.0000000	2.5375998	-12.5401411
1180.0000000	2.5863998	-10.7028294
1180.0000000	2.6352000	-8.8218498
1180.0000000	2.6840000	-15.5975943
1180.0000000	2.7328000	129.0492401
1180.0000000	2.7815998	152.3881226
1180.0000000	2.8304000	-23.7742634
1180.0000000	2.8792000	-20.6696415
1180.0000000	2.9280000	-22.5630169
1180.0000000	2.9768000	-16.9182491
1180.0000000	3.0256000	-20.5126247
1180.0000000	3.0743999	-29.3416500
1180.0000000	3.1231999	-24.2019634
1180.0000000	3.1719999	-29.4782143
1180.0000000	3.2208002	-500.5768433
1180.0000000	3.2695999	-979.9199219
1180.0000000	3.3183999	-20.4927654
1180.0000000	3.3671999	-3373.2739258
1180.0000000	3.4159999	1209.9830322
1180.0000000	3.4648001	-7.2318478
1180.0000000	3.5135999	-26.9892845

1180.0000000	3.5623999	-319.8752136
1180.0000000	3.6111999	-2009.0550537
1180.0000000	3.6600001	-454.9580688
1180.0000000	3.7088001	-600.6887817
1180.0000000	3.7575998	-940.3812866
1180.0000000	3.8063998	-1706.2987061
1180.0000000	3.8552001	-2477.2265625
1180.0000000	3.9040000	-2428.7534180
1180.0000000	3.9528000	1110.4340820
1180.0000000	4.0015998	-242.8100586
1180.0000000	4.0503998	679.7735596
1180.0000000	4.0991998	86.4357986
1180.0000000	4.1479998	-647.7021484
1180.0000000	4.1967998	2.3061409
1180.0000000	4.2456002	-245.9600677
1180.0000000	4.2943997	-482.7961731
1180.0000000	4.3431997	-13.3879471
1180.0000000	4.3920002	-245.5954132
1180.0000000	4.4407997	-22.4144974
1180.0000000	4.4896002	-15.4971447
1180.0000000	4.5383997	-648.2871704
1180.0000000	4.5871997	-12.8910599
1180.0000000	4.6360002	-21.5809593
1180.0000000	4.6847997	-28.1801949
1180.0000000	4.7336001	-19.5436192
1180.0000000	4.7824001	-16.5242004
1180.0000000	4.8311996	-1460.4793701
1180.0000000	4.8800001	225.9144745
1180.0000000	4.9287996	-199.6926880
1180.0000000	4.9776001	1049.3037109
1180.0000000	5.0264001	85.1932449
1180.0000000	5.0751996	-298.4257813
1180.0000000	5.1240001	-12.3324699
1180.0000000	5.1727996	555.4011230
1180.0000000	5.2216001	31.7978897
1180.0000000	5.2704000	-158.2262421
1180.0000000	5.3191996	-39.0871887
1180.0000000	5.3680000	-3521.3869629
1180.0000000	5.4168000	524.9003906
1180.0000000	5.4656000	-199.9848328
1180.0000000	5.5144000	37.8451309
1180.0000000	5.5631995	486.2738037
1180.0000000	5.6120000	391.4776611
1180.0000000	5.6608000	-36.9997635
1180.0000000	5.7096000	62.2097244
1180.0000000	5.7584000	132.4393005
1180.0000000	5.8071995	-11.5383549
1180.0000000	5.8559999	29.6340084
1180.0000000	5.9047999	-16.6015415
1180.0000000	5.9535999	-39.6786079
1180.0000000	6.0023999	-1566.7235107
1180.0000000	6.0511999	-14.5725174
1180.0000000	6.0999999	-19.3923626
1180.0000000	6.1487999	-13.2827225
1180.0000000	6.1975999	-23.1845379
1180.0000000	6.2463999	-1784.8466797
1180.0000000	6.2951999	-19.0539360
1180.0000000	6.3439999	-341.4271545

1180.0000000	6.3927999	-15.0477552
1180.0000000	6.4416003	-12.3975840
1180.0000000	6.4903998	-11.2769852
1180.0000000	6.5391998	-11.8195295
1180.0000000	6.5879998	371.6574097
1180.0000000	6.6367998	-12.0947399
1180.0000000	6.6856003	-13.8181725
1180.0000000	6.7343998	-13.4597521
1180.0000000	6.7831998	-14.3647480
1180.0000000	6.8319998	58.0830917
1180.0000000	6.8807998	-14.0468483
1180.0000000	6.9296002	-435.5244751
1180.0000000	6.9783998	-5.3181868
1180.0000000	7.0271997	-14.1187592
1180.0000000	7.0760002	10.4318800
1180.0000000	7.1247997	-2061.6191406
1180.0000000	7.1736002	-1653.2949219
1180.0000000	7.2223997	-940.6765137
1180.0000000	7.2711997	-203.1596527
1180.0000000	7.3200002	-18.6731052
1180.0000000	7.3687997	181.3807831
1180.0000000	7.4176002	378.8020935
1180.0000000	7.4663997	297.5282593
1180.0000000	7.5151997	-920.1801147
1180.0000000	7.5640001	-1016.3766479
1180.0000000	7.6127996	-2395.2014160
1180.0000000	7.6616001	-769.1759644
1180.0000000	7.7104001	-395.2403564
1180.0000000	7.7591996	41.4990692
1180.0000000	7.8080001	393.6558533
1180.0000000	7.8567996	518.6782227
1180.0000000	7.9056001	-1075.9375000
1180.0000000	7.9544001	1712.3096924
1180.0000000	8.0031996	2209.4790039
1180.0000000	8.0520000	2205.2866211
1180.0000000	8.1007996	2333.6391602
1180.0000000	8.1496000	2488.7783203
1180.0000000	8.1983995	1548.3426514
1180.0000000	8.2472000	-3542.7888184
1180.0000000	8.2959995	-1255.8811035
1180.0000000	8.3448000	-1000.3858643
1180.0000000	8.3935995	-916.8365479
1180.0000000	8.4423990	-394.9788513
1180.0000000	8.4912004	-922.0028076
1180.0000000	8.5400000	-2487.0195313
1180.0000000	8.5887995	-1205.6021729
1180.0000000	8.6375999	235.3679657
1180.0000000	8.6863995	2127.0961914
1180.0000000	8.7351999	-790.6824951
1180.0000000	8.7840004	903.2896729
1180.0000000	8.8327999	48.7001266
1180.0000000	8.8815994	-11.4993019
1180.0000000	8.9303989	-23.8841782
1180.0000000	8.9792004	-11.5804510
1180.0000000	9.0279999	-5.8657398
1180.0000000	9.0767994	6.9463062
1180.0000000	9.1255999	-17.2142391
1180.0000000	9.1743994	-10.0800915

1180.0000000	9.2231998	36.7576523
1180.0000000	9.2720003	-8.4351826
1180.0000000	9.3207998	-9.8067684
1180.0000000	9.3695993	-10.7674046
1180.0000000	9.4183998	-11.7518845
1180.0000000	9.4672003	-11.1619902
1180.0000000	9.5159998	-14.1499519
1180.0000000	9.5648003	-0.4470406
1180.0000000	9.6135998	-13.3833580
1180.0000000	9.6623993	-10.2257347
1180.0000000	9.7111998	-10.1620512
1180.0000000	9.7600002	-10.8582430
1180.0000000	9.8087997	-10.9949055
1180.0000000	9.8575993	3.1191444
1180.0000000	9.9063997	-7.9836860
1180.0000000	9.9552002	-10.9066706
1180.0000000	10.0039997	-10.0786800
1190.0000000	0.3416000	-36.6341782
1190.0000000	0.3904000	-38.9014740
1190.0000000	0.4392000	-39.6338348
1190.0000000	0.4880000	-39.5707016
1190.0000000	0.5368000	-39.6337128
1190.0000000	0.5856000	-39.3091660
1190.0000000	0.6344000	-38.2307243
1190.0000000	0.6832000	-36.2037277
1190.0000000	0.7320000	-36.0365219
1190.0000000	0.7808000	-36.5332832
1190.0000000	0.8296000	-35.9816971
1190.0000000	0.8784000	-35.1626816
1190.0000000	0.9272000	-32.8639603
1190.0000000	0.9760000	-30.1228580
1190.0000000	1.0247999	-28.3632240
1190.0000000	1.0735999	-28.6987286
1190.0000000	1.1224000	-29.1637211
1190.0000000	1.1711999	-29.2604504
1190.0000000	1.2200000	-29.7127399
1190.0000000	1.2687999	-31.2440529
1190.0000000	1.3176000	-31.0896435
1190.0000000	1.3664000	-30.6919460
1190.0000000	1.4152000	-30.1657906
1190.0000000	1.4640000	-29.5862579
1190.0000000	1.5128000	-37.7232018
1190.0000000	1.5616000	-32.9189796
1190.0000000	1.6104001	-1513.9893799
1190.0000000	1.6592000	-68.2613525
1190.0000000	1.7079999	-27.3212395
1190.0000000	1.7567999	-27.7624683
1190.0000000	1.8055999	-27.6932564
1190.0000000	1.8544000	-27.3746529
1190.0000000	1.9031999	-26.7148399
1190.0000000	1.9520000	-26.3436337
1190.0000000	2.0007999	-26.2732086
1190.0000000	2.0495999	-26.2026234
1190.0000000	2.0983999	-122.8859329
1190.0000000	2.1471999	-6.2181740
1190.0000000	2.1960001	-25.3789539
1190.0000000	2.2448001	-25.5232697
1190.0000000	2.2935998	-26.6668358

1190.0000000	2.3423998	-21.2347279
1190.0000000	2.3912001	21.3142529
1190.0000000	2.4400001	-22.8797340
1190.0000000	2.4888000	-23.4563980
1190.0000000	2.5375998	-12.5572481
1190.0000000	2.5863998	-10.7225885
1190.0000000	2.6352000	-8.8445940
1190.0000000	2.6840000	-15.6095362
1190.0000000	2.7328000	128.7832184
1190.0000000	2.7815998	152.0816956
1190.0000000	2.8304000	-23.7729607
1190.0000000	2.8792000	-20.6729050
1190.0000000	2.9280000	-22.5624485
1190.0000000	2.9768000	-16.9266205
1190.0000000	3.0256000	-20.5152092
1190.0000000	3.0743999	-29.3302631
1190.0000000	3.1231999	-24.1985073
1190.0000000	3.1719999	-29.4699211
1190.0000000	3.2208002	-500.3391113
1190.0000000	3.2695999	-979.4449463
1190.0000000	3.3183999	-20.4929638
1190.0000000	3.3671999	-3371.8122559
1190.0000000	3.4159999	1209.4477539
1190.0000000	3.4648001	-7.2375565
1190.0000000	3.5135999	-26.9862938
1190.0000000	3.5623999	-319.7456360
1190.0000000	3.6111999	-2008.1953125
1190.0000000	3.6600001	-454.7699585
1190.0000000	3.7088001	-600.4373779
1190.0000000	3.7575998	-939.9827271
1190.0000000	3.8063998	-1705.5688477
1190.0000000	3.8552001	-2476.1630859
1190.0000000	3.9040000	-2427.7109375
1190.0000000	3.9528000	1109.9458008
1190.0000000	4.0015998	-242.7129517
1190.0000000	4.0503998	679.4710083
1190.0000000	4.0991998	86.3901138
1190.0000000	4.1479998	-647.4299316
1190.0000000	4.1967998	2.2968845
1190.0000000	4.2456002	-245.8617554
1190.0000000	4.2943997	-482.5956116
1190.0000000	4.3431997	-13.3901281
1190.0000000	4.3920002	-245.4972992
1190.0000000	4.4407997	-22.4127216
1190.0000000	4.4896002	-15.4980268
1190.0000000	4.5383997	-648.0261230
1190.0000000	4.5871997	-12.8930759
1190.0000000	4.6360002	-21.5794163
1190.0000000	4.6847997	-28.1762695
1190.0000000	4.7336001	-19.5428963
1190.0000000	4.7824001	-16.5246563
1190.0000000	4.8311996	-1459.9481201
1190.0000000	4.8800001	225.8249207
1190.0000000	4.9287996	-199.6254120
1190.0000000	4.9776001	1048.9111328
1190.0000000	5.0264001	85.1557312
1190.0000000	5.0751996	-298.3221741
1190.0000000	5.1240001	-12.3333912

1190.0000000	5.1727996	555.2852783
1190.0000000	5.2216001	31.7884445
1190.0000000	5.2704000	-158.1981354
1190.0000000	5.3191996	-39.0826416
1190.0000000	5.3680000	-3520.6945801
1190.0000000	5.4168000	524.7935791
1190.0000000	5.4656000	-199.9486542
1190.0000000	5.5144000	37.8344727
1190.0000000	5.5631995	486.1752319
1190.0000000	5.6120000	391.3976746
1190.0000000	5.6608000	-36.9956741
1190.0000000	5.7096000	62.1942902
1190.0000000	5.7584000	132.4103699
1190.0000000	5.8071995	-11.5392265
1190.0000000	5.8559999	29.6251545
1190.0000000	5.9047999	-16.6013451
1190.0000000	5.9535999	-39.6738701
1190.0000000	6.0023999	-1566.4191895
1190.0000000	6.0511999	-14.5727291
1190.0000000	6.0999999	-19.3915291
1190.0000000	6.1487999	-13.2831860
1190.0000000	6.1975999	-23.1827736
1190.0000000	6.2463999	-1784.4992676
1190.0000000	6.2951999	-19.0531216
1190.0000000	6.3439999	-341.3630981
1190.0000000	6.3927999	-15.0477371
1190.0000000	6.4416003	-12.3981714
1190.0000000	6.4903998	-11.2776613
1190.0000000	6.5391998	-11.8202105
1190.0000000	6.5879998	370.9667053
1190.0000000	6.6367998	-12.0952892
1190.0000000	6.6856003	-13.8183422
1190.0000000	6.7343998	-13.4599676
1190.0000000	6.7831998	-14.3643322
1190.0000000	6.8319998	58.0639725
1190.0000000	6.8807998	-14.0467100
1190.0000000	6.9296002	-434.7630310
1190.0000000	6.9783998	-5.3310099
1190.0000000	7.0271997	-14.1177311
1190.0000000	7.0760002	10.3896933
1190.0000000	7.1247997	-2057.6730957
1190.0000000	7.1736002	-1650.1320801
1190.0000000	7.2223997	-938.8833618
1190.0000000	7.2711997	-202.7918701
1190.0000000	7.3200002	-18.6611176
1190.0000000	7.3687997	181.0062561
1190.0000000	7.4176002	378.0462646
1190.0000000	7.4663997	296.9306335
1190.0000000	7.5151997	-918.4190674
1190.0000000	7.5640001	-1014.4291992
1190.0000000	7.6127996	-2390.5837402
1190.0000000	7.6616001	-767.7045898
1190.0000000	7.7104001	-394.4940796
1190.0000000	7.7591996	41.3980217
1190.0000000	7.8080001	392.8705139
1190.0000000	7.8567996	517.6500854
1190.0000000	7.9056001	-1073.8646240
1190.0000000	7.9544001	1708.9997559

1190.0000000	8.0031996	2205.2092285
1190.0000000	8.0520000	2201.0246582
1190.0000000	8.1007996	2329.1296387
1190.0000000	8.1496000	2483.9702148
1190.0000000	8.1983995	1545.3500977
1190.0000000	8.2472000	-3535.9538574
1190.0000000	8.2959995	-1253.4609375
1190.0000000	8.3448000	-998.4567261
1190.0000000	8.3935995	-915.0688477
1190.0000000	8.4423990	-394.2208252
1190.0000000	8.4912004	-920.2265625
1190.0000000	8.5400000	-2482.2197266
1190.0000000	8.5887995	-1203.2806396
1190.0000000	8.6375999	234.9066467
1190.0000000	8.6863995	2122.9777832
1190.0000000	8.7351999	-789.1582642
1190.0000000	8.7840004	901.5328979
1190.0000000	8.8327999	48.5885201
1190.0000000	8.8815994	-11.4994755
1190.0000000	8.9303989	-23.8804588
1190.0000000	8.9792004	-11.5805330
1190.0000000	9.0279999	-5.8675079
1190.0000000	9.0767994	6.9405899
1190.0000000	9.1255999	-17.2125244
1190.0000000	9.1743994	-10.0804939
1190.0000000	9.2231998	36.7419205
1190.0000000	9.2720003	-8.4361191
1190.0000000	9.3207998	-9.8073082
1190.0000000	9.3695993	-10.7676306
1190.0000000	9.4183998	-11.7517595
1190.0000000	9.4672003	-11.1620312
1190.0000000	9.5159998	-14.1490612
1190.0000000	9.5648003	-0.4504128
1190.0000000	9.6135998	-13.3825674
1190.0000000	9.6623993	-10.2260094
1190.0000000	9.7111998	-10.1623230
1190.0000000	9.7600002	-10.8582850
1190.0000000	9.8087997	-10.9949131
1190.0000000	9.8575993	3.1147223
1190.0000000	9.9063997	-7.9846001
1190.0000000	9.9552002	-10.9066753
1190.0000000	10.0039997	-10.0789270
1200.0000000	0.3416000	-36.6668739
1200.0000000	0.3904000	-39.3465614
1200.0000000	0.4392000	-39.6274910
1200.0000000	0.4880000	-39.5694923
1200.0000000	0.5368000	-39.6348839
1200.0000000	0.5856000	-39.3077850
1200.0000000	0.6344000	-38.2310371
1200.0000000	0.6832000	-36.2059517
1200.0000000	0.7320000	-36.0385742
1200.0000000	0.7808000	-36.5334549
1200.0000000	0.8296000	-35.9798508
1200.0000000	0.8784000	-35.1370049
1200.0000000	0.9272000	-33.0260048
1200.0000000	0.9760000	-30.8689518
1200.0000000	1.0247999	-29.0117607
1200.0000000	1.0735999	-28.7496204

1200.0000000	1.1224000	-29.4498787
1200.0000000	1.1711999	-30.0058689
1200.0000000	1.2200000	-31.0923672
1200.0000000	1.2687999	-31.3803005
1200.0000000	1.3176000	-31.0832577
1200.0000000	1.3664000	-30.6890011
1200.0000000	1.4152000	-30.1659946
1200.0000000	1.4640000	-29.5845490
1200.0000000	1.5128000	-37.6260223
1200.0000000	1.5616000	-32.8771362
1200.0000000	1.6104001	-1511.4465332
1200.0000000	1.6592000	-67.9674225
1200.0000000	1.7079999	-27.3249855
1200.0000000	1.7567999	-27.7624874
1200.0000000	1.8055999	-27.6979580
1200.0000000	1.8544000	-27.3074417
1200.0000000	1.9031999	-26.6295547
1200.0000000	1.9520000	-26.3400631
1200.0000000	2.0007999	-26.2495594
1200.0000000	2.0495999	-26.2046413
1200.0000000	2.0983999	-121.4101563
1200.0000000	2.1471999	-6.4854412
1200.0000000	2.1960001	-25.3748627
1200.0000000	2.2448001	-25.5053577
1200.0000000	2.2935998	-26.6072121
1200.0000000	2.3423998	-21.2491322
1200.0000000	2.3912001	20.6927090
1200.0000000	2.4400001	-22.8822212
1200.0000000	2.4888000	-23.4561806
1200.0000000	2.5375998	-12.5743561
1200.0000000	2.5863998	-10.7423477
1200.0000000	2.6352000	-8.8673382
1200.0000000	2.6840000	-15.6214771
1200.0000000	2.7328000	128.5172119
1200.0000000	2.7815998	151.7752686
1200.0000000	2.8304000	-23.7716560
1200.0000000	2.8792000	-20.6761684
1200.0000000	2.9280000	-22.5618801
1200.0000000	2.9768000	-16.9349899
1200.0000000	3.0256000	-20.5177937
1200.0000000	3.0743999	-29.3188763
1200.0000000	3.1231999	-24.1950531
1200.0000000	3.1719999	-29.4616280
1200.0000000	3.2208002	-500.1013794
1200.0000000	3.2695999	-978.9699707
1200.0000000	3.3183999	-20.4931602
1200.0000000	3.3671999	-3370.3505859
1200.0000000	3.4159999	1208.9124756
1200.0000000	3.4648001	-7.2432642
1200.0000000	3.5135999	-26.9833031
1200.0000000	3.5623999	-319.6160583
1200.0000000	3.6111999	-2007.3355713
1200.0000000	3.6600001	-454.5817871
1200.0000000	3.7088001	-600.1860352
1200.0000000	3.7575998	-939.5841675
1200.0000000	3.8063998	-1704.8389893
1200.0000000	3.8552001	-2475.0996094
1200.0000000	3.9040000	-2426.6684570

1200.0000000	3.9528000	1109.4575195
1200.0000000	4.0015998	-242.6158447
1200.0000000	4.0503998	679.1683960
1200.0000000	4.0991998	86.3444366
1200.0000000	4.1479998	-647.1577148
1200.0000000	4.1967998	2.2876282
1200.0000000	4.2456002	-245.7634583
1200.0000000	4.2943997	-482.3950500
1200.0000000	4.3431997	-13.3923092
1200.0000000	4.3920002	-245.3991852
1200.0000000	4.4407997	-22.4109459
1200.0000000	4.4896002	-15.4989090
1200.0000000	4.5383997	-647.7650146
1200.0000000	4.5871997	-12.8950920
1200.0000000	4.6360002	-21.5778732
1200.0000000	4.6847997	-28.1723442
1200.0000000	4.7336001	-19.5421753
1200.0000000	4.7824001	-16.5251102
1200.0000000	4.8311996	-1459.4168701
1200.0000000	4.8800001	225.7353821
1200.0000000	4.9287996	-199.5581512
1200.0000000	4.9776001	1048.5186768
1200.0000000	5.0264001	85.1182098
1200.0000000	5.0751996	-298.2185364
1200.0000000	5.1240001	-12.3343124
1200.0000000	5.1727996	555.1694336
1200.0000000	5.2216001	31.7789993
1200.0000000	5.2704000	-158.1700287
1200.0000000	5.3191996	-39.0780945
1200.0000000	5.3680000	-3520.0021973
1200.0000000	5.4168000	524.6868286
1200.0000000	5.4656000	-199.9124756
1200.0000000	5.5144000	37.8238144
1200.0000000	5.5631995	486.0766602
1200.0000000	5.6120000	391.3177185
1200.0000000	5.6608000	-36.9915848
1200.0000000	5.7096000	62.1788559
1200.0000000	5.7584000	132.3814392
1200.0000000	5.8071995	-11.5400963
1200.0000000	5.8559999	29.6163044
1200.0000000	5.9047999	-16.6011467
1200.0000000	5.9535999	-39.6691284
1200.0000000	6.0023999	-1566.1147461
1200.0000000	6.0511999	-14.5729408
1200.0000000	6.0999999	-19.3906975
1200.0000000	6.1487999	-13.2836485
1200.0000000	6.1975999	-23.1810074
1200.0000000	6.2463999	-1784.1518555
1200.0000000	6.2951999	-19.0523071
1200.0000000	6.3439999	-341.2990417
1200.0000000	6.3927999	-15.0477190
1200.0000000	6.4416003	-12.3987579
1200.0000000	6.4903998	-11.2783365
1200.0000000	6.5391998	-11.8208914
1200.0000000	6.5879998	370.2760010
1200.0000000	6.6367998	-12.0958385
1200.0000000	6.6856003	-13.8185129
1200.0000000	6.7343998	-13.4601831

1200.0000000	6.7831998	-14.3639164
1200.0000000	6.8319998	58.0448608
1200.0000000	6.8807998	-14.0465708
1200.0000000	6.9296002	-434.0015869
1200.0000000	6.9783998	-5.3438334
1200.0000000	7.0271997	-14.1167030
1200.0000000	7.0760002	10.3475084
1200.0000000	7.1247997	-2053.7272949
1200.0000000	7.1736002	-1646.9691162
1200.0000000	7.2223997	-937.0902100
1200.0000000	7.2711997	-202.4241028
1200.0000000	7.3200002	-18.6491280
1200.0000000	7.3687997	180.6317139
1200.0000000	7.4176002	377.2904358
1200.0000000	7.4663997	296.3329773
1200.0000000	7.5151997	-916.6580200
1200.0000000	7.5640001	-1012.4818115
1200.0000000	7.6127996	-2385.9660645
1200.0000000	7.6616001	-766.2332764
1200.0000000	7.7104001	-393.7478027
1200.0000000	7.7591996	41.2969704
1200.0000000	7.8080001	392.0851440
1200.0000000	7.8567996	516.6220093
1200.0000000	7.9056001	-1071.7917480
1200.0000000	7.9544001	1705.6898193
1200.0000000	8.0031996	2200.9396973
1200.0000000	8.0520000	2196.7626953
1200.0000000	8.1007996	2324.6201172
1200.0000000	8.1496000	2479.1621094
1200.0000000	8.1983995	1542.3576660
1200.0000000	8.2472000	-3529.1191406
1200.0000000	8.2959995	-1251.0407715
1200.0000000	8.3448000	-996.5275269
1200.0000000	8.3935995	-913.3012085
1200.0000000	8.4423990	-393.4628296
1200.0000000	8.4912004	-918.4503784
1200.0000000	8.5400000	-2477.4199219
1200.0000000	8.5887995	-1200.9591064
1200.0000000	8.6375999	234.4453125
1200.0000000	8.6863995	2118.8593750
1200.0000000	8.7351999	-787.6339722
1200.0000000	8.7840004	899.7761841
1200.0000000	8.8327999	48.4769096
1200.0000000	8.8815994	-11.4996500
1200.0000000	8.9303989	-23.8767395
1200.0000000	8.9792004	-11.5806160
1200.0000000	9.0279999	-5.8692760
1200.0000000	9.0767994	6.9348717
1200.0000000	9.1255999	-17.2108116
1200.0000000	9.1743994	-10.0808964
1200.0000000	9.2231998	36.7261887
1200.0000000	9.2720003	-8.4370556
1200.0000000	9.3207998	-9.8078470
1200.0000000	9.3695993	-10.7678566
1200.0000000	9.4183998	-11.7516346
1200.0000000	9.4672003	-11.1620731
1200.0000000	9.5159998	-14.1481705
1200.0000000	9.5648003	-0.4537849

1200.0000000	9.6135998	-13.3817768
1200.0000000	9.6623993	-10.2262840
1200.0000000	9.7111998	-10.1625938
1200.0000000	9.7600002	-10.8583260
1200.0000000	9.8087997	-10.9949217
1200.0000000	9.8575993	3.1103001
1200.0000000	9.9063997	-7.9855137
1200.0000000	9.9552002	-10.9066792
1200.0000000	10.0039997	-10.0791750
1210.0000000	0.3416000	-36.3178444
1210.0000000	0.3904000	-38.7572823
1210.0000000	0.4392000	-39.6211510
1210.0000000	0.4880000	-39.5682831
1210.0000000	0.5368000	-39.6360588
1210.0000000	0.5856000	-39.3064041
1210.0000000	0.6344000	-38.2313461
1210.0000000	0.6832000	-36.2081757
1210.0000000	0.7320000	-36.0406265
1210.0000000	0.7808000	-36.5336227
1210.0000000	0.8296000	-35.9780045
1210.0000000	0.8784000	-35.2581596
1210.0000000	0.9272000	-33.1155663
1210.0000000	0.9760000	-31.0775890
1210.0000000	1.0247999	-29.5150852
1210.0000000	1.0735999	-29.1045609
1210.0000000	1.1224000	-29.7100620
1210.0000000	1.1711999	-30.1973019
1210.0000000	1.2200000	-30.5084209
1210.0000000	1.2687999	-31.2452812
1210.0000000	1.3176000	-31.0768719
1210.0000000	1.3664000	-30.6860580
1210.0000000	1.4152000	-30.1661987
1210.0000000	1.4640000	-29.5828381
1210.0000000	1.5128000	-37.5288467
1210.0000000	1.5616000	-32.8352966
1210.0000000	1.6104001	-1508.9036865
1210.0000000	1.6592000	-67.6734924
1210.0000000	1.7079999	-27.3287334
1210.0000000	1.7567999	-27.7625065
1210.0000000	1.8055999	-27.7026596
1210.0000000	1.8544000	-27.2402325
1210.0000000	1.9031999	-26.5442696
1210.0000000	1.9520000	-26.3364906
1210.0000000	2.0007999	-26.2259083
1210.0000000	2.0495999	-26.2066612
1210.0000000	2.0983999	-119.9343796
1210.0000000	2.1471999	-6.7527084
1210.0000000	2.1960001	-25.3707714
1210.0000000	2.2448001	-25.4874458
1210.0000000	2.2935998	-26.5475883
1210.0000000	2.3423998	-21.2635384
1210.0000000	2.3912001	20.0711651
1210.0000000	2.4400001	-22.8847084
1210.0000000	2.4888000	-23.4559631
1210.0000000	2.5375998	-12.5914640
1210.0000000	2.5863998	-10.7621069
1210.0000000	2.6352000	-8.8900824
1210.0000000	2.6840000	-15.6334190

1210.0000000	2.7328000	128.2511902
1210.0000000	2.7815998	151.4688721
1210.0000000	2.8304000	-23.7703514
1210.0000000	2.8792000	-20.6794319
1210.0000000	2.9280000	-22.5613098
1210.0000000	2.9768000	-16.9433613
1210.0000000	3.0256000	-20.5203781
1210.0000000	3.0743999	-29.3074894
1210.0000000	3.1231999	-24.1915970
1210.0000000	3.1719999	-29.4533348
1210.0000000	3.2208002	-499.8636169
1210.0000000	3.2695999	-978.4949951
1210.0000000	3.3183999	-20.4933586
1210.0000000	3.3671999	-3368.8889160
1210.0000000	3.4159999	1208.3771973
1210.0000000	3.4648001	-7.2489719
1210.0000000	3.5135999	-26.9803123
1210.0000000	3.5623999	-319.4864502
1210.0000000	3.6111999	-2006.4758301
1210.0000000	3.6600001	-454.3936768
1210.0000000	3.7088001	-599.9346313
1210.0000000	3.7575998	-939.1856689
1210.0000000	3.8063998	-1704.1092529
1210.0000000	3.8552001	-2474.0361328
1210.0000000	3.9040000	-2425.6259766
1210.0000000	3.9528000	1108.9692383
1210.0000000	4.0015998	-242.5187378
1210.0000000	4.0503998	678.8658447
1210.0000000	4.0991998	86.2987518
1210.0000000	4.1479998	-646.8854980
1210.0000000	4.1967998	2.2783718
1210.0000000	4.2456002	-245.6651611
1210.0000000	4.2943997	-482.1944275
1210.0000000	4.3431997	-13.3944893
1210.0000000	4.3920002	-245.3010712
1210.0000000	4.4407997	-22.4091702
1210.0000000	4.4896002	-15.4997921
1210.0000000	4.5383997	-647.5039673
1210.0000000	4.5871997	-12.8971081
1210.0000000	4.6360002	-21.5763321
1210.0000000	4.6847997	-28.1684189
1210.0000000	4.7336001	-19.5414524
1210.0000000	4.7824001	-16.5255642
1210.0000000	4.8311996	-1458.8856201
1210.0000000	4.8800001	225.6458282
1210.0000000	4.9287996	-199.4908905
1210.0000000	4.9776001	1048.1262207
1210.0000000	5.0264001	85.0806885
1210.0000000	5.0751996	-298.1148987
1210.0000000	5.1240001	-12.3352337
1210.0000000	5.1727996	555.0535278
1210.0000000	5.2216001	31.7695580
1210.0000000	5.2704000	-158.1419220
1210.0000000	5.3191996	-39.0735435
1210.0000000	5.3680000	-3519.3098145
1210.0000000	5.4168000	524.5800171
1210.0000000	5.4656000	-199.8762817
1210.0000000	5.5144000	37.8131561

1210.0000000	5.5631995	485.9781189
1210.0000000	5.6120000	391.2377319
1210.0000000	5.6608000	-36.9874954
1210.0000000	5.7096000	62.1634293
1210.0000000	5.7584000	132.3525085
1210.0000000	5.8071995	-11.5409679
1210.0000000	5.8559999	29.6074543
1210.0000000	5.9047999	-16.6009483
1210.0000000	5.9535999	-39.6643944
1210.0000000	6.0023999	-1565.8104248
1210.0000000	6.0511999	-14.5731516
1210.0000000	6.0999999	-19.3898659
1210.0000000	6.1487999	-13.2841110
1210.0000000	6.1975999	-23.1792431
1210.0000000	6.2463999	-1783.8045654
1210.0000000	6.2951999	-19.0514927
1210.0000000	6.3439999	-341.2349854
1210.0000000	6.3927999	-15.0476999
1210.0000000	6.4416003	-12.3993454
1210.0000000	6.4903998	-11.2790127
1210.0000000	6.5391998	-11.8215723
1210.0000000	6.5879998	369.5853271
1210.0000000	6.6367998	-12.0963879
1210.0000000	6.6856003	-13.8186827
1210.0000000	6.7343998	-13.4603987
1210.0000000	6.7831998	-14.3635006
1210.0000000	6.8319998	58.0257492
1210.0000000	6.8807998	-14.0464325
1210.0000000	6.9296002	-433.2401123
1210.0000000	6.9783998	-5.3566575
1210.0000000	7.0271997	-14.1156759
1210.0000000	7.0760002	10.3053236
1210.0000000	7.1247997	-2049.7814941
1210.0000000	7.1736002	-1643.8062744
1210.0000000	7.2223997	-935.2969971
1210.0000000	7.2711997	-202.0563202
1210.0000000	7.3200002	-18.6371384
1210.0000000	7.3687997	180.2571869
1210.0000000	7.4176002	376.5346069
1210.0000000	7.4663997	295.7353210
1210.0000000	7.5151997	-914.8969727
1210.0000000	7.5640001	-1010.5344238
1210.0000000	7.6127996	-2381.3486328
1210.0000000	7.6616001	-764.7619019
1210.0000000	7.7104001	-393.0015869
1210.0000000	7.7591996	41.1959229
1210.0000000	7.8080001	391.2997742
1210.0000000	7.8567996	515.5939331
1210.0000000	7.9056001	-1069.7188721
1210.0000000	7.9544001	1702.3798828
1210.0000000	8.0031996	2196.6701660
1210.0000000	8.0520000	2192.5004883
1210.0000000	8.1007996	2320.1105957
1210.0000000	8.1496000	2474.3542480
1210.0000000	8.1983995	1539.3651123
1210.0000000	8.2472000	-3522.2844238
1210.0000000	8.2959995	-1248.6206055
1210.0000000	8.3448000	-994.5983276

1210.0000000	8.3935995	-911.5335083
1210.0000000	8.4423990	-392.7048035
1210.0000000	8.4912004	-916.6741333
1210.0000000	8.5400000	-2472.6201172
1210.0000000	8.5887995	-1198.6375732
1210.0000000	8.6375999	233.9839935
1210.0000000	8.6863995	2114.7412109
1210.0000000	8.7351999	-786.1097412
1210.0000000	8.7840004	898.0194092
1210.0000000	8.8327999	48.3653069
1210.0000000	8.8815994	-11.4998245
1210.0000000	8.9303989	-23.8730202
1210.0000000	8.9792004	-11.5806980
1210.0000000	9.0279999	-5.8710446
1210.0000000	9.0767994	6.9291534
1210.0000000	9.1255999	-17.2090969
1210.0000000	9.1743994	-10.0812979
1210.0000000	9.2231998	36.7104568
1210.0000000	9.2720003	-8.4379921
1210.0000000	9.3207998	-9.8083868
1210.0000000	9.3695993	-10.7680836
1210.0000000	9.4183998	-11.7515106
1210.0000000	9.4672003	-11.1621141
1210.0000000	9.5159998	-14.1472788
1210.0000000	9.5648003	-0.4571581
1210.0000000	9.6135998	-13.3809862
1210.0000000	9.6623993	-10.2265596
1210.0000000	9.7111998	-10.1628656
1210.0000000	9.7600002	-10.8583679
1210.0000000	9.8087997	-10.9949293
1210.0000000	9.8575993	3.1058788
1210.0000000	9.9063997	-7.9864273
1210.0000000	9.9552002	-10.9066839
1210.0000000	10.0039997	-10.0794230
1220.0000000	0.3416000	-36.4346390
1220.0000000	0.3904000	-38.7579460
1220.0000000	0.4392000	-39.6148071
1220.0000000	0.4880000	-39.5670738
1220.0000000	0.5368000	-39.6372337
1220.0000000	0.5856000	-39.3050232
1220.0000000	0.6344000	-38.2316551
1220.0000000	0.6832000	-36.2104034
1220.0000000	0.7320000	-36.0426788
1220.0000000	0.7808000	-36.5337944
1220.0000000	0.8296000	-35.9761581
1220.0000000	0.8784000	-35.2485962
1220.0000000	0.9272000	-33.6502762
1220.0000000	0.9760000	-31.2150135
1220.0000000	1.0247999	-29.9147892
1220.0000000	1.0735999	-29.6384335
1220.0000000	1.1224000	-29.9101372
1220.0000000	1.1711999	-30.4284134
1220.0000000	1.2200000	-31.3550396
1220.0000000	1.2687999	-31.2239552
1220.0000000	1.3176000	-31.0704861
1220.0000000	1.3664000	-30.6831150
1220.0000000	1.4152000	-30.1664028
1220.0000000	1.4640000	-29.5811291

1220.0000000	1.5128000	-37.4316673
1220.0000000	1.5616000	-32.7934570
1220.0000000	1.6104001	-1506.3609619
1220.0000000	1.6592000	-67.3795547
1220.0000000	1.7079999	-27.3324814
1220.0000000	1.7567999	-27.7625256
1220.0000000	1.8055999	-27.7073612
1220.0000000	1.8544000	-27.2981224
1220.0000000	1.9031999	-26.4589844
1220.0000000	1.9520000	-26.3329201
1220.0000000	2.0007999	-26.2022591
1220.0000000	2.0495999	-26.2086792
1220.0000000	2.0983999	-118.4586029
1220.0000000	2.1471999	-7.0199757
1220.0000000	2.1960001	-25.3666801
1220.0000000	2.2448001	-25.4695358
1220.0000000	2.2935998	-26.4879646
1220.0000000	2.3423998	-21.2779427
1220.0000000	2.3912001	19.4496174
1220.0000000	2.4400001	-22.8871937
1220.0000000	2.4888000	-23.4557457
1220.0000000	2.5375998	-12.6085720
1220.0000000	2.5863998	-10.7818642
1220.0000000	2.6352000	-8.9128265
1220.0000000	2.6840000	-15.6453619
1220.0000000	2.7328000	127.9851761
1220.0000000	2.7815998	151.1624451
1220.0000000	2.8304000	-23.7690468
1220.0000000	2.8792000	-20.6826954
1220.0000000	2.9280000	-22.5607414
1220.0000000	2.9768000	-16.9517326
1220.0000000	3.0256000	-20.5229626
1220.0000000	3.0743999	-29.2961025
1220.0000000	3.1231999	-24.1881409
1220.0000000	3.1719999	-29.4450417
1220.0000000	3.2208002	-499.6258850
1220.0000000	3.2695999	-978.0200195
1220.0000000	3.3183999	-20.4935570
1220.0000000	3.3671999	-3367.4272461
1220.0000000	3.4159999	1207.8419189
1220.0000000	3.4648001	-7.2546806
1220.0000000	3.5135999	-26.9773216
1220.0000000	3.5623999	-319.3568726
1220.0000000	3.6111999	-2005.6160889
1220.0000000	3.6600001	-454.2055664
1220.0000000	3.7088001	-599.6832275
1220.0000000	3.7575998	-938.7871094
1220.0000000	3.8063998	-1703.3793945
1220.0000000	3.8552001	-2472.9726563
1220.0000000	3.9040000	-2424.5834961
1220.0000000	3.9528000	1108.4809570
1220.0000000	4.0015998	-242.4216156
1220.0000000	4.0503998	678.5632935
1220.0000000	4.0991998	86.2530670
1220.0000000	4.1479998	-646.6132202
1220.0000000	4.1967998	2.2691154
1220.0000000	4.2456002	-245.5668488
1220.0000000	4.2943997	-481.9938660

1220.0000000	4.3431997	-13.3966694
1220.0000000	4.3920002	-245.2029572
1220.0000000	4.4407997	-22.4073944
1220.0000000	4.4896002	-15.5006752
1220.0000000	4.5383997	-647.2429199
1220.0000000	4.5871997	-12.8991241
1220.0000000	4.6360002	-21.5747890
1220.0000000	4.6847997	-28.1644936
1220.0000000	4.7336001	-19.5407295
1220.0000000	4.7824001	-16.5260181
1220.0000000	4.8311996	-1458.3542480
1220.0000000	4.8800001	225.5562744
1220.0000000	4.9287996	-199.4236298
1220.0000000	4.9776001	1047.7337646
1220.0000000	5.0264001	85.0431671
1220.0000000	5.0751996	-298.0112610
1220.0000000	5.1240001	-12.3361549
1220.0000000	5.1727996	554.9376831
1220.0000000	5.2216001	31.7601128
1220.0000000	5.2704000	-158.1138153
1220.0000000	5.3191996	-39.0689926
1220.0000000	5.3680000	-3518.6174316
1220.0000000	5.4168000	524.4732666
1220.0000000	5.4656000	-199.8401031
1220.0000000	5.5144000	37.8024979
1220.0000000	5.5631995	485.8795471
1220.0000000	5.6120000	391.1577454
1220.0000000	5.6608000	-36.9834099
1220.0000000	5.7096000	62.1480026
1220.0000000	5.7584000	132.3235626
1220.0000000	5.8071995	-11.5418377
1220.0000000	5.8559999	29.5986042
1220.0000000	5.9047999	-16.6007500
1220.0000000	5.9535999	-39.6596527
1220.0000000	6.0023999	-1565.5058594
1220.0000000	6.0511999	-14.5733633
1220.0000000	6.0999999	-19.3890324
1220.0000000	6.1487999	-13.2845745
1220.0000000	6.1975999	-23.1774788
1220.0000000	6.2463999	-1783.4571533
1220.0000000	6.2951999	-19.0506783
1220.0000000	6.3439999	-341.1709595
1220.0000000	6.3927999	-15.0476818
1220.0000000	6.4416003	-12.3999319
1220.0000000	6.4903998	-11.2796888
1220.0000000	6.5391998	-11.8222542
1220.0000000	6.5879998	368.8946228
1220.0000000	6.6367998	-12.0969372
1220.0000000	6.6856003	-13.8188534
1220.0000000	6.7343998	-13.4606152
1220.0000000	6.7831998	-14.3630848
1220.0000000	6.8319998	58.0066299
1220.0000000	6.8807998	-14.0462942
1220.0000000	6.9296002	-432.4786682
1220.0000000	6.9783998	-5.3694811
1220.0000000	7.0271997	-14.1146479
1220.0000000	7.0760002	10.2631369
1220.0000000	7.1247997	-2045.8355713

1220.0000000	7.1736002	-1640.6434326
1220.0000000	7.2223997	-933.5038452
1220.0000000	7.2711997	-201.6885376
1220.0000000	7.3200002	-18.6251507
1220.0000000	7.3687997	179.8826599
1220.0000000	7.4176002	375.7787781
1220.0000000	7.4663997	295.1376648
1220.0000000	7.5151997	-913.1359253
1220.0000000	7.5640001	-1008.5870361
1220.0000000	7.6127996	-2376.7309570
1220.0000000	7.6616001	-763.2905273
1220.0000000	7.7104001	-392.2553101
1220.0000000	7.7591996	41.0948753
1220.0000000	7.8080001	390.5144348
1220.0000000	7.8567996	514.5657959
1220.0000000	7.9056001	-1067.6459961
1220.0000000	7.9544001	1699.0699463
1220.0000000	8.0031996	2192.4006348
1220.0000000	8.0520000	2188.2385254
1220.0000000	8.1007996	2315.6010742
1220.0000000	8.1496000	2469.5461426
1220.0000000	8.1983995	1536.3725586
1220.0000000	8.2472000	-3515.4494629
1220.0000000	8.2959995	-1246.2004395
1220.0000000	8.3448000	-992.6691895
1220.0000000	8.3935995	-909.7658691
1220.0000000	8.4423990	-391.9468079
1220.0000000	8.4912004	-914.8979492
1220.0000000	8.5400000	-2467.8203125
1220.0000000	8.5887995	-1196.3160400
1220.0000000	8.6375999	233.5226746
1220.0000000	8.6863995	2110.6228027
1220.0000000	8.7351999	-784.5854492
1220.0000000	8.7840004	896.2626953
1220.0000000	8.8327999	48.2537003
1220.0000000	8.8815994	-11.4999990
1220.0000000	8.9303989	-23.8693008
1220.0000000	8.9792004	-11.5807800
1220.0000000	9.0279999	-5.8728127
1220.0000000	9.0767994	6.9234352
1220.0000000	9.1255999	-17.2073841
1220.0000000	9.1743994	-10.0817003
1220.0000000	9.2231998	36.6947250
1220.0000000	9.2720003	-8.4389286
1220.0000000	9.3207998	-9.8089256
1220.0000000	9.3695993	-10.7683096
1220.0000000	9.4183998	-11.7513857
1220.0000000	9.4672003	-11.1621552
1220.0000000	9.5159998	-14.1463881
1220.0000000	9.5648003	-0.4605312
1220.0000000	9.6135998	-13.3801956
1220.0000000	9.6623993	-10.2268343
1220.0000000	9.7111998	-10.1631374
1220.0000000	9.7600002	-10.8584099
1220.0000000	9.8087997	-10.9949379
1220.0000000	9.8575993	3.1014566
1220.0000000	9.9063997	-7.9873419
1220.0000000	9.9552002	-10.9066877

1220.0000000	10.0039997	-10.0796700
1230.0000000	0.3416000	-36.4627380
1230.0000000	0.3904000	-38.9926605
1230.0000000	0.4392000	-39.6084633
1230.0000000	0.4880000	-39.5658646
1230.0000000	0.5368000	-39.6384048
1230.0000000	0.5856000	-39.3036385
1230.0000000	0.6344000	-38.2319679
1230.0000000	0.6832000	-36.2126274
1230.0000000	0.7320000	-36.0447311
1230.0000000	0.7808000	-36.5339622
1230.0000000	0.8296000	-35.9743156
1230.0000000	0.8784000	-35.2390289
1230.0000000	0.9272000	-34.5629730
1230.0000000	0.9760000	-32.2281151
1230.0000000	1.0247999	-30.3515224
1230.0000000	1.0735999	-30.3342094
1230.0000000	1.1224000	-30.4678879
1230.0000000	1.1711999	-30.8087902
1230.0000000	1.2200000	-31.7365627
1230.0000000	1.2687999	-31.2026272
1230.0000000	1.3176000	-31.0641003
1230.0000000	1.3664000	-30.6801701
1230.0000000	1.4152000	-30.1666069
1230.0000000	1.4640000	-29.5794201
1230.0000000	1.5128000	-37.3344917
1230.0000000	1.5616000	-32.7516136
1230.0000000	1.6104001	-1503.8181152
1230.0000000	1.6592000	-67.0856247
1230.0000000	1.7079999	-27.3362274
1230.0000000	1.7567999	-27.7625446
1230.0000000	1.8055999	-27.6647110
1230.0000000	1.8544000	-26.9856796
1230.0000000	1.9031999	-26.3737011
1230.0000000	1.9520000	-26.3293476
1230.0000000	2.0007999	-26.1786079
1230.0000000	2.0495999	-26.2106991
1230.0000000	2.0983999	-116.9828262
1230.0000000	2.1471999	-7.2872429
1230.0000000	2.1960001	-25.3625889
1230.0000000	2.2448001	-25.4516239
1230.0000000	2.2935998	-26.4283409
1230.0000000	2.3423998	-21.2923470
1230.0000000	2.3912001	18.8280697
1230.0000000	2.4400001	-22.8896809
1230.0000000	2.4888000	-23.4555283
1230.0000000	2.5375998	-12.6256800
1230.0000000	2.5863998	-10.8016233
1230.0000000	2.6352000	-8.9355707
1230.0000000	2.6840000	-15.6573029
1230.0000000	2.7328000	127.7191696
1230.0000000	2.7815998	150.8560181
1230.0000000	2.8304000	-23.7677441
1230.0000000	2.8792000	-20.6859589
1230.0000000	2.9280000	-22.5601711
1230.0000000	2.9768000	-16.9601021
1230.0000000	3.0256000	-20.5255470
1230.0000000	3.0743999	-29.2847157

1230.0000000	3.1231999	-24.1846867
1230.0000000	3.1719999	-29.4367485
1230.0000000	3.2208002	-499.3881531
1230.0000000	3.2695999	-977.5450439
1230.0000000	3.3183999	-20.4937553
1230.0000000	3.3671999	-3365.9655762
1230.0000000	3.4159999	1207.3066406
1230.0000000	3.4648001	-7.2603884
1230.0000000	3.5135999	-26.9743309
1230.0000000	3.5623999	-319.2272644
1230.0000000	3.6111999	-2004.7563477
1230.0000000	3.6600001	-454.0173950
1230.0000000	3.7088001	-599.4318848
1230.0000000	3.7575998	-938.3886108
1230.0000000	3.8063998	-1702.6495361
1230.0000000	3.8552001	-2471.9094238
1230.0000000	3.9040000	-2423.5410156
1230.0000000	3.9528000	1107.9926758
1230.0000000	4.0015998	-242.3245087
1230.0000000	4.0503998	678.2607422
1230.0000000	4.0991998	86.2073822
1230.0000000	4.1479998	-646.3410034
1230.0000000	4.1967998	2.2598591
1230.0000000	4.2456002	-245.4685516
1230.0000000	4.2943997	-481.7933044
1230.0000000	4.3431997	-13.3988504
1230.0000000	4.3920002	-245.1048431
1230.0000000	4.4407997	-22.4056206
1230.0000000	4.4896002	-15.5015574
1230.0000000	4.5383997	-646.9818115
1230.0000000	4.5871997	-12.9011402
1230.0000000	4.6360002	-21.5732460
1230.0000000	4.6847997	-28.1605682
1230.0000000	4.7336001	-19.5400085
1230.0000000	4.7824001	-16.5264740
1230.0000000	4.8311996	-1457.8229980
1230.0000000	4.8800001	225.4667206
1230.0000000	4.9287996	-199.3563690
1230.0000000	4.9776001	1047.3411865
1230.0000000	5.0264001	85.0056458
1230.0000000	5.0751996	-297.9076538
1230.0000000	5.1240001	-12.3370762
1230.0000000	5.1727996	554.8218384
1230.0000000	5.2216001	31.7506676
1230.0000000	5.2704000	-158.0857086
1230.0000000	5.3191996	-39.0644455
1230.0000000	5.3680000	-3517.9250488
1230.0000000	5.4168000	524.3664551
1230.0000000	5.4656000	-199.8039246
1230.0000000	5.5144000	37.7918472
1230.0000000	5.5631995	485.7809753
1230.0000000	5.6120000	391.0777893
1230.0000000	5.6608000	-36.9793205
1230.0000000	5.7096000	62.1325684
1230.0000000	5.7584000	132.2946320
1230.0000000	5.8071995	-11.5427094
1230.0000000	5.8559999	29.5897541
1230.0000000	5.9047999	-16.6005535

1230.0000000	5.9535999	-39.6549149
1230.0000000	6.0023999	-1565.2014160
1230.0000000	6.0511999	-14.5735741
1230.0000000	6.0999999	-19.3882008
1230.0000000	6.1487999	-13.2850370
1230.0000000	6.1975999	-23.1757126
1230.0000000	6.2463999	-1783.1097412
1230.0000000	6.2951999	-19.0498638
1230.0000000	6.3439999	-341.1068726
1230.0000000	6.3927999	-15.0476637
1230.0000000	6.4416003	-12.4005184
1230.0000000	6.4903998	-11.2803650
1230.0000000	6.5391998	-11.8229351
1230.0000000	6.5879998	368.2039185
1230.0000000	6.6367998	-12.0974865
1230.0000000	6.6856003	-13.8190231
1230.0000000	6.7343998	-13.4608307
1230.0000000	6.7831998	-14.3626690
1230.0000000	6.8319998	57.9875183
1230.0000000	6.8807998	-14.0461550
1230.0000000	6.9296002	-431.7172241
1230.0000000	6.9783998	-5.3823051
1230.0000000	7.0271997	-14.1136198
1230.0000000	7.0760002	10.2209520
1230.0000000	7.1247997	-2041.8896484
1230.0000000	7.1736002	-1637.4804688
1230.0000000	7.2223997	-931.7106934
1230.0000000	7.2711997	-201.3207550
1230.0000000	7.3200002	-18.6131630
1230.0000000	7.3687997	179.5081329
1230.0000000	7.4176002	375.0229492
1230.0000000	7.4663997	294.5400085
1230.0000000	7.5151997	-911.3748779
1230.0000000	7.5640001	-1006.6396484
1230.0000000	7.6127996	-2372.1132813
1230.0000000	7.6616001	-761.8192139
1230.0000000	7.7104001	-391.5090332
1230.0000000	7.7591996	40.9938240
1230.0000000	7.8080001	389.7290649
1230.0000000	7.8567996	513.5377197
1230.0000000	7.9056001	-1065.5729980
1230.0000000	7.9544001	1695.7600098
1230.0000000	8.0031996	2188.1308594
1230.0000000	8.0520000	2183.9765625
1230.0000000	8.1007996	2311.0915527
1230.0000000	8.1496000	2464.7382813
1230.0000000	8.1983995	1533.3801270
1230.0000000	8.2472000	-3508.6147461
1230.0000000	8.2959995	-1243.7802734
1230.0000000	8.3448000	-990.7399902
1230.0000000	8.3935995	-907.9981689
1230.0000000	8.4423990	-391.1887817
1230.0000000	8.4912004	-913.1217041
1230.0000000	8.5400000	-2463.0205078
1230.0000000	8.5887995	-1193.9945068
1230.0000000	8.6375999	233.0613556
1230.0000000	8.6863995	2106.5043945
1230.0000000	8.7351999	-783.0612183

1230.0000000	8.7840004	894.5059204
1230.0000000	8.8327999	48.1420898
1230.0000000	8.8815994	-11.5001726
1230.0000000	8.9303989	-23.8655796
1230.0000000	8.9792004	-11.5808620
1230.0000000	9.0279999	-5.8745813
1230.0000000	9.0767994	6.9177189
1230.0000000	9.1255999	-17.2056694
1230.0000000	9.1743994	-10.0821018
1230.0000000	9.2231998	36.6789932
1230.0000000	9.2720003	-8.4398642
1230.0000000	9.3207998	-9.8094654
1230.0000000	9.3695993	-10.7685366
1230.0000000	9.4183998	-11.7512617
1230.0000000	9.4672003	-11.1621962
1230.0000000	9.5159998	-14.1454964
1230.0000000	9.5648003	-0.4639034
1230.0000000	9.6135998	-13.3794050
1230.0000000	9.6623993	-10.2271099
1230.0000000	9.7111998	-10.1634092
1230.0000000	9.7600002	-10.8584518
1230.0000000	9.8087997	-10.9949455
1230.0000000	9.8575993	3.0970325
1230.0000000	9.9063997	-7.9882555
1230.0000000	9.9552002	-10.9066925
1230.0000000	10.0039997	-10.0799179
1240.0000000	0.3416000	-36.6127968
1240.0000000	0.3904000	-39.0796700
1240.0000000	0.4392000	-39.6021194
1240.0000000	0.4880000	-39.5646553
1240.0000000	0.5368000	-39.6395798
1240.0000000	0.5856000	-39.3022575
1240.0000000	0.6344000	-38.2322769
1240.0000000	0.6832000	-36.2148552
1240.0000000	0.7320000	-36.0467834
1240.0000000	0.7808000	-36.5341301
1240.0000000	0.8296000	-35.9724693
1240.0000000	0.8784000	-35.2294655
1240.0000000	0.9272000	-34.5442543
1240.0000000	0.9760000	-32.3090782
1240.0000000	1.0247999	-30.8494663
1240.0000000	1.0735999	-30.1702175
1240.0000000	1.1224000	-29.9996185
1240.0000000	1.1711999	-30.6225204
1240.0000000	1.2200000	-31.2874355
1240.0000000	1.2687999	-31.1813011
1240.0000000	1.3176000	-31.0577164
1240.0000000	1.3664000	-30.6772270
1240.0000000	1.4152000	-30.1668129
1240.0000000	1.4640000	-29.5777092
1240.0000000	1.5128000	-37.2373123
1240.0000000	1.5616000	-32.7097740
1240.0000000	1.6104001	-1501.2752686
1240.0000000	1.6592000	-66.7916946
1240.0000000	1.7079999	-27.3399754
1240.0000000	1.7567999	-27.7625656
1240.0000000	1.8055999	-27.6220627
1240.0000000	1.8544000	-26.6732368

1240.0000000	1.9031999	-26.2884159
1240.0000000	1.9520000	-26.0187302
1240.0000000	2.0007999	-26.1549587
1240.0000000	2.0495999	-26.2127171
1240.0000000	2.0983999	-115.5070419
1240.0000000	2.1471999	-7.5545101
1240.0000000	2.1960001	-25.3584976
1240.0000000	2.2448001	-25.4337120
1240.0000000	2.2935998	-26.3687172
1240.0000000	2.3423998	-21.3067513
1240.0000000	2.3912001	18.2065258
1240.0000000	2.4400001	-22.8921661
1240.0000000	2.4888000	-23.4553108
1240.0000000	2.5375998	-12.6427889
1240.0000000	2.5863998	-10.8213825
1240.0000000	2.6352000	-8.9583149
1240.0000000	2.6840000	-15.6692448
1240.0000000	2.7328000	127.4531479
1240.0000000	2.7815998	150.5496216
1240.0000000	2.8304000	-23.7664394
1240.0000000	2.8792000	-20.6892223
1240.0000000	2.9280000	-22.5596027
1240.0000000	2.9768000	-16.9684715
1240.0000000	3.0256000	-20.5281315
1240.0000000	3.0743999	-29.2733269
1240.0000000	3.1231999	-24.1812305
1240.0000000	3.1719999	-29.4284554
1240.0000000	3.2208002	-499.1504211
1240.0000000	3.2695999	-977.0700684
1240.0000000	3.3183999	-20.4939537
1240.0000000	3.3671999	-3364.5039063
1240.0000000	3.4159999	1206.7713623
1240.0000000	3.4648001	-7.2660971
1240.0000000	3.5135999	-26.9713402
1240.0000000	3.5623999	-319.0976868
1240.0000000	3.6111999	-2003.8964844
1240.0000000	3.6600001	-453.8292847
1240.0000000	3.7088001	-599.1804810
1240.0000000	3.7575998	-937.9900513
1240.0000000	3.8063998	-1701.9196777
1240.0000000	3.8552001	-2470.8459473
1240.0000000	3.9040000	-2422.4985352
1240.0000000	3.9528000	1107.5042725
1240.0000000	4.0015998	-242.2274017
1240.0000000	4.0503998	677.9581909
1240.0000000	4.0991998	86.1616974
1240.0000000	4.1479998	-646.0687866
1240.0000000	4.1967998	2.2506027
1240.0000000	4.2456002	-245.3702545
1240.0000000	4.2943997	-481.5927429
1240.0000000	4.3431997	-13.4010315
1240.0000000	4.3920002	-245.0067291
1240.0000000	4.4407997	-22.4038429
1240.0000000	4.4896002	-15.5024395
1240.0000000	4.5383997	-646.7207642
1240.0000000	4.5871997	-12.9031572
1240.0000000	4.6360002	-21.5717049
1240.0000000	4.6847997	-28.1566429

1240.0000000	4.7336001	-19.5392857
1240.0000000	4.7824001	-16.5269279
1240.0000000	4.8311996	-1457.2917480
1240.0000000	4.8800001	225.3771667
1240.0000000	4.9287996	-199.2891083
1240.0000000	4.9776001	1046.9487305
1240.0000000	5.0264001	84.9681244
1240.0000000	5.0751996	-297.8040161
1240.0000000	5.1240001	-12.3379974
1240.0000000	5.1727996	554.7059326
1240.0000000	5.2216001	31.7412262
1240.0000000	5.2704000	-158.0576019
1240.0000000	5.3191996	-39.0598946
1240.0000000	5.3680000	-3517.2329102
1240.0000000	5.4168000	524.2597046
1240.0000000	5.4656000	-199.7677307
1240.0000000	5.5144000	37.7811890
1240.0000000	5.5631995	485.6824036
1240.0000000	5.6120000	390.9978027
1240.0000000	5.6608000	-36.9752312
1240.0000000	5.7096000	62.1171341
1240.0000000	5.7584000	132.2657013
1240.0000000	5.8071995	-11.5435791
1240.0000000	5.8559999	29.5809002
1240.0000000	5.9047999	-16.6003551
1240.0000000	5.9535999	-39.6501770
1240.0000000	6.0023999	-1564.8969727
1240.0000000	6.0511999	-14.5737858
1240.0000000	6.0999999	-19.3873672
1240.0000000	6.1487999	-13.2854996
1240.0000000	6.1975999	-23.1739464
1240.0000000	6.2463999	-1782.7624512
1240.0000000	6.2951999	-19.0490494
1240.0000000	6.3439999	-341.0428467
1240.0000000	6.3927999	-15.0476456
1240.0000000	6.4416003	-12.4011059
1240.0000000	6.4903998	-11.2810402
1240.0000000	6.5391998	-11.8236160
1240.0000000	6.5879998	367.5132141
1240.0000000	6.6367998	-12.0980349
1240.0000000	6.6856003	-13.8191938
1240.0000000	6.7343998	-13.4610462
1240.0000000	6.7831998	-14.3622532
1240.0000000	6.8319998	57.9683990
1240.0000000	6.8807998	-14.0460167
1240.0000000	6.9296002	-430.9557800
1240.0000000	6.9783998	-5.3951283
1240.0000000	7.0271997	-14.1125917
1240.0000000	7.0760002	10.1787672
1240.0000000	7.1247997	-2037.9438477
1240.0000000	7.1736002	-1634.3176270
1240.0000000	7.2223997	-929.9175415
1240.0000000	7.2711997	-200.9529724
1240.0000000	7.3200002	-18.6011734
1240.0000000	7.3687997	179.1336060
1240.0000000	7.4176002	374.2671204
1240.0000000	7.4663997	293.9423523
1240.0000000	7.5151997	-909.6138306

1240.0000000	7.5640001	-1004.6921997
1240.0000000	7.6127996	-2367.4958496
1240.0000000	7.6616001	-760.3479004
1240.0000000	7.7104001	-390.7627563
1240.0000000	7.7591996	40.8927765
1240.0000000	7.8080001	388.9436951
1240.0000000	7.8567996	512.5096436
1240.0000000	7.9056001	-1063.5001221
1240.0000000	7.9544001	1692.4500732
1240.0000000	8.0031996	2183.8613281
1240.0000000	8.0520000	2179.7145996
1240.0000000	8.1007996	2306.5820313
1240.0000000	8.1496000	2459.9301758
1240.0000000	8.1983995	1530.3875732
1240.0000000	8.2472000	-3501.7800293
1240.0000000	8.2959995	-1241.3601074
1240.0000000	8.3448000	-988.8107910
1240.0000000	8.3935995	-906.2304688
1240.0000000	8.4423990	-390.4307556
1240.0000000	8.4912004	-911.3454590
1240.0000000	8.5400000	-2458.2207031
1240.0000000	8.5887995	-1191.6729736
1240.0000000	8.6375999	232.6000366
1240.0000000	8.6863995	2102.3859863
1240.0000000	8.7351999	-781.5369873
1240.0000000	8.7840004	892.7491455
1240.0000000	8.8327999	48.0304794
1240.0000000	8.8815994	-11.5003471
1240.0000000	8.9303989	-23.8618584
1240.0000000	8.9792004	-11.5809450
1240.0000000	9.0279999	-5.8763494
1240.0000000	9.0767994	6.9120007
1240.0000000	9.1255999	-17.2039547
1240.0000000	9.1743994	-10.0825043
1240.0000000	9.2231998	36.6632614
1240.0000000	9.2720003	-8.4408007
1240.0000000	9.3207998	-9.8100052
1240.0000000	9.3695993	-10.7687626
1240.0000000	9.4183998	-11.7511368
1240.0000000	9.4672003	-11.1622372
1240.0000000	9.5159998	-14.1446056
1240.0000000	9.5648003	-0.4672756
1240.0000000	9.6135998	-13.3786144
1240.0000000	9.6623993	-10.2273846
1240.0000000	9.7111998	-10.1636801
1240.0000000	9.7600002	-10.8584929
1240.0000000	9.8087997	-10.9949532
1240.0000000	9.8575993	3.0926113
1240.0000000	9.9063997	-7.9891691
1240.0000000	9.9552002	-10.9066963
1240.0000000	10.0039997	-10.0801649
1250.0000000	0.3416000	-36.8899117
1250.0000000	0.3904000	-39.3772583
1250.0000000	0.4392000	-39.5957756
1250.0000000	0.4880000	-39.5634460
1250.0000000	0.5368000	-39.6407547
1250.0000000	0.5856000	-39.3008766
1250.0000000	0.6344000	-38.2325859

1250.0000000	0.6832000	-36.2170792
1250.0000000	0.7320000	-36.0488358
1250.0000000	0.7808000	-36.5343018
1250.0000000	0.8296000	-35.9706230
1250.0000000	0.8784000	-35.2199020
1250.0000000	0.9272000	-34.3290253
1250.0000000	0.9760000	-32.2110710
1250.0000000	1.0247999	-30.7747231
1250.0000000	1.0735999	-30.6254082
1250.0000000	1.1224000	-30.8723469
1250.0000000	1.1711999	-31.5037556
1250.0000000	1.2200000	-31.9310131
1250.0000000	1.2687999	-31.1599731
1250.0000000	1.3176000	-31.0513306
1250.0000000	1.3664000	-30.6742840
1250.0000000	1.4152000	-30.1670170
1250.0000000	1.4640000	-29.5760002
1250.0000000	1.5128000	-37.1401329
1250.0000000	1.5616000	-32.6679306
1250.0000000	1.6104001	-1498.7325439
1250.0000000	1.6592000	-66.4977646
1250.0000000	1.7079999	-27.3437214
1250.0000000	1.7567999	-27.7625847
1250.0000000	1.8055999	-27.5794125
1250.0000000	1.8544000	-27.2369232
1250.0000000	1.9031999	-26.0899734
1250.0000000	1.9520000	-25.2673931
1250.0000000	2.0007999	-25.8654003
1250.0000000	2.0495999	-26.2147369
1250.0000000	2.0983999	-114.0312653
1250.0000000	2.1471999	-7.8217773
1250.0000000	2.1960001	-25.3544064
1250.0000000	2.2448001	-25.4158020
1250.0000000	2.2935998	-26.3090954
1250.0000000	2.3423998	-21.3211555
1250.0000000	2.3912001	17.5849819
1250.0000000	2.4400001	-22.8946533
1250.0000000	2.4888000	-23.4550934
1250.0000000	2.5375998	-12.6598969
1250.0000000	2.5863998	-10.8411417
1250.0000000	2.6352000	-8.9810591
1250.0000000	2.6840000	-15.6811867
1250.0000000	2.7328000	127.1871262
1250.0000000	2.7815998	150.2431946
1250.0000000	2.8304000	-23.7651348
1250.0000000	2.8792000	-20.6924858
1250.0000000	2.9280000	-22.5590343
1250.0000000	2.9768000	-16.9768429
1250.0000000	3.0256000	-20.5307159
1250.0000000	3.0743999	-29.2619400
1250.0000000	3.1231999	-24.1777763
1250.0000000	3.1719999	-29.4201622
1250.0000000	3.2208002	-498.9126892
1250.0000000	3.2695999	-976.5950928
1250.0000000	3.3183999	-20.4941502
1250.0000000	3.3671999	-3363.0422363
1250.0000000	3.4159999	1206.2360840
1250.0000000	3.4648001	-7.2718048

1250.0000000	3.5135999	-26.9683495
1250.0000000	3.5623999	-318.9680786
1250.0000000	3.6111999	-2003.0367432
1250.0000000	3.6600001	-453.6411743
1250.0000000	3.7088001	-598.9290771
1250.0000000	3.7575998	-937.5914917
1250.0000000	3.8063998	-1701.1898193
1250.0000000	3.8552001	-2469.7824707
1250.0000000	3.9040000	-2421.4560547
1250.0000000	3.9528000	1107.0159912
1250.0000000	4.0015998	-242.1302948
1250.0000000	4.0503998	677.6556396
1250.0000000	4.0991998	86.1160126
1250.0000000	4.1479998	-645.7965698
1250.0000000	4.1967998	2.2413464
1250.0000000	4.2456002	-245.2719421
1250.0000000	4.2943997	-481.3921509
1250.0000000	4.3431997	-13.4032116
1250.0000000	4.3920002	-244.9086151
1250.0000000	4.4407997	-22.4020691
1250.0000000	4.4896002	-15.5033226
1250.0000000	4.5383997	-646.4597168
1250.0000000	4.5871997	-12.9051723
1250.0000000	4.6360002	-21.5701618
1250.0000000	4.6847997	-28.1527176
1250.0000000	4.7336001	-19.5385628
1250.0000000	4.7824001	-16.5273819
1250.0000000	4.8311996	-1456.7604980
1250.0000000	4.8800001	225.2876282
1250.0000000	4.9287996	-199.2218475
1250.0000000	4.9776001	1046.5562744
1250.0000000	5.0264001	84.9306030
1250.0000000	5.0751996	-297.7003784
1250.0000000	5.1240001	-12.3389187
1250.0000000	5.1727996	554.5900879
1250.0000000	5.2216001	31.7317810
1250.0000000	5.2704000	-158.0294952
1250.0000000	5.3191996	-39.0553474
1250.0000000	5.3680000	-3516.5405273
1250.0000000	5.4168000	524.1528931
1250.0000000	5.4656000	-199.7315521
1250.0000000	5.5144000	37.7705307
1250.0000000	5.5631995	485.5838318
1250.0000000	5.6120000	390.9178467
1250.0000000	5.6608000	-36.9711418
1250.0000000	5.7096000	62.1017075
1250.0000000	5.7584000	132.2367706
1250.0000000	5.8071995	-11.5444498
1250.0000000	5.8559999	29.5720501
1250.0000000	5.9047999	-16.6001568
1250.0000000	5.9535999	-39.6454353
1250.0000000	6.0023999	-1564.5925293
1250.0000000	6.0511999	-14.5739975
1250.0000000	6.0999999	-19.3865356
1250.0000000	6.1487999	-13.2859621
1250.0000000	6.1975999	-23.1721821
1250.0000000	6.2463999	-1782.4150391
1250.0000000	6.2951999	-19.0482349

1250.0000000	6.3439999	-340.9787903
1250.0000000	6.3927999	-15.0476265
1250.0000000	6.4416003	-12.4016924
1250.0000000	6.4903998	-11.2817163
1250.0000000	6.5391998	-11.8242970
1250.0000000	6.5879998	366.8225098
1250.0000000	6.6367998	-12.0985842
1250.0000000	6.6856003	-13.8193636
1250.0000000	6.7343998	-13.4612617
1250.0000000	6.7831998	-14.3618374
1250.0000000	6.8319998	57.9492874
1250.0000000	6.8807998	-14.0458775
1250.0000000	6.9296002	-430.1943359
1250.0000000	6.9783998	-5.4079518
1250.0000000	7.0271997	-14.1115637
1250.0000000	7.0760002	10.1365824
1250.0000000	7.1247997	-2033.9979248
1250.0000000	7.1736002	-1631.1547852
1250.0000000	7.2223997	-928.1243896
1250.0000000	7.2711997	-200.5852051
1250.0000000	7.3200002	-18.5891838
1250.0000000	7.3687997	178.7590637
1250.0000000	7.4176002	373.5112915
1250.0000000	7.4663997	293.3446960
1250.0000000	7.5151997	-907.8528442
1250.0000000	7.5640001	-1002.7448120
1250.0000000	7.6127996	-2362.8781738
1250.0000000	7.6616001	-758.8765869
1250.0000000	7.7104001	-390.0164795
1250.0000000	7.7591996	40.7917290
1250.0000000	7.8080001	388.1583557
1250.0000000	7.8567996	511.4815979
1250.0000000	7.9056001	-1061.4272461
1250.0000000	7.9544001	1689.1401367
1250.0000000	8.0031996	2179.5917969
1250.0000000	8.0520000	2175.4526367
1250.0000000	8.1007996	2302.0725098
1250.0000000	8.1496000	2455.1220703
1250.0000000	8.1983995	1527.3950195
1250.0000000	8.2472000	-3494.9450684
1250.0000000	8.2959995	-1238.9399414
1250.0000000	8.3448000	-986.8816528
1250.0000000	8.3935995	-904.4628296
1250.0000000	8.4423990	-389.6727600
1250.0000000	8.4912004	-909.5692749
1250.0000000	8.5400000	-2453.4208984
1250.0000000	8.5887995	-1189.3514404
1250.0000000	8.6375999	232.1387177
1250.0000000	8.6863995	2098.2675781
1250.0000000	8.7351999	-780.0126953
1250.0000000	8.7840004	890.9924316
1250.0000000	8.8327999	47.9188728
1250.0000000	8.8815994	-11.5005217
1250.0000000	8.9303989	-23.8581390
1250.0000000	8.9792004	-11.5810270
1250.0000000	9.0279999	-5.8781176
1250.0000000	9.0767994	6.9062824
1250.0000000	9.1255999	-17.2022419

1250.0000000	9.1743994	-10.0829067
1250.0000000	9.2231998	36.6475296
1250.0000000	9.2720003	-8.4417372
1250.0000000	9.3207998	-9.8105440
1250.0000000	9.3695993	-10.7689896
1250.0000000	9.4183998	-11.7510128
1250.0000000	9.4672003	-11.1622791
1250.0000000	9.5159998	-14.1437149
1250.0000000	9.5648003	-0.4706488
1250.0000000	9.6135998	-13.3778229
1250.0000000	9.6623993	-10.2276592
1250.0000000	9.7111998	-10.1639519
1250.0000000	9.7600002	-10.8585348
1250.0000000	9.8087997	-10.9949617
1250.0000000	9.8575993	3.0881891
1250.0000000	9.9063997	-7.9900832
1250.0000000	9.9552002	-10.9067011
1250.0000000	10.0039997	-10.0804129
1260.0000000	0.3416000	-36.5343246
1260.0000000	0.3904000	-38.7603722
1260.0000000	0.4392000	-39.5894318
1260.0000000	0.4880000	-39.5622368
1260.0000000	0.5368000	-39.6419258
1260.0000000	0.5856000	-39.2994957
1260.0000000	0.6344000	-38.2328987
1260.0000000	0.6832000	-36.2193031
1260.0000000	0.7320000	-36.0508881
1260.0000000	0.7808000	-36.5344696
1260.0000000	0.8296000	-35.9687805
1260.0000000	0.8784000	-35.2103348
1260.0000000	0.9272000	-34.2391396
1260.0000000	0.9760000	-31.9693375
1260.0000000	1.0247999	-30.2939625
1260.0000000	1.0735999	-29.8379631
1260.0000000	1.1224000	-30.7524586
1260.0000000	1.1711999	-31.4406090
1260.0000000	1.2200000	-31.5000992
1260.0000000	1.2687999	-31.1386471
1260.0000000	1.3176000	-31.0449448
1260.0000000	1.3664000	-30.6713390
1260.0000000	1.4152000	-30.1672211
1260.0000000	1.4640000	-29.5742912
1260.0000000	1.5128000	-37.0429573
1260.0000000	1.5616000	-32.6260910
1260.0000000	1.6104001	-1496.1896973
1260.0000000	1.6592000	-66.2038269
1260.0000000	1.7079999	-27.3474693
1260.0000000	1.7567999	-27.7626038
1260.0000000	1.8055999	-27.5367622
1260.0000000	1.8544000	-26.9431019
1260.0000000	1.9031999	-26.2922974
1260.0000000	1.9520000	-26.3280392
1260.0000000	2.0007999	-25.8707809
1260.0000000	2.0495999	-26.2167549
1260.0000000	2.0983999	-112.5554886
1260.0000000	2.1471999	-8.0890446
1260.0000000	2.1960001	-25.3503151
1260.0000000	2.2448001	-25.3978901

1260.0000000	2.2935998	-26.2494717
1260.0000000	2.3423998	-21.3355598
1260.0000000	2.3912001	16.9634342
1260.0000000	2.4400001	-22.8971405
1260.0000000	2.4888000	-23.4548759
1260.0000000	2.5375998	-12.6770048
1260.0000000	2.5863998	-10.8609009
1260.0000000	2.6352000	-9.0038033
1260.0000000	2.6840000	-15.6931286
1260.0000000	2.7328000	126.9211197
1260.0000000	2.7815998	149.9367676
1260.0000000	2.8304000	-23.7638302
1260.0000000	2.8792000	-20.6957493
1260.0000000	2.9280000	-22.5584641
1260.0000000	2.9768000	-16.9852142
1260.0000000	3.0256000	-20.5333004
1260.0000000	3.0743999	-29.2505531
1260.0000000	3.1231999	-24.1743202
1260.0000000	3.1719999	-29.4118690
1260.0000000	3.2208002	-498.6749573
1260.0000000	3.2695999	-976.1201172
1260.0000000	3.3183999	-20.4943485
1260.0000000	3.3671999	-3361.5808105
1260.0000000	3.4159999	1205.7008057
1260.0000000	3.4648001	-7.2775135
1260.0000000	3.5135999	-26.9653587
1260.0000000	3.5623999	-318.8385010
1260.0000000	3.6111999	-2002.1770020
1260.0000000	3.6600001	-453.4530029
1260.0000000	3.7088001	-598.6777344
1260.0000000	3.7575998	-937.1929932
1260.0000000	3.8063998	-1700.4599609
1260.0000000	3.8552001	-2468.7189941
1260.0000000	3.9040000	-2420.4135742
1260.0000000	3.9528000	1106.5277100
1260.0000000	4.0015998	-242.0331879
1260.0000000	4.0503998	677.3530884
1260.0000000	4.0991998	86.0703278
1260.0000000	4.1479998	-645.5243530
1260.0000000	4.1967998	2.2320900
1260.0000000	4.2456002	-245.1736450
1260.0000000	4.2943997	-481.1915894
1260.0000000	4.3431997	-13.4053917
1260.0000000	4.3920002	-244.8105011
1260.0000000	4.4407997	-22.4002934
1260.0000000	4.4896002	-15.5042057
1260.0000000	4.5383997	-646.1986084
1260.0000000	4.5871997	-12.9071894
1260.0000000	4.6360002	-21.5686188
1260.0000000	4.6847997	-28.1487923
1260.0000000	4.7336001	-19.5378418
1260.0000000	4.7824001	-16.5278358
1260.0000000	4.8311996	-1456.2292480
1260.0000000	4.8800001	225.1980743
1260.0000000	4.9287996	-199.1545715
1260.0000000	4.9776001	1046.1636963
1260.0000000	5.0264001	84.8930893
1260.0000000	5.0751996	-297.5967712

1260.0000000	5.1240001	-12.3398399
1260.0000000	5.1727996	554.4742432
1260.0000000	5.2216001	31.7223358
1260.0000000	5.2704000	-158.0013885
1260.0000000	5.3191996	-39.0507965
1260.0000000	5.3680000	-3515.8481445
1260.0000000	5.4168000	524.0460815
1260.0000000	5.4656000	-199.6953735
1260.0000000	5.5144000	37.7598724
1260.0000000	5.5631995	485.4852600
1260.0000000	5.6120000	390.8378601
1260.0000000	5.6608000	-36.9670563
1260.0000000	5.7096000	62.0862732
1260.0000000	5.7584000	132.2078247
1260.0000000	5.8071995	-11.5453205
1260.0000000	5.8559999	29.5632000
1260.0000000	5.9047999	-16.5999603
1260.0000000	5.9535999	-39.6406975
1260.0000000	6.0023999	-1564.2882080
1260.0000000	6.0511999	-14.5742083
1260.0000000	6.0999999	-19.3857040
1260.0000000	6.1487999	-13.2864246
1260.0000000	6.1975999	-23.1704159
1260.0000000	6.2463999	-1782.0676270
1260.0000000	6.2951999	-19.0474205
1260.0000000	6.3439999	-340.9147339
1260.0000000	6.3927999	-15.0476084
1260.0000000	6.4416003	-12.4022799
1260.0000000	6.4903998	-11.2823925
1260.0000000	6.5391998	-11.8249779
1260.0000000	6.5879998	366.1318359
1260.0000000	6.6367998	-12.0991335
1260.0000000	6.6856003	-13.8195343
1260.0000000	6.7343998	-13.4614782
1260.0000000	6.7831998	-14.3614216
1260.0000000	6.8319998	57.9301682
1260.0000000	6.8807998	-14.0457392
1260.0000000	6.9296002	-429.4328613
1260.0000000	6.9783998	-5.4207759
1260.0000000	7.0271997	-14.1105366
1260.0000000	7.0760002	10.0943956
1260.0000000	7.1247997	-2030.0520020
1260.0000000	7.1736002	-1627.9919434
1260.0000000	7.2223997	-926.3311768
1260.0000000	7.2711997	-200.2174225
1260.0000000	7.3200002	-18.5771961
1260.0000000	7.3687997	178.3845367
1260.0000000	7.4176002	372.7554626
1260.0000000	7.4663997	292.7470703
1260.0000000	7.5151997	-906.0917969
1260.0000000	7.5640001	-1000.7974243
1260.0000000	7.6127996	-2358.2604980
1260.0000000	7.6616001	-757.4052124
1260.0000000	7.7104001	-389.2702637
1260.0000000	7.7591996	40.6906776
1260.0000000	7.8080001	387.3729858
1260.0000000	7.8567996	510.4535217
1260.0000000	7.9056001	-1059.3543701

1260.0000000	7.9544001	1685.8302002
1260.0000000	8.0031996	2175.3220215
1260.0000000	8.0520000	2171.1906738
1260.0000000	8.1007996	2297.5629883
1260.0000000	8.1496000	2450.3142090
1260.0000000	8.1983995	1524.4025879
1260.0000000	8.2472000	-3488.1103516
1260.0000000	8.2959995	-1236.5197754
1260.0000000	8.3448000	-984.9524536
1260.0000000	8.3935995	-902.6951294
1260.0000000	8.4423990	-388.9147339
1260.0000000	8.4912004	-907.7930298
1260.0000000	8.5400000	-2448.6210938
1260.0000000	8.5887995	-1187.0299072
1260.0000000	8.6375999	231.6773987
1260.0000000	8.6863995	2094.1494141
1260.0000000	8.7351999	-778.4884644
1260.0000000	8.7840004	889.2356567
1260.0000000	8.8327999	47.8072701
1260.0000000	8.8815994	-11.5006962
1260.0000000	8.9303989	-23.8544197
1260.0000000	8.9792004	-11.5811090
1260.0000000	9.0279999	-5.8798862
1260.0000000	9.0767994	6.9005642
1260.0000000	9.1255999	-17.2005272
1260.0000000	9.1743994	-10.0833082
1260.0000000	9.2231998	36.6317902
1260.0000000	9.2720003	-8.4426727
1260.0000000	9.3207998	-9.8110828
1260.0000000	9.3695993	-10.7692156
1260.0000000	9.4183998	-11.7508879
1260.0000000	9.4672003	-11.1623201
1260.0000000	9.5159998	-14.1428242
1260.0000000	9.5648003	-0.4740219
1260.0000000	9.6135998	-13.3770323
1260.0000000	9.6623993	-10.2279348
1260.0000000	9.7111998	-10.1642237
1260.0000000	9.7600002	-10.8585768
1260.0000000	9.8087997	-10.9949694
1260.0000000	9.8575993	3.0837650
1260.0000000	9.9063997	-7.9909968
1260.0000000	9.9552002	-10.9067049
1260.0000000	10.0039997	-10.0806599
1270.0000000	0.3416000	-37.3672638
1270.0000000	0.3904000	-39.0029297
1270.0000000	0.4392000	-39.5830879
1270.0000000	0.4880000	-39.5610275
1270.0000000	0.5368000	-39.6431007
1270.0000000	0.5856000	-39.2981148
1270.0000000	0.6344000	-38.2332077
1270.0000000	0.6832000	-36.2215309
1270.0000000	0.7320000	-36.0529404
1270.0000000	0.7808000	-36.5346375
1270.0000000	0.8296000	-35.9669342
1270.0000000	0.8784000	-35.2007713
1270.0000000	0.9272000	-34.2397804
1270.0000000	0.9760000	-31.9668140
1270.0000000	1.0247999	-30.2951984

1270.0000000	1.0735999	-29.8515644
1270.0000000	1.1224000	-30.7505856
1270.0000000	1.1711999	-31.4343071
1270.0000000	1.2200000	-31.4563007
1270.0000000	1.2687999	-31.1173191
1270.0000000	1.3176000	-31.0385590
1270.0000000	1.3664000	-30.6683960
1270.0000000	1.4152000	-30.1674252
1270.0000000	1.4640000	-29.5725822
1270.0000000	1.5128000	-36.9457779
1270.0000000	1.5616000	-32.5842514
1270.0000000	1.6104001	-1493.6469727
1270.0000000	1.6592000	-65.9098969
1270.0000000	1.7079999	-27.3512173
1270.0000000	1.7567999	-27.7626228
1270.0000000	1.8055999	-27.4941139
1270.0000000	1.8544000	-26.7694855
1270.0000000	1.9031999	-25.7265415
1270.0000000	1.9520000	-26.4093361
1270.0000000	2.0007999	-25.8761616
1270.0000000	2.0495999	-26.2187748
1270.0000000	2.0983999	-111.0797043
1270.0000000	2.1471999	-8.3563118
1270.0000000	2.1960001	-25.3462238
1270.0000000	2.2448001	-25.3799782
1270.0000000	2.2935998	-26.1898479
1270.0000000	2.3423998	-21.3499660
1270.0000000	2.3912001	16.3418865
1270.0000000	2.4400001	-22.8996258
1270.0000000	2.4888000	-23.4546585
1270.0000000	2.5375998	-12.6941128
1270.0000000	2.5863998	-10.8806591
1270.0000000	2.6352000	-9.0265474
1270.0000000	2.6840000	-15.7050705
1270.0000000	2.7328000	126.6550980
1270.0000000	2.7815998	149.6303711
1270.0000000	2.8304000	-23.7625256
1270.0000000	2.8792000	-20.6990128
1270.0000000	2.9280000	-22.5578957
1270.0000000	2.9768000	-16.9935837
1270.0000000	3.0256000	-20.5358849
1270.0000000	3.0743999	-29.2391663
1270.0000000	3.1231999	-24.1708641
1270.0000000	3.1719999	-29.4035759
1270.0000000	3.2208002	-498.4371948
1270.0000000	3.2695999	-975.6451416
1270.0000000	3.3183999	-20.4945469
1270.0000000	3.3671999	-3360.1191406
1270.0000000	3.4159999	1205.1655273
1270.0000000	3.4648001	-7.2832222
1270.0000000	3.5135999	-26.9623680
1270.0000000	3.5623999	-318.7088928
1270.0000000	3.6111999	-2001.3172607
1270.0000000	3.6600001	-453.2648926
1270.0000000	3.7088001	-598.4263306
1270.0000000	3.7575998	-936.7944336
1270.0000000	3.8063998	-1699.7301025
1270.0000000	3.8552001	-2467.6555176

1270.0000000	3.9040000	-2419.3710938
1270.0000000	3.9528000	1106.0394287
1270.0000000	4.0015998	-241.9360809
1270.0000000	4.0503998	677.0505371
1270.0000000	4.0991998	86.0246429
1270.0000000	4.1479998	-645.2520752
1270.0000000	4.1967998	2.2228336
1270.0000000	4.2456002	-245.0753479
1270.0000000	4.2943997	-480.9910278
1270.0000000	4.3431997	-13.4075727
1270.0000000	4.3920002	-244.7123871
1270.0000000	4.4407997	-22.3985176
1270.0000000	4.4896002	-15.5050879
1270.0000000	4.5383997	-645.9375610
1270.0000000	4.5871997	-12.9092054
1270.0000000	4.6360002	-21.5670757
1270.0000000	4.6847997	-28.1448669
1270.0000000	4.7336001	-19.5371189
1270.0000000	4.7824001	-16.5282917
1270.0000000	4.8311996	-1455.6979980
1270.0000000	4.8800001	225.1085205
1270.0000000	4.9287996	-199.0873108
1270.0000000	4.9776001	1045.7712402
1270.0000000	5.0264001	84.8555679
1270.0000000	5.0751996	-297.4931335
1270.0000000	5.1240001	-12.3407612
1270.0000000	5.1727996	554.3583374
1270.0000000	5.2216001	31.7128944
1270.0000000	5.2704000	-157.9732819
1270.0000000	5.3191996	-39.0462494
1270.0000000	5.3680000	-3515.1557617
1270.0000000	5.4168000	523.9393311
1270.0000000	5.4656000	-199.6591797
1270.0000000	5.5144000	37.7492142
1270.0000000	5.5631995	485.3866882
1270.0000000	5.6120000	390.7578735
1270.0000000	5.6608000	-36.9629669
1270.0000000	5.7096000	62.0708389
1270.0000000	5.7584000	132.1788940
1270.0000000	5.8071995	-11.5461912
1270.0000000	5.8559999	29.5543461
1270.0000000	5.9047999	-16.5997620
1270.0000000	5.9535999	-39.6359596
1270.0000000	6.0023999	-1563.9836426
1270.0000000	6.0511999	-14.5744200
1270.0000000	6.0999999	-19.3848705
1270.0000000	6.1487999	-13.2868881
1270.0000000	6.1975999	-23.1686516
1270.0000000	6.2463999	-1781.7203369
1270.0000000	6.2951999	-19.0466061
1270.0000000	6.3439999	-340.8506775
1270.0000000	6.3927999	-15.0475903
1270.0000000	6.4416003	-12.4028664
1270.0000000	6.4903998	-11.2830687
1270.0000000	6.5391998	-11.8256598
1270.0000000	6.5879998	365.4411316
1270.0000000	6.6367998	-12.0996828
1270.0000000	6.6856003	-13.8197041

1270.0000000	6.7343998	-13.4616938
1270.0000000	6.7831998	-14.3610058
1270.0000000	6.8319998	57.9110565
1270.0000000	6.8807998	-14.0456009
1270.0000000	6.9296002	-428.6714172
1270.0000000	6.9783998	-5.4335985
1270.0000000	7.0271997	-14.1095085
1270.0000000	7.0760002	10.0522108
1270.0000000	7.1247997	-2026.1062012
1270.0000000	7.1736002	-1624.8291016
1270.0000000	7.2223997	-924.5380249
1270.0000000	7.2711997	-199.8496399
1270.0000000	7.3200002	-18.5652084
1270.0000000	7.3687997	178.0100098
1270.0000000	7.4176002	371.9996338
1270.0000000	7.4663997	292.1494141
1270.0000000	7.5151997	-904.3307495
1270.0000000	7.5640001	-998.8500366
1270.0000000	7.6127996	-2353.6428223
1270.0000000	7.6616001	-755.9338379
1270.0000000	7.7104001	-388.5239868
1270.0000000	7.7591996	40.5896301
1270.0000000	7.8080001	386.5876160
1270.0000000	7.8567996	509.4254456
1270.0000000	7.9056001	-1057.2813721
1270.0000000	7.9544001	1682.5202637
1270.0000000	8.0031996	2171.0524902
1270.0000000	8.0520000	2166.9287109
1270.0000000	8.1007996	2293.0534668
1270.0000000	8.1496000	2445.5061035
1270.0000000	8.1983995	1521.4100342
1270.0000000	8.2472000	-3481.2756348
1270.0000000	8.2959995	-1234.0996094
1270.0000000	8.3448000	-983.0232544
1270.0000000	8.3935995	-900.9274902
1270.0000000	8.4423990	-388.1567383
1270.0000000	8.4912004	-906.0168457
1270.0000000	8.5400000	-2443.8212891
1270.0000000	8.5887995	-1184.7083740
1270.0000000	8.6375999	231.2160645
1270.0000000	8.6863995	2090.0310059
1270.0000000	8.7351999	-776.9641724
1270.0000000	8.7840004	887.4789429
1270.0000000	8.8327999	47.6956596
1270.0000000	8.8815994	-11.5008698
1270.0000000	8.9303989	-23.8507004
1270.0000000	8.9792004	-11.5811920
1270.0000000	9.0279999	-5.8816543
1270.0000000	9.0767994	6.8948479
1270.0000000	9.1255999	-17.1988144
1270.0000000	9.1743994	-10.0837107
1270.0000000	9.2231998	36.6160583
1270.0000000	9.2720003	-8.4436092
1270.0000000	9.3207998	-9.8116226
1270.0000000	9.3695993	-10.7694416
1270.0000000	9.4183998	-11.7507629
1270.0000000	9.4672003	-11.1623611
1270.0000000	9.5159998	-14.1419334

1270.0000000	9.5648003	-0.4773941
1270.0000000	9.6135998	-13.3762417
1270.0000000	9.6623993	-10.2282095
1270.0000000	9.7111998	-10.1644955
1270.0000000	9.7600002	-10.8586187
1270.0000000	9.8087997	-10.9949770
1270.0000000	9.8575993	3.0793438
1270.0000000	9.9063997	-7.9919109
1270.0000000	9.9552002	-10.9067097
1270.0000000	10.0039997	-10.0809078
1280.0000000	0.3416000	-37.5220108
1280.0000000	0.3904000	-39.2454872
1280.0000000	0.4392000	-39.5767441
1280.0000000	0.4880000	-39.5598183
1280.0000000	0.5368000	-39.6442757
1280.0000000	0.5856000	-39.2967339
1280.0000000	0.6344000	-38.2335167
1280.0000000	0.6832000	-36.2237549
1280.0000000	0.7320000	-36.0549927
1280.0000000	0.7808000	-36.5348091
1280.0000000	0.8296000	-35.9650879
1280.0000000	0.8784000	-35.1912041
1280.0000000	0.9272000	-34.2404251
1280.0000000	0.9760000	-31.9642906
1280.0000000	1.0247999	-30.2964344
1280.0000000	1.0735999	-29.8651638
1280.0000000	1.1224000	-30.7487144
1280.0000000	1.1711999	-31.4280033
1280.0000000	1.2200000	-31.4125023
1280.0000000	1.2687999	-31.0959930
1280.0000000	1.3176000	-31.0321732
1280.0000000	1.3664000	-30.6654530
1280.0000000	1.4152000	-30.1676292
1280.0000000	1.4640000	-29.5708714
1280.0000000	1.5128000	-36.8486023
1280.0000000	1.5616000	-32.5424080
1280.0000000	1.6104001	-1491.1041260
1280.0000000	1.6592000	-65.6159668
1280.0000000	1.7079999	-27.3549633
1280.0000000	1.7567999	-27.7626419
1280.0000000	1.8055999	-27.4514637
1280.0000000	1.8544000	-26.7646980
1280.0000000	1.9031999	-25.8958778
1280.0000000	1.9520000	-25.9605846
1280.0000000	2.0007999	-25.8815403
1280.0000000	2.0495999	-26.2207928
1280.0000000	2.0983999	-109.6039276
1280.0000000	2.1471999	-8.6235790
1280.0000000	2.1960001	-25.3421326
1280.0000000	2.2448001	-25.3620682
1280.0000000	2.2935998	-26.1302242
1280.0000000	2.3423998	-21.3643703
1280.0000000	2.3912001	15.7203426
1280.0000000	2.4400001	-22.9021130
1280.0000000	2.4888000	-23.4544430
1280.0000000	2.5375998	-12.7112207
1280.0000000	2.5863998	-10.9004183
1280.0000000	2.6352000	-9.0492916

1280.0000000	2.6840000	-15.7170124
1280.0000000	2.7328000	126.3890915
1280.0000000	2.7815998	149.3239441
1280.0000000	2.8304000	-23.7612228
1280.0000000	2.8792000	-20.7022762
1280.0000000	2.9280000	-22.5573273
1280.0000000	2.9768000	-17.0019550
1280.0000000	3.0256000	-20.5384693
1280.0000000	3.0743999	-29.2277794
1280.0000000	3.1231999	-24.1674099
1280.0000000	3.1719999	-29.3952827
1280.0000000	3.2208002	-498.1994629
1280.0000000	3.2695999	-975.1702881
1280.0000000	3.3183999	-20.4947453
1280.0000000	3.3671999	-3358.6574707
1280.0000000	3.4159999	1204.6302490
1280.0000000	3.4648001	-7.2889290
1280.0000000	3.5135999	-26.9593773
1280.0000000	3.5623999	-318.5793152
1280.0000000	3.6111999	-2000.4575195
1280.0000000	3.6600001	-453.0767822
1280.0000000	3.7088001	-598.1749268
1280.0000000	3.7575998	-936.3959351
1280.0000000	3.8063998	-1699.0002441
1280.0000000	3.8552001	-2466.5922852
1280.0000000	3.9040000	-2418.3286133
1280.0000000	3.9528000	1105.5511475
1280.0000000	4.0015998	-241.8389740
1280.0000000	4.0503998	676.7479858
1280.0000000	4.0991998	85.9789581
1280.0000000	4.1479998	-644.9798584
1280.0000000	4.1967998	2.2135773
1280.0000000	4.2456002	-244.9770355
1280.0000000	4.2943997	-480.7904663
1280.0000000	4.3431997	-13.4097538
1280.0000000	4.3920002	-244.6142731
1280.0000000	4.4407997	-22.3967419
1280.0000000	4.4896002	-15.5059700
1280.0000000	4.5383997	-645.6765137
1280.0000000	4.5871997	-12.9112215
1280.0000000	4.6360002	-21.5655346
1280.0000000	4.6847997	-28.1409416
1280.0000000	4.7336001	-19.5363960
1280.0000000	4.7824001	-16.5287457
1280.0000000	4.8311996	-1455.1666260
1280.0000000	4.8800001	225.0189819
1280.0000000	4.9287996	-199.0200500
1280.0000000	4.9776001	1045.3787842
1280.0000000	5.0264001	84.8180466
1280.0000000	5.0751996	-297.3894958
1280.0000000	5.1240001	-12.3416824
1280.0000000	5.1727996	554.2424927
1280.0000000	5.2216001	31.7034492
1280.0000000	5.2704000	-157.9451904
1280.0000000	5.3191996	-39.0416985
1280.0000000	5.3680000	-3514.4633789
1280.0000000	5.4168000	523.8325806
1280.0000000	5.4656000	-199.6230011

1280.0000000	5.5144000	37.7385635
1280.0000000	5.5631995	485.2881165
1280.0000000	5.6120000	390.6779175
1280.0000000	5.6608000	-36.9588776
1280.0000000	5.7096000	62.0554123
1280.0000000	5.7584000	132.1499634
1280.0000000	5.8071995	-11.5470619
1280.0000000	5.8559999	29.5454960
1280.0000000	5.9047999	-16.5995636
1280.0000000	5.9535999	-39.6312218
1280.0000000	6.0023999	-1563.6793213
1280.0000000	6.0511999	-14.5746317
1280.0000000	6.0999999	-19.3840389
1280.0000000	6.1487999	-13.2873507
1280.0000000	6.1975999	-23.1668873
1280.0000000	6.2463999	-1781.3729248
1280.0000000	6.2951999	-19.0457916
1280.0000000	6.3439999	-340.7866516
1280.0000000	6.3927999	-15.0475721
1280.0000000	6.4416003	-12.4034538
1280.0000000	6.4903998	-11.2837439
1280.0000000	6.5391998	-11.8263407
1280.0000000	6.5879998	364.7504272
1280.0000000	6.6367998	-12.1002321
1280.0000000	6.6856003	-13.8198748
1280.0000000	6.7343998	-13.4619093
1280.0000000	6.7831998	-14.3605900
1280.0000000	6.8319998	57.8919449
1280.0000000	6.8807998	-14.0454617
1280.0000000	6.9296002	-427.9099731
1280.0000000	6.9783998	-5.4464226
1280.0000000	7.0271997	-14.1084805
1280.0000000	7.0760002	10.0100260
1280.0000000	7.1247997	-2022.1602783
1280.0000000	7.1736002	-1621.6661377
1280.0000000	7.2223997	-922.7448730
1280.0000000	7.2711997	-199.4818726
1280.0000000	7.3200002	-18.5532188
1280.0000000	7.3687997	177.6354675
1280.0000000	7.4176002	371.2438049
1280.0000000	7.4663997	291.5517578
1280.0000000	7.5151997	-902.5697021
1280.0000000	7.5640001	-996.9025879
1280.0000000	7.6127996	-2349.0253906
1280.0000000	7.6616001	-754.4625244
1280.0000000	7.7104001	-387.7777100
1280.0000000	7.7591996	40.4885826
1280.0000000	7.8080001	385.8022766
1280.0000000	7.8567996	508.3973083
1280.0000000	7.9056001	-1055.2084961
1280.0000000	7.9544001	1679.2103271
1280.0000000	8.0031996	2166.7829590
1280.0000000	8.0520000	2162.6665039
1280.0000000	8.1007996	2288.5439453
1280.0000000	8.1496000	2440.6982422
1280.0000000	8.1983995	1518.4174805
1280.0000000	8.2472000	-3474.4406738
1280.0000000	8.2959995	-1231.6794434

1280.0000000	8.3448000	-981.0941162
1280.0000000	8.3935995	-899.1597900
1280.0000000	8.4423990	-387.3987122
1280.0000000	8.4912004	-904.2406006
1280.0000000	8.5400000	-2439.0214844
1280.0000000	8.5887995	-1182.3868408
1280.0000000	8.6375999	230.7547455
1280.0000000	8.6863995	2085.9125977
1280.0000000	8.7351999	-775.4399414
1280.0000000	8.7840004	885.7221680
1280.0000000	8.8327999	47.5840530
1280.0000000	8.8815994	-11.5010443
1280.0000000	8.9303989	-23.8469810
1280.0000000	8.9792004	-11.5812740
1280.0000000	9.0279999	-5.8834229
1280.0000000	9.0767994	6.8891296
1280.0000000	9.1255999	-17.1970997
1280.0000000	9.1743994	-10.0841122
1280.0000000	9.2231998	36.6003265
1280.0000000	9.2720003	-8.4445457
1280.0000000	9.3207998	-9.8121624
1280.0000000	9.3695993	-10.7696686
1280.0000000	9.4183998	-11.7506390
1280.0000000	9.4672003	-11.1624022
1280.0000000	9.5159998	-14.1410418
1280.0000000	9.5648003	-0.4807663
1280.0000000	9.6135998	-13.3754511
1280.0000000	9.6623993	-10.2284851
1280.0000000	9.7111998	-10.1647663
1280.0000000	9.7600002	-10.8586597
1280.0000000	9.8087997	-10.9949856
1280.0000000	9.8575993	3.0749216
1280.0000000	9.9063997	-7.9928246
1280.0000000	9.9552002	-10.9067135
1280.0000000	10.0039997	-10.0811558
1290.0000000	0.3416000	-37.0644989
1290.0000000	0.3904000	-39.4880447
1290.0000000	0.4392000	-39.5704041
1290.0000000	0.4880000	-39.5586090
1290.0000000	0.5368000	-39.6454468
1290.0000000	0.5856000	-39.2953529
1290.0000000	0.6344000	-38.2338295
1290.0000000	0.6832000	-36.2259827
1290.0000000	0.7320000	-36.0570488
1290.0000000	0.7808000	-36.5349770
1290.0000000	0.8296000	-35.9632416
1290.0000000	0.8784000	-35.1816406
1290.0000000	0.9272000	-34.2410660
1290.0000000	0.9760000	-31.9617672
1290.0000000	1.0247999	-30.2976685
1290.0000000	1.0735999	-29.8787651
1290.0000000	1.1224000	-30.7468414
1290.0000000	1.1711999	-31.4217014
1290.0000000	1.2200000	-31.3687038
1290.0000000	1.2687999	-31.0746651
1290.0000000	1.3176000	-31.0257874
1290.0000000	1.3664000	-30.6625080
1290.0000000	1.4152000	-30.1678352

1290.0000000	1.4640000	-29.5691624
1290.0000000	1.5128000	-36.7514229
1290.0000000	1.5616000	-32.5005684
1290.0000000	1.6104001	-1488.5612793
1290.0000000	1.6592000	-65.3220367
1290.0000000	1.7079999	-27.3587112
1290.0000000	1.7567999	-27.7626610
1290.0000000	1.8055999	-27.4088135
1290.0000000	1.8544000	-26.3133698
1290.0000000	1.9031999	-25.6074982
1290.0000000	1.9520000	-26.2172527
1290.0000000	2.0007999	-25.8869209
1290.0000000	2.0495999	-26.2228127
1290.0000000	2.0983999	-108.1281509
1290.0000000	2.1471999	-8.8908463
1290.0000000	2.1960001	-25.3380413
1290.0000000	2.2448001	-25.3441563
1290.0000000	2.2935998	-26.0706005
1290.0000000	2.3423998	-21.3787746
1290.0000000	2.3912001	15.0987988
1290.0000000	2.4400001	-22.9046001
1290.0000000	2.4888000	-23.4542255
1290.0000000	2.5375998	-12.7283278
1290.0000000	2.5863998	-10.9201775
1290.0000000	2.6352000	-9.0720358
1290.0000000	2.6840000	-15.7289543
1290.0000000	2.7328000	126.1230698
1290.0000000	2.7815998	149.0175171
1290.0000000	2.8304000	-23.7599182
1290.0000000	2.8792000	-20.7055397
1290.0000000	2.9280000	-22.5567570
1290.0000000	2.9768000	-17.0103264
1290.0000000	3.0256000	-20.5410538
1290.0000000	3.0743999	-29.2163925
1290.0000000	3.1231999	-24.1639538
1290.0000000	3.1719999	-29.3869896
1290.0000000	3.2208002	-497.9617310
1290.0000000	3.2695999	-974.6951904
1290.0000000	3.3183999	-20.4949436
1290.0000000	3.3671999	-3357.1958008
1290.0000000	3.4159999	1204.0949707
1290.0000000	3.4648001	-7.2946377
1290.0000000	3.5135999	-26.9563866
1290.0000000	3.5623999	-318.4497375
1290.0000000	3.6111999	-1999.5977783
1290.0000000	3.6600001	-452.8886719
1290.0000000	3.7088001	-597.9235840
1290.0000000	3.7575998	-935.9973755
1290.0000000	3.8063998	-1698.2703857
1290.0000000	3.8552001	-2465.5288086
1290.0000000	3.9040000	-2417.2861328
1290.0000000	3.9528000	1105.0627441
1290.0000000	4.0015998	-241.7418671
1290.0000000	4.0503998	676.4453735
1290.0000000	4.0991998	85.9332809
1290.0000000	4.1479998	-644.7076416
1290.0000000	4.1967998	2.2043209
1290.0000000	4.2456002	-244.8787384

1290.0000000	4.2943997	-480.5899048
1290.0000000	4.3431997	-13.4119339
1290.0000000	4.3920002	-244.5161591
1290.0000000	4.4407997	-22.3949661
1290.0000000	4.4896002	-15.5068531
1290.0000000	4.5383997	-645.4154053
1290.0000000	4.5871997	-12.9132385
1290.0000000	4.6360002	-21.5639915
1290.0000000	4.6847997	-28.1370163
1290.0000000	4.7336001	-19.5356750
1290.0000000	4.7824001	-16.5291996
1290.0000000	4.8311996	-1454.6353760
1290.0000000	4.8800001	224.9294281
1290.0000000	4.9287996	-198.9527893
1290.0000000	4.9776001	1044.9862061
1290.0000000	5.0264001	84.7805252
1290.0000000	5.0751996	-297.2858887
1290.0000000	5.1240001	-12.3426037
1290.0000000	5.1727996	554.1265869
1290.0000000	5.2216001	31.6940079
1290.0000000	5.2704000	-157.9170685
1290.0000000	5.3191996	-39.0371475
1290.0000000	5.3680000	-3513.7709961
1290.0000000	5.4168000	523.7257690
1290.0000000	5.4656000	-199.5868225
1290.0000000	5.5144000	37.7279053
1290.0000000	5.5631995	485.1895447
1290.0000000	5.6120000	390.5979309
1290.0000000	5.6608000	-36.9547882
1290.0000000	5.7096000	62.0399857
1290.0000000	5.7584000	132.1210327
1290.0000000	5.8071995	-11.5479326
1290.0000000	5.8559999	29.5366459
1290.0000000	5.9047999	-16.5993671
1290.0000000	5.9535999	-39.6264839
1290.0000000	6.0023999	-1563.3748779
1290.0000000	6.0511999	-14.5748425
1290.0000000	6.0999999	-19.3832054
1290.0000000	6.1487999	-13.2878132
1290.0000000	6.1975999	-23.1651211
1290.0000000	6.2463999	-1781.0255127
1290.0000000	6.2951999	-19.0449791
1290.0000000	6.3439999	-340.7225952
1290.0000000	6.3927999	-15.0475531
1290.0000000	6.4416003	-12.4040403
1290.0000000	6.4903998	-11.2844200
1290.0000000	6.5391998	-11.8270216
1290.0000000	6.5879998	364.0597534
1290.0000000	6.6367998	-12.1007814
1290.0000000	6.6856003	-13.8200445
1290.0000000	6.7343998	-13.4621258
1290.0000000	6.7831998	-14.3601751
1290.0000000	6.8319998	57.8728256
1290.0000000	6.8807998	-14.0453234
1290.0000000	6.9296002	-427.1485291
1290.0000000	6.9783998	-5.4592462
1290.0000000	7.0271997	-14.1074524
1290.0000000	7.0760002	9.9678392

1290.0000000	7.1247997	-2018.2144775
1290.0000000	7.1736002	-1618.5032959
1290.0000000	7.2223997	-920.9517212
1290.0000000	7.2711997	-199.1140900
1290.0000000	7.3200002	-18.5412292
1290.0000000	7.3687997	177.2609406
1290.0000000	7.4176002	370.4879761
1290.0000000	7.4663997	290.9541016
1290.0000000	7.5151997	-900.8086548
1290.0000000	7.5640001	-994.9552002
1290.0000000	7.6127996	-2344.4077148
1290.0000000	7.6616001	-752.9911499
1290.0000000	7.7104001	-387.0314331
1290.0000000	7.7591996	40.3875275
1290.0000000	7.8080001	385.0169067
1290.0000000	7.8567996	507.3692322
1290.0000000	7.9056001	-1053.1356201
1290.0000000	7.9544001	1675.9003906
1290.0000000	8.0031996	2162.5134277
1290.0000000	8.0520000	2158.4045410
1290.0000000	8.1007996	2284.0344238
1290.0000000	8.1496000	2435.8901367
1290.0000000	8.1983995	1515.4250488
1290.0000000	8.2472000	-3467.6059570
1290.0000000	8.2959995	-1229.2592773
1290.0000000	8.3448000	-979.1649170
1290.0000000	8.3935995	-897.3921509
1290.0000000	8.4423990	-386.6407166
1290.0000000	8.4912004	-902.4644165
1290.0000000	8.5400000	-2434.2216797
1290.0000000	8.5887995	-1180.0653076
1290.0000000	8.6375999	230.2934265
1290.0000000	8.6863995	2081.7941895
1290.0000000	8.7351999	-773.9157104
1290.0000000	8.7840004	883.9654541
1290.0000000	8.8327999	47.4724426
1290.0000000	8.8815994	-11.5012188
1290.0000000	8.9303989	-23.8432598
1290.0000000	8.9792004	-11.5813560
1290.0000000	9.0279999	-5.8851905
1290.0000000	9.0767994	6.8834114
1290.0000000	9.1255999	-17.1953850
1290.0000000	9.1743994	-10.0845146
1290.0000000	9.2231998	36.5845947
1290.0000000	9.2720003	-8.4454823
1290.0000000	9.3207998	-9.8127012
1290.0000000	9.3695993	-10.7698946
1290.0000000	9.4183998	-11.7505140
1290.0000000	9.4672003	-11.1624432
1290.0000000	9.5159998	-14.1401510
1290.0000000	9.5648003	-0.4841394
1290.0000000	9.6135998	-13.3746605
1290.0000000	9.6623993	-10.2287598
1290.0000000	9.7111998	-10.1650381
1290.0000000	9.7600002	-10.8587017
1290.0000000	9.8087997	-10.9949932
1290.0000000	9.8575993	3.0704994
1290.0000000	9.9063997	-7.9937391

1290.0000000	9.9552002	-10.9067183
1290.0000000	10.0039997	-10.0814028
1300.0000000	0.3416000	-37.4571228
1300.0000000	0.3904000	-39.7306023
1300.0000000	0.4392000	-39.5640602
1300.0000000	0.4880000	-39.5573997
1300.0000000	0.5368000	-39.6466217
1300.0000000	0.5856000	-39.2939682
1300.0000000	0.6344000	-38.2341385
1300.0000000	0.6832000	-36.2282066
1300.0000000	0.7320000	-36.0591011
1300.0000000	0.7808000	-36.5351486
1300.0000000	0.8296000	-35.9613991
1300.0000000	0.8784000	-35.1720772
1300.0000000	0.9272000	-34.2417068
1300.0000000	0.9760000	-31.9592438
1300.0000000	1.0247999	-30.2989044
1300.0000000	1.0735999	-29.8923645
1300.0000000	1.1224000	-30.7449703
1300.0000000	1.1711999	-31.4153976
1300.0000000	1.2200000	-31.3249054
1300.0000000	1.2687999	-31.0533390
1300.0000000	1.3176000	-31.0194016
1300.0000000	1.3664000	-30.6595650
1300.0000000	1.4152000	-30.1680393
1300.0000000	1.4640000	-29.5674534
1300.0000000	1.5128000	-36.6542473
1300.0000000	1.5616000	-32.4587250
1300.0000000	1.6104001	-1486.0185547
1300.0000000	1.6592000	-65.0280991
1300.0000000	1.7079999	-27.3624573
1300.0000000	1.7567999	-27.7626801
1300.0000000	1.8055999	-27.3661652
1300.0000000	1.8544000	-26.7339821
1300.0000000	1.9031999	-25.7775383
1300.0000000	1.9520000	-26.2759304
1300.0000000	2.0007999	-25.8923016
1300.0000000	2.0495999	-26.2248306
1300.0000000	2.0983999	-106.6523743
1300.0000000	2.1471999	-9.1581135
1300.0000000	2.1960001	-25.3339500
1300.0000000	2.2448001	-25.3262444
1300.0000000	2.2935998	-26.0109768
1300.0000000	2.3423998	-21.3931789
1300.0000000	2.3912001	14.4772511
1300.0000000	2.4400001	-22.9070854
1300.0000000	2.4888000	-23.4540081
1300.0000000	2.5375998	-12.7454357
1300.0000000	2.5863998	-10.9399366
1300.0000000	2.6352000	-9.0947800
1300.0000000	2.6840000	-15.7408962
1300.0000000	2.7328000	125.8570480
1300.0000000	2.7815998	148.7111206
1300.0000000	2.8304000	-23.7586136
1300.0000000	2.8792000	-20.7088032
1300.0000000	2.9280000	-22.5561886
1300.0000000	2.9768000	-17.0186958
1300.0000000	3.0256000	-20.5436382

1300.0000000	3.0743999	-29.2050056
1300.0000000	3.1231999	-24.1604996
1300.0000000	3.1719999	-29.3786964
1300.0000000	3.2208002	-497.7239990
1300.0000000	3.2695999	-974.2203369
1300.0000000	3.3183999	-20.4951401
1300.0000000	3.3671999	-3355.7341309
1300.0000000	3.4159999	1203.5596924
1300.0000000	3.4648001	-7.3003464
1300.0000000	3.5135999	-26.9533958
1300.0000000	3.5623999	-318.3201294
1300.0000000	3.6111999	-1998.7380371
1300.0000000	3.6600001	-452.7005005
1300.0000000	3.7088001	-597.6721802
1300.0000000	3.7575998	-935.5988159
1300.0000000	3.8063998	-1697.5406494
1300.0000000	3.8552001	-2464.4653320
1300.0000000	3.9040000	-2416.2436523
1300.0000000	3.9528000	1104.5744629
1300.0000000	4.0015998	-241.6447601
1300.0000000	4.0503998	676.1428223
1300.0000000	4.0991998	85.8875961
1300.0000000	4.1479998	-644.4354248
1300.0000000	4.1967998	2.1950645
1300.0000000	4.2456002	-244.7804413
1300.0000000	4.2943997	-480.3893127
1300.0000000	4.3431997	-13.4141140
1300.0000000	4.3920002	-244.4180450
1300.0000000	4.4407997	-22.3931904
1300.0000000	4.4896002	-15.5077362
1300.0000000	4.5383997	-645.1543579
1300.0000000	4.5871997	-12.9152546
1300.0000000	4.6360002	-21.5624485
1300.0000000	4.6847997	-28.1330910
1300.0000000	4.7336001	-19.5349522
1300.0000000	4.7824001	-16.5296555
1300.0000000	4.8311996	-1454.1041260
1300.0000000	4.8800001	224.8398743
1300.0000000	4.9287996	-198.8855286
1300.0000000	4.9776001	1044.5937500
1300.0000000	5.0264001	84.7430038
1300.0000000	5.0751996	-297.1822510
1300.0000000	5.1240001	-12.3435249
1300.0000000	5.1727996	554.0107422
1300.0000000	5.2216001	31.6845589
1300.0000000	5.2704000	-157.8889771
1300.0000000	5.3191996	-39.0326004
1300.0000000	5.3680000	-3513.0786133
1300.0000000	5.4168000	523.6189575
1300.0000000	5.4656000	-199.5506287
1300.0000000	5.5144000	37.7172470
1300.0000000	5.5631995	485.0909729
1300.0000000	5.6120000	390.5179749
1300.0000000	5.6608000	-36.9506989
1300.0000000	5.7096000	62.0245514
1300.0000000	5.7584000	132.0921021
1300.0000000	5.8071995	-11.5488033
1300.0000000	5.8559999	29.5277920

1300.0000000	5.9047999	-16.5991688
1300.0000000	5.9535999	-39.6217422
1300.0000000	6.0023999	-1563.0705566
1300.0000000	6.0511999	-14.5750542
1300.0000000	6.0999999	-19.3823738
1300.0000000	6.1487999	-13.2882767
1300.0000000	6.1975999	-23.1633568
1300.0000000	6.2463999	-1780.6782227
1300.0000000	6.2951999	-19.0441647
1300.0000000	6.3439999	-340.6585388
1300.0000000	6.3927999	-15.0475349
1300.0000000	6.4416003	-12.4046268
1300.0000000	6.4903998	-11.2850962
1300.0000000	6.5391998	-11.8277025
1300.0000000	6.5879998	363.3690491
1300.0000000	6.6367998	-12.1013298
1300.0000000	6.6856003	-13.8202152
1300.0000000	6.7343998	-13.4623413
1300.0000000	6.7831998	-14.3597593
1300.0000000	6.8319998	57.8537064
1300.0000000	6.8807998	-14.0451841
1300.0000000	6.9296002	-426.3870850
1300.0000000	6.9783998	-5.4720702
1300.0000000	7.0271997	-14.1064243
1300.0000000	7.0760002	9.9256563
1300.0000000	7.1247997	-2014.2685547
1300.0000000	7.1736002	-1615.3404541
1300.0000000	7.2223997	-919.1585693
1300.0000000	7.2711997	-198.7463074
1300.0000000	7.3200002	-18.5292416
1300.0000000	7.3687997	176.8864136
1300.0000000	7.4176002	369.7321472
1300.0000000	7.4663997	290.3564453
1300.0000000	7.5151997	-899.0476074
1300.0000000	7.5640001	-993.0078125
1300.0000000	7.6127996	-2339.7900391
1300.0000000	7.6616001	-751.5197754
1300.0000000	7.7104001	-386.2851563
1300.0000000	7.7591996	40.2864799
1300.0000000	7.8080001	384.2315369
1300.0000000	7.8567996	506.3411560
1300.0000000	7.9056001	-1051.0627441
1300.0000000	7.9544001	1672.5904541
1300.0000000	8.0031996	2158.2436523
1300.0000000	8.0520000	2154.1425781
1300.0000000	8.1007996	2279.5249023
1300.0000000	8.1496000	2431.0820313
1300.0000000	8.1983995	1512.4324951
1300.0000000	8.2472000	-3460.7712402
1300.0000000	8.2959995	-1226.8391113
1300.0000000	8.3448000	-977.2357178
1300.0000000	8.3935995	-895.6244507
1300.0000000	8.4423990	-385.8826904
1300.0000000	8.4912004	-900.6881714
1300.0000000	8.5400000	-2429.4218750
1300.0000000	8.5887995	-1177.7437744
1300.0000000	8.6375999	229.8321075
1300.0000000	8.6863995	2077.6760254

1300.0000000	8.7351999	-772.3914185
1300.0000000	8.7840004	882.2086792
1300.0000000	8.8327999	47.3608398
1300.0000000	8.8815994	-11.5013933
1300.0000000	8.9303989	-23.8395386
1300.0000000	8.9792004	-11.5814390
1300.0000000	9.0279999	-5.8869591
1300.0000000	9.0767994	6.8776951
1300.0000000	9.1255999	-17.1936722
1300.0000000	9.1743994	-10.0849161
1300.0000000	9.2231998	36.5688629
1300.0000000	9.2720003	-8.4464188
1300.0000000	9.3207998	-9.8132410
1300.0000000	9.3695993	-10.7701216
1300.0000000	9.4183998	-11.7503891
1300.0000000	9.4672003	-11.1624851
1300.0000000	9.5159998	-14.1392593
1300.0000000	9.5648003	-0.4875126
1300.0000000	9.6135998	-13.3738699
1300.0000000	9.6623993	-10.2290344
1300.0000000	9.7111998	-10.1653099
1300.0000000	9.7600002	-10.8587437
1300.0000000	9.8087997	-10.9950008
1300.0000000	9.8575993	3.0660772
1300.0000000	9.9063997	-7.9946527
1300.0000000	9.9552002	-10.9067221
1300.0000000	10.0039997	-10.0816507
1310.0000000	0.3416000	-36.7571564
1310.0000000	0.3904000	-39.9731598
1310.0000000	0.4392000	-39.5577164
1310.0000000	0.4880000	-39.5561905
1310.0000000	0.5368000	-39.6477966
1310.0000000	0.5856000	-39.2925873
1310.0000000	0.6344000	-38.2344513
1310.0000000	0.6832000	-36.2304306
1310.0000000	0.7320000	-36.0611534
1310.0000000	0.7808000	-36.5353165
1310.0000000	0.8296000	-35.9595528
1310.0000000	0.8784000	-35.1625099
1310.0000000	0.9272000	-34.2423515
1310.0000000	0.9760000	-31.9567204
1310.0000000	1.0247999	-30.3001404
1310.0000000	1.0735999	-29.9059658
1310.0000000	1.1224000	-30.7430973
1310.0000000	1.1711999	-31.4090958
1310.0000000	1.2200000	-31.2811069
1310.0000000	1.2687999	-31.0320110
1310.0000000	1.3176000	-31.0130157
1310.0000000	1.3664000	-30.6566219
1310.0000000	1.4152000	-30.1682434
1310.0000000	1.4640000	-29.5657444
1310.0000000	1.5128000	-36.5570679
1310.0000000	1.5616000	-32.4168854
1310.0000000	1.6104001	-1483.4757080
1310.0000000	1.6592000	-64.7341690
1310.0000000	1.7079999	-27.3662052
1310.0000000	1.7567999	-27.7626991
1310.0000000	1.8055999	-27.3235149

1310.0000000	1.8544000	-26.8508396
1310.0000000	1.9031999	-26.9405174
1310.0000000	1.9520000	-26.3346062
1310.0000000	2.0007999	-25.8976822
1310.0000000	2.0495999	-26.2268505
1310.0000000	2.0983999	-105.1765976
1310.0000000	2.1471999	-9.4253807
1310.0000000	2.1960001	-25.3298588
1310.0000000	2.2448001	-25.3083344
1310.0000000	2.2935998	-25.9513531
1310.0000000	2.3423998	-21.4075832
1310.0000000	2.3912001	13.8557034
1310.0000000	2.4400001	-22.9095726
1310.0000000	2.4888000	-23.4537907
1310.0000000	2.5375998	-12.7625437
1310.0000000	2.5863998	-10.9596958
1310.0000000	2.6352000	-9.1175241
1310.0000000	2.6840000	-15.7528381
1310.0000000	2.7328000	125.5910416
1310.0000000	2.7815998	148.4046936
1310.0000000	2.8304000	-23.7573090
1310.0000000	2.8792000	-20.7120667
1310.0000000	2.9280000	-22.5556183
1310.0000000	2.9768000	-17.0270653
1310.0000000	3.0256000	-20.5462227
1310.0000000	3.0743999	-29.1936188
1310.0000000	3.1231999	-24.1570435
1310.0000000	3.1719999	-29.3704014
1310.0000000	3.2208002	-497.4862671
1310.0000000	3.2695999	-973.7452393
1310.0000000	3.3183999	-20.4953384
1310.0000000	3.3671999	-3354.2724609
1310.0000000	3.4159999	1203.0242920
1310.0000000	3.4648001	-7.3060541
1310.0000000	3.5135999	-26.9504051
1310.0000000	3.5623999	-318.1905518
1310.0000000	3.6111999	-1997.8781738
1310.0000000	3.6600001	-452.5123901
1310.0000000	3.7088001	-597.4207764
1310.0000000	3.7575998	-935.2003174
1310.0000000	3.8063998	-1696.8107910
1310.0000000	3.8552001	-2463.4018555
1310.0000000	3.9040000	-2415.2011719
1310.0000000	3.9528000	1104.0861816
1310.0000000	4.0015998	-241.5476379
1310.0000000	4.0503998	675.8402710
1310.0000000	4.0991998	85.8419113
1310.0000000	4.1479998	-644.1632080
1310.0000000	4.1967998	2.1858082
1310.0000000	4.2456002	-244.6821289
1310.0000000	4.2943997	-480.1887512
1310.0000000	4.3431997	-13.4162951
1310.0000000	4.3920002	-244.3199310
1310.0000000	4.4407997	-22.3914146
1310.0000000	4.4896002	-15.5086184
1310.0000000	4.5383997	-644.8932495
1310.0000000	4.5871997	-12.9172707
1310.0000000	4.6360002	-21.5609074

1310.0000000	4.6847997	-28.1291656
1310.0000000	4.7336001	-19.5342293
1310.0000000	4.7824001	-16.5301094
1310.0000000	4.8311996	-1453.5728760
1310.0000000	4.8800001	224.7503204
1310.0000000	4.9287996	-198.8182678
1310.0000000	4.9776001	1044.2012939
1310.0000000	5.0264001	84.7054825
1310.0000000	5.0751996	-297.0786133
1310.0000000	5.1240001	-12.3444462
1310.0000000	5.1727996	553.8948975
1310.0000000	5.2216001	31.6751175
1310.0000000	5.2704000	-157.8608551
1310.0000000	5.3191996	-39.0280533
1310.0000000	5.3680000	-3512.3862305
1310.0000000	5.4168000	523.5122070
1310.0000000	5.4656000	-199.5144501
1310.0000000	5.5144000	37.7065964
1310.0000000	5.5631995	484.9924011
1310.0000000	5.6120000	390.4379883
1310.0000000	5.6608000	-36.9466133
1310.0000000	5.7096000	62.0091171
1310.0000000	5.7584000	132.0631714
1310.0000000	5.8071995	-11.5496731
1310.0000000	5.8559999	29.5189419
1310.0000000	5.9047999	-16.5989704
1310.0000000	5.9535999	-39.6170044
1310.0000000	6.0023999	-1562.7659912
1310.0000000	6.0511999	-14.5752659
1310.0000000	6.0999999	-19.3815422
1310.0000000	6.1487999	-13.2887392
1310.0000000	6.1975999	-23.1615906
1310.0000000	6.2463999	-1780.3308105
1310.0000000	6.2951999	-19.0433502
1310.0000000	6.3439999	-340.5944824
1310.0000000	6.3927999	-15.0475168
1310.0000000	6.4416003	-12.4052143
1310.0000000	6.4903998	-11.2857723
1310.0000000	6.5391998	-11.8283834
1310.0000000	6.5879998	362.6783447
1310.0000000	6.6367998	-12.1018791
1310.0000000	6.6856003	-13.8203850
1310.0000000	6.7343998	-13.4625568
1310.0000000	6.7831998	-14.3593435
1310.0000000	6.8319998	57.8345947
1310.0000000	6.8807998	-14.0450459
1310.0000000	6.9296002	-425.6256104
1310.0000000	6.9783998	-5.4848938
1310.0000000	7.0271997	-14.1053972
1310.0000000	7.0760002	9.8834696
1310.0000000	7.1247997	-2010.3227539
1310.0000000	7.1736002	-1612.1774902
1310.0000000	7.2223997	-917.3653564
1310.0000000	7.2711997	-198.3785248
1310.0000000	7.3200002	-18.5172539
1310.0000000	7.3687997	176.5118866
1310.0000000	7.4176002	368.9763184
1310.0000000	7.4663997	289.7587891

1310.0000000	7.5151997	-897.2865601
1310.0000000	7.5640001	-991.0604248
1310.0000000	7.6127996	-2335.1723633
1310.0000000	7.6616001	-750.0485229
1310.0000000	7.7104001	-385.5389404
1310.0000000	7.7591996	40.1854286
1310.0000000	7.8080001	383.4461975
1310.0000000	7.8567996	505.3130188
1310.0000000	7.9056001	-1048.9897461
1310.0000000	7.9544001	1669.2805176
1310.0000000	8.0031996	2153.9741211
1310.0000000	8.0520000	2149.8806152
1310.0000000	8.1007996	2275.0153809
1310.0000000	8.1496000	2426.2741699
1310.0000000	8.1983995	1509.4399414
1310.0000000	8.2472000	-3453.9362793
1310.0000000	8.2959995	-1224.4188232
1310.0000000	8.3448000	-975.3065796
1310.0000000	8.3935995	-893.8568115
1310.0000000	8.4423990	-385.1246948
1310.0000000	8.4912004	-898.9119263
1310.0000000	8.5400000	-2424.6220703
1310.0000000	8.5887995	-1175.4222412
1310.0000000	8.6375999	229.3707886
1310.0000000	8.6863995	2073.5576172
1310.0000000	8.7351999	-770.8671875
1310.0000000	8.7840004	880.4519653
1310.0000000	8.8327999	47.2492294
1310.0000000	8.8815994	-11.5015678
1310.0000000	8.9303989	-23.8358192
1310.0000000	8.9792004	-11.5815210
1310.0000000	9.0279999	-5.8887277
1310.0000000	9.0767994	6.8719769
1310.0000000	9.1255999	-17.1919575
1310.0000000	9.1743994	-10.0853186
1310.0000000	9.2231998	36.5531311
1310.0000000	9.2720003	-8.4473553
1310.0000000	9.3207998	-9.8137798
1310.0000000	9.3695993	-10.7703476
1310.0000000	9.4183998	-11.7502651
1310.0000000	9.4672003	-11.1625261
1310.0000000	9.5159998	-14.1383686
1310.0000000	9.5648003	-0.4908848
1310.0000000	9.6135998	-13.3730793
1310.0000000	9.6623993	-10.2293100
1310.0000000	9.7111998	-10.1655817
1310.0000000	9.7600002	-10.8587856
1310.0000000	9.8087997	-10.9950094
1310.0000000	9.8575993	3.0616541
1310.0000000	9.9063997	-7.9955664
1310.0000000	9.9552002	-10.9067268
1310.0000000	10.0039997	-10.0818977
1320.0000000	0.3416000	-37.0484009
1320.0000000	0.3904000	-39.9646034
1320.0000000	0.4392000	-39.5513725
1320.0000000	0.4880000	-39.5549812
1320.0000000	0.5368000	-39.6489677
1320.0000000	0.5856000	-39.2912064

1320.0000000	0.6344000	-38.2347603
1320.0000000	0.6832000	-36.2326584
1320.0000000	0.7320000	-36.0632057
1320.0000000	0.7808000	-36.5354843
1320.0000000	0.8296000	-35.9577065
1320.0000000	0.8784000	-35.1529465
1320.0000000	0.9272000	-34.2429924
1320.0000000	0.9760000	-31.9541969
1320.0000000	1.0247999	-30.3013763
1320.0000000	1.0735999	-29.9195652
1320.0000000	1.1224000	-30.7412262
1320.0000000	1.1711999	-31.4027920
1320.0000000	1.2200000	-31.2373085
1320.0000000	1.2687999	-31.0106850
1320.0000000	1.3176000	-31.0066299
1320.0000000	1.3664000	-30.6536770
1320.0000000	1.4152000	-30.1684475
1320.0000000	1.4640000	-29.5640335
1320.0000000	1.5128000	-36.4598923
1320.0000000	1.5616000	-32.3750458
1320.0000000	1.6104001	-1480.9329834
1320.0000000	1.6592000	-64.4402390
1320.0000000	1.7079999	-27.3699532
1320.0000000	1.7567999	-27.7627182
1320.0000000	1.8055999	-27.2808647
1320.0000000	1.8544000	-26.5777302
1320.0000000	1.9031999	-26.1020889
1320.0000000	1.9520000	-26.3932838
1320.0000000	2.0007999	-25.9030628
1320.0000000	2.0495999	-26.2288685
1320.0000000	2.0983999	-103.7008133
1320.0000000	2.1471999	-9.6926479
1320.0000000	2.1960001	-25.3257675
1320.0000000	2.2448001	-25.2904224
1320.0000000	2.2935998	-25.8917294
1320.0000000	2.3423998	-21.4219875
1320.0000000	2.3912001	13.2341595
1320.0000000	2.4400001	-22.9120598
1320.0000000	2.4888000	-23.4535732
1320.0000000	2.5375998	-12.7796516
1320.0000000	2.5863998	-10.9794550
1320.0000000	2.6352000	-9.1402683
1320.0000000	2.6840000	-15.7647800
1320.0000000	2.7328000	125.3250198
1320.0000000	2.7815998	148.0982666
1320.0000000	2.8304000	-23.7560062
1320.0000000	2.8792000	-20.7153301
1320.0000000	2.9280000	-22.5550499
1320.0000000	2.9768000	-17.0354366
1320.0000000	3.0256000	-20.5488071
1320.0000000	3.0743999	-29.1822319
1320.0000000	3.1231999	-24.1535873
1320.0000000	3.1719999	-29.3621082
1320.0000000	3.2208002	-497.2485352
1320.0000000	3.2695999	-973.2703857
1320.0000000	3.3183999	-20.4955368
1320.0000000	3.3671999	-3352.8107910
1320.0000000	3.4159999	1202.4890137

1320.0000000	3.4648001	-7.3117619
1320.0000000	3.5135999	-26.9474144
1320.0000000	3.5623999	-318.0609436
1320.0000000	3.6111999	-1997.0184326
1320.0000000	3.6600001	-452.3242798
1320.0000000	3.7088001	-597.1694336
1320.0000000	3.7575998	-934.8017578
1320.0000000	3.8063998	-1696.0809326
1320.0000000	3.8552001	-2462.3383789
1320.0000000	3.9040000	-2414.1586914
1320.0000000	3.9528000	1103.5979004
1320.0000000	4.0015998	-241.4505310
1320.0000000	4.0503998	675.5377197
1320.0000000	4.0991998	85.7962265
1320.0000000	4.1479998	-643.8909912
1320.0000000	4.1967998	2.1765518
1320.0000000	4.2456002	-244.5838318
1320.0000000	4.2943997	-479.9881897
1320.0000000	4.3431997	-13.4184761
1320.0000000	4.3920002	-244.2218170
1320.0000000	4.4407997	-22.3896389
1320.0000000	4.4896002	-15.5095015
1320.0000000	4.5383997	-644.6322021
1320.0000000	4.5871997	-12.9192867
1320.0000000	4.6360002	-21.5593643
1320.0000000	4.6847997	-28.1252403
1320.0000000	4.7336001	-19.5335064
1320.0000000	4.7824001	-16.5305634
1320.0000000	4.8311996	-1453.0416260
1320.0000000	4.8800001	224.6607666
1320.0000000	4.9287996	-198.7510071
1320.0000000	4.9776001	1043.8087158
1320.0000000	5.0264001	84.6679611
1320.0000000	5.0751996	-296.9750061
1320.0000000	5.1240001	-12.3453674
1320.0000000	5.1727996	553.7789917
1320.0000000	5.2216001	31.6656761
1320.0000000	5.2704000	-157.8327637
1320.0000000	5.3191996	-39.0234985
1320.0000000	5.3680000	-3511.6938477
1320.0000000	5.4168000	523.4054565
1320.0000000	5.4656000	-199.4782715
1320.0000000	5.5144000	37.6959381
1320.0000000	5.5631995	484.8938599
1320.0000000	5.6120000	390.3580017
1320.0000000	5.6608000	-36.9425278
1320.0000000	5.7096000	61.9936905
1320.0000000	5.7584000	132.0342407
1320.0000000	5.8071995	-11.5505447
1320.0000000	5.8559999	29.5100918
1320.0000000	5.9047999	-16.5987740
1320.0000000	5.9535999	-39.6122665
1320.0000000	6.0023999	-1562.4616699
1320.0000000	6.0511999	-14.5754766
1320.0000000	6.0999999	-19.3807087
1320.0000000	6.1487999	-13.2892017
1320.0000000	6.1975999	-23.1598244
1320.0000000	6.2463999	-1779.9833984

1320.0000000	6.2951999	-19.0425358
1320.0000000	6.3439999	-340.5304565
1320.0000000	6.3927999	-15.0474987
1320.0000000	6.4416003	-12.4058008
1320.0000000	6.4903998	-11.2864475
1320.0000000	6.5391998	-11.8290653
1320.0000000	6.5879998	361.9876404
1320.0000000	6.6367998	-12.1024284
1320.0000000	6.6856003	-13.8205557
1320.0000000	6.7343998	-13.4627724
1320.0000000	6.7831998	-14.3589277
1320.0000000	6.8319998	57.8154831
1320.0000000	6.8807998	-14.0449076
1320.0000000	6.9296002	-424.8641663
1320.0000000	6.9783998	-5.4977169
1320.0000000	7.0271997	-14.1043692
1320.0000000	7.0760002	9.8412828
1320.0000000	7.1247997	-2006.3768311
1320.0000000	7.1736002	-1609.0146484
1320.0000000	7.2223997	-915.5722046
1320.0000000	7.2711997	-198.0107422
1320.0000000	7.3200002	-18.5052643
1320.0000000	7.3687997	176.1373596
1320.0000000	7.4176002	368.2204895
1320.0000000	7.4663997	289.1611633
1320.0000000	7.5151997	-895.5255737
1320.0000000	7.5640001	-989.1130371
1320.0000000	7.6127996	-2330.5549316
1320.0000000	7.6616001	-748.5771484
1320.0000000	7.7104001	-384.7926636
1320.0000000	7.7591996	40.0843811
1320.0000000	7.8080001	382.6608276
1320.0000000	7.8567996	504.2849426
1320.0000000	7.9056001	-1046.9168701
1320.0000000	7.9544001	1665.9705811
1320.0000000	8.0031996	2149.7045898
1320.0000000	8.0520000	2145.6186523
1320.0000000	8.1007996	2270.5058594
1320.0000000	8.1496000	2421.4660645
1320.0000000	8.1983995	1506.4475098
1320.0000000	8.2472000	-3447.1015625
1320.0000000	8.2959995	-1221.9986572
1320.0000000	8.3448000	-973.3773804
1320.0000000	8.3935995	-892.0891113
1320.0000000	8.4423990	-384.3666687
1320.0000000	8.4912004	-897.1357422
1320.0000000	8.5400000	-2419.8222656
1320.0000000	8.5887995	-1173.1005859
1320.0000000	8.6375999	228.9094696
1320.0000000	8.6863995	2069.4392090
1320.0000000	8.7351999	-769.3428955
1320.0000000	8.7840004	878.6951904
1320.0000000	8.8327999	47.1376228
1320.0000000	8.8815994	-11.5017414
1320.0000000	8.9303989	-23.8320999
1320.0000000	8.9792004	-11.5816031
1320.0000000	9.0279999	-5.8904958
1320.0000000	9.0767994	6.8662586

1320.0000000	9.1255999	-17.1902428
1320.0000000	9.1743994	-10.0857201
1320.0000000	9.2231998	36.5373993
1320.0000000	9.2720003	-8.4482918
1320.0000000	9.3207998	-9.8143196
1320.0000000	9.3695993	-10.7705746
1320.0000000	9.4183998	-11.7501402
1320.0000000	9.4672003	-11.1625671
1320.0000000	9.5159998	-14.1374779
1320.0000000	9.5648003	-0.4942570
1320.0000000	9.6135998	-13.3722887
1320.0000000	9.6623993	-10.2295847
1320.0000000	9.7111998	-10.1658535
1320.0000000	9.7600002	-10.8588266
1320.0000000	9.8087997	-10.9950171
1320.0000000	9.8575993	3.0572319
1320.0000000	9.9063997	-7.9964805
1320.0000000	9.9552002	-10.9067307
1320.0000000	10.0039997	-10.0821457
1330.0000000	0.3416000	-36.7299194
1330.0000000	0.3904000	-39.9560509
1330.0000000	0.4392000	-39.5450287
1330.0000000	0.4880000	-39.5537720
1330.0000000	0.5368000	-39.6501427
1330.0000000	0.5856000	-39.2898254
1330.0000000	0.6344000	-38.2350693
1330.0000000	0.6832000	-36.2348824
1330.0000000	0.7320000	-36.0652580
1330.0000000	0.7808000	-36.5356560
1330.0000000	0.8296000	-35.9558640
1330.0000000	0.8784000	-35.1433830
1330.0000000	0.9272000	-34.2436333
1330.0000000	0.9760000	-31.9516735
1330.0000000	1.0247999	-30.3026104
1330.0000000	1.0735999	-29.9331665
1330.0000000	1.1224000	-30.7393532
1330.0000000	1.1711999	-31.3964901
1330.0000000	1.2200000	-31.1935101
1330.0000000	1.2687999	-30.9893589
1330.0000000	1.3176000	-31.0002441
1330.0000000	1.3664000	-30.6507339
1330.0000000	1.4152000	-30.1686516
1330.0000000	1.4640000	-29.5623245
1330.0000000	1.5128000	-36.3627129
1330.0000000	1.5616000	-32.3332024
1330.0000000	1.6104001	-1478.3901367
1330.0000000	1.6592000	-64.1463089
1330.0000000	1.7079999	-27.3736992
1330.0000000	1.7567999	-27.7627373
1330.0000000	1.8055999	-27.2382164
1330.0000000	1.8544000	-26.7340088
1330.0000000	1.9031999	-26.6724033
1330.0000000	1.9520000	-26.4519596
1330.0000000	2.0007999	-25.9084415
1330.0000000	2.0495999	-26.2308884
1330.0000000	2.0983999	-102.2250366
1330.0000000	2.1471999	-9.9599152
1330.0000000	2.1960001	-25.3216763

1330.0000000	2.2448001	-25.2725105
1330.0000000	2.2935998	-25.8321056
1330.0000000	2.3423998	-21.4363937
1330.0000000	2.3912001	12.6126156
1330.0000000	2.4400001	-22.9145451
1330.0000000	2.4888000	-23.4533558
1330.0000000	2.5375998	-12.7967606
1330.0000000	2.5863998	-10.9992132
1330.0000000	2.6352000	-9.1630125
1330.0000000	2.6840000	-15.7767220
1330.0000000	2.7328000	125.0590134
1330.0000000	2.7815998	147.7918396
1330.0000000	2.8304000	-23.7547016
1330.0000000	2.8792000	-20.7185936
1330.0000000	2.9280000	-22.5544815
1330.0000000	2.9768000	-17.0438080
1330.0000000	3.0256000	-20.5513916
1330.0000000	3.0743999	-29.1708450
1330.0000000	3.1231999	-24.1501331
1330.0000000	3.1719999	-29.3538151
1330.0000000	3.2208002	-497.0107727
1330.0000000	3.2695999	-972.7952881
1330.0000000	3.3183999	-20.4957352
1330.0000000	3.3671999	-3351.3491211
1330.0000000	3.4159999	1201.9537354
1330.0000000	3.4648001	-7.3174706
1330.0000000	3.5135999	-26.9444237
1330.0000000	3.5623999	-317.9313660
1330.0000000	3.6111999	-1996.1586914
1330.0000000	3.6600001	-452.1361084
1330.0000000	3.7088001	-596.9180298
1330.0000000	3.7575998	-934.4032593
1330.0000000	3.8063998	-1695.3510742
1330.0000000	3.8552001	-2461.2751465
1330.0000000	3.9040000	-2413.1162109
1330.0000000	3.9528000	1103.1096191
1330.0000000	4.0015998	-241.3534241
1330.0000000	4.0503998	675.2351685
1330.0000000	4.0991998	85.7505417
1330.0000000	4.1479998	-643.6187744
1330.0000000	4.1967998	2.1672955
1330.0000000	4.2456002	-244.4855347
1330.0000000	4.2943997	-479.7876282
1330.0000000	4.3431997	-13.4206562
1330.0000000	4.3920002	-244.1237030
1330.0000000	4.4407997	-22.3878632
1330.0000000	4.4896002	-15.5103836
1330.0000000	4.5383997	-644.3711548
1330.0000000	4.5871997	-12.9213028
1330.0000000	4.6360002	-21.5578213
1330.0000000	4.6847997	-28.1213150
1330.0000000	4.7336001	-19.5327854
1330.0000000	4.7824001	-16.5310173
1330.0000000	4.8311996	-1452.5102539
1330.0000000	4.8800001	224.5712280
1330.0000000	4.9287996	-198.6837463
1330.0000000	4.9776001	1043.4162598
1330.0000000	5.0264001	84.6304398

1330.0000000	5.0751996	-296.8713684
1330.0000000	5.1240001	-12.3462887
1330.0000000	5.1727996	553.6631470
1330.0000000	5.2216001	31.6562271
1330.0000000	5.2704000	-157.8046417
1330.0000000	5.3191996	-39.0189514
1330.0000000	5.3680000	-3511.0014648
1330.0000000	5.4168000	523.2986450
1330.0000000	5.4656000	-199.4420776
1330.0000000	5.5144000	37.6852875
1330.0000000	5.5631995	484.7952881
1330.0000000	5.6120000	390.2780457
1330.0000000	5.6608000	-36.9384384
1330.0000000	5.7096000	61.9782562
1330.0000000	5.7584000	132.0052948
1330.0000000	5.8071995	-11.5514145
1330.0000000	5.8559999	29.5012379
1330.0000000	5.9047999	-16.5985756
1330.0000000	5.9535999	-39.6075249
1330.0000000	6.0023999	-1562.1571045
1330.0000000	6.0511999	-14.5756884
1330.0000000	6.0999999	-19.3798771
1330.0000000	6.1487999	-13.2896652
1330.0000000	6.1975999	-23.1580601
1330.0000000	6.2463999	-1779.6359863
1330.0000000	6.2951999	-19.0417213
1330.0000000	6.3439999	-340.4663696
1330.0000000	6.3927999	-15.0474796
1330.0000000	6.4416003	-12.4063883
1330.0000000	6.4903998	-11.2871237
1330.0000000	6.5391998	-11.8297462
1330.0000000	6.5879998	361.2969360
1330.0000000	6.6367998	-12.1029778
1330.0000000	6.6856003	-13.8207254
1330.0000000	6.7343998	-13.4629889
1330.0000000	6.7831998	-14.3585119
1330.0000000	6.8319998	57.7963638
1330.0000000	6.8807998	-14.0447683
1330.0000000	6.9296002	-424.1027222
1330.0000000	6.9783998	-5.5105410
1330.0000000	7.0271997	-14.1033411
1330.0000000	7.0760002	9.7990999
1330.0000000	7.1247997	-2002.4310303
1330.0000000	7.1736002	-1605.8518066
1330.0000000	7.2223997	-913.7790527
1330.0000000	7.2711997	-197.6429749
1330.0000000	7.3200002	-18.4932747
1330.0000000	7.3687997	175.7628174
1330.0000000	7.4176002	367.4646606
1330.0000000	7.4663997	288.5635071
1330.0000000	7.5151997	-893.7645264
1330.0000000	7.5640001	-987.1655884
1330.0000000	7.6127996	-2325.9372559
1330.0000000	7.6616001	-747.1058350
1330.0000000	7.7104001	-384.0463867
1330.0000000	7.7591996	39.9833336
1330.0000000	7.8080001	381.8754578
1330.0000000	7.8567996	503.2568665

1330.0000000	7.9056001	-1044.8439941
1330.0000000	7.9544001	1662.6606445
1330.0000000	8.0031996	2145.4348145
1330.0000000	8.0520000	2141.3566895
1330.0000000	8.1007996	2265.9963379
1330.0000000	8.1496000	2416.6582031
1330.0000000	8.1983995	1503.4549561
1330.0000000	8.2472000	-3440.2666016
1330.0000000	8.2959995	-1219.5784912
1330.0000000	8.3448000	-971.4481812
1330.0000000	8.3935995	-890.3214722
1330.0000000	8.4423990	-383.6086731
1330.0000000	8.4912004	-895.3594971
1330.0000000	8.5400000	-2415.0224609
1330.0000000	8.5887995	-1170.7790527
1330.0000000	8.6375999	228.4481354
1330.0000000	8.6863995	2065.3208008
1330.0000000	8.7351999	-767.8186646
1330.0000000	8.7840004	876.9384766
1330.0000000	8.8327999	47.0260124
1330.0000000	8.8815994	-11.5019159
1330.0000000	8.9303989	-23.8283806
1330.0000000	8.9792004	-11.5816860
1330.0000000	9.0279999	-5.8922644
1330.0000000	9.0767994	6.8605404
1330.0000000	9.1255999	-17.1885300
1330.0000000	9.1743994	-10.0861225
1330.0000000	9.2231998	36.5216675
1330.0000000	9.2720003	-8.4492273
1330.0000000	9.3207998	-9.8148584
1330.0000000	9.3695993	-10.7708006
1330.0000000	9.4183998	-11.7500162
1330.0000000	9.4672003	-11.1626081
1330.0000000	9.5159998	-14.1365871
1330.0000000	9.5648003	-0.4976301
1330.0000000	9.6135998	-13.3714981
1330.0000000	9.6623993	-10.2298603
1330.0000000	9.7111998	-10.1661243
1330.0000000	9.7600002	-10.8588686
1330.0000000	9.8087997	-10.9950247
1330.0000000	9.8575993	3.0528097
1330.0000000	9.9063997	-7.9973941
1330.0000000	9.9552002	-10.9067354
1330.0000000	10.0039997	-10.0823927
1340.0000000	0.3416000	-36.9206429
1340.0000000	0.3904000	-39.9474945
1340.0000000	0.4392000	-39.5386848
1340.0000000	0.4880000	-39.5525627
1340.0000000	0.5368000	-39.6513176
1340.0000000	0.5856000	-39.2884445
1340.0000000	0.6344000	-38.2353821
1340.0000000	0.6832000	-36.2371101
1340.0000000	0.7320000	-36.0673103
1340.0000000	0.7808000	-36.5358238
1340.0000000	0.8296000	-35.9540176
1340.0000000	0.8784000	-35.1338158
1340.0000000	0.9272000	-34.2442741
1340.0000000	0.9760000	-31.9491501

1340.0000000	1.0247999	-30.3038464
1340.0000000	1.0735999	-29.9467659
1340.0000000	1.1224000	-30.7374821
1340.0000000	1.1711999	-31.3901863
1340.0000000	1.2200000	-31.1497135
1340.0000000	1.2687999	-30.9680309
1340.0000000	1.3176000	-30.9938583
1340.0000000	1.3664000	-30.6477909
1340.0000000	1.4152000	-30.1688557
1340.0000000	1.4640000	-29.5606155
1340.0000000	1.5128000	-36.2655334
1340.0000000	1.5616000	-32.2913628
1340.0000000	1.6104001	-1475.8472900
1340.0000000	1.6592000	-63.8523712
1340.0000000	1.7079999	-27.3774471
1340.0000000	1.7567999	-27.7627563
1340.0000000	1.8055999	-27.1955662
1340.0000000	1.8544000	-26.6325836
1340.0000000	1.9031999	-26.2406139
1340.0000000	1.9520000	-26.5106373
1340.0000000	2.0007999	-25.9138222
1340.0000000	2.0495999	-26.2329063
1340.0000000	2.0983999	-100.7492599
1340.0000000	2.1471999	-10.2271824
1340.0000000	2.1960001	-25.3175850
1340.0000000	2.2448001	-25.2546005
1340.0000000	2.2935998	-25.7724838
1340.0000000	2.3423998	-21.4507980
1340.0000000	2.3912001	11.9910679
1340.0000000	2.4400001	-22.9170322
1340.0000000	2.4888000	-23.4531384
1340.0000000	2.5375998	-12.8138685
1340.0000000	2.5863998	-11.0189724
1340.0000000	2.6352000	-9.1857567
1340.0000000	2.6840000	-15.7886639
1340.0000000	2.7328000	124.7929916
1340.0000000	2.7815998	147.4854431
1340.0000000	2.8304000	-23.7533970
1340.0000000	2.8792000	-20.7218571
1340.0000000	2.9280000	-22.5539112
1340.0000000	2.9768000	-17.0521774
1340.0000000	3.0256000	-20.5539761
1340.0000000	3.0743999	-29.1594582
1340.0000000	3.1231999	-24.1466770
1340.0000000	3.1719999	-29.3455219
1340.0000000	3.2208002	-496.7730408
1340.0000000	3.2695999	-972.3204346
1340.0000000	3.3183999	-20.4959335
1340.0000000	3.3671999	-3349.8874512
1340.0000000	3.4159999	1201.4184570
1340.0000000	3.4648001	-7.3231792
1340.0000000	3.5135999	-26.9414330
1340.0000000	3.5623999	-317.8017578
1340.0000000	3.6111999	-1995.2989502
1340.0000000	3.6600001	-451.9479980
1340.0000000	3.7088001	-596.6666260
1340.0000000	3.7575998	-934.0046997
1340.0000000	3.8063998	-1694.6212158

1340.0000000	3.8552001	-2460.2116699
1340.0000000	3.9040000	-2412.0737305
1340.0000000	3.9528000	1102.6213379
1340.0000000	4.0015998	-241.2563171
1340.0000000	4.0503998	674.9326172
1340.0000000	4.0991998	85.7048569
1340.0000000	4.1479998	-643.3465576
1340.0000000	4.1967998	2.1580391
1340.0000000	4.2456002	-244.3872223
1340.0000000	4.2943997	-479.5870056
1340.0000000	4.3431997	-13.4228363
1340.0000000	4.3920002	-244.0255890
1340.0000000	4.4407997	-22.3860893
1340.0000000	4.4896002	-15.5112667
1340.0000000	4.5383997	-644.1100464
1340.0000000	4.5871997	-12.9233189
1340.0000000	4.6360002	-21.5562801
1340.0000000	4.6847997	-28.1173897
1340.0000000	4.7336001	-19.5320625
1340.0000000	4.7824001	-16.5314732
1340.0000000	4.8311996	-1451.9790039
1340.0000000	4.8800001	224.4816742
1340.0000000	4.9287996	-198.6164856
1340.0000000	4.9776001	1043.0238037
1340.0000000	5.0264001	84.5929184
1340.0000000	5.0751996	-296.7677307
1340.0000000	5.1240001	-12.3472099
1340.0000000	5.1727996	553.5473022
1340.0000000	5.2216001	31.6467857
1340.0000000	5.2704000	-157.7765503
1340.0000000	5.3191996	-39.0144043
1340.0000000	5.3680000	-3510.3090820
1340.0000000	5.4168000	523.1918335
1340.0000000	5.4656000	-199.4058990
1340.0000000	5.5144000	37.6746292
1340.0000000	5.5631995	484.6967163
1340.0000000	5.6120000	390.1980591
1340.0000000	5.6608000	-36.9343491
1340.0000000	5.7096000	61.9628220
1340.0000000	5.7584000	131.9763641
1340.0000000	5.8071995	-11.5522861
1340.0000000	5.8559999	29.4923878
1340.0000000	5.9047999	-16.5983772
1340.0000000	5.9535999	-39.6027870
1340.0000000	6.0023999	-1561.8527832
1340.0000000	6.0511999	-14.5759001
1340.0000000	6.0999999	-19.3790436
1340.0000000	6.1487999	-13.2901278
1340.0000000	6.1975999	-23.1562958
1340.0000000	6.2463999	-1779.2886963
1340.0000000	6.2951999	-19.0409069
1340.0000000	6.3439999	-340.4023438
1340.0000000	6.3927999	-15.0474615
1340.0000000	6.4416003	-12.4069748
1340.0000000	6.4903998	-11.2877998
1340.0000000	6.5391998	-11.8304272
1340.0000000	6.5879998	360.6062622
1340.0000000	6.6367998	-12.1035271

1340.0000000	6.6856003	-13.8208961
1340.0000000	6.7343998	-13.4632044
1340.0000000	6.7831998	-14.3580961
1340.0000000	6.8319998	57.7772522
1340.0000000	6.8807998	-14.0446301
1340.0000000	6.9296002	-423.3412781
1340.0000000	6.9783998	-5.5233645
1340.0000000	7.0271997	-14.1023130
1340.0000000	7.0760002	9.7569132
1340.0000000	7.1247997	-1998.4851074
1340.0000000	7.1736002	-1602.6889648
1340.0000000	7.2223997	-911.9859009
1340.0000000	7.2711997	-197.2751923
1340.0000000	7.3200002	-18.4812870
1340.0000000	7.3687997	175.3882904
1340.0000000	7.4176002	366.7088318
1340.0000000	7.4663997	287.9658508
1340.0000000	7.5151997	-892.0034790
1340.0000000	7.5640001	-985.2182007
1340.0000000	7.6127996	-2321.3195801
1340.0000000	7.6616001	-745.6344604
1340.0000000	7.7104001	-383.3001099
1340.0000000	7.7591996	39.8822823
1340.0000000	7.8080001	381.0901184
1340.0000000	7.8567996	502.2287598
1340.0000000	7.9056001	-1042.7711182
1340.0000000	7.9544001	1659.3507080
1340.0000000	8.0031996	2141.1652832
1340.0000000	8.0520000	2137.0947266
1340.0000000	8.1007996	2261.4868164
1340.0000000	8.1496000	2411.8500977
1340.0000000	8.1983995	1500.4624023
1340.0000000	8.2472000	-3433.4318848
1340.0000000	8.2959995	-1217.1583252
1340.0000000	8.3448000	-969.5190430
1340.0000000	8.3935995	-888.5537720
1340.0000000	8.4423990	-382.8506470
1340.0000000	8.4912004	-893.5833130
1340.0000000	8.5400000	-2410.2226563
1340.0000000	8.5887995	-1168.4575195
1340.0000000	8.6375999	227.9868164
1340.0000000	8.6863995	2061.2023926
1340.0000000	8.7351999	-766.2944336
1340.0000000	8.7840004	875.1817017
1340.0000000	8.8327999	46.9144058
1340.0000000	8.8815994	-11.5020905
1340.0000000	8.9303989	-23.8246593
1340.0000000	8.9792004	-11.5817680
1340.0000000	9.0279999	-5.8940320
1340.0000000	9.0767994	6.8548241
1340.0000000	9.1255999	-17.1868172
1340.0000000	9.1743994	-10.0865250
1340.0000000	9.2231998	36.5059280
1340.0000000	9.2720003	-8.4501638
1340.0000000	9.3207998	-9.8153982
1340.0000000	9.3695993	-10.7710266
1340.0000000	9.4183998	-11.7498913
1340.0000000	9.4672003	-11.1626492

1340.0000000	9.5159998	-14.1356964
1340.0000000	9.5648003	-0.5010033
1340.0000000	9.6135998	-13.3707066
1340.0000000	9.6623993	-10.2301350
1340.0000000	9.7111998	-10.1663961
1340.0000000	9.7600002	-10.8589106
1340.0000000	9.8087997	-10.9950333
1340.0000000	9.8575993	3.0483866
1340.0000000	9.9063997	-7.9983082
1340.0000000	9.9552002	-10.9067392
1340.0000000	10.0039997	-10.0826406
1350.0000000	0.3416000	-36.9955215
1350.0000000	0.3904000	-39.9389381
1350.0000000	0.4392000	-39.5323410
1350.0000000	0.4880000	-39.5513535
1350.0000000	0.5368000	-39.6524887
1350.0000000	0.5856000	-39.2870636
1350.0000000	0.6344000	-38.2356911
1350.0000000	0.6832000	-36.2393341
1350.0000000	0.7320000	-36.0693626
1350.0000000	0.7808000	-36.5359917
1350.0000000	0.8296000	-35.9521713
1350.0000000	0.8784000	-35.1242523
1350.0000000	0.9272000	-34.2449188
1350.0000000	0.9760000	-31.9466267
1350.0000000	1.0247999	-30.3050823
1350.0000000	1.0735999	-29.9603672
1350.0000000	1.1224000	-30.7356091
1350.0000000	1.1711999	-31.3838844
1350.0000000	1.2200000	-31.1059151
1350.0000000	1.2687999	-30.9467049
1350.0000000	1.3176000	-30.9874744
1350.0000000	1.3664000	-30.6448460
1350.0000000	1.4152000	-30.1690617
1350.0000000	1.4640000	-29.5589066
1350.0000000	1.5128000	-36.1683578
1350.0000000	1.5616000	-32.2495193
1350.0000000	1.6104001	-1473.3045654
1350.0000000	1.6592000	-63.5584412
1350.0000000	1.7079999	-27.3811932
1350.0000000	1.7567999	-27.7627754
1350.0000000	1.8055999	-27.1529160
1350.0000000	1.8544000	-26.8671913
1350.0000000	1.9031999	-26.8192081
1350.0000000	1.9520000	-26.5693150
1350.0000000	2.0007999	-25.9192028
1350.0000000	2.0495999	-26.2349262
1350.0000000	2.0983999	-99.2734756
1350.0000000	2.1471999	-10.4944496
1350.0000000	2.1960001	-25.3134937
1350.0000000	2.2448001	-25.2366886
1350.0000000	2.2935998	-25.7128601
1350.0000000	2.3423998	-21.4652023
1350.0000000	2.3912001	11.3695202
1350.0000000	2.4400001	-22.9195175
1350.0000000	2.4888000	-23.4529209
1350.0000000	2.5375998	-12.8309765
1350.0000000	2.5863998	-11.0387316

1350.0000000	2.6352000	-9.2085009
1350.0000000	2.6840000	-15.8006058
1350.0000000	2.7328000	124.5269699
1350.0000000	2.7815998	147.1790161
1350.0000000	2.8304000	-23.7520924
1350.0000000	2.8792000	-20.7251205
1350.0000000	2.9280000	-22.5533428
1350.0000000	2.9768000	-17.0605488
1350.0000000	3.0256000	-20.5565605
1350.0000000	3.0743999	-29.1480713
1350.0000000	3.1231999	-24.1432209
1350.0000000	3.1719999	-29.3372288
1350.0000000	3.2208002	-496.5353088
1350.0000000	3.2695999	-971.8453369
1350.0000000	3.3183999	-20.4961300
1350.0000000	3.3671999	-3348.4257813
1350.0000000	3.4159999	1200.8831787
1350.0000000	3.4648001	-7.3288870
1350.0000000	3.5135999	-26.9384422
1350.0000000	3.5623999	-317.6721802
1350.0000000	3.6111999	-1994.4392090
1350.0000000	3.6600001	-451.7598877
1350.0000000	3.7088001	-596.4152832
1350.0000000	3.7575998	-933.6061401
1350.0000000	3.8063998	-1693.8913574
1350.0000000	3.8552001	-2459.1481934
1350.0000000	3.9040000	-2411.0312500
1350.0000000	3.9528000	1102.1329346
1350.0000000	4.0015998	-241.1592102
1350.0000000	4.0503998	674.6300659
1350.0000000	4.0991998	85.6591721
1350.0000000	4.1479998	-643.0742798
1350.0000000	4.1967998	2.1487827
1350.0000000	4.2456002	-244.2889252
1350.0000000	4.2943997	-479.3864441
1350.0000000	4.3431997	-13.4250174
1350.0000000	4.3920002	-243.9274750
1350.0000000	4.4407997	-22.3843117
1350.0000000	4.4896002	-15.5121489
1350.0000000	4.5383997	-643.8489990
1350.0000000	4.5871997	-12.9253349
1350.0000000	4.6360002	-21.5547371
1350.0000000	4.6847997	-28.1134644
1350.0000000	4.7336001	-19.5313396
1350.0000000	4.7824001	-16.5319271
1350.0000000	4.8311996	-1451.4477539
1350.0000000	4.8800001	224.3921204
1350.0000000	4.9287996	-198.5492096
1350.0000000	4.9776001	1042.6312256
1350.0000000	5.0264001	84.5554047
1350.0000000	5.0751996	-296.6641235
1350.0000000	5.1240001	-12.3481312
1350.0000000	5.1727996	553.4313965
1350.0000000	5.2216001	31.6373444
1350.0000000	5.2704000	-157.7484283
1350.0000000	5.3191996	-39.0098572
1350.0000000	5.3680000	-3509.6166992
1350.0000000	5.4168000	523.0850830

1350.0000000	5.4656000	-199.3697205
1350.0000000	5.5144000	37.6639709
1350.0000000	5.5631995	484.5981445
1350.0000000	5.6120000	390.1180725
1350.0000000	5.6608000	-36.9302597
1350.0000000	5.7096000	61.9473953
1350.0000000	5.7584000	131.9474335
1350.0000000	5.8071995	-11.5531559
1350.0000000	5.8559999	29.4835377
1350.0000000	5.9047999	-16.5981789
1350.0000000	5.9535999	-39.5980492
1350.0000000	6.0023999	-1561.5483398
1350.0000000	6.0511999	-14.5761108
1350.0000000	6.0999999	-19.3782120
1350.0000000	6.1487999	-13.2905903
1350.0000000	6.1975999	-23.1545296
1350.0000000	6.2463999	-1778.9412842
1350.0000000	6.2951999	-19.0400925
1350.0000000	6.3439999	-340.3382874
1350.0000000	6.3927999	-15.0474434
1350.0000000	6.4416003	-12.4075613
1350.0000000	6.4903998	-11.2884760
1350.0000000	6.5391998	-11.8311081
1350.0000000	6.5879998	359.9155579
1350.0000000	6.6367998	-12.1040764
1350.0000000	6.6856003	-13.8210659
1350.0000000	6.7343998	-13.4634199
1350.0000000	6.7831998	-14.3576803
1350.0000000	6.8319998	57.7581406
1350.0000000	6.8807998	-14.0444908
1350.0000000	6.9296002	-422.5798340
1350.0000000	6.9783998	-5.5361876
1350.0000000	7.0271997	-14.1012850
1350.0000000	7.0760002	9.7147264
1350.0000000	7.1247997	-1994.5393066
1350.0000000	7.1736002	-1599.5261230
1350.0000000	7.2223997	-910.1927490
1350.0000000	7.2711997	-196.9074097
1350.0000000	7.3200002	-18.4692993
1350.0000000	7.3687997	175.0137634
1350.0000000	7.4176002	365.9530029
1350.0000000	7.4663997	287.3681946
1350.0000000	7.5151997	-890.2424316
1350.0000000	7.5640001	-983.2708130
1350.0000000	7.6127996	-2316.7019043
1350.0000000	7.6616001	-744.1630859
1350.0000000	7.7104001	-382.5538330
1350.0000000	7.7591996	39.7812347
1350.0000000	7.8080001	380.3047485
1350.0000000	7.8567996	501.2006836
1350.0000000	7.9056001	-1040.6981201
1350.0000000	7.9544001	1656.0408936
1350.0000000	8.0031996	2136.8957520
1350.0000000	8.0520000	2132.8325195
1350.0000000	8.1007996	2256.9772949
1350.0000000	8.1496000	2407.0422363
1350.0000000	8.1983995	1497.4699707
1350.0000000	8.2472000	-3426.5971680

1350.0000000	8.2959995	-1214.7381592
1350.0000000	8.3448000	-967.5898438
1350.0000000	8.3935995	-886.7860718
1350.0000000	8.4423990	-382.0926514
1350.0000000	8.4912004	-891.8070679
1350.0000000	8.5400000	-2405.4228516
1350.0000000	8.5887995	-1166.1359863
1350.0000000	8.6375999	227.5254974
1350.0000000	8.6863995	2057.0842285
1350.0000000	8.7351999	-764.7701416
1350.0000000	8.7840004	873.4249878
1350.0000000	8.8327999	46.8028030
1350.0000000	8.8815994	-11.5022650
1350.0000000	8.9303989	-23.8209400
1350.0000000	8.9792004	-11.5818501
1350.0000000	9.0279999	-5.8958006
1350.0000000	9.0767994	6.8491058
1350.0000000	9.1255999	-17.1851025
1350.0000000	9.1743994	-10.0869265
1350.0000000	9.2231998	36.4901962
1350.0000000	9.2720003	-8.4511003
1350.0000000	9.3207998	-9.8159370
1350.0000000	9.3695993	-10.7712536
1350.0000000	9.4183998	-11.7497673
1350.0000000	9.4672003	-11.1626902
1350.0000000	9.5159998	-14.1348047
1350.0000000	9.5648003	-0.5043755
1350.0000000	9.6135998	-13.3699169
1350.0000000	9.6623993	-10.2304096
1350.0000000	9.7111998	-10.1666679
1350.0000000	9.7600002	-10.8589516
1350.0000000	9.8087997	-10.9950409
1350.0000000	9.8575993	3.0439644
1350.0000000	9.9063997	-7.9992218
1350.0000000	9.9552002	-10.9067440
1350.0000000	10.0039997	-10.0828886
1360.0000000	0.3416000	-37.0698280
1360.0000000	0.3904000	-39.9303818
1360.0000000	0.4392000	-39.5260010
1360.0000000	0.4880000	-39.5501442
1360.0000000	0.5368000	-39.6536636
1360.0000000	0.5856000	-39.2856827
1360.0000000	0.6344000	-38.2360001
1360.0000000	0.6832000	-36.2415581
1360.0000000	0.7320000	-36.0714149
1360.0000000	0.7808000	-36.5361633
1360.0000000	0.8296000	-35.9503288
1360.0000000	0.8784000	-35.1146851
1360.0000000	0.9272000	-34.2455597
1360.0000000	0.9760000	-31.9441032
1360.0000000	1.0247999	-30.3063183
1360.0000000	1.0735999	-29.9739666
1360.0000000	1.1224000	-30.7337379
1360.0000000	1.1711999	-31.3775806
1360.0000000	1.2200000	-31.0621166
1360.0000000	1.2687999	-30.9253769
1360.0000000	1.3176000	-30.9810886
1360.0000000	1.3664000	-30.6419029

1360.0000000	1.4152000	-30.1692657
1360.0000000	1.4640000	-29.5571957
1360.0000000	1.5128000	-36.0711784
1360.0000000	1.5616000	-32.2076797
1360.0000000	1.6104001	-1470.7617188
1360.0000000	1.6592000	-63.2645111
1360.0000000	1.7079999	-27.3849411
1360.0000000	1.7567999	-27.7627945
1360.0000000	1.8055999	-27.1102676
1360.0000000	1.8544000	-26.7978973
1360.0000000	1.9031999	-26.8846588
1360.0000000	1.9520000	-26.6279907
1360.0000000	2.0007999	-25.9245834
1360.0000000	2.0495999	-26.2369442
1360.0000000	2.0983999	-97.7976990
1360.0000000	2.1471999	-10.7617168
1360.0000000	2.1960001	-25.3094025
1360.0000000	2.2448001	-25.2187767
1360.0000000	2.2935998	-25.6532364
1360.0000000	2.3423998	-21.4796066
1360.0000000	2.3912001	10.7479763
1360.0000000	2.4400001	-22.9220047
1360.0000000	2.4888000	-23.4527035
1360.0000000	2.5375998	-12.8480844
1360.0000000	2.5863998	-11.0584908
1360.0000000	2.6352000	-9.2312450
1360.0000000	2.6840000	-15.8125477
1360.0000000	2.7328000	124.2609634
1360.0000000	2.7815998	146.8725891
1360.0000000	2.8304000	-23.7507896
1360.0000000	2.8792000	-20.7283840
1360.0000000	2.9280000	-22.5527744
1360.0000000	2.9768000	-17.0689201
1360.0000000	3.0256000	-20.5591450
1360.0000000	3.0743999	-29.1366844
1360.0000000	3.1231999	-24.1397667
1360.0000000	3.1719999	-29.3289356
1360.0000000	3.2208002	-496.2975769
1360.0000000	3.2695999	-971.3704834
1360.0000000	3.3183999	-20.4963284
1360.0000000	3.3671999	-3346.9641113
1360.0000000	3.4159999	1200.3479004
1360.0000000	3.4648001	-7.3345947
1360.0000000	3.5135999	-26.9354515
1360.0000000	3.5623999	-317.5425720
1360.0000000	3.6111999	-1993.5794678
1360.0000000	3.6600001	-451.5717163
1360.0000000	3.7088001	-596.1638794
1360.0000000	3.7575998	-933.2076416
1360.0000000	3.8063998	-1693.1614990
1360.0000000	3.8552001	-2458.0847168
1360.0000000	3.9040000	-2409.9887695
1360.0000000	3.9528000	1101.6446533
1360.0000000	4.0015998	-241.0621033
1360.0000000	4.0503998	674.3274536
1360.0000000	4.0991998	85.6134949
1360.0000000	4.1479998	-642.8020630
1360.0000000	4.1967998	2.1395264

1360.0000000	4.2456002	-244.1906281
1360.0000000	4.2943997	-479.1858826
1360.0000000	4.3431997	-13.4271984
1360.0000000	4.3920002	-243.8293610
1360.0000000	4.4407997	-22.3825378
1360.0000000	4.4896002	-15.5130320
1360.0000000	4.5383997	-643.5879517
1360.0000000	4.5871997	-12.9273510
1360.0000000	4.6360002	-21.5531940
1360.0000000	4.6847997	-28.1095390
1360.0000000	4.7336001	-19.5306187
1360.0000000	4.7824001	-16.5323811
1360.0000000	4.8311996	-1450.9165039
1360.0000000	4.8800001	224.3025818
1360.0000000	4.9287996	-198.4819489
1360.0000000	4.9776001	1042.2387695
1360.0000000	5.0264001	84.5178833
1360.0000000	5.0751996	-296.5604858
1360.0000000	5.1240001	-12.3490524
1360.0000000	5.1727996	553.3155518
1360.0000000	5.2216001	31.6278954
1360.0000000	5.2704000	-157.7203369
1360.0000000	5.3191996	-39.0053024
1360.0000000	5.3680000	-3508.9243164
1360.0000000	5.4168000	522.9783325
1360.0000000	5.4656000	-199.3335266
1360.0000000	5.5144000	37.6533127
1360.0000000	5.5631995	484.4995728
1360.0000000	5.6120000	390.0381165
1360.0000000	5.6608000	-36.9261703
1360.0000000	5.7096000	61.9319687
1360.0000000	5.7584000	131.9185028
1360.0000000	5.8071995	-11.5540266
1360.0000000	5.8559999	29.4746876
1360.0000000	5.9047999	-16.5979824
1360.0000000	5.9535999	-39.5933113
1360.0000000	6.0023999	-1561.2440186
1360.0000000	6.0511999	-14.5763226
1360.0000000	6.0999999	-19.3773804
1360.0000000	6.1487999	-13.2910538
1360.0000000	6.1975999	-23.1527653
1360.0000000	6.2463999	-1778.5938721
1360.0000000	6.2951999	-19.0392780
1360.0000000	6.3439999	-340.2742310
1360.0000000	6.3927999	-15.0474243
1360.0000000	6.4416003	-12.4081488
1360.0000000	6.4903998	-11.2891512
1360.0000000	6.5391998	-11.8317890
1360.0000000	6.5879998	359.2248535
1360.0000000	6.6367998	-12.1046247
1360.0000000	6.6856003	-13.8212366
1360.0000000	6.7343998	-13.4636354
1360.0000000	6.7831998	-14.3572645
1360.0000000	6.8319998	57.7390213
1360.0000000	6.8807998	-14.0443525
1360.0000000	6.9296002	-421.8183594
1360.0000000	6.9783998	-5.5490112
1360.0000000	7.0271997	-14.1002579

1360.0000000	7.0760002	9.6725435
1360.0000000	7.1247997	-1990.5933838
1360.0000000	7.1736002	-1596.3631592
1360.0000000	7.2223997	-908.3995972
1360.0000000	7.2711997	-196.5396423
1360.0000000	7.3200002	-18.4573097
1360.0000000	7.3687997	174.6392212
1360.0000000	7.4176002	365.1971741
1360.0000000	7.4663997	286.7705383
1360.0000000	7.5151997	-888.4813843
1360.0000000	7.5640001	-981.3234253
1360.0000000	7.6127996	-2312.0844727
1360.0000000	7.6616001	-742.6917725
1360.0000000	7.7104001	-381.8076172
1360.0000000	7.7591996	39.6801872
1360.0000000	7.8080001	379.5193787
1360.0000000	7.8567996	500.1726074
1360.0000000	7.9056001	-1038.6252441
1360.0000000	7.9544001	1652.7309570
1360.0000000	8.0031996	2132.6262207
1360.0000000	8.0520000	2128.5705566
1360.0000000	8.1007996	2252.4680176
1360.0000000	8.1496000	2402.2341309
1360.0000000	8.1983995	1494.4774170
1360.0000000	8.2472000	-3419.7622070
1360.0000000	8.2959995	-1212.3179932
1360.0000000	8.3448000	-965.6606445
1360.0000000	8.3935995	-885.0184326
1360.0000000	8.4423990	-381.3346252
1360.0000000	8.4912004	-890.0308228
1360.0000000	8.5400000	-2400.6230469
1360.0000000	8.5887995	-1163.8144531
1360.0000000	8.6375999	227.0641785
1360.0000000	8.6863995	2052.9658203
1360.0000000	8.7351999	-763.2459106
1360.0000000	8.7840004	871.6682129
1360.0000000	8.8327999	46.6911926
1360.0000000	8.8815994	-11.5024385
1360.0000000	8.9303989	-23.8172188
1360.0000000	8.9792004	-11.5819330
1360.0000000	9.0279999	-5.8975687
1360.0000000	9.0767994	6.8433876
1360.0000000	9.1255999	-17.1833878
1360.0000000	9.1743994	-10.0873289
1360.0000000	9.2231998	36.4744644
1360.0000000	9.2720003	-8.4520369
1360.0000000	9.3207998	-9.8164768
1360.0000000	9.3695993	-10.7714796
1360.0000000	9.4183998	-11.7496424
1360.0000000	9.4672003	-11.1627321
1360.0000000	9.5159998	-14.1339140
1360.0000000	9.5648003	-0.5077477
1360.0000000	9.6135998	-13.3691254
1360.0000000	9.6623993	-10.2306852
1360.0000000	9.7111998	-10.1669397
1360.0000000	9.7600002	-10.8589935
1360.0000000	9.8087997	-10.9950485
1360.0000000	9.8575993	3.0395422

1360.0000000	9.9063997	-8.0001354
1360.0000000	9.9552002	-10.9067478
1360.0000000	10.0039997	-10.0831356
1370.0000000	0.3416000	-36.9064331
1370.0000000	0.3904000	-39.9218292
1370.0000000	0.4392000	-39.5196571
1370.0000000	0.4880000	-39.5489388
1370.0000000	0.5368000	-39.6548386
1370.0000000	0.5856000	-39.2842979
1370.0000000	0.6344000	-38.2363129
1370.0000000	0.6832000	-36.2437859
1370.0000000	0.7320000	-36.0734673
1370.0000000	0.7808000	-36.5363312
1370.0000000	0.8296000	-35.9484825
1370.0000000	0.8784000	-35.1051216
1370.0000000	0.9272000	-34.2462006
1370.0000000	0.9760000	-31.9415798
1370.0000000	1.0247999	-30.3075523
1370.0000000	1.0735999	-29.9875679
1370.0000000	1.1224000	-30.7318649
1370.0000000	1.1711999	-31.3712788
1370.0000000	1.2200000	-31.0183182
1370.0000000	1.2687999	-30.9040508
1370.0000000	1.3176000	-30.9747028
1370.0000000	1.3664000	-30.6389599
1370.0000000	1.4152000	-30.1694698
1370.0000000	1.4640000	-29.5554867
1370.0000000	1.5128000	-35.9740028
1370.0000000	1.5616000	-32.1658401
1370.0000000	1.6104001	-1468.2188721
1370.0000000	1.6592000	-62.9705811
1370.0000000	1.7079999	-27.3886890
1370.0000000	1.7567999	-27.7628136
1370.0000000	1.8055999	-27.0676174
1370.0000000	1.8544000	-26.7286015
1370.0000000	1.9031999	-26.9501095
1370.0000000	1.9520000	-26.6866684
1370.0000000	2.0007999	-25.9299622
1370.0000000	2.0495999	-26.2389641
1370.0000000	2.0983999	-96.3219223
1370.0000000	2.1471999	-11.0289841
1370.0000000	2.1960001	-25.3053131
1370.0000000	2.2448001	-25.2008667
1370.0000000	2.2935998	-25.5936127
1370.0000000	2.3423998	-21.4940109
1370.0000000	2.3912001	10.1264324
1370.0000000	2.4400001	-22.9244919
1370.0000000	2.4888000	-23.4524860
1370.0000000	2.5375998	-12.8651924
1370.0000000	2.5863998	-11.0782490
1370.0000000	2.6352000	-9.2539892
1370.0000000	2.6840000	-15.8244896
1370.0000000	2.7328000	123.9949417
1370.0000000	2.7815998	146.5661926
1370.0000000	2.8304000	-23.7494850
1370.0000000	2.8792000	-20.7316475
1370.0000000	2.9280000	-22.5522041
1370.0000000	2.9768000	-17.0772896

1370.0000000	3.0256000	-20.5617294
1370.0000000	3.0743999	-29.1252975
1370.0000000	3.1231999	-24.1363106
1370.0000000	3.1719999	-29.3206425
1370.0000000	3.2208002	-496.0598450
1370.0000000	3.2695999	-970.8953857
1370.0000000	3.3183999	-20.4965267
1370.0000000	3.3671999	-3345.5024414
1370.0000000	3.4159999	1199.8126221
1370.0000000	3.4648001	-7.3403034
1370.0000000	3.5135999	-26.9324608
1370.0000000	3.5623999	-317.4129944
1370.0000000	3.6111999	-1992.7196045
1370.0000000	3.6600001	-451.3836060
1370.0000000	3.7088001	-595.9124756
1370.0000000	3.7575998	-932.8090820
1370.0000000	3.8063998	-1692.4317627
1370.0000000	3.8552001	-2457.0214844
1370.0000000	3.9040000	-2408.9462891
1370.0000000	3.9528000	1101.1563721
1370.0000000	4.0015998	-240.9649963
1370.0000000	4.0503998	674.0249023
1370.0000000	4.0991998	85.5678101
1370.0000000	4.1479998	-642.5298462
1370.0000000	4.1967998	2.1302700
1370.0000000	4.2456002	-244.0923157
1370.0000000	4.2943997	-478.9853210
1370.0000000	4.3431997	-13.4293785
1370.0000000	4.3920002	-243.7312469
1370.0000000	4.4407997	-22.3807621
1370.0000000	4.4896002	-15.5139141
1370.0000000	4.5383997	-643.3268433
1370.0000000	4.5871997	-12.9293680
1370.0000000	4.6360002	-21.5516510
1370.0000000	4.6847997	-28.1056137
1370.0000000	4.7336001	-19.5298958
1370.0000000	4.7824001	-16.5328369
1370.0000000	4.8311996	-1450.3852539
1370.0000000	4.8800001	224.2130280
1370.0000000	4.9287996	-198.4146881
1370.0000000	4.9776001	1041.8463135
1370.0000000	5.0264001	84.4803619
1370.0000000	5.0751996	-296.4568481
1370.0000000	5.1240001	-12.3499737
1370.0000000	5.1727996	553.1996460
1370.0000000	5.2216001	31.6184540
1370.0000000	5.2704000	-157.6922150
1370.0000000	5.3191996	-39.0007553
1370.0000000	5.3680000	-3508.2319336
1370.0000000	5.4168000	522.8715210
1370.0000000	5.4656000	-199.2973480
1370.0000000	5.5144000	37.6426544
1370.0000000	5.5631995	484.4010010
1370.0000000	5.6120000	389.9581299
1370.0000000	5.6608000	-36.9220848
1370.0000000	5.7096000	61.9165344
1370.0000000	5.7584000	131.8895569
1370.0000000	5.8071995	-11.5548973

1370.0000000	5.8559999	29.4658375
1370.0000000	5.9047999	-16.5977840
1370.0000000	5.9535999	-39.5885735
1370.0000000	6.0023999	-1560.9394531
1370.0000000	6.0511999	-14.5765333
1370.0000000	6.0999999	-19.3765469
1370.0000000	6.1487999	-13.2915163
1370.0000000	6.1975999	-23.1509991
1370.0000000	6.2463999	-1778.2465820
1370.0000000	6.2951999	-19.0384636
1370.0000000	6.3439999	-340.2101746
1370.0000000	6.3927999	-15.0474062
1370.0000000	6.4416003	-12.4087353
1370.0000000	6.4903998	-11.2898273
1370.0000000	6.5391998	-11.8324709
1370.0000000	6.5879998	358.5341797
1370.0000000	6.6367998	-12.1051741
1370.0000000	6.6856003	-13.8214064
1370.0000000	6.7343998	-13.4638519
1370.0000000	6.7831998	-14.3568487
1370.0000000	6.8319998	57.7199020
1370.0000000	6.8807998	-14.0442133
1370.0000000	6.9296002	-421.0569153
1370.0000000	6.9783998	-5.5618353
1370.0000000	7.0271997	-14.0992298
1370.0000000	7.0760002	9.6303568
1370.0000000	7.1247997	-1986.6475830
1370.0000000	7.1736002	-1593.2003174
1370.0000000	7.2223997	-906.6063843
1370.0000000	7.2711997	-196.1718597
1370.0000000	7.3200002	-18.4453201
1370.0000000	7.3687997	174.2646942
1370.0000000	7.4176002	364.4413452
1370.0000000	7.4663997	286.1728821
1370.0000000	7.5151997	-886.7203369
1370.0000000	7.5640001	-979.3759766
1370.0000000	7.6127996	-2307.4667969
1370.0000000	7.6616001	-741.2203979
1370.0000000	7.7104001	-381.0613403
1370.0000000	7.7591996	39.5791359
1370.0000000	7.8080001	378.7340393
1370.0000000	7.8567996	499.1445007
1370.0000000	7.9056001	-1036.5523682
1370.0000000	7.9544001	1649.4210205
1370.0000000	8.0031996	2128.3564453
1370.0000000	8.0520000	2124.3085938
1370.0000000	8.1007996	2247.9584961
1370.0000000	8.1496000	2397.4260254
1370.0000000	8.1983995	1491.4849854
1370.0000000	8.2472000	-3412.9274902
1370.0000000	8.2959995	-1209.8978271
1370.0000000	8.3448000	-963.7315063
1370.0000000	8.3935995	-883.2507324
1370.0000000	8.4423990	-380.5766296
1370.0000000	8.4912004	-888.2546387
1370.0000000	8.5400000	-2395.8232422
1370.0000000	8.5887995	-1161.4929199
1370.0000000	8.6375999	226.6028595

1370.0000000	8.6863995	2048.8474121
1370.0000000	8.7351999	-761.7216187
1370.0000000	8.7840004	869.9114990
1370.0000000	8.8327999	46.5795822
1370.0000000	8.8815994	-11.5026131
1370.0000000	8.9303989	-23.8134995
1370.0000000	8.9792004	-11.5820150
1370.0000000	9.0279999	-5.8993373
1370.0000000	9.0767994	6.8376694
1370.0000000	9.1255999	-17.1816750
1370.0000000	9.1743994	-10.0877304
1370.0000000	9.2231998	36.4587326
1370.0000000	9.2720003	-8.4529724
1370.0000000	9.3207998	-9.8170156
1370.0000000	9.3695993	-10.7717066
1370.0000000	9.4183998	-11.7495174
1370.0000000	9.4672003	-11.1627731
1370.0000000	9.5159998	-14.1330223
1370.0000000	9.5648003	-0.5111208
1370.0000000	9.6135998	-13.3683348
1370.0000000	9.6623993	-10.2309599
1370.0000000	9.7111998	-10.1672106
1370.0000000	9.7600002	-10.8590355
1370.0000000	9.8087997	-10.9950571
1370.0000000	9.8575993	3.0351200
1370.0000000	9.9063997	-8.0010490
1370.0000000	9.9552002	-10.9067526
1370.0000000	10.0039997	-10.0833836
1380.0000000	0.3416000	-37.3705673
1380.0000000	0.3904000	-39.9132729
1380.0000000	0.4392000	-39.5133133
1380.0000000	0.4880000	-39.5477295
1380.0000000	0.5368000	-39.6560097
1380.0000000	0.5856000	-39.2829170
1380.0000000	0.6344000	-38.2366219
1380.0000000	0.6832000	-36.2460098
1380.0000000	0.7320000	-36.0755196
1380.0000000	0.7808000	-36.5365028
1380.0000000	0.8296000	-35.9466362
1380.0000000	0.8784000	-35.0955582
1380.0000000	0.9272000	-34.2468452
1380.0000000	0.9760000	-31.9390564
1380.0000000	1.0247999	-30.3087883
1380.0000000	1.0735999	-30.0011673
1380.0000000	1.1224000	-30.7299938
1380.0000000	1.1711999	-31.3649750
1380.0000000	1.2200000	-30.9745197
1380.0000000	1.2687999	-30.8827229
1380.0000000	1.3176000	-30.9683170
1380.0000000	1.3664000	-30.6360149
1380.0000000	1.4152000	-30.1696739
1380.0000000	1.4640000	-29.5537777
1380.0000000	1.5128000	-35.8768234
1380.0000000	1.5616000	-32.1239967
1380.0000000	1.6104001	-1465.6761475
1380.0000000	1.6592000	-62.6766434
1380.0000000	1.7079999	-27.3924351
1380.0000000	1.7567999	-27.7628326

1380.0000000	1.8055999	-27.2345276
1380.0000000	1.8544000	-26.8985481
1380.0000000	1.9031999	-26.8599548
1380.0000000	1.9520000	-26.7453442
1380.0000000	2.0007999	-25.9353428
1380.0000000	2.0495999	-26.2409821
1380.0000000	2.0983999	-94.8461456
1380.0000000	2.1471999	-11.2962513
1380.0000000	2.1960001	-25.3012218
1380.0000000	2.2448001	-25.1829548
1380.0000000	2.2935998	-25.5339890
1380.0000000	2.3423998	-21.5084152
1380.0000000	2.3912001	9.5048847
1380.0000000	2.4400001	-22.9269772
1380.0000000	2.4888000	-23.4522705
1380.0000000	2.5375998	-12.8823004
1380.0000000	2.5863998	-11.0980072
1380.0000000	2.6352000	-9.2767334
1380.0000000	2.6840000	-15.8364315
1380.0000000	2.7328000	123.7289200
1380.0000000	2.7815998	146.2597656
1380.0000000	2.8304000	-23.7481804
1380.0000000	2.8792000	-20.7349110
1380.0000000	2.9280000	-22.5516357
1380.0000000	2.9768000	-17.0856590
1380.0000000	3.0256000	-20.5643139
1380.0000000	3.0743999	-29.1139107
1380.0000000	3.1231999	-24.1328564
1380.0000000	3.1719999	-29.3123493
1380.0000000	3.2208002	-495.8221130
1380.0000000	3.2695999	-970.4205322
1380.0000000	3.3183999	-20.4967251
1380.0000000	3.3671999	-3344.0410156
1380.0000000	3.4159999	1199.2773438
1380.0000000	3.4648001	-7.3460121
1380.0000000	3.5135999	-26.9294701
1380.0000000	3.5623999	-317.2834167
1380.0000000	3.6111999	-1991.8598633
1380.0000000	3.6600001	-451.1954956
1380.0000000	3.7088001	-595.6611328
1380.0000000	3.7575998	-932.4105835
1380.0000000	3.8063998	-1691.7019043
1380.0000000	3.8552001	-2455.9580078
1380.0000000	3.9040000	-2407.9038086
1380.0000000	3.9528000	1100.6680908
1380.0000000	4.0015998	-240.8678741
1380.0000000	4.0503998	673.7223511
1380.0000000	4.0991998	85.5221252
1380.0000000	4.1479998	-642.2575684
1380.0000000	4.1967998	2.1210136
1380.0000000	4.2456002	-243.9940186
1380.0000000	4.2943997	-478.7847595
1380.0000000	4.3431997	-13.4315586
1380.0000000	4.3920002	-243.6331329
1380.0000000	4.4407997	-22.3789864
1380.0000000	4.4896002	-15.5147972
1380.0000000	4.5383997	-643.0657959
1380.0000000	4.5871997	-12.9313841

1380.0000000	4.6360002	-21.5501099
1380.0000000	4.6847997	-28.1016884
1380.0000000	4.7336001	-19.5291729
1380.0000000	4.7824001	-16.5332909
1380.0000000	4.8311996	-1449.8540039
1380.0000000	4.8800001	224.1234741
1380.0000000	4.9287996	-198.3474274
1380.0000000	4.9776001	1041.4537354
1380.0000000	5.0264001	84.4428406
1380.0000000	5.0751996	-296.3532410
1380.0000000	5.1240001	-12.3508949
1380.0000000	5.1727996	553.0838013
1380.0000000	5.2216001	31.6090126
1380.0000000	5.2704000	-157.6641235
1380.0000000	5.3191996	-38.9962082
1380.0000000	5.3680000	-3507.5395508
1380.0000000	5.4168000	522.7647095
1380.0000000	5.4656000	-199.2611694
1380.0000000	5.5144000	37.6320038
1380.0000000	5.5631995	484.3024292
1380.0000000	5.6120000	389.8781433
1380.0000000	5.6608000	-36.9179955
1380.0000000	5.7096000	61.9011002
1380.0000000	5.7584000	131.8606262
1380.0000000	5.8071995	-11.5557680
1380.0000000	5.8559999	29.4569836
1380.0000000	5.9047999	-16.5975857
1380.0000000	5.9535999	-39.5838318
1380.0000000	6.0023999	-1560.6350098
1380.0000000	6.0511999	-14.5767450
1380.0000000	6.0999999	-19.3757153
1380.0000000	6.1487999	-13.2919788
1380.0000000	6.1975999	-23.1492348
1380.0000000	6.2463999	-1777.8991699
1380.0000000	6.2951999	-19.0376492
1380.0000000	6.3439999	-340.1461487
1380.0000000	6.3927999	-15.0473881
1380.0000000	6.4416003	-12.4093227
1380.0000000	6.4903998	-11.2905035
1380.0000000	6.5391998	-11.8331518
1380.0000000	6.5879998	357.8434753
1380.0000000	6.6367998	-12.1057234
1380.0000000	6.6856003	-13.8215771
1380.0000000	6.7343998	-13.4640675
1380.0000000	6.7831998	-14.3564329
1380.0000000	6.8319998	57.7007904
1380.0000000	6.8807998	-14.0440750
1380.0000000	6.9296002	-420.2954712
1380.0000000	6.9783998	-5.5746589
1380.0000000	7.0271997	-14.0982018
1380.0000000	7.0760002	9.5881701
1380.0000000	7.1247997	-1982.7016602
1380.0000000	7.1736002	-1590.0374756
1380.0000000	7.2223997	-904.8132324
1380.0000000	7.2711997	-195.8040771
1380.0000000	7.3200002	-18.4333324
1380.0000000	7.3687997	173.8901672
1380.0000000	7.4176002	363.6855164

1380.0000000	7.4663997	285.5752258
1380.0000000	7.5151997	-884.9592896
1380.0000000	7.5640001	-977.4285889
1380.0000000	7.6127996	-2302.8491211
1380.0000000	7.6616001	-739.7491455
1380.0000000	7.7104001	-380.3150635
1380.0000000	7.7591996	39.4780884
1380.0000000	7.8080001	377.9486694
1380.0000000	7.8567996	498.1164246
1380.0000000	7.9056001	-1034.4794922
1380.0000000	7.9544001	1646.1110840
1380.0000000	8.0031996	2124.0869141
1380.0000000	8.0520000	2120.0466309
1380.0000000	8.1007996	2243.4489746
1380.0000000	8.1496000	2392.6181641
1380.0000000	8.1983995	1488.4924316
1380.0000000	8.2472000	-3406.0927734
1380.0000000	8.2959995	-1207.4776611
1380.0000000	8.3448000	-961.8023071
1380.0000000	8.3935995	-881.4830933
1380.0000000	8.4423990	-379.8186035
1380.0000000	8.4912004	-886.4783936
1380.0000000	8.5400000	-2391.0234375
1380.0000000	8.5887995	-1159.1713867
1380.0000000	8.6375999	226.1415405
1380.0000000	8.6863995	2044.7291260
1380.0000000	8.7351999	-760.1973877
1380.0000000	8.7840004	868.1547241
1380.0000000	8.8327999	46.4679756
1380.0000000	8.8815994	-11.5027876
1380.0000000	8.9303989	-23.8097801
1380.0000000	8.9792004	-11.5820971
1380.0000000	9.0279999	-5.9011059
1380.0000000	9.0767994	6.8319530
1380.0000000	9.1255999	-17.1799603
1380.0000000	9.1743994	-10.0881329
1380.0000000	9.2231998	36.4430008
1380.0000000	9.2720003	-8.4539089
1380.0000000	9.3207998	-9.8175554
1380.0000000	9.3695993	-10.7719326
1380.0000000	9.4183998	-11.7493935
1380.0000000	9.4672003	-11.1628141
1380.0000000	9.5159998	-14.1321316
1380.0000000	9.5648003	-0.5144939
1380.0000000	9.6135998	-13.3675442
1380.0000000	9.6623993	-10.2312355
1380.0000000	9.7111998	-10.1674824
1380.0000000	9.7600002	-10.8590775
1380.0000000	9.8087997	-10.9950647
1380.0000000	9.8575993	3.0306988
1380.0000000	9.9063997	-8.0019636
1380.0000000	9.9552002	-10.9067564
1380.0000000	10.0039997	-10.0836306
1390.0000000	0.3416000	-37.1311302
1390.0000000	0.3904000	-39.9047165
1390.0000000	0.4392000	-39.5069695
1390.0000000	0.4880000	-39.5465202
1390.0000000	0.5368000	-39.6571846

1390.0000000	0.5856000	-39.2815361
1390.0000000	0.6344000	-38.2369347
1390.0000000	0.6832000	-36.2482376
1390.0000000	0.7320000	-36.0775719
1390.0000000	0.7808000	-36.5366707
1390.0000000	0.8296000	-35.9447899
1390.0000000	0.8784000	-35.0859909
1390.0000000	0.9272000	-34.2474861
1390.0000000	0.9760000	-31.9365330
1390.0000000	1.0247999	-30.3100243
1390.0000000	1.0735999	-30.0147686
1390.0000000	1.1224000	-30.7281208
1390.0000000	1.1711999	-31.3586731
1390.0000000	1.2200000	-30.9307213
1390.0000000	1.2687999	-30.8613968
1390.0000000	1.3176000	-30.9619312
1390.0000000	1.3664000	-30.6330719
1390.0000000	1.4152000	-30.1698780
1390.0000000	1.4640000	-29.5520687
1390.0000000	1.5128000	-35.7796478
1390.0000000	1.5616000	-32.0821571
1390.0000000	1.6104001	-1463.1333008
1390.0000000	1.6592000	-62.3827133
1390.0000000	1.7079999	-27.3961830
1390.0000000	1.7567999	-27.7628517
1390.0000000	1.8055999	-27.4014397
1390.0000000	1.8544000	-26.3176212
1390.0000000	1.9031999	-26.2273121
1390.0000000	1.9520000	-26.8040218
1390.0000000	2.0007999	-25.9407234
1390.0000000	2.0495999	-26.2430000
1390.0000000	2.0983999	-93.3703690
1390.0000000	2.1471999	-11.5635185
1390.0000000	2.1960001	-25.2971306
1390.0000000	2.2448001	-25.1650429
1390.0000000	2.2935998	-25.4743652
1390.0000000	2.3423998	-21.5228195
1390.0000000	2.3912001	8.8833370
1390.0000000	2.4400001	-22.9294643
1390.0000000	2.4888000	-23.4520531
1390.0000000	2.5375998	-12.8994083
1390.0000000	2.5863998	-11.1177664
1390.0000000	2.6352000	-9.2994776
1390.0000000	2.6840000	-15.8483725
1390.0000000	2.7328000	123.4629135
1390.0000000	2.7815998	145.9533386
1390.0000000	2.8304000	-23.7468758
1390.0000000	2.8792000	-20.7381744
1390.0000000	2.9280000	-22.5510674
1390.0000000	2.9768000	-17.0940304
1390.0000000	3.0256000	-20.5668983
1390.0000000	3.0743999	-29.1025238
1390.0000000	3.1231999	-24.1294003
1390.0000000	3.1719999	-29.3040562
1390.0000000	3.2208002	-495.5843811
1390.0000000	3.2695999	-969.9454346
1390.0000000	3.3183999	-20.4969215
1390.0000000	3.3671999	-3342.5793457

1390.0000000	3.4159999	1198.7420654
1390.0000000	3.4648001	-7.3517189
1390.0000000	3.5135999	-26.9264793
1390.0000000	3.5623999	-317.1538086
1390.0000000	3.6111999	-1991.0001221
1390.0000000	3.6600001	-451.0073242
1390.0000000	3.7088001	-595.4097290
1390.0000000	3.7575998	-932.0120239
1390.0000000	3.8063998	-1690.9720459
1390.0000000	3.8552001	-2454.8945313
1390.0000000	3.9040000	-2406.8613281
1390.0000000	3.9528000	1100.1798096
1390.0000000	4.0015998	-240.7707672
1390.0000000	4.0503998	673.4197998
1390.0000000	4.0991998	85.4764404
1390.0000000	4.1479998	-641.9853516
1390.0000000	4.1967998	2.1117573
1390.0000000	4.2456002	-243.8957214
1390.0000000	4.2943997	-478.5841675
1390.0000000	4.3431997	-13.4337397
1390.0000000	4.3920002	-243.5350189
1390.0000000	4.4407997	-22.3772106
1390.0000000	4.4896002	-15.5156794
1390.0000000	4.5383997	-642.8047485
1390.0000000	4.5871997	-12.9334002
1390.0000000	4.6360002	-21.5485668
1390.0000000	4.6847997	-28.0977631
1390.0000000	4.7336001	-19.5284500
1390.0000000	4.7824001	-16.5337448
1390.0000000	4.8311996	-1449.3226318
1390.0000000	4.8800001	224.0339203
1390.0000000	4.9287996	-198.2801666
1390.0000000	4.9776001	1041.0612793
1390.0000000	5.0264001	84.4053192
1390.0000000	5.0751996	-296.2496033
1390.0000000	5.1240001	-12.3518162
1390.0000000	5.1727996	552.9679565
1390.0000000	5.2216001	31.5995636
1390.0000000	5.2704000	-157.6360016
1390.0000000	5.3191996	-38.9916573
1390.0000000	5.3680000	-3506.8471680
1390.0000000	5.4168000	522.6579590
1390.0000000	5.4656000	-199.2249756
1390.0000000	5.5144000	37.6213455
1390.0000000	5.5631995	484.2038574
1390.0000000	5.6120000	389.7981873
1390.0000000	5.6608000	-36.9139061
1390.0000000	5.7096000	61.8856735
1390.0000000	5.7584000	131.8316956
1390.0000000	5.8071995	-11.5566387
1390.0000000	5.8559999	29.4481335
1390.0000000	5.9047999	-16.5973892
1390.0000000	5.9535999	-39.5790939
1390.0000000	6.0023999	-1560.3305664
1390.0000000	6.0511999	-14.5769567
1390.0000000	6.0999999	-19.3748817
1390.0000000	6.1487999	-13.2924423
1390.0000000	6.1975999	-23.1474686

1390.0000000	6.2463999	-1777.5517578
1390.0000000	6.2951999	-19.0368347
1390.0000000	6.3439999	-340.0820923
1390.0000000	6.3927999	-15.0473700
1390.0000000	6.4416003	-12.4099092
1390.0000000	6.4903998	-11.2911797
1390.0000000	6.5391998	-11.8338327
1390.0000000	6.5879998	357.1527710
1390.0000000	6.6367998	-12.1062727
1390.0000000	6.6856003	-13.8217468
1390.0000000	6.7343998	-13.4642830
1390.0000000	6.7831998	-14.3560171
1390.0000000	6.8319998	57.6816788
1390.0000000	6.8807998	-14.0439367
1390.0000000	6.9296002	-419.5340271
1390.0000000	6.9783998	-5.5874829
1390.0000000	7.0271997	-14.0971737
1390.0000000	7.0760002	9.5459871
1390.0000000	7.1247997	-1978.7557373
1390.0000000	7.1736002	-1586.8745117
1390.0000000	7.2223997	-903.0200806
1390.0000000	7.2711997	-195.4362946
1390.0000000	7.3200002	-18.4213448
1390.0000000	7.3687997	173.5156403
1390.0000000	7.4176002	362.9296875
1390.0000000	7.4663997	284.9776001
1390.0000000	7.5151997	-883.1983032
1390.0000000	7.5640001	-975.4812012
1390.0000000	7.6127996	-2298.2316895
1390.0000000	7.6616001	-738.2777710
1390.0000000	7.7104001	-379.5688477
1390.0000000	7.7591996	39.3770409
1390.0000000	7.8080001	377.1632996
1390.0000000	7.8567996	497.0883484
1390.0000000	7.9056001	-1032.4064941
1390.0000000	7.9544001	1642.8011475
1390.0000000	8.0031996	2119.8173828
1390.0000000	8.0520000	2115.7846680
1390.0000000	8.1007996	2238.9394531
1390.0000000	8.1496000	2387.8100586
1390.0000000	8.1983995	1485.4998779
1390.0000000	8.2472000	-3399.2578125
1390.0000000	8.2959995	-1205.0574951
1390.0000000	8.3448000	-959.8731079
1390.0000000	8.3935995	-879.7153931
1390.0000000	8.4423990	-379.0606079
1390.0000000	8.4912004	-884.7022095
1390.0000000	8.5400000	-2386.2236328
1390.0000000	8.5887995	-1156.8498535
1390.0000000	8.6375999	225.6802063
1390.0000000	8.6863995	2040.6107178
1390.0000000	8.7351999	-758.6731567
1390.0000000	8.7840004	866.3980103
1390.0000000	8.8327999	46.3563728
1390.0000000	8.8815994	-11.5029621
1390.0000000	8.9303989	-23.8060608
1390.0000000	8.9792004	-11.5821800
1390.0000000	9.0279999	-5.9028735

1390.0000000	9.0767994	6.8262348
1390.0000000	9.1255999	-17.1782455
1390.0000000	9.1743994	-10.0885353
1390.0000000	9.2231998	36.4272690
1390.0000000	9.2720003	-8.4548454
1390.0000000	9.3207998	-9.8180943
1390.0000000	9.3695993	-10.7721596
1390.0000000	9.4183998	-11.7492685
1390.0000000	9.4672003	-11.1628551
1390.0000000	9.5159998	-14.1312408
1390.0000000	9.5648003	-0.5178661
1390.0000000	9.6135998	-13.3667536
1390.0000000	9.6623993	-10.2315102
1390.0000000	9.7111998	-10.1677542
1390.0000000	9.7600002	-10.8591185
1390.0000000	9.8087997	-10.9950724
1390.0000000	9.8575993	3.0262747
1390.0000000	9.9063997	-8.0028772
1390.0000000	9.9552002	-10.9067612
1390.0000000	10.0039997	-10.0838785
1400.0000000	0.3416000	-37.1655159
1400.0000000	0.3904000	-39.8961639
1400.0000000	0.4392000	-39.5006256
1400.0000000	0.4880000	-39.5453110
1400.0000000	0.5368000	-39.6583595
1400.0000000	0.5856000	-39.2801552
1400.0000000	0.6344000	-38.2372437
1400.0000000	0.6832000	-36.2504616
1400.0000000	0.7320000	-36.0796242
1400.0000000	0.7808000	-36.5368385
1400.0000000	0.8296000	-35.9429474
1400.0000000	0.8784000	-35.0764275
1400.0000000	0.9272000	-34.2481270
1400.0000000	0.9760000	-31.9340096
1400.0000000	1.0247999	-30.3112602
1400.0000000	1.0735999	-30.0283680
1400.0000000	1.1224000	-30.7262497
1400.0000000	1.1711999	-31.3523693
1400.0000000	1.2200000	-30.8869228
1400.0000000	1.2687999	-30.8400688
1400.0000000	1.3176000	-30.9555454
1400.0000000	1.3664000	-30.6301289
1400.0000000	1.4152000	-30.1700821
1400.0000000	1.4640000	-29.5503578
1400.0000000	1.5128000	-35.6824684
1400.0000000	1.5616000	-32.0403137
1400.0000000	1.6104001	-1460.5904541
1400.0000000	1.6592000	-62.0887833
1400.0000000	1.7079999	-27.3999290
1400.0000000	1.7567999	-27.7628708
1400.0000000	1.8055999	-27.5683498
1400.0000000	1.8544000	-26.2968102
1400.0000000	1.9031999	-27.1286926
1400.0000000	1.9520000	-26.7713890
1400.0000000	2.0007999	-25.9461040
1400.0000000	2.0495999	-26.2450199
1400.0000000	2.0983999	-91.8945847
1400.0000000	2.1471999	-11.8307858

1400.0000000	2.1960001	-25.2930393
1400.0000000	2.2448001	-25.1471329
1400.0000000	2.2935998	-25.4147415
1400.0000000	2.3423998	-21.5372257
1400.0000000	2.3912001	8.2617931
1400.0000000	2.4400001	-22.9319515
1400.0000000	2.4888000	-23.4518356
1400.0000000	2.5375998	-12.9165154
1400.0000000	2.5863998	-11.1375256
1400.0000000	2.6352000	-9.3222218
1400.0000000	2.6840000	-15.8603144
1400.0000000	2.7328000	123.1968918
1400.0000000	2.7815998	145.6469421
1400.0000000	2.8304000	-23.7455711
1400.0000000	2.8792000	-20.7414379
1400.0000000	2.9280000	-22.5504971
1400.0000000	2.9768000	-17.1024017
1400.0000000	3.0256000	-20.5694828
1400.0000000	3.0743999	-29.0911369
1400.0000000	3.1231999	-24.1259441
1400.0000000	3.1719999	-29.2957630
1400.0000000	3.2208002	-495.3466187
1400.0000000	3.2695999	-969.4705811
1400.0000000	3.3183999	-20.4971199
1400.0000000	3.3671999	-3341.1176758
1400.0000000	3.4159999	1198.2067871
1400.0000000	3.4648001	-7.3574276
1400.0000000	3.5135999	-26.9234886
1400.0000000	3.5623999	-317.0242310
1400.0000000	3.6111999	-1990.1403809
1400.0000000	3.6600001	-450.8192139
1400.0000000	3.7088001	-595.1583862
1400.0000000	3.7575998	-931.6134644
1400.0000000	3.8063998	-1690.2421875
1400.0000000	3.8552001	-2453.8310547
1400.0000000	3.9040000	-2405.8188477
1400.0000000	3.9528000	1099.6915283
1400.0000000	4.0015998	-240.6736603
1400.0000000	4.0503998	673.1172485
1400.0000000	4.0991998	85.4307556
1400.0000000	4.1479998	-641.7131348
1400.0000000	4.1967998	2.1025009
1400.0000000	4.2456002	-243.7974091
1400.0000000	4.2943997	-478.3836060
1400.0000000	4.3431997	-13.4359207
1400.0000000	4.3920002	-243.4369049
1400.0000000	4.4407997	-22.3754349
1400.0000000	4.4896002	-15.5165625
1400.0000000	4.5383997	-642.5436401
1400.0000000	4.5871997	-12.9354162
1400.0000000	4.6360002	-21.5470238
1400.0000000	4.6847997	-28.0938377
1400.0000000	4.7336001	-19.5277290
1400.0000000	4.7824001	-16.5341988
1400.0000000	4.8311996	-1448.7913818
1400.0000000	4.8800001	223.9443665
1400.0000000	4.9287996	-198.2129059
1400.0000000	4.9776001	1040.6688232

1400.0000000	5.0264001	84.3677979
1400.0000000	5.0751996	-296.1459656
1400.0000000	5.1240001	-12.3527374
1400.0000000	5.1727996	552.8520508
1400.0000000	5.2216001	31.5901222
1400.0000000	5.2704000	-157.6079102
1400.0000000	5.3191996	-38.9871063
1400.0000000	5.3680000	-3506.1547852
1400.0000000	5.4168000	522.5512085
1400.0000000	5.4656000	-199.1887970
1400.0000000	5.5144000	37.6106873
1400.0000000	5.5631995	484.1052856
1400.0000000	5.6120000	389.7182007
1400.0000000	5.6608000	-36.9098206
1400.0000000	5.7096000	61.8702393
1400.0000000	5.7584000	131.8027649
1400.0000000	5.8071995	-11.5575094
1400.0000000	5.8559999	29.4392796
1400.0000000	5.9047999	-16.5971909
1400.0000000	5.9535999	-39.5743561
1400.0000000	6.0023999	-1560.0261230
1400.0000000	6.0511999	-14.5771675
1400.0000000	6.0999999	-19.3740501
1400.0000000	6.1487999	-13.2929049
1400.0000000	6.1975999	-23.1457024
1400.0000000	6.2463999	-1777.2044678
1400.0000000	6.2951999	-19.0360203
1400.0000000	6.3439999	-340.0180359
1400.0000000	6.3927999	-15.0473509
1400.0000000	6.4416003	-12.4104967
1400.0000000	6.4903998	-11.2918549
1400.0000000	6.5391998	-11.8345137
1400.0000000	6.5879998	356.4620667
1400.0000000	6.6367998	-12.1068220
1400.0000000	6.6856003	-13.8219175
1400.0000000	6.7343998	-13.4644985
1400.0000000	6.7831998	-14.3556013
1400.0000000	6.8319998	57.6625595
1400.0000000	6.8807998	-14.0437975
1400.0000000	6.9296002	-418.7725830
1400.0000000	6.9783998	-5.6003060
1400.0000000	7.0271997	-14.0961456
1400.0000000	7.0760002	9.5038004
1400.0000000	7.1247997	-1974.8099365
1400.0000000	7.1736002	-1583.7116699
1400.0000000	7.2223997	-901.2269287
1400.0000000	7.2711997	-195.0685120
1400.0000000	7.3200002	-18.4093552
1400.0000000	7.3687997	173.1411133
1400.0000000	7.4176002	362.1738586
1400.0000000	7.4663997	284.3799438
1400.0000000	7.5151997	-881.4372559
1400.0000000	7.5640001	-973.5338135
1400.0000000	7.6127996	-2293.6140137
1400.0000000	7.6616001	-736.8063965
1400.0000000	7.7104001	-378.8225098
1400.0000000	7.7591996	39.2759895
1400.0000000	7.8080001	376.3779602

1400.0000000	7.8567996	496.0602417
1400.0000000	7.9056001	-1030.3336182
1400.0000000	7.9544001	1639.4912109
1400.0000000	8.0031996	2115.5476074
1400.0000000	8.0520000	2111.5227051
1400.0000000	8.1007996	2234.4299316
1400.0000000	8.1496000	2383.0021973
1400.0000000	8.1983995	1482.5074463
1400.0000000	8.2472000	-3392.4230957
1400.0000000	8.2959995	-1202.6373291
1400.0000000	8.3448000	-957.9439697
1400.0000000	8.3935995	-877.9477539
1400.0000000	8.4423990	-378.3025818
1400.0000000	8.4912004	-882.9259644
1400.0000000	8.5400000	-2381.4235840
1400.0000000	8.5887995	-1154.5283203
1400.0000000	8.6375999	225.2188873
1400.0000000	8.6863995	2036.4924316
1400.0000000	8.7351999	-757.1488647
1400.0000000	8.7840004	864.6412354
1400.0000000	8.8327999	46.2447624
1400.0000000	8.8815994	-11.5031357
1400.0000000	8.9303989	-23.8023396
1400.0000000	8.9792004	-11.5822620
1400.0000000	9.0279999	-5.9046421
1400.0000000	9.0767994	6.8205166
1400.0000000	9.1255999	-17.1765327
1400.0000000	9.1743994	-10.0889368
1400.0000000	9.2231998	36.4115372
1400.0000000	9.2720003	-8.4557819
1400.0000000	9.3207998	-9.8186340
1400.0000000	9.3695993	-10.7723856
1400.0000000	9.4183998	-11.7491446
1400.0000000	9.4672003	-11.1628962
1400.0000000	9.5159998	-14.1303501
1400.0000000	9.5648003	-0.5212383
1400.0000000	9.6135998	-13.3659630
1400.0000000	9.6623993	-10.2317848
1400.0000000	9.7111998	-10.1680260
1400.0000000	9.7600002	-10.8591604
1400.0000000	9.8087997	-10.9950809
1400.0000000	9.8575993	3.0218525
1400.0000000	9.9063997	-8.0037918
1400.0000000	9.9552002	-10.9067659
1400.0000000	10.0039997	-10.0841255
1410.0000000	0.3416000	-37.0088501
1410.0000000	0.3904000	-39.8876076
1410.0000000	0.4392000	-39.4942818
1410.0000000	0.4880000	-39.5441017
1410.0000000	0.5368000	-39.6595306
1410.0000000	0.5856000	-39.2787743
1410.0000000	0.6344000	-38.2375526
1410.0000000	0.6832000	-36.2526855
1410.0000000	0.7320000	-36.0816765
1410.0000000	0.7808000	-36.5370102
1410.0000000	0.8296000	-35.9411011
1410.0000000	0.8784000	-35.0668640
1410.0000000	0.9272000	-34.2487717

1410.0000000	0.9760000	-31.9314861
1410.0000000	1.0247999	-30.3124943
1410.0000000	1.0735999	-30.0419693
1410.0000000	1.1224000	-30.7243767
1410.0000000	1.1711999	-31.3460674
1410.0000000	1.2200000	-30.8431244
1410.0000000	1.2687999	-30.8187428
1410.0000000	1.3176000	-30.9491596
1410.0000000	1.3664000	-30.6271839
1410.0000000	1.4152000	-30.1702881
1410.0000000	1.4640000	-29.5486488
1410.0000000	1.5128000	-35.5852928
1410.0000000	1.5616000	-31.9984741
1410.0000000	1.6104001	-1458.0477295
1410.0000000	1.6592000	-61.7948532
1410.0000000	1.7079999	-27.4036770
1410.0000000	1.7567999	-27.7628899
1410.0000000	1.8055999	-27.4381618
1410.0000000	1.8544000	-27.2043018
1410.0000000	1.9031999	-27.0098019
1410.0000000	1.9520000	-26.7387581
1410.0000000	2.0007999	-25.9514847
1410.0000000	2.0495999	-26.2470379
1410.0000000	2.0983999	-90.4188080
1410.0000000	2.1471999	-12.0980530
1410.0000000	2.1960001	-25.2889481
1410.0000000	2.2448001	-25.1292210
1410.0000000	2.2935998	-25.3551178
1410.0000000	2.3423998	-21.5516300
1410.0000000	2.3912001	7.6402473
1410.0000000	2.4400001	-22.9344368
1410.0000000	2.4888000	-23.4516182
1410.0000000	2.5375998	-12.9336233
1410.0000000	2.5863998	-11.1572847
1410.0000000	2.6352000	-9.3449659
1410.0000000	2.6840000	-15.8722572
1410.0000000	2.7328000	122.9308701
1410.0000000	2.7815998	145.3405151
1410.0000000	2.8304000	-23.7442684
1410.0000000	2.8792000	-20.7447014
1410.0000000	2.9280000	-22.5499287
1410.0000000	2.9768000	-17.1107712
1410.0000000	3.0256000	-20.5720673
1410.0000000	3.0743999	-29.0797501
1410.0000000	3.1231999	-24.1224899
1410.0000000	3.1719999	-29.2874699
1410.0000000	3.2208002	-495.1088867
1410.0000000	3.2695999	-968.9956055
1410.0000000	3.3183999	-20.4973183
1410.0000000	3.3671999	-3339.6560059
1410.0000000	3.4159999	1197.6715088
1410.0000000	3.4648001	-7.3631363
1410.0000000	3.5135999	-26.9204979
1410.0000000	3.5623999	-316.8946228
1410.0000000	3.6111999	-1989.2806396
1410.0000000	3.6600001	-450.6311035
1410.0000000	3.7088001	-594.9069824
1410.0000000	3.7575998	-931.2149658

1410.0000000	3.8063998	-1689.5123291
1410.0000000	3.8552001	-2452.7675781
1410.0000000	3.9040000	-2404.7763672
1410.0000000	3.9528000	1099.2031250
1410.0000000	4.0015998	-240.5765533
1410.0000000	4.0503998	672.8146973
1410.0000000	4.0991998	85.3850708
1410.0000000	4.1479998	-641.4409180
1410.0000000	4.1967998	2.0932446
1410.0000000	4.2456002	-243.6991119
1410.0000000	4.2943997	-478.1830444
1410.0000000	4.3431997	-13.4381008
1410.0000000	4.3920002	-243.3387909
1410.0000000	4.4407997	-22.3736591
1410.0000000	4.4896002	-15.5174446
1410.0000000	4.5383997	-642.2825928
1410.0000000	4.5871997	-12.9374332
1410.0000000	4.6360002	-21.5454826
1410.0000000	4.6847997	-28.0899124
1410.0000000	4.7336001	-19.5270061
1410.0000000	4.7824001	-16.5346546
1410.0000000	4.8311996	-1448.2601318
1410.0000000	4.8800001	223.8548279
1410.0000000	4.9287996	-198.1456451
1410.0000000	4.9776001	1040.2763672
1410.0000000	5.0264001	84.3302765
1410.0000000	5.0751996	-296.0423279
1410.0000000	5.1240001	-12.3536587
1410.0000000	5.1727996	552.7362061
1410.0000000	5.2216001	31.5806770
1410.0000000	5.2704000	-157.5797882
1410.0000000	5.3191996	-38.9825592
1410.0000000	5.3680000	-3505.4624023
1410.0000000	5.4168000	522.4443970
1410.0000000	5.4656000	-199.1526184
1410.0000000	5.5144000	37.6000290
1410.0000000	5.5631995	484.0067139
1410.0000000	5.6120000	389.6382446
1410.0000000	5.6608000	-36.9057312
1410.0000000	5.7096000	61.8548050
1410.0000000	5.7584000	131.7738342
1410.0000000	5.8071995	-11.5583801
1410.0000000	5.8559999	29.4304295
1410.0000000	5.9047999	-16.5969925
1410.0000000	5.9535999	-39.5696144
1410.0000000	6.0023999	-1559.7218018
1410.0000000	6.0511999	-14.5773792
1410.0000000	6.0999999	-19.3732185
1410.0000000	6.1487999	-13.2933674
1410.0000000	6.1975999	-23.1439381
1410.0000000	6.2463999	-1776.8570557
1410.0000000	6.2951999	-19.0352058
1410.0000000	6.3439999	-339.9539795
1410.0000000	6.3927999	-15.0473328
1410.0000000	6.4416003	-12.4110832
1410.0000000	6.4903998	-11.2925310
1410.0000000	6.5391998	-11.8351946
1410.0000000	6.5879998	355.7713623

1410.0000000	6.6367998	-12.1073704
1410.0000000	6.6856003	-13.8220873
1410.0000000	6.7343998	-13.4647150
1410.0000000	6.7831998	-14.3551855
1410.0000000	6.8319998	57.6434479
1410.0000000	6.8807998	-14.0436592
1410.0000000	6.9296002	-418.0111389
1410.0000000	6.9783998	-5.6131296
1410.0000000	7.0271997	-14.0951185
1410.0000000	7.0760002	9.4616156
1410.0000000	7.1247997	-1970.8640137
1410.0000000	7.1736002	-1580.5488281
1410.0000000	7.2223997	-899.4337769
1410.0000000	7.2711997	-194.7007446
1410.0000000	7.3200002	-18.3973656
1410.0000000	7.3687997	172.7665710
1410.0000000	7.4176002	361.4180298
1410.0000000	7.4663997	283.7822876
1410.0000000	7.5151997	-879.6762085
1410.0000000	7.5640001	-971.5864258
1410.0000000	7.6127996	-2288.9963379
1410.0000000	7.6616001	-735.3350830
1410.0000000	7.7104001	-378.0762939
1410.0000000	7.7591996	39.1749420
1410.0000000	7.8080001	375.5925903
1410.0000000	7.8567996	495.0321655
1410.0000000	7.9056001	-1028.2607422
1410.0000000	7.9544001	1636.1812744
1410.0000000	8.0031996	2111.2780762
1410.0000000	8.0520000	2107.2607422
1410.0000000	8.1007996	2229.9204102
1410.0000000	8.1496000	2378.1940918
1410.0000000	8.1983995	1479.5148926
1410.0000000	8.2472000	-3385.5883789
1410.0000000	8.2959995	-1200.2171631
1410.0000000	8.3448000	-956.0147705
1410.0000000	8.3935995	-876.1800537
1410.0000000	8.4423990	-377.5445862
1410.0000000	8.4912004	-881.1497803
1410.0000000	8.5400000	-2376.6237793
1410.0000000	8.5887995	-1152.2067871
1410.0000000	8.6375999	224.7575684
1410.0000000	8.6863995	2032.3740234
1410.0000000	8.7351999	-755.6246338
1410.0000000	8.7840004	862.8845215
1410.0000000	8.8327999	46.1331558
1410.0000000	8.8815994	-11.5033102
1410.0000000	8.9303989	-23.7986183
1410.0000000	8.9792004	-11.5823441
1410.0000000	9.0279999	-5.9064102
1410.0000000	9.0767994	6.8147984
1410.0000000	9.1255999	-17.1748180
1410.0000000	9.1743994	-10.0893393
1410.0000000	9.2231998	36.3958054
1410.0000000	9.2720003	-8.4567184
1410.0000000	9.3207998	-9.8191729
1410.0000000	9.3695993	-10.7726116
1410.0000000	9.4183998	-11.7490196

1410.0000000	9.4672003	-11.1629381
1410.0000000	9.5159998	-14.1294584
1410.0000000	9.5648003	-0.5246115
1410.0000000	9.6135998	-13.3651724
1410.0000000	9.6623993	-10.2320604
1410.0000000	9.7111998	-10.1682968
1410.0000000	9.7600002	-10.8592024
1410.0000000	9.8087997	-10.9950886
1410.0000000	9.8575993	3.0174313
1410.0000000	9.9063997	-8.0047054
1410.0000000	9.9552002	-10.9067698
1410.0000000	10.0039997	-10.0843735
1420.0000000	0.3416000	-37.0269661
1420.0000000	0.3904000	-39.8661804
1420.0000000	0.4392000	-39.4879379
1420.0000000	0.4880000	-39.5428925
1420.0000000	0.5368000	-39.6607056
1420.0000000	0.5856000	-39.2773933
1420.0000000	0.6344000	-38.2378654
1420.0000000	0.6832000	-36.2549133
1420.0000000	0.7320000	-36.0837288
1420.0000000	0.7808000	-36.5371780
1420.0000000	0.8296000	-35.9392548
1420.0000000	0.8784000	-35.0572968
1420.0000000	0.9272000	-34.2494125
1420.0000000	0.9760000	-31.9289627
1420.0000000	1.0247999	-30.3137302
1420.0000000	1.0735999	-30.0555687
1420.0000000	1.1224000	-30.7225037
1420.0000000	1.1711999	-31.3397636
1420.0000000	1.2200000	-30.7993259
1420.0000000	1.2687999	-30.7974167
1420.0000000	1.3176000	-30.9427738
1420.0000000	1.3664000	-30.6242409
1420.0000000	1.4152000	-30.1704922
1420.0000000	1.4640000	-29.5469398
1420.0000000	1.5128000	-35.4881134
1420.0000000	1.5616000	-31.9566326
1420.0000000	1.6104001	-1455.5048828
1420.0000000	1.6592000	-61.5009155
1420.0000000	1.7079999	-27.4074249
1420.0000000	1.7567999	-27.7629089
1420.0000000	1.8055999	-27.3079720
1420.0000000	1.8544000	-27.2203293
1420.0000000	1.9031999	-26.8909111
1420.0000000	1.9520000	-26.7061272
1420.0000000	2.0007999	-25.9568634
1420.0000000	2.0495999	-26.2490578
1420.0000000	2.0983999	-88.9430313
1420.0000000	2.1471999	-12.3653202
1420.0000000	2.1960001	-25.2848568
1420.0000000	2.2448001	-25.1113091
1420.0000000	2.2935998	-25.2954941
1420.0000000	2.3423998	-21.5660343
1420.0000000	2.3912001	7.0187016
1420.0000000	2.4400001	-22.9369240
1420.0000000	2.4888000	-23.4514008
1420.0000000	2.5375998	-12.9507322

1420.0000000	2.5863998	-11.1770439
1420.0000000	2.6352000	-9.3677101
1420.0000000	2.6840000	-15.8841982
1420.0000000	2.7328000	122.6648636
1420.0000000	2.7815998	145.0340881
1420.0000000	2.8304000	-23.7429638
1420.0000000	2.8792000	-20.7479649
1420.0000000	2.9280000	-22.5493584
1420.0000000	2.9768000	-17.1191425
1420.0000000	3.0256000	-20.5746517
1420.0000000	3.0743999	-29.0683632
1420.0000000	3.1231999	-24.1190338
1420.0000000	3.1719999	-29.2791767
1420.0000000	3.2208002	-494.8711548
1420.0000000	3.2695999	-968.5206299
1420.0000000	3.3183999	-20.4975166
1420.0000000	3.3671999	-3338.1943359
1420.0000000	3.4159999	1197.1362305
1420.0000000	3.4648001	-7.3688440
1420.0000000	3.5135999	-26.9175072
1420.0000000	3.5623999	-316.7650452
1420.0000000	3.6111999	-1988.4208984
1420.0000000	3.6600001	-450.4429321
1420.0000000	3.7088001	-594.6555786
1420.0000000	3.7575998	-930.8164063
1420.0000000	3.8063998	-1688.7824707
1420.0000000	3.8552001	-2451.7043457
1420.0000000	3.9040000	-2403.7338867
1420.0000000	3.9528000	1098.7148438
1420.0000000	4.0015998	-240.4794464
1420.0000000	4.0503998	672.5121460
1420.0000000	4.0991998	85.3393860
1420.0000000	4.1479998	-641.1687012
1420.0000000	4.1967998	2.0839882
1420.0000000	4.2456002	-243.6007996
1420.0000000	4.2943997	-477.9824829
1420.0000000	4.3431997	-13.4402809
1420.0000000	4.3920002	-243.2406769
1420.0000000	4.4407997	-22.3718834
1420.0000000	4.4896002	-15.5183277
1420.0000000	4.5383997	-642.0215454
1420.0000000	4.5871997	-12.9394493
1420.0000000	4.6360002	-21.5439396
1420.0000000	4.6847997	-28.0859871
1420.0000000	4.7336001	-19.5262833
1420.0000000	4.7824001	-16.5351086
1420.0000000	4.8311996	-1447.7288818
1420.0000000	4.8800001	223.7652740
1420.0000000	4.9287996	-198.0783691
1420.0000000	4.9776001	1039.8837891
1420.0000000	5.0264001	84.2927628
1420.0000000	5.0751996	-295.9387207
1420.0000000	5.1240001	-12.3545799
1420.0000000	5.1727996	552.6203613
1420.0000000	5.2216001	31.5712318
1420.0000000	5.2704000	-157.5516968
1420.0000000	5.3191996	-38.9780083
1420.0000000	5.3680000	-3504.7700195

1420.0000000	5.4168000	522.3375854
1420.0000000	5.4656000	-199.1164246
1420.0000000	5.5144000	37.5893707
1420.0000000	5.5631995	483.9081421
1420.0000000	5.6120000	389.5582581
1420.0000000	5.6608000	-36.9016418
1420.0000000	5.7096000	61.8393784
1420.0000000	5.7584000	131.7449036
1420.0000000	5.8071995	-11.5592499
1420.0000000	5.8559999	29.4215794
1420.0000000	5.9047999	-16.5967960
1420.0000000	5.9535999	-39.5648804
1420.0000000	6.0023999	-1559.4172363
1420.0000000	6.0511999	-14.5775909
1420.0000000	6.0999999	-19.3723850
1420.0000000	6.1487999	-13.2938299
1420.0000000	6.1975999	-23.1421738
1420.0000000	6.2463999	-1776.5096436
1420.0000000	6.2951999	-19.0343933
1420.0000000	6.3439999	-339.8899536
1420.0000000	6.3927999	-15.0473146
1420.0000000	6.4416003	-12.4116697
1420.0000000	6.4903998	-11.2932072
1420.0000000	6.5391998	-11.8358765
1420.0000000	6.5879998	355.0806885
1420.0000000	6.6367998	-12.1079197
1420.0000000	6.6856003	-13.8222580
1420.0000000	6.7343998	-13.4649305
1420.0000000	6.7831998	-14.3547697
1420.0000000	6.8319998	57.6243362
1420.0000000	6.8807998	-14.0435200
1420.0000000	6.9296002	-417.2496643
1420.0000000	6.9783998	-5.6259532
1420.0000000	7.0271997	-14.0940905
1420.0000000	7.0760002	9.4194307
1420.0000000	7.1247997	-1966.9182129
1420.0000000	7.1736002	-1577.3859863
1420.0000000	7.2223997	-897.6405640
1420.0000000	7.2711997	-194.3329620
1420.0000000	7.3200002	-18.3853779
1420.0000000	7.3687997	172.3920441
1420.0000000	7.4176002	360.6622009
1420.0000000	7.4663997	283.1846313
1420.0000000	7.5151997	-877.9151611
1420.0000000	7.5640001	-969.6389771
1420.0000000	7.6127996	-2284.3786621
1420.0000000	7.6616001	-733.8637085
1420.0000000	7.7104001	-377.3300171
1420.0000000	7.7591996	39.0738945
1420.0000000	7.8080001	374.8072205
1420.0000000	7.8567996	494.0040894
1420.0000000	7.9056001	-1026.1877441
1420.0000000	7.9544001	1632.8713379
1420.0000000	8.0031996	2107.0085449
1420.0000000	8.0520000	2102.9985352
1420.0000000	8.1007996	2225.4108887
1420.0000000	8.1496000	2373.3859863
1420.0000000	8.1983995	1476.5223389

1420.0000000	8.2472000	-3378.7534180
1420.0000000	8.2959995	-1197.7969971
1420.0000000	8.3448000	-954.0855713
1420.0000000	8.3935995	-874.4124146
1420.0000000	8.4423990	-376.7865601
1420.0000000	8.4912004	-879.3735352
1420.0000000	8.5400000	-2371.8239746
1420.0000000	8.5887995	-1149.8852539
1420.0000000	8.6375999	224.2962494
1420.0000000	8.6863995	2028.2557373
1420.0000000	8.7351999	-754.1003418
1420.0000000	8.7840004	861.1277466
1420.0000000	8.8327999	46.0215454
1420.0000000	8.8815994	-11.5034847
1420.0000000	8.9303989	-23.7948990
1420.0000000	8.9792004	-11.5824270
1420.0000000	9.0279999	-5.9081788
1420.0000000	9.0767994	6.8090820
1420.0000000	9.1255999	-17.1731033
1420.0000000	9.1743994	-10.0897408
1420.0000000	9.2231998	36.3800659
1420.0000000	9.2720003	-8.4576550
1420.0000000	9.3207998	-9.8197126
1420.0000000	9.3695993	-10.7728386
1420.0000000	9.4183998	-11.7488947
1420.0000000	9.4672003	-11.1629791
1420.0000000	9.5159998	-14.1285677
1420.0000000	9.5648003	-0.5279846
1420.0000000	9.6135998	-13.3643818
1420.0000000	9.6623993	-10.2323351
1420.0000000	9.7111998	-10.1685686
1420.0000000	9.7600002	-10.8592443
1420.0000000	9.8087997	-10.9950962
1420.0000000	9.8575993	3.0130072
1420.0000000	9.9063997	-8.0056190
1420.0000000	9.9552002	-10.9067745
1420.0000000	10.0039997	-10.0846214
1430.0000000	0.3416000	-36.8538475
1430.0000000	0.3904000	-39.8447495
1430.0000000	0.4392000	-39.4815941
1430.0000000	0.4880000	-39.5416832
1430.0000000	0.5368000	-39.6618805
1430.0000000	0.5856000	-39.2760124
1430.0000000	0.6344000	-38.2381744
1430.0000000	0.6832000	-36.2571373
1430.0000000	0.7320000	-36.0857811
1430.0000000	0.7808000	-36.5373459
1430.0000000	0.8296000	-35.9374123
1430.0000000	0.8784000	-35.0477333
1430.0000000	0.9272000	-34.2500534
1430.0000000	0.9760000	-31.9264393
1430.0000000	1.0247999	-30.3149662
1430.0000000	1.0735999	-30.0691700
1430.0000000	1.1224000	-30.7206326
1430.0000000	1.1711999	-31.3334618
1430.0000000	1.2200000	-30.7555275
1430.0000000	1.2687999	-30.7760887
1430.0000000	1.3176000	-30.9363880

1430.0000000	1.3664000	-30.6212978
1430.0000000	1.4152000	-30.1706963
1430.0000000	1.4640000	-29.5452309
1430.0000000	1.5128000	-35.3909378
1430.0000000	1.5616000	-31.9147911
1430.0000000	1.6104001	-1452.9621582
1430.0000000	1.6592000	-61.2069855
1430.0000000	1.7079999	-27.4111710
1430.0000000	1.7567999	-27.7629280
1430.0000000	1.8055999	-27.1777840
1430.0000000	1.8544000	-27.2363586
1430.0000000	1.9031999	-26.7720222
1430.0000000	1.9520000	-26.6734943
1430.0000000	2.0007999	-25.9622440
1430.0000000	2.0495999	-26.2510757
1430.0000000	2.0983999	-87.4672546
1430.0000000	2.1471999	-12.6325874
1430.0000000	2.1960001	-25.2807655
1430.0000000	2.2448001	-25.0933990
1430.0000000	2.2935998	-25.2358723
1430.0000000	2.3423998	-21.5804386
1430.0000000	2.3912001	6.3971558
1430.0000000	2.4400001	-22.9394093
1430.0000000	2.4888000	-23.4511833
1430.0000000	2.5375998	-12.9678402
1430.0000000	2.5863998	-11.1968021
1430.0000000	2.6352000	-9.3904543
1430.0000000	2.6840000	-15.8961401
1430.0000000	2.7328000	122.3988419
1430.0000000	2.7815998	144.7276917
1430.0000000	2.8304000	-23.7416592
1430.0000000	2.8792000	-20.7512283
1430.0000000	2.9280000	-22.5487900
1430.0000000	2.9768000	-17.1275139
1430.0000000	3.0256000	-20.5772362
1430.0000000	3.0743999	-29.0569763
1430.0000000	3.1231999	-24.1155777
1430.0000000	3.1719999	-29.2708836
1430.0000000	3.2208002	-494.6334229
1430.0000000	3.2695999	-968.0456543
1430.0000000	3.3183999	-20.4977150
1430.0000000	3.3671999	-3336.7326660
1430.0000000	3.4159999	1196.6009521
1430.0000000	3.4648001	-7.3745527
1430.0000000	3.5135999	-26.9145164
1430.0000000	3.5623999	-316.6354370
1430.0000000	3.6111999	-1987.5611572
1430.0000000	3.6600001	-450.2548218
1430.0000000	3.7088001	-594.4042358
1430.0000000	3.7575998	-930.4179077
1430.0000000	3.8063998	-1688.0526123
1430.0000000	3.8552001	-2450.6408691
1430.0000000	3.9040000	-2402.6914063
1430.0000000	3.9528000	1098.2265625
1430.0000000	4.0015998	-240.3823395
1430.0000000	4.0503998	672.2095947
1430.0000000	4.0991998	85.2937012
1430.0000000	4.1479998	-640.8964844

1430.0000000	4.1967998	2.0747318
1430.0000000	4.2456002	-243.5025024
1430.0000000	4.2943997	-477.7818909
1430.0000000	4.3431997	-13.4424620
1430.0000000	4.3920002	-243.1425629
1430.0000000	4.4407997	-22.3701077
1430.0000000	4.4896002	-15.5192099
1430.0000000	4.5383997	-641.7604370
1430.0000000	4.5871997	-12.9414654
1430.0000000	4.6360002	-21.5423965
1430.0000000	4.6847997	-28.0820618
1430.0000000	4.7336001	-19.5255623
1430.0000000	4.7824001	-16.5355625
1430.0000000	4.8311996	-1447.1976318
1430.0000000	4.8800001	223.6757202
1430.0000000	4.9287996	-198.0111084
1430.0000000	4.9776001	1039.4913330
1430.0000000	5.0264001	84.2552414
1430.0000000	5.0751996	-295.8350830
1430.0000000	5.1240001	-12.3555012
1430.0000000	5.1727996	552.5044556
1430.0000000	5.2216001	31.5617905
1430.0000000	5.2704000	-157.5235901
1430.0000000	5.3191996	-38.9734612
1430.0000000	5.3680000	-3504.0776367
1430.0000000	5.4168000	522.2308350
1430.0000000	5.4656000	-199.0802460
1430.0000000	5.5144000	37.5787201
1430.0000000	5.5631995	483.8096008
1430.0000000	5.6120000	389.4782715
1430.0000000	5.6608000	-36.8975525
1430.0000000	5.7096000	61.8239517
1430.0000000	5.7584000	131.7159729
1430.0000000	5.8071995	-11.5601215
1430.0000000	5.8559999	29.4127254
1430.0000000	5.9047999	-16.5965977
1430.0000000	5.9535999	-39.5601387
1430.0000000	6.0023999	-1559.1129150
1430.0000000	6.0511999	-14.5778017
1430.0000000	6.0999999	-19.3715534
1430.0000000	6.1487999	-13.2942924
1430.0000000	6.1975999	-23.1404076
1430.0000000	6.2463999	-1776.1623535
1430.0000000	6.2951999	-19.0335789
1430.0000000	6.3439999	-339.8258667
1430.0000000	6.3927999	-15.0472965
1430.0000000	6.4416003	-12.4122572
1430.0000000	6.4903998	-11.2938833
1430.0000000	6.5391998	-11.8365574
1430.0000000	6.5879998	354.3899841
1430.0000000	6.6367998	-12.1084690
1430.0000000	6.6856003	-13.8224277
1430.0000000	6.7343998	-13.4651461
1430.0000000	6.7831998	-14.3543549
1430.0000000	6.8319998	57.6052170
1430.0000000	6.8807998	-14.0433817
1430.0000000	6.9296002	-416.4882202
1430.0000000	6.9783998	-5.6387763

1430.0000000	7.0271997	-14.0930624
1430.0000000	7.0760002	9.3772440
1430.0000000	7.1247997	-1962.9722900
1430.0000000	7.1736002	-1574.2231445
1430.0000000	7.2223997	-895.8474121
1430.0000000	7.2711997	-193.9651794
1430.0000000	7.3200002	-18.3733902
1430.0000000	7.3687997	172.0175171
1430.0000000	7.4176002	359.9063721
1430.0000000	7.4663997	282.5869751
1430.0000000	7.5151997	-876.1541138
1430.0000000	7.5640001	-967.6915894
1430.0000000	7.6127996	-2279.7612305
1430.0000000	7.6616001	-732.3923340
1430.0000000	7.7104001	-376.5837402
1430.0000000	7.7591996	38.9728432
1430.0000000	7.8080001	374.0218811
1430.0000000	7.8567996	492.9759521
1430.0000000	7.9056001	-1024.1148682
1430.0000000	7.9544001	1629.5614014
1430.0000000	8.0031996	2102.7390137
1430.0000000	8.0520000	2098.7365723
1430.0000000	8.1007996	2220.9013672
1430.0000000	8.1496000	2368.5781250
1430.0000000	8.1983995	1473.5299072
1430.0000000	8.2472000	-3371.9187012
1430.0000000	8.2959995	-1195.3768311
1430.0000000	8.3448000	-952.1564331
1430.0000000	8.3935995	-872.6447144
1430.0000000	8.4423990	-376.0285645
1430.0000000	8.4912004	-877.5972900
1430.0000000	8.5400000	-2367.0241699
1430.0000000	8.5887995	-1147.5637207
1430.0000000	8.6375999	223.8349304
1430.0000000	8.6863995	2024.1373291
1430.0000000	8.7351999	-752.5761108
1430.0000000	8.7840004	859.3710327
1430.0000000	8.8327999	45.9099350
1430.0000000	8.8815994	-11.5036592
1430.0000000	8.9303989	-23.7911797
1430.0000000	8.9792004	-11.5825090
1430.0000000	9.0279999	-5.9099474
1430.0000000	9.0767994	6.8033638
1430.0000000	9.1255999	-17.1713905
1430.0000000	9.1743994	-10.0901432
1430.0000000	9.2231998	36.3643341
1430.0000000	9.2720003	-8.4585915
1430.0000000	9.3207998	-9.8202515
1430.0000000	9.3695993	-10.7730646
1430.0000000	9.4183998	-11.7487707
1430.0000000	9.4672003	-11.1630201
1430.0000000	9.5159998	-14.1276760
1430.0000000	9.5648003	-0.5313568
1430.0000000	9.6135998	-13.3635902
1430.0000000	9.6623993	-10.2326107
1430.0000000	9.7111998	-10.1688404
1430.0000000	9.7600002	-10.8592854
1430.0000000	9.8087997	-10.9951048

1430.0000000	9.8575993	3.0085850
1430.0000000	9.9063997	-8.0065327
1430.0000000	9.9552002	-10.9067783
1430.0000000	10.0039997	-10.0848684
1440.0000000	0.3416000	-36.5822334
1440.0000000	0.3904000	-39.5882645
1440.0000000	0.4392000	-39.4752541
1440.0000000	0.4880000	-39.5404739
1440.0000000	0.5368000	-39.6630516
1440.0000000	0.5856000	-39.2746277
1440.0000000	0.6344000	-38.2384834
1440.0000000	0.6832000	-36.2593651
1440.0000000	0.7320000	-36.0878372
1440.0000000	0.7808000	-36.5375175
1440.0000000	0.8296000	-35.9355659
1440.0000000	0.8784000	-35.0381660
1440.0000000	0.9272000	-34.2506981
1440.0000000	0.9760000	-31.9239159
1440.0000000	1.0247999	-30.3162022
1440.0000000	1.0735999	-30.0827694
1440.0000000	1.1224000	-30.7187595
1440.0000000	1.1711999	-31.3271580
1440.0000000	1.2200000	-30.7117290
1440.0000000	1.2687999	-30.7547626
1440.0000000	1.3176000	-30.9300022
1440.0000000	1.3664000	-30.6183529
1440.0000000	1.4152000	-30.1709003
1440.0000000	1.4640000	-29.5435200
1440.0000000	1.5128000	-35.2937584
1440.0000000	1.5616000	-31.8729515
1440.0000000	1.6104001	-1450.4193115
1440.0000000	1.6592000	-60.9130554
1440.0000000	1.7079999	-27.4149189
1440.0000000	1.7567999	-27.7629471
1440.0000000	1.8055999	-27.0475941
1440.0000000	1.8544000	-26.6347160
1440.0000000	1.9031999	-26.6531315
1440.0000000	1.9520000	-26.6408615
1440.0000000	2.0007999	-25.9676247
1440.0000000	2.0495999	-26.2530956
1440.0000000	2.0983999	-85.9914703
1440.0000000	2.1471999	-12.8998547
1440.0000000	2.1960001	-25.2766743
1440.0000000	2.2448001	-25.0754871
1440.0000000	2.2935998	-25.1762486
1440.0000000	2.3423998	-21.5948429
1440.0000000	2.3912001	5.7756100
1440.0000000	2.4400001	-22.9418964
1440.0000000	2.4888000	-23.4509659
1440.0000000	2.5375998	-12.9849482
1440.0000000	2.5863998	-11.2165613
1440.0000000	2.6352000	-9.4131994
1440.0000000	2.6840000	-15.9080811
1440.0000000	2.7328000	122.1328354
1440.0000000	2.7815998	144.4212646
1440.0000000	2.8304000	-23.7403545
1440.0000000	2.8792000	-20.7544918
1440.0000000	2.9280000	-22.5482216

1440.0000000	2.9768000	-17.1358833
1440.0000000	3.0256000	-20.5798206
1440.0000000	3.0743999	-29.0455894
1440.0000000	3.1231999	-24.1121235
1440.0000000	3.1719999	-29.2625904
1440.0000000	3.2208002	-494.3956909
1440.0000000	3.2695999	-967.5706787
1440.0000000	3.3183999	-20.4979115
1440.0000000	3.3671999	-3335.2709961
1440.0000000	3.4159999	1196.0656738
1440.0000000	3.4648001	-7.3802605
1440.0000000	3.5135999	-26.9115257
1440.0000000	3.5623999	-316.5058594
1440.0000000	3.6111999	-1986.7012939
1440.0000000	3.6600001	-450.0667114
1440.0000000	3.7088001	-594.1528320
1440.0000000	3.7575998	-930.0193481
1440.0000000	3.8063998	-1687.3227539
1440.0000000	3.8552001	-2449.5773926
1440.0000000	3.9040000	-2401.6489258
1440.0000000	3.9528000	1097.7382813
1440.0000000	4.0015998	-240.2852325
1440.0000000	4.0503998	671.9070435
1440.0000000	4.0991998	85.2480164
1440.0000000	4.1479998	-640.6242676
1440.0000000	4.1967998	2.0654755
1440.0000000	4.2456002	-243.4042053
1440.0000000	4.2943997	-477.5813293
1440.0000000	4.3431997	-13.4446430
1440.0000000	4.3920002	-243.0444489
1440.0000000	4.4407997	-22.3683319
1440.0000000	4.4896002	-15.5200930
1440.0000000	4.5383997	-641.4993896
1440.0000000	4.5871997	-12.9434814
1440.0000000	4.6360002	-21.5408554
1440.0000000	4.6847997	-28.0781364
1440.0000000	4.7336001	-19.5248394
1440.0000000	4.7824001	-16.5360165
1440.0000000	4.8311996	-1446.6662598
1440.0000000	4.8800001	223.5861816
1440.0000000	4.9287996	-197.9438477
1440.0000000	4.9776001	1039.0988770
1440.0000000	5.0264001	84.2177200
1440.0000000	5.0751996	-295.7314453
1440.0000000	5.1240001	-12.3564224
1440.0000000	5.1727996	552.3886108
1440.0000000	5.2216001	31.5523453
1440.0000000	5.2704000	-157.4954834
1440.0000000	5.3191996	-38.9689102
1440.0000000	5.3680000	-3503.3852539
1440.0000000	5.4168000	522.1240845
1440.0000000	5.4656000	-199.0440674
1440.0000000	5.5144000	37.5680618
1440.0000000	5.5631995	483.7110291
1440.0000000	5.6120000	389.3983154
1440.0000000	5.6608000	-36.8934631
1440.0000000	5.7096000	61.8085175
1440.0000000	5.7584000	131.6870270

1440.0000000	5.8071995	-11.5609913
1440.0000000	5.8559999	29.4038754
1440.0000000	5.9047999	-16.5963993
1440.0000000	5.9535999	-39.5554008
1440.0000000	6.0023999	-1558.8084717
1440.0000000	6.0511999	-14.5780134
1440.0000000	6.0999999	-19.3707199
1440.0000000	6.1487999	-13.2947559
1440.0000000	6.1975999	-23.1386433
1440.0000000	6.2463999	-1775.8149414
1440.0000000	6.2951999	-19.0327644
1440.0000000	6.3439999	-339.7618408
1440.0000000	6.3927999	-15.0472775
1440.0000000	6.4416003	-12.4128437
1440.0000000	6.4903998	-11.2945585
1440.0000000	6.5391998	-11.8372383
1440.0000000	6.5879998	353.6992798
1440.0000000	6.6367998	-12.1090183
1440.0000000	6.6856003	-13.8225985
1440.0000000	6.7343998	-13.4653625
1440.0000000	6.7831998	-14.3539391
1440.0000000	6.8319998	57.5860977
1440.0000000	6.8807998	-14.0432434
1440.0000000	6.9296002	-415.7267761
1440.0000000	6.9783998	-5.6516004
1440.0000000	7.0271997	-14.0920343
1440.0000000	7.0760002	9.3350592
1440.0000000	7.1247997	-1959.0264893
1440.0000000	7.1736002	-1571.0601807
1440.0000000	7.2223997	-894.0542603
1440.0000000	7.2711997	-193.5974121
1440.0000000	7.3200002	-18.3614006
1440.0000000	7.3687997	171.6429749
1440.0000000	7.4176002	359.1505432
1440.0000000	7.4663997	281.9893188
1440.0000000	7.5151997	-874.3930664
1440.0000000	7.5640001	-965.7442017
1440.0000000	7.6127996	-2275.1435547
1440.0000000	7.6616001	-730.9210205
1440.0000000	7.7104001	-375.8375244
1440.0000000	7.7591996	38.8717918
1440.0000000	7.8080001	373.2365112
1440.0000000	7.8567996	491.9478760
1440.0000000	7.9056001	-1022.0419922
1440.0000000	7.9544001	1626.2514648
1440.0000000	8.0031996	2098.4692383
1440.0000000	8.0520000	2094.4746094
1440.0000000	8.1007996	2216.3918457
1440.0000000	8.1496000	2363.7700195
1440.0000000	8.1983995	1470.5373535
1440.0000000	8.2472000	-3365.0839844
1440.0000000	8.2959995	-1192.9566650
1440.0000000	8.3448000	-950.2272339
1440.0000000	8.3935995	-870.8770752
1440.0000000	8.4423990	-375.2705383
1440.0000000	8.4912004	-875.8211060
1440.0000000	8.5400000	-2362.2243652
1440.0000000	8.5887995	-1145.2421875

1440.0000000	8.6375999	223.3736115
1440.0000000	8.6863995	2020.0189209
1440.0000000	8.7351999	-751.0518799
1440.0000000	8.7840004	857.6142578
1440.0000000	8.8327999	45.7983322
1440.0000000	8.8815994	-11.5038338
1440.0000000	8.9303989	-23.7874603
1440.0000000	8.9792004	-11.5825911
1440.0000000	9.0279999	-5.9117150
1440.0000000	9.0767994	6.7976456
1440.0000000	9.1255999	-17.1696758
1440.0000000	9.1743994	-10.0905447
1440.0000000	9.2231998	36.3486023
1440.0000000	9.2720003	-8.4595270
1440.0000000	9.3207998	-9.8207912
1440.0000000	9.3695993	-10.7732916
1440.0000000	9.4183998	-11.7486458
1440.0000000	9.4672003	-11.1630611
1440.0000000	9.5159998	-14.1267853
1440.0000000	9.5648003	-0.5347290
1440.0000000	9.6135998	-13.3628006
1440.0000000	9.6623993	-10.2328854
1440.0000000	9.7111998	-10.1691122
1440.0000000	9.7600002	-10.8593273
1440.0000000	9.8087997	-10.9951124
1440.0000000	9.8575993	3.0041637
1440.0000000	9.9063997	-8.0074463
1440.0000000	9.9552002	-10.9067831
1440.0000000	10.0039997	-10.0851164
1450.0000000	0.3416000	-36.5993156
1450.0000000	0.3904000	-39.7750664
1450.0000000	0.4392000	-39.4689102
1450.0000000	0.4880000	-39.5392647
1450.0000000	0.5368000	-39.6642265
1450.0000000	0.5856000	-39.2732468
1450.0000000	0.6344000	-38.2387962
1450.0000000	0.6832000	-36.2615891
1450.0000000	0.7320000	-36.0898895
1450.0000000	0.7808000	-36.5376854
1450.0000000	0.8296000	-35.9337196
1450.0000000	0.8784000	-35.0286026
1450.0000000	0.9272000	-34.2513390
1450.0000000	0.9760000	-31.9213924
1450.0000000	1.0247999	-30.3174362
1450.0000000	1.0735999	-30.0963707
1450.0000000	1.1224000	-30.7168884
1450.0000000	1.1711999	-31.3208561
1450.0000000	1.2200000	-30.6679306
1450.0000000	1.2687999	-30.7334347
1450.0000000	1.3176000	-30.9236183
1450.0000000	1.3664000	-30.6154099
1450.0000000	1.4152000	-30.1711044
1450.0000000	1.4640000	-29.5418110
1450.0000000	1.5128000	-35.1965790
1450.0000000	1.5616000	-31.8311100
1450.0000000	1.6104001	-1447.8764648
1450.0000000	1.6592000	-60.6191254
1450.0000000	1.7079999	-27.4186668

1450.0000000	1.7567999	-27.7629662
1450.0000000	1.8055999	-26.9174061
1450.0000000	1.8544000	-26.0072098
1450.0000000	1.9031999	-26.4304714
1450.0000000	1.9520000	-26.6082306
1450.0000000	2.0007999	-25.9730053
1450.0000000	2.0495999	-26.2551136
1450.0000000	2.0983999	-84.5156937
1450.0000000	2.1471999	-13.1671219
1450.0000000	2.1960001	-25.2725830
1450.0000000	2.2448001	-25.0575752
1450.0000000	2.2935998	-25.1166248
1450.0000000	2.3423998	-21.6092472
1450.0000000	2.3912001	5.1540642
1450.0000000	2.4400001	-22.9443836
1450.0000000	2.4888000	-23.4507484
1450.0000000	2.5375998	-13.0020561
1450.0000000	2.5863998	-11.2363205
1450.0000000	2.6352000	-9.4359436
1450.0000000	2.6840000	-15.9200230
1450.0000000	2.7328000	121.8668137
1450.0000000	2.7815998	144.1148376
1450.0000000	2.8304000	-23.7390518
1450.0000000	2.8792000	-20.7577553
1450.0000000	2.9280000	-22.5476513
1450.0000000	2.9768000	-17.1442528
1450.0000000	3.0256000	-20.5824051
1450.0000000	3.0743999	-29.0342026
1450.0000000	3.1231999	-24.1086674
1450.0000000	3.1719999	-29.2542973
1450.0000000	3.2208002	-494.1579590
1450.0000000	3.2695999	-967.0957031
1450.0000000	3.3183999	-20.4981098
1450.0000000	3.3671999	-3333.8093262
1450.0000000	3.4159999	1195.5303955
1450.0000000	3.4648001	-7.3859692
1450.0000000	3.5135999	-26.9085350
1450.0000000	3.5623999	-316.3762817
1450.0000000	3.6111999	-1985.8415527
1450.0000000	3.6600001	-449.8786011
1450.0000000	3.7088001	-593.9014282
1450.0000000	3.7575998	-929.6208496
1450.0000000	3.8063998	-1686.5928955
1450.0000000	3.8552001	-2448.5139160
1450.0000000	3.9040000	-2400.6064453
1450.0000000	3.9528000	1097.2500000
1450.0000000	4.0015998	-240.1881256
1450.0000000	4.0503998	671.6044312
1450.0000000	4.0991998	85.2023392
1450.0000000	4.1479998	-640.3520508
1450.0000000	4.1967998	2.0562191
1450.0000000	4.2456002	-243.3058929
1450.0000000	4.2943997	-477.3807678
1450.0000000	4.3431997	-13.4468231
1450.0000000	4.3920002	-242.9463348
1450.0000000	4.4407997	-22.3665581
1450.0000000	4.4896002	-15.5209751
1450.0000000	4.5383997	-641.2382813

1450.0000000	4.5871997	-12.9454975
1450.0000000	4.6360002	-21.5393124
1450.0000000	4.6847997	-28.0742111
1450.0000000	4.7336001	-19.5241165
1450.0000000	4.7824001	-16.5364723
1450.0000000	4.8311996	-1446.1350098
1450.0000000	4.8800001	223.4966278
1450.0000000	4.9287996	-197.8765869
1450.0000000	4.9776001	1038.7062988
1450.0000000	5.0264001	84.1801987
1450.0000000	5.0751996	-295.6278381
1450.0000000	5.1240001	-12.3573437
1450.0000000	5.1727996	552.2727661
1450.0000000	5.2216001	31.5429001
1450.0000000	5.2704000	-157.4673767
1450.0000000	5.3191996	-38.9643631
1450.0000000	5.3680000	-3502.6928711
1450.0000000	5.4168000	522.0172729
1450.0000000	5.4656000	-199.0078735
1450.0000000	5.5144000	37.5574112
1450.0000000	5.5631995	483.6124573
1450.0000000	5.6120000	389.3183289
1450.0000000	5.6608000	-36.8893776
1450.0000000	5.7096000	61.7930832
1450.0000000	5.7584000	131.6580963
1450.0000000	5.8071995	-11.5618629
1450.0000000	5.8559999	29.3950253
1450.0000000	5.9047999	-16.5962029
1450.0000000	5.9535999	-39.5506630
1450.0000000	6.0023999	-1558.5041504
1450.0000000	6.0511999	-14.5782251
1450.0000000	6.0999999	-19.3698883
1450.0000000	6.1487999	-13.2952185
1450.0000000	6.1975999	-23.1368771
1450.0000000	6.2463999	-1775.4675293
1450.0000000	6.2951999	-19.0319500
1450.0000000	6.3439999	-339.6977844
1450.0000000	6.3927999	-15.0472593
1450.0000000	6.4416003	-12.4134312
1450.0000000	6.4903998	-11.2952347
1450.0000000	6.5391998	-11.8379192
1450.0000000	6.5879998	353.0086060
1450.0000000	6.6367998	-12.1095676
1450.0000000	6.6856003	-13.8227682
1450.0000000	6.7343998	-13.4655781
1450.0000000	6.7831998	-14.3535233
1450.0000000	6.8319998	57.5669861
1450.0000000	6.8807998	-14.0431042
1450.0000000	6.9296002	-414.9653320
1450.0000000	6.9783998	-5.6644239
1450.0000000	7.0271997	-14.0910063
1450.0000000	7.0760002	9.2928743
1450.0000000	7.1247997	-1955.0805664
1450.0000000	7.1736002	-1567.8973389
1450.0000000	7.2223997	-892.2611084
1450.0000000	7.2711997	-193.2296295
1450.0000000	7.3200002	-18.3494110
1450.0000000	7.3687997	171.2684479

1450.0000000	7.4176002	358.3947144
1450.0000000	7.4663997	281.3916931
1450.0000000	7.5151997	-872.6320190
1450.0000000	7.5640001	-963.7968140
1450.0000000	7.6127996	-2270.5258789
1450.0000000	7.6616001	-729.4497070
1450.0000000	7.7104001	-375.0912476
1450.0000000	7.7591996	38.7707405
1450.0000000	7.8080001	372.4511414
1450.0000000	7.8567996	490.9197998
1450.0000000	7.9056001	-1019.9691162
1450.0000000	7.9544001	1622.9415283
1450.0000000	8.0031996	2094.1997070
1450.0000000	8.0520000	2090.2126465
1450.0000000	8.1007996	2211.8823242
1450.0000000	8.1496000	2358.9621582
1450.0000000	8.1983995	1467.5447998
1450.0000000	8.2472000	-3358.2490234
1450.0000000	8.2959995	-1190.5363770
1450.0000000	8.3448000	-948.2980347
1450.0000000	8.3935995	-869.1093750
1450.0000000	8.4423990	-374.5125122
1450.0000000	8.4912004	-874.0448608
1450.0000000	8.5400000	-2357.4245605
1450.0000000	8.5887995	-1142.9206543
1450.0000000	8.6375999	222.9122772
1450.0000000	8.6863995	2015.9006348
1450.0000000	8.7351999	-749.5275879
1450.0000000	8.7840004	855.8575439
1450.0000000	8.8327999	45.6867256
1450.0000000	8.8815994	-11.5040073
1450.0000000	8.9303989	-23.7837410
1450.0000000	8.9792004	-11.5826740
1450.0000000	9.0279999	-5.9134836
1450.0000000	9.0767994	6.7919273
1450.0000000	9.1255999	-17.1679611
1450.0000000	9.1743994	-10.0909472
1450.0000000	9.2231998	36.3328705
1450.0000000	9.2720003	-8.4604635
1450.0000000	9.3207998	-9.8213310
1450.0000000	9.3695993	-10.7735176
1450.0000000	9.4183998	-11.7485218
1450.0000000	9.4672003	-11.1631021
1450.0000000	9.5159998	-14.1258945
1450.0000000	9.5648003	-0.5381021
1450.0000000	9.6135998	-13.3620090
1450.0000000	9.6623993	-10.2331600
1450.0000000	9.7111998	-10.1693840
1450.0000000	9.7600002	-10.8593693
1450.0000000	9.8087997	-10.9951200
1450.0000000	9.8575993	2.9997416
1450.0000000	9.9063997	-8.0083609
1450.0000000	9.9552002	-10.9067869
1450.0000000	10.0039997	-10.0853634
1460.0000000	0.3416000	-36.9497528
1460.0000000	0.3904000	-39.7897568
1460.0000000	0.4392000	-39.4625664
1460.0000000	0.4880000	-39.5380554

1460.0000000	0.5368000	-39.6654015
1460.0000000	0.5856000	-39.2718658
1460.0000000	0.6344000	-38.2391052
1460.0000000	0.6832000	-36.2638130
1460.0000000	0.7320000	-36.0919418
1460.0000000	0.7808000	-36.5378571
1460.0000000	0.8296000	-35.9318733
1460.0000000	0.8784000	-35.0190392
1460.0000000	0.9272000	-34.2519798
1460.0000000	0.9760000	-31.9188690
1460.0000000	1.0247999	-30.3186722
1460.0000000	1.0735999	-30.1099701
1460.0000000	1.1224000	-30.7150154
1460.0000000	1.1711999	-31.3145523
1460.0000000	1.2200000	-30.6241322
1460.0000000	1.2687999	-30.7121086
1460.0000000	1.3176000	-30.9172325
1460.0000000	1.3664000	-30.6124668
1460.0000000	1.4152000	-30.1713085
1460.0000000	1.4640000	-29.5401020
1460.0000000	1.5128000	-35.0994034
1460.0000000	1.5616000	-31.7892685
1460.0000000	1.6104001	-1445.3337402
1460.0000000	1.6592000	-60.3251877
1460.0000000	1.7079999	-27.4224129
1460.0000000	1.7567999	-27.7629852
1460.0000000	1.8055999	-26.9940338
1460.0000000	1.8544000	-25.8158417
1460.0000000	1.9031999	-26.0372677
1460.0000000	1.9520000	-26.5755997
1460.0000000	2.0007999	-25.9783859
1460.0000000	2.0495999	-26.2571335
1460.0000000	2.0983999	-83.0399170
1460.0000000	2.1471999	-13.4343891
1460.0000000	2.1960001	-25.2684917
1460.0000000	2.2448001	-25.0396652
1460.0000000	2.2935998	-25.0570011
1460.0000000	2.3423998	-21.6236534
1460.0000000	2.3912001	4.5325184
1460.0000000	2.4400001	-22.9468689
1460.0000000	2.4888000	-23.4505310
1460.0000000	2.5375998	-13.0191641
1460.0000000	2.5863998	-11.2560797
1460.0000000	2.6352000	-9.4586878
1460.0000000	2.6840000	-15.9319658
1460.0000000	2.7328000	121.6007919
1460.0000000	2.7815998	143.8084412
1460.0000000	2.8304000	-23.7377472
1460.0000000	2.8792000	-20.7610188
1460.0000000	2.9280000	-22.5470829
1460.0000000	2.9768000	-17.1526241
1460.0000000	3.0256000	-20.5849895
1460.0000000	3.0743999	-29.0228157
1460.0000000	3.1231999	-24.1052132
1460.0000000	3.1719999	-29.2460041
1460.0000000	3.2208002	-493.9201965
1460.0000000	3.2695999	-966.6207275
1460.0000000	3.3183999	-20.4983082

1460.0000000	3.3671999	-3332.3476563
1460.0000000	3.4159999	1194.9951172
1460.0000000	3.4648001	-7.3916769
1460.0000000	3.5135999	-26.9055443
1460.0000000	3.5623999	-316.2466736
1460.0000000	3.6111999	-1984.9818115
1460.0000000	3.6600001	-449.6904297
1460.0000000	3.7088001	-593.6500854
1460.0000000	3.7575998	-929.2222900
1460.0000000	3.8063998	-1685.8631592
1460.0000000	3.8552001	-2447.4504395
1460.0000000	3.9040000	-2399.5639648
1460.0000000	3.9528000	1096.7617188
1460.0000000	4.0015998	-240.0910187
1460.0000000	4.0503998	671.3018799
1460.0000000	4.0991998	85.1566544
1460.0000000	4.1479998	-640.0797729
1460.0000000	4.1967998	2.0469627
1460.0000000	4.2456002	-243.2075958
1460.0000000	4.2943997	-477.1802063
1460.0000000	4.3431997	-13.4490032
1460.0000000	4.3920002	-242.8482208
1460.0000000	4.4407997	-22.3647804
1460.0000000	4.4896002	-15.5218582
1460.0000000	4.5383997	-640.9772339
1460.0000000	4.5871997	-12.9475136
1460.0000000	4.6360002	-21.5377693
1460.0000000	4.6847997	-28.0702858
1460.0000000	4.7336001	-19.5233955
1460.0000000	4.7824001	-16.5369263
1460.0000000	4.8311996	-1445.6037598
1460.0000000	4.8800001	223.4070740
1460.0000000	4.9287996	-197.8093262
1460.0000000	4.9776001	1038.3138428
1460.0000000	5.0264001	84.1426773
1460.0000000	5.0751996	-295.5242004
1460.0000000	5.1240001	-12.3582649
1460.0000000	5.1727996	552.1568604
1460.0000000	5.2216001	31.5334587
1460.0000000	5.2704000	-157.4392700
1460.0000000	5.3191996	-38.9598122
1460.0000000	5.3680000	-3502.0004883
1460.0000000	5.4168000	521.9104614
1460.0000000	5.4656000	-198.9716949
1460.0000000	5.5144000	37.5467529
1460.0000000	5.5631995	483.5138855
1460.0000000	5.6120000	389.2383728
1460.0000000	5.6608000	-36.8852882
1460.0000000	5.7096000	61.7776566
1460.0000000	5.7584000	131.6291656
1460.0000000	5.8071995	-11.5627327
1460.0000000	5.8559999	29.3861713
1460.0000000	5.9047999	-16.5960045
1460.0000000	5.9535999	-39.5459251
1460.0000000	6.0023999	-1558.1995850
1460.0000000	6.0511999	-14.5784359
1460.0000000	6.0999999	-19.3690567
1460.0000000	6.1487999	-13.2956810

1460.0000000	6.1975999	-23.1351128
1460.0000000	6.2463999	-1775.1202393
1460.0000000	6.2951999	-19.0311356
1460.0000000	6.3439999	-339.6337280
1460.0000000	6.3927999	-15.0472412
1460.0000000	6.4416003	-12.4140177
1460.0000000	6.4903998	-11.2959108
1460.0000000	6.5391998	-11.8386002
1460.0000000	6.5879998	352.3179016
1460.0000000	6.6367998	-12.1101170
1460.0000000	6.6856003	-13.8229389
1460.0000000	6.7343998	-13.4657936
1460.0000000	6.7831998	-14.3531075
1460.0000000	6.8319998	57.5478745
1460.0000000	6.8807998	-14.0429659
1460.0000000	6.9296002	-414.2038879
1460.0000000	6.9783998	-5.6772480
1460.0000000	7.0271997	-14.0899792
1460.0000000	7.0760002	9.2506895
1460.0000000	7.1247997	-1951.1347656
1460.0000000	7.1736002	-1564.7344971
1460.0000000	7.2223997	-890.4679565
1460.0000000	7.2711997	-192.8618469
1460.0000000	7.3200002	-18.3374233
1460.0000000	7.3687997	170.8939209
1460.0000000	7.4176002	357.6388855
1460.0000000	7.4663997	280.7940369
1460.0000000	7.5151997	-870.8710327
1460.0000000	7.5640001	-961.8494263
1460.0000000	7.6127996	-2265.9082031
1460.0000000	7.6616001	-727.9783936
1460.0000000	7.7104001	-374.3449707
1460.0000000	7.7591996	38.6696930
1460.0000000	7.8080001	371.6658020
1460.0000000	7.8567996	489.8916931
1460.0000000	7.9056001	-1017.8961792
1460.0000000	7.9544001	1619.6315918
1460.0000000	8.0031996	2089.9301758
1460.0000000	8.0520000	2085.9506836
1460.0000000	8.1007996	2207.3728027
1460.0000000	8.1496000	2354.1540527
1460.0000000	8.1983995	1464.5523682
1460.0000000	8.2472000	-3351.4143066
1460.0000000	8.2959995	-1188.1162109
1460.0000000	8.3448000	-946.3688965
1460.0000000	8.3935995	-867.3417358
1460.0000000	8.4423990	-373.7545166
1460.0000000	8.4912004	-872.2686768
1460.0000000	8.5400000	-2352.6247559
1460.0000000	8.5887995	-1140.5991211
1460.0000000	8.6375999	222.4509583
1460.0000000	8.6863995	2011.7822266
1460.0000000	8.7351999	-748.0033569
1460.0000000	8.7840004	854.1007690
1460.0000000	8.8327999	45.5751152
1460.0000000	8.8815994	-11.5041819
1460.0000000	8.9303989	-23.7800198
1460.0000000	8.9792004	-11.5827560

1460.0000000	9.0279999	-5.9152517
1460.0000000	9.0767994	6.7862110
1460.0000000	9.1255999	-17.1662483
1460.0000000	9.1743994	-10.0913486
1460.0000000	9.2231998	36.3171387
1460.0000000	9.2720003	-8.4614000
1460.0000000	9.3207998	-9.8218699
1460.0000000	9.3695993	-10.7737446
1460.0000000	9.4183998	-11.7483969
1460.0000000	9.4672003	-11.1631441
1460.0000000	9.5159998	-14.1250038
1460.0000000	9.5648003	-0.5414753
1460.0000000	9.6135998	-13.3612185
1460.0000000	9.6623993	-10.2334356
1460.0000000	9.7111998	-10.1696548
1460.0000000	9.7600002	-10.8594103
1460.0000000	9.8087997	-10.9951286
1460.0000000	9.8575993	2.9953194
1460.0000000	9.9063997	-8.0092745
1460.0000000	9.9552002	-10.9067917
1460.0000000	10.0039997	-10.0856113
1470.0000000	0.3416000	-36.8214455
1470.0000000	0.3904000	-39.8044472
1470.0000000	0.4392000	-39.4562225
1470.0000000	0.4880000	-39.5368462
1470.0000000	0.5368000	-39.6665726
1470.0000000	0.5856000	-39.2704849
1470.0000000	0.6344000	-38.2394180
1470.0000000	0.6832000	-36.2660408
1470.0000000	0.7320000	-36.0939941
1470.0000000	0.7808000	-36.5380249
1470.0000000	0.8296000	-35.9300308
1470.0000000	0.8784000	-35.0094719
1470.0000000	0.9272000	-34.2526245
1470.0000000	0.9760000	-31.9163456
1470.0000000	1.0247999	-30.3199081
1470.0000000	1.0735999	-30.1235714
1470.0000000	1.1224000	-30.7131443
1470.0000000	1.1711999	-31.3082504
1470.0000000	1.2200000	-30.5803337
1470.0000000	1.2687999	-30.6907806
1470.0000000	1.3176000	-30.9108467
1470.0000000	1.3664000	-30.6095238
1470.0000000	1.4152000	-30.1715145
1470.0000000	1.4640000	-29.5383911
1470.0000000	1.5128000	-35.0022240
1470.0000000	1.5616000	-31.7474270
1470.0000000	1.6104001	-1442.7908936
1470.0000000	1.6592000	-60.0312576
1470.0000000	1.7079999	-27.4261608
1470.0000000	1.7567999	-27.7630043
1470.0000000	1.8055999	-26.9112530
1470.0000000	1.8544000	-26.2541180
1470.0000000	1.9031999	-25.4310074
1470.0000000	1.9520000	-26.5429668
1470.0000000	2.0007999	-25.9837646
1470.0000000	2.0495999	-26.2591515
1470.0000000	2.0983999	-81.5641327

1470.0000000	2.1471999	-13.7016563
1470.0000000	2.1960001	-25.2644005
1470.0000000	2.2448001	-25.0217533
1470.0000000	2.2935998	-24.9973774
1470.0000000	2.3423998	-21.6380577
1470.0000000	2.3912001	3.9109726
1470.0000000	2.4400001	-22.9493561
1470.0000000	2.4888000	-23.4503136
1470.0000000	2.5375998	-13.0362720
1470.0000000	2.5863998	-11.2758389
1470.0000000	2.6352000	-9.4814320
1470.0000000	2.6840000	-15.9439068
1470.0000000	2.7328000	121.3347855
1470.0000000	2.7815998	143.5020142
1470.0000000	2.8304000	-23.7364426
1470.0000000	2.8792000	-20.7642822
1470.0000000	2.9280000	-22.5465145
1470.0000000	2.9768000	-17.1609955
1470.0000000	3.0256000	-20.5875740
1470.0000000	3.0743999	-29.0114269
1470.0000000	3.1231999	-24.1017570
1470.0000000	3.1719999	-29.2377090
1470.0000000	3.2208002	-493.6824646
1470.0000000	3.2695999	-966.1457520
1470.0000000	3.3183999	-20.4985065
1470.0000000	3.3671999	-3330.8859863
1470.0000000	3.4159999	1194.4598389
1470.0000000	3.4648001	-7.3973846
1470.0000000	3.5135999	-26.9025536
1470.0000000	3.5623999	-316.1170959
1470.0000000	3.6111999	-1984.1220703
1470.0000000	3.6600001	-449.5023193
1470.0000000	3.7088001	-593.3986816
1470.0000000	3.7575998	-928.8237305
1470.0000000	3.8063998	-1685.1333008
1470.0000000	3.8552001	-2446.3872070
1470.0000000	3.9040000	-2398.5214844
1470.0000000	3.9528000	1096.2733154
1470.0000000	4.0015998	-239.9938965
1470.0000000	4.0503998	670.9993286
1470.0000000	4.0991998	85.1109695
1470.0000000	4.1479998	-639.8075562
1470.0000000	4.1967998	2.0377064
1470.0000000	4.2456002	-243.1092987
1470.0000000	4.2943997	-476.9796448
1470.0000000	4.3431997	-13.4511843
1470.0000000	4.3920002	-242.7501068
1470.0000000	4.4407997	-22.3630066
1470.0000000	4.4896002	-15.5227404
1470.0000000	4.5383997	-640.7161865
1470.0000000	4.5871997	-12.9495296
1470.0000000	4.6360002	-21.5362282
1470.0000000	4.6847997	-28.0663605
1470.0000000	4.7336001	-19.5226727
1470.0000000	4.7824001	-16.5373802
1470.0000000	4.8311996	-1445.0725098
1470.0000000	4.8800001	223.3175201
1470.0000000	4.9287996	-197.7420654

1470.0000000	4.9776001	1037.9213867
1470.0000000	5.0264001	84.1051559
1470.0000000	5.0751996	-295.4205627
1470.0000000	5.1240001	-12.3591862
1470.0000000	5.1727996	552.0410156
1470.0000000	5.2216001	31.5240135
1470.0000000	5.2704000	-157.4111633
1470.0000000	5.3191996	-38.9552612
1470.0000000	5.3680000	-3501.3081055
1470.0000000	5.4168000	521.8037109
1470.0000000	5.4656000	-198.9355011
1470.0000000	5.5144000	37.5360947
1470.0000000	5.5631995	483.4153137
1470.0000000	5.6120000	389.1583862
1470.0000000	5.6608000	-36.8812027
1470.0000000	5.7096000	61.7622223
1470.0000000	5.7584000	131.6002350
1470.0000000	5.8071995	-11.5636044
1470.0000000	5.8559999	29.3773212
1470.0000000	5.9047999	-16.5958061
1470.0000000	5.9535999	-39.5411835
1470.0000000	6.0023999	-1557.8952637
1470.0000000	6.0511999	-14.5786476
1470.0000000	6.0999999	-19.3682232
1470.0000000	6.1487999	-13.2961445
1470.0000000	6.1975999	-23.1333466
1470.0000000	6.2463999	-1774.7728271
1470.0000000	6.2951999	-19.0303211
1470.0000000	6.3439999	-339.5696716
1470.0000000	6.3927999	-15.0472231
1470.0000000	6.4416003	-12.4146042
1470.0000000	6.4903998	-11.2965870
1470.0000000	6.5391998	-11.8392811
1470.0000000	6.5879998	351.6271973
1470.0000000	6.6367998	-12.1106653
1470.0000000	6.6856003	-13.8231087
1470.0000000	6.7343998	-13.4660091
1470.0000000	6.7831998	-14.3526917
1470.0000000	6.8319998	57.5287552
1470.0000000	6.8807998	-14.0428267
1470.0000000	6.9296002	-413.4424133
1470.0000000	6.9783998	-5.6900716
1470.0000000	7.0271997	-14.0889511
1470.0000000	7.0760002	9.2085028
1470.0000000	7.1247997	-1947.1888428
1470.0000000	7.1736002	-1561.5715332
1470.0000000	7.2223997	-888.6748047
1470.0000000	7.2711997	-192.4940643
1470.0000000	7.3200002	-18.3254356
1470.0000000	7.3687997	170.5193939
1470.0000000	7.4176002	356.8830566
1470.0000000	7.4663997	280.1963806
1470.0000000	7.5151997	-869.1099854
1470.0000000	7.5640001	-959.9019775
1470.0000000	7.6127996	-2261.2907715
1470.0000000	7.6616001	-726.5070190
1470.0000000	7.7104001	-373.5986938
1470.0000000	7.7591996	38.5686455

1470.0000000	7.8080001	370.8804321
1470.0000000	7.8567996	488.8636169
1470.0000000	7.9056001	-1015.8233032
1470.0000000	7.9544001	1616.3216553
1470.0000000	8.0031996	2085.6604004
1470.0000000	8.0520000	2081.6887207
1470.0000000	8.1007996	2202.8632813
1470.0000000	8.1496000	2349.3459473
1470.0000000	8.1983995	1461.5598145
1470.0000000	8.2472000	-3344.5793457
1470.0000000	8.2959995	-1185.6960449
1470.0000000	8.3448000	-944.4396973
1470.0000000	8.3935995	-865.5740356
1470.0000000	8.4423990	-372.9964905
1470.0000000	8.4912004	-870.4924316
1470.0000000	8.5400000	-2347.8249512
1470.0000000	8.5887995	-1138.2775879
1470.0000000	8.6375999	221.9896393
1470.0000000	8.6863995	2007.6639404
1470.0000000	8.7351999	-746.4790649
1470.0000000	8.7840004	852.3440552
1470.0000000	8.8327999	45.4635086
1470.0000000	8.8815994	-11.5043564
1470.0000000	8.9303989	-23.7762985
1470.0000000	8.9792004	-11.5828381
1470.0000000	9.0279999	-5.9170203
1470.0000000	9.0767994	6.7804928
1470.0000000	9.1255999	-17.1645336
1470.0000000	9.1743994	-10.0917511
1470.0000000	9.2231998	36.3014069
1470.0000000	9.2720003	-8.4623356
1470.0000000	9.3207998	-9.8224096
1470.0000000	9.3695993	-10.7739706
1470.0000000	9.4183998	-11.7482719
1470.0000000	9.4672003	-11.1631851
1470.0000000	9.5159998	-14.1241121
1470.0000000	9.5648003	-0.5448475
1470.0000000	9.6135998	-13.3604279
1470.0000000	9.6623993	-10.2337103
1470.0000000	9.7111998	-10.1699266
1470.0000000	9.7600002	-10.8594522
1470.0000000	9.8087997	-10.9951363
1470.0000000	9.8575993	2.9908962
1470.0000000	9.9063997	-8.0101881
1470.0000000	9.9552002	-10.9067955
1470.0000000	10.0039997	-10.0858583
1480.0000000	0.3416000	-36.8545074
1480.0000000	0.3904000	-39.8191376
1480.0000000	0.4392000	-39.4498787
1480.0000000	0.4880000	-39.5356369
1480.0000000	0.5368000	-39.6677475
1480.0000000	0.5856000	-39.2691040
1480.0000000	0.6344000	-38.2397270
1480.0000000	0.6832000	-36.2682648
1480.0000000	0.7320000	-36.0960464
1480.0000000	0.7808000	-36.5381927
1480.0000000	0.8296000	-35.9281845
1480.0000000	0.8784000	-34.9999084

1480.0000000	0.9272000	-34.2532654
1480.0000000	0.9760000	-31.9138222
1480.0000000	1.0247999	-30.3211441
1480.0000000	1.0735999	-30.1371708
1480.0000000	1.1224000	-30.7112713
1480.0000000	1.1711999	-31.3019466
1480.0000000	1.2200000	-30.5365353
1480.0000000	1.2687999	-30.6694546
1480.0000000	1.3176000	-30.9044609
1480.0000000	1.3664000	-30.6065788
1480.0000000	1.4152000	-30.1717186
1480.0000000	1.4640000	-29.5366821
1480.0000000	1.5128000	-34.9050484
1480.0000000	1.5616000	-31.7055855
1480.0000000	1.6104001	-1440.2481689
1480.0000000	1.6592000	-59.7373276
1480.0000000	1.7079999	-27.4299068
1480.0000000	1.7567999	-27.7630234
1480.0000000	1.8055999	-26.5712414
1480.0000000	1.8544000	-25.2287025
1480.0000000	1.9031999	-25.2796669
1480.0000000	1.9520000	-26.5247822
1480.0000000	2.0007999	-25.9891453
1480.0000000	2.0495999	-26.2611713
1480.0000000	2.0983999	-80.0883560
1480.0000000	2.1471999	-13.9689236
1480.0000000	2.1960001	-25.2603092
1480.0000000	2.2448001	-25.0038414
1480.0000000	2.2935998	-24.9377537
1480.0000000	2.3423998	-21.6524620
1480.0000000	2.3912001	3.2894268
1480.0000000	2.4400001	-22.9518433
1480.0000000	2.4888000	-23.4500980
1480.0000000	2.5375998	-13.0533800
1480.0000000	2.5863998	-11.2955971
1480.0000000	2.6352000	-9.5041761
1480.0000000	2.6840000	-15.9558487
1480.0000000	2.7328000	121.0687637
1480.0000000	2.7815998	143.1955872
1480.0000000	2.8304000	-23.7351379
1480.0000000	2.8792000	-20.7675457
1480.0000000	2.9280000	-22.5459442
1480.0000000	2.9768000	-17.1693649
1480.0000000	3.0256000	-20.5901585
1480.0000000	3.0743999	-29.0000401
1480.0000000	3.1231999	-24.0983009
1480.0000000	3.1719999	-29.2294159
1480.0000000	3.2208002	-493.4447327
1480.0000000	3.2695999	-965.6707764
1480.0000000	3.3183999	-20.4987049
1480.0000000	3.3671999	-3329.4243164
1480.0000000	3.4159999	1193.9245605
1480.0000000	3.4648001	-7.4030933
1480.0000000	3.5135999	-26.8995628
1480.0000000	3.5623999	-315.9874878
1480.0000000	3.6111999	-1983.2623291
1480.0000000	3.6600001	-449.3142090
1480.0000000	3.7088001	-593.1472778

1480.0000000	3.7575998	-928.4252319
1480.0000000	3.8063998	-1684.4034424
1480.0000000	3.8552001	-2445.3237305
1480.0000000	3.9040000	-2397.4790039
1480.0000000	3.9528000	1095.7850342
1480.0000000	4.0015998	-239.8967896
1480.0000000	4.0503998	670.6967773
1480.0000000	4.0991998	85.0652847
1480.0000000	4.1479998	-639.5353394
1480.0000000	4.1967998	2.0284500
1480.0000000	4.2456002	-243.0109863
1480.0000000	4.2943997	-476.7790222
1480.0000000	4.3431997	-13.4533653
1480.0000000	4.3920002	-242.6519928
1480.0000000	4.4407997	-22.3612309
1480.0000000	4.4896002	-15.5236235
1480.0000000	4.5383997	-640.4550781
1480.0000000	4.5871997	-12.9515457
1480.0000000	4.6360002	-21.5346851
1480.0000000	4.6847997	-28.0624352
1480.0000000	4.7336001	-19.5219498
1480.0000000	4.7824001	-16.5378342
1480.0000000	4.8311996	-1444.5412598
1480.0000000	4.8800001	223.2279663
1480.0000000	4.9287996	-197.6748047
1480.0000000	4.9776001	1037.5288086
1480.0000000	5.0264001	84.0676346
1480.0000000	5.0751996	-295.3169556
1480.0000000	5.1240001	-12.3601074
1480.0000000	5.1727996	551.9251099
1480.0000000	5.2216001	31.5145683
1480.0000000	5.2704000	-157.3830566
1480.0000000	5.3191996	-38.9507141
1480.0000000	5.3680000	-3500.6157227
1480.0000000	5.4168000	521.6969604
1480.0000000	5.4656000	-198.8993225
1480.0000000	5.5144000	37.5254440
1480.0000000	5.5631995	483.3167419
1480.0000000	5.6120000	389.0783997
1480.0000000	5.6608000	-36.8771133
1480.0000000	5.7096000	61.7467880
1480.0000000	5.7584000	131.5712891
1480.0000000	5.8071995	-11.5644741
1480.0000000	5.8559999	29.3684711
1480.0000000	5.9047999	-16.5956078
1480.0000000	5.9535999	-39.5364456
1480.0000000	6.0023999	-1557.5906982
1480.0000000	6.0511999	-14.5788593
1480.0000000	6.0999999	-19.3673916
1480.0000000	6.1487999	-13.2966070
1480.0000000	6.1975999	-23.1315823
1480.0000000	6.2463999	-1774.4254150
1480.0000000	6.2951999	-19.0295067
1480.0000000	6.3439999	-339.5056458
1480.0000000	6.3927999	-15.0472040
1480.0000000	6.4416003	-12.4151917
1480.0000000	6.4903998	-11.2972622
1480.0000000	6.5391998	-11.8399630

1480.0000000	6.5879998	350.9364929
1480.0000000	6.6367998	-12.1112146
1480.0000000	6.6856003	-13.8232794
1480.0000000	6.7343998	-13.4662256
1480.0000000	6.7831998	-14.3522758
1480.0000000	6.8319998	57.5096436
1480.0000000	6.8807998	-14.0426884
1480.0000000	6.9296002	-412.6809692
1480.0000000	6.9783998	-5.7028947
1480.0000000	7.0271997	-14.0879230
1480.0000000	7.0760002	9.1663179
1480.0000000	7.1247997	-1943.2430420
1480.0000000	7.1736002	-1558.4086914
1480.0000000	7.2223997	-886.8815918
1480.0000000	7.2711997	-192.1262817
1480.0000000	7.3200002	-18.3134460
1480.0000000	7.3687997	170.1448669
1480.0000000	7.4176002	356.1272278
1480.0000000	7.4663997	279.5987244
1480.0000000	7.5151997	-867.3489380
1480.0000000	7.5640001	-957.9545898
1480.0000000	7.6127996	-2256.6730957
1480.0000000	7.6616001	-725.0356445
1480.0000000	7.7104001	-372.8524170
1480.0000000	7.7591996	38.4675941
1480.0000000	7.8080001	370.0950623
1480.0000000	7.8567996	487.8355408
1480.0000000	7.9056001	-1013.7503662
1480.0000000	7.9544001	1613.0117188
1480.0000000	8.0031996	2081.3908691
1480.0000000	8.0520000	2077.4267578
1480.0000000	8.1007996	2198.3537598
1480.0000000	8.1496000	2344.5380859
1480.0000000	8.1983995	1458.5672607
1480.0000000	8.2472000	-3337.7446289
1480.0000000	8.2959995	-1183.2758789
1480.0000000	8.3448000	-942.5104980
1480.0000000	8.3935995	-863.8063354
1480.0000000	8.4423990	-372.2384949
1480.0000000	8.4912004	-868.7161865
1480.0000000	8.5400000	-2343.0251465
1480.0000000	8.5887995	-1135.9560547
1480.0000000	8.6375999	221.5283203
1480.0000000	8.6863995	2003.5455322
1480.0000000	8.7351999	-744.9548340
1480.0000000	8.7840004	850.5872803
1480.0000000	8.8327999	45.3518982
1480.0000000	8.8815994	-11.5045309
1480.0000000	8.9303989	-23.7725792
1480.0000000	8.9792004	-11.5829210
1480.0000000	9.0279999	-5.9187884
1480.0000000	9.0767994	6.7747746
1480.0000000	9.1255999	-17.1628189
1480.0000000	9.1743994	-10.0921535
1480.0000000	9.2231998	36.2856750
1480.0000000	9.2720003	-8.4632721
1480.0000000	9.3207998	-9.8229485
1480.0000000	9.3695993	-10.7741966

1480.0000000	9.4183998	-11.7481480
1480.0000000	9.4672003	-11.1632261
1480.0000000	9.5159998	-14.1232214
1480.0000000	9.5648003	-0.5482197
1480.0000000	9.6135998	-13.3596373
1480.0000000	9.6623993	-10.2339859
1480.0000000	9.7111998	-10.1701984
1480.0000000	9.7600002	-10.8594942
1480.0000000	9.8087997	-10.9951439
1480.0000000	9.8575993	2.9864740
1480.0000000	9.9063997	-8.0111027
1480.0000000	9.9552002	-10.9068003
1480.0000000	10.0039997	-10.0861063
1490.0000000	0.3416000	-36.6888390
1490.0000000	0.3904000	-39.8338280
1490.0000000	0.4392000	-39.4435349
1490.0000000	0.4880000	-39.5344276
1490.0000000	0.5368000	-39.6689224
1490.0000000	0.5856000	-39.2677231
1490.0000000	0.6344000	-38.2400360
1490.0000000	0.6832000	-36.2704926
1490.0000000	0.7320000	-36.0980988
1490.0000000	0.7808000	-36.5383644
1490.0000000	0.8296000	-35.9263382
1490.0000000	0.8784000	-34.9903450
1490.0000000	0.9272000	-34.2539063
1490.0000000	0.9760000	-31.9112988
1490.0000000	1.0247999	-30.3223782
1490.0000000	1.0735999	-30.1507721
1490.0000000	1.1224000	-30.7094002
1490.0000000	1.1711999	-31.2956448
1490.0000000	1.2200000	-30.4927368
1490.0000000	1.2687999	-30.6481266
1490.0000000	1.3176000	-30.8980751
1490.0000000	1.3664000	-30.6036358
1490.0000000	1.4152000	-30.1719227
1490.0000000	1.4640000	-29.5349731
1490.0000000	1.5128000	-34.8078690
1490.0000000	1.5616000	-31.6637459
1490.0000000	1.6104001	-1437.7053223
1490.0000000	1.6592000	-59.4433975
1490.0000000	1.7079999	-27.4336548
1490.0000000	1.7567999	-27.7630424
1490.0000000	1.8055999	-26.3721676
1490.0000000	1.8544000	-25.2856026
1490.0000000	1.9031999	-26.1166286
1490.0000000	1.9520000	-26.4451370
1490.0000000	2.0007999	-25.9945259
1490.0000000	2.0495999	-26.2631893
1490.0000000	2.0983999	-78.6125793
1490.0000000	2.1471999	-14.2361908
1490.0000000	2.1960001	-25.2562180
1490.0000000	2.2448001	-24.9859314
1490.0000000	2.2935998	-24.8781300
1490.0000000	2.3423998	-21.6668663
1490.0000000	2.3912001	2.6678810
1490.0000000	2.4400001	-22.9543285
1490.0000000	2.4888000	-23.4498806

1490.0000000	2.5375998	-13.0704880
1490.0000000	2.5863998	-11.3153563
1490.0000000	2.6352000	-9.5269203
1490.0000000	2.6840000	-15.9677906
1490.0000000	2.7328000	120.8027573
1490.0000000	2.7815998	142.8891602
1490.0000000	2.8304000	-23.7338352
1490.0000000	2.8792000	-20.7708092
1490.0000000	2.9280000	-22.5453758
1490.0000000	2.9768000	-17.1777363
1490.0000000	3.0256000	-20.5927429
1490.0000000	3.0743999	-28.9886532
1490.0000000	3.1231999	-24.0948467
1490.0000000	3.1719999	-29.2211227
1490.0000000	3.2208002	-493.2070007
1490.0000000	3.2695999	-965.1958008
1490.0000000	3.3183999	-20.4989014
1490.0000000	3.3671999	-3327.9628906
1490.0000000	3.4159999	1193.3892822
1490.0000000	3.4648001	-7.4088020
1490.0000000	3.5135999	-26.8965721
1490.0000000	3.5623999	-315.8579102
1490.0000000	3.6111999	-1982.4025879
1490.0000000	3.6600001	-449.1260376
1490.0000000	3.7088001	-592.8959351
1490.0000000	3.7575998	-928.0266724
1490.0000000	3.8063998	-1683.6735840
1490.0000000	3.8552001	-2444.2602539
1490.0000000	3.9040000	-2396.4365234
1490.0000000	3.9528000	1095.2967529
1490.0000000	4.0015998	-239.7996826
1490.0000000	4.0503998	670.3942261
1490.0000000	4.0991998	85.0195999
1490.0000000	4.1479998	-639.2631226
1490.0000000	4.1967998	2.0191936
1490.0000000	4.2456002	-242.9126892
1490.0000000	4.2943997	-476.5784607
1490.0000000	4.3431997	-13.4555454
1490.0000000	4.3920002	-242.5538788
1490.0000000	4.4407997	-22.3594551
1490.0000000	4.4896002	-15.5245056
1490.0000000	4.5383997	-640.1940308
1490.0000000	4.5871997	-12.9535618
1490.0000000	4.6360002	-21.5331421
1490.0000000	4.6847997	-28.0585098
1490.0000000	4.7336001	-19.5212288
1490.0000000	4.7824001	-16.5382900
1490.0000000	4.8311996	-1444.0098877
1490.0000000	4.8800001	223.1384277
1490.0000000	4.9287996	-197.6075439
1490.0000000	4.9776001	1037.1363525
1490.0000000	5.0264001	84.0301132
1490.0000000	5.0751996	-295.2133179
1490.0000000	5.1240001	-12.3610287
1490.0000000	5.1727996	551.8092651
1490.0000000	5.2216001	31.5051270
1490.0000000	5.2704000	-157.3549500
1490.0000000	5.3191996	-38.9461670

1490.0000000	5.3680000	-3499.9233398
1490.0000000	5.4168000	521.5901489
1490.0000000	5.4656000	-198.8631439
1490.0000000	5.5144000	37.5147858
1490.0000000	5.5631995	483.2181702
1490.0000000	5.6120000	388.9984436
1490.0000000	5.6608000	-36.8730240
1490.0000000	5.7096000	61.7313614
1490.0000000	5.7584000	131.5423584
1490.0000000	5.8071995	-11.5653448
1490.0000000	5.8559999	29.3596210
1490.0000000	5.9047999	-16.5954113
1490.0000000	5.9535999	-39.5317078
1490.0000000	6.0023999	-1557.2863770
1490.0000000	6.0511999	-14.5790701
1490.0000000	6.0999999	-19.3665581
1490.0000000	6.1487999	-13.2970695
1490.0000000	6.1975999	-23.1298180
1490.0000000	6.2463999	-1774.0781250
1490.0000000	6.2951999	-19.0286922
1490.0000000	6.3439999	-339.4415894
1490.0000000	6.3927999	-15.0471859
1490.0000000	6.4416003	-12.4157782
1490.0000000	6.4903998	-11.2979383
1490.0000000	6.5391998	-11.8406439
1490.0000000	6.5879998	350.2457886
1490.0000000	6.6367998	-12.1117640
1490.0000000	6.6856003	-13.8234491
1490.0000000	6.7343998	-13.4664412
1490.0000000	6.7831998	-14.3518600
1490.0000000	6.8319998	57.4905243
1490.0000000	6.8807998	-14.0425491
1490.0000000	6.9296002	-411.9195251
1490.0000000	6.9783998	-5.7157183
1490.0000000	7.0271997	-14.0868950
1490.0000000	7.0760002	9.1241331
1490.0000000	7.1247997	-1939.2971191
1490.0000000	7.1736002	-1555.2458496
1490.0000000	7.2223997	-885.0884399
1490.0000000	7.2711997	-191.7585144
1490.0000000	7.3200002	-18.3014565
1490.0000000	7.3687997	169.7703247
1490.0000000	7.4176002	355.3713989
1490.0000000	7.4663997	279.0010681
1490.0000000	7.5151997	-865.5878906
1490.0000000	7.5640001	-956.0072021
1490.0000000	7.6127996	-2252.0554199
1490.0000000	7.6616001	-723.5643311
1490.0000000	7.7104001	-372.1062012
1490.0000000	7.7591996	38.3665466
1490.0000000	7.8080001	369.3097229
1490.0000000	7.8567996	486.8074341
1490.0000000	7.9056001	-1011.6774902
1490.0000000	7.9544001	1609.7017822
1490.0000000	8.0031996	2077.1213379
1490.0000000	8.0520000	2073.1645508
1490.0000000	8.1007996	2193.8442383
1490.0000000	8.1496000	2339.7299805

1490.0000000	8.1983995	1455.5748291
1490.0000000	8.2472000	-3330.9099121
1490.0000000	8.2959995	-1180.8557129
1490.0000000	8.3448000	-940.5813599
1490.0000000	8.3935995	-862.0386963
1490.0000000	8.4423990	-371.4804688
1490.0000000	8.4912004	-866.9400024
1490.0000000	8.5400000	-2338.2253418
1490.0000000	8.5887995	-1133.6343994
1490.0000000	8.6375999	221.0670013
1490.0000000	8.6863995	1999.4272461
1490.0000000	8.7351999	-743.4306030
1490.0000000	8.7840004	848.8305664
1490.0000000	8.8327999	45.2402954
1490.0000000	8.8815994	-11.5047045
1490.0000000	8.9303989	-23.7688599
1490.0000000	8.9792004	-11.5830030
1490.0000000	9.0279999	-5.9205565
1490.0000000	9.0767994	6.7690582
1490.0000000	9.1255999	-17.1611061
1490.0000000	9.1743994	-10.0925550
1490.0000000	9.2231998	36.2699432
1490.0000000	9.2720003	-8.4642086
1490.0000000	9.3207998	-9.8234882
1490.0000000	9.3695993	-10.7744236
1490.0000000	9.4183998	-11.7480230
1490.0000000	9.4672003	-11.1632671
1490.0000000	9.5159998	-14.1223307
1490.0000000	9.5648003	-0.5515928
1490.0000000	9.6135998	-13.3588467
1490.0000000	9.6623993	-10.2342606
1490.0000000	9.7111998	-10.1704702
1490.0000000	9.7600002	-10.8595362
1490.0000000	9.8087997	-10.9951525
1490.0000000	9.8575993	2.9820518
1490.0000000	9.9063997	-8.0120163
1490.0000000	9.9552002	-10.9068041
1490.0000000	10.0039997	-10.0863543
1500.0000000	0.3416000	-37.1661644
1500.0000000	0.3904000	-39.8788414
1500.0000000	0.4392000	-39.4371910
1500.0000000	0.4880000	-39.5332184
1500.0000000	0.5368000	-39.6700935
1500.0000000	0.5856000	-39.2663422
1500.0000000	0.6344000	-38.2403488
1500.0000000	0.6832000	-36.2727165
1500.0000000	0.7320000	-36.1001511
1500.0000000	0.7808000	-36.5385323
1500.0000000	0.8296000	-35.9244957
1500.0000000	0.8784000	-34.9807777
1500.0000000	0.9272000	-34.2545471
1500.0000000	0.9760000	-31.9087753
1500.0000000	1.0247999	-30.3236141
1500.0000000	1.0735999	-30.1643715
1500.0000000	1.1224000	-30.7075272
1500.0000000	1.1711999	-31.2893410
1500.0000000	1.2200000	-30.4489403
1500.0000000	1.2687999	-30.6268005

1500.0000000	1.3176000	-30.8916893
1500.0000000	1.3664000	-30.6006927
1500.0000000	1.4152000	-30.1721268
1500.0000000	1.4640000	-29.5332642
1500.0000000	1.5128000	-34.7106934
1500.0000000	1.5616000	-31.6219044
1500.0000000	1.6104001	-1435.1624756
1500.0000000	1.6592000	-59.1494637
1500.0000000	1.7079999	-27.4374008
1500.0000000	1.7567999	-27.7630615
1500.0000000	1.8055999	-25.8715763
1500.0000000	1.8544000	-24.8009396
1500.0000000	1.9031999	-25.5832081
1500.0000000	1.9520000	-26.3654938
1500.0000000	2.0007999	-25.9999065
1500.0000000	2.0495999	-26.2652092
1500.0000000	2.0983999	-77.1368027
1500.0000000	2.1471999	-14.5034580
1500.0000000	2.1960001	-25.2521267
1500.0000000	2.2448001	-24.9680195
1500.0000000	2.2935998	-24.8185062
1500.0000000	2.3423998	-21.6812706
1500.0000000	2.3912001	2.0463352
1500.0000000	2.4400001	-22.9568157
1500.0000000	2.4888000	-23.4496632
1500.0000000	2.5375998	-13.0875959
1500.0000000	2.5863998	-11.3351154
1500.0000000	2.6352000	-9.5496645
1500.0000000	2.6840000	-15.9797325
1500.0000000	2.7328000	120.5367355
1500.0000000	2.7815998	142.5827637
1500.0000000	2.8304000	-23.7325306
1500.0000000	2.8792000	-20.7740726
1500.0000000	2.9280000	-22.5448055
1500.0000000	2.9768000	-17.1861076
1500.0000000	3.0256000	-20.5953274
1500.0000000	3.0743999	-28.9772663
1500.0000000	3.1231999	-24.0913906
1500.0000000	3.1719999	-29.2128296
1500.0000000	3.2208002	-492.9692688
1500.0000000	3.2695999	-964.7208252
1500.0000000	3.3183999	-20.4990997
1500.0000000	3.3671999	-3326.5012207
1500.0000000	3.4159999	1192.8540039
1500.0000000	3.4648001	-7.4145098
1500.0000000	3.5135999	-26.8935814
1500.0000000	3.5623999	-315.7283020
1500.0000000	3.6111999	-1981.5427246
1500.0000000	3.6600001	-448.9379272
1500.0000000	3.7088001	-592.6445313
1500.0000000	3.7575998	-927.6281738
1500.0000000	3.8063998	-1682.9437256
1500.0000000	3.8552001	-2443.1967773
1500.0000000	3.9040000	-2395.3940430
1500.0000000	3.9528000	1094.8084717
1500.0000000	4.0015998	-239.7025757
1500.0000000	4.0503998	670.0916748
1500.0000000	4.0991998	84.9739151

1500.0000000	4.1479998	-638.9909058
1500.0000000	4.1967998	2.0099373
1500.0000000	4.2456002	-242.8143921
1500.0000000	4.2943997	-476.3778992
1500.0000000	4.3431997	-13.4577265
1500.0000000	4.3920002	-242.4557648
1500.0000000	4.4407997	-22.3576794
1500.0000000	4.4896002	-15.5253887
1500.0000000	4.5383997	-639.9329834
1500.0000000	4.5871997	-12.9555779
1500.0000000	4.6360002	-21.5316010
1500.0000000	4.6847997	-28.0545845
1500.0000000	4.7336001	-19.5205059
1500.0000000	4.7824001	-16.5387440
1500.0000000	4.8311996	-1443.4786377
1500.0000000	4.8800001	223.0488739
1500.0000000	4.9287996	-197.5402832
1500.0000000	4.9776001	1036.7438965
1500.0000000	5.0264001	83.9925919
1500.0000000	5.0751996	-295.1096802
1500.0000000	5.1240001	-12.3619499
1500.0000000	5.1727996	551.6934204
1500.0000000	5.2216001	31.4956818
1500.0000000	5.2704000	-157.3268433
1500.0000000	5.3191996	-38.9416161
1500.0000000	5.3680000	-3499.2309570
1500.0000000	5.4168000	521.4833374
1500.0000000	5.4656000	-198.8269501
1500.0000000	5.5144000	37.5041275
1500.0000000	5.5631995	483.1195984
1500.0000000	5.6120000	388.9184570
1500.0000000	5.6608000	-36.8689346
1500.0000000	5.7096000	61.7159348
1500.0000000	5.7584000	131.5134277
1500.0000000	5.8071995	-11.5662155
1500.0000000	5.8559999	29.3507710
1500.0000000	5.9047999	-16.5952129
1500.0000000	5.9535999	-39.5269699
1500.0000000	6.0023999	-1556.9819336
1500.0000000	6.0511999	-14.5792818
1500.0000000	6.0999999	-19.3657265
1500.0000000	6.1487999	-13.2975330
1500.0000000	6.1975999	-23.1280518
1500.0000000	6.2463999	-1773.7307129
1500.0000000	6.2951999	-19.0278778
1500.0000000	6.3439999	-339.3775330
1500.0000000	6.3927999	-15.0471678
1500.0000000	6.4416003	-12.4163656
1500.0000000	6.4903998	-11.2986145
1500.0000000	6.5391998	-11.8413248
1500.0000000	6.5879998	349.5551147
1500.0000000	6.6367998	-12.1123133
1500.0000000	6.6856003	-13.8236198
1500.0000000	6.7343998	-13.4666567
1500.0000000	6.7831998	-14.3514442
1500.0000000	6.8319998	57.4714127
1500.0000000	6.8807998	-14.0424109
1500.0000000	6.9296002	-411.1580811

1500.0000000	6.9783998	-5.7285423
1500.0000000	7.0271997	-14.0858669
1500.0000000	7.0760002	9.0819464
1500.0000000	7.1247997	-1935.3513184
1500.0000000	7.1736002	-1552.0830078
1500.0000000	7.2223997	-883.2952881
1500.0000000	7.2711997	-191.3907318
1500.0000000	7.3200002	-18.2894688
1500.0000000	7.3687997	169.3957977
1500.0000000	7.4176002	354.6155701
1500.0000000	7.4663997	278.4034119
1500.0000000	7.5151997	-863.8268433
1500.0000000	7.5640001	-954.0598145
1500.0000000	7.6127996	-2247.4377441
1500.0000000	7.6616001	-722.0929565
1500.0000000	7.7104001	-371.3599243
1500.0000000	7.7591996	38.2654991
1500.0000000	7.8080001	368.5243530
1500.0000000	7.8567996	485.7793579
1500.0000000	7.9056001	-1009.6045532
1500.0000000	7.9544001	1606.3918457
1500.0000000	8.0031996	2072.8518066
1500.0000000	8.0520000	2068.9025879
1500.0000000	8.1007996	2189.3347168
1500.0000000	8.1496000	2334.9221191
1500.0000000	8.1983995	1452.5822754
1500.0000000	8.2472000	-3324.0749512
1500.0000000	8.2959995	-1178.4355469
1500.0000000	8.3448000	-938.6521606
1500.0000000	8.3935995	-860.2709961
1500.0000000	8.4423990	-370.7224731
1500.0000000	8.4912004	-865.1637573
1500.0000000	8.5400000	-2333.4255371
1500.0000000	8.5887995	-1131.3128662
1500.0000000	8.6375999	220.6056824
1500.0000000	8.6863995	1995.3088379
1500.0000000	8.7351999	-741.9063110
1500.0000000	8.7840004	847.0737915
1500.0000000	8.8327999	45.1286850
1500.0000000	8.8815994	-11.5048790
1500.0000000	8.9303989	-23.7651405
1500.0000000	8.9792004	-11.5830851
1500.0000000	9.0279999	-5.9223247
1500.0000000	9.0767994	6.7633400
1500.0000000	9.1255999	-17.1593933
1500.0000000	9.1743994	-10.0929575
1500.0000000	9.2231998	36.2542038
1500.0000000	9.2720003	-8.4651451
1500.0000000	9.3207998	-9.8240271
1500.0000000	9.3695993	-10.7746496
1500.0000000	9.4183998	-11.7478991
1500.0000000	9.4672003	-11.1633081
1500.0000000	9.5159998	-14.1214390
1500.0000000	9.5648003	-0.5549660
1500.0000000	9.6135998	-13.3580561
1500.0000000	9.6623993	-10.2345362
1500.0000000	9.7111998	-10.1707411
1500.0000000	9.7600002	-10.8595772

1500.0000000	9.8087997	-10.9951601
1500.0000000	9.8575993	2.9776287
1500.0000000	9.9063997	-8.0129299
1500.0000000	9.9552002	-10.9068089
1500.0000000	10.0039997	-10.0866013
1510.0000000	0.3416000	-37.1954727
1510.0000000	0.3904000	-39.9238510
1510.0000000	0.4392000	-39.4308472
1510.0000000	0.4880000	-39.5320091
1510.0000000	0.5368000	-39.6712685
1510.0000000	0.5856000	-39.2649574
1510.0000000	0.6344000	-38.2406578
1510.0000000	0.6832000	-36.2749405
1510.0000000	0.7320000	-36.1022034
1510.0000000	0.7808000	-36.5387001
1510.0000000	0.8296000	-35.9226494
1510.0000000	0.8784000	-34.9712143
1510.0000000	0.9272000	-34.2551918
1510.0000000	0.9760000	-31.9062519
1510.0000000	1.0247999	-30.3248501
1510.0000000	1.0735999	-30.1779728
1510.0000000	1.1224000	-30.7056561
1510.0000000	1.1711999	-31.2830391
1510.0000000	1.2200000	-30.4051418
1510.0000000	1.2687999	-30.6054726
1510.0000000	1.3176000	-30.8853035
1510.0000000	1.3664000	-30.5977478
1510.0000000	1.4152000	-30.1723309
1510.0000000	1.4640000	-29.5315533
1510.0000000	1.5128000	-34.6135139
1510.0000000	1.5616000	-31.5800629
1510.0000000	1.6104001	-1432.6197510
1510.0000000	1.6592000	-58.8555298
1510.0000000	1.7079999	-27.4411488
1510.0000000	1.7567999	-27.7630806
1510.0000000	1.8055999	-26.4738903
1510.0000000	1.8544000	-25.5814514
1510.0000000	1.9031999	-26.0559750
1510.0000000	1.9520000	-26.2858505
1510.0000000	2.0007999	-26.0052853
1510.0000000	2.0495999	-26.2672272
1510.0000000	2.0983999	-75.6610260
1510.0000000	2.1471999	-14.7707253
1510.0000000	2.1960001	-25.2480354
1510.0000000	2.2448001	-24.9501076
1510.0000000	2.2935998	-24.7588825
1510.0000000	2.3423998	-21.6956749
1510.0000000	2.3912001	1.4247894
1510.0000000	2.4400001	-22.9593029
1510.0000000	2.4888000	-23.4494457
1510.0000000	2.5375998	-13.1047039
1510.0000000	2.5863998	-11.3548746
1510.0000000	2.6352000	-9.5724087
1510.0000000	2.6840000	-15.9916744
1510.0000000	2.7328000	120.2707138
1510.0000000	2.7815998	142.2763367
1510.0000000	2.8304000	-23.7312260
1510.0000000	2.8792000	-20.7773361

1510.0000000	2.9280000	-22.5442371
1510.0000000	2.9768000	-17.1944771
1510.0000000	3.0256000	-20.5979118
1510.0000000	3.0743999	-28.9658794
1510.0000000	3.1231999	-24.0879364
1510.0000000	3.1719999	-29.2045364
1510.0000000	3.2208002	-492.7315369
1510.0000000	3.2695999	-964.2458496
1510.0000000	3.3183999	-20.4992981
1510.0000000	3.3671999	-3325.0395508
1510.0000000	3.4159999	1192.3187256
1510.0000000	3.4648001	-7.4202185
1510.0000000	3.5135999	-26.8905907
1510.0000000	3.5623999	-315.5987244
1510.0000000	3.6111999	-1980.6829834
1510.0000000	3.6600001	-448.7498169
1510.0000000	3.7088001	-592.3931274
1510.0000000	3.7575998	-927.2296143
1510.0000000	3.8063998	-1682.2138672
1510.0000000	3.8552001	-2442.1333008
1510.0000000	3.9040000	-2394.3515625
1510.0000000	3.9528000	1094.3201904
1510.0000000	4.0015998	-239.6054688
1510.0000000	4.0503998	669.7891235
1510.0000000	4.0991998	84.9282303
1510.0000000	4.1479998	-638.7186279
1510.0000000	4.1967998	2.0006809
1510.0000000	4.2456002	-242.7160797
1510.0000000	4.2943997	-476.1773376
1510.0000000	4.3431997	-13.4599066
1510.0000000	4.3920002	-242.3576508
1510.0000000	4.4407997	-22.3559036
1510.0000000	4.4896002	-15.5262718
1510.0000000	4.5383997	-639.6718750
1510.0000000	4.5871997	-12.9575949
1510.0000000	4.6360002	-21.5300579
1510.0000000	4.6847997	-28.0506592
1510.0000000	4.7336001	-19.5197830
1510.0000000	4.7824001	-16.5391979
1510.0000000	4.8311996	-1442.9473877
1510.0000000	4.8800001	222.9593201
1510.0000000	4.9287996	-197.4730072
1510.0000000	4.9776001	1036.3513184
1510.0000000	5.0264001	83.9550781
1510.0000000	5.0751996	-295.0060730
1510.0000000	5.1240001	-12.3628712
1510.0000000	5.1727996	551.5775146
1510.0000000	5.2216001	31.4862366
1510.0000000	5.2704000	-157.2987366
1510.0000000	5.3191996	-38.9370651
1510.0000000	5.3680000	-3498.5385742
1510.0000000	5.4168000	521.3765869
1510.0000000	5.4656000	-198.7907715
1510.0000000	5.5144000	37.4934692
1510.0000000	5.5631995	483.0210266
1510.0000000	5.6120000	388.8384705
1510.0000000	5.6608000	-36.8648491
1510.0000000	5.7096000	61.7005005

1510.0000000	5.7584000	131.4844971
1510.0000000	5.8071995	-11.5670862
1510.0000000	5.8559999	29.3419170
1510.0000000	5.9047999	-16.5950146
1510.0000000	5.9535999	-39.5222282
1510.0000000	6.0023999	-1556.6773682
1510.0000000	6.0511999	-14.5794926
1510.0000000	6.0999999	-19.3648949
1510.0000000	6.1487999	-13.2979956
1510.0000000	6.1975999	-23.1262856
1510.0000000	6.2463999	-1773.3833008
1510.0000000	6.2951999	-19.0270634
1510.0000000	6.3439999	-339.3134766
1510.0000000	6.3927999	-15.0471497
1510.0000000	6.4416003	-12.4169521
1510.0000000	6.4903998	-11.2992907
1510.0000000	6.5391998	-11.8420057
1510.0000000	6.5879998	348.8644104
1510.0000000	6.6367998	-12.1128626
1510.0000000	6.6856003	-13.8237896
1510.0000000	6.7343998	-13.4668722
1510.0000000	6.7831998	-14.3510284
1510.0000000	6.8319998	57.4522934
1510.0000000	6.8807998	-14.0422726
1510.0000000	6.9296002	-410.3966370
1510.0000000	6.9783998	-5.7413659
1510.0000000	7.0271997	-14.0848398
1510.0000000	7.0760002	9.0397615
1510.0000000	7.1247997	-1931.4053955
1510.0000000	7.1736002	-1548.9201660
1510.0000000	7.2223997	-881.5021362
1510.0000000	7.2711997	-191.0229492
1510.0000000	7.3200002	-18.2774811
1510.0000000	7.3687997	169.0212708
1510.0000000	7.4176002	353.8597412
1510.0000000	7.4663997	277.8057861
1510.0000000	7.5151997	-862.0657959
1510.0000000	7.5640001	-952.1123657
1510.0000000	7.6127996	-2242.8203125
1510.0000000	7.6616001	-720.6215820
1510.0000000	7.7104001	-370.6136475
1510.0000000	7.7591996	38.1644478
1510.0000000	7.8080001	367.7389832
1510.0000000	7.8567996	484.7512817
1510.0000000	7.9056001	-1007.5316772
1510.0000000	7.9544001	1603.0819092
1510.0000000	8.0031996	2068.5820313
1510.0000000	8.0520000	2064.6406250
1510.0000000	8.1007996	2184.8251953
1510.0000000	8.1496000	2330.1140137
1510.0000000	8.1983995	1449.5897217
1510.0000000	8.2472000	-3317.2402344
1510.0000000	8.2959995	-1176.0153809
1510.0000000	8.3448000	-936.7229614
1510.0000000	8.3935995	-858.5033569
1510.0000000	8.4423990	-369.9644470
1510.0000000	8.4912004	-863.3875732
1510.0000000	8.5400000	-2328.6257324

1510.0000000	8.5887995	-1128.9913330
1510.0000000	8.6375999	220.1443481
1510.0000000	8.6863995	1991.1905518
1510.0000000	8.7351999	-740.3820801
1510.0000000	8.7840004	845.3170776
1510.0000000	8.8327999	45.0170784
1510.0000000	8.8815994	-11.5050535
1510.0000000	8.9303989	-23.7614212
1510.0000000	8.9792004	-11.5831680
1510.0000000	9.0279999	-5.9240932
1510.0000000	9.0767994	6.7576218
1510.0000000	9.1255999	-17.1576786
1510.0000000	9.1743994	-10.0933590
1510.0000000	9.2231998	36.2384720
1510.0000000	9.2720003	-8.4660816
1510.0000000	9.3207998	-9.8245668
1510.0000000	9.3695993	-10.7748766
1510.0000000	9.4183998	-11.7477741
1510.0000000	9.4672003	-11.1633501
1510.0000000	9.5159998	-14.1205482
1510.0000000	9.5648003	-0.5583382
1510.0000000	9.6135998	-13.3572655
1510.0000000	9.6623993	-10.2348108
1510.0000000	9.7111998	-10.1710129
1510.0000000	9.7600002	-10.8596191
1510.0000000	9.8087997	-10.9951677
1510.0000000	9.8575993	2.9732065
1510.0000000	9.9063997	-8.0138435
1510.0000000	9.9552002	-10.9068127
1510.0000000	10.0039997	-10.0868492
1520.0000000	0.3416000	-36.8961296
1520.0000000	0.3904000	-39.9688644
1520.0000000	0.4392000	-39.4245071
1520.0000000	0.4880000	-39.5307999
1520.0000000	0.5368000	-39.6724434
1520.0000000	0.5856000	-39.2635765
1520.0000000	0.6344000	-38.2409668
1520.0000000	0.6832000	-36.2771683
1520.0000000	0.7320000	-36.1042557
1520.0000000	0.7808000	-36.5388718
1520.0000000	0.8296000	-35.9208031
1520.0000000	0.8784000	-34.9616470
1520.0000000	0.9272000	-34.2558327
1520.0000000	0.9760000	-31.9037285
1520.0000000	1.0247999	-30.3260860
1520.0000000	1.0735999	-30.1915722
1520.0000000	1.1224000	-30.7037830
1520.0000000	1.1711999	-31.2767353
1520.0000000	1.2200000	-30.3613434
1520.0000000	1.2687999	-30.5841465
1520.0000000	1.3176000	-30.8789177
1520.0000000	1.3664000	-30.5948048
1520.0000000	1.4152000	-30.1725368
1520.0000000	1.4640000	-29.5298443
1520.0000000	1.5128000	-34.5163383
1520.0000000	1.5616000	-31.5382214
1520.0000000	1.6104001	-1430.0769043
1520.0000000	1.6592000	-58.5615997

1520.0000000	1.7079999	-27.4448967
1520.0000000	1.7567999	-27.7630997
1520.0000000	1.8055999	-26.9114418
1520.0000000	1.8544000	-25.8799744
1520.0000000	1.9031999	-25.9104671
1520.0000000	1.9520000	-26.2062054
1520.0000000	2.0007999	-26.0106659
1520.0000000	2.0495999	-26.2692471
1520.0000000	2.0983999	-74.1852417
1520.0000000	2.1471999	-15.0379925
1520.0000000	2.1960001	-25.2439442
1520.0000000	2.2448001	-24.9321976
1520.0000000	2.2935998	-24.6992607
1520.0000000	2.3423998	-21.7100811
1520.0000000	2.3912001	0.8032436
1520.0000000	2.4400001	-22.9617882
1520.0000000	2.4888000	-23.4492283
1520.0000000	2.5375998	-13.1218119
1520.0000000	2.5863998	-11.3746338
1520.0000000	2.6352000	-9.5951529
1520.0000000	2.6840000	-16.0036163
1520.0000000	2.7328000	120.0047073
1520.0000000	2.7815998	141.9699097
1520.0000000	2.8304000	-23.7299213
1520.0000000	2.8792000	-20.7805996
1520.0000000	2.9280000	-22.5436687
1520.0000000	2.9768000	-17.2028465
1520.0000000	3.0256000	-20.6004963
1520.0000000	3.0743999	-28.9544926
1520.0000000	3.1231999	-24.0844803
1520.0000000	3.1719999	-29.1962433
1520.0000000	3.2208002	-492.4938049
1520.0000000	3.2695999	-963.7708740
1520.0000000	3.3183999	-20.4994965
1520.0000000	3.3671999	-3323.5778809
1520.0000000	3.4159999	1191.7834473
1520.0000000	3.4648001	-7.4259262
1520.0000000	3.5135999	-26.8875999
1520.0000000	3.5623999	-315.4691467
1520.0000000	3.6111999	-1979.8232422
1520.0000000	3.6600001	-448.5616455
1520.0000000	3.7088001	-592.1417847
1520.0000000	3.7575998	-926.8310547
1520.0000000	3.8063998	-1681.4840088
1520.0000000	3.8552001	-2441.0700684
1520.0000000	3.9040000	-2393.3090820
1520.0000000	3.9528000	1093.8319092
1520.0000000	4.0015998	-239.5083618
1520.0000000	4.0503998	669.4865112
1520.0000000	4.0991998	84.8825531
1520.0000000	4.1479998	-638.4464111
1520.0000000	4.1967998	1.9914246
1520.0000000	4.2456002	-242.6177826
1520.0000000	4.2943997	-475.9767761
1520.0000000	4.3431997	-13.4620876
1520.0000000	4.3920002	-242.2595367
1520.0000000	4.4407997	-22.3541279
1520.0000000	4.4896002	-15.5271540

1520.0000000	4.5383997	-639.4108276
1520.0000000	4.5871997	-12.9596109
1520.0000000	4.6360002	-21.5285149
1520.0000000	4.6847997	-28.0467339
1520.0000000	4.7336001	-19.5190601
1520.0000000	4.7824001	-16.5396538
1520.0000000	4.8311996	-1442.4161377
1520.0000000	4.8800001	222.8697815
1520.0000000	4.9287996	-197.4057465
1520.0000000	4.9776001	1035.9588623
1520.0000000	5.0264001	83.9175568
1520.0000000	5.0751996	-294.9024353
1520.0000000	5.1240001	-12.3637924
1520.0000000	5.1727996	551.4616699
1520.0000000	5.2216001	31.4767952
1520.0000000	5.2704000	-157.2706299
1520.0000000	5.3191996	-38.9325180
1520.0000000	5.3680000	-3497.8461914
1520.0000000	5.4168000	521.2698364
1520.0000000	5.4656000	-198.7545929
1520.0000000	5.5144000	37.4828110
1520.0000000	5.5631995	482.9224548
1520.0000000	5.6120000	388.7585144
1520.0000000	5.6608000	-36.8607597
1520.0000000	5.7096000	61.6850662
1520.0000000	5.7584000	131.4555664
1520.0000000	5.8071995	-11.5679569
1520.0000000	5.8559999	29.3330669
1520.0000000	5.9047999	-16.5948181
1520.0000000	5.9535999	-39.5174904
1520.0000000	6.0023999	-1556.3730469
1520.0000000	6.0511999	-14.5797043
1520.0000000	6.0999999	-19.3640614
1520.0000000	6.1487999	-13.2984581
1520.0000000	6.1975999	-23.1245213
1520.0000000	6.2463999	-1773.0358887
1520.0000000	6.2951999	-19.0262489
1520.0000000	6.3439999	-339.2494507
1520.0000000	6.3927999	-15.0471306
1520.0000000	6.4416003	-12.4175396
1520.0000000	6.4903998	-11.2999659
1520.0000000	6.5391998	-11.8426867
1520.0000000	6.5879998	348.1737061
1520.0000000	6.6367998	-12.1134119
1520.0000000	6.6856003	-13.8239603
1520.0000000	6.7343998	-13.4670887
1520.0000000	6.7831998	-14.3506126
1520.0000000	6.8319998	57.4331818
1520.0000000	6.8807998	-14.0421333
1520.0000000	6.9296002	-409.6351929
1520.0000000	6.9783998	-5.7541890
1520.0000000	7.0271997	-14.0838118
1520.0000000	7.0760002	8.9975767
1520.0000000	7.1247997	-1927.4594727
1520.0000000	7.1736002	-1545.7572021
1520.0000000	7.2223997	-879.7089844
1520.0000000	7.2711997	-190.6551819
1520.0000000	7.3200002	-18.2654915

1520.0000000	7.3687997	168.6467285
1520.0000000	7.4176002	353.1039124
1520.0000000	7.4663997	277.2081299
1520.0000000	7.5151997	-860.3047485
1520.0000000	7.5640001	-950.1649780
1520.0000000	7.6127996	-2238.2026367
1520.0000000	7.6616001	-719.1503296
1520.0000000	7.7104001	-369.8673706
1520.0000000	7.7591996	38.0634003
1520.0000000	7.8080001	366.9536438
1520.0000000	7.8567996	483.7232056
1520.0000000	7.9056001	-1005.4587402
1520.0000000	7.9544001	1599.7719727
1520.0000000	8.0031996	2064.3125000
1520.0000000	8.0520000	2060.3786621
1520.0000000	8.1007996	2180.3156738
1520.0000000	8.1496000	2325.3061523
1520.0000000	8.1983995	1446.5972900
1520.0000000	8.2472000	-3310.4055176
1520.0000000	8.2959995	-1173.5952148
1520.0000000	8.3448000	-934.7938232
1520.0000000	8.3935995	-856.7356567
1520.0000000	8.4423990	-369.2064514
1520.0000000	8.4912004	-861.6113281
1520.0000000	8.5400000	-2323.8259277
1520.0000000	8.5887995	-1126.6697998
1520.0000000	8.6375999	219.6830292
1520.0000000	8.6863995	1987.0721436
1520.0000000	8.7351999	-738.8577881
1520.0000000	8.7840004	843.5603027
1520.0000000	8.8327999	44.9054680
1520.0000000	8.8815994	-11.5052280
1520.0000000	8.9303989	-23.7577000
1520.0000000	8.9792004	-11.5832500
1520.0000000	9.0279999	-5.9258618
1520.0000000	9.0767994	6.7519035
1520.0000000	9.1255999	-17.1559639
1520.0000000	9.1743994	-10.0937614
1520.0000000	9.2231998	36.2227402
1520.0000000	9.2720003	-8.4670181
1520.0000000	9.3207998	-9.8251057
1520.0000000	9.3695993	-10.7751026
1520.0000000	9.4183998	-11.7476492
1520.0000000	9.4672003	-11.1633911
1520.0000000	9.5159998	-14.1196575
1520.0000000	9.5648003	-0.5617104
1520.0000000	9.6135998	-13.3564749
1520.0000000	9.6623993	-10.2350855
1520.0000000	9.7111998	-10.1712847
1520.0000000	9.7600002	-10.8596611
1520.0000000	9.8087997	-10.9951763
1520.0000000	9.8575993	2.9687843
1520.0000000	9.9063997	-8.0147581
1520.0000000	9.9552002	-10.9068174
1520.0000000	10.0039997	-10.0870962
1530.0000000	0.3416000	-36.8306236
1530.0000000	0.3904000	-39.9565620
1530.0000000	0.4392000	-39.4181633

1530.0000000	0.4880000	-39.5295906
1530.0000000	0.5368000	-39.6736145
1530.0000000	0.5856000	-39.2621956
1530.0000000	0.6344000	-38.2412796
1530.0000000	0.6832000	-36.2793922
1530.0000000	0.7320000	-36.1063080
1530.0000000	0.7808000	-36.5390396
1530.0000000	0.8296000	-35.9189568
1530.0000000	0.8784000	-34.9520836
1530.0000000	0.9272000	-34.2564735
1530.0000000	0.9760000	-31.9012051
1530.0000000	1.0247999	-30.3273201
1530.0000000	1.0735999	-30.2051735
1530.0000000	1.1224000	-30.7019119
1530.0000000	1.1711999	-31.2704334
1530.0000000	1.2200000	-30.3175449
1530.0000000	1.2687999	-30.5628204
1530.0000000	1.3176000	-30.8725319
1530.0000000	1.3664000	-30.5918617
1530.0000000	1.4152000	-30.1727409
1530.0000000	1.4640000	-29.5281353
1530.0000000	1.5128000	-34.4191589
1530.0000000	1.5616000	-31.4963799
1530.0000000	1.6104001	-1427.5340576
1530.0000000	1.6592000	-58.2676697
1530.0000000	1.7079999	-27.4486427
1530.0000000	1.7567999	-27.7631187
1530.0000000	1.8055999	-26.8784885
1530.0000000	1.8544000	-25.2285099
1530.0000000	1.9031999	-25.3045769
1530.0000000	1.9520000	-25.8878288
1530.0000000	2.0007999	-26.0160465
1530.0000000	2.0495999	-26.2712650
1530.0000000	2.0983999	-72.7094650
1530.0000000	2.1471999	-15.3052597
1530.0000000	2.1960001	-25.2398529
1530.0000000	2.2448001	-24.9142857
1530.0000000	2.2935998	-24.6396370
1530.0000000	2.3423998	-21.7244854
1530.0000000	2.3912001	0.1816978
1530.0000000	2.4400001	-22.9642754
1530.0000000	2.4888000	-23.4490108
1530.0000000	2.5375998	-13.1389198
1530.0000000	2.5863998	-11.3943920
1530.0000000	2.6352000	-9.6178970
1530.0000000	2.6840000	-16.0155582
1530.0000000	2.7328000	119.7386856
1530.0000000	2.7815998	141.6635132
1530.0000000	2.8304000	-23.7286167
1530.0000000	2.8792000	-20.7838631
1530.0000000	2.9280000	-22.5430984
1530.0000000	2.9768000	-17.2112179
1530.0000000	3.0256000	-20.6030807
1530.0000000	3.0743999	-28.9431057
1530.0000000	3.1231999	-24.0810242
1530.0000000	3.1719999	-29.1879501
1530.0000000	3.2208002	-492.2560425
1530.0000000	3.2695999	-963.2958984

1530.0000000	3.3183999	-20.4996948
1530.0000000	3.3671999	-3322.1162109
1530.0000000	3.4159999	1191.2481689
1530.0000000	3.4648001	-7.4316339
1530.0000000	3.5135999	-26.8846092
1530.0000000	3.5623999	-315.3395386
1530.0000000	3.6111999	-1978.9635010
1530.0000000	3.6600001	-448.3735352
1530.0000000	3.7088001	-591.8903809
1530.0000000	3.7575998	-926.4325562
1530.0000000	3.8063998	-1680.7542725
1530.0000000	3.8552001	-2440.0065918
1530.0000000	3.9040000	-2392.2666016
1530.0000000	3.9528000	1093.3435059
1530.0000000	4.0015998	-239.4112549
1530.0000000	4.0503998	669.1839600
1530.0000000	4.0991998	84.8368683
1530.0000000	4.1479998	-638.1741943
1530.0000000	4.1967998	1.9821682
1530.0000000	4.2456002	-242.5194855
1530.0000000	4.2943997	-475.7761841
1530.0000000	4.3431997	-13.4642677
1530.0000000	4.3920002	-242.1614227
1530.0000000	4.4407997	-22.3523521
1530.0000000	4.4896002	-15.5280361
1530.0000000	4.5383997	-639.1497803
1530.0000000	4.5871997	-12.9616270
1530.0000000	4.6360002	-21.5269718
1530.0000000	4.6847997	-28.0428085
1530.0000000	4.7336001	-19.5183392
1530.0000000	4.7824001	-16.5401077
1530.0000000	4.8311996	-1441.8848877
1530.0000000	4.8800001	222.7802277
1530.0000000	4.9287996	-197.3384857
1530.0000000	4.9776001	1035.5664063
1530.0000000	5.0264001	83.8800354
1530.0000000	5.0751996	-294.7987976
1530.0000000	5.1240001	-12.3647137
1530.0000000	5.1727996	551.3458252
1530.0000000	5.2216001	31.4673500
1530.0000000	5.2704000	-157.2425232
1530.0000000	5.3191996	-38.9279709
1530.0000000	5.3680000	-3497.1538086
1530.0000000	5.4168000	521.1630249
1530.0000000	5.4656000	-198.7183990
1530.0000000	5.5144000	37.4721603
1530.0000000	5.5631995	482.8239136
1530.0000000	5.6120000	388.6785278
1530.0000000	5.6608000	-36.8566704
1530.0000000	5.7096000	61.6696396
1530.0000000	5.7584000	131.4266357
1530.0000000	5.8071995	-11.5688267
1530.0000000	5.8559999	29.3242168
1530.0000000	5.9047999	-16.5946198
1530.0000000	5.9535999	-39.5127525
1530.0000000	6.0023999	-1556.0686035
1530.0000000	6.0511999	-14.5799160
1530.0000000	6.0999999	-19.3632298

1530. 0000000	6. 1487999	-13. 2989216
1530. 0000000	6. 1975999	-23. 1227570
1530. 0000000	6. 2463999	-1772. 6885986
1530. 0000000	6. 2951999	-19. 0254364
1530. 0000000	6. 3439999	-339. 1853638
1530. 0000000	6. 3927999	-15. 0471125
1530. 0000000	6. 4416003	-12. 4181261
1530. 0000000	6. 4903998	-11. 3006420
1530. 0000000	6. 5391998	-11. 8433685
1530. 0000000	6. 5879998	347. 4830322
1530. 0000000	6. 6367998	-12. 1139603
1530. 0000000	6. 6856003	-13. 8241301
1530. 0000000	6. 7343998	-13. 4673042
1530. 0000000	6. 7831998	-14. 3501968
1530. 0000000	6. 8319998	57. 4140701
1530. 0000000	6. 8807998	-14. 0419950
1530. 0000000	6. 9296002	-408. 8737183
1530. 0000000	6. 9783998	-5. 7670131
1530. 0000000	7. 0271997	-14. 0827837
1530. 0000000	7. 0760002	8. 9553900
1530. 0000000	7. 1247997	-1923. 5136719
1530. 0000000	7. 1736002	-1542. 5943604
1530. 0000000	7. 2223997	-877. 9157715
1530. 0000000	7. 2711997	-190. 2873993
1530. 0000000	7. 3200002	-18. 2535019
1530. 0000000	7. 3687997	168. 2722015
1530. 0000000	7. 4176002	352. 3480835
1530. 0000000	7. 4663997	276. 6104736
1530. 0000000	7. 5151997	-858. 5437012
1530. 0000000	7. 5640001	-948. 2175903
1530. 0000000	7. 6127996	-2233. 5849609
1530. 0000000	7. 6616001	-717. 6789551
1530. 0000000	7. 7104001	-369. 1210938
1530. 0000000	7. 7591996	37. 9623528
1530. 0000000	7. 8080001	366. 1682434
1530. 0000000	7. 8567996	482. 6950684
1530. 0000000	7. 9056001	-1003. 3858643
1530. 0000000	7. 9544001	1596. 4620361
1530. 0000000	8. 0031996	2060. 0429688
1530. 0000000	8. 0520000	2056. 1166992
1530. 0000000	8. 1007996	2175. 8061523
1530. 0000000	8. 1496000	2320. 4980469
1530. 0000000	8. 1983995	1443. 6047363
1530. 0000000	8. 2472000	-3303. 5705566
1530. 0000000	8. 2959995	-1171. 1750488
1530. 0000000	8. 3448000	-932. 8646240
1530. 0000000	8. 3935995	-854. 9680176
1530. 0000000	8. 4423990	-368. 4484253
1530. 0000000	8. 4912004	-859. 8351440
1530. 0000000	8. 5400000	-2319. 0261230
1530. 0000000	8. 5887995	-1124. 3482666
1530. 0000000	8. 6375999	219. 2217102
1530. 0000000	8. 6863995	1982. 9537354
1530. 0000000	8. 7351999	-737. 3335571
1530. 0000000	8. 7840004	841. 8035889
1530. 0000000	8. 8327999	44. 7938652
1530. 0000000	8. 8815994	-11. 5054016
1530. 0000000	8. 9303989	-23. 7539787

1530.0000000	8.9792004	-11.5833321
1530.0000000	9.0279999	-5.9276295
1530.0000000	9.0767994	6.7461872
1530.0000000	9.1255999	-17.1542511
1530.0000000	9.1743994	-10.0941639
1530.0000000	9.2231998	36.2070084
1530.0000000	9.2720003	-8.4679537
1530.0000000	9.3207998	-9.8256454
1530.0000000	9.3695993	-10.7753296
1530.0000000	9.4183998	-11.7475252
1530.0000000	9.4672003	-11.1634321
1530.0000000	9.5159998	-14.1187668
1530.0000000	9.5648003	-0.5650835
1530.0000000	9.6135998	-13.3556843
1530.0000000	9.6623993	-10.2353611
1530.0000000	9.7111998	-10.1715565
1530.0000000	9.7600002	-10.8597031
1530.0000000	9.8087997	-10.9951839
1530.0000000	9.8575993	2.9643621
1530.0000000	9.9063997	-8.0156717
1530.0000000	9.9552002	-10.9068213
1530.0000000	10.0039997	-10.0873442
1540.0000000	0.3416000	-37.0591545
1540.0000000	0.3904000	-39.9442596
1540.0000000	0.4392000	-39.4118195
1540.0000000	0.4880000	-39.5283813
1540.0000000	0.5368000	-39.6747894
1540.0000000	0.5856000	-39.2608147
1540.0000000	0.6344000	-38.2415886
1540.0000000	0.6832000	-36.2816200
1540.0000000	0.7320000	-36.1083603
1540.0000000	0.7808000	-36.5392113
1540.0000000	0.8296000	-35.9171143
1540.0000000	0.8784000	-34.9425201
1540.0000000	0.9272000	-34.2571182
1540.0000000	0.9760000	-31.8986816
1540.0000000	1.0247999	-30.3285561
1540.0000000	1.0735999	-30.2187729
1540.0000000	1.1224000	-30.7000389
1540.0000000	1.1711999	-31.2641296
1540.0000000	1.2200000	-30.2737465
1540.0000000	1.2687999	-30.5414925
1540.0000000	1.3176000	-30.8661461
1540.0000000	1.3664000	-30.5889168
1540.0000000	1.4152000	-30.1729450
1540.0000000	1.4640000	-29.5264263
1540.0000000	1.5128000	-34.3219833
1540.0000000	1.5616000	-31.4545403
1540.0000000	1.6104001	-1424.9913330
1540.0000000	1.6592000	-57.9737358
1540.0000000	1.7079999	-27.4523907
1540.0000000	1.7567999	-27.7631378
1540.0000000	1.8055999	-25.9393234
1540.0000000	1.8544000	-25.2104034
1540.0000000	1.9031999	-25.7299728
1540.0000000	1.9520000	-26.0878372
1540.0000000	2.0007999	-26.0214272
1540.0000000	2.0495999	-26.2732849

1540.0000000	2.0983999	-71.2336884
1540.0000000	2.1471999	-15.5725269
1540.0000000	2.1960001	-25.2357616
1540.0000000	2.2448001	-24.8963737
1540.0000000	2.2935998	-24.5800133
1540.0000000	2.3423998	-21.7388897
1540.0000000	2.3912001	-0.4398479
1540.0000000	2.4400001	-22.9667606
1540.0000000	2.4888000	-23.4487934
1540.0000000	2.5375998	-13.1560278
1540.0000000	2.5863998	-11.4141502
1540.0000000	2.6352000	-9.6406412
1540.0000000	2.6840000	-16.0275002
1540.0000000	2.7328000	119.4726639
1540.0000000	2.7815998	141.3570862
1540.0000000	2.8304000	-23.7273140
1540.0000000	2.8792000	-20.7871265
1540.0000000	2.9280000	-22.5425301
1540.0000000	2.9768000	-17.2195892
1540.0000000	3.0256000	-20.6056652
1540.0000000	3.0743999	-28.9317188
1540.0000000	3.1231999	-24.0775700
1540.0000000	3.1719999	-29.1796570
1540.0000000	3.2208002	-492.0183105
1540.0000000	3.2695999	-962.8209229
1540.0000000	3.3183999	-20.4998913
1540.0000000	3.3671999	-3320.6545410
1540.0000000	3.4159999	1190.7128906
1540.0000000	3.4648001	-7.4373426
1540.0000000	3.5135999	-26.8816185
1540.0000000	3.5623999	-315.2099609
1540.0000000	3.6111999	-1978.1037598
1540.0000000	3.6600001	-448.1854248
1540.0000000	3.7088001	-591.6389771
1540.0000000	3.7575998	-926.0339966
1540.0000000	3.8063998	-1680.0244141
1540.0000000	3.8552001	-2438.9431152
1540.0000000	3.9040000	-2391.2241211
1540.0000000	3.9528000	1092.8552246
1540.0000000	4.0015998	-239.3141327
1540.0000000	4.0503998	668.8814087
1540.0000000	4.0991998	84.7911835
1540.0000000	4.1479998	-637.9019775
1540.0000000	4.1967998	1.9729118
1540.0000000	4.2456002	-242.4211731
1540.0000000	4.2943997	-475.5756226
1540.0000000	4.3431997	-13.4664478
1540.0000000	4.3920002	-242.0633087
1540.0000000	4.4407997	-22.3505783
1540.0000000	4.4896002	-15.5289192
1540.0000000	4.5383997	-638.8886719
1540.0000000	4.5871997	-12.9636440
1540.0000000	4.6360002	-21.5254307
1540.0000000	4.6847997	-28.0388832
1540.0000000	4.7336001	-19.5176163
1540.0000000	4.7824001	-16.5405617
1540.0000000	4.8311996	-1441.3535156
1540.0000000	4.8800001	222.6906738

1540.0000000	4.9287996	-197.2712250
1540.0000000	4.9776001	1035.1739502
1540.0000000	5.0264001	83.8425140
1540.0000000	5.0751996	-294.6951599
1540.0000000	5.1240001	-12.3656349
1540.0000000	5.1727996	551.2299194
1540.0000000	5.2216001	31.4579086
1540.0000000	5.2704000	-157.2144165
1540.0000000	5.3191996	-38.9234161
1540.0000000	5.3680000	-3496.4614258
1540.0000000	5.4168000	521.0562134
1540.0000000	5.4656000	-198.6822205
1540.0000000	5.5144000	37.4615021
1540.0000000	5.5631995	482.7253418
1540.0000000	5.6120000	388.5985413
1540.0000000	5.6608000	-36.8525848
1540.0000000	5.7096000	61.6542053
1540.0000000	5.7584000	131.3977051
1540.0000000	5.8071995	-11.5696983
1540.0000000	5.8559999	29.3153629
1540.0000000	5.9047999	-16.5944214
1540.0000000	5.9535999	-39.5080147
1540.0000000	6.0023999	-1555.7641602
1540.0000000	6.0511999	-14.5801268
1540.0000000	6.0999999	-19.3623962
1540.0000000	6.1487999	-13.2993841
1540.0000000	6.1975999	-23.1209908
1540.0000000	6.2463999	-1772.3411865
1540.0000000	6.2951999	-19.0246220
1540.0000000	6.3439999	-339.1213379
1540.0000000	6.3927999	-15.0470943
1540.0000000	6.4416003	-12.4187126
1540.0000000	6.4903998	-11.3013182
1540.0000000	6.5391998	-11.8440495
1540.0000000	6.5879998	346.7923279
1540.0000000	6.6367998	-12.1145096
1540.0000000	6.6856003	-13.8243008
1540.0000000	6.7343998	-13.4675198
1540.0000000	6.7831998	-14.3497810
1540.0000000	6.8319998	57.3949509
1540.0000000	6.8807998	-14.0418558
1540.0000000	6.9296002	-408.1122742
1540.0000000	6.9783998	-5.7798367
1540.0000000	7.0271997	-14.0817556
1540.0000000	7.0760002	8.9132051
1540.0000000	7.1247997	-1919.5677490
1540.0000000	7.1736002	-1539.4315186
1540.0000000	7.2223997	-876.1226196
1540.0000000	7.2711997	-189.9196167
1540.0000000	7.3200002	-18.2415142
1540.0000000	7.3687997	167.8976746
1540.0000000	7.4176002	351.5922546
1540.0000000	7.4663997	276.0128174
1540.0000000	7.5151997	-856.7827148
1540.0000000	7.5640001	-946.2702026
1540.0000000	7.6127996	-2228.9672852
1540.0000000	7.6616001	-716.2076416
1540.0000000	7.7104001	-368.3748779

1540.0000000	7.7591996	37.8613014
1540.0000000	7.8080001	365.3828735
1540.0000000	7.8567996	481.6669922
1540.0000000	7.9056001	-1001.3129272
1540.0000000	7.9544001	1593.1520996
1540.0000000	8.0031996	2055.7731934
1540.0000000	8.0520000	2051.8547363
1540.0000000	8.1007996	2171.2966309
1540.0000000	8.1496000	2315.6899414
1540.0000000	8.1983995	1440.6121826
1540.0000000	8.2472000	-3296.7358398
1540.0000000	8.2959995	-1168.7548828
1540.0000000	8.3448000	-930.9354248
1540.0000000	8.3935995	-853.2003174
1540.0000000	8.4423990	-367.6904297
1540.0000000	8.4912004	-858.0588989
1540.0000000	8.5400000	-2314.2263184
1540.0000000	8.5887995	-1122.0267334
1540.0000000	8.6375999	218.7603912
1540.0000000	8.6863995	1978.8354492
1540.0000000	8.7351999	-735.8093262
1540.0000000	8.7840004	840.0468140
1540.0000000	8.8327999	44.6822586
1540.0000000	8.8815994	-11.5055761
1540.0000000	8.9303989	-23.7502594
1540.0000000	8.9792004	-11.5834150
1540.0000000	9.0279999	-5.9293981
1540.0000000	9.0767994	6.7404690
1540.0000000	9.1255999	-17.1525364
1540.0000000	9.1743994	-10.0945654
1540.0000000	9.2231998	36.1912766
1540.0000000	9.2720003	-8.4688902
1540.0000000	9.3207998	-9.8261843
1540.0000000	9.3695993	-10.7755556
1540.0000000	9.4183998	-11.7474003
1540.0000000	9.4672003	-11.1634731
1540.0000000	9.5159998	-14.1178751
1540.0000000	9.5648003	-0.5684566
1540.0000000	9.6135998	-13.3548927
1540.0000000	9.6623993	-10.2356358
1540.0000000	9.7111998	-10.1718273
1540.0000000	9.7600002	-10.8597441
1540.0000000	9.8087997	-10.9951916
1540.0000000	9.8575993	2.9599400
1540.0000000	9.9063997	-8.0165854
1540.0000000	9.9552002	-10.9068260
1540.0000000	10.0039997	-10.0875912
1550.0000000	0.3416000	-37.4167099
1550.0000000	0.3904000	-39.9319611
1550.0000000	0.4392000	-39.4054756
1550.0000000	0.4880000	-39.5271721
1550.0000000	0.5368000	-39.6759644
1550.0000000	0.5856000	-39.2594337
1550.0000000	0.6344000	-38.2419014
1550.0000000	0.6832000	-36.2838440
1550.0000000	0.7320000	-36.1104126
1550.0000000	0.7808000	-36.5393791
1550.0000000	0.8296000	-35.9152679

1550.0000000	0.8784000	-34.9329529
1550.0000000	0.9272000	-34.2577591
1550.0000000	0.9760000	-31.8961582
1550.0000000	1.0247999	-30.3297920
1550.0000000	1.0735999	-30.2323742
1550.0000000	1.1224000	-30.6981678
1550.0000000	1.1711999	-31.2578278
1550.0000000	1.2200000	-30.2299480
1550.0000000	1.2687999	-30.5201664
1550.0000000	1.3176000	-30.8597603
1550.0000000	1.3664000	-30.5859737
1550.0000000	1.4152000	-30.1731491
1550.0000000	1.4640000	-29.5247154
1550.0000000	1.5128000	-34.2248039
1550.0000000	1.5616000	-31.4126987
1550.0000000	1.6104001	-1422.4484863
1550.0000000	1.6592000	-57.6798019
1550.0000000	1.7079999	-27.4561386
1550.0000000	1.7567999	-27.7631569
1550.0000000	1.8055999	-26.4680595
1550.0000000	1.8544000	-25.3204536
1550.0000000	1.9031999	-26.1361599
1550.0000000	1.9520000	-26.2878456
1550.0000000	2.0007999	-26.0268078
1550.0000000	2.0495999	-26.2753029
1550.0000000	2.0983999	-69.7579041
1550.0000000	2.1471999	-15.8397942
1550.0000000	2.1960001	-25.2316704
1550.0000000	2.2448001	-24.8784637
1550.0000000	2.2935998	-24.5203896
1550.0000000	2.3423998	-21.7532940
1550.0000000	2.3912001	-1.0613937
1550.0000000	2.4400001	-22.9692478
1550.0000000	2.4888000	-23.4485760
1550.0000000	2.5375998	-13.1731358
1550.0000000	2.5863998	-11.4339094
1550.0000000	2.6352000	-9.6633854
1550.0000000	2.6840000	-16.0394421
1550.0000000	2.7328000	119.2066574
1550.0000000	2.7815998	141.0506592
1550.0000000	2.8304000	-23.7260094
1550.0000000	2.8792000	-20.7903900
1550.0000000	2.9280000	-22.5419617
1550.0000000	2.9768000	-17.2279587
1550.0000000	3.0256000	-20.6082497
1550.0000000	3.0743999	-28.9203320
1550.0000000	3.1231999	-24.0741138
1550.0000000	3.1719999	-29.1713638
1550.0000000	3.2208002	-491.7805786
1550.0000000	3.2695999	-962.3459473
1550.0000000	3.3183999	-20.5000896
1550.0000000	3.3671999	-3319.1928711
1550.0000000	3.4159999	1190.1776123
1550.0000000	3.4648001	-7.4430504
1550.0000000	3.5135999	-26.8786278
1550.0000000	3.5623999	-315.0803528
1550.0000000	3.6111999	-1977.2440186
1550.0000000	3.6600001	-447.9972534

1550.0000000	3.7088001	-591.3876343
1550.0000000	3.7575998	-925.6354980
1550.0000000	3.8063998	-1679.2945557
1550.0000000	3.8552001	-2437.8796387
1550.0000000	3.9040000	-2390.1816406
1550.0000000	3.9528000	1092.3669434
1550.0000000	4.0015998	-239.2170258
1550.0000000	4.0503998	668.5788574
1550.0000000	4.0991998	84.7454987
1550.0000000	4.1479998	-637.6297607
1550.0000000	4.1967998	1.9636555
1550.0000000	4.2456002	-242.3228760
1550.0000000	4.2943997	-475.3750610
1550.0000000	4.3431997	-13.4686298
1550.0000000	4.3920002	-241.9651947
1550.0000000	4.4407997	-22.3488007
1550.0000000	4.4896002	-15.5298023
1550.0000000	4.5383997	-638.6276245
1550.0000000	4.5871997	-12.9656601
1550.0000000	4.6360002	-21.5238876
1550.0000000	4.6847997	-28.0349579
1550.0000000	4.7336001	-19.5168934
1550.0000000	4.7824001	-16.5410156
1550.0000000	4.8311996	-1440.8222656
1550.0000000	4.8800001	222.6011200
1550.0000000	4.9287996	-197.2039642
1550.0000000	4.9776001	1034.7813721
1550.0000000	5.0264001	83.8049927
1550.0000000	5.0751996	-294.5915527
1550.0000000	5.1240001	-12.3665562
1550.0000000	5.1727996	551.1140747
1550.0000000	5.2216001	31.4484634
1550.0000000	5.2704000	-157.1863098
1550.0000000	5.3191996	-38.9188690
1550.0000000	5.3680000	-3495.7690430
1550.0000000	5.4168000	520.9494629
1550.0000000	5.4656000	-198.6460419
1550.0000000	5.5144000	37.4508438
1550.0000000	5.5631995	482.6267700
1550.0000000	5.6120000	388.5185852
1550.0000000	5.6608000	-36.8484955
1550.0000000	5.7096000	61.6387711
1550.0000000	5.7584000	131.3687592
1550.0000000	5.8071995	-11.5705681
1550.0000000	5.8559999	29.3065128
1550.0000000	5.9047999	-16.5942249
1550.0000000	5.9535999	-39.5032730
1550.0000000	6.0023999	-1555.4597168
1550.0000000	6.0511999	-14.5803385
1550.0000000	6.0999999	-19.3615646
1550.0000000	6.1487999	-13.2998466
1550.0000000	6.1975999	-23.1192265
1550.0000000	6.2463999	-1771.9937744
1550.0000000	6.2951999	-19.0238075
1550.0000000	6.3439999	-339.0572815
1550.0000000	6.3927999	-15.0470753
1550.0000000	6.4416003	-12.4193001
1550.0000000	6.4903998	-11.3019943

1550.0000000	6.5391998	-11.8447304
1550.0000000	6.5879998	346.1016235
1550.0000000	6.6367998	-12.1150589
1550.0000000	6.6856003	-13.8244705
1550.0000000	6.7343998	-13.4677362
1550.0000000	6.7831998	-14.3493652
1550.0000000	6.8319998	57.3758392
1550.0000000	6.8807998	-14.0417175
1550.0000000	6.9296002	-407.3508301
1550.0000000	6.9783998	-5.7926602
1550.0000000	7.0271997	-14.0807276
1550.0000000	7.0760002	8.8710203
1550.0000000	7.1247997	-1915.6219482
1550.0000000	7.1736002	-1536.2685547
1550.0000000	7.2223997	-874.3294678
1550.0000000	7.2711997	-189.5518341
1550.0000000	7.3200002	-18.2295265
1550.0000000	7.3687997	167.5231476
1550.0000000	7.4176002	350.8364258
1550.0000000	7.4663997	275.4151611
1550.0000000	7.5151997	-855.0216675
1550.0000000	7.5640001	-944.3228149
1550.0000000	7.6127996	-2224.3498535
1550.0000000	7.6616001	-714.7362671
1550.0000000	7.7104001	-367.6286011
1550.0000000	7.7591996	37.7602539
1550.0000000	7.8080001	364.5975037
1550.0000000	7.8567996	480.6389160
1550.0000000	7.9056001	-999.2400513
1550.0000000	7.9544001	1589.8422852
1550.0000000	8.0031996	2051.5036621
1550.0000000	8.0520000	2047.5926514
1550.0000000	8.1007996	2166.7871094
1550.0000000	8.1496000	2310.8820801
1550.0000000	8.1983995	1437.6197510
1550.0000000	8.2472000	-3289.9011230
1550.0000000	8.2959995	-1166.3347168
1550.0000000	8.3448000	-929.0062866
1550.0000000	8.3935995	-851.4326782
1550.0000000	8.4423990	-366.9324036
1550.0000000	8.4912004	-856.2826538
1550.0000000	8.5400000	-2309.4265137
1550.0000000	8.5887995	-1119.7052002
1550.0000000	8.6375999	218.2990723
1550.0000000	8.6863995	1974.7170410
1550.0000000	8.7351999	-734.2850342
1550.0000000	8.7840004	838.2901001
1550.0000000	8.8327999	44.5706482
1550.0000000	8.8815994	-11.5057507
1550.0000000	8.9303989	-23.7465401
1550.0000000	8.9792004	-11.5834970
1550.0000000	9.0279999	-5.9311662
1550.0000000	9.0767994	6.7347507
1550.0000000	9.1255999	-17.1508217
1550.0000000	9.1743994	-10.0949678
1550.0000000	9.2231998	36.1755447
1550.0000000	9.2720003	-8.4698267
1550.0000000	9.3207998	-9.8267241

1550.0000000	9.3695993	-10.7757816
1550.0000000	9.4183998	-11.7472763
1550.0000000	9.4672003	-11.1635141
1550.0000000	9.5159998	-14.1169844
1550.0000000	9.5648003	-0.5718288
1550.0000000	9.6135998	-13.3541021
1550.0000000	9.6623993	-10.2359114
1550.0000000	9.7111998	-10.1720991
1550.0000000	9.7600002	-10.8597860
1550.0000000	9.8087997	-10.9952002
1550.0000000	9.8575993	2.9555168
1550.0000000	9.9063997	-8.0174999
1550.0000000	9.9552002	-10.9068298
1550.0000000	10.0039997	-10.0878391
1560.0000000	0.3416000	-36.6918869
1560.0000000	0.3904000	-39.9196587
1560.0000000	0.4392000	-39.3991318
1560.0000000	0.4880000	-39.5259628
1560.0000000	0.5368000	-39.6771355
1560.0000000	0.5856000	-39.2580528
1560.0000000	0.6344000	-38.2422104
1560.0000000	0.6832000	-36.2860680
1560.0000000	0.7320000	-36.1124649
1560.0000000	0.7808000	-36.5395470
1560.0000000	0.8296000	-35.9134216
1560.0000000	0.8784000	-34.9233894
1560.0000000	0.9272000	-34.2584000
1560.0000000	0.9760000	-31.8936348
1560.0000000	1.0247999	-30.3310280
1560.0000000	1.0735999	-30.2459736
1560.0000000	1.1224000	-30.6962948
1560.0000000	1.1711999	-31.2515240
1560.0000000	1.2200000	-30.1861496
1560.0000000	1.2687999	-30.4988384
1560.0000000	1.3176000	-30.8533764
1560.0000000	1.3664000	-30.5830307
1560.0000000	1.4152000	-30.1733532
1560.0000000	1.4640000	-29.5230064
1560.0000000	1.5128000	-34.1276245
1560.0000000	1.5616000	-31.3708572
1560.0000000	1.6104001	-1419.9056396
1560.0000000	1.6592000	-57.3858719
1560.0000000	1.7079999	-27.4598846
1560.0000000	1.7567999	-27.7631760
1560.0000000	1.8055999	-26.2673779
1560.0000000	1.8544000	-25.9689465
1560.0000000	1.9031999	-26.9795723
1560.0000000	1.9520000	-26.4878559
1560.0000000	2.0007999	-26.0321865
1560.0000000	2.0495999	-26.2773228
1560.0000000	2.0983999	-68.2821274
1560.0000000	2.1471999	-16.1070614
1560.0000000	2.1960001	-25.2275791
1560.0000000	2.2448001	-24.8605518
1560.0000000	2.2935998	-24.4607658
1560.0000000	2.3423998	-21.7676983
1560.0000000	2.3912001	-1.6829395
1560.0000000	2.4400001	-22.9717350

1560.0000000	2.4888000	-23.4483585
1560.0000000	2.5375998	-13.1902437
1560.0000000	2.5863998	-11.4536686
1560.0000000	2.6352000	-9.6861296
1560.0000000	2.6840000	-16.0513840
1560.0000000	2.7328000	118.9406357
1560.0000000	2.7815998	140.7442627
1560.0000000	2.8304000	-23.7247047
1560.0000000	2.8792000	-20.7936535
1560.0000000	2.9280000	-22.5413914
1560.0000000	2.9768000	-17.2363300
1560.0000000	3.0256000	-20.6108341
1560.0000000	3.0743999	-28.9089451
1560.0000000	3.1231999	-24.0706596
1560.0000000	3.1719999	-29.1630707
1560.0000000	3.2208002	-491.5428467
1560.0000000	3.2695999	-961.8709717
1560.0000000	3.3183999	-20.5002880
1560.0000000	3.3671999	-3317.7312012
1560.0000000	3.4159999	1189.6423340
1560.0000000	3.4648001	-7.4487591
1560.0000000	3.5135999	-26.8756371
1560.0000000	3.5623999	-314.9507751
1560.0000000	3.6111999	-1976.3841553
1560.0000000	3.6600001	-447.8091431
1560.0000000	3.7088001	-591.1362305
1560.0000000	3.7575998	-925.2369385
1560.0000000	3.8063998	-1678.5646973
1560.0000000	3.8552001	-2436.8164063
1560.0000000	3.9040000	-2389.1391602
1560.0000000	3.9528000	1091.8786621
1560.0000000	4.0015998	-239.1199188
1560.0000000	4.0503998	668.2763062
1560.0000000	4.0991998	84.6998138
1560.0000000	4.1479998	-637.3575439
1560.0000000	4.1967998	1.9543991
1560.0000000	4.2456002	-242.2245789
1560.0000000	4.2943997	-475.1744995
1560.0000000	4.3431997	-13.4708099
1560.0000000	4.3920002	-241.8670807
1560.0000000	4.4407997	-22.3470268
1560.0000000	4.4896002	-15.5306845
1560.0000000	4.5383997	-638.3665771
1560.0000000	4.5871997	-12.9676762
1560.0000000	4.6360002	-21.5223446
1560.0000000	4.6847997	-28.0310326
1560.0000000	4.7336001	-19.5161724
1560.0000000	4.7824001	-16.5414715
1560.0000000	4.8311996	-1440.2910156
1560.0000000	4.8800001	222.5115662
1560.0000000	4.9287996	-197.1367035
1560.0000000	4.9776001	1034.3889160
1560.0000000	5.0264001	83.7674713
1560.0000000	5.0751996	-294.4879150
1560.0000000	5.1240001	-12.3674774
1560.0000000	5.1727996	550.9981689
1560.0000000	5.2216001	31.4390182
1560.0000000	5.2704000	-157.1582031

1560.0000000	5.3191996	-38.9143219
1560.0000000	5.3680000	-3495.0766602
1560.0000000	5.4168000	520.8427124
1560.0000000	5.4656000	-198.6098480
1560.0000000	5.5144000	37.4401855
1560.0000000	5.5631995	482.5281982
1560.0000000	5.6120000	388.4385986
1560.0000000	5.6608000	-36.8444061
1560.0000000	5.7096000	61.6233444
1560.0000000	5.7584000	131.3398285
1560.0000000	5.8071995	-11.5714397
1560.0000000	5.8559999	29.2976589
1560.0000000	5.9047999	-16.5940266
1560.0000000	5.9535999	-39.4985352
1560.0000000	6.0023999	-1555.1553955
1560.0000000	6.0511999	-14.5805502
1560.0000000	6.0999999	-19.3607330
1560.0000000	6.1487999	-13.3003101
1560.0000000	6.1975999	-23.1174603
1560.0000000	6.2463999	-1771.6464844
1560.0000000	6.2951999	-19.0229931
1560.0000000	6.3439999	-338.9932251
1560.0000000	6.3927999	-15.0470572
1560.0000000	6.4416003	-12.4198866
1560.0000000	6.4903998	-11.3026695
1560.0000000	6.5391998	-11.8454113
1560.0000000	6.5879998	345.4109192
1560.0000000	6.6367998	-12.1156082
1560.0000000	6.6856003	-13.8246412
1560.0000000	6.7343998	-13.4679518
1560.0000000	6.7831998	-14.3489494
1560.0000000	6.8319998	57.3567200
1560.0000000	6.8807998	-14.0415792
1560.0000000	6.9296002	-406.5893860
1560.0000000	6.9783998	-5.8054833
1560.0000000	7.0271997	-14.0797005
1560.0000000	7.0760002	8.8288336
1560.0000000	7.1247997	-1911.6760254
1560.0000000	7.1736002	-1533.1057129
1560.0000000	7.2223997	-872.5363159
1560.0000000	7.2711997	-189.1840515
1560.0000000	7.3200002	-18.2175369
1560.0000000	7.3687997	167.1486206
1560.0000000	7.4176002	350.0805969
1560.0000000	7.4663997	274.8175049
1560.0000000	7.5151997	-853.2606201
1560.0000000	7.5640001	-942.3753662
1560.0000000	7.6127996	-2219.7321777
1560.0000000	7.6616001	-713.2648926
1560.0000000	7.7104001	-366.8823242
1560.0000000	7.7591996	37.6592064
1560.0000000	7.8080001	363.8121643
1560.0000000	7.8567996	479.6108093
1560.0000000	7.9056001	-997.1671143
1560.0000000	7.9544001	1586.5323486
1560.0000000	8.0031996	2047.2341309
1560.0000000	8.0520000	2043.3305664
1560.0000000	8.1007996	2162.2775879

1560.0000000	8.1496000	2306.0739746
1560.0000000	8.1983995	1434.6271973
1560.0000000	8.2472000	-3283.0661621
1560.0000000	8.2959995	-1163.9145508
1560.0000000	8.3448000	-927.0770874
1560.0000000	8.3935995	-849.6649780
1560.0000000	8.4423990	-366.1744080
1560.0000000	8.4912004	-854.5064697
1560.0000000	8.5400000	-2304.6267090
1560.0000000	8.5887995	-1117.3836670
1560.0000000	8.6375999	217.8377533
1560.0000000	8.6863995	1970.5987549
1560.0000000	8.7351999	-732.7608032
1560.0000000	8.7840004	836.5333252
1560.0000000	8.8327999	44.4590378
1560.0000000	8.8815994	-11.5059252
1560.0000000	8.9303989	-23.7428207
1560.0000000	8.9792004	-11.5835791
1560.0000000	9.0279999	-5.9329348
1560.0000000	9.0767994	6.7290325
1560.0000000	9.1255999	-17.1491089
1560.0000000	9.1743994	-10.0953693
1560.0000000	9.2231998	36.1598129
1560.0000000	9.2720003	-8.4707632
1560.0000000	9.3207998	-9.8272629
1560.0000000	9.3695993	-10.7760086
1560.0000000	9.4183998	-11.7471514
1560.0000000	9.4672003	-11.1635551
1560.0000000	9.5159998	-14.1160936
1560.0000000	9.5648003	-0.5752010
1560.0000000	9.6135998	-13.3533115
1560.0000000	9.6623993	-10.2361860
1560.0000000	9.7111998	-10.1723709
1560.0000000	9.7600002	-10.8598280
1560.0000000	9.8087997	-10.9952078
1560.0000000	9.8575993	2.9510946
1560.0000000	9.9063997	-8.0184135
1560.0000000	9.9552002	-10.9068346
1560.0000000	10.0039997	-10.0880871
1570.0000000	0.3416000	-37.1444397
1570.0000000	0.3904000	-39.8748550
1570.0000000	0.4392000	-39.3927879
1570.0000000	0.4880000	-39.5247536
1570.0000000	0.5368000	-39.6783104
1570.0000000	0.5856000	-39.2566719
1570.0000000	0.6344000	-38.2425194
1570.0000000	0.6832000	-36.2882957
1570.0000000	0.7320000	-36.1145172
1570.0000000	0.7808000	-36.5397186
1570.0000000	0.8296000	-35.9115791
1570.0000000	0.8784000	-34.9138260
1570.0000000	0.9272000	-34.2590446
1570.0000000	0.9760000	-31.8911114
1570.0000000	1.0247999	-30.3322620
1570.0000000	1.0735999	-30.2595749
1570.0000000	1.1224000	-30.6944218
1570.0000000	1.1711999	-31.2452221
1570.0000000	1.2200000	-30.1423512

1570.0000000	1.2687999	-30.4775124
1570.0000000	1.3176000	-30.8469906
1570.0000000	1.3664000	-30.5800858
1570.0000000	1.4152000	-30.1735573
1570.0000000	1.4640000	-29.5212975
1570.0000000	1.5128000	-34.0304489
1570.0000000	1.5616000	-31.3290157
1570.0000000	1.6104001	-1417.3629150
1570.0000000	1.6592000	-57.0919418
1570.0000000	1.7079999	-27.4636326
1570.0000000	1.7567999	-27.7631950
1570.0000000	1.8055999	-26.8852177
1570.0000000	1.8544000	-26.3239613
1570.0000000	1.9031999	-26.1797562
1570.0000000	1.9520000	-26.6878643
1570.0000000	2.0007999	-26.0375671
1570.0000000	2.0495999	-26.2793407
1570.0000000	2.0983999	-66.8063507
1570.0000000	2.1471999	-16.3743286
1570.0000000	2.1960001	-25.2234898
1570.0000000	2.2448001	-24.8426399
1570.0000000	2.2935998	-24.4011421
1570.0000000	2.3423998	-21.7821026
1570.0000000	2.3912001	-2.3044853
1570.0000000	2.4400001	-22.9742203
1570.0000000	2.4888000	-23.4481411
1570.0000000	2.5375998	-13.2073517
1570.0000000	2.5863998	-11.4734278
1570.0000000	2.6352000	-9.7088747
1570.0000000	2.6840000	-16.0633259
1570.0000000	2.7328000	118.6746140
1570.0000000	2.7815998	140.4378357
1570.0000000	2.8304000	-23.7234001
1570.0000000	2.8792000	-20.7969170
1570.0000000	2.9280000	-22.5408230
1570.0000000	2.9768000	-17.2447014
1570.0000000	3.0256000	-20.6134186
1570.0000000	3.0743999	-28.8975582
1570.0000000	3.1231999	-24.0672035
1570.0000000	3.1719999	-29.1547775
1570.0000000	3.2208002	-491.3051147
1570.0000000	3.2695999	-961.3959961
1570.0000000	3.3183999	-20.5004864
1570.0000000	3.3671999	-3316.2695313
1570.0000000	3.4159999	1189.1070557
1570.0000000	3.4648001	-7.4544668
1570.0000000	3.5135999	-26.8726463
1570.0000000	3.5623999	-314.8211670
1570.0000000	3.6111999	-1975.5244141
1570.0000000	3.6600001	-447.6210327
1570.0000000	3.7088001	-590.8848267
1570.0000000	3.7575998	-924.8383789
1570.0000000	3.8063998	-1677.8348389
1570.0000000	3.8552001	-2435.7529297
1570.0000000	3.9040000	-2388.0966797
1570.0000000	3.9528000	1091.3903809
1570.0000000	4.0015998	-239.0228119
1570.0000000	4.0503998	667.9737549

1570.0000000	4.0991998	84.6541290
1570.0000000	4.1479998	-637.0853271
1570.0000000	4.1967998	1.9451427
1570.0000000	4.2456002	-242.1262665
1570.0000000	4.2943997	-474.9739075
1570.0000000	4.3431997	-13.4729900
1570.0000000	4.3920002	-241.7689667
1570.0000000	4.4407997	-22.3452511
1570.0000000	4.4896002	-15.5315666
1570.0000000	4.5383997	-638.1054688
1570.0000000	4.5871997	-12.9696922
1570.0000000	4.6360002	-21.5208035
1570.0000000	4.6847997	-28.0271072
1570.0000000	4.7336001	-19.5154495
1570.0000000	4.7824001	-16.5419254
1570.0000000	4.8311996	-1439.7597656
1570.0000000	4.8800001	222.4220276
1570.0000000	4.9287996	-197.0694427
1570.0000000	4.9776001	1033.9964600
1570.0000000	5.0264001	83.7299500
1570.0000000	5.0751996	-294.3842773
1570.0000000	5.1240001	-12.3683987
1570.0000000	5.1727996	550.8823242
1570.0000000	5.2216001	31.4295769
1570.0000000	5.2704000	-157.1300964
1570.0000000	5.3191996	-38.9097710
1570.0000000	5.3680000	-3494.3842773
1570.0000000	5.4168000	520.7359009
1570.0000000	5.4656000	-198.5736694
1570.0000000	5.5144000	37.4295273
1570.0000000	5.5631995	482.4296265
1570.0000000	5.6120000	388.3586426
1570.0000000	5.6608000	-36.8403168
1570.0000000	5.7096000	61.6079178
1570.0000000	5.7584000	131.3108978
1570.0000000	5.8071995	-11.5723095
1570.0000000	5.8559999	29.2888088
1570.0000000	5.9047999	-16.5938282
1570.0000000	5.9535999	-39.4937973
1570.0000000	6.0023999	-1554.8508301
1570.0000000	6.0511999	-14.5807610
1570.0000000	6.0999999	-19.3598995
1570.0000000	6.1487999	-13.3007717
1570.0000000	6.1975999	-23.1156960
1570.0000000	6.2463999	-1771.2990723
1570.0000000	6.2951999	-19.0221786
1570.0000000	6.3439999	-338.9291687
1570.0000000	6.3927999	-15.0470390
1570.0000000	6.4416003	-12.4204741
1570.0000000	6.4903998	-11.3033457
1570.0000000	6.5391998	-11.8460922
1570.0000000	6.5879998	344.7202148
1570.0000000	6.6367998	-12.1161575
1570.0000000	6.6856003	-13.8248110
1570.0000000	6.7343998	-13.4681673
1570.0000000	6.7831998	-14.3485346
1570.0000000	6.8319998	57.3376083
1570.0000000	6.8807998	-14.0414400

1570.0000000	6.9296002	-405.8279419
1570.0000000	6.9783998	-5.8183074
1570.0000000	7.0271997	-14.0786724
1570.0000000	7.0760002	8.7866507
1570.0000000	7.1247997	-1907.7302246
1570.0000000	7.1736002	-1529.9428711
1570.0000000	7.2223997	-870.7431641
1570.0000000	7.2711997	-188.8162842
1570.0000000	7.3200002	-18.2055473
1570.0000000	7.3687997	166.7740784
1570.0000000	7.4176002	349.3247681
1570.0000000	7.4663997	274.2198486
1570.0000000	7.5151997	-851.4995728
1570.0000000	7.5640001	-940.4279785
1570.0000000	7.6127996	-2215.1145020
1570.0000000	7.6616001	-711.7935791
1570.0000000	7.7104001	-366.1360474
1570.0000000	7.7591996	37.5581551
1570.0000000	7.8080001	363.0267944
1570.0000000	7.8567996	478.5827332
1570.0000000	7.9056001	-995.0942383
1570.0000000	7.9544001	1583.2224121
1570.0000000	8.0031996	2042.9644775
1570.0000000	8.0520000	2039.0684814
1570.0000000	8.1007996	2157.7680664
1570.0000000	8.1496000	2301.2661133
1570.0000000	8.1983995	1431.6346436
1570.0000000	8.2472000	-3276.2314453
1570.0000000	8.2959995	-1161.4943848
1570.0000000	8.3448000	-925.1478882
1570.0000000	8.3935995	-847.8973389
1570.0000000	8.4423990	-365.4163818
1570.0000000	8.4912004	-852.7302246
1570.0000000	8.5400000	-2299.8269043
1570.0000000	8.5887995	-1115.0621338
1570.0000000	8.6375999	217.3764191
1570.0000000	8.6863995	1966.4803467
1570.0000000	8.7351999	-731.2365112
1570.0000000	8.7840004	834.7765503
1570.0000000	8.8327999	44.3474312
1570.0000000	8.8815994	-11.5060997
1570.0000000	8.9303989	-23.7390995
1570.0000000	8.9792004	-11.5836620
1570.0000000	9.0279999	-5.9347034
1570.0000000	9.0767994	6.7233162
1570.0000000	9.1255999	-17.1473942
1570.0000000	9.1743994	-10.0957718
1570.0000000	9.2231998	36.1440811
1570.0000000	9.2720003	-8.4716997
1570.0000000	9.3207998	-9.8278027
1570.0000000	9.3695993	-10.7762346
1570.0000000	9.4183998	-11.7470264
1570.0000000	9.4672003	-11.1635971
1570.0000000	9.5159998	-14.1152020
1570.0000000	9.5648003	-0.5785742
1570.0000000	9.6135998	-13.3525209
1570.0000000	9.6623993	-10.2364607
1570.0000000	9.7111998	-10.1726427

1570.0000000	9.7600002	-10.8598690
1570.0000000	9.8087997	-10.9952164
1570.0000000	9.8575993	2.9466724
1570.0000000	9.9063997	-8.0193272
1570.0000000	9.9552002	-10.9068384
1570.0000000	10.0039997	-10.0883341
1580.0000000	0.3416000	-36.8575554
1580.0000000	0.3904000	-39.8300514
1580.0000000	0.4392000	-39.3864441
1580.0000000	0.4880000	-39.5235443
1580.0000000	0.5368000	-39.6794853
1580.0000000	0.5856000	-39.2552872
1580.0000000	0.6344000	-38.2428322
1580.0000000	0.6832000	-36.2905197
1580.0000000	0.7320000	-36.1165695
1580.0000000	0.7808000	-36.5398865
1580.0000000	0.8296000	-35.9097328
1580.0000000	0.8784000	-34.9042587
1580.0000000	0.9272000	-34.2596855
1580.0000000	0.9760000	-31.8885880
1580.0000000	1.0247999	-30.3334980
1580.0000000	1.0735999	-30.2731743
1580.0000000	1.1224000	-30.6925507
1580.0000000	1.1711999	-31.2389183
1580.0000000	1.2200000	-30.0985527
1580.0000000	1.2687999	-30.4561844
1580.0000000	1.3176000	-30.8406048
1580.0000000	1.3664000	-30.5771427
1580.0000000	1.4152000	-30.1737633
1580.0000000	1.4640000	-29.5195885
1580.0000000	1.5128000	-33.9332695
1580.0000000	1.5616000	-31.2871742
1580.0000000	1.6104001	-1414.8200684
1580.0000000	1.6592000	-56.7980080
1580.0000000	1.7079999	-27.4673786
1580.0000000	1.7567999	-27.7632141
1580.0000000	1.8055999	-26.7917881
1580.0000000	1.8544000	-25.7722626
1580.0000000	1.9031999	-25.6175251
1580.0000000	1.9520000	-26.5565929
1580.0000000	2.0007999	-26.0429478
1580.0000000	2.0495999	-26.2813606
1580.0000000	2.0983999	-65.3305740
1580.0000000	2.1471999	-16.6415958
1580.0000000	2.1960001	-25.2193985
1580.0000000	2.2448001	-24.8247299
1580.0000000	2.2935998	-24.3415184
1580.0000000	2.3423998	-21.7965088
1580.0000000	2.3912001	-2.9260311
1580.0000000	2.4400001	-22.9767075
1580.0000000	2.4888000	-23.4479256
1580.0000000	2.5375998	-13.2244596
1580.0000000	2.5863998	-11.4931870
1580.0000000	2.6352000	-9.7316189
1580.0000000	2.6840000	-16.0752678
1580.0000000	2.7328000	118.4086075
1580.0000000	2.7815998	140.1314087
1580.0000000	2.8304000	-23.7220974

1580.0000000	2.8792000	-20.8001804
1580.0000000	2.9280000	-22.5402546
1580.0000000	2.9768000	-17.2530708
1580.0000000	3.0256000	-20.6160030
1580.0000000	3.0743999	-28.8861713
1580.0000000	3.1231999	-24.0637474
1580.0000000	3.1719999	-29.1464844
1580.0000000	3.2208002	-491.0673828
1580.0000000	3.2695999	-960.9210205
1580.0000000	3.3183999	-20.5006847
1580.0000000	3.3671999	-3314.8078613
1580.0000000	3.4159999	1188.5717773
1580.0000000	3.4648001	-7.4601755
1580.0000000	3.5135999	-26.8696556
1580.0000000	3.5623999	-314.6915894
1580.0000000	3.6111999	-1974.6646729
1580.0000000	3.6600001	-447.4328613
1580.0000000	3.7088001	-590.6334839
1580.0000000	3.7575998	-924.4398804
1580.0000000	3.8063998	-1677.1049805
1580.0000000	3.8552001	-2434.6894531
1580.0000000	3.9040000	-2387.0539551
1580.0000000	3.9528000	1090.9019775
1580.0000000	4.0015998	-238.9257050
1580.0000000	4.0503998	667.6712036
1580.0000000	4.0991998	84.6084442
1580.0000000	4.1479998	-636.8131104
1580.0000000	4.1967998	1.9358864
1580.0000000	4.2456002	-242.0279694
1580.0000000	4.2943997	-474.7733459
1580.0000000	4.3431997	-13.4751701
1580.0000000	4.3920002	-241.6708527
1580.0000000	4.4407997	-22.3434753
1580.0000000	4.4896002	-15.5324497
1580.0000000	4.5383997	-637.8444214
1580.0000000	4.5871997	-12.9717083
1580.0000000	4.6360002	-21.5192604
1580.0000000	4.6847997	-28.0231819
1580.0000000	4.7336001	-19.5147266
1580.0000000	4.7824001	-16.5423794
1580.0000000	4.8311996	-1439.2285156
1580.0000000	4.8800001	222.3324738
1580.0000000	4.9287996	-197.0021667
1580.0000000	4.9776001	1033.6038818
1580.0000000	5.0264001	83.6924362
1580.0000000	5.0751996	-294.2806702
1580.0000000	5.1240001	-12.3693199
1580.0000000	5.1727996	550.7664795
1580.0000000	5.2216001	31.4201317
1580.0000000	5.2704000	-157.1019897
1580.0000000	5.3191996	-38.9052200
1580.0000000	5.3680000	-3493.6918945
1580.0000000	5.4168000	520.6290894
1580.0000000	5.4656000	-198.5374908
1580.0000000	5.5144000	37.4188766
1580.0000000	5.5631995	482.3310547
1580.0000000	5.6120000	388.2786560
1580.0000000	5.6608000	-36.8362274

1580.0000000	5.7096000	61.5924835
1580.0000000	5.7584000	131.2819519
1580.0000000	5.8071995	-11.5731812
1580.0000000	5.8559999	29.2799587
1580.0000000	5.9047999	-16.5936317
1580.0000000	5.9535999	-39.4890594
1580.0000000	6.0023999	-1554.5465088
1580.0000000	6.0511999	-14.5809727
1580.0000000	6.0999999	-19.3590679
1580.0000000	6.1487999	-13.3012352
1580.0000000	6.1975999	-23.1139297
1580.0000000	6.2463999	-1770.9516602
1580.0000000	6.2951999	-19.0213642
1580.0000000	6.3439999	-338.8651428
1580.0000000	6.3927999	-15.0470209
1580.0000000	6.4416003	-12.4210606
1580.0000000	6.4903998	-11.3040218
1580.0000000	6.5391998	-11.8467741
1580.0000000	6.5879998	344.0295410
1580.0000000	6.6367998	-12.1167068
1580.0000000	6.6856003	-13.8249817
1580.0000000	6.7343998	-13.4683828
1580.0000000	6.7831998	-14.3481188
1580.0000000	6.8319998	57.3184891
1580.0000000	6.8807998	-14.0413017
1580.0000000	6.9296002	-405.0664673
1580.0000000	6.9783998	-5.8311310
1580.0000000	7.0271997	-14.0776443
1580.0000000	7.0760002	8.7444639
1580.0000000	7.1247997	-1903.7843018
1580.0000000	7.1736002	-1526.7800293
1580.0000000	7.2223997	-868.9499512
1580.0000000	7.2711997	-188.4485016
1580.0000000	7.3200002	-18.1935596
1580.0000000	7.3687997	166.3995514
1580.0000000	7.4176002	348.5689392
1580.0000000	7.4663997	273.6222229
1580.0000000	7.5151997	-849.7385254
1580.0000000	7.5640001	-938.4805908
1580.0000000	7.6127996	-2210.4970703
1580.0000000	7.6616001	-710.3222046
1580.0000000	7.7104001	-365.3897705
1580.0000000	7.7591996	37.4571075
1580.0000000	7.8080001	362.2414246
1580.0000000	7.8567996	477.5546570
1580.0000000	7.9056001	-993.0213013
1580.0000000	7.9544001	1579.9124756
1580.0000000	8.0031996	2038.6949463
1580.0000000	8.0520000	2034.8065186
1580.0000000	8.1007996	2153.2585449
1580.0000000	8.1496000	2296.4580078
1580.0000000	8.1983995	1428.6422119
1580.0000000	8.2472000	-3269.3967285
1580.0000000	8.2959995	-1159.0742188
1580.0000000	8.3448000	-923.2187500
1580.0000000	8.3935995	-846.1296387
1580.0000000	8.4423990	-364.6583862
1580.0000000	8.4912004	-850.9540405

1580.0000000	8.5400000	-2295.0270996
1580.0000000	8.5887995	-1112.7406006
1580.0000000	8.6375999	216.9151001
1580.0000000	8.6863995	1962.3620605
1580.0000000	8.7351999	-729.7122803
1580.0000000	8.7840004	833.0198364
1580.0000000	8.8327999	44.2358284
1580.0000000	8.8815994	-11.5062733
1580.0000000	8.9303989	-23.7353783
1580.0000000	8.9792004	-11.5837440
1580.0000000	9.0279999	-5.9364710
1580.0000000	9.0767994	6.7175980
1580.0000000	9.1255999	-17.1456814
1580.0000000	9.1743994	-10.0961733
1580.0000000	9.2231998	36.1283417
1580.0000000	9.2720003	-8.4726353
1580.0000000	9.3207998	-9.8283415
1580.0000000	9.3695993	-10.7764616
1580.0000000	9.4183998	-11.7469025
1580.0000000	9.4672003	-11.1636381
1580.0000000	9.5159998	-14.1143112
1580.0000000	9.5648003	-0.5819473
1580.0000000	9.6135998	-13.3517303
1580.0000000	9.6623993	-10.2367363
1580.0000000	9.7111998	-10.1729145
1580.0000000	9.7600002	-10.8599110
1580.0000000	9.8087997	-10.9952240
1580.0000000	9.8575993	2.9422493
1580.0000000	9.9063997	-8.0202408
1580.0000000	9.9552002	-10.9068432
1580.0000000	10.0039997	-10.0885820
1590.0000000	0.3416000	-36.9420547
1590.0000000	0.3904000	-39.7852440
1590.0000000	0.4392000	-39.3801041
1590.0000000	0.4880000	-39.5223351
1590.0000000	0.5368000	-39.6806564
1590.0000000	0.5856000	-39.2539063
1590.0000000	0.6344000	-38.2431412
1590.0000000	0.6832000	-36.2927475
1590.0000000	0.7320000	-36.1186256
1590.0000000	0.7808000	-36.5400543
1590.0000000	0.8296000	-35.9078865
1590.0000000	0.8784000	-34.8946953
1590.0000000	0.9272000	-34.2603264
1590.0000000	0.9760000	-31.8860645
1590.0000000	1.0247999	-30.3347340
1590.0000000	1.0735999	-30.2867756
1590.0000000	1.1224000	-30.6906776
1590.0000000	1.1711999	-31.2326164
1590.0000000	1.2200000	-30.0547543
1590.0000000	1.2687999	-30.4348583
1590.0000000	1.3176000	-30.8342190
1590.0000000	1.3664000	-30.5741997
1590.0000000	1.4152000	-30.1739674
1590.0000000	1.4640000	-29.5178776
1590.0000000	1.5128000	-33.8360939
1590.0000000	1.5616000	-31.2453346
1590.0000000	1.6104001	-1412.2773438

1590.0000000	1.6592000	-56.5040741
1590.0000000	1.7079999	-27.4711266
1590.0000000	1.7567999	-27.7632351
1590.0000000	1.8055999	-26.7536850
1590.0000000	1.8544000	-25.8396358
1590.0000000	1.9031999	-25.5170670
1590.0000000	1.9520000	-25.9116592
1590.0000000	2.0007999	-26.0483284
1590.0000000	2.0495999	-26.2833786
1590.0000000	2.0983999	-63.8547935
1590.0000000	2.1471999	-16.9088631
1590.0000000	2.1960001	-25.2153072
1590.0000000	2.2448001	-24.8068180
1590.0000000	2.2935998	-24.2818947
1590.0000000	2.3423998	-21.8109131
1590.0000000	2.3912001	-3.5475769
1590.0000000	2.4400001	-22.9791946
1590.0000000	2.4888000	-23.4477081
1590.0000000	2.5375998	-13.2415676
1590.0000000	2.5863998	-11.5129452
1590.0000000	2.6352000	-9.7543631
1590.0000000	2.6840000	-16.0872097
1590.0000000	2.7328000	118.1425858
1590.0000000	2.7815998	139.8250122
1590.0000000	2.8304000	-23.7207928
1590.0000000	2.8792000	-20.8034439
1590.0000000	2.9280000	-22.5396843
1590.0000000	2.9768000	-17.2614403
1590.0000000	3.0256000	-20.6185875
1590.0000000	3.0743999	-28.8747845
1590.0000000	3.1231999	-24.0602932
1590.0000000	3.1719999	-29.1381912
1590.0000000	3.2208002	-490.8296204
1590.0000000	3.2695999	-960.4460449
1590.0000000	3.3183999	-20.5008812
1590.0000000	3.3671999	-3313.3461914
1590.0000000	3.4159999	1188.0364990
1590.0000000	3.4648001	-7.4658842
1590.0000000	3.5135999	-26.8666649
1590.0000000	3.5623999	-314.5619812
1590.0000000	3.6111999	-1973.8049316
1590.0000000	3.6600001	-447.2447510
1590.0000000	3.7088001	-590.3820801
1590.0000000	3.7575998	-924.0413208
1590.0000000	3.8063998	-1676.3751221
1590.0000000	3.8552001	-2433.6259766
1590.0000000	3.9040000	-2386.0114746
1590.0000000	3.9528000	1090.4136963
1590.0000000	4.0015998	-238.8285980
1590.0000000	4.0503998	667.3686523
1590.0000000	4.0991998	84.5627594
1590.0000000	4.1479998	-636.5408325
1590.0000000	4.1967998	1.9266300
1590.0000000	4.2456002	-241.9296722
1590.0000000	4.2943997	-474.5727844
1590.0000000	4.3431997	-13.4773521
1590.0000000	4.3920002	-241.5727386
1590.0000000	4.4407997	-22.3416996

1590.0000000	4.4896002	-15.5333328
1590.0000000	4.5383997	-637.5833130
1590.0000000	4.5871997	-12.9737244
1590.0000000	4.6360002	-21.5177174
1590.0000000	4.6847997	-28.0192566
1590.0000000	4.7336001	-19.5140038
1590.0000000	4.7824001	-16.5428352
1590.0000000	4.8311996	-1438.6972656
1590.0000000	4.8800001	222.2429199
1590.0000000	4.9287996	-196.9349060
1590.0000000	4.9776001	1033.2114258
1590.0000000	5.0264001	83.6549149
1590.0000000	5.0751996	-294.1770325
1590.0000000	5.1240001	-12.3702412
1590.0000000	5.1727996	550.6505737
1590.0000000	5.2216001	31.4106865
1590.0000000	5.2704000	-157.0738831
1590.0000000	5.3191996	-38.9006729
1590.0000000	5.3680000	-3492.9995117
1590.0000000	5.4168000	520.5223389
1590.0000000	5.4656000	-198.5012970
1590.0000000	5.5144000	37.4082184
1590.0000000	5.5631995	482.2324829
1590.0000000	5.6120000	388.1986694
1590.0000000	5.6608000	-36.8321419
1590.0000000	5.7096000	61.5770493
1590.0000000	5.7584000	131.2530212
1590.0000000	5.8071995	-11.5740509
1590.0000000	5.8559999	29.2711048
1590.0000000	5.9047999	-16.5934334
1590.0000000	5.9535999	-39.4843178
1590.0000000	6.0023999	-1554.2420654
1590.0000000	6.0511999	-14.5811844
1590.0000000	6.0999999	-19.3582344
1590.0000000	6.1487999	-13.3016977
1590.0000000	6.1975999	-23.1121655
1590.0000000	6.2463999	-1770.6043701
1590.0000000	6.2951999	-19.0205498
1590.0000000	6.3439999	-338.8010864
1590.0000000	6.3927999	-15.0470018
1590.0000000	6.4416003	-12.4216480
1590.0000000	6.4903998	-11.3046970
1590.0000000	6.5391998	-11.8474550
1590.0000000	6.5879998	343.3388367
1590.0000000	6.6367998	-12.1172552
1590.0000000	6.6856003	-13.8251514
1590.0000000	6.7343998	-13.4685993
1590.0000000	6.7831998	-14.3477030
1590.0000000	6.8319998	57.2993774
1590.0000000	6.8807998	-14.0411625
1590.0000000	6.9296002	-404.3050232
1590.0000000	6.9783998	-5.8439546
1590.0000000	7.0271997	-14.0766163
1590.0000000	7.0760002	8.7022772
1590.0000000	7.1247997	-1899.8385010
1590.0000000	7.1736002	-1523.6171875
1590.0000000	7.2223997	-867.1567993
1590.0000000	7.2711997	-188.0807190

1590.0000000	7.3200002	-18.1815720
1590.0000000	7.3687997	166.0250244
1590.0000000	7.4176002	347.8131104
1590.0000000	7.4663997	273.0245667
1590.0000000	7.5151997	-847.9774780
1590.0000000	7.5640001	-936.5332031
1590.0000000	7.6127996	-2205.8793945
1590.0000000	7.6616001	-708.8509521
1590.0000000	7.7104001	-364.6435547
1590.0000000	7.7591996	37.3560600
1590.0000000	7.8080001	361.4560852
1590.0000000	7.8567996	476.5265503
1590.0000000	7.9056001	-990.9484253
1590.0000000	7.9544001	1576.6025391
1590.0000000	8.0031996	2034.4252930
1590.0000000	8.0520000	2030.5445557
1590.0000000	8.1007996	2148.7492676
1590.0000000	8.1496000	2291.6499023
1590.0000000	8.1983995	1425.6496582
1590.0000000	8.2472000	-3262.5617676
1590.0000000	8.2959995	-1156.6540527
1590.0000000	8.3448000	-921.2895508
1590.0000000	8.3935995	-844.3619385
1590.0000000	8.4423990	-363.9003601
1590.0000000	8.4912004	-849.1777954
1590.0000000	8.5400000	-2290.2272949
1590.0000000	8.5887995	-1110.4190674
1590.0000000	8.6375999	216.4537811
1590.0000000	8.6863995	1958.2436523
1590.0000000	8.7351999	-728.1880493
1590.0000000	8.7840004	831.2630615
1590.0000000	8.8327999	44.1242180
1590.0000000	8.8815994	-11.5064478
1590.0000000	8.9303989	-23.7316589
1590.0000000	8.9792004	-11.5838261
1590.0000000	9.0279999	-5.9382396
1590.0000000	9.0767994	6.7118797
1590.0000000	9.1255999	-17.1439667
1590.0000000	9.1743994	-10.0965757
1590.0000000	9.2231998	36.1126099
1590.0000000	9.2720003	-8.4735718
1590.0000000	9.3207998	-9.8288813
1590.0000000	9.3695993	-10.7766876
1590.0000000	9.4183998	-11.7467775
1590.0000000	9.4672003	-11.1636791
1590.0000000	9.5159998	-14.1134205
1590.0000000	9.5648003	-0.5853195
1590.0000000	9.6135998	-13.3509398
1590.0000000	9.6623993	-10.2370110
1590.0000000	9.7111998	-10.1731853
1590.0000000	9.7600002	-10.8599529
1590.0000000	9.8087997	-10.9952316
1590.0000000	9.8575993	2.9378271
1590.0000000	9.9063997	-8.0211554
1590.0000000	9.9552002	-10.9068470
1590.0000000	10.0039997	-10.0888290
1600.0000000	0.3416000	-36.7820854
1600.0000000	0.3904000	-39.7404404

1600.0000000	0.4392000	-39.3737602
1600.0000000	0.4880000	-39.5211296
1600.0000000	0.5368000	-39.6818314
1600.0000000	0.5856000	-39.2525253
1600.0000000	0.6344000	-38.2434502
1600.0000000	0.6832000	-36.2949715
1600.0000000	0.7320000	-36.1206779
1600.0000000	0.7808000	-36.5402260
1600.0000000	0.8296000	-35.9060440
1600.0000000	0.8784000	-34.8851280
1600.0000000	0.9272000	-34.2609711
1600.0000000	0.9760000	-31.8835411
1600.0000000	1.0247999	-30.3359699
1600.0000000	1.0735999	-30.3003750
1600.0000000	1.1224000	-30.6888065
1600.0000000	1.1711999	-31.2263126
1600.0000000	1.2200000	-30.0109558
1600.0000000	1.2687999	-30.4135303
1600.0000000	1.3176000	-30.8278332
1600.0000000	1.3664000	-30.5712547
1600.0000000	1.4152000	-30.1741714
1600.0000000	1.4640000	-29.5161686
1600.0000000	1.5128000	-33.7389145
1600.0000000	1.5616000	-31.2034931
1600.0000000	1.6104001	-1409.7344971
1600.0000000	1.6592000	-56.2101440
1600.0000000	1.7079999	-27.4748745
1600.0000000	1.7567999	-27.7632542
1600.0000000	1.8055999	-26.7825775
1600.0000000	1.8544000	-26.0301590
1600.0000000	1.9031999	-26.7256145
1600.0000000	1.9520000	-25.9696770
1600.0000000	2.0007999	-26.0537090
1600.0000000	2.0495999	-26.2853985
1600.0000000	2.0983999	-62.3790131
1600.0000000	2.1471999	-17.1761303
1600.0000000	2.1960001	-25.2112160
1600.0000000	2.2448001	-24.7889061
1600.0000000	2.2935998	-24.2222710
1600.0000000	2.3423998	-21.8253174
1600.0000000	2.3912001	-4.1691227
1600.0000000	2.4400001	-22.9816799
1600.0000000	2.4888000	-23.4474907
1600.0000000	2.5375998	-13.2586756
1600.0000000	2.5863998	-11.5327044
1600.0000000	2.6352000	-9.7771072
1600.0000000	2.6840000	-16.0991516
1600.0000000	2.7328000	117.8765793
1600.0000000	2.7815998	139.5185852
1600.0000000	2.8304000	-23.7194881
1600.0000000	2.8792000	-20.8067074
1600.0000000	2.9280000	-22.5391159
1600.0000000	2.9768000	-17.2698116
1600.0000000	3.0256000	-20.6211720
1600.0000000	3.0743999	-28.8633976
1600.0000000	3.1231999	-24.0568371
1600.0000000	3.1719999	-29.1298981
1600.0000000	3.2208002	-490.5918884

1600.0000000	3.2695999	-959.9710693
1600.0000000	3.3183999	-20.5010796
1600.0000000	3.3671999	-3311.8845215
1600.0000000	3.4159999	1187.5012207
1600.0000000	3.4648001	-7.4715919
1600.0000000	3.5135999	-26.8636742
1600.0000000	3.5623999	-314.4324036
1600.0000000	3.6111999	-1972.9451904
1600.0000000	3.6600001	-447.0566406
1600.0000000	3.7088001	-590.1306763
1600.0000000	3.7575998	-923.6428223
1600.0000000	3.8063998	-1675.6452637
1600.0000000	3.8552001	-2432.5625000
1600.0000000	3.9040000	-2384.9689941
1600.0000000	3.9528000	1089.9254150
1600.0000000	4.0015998	-238.7314911
1600.0000000	4.0503998	667.0661011
1600.0000000	4.0991998	84.5170746
1600.0000000	4.1479998	-636.2686157
1600.0000000	4.1967998	1.9173737
1600.0000000	4.2456002	-241.8313599
1600.0000000	4.2943997	-474.3722229
1600.0000000	4.3431997	-13.4795322
1600.0000000	4.3920002	-241.4746246
1600.0000000	4.4407997	-22.3399239
1600.0000000	4.4896002	-15.5342150
1600.0000000	4.5383997	-637.3222656
1600.0000000	4.5871997	-12.9757404
1600.0000000	4.6360002	-21.5161762
1600.0000000	4.6847997	-28.0153313
1600.0000000	4.7336001	-19.5132828
1600.0000000	4.7824001	-16.5432892
1600.0000000	4.8311996	-1438.1658936
1600.0000000	4.8800001	222.1533813
1600.0000000	4.9287996	-196.8676453
1600.0000000	4.9776001	1032.8189697
1600.0000000	5.0264001	83.6173935
1600.0000000	5.0751996	-294.0733948
1600.0000000	5.1240001	-12.3711624
1600.0000000	5.1727996	550.5347290
1600.0000000	5.2216001	31.4012451
1600.0000000	5.2704000	-157.0457916
1600.0000000	5.3191996	-38.8961258
1600.0000000	5.3680000	-3492.3071289
1600.0000000	5.4168000	520.4155884
1600.0000000	5.4656000	-198.4651184
1600.0000000	5.5144000	37.3975677
1600.0000000	5.5631995	482.1339111
1600.0000000	5.6120000	388.1187134
1600.0000000	5.6608000	-36.8280525
1600.0000000	5.7096000	61.5616226
1600.0000000	5.7584000	131.2240906
1600.0000000	5.8071995	-11.5749216
1600.0000000	5.8559999	29.2622547
1600.0000000	5.9047999	-16.5932350
1600.0000000	5.9535999	-39.4795799
1600.0000000	6.0023999	-1553.9377441
1600.0000000	6.0511999	-14.5813951

1600.0000000	6.0999999	-19.3574028
1600.0000000	6.1487999	-13.3021603
1600.0000000	6.1975999	-23.1103992
1600.0000000	6.2463999	-1770.2569580
1600.0000000	6.2951999	-19.0197353
1600.0000000	6.3439999	-338.7370300
1600.0000000	6.3927999	-15.0469837
1600.0000000	6.4416003	-12.4222345
1600.0000000	6.4903998	-11.3053732
1600.0000000	6.5391998	-11.8481359
1600.0000000	6.5879998	342.6481323
1600.0000000	6.6367998	-12.1178045
1600.0000000	6.6856003	-13.8253222
1600.0000000	6.7343998	-13.4688148
1600.0000000	6.7831998	-14.3472872
1600.0000000	6.8319998	57.2802658
1600.0000000	6.8807998	-14.0410242
1600.0000000	6.9296002	-403.5435791
1600.0000000	6.9783998	-5.8567781
1600.0000000	7.0271997	-14.0755882
1600.0000000	7.0760002	8.6600943
1600.0000000	7.1247997	-1895.8925781
1600.0000000	7.1736002	-1520.4542236
1600.0000000	7.2223997	-865.3636475
1600.0000000	7.2711997	-187.7129517
1600.0000000	7.3200002	-18.1695824
1600.0000000	7.3687997	165.6504822
1600.0000000	7.4176002	347.0572815
1600.0000000	7.4663997	272.4269104
1600.0000000	7.5151997	-846.2164307
1600.0000000	7.5640001	-934.5857544
1600.0000000	7.6127996	-2201.2617188
1600.0000000	7.6616001	-707.3795776
1600.0000000	7.7104001	-363.8972778
1600.0000000	7.7591996	37.2550087
1600.0000000	7.8080001	360.6707153
1600.0000000	7.8567996	475.4984741
1600.0000000	7.9056001	-988.8754883
1600.0000000	7.9544001	1573.2926025
1600.0000000	8.0031996	2030.1556396
1600.0000000	8.0520000	2026.2825928
1600.0000000	8.1007996	2144.2397461
1600.0000000	8.1496000	2286.8420410
1600.0000000	8.1983995	1422.6572266
1600.0000000	8.2472000	-3255.7270508
1600.0000000	8.2959995	-1154.2337646
1600.0000000	8.3448000	-919.3603516
1600.0000000	8.3935995	-842.5942993
1600.0000000	8.4423990	-363.1423645
1600.0000000	8.4912004	-847.4016113
1600.0000000	8.5400000	-2285.4274902
1600.0000000	8.5887995	-1108.0975342
1600.0000000	8.6375999	215.9924622
1600.0000000	8.6863995	1954.1253662
1600.0000000	8.7351999	-726.6637573
1600.0000000	8.7840004	829.5063477
1600.0000000	8.8327999	44.0126114
1600.0000000	8.8815994	-11.5066223

1600.0000000	8.9303989	-23.7279396
1600.0000000	8.9792004	-11.5839090
1600.0000000	9.0279999	-5.9400077
1600.0000000	9.0767994	6.7061615
1600.0000000	9.1255999	-17.1422520
1600.0000000	9.1743994	-10.0969772
1600.0000000	9.2231998	36.0968781
1600.0000000	9.2720003	-8.4745083
1600.0000000	9.3207998	-9.8294201
1600.0000000	9.3695993	-10.7769146
1600.0000000	9.4183998	-11.7466536
1600.0000000	9.4672003	-11.1637201
1600.0000000	9.5159998	-14.1125288
1600.0000000	9.5648003	-0.5886917
1600.0000000	9.6135998	-13.3501492
1600.0000000	9.6623993	-10.2372866
1600.0000000	9.7111998	-10.1734571
1600.0000000	9.7600002	-10.8599949
1600.0000000	9.8087997	-10.9952402
1600.0000000	9.8575993	2.9334049
1600.0000000	9.9063997	-8.0220690
1600.0000000	9.9552002	-10.9068518
1600.0000000	10.0039997	-10.0890770
1610.0000000	0.3416000	-36.4729996
1610.0000000	0.3904000	-39.6956367
1610.0000000	0.4392000	-39.3674164
1610.0000000	0.4880000	-39.5199203
1610.0000000	0.5368000	-39.6830063
1610.0000000	0.5856000	-39.2511444
1610.0000000	0.6344000	-38.2437630
1610.0000000	0.6832000	-36.2971954
1610.0000000	0.7320000	-36.1227303
1610.0000000	0.7808000	-36.5403938
1610.0000000	0.8296000	-35.9041977
1610.0000000	0.8784000	-34.8755646
1610.0000000	0.9272000	-34.2616119
1610.0000000	0.9760000	-31.8810177
1610.0000000	1.0247999	-30.3372040
1610.0000000	1.0735999	-30.3139763
1610.0000000	1.1224000	-30.6869335
1610.0000000	1.1711999	-31.2200108
1610.0000000	1.2200000	-29.9671574
1610.0000000	1.2687999	-30.3922043
1610.0000000	1.3176000	-30.8214474
1610.0000000	1.3664000	-30.5683117
1610.0000000	1.4152000	-30.1743755
1610.0000000	1.4640000	-29.5144596
1610.0000000	1.5128000	-33.6417389
1610.0000000	1.5616000	-31.1616516
1610.0000000	1.6104001	-1407.1916504
1610.0000000	1.6592000	-55.9162140
1610.0000000	1.7079999	-27.4786205
1610.0000000	1.7567999	-27.7632732
1610.0000000	1.8055999	-26.9922485
1610.0000000	1.8544000	-26.3581543
1610.0000000	1.9031999	-26.9101391
1610.0000000	1.9520000	-26.0276928
1610.0000000	2.0007999	-26.0590878

1610.0000000	2.0495999	-26.2874165
1610.0000000	2.0983999	-60.9032364
1610.0000000	2.1471999	-17.4433975
1610.0000000	2.1960001	-25.2071247
1610.0000000	2.2448001	-24.7709961
1610.0000000	2.2935998	-24.1626492
1610.0000000	2.3423998	-21.8397217
1610.0000000	2.3912001	-4.7906685
1610.0000000	2.4400001	-22.9841671
1610.0000000	2.4888000	-23.4472733
1610.0000000	2.5375998	-13.2757845
1610.0000000	2.5863998	-11.5524635
1610.0000000	2.6352000	-9.7998505
1610.0000000	2.6840000	-16.1110935
1610.0000000	2.7328000	117.6105576
1610.0000000	2.7815998	139.2121582
1610.0000000	2.8304000	-23.7181835
1610.0000000	2.8792000	-20.8099709
1610.0000000	2.9280000	-22.5385456
1610.0000000	2.9768000	-17.2781830
1610.0000000	3.0256000	-20.6237564
1610.0000000	3.0743999	-28.8520107
1610.0000000	3.1231999	-24.0533810
1610.0000000	3.1719999	-29.1216049
1610.0000000	3.2208002	-490.3541565
1610.0000000	3.2695999	-959.4960938
1610.0000000	3.3183999	-20.5012779
1610.0000000	3.3671999	-3310.4230957
1610.0000000	3.4159999	1186.9659424
1610.0000000	3.4648001	-7.4772997
1610.0000000	3.5135999	-26.8606834
1610.0000000	3.5623999	-314.3028259
1610.0000000	3.6111999	-1972.0854492
1610.0000000	3.6600001	-446.8685303
1610.0000000	3.7088001	-589.8793335
1610.0000000	3.7575998	-923.2442627
1610.0000000	3.8063998	-1674.9154053
1610.0000000	3.8552001	-2431.4992676
1610.0000000	3.9040000	-2383.9265137
1610.0000000	3.9528000	1089.4371338
1610.0000000	4.0015998	-238.6343842
1610.0000000	4.0503998	666.7634888
1610.0000000	4.0991998	84.4713974
1610.0000000	4.1479998	-635.9963989
1610.0000000	4.1967998	1.9081173
1610.0000000	4.2456002	-241.7330627
1610.0000000	4.2943997	-474.1716614
1610.0000000	4.3431997	-13.4817123
1610.0000000	4.3920002	-241.3765106
1610.0000000	4.4407997	-22.3381481
1610.0000000	4.4896002	-15.5350971
1610.0000000	4.5383997	-637.0612183
1610.0000000	4.5871997	-12.9777565
1610.0000000	4.6360002	-21.5146332
1610.0000000	4.6847997	-28.0114059
1610.0000000	4.7336001	-19.5125599
1610.0000000	4.7824001	-16.5437431
1610.0000000	4.8311996	-1437.6346436

1610.0000000	4.8800001	222.0638275
1610.0000000	4.9287996	-196.8003845
1610.0000000	4.9776001	1032.4263916
1610.0000000	5.0264001	83.5798721
1610.0000000	5.0751996	-293.9697876
1610.0000000	5.1240001	-12.3720837
1610.0000000	5.1727996	550.4188843
1610.0000000	5.2216001	31.3917999
1610.0000000	5.2704000	-157.0176697
1610.0000000	5.3191996	-38.8915749
1610.0000000	5.3680000	-3491.6147461
1610.0000000	5.4168000	520.3087769
1610.0000000	5.4656000	-198.4289398
1610.0000000	5.5144000	37.3869095
1610.0000000	5.5631995	482.0353394
1610.0000000	5.6120000	388.0387268
1610.0000000	5.6608000	-36.8239670
1610.0000000	5.7096000	61.5461884
1610.0000000	5.7584000	131.1951599
1610.0000000	5.8071995	-11.5757923
1610.0000000	5.8559999	29.2534046
1610.0000000	5.9047999	-16.5930367
1610.0000000	5.9535999	-39.4748421
1610.0000000	6.0023999	-1553.6331787
1610.0000000	6.0511999	-14.5816069
1610.0000000	6.0999999	-19.3565712
1610.0000000	6.1487999	-13.3026237
1610.0000000	6.1975999	-23.1086349
1610.0000000	6.2463999	-1769.9095459
1610.0000000	6.2951999	-19.0189209
1610.0000000	6.3439999	-338.6729736
1610.0000000	6.3927999	-15.0469656
1610.0000000	6.4416003	-12.4228210
1610.0000000	6.4903998	-11.3060493
1610.0000000	6.5391998	-11.8488169
1610.0000000	6.5879998	341.9574585
1610.0000000	6.6367998	-12.1183538
1610.0000000	6.6856003	-13.8254919
1610.0000000	6.7343998	-13.4690304
1610.0000000	6.7831998	-14.3468714
1610.0000000	6.8319998	57.2611465
1610.0000000	6.8807998	-14.0408859
1610.0000000	6.9296002	-402.7821350
1610.0000000	6.9783998	-5.8696017
1610.0000000	7.0271997	-14.0745611
1610.0000000	7.0760002	8.6179075
1610.0000000	7.1247997	-1891.9467773
1610.0000000	7.1736002	-1517.2913818
1610.0000000	7.2223997	-863.5704956
1610.0000000	7.2711997	-187.3451691
1610.0000000	7.3200002	-18.1575928
1610.0000000	7.3687997	165.2759552
1610.0000000	7.4176002	346.3014526
1610.0000000	7.4663997	271.8292542
1610.0000000	7.5151997	-844.4554443
1610.0000000	7.5640001	-932.6383667
1610.0000000	7.6127996	-2196.6440430
1610.0000000	7.6616001	-705.9082031

1610.0000000	7.7104001	-363.1510010
1610.0000000	7.7591996	37.1539574
1610.0000000	7.8080001	359.8853455
1610.0000000	7.8567996	474.4703979
1610.0000000	7.9056001	-986.8026123
1610.0000000	7.9544001	1569.9826660
1610.0000000	8.0031996	2025.8861084
1610.0000000	8.0520000	2022.0205078
1610.0000000	8.1007996	2139.7302246
1610.0000000	8.1496000	2282.0339355
1610.0000000	8.1983995	1419.6646729
1610.0000000	8.2472000	-3248.8920898
1610.0000000	8.2959995	-1151.8135986
1610.0000000	8.3448000	-917.4312134
1610.0000000	8.3935995	-840.8265991
1610.0000000	8.4423990	-362.3843384
1610.0000000	8.4912004	-845.6253662
1610.0000000	8.5400000	-2280.6276855
1610.0000000	8.5887995	-1105.7760010
1610.0000000	8.6375999	215.5311432
1610.0000000	8.6863995	1950.0069580
1610.0000000	8.7351999	-725.1395264
1610.0000000	8.7840004	827.7495728
1610.0000000	8.8327999	43.9010010
1610.0000000	8.8815994	-11.5067968
1610.0000000	8.9303989	-23.7242203
1610.0000000	8.9792004	-11.5839911
1610.0000000	9.0279999	-5.9417763
1610.0000000	9.0767994	6.7004452
1610.0000000	9.1255999	-17.1405392
1610.0000000	9.1743994	-10.0973797
1610.0000000	9.2231998	36.0811462
1610.0000000	9.2720003	-8.4754448
1610.0000000	9.3207998	-9.8299599
1610.0000000	9.3695993	-10.7771406
1610.0000000	9.4183998	-11.7465286
1610.0000000	9.4672003	-11.1637611
1610.0000000	9.5159998	-14.1116381
1610.0000000	9.5648003	-0.5920649
1610.0000000	9.6135998	-13.3493586
1610.0000000	9.6623993	-10.2375612
1610.0000000	9.7111998	-10.1737289
1610.0000000	9.7600002	-10.8600359
1610.0000000	9.8087997	-10.9952478
1610.0000000	9.8575993	2.9289837
1610.0000000	9.9063997	-8.0229826
1610.0000000	9.9552002	-10.9068556
1610.0000000	10.0039997	-10.0893240
1620.0000000	0.3416000	-36.7618790
1620.0000000	0.3904000	-39.5993538
1620.0000000	0.4392000	-39.3610725
1620.0000000	0.4880000	-39.5187111
1620.0000000	0.5368000	-39.6841774
1620.0000000	0.5856000	-39.2497635
1620.0000000	0.6344000	-38.2440720
1620.0000000	0.6832000	-36.2994232
1620.0000000	0.7320000	-36.1247826
1620.0000000	0.7808000	-36.5405655

1620.0000000	0.8296000	-35.9023514
1620.0000000	0.8784000	-34.8660011
1620.0000000	0.9272000	-34.2622528
1620.0000000	0.9760000	-31.8784943
1620.0000000	1.0247999	-30.3384399
1620.0000000	1.0735999	-30.3275757
1620.0000000	1.1224000	-30.6850624
1620.0000000	1.1711999	-31.2137070
1620.0000000	1.2200000	-29.9233589
1620.0000000	1.2687999	-30.3708763
1620.0000000	1.3176000	-30.8150616
1620.0000000	1.3664000	-30.5653687
1620.0000000	1.4152000	-30.1745796
1620.0000000	1.4640000	-29.5127506
1620.0000000	1.5128000	-33.5445595
1620.0000000	1.5616000	-31.1198101
1620.0000000	1.6104001	-1404.6489258
1620.0000000	1.6592000	-55.6222801
1620.0000000	1.7079999	-27.4823685
1620.0000000	1.7567999	-27.7632923
1620.0000000	1.8055999	-26.7198048
1620.0000000	1.8544000	-26.1954479
1620.0000000	1.9031999	-26.5372448
1620.0000000	1.9520000	-26.0857105
1620.0000000	2.0007999	-26.0644684
1620.0000000	2.0495999	-26.2894363
1620.0000000	2.0983999	-59.4274597
1620.0000000	2.1471999	-17.7106647
1620.0000000	2.1960001	-25.2030334
1620.0000000	2.2448001	-24.7530842
1620.0000000	2.2935998	-24.1030254
1620.0000000	2.3423998	-21.8541260
1620.0000000	2.3912001	-5.4122143
1620.0000000	2.4400001	-22.9866524
1620.0000000	2.4888000	-23.4470558
1620.0000000	2.5375998	-13.2928915
1620.0000000	2.5863998	-11.5722227
1620.0000000	2.6352000	-9.8225946
1620.0000000	2.6840000	-16.1230354
1620.0000000	2.7328000	117.3445358
1620.0000000	2.7815998	138.9057617
1620.0000000	2.8304000	-23.7168808
1620.0000000	2.8792000	-20.8132343
1620.0000000	2.9280000	-22.5379772
1620.0000000	2.9768000	-17.2865524
1620.0000000	3.0256000	-20.6263409
1620.0000000	3.0743999	-28.8406239
1620.0000000	3.1231999	-24.0499268
1620.0000000	3.1719999	-29.1133118
1620.0000000	3.2208002	-490.1164246
1620.0000000	3.2695999	-959.0211182
1620.0000000	3.3183999	-20.5014763
1620.0000000	3.3671999	-3308.9614258
1620.0000000	3.4159999	1186.4306641
1620.0000000	3.4648001	-7.4830084
1620.0000000	3.5135999	-26.8576927
1620.0000000	3.5623999	-314.1732178
1620.0000000	3.6111999	-1971.2257080

1620.0000000	3.6600001	-446.6803589
1620.0000000	3.7088001	-589.6279297
1620.0000000	3.7575998	-922.8457031
1620.0000000	3.8063998	-1674.1856689
1620.0000000	3.8552001	-2430.4357910
1620.0000000	3.9040000	-2382.8840332
1620.0000000	3.9528000	1088.9488525
1620.0000000	4.0015998	-238.5372772
1620.0000000	4.0503998	666.4609375
1620.0000000	4.0991998	84.4257126
1620.0000000	4.1479998	-635.7241211
1620.0000000	4.1967998	1.8988609
1620.0000000	4.2456002	-241.6347656
1620.0000000	4.2943997	-473.9710388
1620.0000000	4.3431997	-13.4838934
1620.0000000	4.3920002	-241.2783966
1620.0000000	4.4407997	-22.3363724
1620.0000000	4.4896002	-15.5359802
1620.0000000	4.5383997	-636.8001099
1620.0000000	4.5871997	-12.9797735
1620.0000000	4.6360002	-21.5130901
1620.0000000	4.6847997	-28.0074806
1620.0000000	4.7336001	-19.5118370
1620.0000000	4.7824001	-16.5441971
1620.0000000	4.8311996	-1437.1033936
1620.0000000	4.8800001	221.9742737
1620.0000000	4.9287996	-196.7331238
1620.0000000	4.9776001	1032.0339355
1620.0000000	5.0264001	83.5423508
1620.0000000	5.0751996	-293.8661499
1620.0000000	5.1240001	-12.3730049
1620.0000000	5.1727996	550.3029785
1620.0000000	5.2216001	31.3823547
1620.0000000	5.2704000	-156.9895782
1620.0000000	5.3191996	-38.8870239
1620.0000000	5.3680000	-3490.9223633
1620.0000000	5.4168000	520.2019653
1620.0000000	5.4656000	-198.3927460
1620.0000000	5.5144000	37.3762512
1620.0000000	5.5631995	481.9367676
1620.0000000	5.6120000	387.9587708
1620.0000000	5.6608000	-36.8198776
1620.0000000	5.7096000	61.5307541
1620.0000000	5.7584000	131.1662292
1620.0000000	5.8071995	-11.5766630
1620.0000000	5.8559999	29.2445545
1620.0000000	5.9047999	-16.5928402
1620.0000000	5.9535999	-39.4701042
1620.0000000	6.0023999	-1553.3288574
1620.0000000	6.0511999	-14.5818176
1620.0000000	6.0999999	-19.3557377
1620.0000000	6.1487999	-13.3030863
1620.0000000	6.1975999	-23.1068687
1620.0000000	6.2463999	-1769.5622559
1620.0000000	6.2951999	-19.0181065
1620.0000000	6.3439999	-338.6089478
1620.0000000	6.3927999	-15.0469475
1620.0000000	6.4416003	-12.4234085

1620.0000000	6.4903998	-11.3067255
1620.0000000	6.5391998	-11.8494978
1620.0000000	6.5879998	341.2667542
1620.0000000	6.6367998	-12.1189032
1620.0000000	6.6856003	-13.8256626
1620.0000000	6.7343998	-13.4692459
1620.0000000	6.7831998	-14.3464556
1620.0000000	6.8319998	57.2420273
1620.0000000	6.8807998	-14.0407467
1620.0000000	6.9296002	-402.0206909
1620.0000000	6.9783998	-5.8824258
1620.0000000	7.0271997	-14.0735331
1620.0000000	7.0760002	8.5757246
1620.0000000	7.1247997	-1888.0008545
1620.0000000	7.1736002	-1514.1285400
1620.0000000	7.2223997	-861.7773438
1620.0000000	7.2711997	-186.9773865
1620.0000000	7.3200002	-18.1456051
1620.0000000	7.3687997	164.9014282
1620.0000000	7.4176002	345.5456238
1620.0000000	7.4663997	271.2315979
1620.0000000	7.5151997	-842.6943970
1620.0000000	7.5640001	-930.6909790
1620.0000000	7.6127996	-2192.0266113
1620.0000000	7.6616001	-704.4368896
1620.0000000	7.7104001	-362.4047241
1620.0000000	7.7591996	37.0529060
1620.0000000	7.8080001	359.1000061
1620.0000000	7.8567996	473.4422607
1620.0000000	7.9056001	-984.7296753
1620.0000000	7.9544001	1566.6727295
1620.0000000	8.0031996	2021.6164551
1620.0000000	8.0520000	2017.7585449
1620.0000000	8.1007996	2135.2207031
1620.0000000	8.1496000	2277.2260742
1620.0000000	8.1983995	1416.6721191
1620.0000000	8.2472000	-3242.0573730
1620.0000000	8.2959995	-1149.3934326
1620.0000000	8.3448000	-915.5020142
1620.0000000	8.3935995	-839.0589600
1620.0000000	8.4423990	-361.6263428
1620.0000000	8.4912004	-843.8491211
1620.0000000	8.5400000	-2275.8278809
1620.0000000	8.5887995	-1103.4544678
1620.0000000	8.6375999	215.0698242
1620.0000000	8.6863995	1945.8886719
1620.0000000	8.7351999	-723.6152344
1620.0000000	8.7840004	825.9928589
1620.0000000	8.8327999	43.7893982
1620.0000000	8.8815994	-11.5069704
1620.0000000	8.9303989	-23.7205009
1620.0000000	8.9792004	-11.5840731
1620.0000000	9.0279999	-5.9435444
1620.0000000	9.0767994	6.6947269
1620.0000000	9.1255999	-17.1388245
1620.0000000	9.1743994	-10.0977821
1620.0000000	9.2231998	36.0654144
1620.0000000	9.2720003	-8.4763813

1620.0000000	9.3207998	-9.8304996
1620.0000000	9.3695993	-10.7773666
1620.0000000	9.4183998	-11.7464037
1620.0000000	9.4672003	-11.1638031
1620.0000000	9.5159998	-14.1107464
1620.0000000	9.5648003	-0.5954380
1620.0000000	9.6135998	-13.3485680
1620.0000000	9.6623993	-10.2378359
1620.0000000	9.7111998	-10.1740007
1620.0000000	9.7600002	-10.8600779
1620.0000000	9.8087997	-10.9952555
1620.0000000	9.8575993	2.9245615
1620.0000000	9.9063997	-8.0238972
1620.0000000	9.9552002	-10.9068604
1620.0000000	10.0039997	-10.0895720
1630.0000000	0.3416000	-36.5404549
1630.0000000	0.3904000	-39.5030746
1630.0000000	0.4392000	-39.3547287
1630.0000000	0.4880000	-39.5175018
1630.0000000	0.5368000	-39.6853523
1630.0000000	0.5856000	-39.2483826
1630.0000000	0.6344000	-38.2443848
1630.0000000	0.6832000	-36.3016472
1630.0000000	0.7320000	-36.1268349
1630.0000000	0.7808000	-36.5407333
1630.0000000	0.8296000	-35.9005051
1630.0000000	0.8784000	-34.8564339
1630.0000000	0.9272000	-34.2628975
1630.0000000	0.9760000	-31.8759708
1630.0000000	1.0247999	-30.3396759
1630.0000000	1.0735999	-30.3411770
1630.0000000	1.1224000	-30.6831894
1630.0000000	1.1711999	-31.2074051
1630.0000000	1.2200000	-29.8795605
1630.0000000	1.2687999	-30.3495502
1630.0000000	1.3176000	-30.8086758
1630.0000000	1.3664000	-30.5624237
1630.0000000	1.4152000	-30.1747837
1630.0000000	1.4640000	-29.5110397
1630.0000000	1.5128000	-33.4473839
1630.0000000	1.5616000	-31.0779686
1630.0000000	1.6104001	-1402.1060791
1630.0000000	1.6592000	-55.3283463
1630.0000000	1.7079999	-27.4861145
1630.0000000	1.7567999	-27.7633114
1630.0000000	1.8055999	-27.4799232
1630.0000000	1.8544000	-25.9621601
1630.0000000	1.9031999	-25.9570313
1630.0000000	1.9520000	-26.1437263
1630.0000000	2.0007999	-26.0698490
1630.0000000	2.0495999	-26.2914543
1630.0000000	2.0983999	-57.9516792
1630.0000000	2.1471999	-17.9779320
1630.0000000	2.1960001	-25.1989422
1630.0000000	2.2448001	-24.7351723
1630.0000000	2.2935998	-24.0434017
1630.0000000	2.3423998	-21.8685303
1630.0000000	2.3912001	-6.0337601

1630.0000000	2.4400001	-22.9891396
1630.0000000	2.4888000	-23.4468384
1630.0000000	2.5375998	-13.3099995
1630.0000000	2.5863998	-11.5919819
1630.0000000	2.6352000	-9.8453388
1630.0000000	2.6840000	-16.1349754
1630.0000000	2.7328000	117.0785294
1630.0000000	2.7815998	138.5993347
1630.0000000	2.8304000	-23.7155762
1630.0000000	2.8792000	-20.8164978
1630.0000000	2.9280000	-22.5374088
1630.0000000	2.9768000	-17.2949238
1630.0000000	3.0256000	-20.6289253
1630.0000000	3.0743999	-28.8292370
1630.0000000	3.1231999	-24.0464706
1630.0000000	3.1719999	-29.1050186
1630.0000000	3.2208002	-489.8786926
1630.0000000	3.2695999	-958.5461426
1630.0000000	3.3183999	-20.5016747
1630.0000000	3.3671999	-3307.4997559
1630.0000000	3.4159999	1185.8953857
1630.0000000	3.4648001	-7.4887161
1630.0000000	3.5135999	-26.8547020
1630.0000000	3.5623999	-314.0436401
1630.0000000	3.6111999	-1970.3658447
1630.0000000	3.6600001	-446.4922485
1630.0000000	3.7088001	-589.3765259
1630.0000000	3.7575998	-922.4472046
1630.0000000	3.8063998	-1673.4558105
1630.0000000	3.8552001	-2429.3723145
1630.0000000	3.9040000	-2381.8415527
1630.0000000	3.9528000	1088.4605713
1630.0000000	4.0015998	-238.4401550
1630.0000000	4.0503998	666.1583862
1630.0000000	4.0991998	84.3800278
1630.0000000	4.1479998	-635.4519043
1630.0000000	4.1967998	1.8896046
1630.0000000	4.2456002	-241.5364532
1630.0000000	4.2943997	-473.7704773
1630.0000000	4.3431997	-13.4860735
1630.0000000	4.3920002	-241.1802826
1630.0000000	4.4407997	-22.3345966
1630.0000000	4.4896002	-15.5368633
1630.0000000	4.5383997	-636.5390625
1630.0000000	4.5871997	-12.9817896
1630.0000000	4.6360002	-21.5115471
1630.0000000	4.6847997	-28.0035553
1630.0000000	4.7336001	-19.5111160
1630.0000000	4.7824001	-16.5446529
1630.0000000	4.8311996	-1436.5721436
1630.0000000	4.8800001	221.8847198
1630.0000000	4.9287996	-196.6658630
1630.0000000	4.9776001	1031.6414795
1630.0000000	5.0264001	83.5048294
1630.0000000	5.0751996	-293.7625122
1630.0000000	5.1240001	-12.3739262
1630.0000000	5.1727996	550.1871338
1630.0000000	5.2216001	31.3729134

1630.0000000	5.2704000	-156.9614563
1630.0000000	5.3191996	-38.8824768
1630.0000000	5.3680000	-3490.2299805
1630.0000000	5.4168000	520.0952148
1630.0000000	5.4656000	-198.3565674
1630.0000000	5.5144000	37.3656006
1630.0000000	5.5631995	481.8381958
1630.0000000	5.6120000	387.8787842
1630.0000000	5.6608000	-36.8157883
1630.0000000	5.7096000	61.5153275
1630.0000000	5.7584000	131.1372986
1630.0000000	5.8071995	-11.5775337
1630.0000000	5.8559999	29.2357044
1630.0000000	5.9047999	-16.5926418
1630.0000000	5.9535999	-39.4653625
1630.0000000	6.0023999	-1553.0242920
1630.0000000	6.0511999	-14.5820293
1630.0000000	6.0999999	-19.3549061
1630.0000000	6.1487999	-13.3035488
1630.0000000	6.1975999	-23.1051044
1630.0000000	6.2463999	-1769.2148438
1630.0000000	6.2951999	-19.0172920
1630.0000000	6.3439999	-338.5448608
1630.0000000	6.3927999	-15.0469284
1630.0000000	6.4416003	-12.4239950
1630.0000000	6.4903998	-11.3074007
1630.0000000	6.5391998	-11.8501797
1630.0000000	6.5879998	340.5760498
1630.0000000	6.6367998	-12.1194525
1630.0000000	6.6856003	-13.8258324
1630.0000000	6.7343998	-13.4694624
1630.0000000	6.7831998	-14.3460398
1630.0000000	6.8319998	57.2229156
1630.0000000	6.8807998	-14.0406084
1630.0000000	6.9296002	-401.2592163
1630.0000000	6.9783998	-5.8952489
1630.0000000	7.0271997	-14.0725050
1630.0000000	7.0760002	8.5335379
1630.0000000	7.1247997	-1884.0550537
1630.0000000	7.1736002	-1510.9655762
1630.0000000	7.2223997	-859.9841309
1630.0000000	7.2711997	-186.6096039
1630.0000000	7.3200002	-18.1336174
1630.0000000	7.3687997	164.5269012
1630.0000000	7.4176002	344.7897949
1630.0000000	7.4663997	270.6339417
1630.0000000	7.5151997	-840.9333496
1630.0000000	7.5640001	-928.7435913
1630.0000000	7.6127996	-2187.4089355
1630.0000000	7.6616001	-702.9655151
1630.0000000	7.7104001	-361.6584473
1630.0000000	7.7591996	36.9518585
1630.0000000	7.8080001	358.3146362
1630.0000000	7.8567996	472.4141846
1630.0000000	7.9056001	-982.6567993
1630.0000000	7.9544001	1563.3627930
1630.0000000	8.0031996	2017.3469238
1630.0000000	8.0520000	2013.4965820

1630.0000000	8.1007996	2130.7111816
1630.0000000	8.1496000	2272.4179688
1630.0000000	8.1983995	1413.6796875
1630.0000000	8.2472000	-3235.2226563
1630.0000000	8.2959995	-1146.9732666
1630.0000000	8.3448000	-913.5728149
1630.0000000	8.3935995	-837.2912598
1630.0000000	8.4423990	-360.8683167
1630.0000000	8.4912004	-842.0729370
1630.0000000	8.5400000	-2271.0280762
1630.0000000	8.5887995	-1101.1329346
1630.0000000	8.6375999	214.6084900
1630.0000000	8.6863995	1941.7702637
1630.0000000	8.7351999	-722.0910034
1630.0000000	8.7840004	824.2360840
1630.0000000	8.8327999	43.6777878
1630.0000000	8.8815994	-11.5071449
1630.0000000	8.9303989	-23.7167797
1630.0000000	8.9792004	-11.5841551
1630.0000000	9.0279999	-5.9453125
1630.0000000	9.0767994	6.6890106
1630.0000000	9.1255999	-17.1371117
1630.0000000	9.1743994	-10.0981836
1630.0000000	9.2231998	36.0496826
1630.0000000	9.2720003	-8.4773169
1630.0000000	9.3207998	-9.8310385
1630.0000000	9.3695993	-10.7775936
1630.0000000	9.4183998	-11.7462797
1630.0000000	9.4672003	-11.1638441
1630.0000000	9.5159998	-14.1098557
1630.0000000	9.5648003	-0.5988102
1630.0000000	9.6135998	-13.3477764
1630.0000000	9.6623993	-10.2381115
1630.0000000	9.7111998	-10.1742716
1630.0000000	9.7600002	-10.8601198
1630.0000000	9.8087997	-10.9952641
1630.0000000	9.8575993	2.9201374
1630.0000000	9.9063997	-8.0248108
1630.0000000	9.9552002	-10.9068642
1630.0000000	10.0039997	-10.0898199
1640.0000000	0.3416000	-36.2198982
1640.0000000	0.3904000	-39.4067917
1640.0000000	0.4392000	-39.3483849
1640.0000000	0.4880000	-39.5162926
1640.0000000	0.5368000	-39.6865273
1640.0000000	0.5856000	-39.2470016
1640.0000000	0.6344000	-38.2446938
1640.0000000	0.6832000	-36.3038750
1640.0000000	0.7320000	-36.1288872
1640.0000000	0.7808000	-36.5409012
1640.0000000	0.8296000	-35.8986626
1640.0000000	0.8784000	-34.8468704
1640.0000000	0.9272000	-34.2635384
1640.0000000	0.9760000	-31.8734474
1640.0000000	1.0247999	-30.3409119
1640.0000000	1.0735999	-30.3547764
1640.0000000	1.1224000	-30.6813183
1640.0000000	1.1711999	-31.2011013

1640.0000000	1.2200000	-29.8357620
1640.0000000	1.2687999	-30.3282242
1640.0000000	1.3176000	-30.8022900
1640.0000000	1.3664000	-30.5594807
1640.0000000	1.4152000	-30.1749897
1640.0000000	1.4640000	-29.5093307
1640.0000000	1.5128000	-33.3502045
1640.0000000	1.5616000	-31.0361290
1640.0000000	1.6104001	-1399.5633545
1640.0000000	1.6592000	-55.0344162
1640.0000000	1.7079999	-27.4898624
1640.0000000	1.7567999	-27.7633305
1640.0000000	1.8055999	-27.4843941
1640.0000000	1.8544000	-26.2462368
1640.0000000	1.9031999	-25.7523098
1640.0000000	1.9520000	-26.2017441
1640.0000000	2.0007999	-26.0752296
1640.0000000	2.0495999	-26.2934742
1640.0000000	2.0983999	-56.4758987
1640.0000000	2.1471999	-18.2451992
1640.0000000	2.1960001	-25.1948509
1640.0000000	2.2448001	-24.7172623
1640.0000000	2.2935998	-23.9837780
1640.0000000	2.3423998	-21.8829365
1640.0000000	2.3912001	-6.6553059
1640.0000000	2.4400001	-22.9916267
1640.0000000	2.4888000	-23.4466209
1640.0000000	2.5375998	-13.3271074
1640.0000000	2.5863998	-11.6117401
1640.0000000	2.6352000	-9.8680830
1640.0000000	2.6840000	-16.1469193
1640.0000000	2.7328000	116.8125076
1640.0000000	2.7815998	138.2929077
1640.0000000	2.8304000	-23.7142715
1640.0000000	2.8792000	-20.8197613
1640.0000000	2.9280000	-22.5368385
1640.0000000	2.9768000	-17.3032951
1640.0000000	3.0256000	-20.6315098
1640.0000000	3.0743999	-28.8178501
1640.0000000	3.1231999	-24.0430145
1640.0000000	3.1719999	-29.0967255
1640.0000000	3.2208002	-489.6409607
1640.0000000	3.2695999	-958.0711670
1640.0000000	3.3183999	-20.5018711
1640.0000000	3.3671999	-3306.0380859
1640.0000000	3.4159999	1185.3601074
1640.0000000	3.4648001	-7.4944239
1640.0000000	3.5135999	-26.8517113
1640.0000000	3.5623999	-313.9140320
1640.0000000	3.6111999	-1969.5061035
1640.0000000	3.6600001	-446.3041382
1640.0000000	3.7088001	-589.1251831
1640.0000000	3.7575998	-922.0486450
1640.0000000	3.8063998	-1672.7259521
1640.0000000	3.8552001	-2428.3088379
1640.0000000	3.9040000	-2380.7990723
1640.0000000	3.9528000	1087.9721680
1640.0000000	4.0015998	-238.3430481

1640.0000000	4.0503998	665.8558350
1640.0000000	4.0991998	84.3343430
1640.0000000	4.1479998	-635.1796875
1640.0000000	4.1967998	1.8803482
1640.0000000	4.2456002	-241.4381561
1640.0000000	4.2943997	-473.5699158
1640.0000000	4.3431997	-13.4882545
1640.0000000	4.3920002	-241.0821686
1640.0000000	4.4407997	-22.3328209
1640.0000000	4.4896002	-15.5377455
1640.0000000	4.5383997	-636.2780151
1640.0000000	4.5871997	-12.9838057
1640.0000000	4.6360002	-21.5100060
1640.0000000	4.6847997	-27.9996300
1640.0000000	4.7336001	-19.5103931
1640.0000000	4.7824001	-16.5451069
1640.0000000	4.8311996	-1436.0408936
1640.0000000	4.8800001	221.7951660
1640.0000000	4.9287996	-196.5986023
1640.0000000	4.9776001	1031.2489014
1640.0000000	5.0264001	83.4673080
1640.0000000	5.0751996	-293.6589050
1640.0000000	5.1240001	-12.3748474
1640.0000000	5.1727996	550.0712891
1640.0000000	5.2216001	31.3634682
1640.0000000	5.2704000	-156.9333649
1640.0000000	5.3191996	-38.8779259
1640.0000000	5.3680000	-3489.5375977
1640.0000000	5.4168000	519.9884644
1640.0000000	5.4656000	-198.3203888
1640.0000000	5.5144000	37.3549423
1640.0000000	5.5631995	481.7396545
1640.0000000	5.6120000	387.7987976
1640.0000000	5.6608000	-36.8116989
1640.0000000	5.7096000	61.4999008
1640.0000000	5.7584000	131.1083679
1640.0000000	5.8071995	-11.5784035
1640.0000000	5.8559999	29.2268543
1640.0000000	5.9047999	-16.5924435
1640.0000000	5.9535999	-39.4606247
1640.0000000	6.0023999	-1552.7199707
1640.0000000	6.0511999	-14.5822411
1640.0000000	6.0999999	-19.3540726
1640.0000000	6.1487999	-13.3040123
1640.0000000	6.1975999	-23.1033401
1640.0000000	6.2463999	-1768.8674316
1640.0000000	6.2951999	-19.0164776
1640.0000000	6.3439999	-338.4808350
1640.0000000	6.3927999	-15.0469103
1640.0000000	6.4416003	-12.4245825
1640.0000000	6.4903998	-11.3080769
1640.0000000	6.5391998	-11.8508606
1640.0000000	6.5879998	339.8853455
1640.0000000	6.6367998	-12.1200018
1640.0000000	6.6856003	-13.8260031
1640.0000000	6.7343998	-13.4696779
1640.0000000	6.7831998	-14.3456240
1640.0000000	6.8319998	57.2038040

1640.0000000	6.8807998	-14.0404692
1640.0000000	6.9296002	-400.4977722
1640.0000000	6.9783998	-5.9080725
1640.0000000	7.0271997	-14.0714769
1640.0000000	7.0760002	8.4913511
1640.0000000	7.1247997	-1880.1091309
1640.0000000	7.1736002	-1507.8027344
1640.0000000	7.2223997	-858.1909790
1640.0000000	7.2711997	-186.2418213
1640.0000000	7.3200002	-18.1216278
1640.0000000	7.3687997	164.1523743
1640.0000000	7.4176002	344.0339661
1640.0000000	7.4663997	270.0363159
1640.0000000	7.5151997	-839.1723022
1640.0000000	7.5640001	-926.7962036
1640.0000000	7.6127996	-2182.7912598
1640.0000000	7.6616001	-701.4941406
1640.0000000	7.7104001	-360.9122314
1640.0000000	7.7591996	36.8508110
1640.0000000	7.8080001	357.5292664
1640.0000000	7.8567996	471.3861084
1640.0000000	7.9056001	-980.5838623
1640.0000000	7.9544001	1560.0528564
1640.0000000	8.0031996	2013.0772705
1640.0000000	8.0520000	2009.2344971
1640.0000000	8.1007996	2126.2016602
1640.0000000	8.1496000	2267.6098633
1640.0000000	8.1983995	1410.6871338
1640.0000000	8.2472000	-3228.3876953
1640.0000000	8.2959995	-1144.5531006
1640.0000000	8.3448000	-911.6436768
1640.0000000	8.3935995	-835.5236206
1640.0000000	8.4423990	-360.1103210
1640.0000000	8.4912004	-840.2966919
1640.0000000	8.5400000	-2266.2282715
1640.0000000	8.5887995	-1098.8114014
1640.0000000	8.6375999	214.1471710
1640.0000000	8.6863995	1937.6518555
1640.0000000	8.7351999	-720.5667725
1640.0000000	8.7840004	822.4793701
1640.0000000	8.8327999	43.5661812
1640.0000000	8.8815994	-11.5073195
1640.0000000	8.9303989	-23.7130585
1640.0000000	8.9792004	-11.5842381
1640.0000000	9.0279999	-5.9470811
1640.0000000	9.0767994	6.6832924
1640.0000000	9.1255999	-17.1353970
1640.0000000	9.1743994	-10.0985861
1640.0000000	9.2231998	36.0339508
1640.0000000	9.2720003	-8.4782534
1640.0000000	9.3207998	-9.8315773
1640.0000000	9.3695993	-10.7778196
1640.0000000	9.4183998	-11.7461548
1640.0000000	9.4672003	-11.1638851
1640.0000000	9.5159998	-14.1089649
1640.0000000	9.5648003	-0.6021824
1640.0000000	9.6135998	-13.3469868
1640.0000000	9.6623993	-10.2383862

1640.0000000	9.7111998	-10.1745434
1640.0000000	9.7600002	-10.8601618
1640.0000000	9.8087997	-10.9952717
1640.0000000	9.8575993	2.9157162
1640.0000000	9.9063997	-8.0257244
1640.0000000	9.9552002	-10.9068689
1640.0000000	10.0039997	-10.0900669
1650.0000000	0.3416000	-36.9996185
1650.0000000	0.3904000	-39.5280418
1650.0000000	0.4392000	-39.3420410
1650.0000000	0.4880000	-39.5150833
1650.0000000	0.5368000	-39.6876984
1650.0000000	0.5856000	-39.2456169
1650.0000000	0.6344000	-38.2450027
1650.0000000	0.6832000	-36.3060989
1650.0000000	0.7320000	-36.1309395
1650.0000000	0.7808000	-36.5410728
1650.0000000	0.8296000	-35.8968163
1650.0000000	0.8784000	-34.8373070
1650.0000000	0.9272000	-34.2641792
1650.0000000	0.9760000	-31.8709240
1650.0000000	1.0247999	-30.3421459
1650.0000000	1.0735999	-30.3683777
1650.0000000	1.1224000	-30.6794453
1650.0000000	1.1711999	-31.1947994
1650.0000000	1.2200000	-29.7919655
1650.0000000	1.2687999	-30.3068962
1650.0000000	1.3176000	-30.7959042
1650.0000000	1.3664000	-30.5565376
1650.0000000	1.4152000	-30.1751938
1650.0000000	1.4640000	-29.5076218
1650.0000000	1.5128000	-33.2530251
1650.0000000	1.5616000	-30.9942875
1650.0000000	1.6104001	-1397.0205078
1650.0000000	1.6592000	-54.7404861
1650.0000000	1.7079999	-27.4936104
1650.0000000	1.7567999	-27.7633495
1650.0000000	1.8055999	-27.4888649
1650.0000000	1.8544000	-26.4228954
1650.0000000	1.9031999	-25.8586750
1650.0000000	1.9520000	-26.2763481
1650.0000000	2.0007999	-26.0806084
1650.0000000	2.0495999	-26.2954922
1650.0000000	2.0983999	-55.0001221
1650.0000000	2.1471999	-18.5124664
1650.0000000	2.1960001	-25.1907597
1650.0000000	2.2448001	-24.6993504
1650.0000000	2.2935998	-23.9241543
1650.0000000	2.3423998	-21.8973408
1650.0000000	2.3912001	-7.2768517
1650.0000000	2.4400001	-22.9941120
1650.0000000	2.4888000	-23.4464035
1650.0000000	2.5375998	-13.3442154
1650.0000000	2.5863998	-11.6314993
1650.0000000	2.6352000	-9.8908272
1650.0000000	2.6840000	-16.1588593
1650.0000000	2.7328000	116.5465012
1650.0000000	2.7815998	137.9864807

1650.0000000	2.8304000	-23.7129669
1650.0000000	2.8792000	-20.8230247
1650.0000000	2.9280000	-22.5362701
1650.0000000	2.9768000	-17.3116646
1650.0000000	3.0256000	-20.6340942
1650.0000000	3.0743999	-28.8064632
1650.0000000	3.1231999	-24.0395603
1650.0000000	3.1719999	-29.0884323
1650.0000000	3.2208002	-489.4032288
1650.0000000	3.2695999	-957.5963135
1650.0000000	3.3183999	-20.5020695
1650.0000000	3.3671999	-3304.5764160
1650.0000000	3.4159999	1184.8248291
1650.0000000	3.4648001	-7.5001326
1650.0000000	3.5135999	-26.8487206
1650.0000000	3.5623999	-313.7844543
1650.0000000	3.6111999	-1968.6463623
1650.0000000	3.6600001	-446.1159668
1650.0000000	3.7088001	-588.8737793
1650.0000000	3.7575998	-921.6501465
1650.0000000	3.8063998	-1671.9960938
1650.0000000	3.8552001	-2427.2453613
1650.0000000	3.9040000	-2379.7565918
1650.0000000	3.9528000	1087.4838867
1650.0000000	4.0015998	-238.2459412
1650.0000000	4.0503998	665.5532837
1650.0000000	4.0991998	84.2886581
1650.0000000	4.1479998	-634.9074707
1650.0000000	4.1967998	1.8710918
1650.0000000	4.2456002	-241.3398590
1650.0000000	4.2943997	-473.3693542
1650.0000000	4.3431997	-13.4904346
1650.0000000	4.3920002	-240.9840546
1650.0000000	4.4407997	-22.3310471
1650.0000000	4.4896002	-15.5386276
1650.0000000	4.5383997	-636.0169067
1650.0000000	4.5871997	-12.9858217
1650.0000000	4.6360002	-21.5084629
1650.0000000	4.6847997	-27.9957047
1650.0000000	4.7336001	-19.5096703
1650.0000000	4.7824001	-16.5455608
1650.0000000	4.8311996	-1435.5095215
1650.0000000	4.8800001	221.7056274
1650.0000000	4.9287996	-196.5313416
1650.0000000	4.9776001	1030.8564453
1650.0000000	5.0264001	83.4297867
1650.0000000	5.0751996	-293.5552673
1650.0000000	5.1240001	-12.3757687
1650.0000000	5.1727996	549.9553833
1650.0000000	5.2216001	31.3540230
1650.0000000	5.2704000	-156.9052429
1650.0000000	5.3191996	-38.8733788
1650.0000000	5.3680000	-3488.8452148
1650.0000000	5.4168000	519.8816528
1650.0000000	5.4656000	-198.2841949
1650.0000000	5.5144000	37.3442841
1650.0000000	5.5631995	481.6410828
1650.0000000	5.6120000	387.7188416

1650.0000000	5.6608000	-36.8076096
1650.0000000	5.7096000	61.4844666
1650.0000000	5.7584000	131.0794373
1650.0000000	5.8071995	-11.5792751
1650.0000000	5.8559999	29.2180004
1650.0000000	5.9047999	-16.5922470
1650.0000000	5.9535999	-39.4558868
1650.0000000	6.0023999	-1552.4155273
1650.0000000	6.0511999	-14.5824518
1650.0000000	6.0999999	-19.3532410
1650.0000000	6.1487999	-13.3044748
1650.0000000	6.1975999	-23.1015739
1650.0000000	6.2463999	-1768.5200195
1650.0000000	6.2951999	-19.0156631
1650.0000000	6.3439999	-338.4167786
1650.0000000	6.3927999	-15.0468922
1650.0000000	6.4416003	-12.4251690
1650.0000000	6.4903998	-11.3087530
1650.0000000	6.5391998	-11.8515415
1650.0000000	6.5879998	339.1946411
1650.0000000	6.6367998	-12.1205502
1650.0000000	6.6856003	-13.8261728
1650.0000000	6.7343998	-13.4698935
1650.0000000	6.7831998	-14.3452082
1650.0000000	6.8319998	57.1846848
1650.0000000	6.8807998	-14.0403309
1650.0000000	6.9296002	-399.7363281
1650.0000000	6.9783998	-5.9208961
1650.0000000	7.0271997	-14.0704489
1650.0000000	7.0760002	8.4491682
1650.0000000	7.1247997	-1876.1632080
1650.0000000	7.1736002	-1504.6398926
1650.0000000	7.2223997	-856.3978271
1650.0000000	7.2711997	-185.8740540
1650.0000000	7.3200002	-18.1096382
1650.0000000	7.3687997	163.7778320
1650.0000000	7.4176002	343.2781372
1650.0000000	7.4663997	269.4386597
1650.0000000	7.5151997	-837.4112549
1650.0000000	7.5640001	-924.8487549
1650.0000000	7.6127996	-2178.1735840
1650.0000000	7.6616001	-700.0228882
1650.0000000	7.7104001	-360.1659546
1650.0000000	7.7591996	36.7497597
1650.0000000	7.8080001	356.7439270
1650.0000000	7.8567996	470.3580017
1650.0000000	7.9056001	-978.5109863
1650.0000000	7.9544001	1556.7429199
1650.0000000	8.0031996	2008.8077393
1650.0000000	8.0520000	2004.9725342
1650.0000000	8.1007996	2121.6921387
1650.0000000	8.1496000	2262.8020020
1650.0000000	8.1983995	1407.6945801
1650.0000000	8.2472000	-3221.5529785
1650.0000000	8.2959995	-1142.1329346
1650.0000000	8.3448000	-909.7144775
1650.0000000	8.3935995	-833.7559204
1650.0000000	8.4423990	-359.3522949

1650.0000000	8.4912004	-838.5205078
1650.0000000	8.5400000	-2261.4284668
1650.0000000	8.5887995	-1096.4898682
1650.0000000	8.6375999	213.6858521
1650.0000000	8.6863995	1933.5335693
1650.0000000	8.7351999	-719.0424805
1650.0000000	8.7840004	820.7225952
1650.0000000	8.8327999	43.4545708
1650.0000000	8.8815994	-11.5074940
1650.0000000	8.9303989	-23.7093391
1650.0000000	8.9792004	-11.5843201
1650.0000000	9.0279999	-5.9488492
1650.0000000	9.0767994	6.6775742
1650.0000000	9.1255999	-17.1336823
1650.0000000	9.1743994	-10.0989876
1650.0000000	9.2231998	36.0182190
1650.0000000	9.2720003	-8.4791899
1650.0000000	9.3207998	-9.8321171
1650.0000000	9.3695993	-10.7780466
1650.0000000	9.4183998	-11.7460308
1650.0000000	9.4672003	-11.1639261
1650.0000000	9.5159998	-14.1080742
1650.0000000	9.5648003	-0.6055555
1650.0000000	9.6135998	-13.3461952
1650.0000000	9.6623993	-10.2386618
1650.0000000	9.7111998	-10.1748152
1650.0000000	9.7600002	-10.8602028
1650.0000000	9.8087997	-10.9952793
1650.0000000	9.8575993	2.9112940
1650.0000000	9.9063997	-8.0266380
1650.0000000	9.9552002	-10.9068737
1650.0000000	10.0039997	-10.0903149
1660.0000000	0.3416000	-37.0131950
1660.0000000	0.3904000	-39.6492920
1660.0000000	0.4392000	-39.3356972
1660.0000000	0.4880000	-39.5138741
1660.0000000	0.5368000	-39.6888733
1660.0000000	0.5856000	-39.2442360
1660.0000000	0.6344000	-38.2453156
1660.0000000	0.6832000	-36.3083229
1660.0000000	0.7320000	-36.1329918
1660.0000000	0.7808000	-36.5412407
1660.0000000	0.8296000	-35.8949699
1660.0000000	0.8784000	-34.8277397
1660.0000000	0.9272000	-34.2648201
1660.0000000	0.9760000	-31.8684006
1660.0000000	1.0247999	-30.3433819
1660.0000000	1.0735999	-30.3819771
1660.0000000	1.1224000	-30.6775742
1660.0000000	1.1711999	-31.1884956
1660.0000000	1.2200000	-29.7481670
1660.0000000	1.2687999	-30.2855701
1660.0000000	1.3176000	-30.7895184
1660.0000000	1.3664000	-30.5535927
1660.0000000	1.4152000	-30.1753979
1660.0000000	1.4640000	-29.5059128
1660.0000000	1.5128000	-33.1558495
1660.0000000	1.5616000	-30.9524460

1660.0000000	1.6104001	-1394.4776611
1660.0000000	1.6592000	-54.4465523
1660.0000000	1.7079999	-27.4973564
1660.0000000	1.7567999	-27.7633686
1660.0000000	1.8055999	-27.4933357
1660.0000000	1.8544000	-26.4221897
1660.0000000	1.9031999	-26.1770611
1660.0000000	1.9520000	-26.3509521
1660.0000000	2.0007999	-26.0859890
1660.0000000	2.0495999	-26.2975121
1660.0000000	2.0983999	-53.5243454
1660.0000000	2.1471999	-18.7797337
1660.0000000	2.1960001	-25.1866684
1660.0000000	2.2448001	-24.6814384
1660.0000000	2.2935998	-23.8645306
1660.0000000	2.3423998	-21.9117451
1660.0000000	2.3912001	-7.8983974
1660.0000000	2.4400001	-22.9965992
1660.0000000	2.4888000	-23.4461861
1660.0000000	2.5375998	-13.3613234
1660.0000000	2.5863998	-11.6512585
1660.0000000	2.6352000	-9.9135714
1660.0000000	2.6840000	-16.1708031
1660.0000000	2.7328000	116.2804794
1660.0000000	2.7815998	137.6800842
1660.0000000	2.8304000	-23.7116623
1660.0000000	2.8792000	-20.8262882
1660.0000000	2.9280000	-22.5357018
1660.0000000	2.9768000	-17.3200340
1660.0000000	3.0256000	-20.6366787
1660.0000000	3.0743999	-28.7950764
1660.0000000	3.1231999	-24.0361042
1660.0000000	3.1719999	-29.0801392
1660.0000000	3.2208002	-489.1654663
1660.0000000	3.2695999	-957.1212158
1660.0000000	3.3183999	-20.5022678
1660.0000000	3.3671999	-3303.1147461
1660.0000000	3.4159999	1184.2895508
1660.0000000	3.4648001	-7.5058413
1660.0000000	3.5135999	-26.8457298
1660.0000000	3.5623999	-313.6548462
1660.0000000	3.6111999	-1967.7866211
1660.0000000	3.6600001	-445.9278564
1660.0000000	3.7088001	-588.6223755
1660.0000000	3.7575998	-921.2515869
1660.0000000	3.8063998	-1671.2662354
1660.0000000	3.8552001	-2426.1821289
1660.0000000	3.9040000	-2378.7141113
1660.0000000	3.9528000	1086.9956055
1660.0000000	4.0015998	-238.1488342
1660.0000000	4.0503998	665.2507324
1660.0000000	4.0991998	84.2429733
1660.0000000	4.1479998	-634.6352539
1660.0000000	4.1967998	1.8618355
1660.0000000	4.2456002	-241.2415466
1660.0000000	4.2943997	-473.1687622
1660.0000000	4.3431997	-13.4926157
1660.0000000	4.3920002	-240.8859406

1660.0000000	4.4407997	-22.3292694
1660.0000000	4.4896002	-15.5395107
1660.0000000	4.5383997	-635.7558594
1660.0000000	4.5871997	-12.9878387
1660.0000000	4.6360002	-21.5069199
1660.0000000	4.6847997	-27.9917793
1660.0000000	4.7336001	-19.5089493
1660.0000000	4.7824001	-16.5460148
1660.0000000	4.8311996	-1434.9782715
1660.0000000	4.8800001	221.6160736
1660.0000000	4.9287996	-196.4640808
1660.0000000	4.9776001	1030.4639893
1660.0000000	5.0264001	83.3922653
1660.0000000	5.0751996	-293.4516296
1660.0000000	5.1240001	-12.3766899
1660.0000000	5.1727996	549.8395386
1660.0000000	5.2216001	31.3445816
1660.0000000	5.2704000	-156.8771515
1660.0000000	5.3191996	-38.8688278
1660.0000000	5.3680000	-3488.1528320
1660.0000000	5.4168000	519.7748413
1660.0000000	5.4656000	-198.2480164
1660.0000000	5.5144000	37.3336258
1660.0000000	5.5631995	481.5425110
1660.0000000	5.6120000	387.6388550
1660.0000000	5.6608000	-36.8035240
1660.0000000	5.7096000	61.4690323
1660.0000000	5.7584000	131.0504913
1660.0000000	5.8071995	-11.5801449
1660.0000000	5.8559999	29.2091503
1660.0000000	5.9047999	-16.5920486
1660.0000000	5.9535999	-39.4511490
1660.0000000	6.0023999	-1552.1109619
1660.0000000	6.0511999	-14.5826635
1660.0000000	6.0999999	-19.3524094
1660.0000000	6.1487999	-13.3049374
1660.0000000	6.1975999	-23.0998077
1660.0000000	6.2463999	-1768.1727295
1660.0000000	6.2951999	-19.0148506
1660.0000000	6.3439999	-338.3527222
1660.0000000	6.3927999	-15.0468740
1660.0000000	6.4416003	-12.4257555
1660.0000000	6.4903998	-11.3094292
1660.0000000	6.5391998	-11.8522224
1660.0000000	6.5879998	338.5039673
1660.0000000	6.6367998	-12.1210995
1660.0000000	6.6856003	-13.8263435
1660.0000000	6.7343998	-13.4701090
1660.0000000	6.7831998	-14.3447924
1660.0000000	6.8319998	57.1655731
1660.0000000	6.8807998	-14.0401917
1660.0000000	6.9296002	-398.9748840
1660.0000000	6.9783998	-5.9337201
1660.0000000	7.0271997	-14.0694218
1660.0000000	7.0760002	8.4069815
1660.0000000	7.1247997	-1872.2174072
1660.0000000	7.1736002	-1501.4770508
1660.0000000	7.2223997	-854.6046753

1660.0000000	7.2711997	-185.5062714
1660.0000000	7.3200002	-18.0976505
1660.0000000	7.3687997	163.4033051
1660.0000000	7.4176002	342.5223083
1660.0000000	7.4663997	268.8410034
1660.0000000	7.5151997	-835.6502075
1660.0000000	7.5640001	-922.9013672
1660.0000000	7.6127996	-2173.5561523
1660.0000000	7.6616001	-698.5515137
1660.0000000	7.7104001	-359.4196777
1660.0000000	7.7591996	36.6487122
1660.0000000	7.8080001	355.9585571
1660.0000000	7.8567996	469.3299255
1660.0000000	7.9056001	-976.4380493
1660.0000000	7.9544001	1553.4329834
1660.0000000	8.0031996	2004.5380859
1660.0000000	8.0520000	2000.7105713
1660.0000000	8.1007996	2117.1826172
1660.0000000	8.1496000	2257.9938965
1660.0000000	8.1983995	1404.7021484
1660.0000000	8.2472000	-3214.7182617
1660.0000000	8.2959995	-1139.7127686
1660.0000000	8.3448000	-907.7852783
1660.0000000	8.3935995	-831.9882813
1660.0000000	8.4423990	-358.5942688
1660.0000000	8.4912004	-836.7442627
1660.0000000	8.5400000	-2256.6286621
1660.0000000	8.5887995	-1094.1682129
1660.0000000	8.6375999	213.2245331
1660.0000000	8.6863995	1929.4151611
1660.0000000	8.7351999	-717.5182495
1660.0000000	8.7840004	818.9658813
1660.0000000	8.8327999	43.3429642
1660.0000000	8.8815994	-11.5076675
1660.0000000	8.9303989	-23.7056198
1660.0000000	8.9792004	-11.5844021
1660.0000000	9.0279999	-5.9506178
1660.0000000	9.0767994	6.6718578
1660.0000000	9.1255999	-17.1319695
1660.0000000	9.1743994	-10.0993900
1660.0000000	9.2231998	36.0024796
1660.0000000	9.2720003	-8.4801264
1660.0000000	9.3207998	-9.8326569
1660.0000000	9.3695993	-10.7782726
1660.0000000	9.4183998	-11.7459059
1660.0000000	9.4672003	-11.1639671
1660.0000000	9.5159998	-14.1071835
1660.0000000	9.5648003	-0.6089287
1660.0000000	9.6135998	-13.3454046
1660.0000000	9.6623993	-10.2389364
1660.0000000	9.7111998	-10.1750870
1660.0000000	9.7600002	-10.8602448
1660.0000000	9.8087997	-10.9952879
1660.0000000	9.8575993	2.9068699
1660.0000000	9.9063997	-8.0275517
1660.0000000	9.9552002	-10.9068775
1660.0000000	10.0039997	-10.0905619
1670.0000000	0.3416000	-36.4956779

1670.0000000	0.3904000	-39.7705383
1670.0000000	0.4392000	-39.3293571
1670.0000000	0.4880000	-39.5126648
1670.0000000	0.5368000	-39.6900482
1670.0000000	0.5856000	-39.2428551
1670.0000000	0.6344000	-38.2456245
1670.0000000	0.6832000	-36.3105507
1670.0000000	0.7320000	-36.1350441
1670.0000000	0.7808000	-36.5414085
1670.0000000	0.8296000	-35.8931274
1670.0000000	0.8784000	-34.8181763
1670.0000000	0.9272000	-34.2654648
1670.0000000	0.9760000	-31.8658772
1670.0000000	1.0247999	-30.3446178
1670.0000000	1.0735999	-30.3955784
1670.0000000	1.1224000	-30.6757011
1670.0000000	1.1711999	-31.1821938
1670.0000000	1.2200000	-29.7043686
1670.0000000	1.2687999	-30.2642422
1670.0000000	1.3176000	-30.7831345
1670.0000000	1.3664000	-30.5506496
1670.0000000	1.4152000	-30.1756020
1670.0000000	1.4640000	-29.5042019
1670.0000000	1.5128000	-33.0586700
1670.0000000	1.5616000	-30.9106045
1670.0000000	1.6104001	-1391.9349365
1670.0000000	1.6592000	-54.1526184
1670.0000000	1.7079999	-27.5011044
1670.0000000	1.7567999	-27.7633877
1670.0000000	1.8055999	-27.4978085
1670.0000000	1.8544000	-26.8919849
1670.0000000	1.9031999	-26.7521477
1670.0000000	1.9520000	-26.4255581
1670.0000000	2.0007999	-26.0913696
1670.0000000	2.0495999	-26.2995300
1670.0000000	2.0983999	-52.0485649
1670.0000000	2.1471999	-19.0470009
1670.0000000	2.1960001	-25.1825771
1670.0000000	2.2448001	-24.6635284
1670.0000000	2.2935998	-23.8049068
1670.0000000	2.3423998	-21.9261494
1670.0000000	2.3912001	-8.5199432
1670.0000000	2.4400001	-22.9990864
1670.0000000	2.4888000	-23.4459686
1670.0000000	2.5375998	-13.3784313
1670.0000000	2.5863998	-11.6710176
1670.0000000	2.6352000	-9.9363155
1670.0000000	2.6840000	-16.1827431
1670.0000000	2.7328000	116.0144577
1670.0000000	2.7815998	137.3736572
1670.0000000	2.8304000	-23.7103596
1670.0000000	2.8792000	-20.8295517
1670.0000000	2.9280000	-22.5351315
1670.0000000	2.9768000	-17.3284054
1670.0000000	3.0256000	-20.6392632
1670.0000000	3.0743999	-28.7836895
1670.0000000	3.1231999	-24.0326500
1670.0000000	3.1719999	-29.0718460

1670.0000000	3.2208002	-488.9277344
1670.0000000	3.2695999	-956.6463623
1670.0000000	3.3183999	-20.5024662
1670.0000000	3.3671999	-3301.6530762
1670.0000000	3.4159999	1183.7542725
1670.0000000	3.4648001	-7.5115499
1670.0000000	3.5135999	-26.8427391
1670.0000000	3.5623999	-313.5252686
1670.0000000	3.6111999	-1966.9268799
1670.0000000	3.6600001	-445.7397461
1670.0000000	3.7088001	-588.3710327
1670.0000000	3.7575998	-920.8530273
1670.0000000	3.8063998	-1670.5363770
1670.0000000	3.8552001	-2425.1186523
1670.0000000	3.9040000	-2377.6716309
1670.0000000	3.9528000	1086.5073242
1670.0000000	4.0015998	-238.0517273
1670.0000000	4.0503998	664.9481812
1670.0000000	4.0991998	84.1972885
1670.0000000	4.1479998	-634.3630371
1670.0000000	4.1967998	1.8525791
1670.0000000	4.2456002	-241.1432495
1670.0000000	4.2943997	-472.9682007
1670.0000000	4.3431997	-13.4947968
1670.0000000	4.3920002	-240.7878265
1670.0000000	4.4407997	-22.3274956
1670.0000000	4.4896002	-15.5403938
1670.0000000	4.5383997	-635.4948120
1670.0000000	4.5871997	-12.9898548
1670.0000000	4.6360002	-21.5053787
1670.0000000	4.6847997	-27.9878540
1670.0000000	4.7336001	-19.5082264
1670.0000000	4.7824001	-16.5464706
1670.0000000	4.8311996	-1434.4470215
1670.0000000	4.8800001	221.5265198
1670.0000000	4.9287996	-196.3968048
1670.0000000	4.9776001	1030.0714111
1670.0000000	5.0264001	83.3547516
1670.0000000	5.0751996	-293.3480225
1670.0000000	5.1240001	-12.3776112
1670.0000000	5.1727996	549.7236328
1670.0000000	5.2216001	31.3351326
1670.0000000	5.2704000	-156.8490295
1670.0000000	5.3191996	-38.8642807
1670.0000000	5.3680000	-3487.4606934
1670.0000000	5.4168000	519.6680908
1670.0000000	5.4656000	-198.2118378
1670.0000000	5.5144000	37.3229752
1670.0000000	5.5631995	481.4439392
1670.0000000	5.6120000	387.5588684
1670.0000000	5.6608000	-36.7994347
1670.0000000	5.7096000	61.4536057
1670.0000000	5.7584000	131.0215607
1670.0000000	5.8071995	-11.5810165
1670.0000000	5.8559999	29.2002964
1670.0000000	5.9047999	-16.5918503
1670.0000000	5.9535999	-39.4464111
1670.0000000	6.0023999	-1551.8066406

1670.0000000	6.0511999	-14.5828753
1670.0000000	6.0999999	-19.3515759
1670.0000000	6.1487999	-13.3054008
1670.0000000	6.1975999	-23.0980434
1670.0000000	6.2463999	-1767.8253174
1670.0000000	6.2951999	-19.0140343
1670.0000000	6.3439999	-338.2886658
1670.0000000	6.3927999	-15.0468550
1670.0000000	6.4416003	-12.4263430
1670.0000000	6.4903998	-11.3101044
1670.0000000	6.5391998	-11.8529034
1670.0000000	6.5879998	337.8132629
1670.0000000	6.6367998	-12.1216488
1670.0000000	6.6856003	-13.8265133
1670.0000000	6.7343998	-13.4703255
1670.0000000	6.7831998	-14.3443766
1670.0000000	6.8319998	57.1464615
1670.0000000	6.8807998	-14.0400534
1670.0000000	6.9296002	-398.2134399
1670.0000000	6.9783998	-5.9465432
1670.0000000	7.0271997	-14.0683937
1670.0000000	7.0760002	8.3647947
1670.0000000	7.1247997	-1868.2714844
1670.0000000	7.1736002	-1498.3142090
1670.0000000	7.2223997	-852.8115234
1670.0000000	7.2711997	-185.1384888
1670.0000000	7.3200002	-18.0856628
1670.0000000	7.3687997	163.0287781
1670.0000000	7.4176002	341.7664795
1670.0000000	7.4663997	268.2433472
1670.0000000	7.5151997	-833.8891602
1670.0000000	7.5640001	-920.9539795
1670.0000000	7.6127996	-2168.9384766
1670.0000000	7.6616001	-697.0802002
1670.0000000	7.7104001	-358.6734619
1670.0000000	7.7591996	36.5476646
1670.0000000	7.8080001	355.1731873
1670.0000000	7.8567996	468.3018494
1670.0000000	7.9056001	-974.3651733
1670.0000000	7.9544001	1550.1230469
1670.0000000	8.0031996	2000.2684326
1670.0000000	8.0520000	1996.4486084
1670.0000000	8.1007996	2112.6730957
1670.0000000	8.1496000	2253.1860352
1670.0000000	8.1983995	1401.7095947
1670.0000000	8.2472000	-3207.8833008
1670.0000000	8.2959995	-1137.2926025
1670.0000000	8.3448000	-905.8561401
1670.0000000	8.3935995	-830.2205811
1670.0000000	8.4423990	-357.8362732
1670.0000000	8.4912004	-834.9680176
1670.0000000	8.5400000	-2251.8288574
1670.0000000	8.5887995	-1091.8466797
1670.0000000	8.6375999	212.7632141
1670.0000000	8.6863995	1925.2968750
1670.0000000	8.7351999	-715.9939575
1670.0000000	8.7840004	817.2091064
1670.0000000	8.8327999	43.2313614

1670.0000000	8.8815994	-11.5078421
1670.0000000	8.9303989	-23.7019005
1670.0000000	8.9792004	-11.5844851
1670.0000000	9.0279999	-5.9523859
1670.0000000	9.0767994	6.6661396
1670.0000000	9.1255999	-17.1302547
1670.0000000	9.1743994	-10.0997925
1670.0000000	9.2231998	35.9867477
1670.0000000	9.2720003	-8.4810629
1670.0000000	9.3207998	-9.8331957
1670.0000000	9.3695993	-10.7784996
1670.0000000	9.4183998	-11.7457809
1670.0000000	9.4672003	-11.1640091
1670.0000000	9.5159998	-14.1062918
1670.0000000	9.5648003	-0.6123009
1670.0000000	9.6135998	-13.3446140
1670.0000000	9.6623993	-10.2392111
1670.0000000	9.7111998	-10.1753578
1670.0000000	9.7600002	-10.8602867
1670.0000000	9.8087997	-10.9952955
1670.0000000	9.8575993	2.9024487
1670.0000000	9.9063997	-8.0284662
1670.0000000	9.9552002	-10.9068823
1670.0000000	10.0039997	-10.0908098
1680.0000000	0.3416000	-36.6437073
1680.0000000	0.3904000	-39.8917885
1680.0000000	0.4392000	-39.3230133
1680.0000000	0.4880000	-39.5114555
1680.0000000	0.5368000	-39.6912231
1680.0000000	0.5856000	-39.2414742
1680.0000000	0.6344000	-38.2459335
1680.0000000	0.6832000	-36.3127747
1680.0000000	0.7320000	-36.1370964
1680.0000000	0.7808000	-36.5415802
1680.0000000	0.8296000	-35.8912811
1680.0000000	0.8784000	-34.8086090
1680.0000000	0.9272000	-34.2661057
1680.0000000	0.9760000	-31.8633537
1680.0000000	1.0247999	-30.3458538
1680.0000000	1.0735999	-30.4091778
1680.0000000	1.1224000	-30.6738300
1680.0000000	1.1711999	-31.1758900
1680.0000000	1.2200000	-29.6605701
1680.0000000	1.2687999	-30.2429161
1680.0000000	1.3176000	-30.7767487
1680.0000000	1.3664000	-30.5477066
1680.0000000	1.4152000	-30.1758060
1680.0000000	1.4640000	-29.5024929
1680.0000000	1.5128000	-32.9614944
1680.0000000	1.5616000	-30.8687630
1680.0000000	1.6104001	-1389.3920898
1680.0000000	1.6592000	-53.8586884
1680.0000000	1.7079999	-27.5048504
1680.0000000	1.7567999	-27.7634068
1680.0000000	1.8055999	-27.5022793
1680.0000000	1.8544000	-26.7682343
1680.0000000	1.9031999	-26.5058136
1680.0000000	1.9520000	-26.5001621

1680.0000000	2.0007999	-26.0967503
1680.0000000	2.0495999	-26.3015499
1680.0000000	2.0983999	-50.5727844
1680.0000000	2.1471999	-19.3142681
1680.0000000	2.1960001	-25.1784859
1680.0000000	2.2448001	-24.6456165
1680.0000000	2.2935998	-23.7452831
1680.0000000	2.3423998	-21.9405537
1680.0000000	2.3912001	-9.1414890
1680.0000000	2.4400001	-23.0015717
1680.0000000	2.4888000	-23.4457531
1680.0000000	2.5375998	-13.3955393
1680.0000000	2.5863998	-11.6907768
1680.0000000	2.6352000	-9.9590597
1680.0000000	2.6840000	-16.1946850
1680.0000000	2.7328000	115.7484512
1680.0000000	2.7815998	137.0672302
1680.0000000	2.8304000	-23.7090549
1680.0000000	2.8792000	-20.8328152
1680.0000000	2.9280000	-22.5345631
1680.0000000	2.9768000	-17.3367767
1680.0000000	3.0256000	-20.6418476
1680.0000000	3.0743999	-28.7723026
1680.0000000	3.1231999	-24.0291939
1680.0000000	3.1719999	-29.0635529
1680.0000000	3.2208002	-488.6900024
1680.0000000	3.2695999	-956.1712646
1680.0000000	3.3183999	-20.5026646
1680.0000000	3.3671999	-3300.1914063
1680.0000000	3.4159999	1183.2189941
1680.0000000	3.4648001	-7.5172567
1680.0000000	3.5135999	-26.8397484
1680.0000000	3.5623999	-313.3956604
1680.0000000	3.6111999	-1966.0671387
1680.0000000	3.6600001	-445.5515747
1680.0000000	3.7088001	-588.1196289
1680.0000000	3.7575998	-920.4545288
1680.0000000	3.8063998	-1669.8065186
1680.0000000	3.8552001	-2424.0551758
1680.0000000	3.9040000	-2376.6291504
1680.0000000	3.9528000	1086.0190430
1680.0000000	4.0015998	-237.9546204
1680.0000000	4.0503998	664.6455688
1680.0000000	4.0991998	84.1516113
1680.0000000	4.1479998	-634.0908203
1680.0000000	4.1967998	1.8433228
1680.0000000	4.2456002	-241.0449524
1680.0000000	4.2943997	-472.7676392
1680.0000000	4.3431997	-13.4969769
1680.0000000	4.3920002	-240.6897125
1680.0000000	4.4407997	-22.3257198
1680.0000000	4.4896002	-15.5412760
1680.0000000	4.5383997	-635.2337036
1680.0000000	4.5871997	-12.9918709
1680.0000000	4.6360002	-21.5038357
1680.0000000	4.6847997	-27.9839287
1680.0000000	4.7336001	-19.5075035
1680.0000000	4.7824001	-16.5469246

1680.0000000	4.8311996	-1433.9157715
1680.0000000	4.8800001	221.4369812
1680.0000000	4.9287996	-196.3295441
1680.0000000	4.9776001	1029.6789551
1680.0000000	5.0264001	83.3172302
1680.0000000	5.0751996	-293.2443848
1680.0000000	5.1240001	-12.3785324
1680.0000000	5.1727996	549.6077881
1680.0000000	5.2216001	31.3256912
1680.0000000	5.2704000	-156.8209381
1680.0000000	5.3191996	-38.8597298
1680.0000000	5.3680000	-3486.7683105
1680.0000000	5.4168000	519.5613403
1680.0000000	5.4656000	-198.1756439
1680.0000000	5.5144000	37.3123169
1680.0000000	5.5631995	481.3453674
1680.0000000	5.6120000	387.4789124
1680.0000000	5.6608000	-36.7953453
1680.0000000	5.7096000	61.4381714
1680.0000000	5.7584000	130.9926300
1680.0000000	5.8071995	-11.5818863
1680.0000000	5.8559999	29.1914463
1680.0000000	5.9047999	-16.5916538
1680.0000000	5.9535999	-39.4416695
1680.0000000	6.0023999	-1551.5021973
1680.0000000	6.0511999	-14.5830860
1680.0000000	6.0999999	-19.3507442
1680.0000000	6.1487999	-13.3058634
1680.0000000	6.1975999	-23.0962772
1680.0000000	6.2463999	-1767.4779053
1680.0000000	6.2951999	-19.0132217
1680.0000000	6.3439999	-338.2246399
1680.0000000	6.3927999	-15.0468369
1680.0000000	6.4416003	-12.4269295
1680.0000000	6.4903998	-11.3107805
1680.0000000	6.5391998	-11.8535852
1680.0000000	6.5879998	337.1225586
1680.0000000	6.6367998	-12.1221981
1680.0000000	6.6856003	-13.8266840
1680.0000000	6.7343998	-13.4705410
1680.0000000	6.7831998	-14.3439608
1680.0000000	6.8319998	57.1273422
1680.0000000	6.8807998	-14.0399151
1680.0000000	6.9296002	-397.4519653
1680.0000000	6.9783998	-5.9593668
1680.0000000	7.0271997	-14.0673656
1680.0000000	7.0760002	8.3226118
1680.0000000	7.1247997	-1864.3256836
1680.0000000	7.1736002	-1495.1512451
1680.0000000	7.2223997	-851.0183716
1680.0000000	7.2711997	-184.7707214
1680.0000000	7.3200002	-18.0736732
1680.0000000	7.3687997	162.6542358
1680.0000000	7.4176002	341.0106506
1680.0000000	7.4663997	267.6456909
1680.0000000	7.5151997	-832.1281738
1680.0000000	7.5640001	-919.0065918
1680.0000000	7.6127996	-2164.3208008

1680.0000000	7.6616001	-695.6088257
1680.0000000	7.7104001	-357.9271240
1680.0000000	7.7591996	36.4466133
1680.0000000	7.8080001	354.3878479
1680.0000000	7.8567996	467.2737427
1680.0000000	7.9056001	-972.2922363
1680.0000000	7.9544001	1546.8131104
1680.0000000	8.0031996	1995.9989014
1680.0000000	8.0520000	1992.1865234
1680.0000000	8.1007996	2108.1635742
1680.0000000	8.1496000	2248.3779297
1680.0000000	8.1983995	1398.7170410
1680.0000000	8.2472000	-3201.0485840
1680.0000000	8.2959995	-1134.8724365
1680.0000000	8.3448000	-903.9269409
1680.0000000	8.3935995	-828.4529419
1680.0000000	8.4423990	-357.0782471
1680.0000000	8.4912004	-833.1918335
1680.0000000	8.5400000	-2247.0290527
1680.0000000	8.5887995	-1089.5251465
1680.0000000	8.6375999	212.3018951
1680.0000000	8.6863995	1921.1784668
1680.0000000	8.7351999	-714.4697266
1680.0000000	8.7840004	815.4523926
1680.0000000	8.8327999	43.1197510
1680.0000000	8.8815994	-11.5080166
1680.0000000	8.9303989	-23.6981812
1680.0000000	8.9792004	-11.5845671
1680.0000000	9.0279999	-5.9541540
1680.0000000	9.0767994	6.6604214
1680.0000000	9.1255999	-17.1285400
1680.0000000	9.1743994	-10.1001940
1680.0000000	9.2231998	35.9710159
1680.0000000	9.2720003	-8.4819984
1680.0000000	9.3207998	-9.8337355
1680.0000000	9.3695993	-10.7787256
1680.0000000	9.4183998	-11.7456570
1680.0000000	9.4672003	-11.1640501
1680.0000000	9.5159998	-14.1054010
1680.0000000	9.5648003	-0.6156731
1680.0000000	9.6135998	-13.3438234
1680.0000000	9.6623993	-10.2394867
1680.0000000	9.7111998	-10.1756296
1680.0000000	9.7600002	-10.8603277
1680.0000000	9.8087997	-10.9953032
1680.0000000	9.8575993	2.8980265
1680.0000000	9.9063997	-8.0293798
1680.0000000	9.9552002	-10.9068861
1680.0000000	10.0039997	-10.0910568
1690.0000000	0.3416000	-36.8534966
1690.0000000	0.3904000	-39.4375534
1690.0000000	0.4392000	-39.3166695
1690.0000000	0.4880000	-39.5102463
1690.0000000	0.5368000	-39.6923943
1690.0000000	0.5856000	-39.2400932
1690.0000000	0.6344000	-38.2462463
1690.0000000	0.6832000	-36.3150024
1690.0000000	0.7320000	-36.1391487

1690.0000000	0.7808000	-36.5417480
1690.0000000	0.8296000	-35.8894348
1690.0000000	0.8784000	-34.7990456
1690.0000000	0.9272000	-34.2667465
1690.0000000	0.9760000	-31.8608303
1690.0000000	1.0247999	-30.3470879
1690.0000000	1.0735999	-30.4227791
1690.0000000	1.1224000	-30.6719570
1690.0000000	1.1711999	-31.1695881
1690.0000000	1.2200000	-29.6167717
1690.0000000	1.2687999	-30.2215881
1690.0000000	1.3176000	-30.7703629
1690.0000000	1.3664000	-30.5447617
1690.0000000	1.4152000	-30.1760101
1690.0000000	1.4640000	-29.5007839
1690.0000000	1.5128000	-32.8643150
1690.0000000	1.5616000	-30.8269234
1690.0000000	1.6104001	-1386.8492432
1690.0000000	1.6592000	-53.5647583
1690.0000000	1.7079999	-27.5085983
1690.0000000	1.7567999	-27.7634258
1690.0000000	1.8055999	-27.5067501
1690.0000000	1.8544000	-26.2937317
1690.0000000	1.9031999	-26.8261814
1690.0000000	1.9520000	-26.5747662
1690.0000000	2.0007999	-26.1021309
1690.0000000	2.0495999	-26.3035679
1690.0000000	2.0983999	-49.0970078
1690.0000000	2.1471999	-19.5815353
1690.0000000	2.1960001	-25.1743946
1690.0000000	2.2448001	-24.6277046
1690.0000000	2.2935998	-23.6856594
1690.0000000	2.3423998	-21.9549580
1690.0000000	2.3912001	-9.7630348
1690.0000000	2.4400001	-23.0040588
1690.0000000	2.4888000	-23.4455357
1690.0000000	2.5375998	-13.4126472
1690.0000000	2.5863998	-11.7105341
1690.0000000	2.6352000	-9.9818039
1690.0000000	2.6840000	-16.2066269
1690.0000000	2.7328000	115.4824295
1690.0000000	2.7815998	136.7608337
1690.0000000	2.8304000	-23.7077503
1690.0000000	2.8792000	-20.8360786
1690.0000000	2.9280000	-22.5339928
1690.0000000	2.9768000	-17.3451462
1690.0000000	3.0256000	-20.6444321
1690.0000000	3.0743999	-28.7609158
1690.0000000	3.1231999	-24.0257378
1690.0000000	3.1719999	-29.0552597
1690.0000000	3.2208002	-488.4522705
1690.0000000	3.2695999	-955.6964111
1690.0000000	3.3183999	-20.5028610
1690.0000000	3.3671999	-3298.7297363
1690.0000000	3.4159999	1182.6837158
1690.0000000	3.4648001	-7.5229654
1690.0000000	3.5135999	-26.8367577
1690.0000000	3.5623999	-313.2660828

1690.0000000	3.6111999	-1965.2072754
1690.0000000	3.6600001	-445.3634644
1690.0000000	3.7088001	-587.8682251
1690.0000000	3.7575998	-920.0559692
1690.0000000	3.8063998	-1669.0767822
1690.0000000	3.8552001	-2422.9916992
1690.0000000	3.9040000	-2375.5866699
1690.0000000	3.9528000	1085.5307617
1690.0000000	4.0015998	-237.8575134
1690.0000000	4.0503998	664.3430176
1690.0000000	4.0991998	84.1059265
1690.0000000	4.1479998	-633.8186035
1690.0000000	4.1967998	1.8340664
1690.0000000	4.2456002	-240.9466400
1690.0000000	4.2943997	-472.5670776
1690.0000000	4.3431997	-13.4991570
1690.0000000	4.3920002	-240.5915985
1690.0000000	4.4407997	-22.3239441
1690.0000000	4.4896002	-15.5421591
1690.0000000	4.5383997	-634.9726563
1690.0000000	4.5871997	-12.9938869
1690.0000000	4.6360002	-21.5022926
1690.0000000	4.6847997	-27.9800034
1690.0000000	4.7336001	-19.5067825
1690.0000000	4.7824001	-16.5473785
1690.0000000	4.8311996	-1433.3845215
1690.0000000	4.8800001	221.3474274
1690.0000000	4.9287996	-196.2622833
1690.0000000	4.9776001	1029.2864990
1690.0000000	5.0264001	83.2797089
1690.0000000	5.0751996	-293.1407471
1690.0000000	5.1240001	-12.3794537
1690.0000000	5.1727996	549.4919434
1690.0000000	5.2216001	31.3162498
1690.0000000	5.2704000	-156.7928162
1690.0000000	5.3191996	-38.8551788
1690.0000000	5.3680000	-3486.0759277
1690.0000000	5.4168000	519.4545288
1690.0000000	5.4656000	-198.1394653
1690.0000000	5.5144000	37.3016586
1690.0000000	5.5631995	481.2467957
1690.0000000	5.6120000	387.3989258
1690.0000000	5.6608000	-36.7912598
1690.0000000	5.7096000	61.4227448
1690.0000000	5.7584000	130.9636841
1690.0000000	5.8071995	-11.5827579
1690.0000000	5.8559999	29.1825962
1690.0000000	5.9047999	-16.5914555
1690.0000000	5.9535999	-39.4369316
1690.0000000	6.0023999	-1551.1977539
1690.0000000	6.0511999	-14.5832977
1690.0000000	6.0999999	-19.3499107
1690.0000000	6.1487999	-13.3063259
1690.0000000	6.1975999	-23.0945129
1690.0000000	6.2463999	-1767.1306152
1690.0000000	6.2951999	-19.0124073
1690.0000000	6.3439999	-338.1605835
1690.0000000	6.3927999	-15.0468187

1690.0000000	6.4416003	-12.4275169
1690.0000000	6.4903998	-11.3114567
1690.0000000	6.5391998	-11.8542662
1690.0000000	6.5879998	336.4318848
1690.0000000	6.6367998	-12.1227474
1690.0000000	6.6856003	-13.8268547
1690.0000000	6.7343998	-13.4707565
1690.0000000	6.7831998	-14.3435450
1690.0000000	6.8319998	57.1082230
1690.0000000	6.8807998	-14.0397758
1690.0000000	6.9296002	-396.6905212
1690.0000000	6.9783998	-5.9721904
1690.0000000	7.0271997	-14.0663376
1690.0000000	7.0760002	8.2804251
1690.0000000	7.1247997	-1860.3797607
1690.0000000	7.1736002	-1491.9884033
1690.0000000	7.2223997	-849.2251587
1690.0000000	7.2711997	-184.4029388
1690.0000000	7.3200002	-18.0616837
1690.0000000	7.3687997	162.2797089
1690.0000000	7.4176002	340.2548218
1690.0000000	7.4663997	267.0480347
1690.0000000	7.5151997	-830.3671265
1690.0000000	7.5640001	-917.0592041
1690.0000000	7.6127996	-2159.7031250
1690.0000000	7.6616001	-694.1374512
1690.0000000	7.7104001	-357.1809082
1690.0000000	7.7591996	36.3455658
1690.0000000	7.8080001	353.6024780
1690.0000000	7.8567996	466.2456665
1690.0000000	7.9056001	-970.2193604
1690.0000000	7.9544001	1543.5031738
1690.0000000	8.0031996	1991.7292480
1690.0000000	8.0520000	1987.9245605
1690.0000000	8.1007996	2103.6540527
1690.0000000	8.1496000	2243.5700684
1690.0000000	8.1983995	1395.7246094
1690.0000000	8.2472000	-3194.2138672
1690.0000000	8.2959995	-1132.4522705
1690.0000000	8.3448000	-901.9977417
1690.0000000	8.3935995	-826.6852417
1690.0000000	8.4423990	-356.3202515
1690.0000000	8.4912004	-831.4155884
1690.0000000	8.5400000	-2242.2292480
1690.0000000	8.5887995	-1087.2036133
1690.0000000	8.6375999	211.8405609
1690.0000000	8.6863995	1917.0601807
1690.0000000	8.7351999	-712.9454956
1690.0000000	8.7840004	813.6956177
1690.0000000	8.8327999	43.0081406
1690.0000000	8.8815994	-11.5081911
1690.0000000	8.9303989	-23.6944599
1690.0000000	8.9792004	-11.5846491
1690.0000000	9.0279999	-5.9559221
1690.0000000	9.0767994	6.6547031
1690.0000000	9.1255999	-17.1268272
1690.0000000	9.1743994	-10.1005964
1690.0000000	9.2231998	35.9552841

1690.0000000	9.2720003	-8.4829350
1690.0000000	9.3207998	-9.8342743
1690.0000000	9.3695993	-10.7789516
1690.0000000	9.4183998	-11.7455320
1690.0000000	9.4672003	-11.1640911
1690.0000000	9.5159998	-14.1045094
1690.0000000	9.5648003	-0.6190462
1690.0000000	9.6135998	-13.3430328
1690.0000000	9.6623993	-10.2397614
1690.0000000	9.7111998	-10.1759014
1690.0000000	9.7600002	-10.8603697
1690.0000000	9.8087997	-10.9953117
1690.0000000	9.8575993	2.8936043
1690.0000000	9.9063997	-8.0302935
1690.0000000	9.9552002	-10.9068909
1690.0000000	10.0039997	-10.0913048
1700.0000000	0.3416000	-36.8028679
1700.0000000	0.3904000	-38.9833145
1700.0000000	0.4392000	-39.3103256
1700.0000000	0.4880000	-39.5090370
1700.0000000	0.5368000	-39.6935692
1700.0000000	0.5856000	-39.2387123
1700.0000000	0.6344000	-38.2465553
1700.0000000	0.6832000	-36.3172264
1700.0000000	0.7320000	-36.1412010
1700.0000000	0.7808000	-36.5419197
1700.0000000	0.8296000	-35.8875885
1700.0000000	0.8784000	-34.7894821
1700.0000000	0.9272000	-34.2673912
1700.0000000	0.9760000	-31.8583069
1700.0000000	1.0247999	-30.3483238
1700.0000000	1.0735999	-30.4363785
1700.0000000	1.1224000	-30.6700859
1700.0000000	1.1711999	-31.1632843
1700.0000000	1.2200000	-29.5729733
1700.0000000	1.2687999	-30.2002621
1700.0000000	1.3176000	-30.7639771
1700.0000000	1.3664000	-30.5418186
1700.0000000	1.4152000	-30.1762161
1700.0000000	1.4640000	-29.4990730
1700.0000000	1.5128000	-32.7671394
1700.0000000	1.5616000	-30.7850819
1700.0000000	1.6104001	-1384.3065186
1700.0000000	1.6592000	-53.2708244
1700.0000000	1.7079999	-27.5123444
1700.0000000	1.7567999	-27.7634449
1700.0000000	1.8055999	-27.5358276
1700.0000000	1.8544000	-26.5305271
1700.0000000	1.9031999	-26.1028214
1700.0000000	1.9520000	-26.6493721
1700.0000000	2.0007999	-26.1075096
1700.0000000	2.0495999	-26.3055878
1700.0000000	2.0983999	-47.6212311
1700.0000000	2.1471999	-19.8488026
1700.0000000	2.1960001	-25.1703033
1700.0000000	2.2448001	-24.6097946
1700.0000000	2.2935998	-23.6260376
1700.0000000	2.3423998	-21.9693623

1700.0000000	2.3912001	-10.3845806
1700.0000000	2.4400001	-23.0065441
1700.0000000	2.4888000	-23.4453182
1700.0000000	2.5375998	-13.4297562
1700.0000000	2.5863998	-11.7302933
1700.0000000	2.6352000	-10.0045490
1700.0000000	2.6840000	-16.2185707
1700.0000000	2.7328000	115.2164078
1700.0000000	2.7815998	136.4544067
1700.0000000	2.8304000	-23.7064457
1700.0000000	2.8792000	-20.8393421
1700.0000000	2.9280000	-22.5334244
1700.0000000	2.9768000	-17.3535175
1700.0000000	3.0256000	-20.6470165
1700.0000000	3.0743999	-28.7495289
1700.0000000	3.1231999	-24.0222836
1700.0000000	3.1719999	-29.0469666
1700.0000000	3.2208002	-488.2145386
1700.0000000	3.2695999	-955.2213135
1700.0000000	3.3183999	-20.5030594
1700.0000000	3.3671999	-3297.2680664
1700.0000000	3.4159999	1182.1484375
1700.0000000	3.4648001	-7.5286741
1700.0000000	3.5135999	-26.8337669
1700.0000000	3.5623999	-313.1365051
1700.0000000	3.6111999	-1964.3475342
1700.0000000	3.6600001	-445.1753540
1700.0000000	3.7088001	-587.6168823
1700.0000000	3.7575998	-919.6574707
1700.0000000	3.8063998	-1668.3469238
1700.0000000	3.8552001	-2421.9282227
1700.0000000	3.9040000	-2374.5441895
1700.0000000	3.9528000	1085.0423584
1700.0000000	4.0015998	-237.7603912
1700.0000000	4.0503998	664.0404663
1700.0000000	4.0991998	84.0602417
1700.0000000	4.1479998	-633.5463257
1700.0000000	4.1967998	1.8248100
1700.0000000	4.2456002	-240.8483429
1700.0000000	4.2943997	-472.3665161
1700.0000000	4.3431997	-13.5013380
1700.0000000	4.3920002	-240.4934845
1700.0000000	4.4407997	-22.3221684
1700.0000000	4.4896002	-15.5430412
1700.0000000	4.5383997	-634.7115479
1700.0000000	4.5871997	-12.9959030
1700.0000000	4.6360002	-21.5007515
1700.0000000	4.6847997	-27.9760780
1700.0000000	4.7336001	-19.5060596
1700.0000000	4.7824001	-16.5478344
1700.0000000	4.8311996	-1432.8532715
1700.0000000	4.8800001	221.2578735
1700.0000000	4.9287996	-196.1950226
1700.0000000	4.9776001	1028.8939209
1700.0000000	5.0264001	83.2421875
1700.0000000	5.0751996	-293.0371399
1700.0000000	5.1240001	-12.3803749
1700.0000000	5.1727996	549.3760376

1700.0000000	5.2216001	31.3068008
1700.0000000	5.2704000	-156.7647247
1700.0000000	5.3191996	-38.8506317
1700.0000000	5.3680000	-3485.3835449
1700.0000000	5.4168000	519.3477173
1700.0000000	5.4656000	-198.1032867
1700.0000000	5.5144000	37.2910004
1700.0000000	5.5631995	481.1482239
1700.0000000	5.6120000	387.3189392
1700.0000000	5.6608000	-36.7871704
1700.0000000	5.7096000	61.4073105
1700.0000000	5.7584000	130.9347534
1700.0000000	5.8071995	-11.5836277
1700.0000000	5.8559999	29.1737423
1700.0000000	5.9047999	-16.5912571
1700.0000000	5.9535999	-39.4321938
1700.0000000	6.0023999	-1550.8933105
1700.0000000	6.0511999	-14.5835094
1700.0000000	6.0999999	-19.3490791
1700.0000000	6.1487999	-13.3067894
1700.0000000	6.1975999	-23.0927486
1700.0000000	6.2463999	-1766.7832031
1700.0000000	6.2951999	-19.0115929
1700.0000000	6.3439999	-338.0965271
1700.0000000	6.3927999	-15.0467997
1700.0000000	6.4416003	-12.4281034
1700.0000000	6.4903998	-11.3121328
1700.0000000	6.5391998	-11.8549471
1700.0000000	6.5879998	335.7411804
1700.0000000	6.6367998	-12.1232967
1700.0000000	6.6856003	-13.8270245
1700.0000000	6.7343998	-13.4709730
1700.0000000	6.7831998	-14.3431292
1700.0000000	6.8319998	57.0891113
1700.0000000	6.8807998	-14.0396376
1700.0000000	6.9296002	-395.9290771
1700.0000000	6.9783998	-5.9850144
1700.0000000	7.0271997	-14.0653095
1700.0000000	7.0760002	8.2382383
1700.0000000	7.1247997	-1856.4339600
1700.0000000	7.1736002	-1488.8255615
1700.0000000	7.2223997	-847.4320068
1700.0000000	7.2711997	-184.0351563
1700.0000000	7.3200002	-18.0496960
1700.0000000	7.3687997	161.9051819
1700.0000000	7.4176002	339.4989929
1700.0000000	7.4663997	266.4504089
1700.0000000	7.5151997	-828.6060791
1700.0000000	7.5640001	-915.1117554
1700.0000000	7.6127996	-2155.0856934
1700.0000000	7.6616001	-692.6661377
1700.0000000	7.7104001	-356.4346313
1700.0000000	7.7591996	36.2445183
1700.0000000	7.8080001	352.8171082
1700.0000000	7.8567996	465.2175903
1700.0000000	7.9056001	-968.1464233
1700.0000000	7.9544001	1540.1932373
1700.0000000	8.0031996	1987.4597168

1700.0000000	8.0520000	1983.6625977
1700.0000000	8.1007996	2099.1445313
1700.0000000	8.1496000	2238.7619629
1700.0000000	8.1983995	1392.7320557
1700.0000000	8.2472000	-3187.3789063
1700.0000000	8.2959995	-1130.0321045
1700.0000000	8.3448000	-900.0686035
1700.0000000	8.3935995	-824.9175415
1700.0000000	8.4423990	-355.5622253
1700.0000000	8.4912004	-829.6394043
1700.0000000	8.5400000	-2237.4294434
1700.0000000	8.5887995	-1084.8820801
1700.0000000	8.6375999	211.3792419
1700.0000000	8.6863995	1912.9417725
1700.0000000	8.7351999	-711.4212036
1700.0000000	8.7840004	811.9389038
1700.0000000	8.8327999	42.8965340
1700.0000000	8.8815994	-11.5083656
1700.0000000	8.9303989	-23.6907387
1700.0000000	8.9792004	-11.5847321
1700.0000000	9.0279999	-5.9576907
1700.0000000	9.0767994	6.6489868
1700.0000000	9.1255999	-17.1251125
1700.0000000	9.1743994	-10.1009979
1700.0000000	9.2231998	35.9395523
1700.0000000	9.2720003	-8.4838715
1700.0000000	9.3207998	-9.8348141
1700.0000000	9.3695993	-10.7791786
1700.0000000	9.4183998	-11.7454081
1700.0000000	9.4672003	-11.1641321
1700.0000000	9.5159998	-14.1036186
1700.0000000	9.5648003	-0.6224194
1700.0000000	9.6135998	-13.3422422
1700.0000000	9.6623993	-10.2400370
1700.0000000	9.7111998	-10.1761732
1700.0000000	9.7600002	-10.8604116
1700.0000000	9.8087997	-10.9953194
1700.0000000	9.8575993	2.8891821
1700.0000000	9.9063997	-8.0312080
1700.0000000	9.9552002	-10.9068947
1700.0000000	10.0039997	-10.0915527
1710.0000000	0.3416000	-36.1150055
1710.0000000	0.3904000	-38.5290794
1710.0000000	0.4392000	-39.3039818
1710.0000000	0.4880000	-39.5078278
1710.0000000	0.5368000	-39.6947441
1710.0000000	0.5856000	-39.2373314
1710.0000000	0.6344000	-38.2468681
1710.0000000	0.6832000	-36.3194504
1710.0000000	0.7320000	-36.1432533
1710.0000000	0.7808000	-36.5420876
1710.0000000	0.8296000	-35.8857460
1710.0000000	0.8784000	-34.7799149
1710.0000000	0.9272000	-34.2680321
1710.0000000	0.9760000	-31.8557835
1710.0000000	1.0247999	-30.3495598
1710.0000000	1.0735999	-30.4499798
1710.0000000	1.1224000	-30.6682129

1710.0000000	1.1711999	-31.1569824
1710.0000000	1.2200000	-29.5291748
1710.0000000	1.2687999	-30.1789341
1710.0000000	1.3176000	-30.7575912
1710.0000000	1.3664000	-30.5388756
1710.0000000	1.4152000	-30.1764202
1710.0000000	1.4640000	-29.4973640
1710.0000000	1.5128000	-32.6699600
1710.0000000	1.5616000	-30.7432404
1710.0000000	1.6104001	-1381.7636719
1710.0000000	1.6592000	-52.9768906
1710.0000000	1.7079999	-27.5160923
1710.0000000	1.7567999	-27.7634640
1710.0000000	1.8055999	-27.5649071
1710.0000000	1.8544000	-25.9490623
1710.0000000	1.9031999	-25.8159046
1710.0000000	1.9520000	-26.7239761
1710.0000000	2.0007999	-26.1128902
1710.0000000	2.0495999	-26.3076057
1710.0000000	2.0983999	-46.1454506
1710.0000000	2.1471999	-20.1160698
1710.0000000	2.1960001	-25.1662121
1710.0000000	2.2448001	-24.5918827
1710.0000000	2.2935998	-23.5664139
1710.0000000	2.3423998	-21.9837685
1710.0000000	2.3912001	-11.0061264
1710.0000000	2.4400001	-23.0090313
1710.0000000	2.4888000	-23.4451008
1710.0000000	2.5375998	-13.4468641
1710.0000000	2.5863998	-11.7500525
1710.0000000	2.6352000	-10.0272932
1710.0000000	2.6840000	-16.2305107
1710.0000000	2.7328000	114.9504013
1710.0000000	2.7815998	136.1479797
1710.0000000	2.8304000	-23.7051430
1710.0000000	2.8792000	-20.8426056
1710.0000000	2.9280000	-22.5328560
1710.0000000	2.9768000	-17.3618889
1710.0000000	3.0256000	-20.6496010
1710.0000000	3.0743999	-28.7381420
1710.0000000	3.1231999	-24.0188274
1710.0000000	3.1719999	-29.0386734
1710.0000000	3.2208002	-487.9768066
1710.0000000	3.2695999	-954.7464600
1710.0000000	3.3183999	-20.5032578
1710.0000000	3.3671999	-3295.8063965
1710.0000000	3.4159999	1181.6131592
1710.0000000	3.4648001	-7.5343819
1710.0000000	3.5135999	-26.8307762
1710.0000000	3.5623999	-313.0068970
1710.0000000	3.6111999	-1963.4877930
1710.0000000	3.6600001	-444.9871826
1710.0000000	3.7088001	-587.3654785
1710.0000000	3.7575998	-919.2589111
1710.0000000	3.8063998	-1667.6170654
1710.0000000	3.8552001	-2420.8649902
1710.0000000	3.9040000	-2373.5017090
1710.0000000	3.9528000	1084.5540771

1710.0000000	4.0015998	-237.6632843
1710.0000000	4.0503998	663.7379150
1710.0000000	4.0991998	84.0145569
1710.0000000	4.1479998	-633.2741089
1710.0000000	4.1967998	1.8155537
1710.0000000	4.2456002	-240.7500458
1710.0000000	4.2943997	-472.1659241
1710.0000000	4.3431997	-13.5035191
1710.0000000	4.3920002	-240.3953705
1710.0000000	4.4407997	-22.3203926
1710.0000000	4.4896002	-15.5439243
1710.0000000	4.5383997	-634.4505005
1710.0000000	4.5871997	-12.9979191
1710.0000000	4.6360002	-21.4992085
1710.0000000	4.6847997	-27.9721527
1710.0000000	4.7336001	-19.5053368
1710.0000000	4.7824001	-16.5482883
1710.0000000	4.8311996	-1432.3218994
1710.0000000	4.8800001	221.1683197
1710.0000000	4.9287996	-196.1277618
1710.0000000	4.9776001	1028.5014648
1710.0000000	5.0264001	83.2046661
1710.0000000	5.0751996	-292.9335022
1710.0000000	5.1240001	-12.3812962
1710.0000000	5.1727996	549.2601929
1710.0000000	5.2216001	31.2973595
1710.0000000	5.2704000	-156.7366028
1710.0000000	5.3191996	-38.8460846
1710.0000000	5.3680000	-3484.6911621
1710.0000000	5.4168000	519.2409668
1710.0000000	5.4656000	-198.0670929
1710.0000000	5.5144000	37.2803421
1710.0000000	5.5631995	481.0496521
1710.0000000	5.6120000	387.2389832
1710.0000000	5.6608000	-36.7830811
1710.0000000	5.7096000	61.3918839
1710.0000000	5.7584000	130.9058228
1710.0000000	5.8071995	-11.5844984
1710.0000000	5.8559999	29.1648922
1710.0000000	5.9047999	-16.5910606
1710.0000000	5.9535999	-39.4274559
1710.0000000	6.0023999	-1550.5889893
1710.0000000	6.0511999	-14.5837202
1710.0000000	6.0999999	-19.3482475
1710.0000000	6.1487999	-13.3072519
1710.0000000	6.1975999	-23.0909824
1710.0000000	6.2463999	-1766.4357910
1710.0000000	6.2951999	-19.0107784
1710.0000000	6.3439999	-338.0324707
1710.0000000	6.3927999	-15.0467815
1710.0000000	6.4416003	-12.4286909
1710.0000000	6.4903998	-11.3128080
1710.0000000	6.5391998	-11.8556280
1710.0000000	6.5879998	335.0504761
1710.0000000	6.6367998	-12.1238451
1710.0000000	6.6856003	-13.8271952
1710.0000000	6.7343998	-13.4711885
1710.0000000	6.7831998	-14.3427143

1710.0000000	6.8319998	57.0699997
1710.0000000	6.8807998	-14.0394983
1710.0000000	6.9296002	-395.1676331
1710.0000000	6.9783998	-5.9978380
1710.0000000	7.0271997	-14.0642824
1710.0000000	7.0760002	8.1960554
1710.0000000	7.1247997	-1852.4880371
1710.0000000	7.1736002	-1485.6625977
1710.0000000	7.2223997	-845.6388550
1710.0000000	7.2711997	-183.6673737
1710.0000000	7.3200002	-18.0377083
1710.0000000	7.3687997	161.5306549
1710.0000000	7.4176002	338.7431641
1710.0000000	7.4663997	265.8527527
1710.0000000	7.5151997	-826.8450317
1710.0000000	7.5640001	-913.1643677
1710.0000000	7.6127996	-2150.4680176
1710.0000000	7.6616001	-691.1948242
1710.0000000	7.7104001	-355.6883545
1710.0000000	7.7591996	36.1434669
1710.0000000	7.8080001	352.0317688
1710.0000000	7.8567996	464.1894531
1710.0000000	7.9056001	-966.0735474
1710.0000000	7.9544001	1536.8833008
1710.0000000	8.0031996	1983.1900635
1710.0000000	8.0520000	1979.4005127
1710.0000000	8.1007996	2094.6350098
1710.0000000	8.1496000	2233.9538574
1710.0000000	8.1983995	1389.7395020
1710.0000000	8.2472000	-3180.5441895
1710.0000000	8.2959995	-1127.6119385
1710.0000000	8.3448000	-898.1394043
1710.0000000	8.3935995	-823.1499023
1710.0000000	8.4423990	-354.8042297
1710.0000000	8.4912004	-827.8631592
1710.0000000	8.5400000	-2232.6293945
1710.0000000	8.5887995	-1082.5605469
1710.0000000	8.6375999	210.9179230
1710.0000000	8.6863995	1908.8234863
1710.0000000	8.7351999	-709.8969727
1710.0000000	8.7840004	810.1821289
1710.0000000	8.8327999	42.7849236
1710.0000000	8.8815994	-11.5085392
1710.0000000	8.9303989	-23.6870193
1710.0000000	8.9792004	-11.5848141
1710.0000000	9.0279999	-5.9594593
1710.0000000	9.0767994	6.6432686
1710.0000000	9.1255999	-17.1233978
1710.0000000	9.1743994	-10.1014004
1710.0000000	9.2231998	35.9238205
1710.0000000	9.2720003	-8.4848080
1710.0000000	9.3207998	-9.8353529
1710.0000000	9.3695993	-10.7794046
1710.0000000	9.4183998	-11.7452831
1710.0000000	9.4672003	-11.1641731
1710.0000000	9.5159998	-14.1027279
1710.0000000	9.5648003	-0.6257915
1710.0000000	9.6135998	-13.3414516

1710.0000000	9.6623993	-10.2403116
1710.0000000	9.7111998	-10.1764441
1710.0000000	9.7600002	-10.8604536
1710.0000000	9.8087997	-10.9953270
1710.0000000	9.8575993	2.8847589
1710.0000000	9.9063997	-8.0321217
1710.0000000	9.9552002	-10.9068995
1710.0000000	10.0039997	-10.0917997
1720.0000000	0.3416000	-35.7417107
1720.0000000	0.3904000	-37.8582840
1720.0000000	0.4392000	-39.2976379
1720.0000000	0.4880000	-39.5066185
1720.0000000	0.5368000	-39.6959152
1720.0000000	0.5856000	-39.2359467
1720.0000000	0.6344000	-38.2471771
1720.0000000	0.6832000	-36.3216782
1720.0000000	0.7320000	-36.1453056
1720.0000000	0.7808000	-36.5422554
1720.0000000	0.8296000	-35.8838997
1720.0000000	0.8784000	-34.7703514
1720.0000000	0.9272000	-32.4715614
1720.0000000	0.9760000	-30.1192932
1720.0000000	1.0247999	-28.6437721
1720.0000000	1.0735999	-28.0966339
1720.0000000	1.1224000	-28.6740360
1720.0000000	1.1711999	-29.6857166
1720.0000000	1.2200000	-29.4853764
1720.0000000	1.2687999	-30.1576080
1720.0000000	1.3176000	-30.7512054
1720.0000000	1.3664000	-30.5359306
1720.0000000	1.4152000	-30.1766243
1720.0000000	1.4640000	-29.4956551
1720.0000000	1.5128000	-32.5727844
1720.0000000	1.5616000	-30.7013988
1720.0000000	1.6104001	-1379.2208252
1720.0000000	1.6592000	-52.6829605
1720.0000000	1.7079999	-27.5198402
1720.0000000	1.7567999	-27.7634830
1720.0000000	1.8055999	-27.1423855
1720.0000000	1.8544000	-25.4392395
1720.0000000	1.9031999	-25.1846333
1720.0000000	1.9520000	-26.7985802
1720.0000000	2.0007999	-26.1182709
1720.0000000	2.0495999	-26.3096256
1720.0000000	2.0983999	-44.6696701
1720.0000000	2.1471999	-20.3833370
1720.0000000	2.1960001	-25.1621208
1720.0000000	2.2448001	-24.5739708
1720.0000000	2.2935998	-23.5067902
1720.0000000	2.3423998	-21.9981728
1720.0000000	2.3912001	-11.6276722
1720.0000000	2.4400001	-23.0115185
1720.0000000	2.4888000	-23.4448833
1720.0000000	2.5375998	-13.4639711
1720.0000000	2.5863998	-11.7698116
1720.0000000	2.6352000	-10.0500374
1720.0000000	2.6840000	-16.2424526
1720.0000000	2.7328000	114.6843796

1720.0000000	2.7815998	135.8415833
1720.0000000	2.8304000	-23.7038383
1720.0000000	2.8792000	-20.8458691
1720.0000000	2.9280000	-22.5322857
1720.0000000	2.9768000	-17.3702583
1720.0000000	3.0256000	-20.6521854
1720.0000000	3.0743999	-28.7267532
1720.0000000	3.1231999	-24.0153732
1720.0000000	3.1719999	-29.0303783
1720.0000000	3.2208002	-487.7390442
1720.0000000	3.2695999	-954.2713623
1720.0000000	3.3183999	-20.5034561
1720.0000000	3.3671999	-3294.3449707
1720.0000000	3.4159999	1181.0778809
1720.0000000	3.4648001	-7.5400896
1720.0000000	3.5135999	-26.8277855
1720.0000000	3.5623999	-312.8773193
1720.0000000	3.6111999	-1962.6280518
1720.0000000	3.6600001	-444.7990723
1720.0000000	3.7088001	-587.1140747
1720.0000000	3.7575998	-918.8604126
1720.0000000	3.8063998	-1666.8872070
1720.0000000	3.8552001	-2419.8015137
1720.0000000	3.9040000	-2372.4592285
1720.0000000	3.9528000	1084.0657959
1720.0000000	4.0015998	-237.5661774
1720.0000000	4.0503998	663.4353638
1720.0000000	4.0991998	83.9688721
1720.0000000	4.1479998	-633.0018921
1720.0000000	4.1967998	1.8062973
1720.0000000	4.2456002	-240.6517334
1720.0000000	4.2943997	-471.9653625
1720.0000000	4.3431997	-13.5056992
1720.0000000	4.3920002	-240.2972565
1720.0000000	4.4407997	-22.3186169
1720.0000000	4.4896002	-15.5448065
1720.0000000	4.5383997	-634.1894531
1720.0000000	4.5871997	-12.9999352
1720.0000000	4.6360002	-21.4976654
1720.0000000	4.6847997	-27.9682274
1720.0000000	4.7336001	-19.5046139
1720.0000000	4.7824001	-16.5487423
1720.0000000	4.8311996	-1431.7906494
1720.0000000	4.8800001	221.0787659
1720.0000000	4.9287996	-196.0605011
1720.0000000	4.9776001	1028.1090088
1720.0000000	5.0264001	83.1671448
1720.0000000	5.0751996	-292.8298645
1720.0000000	5.1240001	-12.3822174
1720.0000000	5.1727996	549.1443481
1720.0000000	5.2216001	31.2879181
1720.0000000	5.2704000	-156.7085114
1720.0000000	5.3191996	-38.8415298
1720.0000000	5.3680000	-3483.9987793
1720.0000000	5.4168000	519.1342163
1720.0000000	5.4656000	-198.0309143
1720.0000000	5.5144000	37.2696915
1720.0000000	5.5631995	480.9510803

1720.0000000	5.6120000	387.1589966
1720.0000000	5.6608000	-36.7789917
1720.0000000	5.7096000	61.3764496
1720.0000000	5.7584000	130.8768921
1720.0000000	5.8071995	-11.5853691
1720.0000000	5.8559999	29.1560383
1720.0000000	5.9047999	-16.5908623
1720.0000000	5.9535999	-39.4227142
1720.0000000	6.0023999	-1550.2844238
1720.0000000	6.0511999	-14.5839319
1720.0000000	6.0999999	-19.3474140
1720.0000000	6.1487999	-13.3077145
1720.0000000	6.1975999	-23.0892181
1720.0000000	6.2463999	-1766.0885010
1720.0000000	6.2951999	-19.0099640
1720.0000000	6.3439999	-337.9684448
1720.0000000	6.3927999	-15.0467634
1720.0000000	6.4416003	-12.4292774
1720.0000000	6.4903998	-11.3134842
1720.0000000	6.5391998	-11.8563089
1720.0000000	6.5879998	334.3597717
1720.0000000	6.6367998	-12.1243944
1720.0000000	6.6856003	-13.8273649
1720.0000000	6.7343998	-13.4714041
1720.0000000	6.7831998	-14.3422985
1720.0000000	6.8319998	57.0508804
1720.0000000	6.8807998	-14.0393600
1720.0000000	6.9296002	-394.4061890
1720.0000000	6.9783998	-6.0106611
1720.0000000	7.0271997	-14.0632544
1720.0000000	7.0760002	8.1538687
1720.0000000	7.1247997	-1848.5422363
1720.0000000	7.1736002	-1482.4997559
1720.0000000	7.2223997	-843.8457031
1720.0000000	7.2711997	-183.2995911
1720.0000000	7.3200002	-18.0257187
1720.0000000	7.3687997	161.1561279
1720.0000000	7.4176002	337.9873352
1720.0000000	7.4663997	265.2550964
1720.0000000	7.5151997	-825.0839844
1720.0000000	7.5640001	-911.2169800
1720.0000000	7.6127996	-2145.8503418
1720.0000000	7.6616001	-689.7234497
1720.0000000	7.7104001	-354.9421387
1720.0000000	7.7591996	36.0424194
1720.0000000	7.8080001	351.2463989
1720.0000000	7.8567996	463.1613770
1720.0000000	7.9056001	-964.0006104
1720.0000000	7.9544001	1533.5733643
1720.0000000	8.0031996	1978.9205322
1720.0000000	8.0520000	1975.1385498
1720.0000000	8.1007996	2090.1254883
1720.0000000	8.1496000	2229.1459961
1720.0000000	8.1983995	1386.7470703
1720.0000000	8.2472000	-3173.7094727
1720.0000000	8.2959995	-1125.1917725
1720.0000000	8.3448000	-896.2102051
1720.0000000	8.3935995	-821.3822021

1720.0000000	8.4423990	-354.0462036
1720.0000000	8.4912004	-826.0869751
1720.0000000	8.5400000	-2227.8295898
1720.0000000	8.5887995	-1080.2390137
1720.0000000	8.6375999	210.4566040
1720.0000000	8.6863995	1904.7050781
1720.0000000	8.7351999	-708.3726807
1720.0000000	8.7840004	808.4254150
1720.0000000	8.8327999	42.6733208
1720.0000000	8.8815994	-11.5087137
1720.0000000	8.9303989	-23.6833000
1720.0000000	8.9792004	-11.5848961
1720.0000000	9.0279999	-5.9612274
1720.0000000	9.0767994	6.6375504
1720.0000000	9.1255999	-17.1216850
1720.0000000	9.1743994	-10.1018019
1720.0000000	9.2231998	35.9080887
1720.0000000	9.2720003	-8.4857445
1720.0000000	9.3207998	-9.8358927
1720.0000000	9.3695993	-10.7796316
1720.0000000	9.4183998	-11.7451582
1720.0000000	9.4672003	-11.1642151
1720.0000000	9.5159998	-14.1018372
1720.0000000	9.5648003	-0.6291637
1720.0000000	9.6135998	-13.3406601
1720.0000000	9.6623993	-10.2405863
1720.0000000	9.7111998	-10.1767159
1720.0000000	9.7600002	-10.8604946
1720.0000000	9.8087997	-10.9953356
1720.0000000	9.8575993	2.8803368
1720.0000000	9.9063997	-8.0330353
1720.0000000	9.9552002	-10.9069033
1720.0000000	10.0039997	-10.0920477
1730.0000000	0.3416000	-35.9812355
1730.0000000	0.3904000	-37.8536491
1730.0000000	0.4392000	-39.2912941
1730.0000000	0.4880000	-39.5054092
1730.0000000	0.5368000	-39.6970901
1730.0000000	0.5856000	-39.2345657
1730.0000000	0.6344000	-38.2474861
1730.0000000	0.6832000	-36.3239021
1730.0000000	0.7320000	-36.1473618
1730.0000000	0.7808000	-36.5424271
1730.0000000	0.8296000	-35.7895470
1730.0000000	0.8784000	-34.9014511
1730.0000000	0.9272000	-31.8220406
1730.0000000	0.9760000	-29.8887444
1730.0000000	1.0247999	-28.1245613
1730.0000000	1.0735999	-27.3908501
1730.0000000	1.1224000	-27.9659615
1730.0000000	1.1711999	-28.7781448
1730.0000000	1.2200000	-28.9101467
1730.0000000	1.2687999	-30.2519703
1730.0000000	1.3176000	-30.6647797
1730.0000000	1.3664000	-30.5329876
1730.0000000	1.4152000	-30.1768284
1730.0000000	1.4640000	-29.4939461
1730.0000000	1.5128000	-32.4756050

1730.0000000	1.5616000	-30.6595573
1730.0000000	1.6104001	-1376.6781006
1730.0000000	1.6592000	-52.3890305
1730.0000000	1.7079999	-27.5235863
1730.0000000	1.7567999	-27.7635021
1730.0000000	1.8055999	-26.7198639
1730.0000000	1.8544000	-25.3570004
1730.0000000	1.9031999	-25.6797409
1730.0000000	1.9520000	-26.8013630
1730.0000000	2.0007999	-26.1236515
1730.0000000	2.0495999	-26.3116436
1730.0000000	2.0983999	-43.1938934
1730.0000000	2.1471999	-20.6506042
1730.0000000	2.1960001	-25.1580296
1730.0000000	2.2448001	-24.5560608
1730.0000000	2.2935998	-23.4471664
1730.0000000	2.3423998	-22.0125771
1730.0000000	2.3912001	-12.2492180
1730.0000000	2.4400001	-23.0140038
1730.0000000	2.4888000	-23.4446659
1730.0000000	2.5375998	-13.4810791
1730.0000000	2.5863998	-11.7895708
1730.0000000	2.6352000	-10.0727816
1730.0000000	2.6840000	-16.2543945
1730.0000000	2.7328000	114.4183578
1730.0000000	2.7815998	135.5351563
1730.0000000	2.8304000	-23.7025337
1730.0000000	2.8792000	-20.8491325
1730.0000000	2.9280000	-22.5317173
1730.0000000	2.9768000	-17.3786278
1730.0000000	3.0256000	-20.6547699
1730.0000000	3.0743999	-28.7153664
1730.0000000	3.1231999	-24.0119171
1730.0000000	3.1719999	-29.0220852
1730.0000000	3.2208002	-487.5013123
1730.0000000	3.2695999	-953.7965088
1730.0000000	3.3183999	-20.5036545
1730.0000000	3.3671999	-3292.8833008
1730.0000000	3.4159999	1180.5424805
1730.0000000	3.4648001	-7.5457983
1730.0000000	3.5135999	-26.8247948
1730.0000000	3.5623999	-312.7477112
1730.0000000	3.6111999	-1961.7683105
1730.0000000	3.6600001	-444.6109619
1730.0000000	3.7088001	-586.8627319
1730.0000000	3.7575998	-918.4618530
1730.0000000	3.8063998	-1666.1573486
1730.0000000	3.8552001	-2418.7380371
1730.0000000	3.9040000	-2371.4167480
1730.0000000	3.9528000	1083.5775146
1730.0000000	4.0015998	-237.4690704
1730.0000000	4.0503998	663.1328125
1730.0000000	4.0991998	83.9231873
1730.0000000	4.1479998	-632.7296753
1730.0000000	4.1967998	1.7970409
1730.0000000	4.2456002	-240.5534363
1730.0000000	4.2943997	-471.7648010
1730.0000000	4.3431997	-13.5078793

1730.0000000	4.3920002	-240.1991425
1730.0000000	4.4407997	-22.3168411
1730.0000000	4.4896002	-15.5456896
1730.0000000	4.5383997	-633.9283447
1730.0000000	4.5871997	-13.0019512
1730.0000000	4.6360002	-21.4961243
1730.0000000	4.6847997	-27.9643021
1730.0000000	4.7336001	-19.5038929
1730.0000000	4.7824001	-16.5491962
1730.0000000	4.8311996	-1431.2593994
1730.0000000	4.8800001	220.9892273
1730.0000000	4.9287996	-195.9932404
1730.0000000	4.9776001	1027.7165527
1730.0000000	5.0264001	83.1296234
1730.0000000	5.0751996	-292.7262268
1730.0000000	5.1240001	-12.3831387
1730.0000000	5.1727996	549.0284424
1730.0000000	5.2216001	31.2784691
1730.0000000	5.2704000	-156.6803894
1730.0000000	5.3191996	-38.8369827
1730.0000000	5.3680000	-3483.3063965
1730.0000000	5.4168000	519.0274048
1730.0000000	5.4656000	-197.9947357
1730.0000000	5.5144000	37.2590332
1730.0000000	5.5631995	480.8525085
1730.0000000	5.6120000	387.0790405
1730.0000000	5.6608000	-36.7749023
1730.0000000	5.7096000	61.3610153
1730.0000000	5.7584000	130.8479614
1730.0000000	5.8071995	-11.5862398
1730.0000000	5.8559999	29.1471882
1730.0000000	5.9047999	-16.5906639
1730.0000000	5.9535999	-39.4179764
1730.0000000	6.0023999	-1549.9801025
1730.0000000	6.0511999	-14.5841427
1730.0000000	6.0999999	-19.3465824
1730.0000000	6.1487999	-13.3081770
1730.0000000	6.1975999	-23.0874519
1730.0000000	6.2463999	-1765.7410889
1730.0000000	6.2951999	-19.0091496
1730.0000000	6.3439999	-337.9043579
1730.0000000	6.3927999	-15.0467453
1730.0000000	6.4416003	-12.4298639
1730.0000000	6.4903998	-11.3141603
1730.0000000	6.5391998	-11.8569908
1730.0000000	6.5879998	333.6690674
1730.0000000	6.6367998	-12.1249437
1730.0000000	6.6856003	-13.8275356
1730.0000000	6.7343998	-13.4716196
1730.0000000	6.7831998	-14.3418827
1730.0000000	6.8319998	57.0317688
1730.0000000	6.8807998	-14.0392218
1730.0000000	6.9296002	-393.6447449
1730.0000000	6.9783998	-6.0234852
1730.0000000	7.0271997	-14.0622263
1730.0000000	7.0760002	8.1116838
1730.0000000	7.1247997	-1844.5963135
1730.0000000	7.1736002	-1479.3369141

1730.0000000	7.2223997	-842.0525513
1730.0000000	7.2711997	-182.9318237
1730.0000000	7.3200002	-18.0137291
1730.0000000	7.3687997	160.7815857
1730.0000000	7.4176002	337.2315063
1730.0000000	7.4663997	264.6574402
1730.0000000	7.5151997	-823.3229370
1730.0000000	7.5640001	-909.2695923
1730.0000000	7.6127996	-2141.2329102
1730.0000000	7.6616001	-688.2520752
1730.0000000	7.7104001	-354.1958008
1730.0000000	7.7591996	35.9413719
1730.0000000	7.8080001	350.4610291
1730.0000000	7.8567996	462.1333008
1730.0000000	7.9056001	-961.9277344
1730.0000000	7.9544001	1530.2634277
1730.0000000	8.0031996	1974.6508789
1730.0000000	8.0520000	1970.8765869
1730.0000000	8.1007996	2085.6159668
1730.0000000	8.1496000	2224.3378906
1730.0000000	8.1983995	1383.7545166
1730.0000000	8.2472000	-3166.8745117
1730.0000000	8.2959995	-1122.7716064
1730.0000000	8.3448000	-894.2810669
1730.0000000	8.3935995	-819.6145630
1730.0000000	8.4423990	-353.2882080
1730.0000000	8.4912004	-824.3107300
1730.0000000	8.5400000	-2223.0297852
1730.0000000	8.5887995	-1077.9174805
1730.0000000	8.6375999	209.9952850
1730.0000000	8.6863995	1900.5866699
1730.0000000	8.7351999	-706.8484497
1730.0000000	8.7840004	806.6686401
1730.0000000	8.8327999	42.5617142
1730.0000000	8.8815994	-11.5088882
1730.0000000	8.9303989	-23.6795807
1730.0000000	8.9792004	-11.5849791
1730.0000000	9.0279999	-5.9629955
1730.0000000	9.0767994	6.6318321
1730.0000000	9.1255999	-17.1199703
1730.0000000	9.1743994	-10.1022043
1730.0000000	9.2231998	35.8923569
1730.0000000	9.2720003	-8.4866810
1730.0000000	9.3207998	-9.8364315
1730.0000000	9.3695993	-10.7798576
1730.0000000	9.4183998	-11.7450342
1730.0000000	9.4672003	-11.1642561
1730.0000000	9.5159998	-14.1009464
1730.0000000	9.5648003	-0.6325369
1730.0000000	9.6135998	-13.3398705
1730.0000000	9.6623993	-10.2408619
1730.0000000	9.7111998	-10.1769876
1730.0000000	9.7600002	-10.8605366
1730.0000000	9.8087997	-10.9953432
1730.0000000	9.8575993	2.8759146
1730.0000000	9.9063997	-8.0339489
1730.0000000	9.9552002	-10.9069080
1730.0000000	10.0039997	-10.0922947

1740.0000000	0.3416000	-36.1512260
1740.0000000	0.3904000	-38.1058044
1740.0000000	0.4392000	-39.2849503
1740.0000000	0.4880000	-39.5042000
1740.0000000	0.5368000	-39.6982651
1740.0000000	0.5856000	-39.2331848
1740.0000000	0.6344000	-38.2477989
1740.0000000	0.6832000	-36.3261299
1740.0000000	0.7320000	-36.1494141
1740.0000000	0.7808000	-36.5425949
1740.0000000	0.8296000	-35.6951942
1740.0000000	0.8784000	-33.8433456
1740.0000000	0.9272000	-31.5766582
1740.0000000	0.9760000	-29.0839157
1740.0000000	1.0247999	-28.0802460
1740.0000000	1.0735999	-27.5346260
1740.0000000	1.1224000	-27.7810020
1740.0000000	1.1711999	-28.8196678
1740.0000000	1.2200000	-29.3776855
1740.0000000	1.2687999	-30.3810825
1740.0000000	1.3176000	-30.4554386
1740.0000000	1.3664000	-30.6251869
1740.0000000	1.4152000	-30.1770325
1740.0000000	1.4640000	-29.4922352
1740.0000000	1.5128000	-32.3784256
1740.0000000	1.5616000	-30.6177177
1740.0000000	1.6104001	-1374.1352539
1740.0000000	1.6592000	-52.0950966
1740.0000000	1.7079999	-27.5273342
1740.0000000	1.7567999	-27.7635212
1740.0000000	1.8055999	-25.8563957
1740.0000000	1.8544000	-25.0741787
1740.0000000	1.9031999	-26.6679134
1740.0000000	1.9520000	-26.8041439
1740.0000000	2.0007999	-26.1290321
1740.0000000	2.0495999	-26.3136635
1740.0000000	2.0983999	-41.7181168
1740.0000000	2.1471999	-20.9178715
1740.0000000	2.1960001	-25.1539383
1740.0000000	2.2448001	-24.5381489
1740.0000000	2.2935998	-23.3875427
1740.0000000	2.3423998	-22.0269814
1740.0000000	2.3912001	-12.8707638
1740.0000000	2.4400001	-23.0164909
1740.0000000	2.4888000	-23.4444485
1740.0000000	2.5375998	-13.4981871
1740.0000000	2.5863998	-11.8093290
1740.0000000	2.6352000	-10.0955257
1740.0000000	2.6840000	-16.2663364
1740.0000000	2.7328000	114.1523514
1740.0000000	2.7815998	135.2287292
1740.0000000	2.8304000	-23.7012291
1740.0000000	2.8792000	-20.8523960
1740.0000000	2.9280000	-22.5311489
1740.0000000	2.9768000	-17.3869991
1740.0000000	3.0256000	-20.6573544
1740.0000000	3.0743999	-28.7039795
1740.0000000	3.1231999	-24.0084610

1740.0000000	3.1719999	-29.0137920
1740.0000000	3.2208002	-487.2635803
1740.0000000	3.2695999	-953.3214111
1740.0000000	3.3183999	-20.5038509
1740.0000000	3.3671999	-3291.4216309
1740.0000000	3.4159999	1180.0072021
1740.0000000	3.4648001	-7.5515070
1740.0000000	3.5135999	-26.8218040
1740.0000000	3.5623999	-312.6181335
1740.0000000	3.6111999	-1960.9085693
1740.0000000	3.6600001	-444.4227905
1740.0000000	3.7088001	-586.6113281
1740.0000000	3.7575998	-918.0632935
1740.0000000	3.8063998	-1665.4274902
1740.0000000	3.8552001	-2417.6745605
1740.0000000	3.9040000	-2370.3742676
1740.0000000	3.9528000	1083.0892334
1740.0000000	4.0015998	-237.3719635
1740.0000000	4.0503998	662.8302612
1740.0000000	4.0991998	83.8775024
1740.0000000	4.1479998	-632.4574585
1740.0000000	4.1967998	1.7877846
1740.0000000	4.2456002	-240.4551392
1740.0000000	4.2943997	-471.5642395
1740.0000000	4.3431997	-13.5100603
1740.0000000	4.3920002	-240.1010284
1740.0000000	4.4407997	-22.3150673
1740.0000000	4.4896002	-15.5465717
1740.0000000	4.5383997	-633.6672974
1740.0000000	4.5871997	-13.0039673
1740.0000000	4.6360002	-21.4945812
1740.0000000	4.6847997	-27.9603767
1740.0000000	4.7336001	-19.5031700
1740.0000000	4.7824001	-16.5496521
1740.0000000	4.8311996	-1430.7281494
1740.0000000	4.8800001	220.8996735
1740.0000000	4.9287996	-195.9259644
1740.0000000	4.9776001	1027.3239746
1740.0000000	5.0264001	83.0921097
1740.0000000	5.0751996	-292.6226196
1740.0000000	5.1240001	-12.3840599
1740.0000000	5.1727996	548.9125977
1740.0000000	5.2216001	31.2690277
1740.0000000	5.2704000	-156.6522980
1740.0000000	5.3191996	-38.8324356
1740.0000000	5.3680000	-3482.6140137
1740.0000000	5.4168000	518.9205933
1740.0000000	5.4656000	-197.9585419
1740.0000000	5.5144000	37.2483749
1740.0000000	5.5631995	480.7539673
1740.0000000	5.6120000	386.9990540
1740.0000000	5.6608000	-36.7708168
1740.0000000	5.7096000	61.3455887
1740.0000000	5.7584000	130.8190308
1740.0000000	5.8071995	-11.5871105
1740.0000000	5.8559999	29.1383381
1740.0000000	5.9047999	-16.5904655
1740.0000000	5.9535999	-39.4132385

1740.0000000	6.0023999	-1549.6756592
1740.0000000	6.0511999	-14.5843544
1740.0000000	6.0999999	-19.3457489
1740.0000000	6.1487999	-13.3086395
1740.0000000	6.1975999	-23.0856857
1740.0000000	6.2463999	-1765.3936768
1740.0000000	6.2951999	-19.0083351
1740.0000000	6.3439999	-337.8403320
1740.0000000	6.3927999	-15.0467262
1740.0000000	6.4416003	-12.4304514
1740.0000000	6.4903998	-11.3148365
1740.0000000	6.5391998	-11.8576717
1740.0000000	6.5879998	332.9783936
1740.0000000	6.6367998	-12.1254930
1740.0000000	6.6856003	-13.8277054
1740.0000000	6.7343998	-13.4718361
1740.0000000	6.7831998	-14.3414669
1740.0000000	6.8319998	57.0126572
1740.0000000	6.8807998	-14.0390825
1740.0000000	6.9296002	-392.8832703
1740.0000000	6.9783998	-6.0363088
1740.0000000	7.0271997	-14.0611982
1740.0000000	7.0760002	8.0694990
1740.0000000	7.1247997	-1840.6505127
1740.0000000	7.1736002	-1476.1740723
1740.0000000	7.2223997	-840.2593384
1740.0000000	7.2711997	-182.5640411
1740.0000000	7.3200002	-18.0017414
1740.0000000	7.3687997	160.4070587
1740.0000000	7.4176002	336.4756775
1740.0000000	7.4663997	264.0597839
1740.0000000	7.5151997	-821.5618896
1740.0000000	7.5640001	-907.3221436
1740.0000000	7.6127996	-2136.6152344
1740.0000000	7.6616001	-686.7807617
1740.0000000	7.7104001	-353.4495850
1740.0000000	7.7591996	35.8403206
1740.0000000	7.8080001	349.6756897
1740.0000000	7.8567996	461.1051941
1740.0000000	7.9056001	-959.8547974
1740.0000000	7.9544001	1526.9534912
1740.0000000	8.0031996	1970.3812256
1740.0000000	8.0520000	1966.6146240
1740.0000000	8.1007996	2081.1064453
1740.0000000	8.1496000	2219.5300293
1740.0000000	8.1983995	1380.7619629
1740.0000000	8.2472000	-3160.0397949
1740.0000000	8.2959995	-1120.3514404
1740.0000000	8.3448000	-892.3518677
1740.0000000	8.3935995	-817.8468628
1740.0000000	8.4423990	-352.5301819
1740.0000000	8.4912004	-822.5344849
1740.0000000	8.5400000	-2218.2299805
1740.0000000	8.5887995	-1075.5959473
1740.0000000	8.6375999	209.5339661
1740.0000000	8.6863995	1896.4683838
1740.0000000	8.7351999	-705.3242188
1740.0000000	8.7840004	804.9119263

1740.0000000	8.8327999	42.4501038
1740.0000000	8.8815994	-11.5090628
1740.0000000	8.9303989	-23.6758595
1740.0000000	8.9792004	-11.5850611
1740.0000000	9.0279999	-5.9647636
1740.0000000	9.0767994	6.6261158
1740.0000000	9.1255999	-17.1182575
1740.0000000	9.1743994	-10.1026058
1740.0000000	9.2231998	35.8766174
1740.0000000	9.2720003	-8.4876165
1740.0000000	9.3207998	-9.8369713
1740.0000000	9.3695993	-10.7800846
1740.0000000	9.4183998	-11.7449093
1740.0000000	9.4672003	-11.1642971
1740.0000000	9.5159998	-14.1000547
1740.0000000	9.5648003	-0.6359100
1740.0000000	9.6135998	-13.3390789
1740.0000000	9.6623993	-10.2411366
1740.0000000	9.7111998	-10.1772594
1740.0000000	9.7600002	-10.8605785
1740.0000000	9.8087997	-10.9953508
1740.0000000	9.8575993	2.8714914
1740.0000000	9.9063997	-8.0348625
1740.0000000	9.9552002	-10.9069118
1740.0000000	10.0039997	-10.0925426
1750.0000000	0.3416000	-36.5276909
1750.0000000	0.3904000	-38.4295883
1750.0000000	0.4392000	-39.2786102
1750.0000000	0.4880000	-39.5029907
1750.0000000	0.5368000	-39.6994362
1750.0000000	0.5856000	-39.2318039
1750.0000000	0.6344000	-38.2481079
1750.0000000	0.6832000	-36.3283539
1750.0000000	0.7320000	-36.1514664
1750.0000000	0.7808000	-36.5427628
1750.0000000	0.8296000	-36.0513000
1750.0000000	0.8784000	-35.0191879
1750.0000000	0.9272000	-33.0510788
1750.0000000	0.9760000	-29.6644325
1750.0000000	1.0247999	-27.7989292
1750.0000000	1.0735999	-28.3069305
1750.0000000	1.1224000	-29.1548824
1750.0000000	1.1711999	-28.9172401
1750.0000000	1.2200000	-29.0914783
1750.0000000	1.2687999	-30.3133850
1750.0000000	1.3176000	-30.8268814
1750.0000000	1.3664000	-30.7173862
1750.0000000	1.4152000	-30.1772385
1750.0000000	1.4640000	-29.4905262
1750.0000000	1.5128000	-32.2812500
1750.0000000	1.5616000	-30.5758762
1750.0000000	1.6104001	-1371.5925293
1750.0000000	1.6592000	-51.8011627
1750.0000000	1.7079999	-27.5310822
1750.0000000	1.7567999	-27.7635403
1750.0000000	1.8055999	-25.6851444
1750.0000000	1.8544000	-25.9888496
1750.0000000	1.9031999	-26.7843742

1750.0000000	1.9520000	-26.8069267
1750.0000000	2.0007999	-26.1344109
1750.0000000	2.0495999	-26.3156815
1750.0000000	2.0983999	-40.2423363
1750.0000000	2.1471999	-21.1851387
1750.0000000	2.1960001	-25.1498470
1750.0000000	2.2448001	-24.5202370
1750.0000000	2.2935998	-23.3279190
1750.0000000	2.3423998	-22.0413857
1750.0000000	2.3912001	-13.4923096
1750.0000000	2.4400001	-23.0189781
1750.0000000	2.4888000	-23.4442310
1750.0000000	2.5375998	-13.5152950
1750.0000000	2.5863998	-11.8290882
1750.0000000	2.6352000	-10.1182699
1750.0000000	2.6840000	-16.2782784
1750.0000000	2.7328000	113.8863297
1750.0000000	2.7815998	134.9223328
1750.0000000	2.8304000	-23.6999264
1750.0000000	2.8792000	-20.8556595
1750.0000000	2.9280000	-22.5305786
1750.0000000	2.9768000	-17.3953705
1750.0000000	3.0256000	-20.6599388
1750.0000000	3.0743999	-28.6925926
1750.0000000	3.1231999	-24.0050068
1750.0000000	3.1719999	-29.0054989
1750.0000000	3.2208002	-487.0258484
1750.0000000	3.2695999	-952.8465576
1750.0000000	3.3183999	-20.5040493
1750.0000000	3.3671999	-3289.9599609
1750.0000000	3.4159999	1179.4719238
1750.0000000	3.4648001	-7.5572147
1750.0000000	3.5135999	-26.8188133
1750.0000000	3.5623999	-312.4885254
1750.0000000	3.6111999	-1960.0487061
1750.0000000	3.6600001	-444.2346802
1750.0000000	3.7088001	-586.3599854
1750.0000000	3.7575998	-917.6647949
1750.0000000	3.8063998	-1664.6976318
1750.0000000	3.8552001	-2416.6113281
1750.0000000	3.9040000	-2369.3317871
1750.0000000	3.9528000	1082.6009521
1750.0000000	4.0015998	-237.2748566
1750.0000000	4.0503998	662.5277100
1750.0000000	4.0991998	83.8318176
1750.0000000	4.1479998	-632.1851807
1750.0000000	4.1967998	1.7785282
1750.0000000	4.2456002	-240.3568268
1750.0000000	4.2943997	-471.3636780
1750.0000000	4.3431997	-13.5122414
1750.0000000	4.3920002	-240.0029144
1750.0000000	4.4407997	-22.3132896
1750.0000000	4.4896002	-15.5474548
1750.0000000	4.5383997	-633.4062500
1750.0000000	4.5871997	-13.0059843
1750.0000000	4.6360002	-21.4930382
1750.0000000	4.6847997	-27.9564514
1750.0000000	4.7336001	-19.5024471

1750.0000000	4.7824001	-16.5501060
1750.0000000	4.8311996	-1430.1968994
1750.0000000	4.8800001	220.8101196
1750.0000000	4.9287996	-195.8587036
1750.0000000	4.9776001	1026.9315186
1750.0000000	5.0264001	83.0545883
1750.0000000	5.0751996	-292.5189819
1750.0000000	5.1240001	-12.3849812
1750.0000000	5.1727996	548.7967529
1750.0000000	5.2216001	31.2595863
1750.0000000	5.2704000	-156.6241760
1750.0000000	5.3191996	-38.8278885
1750.0000000	5.3680000	-3481.9216309
1750.0000000	5.4168000	518.8138428
1750.0000000	5.4656000	-197.9223633
1750.0000000	5.5144000	37.2377243
1750.0000000	5.5631995	480.6553955
1750.0000000	5.6120000	386.9190674
1750.0000000	5.6608000	-36.7667313
1750.0000000	5.7096000	61.3301544
1750.0000000	5.7584000	130.7901001
1750.0000000	5.8071995	-11.5879803
1750.0000000	5.8559999	29.1294880
1750.0000000	5.9047999	-16.5902691
1750.0000000	5.9535999	-39.4085007
1750.0000000	6.0023999	-1549.3713379
1750.0000000	6.0511999	-14.5845661
1750.0000000	6.0999999	-19.3449173
1750.0000000	6.1487999	-13.3091030
1750.0000000	6.1975999	-23.0839214
1750.0000000	6.2463999	-1765.0463867
1750.0000000	6.2951999	-19.0075207
1750.0000000	6.3439999	-337.7762756
1750.0000000	6.3927999	-15.0467081
1750.0000000	6.4416003	-12.4310379
1750.0000000	6.4903998	-11.3155117
1750.0000000	6.5391998	-11.8583527
1750.0000000	6.5879998	332.2876892
1750.0000000	6.6367998	-12.1260424
1750.0000000	6.6856003	-13.8278761
1750.0000000	6.7343998	-13.4720516
1750.0000000	6.7831998	-14.3410511
1750.0000000	6.8319998	56.9935379
1750.0000000	6.8807998	-14.0389442
1750.0000000	6.9296002	-392.1218262
1750.0000000	6.9783998	-6.0491323
1750.0000000	7.0271997	-14.0601702
1750.0000000	7.0760002	8.0273123
1750.0000000	7.1247997	-1836.7045898
1750.0000000	7.1736002	-1473.0112305
1750.0000000	7.2223997	-838.4661865
1750.0000000	7.2711997	-182.1962585
1750.0000000	7.3200002	-17.9897537
1750.0000000	7.3687997	160.0325317
1750.0000000	7.4176002	335.7198486
1750.0000000	7.4663997	263.4621277
1750.0000000	7.5151997	-819.8009033
1750.0000000	7.5640001	-905.3747559

1750.0000000	7.6127996	-2131.9975586
1750.0000000	7.6616001	-685.3094482
1750.0000000	7.7104001	-352.7033081
1750.0000000	7.7591996	35.7392731
1750.0000000	7.8080001	348.8903198
1750.0000000	7.8567996	460.0771179
1750.0000000	7.9056001	-957.7819214
1750.0000000	7.9544001	1523.6435547
1750.0000000	8.0031996	1966.1116943
1750.0000000	8.0520000	1962.3525391
1750.0000000	8.1007996	2076.5969238
1750.0000000	8.1496000	2214.7219238
1750.0000000	8.1983995	1377.7695313
1750.0000000	8.2472000	-3153.2050781
1750.0000000	8.2959995	-1117.9311523
1750.0000000	8.3448000	-890.4226685
1750.0000000	8.3935995	-816.0792236
1750.0000000	8.4423990	-351.7721863
1750.0000000	8.4912004	-820.7583008
1750.0000000	8.5400000	-2213.4301758
1750.0000000	8.5887995	-1073.2744141
1750.0000000	8.6375999	209.0726318
1750.0000000	8.6863995	1892.3499756
1750.0000000	8.7351999	-703.7999268
1750.0000000	8.7840004	803.1551514
1750.0000000	8.8327999	42.3384933
1750.0000000	8.8815994	-11.5092363
1750.0000000	8.9303989	-23.6721382
1750.0000000	8.9792004	-11.5851431
1750.0000000	9.0279999	-5.9665322
1750.0000000	9.0767994	6.6203976
1750.0000000	9.1255999	-17.1165428
1750.0000000	9.1743994	-10.1030083
1750.0000000	9.2231998	35.8608856
1750.0000000	9.2720003	-8.4885530
1750.0000000	9.3207998	-9.8375101
1750.0000000	9.3695993	-10.7803106
1750.0000000	9.4183998	-11.7447853
1750.0000000	9.4672003	-11.1643381
1750.0000000	9.5159998	-14.0991640
1750.0000000	9.5648003	-0.6392822
1750.0000000	9.6135998	-13.3382883
1750.0000000	9.6623993	-10.2414122
1750.0000000	9.7111998	-10.1775312
1750.0000000	9.7600002	-10.8606205
1750.0000000	9.8087997	-10.9953594
1750.0000000	9.8575993	2.8670692
1750.0000000	9.9063997	-8.0357771
1750.0000000	9.9552002	-10.9069166
1750.0000000	10.0039997	-10.0927896
1760.0000000	0.3416000	-36.4147530
1760.0000000	0.3904000	-39.1536713
1760.0000000	0.4392000	-39.2722664
1760.0000000	0.4880000	-39.5017815
1760.0000000	0.5368000	-39.7006111
1760.0000000	0.5856000	-39.2304230
1760.0000000	0.6344000	-38.2484169
1760.0000000	0.6832000	-36.3305779

1760.0000000	0.7320000	-36.1535187
1760.0000000	0.7808000	-36.5429344
1760.0000000	0.8296000	-36.0473938
1760.0000000	0.8784000	-34.8318443
1760.0000000	0.9272000	-32.7909927
1760.0000000	0.9760000	-30.7129364
1760.0000000	1.0247999	-28.8659401
1760.0000000	1.0735999	-28.1510925
1760.0000000	1.1224000	-28.4000549
1760.0000000	1.1711999	-29.4043121
1760.0000000	1.2200000	-30.2598572
1760.0000000	1.2687999	-30.5076504
1760.0000000	1.3176000	-30.7329636
1760.0000000	1.3664000	-30.7169628
1760.0000000	1.4152000	-30.1774426
1760.0000000	1.4640000	-29.4888172
1760.0000000	1.5128000	-32.1840706
1760.0000000	1.5616000	-30.5340347
1760.0000000	1.6104001	-1369.0496826
1760.0000000	1.6592000	-51.5072327
1760.0000000	1.7079999	-27.5348282
1760.0000000	1.7567999	-27.7928772
1760.0000000	1.8055999	-27.0892200
1760.0000000	1.8544000	-27.3310947
1760.0000000	1.9031999	-26.9008350
1760.0000000	1.9520000	-26.8097076
1760.0000000	2.0007999	-26.1397915
1760.0000000	2.0495999	-26.3177013
1760.0000000	2.0983999	-38.7665558
1760.0000000	2.1471999	-21.4524059
1760.0000000	2.1960001	-25.1457558
1760.0000000	2.2448001	-24.5023270
1760.0000000	2.2935998	-23.2682953
1760.0000000	2.3423998	-22.0557899
1760.0000000	2.3912001	-14.1138554
1760.0000000	2.4400001	-23.0214634
1760.0000000	2.4888000	-23.4440136
1760.0000000	2.5375998	-13.5324030
1760.0000000	2.5863998	-11.8488474
1760.0000000	2.6352000	-10.1410141
1760.0000000	2.6840000	-16.2902203
1760.0000000	2.7328000	113.6203232
1760.0000000	2.7815998	134.6159058
1760.0000000	2.8304000	-23.6986217
1760.0000000	2.8792000	-20.8589230
1760.0000000	2.9280000	-22.5300102
1760.0000000	2.9768000	-17.4037399
1760.0000000	3.0256000	-20.6625233
1760.0000000	3.0743999	-28.6812057
1760.0000000	3.1231999	-24.0015507
1760.0000000	3.1719999	-28.9972057
1760.0000000	3.2208002	-486.7881165
1760.0000000	3.2695999	-952.3714600
1760.0000000	3.3183999	-20.5042477
1760.0000000	3.3671999	-3288.4982910
1760.0000000	3.4159999	1178.9366455
1760.0000000	3.4648001	-7.5629225
1760.0000000	3.5135999	-26.8158226

1760.0000000	3.5623999	-312.3589478
1760.0000000	3.6111999	-1959.1889648
1760.0000000	3.6600001	-444.0465698
1760.0000000	3.7088001	-586.1085815
1760.0000000	3.7575998	-917.2662354
1760.0000000	3.8063998	-1663.9677734
1760.0000000	3.8552001	-2415.5478516
1760.0000000	3.9040000	-2368.2893066
1760.0000000	3.9528000	1082.1125488
1760.0000000	4.0015998	-237.1777496
1760.0000000	4.0503998	662.2251587
1760.0000000	4.0991998	83.7861328
1760.0000000	4.1479998	-631.9129639
1760.0000000	4.1967998	1.7692719
1760.0000000	4.2456002	-240.2585297
1760.0000000	4.2943997	-471.1630554
1760.0000000	4.3431997	-13.5144215
1760.0000000	4.3920002	-239.9048004
1760.0000000	4.4407997	-22.3115158
1760.0000000	4.4896002	-15.5483370
1760.0000000	4.5383997	-633.1451416
1760.0000000	4.5871997	-13.0080004
1760.0000000	4.6360002	-21.4914970
1760.0000000	4.6847997	-27.9525261
1760.0000000	4.7336001	-19.5017262
1760.0000000	4.7824001	-16.5505600
1760.0000000	4.8311996	-1429.6655273
1760.0000000	4.8800001	220.7205811
1760.0000000	4.9287996	-195.7914429
1760.0000000	4.9776001	1026.5390625
1760.0000000	5.0264001	83.0170670
1760.0000000	5.0751996	-292.4153442
1760.0000000	5.1240001	-12.3859024
1760.0000000	5.1727996	548.6808472
1760.0000000	5.2216001	31.2501373
1760.0000000	5.2704000	-156.5960846
1760.0000000	5.3191996	-38.8233337
1760.0000000	5.3680000	-3481.2292480
1760.0000000	5.4168000	518.7070923
1760.0000000	5.4656000	-197.8861847
1760.0000000	5.5144000	37.2270660
1760.0000000	5.5631995	480.5568237
1760.0000000	5.6120000	386.8391113
1760.0000000	5.6608000	-36.7626419
1760.0000000	5.7096000	61.3147278
1760.0000000	5.7584000	130.7611694
1760.0000000	5.8071995	-11.5888519
1760.0000000	5.8559999	29.1206379
1760.0000000	5.9047999	-16.5900707
1760.0000000	5.9535999	-39.4037590
1760.0000000	6.0023999	-1549.0667725
1760.0000000	6.0511999	-14.5847769
1760.0000000	6.0999999	-19.3440857
1760.0000000	6.1487999	-13.3095655
1760.0000000	6.1975999	-23.0821571
1760.0000000	6.2463999	-1764.6989746
1760.0000000	6.2951999	-19.0067062
1760.0000000	6.3439999	-337.7122192

1760.0000000	6.3927999	-15.0466900
1760.0000000	6.4416003	-12.4316254
1760.0000000	6.4903998	-11.3161879
1760.0000000	6.5391998	-11.8590336
1760.0000000	6.5879998	331.5969849
1760.0000000	6.6367998	-12.1265917
1760.0000000	6.6856003	-13.8280458
1760.0000000	6.7343998	-13.4722672
1760.0000000	6.7831998	-14.3406353
1760.0000000	6.8319998	56.9744186
1760.0000000	6.8807998	-14.0388050
1760.0000000	6.9296002	-391.3603821
1760.0000000	6.9783998	-6.0619555
1760.0000000	7.0271997	-14.0591431
1760.0000000	7.0760002	7.9851274
1760.0000000	7.1247997	-1832.7587891
1760.0000000	7.1736002	-1469.8482666
1760.0000000	7.2223997	-836.6730347
1760.0000000	7.2711997	-181.8284912
1760.0000000	7.3200002	-17.9777641
1760.0000000	7.3687997	159.6579895
1760.0000000	7.4176002	334.9640198
1760.0000000	7.4663997	262.8644714
1760.0000000	7.5151997	-818.0398560
1760.0000000	7.5640001	-903.4273682
1760.0000000	7.6127996	-2127.3798828
1760.0000000	7.6616001	-683.8380737
1760.0000000	7.7104001	-351.9570313
1760.0000000	7.7591996	35.6382179
1760.0000000	7.8080001	348.1049500
1760.0000000	7.8567996	459.0490417
1760.0000000	7.9056001	-955.7089844
1760.0000000	7.9544001	1520.3337402
1760.0000000	8.0031996	1961.8420410
1760.0000000	8.0520000	1958.0905762
1760.0000000	8.1007996	2072.0874023
1760.0000000	8.1496000	2209.9138184
1760.0000000	8.1983995	1374.7769775
1760.0000000	8.2472000	-3146.3701172
1760.0000000	8.2959995	-1115.5109863
1760.0000000	8.3448000	-888.4935303
1760.0000000	8.3935995	-814.3115234
1760.0000000	8.4423990	-351.0141602
1760.0000000	8.4912004	-818.9820557
1760.0000000	8.5400000	-2208.6303711
1760.0000000	8.5887995	-1070.9528809
1760.0000000	8.6375999	208.6113129
1760.0000000	8.6863995	1888.2316895
1760.0000000	8.7351999	-702.2756958
1760.0000000	8.7840004	801.3984375
1760.0000000	8.8327999	42.2268906
1760.0000000	8.8815994	-11.5094109
1760.0000000	8.9303989	-23.6684189
1760.0000000	8.9792004	-11.5852261
1760.0000000	9.0279999	-5.9683003
1760.0000000	9.0767994	6.6146793
1760.0000000	9.1255999	-17.1148281
1760.0000000	9.1743994	-10.1034107

1760.0000000	9.2231998	35.8451538
1760.0000000	9.2720003	-8.4894896
1760.0000000	9.3207998	-9.8380499
1760.0000000	9.3695993	-10.7805367
1760.0000000	9.4183998	-11.7446604
1760.0000000	9.4672003	-11.1643791
1760.0000000	9.5159998	-14.0982723
1760.0000000	9.5648003	-0.6426544
1760.0000000	9.6135998	-13.3374977
1760.0000000	9.6623993	-10.2416868
1760.0000000	9.7111998	-10.1778021
1760.0000000	9.7600002	-10.8606615
1760.0000000	9.8087997	-10.9953671
1760.0000000	9.8575993	2.8626471
1760.0000000	9.9063997	-8.0366907
1760.0000000	9.9552002	-10.9069204
1760.0000000	10.0039997	-10.0930376
1770.0000000	0.3416000	-36.2179260
1770.0000000	0.3904000	-38.5896568
1770.0000000	0.4392000	-39.2659225
1770.0000000	0.4880000	-39.5005722
1770.0000000	0.5368000	-39.7017860
1770.0000000	0.5856000	-39.2290421
1770.0000000	0.6344000	-38.2487297
1770.0000000	0.6832000	-36.3328056
1770.0000000	0.7320000	-36.1555710
1770.0000000	0.7808000	-36.5431023
1770.0000000	0.8296000	-36.0434875
1770.0000000	0.8784000	-34.9372368
1770.0000000	0.9272000	-33.0543213
1770.0000000	0.9760000	-30.8550777
1770.0000000	1.0247999	-29.2054234
1770.0000000	1.0735999	-28.9008217
1770.0000000	1.1224000	-29.7034817
1770.0000000	1.1711999	-30.3742561
1770.0000000	1.2200000	-30.4604816
1770.0000000	1.2687999	-30.9743214
1770.0000000	1.3176000	-30.7347717
1770.0000000	1.3664000	-30.7165394
1770.0000000	1.4152000	-30.1776466
1770.0000000	1.4640000	-29.4871082
1770.0000000	1.5128000	-32.0868950
1770.0000000	1.5616000	-30.4921932
1770.0000000	1.6104001	-1366.5068359
1770.0000000	1.6592000	-51.2133026
1770.0000000	1.7079999	-27.5385761
1770.0000000	1.7567999	-27.8222141
1770.0000000	1.8055999	-26.5195084
1770.0000000	1.8544000	-26.1806393
1770.0000000	1.9031999	-27.0172958
1770.0000000	1.9520000	-26.8124905
1770.0000000	2.0007999	-26.1451721
1770.0000000	2.0495999	-26.3197193
1770.0000000	2.0983999	-37.2907791
1770.0000000	2.1471999	-21.7196732
1770.0000000	2.1960001	-25.1416664
1770.0000000	2.2448001	-24.4844151
1770.0000000	2.2935998	-23.2086716

1770.0000000	2.3423998	-22.0701962
1770.0000000	2.3912001	-14.7354012
1770.0000000	2.4400001	-23.0239506
1770.0000000	2.4888000	-23.4437962
1770.0000000	2.5375998	-13.5495110
1770.0000000	2.5863998	-11.8686066
1770.0000000	2.6352000	-10.1637583
1770.0000000	2.6840000	-16.3021622
1770.0000000	2.7328000	113.3543015
1770.0000000	2.7815998	134.3094788
1770.0000000	2.8304000	-23.6973171
1770.0000000	2.8792000	-20.8621864
1770.0000000	2.9280000	-22.5294418
1770.0000000	2.9768000	-17.4121113
1770.0000000	3.0256000	-20.6651077
1770.0000000	3.0743999	-28.6698189
1770.0000000	3.1231999	-23.9980965
1770.0000000	3.1719999	-28.9889126
1770.0000000	3.2208002	-486.5503845
1770.0000000	3.2695999	-951.8966064
1770.0000000	3.3183999	-20.5044460
1770.0000000	3.3671999	-3287.0366211
1770.0000000	3.4159999	1178.4013672
1770.0000000	3.4648001	-7.5686312
1770.0000000	3.5135999	-26.8128319
1770.0000000	3.5623999	-312.2293701
1770.0000000	3.6111999	-1958.3292236
1770.0000000	3.6600001	-443.8584595
1770.0000000	3.7088001	-585.8571777
1770.0000000	3.7575998	-916.8677368
1770.0000000	3.8063998	-1663.2379150
1770.0000000	3.8552001	-2414.4843750
1770.0000000	3.9040000	-2367.2468262
1770.0000000	3.9528000	1081.6242676
1770.0000000	4.0015998	-237.0806427
1770.0000000	4.0503998	661.9225464
1770.0000000	4.0991998	83.7404556
1770.0000000	4.1479998	-631.6407471
1770.0000000	4.1967998	1.7600155
1770.0000000	4.2456002	-240.1602173
1770.0000000	4.2943997	-470.9624939
1770.0000000	4.3431997	-13.5166016
1770.0000000	4.3920002	-239.8066864
1770.0000000	4.4407997	-22.3097401
1770.0000000	4.4896002	-15.5492191
1770.0000000	4.5383997	-632.8840942
1770.0000000	4.5871997	-13.0100174
1770.0000000	4.6360002	-21.4899540
1770.0000000	4.6847997	-27.9486008
1770.0000000	4.7336001	-19.5010033
1770.0000000	4.7824001	-16.5510139
1770.0000000	4.8311996	-1429.1342773
1770.0000000	4.8800001	220.6310272
1770.0000000	4.9287996	-195.7241821
1770.0000000	4.9776001	1026.1464844
1770.0000000	5.0264001	82.9795456
1770.0000000	5.0751996	-292.3117371
1770.0000000	5.1240001	-12.3868237

1770.0000000	5.1727996	548.5650024
1770.0000000	5.2216001	31.2406960
1770.0000000	5.2704000	-156.5679779
1770.0000000	5.3191996	-38.8187866
1770.0000000	5.3680000	-3480.5368652
1770.0000000	5.4168000	518.6002808
1770.0000000	5.4656000	-197.8499908
1770.0000000	5.5144000	37.2164154
1770.0000000	5.5631995	480.4582520
1770.0000000	5.6120000	386.7591248
1770.0000000	5.6608000	-36.7585526
1770.0000000	5.7096000	61.2992935
1770.0000000	5.7584000	130.7322235
1770.0000000	5.8071995	-11.5897217
1770.0000000	5.8559999	29.1117878
1770.0000000	5.9047999	-16.5898724
1770.0000000	5.9535999	-39.3990211
1770.0000000	6.0023999	-1548.7624512
1770.0000000	6.0511999	-14.5849886
1770.0000000	6.0999999	-19.3432522
1770.0000000	6.1487999	-13.3100281
1770.0000000	6.1975999	-23.0803909
1770.0000000	6.2463999	-1764.3515625
1770.0000000	6.2951999	-19.0058937
1770.0000000	6.3439999	-337.6481628
1770.0000000	6.3927999	-15.0466719
1770.0000000	6.4416003	-12.4322119
1770.0000000	6.4903998	-11.3168640
1770.0000000	6.5391998	-11.8597145
1770.0000000	6.5879998	330.9063110
1770.0000000	6.6367998	-12.1271400
1770.0000000	6.6856003	-13.8282166
1770.0000000	6.7343998	-13.4724827
1770.0000000	6.7831998	-14.3402195
1770.0000000	6.8319998	56.9553070
1770.0000000	6.8807998	-14.0386667
1770.0000000	6.9296002	-390.5989380
1770.0000000	6.9783998	-6.0747795
1770.0000000	7.0271997	-14.0581150
1770.0000000	7.0760002	7.9429426
1770.0000000	7.1247997	-1828.8128662
1770.0000000	7.1736002	-1466.6854248
1770.0000000	7.2223997	-834.8798828
1770.0000000	7.2711997	-181.4607086
1770.0000000	7.3200002	-17.9657745
1770.0000000	7.3687997	159.2834625
1770.0000000	7.4176002	334.2081909
1770.0000000	7.4663997	262.2668457
1770.0000000	7.5151997	-816.2788086
1770.0000000	7.5640001	-901.4799805
1770.0000000	7.6127996	-2122.7624512
1770.0000000	7.6616001	-682.3666992
1770.0000000	7.7104001	-351.2108154
1770.0000000	7.7591996	35.5371704
1770.0000000	7.8080001	347.3196106
1770.0000000	7.8567996	458.0209351
1770.0000000	7.9056001	-953.6361084
1770.0000000	7.9544001	1517.0238037

1770.0000000	8.0031996	1957.5725098
1770.0000000	8.0520000	1953.8286133
1770.0000000	8.1007996	2067.5778809
1770.0000000	8.1496000	2205.1059570
1770.0000000	8.1983995	1371.7844238
1770.0000000	8.2472000	-3139.5354004
1770.0000000	8.2959995	-1113.0908203
1770.0000000	8.3448000	-886.5643311
1770.0000000	8.3935995	-812.5438843
1770.0000000	8.4423990	-350.2561646
1770.0000000	8.4912004	-817.2058716
1770.0000000	8.5400000	-2203.8305664
1770.0000000	8.5887995	-1068.6313477
1770.0000000	8.6375999	208.1499939
1770.0000000	8.6863995	1884.1132813
1770.0000000	8.7351999	-700.7514038
1770.0000000	8.7840004	799.6416626
1770.0000000	8.8327999	42.1152840
1770.0000000	8.8815994	-11.5095854
1770.0000000	8.9303989	-23.6646996
1770.0000000	8.9792004	-11.5853081
1770.0000000	9.0279999	-5.9700689
1770.0000000	9.0767994	6.6089630
1770.0000000	9.1255999	-17.1131153
1770.0000000	9.1743994	-10.1038122
1770.0000000	9.2231998	35.8294220
1770.0000000	9.2720003	-8.4904251
1770.0000000	9.3207998	-9.8385887
1770.0000000	9.3695993	-10.7807636
1770.0000000	9.4183998	-11.7445354
1770.0000000	9.4672003	-11.1644211
1770.0000000	9.5159998	-14.0973816
1770.0000000	9.5648003	-0.6460276
1770.0000000	9.6135998	-13.3367071
1770.0000000	9.6623993	-10.2419615
1770.0000000	9.7111998	-10.1780739
1770.0000000	9.7600002	-10.8607035
1770.0000000	9.8087997	-10.9953747
1770.0000000	9.8575993	2.8582249
1770.0000000	9.9063997	-8.0376053
1770.0000000	9.9552002	-10.9069252
1770.0000000	10.0039997	-10.0932856
1780.0000000	0.3416000	-36.2214088
1780.0000000	0.3904000	-38.4303932
1780.0000000	0.4392000	-39.2595787
1780.0000000	0.4880000	-39.4993629
1780.0000000	0.5368000	-39.7029572
1780.0000000	0.5856000	-39.2276611
1780.0000000	0.6344000	-38.2490387
1780.0000000	0.6832000	-36.3350296
1780.0000000	0.7320000	-36.1576233
1780.0000000	0.7808000	-36.5432739
1780.0000000	0.8296000	-36.0395813
1780.0000000	0.8784000	-35.0318413
1780.0000000	0.9272000	-33.6144981
1780.0000000	0.9760000	-31.1636772
1780.0000000	1.0247999	-29.4117279
1780.0000000	1.0735999	-29.2309895

1780.0000000	1.1224000	-29.7400799
1780.0000000	1.1711999	-30.1442719
1780.0000000	1.2200000	-30.7030468
1780.0000000	1.2687999	-31.2302341
1780.0000000	1.3176000	-30.7365780
1780.0000000	1.3664000	-30.7161179
1780.0000000	1.4152000	-30.1778507
1780.0000000	1.4640000	-29.4853973
1780.0000000	1.5128000	-31.9897175
1780.0000000	1.5616000	-30.4503517
1780.0000000	1.6104001	-1363.9641113
1780.0000000	1.6592000	-50.9193687
1780.0000000	1.7079999	-27.5423222
1780.0000000	1.7567999	-27.8515511
1780.0000000	1.8055999	-26.7168484
1780.0000000	1.8544000	-26.7070255
1780.0000000	1.9031999	-26.8857212
1780.0000000	1.9520000	-26.8152714
1780.0000000	2.0007999	-26.1505527
1780.0000000	2.0495999	-26.3217392
1780.0000000	2.0983999	-35.8150024
1780.0000000	2.1471999	-21.9869404
1780.0000000	2.1960001	-25.1375751
1780.0000000	2.2448001	-24.4665031
1780.0000000	2.2935998	-23.1490479
1780.0000000	2.3423998	-22.0846004
1780.0000000	2.3912001	-15.3569469
1780.0000000	2.4400001	-23.0264378
1780.0000000	2.4888000	-23.4435806
1780.0000000	2.5375998	-13.5666189
1780.0000000	2.5863998	-11.8883657
1780.0000000	2.6352000	-10.1865025
1780.0000000	2.6840000	-16.3141041
1780.0000000	2.7328000	113.0882797
1780.0000000	2.7815998	134.0030823
1780.0000000	2.8304000	-23.6960125
1780.0000000	2.8792000	-20.8654499
1780.0000000	2.9280000	-22.5288715
1780.0000000	2.9768000	-17.4204826
1780.0000000	3.0256000	-20.6676922
1780.0000000	3.0743999	-28.6584320
1780.0000000	3.1231999	-23.9946404
1780.0000000	3.1719999	-28.9806194
1780.0000000	3.2208002	-486.3126221
1780.0000000	3.2695999	-951.4216309
1780.0000000	3.3183999	-20.5046444
1780.0000000	3.3671999	-3285.5749512
1780.0000000	3.4159999	1177.8660889
1780.0000000	3.4648001	-7.5743399
1780.0000000	3.5135999	-26.8098412
1780.0000000	3.5623999	-312.0997620
1780.0000000	3.6111999	-1957.4694824
1780.0000000	3.6600001	-443.6702881
1780.0000000	3.7088001	-585.6058350
1780.0000000	3.7575998	-916.4691772
1780.0000000	3.8063998	-1662.5081787
1780.0000000	3.8552001	-2413.4208984
1780.0000000	3.9040000	-2366.2043457

1780.0000000	3.9528000	1081.1359863
1780.0000000	4.0015998	-236.9835358
1780.0000000	4.0503998	661.6199951
1780.0000000	4.0991998	83.6947708
1780.0000000	4.1479998	-631.3685303
1780.0000000	4.1967998	1.7507591
1780.0000000	4.2456002	-240.0619202
1780.0000000	4.2943997	-470.7619324
1780.0000000	4.3431997	-13.5187826
1780.0000000	4.3920002	-239.7085724
1780.0000000	4.4407997	-22.3079643
1780.0000000	4.4896002	-15.5501022
1780.0000000	4.5383997	-632.6230469
1780.0000000	4.5871997	-13.0120325
1780.0000000	4.6360002	-21.4884109
1780.0000000	4.6847997	-27.9446754
1780.0000000	4.7336001	-19.5002804
1780.0000000	4.7824001	-16.5514698
1780.0000000	4.8311996	-1428.6030273
1780.0000000	4.8800001	220.5414734
1780.0000000	4.9287996	-195.6569214
1780.0000000	4.9776001	1025.7540283
1780.0000000	5.0264001	82.9420242
1780.0000000	5.0751996	-292.2080994
1780.0000000	5.1240001	-12.3877449
1780.0000000	5.1727996	548.4490967
1780.0000000	5.2216001	31.2312508
1780.0000000	5.2704000	-156.5398712
1780.0000000	5.3191996	-38.8142395
1780.0000000	5.3680000	-3479.8444824
1780.0000000	5.4168000	518.4934692
1780.0000000	5.4656000	-197.8138123
1780.0000000	5.5144000	37.2057571
1780.0000000	5.5631995	480.3596802
1780.0000000	5.6120000	386.6791687
1780.0000000	5.6608000	-36.7544632
1780.0000000	5.7096000	61.2838669
1780.0000000	5.7584000	130.7032928
1780.0000000	5.8071995	-11.5905933
1780.0000000	5.8559999	29.1029339
1780.0000000	5.9047999	-16.5896759
1780.0000000	5.9535999	-39.3942833
1780.0000000	6.0023999	-1548.4578857
1780.0000000	6.0511999	-14.5852003
1780.0000000	6.0999999	-19.3424206
1780.0000000	6.1487999	-13.3104916
1780.0000000	6.1975999	-23.0786266
1780.0000000	6.2463999	-1764.0042725
1780.0000000	6.2951999	-19.0050793
1780.0000000	6.3439999	-337.5841370
1780.0000000	6.3927999	-15.0466528
1780.0000000	6.4416003	-12.4327984
1780.0000000	6.4903998	-11.3175402
1780.0000000	6.5391998	-11.8603954
1780.0000000	6.5879998	330.2156067
1780.0000000	6.6367998	-12.1276894
1780.0000000	6.6856003	-13.8283863
1780.0000000	6.7343998	-13.4726992

1780.0000000	6.7831998	-14.3398037
1780.0000000	6.8319998	56.9361954
1780.0000000	6.8807998	-14.0385275
1780.0000000	6.9296002	-389.8374939
1780.0000000	6.9783998	-6.0876031
1780.0000000	7.0271997	-14.0570869
1780.0000000	7.0760002	7.9007578
1780.0000000	7.1247997	-1824.8669434
1780.0000000	7.1736002	-1463.5225830
1780.0000000	7.2223997	-833.0867310
1780.0000000	7.2711997	-181.0929260
1780.0000000	7.3200002	-17.9537868
1780.0000000	7.3687997	158.9089355
1780.0000000	7.4176002	333.4523621
1780.0000000	7.4663997	261.6691895
1780.0000000	7.5151997	-814.5177612
1780.0000000	7.5640001	-899.5325928
1780.0000000	7.6127996	-2118.1447754
1780.0000000	7.6616001	-680.8953857
1780.0000000	7.7104001	-350.4645386
1780.0000000	7.7591996	35.4361229
1780.0000000	7.8080001	346.5342407
1780.0000000	7.8567996	456.9928589
1780.0000000	7.9056001	-951.5631714
1780.0000000	7.9544001	1513.7138672
1780.0000000	8.0031996	1953.3028564
1780.0000000	8.0520000	1949.5665283
1780.0000000	8.1007996	2063.0683594
1780.0000000	8.1496000	2200.2978516
1780.0000000	8.1983995	1368.7919922
1780.0000000	8.2472000	-3132.7004395
1780.0000000	8.2959995	-1110.6706543
1780.0000000	8.3448000	-884.6351318
1780.0000000	8.3935995	-810.7761841
1780.0000000	8.4423990	-349.4981384
1780.0000000	8.4912004	-815.4296265
1780.0000000	8.5400000	-2199.0307617
1780.0000000	8.5887995	-1066.3098145
1780.0000000	8.6375999	207.6886749
1780.0000000	8.6863995	1879.9949951
1780.0000000	8.7351999	-699.2271729
1780.0000000	8.7840004	797.8849487
1780.0000000	8.8327999	42.0036736
1780.0000000	8.8815994	-11.5097599
1780.0000000	8.9303989	-23.6609802
1780.0000000	8.9792004	-11.5853901
1780.0000000	9.0279999	-5.9718370
1780.0000000	9.0767994	6.6032448
1780.0000000	9.1255999	-17.1114006
1780.0000000	9.1743994	-10.1042147
1780.0000000	9.2231998	35.8136902
1780.0000000	9.2720003	-8.4913626
1780.0000000	9.3207998	-9.8391285
1780.0000000	9.3695993	-10.7809896
1780.0000000	9.4183998	-11.7444115
1780.0000000	9.4672003	-11.1644621
1780.0000000	9.5159998	-14.0964909
1780.0000000	9.5648003	-0.6494007

1780.0000000	9.6135998	-13.3359165
1780.0000000	9.6623993	-10.2422371
1780.0000000	9.7111998	-10.1783457
1780.0000000	9.7600002	-10.8607454
1780.0000000	9.8087997	-10.9953833
1780.0000000	9.8575993	2.8538036
1780.0000000	9.9063997	-8.0385189
1780.0000000	9.9552002	-10.9069290
1780.0000000	10.0039997	-10.0935326
1790.0000000	0.3416000	-36.0960503
1790.0000000	0.3904000	-38.6514740
1790.0000000	0.4392000	-39.2532349
1790.0000000	0.4880000	-39.4981537
1790.0000000	0.5368000	-39.7041321
1790.0000000	0.5856000	-39.2262764
1790.0000000	0.6344000	-38.2493515
1790.0000000	0.6832000	-36.3372574
1790.0000000	0.7320000	-36.1596756
1790.0000000	0.7808000	-36.5434418
1790.0000000	0.8296000	-36.0356712
1790.0000000	0.8784000	-35.1264458
1790.0000000	0.9272000	-33.8056412
1790.0000000	0.9760000	-31.5141373
1790.0000000	1.0247999	-29.9110489
1790.0000000	1.0735999	-29.5548840
1790.0000000	1.1224000	-30.0054417
1790.0000000	1.1711999	-30.7562084
1790.0000000	1.2200000	-31.1259747
1790.0000000	1.2687999	-31.2092724
1790.0000000	1.3176000	-30.7383862
1790.0000000	1.3664000	-30.7156944
1790.0000000	1.4152000	-30.1780548
1790.0000000	1.4640000	-29.4836884
1790.0000000	1.5128000	-31.8925400
1790.0000000	1.5616000	-30.4085121
1790.0000000	1.6104001	-1361.4212646
1790.0000000	1.6592000	-50.6254349
1790.0000000	1.7079999	-27.5460701
1790.0000000	1.7567999	-27.8408833
1790.0000000	1.8055999	-26.6549969
1790.0000000	1.8544000	-26.3234348
1790.0000000	1.9031999	-26.7541466
1790.0000000	1.9520000	-26.8180542
1790.0000000	2.0007999	-26.1559315
1790.0000000	2.0495999	-26.3237572
1790.0000000	2.0983999	-34.3392220
1790.0000000	2.1471999	-22.2542076
1790.0000000	2.1960001	-25.1334839
1790.0000000	2.2448001	-24.4485931
1790.0000000	2.2935998	-23.0894260
1790.0000000	2.3423998	-22.0990047
1790.0000000	2.3912001	-15.9784927
1790.0000000	2.4400001	-23.0289230
1790.0000000	2.4888000	-23.4433632
1790.0000000	2.5375998	-13.5837278
1790.0000000	2.5863998	-11.9081249
1790.0000000	2.6352000	-10.2092466
1790.0000000	2.6840000	-16.3260460

1790.0000000	2.7328000	112.8222733
1790.0000000	2.7815998	133.6966553
1790.0000000	2.8304000	-23.6947098
1790.0000000	2.8792000	-20.8687134
1790.0000000	2.9280000	-22.5283031
1790.0000000	2.9768000	-17.4288521
1790.0000000	3.0256000	-20.6702766
1790.0000000	3.0743999	-28.6470451
1790.0000000	3.1231999	-23.9911842
1790.0000000	3.1719999	-28.9723263
1790.0000000	3.2208002	-486.0748901
1790.0000000	3.2695999	-950.9466553
1790.0000000	3.3183999	-20.5048409
1790.0000000	3.3671999	-3284.1132813
1790.0000000	3.4159999	1177.3308105
1790.0000000	3.4648001	-7.5800467
1790.0000000	3.5135999	-26.8068504
1790.0000000	3.5623999	-311.9701843
1790.0000000	3.6111999	-1956.6097412
1790.0000000	3.6600001	-443.4821777
1790.0000000	3.7088001	-585.3544312
1790.0000000	3.7575998	-916.0706177
1790.0000000	3.8063998	-1661.7783203
1790.0000000	3.8552001	-2412.3574219
1790.0000000	3.9040000	-2365.1618652
1790.0000000	3.9528000	1080.6477051
1790.0000000	4.0015998	-236.8864136
1790.0000000	4.0503998	661.3174438
1790.0000000	4.0991998	83.6490860
1790.0000000	4.1479998	-631.0963135
1790.0000000	4.1967998	1.7415028
1790.0000000	4.2456002	-239.9636230
1790.0000000	4.2943997	-470.5613708
1790.0000000	4.3431997	-13.5209637
1790.0000000	4.3920002	-239.6104584
1790.0000000	4.4407997	-22.3061886
1790.0000000	4.4896002	-15.5509853
1790.0000000	4.5383997	-632.3619385
1790.0000000	4.5871997	-13.0140495
1790.0000000	4.6360002	-21.4868698
1790.0000000	4.6847997	-27.9407501
1790.0000000	4.7336001	-19.4995575
1790.0000000	4.7824001	-16.5519238
1790.0000000	4.8311996	-1428.0717773
1790.0000000	4.8800001	220.4519196
1790.0000000	4.9287996	-195.5896606
1790.0000000	4.9776001	1025.3615723
1790.0000000	5.0264001	82.9045029
1790.0000000	5.0751996	-292.1044617
1790.0000000	5.1240001	-12.3886662
1790.0000000	5.1727996	548.3332520
1790.0000000	5.2216001	31.2218056
1790.0000000	5.2704000	-156.5117645
1790.0000000	5.3191996	-38.8096886
1790.0000000	5.3680000	-3479.1520996
1790.0000000	5.4168000	518.3867188
1790.0000000	5.4656000	-197.7776337
1790.0000000	5.5144000	37.1950989

1790.0000000	5.5631995	480.2611084
1790.0000000	5.6120000	386.5991821
1790.0000000	5.6608000	-36.7503738
1790.0000000	5.7096000	61.2684326
1790.0000000	5.7584000	130.6743622
1790.0000000	5.8071995	-11.5914631
1790.0000000	5.8559999	29.0940838
1790.0000000	5.9047999	-16.5894775
1790.0000000	5.9535999	-39.3895454
1790.0000000	6.0023999	-1548.1535645
1790.0000000	6.0511999	-14.5854111
1790.0000000	6.0999999	-19.3415871
1790.0000000	6.1487999	-13.3109541
1790.0000000	6.1975999	-23.0768604
1790.0000000	6.2463999	-1763.6568604
1790.0000000	6.2951999	-19.0042648
1790.0000000	6.3439999	-337.5200806
1790.0000000	6.3927999	-15.0466347
1790.0000000	6.4416003	-12.4333858
1790.0000000	6.4903998	-11.3182154
1790.0000000	6.5391998	-11.8610773
1790.0000000	6.5879998	329.5249023
1790.0000000	6.6367998	-12.1282387
1790.0000000	6.6856003	-13.8285570
1790.0000000	6.7343998	-13.4729147
1790.0000000	6.7831998	-14.3393879
1790.0000000	6.8319998	56.9170761
1790.0000000	6.8807998	-14.0383892
1790.0000000	6.9296002	-389.0760193
1790.0000000	6.9783998	-6.1004267
1790.0000000	7.0271997	-14.0560589
1790.0000000	7.0760002	7.8585711
1790.0000000	7.1247997	-1820.9211426
1790.0000000	7.1736002	-1460.3596191
1790.0000000	7.2223997	-831.2935791
1790.0000000	7.2711997	-180.7251434
1790.0000000	7.3200002	-17.9417992
1790.0000000	7.3687997	158.5344086
1790.0000000	7.4176002	332.6965332
1790.0000000	7.4663997	261.0715332
1790.0000000	7.5151997	-812.7567139
1790.0000000	7.5640001	-897.5851440
1790.0000000	7.6127996	-2113.5270996
1790.0000000	7.6616001	-679.4240723
1790.0000000	7.7104001	-349.7182617
1790.0000000	7.7591996	35.3350716
1790.0000000	7.8080001	345.7488708
1790.0000000	7.8567996	455.9647827
1790.0000000	7.9056001	-949.4902954
1790.0000000	7.9544001	1510.4039307
1790.0000000	8.0031996	1949.0333252
1790.0000000	8.0520000	1945.3045654
1790.0000000	8.1007996	2058.5588379
1790.0000000	8.1496000	2195.4899902
1790.0000000	8.1983995	1365.7994385
1790.0000000	8.2472000	-3125.8657227
1790.0000000	8.2959995	-1108.2504883
1790.0000000	8.3448000	-882.7059937

1790. 0000000	8. 3935995	-809. 0085449
1790. 0000000	8. 4423990	-348. 7401428
1790. 0000000	8. 4912004	-813. 6534424
1790. 0000000	8. 5400000	-2194. 2309570
1790. 0000000	8. 5887995	-1063. 9882813
1790. 0000000	8. 6375999	207. 2273560
1790. 0000000	8. 6863995	1875. 8765869
1790. 0000000	8. 7351999	-697. 7029419
1790. 0000000	8. 7840004	796. 1281738
1790. 0000000	8. 8327999	41. 8920670
1790. 0000000	8. 8815994	-11. 5099335
1790. 0000000	8. 9303989	-23. 6572609
1790. 0000000	8. 9792004	-11. 5854731
1790. 0000000	9. 0279999	-5. 9736052
1790. 0000000	9. 0767994	6. 5975266
1790. 0000000	9. 1255999	-17. 1096878
1790. 0000000	9. 1743994	-10. 1046162
1790. 0000000	9. 2231998	35. 7979584
1790. 0000000	9. 2720003	-8. 4922981
1790. 0000000	9. 3207998	-9. 8396683
1790. 0000000	9. 3695993	-10. 7812166
1790. 0000000	9. 4183998	-11. 7442865
1790. 0000000	9. 4672003	-11. 1645031
1790. 0000000	9. 5159998	-14. 0956001
1790. 0000000	9. 5648003	-0. 6527729
1790. 0000000	9. 6135998	-13. 3351259
1790. 0000000	9. 6623993	-10. 2425117
1790. 0000000	9. 7111998	-10. 1786175
1790. 0000000	9. 7600002	-10. 8607864
1790. 0000000	9. 8087997	-10. 9953909
1790. 0000000	9. 8575993	2. 8493795
1790. 0000000	9. 9063997	-8. 0394325
1790. 0000000	9. 9552002	-10. 9069338
1790. 0000000	10. 0039997	-10. 0937805
1800. 0000000	0. 3416000	-36. 6236229
1800. 0000000	0. 3904000	-39. 0822296
1800. 0000000	0. 4392000	-39. 2468910
1800. 0000000	0. 4880000	-39. 4969444
1800. 0000000	0. 5368000	-39. 7053070
1800. 0000000	0. 5856000	-39. 2248955
1800. 0000000	0. 6344000	-38. 2496605
1800. 0000000	0. 6832000	-36. 3394814
1800. 0000000	0. 7320000	-36. 1617279
1800. 0000000	0. 7808000	-36. 5436096
1800. 0000000	0. 8296000	-36. 0317650
1800. 0000000	0. 8784000	-35. 2210464
1800. 0000000	0. 9272000	-34. 1622696
1800. 0000000	0. 9760000	-31. 4652882
1800. 0000000	1. 0247999	-30. 0031090
1800. 0000000	1. 0735999	-30. 1370850
1800. 0000000	1. 1224000	-30. 6634998
1800. 0000000	1. 1711999	-30. 9338665
1800. 0000000	1. 2200000	-31. 3577499
1800. 0000000	1. 2687999	-31. 1504040
1800. 0000000	1. 3176000	-30. 7401943
1800. 0000000	1. 3664000	-30. 7152710
1800. 0000000	1. 4152000	-30. 1782589
1800. 0000000	1. 4640000	-29. 4819794

1800.0000000	1.5128000	-31.7953606
1800.0000000	1.5616000	-30.3666706
1800.0000000	1.6104001	-1358.8785400
1800.0000000	1.6592000	-50.3315048
1800.0000000	1.7079999	-27.5498180
1800.0000000	1.7567999	-27.8302174
1800.0000000	1.8055999	-26.6493092
1800.0000000	1.8544000	-26.5595074
1800.0000000	1.9031999	-26.6225719
1800.0000000	1.9520000	-26.8208351
1800.0000000	2.0007999	-26.1613121
1800.0000000	2.0495999	-26.3257771
1800.0000000	2.0983999	-32.8634415
1800.0000000	2.1471999	-22.5214748
1800.0000000	2.1960001	-25.1293926
1800.0000000	2.2448001	-24.4306812
1800.0000000	2.2935998	-23.0298023
1800.0000000	2.3423998	-22.1134090
1800.0000000	2.3912001	-16.6000385
1800.0000000	2.4400001	-23.0314102
1800.0000000	2.4888000	-23.4431458
1800.0000000	2.5375998	-13.6008358
1800.0000000	2.5863998	-11.9278831
1800.0000000	2.6352000	-10.2319908
1800.0000000	2.6840000	-16.3379879
1800.0000000	2.7328000	112.5562515
1800.0000000	2.7815998	133.3902283
1800.0000000	2.8304000	-23.6934052
1800.0000000	2.8792000	-20.8719769
1800.0000000	2.9280000	-22.5277328
1800.0000000	2.9768000	-17.4372215
1800.0000000	3.0256000	-20.6728611
1800.0000000	3.0743999	-28.6356583
1800.0000000	3.1231999	-23.9877300
1800.0000000	3.1719999	-28.9640331
1800.0000000	3.2208002	-485.8371582
1800.0000000	3.2695999	-950.4716797
1800.0000000	3.3183999	-20.5050392
1800.0000000	3.3671999	-3282.6516113
1800.0000000	3.4159999	1176.7955322
1800.0000000	3.4648001	-7.5857553
1800.0000000	3.5135999	-26.8038597
1800.0000000	3.5623999	-311.8405762
1800.0000000	3.6111999	-1955.7500000
1800.0000000	3.6600001	-443.2940674
1800.0000000	3.7088001	-585.1030273
1800.0000000	3.7575998	-915.6721191
1800.0000000	3.8063998	-1661.0484619
1800.0000000	3.8552001	-2411.2941895
1800.0000000	3.9040000	-2364.1193848
1800.0000000	3.9528000	1080.1594238
1800.0000000	4.0015998	-236.7893066
1800.0000000	4.0503998	661.0148926
1800.0000000	4.0991998	83.6034012
1800.0000000	4.1479998	-630.8240967
1800.0000000	4.1967998	1.7322464
1800.0000000	4.2456002	-239.8653107
1800.0000000	4.2943997	-470.3607788

1800.0000000	4.3431997	-13.5231438
1800.0000000	4.3920002	-239.5123444
1800.0000000	4.4407997	-22.3044128
1800.0000000	4.4896002	-15.5518675
1800.0000000	4.5383997	-632.1008911
1800.0000000	4.5871997	-13.0160656
1800.0000000	4.6360002	-21.4853268
1800.0000000	4.6847997	-27.9368248
1800.0000000	4.7336001	-19.4988365
1800.0000000	4.7824001	-16.5523777
1800.0000000	4.8311996	-1427.5405273
1800.0000000	4.8800001	220.3623657
1800.0000000	4.9287996	-195.5223999
1800.0000000	4.9776001	1024.9689941
1800.0000000	5.0264001	82.8669815
1800.0000000	5.0751996	-292.0008545
1800.0000000	5.1240001	-12.3895874
1800.0000000	5.1727996	548.2174072
1800.0000000	5.2216001	31.2123642
1800.0000000	5.2704000	-156.4836578
1800.0000000	5.3191996	-38.8051376
1800.0000000	5.3680000	-3478.4597168
1800.0000000	5.4168000	518.2799683
1800.0000000	5.4656000	-197.7414398
1800.0000000	5.5144000	37.1844406
1800.0000000	5.5631995	480.1625366
1800.0000000	5.6120000	386.5191956
1800.0000000	5.6608000	-36.7462883
1800.0000000	5.7096000	61.2529984
1800.0000000	5.7584000	130.6454163
1800.0000000	5.8071995	-11.5923347
1800.0000000	5.8559999	29.0852337
1800.0000000	5.9047999	-16.5892792
1800.0000000	5.9535999	-39.3848038
1800.0000000	6.0023999	-1547.8491211
1800.0000000	6.0511999	-14.5856228
1800.0000000	6.0999999	-19.3407555
1800.0000000	6.1487999	-13.3114166
1800.0000000	6.1975999	-23.0750961
1800.0000000	6.2463999	-1763.3094482
1800.0000000	6.2951999	-19.0034504
1800.0000000	6.3439999	-337.4560242
1800.0000000	6.3927999	-15.0466166
1800.0000000	6.4416003	-12.4339724
1800.0000000	6.4903998	-11.3188915
1800.0000000	6.5391998	-11.8617582
1800.0000000	6.5879998	328.8341980
1800.0000000	6.6367998	-12.1287880
1800.0000000	6.6856003	-13.8287268
1800.0000000	6.7343998	-13.4731302
1800.0000000	6.7831998	-14.3389721
1800.0000000	6.8319998	56.8979645
1800.0000000	6.8807998	-14.0382509
1800.0000000	6.9296002	-388.3145752
1800.0000000	6.9783998	-6.1132503
1800.0000000	7.0271997	-14.0550308
1800.0000000	7.0760002	7.8163862
1800.0000000	7.1247997	-1816.9752197

1800.0000000	7.1736002	-1457.1967773
1800.0000000	7.2223997	-829.5003662
1800.0000000	7.2711997	-180.3573608
1800.0000000	7.3200002	-17.9298096
1800.0000000	7.3687997	158.1598816
1800.0000000	7.4176002	331.9407043
1800.0000000	7.4663997	260.4738770
1800.0000000	7.5151997	-810.9956665
1800.0000000	7.5640001	-895.6377563
1800.0000000	7.6127996	-2108.9094238
1800.0000000	7.6616001	-677.9526978
1800.0000000	7.7104001	-348.9719849
1800.0000000	7.7591996	35.2340240
1800.0000000	7.8080001	344.9635315
1800.0000000	7.8567996	454.9366455
1800.0000000	7.9056001	-947.4173584
1800.0000000	7.9544001	1507.0939941
1800.0000000	8.0031996	1944.7636719
1800.0000000	8.0520000	1941.0426025
1800.0000000	8.1007996	2054.0493164
1800.0000000	8.1496000	2190.6818848
1800.0000000	8.1983995	1362.8068848
1800.0000000	8.2472000	-3119.0310059
1800.0000000	8.2959995	-1105.8303223
1800.0000000	8.3448000	-880.7767944
1800.0000000	8.3935995	-807.2408447
1800.0000000	8.4423990	-347.9821167
1800.0000000	8.4912004	-811.8771973
1800.0000000	8.5400000	-2189.4311523
1800.0000000	8.5887995	-1061.6667480
1800.0000000	8.6375999	206.7660370
1800.0000000	8.6863995	1871.7583008
1800.0000000	8.7351999	-696.1786499
1800.0000000	8.7840004	794.3714600
1800.0000000	8.8327999	41.7804604
1800.0000000	8.8815994	-11.5101080
1800.0000000	8.9303989	-23.6535397
1800.0000000	8.9792004	-11.5855551
1800.0000000	9.0279999	-5.9753737
1800.0000000	9.0767994	6.5918083
1800.0000000	9.1255999	-17.1079731
1800.0000000	9.1743994	-10.1050186
1800.0000000	9.2231998	35.7822266
1800.0000000	9.2720003	-8.4932346
1800.0000000	9.3207998	-9.8402071
1800.0000000	9.3695993	-10.7814426
1800.0000000	9.4183998	-11.7441626
1800.0000000	9.4672003	-11.1645441
1800.0000000	9.5159998	-14.0947084
1800.0000000	9.5648003	-0.6561451
1800.0000000	9.6135998	-13.3343353
1800.0000000	9.6623993	-10.2427874
1800.0000000	9.7111998	-10.1788883
1800.0000000	9.7600002	-10.8608284
1800.0000000	9.8087997	-10.9953985
1800.0000000	9.8575993	2.8449574
1800.0000000	9.9063997	-8.0403461
1800.0000000	9.9552002	-10.9069376

1800.0000000	10.0039997	-10.0940275
1810.0000000	0.3416000	-36.4009209
1810.0000000	0.3904000	-38.6148148
1810.0000000	0.4392000	-39.2405472
1810.0000000	0.4880000	-39.4957352
1810.0000000	0.5368000	-39.7064781
1810.0000000	0.5856000	-39.2235146
1810.0000000	0.6344000	-38.2499695
1810.0000000	0.6832000	-36.3417053
1810.0000000	0.7320000	-36.1637802
1810.0000000	0.7808000	-36.5437813
1810.0000000	0.8296000	-36.0278587
1810.0000000	0.8784000	-35.3156509
1810.0000000	0.9272000	-34.3775940
1810.0000000	0.9760000	-32.3230820
1810.0000000	1.0247999	-30.6226406
1810.0000000	1.0735999	-30.1412125
1810.0000000	1.1224000	-30.3955593
1810.0000000	1.1711999	-31.0334606
1810.0000000	1.2200000	-31.4666824
1810.0000000	1.2687999	-31.0915375
1810.0000000	1.3176000	-30.7420006
1810.0000000	1.3664000	-30.7148476
1810.0000000	1.4152000	-30.1784649
1810.0000000	1.4640000	-29.4802704
1810.0000000	1.5128000	-31.6981831
1810.0000000	1.5616000	-30.3248291
1810.0000000	1.6104001	-1356.3356934
1810.0000000	1.6592000	-50.0375748
1810.0000000	1.7079999	-27.5535641
1810.0000000	1.7567999	-27.8195496
1810.0000000	1.8055999	-26.8603249
1810.0000000	1.8544000	-26.0923061
1810.0000000	1.9031999	-26.4909973
1810.0000000	1.9520000	-26.8236179
1810.0000000	2.0007999	-26.1666927
1810.0000000	2.0495999	-26.3277950
1810.0000000	2.0983999	-31.3876648
1810.0000000	2.1471999	-22.7887421
1810.0000000	2.1960001	-25.1253014
1810.0000000	2.2448001	-24.4127693
1810.0000000	2.2935998	-22.9701786
1810.0000000	2.3423998	-22.1278133
1810.0000000	2.3912001	-17.2215843
1810.0000000	2.4400001	-23.0338955
1810.0000000	2.4888000	-23.4429283
1810.0000000	2.5375998	-13.6179438
1810.0000000	2.5863998	-11.9476423
1810.0000000	2.6352000	-10.2547350
1810.0000000	2.6840000	-16.3499298
1810.0000000	2.7328000	112.2902451
1810.0000000	2.7815998	133.0838013
1810.0000000	2.8304000	-23.6921005
1810.0000000	2.8792000	-20.8752403
1810.0000000	2.9280000	-22.5271645
1810.0000000	2.9768000	-17.4455929
1810.0000000	3.0256000	-20.6754456
1810.0000000	3.0743999	-28.6242714

1810.0000000	3.1231999	-23.9842739
1810.0000000	3.1719999	-28.9557400
1810.0000000	3.2208002	-485.5994263
1810.0000000	3.2695999	-949.9967041
1810.0000000	3.3183999	-20.5052376
1810.0000000	3.3671999	-3281.1899414
1810.0000000	3.4159999	1176.2602539
1810.0000000	3.4648001	-7.5914640
1810.0000000	3.5135999	-26.8008690
1810.0000000	3.5623999	-311.7109985
1810.0000000	3.6111999	-1954.8902588
1810.0000000	3.6600001	-443.1058960
1810.0000000	3.7088001	-584.8516846
1810.0000000	3.7575998	-915.2735596
1810.0000000	3.8063998	-1660.3186035
1810.0000000	3.8552001	-2410.2307129
1810.0000000	3.9040000	-2363.0769043
1810.0000000	3.9528000	1079.6711426
1810.0000000	4.0015998	-236.6921997
1810.0000000	4.0503998	660.7123413
1810.0000000	4.0991998	83.5577164
1810.0000000	4.1479998	-630.5518799
1810.0000000	4.1967998	1.7229900
1810.0000000	4.2456002	-239.7670135
1810.0000000	4.2943997	-470.1602173
1810.0000000	4.3431997	-13.5253239
1810.0000000	4.3920002	-239.4142303
1810.0000000	4.4407997	-22.3026371
1810.0000000	4.4896002	-15.5527506
1810.0000000	4.5383997	-631.8398438
1810.0000000	4.5871997	-13.0180817
1810.0000000	4.6360002	-21.4837837
1810.0000000	4.6847997	-27.9328995
1810.0000000	4.7336001	-19.4981136
1810.0000000	4.7824001	-16.5528336
1810.0000000	4.8311996	-1427.0091553
1810.0000000	4.8800001	220.2728271
1810.0000000	4.9287996	-195.4551392
1810.0000000	4.9776001	1024.5765381
1810.0000000	5.0264001	82.8294601
1810.0000000	5.0751996	-291.8972168
1810.0000000	5.1240001	-12.3905087
1810.0000000	5.1727996	548.1015015
1810.0000000	5.2216001	31.2029190
1810.0000000	5.2704000	-156.4555511
1810.0000000	5.3191996	-38.8005905
1810.0000000	5.3680000	-3477.7673340
1810.0000000	5.4168000	518.1731567
1810.0000000	5.4656000	-197.7052612
1810.0000000	5.5144000	37.1737823
1810.0000000	5.5631995	480.0639648
1810.0000000	5.6120000	386.4392395
1810.0000000	5.6608000	-36.7421989
1810.0000000	5.7096000	61.2375717
1810.0000000	5.7584000	130.6164856
1810.0000000	5.8071995	-11.5932045
1810.0000000	5.8559999	29.0763798
1810.0000000	5.9047999	-16.5890827

1810.0000000	5.9535999	-39.3800659
1810.0000000	6.0023999	-1547.5445557
1810.0000000	6.0511999	-14.5858345
1810.0000000	6.0999999	-19.3399239
1810.0000000	6.1487999	-13.3118801
1810.0000000	6.1975999	-23.0733299
1810.0000000	6.2463999	-1762.9621582
1810.0000000	6.2951999	-19.0026360
1810.0000000	6.3439999	-337.3919678
1810.0000000	6.3927999	-15.0465984
1810.0000000	6.4416003	-12.4345598
1810.0000000	6.4903998	-11.3195677
1810.0000000	6.5391998	-11.8624392
1810.0000000	6.5879998	328.1434937
1810.0000000	6.6367998	-12.1293373
1810.0000000	6.6856003	-13.8288975
1810.0000000	6.7343998	-13.4733467
1810.0000000	6.7831998	-14.3385563
1810.0000000	6.8319998	56.8788452
1810.0000000	6.8807998	-14.0381117
1810.0000000	6.9296002	-387.5531311
1810.0000000	6.9783998	-6.1260738
1810.0000000	7.0271997	-14.0540037
1810.0000000	7.0760002	7.7742014
1810.0000000	7.1247997	-1813.0294189
1810.0000000	7.1736002	-1454.0339355
1810.0000000	7.2223997	-827.7072144
1810.0000000	7.2711997	-179.9895935
1810.0000000	7.3200002	-17.9178200
1810.0000000	7.3687997	157.7853394
1810.0000000	7.4176002	331.1848755
1810.0000000	7.4663997	259.8762207
1810.0000000	7.5151997	-809.2346191
1810.0000000	7.5640001	-893.6903687
1810.0000000	7.6127996	-2104.2919922
1810.0000000	7.6616001	-676.4813843
1810.0000000	7.7104001	-348.2257080
1810.0000000	7.7591996	35.1329765
1810.0000000	7.8080001	344.1781616
1810.0000000	7.8567996	453.9085693
1810.0000000	7.9056001	-945.3444824
1810.0000000	7.9544001	1503.7840576
1810.0000000	8.0031996	1940.4940186
1810.0000000	8.0520000	1936.7806396
1810.0000000	8.1007996	2049.5397949
1810.0000000	8.1496000	2185.8737793
1810.0000000	8.1983995	1359.8144531
1810.0000000	8.2472000	-3112.1960449
1810.0000000	8.2959995	-1103.4101563
1810.0000000	8.3448000	-878.8475952
1810.0000000	8.3935995	-805.4732056
1810.0000000	8.4423990	-347.2241211
1810.0000000	8.4912004	-810.1009521
1810.0000000	8.5400000	-2184.6313477
1810.0000000	8.5887995	-1059.3452148
1810.0000000	8.6375999	206.3047028
1810.0000000	8.6863995	1867.6398926
1810.0000000	8.7351999	-694.6544189

1810.0000000	8.7840004	792.6146851
1810.0000000	8.8327999	41.6688499
1810.0000000	8.8815994	-11.5102825
1810.0000000	8.9303989	-23.6498184
1810.0000000	8.9792004	-11.5856371
1810.0000000	9.0279999	-5.9771419
1810.0000000	9.0767994	6.5860920
1810.0000000	9.1255999	-17.1062584
1810.0000000	9.1743994	-10.1054211
1810.0000000	9.2231998	35.7664948
1810.0000000	9.2720003	-8.4941711
1810.0000000	9.3207998	-9.8407469
1810.0000000	9.3695993	-10.7816696
1810.0000000	9.4183998	-11.7440376
1810.0000000	9.4672003	-11.1645851
1810.0000000	9.5159998	-14.0938177
1810.0000000	9.5648003	-0.6595182
1810.0000000	9.6135998	-13.3335447
1810.0000000	9.6623993	-10.2430620
1810.0000000	9.7111998	-10.1791601
1810.0000000	9.7600002	-10.8608704
1810.0000000	9.8087997	-10.9954071
1810.0000000	9.8575993	2.8405361
1810.0000000	9.9063997	-8.0412598
1810.0000000	9.9552002	-10.9069424
1810.0000000	10.0039997	-10.0942755
1820.0000000	0.3416000	-36.4964447
1820.0000000	0.3904000	-39.0021820
1820.0000000	0.4392000	-39.2342033
1820.0000000	0.4880000	-39.4945259
1820.0000000	0.5368000	-39.7076530
1820.0000000	0.5856000	-39.2221336
1820.0000000	0.6344000	-38.2502823
1820.0000000	0.6832000	-36.3439331
1820.0000000	0.7320000	-36.1658325
1820.0000000	0.7808000	-36.5439491
1820.0000000	0.8296000	-36.0239525
1820.0000000	0.8784000	-35.4102554
1820.0000000	0.9272000	-33.5569420
1820.0000000	0.9760000	-31.1857815
1820.0000000	1.0247999	-29.8544407
1820.0000000	1.0735999	-29.8119793
1820.0000000	1.1224000	-30.2684669
1820.0000000	1.1711999	-30.9072800
1820.0000000	1.2200000	-31.1939602
1820.0000000	1.2687999	-31.0326691
1820.0000000	1.3176000	-30.7438087
1820.0000000	1.3664000	-30.7144241
1820.0000000	1.4152000	-30.1786690
1820.0000000	1.4640000	-29.4785595
1820.0000000	1.5128000	-31.6010056
1820.0000000	1.5616000	-30.2829876
1820.0000000	1.6104001	-1353.7928467
1820.0000000	1.6592000	-49.7436409
1820.0000000	1.7079999	-27.5573120
1820.0000000	1.7567999	-27.8088837
1820.0000000	1.8055999	-26.6663055
1820.0000000	1.8544000	-26.4930496

1820.0000000	1.9031999	-26.8074226
1820.0000000	1.9520000	-26.8264008
1820.0000000	2.0007999	-26.1720734
1820.0000000	2.0495999	-26.3298149
1820.0000000	2.0983999	-29.9118862
1820.0000000	2.1471999	-23.0560093
1820.0000000	2.1960001	-25.1212101
1820.0000000	2.2448001	-24.3948593
1820.0000000	2.2935998	-22.9105549
1820.0000000	2.3423998	-22.1422176
1820.0000000	2.3912001	-17.8431301
1820.0000000	2.4400001	-23.0363827
1820.0000000	2.4888000	-23.4427109
1820.0000000	2.5375998	-13.6350517
1820.0000000	2.5863998	-11.9674015
1820.0000000	2.6352000	-10.2774792
1820.0000000	2.6840000	-16.3618717
1820.0000000	2.7328000	112.0242233
1820.0000000	2.7815998	132.7774048
1820.0000000	2.8304000	-23.6907959
1820.0000000	2.8792000	-20.8785038
1820.0000000	2.9280000	-22.5265961
1820.0000000	2.9768000	-17.4539642
1820.0000000	3.0256000	-20.6780300
1820.0000000	3.0743999	-28.6128845
1820.0000000	3.1231999	-23.9808197
1820.0000000	3.1719999	-28.9474468
1820.0000000	3.2208002	-485.3616943
1820.0000000	3.2695999	-949.5217285
1820.0000000	3.3183999	-20.5054359
1820.0000000	3.3671999	-3279.7282715
1820.0000000	3.4159999	1175.7249756
1820.0000000	3.4648001	-7.5971718
1820.0000000	3.5135999	-26.7978783
1820.0000000	3.5623999	-311.5813904
1820.0000000	3.6111999	-1954.0303955
1820.0000000	3.6600001	-442.9177856
1820.0000000	3.7088001	-584.6002808
1820.0000000	3.7575998	-914.8750610
1820.0000000	3.8063998	-1659.5887451
1820.0000000	3.8552001	-2409.1672363
1820.0000000	3.9040000	-2362.0344238
1820.0000000	3.9528000	1079.1827393
1820.0000000	4.0015998	-236.5950928
1820.0000000	4.0503998	660.4097900
1820.0000000	4.0991998	83.5120316
1820.0000000	4.1479998	-630.2796631
1820.0000000	4.1967998	1.7137337
1820.0000000	4.2456002	-239.6687164
1820.0000000	4.2943997	-469.9596558
1820.0000000	4.3431997	-13.5275049
1820.0000000	4.3920002	-239.3161163
1820.0000000	4.4407997	-22.3008614
1820.0000000	4.4896002	-15.5536327
1820.0000000	4.5383997	-631.5787354
1820.0000000	4.5871997	-13.0200977
1820.0000000	4.6360002	-21.4822407
1820.0000000	4.6847997	-27.9289742

1820.0000000	4.7336001	-19.4973907
1820.0000000	4.7824001	-16.5532875
1820.0000000	4.8311996	-1426.4779053
1820.0000000	4.8800001	220.1832733
1820.0000000	4.9287996	-195.3878784
1820.0000000	4.9776001	1024.1840820
1820.0000000	5.0264001	82.7919388
1820.0000000	5.0751996	-291.7935791
1820.0000000	5.1240001	-12.3914299
1820.0000000	5.1727996	547.9856567
1820.0000000	5.2216001	31.1934776
1820.0000000	5.2704000	-156.4274445
1820.0000000	5.3191996	-38.7960396
1820.0000000	5.3680000	-3477.0749512
1820.0000000	5.4168000	518.0663452
1820.0000000	5.4656000	-197.6690674
1820.0000000	5.5144000	37.1631317
1820.0000000	5.5631995	479.9653931
1820.0000000	5.6120000	386.3592529
1820.0000000	5.6608000	-36.7381096
1820.0000000	5.7096000	61.2221375
1820.0000000	5.7584000	130.5875549
1820.0000000	5.8071995	-11.5940752
1820.0000000	5.8559999	29.0675297
1820.0000000	5.9047999	-16.5888844
1820.0000000	5.9535999	-39.3753281
1820.0000000	6.0023999	-1547.2402344
1820.0000000	6.0511999	-14.5860453
1820.0000000	6.0999999	-19.3390903
1820.0000000	6.1487999	-13.3123426
1820.0000000	6.1975999	-23.0715637
1820.0000000	6.2463999	-1762.6147461
1820.0000000	6.2951999	-19.0018215
1820.0000000	6.3439999	-337.3279419
1820.0000000	6.3927999	-15.0465794
1820.0000000	6.4416003	-12.4351463
1820.0000000	6.4903998	-11.3202438
1820.0000000	6.5391998	-11.8631201
1820.0000000	6.5879998	327.4528198
1820.0000000	6.6367998	-12.1298866
1820.0000000	6.6856003	-13.8290672
1820.0000000	6.7343998	-13.4735622
1820.0000000	6.7831998	-14.3381405
1820.0000000	6.8319998	56.8597336
1820.0000000	6.8807998	-14.0379734
1820.0000000	6.9296002	-386.7916870
1820.0000000	6.9783998	-6.1388979
1820.0000000	7.0271997	-14.0529757
1820.0000000	7.0760002	7.7320147
1820.0000000	7.1247997	-1809.0834961
1820.0000000	7.1736002	-1450.8710938
1820.0000000	7.2223997	-825.9140625
1820.0000000	7.2711997	-179.6218109
1820.0000000	7.3200002	-17.9058323
1820.0000000	7.3687997	157.4108124
1820.0000000	7.4176002	330.4290466
1820.0000000	7.4663997	259.2785645
1820.0000000	7.5151997	-807.4736328

1820.0000000	7.5640001	-891.7429810
1820.0000000	7.6127996	-2099.6743164
1820.0000000	7.6616001	-675.0100098
1820.0000000	7.7104001	-347.4794922
1820.0000000	7.7591996	35.0319252
1820.0000000	7.8080001	343.3927917
1820.0000000	7.8567996	452.8804932
1820.0000000	7.9056001	-943.2715454
1820.0000000	7.9544001	1500.4741211
1820.0000000	8.0031996	1936.2244873
1820.0000000	8.0520000	1932.5185547
1820.0000000	8.1007996	2045.0303955
1820.0000000	8.1496000	2181.0659180
1820.0000000	8.1983995	1356.8218994
1820.0000000	8.2472000	-3105.3613281
1820.0000000	8.2959995	-1100.9899902
1820.0000000	8.3448000	-876.9184570
1820.0000000	8.3935995	-803.7055054
1820.0000000	8.4423990	-346.4660950
1820.0000000	8.4912004	-808.3247681
1820.0000000	8.5400000	-2179.8315430
1820.0000000	8.5887995	-1057.0236816
1820.0000000	8.6375999	205.8433838
1820.0000000	8.6863995	1863.5216064
1820.0000000	8.7351999	-693.1301270
1820.0000000	8.7840004	790.8579712
1820.0000000	8.8327999	41.5572433
1820.0000000	8.8815994	-11.5104570
1820.0000000	8.9303989	-23.6460991
1820.0000000	8.9792004	-11.5857201
1820.0000000	9.0279999	-5.9789104
1820.0000000	9.0767994	6.5803738
1820.0000000	9.1255999	-17.1045456
1820.0000000	9.1743994	-10.1058226
1820.0000000	9.2231998	35.7507553
1820.0000000	9.2720003	-8.4951077
1820.0000000	9.3207998	-9.8412857
1820.0000000	9.3695993	-10.7818956
1820.0000000	9.4183998	-11.7439127
1820.0000000	9.4672003	-11.1646261
1820.0000000	9.5159998	-14.0929260
1820.0000000	9.5648003	-0.6628914
1820.0000000	9.6135998	-13.3327541
1820.0000000	9.6623993	-10.2433376
1820.0000000	9.7111998	-10.1794319
1820.0000000	9.7600002	-10.8609123
1820.0000000	9.8087997	-10.9954147
1820.0000000	9.8575993	2.8361120
1820.0000000	9.9063997	-8.0421743
1820.0000000	9.9552002	-10.9069462
1820.0000000	10.0039997	-10.0945225
1830.0000000	0.3416000	-36.7719803
1830.0000000	0.3904000	-39.0188675
1830.0000000	0.4392000	-39.2278633
1830.0000000	0.4880000	-39.4933205
1830.0000000	0.5368000	-39.7088280
1830.0000000	0.5856000	-39.2207527
1830.0000000	0.6344000	-38.2505913

1830.0000000	0.6832000	-36.3461571
1830.0000000	0.7320000	-36.1678848
1830.0000000	0.7808000	-36.5441170
1830.0000000	0.8296000	-36.0200462
1830.0000000	0.8784000	-35.4071236
1830.0000000	0.9272000	-33.5413628
1830.0000000	0.9760000	-31.1809654
1830.0000000	1.0247999	-29.8599491
1830.0000000	1.0735999	-29.7972565
1830.0000000	1.1224000	-30.2501907
1830.0000000	1.1711999	-30.9004288
1830.0000000	1.2200000	-31.1957359
1830.0000000	1.2687999	-31.0348377
1830.0000000	1.3176000	-30.7401276
1830.0000000	1.3664000	-30.7140026
1830.0000000	1.4152000	-30.1788731
1830.0000000	1.4640000	-29.4768505
1830.0000000	1.5128000	-31.5038280
1830.0000000	1.5616000	-30.2411461
1830.0000000	1.6104001	-1351.2501221
1830.0000000	1.6592000	-49.4497070
1830.0000000	1.7079999	-27.5610580
1830.0000000	1.7567999	-27.7982159
1830.0000000	1.8055999	-27.0633469
1830.0000000	1.8544000	-27.2452316
1830.0000000	1.9031999	-27.1238480
1830.0000000	1.9520000	-26.8291817
1830.0000000	2.0007999	-26.1774540
1830.0000000	2.0495999	-26.3318329
1830.0000000	2.0983999	-28.4361076
1830.0000000	2.1471999	-23.3232765
1830.0000000	2.1960001	-25.1171188
1830.0000000	2.2448001	-24.3769474
1830.0000000	2.2935998	-22.8509312
1830.0000000	2.3423998	-22.1566238
1830.0000000	2.3912001	-18.4646759
1830.0000000	2.4400001	-23.0388699
1830.0000000	2.4888000	-23.4424934
1830.0000000	2.5375998	-13.6521587
1830.0000000	2.5863998	-11.9871607
1830.0000000	2.6352000	-10.3002243
1830.0000000	2.6840000	-16.3738136
1830.0000000	2.7328000	111.7582016
1830.0000000	2.7815998	132.4709778
1830.0000000	2.8304000	-23.6894913
1830.0000000	2.8792000	-20.8817673
1830.0000000	2.9280000	-22.5260258
1830.0000000	2.9768000	-17.4623337
1830.0000000	3.0256000	-20.6806145
1830.0000000	3.0743999	-28.6014977
1830.0000000	3.1231999	-23.9773636
1830.0000000	3.1719999	-28.9391537
1830.0000000	3.2208002	-485.1239624
1830.0000000	3.2695999	-949.0467529
1830.0000000	3.3183999	-20.5056343
1830.0000000	3.3671999	-3278.2666016
1830.0000000	3.4159999	1175.1896973
1830.0000000	3.4648001	-7.6028805

1830.0000000	3.5135999	-26.7948875
1830.0000000	3.5623999	-311.4518127
1830.0000000	3.6111999	-1953.1706543
1830.0000000	3.6600001	-442.7296753
1830.0000000	3.7088001	-584.3488770
1830.0000000	3.7575998	-914.4765015
1830.0000000	3.8063998	-1658.8588867
1830.0000000	3.8552001	-2408.1037598
1830.0000000	3.9040000	-2360.9919434
1830.0000000	3.9528000	1078.6944580
1830.0000000	4.0015998	-236.4979858
1830.0000000	4.0503998	660.1072388
1830.0000000	4.0991998	83.4663467
1830.0000000	4.1479998	-630.0073853
1830.0000000	4.1967998	1.7044773
1830.0000000	4.2456002	-239.5704041
1830.0000000	4.2943997	-469.7590942
1830.0000000	4.3431997	-13.5296860
1830.0000000	4.3920002	-239.2180023
1830.0000000	4.4407997	-22.2990856
1830.0000000	4.4896002	-15.5545158
1830.0000000	4.5383997	-631.3176880
1830.0000000	4.5871997	-13.0221138
1830.0000000	4.6360002	-21.4806995
1830.0000000	4.6847997	-27.9250488
1830.0000000	4.7336001	-19.4966698
1830.0000000	4.7824001	-16.5537415
1830.0000000	4.8311996	-1425.9466553
1830.0000000	4.8800001	220.0937195
1830.0000000	4.9287996	-195.3206024
1830.0000000	4.9776001	1023.7915649
1830.0000000	5.0264001	82.7544250
1830.0000000	5.0751996	-291.6899719
1830.0000000	5.1240001	-12.3923512
1830.0000000	5.1727996	547.8698120
1830.0000000	5.2216001	31.1840324
1830.0000000	5.2704000	-156.3993378
1830.0000000	5.3191996	-38.7914925
1830.0000000	5.3680000	-3476.3825684
1830.0000000	5.4168000	517.9595947
1830.0000000	5.4656000	-197.6328888
1830.0000000	5.5144000	37.1524734
1830.0000000	5.5631995	479.8668213
1830.0000000	5.6120000	386.2792664
1830.0000000	5.6608000	-36.7340240
1830.0000000	5.7096000	61.2067108
1830.0000000	5.7584000	130.5586243
1830.0000000	5.8071995	-11.5949459
1830.0000000	5.8559999	29.0586758
1830.0000000	5.9047999	-16.5886860
1830.0000000	5.9535999	-39.3705902
1830.0000000	6.0023999	-1546.9357910
1830.0000000	6.0511999	-14.5862570
1830.0000000	6.0999999	-19.3382587
1830.0000000	6.1487999	-13.3128052
1830.0000000	6.1975999	-23.0698013
1830.0000000	6.2463999	-1762.2673340
1830.0000000	6.2951999	-19.0010071

1830.0000000	6.3439999	-337.2638550
1830.0000000	6.3927999	-15.0465612
1830.0000000	6.4416003	-12.4357338
1830.0000000	6.4903998	-11.3209190
1830.0000000	6.5391998	-11.8638010
1830.0000000	6.5879998	326.7621155
1830.0000000	6.6367998	-12.1304350
1830.0000000	6.6856003	-13.8292379
1830.0000000	6.7343998	-13.4737778
1830.0000000	6.7831998	-14.3377247
1830.0000000	6.8319998	56.8406143
1830.0000000	6.8807998	-14.0378342
1830.0000000	6.9296002	-386.0302429
1830.0000000	6.9783998	-6.1517210
1830.0000000	7.0271997	-14.0519476
1830.0000000	7.0760002	7.6898298
1830.0000000	7.1247997	-1805.1376953
1830.0000000	7.1736002	-1447.7082520
1830.0000000	7.2223997	-824.1209106
1830.0000000	7.2711997	-179.2540283
1830.0000000	7.3200002	-17.8938446
1830.0000000	7.3687997	157.0362854
1830.0000000	7.4176002	329.6732178
1830.0000000	7.4663997	258.6809387
1830.0000000	7.5151997	-805.7125854
1830.0000000	7.5640001	-889.7955322
1830.0000000	7.6127996	-2095.0566406
1830.0000000	7.6616001	-673.5386963
1830.0000000	7.7104001	-346.7332153
1830.0000000	7.7591996	34.9308777
1830.0000000	7.8080001	342.6074524
1830.0000000	7.8567996	451.8523865
1830.0000000	7.9056001	-941.1986694
1830.0000000	7.9544001	1497.1641846
1830.0000000	8.0031996	1931.9548340
1830.0000000	8.0520000	1928.2565918
1830.0000000	8.1007996	2040.5208740
1830.0000000	8.1496000	2176.2578125
1830.0000000	8.1983995	1353.8294678
1830.0000000	8.2472000	-3098.5266113
1830.0000000	8.2959995	-1098.5698242
1830.0000000	8.3448000	-874.9892578
1830.0000000	8.3935995	-801.9378052
1830.0000000	8.4423990	-345.7080994
1830.0000000	8.4912004	-806.5485229
1830.0000000	8.5400000	-2175.0317383
1830.0000000	8.5887995	-1054.7020264
1830.0000000	8.6375999	205.3820648
1830.0000000	8.6863995	1859.4031982
1830.0000000	8.7351999	-691.6058960
1830.0000000	8.7840004	789.1011963
1830.0000000	8.8327999	41.4456367
1830.0000000	8.8815994	-11.5106316
1830.0000000	8.9303989	-23.6423798
1830.0000000	8.9792004	-11.5858021
1830.0000000	9.0279999	-5.9806781
1830.0000000	9.0767994	6.5746555
1830.0000000	9.1255999	-17.1028309

1830.0000000	9.1743994	-10.1062241
1830.0000000	9.2231998	35.7350235
1830.0000000	9.2720003	-8.4960442
1830.0000000	9.3207998	-9.8418255
1830.0000000	9.3695993	-10.7821217
1830.0000000	9.4183998	-11.7437887
1830.0000000	9.4672003	-11.1646681
1830.0000000	9.5159998	-14.0920353
1830.0000000	9.5648003	-0.6662636
1830.0000000	9.6135998	-13.3319626
1830.0000000	9.6623993	-10.2436123
1830.0000000	9.7111998	-10.1797037
1830.0000000	9.7600002	-10.8609533
1830.0000000	9.8087997	-10.9954224
1830.0000000	9.8575993	2.8316898
1830.0000000	9.9063997	-8.0430880
1830.0000000	9.9552002	-10.9069510
1830.0000000	10.0039997	-10.0947704
1840.0000000	0.3416000	-37.0772133
1840.0000000	0.3904000	-39.0355530
1840.0000000	0.4392000	-39.2215195
1840.0000000	0.4880000	-39.4921112
1840.0000000	0.5368000	-39.7099991
1840.0000000	0.5856000	-39.2193718
1840.0000000	0.6344000	-38.2509003
1840.0000000	0.6832000	-36.3483849
1840.0000000	0.7320000	-36.1699371
1840.0000000	0.7808000	-36.5442886
1840.0000000	0.8296000	-36.0161400
1840.0000000	0.8784000	-35.4039917
1840.0000000	0.9272000	-33.5257835
1840.0000000	0.9760000	-31.1761475
1840.0000000	1.0247999	-29.8654575
1840.0000000	1.0735999	-29.7825336
1840.0000000	1.1224000	-30.2319145
1840.0000000	1.1711999	-30.8935757
1840.0000000	1.2200000	-31.1975098
1840.0000000	1.2687999	-31.0370064
1840.0000000	1.3176000	-30.7364445
1840.0000000	1.3664000	-30.7135792
1840.0000000	1.4152000	-30.1790771
1840.0000000	1.4640000	-29.4751415
1840.0000000	1.5128000	-31.4066505
1840.0000000	1.5616000	-30.1993065
1840.0000000	1.6104001	-1348.7072754
1840.0000000	1.6592000	-49.1557770
1840.0000000	1.7079999	-27.5648060
1840.0000000	1.7567999	-27.7875481
1840.0000000	1.8055999	-27.5859261
1840.0000000	1.8544000	-26.7011719
1840.0000000	1.9031999	-26.6712132
1840.0000000	1.9520000	-26.8319645
1840.0000000	2.0007999	-26.1828327
1840.0000000	2.0495999	-26.3338528
1840.0000000	2.0983999	-26.9603291
1840.0000000	2.1471999	-23.5905437
1840.0000000	2.1960001	-25.1130276
1840.0000000	2.2448001	-24.3590355

1840.0000000	2.2935998	-22.7913074
1840.0000000	2.3423998	-22.1710281
1840.0000000	2.3912001	-19.0862217
1840.0000000	2.4400001	-23.0413551
1840.0000000	2.4888000	-23.4422760
1840.0000000	2.5375998	-13.6692667
1840.0000000	2.5863998	-12.0069199
1840.0000000	2.6352000	-10.3229685
1840.0000000	2.6840000	-16.3857555
1840.0000000	2.7328000	111.4921951
1840.0000000	2.7815998	132.1645508
1840.0000000	2.8304000	-23.6881886
1840.0000000	2.8792000	-20.8850307
1840.0000000	2.9280000	-22.5254574
1840.0000000	2.9768000	-17.4707050
1840.0000000	3.0256000	-20.6831989
1840.0000000	3.0743999	-28.5901108
1840.0000000	3.1231999	-23.9739075
1840.0000000	3.1719999	-28.9308605
1840.0000000	3.2208002	-484.8862305
1840.0000000	3.2695999	-948.5717773
1840.0000000	3.3183999	-20.5058308
1840.0000000	3.3671999	-3276.8051758
1840.0000000	3.4159999	1174.6544189
1840.0000000	3.4648001	-7.6085882
1840.0000000	3.5135999	-26.7918968
1840.0000000	3.5623999	-311.3222351
1840.0000000	3.6111999	-1952.3109131
1840.0000000	3.6600001	-442.5415039
1840.0000000	3.7088001	-584.0975342
1840.0000000	3.7575998	-914.0779419
1840.0000000	3.8063998	-1658.1290283
1840.0000000	3.8552001	-2407.0402832
1840.0000000	3.9040000	-2359.9494629
1840.0000000	3.9528000	1078.2061768
1840.0000000	4.0015998	-236.4008789
1840.0000000	4.0503998	659.8046265
1840.0000000	4.0991998	83.4206696
1840.0000000	4.1479998	-629.7351685
1840.0000000	4.1967998	1.6952209
1840.0000000	4.2456002	-239.4721069
1840.0000000	4.2943997	-469.5585327
1840.0000000	4.3431997	-13.5318661
1840.0000000	4.3920002	-239.1198883
1840.0000000	4.4407997	-22.2973099
1840.0000000	4.4896002	-15.5553980
1840.0000000	4.5383997	-631.0565796
1840.0000000	4.5871997	-13.0241299
1840.0000000	4.6360002	-21.4791565
1840.0000000	4.6847997	-27.9211235
1840.0000000	4.7336001	-19.4959469
1840.0000000	4.7824001	-16.5541954
1840.0000000	4.8311996	-1425.4154053
1840.0000000	4.8800001	220.0041809
1840.0000000	4.9287996	-195.2533417
1840.0000000	4.9776001	1023.3991089
1840.0000000	5.0264001	82.7169037
1840.0000000	5.0751996	-291.5863342

1840.0000000	5.1240001	-12.3932724
1840.0000000	5.1727996	547.7539063
1840.0000000	5.2216001	31.1745872
1840.0000000	5.2704000	-156.3712311
1840.0000000	5.3191996	-38.7869415
1840.0000000	5.3680000	-3475.6901855
1840.0000000	5.4168000	517.8528442
1840.0000000	5.4656000	-197.5967102
1840.0000000	5.5144000	37.1418152
1840.0000000	5.5631995	479.7682495
1840.0000000	5.6120000	386.1993103
1840.0000000	5.6608000	-36.7299347
1840.0000000	5.7096000	61.1912766
1840.0000000	5.7584000	130.5296936
1840.0000000	5.8071995	-11.5958166
1840.0000000	5.8559999	29.0498257
1840.0000000	5.9047999	-16.5884895
1840.0000000	5.9535999	-39.3658485
1840.0000000	6.0023999	-1546.6313477
1840.0000000	6.0511999	-14.5864677
1840.0000000	6.0999999	-19.3374252
1840.0000000	6.1487999	-13.3132687
1840.0000000	6.1975999	-23.0680351
1840.0000000	6.2463999	-1761.9199219
1840.0000000	6.2951999	-19.0001926
1840.0000000	6.3439999	-337.1998291
1840.0000000	6.3927999	-15.0465431
1840.0000000	6.4416003	-12.4363203
1840.0000000	6.4903998	-11.3215952
1840.0000000	6.5391998	-11.8644829
1840.0000000	6.5879998	326.0714111
1840.0000000	6.6367998	-12.1309843
1840.0000000	6.6856003	-13.8294077
1840.0000000	6.7343998	-13.4739933
1840.0000000	6.7831998	-14.3373089
1840.0000000	6.8319998	56.8215027
1840.0000000	6.8807998	-14.0376959
1840.0000000	6.9296002	-385.2687988
1840.0000000	6.9783998	-6.1645446
1840.0000000	7.0271997	-14.0509195
1840.0000000	7.0760002	7.6476450
1840.0000000	7.1247997	-1801.1917725
1840.0000000	7.1736002	-1444.5452881
1840.0000000	7.2223997	-822.3277588
1840.0000000	7.2711997	-178.8862610
1840.0000000	7.3200002	-17.8818550
1840.0000000	7.3687997	156.6617432
1840.0000000	7.4176002	328.9173889
1840.0000000	7.4663997	258.0832825
1840.0000000	7.5151997	-803.9515381
1840.0000000	7.5640001	-887.8481445
1840.0000000	7.6127996	-2090.4389648
1840.0000000	7.6616001	-672.0673218
1840.0000000	7.7104001	-345.9869385
1840.0000000	7.7591996	34.8298302
1840.0000000	7.8080001	341.8220825
1840.0000000	7.8567996	450.8243103
1840.0000000	7.9056001	-939.1257324

1840.0000000	7.9544001	1493.8542480
1840.0000000	8.0031996	1927.6853027
1840.0000000	8.0520000	1923.9946289
1840.0000000	8.1007996	2036.0113525
1840.0000000	8.1496000	2171.4499512
1840.0000000	8.1983995	1350.8369141
1840.0000000	8.2472000	-3091.6916504
1840.0000000	8.2959995	-1096.1496582
1840.0000000	8.3448000	-873.0600586
1840.0000000	8.3935995	-800.1701660
1840.0000000	8.4423990	-344.9500732
1840.0000000	8.4912004	-804.7723389
1840.0000000	8.5400000	-2170.2319336
1840.0000000	8.5887995	-1052.3804932
1840.0000000	8.6375999	204.9207458
1840.0000000	8.6863995	1855.2847900
1840.0000000	8.7351999	-690.0816650
1840.0000000	8.7840004	787.3444824
1840.0000000	8.8327999	41.3340302
1840.0000000	8.8815994	-11.5108051
1840.0000000	8.9303989	-23.6386604
1840.0000000	8.9792004	-11.5858841
1840.0000000	9.0279999	-5.9824467
1840.0000000	9.0767994	6.5689373
1840.0000000	9.1255999	-17.1011162
1840.0000000	9.1743994	-10.1066265
1840.0000000	9.2231998	35.7192917
1840.0000000	9.2720003	-8.4969797
1840.0000000	9.3207998	-9.8423643
1840.0000000	9.3695993	-10.7823486
1840.0000000	9.4183998	-11.7436638
1840.0000000	9.4672003	-11.1647091
1840.0000000	9.5159998	-14.0911446
1840.0000000	9.5648003	-0.6696358
1840.0000000	9.6135998	-13.3311720
1840.0000000	9.6623993	-10.2438869
1840.0000000	9.7111998	-10.1799755
1840.0000000	9.7600002	-10.8609953
1840.0000000	9.8087997	-10.9954309
1840.0000000	9.8575993	2.8272686
1840.0000000	9.9063997	-8.0440016
1840.0000000	9.9552002	-10.9069548
1840.0000000	10.0039997	-10.0950184
1850.0000000	0.3416000	-36.8387985
1850.0000000	0.3904000	-39.0522346
1850.0000000	0.4392000	-39.2151756
1850.0000000	0.4880000	-39.4909019
1850.0000000	0.5368000	-39.7111740
1850.0000000	0.5856000	-39.2179909
1850.0000000	0.6344000	-38.2512131
1850.0000000	0.6832000	-36.3506088
1850.0000000	0.7320000	-36.1719894
1850.0000000	0.7808000	-36.5444565
1850.0000000	0.8296000	-36.0122299
1850.0000000	0.8784000	-35.4008598
1850.0000000	0.9272000	-33.5102043
1850.0000000	0.9760000	-31.1713314
1850.0000000	1.0247999	-29.8709660

1850.0000000	1.0735999	-29.7678108
1850.0000000	1.1224000	-30.2136364
1850.0000000	1.1711999	-30.8867245
1850.0000000	1.2200000	-31.1992855
1850.0000000	1.2687999	-31.0391731
1850.0000000	1.3176000	-30.7327633
1850.0000000	1.3664000	-30.7131557
1850.0000000	1.4152000	-30.1792812
1850.0000000	1.4640000	-29.4734325
1850.0000000	1.5128000	-31.3094730
1850.0000000	1.5616000	-30.1574650
1850.0000000	1.6104001	-1346.1644287
1850.0000000	1.6592000	-48.8618469
1850.0000000	1.7079999	-27.5685539
1850.0000000	1.7567999	-27.7768822
1850.0000000	1.8055999	-27.7117195
1850.0000000	1.8544000	-27.2918262
1850.0000000	1.9031999	-26.7891560
1850.0000000	1.9520000	-26.8347454
1850.0000000	2.0007999	-26.1882133
1850.0000000	2.0495999	-26.3358707
1850.0000000	2.0983999	-25.4845505
1850.0000000	2.1471999	-23.8578110
1850.0000000	2.1960001	-25.1089363
1850.0000000	2.2448001	-24.3411255
1850.0000000	2.2935998	-22.7316837
1850.0000000	2.3423998	-22.1854324
1850.0000000	2.3912001	-19.7077675
1850.0000000	2.4400001	-23.0438423
1850.0000000	2.4888000	-23.4420586
1850.0000000	2.5375998	-13.6863747
1850.0000000	2.5863998	-12.0266781
1850.0000000	2.6352000	-10.3457127
1850.0000000	2.6840000	-16.3976974
1850.0000000	2.7328000	111.2261734
1850.0000000	2.7815998	131.8581543
1850.0000000	2.8304000	-23.6868839
1850.0000000	2.8792000	-20.8882942
1850.0000000	2.9280000	-22.5248890
1850.0000000	2.9768000	-17.4790764
1850.0000000	3.0256000	-20.6857834
1850.0000000	3.0743999	-28.5787239
1850.0000000	3.1231999	-23.9704533
1850.0000000	3.1719999	-28.9225674
1850.0000000	3.2208002	-484.6484680
1850.0000000	3.2695999	-948.0968018
1850.0000000	3.3183999	-20.5060291
1850.0000000	3.3671999	-3275.3435059
1850.0000000	3.4159999	1174.1191406
1850.0000000	3.4648001	-7.6142969
1850.0000000	3.5135999	-26.7889061
1850.0000000	3.5623999	-311.1926270
1850.0000000	3.6111999	-1951.4511719
1850.0000000	3.6600001	-442.3533936
1850.0000000	3.7088001	-583.8461304
1850.0000000	3.7575998	-913.6794434
1850.0000000	3.8063998	-1657.3992920
1850.0000000	3.8552001	-2405.9770508

1850.0000000	3.9040000	-2358.9069824
1850.0000000	3.9528000	1077.7178955
1850.0000000	4.0015998	-236.3037720
1850.0000000	4.0503998	659.5020752
1850.0000000	4.0991998	83.3749847
1850.0000000	4.1479998	-629.4629517
1850.0000000	4.1967998	1.6859646
1850.0000000	4.2456002	-239.3738098
1850.0000000	4.2943997	-469.3579407
1850.0000000	4.3431997	-13.5340462
1850.0000000	4.3920002	-239.0217743
1850.0000000	4.4407997	-22.2955360
1850.0000000	4.4896002	-15.5562811
1850.0000000	4.5383997	-630.7955322
1850.0000000	4.5871997	-13.0261459
1850.0000000	4.6360002	-21.4776134
1850.0000000	4.6847997	-27.9171982
1850.0000000	4.7336001	-19.4952240
1850.0000000	4.7824001	-16.5546513
1850.0000000	4.8311996	-1424.8841553
1850.0000000	4.8800001	219.9146271
1850.0000000	4.9287996	-195.1860809
1850.0000000	4.9776001	1023.0066528
1850.0000000	5.0264001	82.6793823
1850.0000000	5.0751996	-291.4826965
1850.0000000	5.1240001	-12.3941936
1850.0000000	5.1727996	547.6380615
1850.0000000	5.2216001	31.1651459
1850.0000000	5.2704000	-156.3431244
1850.0000000	5.3191996	-38.7823944
1850.0000000	5.3680000	-3474.9978027
1850.0000000	5.4168000	517.7460327
1850.0000000	5.4656000	-197.5605164
1850.0000000	5.5144000	37.1311569
1850.0000000	5.5631995	479.6697083
1850.0000000	5.6120000	386.1193237
1850.0000000	5.6608000	-36.7258453
1850.0000000	5.7096000	61.1758499
1850.0000000	5.7584000	130.5007629
1850.0000000	5.8071995	-11.5966873
1850.0000000	5.8559999	29.0409756
1850.0000000	5.9047999	-16.5882912
1850.0000000	5.9535999	-39.3611145
1850.0000000	6.0023999	-1546.3269043
1850.0000000	6.0511999	-14.5866795
1850.0000000	6.0999999	-19.3365936
1850.0000000	6.1487999	-13.3137312
1850.0000000	6.1975999	-23.0662689
1850.0000000	6.2463999	-1761.5726318
1850.0000000	6.2951999	-18.9993782
1850.0000000	6.3439999	-337.1357727
1850.0000000	6.3927999	-15.0465250
1850.0000000	6.4416003	-12.4369068
1850.0000000	6.4903998	-11.3222713
1850.0000000	6.5391998	-11.8651638
1850.0000000	6.5879998	325.3807373
1850.0000000	6.6367998	-12.1315336
1850.0000000	6.6856003	-13.8295784

1850.0000000	6.7343998	-13.4742098
1850.0000000	6.7831998	-14.3368931
1850.0000000	6.8319998	56.8023911
1850.0000000	6.8807998	-14.0375576
1850.0000000	6.9296002	-384.5073242
1850.0000000	6.9783998	-6.1773682
1850.0000000	7.0271997	-14.0498915
1850.0000000	7.0760002	7.6054583
1850.0000000	7.1247997	-1797.2459717
1850.0000000	7.1736002	-1441.3824463
1850.0000000	7.2223997	-820.5345459
1850.0000000	7.2711997	-178.5184784
1850.0000000	7.3200002	-17.8698654
1850.0000000	7.3687997	156.2872162
1850.0000000	7.4176002	328.1615601
1850.0000000	7.4663997	257.4856262
1850.0000000	7.5151997	-802.1904907
1850.0000000	7.5640001	-885.9007568
1850.0000000	7.6127996	-2085.8215332
1850.0000000	7.6616001	-670.5960083
1850.0000000	7.7104001	-345.2406616
1850.0000000	7.7591996	34.7287788
1850.0000000	7.8080001	341.0367126
1850.0000000	7.8567996	449.7962341
1850.0000000	7.9056001	-937.0528564
1850.0000000	7.9544001	1490.5443115
1850.0000000	8.0031996	1923.4156494
1850.0000000	8.0520000	1919.7325439
1850.0000000	8.1007996	2031.5018311
1850.0000000	8.1496000	2166.6418457
1850.0000000	8.1983995	1347.8443604
1850.0000000	8.2472000	-3084.8569336
1850.0000000	8.2959995	-1093.7294922
1850.0000000	8.3448000	-871.1309204
1850.0000000	8.3935995	-798.4024658
1850.0000000	8.4423990	-344.1920776
1850.0000000	8.4912004	-802.9960938
1850.0000000	8.5400000	-2165.4321289
1850.0000000	8.5887995	-1050.0589600
1850.0000000	8.6375999	204.4594269
1850.0000000	8.6863995	1851.1665039
1850.0000000	8.7351999	-688.5573730
1850.0000000	8.7840004	785.5877075
1850.0000000	8.8327999	41.2224197
1850.0000000	8.8815994	-11.5109797
1850.0000000	8.9303989	-23.6349411
1850.0000000	8.9792004	-11.5859671
1850.0000000	9.0279999	-5.9842153
1850.0000000	9.0767994	6.5632210
1850.0000000	9.1255999	-17.0994034
1850.0000000	9.1743994	-10.1070290
1850.0000000	9.2231998	35.7035599
1850.0000000	9.2720003	-8.4979162
1850.0000000	9.3207998	-9.8429041
1850.0000000	9.3695993	-10.7825747
1850.0000000	9.4183998	-11.7435398
1850.0000000	9.4672003	-11.1647501
1850.0000000	9.5159998	-14.0902538

1850.0000000	9.5648003	-0.6730089
1850.0000000	9.6135998	-13.3303814
1850.0000000	9.6623993	-10.2441626
1850.0000000	9.7111998	-10.1802464
1850.0000000	9.7600002	-10.8610373
1850.0000000	9.8087997	-10.9954386
1850.0000000	9.8575993	2.8228464
1850.0000000	9.9063997	-8.0449162
1850.0000000	9.9552002	-10.9069595
1850.0000000	10.0039997	-10.0952654
1860.0000000	0.3416000	-36.7633705
1860.0000000	0.3904000	-39.0689201
1860.0000000	0.4392000	-39.2088318
1860.0000000	0.4880000	-39.4896927
1860.0000000	0.5368000	-39.7123489
1860.0000000	0.5856000	-39.2166061
1860.0000000	0.6344000	-38.2515221
1860.0000000	0.6832000	-36.3528328
1860.0000000	0.7320000	-36.1740417
1860.0000000	0.7808000	-36.5446281
1860.0000000	0.8296000	-36.0083237
1860.0000000	0.8784000	-35.3977280
1860.0000000	0.9272000	-33.4946213
1860.0000000	0.9760000	-31.1665134
1860.0000000	1.0247999	-29.8764744
1860.0000000	1.0735999	-29.7530880
1860.0000000	1.1224000	-30.1953602
1860.0000000	1.1711999	-30.8798714
1860.0000000	1.2200000	-31.2010612
1860.0000000	1.2687999	-31.0413418
1860.0000000	1.3176000	-30.7290821
1860.0000000	1.3664000	-30.7127323
1860.0000000	1.4152000	-30.1794853
1860.0000000	1.4640000	-29.4717216
1860.0000000	1.5128000	-31.2122955
1860.0000000	1.5616000	-30.1156235
1860.0000000	1.6104001	-1343.6217041
1860.0000000	1.6592000	-48.5679131
1860.0000000	1.7079999	-27.5723000
1860.0000000	1.7567999	-27.7662144
1860.0000000	1.8055999	-27.3104153
1860.0000000	1.8544000	-26.9664116
1860.0000000	1.9031999	-26.9070969
1860.0000000	1.9520000	-26.8375282
1860.0000000	2.0007999	-26.1935940
1860.0000000	2.0495999	-23.8327045
1860.0000000	2.0983999	-24.0087719
1860.0000000	2.1471999	-24.1250782
1860.0000000	2.1960001	-25.1048450
1860.0000000	2.2448001	-24.3232136
1860.0000000	2.2935998	-22.6720600
1860.0000000	2.3423998	-22.1998367
1860.0000000	2.3912001	-20.3293133
1860.0000000	2.4400001	-23.0463295
1860.0000000	2.4888000	-23.4418411
1860.0000000	2.5375998	-13.7034826
1860.0000000	2.5863998	-12.0464363
1860.0000000	2.6352000	-10.3684568

1860.0000000	2.6840000	-16.4096394
1860.0000000	2.7328000	110.9601517
1860.0000000	2.7815998	131.5517273
1860.0000000	2.8304000	-23.6855793
1860.0000000	2.8792000	-20.8915577
1860.0000000	2.9280000	-22.5243187
1860.0000000	2.9768000	-17.4874458
1860.0000000	3.0256000	-20.6883678
1860.0000000	3.0743999	-28.5673370
1860.0000000	3.1231999	-23.9669971
1860.0000000	3.1719999	-28.9142742
1860.0000000	3.2208002	-484.4107361
1860.0000000	3.2695999	-947.6218262
1860.0000000	3.3183999	-20.5062275
1860.0000000	3.3671999	-3273.8818359
1860.0000000	3.4159999	1173.5838623
1860.0000000	3.4648001	-7.6200047
1860.0000000	3.5135999	-26.7859154
1860.0000000	3.5623999	-311.0630493
1860.0000000	3.6111999	-1950.5914307
1860.0000000	3.6600001	-442.1652832
1860.0000000	3.7088001	-583.5947266
1860.0000000	3.7575998	-913.2808838
1860.0000000	3.8063998	-1656.6694336
1860.0000000	3.8552001	-2404.9135742
1860.0000000	3.9040000	-2357.8645020
1860.0000000	3.9528000	1077.2296143
1860.0000000	4.0015998	-236.2066498
1860.0000000	4.0503998	659.1995239
1860.0000000	4.0991998	83.3292999
1860.0000000	4.1479998	-629.1906738
1860.0000000	4.1967998	1.6767082
1860.0000000	4.2456002	-239.2754974
1860.0000000	4.2943997	-469.1573792
1860.0000000	4.3431997	-13.5362272
1860.0000000	4.3920002	-238.9236603
1860.0000000	4.4407997	-22.2937603
1860.0000000	4.4896002	-15.5571632
1860.0000000	4.5383997	-630.5344849
1860.0000000	4.5871997	-13.0281620
1860.0000000	4.6360002	-21.4760723
1860.0000000	4.6847997	-27.9132729
1860.0000000	4.7336001	-19.4945030
1860.0000000	4.7824001	-16.5551052
1860.0000000	4.8311996	-1424.3527832
1860.0000000	4.8800001	219.8250732
1860.0000000	4.9287996	-195.1188202
1860.0000000	4.9776001	1022.6141968
1860.0000000	5.0264001	82.6418610
1860.0000000	5.0751996	-291.3790588
1860.0000000	5.1240001	-12.3951149
1860.0000000	5.1727996	547.5221558
1860.0000000	5.2216001	31.1557007
1860.0000000	5.2704000	-156.3150177
1860.0000000	5.3191996	-38.7778435
1860.0000000	5.3680000	-3474.3054199
1860.0000000	5.4168000	517.6392212
1860.0000000	5.4656000	-197.5243378

1860.0000000	5.5144000	37.1204987
1860.0000000	5.5631995	479.5711365
1860.0000000	5.6120000	386.0393372
1860.0000000	5.6608000	-36.7217560
1860.0000000	5.7096000	61.1604156
1860.0000000	5.7584000	130.4718323
1860.0000000	5.8071995	-11.5975580
1860.0000000	5.8559999	29.0321217
1860.0000000	5.9047999	-16.5880928
1860.0000000	5.9535999	-39.3563728
1860.0000000	6.0023999	-1546.0225830
1860.0000000	6.0511999	-14.5868912
1860.0000000	6.0999999	-19.3357620
1860.0000000	6.1487999	-13.3141937
1860.0000000	6.1975999	-23.0645046
1860.0000000	6.2463999	-1761.2252197
1860.0000000	6.2951999	-18.9985638
1860.0000000	6.3439999	-337.0717163
1860.0000000	6.3927999	-15.0465059
1860.0000000	6.4416003	-12.4374943
1860.0000000	6.4903998	-11.3229475
1860.0000000	6.5391998	-11.8658447
1860.0000000	6.5879998	324.6900330
1860.0000000	6.6367998	-12.1320829
1860.0000000	6.6856003	-13.8297482
1860.0000000	6.7343998	-13.4744253
1860.0000000	6.7831998	-14.3364782
1860.0000000	6.8319998	56.7832718
1860.0000000	6.8807998	-14.0374184
1860.0000000	6.9296002	-383.7458801
1860.0000000	6.9783998	-6.1901922
1860.0000000	7.0271997	-14.0488644
1860.0000000	7.0760002	7.5632734
1860.0000000	7.1247997	-1793.3000488
1860.0000000	7.1736002	-1438.2196045
1860.0000000	7.2223997	-818.7413940
1860.0000000	7.2711997	-178.1506958
1860.0000000	7.3200002	-17.8578777
1860.0000000	7.3687997	155.9126892
1860.0000000	7.4176002	327.4057312
1860.0000000	7.4663997	256.8879700
1860.0000000	7.5151997	-800.4294434
1860.0000000	7.5640001	-883.9533691
1860.0000000	7.6127996	-2081.2038574
1860.0000000	7.6616001	-669.1246338
1860.0000000	7.7104001	-344.4943848
1860.0000000	7.7591996	34.6277313
1860.0000000	7.8080001	340.2513733
1860.0000000	7.8567996	448.7681274
1860.0000000	7.9056001	-934.9799805
1860.0000000	7.9544001	1487.2343750
1860.0000000	8.0031996	1919.1461182
1860.0000000	8.0520000	1915.4705811
1860.0000000	8.1007996	2026.9923096
1860.0000000	8.1496000	2161.8339844
1860.0000000	8.1983995	1344.8519287
1860.0000000	8.2472000	-3078.0222168
1860.0000000	8.2959995	-1091.3093262

1860.0000000	8.3448000	-869.2017212
1860.0000000	8.3935995	-796.6348267
1860.0000000	8.4423990	-343.4340515
1860.0000000	8.4912004	-801.2198486
1860.0000000	8.5400000	-2160.6323242
1860.0000000	8.5887995	-1047.7374268
1860.0000000	8.6375999	203.9981079
1860.0000000	8.6863995	1847.0480957
1860.0000000	8.7351999	-687.0331421
1860.0000000	8.7840004	783.8309937
1860.0000000	8.8327999	41.1108170
1860.0000000	8.8815994	-11.5111542
1860.0000000	8.9303989	-23.6312199
1860.0000000	8.9792004	-11.5860491
1860.0000000	9.0279999	-5.9859834
1860.0000000	9.0767994	6.5575027
1860.0000000	9.1255999	-17.0976906
1860.0000000	9.1743994	-10.1074305
1860.0000000	9.2231998	35.6878281
1860.0000000	9.2720003	-8.4988527
1860.0000000	9.3207998	-9.8434429
1860.0000000	9.3695993	-10.7828016
1860.0000000	9.4183998	-11.7434149
1860.0000000	9.4672003	-11.1647911
1860.0000000	9.5159998	-14.0893631
1860.0000000	9.5648003	-0.6763821
1860.0000000	9.6135998	-13.3295908
1860.0000000	9.6623993	-10.2444372
1860.0000000	9.7111998	-10.1805182
1860.0000000	9.7600002	-10.8610792
1860.0000000	9.8087997	-10.9954462
1860.0000000	9.8575993	2.8184242
1860.0000000	9.9063997	-8.0458298
1860.0000000	9.9552002	-10.9069633
1860.0000000	10.0039997	-10.0955133
1870.0000000	0.3416000	-36.8250542
1870.0000000	0.3904000	-39.0630035
1870.0000000	0.4392000	-39.2097931
1870.0000000	0.4880000	-39.4884834
1870.0000000	0.5368000	-39.7135201
1870.0000000	0.5856000	-39.2152252
1870.0000000	0.6344000	-38.2518349
1870.0000000	0.6832000	-36.3550606
1870.0000000	0.7320000	-36.1760941
1870.0000000	0.7808000	-36.5447960
1870.0000000	0.8296000	-36.0044174
1870.0000000	0.8784000	-35.3945999
1870.0000000	0.9272000	-33.4790421
1870.0000000	0.9760000	-31.1616974
1870.0000000	1.0247999	-29.8819828
1870.0000000	1.0735999	-29.7383652
1870.0000000	1.1224000	-30.1770840
1870.0000000	1.1711999	-30.8730202
1870.0000000	1.2200000	-31.2028370
1870.0000000	1.2687999	-31.0435104
1870.0000000	1.3176000	-30.7253990
1870.0000000	1.3664000	-30.7123089
1870.0000000	1.4152000	-30.1796913

1870.0000000	1.4640000	-29.4700127
1870.0000000	1.5128000	-31.1151180
1870.0000000	1.5616000	-30.0737820
1870.0000000	1.6104001	-1341.0788574
1870.0000000	1.6592000	-48.2739792
1870.0000000	1.7079999	-27.5760479
1870.0000000	1.7567999	-27.7555485
1870.0000000	1.8055999	-27.3764477
1870.0000000	1.8544000	-27.2827129
1870.0000000	1.9031999	-27.0250397
1870.0000000	1.9520000	-26.8403091
1870.0000000	2.0007999	-26.2691040
1870.0000000	2.0495999	-26.3214951
1870.0000000	2.0983999	-24.4152565
1870.0000000	2.1471999	-24.3923454
1870.0000000	2.1960001	-25.1007538
1870.0000000	2.2448001	-24.3053017
1870.0000000	2.2935998	-22.6124382
1870.0000000	2.3423998	-22.2142410
1870.0000000	2.3912001	-20.9508591
1870.0000000	2.4400001	-23.0488148
1870.0000000	2.4888000	-23.4416237
1870.0000000	2.5375998	-13.7205906
1870.0000000	2.5863998	-12.0661955
1870.0000000	2.6352000	-10.3912010
1870.0000000	2.6840000	-16.4215813
1870.0000000	2.7328000	110.6941452
1870.0000000	2.7815998	131.2453003
1870.0000000	2.8304000	-23.6842747
1870.0000000	2.8792000	-20.8948212
1870.0000000	2.9280000	-22.5237503
1870.0000000	2.9768000	-17.4958153
1870.0000000	3.0256000	-20.6909523
1870.0000000	3.0743999	-28.5559502
1870.0000000	3.1231999	-23.9635410
1870.0000000	3.1719999	-28.9059811
1870.0000000	3.2208002	-484.1730042
1870.0000000	3.2695999	-947.1468506
1870.0000000	3.3183999	-20.5064259
1870.0000000	3.3671999	-3272.4201660
1870.0000000	3.4159999	1173.0485840
1870.0000000	3.4648001	-7.6257124
1870.0000000	3.5135999	-26.7829247
1870.0000000	3.5623999	-310.9334412
1870.0000000	3.6111999	-1949.7316895
1870.0000000	3.6600001	-441.9771118
1870.0000000	3.7088001	-583.3433838
1870.0000000	3.7575998	-912.8823853
1870.0000000	3.8063998	-1655.9395752
1870.0000000	3.8552001	-2403.8500977
1870.0000000	3.9040000	-2356.8220215
1870.0000000	3.9528000	1076.7413330
1870.0000000	4.0015998	-236.1095428
1870.0000000	4.0503998	658.8969727
1870.0000000	4.0991998	83.2836151
1870.0000000	4.1479998	-628.9184570
1870.0000000	4.1967998	1.6674519
1870.0000000	4.2456002	-239.1772003

1870.0000000	4.2943997	-468.9568176
1870.0000000	4.3431997	-13.5384083
1870.0000000	4.3920002	-238.8255463
1870.0000000	4.4407997	-22.2919846
1870.0000000	4.4896002	-15.5580463
1870.0000000	4.5383997	-630.2733765
1870.0000000	4.5871997	-13.0301790
1870.0000000	4.6360002	-21.4745293
1870.0000000	4.6847997	-27.9093475
1870.0000000	4.7336001	-19.4937801
1870.0000000	4.7824001	-16.5555592
1870.0000000	4.8311996	-1423.8215332
1870.0000000	4.8800001	219.7355194
1870.0000000	4.9287996	-195.0515594
1870.0000000	4.9776001	1022.2216187
1870.0000000	5.0264001	82.6043396
1870.0000000	5.0751996	-291.2754517
1870.0000000	5.1240001	-12.3960361
1870.0000000	5.1727996	547.4063110
1870.0000000	5.2216001	31.1462555
1870.0000000	5.2704000	-156.2869110
1870.0000000	5.3191996	-38.7732925
1870.0000000	5.3680000	-3473.6130371
1870.0000000	5.4168000	517.5324707
1870.0000000	5.4656000	-197.4881592
1870.0000000	5.5144000	37.1098480
1870.0000000	5.5631995	479.4725647
1870.0000000	5.6120000	385.9593811
1870.0000000	5.6608000	-36.7176666
1870.0000000	5.7096000	61.1449814
1870.0000000	5.7584000	130.4429016
1870.0000000	5.8071995	-11.5984287
1870.0000000	5.8559999	29.0232716
1870.0000000	5.9047999	-16.5878944
1870.0000000	5.9535999	-39.3516350
1870.0000000	6.0023999	-1545.7180176
1870.0000000	6.0511999	-14.5871019
1870.0000000	6.0999999	-19.3349285
1870.0000000	6.1487999	-13.3146572
1870.0000000	6.1975999	-23.0627384
1870.0000000	6.2463999	-1760.8778076
1870.0000000	6.2951999	-18.9977493
1870.0000000	6.3439999	-337.0076599
1870.0000000	6.3927999	-15.0464878
1870.0000000	6.4416003	-12.4380808
1870.0000000	6.4903998	-11.3236227
1870.0000000	6.5391998	-11.8665257
1870.0000000	6.5879998	323.9993286
1870.0000000	6.6367998	-12.1326323
1870.0000000	6.6856003	-13.8299189
1870.0000000	6.7343998	-13.4746408
1870.0000000	6.7831998	-14.3360624
1870.0000000	6.8319998	56.7641602
1870.0000000	6.8807998	-14.0372801
1870.0000000	6.9296002	-382.9844360
1870.0000000	6.9783998	-6.2030153
1870.0000000	7.0271997	-14.0478363
1870.0000000	7.0760002	7.5210886

1870.0000000	7.1247997	-1789.3542480
1870.0000000	7.1736002	-1435.0566406
1870.0000000	7.2223997	-816.9482422
1870.0000000	7.2711997	-177.7829132
1870.0000000	7.3200002	-17.8458900
1870.0000000	7.3687997	155.5381622
1870.0000000	7.4176002	326.6499023
1870.0000000	7.4663997	256.2903137
1870.0000000	7.5151997	-798.6683960
1870.0000000	7.5640001	-882.0059814
1870.0000000	7.6127996	-2076.5861816
1870.0000000	7.6616001	-667.6533203
1870.0000000	7.7104001	-343.7481689
1870.0000000	7.7591996	34.5266838
1870.0000000	7.8080001	339.4660034
1870.0000000	7.8567996	447.7400513
1870.0000000	7.9056001	-932.9070435
1870.0000000	7.9544001	1483.9244385
1870.0000000	8.0031996	1914.8764648
1870.0000000	8.0520000	1911.2086182
1870.0000000	8.1007996	2022.4827881
1870.0000000	8.1496000	2157.0258789
1870.0000000	8.1983995	1341.8593750
1870.0000000	8.2472000	-3071.1872559
1870.0000000	8.2959995	-1088.8891602
1870.0000000	8.3448000	-867.2725220
1870.0000000	8.3935995	-794.8671265
1870.0000000	8.4423990	-342.6760254
1870.0000000	8.4912004	-799.4436646
1870.0000000	8.5400000	-2155.8325195
1870.0000000	8.5887995	-1045.4158936
1870.0000000	8.6375999	203.5367889
1870.0000000	8.6863995	1842.9298096
1870.0000000	8.7351999	-685.5088501
1870.0000000	8.7840004	782.0742188
1870.0000000	8.8327999	40.9992065
1870.0000000	8.8815994	-11.5113287
1870.0000000	8.9303989	-23.6274986
1870.0000000	8.9792004	-11.5861311
1870.0000000	9.0279999	-5.9877520
1870.0000000	9.0767994	6.5517845
1870.0000000	9.1255999	-17.0959759
1870.0000000	9.1743994	-10.1078329
1870.0000000	9.2231998	35.6720963
1870.0000000	9.2720003	-8.4997892
1870.0000000	9.3207998	-9.8439827
1870.0000000	9.3695993	-10.7830276
1870.0000000	9.4183998	-11.7432899
1870.0000000	9.4672003	-11.1648321
1870.0000000	9.5159998	-14.0884714
1870.0000000	9.5648003	-0.6797543
1870.0000000	9.6135998	-13.3288002
1870.0000000	9.6623993	-10.2447128
1870.0000000	9.7111998	-10.1807899
1870.0000000	9.7600002	-10.8611202
1870.0000000	9.8087997	-10.9954548
1870.0000000	9.8575993	2.8140011
1870.0000000	9.9063997	-8.0467434

1870.0000000	9.9552002	-10.9069681
1870.0000000	10.0039997	-10.0957603
1880.0000000	0.3416000	-36.8857880
1880.0000000	0.3904000	-39.0570908
1880.0000000	0.4392000	-39.2107506
1880.0000000	0.4880000	-39.4872742
1880.0000000	0.5368000	-39.7146950
1880.0000000	0.5856000	-39.2138443
1880.0000000	0.6344000	-38.2521439
1880.0000000	0.6832000	-36.3572845
1880.0000000	0.7320000	-36.1781502
1880.0000000	0.7808000	-36.5449638
1880.0000000	0.8296000	-36.0005112
1880.0000000	0.8784000	-35.3914680
1880.0000000	0.9272000	-33.4634628
1880.0000000	0.9760000	-31.1568794
1880.0000000	1.0247999	-29.8874912
1880.0000000	1.0735999	-29.7236423
1880.0000000	1.1224000	-30.1588078
1880.0000000	1.1711999	-30.8661671
1880.0000000	1.2200000	-31.2046108
1880.0000000	1.2687999	-31.0456791
1880.0000000	1.3176000	-30.7217178
1880.0000000	1.3664000	-30.7118874
1880.0000000	1.4152000	-30.1798954
1880.0000000	1.4640000	-29.4683037
1880.0000000	1.5128000	-31.0179405
1880.0000000	1.5616000	-30.0319405
1880.0000000	1.6104001	-1338.5360107
1880.0000000	1.6592000	-47.9800491
1880.0000000	1.7079999	-27.5797939
1880.0000000	1.7567999	-27.7448807
1880.0000000	1.8055999	-27.4424801
1880.0000000	1.8544000	-27.0126534
1880.0000000	1.9031999	-27.1429825
1880.0000000	1.9520000	-26.8430920
1880.0000000	2.0007999	-26.3446159
1880.0000000	2.0495999	-26.2853489
1880.0000000	2.0983999	-24.6345501
1880.0000000	2.1471999	-24.6596127
1880.0000000	2.1960001	-25.0049381
1880.0000000	2.2448001	-24.2873917
1880.0000000	2.2935998	-22.5528145
1880.0000000	2.3423998	-22.2286453
1880.0000000	2.3912001	-21.5724049
1880.0000000	2.4400001	-23.0513020
1880.0000000	2.4888000	-23.4414082
1880.0000000	2.5375998	-13.7376995
1880.0000000	2.5863998	-12.0859547
1880.0000000	2.6352000	-10.4139452
1880.0000000	2.6840000	-16.4335213
1880.0000000	2.7328000	110.4281235
1880.0000000	2.7815998	130.9389038
1880.0000000	2.8304000	-23.6829720
1880.0000000	2.8792000	-20.8980846
1880.0000000	2.9280000	-22.5231800
1880.0000000	2.9768000	-17.5041866
1880.0000000	3.0256000	-20.6935368

1880.0000000	3.0743999	-28.5445633
1880.0000000	3.1231999	-23.9600868
1880.0000000	3.1719999	-28.8976879
1880.0000000	3.2208002	-483.9352722
1880.0000000	3.2695999	-946.6718750
1880.0000000	3.3183999	-20.5066242
1880.0000000	3.3671999	-3270.9584961
1880.0000000	3.4159999	1172.5133057
1880.0000000	3.4648001	-7.6314211
1880.0000000	3.5135999	-26.7799339
1880.0000000	3.5623999	-310.8038635
1880.0000000	3.6111999	-1948.8718262
1880.0000000	3.6600001	-441.7890015
1880.0000000	3.7088001	-583.0919800
1880.0000000	3.7575998	-912.4838257
1880.0000000	3.8063998	-1655.2097168
1880.0000000	3.8552001	-2402.7866211
1880.0000000	3.9040000	-2355.7795410
1880.0000000	3.9528000	1076.2529297
1880.0000000	4.0015998	-236.0124359
1880.0000000	4.0503998	658.5944214
1880.0000000	4.0991998	83.2379303
1880.0000000	4.1479998	-628.6462402
1880.0000000	4.1967998	1.6581955
1880.0000000	4.2456002	-239.0789032
1880.0000000	4.2943997	-468.7562561
1880.0000000	4.3431997	-13.5405884
1880.0000000	4.3920002	-238.7274323
1880.0000000	4.4407997	-22.2902088
1880.0000000	4.4896002	-15.5589294
1880.0000000	4.5383997	-630.0123291
1880.0000000	4.5871997	-13.0321951
1880.0000000	4.6360002	-21.4729862
1880.0000000	4.6847997	-27.9054222
1880.0000000	4.7336001	-19.4930573
1880.0000000	4.7824001	-16.5560131
1880.0000000	4.8311996	-1423.2902832
1880.0000000	4.8800001	219.6459656
1880.0000000	4.9287996	-194.9842987
1880.0000000	4.9776001	1021.8291626
1880.0000000	5.0264001	82.5668182
1880.0000000	5.0751996	-291.1718140
1880.0000000	5.1240001	-12.3969574
1880.0000000	5.1727996	547.2904663
1880.0000000	5.2216001	31.1368141
1880.0000000	5.2704000	-156.2588043
1880.0000000	5.3191996	-38.7687454
1880.0000000	5.3680000	-3472.9206543
1880.0000000	5.4168000	517.4257202
1880.0000000	5.4656000	-197.4519653
1880.0000000	5.5144000	37.0991898
1880.0000000	5.5631995	479.3739929
1880.0000000	5.6120000	385.8793945
1880.0000000	5.6608000	-36.7135811
1880.0000000	5.7096000	61.1295547
1880.0000000	5.7584000	130.4139557
1880.0000000	5.8071995	-11.5992985
1880.0000000	5.8559999	29.0144215

1880.0000000	5.9047999	-16.5876980
1880.0000000	5.9535999	-39.3468933
1880.0000000	6.0023999	-1545.4136963
1880.0000000	6.0511999	-14.5873137
1880.0000000	6.0999999	-19.3340969
1880.0000000	6.1487999	-13.3151197
1880.0000000	6.1975999	-23.0609741
1880.0000000	6.2463999	-1760.5305176
1880.0000000	6.2951999	-18.9969349
1880.0000000	6.3439999	-336.9436340
1880.0000000	6.3927999	-15.0464697
1880.0000000	6.4416003	-12.4386683
1880.0000000	6.4903998	-11.3242989
1880.0000000	6.5391998	-11.8672066
1880.0000000	6.5879998	323.3086243
1880.0000000	6.6367998	-12.1331816
1880.0000000	6.6856003	-13.8300886
1880.0000000	6.7343998	-13.4748564
1880.0000000	6.7831998	-14.3356466
1880.0000000	6.8319998	56.7450409
1880.0000000	6.8807998	-14.0371408
1880.0000000	6.9296002	-382.2229919
1880.0000000	6.9783998	-6.2158389
1880.0000000	7.0271997	-14.0468082
1880.0000000	7.0760002	7.4789019
1880.0000000	7.1247997	-1785.4083252
1880.0000000	7.1736002	-1431.8937988
1880.0000000	7.2223997	-815.1550903
1880.0000000	7.2711997	-177.4151306
1880.0000000	7.3200002	-17.8339005
1880.0000000	7.3687997	155.1636353
1880.0000000	7.4176002	325.8940735
1880.0000000	7.4663997	255.6926727
1880.0000000	7.5151997	-796.9073486
1880.0000000	7.5640001	-880.0585327
1880.0000000	7.6127996	-2071.9687500
1880.0000000	7.6616001	-666.1820068
1880.0000000	7.7104001	-343.0018921
1880.0000000	7.7591996	34.4256325
1880.0000000	7.8080001	338.6806335
1880.0000000	7.8567996	446.7119751
1880.0000000	7.9056001	-930.8341675
1880.0000000	7.9544001	1480.6145020
1880.0000000	8.0031996	1910.6069336
1880.0000000	8.0520000	1906.9466553
1880.0000000	8.1007996	2017.9733887
1880.0000000	8.1496000	2152.2177734
1880.0000000	8.1983995	1338.8668213
1880.0000000	8.2472000	-3064.3525391
1880.0000000	8.2959995	-1086.4689941
1880.0000000	8.3448000	-865.3433838
1880.0000000	8.3935995	-793.0994873
1880.0000000	8.4423990	-341.9180298
1880.0000000	8.4912004	-797.6674194
1880.0000000	8.5400000	-2151.0327148
1880.0000000	8.5887995	-1043.0943604
1880.0000000	8.6375999	203.0754547
1880.0000000	8.6863995	1838.8114014

1880.0000000	8.7351999	-683.9846191
1880.0000000	8.7840004	780.3174438
1880.0000000	8.8327999	40.8875961
1880.0000000	8.8815994	-11.5115023
1880.0000000	8.9303989	-23.6237793
1880.0000000	8.9792004	-11.5862141
1880.0000000	9.0279999	-5.9895196
1880.0000000	9.0767994	6.5460663
1880.0000000	9.1255999	-17.0942612
1880.0000000	9.1743994	-10.1082344
1880.0000000	9.2231998	35.6563644
1880.0000000	9.2720003	-8.5007248
1880.0000000	9.3207998	-9.8445215
1880.0000000	9.3695993	-10.7832546
1880.0000000	9.4183998	-11.7431660
1880.0000000	9.4672003	-11.1648741
1880.0000000	9.5159998	-14.0875807
1880.0000000	9.5648003	-0.6831264
1880.0000000	9.6135998	-13.3280096
1880.0000000	9.6623993	-10.2449875
1880.0000000	9.7111998	-10.1810608
1880.0000000	9.7600002	-10.8611622
1880.0000000	9.8087997	-10.9954624
1880.0000000	9.8575993	2.8095789
1880.0000000	9.9063997	-8.0476570
1880.0000000	9.9552002	-10.9069719
1880.0000000	10.0039997	-10.0960083
1890.0000000	0.3416000	-36.8882828
1890.0000000	0.3904000	-39.0511742
1890.0000000	0.4392000	-39.2117119
1890.0000000	0.4880000	-39.4860649
1890.0000000	0.5368000	-39.7158699
1890.0000000	0.5856000	-39.2124634
1890.0000000	0.6344000	-38.2524529
1890.0000000	0.6832000	-36.3595123
1890.0000000	0.7320000	-36.1802025
1890.0000000	0.7808000	-36.5451355
1890.0000000	0.8296000	-35.9966049
1890.0000000	0.8784000	-35.3883362
1890.0000000	0.9272000	-33.4478836
1890.0000000	0.9760000	-31.1520634
1890.0000000	1.0247999	-29.8929996
1890.0000000	1.0735999	-29.7089195
1890.0000000	1.1224000	-30.1405315
1890.0000000	1.1711999	-30.8593159
1890.0000000	1.2200000	-31.2063866
1890.0000000	1.2687999	-31.0478477
1890.0000000	1.3176000	-30.7180367
1890.0000000	1.3664000	-30.7114639
1890.0000000	1.4152000	-30.1800995
1890.0000000	1.4640000	-29.4665947
1890.0000000	1.5128000	-30.9207611
1890.0000000	1.5616000	-29.9901009
1890.0000000	1.6104001	-1335.9932861
1890.0000000	1.6592000	-47.6861191
1890.0000000	1.7079999	-27.5835419
1890.0000000	1.7567999	-27.7342129
1890.0000000	1.8055999	-27.5442734

1890.0000000	1.8544000	-26.7792149
1890.0000000	1.9031999	-26.9913177
1890.0000000	1.9520000	-26.8458729
1890.0000000	2.0007999	-26.4201279
1890.0000000	2.0495999	-26.2492027
1890.0000000	2.0983999	-24.6980286
1890.0000000	2.1471999	-24.3497295
1890.0000000	2.1960001	-24.9091225
1890.0000000	2.2448001	-24.2694798
1890.0000000	2.2935998	-22.4931908
1890.0000000	2.3423998	-22.2430515
1890.0000000	2.3912001	-22.1939507
1890.0000000	2.4400001	-23.0537872
1890.0000000	2.4888000	-23.4411907
1890.0000000	2.5375998	-13.7548075
1890.0000000	2.5863998	-12.1057138
1890.0000000	2.6352000	-10.4366894
1890.0000000	2.6840000	-16.4454651
1890.0000000	2.7328000	110.1621017
1890.0000000	2.7815998	130.6324768
1890.0000000	2.8304000	-23.6816673
1890.0000000	2.8792000	-20.9013481
1890.0000000	2.9280000	-22.5226116
1890.0000000	2.9768000	-17.5125580
1890.0000000	3.0256000	-20.6961212
1890.0000000	3.0743999	-28.5331764
1890.0000000	3.1231999	-23.9566307
1890.0000000	3.1719999	-28.8893948
1890.0000000	3.2208002	-483.6975403
1890.0000000	3.2695999	-946.1968994
1890.0000000	3.3183999	-20.5068207
1890.0000000	3.3671999	-3269.4968262
1890.0000000	3.4159999	1171.9780273
1890.0000000	3.4648001	-7.6371298
1890.0000000	3.5135999	-26.7769432
1890.0000000	3.5623999	-310.6742554
1890.0000000	3.6111999	-1948.0120850
1890.0000000	3.6600001	-441.6008911
1890.0000000	3.7088001	-582.8405762
1890.0000000	3.7575998	-912.0852661
1890.0000000	3.8063998	-1654.4798584
1890.0000000	3.8552001	-2401.7233887
1890.0000000	3.9040000	-2354.7370605
1890.0000000	3.9528000	1075.7646484
1890.0000000	4.0015998	-235.9153290
1890.0000000	4.0503998	658.2918701
1890.0000000	4.0991998	83.1922455
1890.0000000	4.1479998	-628.3740234
1890.0000000	4.1967998	1.6489391
1890.0000000	4.2456002	-238.9805908
1890.0000000	4.2943997	-468.5556335
1890.0000000	4.3431997	-13.5427685
1890.0000000	4.3920002	-238.6293182
1890.0000000	4.4407997	-22.2884331
1890.0000000	4.4896002	-15.5598116
1890.0000000	4.5383997	-629.7512817
1890.0000000	4.5871997	-13.0342112
1890.0000000	4.6360002	-21.4714451

1890.0000000	4.6847997	-27.9014969
1890.0000000	4.7336001	-19.4923363
1890.0000000	4.7824001	-16.5564690
1890.0000000	4.8311996	-1422.7590332
1890.0000000	4.8800001	219.5564270
1890.0000000	4.9287996	-194.9170380
1890.0000000	4.9776001	1021.4367065
1890.0000000	5.0264001	82.5292969
1890.0000000	5.0751996	-291.0681763
1890.0000000	5.1240001	-12.3978786
1890.0000000	5.1727996	547.1745605
1890.0000000	5.2216001	31.1273689
1890.0000000	5.2704000	-156.2306976
1890.0000000	5.3191996	-38.7641983
1890.0000000	5.3680000	-3472.2282715
1890.0000000	5.4168000	517.3189087
1890.0000000	5.4656000	-197.4157867
1890.0000000	5.5144000	37.0885391
1890.0000000	5.5631995	479.2754211
1890.0000000	5.6120000	385.7994385
1890.0000000	5.6608000	-36.7094917
1890.0000000	5.7096000	61.1141205
1890.0000000	5.7584000	130.3850250
1890.0000000	5.8071995	-11.6001701
1890.0000000	5.8559999	29.0055714
1890.0000000	5.9047999	-16.5874996
1890.0000000	5.9535999	-39.3421555
1890.0000000	6.0023999	-1545.1092529
1890.0000000	6.0511999	-14.5875254
1890.0000000	6.0999999	-19.3332634
1890.0000000	6.1487999	-13.3155823
1890.0000000	6.1975999	-23.0592079
1890.0000000	6.2463999	-1760.1831055
1890.0000000	6.2951999	-18.9961205
1890.0000000	6.3439999	-336.8795776
1890.0000000	6.3927999	-15.0464506
1890.0000000	6.4416003	-12.4392548
1890.0000000	6.4903998	-11.3249750
1890.0000000	6.5391998	-11.8678885
1890.0000000	6.5879998	322.6179199
1890.0000000	6.6367998	-12.1337299
1890.0000000	6.6856003	-13.8302593
1890.0000000	6.7343998	-13.4750729
1890.0000000	6.7831998	-14.3352308
1890.0000000	6.8319998	56.7259293
1890.0000000	6.8807998	-14.0370026
1890.0000000	6.9296002	-381.4615479
1890.0000000	6.9783998	-6.2286630
1890.0000000	7.0271997	-14.0457802
1890.0000000	7.0760002	7.4367189
1890.0000000	7.1247997	-1781.4625244
1890.0000000	7.1736002	-1428.7309570
1890.0000000	7.2223997	-813.3619385
1890.0000000	7.2711997	-177.0473633
1890.0000000	7.3200002	-17.8219109
1890.0000000	7.3687997	154.7890930
1890.0000000	7.4176002	325.1382446
1890.0000000	7.4663997	255.0950165

1890.0000000	7.5151997	-795.1463623
1890.0000000	7.5640001	-878.1111450
1890.0000000	7.6127996	-2067.3510742
1890.0000000	7.6616001	-664.7106323
1890.0000000	7.7104001	-342.2556152
1890.0000000	7.7591996	34.3245850
1890.0000000	7.8080001	337.8952942
1890.0000000	7.8567996	445.6838379
1890.0000000	7.9056001	-928.7612305
1890.0000000	7.9544001	1477.3045654
1890.0000000	8.0031996	1906.3372803
1890.0000000	8.0520000	1902.6845703
1890.0000000	8.1007996	2013.4638672
1890.0000000	8.1496000	2147.4099121
1890.0000000	8.1983995	1335.8743896
1890.0000000	8.2472000	-3057.5178223
1890.0000000	8.2959995	-1084.0487061
1890.0000000	8.3448000	-863.4141846
1890.0000000	8.3935995	-791.3317871
1890.0000000	8.4423990	-341.1600037
1890.0000000	8.4912004	-795.8912354
1890.0000000	8.5400000	-2146.2329102
1890.0000000	8.5887995	-1040.7728271
1890.0000000	8.6375999	202.6141357
1890.0000000	8.6863995	1834.6931152
1890.0000000	8.7351999	-682.4603882
1890.0000000	8.7840004	778.5607300
1890.0000000	8.8327999	40.7759933
1890.0000000	8.8815994	-11.5116768
1890.0000000	8.9303989	-23.6200600
1890.0000000	8.9792004	-11.5862961
1890.0000000	9.0279999	-5.9912882
1890.0000000	9.0767994	6.5403500
1890.0000000	9.1255999	-17.0925484
1890.0000000	9.1743994	-10.1086369
1890.0000000	9.2231998	35.6406326
1890.0000000	9.2720003	-8.5016613
1890.0000000	9.3207998	-9.8450613
1890.0000000	9.3695993	-10.7834806
1890.0000000	9.4183998	-11.7430410
1890.0000000	9.4672003	-11.1649151
1890.0000000	9.5159998	-14.0866890
1890.0000000	9.5648003	-0.6864996
1890.0000000	9.6135998	-13.3272190
1890.0000000	9.6623993	-10.2452621
1890.0000000	9.7111998	-10.1813326
1890.0000000	9.7600002	-10.8612041
1890.0000000	9.8087997	-10.9954700
1890.0000000	9.8575993	2.8051567
1890.0000000	9.9063997	-8.0485716
1890.0000000	9.9552002	-10.9069767
1890.0000000	10.0039997	-10.0962553
1900.0000000	0.3416000	-37.6148109
1900.0000000	0.3904000	-39.0452576
1900.0000000	0.4392000	-39.2126694
1900.0000000	0.4880000	-39.4848557
1900.0000000	0.5368000	-39.7170410
1900.0000000	0.5856000	-39.2110825

1900.0000000	0.6344000	-38.2527657
1900.0000000	0.6832000	-36.3617363
1900.0000000	0.7320000	-36.1822548
1900.0000000	0.7808000	-36.5453033
1900.0000000	0.8296000	-35.9926987
1900.0000000	0.8784000	-35.3852043
1900.0000000	0.9272000	-33.4323044
1900.0000000	0.9760000	-31.1472454
1900.0000000	1.0247999	-29.8985081
1900.0000000	1.0735999	-29.6941967
1900.0000000	1.1224000	-30.1222553
1900.0000000	1.1711999	-30.8524628
1900.0000000	1.2200000	-31.2081623
1900.0000000	1.2687999	-31.0500164
1900.0000000	1.3176000	-30.7143536
1900.0000000	1.3664000	-30.7110405
1900.0000000	1.4152000	-30.1803036
1900.0000000	1.4640000	-29.4648838
1900.0000000	1.5128000	-30.8235836
1900.0000000	1.5616000	-29.9482594
1900.0000000	1.6104001	-1333.4504395
1900.0000000	1.6592000	-47.3921852
1900.0000000	1.7079999	-27.5872898
1900.0000000	1.7567999	-27.7235470
1900.0000000	1.8055999	-27.6460667
1900.0000000	1.8544000	-26.5457764
1900.0000000	1.9031999	-26.8396511
1900.0000000	1.9520000	-26.8486557
1900.0000000	2.0007999	-26.4956379
1900.0000000	2.0495999	-26.2130566
1900.0000000	2.0983999	-24.7615070
1900.0000000	2.1471999	-24.0398483
1900.0000000	2.1960001	-24.8133087
1900.0000000	2.2448001	-24.2515678
1900.0000000	2.2935998	-22.4335670
1900.0000000	2.3423998	-22.2574558
1900.0000000	2.3912001	-22.8154964
1900.0000000	2.4400001	-23.0562744
1900.0000000	2.4888000	-23.4409733
1900.0000000	2.5375998	-13.7719154
1900.0000000	2.5863998	-12.1254721
1900.0000000	2.6352000	-10.4594336
1900.0000000	2.6840000	-16.4574051
1900.0000000	2.7328000	109.8960953
1900.0000000	2.7815998	130.3260498
1900.0000000	2.8304000	-23.6803627
1900.0000000	2.8792000	-20.9046116
1900.0000000	2.9280000	-22.5220432
1900.0000000	2.9768000	-17.5209274
1900.0000000	3.0256000	-20.6987057
1900.0000000	3.0743999	-28.5217896
1900.0000000	3.1231999	-23.9531746
1900.0000000	3.1719999	-28.8811016
1900.0000000	3.2208002	-483.4598083
1900.0000000	3.2695999	-945.7219238
1900.0000000	3.3183999	-20.5070190
1900.0000000	3.3671999	-3268.0351563
1900.0000000	3.4159999	1171.4427490

1900.0000000	3.4648001	-7.6428375
1900.0000000	3.5135999	-26.7739525
1900.0000000	3.5623999	-310.5446777
1900.0000000	3.6111999	-1947.1523438
1900.0000000	3.6600001	-441.4127197
1900.0000000	3.7088001	-582.5892334
1900.0000000	3.7575998	-911.6867676
1900.0000000	3.8063998	-1653.7500000
1900.0000000	3.8552001	-2400.6599121
1900.0000000	3.9040000	-2353.6945801
1900.0000000	3.9528000	1075.2763672
1900.0000000	4.0015998	-235.8182220
1900.0000000	4.0503998	657.9893188
1900.0000000	4.0991998	83.1465607
1900.0000000	4.1479998	-628.1018066
1900.0000000	4.1967998	1.6396828
1900.0000000	4.2456002	-238.8822937
1900.0000000	4.2943997	-468.3550720
1900.0000000	4.3431997	-13.5449495
1900.0000000	4.3920002	-238.5312042
1900.0000000	4.4407997	-22.2866573
1900.0000000	4.4896002	-15.5606937
1900.0000000	4.5383997	-629.4901733
1900.0000000	4.5871997	-13.0362272
1900.0000000	4.6360002	-21.4699020
1900.0000000	4.6847997	-27.8975716
1900.0000000	4.7336001	-19.4916134
1900.0000000	4.7824001	-16.5569229
1900.0000000	4.8311996	-1422.2277832
1900.0000000	4.8800001	219.4668732
1900.0000000	4.9287996	-194.8497620
1900.0000000	4.9776001	1021.0441284
1900.0000000	5.0264001	82.4917831
1900.0000000	5.0751996	-290.9645691
1900.0000000	5.1240001	-12.3987999
1900.0000000	5.1727996	547.0587158
1900.0000000	5.2216001	31.1179237
1900.0000000	5.2704000	-156.2025909
1900.0000000	5.3191996	-38.7596474
1900.0000000	5.3680000	-3471.5358887
1900.0000000	5.4168000	517.2120972
1900.0000000	5.4656000	-197.3796082
1900.0000000	5.5144000	37.0778809
1900.0000000	5.5631995	479.1768494
1900.0000000	5.6120000	385.7194519
1900.0000000	5.6608000	-36.7054062
1900.0000000	5.7096000	61.0986938
1900.0000000	5.7584000	130.3560944
1900.0000000	5.8071995	-11.6010399
1900.0000000	5.8559999	28.9967213
1900.0000000	5.9047999	-16.5873013
1900.0000000	5.9535999	-39.3374176
1900.0000000	6.0023999	-1544.8049316
1900.0000000	6.0511999	-14.5877361
1900.0000000	6.0999999	-19.3324318
1900.0000000	6.1487999	-13.3160448
1900.0000000	6.1975999	-23.0574436
1900.0000000	6.2463999	-1759.8356934

1900.0000000	6.2951999	-18.9953079
1900.0000000	6.3439999	-336.8155212
1900.0000000	6.3927999	-15.0464325
1900.0000000	6.4416003	-12.4398413
1900.0000000	6.4903998	-11.3256512
1900.0000000	6.5391998	-11.8685694
1900.0000000	6.5879998	321.9272461
1900.0000000	6.6367998	-12.1342793
1900.0000000	6.6856003	-13.8304291
1900.0000000	6.7343998	-13.4752884
1900.0000000	6.7831998	-14.3348150
1900.0000000	6.8319998	56.7068100
1900.0000000	6.8807998	-14.0368643
1900.0000000	6.9296002	-380.7000732
1900.0000000	6.9783998	-6.2414865
1900.0000000	7.0271997	-14.0447521
1900.0000000	7.0760002	7.3945322
1900.0000000	7.1247997	-1777.5166016
1900.0000000	7.1736002	-1425.5681152
1900.0000000	7.2223997	-811.5687256
1900.0000000	7.2711997	-176.6795807
1900.0000000	7.3200002	-17.8099232
1900.0000000	7.3687997	154.4145660
1900.0000000	7.4176002	324.3824158
1900.0000000	7.4663997	254.4973907
1900.0000000	7.5151997	-793.3853149
1900.0000000	7.5640001	-876.1637573
1900.0000000	7.6127996	-2062.7333984
1900.0000000	7.6616001	-663.2392578
1900.0000000	7.7104001	-341.5093384
1900.0000000	7.7591996	34.2235374
1900.0000000	7.8080001	337.1099243
1900.0000000	7.8567996	444.6557617
1900.0000000	7.9056001	-926.6883545
1900.0000000	7.9544001	1473.9946289
1900.0000000	8.0031996	1902.0676270
1900.0000000	8.0520000	1898.4226074
1900.0000000	8.1007996	2008.9543457
1900.0000000	8.1496000	2142.6018066
1900.0000000	8.1983995	1332.8818359
1900.0000000	8.2472000	-3050.6828613
1900.0000000	8.2959995	-1081.6285400
1900.0000000	8.3448000	-861.4849854
1900.0000000	8.3935995	-789.5641479
1900.0000000	8.4423990	-340.4020081
1900.0000000	8.4912004	-794.1149902
1900.0000000	8.5400000	-2141.4331055
1900.0000000	8.5887995	-1038.4512939
1900.0000000	8.6375999	202.1528168
1900.0000000	8.6863995	1830.5747070
1900.0000000	8.7351999	-680.9360962
1900.0000000	8.7840004	776.8039551
1900.0000000	8.8327999	40.6643829
1900.0000000	8.8815994	-11.5118513
1900.0000000	8.9303989	-23.6163406
1900.0000000	8.9792004	-11.5863781
1900.0000000	9.0279999	-5.9930563
1900.0000000	9.0767994	6.5346317

1900.0000000	9.1255999	-17.0908337
1900.0000000	9.1743994	-10.1090393
1900.0000000	9.2231998	35.6248932
1900.0000000	9.2720003	-8.5025978
1900.0000000	9.3207998	-9.8456001
1900.0000000	9.3695993	-10.7837067
1900.0000000	9.4183998	-11.7429171
1900.0000000	9.4672003	-11.1649561
1900.0000000	9.5159998	-14.0857983
1900.0000000	9.5648003	-0.6898727
1900.0000000	9.6135998	-13.3264275
1900.0000000	9.6623993	-10.2455378
1900.0000000	9.7111998	-10.1816044
1900.0000000	9.7600002	-10.8612461
1900.0000000	9.8087997	-10.9954786
1900.0000000	9.8575993	2.8007336
1900.0000000	9.9063997	-8.0494852
1900.0000000	9.9552002	-10.9069815
1900.0000000	10.0039997	-10.0965033
1910.0000000	0.3416000	-37.0778694
1910.0000000	0.3904000	-39.0393448
1910.0000000	0.4392000	-39.2136307
1910.0000000	0.4880000	-39.4836464
1910.0000000	0.5368000	-39.7182159
1910.0000000	0.5856000	-39.2097015
1910.0000000	0.6344000	-38.2530746
1910.0000000	0.6832000	-36.3639603
1910.0000000	0.7320000	-36.1843071
1910.0000000	0.7808000	-36.5454712
1910.0000000	0.8296000	-35.9887886
1910.0000000	0.8784000	-35.3820724
1910.0000000	0.9272000	-33.4167252
1910.0000000	0.9760000	-31.1424294
1910.0000000	1.0247999	-29.9040165
1910.0000000	1.0735999	-29.6794739
1910.0000000	1.1224000	-30.1039772
1910.0000000	1.1711999	-30.8456116
1910.0000000	1.2200000	-31.2099380
1910.0000000	1.2687999	-31.0521832
1910.0000000	1.3176000	-30.7106724
1910.0000000	1.3664000	-30.7106171
1910.0000000	1.4152000	-30.1805077
1910.0000000	1.4640000	-29.4631748
1910.0000000	1.5128000	-30.7264061
1910.0000000	1.5616000	-29.9064178
1910.0000000	1.6104001	-1330.9077148
1910.0000000	1.6592000	-47.0982513
1910.0000000	1.7079999	-27.5910358
1910.0000000	1.7567999	-27.7128792
1910.0000000	1.8055999	-27.6232014
1910.0000000	1.8544000	-27.0608253
1910.0000000	1.9031999	-26.8435745
1910.0000000	1.9520000	-26.8003311
1910.0000000	2.0007999	-26.0650482
1910.0000000	2.0495999	-25.2605438
1910.0000000	2.0983999	-24.8249836
1910.0000000	2.1471999	-23.7299652
1910.0000000	2.1960001	-24.7174931

1910.0000000	2.2448001	-24.2336578
1910.0000000	2.2935998	-22.3739433
1910.0000000	2.3423998	-22.2718601
1910.0000000	2.3912001	-23.4370422
1910.0000000	2.4400001	-23.0587616
1910.0000000	2.4888000	-23.4407558
1910.0000000	2.5375998	-13.7890234
1910.0000000	2.5863998	-12.1452312
1910.0000000	2.6352000	-10.4821777
1910.0000000	2.6840000	-16.4693489
1910.0000000	2.7328000	109.6300735
1910.0000000	2.7815998	130.0196533
1910.0000000	2.8304000	-23.6790581
1910.0000000	2.8792000	-20.9078751
1910.0000000	2.9280000	-22.5214729
1910.0000000	2.9768000	-17.5292988
1910.0000000	3.0256000	-20.7012901
1910.0000000	3.0743999	-28.5104027
1910.0000000	3.1231999	-23.9497204
1910.0000000	3.1719999	-28.8728085
1910.0000000	3.2208002	-483.2220459
1910.0000000	3.2695999	-945.2469482
1910.0000000	3.3183999	-20.5072174
1910.0000000	3.3671999	-3266.5734863
1910.0000000	3.4159999	1170.9074707
1910.0000000	3.4648001	-7.6485462
1910.0000000	3.5135999	-26.7709618
1910.0000000	3.5623999	-310.4150696
1910.0000000	3.6111999	-1946.2926025
1910.0000000	3.6600001	-441.2246094
1910.0000000	3.7088001	-582.3378296
1910.0000000	3.7575998	-911.2882080
1910.0000000	3.8063998	-1653.0201416
1910.0000000	3.8552001	-2399.5964355
1910.0000000	3.9040000	-2352.6520996
1910.0000000	3.9528000	1074.7880859
1910.0000000	4.0015998	-235.7211151
1910.0000000	4.0503998	657.6867676
1910.0000000	4.0991998	83.1008759
1910.0000000	4.1479998	-627.8295898
1910.0000000	4.1967998	1.6304264
1910.0000000	4.2456002	-238.7839966
1910.0000000	4.2943997	-468.1545105
1910.0000000	4.3431997	-13.5471306
1910.0000000	4.3920002	-238.4330902
1910.0000000	4.4407997	-22.2848816
1910.0000000	4.4896002	-15.5615768
1910.0000000	4.5383997	-629.2291260
1910.0000000	4.5871997	-13.0382442
1910.0000000	4.6360002	-21.4683590
1910.0000000	4.6847997	-27.8936462
1910.0000000	4.7336001	-19.4908905
1910.0000000	4.7824001	-16.5573769
1910.0000000	4.8311996	-1421.6965332
1910.0000000	4.8800001	219.3773193
1910.0000000	4.9287996	-194.7825012
1910.0000000	4.9776001	1020.6516724
1910.0000000	5.0264001	82.4542618

1910.0000000	5.0751996	-290.8609314
1910.0000000	5.1240001	-12.3997211
1910.0000000	5.1727996	546.9428711
1910.0000000	5.2216001	31.1084824
1910.0000000	5.2704000	-156.1744843
1910.0000000	5.3191996	-38.7550964
1910.0000000	5.3680000	-3470.8435059
1910.0000000	5.4168000	517.1053467
1910.0000000	5.4656000	-197.3434143
1910.0000000	5.5144000	37.0672226
1910.0000000	5.5631995	479.0782776
1910.0000000	5.6120000	385.6394653
1910.0000000	5.6608000	-36.7013168
1910.0000000	5.7096000	61.0832596
1910.0000000	5.7584000	130.3271484
1910.0000000	5.8071995	-11.6019115
1910.0000000	5.8559999	28.9878674
1910.0000000	5.9047999	-16.5871048
1910.0000000	5.9535999	-39.3326797
1910.0000000	6.0023999	-1544.5003662
1910.0000000	6.0511999	-14.5879478
1910.0000000	6.0999999	-19.3316002
1910.0000000	6.1487999	-13.3165073
1910.0000000	6.1975999	-23.0556793
1910.0000000	6.2463999	-1759.4884033
1910.0000000	6.2951999	-18.9944916
1910.0000000	6.3439999	-336.7514648
1910.0000000	6.3927999	-15.0464144
1910.0000000	6.4416003	-12.4404287
1910.0000000	6.4903998	-11.3263264
1910.0000000	6.5391998	-11.8692503
1910.0000000	6.5879998	321.2365417
1910.0000000	6.6367998	-12.1348286
1910.0000000	6.6856003	-13.8305998
1910.0000000	6.7343998	-13.4755039
1910.0000000	6.7831998	-14.3343992
1910.0000000	6.8319998	56.6876984
1910.0000000	6.8807998	-14.0367250
1910.0000000	6.9296002	-379.9386292
1910.0000000	6.9783998	-6.2543101
1910.0000000	7.0271997	-14.0437250
1910.0000000	7.0760002	7.3523455
1910.0000000	7.1247997	-1773.5706787
1910.0000000	7.1736002	-1422.4052734
1910.0000000	7.2223997	-809.7755737
1910.0000000	7.2711997	-176.3117981
1910.0000000	7.3200002	-17.7979355
1910.0000000	7.3687997	154.0400391
1910.0000000	7.4176002	323.6265869
1910.0000000	7.4663997	253.8997345
1910.0000000	7.5151997	-791.6242676
1910.0000000	7.5640001	-874.2163696
1910.0000000	7.6127996	-2058.1157227
1910.0000000	7.6616001	-661.7679443
1910.0000000	7.7104001	-340.7630615
1910.0000000	7.7591996	34.1224861
1910.0000000	7.8080001	336.3245544
1910.0000000	7.8567996	443.6276855

1910.0000000	7.9056001	-924.6154175
1910.0000000	7.9544001	1470.6846924
1910.0000000	8.0031996	1897.7980957
1910.0000000	8.0520000	1894.1606445
1910.0000000	8.1007996	2004.4448242
1910.0000000	8.1496000	2137.7939453
1910.0000000	8.1983995	1329.8892822
1910.0000000	8.2472000	-3043.8481445
1910.0000000	8.2959995	-1079.2083740
1910.0000000	8.3448000	-859.5558472
1910.0000000	8.3935995	-787.7964478
1910.0000000	8.4423990	-339.6439819
1910.0000000	8.4912004	-792.3388062
1910.0000000	8.5400000	-2136.6333008
1910.0000000	8.5887995	-1036.1297607
1910.0000000	8.6375999	201.6914978
1910.0000000	8.6863995	1826.4564209
1910.0000000	8.7351999	-679.4118652
1910.0000000	8.7840004	775.0472412
1910.0000000	8.8327999	40.5527763
1910.0000000	8.8815994	-11.5120258
1910.0000000	8.9303989	-23.6126194
1910.0000000	8.9792004	-11.5864611
1910.0000000	9.0279999	-5.9948249
1910.0000000	9.0767994	6.5289135
1910.0000000	9.1255999	-17.0891190
1910.0000000	9.1743994	-10.1094408
1910.0000000	9.2231998	35.6091614
1910.0000000	9.2720003	-8.5035343
1910.0000000	9.3207998	-9.8461399
1910.0000000	9.3695993	-10.7839336
1910.0000000	9.4183998	-11.7427921
1910.0000000	9.4672003	-11.1649971
1910.0000000	9.5159998	-14.0849075
1910.0000000	9.5648003	-0.6932449
1910.0000000	9.6135998	-13.3256378
1910.0000000	9.6623993	-10.2458124
1910.0000000	9.7111998	-10.1818762
1910.0000000	9.7600002	-10.8612871
1910.0000000	9.8087997	-10.9954863
1910.0000000	9.8575993	2.7963114
1910.0000000	9.9063997	-8.0503988
1910.0000000	9.9552002	-10.9069853
1910.0000000	10.0039997	-10.0967512
1920.0000000	0.3416000	-36.8014717
1920.0000000	0.3904000	-39.0334282
1920.0000000	0.4392000	-39.2145920
1920.0000000	0.4880000	-39.4824371
1920.0000000	0.5368000	-39.7193909
1920.0000000	0.5856000	-39.2083206
1920.0000000	0.6344000	-38.2533836
1920.0000000	0.6832000	-36.3661880
1920.0000000	0.7320000	-36.1863594
1920.0000000	0.7808000	-36.5456429
1920.0000000	0.8296000	-35.9848824
1920.0000000	0.8784000	-35.3789406
1920.0000000	0.9272000	-33.4011421
1920.0000000	0.9760000	-31.1376114

1920.0000000	1.0247999	-29.9095249
1920.0000000	1.0735999	-29.6647511
1920.0000000	1.1224000	-30.0857010
1920.0000000	1.1711999	-30.8387585
1920.0000000	1.2200000	-31.2117119
1920.0000000	1.2687999	-31.0543518
1920.0000000	1.3176000	-30.7069912
1920.0000000	1.3664000	-30.7101936
1920.0000000	1.4152000	-30.1807117
1920.0000000	1.4640000	-29.4614658
1920.0000000	1.5128000	-30.6292286
1920.0000000	1.5616000	-29.8645763
1920.0000000	1.6104001	-1328.3648682
1920.0000000	1.6592000	-46.8043213
1920.0000000	1.7079999	-27.5947838
1920.0000000	1.7567999	-27.7022133
1920.0000000	1.8055999	-27.6003380
1920.0000000	1.8544000	-26.6411839
1920.0000000	1.9031999	-26.4137421
1920.0000000	1.9520000	-26.7520065
1920.0000000	2.0007999	-25.6344566
1920.0000000	2.0495999	-24.3080330
1920.0000000	2.0983999	-24.8884621
1920.0000000	2.1471999	-23.4200840
1920.0000000	2.1960001	-24.6216774
1920.0000000	2.2448001	-24.2157459
1920.0000000	2.2935998	-22.3143196
1920.0000000	2.3423998	-22.2862644
1920.0000000	2.3912001	-24.0585880
1920.0000000	2.4400001	-23.0612469
1920.0000000	2.4888000	-23.4405384
1920.0000000	2.5375998	-13.8061314
1920.0000000	2.5863998	-12.1649904
1920.0000000	2.6352000	-10.5049219
1920.0000000	2.6840000	-16.4812889
1920.0000000	2.7328000	109.3640671
1920.0000000	2.7815998	129.7132263
1920.0000000	2.8304000	-23.6777554
1920.0000000	2.8792000	-20.9111385
1920.0000000	2.9280000	-22.5209045
1920.0000000	2.9768000	-17.5376701
1920.0000000	3.0256000	-20.7038746
1920.0000000	3.0743999	-28.4990158
1920.0000000	3.1231999	-23.9462643
1920.0000000	3.1719999	-28.8645153
1920.0000000	3.2208002	-482.9843140
1920.0000000	3.2695999	-944.7719727
1920.0000000	3.3183999	-20.5074158
1920.0000000	3.3671999	-3265.1118164
1920.0000000	3.4159999	1170.3721924
1920.0000000	3.4648001	-7.6542540
1920.0000000	3.5135999	-26.7679710
1920.0000000	3.5623999	-310.2854919
1920.0000000	3.6111999	-1945.4328613
1920.0000000	3.6600001	-441.0364990
1920.0000000	3.7088001	-582.0864258
1920.0000000	3.7575998	-910.8897095
1920.0000000	3.8063998	-1652.2902832

1920.0000000	3.8552001	-2398.5329590
1920.0000000	3.9040000	-2351.6096191
1920.0000000	3.9528000	1074.2998047
1920.0000000	4.0015998	-235.6240082
1920.0000000	4.0503998	657.3842163
1920.0000000	4.0991998	83.0551910
1920.0000000	4.1479998	-627.5573730
1920.0000000	4.1967998	1.6211700
1920.0000000	4.2456002	-238.6856842
1920.0000000	4.2943997	-467.9539490
1920.0000000	4.3431997	-13.5493107
1920.0000000	4.3920002	-238.3349762
1920.0000000	4.4407997	-22.2831059
1920.0000000	4.4896002	-15.5624590
1920.0000000	4.5383997	-628.9680786
1920.0000000	4.5871997	-13.0402603
1920.0000000	4.6360002	-21.4668159
1920.0000000	4.6847997	-27.8897209
1920.0000000	4.7336001	-19.4901676
1920.0000000	4.7824001	-16.5578308
1920.0000000	4.8311996	-1421.1651611
1920.0000000	4.8800001	219.2877808
1920.0000000	4.9287996	-194.7152405
1920.0000000	4.9776001	1020.2592163
1920.0000000	5.0264001	82.4167404
1920.0000000	5.0751996	-290.7572937
1920.0000000	5.1240001	-12.4006424
1920.0000000	5.1727996	546.8269653
1920.0000000	5.2216001	31.0990372
1920.0000000	5.2704000	-156.1463776
1920.0000000	5.3191996	-38.7505493
1920.0000000	5.3680000	-3470.1511230
1920.0000000	5.4168000	516.9985962
1920.0000000	5.4656000	-197.3072357
1920.0000000	5.5144000	37.0565720
1920.0000000	5.5631995	478.9797058
1920.0000000	5.6120000	385.5595093
1920.0000000	5.6608000	-36.6972275
1920.0000000	5.7096000	61.0678329
1920.0000000	5.7584000	130.2982178
1920.0000000	5.8071995	-11.6027813
1920.0000000	5.8559999	28.9790173
1920.0000000	5.9047999	-16.5869064
1920.0000000	5.9535999	-39.3279419
1920.0000000	6.0023999	-1544.1960449
1920.0000000	6.0511999	-14.5881596
1920.0000000	6.0999999	-19.3307667
1920.0000000	6.1487999	-13.3169708
1920.0000000	6.1975999	-23.0539131
1920.0000000	6.2463999	-1759.1409912
1920.0000000	6.2951999	-18.9936790
1920.0000000	6.3439999	-336.6874390
1920.0000000	6.3927999	-15.0463963
1920.0000000	6.4416003	-12.4410152
1920.0000000	6.4903998	-11.3270025
1920.0000000	6.5391998	-11.8699312
1920.0000000	6.5879998	320.5458374
1920.0000000	6.6367998	-12.1353779

1920.0000000	6.6856003	-13.8307695
1920.0000000	6.7343998	-13.4757204
1920.0000000	6.7831998	-14.3339834
1920.0000000	6.8319998	56.6685867
1920.0000000	6.8807998	-14.0365868
1920.0000000	6.9296002	-379.1771851
1920.0000000	6.9783998	-6.2671332
1920.0000000	7.0271997	-14.0426970
1920.0000000	7.0760002	7.3101625
1920.0000000	7.1247997	-1769.6248779
1920.0000000	7.1736002	-1419.2423096
1920.0000000	7.2223997	-807.9824219
1920.0000000	7.2711997	-175.9440308
1920.0000000	7.3200002	-17.7859459
1920.0000000	7.3687997	153.6654968
1920.0000000	7.4176002	322.8707581
1920.0000000	7.4663997	253.3020782
1920.0000000	7.5151997	-789.8632202
1920.0000000	7.5640001	-872.2689209
1920.0000000	7.6127996	-2053.4980469
1920.0000000	7.6616001	-660.2966309
1920.0000000	7.7104001	-340.0168457
1920.0000000	7.7591996	34.0214386
1920.0000000	7.8080001	335.5392151
1920.0000000	7.8567996	442.5995789
1920.0000000	7.9056001	-922.5425415
1920.0000000	7.9544001	1467.3747559
1920.0000000	8.0031996	1893.5284424
1920.0000000	8.0520000	1889.8985596
1920.0000000	8.1007996	1999.9353027
1920.0000000	8.1496000	2132.9858398
1920.0000000	8.1983995	1326.8968506
1920.0000000	8.2472000	-3037.0131836
1920.0000000	8.2959995	-1076.7882080
1920.0000000	8.3448000	-857.6266479
1920.0000000	8.3935995	-786.0288086
1920.0000000	8.4423990	-338.8859863
1920.0000000	8.4912004	-790.5625610
1920.0000000	8.5400000	-2131.8334961
1920.0000000	8.5887995	-1033.8082275
1920.0000000	8.6375999	201.2301788
1920.0000000	8.6863995	1822.3380127
1920.0000000	8.7351999	-677.8876343
1920.0000000	8.7840004	773.2904663
1920.0000000	8.8327999	40.4411697
1920.0000000	8.8815994	-11.5121994
1920.0000000	8.9303989	-23.6089001
1920.0000000	8.9792004	-11.5865431
1920.0000000	9.0279999	-5.9965935
1920.0000000	9.0767994	6.5231972
1920.0000000	9.1255999	-17.0874062
1920.0000000	9.1743994	-10.1098433
1920.0000000	9.2231998	35.5934296
1920.0000000	9.2720003	-8.5044708
1920.0000000	9.3207998	-9.8466787
1920.0000000	9.3695993	-10.7841597
1920.0000000	9.4183998	-11.7426672
1920.0000000	9.4672003	-11.1650381

1920.0000000	9.5159998	-14.0840168
1920.0000000	9.5648003	-0.6966171
1920.0000000	9.6135998	-13.3248463
1920.0000000	9.6623993	-10.2460880
1920.0000000	9.7111998	-10.1821480
1920.0000000	9.7600002	-10.8613291
1920.0000000	9.8087997	-10.9954939
1920.0000000	9.8575993	2.7918892
1920.0000000	9.9063997	-8.0513134
1920.0000000	9.9552002	-10.9069901
1920.0000000	10.0039997	-10.0969982
1930.0000000	0.3416000	-37.0545273
1930.0000000	0.3904000	-39.0275116
1930.0000000	0.4392000	-39.2155495
1930.0000000	0.4880000	-39.4812279
1930.0000000	0.5368000	-39.7205620
1930.0000000	0.5856000	-39.2069359
1930.0000000	0.6344000	-38.2536964
1930.0000000	0.6832000	-36.3684120
1930.0000000	0.7320000	-36.1884117
1930.0000000	0.7808000	-36.5458107
1930.0000000	0.8296000	-35.9809761
1930.0000000	0.8784000	-35.3758087
1930.0000000	0.9272000	-33.3855629
1930.0000000	0.9760000	-31.1327953
1930.0000000	1.0247999	-29.9150333
1930.0000000	1.0735999	-29.6500282
1930.0000000	1.1224000	-30.0674248
1930.0000000	1.1711999	-30.8319073
1930.0000000	1.2200000	-31.2134876
1930.0000000	1.2687999	-31.0565205
1930.0000000	1.3176000	-30.7033081
1930.0000000	1.3664000	-30.7097721
1930.0000000	1.4152000	-30.1809177
1930.0000000	1.4640000	-29.4597549
1930.0000000	1.5128000	-30.5320511
1930.0000000	1.5616000	-29.8227348
1930.0000000	1.6104001	-1325.8220215
1930.0000000	1.6592000	-46.5103912
1930.0000000	1.7079999	-27.5985298
1930.0000000	1.7567999	-27.6915455
1930.0000000	1.8055999	-27.5774727
1930.0000000	1.8544000	-27.0573101
1930.0000000	1.9031999	-25.9805813
1930.0000000	1.9520000	-26.7036800
1930.0000000	2.0007999	-25.2038670
1930.0000000	2.0495999	-23.3555222
1930.0000000	2.0983999	-24.9519405
1930.0000000	2.1471999	-23.1102009
1930.0000000	2.1960001	-24.5258617
1930.0000000	2.2448001	-24.1978340
1930.0000000	2.2935998	-23.1444931
1930.0000000	2.3423998	-22.3006687
1930.0000000	2.3912001	-24.6801338
1930.0000000	2.4400001	-23.0637341
1930.0000000	2.4888000	-23.4403210
1930.0000000	2.5375998	-13.8232384
1930.0000000	2.5863998	-12.1847496

1930.0000000	2.6352000	-10.5276661
1930.0000000	2.6840000	-16.4932327
1930.0000000	2.7328000	109.0980453
1930.0000000	2.7815998	129.4067993
1930.0000000	2.8304000	-23.6764507
1930.0000000	2.8792000	-20.9144020
1930.0000000	2.9280000	-22.5203362
1930.0000000	2.9768000	-17.5460396
1930.0000000	3.0256000	-20.7064590
1930.0000000	3.0743999	-28.4876289
1930.0000000	3.1231999	-23.9428101
1930.0000000	3.1719999	-28.8562222
1930.0000000	3.2208002	-482.7465820
1930.0000000	3.2695999	-944.2969971
1930.0000000	3.3183999	-20.5076141
1930.0000000	3.3671999	-3263.6501465
1930.0000000	3.4159999	1169.8369141
1930.0000000	3.4648001	-7.6599617
1930.0000000	3.5135999	-26.7649803
1930.0000000	3.5623999	-310.1559143
1930.0000000	3.6111999	-1944.5731201
1930.0000000	3.6600001	-440.8483887
1930.0000000	3.7088001	-581.8350830
1930.0000000	3.7575998	-910.4911499
1930.0000000	3.8063998	-1651.5604248
1930.0000000	3.8552001	-2397.4694824
1930.0000000	3.9040000	-2350.5671387
1930.0000000	3.9528000	1073.8114014
1930.0000000	4.0015998	-235.5269012
1930.0000000	4.0503998	657.0816040
1930.0000000	4.0991998	83.0095139
1930.0000000	4.1479998	-627.2851563
1930.0000000	4.1967998	1.6119137
1930.0000000	4.2456002	-238.5873871
1930.0000000	4.2943997	-467.7533875
1930.0000000	4.3431997	-13.5514917
1930.0000000	4.3920002	-238.2368622
1930.0000000	4.4407997	-22.2813301
1930.0000000	4.4896002	-15.5633421
1930.0000000	4.5383997	-628.7069702
1930.0000000	4.5871997	-13.0422764
1930.0000000	4.6360002	-21.4652748
1930.0000000	4.6847997	-27.8857956
1930.0000000	4.7336001	-19.4894466
1930.0000000	4.7824001	-16.5582867
1930.0000000	4.8311996	-1420.6339111
1930.0000000	4.8800001	219.1982269
1930.0000000	4.9287996	-194.6479797
1930.0000000	4.9776001	1019.8666382
1930.0000000	5.0264001	82.3792191
1930.0000000	5.0751996	-290.6536865
1930.0000000	5.1240001	-12.4015636
1930.0000000	5.1727996	546.7111206
1930.0000000	5.2216001	31.0895920
1930.0000000	5.2704000	-156.1182709
1930.0000000	5.3191996	-38.7460022
1930.0000000	5.3680000	-3469.4587402
1930.0000000	5.4168000	516.8917847

1930.0000000	5.4656000	-197.2710571
1930.0000000	5.5144000	37.0459137
1930.0000000	5.5631995	478.8811340
1930.0000000	5.6120000	385.4795227
1930.0000000	5.6608000	-36.6931381
1930.0000000	5.7096000	61.0523987
1930.0000000	5.7584000	130.2692871
1930.0000000	5.8071995	-11.6036530
1930.0000000	5.8559999	28.9701672
1930.0000000	5.9047999	-16.5867081
1930.0000000	5.9535999	-39.3232040
1930.0000000	6.0023999	-1543.8914795
1930.0000000	6.0511999	-14.5883703
1930.0000000	6.0999999	-19.3299351
1930.0000000	6.1487999	-13.3174334
1930.0000000	6.1975999	-23.0521469
1930.0000000	6.2463999	-1758.7935791
1930.0000000	6.2951999	-18.9928646
1930.0000000	6.3439999	-336.6233521
1930.0000000	6.3927999	-15.0463772
1930.0000000	6.4416003	-12.4416027
1930.0000000	6.4903998	-11.3276787
1930.0000000	6.5391998	-11.8706121
1930.0000000	6.5879998	319.8551636
1930.0000000	6.6367998	-12.1359272
1930.0000000	6.6856003	-13.8309402
1930.0000000	6.7343998	-13.4759359
1930.0000000	6.7831998	-14.3335676
1930.0000000	6.8319998	56.6494675
1930.0000000	6.8807998	-14.0364475
1930.0000000	6.9296002	-378.4157410
1930.0000000	6.9783998	-6.2799573
1930.0000000	7.0271997	-14.0416689
1930.0000000	7.0760002	7.2679758
1930.0000000	7.1247997	-1765.6789551
1930.0000000	7.1736002	-1416.0794678
1930.0000000	7.2223997	-806.1892700
1930.0000000	7.2711997	-175.5762482
1930.0000000	7.3200002	-17.7739563
1930.0000000	7.3687997	153.2909698
1930.0000000	7.4176002	322.1149292
1930.0000000	7.4663997	252.7044220
1930.0000000	7.5151997	-788.1021729
1930.0000000	7.5640001	-870.3215332
1930.0000000	7.6127996	-2048.8806152
1930.0000000	7.6616001	-658.8252563
1930.0000000	7.7104001	-339.2705688
1930.0000000	7.7591996	33.9203835
1930.0000000	7.8080001	334.7538452
1930.0000000	7.8567996	441.5715027
1930.0000000	7.9056001	-920.4696045
1930.0000000	7.9544001	1464.0648193
1930.0000000	8.0031996	1889.2589111
1930.0000000	8.0520000	1885.6365967
1930.0000000	8.1007996	1995.4257813
1930.0000000	8.1496000	2128.1777344
1930.0000000	8.1983995	1323.9042969
1930.0000000	8.2472000	-3030.1784668

1930.0000000	8.2959995	-1074.3680420
1930.0000000	8.3448000	-855.6974487
1930.0000000	8.3935995	-784.2611084
1930.0000000	8.4423990	-338.1279602
1930.0000000	8.4912004	-788.7863159
1930.0000000	8.5400000	-2127.0336914
1930.0000000	8.5887995	-1031.4866943
1930.0000000	8.6375999	200.7688599
1930.0000000	8.6863995	1818.2196045
1930.0000000	8.7351999	-676.3633423
1930.0000000	8.7840004	771.5337524
1930.0000000	8.8327999	40.3295631
1930.0000000	8.8815994	-11.5123739
1930.0000000	8.9303989	-23.6051788
1930.0000000	8.9792004	-11.5866251
1930.0000000	9.0279999	-5.9983611
1930.0000000	9.0767994	6.5174789
1930.0000000	9.1255999	-17.0856915
1930.0000000	9.1743994	-10.1102448
1930.0000000	9.2231998	35.5776978
1930.0000000	9.2720003	-8.5054073
1930.0000000	9.3207998	-9.8472185
1930.0000000	9.3695993	-10.7843866
1930.0000000	9.4183998	-11.7425432
1930.0000000	9.4672003	-11.1650801
1930.0000000	9.5159998	-14.0831251
1930.0000000	9.5648003	-0.6999903
1930.0000000	9.6135998	-13.3240566
1930.0000000	9.6623993	-10.2463627
1930.0000000	9.7111998	-10.1824188
1930.0000000	9.7600002	-10.8613710
1930.0000000	9.8087997	-10.9955025
1930.0000000	9.8575993	2.7874670
1930.0000000	9.9063997	-8.0522270
1930.0000000	9.9552002	-10.9069939
1930.0000000	10.0039997	-10.0972462
1940.0000000	0.3416000	-37.2049942
1940.0000000	0.3904000	-39.0215950
1940.0000000	0.4392000	-39.2165108
1940.0000000	0.4880000	-39.4800186
1940.0000000	0.5368000	-39.7217369
1940.0000000	0.5856000	-39.2055550
1940.0000000	0.6344000	-38.2540054
1940.0000000	0.6832000	-36.3706398
1940.0000000	0.7320000	-36.1904640
1940.0000000	0.7808000	-36.5459824
1940.0000000	0.8296000	-35.9770699
1940.0000000	0.8784000	-35.3726768
1940.0000000	0.9272000	-33.3699837
1940.0000000	0.9760000	-31.1279793
1940.0000000	1.0247999	-29.9205418
1940.0000000	1.0735999	-29.6353054
1940.0000000	1.1224000	-30.0491486
1940.0000000	1.1711999	-30.8250561
1940.0000000	1.2200000	-31.2152634
1940.0000000	1.2687999	-31.0586891
1940.0000000	1.3176000	-30.6996269
1940.0000000	1.3664000	-30.7093487

1940.0000000	1.4152000	-30.1811218
1940.0000000	1.4640000	-29.4580460
1940.0000000	1.5128000	-30.4348736
1940.0000000	1.5616000	-29.7808952
1940.0000000	1.6104001	-1323.2792969
1940.0000000	1.6592000	-46.2164574
1940.0000000	1.7079999	-27.6022778
1940.0000000	1.7567999	-27.6808777
1940.0000000	1.8055999	-27.5546074
1940.0000000	1.8544000	-27.0741425
1940.0000000	1.9031999	-25.5474186
1940.0000000	1.9520000	-26.6553555
1940.0000000	2.0007999	-24.7732773
1940.0000000	2.0495999	-22.4030094
1940.0000000	2.0983999	-21.0897350
1940.0000000	2.1471999	-22.2851887
1940.0000000	2.1960001	-24.9095020
1940.0000000	2.2448001	-24.1799240
1940.0000000	2.2935998	-23.9746647
1940.0000000	2.3423998	-22.3150730
1940.0000000	2.3912001	-24.6784973
1940.0000000	2.4400001	-23.0662212
1940.0000000	2.4888000	-23.4401035
1940.0000000	2.5375998	-13.8403463
1940.0000000	2.5863998	-12.2045088
1940.0000000	2.6352000	-10.5504093
1940.0000000	2.6840000	-16.5051727
1940.0000000	2.7328000	108.8320236
1940.0000000	2.7815998	129.1004028
1940.0000000	2.8304000	-23.6751461
1940.0000000	2.8792000	-20.9176655
1940.0000000	2.9280000	-22.5197659
1940.0000000	2.9768000	-17.5544090
1940.0000000	3.0256000	-20.7090435
1940.0000000	3.0743999	-28.4762421
1940.0000000	3.1231999	-23.9393539
1940.0000000	3.1719999	-28.8479290
1940.0000000	3.2208002	-482.5088501
1940.0000000	3.2695999	-943.8220215
1940.0000000	3.3183999	-20.5078106
1940.0000000	3.3671999	-3262.1884766
1940.0000000	3.4159999	1169.3016357
1940.0000000	3.4648001	-7.6656704
1940.0000000	3.5135999	-26.7619896
1940.0000000	3.5623999	-310.0263062
1940.0000000	3.6111999	-1943.7133789
1940.0000000	3.6600001	-440.6602173
1940.0000000	3.7088001	-581.5836792
1940.0000000	3.7575998	-910.0925903
1940.0000000	3.8063998	-1650.8306885
1940.0000000	3.8552001	-2396.4062500
1940.0000000	3.9040000	-2349.5246582
1940.0000000	3.9528000	1073.3231201
1940.0000000	4.0015998	-235.4297943
1940.0000000	4.0503998	656.7790527
1940.0000000	4.0991998	82.9638290
1940.0000000	4.1479998	-627.0128784
1940.0000000	4.1967998	1.6026573

1940.0000000	4.2456002	-238.4890900
1940.0000000	4.2943997	-467.5527954
1940.0000000	4.3431997	-13.5536718
1940.0000000	4.3920002	-238.1387482
1940.0000000	4.4407997	-22.2795544
1940.0000000	4.4896002	-15.5642242
1940.0000000	4.5383997	-628.4459229
1940.0000000	4.5871997	-13.0442924
1940.0000000	4.6360002	-21.4637318
1940.0000000	4.6847997	-27.8818703
1940.0000000	4.7336001	-19.4887238
1940.0000000	4.7824001	-16.5587406
1940.0000000	4.8311996	-1420.1026611
1940.0000000	4.8800001	219.1086731
1940.0000000	4.9287996	-194.5807190
1940.0000000	4.9776001	1019.4741821
1940.0000000	5.0264001	82.3416977
1940.0000000	5.0751996	-290.5500488
1940.0000000	5.1240001	-12.4024849
1940.0000000	5.1727996	546.5952759
1940.0000000	5.2216001	31.0801506
1940.0000000	5.2704000	-156.0901642
1940.0000000	5.3191996	-38.7414474
1940.0000000	5.3680000	-3468.7663574
1940.0000000	5.4168000	516.7849731
1940.0000000	5.4656000	-197.2348633
1940.0000000	5.5144000	37.0352554
1940.0000000	5.5631995	478.7825623
1940.0000000	5.6120000	385.3995667
1940.0000000	5.6608000	-36.6890488
1940.0000000	5.7096000	61.0369644
1940.0000000	5.7584000	130.2403564
1940.0000000	5.8071995	-11.6045227
1940.0000000	5.8559999	28.9613132
1940.0000000	5.9047999	-16.5865116
1940.0000000	5.9535999	-39.3184624
1940.0000000	6.0023999	-1543.5870361
1940.0000000	6.0511999	-14.5885820
1940.0000000	6.0999999	-19.3291016
1940.0000000	6.1487999	-13.3178959
1940.0000000	6.1975999	-23.0503826
1940.0000000	6.2463999	-1758.4462891
1940.0000000	6.2951999	-18.9920502
1940.0000000	6.3439999	-336.5593262
1940.0000000	6.3927999	-15.0463591
1940.0000000	6.4416003	-12.4421892
1940.0000000	6.4903998	-11.3283548
1940.0000000	6.5391998	-11.8712940
1940.0000000	6.5879998	319.1644592
1940.0000000	6.6367998	-12.1364765
1940.0000000	6.6856003	-13.8311100
1940.0000000	6.7343998	-13.4761515
1940.0000000	6.7831998	-14.3331518
1940.0000000	6.8319998	56.6303482
1940.0000000	6.8807998	-14.0363092
1940.0000000	6.9296002	-377.6542969
1940.0000000	6.9783998	-6.2927809
1940.0000000	7.0271997	-14.0406408

1940.0000000	7.0760002	7.2257910
1940.0000000	7.1247997	-1761.7331543
1940.0000000	7.1736002	-1412.9166260
1940.0000000	7.2223997	-804.3961182
1940.0000000	7.2711997	-175.2084656
1940.0000000	7.3200002	-17.7619686
1940.0000000	7.3687997	152.9164429
1940.0000000	7.4176002	321.3591003
1940.0000000	7.4663997	252.1067657
1940.0000000	7.5151997	-786.3411255
1940.0000000	7.5640001	-868.3741455
1940.0000000	7.6127996	-2044.2628174
1940.0000000	7.6616001	-657.3538818
1940.0000000	7.7104001	-338.5242920
1940.0000000	7.7591996	33.8193359
1940.0000000	7.8080001	333.9684753
1940.0000000	7.8567996	440.5434265
1940.0000000	7.9056001	-918.3967285
1940.0000000	7.9544001	1460.7548828
1940.0000000	8.0031996	1884.9892578
1940.0000000	8.0520000	1881.3746338
1940.0000000	8.1007996	1990.9162598
1940.0000000	8.1496000	2123.3698730
1940.0000000	8.1983995	1320.9117432
1940.0000000	8.2472000	-3023.3437500
1940.0000000	8.2959995	-1071.9478760
1940.0000000	8.3448000	-853.7683105
1940.0000000	8.3935995	-782.4934082
1940.0000000	8.4423990	-337.3699646
1940.0000000	8.4912004	-787.0101318
1940.0000000	8.5400000	-2122.2338867
1940.0000000	8.5887995	-1029.1651611
1940.0000000	8.6375999	200.3075256
1940.0000000	8.6863995	1814.1013184
1940.0000000	8.7351999	-674.8391113
1940.0000000	8.7840004	769.7769775
1940.0000000	8.8327999	40.2179527
1940.0000000	8.8815994	-11.5125484
1940.0000000	8.9303989	-23.6014595
1940.0000000	8.9792004	-11.5867081
1940.0000000	9.0279999	-6.0001297
1940.0000000	9.0767994	6.5117607
1940.0000000	9.1255999	-17.0839767
1940.0000000	9.1743994	-10.1106472
1940.0000000	9.2231998	35.5619659
1940.0000000	9.2720003	-8.5063438
1940.0000000	9.3207998	-9.8477573
1940.0000000	9.3695993	-10.7846127
1940.0000000	9.4183998	-11.7424183
1940.0000000	9.4672003	-11.1651211
1940.0000000	9.5159998	-14.0822344
1940.0000000	9.5648003	-0.7033634
1940.0000000	9.6135998	-13.3232651
1940.0000000	9.6623993	-10.2466373
1940.0000000	9.7111998	-10.1826906
1940.0000000	9.7600002	-10.8614120
1940.0000000	9.8087997	-10.9955101
1940.0000000	9.8575993	2.7830448

1940.0000000	9.9063997	-8.0531406
1940.0000000	9.9552002	-10.9069986
1940.0000000	10.0039997	-10.0974932
1950.0000000	0.3416000	-37.5747108
1950.0000000	0.3904000	-39.0156822
1950.0000000	0.4392000	-39.2174683
1950.0000000	0.4880000	-39.4788094
1950.0000000	0.5368000	-39.7229118
1950.0000000	0.5856000	-39.2041740
1950.0000000	0.6344000	-38.2543144
1950.0000000	0.6832000	-36.3728638
1950.0000000	0.7320000	-36.1925163
1950.0000000	0.7808000	-36.5461502
1950.0000000	0.8296000	-35.9731636
1950.0000000	0.8784000	-35.3695450
1950.0000000	0.9272000	-33.3544044
1950.0000000	0.9760000	-31.1231613
1950.0000000	1.0247999	-29.9260502
1950.0000000	1.0735999	-29.6205826
1950.0000000	1.1224000	-30.0308723
1950.0000000	1.1711999	-30.8182030
1950.0000000	1.2200000	-31.2170372
1950.0000000	1.2687999	-31.0608578
1950.0000000	1.3176000	-30.6959438
1950.0000000	1.3664000	-30.7089252
1950.0000000	1.4152000	-30.1813259
1950.0000000	1.4640000	-29.4563370
1950.0000000	1.5128000	-30.3376961
1950.0000000	1.5616000	-29.7390537
1950.0000000	1.6104001	-1320.7364502
1950.0000000	1.6592000	-45.9225235
1950.0000000	1.7079999	-27.6060257
1950.0000000	1.7567999	-27.6702118
1950.0000000	1.8055999	-26.6006298
1950.0000000	1.8544000	-27.3863678
1950.0000000	1.9031999	-25.1142578
1950.0000000	1.9520000	-23.2894802
1950.0000000	2.0007999	-21.7037296
1950.0000000	2.0495999	-21.1382828
1950.0000000	2.0983999	-21.6263218
1950.0000000	2.1471999	-22.8371067
1950.0000000	2.1960001	-23.9258289
1950.0000000	2.2448001	-24.1620121
1950.0000000	2.2935998	-24.8048382
1950.0000000	2.3423998	-22.3294773
1950.0000000	2.3912001	-24.6768608
1950.0000000	2.4400001	-23.0687065
1950.0000000	2.4888000	-23.4398861
1950.0000000	2.5375998	-13.8574543
1950.0000000	2.5863998	-12.2242670
1950.0000000	2.6352000	-10.5731535
1950.0000000	2.6840000	-16.5171146
1950.0000000	2.7328000	108.5660172
1950.0000000	2.7815998	128.7939758
1950.0000000	2.8304000	-23.6738415
1950.0000000	2.8792000	-20.9209290
1950.0000000	2.9280000	-22.5191975
1950.0000000	2.9768000	-17.5627804

1950.0000000	3.0256000	-20.7116280
1950.0000000	3.0743999	-28.4648533
1950.0000000	3.1231999	-23.9358978
1950.0000000	3.1719999	-28.8396339
1950.0000000	3.2208002	-482.2711182
1950.0000000	3.2695999	-943.3470459
1950.0000000	3.3183999	-20.5080090
1950.0000000	3.3671999	-3260.7268066
1950.0000000	3.4159999	1168.7663574
1950.0000000	3.4648001	-7.6713781
1950.0000000	3.5135999	-26.7589989
1950.0000000	3.5623999	-309.8967285
1950.0000000	3.6111999	-1942.8535156
1950.0000000	3.6600001	-440.4721069
1950.0000000	3.7088001	-581.3322754
1950.0000000	3.7575998	-909.6940918
1950.0000000	3.8063998	-1650.1008301
1950.0000000	3.8552001	-2395.3427734
1950.0000000	3.9040000	-2348.4821777
1950.0000000	3.9528000	1072.8348389
1950.0000000	4.0015998	-235.3326721
1950.0000000	4.0503998	656.4765015
1950.0000000	4.0991998	82.9181442
1950.0000000	4.1479998	-626.7406616
1950.0000000	4.1967998	1.5934010
1950.0000000	4.2456002	-238.3907776
1950.0000000	4.2943997	-467.3522339
1950.0000000	4.3431997	-13.5558529
1950.0000000	4.3920002	-238.0406342
1950.0000000	4.4407997	-22.2777786
1950.0000000	4.4896002	-15.5651073
1950.0000000	4.5383997	-628.1848755
1950.0000000	4.5871997	-13.0463085
1950.0000000	4.6360002	-21.4621887
1950.0000000	4.6847997	-27.8779449
1950.0000000	4.7336001	-19.4880009
1950.0000000	4.7824001	-16.5591946
1950.0000000	4.8311996	-1419.5714111
1950.0000000	4.8800001	219.0191193
1950.0000000	4.9287996	-194.5134583
1950.0000000	4.9776001	1019.0817261
1950.0000000	5.0264001	82.3041763
1950.0000000	5.0751996	-290.4464111
1950.0000000	5.1240001	-12.4034061
1950.0000000	5.1727996	546.4793701
1950.0000000	5.2216001	31.0707054
1950.0000000	5.2704000	-156.0620575
1950.0000000	5.3191996	-38.7369003
1950.0000000	5.3680000	-3468.0739746
1950.0000000	5.4168000	516.6782227
1950.0000000	5.4656000	-197.1986847
1950.0000000	5.5144000	37.0245972
1950.0000000	5.5631995	478.6839905
1950.0000000	5.6120000	385.3195801
1950.0000000	5.6608000	-36.6849632
1950.0000000	5.7096000	61.0215378
1950.0000000	5.7584000	130.2114258
1950.0000000	5.8071995	-11.6053934

1950.0000000	5.8559999	28.9524632
1950.0000000	5.9047999	-16.5863132
1950.0000000	5.9535999	-39.3137245
1950.0000000	6.0023999	-1543.2827148
1950.0000000	6.0511999	-14.5887938
1950.0000000	6.0999999	-19.3282700
1950.0000000	6.1487999	-13.3183594
1950.0000000	6.1975999	-23.0486183
1950.0000000	6.2463999	-1758.0988770
1950.0000000	6.2951999	-18.9912357
1950.0000000	6.3439999	-336.4952698
1950.0000000	6.3927999	-15.0463409
1950.0000000	6.4416003	-12.4427767
1950.0000000	6.4903998	-11.3290300
1950.0000000	6.5391998	-11.8719749
1950.0000000	6.5879998	318.4737549
1950.0000000	6.6367998	-12.1370249
1950.0000000	6.6856003	-13.8312807
1950.0000000	6.7343998	-13.4763670
1950.0000000	6.7831998	-14.3327360
1950.0000000	6.8319998	56.6112366
1950.0000000	6.8807998	-14.0361700
1950.0000000	6.9296002	-376.8928223
1950.0000000	6.9783998	-6.3056045
1950.0000000	7.0271997	-14.0396128
1950.0000000	7.0760002	7.1836061
1950.0000000	7.1247997	-1757.7872314
1950.0000000	7.1736002	-1409.7536621
1950.0000000	7.2223997	-802.6029053
1950.0000000	7.2711997	-174.8406830
1950.0000000	7.3200002	-17.7499809
1950.0000000	7.3687997	152.5419159
1950.0000000	7.4176002	320.6032715
1950.0000000	7.4663997	251.5091095
1950.0000000	7.5151997	-784.5800781
1950.0000000	7.5640001	-866.4267578
1950.0000000	7.6127996	-2039.6452637
1950.0000000	7.6616001	-655.8825684
1950.0000000	7.7104001	-337.7780151
1950.0000000	7.7591996	33.7182884
1950.0000000	7.8080001	333.1831360
1950.0000000	7.8567996	439.5153198
1950.0000000	7.9056001	-916.3237915
1950.0000000	7.9544001	1457.4449463
1950.0000000	8.0031996	1880.7197266
1950.0000000	8.0520000	1877.1125488
1950.0000000	8.1007996	1986.4067383
1950.0000000	8.1496000	2118.5617676
1950.0000000	8.1983995	1317.9193115
1950.0000000	8.2472000	-3016.5087891
1950.0000000	8.2959995	-1069.5277100
1950.0000000	8.3448000	-851.8391113
1950.0000000	8.3935995	-780.7257690
1950.0000000	8.4423990	-336.6119385
1950.0000000	8.4912004	-785.2338867
1950.0000000	8.5400000	-2117.4340820
1950.0000000	8.5887995	-1026.8436279
1950.0000000	8.6375999	199.8462067

1950.0000000	8.6863995	1809.9829102
1950.0000000	8.7351999	-673.3148193
1950.0000000	8.7840004	768.0202637
1950.0000000	8.8327999	40.1063461
1950.0000000	8.8815994	-11.5127230
1950.0000000	8.9303989	-23.5977402
1950.0000000	8.9792004	-11.5867901
1950.0000000	9.0279999	-6.0018978
1950.0000000	9.0767994	6.5060425
1950.0000000	9.1255999	-17.0822639
1950.0000000	9.1743994	-10.1110487
1950.0000000	9.2231998	35.5462341
1950.0000000	9.2720003	-8.5072794
1950.0000000	9.3207998	-9.8482971
1950.0000000	9.3695993	-10.7848396
1950.0000000	9.4183998	-11.7422943
1950.0000000	9.4672003	-11.1651621
1950.0000000	9.5159998	-14.0813437
1950.0000000	9.5648003	-0.7067356
1950.0000000	9.6135998	-13.3224745
1950.0000000	9.6623993	-10.2469130
1950.0000000	9.7111998	-10.1829624
1950.0000000	9.7600002	-10.8614540
1950.0000000	9.8087997	-10.9955187
1950.0000000	9.8575993	2.7786217
1950.0000000	9.9063997	-8.0540543
1950.0000000	9.9552002	-10.9070024
1950.0000000	10.0039997	-10.0977411
1960.0000000	0.3416000	-37.2975578
1960.0000000	0.3904000	-39.0097656
1960.0000000	0.4392000	-39.2184296
1960.0000000	0.4880000	-39.4776001
1960.0000000	0.5368000	-39.7240829
1960.0000000	0.5856000	-39.2027931
1960.0000000	0.6344000	-38.2546272
1960.0000000	0.6832000	-36.3750916
1960.0000000	0.7320000	-36.1945686
1960.0000000	0.7808000	-36.5463181
1960.0000000	0.8296000	-35.9692574
1960.0000000	0.8784000	-35.3664169
1960.0000000	0.9272000	-33.3388252
1960.0000000	0.9760000	-31.1183453
1960.0000000	1.0247999	-29.9315586
1960.0000000	1.0735999	-29.6058598
1960.0000000	1.1224000	-30.0125942
1960.0000000	1.1711999	-30.8113518
1960.0000000	1.2200000	-31.2188129
1960.0000000	1.2687999	-31.0630245
1960.0000000	1.3176000	-30.6922626
1960.0000000	1.3664000	-30.7085018
1960.0000000	1.4152000	-30.1815300
1960.0000000	1.4640000	-29.4546280
1960.0000000	1.5128000	-30.2405186
1960.0000000	1.5616000	-29.6972122
1960.0000000	1.6104001	-1318.1937256
1960.0000000	1.6592000	-45.6285934
1960.0000000	1.7079999	-27.6097717
1960.0000000	1.7567999	-27.6595440

1960.0000000	1.8055999	-25.6466522
1960.0000000	1.8544000	-24.0568199
1960.0000000	1.9031999	-23.8943424
1960.0000000	1.9520000	-23.1615276
1960.0000000	2.0007999	-21.9412785
1960.0000000	2.0495999	-23.1752892
1960.0000000	2.0983999	-25.7477379
1960.0000000	2.1471999	-22.9237671
1960.0000000	2.1960001	-23.9294834
1960.0000000	2.2448001	-24.2015820
1960.0000000	2.2935998	-24.8027267
1960.0000000	2.3423998	-22.3438835
1960.0000000	2.3912001	-24.6752243
1960.0000000	2.4400001	-23.0711937
1960.0000000	2.4888000	-23.4396687
1960.0000000	2.5375998	-13.8745623
1960.0000000	2.5863998	-12.2440262
1960.0000000	2.6352000	-10.5958986
1960.0000000	2.6840000	-16.5290565
1960.0000000	2.7328000	108.2999954
1960.0000000	2.7815998	128.4875488
1960.0000000	2.8304000	-23.6725368
1960.0000000	2.8792000	-20.9241924
1960.0000000	2.9280000	-22.5186272
1960.0000000	2.9768000	-17.5711517
1960.0000000	3.0256000	-20.7142124
1960.0000000	3.0743999	-28.4534664
1960.0000000	3.1231999	-23.9324436
1960.0000000	3.1719999	-28.8313408
1960.0000000	3.2208002	-482.0333862
1960.0000000	3.2695999	-942.8720703
1960.0000000	3.3183999	-20.5082073
1960.0000000	3.3671999	-3259.2653809
1960.0000000	3.4159999	1168.2310791
1960.0000000	3.4648001	-7.6770868
1960.0000000	3.5135999	-26.7560081
1960.0000000	3.5623999	-309.7671204
1960.0000000	3.6111999	-1941.9937744
1960.0000000	3.6600001	-440.2839966
1960.0000000	3.7088001	-581.0809326
1960.0000000	3.7575998	-909.2955322
1960.0000000	3.8063998	-1649.3709717
1960.0000000	3.8552001	-2394.2792969
1960.0000000	3.9040000	-2347.4396973
1960.0000000	3.9528000	1072.3465576
1960.0000000	4.0015998	-235.2355652
1960.0000000	4.0503998	656.1739502
1960.0000000	4.0991998	82.8724594
1960.0000000	4.1479998	-626.4684448
1960.0000000	4.1967998	1.5841446
1960.0000000	4.2456002	-238.2924805
1960.0000000	4.2943997	-467.1516724
1960.0000000	4.3431997	-13.5580330
1960.0000000	4.3920002	-237.9425201
1960.0000000	4.4407997	-22.2760048
1960.0000000	4.4896002	-15.5659904
1960.0000000	4.5383997	-627.9237671
1960.0000000	4.5871997	-13.0483246

1960.0000000	4.6360002	-21.4606476
1960.0000000	4.6847997	-27.8740196
1960.0000000	4.7336001	-19.4872799
1960.0000000	4.7824001	-16.5596504
1960.0000000	4.8311996	-1419.0401611
1960.0000000	4.8800001	218.9295654
1960.0000000	4.9287996	-194.4461975
1960.0000000	4.9776001	1018.6891479
1960.0000000	5.0264001	82.2666550
1960.0000000	5.0751996	-290.3428040
1960.0000000	5.1240001	-12.4043274
1960.0000000	5.1727996	546.3635254
1960.0000000	5.2216001	31.0612602
1960.0000000	5.2704000	-156.0339508
1960.0000000	5.3191996	-38.7323532
1960.0000000	5.3680000	-3467.3815918
1960.0000000	5.4168000	516.5714722
1960.0000000	5.4656000	-197.1625061
1960.0000000	5.5144000	37.0139389
1960.0000000	5.5631995	478.5854492
1960.0000000	5.6120000	385.2395935
1960.0000000	5.6608000	-36.6808739
1960.0000000	5.7096000	61.0061111
1960.0000000	5.7584000	130.1824951
1960.0000000	5.8071995	-11.6062641
1960.0000000	5.8559999	28.9436131
1960.0000000	5.9047999	-16.5861149
1960.0000000	5.9535999	-39.3089867
1960.0000000	6.0023999	-1542.9781494
1960.0000000	6.0511999	-14.5890045
1960.0000000	6.0999999	-19.3274384
1960.0000000	6.1487999	-13.3188219
1960.0000000	6.1975999	-23.0468521
1960.0000000	6.2463999	-1757.7514648
1960.0000000	6.2951999	-18.9904213
1960.0000000	6.3439999	-336.4312134
1960.0000000	6.3927999	-15.0463228
1960.0000000	6.4416003	-12.4433632
1960.0000000	6.4903998	-11.3297062
1960.0000000	6.5391998	-11.8726559
1960.0000000	6.5879998	317.7830505
1960.0000000	6.6367998	-12.1375742
1960.0000000	6.6856003	-13.8314505
1960.0000000	6.7343998	-13.4765835
1960.0000000	6.7831998	-14.3323202
1960.0000000	6.8319998	56.5921249
1960.0000000	6.8807998	-14.0360317
1960.0000000	6.9296002	-376.1313782
1960.0000000	6.9783998	-6.3184276
1960.0000000	7.0271997	-14.0385857
1960.0000000	7.0760002	7.1414194
1960.0000000	7.1247997	-1753.8414307
1960.0000000	7.1736002	-1406.5908203
1960.0000000	7.2223997	-800.8097534
1960.0000000	7.2711997	-174.4729004
1960.0000000	7.3200002	-17.7379913
1960.0000000	7.3687997	152.1673889
1960.0000000	7.4176002	319.8474426

1960.0000000	7.4663997	250.9114838
1960.0000000	7.5151997	-782.8190918
1960.0000000	7.5640001	-864.4793701
1960.0000000	7.6127996	-2035.0275879
1960.0000000	7.6616001	-654.4112549
1960.0000000	7.7104001	-337.0317993
1960.0000000	7.7591996	33.6172371
1960.0000000	7.8080001	332.3977661
1960.0000000	7.8567996	438.4872437
1960.0000000	7.9056001	-914.2509155
1960.0000000	7.9544001	1454.1351318
1960.0000000	8.0031996	1876.4500732
1960.0000000	8.0520000	1872.8505859
1960.0000000	8.1007996	1981.8972168
1960.0000000	8.1496000	2113.7539063
1960.0000000	8.1983995	1314.9267578
1960.0000000	8.2472000	-3009.6740723
1960.0000000	8.2959995	-1067.1075439
1960.0000000	8.3448000	-849.9099121
1960.0000000	8.3935995	-778.9580688
1960.0000000	8.4423990	-335.8539429
1960.0000000	8.4912004	-783.4577026
1960.0000000	8.5400000	-2112.6342773
1960.0000000	8.5887995	-1024.5219727
1960.0000000	8.6375999	199.3848877
1960.0000000	8.6863995	1805.8646240
1960.0000000	8.7351999	-671.7905884
1960.0000000	8.7840004	766.2634888
1960.0000000	8.8327999	39.9947395
1960.0000000	8.8815994	-11.5128975
1960.0000000	8.9303989	-23.5940208
1960.0000000	8.9792004	-11.5868721
1960.0000000	9.0279999	-6.0036664
1960.0000000	9.0767994	6.5003262
1960.0000000	9.1255999	-17.0805492
1960.0000000	9.1743994	-10.1114511
1960.0000000	9.2231998	35.5305023
1960.0000000	9.2720003	-8.5082159
1960.0000000	9.3207998	-9.8488359
1960.0000000	9.3695993	-10.7850657
1960.0000000	9.4183998	-11.7421694
1960.0000000	9.4672003	-11.1652031
1960.0000000	9.5159998	-14.0804520
1960.0000000	9.5648003	-0.7101078
1960.0000000	9.6135998	-13.3216839
1960.0000000	9.6623993	-10.2471876
1960.0000000	9.7111998	-10.1832342
1960.0000000	9.7600002	-10.8614960
1960.0000000	9.8087997	-10.9955263
1960.0000000	9.8575993	2.7741995
1960.0000000	9.9063997	-8.0549688
1960.0000000	9.9552002	-10.9070072
1960.0000000	10.0039997	-10.0979881
1970.0000000	0.3416000	-37.0282669
1970.0000000	0.3904000	-39.0038490
1970.0000000	0.4392000	-39.2193909
1970.0000000	0.4880000	-39.4763908
1970.0000000	0.5368000	-39.7252579

1970.0000000	0.5856000	-39.2014122
1970.0000000	0.6344000	-38.2549362
1970.0000000	0.6832000	-36.3773155
1970.0000000	0.7320000	-36.1966209
1970.0000000	0.7808000	-36.5464897
1970.0000000	0.8296000	-35.9653511
1970.0000000	0.8784000	-35.3632851
1970.0000000	0.9272000	-33.3232460
1970.0000000	0.9760000	-31.1135273
1970.0000000	1.0247999	-29.9370670
1970.0000000	1.0735999	-29.5911369
1970.0000000	1.1224000	-29.9943180
1970.0000000	1.1711999	-30.8044987
1970.0000000	1.2200000	-31.2205887
1970.0000000	1.2687999	-31.0651932
1970.0000000	1.3176000	-30.6885815
1970.0000000	1.3664000	-30.7080784
1970.0000000	1.4152000	-30.1817341
1970.0000000	1.4640000	-29.4529171
1970.0000000	1.5128000	-30.1433411
1970.0000000	1.5616000	-29.6553707
1970.0000000	1.6104001	-1315.6508789
1970.0000000	1.6592000	-45.3346634
1970.0000000	1.7079999	-27.6135197
1970.0000000	1.7567999	-27.6488762
1970.0000000	1.8055999	-26.2108440
1970.0000000	1.8544000	-26.1128502
1970.0000000	1.9031999	-26.4695950
1970.0000000	1.9520000	-25.5551567
1970.0000000	2.0007999	-24.6258125
1970.0000000	2.0495999	-24.7574425
1970.0000000	2.0983999	-25.7601948
1970.0000000	2.1471999	-23.0104275
1970.0000000	2.1960001	-23.9331379
1970.0000000	2.2448001	-24.2411518
1970.0000000	2.2935998	-24.8006134
1970.0000000	2.3423998	-22.3582878
1970.0000000	2.3912001	-24.6735897
1970.0000000	2.4400001	-23.0736809
1970.0000000	2.4888000	-23.4394512
1970.0000000	2.5375998	-13.8916712
1970.0000000	2.5863998	-12.2637854
1970.0000000	2.6352000	-10.6186428
1970.0000000	2.6840000	-16.5409985
1970.0000000	2.7328000	108.0339890
1970.0000000	2.7815998	128.1811218
1970.0000000	2.8304000	-23.6712341
1970.0000000	2.8792000	-20.9274559
1970.0000000	2.9280000	-22.5180588
1970.0000000	2.9768000	-17.5795212
1970.0000000	3.0256000	-20.7167969
1970.0000000	3.0743999	-28.4420795
1970.0000000	3.1231999	-23.9289875
1970.0000000	3.1719999	-28.8230476
1970.0000000	3.2208002	-481.7956238
1970.0000000	3.2695999	-942.3970947
1970.0000000	3.3183999	-20.5084057
1970.0000000	3.3671999	-3257.8037109

1970.0000000	3.4159999	1167.6958008
1970.0000000	3.4648001	-7.6827946
1970.0000000	3.5135999	-26.7530174
1970.0000000	3.5623999	-309.6375427
1970.0000000	3.6111999	-1941.1340332
1970.0000000	3.6600001	-440.0958252
1970.0000000	3.7088001	-580.8295288
1970.0000000	3.7575998	-908.8970337
1970.0000000	3.8063998	-1648.6411133
1970.0000000	3.8552001	-2393.2158203
1970.0000000	3.9040000	-2346.3972168
1970.0000000	3.9528000	1071.8582764
1970.0000000	4.0015998	-235.1384583
1970.0000000	4.0503998	655.8713989
1970.0000000	4.0991998	82.8267746
1970.0000000	4.1479998	-626.1962280
1970.0000000	4.1967998	1.5748882
1970.0000000	4.2456002	-238.1941833
1970.0000000	4.2943997	-466.9511108
1970.0000000	4.3431997	-13.5602131
1970.0000000	4.3920002	-237.8444061
1970.0000000	4.4407997	-22.2742290
1970.0000000	4.4896002	-15.5668726
1970.0000000	4.5383997	-627.6627197
1970.0000000	4.5871997	-13.0503407
1970.0000000	4.6360002	-21.4591045
1970.0000000	4.6847997	-27.8700943
1970.0000000	4.7336001	-19.4865570
1970.0000000	4.7824001	-16.5601044
1970.0000000	4.8311996	-1418.5087891
1970.0000000	4.8800001	218.8400269
1970.0000000	4.9287996	-194.3789368
1970.0000000	4.9776001	1018.2966919
1970.0000000	5.0264001	82.2291336
1970.0000000	5.0751996	-290.2391663
1970.0000000	5.1240001	-12.4052486
1970.0000000	5.1727996	546.2476196
1970.0000000	5.2216001	31.0518188
1970.0000000	5.2704000	-156.0058441
1970.0000000	5.3191996	-38.7277985
1970.0000000	5.3680000	-3466.6892090
1970.0000000	5.4168000	516.4646606
1970.0000000	5.4656000	-197.1263123
1970.0000000	5.5144000	37.0032883
1970.0000000	5.5631995	478.4868774
1970.0000000	5.6120000	385.1596375
1970.0000000	5.6608000	-36.6767845
1970.0000000	5.7096000	60.9906769
1970.0000000	5.7584000	130.1535645
1970.0000000	5.8071995	-11.6071348
1970.0000000	5.8559999	28.9347591
1970.0000000	5.9047999	-16.5859184
1970.0000000	5.9535999	-39.3042450
1970.0000000	6.0023999	-1542.6738281
1970.0000000	6.0511999	-14.5892162
1970.0000000	6.0999999	-19.3266048
1970.0000000	6.1487999	-13.3192844
1970.0000000	6.1975999	-23.0450878

1970.0000000	6.2463999	-1757.4040527
1970.0000000	6.2951999	-18.9896069
1970.0000000	6.3439999	-336.3671570
1970.0000000	6.3927999	-15.0463037
1970.0000000	6.4416003	-12.4439497
1970.0000000	6.4903998	-11.3303823
1970.0000000	6.5391998	-11.8733368
1970.0000000	6.5879998	317.0923462
1970.0000000	6.6367998	-12.1381235
1970.0000000	6.6856003	-13.8316212
1970.0000000	6.7343998	-13.4767990
1970.0000000	6.7831998	-14.3319044
1970.0000000	6.8319998	56.5730057
1970.0000000	6.8807998	-14.0358934
1970.0000000	6.9296002	-375.3699341
1970.0000000	6.9783998	-6.3312516
1970.0000000	7.0271997	-14.0375576
1970.0000000	7.0760002	7.0992346
1970.0000000	7.1247997	-1749.8955078
1970.0000000	7.1736002	-1403.4279785
1970.0000000	7.2223997	-799.0166016
1970.0000000	7.2711997	-174.1051331
1970.0000000	7.3200002	-17.7260017
1970.0000000	7.3687997	151.7928467
1970.0000000	7.4176002	319.0916138
1970.0000000	7.4663997	250.3138275
1970.0000000	7.5151997	-781.0580444
1970.0000000	7.5640001	-862.5319214
1970.0000000	7.6127996	-2030.4100342
1970.0000000	7.6616001	-652.9398804
1970.0000000	7.7104001	-336.2855225
1970.0000000	7.7591996	33.5161896
1970.0000000	7.8080001	331.6123962
1970.0000000	7.8567996	437.4591675
1970.0000000	7.9056001	-912.1779785
1970.0000000	7.9544001	1450.8251953
1970.0000000	8.0031996	1872.1804199
1970.0000000	8.0520000	1868.5886230
1970.0000000	8.1007996	1977.3876953
1970.0000000	8.1496000	2108.9458008
1970.0000000	8.1983995	1311.9342041
1970.0000000	8.2472000	-3002.8393555
1970.0000000	8.2959995	-1064.6873779
1970.0000000	8.3448000	-847.9807739
1970.0000000	8.3935995	-777.1904297
1970.0000000	8.4423990	-335.0959167
1970.0000000	8.4912004	-781.6814575
1970.0000000	8.5400000	-2107.8344727
1970.0000000	8.5887995	-1022.2004395
1970.0000000	8.6375999	198.9235687
1970.0000000	8.6863995	1801.7462158
1970.0000000	8.7351999	-670.2663574
1970.0000000	8.7840004	764.5067749
1970.0000000	8.8327999	39.8831291
1970.0000000	8.8815994	-11.5130711
1970.0000000	8.9303989	-23.5902996
1970.0000000	8.9792004	-11.5869551
1970.0000000	9.0279999	-6.0054350

1970.0000000	9.0767994	6.4946079
1970.0000000	9.1255999	-17.0788345
1970.0000000	9.1743994	-10.1118526
1970.0000000	9.2231998	35.5147705
1970.0000000	9.2720003	-8.5091524
1970.0000000	9.3207998	-9.8493757
1970.0000000	9.3695993	-10.7852917
1970.0000000	9.4183998	-11.7420444
1970.0000000	9.4672003	-11.1652441
1970.0000000	9.5159998	-14.0795612
1970.0000000	9.5648003	-0.7134809
1970.0000000	9.6135998	-13.3208933
1970.0000000	9.6623993	-10.2474632
1970.0000000	9.7111998	-10.1835051
1970.0000000	9.7600002	-10.8615379
1970.0000000	9.8087997	-10.9955339
1970.0000000	9.8575993	2.7697773
1970.0000000	9.9063997	-8.0558825
1970.0000000	9.9552002	-10.9070110
1970.0000000	10.0039997	-10.0982361
1980.0000000	0.3416000	-37.2674789
1980.0000000	0.3904000	-38.9979362
1980.0000000	0.4392000	-39.2203484
1980.0000000	0.4880000	-39.4751816
1980.0000000	0.5368000	-39.7264328
1980.0000000	0.5856000	-39.2000313
1980.0000000	0.6344000	-38.2552490
1980.0000000	0.6832000	-36.3795395
1980.0000000	0.7320000	-36.1986732
1980.0000000	0.7808000	-36.5466576
1980.0000000	0.8296000	-35.9614410
1980.0000000	0.8784000	-35.3601532
1980.0000000	0.9272000	-33.3076630
1980.0000000	0.9760000	-31.1087112
1980.0000000	1.0247999	-29.9425755
1980.0000000	1.0735999	-29.5764141
1980.0000000	1.1224000	-29.9760418
1980.0000000	1.1711999	-30.7976475
1980.0000000	1.2200000	-31.2223644
1980.0000000	1.2687999	-31.0673618
1980.0000000	1.3176000	-30.6848984
1980.0000000	1.3664000	-30.7076569
1980.0000000	1.4152000	-30.1819401
1980.0000000	1.4640000	-29.4512081
1980.0000000	1.5128000	-30.0461636
1980.0000000	1.5616000	-29.6135292
1980.0000000	1.6104001	-1313.1080322
1980.0000000	1.6592000	-45.0407295
1980.0000000	1.7079999	-27.6172657
1980.0000000	1.7567999	-27.6382103
1980.0000000	1.8055999	-26.2251396
1980.0000000	1.8544000	-26.1103344
1980.0000000	1.9031999	-26.4806347
1980.0000000	1.9520000	-25.5803299
1980.0000000	2.0007999	-24.6551838
1980.0000000	2.0495999	-24.7608471
1980.0000000	2.0983999	-25.7726517
1980.0000000	2.1471999	-23.0970879

1980.0000000	2.1960001	-23.9367924
1980.0000000	2.2448001	-24.2807217
1980.0000000	2.2935998	-24.7985020
1980.0000000	2.3423998	-22.3726921
1980.0000000	2.3912001	-24.6719532
1980.0000000	2.4400001	-23.0761662
1980.0000000	2.4888000	-23.4392338
1980.0000000	2.5375998	-13.9087791
1980.0000000	2.5863998	-12.2835445
1980.0000000	2.6352000	-10.6413870
1980.0000000	2.6840000	-16.5529404
1980.0000000	2.7328000	107.7679672
1980.0000000	2.7815998	127.8747177
1980.0000000	2.8304000	-23.6699295
1980.0000000	2.8792000	-20.9307194
1980.0000000	2.9280000	-22.5174904
1980.0000000	2.9768000	-17.5878925
1980.0000000	3.0256000	-20.7193813
1980.0000000	3.0743999	-28.4306927
1980.0000000	3.1231999	-23.9255333
1980.0000000	3.1719999	-28.8147545
1980.0000000	3.2208002	-481.5578918
1980.0000000	3.2695999	-941.9221191
1980.0000000	3.3183999	-20.5086040
1980.0000000	3.3671999	-3256.3420410
1980.0000000	3.4159999	1167.1605225
1980.0000000	3.4648001	-7.6885033
1980.0000000	3.5135999	-26.7500267
1980.0000000	3.5623999	-309.5079346
1980.0000000	3.6111999	-1940.2742920
1980.0000000	3.6600001	-439.9077148
1980.0000000	3.7088001	-580.5781250
1980.0000000	3.7575998	-908.4984741
1980.0000000	3.8063998	-1647.9112549
1980.0000000	3.8552001	-2392.1523438
1980.0000000	3.9040000	-2345.3547363
1980.0000000	3.9528000	1071.3699951
1980.0000000	4.0015998	-235.0413513
1980.0000000	4.0503998	655.5688477
1980.0000000	4.0991998	82.7810898
1980.0000000	4.1479998	-625.9240112
1980.0000000	4.1967998	1.5656319
1980.0000000	4.2456002	-238.0958710
1980.0000000	4.2943997	-466.7505188
1980.0000000	4.3431997	-13.5623951
1980.0000000	4.3920002	-237.7462921
1980.0000000	4.4407997	-22.2724533
1980.0000000	4.4896002	-15.5677547
1980.0000000	4.5383997	-627.4016113
1980.0000000	4.5871997	-13.0523567
1980.0000000	4.6360002	-21.4575615
1980.0000000	4.6847997	-27.8661690
1980.0000000	4.7336001	-19.4858341
1980.0000000	4.7824001	-16.5605583
1980.0000000	4.8311996	-1417.9775391
1980.0000000	4.8800001	218.7504730
1980.0000000	4.9287996	-194.3116760
1980.0000000	4.9776001	1017.9042358

1980.0000000	5.0264001	82.1916122
1980.0000000	5.0751996	-290.1355286
1980.0000000	5.1240001	-12.4061699
1980.0000000	5.1727996	546.1317749
1980.0000000	5.2216001	31.0423737
1980.0000000	5.2704000	-155.9777374
1980.0000000	5.3191996	-38.7232513
1980.0000000	5.3680000	-3465.9968262
1980.0000000	5.4168000	516.3578491
1980.0000000	5.4656000	-197.0901337
1980.0000000	5.5144000	36.9926300
1980.0000000	5.5631995	478.3883057
1980.0000000	5.6120000	385.0796509
1980.0000000	5.6608000	-36.6726990
1980.0000000	5.7096000	60.9752426
1980.0000000	5.7584000	130.1246338
1980.0000000	5.8071995	-11.6080055
1980.0000000	5.8559999	28.9259090
1980.0000000	5.9047999	-16.5857201
1980.0000000	5.9535999	-39.2995071
1980.0000000	6.0023999	-1542.3693848
1980.0000000	6.0511999	-14.5894279
1980.0000000	6.0999999	-19.3257732
1980.0000000	6.1487999	-13.3197479
1980.0000000	6.1975999	-23.0433216
1980.0000000	6.2463999	-1757.0567627
1980.0000000	6.2951999	-18.9887924
1980.0000000	6.3439999	-336.3031311
1980.0000000	6.3927999	-15.0462856
1980.0000000	6.4416003	-12.4445372
1980.0000000	6.4903998	-11.3310585
1980.0000000	6.5391998	-11.8740177
1980.0000000	6.5879998	316.4016724
1980.0000000	6.6367998	-12.1386728
1980.0000000	6.6856003	-13.8317909
1980.0000000	6.7343998	-13.4770145
1980.0000000	6.7831998	-14.3314886
1980.0000000	6.8319998	56.5538940
1980.0000000	6.8807998	-14.0357542
1980.0000000	6.9296002	-374.6084900
1980.0000000	6.9783998	-6.3440752
1980.0000000	7.0271997	-14.0365295
1980.0000000	7.0760002	7.0570498
1980.0000000	7.1247997	-1745.9497070
1980.0000000	7.1736002	-1400.2651367
1980.0000000	7.2223997	-797.2234497
1980.0000000	7.2711997	-173.7373505
1980.0000000	7.3200002	-17.7140141
1980.0000000	7.3687997	151.4183197
1980.0000000	7.4176002	318.3357849
1980.0000000	7.4663997	249.7161713
1980.0000000	7.5151997	-779.2969971
1980.0000000	7.5640001	-860.5845337
1980.0000000	7.6127996	-2025.7923584
1980.0000000	7.6616001	-651.4685669
1980.0000000	7.7104001	-335.5392456
1980.0000000	7.7591996	33.4151421
1980.0000000	7.8080001	330.8270569

1980.0000000	7.8567996	436.4310303
1980.0000000	7.9056001	-910.1051025
1980.0000000	7.9544001	1447.5152588
1980.0000000	8.0031996	1867.9108887
1980.0000000	8.0520000	1864.3266602
1980.0000000	8.1007996	1972.8781738
1980.0000000	8.1496000	2104.1376953
1980.0000000	8.1983995	1308.9417725
1980.0000000	8.2472000	-2996.0043945
1980.0000000	8.2959995	-1062.2672119
1980.0000000	8.3448000	-846.0515747
1980.0000000	8.3935995	-775.4227295
1980.0000000	8.4423990	-334.3379211
1980.0000000	8.4912004	-779.9052124
1980.0000000	8.5400000	-2103.0346680
1980.0000000	8.5887995	-1019.8789063
1980.0000000	8.6375999	198.4622498
1980.0000000	8.6863995	1797.6279297
1980.0000000	8.7351999	-668.7420654
1980.0000000	8.7840004	762.7500000
1980.0000000	8.8327999	39.7715225
1980.0000000	8.8815994	-11.5132456
1980.0000000	8.9303989	-23.5865784
1980.0000000	8.9792004	-11.5870371
1980.0000000	9.0279999	-6.0072026
1980.0000000	9.0767994	6.4888897
1980.0000000	9.1255999	-17.0771217
1980.0000000	9.1743994	-10.1122551
1980.0000000	9.2231998	35.4990311
1980.0000000	9.2720003	-8.5100880
1980.0000000	9.3207998	-9.8499155
1980.0000000	9.3695993	-10.7855186
1980.0000000	9.4183998	-11.7419205
1980.0000000	9.4672003	-11.1652851
1980.0000000	9.5159998	-14.0786705
1980.0000000	9.5648003	-0.7168541
1980.0000000	9.6135998	-13.3201027
1980.0000000	9.6623993	-10.2477379
1980.0000000	9.7111998	-10.1837769
1980.0000000	9.7600002	-10.8615789
1980.0000000	9.8087997	-10.9955425
1980.0000000	9.8575993	2.7653542
1980.0000000	9.9063997	-8.0567961
1980.0000000	9.9552002	-10.9070158
1980.0000000	10.0039997	-10.0984840
1990.0000000	0.3416000	-37.0981178
1990.0000000	0.3904000	-38.9920197
1990.0000000	0.4392000	-39.2213097
1990.0000000	0.4880000	-39.4739723
1990.0000000	0.5368000	-39.7276039
1990.0000000	0.5856000	-39.1986504
1990.0000000	0.6344000	-38.2555580
1990.0000000	0.6832000	-36.3817673
1990.0000000	0.7320000	-36.2007256
1990.0000000	0.7808000	-36.5468254
1990.0000000	0.8296000	-35.9575348
1990.0000000	0.8784000	-35.3570213
1990.0000000	0.9272000	-33.2920837

1990.0000000	0.9760000	-31.1038933
1990.0000000	1.0247999	-29.9480839
1990.0000000	1.0735999	-29.5616913
1990.0000000	1.1224000	-29.9577656
1990.0000000	1.1711999	-30.7907944
1990.0000000	1.2200000	-31.2241383
1990.0000000	1.2687999	-31.0695305
1990.0000000	1.3176000	-30.6812172
1990.0000000	1.3664000	-30.7072334
1990.0000000	1.4152000	-30.1821442
1990.0000000	1.4640000	-29.4494991
1990.0000000	1.5128000	-29.9489861
1990.0000000	1.5616000	-29.5716896
1990.0000000	1.6104001	-1310.5653076
1990.0000000	1.6592000	-44.7467957
1990.0000000	1.7079999	-27.6210136
1990.0000000	1.7567999	-27.6275425
1990.0000000	1.8055999	-26.2394352
1990.0000000	1.8544000	-26.1078167
1990.0000000	1.9031999	-26.4916744
1990.0000000	1.9520000	-25.6055031
1990.0000000	2.0007999	-24.6845570
1990.0000000	2.0495999	-24.7642517
1990.0000000	2.0983999	-25.7851086
1990.0000000	2.1471999	-23.1837482
1990.0000000	2.1960001	-23.9404488
1990.0000000	2.2448001	-24.3202915
1990.0000000	2.2935998	-24.7963905
1990.0000000	2.3423998	-22.3870964
1990.0000000	2.3912001	-24.6703167
1990.0000000	2.4400001	-23.0786533
1990.0000000	2.4888000	-23.4390182
1990.0000000	2.5375998	-13.9258871
1990.0000000	2.5863998	-12.3033037
1990.0000000	2.6352000	-10.6641312
1990.0000000	2.6840000	-16.5648823
1990.0000000	2.7328000	107.5019455
1990.0000000	2.7815998	127.5683060
1990.0000000	2.8304000	-23.6686249
1990.0000000	2.8792000	-20.9339828
1990.0000000	2.9280000	-22.5169201
1990.0000000	2.9768000	-17.5962639
1990.0000000	3.0256000	-20.7219658
1990.0000000	3.0743999	-28.4193058
1990.0000000	3.1231999	-23.9220772
1990.0000000	3.1719999	-28.8064613
1990.0000000	3.2208002	-481.3201599
1990.0000000	3.2695999	-941.4471436
1990.0000000	3.3183999	-20.5088005
1990.0000000	3.3671999	-3254.8803711
1990.0000000	3.4159999	1166.6252441
1990.0000000	3.4648001	-7.6942120
1990.0000000	3.5135999	-26.7470360
1990.0000000	3.5623999	-309.3783569
1990.0000000	3.6111999	-1939.4145508
1990.0000000	3.6600001	-439.7196045
1990.0000000	3.7088001	-580.3267822
1990.0000000	3.7575998	-908.0999146

1990.0000000	3.8063998	-1647.1813965
1990.0000000	3.8552001	-2391.0891113
1990.0000000	3.9040000	-2344.3122559
1990.0000000	3.9528000	1070.8815918
1990.0000000	4.0015998	-234.9442444
1990.0000000	4.0503998	655.2662964
1990.0000000	4.0991998	82.7354050
1990.0000000	4.1479998	-625.6517334
1990.0000000	4.1967998	1.5563755
1990.0000000	4.2456002	-237.9975739
1990.0000000	4.2943997	-466.5499573
1990.0000000	4.3431997	-13.5645752
1990.0000000	4.3920002	-237.6481781
1990.0000000	4.4407997	-22.2706776
1990.0000000	4.4896002	-15.5686378
1990.0000000	4.5383997	-627.1405640
1990.0000000	4.5871997	-13.0543728
1990.0000000	4.6360002	-21.4560204
1990.0000000	4.6847997	-27.8622437
1990.0000000	4.7336001	-19.4851112
1990.0000000	4.7824001	-16.5610123
1990.0000000	4.8311996	-1417.4462891
1990.0000000	4.8800001	218.6609192
1990.0000000	4.9287996	-194.2444000
1990.0000000	4.9776001	1017.5116577
1990.0000000	5.0264001	82.1540985
1990.0000000	5.0751996	-290.0319214
1990.0000000	5.1240001	-12.4070911
1990.0000000	5.1727996	546.0159302
1990.0000000	5.2216001	31.0329285
1990.0000000	5.2704000	-155.9496307
1990.0000000	5.3191996	-38.7187042
1990.0000000	5.3680000	-3465.3044434
1990.0000000	5.4168000	516.2510986
1990.0000000	5.4656000	-197.0539551
1990.0000000	5.5144000	36.9819717
1990.0000000	5.5631995	478.2897339
1990.0000000	5.6120000	384.9996643
1990.0000000	5.6608000	-36.6686096
1990.0000000	5.7096000	60.9598160
1990.0000000	5.7584000	130.0956879
1990.0000000	5.8071995	-11.6088753
1990.0000000	5.8559999	28.9170551
1990.0000000	5.9047999	-16.5855217
1990.0000000	5.9535999	-39.2947693
1990.0000000	6.0023999	-1542.0649414
1990.0000000	6.0511999	-14.5896387
1990.0000000	6.0999999	-19.3249397
1990.0000000	6.1487999	-13.3202105
1990.0000000	6.1975999	-23.0415573
1990.0000000	6.2463999	-1756.7093506
1990.0000000	6.2951999	-18.9879780
1990.0000000	6.3439999	-336.2390747
1990.0000000	6.3927999	-15.0462675
1990.0000000	6.4416003	-12.4451237
1990.0000000	6.4903998	-11.3317337
1990.0000000	6.5391998	-11.8746996
1990.0000000	6.5879998	315.7109680

1990.0000000	6.6367998	-12.1392221
1990.0000000	6.6856003	-13.8319616
1990.0000000	6.7343998	-13.4772301
1990.0000000	6.7831998	-14.3310728
1990.0000000	6.8319998	56.5347824
1990.0000000	6.8807998	-14.0356159
1990.0000000	6.9296002	-373.8470459
1990.0000000	6.9783998	-6.3568988
1990.0000000	7.0271997	-14.0355015
1990.0000000	7.0760002	7.0148630
1990.0000000	7.1247997	-1742.0037842
1990.0000000	7.1736002	-1397.1022949
1990.0000000	7.2223997	-795.4302979
1990.0000000	7.2711997	-173.3695679
1990.0000000	7.3200002	-17.7020264
1990.0000000	7.3687997	151.0437927
1990.0000000	7.4176002	317.5799561
1990.0000000	7.4663997	249.1185150
1990.0000000	7.5151997	-777.5359497
1990.0000000	7.5640001	-858.6371460
1990.0000000	7.6127996	-2021.1748047
1990.0000000	7.6616001	-649.9971924
1990.0000000	7.7104001	-334.7929688
1990.0000000	7.7591996	33.3140907
1990.0000000	7.8080001	330.0416870
1990.0000000	7.8567996	435.4029541
1990.0000000	7.9056001	-908.0321655
1990.0000000	7.9544001	1444.2053223
1990.0000000	8.0031996	1863.6412354
1990.0000000	8.0520000	1860.0645752
1990.0000000	8.1007996	1968.3686523
1990.0000000	8.1496000	2099.3298340
1990.0000000	8.1983995	1305.9492188
1990.0000000	8.2472000	-2989.1696777
1990.0000000	8.2959995	-1059.8470459
1990.0000000	8.3448000	-844.1223755
1990.0000000	8.3935995	-773.6550903
1990.0000000	8.4423990	-333.5798950
1990.0000000	8.4912004	-778.1290283
1990.0000000	8.5400000	-2098.2348633
1990.0000000	8.5887995	-1017.5573730
1990.0000000	8.6375999	198.0009308
1990.0000000	8.6863995	1793.5095215
1990.0000000	8.7351999	-667.2178345
1990.0000000	8.7840004	760.9932861
1990.0000000	8.8327999	39.6599159
1990.0000000	8.8815994	-11.5134201
1990.0000000	8.9303989	-23.5828590
1990.0000000	8.9792004	-11.5871191
1990.0000000	9.0279999	-6.0089712
1990.0000000	9.0767994	6.4831715
1990.0000000	9.1255999	-17.0754070
1990.0000000	9.1743994	-10.1126575
1990.0000000	9.2231998	35.4832993
1990.0000000	9.2720003	-8.5110245
1990.0000000	9.3207998	-9.8504543
1990.0000000	9.3695993	-10.7857447
1990.0000000	9.4183998	-11.7417955

1990.0000000	9.4672003	-11.1653271
1990.0000000	9.5159998	-14.0777788
1990.0000000	9.5648003	-0.7202263
1990.0000000	9.6135998	-13.3193121
1990.0000000	9.6623993	-10.2480125
1990.0000000	9.7111998	-10.1840487
1990.0000000	9.7600002	-10.8616209
1990.0000000	9.8087997	-10.9955502
1990.0000000	9.8575993	2.7609320
1990.0000000	9.9063997	-8.0577106
1990.0000000	9.9552002	-10.9070196
1990.0000000	10.0039997	-10.0987310
2000.0000000	0.3416000	-37.1380730
2000.0000000	0.3904000	-38.9861031
2000.0000000	0.4392000	-39.2222672
2000.0000000	0.4880000	-39.4727631
2000.0000000	0.5368000	-39.7287788
2000.0000000	0.5856000	-39.1972656
2000.0000000	0.6344000	-38.2558670
2000.0000000	0.6832000	-36.3839912
2000.0000000	0.7320000	-36.2027779
2000.0000000	0.7808000	-36.5469971
2000.0000000	0.8296000	-35.9536285
2000.0000000	0.8784000	-35.3538895
2000.0000000	0.9272000	-33.2765045
2000.0000000	0.9760000	-31.0990772
2000.0000000	1.0247999	-29.9535923
2000.0000000	1.0735999	-29.5469685
2000.0000000	1.1224000	-29.9394894
2000.0000000	1.1711999	-30.7839432
2000.0000000	1.2200000	-31.2259140
2000.0000000	1.2687999	-31.0716991
2000.0000000	1.3176000	-30.6775360
2000.0000000	1.3664000	-30.7068100
2000.0000000	1.4152000	-30.1823483
2000.0000000	1.4640000	-29.4477901
2000.0000000	1.5128000	-29.8518066
2000.0000000	1.5616000	-29.5298481
2000.0000000	1.6104001	-1308.0224609
2000.0000000	1.6592000	-44.4528656
2000.0000000	1.7079999	-27.6247616
2000.0000000	1.7567999	-27.6168766
2000.0000000	1.8055999	-26.2537308
2000.0000000	1.8544000	-26.1053009
2000.0000000	1.9031999	-26.5027142
2000.0000000	1.9520000	-25.6306763
2000.0000000	2.0007999	-24.7139282
2000.0000000	2.0495999	-24.7676563
2000.0000000	2.0983999	-25.7975655
2000.0000000	2.1471999	-23.2704086
2000.0000000	2.1960001	-23.9441032
2000.0000000	2.2448001	-24.3598614
2000.0000000	2.2935998	-24.7942772
2000.0000000	2.3423998	-22.4015007
2000.0000000	2.3912001	-24.6686802
2000.0000000	2.4400001	-23.0811386
2000.0000000	2.4888000	-23.4388008
2000.0000000	2.5375998	-13.9429951

2000.0000000	2.5863998	-12.3230619
2000.0000000	2.6352000	-10.6868753
2000.0000000	2.6840000	-16.5768242
2000.0000000	2.7328000	107.2359390
2000.0000000	2.7815998	127.2618790
2000.0000000	2.8304000	-23.6673203
2000.0000000	2.8792000	-20.9372463
2000.0000000	2.9280000	-22.5163517
2000.0000000	2.9768000	-17.6046333
2000.0000000	3.0256000	-20.7245502
2000.0000000	3.0743999	-28.4079189
2000.0000000	3.1231999	-23.9186211
2000.0000000	3.1719999	-28.7981682
2000.0000000	3.2208002	-481.0824280
2000.0000000	3.2695999	-940.9721680
2000.0000000	3.3183999	-20.5089989
2000.0000000	3.3671999	-3253.4187012
2000.0000000	3.4159999	1166.0899658
2000.0000000	3.4648001	-7.6999197
2000.0000000	3.5135999	-26.7440453
2000.0000000	3.5623999	-309.2487488
2000.0000000	3.6111999	-1938.5548096
2000.0000000	3.6600001	-439.5314331
2000.0000000	3.7088001	-580.0753784
2000.0000000	3.7575998	-907.7014160
2000.0000000	3.8063998	-1646.4515381
2000.0000000	3.8552001	-2390.0256348
2000.0000000	3.9040000	-2343.2697754
2000.0000000	3.9528000	1070.3933105
2000.0000000	4.0015998	-234.8471375
2000.0000000	4.0503998	654.9636841
2000.0000000	4.0991998	82.6897278
2000.0000000	4.1479998	-625.3795166
2000.0000000	4.1967998	1.5471191
2000.0000000	4.2456002	-237.8992767
2000.0000000	4.2943997	-466.3493958
2000.0000000	4.3431997	-13.5667553
2000.0000000	4.3920002	-237.5500641
2000.0000000	4.4407997	-22.2689018
2000.0000000	4.4896002	-15.5695210
2000.0000000	4.5383997	-626.8795166
2000.0000000	4.5871997	-13.0563898
2000.0000000	4.6360002	-21.4544773
2000.0000000	4.6847997	-27.8583183
2000.0000000	4.7336001	-19.4843903
2000.0000000	4.7824001	-16.5614681
2000.0000000	4.8311996	-1416.9150391
2000.0000000	4.8800001	218.5713806
2000.0000000	4.9287996	-194.1771393
2000.0000000	4.9776001	1017.1192017
2000.0000000	5.0264001	82.1165771
2000.0000000	5.0751996	-289.9282837
2000.0000000	5.1240001	-12.4080124
2000.0000000	5.1727996	545.9000244
2000.0000000	5.2216001	31.0234871
2000.0000000	5.2704000	-155.9215240
2000.0000000	5.3191996	-38.7141571
2000.0000000	5.3680000	-3464.6120605

2000.0000000	5.4168000	516.1443481
2000.0000000	5.4656000	-197.0177612
2000.0000000	5.5144000	36.9713135
2000.0000000	5.5631995	478.1911621
2000.0000000	5.6120000	384.9197083
2000.0000000	5.6608000	-36.6645203
2000.0000000	5.7096000	60.9443817
2000.0000000	5.7584000	130.0667572
2000.0000000	5.8071995	-11.6097469
2000.0000000	5.8559999	28.9082050
2000.0000000	5.9047999	-16.5853233
2000.0000000	5.9535999	-39.2900314
2000.0000000	6.0023999	-1541.7604980
2000.0000000	6.0511999	-14.5898504
2000.0000000	6.0999999	-19.3241081
2000.0000000	6.1487999	-13.3206730
2000.0000000	6.1975999	-23.0397911
2000.0000000	6.2463999	-1756.3619385
2000.0000000	6.2951999	-18.9871635
2000.0000000	6.3439999	-336.1750183
2000.0000000	6.3927999	-15.0462494
2000.0000000	6.4416003	-12.4457111
2000.0000000	6.4903998	-11.3324099
2000.0000000	6.5391998	-11.8753805
2000.0000000	6.5879998	315.0202637
2000.0000000	6.6367998	-12.1397705
2000.0000000	6.6856003	-13.8321314
2000.0000000	6.7343998	-13.4774466
2000.0000000	6.7831998	-14.3306580
2000.0000000	6.8319998	56.5156631
2000.0000000	6.8807998	-14.0354767
2000.0000000	6.9296002	-373.0855713
2000.0000000	6.9783998	-6.3697224
2000.0000000	7.0271997	-14.0344734
2000.0000000	7.0760002	6.9726782
2000.0000000	7.1247997	-1738.0579834
2000.0000000	7.1736002	-1393.9393311
2000.0000000	7.2223997	-793.6371460
2000.0000000	7.2711997	-173.0018005
2000.0000000	7.3200002	-17.6900368
2000.0000000	7.3687997	150.6692505
2000.0000000	7.4176002	316.8241272
2000.0000000	7.4663997	248.5208588
2000.0000000	7.5151997	-775.7749023
2000.0000000	7.5640001	-856.6897583
2000.0000000	7.6127996	-2016.5571289
2000.0000000	7.6616001	-648.5258789
2000.0000000	7.7104001	-334.0466919
2000.0000000	7.7591996	33.2130432
2000.0000000	7.8080001	329.2563171
2000.0000000	7.8567996	434.3748779
2000.0000000	7.9056001	-905.9592896
2000.0000000	7.9544001	1440.8953857
2000.0000000	8.0031996	1859.3717041
2000.0000000	8.0520000	1855.8026123
2000.0000000	8.1007996	1963.8592529
2000.0000000	8.1496000	2094.5217285
2000.0000000	8.1983995	1302.9566650

2000.0000000	8.2472000	-2982.3349609
2000.0000000	8.2959995	-1057.4268799
2000.0000000	8.3448000	-842.1932373
2000.0000000	8.3935995	-771.8873901
2000.0000000	8.4423990	-332.8218994
2000.0000000	8.4912004	-776.3527832
2000.0000000	8.5400000	-2093.4350586
2000.0000000	8.5887995	-1015.2358398
2000.0000000	8.6375999	197.5395966
2000.0000000	8.6863995	1789.3912354
2000.0000000	8.7351999	-665.6935425
2000.0000000	8.7840004	759.2365112
2000.0000000	8.8327999	39.5483093
2000.0000000	8.8815994	-11.5135946
2000.0000000	8.9303989	-23.5791397
2000.0000000	8.9792004	-11.5872021
2000.0000000	9.0279999	-6.0107393
2000.0000000	9.0767994	6.4774551
2000.0000000	9.1255999	-17.0736923
2000.0000000	9.1743994	-10.1130590
2000.0000000	9.2231998	35.4675674
2000.0000000	9.2720003	-8.5119610
2000.0000000	9.3207998	-9.8509941
2000.0000000	9.3695993	-10.7859716
2000.0000000	9.4183998	-11.7416716
2000.0000000	9.4672003	-11.1653681
2000.0000000	9.5159998	-14.0768881
2000.0000000	9.5648003	-0.7235985
2000.0000000	9.6135998	-13.3185215
2000.0000000	9.6623993	-10.2482882
2000.0000000	9.7111998	-10.1843204
2000.0000000	9.7600002	-10.8616629
2000.0000000	9.8087997	-10.9955578
2000.0000000	9.8575993	2.7565098
2000.0000000	9.9063997	-8.0586243
2000.0000000	9.9552002	-10.9070244
2000.0000000	10.0039997	-10.0989790
2010.0000000	0.3416000	-37.1826859
2010.0000000	0.3904000	-38.9801865
2010.0000000	0.4392000	-39.2232285
2010.0000000	0.4880000	-39.4715538
2010.0000000	0.5368000	-39.7299538
2010.0000000	0.5856000	-39.1958847
2010.0000000	0.6344000	-38.2561798
2010.0000000	0.6832000	-36.3862190
2010.0000000	0.7320000	-36.2048302
2010.0000000	0.7808000	-36.5471649
2010.0000000	0.8296000	-35.9497223
2010.0000000	0.8784000	-35.3507576
2010.0000000	0.9272000	-33.2609253
2010.0000000	0.9760000	-31.0942593
2010.0000000	1.0247999	-29.9591007
2010.0000000	1.0735999	-29.5322456
2010.0000000	1.1224000	-29.9212132
2010.0000000	1.1711999	-30.7770901
2010.0000000	1.2200000	-31.2276897
2010.0000000	1.2687999	-31.0738678
2010.0000000	1.3176000	-30.6738529

2010.0000000	1.3664000	-30.7063866
2010.0000000	1.4152000	-30.1825523
2010.0000000	1.4640000	-29.4460793
2010.0000000	1.5128000	-29.7546291
2010.0000000	1.5616000	-29.4880066
2010.0000000	1.6104001	-1305.4796143
2010.0000000	1.6592000	-44.1589355
2010.0000000	1.7079999	-27.6285076
2010.0000000	1.7567999	-27.6062088
2010.0000000	1.8055999	-26.2680264
2010.0000000	1.8544000	-26.1027851
2010.0000000	1.9031999	-26.5137539
2010.0000000	1.9520000	-25.6558495
2010.0000000	2.0007999	-24.7433014
2010.0000000	2.0495999	-24.7710609
2010.0000000	2.0983999	-25.8100224
2010.0000000	2.1471999	-23.3570690
2010.0000000	2.1960001	-23.9477577
2010.0000000	2.2448001	-24.3994312
2010.0000000	2.2935998	-24.7921658
2010.0000000	2.3423998	-22.4159050
2010.0000000	2.3912001	-24.6670437
2010.0000000	2.4400001	-23.0836258
2010.0000000	2.4888000	-23.4385834
2010.0000000	2.5375998	-13.9601030
2010.0000000	2.5863998	-12.3428202
2010.0000000	2.6352000	-10.7096195
2010.0000000	2.6840000	-16.5887661
2010.0000000	2.7328000	106.9699173
2010.0000000	2.7815998	126.9554672
2010.0000000	2.8304000	-23.6660175
2010.0000000	2.8792000	-20.9405098
2010.0000000	2.9280000	-22.5157833
2010.0000000	2.9768000	-17.6130028
2010.0000000	3.0256000	-20.7271347
2010.0000000	3.0743999	-28.3965321
2010.0000000	3.1231999	-23.9151669
2010.0000000	3.1719999	-28.7898750
2010.0000000	3.2208002	-480.8446960
2010.0000000	3.2695999	-940.4971924
2010.0000000	3.3183999	-20.5091972
2010.0000000	3.3671999	-3251.9570313
2010.0000000	3.4159999	1165.5546875
2010.0000000	3.4648001	-7.7056274
2010.0000000	3.5135999	-26.7410545
2010.0000000	3.5623999	-309.1191711
2010.0000000	3.6111999	-1937.6949463
2010.0000000	3.6600001	-439.3433228
2010.0000000	3.7088001	-579.8239746
2010.0000000	3.7575998	-907.3028564
2010.0000000	3.8063998	-1645.7218018
2010.0000000	3.8552001	-2388.9621582
2010.0000000	3.9040000	-2342.2272949
2010.0000000	3.9528000	1069.9050293
2010.0000000	4.0015998	-234.7500305
2010.0000000	4.0503998	654.6611328
2010.0000000	4.0991998	82.6440430
2010.0000000	4.1479998	-625.1072998

2010.0000000	4.1967998	1.5378628
2010.0000000	4.2456002	-237.8009644
2010.0000000	4.2943997	-466.1488342
2010.0000000	4.3431997	-13.5689354
2010.0000000	4.3920002	-237.4519501
2010.0000000	4.4407997	-22.2671261
2010.0000000	4.4896002	-15.5704031
2010.0000000	4.5383997	-626.6184082
2010.0000000	4.5871997	-13.0584059
2010.0000000	4.6360002	-21.4529343
2010.0000000	4.6847997	-27.8543930
2010.0000000	4.7336001	-19.4836674
2010.0000000	4.7824001	-16.5619221
2010.0000000	4.8311996	-1416.3837891
2010.0000000	4.8800001	218.4818268
2010.0000000	4.9287996	-194.1098785
2010.0000000	4.9776001	1016.7267456
2010.0000000	5.0264001	82.0790558
2010.0000000	5.0751996	-289.8246460
2010.0000000	5.1240001	-12.4089336
2010.0000000	5.1727996	545.7841797
2010.0000000	5.2216001	31.0140419
2010.0000000	5.2704000	-155.8934174
2010.0000000	5.3191996	-38.7096024
2010.0000000	5.3680000	-3463.9196777
2010.0000000	5.4168000	516.0375366
2010.0000000	5.4656000	-196.9815826
2010.0000000	5.5144000	36.9606552
2010.0000000	5.5631995	478.0925903
2010.0000000	5.6120000	384.8397217
2010.0000000	5.6608000	-36.6604309
2010.0000000	5.7096000	60.9289474
2010.0000000	5.7584000	130.0378265
2010.0000000	5.8071995	-11.6106167
2010.0000000	5.8559999	28.8993549
2010.0000000	5.9047999	-16.5851269
2010.0000000	5.9535999	-39.2852936
2010.0000000	6.0023999	-1541.4561768
2010.0000000	6.0511999	-14.5900612
2010.0000000	6.0999999	-19.3232765
2010.0000000	6.1487999	-13.3211365
2010.0000000	6.1975999	-23.0380268
2010.0000000	6.2463999	-1756.0146484
2010.0000000	6.2951999	-18.9863510
2010.0000000	6.3439999	-336.1109619
2010.0000000	6.3927999	-15.0462303
2010.0000000	6.4416003	-12.4462976
2010.0000000	6.4903998	-11.3330860
2010.0000000	6.5391998	-11.8760614
2010.0000000	6.5879998	314.3295898
2010.0000000	6.6367998	-12.1403198
2010.0000000	6.6856003	-13.8323021
2010.0000000	6.7343998	-13.4776621
2010.0000000	6.7831998	-14.3302422
2010.0000000	6.8319998	56.4965439
2010.0000000	6.8807998	-14.0353384
2010.0000000	6.9296002	-372.3241272
2010.0000000	6.9783998	-6.3825459

2010.0000000	7.0271997	-14.0334463
2010.0000000	7.0760002	6.9304934
2010.0000000	7.1247997	-1734.1120605
2010.0000000	7.1736002	-1390.7764893
2010.0000000	7.2223997	-791.8439331
2010.0000000	7.2711997	-172.6340179
2010.0000000	7.3200002	-17.6780472
2010.0000000	7.3687997	150.2947235
2010.0000000	7.4176002	316.0682983
2010.0000000	7.4663997	247.9232025
2010.0000000	7.5151997	-774.0138550
2010.0000000	7.5640001	-854.7423096
2010.0000000	7.6127996	-2011.9395752
2010.0000000	7.6616001	-647.0545044
2010.0000000	7.7104001	-333.3004761
2010.0000000	7.7591996	33.1119957
2010.0000000	7.8080001	328.4709473
2010.0000000	7.8567996	433.3467712
2010.0000000	7.9056001	-903.8863525
2010.0000000	7.9544001	1437.5854492
2010.0000000	8.0031996	1855.1020508
2010.0000000	8.0520000	1851.5406494
2010.0000000	8.1007996	1959.3497314
2010.0000000	8.1496000	2089.7138672
2010.0000000	8.1983995	1299.9642334
2010.0000000	8.2472000	-2975.5000000
2010.0000000	8.2959995	-1055.0067139
2010.0000000	8.3448000	-840.2640381
2010.0000000	8.3935995	-770.1197510
2010.0000000	8.4423990	-332.0638733
2010.0000000	8.4912004	-774.5765991
2010.0000000	8.5400000	-2088.6352539
2010.0000000	8.5887995	-1012.9143066
2010.0000000	8.6375999	197.0782776
2010.0000000	8.6863995	1785.2728271
2010.0000000	8.7351999	-664.1693115
2010.0000000	8.7840004	757.4797974
2010.0000000	8.8327999	39.4366989
2010.0000000	8.8815994	-11.5137682
2010.0000000	8.9303989	-23.5754204
2010.0000000	8.9792004	-11.5872841
2010.0000000	9.0279999	-6.0125079
2010.0000000	9.0767994	6.4717369
2010.0000000	9.1255999	-17.0719795
2010.0000000	9.1743994	-10.1134615
2010.0000000	9.2231998	35.4518356
2010.0000000	9.2720003	-8.5128975
2010.0000000	9.3207998	-9.8515329
2010.0000000	9.3695993	-10.7861977
2010.0000000	9.4183998	-11.7415466
2010.0000000	9.4672003	-11.1654091
2010.0000000	9.5159998	-14.0759964
2010.0000000	9.5648003	-0.7269716
2010.0000000	9.6135998	-13.3177299
2010.0000000	9.6623993	-10.2485628
2010.0000000	9.7111998	-10.1845913
2010.0000000	9.7600002	-10.8617048
2010.0000000	9.8087997	-10.9955664

2010.0000000	9.8575993	2.7520885
2010.0000000	9.9063997	-8.0595379
2010.0000000	9.9552002	-10.9070282
2010.0000000	10.0039997	-10.0992260
2020.0000000	0.3416000	-37.0426521
2020.0000000	0.3904000	-38.9742737
2020.0000000	0.4392000	-39.2241898
2020.0000000	0.4880000	-39.4703445
2020.0000000	0.5368000	-39.7311249
2020.0000000	0.5856000	-39.1945038
2020.0000000	0.6344000	-38.2564888
2020.0000000	0.6832000	-36.3884430
2020.0000000	0.7320000	-36.2068825
2020.0000000	0.7808000	-36.5473366
2020.0000000	0.8296000	-35.9458160
2020.0000000	0.8784000	-35.3476257
2020.0000000	0.9272000	-33.2453461
2020.0000000	0.9760000	-31.0894432
2020.0000000	1.0247999	-29.9646091
2020.0000000	1.0735999	-29.5175228
2020.0000000	1.1224000	-29.9029350
2020.0000000	1.1711999	-30.7702389
2020.0000000	1.2200000	-31.2294655
2020.0000000	1.2687999	-31.0760345
2020.0000000	1.3176000	-30.6701717
2020.0000000	1.3664000	-30.7059631
2020.0000000	1.4152000	-30.1827564
2020.0000000	1.4640000	-29.4443703
2020.0000000	1.5128000	-29.6574516
2020.0000000	1.5616000	-29.4461651
2020.0000000	1.6104001	-1302.9368896
2020.0000000	1.6592000	-43.8650017
2020.0000000	1.7079999	-27.6322556
2020.0000000	1.7567999	-27.5955410
2020.0000000	1.8055999	-26.2823238
2020.0000000	1.8544000	-26.1002693
2020.0000000	1.9031999	-26.5247936
2020.0000000	1.9520000	-25.6810226
2020.0000000	2.0007999	-24.7726727
2020.0000000	2.0495999	-24.7744656
2020.0000000	2.0983999	-25.8224792
2020.0000000	2.1471999	-23.4437294
2020.0000000	2.1960001	-23.9514122
2020.0000000	2.2448001	-24.4389992
2020.0000000	2.2935998	-24.7900543
2020.0000000	2.3423998	-22.4303112
2020.0000000	2.3912001	-24.6654072
2020.0000000	2.4400001	-23.0861130
2020.0000000	2.4888000	-23.4383659
2020.0000000	2.5375998	-13.9772110
2020.0000000	2.5863998	-12.3625793
2020.0000000	2.6352000	-10.7323637
2020.0000000	2.6840000	-16.6007080
2020.0000000	2.7328000	106.7038956
2020.0000000	2.7815998	126.6490555
2020.0000000	2.8304000	-23.6647129
2020.0000000	2.8792000	-20.9437733
2020.0000000	2.9280000	-22.5152130

2020.0000000	2.9768000	-17.6213741
2020.0000000	3.0256000	-20.7297192
2020.0000000	3.0743999	-28.3851452
2020.0000000	3.1231999	-23.9117107
2020.0000000	3.1719999	-28.7815819
2020.0000000	3.2208002	-480.6069641
2020.0000000	3.2695999	-940.0222168
2020.0000000	3.3183999	-20.5093956
2020.0000000	3.3671999	-3250.4953613
2020.0000000	3.4159999	1165.0194092
2020.0000000	3.4648001	-7.7113361
2020.0000000	3.5135999	-26.7380638
2020.0000000	3.5623999	-308.9895935
2020.0000000	3.6111999	-1936.8352051
2020.0000000	3.6600001	-439.1552124
2020.0000000	3.7088001	-579.5726318
2020.0000000	3.7575998	-906.9043579
2020.0000000	3.8063998	-1644.9919434
2020.0000000	3.8552001	-2387.8986816
2020.0000000	3.9040000	-2341.1848145
2020.0000000	3.9528000	1069.4167480
2020.0000000	4.0015998	-234.6529083
2020.0000000	4.0503998	654.3585815
2020.0000000	4.0991998	82.5983582
2020.0000000	4.1479998	-624.8350830
2020.0000000	4.1967998	1.5286064
2020.0000000	4.2456002	-237.7026672
2020.0000000	4.2943997	-465.9482727
2020.0000000	4.3431997	-13.5711174
2020.0000000	4.3920002	-237.3538361
2020.0000000	4.4407997	-22.2653503
2020.0000000	4.4896002	-15.5712852
2020.0000000	4.5383997	-626.3573608
2020.0000000	4.5871997	-13.0604229
2020.0000000	4.6360002	-21.4513931
2020.0000000	4.6847997	-27.8504677
2020.0000000	4.7336001	-19.4829445
2020.0000000	4.7824001	-16.5623760
2020.0000000	4.8311996	-1415.8525391
2020.0000000	4.8800001	218.3922729
2020.0000000	4.9287996	-194.0426178
2020.0000000	4.9776001	1016.3341675
2020.0000000	5.0264001	82.0415344
2020.0000000	5.0751996	-289.7210388
2020.0000000	5.1240001	-12.4098549
2020.0000000	5.1727996	545.6683350
2020.0000000	5.2216001	31.0045967
2020.0000000	5.2704000	-155.8653107
2020.0000000	5.3191996	-38.7050552
2020.0000000	5.3680000	-3463.2272949
2020.0000000	5.4168000	515.9307251
2020.0000000	5.4656000	-196.9454041
2020.0000000	5.5144000	36.9500046
2020.0000000	5.5631995	477.9940186
2020.0000000	5.6120000	384.7597351
2020.0000000	5.6608000	-36.6563454
2020.0000000	5.7096000	60.9135208
2020.0000000	5.7584000	130.0088806

2020.0000000	5.8071995	-11.6114883
2020.0000000	5.8559999	28.8905048
2020.0000000	5.9047999	-16.5849285
2020.0000000	5.9535999	-39.2805519
2020.0000000	6.0023999	-1541.1516113
2020.0000000	6.0511999	-14.5902729
2020.0000000	6.0999999	-19.3224430
2020.0000000	6.1487999	-13.3215990
2020.0000000	6.1975999	-23.0362606
2020.0000000	6.2463999	-1755.6672363
2020.0000000	6.2951999	-18.9855347
2020.0000000	6.3439999	-336.0469360
2020.0000000	6.3927999	-15.0462122
2020.0000000	6.4416003	-12.4468842
2020.0000000	6.4903998	-11.3337622
2020.0000000	6.5391998	-11.8767424
2020.0000000	6.5879998	313.6388855
2020.0000000	6.6367998	-12.1408691
2020.0000000	6.6856003	-13.8324718
2020.0000000	6.7343998	-13.4778776
2020.0000000	6.7831998	-14.3298264
2020.0000000	6.8319998	56.4774323
2020.0000000	6.8807998	-14.0352001
2020.0000000	6.9296002	-371.5626831
2020.0000000	6.9783998	-6.3953700
2020.0000000	7.0271997	-14.0324183
2020.0000000	7.0760002	6.8883066
2020.0000000	7.1247997	-1730.1661377
2020.0000000	7.1736002	-1387.6136475
2020.0000000	7.2223997	-790.0507813
2020.0000000	7.2711997	-172.2662354
2020.0000000	7.3200002	-17.6660595
2020.0000000	7.3687997	149.9201965
2020.0000000	7.4176002	315.3124695
2020.0000000	7.4663997	247.3255463
2020.0000000	7.5151997	-772.2528076
2020.0000000	7.5640001	-852.7949219
2020.0000000	7.6127996	-2007.3218994
2020.0000000	7.6616001	-645.5831909
2020.0000000	7.7104001	-332.5541992
2020.0000000	7.7591996	33.0109444
2020.0000000	7.8080001	327.6855774
2020.0000000	7.8567996	432.3186951
2020.0000000	7.9056001	-901.8134766
2020.0000000	7.9544001	1434.2755127
2020.0000000	8.0031996	1850.8325195
2020.0000000	8.0520000	1847.2785645
2020.0000000	8.1007996	1954.8402100
2020.0000000	8.1496000	2084.9057617
2020.0000000	8.1983995	1296.9716797
2020.0000000	8.2472000	-2968.6652832
2020.0000000	8.2959995	-1052.5865479
2020.0000000	8.3448000	-838.3348389
2020.0000000	8.3935995	-768.3520508
2020.0000000	8.4423990	-331.3058777
2020.0000000	8.4912004	-772.8003540
2020.0000000	8.5400000	-2083.8354492
2020.0000000	8.5887995	-1010.5927734

2020.0000000	8.6375999	196.6169586
2020.0000000	8.6863995	1781.1544189
2020.0000000	8.7351999	-662.6450806
2020.0000000	8.7840004	755.7230225
2020.0000000	8.8327999	39.3250961
2020.0000000	8.8815994	-11.5139427
2020.0000000	8.9303989	-23.5717010
2020.0000000	8.9792004	-11.5873661
2020.0000000	9.0279999	-6.0142760
2020.0000000	9.0767994	6.4660187
2020.0000000	9.1255999	-17.0702667
2020.0000000	9.1743994	-10.1138630
2020.0000000	9.2231998	35.4361038
2020.0000000	9.2720003	-8.5138340
2020.0000000	9.3207998	-9.8520727
2020.0000000	9.3695993	-10.7864246
2020.0000000	9.4183998	-11.7414217
2020.0000000	9.4672003	-11.1654501
2020.0000000	9.5159998	-14.0751057
2020.0000000	9.5648003	-0.7303448
2020.0000000	9.6135998	-13.3169394
2020.0000000	9.6623993	-10.2488384
2020.0000000	9.7111998	-10.1848631
2020.0000000	9.7600002	-10.8617458
2020.0000000	9.8087997	-10.9955740
2020.0000000	9.8575993	2.7476664
2020.0000000	9.9063997	-8.0604515
2020.0000000	9.9552002	-10.9070330
2020.0000000	10.0039997	-10.0994740
2030.0000000	0.3416000	-36.9444199
2030.0000000	0.3904000	-38.9683571
2030.0000000	0.4392000	-39.2251472
2030.0000000	0.4880000	-39.4691353
2030.0000000	0.5368000	-39.7322998
2030.0000000	0.5856000	-39.1931229
2030.0000000	0.6344000	-38.2567978
2030.0000000	0.6832000	-36.3906670
2030.0000000	0.7320000	-36.2089386
2030.0000000	0.7808000	-36.5475044
2030.0000000	0.8296000	-35.9419098
2030.0000000	0.8784000	-35.3444939
2030.0000000	0.9272000	-33.2297668
2030.0000000	0.9760000	-31.0846252
2030.0000000	1.0247999	-29.9701176
2030.0000000	1.0735999	-29.5028000
2030.0000000	1.1224000	-29.8846588
2030.0000000	1.1711999	-30.7633858
2030.0000000	1.2200000	-31.2312393
2030.0000000	1.2687999	-31.0782032
2030.0000000	1.3176000	-30.6664906
2030.0000000	1.3664000	-30.7055416
2030.0000000	1.4152000	-30.1829605
2030.0000000	1.4640000	-29.4426613
2030.0000000	1.5128000	-29.5602741
2030.0000000	1.5616000	-29.4043236
2030.0000000	1.6104001	-1300.3940430
2030.0000000	1.6592000	-43.5710678
2030.0000000	1.7079999	-27.6360016

2030.0000000	1.7567999	-27.5848751
2030.0000000	1.8055999	-26.2966194
2030.0000000	1.8544000	-26.0977516
2030.0000000	1.9031999	-26.5358334
2030.0000000	1.9520000	-25.7061958
2030.0000000	2.0007999	-24.8020458
2030.0000000	2.0495999	-24.7778702
2030.0000000	2.0983999	-25.8349361
2030.0000000	2.1471999	-23.5303898
2030.0000000	2.1960001	-23.9550667
2030.0000000	2.2448001	-24.4785690
2030.0000000	2.2935998	-24.7879410
2030.0000000	2.3423998	-22.4447155
2030.0000000	2.3912001	-24.6637707
2030.0000000	2.4400001	-23.0885983
2030.0000000	2.4888000	-23.4381485
2030.0000000	2.5375998	-13.9943190
2030.0000000	2.5863998	-12.3823385
2030.0000000	2.6352000	-10.7551079
2030.0000000	2.6840000	-16.6126499
2030.0000000	2.7328000	106.4378891
2030.0000000	2.7815998	126.3426285
2030.0000000	2.8304000	-23.6634083
2030.0000000	2.8792000	-20.9470367
2030.0000000	2.9280000	-22.5146446
2030.0000000	2.9768000	-17.6297455
2030.0000000	3.0256000	-20.7323036
2030.0000000	3.0743999	-28.3737583
2030.0000000	3.1231999	-23.9082565
2030.0000000	3.1719999	-28.7732887
2030.0000000	3.2208002	-480.3692322
2030.0000000	3.2695999	-939.5472412
2030.0000000	3.3183999	-20.5095940
2030.0000000	3.3671999	-3249.0336914
2030.0000000	3.4159999	1164.4841309
2030.0000000	3.4648001	-7.7170439
2030.0000000	3.5135999	-26.7350731
2030.0000000	3.5623999	-308.8599854
2030.0000000	3.6111999	-1935.9754639
2030.0000000	3.6600001	-438.9670410
2030.0000000	3.7088001	-579.3212280
2030.0000000	3.7575998	-906.5057983
2030.0000000	3.8063998	-1644.2620850
2030.0000000	3.8552001	-2386.8352051
2030.0000000	3.9040000	-2340.1423340
2030.0000000	3.9528000	1068.9284668
2030.0000000	4.0015998	-234.5558014
2030.0000000	4.0503998	654.0560303
2030.0000000	4.0991998	82.5526733
2030.0000000	4.1479998	-624.5628662
2030.0000000	4.1967998	1.5193501
2030.0000000	4.2456002	-237.6043701
2030.0000000	4.2943997	-465.7476501
2030.0000000	4.3431997	-13.5732975
2030.0000000	4.3920002	-237.2557220
2030.0000000	4.4407997	-22.2635746
2030.0000000	4.4896002	-15.5721684
2030.0000000	4.5383997	-626.0963135

2030.0000000	4.5871997	-13.0624380
2030.0000000	4.6360002	-21.4498501
2030.0000000	4.6847997	-27.8465424
2030.0000000	4.7336001	-19.4822235
2030.0000000	4.7824001	-16.5628319
2030.0000000	4.8311996	-1415.3211670
2030.0000000	4.8800001	218.3027191
2030.0000000	4.9287996	-193.9753571
2030.0000000	4.9776001	1015.9417114
2030.0000000	5.0264001	82.0040131
2030.0000000	5.0751996	-289.6174011
2030.0000000	5.1240001	-12.4107761
2030.0000000	5.1727996	545.5524292
2030.0000000	5.2216001	30.9951553
2030.0000000	5.2704000	-155.8372040
2030.0000000	5.3191996	-38.7005081
2030.0000000	5.3680000	-3462.5349121
2030.0000000	5.4168000	515.8239746
2030.0000000	5.4656000	-196.9092102
2030.0000000	5.5144000	36.9393463
2030.0000000	5.5631995	477.8954468
2030.0000000	5.6120000	384.6797791
2030.0000000	5.6608000	-36.6522560
2030.0000000	5.7096000	60.8980942
2030.0000000	5.7584000	129.9799500
2030.0000000	5.8071995	-11.6123581
2030.0000000	5.8559999	28.8816547
2030.0000000	5.9047999	-16.5847301
2030.0000000	5.9535999	-39.2758141
2030.0000000	6.0023999	-1540.8472900
2030.0000000	6.0511999	-14.5904846
2030.0000000	6.0999999	-19.3216114
2030.0000000	6.1487999	-13.3220615
2030.0000000	6.1975999	-23.0344963
2030.0000000	6.2463999	-1755.3198242
2030.0000000	6.2951999	-18.9847221
2030.0000000	6.3439999	-335.9828491
2030.0000000	6.3927999	-15.0461941
2030.0000000	6.4416003	-12.4474716
2030.0000000	6.4903998	-11.3344374
2030.0000000	6.5391998	-11.8774233
2030.0000000	6.5879998	312.9481812
2030.0000000	6.6367998	-12.1414185
2030.0000000	6.6856003	-13.8326426
2030.0000000	6.7343998	-13.4780931
2030.0000000	6.7831998	-14.3294106
2030.0000000	6.8319998	56.4583206
2030.0000000	6.8807998	-14.0350609
2030.0000000	6.9296002	-370.8012390
2030.0000000	6.9783998	-6.4081931
2030.0000000	7.0271997	-14.0313902
2030.0000000	7.0760002	6.8461218
2030.0000000	7.1247997	-1726.2203369
2030.0000000	7.1736002	-1384.4506836
2030.0000000	7.2223997	-788.2576294
2030.0000000	7.2711997	-171.8984528
2030.0000000	7.3200002	-17.6540718
2030.0000000	7.3687997	149.5456696

2030.0000000	7.4176002	314.5566406
2030.0000000	7.4663997	246.7279205
2030.0000000	7.5151997	-770.4918213
2030.0000000	7.5640001	-850.8475342
2030.0000000	7.6127996	-2002.7043457
2030.0000000	7.6616001	-644.1118164
2030.0000000	7.7104001	-331.8079224
2030.0000000	7.7591996	32.9098969
2030.0000000	7.8080001	326.9002380
2030.0000000	7.8567996	431.2906189
2030.0000000	7.9056001	-899.7405396
2030.0000000	7.9544001	1430.9655762
2030.0000000	8.0031996	1846.5628662
2030.0000000	8.0520000	1843.0166016
2030.0000000	8.1007996	1950.3306885
2030.0000000	8.1496000	2080.0979004
2030.0000000	8.1983995	1293.9791260
2030.0000000	8.2472000	-2961.8305664
2030.0000000	8.2959995	-1050.1663818
2030.0000000	8.3448000	-836.4057007
2030.0000000	8.3935995	-766.5844116
2030.0000000	8.4423990	-330.5478516
2030.0000000	8.4912004	-771.0241699
2030.0000000	8.5400000	-2079.0356445
2030.0000000	8.5887995	-1008.2712402
2030.0000000	8.6375999	196.1556396
2030.0000000	8.6863995	1777.0361328
2030.0000000	8.7351999	-661.1207886
2030.0000000	8.7840004	753.9663086
2030.0000000	8.8327999	39.2134857
2030.0000000	8.8815994	-11.5141172
2030.0000000	8.9303989	-23.5679798
2030.0000000	8.9792004	-11.5874481
2030.0000000	9.0279999	-6.0160441
2030.0000000	9.0767994	6.4603004
2030.0000000	9.1255999	-17.0685520
2030.0000000	9.1743994	-10.1142654
2030.0000000	9.2231998	35.4203720
2030.0000000	9.2720003	-8.5147705
2030.0000000	9.3207998	-9.8526115
2030.0000000	9.3695993	-10.7866507
2030.0000000	9.4183998	-11.7412977
2030.0000000	9.4672003	-11.1654911
2030.0000000	9.5159998	-14.0742149
2030.0000000	9.5648003	-0.7337170
2030.0000000	9.6135998	-13.3161488
2030.0000000	9.6623993	-10.2491131
2030.0000000	9.7111998	-10.1851349
2030.0000000	9.7600002	-10.8617878
2030.0000000	9.8087997	-10.9955816
2030.0000000	9.8575993	2.7432423
2030.0000000	9.9063997	-8.0613651
2030.0000000	9.9552002	-10.9070368
2030.0000000	10.0039997	-10.0997210
2040.0000000	0.3416000	-37.1301880
2040.0000000	0.3904000	-38.9624405
2040.0000000	0.4392000	-39.2261086
2040.0000000	0.4880000	-39.4679260

2040.0000000	0.5368000	-39.7334747
2040.0000000	0.5856000	-39.1917419
2040.0000000	0.6344000	-38.2571106
2040.0000000	0.6832000	-36.3928947
2040.0000000	0.7320000	-36.2109909
2040.0000000	0.7808000	-36.5476723
2040.0000000	0.8296000	-35.9379997
2040.0000000	0.8784000	-35.3413620
2040.0000000	0.9272000	-33.2141876
2040.0000000	0.9760000	-31.0798092
2040.0000000	1.0247999	-29.9756279
2040.0000000	1.0735999	-29.4880753
2040.0000000	1.1224000	-29.8663826
2040.0000000	1.1711999	-30.7565346
2040.0000000	1.2200000	-31.2330151
2040.0000000	1.2687999	-31.0803719
2040.0000000	1.3176000	-30.6628075
2040.0000000	1.3664000	-30.7051182
2040.0000000	1.4152000	-30.1831665
2040.0000000	1.4640000	-29.4409523
2040.0000000	1.5128000	-29.4630966
2040.0000000	1.5616000	-29.3624840
2040.0000000	1.6104001	-1297.8511963
2040.0000000	1.6592000	-43.2771378
2040.0000000	1.7079999	-27.6397495
2040.0000000	1.7567999	-27.5742073
2040.0000000	1.8055999	-26.3109150
2040.0000000	1.8544000	-26.0952358
2040.0000000	1.9031999	-26.5468731
2040.0000000	1.9520000	-25.7313690
2040.0000000	2.0007999	-24.8314171
2040.0000000	2.0495999	-24.7812729
2040.0000000	2.0983999	-25.8473930
2040.0000000	2.1471999	-23.6170502
2040.0000000	2.1960001	-23.9587212
2040.0000000	2.2448001	-24.5181389
2040.0000000	2.2935998	-24.7858295
2040.0000000	2.3423998	-22.4591198
2040.0000000	2.3912001	-24.6621342
2040.0000000	2.4400001	-23.0910854
2040.0000000	2.4888000	-23.4379311
2040.0000000	2.5375998	-14.0114260
2040.0000000	2.5863998	-12.4020977
2040.0000000	2.6352000	-10.7778521
2040.0000000	2.6840000	-16.6245918
2040.0000000	2.7328000	106.1718674
2040.0000000	2.7815998	126.0362167
2040.0000000	2.8304000	-23.6621037
2040.0000000	2.8792000	-20.9503002
2040.0000000	2.9280000	-22.5140762
2040.0000000	2.9768000	-17.6381149
2040.0000000	3.0256000	-20.7348881
2040.0000000	3.0743999	-28.3623714
2040.0000000	3.1231999	-23.9048004
2040.0000000	3.1719999	-28.7649956
2040.0000000	3.2208002	-480.1314697
2040.0000000	3.2695999	-939.0723877
2040.0000000	3.3183999	-20.5097904

2040.0000000	3.3671999	-3247.5720215
2040.0000000	3.4159999	1163.9488525
2040.0000000	3.4648001	-7.7227516
2040.0000000	3.5135999	-26.7320824
2040.0000000	3.5623999	-308.7304077
2040.0000000	3.6111999	-1935.1157227
2040.0000000	3.6600001	-438.7789307
2040.0000000	3.7088001	-579.0698853
2040.0000000	3.7575998	-906.1072388
2040.0000000	3.8063998	-1643.5322266
2040.0000000	3.8552001	-2385.7719727
2040.0000000	3.9040000	-2339.0998535
2040.0000000	3.9528000	1068.4401855
2040.0000000	4.0015998	-234.4586945
2040.0000000	4.0503998	653.7534790
2040.0000000	4.0991998	82.5069885
2040.0000000	4.1479998	-624.2906494
2040.0000000	4.1967998	1.5100937
2040.0000000	4.2456002	-237.5060577
2040.0000000	4.2943997	-465.5470886
2040.0000000	4.3431997	-13.5754776
2040.0000000	4.3920002	-237.1576080
2040.0000000	4.4407997	-22.2617989
2040.0000000	4.4896002	-15.5730515
2040.0000000	4.5383997	-625.8352051
2040.0000000	4.5871997	-13.0644550
2040.0000000	4.6360002	-21.4483070
2040.0000000	4.6847997	-27.8426170
2040.0000000	4.7336001	-19.4815006
2040.0000000	4.7824001	-16.5632858
2040.0000000	4.8311996	-1414.7899170
2040.0000000	4.8800001	218.2131653
2040.0000000	4.9287996	-193.9080963
2040.0000000	4.9776001	1015.5492554
2040.0000000	5.0264001	81.9664917
2040.0000000	5.0751996	-289.5137634
2040.0000000	5.1240001	-12.4116974
2040.0000000	5.1727996	545.4365845
2040.0000000	5.2216001	30.9857063
2040.0000000	5.2704000	-155.8090973
2040.0000000	5.3191996	-38.6959572
2040.0000000	5.3680000	-3461.8425293
2040.0000000	5.4168000	515.7172241
2040.0000000	5.4656000	-196.8730316
2040.0000000	5.5144000	36.9286957
2040.0000000	5.5631995	477.7968750
2040.0000000	5.6120000	384.5997925
2040.0000000	5.6608000	-36.6481705
2040.0000000	5.7096000	60.8826599
2040.0000000	5.7584000	129.9510193
2040.0000000	5.8071995	-11.6132298
2040.0000000	5.8559999	28.8728008
2040.0000000	5.9047999	-16.5845337
2040.0000000	5.9535999	-39.2710762
2040.0000000	6.0023999	-1540.5428467
2040.0000000	6.0511999	-14.5906954
2040.0000000	6.0999999	-19.3207779
2040.0000000	6.1487999	-13.3225241

2040.0000000	6.1975999	-23.0327301
2040.0000000	6.2463999	-1754.9725342
2040.0000000	6.2951999	-18.9839077
2040.0000000	6.3439999	-335.9188232
2040.0000000	6.3927999	-15.0461760
2040.0000000	6.4416003	-12.4480581
2040.0000000	6.4903998	-11.3351135
2040.0000000	6.5391998	-11.8781052
2040.0000000	6.5879998	312.2574768
2040.0000000	6.6367998	-12.1419678
2040.0000000	6.6856003	-13.8328123
2040.0000000	6.7343998	-13.4783096
2040.0000000	6.7831998	-14.3289948
2040.0000000	6.8319998	56.4392014
2040.0000000	6.8807998	-14.0349226
2040.0000000	6.9296002	-370.0397949
2040.0000000	6.9783998	-6.4210167
2040.0000000	7.0271997	-14.0303621
2040.0000000	7.0760002	6.8039370
2040.0000000	7.1247997	-1722.2744141
2040.0000000	7.1736002	-1381.2878418
2040.0000000	7.2223997	-786.4644775
2040.0000000	7.2711997	-171.5306702
2040.0000000	7.3200002	-17.6420822
2040.0000000	7.3687997	149.1711426
2040.0000000	7.4176002	313.8008118
2040.0000000	7.4663997	246.1302643
2040.0000000	7.5151997	-768.7307739
2040.0000000	7.5640001	-848.9001465
2040.0000000	7.6127996	-1998.0867920
2040.0000000	7.6616001	-642.6405029
2040.0000000	7.7104001	-331.0616455
2040.0000000	7.7591996	32.8088493
2040.0000000	7.8080001	326.1148682
2040.0000000	7.8567996	430.2625122
2040.0000000	7.9056001	-897.6676636
2040.0000000	7.9544001	1427.6556396
2040.0000000	8.0031996	1842.2932129
2040.0000000	8.0520000	1838.7546387
2040.0000000	8.1007996	1945.8211670
2040.0000000	8.1496000	2075.2897949
2040.0000000	8.1983995	1290.9866943
2040.0000000	8.2472000	-2954.9956055
2040.0000000	8.2959995	-1047.7460938
2040.0000000	8.3448000	-834.4765015
2040.0000000	8.3935995	-764.8167114
2040.0000000	8.4423990	-329.7898560
2040.0000000	8.4912004	-769.2479248
2040.0000000	8.5400000	-2074.2355957
2040.0000000	8.5887995	-1005.9497070
2040.0000000	8.6375999	195.6943207
2040.0000000	8.6863995	1772.9177246
2040.0000000	8.7351999	-659.5965576
2040.0000000	8.7840004	752.2095337
2040.0000000	8.8327999	39.1018753
2040.0000000	8.8815994	-11.5142918
2040.0000000	8.9303989	-23.5642586
2040.0000000	8.9792004	-11.5875311

2040.0000000	9.0279999	-6.0178127
2040.0000000	9.0767994	6.4545841
2040.0000000	9.1255999	-17.0668373
2040.0000000	9.1743994	-10.1146679
2040.0000000	9.2231998	35.4046402
2040.0000000	9.2720003	-8.5157061
2040.0000000	9.3207998	-9.8531513
2040.0000000	9.3695993	-10.7868767
2040.0000000	9.4183998	-11.7411728
2040.0000000	9.4672003	-11.1655331
2040.0000000	9.5159998	-14.0733242
2040.0000000	9.5648003	-0.7370892
2040.0000000	9.6135998	-13.3153582
2040.0000000	9.6623993	-10.2493877
2040.0000000	9.7111998	-10.1854067
2040.0000000	9.7600002	-10.8618298
2040.0000000	9.8087997	-10.9955902
2040.0000000	9.8575993	2.7388210
2040.0000000	9.9063997	-8.0622797
2040.0000000	9.9552002	-10.9070415
2040.0000000	10.0039997	-10.0999689
2050.0000000	0.3416000	-36.8344765
2050.0000000	0.3904000	-38.9565277
2050.0000000	0.4392000	-39.2270660
2050.0000000	0.4880000	-39.4667206
2050.0000000	0.5368000	-39.7346458
2050.0000000	0.5856000	-39.1903610
2050.0000000	0.6344000	-38.2574196
2050.0000000	0.6832000	-36.3951187
2050.0000000	0.7320000	-36.2130432
2050.0000000	0.7808000	-36.5478439
2050.0000000	0.8296000	-35.9340935
2050.0000000	0.8784000	-35.3382339
2050.0000000	0.9272000	-33.1986046
2050.0000000	0.9760000	-31.0749931
2050.0000000	1.0247999	-29.9811363
2050.0000000	1.0735999	-29.4733524
2050.0000000	1.1224000	-29.8481064
2050.0000000	1.1711999	-30.7496834
2050.0000000	1.2200000	-31.2347908
2050.0000000	1.2687999	-31.0825405
2050.0000000	1.3176000	-30.6591263
2050.0000000	1.3664000	-30.7046947
2050.0000000	1.4152000	-30.1833706
2050.0000000	1.4640000	-29.4392414
2050.0000000	1.5128000	-29.3659191
2050.0000000	1.5616000	-29.3206425
2050.0000000	1.6104001	-1295.3084717
2050.0000000	1.6592000	-42.9832077
2050.0000000	1.7079999	-27.6434975
2050.0000000	1.7567999	-27.5635414
2050.0000000	1.8055999	-26.3252106
2050.0000000	1.8544000	-26.0927200
2050.0000000	1.9031999	-26.5579128
2050.0000000	1.9520000	-25.7565422
2050.0000000	2.0007999	-24.8607903
2050.0000000	2.0495999	-24.7846775
2050.0000000	2.0983999	-25.8598499

2050.0000000	2.1471999	-23.7037106
2050.0000000	2.1960001	-23.9623775
2050.0000000	2.2448001	-24.5577087
2050.0000000	2.2935998	-24.7837181
2050.0000000	2.3423998	-22.4735241
2050.0000000	2.3912001	-24.6604996
2050.0000000	2.4400001	-23.0935726
2050.0000000	2.4888000	-23.4377136
2050.0000000	2.5375998	-14.0285339
2050.0000000	2.5863998	-12.4218569
2050.0000000	2.6352000	-10.8005962
2050.0000000	2.6840000	-16.6365337
2050.0000000	2.7328000	105.9058456
2050.0000000	2.7815998	125.7298050
2050.0000000	2.8304000	-23.6608009
2050.0000000	2.8792000	-20.9535637
2050.0000000	2.9280000	-22.5135059
2050.0000000	2.9768000	-17.6464863
2050.0000000	3.0256000	-20.7374725
2050.0000000	3.0743999	-28.3509846
2050.0000000	3.1231999	-23.9013443
2050.0000000	3.1719999	-28.7567024
2050.0000000	3.2208002	-479.8937378
2050.0000000	3.2695999	-938.5972900
2050.0000000	3.3183999	-20.5099888
2050.0000000	3.3671999	-3246.1103516
2050.0000000	3.4159999	1163.4135742
2050.0000000	3.4648001	-7.7284603
2050.0000000	3.5135999	-26.7290916
2050.0000000	3.5623999	-308.6007996
2050.0000000	3.6111999	-1934.2559814
2050.0000000	3.6600001	-438.5908203
2050.0000000	3.7088001	-578.8184814
2050.0000000	3.7575998	-905.7087402
2050.0000000	3.8063998	-1642.8023682
2050.0000000	3.8552001	-2384.7084961
2050.0000000	3.9040000	-2338.0573730
2050.0000000	3.9528000	1067.9517822
2050.0000000	4.0015998	-234.3615875
2050.0000000	4.0503998	653.4509277
2050.0000000	4.0991998	82.4613037
2050.0000000	4.1479998	-624.0184326
2050.0000000	4.1967998	1.5008373
2050.0000000	4.2456002	-237.4077606
2050.0000000	4.2943997	-465.3465271
2050.0000000	4.3431997	-13.5776587
2050.0000000	4.3920002	-237.0594940
2050.0000000	4.4407997	-22.2600250
2050.0000000	4.4896002	-15.5739336
2050.0000000	4.5383997	-625.5741577
2050.0000000	4.5871997	-13.0664711
2050.0000000	4.6360002	-21.4467659
2050.0000000	4.6847997	-27.8386917
2050.0000000	4.7336001	-19.4807777
2050.0000000	4.7824001	-16.5637398
2050.0000000	4.8311996	-1414.2586670
2050.0000000	4.8800001	218.1236267
2050.0000000	4.9287996	-193.8408356

2050.0000000	4.9776001	1015.1567993
2050.0000000	5.0264001	81.9289703
2050.0000000	5.0751996	-289.4101257
2050.0000000	5.1240001	-12.4126186
2050.0000000	5.1727996	545.3206787
2050.0000000	5.2216001	30.9762650
2050.0000000	5.2704000	-155.7809906
2050.0000000	5.3191996	-38.6914101
2050.0000000	5.3680000	-3461.1501465
2050.0000000	5.4168000	515.6104126
2050.0000000	5.4656000	-196.8368530
2050.0000000	5.5144000	36.9180374
2050.0000000	5.5631995	477.6983032
2050.0000000	5.6120000	384.5198364
2050.0000000	5.6608000	-36.6440811
2050.0000000	5.7096000	60.8672256
2050.0000000	5.7584000	129.9220886
2050.0000000	5.8071995	-11.6140995
2050.0000000	5.8559999	28.8639507
2050.0000000	5.9047999	-16.5843353
2050.0000000	5.9535999	-39.2663345
2050.0000000	6.0023999	-1540.2385254
2050.0000000	6.0511999	-14.5909071
2050.0000000	6.0999999	-19.3199463
2050.0000000	6.1487999	-13.3229866
2050.0000000	6.1975999	-23.0309658
2050.0000000	6.2463999	-1754.6251221
2050.0000000	6.2951999	-18.9830933
2050.0000000	6.3439999	-335.8547668
2050.0000000	6.3927999	-15.0461569
2050.0000000	6.4416003	-12.4486456
2050.0000000	6.4903998	-11.3357897
2050.0000000	6.5391998	-11.8787861
2050.0000000	6.5879998	311.5667725
2050.0000000	6.6367998	-12.1425171
2050.0000000	6.6856003	-13.8329830
2050.0000000	6.7343998	-13.4785252
2050.0000000	6.7831998	-14.3285789
2050.0000000	6.8319998	56.4200897
2050.0000000	6.8807998	-14.0347834
2050.0000000	6.9296002	-369.2783508
2050.0000000	6.9783998	-6.4338403
2050.0000000	7.0271997	-14.0293341
2050.0000000	7.0760002	6.7617521
2050.0000000	7.1247997	-1718.3286133
2050.0000000	7.1736002	-1378.1250000
2050.0000000	7.2223997	-784.6713257
2050.0000000	7.2711997	-171.1629028
2050.0000000	7.3200002	-17.6300926
2050.0000000	7.3687997	148.7966003
2050.0000000	7.4176002	313.0449829
2050.0000000	7.4663997	245.5326080
2050.0000000	7.5151997	-766.9697266
2050.0000000	7.5640001	-846.9527588
2050.0000000	7.6127996	-1993.4691162
2050.0000000	7.6616001	-641.1691895
2050.0000000	7.7104001	-330.3153687
2050.0000000	7.7591996	32.7077980

2050.0000000	7.8080001	325.3294983
2050.0000000	7.8567996	429.2344360
2050.0000000	7.9056001	-895.5947266
2050.0000000	7.9544001	1424.3457031
2050.0000000	8.0031996	1838.0236816
2050.0000000	8.0520000	1834.4926758
2050.0000000	8.1007996	1941.3116455
2050.0000000	8.1496000	2070.4816895
2050.0000000	8.1983995	1287.9941406
2050.0000000	8.2472000	-2948.1608887
2050.0000000	8.2959995	-1045.3259277
2050.0000000	8.3448000	-832.5473022
2050.0000000	8.3935995	-763.0490112
2050.0000000	8.4423990	-329.0318298
2050.0000000	8.4912004	-767.4716797
2050.0000000	8.5400000	-2069.4357910
2050.0000000	8.5887995	-1003.6281128
2050.0000000	8.6375999	195.2330017
2050.0000000	8.6863995	1768.7994385
2050.0000000	8.7351999	-658.0722656
2050.0000000	8.7840004	750.4528198
2050.0000000	8.8327999	38.9902725
2050.0000000	8.8815994	-11.5144653
2050.0000000	8.9303989	-23.5605392
2050.0000000	8.9792004	-11.5876131
2050.0000000	9.0279999	-6.0195808
2050.0000000	9.0767994	6.4488659
2050.0000000	9.1255999	-17.0651245
2050.0000000	9.1743994	-10.1150694
2050.0000000	9.2231998	35.3889084
2050.0000000	9.2720003	-8.5166426
2050.0000000	9.3207998	-9.8536901
2050.0000000	9.3695993	-10.7871037
2050.0000000	9.4183998	-11.7410488
2050.0000000	9.4672003	-11.1655741
2050.0000000	9.5159998	-14.0724335
2050.0000000	9.5648003	-0.7404623
2050.0000000	9.6135998	-13.3145676
2050.0000000	9.6623993	-10.2496634
2050.0000000	9.7111998	-10.1856785
2050.0000000	9.7600002	-10.8618708
2050.0000000	9.8087997	-10.9955978
2050.0000000	9.8575993	2.7343988
2050.0000000	9.9063997	-8.0631933
2050.0000000	9.9552002	-10.9070454
2050.0000000	10.0039997	-10.1002169
2060.0000000	0.3416000	-36.8736572
2060.0000000	0.3904000	-38.9506111
2060.0000000	0.4392000	-39.2280273
2060.0000000	0.4880000	-39.4655113
2060.0000000	0.5368000	-39.7358208
2060.0000000	0.5856000	-39.1889801
2060.0000000	0.6344000	-38.2577324
2060.0000000	0.6832000	-36.3973465
2060.0000000	0.7320000	-36.2150955
2060.0000000	0.7808000	-36.5480118
2060.0000000	0.8296000	-35.9301872
2060.0000000	0.8784000	-35.3351021

2060.0000000	0.9272000	-33.1830254
2060.0000000	0.9760000	-31.0701752
2060.0000000	1.0247999	-29.9866447
2060.0000000	1.0735999	-29.4586296
2060.0000000	1.1224000	-29.8298302
2060.0000000	1.1711999	-30.7428303
2060.0000000	1.2200000	-31.2365646
2060.0000000	1.2687999	-31.0847092
2060.0000000	1.3176000	-30.6554451
2060.0000000	1.3664000	-30.7042713
2060.0000000	1.4152000	-30.1835747
2060.0000000	1.4640000	-29.4375324
2060.0000000	1.5128000	-29.2687416
2060.0000000	1.5616000	-29.2788010
2060.0000000	1.6104001	-1292.7656250
2060.0000000	1.6592000	-42.6892738
2060.0000000	1.7079999	-27.6472435
2060.0000000	1.7567999	-27.5528736
2060.0000000	1.8055999	-26.3395061
2060.0000000	1.8544000	-26.0902042
2060.0000000	1.9031999	-26.5689526
2060.0000000	1.9520000	-25.7817135
2060.0000000	2.0007999	-24.8901615
2060.0000000	2.0495999	-24.7880821
2060.0000000	2.0983999	-25.8723068
2060.0000000	2.1471999	-23.7903709
2060.0000000	2.1960001	-23.9660320
2060.0000000	2.2448001	-24.5972786
2060.0000000	2.2935998	-24.7816048
2060.0000000	2.3423998	-22.4879284
2060.0000000	2.3912001	-24.6588631
2060.0000000	2.4400001	-23.0960579
2060.0000000	2.4888000	-23.4374962
2060.0000000	2.5375998	-14.0456429
2060.0000000	2.5863998	-12.4416151
2060.0000000	2.6352000	-10.8233404
2060.0000000	2.6840000	-16.6484756
2060.0000000	2.7328000	105.6398392
2060.0000000	2.7815998	125.4233780
2060.0000000	2.8304000	-23.6594963
2060.0000000	2.8792000	-20.9568272
2060.0000000	2.9280000	-22.5129375
2060.0000000	2.9768000	-17.6548576
2060.0000000	3.0256000	-20.7400570
2060.0000000	3.0743999	-28.3395977
2060.0000000	3.1231999	-23.8978901
2060.0000000	3.1719999	-28.7484093
2060.0000000	3.2208002	-479.6560059
2060.0000000	3.2695999	-938.1224365
2060.0000000	3.3183999	-20.5101871
2060.0000000	3.3671999	-3244.6486816
2060.0000000	3.4159999	1162.8782959
2060.0000000	3.4648001	-7.7341690
2060.0000000	3.5135999	-26.7261009
2060.0000000	3.5623999	-308.4712219
2060.0000000	3.6111999	-1933.3962402
2060.0000000	3.6600001	-438.4026489
2060.0000000	3.7088001	-578.5670776

2060.0000000	3.7575998	-905.3101807
2060.0000000	3.8063998	-1642.0725098
2060.0000000	3.8552001	-2383.6450195
2060.0000000	3.9040000	-2337.0148926
2060.0000000	3.9528000	1067.4635010
2060.0000000	4.0015998	-234.2644806
2060.0000000	4.0503998	653.1483765
2060.0000000	4.0991998	82.4156189
2060.0000000	4.1479998	-623.7462158
2060.0000000	4.1967998	1.4915810
2060.0000000	4.2456002	-237.3094482
2060.0000000	4.2943997	-465.1459656
2060.0000000	4.3431997	-13.5798388
2060.0000000	4.3920002	-236.9613800
2060.0000000	4.4407997	-22.2582493
2060.0000000	4.4896002	-15.5748158
2060.0000000	4.5383997	-625.3131104
2060.0000000	4.5871997	-13.0684872
2060.0000000	4.6360002	-21.4452229
2060.0000000	4.6847997	-27.8347664
2060.0000000	4.7336001	-19.4800568
2060.0000000	4.7824001	-16.5641937
2060.0000000	4.8311996	-1413.7274170
2060.0000000	4.8800001	218.0340729
2060.0000000	4.9287996	-193.7735596
2060.0000000	4.9776001	1014.7642212
2060.0000000	5.0264001	81.8914566
2060.0000000	5.0751996	-289.3065186
2060.0000000	5.1240001	-12.4135399
2060.0000000	5.1727996	545.2048340
2060.0000000	5.2216001	30.9668236
2060.0000000	5.2704000	-155.7528839
2060.0000000	5.3191996	-38.6868591
2060.0000000	5.3680000	-3460.4577637
2060.0000000	5.4168000	515.5036011
2060.0000000	5.4656000	-196.8006592
2060.0000000	5.5144000	36.9073792
2060.0000000	5.5631995	477.5997314
2060.0000000	5.6120000	384.4398499
2060.0000000	5.6608000	-36.6399918
2060.0000000	5.7096000	60.8517990
2060.0000000	5.7584000	129.8931580
2060.0000000	5.8071995	-11.6149702
2060.0000000	5.8559999	28.8551006
2060.0000000	5.9047999	-16.5841370
2060.0000000	5.9535999	-39.2615967
2060.0000000	6.0023999	-1539.9339600
2060.0000000	6.0511999	-14.5911188
2060.0000000	6.0999999	-19.3191147
2060.0000000	6.1487999	-13.3234501
2060.0000000	6.1975999	-23.0292015
2060.0000000	6.2463999	-1754.2777100
2060.0000000	6.2951999	-18.9822788
2060.0000000	6.3439999	-335.7907104
2060.0000000	6.3927999	-15.0461388
2060.0000000	6.4416003	-12.4492321
2060.0000000	6.4903998	-11.3364658
2060.0000000	6.5391998	-11.8794670

2060.0000000	6.5879998	310.8760986
2060.0000000	6.6367998	-12.1430655
2060.0000000	6.6856003	-13.8331528
2060.0000000	6.7343998	-13.4787407
2060.0000000	6.7831998	-14.3281631
2060.0000000	6.8319998	56.4009781
2060.0000000	6.8807998	-14.0346451
2060.0000000	6.9296002	-368.5168762
2060.0000000	6.9783998	-6.4466643
2060.0000000	7.0271997	-14.0283070
2060.0000000	7.0760002	6.7195654
2060.0000000	7.1247997	-1714.3826904
2060.0000000	7.1736002	-1374.9621582
2060.0000000	7.2223997	-782.8781128
2060.0000000	7.2711997	-170.7951202
2060.0000000	7.3200002	-17.6181049
2060.0000000	7.3687997	148.4220734
2060.0000000	7.4176002	312.2891541
2060.0000000	7.4663997	244.9349518
2060.0000000	7.5151997	-765.2086792
2060.0000000	7.5640001	-845.0053101
2060.0000000	7.6127996	-1988.8515625
2060.0000000	7.6616001	-639.6978149
2060.0000000	7.7104001	-329.5691528
2060.0000000	7.7591996	32.6067505
2060.0000000	7.8080001	324.5441589
2060.0000000	7.8567996	428.2063599
2060.0000000	7.9056001	-893.5218506
2060.0000000	7.9544001	1421.0357666
2060.0000000	8.0031996	1833.7540283
2060.0000000	8.0520000	1830.2305908
2060.0000000	8.1007996	1936.8021240
2060.0000000	8.1496000	2065.6738281
2060.0000000	8.1983995	1285.0017090
2060.0000000	8.2472000	-2941.3259277
2060.0000000	8.2959995	-1042.9057617
2060.0000000	8.3448000	-830.6181641
2060.0000000	8.3935995	-761.2813721
2060.0000000	8.4423990	-328.2738342
2060.0000000	8.4912004	-765.6954956
2060.0000000	8.5400000	-2064.6359863
2060.0000000	8.5887995	-1001.3065796
2060.0000000	8.6375999	194.7716675
2060.0000000	8.6863995	1764.6810303
2060.0000000	8.7351999	-656.5480347
2060.0000000	8.7840004	748.6960449
2060.0000000	8.8327999	38.8786621
2060.0000000	8.8815994	-11.5146399
2060.0000000	8.9303989	-23.5568199
2060.0000000	8.9792004	-11.5876951
2060.0000000	9.0279999	-6.0213494
2060.0000000	9.0767994	6.4431477
2060.0000000	9.1255999	-17.0634098
2060.0000000	9.1743994	-10.1154718
2060.0000000	9.2231998	35.3731689
2060.0000000	9.2720003	-8.5175791
2060.0000000	9.3207998	-9.8542299
2060.0000000	9.3695993	-10.7873297

2060.0000000	9.4183998	-11.7409239
2060.0000000	9.4672003	-11.1656151
2060.0000000	9.5159998	-14.0715418
2060.0000000	9.5648003	-0.7438354
2060.0000000	9.6135998	-13.3137770
2060.0000000	9.6623993	-10.2499380
2060.0000000	9.7111998	-10.1859493
2060.0000000	9.7600002	-10.8619127
2060.0000000	9.8087997	-10.9956055
2060.0000000	9.8575993	2.7299747
2060.0000000	9.9063997	-8.0641069
2060.0000000	9.9552002	-10.9070501
2060.0000000	10.0039997	-10.1004639
2070.0000000	0.3416000	-37.1298332
2070.0000000	0.3904000	-38.9446945
2070.0000000	0.4392000	-39.2289886
2070.0000000	0.4880000	-39.4643021
2070.0000000	0.5368000	-39.7369957
2070.0000000	0.5856000	-39.1875954
2070.0000000	0.6344000	-38.2580414
2070.0000000	0.6832000	-36.3995705
2070.0000000	0.7320000	-36.2171478
2070.0000000	0.7808000	-36.5481796
2070.0000000	0.8296000	-35.9262810
2070.0000000	0.8784000	-35.3319702
2070.0000000	0.9272000	-33.1674461
2070.0000000	0.9760000	-31.0653591
2070.0000000	1.0247999	-29.9921532
2070.0000000	1.0735999	-29.4439068
2070.0000000	1.1224000	-29.8115520
2070.0000000	1.1711999	-30.7359791
2070.0000000	1.2200000	-31.2383404
2070.0000000	1.2687999	-31.0868759
2070.0000000	1.3176000	-30.6517620
2070.0000000	1.3664000	-30.7038479
2070.0000000	1.4152000	-30.1837788
2070.0000000	1.4640000	-29.4358234
2070.0000000	1.5128000	-29.1715641
2070.0000000	1.5616000	-29.2369595
2070.0000000	1.6104001	-1290.2229004
2070.0000000	1.6592000	-42.3953400
2070.0000000	1.7079999	-27.6509914
2070.0000000	1.7567999	-27.5422058
2070.0000000	1.8055999	-26.3538017
2070.0000000	1.8544000	-26.0876865
2070.0000000	1.9031999	-26.5799923
2070.0000000	1.9520000	-25.8068867
2070.0000000	2.0007999	-24.9195328
2070.0000000	2.0495999	-24.7914867
2070.0000000	2.0983999	-25.8847637
2070.0000000	2.1471999	-23.8770313
2070.0000000	2.1960001	-23.9696865
2070.0000000	2.2448001	-24.6368484
2070.0000000	2.2935998	-24.7794933
2070.0000000	2.3423998	-22.5023327
2070.0000000	2.3912001	-24.6572266
2070.0000000	2.4400001	-23.0985451
2070.0000000	2.4888000	-23.4372787

2070.0000000	2.5375998	-14.0627508
2070.0000000	2.5863998	-12.4613743
2070.0000000	2.6352000	-10.8460846
2070.0000000	2.6840000	-16.6604176
2070.0000000	2.7328000	105.3738174
2070.0000000	2.7815998	125.1169662
2070.0000000	2.8304000	-23.6581917
2070.0000000	2.8792000	-20.9600906
2070.0000000	2.9280000	-22.5123672
2070.0000000	2.9768000	-17.6632271
2070.0000000	3.0256000	-20.7426414
2070.0000000	3.0743999	-28.3282108
2070.0000000	3.1231999	-23.8944340
2070.0000000	3.1719999	-28.7401161
2070.0000000	3.2208002	-479.4182739
2070.0000000	3.2695999	-937.6473389
2070.0000000	3.3183999	-20.5103855
2070.0000000	3.3671999	-3243.1872559
2070.0000000	3.4159999	1162.3430176
2070.0000000	3.4648001	-7.7398767
2070.0000000	3.5135999	-26.7231102
2070.0000000	3.5623999	-308.3416138
2070.0000000	3.6111999	-1932.5364990
2070.0000000	3.6600001	-438.2145386
2070.0000000	3.7088001	-578.3157349
2070.0000000	3.7575998	-904.9116821
2070.0000000	3.8063998	-1641.3426514
2070.0000000	3.8552001	-2382.5815430
2070.0000000	3.9040000	-2335.9724121
2070.0000000	3.9528000	1066.9752197
2070.0000000	4.0015998	-234.1673737
2070.0000000	4.0503998	652.8458252
2070.0000000	4.0991998	82.3699341
2070.0000000	4.1479998	-623.4739380
2070.0000000	4.1967998	1.4823246
2070.0000000	4.2456002	-237.2111511
2070.0000000	4.2943997	-464.9453735
2070.0000000	4.3431997	-13.5820198
2070.0000000	4.3920002	-236.8632660
2070.0000000	4.4407997	-22.2564735
2070.0000000	4.4896002	-15.5756989
2070.0000000	4.5383997	-625.0520020
2070.0000000	4.5871997	-13.0705032
2070.0000000	4.6360002	-21.4436798
2070.0000000	4.6847997	-27.8308411
2070.0000000	4.7336001	-19.4793339
2070.0000000	4.7824001	-16.5646496
2070.0000000	4.8311996	-1413.1961670
2070.0000000	4.8800001	217.9445190
2070.0000000	4.9287996	-193.7062988
2070.0000000	4.9776001	1014.3717651
2070.0000000	5.0264001	81.8539352
2070.0000000	5.0751996	-289.2028809
2070.0000000	5.1240001	-12.4144611
2070.0000000	5.1727996	545.0889893
2070.0000000	5.2216001	30.9573784
2070.0000000	5.2704000	-155.7247925
2070.0000000	5.3191996	-38.6823082

2070.0000000	5.3680000	-3459.7653809
2070.0000000	5.4168000	515.3968506
2070.0000000	5.4656000	-196.7644806
2070.0000000	5.5144000	36.8967285
2070.0000000	5.5631995	477.5011902
2070.0000000	5.6120000	384.3598633
2070.0000000	5.6608000	-36.6359024
2070.0000000	5.7096000	60.8363647
2070.0000000	5.7584000	129.8642273
2070.0000000	5.8071995	-11.6158409
2070.0000000	5.8559999	28.8462467
2070.0000000	5.9047999	-16.5839405
2070.0000000	5.9535999	-39.2568588
2070.0000000	6.0023999	-1539.6296387
2070.0000000	6.0511999	-14.5913296
2070.0000000	6.0999999	-19.3182812
2070.0000000	6.1487999	-13.3239126
2070.0000000	6.1975999	-23.0274353
2070.0000000	6.2463999	-1753.9304199
2070.0000000	6.2951999	-18.9814644
2070.0000000	6.3439999	-335.7266541
2070.0000000	6.3927999	-15.0461206
2070.0000000	6.4416003	-12.4498196
2070.0000000	6.4903998	-11.3371410
2070.0000000	6.5391998	-11.8801479
2070.0000000	6.5879998	310.1853943
2070.0000000	6.6367998	-12.1436148
2070.0000000	6.6856003	-13.8333235
2070.0000000	6.7343998	-13.4789572
2070.0000000	6.7831998	-14.3277473
2070.0000000	6.8319998	56.3818588
2070.0000000	6.8807998	-14.0345058
2070.0000000	6.9296002	-367.7554321
2070.0000000	6.9783998	-6.4594874
2070.0000000	7.0271997	-14.0272789
2070.0000000	7.0760002	6.6773806
2070.0000000	7.1247997	-1710.4368896
2070.0000000	7.1736002	-1371.7993164
2070.0000000	7.2223997	-781.0849609
2070.0000000	7.2711997	-170.4273376
2070.0000000	7.3200002	-17.6061172
2070.0000000	7.3687997	148.0475464
2070.0000000	7.4176002	311.5333252
2070.0000000	7.4663997	244.3373108
2070.0000000	7.5151997	-763.4476318
2070.0000000	7.5640001	-843.0579224
2070.0000000	7.6127996	-1984.2338867
2070.0000000	7.6616001	-638.2264404
2070.0000000	7.7104001	-328.8228760
2070.0000000	7.7591996	32.5057030
2070.0000000	7.8080001	323.7587891
2070.0000000	7.8567996	427.1782227
2070.0000000	7.9056001	-891.4489136
2070.0000000	7.9544001	1417.7258301
2070.0000000	8.0031996	1829.4844971
2070.0000000	8.0520000	1825.9686279
2070.0000000	8.1007996	1932.2926025
2070.0000000	8.1496000	2060.8657227

2070.0000000	8.1983995	1282.0091553
2070.0000000	8.2472000	-2934.4912109
2070.0000000	8.2959995	-1040.4855957
2070.0000000	8.3448000	-828.6889648
2070.0000000	8.3935995	-759.5136719
2070.0000000	8.4423990	-327.5158081
2070.0000000	8.4912004	-763.9192505
2070.0000000	8.5400000	-2059.8361816
2070.0000000	8.5887995	-998.9850464
2070.0000000	8.6375999	194.3103485
2070.0000000	8.6863995	1760.5627441
2070.0000000	8.7351999	-655.0238037
2070.0000000	8.7840004	746.9393311
2070.0000000	8.8327999	38.7670555
2070.0000000	8.8815994	-11.5148144
2070.0000000	8.9303989	-23.5531006
2070.0000000	8.9792004	-11.5877781
2070.0000000	9.0279999	-6.0231175
2070.0000000	9.0767994	6.4374294
2070.0000000	9.1255999	-17.0616951
2070.0000000	9.1743994	-10.1158733
2070.0000000	9.2231998	35.3574371
2070.0000000	9.2720003	-8.5185156
2070.0000000	9.3207998	-9.8547688
2070.0000000	9.3695993	-10.7875566
2070.0000000	9.4183998	-11.7407990
2070.0000000	9.4672003	-11.1656561
2070.0000000	9.5159998	-14.0706511
2070.0000000	9.5648003	-0.7472076
2070.0000000	9.6135998	-13.3129864
2070.0000000	9.6623993	-10.2502136
2070.0000000	9.7111998	-10.1862211
2070.0000000	9.7600002	-10.8619547
2070.0000000	9.8087997	-10.9956141
2070.0000000	9.8575993	2.7255535
2070.0000000	9.9063997	-8.0650215
2070.0000000	9.9552002	-10.9070539
2070.0000000	10.0039997	-10.1007118
2080.0000000	0.3416000	-36.9705124
2080.0000000	0.3904000	-38.9387817
2080.0000000	0.4392000	-39.2299461
2080.0000000	0.4880000	-39.4630928
2080.0000000	0.5368000	-39.7381668
2080.0000000	0.5856000	-39.1862144
2080.0000000	0.6344000	-38.2583504
2080.0000000	0.6832000	-36.4017944
2080.0000000	0.7320000	-36.2192001
2080.0000000	0.7808000	-36.5483513
2080.0000000	0.8296000	-35.9223747
2080.0000000	0.8784000	-35.3288383
2080.0000000	0.9272000	-33.1518669
2080.0000000	0.9760000	-31.0605412
2080.0000000	1.0247999	-29.9976616
2080.0000000	1.0735999	-29.4291840
2080.0000000	1.1224000	-29.7932758
2080.0000000	1.1711999	-30.7291260
2080.0000000	1.2200000	-31.2401161
2080.0000000	1.2687999	-31.0890446

2080.0000000	1.3176000	-30.6480808
2080.0000000	1.3664000	-30.7034264
2080.0000000	1.4152000	-30.1839828
2080.0000000	1.4640000	-29.4341145
2080.0000000	1.5128000	-29.0743866
2080.0000000	1.5616000	-29.1951180
2080.0000000	1.6104001	-1287.6800537
2080.0000000	1.6592000	-42.1014099
2080.0000000	1.7079999	-27.6547375
2080.0000000	1.7567999	-27.5315399
2080.0000000	1.8055999	-26.3680973
2080.0000000	1.8544000	-26.0851707
2080.0000000	1.9031999	-26.5910320
2080.0000000	1.9520000	-25.8320599
2080.0000000	2.0007999	-24.9489059
2080.0000000	2.0495999	-24.7948914
2080.0000000	2.0983999	-25.8972206
2080.0000000	2.1471999	-23.9636917
2080.0000000	2.1960001	-23.9733410
2080.0000000	2.2448001	-24.6764183
2080.0000000	2.2935998	-24.7773819
2080.0000000	2.3423998	-22.5167389
2080.0000000	2.3912001	-24.6555901
2080.0000000	2.4400001	-23.1010303
2080.0000000	2.4888000	-23.4370613
2080.0000000	2.5375998	-14.0798588
2080.0000000	2.5863998	-12.4811335
2080.0000000	2.6352000	-10.8688288
2080.0000000	2.6840000	-16.6723595
2080.0000000	2.7328000	105.1078033
2080.0000000	2.7815998	124.8105392
2080.0000000	2.8304000	-23.6568871
2080.0000000	2.8792000	-20.9633541
2080.0000000	2.9280000	-22.5117989
2080.0000000	2.9768000	-17.6715965
2080.0000000	3.0256000	-20.7452259
2080.0000000	3.0743999	-28.3168240
2080.0000000	3.1231999	-23.8909798
2080.0000000	3.1719999	-28.7318230
2080.0000000	3.2208002	-479.1805420
2080.0000000	3.2695999	-937.1724854
2080.0000000	3.3183999	-20.5105839
2080.0000000	3.3671999	-3241.7255859
2080.0000000	3.4159999	1161.8077393
2080.0000000	3.4648001	-7.7455845
2080.0000000	3.5135999	-26.7201195
2080.0000000	3.5623999	-308.2120361
2080.0000000	3.6111999	-1931.6766357
2080.0000000	3.6600001	-438.0264282
2080.0000000	3.7088001	-578.0643311
2080.0000000	3.7575998	-904.5131226
2080.0000000	3.8063998	-1640.6127930
2080.0000000	3.8552001	-2381.5183105
2080.0000000	3.9040000	-2334.9299316
2080.0000000	3.9528000	1066.4869385
2080.0000000	4.0015998	-234.0702667
2080.0000000	4.0503998	652.5432739
2080.0000000	4.0991998	82.3242493

2080.0000000	4.1479998	-623.2017212
2080.0000000	4.1967998	1.4730682
2080.0000000	4.2456002	-237.1128540
2080.0000000	4.2943997	-464.7448120
2080.0000000	4.3431997	-13.5841999
2080.0000000	4.3920002	-236.7651520
2080.0000000	4.4407997	-22.2546978
2080.0000000	4.4896002	-15.5765820
2080.0000000	4.5383997	-624.7909546
2080.0000000	4.5871997	-13.0725193
2080.0000000	4.6360002	-21.4421368
2080.0000000	4.6847997	-27.8269157
2080.0000000	4.7336001	-19.4786110
2080.0000000	4.7824001	-16.5651035
2080.0000000	4.8311996	-1412.6647949
2080.0000000	4.8800001	217.8549805
2080.0000000	4.9287996	-193.6390381
2080.0000000	4.9776001	1013.9793091
2080.0000000	5.0264001	81.8164139
2080.0000000	5.0751996	-289.0992432
2080.0000000	5.1240001	-12.4153824
2080.0000000	5.1727996	544.9730835
2080.0000000	5.2216001	30.9479332
2080.0000000	5.2704000	-155.6966705
2080.0000000	5.3191996	-38.6777611
2080.0000000	5.3680000	-3459.0729980
2080.0000000	5.4168000	515.2901001
2080.0000000	5.4656000	-196.7283020
2080.0000000	5.5144000	36.8860703
2080.0000000	5.5631995	477.4026184
2080.0000000	5.6120000	384.2799072
2080.0000000	5.6608000	-36.6318130
2080.0000000	5.7096000	60.8209305
2080.0000000	5.7584000	129.8352966
2080.0000000	5.8071995	-11.6167116
2080.0000000	5.8559999	28.8373966
2080.0000000	5.9047999	-16.5837421
2080.0000000	5.9535999	-39.2521210
2080.0000000	6.0023999	-1539.3250732
2080.0000000	6.0511999	-14.5915413
2080.0000000	6.0999999	-19.3174496
2080.0000000	6.1487999	-13.3243752
2080.0000000	6.1975999	-23.0256691
2080.0000000	6.2463999	-1753.5830078
2080.0000000	6.2951999	-18.9806499
2080.0000000	6.3439999	-335.6626282
2080.0000000	6.3927999	-15.0461016
2080.0000000	6.4416003	-12.4504061
2080.0000000	6.4903998	-11.3378172
2080.0000000	6.5391998	-11.8808289
2080.0000000	6.5879998	309.4946899
2080.0000000	6.6367998	-12.1441641
2080.0000000	6.6856003	-13.8334932
2080.0000000	6.7343998	-13.4791727
2080.0000000	6.7831998	-14.3273315
2080.0000000	6.8319998	56.3627396
2080.0000000	6.8807998	-14.0343676
2080.0000000	6.9296002	-366.9939880

2080.0000000	6.9783998	-6.4723110
2080.0000000	7.0271997	-14.0262508
2080.0000000	7.0760002	6.6351957
2080.0000000	7.1247997	-1706.4909668
2080.0000000	7.1736002	-1368.6363525
2080.0000000	7.2223997	-779.2918091
2080.0000000	7.2711997	-170.0595703
2080.0000000	7.3200002	-17.5941277
2080.0000000	7.3687997	147.6730042
2080.0000000	7.4176002	310.7774963
2080.0000000	7.4663997	243.7396545
2080.0000000	7.5151997	-761.6865845
2080.0000000	7.5640001	-841.1105347
2080.0000000	7.6127996	-1979.6163330
2080.0000000	7.6616001	-636.7551270
2080.0000000	7.7104001	-328.0765991
2080.0000000	7.7591996	32.4046478
2080.0000000	7.8080001	322.9734192
2080.0000000	7.8567996	426.1501465
2080.0000000	7.9056001	-889.3760376
2080.0000000	7.9544001	1414.4158936
2080.0000000	8.0031996	1825.2148438
2080.0000000	8.0520000	1821.7066650
2080.0000000	8.1007996	1927.7830811
2080.0000000	8.1496000	2056.0578613
2080.0000000	8.1983995	1279.0166016
2080.0000000	8.2472000	-2927.6564941
2080.0000000	8.2959995	-1038.0654297
2080.0000000	8.3448000	-826.7597656
2080.0000000	8.3935995	-757.7460327
2080.0000000	8.4423990	-326.7578125
2080.0000000	8.4912004	-762.1430664
2080.0000000	8.5400000	-2055.0363770
2080.0000000	8.5887995	-996.6635132
2080.0000000	8.6375999	193.8490295
2080.0000000	8.6863995	1756.4443359
2080.0000000	8.7351999	-653.4995117
2080.0000000	8.7840004	745.1825562
2080.0000000	8.8327999	38.6554489
2080.0000000	8.8815994	-11.5149889
2080.0000000	8.9303989	-23.5493813
2080.0000000	8.9792004	-11.5878601
2080.0000000	9.0279999	-6.0248857
2080.0000000	9.0767994	6.4317131
2080.0000000	9.1255999	-17.0599823
2080.0000000	9.1743994	-10.1162758
2080.0000000	9.2231998	35.3417053
2080.0000000	9.2720003	-8.5194521
2080.0000000	9.3207998	-9.8553085
2080.0000000	9.3695993	-10.7877827
2080.0000000	9.4183998	-11.7406750
2080.0000000	9.4672003	-11.1656971
2080.0000000	9.5159998	-14.0697594
2080.0000000	9.5648003	-0.7505798
2080.0000000	9.6135998	-13.3121958
2080.0000000	9.6623993	-10.2504883
2080.0000000	9.7111998	-10.1864929
2080.0000000	9.7600002	-10.8619967

2080.0000000	9.8087997	-10.9956217
2080.0000000	9.8575993	2.7211313
2080.0000000	9.9063997	-8.0659351
2080.0000000	9.9552002	-10.9070587
2080.0000000	10.0039997	-10.1009588
2090.0000000	0.3416000	-37.0116386
2090.0000000	0.3904000	-38.9328651
2090.0000000	0.4392000	-39.2309074
2090.0000000	0.4880000	-39.4618835
2090.0000000	0.5368000	-39.7393417
2090.0000000	0.5856000	-39.1848335
2090.0000000	0.6344000	-38.2586632
2090.0000000	0.6832000	-36.4040222
2090.0000000	0.7320000	-36.2212524
2090.0000000	0.7808000	-36.5485191
2090.0000000	0.8296000	-35.9184685
2090.0000000	0.8784000	-35.3257065
2090.0000000	0.9272000	-33.1362877
2090.0000000	0.9760000	-31.0557251
2090.0000000	1.0247999	-30.0031700
2090.0000000	1.0735999	-29.4144611
2090.0000000	1.1224000	-29.7749996
2090.0000000	1.1711999	-30.7222748
2090.0000000	1.2200000	-31.2418919
2090.0000000	1.2687999	-31.0912132
2090.0000000	1.3176000	-30.6443996
2090.0000000	1.3664000	-30.7030029
2090.0000000	1.4152000	-30.1841869
2090.0000000	1.4640000	-29.4324036
2090.0000000	1.5128000	-28.9772072
2090.0000000	1.5616000	-29.1532784
2090.0000000	1.6104001	-1285.1372070
2090.0000000	1.6592000	-41.8074799
2090.0000000	1.7079999	-27.6584854
2090.0000000	1.7567999	-27.5208721
2090.0000000	1.8055999	-26.3823929
2090.0000000	1.8544000	-26.0826550
2090.0000000	1.9031999	-26.6020718
2090.0000000	1.9520000	-25.8572330
2090.0000000	2.0007999	-24.9782772
2090.0000000	2.0495999	-24.7982960
2090.0000000	2.0983999	-25.9096775
2090.0000000	2.1471999	-24.0503521
2090.0000000	2.1960001	-23.9769955
2090.0000000	2.2448001	-24.7159882
2090.0000000	2.2935998	-24.7752686
2090.0000000	2.3423998	-22.5311432
2090.0000000	2.3912001	-24.6539536
2090.0000000	2.4400001	-23.1035175
2090.0000000	2.4888000	-23.4368458
2090.0000000	2.5375998	-14.0969667
2090.0000000	2.5863998	-12.5008926
2090.0000000	2.6352000	-10.8915739
2090.0000000	2.6840000	-16.6843014
2090.0000000	2.7328000	104.8417892
2090.0000000	2.7815998	124.5041275
2090.0000000	2.8304000	-23.6555824
2090.0000000	2.8792000	-20.9666176

2090.0000000	2.9280000	-22.5112305
2090.0000000	2.9768000	-17.6799679
2090.0000000	3.0256000	-20.7478104
2090.0000000	3.0743999	-28.3054371
2090.0000000	3.1231999	-23.8875237
2090.0000000	3.1719999	-28.7235298
2090.0000000	3.2208002	-478.9428101
2090.0000000	3.2695999	-936.6973877
2090.0000000	3.3183999	-20.5107803
2090.0000000	3.3671999	-3240.2639160
2090.0000000	3.4159999	1161.2724609
2090.0000000	3.4648001	-7.7512932
2090.0000000	3.5135999	-26.7171288
2090.0000000	3.5623999	-308.0824585
2090.0000000	3.6111999	-1930.8168945
2090.0000000	3.6600001	-437.8383179
2090.0000000	3.7088001	-577.8129272
2090.0000000	3.7575998	-904.1146240
2090.0000000	3.8063998	-1639.8829346
2090.0000000	3.8552001	-2380.4548340
2090.0000000	3.9040000	-2333.8874512
2090.0000000	3.9528000	1065.9986572
2090.0000000	4.0015998	-233.9731598
2090.0000000	4.0503998	652.2406616
2090.0000000	4.0991998	82.2785721
2090.0000000	4.1479998	-622.9295044
2090.0000000	4.1967998	1.4638119
2090.0000000	4.2456002	-237.0145416
2090.0000000	4.2943997	-464.5442505
2090.0000000	4.3431997	-13.5863810
2090.0000000	4.3920002	-236.6670380
2090.0000000	4.4407997	-22.2529221
2090.0000000	4.4896002	-15.5774641
2090.0000000	4.5383997	-624.5298462
2090.0000000	4.5871997	-13.0745354
2090.0000000	4.6360002	-21.4405956
2090.0000000	4.6847997	-27.8229904
2090.0000000	4.7336001	-19.4778900
2090.0000000	4.7824001	-16.5655575
2090.0000000	4.8311996	-1412.1335449
2090.0000000	4.8800001	217.7654266
2090.0000000	4.9287996	-193.5717773
2090.0000000	4.9776001	1013.5867310
2090.0000000	5.0264001	81.7788925
2090.0000000	5.0751996	-288.9956360
2090.0000000	5.1240001	-12.4163036
2090.0000000	5.1727996	544.8572388
2090.0000000	5.2216001	30.9384918
2090.0000000	5.2704000	-155.6685791
2090.0000000	5.3191996	-38.6732101
2090.0000000	5.3680000	-3458.3806152
2090.0000000	5.4168000	515.1832886
2090.0000000	5.4656000	-196.6921082
2090.0000000	5.5144000	36.8754120
2090.0000000	5.5631995	477.3040466
2090.0000000	5.6120000	384.1999207
2090.0000000	5.6608000	-36.6277275
2090.0000000	5.7096000	60.8055038

2090.0000000	5.7584000	129.8063660
2090.0000000	5.8071995	-11.6175823
2090.0000000	5.8559999	28.8285465
2090.0000000	5.9047999	-16.5835438
2090.0000000	5.9535999	-39.2473831
2090.0000000	6.0023999	-1539.0206299
2090.0000000	6.0511999	-14.5917530
2090.0000000	6.0999999	-19.3166161
2090.0000000	6.1487999	-13.3248386
2090.0000000	6.1975999	-23.0239048
2090.0000000	6.2463999	-1753.2355957
2090.0000000	6.2951999	-18.9798355
2090.0000000	6.3439999	-335.5985718
2090.0000000	6.3927999	-15.0460835
2090.0000000	6.4416003	-12.4509926
2090.0000000	6.4903998	-11.3384933
2090.0000000	6.5391998	-11.8815098
2090.0000000	6.5879998	308.8040161
2090.0000000	6.6367998	-12.1447134
2090.0000000	6.6856003	-13.8336639
2090.0000000	6.7343998	-13.4793882
2090.0000000	6.7831998	-14.3269157
2090.0000000	6.8319998	56.3436279
2090.0000000	6.8807998	-14.0342293
2090.0000000	6.9296002	-366.2325439
2090.0000000	6.9783998	-6.4851351
2090.0000000	7.0271997	-14.0252228
2090.0000000	7.0760002	6.5930090
2090.0000000	7.1247997	-1702.5451660
2090.0000000	7.1736002	-1365.4735107
2090.0000000	7.2223997	-777.4986572
2090.0000000	7.2711997	-169.6917877
2090.0000000	7.3200002	-17.5821381
2090.0000000	7.3687997	147.2984772
2090.0000000	7.4176002	310.0216675
2090.0000000	7.4663997	243.1419983
2090.0000000	7.5151997	-759.9255371
2090.0000000	7.5640001	-839.1631470
2090.0000000	7.6127996	-1974.9986572
2090.0000000	7.6616001	-635.2838135
2090.0000000	7.7104001	-327.3303223
2090.0000000	7.7591996	32.3036003
2090.0000000	7.8080001	322.1880798
2090.0000000	7.8567996	425.1220703
2090.0000000	7.9056001	-887.3031006
2090.0000000	7.9544001	1411.1059570
2090.0000000	8.0031996	1820.9453125
2090.0000000	8.0520000	1817.4445801
2090.0000000	8.1007996	1923.2735596
2090.0000000	8.1496000	2051.2497559
2090.0000000	8.1983995	1276.0241699
2090.0000000	8.2472000	-2920.8215332
2090.0000000	8.2959995	-1035.6452637
2090.0000000	8.3448000	-824.8306274
2090.0000000	8.3935995	-755.9783325
2090.0000000	8.4423990	-325.9997864
2090.0000000	8.4912004	-760.3668213
2090.0000000	8.5400000	-2050.2368164

2090.0000000	8.5887995	-994.3419800
2090.0000000	8.6375999	193.3877106
2090.0000000	8.6863995	1752.3260498
2090.0000000	8.7351999	-651.9752808
2090.0000000	8.7840004	743.4258423
2090.0000000	8.8327999	38.5438423
2090.0000000	8.8815994	-11.5151634
2090.0000000	8.9303989	-23.5456600
2090.0000000	8.9792004	-11.5879421
2090.0000000	9.0279999	-6.0266538
2090.0000000	9.0767994	6.4259949
2090.0000000	9.1255999	-17.0582676
2090.0000000	9.1743994	-10.1166773
2090.0000000	9.2231998	35.3259735
2090.0000000	9.2720003	-8.5203876
2090.0000000	9.3207998	-9.8558474
2090.0000000	9.3695993	-10.7880096
2090.0000000	9.4183998	-11.7405500
2090.0000000	9.4672003	-11.1657391
2090.0000000	9.5159998	-14.0688686
2090.0000000	9.5648003	-0.7539530
2090.0000000	9.6135998	-13.3114052
2090.0000000	9.6623993	-10.2507629
2090.0000000	9.7111998	-10.1867647
2090.0000000	9.7600002	-10.8620377
2090.0000000	9.8087997	-10.9956293
2090.0000000	9.8575993	2.7167091
2090.0000000	9.9063997	-8.0668488
2090.0000000	9.9552002	-10.9070625
2090.0000000	10.0039997	-10.1012068
2100.0000000	0.3416000	-37.1065865
2100.0000000	0.3904000	-38.9269485
2100.0000000	0.4392000	-39.2318649
2100.0000000	0.4880000	-39.4606743
2100.0000000	0.5368000	-39.7405167
2100.0000000	0.5856000	-39.1834526
2100.0000000	0.6344000	-38.2589722
2100.0000000	0.6832000	-36.4062462
2100.0000000	0.7320000	-36.2233047
2100.0000000	0.7808000	-36.5486908
2100.0000000	0.8296000	-35.9145584
2100.0000000	0.8784000	-35.3225746
2100.0000000	0.9272000	-33.1207085
2100.0000000	0.9760000	-31.0509071
2100.0000000	1.0247999	-30.0086784
2100.0000000	1.0735999	-29.3997383
2100.0000000	1.1224000	-29.7567234
2100.0000000	1.1711999	-30.7154217
2100.0000000	1.2200000	-31.2436657
2100.0000000	1.2687999	-31.0933819
2100.0000000	1.3176000	-30.6407166
2100.0000000	1.3664000	-30.7025795
2100.0000000	1.4152000	-30.1843929
2100.0000000	1.4640000	-29.4306946
2100.0000000	1.5128000	-28.8800297
2100.0000000	1.5616000	-29.1114368
2100.0000000	1.6104001	-1282.5944824
2100.0000000	1.6592000	-41.5135460

2100.0000000	1.7079999	-27.6622334
2100.0000000	1.7567999	-27.5102062
2100.0000000	1.8055999	-26.3966885
2100.0000000	1.8544000	-26.0801392
2100.0000000	1.9031999	-26.6131096
2100.0000000	1.9520000	-25.8824062
2100.0000000	2.0007999	-25.0076504
2100.0000000	2.0495999	-24.8017006
2100.0000000	2.0983999	-25.9221344
2100.0000000	2.1471999	-24.1370125
2100.0000000	2.1960001	-23.9806499
2100.0000000	2.2448001	-24.7555580
2100.0000000	2.2935998	-24.7731571
2100.0000000	2.3423998	-22.5455475
2100.0000000	2.3912001	-24.6523170
2100.0000000	2.4400001	-23.1060047
2100.0000000	2.4888000	-23.4366283
2100.0000000	2.5375998	-14.1140747
2100.0000000	2.5863998	-12.5206518
2100.0000000	2.6352000	-10.9143181
2100.0000000	2.6840000	-16.6962433
2100.0000000	2.7328000	104.5757751
2100.0000000	2.7815998	124.1977158
2100.0000000	2.8304000	-23.6542797
2100.0000000	2.8792000	-20.9698811
2100.0000000	2.9280000	-22.5106602
2100.0000000	2.9768000	-17.6883392
2100.0000000	3.0256000	-20.7503948
2100.0000000	3.0743999	-28.2940502
2100.0000000	3.1231999	-23.8840675
2100.0000000	3.1719999	-28.7152367
2100.0000000	3.2208002	-478.7050476
2100.0000000	3.2695999	-936.2225342
2100.0000000	3.3183999	-20.5109787
2100.0000000	3.3671999	-3238.8022461
2100.0000000	3.4159999	1160.7371826
2100.0000000	3.4648001	-7.7570019
2100.0000000	3.5135999	-26.7141380
2100.0000000	3.5623999	-307.9528503
2100.0000000	3.6111999	-1929.9571533
2100.0000000	3.6600001	-437.6501465
2100.0000000	3.7088001	-577.5615845
2100.0000000	3.7575998	-903.7160645
2100.0000000	3.8063998	-1639.1531982
2100.0000000	3.8552001	-2379.3913574
2100.0000000	3.9040000	-2332.8449707
2100.0000000	3.9528000	1065.5103760
2100.0000000	4.0015998	-233.8760529
2100.0000000	4.0503998	651.9381104
2100.0000000	4.0991998	82.2328873
2100.0000000	4.1479998	-622.6572266
2100.0000000	4.1967998	1.4545555
2100.0000000	4.2456002	-236.9162445
2100.0000000	4.2943997	-464.3436890
2100.0000000	4.3431997	-13.5885620
2100.0000000	4.3920002	-236.5689240
2100.0000000	4.4407997	-22.2511463
2100.0000000	4.4896002	-15.5783472

2100.0000000	4.5383997	-624.2687988
2100.0000000	4.5871997	-13.0765514
2100.0000000	4.6360002	-21.4390526
2100.0000000	4.6847997	-27.8190651
2100.0000000	4.7336001	-19.4771671
2100.0000000	4.7824001	-16.5660114
2100.0000000	4.8311996	-1411.6022949
2100.0000000	4.8800001	217.6758728
2100.0000000	4.9287996	-193.5045166
2100.0000000	4.9776001	1013.1942749
2100.0000000	5.0264001	81.7413712
2100.0000000	5.0751996	-288.8919983
2100.0000000	5.1240001	-12.4172249
2100.0000000	5.1727996	544.7413940
2100.0000000	5.2216001	30.9290466
2100.0000000	5.2704000	-155.6404572
2100.0000000	5.3191996	-38.6686630
2100.0000000	5.3680000	-3457.6884766
2100.0000000	5.4168000	515.0764771
2100.0000000	5.4656000	-196.6559296
2100.0000000	5.5144000	36.8647537
2100.0000000	5.5631995	477.2054749
2100.0000000	5.6120000	384.1199646
2100.0000000	5.6608000	-36.6236382
2100.0000000	5.7096000	60.7900772
2100.0000000	5.7584000	129.7774200
2100.0000000	5.8071995	-11.6184521
2100.0000000	5.8559999	28.8196926
2100.0000000	5.9047999	-16.5833473
2100.0000000	5.9535999	-39.2426453
2100.0000000	6.0023999	-1538.7163086
2100.0000000	6.0511999	-14.5919638
2100.0000000	6.0999999	-19.3157845
2100.0000000	6.1487999	-13.3253012
2100.0000000	6.1975999	-23.0221386
2100.0000000	6.2463999	-1752.8883057
2100.0000000	6.2951999	-18.9790211
2100.0000000	6.3439999	-335.5345154
2100.0000000	6.3927999	-15.0460653
2100.0000000	6.4416003	-12.4515800
2100.0000000	6.4903998	-11.3391695
2100.0000000	6.5391998	-11.8821917
2100.0000000	6.5879998	308.1133118
2100.0000000	6.6367998	-12.1452627
2100.0000000	6.6856003	-13.8338337
2100.0000000	6.7343998	-13.4796038
2100.0000000	6.7831998	-14.3264999
2100.0000000	6.8319998	56.3245163
2100.0000000	6.8807998	-14.0340900
2100.0000000	6.9296002	-365.4710999
2100.0000000	6.9783998	-6.4979587
2100.0000000	7.0271997	-14.0241947
2100.0000000	7.0760002	6.5508242
2100.0000000	7.1247997	-1698.5992432
2100.0000000	7.1736002	-1362.3106689
2100.0000000	7.2223997	-775.7055054
2100.0000000	7.2711997	-169.3240051
2100.0000000	7.3200002	-17.5701504

2100.0000000	7.3687997	146.9239502
2100.0000000	7.4176002	309.2658386
2100.0000000	7.4663997	242.5443573
2100.0000000	7.5151997	-758.1645508
2100.0000000	7.5640001	-837.2157593
2100.0000000	7.6127996	-1970.3811035
2100.0000000	7.6616001	-633.8124390
2100.0000000	7.7104001	-326.5841064
2100.0000000	7.7591996	32.2025528
2100.0000000	7.8080001	321.4027100
2100.0000000	7.8567996	424.0939636
2100.0000000	7.9056001	-885.2302246
2100.0000000	7.9544001	1407.7960205
2100.0000000	8.0031996	1816.6756592
2100.0000000	8.0520000	1813.1826172
2100.0000000	8.1007996	1918.7640381
2100.0000000	8.1496000	2046.4416504
2100.0000000	8.1983995	1273.0316162
2100.0000000	8.2472000	-2913.9868164
2100.0000000	8.2959995	-1033.2250977
2100.0000000	8.3448000	-822.9014282
2100.0000000	8.3935995	-754.2106934
2100.0000000	8.4423990	-325.2417603
2100.0000000	8.4912004	-758.5906372
2100.0000000	8.5400000	-2045.4370117
2100.0000000	8.5887995	-992.0204468
2100.0000000	8.6375999	192.9263916
2100.0000000	8.6863995	1748.2076416
2100.0000000	8.7351999	-650.4509888
2100.0000000	8.7840004	741.6690674
2100.0000000	8.8327999	38.4322319
2100.0000000	8.8815994	-11.5153370
2100.0000000	8.9303989	-23.5419388
2100.0000000	8.9792004	-11.5880251
2100.0000000	9.0279999	-6.0284224
2100.0000000	9.0767994	6.4202766
2100.0000000	9.1255999	-17.0565548
2100.0000000	9.1743994	-10.1170797
2100.0000000	9.2231998	35.3102417
2100.0000000	9.2720003	-8.5213242
2100.0000000	9.3207998	-9.8563871
2100.0000000	9.3695993	-10.7882357
2100.0000000	9.4183998	-11.7404261
2100.0000000	9.4672003	-11.1657801
2100.0000000	9.5159998	-14.0679779
2100.0000000	9.5648003	-0.7573261
2100.0000000	9.6135998	-13.3106136
2100.0000000	9.6623993	-10.2510386
2100.0000000	9.7111998	-10.1870356
2100.0000000	9.7600002	-10.8620796
2100.0000000	9.8087997	-10.9956379
2100.0000000	9.8575993	2.7122869
2100.0000000	9.9063997	-8.0677624
2100.0000000	9.9552002	-10.9070673
2100.0000000	10.0039997	-10.1014538
2110.0000000	0.3416000	-37.3834457
2110.0000000	0.3904000	-38.9210358
2110.0000000	0.4392000	-39.2328262

2110.0000000	0.4880000	-39.4594650
2110.0000000	0.5368000	-39.7416878
2110.0000000	0.5856000	-39.1820717
2110.0000000	0.6344000	-38.2592812
2110.0000000	0.6832000	-36.4084740
2110.0000000	0.7320000	-36.2253571
2110.0000000	0.7808000	-36.5488586
2110.0000000	0.8296000	-35.9106522
2110.0000000	0.8784000	-35.3194427
2110.0000000	0.9272000	-33.1051254
2110.0000000	0.9760000	-31.0460911
2110.0000000	1.0247999	-30.0141869
2110.0000000	1.0735999	-29.3850155
2110.0000000	1.1224000	-29.7384472
2110.0000000	1.1711999	-30.7085705
2110.0000000	1.2200000	-31.2454414
2110.0000000	1.2687999	-31.0955505
2110.0000000	1.3176000	-30.6370354
2110.0000000	1.3664000	-30.7021561
2110.0000000	1.4152000	-30.1845970
2110.0000000	1.4640000	-29.4289856
2110.0000000	1.5128000	-28.7828522
2110.0000000	1.5616000	-29.0695953
2110.0000000	1.6104001	-1280.0516357
2110.0000000	1.6592000	-41.2196121
2110.0000000	1.7079999	-27.6659794
2110.0000000	1.7567999	-27.4995384
2110.0000000	1.8055999	-26.4109859
2110.0000000	1.8544000	-26.0776215
2110.0000000	1.9031999	-26.6241493
2110.0000000	1.9520000	-25.9075794
2110.0000000	2.0007999	-25.0370216
2110.0000000	2.0495999	-24.8051052
2110.0000000	2.0983999	-25.9345932
2110.0000000	2.1471999	-24.2236729
2110.0000000	2.1960001	-23.9843063
2110.0000000	2.2448001	-24.7951279
2110.0000000	2.2935998	-24.7710438
2110.0000000	2.3423998	-22.5599518
2110.0000000	2.3912001	-24.6506805
2110.0000000	2.4400001	-23.1084900
2110.0000000	2.4888000	-23.4364109
2110.0000000	2.5375998	-14.1311827
2110.0000000	2.5863998	-12.5404100
2110.0000000	2.6352000	-10.9370623
2110.0000000	2.6840000	-16.7081833
2110.0000000	2.7328000	104.3097610
2110.0000000	2.7815998	123.8912888
2110.0000000	2.8304000	-23.6529751
2110.0000000	2.8792000	-20.9731445
2110.0000000	2.9280000	-22.5100918
2110.0000000	2.9768000	-17.6967087
2110.0000000	3.0256000	-20.7529793
2110.0000000	3.0743999	-28.2826633
2110.0000000	3.1231999	-23.8806133
2110.0000000	3.1719999	-28.7069435
2110.0000000	3.2208002	-478.4673157
2110.0000000	3.2695999	-935.7474365

2110.0000000	3.3183999	-20.5111771
2110.0000000	3.3671999	-3237.3405762
2110.0000000	3.4159999	1160.2019043
2110.0000000	3.4648001	-7.7627096
2110.0000000	3.5135999	-26.7111473
2110.0000000	3.5623999	-307.8232727
2110.0000000	3.6111999	-1929.0974121
2110.0000000	3.6600001	-437.4620361
2110.0000000	3.7088001	-577.3101807
2110.0000000	3.7575998	-903.3175049
2110.0000000	3.8063998	-1638.4233398
2110.0000000	3.8552001	-2378.3278809
2110.0000000	3.9040000	-2331.8024902
2110.0000000	3.9528000	1065.0219727
2110.0000000	4.0015998	-233.7789307
2110.0000000	4.0503998	651.6355591
2110.0000000	4.0991998	82.1872025
2110.0000000	4.1479998	-622.3850098
2110.0000000	4.1967998	1.4452991
2110.0000000	4.2456002	-236.8179474
2110.0000000	4.2943997	-464.1431274
2110.0000000	4.3431997	-13.5907421
2110.0000000	4.3920002	-236.4708099
2110.0000000	4.4407997	-22.2493706
2110.0000000	4.4896002	-15.5792294
2110.0000000	4.5383997	-624.0077515
2110.0000000	4.5871997	-13.0785675
2110.0000000	4.6360002	-21.4375114
2110.0000000	4.6847997	-27.8151398
2110.0000000	4.7336001	-19.4764442
2110.0000000	4.7824001	-16.5664673
2110.0000000	4.8311996	-1411.0710449
2110.0000000	4.8800001	217.5863190
2110.0000000	4.9287996	-193.4372559
2110.0000000	4.9776001	1012.8018188
2110.0000000	5.0264001	81.7038498
2110.0000000	5.0751996	-288.7883606
2110.0000000	5.1240001	-12.4181461
2110.0000000	5.1727996	544.6254883
2110.0000000	5.2216001	30.9196014
2110.0000000	5.2704000	-155.6123657
2110.0000000	5.3191996	-38.6641121
2110.0000000	5.3680000	-3456.9960938
2110.0000000	5.4168000	514.9697266
2110.0000000	5.4656000	-196.6197357
2110.0000000	5.5144000	36.8540955
2110.0000000	5.5631995	477.1069031
2110.0000000	5.6120000	384.0399780
2110.0000000	5.6608000	-36.6195488
2110.0000000	5.7096000	60.7746429
2110.0000000	5.7584000	129.7484894
2110.0000000	5.8071995	-11.6193237
2110.0000000	5.8559999	28.8108425
2110.0000000	5.9047999	-16.5831490
2110.0000000	5.9535999	-39.2379036
2110.0000000	6.0023999	-1538.4117432
2110.0000000	6.0511999	-14.5921755
2110.0000000	6.0999999	-19.3149529

2110.0000000	6.1487999	-13.3257637
2110.0000000	6.1975999	-23.0203743
2110.0000000	6.2463999	-1752.5408936
2110.0000000	6.2951999	-18.9782066
2110.0000000	6.3439999	-335.4704590
2110.0000000	6.3927999	-15.0460472
2110.0000000	6.4416003	-12.4521666
2110.0000000	6.4903998	-11.3398447
2110.0000000	6.5391998	-11.8828726
2110.0000000	6.5879998	307.4226074
2110.0000000	6.6367998	-12.1458120
2110.0000000	6.6856003	-13.8340044
2110.0000000	6.7343998	-13.4798203
2110.0000000	6.7831998	-14.3260841
2110.0000000	6.8319998	56.3053970
2110.0000000	6.8807998	-14.0339518
2110.0000000	6.9296002	-364.7096252
2110.0000000	6.9783998	-6.5107822
2110.0000000	7.0271997	-14.0231676
2110.0000000	7.0760002	6.5086393
2110.0000000	7.1247997	-1694.6534424
2110.0000000	7.1736002	-1359.1477051
2110.0000000	7.2223997	-773.9123535
2110.0000000	7.2711997	-168.9562225
2110.0000000	7.3200002	-17.5581627
2110.0000000	7.3687997	146.5494232
2110.0000000	7.4176002	308.5100098
2110.0000000	7.4663997	241.9467010
2110.0000000	7.5151997	-756.4035034
2110.0000000	7.5640001	-835.2683105
2110.0000000	7.6127996	-1965.7634277
2110.0000000	7.6616001	-632.3410645
2110.0000000	7.7104001	-325.8378296
2110.0000000	7.7591996	32.1015053
2110.0000000	7.8080001	320.6173401
2110.0000000	7.8567996	423.0658875
2110.0000000	7.9056001	-883.1572876
2110.0000000	7.9544001	1404.4860840
2110.0000000	8.0031996	1812.4060059
2110.0000000	8.0520000	1808.9206543
2110.0000000	8.1007996	1914.2545166
2110.0000000	8.1496000	2041.6337891
2110.0000000	8.1983995	1270.0390625
2110.0000000	8.2472000	-2907.1520996
2110.0000000	8.2959995	-1030.8049316
2110.0000000	8.3448000	-820.9722290
2110.0000000	8.3935995	-752.4429932
2110.0000000	8.4423990	-324.4837646
2110.0000000	8.4912004	-756.8143921
2110.0000000	8.5400000	-2040.6370850
2110.0000000	8.5887995	-989.6989136
2110.0000000	8.6375999	192.4650726
2110.0000000	8.6863995	1744.0893555
2110.0000000	8.7351999	-648.9267578
2110.0000000	8.7840004	739.9123535
2110.0000000	8.8327999	38.3206253
2110.0000000	8.8815994	-11.5155115
2110.0000000	8.9303989	-23.5382195

2110.0000000	8.9792004	-11.5881071
2110.0000000	9.0279999	-6.0301909
2110.0000000	9.0767994	6.4145603
2110.0000000	9.1255999	-17.0548401
2110.0000000	9.1743994	-10.1174812
2110.0000000	9.2231998	35.2945099
2110.0000000	9.2720003	-8.5222607
2110.0000000	9.3207998	-9.8569260
2110.0000000	9.3695993	-10.7884617
2110.0000000	9.4183998	-11.7403011
2110.0000000	9.4672003	-11.1658211
2110.0000000	9.5159998	-14.0670872
2110.0000000	9.5648003	-0.7606983
2110.0000000	9.6135998	-13.3098240
2110.0000000	9.6623993	-10.2513132
2110.0000000	9.7111998	-10.1873074
2110.0000000	9.7600002	-10.8621216
2110.0000000	9.8087997	-10.9956455
2110.0000000	9.8575993	2.7078638
2110.0000000	9.9063997	-8.0686760
2110.0000000	9.9552002	-10.9070711
2110.0000000	10.0039997	-10.1017017
2120.0000000	0.3416000	-37.0529251
2120.0000000	0.3904000	-38.9151192
2120.0000000	0.4392000	-39.2337875
2120.0000000	0.4880000	-39.4582558
2120.0000000	0.5368000	-39.7428627
2120.0000000	0.5856000	-39.1806908
2120.0000000	0.6344000	-38.2595940
2120.0000000	0.6832000	-36.4106979
2120.0000000	0.7320000	-36.2274094
2120.0000000	0.7808000	-36.5490265
2120.0000000	0.8296000	-35.9067459
2120.0000000	0.8784000	-35.3163109
2120.0000000	0.9272000	-33.0895462
2120.0000000	0.9760000	-31.0412731
2120.0000000	1.0247999	-30.0196953
2120.0000000	1.0735999	-29.3702927
2120.0000000	1.1224000	-29.7201710
2120.0000000	1.1711999	-30.7017174
2120.0000000	1.2200000	-31.2472172
2120.0000000	1.2687999	-31.0977192
2120.0000000	1.3176000	-30.6333542
2120.0000000	1.3664000	-30.7017326
2120.0000000	1.4152000	-30.1848011
2120.0000000	1.4640000	-29.4272766
2120.0000000	1.5128000	-28.6856747
2120.0000000	1.5616000	-29.1766510
2120.0000000	1.6104001	-1277.5089111
2120.0000000	1.6592000	-40.9256821
2120.0000000	1.7079999	-27.6697273
2120.0000000	1.7567999	-27.4888706
2120.0000000	1.8055999	-26.4252815
2120.0000000	1.8544000	-26.0751057
2120.0000000	1.9031999	-26.6351891
2120.0000000	1.9520000	-25.9327526
2120.0000000	2.0007999	-25.0663948
2120.0000000	2.0495999	-24.8085098

2120.0000000	2.0983999	-25.9470501
2120.0000000	2.1471999	-24.3103333
2120.0000000	2.1960001	-23.9879608
2120.0000000	2.2448001	-24.8346977
2120.0000000	2.2935998	-24.7689323
2120.0000000	2.3423998	-22.5743561
2120.0000000	2.3912001	-24.6490459
2120.0000000	2.4400001	-23.1109772
2120.0000000	2.4888000	-23.4361935
2120.0000000	2.5375998	-14.1482906
2120.0000000	2.5863998	-12.5601692
2120.0000000	2.6352000	-10.9598064
2120.0000000	2.6840000	-16.7201271
2120.0000000	2.7328000	104.0437393
2120.0000000	2.7815998	123.5848770
2120.0000000	2.8304000	-23.6516705
2120.0000000	2.8792000	-20.9764080
2120.0000000	2.9280000	-22.5095234
2120.0000000	2.9768000	-17.7050800
2120.0000000	3.0256000	-20.7555637
2120.0000000	3.0743999	-28.2712765
2120.0000000	3.1231999	-23.8771572
2120.0000000	3.1719999	-28.6986504
2120.0000000	3.2208002	-478.2295837
2120.0000000	3.2695999	-935.2725830
2120.0000000	3.3183999	-20.5113754
2120.0000000	3.3671999	-3235.8789063
2120.0000000	3.4159999	1159.6666260
2120.0000000	3.4648001	-7.7684174
2120.0000000	3.5135999	-26.7081566
2120.0000000	3.5623999	-307.6936646
2120.0000000	3.6111999	-1928.2376709
2120.0000000	3.6600001	-437.2739258
2120.0000000	3.7088001	-577.0587769
2120.0000000	3.7575998	-902.9190063
2120.0000000	3.8063998	-1637.6934814
2120.0000000	3.8552001	-2377.2644043
2120.0000000	3.9040000	-2330.7600098
2120.0000000	3.9528000	1064.5336914
2120.0000000	4.0015998	-233.6818237
2120.0000000	4.0503998	651.3330078
2120.0000000	4.0991998	82.1415176
2120.0000000	4.1479998	-622.1127930
2120.0000000	4.1967998	1.4360428
2120.0000000	4.2456002	-236.7196350
2120.0000000	4.2943997	-463.9425354
2120.0000000	4.3431997	-13.5929222
2120.0000000	4.3920002	-236.3726959
2120.0000000	4.4407997	-22.2475948
2120.0000000	4.4896002	-15.5801125
2120.0000000	4.5383997	-623.7466431
2120.0000000	4.5871997	-13.0805845
2120.0000000	4.6360002	-21.4359684
2120.0000000	4.6847997	-27.8112144
2120.0000000	4.7336001	-19.4757214
2120.0000000	4.7824001	-16.5669212
2120.0000000	4.8311996	-1410.5397949
2120.0000000	4.8800001	217.4967651

2120.0000000	4.9287996	-193.3699951
2120.0000000	4.9776001	1012.4092407
2120.0000000	5.0264001	81.6663284
2120.0000000	5.0751996	-288.6847534
2120.0000000	5.1240001	-12.4190674
2120.0000000	5.1727996	544.5096436
2120.0000000	5.2216001	30.9101601
2120.0000000	5.2704000	-155.5842438
2120.0000000	5.3191996	-38.6595612
2120.0000000	5.3680000	-3456.3037109
2120.0000000	5.4168000	514.8629761
2120.0000000	5.4656000	-196.5835571
2120.0000000	5.5144000	36.8434448
2120.0000000	5.5631995	477.0083313
2120.0000000	5.6120000	383.9599915
2120.0000000	5.6608000	-36.6154633
2120.0000000	5.7096000	60.7592087
2120.0000000	5.7584000	129.7195587
2120.0000000	5.8071995	-11.6201935
2120.0000000	5.8559999	28.8019924
2120.0000000	5.9047999	-16.5829506
2120.0000000	5.9535999	-39.2331657
2120.0000000	6.0023999	-1538.1074219
2120.0000000	6.0511999	-14.5923862
2120.0000000	6.0999999	-19.3141193
2120.0000000	6.1487999	-13.3262272
2120.0000000	6.1975999	-23.0186100
2120.0000000	6.2463999	-1752.1934814
2120.0000000	6.2951999	-18.9773941
2120.0000000	6.3439999	-335.4064331
2120.0000000	6.3927999	-15.0460281
2120.0000000	6.4416003	-12.4527540
2120.0000000	6.4903998	-11.3405209
2120.0000000	6.5391998	-11.8835535
2120.0000000	6.5879998	306.7319031
2120.0000000	6.6367998	-12.1463604
2120.0000000	6.6856003	-13.8341742
2120.0000000	6.7343998	-13.4800358
2120.0000000	6.7831998	-14.3256683
2120.0000000	6.8319998	56.2862854
2120.0000000	6.8807998	-14.0338125
2120.0000000	6.9296002	-363.9481812
2120.0000000	6.9783998	-6.5236053
2120.0000000	7.0271997	-14.0221395
2120.0000000	7.0760002	6.4664526
2120.0000000	7.1247997	-1690.7075195
2120.0000000	7.1736002	-1355.9848633
2120.0000000	7.2223997	-772.1191406
2120.0000000	7.2711997	-168.5884399
2120.0000000	7.3200002	-17.5461731
2120.0000000	7.3687997	146.1748962
2120.0000000	7.4176002	307.7541809
2120.0000000	7.4663997	241.3490448
2120.0000000	7.5151997	-754.6424561
2120.0000000	7.5640001	-833.3209229
2120.0000000	7.6127996	-1961.1458740
2120.0000000	7.6616001	-630.8697510
2120.0000000	7.7104001	-325.0915527

2120.0000000	7.7591996	32.0004539
2120.0000000	7.8080001	319.8320007
2120.0000000	7.8567996	422.0378113
2120.0000000	7.9056001	-881.0844116
2120.0000000	7.9544001	1401.1761475
2120.0000000	8.0031996	1808.1364746
2120.0000000	8.0520000	1804.6586914
2120.0000000	8.1007996	1909.7451172
2120.0000000	8.1496000	2036.8258057
2120.0000000	8.1983995	1267.0466309
2120.0000000	8.2472000	-2900.3171387
2120.0000000	8.2959995	-1028.3847656
2120.0000000	8.3448000	-819.0430908
2120.0000000	8.3935995	-750.6753540
2120.0000000	8.4423990	-323.7257385
2120.0000000	8.4912004	-755.0381470
2120.0000000	8.5400000	-2035.8372803
2120.0000000	8.5887995	-987.3773804
2120.0000000	8.6375999	192.0037384
2120.0000000	8.6863995	1739.9709473
2120.0000000	8.7351999	-647.4025269
2120.0000000	8.7840004	738.1555786
2120.0000000	8.8327999	38.2090187
2120.0000000	8.8815994	-11.5156860
2120.0000000	8.9303989	-23.5345001
2120.0000000	8.9792004	-11.5881891
2120.0000000	9.0279999	-6.0319591
2120.0000000	9.0767994	6.4088421
2120.0000000	9.1255999	-17.0531254
2120.0000000	9.1743994	-10.1178837
2120.0000000	9.2231998	35.2787781
2120.0000000	9.2720003	-8.5231972
2120.0000000	9.3207998	-9.8574657
2120.0000000	9.3695993	-10.7886887
2120.0000000	9.4183998	-11.7401762
2120.0000000	9.4672003	-11.1658621
2120.0000000	9.5159998	-14.0661955
2120.0000000	9.5648003	-0.7640705
2120.0000000	9.6135998	-13.3090324
2120.0000000	9.6623993	-10.2515888
2120.0000000	9.7111998	-10.1875792
2120.0000000	9.7600002	-10.8621635
2120.0000000	9.8087997	-10.9956532
2120.0000000	9.8575993	2.7034416
2120.0000000	9.9063997	-8.0695906
2120.0000000	9.9552002	-10.9070759
2120.0000000	10.0039997	-10.1019497
2130.0000000	0.3416000	-37.2779961
2130.0000000	0.3904000	-38.9092026
2130.0000000	0.4392000	-39.2347450
2130.0000000	0.4880000	-39.4570465
2130.0000000	0.5368000	-39.7440376
2130.0000000	0.5856000	-39.1793060
2130.0000000	0.6344000	-38.2599030
2130.0000000	0.6832000	-36.4129219
2130.0000000	0.7320000	-36.2294617
2130.0000000	0.7808000	-36.5491982
2130.0000000	0.8296000	-35.9028397

2130.0000000	0.8784000	-35.3131790
2130.0000000	0.9272000	-33.0739670
2130.0000000	0.9760000	-31.0364571
2130.0000000	1.0247999	-30.0252037
2130.0000000	1.0735999	-29.3555698
2130.0000000	1.1224000	-29.7018929
2130.0000000	1.1711999	-30.6948662
2130.0000000	1.2200000	-31.2489929
2130.0000000	1.2687999	-31.0998859
2130.0000000	1.3176000	-30.6296711
2130.0000000	1.3664000	-30.7013092
2130.0000000	1.4152000	-30.1850052
2130.0000000	1.4640000	-29.4255657
2130.0000000	1.5128000	-28.5884972
2130.0000000	1.5616000	-29.0618877
2130.0000000	1.6104001	-1274.9660645
2130.0000000	1.6592000	-40.6317520
2130.0000000	1.7079999	-27.6734734
2130.0000000	1.7567999	-27.4782047
2130.0000000	1.8055999	-26.4395771
2130.0000000	1.8544000	-26.0725899
2130.0000000	1.9031999	-26.6462288
2130.0000000	1.9520000	-25.9579258
2130.0000000	2.0007999	-25.0957661
2130.0000000	2.0495999	-24.8119144
2130.0000000	2.0983999	-25.9595070
2130.0000000	2.1471999	-24.3969936
2130.0000000	2.1960001	-23.9916153
2130.0000000	2.2448001	-24.8742676
2130.0000000	2.2935998	-24.7668209
2130.0000000	2.3423998	-22.5887604
2130.0000000	2.3912001	-24.6474094
2130.0000000	2.4400001	-23.1134644
2130.0000000	2.4888000	-23.4359760
2130.0000000	2.5375998	-14.1653986
2130.0000000	2.5863998	-12.5799284
2130.0000000	2.6352000	-10.9825506
2130.0000000	2.6840000	-16.7320671
2130.0000000	2.7328000	103.7777252
2130.0000000	2.7815998	123.2784500
2130.0000000	2.8304000	-23.6503658
2130.0000000	2.8792000	-20.9796715
2130.0000000	2.9280000	-22.5089531
2130.0000000	2.9768000	-17.7134514
2130.0000000	3.0256000	-20.7581482
2130.0000000	3.0743999	-28.2598896
2130.0000000	3.1231999	-23.8737011
2130.0000000	3.1719999	-28.6903572
2130.0000000	3.2208002	-477.9918518
2130.0000000	3.2695999	-934.7974854
2130.0000000	3.3183999	-20.5115738
2130.0000000	3.3671999	-3234.4172363
2130.0000000	3.4159999	1159.1313477
2130.0000000	3.4648001	-7.7741261
2130.0000000	3.5135999	-26.7051659
2130.0000000	3.5623999	-307.5640869
2130.0000000	3.6111999	-1927.3779297
2130.0000000	3.6600001	-437.0857544

2130.0000000	3.7088001	-576.8074341
2130.0000000	3.7575998	-902.5204468
2130.0000000	3.8063998	-1636.9636230
2130.0000000	3.8552001	-2376.2011719
2130.0000000	3.9040000	-2329.7175293
2130.0000000	3.9528000	1064.0454102
2130.0000000	4.0015998	-233.5847168
2130.0000000	4.0503998	651.0304565
2130.0000000	4.0991998	82.0958328
2130.0000000	4.1479998	-621.8405762
2130.0000000	4.1967998	1.4267864
2130.0000000	4.2456002	-236.6213379
2130.0000000	4.2943997	-463.7419739
2130.0000000	4.3431997	-13.5951023
2130.0000000	4.3920002	-236.2745819
2130.0000000	4.4407997	-22.2458191
2130.0000000	4.4896002	-15.5809946
2130.0000000	4.5383997	-623.4855957
2130.0000000	4.5871997	-13.0826006
2130.0000000	4.6360002	-21.4344254
2130.0000000	4.6847997	-27.8072891
2130.0000000	4.7336001	-19.4750004
2130.0000000	4.7824001	-16.5673752
2130.0000000	4.8311996	-1410.0084229
2130.0000000	4.8800001	217.4072266
2130.0000000	4.9287996	-193.3027344
2130.0000000	4.9776001	1012.0167847
2130.0000000	5.0264001	81.6288071
2130.0000000	5.0751996	-288.5811157
2130.0000000	5.1240001	-12.4199886
2130.0000000	5.1727996	544.3937988
2130.0000000	5.2216001	30.9007149
2130.0000000	5.2704000	-155.5561523
2130.0000000	5.3191996	-38.6550140
2130.0000000	5.3680000	-3455.6113281
2130.0000000	5.4168000	514.7561646
2130.0000000	5.4656000	-196.5473785
2130.0000000	5.5144000	36.8327866
2130.0000000	5.5631995	476.9097595
2130.0000000	5.6120000	383.8800354
2130.0000000	5.6608000	-36.6113739
2130.0000000	5.7096000	60.7437820
2130.0000000	5.7584000	129.6906128
2130.0000000	5.8071995	-11.6210651
2130.0000000	5.8559999	28.7931385
2130.0000000	5.9047999	-16.5827522
2130.0000000	5.9535999	-39.2284241
2130.0000000	6.0023999	-1537.8029785
2130.0000000	6.0511999	-14.5925980
2130.0000000	6.0999999	-19.3132877
2130.0000000	6.1487999	-13.3266897
2130.0000000	6.1975999	-23.0168438
2130.0000000	6.2463999	-1751.8461914
2130.0000000	6.2951999	-18.9765778
2130.0000000	6.3439999	-335.3423462
2130.0000000	6.3927999	-15.0460100
2130.0000000	6.4416003	-12.4533405
2130.0000000	6.4903998	-11.3411970

2130.0000000	6.5391998	-11.8842344
2130.0000000	6.5879998	306.0411987
2130.0000000	6.6367998	-12.1469097
2130.0000000	6.6856003	-13.8343449
2130.0000000	6.7343998	-13.4802513
2130.0000000	6.7831998	-14.3252525
2130.0000000	6.8319998	56.2671661
2130.0000000	6.8807998	-14.0336742
2130.0000000	6.9296002	-363.1867371
2130.0000000	6.9783998	-6.5364294
2130.0000000	7.0271997	-14.0211115
2130.0000000	7.0760002	6.4242678
2130.0000000	7.1247997	-1686.7617188
2130.0000000	7.1736002	-1352.8220215
2130.0000000	7.2223997	-770.3259888
2130.0000000	7.2711997	-168.2206726
2130.0000000	7.3200002	-17.5341835
2130.0000000	7.3687997	145.8003540
2130.0000000	7.4176002	306.9983521
2130.0000000	7.4663997	240.7513885
2130.0000000	7.5151997	-752.8814087
2130.0000000	7.5640001	-831.3735352
2130.0000000	7.6127996	-1956.5281982
2130.0000000	7.6616001	-629.3984375
2130.0000000	7.7104001	-324.3452759
2130.0000000	7.7591996	31.8994026
2130.0000000	7.8080001	319.0466309
2130.0000000	7.8567996	421.0097046
2130.0000000	7.9056001	-879.0114746
2130.0000000	7.9544001	1397.8662109
2130.0000000	8.0031996	1803.8668213
2130.0000000	8.0520000	1800.3966064
2130.0000000	8.1007996	1905.2355957
2130.0000000	8.1496000	2032.0177002
2130.0000000	8.1983995	1264.0540771
2130.0000000	8.2472000	-2893.4824219
2130.0000000	8.2959995	-1025.9645996
2130.0000000	8.3448000	-817.1138916
2130.0000000	8.3935995	-748.9076538
2130.0000000	8.4423990	-322.9677429
2130.0000000	8.4912004	-753.2619629
2130.0000000	8.5400000	-2031.0374756
2130.0000000	8.5887995	-985.0557861
2130.0000000	8.6375999	191.5424194
2130.0000000	8.6863995	1735.8525391
2130.0000000	8.7351999	-645.8782349
2130.0000000	8.7840004	736.3988647
2130.0000000	8.8327999	38.0974083
2130.0000000	8.8815994	-11.5158606
2130.0000000	8.9303989	-23.5307808
2130.0000000	8.9792004	-11.5882721
2130.0000000	9.0279999	-6.0337272
2130.0000000	9.0767994	6.4031239
2130.0000000	9.1255999	-17.0514126
2130.0000000	9.1743994	-10.1182861
2130.0000000	9.2231998	35.2630463
2130.0000000	9.2720003	-8.5241337
2130.0000000	9.3207998	-9.8580055

2130.0000000	9.3695993	-10.7889147
2130.0000000	9.4183998	-11.7400522
2130.0000000	9.4672003	-11.1659031
2130.0000000	9.5159998	-14.0653048
2130.0000000	9.5648003	-0.7674437
2130.0000000	9.6135998	-13.3082418
2130.0000000	9.6623993	-10.2518635
2130.0000000	9.7111998	-10.1878510
2130.0000000	9.7600002	-10.8622046
2130.0000000	9.8087997	-10.9956617
2130.0000000	9.8575993	2.6990194
2130.0000000	9.9063997	-8.0705042
2130.0000000	9.9552002	-10.9070797
2130.0000000	10.0039997	-10.1021967
2140.0000000	0.3416000	-36.6196327
2140.0000000	0.3904000	-38.9032860
2140.0000000	0.4392000	-39.2357063
2140.0000000	0.4880000	-39.4558372
2140.0000000	0.5368000	-39.7452087
2140.0000000	0.5856000	-39.1779251
2140.0000000	0.6344000	-38.2602158
2140.0000000	0.6832000	-36.4151497
2140.0000000	0.7320000	-36.2315140
2140.0000000	0.7808000	-36.5493660
2140.0000000	0.8296000	-35.8989334
2140.0000000	0.8784000	-35.3100471
2140.0000000	0.9272000	-33.0583878
2140.0000000	0.9760000	-31.0316391
2140.0000000	1.0247999	-30.0307121
2140.0000000	1.0735999	-29.3408470
2140.0000000	1.1224000	-29.6836166
2140.0000000	1.1711999	-30.6880131
2140.0000000	1.2200000	-31.2507668
2140.0000000	1.2687999	-31.1020546
2140.0000000	1.3176000	-30.6259899
2140.0000000	1.3664000	-30.7008877
2140.0000000	1.4152000	-30.1852093
2140.0000000	1.4640000	-29.4238567
2140.0000000	1.5128000	-28.4913197
2140.0000000	1.5616000	-28.9471245
2140.0000000	1.6104001	-1272.4232178
2140.0000000	1.6592000	-40.3378181
2140.0000000	1.7079999	-27.6772213
2140.0000000	1.7567999	-27.4675369
2140.0000000	1.8055999	-26.4538727
2140.0000000	1.8544000	-26.0700741
2140.0000000	1.9031999	-26.6572685
2140.0000000	1.9520000	-25.9830990
2140.0000000	2.0007999	-25.1251392
2140.0000000	2.0495999	-24.8153191
2140.0000000	2.0983999	-25.9719639
2140.0000000	2.1471999	-24.4836540
2140.0000000	2.1960001	-23.9952698
2140.0000000	2.2448001	-24.9138374
2140.0000000	2.2935998	-24.7647076
2140.0000000	2.3423998	-22.6031666
2140.0000000	2.3912001	-24.6457729
2140.0000000	2.4400001	-23.1159496

2140.0000000	2.4888000	-23.4357586
2140.0000000	2.5375998	-14.1825066
2140.0000000	2.5863998	-12.5996876
2140.0000000	2.6352000	-11.0052948
2140.0000000	2.6840000	-16.7440109
2140.0000000	2.7328000	103.5117111
2140.0000000	2.7815998	122.9720383
2140.0000000	2.8304000	-23.6490631
2140.0000000	2.8792000	-20.9829350
2140.0000000	2.9280000	-22.5083847
2140.0000000	2.9768000	-17.7218208
2140.0000000	3.0256000	-20.7607327
2140.0000000	3.0743999	-28.2485027
2140.0000000	3.1231999	-23.8702469
2140.0000000	3.1719999	-28.6820641
2140.0000000	3.2208002	-477.7541199
2140.0000000	3.2695999	-934.3226318
2140.0000000	3.3183999	-20.5117702
2140.0000000	3.3671999	-3232.9555664
2140.0000000	3.4159999	1158.5960693
2140.0000000	3.4648001	-7.7798347
2140.0000000	3.5135999	-26.7021751
2140.0000000	3.5623999	-307.4344788
2140.0000000	3.6111999	-1926.5180664
2140.0000000	3.6600001	-436.8976440
2140.0000000	3.7088001	-576.5560303
2140.0000000	3.7575998	-902.1219482
2140.0000000	3.8063998	-1636.2337646
2140.0000000	3.8552001	-2375.1376953
2140.0000000	3.9040000	-2328.6750488
2140.0000000	3.9528000	1063.5571289
2140.0000000	4.0015998	-233.4876099
2140.0000000	4.0503998	650.7279053
2140.0000000	4.0991998	82.0501480
2140.0000000	4.1479998	-621.5683594
2140.0000000	4.1967998	1.4175301
2140.0000000	4.2456002	-236.5230408
2140.0000000	4.2943997	-463.5414124
2140.0000000	4.3431997	-13.5972843
2140.0000000	4.3920002	-236.1764679
2140.0000000	4.4407997	-22.2440434
2140.0000000	4.4896002	-15.5818768
2140.0000000	4.5383997	-623.2245483
2140.0000000	4.5871997	-13.0846167
2140.0000000	4.6360002	-21.4328823
2140.0000000	4.6847997	-27.8033638
2140.0000000	4.7336001	-19.4742775
2140.0000000	4.7824001	-16.5678310
2140.0000000	4.8311996	-1409.4771729
2140.0000000	4.8800001	217.3176727
2140.0000000	4.9287996	-193.2354736
2140.0000000	4.9776001	1011.6243286
2140.0000000	5.0264001	81.5912857
2140.0000000	5.0751996	-288.4774780
2140.0000000	5.1240001	-12.4209099
2140.0000000	5.1727996	544.2778931
2140.0000000	5.2216001	30.8912697
2140.0000000	5.2704000	-155.5280304

2140.0000000	5.3191996	-38.6504669
2140.0000000	5.3680000	-3454.9189453
2140.0000000	5.4168000	514.6493530
2140.0000000	5.4656000	-196.5111847
2140.0000000	5.5144000	36.8221283
2140.0000000	5.5631995	476.8111877
2140.0000000	5.6120000	383.8000488
2140.0000000	5.6608000	-36.6072845
2140.0000000	5.7096000	60.7283478
2140.0000000	5.7584000	129.6616821
2140.0000000	5.8071995	-11.6219349
2140.0000000	5.8559999	28.7842884
2140.0000000	5.9047999	-16.5825558
2140.0000000	5.9535999	-39.2236900
2140.0000000	6.0023999	-1537.4985352
2140.0000000	6.0511999	-14.5928097
2140.0000000	6.0999999	-19.3124542
2140.0000000	6.1487999	-13.3271523
2140.0000000	6.1975999	-23.0150795
2140.0000000	6.2463999	-1751.4987793
2140.0000000	6.2951999	-18.9757652
2140.0000000	6.3439999	-335.2783203
2140.0000000	6.3927999	-15.0459919
2140.0000000	6.4416003	-12.4539280
2140.0000000	6.4903998	-11.3418732
2140.0000000	6.5391998	-11.8849154
2140.0000000	6.5879998	305.3505249
2140.0000000	6.6367998	-12.1474590
2140.0000000	6.6856003	-13.8345146
2140.0000000	6.7343998	-13.4804668
2140.0000000	6.7831998	-14.3248377
2140.0000000	6.8319998	56.2480545
2140.0000000	6.8807998	-14.0335360
2140.0000000	6.9296002	-362.4252930
2140.0000000	6.9783998	-6.5492530
2140.0000000	7.0271997	-14.0200834
2140.0000000	7.0760002	6.3820829
2140.0000000	7.1247997	-1682.8157959
2140.0000000	7.1736002	-1349.6591797
2140.0000000	7.2223997	-768.5328369
2140.0000000	7.2711997	-167.8528900
2140.0000000	7.3200002	-17.5221958
2140.0000000	7.3687997	145.4258270
2140.0000000	7.4176002	306.2425232
2140.0000000	7.4663997	240.1537476
2140.0000000	7.5151997	-751.1203613
2140.0000000	7.5640001	-829.4261475
2140.0000000	7.6127996	-1951.9106445
2140.0000000	7.6616001	-627.9270630
2140.0000000	7.7104001	-323.5989990
2140.0000000	7.7591996	31.7983551
2140.0000000	7.8080001	318.2612610
2140.0000000	7.8567996	419.9816284
2140.0000000	7.9056001	-876.9385986
2140.0000000	7.9544001	1394.5562744
2140.0000000	8.0031996	1799.5972900
2140.0000000	8.0520000	1796.1346436
2140.0000000	8.1007996	1900.7260742

2140.0000000	8.1496000	2027.2097168
2140.0000000	8.1983995	1261.0615234
2140.0000000	8.2472000	-2886.6477051
2140.0000000	8.2959995	-1023.5444336
2140.0000000	8.3448000	-815.1846924
2140.0000000	8.3935995	-747.1400146
2140.0000000	8.4423990	-322.2097168
2140.0000000	8.4912004	-751.4857178
2140.0000000	8.5400000	-2026.2376709
2140.0000000	8.5887995	-982.7342529
2140.0000000	8.6375999	191.0811005
2140.0000000	8.6863995	1731.7342529
2140.0000000	8.7351999	-644.3540039
2140.0000000	8.7840004	734.6420898
2140.0000000	8.8327999	37.9858017
2140.0000000	8.8815994	-11.5160341
2140.0000000	8.9303989	-23.5270596
2140.0000000	8.9792004	-11.5883541
2140.0000000	9.0279999	-6.0354953
2140.0000000	9.0767994	6.3974056
2140.0000000	9.1255999	-17.0496979
2140.0000000	9.1743994	-10.1186876
2140.0000000	9.2231998	35.2473068
2140.0000000	9.2720003	-8.5250702
2140.0000000	9.3207998	-9.8585443
2140.0000000	9.3695993	-10.7891417
2140.0000000	9.4183998	-11.7399273
2140.0000000	9.4672003	-11.1659451
2140.0000000	9.5159998	-14.0644140
2140.0000000	9.5648003	-0.7708168
2140.0000000	9.6135998	-13.3074512
2140.0000000	9.6623993	-10.2521381
2140.0000000	9.7111998	-10.1881218
2140.0000000	9.7600002	-10.8622465
2140.0000000	9.8087997	-10.9956694
2140.0000000	9.8575993	2.6945963
2140.0000000	9.9063997	-8.0714178
2140.0000000	9.9552002	-10.9070845
2140.0000000	10.0039997	-10.1024446
2150.0000000	0.3416000	-37.3784866
2150.0000000	0.3904000	-38.8973732
2150.0000000	0.4392000	-39.2366638
2150.0000000	0.4880000	-39.4546280
2150.0000000	0.5368000	-39.7463837
2150.0000000	0.5856000	-39.1765442
2150.0000000	0.6344000	-38.2605247
2150.0000000	0.6832000	-36.4173737
2150.0000000	0.7320000	-36.2335663
2150.0000000	0.7808000	-36.5495338
2150.0000000	0.8296000	-35.8950272
2150.0000000	0.8784000	-35.3069191
2150.0000000	0.9272000	-33.0428085
2150.0000000	0.9760000	-31.0268230
2150.0000000	1.0247999	-30.0362206
2150.0000000	1.0735999	-29.3261242
2150.0000000	1.1224000	-29.6653404
2150.0000000	1.1711999	-30.6811619
2150.0000000	1.2200000	-31.2525425

2150.0000000	1.2687999	-31.1042233
2150.0000000	1.3176000	-30.6223068
2150.0000000	1.3664000	-30.7004642
2150.0000000	1.4152000	-30.1854134
2150.0000000	1.4640000	-29.4914837
2150.0000000	1.5128000	-29.0716782
2150.0000000	1.5616000	-28.9414177
2150.0000000	1.6104001	-1269.8804932
2150.0000000	1.6592000	-40.0438843
2150.0000000	1.7079999	-27.6809692
2150.0000000	1.7567999	-27.4568710
2150.0000000	1.8055999	-26.4681683
2150.0000000	1.8544000	-26.0675564
2150.0000000	1.9031999	-26.6683083
2150.0000000	1.9520000	-26.0082722
2150.0000000	2.0007999	-25.1545105
2150.0000000	2.0495999	-24.8187237
2150.0000000	2.0983999	-25.9844208
2150.0000000	2.1471999	-24.5703144
2150.0000000	2.1960001	-23.9989243
2150.0000000	2.2448001	-24.9534073
2150.0000000	2.2935998	-24.7625961
2150.0000000	2.3423998	-22.6175709
2150.0000000	2.3912001	-24.6441364
2150.0000000	2.4400001	-23.1184368
2150.0000000	2.4888000	-23.4355412
2150.0000000	2.5375998	-14.1996145
2150.0000000	2.5863998	-12.6194468
2150.0000000	2.6352000	-11.0280390
2150.0000000	2.6840000	-16.7559509
2150.0000000	2.7328000	103.2456894
2150.0000000	2.7815998	122.6656265
2150.0000000	2.8304000	-23.6477585
2150.0000000	2.8792000	-20.9861984
2150.0000000	2.9280000	-22.5078144
2150.0000000	2.9768000	-17.7301903
2150.0000000	3.0256000	-20.7633171
2150.0000000	3.0743999	-28.2371159
2150.0000000	3.1231999	-23.8667908
2150.0000000	3.1719999	-28.6737709
2150.0000000	3.2208002	-477.5163879
2150.0000000	3.2695999	-933.8475342
2150.0000000	3.3183999	-20.5119686
2150.0000000	3.3671999	-3231.4938965
2150.0000000	3.4159999	1158.0607910
2150.0000000	3.4648001	-7.7855425
2150.0000000	3.5135999	-26.6991844
2150.0000000	3.5623999	-307.3049011
2150.0000000	3.6111999	-1925.6583252
2150.0000000	3.6600001	-436.7095337
2150.0000000	3.7088001	-576.3046265
2150.0000000	3.7575998	-901.7233887
2150.0000000	3.8063998	-1635.5039063
2150.0000000	3.8552001	-2374.0742188
2150.0000000	3.9040000	-2327.6325684
2150.0000000	3.9528000	1063.0688477
2150.0000000	4.0015998	-233.3905029
2150.0000000	4.0503998	650.4253540

2150.0000000	4.0991998	82.0044632
2150.0000000	4.1479998	-621.2961426
2150.0000000	4.1967998	1.4082737
2150.0000000	4.2456002	-236.4247284
2150.0000000	4.2943997	-463.3408508
2150.0000000	4.3431997	-13.5994644
2150.0000000	4.3920002	-236.0783539
2150.0000000	4.4407997	-22.2422676
2150.0000000	4.4896002	-15.5827599
2150.0000000	4.5383997	-622.9634399
2150.0000000	4.5871997	-13.0866337
2150.0000000	4.6360002	-21.4313412
2150.0000000	4.6847997	-27.7994385
2150.0000000	4.7336001	-19.4735546
2150.0000000	4.7824001	-16.5682850
2150.0000000	4.8311996	-1408.9459229
2150.0000000	4.8800001	217.2281189
2150.0000000	4.9287996	-193.1681976
2150.0000000	4.9776001	1011.2317505
2150.0000000	5.0264001	81.5537720
2150.0000000	5.0751996	-288.3738708
2150.0000000	5.1240001	-12.4218311
2150.0000000	5.1727996	544.1620483
2150.0000000	5.2216001	30.8818245
2150.0000000	5.2704000	-155.4999390
2150.0000000	5.3191996	-38.6459160
2150.0000000	5.3680000	-3454.2265625
2150.0000000	5.4168000	514.5426025
2150.0000000	5.4656000	-196.4750061
2150.0000000	5.5144000	36.8114700
2150.0000000	5.5631995	476.7126160
2150.0000000	5.6120000	383.7200623
2150.0000000	5.6608000	-36.6031952
2150.0000000	5.7096000	60.7129135
2150.0000000	5.7584000	129.6327515
2150.0000000	5.8071995	-11.6228065
2150.0000000	5.8559999	28.7754383
2150.0000000	5.9047999	-16.5823574
2150.0000000	5.9535999	-39.2189484
2150.0000000	6.0023999	-1537.1940918
2150.0000000	6.0511999	-14.5930204
2150.0000000	6.0999999	-19.3116226
2150.0000000	6.1487999	-13.3276157
2150.0000000	6.1975999	-23.0133133
2150.0000000	6.2463999	-1751.1513672
2150.0000000	6.2951999	-18.9749508
2150.0000000	6.3439999	-335.2142639
2150.0000000	6.3927999	-15.0459738
2150.0000000	6.4416003	-12.4545145
2150.0000000	6.4903998	-11.3425484
2150.0000000	6.5391998	-11.8855972
2150.0000000	6.5879998	304.6598206
2150.0000000	6.6367998	-12.1480083
2150.0000000	6.6856003	-13.8346853
2150.0000000	6.7343998	-13.4806833
2150.0000000	6.7831998	-14.3244219
2150.0000000	6.8319998	56.2289352
2150.0000000	6.8807998	-14.0333967

2150.0000000	6.9296002	-361.6638489
2150.0000000	6.9783998	-6.5620766
2150.0000000	7.0271997	-14.0190554
2150.0000000	7.0760002	6.3398981
2150.0000000	7.1247997	-1678.8698730
2150.0000000	7.1736002	-1346.4963379
2150.0000000	7.2223997	-766.7396851
2150.0000000	7.2711997	-167.4851074
2150.0000000	7.3200002	-17.5102081
2150.0000000	7.3687997	145.0513000
2150.0000000	7.4176002	305.4866943
2150.0000000	7.4663997	239.5560913
2150.0000000	7.5151997	-749.3593140
2150.0000000	7.5640001	-827.4786987
2150.0000000	7.6127996	-1947.2929688
2150.0000000	7.6616001	-626.4556885
2150.0000000	7.7104001	-322.8527832
2150.0000000	7.7591996	31.6973076
2150.0000000	7.8080001	317.4759216
2150.0000000	7.8567996	418.9535522
2150.0000000	7.9056001	-874.8656616
2150.0000000	7.9544001	1391.2463379
2150.0000000	8.0031996	1795.3276367
2150.0000000	8.0520000	1791.8726807
2150.0000000	8.1007996	1896.2165527
2150.0000000	8.1496000	2022.4017334
2150.0000000	8.1983995	1258.0690918
2150.0000000	8.2472000	-2879.8127441
2150.0000000	8.2959995	-1021.1242065
2150.0000000	8.3448000	-813.2555542
2150.0000000	8.3935995	-745.3723145
2150.0000000	8.4423990	-321.4517212
2150.0000000	8.4912004	-749.7095337
2150.0000000	8.5400000	-2021.4378662
2150.0000000	8.5887995	-980.4127197
2150.0000000	8.6375999	190.6197815
2150.0000000	8.6863995	1727.6158447
2150.0000000	8.7351999	-642.8297119
2150.0000000	8.7840004	732.8853760
2150.0000000	8.8327999	37.8741951
2150.0000000	8.8815994	-11.5162086
2150.0000000	8.9303989	-23.5233402
2150.0000000	8.9792004	-11.5884361
2150.0000000	9.0279999	-6.0372639
2150.0000000	9.0767994	6.3916893
2150.0000000	9.1255999	-17.0479851
2150.0000000	9.1743994	-10.1190901
2150.0000000	9.2231998	35.2315750
2150.0000000	9.2720003	-8.5260057
2150.0000000	9.3207998	-9.8590841
2150.0000000	9.3695993	-10.7893677
2150.0000000	9.4183998	-11.7398033
2150.0000000	9.4672003	-11.1659861
2150.0000000	9.5159998	-14.0635223
2150.0000000	9.5648003	-0.7741890
2150.0000000	9.6135998	-13.3066607
2150.0000000	9.6623993	-10.2524137
2150.0000000	9.7111998	-10.1883936

2150.0000000	9.7600002	-10.8622885
2150.0000000	9.8087997	-10.9956770
2150.0000000	9.8575993	2.6901741
2150.0000000	9.9063997	-8.0723324
2150.0000000	9.9552002	-10.9070892
2150.0000000	10.0039997	-10.1026917
2160.0000000	0.3416000	-37.2842331
2160.0000000	0.3904000	-38.8914566
2160.0000000	0.4392000	-39.2376251
2160.0000000	0.4880000	-39.4534187
2160.0000000	0.5368000	-39.7475586
2160.0000000	0.5856000	-39.1751633
2160.0000000	0.6344000	-38.2608337
2160.0000000	0.6832000	-36.4196014
2160.0000000	0.7320000	-36.2356186
2160.0000000	0.7808000	-36.5497055
2160.0000000	0.8296000	-35.8911209
2160.0000000	0.8784000	-35.3037872
2160.0000000	0.9272000	-33.0272293
2160.0000000	0.9760000	-31.0220070
2160.0000000	1.0247999	-30.0417290
2160.0000000	1.0735999	-29.3114014
2160.0000000	1.1224000	-29.6470642
2160.0000000	1.1711999	-30.6743107
2160.0000000	1.2200000	-31.2543182
2160.0000000	1.2687999	-31.1063919
2160.0000000	1.3176000	-30.6186256
2160.0000000	1.3664000	-30.7000408
2160.0000000	1.4152000	-30.1856194
2160.0000000	1.4640000	-29.0009537
2160.0000000	1.5128000	-28.4076099
2160.0000000	1.5616000	-28.9357109
2160.0000000	1.6104001	-1267.3376465
2160.0000000	1.6592000	-39.7499542
2160.0000000	1.7079999	-27.6847153
2160.0000000	1.7567999	-27.4462032
2160.0000000	1.8055999	-26.4824638
2160.0000000	1.8544000	-26.0650406
2160.0000000	1.9031999	-26.6793480
2160.0000000	1.9520000	-26.0334454
2160.0000000	2.0007999	-25.1838818
2160.0000000	2.0495999	-24.8221283
2160.0000000	2.0983999	-25.9968777
2160.0000000	2.1471999	-24.6569748
2160.0000000	2.1960001	-24.0025787
2160.0000000	2.2448001	-24.9929752
2160.0000000	2.2935998	-24.7604847
2160.0000000	2.3423998	-22.6319752
2160.0000000	2.3912001	-24.6424999
2160.0000000	2.4400001	-23.1209240
2160.0000000	2.4888000	-23.4353237
2160.0000000	2.5375998	-14.2167225
2160.0000000	2.5863998	-12.6392050
2160.0000000	2.6352000	-11.0507832
2160.0000000	2.6840000	-16.7678928
2160.0000000	2.7328000	102.9796753
2160.0000000	2.7815998	122.3591995
2160.0000000	2.8304000	-23.6464539

2160.0000000	2.8792000	-20.9894619
2160.0000000	2.9280000	-22.5072460
2160.0000000	2.9768000	-17.7385616
2160.0000000	3.0256000	-20.7659016
2160.0000000	3.0743999	-28.2257290
2160.0000000	3.1231999	-23.8633347
2160.0000000	3.1719999	-28.6654778
2160.0000000	3.2208002	-477.2786560
2160.0000000	3.2695999	-933.3726807
2160.0000000	3.3183999	-20.5121670
2160.0000000	3.3671999	-3230.0322266
2160.0000000	3.4159999	1157.5253906
2160.0000000	3.4648001	-7.7912502
2160.0000000	3.5135999	-26.6961937
2160.0000000	3.5623999	-307.1753235
2160.0000000	3.6111999	-1924.7985840
2160.0000000	3.6600001	-436.5213623
2160.0000000	3.7088001	-576.0532837
2160.0000000	3.7575998	-901.3248291
2160.0000000	3.8063998	-1634.7740479
2160.0000000	3.8552001	-2373.0107422
2160.0000000	3.9040000	-2326.5900879
2160.0000000	3.9528000	1062.5805664
2160.0000000	4.0015998	-233.2933960
2160.0000000	4.0503998	650.1227417
2160.0000000	4.0991998	81.9587860
2160.0000000	4.1479998	-621.0239258
2160.0000000	4.1967998	1.3990173
2160.0000000	4.2456002	-236.3264313
2160.0000000	4.2943997	-463.1402893
2160.0000000	4.3431997	-13.6016445
2160.0000000	4.3920002	-235.9802399
2160.0000000	4.4407997	-22.2404938
2160.0000000	4.4896002	-15.5836430
2160.0000000	4.5383997	-622.7023926
2160.0000000	4.5871997	-13.0886497
2160.0000000	4.6360002	-21.4297981
2160.0000000	4.6847997	-27.7955132
2160.0000000	4.7336001	-19.4728336
2160.0000000	4.7824001	-16.5687389
2160.0000000	4.8311996	-1408.4146729
2160.0000000	4.8800001	217.1385803
2160.0000000	4.9287996	-193.1009369
2160.0000000	4.9776001	1010.8392944
2160.0000000	5.0264001	81.5162506
2160.0000000	5.0751996	-288.2702332
2160.0000000	5.1240001	-12.4227524
2160.0000000	5.1727996	544.0461426
2160.0000000	5.2216001	30.8723831
2160.0000000	5.2704000	-155.4718170
2160.0000000	5.3191996	-38.6413651
2160.0000000	5.3680000	-3453.5341797
2160.0000000	5.4168000	514.4358521
2160.0000000	5.4656000	-196.4388275
2160.0000000	5.5144000	36.8008194
2160.0000000	5.5631995	476.6140442
2160.0000000	5.6120000	383.6401062
2160.0000000	5.6608000	-36.5991058

2160.0000000	5.7096000	60.6974869
2160.0000000	5.7584000	129.6038208
2160.0000000	5.8071995	-11.6236763
2160.0000000	5.8559999	28.7665882
2160.0000000	5.9047999	-16.5821590
2160.0000000	5.9535999	-39.2142105
2160.0000000	6.0023999	-1536.8897705
2160.0000000	6.0511999	-14.5932322
2160.0000000	6.0999999	-19.3107910
2160.0000000	6.1487999	-13.3280783
2160.0000000	6.1975999	-23.0115471
2160.0000000	6.2463999	-1750.8039551
2160.0000000	6.2951999	-18.9741364
2160.0000000	6.3439999	-335.1502075
2160.0000000	6.3927999	-15.0459547
2160.0000000	6.4416003	-12.4551010
2160.0000000	6.4903998	-11.3432245
2160.0000000	6.5391998	-11.8862782
2160.0000000	6.5879998	303.9691162
2160.0000000	6.6367998	-12.1485577
2160.0000000	6.6856003	-13.8348551
2160.0000000	6.7343998	-13.4808989
2160.0000000	6.7831998	-14.3240061
2160.0000000	6.8319998	56.2098236
2160.0000000	6.8807998	-14.0332584
2160.0000000	6.9296002	-360.9024048
2160.0000000	6.9783998	-6.5749002
2160.0000000	7.0271997	-14.0180283
2160.0000000	7.0760002	6.2977133
2160.0000000	7.1247997	-1674.9240723
2160.0000000	7.1736002	-1343.3333740
2160.0000000	7.2223997	-764.9465332
2160.0000000	7.2711997	-167.1173401
2160.0000000	7.3200002	-17.4982185
2160.0000000	7.3687997	144.6767578
2160.0000000	7.4176002	304.7308655
2160.0000000	7.4663997	238.9584351
2160.0000000	7.5151997	-747.5982666
2160.0000000	7.5640001	-825.5313110
2160.0000000	7.6127996	-1942.6754150
2160.0000000	7.6616001	-624.9843750
2160.0000000	7.7104001	-322.1065063
2160.0000000	7.7591996	31.5962563
2160.0000000	7.8080001	316.6905518
2160.0000000	7.8567996	417.9254150
2160.0000000	7.9056001	-872.7927856
2160.0000000	7.9544001	1387.9364014
2160.0000000	8.0031996	1791.0581055
2160.0000000	8.0520000	1787.6105957
2160.0000000	8.1007996	1891.7070313
2160.0000000	8.1496000	2017.5937500
2160.0000000	8.1983995	1255.0765381
2160.0000000	8.2472000	-2872.9780273
2160.0000000	8.2959995	-1018.7040405
2160.0000000	8.3448000	-811.3263550
2160.0000000	8.3935995	-743.6046753
2160.0000000	8.4423990	-320.6936951
2160.0000000	8.4912004	-747.9332886

2160.0000000	8.5400000	-2016.6380615
2160.0000000	8.5887995	-978.0911865
2160.0000000	8.6375999	190.1584625
2160.0000000	8.6863995	1723.4975586
2160.0000000	8.7351999	-641.3054810
2160.0000000	8.7840004	731.1286011
2160.0000000	8.8327999	37.7625885
2160.0000000	8.8815994	-11.5163832
2160.0000000	8.9303989	-23.5196190
2160.0000000	8.9792004	-11.5885191
2160.0000000	9.0279999	-6.0390320
2160.0000000	9.0767994	6.3859711
2160.0000000	9.1255999	-17.0462704
2160.0000000	9.1743994	-10.1194916
2160.0000000	9.2231998	35.2158432
2160.0000000	9.2720003	-8.5269423
2160.0000000	9.3207998	-9.8596230
2160.0000000	9.3695993	-10.7895947
2160.0000000	9.4183998	-11.7396784
2160.0000000	9.4672003	-11.1660271
2160.0000000	9.5159998	-14.0626316
2160.0000000	9.5648003	-0.7775612
2160.0000000	9.6135998	-13.3058701
2160.0000000	9.6623993	-10.2526884
2160.0000000	9.7111998	-10.1886654
2160.0000000	9.7600002	-10.8623295
2160.0000000	9.8087997	-10.9956856
2160.0000000	9.8575993	2.6857519
2160.0000000	9.9063997	-8.0732460
2160.0000000	9.9552002	-10.9070930
2160.0000000	10.0039997	-10.1029396
2170.0000000	0.3416000	-37.4262733
2170.0000000	0.3904000	-38.8855400
2170.0000000	0.4392000	-39.2385864
2170.0000000	0.4880000	-39.4522095
2170.0000000	0.5368000	-39.7487297
2170.0000000	0.5856000	-39.1737823
2170.0000000	0.6344000	-38.2611465
2170.0000000	0.6832000	-36.4218254
2170.0000000	0.7320000	-36.2376747
2170.0000000	0.7808000	-36.5498734
2170.0000000	0.8296000	-35.8872108
2170.0000000	0.8784000	-35.3006554
2170.0000000	0.9272000	-33.0116463
2170.0000000	0.9760000	-31.0171890
2170.0000000	1.0247999	-30.0472374
2170.0000000	1.0735999	-29.2966785
2170.0000000	1.1224000	-29.6287880
2170.0000000	1.1711999	-30.6674576
2170.0000000	1.2200000	-31.2560921
2170.0000000	1.2687999	-31.1085606
2170.0000000	1.3176000	-30.6149445
2170.0000000	1.3664000	-30.6996174
2170.0000000	1.4152000	-30.1858234
2170.0000000	1.4640000	-28.4167366
2170.0000000	1.5128000	-28.0790043
2170.0000000	1.5616000	-28.9300060
2170.0000000	1.6104001	-1264.7947998

2170.0000000	1.6592000	-39.4560242
2170.0000000	1.7079999	-27.6884632
2170.0000000	1.7567999	-27.4355354
2170.0000000	1.8055999	-26.4967594
2170.0000000	1.8544000	-26.0625248
2170.0000000	1.9031999	-26.6903877
2170.0000000	1.9520000	-26.0586185
2170.0000000	2.0007999	-25.2132549
2170.0000000	2.0495999	-24.8255329
2170.0000000	2.0983999	-26.0093346
2170.0000000	2.1471999	-24.7436352
2170.0000000	2.1960001	-24.0062332
2170.0000000	2.2448001	-25.0325451
2170.0000000	2.2935998	-24.7583714
2170.0000000	2.3423998	-22.6463795
2170.0000000	2.3912001	-24.6408634
2170.0000000	2.4400001	-23.1234093
2170.0000000	2.4888000	-23.4351063
2170.0000000	2.5375998	-14.2338305
2170.0000000	2.5863998	-12.6589642
2170.0000000	2.6352000	-11.0735273
2170.0000000	2.6840000	-16.7798347
2170.0000000	2.7328000	102.7136612
2170.0000000	2.7815998	122.0527878
2170.0000000	2.8304000	-23.6451492
2170.0000000	2.8792000	-20.9927254
2170.0000000	2.9280000	-22.5066776
2170.0000000	2.9768000	-17.7469330
2170.0000000	3.0256000	-20.7684860
2170.0000000	3.0743999	-28.2143421
2170.0000000	3.1231999	-23.8598804
2170.0000000	3.1719999	-28.6571846
2170.0000000	3.2208002	-477.0408936
2170.0000000	3.2695999	-932.8977051
2170.0000000	3.3183999	-20.5123653
2170.0000000	3.3671999	-3228.5705566
2170.0000000	3.4159999	1156.9901123
2170.0000000	3.4648001	-7.7969589
2170.0000000	3.5135999	-26.6932030
2170.0000000	3.5623999	-307.0457153
2170.0000000	3.6111999	-1923.9388428
2170.0000000	3.6600001	-436.3332520
2170.0000000	3.7088001	-575.8018799
2170.0000000	3.7575998	-900.9263306
2170.0000000	3.8063998	-1634.0443115
2170.0000000	3.8552001	-2371.9472656
2170.0000000	3.9040000	-2325.5476074
2170.0000000	3.9528000	1062.0921631
2170.0000000	4.0015998	-233.1962891
2170.0000000	4.0503998	649.8201904
2170.0000000	4.0991998	81.9131012
2170.0000000	4.1479998	-620.7517090
2170.0000000	4.1967998	1.3897610
2170.0000000	4.2456002	-236.2281342
2170.0000000	4.2943997	-462.9396667
2170.0000000	4.3431997	-13.6038256
2170.0000000	4.3920002	-235.8821259
2170.0000000	4.4407997	-22.2387180

2170.0000000	4.4896002	-15.5845251
2170.0000000	4.5383997	-622.4413452
2170.0000000	4.5871997	-13.0906658
2170.0000000	4.6360002	-21.4282551
2170.0000000	4.6847997	-27.7915878
2170.0000000	4.7336001	-19.4721107
2170.0000000	4.7824001	-16.5691929
2170.0000000	4.8311996	-1407.8834229
2170.0000000	4.8800001	217.0490265
2170.0000000	4.9287996	-193.0336761
2170.0000000	4.9776001	1010.4468384
2170.0000000	5.0264001	81.4787292
2170.0000000	5.0751996	-288.1665955
2170.0000000	5.1240001	-12.4236736
2170.0000000	5.1727996	543.9302979
2170.0000000	5.2216001	30.8629379
2170.0000000	5.2704000	-155.4437256
2170.0000000	5.3191996	-38.6368179
2170.0000000	5.3680000	-3452.8417969
2170.0000000	5.4168000	514.3290405
2170.0000000	5.4656000	-196.4026337
2170.0000000	5.5144000	36.7901611
2170.0000000	5.5631995	476.5155029
2170.0000000	5.6120000	383.5601196
2170.0000000	5.6608000	-36.5950203
2170.0000000	5.7096000	60.6820602
2170.0000000	5.7584000	129.5748901
2170.0000000	5.8071995	-11.6245470
2170.0000000	5.8559999	28.7577381
2170.0000000	5.9047999	-16.5819626
2170.0000000	5.9535999	-39.2094727
2170.0000000	6.0023999	-1536.5852051
2170.0000000	6.0511999	-14.5934439
2170.0000000	6.0999999	-19.3099575
2170.0000000	6.1487999	-13.3285408
2170.0000000	6.1975999	-23.0097828
2170.0000000	6.2463999	-1750.4566650
2170.0000000	6.2951999	-18.9733219
2170.0000000	6.3439999	-335.0861511
2170.0000000	6.3927999	-15.0459366
2170.0000000	6.4416003	-12.4556885
2170.0000000	6.4903998	-11.3439007
2170.0000000	6.5391998	-11.8869591
2170.0000000	6.5879998	303.2784424
2170.0000000	6.6367998	-12.1491070
2170.0000000	6.6856003	-13.8350258
2170.0000000	6.7343998	-13.4811144
2170.0000000	6.7831998	-14.3235903
2170.0000000	6.8319998	56.1907120
2170.0000000	6.8807998	-14.0331192
2170.0000000	6.9296002	-360.1409302
2170.0000000	6.9783998	-6.5877237
2170.0000000	7.0271997	-14.0170002
2170.0000000	7.0760002	6.2555265
2170.0000000	7.1247997	-1670.9781494
2170.0000000	7.1736002	-1340.1705322
2170.0000000	7.2223997	-763.1533203
2170.0000000	7.2711997	-166.7495575

2170.0000000	7.3200002	-17.4862289
2170.0000000	7.3687997	144.3022308
2170.0000000	7.4176002	303.9750366
2170.0000000	7.4663997	238.3607941
2170.0000000	7.5151997	-745.8372803
2170.0000000	7.5640001	-823.5839233
2170.0000000	7.6127996	-1938.0577393
2170.0000000	7.6616001	-623.5130615
2170.0000000	7.7104001	-321.3602295
2170.0000000	7.7591996	31.4952087
2170.0000000	7.8080001	315.9051819
2170.0000000	7.8567996	416.8973389
2170.0000000	7.9056001	-870.7198486
2170.0000000	7.9544001	1384.6265869
2170.0000000	8.0031996	1786.7884521
2170.0000000	8.0520000	1783.3486328
2170.0000000	8.1007996	1887.1975098
2170.0000000	8.1496000	2012.7857666
2170.0000000	8.1983995	1252.0839844
2170.0000000	8.2472000	-2866.1433105
2170.0000000	8.2959995	-1016.2838745
2170.0000000	8.3448000	-809.3971558
2170.0000000	8.3935995	-741.8369751
2170.0000000	8.4423990	-319.9356995
2170.0000000	8.4912004	-746.1570435
2170.0000000	8.5400000	-2011.8382568
2170.0000000	8.5887995	-975.7696533
2170.0000000	8.6375999	189.6971436
2170.0000000	8.6863995	1719.3791504
2170.0000000	8.7351999	-639.7812500
2170.0000000	8.7840004	729.3718872
2170.0000000	8.8327999	37.6509781
2170.0000000	8.8815994	-11.5165577
2170.0000000	8.9303989	-23.5158997
2170.0000000	8.9792004	-11.5886011
2170.0000000	9.0279999	-6.0408006
2170.0000000	9.0767994	6.3802528
2170.0000000	9.1255999	-17.0445557
2170.0000000	9.1743994	-10.1198940
2170.0000000	9.2231998	35.2001114
2170.0000000	9.2720003	-8.5278788
2170.0000000	9.3207998	-9.8601627
2170.0000000	9.3695993	-10.7898207
2170.0000000	9.4183998	-11.7395535
2170.0000000	9.4672003	-11.1660681
2170.0000000	9.5159998	-14.0617409
2170.0000000	9.5648003	-0.7809343
2170.0000000	9.6135998	-13.3050795
2170.0000000	9.6623993	-10.2529640
2170.0000000	9.7111998	-10.1889372
2170.0000000	9.7600002	-10.8623714
2170.0000000	9.8087997	-10.9956932
2170.0000000	9.8575993	2.6813297
2170.0000000	9.9063997	-8.0741596
2170.0000000	9.9552002	-10.9070978
2170.0000000	10.0039997	-10.1031866
2180.0000000	0.3416000	-36.8705444
2180.0000000	0.3904000	-38.8796272

2180.0000000	0.4392000	-39.2395439
2180.0000000	0.4880000	-39.4510002
2180.0000000	0.5368000	-39.7499046
2180.0000000	0.5856000	-39.1724014
2180.0000000	0.6344000	-38.2614555
2180.0000000	0.6832000	-36.4240494
2180.0000000	0.7320000	-36.2397270
2180.0000000	0.7808000	-36.5500450
2180.0000000	0.8296000	-35.8833046
2180.0000000	0.8784000	-35.2975235
2180.0000000	0.9272000	-32.9960670
2180.0000000	0.9760000	-31.0123730
2180.0000000	1.0247999	-30.0527458
2180.0000000	1.0735999	-29.2819557
2180.0000000	1.1224000	-29.6105099
2180.0000000	1.1711999	-30.6606064
2180.0000000	1.2200000	-31.2578678
2180.0000000	1.2687999	-31.1107273
2180.0000000	1.3176000	-30.6112614
2180.0000000	1.3664000	-30.6991940
2180.0000000	1.4152000	-30.1860275
2180.0000000	1.4640000	-29.4826717
2180.0000000	1.5128000	-28.8433228
2180.0000000	1.5616000	-28.9242992
2180.0000000	1.6104001	-1262.2520752
2180.0000000	1.6592000	-39.1620903
2180.0000000	1.7079999	-27.6922092
2180.0000000	1.7567999	-27.4248695
2180.0000000	1.8055999	-26.5110550
2180.0000000	1.8544000	-26.0600090
2180.0000000	1.9031999	-26.7014275
2180.0000000	1.9520000	-26.0837917
2180.0000000	2.0007999	-25.2426262
2180.0000000	2.0495999	-24.8289356
2180.0000000	2.0983999	-26.0217915
2180.0000000	2.1471999	-24.8302956
2180.0000000	2.1960001	-24.0098896
2180.0000000	2.2448001	-25.0721149
2180.0000000	2.2935998	-24.7562599
2180.0000000	2.3423998	-22.6607838
2180.0000000	2.3912001	-24.6392269
2180.0000000	2.4400001	-23.1258965
2180.0000000	2.4888000	-23.4348888
2180.0000000	2.5375998	-14.2509384
2180.0000000	2.5863998	-12.6787224
2180.0000000	2.6352000	-11.0962715
2180.0000000	2.6840000	-16.7917786
2180.0000000	2.7328000	102.4476471
2180.0000000	2.7815998	121.7463760
2180.0000000	2.8304000	-23.6438465
2180.0000000	2.8792000	-20.9959888
2180.0000000	2.9280000	-22.5061073
2180.0000000	2.9768000	-17.7553024
2180.0000000	3.0256000	-20.7710705
2180.0000000	3.0743999	-28.2029552
2180.0000000	3.1231999	-23.8564243
2180.0000000	3.1719999	-28.6488914
2180.0000000	3.2208002	-476.8031616

2180.0000000	3.2695999	-932.4227295
2180.0000000	3.3183999	-20.5125637
2180.0000000	3.3671999	-3227.1088867
2180.0000000	3.4159999	1156.4548340
2180.0000000	3.4648001	-7.8026667
2180.0000000	3.5135999	-26.6902122
2180.0000000	3.5623999	-306.9161377
2180.0000000	3.6111999	-1923.0791016
2180.0000000	3.6600001	-436.1451416
2180.0000000	3.7088001	-575.5504761
2180.0000000	3.7575998	-900.5277710
2180.0000000	3.8063998	-1633.3144531
2180.0000000	3.8552001	-2370.8840332
2180.0000000	3.9040000	-2324.5051270
2180.0000000	3.9528000	1061.6038818
2180.0000000	4.0015998	-233.0991669
2180.0000000	4.0503998	649.5176392
2180.0000000	4.0991998	81.8674164
2180.0000000	4.1479998	-620.4794312
2180.0000000	4.1967998	1.3805046
2180.0000000	4.2456002	-236.1298218
2180.0000000	4.2943997	-462.7391052
2180.0000000	4.3431997	-13.6060057
2180.0000000	4.3920002	-235.7840118
2180.0000000	4.4407997	-22.2369423
2180.0000000	4.4896002	-15.5854082
2180.0000000	4.5383997	-622.1802368
2180.0000000	4.5871997	-13.0926819
2180.0000000	4.6360002	-21.4267120
2180.0000000	4.6847997	-27.7876625
2180.0000000	4.7336001	-19.4713879
2180.0000000	4.7824001	-16.5696487
2180.0000000	4.8311996	-1407.3520508
2180.0000000	4.8800001	216.9594727
2180.0000000	4.9287996	-192.9664154
2180.0000000	4.9776001	1010.0543823
2180.0000000	5.0264001	81.4412079
2180.0000000	5.0751996	-288.0629578
2180.0000000	5.1240001	-12.4245949
2180.0000000	5.1727996	543.8144531
2180.0000000	5.2216001	30.8534927
2180.0000000	5.2704000	-155.4156036
2180.0000000	5.3191996	-38.6322708
2180.0000000	5.3680000	-3452.1494141
2180.0000000	5.4168000	514.2222290
2180.0000000	5.4656000	-196.3664551
2180.0000000	5.5144000	36.7795029
2180.0000000	5.5631995	476.4169312
2180.0000000	5.6120000	383.4801331
2180.0000000	5.6608000	-36.5909348
2180.0000000	5.7096000	60.6666260
2180.0000000	5.7584000	129.5459595
2180.0000000	5.8071995	-11.6254177
2180.0000000	5.8559999	28.7488842
2180.0000000	5.9047999	-16.5817642
2180.0000000	5.9535999	-39.2047348
2180.0000000	6.0023999	-1536.2808838
2180.0000000	6.0511999	-14.5936546

2180.0000000	6.0999999	-19.3091259
2180.0000000	6.1487999	-13.3290043
2180.0000000	6.1975999	-23.0080185
2180.0000000	6.2463999	-1750.1092529
2180.0000000	6.2951999	-18.9725075
2180.0000000	6.3439999	-335.0221252
2180.0000000	6.3927999	-15.0459185
2180.0000000	6.4416003	-12.4562750
2180.0000000	6.4903998	-11.3445759
2180.0000000	6.5391998	-11.8876400
2180.0000000	6.5879998	302.5877380
2180.0000000	6.6367998	-12.1496553
2180.0000000	6.6856003	-13.8351955
2180.0000000	6.7343998	-13.4813309
2180.0000000	6.7831998	-14.3231745
2180.0000000	6.8319998	56.1715927
2180.0000000	6.8807998	-14.0329809
2180.0000000	6.9296002	-359.3794861
2180.0000000	6.9783998	-6.6005473
2180.0000000	7.0271997	-14.0159721
2180.0000000	7.0760002	6.2133417
2180.0000000	7.1247997	-1667.0323486
2180.0000000	7.1736002	-1337.0076904
2180.0000000	7.2223997	-761.3601685
2180.0000000	7.2711997	-166.3817749
2180.0000000	7.3200002	-17.4742413
2180.0000000	7.3687997	143.9277039
2180.0000000	7.4176002	303.2192078
2180.0000000	7.4663997	237.7631378
2180.0000000	7.5151997	-744.0762329
2180.0000000	7.5640001	-821.6365356
2180.0000000	7.6127996	-1933.4401855
2180.0000000	7.6616001	-622.0416870
2180.0000000	7.7104001	-320.6139526
2180.0000000	7.7591996	31.3941612
2180.0000000	7.8080001	315.1198425
2180.0000000	7.8567996	415.8692627
2180.0000000	7.9056001	-868.6469727
2180.0000000	7.9544001	1381.3166504
2180.0000000	8.0031996	1782.5187988
2180.0000000	8.0520000	1779.0866699
2180.0000000	8.1007996	1882.6879883
2180.0000000	8.1496000	2007.9776611
2180.0000000	8.1983995	1249.0915527
2180.0000000	8.2472000	-2859.3083496
2180.0000000	8.2959995	-1013.8637085
2180.0000000	8.3448000	-807.4680176
2180.0000000	8.3935995	-740.0692749
2180.0000000	8.4423990	-319.1776733
2180.0000000	8.4912004	-744.3808594
2180.0000000	8.5400000	-2007.0384521
2180.0000000	8.5887995	-973.4481201
2180.0000000	8.6375999	189.2358093
2180.0000000	8.6863995	1715.2608643
2180.0000000	8.7351999	-638.2569580
2180.0000000	8.7840004	727.6151123
2180.0000000	8.8327999	37.5393753
2180.0000000	8.8815994	-11.5167313

2180.0000000	8.9303989	-23.5121803
2180.0000000	8.9792004	-11.5886831
2180.0000000	9.0279999	-6.0425687
2180.0000000	9.0767994	6.3745346
2180.0000000	9.1255999	-17.0428429
2180.0000000	9.1743994	-10.1202965
2180.0000000	9.2231998	35.1843796
2180.0000000	9.2720003	-8.5288153
2180.0000000	9.3207998	-9.8607016
2180.0000000	9.3695993	-10.7900467
2180.0000000	9.4183998	-11.7394295
2180.0000000	9.4672003	-11.1661091
2180.0000000	9.5159998	-14.0608501
2180.0000000	9.5648003	-0.7843075
2180.0000000	9.6135998	-13.3042889
2180.0000000	9.6623993	-10.2532387
2180.0000000	9.7111998	-10.1892090
2180.0000000	9.7600002	-10.8624134
2180.0000000	9.8087997	-10.9957008
2180.0000000	9.8575993	2.6769085
2180.0000000	9.9063997	-8.0750732
2180.0000000	9.9552002	-10.9071016
2180.0000000	10.0039997	-10.1034346
2190.0000000	0.3416000	-37.3038521
2190.0000000	0.3904000	-38.8737106
2190.0000000	0.4392000	-39.2405052
2190.0000000	0.4880000	-39.4497910
2190.0000000	0.5368000	-39.7510796
2190.0000000	0.5856000	-39.1710205
2190.0000000	0.6344000	-38.2617645
2190.0000000	0.6832000	-36.4262772
2190.0000000	0.7320000	-36.2417793
2190.0000000	0.7808000	-36.5502129
2190.0000000	0.8296000	-35.8793983
2190.0000000	0.8784000	-35.2943916
2190.0000000	0.9272000	-32.9804878
2190.0000000	0.9760000	-31.0075550
2190.0000000	1.0247999	-30.0582542
2190.0000000	1.0735999	-29.2672329
2190.0000000	1.1224000	-29.5922337
2190.0000000	1.1711999	-30.6537533
2190.0000000	1.2200000	-31.2596436
2190.0000000	1.2687999	-31.1128960
2190.0000000	1.3176000	-30.6075802
2190.0000000	1.3664000	-30.6987724
2190.0000000	1.4152000	-30.1862316
2190.0000000	1.4640000	-29.4833546
2190.0000000	1.5128000	-28.8425522
2190.0000000	1.5616000	-28.9185925
2190.0000000	1.6104001	-1259.7092285
2190.0000000	1.6592000	-38.8681564
2190.0000000	1.7079999	-27.6959572
2190.0000000	1.7567999	-27.4142017
2190.0000000	1.8055999	-26.5253506
2190.0000000	1.8544000	-26.0574913
2190.0000000	1.9031999	-26.7124672
2190.0000000	1.9520000	-26.1089649
2190.0000000	2.0007999	-25.2719994

2190.0000000	2.0495999	-24.8323402
2190.0000000	2.0983999	-26.0342484
2190.0000000	2.1471999	-24.9169559
2190.0000000	2.1960001	-24.0135441
2190.0000000	2.2448001	-25.1116848
2190.0000000	2.2935998	-24.7541485
2190.0000000	2.3423998	-22.6751881
2190.0000000	2.3912001	-24.6375904
2190.0000000	2.4400001	-23.1283817
2190.0000000	2.4888000	-23.4346733
2190.0000000	2.5375998	-14.2680464
2190.0000000	2.5863998	-12.6984816
2190.0000000	2.6352000	-11.1190157
2190.0000000	2.6840000	-16.8037186
2190.0000000	2.7328000	102.1816330
2190.0000000	2.7815998	121.4399490
2190.0000000	2.8304000	-23.6425419
2190.0000000	2.8792000	-20.9992523
2190.0000000	2.9280000	-22.5055389
2190.0000000	2.9768000	-17.7636738
2190.0000000	3.0256000	-20.7736549
2190.0000000	3.0743999	-28.1915684
2190.0000000	3.1231999	-23.8529701
2190.0000000	3.1719999	-28.6405983
2190.0000000	3.2208002	-476.5654297
2190.0000000	3.2695999	-931.9477539
2190.0000000	3.3183999	-20.5127602
2190.0000000	3.3671999	-3225.6474609
2190.0000000	3.4159999	1155.9195557
2190.0000000	3.4648001	-7.8083744
2190.0000000	3.5135999	-26.6872215
2190.0000000	3.5623999	-306.7865295
2190.0000000	3.6111999	-1922.2193604
2190.0000000	3.6600001	-435.9569702
2190.0000000	3.7088001	-575.2991333
2190.0000000	3.7575998	-900.1292725
2190.0000000	3.8063998	-1632.5845947
2190.0000000	3.8552001	-2369.8205566
2190.0000000	3.9040000	-2323.4626465
2190.0000000	3.9528000	1061.1156006
2190.0000000	4.0015998	-233.0020599
2190.0000000	4.0503998	649.2150879
2190.0000000	4.0991998	81.8217316
2190.0000000	4.1479998	-620.2072144
2190.0000000	4.1967998	1.3712482
2190.0000000	4.2456002	-236.0315247
2190.0000000	4.2943997	-462.5385437
2190.0000000	4.3431997	-13.6081867
2190.0000000	4.3920002	-235.6858978
2190.0000000	4.4407997	-22.2351665
2190.0000000	4.4896002	-15.5862904
2190.0000000	4.5383997	-621.9191895
2190.0000000	4.5871997	-13.0946980
2190.0000000	4.6360002	-21.4251709
2190.0000000	4.6847997	-27.7837372
2190.0000000	4.7336001	-19.4706650
2190.0000000	4.7824001	-16.5701027
2190.0000000	4.8311996	-1406.8208008

2190.0000000	4.8800001	216.8699188
2190.0000000	4.9287996	-192.8991547
2190.0000000	4.9776001	1009.6618042
2190.0000000	5.0264001	81.4036865
2190.0000000	5.0751996	-287.9593506
2190.0000000	5.1240001	-12.4255161
2190.0000000	5.1727996	543.6985474
2190.0000000	5.2216001	30.8440514
2190.0000000	5.2704000	-155.3875122
2190.0000000	5.3191996	-38.6277161
2190.0000000	5.3680000	-3451.4570313
2190.0000000	5.4168000	514.1154785
2190.0000000	5.4656000	-196.3302765
2190.0000000	5.5144000	36.7688522
2190.0000000	5.5631995	476.3183594
2190.0000000	5.6120000	383.4001770
2190.0000000	5.6608000	-36.5868454
2190.0000000	5.7096000	60.6511917
2190.0000000	5.7584000	129.5170288
2190.0000000	5.8071995	-11.6262884
2190.0000000	5.8559999	28.7400341
2190.0000000	5.9047999	-16.5815659
2190.0000000	5.9535999	-39.1999931
2190.0000000	6.0023999	-1535.9764404
2190.0000000	6.0511999	-14.5938663
2190.0000000	6.0999999	-19.3082924
2190.0000000	6.1487999	-13.3294668
2190.0000000	6.1975999	-23.0062523
2190.0000000	6.2463999	-1749.7618408
2190.0000000	6.2951999	-18.9716930
2190.0000000	6.3439999	-334.9580688
2190.0000000	6.3927999	-15.0459003
2190.0000000	6.4416003	-12.4568624
2190.0000000	6.4903998	-11.3452520
2190.0000000	6.5391998	-11.8883209
2190.0000000	6.5879998	301.8970337
2190.0000000	6.6367998	-12.1502047
2190.0000000	6.6856003	-13.8353662
2190.0000000	6.7343998	-13.4815464
2190.0000000	6.7831998	-14.3227587
2190.0000000	6.8319998	56.1524811
2190.0000000	6.8807998	-14.0328426
2190.0000000	6.9296002	-358.6180420
2190.0000000	6.9783998	-6.6133709
2190.0000000	7.0271997	-14.0149441
2190.0000000	7.0760002	6.1711569
2190.0000000	7.1247997	-1663.0864258
2190.0000000	7.1736002	-1333.8447266
2190.0000000	7.2223997	-759.5670166
2190.0000000	7.2711997	-166.0139923
2190.0000000	7.3200002	-17.4622536
2190.0000000	7.3687997	143.5531769
2190.0000000	7.4176002	302.4633789
2190.0000000	7.4663997	237.1654816
2190.0000000	7.5151997	-742.3151855
2190.0000000	7.5640001	-819.6891479
2190.0000000	7.6127996	-1928.8226318
2190.0000000	7.6616001	-620.5703735

2190.0000000	7.7104001	-319.8676758
2190.0000000	7.7591996	31.2931099
2190.0000000	7.8080001	314.3344727
2190.0000000	7.8567996	414.8411865
2190.0000000	7.9056001	-866.5740356
2190.0000000	7.9544001	1378.0067139
2190.0000000	8.0031996	1778.2492676
2190.0000000	8.0520000	1774.8247070
2190.0000000	8.1007996	1878.1784668
2190.0000000	8.1496000	2003.1696777
2190.0000000	8.1983995	1246.0989990
2190.0000000	8.2472000	-2852.4736328
2190.0000000	8.2959995	-1011.4435425
2190.0000000	8.3448000	-805.5388184
2190.0000000	8.3935995	-738.3016357
2190.0000000	8.4423990	-318.4196777
2190.0000000	8.4912004	-742.6046143
2190.0000000	8.5400000	-2002.2386475
2190.0000000	8.5887995	-971.1265869
2190.0000000	8.6375999	188.7744904
2190.0000000	8.6863995	1711.1424561
2190.0000000	8.7351999	-636.7327271
2190.0000000	8.7840004	725.8583374
2190.0000000	8.8327999	37.4277649
2190.0000000	8.8815994	-11.5169058
2190.0000000	8.9303989	-23.5084610
2190.0000000	8.9792004	-11.5887661
2190.0000000	9.0279999	-6.0443368
2190.0000000	9.0767994	6.3688183
2190.0000000	9.1255999	-17.0411282
2190.0000000	9.1743994	-10.1206980
2190.0000000	9.2231998	35.1686478
2190.0000000	9.2720003	-8.5297508
2190.0000000	9.3207998	-9.8612413
2190.0000000	9.3695993	-10.7902737
2190.0000000	9.4183998	-11.7393045
2190.0000000	9.4672003	-11.1661510
2190.0000000	9.5159998	-14.0599585
2190.0000000	9.5648003	-0.7876797
2190.0000000	9.6135998	-13.3034973
2190.0000000	9.6623993	-10.2535133
2190.0000000	9.7111998	-10.1894798
2190.0000000	9.7600002	-10.8624554
2190.0000000	9.8087997	-10.9957094
2190.0000000	9.8575993	2.6724844
2190.0000000	9.9063997	-8.0759878
2190.0000000	9.9552002	-10.9071064
2190.0000000	10.0039997	-10.1036825
2200.0000000	0.3416000	-37.2832832
2200.0000000	0.3904000	-38.8677940
2200.0000000	0.4392000	-39.2414627
2200.0000000	0.4880000	-39.4485817
2200.0000000	0.5368000	-39.7522507
2200.0000000	0.5856000	-39.1696358
2200.0000000	0.6344000	-38.2620773
2200.0000000	0.6832000	-36.4285011
2200.0000000	0.7320000	-36.2438316
2200.0000000	0.7808000	-36.5503807

2200.0000000	0.8296000	-35.8754921
2200.0000000	0.8784000	-35.2912598
2200.0000000	0.9272000	-32.9649086
2200.0000000	0.9760000	-31.0027390
2200.0000000	1.0247999	-30.0637627
2200.0000000	1.0735999	-29.2525101
2200.0000000	1.1224000	-29.5739574
2200.0000000	1.1711999	-30.6469021
2200.0000000	1.2200000	-31.2614193
2200.0000000	1.2687999	-31.1150646
2200.0000000	1.3176000	-30.6038990
2200.0000000	1.3664000	-30.6983490
2200.0000000	1.4152000	-30.1864357
2200.0000000	1.4640000	-29.4840374
2200.0000000	1.5128000	-28.8417835
2200.0000000	1.5616000	-28.9128857
2200.0000000	1.6104001	-1257.1663818
2200.0000000	1.6592000	-38.5742264
2200.0000000	1.7079999	-27.6997051
2200.0000000	1.7567999	-27.4035358
2200.0000000	1.8055999	-26.5396481
2200.0000000	1.8544000	-26.0549755
2200.0000000	1.9031999	-26.7235069
2200.0000000	1.9520000	-26.1341381
2200.0000000	2.0007999	-25.3013706
2200.0000000	2.0495999	-24.8357449
2200.0000000	2.0983999	-26.0467052
2200.0000000	2.1471999	-25.0036163
2200.0000000	2.1960001	-24.0171986
2200.0000000	2.2448001	-25.1512547
2200.0000000	2.2935998	-24.7520351
2200.0000000	2.3423998	-22.6895924
2200.0000000	2.3912001	-24.6359558
2200.0000000	2.4400001	-23.1308689
2200.0000000	2.4888000	-23.4344559
2200.0000000	2.5375998	-14.2851543
2200.0000000	2.5863998	-12.7182407
2200.0000000	2.6352000	-11.1417599
2200.0000000	2.6840000	-16.8156605
2200.0000000	2.7328000	101.9156113
2200.0000000	2.7815998	121.1335373
2200.0000000	2.8304000	-23.6412373
2200.0000000	2.8792000	-21.0025158
2200.0000000	2.9280000	-22.5049706
2200.0000000	2.9768000	-17.7720451
2200.0000000	3.0256000	-20.7762394
2200.0000000	3.0743999	-28.1801796
2200.0000000	3.1231999	-23.8495140
2200.0000000	3.1719999	-28.6323032
2200.0000000	3.2208002	-476.3276978
2200.0000000	3.2695999	-931.4727783
2200.0000000	3.3183999	-20.5129585
2200.0000000	3.3671999	-3224.1857910
2200.0000000	3.4159999	1155.3842773
2200.0000000	3.4648001	-7.8140831
2200.0000000	3.5135999	-26.6842308
2200.0000000	3.5623999	-306.6569519
2200.0000000	3.6111999	-1921.3594971

2200.0000000	3.6600001	-435.7688599
2200.0000000	3.7088001	-575.0477295
2200.0000000	3.7575998	-899.7307129
2200.0000000	3.8063998	-1631.8547363
2200.0000000	3.8552001	-2368.7570801
2200.0000000	3.9040000	-2322.4201660
2200.0000000	3.9528000	1060.6273193
2200.0000000	4.0015998	-232.9049530
2200.0000000	4.0503998	648.9125366
2200.0000000	4.0991998	81.7760468
2200.0000000	4.1479998	-619.9349976
2200.0000000	4.1967998	1.3619919
2200.0000000	4.2456002	-235.9332275
2200.0000000	4.2943997	-462.3379822
2200.0000000	4.3431997	-13.6103668
2200.0000000	4.3920002	-235.5877838
2200.0000000	4.4407997	-22.2333908
2200.0000000	4.4896002	-15.5871735
2200.0000000	4.5383997	-621.6581421
2200.0000000	4.5871997	-13.0967140
2200.0000000	4.6360002	-21.4236279
2200.0000000	4.6847997	-27.7798119
2200.0000000	4.7336001	-19.4699440
2200.0000000	4.7824001	-16.5705566
2200.0000000	4.8311996	-1406.2895508
2200.0000000	4.8800001	216.7803650
2200.0000000	4.9287996	-192.8318939
2200.0000000	4.9776001	1009.2693481
2200.0000000	5.0264001	81.3661652
2200.0000000	5.0751996	-287.8557129
2200.0000000	5.1240001	-12.4264374
2200.0000000	5.1727996	543.5827026
2200.0000000	5.2216001	30.8346062
2200.0000000	5.2704000	-155.3593903
2200.0000000	5.3191996	-38.6231689
2200.0000000	5.3680000	-3450.7646484
2200.0000000	5.4168000	514.0087280
2200.0000000	5.4656000	-196.2940826
2200.0000000	5.5144000	36.7581940
2200.0000000	5.5631995	476.2197876
2200.0000000	5.6120000	383.3201904
2200.0000000	5.6608000	-36.5827560
2200.0000000	5.7096000	60.6357651
2200.0000000	5.7584000	129.4880981
2200.0000000	5.8071995	-11.6271591
2200.0000000	5.8559999	28.7311802
2200.0000000	5.9047999	-16.5813694
2200.0000000	5.9535999	-39.1952553
2200.0000000	6.0023999	-1535.6721191
2200.0000000	6.0511999	-14.5940781
2200.0000000	6.0999999	-19.3074608
2200.0000000	6.1487999	-13.3299294
2200.0000000	6.1975999	-23.0044880
2200.0000000	6.2463999	-1749.4145508
2200.0000000	6.2951999	-18.9708786
2200.0000000	6.3439999	-334.8940125
2200.0000000	6.3927999	-15.0458813
2200.0000000	6.4416003	-12.4574490

2200.0000000	6.4903998	-11.3459282
2200.0000000	6.5391998	-11.8890028
2200.0000000	6.5879998	301.2063293
2200.0000000	6.6367998	-12.1507540
2200.0000000	6.6856003	-13.8355360
2200.0000000	6.7343998	-13.4817619
2200.0000000	6.7831998	-14.3223429
2200.0000000	6.8319998	56.1333618
2200.0000000	6.8807998	-14.0327034
2200.0000000	6.9296002	-357.8565979
2200.0000000	6.9783998	-6.6261945
2200.0000000	7.0271997	-14.0139160
2200.0000000	7.0760002	6.1289701
2200.0000000	7.1247997	-1659.1406250
2200.0000000	7.1736002	-1330.6818848
2200.0000000	7.2223997	-757.7738647
2200.0000000	7.2711997	-165.6462097
2200.0000000	7.3200002	-17.4502640
2200.0000000	7.3687997	143.1786499
2200.0000000	7.4176002	301.7075500
2200.0000000	7.4663997	236.5678406
2200.0000000	7.5151997	-740.5541382
2200.0000000	7.5640001	-817.7416992
2200.0000000	7.6127996	-1924.2049561
2200.0000000	7.6616001	-619.0989990
2200.0000000	7.7104001	-319.1214600
2200.0000000	7.7591996	31.1920624
2200.0000000	7.8080001	313.5491028
2200.0000000	7.8567996	413.8130798
2200.0000000	7.9056001	-864.5011597
2200.0000000	7.9544001	1374.6967773
2200.0000000	8.0031996	1773.9796143
2200.0000000	8.0520000	1770.5626221
2200.0000000	8.1007996	1873.6689453
2200.0000000	8.1496000	1998.3616943
2200.0000000	8.1983995	1243.1064453
2200.0000000	8.2472000	-2845.6389160
2200.0000000	8.2959995	-1009.0233765
2200.0000000	8.3448000	-803.6096191
2200.0000000	8.3935995	-736.5339355
2200.0000000	8.4423990	-317.6616516
2200.0000000	8.4912004	-740.8284302
2200.0000000	8.5400000	-1997.4388428
2200.0000000	8.5887995	-968.8050537
2200.0000000	8.6375999	188.3131714
2200.0000000	8.6863995	1707.0241699
2200.0000000	8.7351999	-635.2084351
2200.0000000	8.7840004	724.1016235
2200.0000000	8.8327999	37.3161545
2200.0000000	8.8815994	-11.5170803
2200.0000000	8.9303989	-23.5047398
2200.0000000	8.9792004	-11.5888481
2200.0000000	9.0279999	-6.0461054
2200.0000000	9.0767994	6.3631001
2200.0000000	9.1255999	-17.0394135
2200.0000000	9.1743994	-10.1211004
2200.0000000	9.2231998	35.1529160
2200.0000000	9.2720003	-8.5306873

2200.0000000	9.3207998	-9.8617802
2200.0000000	9.3695993	-10.7904997
2200.0000000	9.4183998	-11.7391806
2200.0000000	9.4672003	-11.1661921
2200.0000000	9.5159998	-14.0590677
2200.0000000	9.5648003	-0.7910519
2200.0000000	9.6135998	-13.3027077
2200.0000000	9.6623993	-10.2537889
2200.0000000	9.7111998	-10.1897516
2200.0000000	9.7600002	-10.8624964
2200.0000000	9.8087997	-10.9957170
2200.0000000	9.8575993	2.6680622
2200.0000000	9.9063997	-8.0769014
2200.0000000	9.9552002	-10.9071102
2200.0000000	10.0039997	-10.1039295
2210.0000000	0.3416000	-37.0663033
2210.0000000	0.3904000	-38.8618774
2210.0000000	0.4392000	-39.2424240
2210.0000000	0.4880000	-39.4473724
2210.0000000	0.5368000	-39.7534256
2210.0000000	0.5856000	-39.1682549
2210.0000000	0.6344000	-38.2623863
2210.0000000	0.6832000	-36.4307289
2210.0000000	0.7320000	-36.2458839
2210.0000000	0.7808000	-36.5505524
2210.0000000	0.8296000	-35.8715858
2210.0000000	0.8784000	-35.2881279
2210.0000000	0.9272000	-32.9493294
2210.0000000	0.9760000	-30.9979210
2210.0000000	1.0247999	-30.0692711
2210.0000000	1.0735999	-29.2377872
2210.0000000	1.1224000	-29.5556812
2210.0000000	1.1711999	-30.6400490
2210.0000000	1.2200000	-31.2631931
2210.0000000	1.2687999	-31.1172333
2210.0000000	1.3176000	-30.6002159
2210.0000000	1.3664000	-30.6979256
2210.0000000	1.4152000	-30.1866417
2210.0000000	1.4640000	-29.4847202
2210.0000000	1.5128000	-28.8410130
2210.0000000	1.5616000	-28.9071808
2210.0000000	1.6104001	-1254.6236572
2210.0000000	1.6592000	-38.2802963
2210.0000000	1.7079999	-27.7034512
2210.0000000	1.7567999	-27.3928680
2210.0000000	1.8055999	-26.5539436
2210.0000000	1.8544000	-26.0524597
2210.0000000	1.9031999	-26.7345467
2210.0000000	1.9520000	-26.1593113
2210.0000000	2.0007999	-25.3307438
2210.0000000	2.0495999	-24.8391495
2210.0000000	2.0983999	-26.0591621
2210.0000000	2.1471999	-25.0902767
2210.0000000	2.1960001	-24.0208530
2210.0000000	2.2448001	-25.1908245
2210.0000000	2.2935998	-24.7499237
2210.0000000	2.3423998	-22.7039986
2210.0000000	2.3912001	-24.6343193

2210.0000000	2.4400001	-23.1333561
2210.0000000	2.4888000	-23.4342384
2210.0000000	2.5375998	-14.3022623
2210.0000000	2.5863998	-12.7379999
2210.0000000	2.6352000	-11.1645041
2210.0000000	2.6840000	-16.8276024
2210.0000000	2.7328000	101.6495972
2210.0000000	2.7815998	120.8271255
2210.0000000	2.8304000	-23.6399326
2210.0000000	2.8792000	-21.0057793
2210.0000000	2.9280000	-22.5044003
2210.0000000	2.9768000	-17.7804146
2210.0000000	3.0256000	-20.7788239
2210.0000000	3.0743999	-28.1687927
2210.0000000	3.1231999	-23.8460579
2210.0000000	3.1719999	-28.6240101
2210.0000000	3.2208002	-476.0899658
2210.0000000	3.2695999	-930.9978027
2210.0000000	3.3183999	-20.5131569
2210.0000000	3.3671999	-3222.7241211
2210.0000000	3.4159999	1154.8489990
2210.0000000	3.4648001	-7.8197918
2210.0000000	3.5135999	-26.6812401
2210.0000000	3.5623999	-306.5273438
2210.0000000	3.6111999	-1920.4997559
2210.0000000	3.6600001	-435.5807495
2210.0000000	3.7088001	-574.7963257
2210.0000000	3.7575998	-899.3321533
2210.0000000	3.8063998	-1631.1248779
2210.0000000	3.8552001	-2367.6936035
2210.0000000	3.9040000	-2321.3774414
2210.0000000	3.9528000	1060.1390381
2210.0000000	4.0015998	-232.8078461
2210.0000000	4.0503998	648.6099854
2210.0000000	4.0991998	81.7303619
2210.0000000	4.1479998	-619.6627808
2210.0000000	4.1967998	1.3527355
2210.0000000	4.2456002	-235.8349152
2210.0000000	4.2943997	-462.1373901
2210.0000000	4.3431997	-13.6125479
2210.0000000	4.3920002	-235.4896698
2210.0000000	4.4407997	-22.2316151
2210.0000000	4.4896002	-15.5880556
2210.0000000	4.5383997	-621.3970337
2210.0000000	4.5871997	-13.0987301
2210.0000000	4.6360002	-21.4220848
2210.0000000	4.6847997	-27.7758865
2210.0000000	4.7336001	-19.4692211
2210.0000000	4.7824001	-16.5710106
2210.0000000	4.8311996	-1405.7583008
2210.0000000	4.8800001	216.6908264
2210.0000000	4.9287996	-192.7646332
2210.0000000	4.9776001	1008.8768921
2210.0000000	5.0264001	81.3286438
2210.0000000	5.0751996	-287.7520752
2210.0000000	5.1240001	-12.4273586
2210.0000000	5.1727996	543.4668579
2210.0000000	5.2216001	30.8251610

2210.0000000	5.2704000	-155.3312988
2210.0000000	5.3191996	-38.6186218
2210.0000000	5.3680000	-3450.0722656
2210.0000000	5.4168000	513.9019165
2210.0000000	5.4656000	-196.2579041
2210.0000000	5.5144000	36.7475357
2210.0000000	5.5631995	476.1212158
2210.0000000	5.6120000	383.2402344
2210.0000000	5.6608000	-36.5786667
2210.0000000	5.7096000	60.6203308
2210.0000000	5.7584000	129.4591522
2210.0000000	5.8071995	-11.6280289
2210.0000000	5.8559999	28.7223301
2210.0000000	5.9047999	-16.5811710
2210.0000000	5.9535999	-39.1905174
2210.0000000	6.0023999	-1535.3675537
2210.0000000	6.0511999	-14.5942888
2210.0000000	6.0999999	-19.3066292
2210.0000000	6.1487999	-13.3303919
2210.0000000	6.1975999	-23.0027218
2210.0000000	6.2463999	-1749.0671387
2210.0000000	6.2951999	-18.9700642
2210.0000000	6.3439999	-334.8299561
2210.0000000	6.3927999	-15.0458632
2210.0000000	6.4416003	-12.4580355
2210.0000000	6.4903998	-11.3466043
2210.0000000	6.5391998	-11.8896837
2210.0000000	6.5879998	300.5156250
2210.0000000	6.6367998	-12.1513033
2210.0000000	6.6856003	-13.8357067
2210.0000000	6.7343998	-13.4819775
2210.0000000	6.7831998	-14.3219271
2210.0000000	6.8319998	56.1142502
2210.0000000	6.8807998	-14.0325651
2210.0000000	6.9296002	-357.0951538
2210.0000000	6.9783998	-6.6390181
2210.0000000	7.0271997	-14.0128889
2210.0000000	7.0760002	6.0867853
2210.0000000	7.1247997	-1655.1947021
2210.0000000	7.1736002	-1327.5190430
2210.0000000	7.2223997	-755.9807129
2210.0000000	7.2711997	-165.2784424
2210.0000000	7.3200002	-17.4382744
2210.0000000	7.3687997	142.8041077
2210.0000000	7.4176002	300.9517212
2210.0000000	7.4663997	235.9701843
2210.0000000	7.5151997	-738.7930908
2210.0000000	7.5640001	-815.7943115
2210.0000000	7.6127996	-1919.5874023
2210.0000000	7.6616001	-617.6276855
2210.0000000	7.7104001	-318.3751831
2210.0000000	7.7591996	31.0910110
2210.0000000	7.8080001	312.7637634
2210.0000000	7.8567996	412.7850037
2210.0000000	7.9056001	-862.4282227
2210.0000000	7.9544001	1371.3868408
2210.0000000	8.0031996	1769.7100830
2210.0000000	8.0520000	1766.3006592

2210.0000000	8.1007996	1869.1594238
2210.0000000	8.1496000	1993.5537109
2210.0000000	8.1983995	1240.1140137
2210.0000000	8.2472000	-2838.8039551
2210.0000000	8.2959995	-1006.6032104
2210.0000000	8.3448000	-801.6804810
2210.0000000	8.3935995	-734.7662964
2210.0000000	8.4423990	-316.9036560
2210.0000000	8.4912004	-739.0521851
2210.0000000	8.5400000	-1992.6390381
2210.0000000	8.5887995	-966.4835205
2210.0000000	8.6375999	187.8518524
2210.0000000	8.6863995	1702.9057617
2210.0000000	8.7351999	-633.6842041
2210.0000000	8.7840004	722.3448486
2210.0000000	8.8327999	37.2045517
2210.0000000	8.8815994	-11.5172548
2210.0000000	8.9303989	-23.5010185
2210.0000000	8.9792004	-11.5889301
2210.0000000	9.0279999	-6.0478735
2210.0000000	9.0767994	6.3573818
2210.0000000	9.1255999	-17.0377007
2210.0000000	9.1743994	-10.1215019
2210.0000000	9.2231998	35.1371841
2210.0000000	9.2720003	-8.5316238
2210.0000000	9.3207998	-9.8623199
2210.0000000	9.3695993	-10.7907267
2210.0000000	9.4183998	-11.7390556
2210.0000000	9.4672003	-11.1662331
2210.0000000	9.5159998	-14.0581760
2210.0000000	9.5648003	-0.7944250
2210.0000000	9.6135998	-13.3019161
2210.0000000	9.6623993	-10.2540636
2210.0000000	9.7111998	-10.1900234
2210.0000000	9.7600002	-10.8625383
2210.0000000	9.8087997	-10.9957247
2210.0000000	9.8575993	2.6636410
2210.0000000	9.9063997	-8.0778151
2210.0000000	9.9552002	-10.9071150
2210.0000000	10.0039997	-10.1041775
2220.0000000	0.3416000	-37.1142769
2220.0000000	0.3904000	-38.8559647
2220.0000000	0.4392000	-39.2433853
2220.0000000	0.4880000	-39.4461632
2220.0000000	0.5368000	-39.7546005
2220.0000000	0.5856000	-39.1668739
2220.0000000	0.6344000	-38.2626991
2220.0000000	0.6832000	-36.4329529
2220.0000000	0.7320000	-36.2479362
2220.0000000	0.7808000	-36.5507202
2220.0000000	0.8296000	-35.8676796
2220.0000000	0.8784000	-35.2849960
2220.0000000	0.9272000	-32.9337502
2220.0000000	0.9760000	-30.9931049
2220.0000000	1.0247999	-30.0747795
2220.0000000	1.0735999	-29.2230644
2220.0000000	1.1224000	-29.5374050
2220.0000000	1.1711999	-30.6331978

2220.0000000	1.2200000	-31.2649689
2220.0000000	1.2687999	-31.1194019
2220.0000000	1.3176000	-30.5965347
2220.0000000	1.3664000	-30.6975021
2220.0000000	1.4152000	-30.1868458
2220.0000000	1.4640000	-29.4854050
2220.0000000	1.5128000	-28.8402424
2220.0000000	1.5616000	-28.9014740
2220.0000000	1.6104001	-1252.0808105
2220.0000000	1.6592000	-37.9863625
2220.0000000	1.7079999	-27.7071991
2220.0000000	1.7567999	-27.3822002
2220.0000000	1.8055999	-26.5682392
2220.0000000	1.8544000	-26.0499439
2220.0000000	1.9031999	-26.7455864
2220.0000000	1.9520000	-26.1844826
2220.0000000	2.0007999	-25.3601151
2220.0000000	2.0495999	-24.8425541
2220.0000000	2.0983999	-26.0716190
2220.0000000	2.1471999	-25.1769371
2220.0000000	2.1960001	-24.0245075
2220.0000000	2.2448001	-25.2303944
2220.0000000	2.2935998	-24.7478123
2220.0000000	2.3423998	-22.7184029
2220.0000000	2.3912001	-24.6326828
2220.0000000	2.4400001	-23.1358414
2220.0000000	2.4888000	-23.4340210
2220.0000000	2.5375998	-14.3193703
2220.0000000	2.5863998	-12.7577581
2220.0000000	2.6352000	-11.1872482
2220.0000000	2.6840000	-16.8395443
2220.0000000	2.7328000	101.3835831
2220.0000000	2.7815998	120.5206985
2220.0000000	2.8304000	-23.6386280
2220.0000000	2.8792000	-21.0090427
2220.0000000	2.9280000	-22.5038319
2220.0000000	2.9768000	-17.7887840
2220.0000000	3.0256000	-20.7814083
2220.0000000	3.0743999	-28.1574059
2220.0000000	3.1231999	-23.8426037
2220.0000000	3.1719999	-28.6157169
2220.0000000	3.2208002	-475.8522339
2220.0000000	3.2695999	-930.5228271
2220.0000000	3.3183999	-20.5133553
2220.0000000	3.3671999	-3221.2624512
2220.0000000	3.4159999	1154.3137207
2220.0000000	3.4648001	-7.8254995
2220.0000000	3.5135999	-26.6782494
2220.0000000	3.5623999	-306.3977661
2220.0000000	3.6111999	-1919.6400146
2220.0000000	3.6600001	-435.3925781
2220.0000000	3.7088001	-574.5449829
2220.0000000	3.7575998	-898.9336548
2220.0000000	3.8063998	-1630.3950195
2220.0000000	3.8552001	-2366.6301270
2220.0000000	3.9040000	-2320.3349609
2220.0000000	3.9528000	1059.6507568
2220.0000000	4.0015998	-232.7107391

2220.0000000	4.0503998	648.3074341
2220.0000000	4.0991998	81.6846771
2220.0000000	4.1479998	-619.3905640
2220.0000000	4.1967998	1.3434792
2220.0000000	4.2456002	-235.7366180
2220.0000000	4.2943997	-461.9368286
2220.0000000	4.3431997	-13.6147289
2220.0000000	4.3920002	-235.3915558
2220.0000000	4.4407997	-22.2298393
2220.0000000	4.4896002	-15.5889387
2220.0000000	4.5383997	-621.1359863
2220.0000000	4.5871997	-13.1007462
2220.0000000	4.6360002	-21.4205437
2220.0000000	4.6847997	-27.7719612
2220.0000000	4.7336001	-19.4684982
2220.0000000	4.7824001	-16.5714664
2220.0000000	4.8311996	-1405.2270508
2220.0000000	4.8800001	216.6012726
2220.0000000	4.9287996	-192.6973572
2220.0000000	4.9776001	1008.4843140
2220.0000000	5.0264001	81.2911301
2220.0000000	5.0751996	-287.6484680
2220.0000000	5.1240001	-12.4282799
2220.0000000	5.1727996	543.3509521
2220.0000000	5.2216001	30.8157196
2220.0000000	5.2704000	-155.3031769
2220.0000000	5.3191996	-38.6140709
2220.0000000	5.3680000	-3449.3798828
2220.0000000	5.4168000	513.7951050
2220.0000000	5.4656000	-196.2217255
2220.0000000	5.5144000	36.7368851
2220.0000000	5.5631995	476.0226440
2220.0000000	5.6120000	383.1602478
2220.0000000	5.6608000	-36.5745773
2220.0000000	5.7096000	60.6048965
2220.0000000	5.7584000	129.4302216
2220.0000000	5.8071995	-11.6289005
2220.0000000	5.8559999	28.7134800
2220.0000000	5.9047999	-16.5809727
2220.0000000	5.9535999	-39.1857796
2220.0000000	6.0023999	-1535.0632324
2220.0000000	6.0511999	-14.5945005
2220.0000000	6.0999999	-19.3057957
2220.0000000	6.1487999	-13.3308544
2220.0000000	6.1975999	-23.0009575
2220.0000000	6.2463999	-1748.7197266
2220.0000000	6.2951999	-18.9692497
2220.0000000	6.3439999	-334.7659302
2220.0000000	6.3927999	-15.0458450
2220.0000000	6.4416003	-12.4586229
2220.0000000	6.4903998	-11.3472805
2220.0000000	6.5391998	-11.8903646
2220.0000000	6.5879998	299.8249512
2220.0000000	6.6367998	-12.1518526
2220.0000000	6.6856003	-13.8358765
2220.0000000	6.7343998	-13.4821939
2220.0000000	6.7831998	-14.3215113
2220.0000000	6.8319998	56.0951309

2220.0000000	6.8807998	-14.0324259
2220.0000000	6.9296002	-356.3336792
2220.0000000	6.9783998	-6.6518421
2220.0000000	7.0271997	-14.0118608
2220.0000000	7.0760002	6.0446005
2220.0000000	7.1247997	-1651.2489014
2220.0000000	7.1736002	-1324.3562012
2220.0000000	7.2223997	-754.1875000
2220.0000000	7.2711997	-164.9106598
2220.0000000	7.3200002	-17.4262867
2220.0000000	7.3687997	142.4295807
2220.0000000	7.4176002	300.1958923
2220.0000000	7.4663997	235.3725281
2220.0000000	7.5151997	-737.0320435
2220.0000000	7.5640001	-813.8469238
2220.0000000	7.6127996	-1914.9697266
2220.0000000	7.6616001	-616.1563110
2220.0000000	7.7104001	-317.6289063
2220.0000000	7.7591996	30.9899635
2220.0000000	7.8080001	311.9783936
2220.0000000	7.8567996	411.7569275
2220.0000000	7.9056001	-860.3553467
2220.0000000	7.9544001	1368.0769043
2220.0000000	8.0031996	1765.4404297
2220.0000000	8.0520000	1762.0386963
2220.0000000	8.1007996	1864.6499023
2220.0000000	8.1496000	1988.7457275
2220.0000000	8.1983995	1237.1214600
2220.0000000	8.2472000	-2831.9692383
2220.0000000	8.2959995	-1004.1829834
2220.0000000	8.3448000	-799.7512817
2220.0000000	8.3935995	-732.9985962
2220.0000000	8.4423990	-316.1456299
2220.0000000	8.4912004	-737.2760010
2220.0000000	8.5400000	-1987.8392334
2220.0000000	8.5887995	-964.1619263
2220.0000000	8.6375999	187.3905334
2220.0000000	8.6863995	1698.7873535
2220.0000000	8.7351999	-632.1599731
2220.0000000	8.7840004	720.5881348
2220.0000000	8.8327999	37.0929413
2220.0000000	8.8815994	-11.5174294
2220.0000000	8.9303989	-23.4972992
2220.0000000	8.9792004	-11.5890131
2220.0000000	9.0279999	-6.0496416
2220.0000000	9.0767994	6.3516636
2220.0000000	9.1255999	-17.0359859
2220.0000000	9.1743994	-10.1219044
2220.0000000	9.2231998	35.1214447
2220.0000000	9.2720003	-8.5325603
2220.0000000	9.3207998	-9.8628588
2220.0000000	9.3695993	-10.7909527
2220.0000000	9.4183998	-11.7389307
2220.0000000	9.4672003	-11.1662741
2220.0000000	9.5159998	-14.0572853
2220.0000000	9.5648003	-0.7977982
2220.0000000	9.6135998	-13.3011255
2220.0000000	9.6623993	-10.2543392

2220.0000000	9.7111998	-10.1902952
2220.0000000	9.7600002	-10.8625803
2220.0000000	9.8087997	-10.9957333
2220.0000000	9.8575993	2.6592169
2220.0000000	9.9063997	-8.0787296
2220.0000000	9.9552002	-10.9071188
2220.0000000	10.0039997	-10.1044245
2230.0000000	0.3416000	-37.0054741
2230.0000000	0.3904000	-38.8500481
2230.0000000	0.4392000	-39.2443428
2230.0000000	0.4880000	-39.4449539
2230.0000000	0.5368000	-39.7557716
2230.0000000	0.5856000	-39.1654930
2230.0000000	0.6344000	-38.2630081
2230.0000000	0.6832000	-36.4351768
2230.0000000	0.7320000	-36.2499886
2230.0000000	0.7808000	-36.5508881
2230.0000000	0.8296000	-35.8637695
2230.0000000	0.8784000	-35.2818642
2230.0000000	0.9272000	-32.9181671
2230.0000000	0.9760000	-30.9882870
2230.0000000	1.0247999	-30.0802879
2230.0000000	1.0735999	-29.2083416
2230.0000000	1.1224000	-29.5191288
2230.0000000	1.1711999	-30.6263447
2230.0000000	1.2200000	-31.2667446
2230.0000000	1.2687999	-31.1215706
2230.0000000	1.3176000	-30.5928535
2230.0000000	1.3664000	-30.6970787
2230.0000000	1.4152000	-30.1870499
2230.0000000	1.4640000	-29.4860878
2230.0000000	1.5128000	-28.8394737
2230.0000000	1.5616000	-28.8957672
2230.0000000	1.6104001	-1249.5380859
2230.0000000	1.6592000	-37.6924286
2230.0000000	1.7079999	-27.7109451
2230.0000000	1.7567999	-27.3715343
2230.0000000	1.8055999	-26.5825348
2230.0000000	1.8544000	-26.0474262
2230.0000000	1.9031999	-26.7566261
2230.0000000	1.9520000	-26.2096558
2230.0000000	2.0007999	-25.3894882
2230.0000000	2.0495999	-24.8459587
2230.0000000	2.0983999	-26.0840759
2230.0000000	2.1471999	-25.2635975
2230.0000000	2.1960001	-24.0281620
2230.0000000	2.2448001	-25.2272434
2230.0000000	2.2935998	-24.7456989
2230.0000000	2.3423998	-22.7328072
2230.0000000	2.3912001	-24.6310463
2230.0000000	2.4400001	-23.1383286
2230.0000000	2.4888000	-23.4338036
2230.0000000	2.5375998	-14.3364792
2230.0000000	2.5863998	-12.7775173
2230.0000000	2.6352000	-11.2099934
2230.0000000	2.6840000	-16.8514862
2230.0000000	2.7328000	101.1175613
2230.0000000	2.7815998	120.2142868

2230.0000000	2.8304000	-23.6373253
2230.0000000	2.8792000	-21.0123062
2230.0000000	2.9280000	-22.5032635
2230.0000000	2.9768000	-17.7971554
2230.0000000	3.0256000	-20.7839928
2230.0000000	3.0743999	-28.1460190
2230.0000000	3.1231999	-23.8391476
2230.0000000	3.1719999	-28.6074238
2230.0000000	3.2208002	-475.6144714
2230.0000000	3.2695999	-930.0478516
2230.0000000	3.3183999	-20.5135536
2230.0000000	3.3671999	-3219.8007813
2230.0000000	3.4159999	1153.7784424
2230.0000000	3.4648001	-7.8312082
2230.0000000	3.5135999	-26.6752586
2230.0000000	3.5623999	-306.2681580
2230.0000000	3.6111999	-1918.7802734
2230.0000000	3.6600001	-435.2044678
2230.0000000	3.7088001	-574.2935791
2230.0000000	3.7575998	-898.5350952
2230.0000000	3.8063998	-1629.6651611
2230.0000000	3.8552001	-2365.5668945
2230.0000000	3.9040000	-2319.2924805
2230.0000000	3.9528000	1059.1623535
2230.0000000	4.0015998	-232.6136322
2230.0000000	4.0503998	648.0048828
2230.0000000	4.0991998	81.6389923
2230.0000000	4.1479998	-619.1182861
2230.0000000	4.1967998	1.3342228
2230.0000000	4.2456002	-235.6383209
2230.0000000	4.2943997	-461.7362671
2230.0000000	4.3431997	-13.6169090
2230.0000000	4.3920002	-235.2934418
2230.0000000	4.4407997	-22.2280636
2230.0000000	4.4896002	-15.5898209
2230.0000000	4.5383997	-620.8748779
2230.0000000	4.5871997	-13.1027622
2230.0000000	4.6360002	-21.4190006
2230.0000000	4.6847997	-27.7680359
2230.0000000	4.7336001	-19.4677773
2230.0000000	4.7824001	-16.5719204
2230.0000000	4.8311996	-1404.6958008
2230.0000000	4.8800001	216.5117188
2230.0000000	4.9287996	-192.6300964
2230.0000000	4.9776001	1008.0918579
2230.0000000	5.0264001	81.2536087
2230.0000000	5.0751996	-287.5448303
2230.0000000	5.1240001	-12.4292011
2230.0000000	5.1727996	543.2351074
2230.0000000	5.2216001	30.8062744
2230.0000000	5.2704000	-155.2750854
2230.0000000	5.3191996	-38.6095200
2230.0000000	5.3680000	-3448.6875000
2230.0000000	5.4168000	513.6883545
2230.0000000	5.4656000	-196.1855316
2230.0000000	5.5144000	36.7262268
2230.0000000	5.5631995	475.9240723
2230.0000000	5.6120000	383.0802612

2230.0000000	5.6608000	-36.5704918
2230.0000000	5.7096000	60.5894699
2230.0000000	5.7584000	129.4012909
2230.0000000	5.8071995	-11.6297703
2230.0000000	5.8559999	28.7046261
2230.0000000	5.9047999	-16.5807762
2230.0000000	5.9535999	-39.1810379
2230.0000000	6.0023999	-1534.7586670
2230.0000000	6.0511999	-14.5947113
2230.0000000	6.0999999	-19.3049641
2230.0000000	6.1487999	-13.3313179
2230.0000000	6.1975999	-22.9991913
2230.0000000	6.2463999	-1748.3724365
2230.0000000	6.2951999	-18.9684353
2230.0000000	6.3439999	-334.7018433
2230.0000000	6.3927999	-15.0458269
2230.0000000	6.4416003	-12.4592094
2230.0000000	6.4903998	-11.3479557
2230.0000000	6.5391998	-11.8910456
2230.0000000	6.5879998	299.1342468
2230.0000000	6.6367998	-12.1524019
2230.0000000	6.6856003	-13.8360472
2230.0000000	6.7343998	-13.4824095
2230.0000000	6.7831998	-14.3210955
2230.0000000	6.8319998	56.0760193
2230.0000000	6.8807998	-14.0322876
2230.0000000	6.9296002	-355.5722351
2230.0000000	6.9783998	-6.6646652
2230.0000000	7.0271997	-14.0108328
2230.0000000	7.0760002	6.0024157
2230.0000000	7.1247997	-1647.3029785
2230.0000000	7.1736002	-1321.1933594
2230.0000000	7.2223997	-752.3943481
2230.0000000	7.2711997	-164.5428772
2230.0000000	7.3200002	-17.4142990
2230.0000000	7.3687997	142.0550537
2230.0000000	7.4176002	299.4400635
2230.0000000	7.4663997	234.7748871
2230.0000000	7.5151997	-735.2709961
2230.0000000	7.5640001	-811.8995361
2230.0000000	7.6127996	-1910.3521729
2230.0000000	7.6616001	-614.6849976
2230.0000000	7.7104001	-316.8826294
2230.0000000	7.7591996	30.8889122
2230.0000000	7.8080001	311.1930237
2230.0000000	7.8567996	410.7288208
2230.0000000	7.9056001	-858.2824097
2230.0000000	7.9544001	1364.7669678
2230.0000000	8.0031996	1761.1708984
2230.0000000	8.0520000	1757.7766113
2230.0000000	8.1007996	1860.1405029
2230.0000000	8.1496000	1983.9377441
2230.0000000	8.1983995	1234.1289063
2230.0000000	8.2472000	-2825.1342773
2230.0000000	8.2959995	-1001.7628174
2230.0000000	8.3448000	-797.8220825
2230.0000000	8.3935995	-731.2309570
2230.0000000	8.4423990	-315.3876343

2230.0000000	8.4912004	-735.4997559
2230.0000000	8.5400000	-1983.0394287
2230.0000000	8.5887995	-961.8403931
2230.0000000	8.6375999	186.9292145
2230.0000000	8.6863995	1694.6690674
2230.0000000	8.7351999	-630.6356812
2230.0000000	8.7840004	718.8313599
2230.0000000	8.8327999	36.9813347
2230.0000000	8.8815994	-11.5176029
2230.0000000	8.9303989	-23.4935799
2230.0000000	8.9792004	-11.5890951
2230.0000000	9.0279999	-6.0514097
2230.0000000	9.0767994	6.3459473
2230.0000000	9.1255999	-17.0342712
2230.0000000	9.1743994	-10.1223059
2230.0000000	9.2231998	35.1057129
2230.0000000	9.2720003	-8.5334969
2230.0000000	9.3207998	-9.8633986
2230.0000000	9.3695993	-10.7911797
2230.0000000	9.4183998	-11.7388067
2230.0000000	9.4672003	-11.1663151
2230.0000000	9.5159998	-14.0563946
2230.0000000	9.5648003	-0.8011703
2230.0000000	9.6135998	-13.3003349
2230.0000000	9.6623993	-10.2546139
2230.0000000	9.7111998	-10.1905661
2230.0000000	9.7600002	-10.8626223
2230.0000000	9.8087997	-10.9957409
2230.0000000	9.8575993	2.6547947
2230.0000000	9.9063997	-8.0796432
2230.0000000	9.9552002	-10.9071236
2230.0000000	10.0039997	-10.1046724
2240.0000000	0.3416000	-37.3512650
2240.0000000	0.3904000	-38.8441315
2240.0000000	0.4392000	-39.2453041
2240.0000000	0.4880000	-39.4437447
2240.0000000	0.5368000	-39.7569466
2240.0000000	0.5856000	-39.1641121
2240.0000000	0.6344000	-38.2633171
2240.0000000	0.6832000	-36.4374046
2240.0000000	0.7320000	-36.2520409
2240.0000000	0.7808000	-36.5510597
2240.0000000	0.8296000	-35.8598633
2240.0000000	0.8784000	-35.2787361
2240.0000000	0.9272000	-32.9025879
2240.0000000	0.9760000	-30.9834709
2240.0000000	1.0247999	-30.0857964
2240.0000000	1.0735999	-29.1936188
2240.0000000	1.1224000	-29.5008507
2240.0000000	1.1711999	-30.6194935
2240.0000000	1.2200000	-31.2685204
2240.0000000	1.2687999	-31.1237373
2240.0000000	1.3176000	-30.5891705
2240.0000000	1.3664000	-30.6966572
2240.0000000	1.4152000	-30.1872540
2240.0000000	1.4640000	-29.4867706
2240.0000000	1.5128000	-28.8387032
2240.0000000	1.5616000	-28.8900604

2240.0000000	1.6104001	-1246.9952393
2240.0000000	1.6592000	-37.3984985
2240.0000000	1.7079999	-27.7146931
2240.0000000	1.7567999	-27.3608665
2240.0000000	1.8055999	-26.5968304
2240.0000000	1.8544000	-26.0449104
2240.0000000	1.9031999	-26.7676659
2240.0000000	1.9520000	-26.2348289
2240.0000000	2.0007999	-25.4188595
2240.0000000	2.0495999	-24.8493633
2240.0000000	2.0983999	-26.0965328
2240.0000000	2.1471999	-25.3502579
2240.0000000	2.1960001	-24.0318184
2240.0000000	2.2448001	-25.2240925
2240.0000000	2.2935998	-24.7435875
2240.0000000	2.3423998	-22.7472115
2240.0000000	2.3912001	-24.6294098
2240.0000000	2.4400001	-23.1408157
2240.0000000	2.4888000	-23.4335861
2240.0000000	2.5375998	-14.3535862
2240.0000000	2.5863998	-12.7972765
2240.0000000	2.6352000	-11.2327375
2240.0000000	2.6840000	-16.8634281
2240.0000000	2.7328000	100.8515472
2240.0000000	2.7815998	119.9078598
2240.0000000	2.8304000	-23.6360207
2240.0000000	2.8792000	-21.0155697
2240.0000000	2.9280000	-22.5026932
2240.0000000	2.9768000	-17.8055267
2240.0000000	3.0256000	-20.7865772
2240.0000000	3.0743999	-28.1346321
2240.0000000	3.1231999	-23.8356934
2240.0000000	3.1719999	-28.5991306
2240.0000000	3.2208002	-475.3767395
2240.0000000	3.2695999	-929.5728760
2240.0000000	3.3183999	-20.5137501
2240.0000000	3.3671999	-3218.3391113
2240.0000000	3.4159999	1153.2431641
2240.0000000	3.4648001	-7.8369160
2240.0000000	3.5135999	-26.6722679
2240.0000000	3.5623999	-306.1385803
2240.0000000	3.6111999	-1917.9205322
2240.0000000	3.6600001	-435.0163574
2240.0000000	3.7088001	-574.0421753
2240.0000000	3.7575998	-898.1365967
2240.0000000	3.8063998	-1628.9353027
2240.0000000	3.8552001	-2364.5034180
2240.0000000	3.9040000	-2318.2500000
2240.0000000	3.9528000	1058.6740723
2240.0000000	4.0015998	-232.5165253
2240.0000000	4.0503998	647.7023315
2240.0000000	4.0991998	81.5933075
2240.0000000	4.1479998	-618.8460693
2240.0000000	4.1967998	1.3249664
2240.0000000	4.2456002	-235.5400085
2240.0000000	4.2943997	-461.5357056
2240.0000000	4.3431997	-13.6190891
2240.0000000	4.3920002	-235.1953278

2240.0000000	4.4407997	-22.2262878
2240.0000000	4.4896002	-15.5907040
2240.0000000	4.5383997	-620.6138306
2240.0000000	4.5871997	-13.1047783
2240.0000000	4.6360002	-21.4174576
2240.0000000	4.6847997	-27.7641106
2240.0000000	4.7336001	-19.4670544
2240.0000000	4.7824001	-16.5723743
2240.0000000	4.8311996	-1404.1644287
2240.0000000	4.8800001	216.4221802
2240.0000000	4.9287996	-192.5628357
2240.0000000	4.9776001	1007.6994019
2240.0000000	5.0264001	81.2160873
2240.0000000	5.0751996	-287.4411926
2240.0000000	5.1240001	-12.4301224
2240.0000000	5.1727996	543.1192627
2240.0000000	5.2216001	30.7968292
2240.0000000	5.2704000	-155.2469788
2240.0000000	5.3191996	-38.6049728
2240.0000000	5.3680000	-3447.9951172
2240.0000000	5.4168000	513.5816040
2240.0000000	5.4656000	-196.1493530
2240.0000000	5.5144000	36.7155685
2240.0000000	5.5631995	475.8255005
2240.0000000	5.6120000	383.0003052
2240.0000000	5.6608000	-36.5664024
2240.0000000	5.7096000	60.5740433
2240.0000000	5.7584000	129.3723450
2240.0000000	5.8071995	-11.6306410
2240.0000000	5.8559999	28.6957760
2240.0000000	5.9047999	-16.5805779
2240.0000000	5.9535999	-39.1763000
2240.0000000	6.0023999	-1534.4542236
2240.0000000	6.0511999	-14.5949230
2240.0000000	6.0999999	-19.3041306
2240.0000000	6.1487999	-13.3317804
2240.0000000	6.1975999	-22.9974270
2240.0000000	6.2463999	-1748.0250244
2240.0000000	6.2951999	-18.9676208
2240.0000000	6.3439999	-334.6377869
2240.0000000	6.3927999	-15.0458078
2240.0000000	6.4416003	-12.4597969
2240.0000000	6.4903998	-11.3486319
2240.0000000	6.5391998	-11.8917265
2240.0000000	6.5879998	298.4435425
2240.0000000	6.6367998	-12.1529503
2240.0000000	6.6856003	-13.8362169
2240.0000000	6.7343998	-13.4826250
2240.0000000	6.7831998	-14.3206797
2240.0000000	6.8319998	56.0569077
2240.0000000	6.8807998	-14.0321484
2240.0000000	6.9296002	-354.8107910
2240.0000000	6.9783998	-6.6774888
2240.0000000	7.0271997	-14.0098047
2240.0000000	7.0760002	5.9602308
2240.0000000	7.1247997	-1643.3571777
2240.0000000	7.1736002	-1318.0303955
2240.0000000	7.2223997	-750.6011963

2240.0000000	7.2711997	-164.1751099
2240.0000000	7.3200002	-17.4023094
2240.0000000	7.3687997	141.6805115
2240.0000000	7.4176002	298.6842346
2240.0000000	7.4663997	234.1772308
2240.0000000	7.5151997	-733.5099487
2240.0000000	7.5640001	-809.9520874
2240.0000000	7.6127996	-1905.7344971
2240.0000000	7.6616001	-613.2136230
2240.0000000	7.7104001	-316.1364136
2240.0000000	7.7591996	30.7878647
2240.0000000	7.8080001	310.4076843
2240.0000000	7.8567996	409.7007446
2240.0000000	7.9056001	-856.2095337
2240.0000000	7.9544001	1361.4570313
2240.0000000	8.0031996	1756.9012451
2240.0000000	8.0520000	1753.5146484
2240.0000000	8.1007996	1855.6309814
2240.0000000	8.1496000	1979.1296387
2240.0000000	8.1983995	1231.1364746
2240.0000000	8.2472000	-2818.2995605
2240.0000000	8.2959995	-999.3426514
2240.0000000	8.3448000	-795.8929443
2240.0000000	8.3935995	-729.4632568
2240.0000000	8.4423990	-314.6296082
2240.0000000	8.4912004	-733.7235107
2240.0000000	8.5400000	-1978.2396240
2240.0000000	8.5887995	-959.5188599
2240.0000000	8.6375999	186.4678802
2240.0000000	8.6863995	1690.5506592
2240.0000000	8.7351999	-629.1114502
2240.0000000	8.7840004	717.0746460
2240.0000000	8.8327999	36.8697281
2240.0000000	8.8815994	-11.5177774
2240.0000000	8.9303989	-23.4898605
2240.0000000	8.9792004	-11.5891771
2240.0000000	9.0279999	-6.0531783
2240.0000000	9.0767994	6.3402290
2240.0000000	9.1255999	-17.0325584
2240.0000000	9.1743994	-10.1227083
2240.0000000	9.2231998	35.0899811
2240.0000000	9.2720003	-8.5344334
2240.0000000	9.3207998	-9.8639374
2240.0000000	9.3695993	-10.7914057
2240.0000000	9.4183998	-11.7386818
2240.0000000	9.4672003	-11.1663561
2240.0000000	9.5159998	-14.0555038
2240.0000000	9.5648003	-0.8045425
2240.0000000	9.6135998	-13.2995443
2240.0000000	9.6623993	-10.2548885
2240.0000000	9.7111998	-10.1908379
2240.0000000	9.7600002	-10.8626633
2240.0000000	9.8087997	-10.9957485
2240.0000000	9.8575993	2.6503735
2240.0000000	9.9063997	-8.0805569
2240.0000000	9.9552002	-10.9071274
2240.0000000	10.0039997	-10.1049194
2250.0000000	0.3416000	-37.1005020

2250.0000000	0.3904000	-38.8382187
2250.0000000	0.4392000	-39.2462616
2250.0000000	0.4880000	-39.4425354
2250.0000000	0.5368000	-39.7581215
2250.0000000	0.5856000	-39.1627312
2250.0000000	0.6344000	-38.2636299
2250.0000000	0.6832000	-36.4396286
2250.0000000	0.7320000	-36.2540932
2250.0000000	0.7808000	-36.5512276
2250.0000000	0.8296000	-35.8559570
2250.0000000	0.8784000	-35.2756042
2250.0000000	0.9272000	-32.8870087
2250.0000000	0.9760000	-30.9786530
2250.0000000	1.0247999	-30.0913048
2250.0000000	1.0735999	-29.1788960
2250.0000000	1.1224000	-29.4825745
2250.0000000	1.1711999	-30.6126404
2250.0000000	1.2200000	-31.2702942
2250.0000000	1.2687999	-31.1259060
2250.0000000	1.3176000	-30.5854893
2250.0000000	1.3664000	-30.6962337
2250.0000000	1.4152000	-30.1874580
2250.0000000	1.4640000	-29.4874535
2250.0000000	1.5128000	-28.8379326
2250.0000000	1.5616000	-28.8843555
2250.0000000	1.6104001	-1244.4523926
2250.0000000	1.6592000	-37.1045685
2250.0000000	1.7079999	-27.7184410
2250.0000000	1.7567999	-27.3502007
2250.0000000	1.8055999	-26.6111259
2250.0000000	1.8544000	-26.0423946
2250.0000000	1.9031999	-26.7787056
2250.0000000	1.9520000	-26.2600021
2250.0000000	2.0007999	-25.4482307
2250.0000000	2.0495999	-24.8527679
2250.0000000	2.0983999	-26.0963326
2250.0000000	2.1471999	-25.3501778
2250.0000000	2.1960001	-24.0354729
2250.0000000	2.2448001	-25.2209415
2250.0000000	2.2935998	-24.7414761
2250.0000000	2.3423998	-22.7616158
2250.0000000	2.3912001	-24.6277733
2250.0000000	2.4400001	-23.1433010
2250.0000000	2.4888000	-23.4333687
2250.0000000	2.5375998	-14.3706951
2250.0000000	2.5863998	-12.8170357
2250.0000000	2.6352000	-11.2554808
2250.0000000	2.6840000	-16.8753700
2250.0000000	2.7328000	100.5855331
2250.0000000	2.7815998	119.6014481
2250.0000000	2.8304000	-23.6347160
2250.0000000	2.8792000	-21.0188332
2250.0000000	2.9280000	-22.5021248
2250.0000000	2.9768000	-17.8138962
2250.0000000	3.0256000	-20.7891617
2250.0000000	3.0743999	-28.1232452
2250.0000000	3.1231999	-23.8322372
2250.0000000	3.1719999	-28.5908375

2250.0000000	3.2208002	-475.1390076
2250.0000000	3.2695999	-929.0979004
2250.0000000	3.3183999	-20.5139484
2250.0000000	3.3671999	-3216.8774414
2250.0000000	3.4159999	1152.7078857
2250.0000000	3.4648001	-7.8426247
2250.0000000	3.5135999	-26.6692772
2250.0000000	3.5623999	-306.0090027
2250.0000000	3.6111999	-1917.0607910
2250.0000000	3.6600001	-434.8282471
2250.0000000	3.7088001	-573.7908325
2250.0000000	3.7575998	-897.7380371
2250.0000000	3.8063998	-1628.2054443
2250.0000000	3.8552001	-2363.4399414
2250.0000000	3.9040000	-2317.2075195
2250.0000000	3.9528000	1058.1857910
2250.0000000	4.0015998	-232.4194183
2250.0000000	4.0503998	647.3997192
2250.0000000	4.0991998	81.5476303
2250.0000000	4.1479998	-618.5738525
2250.0000000	4.1967998	1.3157101
2250.0000000	4.2456002	-235.4417114
2250.0000000	4.2943997	-461.3351440
2250.0000000	4.3431997	-13.6212702
2250.0000000	4.3920002	-235.0972137
2250.0000000	4.4407997	-22.2245140
2250.0000000	4.4896002	-15.5915861
2250.0000000	4.5383997	-620.3527832
2250.0000000	4.5871997	-13.1067953
2250.0000000	4.6360002	-21.4159164
2250.0000000	4.6847997	-27.7601852
2250.0000000	4.7336001	-19.4663315
2250.0000000	4.7824001	-16.5728302
2250.0000000	4.8311996	-1403.6331787
2250.0000000	4.8800001	216.3326263
2250.0000000	4.9287996	-192.4955750
2250.0000000	4.9776001	1007.3068237
2250.0000000	5.0264001	81.1785660
2250.0000000	5.0751996	-287.3375854
2250.0000000	5.1240001	-12.4310436
2250.0000000	5.1727996	543.0033569
2250.0000000	5.2216001	30.7873878
2250.0000000	5.2704000	-155.2188721
2250.0000000	5.3191996	-38.6004257
2250.0000000	5.3680000	-3447.3027344
2250.0000000	5.4168000	513.4747925
2250.0000000	5.4656000	-196.1131744
2250.0000000	5.5144000	36.7049103
2250.0000000	5.5631995	475.7269287
2250.0000000	5.6120000	382.9203186
2250.0000000	5.6608000	-36.5623131
2250.0000000	5.7096000	60.5586090
2250.0000000	5.7584000	129.3434143
2250.0000000	5.8071995	-11.6315117
2250.0000000	5.8559999	28.6869259
2250.0000000	5.9047999	-16.5803795
2250.0000000	5.9535999	-39.1715622
2250.0000000	6.0023999	-1534.1499023

2250.0000000	6.0511999	-14.5951347
2250.0000000	6.0999999	-19.3032990
2250.0000000	6.1487999	-13.3322430
2250.0000000	6.1975999	-22.9956627
2250.0000000	6.2463999	-1747.6776123
2250.0000000	6.2951999	-18.9668064
2250.0000000	6.3439999	-334.5737610
2250.0000000	6.3927999	-15.0457897
2250.0000000	6.4416003	-12.4603834
2250.0000000	6.4903998	-11.3493080
2250.0000000	6.5391998	-11.8924084
2250.0000000	6.5879998	297.7528687
2250.0000000	6.6367998	-12.1534996
2250.0000000	6.6856003	-13.8363876
2250.0000000	6.7343998	-13.4828405
2250.0000000	6.7831998	-14.3202639
2250.0000000	6.8319998	56.0377884
2250.0000000	6.8807998	-14.0320101
2250.0000000	6.9296002	-354.0493469
2250.0000000	6.9783998	-6.6903124
2250.0000000	7.0271997	-14.0087767
2250.0000000	7.0760002	5.9180441
2250.0000000	7.1247997	-1639.4112549
2250.0000000	7.1736002	-1314.8675537
2250.0000000	7.2223997	-748.8080444
2250.0000000	7.2711997	-163.8073273
2250.0000000	7.3200002	-17.3903198
2250.0000000	7.3687997	141.3059845
2250.0000000	7.4176002	297.9284058
2250.0000000	7.4663997	233.5795746
2250.0000000	7.5151997	-731.7489624
2250.0000000	7.5640001	-808.0046997
2250.0000000	7.6127996	-1901.1169434
2250.0000000	7.6616001	-611.7423096
2250.0000000	7.7104001	-315.3901367
2250.0000000	7.7591996	30.6868172
2250.0000000	7.8080001	309.6223145
2250.0000000	7.8567996	408.6726685
2250.0000000	7.9056001	-854.1365967
2250.0000000	7.9544001	1358.1470947
2250.0000000	8.0031996	1752.6315918
2250.0000000	8.0520000	1749.2526855
2250.0000000	8.1007996	1851.1214600
2250.0000000	8.1496000	1974.3216553
2250.0000000	8.1983995	1228.1439209
2250.0000000	8.2472000	-2811.4648438
2250.0000000	8.2959995	-996.9224854
2250.0000000	8.3448000	-793.9637451
2250.0000000	8.3935995	-727.6956177
2250.0000000	8.4423990	-313.8716125
2250.0000000	8.4912004	-731.9473267
2250.0000000	8.5400000	-1973.4398193
2250.0000000	8.5887995	-957.1973267
2250.0000000	8.6375999	186.0065613
2250.0000000	8.6863995	1686.4323730
2250.0000000	8.7351999	-627.5871582
2250.0000000	8.7840004	715.3178711
2250.0000000	8.8327999	36.7581215

2250.0000000	8.8815994	-11.5179520
2250.0000000	8.9303989	-23.4861412
2250.0000000	8.9792004	-11.5892601
2250.0000000	9.0279999	-6.0549469
2250.0000000	9.0767994	6.3345108
2250.0000000	9.1255999	-17.0308437
2250.0000000	9.1743994	-10.1231098
2250.0000000	9.2231998	35.0742493
2250.0000000	9.2720003	-8.5353689
2250.0000000	9.3207998	-9.8644772
2250.0000000	9.3695993	-10.7916317
2250.0000000	9.4183998	-11.7385578
2250.0000000	9.4672003	-11.1663980
2250.0000000	9.5159998	-14.0546131
2250.0000000	9.5648003	-0.8079157
2250.0000000	9.6135998	-13.2987537
2250.0000000	9.6623993	-10.2551641
2250.0000000	9.7111998	-10.1911097
2250.0000000	9.7600002	-10.8627052
2250.0000000	9.8087997	-10.9957571
2250.0000000	9.8575993	2.6459513
2250.0000000	9.9063997	-8.0814705
2250.0000000	9.9552002	-10.9071321
2250.0000000	10.0039997	-10.1051674
2260.0000000	0.3416000	-36.2002373
2260.0000000	0.3904000	-38.8323021
2260.0000000	0.4392000	-39.2472229
2260.0000000	0.4880000	-39.4413261
2260.0000000	0.5368000	-39.7592926
2260.0000000	0.5856000	-39.1613503
2260.0000000	0.6344000	-38.2639389
2260.0000000	0.6832000	-36.4418564
2260.0000000	0.7320000	-36.2561455
2260.0000000	0.7808000	-36.5513992
2260.0000000	0.8296000	-35.8520508
2260.0000000	0.8784000	-35.2724724
2260.0000000	0.9272000	-32.8714294
2260.0000000	0.9760000	-30.9738369
2260.0000000	1.0247999	-30.0968132
2260.0000000	1.0735999	-29.1641731
2260.0000000	1.1224000	-29.4642982
2260.0000000	1.1711999	-30.6057892
2260.0000000	1.2200000	-31.2720699
2260.0000000	1.2687999	-31.1280746
2260.0000000	1.3176000	-30.5818081
2260.0000000	1.3664000	-30.6958103
2260.0000000	1.4152000	-30.1876621
2260.0000000	1.4640000	-29.4881363
2260.0000000	1.5128000	-28.8371639
2260.0000000	1.5616000	-28.8786488
2260.0000000	1.6104001	-1241.9096680
2260.0000000	1.6592000	-36.8106346
2260.0000000	1.7079999	-27.7221870
2260.0000000	1.7567999	-27.3395329
2260.0000000	1.8055999	-26.6254215
2260.0000000	1.8544000	-26.0398769
2260.0000000	1.9031999	-26.7897453
2260.0000000	1.9520000	-26.2851753

2260.0000000	2.0007999	-25.4776039
2260.0000000	2.0495999	-24.8561726
2260.0000000	2.0983999	-26.0961342
2260.0000000	2.1471999	-25.3500977
2260.0000000	2.1960001	-24.0391273
2260.0000000	2.2448001	-25.2177906
2260.0000000	2.2935998	-24.7393627
2260.0000000	2.3423998	-22.7760201
2260.0000000	2.3912001	-24.6261368
2260.0000000	2.4400001	-23.1457882
2260.0000000	2.4888000	-23.4331512
2260.0000000	2.5375998	-14.3878021
2260.0000000	2.5863998	-12.8367949
2260.0000000	2.6352000	-11.2782249
2260.0000000	2.6840000	-16.8873119
2260.0000000	2.7328000	100.3195190
2260.0000000	2.7815998	119.2950363
2260.0000000	2.8304000	-23.6334114
2260.0000000	2.8792000	-21.0220966
2260.0000000	2.9280000	-22.5015545
2260.0000000	2.9768000	-17.8222675
2260.0000000	3.0256000	-20.7917461
2260.0000000	3.0743999	-28.1118584
2260.0000000	3.1231999	-23.8287811
2260.0000000	3.1719999	-28.5825443
2260.0000000	3.2208002	-474.9012756
2260.0000000	3.2695999	-928.6229248
2260.0000000	3.3183999	-20.5141468
2260.0000000	3.3671999	-3215.4157715
2260.0000000	3.4159999	1152.1726074
2260.0000000	3.4648001	-7.8483324
2260.0000000	3.5135999	-26.6662865
2260.0000000	3.5623999	-305.8793945
2260.0000000	3.6111999	-1916.2010498
2260.0000000	3.6600001	-434.6400757
2260.0000000	3.7088001	-573.5394287
2260.0000000	3.7575998	-897.3394775
2260.0000000	3.8063998	-1627.4757080
2260.0000000	3.8552001	-2362.3764648
2260.0000000	3.9040000	-2316.1650391
2260.0000000	3.9528000	1057.6975098
2260.0000000	4.0015998	-232.3223114
2260.0000000	4.0503998	647.0971680
2260.0000000	4.0991998	81.5019455
2260.0000000	4.1479998	-618.3016357
2260.0000000	4.1967998	1.3064537
2260.0000000	4.2456002	-235.3434143
2260.0000000	4.2943997	-461.1345520
2260.0000000	4.3431997	-13.6234512
2260.0000000	4.3920002	-234.9990997
2260.0000000	4.4407997	-22.2227364
2260.0000000	4.4896002	-15.5924692
2260.0000000	4.5383997	-620.0916748
2260.0000000	4.5871997	-13.1088114
2260.0000000	4.6360002	-21.4143734
2260.0000000	4.6847997	-27.7562599
2260.0000000	4.7336001	-19.4656105
2260.0000000	4.7824001	-16.5732841

2260.0000000	4.8311996	-1403.1019287
2260.0000000	4.8800001	216.2430725
2260.0000000	4.9287996	-192.4283142
2260.0000000	4.9776001	1006.9143677
2260.0000000	5.0264001	81.1410446
2260.0000000	5.0751996	-287.2339478
2260.0000000	5.1240001	-12.4319649
2260.0000000	5.1727996	542.8875122
2260.0000000	5.2216001	30.7779427
2260.0000000	5.2704000	-155.1907654
2260.0000000	5.3191996	-38.5958748
2260.0000000	5.3680000	-3446.6103516
2260.0000000	5.4168000	513.3679810
2260.0000000	5.4656000	-196.0769806
2260.0000000	5.5144000	36.6942520
2260.0000000	5.5631995	475.6283569
2260.0000000	5.6120000	382.8403625
2260.0000000	5.6608000	-36.5582237
2260.0000000	5.7096000	60.5431747
2260.0000000	5.7584000	129.3144836
2260.0000000	5.8071995	-11.6323824
2260.0000000	5.8559999	28.6780720
2260.0000000	5.9047999	-16.5801830
2260.0000000	5.9535999	-39.1668243
2260.0000000	6.0023999	-1533.8453369
2260.0000000	6.0511999	-14.5953455
2260.0000000	6.0999999	-19.3024673
2260.0000000	6.1487999	-13.3327065
2260.0000000	6.1975999	-22.9938965
2260.0000000	6.2463999	-1747.3303223
2260.0000000	6.2951999	-18.9659920
2260.0000000	6.3439999	-334.5097046
2260.0000000	6.3927999	-15.0457716
2260.0000000	6.4416003	-12.4609709
2260.0000000	6.4903998	-11.3499832
2260.0000000	6.5391998	-11.8930893
2260.0000000	6.5879998	297.0621643
2260.0000000	6.6367998	-12.1540489
2260.0000000	6.6856003	-13.8365574
2260.0000000	6.7343998	-13.4830570
2260.0000000	6.7831998	-14.3198481
2260.0000000	6.8319998	56.0186691
2260.0000000	6.8807998	-14.0318718
2260.0000000	6.9296002	-353.2879028
2260.0000000	6.9783998	-6.7031364
2260.0000000	7.0271997	-14.0077496
2260.0000000	7.0760002	5.8758593
2260.0000000	7.1247997	-1635.4654541
2260.0000000	7.1736002	-1311.7047119
2260.0000000	7.2223997	-747.0148926
2260.0000000	7.2711997	-163.4395447
2260.0000000	7.3200002	-17.3783321
2260.0000000	7.3687997	140.9314575
2260.0000000	7.4176002	297.1725769
2260.0000000	7.4663997	232.9819183
2260.0000000	7.5151997	-729.9879150
2260.0000000	7.5640001	-806.0573120
2260.0000000	7.6127996	-1896.4992676

2260.0000000	7.6616001	-610.2709961
2260.0000000	7.7104001	-314.6438599
2260.0000000	7.7591996	30.5857658
2260.0000000	7.8080001	308.8369446
2260.0000000	7.8567996	407.6445313
2260.0000000	7.9056001	-852.0637207
2260.0000000	7.9544001	1354.8371582
2260.0000000	8.0031996	1748.3620605
2260.0000000	8.0520000	1744.9907227
2260.0000000	8.1007996	1846.6119385
2260.0000000	8.1496000	1969.5136719
2260.0000000	8.1983995	1225.1513672
2260.0000000	8.2472000	-2804.6298828
2260.0000000	8.2959995	-994.5023193
2260.0000000	8.3448000	-792.0345459
2260.0000000	8.3935995	-725.9279175
2260.0000000	8.4423990	-313.1135864
2260.0000000	8.4912004	-730.1710815
2260.0000000	8.5400000	-1968.6400146
2260.0000000	8.5887995	-954.8757935
2260.0000000	8.6375999	185.5452423
2260.0000000	8.6863995	1682.3139648
2260.0000000	8.7351999	-626.0629272
2260.0000000	8.7840004	713.5611572
2260.0000000	8.8327999	36.6465111
2260.0000000	8.8815994	-11.5181265
2260.0000000	8.9303989	-23.4824200
2260.0000000	8.9792004	-11.5893421
2260.0000000	9.0279999	-6.0567150
2260.0000000	9.0767994	6.3287945
2260.0000000	9.1255999	-17.0291309
2260.0000000	9.1743994	-10.1235123
2260.0000000	9.2231998	35.0585175
2260.0000000	9.2720003	-8.5363054
2260.0000000	9.3207998	-9.8650160
2260.0000000	9.3695993	-10.7918587
2260.0000000	9.4183998	-11.7384329
2260.0000000	9.4672003	-11.1664391
2260.0000000	9.5159998	-14.0537214
2260.0000000	9.5648003	-0.8112888
2260.0000000	9.6135998	-13.2979631
2260.0000000	9.6623993	-10.2554388
2260.0000000	9.7111998	-10.1913815
2260.0000000	9.7600002	-10.8627472
2260.0000000	9.8087997	-10.9957647
2260.0000000	9.8575993	2.6415291
2260.0000000	9.9063997	-8.0823851
2260.0000000	9.9552002	-10.9071360
2260.0000000	10.0039997	-10.1054153
2270.0000000	0.3416000	-36.3274918
2270.0000000	0.3904000	-38.4756241
2270.0000000	0.4392000	-39.2481842
2270.0000000	0.4880000	-39.4401169
2270.0000000	0.5368000	-39.7604675
2270.0000000	0.5856000	-39.1599655
2270.0000000	0.6344000	-38.2642479
2270.0000000	0.6832000	-36.4440804
2270.0000000	0.7320000	-36.2581978

2270.0000000	0.7808000	-36.5515671
2270.0000000	0.8296000	-35.8481445
2270.0000000	0.8784000	-35.2693405
2270.0000000	0.9272000	-32.9698753
2270.0000000	0.9760000	-30.5965328
2270.0000000	1.0247999	-28.2872353
2270.0000000	1.0735999	-28.2803230
2270.0000000	1.1224000	-29.2245045
2270.0000000	1.1711999	-29.5532589
2270.0000000	1.2200000	-30.4998341
2270.0000000	1.2687999	-30.8553371
2270.0000000	1.3176000	-30.5781250
2270.0000000	1.3664000	-30.6953869
2270.0000000	1.4152000	-30.1878681
2270.0000000	1.4640000	-29.4888191
2270.0000000	1.5128000	-28.8363934
2270.0000000	1.5616000	-28.8729420
2270.0000000	1.6104001	-1239.3668213
2270.0000000	1.6592000	-36.5167007
2270.0000000	1.7079999	-27.7259350
2270.0000000	1.7567999	-27.3288651
2270.0000000	1.8055999	-26.6397171
2270.0000000	1.8544000	-26.0373611
2270.0000000	1.9031999	-26.8007851
2270.0000000	1.9520000	-26.3103485
2270.0000000	2.0007999	-25.5069752
2270.0000000	2.0495999	-24.8595772
2270.0000000	2.0983999	-26.0959339
2270.0000000	2.1471999	-25.3500175
2270.0000000	2.1960001	-24.0427818
2270.0000000	2.2448001	-25.2146397
2270.0000000	2.2935998	-24.7372513
2270.0000000	2.3423998	-22.7904263
2270.0000000	2.3912001	-24.6245022
2270.0000000	2.4400001	-23.1482735
2270.0000000	2.4888000	-23.4329338
2270.0000000	2.5375998	-14.4049101
2270.0000000	2.5863998	-12.8565531
2270.0000000	2.6352000	-11.3009691
2270.0000000	2.6840000	-16.8992538
2270.0000000	2.7328000	100.0535049
2270.0000000	2.7815998	118.9886093
2270.0000000	2.8304000	-23.6321087
2270.0000000	2.8792000	-21.0253601
2270.0000000	2.9280000	-22.5009861
2270.0000000	2.9768000	-17.8306389
2270.0000000	3.0256000	-20.7943306
2270.0000000	3.0743999	-28.1004715
2270.0000000	3.1231999	-23.8253269
2270.0000000	3.1719999	-28.5742512
2270.0000000	3.2208002	-474.6635437
2270.0000000	3.2695999	-928.1479492
2270.0000000	3.3183999	-20.5143452
2270.0000000	3.3671999	-3213.9541016
2270.0000000	3.4159999	1151.6373291
2270.0000000	3.4648001	-7.8540401
2270.0000000	3.5135999	-26.6632957
2270.0000000	3.5623999	-305.7498169

2270.0000000	3.6111999	-1915.3411865
2270.0000000	3.6600001	-434.4519653
2270.0000000	3.7088001	-573.2880249
2270.0000000	3.7575998	-896.9409790
2270.0000000	3.8063998	-1626.7458496
2270.0000000	3.8552001	-2361.3132324
2270.0000000	3.9040000	-2315.1225586
2270.0000000	3.9528000	1057.2092285
2270.0000000	4.0015998	-232.2251892
2270.0000000	4.0503998	646.7946167
2270.0000000	4.0991998	81.4562607
2270.0000000	4.1479998	-618.0294189
2270.0000000	4.1967998	1.2971973
2270.0000000	4.2456002	-235.2451019
2270.0000000	4.2943997	-460.9339905
2270.0000000	4.3431997	-13.6256313
2270.0000000	4.3920002	-234.9009857
2270.0000000	4.4407997	-22.2209625
2270.0000000	4.4896002	-15.5933514
2270.0000000	4.5383997	-619.8306274
2270.0000000	4.5871997	-13.1108284
2270.0000000	4.6360002	-21.4128304
2270.0000000	4.6847997	-27.7523346
2270.0000000	4.7336001	-19.4648876
2270.0000000	4.7824001	-16.5737381
2270.0000000	4.8311996	-1402.5706787
2270.0000000	4.8800001	216.1535187
2270.0000000	4.9287996	-192.3610535
2270.0000000	4.9776001	1006.5218506
2270.0000000	5.0264001	81.1035233
2270.0000000	5.0751996	-287.1303101
2270.0000000	5.1240001	-12.4328861
2270.0000000	5.1727996	542.7716064
2270.0000000	5.2216001	30.7684975
2270.0000000	5.2704000	-155.1626587
2270.0000000	5.3191996	-38.5913239
2270.0000000	5.3680000	-3445.9179688
2270.0000000	5.4168000	513.2612305
2270.0000000	5.4656000	-196.0408020
2270.0000000	5.5144000	36.6836014
2270.0000000	5.5631995	475.5297852
2270.0000000	5.6120000	382.7603760
2270.0000000	5.6608000	-36.5541382
2270.0000000	5.7096000	60.5277481
2270.0000000	5.7584000	129.2855530
2270.0000000	5.8071995	-11.6332531
2270.0000000	5.8559999	28.6692219
2270.0000000	5.9047999	-16.5799847
2270.0000000	5.9535999	-39.1620827
2270.0000000	6.0023999	-1533.5410156
2270.0000000	6.0511999	-14.5955572
2270.0000000	6.0999999	-19.3016338
2270.0000000	6.1487999	-13.3331690
2270.0000000	6.1975999	-22.9921303
2270.0000000	6.2463999	-1746.9829102
2270.0000000	6.2951999	-18.9651794
2270.0000000	6.3439999	-334.4456482
2270.0000000	6.3927999	-15.0457525

2270.0000000	6.4416003	-12.4615574
2270.0000000	6.4903998	-11.3506594
2270.0000000	6.5391998	-11.8937702
2270.0000000	6.5879998	296.3714600
2270.0000000	6.6367998	-12.1545982
2270.0000000	6.6856003	-13.8367281
2270.0000000	6.7343998	-13.4832726
2270.0000000	6.7831998	-14.3194323
2270.0000000	6.8319998	55.9995575
2270.0000000	6.8807998	-14.0317326
2270.0000000	6.9296002	-352.5264282
2270.0000000	6.9783998	-6.7159600
2270.0000000	7.0271997	-14.0067215
2270.0000000	7.0760002	5.8336744
2270.0000000	7.1247997	-1631.5195313
2270.0000000	7.1736002	-1308.5417480
2270.0000000	7.2223997	-745.2216797
2270.0000000	7.2711997	-163.0717621
2270.0000000	7.3200002	-17.3663445
2270.0000000	7.3687997	140.5569305
2270.0000000	7.4176002	296.4167480
2270.0000000	7.4663997	232.3842773
2270.0000000	7.5151997	-728.2268677
2270.0000000	7.5640001	-804.1099243
2270.0000000	7.6127996	-1891.8817139
2270.0000000	7.6616001	-608.7996216
2270.0000000	7.7104001	-313.8975830
2270.0000000	7.7591996	30.4847183
2270.0000000	7.8080001	308.0516052
2270.0000000	7.8567996	406.6164551
2270.0000000	7.9056001	-849.9907837
2270.0000000	7.9544001	1351.5272217
2270.0000000	8.0031996	1744.0924072
2270.0000000	8.0520000	1740.7286377
2270.0000000	8.1007996	1842.1024170
2270.0000000	8.1496000	1964.7056885
2270.0000000	8.1983995	1222.1589355
2270.0000000	8.2472000	-2797.7951660
2270.0000000	8.2959995	-992.0821533
2270.0000000	8.3448000	-790.1054077
2270.0000000	8.3935995	-724.1602783
2270.0000000	8.4423990	-312.3555908
2270.0000000	8.4912004	-728.3948975
2270.0000000	8.5400000	-1963.8402100
2270.0000000	8.5887995	-952.5542603
2270.0000000	8.6375999	185.0839233
2270.0000000	8.6863995	1678.1956787
2270.0000000	8.7351999	-624.5386963
2270.0000000	8.7840004	711.8043823
2270.0000000	8.8327999	36.5349045
2270.0000000	8.8815994	-11.5183001
2270.0000000	8.9303989	-23.4786987
2270.0000000	8.9792004	-11.5894241
2270.0000000	9.0279999	-6.0584831
2270.0000000	9.0767994	6.3230762
2270.0000000	9.1255999	-17.0274162
2270.0000000	9.1743994	-10.1239147
2270.0000000	9.2231998	35.0427856

2270.0000000	9.2720003	-8.5372419
2270.0000000	9.3207998	-9.8655558
2270.0000000	9.3695993	-10.7920847
2270.0000000	9.4183998	-11.7383080
2270.0000000	9.4672003	-11.1664801
2270.0000000	9.5159998	-14.0528307
2270.0000000	9.5648003	-0.8146610
2270.0000000	9.6135998	-13.2971725
2270.0000000	9.6623993	-10.2557144
2270.0000000	9.7111998	-10.1916523
2270.0000000	9.7600002	-10.8627892
2270.0000000	9.8087997	-10.9957724
2270.0000000	9.8575993	2.6371059
2270.0000000	9.9063997	-8.0832987
2270.0000000	9.9552002	-10.9071407
2270.0000000	10.0039997	-10.1056623
2280.0000000	0.3416000	-36.6869278
2280.0000000	0.3904000	-39.4374580
2280.0000000	0.4392000	-39.2491417
2280.0000000	0.4880000	-39.4389114
2280.0000000	0.5368000	-39.7616425
2280.0000000	0.5856000	-39.1585846
2280.0000000	0.6344000	-38.2645607
2280.0000000	0.6832000	-36.4463043
2280.0000000	0.7320000	-36.2602501
2280.0000000	0.7808000	-36.5517349
2280.0000000	0.8296000	-35.8442383
2280.0000000	0.8784000	-35.2662086
2280.0000000	0.9272000	-32.9559212
2280.0000000	0.9760000	-30.2271652
2280.0000000	1.0247999	-28.5057354
2280.0000000	1.0735999	-28.0645409
2280.0000000	1.1224000	-28.2348976
2280.0000000	1.1711999	-29.0916672
2280.0000000	1.2200000	-29.9724083
2280.0000000	1.2687999	-30.6633110
2280.0000000	1.3176000	-30.5744438
2280.0000000	1.3664000	-30.6949635
2280.0000000	1.4152000	-30.1880722
2280.0000000	1.4640000	-29.4895039
2280.0000000	1.5128000	-28.8356228
2280.0000000	1.5616000	-28.8672352
2280.0000000	1.6104001	-1236.8240967
2280.0000000	1.6592000	-36.2227707
2280.0000000	1.7079999	-27.7296810
2280.0000000	1.7567999	-27.3181992
2280.0000000	1.8055999	-26.6540127
2280.0000000	1.8544000	-26.0348454
2280.0000000	1.9031999	-26.8118248
2280.0000000	1.9520000	-26.3355217
2280.0000000	2.0007999	-25.5363483
2280.0000000	2.0495999	-24.8629818
2280.0000000	2.0983999	-26.0957336
2280.0000000	2.1471999	-25.3499374
2280.0000000	2.1960001	-24.0464363
2280.0000000	2.2448001	-25.2114906
2280.0000000	2.2935998	-24.7351398
2280.0000000	2.3423998	-22.8048306

2280.0000000	2.3912001	-24.6228657
2280.0000000	2.4400001	-23.1507607
2280.0000000	2.4888000	-23.4327164
2280.0000000	2.5375998	-14.4220181
2280.0000000	2.5863998	-12.8763123
2280.0000000	2.6352000	-11.3237133
2280.0000000	2.6840000	-16.9111958
2280.0000000	2.7328000	99.7874832
2280.0000000	2.7815998	118.6821976
2280.0000000	2.8304000	-23.6308041
2280.0000000	2.8792000	-21.0286236
2280.0000000	2.9280000	-22.5004177
2280.0000000	2.9768000	-17.8390083
2280.0000000	3.0256000	-20.7969151
2280.0000000	3.0743999	-28.0890846
2280.0000000	3.1231999	-23.8218708
2280.0000000	3.1719999	-28.5659580
2280.0000000	3.2208002	-474.4258118
2280.0000000	3.2695999	-927.6729736
2280.0000000	3.3183999	-20.5145416
2280.0000000	3.3671999	-3212.4924316
2280.0000000	3.4159999	1151.1020508
2280.0000000	3.4648001	-7.8597488
2280.0000000	3.5135999	-26.6603050
2280.0000000	3.5623999	-305.6202087
2280.0000000	3.6111999	-1914.4814453
2280.0000000	3.6600001	-434.2638550
2280.0000000	3.7088001	-573.0366821
2280.0000000	3.7575998	-896.5424194
2280.0000000	3.8063998	-1626.0159912
2280.0000000	3.8552001	-2360.2497559
2280.0000000	3.9040000	-2314.0800781
2280.0000000	3.9528000	1056.7208252
2280.0000000	4.0015998	-232.1280823
2280.0000000	4.0503998	646.4920654
2280.0000000	4.0991998	81.4105759
2280.0000000	4.1479998	-617.7572021
2280.0000000	4.1967998	1.2879410
2280.0000000	4.2456002	-235.1468048
2280.0000000	4.2943997	-460.7334290
2280.0000000	4.3431997	-13.6278114
2280.0000000	4.3920002	-234.8028717
2280.0000000	4.4407997	-22.2191868
2280.0000000	4.4896002	-15.5942345
2280.0000000	4.5383997	-619.5695801
2280.0000000	4.5871997	-13.1128445
2280.0000000	4.6360002	-21.4112892
2280.0000000	4.6847997	-27.7484093
2280.0000000	4.7336001	-19.4641647
2280.0000000	4.7824001	-16.5741920
2280.0000000	4.8311996	-1402.0394287
2280.0000000	4.8800001	216.0639648
2280.0000000	4.9287996	-192.2937927
2280.0000000	4.9776001	1006.1293945
2280.0000000	5.0264001	81.0660019
2280.0000000	5.0751996	-287.0267029
2280.0000000	5.1240001	-12.4338074
2280.0000000	5.1727996	542.6557617

2280.0000000	5.2216001	30.7590561
2280.0000000	5.2704000	-155.1345520
2280.0000000	5.3191996	-38.5867767
2280.0000000	5.3680000	-3445.2255859
2280.0000000	5.4168000	513.1544800
2280.0000000	5.4656000	-196.0046234
2280.0000000	5.5144000	36.6729431
2280.0000000	5.5631995	475.4312439
2280.0000000	5.6120000	382.6803894
2280.0000000	5.6608000	-36.5500488
2280.0000000	5.7096000	60.5123138
2280.0000000	5.7584000	129.2566223
2280.0000000	5.8071995	-11.6341238
2280.0000000	5.8559999	28.6603718
2280.0000000	5.9047999	-16.5797863
2280.0000000	5.9535999	-39.1573448
2280.0000000	6.0023999	-1533.2365723
2280.0000000	6.0511999	-14.5957689
2280.0000000	6.0999999	-19.3008022
2280.0000000	6.1487999	-13.3336315
2280.0000000	6.1975999	-22.9903660
2280.0000000	6.2463999	-1746.6354980
2280.0000000	6.2951999	-18.9643650
2280.0000000	6.3439999	-334.3815918
2280.0000000	6.3927999	-15.0457344
2280.0000000	6.4416003	-12.4621439
2280.0000000	6.4903998	-11.3513355
2280.0000000	6.5391998	-11.8944511
2280.0000000	6.5879998	295.6807556
2280.0000000	6.6367998	-12.1551476
2280.0000000	6.6856003	-13.8368979
2280.0000000	6.7343998	-13.4834881
2280.0000000	6.7831998	-14.3190174
2280.0000000	6.8319998	55.9804459
2280.0000000	6.8807998	-14.0315943
2280.0000000	6.9296002	-351.7649841
2280.0000000	6.9783998	-6.7287831
2280.0000000	7.0271997	-14.0056934
2280.0000000	7.0760002	5.7914877
2280.0000000	7.1247997	-1627.5736084
2280.0000000	7.1736002	-1305.3789063
2280.0000000	7.2223997	-743.4285278
2280.0000000	7.2711997	-162.7039795
2280.0000000	7.3200002	-17.3543549
2280.0000000	7.3687997	140.1824036
2280.0000000	7.4176002	295.6609192
2280.0000000	7.4663997	231.7866211
2280.0000000	7.5151997	-726.4658203
2280.0000000	7.5640001	-802.1625366
2280.0000000	7.6127996	-1887.2640381
2280.0000000	7.6616001	-607.3282471
2280.0000000	7.7104001	-313.1513062
2280.0000000	7.7591996	30.3836670
2280.0000000	7.8080001	307.2662354
2280.0000000	7.8567996	405.5883789
2280.0000000	7.9056001	-847.9179077
2280.0000000	7.9544001	1348.2172852
2280.0000000	8.0031996	1739.8228760

2280.0000000	8.0520000	1736.4666748
2280.0000000	8.1007996	1837.5928955
2280.0000000	8.1496000	1959.8977051
2280.0000000	8.1983995	1219.1663818
2280.0000000	8.2472000	-2790.9604492
2280.0000000	8.2959995	-989.6619873
2280.0000000	8.3448000	-788.1762085
2280.0000000	8.3935995	-722.3925781
2280.0000000	8.4423990	-311.5975647
2280.0000000	8.4912004	-726.6186523
2280.0000000	8.5400000	-1959.0402832
2280.0000000	8.5887995	-950.2327271
2280.0000000	8.6375999	184.6226044
2280.0000000	8.6863995	1674.0772705
2280.0000000	8.7351999	-623.0144043
2280.0000000	8.7840004	710.0476685
2280.0000000	8.8327999	36.4232979
2280.0000000	8.8815994	-11.5184746
2280.0000000	8.9303989	-23.4749794
2280.0000000	8.9792004	-11.5895071
2280.0000000	9.0279999	-6.0602512
2280.0000000	9.0767994	6.3173599
2280.0000000	9.1255999	-17.0257015
2280.0000000	9.1743994	-10.1243162
2280.0000000	9.2231998	35.0270538
2280.0000000	9.2720003	-8.5381784
2280.0000000	9.3207998	-9.8660946
2280.0000000	9.3695993	-10.7923117
2280.0000000	9.4183998	-11.7381840
2280.0000000	9.4672003	-11.1665211
2280.0000000	9.5159998	-14.0519390
2280.0000000	9.5648003	-0.8180332
2280.0000000	9.6135998	-13.2963820
2280.0000000	9.6623993	-10.2559891
2280.0000000	9.7111998	-10.1919241
2280.0000000	9.7600002	-10.8628302
2280.0000000	9.8087997	-10.9957809
2280.0000000	9.8575993	2.6326838
2280.0000000	9.9063997	-8.0842123
2280.0000000	9.9552002	-10.9071445
2280.0000000	10.0039997	-10.1059103
2290.0000000	0.3416000	-36.3111649
2290.0000000	0.3904000	-38.4809952
2290.0000000	0.4392000	-39.2501030
2290.0000000	0.4880000	-39.4377022
2290.0000000	0.5368000	-39.7628136
2290.0000000	0.5856000	-39.1572037
2290.0000000	0.6344000	-38.2648697
2290.0000000	0.6832000	-36.4485321
2290.0000000	0.7320000	-36.2623024
2290.0000000	0.7808000	-36.5519066
2290.0000000	0.8296000	-35.8403282
2290.0000000	0.8784000	-35.1257782
2290.0000000	0.9272000	-33.4316063
2290.0000000	0.9760000	-30.0839539
2290.0000000	1.0247999	-28.3644772
2290.0000000	1.0735999	-28.4146328
2290.0000000	1.1224000	-28.5691071

2290.0000000	1.1711999	-29.2195320
2290.0000000	1.2200000	-31.1621723
2290.0000000	1.2687999	-31.4177341
2290.0000000	1.3176000	-30.5707626
2290.0000000	1.3664000	-30.6945419
2290.0000000	1.4152000	-30.1882763
2290.0000000	1.4640000	-29.4901867
2290.0000000	1.5128000	-28.8348541
2290.0000000	1.5616000	-28.8615303
2290.0000000	1.6104001	-1234.2812500
2290.0000000	1.6592000	-35.9288406
2290.0000000	1.7079999	-27.7334290
2290.0000000	1.7567999	-27.3075314
2290.0000000	1.8055999	-26.6683102
2290.0000000	1.8544000	-26.0323296
2290.0000000	1.9031999	-26.8228645
2290.0000000	1.9520000	-26.3606949
2290.0000000	2.0007999	-25.5657196
2290.0000000	2.0495999	-24.8663864
2290.0000000	2.0983999	-26.0955334
2290.0000000	2.1471999	-25.3498573
2290.0000000	2.1960001	-24.0500908
2290.0000000	2.2448001	-25.2083397
2290.0000000	2.2935998	-24.7330265
2290.0000000	2.3423998	-22.8192348
2290.0000000	2.3912001	-24.6212292
2290.0000000	2.4400001	-23.1532478
2290.0000000	2.4888000	-23.4325008
2290.0000000	2.5375998	-14.4391260
2290.0000000	2.5863998	-12.8960714
2290.0000000	2.6352000	-11.3464575
2290.0000000	2.6840000	-16.9231377
2290.0000000	2.7328000	99.5214691
2290.0000000	2.7815998	118.3757706
2290.0000000	2.8304000	-23.6294994
2290.0000000	2.8792000	-21.0318871
2290.0000000	2.9280000	-22.4998474
2290.0000000	2.9768000	-17.8473778
2290.0000000	3.0256000	-20.7994995
2290.0000000	3.0743999	-28.0776978
2290.0000000	3.1231999	-23.8184166
2290.0000000	3.1719999	-28.5576649
2290.0000000	3.2208002	-474.1880798
2290.0000000	3.2695999	-927.1979980
2290.0000000	3.3183999	-20.5147400
2290.0000000	3.3671999	-3211.0307617
2290.0000000	3.4159999	1150.5667725
2290.0000000	3.4648001	-7.8654566
2290.0000000	3.5135999	-26.6573143
2290.0000000	3.5623999	-305.4906311
2290.0000000	3.6111999	-1913.6217041
2290.0000000	3.6600001	-434.0756836
2290.0000000	3.7088001	-572.7852783
2290.0000000	3.7575998	-896.1439209
2290.0000000	3.8063998	-1625.2861328
2290.0000000	3.8552001	-2359.1862793
2290.0000000	3.9040000	-2313.0375977
2290.0000000	3.9528000	1056.2325439

2290.0000000	4.0015998	-232.0309753
2290.0000000	4.0503998	646.1895142
2290.0000000	4.0991998	81.3648911
2290.0000000	4.1479998	-617.4849854
2290.0000000	4.1967998	1.2786846
2290.0000000	4.2456002	-235.0485077
2290.0000000	4.2943997	-460.5328674
2290.0000000	4.3431997	-13.6299925
2290.0000000	4.3920002	-234.7047577
2290.0000000	4.4407997	-22.2174110
2290.0000000	4.4896002	-15.5951166
2290.0000000	4.5383997	-619.3084717
2290.0000000	4.5871997	-13.1148605
2290.0000000	4.6360002	-21.4097462
2290.0000000	4.6847997	-27.7444839
2290.0000000	4.7336001	-19.4634438
2290.0000000	4.7824001	-16.5746479
2290.0000000	4.8311996	-1401.5080566
2290.0000000	4.8800001	215.9744263
2290.0000000	4.9287996	-192.2265320
2290.0000000	4.9776001	1005.7368774
2290.0000000	5.0264001	81.0284805
2290.0000000	5.0751996	-286.9230652
2290.0000000	5.1240001	-12.4347286
2290.0000000	5.1727996	542.5399170
2290.0000000	5.2216001	30.7496109
2290.0000000	5.2704000	-155.1064453
2290.0000000	5.3191996	-38.5822258
2290.0000000	5.3680000	-3444.5332031
2290.0000000	5.4168000	513.0476685
2290.0000000	5.4656000	-195.9684296
2290.0000000	5.5144000	36.6622849
2290.0000000	5.5631995	475.3326721
2290.0000000	5.6120000	382.6004333
2290.0000000	5.6608000	-36.5459595
2290.0000000	5.7096000	60.4968796
2290.0000000	5.7584000	129.2276917
2290.0000000	5.8071995	-11.6349945
2290.0000000	5.8559999	28.6515217
2290.0000000	5.9047999	-16.5795879
2290.0000000	5.9535999	-39.1526070
2290.0000000	6.0023999	-1532.9321289
2290.0000000	6.0511999	-14.5959797
2290.0000000	6.0999999	-19.2999687
2290.0000000	6.1487999	-13.3340950
2290.0000000	6.1975999	-22.9885998
2290.0000000	6.2463999	-1746.2880859
2290.0000000	6.2951999	-18.9635506
2290.0000000	6.3439999	-334.3175659
2290.0000000	6.3927999	-15.0457163
2290.0000000	6.4416003	-12.4627314
2290.0000000	6.4903998	-11.3520117
2290.0000000	6.5391998	-11.8951321
2290.0000000	6.5879998	294.9900513
2290.0000000	6.6367998	-12.1556969
2290.0000000	6.6856003	-13.8370686
2290.0000000	6.7343998	-13.4837046
2290.0000000	6.7831998	-14.3186016

2290.0000000	6.8319998	55.9613266
2290.0000000	6.8807998	-14.0314550
2290.0000000	6.9296002	-351.0035400
2290.0000000	6.9783998	-6.7416072
2290.0000000	7.0271997	-14.0046654
2290.0000000	7.0760002	5.7493029
2290.0000000	7.1247997	-1623.6278076
2290.0000000	7.1736002	-1302.2160645
2290.0000000	7.2223997	-741.6353760
2290.0000000	7.2711997	-162.3362122
2290.0000000	7.3200002	-17.3423653
2290.0000000	7.3687997	139.8078613
2290.0000000	7.4176002	294.9050903
2290.0000000	7.4663997	231.1889648
2290.0000000	7.5151997	-724.7047729
2290.0000000	7.5640001	-800.2150879
2290.0000000	7.6127996	-1882.6464844
2290.0000000	7.6616001	-605.8569336
2290.0000000	7.7104001	-312.4050903
2290.0000000	7.7591996	30.2826195
2290.0000000	7.8080001	306.4808655
2290.0000000	7.8567996	404.5602722
2290.0000000	7.9056001	-845.8449707
2290.0000000	7.9544001	1344.9073486
2290.0000000	8.0031996	1735.5532227
2290.0000000	8.0520000	1732.2047119
2290.0000000	8.1007996	1833.0833740
2290.0000000	8.1496000	1955.0897217
2290.0000000	8.1983995	1216.1739502
2290.0000000	8.2472000	-2784.1254883
2290.0000000	8.2959995	-987.2418213
2290.0000000	8.3448000	-786.2470093
2290.0000000	8.3935995	-720.6248779
2290.0000000	8.4423990	-310.8395691
2290.0000000	8.4912004	-724.8424683
2290.0000000	8.5400000	-1954.2404785
2290.0000000	8.5887995	-947.9111938
2290.0000000	8.6375999	184.1612854
2290.0000000	8.6863995	1669.9589844
2290.0000000	8.7351999	-621.4901733
2290.0000000	8.7840004	708.2908936
2290.0000000	8.8327999	36.3116875
2290.0000000	8.8815994	-11.5186491
2290.0000000	8.9303989	-23.4712601
2290.0000000	8.9792004	-11.5895891
2290.0000000	9.0279999	-6.0620198
2290.0000000	9.0767994	6.3116417
2290.0000000	9.1255999	-17.0239887
2290.0000000	9.1743994	-10.1247187
2290.0000000	9.2231998	35.0113220
2290.0000000	9.2720003	-8.5391150
2290.0000000	9.3207998	-9.8666344
2290.0000000	9.3695993	-10.7925377
2290.0000000	9.4183998	-11.7380590
2290.0000000	9.4672003	-11.1665621
2290.0000000	9.5159998	-14.0510483
2290.0000000	9.5648003	-0.8214064
2290.0000000	9.6135998	-13.2955914

2290.0000000	9.6623993	-10.2562647
2290.0000000	9.7111998	-10.1921959
2290.0000000	9.7600002	-10.8628721
2290.0000000	9.8087997	-10.9957886
2290.0000000	9.8575993	2.6282616
2290.0000000	9.9063997	-8.0851269
2290.0000000	9.9552002	-10.9071493
2290.0000000	10.0039997	-10.1061573
2300.0000000	0.3416000	-36.8151245
2300.0000000	0.3904000	-39.0052338
2300.0000000	0.4392000	-39.2510605
2300.0000000	0.4880000	-39.4364929
2300.0000000	0.5368000	-39.7639885
2300.0000000	0.5856000	-39.1558228
2300.0000000	0.6344000	-38.2651825
2300.0000000	0.6832000	-36.4507561
2300.0000000	0.7320000	-36.2643547
2300.0000000	0.7808000	-36.5520744
2300.0000000	0.8296000	-35.8364220
2300.0000000	0.8784000	-35.1968842
2300.0000000	0.9272000	-34.0369301
2300.0000000	0.9760000	-31.3042755
2300.0000000	1.0247999	-28.5595589
2300.0000000	1.0735999	-27.9370232
2300.0000000	1.1224000	-28.7555676
2300.0000000	1.1711999	-30.4047604
2300.0000000	1.2200000	-31.2987709
2300.0000000	1.2687999	-31.1312504
2300.0000000	1.3176000	-30.5670795
2300.0000000	1.3664000	-30.6941185
2300.0000000	1.4152000	-30.1884804
2300.0000000	1.4640000	-29.4908695
2300.0000000	1.5128000	-28.8340836
2300.0000000	1.5616000	-28.8558235
2300.0000000	1.6104001	-1231.7384033
2300.0000000	1.6592000	-35.6349068
2300.0000000	1.7079999	-27.7371769
2300.0000000	1.7567999	-27.2968655
2300.0000000	1.8055999	-26.6826057
2300.0000000	1.8544000	-26.0298119
2300.0000000	1.9031999	-26.8339043
2300.0000000	1.9520000	-26.3858681
2300.0000000	2.0007999	-25.5950928
2300.0000000	2.0495999	-24.8697910
2300.0000000	2.0983999	-26.0953350
2300.0000000	2.1471999	-25.3497791
2300.0000000	2.1960001	-24.0537453
2300.0000000	2.2448001	-25.2051888
2300.0000000	2.2935998	-24.7309151
2300.0000000	2.3423998	-22.8336391
2300.0000000	2.3912001	-24.6195927
2300.0000000	2.4400001	-23.1557331
2300.0000000	2.4888000	-23.4322834
2300.0000000	2.5375998	-14.4562340
2300.0000000	2.5863998	-12.9158306
2300.0000000	2.6352000	-11.3692017
2300.0000000	2.6840000	-16.9350796
2300.0000000	2.7328000	99.2554550

2300.0000000	2.7815998	118.0693588
2300.0000000	2.8304000	-23.6281948
2300.0000000	2.8792000	-21.0351505
2300.0000000	2.9280000	-22.4992790
2300.0000000	2.9768000	-17.8557491
2300.0000000	3.0256000	-20.8020840
2300.0000000	3.0743999	-28.0663109
2300.0000000	3.1231999	-23.8149605
2300.0000000	3.1719999	-28.5493717
2300.0000000	3.2208002	-473.9503174
2300.0000000	3.2695999	-926.7230225
2300.0000000	3.3183999	-20.5149384
2300.0000000	3.3671999	-3209.5690918
2300.0000000	3.4159999	1150.0314941
2300.0000000	3.4648001	-7.8711653
2300.0000000	3.5135999	-26.6543236
2300.0000000	3.5623999	-305.3610229
2300.0000000	3.6111999	-1912.7619629
2300.0000000	3.6600001	-433.8875732
2300.0000000	3.7088001	-572.5338745
2300.0000000	3.7575998	-895.7453613
2300.0000000	3.8063998	-1624.5562744
2300.0000000	3.8552001	-2358.1228027
2300.0000000	3.9040000	-2311.9951172
2300.0000000	3.9528000	1055.7442627
2300.0000000	4.0015998	-231.9338684
2300.0000000	4.0503998	645.8869629
2300.0000000	4.0991998	81.3192062
2300.0000000	4.1479998	-617.2127686
2300.0000000	4.1967998	1.2694283
2300.0000000	4.2456002	-234.9501953
2300.0000000	4.2943997	-460.3322449
2300.0000000	4.3431997	-13.6321735
2300.0000000	4.3920002	-234.6066437
2300.0000000	4.4407997	-22.2156353
2300.0000000	4.4896002	-15.5959997
2300.0000000	4.5383997	-619.0474243
2300.0000000	4.5871997	-13.1168766
2300.0000000	4.6360002	-21.4082031
2300.0000000	4.6847997	-27.7405586
2300.0000000	4.7336001	-19.4627209
2300.0000000	4.7824001	-16.5751019
2300.0000000	4.8311996	-1400.9768066
2300.0000000	4.8800001	215.8848724
2300.0000000	4.9287996	-192.1592712
2300.0000000	4.9776001	1005.3444214
2300.0000000	5.0264001	80.9909592
2300.0000000	5.0751996	-286.8194275
2300.0000000	5.1240001	-12.4356499
2300.0000000	5.1727996	542.4240112
2300.0000000	5.2216001	30.7401657
2300.0000000	5.2704000	-155.0783386
2300.0000000	5.3191996	-38.5776787
2300.0000000	5.3680000	-3443.8408203
2300.0000000	5.4168000	512.9408569
2300.0000000	5.4656000	-195.9322510
2300.0000000	5.5144000	36.6516266
2300.0000000	5.5631995	475.2341003

2300.0000000	5.6120000	382.5204468
2300.0000000	5.6608000	-36.5418701
2300.0000000	5.7096000	60.4814529
2300.0000000	5.7584000	129.1987610
2300.0000000	5.8071995	-11.6358652
2300.0000000	5.8559999	28.6426716
2300.0000000	5.9047999	-16.5793915
2300.0000000	5.9535999	-39.1478691
2300.0000000	6.0023999	-1532.6276855
2300.0000000	6.0511999	-14.5961914
2300.0000000	6.0999999	-19.2991371
2300.0000000	6.1487999	-13.3345575
2300.0000000	6.1975999	-22.9868355
2300.0000000	6.2463999	-1745.9407959
2300.0000000	6.2951999	-18.9627361
2300.0000000	6.3439999	-334.2534790
2300.0000000	6.3927999	-15.0456982
2300.0000000	6.4416003	-12.4633179
2300.0000000	6.4903998	-11.3526869
2300.0000000	6.5391998	-11.8958139
2300.0000000	6.5879998	294.2993774
2300.0000000	6.6367998	-12.1562452
2300.0000000	6.6856003	-13.8372383
2300.0000000	6.7343998	-13.4839201
2300.0000000	6.7831998	-14.3181858
2300.0000000	6.8319998	55.9422150
2300.0000000	6.8807998	-14.0313168
2300.0000000	6.9296002	-350.2420959
2300.0000000	6.9783998	-6.7544308
2300.0000000	7.0271997	-14.0036373
2300.0000000	7.0760002	5.7071180
2300.0000000	7.1247997	-1619.6818848
2300.0000000	7.1736002	-1299.0532227
2300.0000000	7.2223997	-739.8422241
2300.0000000	7.2711997	-161.9684296
2300.0000000	7.3200002	-17.3303776
2300.0000000	7.3687997	139.4333344
2300.0000000	7.4176002	294.1492615
2300.0000000	7.4663997	230.5913239
2300.0000000	7.5151997	-722.9437256
2300.0000000	7.5640001	-798.2677002
2300.0000000	7.6127996	-1878.0288086
2300.0000000	7.6616001	-604.3856201
2300.0000000	7.7104001	-311.6588135
2300.0000000	7.7591996	30.1815681
2300.0000000	7.8080001	305.6955261
2300.0000000	7.8567996	403.5321960
2300.0000000	7.9056001	-843.7720947
2300.0000000	7.9544001	1341.5974121
2300.0000000	8.0031996	1731.2836914
2300.0000000	8.0520000	1727.9426270
2300.0000000	8.1007996	1828.5738525
2300.0000000	8.1496000	1950.2816162
2300.0000000	8.1983995	1213.1813965
2300.0000000	8.2472000	-2777.2907715
2300.0000000	8.2959995	-984.8215942
2300.0000000	8.3448000	-784.3178711
2300.0000000	8.3935995	-718.8572388

2300.0000000	8.4423990	-310.0815430
2300.0000000	8.4912004	-723.0662231
2300.0000000	8.5400000	-1949.4406738
2300.0000000	8.5887995	-945.5895996
2300.0000000	8.6375999	183.6999512
2300.0000000	8.6863995	1665.8405762
2300.0000000	8.7351999	-619.9658813
2300.0000000	8.7840004	706.5341797
2300.0000000	8.8327999	36.2000809
2300.0000000	8.8815994	-11.5188236
2300.0000000	8.9303989	-23.4675407
2300.0000000	8.9792004	-11.5896711
2300.0000000	9.0279999	-6.0637884
2300.0000000	9.0767994	6.3059235
2300.0000000	9.1255999	-17.0222740
2300.0000000	9.1743994	-10.1251202
2300.0000000	9.2231998	34.9955826
2300.0000000	9.2720003	-8.5400505
2300.0000000	9.3207998	-9.8671741
2300.0000000	9.3695993	-10.7927647
2300.0000000	9.4183998	-11.7379351
2300.0000000	9.4672003	-11.1666040
2300.0000000	9.5159998	-14.0501575
2300.0000000	9.5648003	-0.8247795
2300.0000000	9.6135998	-13.2947998
2300.0000000	9.6623993	-10.2565393
2300.0000000	9.7111998	-10.1924677
2300.0000000	9.7600002	-10.8629141
2300.0000000	9.8087997	-10.9957962
2300.0000000	9.8575993	2.6238384
2300.0000000	9.9063997	-8.0860405
2300.0000000	9.9552002	-10.9071531
2300.0000000	10.0039997	-10.1064053
2310.0000000	0.3416000	-36.8701935
2310.0000000	0.3904000	-39.4974403
2310.0000000	0.4392000	-39.2520218
2310.0000000	0.4880000	-39.4352837
2310.0000000	0.5368000	-39.7651634
2310.0000000	0.5856000	-39.1544418
2310.0000000	0.6344000	-38.2654915
2310.0000000	0.6832000	-36.4529839
2310.0000000	0.7320000	-36.2664070
2310.0000000	0.7808000	-36.5522423
2310.0000000	0.8296000	-35.8325157
2310.0000000	0.8784000	-35.1799164
2310.0000000	0.9272000	-33.9432716
2310.0000000	0.9760000	-31.7183323
2310.0000000	1.0247999	-29.9050751
2310.0000000	1.0735999	-29.1576424
2310.0000000	1.1224000	-29.3727608
2310.0000000	1.1711999	-30.2094975
2310.0000000	1.2200000	-31.4399853
2310.0000000	1.2687999	-31.1249352
2310.0000000	1.3176000	-30.5633984
2310.0000000	1.3664000	-30.6936951
2310.0000000	1.4152000	-30.1886845
2310.0000000	1.4640000	-29.4915524
2310.0000000	1.5128000	-28.8333149

2310.0000000	1.5616000	-28.8501167
2310.0000000	1.6104001	-1229.1956787
2310.0000000	1.6592000	-35.3409729
2310.0000000	1.7079999	-27.7409229
2310.0000000	1.7567999	-27.2861977
2310.0000000	1.8055999	-26.6969013
2310.0000000	1.8544000	-26.0272961
2310.0000000	1.9031999	-26.8449440
2310.0000000	1.9520000	-26.4110413
2310.0000000	2.0007999	-25.6244640
2310.0000000	2.0495999	-24.8731937
2310.0000000	2.0983999	-26.0951347
2310.0000000	2.1471999	-25.3496990
2310.0000000	2.1960001	-24.0574017
2310.0000000	2.2448001	-25.2020378
2310.0000000	2.2935998	-24.7288036
2310.0000000	2.3423998	-22.8480434
2310.0000000	2.3912001	-24.6179562
2310.0000000	2.4400001	-23.1582203
2310.0000000	2.4888000	-23.4320660
2310.0000000	2.5375998	-14.4733419
2310.0000000	2.5863998	-12.9355898
2310.0000000	2.6352000	-11.3919458
2310.0000000	2.6840000	-16.9470215
2310.0000000	2.7328000	98.9894333
2310.0000000	2.7815998	117.7629471
2310.0000000	2.8304000	-23.6268921
2310.0000000	2.8792000	-21.0384140
2310.0000000	2.9280000	-22.4987106
2310.0000000	2.9768000	-17.8641205
2310.0000000	3.0256000	-20.8046684
2310.0000000	3.0743999	-28.0549240
2310.0000000	3.1231999	-23.8115044
2310.0000000	3.1719999	-28.5410786
2310.0000000	3.2208002	-473.7125854
2310.0000000	3.2695999	-926.2480469
2310.0000000	3.3183999	-20.5151367
2310.0000000	3.3671999	-3208.1076660
2310.0000000	3.4159999	1149.4962158
2310.0000000	3.4648001	-7.8768740
2310.0000000	3.5135999	-26.6513329
2310.0000000	3.5623999	-305.2314453
2310.0000000	3.6111999	-1911.9022217
2310.0000000	3.6600001	-433.6994629
2310.0000000	3.7088001	-572.2825317
2310.0000000	3.7575998	-895.3468018
2310.0000000	3.8063998	-1623.8264160
2310.0000000	3.8552001	-2357.0593262
2310.0000000	3.9040000	-2310.9526367
2310.0000000	3.9528000	1055.2559814
2310.0000000	4.0015998	-231.8367615
2310.0000000	4.0503998	645.5844116
2310.0000000	4.0991998	81.2735214
2310.0000000	4.1479998	-616.9404907
2310.0000000	4.1967998	1.2601719
2310.0000000	4.2456002	-234.8518982
2310.0000000	4.2943997	-460.1316833
2310.0000000	4.3431997	-13.6343536

2310.0000000	4.3920002	-234.5085297
2310.0000000	4.4407997	-22.2138596
2310.0000000	4.4896002	-15.5968819
2310.0000000	4.5383997	-618.7863770
2310.0000000	4.5871997	-13.1188927
2310.0000000	4.6360002	-21.4066620
2310.0000000	4.6847997	-27.7366333
2310.0000000	4.7336001	-19.4619980
2310.0000000	4.7824001	-16.5755558
2310.0000000	4.8311996	-1400.4455566
2310.0000000	4.8800001	215.7953186
2310.0000000	4.9287996	-192.0919952
2310.0000000	4.9776001	1004.9519043
2310.0000000	5.0264001	80.9534454
2310.0000000	5.0751996	-286.7158203
2310.0000000	5.1240001	-12.4365711
2310.0000000	5.1727996	542.3081665
2310.0000000	5.2216001	30.7307243
2310.0000000	5.2704000	-155.0502319
2310.0000000	5.3191996	-38.5731277
2310.0000000	5.3680000	-3443.1484375
2310.0000000	5.4168000	512.8341064
2310.0000000	5.4656000	-195.8960724
2310.0000000	5.5144000	36.6409760
2310.0000000	5.5631995	475.1355286
2310.0000000	5.6120000	382.4404602
2310.0000000	5.6608000	-36.5377846
2310.0000000	5.7096000	60.4660263
2310.0000000	5.7584000	129.1698303
2310.0000000	5.8071995	-11.6367359
2310.0000000	5.8559999	28.6338177
2310.0000000	5.9047999	-16.5791931
2310.0000000	5.9535999	-39.1431274
2310.0000000	6.0023999	-1532.3233643
2310.0000000	6.0511999	-14.5964031
2310.0000000	6.0999999	-19.2983055
2310.0000000	6.1487999	-13.3350201
2310.0000000	6.1975999	-22.9850693
2310.0000000	6.2463999	-1745.5933838
2310.0000000	6.2951999	-18.9619217
2310.0000000	6.3439999	-334.1894531
2310.0000000	6.3927999	-15.0456791
2310.0000000	6.4416003	-12.4639053
2310.0000000	6.4903998	-11.3533630
2310.0000000	6.5391998	-11.8964949
2310.0000000	6.5879998	293.6086731
2310.0000000	6.6367998	-12.1567945
2310.0000000	6.6856003	-13.8374090
2310.0000000	6.7343998	-13.4841356
2310.0000000	6.7831998	-14.3177700
2310.0000000	6.8319998	55.9231033
2310.0000000	6.8807998	-14.0311785
2310.0000000	6.9296002	-349.4806519
2310.0000000	6.9783998	-6.7672544
2310.0000000	7.0271997	-14.0026102
2310.0000000	7.0760002	5.6649313
2310.0000000	7.1247997	-1615.7360840
2310.0000000	7.1736002	-1295.8903809

2310.0000000	7.2223997	-738.0490723
2310.0000000	7.2711997	-161.6006470
2310.0000000	7.3200002	-17.3183899
2310.0000000	7.3687997	139.0588074
2310.0000000	7.4176002	293.3934326
2310.0000000	7.4663997	229.9936676
2310.0000000	7.5151997	-721.1826782
2310.0000000	7.5640001	-796.3203125
2310.0000000	7.6127996	-1873.4112549
2310.0000000	7.6616001	-602.9142456
2310.0000000	7.7104001	-310.9125366
2310.0000000	7.7591996	30.0805206
2310.0000000	7.8080001	304.9101563
2310.0000000	7.8567996	402.5041199
2310.0000000	7.9056001	-841.6991577
2310.0000000	7.9544001	1338.2874756
2310.0000000	8.0031996	1727.0140381
2310.0000000	8.0520000	1723.6806641
2310.0000000	8.1007996	1824.0643311
2310.0000000	8.1496000	1945.4736328
2310.0000000	8.1983995	1210.1888428
2310.0000000	8.2472000	-2770.4560547
2310.0000000	8.2959995	-982.4014282
2310.0000000	8.3448000	-782.3886719
2310.0000000	8.3935995	-717.0895386
2310.0000000	8.4423990	-309.3235168
2310.0000000	8.4912004	-721.2899780
2310.0000000	8.5400000	-1944.6408691
2310.0000000	8.5887995	-943.2680664
2310.0000000	8.6375999	183.2386322
2310.0000000	8.6863995	1661.7222900
2310.0000000	8.7351999	-618.4416504
2310.0000000	8.7840004	704.7774048
2310.0000000	8.8327999	36.0884743
2310.0000000	8.8815994	-11.5189972
2310.0000000	8.9303989	-23.4638214
2310.0000000	8.9792004	-11.5897541
2310.0000000	9.0279999	-6.0655565
2310.0000000	9.0767994	6.3002052
2310.0000000	9.1255999	-17.0205612
2310.0000000	9.1743994	-10.1255226
2310.0000000	9.2231998	34.9798508
2310.0000000	9.2720003	-8.5409870
2310.0000000	9.3207998	-9.8677130
2310.0000000	9.3695993	-10.7929907
2310.0000000	9.4183998	-11.7378101
2310.0000000	9.4672003	-11.1666451
2310.0000000	9.5159998	-14.0492668
2310.0000000	9.5648003	-0.8281517
2310.0000000	9.6135998	-13.2940092
2310.0000000	9.6623993	-10.2568140
2310.0000000	9.7111998	-10.1927395
2310.0000000	9.7600002	-10.8629551
2310.0000000	9.8087997	-10.9958048
2310.0000000	9.8575993	2.6194162
2310.0000000	9.9063997	-8.0869541
2310.0000000	9.9552002	-10.9071579
2310.0000000	10.0039997	-10.1066523

2320.0000000	0.3416000	-36.8808632
2320.0000000	0.3904000	-39.5854340
2320.0000000	0.4392000	-39.2529831
2320.0000000	0.4880000	-39.4340744
2320.0000000	0.5368000	-39.7663345
2320.0000000	0.5856000	-39.1530609
2320.0000000	0.6344000	-38.2658005
2320.0000000	0.6832000	-36.4552078
2320.0000000	0.7320000	-36.2684631
2320.0000000	0.7808000	-36.5524139
2320.0000000	0.8296000	-35.8286095
2320.0000000	0.8784000	-35.1629448
2320.0000000	0.9272000	-33.6666641
2320.0000000	0.9760000	-31.0148468
2320.0000000	1.0247999	-29.2508678
2320.0000000	1.0735999	-28.9312210
2320.0000000	1.1224000	-29.5302467
2320.0000000	1.1711999	-30.4720249
2320.0000000	1.2200000	-31.1537800
2320.0000000	1.2687999	-31.1186218
2320.0000000	1.3176000	-30.5597172
2320.0000000	1.3664000	-30.6932716
2320.0000000	1.4152000	-30.1888885
2320.0000000	1.4640000	-29.4922352
2320.0000000	1.5128000	-28.8325443
2320.0000000	1.5616000	-28.8444099
2320.0000000	1.6104001	-1226.6528320
2320.0000000	1.6592000	-35.0470428
2320.0000000	1.7079999	-27.7446709
2320.0000000	1.7567999	-27.2755299
2320.0000000	1.8055999	-26.7111969
2320.0000000	1.8544000	-26.0247803
2320.0000000	1.9031999	-26.8559837
2320.0000000	1.9520000	-26.4362144
2320.0000000	2.0007999	-25.6538353
2320.0000000	2.0495999	-24.8765984
2320.0000000	2.0983999	-26.0949345
2320.0000000	2.1471999	-25.3496189
2320.0000000	2.1960001	-24.0610561
2320.0000000	2.2448001	-25.1988869
2320.0000000	2.2935998	-24.7266903
2320.0000000	2.3423998	-22.8624477
2320.0000000	2.3912001	-24.6163197
2320.0000000	2.4400001	-23.1607075
2320.0000000	2.4888000	-23.4318485
2320.0000000	2.5375998	-14.4904499
2320.0000000	2.5863998	-12.9553480
2320.0000000	2.6352000	-11.4146900
2320.0000000	2.6840000	-16.9589634
2320.0000000	2.7328000	98.7234192
2320.0000000	2.7815998	117.4565201
2320.0000000	2.8304000	-23.6255875
2320.0000000	2.8792000	-21.0416775
2320.0000000	2.9280000	-22.4981403
2320.0000000	2.9768000	-17.8724899
2320.0000000	3.0256000	-20.8072529
2320.0000000	3.0743999	-28.0435371
2320.0000000	3.1231999	-23.8080502

2320.0000000	3.1719999	-28.5327854
2320.0000000	3.2208002	-473.4748535
2320.0000000	3.2695999	-925.7730713
2320.0000000	3.3183999	-20.5153351
2320.0000000	3.3671999	-3206.6459961
2320.0000000	3.4159999	1148.9609375
2320.0000000	3.4648001	-7.8825817
2320.0000000	3.5135999	-26.6483421
2320.0000000	3.5623999	-305.1018372
2320.0000000	3.6111999	-1911.0424805
2320.0000000	3.6600001	-433.5112915
2320.0000000	3.7088001	-572.0311279
2320.0000000	3.7575998	-894.9483032
2320.0000000	3.8063998	-1623.0965576
2320.0000000	3.8552001	-2355.9960938
2320.0000000	3.9040000	-2309.9101563
2320.0000000	3.9528000	1054.7677002
2320.0000000	4.0015998	-231.7396545
2320.0000000	4.0503998	645.2817993
2320.0000000	4.0991998	81.2278442
2320.0000000	4.1479998	-616.6682739
2320.0000000	4.1967998	1.2509155
2320.0000000	4.2456002	-234.7536011
2320.0000000	4.2943997	-459.9311218
2320.0000000	4.3431997	-13.6365337
2320.0000000	4.3920002	-234.4104156
2320.0000000	4.4407997	-22.2120838
2320.0000000	4.4896002	-15.5977650
2320.0000000	4.5383997	-618.5252686
2320.0000000	4.5871997	-13.1209087
2320.0000000	4.6360002	-21.4051189
2320.0000000	4.6847997	-27.7327080
2320.0000000	4.7336001	-19.4612751
2320.0000000	4.7824001	-16.5760098
2320.0000000	4.8311996	-1399.9143066
2320.0000000	4.8800001	215.7057800
2320.0000000	4.9287996	-192.0247345
2320.0000000	4.9776001	1004.5593872
2320.0000000	5.0264001	80.9159241
2320.0000000	5.0751996	-286.6121826
2320.0000000	5.1240001	-12.4374924
2320.0000000	5.1727996	542.1923218
2320.0000000	5.2216001	30.7212791
2320.0000000	5.2704000	-155.0221252
2320.0000000	5.3191996	-38.5685806
2320.0000000	5.3680000	-3442.4560547
2320.0000000	5.4168000	512.7273560
2320.0000000	5.4656000	-195.8598785
2320.0000000	5.5144000	36.6303177
2320.0000000	5.5631995	475.0369568
2320.0000000	5.6120000	382.3605042
2320.0000000	5.6608000	-36.5336952
2320.0000000	5.7096000	60.4505920
2320.0000000	5.7584000	129.1408844
2320.0000000	5.8071995	-11.6376057
2320.0000000	5.8559999	28.6249676
2320.0000000	5.9047999	-16.5789948
2320.0000000	5.9535999	-39.1383896

2320.0000000	6.0023999	-1532.0187988
2320.0000000	6.0511999	-14.5966139
2320.0000000	6.0999999	-19.2974720
2320.0000000	6.1487999	-13.3354836
2320.0000000	6.1975999	-22.9833050
2320.0000000	6.2463999	-1745.2459717
2320.0000000	6.2951999	-18.9611073
2320.0000000	6.3439999	-334.1253967
2320.0000000	6.3927999	-15.0456610
2320.0000000	6.4416003	-12.4644918
2320.0000000	6.4903998	-11.3540392
2320.0000000	6.5391998	-11.8971758
2320.0000000	6.5879998	292.9179688
2320.0000000	6.6367998	-12.1573439
2320.0000000	6.6856003	-13.8375788
2320.0000000	6.7343998	-13.4843512
2320.0000000	6.7831998	-14.3173542
2320.0000000	6.8319998	55.9039841
2320.0000000	6.8807998	-14.0310392
2320.0000000	6.9296002	-348.7191772
2320.0000000	6.9783998	-6.7800775
2320.0000000	7.0271997	-14.0015821
2320.0000000	7.0760002	5.6227465
2320.0000000	7.1247997	-1611.7901611
[truncated: 510,268 more chars]
