# Supplementary material for: Periodic oscillations of atmospheric electric field during snowfall in the Tokyo metropolitan area
Source: Sci Rep. 2021 Jan 26;11:2280. doi: 10.1038/s41598-021-82091-w (PMC7838252; doi:10.1038/s41598-021-82091-w)
Supplement: Supplementary file 3 — Supplementary Information 3. [file 41598_2021_82091_MOESM3_ESM.pdf]

-32400.0000000	1.4150000
-32399.0000000	1.4200000
-32398.0000000	1.4200000
-32397.0000000	1.4250000
-32396.0000000	1.4349999
-32395.0000000	1.4299999
-32394.0000000	1.4349999
-32393.0000000	1.4299999
-32392.0000000	1.4299999
-32391.0000000	1.4333333
-32390.0000000	1.4349999
-32389.0000000	1.4450001
-32388.0000000	1.4400001
-32387.0000000	1.4450001
-32386.0000000	1.4400001
-32385.0000000	1.4450001
-32384.0000000	1.4450001
-32383.0000000	1.4500000
-32382.0000000	1.4450001
-32381.0000000	1.4500000
-32380.0000000	1.4500000
-32379.0000000	1.4450001
-32378.0000000	1.4550000
-32377.0000000	1.4550000
-32376.0000000	1.4550000
-32375.0000000	1.4550000
-32374.0000000	1.4500000
-32373.0000000	1.4500000
-32372.0000000	1.4500000
-32371.0000000	1.4500000
-32370.0000000	1.4500000
-32369.0000000	1.4500000
-32368.0000000	1.4500000
-32367.0000000	1.4450001
-32366.0000000	1.4500000
-32365.0000000	1.4550000
-32364.0000000	1.4500000
-32363.0000000	1.4500000
-32362.0000000	1.4550000
-32361.0000000	1.4550000
-32360.0000000	1.4533334
-32359.0000000	1.4500000
-32358.0000000	1.4500000
-32357.0000000	1.4550000
-32356.0000000	1.4500000
-32355.0000000	1.4500000
-32354.0000000	1.4450001
-32353.0000000	1.4450001
-32352.0000000	1.4450001
-32351.0000000	1.4500000
-32350.0000000	1.4450001
-32349.0000000	1.4500000
-32348.0000000	1.4450001
-32347.0000000	1.4450001
-32346.0000000	1.4550000
-32345.0000000	1.4500000
-32344.0000000	1.4500000

-32343.0000000	1.4500000
-32342.0000000	1.4500000
-32341.0000000	1.4550000
-32340.0000000	1.4550000
-32339.0000000	1.4450001
-32338.0000000	1.4500000
-32337.0000000	1.4450001
-32336.0000000	1.4400001
-32335.0000000	1.4450001
-32334.0000000	1.4450001
-32333.0000000	1.4400001
-32332.0000000	1.4349999
-32331.0000000	1.4349999
-32330.0000000	1.4266666
-32329.0000000	1.4250000
-32328.0000000	1.4200000
-32327.0000000	1.4200000
-32326.0000000	1.4250000
-32325.0000000	1.4250000
-32324.0000000	1.4250000
-32323.0000000	1.4250000
-32322.0000000	1.4250000
-32321.0000000	1.4250000
-32320.0000000	1.4250000
-32319.0000000	1.4250000
-32318.0000000	1.4250000
-32317.0000000	1.4349999
-32316.0000000	1.4299999
-32315.0000000	1.4349999
-32314.0000000	1.4349999
-32313.0000000	1.4400001
-32312.0000000	1.4349999
-32311.0000000	1.4450001
-32310.0000000	1.4450001
-32309.0000000	1.4500000
-32308.0000000	1.4500000
-32307.0000000	1.4450001
-32306.0000000	1.4450001
-32305.0000000	1.4500000
-32304.0000000	1.4550000
-32303.0000000	1.4550000
-32302.0000000	1.4600000
-32301.0000000	1.4600000
-32300.0000000	1.4600000
-32299.0000000	1.4650000
-32298.0000000	1.4650000
-32297.0000000	1.4700000
-32296.0000000	1.4750000
-32295.0000000	1.4800000
-32294.0000000	1.4800000
-32293.0000000	1.4800000
-32292.0000000	1.4800000
-32291.0000000	1.4950000
-32290.0000000	1.4950000
-32289.0000000	1.4950000
-32288.0000000	1.5000000
-32287.0000000	1.5050000

-32286.0000000	1.5100000
-32285.0000000	1.5050000
-32284.0000000	1.5150000
-32283.0000000	1.5150000
-32282.0000000	1.5200000
-32281.0000000	1.5200000
-32280.0000000	1.5200000
-32279.0000000	1.5250000
-32278.0000000	1.5300000
-32277.0000000	1.5300000
-32276.0000000	1.5350000
-32275.0000000	1.5300000
-32274.0000000	1.5450000
-32273.0000000	1.5450000
-32272.0000000	1.5500000
-32271.0000000	1.5566667
-32270.0000000	1.5650001
-32269.0000000	1.5650001
-32268.0000000	1.5699999
-32267.0000000	1.5750000
-32266.0000000	1.5800000
-32265.0000000	1.5800000
-32264.0000000	1.5900000
-32263.0000000	1.5950000
-32262.0000000	1.6000000
-32261.0000000	1.6000000
-32260.0000000	1.6050000
-32259.0000000	1.6100000
-32258.0000000	1.6150000
-32257.0000000	1.6200000
-32256.0000000	1.6200000
-32255.0000000	1.6200000
-32254.0000000	1.6300000
-32253.0000000	1.6300000
-32252.0000000	1.6300000
-32251.0000000	1.6300000
-32250.0000000	1.6350000
-32249.0000000	1.6350000
-32248.0000000	1.6400000
-32247.0000000	1.6350000
-32246.0000000	1.6400000
-32245.0000000	1.6400000
-32244.0000000	1.6400000
-32243.0000000	1.6400000
-32242.0000000	1.6450000
-32241.0000000	1.6450000
-32240.0000000	1.6450000
-32239.0000000	1.6500000
-32238.0000000	1.6550000
-32237.0000000	1.6600000
-32236.0000000	1.6550000
-32235.0000000	1.6650000
-32234.0000000	1.6650000
-32233.0000000	1.6650000
-32232.0000000	1.6650000
-32231.0000000	1.6650000
-32230.0000000	1.6700000

-32229.0000000	1.6700000
-32228.0000000	1.6750000
-32227.0000000	1.6750000
-32226.0000000	1.6849999
-32225.0000000	1.6849999
-32224.0000000	1.6849999
-32223.0000000	1.6900001
-32222.0000000	1.6849999
-32221.0000000	1.6900001
-32220.0000000	1.6950001
-32219.0000000	1.6900001
-32218.0000000	1.6950001
-32217.0000000	1.6950001
-32216.0000000	1.7000000
-32215.0000000	1.7000000
-32214.0000000	1.7050000
-32213.0000000	1.6950001
-32212.0000000	1.7050000
-32211.0000000	1.7000000
-32210.0000000	1.7100000
-32209.0000000	1.7100000
-32208.0000000	1.7100000
-32207.0000000	1.7100000
-32206.0000000	1.7200000
-32205.0000000	1.7150000
-32204.0000000	1.7150000
-32203.0000000	1.7250000
-32202.0000000	1.7200000
-32201.0000000	1.7250000
-32200.0000000	1.7250000
-32199.0000000	1.7250000
-32198.0000000	1.7200000
-32197.0000000	1.7250000
-32196.0000000	1.7250000
-32195.0000000	1.7250000
-32194.0000000	1.7200000
-32193.0000000	1.7250000
-32192.0000000	1.7250000
-32191.0000000	1.7250000
-32190.0000000	1.7300000
-32189.0000000	1.7250000
-32188.0000000	1.7200000
-32187.0000000	1.7250000
-32186.0000000	1.7250000
-32185.0000000	1.7250000
-32184.0000000	1.7300000
-32183.0000000	1.7300000
-32182.0000000	1.7250000
-32181.0000000	1.7150000
-32180.0000000	1.7200000
-32179.0000000	1.7233334
-32178.0000000	1.7200000
-32177.0000000	1.7150000
-32176.0000000	1.7150000
-32175.0000000	1.7250000
-32174.0000000	1.7250000
-32173.0000000	1.7250000

-32172.0000000	1.7200000
-32171.0000000	1.7250000
-32170.0000000	1.7250000
-32169.0000000	1.7300000
-32168.0000000	1.7300000
-32167.0000000	1.7350000
-32166.0000000	1.7350000
-32165.0000000	1.7300000
-32164.0000000	1.7300000
-32163.0000000	1.7300000
-32162.0000000	1.7300000
-32161.0000000	1.7350000
-32160.0000000	1.7300000
-32159.0000000	1.7200000
-32158.0000000	1.7250000
-32157.0000000	1.7200000
-32156.0000000	1.7150000
-32155.0000000	1.7200000
-32154.0000000	1.7150000
-32153.0000000	1.7100000
-32152.0000000	1.7100000
-32151.0000000	1.7050000
-32150.0000000	1.7150000
-32149.0000000	1.7066666
-32148.0000000	1.7000000
-32147.0000000	1.7050000
-32146.0000000	1.7000000
-32145.0000000	1.7000000
-32144.0000000	1.7000000
-32143.0000000	1.7000000
-32142.0000000	1.7000000
-32141.0000000	1.6950001
-32140.0000000	1.6950001
-32139.0000000	1.6950001
-32138.0000000	1.7050000
-32137.0000000	1.7000000
-32136.0000000	1.6950001
-32135.0000000	1.7000000
-32134.0000000	1.7000000
-32133.0000000	1.7000000
-32132.0000000	1.7050000
-32131.0000000	1.7000000
-32130.0000000	1.7050000
-32129.0000000	1.7050000
-32128.0000000	1.7050000
-32127.0000000	1.7050000
-32126.0000000	1.7000000
-32125.0000000	1.7000000
-32124.0000000	1.6950001
-32123.0000000	1.6950001
-32122.0000000	1.6950001
-32121.0000000	1.6950001
-32120.0000000	1.6900001
-32119.0000000	1.6950001
-32118.0000000	1.6849999
-32117.0000000	1.6799999
-32116.0000000	1.6799999

-32115.0000000	1.6750000
-32114.0000000	1.6799999
-32113.0000000	1.6799999
-32112.0000000	1.6799999
-32111.0000000	1.6799999
-32110.0000000	1.6799999
-32109.0000000	1.6849999
-32108.0000000	1.6849999
-32107.0000000	1.6849999
-32106.0000000	1.6900001
-32105.0000000	1.6900001
-32104.0000000	1.6900001
-32103.0000000	1.6950001
-32102.0000000	1.6950001
-32101.0000000	1.7000000
-32100.0000000	1.6950001
-32099.0000000	1.7000000
-32098.0000000	1.7050000
-32097.0000000	1.7000000
-32096.0000000	1.7050000
-32095.0000000	1.7150000
-32094.0000000	1.7150000
-32093.0000000	1.7100000
-32092.0000000	1.7200000
-32091.0000000	1.7200000
-32090.0000000	1.7200000
-32089.0000000	1.7250000
-32088.0000000	1.7266668
-32087.0000000	1.7350000
-32086.0000000	1.7350000
-32085.0000000	1.7350000
-32084.0000000	1.7400000
-32083.0000000	1.7450000
-32082.0000000	1.7450000
-32081.0000000	1.7450000
-32080.0000000	1.7500000
-32079.0000000	1.7500000
-32078.0000000	1.7500000
-32077.0000000	1.7500000
-32076.0000000	1.7550000
-32075.0000000	1.7500000
-32074.0000000	1.7550000
-32073.0000000	1.7550000
-32072.0000000	1.7550000
-32071.0000000	1.7550000
-32070.0000000	1.7550000
-32069.0000000	1.7550000
-32068.0000000	1.7500000
-32067.0000000	1.7500000
-32066.0000000	1.7450000
-32065.0000000	1.7450000
-32064.0000000	1.7450000
-32063.0000000	1.7400000
-32062.0000000	1.7400000
-32061.0000000	1.7450000
-32060.0000000	1.7350000
-32059.0000000	1.7366667

-32058.0000000	1.7350000
-32057.0000000	1.7350000
-32056.0000000	1.7300000
-32055.0000000	1.7300000
-32054.0000000	1.7350000
-32053.0000000	1.7350000
-32052.0000000	1.7350000
-32051.0000000	1.7350000
-32050.0000000	1.7350000
-32049.0000000	1.7400000
-32048.0000000	1.7400000
-32047.0000000	1.7400000
-32046.0000000	1.7400000
-32045.0000000	1.7400000
-32044.0000000	1.7450000
-32043.0000000	1.7500000
-32042.0000000	1.7450000
-32041.0000000	1.7500000
-32040.0000000	1.7500000
-32039.0000000	1.7500000
-32038.0000000	1.7450000
-32037.0000000	1.7500000
-32036.0000000	1.7500000
-32035.0000000	1.7550000
-32034.0000000	1.7500000
-32033.0000000	1.7500000
-32032.0000000	1.7550000
-32031.0000000	1.7566667
-32030.0000000	1.7600000
-32029.0000000	1.7650000
-32028.0000000	1.7650000
-32027.0000000	1.7700000
-32026.0000000	1.7650000
-32025.0000000	1.7700000
-32024.0000000	1.7800000
-32023.0000000	1.7800000
-32022.0000000	1.7800000
-32021.0000000	1.7900000
-32020.0000000	1.7900000
-32019.0000000	1.7950000
-32018.0000000	1.8000000
-32017.0000000	1.8049999
-32016.0000000	1.8049999
-32015.0000000	1.8150001
-32014.0000000	1.8200001
-32013.0000000	1.8200001
-32012.0000000	1.8300000
-32011.0000000	1.8350000
-32010.0000000	1.8350000
-32009.0000000	1.8450000
-32008.0000000	1.8450000
-32007.0000000	1.8450000
-32006.0000000	1.8550000
-32005.0000000	1.8650000
-32004.0000000	1.8600000
-32003.0000000	1.8700000
-32002.0000000	1.8700000

-32001.0000000	1.8750000
-32000.0000000	1.8750000
-31999.0000000	1.8850000
-31998.0000000	1.8866667
-31997.0000000	1.8850000
-31996.0000000	1.8950000
-31995.0000000	1.8950000
-31994.0000000	1.9050000
-31993.0000000	1.9100000
-31992.0000000	1.9100000
-31991.0000000	1.9150000
-31990.0000000	1.9200000
-31989.0000000	1.9250000
-31988.0000000	1.9349999
-31987.0000000	1.9400001
-31986.0000000	1.9400001
-31985.0000000	1.9500000
-31984.0000000	1.9550000
-31983.0000000	1.9650000
-31982.0000000	1.9750000
-31981.0000000	1.9800000
-31980.0000000	1.9850000
-31979.0000000	1.9900000
-31978.0000000	1.9950000
-31977.0000000	2.0000000
-31976.0000000	2.0050001
-31975.0000000	2.0100000
-31974.0000000	2.0250001
-31973.0000000	2.0300000
-31972.0000000	2.0349998
-31971.0000000	2.0450001
-31970.0000000	2.0450001
-31969.0000000	2.0500000
-31968.0000000	2.0549998
-31967.0000000	2.0633333
-31966.0000000	2.0749998
-31965.0000000	2.0749998
-31964.0000000	2.0850000
-31963.0000000	2.0949998
-31962.0000000	2.0949998
-31961.0000000	2.0999999
-31960.0000000	2.1099999
-31959.0000000	2.1149998
-31958.0000000	2.1250000
-31957.0000000	2.1350002
-31956.0000000	2.1400001
-31955.0000000	2.1450000
-31954.0000000	2.1500001
-31953.0000000	2.1600001
-31952.0000000	2.1650000
-31951.0000000	2.1650000
-31950.0000000	2.1849999
-31949.0000000	2.1900001
-31948.0000000	2.1900001
-31947.0000000	2.1950002
-31946.0000000	2.2049999
-31945.0000000	2.2150002

-31944.0000000	2.2150002
-31943.0000000	2.2300000
-31942.0000000	2.2400000
-31941.0000000	2.2400000
-31940.0000000	2.2550001
-31939.0000000	2.2600000
-31938.0000000	2.2666667
-31937.0000000	2.2849998
-31936.0000000	2.2900000
-31935.0000000	2.2950001
-31934.0000000	2.3049998
-31933.0000000	2.3049998
-31932.0000000	2.3150001
-31931.0000000	2.3150001
-31930.0000000	2.3199999
-31929.0000000	2.3249998
-31928.0000000	2.3299999
-31927.0000000	2.3399999
-31926.0000000	2.3499999
-31925.0000000	2.3499999
-31924.0000000	2.3599999
-31923.0000000	2.3649998
-31922.0000000	2.3649998
-31921.0000000	2.3699999
-31920.0000000	2.3800001
-31919.0000000	2.3900001
-31918.0000000	2.3950000
-31917.0000000	2.4000001
-31916.0000000	2.4100001
-31915.0000000	2.4150000
-31914.0000000	2.4300001
-31913.0000000	2.4250002
-31912.0000000	2.4300001
-31911.0000000	2.4400001
-31910.0000000	2.4500000
-31909.0000000	2.4450002
-31908.0000000	2.4600000
-31907.0000000	2.4600000
-31906.0000000	2.4749999
-31905.0000000	2.4749999
-31904.0000000	2.4700000
-31903.0000000	2.4800000
-31902.0000000	2.4850001
-31901.0000000	2.4949999
-31900.0000000	2.4949999
-31899.0000000	2.5000000
-31898.0000000	2.5100000
-31897.0000000	2.5149999
-31896.0000000	2.5149999
-31895.0000000	2.5200000
-31894.0000000	2.5250001
-31893.0000000	2.5400000
-31892.0000000	2.5450001
-31891.0000000	2.5450001
-31890.0000000	2.5500000
-31889.0000000	2.5500000
-31888.0000000	2.5500000

-31887.0000000	2.5650001
-31886.0000000	2.5599999
-31885.0000000	2.5749998
-31884.0000000	2.5799999
-31883.0000000	2.5850000
-31882.0000000	2.5899999
-31881.0000000	2.5999999
-31880.0000000	2.5999999
-31879.0000000	2.6099999
-31878.0000000	2.6099999
-31877.0000000	2.6199999
-31876.0000000	2.6250000
-31875.0000000	2.6400001
-31874.0000000	2.6300001
-31873.0000000	2.6400001
-31872.0000000	2.6400001
-31871.0000000	2.6400001
-31870.0000000	2.6450000
-31869.0000000	2.6500001
-31868.0000000	2.6550002
-31867.0000000	2.6550002
-31866.0000000	2.6550002
-31865.0000000	2.6550002
-31864.0000000	2.6650000
-31863.0000000	2.6650000
-31862.0000000	2.6700001
-31861.0000000	2.6750002
-31860.0000000	2.6800001
-31859.0000000	2.6800001
-31858.0000000	2.6849999
-31857.0000000	2.6849999
-31856.0000000	2.6800001
-31855.0000000	2.6900001
-31854.0000000	2.6900001
-31853.0000000	2.6849999
-31852.0000000	2.6950002
-31851.0000000	2.6950002
-31850.0000000	2.7000000
-31849.0000000	2.7000000
-31848.0000000	2.7033331
-31847.0000000	2.7049999
-31846.0000000	2.7100000
-31845.0000000	2.7049999
-31844.0000000	2.7150002
-31843.0000000	2.7150002
-31842.0000000	2.7100000
-31841.0000000	2.7150002
-31840.0000000	2.7100000
-31839.0000000	2.7049999
-31838.0000000	2.7049999
-31837.0000000	2.7100000
-31836.0000000	2.7150002
-31835.0000000	2.7100000
-31834.0000000	2.7150002
-31833.0000000	2.7100000
-31832.0000000	2.7150002
-31831.0000000	2.7150002

-31830.0000000	2.7200000
-31829.0000000	2.7249999
-31828.0000000	2.7150002
-31827.0000000	2.7249999
-31826.0000000	2.7200000
-31825.0000000	2.7200000
-31824.0000000	2.7249999
-31823.0000000	2.7249999
-31822.0000000	2.7150002
-31821.0000000	2.7200000
-31820.0000000	2.7249999
-31819.0000000	2.7200000
-31818.0000000	2.7233334
-31817.0000000	2.7200000
-31816.0000000	2.7249999
-31815.0000000	2.7249999
-31814.0000000	2.7300000
-31813.0000000	2.7200000
-31812.0000000	2.7249999
-31811.0000000	2.7300000
-31810.0000000	2.7300000
-31809.0000000	2.7249999
-31808.0000000	2.7300000
-31807.0000000	2.7350001
-31806.0000000	2.7249999
-31805.0000000	2.7350001
-31804.0000000	2.7400000
-31803.0000000	2.7300000
-31802.0000000	2.7300000
-31801.0000000	2.7350001
-31800.0000000	2.7249999
-31799.0000000	2.7400000
-31798.0000000	2.7350001
-31797.0000000	2.7350001
-31796.0000000	2.7400000
-31795.0000000	2.7350001
-31794.0000000	2.7350001
-31793.0000000	2.7300000
-31792.0000000	2.7350001
-31791.0000000	2.7300000
-31790.0000000	2.7300000
-31789.0000000	2.7249999
-31788.0000000	2.7249999
-31787.0000000	2.7200000
-31786.0000000	2.7150002
-31785.0000000	2.7150002
-31784.0000000	2.7000000
-31783.0000000	2.7049999
-31782.0000000	2.7000000
-31781.0000000	2.6950002
-31780.0000000	2.7000000
-31779.0000000	2.6900001
-31778.0000000	2.6849999
-31777.0000000	2.6800001
-31776.0000000	2.6800001
-31775.0000000	2.6800001
-31774.0000000	2.6750002

-31773.0000000	2.6800001
-31772.0000000	2.6750002
-31771.0000000	2.6650000
-31770.0000000	2.6700001
-31769.0000000	2.6600001
-31768.0000000	2.6650000
-31767.0000000	2.6550002
-31766.0000000	2.6600001
-31765.0000000	2.6550002
-31764.0000000	2.6550002
-31763.0000000	2.6450000
-31762.0000000	2.6400001
-31761.0000000	2.6450000
-31760.0000000	2.6350002
-31759.0000000	2.6350002
-31758.0000000	2.6333334
-31757.0000000	2.6199999
-31756.0000000	2.6099999
-31755.0000000	2.6149998
-31754.0000000	2.6099999
-31753.0000000	2.6050000
-31752.0000000	2.5999999
-31751.0000000	2.5949998
-31750.0000000	2.5899999
-31749.0000000	2.5850000
-31748.0000000	2.5799999
-31747.0000000	2.5749998
-31746.0000000	2.5699999
-31745.0000000	2.5699999
-31744.0000000	2.5599999
-31743.0000000	2.5549998
-31742.0000000	2.5500000
-31741.0000000	2.5400000
-31740.0000000	2.5400000
-31739.0000000	2.5349998
-31738.0000000	2.5400000
-31737.0000000	2.5300000
-31736.0000000	2.5300000
-31735.0000000	2.5250001
-31734.0000000	2.5250001
-31733.0000000	2.5250001
-31732.0000000	2.5149999
-31731.0000000	2.5149999
-31730.0000000	2.5149999
-31729.0000000	2.5100000
-31728.0000000	2.5000000
-31727.0000000	2.5000000
-31726.0000000	2.5000000
-31725.0000000	2.4949999
-31724.0000000	2.4900000
-31723.0000000	2.4900000
-31722.0000000	2.4900000
-31721.0000000	2.4800000
-31720.0000000	2.4800000
-31719.0000000	2.4749999
-31718.0000000	2.4749999
-31717.0000000	2.4700000

-31716.0000000	2.4700000
-31715.0000000	2.4600000
-31714.0000000	2.4600000
-31713.0000000	2.4600000
-31712.0000000	2.4500000
-31711.0000000	2.4450002
-31710.0000000	2.4400001
-31709.0000000	2.4400001
-31708.0000000	2.4349999
-31707.0000000	2.4349999
-31706.0000000	2.4300001
-31705.0000000	2.4250002
-31704.0000000	2.4300001
-31703.0000000	2.4250002
-31702.0000000	2.4200001
-31701.0000000	2.4100001
-31700.0000000	2.4100001
-31699.0000000	2.4050002
-31698.0000000	2.4033334
-31697.0000000	2.4050002
-31696.0000000	2.3950000
-31695.0000000	2.4000001
-31694.0000000	2.3950000
-31693.0000000	2.3850002
-31692.0000000	2.3850002
-31691.0000000	2.3950000
-31690.0000000	2.3900001
-31689.0000000	2.3800001
-31688.0000000	2.3850002
-31687.0000000	2.3800001
-31686.0000000	2.3750000
-31685.0000000	2.3699999
-31684.0000000	2.3599999
-31683.0000000	2.3599999
-31682.0000000	2.3649998
-31681.0000000	2.3550000
-31680.0000000	2.3550000
-31679.0000000	2.3499999
-31678.0000000	2.3449998
-31677.0000000	2.3449998
-31676.0000000	2.3399999
-31675.0000000	2.3449998
-31674.0000000	2.3350000
-31673.0000000	2.3350000
-31672.0000000	2.3299999
-31671.0000000	2.3299999
-31670.0000000	2.3249998
-31669.0000000	2.3249998
-31668.0000000	2.3199999
-31667.0000000	2.3166666
-31666.0000000	2.3150001
-31665.0000000	2.3099999
-31664.0000000	2.3049998
-31663.0000000	2.3099999
-31662.0000000	2.3049998
-31661.0000000	2.3049998
-31660.0000000	2.2950001

-31659.0000000	2.2950001
-31658.0000000	2.2950001
-31657.0000000	2.2900000
-31656.0000000	2.2849998
-31655.0000000	2.2800000
-31654.0000000	2.2800000
-31653.0000000	2.2700000
-31652.0000000	2.2750001
-31651.0000000	2.2750001
-31650.0000000	2.2700000
-31649.0000000	2.2700000
-31648.0000000	2.2700000
-31647.0000000	2.2649999
-31646.0000000	2.2649999
-31645.0000000	2.2600000
-31644.0000000	2.2550001
-31643.0000000	2.2550001
-31642.0000000	2.2550001
-31641.0000000	2.2449999
-31640.0000000	2.2449999
-31639.0000000	2.2400000
-31638.0000000	2.2449999
-31637.0000000	2.2400000
-31636.0000000	2.2350001
-31635.0000000	2.2350001
-31634.0000000	2.2249999
-31633.0000000	2.2249999
-31632.0000000	2.2249999
-31631.0000000	2.2200000
-31630.0000000	2.2150002
-31629.0000000	2.2200000
-31628.0000000	2.2150002
-31627.0000000	2.2100000
-31626.0000000	2.2049999
-31625.0000000	2.2049999
-31624.0000000	2.2000000
-31623.0000000	2.1950002
-31622.0000000	2.1950002
-31621.0000000	2.1900001
-31620.0000000	2.1800001
-31619.0000000	2.1849999
-31618.0000000	2.1750002
-31617.0000000	2.1750002
-31616.0000000	2.1700001
-31615.0000000	2.1650000
-31614.0000000	2.1600001
-31613.0000000	2.1600001
-31612.0000000	2.1600001
-31611.0000000	2.1550002
-31610.0000000	2.1550002
-31609.0000000	2.1500001
-31608.0000000	2.1400001
-31607.0000000	2.1366670
-31606.0000000	2.1400001
-31605.0000000	2.1300001
-31604.0000000	2.1350002
-31603.0000000	2.1250000

-31602.0000000	2.1300001
-31601.0000000	2.1250000
-31600.0000000	2.1250000
-31599.0000000	2.1149998
-31598.0000000	2.1199999
-31597.0000000	2.1149998
-31596.0000000	2.1099999
-31595.0000000	2.1099999
-31594.0000000	2.1099999
-31593.0000000	2.0999999
-31592.0000000	2.0999999
-31591.0000000	2.0999999
-31590.0000000	2.0899999
-31589.0000000	2.0899999
-31588.0000000	2.0850000
-31587.0000000	2.0850000
-31586.0000000	2.0850000
-31585.0000000	2.0799999
-31584.0000000	2.0749998
-31583.0000000	2.0749998
-31582.0000000	2.0650001
-31581.0000000	2.0599999
-31580.0000000	2.0599999
-31579.0000000	2.0549998
-31578.0000000	2.0500000
-31577.0000000	2.0466666
-31576.0000000	2.0450001
-31575.0000000	2.0450001
-31574.0000000	2.0349998
-31573.0000000	2.0300000
-31572.0000000	2.0250001
-31571.0000000	2.0250001
-31570.0000000	2.0250001
-31569.0000000	2.0200000
-31568.0000000	2.0200000
-31567.0000000	2.0200000
-31566.0000000	2.0100000
-31565.0000000	2.0149999
-31564.0000000	2.0100000
-31563.0000000	2.0050001
-31562.0000000	2.0100000
-31561.0000000	2.0050001
-31560.0000000	2.0050001
-31559.0000000	2.0000000
-31558.0000000	1.9950000
-31557.0000000	1.9900000
-31556.0000000	1.9900000
-31555.0000000	1.9900000
-31554.0000000	1.9900000
-31553.0000000	1.9800000
-31552.0000000	1.9800000
-31551.0000000	1.9800000
-31550.0000000	1.9750000
-31549.0000000	1.9750000
-31548.0000000	1.9750000
-31547.0000000	1.9733334
-31546.0000000	1.9700000

-31545.0000000	1.9650000
-31544.0000000	1.9666667
-31543.0000000	1.9700000
-31542.0000000	1.9600000
-31541.0000000	1.9650000
-31540.0000000	1.9600000
-31539.0000000	1.9600000
-31538.0000000	1.9600000
-31537.0000000	1.9600000
-31536.0000000	1.9600000
-31535.0000000	1.9550000
-31534.0000000	1.9550000
-31533.0000000	1.9550000
-31532.0000000	1.9550000
-31531.0000000	1.9550000
-31530.0000000	1.9500000
-31529.0000000	1.9550000
-31528.0000000	1.9500000
-31527.0000000	1.9550000
-31526.0000000	1.9500000
-31525.0000000	1.9550000
-31524.0000000	1.9550000
-31523.0000000	1.9550000
-31522.0000000	1.9500000
-31521.0000000	1.9600000
-31520.0000000	1.9550000
-31519.0000000	1.9550000
-31518.0000000	1.9550000
-31517.0000000	1.9533334
-31516.0000000	1.9600000
-31515.0000000	1.9600000
-31514.0000000	1.9600000
-31513.0000000	1.9550000
-31512.0000000	1.9600000
-31511.0000000	1.9600000
-31510.0000000	1.9550000
-31509.0000000	1.9600000
-31508.0000000	1.9550000
-31507.0000000	1.9600000
-31506.0000000	1.9600000
-31505.0000000	1.9600000
-31504.0000000	1.9550000
-31503.0000000	1.9600000
-31502.0000000	1.9600000
-31501.0000000	1.9600000
-31500.0000000	1.9600000
-31499.0000000	1.9650000
-31498.0000000	1.9650000
-31497.0000000	1.9550000
-31496.0000000	1.9600000
-31495.0000000	1.9600000
-31494.0000000	1.9650000
-31493.0000000	1.9550000
-31492.0000000	1.9650000
-31491.0000000	1.9650000
-31490.0000000	1.9600000
-31489.0000000	1.9650000

-31488.0000000	1.9550000
-31487.0000000	1.9600000
-31486.0000000	1.9600000
-31485.0000000	1.9600000
-31484.0000000	1.9600000
-31483.0000000	1.9550000
-31482.0000000	1.9600000
-31481.0000000	1.9600000
-31480.0000000	1.9500000
-31479.0000000	1.9550000
-31478.0000000	1.9550000
-31477.0000000	1.9600000
-31476.0000000	1.9550000
-31475.0000000	1.9500000
-31474.0000000	1.9550000
-31473.0000000	1.9550000
-31472.0000000	1.9500000
-31471.0000000	1.9500000
-31470.0000000	1.9400001
-31469.0000000	1.9450001
-31468.0000000	1.9450001
-31467.0000000	1.9400001
-31466.0000000	1.9500000
-31465.0000000	1.9500000
-31464.0000000	1.9500000
-31463.0000000	1.9450001
-31462.0000000	1.9450001
-31461.0000000	1.9500000
-31460.0000000	1.9450001
-31459.0000000	1.9450001
-31458.0000000	1.9450001
-31457.0000000	1.9500000
-31456.0000000	1.9433333
-31455.0000000	1.9450001
-31454.0000000	1.9450001
-31453.0000000	1.9450001
-31452.0000000	1.9450001
-31451.0000000	1.9450001
-31450.0000000	1.9400001
-31449.0000000	1.9400001
-31448.0000000	1.9400001
-31447.0000000	1.9349999
-31446.0000000	1.9349999
-31445.0000000	1.9400001
-31444.0000000	1.9400001
-31443.0000000	1.9349999
-31442.0000000	1.9349999
-31441.0000000	1.9400001
-31440.0000000	1.9349999
-31439.0000000	1.9349999
-31438.0000000	1.9300001
-31437.0000000	1.9349999
-31436.0000000	1.9299999
-31435.0000000	1.9299999
-31434.0000000	1.9250000
-31433.0000000	1.9299999
-31432.0000000	1.9250000

-31431.0000000	1.9250000
-31430.0000000	1.9250000
-31429.0000000	1.9250000
-31428.0000000	1.9200000
-31427.0000000	1.9200000
-31426.0000000	1.9133333
-31425.0000000	1.9150000
-31424.0000000	1.9150000
-31423.0000000	1.9150000
-31422.0000000	1.9150000
-31421.0000000	1.9100000
-31420.0000000	1.9100000
-31419.0000000	1.9100000
-31418.0000000	1.9050000
-31417.0000000	1.9000000
-31416.0000000	1.8950000
-31415.0000000	1.9000000
-31414.0000000	1.9000000
-31413.0000000	1.8900000
-31412.0000000	1.8950000
-31411.0000000	1.8900000
-31410.0000000	1.8950000
-31409.0000000	1.8850000
-31408.0000000	1.8850000
-31407.0000000	1.8800000
-31406.0000000	1.8850000
-31405.0000000	1.8850000
-31404.0000000	1.8850000
-31403.0000000	1.8800000
-31402.0000000	1.8800000
-31401.0000000	1.8750000
-31400.0000000	1.8750000
-31399.0000000	1.8750000
-31398.0000000	1.8700000
-31397.0000000	1.8700000
-31396.0000000	1.8700000
-31395.0000000	1.8750000
-31394.0000000	1.8600000
-31393.0000000	1.8650000
-31392.0000000	1.8650000
-31391.0000000	1.8600000
-31390.0000000	1.8650000
-31389.0000000	1.8600000
-31388.0000000	1.8550000
-31387.0000000	1.8550000
-31386.0000000	1.8550000
-31385.0000000	1.8500000
-31384.0000000	1.8500000
-31383.0000000	1.8500000
-31382.0000000	1.8400000
-31381.0000000	1.8450000
-31380.0000000	1.8350000
-31379.0000000	1.8350000
-31378.0000000	1.8250000
-31377.0000000	1.8250000
-31376.0000000	1.8250000
-31375.0000000	1.8199999

-31374.0000000	1.8150001
-31373.0000000	1.8099999
-31372.0000000	1.8099999
-31371.0000000	1.8099999
-31370.0000000	1.8049999
-31369.0000000	1.7950000
-31368.0000000	1.7900000
-31367.0000000	1.7900000
-31366.0000000	1.7850000
-31365.0000000	1.7800000
-31364.0000000	1.7766666
-31363.0000000	1.7650000
-31362.0000000	1.7600000
-31361.0000000	1.7600000
-31360.0000000	1.7550000
-31359.0000000	1.7550000
-31358.0000000	1.7450000
-31357.0000000	1.7350000
-31356.0000000	1.7300000
-31355.0000000	1.7300000
-31354.0000000	1.7250000
-31353.0000000	1.7200000
-31352.0000000	1.7150000
-31351.0000000	1.7050000
-31350.0000000	1.7050000
-31349.0000000	1.7050000
-31348.0000000	1.7000000
-31347.0000000	1.6950001
-31346.0000000	1.6849999
-31345.0000000	1.6799999
-31344.0000000	1.6849999
-31343.0000000	1.6750000
-31342.0000000	1.6750000
-31341.0000000	1.6650000
-31340.0000000	1.6650000
-31339.0000000	1.6600000
-31338.0000000	1.6550000
-31337.0000000	1.6550000
-31336.0000000	1.6500000
-31335.0000000	1.6450000
-31334.0000000	1.6366667
-31333.0000000	1.6300000
-31332.0000000	1.6300000
-31331.0000000	1.6200000
-31330.0000000	1.6250000
-31329.0000000	1.6150000
-31328.0000000	1.6150000
-31327.0000000	1.6050000
-31326.0000000	1.6000000
-31325.0000000	1.6000000
-31324.0000000	1.6000000
-31323.0000000	1.5900000
-31322.0000000	1.5900000
-31321.0000000	1.5850000
-31320.0000000	1.5800000
-31319.0000000	1.5750000
-31318.0000000	1.5700001

-31317.0000000	1.5700001
-31316.0000000	1.5650001
-31315.0000000	1.5599999
-31314.0000000	1.5650001
-31313.0000000	1.5549999
-31312.0000000	1.5500000
-31311.0000000	1.5500000
-31310.0000000	1.5400000
-31309.0000000	1.5350000
-31308.0000000	1.5300000
-31307.0000000	1.5300000
-31306.0000000	1.5250000
-31305.0000000	1.5200000
-31304.0000000	1.5200000
-31303.0000000	1.5150000
-31302.0000000	1.5050000
-31301.0000000	1.5150000
-31300.0000000	1.5100000
-31299.0000000	1.5050000
-31298.0000000	1.5000000
-31297.0000000	1.5000000
-31296.0000000	1.4950000
-31295.0000000	1.4950000
-31294.0000000	1.4850000
-31293.0000000	1.4850000
-31292.0000000	1.4800000
-31291.0000000	1.4750000
-31290.0000000	1.4750000
-31289.0000000	1.4650000
-31288.0000000	1.4650000
-31287.0000000	1.4650000
-31286.0000000	1.4650000
-31285.0000000	1.4550000
-31284.0000000	1.4550000
-31283.0000000	1.4500000
-31282.0000000	1.4400001
-31281.0000000	1.4400001
-31280.0000000	1.4349999
-31279.0000000	1.4349999
-31278.0000000	1.4250000
-31277.0000000	1.4200000
-31276.0000000	1.4250000
-31275.0000000	1.4100000
-31274.0000000	1.4100000
-31273.0000000	1.4066666
-31272.0000000	1.4000000
-31271.0000000	1.3950000
-31270.0000000	1.3950000
-31269.0000000	1.3850000
-31268.0000000	1.3800000
-31267.0000000	1.3850000
-31266.0000000	1.3750000
-31265.0000000	1.3700000
-31264.0000000	1.3750000
-31263.0000000	1.3650000
-31262.0000000	1.3550000
-31261.0000000	1.3550000

-31260.0000000	1.3500000
-31259.0000000	1.3450000
-31258.0000000	1.3450000
-31257.0000000	1.3400000
-31256.0000000	1.3350000
-31255.0000000	1.3300000
-31254.0000000	1.3300000
-31253.0000000	1.3250000
-31252.0000000	1.3200001
-31251.0000000	1.3200001
-31250.0000000	1.3200001
-31249.0000000	1.3150001
-31248.0000000	1.3099999
-31247.0000000	1.3049999
-31246.0000000	1.3000000
-31245.0000000	1.2933333
-31244.0000000	1.2950000
-31243.0000000	1.2900000
-31242.0000000	1.2850000
-31241.0000000	1.2850000
-31240.0000000	1.2800000
-31239.0000000	1.2800000
-31238.0000000	1.2700000
-31237.0000000	1.2750000
-31236.0000000	1.2700000
-31235.0000000	1.2700000
-31234.0000000	1.2700000
-31233.0000000	1.2650000
-31232.0000000	1.2600000
-31231.0000000	1.2600000
-31230.0000000	1.2550000
-31229.0000000	1.2500000
-31228.0000000	1.2450000
-31227.0000000	1.2400000
-31226.0000000	1.2450000
-31225.0000000	1.2400000
-31224.0000000	1.2400000
-31223.0000000	1.2300000
-31222.0000000	1.2250000
-31221.0000000	1.2150000
-31220.0000000	1.2200000
-31219.0000000	1.2150000
-31218.0000000	1.2100000
-31217.0000000	1.2050000
-31216.0000000	1.2000000
-31215.0000000	1.2000000
-31214.0000000	1.1950001
-31213.0000000	1.1900001
-31212.0000000	1.1950001
-31211.0000000	1.1849999
-31210.0000000	1.1849999
-31209.0000000	1.1799999
-31208.0000000	1.1799999
-31207.0000000	1.1799999
-31206.0000000	1.1750000
-31205.0000000	1.1650000
-31204.0000000	1.1750000

-31203.0000000	1.1700000
-31202.0000000	1.1650000
-31201.0000000	1.1600000
-31200.0000000	1.1600000
-31199.0000000	1.1550000
-31198.0000000	1.1550000
-31197.0000000	1.1550000
-31196.0000000	1.1500000
-31195.0000000	1.1550000
-31194.0000000	1.1550000
-31193.0000000	1.1500000
-31192.0000000	1.1450000
-31191.0000000	1.1450000
-31190.0000000	1.1450000
-31189.0000000	1.1450000
-31188.0000000	1.1450000
-31187.0000000	1.1400000
-31186.0000000	1.1400000
-31185.0000000	1.1400000
-31184.0000000	1.1350000
-31183.0000000	1.1350000
-31182.0000000	1.1450000
-31181.0000000	1.1350000
-31180.0000000	1.1350000
-31179.0000000	1.1400000
-31178.0000000	1.1400000
-31177.0000000	1.1400000
-31176.0000000	1.1350000
-31175.0000000	1.1350000
-31174.0000000	1.1350000
-31173.0000000	1.1350000
-31172.0000000	1.1350000
-31171.0000000	1.1300000
-31170.0000000	1.1350000
-31169.0000000	1.1300000
-31168.0000000	1.1300000
-31167.0000000	1.1350000
-31166.0000000	1.1300000
-31165.0000000	1.1300000
-31164.0000000	1.1300000
-31163.0000000	1.1300000
-31162.0000000	1.1300000
-31161.0000000	1.1250000
-31160.0000000	1.1250000
-31159.0000000	1.1250000
-31158.0000000	1.1250000
-31157.0000000	1.1200000
-31156.0000000	1.1200000
-31155.0000000	1.1200000
-31154.0000000	1.1200000
-31153.0000000	1.1266667
-31152.0000000	1.1250000
-31151.0000000	1.1150000
-31150.0000000	1.1200000
-31149.0000000	1.1200000
-31148.0000000	1.1200000
-31147.0000000	1.1200000

-31146.0000000	1.1200000
-31145.0000000	1.1200000
-31144.0000000	1.1200000
-31143.0000000	1.1200000
-31142.0000000	1.1200000
-31141.0000000	1.1200000
-31140.0000000	1.1200000
-31139.0000000	1.1250000
-31138.0000000	1.1250000
-31137.0000000	1.1250000
-31136.0000000	1.1150000
-31135.0000000	1.1200000
-31134.0000000	1.1150000
-31133.0000000	1.1200000
-31132.0000000	1.1150000
-31131.0000000	1.1150000
-31130.0000000	1.1150000
-31129.0000000	1.1150000
-31128.0000000	1.1150000
-31127.0000000	1.1100000
-31126.0000000	1.1050000
-31125.0000000	1.1000000
-31124.0000000	1.1000000
-31123.0000000	1.0950000
-31122.0000000	1.1000000
-31121.0000000	1.0900000
-31120.0000000	1.0900000
-31119.0000000	1.0850000
-31118.0000000	1.0850000
-31117.0000000	1.0800000
-31116.0000000	1.0750000
-31115.0000000	1.0750000
-31114.0000000	1.0699999
-31113.0000000	1.0650001
-31112.0000000	1.0599999
-31111.0000000	1.0599999
-31110.0000000	1.0549999
-31109.0000000	1.0500000
-31108.0000000	1.0500000
-31107.0000000	1.0450000
-31106.0000000	1.0450000
-31105.0000000	1.0400000
-31104.0000000	1.0400000
-31103.0000000	1.0300000
-31102.0000000	1.0250000
-31101.0000000	1.0250000
-31100.0000000	1.0200000
-31099.0000000	1.0150000
-31098.0000000	1.0150000
-31097.0000000	1.0100000
-31096.0000000	1.0050000
-31095.0000000	1.0000000
-31094.0000000	1.0000000
-31093.0000000	0.9950000
-31092.0000000	0.9950000
-31091.0000000	0.9866667
-31090.0000000	0.9800000

-31089.0000000	0.9800000
-31088.0000000	0.9750000
-31087.0000000	0.9750000
-31086.0000000	0.9700000
-31085.0000000	0.9650000
-31084.0000000	0.9600000
-31083.0000000	0.9550000
-31082.0000000	0.9500000
-31081.0000000	0.9500000
-31080.0000000	0.9450000
-31079.0000000	0.9400000
-31078.0000000	0.9400000
-31077.0000000	0.9300000
-31076.0000000	0.9300000
-31075.0000000	0.9200000
-31074.0000000	0.9250000
-31073.0000000	0.9200000
-31072.0000000	0.9150000
-31071.0000000	0.9100000
-31070.0000000	0.9050000
-31069.0000000	0.9000000
-31068.0000000	0.9050000
-31067.0000000	0.9000000
-31066.0000000	0.9000000
-31065.0000000	0.8900000
-31064.0000000	0.8850000
-31063.0000000	0.8850000
-31062.0000000	0.8850000
-31061.0000000	0.8866666
-31060.0000000	0.8800000
-31059.0000000	0.8800000
-31058.0000000	0.8750000
-31057.0000000	0.8700000
-31056.0000000	0.8650000
-31055.0000000	0.8600000
-31054.0000000	0.8600000
-31053.0000000	0.8550000
-31052.0000000	0.8600000
-31051.0000000	0.8550000
-31050.0000000	0.8550000
-31049.0000000	0.8550000
-31048.0000000	0.8550000
-31047.0000000	0.8550000
-31046.0000000	0.8500000
-31045.0000000	0.8450000
-31044.0000000	0.8500000
-31043.0000000	0.8450000
-31042.0000000	0.8450000
-31041.0000000	0.8450000
-31040.0000000	0.8450000
-31039.0000000	0.8400000
-31038.0000000	0.8400000
-31037.0000000	0.8400000
-31036.0000000	0.8400000
-31035.0000000	0.8350000
-31034.0000000	0.8400000
-31033.0000000	0.8350000

-31032.0000000	0.8333333
-31031.0000000	0.8350000
-31030.0000000	0.8350000
-31029.0000000	0.8350000
-31028.0000000	0.8350000
-31027.0000000	0.8350000
-31026.0000000	0.8350000
-31025.0000000	0.8350000
-31024.0000000	0.8250000
-31023.0000000	0.8300000
-31022.0000000	0.8300000
-31021.0000000	0.8250000
-31020.0000000	0.8250000
-31019.0000000	0.8250000
-31018.0000000	0.8200000
-31017.0000000	0.8200000
-31016.0000000	0.8150000
-31015.0000000	0.8150000
-31014.0000000	0.8100000
-31013.0000000	0.8050000
-31012.0000000	0.8050000
-31011.0000000	0.8100000
-31010.0000000	0.8050000
-31009.0000000	0.8050000
-31008.0000000	0.8000000
-31007.0000000	0.8000000
-31006.0000000	0.7950000
-31005.0000000	0.7900000
-31004.0000000	0.7900000
-31003.0000000	0.7850000
-31002.0000000	0.7800000
-31001.0000000	0.7800000
-31000.0000000	0.7800000
-30999.0000000	0.7750000
-30998.0000000	0.7700000
-30997.0000000	0.7650000
-30996.0000000	0.7650000
-30995.0000000	0.7650000
-30994.0000000	0.7600000
-30993.0000000	0.7550000
-30992.0000000	0.7550000
-30991.0000000	0.7500000
-30990.0000000	0.7450000
-30989.0000000	0.7450000
-30988.0000000	0.7400000
-30987.0000000	0.7350000
-30986.0000000	0.7300000
-30985.0000000	0.7300000
-30984.0000000	0.7250000
-30983.0000000	0.7250000
-30982.0000000	0.7200000
-30981.0000000	0.7150000
-30980.0000000	0.7100000
-30979.0000000	0.7150000
-30978.0000000	0.7050000
-30977.0000000	0.7050000
-30976.0000000	0.7000000

-30975.0000000	0.7000000
-30974.0000000	0.6950000
-30973.0000000	0.6950000
-30972.0000000	0.6900000
-30971.0000000	0.6833333
-30970.0000000	0.6800000
-30969.0000000	0.6750000
-30968.0000000	0.6750000
-30967.0000000	0.6700000
-30966.0000000	0.6650000
-30965.0000000	0.6600000
-30964.0000000	0.6600000
-30963.0000000	0.6500000
-30962.0000000	0.6500000
-30961.0000000	0.6450000
-30960.0000000	0.6400000
-30959.0000000	0.6350000
-30958.0000000	0.6350000
-30957.0000000	0.6350000
-30956.0000000	0.6250000
-30955.0000000	0.6300000
-30954.0000000	0.6200000
-30953.0000000	0.6200000
-30952.0000000	0.6150000
-30951.0000000	0.6050000
-30950.0000000	0.6000000
-30949.0000000	0.5950000
-30948.0000000	0.5900000
-30947.0000000	0.5850000
-30946.0000000	0.5800000
-30945.0000000	0.5750000
-30944.0000000	0.5750000
-30943.0000000	0.5650000
-30942.0000000	0.5600000
-30941.0000000	0.5550000
-30940.0000000	0.5500000
-30939.0000000	0.5450000
-30938.0000000	0.5400000
-30937.0000000	0.5400000
-30936.0000000	0.5350000
-30935.0000000	0.5300000
-30934.0000000	0.5300000
-30933.0000000	0.5200000
-30932.0000000	0.5200000
-30931.0000000	0.5200000
-30930.0000000	0.5150000
-30929.0000000	0.5150000
-30928.0000000	0.5100000
-30927.0000000	0.5100000
-30926.0000000	0.5000000
-30925.0000000	0.5000000
-30924.0000000	0.4950000
-30923.0000000	0.4950000
-30922.0000000	0.4900000
-30921.0000000	0.4900000
-30920.0000000	0.4850000
-30919.0000000	0.4800000

-30918.0000000	0.4800000
-30917.0000000	0.4700000
-30916.0000000	0.4700000
-30915.0000000	0.4650000
-30914.0000000	0.4650000
-30913.0000000	0.4550000
-30912.0000000	0.4550000
-30911.0000000	0.4500000
-30910.0000000	0.4450000
-30909.0000000	0.4450000
-30908.0000000	0.4400000
-30907.0000000	0.4350000
-30906.0000000	0.4350000
-30905.0000000	0.4300000
-30904.0000000	0.4300000
-30903.0000000	0.4200000
-30902.0000000	0.4150000
-30901.0000000	0.4150000
-30900.0000000	0.4150000
-30899.0000000	0.4050000
-30898.0000000	0.4050000
-30897.0000000	0.3950000
-30896.0000000	0.3950000
-30895.0000000	0.3900000
-30894.0000000	0.3900000
-30893.0000000	0.3850000
-30892.0000000	0.3850000
-30891.0000000	0.3800000
-30890.0000000	0.3750000
-30889.0000000	0.3750000
-30888.0000000	0.3700000
-30887.0000000	0.3650000
-30886.0000000	0.3650000
-30885.0000000	0.3650000
-30884.0000000	0.3550000
-30883.0000000	0.3550000
-30882.0000000	0.3500000
-30881.0000000	0.3500000
-30880.0000000	0.3450000
-30879.0000000	0.3400000
-30878.0000000	0.3400000
-30877.0000000	0.3350000
-30876.0000000	0.3400000
-30875.0000000	0.3300000
-30874.0000000	0.3300000
-30873.0000000	0.3300000
-30872.0000000	0.3250000
-30871.0000000	0.3250000
-30870.0000000	0.3200000
-30869.0000000	0.3150000
-30868.0000000	0.3150000
-30867.0000000	0.3100000
-30866.0000000	0.3100000
-30865.0000000	0.3050000
-30864.0000000	0.3050000
-30863.0000000	0.3050000
-30862.0000000	0.3000000

-30861.0000000	0.2950000
-30860.0000000	0.2950000
-30859.0000000	0.2900000
-30858.0000000	0.2900000
-30857.0000000	0.2850000
-30856.0000000	0.2850000
-30855.0000000	0.2800000
-30854.0000000	0.2850000
-30853.0000000	0.2800000
-30852.0000000	0.2700000
-30851.0000000	0.2700000
-30850.0000000	0.2650000
-30849.0000000	0.2650000
-30848.0000000	0.2600000
-30847.0000000	0.2550000
-30846.0000000	0.2550000
-30845.0000000	0.2450000
-30844.0000000	0.2500000
-30843.0000000	0.2450000
-30842.0000000	0.2450000
-30841.0000000	0.2450000
-30840.0000000	0.2350000
-30839.0000000	0.2350000
-30838.0000000	0.2300000
-30837.0000000	0.2300000
-30836.0000000	0.2300000
-30835.0000000	0.2250000
-30834.0000000	0.2200000
-30833.0000000	0.2200000
-30832.0000000	0.2150000
-30831.0000000	0.2100000
-30830.0000000	0.2100000
-30829.0000000	0.2100000
-30828.0000000	0.2050000
-30827.0000000	0.2000000
-30826.0000000	0.2000000
-30825.0000000	0.1950000
-30824.0000000	0.1950000
-30823.0000000	0.1900000
-30822.0000000	0.1900000
-30821.0000000	0.1800000
-30820.0000000	0.1800000
-30819.0000000	0.1750000
-30818.0000000	0.1750000
-30817.0000000	0.1750000
-30816.0000000	0.1700000
-30815.0000000	0.1650000
-30814.0000000	0.1650000
-30813.0000000	0.1600000
-30812.0000000	0.1600000
-30811.0000000	0.1550000
-30810.0000000	0.1500000
-30809.0000000	0.1450000
-30808.0000000	0.1400000
-30807.0000000	0.1400000
-30806.0000000	0.1400000
-30805.0000000	0.1350000

-30804.0000000	0.1350000
-30803.0000000	0.1350000
-30802.0000000	0.1250000
-30801.0000000	0.1250000
-30800.0000000	0.1250000
-30799.0000000	0.1200000
-30798.0000000	0.1150000
-30797.0000000	0.1150000
-30796.0000000	0.1150000
-30795.0000000	0.1050000
-30794.0000000	0.1050000
-30793.0000000	0.1000000
-30792.0000000	0.1000000
-30791.0000000	0.1000000
-30790.0000000	0.0900000
-30789.0000000	0.0850000
-30788.0000000	0.0850000
-30787.0000000	0.0850000
-30786.0000000	0.0800000
-30785.0000000	0.0800000
-30784.0000000	0.0800000
-30783.0000000	0.0750000
-30782.0000000	0.0750000
-30781.0000000	0.0650000
-30780.0000000	0.0650000
-30779.0000000	0.0650000
-30778.0000000	0.0600000
-30777.0000000	0.0600000
-30776.0000000	0.0550000
-30775.0000000	0.0550000
-30774.0000000	0.0550000
-30773.0000000	0.0500000
-30772.0000000	0.0500000
-30771.0000000	0.0450000
-30770.0000000	0.0400000
-30769.0000000	0.0400000
-30768.0000000	0.0400000
-30767.0000000	0.0350000
-30766.0000000	0.0350000
-30765.0000000	0.0300000
-30764.0000000	0.0250000
-30763.0000000	0.0250000
-30762.0000000	0.0250000
-30761.0000000	0.0200000
-30760.0000000	0.0200000
-30759.0000000	0.0133333
-30758.0000000	0.0100000
-30757.0000000	0.0050000
-30756.0000000	0.0050000
-30755.0000000	0.0000000
-30754.0000000	0.0000000
-30753.0000000	0.0000000
-30752.0000000	-0.0050000
-30751.0000000	-0.0100000
-30750.0000000	-0.0050000
-30749.0000000	-0.0150000
-30748.0000000	-0.0150000

-30747.0000000	-0.0150000
-30746.0000000	-0.0200000
-30745.0000000	-0.0250000
-30744.0000000	-0.0250000
-30743.0000000	-0.0250000
-30742.0000000	-0.0300000
-30741.0000000	-0.0350000
-30740.0000000	-0.0350000
-30739.0000000	-0.0400000
-30738.0000000	-0.0400000
-30737.0000000	-0.0450000
-30736.0000000	-0.0450000
-30735.0000000	-0.0500000
-30734.0000000	-0.0500000
-30733.0000000	-0.0550000
-30732.0000000	-0.0550000
-30731.0000000	-0.0600000
-30730.0000000	-0.0600000
-30729.0000000	-0.0633333
-30728.0000000	-0.0700000
-30727.0000000	-0.0700000
-30726.0000000	-0.0750000
-30725.0000000	-0.0800000
-30724.0000000	-0.0800000
-30723.0000000	-0.0850000
-30722.0000000	-0.0850000
-30721.0000000	-0.0850000
-30720.0000000	-0.0900000
-30719.0000000	-0.0900000
-30718.0000000	-0.1000000
-30717.0000000	-0.1000000
-30716.0000000	-0.1000000
-30715.0000000	-0.1050000
-30714.0000000	-0.1050000
-30713.0000000	-0.1100000
-30712.0000000	-0.1150000
-30711.0000000	-0.1150000
-30710.0000000	-0.1200000
-30709.0000000	-0.1250000
-30708.0000000	-0.1250000
-30707.0000000	-0.1250000
-30706.0000000	-0.1350000
-30705.0000000	-0.1350000
-30704.0000000	-0.1350000
-30703.0000000	-0.1400000
-30702.0000000	-0.1466667
-30701.0000000	-0.1500000
-30700.0000000	-0.1550000
-30699.0000000	-0.1550000
-30698.0000000	-0.1550000
-30697.0000000	-0.1650000
-30696.0000000	-0.1700000
-30695.0000000	-0.1700000
-30694.0000000	-0.1750000
-30693.0000000	-0.1750000
-30692.0000000	-0.1800000
-30691.0000000	-0.1850000

-30690.0000000	-0.1850000
-30689.0000000	-0.1900000
-30688.0000000	-0.1900000
-30687.0000000	-0.1950000
-30686.0000000	-0.2050000
-30685.0000000	-0.2050000
-30684.0000000	-0.2050000
-30683.0000000	-0.2100000
-30682.0000000	-0.2150000
-30681.0000000	-0.2200000
-30680.0000000	-0.2200000
-30679.0000000	-0.2300000
-30678.0000000	-0.2300000
-30677.0000000	-0.2350000
-30676.0000000	-0.2400000
-30675.0000000	-0.2400000
-30674.0000000	-0.2450000
-30673.0000000	-0.2500000
-30672.0000000	-0.2550000
-30671.0000000	-0.2600000
-30670.0000000	-0.2550000
-30669.0000000	-0.2633333
-30668.0000000	-0.2700000
-30667.0000000	-0.2800000
-30666.0000000	-0.2800000
-30665.0000000	-0.2850000
-30664.0000000	-0.2850000
-30663.0000000	-0.2850000
-30662.0000000	-0.2950000
-30661.0000000	-0.2950000
-30660.0000000	-0.2950000
-30659.0000000	-0.3050000
-30658.0000000	-0.3100000
-30657.0000000	-0.3100000
-30656.0000000	-0.3150000
-30655.0000000	-0.3200000
-30654.0000000	-0.3200000
-30653.0000000	-0.3300000
-30652.0000000	-0.3300000
-30651.0000000	-0.3350000
-30650.0000000	-0.3400000
-30649.0000000	-0.3450000
-30648.0000000	-0.3450000
-30647.0000000	-0.3500000
-30646.0000000	-0.3550000
-30645.0000000	-0.3550000
-30644.0000000	-0.3600000
-30643.0000000	-0.3600000
-30642.0000000	-0.3700000
-30641.0000000	-0.3700000
-30640.0000000	-0.3750000
-30639.0000000	-0.3833333
-30638.0000000	-0.3850000
-30637.0000000	-0.3900000
-30636.0000000	-0.3950000
-30635.0000000	-0.4000000
-30634.0000000	-0.4050000

-30633.0000000	-0.4050000
-30632.0000000	-0.4100000
-30631.0000000	-0.4150000
-30630.0000000	-0.4150000
-30629.0000000	-0.4200000
-30628.0000000	-0.4250000
-30627.0000000	-0.4300000
-30626.0000000	-0.4350000
-30625.0000000	-0.4350000
-30624.0000000	-0.4450000
-30623.0000000	-0.4450000
-30622.0000000	-0.4500000
-30621.0000000	-0.4500000
-30620.0000000	-0.4550000
-30619.0000000	-0.4600000
-30618.0000000	-0.4700000
-30617.0000000	-0.4650000
-30616.0000000	-0.4750000
-30615.0000000	-0.4800000
-30614.0000000	-0.4850000
-30613.0000000	-0.4850000
-30612.0000000	-0.4900000
-30611.0000000	-0.4950000
-30610.0000000	-0.4950000
-30609.0000000	-0.5033333
-30608.0000000	-0.5100000
-30607.0000000	-0.5100000
-30606.0000000	-0.5150000
-30605.0000000	-0.5150000
-30604.0000000	-0.5200000
-30603.0000000	-0.5250000
-30602.0000000	-0.5350000
-30601.0000000	-0.5350000
-30600.0000000	-0.5350000
-30599.0000000	-0.5400000
-30598.0000000	-0.5400000
-30597.0000000	-0.5450000
-30596.0000000	-0.5550000
-30595.0000000	-0.5500000
-30594.0000000	-0.5600000
-30593.0000000	-0.5600000
-30592.0000000	-0.5650000
-30591.0000000	-0.5700000
-30590.0000000	-0.5650000
-30589.0000000	-0.5750000
-30588.0000000	-0.5750000
-30587.0000000	-0.5800000
-30586.0000000	-0.5850000
-30585.0000000	-0.5900000
-30584.0000000	-0.5900000
-30583.0000000	-0.6000000
-30582.0000000	-0.6000000
-30581.0000000	-0.6050000
-30580.0000000	-0.6050000
-30579.0000000	-0.6100000
-30578.0000000	-0.6200000
-30577.0000000	-0.6200000

-30576.0000000	-0.6200000
-30575.0000000	-0.6300000
-30574.0000000	-0.6250000
-30573.0000000	-0.6350000
-30572.0000000	-0.6350000
-30571.0000000	-0.6400000
-30570.0000000	-0.6400000
-30569.0000000	-0.6450000
-30568.0000000	-0.6500000
-30567.0000000	-0.6550000
-30566.0000000	-0.6550000
-30565.0000000	-0.6550000
-30564.0000000	-0.6600000
-30563.0000000	-0.6650000
-30562.0000000	-0.6600000
-30561.0000000	-0.6700000
-30560.0000000	-0.6750000
-30559.0000000	-0.6750000
-30558.0000000	-0.6800000
-30557.0000000	-0.6800000
-30556.0000000	-0.6800000
-30555.0000000	-0.6850000
-30554.0000000	-0.6900000
-30553.0000000	-0.6900000
-30552.0000000	-0.6900000
-30551.0000000	-0.6950000
-30550.0000000	-0.7000000
-30549.0000000	-0.7000000
-30548.0000000	-0.7100000
-30547.0000000	-0.7100000
-30546.0000000	-0.7150000
-30545.0000000	-0.7150000
-30544.0000000	-0.7150000
-30543.0000000	-0.7200000
-30542.0000000	-0.7200000
-30541.0000000	-0.7250000
-30540.0000000	-0.7250000
-30539.0000000	-0.7250000
-30538.0000000	-0.7250000
-30537.0000000	-0.7350000
-30536.0000000	-0.7350000
-30535.0000000	-0.7350000
-30534.0000000	-0.7400000
-30533.0000000	-0.7400000
-30532.0000000	-0.7400000
-30531.0000000	-0.7450000
-30530.0000000	-0.7450000
-30529.0000000	-0.7500000
-30528.0000000	-0.7500000
-30527.0000000	-0.7550000
-30526.0000000	-0.7550000
-30525.0000000	-0.7500000
-30524.0000000	-0.7550000
-30523.0000000	-0.7600000
-30522.0000000	-0.7600000
-30521.0000000	-0.7600000
-30520.0000000	-0.7650000

-30519.0000000	-0.7666667
-30518.0000000	-0.7700000
-30517.0000000	-0.7700000
-30516.0000000	-0.7750000
-30515.0000000	-0.7750000
-30514.0000000	-0.7750000
-30513.0000000	-0.7750000
-30512.0000000	-0.7800000
-30511.0000000	-0.7800000
-30510.0000000	-0.7800000
-30509.0000000	-0.7800000
-30508.0000000	-0.7850000
-30507.0000000	-0.7850000
-30506.0000000	-0.7850000
-30505.0000000	-0.7850000
-30504.0000000	-0.7900000
-30503.0000000	-0.7850000
-30502.0000000	-0.7900000
-30501.0000000	-0.7900000
-30500.0000000	-0.7850000
-30499.0000000	-0.7900000
-30498.0000000	-0.7900000
-30497.0000000	-0.7950000
-30496.0000000	-0.7950000
-30495.0000000	-0.8000000
-30494.0000000	-0.8000000
-30493.0000000	-0.8000000
-30492.0000000	-0.8000000
-30491.0000000	-0.8050000
-30490.0000000	-0.8000000
-30489.0000000	-0.8000000
-30488.0000000	-0.8050000
-30487.0000000	-0.8000000
-30486.0000000	-0.8000000
-30485.0000000	-0.8050000
-30484.0000000	-0.8000000
-30483.0000000	-0.8050000
-30482.0000000	-0.8100000
-30481.0000000	-0.8050000
-30480.0000000	-0.8000000
-30479.0000000	-0.8100000
-30478.0000000	-0.8050000
-30477.0000000	-0.8050000
-30476.0000000	-0.8100000
-30475.0000000	-0.8050000
-30474.0000000	-0.8100000
-30473.0000000	-0.8100000
-30472.0000000	-0.8100000
-30471.0000000	-0.8100000
-30470.0000000	-0.8050000
-30469.0000000	-0.8100000
-30468.0000000	-0.8100000
-30467.0000000	-0.8050000
-30466.0000000	-0.8100000
-30465.0000000	-0.8100000
-30464.0000000	-0.8100000
-30463.0000000	-0.8100000

-30462.0000000	-0.8050000
-30461.0000000	-0.8100000
-30460.0000000	-0.8050000
-30459.0000000	-0.8100000
-30458.0000000	-0.8050000
-30457.0000000	-0.8033333
-30456.0000000	-0.8050000
-30455.0000000	-0.8050000
-30454.0000000	-0.8050000
-30453.0000000	-0.8050000
-30452.0000000	-0.8000000
-30451.0000000	-0.8050000
-30450.0000000	-0.8000000
-30449.0000000	-0.7950000
-30448.0000000	-0.8000000
-30447.0000000	-0.7950000
-30446.0000000	-0.7950000
-30445.0000000	-0.7950000
-30444.0000000	-0.7900000
-30443.0000000	-0.7900000
-30442.0000000	-0.7900000
-30441.0000000	-0.7900000
-30440.0000000	-0.7900000
-30439.0000000	-0.7850000
-30438.0000000	-0.7800000
-30437.0000000	-0.7800000
-30436.0000000	-0.7800000
-30435.0000000	-0.7800000
-30434.0000000	-0.7800000
-30433.0000000	-0.7800000
-30432.0000000	-0.7700000
-30431.0000000	-0.7750000
-30430.0000000	-0.7750000
-30429.0000000	-0.7650000
-30428.0000000	-0.7700000
-30427.0000000	-0.7700000
-30426.0000000	-0.7700000
-30425.0000000	-0.7700000
-30424.0000000	-0.7650000
-30423.0000000	-0.7600000
-30422.0000000	-0.7600000
-30421.0000000	-0.7600000
-30420.0000000	-0.7600000
-30419.0000000	-0.7600000
-30418.0000000	-0.7500000
-30417.0000000	-0.7500000
-30416.0000000	-0.7450000
-30415.0000000	-0.7500000
-30414.0000000	-0.7500000
-30413.0000000	-0.7450000
-30412.0000000	-0.7400000
-30411.0000000	-0.7400000
-30410.0000000	-0.7400000
-30409.0000000	-0.7350000
-30408.0000000	-0.7300000
-30407.0000000	-0.7300000
-30406.0000000	-0.7250000

-30405.0000000	-0.7250000
-30404.0000000	-0.7200000
-30403.0000000	-0.7200000
-30402.0000000	-0.7150000
-30401.0000000	-0.7150000
-30400.0000000	-0.7150000
-30399.0000000	-0.7100000
-30398.0000000	-0.7100000
-30397.0000000	-0.7033333
-30396.0000000	-0.7000000
-30395.0000000	-0.6950000
-30394.0000000	-0.7000000
-30393.0000000	-0.6950000
-30392.0000000	-0.6850000
-30391.0000000	-0.6850000
-30390.0000000	-0.6800000
-30389.0000000	-0.6800000
-30388.0000000	-0.6750000
-30387.0000000	-0.6750000
-30386.0000000	-0.6700000
-30385.0000000	-0.6600000
-30384.0000000	-0.6600000
-30383.0000000	-0.6600000
-30382.0000000	-0.6500000
-30381.0000000	-0.6550000
-30380.0000000	-0.6450000
-30379.0000000	-0.6400000
-30378.0000000	-0.6350000
-30377.0000000	-0.6350000
-30376.0000000	-0.6300000
-30375.0000000	-0.6300000
-30374.0000000	-0.6250000
-30373.0000000	-0.6200000
-30372.0000000	-0.6150000
-30371.0000000	-0.6100000
-30370.0000000	-0.6050000
-30369.0000000	-0.6000000
-30368.0000000	-0.6000000
-30367.0000000	-0.5900000
-30366.0000000	-0.5900000
-30365.0000000	-0.5800000
-30364.0000000	-0.5800000
-30363.0000000	-0.5750000
-30362.0000000	-0.5650000
-30361.0000000	-0.5650000
-30360.0000000	-0.5600000
-30359.0000000	-0.5550000
-30358.0000000	-0.5450000
-30357.0000000	-0.5450000
-30356.0000000	-0.5450000
-30355.0000000	-0.5350000
-30354.0000000	-0.5250000
-30353.0000000	-0.5250000
-30352.0000000	-0.5200000
-30351.0000000	-0.5150000
-30350.0000000	-0.5100000
-30349.0000000	-0.5000000

-30348.0000000	-0.5000000
-30347.0000000	-0.4900000
-30346.0000000	-0.4900000
-30345.0000000	-0.4800000
-30344.0000000	-0.4750000
-30343.0000000	-0.4700000
-30342.0000000	-0.4650000
-30341.0000000	-0.4650000
-30340.0000000	-0.4600000
-30339.0000000	-0.4500000
-30338.0000000	-0.4466666
-30337.0000000	-0.4350000
-30336.0000000	-0.4250000
-30335.0000000	-0.4250000
-30334.0000000	-0.4200000
-30333.0000000	-0.4150000
-30332.0000000	-0.4050000
-30331.0000000	-0.4050000
-30330.0000000	-0.4000000
-30329.0000000	-0.3900000
-30328.0000000	-0.3900000
-30327.0000000	-0.3750000
-30326.0000000	-0.3750000
-30325.0000000	-0.3700000
-30324.0000000	-0.3600000
-30323.0000000	-0.3550000
-30322.0000000	-0.3500000
-30321.0000000	-0.3450000
-30320.0000000	-0.3350000
-30319.0000000	-0.3350000
-30318.0000000	-0.3300000
-30317.0000000	-0.3250000
-30316.0000000	-0.3150000
-30315.0000000	-0.3100000
-30314.0000000	-0.3100000
-30313.0000000	-0.3000000
-30312.0000000	-0.2900000
-30311.0000000	-0.2850000
-30310.0000000	-0.2800000
-30309.0000000	-0.2733333
-30308.0000000	-0.2700000
-30307.0000000	-0.2600000
-30306.0000000	-0.2500000
-30305.0000000	-0.2500000
-30304.0000000	-0.2400000
-30303.0000000	-0.2350000
-30302.0000000	-0.2300000
-30301.0000000	-0.2200000
-30300.0000000	-0.2150000
-30299.0000000	-0.2100000
-30298.0000000	-0.2050000
-30297.0000000	-0.1950000
-30296.0000000	-0.1950000
-30295.0000000	-0.1850000
-30294.0000000	-0.1850000
-30293.0000000	-0.1800000
-30292.0000000	-0.1700000

-30291.0000000	-0.1600000
-30290.0000000	-0.1550000
-30289.0000000	-0.1550000
-30288.0000000	-0.1500000
-30287.0000000	-0.1400000
-30286.0000000	-0.1300000
-30285.0000000	-0.1300000
-30284.0000000	-0.1250000
-30283.0000000	-0.1150000
-30282.0000000	-0.1150000
-30281.0000000	-0.1050000
-30280.0000000	-0.1050000
-30279.0000000	-0.0950000
-30278.0000000	-0.0866667
-30277.0000000	-0.0850000
-30276.0000000	-0.0750000
-30275.0000000	-0.0700000
-30274.0000000	-0.0650000
-30273.0000000	-0.0650000
-30272.0000000	-0.0600000
-30271.0000000	-0.0500000
-30270.0000000	-0.0500000
-30269.0000000	-0.0400000
-30268.0000000	-0.0350000
-30267.0000000	-0.0300000
-30266.0000000	-0.0250000
-30265.0000000	-0.0200000
-30264.0000000	-0.0150000
-30263.0000000	-0.0100000
-30262.0000000	-0.0050000
-30261.0000000	0.0000000
-30260.0000000	0.0050000
-30259.0000000	0.0100000
-30258.0000000	0.0150000
-30257.0000000	0.0200000
-30256.0000000	0.0200000
-30255.0000000	0.0300000
-30254.0000000	0.0300000
-30253.0000000	0.0350000
-30252.0000000	0.0400000
-30251.0000000	0.0450000
-30250.0000000	0.0500000
-30249.0000000	0.0550000
-30248.0000000	0.0633333
-30247.0000000	0.0700000
-30246.0000000	0.0700000
-30245.0000000	0.0800000
-30244.0000000	0.0850000
-30243.0000000	0.0850000
-30242.0000000	0.0900000
-30241.0000000	0.0950000
-30240.0000000	0.1000000
-30239.0000000	0.1050000
-30238.0000000	0.1100000
-30237.0000000	0.1150000
-30236.0000000	0.1150000
-30235.0000000	0.1200000

-30234.0000000	0.1250000
-30233.0000000	0.1300000
-30232.0000000	0.1300000
-30231.0000000	0.1400000
-30230.0000000	0.1400000
-30229.0000000	0.1450000
-30228.0000000	0.1500000
-30227.0000000	0.1550000
-30226.0000000	0.1600000
-30225.0000000	0.1600000
-30224.0000000	0.1650000
-30223.0000000	0.1750000
-30222.0000000	0.1750000
-30221.0000000	0.1800000
-30220.0000000	0.1800000
-30219.0000000	0.1850000
-30218.0000000	0.1900000
-30217.0000000	0.1950000
-30216.0000000	0.2000000
-30215.0000000	0.2050000
-30214.0000000	0.2100000
-30213.0000000	0.2100000
-30212.0000000	0.2150000
-30211.0000000	0.2200000
-30210.0000000	0.2250000
-30209.0000000	0.2300000
-30208.0000000	0.2350000
-30207.0000000	0.2350000
-30206.0000000	0.2400000
-30205.0000000	0.2450000
-30204.0000000	0.2500000
-30203.0000000	0.2500000
-30202.0000000	0.2600000
-30201.0000000	0.2650000
-30200.0000000	0.2650000
-30199.0000000	0.2650000
-30198.0000000	0.2750000
-30197.0000000	0.2750000
-30196.0000000	0.2750000
-30195.0000000	0.2800000
-30194.0000000	0.2850000
-30193.0000000	0.2850000
-30192.0000000	0.2950000
-30191.0000000	0.3000000
-30190.0000000	0.3000000
-30189.0000000	0.3000000
-30188.0000000	0.3100000
-30187.0000000	0.3150000
-30186.0000000	0.3166667
-30185.0000000	0.3250000
-30184.0000000	0.3250000
-30183.0000000	0.3300000
-30182.0000000	0.3300000
-30181.0000000	0.3400000
-30180.0000000	0.3400000
-30179.0000000	0.3450000
-30178.0000000	0.3450000

-30177.0000000	0.3550000
-30176.0000000	0.3550000
-30175.0000000	0.3650000
-30174.0000000	0.3650000
-30173.0000000	0.3700000
-30172.0000000	0.3750000
-30171.0000000	0.3800000
-30170.0000000	0.3800000
-30169.0000000	0.3850000
-30168.0000000	0.3950000
-30167.0000000	0.4000000
-30166.0000000	0.4000000
-30165.0000000	0.4050000
-30164.0000000	0.4050000
-30163.0000000	0.4150000
-30162.0000000	0.4150000
-30161.0000000	0.4200000
-30160.0000000	0.4250000
-30159.0000000	0.4300000
-30158.0000000	0.4300000
-30157.0000000	0.4400000
-30156.0000000	0.4433333
-30155.0000000	0.4550000
-30154.0000000	0.4550000
-30153.0000000	0.4550000
-30152.0000000	0.4650000
-30151.0000000	0.4700000
-30150.0000000	0.4700000
-30149.0000000	0.4750000
-30148.0000000	0.4800000
-30147.0000000	0.4850000
-30146.0000000	0.4900000
-30145.0000000	0.4900000
-30144.0000000	0.5000000
-30143.0000000	0.5050000
-30142.0000000	0.5100000
-30141.0000000	0.5150000
-30140.0000000	0.5150000
-30139.0000000	0.5150000
-30138.0000000	0.5250000
-30137.0000000	0.5250000
-30136.0000000	0.5300000
-30135.0000000	0.5400000
-30134.0000000	0.5400000
-30133.0000000	0.5450000
-30132.0000000	0.5500000
-30131.0000000	0.5550000
-30130.0000000	0.5600000
-30129.0000000	0.5600000
-30128.0000000	0.5700000
-30127.0000000	0.5700000
-30126.0000000	0.5750000
-30125.0000000	0.5800000
-30124.0000000	0.5850000
-30123.0000000	0.5950000
-30122.0000000	0.5950000
-30121.0000000	0.6000000

-30120.0000000	0.6050000
-30119.0000000	0.6100000
-30118.0000000	0.6100000
-30117.0000000	0.6150000
-30116.0000000	0.6200000
-30115.0000000	0.6250000
-30114.0000000	0.6300000
-30113.0000000	0.6300000
-30112.0000000	0.6400000
-30111.0000000	0.6450000
-30110.0000000	0.6500000
-30109.0000000	0.6550000
-30108.0000000	0.6600000
-30107.0000000	0.6650000
-30106.0000000	0.6700000
-30105.0000000	0.6750000
-30104.0000000	0.6800000
-30103.0000000	0.6850000
-30102.0000000	0.6900000
-30101.0000000	0.6950000
-30100.0000000	0.7050000
-30099.0000000	0.7050000
-30098.0000000	0.7050000
-30097.0000000	0.7100000
-30096.0000000	0.7233334
-30095.0000000	0.7250000
-30094.0000000	0.7250000
-30093.0000000	0.7350000
-30092.0000000	0.7350000
-30091.0000000	0.7450000
-30090.0000000	0.7500000
-30089.0000000	0.7550000
-30088.0000000	0.7550000
-30087.0000000	0.7600000
-30086.0000000	0.7650000
-30085.0000000	0.7650000
-30084.0000000	0.7700000
-30083.0000000	0.7750000
-30082.0000000	0.7750000
-30081.0000000	0.7800000
-30080.0000000	0.7850000
-30079.0000000	0.7900000
-30078.0000000	0.7950000
-30077.0000000	0.7950000
-30076.0000000	0.8000000
-30075.0000000	0.8050000
-30074.0000000	0.8100000
-30073.0000000	0.8150000
-30072.0000000	0.8200000
-30071.0000000	0.8250000
-30070.0000000	0.8250000
-30069.0000000	0.8300000
-30068.0000000	0.8400000
-30067.0000000	0.8350000
-30066.0000000	0.8433333
-30065.0000000	0.8500000
-30064.0000000	0.8550000

-30063.0000000	0.8600000
-30062.0000000	0.8650000
-30061.0000000	0.8700000
-30060.0000000	0.8750000
-30059.0000000	0.8800000
-30058.0000000	0.8850000
-30057.0000000	0.8900000
-30056.0000000	0.8950000
-30055.0000000	0.9000000
-30054.0000000	0.9050000
-30053.0000000	0.9050000
-30052.0000000	0.9100000
-30051.0000000	0.9200000
-30050.0000000	0.9250000
-30049.0000000	0.9300000
-30048.0000000	0.9350000
-30047.0000000	0.9400000
-30046.0000000	0.9400000
-30045.0000000	0.9500000
-30044.0000000	0.9550000
-30043.0000000	0.9550000
-30042.0000000	0.9650000
-30041.0000000	0.9650000
-30040.0000000	0.9650000
-30039.0000000	0.9700000
-30038.0000000	0.9750000
-30037.0000000	0.9800000
-30036.0000000	0.9866667
-30035.0000000	0.9850000
-30034.0000000	0.9950000
-30033.0000000	1.0000000
-30032.0000000	1.0050000
-30031.0000000	1.0050000
-30030.0000000	1.0100000
-30029.0000000	1.0100000
-30028.0000000	1.0150000
-30027.0000000	1.0200000
-30026.0000000	1.0200000
-30025.0000000	1.0300000
-30024.0000000	1.0300000
-30023.0000000	1.0350000
-30022.0000000	1.0400000
-30021.0000000	1.0400000
-30020.0000000	1.0450000
-30019.0000000	1.0450000
-30018.0000000	1.0549999
-30017.0000000	1.0549999
-30016.0000000	1.0650001
-30015.0000000	1.0650001
-30014.0000000	1.0700001
-30013.0000000	1.0750000
-30012.0000000	1.0800000
-30011.0000000	1.0800000
-30010.0000000	1.0850000
-30009.0000000	1.0900000
-30008.0000000	1.1000000
-30007.0000000	1.1000000

-30006.0000000	1.1000000
-30005.0000000	1.1100000
-30004.0000000	1.1100000
-30003.0000000	1.1150000
-30002.0000000	1.1200000
-30001.0000000	1.1250000
-30000.0000000	1.1250000
-29999.0000000	1.1250000
-29998.0000000	1.1300000
-29997.0000000	1.1400000
-29996.0000000	1.1350000
-29995.0000000	1.1350000
-29994.0000000	1.1450000
-29993.0000000	1.1450000
-29992.0000000	1.1400000
-29991.0000000	1.1450000
-29990.0000000	1.1550000
-29989.0000000	1.1550000
-29988.0000000	1.1600000
-29987.0000000	1.1650000
-29986.0000000	1.1650000
-29985.0000000	1.1700000
-29984.0000000	1.1700000
-29983.0000000	1.1700000
-29982.0000000	1.1800001
-29981.0000000	1.1750000
-29980.0000000	1.1750000
-29979.0000000	1.1849999
-29978.0000000	1.1900001
-29977.0000000	1.1900001
-29976.0000000	1.1900001
-29975.0000000	1.1900001
-29974.0000000	1.2000000
-29973.0000000	1.2000000
-29972.0000000	1.2050000
-29971.0000000	1.2050000
-29970.0000000	1.2100000
-29969.0000000	1.2150000
-29968.0000000	1.2100000
-29967.0000000	1.2200000
-29966.0000000	1.2200000
-29965.0000000	1.2200000
-29964.0000000	1.2250000
-29963.0000000	1.2250000
-29962.0000000	1.2300000
-29961.0000000	1.2300000
-29960.0000000	1.2350000
-29959.0000000	1.2350000
-29958.0000000	1.2400000
-29957.0000000	1.2450000
-29956.0000000	1.2450000
-29955.0000000	1.2500000
-29954.0000000	1.2450000
-29953.0000000	1.2500000
-29952.0000000	1.2550000
-29951.0000000	1.2550000
-29950.0000000	1.2550000

-29949.0000000	1.2600000
-29948.0000000	1.2650000
-29947.0000000	1.2650000
-29946.0000000	1.2700000
-29945.0000000	1.2733333
-29944.0000000	1.2750000
-29943.0000000	1.2750000
-29942.0000000	1.2800000
-29941.0000000	1.2800000
-29940.0000000	1.2850000
-29939.0000000	1.2850000
-29938.0000000	1.2900000
-29937.0000000	1.2900000
-29936.0000000	1.2850000
-29935.0000000	1.2900000
-29934.0000000	1.2950000
-29933.0000000	1.2900000
-29932.0000000	1.2900000
-29931.0000000	1.2900000
-29930.0000000	1.2900000
-29929.0000000	1.2900000
-29928.0000000	1.2850000
-29927.0000000	1.2900000
-29926.0000000	1.2950000
-29925.0000000	1.2900000
-29924.0000000	1.2900000
-29923.0000000	1.2900000
-29922.0000000	1.2900000
-29921.0000000	1.2900000
-29920.0000000	1.2850000
-29919.0000000	1.2800000
-29918.0000000	1.2850000
-29917.0000000	1.2800000
-29916.0000000	1.2900000
-29915.0000000	1.2833333
-29914.0000000	1.2850000
-29913.0000000	1.2850000
-29912.0000000	1.2850000
-29911.0000000	1.2850000
-29910.0000000	1.2850000
-29909.0000000	1.2900000
-29908.0000000	1.2900000
-29907.0000000	1.2900000
-29906.0000000	1.2900000
-29905.0000000	1.2900000
-29904.0000000	1.2850000
-29903.0000000	1.2950000
-29902.0000000	1.2900000
-29901.0000000	1.2950000
-29900.0000000	1.2900000
-29899.0000000	1.2900000
-29898.0000000	1.2950000
-29897.0000000	1.2900000
-29896.0000000	1.2950000
-29895.0000000	1.2950000
-29894.0000000	1.2950000
-29893.0000000	1.2950000

-29892.0000000	1.2950000
-29891.0000000	1.3000000
-29890.0000000	1.2950000
-29889.0000000	1.2950000
-29888.0000000	1.2950000
-29887.0000000	1.2950000
-29886.0000000	1.2950000
-29885.0000000	1.2950000
-29884.0000000	1.2966666
-29883.0000000	1.3000000
-29882.0000000	1.3000000
-29881.0000000	1.3000000
-29880.0000000	1.3049999
-29879.0000000	1.3000000
-29878.0000000	1.3000000
-29877.0000000	1.3000000
-29876.0000000	1.3000000
-29875.0000000	1.2900000
-29874.0000000	1.2950000
-29873.0000000	1.2900000
-29872.0000000	1.2900000
-29871.0000000	1.2950000
-29870.0000000	1.2900000
-29869.0000000	1.2900000
-29868.0000000	1.2850000
-29867.0000000	1.2850000
-29866.0000000	1.2850000
-29865.0000000	1.2850000
-29864.0000000	1.2750000
-29863.0000000	1.2850000
-29862.0000000	1.2800000
-29861.0000000	1.2800000
-29860.0000000	1.2750000
-29859.0000000	1.2750000
-29858.0000000	1.2750000
-29857.0000000	1.2750000
-29856.0000000	1.2700000
-29855.0000000	1.2700000
-29854.0000000	1.2700000
-29853.0000000	1.2700000
-29852.0000000	1.2650000
-29851.0000000	1.2650000
-29850.0000000	1.2650000
-29849.0000000	1.2650000
-29848.0000000	1.2600000
-29847.0000000	1.2600000
-29846.0000000	1.2600000
-29845.0000000	1.2600000
-29844.0000000	1.2550000
-29843.0000000	1.2550000
-29842.0000000	1.2550000
-29841.0000000	1.2550000
-29840.0000000	1.2550000
-29839.0000000	1.2550000
-29838.0000000	1.2600000
-29837.0000000	1.2550000
-29836.0000000	1.2500000

-29835.0000000	1.2500000
-29834.0000000	1.2550000
-29833.0000000	1.2550000
-29832.0000000	1.2550000
-29831.0000000	1.2500000
-29830.0000000	1.2500000
-29829.0000000	1.2500000
-29828.0000000	1.2500000
-29827.0000000	1.2450000
-29826.0000000	1.2450000
-29825.0000000	1.2466667
-29824.0000000	1.2400000
-29823.0000000	1.2500000
-29822.0000000	1.2450000
-29821.0000000	1.2450000
-29820.0000000	1.2450000
-29819.0000000	1.2450000
-29818.0000000	1.2450000
-29817.0000000	1.2450000
-29816.0000000	1.2500000
-29815.0000000	1.2500000
-29814.0000000	1.2500000
-29813.0000000	1.2500000
-29812.0000000	1.2500000
-29811.0000000	1.2450000
-29810.0000000	1.2450000
-29809.0000000	1.2450000
-29808.0000000	1.2500000
-29807.0000000	1.2450000
-29806.0000000	1.2450000
-29805.0000000	1.2450000
-29804.0000000	1.2450000
-29803.0000000	1.2450000
-29802.0000000	1.2400000
-29801.0000000	1.2400000
-29800.0000000	1.2400000
-29799.0000000	1.2350000
-29798.0000000	1.2350000
-29797.0000000	1.2300000
-29796.0000000	1.2350000
-29795.0000000	1.2300000
-29794.0000000	1.2300000
-29793.0000000	1.2300000
-29792.0000000	1.2300000
-29791.0000000	1.2350000
-29790.0000000	1.2350000
-29789.0000000	1.2300000
-29788.0000000	1.2300000
-29787.0000000	1.2300000
-29786.0000000	1.2300000
-29785.0000000	1.2300000
-29784.0000000	1.2250000
-29783.0000000	1.2300000
-29782.0000000	1.2300000
-29781.0000000	1.2300000
-29780.0000000	1.2300000
-29779.0000000	1.2300000

-29778.0000000	1.2300000
-29777.0000000	1.2300000
-29776.0000000	1.2300000
-29775.0000000	1.2350000
-29774.0000000	1.2350000
-29773.0000000	1.2350000
-29772.0000000	1.2350000
-29771.0000000	1.2350000
-29770.0000000	1.2300000
-29769.0000000	1.2350000
-29768.0000000	1.2350000
-29767.0000000	1.2300000
-29766.0000000	1.2300000
-29765.0000000	1.2250000
-29764.0000000	1.2266667
-29763.0000000	1.2200000
-29762.0000000	1.2200000
-29761.0000000	1.2150000
-29760.0000000	1.2100000
-29759.0000000	1.2050000
-29758.0000000	1.2050000
-29757.0000000	1.2000000
-29756.0000000	1.2000000
-29755.0000000	1.1950001
-29754.0000000	1.1950001
-29753.0000000	1.1950001
-29752.0000000	1.1900001
-29751.0000000	1.1849999
-29750.0000000	1.1849999
-29749.0000000	1.1799999
-29748.0000000	1.1799999
-29747.0000000	1.1799999
-29746.0000000	1.1700000
-29745.0000000	1.1700000
-29744.0000000	1.1650000
-29743.0000000	1.1650000
-29742.0000000	1.1650000
-29741.0000000	1.1600000
-29740.0000000	1.1600000
-29739.0000000	1.1500000
-29738.0000000	1.1550000
-29737.0000000	1.1550000
-29736.0000000	1.1500000
-29735.0000000	1.1500000
-29734.0000000	1.1433333
-29733.0000000	1.1500000
-29732.0000000	1.1500000
-29731.0000000	1.1450000
-29730.0000000	1.1450000
-29729.0000000	1.1450000
-29728.0000000	1.1450000
-29727.0000000	1.1450000
-29726.0000000	1.1400000
-29725.0000000	1.1400000
-29724.0000000	1.1350000
-29723.0000000	1.1350000
-29722.0000000	1.1300000

-29721.0000000	1.1300000
-29720.0000000	1.1250000
-29719.0000000	1.1250000
-29718.0000000	1.1250000
-29717.0000000	1.1200000
-29716.0000000	1.1200000
-29715.0000000	1.1100000
-29714.0000000	1.1100000
-29713.0000000	1.1050000
-29712.0000000	1.1050000
-29711.0000000	1.1000000
-29710.0000000	1.0950000
-29709.0000000	1.1000000
-29708.0000000	1.0950000
-29707.0000000	1.0950000
-29706.0000000	1.0950000
-29705.0000000	1.0950000
-29704.0000000	1.0900000
-29703.0000000	1.0850000
-29702.0000000	1.0850000
-29701.0000000	1.0850000
-29700.0000000	1.0800000
-29699.0000000	1.0800000
-29698.0000000	1.0750000
-29697.0000000	1.0750000
-29696.0000000	1.0750000
-29695.0000000	1.0699999
-29694.0000000	1.0700001
-29693.0000000	1.0599999
-29692.0000000	1.0599999
-29691.0000000	1.0599999
-29690.0000000	1.0549999
-29689.0000000	1.0549999
-29688.0000000	1.0450000
-29687.0000000	1.0450000
-29686.0000000	1.0350000
-29685.0000000	1.0350000
-29684.0000000	1.0300000
-29683.0000000	1.0250000
-29682.0000000	1.0200000
-29681.0000000	1.0200000
-29680.0000000	1.0150000
-29679.0000000	1.0150000
-29678.0000000	1.0100000
-29677.0000000	1.0050000
-29676.0000000	1.0000000
-29675.0000000	1.0000000
-29674.0000000	0.9966667
-29673.0000000	0.9950000
-29672.0000000	0.9950000
-29671.0000000	0.9900000
-29670.0000000	0.9850000
-29669.0000000	0.9850000
-29668.0000000	0.9850000
-29667.0000000	0.9850000
-29666.0000000	0.9850000
-29665.0000000	0.9800000

-29664.0000000	0.9800000
-29663.0000000	0.9750000
-29662.0000000	0.9750000
-29661.0000000	0.9700000
-29660.0000000	0.9650000
-29659.0000000	0.9650000
-29658.0000000	0.9600000
-29657.0000000	0.9650000
-29656.0000000	0.9550000
-29655.0000000	0.9550000
-29654.0000000	0.9550000
-29653.0000000	0.9500000
-29652.0000000	0.9450000
-29651.0000000	0.9450000
-29650.0000000	0.9400000
-29649.0000000	0.9350000
-29648.0000000	0.9350000
-29647.0000000	0.9300000
-29646.0000000	0.9300000
-29645.0000000	0.9300000
-29644.0000000	0.9350000
-29643.0000000	0.9300000
-29642.0000000	0.9266667
-29641.0000000	0.9250000
-29640.0000000	0.9250000
-29639.0000000	0.9200000
-29638.0000000	0.9200000
-29637.0000000	0.9200000
-29636.0000000	0.9150000
-29635.0000000	0.9200000
-29634.0000000	0.9150000
-29633.0000000	0.9150000
-29632.0000000	0.9100000
-29631.0000000	0.9100000
-29630.0000000	0.9100000
-29629.0000000	0.9050000
-29628.0000000	0.9050000
-29627.0000000	0.8950000
-29626.0000000	0.8900000
-29625.0000000	0.8950000
-29624.0000000	0.8850000
-29623.0000000	0.8800000
-29622.0000000	0.8750000
-29621.0000000	0.8750000
-29620.0000000	0.8750000
-29619.0000000	0.8700000
-29618.0000000	0.8700000
-29617.0000000	0.8650000
-29616.0000000	0.8650000
-29615.0000000	0.8600000
-29614.0000000	0.8600000
-29613.0000000	0.8600000
-29612.0000000	0.8533333
-29611.0000000	0.8550000
-29610.0000000	0.8500000
-29609.0000000	0.8500000
-29608.0000000	0.8500000

-29607.0000000	0.8450000
-29606.0000000	0.8450000
-29605.0000000	0.8400000
-29604.0000000	0.8350000
-29603.0000000	0.8350000
-29602.0000000	0.8300000
-29601.0000000	0.8300000
-29600.0000000	0.8300000
-29599.0000000	0.8250000
-29598.0000000	0.8250000
-29597.0000000	0.8200000
-29596.0000000	0.8150000
-29595.0000000	0.8150000
-29594.0000000	0.8050000
-29593.0000000	0.8050000
-29592.0000000	0.8000000
-29591.0000000	0.8050000
-29590.0000000	0.8000000
-29589.0000000	0.7950000
-29588.0000000	0.7900000
-29587.0000000	0.7900000
-29586.0000000	0.7850000
-29585.0000000	0.7800000
-29584.0000000	0.7800000
-29583.0000000	0.7800000
-29582.0000000	0.7766666
-29581.0000000	0.7750000
-29580.0000000	0.7800000
-29579.0000000	0.7700000
-29578.0000000	0.7700000
-29577.0000000	0.7750000
-29576.0000000	0.7700000
-29575.0000000	0.7750000
-29574.0000000	0.7650000
-29573.0000000	0.7650000
-29572.0000000	0.7700000
-29571.0000000	0.7600000
-29570.0000000	0.7600000
-29569.0000000	0.7650000
-29568.0000000	0.7600000
-29567.0000000	0.7600000
-29566.0000000	0.7600000
-29565.0000000	0.7600000
-29564.0000000	0.7600000
-29563.0000000	0.7550000
-29562.0000000	0.7600000
-29561.0000000	0.7600000
-29560.0000000	0.7600000
-29559.0000000	0.7550000
-29558.0000000	0.7550000
-29557.0000000	0.7550000
-29556.0000000	0.7550000
-29555.0000000	0.7550000
-29554.0000000	0.7550000
-29553.0000000	0.7550000
-29552.0000000	0.7500000
-29551.0000000	0.7500000

-29550.0000000	0.7450000
-29549.0000000	0.7450000
-29548.0000000	0.7450000
-29547.0000000	0.7500000
-29546.0000000	0.7450000
-29545.0000000	0.7450000
-29544.0000000	0.7450000
-29543.0000000	0.7450000
-29542.0000000	0.7450000
-29541.0000000	0.7450000
-29540.0000000	0.7400000
-29539.0000000	0.7450000
-29538.0000000	0.7400000
-29537.0000000	0.7400000
-29536.0000000	0.7450000
-29535.0000000	0.7400000
-29534.0000000	0.7450000
-29533.0000000	0.7400000
-29532.0000000	0.7400000
-29531.0000000	0.7450000
-29530.0000000	0.7450000
-29529.0000000	0.7450000
-29528.0000000	0.7450000
-29527.0000000	0.7450000
-29526.0000000	0.7450000
-29525.0000000	0.7450000
-29524.0000000	0.7400000
-29523.0000000	0.7433333
-29522.0000000	0.7450000
-29521.0000000	0.7450000
-29520.0000000	0.7450000
-29519.0000000	0.7450000
-29518.0000000	0.7500000
-29517.0000000	0.7450000
-29516.0000000	0.7500000
-29515.0000000	0.7450000
-29514.0000000	0.7500000
-29513.0000000	0.7550000
-29512.0000000	0.7550000
-29511.0000000	0.7600000
-29510.0000000	0.7600000
-29509.0000000	0.7600000
-29508.0000000	0.7600000
-29507.0000000	0.7650000
-29506.0000000	0.7650000
-29505.0000000	0.7700000
-29504.0000000	0.7700000
-29503.0000000	0.7700000
-29502.0000000	0.7700000
-29501.0000000	0.7700000
-29500.0000000	0.7750000
-29499.0000000	0.7750000
-29498.0000000	0.7750000
-29497.0000000	0.7850000
-29496.0000000	0.7800000
-29495.0000000	0.7900000
-29494.0000000	0.7800000

-29493.0000000	0.7850000
-29492.0000000	0.7850000
-29491.0000000	0.7833333
-29490.0000000	0.7900000
-29489.0000000	0.7850000
-29488.0000000	0.7900000
-29487.0000000	0.7900000
-29486.0000000	0.7900000
-29485.0000000	0.7900000
-29484.0000000	0.7950000
-29483.0000000	0.7950000
-29482.0000000	0.7950000
-29481.0000000	0.7900000
-29480.0000000	0.7900000
-29479.0000000	0.7900000
-29478.0000000	0.7900000
-29477.0000000	0.7900000
-29476.0000000	0.7950000
-29475.0000000	0.7900000
-29474.0000000	0.7850000
-29473.0000000	0.7850000
-29472.0000000	0.7850000
-29471.0000000	0.7800000
-29470.0000000	0.7850000
-29469.0000000	0.7800000
-29468.0000000	0.7800000
-29467.0000000	0.7750000
-29466.0000000	0.7800000
-29465.0000000	0.7800000
-29464.0000000	0.7800000
-29463.0000000	0.7800000
-29462.0000000	0.7800000
-29461.0000000	0.7800000
-29460.0000000	0.7866666
-29459.0000000	0.7850000
-29458.0000000	0.7850000
-29457.0000000	0.7800000
-29456.0000000	0.7850000
-29455.0000000	0.7850000
-29454.0000000	0.7850000
-29453.0000000	0.7900000
-29452.0000000	0.7900000
-29451.0000000	0.7950000
-29450.0000000	0.7900000
-29449.0000000	0.8000000
-29448.0000000	0.7950000
-29447.0000000	0.7900000
-29446.0000000	0.8000000
-29445.0000000	0.7950000
-29444.0000000	0.8000000
-29443.0000000	0.7950000
-29442.0000000	0.7950000
-29441.0000000	0.7950000
-29440.0000000	0.8000000
-29439.0000000	0.8000000
-29438.0000000	0.8000000
-29437.0000000	0.8050000

-29436.0000000	0.8050000
-29435.0000000	0.8050000
-29434.0000000	0.8100000
-29433.0000000	0.8100000
-29432.0000000	0.8100000
-29431.0000000	0.8150000
-29430.0000000	0.8133333
-29429.0000000	0.8150000
-29428.0000000	0.8100000
-29427.0000000	0.8150000
-29426.0000000	0.8150000
-29425.0000000	0.8150000
-29424.0000000	0.8200000
-29423.0000000	0.8200000
-29422.0000000	0.8250000
-29421.0000000	0.8200000
-29420.0000000	0.8250000
-29419.0000000	0.8250000
-29418.0000000	0.8250000
-29417.0000000	0.8250000
-29416.0000000	0.8300000
-29415.0000000	0.8300000
-29414.0000000	0.8350000
-29413.0000000	0.8350000
-29412.0000000	0.8400000
-29411.0000000	0.8400000
-29410.0000000	0.8450000
-29409.0000000	0.8450000
-29408.0000000	0.8500000
-29407.0000000	0.8500000
-29406.0000000	0.8500000
-29405.0000000	0.8550000
-29404.0000000	0.8550000
-29403.0000000	0.8550000
-29402.0000000	0.8550000
-29401.0000000	0.8600000
-29400.0000000	0.8633334
-29399.0000000	0.8650000
-29398.0000000	0.8650000
-29397.0000000	0.8700000
-29396.0000000	0.8700000
-29395.0000000	0.8700000
-29394.0000000	0.8700000
-29393.0000000	0.8700000
-29392.0000000	0.8700000
-29391.0000000	0.8750000
-29390.0000000	0.8800000
-29389.0000000	0.8800000
-29388.0000000	0.8850000
-29387.0000000	0.8850000
-29386.0000000	0.8800000
-29385.0000000	0.8850000
-29384.0000000	0.8850000
-29383.0000000	0.8850000
-29382.0000000	0.8900000
-29381.0000000	0.8900000
-29380.0000000	0.8950000

-29379.0000000	0.8950000
-29378.0000000	0.8950000
-29377.0000000	0.8900000
-29376.0000000	0.9000000
-29375.0000000	0.9000000
-29374.0000000	0.9050000
-29373.0000000	0.9050000
-29372.0000000	0.9050000
-29371.0000000	0.9000000
-29370.0000000	0.9050000
-29369.0000000	0.9100000
-29368.0000000	0.9100000
-29367.0000000	0.9150000
-29366.0000000	0.9150000
-29365.0000000	0.9150000
-29364.0000000	0.9150000
-29363.0000000	0.9100000
-29362.0000000	0.9200000
-29361.0000000	0.9200000
-29360.0000000	0.9150000
-29359.0000000	0.9200000
-29358.0000000	0.9200000
-29357.0000000	0.9200000
-29356.0000000	0.9250000
-29355.0000000	0.9200000
-29354.0000000	0.9200000
-29353.0000000	0.9250000
-29352.0000000	0.9200000
-29351.0000000	0.9250000
-29350.0000000	0.9250000
-29349.0000000	0.9200000
-29348.0000000	0.9250000
-29347.0000000	0.9250000
-29346.0000000	0.9250000
-29345.0000000	0.9300000
-29344.0000000	0.9250000
-29343.0000000	0.9250000
-29342.0000000	0.9233333
-29341.0000000	0.9300000
-29340.0000000	0.9300000
-29339.0000000	0.9300000
-29338.0000000	0.9300000
-29337.0000000	0.9300000
-29336.0000000	0.9350000
-29335.0000000	0.9350000
-29334.0000000	0.9350000
-29333.0000000	0.9350000
-29332.0000000	0.9350000
-29331.0000000	0.9400000
-29330.0000000	0.9400000
-29329.0000000	0.9400000
-29328.0000000	0.9450000
-29327.0000000	0.9450000
-29326.0000000	0.9500000
-29325.0000000	0.9450000
-29324.0000000	0.9500000
-29323.0000000	0.9450000

-29322.0000000	0.9500000
-29321.0000000	0.9500000
-29320.0000000	0.9500000
-29319.0000000	0.9500000
-29318.0000000	0.9500000
-29317.0000000	0.9450000
-29316.0000000	0.9450000
-29315.0000000	0.9450000
-29314.0000000	0.9450000
-29313.0000000	0.9450000
-29312.0000000	0.9466667
-29311.0000000	0.9450000
-29310.0000000	0.9450000
-29309.0000000	0.9400000
-29308.0000000	0.9400000
-29307.0000000	0.9400000
-29306.0000000	0.9400000
-29305.0000000	0.9400000
-29304.0000000	0.9350000
-29303.0000000	0.9350000
-29302.0000000	0.9400000
-29301.0000000	0.9350000
-29300.0000000	0.9400000
-29299.0000000	0.9400000
-29298.0000000	0.9400000
-29297.0000000	0.9400000
-29296.0000000	0.9400000
-29295.0000000	0.9400000
-29294.0000000	0.9350000
-29293.0000000	0.9350000
-29292.0000000	0.9350000
-29291.0000000	0.9350000
-29290.0000000	0.9350000
-29289.0000000	0.9400000
-29288.0000000	0.9400000
-29287.0000000	0.9400000
-29286.0000000	0.9400000
-29285.0000000	0.9400000
-29284.0000000	0.9350000
-29283.0000000	0.9350000
-29282.0000000	0.9350000
-29281.0000000	0.9400000
-29280.0000000	0.9333333
-29279.0000000	0.9400000
-29278.0000000	0.9400000
-29277.0000000	0.9400000
-29276.0000000	0.9400000
-29275.0000000	0.9400000
-29274.0000000	0.9350000
-29273.0000000	0.9350000
-29272.0000000	0.9350000
-29271.0000000	0.9400000
-29270.0000000	0.9400000
-29269.0000000	0.9400000
-29268.0000000	0.9400000
-29267.0000000	0.9400000
-29266.0000000	0.9450000

-29265.0000000	0.9450000
-29264.0000000	0.9450000
-29263.0000000	0.9450000
-29262.0000000	0.9450000
-29261.0000000	0.9500000
-29260.0000000	0.9500000
-29259.0000000	0.9500000
-29258.0000000	0.9500000
-29257.0000000	0.9500000
-29256.0000000	0.9550000
-29255.0000000	0.9500000
-29254.0000000	0.9600000
-29253.0000000	0.9600000
-29252.0000000	0.9600000
-29251.0000000	0.9650000
-29250.0000000	0.9650000
-29249.0000000	0.9600000
-29248.0000000	0.9633334
-29247.0000000	0.9700000
-29246.0000000	0.9650000
-29245.0000000	0.9650000
-29244.0000000	0.9650000
-29243.0000000	0.9650000
-29242.0000000	0.9650000
-29241.0000000	0.9700000
-29240.0000000	0.9700000
-29239.0000000	0.9650000
-29238.0000000	0.9750000
-29237.0000000	0.9700000
-29236.0000000	0.9650000
-29235.0000000	0.9650000
-29234.0000000	0.9700000
-29233.0000000	0.9700000
-29232.0000000	0.9650000
-29231.0000000	0.9700000
-29230.0000000	0.9650000
-29229.0000000	0.9700000
-29228.0000000	0.9650000
-29227.0000000	0.9650000
-29226.0000000	0.9650000
-29225.0000000	0.9650000
-29224.0000000	0.9600000
-29223.0000000	0.9650000
-29222.0000000	0.9600000
-29221.0000000	0.9650000
-29220.0000000	0.9566666
-29219.0000000	0.9650000
-29218.0000000	0.9600000
-29217.0000000	0.9600000
-29216.0000000	0.9600000
-29215.0000000	0.9650000
-29214.0000000	0.9550000
-29213.0000000	0.9550000
-29212.0000000	0.9550000
-29211.0000000	0.9550000
-29210.0000000	0.9550000
-29209.0000000	0.9500000

-29208.0000000	0.9600000
-29207.0000000	0.9550000
-29206.0000000	0.9550000
-29205.0000000	0.9550000
-29204.0000000	0.9550000
-29203.0000000	0.9550000
-29202.0000000	0.9550000
-29201.0000000	0.9600000
-29200.0000000	0.9600000
-29199.0000000	0.9600000
-29198.0000000	0.9600000
-29197.0000000	0.9650000
-29196.0000000	0.9650000
-29195.0000000	0.9650000
-29194.0000000	0.9600000
-29193.0000000	0.9650000
-29192.0000000	0.9650000
-29191.0000000	0.9700000
-29190.0000000	0.9766667
-29189.0000000	0.9750000
-29188.0000000	0.9800000
-29187.0000000	0.9800000
-29186.0000000	0.9800000
-29185.0000000	0.9800000
-29184.0000000	0.9850000
-29183.0000000	0.9850000
-29182.0000000	0.9900000
-29181.0000000	0.9900000
-29180.0000000	0.9900000
-29179.0000000	1.0000000
-29178.0000000	1.0000000
-29177.0000000	1.0000000
-29176.0000000	1.0000000
-29175.0000000	1.0050000
-29174.0000000	1.0050000
-29173.0000000	1.0050000
-29172.0000000	1.0150000
-29171.0000000	1.0150000
-29170.0000000	1.0150000
-29169.0000000	1.0150000
-29168.0000000	1.0200000
-29167.0000000	1.0200000
-29166.0000000	1.0250000
-29165.0000000	1.0200000
-29164.0000000	1.0200000
-29163.0000000	1.0200000
-29162.0000000	1.0300000
-29161.0000000	1.0200000
-29160.0000000	1.0233333
-29159.0000000	1.0250000
-29158.0000000	1.0250000
-29157.0000000	1.0300000
-29156.0000000	1.0200000
-29155.0000000	1.0200000
-29154.0000000	1.0250000
-29153.0000000	1.0250000
-29152.0000000	1.0200000

-29151.0000000	1.0200000
-29150.0000000	1.0250000
-29149.0000000	1.0200000
-29148.0000000	1.0200000
-29147.0000000	1.0200000
-29146.0000000	1.0200000
-29145.0000000	1.0250000
-29144.0000000	1.0200000
-29143.0000000	1.0200000
-29142.0000000	1.0200000
-29141.0000000	1.0200000
-29140.0000000	1.0200000
-29139.0000000	1.0200000
-29138.0000000	1.0150000
-29137.0000000	1.0150000
-29136.0000000	1.0150000
-29135.0000000	1.0150000
-29134.0000000	1.0150000
-29133.0000000	1.0150000
-29132.0000000	1.0200000
-29131.0000000	1.0150000
-29130.0000000	1.0200000
-29129.0000000	1.0150000
-29128.0000000	1.0200000
-29127.0000000	1.0150000
-29126.0000000	1.0150000
-29125.0000000	1.0200000
-29124.0000000	1.0150000
-29123.0000000	1.0150000
-29122.0000000	1.0200000
-29121.0000000	1.0200000
-29120.0000000	1.0200000
-29119.0000000	1.0200000
-29118.0000000	1.0200000
-29117.0000000	1.0150000
-29116.0000000	1.0150000
-29115.0000000	1.0150000
-29114.0000000	1.0150000
-29113.0000000	1.0150000
-29112.0000000	1.0150000
-29111.0000000	1.0150000
-29110.0000000	1.0100000
-29109.0000000	1.0100000
-29108.0000000	1.0100000
-29107.0000000	1.0150000
-29106.0000000	1.0100000
-29105.0000000	1.0100000
-29104.0000000	1.0100000
-29103.0000000	1.0100000
-29102.0000000	1.0100000
-29101.0000000	1.0050000
-29100.0000000	1.0050000
-29099.0000000	1.0050000
-29098.0000000	1.0033333
-29097.0000000	1.0000000
-29096.0000000	1.0000000
-29095.0000000	0.9950000

-29094.0000000	0.9950000
-29093.0000000	0.9950000
-29092.0000000	0.9900000
-29091.0000000	0.9850000
-29090.0000000	0.9800000
-29089.0000000	0.9800000
-29088.0000000	0.9750000
-29087.0000000	0.9750000
-29086.0000000	0.9750000
-29085.0000000	0.9750000
-29084.0000000	0.9700000
-29083.0000000	0.9650000
-29082.0000000	0.9650000
-29081.0000000	0.9600000
-29080.0000000	0.9600000
-29079.0000000	0.9550000
-29078.0000000	0.9500000
-29077.0000000	0.9450000
-29076.0000000	0.9450000
-29075.0000000	0.9350000
-29074.0000000	0.9300000
-29073.0000000	0.9250000
-29072.0000000	0.9250000
-29071.0000000	0.9150000
-29070.0000000	0.9050000
-29069.0000000	0.9000000
-29068.0000000	0.8900000
-29067.0000000	0.8833334
-29066.0000000	0.8700000
-29065.0000000	0.8650000
-29064.0000000	0.8550000
-29063.0000000	0.8450000
-29062.0000000	0.8450000
-29061.0000000	0.8300000
-29060.0000000	0.8250000
-29059.0000000	0.8150000
-29058.0000000	0.8050000
-29057.0000000	0.8000000
-29056.0000000	0.7950000
-29055.0000000	0.7900000
-29054.0000000	0.7850000
-29053.0000000	0.7750000
-29052.0000000	0.7700000
-29051.0000000	0.7700000
-29050.0000000	0.7700000
-29049.0000000	0.7650000
-29048.0000000	0.7600000
-29047.0000000	0.7550000
-29046.0000000	0.7550000
-29045.0000000	0.7500000
-29044.0000000	0.7500000
-29043.0000000	0.7400000
-29042.0000000	0.7500000
-29041.0000000	0.7450000
-29040.0000000	0.7366667
-29039.0000000	0.7350000
-29038.0000000	0.7350000

-29037.0000000	0.7300000
-29036.0000000	0.7350000
-29035.0000000	0.7250000
-29034.0000000	0.7200000
-29033.0000000	0.7200000
-29032.0000000	0.7200000
-29031.0000000	0.7150000
-29030.0000000	0.7100000
-29029.0000000	0.7100000
-29028.0000000	0.7100000
-29027.0000000	0.7050000
-29026.0000000	0.7100000
-29025.0000000	0.7100000
-29024.0000000	0.7050000
-29023.0000000	0.7050000
-29022.0000000	0.7050000
-29021.0000000	0.7100000
-29020.0000000	0.7100000
-29019.0000000	0.7100000
-29018.0000000	0.7100000
-29017.0000000	0.7150000
-29016.0000000	0.7150000
-29015.0000000	0.7100000
-29014.0000000	0.7100000
-29013.0000000	0.7100000
-29012.0000000	0.7100000
-29011.0000000	0.7150000
-29010.0000000	0.7100000
-29009.0000000	0.7150000
-29008.0000000	0.7150000
-29007.0000000	0.7150000
-29006.0000000	0.7100000
-29005.0000000	0.7100000
-29004.0000000	0.7100000
-29003.0000000	0.7100000
-29002.0000000	0.7100000
-29001.0000000	0.7050000
-29000.0000000	0.7050000
-28999.0000000	0.7050000
-28998.0000000	0.7000000
-28997.0000000	0.7000000
-28996.0000000	0.6900000
-28995.0000000	0.6900000
-28994.0000000	0.6850000
-28993.0000000	0.6850000
-28992.0000000	0.6850000
-28991.0000000	0.6800000
-28990.0000000	0.6750000
-28989.0000000	0.6800000
-28988.0000000	0.6750000
-28987.0000000	0.6750000
-28986.0000000	0.6750000
-28985.0000000	0.6750000
-28984.0000000	0.6700000
-28983.0000000	0.6700000
-28982.0000000	0.6750000
-28981.0000000	0.6700000

-28980.0000000	0.6750000
-28979.0000000	0.6733333
-28978.0000000	0.6750000
-28977.0000000	0.6750000
-28976.0000000	0.6750000
-28975.0000000	0.6750000
-28974.0000000	0.6800000
-28973.0000000	0.6800000
-28972.0000000	0.6750000
-28971.0000000	0.6750000
-28970.0000000	0.6750000
-28969.0000000	0.6750000
-28968.0000000	0.6700000
-28967.0000000	0.6700000
-28966.0000000	0.6750000
-28965.0000000	0.6750000
-28964.0000000	0.6750000
-28963.0000000	0.6750000
-28962.0000000	0.6750000
-28961.0000000	0.6800000
-28960.0000000	0.6800000
-28959.0000000	0.6900000
-28958.0000000	0.6950000
-28957.0000000	0.7000000
-28956.0000000	0.7050000
-28955.0000000	0.7050000
-28954.0000000	0.7150000
-28953.0000000	0.7150000
-28952.0000000	0.7200000
-28951.0000000	0.7200000
-28950.0000000	0.7300000
-28949.0000000	0.7366667
-28948.0000000	0.7450000
-28947.0000000	0.7550000
-28946.0000000	0.7600000
-28945.0000000	0.7650000
-28944.0000000	0.7800000
-28943.0000000	0.7800000
-28942.0000000	0.7900000
-28941.0000000	0.7950000
-28940.0000000	0.8100000
-28939.0000000	0.8150000
-28938.0000000	0.8200000
-28937.0000000	0.8300000
-28936.0000000	0.8400000
-28935.0000000	0.8500000
-28934.0000000	0.8550000
-28933.0000000	0.8650000
-28932.0000000	0.8700000
-28931.0000000	0.8800000
-28930.0000000	0.8900000
-28929.0000000	0.8900000
-28928.0000000	0.9000000
-28927.0000000	0.9050000
-28926.0000000	0.9150000
-28925.0000000	0.9200000
-28924.0000000	0.9250000

-28923.0000000	0.9300000
-28922.0000000	0.9350000
-28921.0000000	0.9450000
-28920.0000000	0.9500000
-28919.0000000	0.9550000
-28918.0000000	0.9650000
-28917.0000000	0.9633334
-28916.0000000	0.9700000
-28915.0000000	0.9700000
-28914.0000000	0.9800000
-28913.0000000	0.9850000
-28912.0000000	0.9900000
-28911.0000000	0.9900000
-28910.0000000	0.9950000
-28909.0000000	1.0050000
-28908.0000000	1.0050000
-28907.0000000	1.0050000
-28906.0000000	1.0100000
-28905.0000000	1.0200000
-28904.0000000	1.0250000
-28903.0000000	1.0250000
-28902.0000000	1.0350000
-28901.0000000	1.0300000
-28900.0000000	1.0350000
-28899.0000000	1.0400000
-28898.0000000	1.0400000
-28897.0000000	1.0350000
-28896.0000000	1.0400000
-28895.0000000	1.0450000
-28894.0000000	1.0450000
-28893.0000000	1.0500000
-28892.0000000	1.0500000
-28891.0000000	1.0500000
-28890.0000000	1.0549999
-28889.0000000	1.0599999
-28888.0000000	1.0666667
-28887.0000000	1.0650001
-28886.0000000	1.0750000
-28885.0000000	1.0700001
-28884.0000000	1.0750000
-28883.0000000	1.0800000
-28882.0000000	1.0800000
-28881.0000000	1.0850000
-28880.0000000	1.0850000
-28879.0000000	1.0900000
-28878.0000000	1.0900000
-28877.0000000	1.0950000
-28876.0000000	1.0950000
-28875.0000000	1.1000000
-28874.0000000	1.1050000
-28873.0000000	1.1050000
-28872.0000000	1.1100000
-28871.0000000	1.1050000
-28870.0000000	1.1100000
-28869.0000000	1.1150000
-28868.0000000	1.1200000
-28867.0000000	1.1150000

-28866.0000000	1.1200000
-28865.0000000	1.1250000
-28864.0000000	1.1300000
-28863.0000000	1.1300000
-28862.0000000	1.1300000
-28861.0000000	1.1300000
-28860.0000000	1.1400000
-28859.0000000	1.1400000
-28858.0000000	1.1333333
-28857.0000000	1.1450000
-28856.0000000	1.1500000
-28855.0000000	1.1500000
-28854.0000000	1.1500000
-28853.0000000	1.1550000
-28852.0000000	1.1550000
-28851.0000000	1.1650000
-28850.0000000	1.1650000
-28849.0000000	1.1650000
-28848.0000000	1.1750000
-28847.0000000	1.1750000
-28846.0000000	1.1799999
-28845.0000000	1.1799999
-28844.0000000	1.1799999
-28843.0000000	1.1799999
-28842.0000000	1.1849999
-28841.0000000	1.1849999
-28840.0000000	1.1950001
-28839.0000000	1.1900001
-28838.0000000	1.1900001
-28837.0000000	1.1900001
-28836.0000000	1.1900001
-28835.0000000	1.1950001
-28834.0000000	1.1950001
-28833.0000000	1.1950001
-28832.0000000	1.1950001
-28831.0000000	1.2000000
-28830.0000000	1.2000000
-28829.0000000	1.2000000
-28828.0000000	1.2033334
-28827.0000000	1.2050000
-28826.0000000	1.2100000
-28825.0000000	1.2050000
-28824.0000000	1.2100000
-28823.0000000	1.2150000
-28822.0000000	1.2150000
-28821.0000000	1.2150000
-28820.0000000	1.2150000
-28819.0000000	1.2200000
-28818.0000000	1.2250000
-28817.0000000	1.2250000
-28816.0000000	1.2350000
-28815.0000000	1.2350000
-28814.0000000	1.2350000
-28813.0000000	1.2350000
-28812.0000000	1.2400000
-28811.0000000	1.2450000
-28810.0000000	1.2450000

-28809.0000000	1.2500000
-28808.0000000	1.2500000
-28807.0000000	1.2500000
-28806.0000000	1.2550000
-28805.0000000	1.2600000
-28804.0000000	1.2650000
-28803.0000000	1.2650000
-28802.0000000	1.2650000
-28801.0000000	1.2650000
-28800.0000000	1.2750000
-28799.0000000	1.2750000
-28798.0000000	1.2766666
-28797.0000000	1.2900000
-28796.0000000	1.2866666
-28795.0000000	1.2900000
-28794.0000000	1.2900000
-28793.0000000	1.3000000
-28792.0000000	1.2950000
-28791.0000000	1.3000000
-28790.0000000	1.3049999
-28789.0000000	1.3049999
-28788.0000000	1.3049999
-28787.0000000	1.3099999
-28786.0000000	1.3099999
-28785.0000000	1.3200001
-28784.0000000	1.3150001
-28783.0000000	1.3200001
-28782.0000000	1.3200001
-28781.0000000	1.3200001
-28780.0000000	1.3250000
-28779.0000000	1.3200001
-28778.0000000	1.3250000
-28777.0000000	1.3300000
-28776.0000000	1.3300000
-28775.0000000	1.3300000
-28774.0000000	1.3350000
-28773.0000000	1.3350000
-28772.0000000	1.3350000
-28771.0000000	1.3300000
-28770.0000000	1.3350000
-28769.0000000	1.3400000
-28768.0000000	1.3450000
-28767.0000000	1.3450000
-28766.0000000	1.3466667
-28765.0000000	1.3500000
-28764.0000000	1.3450000
-28763.0000000	1.3500000
-28762.0000000	1.3450000
-28761.0000000	1.3550000
-28760.0000000	1.3500000
-28759.0000000	1.3550000
-28758.0000000	1.3550000
-28757.0000000	1.3550000
-28756.0000000	1.3550000
-28755.0000000	1.3600000
-28754.0000000	1.3600000
-28753.0000000	1.3650000

-28752.0000000	1.3600000
-28751.0000000	1.3650000
-28750.0000000	1.3650000
-28749.0000000	1.3700000
-28748.0000000	1.3700000
-28747.0000000	1.3700000
-28746.0000000	1.3750000
-28745.0000000	1.3700000
-28744.0000000	1.3750000
-28743.0000000	1.3750000
-28742.0000000	1.3800000
-28741.0000000	1.3800000
-28740.0000000	1.3800000
-28739.0000000	1.3850000
-28738.0000000	1.3850000
-28737.0000000	1.3850000
-28736.0000000	1.3833333
-28735.0000000	1.3850000
-28734.0000000	1.3850000
-28733.0000000	1.3850000
-28732.0000000	1.3850000
-28731.0000000	1.3800000
-28730.0000000	1.3850000
-28729.0000000	1.3850000
-28728.0000000	1.3900000
-28727.0000000	1.3900000
-28726.0000000	1.3900000
-28725.0000000	1.3900000
-28724.0000000	1.3900000
-28723.0000000	1.3950000
-28722.0000000	1.3950000
-28721.0000000	1.3950000
-28720.0000000	1.3950000
-28719.0000000	1.3950000
-28718.0000000	1.3900000
-28717.0000000	1.3900000
-28716.0000000	1.3900000
-28715.0000000	1.3950000
-28714.0000000	1.3950000
-28713.0000000	1.3950000
-28712.0000000	1.3950000
-28711.0000000	1.3950000
-28710.0000000	1.3950000
-28709.0000000	1.3950000
-28708.0000000	1.3900000
-28707.0000000	1.3933333
-28706.0000000	1.3950000
-28705.0000000	1.3950000
-28704.0000000	1.3900000
-28703.0000000	1.4000000
-28702.0000000	1.4000000
-28701.0000000	1.3950000
-28700.0000000	1.3950000
-28699.0000000	1.4000000
-28698.0000000	1.3950000
-28697.0000000	1.3950000
-28696.0000000	1.3900000

-28695.0000000	1.3900000
-28694.0000000	1.3950000
-28693.0000000	1.3900000
-28692.0000000	1.3850000
-28691.0000000	1.3850000
-28690.0000000	1.3800000
-28689.0000000	1.3850000
-28688.0000000	1.3800000
-28687.0000000	1.3750000
-28686.0000000	1.3800000
-28685.0000000	1.3750000
-28684.0000000	1.3750000
-28683.0000000	1.3750000
-28682.0000000	1.3700000
-28681.0000000	1.3700000
-28680.0000000	1.3750000
-28679.0000000	1.3750000
-28678.0000000	1.3750000
-28677.0000000	1.3700000
-28676.0000000	1.3650000
-28675.0000000	1.3650000
-28674.0000000	1.3600000
-28673.0000000	1.3600000
-28672.0000000	1.3600000
-28671.0000000	1.3600000
-28670.0000000	1.3650000
-28669.0000000	1.3600000
-28668.0000000	1.3600000
-28667.0000000	1.3600000
-28666.0000000	1.3600000
-28665.0000000	1.3550000
-28664.0000000	1.3500000
-28663.0000000	1.3500000
-28662.0000000	1.3550000
-28661.0000000	1.3600000
-28660.0000000	1.3600000
-28659.0000000	1.3600000
-28658.0000000	1.3550000
-28657.0000000	1.3550000
-28656.0000000	1.3550000
-28655.0000000	1.3500000
-28654.0000000	1.3500000
-28653.0000000	1.3550000
-28652.0000000	1.3350000
-28651.0000000	1.3450000
-28650.0000000	1.3550000
-28649.0000000	1.3550000
-28648.0000000	1.3450000
-28647.0000000	1.3466667
-28646.0000000	1.3450000
-28645.0000000	1.3400000
-28644.0000000	1.3400000
-28643.0000000	1.3450000
-28642.0000000	1.3400000
-28641.0000000	1.3400000
-28640.0000000	1.3450000
-28639.0000000	1.3450000

-28638.0000000	1.3450000
-28637.0000000	1.3450000
-28636.0000000	1.3400000
-28635.0000000	1.3400000
-28634.0000000	1.3400000
-28633.0000000	1.3400000
-28632.0000000	1.3350000
-28631.0000000	1.3350000
-28630.0000000	1.3350000
-28629.0000000	1.3400000
-28628.0000000	1.3350000
-28627.0000000	1.3350000
-28626.0000000	1.3400000
-28625.0000000	1.3350000
-28624.0000000	1.3350000
-28623.0000000	1.3350000
-28622.0000000	1.3350000
-28621.0000000	1.3300000
-28620.0000000	1.3300000
-28619.0000000	1.3300000
-28618.0000000	1.3300000
-28617.0000000	1.3300000
-28616.0000000	1.3300000
-28615.0000000	1.3300000
-28614.0000000	1.3300000
-28613.0000000	1.3300000
-28612.0000000	1.3300000
-28611.0000000	1.3300000
-28610.0000000	1.3300000
-28609.0000000	1.3350000
-28608.0000000	1.3350000
-28607.0000000	1.3350000
-28606.0000000	1.3350000
-28605.0000000	1.3300000
-28604.0000000	1.3300000
-28603.0000000	1.3300000
-28602.0000000	1.3300000
-28601.0000000	1.3250000
-28600.0000000	1.3250000
-28599.0000000	1.3250000
-28598.0000000	1.3250000
-28597.0000000	1.3250000
-28596.0000000	1.3300000
-28595.0000000	1.3300000
-28594.0000000	1.3300000
-28593.0000000	1.3250000
-28592.0000000	1.3300000
-28591.0000000	1.3250000
-28590.0000000	1.3250000
-28589.0000000	1.3300000
-28588.0000000	1.3300000
-28587.0000000	1.3300000
-28586.0000000	1.3300000
-28585.0000000	1.3250000
-28584.0000000	1.3350000
-28583.0000000	1.3350000
-28582.0000000	1.3350000

-28581.0000000	1.3300000
-28580.0000000	1.3250000
-28579.0000000	1.3300000
-28578.0000000	1.3350000
-28577.0000000	1.3350000
-28576.0000000	1.3350000
-28575.0000000	1.3400000
-28574.0000000	1.3350000
-28573.0000000	1.3400000
-28572.0000000	1.3450000
-28571.0000000	1.3450000
-28570.0000000	1.3450000
-28569.0000000	1.3500000
-28568.0000000	1.3550000
-28567.0000000	1.3500000
-28566.0000000	1.3550000
-28565.0000000	1.3600000
-28564.0000000	1.3600000
-28563.0000000	1.3550000
-28562.0000000	1.3650000
-28561.0000000	1.3750000
-28560.0000000	1.3750000
-28559.0000000	1.3700000
-28558.0000000	1.3700000
-28557.0000000	1.3750000
-28556.0000000	1.3800000
-28555.0000000	1.3800000
-28554.0000000	1.3800000
-28553.0000000	1.3850000
-28552.0000000	1.3850000
-28551.0000000	1.3800000
-28550.0000000	1.3900000
-28549.0000000	1.3950000
-28548.0000000	1.3900000
-28547.0000000	1.3950000
-28546.0000000	1.3850000
-28545.0000000	1.3900000
-28544.0000000	1.3900000
-28543.0000000	1.3900000
-28542.0000000	1.3900000
-28541.0000000	1.3950000
-28540.0000000	1.3900000
-28539.0000000	1.3950000
-28538.0000000	1.3950000
-28537.0000000	1.3950000
-28536.0000000	1.3950000
-28535.0000000	1.3900000
-28534.0000000	1.3900000
-28533.0000000	1.3950000
-28532.0000000	1.3950000
-28531.0000000	1.3900000
-28530.0000000	1.3900000
-28529.0000000	1.3900000
-28528.0000000	1.3950000
-28527.0000000	1.4000000
-28526.0000000	1.4000000
-28525.0000000	1.4000000

-28524.0000000	1.3966666
-28523.0000000	1.3850000
-28522.0000000	1.3950000
-28521.0000000	1.3900000
-28520.0000000	1.3900000
-28519.0000000	1.3900000
-28518.0000000	1.3850000
-28517.0000000	1.3900000
-28516.0000000	1.3850000
-28515.0000000	1.3850000
-28514.0000000	1.3850000
-28513.0000000	1.3800000
-28512.0000000	1.3850000
-28511.0000000	1.3800000
-28510.0000000	1.3850000
-28509.0000000	1.3800000
-28508.0000000	1.3750000
-28507.0000000	1.3700000
-28506.0000000	1.3750000
-28505.0000000	1.3800000
-28504.0000000	1.3750000
-28503.0000000	1.3750000
-28502.0000000	1.3750000
-28501.0000000	1.3800000
-28500.0000000	1.3850000
-28499.0000000	1.3800000
-28498.0000000	1.3800000
-28497.0000000	1.3800000
-28496.0000000	1.3800000
-28495.0000000	1.3750000
-28494.0000000	1.3766667
-28493.0000000	1.3800000
-28492.0000000	1.3750000
-28491.0000000	1.3850000
-28490.0000000	1.3800000
-28489.0000000	1.3750000
-28488.0000000	1.3750000
-28487.0000000	1.3750000
-28486.0000000	1.3750000
-28485.0000000	1.3750000
-28484.0000000	1.3750000
-28483.0000000	1.3750000
-28482.0000000	1.3700000
-28481.0000000	1.3700000
-28480.0000000	1.3700000
-28479.0000000	1.3700000
-28478.0000000	1.3700000
-28477.0000000	1.3650000
-28476.0000000	1.3650000
-28475.0000000	1.3700000
-28474.0000000	1.3700000
-28473.0000000	1.3750000
-28472.0000000	1.3750000
-28471.0000000	1.3750000
-28470.0000000	1.3750000
-28469.0000000	1.3750000
-28468.0000000	1.3700000

-28467.0000000	1.3750000
-28466.0000000	1.3700000
-28465.0000000	1.3750000
-28464.0000000	1.3766667
-28463.0000000	1.3750000
-28462.0000000	1.3750000
-28461.0000000	1.3750000
-28460.0000000	1.3750000
-28459.0000000	1.3800000
-28458.0000000	1.3750000
-28457.0000000	1.3750000
-28456.0000000	1.3700000
-28455.0000000	1.3700000
-28454.0000000	1.3700000
-28453.0000000	1.3750000
-28452.0000000	1.3750000
-28451.0000000	1.3800000
-28450.0000000	1.3750000
-28449.0000000	1.3750000
-28448.0000000	1.3750000
-28447.0000000	1.3750000
-28446.0000000	1.3750000
-28445.0000000	1.3750000
-28444.0000000	1.3800000
-28443.0000000	1.3800000
-28442.0000000	1.3800000
-28441.0000000	1.3800000
-28440.0000000	1.3850000
-28439.0000000	1.3850000
-28438.0000000	1.3850000
-28437.0000000	1.3850000
-28436.0000000	1.3900000
-28435.0000000	1.3950000
-28434.0000000	1.3933333
-28433.0000000	1.3950000
-28432.0000000	1.3950000
-28431.0000000	1.3950000
-28430.0000000	1.3950000
-28429.0000000	1.3900000
-28428.0000000	1.4000000
-28427.0000000	1.3950000
-28426.0000000	1.3900000
-28425.0000000	1.3950000
-28424.0000000	1.4000000
-28423.0000000	1.4050000
-28422.0000000	1.4050000
-28421.0000000	1.4050000
-28420.0000000	1.4100000
-28419.0000000	1.4100000
-28418.0000000	1.4050000
-28417.0000000	1.4150000
-28416.0000000	1.4050000
-28415.0000000	1.4100000
-28414.0000000	1.4100000
-28413.0000000	1.4200000
-28412.0000000	1.4200000
-28411.0000000	1.4200000

-28410.0000000	1.4200000
-28409.0000000	1.4150000
-28408.0000000	1.4200000
-28407.0000000	1.4150000
-28406.0000000	1.4100000
-28405.0000000	1.4200000
-28404.0000000	1.4200000
-28403.0000000	1.4266666
-28402.0000000	1.4250000
-28401.0000000	1.4250000
-28400.0000000	1.4200000
-28399.0000000	1.4200000
-28398.0000000	1.4250000
-28397.0000000	1.4299999
-28396.0000000	1.4299999
-28395.0000000	1.4299999
-28394.0000000	1.4349999
-28393.0000000	1.4299999
-28392.0000000	1.4299999
-28391.0000000	1.4299999
-28390.0000000	1.4299999
-28389.0000000	1.4400001
-28388.0000000	1.4450001
-28387.0000000	1.4450001
-28386.0000000	1.4450001
-28385.0000000	1.4450001
-28384.0000000	1.4450001
-28383.0000000	1.4450001
-28382.0000000	1.4450001
-28381.0000000	1.4450001
-28380.0000000	1.4550000
-28379.0000000	1.4550000
-28378.0000000	1.4550000
-28377.0000000	1.4550000
-28376.0000000	1.4600000
-28375.0000000	1.4650000
-28374.0000000	1.4600000
-28373.0000000	1.4633335
-28372.0000000	1.4650000
-28371.0000000	1.4650000
-28370.0000000	1.4750000
-28369.0000000	1.4750000
-28368.0000000	1.4750000
-28367.0000000	1.4800000
-28366.0000000	1.4800000
-28365.0000000	1.4800000
-28364.0000000	1.4850000
-28363.0000000	1.4800000
-28362.0000000	1.4900000
-28361.0000000	1.4900000
-28360.0000000	1.5000000
-28359.0000000	1.5000000
-28358.0000000	1.4950000
-28357.0000000	1.4950000
-28356.0000000	1.5000000
-28355.0000000	1.5000000
-28354.0000000	1.5050000

-28353.0000000	1.5150000
-28352.0000000	1.5050000
-28351.0000000	1.5150000
-28350.0000000	1.5150000
-28349.0000000	1.5250000
-28348.0000000	1.5250000
-28347.0000000	1.5250000
-28346.0000000	1.5350000
-28345.0000000	1.5350000
-28344.0000000	1.5350000
-28343.0000000	1.5400000
-28342.0000000	1.5500000
-28341.0000000	1.5599999
-28340.0000000	1.5549999
-28339.0000000	1.5599999
-28338.0000000	1.5650001
-28337.0000000	1.5650001
-28336.0000000	1.5750000
-28335.0000000	1.5750000
-28334.0000000	1.5800000
-28333.0000000	1.5850000
-28332.0000000	1.5850000
-28331.0000000	1.5900000
-28330.0000000	1.5950000
-28329.0000000	1.6000000
-28328.0000000	1.6050000
-28327.0000000	1.6000000
-28326.0000000	1.6050000
-28325.0000000	1.6100000
-28324.0000000	1.6100000
-28323.0000000	1.6150000
-28322.0000000	1.6200000
-28321.0000000	1.6250000
-28320.0000000	1.6250000
-28319.0000000	1.6300000
-28318.0000000	1.6300000
-28317.0000000	1.6300000
-28316.0000000	1.6300000
-28315.0000000	1.6250000
-28314.0000000	1.6300000
-28313.0000000	1.6433333
-28312.0000000	1.6500000
-28311.0000000	1.6500000
-28310.0000000	1.6500000
-28309.0000000	1.6550000
-28308.0000000	1.6600000
-28307.0000000	1.6650000
-28306.0000000	1.6650000
-28305.0000000	1.6700000
-28304.0000000	1.6650000
-28303.0000000	1.6700000
-28302.0000000	1.6600000
-28301.0000000	1.6600000
-28300.0000000	1.6650000
-28299.0000000	1.6650000
-28298.0000000	1.6650000
-28297.0000000	1.6750000

-28296.0000000	1.6750000
-28295.0000000	1.6750000
-28294.0000000	1.6750000
-28293.0000000	1.6849999
-28292.0000000	1.6900001
-28291.0000000	1.6900001
-28290.0000000	1.6950001
-28289.0000000	1.7000000
-28288.0000000	1.7050000
-28287.0000000	1.7150000
-28286.0000000	1.7200000
-28285.0000000	1.7250000
-28284.0000000	1.7200000
-28283.0000000	1.7199999
-28282.0000000	1.7150000
-28281.0000000	1.7200000
-28280.0000000	1.7250000
-28279.0000000	1.7250000
-28278.0000000	1.7200000
-28277.0000000	1.7150000
-28276.0000000	1.7200000
-28275.0000000	1.7150000
-28274.0000000	1.7150000
-28273.0000000	1.7100000
-28272.0000000	1.7150000
-28271.0000000	1.7200000
-28270.0000000	1.7350000
-28269.0000000	1.7500000
-28268.0000000	1.7350000
-28267.0000000	1.7500000
-28266.0000000	1.7450000
-28265.0000000	1.7500000
-28264.0000000	1.7500000
-28263.0000000	1.7550000
-28262.0000000	1.7550000
-28261.0000000	1.7500000
-28260.0000000	1.7550000
-28259.0000000	1.7650000
-28258.0000000	1.7550000
-28257.0000000	1.7500000
-28256.0000000	1.7600000
-28255.0000000	1.7600000
-28254.0000000	1.7600000
-28253.0000000	1.7533334
-28252.0000000	1.7550000
-28251.0000000	1.7550000
-28250.0000000	1.7600000
-28249.0000000	1.7600000
-28248.0000000	1.7650000
-28247.0000000	1.7600000
-28246.0000000	1.7650000
-28245.0000000	1.7600000
-28244.0000000	1.7600000
-28243.0000000	1.7700000
-28242.0000000	1.7650000
-28241.0000000	1.7650000
-28240.0000000	1.7750000

-28239.0000000	1.7750000
-28238.0000000	1.7700000
-28237.0000000	1.7700000
-28236.0000000	1.7750000
-28235.0000000	1.7750000
-28234.0000000	1.7700000
-28233.0000000	1.7750000
-28232.0000000	1.7800000
-28231.0000000	1.7750000
-28230.0000000	1.7750000
-28229.0000000	1.7750000
-28228.0000000	1.7850000
-28227.0000000	1.7900000
-28226.0000000	1.7800000
-28225.0000000	1.7800000
-28224.0000000	1.7900000
-28223.0000000	1.7850000
-28222.0000000	1.7933334
-28221.0000000	1.8000000
-28220.0000000	1.8150001
-28219.0000000	1.8200001
-28218.0000000	1.8200001
-28217.0000000	1.8200001
-28216.0000000	1.8250000
-28215.0000000	1.8200001
-28214.0000000	1.8200001
-28213.0000000	1.8250000
-28212.0000000	1.8199999
-28211.0000000	1.8200001
-28210.0000000	1.8200001
-28209.0000000	1.8099999
-28208.0000000	1.8000000
-28207.0000000	1.8000000
-28206.0000000	1.8049999
-28205.0000000	1.7950000
-28204.0000000	1.7950000
-28203.0000000	1.7900000
-28202.0000000	1.7900000
-28201.0000000	1.7850000
-28200.0000000	1.7850000
-28199.0000000	1.7850000
-28198.0000000	1.7800000
-28197.0000000	1.7850000
-28196.0000000	1.7900000
-28195.0000000	1.7750000
-28194.0000000	1.7700000
-28193.0000000	1.7750000
-28192.0000000	1.7766666
-28191.0000000	1.7750000
-28190.0000000	1.7750000
-28189.0000000	1.7750000
-28188.0000000	1.7700000
-28187.0000000	1.7750000
-28186.0000000	1.7800000
-28185.0000000	1.7800000
-28184.0000000	1.7800000
-28183.0000000	1.7850000

-28182.0000000	1.7750000
-28181.0000000	1.7850000
-28180.0000000	1.7800000
-28179.0000000	1.7850000
-28178.0000000	1.8099999
-28177.0000000	1.8000000
-28176.0000000	1.7900000
-28175.0000000	1.7850000
-28174.0000000	1.7850000
-28173.0000000	1.7850000
-28172.0000000	1.7900000
-28171.0000000	1.8049999
-28170.0000000	1.8049999
-28169.0000000	1.8049999
-28168.0000000	1.8150001
-28167.0000000	1.8049999
-28166.0000000	1.8099999
-28165.0000000	1.8099999
-28164.0000000	1.8099999
-28163.0000000	1.8049999
-28162.0000000	1.8099999
-28161.0000000	1.8099999
-28160.0000000	1.8200001
-28159.0000000	1.8150001
-28158.0000000	1.8150001
-28157.0000000	1.8250000
-28156.0000000	1.8300000
-28155.0000000	1.8300000
-28154.0000000	1.8400000
-28153.0000000	1.8350000
-28152.0000000	1.8350000
-28151.0000000	1.8400000
-28150.0000000	1.8450000
-28149.0000000	1.8500000
-28148.0000000	1.8550000
-28147.0000000	1.8650000
-28146.0000000	1.8650000
-28145.0000000	1.8750000
-28144.0000000	1.8750000
-28143.0000000	1.8750000
-28142.0000000	1.8900000
-28141.0000000	1.8900000
-28140.0000000	1.9000000
-28139.0000000	1.9000000
-28138.0000000	1.9050000
-28137.0000000	1.9100000
-28136.0000000	1.9150000
-28135.0000000	1.9200000
-28134.0000000	1.9200000
-28133.0000000	1.9200000
-28132.0000000	1.9250000
-28131.0000000	1.9200000
-28130.0000000	1.9200000
-28129.0000000	1.9200000
-28128.0000000	1.9400001
-28127.0000000	1.9400001
-28126.0000000	1.9400001

-28125.0000000	1.9400001
-28124.0000000	1.9450001
-28123.0000000	1.9450001
-28122.0000000	1.9450001
-28121.0000000	1.9500000
-28120.0000000	1.9550000
-28119.0000000	1.9550000
-28118.0000000	1.9600000
-28117.0000000	1.9500000
-28116.0000000	1.9500000
-28115.0000000	1.9550000
-28114.0000000	1.9550000
-28113.0000000	1.9650000
-28112.0000000	1.9550000
-28111.0000000	1.9500000
-28110.0000000	1.9550000
-28109.0000000	1.9600000
-28108.0000000	1.9550000
-28107.0000000	1.9550000
-28106.0000000	1.9550000
-28105.0000000	1.9650000
-28104.0000000	1.9650000
-28103.0000000	1.9600000
-28102.0000000	1.9650000
-28101.0000000	1.9600000
-28100.0000000	1.9600000
-28099.0000000	1.9600000
-28098.0000000	1.9600000
-28097.0000000	1.9650000
-28096.0000000	1.9600000
-28095.0000000	1.9600000
-28094.0000000	1.9650000
-28093.0000000	1.9600000
-28092.0000000	1.9600000
-28091.0000000	1.9500000
-28090.0000000	1.9550000
-28089.0000000	1.9550000
-28088.0000000	1.9550000
-28087.0000000	1.9650000
-28086.0000000	1.9600000
-28085.0000000	1.9550000
-28084.0000000	1.9550000
-28083.0000000	1.9600000
-28082.0000000	1.9600000
-28081.0000000	1.9650000
-28080.0000000	1.9650000
-28079.0000000	1.9700000
-28078.0000000	1.9650000
-28077.0000000	1.9700000
-28076.0000000	1.9650000
-28075.0000000	1.9700000
-28074.0000000	1.9650000
-28073.0000000	1.9600000
-28072.0000000	1.9650000
-28071.0000000	1.9633335
-28070.0000000	1.9600000
-28069.0000000	1.9550000

-28068.0000000	1.9550000
-28067.0000000	1.9550000
-28066.0000000	1.9450001
-28065.0000000	1.9400001
-28064.0000000	1.9450001
-28063.0000000	1.9400001
-28062.0000000	1.9400001
-28061.0000000	1.9250000
-28060.0000000	1.9250000
-28059.0000000	1.9250000
-28058.0000000	1.9200000
-28057.0000000	1.9250000
-28056.0000000	1.9150000
-28055.0000000	1.9150000
-28054.0000000	1.9100000
-28053.0000000	1.9100000
-28052.0000000	1.9100000
-28051.0000000	1.9050000
-28050.0000000	1.9100000
-28049.0000000	1.9100000
-28048.0000000	1.9050000
-28047.0000000	1.9050000
-28046.0000000	1.9100000
-28045.0000000	1.9000000
-28044.0000000	1.9050000
-28043.0000000	1.9000000
-28042.0000000	1.8950000
-28041.0000000	1.9033333
-28040.0000000	1.9000000
-28039.0000000	1.8900000
-28038.0000000	1.8800000
-28037.0000000	1.8850000
-28036.0000000	1.8850000
-28035.0000000	1.8850000
-28034.0000000	1.8800000
-28033.0000000	1.8850000
-28032.0000000	1.8800000
-28031.0000000	1.8850000
-28030.0000000	1.8800000
-28029.0000000	1.8800000
-28028.0000000	1.8800000
-28027.0000000	1.8750000
-28026.0000000	1.8750000
-28025.0000000	1.8750000
-28024.0000000	1.8750000
-28023.0000000	1.8800000
-28022.0000000	1.8750000
-28021.0000000	1.8750000
-28020.0000000	1.8750000
-28019.0000000	1.8750000
-28018.0000000	1.8750000
-28017.0000000	1.8750000
-28016.0000000	1.8700000
-28015.0000000	1.8650000
-28014.0000000	1.8700000
-28013.0000000	1.8650000
-28012.0000000	1.8650000

-28011.0000000	1.8666667
-28010.0000000	1.8600000
-28009.0000000	1.8650000
-28008.0000000	1.8600000
-28007.0000000	1.8650000
-28006.0000000	1.8650000
-28005.0000000	1.8600000
-28004.0000000	1.8650000
-28003.0000000	1.8650000
-28002.0000000	1.8700000
-28001.0000000	1.8750000
-28000.0000000	1.8700000
-27999.0000000	1.8800000
-27998.0000000	1.8800000
-27997.0000000	1.8800000
-27996.0000000	1.8850000
-27995.0000000	1.8750000
-27994.0000000	1.8850000
-27993.0000000	1.8800000
-27992.0000000	1.8850000
-27991.0000000	1.8850000
-27990.0000000	1.8900000
-27989.0000000	1.8850000
-27988.0000000	1.8850000
-27987.0000000	1.8850000
-27986.0000000	1.8850000
-27985.0000000	1.8950000
-27984.0000000	1.8900000
-27983.0000000	1.8950000
-27982.0000000	1.8950000
-27981.0000000	1.8933333
-27980.0000000	1.8800000
-27979.0000000	1.8800000
-27978.0000000	1.8800000
-27977.0000000	1.8800000
-27976.0000000	1.8850000
-27975.0000000	1.8800000
-27974.0000000	1.8750000
-27973.0000000	1.8800000
-27972.0000000	1.8750000
-27971.0000000	1.8750000
-27970.0000000	1.8800000
-27969.0000000	1.8750000
-27968.0000000	1.8700000
-27967.0000000	1.8650000
-27966.0000000	1.8550000
-27965.0000000	1.8550000
-27964.0000000	1.8650000
-27963.0000000	1.8550000
-27962.0000000	1.8550000
-27961.0000000	1.8500000
-27960.0000000	1.8650000
-27959.0000000	1.8650000
-27958.0000000	1.8600000
-27957.0000000	1.8600000
-27956.0000000	1.8550000
-27955.0000000	1.8650000

-27954.0000000	1.8650000
-27953.0000000	1.8550000
-27952.0000000	1.8550000
-27951.0000000	1.8550000
-27950.0000000	1.8500000
-27949.0000000	1.8650000
-27948.0000000	1.8650000
-27947.0000000	1.8650000
-27946.0000000	1.8650000
-27945.0000000	1.8700000
-27944.0000000	1.8650000
-27943.0000000	1.8700000
-27942.0000000	1.8700000
-27941.0000000	1.8750000
-27940.0000000	1.8750000
-27939.0000000	1.8800000
-27938.0000000	1.8800000
-27937.0000000	1.8800000
-27936.0000000	1.8850000
-27935.0000000	1.8800000
-27934.0000000	1.8850000
-27933.0000000	1.8850000
-27932.0000000	1.8800000
-27931.0000000	1.8850000
-27930.0000000	1.8850000
-27929.0000000	1.8850000
-27928.0000000	1.8850000
-27927.0000000	1.8850000
-27926.0000000	1.8850000
-27925.0000000	1.8850000
-27924.0000000	1.8850000
-27923.0000000	1.8850000
-27922.0000000	1.8800000
-27921.0000000	1.8800000
-27920.0000000	1.8866667
-27919.0000000	1.8850000
-27918.0000000	1.8800000
-27917.0000000	1.8800000
-27916.0000000	1.8850000
-27915.0000000	1.8850000
-27914.0000000	1.8850000
-27913.0000000	1.8850000
-27912.0000000	1.8850000
-27911.0000000	1.8850000
-27910.0000000	1.8900000
-27909.0000000	1.8950000
-27908.0000000	1.8900000
-27907.0000000	1.8950000
-27906.0000000	1.9000000
-27905.0000000	1.9000000
-27904.0000000	1.9050000
-27903.0000000	1.9050000
-27902.0000000	1.9050000
-27901.0000000	1.9050000
-27900.0000000	1.9000000
-27899.0000000	1.9000000
-27898.0000000	1.9050000

-27897.0000000	1.9050000
-27896.0000000	1.9050000
-27895.0000000	1.9100000
-27894.0000000	1.9050000
-27893.0000000	1.9100000
-27892.0000000	1.9150000
-27891.0000000	1.9100000
-27890.0000000	1.9150000
-27889.0000000	1.9166666
-27888.0000000	1.9200000
-27887.0000000	1.9250000
-27886.0000000	1.9250000
-27885.0000000	1.9250000
-27884.0000000	1.9250000
-27883.0000000	1.9250000
-27882.0000000	1.9250000
-27881.0000000	1.9300001
-27880.0000000	1.9200000
-27879.0000000	1.9200000
-27878.0000000	1.9450001
-27877.0000000	1.9400001
-27876.0000000	1.9500000
-27875.0000000	1.9450001
-27874.0000000	1.9550000
-27873.0000000	1.9550000
-27872.0000000	1.9500000
-27871.0000000	1.9600000
-27870.0000000	1.9650000
-27869.0000000	1.9650000
-27868.0000000	1.9700000
-27867.0000000	1.9650000
-27866.0000000	1.9750000
-27865.0000000	1.9750000
-27864.0000000	1.9750000
-27863.0000000	1.9800000
-27862.0000000	1.9850000
-27861.0000000	1.9850000
-27860.0000000	1.9950000
-27859.0000000	2.0000000
-27858.0000000	2.0050001
-27857.0000000	2.0050001
-27856.0000000	2.0050001
-27855.0000000	2.0149999
-27854.0000000	2.0149999
-27853.0000000	2.0200000
-27852.0000000	2.0300000
-27851.0000000	2.0250001
-27850.0000000	2.0349998
-27849.0000000	2.0349998
-27848.0000000	2.0349998
-27847.0000000	2.0400000
-27846.0000000	2.0450001
-27845.0000000	2.0450001
-27844.0000000	2.0349998
-27843.0000000	2.0450001
-27842.0000000	2.0500000
-27841.0000000	2.0500000

-27840.0000000	2.0549998
-27839.0000000	2.0549998
-27838.0000000	2.0599999
-27837.0000000	2.0549998
-27836.0000000	2.0599999
-27835.0000000	2.0599999
-27834.0000000	2.0599999
-27833.0000000	2.0549998
-27832.0000000	2.0549998
-27831.0000000	2.0666666
-27830.0000000	2.0650001
-27829.0000000	2.0650001
-27828.0000000	2.0599999
-27827.0000000	2.0650001
-27826.0000000	2.0650001
-27825.0000000	2.0599999
-27824.0000000	2.0749998
-27823.0000000	2.0650001
-27822.0000000	2.0699999
-27821.0000000	2.0749998
-27820.0000000	2.0749998
-27819.0000000	2.0749998
-27818.0000000	2.0749998
-27817.0000000	2.0650001
-27816.0000000	2.0699999
-27815.0000000	2.0699999
-27814.0000000	2.0799999
-27813.0000000	2.0749998
-27812.0000000	2.0699999
-27811.0000000	2.0749998
-27810.0000000	2.0799999
-27809.0000000	2.0899999
-27808.0000000	2.0850000
-27807.0000000	2.0850000
-27806.0000000	2.0899999
-27805.0000000	2.0899999
-27804.0000000	2.0999999
-27803.0000000	2.0949998
-27802.0000000	2.0949998
-27801.0000000	2.0966666
-27800.0000000	2.0999999
-27799.0000000	2.0999999
-27798.0000000	2.0999999
-27797.0000000	2.0999999
-27796.0000000	2.1099999
-27795.0000000	2.1149998
-27794.0000000	2.1149998
-27793.0000000	2.1050000
-27792.0000000	2.1099999
-27791.0000000	2.1149998
-27790.0000000	2.1250000
-27789.0000000	2.1250000
-27788.0000000	2.1250000
-27787.0000000	2.1250000
-27786.0000000	2.1149998
-27785.0000000	2.1300001
-27784.0000000	2.1400001

-27783.0000000	2.1450000
-27782.0000000	2.1450000
-27781.0000000	2.1400001
-27780.0000000	2.1450000
-27779.0000000	2.1500001
-27778.0000000	2.1550002
-27777.0000000	2.1600001
-27776.0000000	2.1650000
-27775.0000000	2.1700001
-27774.0000000	2.1650000
-27773.0000000	2.1750002
-27772.0000000	2.1800001
-27771.0000000	2.1800001
-27770.0000000	2.1900001
-27769.0000000	2.1950002
-27768.0000000	2.2000000
-27767.0000000	2.2049999
-27766.0000000	2.2049999
-27765.0000000	2.2100000
-27764.0000000	2.2150002
-27763.0000000	2.2150002
-27762.0000000	2.2249999
-27761.0000000	2.2350001
-27760.0000000	2.2350001
-27759.0000000	2.2400000
-27758.0000000	2.2500000
-27757.0000000	2.2600000
-27756.0000000	2.2550001
-27755.0000000	2.2400000
-27754.0000000	2.2550001
-27753.0000000	2.2500000
-27752.0000000	2.2600000
-27751.0000000	2.2700000
-27750.0000000	2.2600000
-27749.0000000	2.2700000
-27748.0000000	2.2800000
-27747.0000000	2.2800000
-27746.0000000	2.2800000
-27745.0000000	2.2900000
-27744.0000000	2.2900000
-27743.0000000	2.2849998
-27742.0000000	2.2849998
-27741.0000000	2.2849998
-27740.0000000	2.2700000
-27739.0000000	2.2750001
-27738.0000000	2.2733333
-27737.0000000	2.2800000
-27736.0000000	2.2800000
-27735.0000000	2.2900000
-27734.0000000	2.2900000
-27733.0000000	2.3000000
-27732.0000000	2.2950001
-27731.0000000	2.2950001
-27730.0000000	2.2950001
-27729.0000000	2.3049998
-27728.0000000	2.2950001
-27727.0000000	2.2900000

-27726.0000000	2.2900000
-27725.0000000	2.2849998
-27724.0000000	2.2900000
-27723.0000000	2.2950001
-27722.0000000	2.2950001
-27721.0000000	2.3000000
-27720.0000000	2.2950001
-27719.0000000	2.2950001
-27718.0000000	2.2950001
-27717.0000000	2.2950001
-27716.0000000	2.2950001
-27715.0000000	2.3000000
-27714.0000000	2.3049998
-27713.0000000	2.3049998
-27712.0000000	2.3150001
-27711.0000000	2.3249998
-27710.0000000	2.3299999
-27709.0000000	2.3350000
-27708.0000000	2.3399999
-27707.0000000	2.3449998
-27706.0000000	2.3499999
-27705.0000000	2.3550000
-27704.0000000	2.3550000
-27703.0000000	2.3649998
-27702.0000000	2.3550000
-27701.0000000	2.3649998
-27700.0000000	2.3599999
-27699.0000000	2.3599999
-27698.0000000	2.3649998
-27697.0000000	2.3599999
-27696.0000000	2.3599999
-27695.0000000	2.3599999
-27694.0000000	2.3649998
-27693.0000000	2.3599999
-27692.0000000	2.3649998
-27691.0000000	2.3599999
-27690.0000000	2.3699999
-27689.0000000	2.3599999
-27688.0000000	2.3550000
-27687.0000000	2.3550000
-27686.0000000	2.3449998
-27685.0000000	2.3449998
-27684.0000000	2.3399999
-27683.0000000	2.3550000
-27682.0000000	2.3499999
-27681.0000000	2.3499999
-27680.0000000	2.3466666
-27679.0000000	2.3449998
-27678.0000000	2.3449998
-27677.0000000	2.3399999
-27676.0000000	2.3399999
-27675.0000000	2.3499999
-27674.0000000	2.3550000
-27673.0000000	2.3599999
-27672.0000000	2.3649998
-27671.0000000	2.3649998
-27670.0000000	2.3699999

-27669.0000000	2.3750000
-27668.0000000	2.3699999
-27667.0000000	2.3599999
-27666.0000000	2.3599999
-27665.0000000	2.3649998
-27664.0000000	2.3699999
-27663.0000000	2.3699999
-27662.0000000	2.3750000
-27661.0000000	2.3750000
-27660.0000000	2.3699999
-27659.0000000	2.3750000
-27658.0000000	2.3750000
-27657.0000000	2.3850002
-27656.0000000	2.3850002
-27655.0000000	2.3850002
-27654.0000000	2.3800001
-27653.0000000	2.3699999
-27652.0000000	2.3699999
-27651.0000000	2.3800001
-27650.0000000	2.3850002
-27649.0000000	2.3833334
-27648.0000000	2.3750000
-27647.0000000	2.3800001
-27646.0000000	2.3750000
-27645.0000000	2.3800001
-27644.0000000	2.3699999
-27643.0000000	2.3750000
-27642.0000000	2.3750000
-27641.0000000	2.3750000
-27640.0000000	2.3850002
-27639.0000000	2.3750000
-27638.0000000	2.3750000
-27637.0000000	2.3750000
-27636.0000000	2.3750000
-27635.0000000	2.3649998
-27634.0000000	2.3850002
-27633.0000000	2.3900001
-27632.0000000	2.3800001
-27631.0000000	2.3800001
-27630.0000000	2.3699999
-27629.0000000	2.3800001
-27628.0000000	2.3649998
-27627.0000000	2.3800001
-27626.0000000	2.3800001
-27625.0000000	2.3800001
-27624.0000000	2.3750000
-27623.0000000	2.3699999
-27622.0000000	2.3750000
-27621.0000000	2.3800001
-27620.0000000	2.3666666
-27619.0000000	2.3649998
-27618.0000000	2.3599999
-27617.0000000	2.3699999
-27616.0000000	2.3750000
-27615.0000000	2.3750000
-27614.0000000	2.3699999
-27613.0000000	2.3850002

-27612.0000000	2.3900001
-27611.0000000	2.3900001
-27610.0000000	2.3900001
-27609.0000000	2.3900001
-27608.0000000	2.3950000
-27607.0000000	2.3900001
-27606.0000000	2.3950000
-27605.0000000	2.3900001
-27604.0000000	2.3950000
-27603.0000000	2.3900001
-27602.0000000	2.3950000
-27601.0000000	2.4000001
-27600.0000000	2.4050002
-27599.0000000	2.4050002
-27598.0000000	2.4050002
-27597.0000000	2.4050002
-27596.0000000	2.4150000
-27595.0000000	2.4150000
-27594.0000000	2.4150000
-27593.0000000	2.4200001
-27592.0000000	2.4150000
-27591.0000000	2.4150000
-27590.0000000	2.4250002
-27589.0000000	2.4233334
-27588.0000000	2.4349999
-27587.0000000	2.4250002
-27586.0000000	2.4300001
-27585.0000000	2.4349999
-27584.0000000	2.4400001
-27583.0000000	2.4400001
-27582.0000000	2.4400001
-27581.0000000	2.4450002
-27580.0000000	2.4500000
-27579.0000000	2.4600000
-27578.0000000	2.4600000
-27577.0000000	2.4650002
-27576.0000000	2.4700000
-27575.0000000	2.4700000
-27574.0000000	2.4700000
-27573.0000000	2.4749999
-27572.0000000	2.4850001
-27571.0000000	2.4900000
-27570.0000000	2.4900000
-27569.0000000	2.4850001
-27568.0000000	2.4900000
-27567.0000000	2.4850001
-27566.0000000	2.4949999
-27565.0000000	2.5000000
-27564.0000000	2.5050001
-27563.0000000	2.5050001
-27562.0000000	2.5050001
-27561.0000000	2.5050001
-27560.0000000	2.5000000
-27559.0000000	2.5000000
-27558.0000000	2.5000000
-27557.0000000	2.5000000
-27556.0000000	2.4949999

-27555.0000000	2.5000000
-27554.0000000	2.5000000
-27553.0000000	2.5100000
-27552.0000000	2.5050001
-27551.0000000	2.5200000
-27550.0000000	2.5250001
-27549.0000000	2.5200000
-27548.0000000	2.5300000
-27547.0000000	2.5300000
-27546.0000000	2.5300000
-27545.0000000	2.5349998
-27544.0000000	2.5349998
-27543.0000000	2.5400000
-27542.0000000	2.5500000
-27541.0000000	2.5450001
-27540.0000000	2.5450001
-27539.0000000	2.5450001
-27538.0000000	2.5500000
-27537.0000000	2.5400000
-27536.0000000	2.5400000
-27535.0000000	2.5400000
-27534.0000000	2.5400000
-27533.0000000	2.5349998
-27532.0000000	2.5400000
-27531.0000000	2.5400000
-27530.0000000	2.5450001
-27529.0000000	2.5450001
-27528.0000000	2.5400000
-27527.0000000	2.5400000
-27526.0000000	2.5349998
-27525.0000000	2.5450001
-27524.0000000	2.5500000
-27523.0000000	2.5549998
-27522.0000000	2.5500000
-27521.0000000	2.5599999
-27520.0000000	2.5599999
-27519.0000000	2.5549998
-27518.0000000	2.5549998
-27517.0000000	2.5500000
-27516.0000000	2.5500000
-27515.0000000	2.5599999
-27514.0000000	2.5500000
-27513.0000000	2.5500000
-27512.0000000	2.5599999
-27511.0000000	2.5500000
-27510.0000000	2.5549998
-27509.0000000	2.5599999
-27508.0000000	2.5599999
-27507.0000000	2.5500000
-27506.0000000	2.5599999
-27505.0000000	2.5549998
-27504.0000000	2.5549998
-27503.0000000	2.5549998
-27502.0000000	2.5749998
-27501.0000000	2.5699999
-27500.0000000	2.5699999
-27499.0000000	2.5699999

-27498.0000000	2.5749998
-27497.0000000	2.5699999
-27496.0000000	2.5850000
-27495.0000000	2.5799999
-27494.0000000	2.5899999
-27493.0000000	2.5949998
-27492.0000000	2.5999999
-27491.0000000	2.5949998
-27490.0000000	2.5949998
-27489.0000000	2.6050000
-27488.0000000	2.6099999
-27487.0000000	2.6050000
-27486.0000000	2.6050000
-27485.0000000	2.6149998
-27484.0000000	2.6149998
-27483.0000000	2.6250000
-27482.0000000	2.6199999
-27481.0000000	2.6250000
-27480.0000000	2.6199999
-27479.0000000	2.6199999
-27478.0000000	2.6250000
-27477.0000000	2.6350002
-27476.0000000	2.6500001
-27475.0000000	2.6550002
-27474.0000000	2.6400001
-27473.0000000	2.6450000
-27472.0000000	2.6450000
-27471.0000000	2.6450000
-27470.0000000	2.6450000
-27469.0000000	2.6450000
-27468.0000000	2.6450000
-27467.0000000	2.6499999
-27466.0000000	2.6700001
-27465.0000000	2.6550002
-27464.0000000	2.6450000
-27463.0000000	2.6500001
-27462.0000000	2.6600001
-27461.0000000	2.6750002
-27460.0000000	2.6800001
-27459.0000000	2.6800001
-27458.0000000	2.6750002
-27457.0000000	2.6700001
-27456.0000000	2.6650000
-27455.0000000	2.6700001
-27454.0000000	2.6849999
-27453.0000000	2.6900001
-27452.0000000	2.6900001
-27451.0000000	2.6849999
-27450.0000000	2.6950002
-27449.0000000	2.6950002
-27448.0000000	2.7049999
-27447.0000000	2.6950002
-27446.0000000	2.7000000
-27445.0000000	2.6950002
-27444.0000000	2.6900001
-27443.0000000	2.6950002
-27442.0000000	2.7000000

-27441.0000000	2.7150002
-27440.0000000	2.7200000
-27439.0000000	2.7249999
-27438.0000000	2.7200000
-27437.0000000	2.7300003
-27436.0000000	2.7249999
-27435.0000000	2.7400000
-27434.0000000	2.7400000
-27433.0000000	2.7500000
-27432.0000000	2.7649999
-27431.0000000	2.7600000
-27430.0000000	2.7649999
-27429.0000000	2.7649999
-27428.0000000	2.7700000
-27427.0000000	2.7700000
-27426.0000000	2.7750001
-27425.0000000	2.7849998
-27424.0000000	2.7900000
-27423.0000000	2.7800000
-27422.0000000	2.7950001
-27421.0000000	2.8150001
-27420.0000000	2.8150001
-27419.0000000	2.8150001
-27418.0000000	2.8150001
-27417.0000000	2.8150001
-27416.0000000	2.8199999
-27415.0000000	2.8249998
-27414.0000000	2.8249998
-27413.0000000	2.8350000
-27412.0000000	2.8350000
-27411.0000000	2.8299999
-27410.0000000	2.8299999
-27409.0000000	2.8399999
-27408.0000000	2.8399999
-27407.0000000	2.8499999
-27406.0000000	2.8499999
-27405.0000000	2.8499999
-27404.0000000	2.8599999
-27403.0000000	2.8550000
-27402.0000000	2.8649998
-27401.0000000	2.8649998
-27400.0000000	2.8649998
-27399.0000000	2.8599999
-27398.0000000	2.8599999
-27397.0000000	2.8649998
-27396.0000000	2.8750000
-27395.0000000	2.8800001
-27394.0000000	2.8800001
-27393.0000000	2.8850002
-27392.0000000	2.8900001
-27391.0000000	2.8950000
-27390.0000000	2.9000001
-27389.0000000	2.8950000
-27388.0000000	2.9000001
-27387.0000000	2.9050002
-27386.0000000	2.9050002
-27385.0000000	2.9050002

-27384.0000000	2.9000001
-27383.0000000	2.9000001
-27382.0000000	2.9000001
-27381.0000000	2.8950000
-27380.0000000	2.8950000
-27379.0000000	2.9050002
-27378.0000000	2.9050002
-27377.0000000	2.9166667
-27376.0000000	2.9300001
-27375.0000000	2.9250002
-27374.0000000	2.9300001
-27373.0000000	2.9300001
-27372.0000000	2.9300001
-27371.0000000	2.9300001
-27370.0000000	2.9300001
-27369.0000000	2.9450002
-27368.0000000	2.9349999
-27367.0000000	2.9300001
-27366.0000000	2.9349999
-27365.0000000	2.9300001
-27364.0000000	2.9400001
-27363.0000000	2.9300001
-27362.0000000	2.9250002
-27361.0000000	2.9349999
-27360.0000000	2.9200001
-27359.0000000	2.9250002
-27358.0000000	2.9300001
-27357.0000000	2.9300001
-27356.0000000	2.9250002
-27355.0000000	2.9300001
-27354.0000000	2.9349999
-27353.0000000	2.9349999
-27352.0000000	2.9500000
-27351.0000000	2.9600000
-27350.0000000	2.9650002
-27349.0000000	2.9700000
-27348.0000000	2.9650002
-27347.0000000	2.9766667
-27346.0000000	2.9749999
-27345.0000000	2.9749999
-27344.0000000	2.9800000
-27343.0000000	2.9800000
-27342.0000000	2.9749999
-27341.0000000	2.9700000
-27340.0000000	2.9800000
-27339.0000000	2.9800000
-27338.0000000	2.9949999
-27337.0000000	2.9900000
-27336.0000000	2.9900000
-27335.0000000	2.9949999
-27334.0000000	2.9850001
-27333.0000000	2.9850001
-27332.0000000	2.9800000
-27331.0000000	2.9850001
-27330.0000000	2.9749999
-27329.0000000	2.9850001
-27328.0000000	2.9800000

-27327.0000000	2.9700000
-27326.0000000	2.9749999
-27325.0000000	2.9650002
-27324.0000000	2.9749999
-27323.0000000	2.9800000
-27322.0000000	2.9749999
-27321.0000000	2.9749999
-27320.0000000	2.9749999
-27319.0000000	2.9650002
-27318.0000000	2.9650002
-27317.0000000	2.9600000
-27316.0000000	2.9600000
-27315.0000000	2.9600000
-27314.0000000	2.9500000
-27313.0000000	2.9500000
-27312.0000000	2.9500000
-27311.0000000	2.9549999
-27310.0000000	2.9450002
-27309.0000000	2.9500000
-27308.0000000	2.9400001
-27307.0000000	2.9450002
-27306.0000000	2.9400001
-27305.0000000	2.9450002
-27304.0000000	2.9400001
-27303.0000000	2.9400001
-27302.0000000	2.9500000
-27301.0000000	2.9600000
-27300.0000000	2.9600000
-27299.0000000	2.9650002
-27298.0000000	2.9600000
-27297.0000000	2.9600000
-27296.0000000	2.9600000
-27295.0000000	2.9650002
-27294.0000000	2.9700000
-27293.0000000	2.9700000
-27292.0000000	2.9749999
-27291.0000000	2.9749999
-27290.0000000	2.9749999
-27289.0000000	2.9800000
-27288.0000000	2.9800000
-27287.0000000	2.9900000
-27286.0000000	2.9966667
-27285.0000000	2.9900000
-27284.0000000	2.9900000
-27283.0000000	2.9949999
-27282.0000000	2.9900000
-27281.0000000	2.9800000
-27280.0000000	2.9749999
-27279.0000000	2.9850001
-27278.0000000	2.9749999
-27277.0000000	2.9749999
-27276.0000000	2.9749999
-27275.0000000	2.9650002
-27274.0000000	2.9549999
-27273.0000000	2.9500000
-27272.0000000	2.9500000
-27271.0000000	2.9549999

-27270.0000000	2.9600000
-27269.0000000	2.9549999
-27268.0000000	2.9500000
-27267.0000000	2.9450002
-27266.0000000	2.9400001
-27265.0000000	2.9400001
-27264.0000000	2.9400001
-27263.0000000	2.9300001
-27262.0000000	2.9349999
-27261.0000000	2.9300001
-27260.0000000	2.9349999
-27259.0000000	2.9349999
-27258.0000000	2.9250002
-27257.0000000	2.9300001
-27256.0000000	2.9300001
-27255.0000000	2.9300001
-27254.0000000	2.9349999
-27253.0000000	2.9349999
-27252.0000000	2.9250002
-27251.0000000	2.9250002
-27250.0000000	2.9250002
-27249.0000000	2.9250002
-27248.0000000	2.9250002
-27247.0000000	2.9150000
-27246.0000000	2.9250002
-27245.0000000	2.9150000
-27244.0000000	2.9100001
-27243.0000000	2.9100001
-27242.0000000	2.9000001
-27241.0000000	2.9000001
-27240.0000000	2.9050002
-27239.0000000	2.9000001
-27238.0000000	2.9100001
-27237.0000000	2.9000001
-27236.0000000	2.9000001
-27235.0000000	2.9000001
-27234.0000000	2.8850002
-27233.0000000	2.8699999
-27232.0000000	2.8699999
-27231.0000000	2.8599999
-27230.0000000	2.8599999
-27229.0000000	2.8699999
-27228.0000000	2.8550000
-27227.0000000	2.8649998
-27226.0000000	2.8499999
-27225.0000000	2.8566666
-27224.0000000	2.8599999
-27223.0000000	2.8550000
-27222.0000000	2.8550000
-27221.0000000	2.8599999
-27220.0000000	2.8550000
-27219.0000000	2.8550000
-27218.0000000	2.8499999
-27217.0000000	2.8449998
-27216.0000000	2.8350000
-27215.0000000	2.8350000
-27214.0000000	2.8399999

-27213.0000000	2.8350000
-27212.0000000	2.8449998
-27211.0000000	2.8499999
-27210.0000000	2.8499999
-27209.0000000	2.8499999
-27208.0000000	2.8550000
-27207.0000000	2.8499999
-27206.0000000	2.8499999
-27205.0000000	2.8499999
-27204.0000000	2.8499999
-27203.0000000	2.8550000
-27202.0000000	2.8449998
-27201.0000000	2.8499999
-27200.0000000	2.8399999
-27199.0000000	2.8350000
-27198.0000000	2.8350000
-27197.0000000	2.8350000
-27196.0000000	2.8399999
-27195.0000000	2.8366668
-27194.0000000	2.8350000
-27193.0000000	2.8350000
-27192.0000000	2.8249998
-27191.0000000	2.8150001
-27190.0000000	2.8150001
-27189.0000000	2.8099999
-27188.0000000	2.8150001
-27187.0000000	2.8199999
-27186.0000000	2.8199999
-27185.0000000	2.8249998
-27184.0000000	2.8199999
-27183.0000000	2.8150001
-27182.0000000	2.8199999
-27181.0000000	2.8150001
-27180.0000000	2.8199999
-27179.0000000	2.8199999
-27178.0000000	2.8199999
-27177.0000000	2.8199999
-27176.0000000	2.8199999
-27175.0000000	2.8150001
-27174.0000000	2.8150001
-27173.0000000	2.8249998
-27172.0000000	2.8199999
-27171.0000000	2.8199999
-27170.0000000	2.8150001
-27169.0000000	2.8150001
-27168.0000000	2.8249998
-27167.0000000	2.8249998
-27166.0000000	2.8233330
-27165.0000000	2.8150001
-27164.0000000	2.8199999
-27163.0000000	2.8150001
-27162.0000000	2.8049998
-27161.0000000	2.8099999
-27160.0000000	2.8049998
-27159.0000000	2.7950001
-27158.0000000	2.7950001
-27157.0000000	2.8000000

-27156.0000000	2.8000000
-27155.0000000	2.7950001
-27154.0000000	2.7900000
-27153.0000000	2.7849998
-27152.0000000	2.7800000
-27151.0000000	2.7750001
-27150.0000000	2.7750001
-27149.0000000	2.7700000
-27148.0000000	2.7649999
-27147.0000000	2.7649999
-27146.0000000	2.7600000
-27145.0000000	2.7600000
-27144.0000000	2.7600000
-27143.0000000	2.7500000
-27142.0000000	2.7449999
-27141.0000000	2.7500000
-27140.0000000	2.7449999
-27139.0000000	2.7400000
-27138.0000000	2.7400000
-27137.0000000	2.7449999
-27136.0000000	2.7449999
-27135.0000000	2.7366667
-27134.0000000	2.7350001
-27133.0000000	2.7449999
-27132.0000000	2.7350001
-27131.0000000	2.7350001
-27130.0000000	2.7350001
-27129.0000000	2.7350001
-27128.0000000	2.7249999
-27127.0000000	2.7200000
-27126.0000000	2.7200000
-27125.0000000	2.7100000
-27124.0000000	2.7100000
-27123.0000000	2.7150002
-27122.0000000	2.7150002
-27121.0000000	2.7049999
-27120.0000000	2.7100000
-27119.0000000	2.7049999
-27118.0000000	2.7049999
-27117.0000000	2.7049999
-27116.0000000	2.7000000
-27115.0000000	2.7000000
-27114.0000000	2.6900001
-27113.0000000	2.6900001
-27112.0000000	2.6849999
-27111.0000000	2.6750002
-27110.0000000	2.6700001
-27109.0000000	2.6600001
-27108.0000000	2.6600001
-27107.0000000	2.6650000
-27106.0000000	2.6600001
-27105.0000000	2.6550002
-27104.0000000	2.6533334
-27103.0000000	2.6550002
-27102.0000000	2.6450000
-27101.0000000	2.6450000
-27100.0000000	2.6450000

-27099.0000000	2.6400001
-27098.0000000	2.6450000
-27097.0000000	2.6350002
-27096.0000000	2.6450000
-27095.0000000	2.6400001
-27094.0000000	2.6350002
-27093.0000000	2.6300001
-27092.0000000	2.6300001
-27091.0000000	2.6300001
-27090.0000000	2.6350002
-27089.0000000	2.6199999
-27088.0000000	2.6199999
-27087.0000000	2.6199999
-27086.0000000	2.6149998
-27085.0000000	2.6149998
-27084.0000000	2.6149998
-27083.0000000	2.6099999
-27082.0000000	2.6099999
-27081.0000000	2.6050000
-27080.0000000	2.6149998
-27079.0000000	2.6099999
-27078.0000000	2.6099999
-27077.0000000	2.6099999
-27076.0000000	2.6199999
-27075.0000000	2.6149998
-27074.0000000	2.6099999
-27073.0000000	2.6199999
-27072.0000000	2.6149998
-27071.0000000	2.6149998
-27070.0000000	2.6199999
-27069.0000000	2.6250000
-27068.0000000	2.6250000
-27067.0000000	2.6300001
-27066.0000000	2.6250000
-27065.0000000	2.6300001
-27064.0000000	2.6300001
-27063.0000000	2.6350002
-27062.0000000	2.6350002
-27061.0000000	2.6350002
-27060.0000000	2.6350002
-27059.0000000	2.6350002
-27058.0000000	2.6400001
-27057.0000000	2.6450000
-27056.0000000	2.6550002
-27055.0000000	2.6550002
-27054.0000000	2.6650000
-27053.0000000	2.6650000
-27052.0000000	2.6600001
-27051.0000000	2.6600001
-27050.0000000	2.6550002
-27049.0000000	2.6550002
-27048.0000000	2.6550002
-27047.0000000	2.6550002
-27046.0000000	2.6500001
-27045.0000000	2.6500001
-27044.0000000	2.6466668
-27043.0000000	2.6450000

-27042.0000000	2.6300001
-27041.0000000	2.6350002
-27040.0000000	2.6199999
-27039.0000000	2.6199999
-27038.0000000	2.6199999
-27037.0000000	2.6149998
-27036.0000000	2.6099999
-27035.0000000	2.5999999
-27034.0000000	2.5949998
-27033.0000000	2.5949998
-27032.0000000	2.5949998
-27031.0000000	2.5899999
-27030.0000000	2.5899999
-27029.0000000	2.5799999
-27028.0000000	2.5749998
-27027.0000000	2.5699999
-27026.0000000	2.5599999
-27025.0000000	2.5549998
-27024.0000000	2.5500000
-27023.0000000	2.5500000
-27022.0000000	2.5450001
-27021.0000000	2.5500000
-27020.0000000	2.5450001
-27019.0000000	2.5400000
-27018.0000000	2.5300000
-27017.0000000	2.5250001
-27016.0000000	2.5250001
-27015.0000000	2.5149999
-27014.0000000	2.5200000
-27013.0000000	2.5066667
-27012.0000000	2.5050001
-27011.0000000	2.5050001
-27010.0000000	2.5000000
-27009.0000000	2.4949999
-27008.0000000	2.4900000
-27007.0000000	2.4800000
-27006.0000000	2.4749999
-27005.0000000	2.4749999
-27004.0000000	2.4650002
-27003.0000000	2.4600000
-27002.0000000	2.4549999
-27001.0000000	2.4500000
-27000.0000000	2.4400001
-26999.0000000	2.4349999
-26998.0000000	2.4300001
-26997.0000000	2.4300001
-26996.0000000	2.4349999
-26995.0000000	2.4300001
-26994.0000000	2.4400001
-26993.0000000	2.4400001
-26992.0000000	2.4400001
-26991.0000000	2.4349999
-26990.0000000	2.4349999
-26989.0000000	2.4300001
-26988.0000000	2.4349999
-26987.0000000	2.4300001
-26986.0000000	2.4450002

-26985.0000000	2.4450002
-26984.0000000	2.4400001
-26983.0000000	2.4433334
-26982.0000000	2.4450002
-26981.0000000	2.4400001
-26980.0000000	2.4349999
-26979.0000000	2.4400001
-26978.0000000	2.4300001
-26977.0000000	2.4300001
-26976.0000000	2.4349999
-26975.0000000	2.4300001
-26974.0000000	2.4250002
-26973.0000000	2.4250002
-26972.0000000	2.4200001
-26971.0000000	2.4200001
-26970.0000000	2.4050002
-26969.0000000	2.4150000
-26968.0000000	2.4100001
-26967.0000000	2.4100001
-26966.0000000	2.4150000
-26965.0000000	2.4150000
-26964.0000000	2.4150000
-26963.0000000	2.4100001
-26962.0000000	2.4050002
-26961.0000000	2.4000001
-26960.0000000	2.4050002
-26959.0000000	2.4100001
-26958.0000000	2.4050002
-26957.0000000	2.4000001
-26956.0000000	2.3900001
-26955.0000000	2.4000001
-26954.0000000	2.3966668
-26953.0000000	2.4000001
-26952.0000000	2.4000001
-26951.0000000	2.3900001
-26950.0000000	2.3950000
-26949.0000000	2.3950000
-26948.0000000	2.4000001
-26947.0000000	2.4000001
-26946.0000000	2.4000001
-26945.0000000	2.4000001
-26944.0000000	2.3900001
-26943.0000000	2.3950000
-26942.0000000	2.3950000
-26941.0000000	2.3950000
-26940.0000000	2.3950000
-26939.0000000	2.3950000
-26938.0000000	2.3950000
-26937.0000000	2.4000001
-26936.0000000	2.4000001
-26935.0000000	2.4050002
-26934.0000000	2.4100001
-26933.0000000	2.4150000
-26932.0000000	2.4100001
-26931.0000000	2.4150000
-26930.0000000	2.4000001
-26929.0000000	2.3950000

-26928.0000000	2.4000001
-26927.0000000	2.3950000
-26926.0000000	2.4000001
-26925.0000000	2.4000001
-26924.0000000	2.3950000
-26923.0000000	2.3900001
-26922.0000000	2.3900001
-26921.0000000	2.3800001
-26920.0000000	2.3850002
-26919.0000000	2.3750000
-26918.0000000	2.3599999
-26917.0000000	2.3599999
-26916.0000000	2.3599999
-26915.0000000	2.3499999
-26914.0000000	2.3449998
-26913.0000000	2.3399999
-26912.0000000	2.3449998
-26911.0000000	2.3299999
-26910.0000000	2.3199999
-26909.0000000	2.3249998
-26908.0000000	2.3299999
-26907.0000000	2.3150001
-26906.0000000	2.3249998
-26905.0000000	2.3299999
-26904.0000000	2.3299999
-26903.0000000	2.3249998
-26902.0000000	2.3199999
-26901.0000000	2.3249998
-26900.0000000	2.3299999
-26899.0000000	2.3249998
-26898.0000000	2.3249998
-26897.0000000	2.3199999
-26896.0000000	2.3150001
-26895.0000000	2.3150001
-26894.0000000	2.3199999
-26893.0000000	2.3066666
-26892.0000000	2.3049998
-26891.0000000	2.3049998
-26890.0000000	2.3049998
-26889.0000000	2.3049998
-26888.0000000	2.3099999
-26887.0000000	2.3049998
-26886.0000000	2.3049998
-26885.0000000	2.3049998
-26884.0000000	2.3199999
-26883.0000000	2.3249998
-26882.0000000	2.3199999
-26881.0000000	2.3199999
-26880.0000000	2.3150001
-26879.0000000	2.3150001
-26878.0000000	2.3150001
-26877.0000000	2.3199999
-26876.0000000	2.3199999
-26875.0000000	2.3199999
-26874.0000000	2.3199999
-26873.0000000	2.3150001
-26872.0000000	2.3150001

-26871.0000000	2.3099999
-26870.0000000	2.3150001
-26869.0000000	2.3150001
-26868.0000000	2.3150001
-26867.0000000	2.3150001
-26866.0000000	2.3150001
-26865.0000000	2.3199999
-26864.0000000	2.3199999
-26863.0000000	2.3199999
-26862.0000000	2.3199999
-26861.0000000	2.3249998
-26860.0000000	2.3150001
-26859.0000000	2.3099999
-26858.0000000	2.3150001
-26857.0000000	2.3199999
-26856.0000000	2.3199999
-26855.0000000	2.3249998
-26854.0000000	2.3199999
-26853.0000000	2.3150001
-26852.0000000	2.3199999
-26851.0000000	2.3249998
-26850.0000000	2.3249998
-26849.0000000	2.3350000
-26848.0000000	2.3350000
-26847.0000000	2.3350000
-26846.0000000	2.3350000
-26845.0000000	2.3399999
-26844.0000000	2.3449998
-26843.0000000	2.3449998
-26842.0000000	2.3399999
-26841.0000000	2.3350000
-26840.0000000	2.3350000
-26839.0000000	2.3249998
-26838.0000000	2.3299999
-26837.0000000	2.3350000
-26836.0000000	2.3350000
-26835.0000000	2.3399999
-26834.0000000	2.3350000
-26833.0000000	2.3433332
-26832.0000000	2.3599999
-26831.0000000	2.3599999
-26830.0000000	2.3649998
-26829.0000000	2.3750000
-26828.0000000	2.3800001
-26827.0000000	2.3850002
-26826.0000000	2.3900001
-26825.0000000	2.3950000
-26824.0000000	2.3950000
-26823.0000000	2.4050002
-26822.0000000	2.4100001
-26821.0000000	2.4100001
-26820.0000000	2.4250002
-26819.0000000	2.4200001
-26818.0000000	2.4300001
-26817.0000000	2.4349999
-26816.0000000	2.4349999
-26815.0000000	2.4400001

-26814.0000000	2.4500000
-26813.0000000	2.4549999
-26812.0000000	2.4600000
-26811.0000000	2.4549999
-26810.0000000	2.4549999
-26809.0000000	2.4600000
-26808.0000000	2.4700000
-26807.0000000	2.4650002
-26806.0000000	2.4749999
-26805.0000000	2.4800000
-26804.0000000	2.4833333
-26803.0000000	2.4850001
-26802.0000000	2.4900000
-26801.0000000	2.4900000
-26800.0000000	2.4949999
-26799.0000000	2.4900000
-26798.0000000	2.4949999
-26797.0000000	2.5000000
-26796.0000000	2.5000000
-26795.0000000	2.5000000
-26794.0000000	2.5100000
-26793.0000000	2.5100000
-26792.0000000	2.5050001
-26791.0000000	2.5100000
-26790.0000000	2.5200000
-26789.0000000	2.5200000
-26788.0000000	2.5250001
-26787.0000000	2.5200000
-26786.0000000	2.5149999
-26785.0000000	2.5100000
-26784.0000000	2.5149999
-26783.0000000	2.5149999
-26782.0000000	2.5149999
-26781.0000000	2.5149999
-26780.0000000	2.5100000
-26779.0000000	2.5100000
-26778.0000000	2.5100000
-26777.0000000	2.5050001
-26776.0000000	2.5000000
-26775.0000000	2.5100000
-26774.0000000	2.5050001
-26773.0000000	2.5050001
-26772.0000000	2.5033333
-26771.0000000	2.5000000
-26770.0000000	2.4949999
-26769.0000000	2.5100000
-26768.0000000	2.5000000
-26767.0000000	2.4900000
-26766.0000000	2.4949999
-26765.0000000	2.4900000
-26764.0000000	2.4900000
-26763.0000000	2.4900000
-26762.0000000	2.4949999
-26761.0000000	2.5000000
-26760.0000000	2.5000000
-26759.0000000	2.5000000
-26758.0000000	2.5000000

-26757.0000000	2.5050001
-26756.0000000	2.5050001
-26755.0000000	2.5149999
-26754.0000000	2.5100000
-26753.0000000	2.5149999
-26752.0000000	2.5149999
-26751.0000000	2.5100000
-26750.0000000	2.5000000
-26749.0000000	2.5000000
-26748.0000000	2.4949999
-26747.0000000	2.4900000
-26746.0000000	2.4900000
-26745.0000000	2.4900000
-26744.0000000	2.4766667
-26743.0000000	2.4749999
-26742.0000000	2.4700000
-26741.0000000	2.4800000
-26740.0000000	2.4700000
-26739.0000000	2.4700000
-26738.0000000	2.4650002
-26737.0000000	2.4650002
-26736.0000000	2.4700000
-26735.0000000	2.4700000
-26734.0000000	2.4600000
-26733.0000000	2.4700000
-26732.0000000	2.4749999
-26731.0000000	2.4800000
-26730.0000000	2.4850001
-26729.0000000	2.4949999
-26728.0000000	2.4949999
-26727.0000000	2.4949999
-26726.0000000	2.4949999
-26725.0000000	2.4900000
-26724.0000000	2.4900000
-26723.0000000	2.4900000
-26722.0000000	2.4850001
-26721.0000000	2.4900000
-26720.0000000	2.4900000
-26719.0000000	2.4900000
-26718.0000000	2.4900000
-26717.0000000	2.4900000
-26716.0000000	2.4800000
-26715.0000000	2.4900000
-26714.0000000	2.4850001
-26713.0000000	2.4850001
-26712.0000000	2.4833333
-26711.0000000	2.4900000
-26710.0000000	2.4850001
-26709.0000000	2.4850001
-26708.0000000	2.4749999
-26707.0000000	2.4749999
-26706.0000000	2.4600000
-26705.0000000	2.4650002
-26704.0000000	2.4650002
-26703.0000000	2.4650002
-26702.0000000	2.4700000
-26701.0000000	2.4700000

-26700.0000000	2.4800000
-26699.0000000	2.4800000
-26698.0000000	2.4650002
-26697.0000000	2.4700000
-26696.0000000	2.4749999
-26695.0000000	2.4800000
-26694.0000000	2.4749999
-26693.0000000	2.4800000
-26692.0000000	2.4850001
-26691.0000000	2.4850001
-26690.0000000	2.4700000
-26689.0000000	2.4700000
-26688.0000000	2.4650002
-26687.0000000	2.4650002
-26686.0000000	2.4650002
-26685.0000000	2.4549999
-26684.0000000	2.4500000
-26683.0000000	2.4266667
-26682.0000000	2.4349999
-26681.0000000	2.4400001
-26680.0000000	2.4349999
-26679.0000000	2.4349999
-26678.0000000	2.4349999
-26677.0000000	2.4300001
-26676.0000000	2.4250002
-26675.0000000	2.4300001
-26674.0000000	2.4400001
-26673.0000000	2.4400001
-26672.0000000	2.4450002
-26671.0000000	2.4349999
-26670.0000000	2.4500000
-26669.0000000	2.4549999
-26668.0000000	2.4549999
-26667.0000000	2.4600000
-26666.0000000	2.4600000
-26665.0000000	2.4650002
-26664.0000000	2.4650002
-26663.0000000	2.4650002
-26662.0000000	2.4650002
-26661.0000000	2.4650002
-26660.0000000	2.4650002
-26659.0000000	2.4600000
-26658.0000000	2.4650002
-26657.0000000	2.4549999
-26656.0000000	2.4700000
-26655.0000000	2.4600000
-26654.0000000	2.4700000
-26653.0000000	2.4650002
-26652.0000000	2.4666669
-26651.0000000	2.4749999
-26650.0000000	2.4749999
-26649.0000000	2.4700000
-26648.0000000	2.4650002
-26647.0000000	2.4650002
-26646.0000000	2.4650002
-26645.0000000	2.4650002
-26644.0000000	2.4600000

-26643.0000000	2.4600000
-26642.0000000	2.4650002
-26641.0000000	2.4700000
-26640.0000000	2.4650002
-26639.0000000	2.4600000
-26638.0000000	2.4600000
-26637.0000000	2.4650002
-26636.0000000	2.4600000
-26635.0000000	2.4600000
-26634.0000000	2.4549999
-26633.0000000	2.4650002
-26632.0000000	2.4700000
-26631.0000000	2.4650002
-26630.0000000	2.4749999
-26629.0000000	2.4850001
-26628.0000000	2.4850001
-26627.0000000	2.4749999
-26626.0000000	2.4800000
-26625.0000000	2.4650002
-26624.0000000	2.4650002
-26623.0000000	2.4549999
-26622.0000000	2.4466667
-26621.0000000	2.4349999
-26620.0000000	2.4150000
-26619.0000000	2.4200001
-26618.0000000	2.4050002
-26617.0000000	2.4200001
-26616.0000000	2.4200001
-26615.0000000	2.4150000
-26614.0000000	2.4100001
-26613.0000000	2.4000001
-26612.0000000	2.3900001
-26611.0000000	2.3900001
-26610.0000000	2.3900001
-26609.0000000	2.3850002
-26608.0000000	2.3950000
-26607.0000000	2.3850002
-26606.0000000	2.3750000
-26605.0000000	2.3800001
-26604.0000000	2.3800001
-26603.0000000	2.3699999
-26602.0000000	2.3750000
-26601.0000000	2.3750000
-26600.0000000	2.3750000
-26599.0000000	2.3750000
-26598.0000000	2.3850002
-26597.0000000	2.4000001
-26596.0000000	2.4000001
-26595.0000000	2.4000001
-26594.0000000	2.4050002
-26593.0000000	2.4000001
-26592.0000000	2.4000003
-26591.0000000	2.4050002
-26590.0000000	2.4050002
-26589.0000000	2.4050002
-26588.0000000	2.4050002
-26587.0000000	2.4100001

-26586.0000000	2.4100001
-26585.0000000	2.4100001
-26584.0000000	2.4150000
-26583.0000000	2.4100001
-26582.0000000	2.4150000
-26581.0000000	2.4100001
-26580.0000000	2.4200001
-26579.0000000	2.4250002
-26578.0000000	2.4200001
-26577.0000000	2.4250002
-26576.0000000	2.4200001
-26575.0000000	2.4300001
-26574.0000000	2.4300001
-26573.0000000	2.4300001
-26572.0000000	2.4250002
-26571.0000000	2.4150000
-26570.0000000	2.4200001
-26569.0000000	2.4349999
-26568.0000000	2.4549999
-26567.0000000	2.4600000
-26566.0000000	2.4700000
-26565.0000000	2.4700000
-26564.0000000	2.4800000
-26563.0000000	2.4850001
-26562.0000000	2.4900000
-26561.0000000	2.4966667
-26560.0000000	2.5000000
-26559.0000000	2.5050001
-26558.0000000	2.5149999
-26557.0000000	2.5149999
-26556.0000000	2.5149999
-26555.0000000	2.5100000
-26554.0000000	2.5149999
-26553.0000000	2.5149999
-26552.0000000	2.5149999
-26551.0000000	2.5200000
-26550.0000000	2.5300000
-26549.0000000	2.5300000
-26548.0000000	2.5349998
-26547.0000000	2.5349998
-26546.0000000	2.5300000
-26545.0000000	2.5349998
-26544.0000000	2.5400000
-26543.0000000	2.5250001
-26542.0000000	2.5250001
-26541.0000000	2.5250001
-26540.0000000	2.5149999
-26539.0000000	2.5149999
-26538.0000000	2.5100000
-26537.0000000	2.5100000
-26536.0000000	2.5100000
-26535.0000000	2.5149999
-26534.0000000	2.5100000
-26533.0000000	2.5050001
-26532.0000000	2.5000000
-26531.0000000	2.5033333
-26530.0000000	2.5050001

-26529.0000000	2.5100000
-26528.0000000	2.4949999
-26527.0000000	2.5000000
-26526.0000000	2.4949999
-26525.0000000	2.4949999
-26524.0000000	2.4900000
-26523.0000000	2.4900000
-26522.0000000	2.4850001
-26521.0000000	2.4850001
-26520.0000000	2.4850001
-26519.0000000	2.4949999
-26518.0000000	2.4949999
-26517.0000000	2.4900000
-26516.0000000	2.4900000
-26515.0000000	2.4900000
-26514.0000000	2.4949999
-26513.0000000	2.4949999
-26512.0000000	2.4949999
-26511.0000000	2.4850001
-26510.0000000	2.4900000
-26509.0000000	2.4850001
-26508.0000000	2.4749999
-26507.0000000	2.4749999
-26506.0000000	2.4949999
-26505.0000000	2.5050001
-26504.0000000	2.5050001
-26503.0000000	2.5066667
-26502.0000000	2.5050001
-26501.0000000	2.5100000
-26500.0000000	2.5050001
-26499.0000000	2.5149999
-26498.0000000	2.5149999
-26497.0000000	2.5050001
-26496.0000000	2.5050001
-26495.0000000	2.5100000
-26494.0000000	2.5050001
-26493.0000000	2.5000000
-26492.0000000	2.4900000
-26491.0000000	2.4900000
-26490.0000000	2.4749999
-26489.0000000	2.4700000
-26488.0000000	2.4650002
-26487.0000000	2.4450002
-26486.0000000	2.4450002
-26485.0000000	2.4349999
-26484.0000000	2.4349999
-26483.0000000	2.4300001
-26482.0000000	2.4200001
-26481.0000000	2.4100001
-26480.0000000	2.4000001
-26479.0000000	2.3950000
-26478.0000000	2.3950000
-26477.0000000	2.3800001
-26476.0000000	2.4000001
-26475.0000000	2.3950000
-26474.0000000	2.3900001
-26473.0000000	2.3950000

-26472.0000000	2.3900001
-26471.0000000	2.3850002
-26470.0000000	2.3850002
-26469.0000000	2.3850002
-26468.0000000	2.3850002
-26467.0000000	2.3900001
-26466.0000000	2.3950000
-26465.0000000	2.4050002
-26464.0000000	2.4000001
-26463.0000000	2.4000001
-26462.0000000	2.4100001
-26461.0000000	2.4100001
-26460.0000000	2.4100001
-26459.0000000	2.4050002
-26458.0000000	2.4150000
-26457.0000000	2.4100001
-26456.0000000	2.4200001
-26455.0000000	2.4200001
-26454.0000000	2.4200001
-26453.0000000	2.4150000
-26452.0000000	2.4250002
-26451.0000000	2.4250002
-26450.0000000	2.4200001
-26449.0000000	2.4250002
-26448.0000000	2.4250002
-26447.0000000	2.4250002
-26446.0000000	2.4300001
-26445.0000000	2.4200001
-26444.0000000	2.4200001
-26443.0000000	2.4200001
-26442.0000000	2.4166667
-26441.0000000	2.4150000
-26440.0000000	2.4150000
-26439.0000000	2.4050002
-26438.0000000	2.4100001
-26437.0000000	2.4150000
-26436.0000000	2.4100001
-26435.0000000	2.4100001
-26434.0000000	2.4100001
-26433.0000000	2.4150000
-26432.0000000	2.4050002
-26431.0000000	2.4050002
-26430.0000000	2.4050002
-26429.0000000	2.4100001
-26428.0000000	2.4100001
-26427.0000000	2.4100001
-26426.0000000	2.4100001
-26425.0000000	2.4100001
-26424.0000000	2.4100001
-26423.0000000	2.4150000
-26422.0000000	2.4150000
-26421.0000000	2.4100001
-26420.0000000	2.4100001
-26419.0000000	2.4050002
-26418.0000000	2.4100001
-26417.0000000	2.4000001
-26416.0000000	2.4000001

-26415.0000000	2.3950000
-26414.0000000	2.3800001
-26413.0000000	2.3850002
-26412.0000000	2.3800001
-26411.0000000	2.3800001
-26410.0000000	2.3750000
-26409.0000000	2.3800001
-26408.0000000	2.3800001
-26407.0000000	2.3800001
-26406.0000000	2.3850002
-26405.0000000	2.3850002
-26404.0000000	2.3850002
-26403.0000000	2.3800001
-26402.0000000	2.3800001
-26401.0000000	2.3850002
-26400.0000000	2.3850002
-26399.0000000	2.3950000
-26398.0000000	2.3900001
-26397.0000000	2.3900001
-26396.0000000	2.3950000
-26395.0000000	2.4000001
-26394.0000000	2.4100001
-26393.0000000	2.4100001
-26392.0000000	2.4150000
-26391.0000000	2.4200001
-26390.0000000	2.4100001
-26389.0000000	2.4150000
-26388.0000000	2.4150000
-26387.0000000	2.4100001
-26386.0000000	2.4100001
-26385.0000000	2.4100001
-26384.0000000	2.4150000
-26383.0000000	2.4150000
-26382.0000000	2.4150000
-26381.0000000	2.4166667
-26380.0000000	2.4150000
-26379.0000000	2.4150000
-26378.0000000	2.4150000
-26377.0000000	2.4200001
-26376.0000000	2.4200001
-26375.0000000	2.4250002
-26374.0000000	2.4250002
-26373.0000000	2.4300001
-26372.0000000	2.4300001
-26371.0000000	2.4349999
-26370.0000000	2.4349999
-26369.0000000	2.4349999
-26368.0000000	2.4400001
-26367.0000000	2.4400001
-26366.0000000	2.4400001
-26365.0000000	2.4400001
-26364.0000000	2.4349999
-26363.0000000	2.4400001
-26362.0000000	2.4400001
-26361.0000000	2.4400001
-26360.0000000	2.4450002
-26359.0000000	2.4450002

-26358.0000000	2.4450002
-26357.0000000	2.4500000
-26356.0000000	2.4600000
-26355.0000000	2.4549999
-26354.0000000	2.4600000
-26353.0000000	2.4549999
-26352.0000000	2.4650002
-26351.0000000	2.4633334
-26350.0000000	2.4700000
-26349.0000000	2.4650002
-26348.0000000	2.4700000
-26347.0000000	2.4700000
-26346.0000000	2.4800000
-26345.0000000	2.4850001
-26344.0000000	2.4749999
-26343.0000000	2.4850001
-26342.0000000	2.4749999
-26341.0000000	2.4800000
-26340.0000000	2.4800000
-26339.0000000	2.4850001
-26338.0000000	2.4749999
-26337.0000000	2.4700000
-26336.0000000	2.4800000
-26335.0000000	2.4850001
-26334.0000000	2.4800000
-26333.0000000	2.4749999
-26332.0000000	2.4700000
-26331.0000000	2.4600000
-26330.0000000	2.4600000
-26329.0000000	2.4549999
-26328.0000000	2.4500000
-26327.0000000	2.4450002
-26326.0000000	2.4400001
-26325.0000000	2.4450002
-26324.0000000	2.4400001
-26323.0000000	2.4400001
-26322.0000000	2.4349999
-26321.0000000	2.4400001
-26320.0000000	2.4400001
-26319.0000000	2.4450002
-26318.0000000	2.4400001
-26317.0000000	2.4450002
-26316.0000000	2.4400001
-26315.0000000	2.4400001
-26314.0000000	2.4400001
-26313.0000000	2.4400001
-26312.0000000	2.4400001
-26311.0000000	2.4400001
-26310.0000000	2.4400001
-26309.0000000	2.4349999
-26308.0000000	2.4400001
-26307.0000000	2.4549999
-26306.0000000	2.4549999
-26305.0000000	2.4700000
-26304.0000000	2.4749999
-26303.0000000	2.4700000
-26302.0000000	2.4749999

-26301.0000000	2.4850001
-26300.0000000	2.4850001
-26299.0000000	2.4949999
-26298.0000000	2.4949999
-26297.0000000	2.4900000
-26296.0000000	2.4850001
-26295.0000000	2.4800000
-26294.0000000	2.4800000
-26293.0000000	2.4850001
-26292.0000000	2.4800000
-26291.0000000	2.4800000
-26290.0000000	2.4749999
-26289.0000000	2.4700000
-26288.0000000	2.4733334
-26287.0000000	2.4650002
-26286.0000000	2.4650002
-26285.0000000	2.4600000
-26284.0000000	2.4549999
-26283.0000000	2.4450002
-26282.0000000	2.4450002
-26281.0000000	2.4400001
-26280.0000000	2.4349999
-26279.0000000	2.4400001
-26278.0000000	2.4400001
-26277.0000000	2.4450002
-26276.0000000	2.4500000
-26275.0000000	2.4400001
-26274.0000000	2.4400001
-26273.0000000	2.4450002
-26272.0000000	2.4450002
-26271.0000000	2.4450002
-26270.0000000	2.4400001
-26269.0000000	2.4349999
-26268.0000000	2.4400001
-26267.0000000	2.4300001
-26266.0000000	2.4349999
-26265.0000000	2.4349999
-26264.0000000	2.4250002
-26263.0000000	2.4200001
-26262.0000000	2.4150000
-26261.0000000	2.4050002
-26260.0000000	2.3950000
-26259.0000000	2.3850002
-26258.0000000	2.3733332
-26257.0000000	2.3699999
-26256.0000000	2.3599999
-26255.0000000	2.3649998
-26254.0000000	2.3599999
-26253.0000000	2.3499999
-26252.0000000	2.3499999
-26251.0000000	2.3350000
-26250.0000000	2.3299999
-26249.0000000	2.3299999
-26248.0000000	2.3299999
-26247.0000000	2.3299999
-26246.0000000	2.3249998
-26245.0000000	2.3249998

-26244.0000000	2.3199999
-26243.0000000	2.3199999
-26242.0000000	2.3150001
-26241.0000000	2.3199999
-26240.0000000	2.3099999
-26239.0000000	2.3099999
-26238.0000000	2.3049998
-26237.0000000	2.3049998
-26236.0000000	2.3049998
-26235.0000000	2.2950001
-26234.0000000	2.2900000
-26233.0000000	2.2800000
-26232.0000000	2.2800000
-26231.0000000	2.2800000
-26230.0000000	2.2800000
-26229.0000000	2.2800000
-26228.0000000	2.2700000
-26227.0000000	2.2700000
-26226.0000000	2.2700000
-26225.0000000	2.2649999
-26224.0000000	2.2649999
-26223.0000000	2.2649999
-26222.0000000	2.2649999
-26221.0000000	2.2600000
-26220.0000000	2.2649999
-26219.0000000	2.2649999
-26218.0000000	2.2500000
-26217.0000000	2.2500000
-26216.0000000	2.2449999
-26215.0000000	2.2449999
-26214.0000000	2.2449999
-26213.0000000	2.2350001
-26212.0000000	2.2350001
-26211.0000000	2.2249999
-26210.0000000	2.2200000
-26209.0000000	2.2200000
-26208.0000000	2.2150002
-26207.0000000	2.2100000
-26206.0000000	2.2049999
-26205.0000000	2.2049999
-26204.0000000	2.2000000
-26203.0000000	2.2000000
-26202.0000000	2.2049999
-26201.0000000	2.2049999
-26200.0000000	2.1950002
-26199.0000000	2.1950002
-26198.0000000	2.1900001
-26197.0000000	2.1950002
-26196.0000000	2.1849999
-26195.0000000	2.1849999
-26194.0000000	2.1800001
-26193.0000000	2.1750002
-26192.0000000	2.1700001
-26191.0000000	2.1700001
-26190.0000000	2.1750002
-26189.0000000	2.1800001
-26188.0000000	2.1750002

-26187.0000000	2.1800001
-26186.0000000	2.1750002
-26185.0000000	2.1750002
-26184.0000000	2.1700001
-26183.0000000	2.1750002
-26182.0000000	2.1750002
-26181.0000000	2.1800001
-26180.0000000	2.1800001
-26179.0000000	2.1849999
-26178.0000000	2.1800001
-26177.0000000	2.1800001
-26176.0000000	2.1700001
-26175.0000000	2.1750002
-26174.0000000	2.1750002
-26173.0000000	2.1650000
-26172.0000000	2.1700001
-26171.0000000	2.1750002
-26170.0000000	2.1750002
-26169.0000000	2.1766670
-26168.0000000	2.1800001
-26167.0000000	2.1700001
-26166.0000000	2.1800001
-26165.0000000	2.1800001
-26164.0000000	2.1800001
-26163.0000000	2.1800001
-26162.0000000	2.1849999
-26161.0000000	2.1750002
-26160.0000000	2.1700001
-26159.0000000	2.1800001
-26158.0000000	2.1800001
-26157.0000000	2.1700001
-26156.0000000	2.1750002
-26155.0000000	2.1650000
-26154.0000000	2.1600001
-26153.0000000	2.1600001
-26152.0000000	2.1600001
-26151.0000000	2.1500001
-26150.0000000	2.1550002
-26149.0000000	2.1550002
-26148.0000000	2.1450000
-26147.0000000	2.1450000
-26146.0000000	2.1350002
-26145.0000000	2.1350002
-26144.0000000	2.1250000
-26143.0000000	2.1250000
-26142.0000000	2.1199999
-26141.0000000	2.1199999
-26140.0000000	2.1199999
-26139.0000000	2.1133330
-26138.0000000	2.1099999
-26137.0000000	2.1099999
-26136.0000000	2.1099999
-26135.0000000	2.1099999
-26134.0000000	2.1149998
-26133.0000000	2.1099999
-26132.0000000	2.1099999
-26131.0000000	2.1099999

-26130.0000000	2.1050000
-26129.0000000	2.1050000
-26128.0000000	2.1050000
-26127.0000000	2.0949998
-26126.0000000	2.1050000
-26125.0000000	2.0999999
-26124.0000000	2.0999999
-26123.0000000	2.0949998
-26122.0000000	2.0949998
-26121.0000000	2.0949998
-26120.0000000	2.0899999
-26119.0000000	2.0850000
-26118.0000000	2.0799999
-26117.0000000	2.0749998
-26116.0000000	2.0699999
-26115.0000000	2.0599999
-26114.0000000	2.0599999
-26113.0000000	2.0599999
-26112.0000000	2.0549998
-26111.0000000	2.0500000
-26110.0000000	2.0466669
-26109.0000000	2.0400000
-26108.0000000	2.0400000
-26107.0000000	2.0400000
-26106.0000000	2.0450001
-26105.0000000	2.0400000
-26104.0000000	2.0450001
-26103.0000000	2.0450001
-26102.0000000	2.0450001
-26101.0000000	2.0450001
-26100.0000000	2.0500000
-26099.0000000	2.0450001
-26098.0000000	2.0450001
-26097.0000000	2.0450001
-26096.0000000	2.0400000
-26095.0000000	2.0349998
-26094.0000000	2.0400000
-26093.0000000	2.0300000
-26092.0000000	2.0250001
-26091.0000000	2.0250001
-26090.0000000	2.0300000
-26089.0000000	2.0149999
-26088.0000000	2.0200000
-26087.0000000	2.0149999
-26086.0000000	2.0050001
-26085.0000000	2.0050001
-26084.0000000	2.0050001
-26083.0000000	2.0000000
-26082.0000000	2.0000000
-26081.0000000	1.9950000
-26080.0000000	1.9933333
-26079.0000000	1.9950000
-26078.0000000	1.9950000
-26077.0000000	1.9950000
-26076.0000000	1.9850000
-26075.0000000	1.9900000
-26074.0000000	1.9900000

-26073.0000000	1.9900000
-26072.0000000	1.9900000
-26071.0000000	1.9950000
-26070.0000000	1.9950000
-26069.0000000	1.9900000
-26068.0000000	1.9900000
-26067.0000000	1.9950000
-26066.0000000	2.0050001
-26065.0000000	1.9950000
-26064.0000000	1.9950000
-26063.0000000	2.0000000
-26062.0000000	1.9950000
-26061.0000000	1.9950000
-26060.0000000	1.9950000
-26059.0000000	1.9950000
-26058.0000000	2.0050001
-26057.0000000	2.0050001
-26056.0000000	2.0100000
-26055.0000000	2.0149999
-26054.0000000	2.0200000
-26053.0000000	2.0349998
-26052.0000000	2.0300000
-26051.0000000	2.0400000
-26050.0000000	2.0400000
-26049.0000000	2.0450001
-26048.0000000	2.0400000
-26047.0000000	2.0400000
-26046.0000000	2.0450001
-26045.0000000	2.0349998
-26044.0000000	2.0300000
-26043.0000000	2.0300000
-26042.0000000	2.0400000
-26041.0000000	2.0300000
-26040.0000000	2.0349998
-26039.0000000	2.0250001
-26038.0000000	2.0250001
-26037.0000000	2.0100000
-26036.0000000	2.0149999
-26035.0000000	2.0050001
-26034.0000000	2.0050001
-26033.0000000	2.0050001
-26032.0000000	2.0050001
-26031.0000000	2.0000000
-26030.0000000	2.0000000
-26029.0000000	1.9950000
-26028.0000000	1.9950000
-26027.0000000	2.0000000
-26026.0000000	1.9950000
-26025.0000000	1.9950000
-26024.0000000	1.9950000
-26023.0000000	1.9950000
-26022.0000000	1.9900000
-26021.0000000	1.9950000
-26020.0000000	2.0000000
-26019.0000000	1.9966666
-26018.0000000	1.9950000
-26017.0000000	1.9950000

-26016.0000000	2.0000000
-26015.0000000	2.0000000
-26014.0000000	2.0000000
-26013.0000000	2.0000000
-26012.0000000	2.0050001
-26011.0000000	2.0050001
-26010.0000000	2.0050001
-26009.0000000	2.0050001
-26008.0000000	2.0000000
-26007.0000000	2.0000000
-26006.0000000	1.9950000
-26005.0000000	1.9950000
-26004.0000000	1.9900000
-26003.0000000	2.0000000
-26002.0000000	1.9900000
-26001.0000000	1.9900000
-26000.0000000	1.9850000
-25999.0000000	1.9850000
-25998.0000000	1.9800000
-25997.0000000	1.9700000
-25996.0000000	1.9750000
-25995.0000000	1.9750000
-25994.0000000	1.9700000
-25993.0000000	1.9700000
-25992.0000000	1.9700000
-25991.0000000	1.9700000
-25990.0000000	1.9700000
-25989.0000000	1.9650000
-25988.0000000	1.9633335
-25987.0000000	1.9600000
-25986.0000000	1.9700000
-25985.0000000	1.9700000
-25984.0000000	1.9700000
-25983.0000000	1.9750000
-25982.0000000	1.9750000
-25981.0000000	1.9800000
-25980.0000000	1.9800000
-25979.0000000	1.9850000
-25978.0000000	1.9800000
-25977.0000000	1.9800000
-25976.0000000	1.9900000
-25975.0000000	1.9900000
-25974.0000000	1.9950000
-25973.0000000	2.0000000
-25972.0000000	1.9950000
-25971.0000000	2.0000000
-25970.0000000	1.9950000
-25969.0000000	2.0000000
-25968.0000000	1.9950000
-25967.0000000	2.0000000
-25966.0000000	2.0000000
-25965.0000000	1.9950000
-25964.0000000	1.9950000
-25963.0000000	1.9900000
-25962.0000000	1.9800000
-25961.0000000	1.9800000
-25960.0000000	1.9850000

-25959.0000000	1.9850000
-25958.0000000	1.9850000
-25957.0000000	1.9850000
-25956.0000000	1.9800000
-25955.0000000	1.9800000
-25954.0000000	1.9800000
-25953.0000000	1.9750000
-25952.0000000	1.9750000
-25951.0000000	1.9700000
-25950.0000000	1.9750000
-25949.0000000	1.9700000
-25948.0000000	1.9600000
-25947.0000000	1.9600000
-25946.0000000	1.9550000
-25945.0000000	1.9550000
-25944.0000000	1.9450001
-25943.0000000	1.9349999
-25942.0000000	1.9400001
-25941.0000000	1.9299999
-25940.0000000	1.9349999
-25939.0000000	1.9250000
-25938.0000000	1.9250000
-25937.0000000	1.9250000
-25936.0000000	1.9200000
-25935.0000000	1.9200000
-25934.0000000	1.9200000
-25933.0000000	1.9150000
-25932.0000000	1.9150000
-25931.0000000	1.9100000
-25930.0000000	1.9050000
-25929.0000000	1.9050000
-25928.0000000	1.9000000
-25927.0000000	1.9000000
-25926.0000000	1.9000000
-25925.0000000	1.9050000
-25924.0000000	1.9050000
-25923.0000000	1.8950000
-25922.0000000	1.9000000
-25921.0000000	1.9000000
-25920.0000000	1.9100000
-25919.0000000	1.9100000
-25918.0000000	1.9100000
-25917.0000000	1.9100000
-25916.0000000	1.9150000
-25915.0000000	1.9100000
-25914.0000000	1.9100000
-25913.0000000	1.9050000
-25912.0000000	1.9050000
-25911.0000000	1.9050000
-25910.0000000	1.9100000
-25909.0000000	1.9000000
-25908.0000000	1.9050000
-25907.0000000	1.9000000
-25906.0000000	1.9000000
-25905.0000000	1.9000000
-25904.0000000	1.9000000
-25903.0000000	1.9000000

-25902.0000000	1.9000000
-25901.0000000	1.9050000
-25900.0000000	1.9050000
-25899.0000000	1.9000000
-25898.0000000	1.9000000
-25897.0000000	1.9000000
-25896.0000000	1.9050000
-25895.0000000	1.9050000
-25894.0000000	1.9050000
-25893.0000000	1.9000000
-25892.0000000	1.9050000
-25891.0000000	1.9100000
-25890.0000000	1.9100000
-25889.0000000	1.9050000
-25888.0000000	1.9050000
-25887.0000000	1.9050000
-25886.0000000	1.9100000
-25885.0000000	1.9050000
-25884.0000000	1.9050000
-25883.0000000	1.9100000
-25882.0000000	1.9100000
-25881.0000000	1.9050000
-25880.0000000	1.9000000
-25879.0000000	1.9050000
-25878.0000000	1.9050000
-25877.0000000	1.9100000
-25876.0000000	1.9100000
-25875.0000000	1.9050000
-25874.0000000	1.9050000
-25873.0000000	1.9050000
-25872.0000000	1.9050000
-25871.0000000	1.9000000
-25870.0000000	1.9050000
-25869.0000000	1.9000000
-25868.0000000	1.9050000
-25867.0000000	1.9033333
-25866.0000000	1.9050000
-25865.0000000	1.9100000
-25864.0000000	1.9050000
-25863.0000000	1.9000000
-25862.0000000	1.9050000
-25861.0000000	1.9000000
-25860.0000000	1.9050000
-25859.0000000	1.9000000
-25858.0000000	1.9050000
-25857.0000000	1.9050000
-25856.0000000	1.9000000
-25855.0000000	1.9050000
-25854.0000000	1.9000000
-25853.0000000	1.9000000
-25852.0000000	1.9050000
-25851.0000000	1.9050000
-25850.0000000	1.9100000
-25849.0000000	1.9000000
-25848.0000000	1.9000000
-25847.0000000	1.9050000
-25846.0000000	1.9100000

-25845.0000000	1.9100000
-25844.0000000	1.9100000
-25843.0000000	1.9050000
-25842.0000000	1.9050000
-25841.0000000	1.9100000
-25840.0000000	1.9150000
-25839.0000000	1.9150000
-25838.0000000	1.9150000
-25837.0000000	1.9133333
-25836.0000000	1.9200000
-25835.0000000	1.9250000
-25834.0000000	1.9250000
-25833.0000000	1.9250000
-25832.0000000	1.9299999
-25831.0000000	1.9299999
-25830.0000000	1.9250000
-25829.0000000	1.9299999
-25828.0000000	1.9349999
-25827.0000000	1.9400001
-25826.0000000	1.9500000
-25825.0000000	1.9500000
-25824.0000000	1.9500000
-25823.0000000	1.9600000
-25822.0000000	1.9550000
-25821.0000000	1.9600000
-25820.0000000	1.9600000
-25819.0000000	1.9600000
-25818.0000000	1.9600000
-25817.0000000	1.9650000
-25816.0000000	1.9650000
-25815.0000000	1.9700000
-25814.0000000	1.9650000
-25813.0000000	1.9650000
-25812.0000000	1.9700000
-25811.0000000	1.9700000
-25810.0000000	1.9800000
-25809.0000000	1.9750000
-25808.0000000	1.9800000
-25807.0000000	1.9833332
-25806.0000000	1.9850000
-25805.0000000	1.9900000
-25804.0000000	1.9900000
-25803.0000000	1.9900000
-25802.0000000	1.9850000
-25801.0000000	1.9850000
-25800.0000000	1.9850000
-25799.0000000	1.9850000
-25798.0000000	1.9850000
-25797.0000000	1.9800000
-25796.0000000	1.9800000
-25795.0000000	1.9900000
-25794.0000000	1.9900000
-25793.0000000	1.9850000
-25792.0000000	1.9850000
-25791.0000000	1.9950000
-25790.0000000	1.9900000
-25789.0000000	1.9900000

-25788.0000000	1.9950000
-25787.0000000	1.9900000
-25786.0000000	1.9950000
-25785.0000000	1.9950000
-25784.0000000	2.0000000
-25783.0000000	2.0050001
-25782.0000000	2.0050001
-25781.0000000	2.0050001
-25780.0000000	2.0050001
-25779.0000000	2.0100000
-25778.0000000	2.0050001
-25777.0000000	2.0133333
-25776.0000000	2.0149999
-25775.0000000	2.0100000
-25774.0000000	2.0100000
-25773.0000000	2.0100000
-25772.0000000	2.0149999
-25771.0000000	2.0149999
-25770.0000000	2.0200000
-25769.0000000	2.0200000
-25768.0000000	2.0200000
-25767.0000000	2.0250001
-25766.0000000	2.0250001
-25765.0000000	2.0250001
-25764.0000000	2.0300000
-25763.0000000	2.0300000
-25762.0000000	2.0450001
-25761.0000000	2.0400000
-25760.0000000	2.0400000
-25759.0000000	2.0500000
-25758.0000000	2.0450001
-25757.0000000	2.0450001
-25756.0000000	2.0400000
-25755.0000000	2.0349998
-25754.0000000	2.0250001
-25753.0000000	2.0200000
-25752.0000000	2.0200000
-25751.0000000	2.0100000
-25750.0000000	2.0149999
-25749.0000000	2.0050001
-25748.0000000	2.0100000
-25747.0000000	2.0050001
-25746.0000000	2.0000000
-25745.0000000	1.9900000
-25744.0000000	1.9766668
-25743.0000000	1.9750000
-25742.0000000	1.9750000
-25741.0000000	1.9650000
-25740.0000000	1.9600000
-25739.0000000	1.9550000
-25738.0000000	1.9450001
-25737.0000000	1.9400001
-25736.0000000	1.9349999
-25735.0000000	1.9299999
-25734.0000000	1.9299999
-25733.0000000	1.9250000
-25732.0000000	1.9150000

-25731.0000000	1.9100000
-25730.0000000	1.9000000
-25729.0000000	1.8950000
-25728.0000000	1.8950000
-25727.0000000	1.8900000
-25726.0000000	1.8850000
-25725.0000000	1.8750000
-25724.0000000	1.8650000
-25723.0000000	1.8500000
-25722.0000000	1.8450000
-25721.0000000	1.8400000
-25720.0000000	1.8300000
-25719.0000000	1.8250000
-25718.0000000	1.8150001
-25717.0000000	1.8099999
-25716.0000000	1.8000000
-25715.0000000	1.7933334
-25714.0000000	1.7850000
-25713.0000000	1.7800000
-25712.0000000	1.7700000
-25711.0000000	1.7700000
-25710.0000000	1.7550000
-25709.0000000	1.7550000
-25708.0000000	1.7500000
-25707.0000000	1.7450000
-25706.0000000	1.7450000
-25705.0000000	1.7400000
-25704.0000000	1.7350000
-25703.0000000	1.7350000
-25702.0000000	1.7250000
-25701.0000000	1.7250000
-25700.0000000	1.7200000
-25699.0000000	1.7200000
-25698.0000000	1.7200000
-25697.0000000	1.7150000
-25696.0000000	1.7150000
-25695.0000000	1.7000000
-25694.0000000	1.7000000
-25693.0000000	1.6950001
-25692.0000000	1.6900001
-25691.0000000	1.6799999
-25690.0000000	1.6800001
-25689.0000000	1.6750000
-25688.0000000	1.6700000
-25687.0000000	1.6650000
-25686.0000000	1.6600000
-25685.0000000	1.6600000
-25684.0000000	1.6600000
-25683.0000000	1.6600000
-25682.0000000	1.6650000
-25681.0000000	1.6650000
-25680.0000000	1.6650000
-25679.0000000	1.6700000
-25678.0000000	1.6700000
-25677.0000000	1.6700000
-25676.0000000	1.6750000
-25675.0000000	1.6750000

-25674.0000000	1.6750000
-25673.0000000	1.6750000
-25672.0000000	1.6799999
-25671.0000000	1.6799999
-25670.0000000	1.6799999
-25669.0000000	1.6849999
-25668.0000000	1.6849999
-25667.0000000	1.6900001
-25666.0000000	1.6950001
-25665.0000000	1.6950001
-25664.0000000	1.6950001
-25663.0000000	1.7000000
-25662.0000000	1.7000000
-25661.0000000	1.6950001
-25660.0000000	1.7050000
-25659.0000000	1.7000000
-25658.0000000	1.7050000
-25657.0000000	1.7000000
-25656.0000000	1.7050000
-25655.0000000	1.7050000
-25654.0000000	1.7000002
-25653.0000000	1.7000000
-25652.0000000	1.7000000
-25651.0000000	1.7050000
-25650.0000000	1.7100000
-25649.0000000	1.7050000
-25648.0000000	1.7050000
-25647.0000000	1.7150000
-25646.0000000	1.7050000
-25645.0000000	1.7100000
-25644.0000000	1.7100000
-25643.0000000	1.7150000
-25642.0000000	1.7200000
-25641.0000000	1.7200000
-25640.0000000	1.7250000
-25639.0000000	1.7350000
-25638.0000000	1.7350000
-25637.0000000	1.7350000
-25636.0000000	1.7450000
-25635.0000000	1.7500000
-25634.0000000	1.7550000
-25633.0000000	1.7600000
-25632.0000000	1.7700000
-25631.0000000	1.7700000
-25630.0000000	1.7750000
-25629.0000000	1.7800000
-25628.0000000	1.7850000
-25627.0000000	1.7850000
-25626.0000000	1.7950000
-25625.0000000	1.7950000
-25624.0000000	1.7900000
-25623.0000000	1.7900000
-25622.0000000	1.7900000
-25621.0000000	1.7900000
-25620.0000000	1.8000000
-25619.0000000	1.7950000
-25618.0000000	1.8049999

-25617.0000000	1.8049999
-25616.0000000	1.8000000
-25615.0000000	1.8000000
-25614.0000000	1.8000000
-25613.0000000	1.8000000
-25612.0000000	1.7950000
-25611.0000000	1.7950000
-25610.0000000	1.7900000
-25609.0000000	1.7950000
-25608.0000000	1.7950000
-25607.0000000	1.7950000
-25606.0000000	1.8000000
-25605.0000000	1.8049999
-25604.0000000	1.8049999
-25603.0000000	1.8049999
-25602.0000000	1.8099999
-25601.0000000	1.8099999
-25600.0000000	1.8150001
-25599.0000000	1.8150001
-25598.0000000	1.8099999
-25597.0000000	1.8200001
-25596.0000000	1.8250000
-25595.0000000	1.8366667
-25594.0000000	1.8300000
-25593.0000000	1.8350000
-25592.0000000	1.8300000
-25591.0000000	1.8300000
-25590.0000000	1.8350000
-25589.0000000	1.8400000
-25588.0000000	1.8400000
-25587.0000000	1.8500000
-25586.0000000	1.8550000
-25585.0000000	1.8600000
-25584.0000000	1.8600000
-25583.0000000	1.8650000
-25582.0000000	1.8700000
-25581.0000000	1.8700000
-25580.0000000	1.8750000
-25579.0000000	1.8800000
-25578.0000000	1.8800000
-25577.0000000	1.8800000
-25576.0000000	1.8850000
-25575.0000000	1.8850000
-25574.0000000	1.8900000
-25573.0000000	1.8950000
-25572.0000000	1.8950000
-25571.0000000	1.9000000
-25570.0000000	1.9000000
-25569.0000000	1.9050000
-25568.0000000	1.9050000
-25567.0000000	1.9000000
-25566.0000000	1.9000000
-25565.0000000	1.9033333
-25564.0000000	1.9000000
-25563.0000000	1.9100000
-25562.0000000	1.9050000
-25561.0000000	1.9150000

-25560.0000000	1.9250000
-25559.0000000	1.9200000
-25558.0000000	1.9250000
-25557.0000000	1.9200000
-25556.0000000	1.9150000
-25555.0000000	1.9200000
-25554.0000000	1.9200000
-25553.0000000	1.9200000
-25552.0000000	1.9349999
-25551.0000000	1.9299999
-25550.0000000	1.9299999
-25549.0000000	1.9299999
-25548.0000000	1.9400001
-25547.0000000	1.9349999
-25546.0000000	1.9349999
-25545.0000000	1.9400001
-25544.0000000	1.9400001
-25543.0000000	1.9400001
-25542.0000000	1.9450001
-25541.0000000	1.9400001
-25540.0000000	1.9450001
-25539.0000000	1.9400001
-25538.0000000	1.9450001
-25537.0000000	1.9400001
-25536.0000000	1.9450001
-25535.0000000	1.9400001
-25534.0000000	1.9400001
-25533.0000000	1.9450001
-25532.0000000	1.9400001
-25531.0000000	1.9450001
-25530.0000000	1.9400001
-25529.0000000	1.9299999
-25528.0000000	1.9349999
-25527.0000000	1.9299999
-25526.0000000	1.9349999
-25525.0000000	1.9299999
-25524.0000000	1.9299999
-25523.0000000	1.9200000
-25522.0000000	1.9200000
-25521.0000000	1.9250000
-25520.0000000	1.9200000
-25519.0000000	1.9200000
-25518.0000000	1.9150000
-25517.0000000	1.9200000
-25516.0000000	1.9150000
-25515.0000000	1.9150000
-25514.0000000	1.9150000
-25513.0000000	1.9050000
-25512.0000000	1.8950000
-25511.0000000	1.9000000
-25510.0000000	1.9000000
-25509.0000000	1.8950000
-25508.0000000	1.8900000
-25507.0000000	1.8900000
-25506.0000000	1.8800000
-25505.0000000	1.8750000
-25504.0000000	1.8766667

-25503.0000000	1.8750000
-25502.0000000	1.8650000
-25501.0000000	1.8650000
-25500.0000000	1.8550000
-25499.0000000	1.8500000
-25498.0000000	1.8450000
-25497.0000000	1.8400000
-25496.0000000	1.8350000
-25495.0000000	1.8300000
-25494.0000000	1.8250000
-25493.0000000	1.8250000
-25492.0000000	1.8150001
-25491.0000000	1.8150001
-25490.0000000	1.8150001
-25489.0000000	1.8099999
-25488.0000000	1.8049999
-25487.0000000	1.8049999
-25486.0000000	1.8000000
-25485.0000000	1.7950000
-25484.0000000	1.7900000
-25483.0000000	1.7900000
-25482.0000000	1.7850000
-25481.0000000	1.7750000
-25480.0000000	1.7750000
-25479.0000000	1.7750000
-25478.0000000	1.7650000
-25477.0000000	1.7650000
-25476.0000000	1.7600000
-25475.0000000	1.7550000
-25474.0000000	1.7466666
-25473.0000000	1.7500000
-25472.0000000	1.7400000
-25471.0000000	1.7450000
-25470.0000000	1.7450000
-25469.0000000	1.7450000
-25468.0000000	1.7450000
-25467.0000000	1.7400000
-25466.0000000	1.7350000
-25465.0000000	1.7350000
-25464.0000000	1.7350000
-25463.0000000	1.7350000
-25462.0000000	1.7250000
-25461.0000000	1.7250000
-25460.0000000	1.7250000
-25459.0000000	1.7250000
-25458.0000000	1.7250000
-25457.0000000	1.7250000
-25456.0000000	1.7150000
-25455.0000000	1.7150000
-25454.0000000	1.7050000
-25453.0000000	1.7050000
-25452.0000000	1.7150000
-25451.0000000	1.7100000
-25450.0000000	1.7050000
-25449.0000000	1.7050000
-25448.0000000	1.7050000
-25447.0000000	1.7050000

-25446.0000000	1.7000000
-25445.0000000	1.7050000
-25444.0000000	1.6966667
-25443.0000000	1.7000000
-25442.0000000	1.6950001
-25441.0000000	1.6900001
-25440.0000000	1.6900001
-25439.0000000	1.6900001
-25438.0000000	1.6849999
-25437.0000000	1.6849999
-25436.0000000	1.6849999
-25435.0000000	1.6849999
-25434.0000000	1.6849999
-25433.0000000	1.6900001
-25432.0000000	1.6849999
-25431.0000000	1.6900001
-25430.0000000	1.6849999
-25429.0000000	1.6849999
-25428.0000000	1.6849999
-25427.0000000	1.6799999
-25426.0000000	1.6750000
-25425.0000000	1.6700000
-25424.0000000	1.6700000
-25423.0000000	1.6650000
-25422.0000000	1.6650000
-25421.0000000	1.6650000
-25420.0000000	1.6600000
-25419.0000000	1.6550000
-25418.0000000	1.6550000
-25417.0000000	1.6550000
-25416.0000000	1.6500000
-25415.0000000	1.6550000
-25414.0000000	1.6500000
-25413.0000000	1.6550000
-25412.0000000	1.6500000
-25411.0000000	1.6500000
-25410.0000000	1.6500000
-25409.0000000	1.6500000
-25408.0000000	1.6450000
-25407.0000000	1.6550000
-25406.0000000	1.6550000
-25405.0000000	1.6550000
-25404.0000000	1.6550000
-25403.0000000	1.6500000
-25402.0000000	1.6550000
-25401.0000000	1.6550000
-25400.0000000	1.6550000
-25399.0000000	1.6550000
-25398.0000000	1.6500000
-25397.0000000	1.6500000
-25396.0000000	1.6500000
-25395.0000000	1.6450000
-25394.0000000	1.6400000
-25393.0000000	1.6450000
-25392.0000000	1.6400000
-25391.0000000	1.6350000
-25390.0000000	1.6350000

-25389.0000000	1.6300000
-25388.0000000	1.6250000
-25387.0000000	1.6250000
-25386.0000000	1.6250000
-25385.0000000	1.6250000
-25384.0000000	1.6250000
-25383.0000000	1.6250000
-25382.0000000	1.6266667
-25381.0000000	1.6200000
-25380.0000000	1.6150000
-25379.0000000	1.6150000
-25378.0000000	1.6150000
-25377.0000000	1.6150000
-25376.0000000	1.6150000
-25375.0000000	1.6200000
-25374.0000000	1.6200000
-25373.0000000	1.6150000
-25372.0000000	1.6150000
-25371.0000000	1.6150000
-25370.0000000	1.6100000
-25369.0000000	1.6100000
-25368.0000000	1.6100000
-25367.0000000	1.6050000
-25366.0000000	1.6050000
-25365.0000000	1.6000000
-25364.0000000	1.5950000
-25363.0000000	1.5950000
-25362.0000000	1.5900000
-25361.0000000	1.5900000
-25360.0000000	1.5900000
-25359.0000000	1.5950000
-25358.0000000	1.5900000
-25357.0000000	1.5900000
-25356.0000000	1.5950000
-25355.0000000	1.5900000
-25354.0000000	1.5900000
-25353.0000000	1.5900000
-25352.0000000	1.5866667
-25351.0000000	1.5850000
-25350.0000000	1.5950000
-25349.0000000	1.5950000
-25348.0000000	1.5900000
-25347.0000000	1.5900000
-25346.0000000	1.5900000
-25345.0000000	1.5850000
-25344.0000000	1.5850000
-25343.0000000	1.5800000
-25342.0000000	1.5800000
-25341.0000000	1.5800000
-25340.0000000	1.5800000
-25339.0000000	1.5750000
-25338.0000000	1.5700001
-25337.0000000	1.5650001
-25336.0000000	1.5650001
-25335.0000000	1.5699999
-25334.0000000	1.5650001
-25333.0000000	1.5599999

-25332.0000000	1.5599999
-25331.0000000	1.5549999
-25330.0000000	1.5500000
-25329.0000000	1.5549999
-25328.0000000	1.5500000
-25327.0000000	1.5500000
-25326.0000000	1.5500000
-25325.0000000	1.5549999
-25324.0000000	1.5500000
-25323.0000000	1.5499998
-25322.0000000	1.5549999
-25321.0000000	1.5549999
-25320.0000000	1.5549999
-25319.0000000	1.5500000
-25318.0000000	1.5549999
-25317.0000000	1.5500000
-25316.0000000	1.5500000
-25315.0000000	1.5500000
-25314.0000000	1.5450000
-25313.0000000	1.5450000
-25312.0000000	1.5400000
-25311.0000000	1.5400000
-25310.0000000	1.5400000
-25309.0000000	1.5400000
-25308.0000000	1.5350000
-25307.0000000	1.5350000
-25306.0000000	1.5350000
-25305.0000000	1.5350000
-25304.0000000	1.5350000
-25303.0000000	1.5300000
-25302.0000000	1.5300000
-25301.0000000	1.5300000
-25300.0000000	1.5350000
-25299.0000000	1.5300000
-25298.0000000	1.5250000
-25297.0000000	1.5200000
-25296.0000000	1.5200000
-25295.0000000	1.5200000
-25294.0000000	1.5200000
-25293.0000000	1.5133333
-25292.0000000	1.5100000
-25291.0000000	1.5050000
-25290.0000000	1.5050000
-25289.0000000	1.5050000
-25288.0000000	1.5000000
-25287.0000000	1.5050000
-25286.0000000	1.5000000
-25285.0000000	1.5050000
-25284.0000000	1.5050000
-25283.0000000	1.5100000
-25282.0000000	1.5200000
-25281.0000000	1.5150000
-25280.0000000	1.5250000
-25279.0000000	1.5250000
-25278.0000000	1.5250000
-25277.0000000	1.5300000
-25276.0000000	1.5250000

-25275.0000000	1.5350000
-25274.0000000	1.5300000
-25273.0000000	1.5300000
-25272.0000000	1.5350000
-25271.0000000	1.5400000
-25270.0000000	1.5400000
-25269.0000000	1.5450000
-25268.0000000	1.5450000
-25267.0000000	1.5450000
-25266.0000000	1.5450000
-25265.0000000	1.5450000
-25264.0000000	1.5400000
-25263.0000000	1.5400000
-25262.0000000	1.5350000
-25261.0000000	1.5350000
-25260.0000000	1.5350000
-25259.0000000	1.5350000
-25258.0000000	1.5350000
-25257.0000000	1.5350000
-25256.0000000	1.5300000
-25255.0000000	1.5300000
-25254.0000000	1.5300000
-25253.0000000	1.5350000
-25252.0000000	1.5300000
-25251.0000000	1.5300000
-25250.0000000	1.5300000
-25249.0000000	1.5350000
-25248.0000000	1.5350000
-25247.0000000	1.5300000
-25246.0000000	1.5250000
-25245.0000000	1.5200000
-25244.0000000	1.5250000
-25243.0000000	1.5200000
-25242.0000000	1.5100000
-25241.0000000	1.5100000
-25240.0000000	1.5050000
-25239.0000000	1.4950000
-25238.0000000	1.4850000
-25237.0000000	1.4750000
-25236.0000000	1.4750000
-25235.0000000	1.4750000
-25234.0000000	1.4700000
-25233.0000000	1.4666667
-25232.0000000	1.4650000
-25231.0000000	1.4600000
-25230.0000000	1.4650000
-25229.0000000	1.4600000
-25228.0000000	1.4600000
-25227.0000000	1.4650000
-25226.0000000	1.4650000
-25225.0000000	1.4700000
-25224.0000000	1.4700000
-25223.0000000	1.4750000
-25222.0000000	1.4700000
-25221.0000000	1.4700000
-25220.0000000	1.4750000
-25219.0000000	1.4700000

-25218.0000000	1.4700000
-25217.0000000	1.4700000
-25216.0000000	1.4750000
-25215.0000000	1.4750000
-25214.0000000	1.4800000
-25213.0000000	1.4800000
-25212.0000000	1.4750000
-25211.0000000	1.4800000
-25210.0000000	1.4800000
-25209.0000000	1.4800000
-25208.0000000	1.4800000
-25207.0000000	1.4750000
-25206.0000000	1.4750000
-25205.0000000	1.4800000
-25204.0000000	1.4750000
-25203.0000000	1.4750000
-25202.0000000	1.4700000
-25201.0000000	1.4800000
-25200.0000000	1.4800000
-25199.0000000	1.4850000
-25198.0000000	1.4800000
-25197.0000000	1.4850000
-25196.0000000	1.4850000
-25195.0000000	1.4850000
-25194.0000000	1.4900000
-25193.0000000	1.4950000
-25192.0000000	1.4950000
-25191.0000000	1.4950000
-25190.0000000	1.5000000
-25189.0000000	1.4950000
-25188.0000000	1.5000000
-25187.0000000	1.5000000
-25186.0000000	1.4950000
-25185.0000000	1.5000000
-25184.0000000	1.5000000
-25183.0000000	1.5000000
-25182.0000000	1.4950000
-25181.0000000	1.4950000
-25180.0000000	1.5000000
-25179.0000000	1.4950000
-25178.0000000	1.5050000
-25177.0000000	1.5050000
-25176.0000000	1.5000000
-25175.0000000	1.5000000
-25174.0000000	1.5050000
-25173.0000000	1.5000000
-25172.0000000	1.5050000
-25171.0000000	1.5033334
-25170.0000000	1.5050000
-25169.0000000	1.5050000
-25168.0000000	1.4950000
-25167.0000000	1.5000000
-25166.0000000	1.5000000
-25165.0000000	1.4950000
-25164.0000000	1.4900000
-25163.0000000	1.4950000
-25162.0000000	1.4950000

-25161.0000000	1.4900000
-25160.0000000	1.4850000
-25159.0000000	1.4850000
-25158.0000000	1.4850000
-25157.0000000	1.4850000
-25156.0000000	1.4850000
-25155.0000000	1.4850000
-25154.0000000	1.4750000
-25153.0000000	1.4850000
-25152.0000000	1.4800000
-25151.0000000	1.4750000
-25150.0000000	1.4750000
-25149.0000000	1.4750000
-25148.0000000	1.4750000
-25147.0000000	1.4800000
-25146.0000000	1.4700000
-25145.0000000	1.4750000
-25144.0000000	1.4800000
-25143.0000000	1.4800000
-25142.0000000	1.4800000
-25141.0000000	1.4800000
-25140.0000000	1.4800000
-25139.0000000	1.4800000
-25138.0000000	1.4800000
-25137.0000000	1.4900000
-25136.0000000	1.4800000
-25135.0000000	1.4850000
-25134.0000000	1.4900000
-25133.0000000	1.4800000
-25132.0000000	1.4850000
-25131.0000000	1.4850000
-25130.0000000	1.4900000
-25129.0000000	1.4850000
-25128.0000000	1.4850000
-25127.0000000	1.4800000
-25126.0000000	1.4850000
-25125.0000000	1.4800000
-25124.0000000	1.4800000
-25123.0000000	1.4800000
-25122.0000000	1.4800000
-25121.0000000	1.4850000
-25120.0000000	1.4850000
-25119.0000000	1.4850000
-25118.0000000	1.4850000
-25117.0000000	1.4850000
-25116.0000000	1.4850000
-25115.0000000	1.4800000
-25114.0000000	1.4800000
-25113.0000000	1.4800000
-25112.0000000	1.4800000
-25111.0000000	1.4850000
-25110.0000000	1.4833332
-25109.0000000	1.4900000
-25108.0000000	1.4900000
-25107.0000000	1.4900000
-25106.0000000	1.4800000
-25105.0000000	1.4850000

-25104.0000000	1.4850000
-25103.0000000	1.4750000
-25102.0000000	1.4750000
-25101.0000000	1.4800000
-25100.0000000	1.4750000
-25099.0000000	1.4700000
-25098.0000000	1.4750000
-25097.0000000	1.4750000
-25096.0000000	1.4750000
-25095.0000000	1.4700000
-25094.0000000	1.4700000
-25093.0000000	1.4750000
-25092.0000000	1.4750000
-25091.0000000	1.4750000
-25090.0000000	1.4750000
-25089.0000000	1.4700000
-25088.0000000	1.4750000
-25087.0000000	1.4700000
-25086.0000000	1.4750000
-25085.0000000	1.4700000
-25084.0000000	1.4700000
-25083.0000000	1.4700000
-25082.0000000	1.4750000
-25081.0000000	1.4750000
-25080.0000000	1.4699999
-25079.0000000	1.4650000
-25078.0000000	1.4600000
-25077.0000000	1.4600000
-25076.0000000	1.4550000
-25075.0000000	1.4550000
-25074.0000000	1.4550000
-25073.0000000	1.4450001
-25072.0000000	1.4450001
-25071.0000000	1.4400001
-25070.0000000	1.4400001
-25069.0000000	1.4400001
-25068.0000000	1.4349999
-25067.0000000	1.4349999
-25066.0000000	1.4349999
-25065.0000000	1.4400001
-25064.0000000	1.4349999
-25063.0000000	1.4400001
-25062.0000000	1.4400001
-25061.0000000	1.4450001
-25060.0000000	1.4349999
-25059.0000000	1.4349999
-25058.0000000	1.4250000
-25057.0000000	1.4200000
-25056.0000000	1.4200000
-25055.0000000	1.4100000
-25054.0000000	1.4100000
-25053.0000000	1.4050000
-25052.0000000	1.4033333
-25051.0000000	1.4000000
-25050.0000000	1.4050000
-25049.0000000	1.4000000
-25048.0000000	1.4000000

-25047.0000000	1.4000000
-25046.0000000	1.4000000
-25045.0000000	1.4050000
-25044.0000000	1.4100000
-25043.0000000	1.4100000
-25042.0000000	1.4150000
-25041.0000000	1.4150000
-25040.0000000	1.4200000
-25039.0000000	1.4250000
-25038.0000000	1.4250000
-25037.0000000	1.4200000
-25036.0000000	1.4299999
-25035.0000000	1.4299999
-25034.0000000	1.4299999
-25033.0000000	1.4349999
-25032.0000000	1.4349999
-25031.0000000	1.4349999
-25030.0000000	1.4349999
-25029.0000000	1.4349999
-25028.0000000	1.4400001
-25027.0000000	1.4400001
-25026.0000000	1.4400001
-25025.0000000	1.4500000
-25024.0000000	1.4450001
-25023.0000000	1.4450001
-25022.0000000	1.4550000
-25021.0000000	1.4500000
-25020.0000000	1.4500000
-25019.0000000	1.4600000
-25018.0000000	1.4650000
-25017.0000000	1.4650000
-25016.0000000	1.4650000
-25015.0000000	1.4650000
-25014.0000000	1.4700000
-25013.0000000	1.4700000
-25012.0000000	1.4800000
-25011.0000000	1.4800000
-25010.0000000	1.4800000
-25009.0000000	1.4850000
-25008.0000000	1.4900000
-25007.0000000	1.4850000
-25006.0000000	1.4950000
-25005.0000000	1.4900000
-25004.0000000	1.4900000
-25003.0000000	1.5000000
-25002.0000000	1.5000000
-25001.0000000	1.5000000
-25000.0000000	1.5050000
-24999.0000000	1.5000000
-24998.0000000	1.5050000
-24997.0000000	1.5100000
-24996.0000000	1.5000000
-24995.0000000	1.5000000
-24994.0000000	1.5050000
-24993.0000000	1.5000000
-24992.0000000	1.4950000
-24991.0000000	1.4950000

-24990.0000000	1.4900000
-24989.0000000	1.4833332
-24988.0000000	1.4800000
-24987.0000000	1.4800000
-24986.0000000	1.4750000
-24985.0000000	1.4750000
-24984.0000000	1.4700000
-24983.0000000	1.4700000
-24982.0000000	1.4650000
-24981.0000000	1.4650000
-24980.0000000	1.4650000
-24979.0000000	1.4650000
-24978.0000000	1.4600000
-24977.0000000	1.4600000
-24976.0000000	1.4550000
-24975.0000000	1.4500000
-24974.0000000	1.4500000
-24973.0000000	1.4550000
-24972.0000000	1.4500000
-24971.0000000	1.4450001
-24970.0000000	1.4500000
-24969.0000000	1.4400001
-24968.0000000	1.4349999
-24967.0000000	1.4299999
-24966.0000000	1.4299999
-24965.0000000	1.4250000
-24964.0000000	1.4250000
-24963.0000000	1.4299999
-24962.0000000	1.4250000
-24961.0000000	1.4299999
-24960.0000000	1.4299999
-24959.0000000	1.4299999
-24958.0000000	1.4250000
-24957.0000000	1.4250000
-24956.0000000	1.4250000
-24955.0000000	1.4200000
-24954.0000000	1.4250000
-24953.0000000	1.4200000
-24952.0000000	1.4150000
-24951.0000000	1.4150000
-24950.0000000	1.4200000
-24949.0000000	1.4200000
-24948.0000000	1.4100000
-24947.0000000	1.4100000
-24946.0000000	1.4150000
-24945.0000000	1.4050000
-24944.0000000	1.4050000
-24943.0000000	1.4000000
-24942.0000000	1.3950000
-24941.0000000	1.4000000
-24940.0000000	1.3950000
-24939.0000000	1.4000000
-24938.0000000	1.3950000
-24937.0000000	1.3950000
-24936.0000000	1.3900000
-24935.0000000	1.3850000
-24934.0000000	1.3900000

-24933.0000000	1.3800000
-24932.0000000	1.3750000
-24931.0000000	1.3800000
-24930.0000000	1.3700000
-24929.0000000	1.3650000
-24928.0000000	1.3600000
-24927.0000000	1.3600000
-24926.0000000	1.3500000
-24925.0000000	1.3450000
-24924.0000000	1.3450000
-24923.0000000	1.3350000
-24922.0000000	1.3350000
-24921.0000000	1.3300000
-24920.0000000	1.3300000
-24919.0000000	1.3300000
-24918.0000000	1.3300000
-24917.0000000	1.3200001
-24916.0000000	1.3250000
-24915.0000000	1.3200001
-24914.0000000	1.3150001
-24913.0000000	1.3200001
-24912.0000000	1.3150001
-24911.0000000	1.3200001
-24910.0000000	1.3150001
-24909.0000000	1.3099999
-24908.0000000	1.3099999
-24907.0000000	1.3150001
-24906.0000000	1.3049999
-24905.0000000	1.3000000
-24904.0000000	1.3000000
-24903.0000000	1.2950000
-24902.0000000	1.2850000
-24901.0000000	1.2800000
-24900.0000000	1.2700000
-24899.0000000	1.2650000
-24898.0000000	1.2600000
-24897.0000000	1.2500000
-24896.0000000	1.2400000
-24895.0000000	1.2350000
-24894.0000000	1.2300000
-24893.0000000	1.2250000
-24892.0000000	1.2250000
-24891.0000000	1.2200000
-24890.0000000	1.2150000
-24889.0000000	1.2200000
-24888.0000000	1.2150000
-24887.0000000	1.2050000
-24886.0000000	1.2000000
-24885.0000000	1.2000000
-24884.0000000	1.1950001
-24883.0000000	1.1900001
-24882.0000000	1.1900001
-24881.0000000	1.1900001
-24880.0000000	1.1799999
-24879.0000000	1.1799999
-24878.0000000	1.1750000
-24877.0000000	1.1700000

-24876.0000000	1.1600000
-24875.0000000	1.1600000
-24874.0000000	1.1650000
-24873.0000000	1.1600000
-24872.0000000	1.1550000
-24871.0000000	1.1566666
-24870.0000000	1.1400000
-24869.0000000	1.1400000
-24868.0000000	1.1300000
-24867.0000000	1.1250000
-24866.0000000	1.1200000
-24865.0000000	1.1150000
-24864.0000000	1.1150000
-24863.0000000	1.1050000
-24862.0000000	1.1000000
-24861.0000000	1.1000000
-24860.0000000	1.0950000
-24859.0000000	1.0900000
-24858.0000000	1.0950000
-24857.0000000	1.0900000
-24856.0000000	1.0900000
-24855.0000000	1.0900000
-24854.0000000	1.0900000
-24853.0000000	1.0900000
-24852.0000000	1.0850000
-24851.0000000	1.0800000
-24850.0000000	1.0800000
-24849.0000000	1.0850000
-24848.0000000	1.0800000
-24847.0000000	1.0750000
-24846.0000000	1.0750000
-24845.0000000	1.0750000
-24844.0000000	1.0650001
-24843.0000000	1.0650001
-24842.0000000	1.0650001
-24841.0000000	1.0599999
-24840.0000000	1.0599999
-24839.0000000	1.0599999
-24838.0000000	1.0599999
-24837.0000000	1.0549999
-24836.0000000	1.0549999
-24835.0000000	1.0500000
-24834.0000000	1.0500000
-24833.0000000	1.0450000
-24832.0000000	1.0400000
-24831.0000000	1.0350000
-24830.0000000	1.0300000
-24829.0000000	1.0250000
-24828.0000000	1.0150000
-24827.0000000	1.0150000
-24826.0000000	1.0050000
-24825.0000000	1.0000000
-24824.0000000	1.0000000
-24823.0000000	1.0000000
-24822.0000000	0.9950000
-24821.0000000	0.9950000
-24820.0000000	0.9950000

-24819.0000000	1.0000000
-24818.0000000	1.0000000
-24817.0000000	1.0000000
-24816.0000000	1.0000000
-24815.0000000	0.9950000
-24814.0000000	0.9950000
-24813.0000000	0.9950000
-24812.0000000	0.9950000
-24811.0000000	0.9900000
-24810.0000000	0.9866667
-24809.0000000	0.9850000
-24808.0000000	0.9850000
-24807.0000000	0.9850000
-24806.0000000	0.9800000
-24805.0000000	0.9750000
-24804.0000000	0.9700000
-24803.0000000	0.9700000
-24802.0000000	0.9700000
-24801.0000000	0.9650000
-24800.0000000	0.9650000
-24799.0000000	0.9650000
-24798.0000000	0.9600000
-24797.0000000	0.9600000
-24796.0000000	0.9600000
-24795.0000000	0.9550000
-24794.0000000	0.9550000
-24793.0000000	0.9500000
-24792.0000000	0.9450000
-24791.0000000	0.9450000
-24790.0000000	0.9450000
-24789.0000000	0.9350000
-24788.0000000	0.9300000
-24787.0000000	0.9250000
-24786.0000000	0.9250000
-24785.0000000	0.9300000
-24784.0000000	0.9250000
-24783.0000000	0.9150000
-24782.0000000	0.9150000
-24781.0000000	0.9150000
-24780.0000000	0.9133334
-24779.0000000	0.9100000
-24778.0000000	0.9100000
-24777.0000000	0.9050000
-24776.0000000	0.9050000
-24775.0000000	0.9000000
-24774.0000000	0.9000000
-24773.0000000	0.9000000
-24772.0000000	0.8950000
-24771.0000000	0.9000000
-24770.0000000	0.8950000
-24769.0000000	0.8900000
-24768.0000000	0.8900000
-24767.0000000	0.8900000
-24766.0000000	0.8900000
-24765.0000000	0.8850000
-24764.0000000	0.8850000
-24763.0000000	0.8850000

-24762.0000000	0.8800000
-24761.0000000	0.8750000
-24760.0000000	0.8750000
-24759.0000000	0.8700000
-24758.0000000	0.8700000
-24757.0000000	0.8650000
-24756.0000000	0.8600000
-24755.0000000	0.8550000
-24754.0000000	0.8500000
-24753.0000000	0.8500000
-24752.0000000	0.8450000
-24751.0000000	0.8400000
-24750.0000000	0.8400000
-24749.0000000	0.8350000
-24748.0000000	0.8300000
-24747.0000000	0.8300000
-24746.0000000	0.8350000
-24745.0000000	0.8300000
-24744.0000000	0.8300000
-24743.0000000	0.8250000
-24742.0000000	0.8250000
-24741.0000000	0.8200000
-24740.0000000	0.8150000
-24739.0000000	0.8150000
-24738.0000000	0.8100000
-24737.0000000	0.8100000
-24736.0000000	0.8100000
-24735.0000000	0.8050000
-24734.0000000	0.8050000
-24733.0000000	0.8050000
-24732.0000000	0.8050000
-24731.0000000	0.8050000
-24730.0000000	0.8100000
-24729.0000000	0.8050000
-24728.0000000	0.8050000
-24727.0000000	0.8100000
-24726.0000000	0.8150000
-24725.0000000	0.8100000
-24724.0000000	0.8100000
-24723.0000000	0.8150000
-24722.0000000	0.8200000
-24721.0000000	0.8200000
-24720.0000000	0.8166667
-24719.0000000	0.8150000
-24718.0000000	0.8200000
-24717.0000000	0.8200000
-24716.0000000	0.8200000
-24715.0000000	0.8200000
-24714.0000000	0.8200000
-24713.0000000	0.8250000
-24712.0000000	0.8250000
-24711.0000000	0.8200000
-24710.0000000	0.8200000
-24709.0000000	0.8200000
-24708.0000000	0.8250000
-24707.0000000	0.8250000
-24706.0000000	0.8300000

-24705.0000000	0.8300000
-24704.0000000	0.8300000
-24703.0000000	0.8300000
-24702.0000000	0.8300000
-24701.0000000	0.8300000
-24700.0000000	0.8300000
-24699.0000000	0.8350000
-24698.0000000	0.8300000
-24697.0000000	0.8300000
-24696.0000000	0.8300000
-24695.0000000	0.8300000
-24694.0000000	0.8300000
-24693.0000000	0.8250000
-24692.0000000	0.8250000
-24691.0000000	0.8200000
-24690.0000000	0.8150000
-24689.0000000	0.8200000
-24688.0000000	0.8166667
-24687.0000000	0.8200000
-24686.0000000	0.8150000
-24685.0000000	0.8150000
-24684.0000000	0.8150000
-24683.0000000	0.8150000
-24682.0000000	0.8150000
-24681.0000000	0.8100000
-24680.0000000	0.8150000
-24679.0000000	0.8150000
-24678.0000000	0.8100000
-24677.0000000	0.8150000
-24676.0000000	0.8050000
-24675.0000000	0.8050000
-24674.0000000	0.8100000
-24673.0000000	0.8100000
-24672.0000000	0.8050000
-24671.0000000	0.8050000
-24670.0000000	0.8050000
-24669.0000000	0.8050000
-24668.0000000	0.8050000
-24667.0000000	0.8050000
-24666.0000000	0.8050000
-24665.0000000	0.8050000
-24664.0000000	0.8050000
-24663.0000000	0.8050000
-24662.0000000	0.8050000
-24661.0000000	0.8050000
-24660.0000000	0.8100000
-24659.0000000	0.8100000
-24658.0000000	0.8150000
-24657.0000000	0.8200000
-24656.0000000	0.8200000
-24655.0000000	0.8200000
-24654.0000000	0.8250000
-24653.0000000	0.8300000
-24652.0000000	0.8300000
-24651.0000000	0.8350000
-24650.0000000	0.8350000
-24649.0000000	0.8350000

-24648.0000000	0.8350000
-24647.0000000	0.8400000
-24646.0000000	0.8400000
-24645.0000000	0.8450000
-24644.0000000	0.8500000
-24643.0000000	0.8550000
-24642.0000000	0.8550000
-24641.0000000	0.8600000
-24640.0000000	0.8600000
-24639.0000000	0.8600000
-24638.0000000	0.8650000
-24637.0000000	0.8700000
-24636.0000000	0.8700000
-24635.0000000	0.8750000
-24634.0000000	0.8750000
-24633.0000000	0.8700000
-24632.0000000	0.8700000
-24631.0000000	0.8700000
-24630.0000000	0.8700000
-24629.0000000	0.8700001
-24628.0000000	0.8650000
-24627.0000000	0.8650000
-24626.0000000	0.8650000
-24625.0000000	0.8600000
-24624.0000000	0.8550000
-24623.0000000	0.8550000
-24622.0000000	0.8550000
-24621.0000000	0.8500000
-24620.0000000	0.8500000
-24619.0000000	0.8500000
-24618.0000000	0.8450000
-24617.0000000	0.8400000
-24616.0000000	0.8400000
-24615.0000000	0.8450000
-24614.0000000	0.8450000
-24613.0000000	0.8450000
-24612.0000000	0.8450000
-24611.0000000	0.8400000
-24610.0000000	0.8400000
-24609.0000000	0.8400000
-24608.0000000	0.8400000
-24607.0000000	0.8450000
-24606.0000000	0.8350000
-24605.0000000	0.8400000
-24604.0000000	0.8400000
-24603.0000000	0.8400000
-24602.0000000	0.8400000
-24601.0000000	0.8450000
-24600.0000000	0.8450000
-24599.0000000	0.8400000
-24598.0000000	0.8400000
-24597.0000000	0.8400000
-24596.0000000	0.8400000
-24595.0000000	0.8350000
-24594.0000000	0.8400000
-24593.0000000	0.8400000
-24592.0000000	0.8350000

-24591.0000000	0.8400000
-24590.0000000	0.8350000
-24589.0000000	0.8300000
-24588.0000000	0.8300000
-24587.0000000	0.8300000
-24586.0000000	0.8250000
-24585.0000000	0.8250000
-24584.0000000	0.8250000
-24583.0000000	0.8250000
-24582.0000000	0.8200000
-24581.0000000	0.8200000
-24580.0000000	0.8200000
-24579.0000000	0.8150000
-24578.0000000	0.8150000
-24577.0000000	0.8150000
-24576.0000000	0.8100000
-24575.0000000	0.8100000
-24574.0000000	0.8100000
-24573.0000000	0.8050000
-24572.0000000	0.8050000
-24571.0000000	0.8050000
-24570.0000000	0.8050000
-24569.0000000	0.8000000
-24568.0000000	0.8100000
-24567.0000000	0.8000000
-24566.0000000	0.7950000
-24565.0000000	0.8000000
-24564.0000000	0.7950000
-24563.0000000	0.7950000
-24562.0000000	0.7950000
-24561.0000000	0.7950000
-24560.0000000	0.7950000
-24559.0000000	0.7950000
-24558.0000000	0.7950000
-24557.0000000	0.7950000
-24556.0000000	0.7950000
-24555.0000000	0.8000000
-24554.0000000	0.8000000
-24553.0000000	0.8050000
-24552.0000000	0.8000000
-24551.0000000	0.8050000
-24550.0000000	0.8050000
-24549.0000000	0.8050000
-24548.0000000	0.8100000
-24547.0000000	0.8100000
-24546.0000000	0.8100000
-24545.0000000	0.8150000
-24544.0000000	0.8100000
-24543.0000000	0.8150000
-24542.0000000	0.8150000
-24541.0000000	0.8150000
-24540.0000000	0.8200000
-24539.0000000	0.8200000
-24538.0000000	0.8150000
-24537.0000000	0.8133333
-24536.0000000	0.8150000
-24535.0000000	0.8150000

-24534.0000000	0.8100000
-24533.0000000	0.8100000
-24532.0000000	0.8050000
-24531.0000000	0.8000000
-24530.0000000	0.8050000
-24529.0000000	0.7950000
-24528.0000000	0.7950000
-24527.0000000	0.7900000
-24526.0000000	0.7950000
-24525.0000000	0.7900000
-24524.0000000	0.8000000
-24523.0000000	0.7950000
-24522.0000000	0.7950000
-24521.0000000	0.8000000
-24520.0000000	0.7950000
-24519.0000000	0.7950000
-24518.0000000	0.7950000
-24517.0000000	0.8000000
-24516.0000000	0.8000000
-24515.0000000	0.8000000
-24514.0000000	0.8050000
-24513.0000000	0.8050000
-24512.0000000	0.8000000
-24511.0000000	0.8050000
-24510.0000000	0.8050000
-24509.0000000	0.8050000
-24508.0000000	0.8033333
-24507.0000000	0.8000000
-24506.0000000	0.8050000
-24505.0000000	0.8050000
-24504.0000000	0.8100000
-24503.0000000	0.8100000
-24502.0000000	0.8100000
-24501.0000000	0.8050000
-24500.0000000	0.8050000
-24499.0000000	0.8050000
-24498.0000000	0.8050000
-24497.0000000	0.8000000
-24496.0000000	0.8000000
-24495.0000000	0.7950000
-24494.0000000	0.7950000
-24493.0000000	0.7950000
-24492.0000000	0.7950000
-24491.0000000	0.7950000
-24490.0000000	0.7950000
-24489.0000000	0.7900000
-24488.0000000	0.7900000
-24487.0000000	0.7850000
-24486.0000000	0.7850000
-24485.0000000	0.7800000
-24484.0000000	0.7800000
-24483.0000000	0.7800000
-24482.0000000	0.7800000
-24481.0000000	0.7750000
-24480.0000000	0.7750000
-24479.0000000	0.7700000
-24478.0000000	0.7750000

-24477.0000000	0.7750000
-24476.0000000	0.7733333
-24475.0000000	0.7700000
-24474.0000000	0.7750000
-24473.0000000	0.7700000
-24472.0000000	0.7700000
-24471.0000000	0.7750000
-24470.0000000	0.7800000
-24469.0000000	0.7750000
-24468.0000000	0.7800000
-24467.0000000	0.7750000
-24466.0000000	0.7750000
-24465.0000000	0.7800000
-24464.0000000	0.7850000
-24463.0000000	0.7800000
-24462.0000000	0.7800000
-24461.0000000	0.7800000
-24460.0000000	0.7850000
-24459.0000000	0.7850000
-24458.0000000	0.7950000
-24457.0000000	0.7900000
-24456.0000000	0.7950000
-24455.0000000	0.7950000
-24454.0000000	0.7900000
-24453.0000000	0.7900000
-24452.0000000	0.7900000
-24451.0000000	0.7950000
-24450.0000000	0.7950000
-24449.0000000	0.7950000
-24448.0000000	0.7900000
-24447.0000000	0.7966667
-24446.0000000	0.7950000
-24445.0000000	0.7950000
-24444.0000000	0.7950000
-24443.0000000	0.7950000
-24442.0000000	0.7950000
-24441.0000000	0.8000000
-24440.0000000	0.7950000
-24439.0000000	0.7950000
-24438.0000000	0.7950000
-24437.0000000	0.7950000
-24436.0000000	0.7950000
-24435.0000000	0.7900000
-24434.0000000	0.7900000
-24433.0000000	0.7900000
-24432.0000000	0.7900000
-24431.0000000	0.7850000
-24430.0000000	0.7850000
-24429.0000000	0.7850000
-24428.0000000	0.7800000
-24427.0000000	0.7800000
-24426.0000000	0.7800000
-24425.0000000	0.7800000
-24424.0000000	0.7800000
-24423.0000000	0.7800000
-24422.0000000	0.7750000
-24421.0000000	0.7750000

-24420.0000000	0.7800000
-24419.0000000	0.7750000
-24418.0000000	0.7750000
-24417.0000000	0.7800000
-24416.0000000	0.7733333
-24415.0000000	0.7750000
-24414.0000000	0.7750000
-24413.0000000	0.7750000
-24412.0000000	0.7700000
-24411.0000000	0.7700000
-24410.0000000	0.7700000
-24409.0000000	0.7700000
-24408.0000000	0.7750000
-24407.0000000	0.7750000
-24406.0000000	0.7700000
-24405.0000000	0.7750000
-24404.0000000	0.7700000
-24403.0000000	0.7750000
-24402.0000000	0.7750000
-24401.0000000	0.7700000
-24400.0000000	0.7750000
-24399.0000000	0.7750000
-24398.0000000	0.7750000
-24397.0000000	0.7800000
-24396.0000000	0.7800000
-24395.0000000	0.7800000
-24394.0000000	0.7800000
-24393.0000000	0.7800000
-24392.0000000	0.7750000
-24391.0000000	0.7750000
-24390.0000000	0.7750000
-24389.0000000	0.7700000
-24388.0000000	0.7750000
-24387.0000000	0.7700000
-24386.0000000	0.7766666
-24385.0000000	0.7750000
-24384.0000000	0.7800000
-24383.0000000	0.7800000
-24382.0000000	0.7800000
-24381.0000000	0.7800000
-24380.0000000	0.7800000
-24379.0000000	0.7850000
-24378.0000000	0.7850000
-24377.0000000	0.7900000
-24376.0000000	0.7950000
-24375.0000000	0.8000000
-24374.0000000	0.8000000
-24373.0000000	0.7950000
-24372.0000000	0.7950000
-24371.0000000	0.8000000
-24370.0000000	0.7950000
-24369.0000000	0.8050000
-24368.0000000	0.8000000
-24367.0000000	0.8050000
-24366.0000000	0.8100000
-24365.0000000	0.8100000
-24364.0000000	0.8150000

-24363.0000000	0.8100000
-24362.0000000	0.8200000
-24361.0000000	0.8200000
-24360.0000000	0.8250000
-24359.0000000	0.8250000
-24358.0000000	0.8300000
-24357.0000000	0.8300000
-24356.0000000	0.8400000
-24355.0000000	0.8400000
-24354.0000000	0.8400000
-24353.0000000	0.8400000
-24352.0000000	0.8400000
-24351.0000000	0.8400000
-24350.0000000	0.8450000
-24349.0000000	0.8450000
-24348.0000000	0.8450000
-24347.0000000	0.8500000
-24346.0000000	0.8500000
-24345.0000000	0.8500000
-24344.0000000	0.8550000
-24343.0000000	0.8550000
-24342.0000000	0.8600000
-24341.0000000	0.8600000
-24340.0000000	0.8600000
-24339.0000000	0.8600000
-24338.0000000	0.8650000
-24337.0000000	0.8650000
-24336.0000000	0.8650000
-24335.0000000	0.8700000
-24334.0000000	0.8650000
-24333.0000000	0.8700000
-24332.0000000	0.8700000
-24331.0000000	0.8750000
-24330.0000000	0.8750000
-24329.0000000	0.8750000
-24328.0000000	0.8800000
-24327.0000000	0.8866666
-24326.0000000	0.8850000
-24325.0000000	0.8900000
-24324.0000000	0.8900000
-24323.0000000	0.8900000
-24322.0000000	0.8950000
-24321.0000000	0.8950000
-24320.0000000	0.8950000
-24319.0000000	0.9000000
-24318.0000000	0.9000000
-24317.0000000	0.9000000
-24316.0000000	0.9000000
-24315.0000000	0.9000000
-24314.0000000	0.9000000
-24313.0000000	0.9000000
-24312.0000000	0.9050000
-24311.0000000	0.9000000
-24310.0000000	0.9000000
-24309.0000000	0.9000000
-24308.0000000	0.9050000
-24307.0000000	0.9000000

-24306.0000000	0.9050000
-24305.0000000	0.9050000
-24304.0000000	0.9050000
-24303.0000000	0.9050000
-24302.0000000	0.9100000
-24301.0000000	0.9100000
-24300.0000000	0.9100000
-24299.0000000	0.9150000
-24298.0000000	0.9150000
-24297.0000000	0.9150000
-24296.0000000	0.9166667
-24295.0000000	0.9200000
-24294.0000000	0.9200000
-24293.0000000	0.9200000
-24292.0000000	0.9250000
-24291.0000000	0.9250000
-24290.0000000	0.9250000
-24289.0000000	0.9200000
-24288.0000000	0.9250000
-24287.0000000	0.9250000
-24286.0000000	0.9250000
-24285.0000000	0.9250000
-24284.0000000	0.9200000
-24283.0000000	0.9200000
-24282.0000000	0.9200000
-24281.0000000	0.9200000
-24280.0000000	0.9200000
-24279.0000000	0.9200000
-24278.0000000	0.9200000
-24277.0000000	0.9150000
-24276.0000000	0.9200000
-24275.0000000	0.9200000
-24274.0000000	0.9250000
-24273.0000000	0.9300000
-24272.0000000	0.9300000
-24271.0000000	0.9300000
-24270.0000000	0.9350000
-24269.0000000	0.9400000
-24268.0000000	0.9400000
-24267.0000000	0.9400000
-24266.0000000	0.9450000
-24265.0000000	0.9433333
-24264.0000000	0.9450000
-24263.0000000	0.9500000
-24262.0000000	0.9500000
-24261.0000000	0.9550000
-24260.0000000	0.9550000
-24259.0000000	0.9500000
-24258.0000000	0.9550000
-24257.0000000	0.9550000
-24256.0000000	0.9550000
-24255.0000000	0.9600000
-24254.0000000	0.9600000
-24253.0000000	0.9600000
-24252.0000000	0.9600000
-24251.0000000	0.9600000
-24250.0000000	0.9600000

-24249.0000000	0.9550000
-24248.0000000	0.9600000
-24247.0000000	0.9600000
-24246.0000000	0.9550000
-24245.0000000	0.9550000
-24244.0000000	0.9550000
-24243.0000000	0.9550000
-24242.0000000	0.9550000
-24241.0000000	0.9550000
-24240.0000000	0.9550000
-24239.0000000	0.9550000
-24238.0000000	0.9600000
-24237.0000000	0.9600000
-24236.0000000	0.9600000
-24235.0000000	0.9650000
-24234.0000000	0.9650000
-24233.0000000	0.9650000
-24232.0000000	0.9600000
-24231.0000000	0.9600000
-24230.0000000	0.9650000
-24229.0000000	0.9700000
-24228.0000000	0.9700000
-24227.0000000	0.9750000
-24226.0000000	0.9750000
-24225.0000000	0.9800000
-24224.0000000	0.9850000
-24223.0000000	0.9850000
-24222.0000000	0.9900000
-24221.0000000	0.9950000
-24220.0000000	0.9950000
-24219.0000000	0.9950000
-24218.0000000	1.0000000
-24217.0000000	0.9950000
-24216.0000000	0.9950000
-24215.0000000	0.9950000
-24214.0000000	0.9950000
-24213.0000000	0.9950000
-24212.0000000	0.9950000
-24211.0000000	0.9950000
-24210.0000000	1.0000000
-24209.0000000	1.0000000
-24208.0000000	1.0000000
-24207.0000000	1.0000000
-24206.0000000	1.0033333
-24205.0000000	1.0050000
-24204.0000000	1.0050000
-24203.0000000	1.0100000
-24202.0000000	1.0050000
-24201.0000000	1.0050000
-24200.0000000	1.0100000
-24199.0000000	1.0100000
-24198.0000000	1.0100000
-24197.0000000	1.0100000
-24196.0000000	1.0100000
-24195.0000000	1.0100000
-24194.0000000	1.0150000
-24193.0000000	1.0100000

-24192.0000000	1.0100000
-24191.0000000	1.0150000
-24190.0000000	1.0100000
-24189.0000000	1.0100000
-24188.0000000	1.0200000
-24187.0000000	1.0150000
-24186.0000000	1.0200000
-24185.0000000	1.0200000
-24184.0000000	1.0200000
-24183.0000000	1.0300000
-24182.0000000	1.0300000
-24181.0000000	1.0300000
-24180.0000000	1.0300000
-24179.0000000	1.0350000
-24178.0000000	1.0400000
-24177.0000000	1.0450000
-24176.0000000	1.0433333
-24175.0000000	1.0400000
-24174.0000000	1.0400000
-24173.0000000	1.0450000
-24172.0000000	1.0500000
-24171.0000000	1.0500000
-24170.0000000	1.0549999
-24169.0000000	1.0599999
-24168.0000000	1.0599999
-24167.0000000	1.0599999
-24166.0000000	1.0599999
-24165.0000000	1.0650001
-24164.0000000	1.0650001
-24163.0000000	1.0650001
-24162.0000000	1.0700001
-24161.0000000	1.0750000
-24160.0000000	1.0700001
-24159.0000000	1.0800000
-24158.0000000	1.0850000
-24157.0000000	1.0850000
-24156.0000000	1.0850000
-24155.0000000	1.0850000
-24154.0000000	1.0900000
-24153.0000000	1.0950000
-24152.0000000	1.1000000
-24151.0000000	1.0950000
-24150.0000000	1.0900000
-24149.0000000	1.0950000
-24148.0000000	1.1000000
-24147.0000000	1.1000000
-24146.0000000	1.1000000
-24145.0000000	1.1000000
-24144.0000000	1.0950000
-24143.0000000	1.0950000
-24142.0000000	1.1050000
-24141.0000000	1.0950000
-24140.0000000	1.1000000
-24139.0000000	1.1000000
-24138.0000000	1.1000000
-24137.0000000	1.1000000
-24136.0000000	1.1000000

-24135.0000000	1.1000000
-24134.0000000	1.1050000
-24133.0000000	1.1050000
-24132.0000000	1.1050000
-24131.0000000	1.1100000
-24130.0000000	1.1150000
-24129.0000000	1.1150000
-24128.0000000	1.1150000
-24127.0000000	1.1150000
-24126.0000000	1.1150000
-24125.0000000	1.1200000
-24124.0000000	1.1200000
-24123.0000000	1.1150000
-24122.0000000	1.1250000
-24121.0000000	1.1200000
-24120.0000000	1.1200000
-24119.0000000	1.1250000
-24118.0000000	1.1300000
-24117.0000000	1.1350000
-24116.0000000	1.1333333
-24115.0000000	1.1300000
-24114.0000000	1.1350000
-24113.0000000	1.1450000
-24112.0000000	1.1400000
-24111.0000000	1.1400000
-24110.0000000	1.1500000
-24109.0000000	1.1600000
-24108.0000000	1.1550000
-24107.0000000	1.1600000
-24106.0000000	1.1650000
-24105.0000000	1.1650000
-24104.0000000	1.1700000
-24103.0000000	1.1700000
-24102.0000000	1.1750000
-24101.0000000	1.1750000
-24100.0000000	1.1799999
-24099.0000000	1.1799999
-24098.0000000	1.1849999
-24097.0000000	1.1900001
-24096.0000000	1.1900001
-24095.0000000	1.1900001
-24094.0000000	1.1950001
-24093.0000000	1.1950001
-24092.0000000	1.2050000
-24091.0000000	1.2050000
-24090.0000000	1.2000000
-24089.0000000	1.2000000
-24088.0000000	1.2000000
-24087.0000000	1.2100000
-24086.0000000	1.2050000
-24085.0000000	1.2133334
-24084.0000000	1.2100000
-24083.0000000	1.2150000
-24082.0000000	1.2100000
-24081.0000000	1.2100000
-24080.0000000	1.2100000
-24079.0000000	1.2100000

-24078.0000000	1.2100000
-24077.0000000	1.2100000
-24076.0000000	1.2150000
-24075.0000000	1.2150000
-24074.0000000	1.2150000
-24073.0000000	1.2150000
-24072.0000000	1.2100000
-24071.0000000	1.2050000
-24070.0000000	1.2050000
-24069.0000000	1.2050000
-24068.0000000	1.2100000
-24067.0000000	1.2050000
-24066.0000000	1.2050000
-24065.0000000	1.2050000
-24064.0000000	1.2050000
-24063.0000000	1.2050000
-24062.0000000	1.2000000
-24061.0000000	1.2100000
-24060.0000000	1.2000000
-24059.0000000	1.2100000
-24058.0000000	1.2150000
-24057.0000000	1.2100000
-24056.0000000	1.2100000
-24055.0000000	1.2133334
-24054.0000000	1.2150000
-24053.0000000	1.2150000
-24052.0000000	1.2200000
-24051.0000000	1.2150000
-24050.0000000	1.2150000
-24049.0000000	1.2150000
-24048.0000000	1.2100000
-24047.0000000	1.2100000
-24046.0000000	1.2100000
-24045.0000000	1.2100000
-24044.0000000	1.2050000
-24043.0000000	1.2050000
-24042.0000000	1.2050000
-24041.0000000	1.2000000
-24040.0000000	1.2000000
-24039.0000000	1.2000000
-24038.0000000	1.1950001
-24037.0000000	1.2000000
-24036.0000000	1.2000000
-24035.0000000	1.2000000
-24034.0000000	1.2050000
-24033.0000000	1.2050000
-24032.0000000	1.2100000
-24031.0000000	1.2100000
-24030.0000000	1.2150000
-24029.0000000	1.2200000
-24028.0000000	1.2200000
-24027.0000000	1.2200000
-24026.0000000	1.2150000
-24025.0000000	1.2100000
-24024.0000000	1.2133334
-24023.0000000	1.2150000
-24022.0000000	1.2100000

-24021.0000000	1.2150000
-24020.0000000	1.2150000
-24019.0000000	1.2150000
-24018.0000000	1.2200000
-24017.0000000	1.2150000
-24016.0000000	1.2200000
-24015.0000000	1.2250000
-24014.0000000	1.2300000
-24013.0000000	1.2350000
-24012.0000000	1.2350000
-24011.0000000	1.2400000
-24010.0000000	1.2450000
-24009.0000000	1.2450000
-24008.0000000	1.2450000
-24007.0000000	1.2550000
-24006.0000000	1.2500000
-24005.0000000	1.2550000
-24004.0000000	1.2600000
-24003.0000000	1.2600000
-24002.0000000	1.2550000
-24001.0000000	1.2650000
-24000.0000000	1.2600000
-23999.0000000	1.2600000
-23998.0000000	1.2600000
-23997.0000000	1.2650000
-23996.0000000	1.2600000
-23995.0000000	1.2600000
-23994.0000000	1.2633333
-23993.0000000	1.2650000
-23992.0000000	1.2750000
-23991.0000000	1.2750000
-23990.0000000	1.2750000
-23989.0000000	1.2800000
-23988.0000000	1.2850000
-23987.0000000	1.2900000
-23986.0000000	1.2900000
-23985.0000000	1.3000000
-23984.0000000	1.3000000
-23983.0000000	1.3000000
-23982.0000000	1.3000000
-23981.0000000	1.3000000
-23980.0000000	1.3049999
-23979.0000000	1.3099999
-23978.0000000	1.3049999
-23977.0000000	1.3150001
-23976.0000000	1.3150001
-23975.0000000	1.3150001
-23974.0000000	1.3200001
-23973.0000000	1.3200001
-23972.0000000	1.3300000
-23971.0000000	1.3400000
-23970.0000000	1.3400000
-23969.0000000	1.3450000
-23968.0000000	1.3450000
-23967.0000000	1.3450000
-23966.0000000	1.3550000
-23965.0000000	1.3550000

-23964.0000000	1.3600000
-23963.0000000	1.3633333
-23962.0000000	1.3750000
-23961.0000000	1.3750000
-23960.0000000	1.3750000
-23959.0000000	1.3800000
-23958.0000000	1.3800000
-23957.0000000	1.3800000
-23956.0000000	1.3850000
-23955.0000000	1.3800000
-23954.0000000	1.3850000
-23953.0000000	1.3900000
-23952.0000000	1.3900000
-23951.0000000	1.4000000
-23950.0000000	1.3950000
-23949.0000000	1.4050000
-23948.0000000	1.4100000
-23947.0000000	1.4100000
-23946.0000000	1.4200000
-23945.0000000	1.4299999
-23944.0000000	1.4349999
-23943.0000000	1.4450001
-23942.0000000	1.4450001
-23941.0000000	1.4500000
-23940.0000000	1.4550000
-23939.0000000	1.4500000
-23938.0000000	1.4550000
-23937.0000000	1.4600000
-23936.0000000	1.4650000
-23935.0000000	1.4650000
-23934.0000000	1.4699999
-23933.0000000	1.4800000
-23932.0000000	1.4800000
-23931.0000000	1.4800000
-23930.0000000	1.4850000
-23929.0000000	1.4850000
-23928.0000000	1.4900000
-23927.0000000	1.4900000
-23926.0000000	1.4900000
-23925.0000000	1.4900000
-23924.0000000	1.4900000
-23923.0000000	1.4900000
-23922.0000000	1.4900000
-23921.0000000	1.4950000
-23920.0000000	1.4950000
-23919.0000000	1.4950000
-23918.0000000	1.4950000
-23917.0000000	1.5000000
-23916.0000000	1.5050000
-23915.0000000	1.5000000
-23914.0000000	1.5050000
-23913.0000000	1.5050000
-23912.0000000	1.5050000
-23911.0000000	1.5100000
-23910.0000000	1.5100000
-23909.0000000	1.5100000
-23908.0000000	1.5050000

-23907.0000000	1.5050000
-23906.0000000	1.5050000
-23905.0000000	1.5050000
-23904.0000000	1.5066667
-23903.0000000	1.5050000
-23902.0000000	1.5050000
-23901.0000000	1.5100000
-23900.0000000	1.5100000
-23899.0000000	1.5100000
-23898.0000000	1.5150000
-23897.0000000	1.5050000
-23896.0000000	1.5000000
-23895.0000000	1.5000000
-23894.0000000	1.5000000
-23893.0000000	1.5000000
-23892.0000000	1.4950000
-23891.0000000	1.4950000
-23890.0000000	1.4950000
-23889.0000000	1.5000000
-23888.0000000	1.4950000
-23887.0000000	1.5000000
-23886.0000000	1.5000000
-23885.0000000	1.4950000
-23884.0000000	1.4950000
-23883.0000000	1.4900000
-23882.0000000	1.4900000
-23881.0000000	1.4850000
-23880.0000000	1.4900000
-23879.0000000	1.4900000
-23878.0000000	1.4950000
-23877.0000000	1.4950000
-23876.0000000	1.4950000
-23875.0000000	1.4950000
-23874.0000000	1.5000000
-23873.0000000	1.5000000
-23872.0000000	1.4966666
-23871.0000000	1.4950000
-23870.0000000	1.4900000
-23869.0000000	1.4900000
-23868.0000000	1.4900000
-23867.0000000	1.4850000
-23866.0000000	1.4900000
-23865.0000000	1.4900000
-23864.0000000	1.4900000
-23863.0000000	1.4950000
-23862.0000000	1.4950000
-23861.0000000	1.4900000
-23860.0000000	1.4900000
-23859.0000000	1.4900000
-23858.0000000	1.4900000
-23857.0000000	1.4900000
-23856.0000000	1.4900000
-23855.0000000	1.4950000
-23854.0000000	1.4900000
-23853.0000000	1.4850000
-23852.0000000	1.4850000
-23851.0000000	1.4900000

-23850.0000000	1.4900000
-23849.0000000	1.4850000
-23848.0000000	1.4900000
-23847.0000000	1.4850000
-23846.0000000	1.4900000
-23845.0000000	1.4900000
-23844.0000000	1.4900000
-23843.0000000	1.4900000
-23842.0000000	1.4850000
-23841.0000000	1.4933333
-23840.0000000	1.4900000
-23839.0000000	1.4900000
-23838.0000000	1.4900000
-23837.0000000	1.4900000
-23836.0000000	1.4900000
-23835.0000000	1.4950000
-23834.0000000	1.4850000
-23833.0000000	1.4850000
-23832.0000000	1.4900000
-23831.0000000	1.4900000
-23830.0000000	1.4900000
-23829.0000000	1.4900000
-23828.0000000	1.4900000
-23827.0000000	1.4900000
-23826.0000000	1.4900000
-23825.0000000	1.4800000
-23824.0000000	1.4850000
-23823.0000000	1.4800000
-23822.0000000	1.4750000
-23821.0000000	1.4750000
-23820.0000000	1.4750000
-23819.0000000	1.4650000
-23818.0000000	1.4600000
-23817.0000000	1.4600000
-23816.0000000	1.4600000
-23815.0000000	1.4600000
-23814.0000000	1.4650000
-23813.0000000	1.4550000
-23812.0000000	1.4650000
-23811.0000000	1.4566666
-23810.0000000	1.4650000
-23809.0000000	1.4650000
-23808.0000000	1.4600000
-23807.0000000	1.4650000
-23806.0000000	1.4600000
-23805.0000000	1.4650000
-23804.0000000	1.4700000
-23803.0000000	1.4650000
-23802.0000000	1.4750000
-23801.0000000	1.4700000
-23800.0000000	1.4800000
-23799.0000000	1.4900000
-23798.0000000	1.4950000
-23797.0000000	1.5000000
-23796.0000000	1.5000000
-23795.0000000	1.5050000
-23794.0000000	1.5100000

-23793.0000000	1.5100000
-23792.0000000	1.5150000
-23791.0000000	1.5100000
-23790.0000000	1.5150000
-23789.0000000	1.5100000
-23788.0000000	1.5100000
-23787.0000000	1.5050000
-23786.0000000	1.5050000
-23785.0000000	1.5050000
-23784.0000000	1.5000000
-23783.0000000	1.5033334
-23782.0000000	1.4950000
-23781.0000000	1.4900000
-23780.0000000	1.4850000
-23779.0000000	1.4850000
-23778.0000000	1.4850000
-23777.0000000	1.4850000
-23776.0000000	1.4850000
-23775.0000000	1.4750000
-23774.0000000	1.4800000
-23773.0000000	1.4800000
-23772.0000000	1.4800000
-23771.0000000	1.4850000
-23770.0000000	1.4850000
-23769.0000000	1.4900000
-23768.0000000	1.4850000
-23767.0000000	1.4850000
-23766.0000000	1.4850000
-23765.0000000	1.4850000
-23764.0000000	1.4900000
-23763.0000000	1.4850000
-23762.0000000	1.4900000
-23761.0000000	1.4950000
-23760.0000000	1.4950000
-23759.0000000	1.5000000
-23758.0000000	1.5100000
-23757.0000000	1.5100000
-23756.0000000	1.5100000
-23755.0000000	1.5100000
-23754.0000000	1.5150000
-23753.0000000	1.5166668
-23752.0000000	1.5200000
-23751.0000000	1.5250000
-23750.0000000	1.5250000
-23749.0000000	1.5300000
-23748.0000000	1.5350000
-23747.0000000	1.5350000
-23746.0000000	1.5400000
-23745.0000000	1.5400000
-23744.0000000	1.5400000
-23743.0000000	1.5350000
-23742.0000000	1.5400000
-23741.0000000	1.5250000
-23740.0000000	1.5250000
-23739.0000000	1.5150000
-23738.0000000	1.5000000
-23737.0000000	1.4900000

-23736.0000000	1.4800000
-23735.0000000	1.4800000
-23734.0000000	1.4750000
-23733.0000000	1.4750000
-23732.0000000	1.4750000
-23731.0000000	1.4650000
-23730.0000000	1.4600000
-23729.0000000	1.4600000
-23728.0000000	1.4600000
-23727.0000000	1.4600000
-23726.0000000	1.4600000
-23725.0000000	1.4600000
-23724.0000000	1.4650000
-23723.0000000	1.4600000
-23722.0000000	1.4650000
-23721.0000000	1.4650000
-23720.0000000	1.4650000
-23719.0000000	1.4750000
-23718.0000000	1.4750000
-23717.0000000	1.4750000
-23716.0000000	1.4650000
-23715.0000000	1.4650000
-23714.0000000	1.4700000
-23713.0000000	1.4700000
-23712.0000000	1.4750000
-23711.0000000	1.4800000
-23710.0000000	1.4850000
-23709.0000000	1.4850000
-23708.0000000	1.4850000
-23707.0000000	1.4850000
-23706.0000000	1.4800000
-23705.0000000	1.4900000
-23704.0000000	1.5000000
-23703.0000000	1.5050000
-23702.0000000	1.5100000
-23701.0000000	1.5150000
-23700.0000000	1.5150000
-23699.0000000	1.5200000
-23698.0000000	1.5300000
-23697.0000000	1.5300000
-23696.0000000	1.5300000
-23695.0000000	1.5350000
-23694.0000000	1.5250000
-23693.0000000	1.5250000
-23692.0000000	1.5200000
-23691.0000000	1.5200000
-23690.0000000	1.5099999
-23689.0000000	1.5000000
-23688.0000000	1.4950000
-23687.0000000	1.4950000
-23686.0000000	1.5000000
-23685.0000000	1.5000000
-23684.0000000	1.5050000
-23683.0000000	1.5100000
-23682.0000000	1.5150000
-23681.0000000	1.5100000
-23680.0000000	1.5100000

-23679.0000000	1.5150000
-23678.0000000	1.5150000
-23677.0000000	1.5100000
-23676.0000000	1.5150000
-23675.0000000	1.5150000
-23674.0000000	1.5150000
-23673.0000000	1.5100000
-23672.0000000	1.5150000
-23671.0000000	1.5000000
-23670.0000000	1.5000000
-23669.0000000	1.4950000
-23668.0000000	1.4850000
-23667.0000000	1.4900000
-23666.0000000	1.4850000
-23665.0000000	1.4900000
-23664.0000000	1.4900000
-23663.0000000	1.4900000
-23662.0000000	1.4966666
-23661.0000000	1.5000000
-23660.0000000	1.5050000
-23659.0000000	1.5050000
-23658.0000000	1.5050000
-23657.0000000	1.5100000
-23656.0000000	1.5100000
-23655.0000000	1.5100000
-23654.0000000	1.5200000
-23653.0000000	1.5200000
-23652.0000000	1.5200000
-23651.0000000	1.5150000
-23650.0000000	1.5200000
-23649.0000000	1.5250000
-23648.0000000	1.5200000
-23647.0000000	1.5200000
-23646.0000000	1.5200000
-23645.0000000	1.5150000
-23644.0000000	1.5150000
-23643.0000000	1.5200000
-23642.0000000	1.5200000
-23641.0000000	1.5250000
-23640.0000000	1.5200000
-23639.0000000	1.5150000
-23638.0000000	1.5150000
-23637.0000000	1.5200000
-23636.0000000	1.5150000
-23635.0000000	1.5150000
-23634.0000000	1.5150000
-23633.0000000	1.5150000
-23632.0000000	1.5200000
-23631.0000000	1.5200000
-23630.0000000	1.5200000
-23629.0000000	1.5250000
-23628.0000000	1.5250000
-23627.0000000	1.5250000
-23626.0000000	1.5250000
-23625.0000000	1.5350000
-23624.0000000	1.5400000
-23623.0000000	1.5400000

-23622.0000000	1.5450000
-23621.0000000	1.5450000
-23620.0000000	1.5500000
-23619.0000000	1.5500000
-23618.0000000	1.5549999
-23617.0000000	1.5650001
-23616.0000000	1.5650001
-23615.0000000	1.5650001
-23614.0000000	1.5650001
-23613.0000000	1.5699999
-23612.0000000	1.5700001
-23611.0000000	1.5700001
-23610.0000000	1.5700001
-23609.0000000	1.5650001
-23608.0000000	1.5650001
-23607.0000000	1.5599999
-23606.0000000	1.5650001
-23605.0000000	1.5650001
-23604.0000000	1.5650001
-23603.0000000	1.5700001
-23602.0000000	1.5599999
-23601.0000000	1.5599999
-23600.0000000	1.5500000
-23599.0000000	1.5433334
-23598.0000000	1.5400000
-23597.0000000	1.5400000
-23596.0000000	1.5400000
-23595.0000000	1.5350000
-23594.0000000	1.5400000
-23593.0000000	1.5450000
-23592.0000000	1.5400000
-23591.0000000	1.5400000
-23590.0000000	1.5450000
-23589.0000000	1.5500000
-23588.0000000	1.5450000
-23587.0000000	1.5500000
-23586.0000000	1.5549999
-23585.0000000	1.5549999
-23584.0000000	1.5500000
-23583.0000000	1.5450000
-23582.0000000	1.5400000
-23581.0000000	1.5350000
-23580.0000000	1.5350000
-23579.0000000	1.5350000
-23578.0000000	1.5350000
-23577.0000000	1.5350000
-23576.0000000	1.5300000
-23575.0000000	1.5350000
-23574.0000000	1.5350000
-23573.0000000	1.5400000
-23572.0000000	1.5400000
-23571.0000000	1.5400000
-23570.0000000	1.5433334
-23569.0000000	1.5500000
-23568.0000000	1.5500000
-23567.0000000	1.5450000
-23566.0000000	1.5450000

-23565.0000000	1.5500000
-23564.0000000	1.5549999
-23563.0000000	1.5549999
-23562.0000000	1.5549999
-23561.0000000	1.5599999
-23560.0000000	1.5650001
-23559.0000000	1.5700001
-23558.0000000	1.5700001
-23557.0000000	1.5700001
-23556.0000000	1.5750000
-23555.0000000	1.5750000
-23554.0000000	1.5800000
-23553.0000000	1.5800000
-23552.0000000	1.5850000
-23551.0000000	1.5900000
-23550.0000000	1.5900000
-23549.0000000	1.5900000
-23548.0000000	1.5950000
-23547.0000000	1.6000000
-23546.0000000	1.5950000
-23545.0000000	1.6000000
-23544.0000000	1.6000000
-23543.0000000	1.6050000
-23542.0000000	1.6050000
-23541.0000000	1.6100000
-23540.0000000	1.6150000
-23539.0000000	1.6150000
-23538.0000000	1.6200000
-23537.0000000	1.6250000
-23536.0000000	1.6250000
-23535.0000000	1.6250000
-23534.0000000	1.6300000
-23533.0000000	1.6300000
-23532.0000000	1.6350000
-23531.0000000	1.6400000
-23530.0000000	1.6400000
-23529.0000000	1.6500000
-23528.0000000	1.6500000
-23527.0000000	1.6550000
-23526.0000000	1.6550000
-23525.0000000	1.6550000
-23524.0000000	1.6600000
-23523.0000000	1.6650000
-23522.0000000	1.6550000
-23521.0000000	1.6650000
-23520.0000000	1.6700000
-23519.0000000	1.6700000
-23518.0000000	1.6700000
-23517.0000000	1.6799999
-23516.0000000	1.6799999
-23515.0000000	1.6750000
-23514.0000000	1.6750000
-23513.0000000	1.6799999
-23512.0000000	1.6750000
-23511.0000000	1.6799999
-23510.0000000	1.6750000
-23509.0000000	1.6733333

-23508.0000000	1.6700000
-23507.0000000	1.6650000
-23506.0000000	1.6650000
-23505.0000000	1.6650000
-23504.0000000	1.6600000
-23503.0000000	1.6650000
-23502.0000000	1.6700000
-23501.0000000	1.6650000
-23500.0000000	1.6650000
-23499.0000000	1.6600000
-23498.0000000	1.6600000
-23497.0000000	1.6600000
-23496.0000000	1.6650000
-23495.0000000	1.6650000
-23494.0000000	1.6700000
-23493.0000000	1.6700000
-23492.0000000	1.6700000
-23491.0000000	1.6700000
-23490.0000000	1.6700000
-23489.0000000	1.6750000
-23488.0000000	1.6750000
-23487.0000000	1.6700000
-23486.0000000	1.6700000
-23485.0000000	1.6650000
-23484.0000000	1.6600000
-23483.0000000	1.6600000
-23482.0000000	1.6600000
-23481.0000000	1.6600000
-23480.0000000	1.6500000
-23479.0000000	1.6533333
-23478.0000000	1.6500000
-23477.0000000	1.6550000
-23476.0000000	1.6500000
-23475.0000000	1.6450000
-23474.0000000	1.6450000
-23473.0000000	1.6400000
-23472.0000000	1.6350000
-23471.0000000	1.6350000
-23470.0000000	1.6350000
-23469.0000000	1.6400000
-23468.0000000	1.6450000
-23467.0000000	1.6450000
-23466.0000000	1.6450000
-23465.0000000	1.6450000
-23464.0000000	1.6500000
-23463.0000000	1.6550000
-23462.0000000	1.6600000
-23461.0000000	1.6600000
-23460.0000000	1.6650000
-23459.0000000	1.6650000
-23458.0000000	1.6700000
-23457.0000000	1.6700000
-23456.0000000	1.6650000
-23455.0000000	1.6750000
-23454.0000000	1.6750000
-23453.0000000	1.6799999
-23452.0000000	1.6799999

-23451.0000000	1.6799999
-23450.0000000	1.6700000
-23449.0000000	1.6700000
-23448.0000000	1.6700000
-23447.0000000	1.6700000
-23446.0000000	1.6750000
-23445.0000000	1.6750000
-23444.0000000	1.6750000
-23443.0000000	1.6700000
-23442.0000000	1.6750000
-23441.0000000	1.6750000
-23440.0000000	1.6700000
-23439.0000000	1.6700000
-23438.0000000	1.6650000
-23437.0000000	1.6650000
-23436.0000000	1.6600000
-23435.0000000	1.6550000
-23434.0000000	1.6550000
-23433.0000000	1.6500000
-23432.0000000	1.6500000
-23431.0000000	1.6450000
-23430.0000000	1.6450000
-23429.0000000	1.6450000
-23428.0000000	1.6450000
-23427.0000000	1.6450000
-23426.0000000	1.6400000
-23425.0000000	1.6450000
-23424.0000000	1.6400000
-23423.0000000	1.6450000
-23422.0000000	1.6450000
-23421.0000000	1.6500000
-23420.0000000	1.6600000
-23419.0000000	1.6600000
-23418.0000000	1.6633333
-23417.0000000	1.6750000
-23416.0000000	1.6799999
-23415.0000000	1.6799999
-23414.0000000	1.6799999
-23413.0000000	1.6750000
-23412.0000000	1.6750000
-23411.0000000	1.6750000
-23410.0000000	1.6750000
-23409.0000000	1.6750000
-23408.0000000	1.6700000
-23407.0000000	1.6700000
-23406.0000000	1.6650000
-23405.0000000	1.6600000
-23404.0000000	1.6600000
-23403.0000000	1.6500000
-23402.0000000	1.6550000
-23401.0000000	1.6500000
-23400.0000000	1.6500000
-23399.0000000	1.6500000
-23398.0000000	1.6450000
-23397.0000000	1.6450000
-23396.0000000	1.6350000
-23395.0000000	1.6400000

-23394.0000000	1.6350000
-23393.0000000	1.6350000
-23392.0000000	1.6350000
-23391.0000000	1.6250000
-23390.0000000	1.6250000
-23389.0000000	1.6266667
-23388.0000000	1.6200000
-23387.0000000	1.6250000
-23386.0000000	1.6250000
-23385.0000000	1.6200000
-23384.0000000	1.6200000
-23383.0000000	1.6150000
-23382.0000000	1.6100000
-23381.0000000	1.6150000
-23380.0000000	1.6050000
-23379.0000000	1.6000000
-23378.0000000	1.6050000
-23377.0000000	1.6050000
-23376.0000000	1.6000000
-23375.0000000	1.6000000
-23374.0000000	1.6050000
-23373.0000000	1.6050000
-23372.0000000	1.6000000
-23371.0000000	1.6100000
-23370.0000000	1.6100000
-23369.0000000	1.6150000
-23368.0000000	1.6150000
-23367.0000000	1.6200000
-23366.0000000	1.6200000
-23365.0000000	1.6250000
-23364.0000000	1.6250000
-23363.0000000	1.6300000
-23362.0000000	1.6300000
-23361.0000000	1.6350000
-23360.0000000	1.6333333
-23359.0000000	1.6400000
-23358.0000000	1.6450000
-23357.0000000	1.6550000
-23356.0000000	1.6550000
-23355.0000000	1.6600000
-23354.0000000	1.6600000
-23353.0000000	1.6600000
-23352.0000000	1.6650000
-23351.0000000	1.6650000
-23350.0000000	1.6650000
-23349.0000000	1.6650000
-23348.0000000	1.6700000
-23347.0000000	1.6750000
-23346.0000000	1.6700000
-23345.0000000	1.6700000
-23344.0000000	1.6700000
-23343.0000000	1.6750000
-23342.0000000	1.6750000
-23341.0000000	1.6799999
-23340.0000000	1.6849999
-23339.0000000	1.6900001
-23338.0000000	1.6900001

-23337.0000000	1.6950001
-23336.0000000	1.7000000
-23335.0000000	1.7050000
-23334.0000000	1.7100000
-23333.0000000	1.7250000
-23332.0000000	1.7300000
-23331.0000000	1.7300000
-23330.0000000	1.7366667
-23329.0000000	1.7300000
-23328.0000000	1.7433333
-23327.0000000	1.7450000
-23326.0000000	1.7500000
-23325.0000000	1.7450000
-23324.0000000	1.7550000
-23323.0000000	1.7500000
-23322.0000000	1.7550000
-23321.0000000	1.7600000
-23320.0000000	1.7550000
-23319.0000000	1.7500000
-23318.0000000	1.7450000
-23317.0000000	1.7500000
-23316.0000000	1.7500000
-23315.0000000	1.7400000
-23314.0000000	1.7450000
-23313.0000000	1.7450000
-23312.0000000	1.7400000
-23311.0000000	1.7400000
-23310.0000000	1.7350000
-23309.0000000	1.7400000
-23308.0000000	1.7400000
-23307.0000000	1.7400000
-23306.0000000	1.7450000
-23305.0000000	1.7500000
-23304.0000000	1.7500000
-23303.0000000	1.7550000
-23302.0000000	1.7600000
-23301.0000000	1.7600000
-23300.0000000	1.7600000
-23299.0000000	1.7650000
-23298.0000000	1.7600000
-23297.0000000	1.7666668
-23296.0000000	1.7600000
-23295.0000000	1.7700000
-23294.0000000	1.7650000
-23293.0000000	1.7650000
-23292.0000000	1.7650000
-23291.0000000	1.7600000
-23290.0000000	1.7600000
-23289.0000000	1.7700000
-23288.0000000	1.7700000
-23287.0000000	1.7700000
-23286.0000000	1.7750000
-23285.0000000	1.7800000
-23284.0000000	1.7850000
-23283.0000000	1.7900000
-23282.0000000	1.8000000
-23281.0000000	1.8099999

-23280.0000000	1.8150001
-23279.0000000	1.8250000
-23278.0000000	1.8350000
-23277.0000000	1.8400000
-23276.0000000	1.8550000
-23275.0000000	1.8600000
-23274.0000000	1.8700000
-23273.0000000	1.8850000
-23272.0000000	1.8900000
-23271.0000000	1.9050000
-23270.0000000	1.9100000
-23269.0000000	1.9200000
-23268.0000000	1.9200000
-23267.0000000	1.9266666
-23266.0000000	1.9299999
-23265.0000000	1.9400001
-23264.0000000	1.9400001
-23263.0000000	1.9500000
-23262.0000000	1.9600000
-23261.0000000	1.9600000
-23260.0000000	1.9700000
-23259.0000000	1.9850000
-23258.0000000	2.0000000
-23257.0000000	2.0050001
-23256.0000000	2.0200000
-23255.0000000	2.0300000
-23254.0000000	2.0400000
-23253.0000000	2.0450001
-23252.0000000	2.0500000
-23251.0000000	2.0549998
-23250.0000000	2.0699999
-23249.0000000	2.0699999
-23248.0000000	2.0799999
-23247.0000000	2.0850000
-23246.0000000	2.0899999
-23245.0000000	2.0899999
-23244.0000000	2.0899999
-23243.0000000	2.0850000
-23242.0000000	2.0850000
-23241.0000000	2.0850000
-23240.0000000	2.0899999
-23239.0000000	2.0949998
-23238.0000000	2.0949998
-23237.0000000	2.0999999
-23236.0000000	2.1099999
-23235.0000000	2.1149998
-23234.0000000	2.1149998
-23233.0000000	2.1300001
-23232.0000000	2.1400001
-23231.0000000	2.1400001
-23230.0000000	2.1450000
-23229.0000000	2.1500001
-23228.0000000	2.1550002
-23227.0000000	2.1450000
-23226.0000000	2.1550002
-23225.0000000	2.1500001
-23224.0000000	2.1550002

-23223.0000000	2.1550002
-23222.0000000	2.1450000
-23221.0000000	2.1550002
-23220.0000000	2.1550002
-23219.0000000	2.1550002
-23218.0000000	2.1550002
-23217.0000000	2.1450000
-23216.0000000	2.1450000
-23215.0000000	2.1450000
-23214.0000000	2.1450000
-23213.0000000	2.1350002
-23212.0000000	2.1250000
-23211.0000000	2.1149998
-23210.0000000	2.0999999
-23209.0000000	2.0899999
-23208.0000000	2.0850000
-23207.0000000	2.0799999
-23206.0000000	2.0749998
-23205.0000000	2.0749998
-23204.0000000	2.0799999
-23203.0000000	2.0749998
-23202.0000000	2.0799999
-23201.0000000	2.0749998
-23200.0000000	2.0799999
-23199.0000000	2.0749998
-23198.0000000	2.0850000
-23197.0000000	2.0799999
-23196.0000000	2.0799999
-23195.0000000	2.0899999
-23194.0000000	2.0850000
-23193.0000000	2.0949998
-23192.0000000	2.0999999
-23191.0000000	2.0949998
-23190.0000000	2.0949998
-23189.0000000	2.0999999
-23188.0000000	2.0949998
-23187.0000000	2.0999999
-23186.0000000	2.0999999
-23185.0000000	2.1050000
-23184.0000000	2.1050000
-23183.0000000	2.1149998
-23182.0000000	2.1199999
-23181.0000000	2.1199999
-23180.0000000	2.1250000
-23179.0000000	2.1350002
-23178.0000000	2.1350002
-23177.0000000	2.1433334
-23176.0000000	2.1450000
-23175.0000000	2.1550002
-23174.0000000	2.1700001
-23173.0000000	2.1750002
-23172.0000000	2.1800001
-23171.0000000	2.1950002
-23170.0000000	2.1950002
-23169.0000000	2.2049999
-23168.0000000	2.2100000
-23167.0000000	2.2150002

-23166.0000000	2.2200000
-23165.0000000	2.2249999
-23164.0000000	2.2300000
-23163.0000000	2.2400000
-23162.0000000	2.2500000
-23161.0000000	2.2449999
-23160.0000000	2.2550001
-23159.0000000	2.2600000
-23158.0000000	2.2600000
-23157.0000000	2.2600000
-23156.0000000	2.2600000
-23155.0000000	2.2500000
-23154.0000000	2.2550001
-23153.0000000	2.2400000
-23152.0000000	2.2400000
-23151.0000000	2.2350001
-23150.0000000	2.2300000
-23149.0000000	2.2200000
-23148.0000000	2.2249999
-23147.0000000	2.2150002
-23146.0000000	2.2066667
-23145.0000000	2.1950002
-23144.0000000	2.1900001
-23143.0000000	2.1750002
-23142.0000000	2.1650000
-23141.0000000	2.1600001
-23140.0000000	2.1500001
-23139.0000000	2.1450000
-23138.0000000	2.1400001
-23137.0000000	2.1400001
-23136.0000000	2.1400001
-23135.0000000	2.1400001
-23134.0000000	2.1350002
-23133.0000000	2.1350002
-23132.0000000	2.1350002
-23131.0000000	2.1250000
-23130.0000000	2.1250000
-23129.0000000	2.1250000
-23128.0000000	2.1199999
-23127.0000000	2.1199999
-23126.0000000	2.1149998
-23125.0000000	2.1099999
-23124.0000000	2.1099999
-23123.0000000	2.1099999
-23122.0000000	2.0999999
-23121.0000000	2.0850000
-23120.0000000	2.0799999
-23119.0000000	2.0749998
-23118.0000000	2.0599999
-23117.0000000	2.0599999
-23116.0000000	2.0549998
-23115.0000000	2.0450001
-23114.0000000	2.0349998
-23113.0000000	2.0300000
-23112.0000000	2.0349998
-23111.0000000	2.0250001
-23110.0000000	2.0300000

-23109.0000000	2.0300000
-23108.0000000	2.0149999
-23107.0000000	2.0149999
-23106.0000000	2.0050001
-23105.0000000	1.9950000
-23104.0000000	1.9900000
-23103.0000000	1.9850000
-23102.0000000	1.9850000
-23101.0000000	1.9750000
-23100.0000000	1.9800000
-23099.0000000	1.9750000
-23098.0000000	1.9750000
-23097.0000000	1.9700000
-23096.0000000	1.9650000
-23095.0000000	1.9700000
-23094.0000000	1.9600000
-23093.0000000	1.9600000
-23092.0000000	1.9550000
-23091.0000000	1.9500000
-23090.0000000	1.9450001
-23089.0000000	1.9400001
-23088.0000000	1.9400001
-23087.0000000	1.9450001
-23086.0000000	1.9433333
-23085.0000000	1.9450001
-23084.0000000	1.9450001
-23083.0000000	1.9450001
-23082.0000000	1.9450001
-23081.0000000	1.9400001
-23080.0000000	1.9300001
-23079.0000000	1.9150000
-23078.0000000	1.9150000
-23077.0000000	1.9000000
-23076.0000000	1.8950000
-23075.0000000	1.8850000
-23074.0000000	1.8750000
-23073.0000000	1.8650000
-23072.0000000	1.8550000
-23071.0000000	1.8500000
-23070.0000000	1.8500000
-23069.0000000	1.8450000
-23068.0000000	1.8450000
-23067.0000000	1.8400000
-23066.0000000	1.8450000
-23065.0000000	1.8450000
-23064.0000000	1.8450000
-23063.0000000	1.8400000
-23062.0000000	1.8500000
-23061.0000000	1.8550000
-23060.0000000	1.8550000
-23059.0000000	1.8600000
-23058.0000000	1.8550000
-23057.0000000	1.8550000
-23056.0000000	1.8533334
-23055.0000000	1.8500000
-23054.0000000	1.8500000
-23053.0000000	1.8500000

-23052.0000000	1.8500000
-23051.0000000	1.8500000
-23050.0000000	1.8550000
-23049.0000000	1.8550000
-23048.0000000	1.8500000
-23047.0000000	1.8650000
-23046.0000000	1.8550000
-23045.0000000	1.8550000
-23044.0000000	1.8600000
-23043.0000000	1.8550000
-23042.0000000	1.8550000
-23041.0000000	1.8550000
-23040.0000000	1.8550000
-23039.0000000	1.8600000
-23038.0000000	1.8550000
-23037.0000000	1.8600000
-23036.0000000	1.8650000
-23035.0000000	1.8700000
-23034.0000000	1.8700000
-23033.0000000	1.8700000
-23032.0000000	1.8750000
-23031.0000000	1.8750000
-23030.0000000	1.8700000
-23029.0000000	1.8650000
-23028.0000000	1.8533334
-23027.0000000	1.8500000
-23026.0000000	1.8400000
-23025.0000000	1.8300000
-23024.0000000	1.8250000
-23023.0000000	1.8199999
-23022.0000000	1.8150001
-23021.0000000	1.8200001
-23020.0000000	1.8200001
-23019.0000000	1.8200001
-23018.0000000	1.8200001
-23017.0000000	1.8200001
-23016.0000000	1.8150001
-23015.0000000	1.8049999
-23014.0000000	1.8150001
-23013.0000000	1.8049999
-23012.0000000	1.8049999
-23011.0000000	1.8000000
-23010.0000000	1.8000000
-23009.0000000	1.8049999
-23008.0000000	1.8000000
-23007.0000000	1.8000000
-23006.0000000	1.8049999
-23005.0000000	1.8049999
-23004.0000000	1.8049999
-23003.0000000	1.8099999
-23002.0000000	1.8099999
-23001.0000000	1.8049999
-23000.0000000	1.8049999
-22999.0000000	1.8000000
-22998.0000000	1.8000000
-22997.0000000	1.8133334
-22996.0000000	1.8150001

-22995.0000000	1.8200001
-22994.0000000	1.8250000
-22993.0000000	1.8250000
-22992.0000000	1.8350000
-22991.0000000	1.8450000
-22990.0000000	1.8450000
-22989.0000000	1.8500000
-22988.0000000	1.8600000
-22987.0000000	1.8600000
-22986.0000000	1.8700000
-22985.0000000	1.8750000
-22984.0000000	1.8800000
-22983.0000000	1.8900000
-22982.0000000	1.8900000
-22981.0000000	1.8950000
-22980.0000000	1.9050000
-22979.0000000	1.9000000
-22978.0000000	1.9050000
-22977.0000000	1.9050000
-22976.0000000	1.9100000
-22975.0000000	1.9150000
-22974.0000000	1.9200000
-22973.0000000	1.9250000
-22972.0000000	1.9299999
-22971.0000000	1.9400001
-22970.0000000	1.9450001
-22969.0000000	1.9550000
-22968.0000000	1.9600000
-22967.0000000	1.9650000
-22966.0000000	1.9733334
-22965.0000000	1.9800000
-22964.0000000	1.9850000
-22963.0000000	1.9850000
-22962.0000000	1.9900000
-22961.0000000	1.9950000
-22960.0000000	1.9950000
-22959.0000000	2.0000000
-22958.0000000	2.0000000
-22957.0000000	2.0050001
-22956.0000000	2.0050001
-22955.0000000	2.0000000
-22954.0000000	1.9950000
-22953.0000000	2.0000000
-22952.0000000	1.9900000
-22951.0000000	1.9900000
-22950.0000000	1.9900000
-22949.0000000	1.9900000
-22948.0000000	1.9800000
-22947.0000000	1.9850000
-22946.0000000	1.9800000
-22945.0000000	1.9800000
-22944.0000000	1.9750000
-22943.0000000	1.9750000
-22942.0000000	1.9750000
-22941.0000000	1.9700000
-22940.0000000	1.9750000
-22939.0000000	1.9750000

-22938.0000000	1.9750000
-22937.0000000	1.9800000
-22936.0000000	1.9900000
-22935.0000000	1.9850000
-22934.0000000	1.9950000
-22933.0000000	1.9950000
-22932.0000000	2.0000000
-22931.0000000	2.0100000
-22930.0000000	2.0050001
-22929.0000000	2.0050001
-22928.0000000	1.9950000
-22927.0000000	1.9950000
-22926.0000000	1.9900000
-22925.0000000	1.9800000
-22924.0000000	1.9750000
-22923.0000000	1.9650000
-22922.0000000	1.9500000
-22921.0000000	1.9500000
-22920.0000000	1.9450001
-22919.0000000	1.9349999
-22918.0000000	1.9250000
-22917.0000000	1.9150000
-22916.0000000	1.9050000
-22915.0000000	1.9050000
-22914.0000000	1.9000000
-22913.0000000	1.9000000
-22912.0000000	1.8950000
-22911.0000000	1.9000000
-22910.0000000	1.9000000
-22909.0000000	1.9000000
-22908.0000000	1.9000000
-22907.0000000	1.9100000
-22906.0000000	1.9150000
-22905.0000000	1.9150000
-22904.0000000	1.9200000
-22903.0000000	1.9299999
-22902.0000000	1.9250000
-22901.0000000	1.9349999
-22900.0000000	1.9400001
-22899.0000000	1.9400001
-22898.0000000	1.9550000
-22897.0000000	1.9500000
-22896.0000000	1.9550000
-22895.0000000	1.9650000
-22894.0000000	1.9650000
-22893.0000000	1.9550000
-22892.0000000	1.9500000
-22891.0000000	1.9550000
-22890.0000000	1.9500000
-22889.0000000	1.9500000
-22888.0000000	1.9500000
-22887.0000000	1.9400001
-22886.0000000	1.9299999
-22885.0000000	1.9250000
-22884.0000000	1.9150000
-22883.0000000	1.9100000
-22882.0000000	1.9100000

-22881.0000000	1.9050000
-22880.0000000	1.9000000
-22879.0000000	1.9000000
-22878.0000000	1.8950000
-22877.0000000	1.9000000
-22876.0000000	1.9000000
-22875.0000000	1.9100000
-22874.0000000	1.9050000
-22873.0000000	1.9050000
-22872.0000000	1.9200000
-22871.0000000	1.9150000
-22870.0000000	1.9250000
-22869.0000000	1.9200000
-22868.0000000	1.9150000
-22867.0000000	1.9050000
-22866.0000000	1.9100000
-22865.0000000	1.9050000
-22864.0000000	1.9100000
-22863.0000000	1.9000000
-22862.0000000	1.9150000
-22861.0000000	1.9100000
-22860.0000000	1.9200000
-22859.0000000	1.9250000
-22858.0000000	1.9250000
-22857.0000000	1.9349999
-22856.0000000	1.9349999
-22855.0000000	1.9400001
-22854.0000000	1.9450001
-22853.0000000	1.9450001
-22852.0000000	1.9550000
-22851.0000000	1.9550000
-22850.0000000	1.9650000
-22849.0000000	1.9600000
-22848.0000000	1.9700000
-22847.0000000	1.9700000
-22846.0000000	1.9800000
-22845.0000000	1.9800000
-22844.0000000	1.9850000
-22843.0000000	1.9900000
-22842.0000000	1.9950000
-22841.0000000	2.0050001
-22840.0000000	2.0100000
-22839.0000000	2.0149999
-22838.0000000	2.0200000
-22837.0000000	2.0349998
-22836.0000000	2.0349998
-22835.0000000	2.0400000
-22834.0000000	2.0450001
-22833.0000000	2.0450001
-22832.0000000	2.0549998
-22831.0000000	2.0549998
-22830.0000000	2.0549998
-22829.0000000	2.0650001
-22828.0000000	2.0650001
-22827.0000000	2.0699999
-22826.0000000	2.0650001
-22825.0000000	2.0599999

-22824.0000000	2.0650001
-22823.0000000	2.0650001
-22822.0000000	2.0599999
-22821.0000000	2.0650001
-22820.0000000	2.0650001
-22819.0000000	2.0699999
-22818.0000000	2.0650001
-22817.0000000	2.0699999
-22816.0000000	2.0733330
-22815.0000000	2.0749998
-22814.0000000	2.0850000
-22813.0000000	2.0850000
-22812.0000000	2.0850000
-22811.0000000	2.0850000
-22810.0000000	2.0899999
-22809.0000000	2.0899999
-22808.0000000	2.0850000
-22807.0000000	2.0799999
-22806.0000000	2.0699999
-22805.0000000	2.0650001
-22804.0000000	2.0749998
-22803.0000000	2.0749998
-22802.0000000	2.0799999
-22801.0000000	2.0850000
-22800.0000000	2.0899999
-22799.0000000	2.0850000
-22798.0000000	2.0799999
-22797.0000000	2.0850000
-22796.0000000	2.0899999
-22795.0000000	2.0949998
-22794.0000000	2.0949998
-22793.0000000	2.0999999
-22792.0000000	2.1050000
-22791.0000000	2.0999999
-22790.0000000	2.1099999
-22789.0000000	2.1050000
-22788.0000000	2.1149998
-22787.0000000	2.1199999
-22786.0000000	2.1250000
-22785.0000000	2.1333334
-22784.0000000	2.1400001
-22783.0000000	2.1450000
-22782.0000000	2.1500001
-22781.0000000	2.1550002
-22780.0000000	2.1650000
-22779.0000000	2.1650000
-22778.0000000	2.1750002
-22777.0000000	2.1800001
-22776.0000000	2.1849999
-22775.0000000	2.1900001
-22774.0000000	2.1950002
-22773.0000000	2.2000000
-22772.0000000	2.2150002
-22771.0000000	2.2100000
-22770.0000000	2.2200000
-22769.0000000	2.2200000
-22768.0000000	2.2249999

-22767.0000000	2.2249999
-22766.0000000	2.2350001
-22765.0000000	2.2400000
-22764.0000000	2.2400000
-22763.0000000	2.2449999
-22762.0000000	2.2500000
-22761.0000000	2.2550001
-22760.0000000	2.2550001
-22759.0000000	2.2600000
-22758.0000000	2.2649999
-22757.0000000	2.2649999
-22756.0000000	2.2666667
-22755.0000000	2.2649999
-22754.0000000	2.2600000
-22753.0000000	2.2649999
-22752.0000000	2.2600000
-22751.0000000	2.2550001
-22750.0000000	2.2550001
-22749.0000000	2.2500000
-22748.0000000	2.2449999
-22747.0000000	2.2449999
-22746.0000000	2.2500000
-22745.0000000	2.2550001
-22744.0000000	2.2550001
-22743.0000000	2.2550001
-22742.0000000	2.2550001
-22741.0000000	2.2600000
-22740.0000000	2.2550001
-22739.0000000	2.2649999
-22738.0000000	2.2550001
-22737.0000000	2.2550001
-22736.0000000	2.2449999
-22735.0000000	2.2400000
-22734.0000000	2.2400000
-22733.0000000	2.2400000
-22732.0000000	2.2400000
-22731.0000000	2.2449999
-22730.0000000	2.2400000
-22729.0000000	2.2400000
-22728.0000000	2.2500000
-22727.0000000	2.2500000
-22726.0000000	2.2500000
-22725.0000000	2.2500000
-22724.0000000	2.2550001
-22723.0000000	2.2449999
-22722.0000000	2.2550001
-22721.0000000	2.2600000
-22720.0000000	2.2550001
-22719.0000000	2.2600000
-22718.0000000	2.2700000
-22717.0000000	2.2750001
-22716.0000000	2.2800000
-22715.0000000	2.2849998
-22714.0000000	2.2900000
-22713.0000000	2.2950001
-22712.0000000	2.3049998
-22711.0000000	2.3049998

-22710.0000000	2.3099999
-22709.0000000	2.3150001
-22708.0000000	2.3199999
-22707.0000000	2.3150001
-22706.0000000	2.3150001
-22705.0000000	2.3049998
-22704.0000000	2.3049998
-22703.0000000	2.3049998
-22702.0000000	2.3000000
-22701.0000000	2.3049998
-22700.0000000	2.3099999
-22699.0000000	2.3099999
-22698.0000000	2.3199999
-22697.0000000	2.3299999
-22696.0000000	2.3249998
-22695.0000000	2.3299999
-22694.0000000	2.3399999
-22693.0000000	2.3449998
-22692.0000000	2.3449998
-22691.0000000	2.3399999
-22690.0000000	2.3449998
-22689.0000000	2.3499999
-22688.0000000	2.3499999
-22687.0000000	2.3499999
-22686.0000000	2.3499999
-22685.0000000	2.3449998
-22684.0000000	2.3449998
-22683.0000000	2.3449998
-22682.0000000	2.3399999
-22681.0000000	2.3449998
-22680.0000000	2.3299999
-22679.0000000	2.3299999
-22678.0000000	2.3249998
-22677.0000000	2.3199999
-22676.0000000	2.3150001
-22675.0000000	2.3099999
-22674.0000000	2.3150001
-22673.0000000	2.3150001
-22672.0000000	2.3099999
-22671.0000000	2.3099999
-22670.0000000	2.3000000
-22669.0000000	2.3049998
-22668.0000000	2.3049998
-22667.0000000	2.3049998
-22666.0000000	2.3000000
-22665.0000000	2.3049998
-22664.0000000	2.3133333
-22663.0000000	2.3150001
-22662.0000000	2.3199999
-22661.0000000	2.3199999
-22660.0000000	2.3099999
-22659.0000000	2.3199999
-22658.0000000	2.3150001
-22657.0000000	2.3150001
-22656.0000000	2.3199999
-22655.0000000	2.3249998
-22654.0000000	2.3299999

-22653.0000000	2.3249998
-22652.0000000	2.3350000
-22651.0000000	2.3350000
-22650.0000000	2.3350000
-22649.0000000	2.3499999
-22648.0000000	2.3449998
-22647.0000000	2.3449998
-22646.0000000	2.3550000
-22645.0000000	2.3599999
-22644.0000000	2.3649998
-22643.0000000	2.3649998
-22642.0000000	2.3750000
-22641.0000000	2.3800001
-22640.0000000	2.3800001
-22639.0000000	2.3800001
-22638.0000000	2.3850002
-22637.0000000	2.3850002
-22636.0000000	2.3800001
-22635.0000000	2.3800001
-22634.0000000	2.3750000
-22633.0000000	2.3800001
-22632.0000000	2.3750000
-22631.0000000	2.3649998
-22630.0000000	2.3599999
-22629.0000000	2.3550000
-22628.0000000	2.3550000
-22627.0000000	2.3550000
-22626.0000000	2.3550000
-22625.0000000	2.3399999
-22624.0000000	2.3399999
-22623.0000000	2.3399999
-22622.0000000	2.3350000
-22621.0000000	2.3350000
-22620.0000000	2.3199999
-22619.0000000	2.3099999
-22618.0000000	2.3049998
-22617.0000000	2.2900000
-22616.0000000	2.2849998
-22615.0000000	2.2900000
-22614.0000000	2.2950001
-22613.0000000	2.3000000
-22612.0000000	2.3150001
-22611.0000000	2.3249998
-22610.0000000	2.3350000
-22609.0000000	2.3499999
-22608.0000000	2.3550000
-22607.0000000	2.3649998
-22606.0000000	2.3699999
-22605.0000000	2.3733332
-22604.0000000	2.3750000
-22603.0000000	2.3750000
-22602.0000000	2.3649998
-22601.0000000	2.3750000
-22600.0000000	2.3750000
-22599.0000000	2.3750000
-22598.0000000	2.3750000
-22597.0000000	2.3699999

-22596.0000000	2.3800001
-22595.0000000	2.3699999
-22594.0000000	2.3750000
-22593.0000000	2.3750000
-22592.0000000	2.3750000
-22591.0000000	2.3699999
-22590.0000000	2.3850002
-22589.0000000	2.3800001
-22588.0000000	2.3800001
-22587.0000000	2.3850002
-22586.0000000	2.3850002
-22585.0000000	2.3950000
-22584.0000000	2.3900001
-22583.0000000	2.3950000
-22582.0000000	2.3900001
-22581.0000000	2.4000001
-22580.0000000	2.4050002
-22579.0000000	2.3950000
-22578.0000000	2.3950000
-22577.0000000	2.4050002
-22576.0000000	2.4050002
-22575.0000000	2.4000001
-22574.0000000	2.4100001
-22573.0000000	2.4100001
-22572.0000000	2.4100001
-22571.0000000	2.4150000
-22570.0000000	2.4200001
-22569.0000000	2.4349999
-22568.0000000	2.4450002
-22567.0000000	2.4650002
-22566.0000000	2.4749999
-22565.0000000	2.4800000
-22564.0000000	2.4850001
-22563.0000000	2.4900000
-22562.0000000	2.4949999
-22561.0000000	2.4900000
-22560.0000000	2.4949999
-22559.0000000	2.5000000
-22558.0000000	2.4900000
-22557.0000000	2.4900000
-22556.0000000	2.4850001
-22555.0000000	2.4900000
-22554.0000000	2.4900000
-22553.0000000	2.4850001
-22552.0000000	2.4900000
-22551.0000000	2.4949999
-22550.0000000	2.4949999
-22549.0000000	2.4900000
-22548.0000000	2.4900000
-22547.0000000	2.4850001
-22546.0000000	2.4850001
-22545.0000000	2.4733334
-22544.0000000	2.4850001
-22543.0000000	2.4949999
-22542.0000000	2.4900000
-22541.0000000	2.5050001
-22540.0000000	2.5100000

-22539.0000000	2.5050001
-22538.0000000	2.5149999
-22537.0000000	2.5100000
-22536.0000000	2.5100000
-22535.0000000	2.5000000
-22534.0000000	2.5100000
-22533.0000000	2.5050001
-22532.0000000	2.5000000
-22531.0000000	2.5000000
-22530.0000000	2.4949999
-22529.0000000	2.4949999
-22528.0000000	2.4900000
-22527.0000000	2.4900000
-22526.0000000	2.4900000
-22525.0000000	2.5000000
-22524.0000000	2.4900000
-22523.0000000	2.4900000
-22522.0000000	2.4949999
-22521.0000000	2.4850001
-22520.0000000	2.4900000
-22519.0000000	2.4850001
-22518.0000000	2.4800000
-22517.0000000	2.4800000
-22516.0000000	2.4850001
-22515.0000000	2.4900000
-22514.0000000	2.4900000
-22513.0000000	2.4966667
-22512.0000000	2.4900000
-22511.0000000	2.5000000
-22510.0000000	2.5100000
-22509.0000000	2.5050001
-22508.0000000	2.5100000
-22507.0000000	2.5100000
-22506.0000000	2.5050001
-22505.0000000	2.5100000
-22504.0000000	2.5149999
-22503.0000000	2.5050001
-22502.0000000	2.5100000
-22501.0000000	2.5149999
-22500.0000000	2.5100000
-22499.0000000	2.5000000
-22498.0000000	2.5050001
-22497.0000000	2.4949999
-22496.0000000	2.4949999
-22495.0000000	2.4850001
-22494.0000000	2.4749999
-22493.0000000	2.4700000
-22492.0000000	2.4549999
-22491.0000000	2.4450002
-22490.0000000	2.4450002
-22489.0000000	2.4450002
-22488.0000000	2.4400001
-22487.0000000	2.4400001
-22486.0000000	2.4400001
-22485.0000000	2.4300001
-22484.0000000	2.4400001
-22483.0000000	2.4400001

-22482.0000000	2.4349999
-22481.0000000	2.4500000
-22480.0000000	2.4450002
-22479.0000000	2.4549999
-22478.0000000	2.4600000
-22477.0000000	2.4650002
-22476.0000000	2.4700000
-22475.0000000	2.4850001
-22474.0000000	2.4900000
-22473.0000000	2.4949999
-22472.0000000	2.5000000
-22471.0000000	2.5050001
-22470.0000000	2.5050001
-22469.0000000	2.5100000
-22468.0000000	2.5100000
-22467.0000000	2.5050001
-22466.0000000	2.5050001
-22465.0000000	2.5000000
-22464.0000000	2.4900000
-22463.0000000	2.4949999
-22462.0000000	2.4949999
-22461.0000000	2.4850001
-22460.0000000	2.4900000
-22459.0000000	2.4749999
-22458.0000000	2.4850001
-22457.0000000	2.4700000
-22456.0000000	2.4749999
-22455.0000000	2.4600000
-22454.0000000	2.4500000
-22453.0000000	2.4349999
-22452.0000000	2.4200001
-22451.0000000	2.4050002
-22450.0000000	2.3850000
-22449.0000000	2.3699999
-22448.0000000	2.3599999
-22447.0000000	2.3599999
-22446.0000000	2.3449998
-22445.0000000	2.3550000
-22444.0000000	2.3550000
-22443.0000000	2.3599999
-22442.0000000	2.3599999
-22441.0000000	2.3750000
-22440.0000000	2.3800001
-22439.0000000	2.3850002
-22438.0000000	2.3900001
-22437.0000000	2.3900001
-22436.0000000	2.4000001
-22435.0000000	2.4050002
-22434.0000000	2.4100001
-22433.0000000	2.4100001
-22432.0000000	2.4200001
-22431.0000000	2.4150000
-22430.0000000	2.4250002
-22429.0000000	2.4400001
-22428.0000000	2.4500000
-22427.0000000	2.4549999
-22426.0000000	2.4600000

-22425.0000000	2.4700000
-22424.0000000	2.4933333
-22423.0000000	2.5000000
-22422.0000000	2.5100000
-22421.0000000	2.5250001
-22420.0000000	2.5200000
-22419.0000000	2.5400000
-22418.0000000	2.5349998
-22417.0000000	2.5450001
-22416.0000000	2.5549998
-22415.0000000	2.5500000
-22414.0000000	2.5549998
-22413.0000000	2.5500000
-22412.0000000	2.5500000
-22411.0000000	2.5450001
-22410.0000000	2.5400000
-22409.0000000	2.5349998
-22408.0000000	2.5250001
-22407.0000000	2.5200000
-22406.0000000	2.5200000
-22405.0000000	2.5200000
-22404.0000000	2.5200000
-22403.0000000	2.5200000
-22402.0000000	2.5100000
-22401.0000000	2.5149999
-22400.0000000	2.5100000
-22399.0000000	2.5050001
-22398.0000000	2.5050001
-22397.0000000	2.5000000
-22396.0000000	2.4949999
-22395.0000000	2.4949999
-22394.0000000	2.4900000
-22393.0000000	2.4966667
-22392.0000000	2.4949999
-22391.0000000	2.4900000
-22390.0000000	2.4900000
-22389.0000000	2.4850001
-22388.0000000	2.4850001
-22387.0000000	2.4749999
-22386.0000000	2.4749999
-22385.0000000	2.4749999
-22384.0000000	2.4749999
-22383.0000000	2.4600000
-22382.0000000	2.4650002
-22381.0000000	2.4600000
-22380.0000000	2.4700000
-22379.0000000	2.4700000
-22378.0000000	2.4800000
-22377.0000000	2.4850001
-22376.0000000	2.4850001
-22375.0000000	2.4650002
-22374.0000000	2.4500000
-22373.0000000	2.4300001
-22372.0000000	2.4150000
-22371.0000000	2.3850002
-22370.0000000	2.3850002
-22369.0000000	2.3850002

-22368.0000000	2.3800001
-22367.0000000	2.3750000
-22366.0000000	2.3750000
-22365.0000000	2.3800001
-22364.0000000	2.3833334
-22363.0000000	2.3950000
-22362.0000000	2.4000001
-22361.0000000	2.4050002
-22360.0000000	2.4100001
-22359.0000000	2.4250002
-22358.0000000	2.4349999
-22357.0000000	2.4400001
-22356.0000000	2.4500000
-22355.0000000	2.4650002
-22354.0000000	2.4700000
-22353.0000000	2.4650002
-22352.0000000	2.4749999
-22351.0000000	2.4650002
-22350.0000000	2.4700000
-22349.0000000	2.4700000
-22348.0000000	2.4700000
-22347.0000000	2.4749999
-22346.0000000	2.4650002
-22345.0000000	2.4700000
-22344.0000000	2.4700000
-22343.0000000	2.4749999
-22342.0000000	2.4749999
-22341.0000000	2.4850001
-22340.0000000	2.4800000
-22339.0000000	2.4850001
-22338.0000000	2.4850001
-22337.0000000	2.4949999
-22336.0000000	2.4900000
-22335.0000000	2.5000000
-22334.0000000	2.4949999
-22333.0000000	2.5000000
-22332.0000000	2.4850001
-22331.0000000	2.4766667
-22330.0000000	2.4749999
-22329.0000000	2.4749999
-22328.0000000	2.4800000
-22327.0000000	2.4850001
-22326.0000000	2.5000000
-22325.0000000	2.5100000
-22324.0000000	2.5200000
-22323.0000000	2.5300000
-22322.0000000	2.5300000
-22321.0000000	2.5400000
-22320.0000000	2.5349998
-22319.0000000	2.5349998
-22318.0000000	2.5349998
-22317.0000000	2.5349998
-22316.0000000	2.5400000
-22315.0000000	2.5300000
-22314.0000000	2.5400000
-22313.0000000	2.5400000
-22312.0000000	2.5549998

-22311.0000000	2.5549998
-22310.0000000	2.5549998
-22309.0000000	2.5650001
-22308.0000000	2.5699999
-22307.0000000	2.5650001
-22306.0000000	2.5749998
-22305.0000000	2.5799999
-22304.0000000	2.5749998
-22303.0000000	2.5749998
-22302.0000000	2.5666666
-22301.0000000	2.5650001
-22300.0000000	2.5599999
-22299.0000000	2.5599999
-22298.0000000	2.5549998
-22297.0000000	2.5549998
-22296.0000000	2.5549998
-22295.0000000	2.5450001
-22294.0000000	2.5549998
-22293.0000000	2.5450001
-22292.0000000	2.5500000
-22291.0000000	2.5450001
-22290.0000000	2.5500000
-22289.0000000	2.5549998
-22288.0000000	2.5549998
-22287.0000000	2.5599999
-22286.0000000	2.5599999
-22285.0000000	2.5650001
-22284.0000000	2.5699999
-22283.0000000	2.5799999
-22282.0000000	2.5749998
-22281.0000000	2.5799999
-22280.0000000	2.5850000
-22279.0000000	2.5799999
-22278.0000000	2.5799999
-22277.0000000	2.5749998
-22276.0000000	2.5699999
-22275.0000000	2.5699999
-22274.0000000	2.5650001
-22273.0000000	2.5599999
-22272.0000000	2.5599999
-22271.0000000	2.5549998
-22270.0000000	2.5599999
-22269.0000000	2.5650001
-22268.0000000	2.5650001
-22267.0000000	2.5699999
-22266.0000000	2.5699999
-22265.0000000	2.5749998
-22264.0000000	2.5749998
-22263.0000000	2.5749998
-22262.0000000	2.5749998
-22261.0000000	2.5850000
-22260.0000000	2.5799999
-22259.0000000	2.5799999
-22258.0000000	2.5799999
-22257.0000000	2.5850000
-22256.0000000	2.5850000
-22255.0000000	2.5899999

-22254.0000000	2.5850000
-22253.0000000	2.5799999
-22252.0000000	2.5749998
-22251.0000000	2.5699999
-22250.0000000	2.5699999
-22249.0000000	2.5549998
-22248.0000000	2.5450001
-22247.0000000	2.5450001
-22246.0000000	2.5400000
-22245.0000000	2.5349998
-22244.0000000	2.5349998
-22243.0000000	2.5250001
-22242.0000000	2.5333333
-22241.0000000	2.5349998
-22240.0000000	2.5400000
-22239.0000000	2.5400000
-22238.0000000	2.5400000
-22237.0000000	2.5400000
-22236.0000000	2.5500000
-22235.0000000	2.5500000
-22234.0000000	2.5549998
-22233.0000000	2.5699999
-22232.0000000	2.5749998
-22231.0000000	2.5749998
-22230.0000000	2.5899999
-22229.0000000	2.5899999
-22228.0000000	2.5899999
-22227.0000000	2.5899999
-22226.0000000	2.5899999
-22225.0000000	2.5899999
-22224.0000000	2.5899999
-22223.0000000	2.5899999
-22222.0000000	2.5999999
-22221.0000000	2.5999999
-22220.0000000	2.6050000
-22219.0000000	2.6050000
-22218.0000000	2.6149998
-22217.0000000	2.6199999
-22216.0000000	2.6250000
-22215.0000000	2.6250000
-22214.0000000	2.6149998
-22213.0000000	2.6199999
-22212.0000000	2.6149998
-22211.0000000	2.6099999
-22210.0000000	2.6066666
-22209.0000000	2.5949998
-22208.0000000	2.5949998
-22207.0000000	2.5949998
-22206.0000000	2.5899999
-22205.0000000	2.5799999
-22204.0000000	2.5749998
-22203.0000000	2.5599999
-22202.0000000	2.5500000
-22201.0000000	2.5400000
-22200.0000000	2.5349998
-22199.0000000	2.5250001
-22198.0000000	2.5149999

-22197.0000000	2.4949999
-22196.0000000	2.4900000
-22195.0000000	2.4800000
-22194.0000000	2.4749999
-22193.0000000	2.4500000
-22192.0000000	2.4450002
-22191.0000000	2.4300001
-22190.0000000	2.4200001
-22189.0000000	2.4000001
-22188.0000000	2.3950000
-22187.0000000	2.3900001
-22186.0000000	2.3800001
-22185.0000000	2.3800001
-22184.0000000	2.3800001
-22183.0000000	2.3800001
-22182.0000000	2.3950000
-22181.0000000	2.3900001
-22180.0000000	2.3900001
-22179.0000000	2.3900001
-22178.0000000	2.3850002
-22177.0000000	2.3900001
-22176.0000000	2.3900001
-22175.0000000	2.4000001
-22174.0000000	2.4000001
-22173.0000000	2.4050002
-22172.0000000	2.4100001
-22171.0000000	2.4100001
-22170.0000000	2.4200001
-22169.0000000	2.4200001
-22168.0000000	2.4250002
-22167.0000000	2.4200001
-22166.0000000	2.4150000
-22165.0000000	2.4050002
-22164.0000000	2.4000001
-22163.0000000	2.3950000
-22162.0000000	2.4050002
-22161.0000000	2.4050002
-22160.0000000	2.4000001
-22159.0000000	2.4100001
-22158.0000000	2.4150000
-22157.0000000	2.4250002
-22156.0000000	2.4300001
-22155.0000000	2.4250002
-22154.0000000	2.4349999
-22153.0000000	2.4300001
-22152.0000000	2.4250002
-22151.0000000	2.4300001
-22150.0000000	2.4200001
-22149.0000000	2.4150000
-22148.0000000	2.4050002
-22147.0000000	2.4000001
-22146.0000000	2.4000001
-22145.0000000	2.3950000
-22144.0000000	2.3950000
-22143.0000000	2.4000001
-22142.0000000	2.4000001
-22141.0000000	2.4000001

-22140.0000000	2.4050002
-22139.0000000	2.4000001
-22138.0000000	2.4050002
-22137.0000000	2.4050002
-22136.0000000	2.4150000
-22135.0000000	2.4150000
-22134.0000000	2.4150000
-22133.0000000	2.4250002
-22132.0000000	2.4400001
-22131.0000000	2.4500000
-22130.0000000	2.4549999
-22129.0000000	2.4700000
-22128.0000000	2.4700000
-22127.0000000	2.4850001
-22126.0000000	2.4949999
-22125.0000000	2.5050001
-22124.0000000	2.5050001
-22123.0000000	2.5050001
-22122.0000000	2.4933331
-22121.0000000	2.4800000
-22120.0000000	2.4700000
-22119.0000000	2.4549999
-22118.0000000	2.4500000
-22117.0000000	2.4450002
-22116.0000000	2.4400001
-22115.0000000	2.4450002
-22114.0000000	2.4400001
-22113.0000000	2.4450002
-22112.0000000	2.4450002
-22111.0000000	2.4400001
-22110.0000000	2.4349999
-22109.0000000	2.4400001
-22108.0000000	2.4349999
-22107.0000000	2.4300001
-22106.0000000	2.4300001
-22105.0000000	2.4300001
-22104.0000000	2.4400001
-22103.0000000	2.4500000
-22102.0000000	2.4450002
-22101.0000000	2.4450002
-22100.0000000	2.4549999
-22099.0000000	2.4600000
-22098.0000000	2.4549999
-22097.0000000	2.4600000
-22096.0000000	2.4700000
-22095.0000000	2.4800000
-22094.0000000	2.4749999
-22093.0000000	2.4800000
-22092.0000000	2.4749999
-22091.0000000	2.4850001
-22090.0000000	2.4833333
-22089.0000000	2.4900000
-22088.0000000	2.4900000
-22087.0000000	2.4900000
-22086.0000000	2.4850001
-22085.0000000	2.4749999
-22084.0000000	2.4700000

-22083.0000000	2.4500000
-22082.0000000	2.4400001
-22081.0000000	2.4200001
-22080.0000000	2.4150000
-22079.0000000	2.4200001
-22078.0000000	2.4150000
-22077.0000000	2.4250002
-22076.0000000	2.4300001
-22075.0000000	2.4349999
-22074.0000000	2.4549999
-22073.0000000	2.4600000
-22072.0000000	2.4549999
-22071.0000000	2.4600000
-22070.0000000	2.4500000
-22069.0000000	2.4500000
-22068.0000000	2.4549999
-22067.0000000	2.4549999
-22066.0000000	2.4650002
-22065.0000000	2.4549999
-22064.0000000	2.4600000
-22063.0000000	2.4650002
-22062.0000000	2.4549999
-22061.0000000	2.4500000
-22060.0000000	2.4500000
-22059.0000000	2.4600000
-22058.0000000	2.4600000
-22057.0000000	2.4549999
-22056.0000000	2.4500000
-22055.0000000	2.4549999
-22054.0000000	2.4500000
-22053.0000000	2.4500000
-22052.0000000	2.4500000
-22051.0000000	2.4500000
-22050.0000000	2.4500000
-22049.0000000	2.4500000
-22048.0000000	2.4500000
-22047.0000000	2.4500000
-22046.0000000	2.4500000
-22045.0000000	2.4500000
-22044.0000000	2.4400001
-22043.0000000	2.4349999
-22042.0000000	2.4400001
-22041.0000000	2.4349999
-22040.0000000	2.4250002
-22039.0000000	2.4200001
-22038.0000000	2.4100001
-22037.0000000	2.4150000
-22036.0000000	2.4050002
-22035.0000000	2.4050002
-22034.0000000	2.4050002
-22033.0000000	2.4000001
-22032.0000000	2.4100001
-22031.0000000	2.4150000
-22030.0000000	2.4266670
-22029.0000000	2.4300001
-22028.0000000	2.4300001
-22027.0000000	2.4200001

-22026.0000000	2.4050002
-22025.0000000	2.3850002
-22024.0000000	2.3649998
-22023.0000000	2.3399999
-22022.0000000	2.3000000
-22021.0000000	2.2700000
-22020.0000000	2.2500000
-22019.0000000	2.2300000
-22018.0000000	2.2300000
-22017.0000000	2.2249999
-22016.0000000	2.2249999
-22015.0000000	2.2200000
-22014.0000000	2.2000000
-22013.0000000	2.2000000
-22012.0000000	2.2049999
-22011.0000000	2.2049999
-22010.0000000	2.2100000
-22009.0000000	2.2100000
-22008.0000000	2.2249999
-22007.0000000	2.2249999
-22006.0000000	2.2400000
-22005.0000000	2.2449999
-22004.0000000	2.2600000
-22003.0000000	2.2700000
-22002.0000000	2.2700000
-22001.0000000	2.2800000
-22000.0000000	2.2849998
-21999.0000000	2.2933333
-21998.0000000	2.2950001
-21997.0000000	2.2950001
-21996.0000000	2.2900000
-21995.0000000	2.2950001
-21994.0000000	2.3000000
-21993.0000000	2.3049998
-21992.0000000	2.3099999
-21991.0000000	2.3150001
-21990.0000000	2.3150001
-21989.0000000	2.3099999
-21988.0000000	2.3049998
-21987.0000000	2.3049998
-21986.0000000	2.3049998
-21985.0000000	2.3049998
-21984.0000000	2.3049998
-21983.0000000	2.3049998
-21982.0000000	2.3199999
-21981.0000000	2.3150001
-21980.0000000	2.3199999
-21979.0000000	2.3199999
-21978.0000000	2.3199999
-21977.0000000	2.3150001
-21976.0000000	2.3199999
-21975.0000000	2.3249998
-21974.0000000	2.3199999
-21973.0000000	2.3249998
-21972.0000000	2.3249998
-21971.0000000	2.3299999
-21970.0000000	2.3350000

-21969.0000000	2.3266666
-21968.0000000	2.3350000
-21967.0000000	2.3350000
-21966.0000000	2.3350000
-21965.0000000	2.3350000
-21964.0000000	2.3399999
-21963.0000000	2.3449998
-21962.0000000	2.3449998
-21961.0000000	2.3449998
-21960.0000000	2.3449998
-21959.0000000	2.3299999
-21958.0000000	2.3249998
-21957.0000000	2.3150001
-21956.0000000	2.3049998
-21955.0000000	2.2900000
-21954.0000000	2.2900000
-21953.0000000	2.2849998
-21952.0000000	2.2849998
-21951.0000000	2.2800000
-21950.0000000	2.2750001
-21949.0000000	2.2700000
-21948.0000000	2.2649999
-21947.0000000	2.2600000
-21946.0000000	2.2550001
-21945.0000000	2.2550001
-21944.0000000	2.2550001
-21943.0000000	2.2550001
-21942.0000000	2.2600000
-21941.0000000	2.2649999
-21940.0000000	2.2649999
-21939.0000000	2.2566669
-21938.0000000	2.2550001
-21937.0000000	2.2500000
-21936.0000000	2.2400000
-21935.0000000	2.2300000
-21934.0000000	2.2350001
-21933.0000000	2.2350001
-21932.0000000	2.2300000
-21931.0000000	2.2200000
-21930.0000000	2.2200000
-21929.0000000	2.2100000
-21928.0000000	2.2000000
-21927.0000000	2.2000000
-21926.0000000	2.1849999
-21925.0000000	2.1700001
-21924.0000000	2.1550002
-21923.0000000	2.1400001
-21922.0000000	2.1300001
-21921.0000000	2.1250000
-21920.0000000	2.1300001
-21919.0000000	2.1400001
-21918.0000000	2.1550002
-21917.0000000	2.1650000
-21916.0000000	2.1600001
-21915.0000000	2.1650000
-21914.0000000	2.1700001
-21913.0000000	2.1650000

-21912.0000000	2.1700001
-21911.0000000	2.1849999
-21910.0000000	2.2000000
-21909.0000000	2.2200000
-21908.0000000	2.2249999
-21907.0000000	2.2300000
-21906.0000000	2.2449999
-21905.0000000	2.2500000
-21904.0000000	2.2550001
-21903.0000000	2.2500000
-21902.0000000	2.2550001
-21901.0000000	2.2300000
-21900.0000000	2.2300000
-21899.0000000	2.1950002
-21898.0000000	2.1700001
-21897.0000000	2.1650000
-21896.0000000	2.1550002
-21895.0000000	2.1550002
-21894.0000000	2.1550002
-21893.0000000	2.1550002
-21892.0000000	2.1550002
-21891.0000000	2.1700001
-21890.0000000	2.1700001
-21889.0000000	2.1950002
-21888.0000000	2.1950002
-21887.0000000	2.1950002
-21886.0000000	2.2150002
-21885.0000000	2.2200000
-21884.0000000	2.2300000
-21883.0000000	2.2400000
-21882.0000000	2.2500000
-21881.0000000	2.2500000
-21880.0000000	2.2550001
-21879.0000000	2.2600000
-21878.0000000	2.2733333
-21877.0000000	2.2700000
-21876.0000000	2.2700000
-21875.0000000	2.2700000
-21874.0000000	2.2649999
-21873.0000000	2.2649999
-21872.0000000	2.2649999
-21871.0000000	2.2700000
-21870.0000000	2.2750001
-21869.0000000	2.2800000
-21868.0000000	2.2900000
-21867.0000000	2.2900000
-21866.0000000	2.2900000
-21865.0000000	2.2900000
-21864.0000000	2.2900000
-21863.0000000	2.2900000
-21862.0000000	2.2950001
-21861.0000000	2.2900000
-21860.0000000	2.2849998
-21859.0000000	2.2900000
-21858.0000000	2.2849998
-21857.0000000	2.2900000
-21856.0000000	2.2849998

-21855.0000000	2.2849998
-21854.0000000	2.2800000
-21853.0000000	2.2750001
-21852.0000000	2.2649999
-21851.0000000	2.2649999
-21850.0000000	2.2566669
-21849.0000000	2.2550001
-21848.0000000	2.2449999
-21847.0000000	2.2350001
-21846.0000000	2.2350001
-21845.0000000	2.2249999
-21844.0000000	2.2200000
-21843.0000000	2.2200000
-21842.0000000	2.2150002
-21841.0000000	2.2200000
-21840.0000000	2.2200000
-21839.0000000	2.2200000
-21838.0000000	2.2300000
-21837.0000000	2.2300000
-21836.0000000	2.2350001
-21835.0000000	2.2400000
-21834.0000000	2.2350001
-21833.0000000	2.2449999
-21832.0000000	2.2350001
-21831.0000000	2.2400000
-21830.0000000	2.2350001
-21829.0000000	2.2300000
-21828.0000000	2.2249999
-21827.0000000	2.2200000
-21826.0000000	2.2100000
-21825.0000000	2.2100000
-21824.0000000	2.2049999
-21823.0000000	2.2100000
-21822.0000000	2.2000000
-21821.0000000	2.1950002
-21820.0000000	2.1950002
-21819.0000000	2.2000000
-21818.0000000	2.1950002
-21817.0000000	2.1900001
-21816.0000000	2.1849999
-21815.0000000	2.1950002
-21814.0000000	2.1900001
-21813.0000000	2.1900001
-21812.0000000	2.1849999
-21811.0000000	2.1750002
-21810.0000000	2.1700001
-21809.0000000	2.1650000
-21808.0000000	2.1600001
-21807.0000000	2.1600001
-21806.0000000	2.1550002
-21805.0000000	2.1450000
-21804.0000000	2.1350002
-21803.0000000	2.1199999
-21802.0000000	2.1199999
-21801.0000000	2.1149998
-21800.0000000	2.1199999
-21799.0000000	2.1149998

-21798.0000000	2.1250000
-21797.0000000	2.1300001
-21796.0000000	2.1300001
-21795.0000000	2.1300001
-21794.0000000	2.1300001
-21793.0000000	2.1350002
-21792.0000000	2.1450000
-21791.0000000	2.1500001
-21790.0000000	2.1550002
-21789.0000000	2.1666667
-21788.0000000	2.1750002
-21787.0000000	2.1700001
-21786.0000000	2.1800001
-21785.0000000	2.1800001
-21784.0000000	2.1800001
-21783.0000000	2.1849999
-21782.0000000	2.1900001
-21781.0000000	2.1800001
-21780.0000000	2.1800001
-21779.0000000	2.1750002
-21778.0000000	2.1750002
-21777.0000000	2.1700001
-21776.0000000	2.1700001
-21775.0000000	2.1700001
-21774.0000000	2.1550002
-21773.0000000	2.1550002
-21772.0000000	2.1500001
-21771.0000000	2.1500001
-21770.0000000	2.1450000
-21769.0000000	2.1500001
-21768.0000000	2.1550002
-21767.0000000	2.1550002
-21766.0000000	2.1450000
-21765.0000000	2.1450000
-21764.0000000	2.1450000
-21763.0000000	2.1400001
-21762.0000000	2.1400001
-21761.0000000	2.1350002
-21760.0000000	2.1350002
-21759.0000000	2.1366670
-21758.0000000	2.1300001
-21757.0000000	2.1266668
-21756.0000000	2.1149998
-21755.0000000	2.1050000
-21754.0000000	2.1050000
-21753.0000000	2.1199999
-21752.0000000	2.1300001
-21751.0000000	2.1450000
-21750.0000000	2.1550002
-21749.0000000	2.1600001
-21748.0000000	2.1650000
-21747.0000000	2.1650000
-21746.0000000	2.1600001
-21745.0000000	2.1700001
-21744.0000000	2.1700001
-21743.0000000	2.1800001
-21742.0000000	2.1900001

-21741.0000000	2.1950002
-21740.0000000	2.2049999
-21739.0000000	2.2100000
-21738.0000000	2.2150002
-21737.0000000	2.2200000
-21736.0000000	2.2249999
-21735.0000000	2.2200000
-21734.0000000	2.2150002
-21733.0000000	2.2150002
-21732.0000000	2.2150002
-21731.0000000	2.2150002
-21730.0000000	2.2150002
-21729.0000000	2.2150002
-21728.0000000	2.2200000
-21727.0000000	2.2150002
-21726.0000000	2.2166669
-21725.0000000	2.2150002
-21724.0000000	2.2100000
-21723.0000000	2.2000000
-21722.0000000	2.2000000
-21721.0000000	2.2000000
-21720.0000000	2.2049999
-21719.0000000	2.2000000
-21718.0000000	2.1950002
-21717.0000000	2.1950002
-21716.0000000	2.1950002
-21715.0000000	2.1950002
-21714.0000000	2.1950002
-21713.0000000	2.1900001
-21712.0000000	2.1900001
-21711.0000000	2.2000000
-21710.0000000	2.1950002
-21709.0000000	2.2000000
-21708.0000000	2.1950002
-21707.0000000	2.1900001
-21706.0000000	2.1950002
-21705.0000000	2.1900001
-21704.0000000	2.1800001
-21703.0000000	2.1800001
-21702.0000000	2.1750002
-21701.0000000	2.1700001
-21700.0000000	2.1650000
-21699.0000000	2.1600001
-21698.0000000	2.1450000
-21697.0000000	2.1233332
-21696.0000000	2.1050000
-21695.0000000	2.0899999
-21694.0000000	2.0749998
-21693.0000000	2.0650001
-21692.0000000	2.0500000
-21691.0000000	2.0300000
-21690.0000000	2.0000000
-21689.0000000	1.9950000
-21688.0000000	1.9850000
-21687.0000000	1.9800000
-21686.0000000	1.9750000
-21685.0000000	1.9850000

-21684.0000000	1.9900000
-21683.0000000	1.9950000
-21682.0000000	2.0050001
-21681.0000000	2.0050001
-21680.0000000	2.0149999
-21679.0000000	2.0149999
-21678.0000000	2.0149999
-21677.0000000	2.0149999
-21676.0000000	2.0200000
-21675.0000000	2.0300000
-21674.0000000	2.0300000
-21673.0000000	2.0500000
-21672.0000000	2.0450001
-21671.0000000	2.0549998
-21670.0000000	2.0650001
-21669.0000000	2.0749998
-21668.0000000	2.0899999
-21667.0000000	2.0966666
-21666.0000000	2.1099999
-21665.0000000	2.1199999
-21664.0000000	2.1250000
-21663.0000000	2.1250000
-21662.0000000	2.1400001
-21661.0000000	2.1500001
-21660.0000000	2.1600001
-21659.0000000	2.1700001
-21658.0000000	2.1750002
-21657.0000000	2.1900001
-21656.0000000	2.1950002
-21655.0000000	2.2100000
-21654.0000000	2.2200000
-21653.0000000	2.2300000
-21652.0000000	2.2350001
-21651.0000000	2.2400000
-21650.0000000	2.2600000
-21649.0000000	2.2649999
-21648.0000000	2.2800000
-21647.0000000	2.2800000
-21646.0000000	2.2950001
-21645.0000000	2.2849998
-21644.0000000	2.2950001
-21643.0000000	2.2950001
-21642.0000000	2.2950001
-21641.0000000	2.2950001
-21640.0000000	2.2950001
-21639.0000000	2.3000000
-21638.0000000	2.3099999
-21637.0000000	2.3133333
-21636.0000000	2.3199999
-21635.0000000	2.3249998
-21634.0000000	2.3249998
-21633.0000000	2.3199999
-21632.0000000	2.3249998
-21631.0000000	2.3299999
-21630.0000000	2.3249998
-21629.0000000	2.3249998
-21628.0000000	2.3199999

-21627.0000000	2.3199999
-21626.0000000	2.3099999
-21625.0000000	2.3099999
-21624.0000000	2.3049998
-21623.0000000	2.3049998
-21622.0000000	2.3049998
-21621.0000000	2.3000000
-21620.0000000	2.3000000
-21619.0000000	2.2950001
-21618.0000000	2.3000000
-21617.0000000	2.3000000
-21616.0000000	2.3000000
-21615.0000000	2.3000000
-21614.0000000	2.3000000
-21613.0000000	2.3099999
-21612.0000000	2.3150001
-21611.0000000	2.3249998
-21610.0000000	2.3299999
-21609.0000000	2.3299999
-21608.0000000	2.3399999
-21607.0000000	2.3399999
-21606.0000000	2.3399999
-21605.0000000	2.3350000
-21604.0000000	2.3299999
-21603.0000000	2.3299999
-21602.0000000	2.3249998
-21601.0000000	2.3249998
-21600.0000000	2.3099999
-21599.0000000	2.3000000
-21598.0000000	2.3000000
-21597.0000000	2.2900000
-21596.0000000	2.2849998
-21595.0000000	2.2950001
-21594.0000000	2.2849998
-21593.0000000	2.2849998
-21592.0000000	2.2950001
-21591.0000000	2.2900000
-21590.0000000	2.2950001
-21589.0000000	2.2950001
-21588.0000000	2.2950001
-21587.0000000	2.2950001
-21586.0000000	2.2950001
-21585.0000000	2.2950001
-21584.0000000	2.3049998
-21583.0000000	2.3000000
-21582.0000000	2.3049998
-21581.0000000	2.3049998
-21580.0000000	2.3000000
-21579.0000000	2.2950001
-21578.0000000	2.2900000
-21577.0000000	2.2950001
-21576.0000000	2.2933333
-21575.0000000	2.2900000
-21574.0000000	2.2950001
-21573.0000000	2.2900000
-21572.0000000	2.2900000
-21571.0000000	2.2950001

-21570.0000000	2.2950001
-21569.0000000	2.2950001
-21568.0000000	2.2900000
-21567.0000000	2.3000000
-21566.0000000	2.2900000
-21565.0000000	2.2900000
-21564.0000000	2.2849998
-21563.0000000	2.2800000
-21562.0000000	2.2600000
-21561.0000000	2.2649999
-21560.0000000	2.2500000
-21559.0000000	2.2550001
-21558.0000000	2.2449999
-21557.0000000	2.2350001
-21556.0000000	2.2249999
-21555.0000000	2.2150002
-21554.0000000	2.2150002
-21553.0000000	2.2100000
-21552.0000000	2.2000000
-21551.0000000	2.1900001
-21550.0000000	2.1750002
-21549.0000000	2.1650000
-21548.0000000	2.1450000
-21547.0000000	2.1350002
-21546.0000000	2.1233332
-21545.0000000	2.0999999
-21544.0000000	2.0799999
-21543.0000000	2.0500000
-21542.0000000	2.0300000
-21541.0000000	2.0250001
-21540.0000000	2.0250001
-21539.0000000	2.0349998
-21538.0000000	2.0400000
-21537.0000000	2.0500000
-21536.0000000	2.0599999
-21535.0000000	2.0650001
-21534.0000000	2.0749998
-21533.0000000	2.0899999
-21532.0000000	2.0899999
-21531.0000000	2.0899999
-21530.0000000	2.0949998
-21529.0000000	2.0949998
-21528.0000000	2.0999999
-21527.0000000	2.0949998
-21526.0000000	2.0949998
-21525.0000000	2.1050000
-21524.0000000	2.0949998
-21523.0000000	2.0999999
-21522.0000000	2.1099999
-21521.0000000	2.1250000
-21520.0000000	2.1300001
-21519.0000000	2.1400001
-21518.0000000	2.1500001
-21517.0000000	2.1566668
-21516.0000000	2.1650000
-21515.0000000	2.1650000
-21514.0000000	2.1600001

-21513.0000000	2.1550002
-21512.0000000	2.1550002
-21511.0000000	2.1650000
-21510.0000000	2.1650000
-21509.0000000	2.1700001
-21508.0000000	2.1700001
-21507.0000000	2.1700001
-21506.0000000	2.1800001
-21505.0000000	2.1849999
-21504.0000000	2.1849999
-21503.0000000	2.1950002
-21502.0000000	2.2000000
-21501.0000000	2.2000000
-21500.0000000	2.2100000
-21499.0000000	2.2200000
-21498.0000000	2.2200000
-21497.0000000	2.2249999
-21496.0000000	2.2300000
-21495.0000000	2.2350001
-21494.0000000	2.2249999
-21493.0000000	2.2249999
-21492.0000000	2.2300000
-21491.0000000	2.2350001
-21490.0000000	2.2350001
-21489.0000000	2.2350001
-21488.0000000	2.2249999
-21487.0000000	2.2200000
-21486.0000000	2.2000000
-21485.0000000	2.2000000
-21484.0000000	2.1950002
-21483.0000000	2.1950002
-21482.0000000	2.1950002
-21481.0000000	2.1900001
-21480.0000000	2.2000000
-21479.0000000	2.2150002
-21478.0000000	2.2200000
-21477.0000000	2.2249999
-21476.0000000	2.2300000
-21475.0000000	2.2300000
-21474.0000000	2.2350001
-21473.0000000	2.2449999
-21472.0000000	2.2449999
-21471.0000000	2.2550001
-21470.0000000	2.2500000
-21469.0000000	2.2550001
-21468.0000000	2.2550001
-21467.0000000	2.2649999
-21466.0000000	2.2750001
-21465.0000000	2.2750001
-21464.0000000	2.2700000
-21463.0000000	2.2750001
-21462.0000000	2.2700000
-21461.0000000	2.2649999
-21460.0000000	2.2550001
-21459.0000000	2.2449999
-21458.0000000	2.2300000
-21457.0000000	2.2249999

-21456.0000000	2.2100000
-21455.0000000	2.2100000
-21454.0000000	2.2100000
-21453.0000000	2.2049999
-21452.0000000	2.2150002
-21451.0000000	2.2049999
-21450.0000000	2.2049999
-21449.0000000	2.2049999
-21448.0000000	2.1950002
-21447.0000000	2.2000000
-21446.0000000	2.1950002
-21445.0000000	2.1950002
-21444.0000000	2.2049999
-21443.0000000	2.2150002
-21442.0000000	2.2200000
-21441.0000000	2.2249999
-21440.0000000	2.2249999
-21439.0000000	2.2300000
-21438.0000000	2.2350001
-21437.0000000	2.2400000
-21436.0000000	2.2400000
-21435.0000000	2.2400000
-21434.0000000	2.2550001
-21433.0000000	2.2550001
-21432.0000000	2.2500000
-21431.0000000	2.2449999
-21430.0000000	2.2400000
-21429.0000000	2.2449999
-21428.0000000	2.2400000
-21427.0000000	2.2350001
-21426.0000000	2.2233334
-21425.0000000	2.2049999
-21424.0000000	2.1950002
-21423.0000000	2.1849999
-21422.0000000	2.1600001
-21421.0000000	2.1550002
-21420.0000000	2.1600001
-21419.0000000	2.1500001
-21418.0000000	2.1550002
-21417.0000000	2.1600001
-21416.0000000	2.1550002
-21415.0000000	2.1550002
-21414.0000000	2.1500001
-21413.0000000	2.1450000
-21412.0000000	2.1400001
-21411.0000000	2.1400001
-21410.0000000	2.1400001
-21409.0000000	2.1450000
-21408.0000000	2.1500001
-21407.0000000	2.1550002
-21406.0000000	2.1650000
-21405.0000000	2.1750002
-21404.0000000	2.1800001
-21403.0000000	2.1900001
-21402.0000000	2.1950002
-21401.0000000	2.1950002
-21400.0000000	2.2000000

-21399.0000000	2.1800001
-21398.0000000	2.1900001
-21397.0000000	2.1950002
-21396.0000000	2.2000000
-21395.0000000	2.2166669
-21394.0000000	2.2350001
-21393.0000000	2.2550001
-21392.0000000	2.2700000
-21391.0000000	2.2849998
-21390.0000000	2.2950001
-21389.0000000	2.3099999
-21388.0000000	2.3150001
-21387.0000000	2.3199999
-21386.0000000	2.3150001
-21385.0000000	2.3249998
-21384.0000000	2.3199999
-21383.0000000	2.3150001
-21382.0000000	2.3150001
-21381.0000000	2.3150001
-21380.0000000	2.3150001
-21379.0000000	2.3099999
-21378.0000000	2.3000000
-21377.0000000	2.2950001
-21376.0000000	2.2900000
-21375.0000000	2.2849998
-21374.0000000	2.2800000
-21373.0000000	2.2750001
-21372.0000000	2.2800000
-21371.0000000	2.2700000
-21370.0000000	2.2800000
-21369.0000000	2.2800000
-21368.0000000	2.2750001
-21367.0000000	2.2700000
-21366.0000000	2.2649999
-21365.0000000	2.2566667
-21364.0000000	2.2400000
-21363.0000000	2.2249999
-21362.0000000	2.2049999
-21361.0000000	2.1800001
-21360.0000000	2.1700001
-21359.0000000	2.1750002
-21358.0000000	2.1700001
-21357.0000000	2.1750002
-21356.0000000	2.1900001
-21355.0000000	2.2049999
-21354.0000000	2.2200000
-21353.0000000	2.2200000
-21352.0000000	2.2249999
-21351.0000000	2.2200000
-21350.0000000	2.2100000
-21349.0000000	2.2000000
-21348.0000000	2.1849999
-21347.0000000	2.1800001
-21346.0000000	2.1700001
-21345.0000000	2.1650000
-21344.0000000	2.1600001
-21343.0000000	2.1650000

-21342.0000000	2.1600001
-21341.0000000	2.1650000
-21340.0000000	2.1700001
-21339.0000000	2.1750002
-21338.0000000	2.1700001
-21337.0000000	2.1750002
-21336.0000000	2.1800001
-21335.0000000	2.1800001
-21334.0000000	2.1833334
-21333.0000000	2.1900001
-21332.0000000	2.1900001
-21331.0000000	2.1950002
-21330.0000000	2.1849999
-21329.0000000	2.1950002
-21328.0000000	2.1950002
-21327.0000000	2.1900001
-21326.0000000	2.1849999
-21325.0000000	2.2000000
-21324.0000000	2.2049999
-21323.0000000	2.2150002
-21322.0000000	2.2200000
-21321.0000000	2.2300000
-21320.0000000	2.2300000
-21319.0000000	2.2400000
-21318.0000000	2.2500000
-21317.0000000	2.2449999
-21316.0000000	2.2550001
-21315.0000000	2.2600000
-21314.0000000	2.2550001
-21313.0000000	2.2550001
-21312.0000000	2.2500000
-21311.0000000	2.2649999
-21310.0000000	2.2649999
-21309.0000000	2.2700000
-21308.0000000	2.2700000
-21307.0000000	2.2700000
-21306.0000000	2.2800000
-21305.0000000	2.2933333
-21304.0000000	2.3000000
-21303.0000000	2.3000000
-21302.0000000	2.3049998
-21301.0000000	2.3199999
-21300.0000000	2.3249998
-21299.0000000	2.3299999
-21298.0000000	2.3350000
-21297.0000000	2.3299999
-21296.0000000	2.3249998
-21295.0000000	2.3299999
-21294.0000000	2.3350000
-21293.0000000	2.3350000
-21292.0000000	2.3299999
-21291.0000000	2.3399999
-21290.0000000	2.3449998
-21289.0000000	2.3499999
-21288.0000000	2.3599999
-21287.0000000	2.3599999
-21286.0000000	2.3649998

-21285.0000000	2.3649998
-21284.0000000	2.3649998
-21283.0000000	2.3599999
-21282.0000000	2.3699999
-21281.0000000	2.3699999
-21280.0000000	2.3750000
-21279.0000000	2.3750000
-21278.0000000	2.3750000
-21277.0000000	2.3649998
-21276.0000000	2.3533332
-21275.0000000	2.3550000
-21274.0000000	2.3649998
-21273.0000000	2.3699999
-21272.0000000	2.3800001
-21271.0000000	2.3800001
-21270.0000000	2.3900001
-21269.0000000	2.3800001
-21268.0000000	2.3800001
-21267.0000000	2.3699999
-21266.0000000	2.3800001
-21265.0000000	2.3800001
-21264.0000000	2.3850002
-21263.0000000	2.3850002
-21262.0000000	2.3800001
-21261.0000000	2.3699999
-21260.0000000	2.3750000
-21259.0000000	2.3699999
-21258.0000000	2.3699999
-21257.0000000	2.3699999
-21256.0000000	2.3699999
-21255.0000000	2.3750000
-21254.0000000	2.3699999
-21253.0000000	2.3850002
-21252.0000000	2.3800001
-21251.0000000	2.3800001
-21250.0000000	2.3750000
-21249.0000000	2.3699999
-21248.0000000	2.3699999
-21247.0000000	2.3699999
-21246.0000000	2.3733332
-21245.0000000	2.3699999
-21244.0000000	2.3800001
-21243.0000000	2.3850002
-21242.0000000	2.3850002
-21241.0000000	2.3900001
-21240.0000000	2.3950000
-21239.0000000	2.3950000
-21238.0000000	2.3950000
-21237.0000000	2.4000001
-21236.0000000	2.4000001
-21235.0000000	2.4050002
-21234.0000000	2.4000001
-21233.0000000	2.4100001
-21232.0000000	2.4050002
-21231.0000000	2.4050002
-21230.0000000	2.4000001
-21229.0000000	2.4050002

-21228.0000000	2.4100001
-21227.0000000	2.4050002
-21226.0000000	2.4050002
-21225.0000000	2.4150000
-21224.0000000	2.4150000
-21223.0000000	2.4100001
-21222.0000000	2.4050002
-21221.0000000	2.4050002
-21220.0000000	2.4050002
-21219.0000000	2.4050002
-21218.0000000	2.4150000
-21217.0000000	2.4150000
-21216.0000000	2.4250002
-21215.0000000	2.4166667
-21214.0000000	2.4250002
-21213.0000000	2.4300001
-21212.0000000	2.4300001
-21211.0000000	2.4349999
-21210.0000000	2.4200001
-21209.0000000	2.4300001
-21208.0000000	2.4200001
-21207.0000000	2.4150000
-21206.0000000	2.4150000
-21205.0000000	2.4250002
-21204.0000000	2.4300001
-21203.0000000	2.4349999
-21202.0000000	2.4250002
-21201.0000000	2.4250002
-21200.0000000	2.4100001
-21199.0000000	2.4200001
-21198.0000000	2.4000001
-21197.0000000	2.3900001
-21196.0000000	2.3750000
-21195.0000000	2.3750000
-21194.0000000	2.3699999
-21193.0000000	2.3599999
-21192.0000000	2.3449998
-21191.0000000	2.3399999
-21190.0000000	2.3299999
-21189.0000000	2.3350000
-21188.0000000	2.3299999
-21187.0000000	2.3299999
-21186.0000000	2.3350000
-21185.0000000	2.3350000
-21184.0000000	2.3299999
-21183.0000000	2.3266666
-21182.0000000	2.3299999
-21181.0000000	2.3350000
-21180.0000000	2.3449998
-21179.0000000	2.3449998
-21178.0000000	2.3649998
-21177.0000000	2.3750000
-21176.0000000	2.3750000
-21175.0000000	2.3900001
-21174.0000000	2.3900001
-21173.0000000	2.3900001
-21172.0000000	2.3950000

-21171.0000000	2.4000001
-21170.0000000	2.4050002
-21169.0000000	2.4050002
-21168.0000000	2.4050002
-21167.0000000	2.4050002
-21166.0000000	2.4100001
-21165.0000000	2.4200001
-21164.0000000	2.4150000
-21163.0000000	2.4150000
-21162.0000000	2.4100001
-21161.0000000	2.4150000
-21160.0000000	2.4100001
-21159.0000000	2.4050002
-21158.0000000	2.4050002
-21157.0000000	2.3950000
-21156.0000000	2.3950000
-21155.0000000	2.3800001
-21154.0000000	2.3699999
-21153.0000000	2.3649998
-21152.0000000	2.3599999
-21151.0000000	2.3449998
-21150.0000000	2.3499999
-21149.0000000	2.3249998
-21148.0000000	2.3099999
-21147.0000000	2.3049998
-21146.0000000	2.3000000
-21145.0000000	2.3049998
-21144.0000000	2.3049998
-21143.0000000	2.3049998
-21142.0000000	2.3049998
-21141.0000000	2.3150001
-21140.0000000	2.3049998
-21139.0000000	2.3049998
-21138.0000000	2.3000000
-21137.0000000	2.3049998
-21136.0000000	2.3099999
-21135.0000000	2.3099999
-21134.0000000	2.3000000
-21133.0000000	2.2950001
-21132.0000000	2.2950001
-21131.0000000	2.2950001
-21130.0000000	2.2849998
-21129.0000000	2.2950001
-21128.0000000	2.2950001
-21127.0000000	2.2800000
-21126.0000000	2.2750001
-21125.0000000	2.2700000
-21124.0000000	2.2600000
-21123.0000000	2.2550001
-21122.0000000	2.2600000
-21121.0000000	2.2600000
-21120.0000000	2.2649999
-21119.0000000	2.2649999
-21118.0000000	2.2750001
-21117.0000000	2.2750001
-21116.0000000	2.2649999
-21115.0000000	2.2500000

-21114.0000000	2.2350001
-21113.0000000	2.2249999
-21112.0000000	2.2150002
-21111.0000000	2.2000000
-21110.0000000	2.1950002
-21109.0000000	2.1950002
-21108.0000000	2.1900001
-21107.0000000	2.1800001
-21106.0000000	2.1800001
-21105.0000000	2.1800001
-21104.0000000	2.1750002
-21103.0000000	2.1600001
-21102.0000000	2.1500001
-21101.0000000	2.1500001
-21100.0000000	2.1400001
-21099.0000000	2.1400001
-21098.0000000	2.1400001
-21097.0000000	2.1450000
-21096.0000000	2.1450000
-21095.0000000	2.1433334
-21094.0000000	2.1400001
-21093.0000000	2.1300001
-21092.0000000	2.1300001
-21091.0000000	2.1250000
-21090.0000000	2.1199999
-21089.0000000	2.1099999
-21088.0000000	2.0949998
-21087.0000000	2.0899999
-21086.0000000	2.0749998
-21085.0000000	2.0650001
-21084.0000000	2.0500000
-21083.0000000	2.0400000
-21082.0000000	2.0200000
-21081.0000000	2.0000000
-21080.0000000	2.0000000
-21079.0000000	2.0100000
-21078.0000000	2.0149999
-21077.0000000	2.0200000
-21076.0000000	2.0300000
-21075.0000000	2.0400000
-21074.0000000	2.0549998
-21073.0000000	2.0599999
-21072.0000000	2.0699999
-21071.0000000	2.0799999
-21070.0000000	2.0899999
-21069.0000000	2.0949998
-21068.0000000	2.1050000
-21067.0000000	2.1099999
-21066.0000000	2.1199999
-21065.0000000	2.1250000
-21064.0000000	2.1300001
-21063.0000000	2.1400001
-21062.0000000	2.1450000
-21061.0000000	2.1500001
-21060.0000000	2.1500001
-21059.0000000	2.1400001
-21058.0000000	2.1099999

-21057.0000000	2.0999999
-21056.0000000	2.0899999
-21055.0000000	2.0799999
-21054.0000000	2.0899999
-21053.0000000	2.0999999
-21052.0000000	2.0999999
-21051.0000000	2.1099999
-21050.0000000	2.1099999
-21049.0000000	2.1199999
-21048.0000000	2.1199999
-21047.0000000	2.1250000
-21046.0000000	2.1400001
-21045.0000000	2.1450000
-21044.0000000	2.1550002
-21043.0000000	2.1500001
-21042.0000000	2.1550002
-21041.0000000	2.1550002
-21040.0000000	2.1550002
-21039.0000000	2.1550002
-21038.0000000	2.1500001
-21037.0000000	2.1500001
-21036.0000000	2.1450000
-21035.0000000	2.1400001
-21034.0000000	2.1500001
-21033.0000000	2.1650000
-21032.0000000	2.1733334
-21031.0000000	2.1950002
-21030.0000000	2.2000000
-21029.0000000	2.2150002
-21028.0000000	2.2300000
-21027.0000000	2.2449999
-21026.0000000	2.2600000
-21025.0000000	2.2700000
-21024.0000000	2.2750001
-21023.0000000	2.2900000
-21022.0000000	2.2900000
-21021.0000000	2.2950001
-21020.0000000	2.3000000
-21019.0000000	2.3000000
-21018.0000000	2.2950001
-21017.0000000	2.2950001
-21016.0000000	2.2950001
-21015.0000000	2.2950001
-21014.0000000	2.3000000
-21013.0000000	2.2900000
-21012.0000000	2.2950001
-21011.0000000	2.2950001
-21010.0000000	2.3000000
-21009.0000000	2.3000000
-21008.0000000	2.3049998
-21007.0000000	2.3049998
-21006.0000000	2.3150001
-21005.0000000	2.3150001
-21004.0000000	2.3049998
-21003.0000000	2.3099999
-21002.0000000	2.3099999
-21001.0000000	2.3150001

-21000.0000000	2.3150001
-20999.0000000	2.3249998
-20998.0000000	2.3150001
-20997.0000000	2.3150001
-20996.0000000	2.3099999
-20995.0000000	2.3099999
-20994.0000000	2.3099999
-20993.0000000	2.2950001
-20992.0000000	2.2900000
-20991.0000000	2.2849998
-20990.0000000	2.2900000
-20989.0000000	2.2950001
-20988.0000000	2.2900000
-20987.0000000	2.2950001
-20986.0000000	2.2900000
-20985.0000000	2.2950001
-20984.0000000	2.2900000
-20983.0000000	2.2849998
-20982.0000000	2.2849998
-20981.0000000	2.2750001
-20980.0000000	2.2600000
-20979.0000000	2.2550001
-20978.0000000	2.2600000
-20977.0000000	2.2550001
-20976.0000000	2.2449999
-20975.0000000	2.2500000
-20974.0000000	2.2500000
-20973.0000000	2.2500000
-20972.0000000	2.2566669
-20971.0000000	2.2550001
-20970.0000000	2.2649999
-20969.0000000	2.2550001
-20968.0000000	2.2500000
-20967.0000000	2.2449999
-20966.0000000	2.2249999
-20965.0000000	2.2249999
-20964.0000000	2.2150002
-20963.0000000	2.2100000
-20962.0000000	2.2000000
-20961.0000000	2.1800001
-20960.0000000	2.1600001
-20959.0000000	2.1400001
-20958.0000000	2.1300001
-20957.0000000	2.1300001
-20956.0000000	2.1250000
-20955.0000000	2.1149998
-20954.0000000	2.1149998
-20953.0000000	2.1050000
-20952.0000000	2.0999999
-20951.0000000	2.1050000
-20950.0000000	2.1050000
-20949.0000000	2.0999999
-20948.0000000	2.0949998
-20947.0000000	2.0999999
-20946.0000000	2.0899999
-20945.0000000	2.0949998
-20944.0000000	2.0949998

-20943.0000000	2.0949998
-20942.0000000	2.1033332
-20941.0000000	2.0949998
-20940.0000000	2.0999999
-20939.0000000	2.0850000
-20938.0000000	2.0850000
-20937.0000000	2.0899999
-20936.0000000	2.0899999
-20935.0000000	2.0999999
-20934.0000000	2.1099999
-20933.0000000	2.1099999
-20932.0000000	2.1099999
-20931.0000000	2.0999999
-20930.0000000	2.0949998
-20929.0000000	2.0949998
-20928.0000000	2.0999999
-20927.0000000	2.0949998
-20926.0000000	2.0949998
-20925.0000000	2.0999999
-20924.0000000	2.0949998
-20923.0000000	2.0999999
-20922.0000000	2.0999999
-20921.0000000	2.1050000
-20920.0000000	2.1149998
-20919.0000000	2.1199999
-20918.0000000	2.1300001
-20917.0000000	2.1350002
-20916.0000000	2.1450000
-20915.0000000	2.1450000
-20914.0000000	2.1566668
-20913.0000000	2.1600001
-20912.0000000	2.1700001
-20911.0000000	2.1650000
-20910.0000000	2.1650000
-20909.0000000	2.1650000
-20908.0000000	2.1550002
-20907.0000000	2.1550002
-20906.0000000	2.1500001
-20905.0000000	2.1450000
-20904.0000000	2.1550002
-20903.0000000	2.1500001
-20902.0000000	2.1500001
-20901.0000000	2.1550002
-20900.0000000	2.1550002
-20899.0000000	2.1500001
-20898.0000000	2.1550002
-20897.0000000	2.1450000
-20896.0000000	2.1500001
-20895.0000000	2.1450000
-20894.0000000	2.1500001
-20893.0000000	2.1450000
-20892.0000000	2.1550002
-20891.0000000	2.1500001
-20890.0000000	2.1500001
-20889.0000000	2.1400001
-20888.0000000	2.1450000
-20887.0000000	2.1500001

-20886.0000000	2.1450000
-20885.0000000	2.1500001
-20884.0000000	2.1550002
-20883.0000000	2.1500001
-20882.0000000	2.1500001
-20881.0000000	2.1466668
-20880.0000000	2.1450000
-20879.0000000	2.1400001
-20878.0000000	2.1550002
-20877.0000000	2.1500001
-20876.0000000	2.1400001
-20875.0000000	2.1300001
-20874.0000000	2.1199999
-20873.0000000	2.1149998
-20872.0000000	2.1099999
-20871.0000000	2.1050000
-20870.0000000	2.0949998
-20869.0000000	2.0999999
-20868.0000000	2.1050000
-20867.0000000	2.1199999
-20866.0000000	2.1199999
-20865.0000000	2.1350002
-20864.0000000	2.1450000
-20863.0000000	2.1550002
-20862.0000000	2.1550002
-20861.0000000	2.1600001
-20860.0000000	2.1600001
-20859.0000000	2.1600001
-20858.0000000	2.1600001
-20857.0000000	2.1650000
-20856.0000000	2.1650000
-20855.0000000	2.1650000
-20854.0000000	2.1500001
-20853.0000000	2.1450000
-20852.0000000	2.1400001
-20851.0000000	2.1350002
-20850.0000000	2.1250000
-20849.0000000	2.1199999
-20848.0000000	2.1149998
-20847.0000000	2.1199999
-20846.0000000	2.1250000
-20845.0000000	2.1250000
-20844.0000000	2.1149998
-20843.0000000	2.1099999
-20842.0000000	2.1050000
-20841.0000000	2.0949998
-20840.0000000	2.0850000
-20839.0000000	2.0850000
-20838.0000000	2.0799999
-20837.0000000	2.0699999
-20836.0000000	2.0749998
-20835.0000000	2.0699999
-20834.0000000	2.0749998
-20833.0000000	2.0699999
-20832.0000000	2.0699999
-20831.0000000	2.0699999
-20830.0000000	2.0650001

-20829.0000000	2.0650001
-20828.0000000	2.0650001
-20827.0000000	2.0599999
-20826.0000000	2.0599999
-20825.0000000	2.0650001
-20824.0000000	2.0749998
-20823.0000000	2.0799999
-20822.0000000	2.0850000
-20821.0000000	2.0899999
-20820.0000000	2.0999999
-20819.0000000	2.0999999
-20818.0000000	2.0999999
-20817.0000000	2.1050000
-20816.0000000	2.1099999
-20815.0000000	2.1099999
-20814.0000000	2.1099999
-20813.0000000	2.1099999
-20812.0000000	2.1099999
-20811.0000000	2.1149998
-20810.0000000	2.1199999
-20809.0000000	2.1250000
-20808.0000000	2.1250000
-20807.0000000	2.1199999
-20806.0000000	2.1250000
-20805.0000000	2.1149998
-20804.0000000	2.1199999
-20803.0000000	2.1149998
-20802.0000000	2.1149998
-20801.0000000	2.1099999
-20800.0000000	2.0999999
-20799.0000000	2.0949998
-20798.0000000	2.0899999
-20797.0000000	2.0799999
-20796.0000000	2.0699999
-20795.0000000	2.0599999
-20794.0000000	2.0500000
-20793.0000000	2.0400000
-20792.0000000	2.0349998
-20791.0000000	2.0400000
-20790.0000000	2.0533333
-20789.0000000	2.0749998
-20788.0000000	2.0749998
-20787.0000000	2.0850000
-20786.0000000	2.0949998
-20785.0000000	2.0999999
-20784.0000000	2.0999999
-20783.0000000	2.0949998
-20782.0000000	2.0899999
-20781.0000000	2.0749998
-20780.0000000	2.0650001
-20779.0000000	2.0549998
-20778.0000000	2.0650001
-20777.0000000	2.0650001
-20776.0000000	2.0549998
-20775.0000000	2.0599999
-20774.0000000	2.0450001
-20773.0000000	2.0400000

-20772.0000000	2.0400000
-20771.0000000	2.0349998
-20770.0000000	2.0349998
-20769.0000000	2.0300000
-20768.0000000	2.0149999
-20767.0000000	2.0050001
-20766.0000000	2.0000000
-20765.0000000	1.9950000
-20764.0000000	1.9900000
-20763.0000000	1.9900000
-20762.0000000	1.9900000
-20761.0000000	2.0066667
-20760.0000000	2.0200000
-20759.0000000	2.0300000
-20758.0000000	2.0250001
-20757.0000000	2.0300000
-20756.0000000	2.0300000
-20755.0000000	2.0300000
-20754.0000000	2.0250001
-20753.0000000	2.0250001
-20752.0000000	2.0250001
-20751.0000000	2.0300000
-20750.0000000	2.0349998
-20749.0000000	2.0349998
-20748.0000000	2.0400000
-20747.0000000	2.0400000
-20746.0000000	2.0250001
-20745.0000000	2.0149999
-20744.0000000	2.0050001
-20743.0000000	1.9850000
-20742.0000000	1.9800000
-20741.0000000	1.9750000
-20740.0000000	1.9900000
-20739.0000000	1.9900000
-20738.0000000	2.0000000
-20737.0000000	2.0100000
-20736.0000000	2.0250001
-20735.0000000	2.0200000
-20734.0000000	2.0200000
-20733.0000000	2.0200000
-20732.0000000	2.0149999
-20731.0000000	2.0050001
-20730.0000000	2.0066669
-20729.0000000	2.0050001
-20728.0000000	2.0100000
-20727.0000000	2.0200000
-20726.0000000	2.0300000
-20725.0000000	2.0450001
-20724.0000000	2.0599999
-20723.0000000	2.0549998
-20722.0000000	2.0549998
-20721.0000000	2.0450001
-20720.0000000	2.0450001
-20719.0000000	2.0400000
-20718.0000000	2.0349998
-20717.0000000	2.0300000
-20716.0000000	2.0200000

-20715.0000000	2.0200000
-20714.0000000	2.0149999
-20713.0000000	2.0200000
-20712.0000000	2.0300000
-20711.0000000	2.0349998
-20710.0000000	2.0400000
-20709.0000000	2.0400000
-20708.0000000	2.0450001
-20707.0000000	2.0500000
-20706.0000000	2.0599999
-20705.0000000	2.0650001
-20704.0000000	2.0699999
-20703.0000000	2.0749998
-20702.0000000	2.0699999
-20701.0000000	2.0749998
-20700.0000000	2.0799999
-20699.0000000	2.0850000
-20698.0000000	2.0850000
-20697.0000000	2.0949998
-20696.0000000	2.0949998
-20695.0000000	2.1099999
-20694.0000000	2.1050000
-20693.0000000	2.1199999
-20692.0000000	2.1250000
-20691.0000000	2.1199999
-20690.0000000	2.1250000
-20689.0000000	2.1250000
-20688.0000000	2.1350002
-20687.0000000	2.1350002
-20686.0000000	2.1350002
-20685.0000000	2.1400001
-20684.0000000	2.1450000
-20683.0000000	2.1450000
-20682.0000000	2.1550002
-20681.0000000	2.1500001
-20680.0000000	2.1550002
-20679.0000000	2.1500001
-20678.0000000	2.1550002
-20677.0000000	2.1500001
-20676.0000000	2.1400001
-20675.0000000	2.1300001
-20674.0000000	2.1350002
-20673.0000000	2.1250000
-20672.0000000	2.1199999
-20671.0000000	2.1149998
-20670.0000000	2.1266668
-20669.0000000	2.1300001
-20668.0000000	2.1350002
-20667.0000000	2.1400001
-20666.0000000	2.1400001
-20665.0000000	2.1350002
-20664.0000000	2.1350002
-20663.0000000	2.1350002
-20662.0000000	2.1250000
-20661.0000000	2.1250000
-20660.0000000	2.1250000
-20659.0000000	2.1149998

-20658.0000000	2.1149998
-20657.0000000	2.0949998
-20656.0000000	2.0949998
-20655.0000000	2.0949998
-20654.0000000	2.0899999
-20653.0000000	2.0949998
-20652.0000000	2.0899999
-20651.0000000	2.0949998
-20650.0000000	2.0850000
-20649.0000000	2.0850000
-20648.0000000	2.0799999
-20647.0000000	2.0749998
-20646.0000000	2.0699999
-20645.0000000	2.0599999
-20644.0000000	2.0549998
-20643.0000000	2.0549998
-20642.0000000	2.0549998
-20641.0000000	2.0450001
-20640.0000000	2.0333331
-20639.0000000	2.0300000
-20638.0000000	2.0300000
-20637.0000000	2.0200000
-20636.0000000	2.0149999
-20635.0000000	1.9950000
-20634.0000000	1.9900000
-20633.0000000	1.9800000
-20632.0000000	1.9650000
-20631.0000000	1.9600000
-20630.0000000	1.9600000
-20629.0000000	1.9600000
-20628.0000000	1.9700000
-20627.0000000	1.9650000
-20626.0000000	1.9750000
-20625.0000000	1.9600000
-20624.0000000	1.9550000
-20623.0000000	1.9500000
-20622.0000000	1.9400001
-20621.0000000	1.9349999
-20620.0000000	1.9450001
-20619.0000000	1.9500000
-20618.0000000	1.9650000
-20617.0000000	1.9750000
-20616.0000000	1.9800000
-20615.0000000	1.9900000
-20614.0000000	2.0000000
-20613.0000000	2.0000000
-20612.0000000	2.0050001
-20611.0000000	2.0100000
-20610.0000000	2.0200000
-20609.0000000	2.0300000
-20608.0000000	2.0300000
-20607.0000000	2.0349998
-20606.0000000	2.0400000
-20605.0000000	2.0400000
-20604.0000000	2.0450001
-20603.0000000	2.0400000
-20602.0000000	2.0500000

-20601.0000000	2.0500000
-20600.0000000	2.0500000
-20599.0000000	2.0500000
-20598.0000000	2.0400000
-20597.0000000	2.0300000
-20596.0000000	2.0200000
-20595.0000000	1.9950000
-20594.0000000	1.9850000
-20593.0000000	1.9900000
-20592.0000000	1.9850000
-20591.0000000	1.9950000
-20590.0000000	2.0050001
-20589.0000000	2.0250001
-20588.0000000	2.0349998
-20587.0000000	2.0400000
-20586.0000000	2.0450001
-20585.0000000	2.0549998
-20584.0000000	2.0599999
-20583.0000000	2.0549998
-20582.0000000	2.0549998
-20581.0000000	2.0450001
-20580.0000000	2.0450001
-20579.0000000	2.0500000
-20578.0000000	2.0500000
-20577.0000000	2.0549998
-20576.0000000	2.0450001
-20575.0000000	2.0400000
-20574.0000000	2.0349998
-20573.0000000	2.0300000
-20572.0000000	2.0349998
-20571.0000000	2.0300000
-20570.0000000	2.0400000
-20569.0000000	2.0450001
-20568.0000000	2.0549998
-20567.0000000	2.0500000
-20566.0000000	2.0500000
-20565.0000000	2.0500000
-20564.0000000	2.0500000
-20563.0000000	2.0400000
-20562.0000000	2.0300000
-20561.0000000	2.0250001
-20560.0000000	2.0250001
-20559.0000000	2.0250001
-20558.0000000	2.0250001
-20557.0000000	2.0349998
-20556.0000000	2.0400000
-20555.0000000	2.0400000
-20554.0000000	2.0500000
-20553.0000000	2.0500000
-20552.0000000	2.0650001
-20551.0000000	2.0666666
-20550.0000000	2.0699999
-20549.0000000	2.0749998
-20548.0000000	2.0749998
-20547.0000000	2.0699999
-20546.0000000	2.0699999
-20545.0000000	2.0749998

-20544.0000000	2.0599999
-20543.0000000	2.0599999
-20542.0000000	2.0450001
-20541.0000000	2.0349998
-20540.0000000	2.0349998
-20539.0000000	2.0300000
-20538.0000000	2.0349998
-20537.0000000	2.0349998
-20536.0000000	2.0450001
-20535.0000000	2.0500000
-20534.0000000	2.0500000
-20533.0000000	2.0549998
-20532.0000000	2.0450001
-20531.0000000	2.0400000
-20530.0000000	2.0450001
-20529.0000000	2.0500000
-20528.0000000	2.0549998
-20527.0000000	2.0500000
-20526.0000000	2.0500000
-20525.0000000	2.0400000
-20524.0000000	2.0250001
-20523.0000000	2.0100000
-20522.0000000	2.0050001
-20521.0000000	1.9900001
-20520.0000000	1.9800000
-20519.0000000	1.9650000
-20518.0000000	1.9600000
-20517.0000000	1.9400001
-20516.0000000	1.9200000
-20515.0000000	1.9150000
-20514.0000000	1.9100000
-20513.0000000	1.9200000
-20512.0000000	1.9299999
-20511.0000000	1.9400001
-20510.0000000	1.9450001
-20509.0000000	1.9500000
-20508.0000000	1.9450001
-20507.0000000	1.9550000
-20506.0000000	1.9600000
-20505.0000000	1.9650000
-20504.0000000	1.9700000
-20503.0000000	1.9750000
-20502.0000000	1.9850000
-20501.0000000	1.9850000
-20500.0000000	1.9850000
-20499.0000000	1.9850000
-20498.0000000	1.9750000
-20497.0000000	1.9800000
-20496.0000000	1.9750000
-20495.0000000	1.9650000
-20494.0000000	1.9550000
-20493.0000000	1.9550000
-20492.0000000	1.9349999
-20491.0000000	1.9299999
-20490.0000000	1.9349999
-20489.0000000	1.9366666
-20488.0000000	1.9400001

-20487.0000000	1.9349999
-20486.0000000	1.9299999
-20485.0000000	1.9250000
-20484.0000000	1.9100000
-20483.0000000	1.8900000
-20482.0000000	1.8700000
-20481.0000000	1.8600000
-20480.0000000	1.8350000
-20479.0000000	1.8300000
-20478.0000000	1.8150001
-20477.0000000	1.8049999
-20476.0000000	1.8150001
-20475.0000000	1.8099999
-20474.0000000	1.8250000
-20473.0000000	1.8250000
-20472.0000000	1.8400000
-20471.0000000	1.8650000
-20470.0000000	1.8900000
-20469.0000000	1.9100000
-20468.0000000	1.9400001
-20467.0000000	1.9450001
-20466.0000000	1.9500000
-20465.0000000	1.9650000
-20464.0000000	1.9700000
-20463.0000000	1.9850000
-20462.0000000	1.9900000
-20461.0000000	1.9900000
-20460.0000000	1.9866667
-20459.0000000	1.9750000
-20458.0000000	1.9700000
-20457.0000000	1.9850000
-20456.0000000	1.9800000
-20455.0000000	1.9900000
-20454.0000000	1.9900000
-20453.0000000	1.9750000
-20452.0000000	1.9650000
-20451.0000000	1.9500000
-20450.0000000	1.9349999
-20449.0000000	1.9250000
-20448.0000000	1.9150000
-20447.0000000	1.8950000
-20446.0000000	1.8800000
-20445.0000000	1.8600000
-20444.0000000	1.8500000
-20443.0000000	1.8450000
-20442.0000000	1.8400000
-20441.0000000	1.8400000
-20440.0000000	1.8350000
-20439.0000000	1.8300000
-20438.0000000	1.8300000
-20437.0000000	1.8350000
-20436.0000000	1.8300000
-20435.0000000	1.8450000
-20434.0000000	1.8300000
-20433.0000000	1.8200001
-20432.0000000	1.8000000
-20431.0000000	1.8000000

-20430.0000000	1.7999998
-20429.0000000	1.8150001
-20428.0000000	1.8250000
-20427.0000000	1.8250000
-20426.0000000	1.8400000
-20425.0000000	1.8450000
-20424.0000000	1.8500000
-20423.0000000	1.8550000
-20422.0000000	1.8550000
-20421.0000000	1.8550000
-20420.0000000	1.8550000
-20419.0000000	1.8550000
-20418.0000000	1.8450000
-20417.0000000	1.8450000
-20416.0000000	1.8300000
-20415.0000000	1.8099999
-20414.0000000	1.7900000
-20413.0000000	1.7550000
-20412.0000000	1.7300000
-20411.0000000	1.7150000
-20410.0000000	1.7100000
-20409.0000000	1.7150000
-20408.0000000	1.7150000
-20407.0000000	1.7150000
-20406.0000000	1.7200000
-20405.0000000	1.7350000
-20404.0000000	1.7500000
-20403.0000000	1.7650000
-20402.0000000	1.7800000
-20401.0000000	1.7800000
-20400.0000000	1.7900000
-20399.0000000	1.7950000
-20398.0000000	1.7966666
-20397.0000000	1.8049999
-20396.0000000	1.8049999
-20395.0000000	1.8099999
-20394.0000000	1.8099999
-20393.0000000	1.8099999
-20392.0000000	1.8099999
-20391.0000000	1.8099999
-20390.0000000	1.8150001
-20389.0000000	1.8250000
-20388.0000000	1.8250000
-20387.0000000	1.8200001
-20386.0000000	1.8250000
-20385.0000000	1.8200001
-20384.0000000	1.8200001
-20383.0000000	1.8300000
-20382.0000000	1.8300000
-20381.0000000	1.8300000
-20380.0000000	1.8350000
-20379.0000000	1.8300000
-20378.0000000	1.8350000
-20377.0000000	1.8300000
-20376.0000000	1.8350000
-20375.0000000	1.8350000
-20374.0000000	1.8450000

-20373.0000000	1.8450000
-20372.0000000	1.8400000
-20371.0000000	1.8400000
-20370.0000000	1.8333334
-20369.0000000	1.8400000
-20368.0000000	1.8450000
-20367.0000000	1.8350000
-20366.0000000	1.8250000
-20365.0000000	1.8200001
-20364.0000000	1.8150001
-20363.0000000	1.8049999
-20362.0000000	1.8099999
-20361.0000000	1.8049999
-20360.0000000	1.8150001
-20359.0000000	1.8099999
-20358.0000000	1.8000000
-20357.0000000	1.8049999
-20356.0000000	1.8000000
-20355.0000000	1.8000000
-20354.0000000	1.8000000
-20353.0000000	1.8000000
-20352.0000000	1.7950000
-20351.0000000	1.7800000
-20350.0000000	1.7700000
-20349.0000000	1.7650000
-20348.0000000	1.7650000
-20347.0000000	1.7550000
-20346.0000000	1.7550000
-20345.0000000	1.7400000
-20344.0000000	1.7350000
-20343.0000000	1.7150000
-20342.0000000	1.7000000
-20341.0000000	1.6750000
-20340.0000000	1.6566666
-20339.0000000	1.6600000
-20338.0000000	1.6600000
-20337.0000000	1.6700000
-20336.0000000	1.6900001
-20335.0000000	1.7000000
-20334.0000000	1.6950001
-20333.0000000	1.7050000
-20332.0000000	1.7250000
-20331.0000000	1.7350000
-20330.0000000	1.7400000
-20329.0000000	1.7500000
-20328.0000000	1.7700000
-20327.0000000	1.7750000
-20326.0000000	1.7950000
-20325.0000000	1.8099999
-20324.0000000	1.8250000
-20323.0000000	1.8400000
-20322.0000000	1.8500000
-20321.0000000	1.8600000
-20320.0000000	1.8600000
-20319.0000000	1.8650000
-20318.0000000	1.8700000
-20317.0000000	1.8800000

-20316.0000000	1.8700000
-20315.0000000	1.8750000
-20314.0000000	1.8750000
-20313.0000000	1.8700000
-20312.0000000	1.8850000
-20311.0000000	1.8850000
-20310.0000000	1.8900000
-20309.0000000	1.9033333
-20308.0000000	1.9150000
-20307.0000000	1.9150000
-20306.0000000	1.9150000
-20305.0000000	1.9050000
-20304.0000000	1.9100000
-20303.0000000	1.9200000
-20302.0000000	1.9250000
-20301.0000000	1.9250000
-20300.0000000	1.9250000
-20299.0000000	1.9200000
-20298.0000000	1.9200000
-20297.0000000	1.9100000
-20296.0000000	1.9000000
-20295.0000000	1.8850000
-20294.0000000	1.8850000
-20293.0000000	1.8850000
-20292.0000000	1.8900000
-20291.0000000	1.8950000
-20290.0000000	1.8950000
-20289.0000000	1.9000000
-20288.0000000	1.9000000
-20287.0000000	1.8900000
-20286.0000000	1.8800000
-20285.0000000	1.8700000
-20284.0000000	1.8500000
-20283.0000000	1.8400000
-20282.0000000	1.8250000
-20281.0000000	1.8099999
-20280.0000000	1.8099999
-20279.0000000	1.8066667
-20278.0000000	1.8099999
-20277.0000000	1.8099999
-20276.0000000	1.8099999
-20275.0000000	1.8200001
-20274.0000000	1.8199999
-20273.0000000	1.8150001
-20272.0000000	1.8250000
-20271.0000000	1.8200001
-20270.0000000	1.8200001
-20269.0000000	1.8099999
-20268.0000000	1.8049999
-20267.0000000	1.8049999
-20266.0000000	1.7850000
-20265.0000000	1.7800000
-20264.0000000	1.7700000
-20263.0000000	1.7700000
-20262.0000000	1.7750000
-20261.0000000	1.7750000
-20260.0000000	1.7800000

-20259.0000000	1.7900000
-20258.0000000	1.7800000
-20257.0000000	1.7900000
-20256.0000000	1.7800000
-20255.0000000	1.7950000
-20254.0000000	1.7900000
-20253.0000000	1.7950000
-20252.0000000	1.7950000
-20251.0000000	1.7950000
-20250.0000000	1.7950000
-20249.0000000	1.7950000
-20248.0000000	1.7866665
-20247.0000000	1.7850000
-20246.0000000	1.7750000
-20245.0000000	1.7750000
-20244.0000000	1.7750000
-20243.0000000	1.7750000
-20242.0000000	1.7750000
-20241.0000000	1.7800000
-20240.0000000	1.7700000
-20239.0000000	1.7700000
-20238.0000000	1.7650000
-20237.0000000	1.7700000
-20236.0000000	1.7650000
-20235.0000000	1.7700000
-20234.0000000	1.7750000
-20233.0000000	1.7850000
-20232.0000000	1.7900000
-20231.0000000	1.8000000
-20230.0000000	1.8000000
-20229.0000000	1.8049999
-20228.0000000	1.8000000
-20227.0000000	1.8000000
-20226.0000000	1.7950000
-20225.0000000	1.7950000
-20224.0000000	1.8049999
-20223.0000000	1.8099999
-20222.0000000	1.8049999
-20221.0000000	1.8000000
-20220.0000000	1.7900000
-20219.0000000	1.7850000
-20218.0000000	1.7850000
-20217.0000000	1.7900000
-20216.0000000	1.7850000
-20215.0000000	1.7850000
-20214.0000000	1.7700000
-20213.0000000	1.7650000
-20212.0000000	1.7650000
-20211.0000000	1.7650000
-20210.0000000	1.7650000
-20209.0000000	1.7750000
-20208.0000000	1.7900000
-20207.0000000	1.7950000
-20206.0000000	1.8000000
-20205.0000000	1.8049999
-20204.0000000	1.8199999
-20203.0000000	1.8300000

-20202.0000000	1.8350000
-20201.0000000	1.8400000
-20200.0000000	1.8300000
-20199.0000000	1.8400000
-20198.0000000	1.8400000
-20197.0000000	1.8400000
-20196.0000000	1.8450000
-20195.0000000	1.8450000
-20194.0000000	1.8450000
-20193.0000000	1.8400000
-20192.0000000	1.8350000
-20191.0000000	1.8300000
-20190.0000000	1.8250000
-20189.0000000	1.8150001
-20188.0000000	1.8133334
-20187.0000000	1.8099999
-20186.0000000	1.8000000
-20185.0000000	1.8000000
-20184.0000000	1.8000000
-20183.0000000	1.8000000
-20182.0000000	1.7950000
-20181.0000000	1.7950000
-20180.0000000	1.8049999
-20179.0000000	1.7950000
-20178.0000000	1.8000000
-20177.0000000	1.7950000
-20176.0000000	1.8000000
-20175.0000000	1.8049999
-20174.0000000	1.8150001
-20173.0000000	1.8200001
-20172.0000000	1.8200001
-20171.0000000	1.8200001
-20170.0000000	1.8150001
-20169.0000000	1.8150001
-20168.0000000	1.8150001
-20167.0000000	1.8150001
-20166.0000000	1.8099999
-20165.0000000	1.8150001
-20164.0000000	1.8150001
-20163.0000000	1.8099999
-20162.0000000	1.8150001
-20161.0000000	1.8150001
-20160.0000000	1.8099999
-20159.0000000	1.8049999
-20158.0000000	1.8000000
-20157.0000000	1.8049999
-20156.0000000	1.7999998
-20155.0000000	1.7950000
-20154.0000000	1.7900000
-20153.0000000	1.7900000
-20152.0000000	1.7900000
-20151.0000000	1.7800000
-20150.0000000	1.7850000
-20149.0000000	1.7850000
-20148.0000000	1.7900000
-20147.0000000	1.7950000
-20146.0000000	1.7950000

-20145.0000000	1.7950000
-20144.0000000	1.7850000
-20143.0000000	1.7900000
-20142.0000000	1.7800000
-20141.0000000	1.7700000
-20140.0000000	1.7650000
-20139.0000000	1.7650000
-20138.0000000	1.7700000
-20137.0000000	1.7750000
-20136.0000000	1.7700000
-20135.0000000	1.7800000
-20134.0000000	1.7800000
-20133.0000000	1.7900000
-20132.0000000	1.7750000
-20131.0000000	1.7700000
-20130.0000000	1.7500000
-20129.0000000	1.7450000
-20128.0000000	1.7400000
-20127.0000000	1.7266668
-20126.0000000	1.7100000
-20125.0000000	1.7000000
-20124.0000000	1.6900001
-20123.0000000	1.6799999
-20122.0000000	1.6799999
-20121.0000000	1.6799999
-20120.0000000	1.6799999
-20119.0000000	1.6849999
-20118.0000000	1.6950001
-20117.0000000	1.6950001
-20116.0000000	1.7000000
-20115.0000000	1.7100000
-20114.0000000	1.7100000
-20113.0000000	1.7050000
-20112.0000000	1.7100000
-20111.0000000	1.7150000
-20110.0000000	1.7400000
-20109.0000000	1.7400000
-20108.0000000	1.7450000
-20107.0000000	1.7450000
-20106.0000000	1.7500000
-20105.0000000	1.7550000
-20104.0000000	1.7500000
-20103.0000000	1.7550000
-20102.0000000	1.7600000
-20101.0000000	1.7650000
-20100.0000000	1.7550000
-20099.0000000	1.7500000
-20098.0000000	1.7350000
-20097.0000000	1.7200000
-20096.0000000	1.7100000
-20095.0000000	1.7000000
-20094.0000000	1.7000000
-20093.0000000	1.6900001
-20092.0000000	1.6750000
-20091.0000000	1.6650000
-20090.0000000	1.6450000
-20089.0000000	1.6250000

-20088.0000000	1.6100000
-20087.0000000	1.5950000
-20086.0000000	1.5900000
-20085.0000000	1.5850000
-20084.0000000	1.5699999
-20083.0000000	1.5750000
-20082.0000000	1.5800000
-20081.0000000	1.5900000
-20080.0000000	1.5950000
-20079.0000000	1.5950000
-20078.0000000	1.6000000
-20077.0000000	1.5950000
-20076.0000000	1.6000000
-20075.0000000	1.6000000
-20074.0000000	1.6000000
-20073.0000000	1.6100000
-20072.0000000	1.6050000
-20071.0000000	1.5950000
-20070.0000000	1.5800000
-20069.0000000	1.5800000
-20068.0000000	1.5850000
-20067.0000000	1.5850000
-20066.0000000	1.5666666
-20065.0000000	1.5549999
-20064.0000000	1.5500000
-20063.0000000	1.5500000
-20062.0000000	1.5549999
-20061.0000000	1.5599999
-20060.0000000	1.5599999
-20059.0000000	1.5650001
-20058.0000000	1.5700001
-20057.0000000	1.5750000
-20056.0000000	1.5750000
-20055.0000000	1.5750000
-20054.0000000	1.5800000
-20053.0000000	1.5800000
-20052.0000000	1.5800000
-20051.0000000	1.5800000
-20050.0000000	1.5900000
-20049.0000000	1.5950000
-20048.0000000	1.6000000
-20047.0000000	1.6100000
-20046.0000000	1.6150000
-20045.0000000	1.6250000
-20044.0000000	1.6250000
-20043.0000000	1.6300000
-20042.0000000	1.6400000
-20041.0000000	1.6550000
-20040.0000000	1.6700000
-20039.0000000	1.6799999
-20038.0000000	1.6950001
-20037.0000000	1.6849999
-20036.0000000	1.6799999
-20035.0000000	1.6799999
-20034.0000000	1.6700000
-20033.0000000	1.6650000
-20032.0000000	1.6550000

-20031.0000000	1.6500000
-20030.0000000	1.6450000
-20029.0000000	1.6400000
-20028.0000000	1.6450000
-20027.0000000	1.6350000
-20026.0000000	1.6350000
-20025.0000000	1.6250000
-20024.0000000	1.6250000
-20023.0000000	1.6250000
-20022.0000000	1.6150000
-20021.0000000	1.6200000
-20020.0000000	1.6050000
-20019.0000000	1.6050000
-20018.0000000	1.5900000
-20017.0000000	1.5650001
-20016.0000000	1.5549999
-20015.0000000	1.5500000
-20014.0000000	1.5450000
-20013.0000000	1.5450000
-20012.0000000	1.5500000
-20011.0000000	1.5599999
-20010.0000000	1.5750000
-20009.0000000	1.5850000
-20008.0000000	1.5950000
-20007.0000000	1.6050000
-20006.0000000	1.6133333
-20005.0000000	1.6400000
-20004.0000000	1.6500000
-20003.0000000	1.6700000
-20002.0000000	1.6799999
-20001.0000000	1.6950001
-20000.0000000	1.6950001
-19999.0000000	1.7050000
-19998.0000000	1.7050000
-19997.0000000	1.7050000
-19996.0000000	1.6950001
-19995.0000000	1.6799999
-19994.0000000	1.6700000
-19993.0000000	1.6700000
-19992.0000000	1.6750000
-19991.0000000	1.6750000
-19990.0000000	1.6849999
-19989.0000000	1.6849999
-19988.0000000	1.6900001
-19987.0000000	1.6849999
-19986.0000000	1.6849999
-19985.0000000	1.6849999
-19984.0000000	1.6950001
-19983.0000000	1.6900001
-19982.0000000	1.6849999
-19981.0000000	1.6849999
-19980.0000000	1.6799999
-19979.0000000	1.6849999
-19978.0000000	1.6750000
-19977.0000000	1.6700000
-19976.0000000	1.6633333
-19975.0000000	1.6550000

-19974.0000000	1.6450000
-19973.0000000	1.6400000
-19972.0000000	1.6300000
-19971.0000000	1.6200000
-19970.0000000	1.6200000
-19969.0000000	1.6250000
-19968.0000000	1.6150000
-19967.0000000	1.6100000
-19966.0000000	1.6050000
-19965.0000000	1.6000000
-19964.0000000	1.6100000
-19963.0000000	1.6150000
-19962.0000000	1.6200000
-19961.0000000	1.6100000
-19960.0000000	1.6100000
-19959.0000000	1.5850000
-19958.0000000	1.5700001
-19957.0000000	1.5450000
-19956.0000000	1.5350000
-19955.0000000	1.5350000
-19954.0000000	1.5450000
-19953.0000000	1.5599999
-19952.0000000	1.5700001
-19951.0000000	1.5750000
-19950.0000000	1.5800000
-19949.0000000	1.5900000
-19948.0000000	1.6050000
-19947.0000000	1.6100000
-19946.0000000	1.6133333
-19945.0000000	1.6100000
-19944.0000000	1.6200000
-19943.0000000	1.6200000
-19942.0000000	1.6200000
-19941.0000000	1.6250000
-19940.0000000	1.6250000
-19939.0000000	1.6250000
-19938.0000000	1.6350000
-19937.0000000	1.6250000
-19936.0000000	1.6300000
-19935.0000000	1.6250000
-19934.0000000	1.6200000
-19933.0000000	1.6150000
-19932.0000000	1.6200000
-19931.0000000	1.6250000
-19930.0000000	1.6200000
-19929.0000000	1.6300000
-19928.0000000	1.6200000
-19927.0000000	1.6050000
-19926.0000000	1.6000000
-19925.0000000	1.5850000
-19924.0000000	1.5850000
-19923.0000000	1.5800000
-19922.0000000	1.5750000
-19921.0000000	1.5800000
-19920.0000000	1.5750000
-19919.0000000	1.5800000
-19918.0000000	1.5850000

-19917.0000000	1.5850000
-19916.0000000	1.5850000
-19915.0000000	1.5833334
-19914.0000000	1.5750000
-19913.0000000	1.5800000
-19912.0000000	1.5900000
-19911.0000000	1.6000000
-19910.0000000	1.6000000
-19909.0000000	1.6050000
-19908.0000000	1.6050000
-19907.0000000	1.6100000
-19906.0000000	1.6050000
-19905.0000000	1.6100000
-19904.0000000	1.6150000
-19903.0000000	1.6050000
-19902.0000000	1.6100000
-19901.0000000	1.6100000
-19900.0000000	1.6100000
-19899.0000000	1.6200000
-19898.0000000	1.6150000
-19897.0000000	1.6150000
-19896.0000000	1.6050000
-19895.0000000	1.6050000
-19894.0000000	1.6050000
-19893.0000000	1.6000000
-19892.0000000	1.6050000
-19891.0000000	1.5950000
-19890.0000000	1.6000000
-19889.0000000	1.5900000
-19888.0000000	1.5850000
-19887.0000000	1.5750000
-19886.0000000	1.5599999
-19885.0000000	1.5633334
-19884.0000000	1.5650001
-19883.0000000	1.5750000
-19882.0000000	1.5800000
-19881.0000000	1.5950000
-19880.0000000	1.5900000
-19879.0000000	1.5950000
-19878.0000000	1.5900000
-19877.0000000	1.5950000
-19876.0000000	1.5900000
-19875.0000000	1.5900000
-19874.0000000	1.5800000
-19873.0000000	1.5700001
-19872.0000000	1.5599999
-19871.0000000	1.5599999
-19870.0000000	1.5549999
-19869.0000000	1.5549999
-19868.0000000	1.5500000
-19867.0000000	1.5450000
-19866.0000000	1.5350000
-19865.0000000	1.5200000
-19864.0000000	1.5200000
-19863.0000000	1.5100000
-19862.0000000	1.5000000
-19861.0000000	1.4950000

-19860.0000000	1.4900000
-19859.0000000	1.4800000
-19858.0000000	1.4800000
-19857.0000000	1.4850000
-19856.0000000	1.4950000
-19855.0000000	1.4950000
-19854.0000000	1.5033334
-19853.0000000	1.5100000
-19852.0000000	1.5200000
-19851.0000000	1.5200000
-19850.0000000	1.5250000
-19849.0000000	1.5200000
-19848.0000000	1.5300000
-19847.0000000	1.5400000
-19846.0000000	1.5650001
-19845.0000000	1.5700001
-19844.0000000	1.5800000
-19843.0000000	1.5850000
-19842.0000000	1.5850000
-19841.0000000	1.5850000
-19840.0000000	1.5850000
-19839.0000000	1.5700001
-19838.0000000	1.5599999
-19837.0000000	1.5599999
-19836.0000000	1.5500000
-19835.0000000	1.5400000
-19834.0000000	1.5400000
-19833.0000000	1.5400000
-19832.0000000	1.5400000
-19831.0000000	1.5300000
-19830.0000000	1.5250000
-19829.0000000	1.5250000
-19828.0000000	1.5350000
-19827.0000000	1.5350000
-19826.0000000	1.5350000
-19825.0000000	1.5300000
-19824.0000000	1.5333333
-19823.0000000	1.5250000
-19822.0000000	1.5250000
-19821.0000000	1.5250000
-19820.0000000	1.5100000
-19819.0000000	1.5100000
-19818.0000000	1.5100000
-19817.0000000	1.5100000
-19816.0000000	1.5050000
-19815.0000000	1.5000000
-19814.0000000	1.4950000
-19813.0000000	1.4950000
-19812.0000000	1.4900000
-19811.0000000	1.4850000
-19810.0000000	1.4800000
-19809.0000000	1.4800000
-19808.0000000	1.4750000
-19807.0000000	1.4850000
-19806.0000000	1.4850000
-19805.0000000	1.4850000
-19804.0000000	1.4850000

-19803.0000000	1.4950000
-19802.0000000	1.4950000
-19801.0000000	1.5000000
-19800.0000000	1.5050000
-19799.0000000	1.5200000
-19798.0000000	1.5250000
-19797.0000000	1.5250000
-19796.0000000	1.5300000
-19795.0000000	1.5450000
-19794.0000000	1.5566667
-19793.0000000	1.5700001
-19792.0000000	1.5800000
-19791.0000000	1.5850000
-19790.0000000	1.5950000
-19789.0000000	1.5900000
-19788.0000000	1.5950000
-19787.0000000	1.6000000
-19786.0000000	1.6000000
-19785.0000000	1.6000000
-19784.0000000	1.6050000
-19783.0000000	1.6200000
-19782.0000000	1.6300000
-19781.0000000	1.6350000
-19780.0000000	1.6400000
-19779.0000000	1.6350000
-19778.0000000	1.6400000
-19777.0000000	1.6450000
-19776.0000000	1.6400000
-19775.0000000	1.6400000
-19774.0000000	1.6400000
-19773.0000000	1.6400000
-19772.0000000	1.6400000
-19771.0000000	1.6350000
-19770.0000000	1.6350000
-19769.0000000	1.6300000
-19768.0000000	1.6400000
-19767.0000000	1.6400000
-19766.0000000	1.6450000
-19765.0000000	1.6500000
-19764.0000000	1.6500000
-19763.0000000	1.6600000
-19762.0000000	1.6600000
-19761.0000000	1.6650000
-19760.0000000	1.6700000
-19759.0000000	1.6700000
-19758.0000000	1.6650000
-19757.0000000	1.6700000
-19756.0000000	1.6700000
-19755.0000000	1.6700000
-19754.0000000	1.6750000
-19753.0000000	1.6700000
-19752.0000000	1.6650000
-19751.0000000	1.6700000
-19750.0000000	1.6700000
-19749.0000000	1.6700000
-19748.0000000	1.6600000
-19747.0000000	1.6600000

-19746.0000000	1.6550000
-19745.0000000	1.6450000
-19744.0000000	1.6400000
-19743.0000000	1.6450000
-19742.0000000	1.6450000
-19741.0000000	1.6350000
-19740.0000000	1.6300000
-19739.0000000	1.6250000
-19738.0000000	1.6150000
-19737.0000000	1.6150000
-19736.0000000	1.6200000
-19735.0000000	1.6200000
-19734.0000000	1.6200000
-19733.0000000	1.6200000
-19732.0000000	1.6250000
-19731.0000000	1.6200000
-19730.0000000	1.6200000
-19729.0000000	1.6150000
-19728.0000000	1.6150000
-19727.0000000	1.6100000
-19726.0000000	1.6050000
-19725.0000000	1.6000000
-19724.0000000	1.6000000
-19723.0000000	1.6000000
-19722.0000000	1.5900000
-19721.0000000	1.5900000
-19720.0000000	1.5750000
-19719.0000000	1.5650001
-19718.0000000	1.5500000
-19717.0000000	1.5350000
-19716.0000000	1.5250000
-19715.0000000	1.5200000
-19714.0000000	1.5100000
-19713.0000000	1.4950000
-19712.0000000	1.4950000
-19711.0000000	1.4950000
-19710.0000000	1.5000000
-19709.0000000	1.5150000
-19708.0000000	1.5250000
-19707.0000000	1.5500000
-19706.0000000	1.5599999
-19705.0000000	1.5766667
-19704.0000000	1.5900000
-19703.0000000	1.5950000
-19702.0000000	1.6050000
-19701.0000000	1.6050000
-19700.0000000	1.6100000
-19699.0000000	1.6100000
-19698.0000000	1.6100000
-19697.0000000	1.6150000
-19696.0000000	1.6150000
-19695.0000000	1.6150000
-19694.0000000	1.6100000
-19693.0000000	1.6050000
-19692.0000000	1.6050000
-19691.0000000	1.5950000
-19690.0000000	1.5900000

-19689.0000000	1.5800000
-19688.0000000	1.5650001
-19687.0000000	1.5549999
-19686.0000000	1.5450000
-19685.0000000	1.5400000
-19684.0000000	1.5250000
-19683.0000000	1.5150000
-19682.0000000	1.5150000
-19681.0000000	1.5150000
-19680.0000000	1.5200000
-19679.0000000	1.5400000
-19678.0000000	1.5450000
-19677.0000000	1.5650001
-19676.0000000	1.5750000
-19675.0000000	1.5900000
-19674.0000000	1.5950000
-19673.0000000	1.5950000
-19672.0000000	1.5800000
-19671.0000000	1.5650001
-19670.0000000	1.5599999
-19669.0000000	1.5500000
-19668.0000000	1.5500000
-19667.0000000	1.5450000
-19666.0000000	1.5350000
-19665.0000000	1.5350000
-19664.0000000	1.5300000
-19663.0000000	1.5250000
-19662.0000000	1.5200000
-19661.0000000	1.5250000
-19660.0000000	1.5250000
-19659.0000000	1.5200000
-19658.0000000	1.5350000
-19657.0000000	1.5400000
-19656.0000000	1.5400000
-19655.0000000	1.5500000
-19654.0000000	1.5549999
-19653.0000000	1.5599999
-19652.0000000	1.5750000
-19651.0000000	1.5800000
-19650.0000000	1.5950000
-19649.0000000	1.6000000
-19648.0000000	1.6100000
-19647.0000000	1.6200000
-19646.0000000	1.6250000
-19645.0000000	1.6300000
-19644.0000000	1.6350000
-19643.0000000	1.6400000
-19642.0000000	1.6500000
-19641.0000000	1.6500000
-19640.0000000	1.6550000
-19639.0000000	1.6550000
-19638.0000000	1.6550000
-19637.0000000	1.6600000
-19636.0000000	1.6650000
-19635.0000000	1.6700000
-19634.0000000	1.6700000
-19633.0000000	1.6700000

-19632.0000000	1.6800001
-19631.0000000	1.6750000
-19630.0000000	1.6799999
-19629.0000000	1.6750000
-19628.0000000	1.6700000
-19627.0000000	1.6650000
-19626.0000000	1.6500000
-19625.0000000	1.6400000
-19624.0000000	1.6200000
-19623.0000000	1.6150000
-19622.0000000	1.6050000
-19621.0000000	1.6050000
-19620.0000000	1.6150000
-19619.0000000	1.6100000
-19618.0000000	1.6100000
-19617.0000000	1.6150000
-19616.0000000	1.6200000
-19615.0000000	1.6200000
-19614.0000000	1.6250000
-19613.0000000	1.6200000
-19612.0000000	1.6233333
-19611.0000000	1.6200000
-19610.0000000	1.6100000
-19609.0000000	1.6100000
-19608.0000000	1.6100000
-19607.0000000	1.6000000
-19606.0000000	1.5950000
-19605.0000000	1.5900000
-19604.0000000	1.5900000
-19603.0000000	1.5950000
-19602.0000000	1.6000000
-19601.0000000	1.5900000
-19600.0000000	1.5900000
-19599.0000000	1.5850000
-19598.0000000	1.5950000
-19597.0000000	1.6000000
-19596.0000000	1.5950000
-19595.0000000	1.5950000
-19594.0000000	1.6000000
-19593.0000000	1.5950000
-19592.0000000	1.5950000
-19591.0000000	1.5900000
-19590.0000000	1.5900000
-19589.0000000	1.5950000
-19588.0000000	1.5900000
-19587.0000000	1.5950000
-19586.0000000	1.5900000
-19585.0000000	1.5950000
-19584.0000000	1.5900000
-19583.0000000	1.5900000
-19582.0000000	1.5900000
-19581.0000000	1.5850000
-19580.0000000	1.5750000
-19579.0000000	1.5549999
-19578.0000000	1.5450000
-19577.0000000	1.5350000
-19576.0000000	1.5400000

-19575.0000000	1.5300000
-19574.0000000	1.5300000
-19573.0000000	1.5300000
-19572.0000000	1.5300000
-19571.0000000	1.5200000
-19570.0000000	1.5250000
-19569.0000000	1.5200000
-19568.0000000	1.5250000
-19567.0000000	1.5200000
-19566.0000000	1.5100000
-19565.0000000	1.5000000
-19564.0000000	1.5000000
-19563.0000000	1.4900000
-19562.0000000	1.4900000
-19561.0000000	1.4850000
-19560.0000000	1.4750000
-19559.0000000	1.4500000
-19558.0000000	1.4300001
-19557.0000000	1.4050000
-19556.0000000	1.4200000
-19555.0000000	1.4200000
-19554.0000000	1.4299999
-19553.0000000	1.4349999
-19552.0000000	1.4500000
-19551.0000000	1.4600000
-19550.0000000	1.4650000
-19549.0000000	1.4750000
-19548.0000000	1.4700000
-19547.0000000	1.4650000
-19546.0000000	1.4600000
-19545.0000000	1.4550000
-19544.0000000	1.4349999
-19543.0000000	1.4300001
-19542.0000000	1.4349999
-19541.0000000	1.4400001
-19540.0000000	1.4349999
-19539.0000000	1.4250000
-19538.0000000	1.4150000
-19537.0000000	1.4250000
-19536.0000000	1.4250000
-19535.0000000	1.4400001
-19534.0000000	1.4500000
-19533.0000000	1.4600000
-19532.0000000	1.4600000
-19531.0000000	1.4650000
-19530.0000000	1.4600000
-19529.0000000	1.4500000
-19528.0000000	1.4349999
-19527.0000000	1.4200000
-19526.0000000	1.4100000
-19525.0000000	1.4100000
-19524.0000000	1.4000000
-19523.0000000	1.3950000
-19522.0000000	1.3800000
-19521.0000000	1.3866667
-19520.0000000	1.3850000
-19519.0000000	1.3950000

-19518.0000000	1.4050000
-19517.0000000	1.4200000
-19516.0000000	1.4500000
-19515.0000000	1.4650000
-19514.0000000	1.4800000
-19513.0000000	1.5000000
-19512.0000000	1.5150000
-19511.0000000	1.5250000
-19510.0000000	1.5450000
-19509.0000000	1.5549999
-19508.0000000	1.5650001
-19507.0000000	1.5800000
-19506.0000000	1.5900000
-19505.0000000	1.5950000
-19504.0000000	1.6000000
-19503.0000000	1.6100000
-19502.0000000	1.6150000
-19501.0000000	1.6250000
-19500.0000000	1.6350000
-19499.0000000	1.6350000
-19498.0000000	1.6450000
-19497.0000000	1.6450000
-19496.0000000	1.6500000
-19495.0000000	1.6500000
-19494.0000000	1.6466666
-19493.0000000	1.6550000
-19492.0000000	1.6550000
-19491.0000000	1.6500000
-19490.0000000	1.6400000
-19489.0000000	1.6200000
-19488.0000000	1.6050000
-19487.0000000	1.5750000
-19486.0000000	1.5450000
-19485.0000000	1.5100000
-19484.0000000	1.4650000
-19483.0000000	1.4349999
-19482.0000000	1.4150000
-19481.0000000	1.4050000
-19480.0000000	1.4050000
-19479.0000000	1.4050000
-19478.0000000	1.4050000
-19477.0000000	1.4050000
-19476.0000000	1.4050000
-19475.0000000	1.4050000
-19474.0000000	1.4200000
-19473.0000000	1.4349999
-19472.0000000	1.4500000
-19471.0000000	1.4600000
-19470.0000000	1.4700000
-19469.0000000	1.4750000
-19468.0000000	1.4900000
-19467.0000000	1.5100000
-19466.0000000	1.5200000
-19465.0000000	1.5300000
-19464.0000000	1.5400000
-19463.0000000	1.5450000
-19462.0000000	1.5466666

-19461.0000000	1.5500000
-19460.0000000	1.5549999
-19459.0000000	1.5500000
-19458.0000000	1.5549999
-19457.0000000	1.5500000
-19456.0000000	1.5599999
-19455.0000000	1.5599999
-19454.0000000	1.5599999
-19453.0000000	1.5650001
-19452.0000000	1.5650001
-19451.0000000	1.5599999
-19450.0000000	1.5650001
-19449.0000000	1.5700001
-19448.0000000	1.5700001
-19447.0000000	1.5650001
-19446.0000000	1.5650001
-19445.0000000	1.5800000
-19444.0000000	1.5850000
-19443.0000000	1.5900000
-19442.0000000	1.5950000
-19441.0000000	1.5900000
-19440.0000000	1.5850000
-19439.0000000	1.5700001
-19438.0000000	1.5750000
-19437.0000000	1.5599999
-19436.0000000	1.5549999
-19435.0000000	1.5500000
-19434.0000000	1.5450000
-19433.0000000	1.5400000
-19432.0000000	1.5400000
-19431.0000000	1.5400000
-19430.0000000	1.5350000
-19429.0000000	1.5300000
-19428.0000000	1.5400000
-19427.0000000	1.5400000
-19426.0000000	1.5450000
-19425.0000000	1.5500000
-19424.0000000	1.5500000
-19423.0000000	1.5599999
-19422.0000000	1.5650001
-19421.0000000	1.5800000
-19420.0000000	1.5850000
-19419.0000000	1.5900000
-19418.0000000	1.5950000
-19417.0000000	1.5900000
-19416.0000000	1.5950000
-19415.0000000	1.5950000
-19414.0000000	1.5850000
-19413.0000000	1.5800000
-19412.0000000	1.5800000
-19411.0000000	1.5800000
-19410.0000000	1.5850000
-19409.0000000	1.5850000
-19408.0000000	1.5850000
-19407.0000000	1.5850000
-19406.0000000	1.5900000
-19405.0000000	1.5900000

-19404.0000000	1.5900000
-19403.0000000	1.5800000
-19402.0000000	1.5800000
-19401.0000000	1.5850000
-19400.0000000	1.5800000
-19399.0000000	1.5750000
-19398.0000000	1.5800000
-19397.0000000	1.5800000
-19396.0000000	1.5850000
-19395.0000000	1.5850000
-19394.0000000	1.5900000
-19393.0000000	1.6000000
-19392.0000000	1.6100000
-19391.0000000	1.6100000
-19390.0000000	1.6150000
-19389.0000000	1.6150000
-19388.0000000	1.6050000
-19387.0000000	1.5950000
-19386.0000000	1.5950000
-19385.0000000	1.6000000
-19384.0000000	1.6000000
-19383.0000000	1.5900000
-19382.0000000	1.5800000
-19381.0000000	1.5750000
-19380.0000000	1.5800000
-19379.0000000	1.5800000
-19378.0000000	1.5800000
-19377.0000000	1.5800000
-19376.0000000	1.5850000
-19375.0000000	1.5850000
-19374.0000000	1.5800000
-19373.0000000	1.5750000
-19372.0000000	1.5750000
-19371.0000000	1.5800000
-19370.0000000	1.5800000
-19369.0000000	1.5750000
-19368.0000000	1.5750000
-19367.0000000	1.5800000
-19366.0000000	1.5599999
-19365.0000000	1.5549999
-19364.0000000	1.5500000
-19363.0000000	1.5549999
-19362.0000000	1.5500000
-19361.0000000	1.5400000
-19360.0000000	1.5350000
-19359.0000000	1.5200000
-19358.0000000	1.5200000
-19357.0000000	1.5050000
-19356.0000000	1.5000000
-19355.0000000	1.4950000
-19354.0000000	1.4750000
-19353.0000000	1.4750000
-19352.0000000	1.4750000
-19351.0000000	1.4800000
-19350.0000000	1.4800000
-19349.0000000	1.4850000
-19348.0000000	1.4900000

-19347.0000000	1.4850000
-19346.0000000	1.4950000
-19345.0000000	1.4900000
-19344.0000000	1.4950000
-19343.0000000	1.4900000
-19342.0000000	1.4900000
-19341.0000000	1.4866667
-19340.0000000	1.4850000
-19339.0000000	1.4950000
-19338.0000000	1.4900000
-19337.0000000	1.5000000
-19336.0000000	1.5000000
-19335.0000000	1.4950000
-19334.0000000	1.4900000
-19333.0000000	1.4900000
-19332.0000000	1.4950000
-19331.0000000	1.4950000
-19330.0000000	1.4900000
-19329.0000000	1.4950000
-19328.0000000	1.4850000
-19327.0000000	1.4850000
-19326.0000000	1.4900000
-19325.0000000	1.5000000
-19324.0000000	1.5100000
-19323.0000000	1.5100000
-19322.0000000	1.5200000
-19321.0000000	1.5200000
-19320.0000000	1.5300000
-19319.0000000	1.5350000
-19318.0000000	1.5350000
-19317.0000000	1.5450000
-19316.0000000	1.5450000
-19315.0000000	1.5500000
-19314.0000000	1.5500000
-19313.0000000	1.5500000
-19312.0000000	1.5400000
-19311.0000000	1.5266666
-19310.0000000	1.5200000
-19309.0000000	1.5250000
-19308.0000000	1.5400000
-19307.0000000	1.5400000
-19306.0000000	1.5450000
-19305.0000000	1.5549999
-19304.0000000	1.5500000
-19303.0000000	1.5400000
-19302.0000000	1.5400000
-19301.0000000	1.5450000
-19300.0000000	1.5500000
-19299.0000000	1.5549999
-19298.0000000	1.5599999
-19297.0000000	1.5650001
-19296.0000000	1.5599999
-19295.0000000	1.5750000
-19294.0000000	1.5700001
-19293.0000000	1.5650001
-19292.0000000	1.5599999
-19291.0000000	1.5650001

-19290.0000000	1.5599999
-19289.0000000	1.5650001
-19288.0000000	1.5700001
-19287.0000000	1.5800000
-19286.0000000	1.5700001
-19285.0000000	1.5800000
-19284.0000000	1.5800000
-19283.0000000	1.5900000
-19282.0000000	1.5900000
-19281.0000000	1.6000000
-19280.0000000	1.6050000
-19279.0000000	1.6000000
-19278.0000000	1.6050000
-19277.0000000	1.6150000
-19276.0000000	1.6200000
-19275.0000000	1.6300000
-19274.0000000	1.6250000
-19273.0000000	1.6400000
-19272.0000000	1.6400000
-19271.0000000	1.6450000
-19270.0000000	1.6450000
-19269.0000000	1.6500000
-19268.0000000	1.6450000
-19267.0000000	1.6350000
-19266.0000000	1.6300000
-19265.0000000	1.6250000
-19264.0000000	1.6250000
-19263.0000000	1.6200000
-19262.0000000	1.6200000
-19261.0000000	1.6200000
-19260.0000000	1.6200000
-19259.0000000	1.6100000
-19258.0000000	1.6100000
-19257.0000000	1.6150000
-19256.0000000	1.6100000
-19255.0000000	1.6150000
-19254.0000000	1.6150000
-19253.0000000	1.6100000
-19252.0000000	1.6150000
-19251.0000000	1.6150000
-19250.0000000	1.6200000
-19249.0000000	1.6250000
-19248.0000000	1.6200000
-19247.0000000	1.6200000
-19246.0000000	1.6200000
-19245.0000000	1.6150000
-19244.0000000	1.6100000
-19243.0000000	1.6100000
-19242.0000000	1.6150000
-19241.0000000	1.6150000
-19240.0000000	1.6150000
-19239.0000000	1.6200000
-19238.0000000	1.6100000
-19237.0000000	1.5950000
-19236.0000000	1.5800000
-19235.0000000	1.5800000
-19234.0000000	1.5700001

-19233.0000000	1.5599999
-19232.0000000	1.5450000
-19231.0000000	1.5350000
-19230.0000000	1.5300000
-19229.0000000	1.5300000
-19228.0000000	1.5250000
-19227.0000000	1.5250000
-19226.0000000	1.5250000
-19225.0000000	1.5300000
-19224.0000000	1.5300000
-19223.0000000	1.5300000
-19222.0000000	1.5250000
-19221.0000000	1.5250000
-19220.0000000	1.5266666
-19219.0000000	1.5300000
-19218.0000000	1.5300000
-19217.0000000	1.5300000
-19216.0000000	1.5250000
-19215.0000000	1.5200000
-19214.0000000	1.5200000
-19213.0000000	1.5050000
-19212.0000000	1.5000000
-19211.0000000	1.4950000
-19210.0000000	1.4850000
-19209.0000000	1.4850000
-19208.0000000	1.4850000
-19207.0000000	1.4850000
-19206.0000000	1.4900000
-19205.0000000	1.4900000
-19204.0000000	1.5000000
-19203.0000000	1.5050000
-19202.0000000	1.5150000
-19201.0000000	1.5200000
-19200.0000000	1.5250000
-19199.0000000	1.5350000
-19198.0000000	1.5450000
-19197.0000000	1.5450000
-19196.0000000	1.5500000
-19195.0000000	1.5500000
-19194.0000000	1.5599999
-19193.0000000	1.5549999
-19192.0000000	1.5533333
-19191.0000000	1.5450000
-19190.0000000	1.5450000
-19189.0000000	1.5350000
-19188.0000000	1.5350000
-19187.0000000	1.5250000
-19186.0000000	1.5100000
-19185.0000000	1.5100000
-19184.0000000	1.5050000
-19183.0000000	1.5100000
-19182.0000000	1.5100000
-19181.0000000	1.5200000
-19180.0000000	1.5350000
-19179.0000000	1.5400000
-19178.0000000	1.5450000
-19177.0000000	1.5450000

-19176.0000000	1.5450000
-19175.0000000	1.5400000
-19174.0000000	1.5500000
-19173.0000000	1.5450000
-19172.0000000	1.5350000
-19171.0000000	1.5350000
-19170.0000000	1.5400000
-19169.0000000	1.5450000
-19168.0000000	1.5350000
-19167.0000000	1.5400000
-19166.0000000	1.5350000
-19165.0000000	1.5350000
-19164.0000000	1.5250000
-19163.0000000	1.5200000
-19162.0000000	1.5150000
-19161.0000000	1.4933333
-19160.0000000	1.4700000
-19159.0000000	1.4450001
-19158.0000000	1.4200000
-19157.0000000	1.3950000
-19156.0000000	1.3900000
-19155.0000000	1.3800000
-19154.0000000	1.3650000
-19153.0000000	1.3500000
-19152.0000000	1.3350000
-19151.0000000	1.3250000
-19150.0000000	1.3200001
-19149.0000000	1.3250000
-19148.0000000	1.3350000
-19147.0000000	1.3400000
-19146.0000000	1.3450000
-19145.0000000	1.3550000
-19144.0000000	1.3550000
-19143.0000000	1.3650000
-19142.0000000	1.3900000
-19141.0000000	1.4050000
-19140.0000000	1.4150000
-19139.0000000	1.4200000
-19138.0000000	1.4200000
-19137.0000000	1.4150000
-19136.0000000	1.4100000
-19135.0000000	1.4200000
-19134.0000000	1.4200000
-19133.0000000	1.4299999
-19132.0000000	1.4349999
-19131.0000000	1.4250000
-19130.0000000	1.4166666
-19129.0000000	1.4050000
-19128.0000000	1.4000000
-19127.0000000	1.3800000
-19126.0000000	1.3700000
-19125.0000000	1.3600000
-19124.0000000	1.3600000
-19123.0000000	1.3600000
-19122.0000000	1.3600000
-19121.0000000	1.3800000
-19120.0000000	1.3900000

-19119.0000000	1.4000000
-19118.0000000	1.4150000
-19117.0000000	1.4150000
-19116.0000000	1.4150000
-19115.0000000	1.4050000
-19114.0000000	1.4050000
-19113.0000000	1.3900000
-19112.0000000	1.3750000
-19111.0000000	1.3600000
-19110.0000000	1.3400000
-19109.0000000	1.3300000
-19108.0000000	1.3150001
-19107.0000000	1.3200001
-19106.0000000	1.3150001
-19105.0000000	1.3099999
-19104.0000000	1.3049999
-19103.0000000	1.3000000
-19102.0000000	1.3099999
-19101.0000000	1.3150001
-19100.0000000	1.3333334
-19099.0000000	1.3450000
-19098.0000000	1.3550000
-19097.0000000	1.3650000
-19096.0000000	1.3650000
-19095.0000000	1.3700000
-19094.0000000	1.3950000
-19093.0000000	1.4100000
-19092.0000000	1.4100000
-19091.0000000	1.4150000
-19090.0000000	1.4200000
-19089.0000000	1.4299999
-19088.0000000	1.4349999
-19087.0000000	1.4450001
-19086.0000000	1.4550000
-19085.0000000	1.4500000
-19084.0000000	1.4450001
-19083.0000000	1.4500000
-19082.0000000	1.4550000
-19081.0000000	1.4650000
-19080.0000000	1.4600000
-19079.0000000	1.4600000
-19078.0000000	1.4600000
-19077.0000000	1.4600000
-19076.0000000	1.4550000
-19075.0000000	1.4550000
-19074.0000000	1.4400001
-19073.0000000	1.4500000
-19072.0000000	1.4550000
-19071.0000000	1.4650000
-19070.0000000	1.4700000
-19069.0000000	1.4833332
-19068.0000000	1.4900000
-19067.0000000	1.4950000
-19066.0000000	1.5000000
-19065.0000000	1.5000000
-19064.0000000	1.4950000
-19063.0000000	1.5000000

-19062.0000000	1.4950000
-19061.0000000	1.5050000
-19060.0000000	1.5100000
-19059.0000000	1.5000000
-19058.0000000	1.5100000
-19057.0000000	1.5050000
-19056.0000000	1.5050000
-19055.0000000	1.5050000
-19054.0000000	1.5000000
-19053.0000000	1.4850000
-19052.0000000	1.4850000
-19051.0000000	1.4750000
-19050.0000000	1.4600000
-19049.0000000	1.4500000
-19048.0000000	1.4400001
-19047.0000000	1.4299999
-19046.0000000	1.4050000
-19045.0000000	1.3850000
-19044.0000000	1.3550000
-19043.0000000	1.3500000
-19042.0000000	1.3550000
-19041.0000000	1.3450000
-19040.0000000	1.3450000
-19039.0000000	1.3533334
-19038.0000000	1.3400000
-19037.0000000	1.3500000
-19036.0000000	1.3600000
-19035.0000000	1.3650000
-19034.0000000	1.3600000
-19033.0000000	1.3450000
-19032.0000000	1.3450000
-19031.0000000	1.3450000
-19030.0000000	1.3500000
-19029.0000000	1.3600000
-19028.0000000	1.3600000
-19027.0000000	1.3550000
-19026.0000000	1.3500000
-19025.0000000	1.3500000
-19024.0000000	1.3450000
-19023.0000000	1.3450000
-19022.0000000	1.3450000
-19021.0000000	1.3600000
-19020.0000000	1.3600000
-19019.0000000	1.3550000
-19018.0000000	1.3450000
-19017.0000000	1.3400000
-19016.0000000	1.3450000
-19015.0000000	1.3350000
-19014.0000000	1.3350000
-19013.0000000	1.3450000
-19012.0000000	1.3500000
-19011.0000000	1.3500000
-19010.0000000	1.3550000
-19009.0000000	1.3600000
-19008.0000000	1.3666667
-19007.0000000	1.3700000
-19006.0000000	1.3800000

-19005.0000000	1.3700000
-19004.0000000	1.3750000
-19003.0000000	1.3750000
-19002.0000000	1.3800000
-19001.0000000	1.3800000
-19000.0000000	1.3800000
-18999.0000000	1.3800000
-18998.0000000	1.3800000
-18997.0000000	1.3650000
-18996.0000000	1.3550000
-18995.0000000	1.3450000
-18994.0000000	1.3400000
-18993.0000000	1.3400000
-18992.0000000	1.3450000
-18991.0000000	1.3500000
-18990.0000000	1.3450000
-18989.0000000	1.3500000
-18988.0000000	1.3600000
-18987.0000000	1.3650000
-18986.0000000	1.3750000
-18985.0000000	1.3850000
-18984.0000000	1.4050000
-18983.0000000	1.4150000
-18982.0000000	1.4349999
-18981.0000000	1.4400001
-18980.0000000	1.4550000
-18979.0000000	1.4500000
-18978.0000000	1.4500002
-18977.0000000	1.4450001
-18976.0000000	1.4349999
-18975.0000000	1.4349999
-18974.0000000	1.4150000
-18973.0000000	1.4000000
-18972.0000000	1.3650000
-18971.0000000	1.3400000
-18970.0000000	1.3250000
-18969.0000000	1.2950000
-18968.0000000	1.2750000
-18967.0000000	1.2700000
-18966.0000000	1.2650000
-18965.0000000	1.2600000
-18964.0000000	1.2700000
-18963.0000000	1.2800000
-18962.0000000	1.2900000
-18961.0000000	1.3000000
-18960.0000000	1.3150001
-18959.0000000	1.3200001
-18958.0000000	1.3400000
-18957.0000000	1.3500000
-18956.0000000	1.3500000
-18955.0000000	1.3650000
-18954.0000000	1.3700000
-18953.0000000	1.3900000
-18952.0000000	1.4100000
-18951.0000000	1.4150000
-18950.0000000	1.4200000
-18949.0000000	1.4299999

-18948.0000000	1.4366666
-18947.0000000	1.4550000
-18946.0000000	1.4550000
-18945.0000000	1.4650000
-18944.0000000	1.4600000
-18943.0000000	1.4500000
-18942.0000000	1.4349999
-18941.0000000	1.4349999
-18940.0000000	1.4299999
-18939.0000000	1.4250000
-18938.0000000	1.4300001
-18937.0000000	1.4349999
-18936.0000000	1.4299999
-18935.0000000	1.4299999
-18934.0000000	1.4349999
-18933.0000000	1.4299999
-18932.0000000	1.4400001
-18931.0000000	1.4349999
-18930.0000000	1.4500000
-18929.0000000	1.4500000
-18928.0000000	1.4500000
-18927.0000000	1.4550000
-18926.0000000	1.4500000
-18925.0000000	1.4450001
-18924.0000000	1.4400001
-18923.0000000	1.4349999
-18922.0000000	1.4200000
-18921.0000000	1.4100000
-18920.0000000	1.4050000
-18919.0000000	1.4000000
-18918.0000000	1.3900000
-18917.0000000	1.3800000
-18916.0000000	1.3650000
-18915.0000000	1.3550000
-18914.0000000	1.3550000
-18913.0000000	1.3600000
-18912.0000000	1.3600000
-18911.0000000	1.3600000
-18910.0000000	1.3650000
-18909.0000000	1.3650000
-18908.0000000	1.3600000
-18907.0000000	1.3550000
-18906.0000000	1.3550000
-18905.0000000	1.3550000
-18904.0000000	1.3600000
-18903.0000000	1.3650000
-18902.0000000	1.3750000
-18901.0000000	1.3800000
-18900.0000000	1.3800000
-18899.0000000	1.3650000
-18898.0000000	1.3550000
-18897.0000000	1.3500000
-18896.0000000	1.3350000
-18895.0000000	1.3200001
-18894.0000000	1.3200001
-18893.0000000	1.3200001
-18892.0000000	1.3150001

-18891.0000000	1.3150001
-18890.0000000	1.3300000
-18889.0000000	1.3250000
-18888.0000000	1.3200001
-18887.0000000	1.3350000
-18886.0000000	1.3450000
-18885.0000000	1.3550000
-18884.0000000	1.3600000
-18883.0000000	1.3700000
-18882.0000000	1.3850000
-18881.0000000	1.3950000
-18880.0000000	1.4000000
-18879.0000000	1.4000000
-18878.0000000	1.4100000
-18877.0000000	1.3900000
-18876.0000000	1.3700000
-18875.0000000	1.3400000
-18874.0000000	1.3099999
-18873.0000000	1.2950000
-18872.0000000	1.2900000
-18871.0000000	1.2950000
-18870.0000000	1.3000000
-18869.0000000	1.3000000
-18868.0000000	1.2950000
-18867.0000000	1.3000000
-18866.0000000	1.3150001
-18865.0000000	1.3200001
-18864.0000000	1.3250000
-18863.0000000	1.3400000
-18862.0000000	1.3500000
-18861.0000000	1.3650000
-18860.0000000	1.3750000
-18859.0000000	1.3900000
-18858.0000000	1.4000000
-18857.0000000	1.4000000
-18856.0000000	1.4100000
-18855.0000000	1.4100000
-18854.0000000	1.4150000
-18853.0000000	1.4250000
-18852.0000000	1.4299999
-18851.0000000	1.4400001
-18850.0000000	1.4400001
-18849.0000000	1.4400001
-18848.0000000	1.4299999
-18847.0000000	1.4400001
-18846.0000000	1.4299999
-18845.0000000	1.4300001
-18844.0000000	1.4300001
-18843.0000000	1.4250000
-18842.0000000	1.4200000
-18841.0000000	1.4250000
-18840.0000000	1.4100000
-18839.0000000	1.4000000
-18838.0000000	1.3900000
-18837.0000000	1.3800000
-18836.0000000	1.3700000
-18835.0000000	1.3600000

-18834.0000000	1.3600000
-18833.0000000	1.3500000
-18832.0000000	1.3450000
-18831.0000000	1.3350000
-18830.0000000	1.3250000
-18829.0000000	1.3150001
-18828.0000000	1.3033333
-18827.0000000	1.2850000
-18826.0000000	1.2850000
-18825.0000000	1.2750000
-18824.0000000	1.2600000
-18823.0000000	1.2350000
-18822.0000000	1.2350000
-18821.0000000	1.2400000
-18820.0000000	1.2450000
-18819.0000000	1.2550000
-18818.0000000	1.2650000
-18817.0000000	1.2700000
-18816.0000000	1.2750000
-18815.0000000	1.3000000
-18814.0000000	1.3000000
-18813.0000000	1.2950000
-18812.0000000	1.2900000
-18811.0000000	1.2750000
-18810.0000000	1.2750000
-18809.0000000	1.2850000
-18808.0000000	1.2950000
-18807.0000000	1.3000000
-18806.0000000	1.3000000
-18805.0000000	1.3000000
-18804.0000000	1.3049999
-18803.0000000	1.3150001
-18802.0000000	1.3200001
-18801.0000000	1.3300000
-18800.0000000	1.3400000
-18799.0000000	1.3400000
-18798.0000000	1.3533334
-18797.0000000	1.3650000
-18796.0000000	1.3650000
-18795.0000000	1.3800000
-18794.0000000	1.3800000
-18793.0000000	1.3850000
-18792.0000000	1.3850000
-18791.0000000	1.3950000
-18790.0000000	1.4000000
-18789.0000000	1.4000000
-18788.0000000	1.4100000
-18787.0000000	1.4100000
-18786.0000000	1.4150000
-18785.0000000	1.4200000
-18784.0000000	1.4250000
-18783.0000000	1.4200000
-18782.0000000	1.4200000
-18781.0000000	1.4200000
-18780.0000000	1.4150000
-18779.0000000	1.4200000
-18778.0000000	1.4100000

-18777.0000000	1.4050000
-18776.0000000	1.3950000
-18775.0000000	1.3950000
-18774.0000000	1.4000000
-18773.0000000	1.4150000
-18772.0000000	1.4200000
-18771.0000000	1.4200000
-18770.0000000	1.4250000
-18769.0000000	1.4299999
-18768.0000000	1.4366666
-18767.0000000	1.4400001
-18766.0000000	1.4450001
-18765.0000000	1.4400001
-18764.0000000	1.4550000
-18763.0000000	1.4600000
-18762.0000000	1.4500000
-18761.0000000	1.4400001
-18760.0000000	1.4349999
-18759.0000000	1.4349999
-18758.0000000	1.4450001
-18757.0000000	1.4450001
-18756.0000000	1.4450001
-18755.0000000	1.4400001
-18754.0000000	1.4349999
-18753.0000000	1.4349999
-18752.0000000	1.4250000
-18751.0000000	1.4150000
-18750.0000000	1.3850000
-18749.0000000	1.3900000
-18748.0000000	1.3850000
-18747.0000000	1.4000000
-18746.0000000	1.4100000
-18745.0000000	1.4250000
-18744.0000000	1.4349999
-18743.0000000	1.4500000
-18742.0000000	1.4550000
-18741.0000000	1.4600000
-18740.0000000	1.4600000
-18739.0000000	1.4600000
-18738.0000000	1.4600000
-18737.0000000	1.4733334
-18736.0000000	1.4700000
-18735.0000000	1.4800000
-18734.0000000	1.4800000
-18733.0000000	1.4800000
-18732.0000000	1.4850000
-18731.0000000	1.4850000
-18730.0000000	1.4900000
-18729.0000000	1.4950000
-18728.0000000	1.5000000
-18727.0000000	1.5000000
-18726.0000000	1.5050000
-18725.0000000	1.5050000
-18724.0000000	1.5100000
-18723.0000000	1.5100000
-18722.0000000	1.5100000
-18721.0000000	1.5150000

-18720.0000000	1.5100000
-18719.0000000	1.5150000
-18718.0000000	1.5100000
-18717.0000000	1.5100000
-18716.0000000	1.5050000
-18715.0000000	1.5000000
-18714.0000000	1.4900000
-18713.0000000	1.4800000
-18712.0000000	1.4650000
-18711.0000000	1.4650000
-18710.0000000	1.4550000
-18709.0000000	1.4600000
-18708.0000000	1.4600000
-18707.0000000	1.4566666
-18706.0000000	1.4500000
-18705.0000000	1.4500000
-18704.0000000	1.4400001
-18703.0000000	1.4349999
-18702.0000000	1.4349999
-18701.0000000	1.4250000
-18700.0000000	1.4250000
-18699.0000000	1.4250000
-18698.0000000	1.4100000
-18697.0000000	1.3950000
-18696.0000000	1.3800000
-18695.0000000	1.3800000
-18694.0000000	1.3700000
-18693.0000000	1.3700000
-18692.0000000	1.3800000
-18691.0000000	1.3600000
-18690.0000000	1.3500000
-18689.0000000	1.3500000
-18688.0000000	1.3550000
-18687.0000000	1.3650000
-18686.0000000	1.3800000
-18685.0000000	1.3950000
-18684.0000000	1.4150000
-18683.0000000	1.4250000
-18682.0000000	1.4400001
-18681.0000000	1.4450001
-18680.0000000	1.4600000
-18679.0000000	1.4600000
-18678.0000000	1.4666667
-18677.0000000	1.4800000
-18676.0000000	1.4850000
-18675.0000000	1.4950000
-18674.0000000	1.4900000
-18673.0000000	1.4950000
-18672.0000000	1.5000000
-18671.0000000	1.5050000
-18670.0000000	1.5100000
-18669.0000000	1.5150000
-18668.0000000	1.5200000
-18667.0000000	1.5250000
-18666.0000000	1.5250000
-18665.0000000	1.5250000
-18664.0000000	1.5150000

-18663.0000000	1.5050000
-18662.0000000	1.5050000
-18661.0000000	1.5050000
-18660.0000000	1.5000000
-18659.0000000	1.5000000
-18658.0000000	1.4950000
-18657.0000000	1.4800000
-18656.0000000	1.4800000
-18655.0000000	1.4600000
-18654.0000000	1.4349999
-18653.0000000	1.4150000
-18652.0000000	1.3900000
-18651.0000000	1.3750000
-18650.0000000	1.3500000
-18649.0000000	1.3350000
-18648.0000000	1.3250000
-18647.0000000	1.3300000
-18646.0000000	1.3400000
-18645.0000000	1.3366667
-18644.0000000	1.3300000
-18643.0000000	1.3300000
-18642.0000000	1.3500000
-18641.0000000	1.3500000
-18640.0000000	1.3550000
-18639.0000000	1.3600000
-18638.0000000	1.3700000
-18637.0000000	1.3750000
-18636.0000000	1.3850000
-18635.0000000	1.3950000
-18634.0000000	1.4100000
-18633.0000000	1.4250000
-18632.0000000	1.4299999
-18631.0000000	1.4200000
-18630.0000000	1.4200000
-18629.0000000	1.4250000
-18628.0000000	1.4299999
-18627.0000000	1.4299999
-18626.0000000	1.4250000
-18625.0000000	1.4250000
-18624.0000000	1.4200000
-18623.0000000	1.4200000
-18622.0000000	1.4299999
-18621.0000000	1.4250000
-18620.0000000	1.4250000
-18619.0000000	1.4300001
-18618.0000000	1.4299999
-18617.0000000	1.4299999
-18616.0000000	1.4349999
-18615.0000000	1.4466667
-18614.0000000	1.4550000
-18613.0000000	1.4700000
-18612.0000000	1.4800000
-18611.0000000	1.4850000
-18610.0000000	1.4900000
-18609.0000000	1.4950000
-18608.0000000	1.4950000
-18607.0000000	1.5000000

-18606.0000000	1.5150000
-18605.0000000	1.5300000
-18604.0000000	1.5300000
-18603.0000000	1.5400000
-18602.0000000	1.5350000
-18601.0000000	1.5300000
-18600.0000000	1.5350000
-18599.0000000	1.5250000
-18598.0000000	1.5200000
-18597.0000000	1.5100000
-18596.0000000	1.5100000
-18595.0000000	1.5100000
-18594.0000000	1.5100000
-18593.0000000	1.5150000
-18592.0000000	1.5100000
-18591.0000000	1.5100000
-18590.0000000	1.5050000
-18589.0000000	1.5100000
-18588.0000000	1.5100000
-18587.0000000	1.5050000
-18586.0000000	1.5000000
-18585.0000000	1.5000000
-18584.0000000	1.4900000
-18583.0000000	1.4900000
-18582.0000000	1.4800000
-18581.0000000	1.4850000
-18580.0000000	1.4750000
-18579.0000000	1.4800000
-18578.0000000	1.4900000
-18577.0000000	1.4850000
-18576.0000000	1.4850000
-18575.0000000	1.4750000
-18574.0000000	1.4750000
-18573.0000000	1.4700000
-18572.0000000	1.4550000
-18571.0000000	1.4400001
-18570.0000000	1.4349999
-18569.0000000	1.4200000
-18568.0000000	1.4050000
-18567.0000000	1.3950000
-18566.0000000	1.3900000
-18565.0000000	1.3850000
-18564.0000000	1.3850000
-18563.0000000	1.3800000
-18562.0000000	1.3850000
-18561.0000000	1.3900000
-18560.0000000	1.3900000
-18559.0000000	1.4100000
-18558.0000000	1.4200000
-18557.0000000	1.4250000
-18556.0000000	1.4366666
-18555.0000000	1.4500000
-18554.0000000	1.4500000
-18553.0000000	1.4450001
-18552.0000000	1.4349999
-18551.0000000	1.4299999
-18550.0000000	1.4299999

-18549.0000000	1.4150000
-18548.0000000	1.4050000
-18547.0000000	1.4000000
-18546.0000000	1.4000000
-18545.0000000	1.4000000
-18544.0000000	1.4100000
-18543.0000000	1.4100000
-18542.0000000	1.4200000
-18541.0000000	1.4250000
-18540.0000000	1.4250000
-18539.0000000	1.4349999
-18538.0000000	1.4550000
-18537.0000000	1.4700000
-18536.0000000	1.4800000
-18535.0000000	1.4900000
-18534.0000000	1.4950000
-18533.0000000	1.4950000
-18532.0000000	1.5100000
-18531.0000000	1.5200000
-18530.0000000	1.5300000
-18529.0000000	1.5400000
-18528.0000000	1.5350000
-18527.0000000	1.5300000
-18526.0000000	1.5400000
-18525.0000000	1.5450000
-18524.0000000	1.5350000
-18523.0000000	1.5250000
-18522.0000000	1.5100000
-18521.0000000	1.4950000
-18520.0000000	1.4950000
-18519.0000000	1.4950000
-18518.0000000	1.5000000
-18517.0000000	1.5100000
-18516.0000000	1.5150000
-18515.0000000	1.5250000
-18514.0000000	1.5200000
-18513.0000000	1.5250000
-18512.0000000	1.5200000
-18511.0000000	1.5200000
-18510.0000000	1.5200000
-18509.0000000	1.5150000
-18508.0000000	1.5100000
-18507.0000000	1.5050000
-18506.0000000	1.4950000
-18505.0000000	1.4850000
-18504.0000000	1.4800000
-18503.0000000	1.4800000
-18502.0000000	1.4800000
-18501.0000000	1.4700000
-18500.0000000	1.4750000
-18499.0000000	1.4700000
-18498.0000000	1.4750000
-18497.0000000	1.4700000
-18496.0000000	1.4699999
-18495.0000000	1.4700000
-18494.0000000	1.4650000
-18493.0000000	1.4700000

-18492.0000000	1.4600000
-18491.0000000	1.4700000
-18490.0000000	1.4600000
-18489.0000000	1.4550000
-18488.0000000	1.4400001
-18487.0000000	1.4299999
-18486.0000000	1.4349999
-18485.0000000	1.4400001
-18484.0000000	1.4500000
-18483.0000000	1.4600000
-18482.0000000	1.4550000
-18481.0000000	1.4600000
-18480.0000000	1.4600000
-18479.0000000	1.4650000
-18478.0000000	1.4800000
-18477.0000000	1.4850000
-18476.0000000	1.5000000
-18475.0000000	1.5100000
-18474.0000000	1.5200000
-18473.0000000	1.5250000
-18472.0000000	1.5150000
-18471.0000000	1.5050000
-18470.0000000	1.5000000
-18469.0000000	1.5000000
-18468.0000000	1.4950000
-18467.0000000	1.5050000
-18466.0000000	1.5050000
-18465.0000000	1.5033334
-18464.0000000	1.5050000
-18463.0000000	1.5200000
-18462.0000000	1.5250000
-18461.0000000	1.5200000
-18460.0000000	1.5200000
-18459.0000000	1.5100000
-18458.0000000	1.4950000
-18457.0000000	1.4750000
-18456.0000000	1.4500000
-18455.0000000	1.4299999
-18454.0000000	1.4100000
-18453.0000000	1.3950000
-18452.0000000	1.3950000
-18451.0000000	1.3950000
-18450.0000000	1.4050000
-18449.0000000	1.3950000
-18448.0000000	1.4000000
-18447.0000000	1.4050000
-18446.0000000	1.4250000
-18445.0000000	1.4250000
-18444.0000000	1.4299999
-18443.0000000	1.4450001
-18442.0000000	1.4450001
-18441.0000000	1.4500000
-18440.0000000	1.4550000
-18439.0000000	1.4650000
-18438.0000000	1.4650000
-18437.0000000	1.4700000
-18436.0000000	1.4750000

-18435.0000000	1.4750000
-18434.0000000	1.4766668
-18433.0000000	1.4850000
-18432.0000000	1.4800000
-18431.0000000	1.4850000
-18430.0000000	1.4750000
-18429.0000000	1.4800000
-18428.0000000	1.4750000
-18427.0000000	1.4700000
-18426.0000000	1.4650000
-18425.0000000	1.4650000
-18424.0000000	1.4550000
-18423.0000000	1.4450001
-18422.0000000	1.4349999
-18421.0000000	1.4200000
-18420.0000000	1.4150000
-18419.0000000	1.4050000
-18418.0000000	1.4000000
-18417.0000000	1.3900000
-18416.0000000	1.4000000
-18415.0000000	1.4000000
-18414.0000000	1.4050000
-18413.0000000	1.4050000
-18412.0000000	1.4150000
-18411.0000000	1.4200000
-18410.0000000	1.4050000
-18409.0000000	1.4000000
-18408.0000000	1.4000000
-18407.0000000	1.4000000
-18406.0000000	1.3950000
-18405.0000000	1.3966666
-18404.0000000	1.3950000
-18403.0000000	1.4100000
-18402.0000000	1.4200000
-18401.0000000	1.4299999
-18400.0000000	1.4349999
-18399.0000000	1.4250000
-18398.0000000	1.4299999
-18397.0000000	1.4400001
-18396.0000000	1.4450001
-18395.0000000	1.4500000
-18394.0000000	1.4600000
-18393.0000000	1.4600000
-18392.0000000	1.4550000
-18391.0000000	1.4500000
-18390.0000000	1.4450001
-18389.0000000	1.4450001
-18388.0000000	1.4550000
-18387.0000000	1.4750000
-18386.0000000	1.4850000
-18385.0000000	1.4950000
-18384.0000000	1.4900000
-18383.0000000	1.4850000
-18382.0000000	1.4800000
-18381.0000000	1.4750000
-18380.0000000	1.4600000
-18379.0000000	1.4400001

-18378.0000000	1.4200000
-18377.0000000	1.4150000
-18376.0000000	1.4150000
-18375.0000000	1.4166666
-18374.0000000	1.4150000
-18373.0000000	1.4299999
-18372.0000000	1.4349999
-18371.0000000	1.4299999
-18370.0000000	1.4200000
-18369.0000000	1.4100000
-18368.0000000	1.4100000
-18367.0000000	1.4150000
-18366.0000000	1.4200000
-18365.0000000	1.4250000
-18364.0000000	1.4299999
-18363.0000000	1.4400001
-18362.0000000	1.4450001
-18361.0000000	1.4500000
-18360.0000000	1.4550000
-18359.0000000	1.4550000
-18358.0000000	1.4600000
-18357.0000000	1.4550000
-18356.0000000	1.4500000
-18355.0000000	1.4650000
-18354.0000000	1.4650000
-18353.0000000	1.4700000
-18352.0000000	1.4800000
-18351.0000000	1.4900000
-18350.0000000	1.5100000
-18349.0000000	1.5150000
-18348.0000000	1.5200000
-18347.0000000	1.5350000
-18346.0000000	1.5266666
-18345.0000000	1.5300000
-18344.0000000	1.5150000
-18343.0000000	1.5050000
-18342.0000000	1.4900000
-18341.0000000	1.4900000
-18340.0000000	1.4850000
-18339.0000000	1.4950000
-18338.0000000	1.5000000
-18337.0000000	1.5150000
-18336.0000000	1.5300000
-18335.0000000	1.5450000
-18334.0000000	1.5650001
-18333.0000000	1.5750000
-18332.0000000	1.5750000
-18331.0000000	1.5699999
-18330.0000000	1.5650001
-18329.0000000	1.5650001
-18328.0000000	1.5599999
-18327.0000000	1.5750000
-18326.0000000	1.5750000
-18325.0000000	1.5850000
-18324.0000000	1.5950000
-18323.0000000	1.6050000
-18322.0000000	1.6100000

-18321.0000000	1.6100000
-18320.0000000	1.6100000
-18319.0000000	1.5950000
-18318.0000000	1.5750000
-18317.0000000	1.5650001
-18316.0000000	1.5766667
-18315.0000000	1.5900000
-18314.0000000	1.6000000
-18313.0000000	1.6000000
-18312.0000000	1.6050000
-18311.0000000	1.6050000
-18310.0000000	1.6100000
-18309.0000000	1.6050000
-18308.0000000	1.6000000
-18307.0000000	1.5950000
-18306.0000000	1.5800000
-18305.0000000	1.5549999
-18304.0000000	1.5300000
-18303.0000000	1.4750000
-18302.0000000	1.4500000
-18301.0000000	1.4400001
-18300.0000000	1.4400001
-18299.0000000	1.4450001
-18298.0000000	1.4450001
-18297.0000000	1.4400001
-18296.0000000	1.4450001
-18295.0000000	1.4349999
-18294.0000000	1.4250000
-18293.0000000	1.4349999
-18292.0000000	1.4500000
-18291.0000000	1.4700000
-18290.0000000	1.4850000
-18289.0000000	1.5000000
-18288.0000000	1.5100000
-18287.0000000	1.5200000
-18286.0000000	1.5450000
-18285.0000000	1.5533333
-18284.0000000	1.5599999
-18283.0000000	1.5599999
-18282.0000000	1.5599999
-18281.0000000	1.5650001
-18280.0000000	1.5650001
-18279.0000000	1.5599999
-18278.0000000	1.5549999
-18277.0000000	1.5500000
-18276.0000000	1.5450000
-18275.0000000	1.5350000
-18274.0000000	1.5200000
-18273.0000000	1.5100000
-18272.0000000	1.5000000
-18271.0000000	1.4900000
-18270.0000000	1.4900000
-18269.0000000	1.4750000
-18268.0000000	1.4650000
-18267.0000000	1.4550000
-18266.0000000	1.4400001
-18265.0000000	1.4500000

-18264.0000000	1.4450001
-18263.0000000	1.4500000
-18262.0000000	1.4400001
-18261.0000000	1.4450001
-18260.0000000	1.4550000
-18259.0000000	1.4600000
-18258.0000000	1.4650000
-18257.0000000	1.4750000
-18256.0000000	1.4850000
-18255.0000000	1.4900000
-18254.0000000	1.5000000
-18253.0000000	1.4866667
-18252.0000000	1.4750000
-18251.0000000	1.4800000
-18250.0000000	1.5000000
-18249.0000000	1.5050000
-18248.0000000	1.5150000
-18247.0000000	1.5150000
-18246.0000000	1.5200000
-18245.0000000	1.5300000
-18244.0000000	1.5450000
-18243.0000000	1.5599999
-18242.0000000	1.5700001
-18241.0000000	1.5850000
-18240.0000000	1.5950000
-18239.0000000	1.5900000
-18238.0000000	1.5900000
-18237.0000000	1.5800000
-18236.0000000	1.5800000
-18235.0000000	1.5700001
-18234.0000000	1.5549999
-18233.0000000	1.5549999
-18232.0000000	1.5599999
-18231.0000000	1.5650001
-18230.0000000	1.5650001
-18229.0000000	1.5650001
-18228.0000000	1.5549999
-18227.0000000	1.5650001
-18226.0000000	1.5699999
-18225.0000000	1.5650001
-18224.0000000	1.5549999
-18223.0000000	1.5433334
-18222.0000000	1.5400000
-18221.0000000	1.5350000
-18220.0000000	1.5350000
-18219.0000000	1.5350000
-18218.0000000	1.5350000
-18217.0000000	1.5300000
-18216.0000000	1.5300000
-18215.0000000	1.5200000
-18214.0000000	1.5100000
-18213.0000000	1.5100000
-18212.0000000	1.5050000
-18211.0000000	1.5150000
-18210.0000000	1.5200000
-18209.0000000	1.5300000
-18208.0000000	1.5400000

-18207.0000000	1.5450000
-18206.0000000	1.5500000
-18205.0000000	1.5549999
-18204.0000000	1.5549999
-18203.0000000	1.5599999
-18202.0000000	1.5599999
-18201.0000000	1.5750000
-18200.0000000	1.5900000
-18199.0000000	1.5950000
-18198.0000000	1.5950000
-18197.0000000	1.5950000
-18196.0000000	1.5900000
-18195.0000000	1.5900000
-18194.0000000	1.5950000
-18193.0000000	1.6033334
-18192.0000000	1.6200000
-18191.0000000	1.6200000
-18190.0000000	1.6200000
-18189.0000000	1.6100000
-18188.0000000	1.6000000
-18187.0000000	1.5800000
-18186.0000000	1.5599999
-18185.0000000	1.5450000
-18184.0000000	1.5300000
-18183.0000000	1.5200000
-18182.0000000	1.5100000
-18181.0000000	1.4850000
-18180.0000000	1.4800000
-18179.0000000	1.4850000
-18178.0000000	1.4900000
-18177.0000000	1.4900000
-18176.0000000	1.5050000
-18175.0000000	1.5200000
-18174.0000000	1.5350000
-18173.0000000	1.5500000
-18172.0000000	1.5650001
-18171.0000000	1.5800000
-18170.0000000	1.5900000
-18169.0000000	1.6050000
-18168.0000000	1.6150000
-18167.0000000	1.6250000
-18166.0000000	1.6150000
-18165.0000000	1.6000000
-18164.0000000	1.5850000
-18163.0000000	1.5733334
-18162.0000000	1.5500000
-18161.0000000	1.5350000
-18160.0000000	1.5300000
-18159.0000000	1.5300000
-18158.0000000	1.5300000
-18157.0000000	1.5400000
-18156.0000000	1.5400000
-18155.0000000	1.5500000
-18154.0000000	1.5599999
-18153.0000000	1.5500000
-18152.0000000	1.5599999
-18151.0000000	1.5549999

-18150.0000000	1.5549999
-18149.0000000	1.5599999
-18148.0000000	1.5650001
-18147.0000000	1.5650001
-18146.0000000	1.5599999
-18145.0000000	1.5450000
-18144.0000000	1.5350000
-18143.0000000	1.5150000
-18142.0000000	1.5200000
-18141.0000000	1.5300000
-18140.0000000	1.5250000
-18139.0000000	1.5150000
-18138.0000000	1.5150000
-18137.0000000	1.5100000
-18136.0000000	1.5100000
-18135.0000000	1.5150000
-18134.0000000	1.5200000
-18133.0000000	1.5250000
-18132.0000000	1.5300000
-18131.0000000	1.5350000
-18130.0000000	1.5450000
-18129.0000000	1.5450000
-18128.0000000	1.5599999
-18127.0000000	1.5599999
-18126.0000000	1.5599999
-18125.0000000	1.5700001
-18124.0000000	1.5650001
-18123.0000000	1.5549999
-18122.0000000	1.5500000
-18121.0000000	1.5400000
-18120.0000000	1.5300000
-18119.0000000	1.5300000
-18118.0000000	1.5250000
-18117.0000000	1.5250000
-18116.0000000	1.5300000
-18115.0000000	1.5300000
-18114.0000000	1.5200000
-18113.0000000	1.5300000
-18112.0000000	1.5300000
-18111.0000000	1.5400000
-18110.0000000	1.5500000
-18109.0000000	1.5549999
-18108.0000000	1.5599999
-18107.0000000	1.5650001
-18106.0000000	1.5650001
-18105.0000000	1.5699999
-18104.0000000	1.5800000
-18103.0000000	1.5866667
-18102.0000000	1.6000000
-18101.0000000	1.6050000
-18100.0000000	1.6000000
-18099.0000000	1.6000000
-18098.0000000	1.5900000
-18097.0000000	1.5800000
-18096.0000000	1.5700001
-18095.0000000	1.5500000
-18094.0000000	1.5350000

-18093.0000000	1.5150000
-18092.0000000	1.5200000
-18091.0000000	1.5250000
-18090.0000000	1.5300000
-18089.0000000	1.5350000
-18088.0000000	1.5450000
-18087.0000000	1.5500000
-18086.0000000	1.5599999
-18085.0000000	1.5650001
-18084.0000000	1.5700001
-18083.0000000	1.5700001
-18082.0000000	1.5700001
-18081.0000000	1.5750000
-18080.0000000	1.5750000
-18079.0000000	1.5800000
-18078.0000000	1.5850000
-18077.0000000	1.5950000
-18076.0000000	1.6000000
-18075.0000000	1.6050000
-18074.0000000	1.6166667
-18073.0000000	1.6200000
-18072.0000000	1.6433333
-18071.0000000	1.6600000
-18070.0000000	1.6799999
-18069.0000000	1.6849999
-18068.0000000	1.7050000
-18067.0000000	1.7150000
-18066.0000000	1.7250000
-18065.0000000	1.7200000
-18064.0000000	1.7200000
-18063.0000000	1.7150000
-18062.0000000	1.7100000
-18061.0000000	1.7150000
-18060.0000000	1.7050000
-18059.0000000	1.7050000
-18058.0000000	1.7050000
-18057.0000000	1.6950001
-18056.0000000	1.6900001
-18055.0000000	1.6950001
-18054.0000000	1.6849999
-18053.0000000	1.6799999
-18052.0000000	1.6849999
-18051.0000000	1.6799999
-18050.0000000	1.6750000
-18049.0000000	1.6550000
-18048.0000000	1.6550000
-18047.0000000	1.6550000
-18046.0000000	1.6500000
-18045.0000000	1.6550000
-18044.0000000	1.6550000
-18043.0000000	1.6500000
-18042.0000000	1.6650000
-18041.0000000	1.6600000
-18040.0000000	1.6550000
-18039.0000000	1.6600000
-18038.0000000	1.6600000
-18037.0000000	1.6700000

-18036.0000000	1.6650000
-18035.0000000	1.6700000
-18034.0000000	1.6650000
-18033.0000000	1.6650000
-18032.0000000	1.6700000
-18031.0000000	1.6700000
-18030.0000000	1.6600000
-18029.0000000	1.6600000
-18028.0000000	1.6600000
-18027.0000000	1.6550000
-18026.0000000	1.6500000
-18025.0000000	1.6450000
-18024.0000000	1.6400000
-18023.0000000	1.6300000
-18022.0000000	1.6100000
-18021.0000000	1.5850000
-18020.0000000	1.5650001
-18019.0000000	1.5450000
-18018.0000000	1.5200000
-18017.0000000	1.4950000
-18016.0000000	1.4900000
-18015.0000000	1.4750000
-18014.0000000	1.4733334
-18013.0000000	1.4600000
-18012.0000000	1.4500000
-18011.0000000	1.4333333
-18010.0000000	1.4200000
-18009.0000000	1.4100000
-18008.0000000	1.4000000
-18007.0000000	1.3950000
-18006.0000000	1.4000000
-18005.0000000	1.4100000
-18004.0000000	1.4250000
-18003.0000000	1.4400001
-18002.0000000	1.4550000
-18001.0000000	1.4550000
-18000.0000000	1.4450001
-17999.0000000	1.4450001
-17998.0000000	1.4450001
-17997.0000000	1.4500000
-17996.0000000	1.4550000
-17995.0000000	1.4650000
-17994.0000000	1.4750000
-17993.0000000	1.4850000
-17992.0000000	1.4950000
-17991.0000000	1.5050000
-17990.0000000	1.5150000
-17989.0000000	1.5100000
-17988.0000000	1.5100000
-17987.0000000	1.5100000
-17986.0000000	1.5100000
-17985.0000000	1.5100000
-17984.0000000	1.5150000
-17983.0000000	1.5200000
-17982.0000000	1.5300000
-17981.0000000	1.5300000
-17980.0000000	1.5200000

-17979.0000000	1.5100000
-17978.0000000	1.4950000
-17977.0000000	1.4750000
-17976.0000000	1.4550000
-17975.0000000	1.4450001
-17974.0000000	1.4299999
-17973.0000000	1.4200000
-17972.0000000	1.4150000
-17971.0000000	1.4200000
-17970.0000000	1.4299999
-17969.0000000	1.4450001
-17968.0000000	1.4600000
-17967.0000000	1.4750000
-17966.0000000	1.4650000
-17965.0000000	1.4600000
-17964.0000000	1.4600000
-17963.0000000	1.4650000
-17962.0000000	1.4800000
-17961.0000000	1.4900000
-17960.0000000	1.4850000
-17959.0000000	1.4650000
-17958.0000000	1.4700000
-17957.0000000	1.4800000
-17956.0000000	1.4850000
-17955.0000000	1.4950000
-17954.0000000	1.5100000
-17953.0000000	1.5250000
-17952.0000000	1.5233332
-17951.0000000	1.5250000
-17950.0000000	1.5350000
-17949.0000000	1.5549999
-17948.0000000	1.5650001
-17947.0000000	1.5750000
-17946.0000000	1.5800000
-17945.0000000	1.5850000
-17944.0000000	1.5850000
-17943.0000000	1.5800000
-17942.0000000	1.5800000
-17941.0000000	1.5900000
-17940.0000000	1.6050000
-17939.0000000	1.6150000
-17938.0000000	1.6100000
-17937.0000000	1.6200000
-17936.0000000	1.6250000
-17935.0000000	1.6350000
-17934.0000000	1.6350000
-17933.0000000	1.6450000
-17932.0000000	1.6500000
-17931.0000000	1.6600000
-17930.0000000	1.6650000
-17929.0000000	1.6550000
-17928.0000000	1.6600000
-17927.0000000	1.6550000
-17926.0000000	1.6550000
-17925.0000000	1.6450000
-17924.0000000	1.6550000
-17923.0000000	1.6450000

-17922.0000000	1.6333333
-17921.0000000	1.6000000
-17920.0000000	1.5750000
-17919.0000000	1.5549999
-17918.0000000	1.5500000
-17917.0000000	1.5599999
-17916.0000000	1.5650001
-17915.0000000	1.5650001
-17914.0000000	1.5549999
-17913.0000000	1.5450000
-17912.0000000	1.5300000
-17911.0000000	1.5300000
-17910.0000000	1.5300000
-17909.0000000	1.5350000
-17908.0000000	1.5500000
-17907.0000000	1.5650001
-17906.0000000	1.5700001
-17905.0000000	1.5750000
-17904.0000000	1.5750000
-17903.0000000	1.5750000
-17902.0000000	1.5800000
-17901.0000000	1.5800000
-17900.0000000	1.5700001
-17899.0000000	1.5700001
-17898.0000000	1.5650001
-17897.0000000	1.5650001
-17896.0000000	1.5700001
-17895.0000000	1.5650001
-17894.0000000	1.5650001
-17893.0000000	1.5700001
-17892.0000000	1.5666667
-17891.0000000	1.5700001
-17890.0000000	1.5850000
-17889.0000000	1.5850000
-17888.0000000	1.5850000
-17887.0000000	1.5950000
-17886.0000000	1.5850000
-17885.0000000	1.5850000
-17884.0000000	1.5700001
-17883.0000000	1.5599999
-17882.0000000	1.5450000
-17881.0000000	1.5350000
-17880.0000000	1.5450000
-17879.0000000	1.5549999
-17878.0000000	1.5500000
-17877.0000000	1.5350000
-17876.0000000	1.5250000
-17875.0000000	1.5250000
-17874.0000000	1.5200000
-17873.0000000	1.5250000
-17872.0000000	1.5400000
-17871.0000000	1.5599999
-17870.0000000	1.5599999
-17869.0000000	1.5650001
-17868.0000000	1.5800000
-17867.0000000	1.5900000
-17866.0000000	1.6000000

-17865.0000000	1.6150000
-17864.0000000	1.6200000
-17863.0000000	1.6166667
-17862.0000000	1.6250000
-17861.0000000	1.6250000
-17860.0000000	1.6250000
-17859.0000000	1.6100000
-17858.0000000	1.6000000
-17857.0000000	1.5950000
-17856.0000000	1.5850000
-17855.0000000	1.5850000
-17854.0000000	1.5750000
-17853.0000000	1.5800000
-17852.0000000	1.5750000
-17851.0000000	1.5800000
-17850.0000000	1.5850000
-17849.0000000	1.5900000
-17848.0000000	1.5900000
-17847.0000000	1.5900000
-17846.0000000	1.5850000
-17845.0000000	1.5750000
-17844.0000000	1.5800000
-17843.0000000	1.5700001
-17842.0000000	1.5750000
-17841.0000000	1.5800000
-17840.0000000	1.5850000
-17839.0000000	1.5900000
-17838.0000000	1.5950000
-17837.0000000	1.5900000
-17836.0000000	1.5850000
-17835.0000000	1.5850000
-17834.0000000	1.5800000
-17833.0000000	1.5850000
-17832.0000000	1.5800000
-17831.0000000	1.5900000
-17830.0000000	1.5950000
-17829.0000000	1.6000000
-17828.0000000	1.5900000
-17827.0000000	1.5900000
-17826.0000000	1.5900000
-17825.0000000	1.5750000
-17824.0000000	1.5650001
-17823.0000000	1.5549999
-17822.0000000	1.5450000
-17821.0000000	1.5300000
-17820.0000000	1.5250000
-17819.0000000	1.5200000
-17818.0000000	1.5100000
-17817.0000000	1.5000000
-17816.0000000	1.4950000
-17815.0000000	1.5000000
-17814.0000000	1.5000000
-17813.0000000	1.5050000
-17812.0000000	1.5000000
-17811.0000000	1.5100000
-17810.0000000	1.4950000
-17809.0000000	1.5000000

-17808.0000000	1.5000000
-17807.0000000	1.5100000
-17806.0000000	1.5100000
-17805.0000000	1.5050000
-17804.0000000	1.5000000
-17803.0000000	1.5000000
-17802.0000000	1.5033334
-17801.0000000	1.5100000
-17800.0000000	1.5200000
-17799.0000000	1.5300000
-17798.0000000	1.5350000
-17797.0000000	1.5350000
-17796.0000000	1.5300000
-17795.0000000	1.5200000
-17794.0000000	1.5100000
-17793.0000000	1.5100000
-17792.0000000	1.4950000
-17791.0000000	1.4950000
-17790.0000000	1.5150000
-17789.0000000	1.5300000
-17788.0000000	1.5300000
-17787.0000000	1.5150000
-17786.0000000	1.5100000
-17785.0000000	1.4850000
-17784.0000000	1.4700000
-17783.0000000	1.4550000
-17782.0000000	1.4450001
-17781.0000000	1.4550000
-17780.0000000	1.4600000
-17779.0000000	1.4750000
-17778.0000000	1.4450001
-17777.0000000	1.4200000
-17776.0000000	1.4200000
-17775.0000000	1.4150000
-17774.0000000	1.4200000
-17773.0000000	1.4200000
-17772.0000000	1.4299999
-17771.0000000	1.4299999
-17770.0000000	1.4100000
-17769.0000000	1.4000000
-17768.0000000	1.3750000
-17767.0000000	1.3750000
-17766.0000000	1.3800000
-17765.0000000	1.3950000
-17764.0000000	1.4150000
-17763.0000000	1.4349999
-17762.0000000	1.4450001
-17761.0000000	1.4650000
-17760.0000000	1.4750000
-17759.0000000	1.4850000
-17758.0000000	1.4850000
-17757.0000000	1.4900000
-17756.0000000	1.4850000
-17755.0000000	1.4850000
-17754.0000000	1.4950000
-17753.0000000	1.4900000
-17752.0000000	1.4800000

-17751.0000000	1.4700000
-17750.0000000	1.4600000
-17749.0000000	1.4550000
-17748.0000000	1.4550000
-17747.0000000	1.4550000
-17746.0000000	1.4650000
-17745.0000000	1.4700000
-17744.0000000	1.4700000
-17743.0000000	1.4700000
-17742.0000000	1.4800000
-17741.0000000	1.4933333
-17740.0000000	1.5050000
-17739.0000000	1.5100000
-17738.0000000	1.5200000
-17737.0000000	1.5250000
-17736.0000000	1.5200000
-17735.0000000	1.5300000
-17734.0000000	1.5400000
-17733.0000000	1.5500000
-17732.0000000	1.5549999
-17731.0000000	1.5599999
-17730.0000000	1.5599999
-17729.0000000	1.5599999
-17728.0000000	1.5650001
-17727.0000000	1.5599999
-17726.0000000	1.5500000
-17725.0000000	1.5500000
-17724.0000000	1.5549999
-17723.0000000	1.5599999
-17722.0000000	1.5650001
-17721.0000000	1.5800000
-17720.0000000	1.5900000
-17719.0000000	1.5950000
-17718.0000000	1.6000000
-17717.0000000	1.5950000
-17716.0000000	1.5900000
-17715.0000000	1.5800000
-17714.0000000	1.5800000
-17713.0000000	1.5900000
-17712.0000000	1.5850000
-17711.0000000	1.5666666
-17710.0000000	1.5450000
-17709.0000000	1.5300000
-17708.0000000	1.5300000
-17707.0000000	1.5300000
-17706.0000000	1.5350000
-17705.0000000	1.5300000
-17704.0000000	1.5250000
-17703.0000000	1.5150000
-17702.0000000	1.4950000
-17701.0000000	1.4750000
-17700.0000000	1.4500000
-17699.0000000	1.4299999
-17698.0000000	1.4100000
-17697.0000000	1.3950000
-17696.0000000	1.3900000
-17695.0000000	1.3750000

-17694.0000000	1.3650000
-17693.0000000	1.3700000
-17692.0000000	1.3750000
-17691.0000000	1.3950000
-17690.0000000	1.4200000
-17689.0000000	1.4349999
-17688.0000000	1.4349999
-17687.0000000	1.4400001
-17686.0000000	1.4349999
-17685.0000000	1.4250000
-17684.0000000	1.4200000
-17683.0000000	1.4150000
-17682.0000000	1.4150000
-17681.0000000	1.4200000
-17680.0000000	1.4200000
-17679.0000000	1.4299999
-17678.0000000	1.4450001
-17677.0000000	1.4650000
-17676.0000000	1.4850000
-17675.0000000	1.4800000
-17674.0000000	1.4950000
-17673.0000000	1.4950000
-17672.0000000	1.5000000
-17671.0000000	1.5000000
-17670.0000000	1.5050000
-17669.0000000	1.5150000
-17668.0000000	1.5150000
-17667.0000000	1.5150000
-17666.0000000	1.5200000
-17665.0000000	1.5250000
-17664.0000000	1.5300000
-17663.0000000	1.5350000
-17662.0000000	1.5450000
-17661.0000000	1.5450000
-17660.0000000	1.5400000
-17659.0000000	1.5400000
-17658.0000000	1.5400000
-17657.0000000	1.5549999
-17656.0000000	1.5599999
-17655.0000000	1.5599999
-17654.0000000	1.5500000
-17653.0000000	1.5549999
-17652.0000000	1.5450000
-17651.0000000	1.5400000
-17650.0000000	1.5400000
-17649.0000000	1.5466666
-17648.0000000	1.5549999
-17647.0000000	1.5549999
-17646.0000000	1.5500000
-17645.0000000	1.5500000
-17644.0000000	1.5400000
-17643.0000000	1.5200000
-17642.0000000	1.5000000
-17641.0000000	1.4800000
-17640.0000000	1.4800000
-17639.0000000	1.4700000
-17638.0000000	1.4600000

-17637.0000000	1.4500000
-17636.0000000	1.4650000
-17635.0000000	1.4650000
-17634.0000000	1.4650000
-17633.0000000	1.4700000
-17632.0000000	1.4750000
-17631.0000000	1.4900000
-17630.0000000	1.5000000
-17629.0000000	1.5300000
-17628.0000000	1.5450000
-17627.0000000	1.5549999
-17626.0000000	1.5599999
-17625.0000000	1.5599999
-17624.0000000	1.5500000
-17623.0000000	1.5400000
-17622.0000000	1.5200000
-17621.0000000	1.4950000
-17620.0000000	1.4850000
-17619.0000000	1.4800000
-17618.0000000	1.4733334
-17617.0000000	1.4550000
-17616.0000000	1.4600000
-17615.0000000	1.4700000
-17614.0000000	1.4800000
-17613.0000000	1.4950000
-17612.0000000	1.5050000
-17611.0000000	1.5100000
-17610.0000000	1.5100000
-17609.0000000	1.5150000
-17608.0000000	1.5250000
-17607.0000000	1.5400000
-17606.0000000	1.5400000
-17605.0000000	1.5400000
-17604.0000000	1.5300000
-17603.0000000	1.5250000
-17602.0000000	1.5200000
-17601.0000000	1.5150000
-17600.0000000	1.5150000
-17599.0000000	1.5050000
-17598.0000000	1.5000000
-17597.0000000	1.5050000
-17596.0000000	1.5050000
-17595.0000000	1.5100000
-17594.0000000	1.5300000
-17593.0000000	1.5300000
-17592.0000000	1.5400000
-17591.0000000	1.5500000
-17590.0000000	1.5549999
-17589.0000000	1.5650001
-17588.0000000	1.5599999
-17587.0000000	1.5700001
-17586.0000000	1.5850000
-17585.0000000	1.5950000
-17584.0000000	1.5950000
-17583.0000000	1.5950000
-17582.0000000	1.5950000
-17581.0000000	1.5950000

-17580.0000000	1.5900000
-17579.0000000	1.5800000
-17578.0000000	1.5800000
-17577.0000000	1.5650001
-17576.0000000	1.5500000
-17575.0000000	1.5350000
-17574.0000000	1.5200000
-17573.0000000	1.5050000
-17572.0000000	1.5050000
-17571.0000000	1.5050000
-17570.0000000	1.5100000
-17569.0000000	1.5150000
-17568.0000000	1.5100000
-17567.0000000	1.5100000
-17566.0000000	1.5050000
-17565.0000000	1.5150000
-17564.0000000	1.5250000
-17563.0000000	1.5350000
-17562.0000000	1.5350000
-17561.0000000	1.5350000
-17560.0000000	1.5400000
-17559.0000000	1.5500000
-17558.0000000	1.5733334
-17557.0000000	1.5900000
-17556.0000000	1.6100000
-17555.0000000	1.6150000
-17554.0000000	1.6250000
-17553.0000000	1.6300000
-17552.0000000	1.6300000
-17551.0000000	1.6250000
-17550.0000000	1.6300000
-17549.0000000	1.6200000
-17548.0000000	1.6100000
-17547.0000000	1.5950000
-17546.0000000	1.6000000
-17545.0000000	1.5950000
-17544.0000000	1.5900000
-17543.0000000	1.5900000
-17542.0000000	1.5900000
-17541.0000000	1.5900000
-17540.0000000	1.5800000
-17539.0000000	1.5750000
-17538.0000000	1.5650001
-17537.0000000	1.5700001
-17536.0000000	1.5650001
-17535.0000000	1.5750000
-17534.0000000	1.5750000
-17533.0000000	1.5800000
-17532.0000000	1.5850000
-17531.0000000	1.5900000
-17530.0000000	1.6000000
-17529.0000000	1.6050000
-17528.0000000	1.6100000
-17527.0000000	1.6166667
-17526.0000000	1.6200000
-17525.0000000	1.6150000
-17524.0000000	1.6100000

-17523.0000000	1.5950000
-17522.0000000	1.5800000
-17521.0000000	1.5599999
-17520.0000000	1.5450000
-17519.0000000	1.5400000
-17518.0000000	1.5350000
-17517.0000000	1.5250000
-17516.0000000	1.5300000
-17515.0000000	1.5350000
-17514.0000000	1.5350000
-17513.0000000	1.5300000
-17512.0000000	1.5300000
-17511.0000000	1.5250000
-17510.0000000	1.5200000
-17509.0000000	1.5150000
-17508.0000000	1.5200000
-17507.0000000	1.5350000
-17506.0000000	1.5400000
-17505.0000000	1.5500000
-17504.0000000	1.5549999
-17503.0000000	1.5549999
-17502.0000000	1.5599999
-17501.0000000	1.5650001
-17500.0000000	1.5599999
-17499.0000000	1.5700001
-17498.0000000	1.5900000
-17497.0000000	1.6033334
-17496.0000000	1.6200000
-17495.0000000	1.6300000
-17494.0000000	1.6250000
-17493.0000000	1.6300000
-17492.0000000	1.6350000
-17491.0000000	1.6250000
-17490.0000000	1.6250000
-17489.0000000	1.6250000
-17488.0000000	1.6200000
-17487.0000000	1.6050000
-17486.0000000	1.6000000
-17485.0000000	1.5950000
-17484.0000000	1.5950000
-17483.0000000	1.5900000
-17482.0000000	1.5800000
-17481.0000000	1.5650001
-17480.0000000	1.5500000
-17479.0000000	1.5450000
-17478.0000000	1.5350000
-17477.0000000	1.5350000
-17476.0000000	1.5250000
-17475.0000000	1.5250000
-17474.0000000	1.5250000
-17473.0000000	1.5100000
-17472.0000000	1.5000000
-17471.0000000	1.5000000
-17470.0000000	1.4950000
-17469.0000000	1.5000000
-17468.0000000	1.5050000
-17467.0000000	1.4966666

-17466.0000000	1.5000000
-17465.0000000	1.5050000
-17464.0000000	1.5100000
-17463.0000000	1.5050000
-17462.0000000	1.5100000
-17461.0000000	1.5150000
-17460.0000000	1.5250000
-17459.0000000	1.5200000
-17458.0000000	1.5350000
-17457.0000000	1.5400000
-17456.0000000	1.5400000
-17455.0000000	1.5450000
-17454.0000000	1.5500000
-17453.0000000	1.5500000
-17452.0000000	1.5549999
-17451.0000000	1.5650001
-17450.0000000	1.5750000
-17449.0000000	1.5700001
-17448.0000000	1.5850000
-17447.0000000	1.5850000
-17446.0000000	1.5900000
-17445.0000000	1.5950000
-17444.0000000	1.6100000
-17443.0000000	1.6100000
-17442.0000000	1.6100000
-17441.0000000	1.6100000
-17440.0000000	1.6050000
-17439.0000000	1.6000000
-17438.0000000	1.5900000
-17437.0000000	1.5850000
-17436.0000000	1.5950000
-17435.0000000	1.5900000
-17434.0000000	1.5900000
-17433.0000000	1.5900000
-17432.0000000	1.6000000
-17431.0000000	1.5850000
-17430.0000000	1.5800000
-17429.0000000	1.5800000
-17428.0000000	1.5549999
-17427.0000000	1.5350000
-17426.0000000	1.5200000
-17425.0000000	1.5000000
-17424.0000000	1.4900000
-17423.0000000	1.4900000
-17422.0000000	1.4900000
-17421.0000000	1.4800000
-17420.0000000	1.4950000
-17419.0000000	1.5100000
-17418.0000000	1.5150000
-17417.0000000	1.5400000
-17416.0000000	1.5500000
-17415.0000000	1.5700001
-17414.0000000	1.5800000
-17413.0000000	1.5900000
-17412.0000000	1.6000000
-17411.0000000	1.6150000
-17410.0000000	1.6250000

-17409.0000000	1.6350000
-17408.0000000	1.6350000
-17407.0000000	1.6433333
-17406.0000000	1.6550000
-17405.0000000	1.6550000
-17404.0000000	1.6550000
-17403.0000000	1.6550000
-17402.0000000	1.6600000
-17401.0000000	1.6650000
-17400.0000000	1.6650000
-17399.0000000	1.6650000
-17398.0000000	1.6700000
-17397.0000000	1.6700000
-17396.0000000	1.6700000
-17395.0000000	1.6700000
-17394.0000000	1.6799999
-17393.0000000	1.6799999
-17392.0000000	1.6950001
-17391.0000000	1.6950001
-17390.0000000	1.6950001
-17389.0000000	1.6950001
-17388.0000000	1.6900001
-17387.0000000	1.6900001
-17386.0000000	1.6900001
-17385.0000000	1.6800001
-17384.0000000	1.6700000
-17383.0000000	1.6700000
-17382.0000000	1.6600000
-17381.0000000	1.6500000
-17380.0000000	1.6400000
-17379.0000000	1.6350000
-17378.0000000	1.6200000
-17377.0000000	1.6166667
-17376.0000000	1.6100000
-17375.0000000	1.6100000
-17374.0000000	1.6100000
-17373.0000000	1.6000000
-17372.0000000	1.5800000
-17371.0000000	1.5650001
-17370.0000000	1.5699999
-17369.0000000	1.5700001
-17368.0000000	1.5750000
-17367.0000000	1.5900000
-17366.0000000	1.6000000
-17365.0000000	1.6100000
-17364.0000000	1.6100000
-17363.0000000	1.6200000
-17362.0000000	1.6250000
-17361.0000000	1.6200000
-17360.0000000	1.6150000
-17359.0000000	1.6200000
-17358.0000000	1.6200000
-17357.0000000	1.6200000
-17356.0000000	1.6150000
-17355.0000000	1.6200000
-17354.0000000	1.6050000
-17353.0000000	1.5800000

-17352.0000000	1.5549999
-17351.0000000	1.5450000
-17350.0000000	1.5400000
-17349.0000000	1.5300000
-17348.0000000	1.5400000
-17347.0000000	1.5466666
-17346.0000000	1.5549999
-17345.0000000	1.5549999
-17344.0000000	1.5450000
-17343.0000000	1.5500000
-17342.0000000	1.5599999
-17341.0000000	1.5599999
-17340.0000000	1.5650001
-17339.0000000	1.5700001
-17338.0000000	1.5700001
-17337.0000000	1.5650001
-17336.0000000	1.5599999
-17335.0000000	1.5549999
-17334.0000000	1.5549999
-17333.0000000	1.5599999
-17332.0000000	1.5500000
-17331.0000000	1.5500000
-17330.0000000	1.5500000
-17329.0000000	1.5650001
-17328.0000000	1.5650001
-17327.0000000	1.5750000
-17326.0000000	1.5750000
-17325.0000000	1.5850000
-17324.0000000	1.5850000
-17323.0000000	1.6000000
-17322.0000000	1.6100000
-17321.0000000	1.6150000
-17320.0000000	1.6200000
-17319.0000000	1.6350000
-17318.0000000	1.6400000
-17317.0000000	1.6433333
-17316.0000000	1.6400000
-17315.0000000	1.6350000
-17314.0000000	1.6350000
-17313.0000000	1.6200000
-17312.0000000	1.6250000
-17311.0000000	1.6100000
-17310.0000000	1.6100000
-17309.0000000	1.6100000
-17308.0000000	1.6150000
-17307.0000000	1.6250000
-17306.0000000	1.6400000
-17305.0000000	1.6500000
-17304.0000000	1.6550000
-17303.0000000	1.6600000
-17302.0000000	1.6600000
-17301.0000000	1.6650000
-17300.0000000	1.6700000
-17299.0000000	1.6700000
-17298.0000000	1.6849999
-17297.0000000	1.7000000
-17296.0000000	1.7050000

-17295.0000000	1.7100000
-17294.0000000	1.7250000
-17293.0000000	1.7150000
-17292.0000000	1.7150000
-17291.0000000	1.7100000
-17290.0000000	1.7050000
-17289.0000000	1.7050000
-17288.0000000	1.6966667
-17287.0000000	1.6900001
-17286.0000000	1.6900001
-17285.0000000	1.6849999
-17284.0000000	1.6750000
-17283.0000000	1.6750000
-17282.0000000	1.6700000
-17281.0000000	1.6799999
-17280.0000000	1.6799999
-17279.0000000	1.6849999
-17278.0000000	1.6950001
-17277.0000000	1.7000000
-17276.0000000	1.6950001
-17275.0000000	1.6849999
-17274.0000000	1.6849999
-17273.0000000	1.6849999
-17272.0000000	1.6700000
-17271.0000000	1.6550000
-17270.0000000	1.6400000
-17269.0000000	1.6300000
-17268.0000000	1.6250000
-17267.0000000	1.6250000
-17266.0000000	1.6200000
-17265.0000000	1.6150000
-17264.0000000	1.6100000
-17263.0000000	1.6100000
-17262.0000000	1.6250000
-17261.0000000	1.6300000
-17260.0000000	1.6300000
-17259.0000000	1.6300000
-17258.0000000	1.6250000
-17257.0000000	1.6133333
-17256.0000000	1.6150000
-17255.0000000	1.6250000
-17254.0000000	1.6250000
-17253.0000000	1.6300000
-17252.0000000	1.6450000
-17251.0000000	1.6550000
-17250.0000000	1.6700000
-17249.0000000	1.6650000
-17248.0000000	1.6650000
-17247.0000000	1.6500000
-17246.0000000	1.6550000
-17245.0000000	1.6550000
-17244.0000000	1.6650000
-17243.0000000	1.6700000
-17242.0000000	1.6700000
-17241.0000000	1.6650000
-17240.0000000	1.6550000
-17239.0000000	1.6500000

-17238.0000000	1.6450000
-17237.0000000	1.6500000
-17236.0000000	1.6400000
-17235.0000000	1.6300000
-17234.0000000	1.6300000
-17233.0000000	1.6400000
-17232.0000000	1.6500000
-17231.0000000	1.6550000
-17230.0000000	1.6500000
-17229.0000000	1.6500000
-17228.0000000	1.6500000
-17227.0000000	1.6533333
-17226.0000000	1.6550000
-17225.0000000	1.6700000
-17224.0000000	1.6750000
-17223.0000000	1.6750000
-17222.0000000	1.6650000
-17221.0000000	1.6600000
-17220.0000000	1.6600000
-17219.0000000	1.6550000
-17218.0000000	1.6550000
-17217.0000000	1.6400000
-17216.0000000	1.6200000
-17215.0000000	1.6150000
-17214.0000000	1.6000000
-17213.0000000	1.5699999
-17212.0000000	1.5549999
-17211.0000000	1.5549999
-17210.0000000	1.5450000
-17209.0000000	1.5450000
-17208.0000000	1.5350000
-17207.0000000	1.5100000
-17206.0000000	1.5050000
-17205.0000000	1.5000000
-17204.0000000	1.5100000
-17203.0000000	1.5250000
-17202.0000000	1.5300000
-17201.0000000	1.5300000
-17200.0000000	1.5300000
-17199.0000000	1.5300000
-17198.0000000	1.5366665
-17197.0000000	1.5500000
-17196.0000000	1.5549999
-17195.0000000	1.5500000
-17194.0000000	1.5599999
-17193.0000000	1.5750000
-17192.0000000	1.5900000
-17191.0000000	1.6050000
-17190.0000000	1.6200000
-17189.0000000	1.6350000
-17188.0000000	1.6550000
-17187.0000000	1.6799999
-17186.0000000	1.6849999
-17185.0000000	1.7000000
-17184.0000000	1.7000000
-17183.0000000	1.7000000
-17182.0000000	1.6849999

-17181.0000000	1.6900001
-17180.0000000	1.7000000
-17179.0000000	1.7050000
-17178.0000000	1.7050000
-17177.0000000	1.7100000
-17176.0000000	1.7200000
-17175.0000000	1.7350000
-17174.0000000	1.7450000
-17173.0000000	1.7550000
-17172.0000000	1.7650000
-17171.0000000	1.7750000
-17170.0000000	1.7800000
-17169.0000000	1.7850000
-17168.0000000	1.7800000
-17167.0000000	1.7800001
-17166.0000000	1.7750000
-17165.0000000	1.7700000
-17164.0000000	1.7650000
-17163.0000000	1.7500000
-17162.0000000	1.7450000
-17161.0000000	1.7350000
-17160.0000000	1.7400000
-17159.0000000	1.7250000
-17158.0000000	1.7150000
-17157.0000000	1.7050000
-17156.0000000	1.7000000
-17155.0000000	1.7000000
-17154.0000000	1.6950001
-17153.0000000	1.6950001
-17152.0000000	1.6950001
-17151.0000000	1.6849999
-17150.0000000	1.6650000
-17149.0000000	1.6500000
-17148.0000000	1.6400000
-17147.0000000	1.6450000
-17146.0000000	1.6450000
-17145.0000000	1.6450000
-17144.0000000	1.6450000
-17143.0000000	1.6450000
-17142.0000000	1.6400000
-17141.0000000	1.6400000
-17140.0000000	1.6400000
-17139.0000000	1.6450000
-17138.0000000	1.6450000
-17137.0000000	1.6566666
-17136.0000000	1.6600000
-17135.0000000	1.6550000
-17134.0000000	1.6400000
-17133.0000000	1.6300000
-17132.0000000	1.6200000
-17131.0000000	1.6100000
-17130.0000000	1.6100000
-17129.0000000	1.6150000
-17128.0000000	1.6100000
-17127.0000000	1.6200000
-17126.0000000	1.6300000
-17125.0000000	1.6450000

-17124.0000000	1.6450000
-17123.0000000	1.6550000
-17122.0000000	1.6500000
-17121.0000000	1.6500000
-17120.0000000	1.6300000
-17119.0000000	1.6150000
-17118.0000000	1.6050000
-17117.0000000	1.6100000
-17116.0000000	1.6100000
-17115.0000000	1.6100000
-17114.0000000	1.6100000
-17113.0000000	1.6000000
-17112.0000000	1.6050000
-17111.0000000	1.6050000
-17110.0000000	1.6100000
-17109.0000000	1.6250000
-17108.0000000	1.6400000
-17107.0000000	1.6400000
-17106.0000000	1.6566666
-17105.0000000	1.6700000
-17104.0000000	1.6750000
-17103.0000000	1.6949999
-17102.0000000	1.7100000
-17101.0000000	1.7200000
-17100.0000000	1.7300000
-17099.0000000	1.7400000
-17098.0000000	1.7350000
-17097.0000000	1.7300000
-17096.0000000	1.7250000
-17095.0000000	1.7300000
-17094.0000000	1.7200000
-17093.0000000	1.7050000
-17092.0000000	1.7150000
-17091.0000000	1.7050000
-17090.0000000	1.7000000
-17089.0000000	1.7050000
-17088.0000000	1.7000000
-17087.0000000	1.6950001
-17086.0000000	1.7000000
-17085.0000000	1.6950001
-17084.0000000	1.7000000
-17083.0000000	1.7100000
-17082.0000000	1.7200000
-17081.0000000	1.7300000
-17080.0000000	1.7350000
-17079.0000000	1.7450000
-17078.0000000	1.7500000
-17077.0000000	1.7650000
-17076.0000000	1.7550000
-17075.0000000	1.7633333
-17074.0000000	1.7650000
-17073.0000000	1.7600000
-17072.0000000	1.7600000
-17071.0000000	1.7500000
-17070.0000000	1.7450000
-17069.0000000	1.7150000
-17068.0000000	1.7000000

-17067.0000000	1.6849999
-17066.0000000	1.6650000
-17065.0000000	1.6550000
-17064.0000000	1.6450000
-17063.0000000	1.6350000
-17062.0000000	1.6250000
-17061.0000000	1.6350000
-17060.0000000	1.6450000
-17059.0000000	1.6450000
-17058.0000000	1.6550000
-17057.0000000	1.6450000
-17056.0000000	1.6550000
-17055.0000000	1.6500000
-17054.0000000	1.6350000
-17053.0000000	1.6400000
-17052.0000000	1.6400000
-17051.0000000	1.6350000
-17050.0000000	1.6450000
-17049.0000000	1.6550000
-17048.0000000	1.6450000
-17047.0000000	1.6450000
-17046.0000000	1.6433333
-17045.0000000	1.6450000
-17044.0000000	1.6400000
-17043.0000000	1.6250000
-17042.0000000	1.6100000
-17041.0000000	1.6000000
-17040.0000000	1.5900000
-17039.0000000	1.5750000
-17038.0000000	1.5549999
-17037.0000000	1.5350000
-17036.0000000	1.5350000
-17035.0000000	1.5300000
-17034.0000000	1.5300000
-17033.0000000	1.5200000
-17032.0000000	1.5050000
-17031.0000000	1.5150000
-17030.0000000	1.5200000
-17029.0000000	1.5350000
-17028.0000000	1.5400000
-17027.0000000	1.5549999
-17026.0000000	1.5650001
-17025.0000000	1.5650001
-17024.0000000	1.5650001
-17023.0000000	1.5700001
-17022.0000000	1.5750000
-17021.0000000	1.5900000
-17020.0000000	1.6000000
-17019.0000000	1.6000000
-17018.0000000	1.6150000
-17017.0000000	1.6150000
-17016.0000000	1.6150000
-17015.0000000	1.6100000
-17014.0000000	1.5900000
-17013.0000000	1.5650001
-17012.0000000	1.5549999
-17011.0000000	1.5650001

-17010.0000000	1.5650001
-17009.0000000	1.5650001
-17008.0000000	1.5500000
-17007.0000000	1.5500000
-17006.0000000	1.5549999
-17005.0000000	1.5599999
-17004.0000000	1.5599999
-17003.0000000	1.5750000
-17002.0000000	1.5800000
-17001.0000000	1.5950000
-17000.0000000	1.5900000
-16999.0000000	1.5900000
-16998.0000000	1.5950000
-16997.0000000	1.6100000
-16996.0000000	1.6300000
-16995.0000000	1.6400000
-16994.0000000	1.6550000
-16993.0000000	1.6550000
-16992.0000000	1.6700000
-16991.0000000	1.6700000
-16990.0000000	1.6650000
-16989.0000000	1.6600000
-16988.0000000	1.6500000
-16987.0000000	1.6650000
-16986.0000000	1.6700000
-16985.0000000	1.6766666
-16984.0000000	1.6849999
-16983.0000000	1.6799999
-16982.0000000	1.6799999
-16981.0000000	1.6750000
-16980.0000000	1.6700000
-16979.0000000	1.6849999
-16978.0000000	1.6849999
-16977.0000000	1.6849999
-16976.0000000	1.6950001
-16975.0000000	1.7000000
-16974.0000000	1.7100000
-16973.0000000	1.7150000
-16972.0000000	1.7250000
-16971.0000000	1.7400000
-16970.0000000	1.7350000
-16969.0000000	1.7450000
-16968.0000000	1.7600000
-16967.0000000	1.7600000
-16966.0000000	1.7650000
-16965.0000000	1.7700000
-16964.0000000	1.7650000
-16963.0000000	1.7550000
-16962.0000000	1.7600000
-16961.0000000	1.7500000
-16960.0000000	1.7500000
-16959.0000000	1.7450000
-16958.0000000	1.7500000
-16957.0000000	1.7650000
-16956.0000000	1.7650000
-16955.0000000	1.7733332
-16954.0000000	1.7700000

-16953.0000000	1.7500000
-16952.0000000	1.7400000
-16951.0000000	1.7350000
-16950.0000000	1.7400000
-16949.0000000	1.7300000
-16948.0000000	1.7200000
-16947.0000000	1.7100000
-16946.0000000	1.7000000
-16945.0000000	1.6950001
-16944.0000000	1.6950001
-16943.0000000	1.6800001
-16942.0000000	1.6700000
-16941.0000000	1.6500000
-16940.0000000	1.6350000
-16939.0000000	1.6250000
-16938.0000000	1.6200000
-16937.0000000	1.6150000
-16936.0000000	1.6250000
-16935.0000000	1.6350000
-16934.0000000	1.6500000
-16933.0000000	1.6650000
-16932.0000000	1.6799999
-16931.0000000	1.6950001
-16930.0000000	1.7000000
-16929.0000000	1.7100000
-16928.0000000	1.7150000
-16927.0000000	1.7100000
-16926.0000000	1.7100000
-16925.0000000	1.7066666
-16924.0000000	1.6950001
-16923.0000000	1.6950001
-16922.0000000	1.6950001
-16921.0000000	1.7150000
-16920.0000000	1.7050000
-16919.0000000	1.6950001
-16918.0000000	1.6750000
-16917.0000000	1.6700000
-16916.0000000	1.6750000
-16915.0000000	1.6900001
-16914.0000000	1.7050000
-16913.0000000	1.7200000
-16912.0000000	1.7300000
-16911.0000000	1.7450000
-16910.0000000	1.7500000
-16909.0000000	1.7550000
-16908.0000000	1.7650000
-16907.0000000	1.7700000
-16906.0000000	1.7650000
-16905.0000000	1.7750000
-16904.0000000	1.7900000
-16903.0000000	1.7850000
-16902.0000000	1.7950000
-16901.0000000	1.8000000
-16900.0000000	1.7950000
-16899.0000000	1.7900000
-16898.0000000	1.7950000
-16897.0000000	1.8049999

-16896.0000000	1.8099999
-16895.0000000	1.8099999
-16894.0000000	1.8033333
-16893.0000000	1.7950000
-16892.0000000	1.8000000
-16891.0000000	1.8000000
-16890.0000000	1.8099999
-16889.0000000	1.8250000
-16888.0000000	1.8250000
-16887.0000000	1.8350000
-16886.0000000	1.8450000
-16885.0000000	1.8550000
-16884.0000000	1.8700000
-16883.0000000	1.8850000
-16882.0000000	1.8950000
-16881.0000000	1.9000000
-16880.0000000	1.9050000
-16879.0000000	1.9050000
-16878.0000000	1.9200000
-16877.0000000	1.9200000
-16876.0000000	1.9200000
-16875.0000000	1.9250000
-16874.0000000	1.9150000
-16873.0000000	1.9000000
-16872.0000000	1.9050000
-16871.0000000	1.9000000
-16870.0000000	1.8900000
-16869.0000000	1.8850000
-16868.0000000	1.8700000
-16867.0000000	1.8650000
-16866.0000000	1.8550000
-16865.0000000	1.8500000
-16864.0000000	1.8200001
-16863.0000000	1.7950000
-16862.0000000	1.7850000
-16861.0000000	1.7750000
-16860.0000000	1.7750000
-16859.0000000	1.7800000
-16858.0000000	1.7800000
-16857.0000000	1.7500000
-16856.0000000	1.7350000
-16855.0000000	1.7400000
-16854.0000000	1.7450000
-16853.0000000	1.7500000
-16852.0000000	1.7600000
-16851.0000000	1.7750000
-16850.0000000	1.7900000
-16849.0000000	1.8049999
-16848.0000000	1.8200001
-16847.0000000	1.8250000
-16846.0000000	1.8300000
-16845.0000000	1.8400000
-16844.0000000	1.8400000
-16843.0000000	1.8550000
-16842.0000000	1.8650000
-16841.0000000	1.8750000
-16840.0000000	1.8850000

-16839.0000000	1.8900000
-16838.0000000	1.8900000
-16837.0000000	1.8850000
-16836.0000000	1.8800000
-16835.0000000	1.8800000
-16834.0000000	1.8800000
-16833.0000000	1.8850000
-16832.0000000	1.8950000
-16831.0000000	1.9100000
-16830.0000000	1.9100000
-16829.0000000	1.9299999
-16828.0000000	1.9299999
-16827.0000000	1.9400001
-16826.0000000	1.9400001
-16825.0000000	1.9500000
-16824.0000000	1.9650000
-16823.0000000	1.9600000
-16822.0000000	1.9800000
-16821.0000000	1.9950000
-16820.0000000	2.0000000
-16819.0000000	2.0050001
-16818.0000000	2.0250001
-16817.0000000	2.0349998
-16816.0000000	2.0450001
-16815.0000000	2.0450001
-16814.0000000	2.0400000
-16813.0000000	2.0450001
-16812.0000000	2.0400000
-16811.0000000	2.0300000
-16810.0000000	2.0349998
-16809.0000000	2.0300000
-16808.0000000	2.0300000
-16807.0000000	2.0300000
-16806.0000000	2.0300000
-16805.0000000	2.0250001
-16804.0000000	2.0100000
-16803.0000000	2.0050001
-16802.0000000	1.9950000
-16801.0000000	1.9950000
-16800.0000000	1.9900000
-16799.0000000	1.9750000
-16798.0000000	1.9850000
-16797.0000000	1.9750000
-16796.0000000	1.9700000
-16795.0000000	1.9700000
-16794.0000000	1.9750000
-16793.0000000	1.9700000
-16792.0000000	1.9650000
-16791.0000000	1.9700000
-16790.0000000	1.9550000
-16789.0000000	1.9500000
-16788.0000000	1.9400001
-16787.0000000	1.9349999
-16786.0000000	1.9200000
-16785.0000000	1.9050000
-16784.0000000	1.8950000
-16783.0000000	1.8900000

-16782.0000000	1.8650000
-16781.0000000	1.8500000
-16780.0000000	1.8450000
-16779.0000000	1.8150001
-16778.0000000	1.8000000
-16777.0000000	1.7700000
-16776.0000000	1.7650000
-16775.0000000	1.7650000
-16774.0000000	1.7650000
-16773.0000000	1.7833333
-16772.0000000	1.7800000
-16771.0000000	1.7650000
-16770.0000000	1.7600000
-16769.0000000	1.7700000
-16768.0000000	1.7750000
-16767.0000000	1.7850000
-16766.0000000	1.7800000
-16765.0000000	1.7850000
-16764.0000000	1.8000000
-16763.0000000	1.8099999
-16762.0000000	1.8200001
-16761.0000000	1.8150001
-16760.0000000	1.8250000
-16759.0000000	1.8300000
-16758.0000000	1.8450000
-16757.0000000	1.8450000
-16756.0000000	1.8600000
-16755.0000000	1.8550000
-16754.0000000	1.8450000
-16753.0000000	1.8250000
-16752.0000000	1.8250000
-16751.0000000	1.8150001
-16750.0000000	1.8000000
-16749.0000000	1.8000000
-16748.0000000	1.8150001
-16747.0000000	1.8250000
-16746.0000000	1.8400000
-16745.0000000	1.8450000
-16744.0000000	1.8450000
-16743.0000000	1.8266667
-16742.0000000	1.8200001
-16741.0000000	1.8250000
-16740.0000000	1.8250000
-16739.0000000	1.8049999
-16738.0000000	1.7950000
-16737.0000000	1.7900000
-16736.0000000	1.7800000
-16735.0000000	1.7950000
-16734.0000000	1.8000000
-16733.0000000	1.8049999
-16732.0000000	1.8150001
-16731.0000000	1.8250000
-16730.0000000	1.8350000
-16729.0000000	1.8600000
-16728.0000000	1.8650000
-16727.0000000	1.8900000
-16726.0000000	1.8950000

-16725.0000000	1.9100000
-16724.0000000	1.9100000
-16723.0000000	1.9200000
-16722.0000000	1.9200000
-16721.0000000	1.9200000
-16720.0000000	1.9200000
-16719.0000000	1.9349999
-16718.0000000	1.9500000
-16717.0000000	1.9600000
-16716.0000000	1.9700000
-16715.0000000	1.9800000
-16714.0000000	1.9850000
-16713.0000000	1.9833332
-16712.0000000	1.9800000
-16711.0000000	1.9750000
-16710.0000000	1.9600000
-16709.0000000	1.9600000
-16708.0000000	1.9550000
-16707.0000000	1.9500000
-16706.0000000	1.9550000
-16705.0000000	1.9600000
-16704.0000000	1.9600000
-16703.0000000	1.9600000
-16702.0000000	1.9550000
-16701.0000000	1.9550000
-16700.0000000	1.9450001
-16699.0000000	1.9299999
-16698.0000000	1.9400001
-16697.0000000	1.9349999
-16696.0000000	1.9150000
-16695.0000000	1.8800000
-16694.0000000	1.8800000
-16693.0000000	1.8800000
-16692.0000000	1.8800000
-16691.0000000	1.8850000
-16690.0000000	1.8900000
-16689.0000000	1.8850000
-16688.0000000	1.8750000
-16687.0000000	1.8750000
-16686.0000000	1.8900000
-16685.0000000	1.8900000
-16684.0000000	1.8850000
-16683.0000000	1.8866667
-16682.0000000	1.8800000
-16681.0000000	1.8750000
-16680.0000000	1.8700000
-16679.0000000	1.8600000
-16678.0000000	1.8500000
-16677.0000000	1.8400000
-16676.0000000	1.8350000
-16675.0000000	1.8350000
-16674.0000000	1.8500000
-16673.0000000	1.8450000
-16672.0000000	1.8500000
-16671.0000000	1.8500000
-16670.0000000	1.8350000
-16669.0000000	1.8200001

-16668.0000000	1.8150001
-16667.0000000	1.8150001
-16666.0000000	1.8150001
-16665.0000000	1.8250000
-16664.0000000	1.8350000
-16663.0000000	1.8350000
-16662.0000000	1.8250000
-16661.0000000	1.8099999
-16660.0000000	1.7950000
-16659.0000000	1.7650000
-16658.0000000	1.7350000
-16657.0000000	1.7150000
-16656.0000000	1.7050000
-16655.0000000	1.7050000
-16654.0000000	1.7150000
-16653.0000000	1.7250000
-16652.0000000	1.7500000
-16651.0000000	1.7633333
-16650.0000000	1.7850000
-16649.0000000	1.7900000
-16648.0000000	1.7900000
-16647.0000000	1.7950000
-16646.0000000	1.7900000
-16645.0000000	1.8150001
-16644.0000000	1.8099999
-16643.0000000	1.8099999
-16642.0000000	1.8150001
-16641.0000000	1.8250000
-16640.0000000	1.8250000
-16639.0000000	1.8300000
-16638.0000000	1.8300000
-16637.0000000	1.8300000
-16636.0000000	1.8400000
-16635.0000000	1.8450000
-16634.0000000	1.8450000
-16633.0000000	1.8450000
-16632.0000000	1.8350000
-16631.0000000	1.8350000
-16630.0000000	1.8400000
-16629.0000000	1.8500000
-16628.0000000	1.8550000
-16627.0000000	1.8500000
-16626.0000000	1.8650000
-16625.0000000	1.8650000
-16624.0000000	1.8750000
-16623.0000000	1.8800000
-16622.0000000	1.8750000
-16621.0000000	1.8833333
-16620.0000000	1.8950000
-16619.0000000	1.9000000
-16618.0000000	1.9050000
-16617.0000000	1.9100000
-16616.0000000	1.9100000
-16615.0000000	1.9150000
-16614.0000000	1.9100000
-16613.0000000	1.9150000
-16612.0000000	1.9100000

-16611.0000000	1.9100000
-16610.0000000	1.9000000
-16609.0000000	1.9000000
-16608.0000000	1.9100000
-16607.0000000	1.9100000
-16606.0000000	1.9100000
-16605.0000000	1.8950000
-16604.0000000	1.8850000
-16603.0000000	1.8750000
-16602.0000000	1.8700000
-16601.0000000	1.8550000
-16600.0000000	1.8600000
-16599.0000000	1.8700000
-16598.0000000	1.8700000
-16597.0000000	1.8700000
-16596.0000000	1.8750000
-16595.0000000	1.8800000
-16594.0000000	1.9000000
-16593.0000000	1.9000000
-16592.0000000	1.9100000
-16591.0000000	1.9266666
-16590.0000000	1.9500000
-16589.0000000	1.9500000
-16588.0000000	1.9550000
-16587.0000000	1.9700000
-16586.0000000	1.9600000
-16585.0000000	1.9750000
-16584.0000000	1.9850000
-16583.0000000	1.9900000
-16582.0000000	1.9900000
-16581.0000000	1.9850000
-16580.0000000	1.9900000
-16579.0000000	1.9900000
-16578.0000000	1.9900000
-16577.0000000	1.9700000
-16576.0000000	1.9650000
-16575.0000000	1.9600000
-16574.0000000	1.9600000
-16573.0000000	1.9600000
-16572.0000000	1.9650000
-16571.0000000	1.9750000
-16570.0000000	1.9750000
-16569.0000000	1.9800000
-16568.0000000	1.9750000
-16567.0000000	1.9750000
-16566.0000000	1.9850000
-16565.0000000	1.9800000
-16564.0000000	1.9950000
-16563.0000000	1.9900000
-16562.0000000	2.0000000
-16561.0000000	2.0033333
-16560.0000000	2.0149999
-16559.0000000	2.0149999
-16558.0000000	2.0100000
-16557.0000000	2.0200000
-16556.0000000	2.0250001
-16555.0000000	2.0250001

-16554.0000000	2.0200000
-16553.0000000	2.0300000
-16552.0000000	2.0200000
-16551.0000000	2.0149999
-16550.0000000	2.0250001
-16549.0000000	2.0349998
-16548.0000000	2.0349998
-16547.0000000	2.0400000
-16546.0000000	2.0400000
-16545.0000000	2.0400000
-16544.0000000	2.0450001
-16543.0000000	2.0349998
-16542.0000000	2.0300000
-16541.0000000	2.0300000
-16540.0000000	2.0250001
-16539.0000000	2.0149999
-16538.0000000	2.0149999
-16537.0000000	2.0050001
-16536.0000000	2.0000000
-16535.0000000	1.9750000
-16534.0000000	1.9650000
-16533.0000000	1.9500000
-16532.0000000	1.9300001
-16531.0000000	1.9100000
-16530.0000000	1.9050000
-16529.0000000	1.9150000
-16528.0000000	1.9050000
-16527.0000000	1.9050000
-16526.0000000	1.9050000
-16525.0000000	1.9000000
-16524.0000000	1.8800000
-16523.0000000	1.8750000
-16522.0000000	1.8550000
-16521.0000000	1.8300000
-16520.0000000	1.8049999
-16519.0000000	1.8049999
-16518.0000000	1.8099999
-16517.0000000	1.8200001
-16516.0000000	1.8350000
-16515.0000000	1.8450000
-16514.0000000	1.8450000
-16513.0000000	1.8400000
-16512.0000000	1.8250000
-16511.0000000	1.8250000
-16510.0000000	1.8000000
-16509.0000000	1.7750000
-16508.0000000	1.7550000
-16507.0000000	1.7700000
-16506.0000000	1.7850000
-16505.0000000	1.8049999
-16504.0000000	1.8250000
-16503.0000000	1.8400000
-16502.0000000	1.8500000
-16501.0000000	1.8650000
-16500.0000000	1.8900000
-16499.0000000	1.9050000
-16498.0000000	1.9150000

-16497.0000000	1.9299999
-16496.0000000	1.9400001
-16495.0000000	1.9400001
-16494.0000000	1.9400001
-16493.0000000	1.9450001
-16492.0000000	1.9400001
-16491.0000000	1.9200000
-16490.0000000	1.9200000
-16489.0000000	1.9050000
-16488.0000000	1.8900000
-16487.0000000	1.8550000
-16486.0000000	1.8400000
-16485.0000000	1.8350000
-16484.0000000	1.8350000
-16483.0000000	1.8550000
-16482.0000000	1.8650000
-16481.0000000	1.8750000
-16480.0000000	1.9000000
-16479.0000000	1.9050000
-16478.0000000	1.9100000
-16477.0000000	1.9150000
-16476.0000000	1.9200000
-16475.0000000	1.9150000
-16474.0000000	1.9250000
-16473.0000000	1.9250000
-16472.0000000	1.9133333
-16471.0000000	1.8950000
-16470.0000000	1.8900000
-16469.0000000	1.8900000
-16468.0000000	1.8750000
-16467.0000000	1.8700000
-16466.0000000	1.8750000
-16465.0000000	1.8650000
-16464.0000000	1.8650000
-16463.0000000	1.8700000
-16462.0000000	1.8800000
-16461.0000000	1.8750000
-16460.0000000	1.8850000
-16459.0000000	1.8900000
-16458.0000000	1.8950000
-16457.0000000	1.8900000
-16456.0000000	1.8950000
-16455.0000000	1.8850000
-16454.0000000	1.8900000
-16453.0000000	1.8850000
-16452.0000000	1.8650000
-16451.0000000	1.8450000
-16450.0000000	1.8300000
-16449.0000000	1.8350000
-16448.0000000	1.8300000
-16447.0000000	1.8500000
-16446.0000000	1.8650000
-16445.0000000	1.8800000
-16444.0000000	1.8950000
-16443.0000000	1.9100000
-16442.0000000	1.9033333
-16441.0000000	1.9050000

-16440.0000000	1.9200000
-16439.0000000	1.9150000
-16438.0000000	1.9150000
-16437.0000000	1.9100000
-16436.0000000	1.9050000
-16435.0000000	1.8800000
-16434.0000000	1.8850000
-16433.0000000	1.8850000
-16432.0000000	1.8750000
-16431.0000000	1.8700000
-16430.0000000	1.8650000
-16429.0000000	1.8750000
-16428.0000000	1.8800000
-16427.0000000	1.9000000
-16426.0000000	1.9100000
-16425.0000000	1.9200000
-16424.0000000	1.9150000
-16423.0000000	1.9150000
-16422.0000000	1.9349999
-16421.0000000	1.9400001
-16420.0000000	1.9349999
-16419.0000000	1.9450001
-16418.0000000	1.9600000
-16417.0000000	1.9600000
-16416.0000000	1.9750000
-16415.0000000	1.9850000
-16414.0000000	2.0000000
-16413.0000000	2.0100000
-16412.0000000	2.0200000
-16411.0000000	2.0133333
-16410.0000000	2.0100000
-16409.0000000	2.0149999
-16408.0000000	2.0200000
-16407.0000000	2.0250001
-16406.0000000	2.0400000
-16405.0000000	2.0450001
-16404.0000000	2.0400000
-16403.0000000	2.0349998
-16402.0000000	2.0200000
-16401.0000000	2.0149999
-16400.0000000	2.0050001
-16399.0000000	1.9950000
-16398.0000000	1.9850000
-16397.0000000	1.9550000
-16396.0000000	1.9500000
-16395.0000000	1.9500000
-16394.0000000	1.9450001
-16393.0000000	1.9600000
-16392.0000000	1.9800000
-16391.0000000	1.9900000
-16390.0000000	2.0050001
-16389.0000000	2.0149999
-16388.0000000	2.0149999
-16387.0000000	2.0000000
-16386.0000000	1.9800000
-16385.0000000	1.9600000
-16384.0000000	1.9650000

-16383.0000000	1.9600000
-16382.0000000	1.9500000
-16381.0000000	1.9500000
-16380.0000000	1.9400001
-16379.0000000	1.9450001
-16378.0000000	1.9550000
-16377.0000000	1.9500000
-16376.0000000	1.9550000
-16375.0000000	1.9550000
-16374.0000000	1.9500000
-16373.0000000	1.9500000
-16372.0000000	1.9500000
-16371.0000000	1.9550000
-16370.0000000	1.9550000
-16369.0000000	1.9500000
-16368.0000000	1.9550000
-16367.0000000	1.9550000
-16366.0000000	1.9450001
-16365.0000000	1.9250000
-16364.0000000	1.9000000
-16363.0000000	1.9000000
-16362.0000000	1.8950000
-16361.0000000	1.9050000
-16360.0000000	1.9050000
-16359.0000000	1.9000000
-16358.0000000	1.9150000
-16357.0000000	1.9250000
-16356.0000000	1.9299999
-16355.0000000	1.9400001
-16354.0000000	1.9500000
-16353.0000000	1.9400001
-16352.0000000	1.9450001
-16351.0000000	1.9550000
-16350.0000000	1.9600000
-16349.0000000	1.9600000
-16348.0000000	1.9600000
-16347.0000000	1.9500000
-16346.0000000	1.9450001
-16345.0000000	1.9550000
-16344.0000000	1.9800000
-16343.0000000	1.9950000
-16342.0000000	2.0149999
-16341.0000000	2.0300000
-16340.0000000	2.0500000
-16339.0000000	2.0799999
-16338.0000000	2.0949998
-16337.0000000	2.1149998
-16336.0000000	2.1300001
-16335.0000000	2.1300001
-16334.0000000	2.1450000
-16333.0000000	2.1500001
-16332.0000000	2.1600001
-16331.0000000	2.1550002
-16330.0000000	2.1500001
-16329.0000000	2.1250000
-16328.0000000	2.1250000
-16327.0000000	2.1149998

-16326.0000000	2.1050000
-16325.0000000	2.0999999
-16324.0000000	2.0999999
-16323.0000000	2.0999999
-16322.0000000	2.1050000
-16321.0000000	2.1099999
-16320.0000000	2.1050000
-16319.0000000	2.0966666
-16318.0000000	2.0999999
-16317.0000000	2.1050000
-16316.0000000	2.1099999
-16315.0000000	2.1149998
-16314.0000000	2.1050000
-16313.0000000	2.0999999
-16312.0000000	2.0850000
-16311.0000000	2.0850000
-16310.0000000	2.0899999
-16309.0000000	2.0899999
-16308.0000000	2.0949998
-16307.0000000	2.0749998
-16306.0000000	2.0650001
-16305.0000000	2.0450001
-16304.0000000	2.0500000
-16303.0000000	2.0599999
-16302.0000000	2.0699999
-16301.0000000	2.0749998
-16300.0000000	2.0899999
-16299.0000000	2.0899999
-16298.0000000	2.1099999
-16297.0000000	2.1099999
-16296.0000000	2.1149998
-16295.0000000	2.1250000
-16294.0000000	2.1250000
-16293.0000000	2.1350002
-16292.0000000	2.1400001
-16291.0000000	2.1450000
-16290.0000000	2.1500001
-16289.0000000	2.1600001
-16288.0000000	2.1650000
-16287.0000000	2.1700001
-16286.0000000	2.1700001
-16285.0000000	2.1750002
-16284.0000000	2.1800001
-16283.0000000	2.1900001
-16282.0000000	2.1950002
-16281.0000000	2.1950002
-16280.0000000	2.2049999
-16279.0000000	2.1900001
-16278.0000000	2.1900001
-16277.0000000	2.1849999
-16276.0000000	2.1950002
-16275.0000000	2.1950002
-16274.0000000	2.2000000
-16273.0000000	2.2150002
-16272.0000000	2.2200000
-16271.0000000	2.2200000
-16270.0000000	2.2300000

-16269.0000000	2.2300000
-16268.0000000	2.2300000
-16267.0000000	2.2350001
-16266.0000000	2.2449999
-16265.0000000	2.2400000
-16264.0000000	2.2400000
-16263.0000000	2.2300000
-16262.0000000	2.2249999
-16261.0000000	2.2049999
-16260.0000000	2.2100000
-16259.0000000	2.2100000
-16258.0000000	2.2150002
-16257.0000000	2.2200000
-16256.0000000	2.2350001
-16255.0000000	2.2350001
-16254.0000000	2.2249999
-16253.0000000	2.2249999
-16252.0000000	2.2300000
-16251.0000000	2.2400000
-16250.0000000	2.2300000
-16249.0000000	2.2249999
-16248.0000000	2.2200000
-16247.0000000	2.2300000
-16246.0000000	2.2350001
-16245.0000000	2.2300000
-16244.0000000	2.2350001
-16243.0000000	2.2400000
-16242.0000000	2.2400000
-16241.0000000	2.2400000
-16240.0000000	2.2350001
-16239.0000000	2.2300000
-16238.0000000	2.2200000
-16237.0000000	2.1800001
-16236.0000000	2.1600001
-16235.0000000	2.1650000
-16234.0000000	2.1600001
-16233.0000000	2.1700001
-16232.0000000	2.1849999
-16231.0000000	2.1950002
-16230.0000000	2.1900001
-16229.0000000	2.2100000
-16228.0000000	2.2150002
-16227.0000000	2.2249999
-16226.0000000	2.2350001
-16225.0000000	2.2400000
-16224.0000000	2.2400000
-16223.0000000	2.2449999
-16222.0000000	2.2500000
-16221.0000000	2.2500000
-16220.0000000	2.2449999
-16219.0000000	2.2600000
-16218.0000000	2.2600000
-16217.0000000	2.2400000
-16216.0000000	2.2350001
-16215.0000000	2.2200000
-16214.0000000	2.2100000
-16213.0000000	2.1950002

-16212.0000000	2.1750002
-16211.0000000	2.1700001
-16210.0000000	2.1500001
-16209.0000000	2.1350002
-16208.0000000	2.1199999
-16207.0000000	2.1250000
-16206.0000000	2.1099999
-16205.0000000	2.1149998
-16204.0000000	2.1050000
-16203.0000000	2.0899999
-16202.0000000	2.0850000
-16201.0000000	2.0699999
-16200.0000000	2.0650001
-16199.0000000	2.0466666
-16198.0000000	2.0349998
-16197.0000000	2.0200000
-16196.0000000	1.9900000
-16195.0000000	1.9349999
-16194.0000000	1.8900000
-16193.0000000	1.8750000
-16192.0000000	1.8850000
-16191.0000000	1.9050000
-16190.0000000	1.9050000
-16189.0000000	1.9050000
-16188.0000000	1.9200000
-16187.0000000	1.9250000
-16186.0000000	1.9349999
-16185.0000000	1.9400001
-16184.0000000	1.9450001
-16183.0000000	1.9400001
-16182.0000000	1.9200000
-16181.0000000	1.9100000
-16180.0000000	1.8800000
-16179.0000000	1.8650000
-16178.0000000	1.8650000
-16177.0000000	1.8650000
-16176.0000000	1.8850000
-16175.0000000	1.8900000
-16174.0000000	1.8950000
-16173.0000000	1.9000000
-16172.0000000	1.8900000
-16171.0000000	1.8850000
-16170.0000000	1.8833333
-16169.0000000	1.8900000
-16168.0000000	1.8900000
-16167.0000000	1.8900000
-16166.0000000	1.8950000
-16165.0000000	1.9000000
-16164.0000000	1.8950000
-16163.0000000	1.8950000
-16162.0000000	1.8800000
-16161.0000000	1.8700000
-16160.0000000	1.8650000
-16159.0000000	1.8800000
-16158.0000000	1.8900000
-16157.0000000	1.9050000
-16156.0000000	1.9100000

-16155.0000000	1.9150000
-16154.0000000	1.9150000
-16153.0000000	1.9250000
-16152.0000000	1.9349999
-16151.0000000	1.9450001
-16150.0000000	1.9500000
-16149.0000000	1.9500000
-16148.0000000	1.9600000
-16147.0000000	1.9700000
-16146.0000000	1.9950000
-16145.0000000	2.0100000
-16144.0000000	2.0349998
-16143.0000000	2.0450001
-16142.0000000	2.0450001
-16141.0000000	2.0450001
-16140.0000000	2.0333331
-16139.0000000	2.0250001
-16138.0000000	2.0100000
-16137.0000000	2.0000000
-16136.0000000	1.9900000
-16135.0000000	1.9950000
-16134.0000000	2.0000000
-16133.0000000	2.0000000
-16132.0000000	2.0050001
-16131.0000000	2.0200000
-16130.0000000	2.0349998
-16129.0000000	2.0400000
-16128.0000000	2.0450001
-16127.0000000	2.0400000
-16126.0000000	2.0250001
-16125.0000000	2.0250001
-16124.0000000	2.0149999
-16123.0000000	2.0149999
-16122.0000000	2.0200000
-16121.0000000	2.0149999
-16120.0000000	2.0300000
-16119.0000000	2.0300000
-16118.0000000	2.0450001
-16117.0000000	2.0450001
-16116.0000000	2.0450001
-16115.0000000	2.0300000
-16114.0000000	2.0100000
-16113.0000000	1.9950000
-16112.0000000	1.9900000
-16111.0000000	1.9850000
-16110.0000000	1.9666667
-16109.0000000	1.9500000
-16108.0000000	1.9500000
-16107.0000000	1.9299999
-16106.0000000	1.9300001
-16105.0000000	1.9299999
-16104.0000000	1.9349999
-16103.0000000	1.9349999
-16102.0000000	1.9500000
-16101.0000000	1.9700000
-16100.0000000	1.9750000
-16099.0000000	1.9900000

-16098.0000000	2.0050001
-16097.0000000	2.0300000
-16096.0000000	2.0400000
-16095.0000000	2.0549998
-16094.0000000	2.0599999
-16093.0000000	2.0599999
-16092.0000000	2.0699999
-16091.0000000	2.0650001
-16090.0000000	2.0699999
-16089.0000000	2.0749998
-16088.0000000	2.0749998
-16087.0000000	2.0850000
-16086.0000000	2.0850000
-16085.0000000	2.0899999
-16084.0000000	2.0949998
-16083.0000000	2.0999999
-16082.0000000	2.0899999
-16081.0000000	2.0899999
-16080.0000000	2.0833333
-16079.0000000	2.0699999
-16078.0000000	2.0599999
-16077.0000000	2.0450001
-16076.0000000	2.0200000
-16075.0000000	2.0050001
-16074.0000000	2.0050001
-16073.0000000	2.0200000
-16072.0000000	2.0250001
-16071.0000000	2.0200000
-16070.0000000	2.0200000
-16069.0000000	2.0100000
-16068.0000000	2.0149999
-16067.0000000	2.0149999
-16066.0000000	2.0250001
-16065.0000000	2.0349998
-16064.0000000	2.0500000
-16063.0000000	2.0650001
-16062.0000000	2.0699999
-16061.0000000	2.0999999
-16060.0000000	2.1050000
-16059.0000000	2.1250000
-16058.0000000	2.1250000
-16057.0000000	2.1300001
-16056.0000000	2.1450000
-16055.0000000	2.1450000
-16054.0000000	2.1550002
-16053.0000000	2.1500001
-16052.0000000	2.1550002
-16051.0000000	2.1500001
-16050.0000000	2.1500001
-16049.0000000	2.1533334
-16048.0000000	2.1500001
-16047.0000000	2.1366668
-16046.0000000	2.1250000
-16045.0000000	2.1099999
-16044.0000000	2.0999999
-16043.0000000	2.0850000
-16042.0000000	2.0799999

-16041.0000000	2.0699999
-16040.0000000	2.0699999
-16039.0000000	2.0749998
-16038.0000000	2.0850000
-16037.0000000	2.0899999
-16036.0000000	2.0899999
-16035.0000000	2.0850000
-16034.0000000	2.0949998
-16033.0000000	2.0949998
-16032.0000000	2.0949998
-16031.0000000	2.0899999
-16030.0000000	2.0949998
-16029.0000000	2.0949998
-16028.0000000	2.0999999
-16027.0000000	2.0949998
-16026.0000000	2.0999999
-16025.0000000	2.0899999
-16024.0000000	2.0850000
-16023.0000000	2.0799999
-16022.0000000	2.0699999
-16021.0000000	2.0549998
-16020.0000000	2.0349998
-16019.0000000	2.0100000
-16018.0000000	2.0033333
-16017.0000000	1.9850000
-16016.0000000	1.9750000
-16015.0000000	1.9850000
-16014.0000000	2.0000000
-16013.0000000	2.0149999
-16012.0000000	2.0250001
-16011.0000000	2.0300000
-16010.0000000	2.0200000
-16009.0000000	2.0250001
-16008.0000000	2.0200000
-16007.0000000	2.0100000
-16006.0000000	2.0000000
-16005.0000000	1.9750000
-16004.0000000	1.9550000
-16003.0000000	1.9500000
-16002.0000000	1.9299999
-16001.0000000	1.9250000
-16000.0000000	1.9200000
-15999.0000000	1.9200000
-15998.0000000	1.9200000
-15997.0000000	1.9349999
-15996.0000000	1.9400001
-15995.0000000	1.9500000
-15994.0000000	1.9600000
-15993.0000000	1.9600000
-15992.0000000	1.9600000
-15991.0000000	1.9600000
-15990.0000000	1.9349999
-15989.0000000	1.9200000
-15988.0000000	1.9200000
-15987.0000000	1.9333334
-15986.0000000	1.9500000
-15985.0000000	1.9650000

-15984.0000000	1.9750000
-15983.0000000	1.9800000
-15982.0000000	1.9800000
-15981.0000000	1.9800000
-15980.0000000	1.9850000
-15979.0000000	1.9900000
-15978.0000000	1.9900000
-15977.0000000	1.9850000
-15976.0000000	1.9800000
-15975.0000000	1.9900000
-15974.0000000	1.9950000
-15973.0000000	1.9950000
-15972.0000000	2.0149999
-15971.0000000	2.0050001
-15970.0000000	1.9900000
-15969.0000000	1.9900000
-15968.0000000	1.9850000
-15967.0000000	1.9750000
-15966.0000000	1.9700000
-15965.0000000	1.9750000
-15964.0000000	1.9750000
-15963.0000000	1.9750000
-15962.0000000	1.9700000
-15961.0000000	1.9650000
-15960.0000000	1.9700000
-15959.0000000	1.9700000
-15958.0000000	1.9766668
-15957.0000000	1.9750000
-15956.0000000	1.9800000
-15955.0000000	1.9800000
-15954.0000000	1.9750000
-15953.0000000	1.9600000
-15952.0000000	1.9500000
-15951.0000000	1.9349999
-15950.0000000	1.9349999
-15949.0000000	1.9400001
-15948.0000000	1.9500000
-15947.0000000	1.9650000
-15946.0000000	1.9750000
-15945.0000000	1.9700000
-15944.0000000	1.9650000
-15943.0000000	1.9600000
-15942.0000000	1.9550000
-15941.0000000	1.9600000
-15940.0000000	1.9650000
-15939.0000000	1.9800000
-15938.0000000	1.9850000
-15937.0000000	1.9900000
-15936.0000000	1.9950000
-15935.0000000	2.0149999
-15934.0000000	2.0300000
-15933.0000000	2.0450001
-15932.0000000	2.0500000
-15931.0000000	2.0549998
-15930.0000000	2.0599999
-15929.0000000	2.0566666
-15928.0000000	2.0500000

-15927.0000000	2.0400000
-15926.0000000	2.0200000
-15925.0000000	2.0050001
-15924.0000000	2.0100000
-15923.0000000	2.0250001
-15922.0000000	2.0349998
-15921.0000000	2.0400000
-15920.0000000	2.0500000
-15919.0000000	2.0450001
-15918.0000000	2.0500000
-15917.0000000	2.0500000
-15916.0000000	2.0500000
-15915.0000000	2.0500000
-15914.0000000	2.0400000
-15913.0000000	2.0250001
-15912.0000000	2.0149999
-15911.0000000	2.0000000
-15910.0000000	1.9850000
-15909.0000000	1.9850000
-15908.0000000	1.9900000
-15907.0000000	1.9700000
-15906.0000000	1.9700000
-15905.0000000	1.9650000
-15904.0000000	1.9700000
-15903.0000000	1.9700000
-15902.0000000	1.9900000
-15901.0000000	2.0000000
-15900.0000000	2.0149999
-15899.0000000	2.0200000
-15898.0000000	2.0300000
-15897.0000000	2.0400000
-15896.0000000	2.0450001
-15895.0000000	2.0500000
-15894.0000000	2.0599999
-15893.0000000	2.0650001
-15892.0000000	2.0400000
-15891.0000000	2.0400000
-15890.0000000	2.0450001
-15889.0000000	2.0400000
-15888.0000000	2.0349998
-15887.0000000	2.0400000
-15886.0000000	2.0650001
-15885.0000000	2.0650001
-15884.0000000	2.0699999
-15883.0000000	2.0799999
-15882.0000000	2.0850000
-15881.0000000	2.0850000
-15880.0000000	2.0799999
-15879.0000000	2.0699999
-15878.0000000	2.0650001
-15877.0000000	2.0349998
-15876.0000000	1.9850000
-15875.0000000	1.9500000
-15874.0000000	1.9250000
-15873.0000000	1.9100000
-15872.0000000	1.9050000
-15871.0000000	1.9050000

-15870.0000000	1.9100000
-15869.0000000	1.9250000
-15868.0000000	1.9399999
-15867.0000000	1.9650000
-15866.0000000	1.9800000
-15865.0000000	1.9950000
-15864.0000000	2.0149999
-15863.0000000	2.0050001
-15862.0000000	2.0000000
-15861.0000000	1.9900000
-15860.0000000	1.9850000
-15859.0000000	1.9950000
-15858.0000000	2.0000000
-15857.0000000	2.0000000
-15856.0000000	2.0050001
-15855.0000000	2.0050001
-15854.0000000	2.0149999
-15853.0000000	2.0050001
-15852.0000000	2.0000000
-15851.0000000	2.0000000
-15850.0000000	1.9900000
-15849.0000000	1.9900000
-15848.0000000	1.9900000
-15847.0000000	2.0050001
-15846.0000000	2.0200000
-15845.0000000	2.0400000
-15844.0000000	2.0599999
-15843.0000000	2.0799999
-15842.0000000	2.0999999
-15841.0000000	2.1250000
-15840.0000000	2.1350002
-15839.0000000	2.1450000
-15838.0000000	2.1450000
-15837.0000000	2.1500001
-15836.0000000	2.1533334
-15835.0000000	2.1400001
-15834.0000000	2.1400001
-15833.0000000	2.1300001
-15832.0000000	2.1199999
-15831.0000000	2.0949998
-15830.0000000	2.0699999
-15829.0000000	2.0650001
-15828.0000000	2.0699999
-15827.0000000	2.0899999
-15826.0000000	2.0999999
-15825.0000000	2.0899999
-15824.0000000	2.1099999
-15823.0000000	2.1300001
-15822.0000000	2.1550002
-15821.0000000	2.1650000
-15820.0000000	2.1650000
-15819.0000000	2.1750002
-15818.0000000	2.1800001
-15817.0000000	2.1950002
-15816.0000000	2.1950002
-15815.0000000	2.1849999
-15814.0000000	2.1700001

-15813.0000000	2.1800001
-15812.0000000	2.1800001
-15811.0000000	2.1700001
-15810.0000000	2.1700001
-15809.0000000	2.1700001
-15808.0000000	2.1650000
-15807.0000000	2.1650000
-15806.0000000	2.1566668
-15805.0000000	2.1650000
-15804.0000000	2.1750002
-15803.0000000	2.1900001
-15802.0000000	2.1950002
-15801.0000000	2.2049999
-15800.0000000	2.2000000
-15799.0000000	2.2049999
-15798.0000000	2.2000000
-15797.0000000	2.1950002
-15796.0000000	2.1950002
-15795.0000000	2.1950002
-15794.0000000	2.1950002
-15793.0000000	2.2049999
-15792.0000000	2.2100000
-15791.0000000	2.2049999
-15790.0000000	2.2100000
-15789.0000000	2.2150002
-15788.0000000	2.2150002
-15787.0000000	2.2200000
-15786.0000000	2.2150002
-15785.0000000	2.2249999
-15784.0000000	2.2350001
-15783.0000000	2.2300000
-15782.0000000	2.2300000
-15781.0000000	2.2249999
-15780.0000000	2.2200000
-15779.0000000	2.2350001
-15778.0000000	2.2350001
-15777.0000000	2.2400000
-15776.0000000	2.2400000
-15775.0000000	2.2300000
-15774.0000000	2.2150002
-15773.0000000	2.2100000
-15772.0000000	2.1900001
-15771.0000000	2.1750002
-15770.0000000	2.1450000
-15769.0000000	2.1199999
-15768.0000000	2.0999999
-15767.0000000	2.0949998
-15766.0000000	2.0850000
-15765.0000000	2.0850000
-15764.0000000	2.0949998
-15763.0000000	2.0949998
-15762.0000000	2.0899999
-15761.0000000	2.0899999
-15760.0000000	2.0850000
-15759.0000000	2.0799999
-15758.0000000	2.0749998
-15757.0000000	2.0450001

-15756.0000000	2.0250001
-15755.0000000	2.0100000
-15754.0000000	2.0149999
-15753.0000000	2.0200000
-15752.0000000	2.0450001
-15751.0000000	2.0549998
-15750.0000000	2.0599999
-15749.0000000	2.0599999
-15748.0000000	2.0699999
-15747.0000000	2.0733330
-15746.0000000	2.0899999
-15745.0000000	2.1050000
-15744.0000000	2.1050000
-15743.0000000	2.1149998
-15742.0000000	2.1149998
-15741.0000000	2.1149998
-15740.0000000	2.1099999
-15739.0000000	2.1050000
-15738.0000000	2.0850000
-15737.0000000	2.0799999
-15736.0000000	2.0749998
-15735.0000000	2.0450001
-15734.0000000	2.0250001
-15733.0000000	2.0149999
-15732.0000000	1.9850000
-15731.0000000	1.9700000
-15730.0000000	1.9750000
-15729.0000000	1.9800000
-15728.0000000	1.9900000
-15727.0000000	1.9950000
-15726.0000000	2.0149999
-15725.0000000	2.0250001
-15724.0000000	2.0400000
-15723.0000000	2.0599999
-15722.0000000	2.0749998
-15721.0000000	2.0949998
-15720.0000000	2.1199999
-15719.0000000	2.1350002
-15718.0000000	2.1500001
-15717.0000000	2.1366668
-15716.0000000	2.1199999
-15715.0000000	2.1149998
-15714.0000000	2.1050000
-15713.0000000	2.1099999
-15712.0000000	2.1199999
-15711.0000000	2.1250000
-15710.0000000	2.1199999
-15709.0000000	2.1350002
-15708.0000000	2.1500001
-15707.0000000	2.1550002
-15706.0000000	2.1650000
-15705.0000000	2.1650000
-15704.0000000	2.1600001
-15703.0000000	2.1500001
-15702.0000000	2.1500001
-15701.0000000	2.1450000
-15700.0000000	2.1400001

-15699.0000000	2.1350002
-15698.0000000	2.1300001
-15697.0000000	2.1400001
-15696.0000000	2.1500001
-15695.0000000	2.1600001
-15694.0000000	2.1700001
-15693.0000000	2.1900001
-15692.0000000	2.2000000
-15691.0000000	2.2049999
-15690.0000000	2.2150002
-15689.0000000	2.2249999
-15688.0000000	2.2350001
-15687.0000000	2.2350001
-15686.0000000	2.2249999
-15685.0000000	2.2350001
-15684.0000000	2.2333333
-15683.0000000	2.2200000
-15682.0000000	2.2249999
-15681.0000000	2.2150002
-15680.0000000	2.2049999
-15679.0000000	2.1950002
-15678.0000000	2.1900001
-15677.0000000	2.1950002
-15676.0000000	2.1950002
-15675.0000000	2.2000000
-15674.0000000	2.2100000
-15673.0000000	2.2150002
-15672.0000000	2.2150002
-15671.0000000	2.2200000
-15670.0000000	2.2150002
-15669.0000000	2.2150002
-15668.0000000	2.2200000
-15667.0000000	2.2200000
-15666.0000000	2.2249999
-15665.0000000	2.2400000
-15664.0000000	2.2350001
-15663.0000000	2.2300000
-15662.0000000	2.2200000
-15661.0000000	2.2150002
-15660.0000000	2.2150002
-15659.0000000	2.2049999
-15658.0000000	2.1849999
-15657.0000000	2.1900001
-15656.0000000	2.1900001
-15655.0000000	2.1849999
-15654.0000000	2.1849999
-15653.0000000	2.1800001
-15652.0000000	2.1700001
-15651.0000000	2.1550002
-15650.0000000	2.1250000
-15649.0000000	2.0799999
-15648.0000000	2.0500000
-15647.0000000	2.0300000
-15646.0000000	2.0200000
-15645.0000000	2.0200000
-15644.0000000	2.0300000
-15643.0000000	2.0400000

-15642.0000000	2.0400000
-15641.0000000	2.0349998
-15640.0000000	2.0300000
-15639.0000000	2.0300000
-15638.0000000	2.0450001
-15637.0000000	2.0549998
-15636.0000000	2.0549998
-15635.0000000	2.0699999
-15634.0000000	2.0699999
-15633.0000000	2.0799999
-15632.0000000	2.0899999
-15631.0000000	2.0899999
-15630.0000000	2.0949998
-15629.0000000	2.0899999
-15628.0000000	2.0799999
-15627.0000000	2.0699999
-15626.0000000	2.0549998
-15625.0000000	2.0400000
-15624.0000000	2.0349998
-15623.0000000	2.0300000
-15622.0000000	2.0250001
-15621.0000000	2.0050001
-15620.0000000	1.9900000
-15619.0000000	1.9850000
-15618.0000000	1.9700000
-15617.0000000	1.9650000
-15616.0000000	1.9750000
-15615.0000000	1.9850000
-15614.0000000	1.9950000
-15613.0000000	2.0200000
-15612.0000000	2.0349998
-15611.0000000	2.0549998
-15610.0000000	2.0699999
-15609.0000000	2.0899999
-15608.0000000	2.1199999
-15607.0000000	2.1300001
-15606.0000000	2.1500001
-15605.0000000	2.1700001
-15604.0000000	2.1900001
-15603.0000000	2.1950002
-15602.0000000	2.2000000
-15601.0000000	2.2049999
-15600.0000000	2.2049999
-15599.0000000	2.2000000
-15598.0000000	2.2000000
-15597.0000000	2.1849999
-15596.0000000	2.1800001
-15595.0000000	2.1650000
-15594.0000000	2.1600001
-15593.0000000	2.1450000
-15592.0000000	2.1350002
-15591.0000000	2.1350002
-15590.0000000	2.1300001
-15589.0000000	2.1300001
-15588.0000000	2.1250000
-15587.0000000	2.1300001
-15586.0000000	2.1400001

-15585.0000000	2.1350002
-15584.0000000	2.1450000
-15583.0000000	2.1500001
-15582.0000000	2.1550002
-15581.0000000	2.1600001
-15580.0000000	2.1500001
-15579.0000000	2.1450000
-15578.0000000	2.1600001
-15577.0000000	2.1650000
-15576.0000000	2.1650000
-15575.0000000	2.1700001
-15574.0000000	2.1900001
-15573.0000000	2.1900001
-15572.0000000	2.2000000
-15571.0000000	2.2100000
-15570.0000000	2.2150002
-15569.0000000	2.2150002
-15568.0000000	2.2100000
-15567.0000000	2.2150002
-15566.0000000	2.2200000
-15565.0000000	2.2233334
-15564.0000000	2.2049999
-15563.0000000	2.2100000
-15562.0000000	2.2100000
-15561.0000000	2.1950002
-15560.0000000	2.1900001
-15559.0000000	2.1950002
-15558.0000000	2.1849999
-15557.0000000	2.1849999
-15556.0000000	2.1849999
-15555.0000000	2.1849999
-15554.0000000	2.1700001
-15553.0000000	2.1550002
-15552.0000000	2.1350000
-15551.0000000	2.1250000
-15550.0000000	2.1050000
-15549.0000000	2.0899999
-15548.0000000	2.0799999
-15547.0000000	2.0650001
-15546.0000000	2.0599999
-15545.0000000	2.0500000
-15544.0000000	2.0349998
-15543.0000000	2.0200000
-15542.0000000	2.0149999
-15541.0000000	2.0200000
-15540.0000000	2.0200000
-15539.0000000	2.0200000
-15538.0000000	2.0349998
-15537.0000000	2.0450001
-15536.0000000	2.0633333
-15535.0000000	2.0749998
-15534.0000000	2.0850000
-15533.0000000	2.0899999
-15532.0000000	2.0949998
-15531.0000000	2.0899999
-15530.0000000	2.0749998
-15529.0000000	2.0699999

-15528.0000000	2.0500000
-15527.0000000	2.0450001
-15526.0000000	2.0349998
-15525.0000000	2.0149999
-15524.0000000	2.0149999
-15523.0000000	2.0149999
-15522.0000000	2.0000000
-15521.0000000	1.9900000
-15520.0000000	1.9750000
-15519.0000000	1.9750000
-15518.0000000	1.9850000
-15517.0000000	1.9900000
-15516.0000000	2.0149999
-15515.0000000	2.0250001
-15514.0000000	2.0400000
-15513.0000000	2.0650001
-15512.0000000	2.0749998
-15511.0000000	2.0749998
-15510.0000000	2.0799999
-15509.0000000	2.0749998
-15508.0000000	2.0699999
-15507.0000000	2.0599999
-15506.0000000	2.0500000
-15505.0000000	2.0466669
-15504.0000000	2.0450001
-15503.0000000	2.0349998
-15502.0000000	2.0349998
-15501.0000000	2.0300000
-15500.0000000	2.0250001
-15499.0000000	2.0200000
-15498.0000000	2.0250001
-15497.0000000	2.0250001
-15496.0000000	2.0200000
-15495.0000000	2.0149999
-15494.0000000	2.0349998
-15493.0000000	2.0250001
-15492.0000000	2.0250001
-15491.0000000	2.0100000
-15490.0000000	2.0200000
-15489.0000000	2.0349998
-15488.0000000	2.0300000
-15487.0000000	2.0349998
-15486.0000000	2.0549998
-15485.0000000	2.0699999
-15484.0000000	2.0799999
-15483.0000000	2.0999999
-15482.0000000	2.1099999
-15481.0000000	2.1099999
-15480.0000000	2.1250000
-15479.0000000	2.1250000
-15478.0000000	2.1250000
-15477.0000000	2.1199999
-15476.0000000	2.1050000
-15475.0000000	2.0666666
-15474.0000000	2.0450001
-15473.0000000	2.0149999
-15472.0000000	2.0000000

-15471.0000000	1.9650000
-15470.0000000	1.9500000
-15469.0000000	1.9250000
-15468.0000000	1.9050000
-15467.0000000	1.8950000
-15466.0000000	1.8850000
-15465.0000000	1.8800000
-15464.0000000	1.8650000
-15463.0000000	1.8600000
-15462.0000000	1.8650000
-15461.0000000	1.8700000
-15460.0000000	1.8700000
-15459.0000000	1.8650000
-15458.0000000	1.8700000
-15457.0000000	1.8850000
-15456.0000000	1.9100000
-15455.0000000	1.9300001
-15454.0000000	1.9299999
-15453.0000000	1.9600000
-15452.0000000	1.9850000
-15451.0000000	1.9900000
-15450.0000000	1.9950000
-15449.0000000	2.0000000
-15448.0000000	2.0100000
-15447.0000000	2.0250001
-15446.0000000	2.0400000
-15445.0000000	2.0400000
-15444.0000000	2.0599999
-15443.0000000	2.0650001
-15442.0000000	2.0749998
-15441.0000000	2.0699999
-15440.0000000	2.0699999
-15439.0000000	2.0650001
-15438.0000000	2.0650001
-15437.0000000	2.0699999
-15436.0000000	2.0850000
-15435.0000000	2.0899999
-15434.0000000	2.1050000
-15433.0000000	2.1050000
-15432.0000000	2.1050000
-15431.0000000	2.0999999
-15430.0000000	2.1099999
-15429.0000000	2.1050000
-15428.0000000	2.0899999
-15427.0000000	2.0799999
-15426.0000000	2.0699999
-15425.0000000	2.0650001
-15424.0000000	2.0699999
-15423.0000000	2.0899999
-15422.0000000	2.0999999
-15421.0000000	2.0999999
-15420.0000000	2.0949998
-15419.0000000	2.0999999
-15418.0000000	2.0949998
-15417.0000000	2.0999999
-15416.0000000	2.0949998
-15415.0000000	2.0966666

-15414.0000000	2.1050000
-15413.0000000	2.0999999
-15412.0000000	2.1050000
-15411.0000000	2.1149998
-15410.0000000	2.0999999
-15409.0000000	2.0899999
-15408.0000000	2.0749998
-15407.0000000	2.0699999
-15406.0000000	2.0500000
-15405.0000000	2.0400000
-15404.0000000	2.0300000
-15403.0000000	2.0149999
-15402.0000000	2.0050001
-15401.0000000	2.0000000
-15400.0000000	1.9900000
-15399.0000000	1.9900000
-15398.0000000	1.9950000
-15397.0000000	2.0000000
-15396.0000000	2.0100000
-15395.0000000	2.0250001
-15394.0000000	2.0349998
-15393.0000000	2.0450001
-15392.0000000	2.0500000
-15391.0000000	2.0450001
-15390.0000000	2.0500000
-15389.0000000	2.0500000
-15388.0000000	2.0400000
-15387.0000000	2.0349998
-15386.0000000	2.0300000
-15385.0000000	2.0400000
-15384.0000000	2.0466669
-15383.0000000	2.0549998
-15382.0000000	2.0699999
-15381.0000000	2.0749998
-15380.0000000	2.0949998
-15379.0000000	2.1050000
-15378.0000000	2.1099999
-15377.0000000	2.1050000
-15376.0000000	2.1050000
-15375.0000000	2.0999999
-15374.0000000	2.0949998
-15373.0000000	2.0899999
-15372.0000000	2.0749998
-15371.0000000	2.0549998
-15370.0000000	2.0349998
-15369.0000000	2.0149999
-15368.0000000	2.0000000
-15367.0000000	2.0050001
-15366.0000000	1.9900000
-15365.0000000	1.9800000
-15364.0000000	1.9750000
-15363.0000000	1.9750000
-15362.0000000	1.9650000
-15361.0000000	1.9650000
-15360.0000000	1.9450001
-15359.0000000	1.9450001
-15358.0000000	1.9500000

-15357.0000000	1.9400001
-15356.0000000	1.9349999
-15355.0000000	1.9299999
-15354.0000000	1.9233333
-15353.0000000	1.9200000
-15352.0000000	1.9150000
-15351.0000000	1.9200000
-15350.0000000	1.9150000
-15349.0000000	1.9150000
-15348.0000000	1.9250000
-15347.0000000	1.9349999
-15346.0000000	1.9500000
-15345.0000000	1.9650000
-15344.0000000	1.9700000
-15343.0000000	1.9700000
-15342.0000000	1.9750000
-15341.0000000	1.9700000
-15340.0000000	1.9500000
-15339.0000000	1.9349999
-15338.0000000	1.9299999
-15337.0000000	1.9349999
-15336.0000000	1.9400001
-15335.0000000	1.9550000
-15334.0000000	1.9500000
-15333.0000000	1.9500000
-15332.0000000	1.9550000
-15331.0000000	1.9550000
-15330.0000000	1.9700000
-15329.0000000	1.9750000
-15328.0000000	1.9700000
-15327.0000000	1.9700000
-15326.0000000	1.9750000
-15325.0000000	1.9950000
-15324.0000000	2.0200000
-15323.0000000	2.0366666
-15322.0000000	2.0400000
-15321.0000000	2.0500000
-15320.0000000	2.0650001
-15319.0000000	2.0650001
-15318.0000000	2.0699999
-15317.0000000	2.0799999
-15316.0000000	2.0899999
-15315.0000000	2.0899999
-15314.0000000	2.0850000
-15313.0000000	2.0899999
-15312.0000000	2.0850000
-15311.0000000	2.0899999
-15310.0000000	2.0850000
-15309.0000000	2.0749998
-15308.0000000	2.0749998
-15307.0000000	2.0749998
-15306.0000000	2.0650001
-15305.0000000	2.0650001
-15304.0000000	2.0549998
-15303.0000000	2.0549998
-15302.0000000	2.0599999
-15301.0000000	2.0599999

-15300.0000000	2.0549998
-15299.0000000	2.0599999
-15298.0000000	2.0650001
-15297.0000000	2.0599999
-15296.0000000	2.0500000
-15295.0000000	2.0500000
-15294.0000000	2.0300000
-15293.0000000	2.0200000
-15292.0000000	2.0149999
-15291.0000000	2.0000000
-15290.0000000	1.9950000
-15289.0000000	1.9900000
-15288.0000000	1.9850000
-15287.0000000	1.9950000
-15286.0000000	1.9950000
-15285.0000000	1.9850000
-15284.0000000	1.9800000
-15283.0000000	1.9800000
-15282.0000000	1.9750000
-15281.0000000	1.9800000
-15280.0000000	1.9800000
-15279.0000000	1.9900000
-15278.0000000	1.9950000
-15277.0000000	2.0149999
-15276.0000000	2.0250001
-15275.0000000	2.0400000
-15274.0000000	2.0300000
-15273.0000000	2.0349998
-15272.0000000	2.0400000
-15271.0000000	2.0400000
-15270.0000000	2.0500000
-15269.0000000	2.0599999
-15268.0000000	2.0799999
-15267.0000000	2.0899999
-15266.0000000	2.0899999
-15265.0000000	2.0899999
-15264.0000000	2.0650001
-15263.0000000	2.0599999
-15262.0000000	2.0633333
-15261.0000000	2.0549998
-15260.0000000	2.0549998
-15259.0000000	2.0599999
-15258.0000000	2.0599999
-15257.0000000	2.0749998
-15256.0000000	2.0899999
-15255.0000000	2.0850000
-15254.0000000	2.0899999
-15253.0000000	2.1050000
-15252.0000000	2.1199999
-15251.0000000	2.1300001
-15250.0000000	2.1350002
-15249.0000000	2.1450000
-15248.0000000	2.1550002
-15247.0000000	2.1800001
-15246.0000000	2.1900001
-15245.0000000	2.2049999
-15244.0000000	2.2200000

-15243.0000000	2.2100000
-15242.0000000	2.2049999
-15241.0000000	2.1950002
-15240.0000000	2.1800001
-15239.0000000	2.1750002
-15238.0000000	2.1550002
-15237.0000000	2.1350002
-15236.0000000	2.1149998
-15235.0000000	2.0949998
-15234.0000000	2.0799999
-15233.0000000	2.0650001
-15232.0000000	2.0433333
-15231.0000000	2.0100000
-15230.0000000	1.9950000
-15229.0000000	1.9950000
-15228.0000000	1.9950000
-15227.0000000	1.9850000
-15226.0000000	1.9850000
-15225.0000000	1.9750000
-15224.0000000	1.9500000
-15223.0000000	1.9600000
-15222.0000000	1.9700000
-15221.0000000	1.9900000
-15220.0000000	2.0000000
-15219.0000000	2.0149999
-15218.0000000	2.0149999
-15217.0000000	2.0250001
-15216.0000000	2.0250001
-15215.0000000	2.0200000
-15214.0000000	2.0200000
-15213.0000000	2.0200000
-15212.0000000	2.0100000
-15211.0000000	2.0200000
-15210.0000000	2.0200000
-15209.0000000	2.0200000
-15208.0000000	2.0200000
-15207.0000000	2.0200000
-15206.0000000	2.0250001
-15205.0000000	2.0300000
-15204.0000000	2.0300000
-15203.0000000	2.0300000
-15202.0000000	2.0333331
-15201.0000000	2.0349998
-15200.0000000	2.0349998
-15199.0000000	2.0300000
-15198.0000000	2.0250001
-15197.0000000	2.0200000
-15196.0000000	2.0200000
-15195.0000000	2.0500000
-15194.0000000	2.0549998
-15193.0000000	2.0549998
-15192.0000000	2.0549998
-15191.0000000	2.0300000
-15190.0000000	2.0200000
-15189.0000000	2.0250001
-15188.0000000	2.0100000
-15187.0000000	1.9850000

-15186.0000000	1.9750000
-15185.0000000	1.9750000
-15184.0000000	1.9850000
-15183.0000000	1.9800000
-15182.0000000	1.9950000
-15181.0000000	2.0050001
-15180.0000000	2.0100000
-15179.0000000	2.0100000
-15178.0000000	2.0250001
-15177.0000000	2.0349998
-15176.0000000	2.0500000
-15175.0000000	2.0349998
-15174.0000000	2.0349998
-15173.0000000	2.0400000
-15172.0000000	2.0566666
-15171.0000000	2.0699999
-15170.0000000	2.0850000
-15169.0000000	2.0999999
-15168.0000000	2.0999999
-15167.0000000	2.1199999
-15166.0000000	2.1199999
-15165.0000000	2.1250000
-15164.0000000	2.1149998
-15163.0000000	2.1099999
-15162.0000000	2.1050000
-15161.0000000	2.1099999
-15160.0000000	2.1149998
-15159.0000000	2.1149998
-15158.0000000	2.1199999
-15157.0000000	2.1300001
-15156.0000000	2.1300001
-15155.0000000	2.1250000
-15154.0000000	2.1300001
-15153.0000000	2.1149998
-15152.0000000	2.0999999
-15151.0000000	2.0799999
-15150.0000000	2.0450001
-15149.0000000	2.0100000
-15148.0000000	1.9850000
-15147.0000000	1.9800000
-15146.0000000	1.9850000
-15145.0000000	2.0100000
-15144.0000000	2.0200000
-15143.0000000	2.0450001
-15142.0000000	2.0599999
-15141.0000000	2.0566666
-15140.0000000	2.0450001
-15139.0000000	2.0250001
-15138.0000000	2.0149999
-15137.0000000	2.0149999
-15136.0000000	1.9900000
-15135.0000000	1.9900000
-15134.0000000	1.9950000
-15133.0000000	2.0050001
-15132.0000000	2.0200000
-15131.0000000	2.0250001
-15130.0000000	2.0400000

-15129.0000000	2.0450001
-15128.0000000	2.0599999
-15127.0000000	2.0749998
-15126.0000000	2.0799999
-15125.0000000	2.0899999
-15124.0000000	2.1050000
-15123.0000000	2.1199999
-15122.0000000	2.1099999
-15121.0000000	2.1149998
-15120.0000000	2.1149998
-15119.0000000	2.1149998
-15118.0000000	2.1050000
-15117.0000000	2.0999999
-15116.0000000	2.0999999
-15115.0000000	2.0949998
-15114.0000000	2.0850000
-15113.0000000	2.0699999
-15112.0000000	2.0400000
-15111.0000000	2.0433333
-15110.0000000	2.0500000
-15109.0000000	2.0549998
-15108.0000000	2.0500000
-15107.0000000	2.0549998
-15106.0000000	2.0400000
-15105.0000000	2.0400000
-15104.0000000	2.0450001
-15103.0000000	2.0549998
-15102.0000000	2.0749998
-15101.0000000	2.0949998
-15100.0000000	2.0999999
-15099.0000000	2.1050000
-15098.0000000	2.1050000
-15097.0000000	2.1149998
-15096.0000000	2.1199999
-15095.0000000	2.1199999
-15094.0000000	2.1199999
-15093.0000000	2.1099999
-15092.0000000	2.1050000
-15091.0000000	2.0949998
-15090.0000000	2.0949998
-15089.0000000	2.0949998
-15088.0000000	2.0949998
-15087.0000000	2.1050000
-15086.0000000	2.1050000
-15085.0000000	2.1050000
-15084.0000000	2.1099999
-15083.0000000	2.1099999
-15082.0000000	2.0999999
-15081.0000000	2.0866666
-15080.0000000	2.0699999
-15079.0000000	2.0599999
-15078.0000000	2.0549998
-15077.0000000	2.0500000
-15076.0000000	2.0500000
-15075.0000000	2.0500000
-15074.0000000	2.0400000
-15073.0000000	2.0450001

-15072.0000000	2.0400000
-15071.0000000	2.0400000
-15070.0000000	2.0549998
-15069.0000000	2.0650001
-15068.0000000	2.0749998
-15067.0000000	2.0799999
-15066.0000000	2.0899999
-15065.0000000	2.0999999
-15064.0000000	2.1199999
-15063.0000000	2.1199999
-15062.0000000	2.1300001
-15061.0000000	2.1450000
-15060.0000000	2.1400001
-15059.0000000	2.1450000
-15058.0000000	2.1450000
-15057.0000000	2.1450000
-15056.0000000	2.1450000
-15055.0000000	2.1400001
-15054.0000000	2.1400001
-15053.0000000	2.1550002
-15052.0000000	2.1550002
-15051.0000000	2.1666667
-15050.0000000	2.1700001
-15049.0000000	2.1800001
-15048.0000000	2.1650000
-15047.0000000	2.1550002
-15046.0000000	2.1450000
-15045.0000000	2.1350002
-15044.0000000	2.1300001
-15043.0000000	2.1300001
-15042.0000000	2.1250000
-15041.0000000	2.1400001
-15040.0000000	2.1400001
-15039.0000000	2.1600001
-15038.0000000	2.1700001
-15037.0000000	2.1950002
-15036.0000000	2.2000000
-15035.0000000	2.2100000
-15034.0000000	2.2150002
-15033.0000000	2.2150002
-15032.0000000	2.2200000
-15031.0000000	2.2249999
-15030.0000000	2.2249999
-15029.0000000	2.2300000
-15028.0000000	2.2350001
-15027.0000000	2.2400000
-15026.0000000	2.2500000
-15025.0000000	2.2600000
-15024.0000000	2.2500000
-15023.0000000	2.2533333
-15022.0000000	2.2500000
-15021.0000000	2.2550001
-15020.0000000	2.2550001
-15019.0000000	2.2550001
-15018.0000000	2.2550001
-15017.0000000	2.2600000
-15016.0000000	2.2500000

-15015.0000000	2.2350001
-15014.0000000	2.2249999
-15013.0000000	2.2249999
-15012.0000000	2.2300000
-15011.0000000	2.2350001
-15010.0000000	2.2400000
-15009.0000000	2.2449999
-15008.0000000	2.2500000
-15007.0000000	2.2449999
-15006.0000000	2.2500000
-15005.0000000	2.2449999
-15004.0000000	2.2449999
-15003.0000000	2.2500000
-15002.0000000	2.2550001
-15001.0000000	2.2600000
-15000.0000000	2.2500000
-14999.0000000	2.2500000
-14998.0000000	2.2449999
-14997.0000000	2.2400000
-14996.0000000	2.2400000
-14995.0000000	2.2400000
-14994.0000000	2.2249999
-14993.0000000	2.2150002
-14992.0000000	2.1966667
-14991.0000000	2.1900001
-14990.0000000	2.1800001
-14989.0000000	2.1700001
-14988.0000000	2.1750002
-14987.0000000	2.1750002
-14986.0000000	2.1700001
-14985.0000000	2.1800001
-14984.0000000	2.1900001
-14983.0000000	2.2100000
-14982.0000000	2.2049999
-14981.0000000	2.1900001
-14980.0000000	2.1800001
-14979.0000000	2.1450000
-14978.0000000	2.1099999
-14977.0000000	2.0850000
-14976.0000000	2.0650001
-14975.0000000	2.0650001
-14974.0000000	2.0699999
-14973.0000000	2.0799999
-14972.0000000	2.1050000
-14971.0000000	2.1099999
-14970.0000000	2.1099999
-14969.0000000	2.1300001
-14968.0000000	2.1199999
-14967.0000000	2.1099999
-14966.0000000	2.0899999
-14965.0000000	2.0850000
-14964.0000000	2.0799999
-14963.0000000	2.0999999
-14962.0000000	2.1199999
-14961.0000000	2.1400001
-14960.0000000	2.1650000
-14959.0000000	2.1950002

-14958.0000000	2.2100000
-14957.0000000	2.2200000
-14956.0000000	2.2249999
-14955.0000000	2.2300000
-14954.0000000	2.2249999
-14953.0000000	2.2150002
-14952.0000000	2.2249999
-14951.0000000	2.2200000
-14950.0000000	2.2049999
-14949.0000000	2.2000000
-14948.0000000	2.1849999
-14947.0000000	2.1600001
-14946.0000000	2.1400001
-14945.0000000	2.1199999
-14944.0000000	2.1050000
-14943.0000000	2.0850000
-14942.0000000	2.0899999
-14941.0000000	2.0699999
-14940.0000000	2.0599999
-14939.0000000	2.0599999
-14938.0000000	2.0699999
-14937.0000000	2.0699999
-14936.0000000	2.0599999
-14935.0000000	2.0650001
-14934.0000000	2.0749998
-14933.0000000	2.0999999
-14932.0000000	2.1050000
-14931.0000000	2.1133332
-14930.0000000	2.1099999
-14929.0000000	2.1050000
-14928.0000000	2.0899999
-14927.0000000	2.0899999
-14926.0000000	2.0949998
-14925.0000000	2.0949998
-14924.0000000	2.0999999
-14923.0000000	2.1149998
-14922.0000000	2.1450000
-14921.0000000	2.1600001
-14920.0000000	2.1700001
-14919.0000000	2.1650000
-14918.0000000	2.1550002
-14917.0000000	2.1500001
-14916.0000000	2.1550002
-14915.0000000	2.1650000
-14914.0000000	2.1800001
-14913.0000000	2.1700001
-14912.0000000	2.1600001
-14911.0000000	2.1450000
-14910.0000000	2.1400001
-14909.0000000	2.1400001
-14908.0000000	2.1400001
-14907.0000000	2.1350002
-14906.0000000	2.1400001
-14905.0000000	2.1350002
-14904.0000000	2.1250000
-14903.0000000	2.1199999
-14902.0000000	2.1199999

-14901.0000000	2.1266668
-14900.0000000	2.1250000
-14899.0000000	2.1300001
-14898.0000000	2.1450000
-14897.0000000	2.1500001
-14896.0000000	2.1550002
-14895.0000000	2.1550002
-14894.0000000	2.1500001
-14893.0000000	2.1450000
-14892.0000000	2.1500001
-14891.0000000	2.1600001
-14890.0000000	2.1700001
-14889.0000000	2.1650000
-14888.0000000	2.1849999
-14887.0000000	2.1900001
-14886.0000000	2.1849999
-14885.0000000	2.1849999
-14884.0000000	2.1800001
-14883.0000000	2.1800001
-14882.0000000	2.1800001
-14881.0000000	2.1700001
-14880.0000000	2.1800001
-14879.0000000	2.1750002
-14878.0000000	2.1650000
-14877.0000000	2.1650000
-14876.0000000	2.1700001
-14875.0000000	2.1600001
-14874.0000000	2.1550002
-14873.0000000	2.1500001
-14872.0000000	2.1400001
-14871.0000000	2.1500001
-14870.0000000	2.1450000
-14869.0000000	2.1400001
-14868.0000000	2.1250000
-14867.0000000	2.1300001
-14866.0000000	2.1300001
-14865.0000000	2.1400001
-14864.0000000	2.1400001
-14863.0000000	2.1400001
-14862.0000000	2.1350002
-14861.0000000	2.1400001
-14860.0000000	2.1400001
-14859.0000000	2.1300001
-14858.0000000	2.1250000
-14857.0000000	2.1199999
-14856.0000000	2.1350002
-14855.0000000	2.1350002
-14854.0000000	2.1300001
-14853.0000000	2.1250000
-14852.0000000	2.1199999
-14851.0000000	2.1199999
-14850.0000000	2.1199999
-14849.0000000	2.1250000
-14848.0000000	2.1300001
-14847.0000000	2.1250000
-14846.0000000	2.1250000
-14845.0000000	2.1199999

-14844.0000000	2.1050000
-14843.0000000	2.0799999
-14842.0000000	2.0650001
-14841.0000000	2.0549998
-14840.0000000	2.0599999
-14839.0000000	2.0733333
-14838.0000000	2.0749998
-14837.0000000	2.0999999
-14836.0000000	2.1199999
-14835.0000000	2.1400001
-14834.0000000	2.1500001
-14833.0000000	2.1650000
-14832.0000000	2.1800001
-14831.0000000	2.1800001
-14830.0000000	2.1950002
-14829.0000000	2.2000000
-14828.0000000	2.2100000
-14827.0000000	2.2100000
-14826.0000000	2.2100000
-14825.0000000	2.2100000
-14824.0000000	2.2200000
-14823.0000000	2.2200000
-14822.0000000	2.2249999
-14821.0000000	2.2300000
-14820.0000000	2.2350001
-14819.0000000	2.2249999
-14818.0000000	2.2400000
-14817.0000000	2.2400000
-14816.0000000	2.2550001
-14815.0000000	2.2649999
-14814.0000000	2.2649999
-14813.0000000	2.2750001
-14812.0000000	2.2750001
-14811.0000000	2.2649999
-14810.0000000	2.2600000
-14809.0000000	2.2633333
-14808.0000000	2.2700000
-14807.0000000	2.2849998
-14806.0000000	2.2900000
-14805.0000000	2.2800000
-14804.0000000	2.2849998
-14803.0000000	2.2750001
-14802.0000000	2.2600000
-14801.0000000	2.2449999
-14800.0000000	2.2150002
-14799.0000000	2.1849999
-14798.0000000	2.1500001
-14797.0000000	2.1199999
-14796.0000000	2.1050000
-14795.0000000	2.0850000
-14794.0000000	2.0500000
-14793.0000000	2.0300000
-14792.0000000	2.0100000
-14791.0000000	2.0050001
-14790.0000000	2.0200000
-14789.0000000	2.0250001
-14788.0000000	2.0549998

-14787.0000000	2.0749998
-14786.0000000	2.0799999
-14785.0000000	2.0899999
-14784.0000000	2.0799999
-14783.0000000	2.0749998
-14782.0000000	2.0699999
-14781.0000000	2.0749998
-14780.0000000	2.0650001
-14779.0000000	2.0566666
-14778.0000000	2.0450001
-14777.0000000	2.0450001
-14776.0000000	2.0599999
-14775.0000000	2.0850000
-14774.0000000	2.0949998
-14773.0000000	2.0949998
-14772.0000000	2.0850000
-14771.0000000	2.0999999
-14770.0000000	2.0899999
-14769.0000000	2.0949998
-14768.0000000	2.1149998
-14767.0000000	2.0999999
-14766.0000000	2.1149998
-14765.0000000	2.1250000
-14764.0000000	2.1250000
-14763.0000000	2.1350002
-14762.0000000	2.1400001
-14761.0000000	2.1300001
-14760.0000000	2.1250000
-14759.0000000	2.1199999
-14758.0000000	2.0999999
-14757.0000000	2.0850000
-14756.0000000	2.0850000
-14755.0000000	2.0850000
-14754.0000000	2.0949998
-14753.0000000	2.0999999
-14752.0000000	2.0999999
-14751.0000000	2.1199999
-14750.0000000	2.1366670
-14749.0000000	2.1350002
-14748.0000000	2.1250000
-14747.0000000	2.1149998
-14746.0000000	2.0999999
-14745.0000000	2.0999999
-14744.0000000	2.1149998
-14743.0000000	2.1149998
-14742.0000000	2.1250000
-14741.0000000	2.1300001
-14740.0000000	2.1400001
-14739.0000000	2.1450000
-14738.0000000	2.1500001
-14737.0000000	2.1550002
-14736.0000000	2.1600001
-14735.0000000	2.1550002
-14734.0000000	2.1500001
-14733.0000000	2.1550002
-14732.0000000	2.1600001
-14731.0000000	2.1600001

-14730.0000000	2.1750002
-14729.0000000	2.2000000
-14728.0000000	2.2200000
-14727.0000000	2.2400000
-14726.0000000	2.2600000
-14725.0000000	2.2800000
-14724.0000000	2.2849998
-14723.0000000	2.3049998
-14722.0000000	2.3150001
-14721.0000000	2.3350000
-14720.0000000	2.3466666
-14719.0000000	2.3350000
-14718.0000000	2.3350000
-14717.0000000	2.3449998
-14716.0000000	2.3449998
-14715.0000000	2.3449998
-14714.0000000	2.3399999
-14713.0000000	2.3449998
-14712.0000000	2.3449998
-14711.0000000	2.3350000
-14710.0000000	2.3199999
-14709.0000000	2.2950001
-14708.0000000	2.2849998
-14707.0000000	2.2950001
-14706.0000000	2.2750001
-14705.0000000	2.2750001
-14704.0000000	2.2649999
-14703.0000000	2.2550001
-14702.0000000	2.2400000
-14701.0000000	2.2200000
-14700.0000000	2.2100000
-14699.0000000	2.2049999
-14698.0000000	2.2100000
-14697.0000000	2.2300000
-14696.0000000	2.2400000
-14695.0000000	2.2500000
-14694.0000000	2.2649999
-14693.0000000	2.2750001
-14692.0000000	2.2849998
-14691.0000000	2.2900000
-14690.0000000	2.3049998
-14689.0000000	2.3233335
-14688.0000000	2.3449998
-14687.0000000	2.3599999
-14686.0000000	2.3750000
-14685.0000000	2.3699999
-14684.0000000	2.3449998
-14683.0000000	2.3449998
-14682.0000000	2.3199999
-14681.0000000	2.3049998
-14680.0000000	2.3000000
-14679.0000000	2.2849998
-14678.0000000	2.2800000
-14677.0000000	2.2800000
-14676.0000000	2.2750001
-14675.0000000	2.2750001
-14674.0000000	2.2900000

-14673.0000000	2.2849998
-14672.0000000	2.2849998
-14671.0000000	2.2950001
-14670.0000000	2.2950001
-14669.0000000	2.2950001
-14668.0000000	2.2849998
-14667.0000000	2.2950001
-14666.0000000	2.2900000
-14665.0000000	2.2849998
-14664.0000000	2.2849998
-14663.0000000	2.2800000
-14662.0000000	2.2800000
-14661.0000000	2.2800000
-14660.0000000	2.2800000
-14659.0000000	2.2833333
-14658.0000000	2.2800000
-14657.0000000	2.2800000
-14656.0000000	2.2800000
-14655.0000000	2.2849998
-14654.0000000	2.2849998
-14653.0000000	2.2800000
-14652.0000000	2.2750001
-14651.0000000	2.2649999
-14650.0000000	2.2649999
-14649.0000000	2.2500000
-14648.0000000	2.2200000
-14647.0000000	2.2049999
-14646.0000000	2.1950002
-14645.0000000	2.1950002
-14644.0000000	2.1900001
-14643.0000000	2.1900001
-14642.0000000	2.1800001
-14641.0000000	2.1849999
-14640.0000000	2.1900001
-14639.0000000	2.2100000
-14638.0000000	2.2049999
-14637.0000000	2.2000000
-14636.0000000	2.1900001
-14635.0000000	2.1950002
-14634.0000000	2.2000000
-14633.0000000	2.2000000
-14632.0000000	2.1849999
-14631.0000000	2.1750002
-14630.0000000	2.1650000
-14629.0000000	2.1600001
-14628.0000000	2.1500001
-14627.0000000	2.1400001
-14626.0000000	2.1450000
-14625.0000000	2.1400001
-14624.0000000	2.1250000
-14623.0000000	2.0999999
-14622.0000000	2.0999999
-14621.0000000	2.0949998
-14620.0000000	2.0899999
-14619.0000000	2.0899999
-14618.0000000	2.1050000
-14617.0000000	2.1099999

-14616.0000000	2.1149998
-14615.0000000	2.1250000
-14614.0000000	2.1199999
-14613.0000000	2.1250000
-14612.0000000	2.1199999
-14611.0000000	2.1199999
-14610.0000000	2.1149998
-14609.0000000	2.1099999
-14608.0000000	2.1050000
-14607.0000000	2.0949998
-14606.0000000	2.0949998
-14605.0000000	2.0899999
-14604.0000000	2.0749998
-14603.0000000	2.0500000
-14602.0000000	2.0549998
-14601.0000000	2.0500000
-14600.0000000	2.0400000
-14599.0000000	2.0300000
-14598.0000000	2.0349998
-14597.0000000	2.0349998
-14596.0000000	2.0300000
-14595.0000000	2.0549998
-14594.0000000	2.0500000
-14593.0000000	2.0599999
-14592.0000000	2.0699999
-14591.0000000	2.0699999
-14590.0000000	2.0699999
-14589.0000000	2.0850000
-14588.0000000	2.0850000
-14587.0000000	2.0850000
-14586.0000000	2.0799999
-14585.0000000	2.0749998
-14584.0000000	2.0799999
-14583.0000000	2.0799999
-14582.0000000	2.0699999
-14581.0000000	2.0799999
-14580.0000000	2.0650001
-14579.0000000	2.0599999
-14578.0000000	2.0549998
-14577.0000000	2.0699999
-14576.0000000	2.0599999
-14575.0000000	2.0650001
-14574.0000000	2.0799999
-14573.0000000	2.0850000
-14572.0000000	2.0850000
-14571.0000000	2.0999999
-14570.0000000	2.0999999
-14569.0000000	2.1099999
-14568.0000000	2.0999999
-14567.0000000	2.0749998
-14566.0000000	2.0450001
-14565.0000000	2.0400000
-14564.0000000	2.0349998
-14563.0000000	2.0400000
-14562.0000000	2.0450001
-14561.0000000	2.0400000
-14560.0000000	2.0500000

-14559.0000000	2.0500000
-14558.0000000	2.0549998
-14557.0000000	2.0650001
-14556.0000000	2.0799999
-14555.0000000	2.0949998
-14554.0000000	2.1050000
-14553.0000000	2.1149998
-14552.0000000	2.1199999
-14551.0000000	2.1350002
-14550.0000000	2.1500001
-14549.0000000	2.1650000
-14548.0000000	2.1700001
-14547.0000000	2.1800001
-14546.0000000	2.1700001
-14545.0000000	2.1650000
-14544.0000000	2.1500001
-14543.0000000	2.1500001
-14542.0000000	2.1700001
-14541.0000000	2.1700001
-14540.0000000	2.1700001
-14539.0000000	2.1833334
-14538.0000000	2.1849999
-14537.0000000	2.1700001
-14536.0000000	2.1450000
-14535.0000000	2.1350002
-14534.0000000	2.1250000
-14533.0000000	2.1250000
-14532.0000000	2.1199999
-14531.0000000	2.1250000
-14530.0000000	2.1149998
-14529.0000000	2.1050000
-14528.0000000	2.0999999
-14527.0000000	2.0899999
-14526.0000000	2.0749998
-14525.0000000	2.0599999
-14524.0000000	2.0749998
-14523.0000000	2.0850000
-14522.0000000	2.0999999
-14521.0000000	2.1050000
-14520.0000000	2.1300001
-14519.0000000	2.1500001
-14518.0000000	2.1600001
-14517.0000000	2.1500001
-14516.0000000	2.1550002
-14515.0000000	2.1500001
-14514.0000000	2.1650000
-14513.0000000	2.1700001
-14512.0000000	2.1650000
-14511.0000000	2.1700001
-14510.0000000	2.1650000
-14509.0000000	2.1550002
-14508.0000000	2.1300001
-14507.0000000	2.1300001
-14506.0000000	2.1300001
-14505.0000000	2.1149998
-14504.0000000	2.1199999
-14503.0000000	2.1099999

-14502.0000000	2.0949998
-14501.0000000	2.0899999
-14500.0000000	2.0799999
-14499.0000000	2.0749998
-14498.0000000	2.0599999
-14497.0000000	2.0599999
-14496.0000000	2.0549998
-14495.0000000	2.0400000
-14494.0000000	2.0349998
-14493.0000000	2.0200000
-14492.0000000	2.0149999
-14491.0000000	2.0100000
-14490.0000000	1.9950000
-14489.0000000	1.9900000
-14488.0000000	1.9950000
-14487.0000000	2.0000000
-14486.0000000	2.0149999
-14485.0000000	2.0349998
-14484.0000000	2.0450001
-14483.0000000	2.0599999
-14482.0000000	2.0749998
-14481.0000000	2.0749998
-14480.0000000	2.0850000
-14479.0000000	2.0799999
-14478.0000000	2.0966666
-14477.0000000	2.1099999
-14476.0000000	2.1149998
-14475.0000000	2.1149998
-14474.0000000	2.1149998
-14473.0000000	2.1099999
-14472.0000000	2.1050000
-14471.0000000	2.0850000
-14470.0000000	2.0749998
-14469.0000000	2.0549998
-14468.0000000	2.0500000
-14467.0000000	2.0599999
-14466.0000000	2.0599999
-14465.0000000	2.0699999
-14464.0000000	2.0850000
-14463.0000000	2.0850000
-14462.0000000	2.1050000
-14461.0000000	2.1149998
-14460.0000000	2.1250000
-14459.0000000	2.1199999
-14458.0000000	2.1149998
-14457.0000000	2.1199999
-14456.0000000	2.1250000
-14455.0000000	2.1400001
-14454.0000000	2.1550002
-14453.0000000	2.1750002
-14452.0000000	2.1750002
-14451.0000000	2.1700001
-14450.0000000	2.1700001
-14449.0000000	2.1650000
-14448.0000000	2.1500001
-14447.0000000	2.1300001
-14446.0000000	2.1199999

-14445.0000000	2.1099999
-14444.0000000	2.0949998
-14443.0000000	2.0949998
-14442.0000000	2.0949998
-14441.0000000	2.1050000
-14440.0000000	2.1149998
-14439.0000000	2.1300001
-14438.0000000	2.1400001
-14437.0000000	2.1450000
-14436.0000000	2.1400001
-14435.0000000	2.1500001
-14434.0000000	2.1500001
-14433.0000000	2.1550002
-14432.0000000	2.1650000
-14431.0000000	2.1700001
-14430.0000000	2.1600001
-14429.0000000	2.1600001
-14428.0000000	2.1500001
-14427.0000000	2.1350002
-14426.0000000	2.1300001
-14425.0000000	2.1199999
-14424.0000000	2.1250000
-14423.0000000	2.1199999
-14422.0000000	2.1149998
-14421.0000000	2.1300001
-14420.0000000	2.1400001
-14419.0000000	2.1600001
-14418.0000000	2.1650000
-14417.0000000	2.1833334
-14416.0000000	2.1849999
-14415.0000000	2.1800001
-14414.0000000	2.1700001
-14413.0000000	2.1650000
-14412.0000000	2.1950002
-14411.0000000	2.2000000
-14410.0000000	2.2100000
-14409.0000000	2.2200000
-14408.0000000	2.2150002
-14407.0000000	2.2000000
-14406.0000000	2.1800001
-14405.0000000	2.1750002
-14404.0000000	2.1849999
-14403.0000000	2.1900001
-14402.0000000	2.1950002
-14401.0000000	2.2049999
-14400.0000000	2.2150002
-14399.0000000	2.2150002
-14398.0000000	2.2049999
-14397.0000000	2.2000000
-14396.0000000	2.2049999
-14395.0000000	2.2049999
-14394.0000000	2.2150002
-14393.0000000	2.2200000
-14392.0000000	2.2300000
-14391.0000000	2.2350001
-14390.0000000	2.2350001
-14389.0000000	2.2300000

-14388.0000000	2.2200000
-14387.0000000	2.2100000
-14386.0000000	2.2033334
-14385.0000000	2.1800001
-14384.0000000	2.1650000
-14383.0000000	2.1600001
-14382.0000000	2.1650000
-14381.0000000	2.1800001
-14380.0000000	2.1900001
-14379.0000000	2.2049999
-14378.0000000	2.1849999
-14377.0000000	2.1700001
-14376.0000000	2.1600001
-14375.0000000	2.1650000
-14374.0000000	2.1600001
-14373.0000000	2.1650000
-14372.0000000	2.1600001
-14371.0000000	2.1550002
-14370.0000000	2.1500001
-14369.0000000	2.1450000
-14368.0000000	2.1400001
-14367.0000000	2.1500001
-14366.0000000	2.1550002
-14365.0000000	2.1500001
-14364.0000000	2.1550002
-14363.0000000	2.1550002
-14362.0000000	2.1500001
-14361.0000000	2.1500001
-14360.0000000	2.1450000
-14359.0000000	2.1550002
-14358.0000000	2.1550002
-14357.0000000	2.1550002
-14356.0000000	2.1533334
-14355.0000000	2.1350002
-14354.0000000	2.1250000
-14353.0000000	2.1300001
-14352.0000000	2.1400001
-14351.0000000	2.1400001
-14350.0000000	2.1600001
-14349.0000000	2.1700001
-14348.0000000	2.1800001
-14347.0000000	2.1800001
-14346.0000000	2.1800001
-14345.0000000	2.1849999
-14344.0000000	2.1849999
-14343.0000000	2.1750002
-14342.0000000	2.1750002
-14341.0000000	2.1849999
-14340.0000000	2.1849999
-14339.0000000	2.2000000
-14338.0000000	2.2049999
-14337.0000000	2.2100000
-14336.0000000	2.2150002
-14335.0000000	2.2249999
-14334.0000000	2.2400000
-14333.0000000	2.2449999
-14332.0000000	2.2550001

-14331.0000000	2.2550001
-14330.0000000	2.2350001
-14329.0000000	2.2249999
-14328.0000000	2.2166669
-14327.0000000	2.2100000
-14326.0000000	2.2100000
-14325.0000000	2.2150002
-14324.0000000	2.2249999
-14323.0000000	2.2249999
-14322.0000000	2.2350001
-14321.0000000	2.2350001
-14320.0000000	2.2350001
-14319.0000000	2.2449999
-14318.0000000	2.2500000
-14317.0000000	2.2600000
-14316.0000000	2.2700000
-14315.0000000	2.2700000
-14314.0000000	2.2700000
-14313.0000000	2.2800000
-14312.0000000	2.2700000
-14311.0000000	2.2750001
-14310.0000000	2.2700000
-14309.0000000	2.2649999
-14308.0000000	2.2449999
-14307.0000000	2.2300000
-14306.0000000	2.2200000
-14305.0000000	2.2200000
-14304.0000000	2.2150002
-14303.0000000	2.2150002
-14302.0000000	2.2150002
-14301.0000000	2.2200000
-14300.0000000	2.2100000
-14299.0000000	2.2049999
-14298.0000000	2.2200000
-14297.0000000	2.2300000
-14296.0000000	2.2400000
-14295.0000000	2.2500000
-14294.0000000	2.2400000
-14293.0000000	2.2300000
-14292.0000000	2.2300000
-14291.0000000	2.2300000
-14290.0000000	2.2200000
-14289.0000000	2.2249999
-14288.0000000	2.2400000
-14287.0000000	2.2400000
-14286.0000000	2.2500000
-14285.0000000	2.2500000
-14284.0000000	2.2500000
-14283.0000000	2.2500000
-14282.0000000	2.2449999
-14281.0000000	2.2300000
-14280.0000000	2.2249999
-14279.0000000	2.2150002
-14278.0000000	2.2100000
-14277.0000000	2.2249999
-14276.0000000	2.2200000
-14275.0000000	2.2100000

-14274.0000000	2.2049999
-14273.0000000	2.2100000
-14272.0000000	2.2049999
-14271.0000000	2.2249999
-14270.0000000	2.2350001
-14269.0000000	2.2449999
-14268.0000000	2.2449999
-14267.0000000	2.2350001
-14266.0000000	2.2466667
-14265.0000000	2.2550001
-14264.0000000	2.2600000
-14263.0000000	2.2550001
-14262.0000000	2.2550001
-14261.0000000	2.2449999
-14260.0000000	2.2249999
-14259.0000000	2.2100000
-14258.0000000	2.1900001
-14257.0000000	2.1800001
-14256.0000000	2.1650000
-14255.0000000	2.1450000
-14254.0000000	2.1450000
-14253.0000000	2.1400001
-14252.0000000	2.1500001
-14251.0000000	2.1600001
-14250.0000000	2.1750002
-14249.0000000	2.1700001
-14248.0000000	2.1500001
-14247.0000000	2.1350002
-14246.0000000	2.1199999
-14245.0000000	2.1149998
-14244.0000000	2.1149998
-14243.0000000	2.1199999
-14242.0000000	2.1300001
-14241.0000000	2.1250000
-14240.0000000	2.1350002
-14239.0000000	2.1500001
-14238.0000000	2.1650000
-14237.0000000	2.1800001
-14236.0000000	2.1950002
-14235.0000000	2.2100000
-14234.0000000	2.2200000
-14233.0000000	2.2249999
-14232.0000000	2.2300000
-14231.0000000	2.2400000
-14230.0000000	2.2350001
-14229.0000000	2.2200000
-14228.0000000	2.2150002
-14227.0000000	2.1950002
-14226.0000000	2.1800001
-14225.0000000	2.1650000
-14224.0000000	2.1250000
-14223.0000000	2.1050000
-14222.0000000	2.1050000
-14221.0000000	2.1149998
-14220.0000000	2.1300001
-14219.0000000	2.1350002
-14218.0000000	2.1450000

-14217.0000000	2.1500001
-14216.0000000	2.1600001
-14215.0000000	2.1650000
-14214.0000000	2.1700001
-14213.0000000	2.1600001
-14212.0000000	2.1650000
-14211.0000000	2.1750002
-14210.0000000	2.1849999
-14209.0000000	2.2000000
-14208.0000000	2.2150002
-14207.0000000	2.2150002
-14206.0000000	2.2200000
-14205.0000000	2.2150002
-14204.0000000	2.2200000
-14203.0000000	2.2200000
-14202.0000000	2.2150002
-14201.0000000	2.2150002
-14200.0000000	2.2100000
-14199.0000000	2.1900001
-14198.0000000	2.1750002
-14197.0000000	2.1600001
-14196.0000000	2.1400001
-14195.0000000	2.0999999
-14194.0000000	2.0799999
-14193.0000000	2.0599999
-14192.0000000	2.0500000
-14191.0000000	2.0599999
-14190.0000000	2.0650001
-14189.0000000	2.0850000
-14188.0000000	2.1250000
-14187.0000000	2.1650000
-14186.0000000	2.1950002
-14185.0000000	2.2200000
-14184.0000000	2.2600000
-14183.0000000	2.2950001
-14182.0000000	2.3099999
-14181.0000000	2.3199999
-14180.0000000	2.3299999
-14179.0000000	2.3399999
-14178.0000000	2.3499999
-14177.0000000	2.3499999
-14176.0000000	2.3499999
-14175.0000000	2.3449998
-14174.0000000	2.3399999
-14173.0000000	2.3350000
-14172.0000000	2.3350000
-14171.0000000	2.3299999
-14170.0000000	2.3350000
-14169.0000000	2.3350000
-14168.0000000	2.3499999
-14167.0000000	2.3599999
-14166.0000000	2.3750000
-14165.0000000	2.3750000
-14164.0000000	2.3850002
-14163.0000000	2.3850002
-14162.0000000	2.3950000
-14161.0000000	2.3950000

-14160.0000000	2.3900001
-14159.0000000	2.3800001
-14158.0000000	2.3649998
-14157.0000000	2.3599999
-14156.0000000	2.3449998
-14155.0000000	2.3399999
-14154.0000000	2.3350000
-14153.0000000	2.3399999
-14152.0000000	2.3299999
-14151.0000000	2.3150001
-14150.0000000	2.3099999
-14149.0000000	2.3099999
-14148.0000000	2.2950001
-14147.0000000	2.3049998
-14146.0000000	2.3150001
-14145.0000000	2.3333333
-14144.0000000	2.3499999
-14143.0000000	2.3499999
-14142.0000000	2.3499999
-14141.0000000	2.3449998
-14140.0000000	2.3399999
-14139.0000000	2.3350000
-14138.0000000	2.3249998
-14137.0000000	2.3249998
-14136.0000000	2.3099999
-14135.0000000	2.3099999
-14134.0000000	2.3049998
-14133.0000000	2.3049998
-14132.0000000	2.3099999
-14131.0000000	2.3099999
-14130.0000000	2.3199999
-14129.0000000	2.3099999
-14128.0000000	2.3000000
-14127.0000000	2.2750001
-14126.0000000	2.2649999
-14125.0000000	2.2550001
-14124.0000000	2.2550001
-14123.0000000	2.2550001
-14122.0000000	2.2649999
-14121.0000000	2.2750001
-14120.0000000	2.2800000
-14119.0000000	2.2849998
-14118.0000000	2.2900000
-14117.0000000	2.3049998
-14116.0000000	2.3099999
-14115.0000000	2.3150001
-14114.0000000	2.3133333
-14113.0000000	2.3299999
-14112.0000000	2.3399999
-14111.0000000	2.3550000
-14110.0000000	2.3800001
-14109.0000000	2.3800001
-14108.0000000	2.3900001
-14107.0000000	2.4000001
-14106.0000000	2.4150000
-14105.0000000	2.4200001
-14104.0000000	2.4050002

-14103.0000000	2.3900001
-14102.0000000	2.3800001
-14101.0000000	2.3900001
-14100.0000000	2.3850002
-14099.0000000	2.3900001
-14098.0000000	2.3850002
-14097.0000000	2.3900001
-14096.0000000	2.3800001
-14095.0000000	2.3800001
-14094.0000000	2.3750000
-14093.0000000	2.3599999
-14092.0000000	2.3350000
-14091.0000000	2.3299999
-14090.0000000	2.3099999
-14089.0000000	2.3049998
-14088.0000000	2.3150001
-14087.0000000	2.3099999
-14086.0000000	2.3099999
-14085.0000000	2.3166666
-14084.0000000	2.3099999
-14083.0000000	2.3000000
-14082.0000000	2.2950001
-14081.0000000	2.2849998
-14080.0000000	2.2800000
-14079.0000000	2.2849998
-14078.0000000	2.2750001
-14077.0000000	2.2649999
-14076.0000000	2.2550001
-14075.0000000	2.2500000
-14074.0000000	2.2500000
-14073.0000000	2.2649999
-14072.0000000	2.2700000
-14071.0000000	2.2800000
-14070.0000000	2.2950001
-14069.0000000	2.3000000
-14068.0000000	2.3199999
-14067.0000000	2.3299999
-14066.0000000	2.3449998
-14065.0000000	2.3599999
-14064.0000000	2.3750000
-14063.0000000	2.3800001
-14062.0000000	2.3850002
-14061.0000000	2.3900001
-14060.0000000	2.3850002
-14059.0000000	2.3800001
-14058.0000000	2.3649998
-14057.0000000	2.3399999
-14056.0000000	2.3099999
-14055.0000000	2.2800000
-14054.0000000	2.2500000
-14053.0000000	2.2200000
-14052.0000000	2.2200000
-14051.0000000	2.2249999
-14050.0000000	2.2400000
-14049.0000000	2.2600000
-14048.0000000	2.2750001
-14047.0000000	2.2849998

-14046.0000000	2.2800000
-14045.0000000	2.2800000
-14044.0000000	2.2849998
-14043.0000000	2.2750001
-14042.0000000	2.2550001
-14041.0000000	2.2150002
-14040.0000000	2.1900001
-14039.0000000	2.2000000
-14038.0000000	2.2200000
-14037.0000000	2.2249999
-14036.0000000	2.2300000
-14035.0000000	2.2300000
-14034.0000000	2.2300000
-14033.0000000	2.2200000
-14032.0000000	2.2049999
-14031.0000000	2.1849999
-14030.0000000	2.1650000
-14029.0000000	2.1650000
-14028.0000000	2.1500001
-14027.0000000	2.1350002
-14026.0000000	2.1400001
-14025.0000000	2.1400001
-14024.0000000	2.1350002
-14023.0000000	2.1366670
-14022.0000000	2.1350002
-14021.0000000	2.1350002
-14020.0000000	2.1300001
-14019.0000000	2.1199999
-14018.0000000	2.1050000
-14017.0000000	2.0999999
-14016.0000000	2.0850000
-14015.0000000	2.0500000
-14014.0000000	2.0500000
-14013.0000000	2.0599999
-14012.0000000	2.0650001
-14011.0000000	2.0799999
-14010.0000000	2.0949998
-14009.0000000	2.0999999
-14008.0000000	2.1050000
-14007.0000000	2.1050000
-14006.0000000	2.1099999
-14005.0000000	2.1050000
-14004.0000000	2.0999999
-14003.0000000	2.0899999
-14002.0000000	2.0850000
-14001.0000000	2.0699999
-14000.0000000	2.0549998
-13999.0000000	2.0349998
-13998.0000000	2.0100000
-13997.0000000	1.9850000
-13996.0000000	1.9650000
-13995.0000000	1.9700000
-13994.0000000	1.9866667
-13993.0000000	1.9950000
-13992.0000000	2.0050001
-13991.0000000	2.0200000
-13990.0000000	2.0349998

-13989.0000000	2.0500000
-13988.0000000	2.0699999
-13987.0000000	2.0799999
-13986.0000000	2.0850000
-13985.0000000	2.1050000
-13984.0000000	2.1099999
-13983.0000000	2.1149998
-13982.0000000	2.0949998
-13981.0000000	2.0949998
-13980.0000000	2.0850000
-13979.0000000	2.0749998
-13978.0000000	2.0749998
-13977.0000000	2.0850000
-13976.0000000	2.0949998
-13975.0000000	2.0999999
-13974.0000000	2.0949998
-13973.0000000	2.0899999
-13972.0000000	2.0650001
-13971.0000000	2.0450001
-13970.0000000	2.0699999
-13969.0000000	2.0650001
-13968.0000000	2.0650001
-13967.0000000	2.0650001
-13966.0000000	2.0850000
-13965.0000000	2.0949998
-13964.0000000	2.0999999
-13963.0000000	2.0999999
-13962.0000000	2.0999999
-13961.0000000	2.1099999
-13960.0000000	2.1250000
-13959.0000000	2.1300001
-13958.0000000	2.1350002
-13957.0000000	2.1500001
-13956.0000000	2.1550002
-13955.0000000	2.1550002
-13954.0000000	2.1500001
-13953.0000000	2.1450000
-13952.0000000	2.1550002
-13951.0000000	2.1550002
-13950.0000000	2.1450000
-13949.0000000	2.1400001
-13948.0000000	2.1300001
-13947.0000000	2.1350002
-13946.0000000	2.1250000
-13945.0000000	2.1250000
-13944.0000000	2.1199999
-13943.0000000	2.1250000
-13942.0000000	2.1250000
-13941.0000000	2.1099999
-13940.0000000	2.0999999
-13939.0000000	2.0949998
-13938.0000000	2.0949998
-13937.0000000	2.0899999
-13936.0000000	2.0799999
-13935.0000000	2.0450001
-13934.0000000	2.0133333
-13933.0000000	2.0000000

-13932.0000000	2.0050001
-13931.0000000	2.0050001
-13930.0000000	2.0000000
-13929.0000000	1.9950000
-13928.0000000	2.0000000
-13927.0000000	1.9900000
-13926.0000000	1.9950000
-13925.0000000	2.0050001
-13924.0000000	1.9950000
-13923.0000000	1.9950000
-13922.0000000	2.0000000
-13921.0000000	2.0100000
-13920.0000000	2.0149999
-13919.0000000	2.0200000
-13918.0000000	2.0250001
-13917.0000000	2.0100000
-13916.0000000	2.0050001
-13915.0000000	2.0149999
-13914.0000000	2.0300000
-13913.0000000	2.0400000
-13912.0000000	2.0400000
-13911.0000000	2.0250001
-13910.0000000	2.0100000
-13909.0000000	2.0050001
-13908.0000000	2.0050001
-13907.0000000	2.0100000
-13906.0000000	2.0050001
-13905.0000000	2.0149999
-13904.0000000	2.0266666
-13903.0000000	2.0549998
-13902.0000000	2.0699999
-13901.0000000	2.0949998
-13900.0000000	2.0999999
-13899.0000000	2.1149998
-13898.0000000	2.1149998
-13897.0000000	2.1350002
-13896.0000000	2.1300001
-13895.0000000	2.1350002
-13894.0000000	2.1450000
-13893.0000000	2.1450000
-13892.0000000	2.1400001
-13891.0000000	2.1450000
-13890.0000000	2.1450000
-13889.0000000	2.1500001
-13888.0000000	2.1500001
-13887.0000000	2.1450000
-13886.0000000	2.1350002
-13885.0000000	2.1250000
-13884.0000000	2.0999999
-13883.0000000	2.0899999
-13882.0000000	2.0799999
-13881.0000000	2.0949998
-13880.0000000	2.0899999
-13879.0000000	2.0899999
-13878.0000000	2.0999999
-13877.0000000	2.1050000
-13876.0000000	2.1250000

-13875.0000000	2.1350002
-13874.0000000	2.1433334
-13873.0000000	2.1450000
-13872.0000000	2.1550002
-13871.0000000	2.1300001
-13870.0000000	2.1300001
-13869.0000000	2.1300001
-13868.0000000	2.1250000
-13867.0000000	2.1199999
-13866.0000000	2.1250000
-13865.0000000	2.1450000
-13864.0000000	2.1400001
-13863.0000000	2.1450000
-13862.0000000	2.1500001
-13861.0000000	2.1550002
-13860.0000000	2.1600001
-13859.0000000	2.1700001
-13858.0000000	2.1800001
-13857.0000000	2.1750002
-13856.0000000	2.1750002
-13855.0000000	2.1750002
-13854.0000000	2.1750002
-13853.0000000	2.1800001
-13852.0000000	2.1600001
-13851.0000000	2.1600001
-13850.0000000	2.1550002
-13849.0000000	2.1600001
-13848.0000000	2.1600001
-13847.0000000	2.1600001
-13846.0000000	2.1400001
-13845.0000000	2.1300001
-13844.0000000	2.1300001
-13843.0000000	2.1433334
-13842.0000000	2.1600001
-13841.0000000	2.1750002
-13840.0000000	2.1849999
-13839.0000000	2.1900001
-13838.0000000	2.1849999
-13837.0000000	2.1800001
-13836.0000000	2.1700001
-13835.0000000	2.1750002
-13834.0000000	2.1700001
-13833.0000000	2.1700001
-13832.0000000	2.1800001
-13831.0000000	2.2000000
-13830.0000000	2.2100000
-13829.0000000	2.2150002
-13828.0000000	2.2300000
-13827.0000000	2.2400000
-13826.0000000	2.2600000
-13825.0000000	2.2649999
-13824.0000000	2.2649999
-13823.0000000	2.2750001
-13822.0000000	2.2800000
-13821.0000000	2.3000000
-13820.0000000	2.3099999
-13819.0000000	2.3049998

-13818.0000000	2.3099999
-13817.0000000	2.3099999
-13816.0000000	2.3049998
-13815.0000000	2.3199999
-13814.0000000	2.3266666
-13813.0000000	2.3449998
-13812.0000000	2.3699999
-13811.0000000	2.3850002
-13810.0000000	2.3850002
-13809.0000000	2.4000001
-13808.0000000	2.4200001
-13807.0000000	2.4200001
-13806.0000000	2.4200001
-13805.0000000	2.4200001
-13804.0000000	2.4250002
-13803.0000000	2.4300001
-13802.0000000	2.4349999
-13801.0000000	2.4300001
-13800.0000000	2.4300001
-13799.0000000	2.4250002
-13798.0000000	2.4349999
-13797.0000000	2.4300001
-13796.0000000	2.4400001
-13795.0000000	2.4300001
-13794.0000000	2.4349999
-13793.0000000	2.4349999
-13792.0000000	2.4300001
-13791.0000000	2.4250002
-13790.0000000	2.4050002
-13789.0000000	2.3900001
-13788.0000000	2.3950000
-13787.0000000	2.3900001
-13786.0000000	2.3900001
-13785.0000000	2.3800001
-13784.0000000	2.3750000
-13783.0000000	2.3733332
-13782.0000000	2.3699999
-13781.0000000	2.3649998
-13780.0000000	2.3599999
-13779.0000000	2.3599999
-13778.0000000	2.3750000
-13777.0000000	2.3699999
-13776.0000000	2.3750000
-13775.0000000	2.3800001
-13774.0000000	2.3800001
-13773.0000000	2.3800001
-13772.0000000	2.3800001
-13771.0000000	2.3850002
-13770.0000000	2.3950000
-13769.0000000	2.3950000
-13768.0000000	2.4050002
-13767.0000000	2.4150000
-13766.0000000	2.4250002
-13765.0000000	2.4100001
-13764.0000000	2.4050002
-13763.0000000	2.3950000
-13762.0000000	2.3950000

-13761.0000000	2.3900001
-13760.0000000	2.3900001
-13759.0000000	2.3900001
-13758.0000000	2.3800001
-13757.0000000	2.3850002
-13756.0000000	2.3699999
-13755.0000000	2.3550000
-13754.0000000	2.3399999
-13753.0000000	2.3299999
-13752.0000000	2.3199999
-13751.0000000	2.3150001
-13750.0000000	2.3000000
-13749.0000000	2.2700000
-13748.0000000	2.2550001
-13747.0000000	2.2500000
-13746.0000000	2.2449999
-13745.0000000	2.2449999
-13744.0000000	2.2350001
-13743.0000000	2.2350001
-13742.0000000	2.2400000
-13741.0000000	2.2449999
-13740.0000000	2.2400000
-13739.0000000	2.2449999
-13738.0000000	2.2449999
-13737.0000000	2.2400000
-13736.0000000	2.2350001
-13735.0000000	2.2249999
-13734.0000000	2.2350001
-13733.0000000	2.2150002
-13732.0000000	2.2100000
-13731.0000000	2.2000000
-13730.0000000	2.1950002
-13729.0000000	2.1900001
-13728.0000000	2.1800001
-13727.0000000	2.1700001
-13726.0000000	2.1350000
-13725.0000000	2.1250000
-13724.0000000	2.1350002
-13723.0000000	2.1433334
-13722.0000000	2.1450000
-13721.0000000	2.1500001
-13720.0000000	2.1550002
-13719.0000000	2.1550002
-13718.0000000	2.1650000
-13717.0000000	2.1800001
-13716.0000000	2.1849999
-13715.0000000	2.1849999
-13714.0000000	2.2000000
-13713.0000000	2.1950002
-13712.0000000	2.2000000
-13711.0000000	2.1950002
-13710.0000000	2.2049999
-13709.0000000	2.2000000
-13708.0000000	2.2000000
-13707.0000000	2.2100000
-13706.0000000	2.2100000
-13705.0000000	2.2200000

-13704.0000000	2.2200000
-13703.0000000	2.2150002
-13702.0000000	2.2150002
-13701.0000000	2.2150002
-13700.0000000	2.2049999
-13699.0000000	2.2000000
-13698.0000000	2.2049999
-13697.0000000	2.2150002
-13696.0000000	2.2100000
-13695.0000000	2.2100000
-13694.0000000	2.2000000
-13693.0000000	2.1900001
-13692.0000000	2.1800001
-13691.0000000	2.1650000
-13690.0000000	2.1650000
-13689.0000000	2.1600001
-13688.0000000	2.1650000
-13687.0000000	2.1650000
-13686.0000000	2.1700001
-13685.0000000	2.1600001
-13684.0000000	2.1600001
-13683.0000000	2.1450000
-13682.0000000	2.1199999
-13681.0000000	2.1050000
-13680.0000000	2.0999999
-13679.0000000	2.0699999
-13678.0000000	2.0599999
-13677.0000000	2.0400000
-13676.0000000	2.0149999
-13675.0000000	2.0100000
-13674.0000000	2.0250001
-13673.0000000	2.0250001
-13672.0000000	2.0300000
-13671.0000000	2.0300000
-13670.0000000	2.0349998
-13669.0000000	2.0400000
-13668.0000000	2.0500000
-13667.0000000	2.0599999
-13666.0000000	2.0599999
-13665.0000000	2.0699999
-13664.0000000	2.0749998
-13663.0000000	2.0799999
-13662.0000000	2.0899999
-13661.0000000	2.0899999
-13660.0000000	2.0899999
-13659.0000000	2.0899999
-13658.0000000	2.0799999
-13657.0000000	2.0749998
-13656.0000000	2.0599999
-13655.0000000	2.0500000
-13654.0000000	2.0599999
-13653.0000000	2.0650001
-13652.0000000	2.0699999
-13651.0000000	2.0749998
-13650.0000000	2.0999999
-13649.0000000	2.1199999
-13648.0000000	2.1199999

-13647.0000000	2.1450000
-13646.0000000	2.1450000
-13645.0000000	2.1500001
-13644.0000000	2.1500001
-13643.0000000	2.1600001
-13642.0000000	2.1750002
-13641.0000000	2.1800001
-13640.0000000	2.1900001
-13639.0000000	2.1849999
-13638.0000000	2.1650000
-13637.0000000	2.1600001
-13636.0000000	2.1550002
-13635.0000000	2.1650000
-13634.0000000	2.1750002
-13633.0000000	2.2000000
-13632.0000000	2.2200000
-13631.0000000	2.2100000
-13630.0000000	2.2200000
-13629.0000000	2.2150002
-13628.0000000	2.2100000
-13627.0000000	2.2100000
-13626.0000000	2.2150002
-13625.0000000	2.2100000
-13624.0000000	2.2100000
-13623.0000000	2.2100000
-13622.0000000	2.2049999
-13621.0000000	2.2049999
-13620.0000000	2.2150002
-13619.0000000	2.2300000
-13618.0000000	2.2350001
-13617.0000000	2.2300000
-13616.0000000	2.2300000
-13615.0000000	2.2350001
-13614.0000000	2.2300000
-13613.0000000	2.2249999
-13612.0000000	2.2300000
-13611.0000000	2.2249999
-13610.0000000	2.2100000
-13609.0000000	2.1950002
-13608.0000000	2.2000000
-13607.0000000	2.1950002
-13606.0000000	2.2000000
-13605.0000000	2.1849999
-13604.0000000	2.1849999
-13603.0000000	2.1849999
-13602.0000000	2.1650000
-13601.0000000	2.1600001
-13600.0000000	2.1533334
-13599.0000000	2.1750002
-13598.0000000	2.1650000
-13597.0000000	2.1800001
-13596.0000000	2.1800001
-13595.0000000	2.1750002
-13594.0000000	2.1800001
-13593.0000000	2.1849999
-13592.0000000	2.1900001
-13591.0000000	2.1950002

-13590.0000000	2.2000000
-13589.0000000	2.2150002
-13588.0000000	2.2150002
-13587.0000000	2.2200000
-13586.0000000	2.2300000
-13585.0000000	2.2350001
-13584.0000000	2.2449999
-13583.0000000	2.2400000
-13582.0000000	2.2400000
-13581.0000000	2.2400000
-13580.0000000	2.2350001
-13579.0000000	2.2300000
-13578.0000000	2.2300000
-13577.0000000	2.2249999
-13576.0000000	2.2249999
-13575.0000000	2.2150002
-13574.0000000	2.2000000
-13573.0000000	2.1800001
-13572.0000000	2.1550002
-13571.0000000	2.1333334
-13570.0000000	2.1050000
-13569.0000000	2.0850000
-13568.0000000	2.0699999
-13567.0000000	2.0599999
-13566.0000000	2.0500000
-13565.0000000	2.0549998
-13564.0000000	2.0650001
-13563.0000000	2.0749998
-13562.0000000	2.0749998
-13561.0000000	2.0799999
-13560.0000000	2.0799999
-13559.0000000	2.0850000
-13558.0000000	2.1099999
-13557.0000000	2.1149998
-13556.0000000	2.1300001
-13555.0000000	2.1300001
-13554.0000000	2.1149998
-13553.0000000	2.1149998
-13552.0000000	2.1099999
-13551.0000000	2.1149998
-13550.0000000	2.1199999
-13549.0000000	2.1250000
-13548.0000000	2.1199999
-13547.0000000	2.1250000
-13546.0000000	2.1199999
-13545.0000000	2.1250000
-13544.0000000	2.1250000
-13543.0000000	2.1300001
-13542.0000000	2.1300001
-13541.0000000	2.1350002
-13540.0000000	2.1333334
-13539.0000000	2.1300001
-13538.0000000	2.1199999
-13537.0000000	2.1199999
-13536.0000000	2.1250000
-13535.0000000	2.1199999
-13534.0000000	2.1300001

-13533.0000000	2.1300001
-13532.0000000	2.1300001
-13531.0000000	2.1400001
-13530.0000000	2.1450000
-13529.0000000	2.1600001
-13528.0000000	2.1550002
-13527.0000000	2.1550002
-13526.0000000	2.1650000
-13525.0000000	2.1700001
-13524.0000000	2.1950002
-13523.0000000	2.2249999
-13522.0000000	2.2500000
-13521.0000000	2.2550001
-13520.0000000	2.2649999
-13519.0000000	2.2649999
-13518.0000000	2.2700000
-13517.0000000	2.2800000
-13516.0000000	2.2950001
-13515.0000000	2.3049998
-13514.0000000	2.3049998
-13513.0000000	2.3099999
-13512.0000000	2.3150001
-13511.0000000	2.3133333
-13510.0000000	2.3199999
-13509.0000000	2.3150001
-13508.0000000	2.3099999
-13507.0000000	2.3000000
-13506.0000000	2.3049998
-13505.0000000	2.2950001
-13504.0000000	2.2849998
-13503.0000000	2.2500000
-13502.0000000	2.2150002
-13501.0000000	2.1700001
-13500.0000000	2.1500001
-13499.0000000	2.1400001
-13498.0000000	2.1199999
-13497.0000000	2.1199999
-13496.0000000	2.1149998
-13495.0000000	2.1099999
-13494.0000000	2.1050000
-13493.0000000	2.1099999
-13492.0000000	2.1250000
-13491.0000000	2.1350002
-13490.0000000	2.1450000
-13489.0000000	2.1400001
-13488.0000000	2.1350002
-13487.0000000	2.1300001
-13486.0000000	2.1300001
-13485.0000000	2.1199999
-13484.0000000	2.0999999
-13483.0000000	2.0749998
-13482.0000000	2.0599999
-13481.0000000	2.0633333
-13480.0000000	2.0650001
-13479.0000000	2.0599999
-13478.0000000	2.0650001
-13477.0000000	2.0799999

-13476.0000000	2.0799999
-13475.0000000	2.0749998
-13474.0000000	2.0549998
-13473.0000000	2.0400000
-13472.0000000	2.0450001
-13471.0000000	2.0549998
-13470.0000000	2.0549998
-13469.0000000	2.0599999
-13468.0000000	2.0650001
-13467.0000000	2.0650001
-13466.0000000	2.0799999
-13465.0000000	2.1050000
-13464.0000000	2.1250000
-13463.0000000	2.1450000
-13462.0000000	2.1600001
-13461.0000000	2.1800001
-13460.0000000	2.1849999
-13459.0000000	2.1849999
-13458.0000000	2.1950002
-13457.0000000	2.1950002
-13456.0000000	2.1900001
-13455.0000000	2.1800001
-13454.0000000	2.1500001
-13453.0000000	2.1149998
-13452.0000000	2.1050000
-13451.0000000	2.0799999
-13450.0000000	2.0733330
-13449.0000000	2.0749998
-13448.0000000	2.0799999
-13447.0000000	2.0650001
-13446.0000000	2.0450001
-13445.0000000	2.0349998
-13444.0000000	2.0300000
-13443.0000000	2.0149999
-13442.0000000	2.0100000
-13441.0000000	2.0200000
-13440.0000000	2.0349998
-13439.0000000	2.0500000
-13438.0000000	2.0699999
-13437.0000000	2.0899999
-13436.0000000	2.0949998
-13435.0000000	2.1050000
-13434.0000000	2.1050000
-13433.0000000	2.1300001
-13432.0000000	2.1400001
-13431.0000000	2.1500001
-13430.0000000	2.1500001
-13429.0000000	2.1550002
-13428.0000000	2.1600001
-13427.0000000	2.1600001
-13426.0000000	2.1550002
-13425.0000000	2.1550002
-13424.0000000	2.1400001
-13423.0000000	2.1400001
-13422.0000000	2.1400001
-13421.0000000	2.1350002
-13420.0000000	2.1366670

-13419.0000000	2.1350002
-13418.0000000	2.1450000
-13417.0000000	2.1450000
-13416.0000000	2.1450000
-13415.0000000	2.1350002
-13414.0000000	2.1300001
-13413.0000000	2.1400001
-13412.0000000	2.1250000
-13411.0000000	2.1199999
-13410.0000000	2.1099999
-13409.0000000	2.0899999
-13408.0000000	2.0799999
-13407.0000000	2.0699999
-13406.0000000	2.0699999
-13405.0000000	2.0749998
-13404.0000000	2.0850000
-13403.0000000	2.0850000
-13402.0000000	2.0799999
-13401.0000000	2.0749998
-13400.0000000	2.0899999
-13399.0000000	2.0949998
-13398.0000000	2.0949998
-13397.0000000	2.0899999
-13396.0000000	2.0850000
-13395.0000000	2.0850000
-13394.0000000	2.0850000
-13393.0000000	2.0749998
-13392.0000000	2.0749998
-13391.0000000	2.0599999
-13390.0000000	2.0533333
-13389.0000000	2.0450001
-13388.0000000	2.0400000
-13387.0000000	2.0300000
-13386.0000000	2.0149999
-13385.0000000	1.9950000
-13384.0000000	1.9750000
-13383.0000000	1.9600000
-13382.0000000	1.9400001
-13381.0000000	1.9050000
-13380.0000000	1.8600000
-13379.0000000	1.8049999
-13378.0000000	1.8049999
-13377.0000000	1.8199999
-13376.0000000	1.8350000
-13375.0000000	1.8350000
-13374.0000000	1.8400000
-13373.0000000	1.8300000
-13372.0000000	1.8250000
-13371.0000000	1.8350000
-13370.0000000	1.8450000
-13369.0000000	1.8650000
-13368.0000000	1.8900000
-13367.0000000	1.9150000
-13366.0000000	1.9449999
-13365.0000000	1.9700000
-13364.0000000	1.9800000
-13363.0000000	2.0000000

-13362.0000000	2.0250001
-13361.0000000	2.0300000
-13360.0000000	2.0450001
-13359.0000000	2.0500000
-13358.0000000	2.0599999
-13357.0000000	2.0599999
-13356.0000000	2.0699999
-13355.0000000	2.0749998
-13354.0000000	2.0749998
-13353.0000000	2.0699999
-13352.0000000	2.0650001
-13351.0000000	2.0450001
-13350.0000000	2.0349998
-13349.0000000	2.0100000
-13348.0000000	1.9950000
-13347.0000000	1.9650000
-13346.0000000	1.9500000
-13345.0000000	1.9400001
-13344.0000000	1.9500000
-13343.0000000	1.9550000
-13342.0000000	1.9700000
-13341.0000000	1.9700000
-13340.0000000	1.9700000
-13339.0000000	1.9750000
-13338.0000000	1.9950000
-13337.0000000	2.0200000
-13336.0000000	2.0450001
-13335.0000000	2.0699999
-13334.0000000	2.0949998
-13333.0000000	2.1149998
-13332.0000000	2.1300001
-13331.0000000	2.1450000
-13330.0000000	2.1500001
-13329.0000000	2.1633334
-13328.0000000	2.1700001
-13327.0000000	2.1650000
-13326.0000000	2.1600001
-13325.0000000	2.1500001
-13324.0000000	2.1199999
-13323.0000000	2.1050000
-13322.0000000	2.0799999
-13321.0000000	2.0549998
-13320.0000000	2.0450001
-13319.0000000	2.0200000
-13318.0000000	2.0200000
-13317.0000000	2.0250001
-13316.0000000	2.0300000
-13315.0000000	2.0500000
-13314.0000000	2.0549998
-13313.0000000	2.0599999
-13312.0000000	2.0650001
-13311.0000000	2.0699999
-13310.0000000	2.0749998
-13309.0000000	2.0799999
-13308.0000000	2.0749998
-13307.0000000	2.0650001
-13306.0000000	2.0599999

-13305.0000000	2.0500000
-13304.0000000	2.0549998
-13303.0000000	2.0549998
-13302.0000000	2.0599999
-13301.0000000	2.0599999
-13300.0000000	2.0466666
-13299.0000000	2.0200000
-13298.0000000	1.9950000
-13297.0000000	1.9750000
-13296.0000000	1.9500000
-13295.0000000	1.9450001
-13294.0000000	1.9500000
-13293.0000000	1.9600000
-13292.0000000	1.9800000
-13291.0000000	2.0050001
-13290.0000000	2.0250001
-13289.0000000	2.0549998
-13288.0000000	2.0749998
-13287.0000000	2.0949998
-13286.0000000	2.1050000
-13285.0000000	2.1199999
-13284.0000000	2.1500001
-13283.0000000	2.1650000
-13282.0000000	2.1550002
-13281.0000000	2.1700001
-13280.0000000	2.1600001
-13279.0000000	2.1550002
-13278.0000000	2.1600001
-13277.0000000	2.1550002
-13276.0000000	2.1550002
-13275.0000000	2.1550002
-13274.0000000	2.1550002
-13273.0000000	2.1550002
-13272.0000000	2.1550002
-13271.0000000	2.1500001
-13270.0000000	2.1450000
-13269.0000000	2.1500001
-13268.0000000	2.1450000
-13267.0000000	2.1350002
-13266.0000000	2.1250000
-13265.0000000	2.1050000
-13264.0000000	2.0799999
-13263.0000000	2.0599999
-13262.0000000	2.0400000
-13261.0000000	2.0450001
-13260.0000000	2.0349998
-13259.0000000	2.0250001
-13258.0000000	2.0250001
-13257.0000000	2.0250001
-13256.0000000	2.0149999
-13255.0000000	2.0149999
-13254.0000000	2.0100000
-13253.0000000	1.9950000
-13252.0000000	2.0050001
-13251.0000000	2.0000000
-13250.0000000	1.9800000
-13249.0000000	1.9800000

-13248.0000000	1.9850000
-13247.0000000	1.9950000
-13246.0000000	2.0100000
-13245.0000000	2.0149999
-13244.0000000	2.0200000
-13243.0000000	2.0349998
-13242.0000000	2.0450001
-13241.0000000	2.0650001
-13240.0000000	2.0699999
-13239.0000000	2.0699999
-13238.0000000	2.0650001
-13237.0000000	2.0549998
-13236.0000000	2.0450001
-13235.0000000	2.0450001
-13234.0000000	2.0500000
-13233.0000000	2.0549998
-13232.0000000	2.0699999
-13231.0000000	2.0699999
-13230.0000000	2.0699999
-13229.0000000	2.0799999
-13228.0000000	2.0899999
-13227.0000000	2.0850000
-13226.0000000	2.0850000
-13225.0000000	2.0799999
-13224.0000000	2.0699999
-13223.0000000	2.0650001
-13222.0000000	2.0500000
-13221.0000000	2.0400000
-13220.0000000	2.0250001
-13219.0000000	2.0000000
-13218.0000000	2.0000000
-13217.0000000	2.0000000
-13216.0000000	1.9800000
-13215.0000000	1.9650000
-13214.0000000	1.9600000
-13213.0000000	1.9650000
-13212.0000000	1.9650000
-13211.0000000	1.9550000
-13210.0000000	1.9400001
-13209.0000000	1.9333333
-13208.0000000	1.9299999
-13207.0000000	1.9200000
-13206.0000000	1.9300001
-13205.0000000	1.9400001
-13204.0000000	1.9450001
-13203.0000000	1.9550000
-13202.0000000	1.9550000
-13201.0000000	1.9500000
-13200.0000000	1.9400001
-13199.0000000	1.9300001
-13198.0000000	1.9200000
-13197.0000000	1.9250000
-13196.0000000	1.9400001
-13195.0000000	1.9550000
-13194.0000000	1.9700000
-13193.0000000	1.9950000
-13192.0000000	2.0050001

-13191.0000000	2.0000000
-13190.0000000	2.0000000
-13189.0000000	2.0100000
-13188.0000000	2.0400000
-13187.0000000	2.0650001
-13186.0000000	2.0899999
-13185.0000000	2.1099999
-13184.0000000	2.1050000
-13183.0000000	2.1050000
-13182.0000000	2.1199999
-13181.0000000	2.1300001
-13180.0000000	2.1450000
-13179.0000000	2.1600001
-13178.0000000	2.1666667
-13177.0000000	2.1950002
-13176.0000000	2.2049999
-13175.0000000	2.2100000
-13174.0000000	2.2100000
-13173.0000000	2.2150002
-13172.0000000	2.2049999
-13171.0000000	2.2100000
-13170.0000000	2.1900001
-13169.0000000	2.1750002
-13168.0000000	2.1450000
-13167.0000000	2.1149998
-13166.0000000	2.1050000
-13165.0000000	2.0749998
-13164.0000000	2.0400000
-13163.0000000	1.9850000
-13162.0000000	1.9550000
-13161.0000000	1.9500000
-13160.0000000	1.9299999
-13159.0000000	1.9200000
-13158.0000000	1.9100000
-13157.0000000	1.9100000
-13156.0000000	1.9100000
-13155.0000000	1.9449999
-13154.0000000	1.9700000
-13153.0000000	1.9950000
-13152.0000000	1.9950000
-13151.0000000	1.9850000
-13150.0000000	2.0149999
-13149.0000000	2.0200000
-13148.0000000	2.0400000
-13147.0000000	2.0200000
-13146.0000000	2.0149999
-13145.0000000	2.0000000
-13144.0000000	1.9950000
-13143.0000000	2.0000000
-13142.0000000	2.0000000
-13141.0000000	1.9800000
-13140.0000000	1.9850000
-13139.0000000	1.9900000
-13138.0000000	1.9900000
-13137.0000000	1.9750000
-13136.0000000	1.9700000
-13135.0000000	1.9600000

-13134.0000000	1.9450001
-13133.0000000	1.9400001
-13132.0000000	1.9450001
-13131.0000000	1.9500000
-13130.0000000	1.9750000
-13129.0000000	2.0000000
-13128.0000000	2.0200000
-13127.0000000	2.0400000
-13126.0000000	2.0500000
-13125.0000000	2.0699999
-13124.0000000	2.0899999
-13123.0000000	2.1149998
-13122.0000000	2.1199999
-13121.0000000	2.1250000
-13120.0000000	2.1350002
-13119.0000000	2.1350002
-13118.0000000	2.1400001
-13117.0000000	2.1233332
-13116.0000000	2.1149998
-13115.0000000	2.1099999
-13114.0000000	2.1199999
-13113.0000000	2.1199999
-13112.0000000	2.1199999
-13111.0000000	2.1149998
-13110.0000000	2.1050000
-13109.0000000	2.1050000
-13108.0000000	2.0899999
-13107.0000000	2.0599999
-13106.0000000	2.0349998
-13105.0000000	2.0250001
-13104.0000000	2.0149999
-13103.0000000	2.0149999
-13102.0000000	2.0100000
-13101.0000000	2.0149999
-13100.0000000	2.0100000
-13099.0000000	2.0000000
-13098.0000000	1.9850000
-13097.0000000	1.9900000
-13096.0000000	1.9950000
-13095.0000000	1.9950000
-13094.0000000	1.9900000
-13093.0000000	1.9850000
-13092.0000000	1.9800000
-13091.0000000	1.9700000
-13090.0000000	1.9600000
-13089.0000000	1.9366666
-13088.0000000	1.9349999
-13087.0000000	1.9400001
-13086.0000000	1.9500000
-13085.0000000	1.9500000
-13084.0000000	1.9500000
-13083.0000000	1.9500000
-13082.0000000	1.9700000
-13081.0000000	1.9750000
-13080.0000000	1.9900000
-13079.0000000	1.9950000
-13078.0000000	2.0050001

-13077.0000000	2.0149999
-13076.0000000	2.0200000
-13075.0000000	2.0200000
-13074.0000000	2.0400000
-13073.0000000	2.0400000
-13072.0000000	2.0500000
-13071.0000000	2.0500000
-13070.0000000	2.0500000
-13069.0000000	2.0450001
-13068.0000000	2.0450001
-13067.0000000	2.0450001
-13066.0000000	2.0549998
-13065.0000000	2.0599999
-13064.0000000	2.0749998
-13063.0000000	2.0850000
-13062.0000000	2.0899999
-13061.0000000	2.0949998
-13060.0000000	2.1099999
-13059.0000000	2.1099999
-13058.0000000	2.1133330
-13057.0000000	2.1199999
-13056.0000000	2.1300001
-13055.0000000	2.1350002
-13054.0000000	2.1400001
-13053.0000000	2.1400001
-13052.0000000	2.1500001
-13051.0000000	2.1450000
-13050.0000000	2.1350002
-13049.0000000	2.0899999
-13048.0000000	2.0650001
-13047.0000000	2.0549998
-13046.0000000	2.0400000
-13045.0000000	2.0349998
-13044.0000000	2.0450001
-13043.0000000	2.0400000
-13042.0000000	2.0450001
-13041.0000000	2.0549998
-13040.0000000	2.0699999
-13039.0000000	2.0699999
-13038.0000000	2.0799999
-13037.0000000	2.0999999
-13036.0000000	2.1149998
-13035.0000000	2.1149998
-13034.0000000	2.1149998
-13033.0000000	2.1149998
-13032.0000000	2.0899999
-13031.0000000	2.0699999
-13030.0000000	2.0599999
-13029.0000000	2.0450001
-13028.0000000	2.0333333
-13027.0000000	2.0549998
-13026.0000000	2.0599999
-13025.0000000	2.0650001
-13024.0000000	2.0699999
-13023.0000000	2.0749998
-13022.0000000	2.0899999
-13021.0000000	2.1099999

-13020.0000000	2.1149998
-13019.0000000	2.1199999
-13018.0000000	2.1350002
-13017.0000000	2.1300001
-13016.0000000	2.1250000
-13015.0000000	2.1300001
-13014.0000000	2.1199999
-13013.0000000	2.1250000
-13012.0000000	2.1149998
-13011.0000000	2.0999999
-13010.0000000	2.1050000
-13009.0000000	2.1199999
-13008.0000000	2.1250000
-13007.0000000	2.1149998
-13006.0000000	2.1149998
-13005.0000000	2.1099999
-13004.0000000	2.0999999
-13003.0000000	2.1099999
-13002.0000000	2.1149998
-13001.0000000	2.1350002
-13000.0000000	2.1450000
-12999.0000000	2.1500001
-12998.0000000	2.1500001
-12997.0000000	2.1300001
-12996.0000000	2.1250000
-12995.0000000	2.1199999
-12994.0000000	2.1149998
-12993.0000000	2.1199999
-12992.0000000	2.1199999
-12991.0000000	2.1149998
-12990.0000000	2.1050000
-12989.0000000	2.0999999
-12988.0000000	2.0949998
-12987.0000000	2.0850000
-12986.0000000	2.0799999
-12985.0000000	2.0799999
-12984.0000000	2.0749998
-12983.0000000	2.0650001
-12982.0000000	2.0699999
-12981.0000000	2.0749998
-12980.0000000	2.0650001
-12979.0000000	2.0599999
-12978.0000000	2.0549998
-12977.0000000	2.0549998
-12976.0000000	2.0549998
-12975.0000000	2.0450001
-12974.0000000	2.0400000
-12973.0000000	2.0349998
-12972.0000000	2.0400000
-12971.0000000	2.0400000
-12970.0000000	2.0400000
-12969.0000000	2.0400000
-12968.0000000	2.0349998
-12967.0000000	2.0349998
-12966.0000000	2.0349998
-12965.0000000	2.0400000
-12964.0000000	2.0149999

-12963.0000000	1.9950000
-12962.0000000	1.9700000
-12961.0000000	1.9600000
-12960.0000000	1.9500000
-12959.0000000	1.9550000
-12958.0000000	1.9600000
-12957.0000000	1.9700000
-12956.0000000	1.9750000
-12955.0000000	1.9800000
-12954.0000000	2.0000000
-12953.0000000	2.0000000
-12952.0000000	2.0050001
-12951.0000000	2.0300000
-12950.0000000	2.0349998
-12949.0000000	2.0549998
-12948.0000000	2.0549998
-12947.0000000	2.0599999
-12946.0000000	2.0699999
-12945.0000000	2.0699999
-12944.0000000	2.0850000
-12943.0000000	2.0899999
-12942.0000000	2.0999999
-12941.0000000	2.0999999
-12940.0000000	2.0949998
-12939.0000000	2.0850000
-12938.0000000	2.0749998
-12937.0000000	2.0549998
-12936.0000000	2.0333331
-12935.0000000	2.0400000
-12934.0000000	2.0549998
-12933.0000000	2.0500000
-12932.0000000	2.0599999
-12931.0000000	2.0749998
-12930.0000000	2.0899999
-12929.0000000	2.0949998
-12928.0000000	2.1099999
-12927.0000000	2.1199999
-12926.0000000	2.1250000
-12925.0000000	2.1500001
-12924.0000000	2.1550002
-12923.0000000	2.1650000
-12922.0000000	2.1800001
-12921.0000000	2.1800001
-12920.0000000	2.1849999
-12919.0000000	2.1849999
-12918.0000000	2.1750002
-12917.0000000	2.1650000
-12916.0000000	2.1500001
-12915.0000000	2.1450000
-12914.0000000	2.1450000
-12913.0000000	2.1500001
-12912.0000000	2.1550002
-12911.0000000	2.1650000
-12910.0000000	2.1600001
-12909.0000000	2.1500001
-12908.0000000	2.1350002
-12907.0000000	2.1400001

-12906.0000000	2.1600001
-12905.0000000	2.1750002
-12904.0000000	2.1849999
-12903.0000000	2.1950002
-12902.0000000	2.1900001
-12901.0000000	2.2000000
-12900.0000000	2.1950002
-12899.0000000	2.1900001
-12898.0000000	2.1800001
-12897.0000000	2.1849999
-12896.0000000	2.1800001
-12895.0000000	2.1900001
-12894.0000000	2.1800001
-12893.0000000	2.1800001
-12892.0000000	2.1849999
-12891.0000000	2.1950002
-12890.0000000	2.1950002
-12889.0000000	2.1950002
-12888.0000000	2.1849999
-12887.0000000	2.1849999
-12886.0000000	2.1650000
-12885.0000000	2.1450000
-12884.0000000	2.1450000
-12883.0000000	2.1300001
-12882.0000000	2.1250000
-12881.0000000	2.1149998
-12880.0000000	2.1199999
-12879.0000000	2.1250000
-12878.0000000	2.1300001
-12877.0000000	2.1233332
-12876.0000000	2.1300001
-12875.0000000	2.1300001
-12874.0000000	2.1199999
-12873.0000000	2.1250000
-12872.0000000	2.1300001
-12871.0000000	2.1350002
-12870.0000000	2.1450000
-12869.0000000	2.1550002
-12868.0000000	2.1650000
-12867.0000000	2.1650000
-12866.0000000	2.1650000
-12865.0000000	2.1550002
-12864.0000000	2.1500001
-12863.0000000	2.1400001
-12862.0000000	2.1300001
-12861.0000000	2.1250000
-12860.0000000	2.1250000
-12859.0000000	2.1300001
-12858.0000000	2.1250000
-12857.0000000	2.1350002
-12856.0000000	2.1400001
-12855.0000000	2.1450000
-12854.0000000	2.1650000
-12853.0000000	2.1750002
-12852.0000000	2.1750002
-12851.0000000	2.1750002
-12850.0000000	2.1800001

-12849.0000000	2.1950002
-12848.0000000	2.2000000
-12847.0000000	2.2100000
-12846.0000000	2.2200000
-12845.0000000	2.2150002
-12844.0000000	2.2150002
-12843.0000000	2.1950002
-12842.0000000	2.1950002
-12841.0000000	2.1800001
-12840.0000000	2.1600001
-12839.0000000	2.1500001
-12838.0000000	2.1300001
-12837.0000000	2.1199999
-12836.0000000	2.0949998
-12835.0000000	2.0799999
-12834.0000000	2.0650001
-12833.0000000	2.0500000
-12832.0000000	2.0300000
-12831.0000000	2.0149999
-12830.0000000	2.0050001
-12829.0000000	2.0250001
-12828.0000000	2.0400000
-12827.0000000	2.0599999
-12826.0000000	2.0699999
-12825.0000000	2.0949998
-12824.0000000	2.1149998
-12823.0000000	2.1300001
-12822.0000000	2.1550002
-12821.0000000	2.1849999
-12820.0000000	2.2049999
-12819.0000000	2.2200000
-12818.0000000	2.2500000
-12817.0000000	2.2700000
-12816.0000000	2.2733333
-12815.0000000	2.2900000
-12814.0000000	2.3000000
-12813.0000000	2.2900000
-12812.0000000	2.2849998
-12811.0000000	2.2950001
-12810.0000000	2.2950001
-12809.0000000	2.3000000
-12808.0000000	2.2900000
-12807.0000000	2.2600000
-12806.0000000	2.2200000
-12805.0000000	2.1750002
-12804.0000000	2.1550002
-12803.0000000	2.1500001
-12802.0000000	2.1500001
-12801.0000000	2.1650000
-12800.0000000	2.1750002
-12799.0000000	2.1800001
-12798.0000000	2.1900001
-12797.0000000	2.2000000
-12796.0000000	2.2200000
-12795.0000000	2.2350001
-12794.0000000	2.2500000
-12793.0000000	2.2700000

-12792.0000000	2.2800000
-12791.0000000	2.2950001
-12790.0000000	2.3000000
-12789.0000000	2.3049998
-12788.0000000	2.3150001
-12787.0000000	2.3199999
-12786.0000000	2.3266666
-12785.0000000	2.3199999
-12784.0000000	2.3249998
-12783.0000000	2.3199999
-12782.0000000	2.3199999
-12781.0000000	2.3150001
-12780.0000000	2.3150001
-12779.0000000	2.2950001
-12778.0000000	2.3000000
-12777.0000000	2.3000000
-12776.0000000	2.2950001
-12775.0000000	2.2849998
-12774.0000000	2.2950001
-12773.0000000	2.3049998
-12772.0000000	2.3199999
-12771.0000000	2.3099999
-12770.0000000	2.3000000
-12769.0000000	2.2950001
-12768.0000000	2.3000000
-12767.0000000	2.2900000
-12766.0000000	2.3000000
-12765.0000000	2.3099999
-12764.0000000	2.3199999
-12763.0000000	2.3299999
-12762.0000000	2.3350000
-12761.0000000	2.3449998
-12760.0000000	2.3550000
-12759.0000000	2.3599999
-12758.0000000	2.3550000
-12757.0000000	2.3499999
-12756.0000000	2.3399999
-12755.0000000	2.3299999
-12754.0000000	2.3199999
-12753.0000000	2.3249998
-12752.0000000	2.3150001
-12751.0000000	2.3199999
-12750.0000000	2.3150001
-12749.0000000	2.3049998
-12748.0000000	2.3049998
-12747.0000000	2.3049998
-12746.0000000	2.3099999
-12745.0000000	2.3150001
-12744.0000000	2.3150001
-12743.0000000	2.3199999
-12742.0000000	2.3299999
-12741.0000000	2.3299999
-12740.0000000	2.3299999
-12739.0000000	2.3150001
-12738.0000000	2.3049998
-12737.0000000	2.2800000
-12736.0000000	2.2649999

-12735.0000000	2.2750001
-12734.0000000	2.2900000
-12733.0000000	2.3049998
-12732.0000000	2.3150001
-12731.0000000	2.3249998
-12730.0000000	2.3249998
-12729.0000000	2.3099999
-12728.0000000	2.2950001
-12727.0000000	2.2849998
-12726.0000000	2.2900000
-12725.0000000	2.3066666
-12724.0000000	2.3099999
-12723.0000000	2.2950001
-12722.0000000	2.2750001
-12721.0000000	2.2600000
-12720.0000000	2.2550001
-12719.0000000	2.2350001
-12718.0000000	2.2150002
-12717.0000000	2.1900001
-12716.0000000	2.1800001
-12715.0000000	2.1600001
-12714.0000000	2.1199999
-12713.0000000	2.0850000
-12712.0000000	2.0650001
-12711.0000000	2.0599999
-12710.0000000	2.0650001
-12709.0000000	2.0999999
-12708.0000000	2.1199999
-12707.0000000	2.1550002
-12706.0000000	2.1900001
-12705.0000000	2.2100000
-12704.0000000	2.2400000
-12703.0000000	2.2500000
-12702.0000000	2.2700000
-12701.0000000	2.2800000
-12700.0000000	2.2900000
-12699.0000000	2.3000000
-12698.0000000	2.3099999
-12697.0000000	2.3199999
-12696.0000000	2.3099999
-12695.0000000	2.3150001
-12694.0000000	2.3399999
-12693.0000000	2.3550000
-12692.0000000	2.3699999
-12691.0000000	2.3800001
-12690.0000000	2.3900001
-12689.0000000	2.3900001
-12688.0000000	2.4000001
-12687.0000000	2.4000001
-12686.0000000	2.3699999
-12685.0000000	2.3699999
-12684.0000000	2.3649998
-12683.0000000	2.3449998
-12682.0000000	2.3649998
-12681.0000000	2.3800001
-12680.0000000	2.4000001
-12679.0000000	2.4200001

-12678.0000000	2.4250002
-12677.0000000	2.4300001
-12676.0000000	2.4349999
-12675.0000000	2.4349999
-12674.0000000	2.4450002
-12673.0000000	2.4450002
-12672.0000000	2.4600000
-12671.0000000	2.4650002
-12670.0000000	2.4600000
-12669.0000000	2.4600000
-12668.0000000	2.4450002
-12667.0000000	2.4400001
-12666.0000000	2.4349999
-12665.0000000	2.4300001
-12664.0000000	2.4400001
-12663.0000000	2.4349999
-12662.0000000	2.4300001
-12661.0000000	2.4200001
-12660.0000000	2.4050002
-12659.0000000	2.4000001
-12658.0000000	2.3900001
-12657.0000000	2.3950000
-12656.0000000	2.4050002
-12655.0000000	2.4200001
-12654.0000000	2.4400001
-12653.0000000	2.4549999
-12652.0000000	2.4700000
-12651.0000000	2.4650002
-12650.0000000	2.4700000
-12649.0000000	2.4800000
-12648.0000000	2.4900000
-12647.0000000	2.4949999
-12646.0000000	2.4900000
-12645.0000000	2.4900000
-12644.0000000	2.4800000
-12643.0000000	2.4700000
-12642.0000000	2.4650002
-12641.0000000	2.4650002
-12640.0000000	2.4749999
-12639.0000000	2.4749999
-12638.0000000	2.4800000
-12637.0000000	2.4800000
-12636.0000000	2.4900000
-12635.0000000	2.5000000
-12634.0000000	2.5266666
-12633.0000000	2.5450001
-12632.0000000	2.5500000
-12631.0000000	2.5549998
-12630.0000000	2.5599999
-12629.0000000	2.5549998
-12628.0000000	2.5500000
-12627.0000000	2.5500000
-12626.0000000	2.5500000
-12625.0000000	2.5450001
-12624.0000000	2.5450001
-12623.0000000	2.5450001
-12622.0000000	2.5300000

-12621.0000000	2.5250001
-12620.0000000	2.4900000
-12619.0000000	2.4800000
-12618.0000000	2.4549999
-12617.0000000	2.4349999
-12616.0000000	2.4150000
-12615.0000000	2.4150000
-12614.0000000	2.4349999
-12613.0000000	2.4549999
-12612.0000000	2.4600000
-12611.0000000	2.4650002
-12610.0000000	2.4650002
-12609.0000000	2.4600000
-12608.0000000	2.4650002
-12607.0000000	2.4549999
-12606.0000000	2.4650002
-12605.0000000	2.4650002
-12604.0000000	2.4799998
-12603.0000000	2.4900000
-12602.0000000	2.5100000
-12601.0000000	2.5200000
-12600.0000000	2.5300000
-12599.0000000	2.5300000
-12598.0000000	2.5349998
-12597.0000000	2.5349998
-12596.0000000	2.5200000
-12595.0000000	2.5250001
-12594.0000000	2.5250001
-12593.0000000	2.5300000
-12592.0000000	2.5100000
-12591.0000000	2.5050001
-12590.0000000	2.5000000
-12589.0000000	2.5000000
-12588.0000000	2.5000000
-12587.0000000	2.5050001
-12586.0000000	2.5149999
-12585.0000000	2.5149999
-12584.0000000	2.5200000
-12583.0000000	2.5250001
-12582.0000000	2.5250001
-12581.0000000	2.5250001
-12580.0000000	2.5200000
-12579.0000000	2.5100000
-12578.0000000	2.5050001
-12577.0000000	2.5050001
-12576.0000000	2.5149999
-12575.0000000	2.5250001
-12574.0000000	2.5300000
-12573.0000000	2.5466666
-12572.0000000	2.5549998
-12571.0000000	2.5599999
-12570.0000000	2.5599999
-12569.0000000	2.5549998
-12568.0000000	2.5599999
-12567.0000000	2.5749998
-12566.0000000	2.5799999
-12565.0000000	2.5799999

-12564.0000000	2.5850000
-12563.0000000	2.5949998
-12562.0000000	2.5850000
-12561.0000000	2.5799999
-12560.0000000	2.5799999
-12559.0000000	2.5799999
-12558.0000000	2.5749998
-12557.0000000	2.5699999
-12556.0000000	2.5599999
-12555.0000000	2.5450001
-12554.0000000	2.5400000
-12553.0000000	2.5349998
-12552.0000000	2.5250001
-12551.0000000	2.5250001
-12550.0000000	2.5149999
-12549.0000000	2.5149999
-12548.0000000	2.5250001
-12547.0000000	2.5250001
-12546.0000000	2.5250001
-12545.0000000	2.5100000
-12544.0000000	2.4800000
-12543.0000000	2.4633334
-12542.0000000	2.4500000
-12541.0000000	2.4300001
-12540.0000000	2.4200001
-12539.0000000	2.4050002
-12538.0000000	2.4100001
-12537.0000000	2.4150000
-12536.0000000	2.4150000
-12535.0000000	2.4200001
-12534.0000000	2.4250002
-12533.0000000	2.4300001
-12532.0000000	2.4500000
-12531.0000000	2.4549999
-12530.0000000	2.4549999
-12529.0000000	2.4600000
-12528.0000000	2.4700000
-12527.0000000	2.4850001
-12526.0000000	2.5050001
-12525.0000000	2.5250001
-12524.0000000	2.5400000
-12523.0000000	2.5749998
-12522.0000000	2.5999999
-12521.0000000	2.6300001
-12520.0000000	2.6500001
-12519.0000000	2.6650000
-12518.0000000	2.6700001
-12517.0000000	2.6800001
-12516.0000000	2.6900001
-12515.0000000	2.7049999
-12514.0000000	2.7249999
-12513.0000000	2.7333336
-12512.0000000	2.7249999
-12511.0000000	2.6900001
-12510.0000000	2.6600001
-12509.0000000	2.6300001
-12508.0000000	2.6099999

-12507.0000000	2.6099999
-12506.0000000	2.6199999
-12505.0000000	2.6300001
-12504.0000000	2.6450000
-12503.0000000	2.6550002
-12502.0000000	2.6550002
-12501.0000000	2.6500001
-12500.0000000	2.6450000
-12499.0000000	2.6350002
-12498.0000000	2.6300001
-12497.0000000	2.6250000
-12496.0000000	2.6300001
-12495.0000000	2.6199999
-12494.0000000	2.6149998
-12493.0000000	2.6199999
-12492.0000000	2.6300001
-12491.0000000	2.6250000
-12490.0000000	2.6300001
-12489.0000000	2.6300001
-12488.0000000	2.6350002
-12487.0000000	2.6350002
-12486.0000000	2.6350002
-12485.0000000	2.6350002
-12484.0000000	2.6199999
-12483.0000000	2.6033332
-12482.0000000	2.5899999
-12481.0000000	2.5699999
-12480.0000000	2.5500000
-12479.0000000	2.5400000
-12478.0000000	2.5450001
-12477.0000000	2.5250001
-12476.0000000	2.5149999
-12475.0000000	2.5000000
-12474.0000000	2.5050001
-12473.0000000	2.4900000
-12472.0000000	2.4900000
-12471.0000000	2.4949999
-12470.0000000	2.4949999
-12469.0000000	2.5000000
-12468.0000000	2.5200000
-12467.0000000	2.5349998
-12466.0000000	2.5400000
-12465.0000000	2.5300000
-12464.0000000	2.5349998
-12463.0000000	2.5149999
-12462.0000000	2.4850001
-12461.0000000	2.4450002
-12460.0000000	2.4050002
-12459.0000000	2.3750000
-12458.0000000	2.3499999
-12457.0000000	2.3249998
-12456.0000000	2.3000000
-12455.0000000	2.2750001
-12454.0000000	2.2350001
-12453.0000000	2.2200000
-12452.0000000	2.2200000
-12451.0000000	2.2100000

-12450.0000000	2.2300000
-12449.0000000	2.2400000
-12448.0000000	2.2649999
-12447.0000000	2.2800000
-12446.0000000	2.3099999
-12445.0000000	2.3150001
-12444.0000000	2.3350000
-12443.0000000	2.3499999
-12442.0000000	2.3599999
-12441.0000000	2.3599999
-12440.0000000	2.3449998
-12439.0000000	2.3299999
-12438.0000000	2.3350000
-12437.0000000	2.3650000
-12436.0000000	2.4000001
-12435.0000000	2.4150000
-12434.0000000	2.4150000
-12433.0000000	2.4050002
-12432.0000000	2.3950000
-12431.0000000	2.3850002
-12430.0000000	2.3750000
-12429.0000000	2.3750000
-12428.0000000	2.3649998
-12427.0000000	2.3599999
-12426.0000000	2.3649998
-12425.0000000	2.3550000
-12424.0000000	2.3499999
-12423.0000000	2.3699999
-12422.0000000	2.3866670
-12421.0000000	2.3950000
-12420.0000000	2.4150000
-12419.0000000	2.4200001
-12418.0000000	2.4450002
-12417.0000000	2.4450002
-12416.0000000	2.4450002
-12415.0000000	2.4500000
-12414.0000000	2.4549999
-12413.0000000	2.4549999
-12412.0000000	2.4500000
-12411.0000000	2.4549999
-12410.0000000	2.4500000
-12409.0000000	2.4500000
-12408.0000000	2.4349999
-12407.0000000	2.4250002
-12406.0000000	2.4000001
-12405.0000000	2.3800001
-12404.0000000	2.3599999
-12403.0000000	2.3550000
-12402.0000000	2.3499999
-12401.0000000	2.3399999
-12400.0000000	2.3350000
-12399.0000000	2.3150001
-12398.0000000	2.3049998
-12397.0000000	2.2950001
-12396.0000000	2.2900000
-12395.0000000	2.2700000
-12394.0000000	2.2449999

-12393.0000000	2.2150002
-12392.0000000	2.2133334
-12391.0000000	2.2350001
-12390.0000000	2.2500000
-12389.0000000	2.2500000
-12388.0000000	2.2400000
-12387.0000000	2.2400000
-12386.0000000	2.2350001
-12385.0000000	2.2400000
-12384.0000000	2.2500000
-12383.0000000	2.2649999
-12382.0000000	2.2600000
-12381.0000000	2.2500000
-12380.0000000	2.2500000
-12379.0000000	2.2350001
-12378.0000000	2.2350001
-12377.0000000	2.2249999
-12376.0000000	2.2150002
-12375.0000000	2.2049999
-12374.0000000	2.2150002
-12373.0000000	2.2300000
-12372.0000000	2.2150002
-12371.0000000	2.2100000
-12370.0000000	2.2300000
-12369.0000000	2.2350001
-12368.0000000	2.2350001
-12367.0000000	2.2550001
-12366.0000000	2.2600000
-12365.0000000	2.2800000
-12364.0000000	2.2849998
-12363.0000000	2.3000000
-12362.0000000	2.3166666
-12361.0000000	2.3299999
-12360.0000000	2.3399999
-12359.0000000	2.3350000
-12358.0000000	2.3249998
-12357.0000000	2.2900000
-12356.0000000	2.2600000
-12355.0000000	2.2150002
-12354.0000000	2.1849999
-12353.0000000	2.1700001
-12352.0000000	2.1450000
-12351.0000000	2.1350002
-12350.0000000	2.1450000
-12349.0000000	2.1800001
-12348.0000000	2.2200000
-12347.0000000	2.2649999
-12346.0000000	2.3049998
-12345.0000000	2.3199999
-12344.0000000	2.3299999
-12343.0000000	2.3299999
-12342.0000000	2.3099999
-12341.0000000	2.3150001
-12340.0000000	2.3299999
-12339.0000000	2.3299999
-12338.0000000	2.3499999
-12337.0000000	2.3750000

-12336.0000000	2.3850002
-12335.0000000	2.4050002
-12334.0000000	2.4100001
-12333.0000000	2.4349999
-12332.0000000	2.4533334
-12331.0000000	2.4749999
-12330.0000000	2.4700000
-12329.0000000	2.4749999
-12328.0000000	2.4600000
-12327.0000000	2.4650002
-12326.0000000	2.4600000
-12325.0000000	2.4749999
-12324.0000000	2.4800000
-12323.0000000	2.4850001
-12322.0000000	2.4800000
-12321.0000000	2.4800000
-12320.0000000	2.4800000
-12319.0000000	2.4749999
-12318.0000000	2.4700000
-12317.0000000	2.4800000
-12316.0000000	2.4800000
-12315.0000000	2.4800000
-12314.0000000	2.4800000
-12313.0000000	2.4700000
-12312.0000000	2.4800000
-12311.0000000	2.4700000
-12310.0000000	2.4650002
-12309.0000000	2.4500000
-12308.0000000	2.4400001
-12307.0000000	2.4300001
-12306.0000000	2.4000001
-12305.0000000	2.3750000
-12304.0000000	2.3800001
-12303.0000000	2.3800001
-12302.0000000	2.3900001
-12301.0000000	2.4000001
-12300.0000000	2.4000001
-12299.0000000	2.4000001
-12298.0000000	2.3950000
-12297.0000000	2.3850002
-12296.0000000	2.3800001
-12295.0000000	2.3649998
-12294.0000000	2.3649998
-12293.0000000	2.3649998
-12292.0000000	2.3699999
-12291.0000000	2.3800001
-12290.0000000	2.3900001
-12289.0000000	2.3900001
-12288.0000000	2.3800001
-12287.0000000	2.3599999
-12286.0000000	2.3399999
-12285.0000000	2.3299999
-12284.0000000	2.3199999
-12283.0000000	2.3249998
-12282.0000000	2.3299999
-12281.0000000	2.3399999
-12280.0000000	2.3550000

-12279.0000000	2.3649998
-12278.0000000	2.3750000
-12277.0000000	2.3850002
-12276.0000000	2.4100001
-12275.0000000	2.4300001
-12274.0000000	2.4500000
-12273.0000000	2.4700000
-12272.0000000	2.4850001
-12271.0000000	2.5000000
-12270.0000000	2.5200000
-12269.0000000	2.5349998
-12268.0000000	2.5599999
-12267.0000000	2.5850000
-12266.0000000	2.6050000
-12265.0000000	2.6199999
-12264.0000000	2.6350002
-12263.0000000	2.6300001
-12262.0000000	2.6450000
-12261.0000000	2.6500001
-12260.0000000	2.6500001
-12259.0000000	2.6500001
-12258.0000000	2.6500001
-12257.0000000	2.6550002
-12256.0000000	2.6400001
-12255.0000000	2.6250000
-12254.0000000	2.6050000
-12253.0000000	2.5799999
-12252.0000000	2.5650001
-12251.0000000	2.5500000
-12250.0000000	2.5349998
-12249.0000000	2.5200000
-12248.0000000	2.5100000
-12247.0000000	2.5000000
-12246.0000000	2.4900000
-12245.0000000	2.4749999
-12244.0000000	2.4749999
-12243.0000000	2.4633334
-12242.0000000	2.4549999
-12241.0000000	2.4650002
-12240.0000000	2.4549999
-12239.0000000	2.4549999
-12238.0000000	2.4500000
-12237.0000000	2.4349999
-12236.0000000	2.4250002
-12235.0000000	2.4150000
-12234.0000000	2.4150000
-12233.0000000	2.4100001
-12232.0000000	2.3850002
-12231.0000000	2.3649998
-12230.0000000	2.3449998
-12229.0000000	2.3299999
-12228.0000000	2.3150001
-12227.0000000	2.3000000
-12226.0000000	2.2800000
-12225.0000000	2.2649999
-12224.0000000	2.2550001
-12223.0000000	2.2500000

-12222.0000000	2.2600000
-12221.0000000	2.2649999
-12220.0000000	2.2750001
-12219.0000000	2.2849998
-12218.0000000	2.2750001
-12217.0000000	2.2600000
-12216.0000000	2.2600000
-12215.0000000	2.2449999
-12214.0000000	2.2500000
-12213.0000000	2.2550001
-12212.0000000	2.2500000
-12211.0000000	2.2699997
-12210.0000000	2.2750001
-12209.0000000	2.2300000
-12208.0000000	2.2200000
-12207.0000000	2.2249999
-12206.0000000	2.2400000
-12205.0000000	2.2550001
-12204.0000000	2.2649999
-12203.0000000	2.2600000
-12202.0000000	2.2300000
-12201.0000000	2.1900001
-12200.0000000	2.1800001
-12199.0000000	2.1700001
-12198.0000000	2.1550002
-12197.0000000	2.1550002
-12196.0000000	2.1600001
-12195.0000000	2.1849999
-12194.0000000	2.2100000
-12193.0000000	2.2400000
-12192.0000000	2.2800000
-12191.0000000	2.3099999
-12190.0000000	2.3499999
-12189.0000000	2.3649998
-12188.0000000	2.3599999
-12187.0000000	2.3649998
-12186.0000000	2.3550000
-12185.0000000	2.3550000
-12184.0000000	2.3550000
-12183.0000000	2.3299999
-12182.0000000	2.2900000
-12181.0000000	2.2766666
-12180.0000000	2.2700000
-12179.0000000	2.2700000
-12178.0000000	2.2849998
-12177.0000000	2.2750001
-12176.0000000	2.2800000
-12175.0000000	2.2950001
-12174.0000000	2.2800000
-12173.0000000	2.2750001
-12172.0000000	2.2550001
-12171.0000000	2.2449999
-12170.0000000	2.2500000
-12169.0000000	2.2750001
-12168.0000000	2.2849998
-12167.0000000	2.3150001
-12166.0000000	2.3249998

-12165.0000000	2.3350000
-12164.0000000	2.3350000
-12163.0000000	2.3350000
-12162.0000000	2.3199999
-12161.0000000	2.2849998
-12160.0000000	2.2600000
-12159.0000000	2.2449999
-12158.0000000	2.2449999
-12157.0000000	2.2449999
-12156.0000000	2.2500000
-12155.0000000	2.2449999
-12154.0000000	2.2649999
-12153.0000000	2.2900000
-12152.0000000	2.3299999
-12151.0000000	2.3566666
-12150.0000000	2.3750000
-12149.0000000	2.3900001
-12148.0000000	2.4150000
-12147.0000000	2.4250002
-12146.0000000	2.4450002
-12145.0000000	2.4700000
-12144.0000000	2.4949999
-12143.0000000	2.5200000
-12142.0000000	2.5200000
-12141.0000000	2.5349998
-12140.0000000	2.5349998
-12139.0000000	2.5250001
-12138.0000000	2.5250001
-12137.0000000	2.5149999
-12136.0000000	2.5100000
-12135.0000000	2.4949999
-12134.0000000	2.4549999
-12133.0000000	2.4349999
-12132.0000000	2.4150000
-12131.0000000	2.3850000
-12130.0000000	2.3599999
-12129.0000000	2.3249998
-12128.0000000	2.2849998
-12127.0000000	2.2649999
-12126.0000000	2.2449999
-12125.0000000	2.2100000
-12124.0000000	2.2100000
-12123.0000000	2.2400000
-12122.0000000	2.2449999
-12121.0000000	2.2700000
-12120.0000000	2.2750001
-12119.0000000	2.2849998
-12118.0000000	2.2950001
-12117.0000000	2.3000000
-12116.0000000	2.2900000
-12115.0000000	2.2950001
-12114.0000000	2.3099999
-12113.0000000	2.3350000
-12112.0000000	2.3249998
-12111.0000000	2.3299999
-12110.0000000	2.3249998
-12109.0000000	2.3199999

-12108.0000000	2.3199999
-12107.0000000	2.3099999
-12106.0000000	2.3049998
-12105.0000000	2.3099999
-12104.0000000	2.3049998
-12103.0000000	2.3000000
-12102.0000000	2.2849998
-12101.0000000	2.2600000
-12100.0000000	2.2449999
-12099.0000000	2.2500000
-12098.0000000	2.2600000
-12097.0000000	2.2750001
-12096.0000000	2.2900000
-12095.0000000	2.3099999
-12094.0000000	2.3299999
-12093.0000000	2.3399999
-12092.0000000	2.3499999
-12091.0000000	2.3550000
-12090.0000000	2.3499999
-12089.0000000	2.3599999
-12088.0000000	2.3699999
-12087.0000000	2.3800001
-12086.0000000	2.3800001
-12085.0000000	2.3900001
-12084.0000000	2.4000001
-12083.0000000	2.4050002
-12082.0000000	2.4100001
-12081.0000000	2.4100001
-12080.0000000	2.4100001
-12079.0000000	2.4100001
-12078.0000000	2.4100001
-12077.0000000	2.4100001
-12076.0000000	2.3950000
-12075.0000000	2.3800001
-12074.0000000	2.3699999
-12073.0000000	2.3850002
-12072.0000000	2.4050002
-12071.0000000	2.4200001
-12070.0000000	2.4300001
-12069.0000000	2.4200001
-12068.0000000	2.4200001
-12067.0000000	2.4050002
-12066.0000000	2.4100001
-12065.0000000	2.4050002
-12064.0000000	2.4000001
-12063.0000000	2.3900001
-12062.0000000	2.4100001
-12061.0000000	2.4150000
-12060.0000000	2.4150000
-12059.0000000	2.4200001
-12058.0000000	2.4250002
-12057.0000000	2.4300001
-12056.0000000	2.4450002
-12055.0000000	2.4500000
-12054.0000000	2.4600000
-12053.0000000	2.4600000
-12052.0000000	2.4549999

-12051.0000000	2.4500000
-12050.0000000	2.4250002
-12049.0000000	2.4050002
-12048.0000000	2.3950000
-12047.0000000	2.3800001
-12046.0000000	2.3599999
-12045.0000000	2.3449998
-12044.0000000	2.3350000
-12043.0000000	2.3399999
-12042.0000000	2.3399999
-12041.0000000	2.3249998
-12040.0000000	2.3099999
-12039.0000000	2.3099999
-12038.0000000	2.3099999
-12037.0000000	2.3000000
-12036.0000000	2.2950001
-12035.0000000	2.2900000
-12034.0000000	2.2950001
-12033.0000000	2.2849998
-12032.0000000	2.2649999
-12031.0000000	2.2649999
-12030.0000000	2.2449999
-12029.0000000	2.2500000
-12028.0000000	2.2550001
-12027.0000000	2.2700000
-12026.0000000	2.2900000
-12025.0000000	2.3049998
-12024.0000000	2.3449998
-12023.0000000	2.3699999
-12022.0000000	2.3950000
-12021.0000000	2.4250002
-12020.0000000	2.4250002
-12019.0000000	2.4349999
-12018.0000000	2.4450002
-12017.0000000	2.4650002
-12016.0000000	2.4700000
-12015.0000000	2.4850001
-12014.0000000	2.5050001
-12013.0000000	2.5100000
-12012.0000000	2.5200000
-12011.0000000	2.5200000
-12010.0000000	2.5149999
-12009.0000000	2.5050001
-12008.0000000	2.5050001
-12007.0000000	2.4900000
-12006.0000000	2.4749999
-12005.0000000	2.4700000
-12004.0000000	2.4549999
-12003.0000000	2.4300001
-12002.0000000	2.4200001
-12001.0000000	2.4150000
-12000.0000000	2.4100001
-11999.0000000	2.4033334
-11998.0000000	2.4050002
-11997.0000000	2.4000001
-11996.0000000	2.3950000
-11995.0000000	2.3950000

-11994.0000000	2.3950000
-11993.0000000	2.4050002
-11992.0000000	2.4000001
-11991.0000000	2.4000001
-11990.0000000	2.4000001
-11989.0000000	2.4000001
-11988.0000000	2.3950000
-11987.0000000	2.3800001
-11986.0000000	2.3599999
-11985.0000000	2.3399999
-11984.0000000	2.3299999
-11983.0000000	2.3449998
-11982.0000000	2.3449998
-11981.0000000	2.3649998
-11980.0000000	2.3699999
-11979.0000000	2.3699999
-11978.0000000	2.3649998
-11977.0000000	2.3750000
-11976.0000000	2.3950000
-11975.0000000	2.4000001
-11974.0000000	2.4250002
-11973.0000000	2.4450002
-11972.0000000	2.4600000
-11971.0000000	2.4650002
-11970.0000000	2.4850001
-11969.0000000	2.5000000
-11968.0000000	2.5100000
-11967.0000000	2.5200000
-11966.0000000	2.5250001
-11965.0000000	2.5200000
-11964.0000000	2.5450001
-11963.0000000	2.5749998
-11962.0000000	2.6099999
-11961.0000000	2.6250000
-11960.0000000	2.6350002
-11959.0000000	2.6450000
-11958.0000000	2.6450000
-11957.0000000	2.6350002
-11956.0000000	2.6250000
-11955.0000000	2.5949998
-11954.0000000	2.5699999
-11953.0000000	2.5400000
-11952.0000000	2.5250001
-11951.0000000	2.5149999
-11950.0000000	2.4800000
-11949.0000000	2.4650002
-11948.0000000	2.4549999
-11947.0000000	2.4400001
-11946.0000000	2.4250002
-11945.0000000	2.4250002
-11944.0000000	2.4200001
-11943.0000000	2.4200001
-11942.0000000	2.4200001
-11941.0000000	2.4150000
-11940.0000000	2.4300001
-11939.0000000	2.4349999
-11938.0000000	2.4400001

-11937.0000000	2.4500000
-11936.0000000	2.4450002
-11935.0000000	2.4349999
-11934.0000000	2.4400001
-11933.0000000	2.4400001
-11932.0000000	2.4400001
-11931.0000000	2.4500000
-11930.0000000	2.4650002
-11929.0000000	2.4749999
-11928.0000000	2.4749999
-11927.0000000	2.4549999
-11926.0000000	2.4349999
-11925.0000000	2.4200001
-11924.0000000	2.4050002
-11923.0000000	2.3900001
-11922.0000000	2.3350000
-11921.0000000	2.2800000
-11920.0000000	2.2649999
-11919.0000000	2.2600000
-11918.0000000	2.2500000
-11917.0000000	2.2700000
-11916.0000000	2.2950001
-11915.0000000	2.3249998
-11914.0000000	2.3350000
-11913.0000000	2.3299999
-11912.0000000	2.3550000
-11911.0000000	2.3599999
-11910.0000000	2.3599999
-11909.0000000	2.3649998
-11908.0000000	2.3800001
-11907.0000000	2.4000001
-11906.0000000	2.4100001
-11905.0000000	2.4250002
-11904.0000000	2.4400001
-11903.0000000	2.4600000
-11902.0000000	2.4749999
-11901.0000000	2.4949999
-11900.0000000	2.4800000
-11899.0000000	2.4850001
-11898.0000000	2.4850001
-11897.0000000	2.4749999
-11896.0000000	2.4600000
-11895.0000000	2.4300001
-11894.0000000	2.3800001
-11893.0000000	2.3599999
-11892.0000000	2.3649998
-11891.0000000	2.3699999
-11890.0000000	2.3499999
-11889.0000000	2.3249998
-11888.0000000	2.3199999
-11887.0000000	2.3099999
-11886.0000000	2.2900000
-11885.0000000	2.2700000
-11884.0000000	2.2449999
-11883.0000000	2.2300000
-11882.0000000	2.2049999
-11881.0000000	2.2150002

-11880.0000000	2.2249999
-11879.0000000	2.2333333
-11878.0000000	2.2449999
-11877.0000000	2.2449999
-11876.0000000	2.2550001
-11875.0000000	2.2600000
-11874.0000000	2.2600000
-11873.0000000	2.2649999
-11872.0000000	2.2600000
-11871.0000000	2.2400000
-11870.0000000	2.2300000
-11869.0000000	2.2049999
-11868.0000000	2.1800001
-11867.0000000	2.1550002
-11866.0000000	2.1400001
-11865.0000000	2.1199999
-11864.0000000	2.1300001
-11863.0000000	2.1400001
-11862.0000000	2.1700001
-11861.0000000	2.1900001
-11860.0000000	2.1950002
-11859.0000000	2.2049999
-11858.0000000	2.1950002
-11857.0000000	2.2000000
-11856.0000000	2.2000000
-11855.0000000	2.2049999
-11854.0000000	2.2100000
-11853.0000000	2.2249999
-11852.0000000	2.2449999
-11851.0000000	2.2600000
-11850.0000000	2.2849998
-11849.0000000	2.2866666
-11848.0000000	2.2900000
-11847.0000000	2.3099999
-11846.0000000	2.3150001
-11845.0000000	2.3299999
-11844.0000000	2.3150001
-11843.0000000	2.3000000
-11842.0000000	2.2900000
-11841.0000000	2.2849998
-11840.0000000	2.2900000
-11839.0000000	2.3000000
-11838.0000000	2.2950001
-11837.0000000	2.2849998
-11836.0000000	2.2800000
-11835.0000000	2.2849998
-11834.0000000	2.2750001
-11833.0000000	2.2750001
-11832.0000000	2.2900000
-11831.0000000	2.2950001
-11830.0000000	2.2950001
-11829.0000000	2.2849998
-11828.0000000	2.2849998
-11827.0000000	2.2700000
-11826.0000000	2.2600000
-11825.0000000	2.2600000
-11824.0000000	2.2700000

-11823.0000000	2.2750001
-11822.0000000	2.2849998
-11821.0000000	2.2950001
-11820.0000000	2.3000000
-11819.0000000	2.3199999
-11818.0000000	2.3249998
-11817.0000000	2.3350000
-11816.0000000	2.3449998
-11815.0000000	2.3649998
-11814.0000000	2.3699999
-11813.0000000	2.3750000
-11812.0000000	2.3750000
-11811.0000000	2.3750000
-11810.0000000	2.3750000
-11809.0000000	2.3850002
-11808.0000000	2.3800001
-11807.0000000	2.3900001
-11806.0000000	2.3900001
-11805.0000000	2.3750000
-11804.0000000	2.3649998
-11803.0000000	2.3649998
-11802.0000000	2.3649998
-11801.0000000	2.3649998
-11800.0000000	2.3750000
-11799.0000000	2.3800001
-11798.0000000	2.3850002
-11797.0000000	2.3850002
-11796.0000000	2.3950000
-11795.0000000	2.3950000
-11794.0000000	2.4050002
-11793.0000000	2.4050002
-11792.0000000	2.4100001
-11791.0000000	2.4150000
-11790.0000000	2.4150000
-11789.0000000	2.4150000
-11788.0000000	2.4066668
-11787.0000000	2.4000001
-11786.0000000	2.4150000
-11785.0000000	2.4150000
-11784.0000000	2.4100001
-11783.0000000	2.4150000
-11782.0000000	2.4050002
-11781.0000000	2.4050002
-11780.0000000	2.3850002
-11779.0000000	2.3750000
-11778.0000000	2.3800001
-11777.0000000	2.3699999
-11776.0000000	2.3750000
-11775.0000000	2.3699999
-11774.0000000	2.3750000
-11773.0000000	2.3800001
-11772.0000000	2.4000001
-11771.0000000	2.4000001
-11770.0000000	2.3900001
-11769.0000000	2.3800001
-11768.0000000	2.3800001
-11767.0000000	2.3850002

-11766.0000000	2.3800001
-11765.0000000	2.3900001
-11764.0000000	2.4150000
-11763.0000000	2.4250002
-11762.0000000	2.4500000
-11761.0000000	2.4800000
-11760.0000000	2.5033333
-11759.0000000	2.5050001
-11758.0000000	2.5000000
-11757.0000000	2.4900000
-11756.0000000	2.4900000
-11755.0000000	2.4800000
-11754.0000000	2.4749999
-11753.0000000	2.4749999
-11752.0000000	2.4749999
-11751.0000000	2.4850001
-11750.0000000	2.4749999
-11749.0000000	2.4850001
-11748.0000000	2.4749999
-11747.0000000	2.4549999
-11746.0000000	2.4400001
-11745.0000000	2.4150000
-11744.0000000	2.3800001
-11743.0000000	2.3399999
-11742.0000000	2.3049998
-11741.0000000	2.2750001
-11740.0000000	2.2550001
-11739.0000000	2.2150002
-11738.0000000	2.1950002
-11737.0000000	2.1950002
-11736.0000000	2.2100000
-11735.0000000	2.2350001
-11734.0000000	2.2600000
-11733.0000000	2.2849998
-11732.0000000	2.2950001
-11731.0000000	2.3199999
-11730.0000000	2.3249998
-11729.0000000	2.3199999
-11728.0000000	2.3249998
-11727.0000000	2.3199999
-11726.0000000	2.2950001
-11725.0000000	2.3099999
-11724.0000000	2.3199999
-11723.0000000	2.3249998
-11722.0000000	2.3399999
-11721.0000000	2.3499999
-11720.0000000	2.3499999
-11719.0000000	2.3550000
-11718.0000000	2.3499999
-11717.0000000	2.3449998
-11716.0000000	2.3150001
-11715.0000000	2.2849998
-11714.0000000	2.2550001
-11713.0000000	2.2350001
-11712.0000000	2.2150002
-11711.0000000	2.1950002
-11710.0000000	2.1700001

-11709.0000000	2.1400001
-11708.0000000	2.1050000
-11707.0000000	2.0850000
-11706.0000000	2.0799999
-11705.0000000	2.0749998
-11704.0000000	2.0699999
-11703.0000000	2.0749998
-11702.0000000	2.0799999
-11701.0000000	2.0949998
-11700.0000000	2.0999999
-11699.0000000	2.1166666
-11698.0000000	2.1099999
-11697.0000000	2.1050000
-11696.0000000	2.1149998
-11695.0000000	2.1250000
-11694.0000000	2.1550002
-11693.0000000	2.1849999
-11692.0000000	2.2200000
-11691.0000000	2.2300000
-11690.0000000	2.2550001
-11689.0000000	2.2750001
-11688.0000000	2.2800000
-11687.0000000	2.2900000
-11686.0000000	2.3049998
-11685.0000000	2.3000000
-11684.0000000	2.2950001
-11683.0000000	2.2950001
-11682.0000000	2.2800000
-11681.0000000	2.2600000
-11680.0000000	2.2500000
-11679.0000000	2.2400000
-11678.0000000	2.2350001
-11677.0000000	2.2350001
-11676.0000000	2.2350001
-11675.0000000	2.2400000
-11674.0000000	2.2449999
-11673.0000000	2.2350001
-11672.0000000	2.2200000
-11671.0000000	2.2150002
-11670.0000000	2.2100000
-11669.0000000	2.2049999
-11668.0000000	2.2100000
-11667.0000000	2.2200000
-11666.0000000	2.2150002
-11665.0000000	2.2400000
-11664.0000000	2.2550001
-11663.0000000	2.2649999
-11662.0000000	2.2849998
-11661.0000000	2.3049998
-11660.0000000	2.3150001
-11659.0000000	2.3049998
-11658.0000000	2.3150001
-11657.0000000	2.3049998
-11656.0000000	2.3150001
-11655.0000000	2.3199999
-11654.0000000	2.3249998
-11653.0000000	2.3299999

-11652.0000000	2.3350000
-11651.0000000	2.3199999
-11650.0000000	2.3049998
-11649.0000000	2.2900000
-11648.0000000	2.2900000
-11647.0000000	2.3049998
-11646.0000000	2.3099999
-11645.0000000	2.3150001
-11644.0000000	2.3350000
-11643.0000000	2.3499999
-11642.0000000	2.3550000
-11641.0000000	2.3649998
-11640.0000000	2.3649998
-11639.0000000	2.3649998
-11638.0000000	2.3649998
-11637.0000000	2.3466666
-11636.0000000	2.3049998
-11635.0000000	2.2800000
-11634.0000000	2.2700000
-11633.0000000	2.2700000
-11632.0000000	2.2700000
-11631.0000000	2.2649999
-11630.0000000	2.2600000
-11629.0000000	2.2649999
-11628.0000000	2.2600000
-11627.0000000	2.2649999
-11626.0000000	2.2600000
-11625.0000000	2.2550001
-11624.0000000	2.2750001
-11623.0000000	2.2849998
-11622.0000000	2.2800000
-11621.0000000	2.2800000
-11620.0000000	2.2900000
-11619.0000000	2.2849998
-11618.0000000	2.2800000
-11617.0000000	2.2900000
-11616.0000000	2.3000000
-11615.0000000	2.2950001
-11614.0000000	2.3049998
-11613.0000000	2.3000000
-11612.0000000	2.2900000
-11611.0000000	2.2800000
-11610.0000000	2.2649999
-11609.0000000	2.2550001
-11608.0000000	2.2300000
-11607.0000000	2.2266667
-11606.0000000	2.2100000
-11605.0000000	2.2049999
-11604.0000000	2.2100000
-11603.0000000	2.2200000
-11602.0000000	2.2350001
-11601.0000000	2.2500000
-11600.0000000	2.2550001
-11599.0000000	2.2550001
-11598.0000000	2.2400000
-11597.0000000	2.2150002
-11596.0000000	2.1849999

-11595.0000000	2.1900001
-11594.0000000	2.1800001
-11593.0000000	2.1849999
-11592.0000000	2.1900001
-11591.0000000	2.1950002
-11590.0000000	2.1900001
-11589.0000000	2.2200000
-11588.0000000	2.2300000
-11587.0000000	2.2400000
-11586.0000000	2.2500000
-11585.0000000	2.2649999
-11584.0000000	2.2800000
-11583.0000000	2.2950001
-11582.0000000	2.2900000
-11581.0000000	2.3049998
-11580.0000000	2.3150001
-11579.0000000	2.3199999
-11578.0000000	2.3350000
-11577.0000000	2.3550000
-11576.0000000	2.3633330
-11575.0000000	2.3649998
-11574.0000000	2.3800001
-11573.0000000	2.3800001
-11572.0000000	2.3800001
-11571.0000000	2.3750000
-11570.0000000	2.3750000
-11569.0000000	2.3699999
-11568.0000000	2.3800001
-11567.0000000	2.3699999
-11566.0000000	2.3650000
-11565.0000000	2.3550000
-11564.0000000	2.3550000
-11563.0000000	2.3499999
-11562.0000000	2.3550000
-11561.0000000	2.3699999
-11560.0000000	2.3750000
-11559.0000000	2.3699999
-11558.0000000	2.3550000
-11557.0000000	2.3350000
-11556.0000000	2.3350000
-11555.0000000	2.3350000
-11554.0000000	2.3350000
-11553.0000000	2.3449998
-11552.0000000	2.3599999
-11551.0000000	2.3649998
-11550.0000000	2.3649998
-11549.0000000	2.3699999
-11548.0000000	2.3599999
-11547.0000000	2.3649998
-11546.0000000	2.3666666
-11545.0000000	2.3699999
-11544.0000000	2.3750000
-11543.0000000	2.3800001
-11542.0000000	2.3900001
-11541.0000000	2.4000001
-11540.0000000	2.4150000
-11539.0000000	2.4150000

-11538.0000000	2.4150000
-11537.0000000	2.3950000
-11536.0000000	2.4050002
-11535.0000000	2.4150000
-11534.0000000	2.4200001
-11533.0000000	2.4349999
-11532.0000000	2.4300001
-11531.0000000	2.4300001
-11530.0000000	2.4250002
-11529.0000000	2.4000001
-11528.0000000	2.3800001
-11527.0000000	2.3699999
-11526.0000000	2.3499999
-11525.0000000	2.3299999
-11524.0000000	2.3299999
-11523.0000000	2.3299999
-11522.0000000	2.3399999
-11521.0000000	2.3550000
-11520.0000000	2.3649998
-11519.0000000	2.3750000
-11518.0000000	2.4000001
-11517.0000000	2.4200001
-11516.0000000	2.4333334
-11515.0000000	2.4500000
-11514.0000000	2.4500000
-11513.0000000	2.4500000
-11512.0000000	2.4400001
-11511.0000000	2.4300001
-11510.0000000	2.4349999
-11509.0000000	2.4349999
-11508.0000000	2.4349999
-11507.0000000	2.4400001
-11506.0000000	2.4300001
-11505.0000000	2.4100001
-11504.0000000	2.3699999
-11503.0000000	2.3249998
-11502.0000000	2.3000000
-11501.0000000	2.2900000
-11500.0000000	2.2800000
-11499.0000000	2.2950001
-11498.0000000	2.3049998
-11497.0000000	2.3350000
-11496.0000000	2.3550000
-11495.0000000	2.3750000
-11494.0000000	2.3850002
-11493.0000000	2.4000001
-11492.0000000	2.4000001
-11491.0000000	2.3800001
-11490.0000000	2.3599999
-11489.0000000	2.3299999
-11488.0000000	2.2900000
-11487.0000000	2.2649999
-11486.0000000	2.2600000
-11485.0000000	2.2600000
-11484.0000000	2.2600000
-11483.0000000	2.2600000
-11482.0000000	2.2300000

-11481.0000000	2.1950002
-11480.0000000	2.1650000
-11479.0000000	2.1450000
-11478.0000000	2.1250000
-11477.0000000	2.1199999
-11476.0000000	2.1149998
-11475.0000000	2.1250000
-11474.0000000	2.1199999
-11473.0000000	2.1099999
-11472.0000000	2.1149998
-11471.0000000	2.1199999
-11470.0000000	2.1450000
-11469.0000000	2.1750002
-11468.0000000	2.2000000
-11467.0000000	2.2000000
-11466.0000000	2.1950002
-11465.0000000	2.2100000
-11464.0000000	2.2200000
-11463.0000000	2.2249999
-11462.0000000	2.2449999
-11461.0000000	2.2649999
-11460.0000000	2.2950001
-11459.0000000	2.3249998
-11458.0000000	2.3649998
-11457.0000000	2.4050002
-11456.0000000	2.4400001
-11455.0000000	2.4466667
-11454.0000000	2.4700000
-11453.0000000	2.4850001
-11452.0000000	2.4949999
-11451.0000000	2.5149999
-11450.0000000	2.5100000
-11449.0000000	2.5149999
-11448.0000000	2.5200000
-11447.0000000	2.5200000
-11446.0000000	2.5300000
-11445.0000000	2.5349998
-11444.0000000	2.5500000
-11443.0000000	2.5650001
-11442.0000000	2.5799999
-11441.0000000	2.5949998
-11440.0000000	2.5899999
-11439.0000000	2.5999999
-11438.0000000	2.5899999
-11437.0000000	2.5749998
-11436.0000000	2.5749998
-11435.0000000	2.5650001
-11434.0000000	2.5549998
-11433.0000000	2.5549998
-11432.0000000	2.5549998
-11431.0000000	2.5549998
-11430.0000000	2.5300000
-11429.0000000	2.5100000
-11428.0000000	2.4900000
-11427.0000000	2.4700000
-11426.0000000	2.4500000
-11425.0000000	2.4433334

-11424.0000000	2.4300001
-11423.0000000	2.4250002
-11422.0000000	2.4000001
-11421.0000000	2.3900001
-11420.0000000	2.3950000
-11419.0000000	2.3850002
-11418.0000000	2.3449998
-11417.0000000	2.3199999
-11416.0000000	2.3150001
-11415.0000000	2.3350000
-11414.0000000	2.3399999
-11413.0000000	2.3650000
-11412.0000000	2.3900001
-11411.0000000	2.4100001
-11410.0000000	2.4500000
-11409.0000000	2.4749999
-11408.0000000	2.5100000
-11407.0000000	2.5400000
-11406.0000000	2.5599999
-11405.0000000	2.5749998
-11404.0000000	2.5799999
-11403.0000000	2.5749998
-11402.0000000	2.5400000
-11401.0000000	2.5100000
-11400.0000000	2.4749999
-11399.0000000	2.4549999
-11398.0000000	2.4500000
-11397.0000000	2.4300001
-11396.0000000	2.4100001
-11395.0000000	2.3700001
-11394.0000000	2.3299999
-11393.0000000	2.3299999
-11392.0000000	2.3550000
-11391.0000000	2.3900001
-11390.0000000	2.4150000
-11389.0000000	2.3850000
-11388.0000000	2.3800001
-11387.0000000	2.3850002
-11386.0000000	2.3900001
-11385.0000000	2.4200001
-11384.0000000	2.4549999
-11383.0000000	2.4850001
-11382.0000000	2.5000000
-11381.0000000	2.5050001
-11380.0000000	2.5050001
-11379.0000000	2.5250001
-11378.0000000	2.5349998
-11377.0000000	2.5300000
-11376.0000000	2.5349998
-11375.0000000	2.5500000
-11374.0000000	2.5500000
-11373.0000000	2.5599999
-11372.0000000	2.5799999
-11371.0000000	2.5899999
-11370.0000000	2.6050000
-11369.0000000	2.6350002
-11368.0000000	2.6550002

-11367.0000000	2.6650000
-11366.0000000	2.6700001
-11365.0000000	2.6766670
-11364.0000000	2.6800001
-11363.0000000	2.6700001
-11362.0000000	2.6550002
-11361.0000000	2.6550002
-11360.0000000	2.6450000
-11359.0000000	2.6400001
-11358.0000000	2.6400001
-11357.0000000	2.6500001
-11356.0000000	2.6450000
-11355.0000000	2.6400001
-11354.0000000	2.6350002
-11353.0000000	2.6400001
-11352.0000000	2.6400001
-11351.0000000	2.6400001
-11350.0000000	2.6400001
-11349.0000000	2.6300001
-11348.0000000	2.6149998
-11347.0000000	2.6099999
-11346.0000000	2.5999999
-11345.0000000	2.5850000
-11344.0000000	2.5799999
-11343.0000000	2.5699999
-11342.0000000	2.5749998
-11341.0000000	2.5850000
-11340.0000000	2.5850000
-11339.0000000	2.5749998
-11338.0000000	2.5899999
-11337.0000000	2.5899999
-11336.0000000	2.5949998
-11335.0000000	2.5999999
-11334.0000000	2.6250000
-11333.0000000	2.6500001
-11332.0000000	2.6600001
-11331.0000000	2.6750002
-11330.0000000	2.6900001
-11329.0000000	2.7000000
-11328.0000000	2.7049999
-11327.0000000	2.7100000
-11326.0000000	2.7049999
-11325.0000000	2.7100000
-11324.0000000	2.7100000
-11323.0000000	2.6900001
-11322.0000000	2.6650000
-11321.0000000	2.6300001
-11320.0000000	2.5949998
-11319.0000000	2.5749998
-11318.0000000	2.5450001
-11317.0000000	2.5250001
-11316.0000000	2.5250001
-11315.0000000	2.5200000
-11314.0000000	2.5200000
-11313.0000000	2.5250001
-11312.0000000	2.5200000
-11311.0000000	2.5200000

-11310.0000000	2.5100000
-11309.0000000	2.5149999
-11308.0000000	2.5200000
-11307.0000000	2.5300000
-11306.0000000	2.5549998
-11305.0000000	2.5699999
-11304.0000000	2.5699999
-11303.0000000	2.5749998
-11302.0000000	2.5599999
-11301.0000000	2.5749998
-11300.0000000	2.5500000
-11299.0000000	2.5349998
-11298.0000000	2.5200000
-11297.0000000	2.5050001
-11296.0000000	2.4949999
-11295.0000000	2.4749999
-11294.0000000	2.4650002
-11293.0000000	2.4500000
-11292.0000000	2.4300001
-11291.0000000	2.4200001
-11290.0000000	2.4000001
-11289.0000000	2.3950000
-11288.0000000	2.4200001
-11287.0000000	2.4450002
-11286.0000000	2.4800000
-11285.0000000	2.4900000
-11284.0000000	2.4850001
-11283.0000000	2.4700000
-11282.0000000	2.4450002
-11281.0000000	2.4400001
-11280.0000000	2.4549999
-11279.0000000	2.4650002
-11278.0000000	2.4700000
-11277.0000000	2.4749999
-11276.0000000	2.4700000
-11275.0000000	2.4549999
-11274.0000000	2.4666669
-11273.0000000	2.4749999
-11272.0000000	2.4800000
-11271.0000000	2.4900000
-11270.0000000	2.4949999
-11269.0000000	2.4850001
-11268.0000000	2.4850001
-11267.0000000	2.4800000
-11266.0000000	2.4749999
-11265.0000000	2.4450002
-11264.0000000	2.4100001
-11263.0000000	2.3750000
-11262.0000000	2.3550000
-11261.0000000	2.3350000
-11260.0000000	2.3199999
-11259.0000000	2.3199999
-11258.0000000	2.3199999
-11257.0000000	2.3299999
-11256.0000000	2.3399999
-11255.0000000	2.3499999
-11254.0000000	2.3649998

-11253.0000000	2.3649998
-11252.0000000	2.3699999
-11251.0000000	2.3599999
-11250.0000000	2.3499999
-11249.0000000	2.3399999
-11248.0000000	2.3249998
-11247.0000000	2.3199999
-11246.0000000	2.3333333
-11245.0000000	2.3699999
-11244.0000000	2.4000001
-11243.0000000	2.4349999
-11242.0000000	2.4650002
-11241.0000000	2.4850001
-11240.0000000	2.4900000
-11239.0000000	2.4949999
-11238.0000000	2.5000000
-11237.0000000	2.5100000
-11236.0000000	2.4949999
-11235.0000000	2.4600000
-11234.0000000	2.4250002
-11233.0000000	2.3950000
-11232.0000000	2.3599999
-11231.0000000	2.3350000
-11230.0000000	2.3099999
-11229.0000000	2.2750001
-11228.0000000	2.2550001
-11227.0000000	2.2449999
-11226.0000000	2.2449999
-11225.0000000	2.2449999
-11224.0000000	2.2449999
-11223.0000000	2.2400000
-11222.0000000	2.2400000
-11221.0000000	2.2449999
-11220.0000000	2.2449999
-11219.0000000	2.2449999
-11218.0000000	2.2350001
-11217.0000000	2.2300000
-11216.0000000	2.2166667
-11215.0000000	2.2100000
-11214.0000000	2.2049999
-11213.0000000	2.1900001
-11212.0000000	2.1650000
-11211.0000000	2.1300001
-11210.0000000	2.1250000
-11209.0000000	2.1199999
-11208.0000000	2.1250000
-11207.0000000	2.1350002
-11206.0000000	2.1300001
-11205.0000000	2.1300001
-11204.0000000	2.1400001
-11203.0000000	2.1450000
-11202.0000000	2.1600001
-11201.0000000	2.1700001
-11200.0000000	2.1900001
-11199.0000000	2.2200000
-11198.0000000	2.2449999
-11197.0000000	2.2550001

-11196.0000000	2.2500000
-11195.0000000	2.2449999
-11194.0000000	2.2449999
-11193.0000000	2.2400000
-11192.0000000	2.2500000
-11191.0000000	2.2500000
-11190.0000000	2.2550001
-11189.0000000	2.2600000
-11188.0000000	2.2649999
-11187.0000000	2.2649999
-11186.0000000	2.2550001
-11185.0000000	2.2500000
-11184.0000000	2.2500000
-11183.0000000	2.2566669
-11182.0000000	2.2600000
-11181.0000000	2.2600000
-11180.0000000	2.2550001
-11179.0000000	2.2649999
-11178.0000000	2.2600000
-11177.0000000	2.2600000
-11176.0000000	2.2400000
-11175.0000000	2.2249999
-11174.0000000	2.2200000
-11173.0000000	2.1950002
-11172.0000000	2.1849999
-11171.0000000	2.1849999
-11170.0000000	2.2000000
-11169.0000000	2.2150002
-11168.0000000	2.2249999
-11167.0000000	2.2400000
-11166.0000000	2.2350001
-11165.0000000	2.2300000
-11164.0000000	2.2350001
-11163.0000000	2.2350001
-11162.0000000	2.2350001
-11161.0000000	2.2400000
-11160.0000000	2.2500000
-11159.0000000	2.2600000
-11158.0000000	2.2750001
-11157.0000000	2.2750001
-11156.0000000	2.2849998
-11155.0000000	2.2800000
-11154.0000000	2.2750001
-11153.0000000	2.2566667
-11152.0000000	2.2350001
-11151.0000000	2.2200000
-11150.0000000	2.2150002
-11149.0000000	2.2150002
-11148.0000000	2.2200000
-11147.0000000	2.2200000
-11146.0000000	2.2049999
-11145.0000000	2.2100000
-11144.0000000	2.2000000
-11143.0000000	2.1849999
-11142.0000000	2.1650000
-11141.0000000	2.1550002
-11140.0000000	2.1550002

-11139.0000000	2.1849999
-11138.0000000	2.1900001
-11137.0000000	2.2000000
-11136.0000000	2.2100000
-11135.0000000	2.2150002
-11134.0000000	2.2200000
-11133.0000000	2.2249999
-11132.0000000	2.2400000
-11131.0000000	2.2350001
-11130.0000000	2.2350001
-11129.0000000	2.2300000
-11128.0000000	2.2150002
-11127.0000000	2.1950002
-11126.0000000	2.1900001
-11125.0000000	2.1800001
-11124.0000000	2.1600001
-11123.0000000	2.1600001
-11122.0000000	2.1800001
-11121.0000000	2.2000000
-11120.0000000	2.2249999
-11119.0000000	2.2350001
-11118.0000000	2.2600000
-11117.0000000	2.2700000
-11116.0000000	2.2700000
-11115.0000000	2.2649999
-11114.0000000	2.2550001
-11113.0000000	2.2649999
-11112.0000000	2.2800000
-11111.0000000	2.2750001
-11110.0000000	2.2800000
-11109.0000000	2.2950001
-11108.0000000	2.3299999
-11107.0000000	2.3599999
-11106.0000000	2.3750000
-11105.0000000	2.3900001
-11104.0000000	2.4100001
-11103.0000000	2.4300001
-11102.0000000	2.4400001
-11101.0000000	2.4400001
-11100.0000000	2.4450002
-11099.0000000	2.4450002
-11098.0000000	2.4450002
-11097.0000000	2.4349999
-11096.0000000	2.4349999
-11095.0000000	2.4300001
-11094.0000000	2.3900001
-11093.0000000	2.3666666
-11092.0000000	2.3599999
-11091.0000000	2.3550000
-11090.0000000	2.3350000
-11089.0000000	2.3299999
-11088.0000000	2.3350000
-11087.0000000	2.3299999
-11086.0000000	2.3350000
-11085.0000000	2.3399999
-11084.0000000	2.3499999
-11083.0000000	2.3599999

-11082.0000000	2.3599999
-11081.0000000	2.3649998
-11080.0000000	2.3550000
-11079.0000000	2.3550000
-11078.0000000	2.3499999
-11077.0000000	2.3399999
-11076.0000000	2.3449998
-11075.0000000	2.3449998
-11074.0000000	2.3550000
-11073.0000000	2.3550000
-11072.0000000	2.3599999
-11071.0000000	2.3850002
-11070.0000000	2.3850002
-11069.0000000	2.3850002
-11068.0000000	2.3750000
-11067.0000000	2.3800001
-11066.0000000	2.3850002
-11065.0000000	2.4050002
-11064.0000000	2.4150000
-11063.0000000	2.4033334
-11062.0000000	2.3900001
-11061.0000000	2.3950000
-11060.0000000	2.3900001
-11059.0000000	2.3850002
-11058.0000000	2.3649998
-11057.0000000	2.3449998
-11056.0000000	2.3249998
-11055.0000000	2.3150001
-11054.0000000	2.3150001
-11053.0000000	2.3000000
-11052.0000000	2.2849998
-11051.0000000	2.2700000
-11050.0000000	2.2350001
-11049.0000000	2.1950002
-11048.0000000	2.1600001
-11047.0000000	2.1250000
-11046.0000000	2.1050000
-11045.0000000	2.0949998
-11044.0000000	2.0949998
-11043.0000000	2.0899999
-11042.0000000	2.1099999
-11041.0000000	2.1199999
-11040.0000000	2.1500001
-11039.0000000	2.1849999
-11038.0000000	2.2049999
-11037.0000000	2.2200000
-11036.0000000	2.2350001
-11035.0000000	2.2433333
-11034.0000000	2.2550001
-11033.0000000	2.2500000
-11032.0000000	2.2500000
-11031.0000000	2.2550001
-11030.0000000	2.2500000
-11029.0000000	2.2400000
-11028.0000000	2.2500000
-11027.0000000	2.2600000
-11026.0000000	2.2600000

-11025.0000000	2.2700000
-11024.0000000	2.2900000
-11023.0000000	2.2950001
-11022.0000000	2.3000000
-11021.0000000	2.3049998
-11020.0000000	2.3049998
-11019.0000000	2.3049998
-11018.0000000	2.2800000
-11017.0000000	2.2500000
-11016.0000000	2.2200000
-11015.0000000	2.2150002
-11014.0000000	2.2249999
-11013.0000000	2.2400000
-11012.0000000	2.2400000
-11011.0000000	2.2500000
-11010.0000000	2.2600000
-11009.0000000	2.2600000
-11008.0000000	2.2700000
-11007.0000000	2.2649999
-11006.0000000	2.2550001
-11005.0000000	2.2400000
-11004.0000000	2.2400000
-11003.0000000	2.2350001
-11002.0000000	2.2350001
-11001.0000000	2.2350001
-11000.0000000	2.2449999
-10999.0000000	2.2550001
-10998.0000000	2.2700000
-10997.0000000	2.2849998
-10996.0000000	2.3000000
-10995.0000000	2.3199999
-10994.0000000	2.3449998
-10993.0000000	2.3649998
-10992.0000000	2.3850002
-10991.0000000	2.3950000
-10990.0000000	2.4200001
-10989.0000000	2.4450002
-10988.0000000	2.4700000
-10987.0000000	2.4900000
-10986.0000000	2.5200000
-10985.0000000	2.5400000
-10984.0000000	2.5650001
-10983.0000000	2.5850000
-10982.0000000	2.6050000
-10981.0000000	2.5999999
-10980.0000000	2.6050000
-10979.0000000	2.6099999
-10978.0000000	2.5999999
-10977.0000000	2.5949998
-10976.0000000	2.5899999
-10975.0000000	2.5766666
-10974.0000000	2.5549998
-10973.0000000	2.5400000
-10972.0000000	2.5599999
-10971.0000000	2.5650001
-10970.0000000	2.5699999
-10969.0000000	2.5899999

-10968.0000000	2.6050000
-10967.0000000	2.6099999
-10966.0000000	2.6199999
-10965.0000000	2.6250000
-10964.0000000	2.6300001
-10963.0000000	2.6400001
-10962.0000000	2.6400001
-10961.0000000	2.6450000
-10960.0000000	2.6550002
-10959.0000000	2.6650000
-10958.0000000	2.6700001
-10957.0000000	2.6600001
-10956.0000000	2.6500001
-10955.0000000	2.6350002
-10954.0000000	2.6350002
-10953.0000000	2.6199999
-10952.0000000	2.6149998
-10951.0000000	2.6099999
-10950.0000000	2.5999999
-10949.0000000	2.6050000
-10948.0000000	2.6050000
-10947.0000000	2.5850000
-10946.0000000	2.5749998
-10945.0000000	2.5500000
-10944.0000000	2.5250001
-10943.0000000	2.4900000
-10942.0000000	2.4800000
-10941.0000000	2.4800000
-10940.0000000	2.4650002
-10939.0000000	2.4600000
-10938.0000000	2.4600000
-10937.0000000	2.4549999
-10936.0000000	2.4549999
-10935.0000000	2.4450002
-10934.0000000	2.4400001
-10933.0000000	2.4400001
-10932.0000000	2.4450002
-10931.0000000	2.4450002
-10930.0000000	2.4450002
-10929.0000000	2.4400001
-10928.0000000	2.4300001
-10927.0000000	2.4250002
-10926.0000000	2.4300001
-10925.0000000	2.4300001
-10924.0000000	2.4150000
-10923.0000000	2.4150000
-10922.0000000	2.4100001
-10921.0000000	2.4150000
-10920.0000000	2.4500000
-10919.0000000	2.4549999
-10918.0000000	2.4800000
-10917.0000000	2.4850001
-10916.0000000	2.5100000
-10915.0000000	2.5349998
-10914.0000000	2.5599999
-10913.0000000	2.5899999
-10912.0000000	2.5999999

-10911.0000000	2.5949998
-10910.0000000	2.5899999
-10909.0000000	2.5850000
-10908.0000000	2.5699999
-10907.0000000	2.5749998
-10906.0000000	2.5850000
-10905.0000000	2.5799999
-10904.0000000	2.5799999
-10903.0000000	2.5650001
-10902.0000000	2.5599999
-10901.0000000	2.5650001
-10900.0000000	2.5599999
-10899.0000000	2.5650001
-10898.0000000	2.5749998
-10897.0000000	2.5799999
-10896.0000000	2.5599999
-10895.0000000	2.5650001
-10894.0000000	2.5599999
-10893.0000000	2.5650001
-10892.0000000	2.5650001
-10891.0000000	2.5799999
-10890.0000000	2.5799999
-10889.0000000	2.5850000
-10888.0000000	2.5749998
-10887.0000000	2.5749998
-10886.0000000	2.5799999
-10885.0000000	2.5799999
-10884.0000000	2.5850000
-10883.0000000	2.5799999
-10882.0000000	2.5766666
-10881.0000000	2.5699999
-10880.0000000	2.5799999
-10879.0000000	2.5799999
-10878.0000000	2.5850000
-10877.0000000	2.5850000
-10876.0000000	2.5949998
-10875.0000000	2.5949998
-10874.0000000	2.5949998
-10873.0000000	2.6099999
-10872.0000000	2.6050000
-10871.0000000	2.6050000
-10870.0000000	2.6099999
-10869.0000000	2.6149998
-10868.0000000	2.6250000
-10867.0000000	2.6199999
-10866.0000000	2.6199999
-10865.0000000	2.6099999
-10864.0000000	2.6050000
-10863.0000000	2.5899999
-10862.0000000	2.5899999
-10861.0000000	2.5799999
-10860.0000000	2.5699999
-10859.0000000	2.5650001
-10858.0000000	2.5500000
-10857.0000000	2.5400000
-10856.0000000	2.5200000
-10855.0000000	2.5000000

-10854.0000000	2.4700000
-10853.0000000	2.4500000
-10852.0000000	2.4300001
-10851.0000000	2.4300001
-10850.0000000	2.4349999
-10849.0000000	2.4349999
-10848.0000000	2.4300001
-10847.0000000	2.4050002
-10846.0000000	2.3950000
-10845.0000000	2.3900001
-10844.0000000	2.3850002
-10843.0000000	2.3850002
-10842.0000000	2.3900001
-10841.0000000	2.3850002
-10840.0000000	2.3850002
-10839.0000000	2.3950000
-10838.0000000	2.3950000
-10837.0000000	2.4000001
-10836.0000000	2.4050002
-10835.0000000	2.4000001
-10834.0000000	2.3900001
-10833.0000000	2.3850002
-10832.0000000	2.3900001
-10831.0000000	2.3850002
-10830.0000000	2.3900001
-10829.0000000	2.3950000
-10828.0000000	2.4050002
-10827.0000000	2.4300001
-10826.0000000	2.4600000
-10825.0000000	2.4900000
-10824.0000000	2.5100000
-10823.0000000	2.5250001
-10822.0000000	2.5333331
-10821.0000000	2.5349998
-10820.0000000	2.5500000
-10819.0000000	2.5450001
-10818.0000000	2.5300000
-10817.0000000	2.5100000
-10816.0000000	2.4900000
-10815.0000000	2.4600000
-10814.0000000	2.4500000
-10813.0000000	2.4700000
-10812.0000000	2.4650002
-10811.0000000	2.4650002
-10810.0000000	2.4549999
-10809.0000000	2.4349999
-10808.0000000	2.4050002
-10807.0000000	2.3800001
-10806.0000000	2.3750000
-10805.0000000	2.3800001
-10804.0000000	2.3850002
-10803.0000000	2.3850002
-10802.0000000	2.3900001
-10801.0000000	2.4100001
-10800.0000000	2.4200001
-10799.0000000	2.4349999
-10798.0000000	2.4500000

-10797.0000000	2.4400001
-10796.0000000	2.4349999
-10795.0000000	2.4300001
-10794.0000000	2.4150000
-10793.0000000	2.3850002
-10792.0000000	2.3466666
-10791.0000000	2.2950001
-10790.0000000	2.2400000
-10789.0000000	2.2049999
-10788.0000000	2.1800001
-10787.0000000	2.1950002
-10786.0000000	2.2000000
-10785.0000000	2.1950002
-10784.0000000	2.1950002
-10783.0000000	2.1950002
-10782.0000000	2.2150002
-10781.0000000	2.2300000
-10780.0000000	2.2400000
-10779.0000000	2.2400000
-10778.0000000	2.2449999
-10777.0000000	2.2700000
-10776.0000000	2.2950001
-10775.0000000	2.3150001
-10774.0000000	2.3199999
-10773.0000000	2.3299999
-10772.0000000	2.3199999
-10771.0000000	2.3249998
-10770.0000000	2.3299999
-10769.0000000	2.3399999
-10768.0000000	2.3550000
-10767.0000000	2.3599999
-10766.0000000	2.3750000
-10765.0000000	2.3800001
-10764.0000000	2.3800001
-10763.0000000	2.3800001
-10762.0000000	2.3750000
-10761.0000000	2.3699999
-10760.0000000	2.3800001
-10759.0000000	2.3800001
-10758.0000000	2.3850002
-10757.0000000	2.3800001
-10756.0000000	2.3649998
-10755.0000000	2.3550000
-10754.0000000	2.3350000
-10753.0000000	2.3249998
-10752.0000000	2.3399999
-10751.0000000	2.3449998
-10750.0000000	2.3499999
-10749.0000000	2.3599999
-10748.0000000	2.3750000
-10747.0000000	2.3850002
-10746.0000000	2.3900001
-10745.0000000	2.3850002
-10744.0000000	2.3850002
-10743.0000000	2.3800001
-10742.0000000	2.3599999
-10741.0000000	2.3499999

-10740.0000000	2.3649998
-10739.0000000	2.3750000
-10738.0000000	2.3900001
-10737.0000000	2.4200001
-10736.0000000	2.4150000
-10735.0000000	2.4150000
-10734.0000000	2.4100001
-10733.0000000	2.4100001
-10732.0000000	2.4000001
-10731.0000000	2.4000003
-10730.0000000	2.3850002
-10729.0000000	2.3699999
-10728.0000000	2.3649998
-10727.0000000	2.3449998
-10726.0000000	2.3199999
-10725.0000000	2.3249998
-10724.0000000	2.3350000
-10723.0000000	2.3249998
-10722.0000000	2.3000000
-10721.0000000	2.2700000
-10720.0000000	2.2550001
-10719.0000000	2.2550001
-10718.0000000	2.2550001
-10717.0000000	2.2500000
-10716.0000000	2.2449999
-10715.0000000	2.2550001
-10714.0000000	2.2800000
-10713.0000000	2.2950001
-10712.0000000	2.3249998
-10711.0000000	2.3249998
-10710.0000000	2.3399999
-10709.0000000	2.3350000
-10708.0000000	2.3399999
-10707.0000000	2.3449998
-10706.0000000	2.3550000
-10705.0000000	2.3649998
-10704.0000000	2.3550000
-10703.0000000	2.3649998
-10702.0000000	2.3699999
-10701.0000000	2.3800001
-10700.0000000	2.4000001
-10699.0000000	2.4150000
-10698.0000000	2.4300001
-10697.0000000	2.4300001
-10696.0000000	2.4349999
-10695.0000000	2.4500000
-10694.0000000	2.4500000
-10693.0000000	2.4500000
-10692.0000000	2.4500000
-10691.0000000	2.4500000
-10690.0000000	2.4600000
-10689.0000000	2.4549999
-10688.0000000	2.4600000
-10687.0000000	2.4700000
-10686.0000000	2.4850001
-10685.0000000	2.4850001
-10684.0000000	2.4900000

-10683.0000000	2.4800000
-10682.0000000	2.4749999
-10681.0000000	2.4600000
-10680.0000000	2.4400001
-10679.0000000	2.4450002
-10678.0000000	2.4400001
-10677.0000000	2.4300001
-10676.0000000	2.4250002
-10675.0000000	2.4349999
-10674.0000000	2.4250002
-10673.0000000	2.4200001
-10672.0000000	2.4100001
-10671.0000000	2.3850002
-10670.0000000	2.3649998
-10669.0000000	2.3266666
-10668.0000000	2.3049998
-10667.0000000	2.2849998
-10666.0000000	2.2700000
-10665.0000000	2.2750001
-10664.0000000	2.2700000
-10663.0000000	2.2649999
-10662.0000000	2.2300000
-10661.0000000	2.1900001
-10660.0000000	2.1650000
-10659.0000000	2.1800001
-10658.0000000	2.2100000
-10657.0000000	2.2000000
-10656.0000000	2.2200000
-10655.0000000	2.2500000
-10654.0000000	2.2849998
-10653.0000000	2.3049998
-10652.0000000	2.3199999
-10651.0000000	2.3350000
-10650.0000000	2.3449998
-10649.0000000	2.3599999
-10648.0000000	2.3649998
-10647.0000000	2.3649998
-10646.0000000	2.3699999
-10645.0000000	2.3649998
-10644.0000000	2.3499999
-10643.0000000	2.3350000
-10642.0000000	2.3249998
-10641.0000000	2.3150001
-10640.0000000	2.3199999
-10639.0000000	2.3166666
-10638.0000000	2.3199999
-10637.0000000	2.3299999
-10636.0000000	2.3449998
-10635.0000000	2.3299999
-10634.0000000	2.3299999
-10633.0000000	2.3299999
-10632.0000000	2.3099999
-10631.0000000	2.3000000
-10630.0000000	2.2849998
-10629.0000000	2.2800000
-10628.0000000	2.2849998
-10627.0000000	2.2900000

-10626.0000000	2.2900000
-10625.0000000	2.2900000
-10624.0000000	2.2900000
-10623.0000000	2.3000000
-10622.0000000	2.3000000
-10621.0000000	2.3249998
-10620.0000000	2.3399999
-10619.0000000	2.3649998
-10618.0000000	2.3800001
-10617.0000000	2.3900001
-10616.0000000	2.4100001
-10615.0000000	2.4200001
-10614.0000000	2.4349999
-10613.0000000	2.4450002
-10612.0000000	2.4549999
-10611.0000000	2.4549999
-10610.0000000	2.4650002
-10609.0000000	2.4700000
-10608.0000000	2.4700000
-10607.0000000	2.4700000
-10606.0000000	2.4549999
-10605.0000000	2.4300001
-10604.0000000	2.4100001
-10603.0000000	2.4050002
-10602.0000000	2.4050002
-10601.0000000	2.4100001
-10600.0000000	2.4250002
-10599.0000000	2.4100001
-10598.0000000	2.3950000
-10597.0000000	2.3699999
-10596.0000000	2.3599999
-10595.0000000	2.3399999
-10594.0000000	2.3299999
-10593.0000000	2.3099999
-10592.0000000	2.2950001
-10591.0000000	2.2900000
-10590.0000000	2.2950001
-10589.0000000	2.3099999
-10588.0000000	2.3299999
-10587.0000000	2.3449998
-10586.0000000	2.3449998
-10585.0000000	2.3350000
-10584.0000000	2.3000000
-10583.0000000	2.2700000
-10582.0000000	2.2500000
-10581.0000000	2.2350001
-10580.0000000	2.2600000
-10579.0000000	2.2750001
-10578.0000000	2.2800000
-10577.0000000	2.2950001
-10576.0000000	2.3199999
-10575.0000000	2.3399999
-10574.0000000	2.3550000
-10573.0000000	2.3699999
-10572.0000000	2.3850002
-10571.0000000	2.4000001
-10570.0000000	2.4150000

-10569.0000000	2.4250002
-10568.0000000	2.4349999
-10567.0000000	2.4450002
-10566.0000000	2.4549999
-10565.0000000	2.4600000
-10564.0000000	2.4749999
-10563.0000000	2.4749999
-10562.0000000	2.4800000
-10561.0000000	2.4900000
-10560.0000000	2.5000000
-10559.0000000	2.4949999
-10558.0000000	2.5000000
-10557.0000000	2.5000000
-10556.0000000	2.5050001
-10555.0000000	2.5050001
-10554.0000000	2.5149999
-10553.0000000	2.5349998
-10552.0000000	2.5549998
-10551.0000000	2.5533333
-10550.0000000	2.5749998
-10549.0000000	2.5749998
-10548.0000000	2.5799999
-10547.0000000	2.5999999
-10546.0000000	2.6099999
-10545.0000000	2.6300001
-10544.0000000	2.6400001
-10543.0000000	2.6550002
-10542.0000000	2.6650000
-10541.0000000	2.6650000
-10540.0000000	2.6650000
-10539.0000000	2.6600001
-10538.0000000	2.6650000
-10537.0000000	2.6650000
-10536.0000000	2.6600001
-10535.0000000	2.6650000
-10534.0000000	2.6650000
-10533.0000000	2.6600001
-10532.0000000	2.6650000
-10531.0000000	2.6500001
-10530.0000000	2.6350002
-10529.0000000	2.6149998
-10528.0000000	2.5949998
-10527.0000000	2.5749998
-10526.0000000	2.5699999
-10525.0000000	2.5549998
-10524.0000000	2.5650001
-10523.0000000	2.5599999
-10522.0000000	2.5450001
-10521.0000000	2.5300000
-10520.0000000	2.5200000
-10519.0000000	2.5166667
-10518.0000000	2.5149999
-10517.0000000	2.5149999
-10516.0000000	2.5200000
-10515.0000000	2.5200000
-10514.0000000	2.5300000
-10513.0000000	2.5450001

-10512.0000000	2.5599999
-10511.0000000	2.5899999
-10510.0000000	2.5999999
-10509.0000000	2.6099999
-10508.0000000	2.6149998
-10507.0000000	2.6149998
-10506.0000000	2.6300001
-10505.0000000	2.6500001
-10504.0000000	2.6600001
-10503.0000000	2.6700001
-10502.0000000	2.6600001
-10501.0000000	2.6450000
-10500.0000000	2.6250000
-10499.0000000	2.6149998
-10498.0000000	2.5850000
-10497.0000000	2.5599999
-10496.0000000	2.5450001
-10495.0000000	2.5500000
-10494.0000000	2.5599999
-10493.0000000	2.5450001
-10492.0000000	2.5400000
-10491.0000000	2.5349998
-10490.0000000	2.5450001
-10489.0000000	2.5366666
-10488.0000000	2.5549998
-10487.0000000	2.5650001
-10486.0000000	2.5699999
-10485.0000000	2.5699999
-10484.0000000	2.5500000
-10483.0000000	2.5050001
-10482.0000000	2.4450002
-10481.0000000	2.4100001
-10480.0000000	2.3950000
-10479.0000000	2.3850002
-10478.0000000	2.3900001
-10477.0000000	2.4050002
-10476.0000000	2.4150000
-10475.0000000	2.4250002
-10474.0000000	2.4300001
-10473.0000000	2.4349999
-10472.0000000	2.4400001
-10471.0000000	2.4250002
-10470.0000000	2.4000001
-10469.0000000	2.3449998
-10468.0000000	2.2950001
-10467.0000000	2.2550001
-10466.0000000	2.2249999
-10465.0000000	2.1950002
-10464.0000000	2.1700001
-10463.0000000	2.1800001
-10462.0000000	2.2100000
-10461.0000000	2.2449999
-10460.0000000	2.2900000
-10459.0000000	2.3266666
-10458.0000000	2.3399999
-10457.0000000	2.3449998
-10456.0000000	2.3550000

-10455.0000000	2.3699999
-10454.0000000	2.3800001
-10453.0000000	2.3900001
-10452.0000000	2.3950000
-10451.0000000	2.4200001
-10450.0000000	2.4200001
-10449.0000000	2.4200001
-10448.0000000	2.4100001
-10447.0000000	2.4000001
-10446.0000000	2.4000001
-10445.0000000	2.3950000
-10444.0000000	2.3750000
-10443.0000000	2.3599999
-10442.0000000	2.3199999
-10441.0000000	2.2900000
-10440.0000000	2.3049998
-10439.0000000	2.3049998
-10438.0000000	2.3049998
-10437.0000000	2.3150001
-10436.0000000	2.3249998
-10435.0000000	2.3199999
-10434.0000000	2.3249998
-10433.0000000	2.3249998
-10432.0000000	2.3150001
-10431.0000000	2.3049998
-10430.0000000	2.2800000
-10429.0000000	2.2550001
-10428.0000000	2.2500000
-10427.0000000	2.2550001
-10426.0000000	2.2750001
-10425.0000000	2.2950001
-10424.0000000	2.3150001
-10423.0000000	2.3350000
-10422.0000000	2.3499999
-10421.0000000	2.3750000
-10420.0000000	2.4000001
-10419.0000000	2.4349999
-10418.0000000	2.4700000
-10417.0000000	2.4800000
-10416.0000000	2.4949999
-10415.0000000	2.4900000
-10414.0000000	2.4650002
-10413.0000000	2.4349999
-10412.0000000	2.4349999
-10411.0000000	2.4300001
-10410.0000000	2.4200001
-10409.0000000	2.4200001
-10408.0000000	2.4250002
-10407.0000000	2.4300001
-10406.0000000	2.4250002
-10405.0000000	2.4349999
-10404.0000000	2.4400001
-10403.0000000	2.4450002
-10402.0000000	2.4500000
-10401.0000000	2.4400001
-10400.0000000	2.4349999
-10399.0000000	2.4500000

-10398.0000000	2.4549999
-10397.0000000	2.4600000
-10396.0000000	2.4600000
-10395.0000000	2.4300001
-10394.0000000	2.4450002
-10393.0000000	2.4500000
-10392.0000000	2.4600000
-10391.0000000	2.4450002
-10390.0000000	2.4349999
-10389.0000000	2.4349999
-10388.0000000	2.4300001
-10387.0000000	2.4400001
-10386.0000000	2.4549999
-10385.0000000	2.4749999
-10384.0000000	2.5000000
-10383.0000000	2.5000000
-10382.0000000	2.5100000
-10381.0000000	2.5100000
-10380.0000000	2.5250001
-10379.0000000	2.5450001
-10378.0000000	2.5699999
-10377.0000000	2.5850000
-10376.0000000	2.5949998
-10375.0000000	2.5999999
-10374.0000000	2.6149998
-10373.0000000	2.6300001
-10372.0000000	2.6350002
-10371.0000000	2.6300001
-10370.0000000	2.6350002
-10369.0000000	2.6366668
-10368.0000000	2.6400001
-10367.0000000	2.6250000
-10366.0000000	2.6199999
-10365.0000000	2.6199999
-10364.0000000	2.5949998
-10363.0000000	2.5850000
-10362.0000000	2.5799999
-10361.0000000	2.5799999
-10360.0000000	2.5850000
-10359.0000000	2.5749998
-10358.0000000	2.5749998
-10357.0000000	2.5699999
-10356.0000000	2.5650001
-10355.0000000	2.5699999
-10354.0000000	2.5650001
-10353.0000000	2.5799999
-10352.0000000	2.5749998
-10351.0000000	2.5899999
-10350.0000000	2.5999999
-10349.0000000	2.6050000
-10348.0000000	2.6199999
-10347.0000000	2.6350002
-10346.0000000	2.6550002
-10345.0000000	2.6750002
-10344.0000000	2.6800001
-10343.0000000	2.6900001
-10342.0000000	2.7049999

-10341.0000000	2.7150002
-10340.0000000	2.7200000
-10339.0000000	2.7400000
-10338.0000000	2.7449999
-10337.0000000	2.7500000
-10336.0000000	2.7500000
-10335.0000000	2.7500000
-10334.0000000	2.7500000
-10333.0000000	2.7600000
-10332.0000000	2.7649999
-10331.0000000	2.7500000
-10330.0000000	2.7400000
-10329.0000000	2.7200000
-10328.0000000	2.7150002
-10327.0000000	2.7100000
-10326.0000000	2.7049999
-10325.0000000	2.7049999
-10324.0000000	2.7100000
-10323.0000000	2.7100000
-10322.0000000	2.7249999
-10321.0000000	2.7249999
-10320.0000000	2.7350001
-10319.0000000	2.7400000
-10318.0000000	2.7400000
-10317.0000000	2.7449999
-10316.0000000	2.7600000
-10315.0000000	2.7600000
-10314.0000000	2.7649999
-10313.0000000	2.7750001
-10312.0000000	2.7750001
-10311.0000000	2.7800000
-10310.0000000	2.7800000
-10309.0000000	2.7849998
-10308.0000000	2.7900000
-10307.0000000	2.8000000
-10306.0000000	2.7950001
-10305.0000000	2.7849998
-10304.0000000	2.7900000
-10303.0000000	2.7750001
-10302.0000000	2.7500000
-10301.0000000	2.7400000
-10300.0000000	2.7400000
-10299.0000000	2.7300000
-10298.0000000	2.7100000
-10297.0000000	2.7049999
-10296.0000000	2.6849999
-10295.0000000	2.6900001
-10294.0000000	2.6849999
-10293.0000000	2.6750002
-10292.0000000	2.6700001
-10291.0000000	2.6400001
-10290.0000000	2.6199999
-10289.0000000	2.6149998
-10288.0000000	2.5999999
-10287.0000000	2.6149998
-10286.0000000	2.6149998
-10285.0000000	2.6149998

-10284.0000000	2.6050000
-10283.0000000	2.6099999
-10282.0000000	2.5949998
-10281.0000000	2.6099999
-10280.0000000	2.5949998
-10279.0000000	2.5899999
-10278.0000000	2.5966666
-10277.0000000	2.6050000
-10276.0000000	2.6149998
-10275.0000000	2.6250000
-10274.0000000	2.6350002
-10273.0000000	2.6550002
-10272.0000000	2.6600001
-10271.0000000	2.6700001
-10270.0000000	2.6800001
-10269.0000000	2.6750002
-10268.0000000	2.6600001
-10267.0000000	2.6450000
-10266.0000000	2.6550002
-10265.0000000	2.6650000
-10264.0000000	2.6800001
-10263.0000000	2.6950002
-10262.0000000	2.6950002
-10261.0000000	2.7100000
-10260.0000000	2.7200000
-10259.0000000	2.7150002
-10258.0000000	2.7150002
-10257.0000000	2.7000000
-10256.0000000	2.6800001
-10255.0000000	2.6600001
-10254.0000000	2.6450000
-10253.0000000	2.6400001
-10252.0000000	2.6300001
-10251.0000000	2.6300001
-10250.0000000	2.6250000
-10249.0000000	2.6300001
-10248.0000000	2.6466668
-10247.0000000	2.6650000
-10246.0000000	2.6750002
-10245.0000000	2.6750002
-10244.0000000	2.6600001
-10243.0000000	2.6400001
-10242.0000000	2.5899999
-10241.0000000	2.5300000
-10240.0000000	2.4800000
-10239.0000000	2.4050002
-10238.0000000	2.3599999
-10237.0000000	2.3449998
-10236.0000000	2.3750000
-10235.0000000	2.3800001
-10234.0000000	2.4050002
-10233.0000000	2.4100001
-10232.0000000	2.4100001
-10231.0000000	2.4200001
-10230.0000000	2.4300001
-10229.0000000	2.4650002
-10228.0000000	2.5000000

-10227.0000000	2.5149999
-10226.0000000	2.5349998
-10225.0000000	2.5650001
-10224.0000000	2.5749998
-10223.0000000	2.5650001
-10222.0000000	2.5699999
-10221.0000000	2.5650001
-10220.0000000	2.5699999
-10219.0000000	2.5833333
-10218.0000000	2.6099999
-10217.0000000	2.6300001
-10216.0000000	2.6400001
-10215.0000000	2.6500001
-10214.0000000	2.6550002
-10213.0000000	2.6600001
-10212.0000000	2.6450000
-10211.0000000	2.6350002
-10210.0000000	2.6099999
-10209.0000000	2.6050000
-10208.0000000	2.5999999
-10207.0000000	2.6099999
-10206.0000000	2.6300001
-10205.0000000	2.6250000
-10204.0000000	2.6199999
-10203.0000000	2.5999999
-10202.0000000	2.5999999
-10201.0000000	2.5899999
-10200.0000000	2.5749998
-10199.0000000	2.5599999
-10198.0000000	2.5650001
-10197.0000000	2.5699999
-10196.0000000	2.5799999
-10195.0000000	2.5799999
-10194.0000000	2.5749998
-10193.0000000	2.5650001
-10192.0000000	2.5699999
-10191.0000000	2.5949998
-10190.0000000	2.6099999
-10189.0000000	2.6050000
-10188.0000000	2.6149998
-10187.0000000	2.6099999
-10186.0000000	2.6033332
-10185.0000000	2.6199999
-10184.0000000	2.6149998
-10183.0000000	2.5899999
-10182.0000000	2.5749998
-10181.0000000	2.5500000
-10180.0000000	2.5400000
-10179.0000000	2.5250001
-10178.0000000	2.5050001
-10177.0000000	2.5000000
-10176.0000000	2.5100000
-10175.0000000	2.5149999
-10174.0000000	2.5200000
-10173.0000000	2.5200000
-10172.0000000	2.5200000
-10171.0000000	2.5100000

-10170.0000000	2.5149999
-10169.0000000	2.5100000
-10168.0000000	2.5100000
-10167.0000000	2.5200000
-10166.0000000	2.5349998
-10165.0000000	2.5400000
-10164.0000000	2.5300000
-10163.0000000	2.5200000
-10162.0000000	2.4850001
-10161.0000000	2.4450002
-10160.0000000	2.3850000
-10159.0000000	2.3049998
-10158.0000000	2.2500000
-10157.0000000	2.2400000
-10156.0000000	2.2249999
-10155.0000000	2.2049999
-10154.0000000	2.1900001
-10153.0000000	2.1950002
-10152.0000000	2.1900001
-10151.0000000	2.1950002
-10150.0000000	2.1950002
-10149.0000000	2.1900001
-10148.0000000	2.1950002
-10147.0000000	2.1900001
-10146.0000000	2.1800001
-10145.0000000	2.1800001
-10144.0000000	2.1849999
-10143.0000000	2.1750002
-10142.0000000	2.1800001
-10141.0000000	2.1650000
-10140.0000000	2.1500001
-10139.0000000	2.1199999
-10138.0000000	2.0899999
-10137.0000000	2.0549998
-10136.0000000	2.0400000
-10135.0000000	2.0349998
-10134.0000000	2.0400000
-10133.0000000	2.0450001
-10132.0000000	2.0650001
-10131.0000000	2.0850000
-10130.0000000	2.0899999
-10129.0000000	2.1050000
-10128.0000000	2.1350002
-10127.0000000	2.1533334
-10126.0000000	2.1550002
-10125.0000000	2.1650000
-10124.0000000	2.1900001
-10123.0000000	2.2100000
-10122.0000000	2.2150002
-10121.0000000	2.2150002
-10120.0000000	2.2100000
-10119.0000000	2.2000000
-10118.0000000	2.2150002
-10117.0000000	2.2400000
-10116.0000000	2.2750001
-10115.0000000	2.3000000
-10114.0000000	2.3049998

-10113.0000000	2.3049998
-10112.0000000	2.3000000
-10111.0000000	2.2849998
-10110.0000000	2.2550001
-10109.0000000	2.2350001
-10108.0000000	2.2200000
-10107.0000000	2.2000000
-10106.0000000	2.1700001
-10105.0000000	2.1500001
-10104.0000000	2.1500001
-10103.0000000	2.1450000
-10102.0000000	2.1099999
-10101.0000000	2.0799999
-10100.0000000	2.0799999
-10099.0000000	2.0749998
-10098.0000000	2.0650001
-10097.0000000	2.0500000
-10096.0000000	2.0633333
-10095.0000000	2.0650001
-10094.0000000	2.0650001
-10093.0000000	2.0850000
-10092.0000000	2.1149998
-10091.0000000	2.1250000
-10090.0000000	2.1400001
-10089.0000000	2.1500001
-10088.0000000	2.1600001
-10087.0000000	2.1800001
-10086.0000000	2.2049999
-10085.0000000	2.2150002
-10084.0000000	2.2449999
-10083.0000000	2.2550001
-10082.0000000	2.2700000
-10081.0000000	2.2800000
-10080.0000000	2.2750001
-10079.0000000	2.2500000
-10078.0000000	2.2400000
-10077.0000000	2.2249999
-10076.0000000	2.2049999
-10075.0000000	2.2200000
-10074.0000000	2.2449999
-10073.0000000	2.2750001
-10072.0000000	2.3049998
-10071.0000000	2.3350000
-10070.0000000	2.3599999
-10069.0000000	2.3699999
-10068.0000000	2.3750000
-10067.0000000	2.3699999
-10066.0000000	2.3933334
-10065.0000000	2.4100001
-10064.0000000	2.4250002
-10063.0000000	2.4250002
-10062.0000000	2.4349999
-10061.0000000	2.4300001
-10060.0000000	2.4250002
-10059.0000000	2.4200001
-10058.0000000	2.4349999
-10057.0000000	2.4450002

-10056.0000000	2.4450002
-10055.0000000	2.4549999
-10054.0000000	2.4450002
-10053.0000000	2.4349999
-10052.0000000	2.4300001
-10051.0000000	2.4349999
-10050.0000000	2.4400001
-10049.0000000	2.4300001
-10048.0000000	2.4250002
-10047.0000000	2.4150000
-10046.0000000	2.4000001
-10045.0000000	2.3900001
-10044.0000000	2.3900001
-10043.0000000	2.3900001
-10042.0000000	2.3750000
-10041.0000000	2.3449998
-10040.0000000	2.3150001
-10039.0000000	2.3049998
-10038.0000000	2.3099999
-10037.0000000	2.3199999
-10036.0000000	2.3399999
-10035.0000000	2.3599999
-10034.0000000	2.3800001
-10033.0000000	2.4000001
-10032.0000000	2.4250002
-10031.0000000	2.4600000
-10030.0000000	2.4700000
-10029.0000000	2.4850001
-10028.0000000	2.5000000
-10027.0000000	2.5149999
-10026.0000000	2.5149999
-10025.0000000	2.5050001
-10024.0000000	2.4850001
-10023.0000000	2.4900000
-10022.0000000	2.5050001
-10021.0000000	2.5050001
-10020.0000000	2.5100000
-10019.0000000	2.5250001
-10018.0000000	2.5300000
-10017.0000000	2.5400000
-10016.0000000	2.5400000
-10015.0000000	2.5400000
-10014.0000000	2.5500000
-10013.0000000	2.5549998
-10012.0000000	2.5500000
-10011.0000000	2.5400000
-10010.0000000	2.5450001
-10009.0000000	2.5349998
-10008.0000000	2.5300000
-10007.0000000	2.5250001
-10006.0000000	2.5100000
-10005.0000000	2.5149999
-10004.0000000	2.5100000
-10003.0000000	2.5050001
-10002.0000000	2.5149999
-10001.0000000	2.5200000
-10000.0000000	2.5349998

-9999.0000000	2.5349998
-9998.0000000	2.5400000
-9997.0000000	2.5450001
-9996.0000000	2.5349998
-9995.0000000	2.5349998
-9994.0000000	2.5349998
-9993.0000000	2.5450001
-9992.0000000	2.5450001
-9991.0000000	2.5599999
-9990.0000000	2.5650001
-9989.0000000	2.5749998
-9988.0000000	2.5749998
-9987.0000000	2.5650001
-9986.0000000	2.5650001
-9985.0000000	2.5699999
-9984.0000000	2.5949998
-9983.0000000	2.6050000
-9982.0000000	2.6149998
-9981.0000000	2.6099999
-9980.0000000	2.6050000
-9979.0000000	2.5949998
-9978.0000000	2.5850000
-9977.0000000	2.5749998
-9976.0000000	2.5599999
-9975.0000000	2.5500000
-9974.0000000	2.5549998
-9973.0000000	2.5450001
-9972.0000000	2.5149999
-9971.0000000	2.4900000
-9970.0000000	2.4600000
-9969.0000000	2.4300001
-9968.0000000	2.4050002
-9967.0000000	2.3699999
-9966.0000000	2.3449998
-9965.0000000	2.3099999
-9964.0000000	2.2849998
-9963.0000000	2.2849998
-9962.0000000	2.2750001
-9961.0000000	2.2700000
-9960.0000000	2.2700000
-9959.0000000	2.2849998
-9958.0000000	2.3000000
-9957.0000000	2.3099999
-9956.0000000	2.3150001
-9955.0000000	2.3150001
-9954.0000000	2.3249998
-9953.0000000	2.3350000
-9952.0000000	2.3499999
-9951.0000000	2.3550000
-9950.0000000	2.3699999
-9949.0000000	2.3850002
-9948.0000000	2.3900001
-9947.0000000	2.4100001
-9946.0000000	2.4300001
-9945.0000000	2.4500000
-9944.0000000	2.4500000
-9943.0000000	2.4600000

-9942.0000000	2.4600000
-9941.0000000	2.4650002
-9940.0000000	2.4749999
-9939.0000000	2.4850001
-9938.0000000	2.5050001
-9937.0000000	2.5050001
-9936.0000000	2.5050001
-9935.0000000	2.5100000
-9934.0000000	2.5149999
-9933.0000000	2.5100000
-9932.0000000	2.5149999
-9931.0000000	2.5149999
-9930.0000000	2.5149999
-9929.0000000	2.5000000
-9928.0000000	2.4949999
-9927.0000000	2.4900000
-9926.0000000	2.4800000
-9925.0000000	2.4700000
-9924.0000000	2.4400001
-9923.0000000	2.4100001
-9922.0000000	2.3900001
-9921.0000000	2.3699999
-9920.0000000	2.3649998
-9919.0000000	2.3550000
-9918.0000000	2.3399999
-9917.0000000	2.3049998
-9916.0000000	2.2900000
-9915.0000000	2.2900000
-9914.0000000	2.2950001
-9913.0000000	2.2800000
-9912.0000000	2.2449999
-9911.0000000	2.2350001
-9910.0000000	2.2350001
-9909.0000000	2.2400000
-9908.0000000	2.2600000
-9907.0000000	2.2800000
-9906.0000000	2.3099999
-9905.0000000	2.3249998
-9904.0000000	2.3449998
-9903.0000000	2.3499999
-9902.0000000	2.3499999
-9901.0000000	2.3599999
-9900.0000000	2.3850000
-9899.0000000	2.4150000
-9898.0000000	2.4450002
-9897.0000000	2.4749999
-9896.0000000	2.4900000
-9895.0000000	2.4949999
-9894.0000000	2.4900000
-9893.0000000	2.4949999
-9892.0000000	2.4900000
-9891.0000000	2.4850001
-9890.0000000	2.4900000
-9889.0000000	2.5000000
-9888.0000000	2.5100000
-9887.0000000	2.5100000
-9886.0000000	2.5200000

-9885.0000000	2.5266666
-9884.0000000	2.5349998
-9883.0000000	2.5349998
-9882.0000000	2.5500000
-9881.0000000	2.5749998
-9880.0000000	2.5749998
-9879.0000000	2.5850000
-9878.0000000	2.5850000
-9877.0000000	2.6050000
-9876.0000000	2.6149998
-9875.0000000	2.6450000
-9874.0000000	2.6800001
-9873.0000000	2.7000000
-9872.0000000	2.7400000
-9871.0000000	2.7649999
-9870.0000000	2.7800000
-9869.0000000	2.7950001
-9868.0000000	2.7950001
-9867.0000000	2.8099999
-9866.0000000	2.8099999
-9865.0000000	2.8199999
-9864.0000000	2.8399999
-9863.0000000	2.8550000
-9862.0000000	2.8599999
-9861.0000000	2.8699999
-9860.0000000	2.8699999
-9859.0000000	2.8699999
-9858.0000000	2.8699999
-9857.0000000	2.8699999
-9856.0000000	2.8649998
-9855.0000000	2.8566666
-9854.0000000	2.8150001
-9853.0000000	2.7950001
-9852.0000000	2.8049998
-9851.0000000	2.8049998
-9850.0000000	2.8150001
-9849.0000000	2.8199999
-9848.0000000	2.8249998
-9847.0000000	2.8299999
-9846.0000000	2.8199999
-9845.0000000	2.8199999
-9844.0000000	2.8299999
-9843.0000000	2.8499999
-9842.0000000	2.8599999
-9841.0000000	2.8750000
-9840.0000000	2.8950000
-9839.0000000	2.9050002
-9838.0000000	2.9200001
-9837.0000000	2.9250002
-9836.0000000	2.9400001
-9835.0000000	2.9400001
-9834.0000000	2.9300001
-9833.0000000	2.9400001
-9832.0000000	2.9250002
-9831.0000000	2.9250002
-9830.0000000	2.9349999
-9829.0000000	2.9250002

-9828.0000000	2.9349999
-9827.0000000	2.9300001
-9826.0000000	2.9300001
-9825.0000000	2.9200001
-9824.0000000	2.9166667
-9823.0000000	2.9050002
-9822.0000000	2.8850002
-9821.0000000	2.8750000
-9820.0000000	2.8599999
-9819.0000000	2.8449998
-9818.0000000	2.8299999
-9817.0000000	2.8249998
-9816.0000000	2.8150001
-9815.0000000	2.8099999
-9814.0000000	2.8049998
-9813.0000000	2.8000000
-9812.0000000	2.8000000
-9811.0000000	2.7950001
-9810.0000000	2.7800000
-9809.0000000	2.7800000
-9808.0000000	2.7700000
-9807.0000000	2.7600000
-9806.0000000	2.7600000
-9805.0000000	2.7500000
-9804.0000000	2.7400000
-9803.0000000	2.7300000
-9802.0000000	2.7249999
-9801.0000000	2.7249999
-9800.0000000	2.7150002
-9799.0000000	2.7150002
-9798.0000000	2.7249999
-9797.0000000	2.7300000
-9796.0000000	2.7400000
-9795.0000000	2.7400000
-9794.0000000	2.7466667
-9793.0000000	2.7550001
-9792.0000000	2.7649999
-9791.0000000	2.7750001
-9790.0000000	2.7750001
-9789.0000000	2.7849998
-9788.0000000	2.7900000
-9787.0000000	2.7950001
-9786.0000000	2.8000000
-9785.0000000	2.8049998
-9784.0000000	2.8150001
-9783.0000000	2.8299999
-9782.0000000	2.8499999
-9781.0000000	2.8599999
-9780.0000000	2.8699999
-9779.0000000	2.8699999
-9778.0000000	2.8750000
-9777.0000000	2.8850002
-9776.0000000	2.8900001
-9775.0000000	2.9000001
-9774.0000000	2.8950000
-9773.0000000	2.8900001
-9772.0000000	2.8800001

-9771.0000000	2.8649998
-9770.0000000	2.8599999
-9769.0000000	2.8449998
-9768.0000000	2.8350000
-9767.0000000	2.8249998
-9766.0000000	2.8199999
-9765.0000000	2.8049998
-9764.0000000	2.7700002
-9763.0000000	2.7400000
-9762.0000000	2.7249999
-9761.0000000	2.6950002
-9760.0000000	2.6950002
-9759.0000000	2.7100000
-9758.0000000	2.7150002
-9757.0000000	2.7300000
-9756.0000000	2.7649999
-9755.0000000	2.7900000
-9754.0000000	2.8099999
-9753.0000000	2.8299999
-9752.0000000	2.8550000
-9751.0000000	2.8850002
-9750.0000000	2.9050002
-9749.0000000	2.9400001
-9748.0000000	2.9650002
-9747.0000000	2.9850001
-9746.0000000	3.0000000
-9745.0000000	3.0149999
-9744.0000000	3.0250001
-9743.0000000	3.0450001
-9742.0000000	3.0400000
-9741.0000000	3.0500000
-9740.0000000	3.0699999
-9739.0000000	3.0799999
-9738.0000000	3.0850000
-9737.0000000	3.0899999
-9736.0000000	3.0850000
-9735.0000000	3.0999999
-9734.0000000	3.1066666
-9733.0000000	3.1099999
-9732.0000000	3.1099999
-9731.0000000	3.0949998
-9730.0000000	3.0549998
-9729.0000000	3.0450001
-9728.0000000	3.0050001
-9727.0000000	3.0000000
-9726.0000000	2.9900000
-9725.0000000	2.9650002
-9724.0000000	2.9400001
-9723.0000000	2.9300001
-9722.0000000	2.9250002
-9721.0000000	2.9150000
-9720.0000000	2.9000001
-9719.0000000	2.8850002
-9718.0000000	2.8750000
-9717.0000000	2.8649998
-9716.0000000	2.8550000
-9715.0000000	2.8449998

-9714.0000000	2.8299999
-9713.0000000	2.8099999
-9712.0000000	2.8000000
-9711.0000000	2.7800000
-9710.0000000	2.7750001
-9709.0000000	2.7700000
-9708.0000000	2.7500000
-9707.0000000	2.7500000
-9706.0000000	2.7500000
-9705.0000000	2.7600000
-9704.0000000	2.7600000
-9703.0000000	2.7750001
-9702.0000000	2.7800000
-9701.0000000	2.7849998
-9700.0000000	2.7950001
-9699.0000000	2.7950001
-9698.0000000	2.7900000
-9697.0000000	2.7800000
-9696.0000000	2.7500000
-9695.0000000	2.7150002
-9694.0000000	2.6849999
-9693.0000000	2.6500001
-9692.0000000	2.6099999
-9691.0000000	2.5949998
-9690.0000000	2.5899999
-9689.0000000	2.5799999
-9688.0000000	2.5899999
-9687.0000000	2.6149998
-9686.0000000	2.6350002
-9685.0000000	2.6500001
-9684.0000000	2.7000000
-9683.0000000	2.7300000
-9682.0000000	2.7449999
-9681.0000000	2.7500000
-9680.0000000	2.7600000
-9679.0000000	2.7550001
-9678.0000000	2.7400000
-9677.0000000	2.7249999
-9676.0000000	2.7249999
-9675.0000000	2.7550001
-9674.0000000	2.7833335
-9673.0000000	2.8000000
-9672.0000000	2.8000000
-9671.0000000	2.8000000
-9670.0000000	2.7849998
-9669.0000000	2.7649999
-9668.0000000	2.7500000
-9667.0000000	2.7249999
-9666.0000000	2.7049999
-9665.0000000	2.7000000
-9664.0000000	2.6900001
-9663.0000000	2.6900001
-9662.0000000	2.6950002
-9661.0000000	2.7100000
-9660.0000000	2.7049999
-9659.0000000	2.6950002
-9658.0000000	2.7000000

-9657.0000000	2.6900001
-9656.0000000	2.6849999
-9655.0000000	2.6800001
-9654.0000000	2.6950002
-9653.0000000	2.7000000
-9652.0000000	2.6950002
-9651.0000000	2.6700001
-9650.0000000	2.6550002
-9649.0000000	2.6400001
-9648.0000000	2.6150000
-9647.0000000	2.5899999
-9646.0000000	2.5699999
-9645.0000000	2.5633333
-9644.0000000	2.5349998
-9643.0000000	2.5250001
-9642.0000000	2.5200000
-9641.0000000	2.5050001
-9640.0000000	2.5000000
-9639.0000000	2.5000000
-9638.0000000	2.4949999
-9637.0000000	2.4749999
-9636.0000000	2.4650002
-9635.0000000	2.4700000
-9634.0000000	2.4900000
-9633.0000000	2.5149999
-9632.0000000	2.5349998
-9631.0000000	2.5699999
-9630.0000000	2.5999999
-9629.0000000	2.6099999
-9628.0000000	2.6250000
-9627.0000000	2.6400001
-9626.0000000	2.6500001
-9625.0000000	2.6650000
-9624.0000000	2.6900001
-9623.0000000	2.7000000
-9622.0000000	2.7100000
-9621.0000000	2.7150002
-9620.0000000	2.7249999
-9619.0000000	2.7249999
-9618.0000000	2.7200000
-9617.0000000	2.6950002
-9616.0000000	2.6849999
-9615.0000000	2.6849999
-9614.0000000	2.6766670
-9613.0000000	2.6800001
-9612.0000000	2.6700001
-9611.0000000	2.6550002
-9610.0000000	2.6150000
-9609.0000000	2.5699999
-9608.0000000	2.5100000
-9607.0000000	2.4500000
-9606.0000000	2.4300001
-9605.0000000	2.4100001
-9604.0000000	2.3900001
-9603.0000000	2.3950000
-9602.0000000	2.4100001
-9601.0000000	2.4050002

-9600.0000000	2.4200001
-9599.0000000	2.4400001
-9598.0000000	2.4600000
-9597.0000000	2.4650002
-9596.0000000	2.4500000
-9595.0000000	2.4100001
-9594.0000000	2.3499999
-9593.0000000	2.2800000
-9592.0000000	2.2249999
-9591.0000000	2.2000000
-9590.0000000	2.2000000
-9589.0000000	2.1800001
-9588.0000000	2.1550002
-9587.0000000	2.1600001
-9586.0000000	2.1500001
-9585.0000000	2.1600001
-9584.0000000	2.1833334
-9583.0000000	2.2150002
-9582.0000000	2.2300000
-9581.0000000	2.2400000
-9580.0000000	2.2500000
-9579.0000000	2.2800000
-9578.0000000	2.2649999
-9577.0000000	2.2700000
-9576.0000000	2.2649999
-9575.0000000	2.2700000
-9574.0000000	2.2750001
-9573.0000000	2.3000000
-9572.0000000	2.3249998
-9571.0000000	2.3449998
-9570.0000000	2.3449998
-9569.0000000	2.3550000
-9568.0000000	2.3649998
-9567.0000000	2.3850002
-9566.0000000	2.4150000
-9565.0000000	2.4300001
-9564.0000000	2.4349999
-9563.0000000	2.4349999
-9562.0000000	2.4500000
-9561.0000000	2.4300001
-9560.0000000	2.3950000
-9559.0000000	2.3499999
-9558.0000000	2.3299999
-9557.0000000	2.3249998
-9556.0000000	2.3350000
-9555.0000000	2.3499999
-9554.0000000	2.3633332
-9553.0000000	2.3699999
-9552.0000000	2.3699999
-9551.0000000	2.3900001
-9550.0000000	2.4200001
-9549.0000000	2.4250002
-9548.0000000	2.4450002
-9547.0000000	2.4650002
-9546.0000000	2.4800000
-9545.0000000	2.4850001
-9544.0000000	2.4850001

-9543.0000000	2.4749999
-9542.0000000	2.4600000
-9541.0000000	2.4400001
-9540.0000000	2.4150000
-9539.0000000	2.3750000
-9538.0000000	2.3249998
-9537.0000000	2.2849998
-9536.0000000	2.2350001
-9535.0000000	2.1950002
-9534.0000000	2.1750002
-9533.0000000	2.1650000
-9532.0000000	2.1550002
-9531.0000000	2.1500001
-9530.0000000	2.1700001
-9529.0000000	2.2049999
-9528.0000000	2.2249999
-9527.0000000	2.2600000
-9526.0000000	2.2849998
-9525.0000000	2.3249998
-9524.0000000	2.3550000
-9523.0000000	2.3800001
-9522.0000000	2.4066668
-9521.0000000	2.4349999
-9520.0000000	2.4500000
-9519.0000000	2.4600000
-9518.0000000	2.4749999
-9517.0000000	2.4749999
-9516.0000000	2.4800000
-9515.0000000	2.4949999
-9514.0000000	2.5100000
-9513.0000000	2.5100000
-9512.0000000	2.5200000
-9511.0000000	2.5250001
-9510.0000000	2.5250001
-9509.0000000	2.5250001
-9508.0000000	2.5250001
-9507.0000000	2.5250001
-9506.0000000	2.5250001
-9505.0000000	2.5300000
-9504.0000000	2.5300000
-9503.0000000	2.5450001
-9502.0000000	2.5500000
-9501.0000000	2.5500000
-9500.0000000	2.5500000
-9499.0000000	2.5450001
-9498.0000000	2.5450001
-9497.0000000	2.5400000
-9496.0000000	2.5300000
-9495.0000000	2.5300000
-9494.0000000	2.5200000
-9493.0000000	2.5100000
-9492.0000000	2.4766667
-9491.0000000	2.4450002
-9490.0000000	2.4150000
-9489.0000000	2.3900001
-9488.0000000	2.3800001
-9487.0000000	2.3649998

-9486.0000000	2.3499999
-9485.0000000	2.3399999
-9484.0000000	2.3399999
-9483.0000000	2.3299999
-9482.0000000	2.3399999
-9481.0000000	2.3350000
-9480.0000000	2.3599999
-9479.0000000	2.4000001
-9478.0000000	2.4200001
-9477.0000000	2.4250002
-9476.0000000	2.4000001
-9475.0000000	2.3950000
-9474.0000000	2.4000001
-9473.0000000	2.4100001
-9472.0000000	2.4250002
-9471.0000000	2.4300001
-9470.0000000	2.4349999
-9469.0000000	2.4250002
-9468.0000000	2.4450002
-9467.0000000	2.4349999
-9466.0000000	2.4450002
-9465.0000000	2.4450002
-9464.0000000	2.4400001
-9463.0000000	2.4400001
-9462.0000000	2.4400003
-9461.0000000	2.4400001
-9460.0000000	2.4400001
-9459.0000000	2.4450002
-9458.0000000	2.4500000
-9457.0000000	2.4500000
-9456.0000000	2.4450002
-9455.0000000	2.4300001
-9454.0000000	2.4200001
-9453.0000000	2.4150000
-9452.0000000	2.4100001
-9451.0000000	2.4050002
-9450.0000000	2.4000001
-9449.0000000	2.3950000
-9448.0000000	2.3900001
-9447.0000000	2.3950000
-9446.0000000	2.3900001
-9445.0000000	2.3850002
-9444.0000000	2.3750000
-9443.0000000	2.3750000
-9442.0000000	2.3900001
-9441.0000000	2.3750000
-9440.0000000	2.3599999
-9439.0000000	2.3599999
-9438.0000000	2.3649998
-9437.0000000	2.3649998
-9436.0000000	2.3649998
-9435.0000000	2.3699999
-9434.0000000	2.3750000
-9433.0000000	2.3850002
-9432.0000000	2.3866668
-9431.0000000	2.4200001
-9430.0000000	2.4300001

-9429.0000000	2.4400001
-9428.0000000	2.4349999
-9427.0000000	2.4349999
-9426.0000000	2.4400001
-9425.0000000	2.4349999
-9424.0000000	2.4200001
-9423.0000000	2.4050002
-9422.0000000	2.4000001
-9421.0000000	2.4050002
-9420.0000000	2.4050002
-9419.0000000	2.4100001
-9418.0000000	2.4250002
-9417.0000000	2.4300001
-9416.0000000	2.4450002
-9415.0000000	2.4450002
-9414.0000000	2.4549999
-9413.0000000	2.4500000
-9412.0000000	2.4549999
-9411.0000000	2.4650002
-9410.0000000	2.4549999
-9409.0000000	2.4450002
-9408.0000000	2.4300001
-9407.0000000	2.4050002
-9406.0000000	2.4000001
-9405.0000000	2.3800001
-9404.0000000	2.3699999
-9403.0000000	2.3599999
-9402.0000000	2.3649998
-9401.0000000	2.3633330
-9400.0000000	2.3649998
-9399.0000000	2.3499999
-9398.0000000	2.3299999
-9397.0000000	2.3150001
-9396.0000000	2.2849998
-9395.0000000	2.2449999
-9394.0000000	2.2400000
-9393.0000000	2.2449999
-9392.0000000	2.2449999
-9391.0000000	2.2500000
-9390.0000000	2.2600000
-9389.0000000	2.2700000
-9388.0000000	2.2700000
-9387.0000000	2.2750001
-9386.0000000	2.2750001
-9385.0000000	2.2500000
-9384.0000000	2.2300000
-9383.0000000	2.2300000
-9382.0000000	2.2400000
-9381.0000000	2.2700000
-9380.0000000	2.2800000
-9379.0000000	2.3000000
-9378.0000000	2.3000000
-9377.0000000	2.3150001
-9376.0000000	2.3249998
-9375.0000000	2.3350000
-9374.0000000	2.3399999
-9373.0000000	2.3550000

-9372.0000000	2.3649998
-9371.0000000	2.3733332
-9370.0000000	2.3850002
-9369.0000000	2.3800001
-9368.0000000	2.3900001
-9367.0000000	2.4000001
-9366.0000000	2.4100001
-9365.0000000	2.4150000
-9364.0000000	2.4250002
-9363.0000000	2.4300001
-9362.0000000	2.4400001
-9361.0000000	2.4500000
-9360.0000000	2.4600000
-9359.0000000	2.4700000
-9358.0000000	2.4650002
-9357.0000000	2.4749999
-9356.0000000	2.4650002
-9355.0000000	2.4700000
-9354.0000000	2.4700000
-9353.0000000	2.4600000
-9352.0000000	2.4450002
-9351.0000000	2.4400001
-9350.0000000	2.4450002
-9349.0000000	2.4349999
-9348.0000000	2.4200001
-9347.0000000	2.3850002
-9346.0000000	2.3649998
-9345.0000000	2.3499999
-9344.0000000	2.3299999
-9343.0000000	2.3099999
-9342.0000000	2.3000000
-9341.0000000	2.2800000
-9340.0000000	2.2550001
-9339.0000000	2.2249999
-9338.0000000	2.2100000
-9337.0000000	2.1950002
-9336.0000000	2.1849999
-9335.0000000	2.2000000
-9334.0000000	2.2150002
-9333.0000000	2.2150002
-9332.0000000	2.2300000
-9331.0000000	2.2550001
-9330.0000000	2.2700000
-9329.0000000	2.2800000
-9328.0000000	2.2849998
-9327.0000000	2.3049998
-9326.0000000	2.3249998
-9325.0000000	2.3399999
-9324.0000000	2.3649998
-9323.0000000	2.3800001
-9322.0000000	2.4050002
-9321.0000000	2.4250002
-9320.0000000	2.4300001
-9319.0000000	2.4349999
-9318.0000000	2.4200001
-9317.0000000	2.4100001
-9316.0000000	2.4050002

-9315.0000000	2.3950000
-9314.0000000	2.3900001
-9313.0000000	2.3766668
-9312.0000000	2.3699999
-9311.0000000	2.3750000
-9310.0000000	2.3649998
-9309.0000000	2.3499999
-9308.0000000	2.3550000
-9307.0000000	2.3800001
-9306.0000000	2.4050002
-9305.0000000	2.4300001
-9304.0000000	2.4500000
-9303.0000000	2.4700000
-9302.0000000	2.5000000
-9301.0000000	2.5149999
-9300.0000000	2.5300000
-9299.0000000	2.5500000
-9298.0000000	2.5549998
-9297.0000000	2.5549998
-9296.0000000	2.5500000
-9295.0000000	2.5450001
-9294.0000000	2.5300000
-9293.0000000	2.5250001
-9292.0000000	2.5250001
-9291.0000000	2.5100000
-9290.0000000	2.5000000
-9289.0000000	2.5050001
-9288.0000000	2.5000000
-9287.0000000	2.4749999
-9286.0000000	2.4749999
-9285.0000000	2.4700000
-9284.0000000	2.4700000
-9283.0000000	2.4666669
-9282.0000000	2.4850001
-9281.0000000	2.5000000
-9280.0000000	2.5100000
-9279.0000000	2.5200000
-9278.0000000	2.5250001
-9277.0000000	2.5300000
-9276.0000000	2.5400000
-9275.0000000	2.5450001
-9274.0000000	2.5250001
-9273.0000000	2.5000000
-9272.0000000	2.4949999
-9271.0000000	2.5100000
-9270.0000000	2.5149999
-9269.0000000	2.5200000
-9268.0000000	2.5250001
-9267.0000000	2.5250001
-9266.0000000	2.5400000
-9265.0000000	2.5400000
-9264.0000000	2.5400000
-9263.0000000	2.5450001
-9262.0000000	2.5300000
-9261.0000000	2.5149999
-9260.0000000	2.5400000
-9259.0000000	2.5450001

-9258.0000000	2.5450001
-9257.0000000	2.5650001
-9256.0000000	2.5850000
-9255.0000000	2.5899999
-9254.0000000	2.6149998
-9253.0000000	2.6400001
-9252.0000000	2.6650000
-9251.0000000	2.6849999
-9250.0000000	2.7049999
-9249.0000000	2.7049999
-9248.0000000	2.7150002
-9247.0000000	2.7249999
-9246.0000000	2.7300000
-9245.0000000	2.7249999
-9244.0000000	2.7249999
-9243.0000000	2.7350001
-9242.0000000	2.7249999
-9241.0000000	2.7249999
-9240.0000000	2.7350001
-9239.0000000	2.7400000
-9238.0000000	2.7600000
-9237.0000000	2.7500000
-9236.0000000	2.7500000
-9235.0000000	2.7350001
-9234.0000000	2.7249999
-9233.0000000	2.7400000
-9232.0000000	2.7400000
-9231.0000000	2.7449999
-9230.0000000	2.7750001
-9229.0000000	2.7950001
-9228.0000000	2.8099999
-9227.0000000	2.8150001
-9226.0000000	2.8049998
-9225.0000000	2.7750001
-9224.0000000	2.7649999
-9223.0000000	2.7449999
-9222.0000000	2.7600000
-9221.0000000	2.7633333
-9220.0000000	2.7500000
-9219.0000000	2.7550001
-9218.0000000	2.7550001
-9217.0000000	2.7550001
-9216.0000000	2.7449999
-9215.0000000	2.7350001
-9214.0000000	2.7249999
-9213.0000000	2.7150002
-9212.0000000	2.7049999
-9211.0000000	2.7049999
-9210.0000000	2.6900001
-9209.0000000	2.6700001
-9208.0000000	2.6600001
-9207.0000000	2.6500001
-9206.0000000	2.6450000
-9205.0000000	2.6500001
-9204.0000000	2.6550002
-9203.0000000	2.6750002
-9202.0000000	2.6849999

-9201.0000000	2.7049999
-9200.0000000	2.7100000
-9199.0000000	2.7300000
-9198.0000000	2.7400000
-9197.0000000	2.7500000
-9196.0000000	2.7649999
-9195.0000000	2.7900000
-9194.0000000	2.8199999
-9193.0000000	2.8499999
-9192.0000000	2.8733332
-9191.0000000	2.8800001
-9190.0000000	2.9050002
-9189.0000000	2.9150000
-9188.0000000	2.9100001
-9187.0000000	2.9150000
-9186.0000000	2.9200001
-9185.0000000	2.9250002
-9184.0000000	2.9250002
-9183.0000000	2.9300001
-9182.0000000	2.9100001
-9181.0000000	2.8950000
-9180.0000000	2.8900001
-9179.0000000	2.8850002
-9178.0000000	2.8800001
-9177.0000000	2.8850002
-9176.0000000	2.9050002
-9175.0000000	2.9400001
-9174.0000000	2.9600000
-9173.0000000	2.9800000
-9172.0000000	2.9949999
-9171.0000000	3.0050001
-9170.0000000	2.9900000
-9169.0000000	2.9700000
-9168.0000000	2.9600000
-9167.0000000	2.9600000
-9166.0000000	2.9600000
-9165.0000000	2.9400001
-9164.0000000	2.9349999
-9163.0000000	2.9300001
-9162.0000000	2.9500000
-9161.0000000	2.9500000
-9160.0000000	2.9600000
-9159.0000000	2.9749999
-9158.0000000	2.9949999
-9157.0000000	3.0100000
-9156.0000000	3.0149999
-9155.0000000	3.0149999
-9154.0000000	3.0300000
-9153.0000000	3.0400000
-9152.0000000	3.0599999
-9151.0000000	3.0549998
-9150.0000000	3.0650001
-9149.0000000	3.0850000
-9148.0000000	3.0899999
-9147.0000000	3.0949998
-9146.0000000	3.1050000
-9145.0000000	3.1199999

-9144.0000000	3.1199999
-9143.0000000	3.1300001
-9142.0000000	3.1250000
-9141.0000000	3.0999999
-9140.0000000	3.0999999
-9139.0000000	3.0749998
-9138.0000000	3.0349998
-9137.0000000	3.0000000
-9136.0000000	2.9900000
-9135.0000000	3.0000000
-9134.0000000	3.0200000
-9133.0000000	3.0450001
-9132.0000000	3.0799999
-9131.0000000	3.0999999
-9130.0000000	3.1000001
-9129.0000000	3.0949998
-9128.0000000	3.0949998
-9127.0000000	3.1099999
-9126.0000000	3.1149998
-9125.0000000	3.1149998
-9124.0000000	3.1350002
-9123.0000000	3.1450000
-9122.0000000	3.1500001
-9121.0000000	3.1550002
-9120.0000000	3.1600001
-9119.0000000	3.1700001
-9118.0000000	3.1700001
-9117.0000000	3.1700001
-9116.0000000	3.1650000
-9115.0000000	3.1650000
-9114.0000000	3.1600001
-9113.0000000	3.1500001
-9112.0000000	3.1450000
-9111.0000000	3.1350002
-9110.0000000	3.1300001
-9109.0000000	3.1250000
-9108.0000000	3.1199999
-9107.0000000	3.1300001
-9106.0000000	3.1250000
-9105.0000000	3.1350002
-9104.0000000	3.1500001
-9103.0000000	3.1450000
-9102.0000000	3.1450000
-9101.0000000	3.1300001
-9100.0000000	3.1250000
-9099.0000000	3.1250000
-9098.0000000	3.1266668
-9097.0000000	3.1149998
-9096.0000000	3.0949998
-9095.0000000	3.0799999
-9094.0000000	3.0699999
-9093.0000000	3.0500000
-9092.0000000	3.0349998
-9091.0000000	3.0149999
-9090.0000000	3.0000000
-9089.0000000	2.9800000
-9088.0000000	2.9749999

-9087.0000000	2.9850001
-9086.0000000	3.0000000
-9085.0000000	3.0000000
-9084.0000000	2.9800000
-9083.0000000	2.9700000
-9082.0000000	2.9450002
-9081.0000000	2.9200001
-9080.0000000	2.9200001
-9079.0000000	2.8950000
-9078.0000000	2.8850002
-9077.0000000	2.8750000
-9076.0000000	2.8350000
-9075.0000000	2.8099999
-9074.0000000	2.7950001
-9073.0000000	2.7800000
-9072.0000000	2.7900000
-9071.0000000	2.7950001
-9070.0000000	2.8000000
-9069.0000000	2.7900000
-9068.0000000	2.7633333
-9067.0000000	2.7500000
-9066.0000000	2.7400000
-9065.0000000	2.7400000
-9064.0000000	2.7500000
-9063.0000000	2.7500000
-9062.0000000	2.7600000
-9061.0000000	2.7750001
-9060.0000000	2.7800000
-9059.0000000	2.7750001
-9058.0000000	2.7849998
-9057.0000000	2.7849998
-9056.0000000	2.7849998
-9055.0000000	2.7700000
-9054.0000000	2.7500000
-9053.0000000	2.7350001
-9052.0000000	2.7200000
-9051.0000000	2.7049999
-9050.0000000	2.6750002
-9049.0000000	2.6600001
-9048.0000000	2.6350000
-9047.0000000	2.6099999
-9046.0000000	2.5899999
-9045.0000000	2.5699999
-9044.0000000	2.5749998
-9043.0000000	2.5850000
-9042.0000000	2.6300001
-9041.0000000	2.6500001
-9040.0000000	2.6800001
-9039.0000000	2.7100000
-9038.0000000	2.7299998
-9037.0000000	2.7500000
-9036.0000000	2.7700000
-9035.0000000	2.7950001
-9034.0000000	2.8150001
-9033.0000000	2.8399999
-9032.0000000	2.8649998
-9031.0000000	2.8900001

-9030.0000000	2.9150000
-9029.0000000	2.9200001
-9028.0000000	2.9200001
-9027.0000000	2.9300001
-9026.0000000	2.9349999
-9025.0000000	2.9450002
-9024.0000000	2.9500000
-9023.0000000	2.9600000
-9022.0000000	2.9600000
-9021.0000000	2.9749999
-9020.0000000	2.9850001
-9019.0000000	2.9749999
-9018.0000000	2.9949999
-9017.0000000	2.9949999
-9016.0000000	2.9900000
-9015.0000000	2.9900000
-9014.0000000	2.9900000
-9013.0000000	2.9949999
-9012.0000000	2.9949999
-9011.0000000	2.9949999
-9010.0000000	2.9949999
-9009.0000000	3.0000000
-9008.0000000	3.0233333
-9007.0000000	3.0450001
-9006.0000000	3.0300000
-9005.0000000	3.0300000
-9004.0000000	3.0250001
-9003.0000000	3.0149999
-9002.0000000	3.0100000
-9001.0000000	2.9800000
-9000.0000000	2.9500000
-8999.0000000	2.9100001
-8998.0000000	2.8750000
-8997.0000000	2.8399999
-8996.0000000	2.8449998
-8995.0000000	2.8499999
-8994.0000000	2.8649998
-8993.0000000	2.8850002
-8992.0000000	2.8800001
-8991.0000000	2.8750000
-8990.0000000	2.8750000
-8989.0000000	2.8599999
-8988.0000000	2.8699999
-8987.0000000	2.9000001
-8986.0000000	2.9300001
-8985.0000000	2.9650002
-8984.0000000	2.9800000
-8983.0000000	3.0000000
-8982.0000000	3.0300000
-8981.0000000	3.0549998
-8980.0000000	3.0850000
-8979.0000000	3.1200001
-8978.0000000	3.1650000
-8977.0000000	3.1650000
-8976.0000000	3.1700001
-8975.0000000	3.1550002
-8974.0000000	3.1500001

-8973.0000000	3.1450000
-8972.0000000	3.1500001
-8971.0000000	3.1600001
-8970.0000000	3.1600001
-8969.0000000	3.1550002
-8968.0000000	3.1550002
-8967.0000000	3.1450000
-8966.0000000	3.1250000
-8965.0000000	3.0949998
-8964.0000000	3.0549998
-8963.0000000	3.0250001
-8962.0000000	2.9850001
-8961.0000000	2.9500000
-8960.0000000	2.9050002
-8959.0000000	2.8699999
-8958.0000000	2.8449998
-8957.0000000	2.8399999
-8956.0000000	2.8399999
-8955.0000000	2.8350000
-8954.0000000	2.8150001
-8953.0000000	2.7849998
-8952.0000000	2.7750001
-8951.0000000	2.7800000
-8950.0000000	2.7600000
-8949.0000000	2.7433331
-8948.0000000	2.7350001
-8947.0000000	2.7200000
-8946.0000000	2.6849999
-8945.0000000	2.6550002
-8944.0000000	2.6350002
-8943.0000000	2.6250000
-8942.0000000	2.6250000
-8941.0000000	2.6300001
-8940.0000000	2.6350002
-8939.0000000	2.6350002
-8938.0000000	2.6149998
-8937.0000000	2.5949998
-8936.0000000	2.5799999
-8935.0000000	2.5500000
-8934.0000000	2.5300000
-8933.0000000	2.5000000
-8932.0000000	2.4600000
-8931.0000000	2.4300001
-8930.0000000	2.4150000
-8929.0000000	2.4250002
-8928.0000000	2.4200001
-8927.0000000	2.4250002
-8926.0000000	2.4300001
-8925.0000000	2.4349999
-8924.0000000	2.4450002
-8923.0000000	2.4400001
-8922.0000000	2.4600000
-8921.0000000	2.4650002
-8920.0000000	2.4949999
-8919.0000000	2.5066667
-8918.0000000	2.5200000
-8917.0000000	2.5250001

-8916.0000000	2.5250001
-8915.0000000	2.5250001
-8914.0000000	2.5450001
-8913.0000000	2.5599999
-8912.0000000	2.5599999
-8911.0000000	2.5599999
-8910.0000000	2.5749998
-8909.0000000	2.5799999
-8908.0000000	2.5699999
-8907.0000000	2.5599999
-8906.0000000	2.5500000
-8905.0000000	2.5349998
-8904.0000000	2.5149999
-8903.0000000	2.4749999
-8902.0000000	2.4450002
-8901.0000000	2.4250002
-8900.0000000	2.4150000
-8899.0000000	2.4050002
-8898.0000000	2.4100001
-8897.0000000	2.4000001
-8896.0000000	2.4000001
-8895.0000000	2.4000001
-8894.0000000	2.4050002
-8893.0000000	2.4050002
-8892.0000000	2.4200001
-8891.0000000	2.4250002
-8890.0000000	2.4349999
-8889.0000000	2.4233334
-8888.0000000	2.4150000
-8887.0000000	2.4000001
-8886.0000000	2.3950000
-8885.0000000	2.3850002
-8884.0000000	2.3699999
-8883.0000000	2.3699999
-8882.0000000	2.3900001
-8881.0000000	2.3900001
-8880.0000000	2.4100001
-8879.0000000	2.4200001
-8878.0000000	2.4450002
-8877.0000000	2.4650002
-8876.0000000	2.4900000
-8875.0000000	2.5200000
-8874.0000000	2.5349998
-8873.0000000	2.5500000
-8872.0000000	2.5699999
-8871.0000000	2.5749998
-8870.0000000	2.5650001
-8869.0000000	2.5400000
-8868.0000000	2.5100000
-8867.0000000	2.5000000
-8866.0000000	2.4800000
-8865.0000000	2.4650002
-8864.0000000	2.4450002
-8863.0000000	2.4150000
-8862.0000000	2.3900001
-8861.0000000	2.3550000
-8860.0000000	2.3299999

-8859.0000000	2.2966664
-8858.0000000	2.2500000
-8857.0000000	2.2449999
-8856.0000000	2.2350001
-8855.0000000	2.2400000
-8854.0000000	2.2750001
-8853.0000000	2.3000000
-8852.0000000	2.3150001
-8851.0000000	2.3199999
-8850.0000000	2.3449998
-8849.0000000	2.3800001
-8848.0000000	2.3900001
-8847.0000000	2.4000001
-8846.0000000	2.4100001
-8845.0000000	2.4250002
-8844.0000000	2.4349999
-8843.0000000	2.4600000
-8842.0000000	2.4749999
-8841.0000000	2.4900000
-8840.0000000	2.5050001
-8839.0000000	2.5200000
-8838.0000000	2.5400000
-8837.0000000	2.5500000
-8836.0000000	2.5650001
-8835.0000000	2.5749998
-8834.0000000	2.5899999
-8833.0000000	2.6050000
-8832.0000000	2.6350002
-8831.0000000	2.6500001
-8830.0000000	2.6650000
-8829.0000000	2.6866665
-8828.0000000	2.7000000
-8827.0000000	2.6849999
-8826.0000000	2.6750002
-8825.0000000	2.6750002
-8824.0000000	2.6600001
-8823.0000000	2.6450000
-8822.0000000	2.6400001
-8821.0000000	2.6300001
-8820.0000000	2.6250000
-8819.0000000	2.6199999
-8818.0000000	2.6149998
-8817.0000000	2.6099999
-8816.0000000	2.6199999
-8815.0000000	2.6250000
-8814.0000000	2.6250000
-8813.0000000	2.6400001
-8812.0000000	2.6500001
-8811.0000000	2.6600001
-8810.0000000	2.6650000
-8809.0000000	2.6950002
-8808.0000000	2.6950002
-8807.0000000	2.6950002
-8806.0000000	2.6849999
-8805.0000000	2.6950002
-8804.0000000	2.6849999
-8803.0000000	2.6700001

-8802.0000000	2.6600001
-8801.0000000	2.6450000
-8800.0000000	2.6250000
-8799.0000000	2.6199999
-8798.0000000	2.6149998
-8797.0000000	2.6350002
-8796.0000000	2.6550002
-8795.0000000	2.6350000
-8794.0000000	2.6099999
-8793.0000000	2.6199999
-8792.0000000	2.6199999
-8791.0000000	2.6300001
-8790.0000000	2.6450000
-8789.0000000	2.6550002
-8788.0000000	2.6700001
-8787.0000000	2.6849999
-8786.0000000	2.6849999
-8785.0000000	2.6900001
-8784.0000000	2.6900001
-8783.0000000	2.7150002
-8782.0000000	2.7300000
-8781.0000000	2.7600000
-8780.0000000	2.7750001
-8779.0000000	2.7950001
-8778.0000000	2.7950001
-8777.0000000	2.8049998
-8776.0000000	2.8150001
-8775.0000000	2.8249998
-8774.0000000	2.8350000
-8773.0000000	2.8399999
-8772.0000000	2.8499999
-8771.0000000	2.8499999
-8770.0000000	2.8449998
-8769.0000000	2.8466663
-8768.0000000	2.8499999
-8767.0000000	2.8550000
-8766.0000000	2.8699999
-8765.0000000	2.8750000
-8764.0000000	2.8850002
-8763.0000000	2.8900001
-8762.0000000	2.8900001
-8761.0000000	2.8850002
-8760.0000000	2.8850002
-8759.0000000	2.8900001
-8758.0000000	2.9050002
-8757.0000000	2.9200001
-8756.0000000	2.9250002
-8755.0000000	2.9400001
-8754.0000000	2.9400001
-8753.0000000	2.9300001
-8752.0000000	2.9300001
-8751.0000000	2.9250002
-8750.0000000	2.9349999
-8749.0000000	2.9250002
-8748.0000000	2.9300001
-8747.0000000	2.9200001
-8746.0000000	2.9050002

-8745.0000000	2.8950000
-8744.0000000	2.8750000
-8743.0000000	2.8750000
-8742.0000000	2.8550000
-8741.0000000	2.8399999
-8740.0000000	2.8150001
-8739.0000000	2.7699997
-8738.0000000	2.7300000
-8737.0000000	2.7000000
-8736.0000000	2.6750002
-8735.0000000	2.6550002
-8734.0000000	2.6500001
-8733.0000000	2.6400001
-8732.0000000	2.6250000
-8731.0000000	2.6099999
-8730.0000000	2.5850000
-8729.0000000	2.5599999
-8728.0000000	2.5450001
-8727.0000000	2.5250001
-8726.0000000	2.4949999
-8725.0000000	2.4949999
-8724.0000000	2.5000000
-8723.0000000	2.4949999
-8722.0000000	2.4900000
-8721.0000000	2.4749999
-8720.0000000	2.4650002
-8719.0000000	2.4500000
-8718.0000000	2.4050002
-8717.0000000	2.3800001
-8716.0000000	2.3800001
-8715.0000000	2.3800001
-8714.0000000	2.3800001
-8713.0000000	2.3599999
-8712.0000000	2.3350000
-8711.0000000	2.3049998
-8710.0000000	2.2849998
-8709.0000000	2.2400000
-8708.0000000	2.2100000
-8707.0000000	2.1800001
-8706.0000000	2.1400001
-8705.0000000	2.1199999
-8704.0000000	2.1149998
-8703.0000000	2.1050000
-8702.0000000	2.1199999
-8701.0000000	2.1500001
-8700.0000000	2.1750002
-8699.0000000	2.2100000
-8698.0000000	2.2200000
-8697.0000000	2.2249999
-8696.0000000	2.2300000
-8695.0000000	2.2249999
-8694.0000000	2.2300000
-8693.0000000	2.2249999
-8692.0000000	2.2200000
-8691.0000000	2.2150002
-8690.0000000	2.2100000
-8689.0000000	2.2000000

-8688.0000000	2.2150002
-8687.0000000	2.2300000
-8686.0000000	2.2400000
-8685.0000000	2.2449999
-8684.0000000	2.2550001
-8683.0000000	2.2700000
-8682.0000000	2.2750001
-8681.0000000	2.2800000
-8680.0000000	2.2800000
-8679.0000000	2.2750001
-8678.0000000	2.2649999
-8677.0000000	2.2766666
-8676.0000000	2.3000000
-8675.0000000	2.2900000
-8674.0000000	2.2800000
-8673.0000000	2.2649999
-8672.0000000	2.2449999
-8671.0000000	2.2100000
-8670.0000000	2.1700001
-8669.0000000	2.1500001
-8668.0000000	2.1400001
-8667.0000000	2.1500001
-8666.0000000	2.1700001
-8665.0000000	2.1800001
-8664.0000000	2.1950002
-8663.0000000	2.2150002
-8662.0000000	2.2150002
-8661.0000000	2.2200000
-8660.0000000	2.2350001
-8659.0000000	2.2500000
-8658.0000000	2.2649999
-8657.0000000	2.2750001
-8656.0000000	2.2800000
-8655.0000000	2.2849998
-8654.0000000	2.2800000
-8653.0000000	2.2700000
-8652.0000000	2.2750001
-8651.0000000	2.2750001
-8650.0000000	2.2700000
-8649.0000000	2.2800000
-8648.0000000	2.2900000
-8647.0000000	2.3199999
-8646.0000000	2.3433332
-8645.0000000	2.3649998
-8644.0000000	2.3649998
-8643.0000000	2.3649998
-8642.0000000	2.3750000
-8641.0000000	2.3850002
-8640.0000000	2.3699999
-8639.0000000	2.3550000
-8638.0000000	2.3699999
-8637.0000000	2.3900001
-8636.0000000	2.3950000
-8635.0000000	2.4000001
-8634.0000000	2.4050002
-8633.0000000	2.4100001
-8632.0000000	2.4200001

-8631.0000000	2.4250002
-8630.0000000	2.4300001
-8629.0000000	2.4349999
-8628.0000000	2.4300001
-8627.0000000	2.4349999
-8626.0000000	2.4349999
-8625.0000000	2.4500000
-8624.0000000	2.4549999
-8623.0000000	2.4600000
-8622.0000000	2.4700000
-8621.0000000	2.4749999
-8620.0000000	2.4749999
-8619.0000000	2.4650002
-8618.0000000	2.4700000
-8617.0000000	2.4749999
-8616.0000000	2.4799998
-8615.0000000	2.4900000
-8614.0000000	2.5000000
-8613.0000000	2.5100000
-8612.0000000	2.5149999
-8611.0000000	2.5149999
-8610.0000000	2.5400000
-8609.0000000	2.5400000
-8608.0000000	2.5599999
-8607.0000000	2.5699999
-8606.0000000	2.5949998
-8605.0000000	2.6150000
-8604.0000000	2.6350002
-8603.0000000	2.6650000
-8602.0000000	2.6800001
-8601.0000000	2.7100000
-8600.0000000	2.7400000
-8599.0000000	2.7649999
-8598.0000000	2.7800000
-8597.0000000	2.8049998
-8596.0000000	2.8099999
-8595.0000000	2.8299999
-8594.0000000	2.8249998
-8593.0000000	2.8399999
-8592.0000000	2.8599999
-8591.0000000	2.8699999
-8590.0000000	2.8499999
-8589.0000000	2.8199999
-8588.0000000	2.8000000
-8587.0000000	2.7700000
-8586.0000000	2.7600000
-8585.0000000	2.7100000
-8584.0000000	2.6650000
-8583.0000000	2.6199999
-8582.0000000	2.5749998
-8581.0000000	2.5450001
-8580.0000000	2.5300000
-8579.0000000	2.5200000
-8578.0000000	2.5450001
-8577.0000000	2.5650001
-8576.0000000	2.5549998
-8575.0000000	2.5549998

-8574.0000000	2.5650001
-8573.0000000	2.5799999
-8572.0000000	2.6099999
-8571.0000000	2.6350002
-8570.0000000	2.6600001
-8569.0000000	2.6800001
-8568.0000000	2.6900001
-8567.0000000	2.6700001
-8566.0000000	2.6550002
-8565.0000000	2.6450000
-8564.0000000	2.6650000
-8563.0000000	2.6650000
-8562.0000000	2.6700001
-8561.0000000	2.6800001
-8560.0000000	2.6849999
-8559.0000000	2.6750002
-8558.0000000	2.6700001
-8557.0000000	2.6250000
-8556.0000000	2.6199999
-8555.0000000	2.6000001
-8554.0000000	2.5549998
-8553.0000000	2.4850001
-8552.0000000	2.4100001
-8551.0000000	2.3499999
-8550.0000000	2.2950001
-8549.0000000	2.2400000
-8548.0000000	2.1950002
-8547.0000000	2.1800001
-8546.0000000	2.1750002
-8545.0000000	2.1800001
-8544.0000000	2.1800001
-8543.0000000	2.1800001
-8542.0000000	2.1750002
-8541.0000000	2.1700001
-8540.0000000	2.1500001
-8539.0000000	2.1400001
-8538.0000000	2.1300001
-8537.0000000	2.1099999
-8536.0000000	2.0850000
-8535.0000000	2.0650001
-8534.0000000	2.0650001
-8533.0000000	2.0799999
-8532.0000000	2.0949998
-8531.0000000	2.1050000
-8530.0000000	2.1149998
-8529.0000000	2.1149998
-8528.0000000	2.1050000
-8527.0000000	2.0999999
-8526.0000000	2.0850000
-8525.0000000	2.0833333
-8524.0000000	2.0949998
-8523.0000000	2.0999999
-8522.0000000	2.1099999
-8521.0000000	2.1099999
-8520.0000000	2.0999999
-8519.0000000	2.0999999
-8518.0000000	2.0899999

-8517.0000000	2.0799999
-8516.0000000	2.0799999
-8515.0000000	2.0949998
-8514.0000000	2.1300001
-8513.0000000	2.1300001
-8512.0000000	2.1450000
-8511.0000000	2.1450000
-8510.0000000	2.1650000
-8509.0000000	2.1900001
-8508.0000000	2.2049999
-8507.0000000	2.2300000
-8506.0000000	2.2400000
-8505.0000000	2.2600000
-8504.0000000	2.2700000
-8503.0000000	2.2750001
-8502.0000000	2.2800000
-8501.0000000	2.2849998
-8500.0000000	2.2700000
-8499.0000000	2.2550001
-8498.0000000	2.2150002
-8497.0000000	2.1750002
-8496.0000000	2.1800001
-8495.0000000	2.1849999
-8494.0000000	2.1866667
-8493.0000000	2.1750002
-8492.0000000	2.1450000
-8491.0000000	2.1250000
-8490.0000000	2.1450000
-8489.0000000	2.1600001
-8488.0000000	2.1800001
-8487.0000000	2.1750002
-8486.0000000	2.1849999
-8485.0000000	2.2000000
-8484.0000000	2.2000000
-8483.0000000	2.2049999
-8482.0000000	2.2200000
-8481.0000000	2.2300000
-8480.0000000	2.2449999
-8479.0000000	2.2300000
-8478.0000000	2.2100000
-8477.0000000	2.1950002
-8476.0000000	2.1849999
-8475.0000000	2.1800001
-8474.0000000	2.1800001
-8473.0000000	2.1800001
-8472.0000000	2.1900001
-8471.0000000	2.1750002
-8470.0000000	2.1849999
-8469.0000000	2.2100000
-8468.0000000	2.2550001
-8467.0000000	2.3000000
-8466.0000000	2.3449998
-8465.0000000	2.3550000
-8464.0000000	2.3833334
-8463.0000000	2.4200001
-8462.0000000	2.4400001
-8461.0000000	2.4549999

-8460.0000000	2.4500000
-8459.0000000	2.4450002
-8458.0000000	2.4500000
-8457.0000000	2.4450002
-8456.0000000	2.4600000
-8455.0000000	2.4650002
-8454.0000000	2.4800000
-8453.0000000	2.4900000
-8452.0000000	2.5050001
-8451.0000000	2.5349998
-8450.0000000	2.5549998
-8449.0000000	2.5599999
-8448.0000000	2.5799999
-8447.0000000	2.5949998
-8446.0000000	2.6050000
-8445.0000000	2.6099999
-8444.0000000	2.5999999
-8443.0000000	2.6199999
-8442.0000000	2.5999999
-8441.0000000	2.6099999
-8440.0000000	2.6149998
-8439.0000000	2.6199999
-8438.0000000	2.6400001
-8437.0000000	2.6500001
-8436.0000000	2.6600001
-8435.0000000	2.6500001
-8434.0000000	2.6499999
-8433.0000000	2.6350002
-8432.0000000	2.6400001
-8431.0000000	2.6550002
-8430.0000000	2.6450000
-8429.0000000	2.6450000
-8428.0000000	2.6500001
-8427.0000000	2.6550002
-8426.0000000	2.6650000
-8425.0000000	2.6500001
-8424.0000000	2.6250000
-8423.0000000	2.5899999
-8422.0000000	2.5599999
-8421.0000000	2.5300000
-8420.0000000	2.4900000
-8419.0000000	2.4650002
-8418.0000000	2.4349999
-8417.0000000	2.4100001
-8416.0000000	2.3900001
-8415.0000000	2.3950000
-8414.0000000	2.3800001
-8413.0000000	2.3800001
-8412.0000000	2.3649998
-8411.0000000	2.3649998
-8410.0000000	2.3599999
-8409.0000000	2.3550000
-8408.0000000	2.3550000
-8407.0000000	2.3199999
-8406.0000000	2.2750001
-8405.0000000	2.2049999
-8404.0000000	2.1133335

-8403.0000000	2.0500000
-8402.0000000	2.0200000
-8401.0000000	2.0050001
-8400.0000000	2.0000000
-8399.0000000	2.0100000
-8398.0000000	2.0100000
-8397.0000000	2.0149999
-8396.0000000	2.0450001
-8395.0000000	2.0999999
-8394.0000000	2.1400001
-8393.0000000	2.1849999
-8392.0000000	2.2300000
-8391.0000000	2.2800000
-8390.0000000	2.3049998
-8389.0000000	2.3249998
-8388.0000000	2.3199999
-8387.0000000	2.3150001
-8386.0000000	2.3199999
-8385.0000000	2.3350000
-8384.0000000	2.3499999
-8383.0000000	2.3550000
-8382.0000000	2.3599999
-8381.0000000	2.3599999
-8380.0000000	2.3399999
-8379.0000000	2.3199999
-8378.0000000	2.2849998
-8377.0000000	2.2500000
-8376.0000000	2.2249999
-8375.0000000	2.2133334
-8374.0000000	2.1950002
-8373.0000000	2.1800001
-8372.0000000	2.1700001
-8371.0000000	2.1750002
-8370.0000000	2.1750002
-8369.0000000	2.1900001
-8368.0000000	2.1950002
-8367.0000000	2.1900001
-8366.0000000	2.1800001
-8365.0000000	2.1550002
-8364.0000000	2.1550002
-8363.0000000	2.1849999
-8362.0000000	2.1950002
-8361.0000000	2.2000000
-8360.0000000	2.2100000
-8359.0000000	2.2200000
-8358.0000000	2.2150002
-8357.0000000	2.2249999
-8356.0000000	2.2249999
-8355.0000000	2.2150002
-8354.0000000	2.2100000
-8353.0000000	2.2049999
-8352.0000000	2.2000000
-8351.0000000	2.1900001
-8350.0000000	2.1600001
-8349.0000000	2.1199999
-8348.0000000	2.0699999
-8347.0000000	2.0400000

-8346.0000000	2.0200000
-8345.0000000	2.0133333
-8344.0000000	2.0200000
-8343.0000000	2.0200000
-8342.0000000	2.0200000
-8341.0000000	2.0349998
-8340.0000000	2.0450001
-8339.0000000	2.0349998
-8338.0000000	2.0349998
-8337.0000000	2.0149999
-8336.0000000	2.0050001
-8335.0000000	2.0000000
-8334.0000000	1.9950000
-8333.0000000	2.0050001
-8332.0000000	2.0149999
-8331.0000000	2.0349998
-8330.0000000	2.0500000
-8329.0000000	2.0599999
-8328.0000000	2.0599999
-8327.0000000	2.0749998
-8326.0000000	2.0949998
-8325.0000000	2.1199999
-8324.0000000	2.1350002
-8323.0000000	2.1550002
-8322.0000000	2.1800001
-8321.0000000	2.2000000
-8320.0000000	2.2249999
-8319.0000000	2.2500000
-8318.0000000	2.2600000
-8317.0000000	2.2849998
-8316.0000000	2.3000000
-8315.0000000	2.3299999
-8314.0000000	2.3399999
-8313.0000000	2.3499999
-8312.0000000	2.3599999
-8311.0000000	2.3699999
-8310.0000000	2.3850002
-8309.0000000	2.4349999
-8308.0000000	2.4600000
-8307.0000000	2.4050002
-8306.0000000	2.3850002
-8305.0000000	2.3499999
-8304.0000000	2.3150001
-8303.0000000	2.2800000
-8302.0000000	2.2400000
-8301.0000000	2.1900001
-8300.0000000	2.1350000
-8299.0000000	2.0599999
-8298.0000000	2.0100000
-8297.0000000	1.9800000
-8296.0000000	1.9450001
-8295.0000000	1.9500000
-8294.0000000	2.0000000
-8293.0000000	2.0250001
-8292.0000000	2.0400000
-8291.0000000	2.0549998
-8290.0000000	2.0749998

-8289.0000000	2.0749998
-8288.0000000	2.0850000
-8287.0000000	2.0899999
-8286.0000000	2.0899999
-8285.0000000	2.0866666
-8284.0000000	2.0549998
-8283.0000000	2.0200000
-8282.0000000	2.0000000
-8281.0000000	1.9950000
-8280.0000000	1.9850000
-8279.0000000	1.9700000
-8278.0000000	1.9900000
-8277.0000000	2.0100000
-8276.0000000	2.0400000
-8275.0000000	2.0650001
-8274.0000000	2.0850000
-8273.0000000	2.1099999
-8272.0000000	2.1350002
-8271.0000000	2.1450000
-8270.0000000	2.1600001
-8269.0000000	2.1849999
-8268.0000000	2.2100000
-8267.0000000	2.2449999
-8266.0000000	2.2600000
-8265.0000000	2.2800000
-8264.0000000	2.2950001
-8263.0000000	2.2950001
-8262.0000000	2.2950001
-8261.0000000	2.3099999
-8260.0000000	2.3099999
-8259.0000000	2.3199999
-8258.0000000	2.3249998
-8257.0000000	2.3299999
-8256.0000000	2.3299999
-8255.0000000	2.3299999
-8254.0000000	2.3233330
-8253.0000000	2.3150001
-8252.0000000	2.3150001
-8251.0000000	2.3099999
-8250.0000000	2.3150001
-8249.0000000	2.3049998
-8248.0000000	2.3049998
-8247.0000000	2.2950001
-8246.0000000	2.2849998
-8245.0000000	2.2649999
-8244.0000000	2.2449999
-8243.0000000	2.2500000
-8242.0000000	2.2550001
-8241.0000000	2.2600000
-8240.0000000	2.2550001
-8239.0000000	2.2500000
-8238.0000000	2.2550001
-8237.0000000	2.2600000
-8236.0000000	2.2649999
-8235.0000000	2.2700000
-8234.0000000	2.2800000
-8233.0000000	2.2900000

-8232.0000000	2.3000000
-8231.0000000	2.3199999
-8230.0000000	2.3350000
-8229.0000000	2.3599999
-8228.0000000	2.3850002
-8227.0000000	2.4000001
-8226.0000000	2.4150000
-8225.0000000	2.4200001
-8224.0000000	2.4399998
-8223.0000000	2.4500000
-8222.0000000	2.4549999
-8221.0000000	2.4600000
-8220.0000000	2.4749999
-8219.0000000	2.4900000
-8218.0000000	2.5000000
-8217.0000000	2.5000000
-8216.0000000	2.4800000
-8215.0000000	2.4800000
-8214.0000000	2.4800000
-8213.0000000	2.5000000
-8212.0000000	2.5100000
-8211.0000000	2.5250001
-8210.0000000	2.5450001
-8209.0000000	2.5500000
-8208.0000000	2.5549998
-8207.0000000	2.5749998
-8206.0000000	2.5899999
-8205.0000000	2.5949998
-8204.0000000	2.5999999
-8203.0000000	2.5999999
-8202.0000000	2.5999999
-8201.0000000	2.6099999
-8200.0000000	2.6199999
-8199.0000000	2.6300001
-8198.0000000	2.6300001
-8197.0000000	2.6350002
-8196.0000000	2.6450000
-8195.0000000	2.6550002
-8194.0000000	2.6633334
-8193.0000000	2.6800001
-8192.0000000	2.6849999
-8191.0000000	2.6849999
-8190.0000000	2.6900001
-8189.0000000	2.7000000
-8188.0000000	2.6950002
-8187.0000000	2.7000000
-8186.0000000	2.7150002
-8185.0000000	2.7350001
-8184.0000000	2.7550001
-8183.0000000	2.7649999
-8182.0000000	2.7849998
-8181.0000000	2.7900000
-8180.0000000	2.7900000
-8179.0000000	2.8099999
-8178.0000000	2.8150001
-8177.0000000	2.8099999
-8176.0000000	2.7900000

-8175.0000000	2.7800000
-8174.0000000	2.7900000
-8173.0000000	2.8099999
-8172.0000000	2.8449998
-8171.0000000	2.8650000
-8170.0000000	2.8800001
-8169.0000000	2.8900001
-8168.0000000	2.9150000
-8167.0000000	2.9150000
-8166.0000000	2.9200001
-8165.0000000	2.9333334
-8164.0000000	2.9700000
-8163.0000000	2.9850001
-8162.0000000	2.9949999
-8161.0000000	2.9850001
-8160.0000000	2.9800000
-8159.0000000	2.9800000
-8158.0000000	2.9900000
-8157.0000000	2.9949999
-8156.0000000	3.0050001
-8155.0000000	3.0100000
-8154.0000000	3.0200000
-8153.0000000	2.9949999
-8152.0000000	2.9749999
-8151.0000000	2.9650002
-8150.0000000	2.9749999
-8149.0000000	2.9700000
-8148.0000000	2.9600000
-8147.0000000	2.9450002
-8146.0000000	2.9300001
-8145.0000000	2.9150000
-8144.0000000	2.9150000
-8143.0000000	2.9000001
-8142.0000000	2.9050002
-8141.0000000	2.9050002
-8140.0000000	2.8950000
-8139.0000000	2.9000001
-8138.0000000	2.8850002
-8137.0000000	2.8850002
-8136.0000000	2.8699999
-8135.0000000	2.8599999
-8134.0000000	2.8599999
-8133.0000000	2.8499999
-8132.0000000	2.8399999
-8131.0000000	2.8249998
-8130.0000000	2.8150001
-8129.0000000	2.8150001
-8128.0000000	2.8099999
-8127.0000000	2.8099999
-8126.0000000	2.7649999
-8125.0000000	2.6950002
-8124.0000000	2.6450000
-8123.0000000	2.5949998
-8122.0000000	2.5200000
-8121.0000000	2.4949999
-8120.0000000	2.4900000
-8119.0000000	2.5050001

-8118.0000000	2.5050001
-8117.0000000	2.5000000
-8116.0000000	2.5000000
-8115.0000000	2.4900000
-8114.0000000	2.4700000
-8113.0000000	2.4400001
-8112.0000000	2.3950000
-8111.0000000	2.3900001
-8110.0000000	2.3750000
-8109.0000000	2.3599999
-8108.0000000	2.3449998
-8107.0000000	2.3249998
-8106.0000000	2.3099999
-8105.0000000	2.3299999
-8104.0000000	2.3766668
-8103.0000000	2.4349999
-8102.0000000	2.4650002
-8101.0000000	2.4949999
-8100.0000000	2.5100000
-8099.0000000	2.5349998
-8098.0000000	2.5599999
-8097.0000000	2.5850000
-8096.0000000	2.6199999
-8095.0000000	2.6450000
-8094.0000000	2.6700001
-8093.0000000	2.6700001
-8092.0000000	2.6500001
-8091.0000000	2.6550002
-8090.0000000	2.6600001
-8089.0000000	2.6700001
-8088.0000000	2.6900001
-8087.0000000	2.7000000
-8086.0000000	2.7300000
-8085.0000000	2.7449999
-8084.0000000	2.7750001
-8083.0000000	2.7849998
-8082.0000000	2.8000000
-8081.0000000	2.8049998
-8080.0000000	2.8049998
-8079.0000000	2.8150001
-8078.0000000	2.8249998
-8077.0000000	2.8299999
-8076.0000000	2.8350000
-8075.0000000	2.8299999
-8074.0000000	2.8299999
-8073.0000000	2.8199999
-8072.0000000	2.8049998
-8071.0000000	2.8000000
-8070.0000000	2.7950001
-8069.0000000	2.8049998
-8068.0000000	2.8000000
-8067.0000000	2.7950001
-8066.0000000	2.8000000
-8065.0000000	2.8249998
-8064.0000000	2.8399999
-8063.0000000	2.8499999
-8062.0000000	2.8550000

-8061.0000000	2.8649998
-8060.0000000	2.8750000
-8059.0000000	2.8699999
-8058.0000000	2.8649998
-8057.0000000	2.8499999
-8056.0000000	2.8049998
-8055.0000000	2.7600000
-8054.0000000	2.7049999
-8053.0000000	2.6750002
-8052.0000000	2.6700001
-8051.0000000	2.6400001
-8050.0000000	2.6150000
-8049.0000000	2.5949998
-8048.0000000	2.5749998
-8047.0000000	2.5549998
-8046.0000000	2.5599999
-8045.0000000	2.5799999
-8044.0000000	2.6250000
-8043.0000000	2.6450000
-8042.0000000	2.6550002
-8041.0000000	2.6833334
-8040.0000000	2.7200000
-8039.0000000	2.7449999
-8038.0000000	2.7700000
-8037.0000000	2.7900000
-8036.0000000	2.7849998
-8035.0000000	2.8000000
-8034.0000000	2.8199999
-8033.0000000	2.8449998
-8032.0000000	2.8599999
-8031.0000000	2.8399999
-8030.0000000	2.8449998
-8029.0000000	2.8699999
-8028.0000000	2.8900001
-8027.0000000	2.9000001
-8026.0000000	2.9250002
-8025.0000000	2.9749999
-8024.0000000	2.9949999
-8023.0000000	3.0000000
-8022.0000000	3.0000000
-8021.0000000	2.9700000
-8020.0000000	2.9300001
-8019.0000000	2.8499999
-8018.0000000	2.7900000
-8017.0000000	2.7350001
-8016.0000000	2.6700001
-8015.0000000	2.6500001
-8014.0000000	2.6849999
-8013.0000000	2.7150002
-8012.0000000	2.7449999
-8011.0000000	2.7600000
-8010.0000000	2.8150001
-8009.0000000	2.8499999
-8008.0000000	2.8649998
-8007.0000000	2.8649998
-8006.0000000	2.8699999
-8005.0000000	2.8649998

-8004.0000000	2.8750000
-8003.0000000	2.8800001
-8002.0000000	2.8950000
-8001.0000000	2.9100001
-8000.0000000	2.9349999
-7999.0000000	2.9700000
-7998.0000000	3.0050001
-7997.0000000	3.0500000
-7996.0000000	3.0650001
-7995.0000000	3.0799999
-7994.0000000	3.0850000
-7993.0000000	3.1050000
-7992.0000000	3.1149998
-7991.0000000	3.1300001
-7990.0000000	3.1450000
-7989.0000000	3.1400001
-7988.0000000	3.1400001
-7987.0000000	3.1300001
-7986.0000000	3.1300001
-7985.0000000	3.1099999
-7984.0000000	3.0999999
-7983.0000000	3.0799999
-7982.0000000	3.0549998
-7981.0000000	3.0466669
-7980.0000000	3.0500000
-7979.0000000	3.0450001
-7978.0000000	3.0250001
-7977.0000000	3.0250001
-7976.0000000	3.0250001
-7975.0000000	3.0200000
-7974.0000000	3.0250001
-7973.0000000	3.0450001
-7972.0000000	3.0699999
-7971.0000000	3.0850000
-7970.0000000	3.0799999
-7969.0000000	3.0799999
-7968.0000000	3.1050000
-7967.0000000	3.1149998
-7966.0000000	3.1400001
-7965.0000000	3.1500001
-7964.0000000	3.1650000
-7963.0000000	3.1600001
-7962.0000000	3.1250000
-7961.0000000	3.0749998
-7960.0000000	3.0250001
-7959.0000000	2.9700000
-7958.0000000	2.9250002
-7957.0000000	2.8650000
-7956.0000000	2.8000000
-7955.0000000	2.7649999
-7954.0000000	2.7449999
-7953.0000000	2.7500000
-7952.0000000	2.7700000
-7951.0000000	2.7866666
-7950.0000000	2.7900000
-7949.0000000	2.7849998
-7948.0000000	2.7800000

-7947.0000000	2.7449999
-7946.0000000	2.7150002
-7945.0000000	2.6900001
-7944.0000000	2.6600001
-7943.0000000	2.6400001
-7942.0000000	2.6250000
-7941.0000000	2.6050000
-7940.0000000	2.5999999
-7939.0000000	2.5899999
-7938.0000000	2.5850000
-7937.0000000	2.6099999
-7936.0000000	2.6500001
-7935.0000000	2.6849999
-7934.0000000	2.7100000
-7933.0000000	2.7249999
-7932.0000000	2.7400000
-7931.0000000	2.7500000
-7930.0000000	2.7400000
-7929.0000000	2.7400000
-7928.0000000	2.7600000
-7927.0000000	2.7649999
-7926.0000000	2.7300000
-7925.0000000	2.7300000
-7924.0000000	2.7249999
-7923.0000000	2.7200000
-7922.0000000	2.7300000
-7921.0000000	2.7566664
-7920.0000000	2.7849998
-7919.0000000	2.8049998
-7918.0000000	2.8150001
-7917.0000000	2.8399999
-7916.0000000	2.8350000
-7915.0000000	2.8249998
-7914.0000000	2.7800000
-7913.0000000	2.7750001
-7912.0000000	2.7649999
-7911.0000000	2.7200000
-7910.0000000	2.6849999
-7909.0000000	2.6500001
-7908.0000000	2.6250000
-7907.0000000	2.5999999
-7906.0000000	2.6199999
-7905.0000000	2.6300001
-7904.0000000	2.6700001
-7903.0000000	2.6950002
-7902.0000000	2.7350001
-7901.0000000	2.7849998
-7900.0000000	2.8150001
-7899.0000000	2.8399999
-7898.0000000	2.8850000
-7897.0000000	2.9000001
-7896.0000000	2.9050002
-7895.0000000	2.9050002
-7894.0000000	2.9100001
-7893.0000000	2.9349999
-7892.0000000	2.9733334
-7891.0000000	3.0149999

-7890.0000000	3.0400000
-7889.0000000	3.0549998
-7888.0000000	3.0699999
-7887.0000000	3.0799999
-7886.0000000	3.0899999
-7885.0000000	3.0899999
-7884.0000000	3.0999999
-7883.0000000	3.1149998
-7882.0000000	3.1400001
-7881.0000000	3.1400001
-7880.0000000	3.1650000
-7879.0000000	3.1950002
-7878.0000000	3.2150002
-7877.0000000	3.2300000
-7876.0000000	3.2500000
-7875.0000000	3.2750001
-7874.0000000	3.2600000
-7873.0000000	3.2150002
-7872.0000000	3.1900001
-7871.0000000	3.1500001
-7870.0000000	3.1250000
-7869.0000000	3.1300001
-7868.0000000	3.1300001
-7867.0000000	3.1250000
-7866.0000000	3.1199999
-7865.0000000	3.1400001
-7864.0000000	3.1300001
-7863.0000000	3.1466668
-7862.0000000	3.1600001
-7861.0000000	3.1750002
-7860.0000000	3.1900001
-7859.0000000	3.2100000
-7858.0000000	3.2400000
-7857.0000000	3.2500000
-7856.0000000	3.2600000
-7855.0000000	3.2500000
-7854.0000000	3.2500000
-7853.0000000	3.2550001
-7852.0000000	3.2600000
-7851.0000000	3.2600000
-7850.0000000	3.2550001
-7849.0000000	3.2600000
-7848.0000000	3.2849998
-7847.0000000	3.2950001
-7846.0000000	3.3099999
-7845.0000000	3.3299999
-7844.0000000	3.3499999
-7843.0000000	3.3249998
-7842.0000000	3.3099999
-7841.0000000	3.3000000
-7840.0000000	3.3049998
-7839.0000000	3.2900000
-7838.0000000	3.3150001
-7837.0000000	3.3249998
-7836.0000000	3.3550000
-7835.0000000	3.3800001
-7834.0000000	3.3900001

-7833.0000000	3.4066668
-7832.0000000	3.4349999
-7831.0000000	3.4500000
-7830.0000000	3.4650002
-7829.0000000	3.4749999
-7828.0000000	3.4949999
-7827.0000000	3.5050001
-7826.0000000	3.5100000
-7825.0000000	3.5100000
-7824.0000000	3.5050001
-7823.0000000	3.5000000
-7822.0000000	3.4800000
-7821.0000000	3.4650002
-7820.0000000	3.4300001
-7819.0000000	3.4150000
-7818.0000000	3.3850002
-7817.0000000	3.3399999
-7816.0000000	3.3099999
-7815.0000000	3.3000000
-7814.0000000	3.2950001
-7813.0000000	3.3099999
-7812.0000000	3.3000000
-7811.0000000	3.2950001
-7810.0000000	3.3000000
-7809.0000000	3.3000000
-7808.0000000	3.3000000
-7807.0000000	3.3099999
-7806.0000000	3.3049998
-7805.0000000	3.3150001
-7804.0000000	3.3150001
-7803.0000000	3.3150001
-7802.0000000	3.3000000
-7801.0000000	3.3100002
-7800.0000000	3.3199999
-7799.0000000	3.3249998
-7798.0000000	3.3150001
-7797.0000000	3.3000000
-7796.0000000	3.2900000
-7795.0000000	3.2800000
-7794.0000000	3.2600000
-7793.0000000	3.2500000
-7792.0000000	3.2350001
-7791.0000000	3.2300000
-7790.0000000	3.2150002
-7789.0000000	3.2049999
-7788.0000000	3.2000000
-7787.0000000	3.1750002
-7786.0000000	3.1650000
-7785.0000000	3.1600001
-7784.0000000	3.1600001
-7783.0000000	3.1600001
-7782.0000000	3.1849999
-7781.0000000	3.2049999
-7780.0000000	3.2400000
-7779.0000000	3.2700000
-7778.0000000	3.3099999
-7777.0000000	3.3350000

-7776.0000000	3.3499999
-7775.0000000	3.3599999
-7774.0000000	3.3800001
-7773.0000000	3.3800001
-7772.0000000	3.4050002
-7771.0000000	3.4150000
-7770.0000000	3.4000003
-7769.0000000	3.3900001
-7768.0000000	3.3850002
-7767.0000000	3.3750000
-7766.0000000	3.3750000
-7765.0000000	3.3850002
-7764.0000000	3.3850002
-7763.0000000	3.3699999
-7762.0000000	3.3649998
-7761.0000000	3.3649998
-7760.0000000	3.3599999
-7759.0000000	3.3449998
-7758.0000000	3.3249998
-7757.0000000	3.3199999
-7756.0000000	3.3000000
-7755.0000000	3.2700000
-7754.0000000	3.2449999
-7753.0000000	3.2249999
-7752.0000000	3.2249999
-7751.0000000	3.2150002
-7750.0000000	3.1950002
-7749.0000000	3.1800001
-7748.0000000	3.1700001
-7747.0000000	3.1600001
-7746.0000000	3.1600001
-7745.0000000	3.1650000
-7744.0000000	3.1700001
-7743.0000000	3.1400001
-7742.0000000	3.1350000
-7741.0000000	3.1450000
-7740.0000000	3.1700001
-7739.0000000	3.1950002
-7738.0000000	3.2150002
-7737.0000000	3.2249999
-7736.0000000	3.2249999
-7735.0000000	3.2249999
-7734.0000000	3.2200000
-7733.0000000	3.2150002
-7732.0000000	3.2150002
-7731.0000000	3.1900001
-7730.0000000	3.1700001
-7729.0000000	3.1700001
-7728.0000000	3.1700001
-7727.0000000	3.1650000
-7726.0000000	3.1450000
-7725.0000000	3.1150000
-7724.0000000	3.0599999
-7723.0000000	3.0200000
-7722.0000000	3.0000000
-7721.0000000	2.9900000
-7720.0000000	2.9549999

-7719.0000000	2.9450002
-7718.0000000	2.9600000
-7717.0000000	2.9400001
-7716.0000000	2.9150000
-7715.0000000	2.9050002
-7714.0000000	2.9000001
-7713.0000000	2.8850002
-7712.0000000	2.8800001
-7711.0000000	2.8900001
-7710.0000000	2.9133337
-7709.0000000	2.9600000
-7708.0000000	2.9800000
-7707.0000000	2.9900000
-7706.0000000	3.0100000
-7705.0000000	3.0349998
-7704.0000000	3.0599999
-7703.0000000	3.0699999
-7702.0000000	3.0799999
-7701.0000000	3.0850000
-7700.0000000	3.0799999
-7699.0000000	3.0599999
-7698.0000000	3.0500000
-7697.0000000	3.0500000
-7696.0000000	3.0400000
-7695.0000000	3.0149999
-7694.0000000	2.9949999
-7693.0000000	2.9749999
-7692.0000000	2.9450002
-7691.0000000	2.9150000
-7690.0000000	2.9150000
-7689.0000000	2.9150000
-7688.0000000	2.9150000
-7687.0000000	2.9150000
-7686.0000000	2.9250002
-7685.0000000	2.9349999
-7684.0000000	2.9349999
-7683.0000000	2.9400001
-7682.0000000	2.9349999
-7681.0000000	2.9349999
-7680.0000000	2.9566667
-7679.0000000	2.9400001
-7678.0000000	2.9250002
-7677.0000000	2.9150000
-7676.0000000	2.9100001
-7675.0000000	2.9150000
-7674.0000000	2.9250002
-7673.0000000	2.9500000
-7672.0000000	2.9549999
-7671.0000000	2.9500000
-7670.0000000	2.9549999
-7669.0000000	2.9549999
-7668.0000000	2.9400001
-7667.0000000	2.9349999
-7666.0000000	2.9450002
-7665.0000000	2.9400001
-7664.0000000	2.9450002
-7663.0000000	2.9349999

-7662.0000000	2.9150000
-7661.0000000	2.8550000
-7660.0000000	2.8049998
-7659.0000000	2.7649999
-7658.0000000	2.7400000
-7657.0000000	2.7049999
-7656.0000000	2.6800001
-7655.0000000	2.6350002
-7654.0000000	2.6400001
-7653.0000000	2.6400001
-7652.0000000	2.6750002
-7651.0000000	2.7200000
-7650.0000000	2.7600000
-7649.0000000	2.7933333
-7648.0000000	2.8350000
-7647.0000000	2.8550000
-7646.0000000	2.8750000
-7645.0000000	2.9000001
-7644.0000000	2.9200001
-7643.0000000	2.9250002
-7642.0000000	2.9400001
-7641.0000000	2.9400001
-7640.0000000	2.9349999
-7639.0000000	2.9300001
-7638.0000000	2.9349999
-7637.0000000	2.9450002
-7636.0000000	2.9749999
-7635.0000000	2.9850001
-7634.0000000	2.9700000
-7633.0000000	2.9549999
-7632.0000000	2.9450002
-7631.0000000	2.9300001
-7630.0000000	2.9050002
-7629.0000000	2.8499999
-7628.0000000	2.8099999
-7627.0000000	2.7700000
-7626.0000000	2.7449999
-7625.0000000	2.7249999
-7624.0000000	2.7049999
-7623.0000000	2.6900001
-7622.0000000	2.6900001
-7621.0000000	2.6849999
-7620.0000000	2.6933334
-7619.0000000	2.6950002
-7618.0000000	2.6950002
-7617.0000000	2.7000000
-7616.0000000	2.7049999
-7615.0000000	2.7000000
-7614.0000000	2.6900001
-7613.0000000	2.7300000
-7612.0000000	2.7800000
-7611.0000000	2.8350000
-7610.0000000	2.8699999
-7609.0000000	2.8950000
-7608.0000000	2.9150000
-7607.0000000	2.9050002
-7606.0000000	2.8900001

-7605.0000000	2.8750000
-7604.0000000	2.8449998
-7603.0000000	2.8000000
-7602.0000000	2.7600000
-7601.0000000	2.7100000
-7600.0000000	2.6550002
-7599.0000000	2.6150000
-7598.0000000	2.5650001
-7597.0000000	2.5200000
-7596.0000000	2.5050001
-7595.0000000	2.4949999
-7594.0000000	2.4900000
-7593.0000000	2.4900000
-7592.0000000	2.5000000
-7591.0000000	2.4800000
-7590.0000000	2.4666667
-7589.0000000	2.4800000
-7588.0000000	2.4850001
-7587.0000000	2.5000000
-7586.0000000	2.5200000
-7585.0000000	2.5549998
-7584.0000000	2.5749998
-7583.0000000	2.5949998
-7582.0000000	2.5999999
-7581.0000000	2.6099999
-7580.0000000	2.6050000
-7579.0000000	2.6300001
-7578.0000000	2.6199999
-7577.0000000	2.6250000
-7576.0000000	2.6300001
-7575.0000000	2.6650000
-7574.0000000	2.6600001
-7573.0000000	2.6600001
-7572.0000000	2.6700001
-7571.0000000	2.6849999
-7570.0000000	2.7049999
-7569.0000000	2.7249999
-7568.0000000	2.7449999
-7567.0000000	2.7649999
-7566.0000000	2.7649999
-7565.0000000	2.7800000
-7564.0000000	2.7900000
-7563.0000000	2.8000000
-7562.0000000	2.8199999
-7561.0000000	2.8099999
-7560.0000000	2.8033333
-7559.0000000	2.7700000
-7558.0000000	2.7350001
-7557.0000000	2.7000000
-7556.0000000	2.6849999
-7555.0000000	2.6950002
-7554.0000000	2.6750002
-7553.0000000	2.6800001
-7552.0000000	2.6950002
-7551.0000000	2.6950002
-7550.0000000	2.6950002
-7549.0000000	2.6900001

-7548.0000000	2.6849999
-7547.0000000	2.7000000
-7546.0000000	2.6950002
-7545.0000000	2.6950002
-7544.0000000	2.6950002
-7543.0000000	2.7049999
-7542.0000000	2.7350001
-7541.0000000	2.7649999
-7540.0000000	2.7700000
-7539.0000000	2.7449999
-7538.0000000	2.7600000
-7537.0000000	2.7750001
-7536.0000000	2.7849998
-7535.0000000	2.7849998
-7534.0000000	2.7950001
-7533.0000000	2.7849998
-7532.0000000	2.8150001
-7531.0000000	2.8399999
-7530.0000000	2.8750000
-7529.0000000	2.9200001
-7528.0000000	2.9800000
-7527.0000000	3.0000000
-7526.0000000	3.0250001
-7525.0000000	3.0300000
-7524.0000000	3.0100000
-7523.0000000	2.9700000
-7522.0000000	2.9200001
-7521.0000000	2.9200001
-7520.0000000	2.9050002
-7519.0000000	2.8950000
-7518.0000000	2.8750000
-7517.0000000	2.8900001
-7516.0000000	2.9050002
-7515.0000000	2.9050002
-7514.0000000	2.9000001
-7513.0000000	2.9000001
-7512.0000000	2.9100001
-7511.0000000	2.9150000
-7510.0000000	2.9200001
-7509.0000000	2.9349999
-7508.0000000	2.9549999
-7507.0000000	2.9650002
-7506.0000000	2.9700000
-7505.0000000	2.9700000
-7504.0000000	2.9500000
-7503.0000000	2.9300001
-7502.0000000	2.8950000
-7501.0000000	2.8449998
-7500.0000000	2.8150001
-7499.0000000	2.7849998
-7498.0000000	2.7733333
-7497.0000000	2.7400000
-7496.0000000	2.6950002
-7495.0000000	2.6650000
-7494.0000000	2.6550002
-7493.0000000	2.6450000
-7492.0000000	2.6400001

-7491.0000000	2.6350002
-7490.0000000	2.6350002
-7489.0000000	2.6550002
-7488.0000000	2.6750002
-7487.0000000	2.6950002
-7486.0000000	2.6849999
-7485.0000000	2.6750002
-7484.0000000	2.6500001
-7483.0000000	2.6650000
-7482.0000000	2.6750002
-7481.0000000	2.6750002
-7480.0000000	2.7000000
-7479.0000000	2.7400000
-7478.0000000	2.7649999
-7477.0000000	2.7750001
-7476.0000000	2.7649999
-7475.0000000	2.7500000
-7474.0000000	2.7300000
-7473.0000000	2.7200000
-7472.0000000	2.7249999
-7471.0000000	2.7300000
-7470.0000000	2.7200000
-7469.0000000	2.6866667
-7468.0000000	2.6700001
-7467.0000000	2.6600001
-7466.0000000	2.6600001
-7465.0000000	2.6500001
-7464.0000000	2.6550002
-7463.0000000	2.6700001
-7462.0000000	2.6849999
-7461.0000000	2.6950002
-7460.0000000	2.6849999
-7459.0000000	2.6800001
-7458.0000000	2.6800001
-7457.0000000	2.6650000
-7456.0000000	2.6550002
-7455.0000000	2.6450000
-7454.0000000	2.6550002
-7453.0000000	2.6400001
-7452.0000000	2.6100001
-7451.0000000	2.5650001
-7450.0000000	2.5250001
-7449.0000000	2.5300000
-7448.0000000	2.5599999
-7447.0000000	2.6050000
-7446.0000000	2.6250000
-7445.0000000	2.6350002
-7444.0000000	2.6400001
-7443.0000000	2.6350002
-7442.0000000	2.6250000
-7441.0000000	2.6250000
-7440.0000000	2.6400001
-7439.0000000	2.6433334
-7438.0000000	2.6600001
-7437.0000000	2.6750002
-7436.0000000	2.6700001
-7435.0000000	2.6600001

-7434.0000000	2.6650000
-7433.0000000	2.6800001
-7432.0000000	2.7000000
-7431.0000000	2.7150002
-7430.0000000	2.7300000
-7429.0000000	2.7649999
-7428.0000000	2.7750001
-7427.0000000	2.7849998
-7426.0000000	2.7900000
-7425.0000000	2.8049998
-7424.0000000	2.8249998
-7423.0000000	2.8350000
-7422.0000000	2.8449998
-7421.0000000	2.8800001
-7420.0000000	2.8800001
-7419.0000000	2.8950000
-7418.0000000	2.8950000
-7417.0000000	2.9200001
-7416.0000000	2.9349999
-7415.0000000	2.9450002
-7414.0000000	2.9549999
-7413.0000000	2.9250002
-7412.0000000	2.9150000
-7411.0000000	2.9000001
-7410.0000000	2.8950000
-7409.0000000	2.8900001
-7408.0000000	2.8750000
-7407.0000000	2.8649998
-7406.0000000	2.8399999
-7405.0000000	2.8299999
-7404.0000000	2.8099999
-7403.0000000	2.8049998
-7402.0000000	2.8049998
-7401.0000000	2.8000000
-7400.0000000	2.8000000
-7399.0000000	2.8049998
-7398.0000000	2.7950001
-7397.0000000	2.7950001
-7396.0000000	2.8000000
-7395.0000000	2.8099999
-7394.0000000	2.8099999
-7393.0000000	2.8150001
-7392.0000000	2.8199999
-7391.0000000	2.8350000
-7390.0000000	2.8550000
-7389.0000000	2.8750000
-7388.0000000	2.8850002
-7387.0000000	2.8900001
-7386.0000000	2.9000001
-7385.0000000	2.9200001
-7384.0000000	2.9200001
-7383.0000000	2.9050002
-7382.0000000	2.8900001
-7381.0000000	2.8900001
-7380.0000000	2.9100001
-7379.0000000	2.9300001
-7378.0000000	2.9500000

-7377.0000000	2.9749999
-7376.0000000	3.0000000
-7375.0000000	3.0149999
-7374.0000000	3.0250001
-7373.0000000	3.0200000
-7372.0000000	3.0100000
-7371.0000000	3.0000000
-7370.0000000	2.9850001
-7369.0000000	2.9800000
-7368.0000000	2.9600000
-7367.0000000	2.9450002
-7366.0000000	2.9300001
-7365.0000000	2.9150000
-7364.0000000	2.9300001
-7363.0000000	2.9250002
-7362.0000000	2.9500000
-7361.0000000	2.9450002
-7360.0000000	2.9450002
-7359.0000000	2.9300001
-7358.0000000	2.9250002
-7357.0000000	2.9200001
-7356.0000000	2.9100001
-7355.0000000	2.9000001
-7354.0000000	2.8950000
-7353.0000000	2.8950000
-7352.0000000	2.9000001
-7351.0000000	2.9000001
-7350.0000000	2.9050002
-7349.0000000	2.8999999
-7348.0000000	2.8950000
-7347.0000000	2.8850002
-7346.0000000	2.8950000
-7345.0000000	2.8699999
-7344.0000000	2.8649998
-7343.0000000	2.8699999
-7342.0000000	2.8800001
-7341.0000000	2.9000001
-7340.0000000	2.9150000
-7339.0000000	2.9450002
-7338.0000000	2.9450002
-7337.0000000	2.9500000
-7336.0000000	2.9450002
-7335.0000000	2.9500000
-7334.0000000	2.9749999
-7333.0000000	2.9800000
-7332.0000000	2.9850001
-7331.0000000	2.9949999
-7330.0000000	3.0050001
-7329.0000000	3.0250001
-7328.0000000	3.0349998
-7327.0000000	3.0349998
-7326.0000000	3.0400000
-7325.0000000	3.0349998
-7324.0000000	3.0250001
-7323.0000000	3.0100000
-7322.0000000	3.0200000
-7321.0000000	3.0100000

-7320.0000000	2.9949999
-7319.0000000	2.9966667
-7318.0000000	2.9850001
-7317.0000000	2.9850001
-7316.0000000	2.9949999
-7315.0000000	2.9800000
-7314.0000000	2.9700000
-7313.0000000	2.9749999
-7312.0000000	2.9749999
-7311.0000000	2.9749999
-7310.0000000	2.9800000
-7309.0000000	2.9850001
-7308.0000000	2.9800000
-7307.0000000	2.9900000
-7306.0000000	3.0050001
-7305.0000000	3.0000000
-7304.0000000	3.0100000
-7303.0000000	3.0050001
-7302.0000000	2.9949999
-7301.0000000	2.9850001
-7300.0000000	2.9700000
-7299.0000000	2.9650002
-7298.0000000	2.9700000
-7297.0000000	2.9650002
-7296.0000000	2.9549999
-7295.0000000	2.9500000
-7294.0000000	2.9300001
-7293.0000000	2.9050002
-7292.0000000	2.8900001
-7291.0000000	2.8750000
-7290.0000000	2.8649998
-7289.0000000	2.8499999
-7288.0000000	2.8499997
-7287.0000000	2.8499999
-7286.0000000	2.7900000
-7285.0000000	2.7500000
-7284.0000000	2.7449999
-7283.0000000	2.7700000
-7282.0000000	2.7649999
-7281.0000000	2.7649999
-7280.0000000	2.7649999
-7279.0000000	2.7900000
-7278.0000000	2.7950001
-7277.0000000	2.8049998
-7276.0000000	2.8350000
-7275.0000000	2.8399999
-7274.0000000	2.8499999
-7273.0000000	2.8649998
-7272.0000000	2.8800001
-7271.0000000	2.8900001
-7270.0000000	2.9000001
-7269.0000000	2.9050002
-7268.0000000	2.8950000
-7267.0000000	2.8850002
-7266.0000000	2.8850002
-7265.0000000	2.8850002
-7264.0000000	2.8599999

-7263.0000000	2.8449998
-7262.0000000	2.8850002
-7261.0000000	2.8950000
-7260.0000000	2.9000001
-7259.0000000	2.9100001
-7258.0000000	2.9050002
-7257.0000000	2.9050002
-7256.0000000	2.9100001
-7255.0000000	2.9349999
-7254.0000000	2.9450002
-7253.0000000	2.9549999
-7252.0000000	2.9600000
-7251.0000000	2.9800000
-7250.0000000	2.9800000
-7249.0000000	2.9850001
-7248.0000000	2.9549999
-7247.0000000	2.9250002
-7246.0000000	2.9100001
-7245.0000000	2.9000001
-7244.0000000	2.8850002
-7243.0000000	2.8750000
-7242.0000000	2.8649998
-7241.0000000	2.8449998
-7240.0000000	2.8449998
-7239.0000000	2.8449998
-7238.0000000	2.8699999
-7237.0000000	2.8800001
-7236.0000000	2.8800001
-7235.0000000	2.8850002
-7234.0000000	2.8950000
-7233.0000000	2.8850002
-7232.0000000	2.8699999
-7231.0000000	2.8399999
-7230.0000000	2.8199999
-7229.0000000	2.8000000
-7228.0000000	2.8049998
-7227.0000000	2.8150001
-7226.0000000	2.8233330
-7225.0000000	2.8249998
-7224.0000000	2.8299999
-7223.0000000	2.8299999
-7222.0000000	2.8150001
-7221.0000000	2.8049998
-7220.0000000	2.8000000
-7219.0000000	2.8049998
-7218.0000000	2.8000000
-7217.0000000	2.7950001
-7216.0000000	2.7900000
-7215.0000000	2.7750001
-7214.0000000	2.7350001
-7213.0000000	2.6750002
-7212.0000000	2.6050000
-7211.0000000	2.5999999
-7210.0000000	2.6250000
-7209.0000000	2.6300001
-7208.0000000	2.6350002
-7207.0000000	2.6550002

-7206.0000000	2.6600001
-7205.0000000	2.6550002
-7204.0000000	2.6450000
-7203.0000000	2.6350002
-7202.0000000	2.6250000
-7201.0000000	2.6350002
-7200.0000000	2.6500001
-7199.0000000	2.6500001
-7198.0000000	2.6666667
-7197.0000000	2.6650000
-7196.0000000	2.6550002
-7195.0000000	2.6400001
-7194.0000000	2.5999999
-7193.0000000	2.5699999
-7192.0000000	2.5250001
-7191.0000000	2.4700000
-7190.0000000	2.4349999
-7189.0000000	2.4200001
-7188.0000000	2.3900001
-7187.0000000	2.4250002
-7186.0000000	2.4800000
-7185.0000000	2.5500000
-7184.0000000	2.6099999
-7183.0000000	2.6400001
-7182.0000000	2.6650000
-7181.0000000	2.6800001
-7180.0000000	2.6750002
-7179.0000000	2.6900001
-7178.0000000	2.6950002
-7177.0000000	2.7049999
-7176.0000000	2.7249999
-7175.0000000	2.7100000
-7174.0000000	2.7049999
-7173.0000000	2.7200000
-7172.0000000	2.7350001
-7171.0000000	2.7550001
-7170.0000000	2.7649999
-7169.0000000	2.7849998
-7168.0000000	2.7950001
-7167.0000000	2.8133333
-7166.0000000	2.8299999
-7165.0000000	2.8449998
-7164.0000000	2.8599999
-7163.0000000	2.8649998
-7162.0000000	2.8599999
-7161.0000000	2.8550000
-7160.0000000	2.8550000
-7159.0000000	2.8550000
-7158.0000000	2.8599999
-7157.0000000	2.8750000
-7156.0000000	2.8800001
-7155.0000000	2.8850002
-7154.0000000	2.8800001
-7153.0000000	2.8850002
-7152.0000000	2.8800001
-7151.0000000	2.8699999
-7150.0000000	2.8599999

-7149.0000000	2.8649998
-7148.0000000	2.8900001
-7147.0000000	2.9100001
-7146.0000000	2.9100001
-7145.0000000	2.9000001
-7144.0000000	2.8750000
-7143.0000000	2.8649998
-7142.0000000	2.8649998
-7141.0000000	2.8750000
-7140.0000000	2.9100001
-7139.0000000	2.9450002
-7138.0000000	2.9650002
-7137.0000000	2.9933331
-7136.0000000	3.0250001
-7135.0000000	3.0549998
-7134.0000000	3.0899999
-7133.0000000	3.1400001
-7132.0000000	3.1650000
-7131.0000000	3.1800001
-7130.0000000	3.2049999
-7129.0000000	3.2049999
-7128.0000000	3.2150002
-7127.0000000	3.2150002
-7126.0000000	3.2150002
-7125.0000000	3.2249999
-7124.0000000	3.2000000
-7123.0000000	3.1750002
-7122.0000000	3.1600001
-7121.0000000	3.1450000
-7120.0000000	3.1199999
-7119.0000000	3.1350000
-7118.0000000	3.1800001
-7117.0000000	3.2000000
-7116.0000000	3.2300000
-7115.0000000	3.2649999
-7114.0000000	3.2550001
-7113.0000000	3.2649999
-7112.0000000	3.2800000
-7111.0000000	3.3249998
-7110.0000000	3.3499999
-7109.0000000	3.3850000
-7108.0000000	3.4200001
-7107.0000000	3.4300001
-7106.0000000	3.4450002
-7105.0000000	3.4733334
-7104.0000000	3.4800000
-7103.0000000	3.4949999
-7102.0000000	3.5050001
-7101.0000000	3.5250001
-7100.0000000	3.5300000
-7099.0000000	3.5250001
-7098.0000000	3.5250001
-7097.0000000	3.5250001
-7096.0000000	3.5149999
-7095.0000000	3.4850001
-7094.0000000	3.4600000
-7093.0000000	3.4500000

-7092.0000000	3.4349999
-7091.0000000	3.4100001
-7090.0000000	3.3900001
-7089.0000000	3.4000001
-7088.0000000	3.4150000
-7087.0000000	3.4349999
-7086.0000000	3.4100001
-7085.0000000	3.4000001
-7084.0000000	3.3850002
-7083.0000000	3.3649998
-7082.0000000	3.3350000
-7081.0000000	3.3049998
-7080.0000000	3.2750001
-7079.0000000	3.2500000
-7078.0000000	3.2350001
-7077.0000000	3.2400000
-7076.0000000	3.2433331
-7075.0000000	3.2550001
-7074.0000000	3.2700000
-7073.0000000	3.2700000
-7072.0000000	3.2750001
-7071.0000000	3.2800000
-7070.0000000	3.2600000
-7069.0000000	3.2700000
-7068.0000000	3.2600000
-7067.0000000	3.2449999
-7066.0000000	3.2350001
-7065.0000000	3.2249999
-7064.0000000	3.2300000
-7063.0000000	3.2500000
-7062.0000000	3.2800000
-7061.0000000	3.3249998
-7060.0000000	3.3650000
-7059.0000000	3.4100001
-7058.0000000	3.4300001
-7057.0000000	3.4450002
-7056.0000000	3.4500000
-7055.0000000	3.4549999
-7054.0000000	3.4500000
-7053.0000000	3.4450002
-7052.0000000	3.4150000
-7051.0000000	3.3950000
-7050.0000000	3.3750000
-7049.0000000	3.3599999
-7048.0000000	3.3399999
-7047.0000000	3.3249998
-7046.0000000	3.2733333
-7045.0000000	3.2200000
-7044.0000000	3.1700001
-7043.0000000	3.0999999
-7042.0000000	3.0549998
-7041.0000000	3.0100000
-7040.0000000	2.9900000
-7039.0000000	2.9749999
-7038.0000000	2.9800000
-7037.0000000	2.9850001
-7036.0000000	3.0100000

-7035.0000000	3.0500000
-7034.0000000	3.0799999
-7033.0000000	3.1050000
-7032.0000000	3.1199999
-7031.0000000	3.1350002
-7030.0000000	3.1150000
-7029.0000000	3.1099999
-7028.0000000	3.1050000
-7027.0000000	3.0949998
-7026.0000000	3.0749998
-7025.0000000	3.0650001
-7024.0000000	3.0650001
-7023.0000000	3.0799999
-7022.0000000	3.1150000
-7021.0000000	3.1650000
-7020.0000000	3.2400000
-7019.0000000	3.2800000
-7018.0000000	3.2800000
-7017.0000000	3.2950001
-7016.0000000	3.3000000
-7015.0000000	3.2933333
-7014.0000000	3.3150001
-7013.0000000	3.3199999
-7012.0000000	3.3399999
-7011.0000000	3.3699999
-7010.0000000	3.4000001
-7009.0000000	3.4300001
-7008.0000000	3.4650002
-7007.0000000	3.4700000
-7006.0000000	3.4700000
-7005.0000000	3.4749999
-7004.0000000	3.4749999
-7003.0000000	3.4450002
-7002.0000000	3.4400001
-7001.0000000	3.4349999
-7000.0000000	3.4100001
-6999.0000000	3.3750000
-6998.0000000	3.3350000
-6997.0000000	3.2900000
-6996.0000000	3.2350001
-6995.0000000	3.1800001
-6994.0000000	3.1399999
-6993.0000000	3.1250000
-6992.0000000	3.1199999
-6991.0000000	3.1300001
-6990.0000000	3.1199999
-6989.0000000	3.1300001
-6988.0000000	3.1250000
-6987.0000000	3.1099999
-6986.0000000	3.0999999
-6985.0000000	3.1166668
-6984.0000000	3.1400001
-6983.0000000	3.1450000
-6982.0000000	3.1550002
-6981.0000000	3.1650000
-6980.0000000	3.1550002
-6979.0000000	3.1450000

-6978.0000000	3.1500001
-6977.0000000	3.1400001
-6976.0000000	3.1500001
-6975.0000000	3.1650000
-6974.0000000	3.1600001
-6973.0000000	3.1450000
-6972.0000000	3.1199999
-6971.0000000	3.1050000
-6970.0000000	3.0899999
-6969.0000000	3.0699999
-6968.0000000	3.0500000
-6967.0000000	3.0200000
-6966.0000000	2.9850001
-6965.0000000	2.9549999
-6964.0000000	2.9400001
-6963.0000000	2.9349999
-6962.0000000	2.9250002
-6961.0000000	2.9100001
-6960.0000000	2.9000001
-6959.0000000	2.9050002
-6958.0000000	2.8850002
-6957.0000000	2.8800001
-6956.0000000	2.8950000
-6955.0000000	2.8999999
-6954.0000000	2.9100001
-6953.0000000	2.9100001
-6952.0000000	2.9050002
-6951.0000000	2.8800001
-6950.0000000	2.8399999
-6949.0000000	2.8199999
-6948.0000000	2.8399999
-6947.0000000	2.8900001
-6946.0000000	2.9600000
-6945.0000000	3.0300000
-6944.0000000	3.0650001
-6943.0000000	3.1099999
-6942.0000000	3.1300001
-6941.0000000	3.1600001
-6940.0000000	3.1849999
-6939.0000000	3.2049999
-6938.0000000	3.2200000
-6937.0000000	3.2300000
-6936.0000000	3.2449999
-6935.0000000	3.2550001
-6934.0000000	3.2849998
-6933.0000000	3.3099999
-6932.0000000	3.3150001
-6931.0000000	3.3350000
-6930.0000000	3.3550000
-6929.0000000	3.3599999
-6928.0000000	3.3850002
-6927.0000000	3.4050002
-6926.0000000	3.4250002
-6925.0000000	3.4349999
-6924.0000000	3.4333334
-6923.0000000	3.4450002
-6922.0000000	3.4700000

-6921.0000000	3.4900000
-6920.0000000	3.5100000
-6919.0000000	3.5200000
-6918.0000000	3.5250001
-6917.0000000	3.5250001
-6916.0000000	3.5200000
-6915.0000000	3.5200000
-6914.0000000	3.5100000
-6913.0000000	3.4949999
-6912.0000000	3.4850001
-6911.0000000	3.4500000
-6910.0000000	3.4349999
-6909.0000000	3.4300001
-6908.0000000	3.4349999
-6907.0000000	3.4349999
-6906.0000000	3.4349999
-6905.0000000	3.4250002
-6904.0000000	3.4150000
-6903.0000000	3.4000001
-6902.0000000	3.3850002
-6901.0000000	3.4000001
-6900.0000000	3.4000001
-6899.0000000	3.4050002
-6898.0000000	3.4000001
-6897.0000000	3.3950000
-6896.0000000	3.4000001
-6895.0000000	3.3966668
-6894.0000000	3.3800001
-6893.0000000	3.3750000
-6892.0000000	3.3649998
-6891.0000000	3.3850002
-6890.0000000	3.3850002
-6889.0000000	3.4050002
-6888.0000000	3.4050002
-6887.0000000	3.4250002
-6886.0000000	3.4300001
-6885.0000000	3.4300001
-6884.0000000	3.4450002
-6883.0000000	3.4650002
-6882.0000000	3.4900000
-6881.0000000	3.5149999
-6880.0000000	3.5349998
-6879.0000000	3.5650001
-6878.0000000	3.5949998
-6877.0000000	3.6350002
-6876.0000000	3.6550002
-6875.0000000	3.6700001
-6874.0000000	3.6950002
-6873.0000000	3.7100000
-6872.0000000	3.7350001
-6871.0000000	3.7300000
-6870.0000000	3.7300000
-6869.0000000	3.7500000
-6868.0000000	3.7500000
-6867.0000000	3.7700000
-6866.0000000	3.8033333
-6865.0000000	3.8249998

-6864.0000000	3.8299999
-6863.0000000	3.8399999
-6862.0000000	3.8350000
-6861.0000000	3.8399999
-6860.0000000	3.8350000
-6859.0000000	3.8249998
-6858.0000000	3.8350000
-6857.0000000	3.8150001
-6856.0000000	3.8000000
-6855.0000000	3.7900000
-6854.0000000	3.7600000
-6853.0000000	3.7350001
-6852.0000000	3.6849999
-6851.0000000	3.6350000
-6850.0000000	3.5699999
-6849.0000000	3.5349998
-6848.0000000	3.5100000
-6847.0000000	3.5250001
-6846.0000000	3.5200000
-6845.0000000	3.5349998
-6844.0000000	3.5349998
-6843.0000000	3.5300000
-6842.0000000	3.5100000
-6841.0000000	3.4800000
-6840.0000000	3.4650002
-6839.0000000	3.4549999
-6838.0000000	3.4349999
-6837.0000000	3.4000001
-6836.0000000	3.3599999
-6835.0000000	3.3299999
-6834.0000000	3.3666668
-6833.0000000	3.4100001
-6832.0000000	3.4200001
-6831.0000000	3.4349999
-6830.0000000	3.4650002
-6829.0000000	3.5200000
-6828.0000000	3.5400000
-6827.0000000	3.5200000
-6826.0000000	3.4900000
-6825.0000000	3.4300001
-6824.0000000	3.3800001
-6823.0000000	3.3399999
-6822.0000000	3.3000000
-6821.0000000	3.2649999
-6820.0000000	3.2400000
-6819.0000000	3.2249999
-6818.0000000	3.2249999
-6817.0000000	3.2200000
-6816.0000000	3.1950002
-6815.0000000	3.1500001
-6814.0000000	3.1250000
-6813.0000000	3.1050000
-6812.0000000	3.0899999
-6811.0000000	3.0699999
-6810.0000000	3.0549998
-6809.0000000	3.0300000
-6808.0000000	3.0149999

-6807.0000000	3.0000000
-6806.0000000	3.0300000
-6805.0000000	3.0349998
-6804.0000000	3.0100000
-6803.0000000	2.9900000
-6802.0000000	3.0050001
-6801.0000000	3.0349998
-6800.0000000	3.0699999
-6799.0000000	3.0699999
-6798.0000000	3.0699999
-6797.0000000	3.0599999
-6796.0000000	3.0850000
-6795.0000000	3.1099999
-6794.0000000	3.1350002
-6793.0000000	3.1750002
-6792.0000000	3.2100000
-6791.0000000	3.2249999
-6790.0000000	3.2500000
-6789.0000000	3.2750001
-6788.0000000	3.3049998
-6787.0000000	3.3150001
-6786.0000000	3.2950001
-6785.0000000	3.2350001
-6784.0000000	3.2500000
-6783.0000000	3.2800000
-6782.0000000	3.2849998
-6781.0000000	3.2849998
-6780.0000000	3.2950001
-6779.0000000	3.3099999
-6778.0000000	3.3150001
-6777.0000000	3.3199999
-6776.0000000	3.3299999
-6775.0000000	3.3249998
-6774.0000000	3.3199999
-6773.0000000	3.3049998
-6772.0000000	3.2849998
-6771.0000000	3.2649999
-6770.0000000	3.2449999
-6769.0000000	3.2350001
-6768.0000000	3.2300000
-6767.0000000	3.2350001
-6766.0000000	3.2350001
-6765.0000000	3.2550001
-6764.0000000	3.2700000
-6763.0000000	3.2750001
-6762.0000000	3.2649999
-6761.0000000	3.2600000
-6760.0000000	3.2400000
-6759.0000000	3.2300000
-6758.0000000	3.2400000
-6757.0000000	3.2449999
-6756.0000000	3.2600000
-6755.0000000	3.2600000
-6754.0000000	3.2750001
-6753.0000000	3.2900000
-6752.0000000	3.2750001
-6751.0000000	3.2750001

-6750.0000000	3.2700000
-6749.0000000	3.2500000
-6748.0000000	3.2150002
-6747.0000000	3.1750002
-6746.0000000	3.1600001
-6745.0000000	3.1500003
-6744.0000000	3.1300001
-6743.0000000	3.1149998
-6742.0000000	3.1050000
-6741.0000000	3.0999999
-6740.0000000	3.0599999
-6739.0000000	3.0400000
-6738.0000000	3.0100000
-6737.0000000	2.9800000
-6736.0000000	2.9400001
-6735.0000000	2.9200001
-6734.0000000	2.9050002
-6733.0000000	2.8900001
-6732.0000000	2.9000001
-6731.0000000	2.9000001
-6730.0000000	2.9300001
-6729.0000000	2.9500000
-6728.0000000	2.9949999
-6727.0000000	3.0200000
-6726.0000000	3.0500000
-6725.0000000	3.0549998
-6724.0000000	3.0450001
-6723.0000000	3.0250001
-6722.0000000	3.0200000
-6721.0000000	3.0250001
-6720.0000000	3.0100000
-6719.0000000	2.9949999
-6718.0000000	2.9700000
-6717.0000000	2.9549999
-6716.0000000	2.9450002
-6715.0000000	2.9300001
-6714.0000000	2.8966668
-6713.0000000	2.8800001
-6712.0000000	2.8950000
-6711.0000000	2.9200001
-6710.0000000	2.9400001
-6709.0000000	2.9349999
-6708.0000000	2.9800000
-6707.0000000	3.0250001
-6706.0000000	3.0450001
-6705.0000000	3.0699999
-6704.0000000	3.0949998
-6703.0000000	3.1399999
-6702.0000000	3.1700001
-6701.0000000	3.2100000
-6700.0000000	3.2400000
-6699.0000000	3.2750001
-6698.0000000	3.3000000
-6697.0000000	3.3150001
-6696.0000000	3.3249998
-6695.0000000	3.3299999
-6694.0000000	3.3550000

-6693.0000000	3.3950000
-6692.0000000	3.4150000
-6691.0000000	3.4349999
-6690.0000000	3.4600000
-6689.0000000	3.4700000
-6688.0000000	3.4900000
-6687.0000000	3.4949999
-6686.0000000	3.5050001
-6685.0000000	3.4949999
-6684.0000000	3.4949999
-6683.0000000	3.4900000
-6682.0000000	3.4549999
-6681.0000000	3.4450002
-6680.0000000	3.4349999
-6679.0000000	3.4100001
-6678.0000000	3.3800001
-6677.0000000	3.3449998
-6676.0000000	3.3249998
-6675.0000000	3.3199999
-6674.0000000	3.3150001
-6673.0000000	3.3049998
-6672.0000000	3.2950001
-6671.0000000	3.3000000
-6670.0000000	3.3049998
-6669.0000000	3.3049998
-6668.0000000	3.3199999
-6667.0000000	3.3099999
-6666.0000000	3.3099999
-6665.0000000	3.2950001
-6664.0000000	3.2849998
-6663.0000000	3.2550001
-6662.0000000	3.2249999
-6661.0000000	3.1800001
-6660.0000000	3.1400001
-6659.0000000	3.1149998
-6658.0000000	3.1099999
-6657.0000000	3.0949998
-6656.0000000	3.0699999
-6655.0000000	3.0300000
-6654.0000000	2.9650002
-6653.0000000	2.8866665
-6652.0000000	2.8099999
-6651.0000000	2.7249999
-6650.0000000	2.6350000
-6649.0000000	2.5500000
-6648.0000000	2.5149999
-6647.0000000	2.4900000
-6646.0000000	2.4949999
-6645.0000000	2.5100000
-6644.0000000	2.5050001
-6643.0000000	2.4800000
-6642.0000000	2.4549999
-6641.0000000	2.4800000
-6640.0000000	2.4900000
-6639.0000000	2.4949999
-6638.0000000	2.5349998
-6637.0000000	2.5549998

-6636.0000000	2.5949998
-6635.0000000	2.5949998
-6634.0000000	2.6450000
-6633.0000000	2.6650000
-6632.0000000	2.6750002
-6631.0000000	2.7049999
-6630.0000000	2.7350001
-6629.0000000	2.7300000
-6628.0000000	2.7550001
-6627.0000000	2.7150002
-6626.0000000	2.6950002
-6625.0000000	2.6950002
-6624.0000000	2.7200000
-6623.0000000	2.7350001
-6622.0000000	2.7166665
-6621.0000000	2.6950002
-6620.0000000	2.7049999
-6619.0000000	2.7249999
-6618.0000000	2.7750001
-6617.0000000	2.7950001
-6616.0000000	2.8449998
-6615.0000000	2.8900001
-6614.0000000	2.8950000
-6613.0000000	2.9050002
-6612.0000000	2.9100001
-6611.0000000	2.8950000
-6610.0000000	2.8950000
-6609.0000000	2.8800001
-6608.0000000	2.8699999
-6607.0000000	2.8699999
-6606.0000000	2.8599999
-6605.0000000	2.8099999
-6604.0000000	2.7400000
-6603.0000000	2.7150002
-6602.0000000	2.7000000
-6601.0000000	2.6950002
-6600.0000000	2.6600001
-6599.0000000	2.6149998
-6598.0000000	2.6300001
-6597.0000000	2.6600001
-6596.0000000	2.6750002
-6595.0000000	2.6800001
-6594.0000000	2.6900001
-6593.0000000	2.7033336
-6592.0000000	2.7049999
-6591.0000000	2.7049999
-6590.0000000	2.6900001
-6589.0000000	2.6800001
-6588.0000000	2.7100000
-6587.0000000	2.7500000
-6586.0000000	2.7900000
-6585.0000000	2.8249998
-6584.0000000	2.8550000
-6583.0000000	2.8599999
-6582.0000000	2.8699999
-6581.0000000	2.8800001
-6580.0000000	2.8800001

-6579.0000000	2.8699999
-6578.0000000	2.8599999
-6577.0000000	2.8399999
-6576.0000000	2.8000000
-6575.0000000	2.7750001
-6574.0000000	2.7550001
-6573.0000000	2.7350001
-6572.0000000	2.6900001
-6571.0000000	2.6600001
-6570.0000000	2.6300001
-6569.0000000	2.6199999
-6568.0000000	2.6350002
-6567.0000000	2.6450000
-6566.0000000	2.6700001
-6565.0000000	2.6800001
-6564.0000000	2.6900001
-6563.0000000	2.6833334
-6562.0000000	2.6900001
-6561.0000000	2.6950002
-6560.0000000	2.7100000
-6559.0000000	2.7350001
-6558.0000000	2.7600000
-6557.0000000	2.7900000
-6556.0000000	2.7849998
-6555.0000000	2.8000000
-6554.0000000	2.8049998
-6553.0000000	2.7950001
-6552.0000000	2.8000000
-6551.0000000	2.8000000
-6550.0000000	2.8049998
-6549.0000000	2.8099999
-6548.0000000	2.7950001
-6547.0000000	2.7400000
-6546.0000000	2.6700001
-6545.0000000	2.6199999
-6544.0000000	2.5899999
-6543.0000000	2.5699999
-6542.0000000	2.5650001
-6541.0000000	2.5599999
-6540.0000000	2.5699999
-6539.0000000	2.5650001
-6538.0000000	2.5400000
-6537.0000000	2.5100000
-6536.0000000	2.5149999
-6535.0000000	2.5100000
-6534.0000000	2.5000000
-6533.0000000	2.5050001
-6532.0000000	2.5200000
-6531.0000000	2.5300000
-6530.0000000	2.5349998
-6529.0000000	2.5450001
-6528.0000000	2.5850000
-6527.0000000	2.6149998
-6526.0000000	2.6550002
-6525.0000000	2.6849999
-6524.0000000	2.7100000
-6523.0000000	2.7400000

-6522.0000000	2.7750001
-6521.0000000	2.8249998
-6520.0000000	2.8599999
-6519.0000000	2.8800001
-6518.0000000	2.8950000
-6517.0000000	2.9050002
-6516.0000000	2.9150000
-6515.0000000	2.9200001
-6514.0000000	2.9200001
-6513.0000000	2.9050002
-6512.0000000	2.9150000
-6511.0000000	2.9250002
-6510.0000000	2.9549999
-6509.0000000	2.9500000
-6508.0000000	2.9700000
-6507.0000000	2.9749999
-6506.0000000	2.9850001
-6505.0000000	3.0000000
-6504.0000000	3.0050001
-6503.0000000	3.0133333
-6502.0000000	3.0300000
-6501.0000000	3.0300000
-6500.0000000	3.0400000
-6499.0000000	3.0400000
-6498.0000000	3.0100000
-6497.0000000	2.9900000
-6496.0000000	2.9700000
-6495.0000000	2.9549999
-6494.0000000	2.9300001
-6493.0000000	2.9100001
-6492.0000000	2.9050002
-6491.0000000	2.9150000
-6490.0000000	2.9150000
-6489.0000000	2.9200001
-6488.0000000	2.9200001
-6487.0000000	2.9200001
-6486.0000000	2.9349999
-6485.0000000	2.9400001
-6484.0000000	2.9400001
-6483.0000000	2.9400001
-6482.0000000	2.9349999
-6481.0000000	2.9349999
-6480.0000000	2.9250002
-6479.0000000	2.9050002
-6478.0000000	2.8800001
-6477.0000000	2.8399999
-6476.0000000	2.8000000
-6475.0000000	2.7700000
-6474.0000000	2.7449999
-6473.0000000	2.7249999
-6472.0000000	2.6849999
-6471.0000000	2.6700001
-6470.0000000	2.6500001
-6469.0000000	2.6450000
-6468.0000000	2.6600001
-6467.0000000	2.6750002
-6466.0000000	2.6900001

-6465.0000000	2.7100000
-6464.0000000	2.7400000
-6463.0000000	2.7600000
-6462.0000000	2.7900000
-6461.0000000	2.8350000
-6460.0000000	2.8800001
-6459.0000000	2.9200001
-6458.0000000	2.9600000
-6457.0000000	3.0050001
-6456.0000000	3.0400000
-6455.0000000	3.0749998
-6454.0000000	3.0999999
-6453.0000000	3.1350002
-6452.0000000	3.1700001
-6451.0000000	3.1849999
-6450.0000000	3.2049999
-6449.0000000	3.2100000
-6448.0000000	3.2300000
-6447.0000000	3.2400000
-6446.0000000	3.2500000
-6445.0000000	3.2400000
-6444.0000000	3.2300000
-6443.0000000	3.2249999
-6442.0000000	3.2150002
-6441.0000000	3.1950002
-6440.0000000	3.1700001
-6439.0000000	3.1550002
-6438.0000000	3.1550002
-6437.0000000	3.1550002
-6436.0000000	3.1650000
-6435.0000000	3.1849999
-6434.0000000	3.2100000
-6433.0000000	3.2150002
-6432.0000000	3.2100000
-6431.0000000	3.2200000
-6430.0000000	3.2249999
-6429.0000000	3.2400000
-6428.0000000	3.2500000
-6427.0000000	3.2600000
-6426.0000000	3.2700000
-6425.0000000	3.2800000
-6424.0000000	3.2900000
-6423.0000000	3.2550001
-6422.0000000	3.2100000
-6421.0000000	3.1849999
-6420.0000000	3.1800001
-6419.0000000	3.1900001
-6418.0000000	3.1900001
-6417.0000000	3.1849999
-6416.0000000	3.1849999
-6415.0000000	3.1800001
-6414.0000000	3.1950002
-6413.0000000	3.2100000
-6412.0000000	3.2200000
-6411.0000000	3.2249999
-6410.0000000	3.2166669
-6409.0000000	3.2200000

-6408.0000000	3.2200000
-6407.0000000	3.2300000
-6406.0000000	3.2350001
-6405.0000000	3.2550001
-6404.0000000	3.2649999
-6403.0000000	3.2950001
-6402.0000000	3.2950001
-6401.0000000	3.3049998
-6400.0000000	3.3150001
-6399.0000000	3.3350000
-6398.0000000	3.3399999
-6397.0000000	3.3449998
-6396.0000000	3.3449998
-6395.0000000	3.3599999
-6394.0000000	3.3900001
-6393.0000000	3.4050002
-6392.0000000	3.4150000
-6391.0000000	3.4250002
-6390.0000000	3.4349999
-6389.0000000	3.4250002
-6388.0000000	3.3950000
-6387.0000000	3.3599999
-6386.0000000	3.3299999
-6385.0000000	3.3049998
-6384.0000000	3.2900000
-6383.0000000	3.2600000
-6382.0000000	3.2300003
-6381.0000000	3.2300000
-6380.0000000	3.2249999
-6379.0000000	3.2300000
-6378.0000000	3.2400000
-6377.0000000	3.2649999
-6376.0000000	3.2900000
-6375.0000000	3.3000000
-6374.0000000	3.3249998
-6373.0000000	3.3449998
-6372.0000000	3.3699999
-6371.0000000	3.3750000
-6370.0000000	3.3900001
-6369.0000000	3.4100001
-6368.0000000	3.4250002
-6367.0000000	3.4300001
-6366.0000000	3.4549999
-6365.0000000	3.4700000
-6364.0000000	3.4949999
-6363.0000000	3.5100000
-6362.0000000	3.5400000
-6361.0000000	3.5650001
-6360.0000000	3.5949998
-6359.0000000	3.6199999
-6358.0000000	3.6450000
-6357.0000000	3.6849999
-6356.0000000	3.7000000
-6355.0000000	3.7400000
-6354.0000000	3.7649999
-6353.0000000	3.7800000
-6352.0000000	3.7950001

-6351.0000000	3.8033333
-6350.0000000	3.8049998
-6349.0000000	3.8150001
-6348.0000000	3.8099999
-6347.0000000	3.8199999
-6346.0000000	3.8049998
-6345.0000000	3.7950001
-6344.0000000	3.7750001
-6343.0000000	3.7550001
-6342.0000000	3.7350001
-6341.0000000	3.6950002
-6340.0000000	3.6500001
-6339.0000000	3.6100001
-6338.0000000	3.5749998
-6337.0000000	3.5250001
-6336.0000000	3.4500000
-6335.0000000	3.3850000
-6334.0000000	3.3000000
-6333.0000000	3.2249999
-6332.0000000	3.1500001
-6331.0000000	3.0999999
-6330.0000000	3.1050000
-6329.0000000	3.0899999
-6328.0000000	3.0850000
-6327.0000000	3.0850000
-6326.0000000	3.0899999
-6325.0000000	3.1150000
-6324.0000000	3.1450000
-6323.0000000	3.1750002
-6322.0000000	3.2150002
-6321.0000000	3.2600000
-6320.0000000	3.3049998
-6319.0000000	3.3350000
-6318.0000000	3.3599999
-6317.0000000	3.3850002
-6316.0000000	3.4000001
-6315.0000000	3.3950000
-6314.0000000	3.3850002
-6313.0000000	3.3850002
-6312.0000000	3.3900001
-6311.0000000	3.3950000
-6310.0000000	3.4150000
-6309.0000000	3.4400001
-6308.0000000	3.4600000
-6307.0000000	3.4650002
-6306.0000000	3.4600000
-6305.0000000	3.4500000
-6304.0000000	3.4450002
-6303.0000000	3.4500000
-6302.0000000	3.4600000
-6301.0000000	3.4500000
-6300.0000000	3.4600000
-6299.0000000	3.4650002
-6298.0000000	3.4500000
-6297.0000000	3.4400001
-6296.0000000	3.4200001
-6295.0000000	3.3850002

-6294.0000000	3.3850002
-6293.0000000	3.3649998
-6292.0000000	3.3550000
-6291.0000000	3.3233335
-6290.0000000	3.3150001
-6289.0000000	3.3099999
-6288.0000000	3.2950001
-6287.0000000	3.2950001
-6286.0000000	3.2950001
-6285.0000000	3.2950001
-6284.0000000	3.2950001
-6283.0000000	3.3000000
-6282.0000000	3.2849998
-6281.0000000	3.2649999
-6280.0000000	3.2550001
-6279.0000000	3.2400000
-6278.0000000	3.2200000
-6277.0000000	3.2049999
-6276.0000000	3.2000000
-6275.0000000	3.1900001
-6274.0000000	3.2049999
-6273.0000000	3.2000000
-6272.0000000	3.2049999
-6271.0000000	3.2000000
-6270.0000000	3.1950002
-6269.0000000	3.1750002
-6268.0000000	3.1500001
-6267.0000000	3.1300001
-6266.0000000	3.0999999
-6265.0000000	3.0899999
-6264.0000000	3.0899999
-6263.0000000	3.0949998
-6262.0000000	3.0999999
-6261.0000000	3.1250000
-6260.0000000	3.1366670
-6259.0000000	3.1250000
-6258.0000000	3.1199999
-6257.0000000	3.1099999
-6256.0000000	3.1300001
-6255.0000000	3.1400001
-6254.0000000	3.1450000
-6253.0000000	3.1550002
-6252.0000000	3.1750002
-6251.0000000	3.1849999
-6250.0000000	3.1950002
-6249.0000000	3.2200000
-6248.0000000	3.2249999
-6247.0000000	3.2400000
-6246.0000000	3.2550001
-6245.0000000	3.2700000
-6244.0000000	3.2649999
-6243.0000000	3.2750001
-6242.0000000	3.2849998
-6241.0000000	3.2900000
-6240.0000000	3.2700000
-6239.0000000	3.2449999
-6238.0000000	3.2049999

-6237.0000000	3.1800001
-6236.0000000	3.1800001
-6235.0000000	3.1800001
-6234.0000000	3.1849999
-6233.0000000	3.2100000
-6232.0000000	3.2200000
-6231.0000000	3.2500000
-6230.0000000	3.3033333
-6229.0000000	3.3350000
-6228.0000000	3.3299999
-6227.0000000	3.3350000
-6226.0000000	3.3399999
-6225.0000000	3.3499999
-6224.0000000	3.3399999
-6223.0000000	3.3249998
-6222.0000000	3.3199999
-6221.0000000	3.3150001
-6220.0000000	3.3000000
-6219.0000000	3.3049998
-6218.0000000	3.3000000
-6217.0000000	3.2750001
-6216.0000000	3.2600000
-6215.0000000	3.2200000
-6214.0000000	3.1750002
-6213.0000000	3.1400001
-6212.0000000	3.1500001
-6211.0000000	3.1600001
-6210.0000000	3.1450000
-6209.0000000	3.1550002
-6208.0000000	3.1400001
-6207.0000000	3.1450000
-6206.0000000	3.2000000
-6205.0000000	3.2249999
-6204.0000000	3.2100000
-6203.0000000	3.2049999
-6202.0000000	3.2300000
-6201.0000000	3.2600000
-6200.0000000	3.2900000
-6199.0000000	3.3233330
-6198.0000000	3.3750000
-6197.0000000	3.3950000
-6196.0000000	3.3950000
-6195.0000000	3.4000001
-6194.0000000	3.3950000
-6193.0000000	3.4000001
-6192.0000000	3.4100001
-6191.0000000	3.4000001
-6190.0000000	3.4000001
-6189.0000000	3.3800001
-6188.0000000	3.3699999
-6187.0000000	3.3750000
-6186.0000000	3.3599999
-6185.0000000	3.3449998
-6184.0000000	3.3299999
-6183.0000000	3.3249998
-6182.0000000	3.3150001
-6181.0000000	3.3099999

-6180.0000000	3.3150001
-6179.0000000	3.3150001
-6178.0000000	3.3049998
-6177.0000000	3.3049998
-6176.0000000	3.3049998
-6175.0000000	3.3150001
-6174.0000000	3.3249998
-6173.0000000	3.3199999
-6172.0000000	3.3399999
-6171.0000000	3.3550000
-6170.0000000	3.3649998
-6169.0000000	3.3900001
-6168.0000000	3.4133332
-6167.0000000	3.4300001
-6166.0000000	3.4400001
-6165.0000000	3.4450002
-6164.0000000	3.4549999
-6163.0000000	3.4600000
-6162.0000000	3.4549999
-6161.0000000	3.4349999
-6160.0000000	3.4100001
-6159.0000000	3.3750000
-6158.0000000	3.3150001
-6157.0000000	3.2550001
-6156.0000000	3.2000000
-6155.0000000	3.1700001
-6154.0000000	3.1400001
-6153.0000000	3.1250000
-6152.0000000	3.1250000
-6151.0000000	3.1099999
-6150.0000000	3.0799999
-6149.0000000	3.0500000
-6148.0000000	3.0050001
-6147.0000000	3.0250001
-6146.0000000	3.0349998
-6145.0000000	3.0200000
-6144.0000000	3.0100000
-6143.0000000	2.9900000
-6142.0000000	3.0149999
-6141.0000000	3.0349998
-6140.0000000	3.0549998
-6139.0000000	3.0899999
-6138.0000000	3.1466668
-6137.0000000	3.1750002
-6136.0000000	3.2100000
-6135.0000000	3.2300000
-6134.0000000	3.2500000
-6133.0000000	3.2600000
-6132.0000000	3.2550001
-6131.0000000	3.2300000
-6130.0000000	3.2249999
-6129.0000000	3.2200000
-6128.0000000	3.2249999
-6127.0000000	3.2500000
-6126.0000000	3.2900000
-6125.0000000	3.3350000
-6124.0000000	3.3649998

-6123.0000000	3.4100001
-6122.0000000	3.4450002
-6121.0000000	3.4749999
-6120.0000000	3.4900000
-6119.0000000	3.5000000
-6118.0000000	3.5250001
-6117.0000000	3.5200000
-6116.0000000	3.5349998
-6115.0000000	3.5450001
-6114.0000000	3.5500000
-6113.0000000	3.5650001
-6112.0000000	3.5549998
-6111.0000000	3.5599999
-6110.0000000	3.5400000
-6109.0000000	3.5100000
-6108.0000000	3.4766667
-6107.0000000	3.4450002
-6106.0000000	3.4300001
-6105.0000000	3.4050002
-6104.0000000	3.3900001
-6103.0000000	3.3550000
-6102.0000000	3.3150001
-6101.0000000	3.2950001
-6100.0000000	3.2550001
-6099.0000000	3.2300000
-6098.0000000	3.2000000
-6097.0000000	3.1700001
-6096.0000000	3.1450000
-6095.0000000	3.1199999
-6094.0000000	3.0949998
-6093.0000000	3.0650001
-6092.0000000	3.0549998
-6091.0000000	3.0549998
-6090.0000000	3.0799999
-6089.0000000	3.1050000
-6088.0000000	3.1450000
-6087.0000000	3.1700001
-6086.0000000	3.2000000
-6085.0000000	3.2249999
-6084.0000000	3.2500000
-6083.0000000	3.2700000
-6082.0000000	3.2550001
-6081.0000000	3.2200000
-6080.0000000	3.1900001
-6079.0000000	3.1800001
-6078.0000000	3.1866665
-6077.0000000	3.2249999
-6076.0000000	3.2449999
-6075.0000000	3.2700000
-6074.0000000	3.2800000
-6073.0000000	3.2900000
-6072.0000000	3.3150001
-6071.0000000	3.3399999
-6070.0000000	3.3699999
-6069.0000000	3.3750000
-6068.0000000	3.3900001
-6067.0000000	3.4100001

-6066.0000000	3.4400001
-6065.0000000	3.4600000
-6064.0000000	3.4600000
-6063.0000000	3.4900000
-6062.0000000	3.5050001
-6061.0000000	3.5250001
-6060.0000000	3.5400000
-6059.0000000	3.5599999
-6058.0000000	3.5749998
-6057.0000000	3.5899999
-6056.0000000	3.6050000
-6055.0000000	3.6199999
-6054.0000000	3.6350000
-6053.0000000	3.6600001
-6052.0000000	3.6800001
-6051.0000000	3.7000000
-6050.0000000	3.7049999
-6049.0000000	3.7150002
-6048.0000000	3.7133334
-6047.0000000	3.7049999
-6046.0000000	3.6950002
-6045.0000000	3.6950002
-6044.0000000	3.6849999
-6043.0000000	3.6849999
-6042.0000000	3.6800001
-6041.0000000	3.6849999
-6040.0000000	3.6900001
-6039.0000000	3.7000000
-6038.0000000	3.7100000
-6037.0000000	3.7200000
-6036.0000000	3.7300000
-6035.0000000	3.7300000
-6034.0000000	3.7300000
-6033.0000000	3.7400000
-6032.0000000	3.7350001
-6031.0000000	3.7449999
-6030.0000000	3.7550001
-6029.0000000	3.7500000
-6028.0000000	3.7200000
-6027.0000000	3.7150002
-6026.0000000	3.7049999
-6025.0000000	3.7000000
-6024.0000000	3.7049999
-6023.0000000	3.6950002
-6022.0000000	3.6800001
-6021.0000000	3.6400001
-6020.0000000	3.6350002
-6019.0000000	3.6233332
-6018.0000000	3.6250000
-6017.0000000	3.6400001
-6016.0000000	3.6500001
-6015.0000000	3.6650000
-6014.0000000	3.6800001
-6013.0000000	3.7150002
-6012.0000000	3.7550001
-6011.0000000	3.7900000
-6010.0000000	3.8249998

-6009.0000000	3.8699999
-6008.0000000	3.8950000
-6007.0000000	3.9150000
-6006.0000000	3.9100001
-6005.0000000	3.9200001
-6004.0000000	3.9400001
-6003.0000000	3.9400001
-6002.0000000	3.9500000
-6001.0000000	3.9400001
-6000.0000000	3.9200001
-5999.0000000	3.9150000
-5998.0000000	3.9050002
-5997.0000000	3.8950000
-5996.0000000	3.8900001
-5995.0000000	3.8350000
-5994.0000000	3.8099999
-5993.0000000	3.7649999
-5992.0000000	3.7500000
-5991.0000000	3.7550001
-5990.0000000	3.7633333
-5989.0000000	3.7550001
-5988.0000000	3.7700000
-5987.0000000	3.7800000
-5986.0000000	3.7700000
-5985.0000000	3.7700000
-5984.0000000	3.7500000
-5983.0000000	3.7350001
-5982.0000000	3.7100000
-5981.0000000	3.6900001
-5980.0000000	3.6849999
-5979.0000000	3.6650000
-5978.0000000	3.6250000
-5977.0000000	3.6099999
-5976.0000000	3.5949998
-5975.0000000	3.5699999
-5974.0000000	3.5699999
-5973.0000000	3.5599999
-5972.0000000	3.5450001
-5971.0000000	3.5149999
-5970.0000000	3.4850001
-5969.0000000	3.4700000
-5968.0000000	3.4749999
-5967.0000000	3.4749999
-5966.0000000	3.4400001
-5965.0000000	3.4150000
-5964.0000000	3.3900001
-5963.0000000	3.3649998
-5962.0000000	3.3150001
-5961.0000000	3.2449999
-5960.0000000	3.2100000
-5959.0000000	3.1633332
-5958.0000000	3.1700001
-5957.0000000	3.2000000
-5956.0000000	3.2100000
-5955.0000000	3.2100000
-5954.0000000	3.2100000
-5953.0000000	3.1849999

-5952.0000000	3.1750002
-5951.0000000	3.1900001
-5950.0000000	3.2000000
-5949.0000000	3.1849999
-5948.0000000	3.1600001
-5947.0000000	3.1149998
-5946.0000000	3.0899999
-5945.0000000	3.0699999
-5944.0000000	3.0450001
-5943.0000000	3.0250001
-5942.0000000	3.0300000
-5941.0000000	3.0400000
-5940.0000000	3.0400000
-5939.0000000	3.0349998
-5938.0000000	3.0250001
-5937.0000000	2.9900000
-5936.0000000	2.9800000
-5935.0000000	2.9650002
-5934.0000000	2.9250002
-5933.0000000	2.9200001
-5932.0000000	2.9500000
-5931.0000000	2.9850001
-5930.0000000	2.9900000
-5929.0000000	3.0000000
-5928.0000000	2.9900000
-5927.0000000	2.9900000
-5926.0000000	2.9800000
-5925.0000000	2.9749999
-5924.0000000	2.9650002
-5923.0000000	2.9600000
-5922.0000000	2.9549999
-5921.0000000	2.9500000
-5920.0000000	2.9450002
-5919.0000000	2.9650002
-5918.0000000	2.9850001
-5917.0000000	3.0050001
-5916.0000000	3.0000000
-5915.0000000	3.0100000
-5914.0000000	3.0000000
-5913.0000000	2.9949999
-5912.0000000	3.0000000
-5911.0000000	2.9900000
-5910.0000000	2.9900000
-5909.0000000	3.0000000
-5908.0000000	3.0100000
-5907.0000000	3.0300000
-5906.0000000	3.0400000
-5905.0000000	3.0400000
-5904.0000000	3.0599999
-5903.0000000	3.0799999
-5902.0000000	3.0799999
-5901.0000000	3.0850000
-5900.0000000	3.0949998
-5899.0000000	3.0999997
-5898.0000000	3.1149998
-5897.0000000	3.1099999
-5896.0000000	3.1149998

-5895.0000000	3.0949998
-5894.0000000	3.1050000
-5893.0000000	3.1050000
-5892.0000000	3.1050000
-5891.0000000	3.1250000
-5890.0000000	3.1199999
-5889.0000000	3.1099999
-5888.0000000	3.1150000
-5887.0000000	3.1350002
-5886.0000000	3.1350002
-5885.0000000	3.1250000
-5884.0000000	3.1250000
-5883.0000000	3.1199999
-5882.0000000	3.1250000
-5881.0000000	3.1199999
-5880.0000000	3.1149998
-5879.0000000	3.1199999
-5878.0000000	3.1250000
-5877.0000000	3.1500001
-5876.0000000	3.1450000
-5875.0000000	3.1500001
-5874.0000000	3.1400001
-5873.0000000	3.1600001
-5872.0000000	3.1900001
-5871.0000000	3.2100000
-5870.0000000	3.2350001
-5869.0000000	3.2366667
-5868.0000000	3.2449999
-5867.0000000	3.2249999
-5866.0000000	3.2300000
-5865.0000000	3.2150002
-5864.0000000	3.2300000
-5863.0000000	3.2449999
-5862.0000000	3.2500000
-5861.0000000	3.2550001
-5860.0000000	3.2700000
-5859.0000000	3.2600000
-5858.0000000	3.2500000
-5857.0000000	3.2500000
-5856.0000000	3.2350001
-5855.0000000	3.2100000
-5854.0000000	3.1600001
-5853.0000000	3.1050000
-5852.0000000	3.0500000
-5851.0000000	2.9949999
-5850.0000000	2.9549999
-5849.0000000	2.9150000
-5848.0000000	2.8900001
-5847.0000000	2.9050002
-5846.0000000	2.9200001
-5845.0000000	2.9300001
-5844.0000000	2.9450002
-5843.0000000	2.9650002
-5842.0000000	2.9949999
-5841.0000000	3.0100000
-5840.0000000	3.0300000
-5839.0000000	3.0533333

-5838.0000000	3.0450001
-5837.0000000	3.0349998
-5836.0000000	3.0300000
-5835.0000000	3.0650001
-5834.0000000	3.1050000
-5833.0000000	3.1550002
-5832.0000000	3.2100000
-5831.0000000	3.2649999
-5830.0000000	3.2900000
-5829.0000000	3.3049998
-5828.0000000	3.3299999
-5827.0000000	3.3750000
-5826.0000000	3.4250002
-5825.0000000	3.4949999
-5824.0000000	3.5400000
-5823.0000000	3.5949998
-5822.0000000	3.6149998
-5821.0000000	3.6400001
-5820.0000000	3.6550002
-5819.0000000	3.6650000
-5818.0000000	3.6849999
-5817.0000000	3.7000000
-5816.0000000	3.7150002
-5815.0000000	3.7350001
-5814.0000000	3.7500000
-5813.0000000	3.7750001
-5812.0000000	3.7900000
-5811.0000000	3.7950001
-5810.0000000	3.8150001
-5809.0000000	3.8199999
-5808.0000000	3.8299999
-5807.0000000	3.8350000
-5806.0000000	3.8599999
-5805.0000000	3.8699999
-5804.0000000	3.8499999
-5803.0000000	3.8499999
-5802.0000000	3.8599999
-5801.0000000	3.8650000
-5800.0000000	3.8699999
-5799.0000000	3.8649998
-5798.0000000	3.8649998
-5797.0000000	3.8649998
-5796.0000000	3.8850000
-5795.0000000	3.9000001
-5794.0000000	3.9150000
-5793.0000000	3.9349999
-5792.0000000	3.9450002
-5791.0000000	3.9600000
-5790.0000000	3.9700000
-5789.0000000	4.0000000
-5788.0000000	4.0050001
-5787.0000000	4.0150003
-5786.0000000	4.0400000
-5785.0000000	4.0500002
-5784.0000000	4.0699997
-5783.0000000	4.0950003
-5782.0000000	4.0950003

-5781.0000000	4.0950003
-5780.0000000	4.0799999
-5779.0000000	4.0600004
-5778.0000000	4.0433335
-5777.0000000	4.0250001
-5776.0000000	4.0000000
-5775.0000000	3.9949999
-5774.0000000	3.9900000
-5773.0000000	3.9900000
-5772.0000000	3.9900000
-5771.0000000	3.9850001
-5770.0000000	3.9600000
-5769.0000000	3.9450002
-5768.0000000	3.9500000
-5767.0000000	3.9349999
-5766.0000000	3.9200001
-5765.0000000	3.9050002
-5764.0000000	3.8899999
-5763.0000000	3.8550000
-5762.0000000	3.8249998
-5761.0000000	3.7900000
-5760.0000000	3.7700000
-5759.0000000	3.7750001
-5758.0000000	3.7500000
-5757.0000000	3.7400000
-5756.0000000	3.7350001
-5755.0000000	3.7100000
-5754.0000000	3.7249999
-5753.0000000	3.7350001
-5752.0000000	3.7600000
-5751.0000000	3.7700000
-5750.0000000	3.7750001
-5749.0000000	3.8000000
-5748.0000000	3.8150001
-5747.0000000	3.8299999
-5746.0000000	3.8249998
-5745.0000000	3.8099999
-5744.0000000	3.7950001
-5743.0000000	3.7849998
-5742.0000000	3.7800000
-5741.0000000	3.7750001
-5740.0000000	3.7500000
-5739.0000000	3.7000000
-5738.0000000	3.6849999
-5737.0000000	3.6800001
-5736.0000000	3.6849999
-5735.0000000	3.7100000
-5734.0000000	3.7550001
-5733.0000000	3.7800000
-5732.0000000	3.8150001
-5731.0000000	3.8449998
-5730.0000000	3.8750000
-5729.0000000	3.8850002
-5728.0000000	3.8850000
-5727.0000000	3.8950000
-5726.0000000	3.9050002
-5725.0000000	3.8950000

-5724.0000000	3.8900001
-5723.0000000	3.8900001
-5722.0000000	3.9100001
-5721.0000000	3.9100001
-5720.0000000	3.9400001
-5719.0000000	3.9600000
-5718.0000000	3.9800000
-5717.0000000	3.9833336
-5716.0000000	4.0150003
-5715.0000000	4.0150003
-5714.0000000	3.9600000
-5713.0000000	3.9200001
-5712.0000000	3.8800001
-5711.0000000	3.8699999
-5710.0000000	3.8350000
-5709.0000000	3.8049998
-5708.0000000	3.7900000
-5707.0000000	3.8000000
-5706.0000000	3.8099999
-5705.0000000	3.8000000
-5704.0000000	3.7950001
-5703.0000000	3.7900000
-5702.0000000	3.7700000
-5701.0000000	3.7449999
-5700.0000000	3.7150002
-5699.0000000	3.6750002
-5698.0000000	3.6350000
-5697.0000000	3.6199999
-5696.0000000	3.5799999
-5695.0000000	3.5549998
-5694.0000000	3.5500000
-5693.0000000	3.5500000
-5692.0000000	3.5450001
-5691.0000000	3.5450001
-5690.0000000	3.5500000
-5689.0000000	3.5400000
-5688.0000000	3.5549998
-5687.0000000	3.5266666
-5686.0000000	3.4850001
-5685.0000000	3.4349999
-5684.0000000	3.3750000
-5683.0000000	3.3099999
-5682.0000000	3.3199999
-5681.0000000	3.3399999
-5680.0000000	3.3649998
-5679.0000000	3.4000001
-5678.0000000	3.4549999
-5677.0000000	3.4700000
-5676.0000000	3.4900000
-5675.0000000	3.5100000
-5674.0000000	3.5450001
-5673.0000000	3.5500000
-5672.0000000	3.5599999
-5671.0000000	3.6050000
-5670.0000000	3.6500001
-5669.0000000	3.6849999
-5668.0000000	3.7000000

-5667.0000000	3.7300000
-5666.0000000	3.7500000
-5665.0000000	3.7800000
-5664.0000000	3.8000000
-5663.0000000	3.8299999
-5662.0000000	3.8499999
-5661.0000000	3.8550000
-5660.0000000	3.8649998
-5659.0000000	3.8599999
-5658.0000000	3.8399999
-5657.0000000	3.8199999
-5656.0000000	3.8099999
-5655.0000000	3.7950001
-5654.0000000	3.7533333
-5653.0000000	3.7049999
-5652.0000000	3.6650000
-5651.0000000	3.6350002
-5650.0000000	3.6250000
-5649.0000000	3.6300001
-5648.0000000	3.6300001
-5647.0000000	3.6149998
-5646.0000000	3.5850000
-5645.0000000	3.5500000
-5644.0000000	3.5100000
-5643.0000000	3.4850001
-5642.0000000	3.4600000
-5641.0000000	3.4150000
-5640.0000000	3.3750000
-5639.0000000	3.3199999
-5638.0000000	3.3000000
-5637.0000000	3.3049998
-5636.0000000	3.3249998
-5635.0000000	3.2800000
-5634.0000000	3.2200000
-5633.0000000	3.2100000
-5632.0000000	3.2249999
-5631.0000000	3.2400000
-5630.0000000	3.2400000
-5629.0000000	3.2600000
-5628.0000000	3.2400000
-5627.0000000	3.2550001
-5626.0000000	3.2550001
-5625.0000000	3.3199999
-5624.0000000	3.3099999
-5623.0000000	3.3049998
-5622.0000000	3.3150001
-5621.0000000	3.3000000
-5620.0000000	3.3049998
-5619.0000000	3.3099999
-5618.0000000	3.3099999
-5617.0000000	3.3150001
-5616.0000000	3.2849998
-5615.0000000	3.3000000
-5614.0000000	3.2849998
-5613.0000000	3.2750001
-5612.0000000	3.2600000
-5611.0000000	3.2900000

-5610.0000000	3.3399999
-5609.0000000	3.3550000
-5608.0000000	3.3599999
-5607.0000000	3.3199999
-5606.0000000	3.3249998
-5605.0000000	3.3499999
-5604.0000000	3.3449998
-5603.0000000	3.3299999
-5602.0000000	3.3350000
-5601.0000000	3.3499999
-5600.0000000	3.3699999
-5599.0000000	3.4000001
-5598.0000000	3.4549999
-5597.0000000	3.5149999
-5596.0000000	3.5799999
-5595.0000000	3.6199999
-5594.0000000	3.6400001
-5593.0000000	3.6900001
-5592.0000000	3.6750002
-5591.0000000	3.6800001
-5590.0000000	3.7000000
-5589.0000000	3.7300000
-5588.0000000	3.7550001
-5587.0000000	3.7750001
-5586.0000000	3.7950001
-5585.0000000	3.7900000
-5584.0000000	3.7700000
-5583.0000000	3.7300000
-5582.0000000	3.6849999
-5581.0000000	3.6250000
-5580.0000000	3.5450001
-5579.0000000	3.4700000
-5578.0000000	3.4250002
-5577.0000000	3.4050002
-5576.0000000	3.3499999
-5575.0000000	3.3249998
-5574.0000000	3.3199999
-5573.0000000	3.3150001
-5572.0000000	3.3299999
-5571.0000000	3.3399999
-5570.0000000	3.3299999
-5569.0000000	3.2750001
-5568.0000000	3.2049999
-5567.0000000	3.1800001
-5566.0000000	3.1849999
-5565.0000000	3.2400000
-5564.0000000	3.2833335
-5563.0000000	3.3099999
-5562.0000000	3.3449998
-5561.0000000	3.3750000
-5560.0000000	3.4000001
-5559.0000000	3.4050002
-5558.0000000	3.4150000
-5557.0000000	3.4200001
-5556.0000000	3.4000001
-5555.0000000	3.3499999
-5554.0000000	3.3049998

-5553.0000000	3.2300000
-5552.0000000	3.1750002
-5551.0000000	3.1050000
-5550.0000000	3.0000000
-5549.0000000	2.8850000
-5548.0000000	2.8150001
-5547.0000000	2.8049998
-5546.0000000	2.8249998
-5545.0000000	2.8199999
-5544.0000000	2.7900000
-5543.0000000	2.8099999
-5542.0000000	2.8249998
-5541.0000000	2.8299999
-5540.0000000	2.8499999
-5539.0000000	2.8249998
-5538.0000000	2.8249998
-5537.0000000	2.8649998
-5536.0000000	2.9150000
-5535.0000000	2.9549999
-5534.0000000	3.0000000
-5533.0000000	3.0466664
-5532.0000000	3.1050000
-5531.0000000	3.1350002
-5530.0000000	3.1300001
-5529.0000000	3.1300001
-5528.0000000	3.1300001
-5527.0000000	3.1550002
-5526.0000000	3.1600001
-5525.0000000	3.1700001
-5524.0000000	3.2100000
-5523.0000000	3.2049999
-5522.0000000	3.2400000
-5521.0000000	3.2849998
-5520.0000000	3.3249998
-5519.0000000	3.3350000
-5518.0000000	3.3550000
-5517.0000000	3.3649998
-5516.0000000	3.3550000
-5515.0000000	3.3550000
-5514.0000000	3.3399999
-5513.0000000	3.3150001
-5512.0000000	3.3049998
-5511.0000000	3.3000000
-5510.0000000	3.2950001
-5509.0000000	3.2900000
-5508.0000000	3.2700000
-5507.0000000	3.2300000
-5506.0000000	3.1950002
-5505.0000000	3.1600001
-5504.0000000	3.1149998
-5503.0000000	3.0766666
-5502.0000000	3.0300000
-5501.0000000	2.9949999
-5500.0000000	2.9949999
-5499.0000000	2.9900000
-5498.0000000	2.9949999
-5497.0000000	3.0050001

-5496.0000000	3.0149999
-5495.0000000	3.0250001
-5494.0000000	3.0450001
-5493.0000000	3.0549998
-5492.0000000	3.0699999
-5491.0000000	3.0999999
-5490.0000000	3.1300001
-5489.0000000	3.1400001
-5488.0000000	3.1550002
-5487.0000000	3.1700001
-5486.0000000	3.1849999
-5485.0000000	3.2000000
-5484.0000000	3.2049999
-5483.0000000	3.1950002
-5482.0000000	3.1950002
-5481.0000000	3.1950002
-5480.0000000	3.2049999
-5479.0000000	3.2000000
-5478.0000000	3.1950002
-5477.0000000	3.2000000
-5476.0000000	3.1950002
-5475.0000000	3.1950002
-5474.0000000	3.1800001
-5473.0000000	3.1400001
-5472.0000000	3.1199999
-5471.0000000	3.0949998
-5470.0000000	3.0699999
-5469.0000000	3.0400000
-5468.0000000	3.0200000
-5467.0000000	3.0300000
-5466.0000000	3.0200000
-5465.0000000	3.0500000
-5464.0000000	3.0949998
-5463.0000000	3.1099999
-5462.0000000	3.1350002
-5461.0000000	3.1400001
-5460.0000000	3.1600001
-5459.0000000	3.1550002
-5458.0000000	3.1600001
-5457.0000000	3.1700001
-5456.0000000	3.1650000
-5455.0000000	3.1550002
-5454.0000000	3.1550002
-5453.0000000	3.1650000
-5452.0000000	3.2049999
-5451.0000000	3.2300000
-5450.0000000	3.2750001
-5449.0000000	3.2800000
-5448.0000000	3.2950001
-5447.0000000	3.2900000
-5446.0000000	3.2449999
-5445.0000000	3.2300000
-5444.0000000	3.2266667
-5443.0000000	3.1800001
-5442.0000000	3.1350000
-5441.0000000	3.0899999
-5440.0000000	3.0349998

-5439.0000000	2.9949999
-5438.0000000	2.9749999
-5437.0000000	2.9600000
-5436.0000000	2.9349999
-5435.0000000	2.9050002
-5434.0000000	2.8850002
-5433.0000000	2.8699999
-5432.0000000	2.8449998
-5431.0000000	2.8350000
-5430.0000000	2.8399999
-5429.0000000	2.8399999
-5428.0000000	2.8299999
-5427.0000000	2.8399999
-5426.0000000	2.8299999
-5425.0000000	2.8350000
-5424.0000000	2.8399999
-5423.0000000	2.8499999
-5422.0000000	2.8699999
-5421.0000000	2.8699999
-5420.0000000	2.8750000
-5419.0000000	2.8599999
-5418.0000000	2.8550000
-5417.0000000	2.8550000
-5416.0000000	2.8599999
-5415.0000000	2.8699999
-5414.0000000	2.8933334
-5413.0000000	2.9600000
-5412.0000000	2.9949999
-5411.0000000	3.0149999
-5410.0000000	3.0250001
-5409.0000000	3.0450001
-5408.0000000	3.0699999
-5407.0000000	3.0749998
-5406.0000000	3.1050000
-5405.0000000	3.1199999
-5404.0000000	3.1250000
-5403.0000000	3.1550002
-5402.0000000	3.1900001
-5401.0000000	3.2100000
-5400.0000000	3.2200000
-5399.0000000	3.2400000
-5398.0000000	3.2449999
-5397.0000000	3.2449999
-5396.0000000	3.2600000
-5395.0000000	3.2600000
-5394.0000000	3.2550001
-5393.0000000	3.2500000
-5392.0000000	3.2449999
-5391.0000000	3.2350001
-5390.0000000	3.2150002
-5389.0000000	3.2000000
-5388.0000000	3.1600001
-5387.0000000	3.1350002
-5386.0000000	3.1149998
-5385.0000000	3.1066666
-5384.0000000	3.0850000
-5383.0000000	3.0749998

-5382.0000000	3.0500000
-5381.0000000	3.0250001
-5380.0000000	3.0100000
-5379.0000000	2.9850001
-5378.0000000	2.9749999
-5377.0000000	2.9850001
-5376.0000000	3.0100000
-5375.0000000	3.0050001
-5374.0000000	3.0000000
-5373.0000000	3.0149999
-5372.0000000	3.0300000
-5371.0000000	3.0300000
-5370.0000000	3.0749998
-5369.0000000	3.0999999
-5368.0000000	3.1350000
-5367.0000000	3.1800001
-5366.0000000	3.2049999
-5365.0000000	3.2300000
-5364.0000000	3.2700000
-5363.0000000	3.3150001
-5362.0000000	3.3750000
-5361.0000000	3.4000001
-5360.0000000	3.4400001
-5359.0000000	3.4749999
-5358.0000000	3.5149999
-5357.0000000	3.5450001
-5356.0000000	3.5599999
-5355.0000000	3.5733330
-5354.0000000	3.5899999
-5353.0000000	3.5899999
-5352.0000000	3.5650001
-5351.0000000	3.5599999
-5350.0000000	3.5500000
-5349.0000000	3.5300000
-5348.0000000	3.5250001
-5347.0000000	3.4949999
-5346.0000000	3.4450002
-5345.0000000	3.4100001
-5344.0000000	3.3850002
-5343.0000000	3.3699999
-5342.0000000	3.3699999
-5341.0000000	3.3750000
-5340.0000000	3.3650000
-5339.0000000	3.3750000
-5338.0000000	3.4000001
-5337.0000000	3.4150000
-5336.0000000	3.4400001
-5335.0000000	3.4749999
-5334.0000000	3.4850001
-5333.0000000	3.4949999
-5332.0000000	3.5050001
-5331.0000000	3.5149999
-5330.0000000	3.5200000
-5329.0000000	3.5050001
-5328.0000000	3.4900000
-5327.0000000	3.4700000
-5326.0000000	3.4400001

-5325.0000000	3.4200001
-5324.0000000	3.3950000
-5323.0000000	3.4000001
-5322.0000000	3.4050002
-5321.0000000	3.4200001
-5320.0000000	3.4349999
-5319.0000000	3.4450002
-5318.0000000	3.4450002
-5317.0000000	3.4400001
-5316.0000000	3.4349999
-5315.0000000	3.4200001
-5314.0000000	3.3850000
-5313.0000000	3.3499999
-5312.0000000	3.2849998
-5311.0000000	3.2350001
-5310.0000000	3.1849999
-5309.0000000	3.1600001
-5308.0000000	3.1650000
-5307.0000000	3.1800001
-5306.0000000	3.2100000
-5305.0000000	3.2249999
-5304.0000000	3.2550001
-5303.0000000	3.2600000
-5302.0000000	3.2350001
-5301.0000000	3.1700001
-5300.0000000	3.1050000
-5299.0000000	3.0549998
-5298.0000000	3.0200000
-5297.0000000	3.0050001
-5296.0000000	3.0149999
-5295.0000000	3.0533333
-5294.0000000	3.0850000
-5293.0000000	3.1199999
-5292.0000000	3.1600001
-5291.0000000	3.1750002
-5290.0000000	3.1750002
-5289.0000000	3.2000000
-5288.0000000	3.2200000
-5287.0000000	3.2350001
-5286.0000000	3.2350001
-5285.0000000	3.2300000
-5284.0000000	3.2150002
-5283.0000000	3.1800001
-5282.0000000	3.1350000
-5281.0000000	3.0999999
-5280.0000000	3.0699999
-5279.0000000	3.0300000
-5278.0000000	3.0100000
-5277.0000000	3.0000000
-5276.0000000	3.0050001
-5275.0000000	3.0050001
-5274.0000000	3.0050001
-5273.0000000	2.9800000
-5272.0000000	2.9850001
-5271.0000000	2.9850001
-5270.0000000	2.9900000
-5269.0000000	3.0000000

-5268.0000000	3.0050001
-5267.0000000	3.0300000
-5266.0000000	3.0450001
-5265.0000000	3.0500000
-5264.0000000	3.0400000
-5263.0000000	3.0450001
-5262.0000000	3.0599999
-5261.0000000	3.0549998
-5260.0000000	3.0549998
-5259.0000000	3.0400000
-5258.0000000	3.0450001
-5257.0000000	3.0450001
-5256.0000000	3.0400000
-5255.0000000	3.0400000
-5254.0000000	3.0200000
-5253.0000000	3.0000000
-5252.0000000	2.9850001
-5251.0000000	2.9700000
-5250.0000000	2.9650002
-5249.0000000	2.9800000
-5248.0000000	3.0050001
-5247.0000000	3.0149999
-5246.0000000	3.0400000
-5245.0000000	3.0400000
-5244.0000000	3.0349998
-5243.0000000	3.0300000
-5242.0000000	3.0300000
-5241.0000000	3.0300000
-5240.0000000	3.0149999
-5239.0000000	3.0149999
-5238.0000000	3.0100000
-5237.0000000	3.0050001
-5236.0000000	3.0000000
-5235.0000000	3.0000000
-5234.0000000	2.9933331
-5233.0000000	2.9700000
-5232.0000000	2.9549999
-5231.0000000	2.9500000
-5230.0000000	2.9549999
-5229.0000000	2.9500000
-5228.0000000	2.9500000
-5227.0000000	2.9700000
-5226.0000000	2.9600000
-5225.0000000	2.9250002
-5224.0000000	2.9200001
-5223.0000000	2.9300001
-5222.0000000	2.9400001
-5221.0000000	2.9300001
-5220.0000000	2.9200001
-5219.0000000	2.9000001
-5218.0000000	2.8850002
-5217.0000000	2.8350000
-5216.0000000	2.7950001
-5215.0000000	2.7950001
-5214.0000000	2.7950001
-5213.0000000	2.8049998
-5212.0000000	2.8049998

-5211.0000000	2.8099999
-5210.0000000	2.8150001
-5209.0000000	2.8299999
-5208.0000000	2.8449998
-5207.0000000	2.8699999
-5206.0000000	2.8800001
-5205.0000000	2.8950000
-5204.0000000	2.9200001
-5203.0000000	2.9549999
-5202.0000000	2.9800000
-5201.0000000	3.0100000
-5200.0000000	3.0300000
-5199.0000000	3.0549998
-5198.0000000	3.0899999
-5197.0000000	3.0799999
-5196.0000000	3.0650001
-5195.0000000	3.0749998
-5194.0000000	3.0749998
-5193.0000000	3.0650001
-5192.0000000	3.0450001
-5191.0000000	3.0250001
-5190.0000000	3.0400000
-5189.0000000	3.0500000
-5188.0000000	3.1099999
-5187.0000000	3.1399999
-5186.0000000	3.1900001
-5185.0000000	3.2200000
-5184.0000000	3.2400000
-5183.0000000	3.2700000
-5182.0000000	3.3150001
-5181.0000000	3.3049998
-5180.0000000	3.3399999
-5179.0000000	3.3550000
-5178.0000000	3.3649998
-5177.0000000	3.3699999
-5176.0000000	3.3499999
-5175.0000000	3.3299999
-5174.0000000	3.3399999
-5173.0000000	3.3599999
-5172.0000000	3.4050002
-5171.0000000	3.4150000
-5170.0000000	3.4250002
-5169.0000000	3.4450002
-5168.0000000	3.4650002
-5167.0000000	3.4700000
-5166.0000000	3.4749999
-5165.0000000	3.5000000
-5164.0000000	3.5050001
-5163.0000000	3.5349998
-5162.0000000	3.5300000
-5161.0000000	3.5400000
-5160.0000000	3.5450001
-5159.0000000	3.5500000
-5158.0000000	3.5799999
-5157.0000000	3.6050000
-5156.0000000	3.6199999
-5155.0000000	3.6450000

-5154.0000000	3.6550002
-5153.0000000	3.6600001
-5152.0000000	3.6600001
-5151.0000000	3.6450000
-5150.0000000	3.6199999
-5149.0000000	3.5999999
-5148.0000000	3.5850000
-5147.0000000	3.5799999
-5146.0000000	3.5549998
-5145.0000000	3.5650001
-5144.0000000	3.5650001
-5143.0000000	3.5733330
-5142.0000000	3.5749998
-5141.0000000	3.5799999
-5140.0000000	3.5850000
-5139.0000000	3.5899999
-5138.0000000	3.5949998
-5137.0000000	3.6050000
-5136.0000000	3.6149998
-5135.0000000	3.6350002
-5134.0000000	3.6500001
-5133.0000000	3.6700001
-5132.0000000	3.7000000
-5131.0000000	3.7049999
-5130.0000000	3.7200000
-5129.0000000	3.7300000
-5128.0000000	3.7449999
-5127.0000000	3.7500000
-5126.0000000	3.7649999
-5125.0000000	3.7700000
-5124.0000000	3.7750001
-5123.0000000	3.7849998
-5122.0000000	3.7600000
-5121.0000000	3.7449999
-5120.0000000	3.7350001
-5119.0000000	3.7400000
-5118.0000000	3.7500000
-5117.0000000	3.7700000
-5116.0000000	3.7849998
-5115.0000000	3.7950001
-5114.0000000	3.8199999
-5113.0000000	3.8433332
-5112.0000000	3.8599999
-5111.0000000	3.8449998
-5110.0000000	3.8249998
-5109.0000000	3.7900000
-5108.0000000	3.7800000
-5107.0000000	3.7700000
-5106.0000000	3.7550001
-5105.0000000	3.7500000
-5104.0000000	3.7300000
-5103.0000000	3.7300000
-5102.0000000	3.7150002
-5101.0000000	3.7000000
-5100.0000000	3.7049999
-5099.0000000	3.6800001
-5098.0000000	3.6849999

-5097.0000000	3.6900001
-5096.0000000	3.7000000
-5095.0000000	3.7249999
-5094.0000000	3.7500000
-5093.0000000	3.7700000
-5092.0000000	3.7900000
-5091.0000000	3.8000000
-5090.0000000	3.8249998
-5089.0000000	3.8499999
-5088.0000000	3.8599999
-5087.0000000	3.8649998
-5086.0000000	3.8599999
-5085.0000000	3.8649998
-5084.0000000	3.8599999
-5083.0000000	3.8733332
-5082.0000000	3.8800001
-5081.0000000	3.8850002
-5080.0000000	3.9050002
-5079.0000000	3.9000001
-5078.0000000	3.9150000
-5077.0000000	3.8950000
-5076.0000000	3.9100001
-5075.0000000	3.9250002
-5074.0000000	3.9250002
-5073.0000000	3.9050002
-5072.0000000	3.8850000
-5071.0000000	3.8399999
-5070.0000000	3.8199999
-5069.0000000	3.8049998
-5068.0000000	3.7800000
-5067.0000000	3.7500000
-5066.0000000	3.7100000
-5065.0000000	3.6600001
-5064.0000000	3.5999999
-5063.0000000	3.5650001
-5062.0000000	3.5349998
-5061.0000000	3.5050001
-5060.0000000	3.4949999
-5059.0000000	3.4949999
-5058.0000000	3.5100000
-5057.0000000	3.5300000
-5056.0000000	3.5549998
-5055.0000000	3.5999999
-5054.0000000	3.6250000
-5053.0000000	3.6550002
-5052.0000000	3.6766670
-5051.0000000	3.6849999
-5050.0000000	3.6849999
-5049.0000000	3.6849999
-5048.0000000	3.6600001
-5047.0000000	3.6300001
-5046.0000000	3.6199999
-5045.0000000	3.5949998
-5044.0000000	3.5949998
-5043.0000000	3.5949998
-5042.0000000	3.5999999
-5041.0000000	3.6149998

-5040.0000000	3.6199999
-5039.0000000	3.6250000
-5038.0000000	3.6250000
-5037.0000000	3.6149998
-5036.0000000	3.5999999
-5035.0000000	3.5699999
-5034.0000000	3.5500000
-5033.0000000	3.5200000
-5032.0000000	3.4600000
-5031.0000000	3.3950000
-5030.0000000	3.3249998
-5029.0000000	3.2849998
-5028.0000000	3.2550001
-5027.0000000	3.2300000
-5026.0000000	3.2100000
-5025.0000000	3.1900001
-5024.0000000	3.1800001
-5023.0000000	3.1966667
-5022.0000000	3.1800001
-5021.0000000	3.1600001
-5020.0000000	3.1500001
-5019.0000000	3.1099999
-5018.0000000	3.0699999
-5017.0000000	3.0400000
-5016.0000000	3.0050001
-5015.0000000	3.0300000
-5014.0000000	3.0500000
-5013.0000000	3.0599999
-5012.0000000	3.0450001
-5011.0000000	3.0300000
-5010.0000000	3.0200000
-5009.0000000	3.0349998
-5008.0000000	3.0599999
-5007.0000000	3.0850000
-5006.0000000	3.1149998
-5005.0000000	3.1450000
-5004.0000000	3.1650000
-5003.0000000	3.1849999
-5002.0000000	3.2000000
-5001.0000000	3.2200000
-5000.0000000	3.2400000
-4999.0000000	3.2600000
-4998.0000000	3.3000000
-4997.0000000	3.3249998
-4996.0000000	3.3649998
-4995.0000000	3.3900001
-4994.0000000	3.3950000
-4993.0000000	3.4033337
-4992.0000000	3.4050002
-4991.0000000	3.4150000
-4990.0000000	3.4200001
-4989.0000000	3.4300001
-4988.0000000	3.4400001
-4987.0000000	3.4600000
-4986.0000000	3.4800000
-4985.0000000	3.5050001
-4984.0000000	3.5400000

-4983.0000000	3.5500000
-4982.0000000	3.5650001
-4981.0000000	3.5749998
-4980.0000000	3.5799999
-4979.0000000	3.5749998
-4978.0000000	3.5699999
-4977.0000000	3.5500000
-4976.0000000	3.5400000
-4975.0000000	3.5050001
-4974.0000000	3.4800000
-4973.0000000	3.4600000
-4972.0000000	3.4650002
-4971.0000000	3.4800000
-4970.0000000	3.4650002
-4969.0000000	3.4300001
-4968.0000000	3.4000001
-4967.0000000	3.3599999
-4966.0000000	3.3199999
-4965.0000000	3.2700000
-4964.0000000	3.2300000
-4963.0000000	3.1750002
-4962.0000000	3.1199999
-4961.0000000	3.0599999
-4960.0000000	3.0349998
-4959.0000000	3.0500000
-4958.0000000	3.0549998
-4957.0000000	3.0599999
-4956.0000000	3.0500000
-4955.0000000	3.0450001
-4954.0000000	3.0650001
-4953.0000000	3.0850000
-4952.0000000	3.0949998
-4951.0000000	3.0999999
-4950.0000000	3.0999999
-4949.0000000	3.0949998
-4948.0000000	3.0899999
-4947.0000000	3.0850000
-4946.0000000	3.0949998
-4945.0000000	3.0699999
-4944.0000000	3.0300000
-4943.0000000	2.9700000
-4942.0000000	2.9150000
-4941.0000000	2.8449998
-4940.0000000	2.7900000
-4939.0000000	2.7600000
-4938.0000000	2.7950001
-4937.0000000	2.8399999
-4936.0000000	2.8699999
-4935.0000000	2.8850002
-4934.0000000	2.8950000
-4933.0000000	2.9349999
-4932.0000000	2.9800003
-4931.0000000	3.0349998
-4930.0000000	3.0749998
-4929.0000000	3.1149998
-4928.0000000	3.1350002
-4927.0000000	3.1500001

-4926.0000000	3.1750002
-4925.0000000	3.1800001
-4924.0000000	3.1550002
-4923.0000000	3.1350002
-4922.0000000	3.1450000
-4921.0000000	3.1500001
-4920.0000000	3.1250000
-4919.0000000	3.1250000
-4918.0000000	3.1450000
-4917.0000000	3.1550002
-4916.0000000	3.1700001
-4915.0000000	3.1600001
-4914.0000000	3.1600001
-4913.0000000	3.1500001
-4912.0000000	3.1400001
-4911.0000000	3.1300001
-4910.0000000	3.1300001
-4909.0000000	3.1600001
-4908.0000000	3.1849999
-4907.0000000	3.2049999
-4906.0000000	3.2300000
-4905.0000000	3.2649999
-4904.0000000	3.2750001
-4903.0000000	3.2700000
-4902.0000000	3.2766666
-4901.0000000	3.2800000
-4900.0000000	3.3000000
-4899.0000000	3.3199999
-4898.0000000	3.3199999
-4897.0000000	3.3299999
-4896.0000000	3.3399999
-4895.0000000	3.3399999
-4894.0000000	3.3449998
-4893.0000000	3.3199999
-4892.0000000	3.3150001
-4891.0000000	3.3150001
-4890.0000000	3.3399999
-4889.0000000	3.3449998
-4888.0000000	3.3550000
-4887.0000000	3.3599999
-4886.0000000	3.3900001
-4885.0000000	3.4150000
-4884.0000000	3.4300001
-4883.0000000	3.4650002
-4882.0000000	3.4900000
-4881.0000000	3.5100000
-4880.0000000	3.5149999
-4879.0000000	3.5250001
-4878.0000000	3.5250001
-4877.0000000	3.5599999
-4876.0000000	3.5949998
-4875.0000000	3.6350002
-4874.0000000	3.6400001
-4873.0000000	3.6550002
-4872.0000000	3.6433334
-4871.0000000	3.6350002
-4870.0000000	3.6199999

-4869.0000000	3.6149998
-4868.0000000	3.5949998
-4867.0000000	3.5749998
-4866.0000000	3.5400000
-4865.0000000	3.4549999
-4864.0000000	3.3750000
-4863.0000000	3.2750001
-4862.0000000	3.1750002
-4861.0000000	3.0799999
-4860.0000000	3.0149999
-4859.0000000	2.9600000
-4858.0000000	2.9300001
-4857.0000000	2.9600000
-4856.0000000	3.0100000
-4855.0000000	3.0549998
-4854.0000000	3.1099999
-4853.0000000	3.1750002
-4852.0000000	3.2350001
-4851.0000000	3.2900000
-4850.0000000	3.3350000
-4849.0000000	3.3800001
-4848.0000000	3.4100001
-4847.0000000	3.4549999
-4846.0000000	3.4700000
-4845.0000000	3.4800000
-4844.0000000	3.5100000
-4843.0000000	3.5450001
-4842.0000000	3.5799999
-4841.0000000	3.5866668
-4840.0000000	3.6300001
-4839.0000000	3.6550002
-4838.0000000	3.6600001
-4837.0000000	3.6550002
-4836.0000000	3.6500001
-4835.0000000	3.6600001
-4834.0000000	3.6700001
-4833.0000000	3.6700001
-4832.0000000	3.6800001
-4831.0000000	3.7049999
-4830.0000000	3.7100000
-4829.0000000	3.7249999
-4828.0000000	3.7350001
-4827.0000000	3.7249999
-4826.0000000	3.7300000
-4825.0000000	3.7249999
-4824.0000000	3.7249999
-4823.0000000	3.7249999
-4822.0000000	3.7350001
-4821.0000000	3.7200000
-4820.0000000	3.7249999
-4819.0000000	3.7249999
-4818.0000000	3.7100000
-4817.0000000	3.7000000
-4816.0000000	3.7150002
-4815.0000000	3.7100000
-4814.0000000	3.7200000
-4813.0000000	3.7150002

-4812.0000000	3.7100000
-4811.0000000	3.7233334
-4810.0000000	3.7400000
-4809.0000000	3.7600000
-4808.0000000	3.7600000
-4807.0000000	3.7750001
-4806.0000000	3.7849998
-4805.0000000	3.7900000
-4804.0000000	3.8049998
-4803.0000000	3.8299999
-4802.0000000	3.8499999
-4801.0000000	3.8499999
-4800.0000000	3.8750000
-4799.0000000	3.8800001
-4798.0000000	3.8900001
-4797.0000000	3.9150000
-4796.0000000	3.9400001
-4795.0000000	3.9450002
-4794.0000000	3.9650002
-4793.0000000	3.9850001
-4792.0000000	4.0150003
-4791.0000000	4.0200005
-4790.0000000	4.0349998
-4789.0000000	4.0500002
-4788.0000000	4.0550003
-4787.0000000	4.0599999
-4786.0000000	4.0749998
-4785.0000000	4.0850000
-4784.0000000	4.0850000
-4783.0000000	4.0850000
-4782.0000000	4.0799999
-4781.0000000	4.0900002
-4780.0000000	4.0799999
-4779.0000000	4.0900002
-4778.0000000	4.0749998
-4777.0000000	4.0799999
-4776.0000000	4.0650001
-4775.0000000	4.0400000
-4774.0000000	4.0000000
-4773.0000000	3.9650002
-4772.0000000	3.9300001
-4771.0000000	3.9000001
-4770.0000000	3.8550000
-4769.0000000	3.8099999
-4768.0000000	3.7950001
-4767.0000000	3.7950001
-4766.0000000	3.7900000
-4765.0000000	3.8099999
-4764.0000000	3.8199999
-4763.0000000	3.8350000
-4762.0000000	3.8599999
-4761.0000000	3.8900001
-4760.0000000	3.8950000
-4759.0000000	3.8900001
-4758.0000000	3.8850002
-4757.0000000	3.8950000
-4756.0000000	3.8950000

-4755.0000000	3.8699999
-4754.0000000	3.8449998
-4753.0000000	3.8150001
-4752.0000000	3.7900000
-4751.0000000	3.7849998
-4750.0000000	3.7633333
-4749.0000000	3.7200000
-4748.0000000	3.6849999
-4747.0000000	3.6399999
-4746.0000000	3.6099999
-4745.0000000	3.5899999
-4744.0000000	3.5899999
-4743.0000000	3.5850000
-4742.0000000	3.5999999
-4741.0000000	3.6150000
-4740.0000000	3.6450000
-4739.0000000	3.6900001
-4738.0000000	3.7200000
-4737.0000000	3.7649999
-4736.0000000	3.7950001
-4735.0000000	3.8199999
-4734.0000000	3.8449998
-4733.0000000	3.8599999
-4732.0000000	3.8900001
-4731.0000000	3.9150000
-4730.0000000	3.9100001
-4729.0000000	3.9100001
-4728.0000000	3.9050002
-4727.0000000	3.9100001
-4726.0000000	3.9150000
-4725.0000000	3.9100001
-4724.0000000	3.9150000
-4723.0000000	3.9000001
-4722.0000000	3.8900001
-4721.0000000	3.8950000
-4720.0000000	3.8633335
-4719.0000000	3.8499999
-4718.0000000	3.8599999
-4717.0000000	3.8900001
-4716.0000000	3.9150000
-4715.0000000	3.9500000
-4714.0000000	3.9749999
-4713.0000000	4.0050001
-4712.0000000	4.0200005
-4711.0000000	4.0349998
-4710.0000000	4.0299997
-4709.0000000	4.0000000
-4708.0000000	3.9749999
-4707.0000000	3.9450002
-4706.0000000	3.9349999
-4705.0000000	3.9050002
-4704.0000000	3.8800001
-4703.0000000	3.8499999
-4702.0000000	3.8449998
-4701.0000000	3.8399999
-4700.0000000	3.8399999
-4699.0000000	3.8399999

-4698.0000000	3.8299999
-4697.0000000	3.8099999
-4696.0000000	3.7849998
-4695.0000000	3.7600000
-4694.0000000	3.7500000
-4693.0000000	3.7449999
-4692.0000000	3.7550001
-4691.0000000	3.7649999
-4690.0000000	3.7449999
-4689.0000000	3.7333336
-4688.0000000	3.7100000
-4687.0000000	3.7150002
-4686.0000000	3.7249999
-4685.0000000	3.7150002
-4684.0000000	3.7150002
-4683.0000000	3.7350001
-4682.0000000	3.7400000
-4681.0000000	3.6950002
-4680.0000000	3.6550002
-4679.0000000	3.6250000
-4678.0000000	3.5949998
-4677.0000000	3.5899999
-4676.0000000	3.5699999
-4675.0000000	3.5549998
-4674.0000000	3.5549998
-4673.0000000	3.5749998
-4672.0000000	3.5899999
-4671.0000000	3.5999999
-4670.0000000	3.5850000
-4669.0000000	3.5999999
-4668.0000000	3.6149998
-4667.0000000	3.6300001
-4666.0000000	3.6500001
-4665.0000000	3.6750002
-4664.0000000	3.7049999
-4663.0000000	3.7249999
-4662.0000000	3.7550001
-4661.0000000	3.7800000
-4660.0000000	3.7900000
-4659.0000000	3.7800000
-4658.0000000	3.7700000
-4657.0000000	3.7600000
-4656.0000000	3.7400000
-4655.0000000	3.7200000
-4654.0000000	3.7100000
-4653.0000000	3.6600001
-4652.0000000	3.6100001
-4651.0000000	3.5149999
-4650.0000000	3.4600000
-4649.0000000	3.4650002
-4648.0000000	3.4549999
-4647.0000000	3.4500000
-4646.0000000	3.4850001
-4645.0000000	3.5000000
-4644.0000000	3.4949999
-4643.0000000	3.4800000
-4642.0000000	3.4650002

-4641.0000000	3.4150000
-4640.0000000	3.3900001
-4639.0000000	3.3750000
-4638.0000000	3.3699999
-4637.0000000	3.3449998
-4636.0000000	3.3199999
-4635.0000000	3.3000000
-4634.0000000	3.2750001
-4633.0000000	3.2550001
-4632.0000000	3.2500000
-4631.0000000	3.2449999
-4630.0000000	3.2333336
-4629.0000000	3.2100000
-4628.0000000	3.2150002
-4627.0000000	3.2200000
-4626.0000000	3.2249999
-4625.0000000	3.2449999
-4624.0000000	3.2750001
-4623.0000000	3.2950001
-4622.0000000	3.3199999
-4621.0000000	3.3449998
-4620.0000000	3.3699999
-4619.0000000	3.4000001
-4618.0000000	3.4300001
-4617.0000000	3.4349999
-4616.0000000	3.4400001
-4615.0000000	3.4500000
-4614.0000000	3.4349999
-4613.0000000	3.4100001
-4612.0000000	3.3950000
-4611.0000000	3.4150000
-4610.0000000	3.4300001
-4609.0000000	3.4250002
-4608.0000000	3.4300001
-4607.0000000	3.4150000
-4606.0000000	3.4200001
-4605.0000000	3.4150000
-4604.0000000	3.4050002
-4603.0000000	3.3850002
-4602.0000000	3.3599999
-4601.0000000	3.3649998
-4600.0000000	3.3499999
-4599.0000000	3.3550000
-4598.0000000	3.3599999
-4597.0000000	3.3599999
-4596.0000000	3.3699999
-4595.0000000	3.3850000
-4594.0000000	3.3850002
-4593.0000000	3.4100001
-4592.0000000	3.4200001
-4591.0000000	3.4200001
-4590.0000000	3.4150000
-4589.0000000	3.4100001
-4588.0000000	3.4200001
-4587.0000000	3.4250002
-4586.0000000	3.4349999
-4585.0000000	3.4300001

-4584.0000000	3.4250002
-4583.0000000	3.4100001
-4582.0000000	3.4150000
-4581.0000000	3.4000001
-4580.0000000	3.4100001
-4579.0000000	3.4150000
-4578.0000000	3.4050002
-4577.0000000	3.3950000
-4576.0000000	3.3850002
-4575.0000000	3.3800001
-4574.0000000	3.3499999
-4573.0000000	3.3049998
-4572.0000000	3.2550001
-4571.0000000	3.2200000
-4570.0000000	3.2049999
-4569.0000000	3.1600001
-4568.0000000	3.1050000
-4567.0000000	3.0400000
-4566.0000000	2.9949999
-4565.0000000	2.9650002
-4564.0000000	2.9000001
-4563.0000000	2.8750000
-4562.0000000	2.8449998
-4561.0000000	2.8199999
-4560.0000000	2.8000000
-4559.0000000	2.8049998
-4558.0000000	2.8299999
-4557.0000000	2.8550000
-4556.0000000	2.8699999
-4555.0000000	2.8699999
-4554.0000000	2.8950000
-4553.0000000	2.9000001
-4552.0000000	2.9200001
-4551.0000000	2.9549999
-4550.0000000	2.9700000
-4549.0000000	3.0000000
-4548.0000000	3.0250001
-4547.0000000	3.0699999
-4546.0000000	3.1149998
-4545.0000000	3.1550002
-4544.0000000	3.1900001
-4543.0000000	3.2300000
-4542.0000000	3.2550001
-4541.0000000	3.2750001
-4540.0000000	3.2950001
-4539.0000000	3.3133333
-4538.0000000	3.3199999
-4537.0000000	3.3000000
-4536.0000000	3.2600000
-4535.0000000	3.2200000
-4534.0000000	3.1750002
-4533.0000000	3.1250000
-4532.0000000	3.0850000
-4531.0000000	3.0450001
-4530.0000000	3.0000000
-4529.0000000	2.9850001
-4528.0000000	2.9400001

-4527.0000000	2.9050002
-4526.0000000	2.8800001
-4525.0000000	2.8399999
-4524.0000000	2.8099999
-4523.0000000	2.7900000
-4522.0000000	2.7849998
-4521.0000000	2.7849998
-4520.0000000	2.7849998
-4519.0000000	2.7950001
-4518.0000000	2.8000000
-4517.0000000	2.8099999
-4516.0000000	2.8299999
-4515.0000000	2.8449998
-4514.0000000	2.8499999
-4513.0000000	2.8550000
-4512.0000000	2.8199999
-4511.0000000	2.7900000
-4510.0000000	2.7550001
-4509.0000000	2.7733333
-4508.0000000	2.8199999
-4507.0000000	2.8449998
-4506.0000000	2.8699999
-4505.0000000	2.9000001
-4504.0000000	2.9450002
-4503.0000000	2.9600000
-4502.0000000	2.9650002
-4501.0000000	2.9650002
-4500.0000000	2.9700000
-4499.0000000	2.9500000
-4498.0000000	2.9400001
-4497.0000000	2.9450002
-4496.0000000	2.9749999
-4495.0000000	3.0149999
-4494.0000000	3.0549998
-4493.0000000	3.0899999
-4492.0000000	3.1400001
-4491.0000000	3.1650000
-4490.0000000	3.1800001
-4489.0000000	3.2100000
-4488.0000000	3.2350001
-4487.0000000	3.2449999
-4486.0000000	3.2600000
-4485.0000000	3.2750001
-4484.0000000	3.2900000
-4483.0000000	3.3199999
-4482.0000000	3.3350000
-4481.0000000	3.3399999
-4480.0000000	3.3350000
-4479.0000000	3.3249998
-4478.0000000	3.2950001
-4477.0000000	3.2900000
-4476.0000000	3.2566669
-4475.0000000	3.2449999
-4474.0000000	3.2550001
-4473.0000000	3.2400000
-4472.0000000	3.2550001
-4471.0000000	3.2849998

-4470.0000000	3.3049998
-4469.0000000	3.3249998
-4468.0000000	3.3550000
-4467.0000000	3.3850002
-4466.0000000	3.4000001
-4465.0000000	3.4050002
-4464.0000000	3.4250002
-4463.0000000	3.4549999
-4462.0000000	3.4700000
-4461.0000000	3.5100000
-4460.0000000	3.5349998
-4459.0000000	3.5599999
-4458.0000000	3.5749998
-4457.0000000	3.5799999
-4456.0000000	3.5899999
-4455.0000000	3.5850000
-4454.0000000	3.5799999
-4453.0000000	3.5850000
-4452.0000000	3.5599999
-4451.0000000	3.5699999
-4450.0000000	3.5500000
-4449.0000000	3.5349998
-4448.0000000	3.5149999
-4447.0000000	3.4966667
-4446.0000000	3.4850001
-4445.0000000	3.4850001
-4444.0000000	3.4949999
-4443.0000000	3.5149999
-4442.0000000	3.5050001
-4441.0000000	3.5100000
-4440.0000000	3.5300000
-4439.0000000	3.5349998
-4438.0000000	3.5400000
-4437.0000000	3.5549998
-4436.0000000	3.5699999
-4435.0000000	3.5999999
-4434.0000000	3.6050000
-4433.0000000	3.6250000
-4432.0000000	3.6350002
-4431.0000000	3.6350002
-4430.0000000	3.6250000
-4429.0000000	3.5999999
-4428.0000000	3.5799999
-4427.0000000	3.5400000
-4426.0000000	3.4749999
-4425.0000000	3.4050002
-4424.0000000	3.3750000
-4423.0000000	3.3449998
-4422.0000000	3.3150001
-4421.0000000	3.2950001
-4420.0000000	3.2550001
-4419.0000000	3.2200000
-4418.0000000	3.1833334
-4417.0000000	3.1650000
-4416.0000000	3.1500001
-4415.0000000	3.1450000
-4414.0000000	3.1450000

-4413.0000000	3.1450000
-4412.0000000	3.1450000
-4411.0000000	3.1550002
-4410.0000000	3.1600001
-4409.0000000	3.1650000
-4408.0000000	3.1650000
-4407.0000000	3.1550002
-4406.0000000	3.1199999
-4405.0000000	3.0899999
-4404.0000000	3.0400000
-4403.0000000	2.9749999
-4402.0000000	2.9300001
-4401.0000000	2.8899999
-4400.0000000	2.8249998
-4399.0000000	2.8099999
-4398.0000000	2.8249998
-4397.0000000	2.8399999
-4396.0000000	2.8850000
-4395.0000000	2.9150000
-4394.0000000	2.9549999
-4393.0000000	3.0050001
-4392.0000000	3.0699999
-4391.0000000	3.1300001
-4390.0000000	3.1800001
-4389.0000000	3.2300000
-4388.0000000	3.2400000
-4387.0000000	3.2600000
-4386.0000000	3.2600000
-4385.0000000	3.2600000
-4384.0000000	3.3000000
-4383.0000000	3.3550000
-4382.0000000	3.3899999
-4381.0000000	3.4250002
-4380.0000000	3.4749999
-4379.0000000	3.5000000
-4378.0000000	3.5450001
-4377.0000000	3.5699999
-4376.0000000	3.6250000
-4375.0000000	3.6550002
-4374.0000000	3.6700001
-4373.0000000	3.6700001
-4372.0000000	3.6250000
-4371.0000000	3.5599999
-4370.0000000	3.4900000
-4369.0000000	3.4800000
-4368.0000000	3.5000000
-4367.0000000	3.5349998
-4366.0000000	3.5400000
-4365.0000000	3.5549998
-4364.0000000	3.5899999
-4363.0000000	3.5999999
-4362.0000000	3.5699999
-4361.0000000	3.5250001
-4360.0000000	3.4749999
-4359.0000000	3.4400001
-4358.0000000	3.3850002
-4357.0000000	3.3399999

-4356.0000000	3.2449999
-4355.0000000	3.1550002
-4354.0000000	3.0899999
-4353.0000000	3.0599999
-4352.0000000	3.0349998
-4351.0000000	3.0100000
-4350.0000000	3.0149999
-4349.0000000	3.0300000
-4348.0000000	3.0250001
-4347.0000000	3.0349998
-4346.0000000	3.0749998
-4345.0000000	3.1150000
-4344.0000000	3.1400001
-4343.0000000	3.1750002
-4342.0000000	3.2000000
-4341.0000000	3.2100000
-4340.0000000	3.2300000
-4339.0000000	3.2550001
-4338.0000000	3.2750001
-4337.0000000	3.2900000
-4336.0000000	3.3099999
-4335.0000000	3.3049998
-4334.0000000	3.3199999
-4333.0000000	3.3150001
-4332.0000000	3.2849998
-4331.0000000	3.2649999
-4330.0000000	3.2600000
-4329.0000000	3.2700000
-4328.0000000	3.2849998
-4327.0000000	3.2800000
-4326.0000000	3.2900000
-4325.0000000	3.2866666
-4324.0000000	3.3049998
-4323.0000000	3.3449998
-4322.0000000	3.3599999
-4321.0000000	3.3750000
-4320.0000000	3.3900001
-4319.0000000	3.3900001
-4318.0000000	3.3599999
-4317.0000000	3.3449998
-4316.0000000	3.3049998
-4315.0000000	3.3099999
-4314.0000000	3.3150001
-4313.0000000	3.3249998
-4312.0000000	3.3399999
-4311.0000000	3.3550000
-4310.0000000	3.3750000
-4309.0000000	3.3850002
-4308.0000000	3.3950000
-4307.0000000	3.4100001
-4306.0000000	3.4200001
-4305.0000000	3.4400001
-4304.0000000	3.4400001
-4303.0000000	3.4600000
-4302.0000000	3.4749999
-4301.0000000	3.5050001
-4300.0000000	3.5149999

-4299.0000000	3.5200000
-4298.0000000	3.5149999
-4297.0000000	3.5149999
-4296.0000000	3.5066669
-4295.0000000	3.4850001
-4294.0000000	3.4650002
-4293.0000000	3.4500000
-4292.0000000	3.4400001
-4291.0000000	3.4100001
-4290.0000000	3.3399999
-4289.0000000	3.2400000
-4288.0000000	3.1650000
-4287.0000000	3.1350002
-4286.0000000	3.1350002
-4285.0000000	3.0850000
-4284.0000000	3.0300000
-4283.0000000	2.9900000
-4282.0000000	2.9850001
-4281.0000000	2.9949999
-4280.0000000	3.0100000
-4279.0000000	3.0000000
-4278.0000000	2.9900000
-4277.0000000	2.9800000
-4276.0000000	2.9900000
-4275.0000000	3.0149999
-4274.0000000	3.0549998
-4273.0000000	3.0899999
-4272.0000000	3.1149998
-4271.0000000	3.1149998
-4270.0000000	3.1400001
-4269.0000000	3.1300001
-4268.0000000	3.0850000
-4267.0000000	3.0799999
-4266.0000000	3.0949998
-4265.0000000	3.1233332
-4264.0000000	3.1550002
-4263.0000000	3.1600001
-4262.0000000	3.1550002
-4261.0000000	3.1500001
-4260.0000000	3.1350002
-4259.0000000	3.1500001
-4258.0000000	3.1800001
-4257.0000000	3.2300000
-4256.0000000	3.2900000
-4255.0000000	3.3199999
-4254.0000000	3.3649998
-4253.0000000	3.4000001
-4252.0000000	3.4450002
-4251.0000000	3.4700000
-4250.0000000	3.4700000
-4249.0000000	3.4600000
-4248.0000000	3.4349999
-4247.0000000	3.3600001
-4246.0000000	3.3000000
-4245.0000000	3.2500000
-4244.0000000	3.1950002
-4243.0000000	3.1550002

-4242.0000000	3.1600001
-4241.0000000	3.1550002
-4240.0000000	3.1700001
-4239.0000000	3.1750002
-4238.0000000	3.1900001
-4237.0000000	3.1933334
-4236.0000000	3.2000000
-4235.0000000	3.2400000
-4234.0000000	3.2550001
-4233.0000000	3.2700000
-4232.0000000	3.2800000
-4231.0000000	3.3249998
-4230.0000000	3.3499999
-4229.0000000	3.3850002
-4228.0000000	3.3950000
-4227.0000000	3.3900001
-4226.0000000	3.3900001
-4225.0000000	3.3950000
-4224.0000000	3.4050002
-4223.0000000	3.4300001
-4222.0000000	3.4400001
-4221.0000000	3.4700000
-4220.0000000	3.4800000
-4219.0000000	3.5149999
-4218.0000000	3.5599999
-4217.0000000	3.6199999
-4216.0000000	3.6600001
-4215.0000000	3.6950002
-4214.0000000	3.7449999
-4213.0000000	3.7700000
-4212.0000000	3.7900000
-4211.0000000	3.8000000
-4210.0000000	3.7900000
-4209.0000000	3.7900000
-4208.0000000	3.7700000
-4207.0000000	3.7750001
-4206.0000000	3.7950001
-4205.0000000	3.7833331
-4204.0000000	3.7550001
-4203.0000000	3.7600000
-4202.0000000	3.7750001
-4201.0000000	3.8099999
-4200.0000000	3.8550000
-4199.0000000	3.8950000
-4198.0000000	3.9250002
-4197.0000000	3.9400001
-4196.0000000	3.9500000
-4195.0000000	3.9600000
-4194.0000000	3.9950001
-4193.0000000	4.0250001
-4192.0000000	4.0550003
-4191.0000000	4.0799999
-4190.0000000	4.0950003
-4189.0000000	4.1099997
-4188.0000000	4.1199999
-4187.0000000	4.1450000
-4186.0000000	4.1549997

-4185.0000000	4.1799998
-4184.0000000	4.1949997
-4183.0000000	4.2100000
-4182.0000000	4.2349997
-4181.0000000	4.2900000
-4180.0000000	4.3400002
-4179.0000000	4.3750000
-4178.0000000	4.3999996
-4177.0000000	4.3999996
-4176.0000000	4.4150000
-4175.0000000	4.4099998
-4174.0000000	4.4150000
-4173.0000000	4.4150000
-4172.0000000	4.4300003
-4171.0000000	4.4499998
-4170.0000000	4.4499998
-4169.0000000	4.4449997
-4168.0000000	4.4349999
-4167.0000000	4.4049997
-4166.0000000	4.3899999
-4165.0000000	4.3800001
-4164.0000000	4.3400002
-4163.0000000	4.3150001
-4162.0000000	4.3000002
-4161.0000000	4.2750001
-4160.0000000	4.2700000
-4159.0000000	4.2399998
-4158.0000000	4.2399998
-4157.0000000	4.2449999
-4156.0000000	4.2500000
-4155.0000000	4.2750001
-4154.0000000	4.2950001
-4153.0000000	4.3099999
-4152.0000000	4.3249998
-4151.0000000	4.3299999
-4150.0000000	4.3450003
-4149.0000000	4.3550000
-4148.0000000	4.3600001
-4147.0000000	4.3800001
-4146.0000000	4.3850002
-4145.0000000	4.4000001
-4144.0000000	4.4049997
-4143.0000000	4.4200001
-4142.0000000	4.4300003
-4141.0000000	4.4250002
-4140.0000000	4.4349999
-4139.0000000	4.4349999
-4138.0000000	4.4200001
-4137.0000000	4.4200001
-4136.0000000	4.3999996
-4135.0000000	4.3649998
-4134.0000000	4.2849998
-4133.0000000	4.2299995
-4132.0000000	4.1900001
-4131.0000000	4.1650000
-4130.0000000	4.1450000
-4129.0000000	4.1650000

-4128.0000000	4.1650000
-4127.0000000	4.1700001
-4126.0000000	4.1750002
-4125.0000000	4.1700001
-4124.0000000	4.1599998
-4123.0000000	4.1399999
-4122.0000000	4.1499996
-4121.0000000	4.1499996
-4120.0000000	4.1650000
-4119.0000000	4.1650000
-4118.0000000	4.1650000
-4117.0000000	4.1650000
-4116.0000000	4.1833329
-4115.0000000	4.1800003
-4114.0000000	4.1999998
-4113.0000000	4.2100000
-4112.0000000	4.2100000
-4111.0000000	4.2100000
-4110.0000000	4.2199998
-4109.0000000	4.2249999
-4108.0000000	4.2100000
-4107.0000000	4.1949997
-4106.0000000	4.1799998
-4105.0000000	4.1700001
-4104.0000000	4.1300001
-4103.0000000	4.1050000
-4102.0000000	4.0650001
-4101.0000000	4.0300002
-4100.0000000	3.9900000
-4099.0000000	3.9650002
-4098.0000000	3.9549999
-4097.0000000	3.9300001
-4096.0000000	3.9400001
-4095.0000000	3.9349999
-4094.0000000	3.9200001
-4093.0000000	3.9300001
-4092.0000000	3.9349999
-4091.0000000	3.9400001
-4090.0000000	3.9400001
-4089.0000000	3.9500000
-4088.0000000	3.9549999
-4087.0000000	3.9700000
-4086.0000000	3.9749999
-4085.0000000	4.0000000
-4084.0000000	4.0499997
-4083.0000000	4.1050000
-4082.0000000	4.1300001
-4081.0000000	4.1500001
-4080.0000000	4.1700001
-4079.0000000	4.2100000
-4078.0000000	4.2550001
-4077.0000000	4.2849998
-4076.0000000	4.3299999
-4075.0000000	4.3550000
-4074.0000000	4.3550000
-4073.0000000	4.3400002
-4072.0000000	4.2950001

-4071.0000000	4.2399998
-4070.0000000	4.2200003
-4069.0000000	4.1849999
-4068.0000000	4.1650000
-4067.0000000	4.1700001
-4066.0000000	4.1750002
-4065.0000000	4.1949997
-4064.0000000	4.2150002
-4063.0000000	4.2449999
-4062.0000000	4.2750001
-4061.0000000	4.2799997
-4060.0000000	4.2849998
-4059.0000000	4.2750001
-4058.0000000	4.2650003
-4057.0000000	4.2299995
-4056.0000000	4.1750002
-4055.0000000	4.1549997
-4054.0000000	4.1250000
-4053.0000000	4.0666666
-4052.0000000	3.9800000
-4051.0000000	3.9450002
-4050.0000000	3.9200001
-4049.0000000	3.9000001
-4048.0000000	3.9100001
-4047.0000000	3.9500000
-4046.0000000	4.0050001
-4045.0000000	4.0150003
-4044.0000000	4.0450001
-4043.0000000	4.0799999
-4042.0000000	4.1199999
-4041.0000000	4.1450000
-4040.0000000	4.1499996
-4039.0000000	4.1650000
-4038.0000000	4.1750002
-4037.0000000	4.1999998
-4036.0000000	4.2199998
-4035.0000000	4.2200003
-4034.0000000	4.1949997
-4033.0000000	4.1650000
-4032.0000000	4.1250000
-4031.0000000	4.0900002
-4030.0000000	4.0550003
-4029.0000000	4.0200000
-4028.0000000	3.9850001
-4027.0000000	3.9749999
-4026.0000000	3.9450002
-4025.0000000	3.9100001
-4024.0000000	3.9050002
-4023.0000000	3.9050002
-4022.0000000	3.9000001
-4021.0000000	3.8850002
-4020.0000000	3.8850002
-4019.0000000	3.9000001
-4018.0000000	3.8900001
-4017.0000000	3.8950000
-4016.0000000	3.8850002
-4015.0000000	3.8750000

-4014.0000000	3.8750000
-4013.0000000	3.8750000
-4012.0000000	3.8750000
-4011.0000000	3.8800001
-4010.0000000	3.8850002
-4009.0000000	3.9000001
-4008.0000000	3.9100001
-4007.0000000	3.8950000
-4006.0000000	3.8950000
-4005.0000000	3.8900001
-4004.0000000	3.8650000
-4003.0000000	3.8150001
-4002.0000000	3.7800000
-4001.0000000	3.7649999
-4000.0000000	3.7400000
-3999.0000000	3.7449999
-3998.0000000	3.7600000
-3997.0000000	3.7750001
-3996.0000000	3.7833331
-3995.0000000	3.8199999
-3994.0000000	3.8299999
-3993.0000000	3.8249998
-3992.0000000	3.8099999
-3991.0000000	3.7800000
-3990.0000000	3.7800000
-3989.0000000	3.7600000
-3988.0000000	3.7750001
-3987.0000000	3.7900000
-3986.0000000	3.8000000
-3985.0000000	3.8150001
-3984.0000000	3.8249998
-3983.0000000	3.8099999
-3982.0000000	3.8150001
-3981.0000000	3.8049998
-3980.0000000	3.8049998
-3979.0000000	3.7700000
-3978.0000000	3.7300000
-3977.0000000	3.7000000
-3976.0000000	3.6650000
-3975.0000000	3.6500001
-3974.0000000	3.6400001
-3973.0000000	3.6550002
-3972.0000000	3.6650000
-3971.0000000	3.6700001
-3970.0000000	3.6900001
-3969.0000000	3.7049999
-3968.0000000	3.7049999
-3967.0000000	3.7300000
-3966.0000000	3.7550001
-3965.0000000	3.7750001
-3964.0000000	3.8199999
-3963.0000000	3.8449998
-3962.0000000	3.8550000
-3961.0000000	3.8800001
-3960.0000000	3.8800001
-3959.0000000	3.8599999
-3958.0000000	3.8199999

-3957.0000000	3.8099999
-3956.0000000	3.7750001
-3955.0000000	3.7350001
-3954.0000000	3.6950002
-3953.0000000	3.6600001
-3952.0000000	3.6099999
-3951.0000000	3.5400000
-3950.0000000	3.4900000
-3949.0000000	3.4200001
-3948.0000000	3.3499999
-3947.0000000	3.3049998
-3946.0000000	3.2200000
-3945.0000000	3.1300001
-3944.0000000	3.0500000
-3943.0000000	2.9749999
-3942.0000000	2.9250002
-3941.0000000	2.9050002
-3940.0000000	2.8750000
-3939.0000000	2.8750000
-3938.0000000	2.9100001
-3937.0000000	2.9749999
-3936.0000000	3.0349998
-3935.0000000	3.0749998
-3934.0000000	3.1066666
-3933.0000000	3.1199999
-3932.0000000	3.1350002
-3931.0000000	3.1450000
-3930.0000000	3.1500001
-3929.0000000	3.1700001
-3928.0000000	3.2200000
-3927.0000000	3.2700000
-3926.0000000	3.2849998
-3925.0000000	3.3099999
-3924.0000000	3.3399999
-3923.0000000	3.3599999
-3922.0000000	3.3699999
-3921.0000000	3.3599999
-3920.0000000	3.3350000
-3919.0000000	3.3049998
-3918.0000000	3.2900000
-3917.0000000	3.2800000
-3916.0000000	3.2950001
-3915.0000000	3.3049998
-3914.0000000	3.3350000
-3913.0000000	3.3599999
-3912.0000000	3.3950000
-3911.0000000	3.4300001
-3910.0000000	3.4800000
-3909.0000000	3.5149999
-3908.0000000	3.5349998
-3907.0000000	3.5650001
-3906.0000000	3.5799999
-3905.0000000	3.5949998
-3904.0000000	3.5999999
-3903.0000000	3.6066666
-3902.0000000	3.6050000
-3901.0000000	3.6099999

-3900.0000000	3.6050000
-3899.0000000	3.6099999
-3898.0000000	3.6250000
-3897.0000000	3.6350002
-3896.0000000	3.6199999
-3895.0000000	3.6050000
-3894.0000000	3.6050000
-3893.0000000	3.6250000
-3892.0000000	3.6350002
-3891.0000000	3.6400001
-3890.0000000	3.6550002
-3889.0000000	3.6750002
-3888.0000000	3.6600001
-3887.0000000	3.6550002
-3886.0000000	3.6500001
-3885.0000000	3.6350002
-3884.0000000	3.6250000
-3883.0000000	3.6099999
-3882.0000000	3.6199999
-3881.0000000	3.6149998
-3880.0000000	3.5999999
-3879.0000000	3.5799999
-3878.0000000	3.5599999
-3877.0000000	3.5300000
-3876.0000000	3.4949999
-3875.0000000	3.4800000
-3874.0000000	3.4233334
-3873.0000000	3.3800001
-3872.0000000	3.3649998
-3871.0000000	3.3399999
-3870.0000000	3.3449998
-3869.0000000	3.3449998
-3868.0000000	3.3350000
-3867.0000000	3.2950001
-3866.0000000	3.2750001
-3865.0000000	3.2449999
-3864.0000000	3.2300000
-3863.0000000	3.2300000
-3862.0000000	3.2049999
-3861.0000000	3.1900001
-3860.0000000	3.1849999
-3859.0000000	3.1750002
-3858.0000000	3.1750002
-3857.0000000	3.1950002
-3856.0000000	3.2100000
-3855.0000000	3.2100000
-3854.0000000	3.2000000
-3853.0000000	3.2049999
-3852.0000000	3.2100000
-3851.0000000	3.2200000
-3850.0000000	3.1900001
-3849.0000000	3.1399999
-3848.0000000	3.1099999
-3847.0000000	3.1199999
-3846.0000000	3.1250000
-3845.0000000	3.1099999
-3844.0000000	3.0999999

-3843.0000000	3.0749998
-3842.0000000	3.0333335
-3841.0000000	2.9700000
-3840.0000000	2.9150000
-3839.0000000	2.9200001
-3838.0000000	2.9549999
-3837.0000000	2.9650002
-3836.0000000	2.9749999
-3835.0000000	3.0100000
-3834.0000000	3.0699999
-3833.0000000	3.1300001
-3832.0000000	3.1700001
-3831.0000000	3.2150002
-3830.0000000	3.2500000
-3829.0000000	3.2649999
-3828.0000000	3.2800000
-3827.0000000	3.3099999
-3826.0000000	3.3249998
-3825.0000000	3.3449998
-3824.0000000	3.3550000
-3823.0000000	3.3750000
-3822.0000000	3.3649998
-3821.0000000	3.3550000
-3820.0000000	3.3399999
-3819.0000000	3.3199999
-3818.0000000	3.3150001
-3817.0000000	3.3249998
-3816.0000000	3.3249998
-3815.0000000	3.3299999
-3814.0000000	3.3299999
-3813.0000000	3.3350000
-3812.0000000	3.3433332
-3811.0000000	3.3399999
-3810.0000000	3.3350000
-3809.0000000	3.3249998
-3808.0000000	3.3049998
-3807.0000000	3.2849998
-3806.0000000	3.2649999
-3805.0000000	3.2500000
-3804.0000000	3.2400000
-3803.0000000	3.2200000
-3802.0000000	3.1800001
-3801.0000000	3.1350002
-3800.0000000	3.0949998
-3799.0000000	3.0400000
-3798.0000000	2.9800000
-3797.0000000	2.9300001
-3796.0000000	2.8899999
-3795.0000000	2.8449998
-3794.0000000	2.8299999
-3793.0000000	2.8000000
-3792.0000000	2.7750001
-3791.0000000	2.7350001
-3790.0000000	2.6900001
-3789.0000000	2.6399999
-3788.0000000	2.5799999
-3787.0000000	2.5250001

-3786.0000000	2.4900000
-3785.0000000	2.4650002
-3784.0000000	2.4700000
-3783.0000000	2.4800000
-3782.0000000	2.4833333
-3781.0000000	2.5200000
-3780.0000000	2.5799999
-3779.0000000	2.6350000
-3778.0000000	2.6800001
-3777.0000000	2.6950002
-3776.0000000	2.7100000
-3775.0000000	2.7300000
-3774.0000000	2.7550001
-3773.0000000	2.7800000
-3772.0000000	2.8049998
-3771.0000000	2.8150001
-3770.0000000	2.8249998
-3769.0000000	2.8350000
-3768.0000000	2.8550000
-3767.0000000	2.8649998
-3766.0000000	2.8750000
-3765.0000000	2.8850002
-3764.0000000	2.8850002
-3763.0000000	2.8850002
-3762.0000000	2.8900001
-3761.0000000	2.8950000
-3760.0000000	2.9000001
-3759.0000000	2.9200001
-3758.0000000	2.9349999
-3757.0000000	2.9700000
-3756.0000000	2.9900000
-3755.0000000	3.0100000
-3754.0000000	3.0300000
-3753.0000000	3.0349998
-3752.0000000	3.0549998
-3751.0000000	3.0966666
-3750.0000000	3.1400001
-3749.0000000	3.1500001
-3748.0000000	3.1500001
-3747.0000000	3.1650000
-3746.0000000	3.1900001
-3745.0000000	3.2150002
-3744.0000000	3.2400000
-3743.0000000	3.2649999
-3742.0000000	3.2800000
-3741.0000000	3.3049998
-3740.0000000	3.3199999
-3739.0000000	3.3350000
-3738.0000000	3.3499999
-3737.0000000	3.3599999
-3736.0000000	3.3599999
-3735.0000000	3.3599999
-3734.0000000	3.3449998
-3733.0000000	3.3399999
-3732.0000000	3.3399999
-3731.0000000	3.3350000
-3730.0000000	3.3249998

-3729.0000000	3.3249998
-3728.0000000	3.3150001
-3727.0000000	3.2900000
-3726.0000000	3.2600000
-3725.0000000	3.2100000
-3724.0000000	3.1849999
-3723.0000000	3.2049999
-3722.0000000	3.2049999
-3721.0000000	3.2300003
-3720.0000000	3.2449999
-3719.0000000	3.2400000
-3718.0000000	3.2200000
-3717.0000000	3.2249999
-3716.0000000	3.2449999
-3715.0000000	3.2750001
-3714.0000000	3.2849998
-3713.0000000	3.2700000
-3712.0000000	3.2700000
-3711.0000000	3.2900000
-3710.0000000	3.3249998
-3709.0000000	3.3350000
-3708.0000000	3.3599999
-3707.0000000	3.3800001
-3706.0000000	3.4100001
-3705.0000000	3.4250002
-3704.0000000	3.4549999
-3703.0000000	3.4900000
-3702.0000000	3.5100000
-3701.0000000	3.5349998
-3700.0000000	3.5549998
-3699.0000000	3.5749998
-3698.0000000	3.5899999
-3697.0000000	3.5999999
-3696.0000000	3.5999999
-3695.0000000	3.6149998
-3694.0000000	3.6300001
-3693.0000000	3.6600001
-3692.0000000	3.6849999
-3691.0000000	3.7133334
-3690.0000000	3.7550001
-3689.0000000	3.7800000
-3688.0000000	3.7900000
-3687.0000000	3.8049998
-3686.0000000	3.8249998
-3685.0000000	3.8399999
-3684.0000000	3.8649998
-3683.0000000	3.8850002
-3682.0000000	3.9050002
-3681.0000000	3.9150000
-3680.0000000	3.9200001
-3679.0000000	3.9000001
-3678.0000000	3.9000001
-3677.0000000	3.9000001
-3676.0000000	3.8950000
-3675.0000000	3.8650000
-3674.0000000	3.8299999
-3673.0000000	3.7849998

-3672.0000000	3.7500000
-3671.0000000	3.7150002
-3670.0000000	3.6849999
-3669.0000000	3.6199999
-3668.0000000	3.6050000
-3667.0000000	3.5899999
-3666.0000000	3.5850000
-3665.0000000	3.5799999
-3664.0000000	3.5799999
-3663.0000000	3.5650001
-3662.0000000	3.5650001
-3661.0000000	3.5500000
-3660.0000000	3.5566666
-3659.0000000	3.5450001
-3658.0000000	3.5149999
-3657.0000000	3.4800000
-3656.0000000	3.4300001
-3655.0000000	3.4050002
-3654.0000000	3.3750000
-3653.0000000	3.3649998
-3652.0000000	3.3499999
-3651.0000000	3.3249998
-3650.0000000	3.3150001
-3649.0000000	3.3099999
-3648.0000000	3.3199999
-3647.0000000	3.3199999
-3646.0000000	3.3350000
-3645.0000000	3.3399999
-3644.0000000	3.3550000
-3643.0000000	3.3399999
-3642.0000000	3.3599999
-3641.0000000	3.3750000
-3640.0000000	3.3750000
-3639.0000000	3.3750000
-3638.0000000	3.4000001
-3637.0000000	3.4300001
-3636.0000000	3.4400001
-3635.0000000	3.4600000
-3634.0000000	3.4900000
-3633.0000000	3.4949999
-3632.0000000	3.5149999
-3631.0000000	3.5149999
-3630.0000000	3.5000000
-3629.0000000	3.4650002
-3628.0000000	3.4150000
-3627.0000000	3.3699999
-3626.0000000	3.3499999
-3625.0000000	3.3150001
-3624.0000000	3.3099999
-3623.0000000	3.2950001
-3622.0000000	3.2900000
-3621.0000000	3.2900000
-3620.0000000	3.2950001
-3619.0000000	3.2849998
-3618.0000000	3.2800000
-3617.0000000	3.3099999
-3616.0000000	3.3249998

-3615.0000000	3.3350000
-3614.0000000	3.3499999
-3613.0000000	3.3449998
-3612.0000000	3.3299999
-3611.0000000	3.3399999
-3610.0000000	3.3199999
-3609.0000000	3.3049998
-3608.0000000	3.2800000
-3607.0000000	3.2700000
-3606.0000000	3.2649999
-3605.0000000	3.2550001
-3604.0000000	3.2400000
-3603.0000000	3.2249999
-3602.0000000	3.2049999
-3601.0000000	3.1766670
-3600.0000000	3.1350000
-3599.0000000	3.1050000
-3598.0000000	3.0699999
-3597.0000000	3.0300000
-3596.0000000	2.9949999
-3595.0000000	2.9600000
-3594.0000000	2.9100001
-3593.0000000	2.8599999
-3592.0000000	2.8449998
-3591.0000000	2.8299999
-3590.0000000	2.8550000
-3589.0000000	2.8800001
-3588.0000000	2.9150000
-3587.0000000	2.9549999
-3586.0000000	3.0000000
-3585.0000000	3.0400000
-3584.0000000	3.0749998
-3583.0000000	3.1150000
-3582.0000000	3.1500001
-3581.0000000	3.1900001
-3580.0000000	3.2249999
-3579.0000000	3.2600000
-3578.0000000	3.2900000
-3577.0000000	3.3099999
-3576.0000000	3.3399999
-3575.0000000	3.3599999
-3574.0000000	3.3950000
-3573.0000000	3.4200001
-3572.0000000	3.4349999
-3571.0000000	3.4633334
-3570.0000000	3.4749999
-3569.0000000	3.4850001
-3568.0000000	3.4800000
-3567.0000000	3.4800000
-3566.0000000	3.4800000
-3565.0000000	3.4749999
-3564.0000000	3.4749999
-3563.0000000	3.4800000
-3562.0000000	3.4850001
-3561.0000000	3.4850001
-3560.0000000	3.4749999
-3559.0000000	3.4749999

-3558.0000000	3.4650002
-3557.0000000	3.4549999
-3556.0000000	3.4450002
-3555.0000000	3.4450002
-3554.0000000	3.4300001
-3553.0000000	3.4150000
-3552.0000000	3.3950000
-3551.0000000	3.3699999
-3550.0000000	3.3350000
-3549.0000000	3.2800000
-3548.0000000	3.2249999
-3547.0000000	3.1700001
-3546.0000000	3.1600001
-3545.0000000	3.1650000
-3544.0000000	3.1750002
-3543.0000000	3.2000000
-3542.0000000	3.2000000
-3541.0000000	3.2150002
-3540.0000000	3.2100000
-3539.0000000	3.2200000
-3538.0000000	3.2249999
-3537.0000000	3.2150002
-3536.0000000	3.1950002
-3535.0000000	3.1950002
-3534.0000000	3.1950002
-3533.0000000	3.1950002
-3532.0000000	3.2100000
-3531.0000000	3.2000000
-3530.0000000	3.2100000
-3529.0000000	3.2100000
-3528.0000000	3.2400000
-3527.0000000	3.2550001
-3526.0000000	3.2649999
-3525.0000000	3.2600000
-3524.0000000	3.2649999
-3523.0000000	3.2750001
-3522.0000000	3.3049998
-3521.0000000	3.3199999
-3520.0000000	3.3299999
-3519.0000000	3.3449998
-3518.0000000	3.3750000
-3517.0000000	3.3850002
-3516.0000000	3.4100001
-3515.0000000	3.4349999
-3514.0000000	3.4500000
-3513.0000000	3.4650002
-3512.0000000	3.4500000
-3511.0000000	3.4450002
-3510.0000000	3.4450002
-3509.0000000	3.4666665
-3508.0000000	3.4900000
-3507.0000000	3.5050001
-3506.0000000	3.5149999
-3505.0000000	3.5200000
-3504.0000000	3.5300000
-3503.0000000	3.5450001
-3502.0000000	3.5699999

-3501.0000000	3.5850000
-3500.0000000	3.6050000
-3499.0000000	3.6050000
-3498.0000000	3.6099999
-3497.0000000	3.6199999
-3496.0000000	3.6300001
-3495.0000000	3.6350002
-3494.0000000	3.6400001
-3493.0000000	3.6350002
-3492.0000000	3.6300001
-3491.0000000	3.6300001
-3490.0000000	3.6400001
-3489.0000000	3.6450000
-3488.0000000	3.6450000
-3487.0000000	3.6350002
-3486.0000000	3.6150000
-3485.0000000	3.5699999
-3484.0000000	3.5349998
-3483.0000000	3.5050001
-3482.0000000	3.4533336
-3481.0000000	3.4250002
-3480.0000000	3.4349999
-3479.0000000	3.4549999
-3478.0000000	3.4500000
-3477.0000000	3.4600000
-3476.0000000	3.4650002
-3475.0000000	3.4800000
-3474.0000000	3.4900000
-3473.0000000	3.4900000
-3472.0000000	3.4900000
-3471.0000000	3.4949999
-3470.0000000	3.5050001
-3469.0000000	3.5100000
-3468.0000000	3.5100000
-3467.0000000	3.5149999
-3466.0000000	3.5149999
-3465.0000000	3.5149999
-3464.0000000	3.5250001
-3463.0000000	3.5250001
-3462.0000000	3.5149999
-3461.0000000	3.5050001
-3460.0000000	3.4800000
-3459.0000000	3.4450002
-3458.0000000	3.4100001
-3457.0000000	3.3650000
-3456.0000000	3.3150001
-3455.0000000	3.2550001
-3454.0000000	3.2449999
-3453.0000000	3.2350001
-3452.0000000	3.2100000
-3451.0000000	3.2300000
-3450.0000000	3.2350001
-3449.0000000	3.2600000
-3448.0000000	3.2800000
-3447.0000000	3.3000000
-3446.0000000	3.3299999
-3445.0000000	3.3649998

-3444.0000000	3.3850002
-3443.0000000	3.4100001
-3442.0000000	3.4200001
-3441.0000000	3.4100001
-3440.0000000	3.3950000
-3439.0000000	3.4000001
-3438.0000000	3.3900001
-3437.0000000	3.4000001
-3436.0000000	3.4150000
-3435.0000000	3.4150000
-3434.0000000	3.4200001
-3433.0000000	3.4250002
-3432.0000000	3.4000001
-3431.0000000	3.4150000
-3430.0000000	3.4000001
-3429.0000000	3.4000001
-3428.0000000	3.4100001
-3427.0000000	3.4050002
-3426.0000000	3.4050002
-3425.0000000	3.4150000
-3424.0000000	3.4100001
-3423.0000000	3.4150000
-3422.0000000	3.4050002
-3421.0000000	3.4150000
-3420.0000000	3.4333334
-3419.0000000	3.4349999
-3418.0000000	3.4349999
-3417.0000000	3.4100001
-3416.0000000	3.3800001
-3415.0000000	3.3249998
-3414.0000000	3.3099999
-3413.0000000	3.2950001
-3412.0000000	3.2950001
-3411.0000000	3.3049998
-3410.0000000	3.3049998
-3409.0000000	3.3150001
-3408.0000000	3.3299999
-3407.0000000	3.3499999
-3406.0000000	3.3499999
-3405.0000000	3.3649998
-3404.0000000	3.3750000
-3403.0000000	3.3900001
-3402.0000000	3.3950000
-3401.0000000	3.4100001
-3400.0000000	3.4200001
-3399.0000000	3.4250002
-3398.0000000	3.4250002
-3397.0000000	3.4400001
-3396.0000000	3.4450002
-3395.0000000	3.4400001
-3394.0000000	3.4450002
-3393.0000000	3.4549999
-3392.0000000	3.4450002
-3391.0000000	3.4333334
-3390.0000000	3.3950000
-3389.0000000	3.3550000
-3388.0000000	3.3199999

-3387.0000000	3.3000000
-3386.0000000	3.2700000
-3385.0000000	3.2400000
-3384.0000000	3.2150002
-3383.0000000	3.2049999
-3382.0000000	3.1800001
-3381.0000000	3.1550002
-3380.0000000	3.1149998
-3379.0000000	3.0650001
-3378.0000000	3.0000000
-3377.0000000	2.9200001
-3376.0000000	2.8499999
-3375.0000000	2.7950001
-3374.0000000	2.7500000
-3373.0000000	2.7150002
-3372.0000000	2.6800001
-3371.0000000	2.6800001
-3370.0000000	2.6849999
-3369.0000000	2.6800001
-3368.0000000	2.6700001
-3367.0000000	2.6400001
-3366.0000000	2.6500001
-3365.0000000	2.6350002
-3364.0000000	2.6050000
-3363.0000000	2.5699999
-3362.0000000	2.5349998
-3361.0000000	2.5100000
-3360.0000000	2.4500000
-3359.0000000	2.4150000
-3358.0000000	2.3699999
-3357.0000000	2.3099999
-3356.0000000	2.2900000
-3355.0000000	2.2849998
-3354.0000000	2.2550001
-3353.0000000	2.2249999
-3352.0000000	2.2000000
-3351.0000000	2.1849999
-3350.0000000	2.1800001
-3349.0000000	2.1849999
-3348.0000000	2.1950002
-3347.0000000	2.2049999
-3346.0000000	2.2049999
-3345.0000000	2.2449999
-3344.0000000	2.2800000
-3343.0000000	2.3150001
-3342.0000000	2.3599999
-3341.0000000	2.3900001
-3340.0000000	2.4150000
-3339.0000000	2.4400001
-3338.0000000	2.4650002
-3337.0000000	2.5050001
-3336.0000000	2.5149999
-3335.0000000	2.5450001
-3334.0000000	2.5549998
-3333.0000000	2.5899999
-3332.0000000	2.6149998
-3331.0000000	2.6550002

-3330.0000000	2.6966667
-3329.0000000	2.7449999
-3328.0000000	2.7900000
-3327.0000000	2.8249998
-3326.0000000	2.8499999
-3325.0000000	2.8900001
-3324.0000000	2.9100001
-3323.0000000	2.9300001
-3322.0000000	2.9300001
-3321.0000000	2.9400001
-3320.0000000	2.9549999
-3319.0000000	2.9450002
-3318.0000000	2.9250002
-3317.0000000	2.9250002
-3316.0000000	2.8950000
-3315.0000000	2.8649998
-3314.0000000	2.8499999
-3313.0000000	2.8449998
-3312.0000000	2.8599999
-3311.0000000	2.8649998
-3310.0000000	2.8699999
-3309.0000000	2.8900001
-3308.0000000	2.8900001
-3307.0000000	2.9250002
-3306.0000000	2.9450002
-3305.0000000	2.9650002
-3304.0000000	2.9850001
-3303.0000000	3.0100000
-3302.0000000	3.0300000
-3301.0000000	3.0466669
-3300.0000000	3.0699999
-3299.0000000	3.0749998
-3298.0000000	3.0899999
-3297.0000000	3.1050000
-3296.0000000	3.1250000
-3295.0000000	3.1450000
-3294.0000000	3.1650000
-3293.0000000	3.1650000
-3292.0000000	3.1750002
-3291.0000000	3.2049999
-3290.0000000	3.2449999
-3289.0000000	3.2649999
-3288.0000000	3.2800000
-3287.0000000	3.3049998
-3286.0000000	3.3199999
-3285.0000000	3.3199999
-3284.0000000	3.3299999
-3283.0000000	3.3299999
-3282.0000000	3.3249998
-3281.0000000	3.3150001
-3280.0000000	3.3000000
-3279.0000000	3.2849998
-3278.0000000	3.2700000
-3277.0000000	3.2400000
-3276.0000000	3.2049999
-3275.0000000	3.1750002
-3274.0000000	3.1500001

-3273.0000000	3.1450000
-3272.0000000	3.1250000
-3271.0000000	3.1250000
-3270.0000000	3.1466668
-3269.0000000	3.1500001
-3268.0000000	3.1650000
-3267.0000000	3.1900001
-3266.0000000	3.2000000
-3265.0000000	3.2150002
-3264.0000000	3.2300000
-3263.0000000	3.2350001
-3262.0000000	3.2400000
-3261.0000000	3.2550001
-3260.0000000	3.2700000
-3259.0000000	3.2950001
-3258.0000000	3.2750001
-3257.0000000	3.2550001
-3256.0000000	3.2350001
-3255.0000000	3.2049999
-3254.0000000	3.1550002
-3253.0000000	3.0999999
-3252.0000000	3.0200000
-3251.0000000	2.9500000
-3250.0000000	2.9150000
-3249.0000000	2.8699999
-3248.0000000	2.8499999
-3247.0000000	2.8199999
-3246.0000000	2.7750001
-3245.0000000	2.7200000
-3244.0000000	2.6650000
-3243.0000000	2.6450000
-3242.0000000	2.6550002
-3241.0000000	2.6500001
-3240.0000000	2.6300001
-3239.0000000	2.6300001
-3238.0000000	2.6350002
-3237.0000000	2.6500001
-3236.0000000	2.6650000
-3235.0000000	2.6950002
-3234.0000000	2.7000000
-3233.0000000	2.7049999
-3232.0000000	2.7000000
-3231.0000000	2.7100000
-3230.0000000	2.7400000
-3229.0000000	2.7350001
-3228.0000000	2.7200000
-3227.0000000	2.7150002
-3226.0000000	2.7100000
-3225.0000000	2.7100000
-3224.0000000	2.7200000
-3223.0000000	2.7449999
-3222.0000000	2.7600000
-3221.0000000	2.8000000
-3220.0000000	2.8299999
-3219.0000000	2.8550000
-3218.0000000	2.8599999
-3217.0000000	2.8750000

-3216.0000000	2.8900001
-3215.0000000	2.9200001
-3214.0000000	2.9450002
-3213.0000000	2.9749999
-3212.0000000	3.0149999
-3211.0000000	3.0650001
-3210.0000000	3.1166668
-3209.0000000	3.1650000
-3208.0000000	3.1800001
-3207.0000000	3.1800001
-3206.0000000	3.1950002
-3205.0000000	3.2100000
-3204.0000000	3.2449999
-3203.0000000	3.2600000
-3202.0000000	3.2649999
-3201.0000000	3.2700000
-3200.0000000	3.2800000
-3199.0000000	3.2800000
-3198.0000000	3.2800000
-3197.0000000	3.2600000
-3196.0000000	3.2500000
-3195.0000000	3.2350001
-3194.0000000	3.2200000
-3193.0000000	3.2100000
-3192.0000000	3.2150002
-3191.0000000	3.2300000
-3190.0000000	3.2200000
-3189.0000000	3.2249999
-3188.0000000	3.2350001
-3187.0000000	3.2449999
-3186.0000000	3.2700000
-3185.0000000	3.2950001
-3184.0000000	3.3099999
-3183.0000000	3.3350000
-3182.0000000	3.3550000
-3181.0000000	3.3800001
-3180.0000000	3.4150000
-3179.0000000	3.4266670
-3178.0000000	3.4500000
-3177.0000000	3.4400001
-3176.0000000	3.4650002
-3175.0000000	3.4949999
-3174.0000000	3.5050001
-3173.0000000	3.5250001
-3172.0000000	3.5250001
-3171.0000000	3.5250001
-3170.0000000	3.5300000
-3169.0000000	3.5549998
-3168.0000000	3.5599999
-3167.0000000	3.5699999
-3166.0000000	3.5650001
-3165.0000000	3.5599999
-3164.0000000	3.5599999
-3163.0000000	3.5599999
-3162.0000000	3.5599999
-3161.0000000	3.5650001
-3160.0000000	3.5650001

-3159.0000000	3.5899999
-3158.0000000	3.5999999
-3157.0000000	3.5799999
-3156.0000000	3.5650001
-3155.0000000	3.5500000
-3154.0000000	3.5250001
-3153.0000000	3.5200000
-3152.0000000	3.5050001
-3151.0000000	3.4900000
-3150.0000000	3.4800000
-3149.0000000	3.4766667
-3148.0000000	3.4700000
-3147.0000000	3.4800000
-3146.0000000	3.4650002
-3145.0000000	3.4549999
-3144.0000000	3.4349999
-3143.0000000	3.4100001
-3142.0000000	3.4100001
-3141.0000000	3.3950000
-3140.0000000	3.3900001
-3139.0000000	3.4050002
-3138.0000000	3.4100001
-3137.0000000	3.4150000
-3136.0000000	3.4250002
-3135.0000000	3.4349999
-3134.0000000	3.4349999
-3133.0000000	3.4400001
-3132.0000000	3.4450002
-3131.0000000	3.4200001
-3130.0000000	3.3699999
-3129.0000000	3.3449998
-3128.0000000	3.2800000
-3127.0000000	3.2200000
-3126.0000000	3.1550002
-3125.0000000	3.0949998
-3124.0000000	3.0250001
-3123.0000000	2.9600000
-3122.0000000	2.8850002
-3121.0000000	2.8449998
-3120.0000000	2.8049998
-3119.0000000	2.7950001
-3118.0000000	2.7699997
-3117.0000000	2.7700000
-3116.0000000	2.7750001
-3115.0000000	2.7849998
-3114.0000000	2.8049998
-3113.0000000	2.8350000
-3112.0000000	2.8199999
-3111.0000000	2.8199999
-3110.0000000	2.8299999
-3109.0000000	2.8499999
-3108.0000000	2.8750000
-3107.0000000	2.9050002
-3106.0000000	2.9300001
-3105.0000000	2.9650002
-3104.0000000	3.0050001
-3103.0000000	3.0349998

-3102.0000000	3.0549998
-3101.0000000	3.0749998
-3100.0000000	3.0949998
-3099.0000000	3.1050000
-3098.0000000	3.1149998
-3097.0000000	3.1400001
-3096.0000000	3.1450000
-3095.0000000	3.1300001
-3094.0000000	3.1350002
-3093.0000000	3.1350002
-3092.0000000	3.1500001
-3091.0000000	3.1600001
-3090.0000000	3.1849999
-3089.0000000	3.2200000
-3088.0000000	3.2699997
-3087.0000000	3.3199999
-3086.0000000	3.3550000
-3085.0000000	3.3800001
-3084.0000000	3.4150000
-3083.0000000	3.4349999
-3082.0000000	3.4700000
-3081.0000000	3.5000000
-3080.0000000	3.5349998
-3079.0000000	3.5599999
-3078.0000000	3.5899999
-3077.0000000	3.6250000
-3076.0000000	3.6350002
-3075.0000000	3.6550002
-3074.0000000	3.6700001
-3073.0000000	3.6849999
-3072.0000000	3.6849999
-3071.0000000	3.7000000
-3070.0000000	3.7100000
-3069.0000000	3.7049999
-3068.0000000	3.6900001
-3067.0000000	3.6750002
-3066.0000000	3.6650000
-3065.0000000	3.6450000
-3064.0000000	3.6149998
-3063.0000000	3.5699999
-3062.0000000	3.5250001
-3061.0000000	3.4900000
-3060.0000000	3.4450002
-3059.0000000	3.4166667
-3058.0000000	3.4000001
-3057.0000000	3.3850002
-3056.0000000	3.3800001
-3055.0000000	3.3850002
-3054.0000000	3.3699999
-3053.0000000	3.3649998
-3052.0000000	3.3699999
-3051.0000000	3.3550000
-3050.0000000	3.3150001
-3049.0000000	3.2649999
-3048.0000000	3.2400000
-3047.0000000	3.2150002
-3046.0000000	3.1950002

-3045.0000000	3.2049999
-3044.0000000	3.2000000
-3043.0000000	3.2049999
-3042.0000000	3.2350001
-3041.0000000	3.2500000
-3040.0000000	3.2700000
-3039.0000000	3.2950001
-3038.0000000	3.3199999
-3037.0000000	3.3499999
-3036.0000000	3.3800001
-3035.0000000	3.3900001
-3034.0000000	3.4000001
-3033.0000000	3.4100001
-3032.0000000	3.4100001
-3031.0000000	3.4200001
-3030.0000000	3.4349999
-3029.0000000	3.4300001
-3028.0000000	3.4000003
-3027.0000000	3.3399999
-3026.0000000	3.3000000
-3025.0000000	3.2700000
-3024.0000000	3.2500000
-3023.0000000	3.2500000
-3022.0000000	3.2049999
-3021.0000000	3.1900001
-3020.0000000	3.1750002
-3019.0000000	3.1650000
-3018.0000000	3.1700001
-3017.0000000	3.1900001
-3016.0000000	3.2100000
-3015.0000000	3.2400000
-3014.0000000	3.2649999
-3013.0000000	3.2900000
-3012.0000000	3.3199999
-3011.0000000	3.3299999
-3010.0000000	3.3599999
-3009.0000000	3.3800001
-3008.0000000	3.3950000
-3007.0000000	3.4100001
-3006.0000000	3.4250002
-3005.0000000	3.4200001
-3004.0000000	3.4200001
-3003.0000000	3.4050002
-3002.0000000	3.4100001
-3001.0000000	3.4100001
-3000.0000000	3.4150000
-2999.0000000	3.4000001
-2998.0000000	3.4000001
-2997.0000000	3.3833332
-2996.0000000	3.3699999
-2995.0000000	3.3550000
-2994.0000000	3.3449998
-2993.0000000	3.3399999
-2992.0000000	3.3350000
-2991.0000000	3.3350000
-2990.0000000	3.3249998
-2989.0000000	3.3299999

-2988.0000000	3.3350000
-2987.0000000	3.3449998
-2986.0000000	3.3599999
-2985.0000000	3.3750000
-2984.0000000	3.4000001
-2983.0000000	3.4050002
-2982.0000000	3.4100001
-2981.0000000	3.4250002
-2980.0000000	3.4450002
-2979.0000000	3.4450002
-2978.0000000	3.4500000
-2977.0000000	3.4450002
-2976.0000000	3.4450002
-2975.0000000	3.4450002
-2974.0000000	3.4450002
-2973.0000000	3.4500000
-2972.0000000	3.4300001
-2971.0000000	3.4150000
-2970.0000000	3.4000001
-2969.0000000	3.4000001
-2968.0000000	3.3699999
-2967.0000000	3.3550000
-2966.0000000	3.3000000
-2965.0000000	3.2400000
-2964.0000000	3.1950002
-2963.0000000	3.1849999
-2962.0000000	3.1750002
-2961.0000000	3.1700001
-2960.0000000	3.1650000
-2959.0000000	3.1400001
-2958.0000000	3.0899999
-2957.0000000	3.0200000
-2956.0000000	2.9500000
-2955.0000000	2.8550000
-2954.0000000	2.7449999
-2953.0000000	2.6900001
-2952.0000000	2.6600001
-2951.0000000	2.6450000
-2950.0000000	2.6500001
-2949.0000000	2.6500001
-2948.0000000	2.6550002
-2947.0000000	2.6900001
-2946.0000000	2.7350001
-2945.0000000	2.7600000
-2944.0000000	2.7849998
-2943.0000000	2.8000000
-2942.0000000	2.8249998
-2941.0000000	2.8299999
-2940.0000000	2.8599999
-2939.0000000	2.8750000
-2938.0000000	2.8449998
-2937.0000000	2.8266666
-2936.0000000	2.8199999
-2935.0000000	2.8049998
-2934.0000000	2.7800000
-2933.0000000	2.7700000
-2932.0000000	2.7750001

-2931.0000000	2.7800000
-2930.0000000	2.7849998
-2929.0000000	2.7750001
-2928.0000000	2.7550001
-2927.0000000	2.7200000
-2926.0000000	2.6800001
-2925.0000000	2.6650000
-2924.0000000	2.6500001
-2923.0000000	2.6450000
-2922.0000000	2.6450000
-2921.0000000	2.6250000
-2920.0000000	2.6250000
-2919.0000000	2.6149998
-2918.0000000	2.5999999
-2917.0000000	2.5749998
-2916.0000000	2.5599999
-2915.0000000	2.5549998
-2914.0000000	2.5749998
-2913.0000000	2.5850000
-2912.0000000	2.5850000
-2911.0000000	2.5949998
-2910.0000000	2.5850000
-2909.0000000	2.5899999
-2908.0000000	2.5749998
-2907.0000000	2.5650001
-2906.0000000	2.5866666
-2905.0000000	2.5899999
-2904.0000000	2.5799999
-2903.0000000	2.5749998
-2902.0000000	2.5749998
-2901.0000000	2.5850000
-2900.0000000	2.5949998
-2899.0000000	2.5949998
-2898.0000000	2.6050000
-2897.0000000	2.6099999
-2896.0000000	2.6199999
-2895.0000000	2.6500001
-2894.0000000	2.6700001
-2893.0000000	2.6900001
-2892.0000000	2.7200000
-2891.0000000	2.7449999
-2890.0000000	2.7600000
-2889.0000000	2.7849998
-2888.0000000	2.7950001
-2887.0000000	2.8099999
-2886.0000000	2.8249998
-2885.0000000	2.8299999
-2884.0000000	2.8299999
-2883.0000000	2.8399999
-2882.0000000	2.8649998
-2881.0000000	2.8699999
-2880.0000000	2.8599999
-2879.0000000	2.8499999
-2878.0000000	2.8299999
-2877.0000000	2.8000000
-2876.0000000	2.7600002
-2875.0000000	2.7049999

-2874.0000000	2.6700001
-2873.0000000	2.6400001
-2872.0000000	2.6149998
-2871.0000000	2.5949998
-2870.0000000	2.5749998
-2869.0000000	2.5650001
-2868.0000000	2.5599999
-2867.0000000	2.5549998
-2866.0000000	2.5599999
-2865.0000000	2.5549998
-2864.0000000	2.5549998
-2863.0000000	2.5549998
-2862.0000000	2.5650001
-2861.0000000	2.5749998
-2860.0000000	2.5599999
-2859.0000000	2.5699999
-2858.0000000	2.5650001
-2857.0000000	2.5549998
-2856.0000000	2.5349998
-2855.0000000	2.5149999
-2854.0000000	2.4800000
-2853.0000000	2.4600000
-2852.0000000	2.4549999
-2851.0000000	2.4500000
-2850.0000000	2.4949999
-2849.0000000	2.5250001
-2848.0000000	2.5650001
-2847.0000000	2.5999999
-2846.0000000	2.6433334
-2845.0000000	2.6750002
-2844.0000000	2.7100000
-2843.0000000	2.7400000
-2842.0000000	2.7600000
-2841.0000000	2.7700000
-2840.0000000	2.7649999
-2839.0000000	2.7800000
-2838.0000000	2.7849998
-2837.0000000	2.7849998
-2836.0000000	2.7800000
-2835.0000000	2.7900000
-2834.0000000	2.7950001
-2833.0000000	2.7900000
-2832.0000000	2.7950001
-2831.0000000	2.7950001
-2830.0000000	2.7800000
-2829.0000000	2.7550001
-2828.0000000	2.7449999
-2827.0000000	2.7150002
-2826.0000000	2.7049999
-2825.0000000	2.6700001
-2824.0000000	2.6450000
-2823.0000000	2.6500001
-2822.0000000	2.6400001
-2821.0000000	2.6450000
-2820.0000000	2.6600001
-2819.0000000	2.6750002
-2818.0000000	2.6900001

-2817.0000000	2.7100000
-2816.0000000	2.7333333
-2815.0000000	2.7649999
-2814.0000000	2.7700000
-2813.0000000	2.7750001
-2812.0000000	2.7849998
-2811.0000000	2.7750001
-2810.0000000	2.7649999
-2809.0000000	2.7700000
-2808.0000000	2.7700000
-2807.0000000	2.7649999
-2806.0000000	2.7750001
-2805.0000000	2.7800000
-2804.0000000	2.7600000
-2803.0000000	2.7400000
-2802.0000000	2.7300000
-2801.0000000	2.7100000
-2800.0000000	2.7150002
-2799.0000000	2.7150002
-2798.0000000	2.7000000
-2797.0000000	2.6750002
-2796.0000000	2.6500001
-2795.0000000	2.6450000
-2794.0000000	2.6450000
-2793.0000000	2.6500001
-2792.0000000	2.6700001
-2791.0000000	2.6900001
-2790.0000000	2.6900001
-2789.0000000	2.6849999
-2788.0000000	2.6900001
-2787.0000000	2.6800001
-2786.0000000	2.6866665
-2785.0000000	2.7049999
-2784.0000000	2.7150002
-2783.0000000	2.7249999
-2782.0000000	2.7249999
-2781.0000000	2.7200000
-2780.0000000	2.7100000
-2779.0000000	2.7000000
-2778.0000000	2.6950002
-2777.0000000	2.6849999
-2776.0000000	2.6750002
-2775.0000000	2.6750002
-2774.0000000	2.6750002
-2773.0000000	2.6849999
-2772.0000000	2.6800001
-2771.0000000	2.6700001
-2770.0000000	2.6600001
-2769.0000000	2.6300001
-2768.0000000	2.6149998
-2767.0000000	2.5999999
-2766.0000000	2.5949998
-2765.0000000	2.5799999
-2764.0000000	2.5699999
-2763.0000000	2.5650001
-2762.0000000	2.5500000
-2761.0000000	2.5500000

-2760.0000000	2.5349998
-2759.0000000	2.5250001
-2758.0000000	2.5149999
-2757.0000000	2.5050001
-2756.0000000	2.4866667
-2755.0000000	2.4800000
-2754.0000000	2.4749999
-2753.0000000	2.4650002
-2752.0000000	2.4650002
-2751.0000000	2.4650002
-2750.0000000	2.4650002
-2749.0000000	2.4700000
-2748.0000000	2.4749999
-2747.0000000	2.4850001
-2746.0000000	2.4850001
-2745.0000000	2.4900000
-2744.0000000	2.4949999
-2743.0000000	2.5050001
-2742.0000000	2.5100000
-2741.0000000	2.5050001
-2740.0000000	2.4900000
-2739.0000000	2.4450002
-2738.0000000	2.4100001
-2737.0000000	2.3900001
-2736.0000000	2.3699999
-2735.0000000	2.3750000
-2734.0000000	2.3900001
-2733.0000000	2.3950000
-2732.0000000	2.4200001
-2731.0000000	2.4300001
-2730.0000000	2.4500000
-2729.0000000	2.4850001
-2728.0000000	2.5050001
-2727.0000000	2.5450001
-2726.0000000	2.5599999
-2725.0000000	2.5799999
-2724.0000000	2.5949998
-2723.0000000	2.5949998
-2722.0000000	2.5999999
-2721.0000000	2.6050000
-2720.0000000	2.6149998
-2719.0000000	2.6250000
-2718.0000000	2.6099999
-2717.0000000	2.5949998
-2716.0000000	2.5650001
-2715.0000000	2.5599999
-2714.0000000	2.5650001
-2713.0000000	2.5650001
-2712.0000000	2.5850000
-2711.0000000	2.5949998
-2710.0000000	2.6250000
-2709.0000000	2.6400001
-2708.0000000	2.6650000
-2707.0000000	2.6750002
-2706.0000000	2.6700001
-2705.0000000	2.6750002
-2704.0000000	2.6900001

-2703.0000000	2.6950002
-2702.0000000	2.6950002
-2701.0000000	2.6650000
-2700.0000000	2.6500001
-2699.0000000	2.6300001
-2698.0000000	2.6199999
-2697.0000000	2.6050000
-2696.0000000	2.6199999
-2695.0000000	2.6199999
-2694.0000000	2.6099999
-2693.0000000	2.5999999
-2692.0000000	2.5850000
-2691.0000000	2.5599999
-2690.0000000	2.5250001
-2689.0000000	2.4600000
-2688.0000000	2.4000001
-2687.0000000	2.3499999
-2686.0000000	2.2950001
-2685.0000000	2.2550001
-2684.0000000	2.2500000
-2683.0000000	2.2600000
-2682.0000000	2.2700000
-2681.0000000	2.2900000
-2680.0000000	2.3150001
-2679.0000000	2.3449998
-2678.0000000	2.3750000
-2677.0000000	2.4150000
-2676.0000000	2.4600000
-2675.0000000	2.4800000
-2674.0000000	2.5200000
-2673.0000000	2.5400000
-2672.0000000	2.5650001
-2671.0000000	2.5899999
-2670.0000000	2.5899999
-2669.0000000	2.6099999
-2668.0000000	2.6300001
-2667.0000000	2.6500001
-2666.0000000	2.6733334
-2665.0000000	2.6800001
-2664.0000000	2.6849999
-2663.0000000	2.6700001
-2662.0000000	2.6750002
-2661.0000000	2.6849999
-2660.0000000	2.6900001
-2659.0000000	2.7000000
-2658.0000000	2.7100000
-2657.0000000	2.7150002
-2656.0000000	2.7300000
-2655.0000000	2.7400000
-2654.0000000	2.7249999
-2653.0000000	2.7249999
-2652.0000000	2.7049999
-2651.0000000	2.6900001
-2650.0000000	2.6800001
-2649.0000000	2.6750002
-2648.0000000	2.6700001
-2647.0000000	2.6550002

-2646.0000000	2.6350002
-2645.0000000	2.6250000
-2644.0000000	2.6199999
-2643.0000000	2.5699999
-2642.0000000	2.5250001
-2641.0000000	2.4900000
-2640.0000000	2.4600000
-2639.0000000	2.4600000
-2638.0000000	2.4600000
-2637.0000000	2.4600000
-2636.0000000	2.4700000
-2635.0000000	2.4900000
-2634.0000000	2.5050001
-2633.0000000	2.5149999
-2632.0000000	2.5250001
-2631.0000000	2.5300000
-2630.0000000	2.5500000
-2629.0000000	2.5650001
-2628.0000000	2.5850000
-2627.0000000	2.6099999
-2626.0000000	2.6300001
-2625.0000000	2.6300001
-2624.0000000	2.6099999
-2623.0000000	2.6450000
-2622.0000000	2.6750002
-2621.0000000	2.7150002
-2620.0000000	2.7500000
-2619.0000000	2.7849998
-2618.0000000	2.8199999
-2617.0000000	2.8399999
-2616.0000000	2.8499999
-2615.0000000	2.8499999
-2614.0000000	2.8550000
-2613.0000000	2.8449998
-2612.0000000	2.8350000
-2611.0000000	2.8299999
-2610.0000000	2.8099999
-2609.0000000	2.8049998
-2608.0000000	2.7900000
-2607.0000000	2.7800000
-2606.0000000	2.7800000
-2605.0000000	2.7849998
-2604.0000000	2.7950001
-2603.0000000	2.8066666
-2602.0000000	2.8099999
-2601.0000000	2.8150001
-2600.0000000	2.8150001
-2599.0000000	2.8000000
-2598.0000000	2.7849998
-2597.0000000	2.7800000
-2596.0000000	2.7700000
-2595.0000000	2.7600000
-2594.0000000	2.7649999
-2593.0000000	2.7700000
-2592.0000000	2.7800000
-2591.0000000	2.8049998
-2590.0000000	2.8150001

-2589.0000000	2.8350000
-2588.0000000	2.8449998
-2587.0000000	2.8550000
-2586.0000000	2.8649998
-2585.0000000	2.8950000
-2584.0000000	2.9250002
-2583.0000000	2.9450002
-2582.0000000	2.9549999
-2581.0000000	2.9850001
-2580.0000000	3.0200000
-2579.0000000	3.0450001
-2578.0000000	3.0799999
-2577.0000000	3.1150000
-2576.0000000	3.1433334
-2575.0000000	3.1700001
-2574.0000000	3.1950002
-2573.0000000	3.2049999
-2572.0000000	3.2249999
-2571.0000000	3.2249999
-2570.0000000	3.2300000
-2569.0000000	3.2150002
-2568.0000000	3.2150002
-2567.0000000	3.1900001
-2566.0000000	3.1849999
-2565.0000000	3.1750002
-2564.0000000	3.1650000
-2563.0000000	3.1700001
-2562.0000000	3.1650000
-2561.0000000	3.1700001
-2560.0000000	3.1750002
-2559.0000000	3.1750002
-2558.0000000	3.1700001
-2557.0000000	3.1600001
-2556.0000000	3.1250000
-2555.0000000	3.0599999
-2554.0000000	3.0200000
-2553.0000000	2.9850001
-2552.0000000	2.9749999
-2551.0000000	2.9400001
-2550.0000000	2.9200001
-2549.0000000	2.9100001
-2548.0000000	2.9200001
-2547.0000000	2.9300001
-2546.0000000	2.9450002
-2545.0000000	2.9500000
-2544.0000000	2.9650002
-2543.0000000	2.9799998
-2542.0000000	3.0050001
-2541.0000000	3.0050001
-2540.0000000	2.9800000
-2539.0000000	2.9900000
-2538.0000000	2.9650002
-2537.0000000	2.9300001
-2536.0000000	2.8950000
-2535.0000000	2.8900001
-2534.0000000	2.9150000
-2533.0000000	2.9450002

-2532.0000000	2.9900000
-2531.0000000	3.0149999
-2530.0000000	3.0500000
-2529.0000000	3.0500000
-2528.0000000	3.0749998
-2527.0000000	3.0850000
-2526.0000000	3.1050000
-2525.0000000	3.1450000
-2524.0000000	3.1600001
-2523.0000000	3.1700001
-2522.0000000	3.1900001
-2521.0000000	3.1800001
-2520.0000000	3.1650000
-2519.0000000	3.1800001
-2518.0000000	3.1650000
-2517.0000000	3.1350002
-2516.0000000	3.1400001
-2515.0000000	3.1600001
-2514.0000000	3.1750002
-2513.0000000	3.2033336
-2512.0000000	3.2000000
-2511.0000000	3.2200000
-2510.0000000	3.2300000
-2509.0000000	3.2400000
-2508.0000000	3.2649999
-2507.0000000	3.2849998
-2506.0000000	3.3049998
-2505.0000000	3.3150001
-2504.0000000	3.3150001
-2503.0000000	3.2950001
-2502.0000000	3.2950001
-2501.0000000	3.2800000
-2500.0000000	3.2550001
-2499.0000000	3.2500000
-2498.0000000	3.2350001
-2497.0000000	3.2150002
-2496.0000000	3.1900001
-2495.0000000	3.2000000
-2494.0000000	3.2049999
-2493.0000000	3.2000000
-2492.0000000	3.1950002
-2491.0000000	3.2000000
-2490.0000000	3.2049999
-2489.0000000	3.1900001
-2488.0000000	3.2049999
-2487.0000000	3.2100000
-2486.0000000	3.1750002
-2485.0000000	3.1650000
-2484.0000000	3.1400001
-2483.0000000	3.1099999
-2482.0000000	3.0699999
-2481.0000000	3.0250001
-2480.0000000	2.9749999
-2479.0000000	2.9150000
-2478.0000000	2.8800001
-2477.0000000	2.8550000
-2476.0000000	2.8299999

-2475.0000000	2.7900000
-2474.0000000	2.7649999
-2473.0000000	2.7249999
-2472.0000000	2.6750002
-2471.0000000	2.6050000
-2470.0000000	2.5349998
-2469.0000000	2.4700000
-2468.0000000	2.4150000
-2467.0000000	2.3600001
-2466.0000000	2.2950001
-2465.0000000	2.2200000
-2464.0000000	2.1849999
-2463.0000000	2.1900001
-2462.0000000	2.2100000
-2461.0000000	2.2350001
-2460.0000000	2.2449999
-2459.0000000	2.2800000
-2458.0000000	2.3049998
-2457.0000000	2.3049998
-2456.0000000	2.3249998
-2455.0000000	2.3666666
-2454.0000000	2.3950000
-2453.0000000	2.4250002
-2452.0000000	2.4400001
-2451.0000000	2.4500000
-2450.0000000	2.4400001
-2449.0000000	2.4150000
-2448.0000000	2.4349999
-2447.0000000	2.4700000
-2446.0000000	2.5050001
-2445.0000000	2.5250001
-2444.0000000	2.5349998
-2443.0000000	2.5450001
-2442.0000000	2.5400000
-2441.0000000	2.5650001
-2440.0000000	2.5599999
-2439.0000000	2.5400000
-2438.0000000	2.5200000
-2437.0000000	2.4949999
-2436.0000000	2.4600000
-2435.0000000	2.4450002
-2434.0000000	2.4800000
-2433.0000000	2.5050001
-2432.0000000	2.5100000
-2431.0000000	2.5100000
-2430.0000000	2.5200000
-2429.0000000	2.5200000
-2428.0000000	2.5149999
-2427.0000000	2.5000000
-2426.0000000	2.4749999
-2425.0000000	2.4349999
-2424.0000000	2.3700001
-2423.0000000	2.3049998
-2422.0000000	2.2550001
-2421.0000000	2.2249999
-2420.0000000	2.1900001
-2419.0000000	2.1550002

-2418.0000000	2.1250000
-2417.0000000	2.1199999
-2416.0000000	2.1050000
-2415.0000000	2.1149998
-2414.0000000	2.1400001
-2413.0000000	2.1800001
-2412.0000000	2.2300000
-2411.0000000	2.2700000
-2410.0000000	2.3099999
-2409.0000000	2.3449998
-2408.0000000	2.3699999
-2407.0000000	2.3800001
-2406.0000000	2.3900001
-2405.0000000	2.4000001
-2404.0000000	2.3900001
-2403.0000000	2.3900001
-2402.0000000	2.4000001
-2401.0000000	2.4300001
-2400.0000000	2.4600000
-2399.0000000	2.4949999
-2398.0000000	2.5300000
-2397.0000000	2.5650001
-2396.0000000	2.6050000
-2395.0000000	2.6300001
-2394.0000000	2.6600001
-2393.0000000	2.6849999
-2392.0000000	2.7366667
-2391.0000000	2.7600000
-2390.0000000	2.7849998
-2389.0000000	2.8099999
-2388.0000000	2.8499999
-2387.0000000	2.8699999
-2386.0000000	2.9000001
-2385.0000000	2.9250002
-2384.0000000	2.9450002
-2383.0000000	2.9650002
-2382.0000000	2.9900000
-2381.0000000	2.9949999
-2380.0000000	3.0100000
-2379.0000000	3.0250001
-2378.0000000	3.0500000
-2377.0000000	3.0599999
-2376.0000000	3.0850000
-2375.0000000	3.1149998
-2374.0000000	3.1250000
-2373.0000000	3.1250000
-2372.0000000	3.1400001
-2371.0000000	3.1650000
-2370.0000000	3.1900001
-2369.0000000	3.2049999
-2368.0000000	3.2249999
-2367.0000000	3.2449999
-2366.0000000	3.2649999
-2365.0000000	3.2750001
-2364.0000000	3.2700000
-2363.0000000	3.2700000
-2362.0000000	3.2666667

-2361.0000000	3.2550001
-2360.0000000	3.2350001
-2359.0000000	3.2300000
-2358.0000000	3.1800001
-2357.0000000	3.1099999
-2356.0000000	3.0549998
-2355.0000000	2.9749999
-2354.0000000	2.8899999
-2353.0000000	2.8150001
-2352.0000000	2.7300000
-2351.0000000	2.6900001
-2350.0000000	2.6650000
-2349.0000000	2.6450000
-2348.0000000	2.5949998
-2347.0000000	2.5650001
-2346.0000000	2.5300000
-2345.0000000	2.4949999
-2344.0000000	2.4400001
-2343.0000000	2.3900001
-2342.0000000	2.3699999
-2341.0000000	2.3499999
-2340.0000000	2.3350000
-2339.0000000	2.3299999
-2338.0000000	2.3150001
-2337.0000000	2.3000000
-2336.0000000	2.2750001
-2335.0000000	2.2350001
-2334.0000000	2.2000000
-2333.0000000	2.1650000
-2332.0000000	2.1566668
-2331.0000000	2.1350002
-2330.0000000	2.1149998
-2329.0000000	2.1050000
-2328.0000000	2.0850000
-2327.0000000	2.0699999
-2326.0000000	2.0349998
-2325.0000000	2.0250001
-2324.0000000	2.0300000
-2323.0000000	2.0549998
-2322.0000000	2.0599999
-2321.0000000	2.0749998
-2320.0000000	2.0799999
-2319.0000000	2.0799999
-2318.0000000	2.0999999
-2317.0000000	2.1300001
-2316.0000000	2.1400001
-2315.0000000	2.1450000
-2314.0000000	2.1450000
-2313.0000000	2.1250000
-2312.0000000	2.1050000
-2311.0000000	2.0899999
-2310.0000000	2.0850000
-2309.0000000	2.0850000
-2308.0000000	2.1050000
-2307.0000000	2.1149998
-2306.0000000	2.1250000
-2305.0000000	2.1149998

-2304.0000000	2.1149998
-2303.0000000	2.1050000
-2302.0000000	2.0666666
-2301.0000000	2.0250001
-2300.0000000	2.0300000
-2299.0000000	2.0500000
-2298.0000000	2.0650001
-2297.0000000	2.0699999
-2296.0000000	2.0999999
-2295.0000000	2.1300001
-2294.0000000	2.1550002
-2293.0000000	2.1750002
-2292.0000000	2.1800001
-2291.0000000	2.1900001
-2290.0000000	2.2000000
-2289.0000000	2.2350001
-2288.0000000	2.2449999
-2287.0000000	2.2550001
-2286.0000000	2.2750001
-2285.0000000	2.2750001
-2284.0000000	2.2649999
-2283.0000000	2.2649999
-2282.0000000	2.2550001
-2281.0000000	2.2550001
-2280.0000000	2.2600000
-2279.0000000	2.2649999
-2278.0000000	2.2800000
-2277.0000000	2.2849998
-2276.0000000	2.3000000
-2275.0000000	2.3150001
-2274.0000000	2.3249998
-2273.0000000	2.3366668
-2272.0000000	2.3599999
-2271.0000000	2.3750000
-2270.0000000	2.3750000
-2269.0000000	2.3950000
-2268.0000000	2.4050002
-2267.0000000	2.4100001
-2266.0000000	2.4300001
-2265.0000000	2.4500000
-2264.0000000	2.4500000
-2263.0000000	2.4600000
-2262.0000000	2.4549999
-2261.0000000	2.4700000
-2260.0000000	2.4650002
-2259.0000000	2.4500000
-2258.0000000	2.4450002
-2257.0000000	2.4450002
-2256.0000000	2.4400001
-2255.0000000	2.4349999
-2254.0000000	2.4250002
-2253.0000000	2.4150000
-2252.0000000	2.4000001
-2251.0000000	2.3750000
-2250.0000000	2.3499999
-2249.0000000	2.3150001
-2248.0000000	2.2950001

-2247.0000000	2.2750001
-2246.0000000	2.2500000
-2245.0000000	2.2550001
-2244.0000000	2.2449999
-2243.0000000	2.2500000
-2242.0000000	2.2500000
-2241.0000000	2.2600000
-2240.0000000	2.2600000
-2239.0000000	2.2750001
-2238.0000000	2.2750001
-2237.0000000	2.2700000
-2236.0000000	2.2649999
-2235.0000000	2.2400000
-2234.0000000	2.2200000
-2233.0000000	2.2150002
-2232.0000000	2.2049999
-2231.0000000	2.2000000
-2230.0000000	2.2100000
-2229.0000000	2.2049999
-2228.0000000	2.2150002
-2227.0000000	2.2200000
-2226.0000000	2.2249999
-2225.0000000	2.2200000
-2224.0000000	2.1950002
-2223.0000000	2.1900001
-2222.0000000	2.2000000
-2221.0000000	2.2300000
-2220.0000000	2.2649999
-2219.0000000	2.3099999
-2218.0000000	2.3350000
-2217.0000000	2.3550000
-2216.0000000	2.3800001
-2215.0000000	2.3900001
-2214.0000000	2.3750000
-2213.0000000	2.3699999
-2212.0000000	2.3800001
-2211.0000000	2.4100001
-2210.0000000	2.4349999
-2209.0000000	2.4749999
-2208.0000000	2.5100000
-2207.0000000	2.5450001
-2206.0000000	2.5949998
-2205.0000000	2.6350002
-2204.0000000	2.6750002
-2203.0000000	2.7200000
-2202.0000000	2.7550001
-2201.0000000	2.7950001
-2200.0000000	2.8299999
-2199.0000000	2.8599999
-2198.0000000	2.8950000
-2197.0000000	2.9200001
-2196.0000000	2.9349999
-2195.0000000	2.9500000
-2194.0000000	2.9749999
-2193.0000000	2.9900000
-2192.0000000	3.0100000
-2191.0000000	3.0349998

-2190.0000000	3.0549998
-2189.0000000	3.0650001
-2188.0000000	3.0500000
-2187.0000000	3.0599999
-2186.0000000	3.0799999
-2185.0000000	3.0850000
-2184.0000000	3.1149998
-2183.0000000	3.1199999
-2182.0000000	3.1400001
-2181.0000000	3.1666667
-2180.0000000	3.1650000
-2179.0000000	3.1800001
-2178.0000000	3.2000000
-2177.0000000	3.2049999
-2176.0000000	3.2049999
-2175.0000000	3.1900001
-2174.0000000	3.1750002
-2173.0000000	3.1500001
-2172.0000000	3.1350002
-2171.0000000	3.1099999
-2170.0000000	3.0850000
-2169.0000000	3.0500000
-2168.0000000	2.9949999
-2167.0000000	2.9349999
-2166.0000000	2.8900001
-2165.0000000	2.8699999
-2164.0000000	2.8599999
-2163.0000000	2.8699999
-2162.0000000	2.8750000
-2161.0000000	2.8699999
-2160.0000000	2.8299999
-2159.0000000	2.7950001
-2158.0000000	2.7550001
-2157.0000000	2.6950002
-2156.0000000	2.6650000
-2155.0000000	2.6700001
-2154.0000000	2.6950002
-2153.0000000	2.7049999
-2152.0000000	2.6800001
-2151.0000000	2.6566668
-2150.0000000	2.6600001
-2149.0000000	2.6600001
-2148.0000000	2.6650000
-2147.0000000	2.6700001
-2146.0000000	2.6849999
-2145.0000000	2.7049999
-2144.0000000	2.7150002
-2143.0000000	2.7249999
-2142.0000000	2.7150002
-2141.0000000	2.7100000
-2140.0000000	2.7049999
-2139.0000000	2.7000000
-2138.0000000	2.7100000
-2137.0000000	2.7100000
-2136.0000000	2.7100000
-2135.0000000	2.7049999
-2134.0000000	2.7000000

-2133.0000000	2.6900001
-2132.0000000	2.6800001
-2131.0000000	2.6750002
-2130.0000000	2.6650000
-2129.0000000	2.6550002
-2128.0000000	2.6400001
-2127.0000000	2.6149998
-2126.0000000	2.6149998
-2125.0000000	2.6450000
-2124.0000000	2.6750002
-2123.0000000	2.7100000
-2122.0000000	2.7100000
-2121.0000000	2.7266667
-2120.0000000	2.7350001
-2119.0000000	2.7550001
-2118.0000000	2.7750001
-2117.0000000	2.8049998
-2116.0000000	2.8199999
-2115.0000000	2.8249998
-2114.0000000	2.8299999
-2113.0000000	2.8399999
-2112.0000000	2.8399999
-2111.0000000	2.8499999
-2110.0000000	2.8599999
-2109.0000000	2.8449998
-2108.0000000	2.8449998
-2107.0000000	2.8550000
-2106.0000000	2.8550000
-2105.0000000	2.8499999
-2104.0000000	2.8649998
-2103.0000000	2.8699999
-2102.0000000	2.8699999
-2101.0000000	2.8900001
-2100.0000000	2.8750000
-2099.0000000	2.8750000
-2098.0000000	2.8649998
-2097.0000000	2.8699999
-2096.0000000	2.8649998
-2095.0000000	2.8699999
-2094.0000000	2.8750000
-2093.0000000	2.8750000
-2092.0000000	2.8800001
-2091.0000000	2.8800001
-2090.0000000	2.8833334
-2089.0000000	2.8850002
-2088.0000000	2.8649998
-2087.0000000	2.8550000
-2086.0000000	2.8449998
-2085.0000000	2.8249998
-2084.0000000	2.8199999
-2083.0000000	2.8049998
-2082.0000000	2.8000000
-2081.0000000	2.8049998
-2080.0000000	2.8099999
-2079.0000000	2.8199999
-2078.0000000	2.8449998
-2077.0000000	2.8699999

-2076.0000000	2.8950000
-2075.0000000	2.9200001
-2074.0000000	2.9400001
-2073.0000000	2.9600000
-2072.0000000	2.9749999
-2071.0000000	2.9749999
-2070.0000000	2.9650002
-2069.0000000	2.9600000
-2068.0000000	2.9450002
-2067.0000000	2.9200001
-2066.0000000	2.8900001
-2065.0000000	2.8750000
-2064.0000000	2.8599999
-2063.0000000	2.8399999
-2062.0000000	2.8299999
-2061.0000000	2.8299999
-2060.0000000	2.8433332
-2059.0000000	2.8550000
-2058.0000000	2.8550000
-2057.0000000	2.8599999
-2056.0000000	2.8499999
-2055.0000000	2.8550000
-2054.0000000	2.8599999
-2053.0000000	2.8649998
-2052.0000000	2.8649998
-2051.0000000	2.8649998
-2050.0000000	2.8649998
-2049.0000000	2.8550000
-2048.0000000	2.8499999
-2047.0000000	2.8449998
-2046.0000000	2.8550000
-2045.0000000	2.8699999
-2044.0000000	2.8900001
-2043.0000000	2.9100001
-2042.0000000	2.9250002
-2041.0000000	2.9349999
-2040.0000000	2.9650002
-2039.0000000	2.9800000
-2038.0000000	3.0100000
-2037.0000000	3.0250001
-2036.0000000	3.0349998
-2035.0000000	3.0450001
-2034.0000000	3.0599999
-2033.0000000	3.0599999
-2032.0000000	3.0549998
-2031.0000000	3.0599999
-2030.0000000	3.0666666
-2029.0000000	3.0799999
-2028.0000000	3.0899999
-2027.0000000	3.0949998
-2026.0000000	3.0899999
-2025.0000000	3.0749998
-2024.0000000	3.0650001
-2023.0000000	3.0799999
-2022.0000000	3.0999999
-2021.0000000	3.1250000
-2020.0000000	3.1400001

-2019.0000000	3.1700001
-2018.0000000	3.1800001
-2017.0000000	3.1800001
-2016.0000000	3.1849999
-2015.0000000	3.1950002
-2014.0000000	3.1950002
-2013.0000000	3.1950002
-2012.0000000	3.1900001
-2011.0000000	3.1800001
-2010.0000000	3.1650000
-2009.0000000	3.1500001
-2008.0000000	3.1450000
-2007.0000000	3.1250000
-2006.0000000	3.1199999
-2005.0000000	3.1199999
-2004.0000000	3.1149998
-2003.0000000	3.1199999
-2002.0000000	3.1099999
-2001.0000000	3.0949998
-2000.0000000	3.0833333
-1999.0000000	3.0799999
-1998.0000000	3.0749998
-1997.0000000	3.0699999
-1996.0000000	3.0450001
-1995.0000000	3.0050001
-1994.0000000	2.9949999
-1993.0000000	2.9850001
-1992.0000000	2.9900000
-1991.0000000	2.9650002
-1990.0000000	2.9450002
-1989.0000000	2.9349999
-1988.0000000	2.9200001
-1987.0000000	2.9250002
-1986.0000000	2.9050002
-1985.0000000	2.8699999
-1984.0000000	2.8299999
-1983.0000000	2.7950001
-1982.0000000	2.7600000
-1981.0000000	2.7400000
-1980.0000000	2.7249999
-1979.0000000	2.6950002
-1978.0000000	2.6600001
-1977.0000000	2.6450000
-1976.0000000	2.6650000
-1975.0000000	2.6700001
-1974.0000000	2.6700001
-1973.0000000	2.6750002
-1972.0000000	2.6500001
-1971.0000000	2.6250000
-1970.0000000	2.6066666
-1969.0000000	2.6700001
-1968.0000000	2.7049999
-1967.0000000	2.7100000
-1966.0000000	2.6800001
-1965.0000000	2.7150002
-1964.0000000	2.7300000
-1963.0000000	2.7300000

-1962.0000000	2.7600000
-1961.0000000	2.7800000
-1960.0000000	2.7950001
-1959.0000000	2.8049998
-1958.0000000	2.8049998
-1957.0000000	2.8199999
-1956.0000000	2.8399999
-1955.0000000	2.8750000
-1954.0000000	2.9150000
-1953.0000000	2.9349999
-1952.0000000	2.9450002
-1951.0000000	2.9549999
-1950.0000000	2.9549999
-1949.0000000	2.9450002
-1948.0000000	2.9400001
-1947.0000000	2.9300001
-1946.0000000	2.9050002
-1945.0000000	2.8800001
-1944.0000000	2.8699999
-1943.0000000	2.8649998
-1942.0000000	2.8599999
-1941.0000000	2.8550000
-1940.0000000	2.8499997
-1939.0000000	2.8249998
-1938.0000000	2.7900000
-1937.0000000	2.7750001
-1936.0000000	2.7500000
-1935.0000000	2.7449999
-1934.0000000	2.7200000
-1933.0000000	2.7150002
-1932.0000000	2.7150002
-1931.0000000	2.7150002
-1930.0000000	2.7150002
-1929.0000000	2.7200000
-1928.0000000	2.7000000
-1927.0000000	2.6650000
-1926.0000000	2.6350002
-1925.0000000	2.6250000
-1924.0000000	2.6199999
-1923.0000000	2.6250000
-1922.0000000	2.6500001
-1921.0000000	2.6900001
-1920.0000000	2.7049999
-1919.0000000	2.7100000
-1918.0000000	2.7100000
-1917.0000000	2.7300000
-1916.0000000	2.7700000
-1915.0000000	2.7750001
-1914.0000000	2.7950001
-1913.0000000	2.8249998
-1912.0000000	2.8449998
-1911.0000000	2.8599999
-1910.0000000	2.8499999
-1909.0000000	2.8299999
-1908.0000000	2.8150001
-1907.0000000	2.8449998
-1906.0000000	2.8550000

-1905.0000000	2.8649998
-1904.0000000	2.8800001
-1903.0000000	2.9000001
-1902.0000000	2.9200001
-1901.0000000	2.9500000
-1900.0000000	2.9600000
-1899.0000000	2.9700000
-1898.0000000	2.9949999
-1897.0000000	3.0050001
-1896.0000000	3.0149999
-1895.0000000	3.0500000
-1894.0000000	3.0599999
-1893.0000000	3.0749998
-1892.0000000	3.0799999
-1891.0000000	3.0799999
-1890.0000000	3.0850000
-1889.0000000	3.0850000
-1888.0000000	3.0749998
-1887.0000000	3.0699999
-1886.0000000	3.0650001
-1885.0000000	3.0549998
-1884.0000000	3.0349998
-1883.0000000	3.0149999
-1882.0000000	2.9800000
-1881.0000000	2.9500000
-1880.0000000	2.9100001
-1879.0000000	2.8599999
-1878.0000000	2.7933333
-1877.0000000	2.7400000
-1876.0000000	2.7049999
-1875.0000000	2.7249999
-1874.0000000	2.7249999
-1873.0000000	2.7300000
-1872.0000000	2.7449999
-1871.0000000	2.7400000
-1870.0000000	2.7700000
-1869.0000000	2.7750001
-1868.0000000	2.8049998
-1867.0000000	2.8249998
-1866.0000000	2.8599999
-1865.0000000	2.8900001
-1864.0000000	2.9100001
-1863.0000000	2.9250002
-1862.0000000	2.9250002
-1861.0000000	2.9250002
-1860.0000000	2.9400001
-1859.0000000	2.9600000
-1858.0000000	2.9700000
-1857.0000000	2.9900000
-1856.0000000	2.9949999
-1855.0000000	3.0050001
-1854.0000000	3.0050001
-1853.0000000	3.0000000
-1852.0000000	2.9949999
-1851.0000000	2.9850001
-1850.0000000	2.9600000
-1849.0000000	2.9050002

-1848.0000000	2.8833332
-1847.0000000	2.8599999
-1846.0000000	2.8599999
-1845.0000000	2.8649998
-1844.0000000	2.8699999
-1843.0000000	2.8750000
-1842.0000000	2.8800001
-1841.0000000	2.8800001
-1840.0000000	2.8800001
-1839.0000000	2.8649998
-1838.0000000	2.8649998
-1837.0000000	2.8599999
-1836.0000000	2.8499999
-1835.0000000	2.8399999
-1834.0000000	2.8249998
-1833.0000000	2.8199999
-1832.0000000	2.8049998
-1831.0000000	2.8049998
-1830.0000000	2.8049998
-1829.0000000	2.8049998
-1828.0000000	2.8299999
-1827.0000000	2.8399999
-1826.0000000	2.8599999
-1825.0000000	2.8550000
-1824.0000000	2.8550000
-1823.0000000	2.8350000
-1822.0000000	2.8099999
-1821.0000000	2.7950001
-1820.0000000	2.7900000
-1819.0000000	2.7966669
-1818.0000000	2.8049998
-1817.0000000	2.8049998
-1816.0000000	2.8099999
-1815.0000000	2.8000000
-1814.0000000	2.7800000
-1813.0000000	2.7750001
-1812.0000000	2.7700000
-1811.0000000	2.7600000
-1810.0000000	2.7550001
-1809.0000000	2.7500000
-1808.0000000	2.7400000
-1807.0000000	2.7000000
-1806.0000000	2.6800001
-1805.0000000	2.6350002
-1804.0000000	2.5899999
-1803.0000000	2.5300000
-1802.0000000	2.4549999
-1801.0000000	2.3899999
-1800.0000000	2.3599999
-1799.0000000	2.3399999
-1798.0000000	2.3399999
-1797.0000000	2.3449998
-1796.0000000	2.3449998
-1795.0000000	2.3599999
-1794.0000000	2.3699999
-1793.0000000	2.3800001
-1792.0000000	2.4150000

-1791.0000000	2.4700000
-1790.0000000	2.5050001
-1789.0000000	2.5650001
-1788.0000000	2.6066666
-1787.0000000	2.6350002
-1786.0000000	2.6450000
-1785.0000000	2.6450000
-1784.0000000	2.6450000
-1783.0000000	2.6400001
-1782.0000000	2.6450000
-1781.0000000	2.6450000
-1780.0000000	2.6350002
-1779.0000000	2.6450000
-1778.0000000	2.6550002
-1777.0000000	2.6500001
-1776.0000000	2.6550002
-1775.0000000	2.6450000
-1774.0000000	2.6400001
-1773.0000000	2.6199999
-1772.0000000	2.5949998
-1771.0000000	2.5650001
-1770.0000000	2.5300000
-1769.0000000	2.5000000
-1768.0000000	2.4800000
-1767.0000000	2.4450002
-1766.0000000	2.4100001
-1765.0000000	2.3599999
-1764.0000000	2.3399999
-1763.0000000	2.3550000
-1762.0000000	2.3900001
-1761.0000000	2.4200001
-1760.0000000	2.4349999
-1759.0000000	2.4433334
-1758.0000000	2.4600000
-1757.0000000	2.4900000
-1756.0000000	2.5100000
-1755.0000000	2.5400000
-1754.0000000	2.5549998
-1753.0000000	2.5699999
-1752.0000000	2.5799999
-1751.0000000	2.5749998
-1750.0000000	2.5650001
-1749.0000000	2.5599999
-1748.0000000	2.5300000
-1747.0000000	2.5100000
-1746.0000000	2.4850001
-1745.0000000	2.4450002
-1744.0000000	2.4250002
-1743.0000000	2.4050002
-1742.0000000	2.3950000
-1741.0000000	2.3600001
-1740.0000000	2.3199999
-1739.0000000	2.3000000
-1738.0000000	2.2900000
-1737.0000000	2.3000000
-1736.0000000	2.3249998
-1735.0000000	2.3449998

-1734.0000000	2.3550000
-1733.0000000	2.3649998
-1732.0000000	2.3900001
-1731.0000000	2.4100001
-1730.0000000	2.4400001
-1729.0000000	2.4450002
-1728.0000000	2.4400001
-1727.0000000	2.4400003
-1726.0000000	2.4450002
-1725.0000000	2.4600000
-1724.0000000	2.4949999
-1723.0000000	2.5200000
-1722.0000000	2.5549998
-1721.0000000	2.5799999
-1720.0000000	2.6149998
-1719.0000000	2.6350002
-1718.0000000	2.6600001
-1717.0000000	2.6849999
-1716.0000000	2.7249999
-1715.0000000	2.7550001
-1714.0000000	2.7750001
-1713.0000000	2.8000000
-1712.0000000	2.8150001
-1711.0000000	2.8399999
-1710.0000000	2.8599999
-1709.0000000	2.8850002
-1708.0000000	2.9100001
-1707.0000000	2.9300001
-1706.0000000	2.9549999
-1705.0000000	2.9800000
-1704.0000000	3.0050001
-1703.0000000	3.0300000
-1702.0000000	3.0349998
-1701.0000000	3.0349998
-1700.0000000	3.0450001
-1699.0000000	3.0133333
-1698.0000000	2.9850001
-1697.0000000	2.9800000
-1696.0000000	2.9900000
-1695.0000000	3.0000000
-1694.0000000	3.0050001
-1693.0000000	3.0300000
-1692.0000000	3.0450001
-1691.0000000	3.0749998
-1690.0000000	3.0850000
-1689.0000000	3.0899999
-1688.0000000	3.0949998
-1687.0000000	3.1099999
-1686.0000000	3.1250000
-1685.0000000	3.1450000
-1684.0000000	3.1650000
-1683.0000000	3.1950002
-1682.0000000	3.2100000
-1681.0000000	3.2200000
-1680.0000000	3.2300000
-1679.0000000	3.2350001
-1678.0000000	3.2449999

-1677.0000000	3.2550001
-1676.0000000	3.2649999
-1675.0000000	3.2600000
-1674.0000000	3.2800000
-1673.0000000	3.3049998
-1672.0000000	3.3249998
-1671.0000000	3.3499999
-1670.0000000	3.3699999
-1669.0000000	3.4000001
-1668.0000000	3.4200001
-1667.0000000	3.4300003
-1666.0000000	3.4600000
-1665.0000000	3.4749999
-1664.0000000	3.4949999
-1663.0000000	3.5100000
-1662.0000000	3.5250001
-1661.0000000	3.5300000
-1660.0000000	3.5450001
-1659.0000000	3.5599999
-1658.0000000	3.5699999
-1657.0000000	3.5699999
-1656.0000000	3.5799999
-1655.0000000	3.5799999
-1654.0000000	3.5799999
-1653.0000000	3.5899999
-1652.0000000	3.5949998
-1651.0000000	3.5999999
-1650.0000000	3.5749998
-1649.0000000	3.5599999
-1648.0000000	3.5500000
-1647.0000000	3.5450001
-1646.0000000	3.5200000
-1645.0000000	3.5100000
-1644.0000000	3.5400000
-1643.0000000	3.5699999
-1642.0000000	3.6100001
-1641.0000000	3.6250000
-1640.0000000	3.6550002
-1639.0000000	3.6750002
-1638.0000000	3.6966667
-1637.0000000	3.7150002
-1636.0000000	3.7200000
-1635.0000000	3.7449999
-1634.0000000	3.7700000
-1633.0000000	3.8000000
-1632.0000000	3.8299999
-1631.0000000	3.8599999
-1630.0000000	3.8900001
-1629.0000000	3.9100001
-1628.0000000	3.9250002
-1627.0000000	3.9450002
-1626.0000000	3.9349999
-1625.0000000	3.9600000
-1624.0000000	3.9850001
-1623.0000000	3.9949999
-1622.0000000	4.0150003
-1621.0000000	4.0400000

-1620.0000000	4.0500002
-1619.0000000	4.0599999
-1618.0000000	4.0700002
-1617.0000000	4.0699997
-1616.0000000	4.0749998
-1615.0000000	4.0749998
-1614.0000000	4.0700002
-1613.0000000	4.0650001
-1612.0000000	4.0450001
-1611.0000000	4.0100002
-1610.0000000	3.9949999
-1609.0000000	3.9800000
-1608.0000000	3.9600000
-1607.0000000	3.9466667
-1606.0000000	3.9200001
-1605.0000000	3.8950000
-1604.0000000	3.8399999
-1603.0000000	3.7950001
-1602.0000000	3.7550001
-1601.0000000	3.7200000
-1600.0000000	3.6950002
-1599.0000000	3.6550002
-1598.0000000	3.6300001
-1597.0000000	3.6050000
-1596.0000000	3.5699999
-1595.0000000	3.5500000
-1594.0000000	3.5599999
-1593.0000000	3.5599999
-1592.0000000	3.5850000
-1591.0000000	3.5949998
-1590.0000000	3.6250000
-1589.0000000	3.6149998
-1588.0000000	3.6300001
-1587.0000000	3.6250000
-1586.0000000	3.6350002
-1585.0000000	3.6450000
-1584.0000000	3.6600001
-1583.0000000	3.6600001
-1582.0000000	3.6650000
-1581.0000000	3.6600001
-1580.0000000	3.6550002
-1579.0000000	3.6300001
-1578.0000000	3.6099999
-1577.0000000	3.5666666
-1576.0000000	3.5149999
-1575.0000000	3.4650002
-1574.0000000	3.4250002
-1573.0000000	3.3950000
-1572.0000000	3.3950000
-1571.0000000	3.3850002
-1570.0000000	3.3850002
-1569.0000000	3.3649998
-1568.0000000	3.3649998
-1567.0000000	3.3850002
-1566.0000000	3.3850002
-1565.0000000	3.4000001
-1564.0000000	3.4100001

-1563.0000000	3.4050002
-1562.0000000	3.4200001
-1561.0000000	3.4300001
-1560.0000000	3.4300001
-1559.0000000	3.4349999
-1558.0000000	3.4300001
-1557.0000000	3.4349999
-1556.0000000	3.3950000
-1555.0000000	3.3249998
-1554.0000000	3.3099999
-1553.0000000	3.3049998
-1552.0000000	3.3199999
-1551.0000000	3.3550000
-1550.0000000	3.3850000
-1549.0000000	3.4500000
-1548.0000000	3.5033333
-1547.0000000	3.5500000
-1546.0000000	3.5799999
-1545.0000000	3.5949998
-1544.0000000	3.6050000
-1543.0000000	3.6250000
-1542.0000000	3.6149998
-1541.0000000	3.6250000
-1540.0000000	3.6300001
-1539.0000000	3.6400001
-1538.0000000	3.6300001
-1537.0000000	3.6149998
-1536.0000000	3.5799999
-1535.0000000	3.5100000
-1534.0000000	3.4349999
-1533.0000000	3.3850000
-1532.0000000	3.3750000
-1531.0000000	3.3850002
-1530.0000000	3.3850002
-1529.0000000	3.3850002
-1528.0000000	3.4000001
-1527.0000000	3.3800001
-1526.0000000	3.3649998
-1525.0000000	3.3550000
-1524.0000000	3.3599999
-1523.0000000	3.3750000
-1522.0000000	3.3750000
-1521.0000000	3.3800001
-1520.0000000	3.3950000
-1519.0000000	3.4200001
-1518.0000000	3.4349999
-1517.0000000	3.4700000
-1516.0000000	3.5066669
-1515.0000000	3.5200000
-1514.0000000	3.5300000
-1513.0000000	3.5149999
-1512.0000000	3.5000000
-1511.0000000	3.4900000
-1510.0000000	3.4650002
-1509.0000000	3.4600000
-1508.0000000	3.4700000
-1507.0000000	3.4749999

-1506.0000000	3.4700000
-1505.0000000	3.4749999
-1504.0000000	3.4850001
-1503.0000000	3.4850001
-1502.0000000	3.4800000
-1501.0000000	3.4749999
-1500.0000000	3.4700000
-1499.0000000	3.4749999
-1498.0000000	3.4800000
-1497.0000000	3.4900000
-1496.0000000	3.5149999
-1495.0000000	3.5349998
-1494.0000000	3.5500000
-1493.0000000	3.5650001
-1492.0000000	3.5699999
-1491.0000000	3.5799999
-1490.0000000	3.5650001
-1489.0000000	3.5799999
-1488.0000000	3.5899999
-1487.0000000	3.6050000
-1486.0000000	3.6350002
-1485.0000000	3.6800001
-1484.0000000	3.7200000
-1483.0000000	3.7500000
-1482.0000000	3.7649999
-1481.0000000	3.7700000
-1480.0000000	3.7700000
-1479.0000000	3.7500000
-1478.0000000	3.7150002
-1477.0000000	3.6900001
-1476.0000000	3.6950002
-1475.0000000	3.6750002
-1474.0000000	3.6350000
-1473.0000000	3.6300001
-1472.0000000	3.6099999
-1471.0000000	3.5999999
-1470.0000000	3.5799999
-1469.0000000	3.5400000
-1468.0000000	3.5450001
-1467.0000000	3.5400000
-1466.0000000	3.5050001
-1465.0000000	3.4800000
-1464.0000000	3.4500000
-1463.0000000	3.4300001
-1462.0000000	3.4150000
-1461.0000000	3.4000001
-1460.0000000	3.3750000
-1459.0000000	3.3499999
-1458.0000000	3.3199999
-1457.0000000	3.3199999
-1456.0000000	3.3199999
-1455.0000000	3.3233330
-1454.0000000	3.2849998
-1453.0000000	3.2400000
-1452.0000000	3.1800001
-1451.0000000	3.1250000
-1450.0000000	3.0799999

-1449.0000000	3.0400000
-1448.0000000	3.0149999
-1447.0000000	2.9749999
-1446.0000000	2.8850000
-1445.0000000	2.8350000
-1444.0000000	2.7900000
-1443.0000000	2.7750001
-1442.0000000	2.7700000
-1441.0000000	2.7550001
-1440.0000000	2.7649999
-1439.0000000	2.7550001
-1438.0000000	2.7600000
-1437.0000000	2.7700000
-1436.0000000	2.7750001
-1435.0000000	2.8000000
-1434.0000000	2.8499999
-1433.0000000	2.8800001
-1432.0000000	2.9150000
-1431.0000000	2.9700000
-1430.0000000	3.0000000
-1429.0000000	3.0450001
-1428.0000000	3.0799999
-1427.0000000	3.1250000
-1426.0000000	3.1550002
-1425.0000000	3.2233334
-1424.0000000	3.2849998
-1423.0000000	3.3449998
-1422.0000000	3.3950000
-1421.0000000	3.4500000
-1420.0000000	3.4900000
-1419.0000000	3.5300000
-1418.0000000	3.5500000
-1417.0000000	3.5799999
-1416.0000000	3.5949998
-1415.0000000	3.6150000
-1414.0000000	3.6300001
-1413.0000000	3.6450000
-1412.0000000	3.6600001
-1411.0000000	3.6700001
-1410.0000000	3.6750002
-1409.0000000	3.6849999
-1408.0000000	3.6900001
-1407.0000000	3.6900001
-1406.0000000	3.6849999
-1405.0000000	3.6700001
-1404.0000000	3.6550002
-1403.0000000	3.6500001
-1402.0000000	3.6199999
-1401.0000000	3.6099999
-1400.0000000	3.5749998
-1399.0000000	3.5500000
-1398.0000000	3.5149999
-1397.0000000	3.4949999
-1396.0000000	3.4500000
-1395.0000000	3.4150000
-1394.0000000	3.3850002
-1393.0000000	3.3750000

-1392.0000000	3.3750000
-1391.0000000	3.3649998
-1390.0000000	3.3649998
-1389.0000000	3.3649998
-1388.0000000	3.3699999
-1387.0000000	3.3649998
-1386.0000000	3.3499999
-1385.0000000	3.3249998
-1384.0000000	3.2849998
-1383.0000000	3.2700000
-1382.0000000	3.2500000
-1381.0000000	3.2449999
-1380.0000000	3.2449999
-1379.0000000	3.2500000
-1378.0000000	3.2600000
-1377.0000000	3.2500000
-1376.0000000	3.2600000
-1375.0000000	3.2750001
-1374.0000000	3.2750001
-1373.0000000	3.2550001
-1372.0000000	3.2200000
-1371.0000000	3.1750002
-1370.0000000	3.1600001
-1369.0000000	3.1700001
-1368.0000000	3.1900001
-1367.0000000	3.2000000
-1366.0000000	3.2366667
-1365.0000000	3.2500000
-1364.0000000	3.2500000
-1363.0000000	3.2400000
-1362.0000000	3.2350001
-1361.0000000	3.2249999
-1360.0000000	3.2300000
-1359.0000000	3.2350001
-1358.0000000	3.2350001
-1357.0000000	3.2449999
-1356.0000000	3.2550001
-1355.0000000	3.2550001
-1354.0000000	3.2849998
-1353.0000000	3.2700000
-1352.0000000	3.2449999
-1351.0000000	3.2550001
-1350.0000000	3.2400000
-1349.0000000	3.2350001
-1348.0000000	3.2300000
-1347.0000000	3.2600000
-1346.0000000	3.2649999
-1345.0000000	3.2750001
-1344.0000000	3.2900000
-1343.0000000	3.3000000
-1342.0000000	3.3000000
-1341.0000000	3.2900000
-1340.0000000	3.2700000
-1339.0000000	3.2750001
-1338.0000000	3.2700000
-1337.0000000	3.2600000
-1336.0000000	3.2699997

-1335.0000000	3.2649999
-1334.0000000	3.2550001
-1333.0000000	3.2400000
-1332.0000000	3.2150002
-1331.0000000	3.2000000
-1330.0000000	3.1700001
-1329.0000000	3.0899999
-1328.0000000	3.0200000
-1327.0000000	2.9850001
-1326.0000000	2.9650002
-1325.0000000	2.9349999
-1324.0000000	2.9000001
-1323.0000000	2.8750000
-1322.0000000	2.8750000
-1321.0000000	2.8850002
-1320.0000000	2.8950000
-1319.0000000	2.9000001
-1318.0000000	2.9000001
-1317.0000000	2.8950000
-1316.0000000	2.8599999
-1315.0000000	2.8499999
-1314.0000000	2.8599999
-1313.0000000	2.8649998
-1312.0000000	2.8699999
-1311.0000000	2.8800001
-1310.0000000	2.8649998
-1309.0000000	2.8699999
-1308.0000000	2.8950000
-1307.0000000	2.9000001
-1306.0000000	2.9200001
-1305.0000000	2.9300001
-1304.0000000	2.9349999
-1303.0000000	2.9400001
-1302.0000000	2.9500000
-1301.0000000	2.9600000
-1300.0000000	2.9549999
-1299.0000000	2.9500000
-1298.0000000	2.9500000
-1297.0000000	2.9300001
-1296.0000000	2.9300001
-1295.0000000	2.9349999
-1294.0000000	2.9500000
-1293.0000000	2.9650002
-1292.0000000	2.9700000
-1291.0000000	2.9600000
-1290.0000000	2.9400001
-1289.0000000	2.9250002
-1288.0000000	2.9200001
-1287.0000000	2.9000001
-1286.0000000	2.8800001
-1285.0000000	2.8649998
-1284.0000000	2.8550000
-1283.0000000	2.8299999
-1282.0000000	2.8000000
-1281.0000000	2.7649999
-1280.0000000	2.7600000
-1279.0000000	2.7449999

-1278.0000000	2.7350001
-1277.0000000	2.7449999
-1276.0000000	2.7550001
-1275.0000000	2.7900000
-1274.0000000	2.8199999
-1273.0000000	2.8399999
-1272.0000000	2.8649998
-1271.0000000	2.8850002
-1270.0000000	2.8950000
-1269.0000000	2.9000001
-1268.0000000	2.9000001
-1267.0000000	2.8950000
-1266.0000000	2.9000001
-1265.0000000	2.8850002
-1264.0000000	2.8900001
-1263.0000000	2.8800001
-1262.0000000	2.8750000
-1261.0000000	2.8699999
-1260.0000000	2.8850002
-1259.0000000	2.8950000
-1258.0000000	2.9150000
-1257.0000000	2.9450002
-1256.0000000	2.9749999
-1255.0000000	2.9800000
-1254.0000000	2.9850001
-1253.0000000	3.0100000
-1252.0000000	3.0349998
-1251.0000000	3.0450001
-1250.0000000	3.0549998
-1249.0000000	3.0749998
-1248.0000000	3.1050000
-1247.0000000	3.1199999
-1246.0000000	3.1500001
-1245.0000000	3.1600001
-1244.0000000	3.1650000
-1243.0000000	3.1700001
-1242.0000000	3.1550002
-1241.0000000	3.1400001
-1240.0000000	3.1250000
-1239.0000000	3.1149998
-1238.0000000	3.1050000
-1237.0000000	3.1050000
-1236.0000000	3.0999999
-1235.0000000	3.0949998
-1234.0000000	3.0949998
-1233.0000000	3.0899999
-1232.0000000	3.0850000
-1231.0000000	3.0650001
-1230.0000000	3.0349998
-1229.0000000	3.0000000
-1228.0000000	2.9500000
-1227.0000000	2.9250002
-1226.0000000	2.9100001
-1225.0000000	2.9000001
-1224.0000000	2.9000001
-1223.0000000	2.9050002
-1222.0000000	2.9100001

-1221.0000000	2.8950000
-1220.0000000	2.8950000
-1219.0000000	2.8850002
-1218.0000000	2.8800001
-1217.0000000	2.8800001
-1216.0000000	2.8800001
-1215.0000000	2.8833334
-1214.0000000	2.8750000
-1213.0000000	2.8599999
-1212.0000000	2.8599999
-1211.0000000	2.8649998
-1210.0000000	2.8800001
-1209.0000000	2.8800001
-1208.0000000	2.8900001
-1207.0000000	2.8850002
-1206.0000000	2.8900001
-1205.0000000	2.9050002
-1204.0000000	2.9150000
-1203.0000000	2.9300001
-1202.0000000	2.9400001
-1201.0000000	2.9549999
-1200.0000000	2.9600000
-1199.0000000	2.9700000
-1198.0000000	2.9850001
-1197.0000000	3.0100000
-1196.0000000	3.0300000
-1195.0000000	3.0450001
-1194.0000000	3.0349998
-1193.0000000	3.0149999
-1192.0000000	2.9949999
-1191.0000000	2.9749999
-1190.0000000	2.9549999
-1189.0000000	2.9450002
-1188.0000000	2.9400001
-1187.0000000	2.9500000
-1186.0000000	2.9700000
-1185.0000000	2.9633334
-1184.0000000	2.9500000
-1183.0000000	2.9650002
-1182.0000000	2.9800000
-1181.0000000	2.9650002
-1180.0000000	2.9500000
-1179.0000000	2.9450002
-1178.0000000	2.9000001
-1177.0000000	2.8950000
-1176.0000000	2.8750000
-1175.0000000	2.8550000
-1174.0000000	2.8550000
-1173.0000000	2.8399999
-1172.0000000	2.8249998
-1171.0000000	2.8449998
-1170.0000000	2.8399999
-1169.0000000	2.8399999
-1168.0000000	2.8399999
-1167.0000000	2.8000000
-1166.0000000	2.7649999
-1165.0000000	2.7400000

-1164.0000000	2.7300000
-1163.0000000	2.7300000
-1162.0000000	2.7400000
-1161.0000000	2.7550001
-1160.0000000	2.7550001
-1159.0000000	2.7700000
-1158.0000000	2.7800000
-1157.0000000	2.7849998
-1156.0000000	2.7950001
-1155.0000000	2.8299999
-1154.0000000	2.8599999
-1153.0000000	2.8900001
-1152.0000000	2.9000001
-1151.0000000	2.9150000
-1150.0000000	2.9150000
-1149.0000000	2.9050002
-1148.0000000	2.9000001
-1147.0000000	2.8800001
-1146.0000000	2.8649998
-1145.0000000	2.8499999
-1144.0000000	2.8150001
-1143.0000000	2.8150001
-1142.0000000	2.7900000
-1141.0000000	2.7750001
-1140.0000000	2.7750001
-1139.0000000	2.7800000
-1138.0000000	2.7600000
-1137.0000000	2.7249999
-1136.0000000	2.6900001
-1135.0000000	2.6750002
-1134.0000000	2.6750002
-1133.0000000	2.6500001
-1132.0000000	2.6400001
-1131.0000000	2.6550002
-1130.0000000	2.6450000
-1129.0000000	2.6400001
-1128.0000000	2.6600001
-1127.0000000	2.6800001
-1126.0000000	2.6900001
-1125.0000000	2.7049999
-1124.0000000	2.7000000
-1123.0000000	2.7000000
-1122.0000000	2.6849999
-1121.0000000	2.6849999
-1120.0000000	2.6800001
-1119.0000000	2.6750002
-1118.0000000	2.6800001
-1117.0000000	2.6700001
-1116.0000000	2.6400001
-1115.0000000	2.6350002
-1114.0000000	2.6199999
-1113.0000000	2.5999999
-1112.0000000	2.5899999
-1111.0000000	2.5850000
-1110.0000000	2.5850000
-1109.0000000	2.5999999
-1108.0000000	2.5999999

-1107.0000000	2.6199999
-1106.0000000	2.6350002
-1105.0000000	2.6500001
-1104.0000000	2.6700001
-1103.0000000	2.6700001
-1102.0000000	2.6650000
-1101.0000000	2.6700001
-1100.0000000	2.6800001
-1099.0000000	2.6800001
-1098.0000000	2.6849999
-1097.0000000	2.6950002
-1096.0000000	2.7200000
-1095.0000000	2.7449999
-1094.0000000	2.7733333
-1093.0000000	2.7900000
-1092.0000000	2.8000000
-1091.0000000	2.8099999
-1090.0000000	2.8199999
-1089.0000000	2.8399999
-1088.0000000	2.8550000
-1087.0000000	2.8750000
-1086.0000000	2.8850000
-1085.0000000	2.9100001
-1084.0000000	2.9300001
-1083.0000000	2.9600000
-1082.0000000	2.9700000
-1081.0000000	2.9700000
-1080.0000000	2.9800000
-1079.0000000	2.9700000
-1078.0000000	2.9600000
-1077.0000000	2.9450002
-1076.0000000	2.8950000
-1075.0000000	2.8599999
-1074.0000000	2.8350000
-1073.0000000	2.8299999
-1072.0000000	2.8399999
-1071.0000000	2.8199999
-1070.0000000	2.8099999
-1069.0000000	2.8049998
-1068.0000000	2.8150001
-1067.0000000	2.8499999
-1066.0000000	2.8699999
-1065.0000000	2.8900001
-1064.0000000	2.9100001
-1063.0000000	2.9500000
-1062.0000000	2.9700000
-1061.0000000	3.0000000
-1060.0000000	3.0250001
-1059.0000000	3.0349998
-1058.0000000	3.0650001
-1057.0000000	3.0699999
-1056.0000000	3.0699999
-1055.0000000	3.0799999
-1054.0000000	3.0899999
-1053.0000000	3.0949998
-1052.0000000	3.0999999
-1051.0000000	3.1099999

-1050.0000000	3.1050000
-1049.0000000	3.1199999
-1048.0000000	3.1300001
-1047.0000000	3.1400001
-1046.0000000	3.1400001
-1045.0000000	3.1550002
-1044.0000000	3.1800001
-1043.0000000	3.2200000
-1042.0000000	3.2449999
-1041.0000000	3.2600000
-1040.0000000	3.2800000
-1039.0000000	3.3000000
-1038.0000000	3.3099999
-1037.0000000	3.3299999
-1036.0000000	3.3499999
-1035.0000000	3.3699999
-1034.0000000	3.3866670
-1033.0000000	3.4050002
-1032.0000000	3.4250002
-1031.0000000	3.4450002
-1030.0000000	3.4700000
-1029.0000000	3.4850001
-1028.0000000	3.4949999
-1027.0000000	3.5050001
-1026.0000000	3.5149999
-1025.0000000	3.5300000
-1024.0000000	3.5250001
-1023.0000000	3.5349998
-1022.0000000	3.5349998
-1021.0000000	3.5349998
-1020.0000000	3.5349998
-1019.0000000	3.5450001
-1018.0000000	3.5500000
-1017.0000000	3.5549998
-1016.0000000	3.5650001
-1015.0000000	3.5650001
-1014.0000000	3.5699999
-1013.0000000	3.5999999
-1012.0000000	3.5949998
-1011.0000000	3.5899999
-1010.0000000	3.5850000
-1009.0000000	3.5699999
-1008.0000000	3.5699999
-1007.0000000	3.5699999
-1006.0000000	3.5549998
-1005.0000000	3.5500000
-1004.0000000	3.5300000
-1003.0000000	3.5166667
-1002.0000000	3.5050001
-1001.0000000	3.4949999
-1000.0000000	3.4749999
-999.0000000	3.4650002
-998.0000000	3.4600000
-997.0000000	3.4450002
-996.0000000	3.4250002
-995.0000000	3.4150000
-994.0000000	3.4050002

-993.0000000	3.4000001
-992.0000000	3.3900001
-991.0000000	3.4000001
-990.0000000	3.3850002
-989.0000000	3.3649998
-988.0000000	3.3750000
-987.0000000	3.3599999
-986.0000000	3.3699999
-985.0000000	3.3900001
-984.0000000	3.4250002
-983.0000000	3.4500000
-982.0000000	3.4700000
-981.0000000	3.4949999
-980.0000000	3.5149999
-979.0000000	3.5349998
-978.0000000	3.5400000
-977.0000000	3.5450001
-976.0000000	3.5300000
-975.0000000	3.5200000
-974.0000000	3.5166667
-973.0000000	3.4949999
-972.0000000	3.4800000
-971.0000000	3.4749999
-970.0000000	3.4650002
-969.0000000	3.4600000
-968.0000000	3.4500000
-967.0000000	3.4400001
-966.0000000	3.4150000
-965.0000000	3.4050002
-964.0000000	3.3950000
-963.0000000	3.3850002
-962.0000000	3.3800001
-961.0000000	3.3599999
-960.0000000	3.3449998
-959.0000000	3.3249998
-958.0000000	3.3150001
-957.0000000	3.3049998
-956.0000000	3.2849998
-955.0000000	3.2750001
-954.0000000	3.2900000
-953.0000000	3.3000000
-952.0000000	3.3199999
-951.0000000	3.3399999
-950.0000000	3.3550000
-949.0000000	3.3800001
-948.0000000	3.3850002
-947.0000000	3.4050002
-946.0000000	3.4000001
-945.0000000	3.4200001
-944.0000000	3.4400001
-943.0000000	3.4700000
-942.0000000	3.4900000
-941.0000000	3.4900000
-940.0000000	3.5100000
-939.0000000	3.5200000
-938.0000000	3.5349998
-937.0000000	3.5450001

-936.0000000	3.5549998
-935.0000000	3.5599999
-934.0000000	3.5650001
-933.0000000	3.5650001
-932.0000000	3.5650001
-931.0000000	3.5450001
-930.0000000	3.5250001
-929.0000000	3.5149999
-928.0000000	3.5200000
-927.0000000	3.5349998
-926.0000000	3.5349998
-925.0000000	3.5500000
-924.0000000	3.5450001
-923.0000000	3.5500000
-922.0000000	3.5599999
-921.0000000	3.5799999
-920.0000000	3.5799999
-919.0000000	3.5949998
-918.0000000	3.6050000
-917.0000000	3.6099999
-916.0000000	3.6149998
-915.0000000	3.6149998
-914.0000000	3.6133335
-913.0000000	3.6050000
-912.0000000	3.5999999
-911.0000000	3.5999999
-910.0000000	3.5949998
-909.0000000	3.5799999
-908.0000000	3.5450001
-907.0000000	3.5400000
-906.0000000	3.5349998
-905.0000000	3.5450001
-904.0000000	3.5650001
-903.0000000	3.5599999
-902.0000000	3.5799999
-901.0000000	3.5749998
-900.0000000	3.5799999
-899.0000000	3.5899999
-898.0000000	3.6050000
-897.0000000	3.6149998
-896.0000000	3.6250000
-895.0000000	3.6099999
-894.0000000	3.6099999
-893.0000000	3.6050000
-892.0000000	3.5949998
-891.0000000	3.5949998
-890.0000000	3.5799999
-889.0000000	3.5650001
-888.0000000	3.5749998
-887.0000000	3.5450001
-886.0000000	3.5450001
-885.0000000	3.5300000
-884.0000000	3.5200000
-883.0000000	3.5100000
-882.0000000	3.5000000
-881.0000000	3.4866667
-880.0000000	3.4700000

-879.0000000	3.4600000
-878.0000000	3.4650002
-877.0000000	3.4650002
-876.0000000	3.4650002
-875.0000000	3.4800000
-874.0000000	3.4900000
-873.0000000	3.4949999
-872.0000000	3.5100000
-871.0000000	3.5050001
-870.0000000	3.5100000
-869.0000000	3.5149999
-868.0000000	3.5100000
-867.0000000	3.4949999
-866.0000000	3.4800000
-865.0000000	3.4700000
-864.0000000	3.4250002
-863.0000000	3.4000001
-862.0000000	3.3750000
-861.0000000	3.3750000
-860.0000000	3.3800001
-859.0000000	3.3900001
-858.0000000	3.3950000
-857.0000000	3.4100001
-856.0000000	3.4050002
-855.0000000	3.3950000
-854.0000000	3.3850002
-853.0000000	3.3499999
-852.0000000	3.3350000
-851.0000000	3.3100002
-850.0000000	3.2900000
-849.0000000	3.2550001
-848.0000000	3.2150002
-847.0000000	3.1700001
-846.0000000	3.1350000
-845.0000000	3.1050000
-844.0000000	3.0500000
-843.0000000	2.9900000
-842.0000000	2.9200001
-841.0000000	2.8750000
-840.0000000	2.8249998
-839.0000000	2.7950001
-838.0000000	2.8049998
-837.0000000	2.8099999
-836.0000000	2.8299999
-835.0000000	2.8199999
-834.0000000	2.8199999
-833.0000000	2.8099999
-832.0000000	2.8000000
-831.0000000	2.7649999
-830.0000000	2.7400000
-829.0000000	2.7449999
-828.0000000	2.7649999
-827.0000000	2.7900000
-826.0000000	2.7800000
-825.0000000	2.7600000
-824.0000000	2.7649999
-823.0000000	2.7900000

-822.0000000	2.8499999
-821.0000000	2.8599999
-820.0000000	2.8866665
-819.0000000	2.9150000
-818.0000000	2.9450002
-817.0000000	2.9700000
-816.0000000	2.9600000
-815.0000000	2.9700000
-814.0000000	2.9900000
-813.0000000	2.9900000
-812.0000000	2.9850001
-811.0000000	2.9850001
-810.0000000	2.9800000
-809.0000000	2.9700000
-808.0000000	2.9549999
-807.0000000	2.9450002
-806.0000000	2.9250002
-805.0000000	2.9150000
-804.0000000	2.9100001
-803.0000000	2.8900001
-802.0000000	2.8699999
-801.0000000	2.8599999
-800.0000000	2.8499999
-799.0000000	2.8399999
-798.0000000	2.8399999
-797.0000000	2.8399999
-796.0000000	2.8599999
-795.0000000	2.8649998
-794.0000000	2.8699999
-793.0000000	2.8550000
-792.0000000	2.8550000
-791.0000000	2.8699999
-790.0000000	2.8750000
-789.0000000	2.8699999
-788.0000000	2.8850002
-787.0000000	2.8950000
-786.0000000	2.9100001
-785.0000000	2.9300001
-784.0000000	2.9450002
-783.0000000	2.9450002
-782.0000000	2.9349999
-781.0000000	2.9450002
-780.0000000	2.9400001
-779.0000000	2.9349999
-778.0000000	2.9100001
-777.0000000	2.8950000
-776.0000000	2.8699999
-775.0000000	2.8649998
-774.0000000	2.8399999
-773.0000000	2.8099999
-772.0000000	2.7900000
-771.0000000	2.7649999
-770.0000000	2.7449999
-769.0000000	2.7249999
-768.0000000	2.7000000
-767.0000000	2.6700001
-766.0000000	2.6600001

-765.0000000	2.6600001
-764.0000000	2.6700001
-763.0000000	2.6849999
-762.0000000	2.7049999
-761.0000000	2.7333333
-760.0000000	2.7400000
-759.0000000	2.7400000
-758.0000000	2.7300000
-757.0000000	2.7350001
-756.0000000	2.7300000
-755.0000000	2.7400000
-754.0000000	2.7550001
-753.0000000	2.7700000
-752.0000000	2.8000000
-751.0000000	2.8299999
-750.0000000	2.8350000
-749.0000000	2.8550000
-748.0000000	2.8699999
-747.0000000	2.8800001
-746.0000000	2.9000001
-745.0000000	2.9000001
-744.0000000	2.9000001
-743.0000000	2.8950000
-742.0000000	2.8950000
-741.0000000	2.8800001
-740.0000000	2.8800001
-739.0000000	2.8399999
-738.0000000	2.8249998
-737.0000000	2.8299999
-736.0000000	2.8550000
-735.0000000	2.8900001
-734.0000000	2.9200001
-733.0000000	2.9450002
-732.0000000	2.9749999
-731.0000000	3.0166667
-730.0000000	3.0650001
-729.0000000	3.0899999
-728.0000000	3.1149998
-727.0000000	3.1500001
-726.0000000	3.1650000
-725.0000000	3.1600001
-724.0000000	3.1700001
-723.0000000	3.1750002
-722.0000000	3.1800001
-721.0000000	3.1750002
-720.0000000	3.1849999
-719.0000000	3.1950002
-718.0000000	3.1849999
-717.0000000	3.1600001
-716.0000000	3.1199999
-715.0000000	3.0850000
-714.0000000	3.0450001
-713.0000000	3.0300000
-712.0000000	3.0000000
-711.0000000	2.9949999
-710.0000000	2.9749999
-709.0000000	2.9650002

-708.0000000	2.9500000
-707.0000000	2.9300001
-706.0000000	2.9300001
-705.0000000	2.9050002
-704.0000000	2.8850002
-703.0000000	2.8599999
-702.0000000	2.8550000
-701.0000000	2.8599999
-700.0000000	2.8699999
-699.0000000	2.8900001
-698.0000000	2.9050002
-697.0000000	2.9150000
-696.0000000	2.9100001
-695.0000000	2.8950000
-694.0000000	2.9050002
-693.0000000	2.9150000
-692.0000000	2.9349999
-691.0000000	2.9250002
-690.0000000	2.9349999
-689.0000000	2.9300001
-688.0000000	2.9250002
-687.0000000	2.9050002
-686.0000000	2.9000001
-685.0000000	2.8900001
-684.0000000	2.8750000
-683.0000000	2.8800001
-682.0000000	2.8699999
-681.0000000	2.8499999
-680.0000000	2.8499999
-679.0000000	2.8350000
-678.0000000	2.8150001
-677.0000000	2.7800000
-676.0000000	2.7449999
-675.0000000	2.7200000
-674.0000000	2.7000000
-673.0000000	2.6700001
-672.0000000	2.6450000
-671.0000000	2.6099999
-670.0000000	2.5899999
-669.0000000	2.5949998
-668.0000000	2.5949998
-667.0000000	2.5999999
-666.0000000	2.5850000
-665.0000000	2.5850000
-664.0000000	2.5799999
-663.0000000	2.5450001
-662.0000000	2.4949999
-661.0000000	2.4600000
-660.0000000	2.4700000
-659.0000000	2.4650002
-658.0000000	2.5000000
-657.0000000	2.5250001
-656.0000000	2.5599999
-655.0000000	2.5699999
-654.0000000	2.5799999
-653.0000000	2.6050000
-652.0000000	2.6149998

-651.0000000	2.6350002
-650.0000000	2.6500001
-649.0000000	2.6500001
-648.0000000	2.6800001
-647.0000000	2.7000000
-646.0000000	2.7300000
-645.0000000	2.7700000
-644.0000000	2.8150001
-643.0000000	2.8350000
-642.0000000	2.8649998
-641.0000000	2.8800001
-640.0000000	2.8900001
-639.0000000	2.8850002
-638.0000000	2.8850002
-637.0000000	2.8800001
-636.0000000	2.8649998
-635.0000000	2.8599999
-634.0000000	2.8350000
-633.0000000	2.8000000
-632.0000000	2.7750001
-631.0000000	2.7400000
-630.0000000	2.6849999
-629.0000000	2.6300001
-628.0000000	2.5699999
-627.0000000	2.5149999
-626.0000000	2.4450002
-625.0000000	2.3800001
-624.0000000	2.3399999
-623.0000000	2.3249998
-622.0000000	2.2950001
-621.0000000	2.2800000
-620.0000000	2.2400000
-619.0000000	2.1950002
-618.0000000	2.1500001
-617.0000000	2.1350002
-616.0000000	2.1199999
-615.0000000	2.1149998
-614.0000000	2.0999999
-613.0000000	2.0999999
-612.0000000	2.0999999
-611.0000000	2.1149998
-610.0000000	2.1400001
-609.0000000	2.1666667
-608.0000000	2.1550002
-607.0000000	2.1500001
-606.0000000	2.1450000
-605.0000000	2.1600001
-604.0000000	2.1600001
-603.0000000	2.1500001
-602.0000000	2.1550002
-601.0000000	2.1500001
-600.0000000	2.1500001
-599.0000000	2.1350002
-598.0000000	2.1050000
-597.0000000	2.0999999
-596.0000000	2.1050000
-595.0000000	2.0999999

-594.0000000	2.0799999
-593.0000000	2.0500000
-592.0000000	2.0599999
-591.0000000	2.0699999
-590.0000000	2.0799999
-589.0000000	2.1050000
-588.0000000	2.1450000
-587.0000000	2.1650000
-586.0000000	2.1950002
-585.0000000	2.2150002
-584.0000000	2.2449999
-583.0000000	2.2600000
-582.0000000	2.2800000
-581.0000000	2.3000000
-580.0000000	2.3249998
-579.0000000	2.3466666
-578.0000000	2.3550000
-577.0000000	2.3649998
-576.0000000	2.3900001
-575.0000000	2.4050002
-574.0000000	2.4150000
-573.0000000	2.4150000
-572.0000000	2.4200001
-571.0000000	2.4349999
-570.0000000	2.4600000
-569.0000000	2.4800000
-568.0000000	2.5000000
-567.0000000	2.5349998
-566.0000000	2.5549998
-565.0000000	2.5650001
-564.0000000	2.5899999
-563.0000000	2.5949998
-562.0000000	2.6099999
-561.0000000	2.6149998
-560.0000000	2.6250000
-559.0000000	2.6300001
-558.0000000	2.6300001
-557.0000000	2.6450000
-556.0000000	2.6650000
-555.0000000	2.6650000
-554.0000000	2.6750002
-553.0000000	2.6950002
-552.0000000	2.7200000
-551.0000000	2.7400000
-550.0000000	2.7550001
-549.0000000	2.7733333
-548.0000000	2.7800000
-547.0000000	2.7849998
-546.0000000	2.7950001
-545.0000000	2.8249998
-544.0000000	2.8399999
-543.0000000	2.8550000
-542.0000000	2.8850002
-541.0000000	2.9100001
-540.0000000	2.9200001
-539.0000000	2.9400001
-538.0000000	2.9450002

-537.0000000	2.9549999
-536.0000000	2.9500000
-535.0000000	2.9600000
-534.0000000	2.9650002
-533.0000000	2.9700000
-532.0000000	2.9800000
-531.0000000	2.9900000
-530.0000000	3.0050001
-529.0000000	3.0200000
-528.0000000	3.0250001
-527.0000000	3.0400000
-526.0000000	3.0450001
-525.0000000	3.0250001
-524.0000000	3.0349998
-523.0000000	3.0349998
-522.0000000	3.0400000
-521.0000000	3.0400000
-520.0000000	3.0450001
-519.0000000	3.0400000
-518.0000000	3.0400000
-517.0000000	3.0500000
-516.0000000	3.0500000
-515.0000000	3.0450001
-514.0000000	3.0500000
-513.0000000	3.0450001
-512.0000000	3.0450001
-511.0000000	3.0549998
-510.0000000	3.0599999
-509.0000000	3.0650001
-508.0000000	3.0699999
-507.0000000	3.0699999
-506.0000000	3.0799999
-505.0000000	3.0949998
-504.0000000	3.0899999
-503.0000000	3.0899999
-502.0000000	3.0850000
-501.0000000	3.0999999
-500.0000000	3.1099999
-499.0000000	3.1099999
-498.0000000	3.1099999
-497.0000000	3.1050000
-496.0000000	3.0999999
-495.0000000	3.0999999
-494.0000000	3.0999999
-493.0000000	3.1050000
-492.0000000	3.0999999
-491.0000000	3.0949998
-490.0000000	3.1099999
-489.0000000	3.0999997
-488.0000000	3.1050000
-487.0000000	3.1099999
-486.0000000	3.1199999
-485.0000000	3.1149998
-484.0000000	3.1199999
-483.0000000	3.1149998
-482.0000000	3.1300001
-481.0000000	3.1500001

-480.0000000	3.1300001
-479.0000000	3.1250000
-478.0000000	3.1400001
-477.0000000	3.1500001
-476.0000000	3.1550002
-475.0000000	3.1650000
-474.0000000	3.1849999
-473.0000000	3.1700001
-472.0000000	3.1750002
-471.0000000	3.1600001
-470.0000000	3.1500001
-469.0000000	3.1400001
-468.0000000	3.1250000
-467.0000000	3.1300001
-466.0000000	3.1300001
-465.0000000	3.1350002
-464.0000000	3.1450000
-463.0000000	3.1450000
-462.0000000	3.1450000
-461.0000000	3.1666667
-460.0000000	3.1700001
-459.0000000	3.1849999
-458.0000000	3.2049999
-457.0000000	3.2150002
-456.0000000	3.2350001
-455.0000000	3.2550001
-454.0000000	3.2800000
-453.0000000	3.3000000
-452.0000000	3.3150001
-451.0000000	3.3299999
-450.0000000	3.3350000
-449.0000000	3.3399999
-448.0000000	3.3350000
-447.0000000	3.3249998
-446.0000000	3.3350000
-445.0000000	3.3350000
-444.0000000	3.3499999
-443.0000000	3.3750000
-442.0000000	3.4000001
-441.0000000	3.4200001
-440.0000000	3.4400001
-439.0000000	3.4549999
-438.0000000	3.4450002
-437.0000000	3.4549999
-436.0000000	3.4700000
-435.0000000	3.4650002
-434.0000000	3.4850001
-433.0000000	3.5050001
-432.0000000	3.5000000
-431.0000000	3.5100000
-430.0000000	3.5200000
-429.0000000	3.5233333
-428.0000000	3.5349998
-427.0000000	3.5250001
-426.0000000	3.5200000
-425.0000000	3.5050001
-424.0000000	3.4900000

-423.0000000	3.4800000
-422.0000000	3.4700000
-421.0000000	3.4549999
-420.0000000	3.4600000
-419.0000000	3.4650002
-418.0000000	3.4850001
-417.0000000	3.5000000
-416.0000000	3.5149999
-415.0000000	3.5200000
-414.0000000	3.5149999
-413.0000000	3.5100000
-412.0000000	3.5149999
-411.0000000	3.5100000
-410.0000000	3.5000000
-409.0000000	3.5100000
-408.0000000	3.5200000
-407.0000000	3.5200000
-406.0000000	3.5300000
-405.0000000	3.5349998
-404.0000000	3.5200000
-403.0000000	3.5050001
-402.0000000	3.5100000
-401.0000000	3.5000000
-400.0000000	3.4749999
-399.0000000	3.4100001
-398.0000000	3.3600001
-397.0000000	3.3249998
-396.0000000	3.2950001
-395.0000000	3.2800000
-394.0000000	3.2400000
-393.0000000	3.1950002
-392.0000000	3.1450000
-391.0000000	3.1199999
-390.0000000	3.0949998
-389.0000000	3.0599999
-388.0000000	3.0100000
-387.0000000	2.9700000
-386.0000000	2.9250002
-385.0000000	2.9000001
-384.0000000	2.8649998
-383.0000000	2.8399999
-382.0000000	2.8199999
-381.0000000	2.8199999
-380.0000000	2.8199999
-379.0000000	2.8350000
-378.0000000	2.8399999
-377.0000000	2.8449998
-376.0000000	2.8699999
-375.0000000	2.8900001
-374.0000000	2.9100001
-373.0000000	2.9300001
-372.0000000	2.9549999
-371.0000000	2.9850001
-370.0000000	3.0100000
-369.0000000	2.9933331
-368.0000000	2.9749999
-367.0000000	2.9500000

-366.0000000	2.9300001
-365.0000000	2.8650000
-364.0000000	2.8249998
-363.0000000	2.8299999
-362.0000000	2.8350000
-361.0000000	2.8699999
-360.0000000	2.8800001
-359.0000000	2.8950000
-358.0000000	2.8950000
-357.0000000	2.9250002
-356.0000000	2.9749999
-355.0000000	3.0100000
-354.0000000	3.0549998
-353.0000000	3.0899999
-352.0000000	3.1199999
-351.0000000	3.1450000
-350.0000000	3.1750002
-349.0000000	3.1800001
-348.0000000	3.1800001
-347.0000000	3.1950002
-346.0000000	3.1950002
-345.0000000	3.2100000
-344.0000000	3.2200000
-343.0000000	3.2049999
-342.0000000	3.1950002
-341.0000000	3.1800001
-340.0000000	3.1900001
-339.0000000	3.2000000
-338.0000000	3.2066667
-337.0000000	3.1950002
-336.0000000	3.2150002
-335.0000000	3.2249999
-334.0000000	3.2449999
-333.0000000	3.2649999
-332.0000000	3.2449999
-331.0000000	3.2300000
-330.0000000	3.2100000
-329.0000000	3.1849999
-328.0000000	3.1600001
-327.0000000	3.1550002
-326.0000000	3.1650000
-325.0000000	3.1650000
-324.0000000	3.1800001
-323.0000000	3.1950002
-322.0000000	3.2000000
-321.0000000	3.2049999
-320.0000000	3.2000000
-319.0000000	3.2249999
-318.0000000	3.2449999
-317.0000000	3.2449999
-316.0000000	3.2400000
-315.0000000	3.2400000
-314.0000000	3.2300000
-313.0000000	3.2400000
-312.0000000	3.2249999
-311.0000000	3.2249999
-310.0000000	3.2300000

-309.0000000	3.2300000
-308.0000000	3.2266667
-307.0000000	3.2150002
-306.0000000	3.2150002
-305.0000000	3.2249999
-304.0000000	3.2249999
-303.0000000	3.2350001
-302.0000000	3.2449999
-301.0000000	3.2550001
-300.0000000	3.2600000
-299.0000000	3.2700000
-298.0000000	3.2750001
-297.0000000	3.2849998
-296.0000000	3.2800000
-295.0000000	3.2849998
-294.0000000	3.2750001
-293.0000000	3.2700000
-292.0000000	3.2649999
-291.0000000	3.2300000
-290.0000000	3.2000000
-289.0000000	3.1849999
-288.0000000	3.1700001
-287.0000000	3.1400001
-286.0000000	3.1199999
-285.0000000	3.0949998
-284.0000000	3.0850000
-283.0000000	3.0749998
-282.0000000	3.0699999
-281.0000000	3.0650001
-280.0000000	3.0450001
-279.0000000	3.0450001
-278.0000000	3.0666666
-277.0000000	3.0850000
-276.0000000	3.0850000
-275.0000000	3.0999999
-274.0000000	3.1250000
-273.0000000	3.1450000
-272.0000000	3.1700001
-271.0000000	3.1650000
-270.0000000	3.1700001
-269.0000000	3.1500001
-268.0000000	3.1450000
-267.0000000	3.1300001
-266.0000000	3.1199999
-265.0000000	3.1199999
-264.0000000	3.1300001
-263.0000000	3.1300001
-262.0000000	3.1300001
-261.0000000	3.1300001
-260.0000000	3.1250000
-259.0000000	3.0799999
-258.0000000	3.0000000
-257.0000000	2.9300001
-256.0000000	2.9000001
-255.0000000	2.8850002
-254.0000000	2.8800001
-253.0000000	2.8950000

-252.0000000	2.9150000
-251.0000000	2.9100001
-250.0000000	2.9050002
-249.0000000	2.8950000
-248.0000000	2.9150000
-247.0000000	2.9500000
-246.0000000	2.9850001
-245.0000000	3.0450001
-244.0000000	3.0899999
-243.0000000	3.1300001
-242.0000000	3.1750002
-241.0000000	3.2249999
-240.0000000	3.2400000
-239.0000000	3.2550001
-238.0000000	3.2700000
-237.0000000	3.2649999
-236.0000000	3.2649999
-235.0000000	3.2600000
-234.0000000	3.2500000
-233.0000000	3.2649999
-232.0000000	3.2700000
-231.0000000	3.2700000
-230.0000000	3.2600000
-229.0000000	3.2600000
-228.0000000	3.2500000
-227.0000000	3.2500000
-226.0000000	3.2550001
-225.0000000	3.2550001
-224.0000000	3.2649999
-223.0000000	3.2600000
-222.0000000	3.2500000
-221.0000000	3.2350001
-220.0000000	3.2150002
-219.0000000	3.2000000
-218.0000000	3.1833334
-217.0000000	3.1900001
-216.0000000	3.1950002
-215.0000000	3.2100000
-214.0000000	3.2100000
-213.0000000	3.2200000
-212.0000000	3.2400000
-211.0000000	3.2500000
-210.0000000	3.2600000
-209.0000000	3.2750001
-208.0000000	3.2649999
-207.0000000	3.2600000
-206.0000000	3.2600000
-205.0000000	3.2600000
-204.0000000	3.2500000
-203.0000000	3.2400000
-202.0000000	3.2300000
-201.0000000	3.2200000
-200.0000000	3.2150002
-199.0000000	3.2100000
-198.0000000	3.1950002
-197.0000000	3.2000000
-196.0000000	3.1950002

-195.0000000	3.2000000
-194.0000000	3.1900001
-193.0000000	3.1900001
-192.0000000	3.1800001
-191.0000000	3.1650000
-190.0000000	3.1450000
-189.0000000	3.1300001
-188.0000000	3.1099999
-187.0000000	3.0899999
-186.0000000	3.0699997
-185.0000000	3.0450001
-184.0000000	3.0349998
-183.0000000	3.0400000
-182.0000000	3.0300000
-181.0000000	3.0300000
-180.0000000	3.0200000
-179.0000000	3.0050001
-178.0000000	2.9850001
-177.0000000	2.9549999
-176.0000000	2.9300001
-175.0000000	2.8950000
-174.0000000	2.8550000
-173.0000000	2.8399999
-172.0000000	2.8299999
-171.0000000	2.8099999
-170.0000000	2.7700000
-169.0000000	2.7600000
-168.0000000	2.7300000
-167.0000000	2.7300000
-166.0000000	2.7249999
-165.0000000	2.7200000
-164.0000000	2.7150002
-163.0000000	2.7150002
-162.0000000	2.7049999
-161.0000000	2.6950002
-160.0000000	2.7000000
-159.0000000	2.7049999
-158.0000000	2.7000000
-157.0000000	2.6900001
-156.0000000	2.6700001
-155.0000000	2.6350002
-154.0000000	2.6250000
-153.0000000	2.6300001
-152.0000000	2.6350000
-151.0000000	2.6600001
-150.0000000	2.6750002
-149.0000000	2.6849999
-148.0000000	2.6950002
-147.0000000	2.7049999
-146.0000000	2.7000000
-145.0000000	2.7000000
-144.0000000	2.7150002
-143.0000000	2.7249999
-142.0000000	2.7249999
-141.0000000	2.7300000
-140.0000000	2.7300000
-139.0000000	2.7449999

-138.0000000	2.7649999
-137.0000000	2.7700000
-136.0000000	2.7700000
-135.0000000	2.7700000
-134.0000000	2.7600000
-133.0000000	2.7449999
-132.0000000	2.7400000
-131.0000000	2.7150002
-130.0000000	2.6800001
-129.0000000	2.6550002
-128.0000000	2.6450000
-127.0000000	2.6466668
-126.0000000	2.6600001
-125.0000000	2.6600001
-124.0000000	2.6700001
-123.0000000	2.6900001
-122.0000000	2.6900001
-121.0000000	2.6849999
-120.0000000	2.6950002
-119.0000000	2.6900001
-118.0000000	2.6950002
-117.0000000	2.7049999
-116.0000000	2.7049999
-115.0000000	2.7200000
-114.0000000	2.7500000
-113.0000000	2.7700000
-112.0000000	2.8049998
-111.0000000	2.8199999
-110.0000000	2.8199999
-109.0000000	2.8399999
-108.0000000	2.8599999
-107.0000000	2.8649998
-106.0000000	2.8750000
-105.0000000	2.8750000
-104.0000000	2.8750000
-103.0000000	2.8850002
-102.0000000	2.8800001
-101.0000000	2.8900001
-100.0000000	2.8750000
-99.0000000	2.8750000
-98.0000000	2.8800001
-97.0000000	2.8633330
-96.0000000	2.8449998
-95.0000000	2.8499999
-94.0000000	2.8449998
-93.0000000	2.8449998
-92.0000000	2.8350000
-91.0000000	2.8299999
-90.0000000	2.8350000
-89.0000000	2.8249998
-88.0000000	2.8199999
-87.0000000	2.8199999
-86.0000000	2.8049998
-85.0000000	2.7700000
-84.0000000	2.7300000
-83.0000000	2.7049999
-82.0000000	2.7000000

-81.0000000	2.6849999
-80.0000000	2.6750002
-79.0000000	2.6700001
-78.0000000	2.6750002
-77.0000000	2.6750002
-76.0000000	2.6849999
-75.0000000	2.6900001
-74.0000000	2.6950002
-73.0000000	2.7000000
-72.0000000	2.7100000
-71.0000000	2.7200000
-70.0000000	2.7400000
-69.0000000	2.7500000
-68.0000000	2.7566669
-67.0000000	2.7600000
-66.0000000	2.7800000
-65.0000000	2.7849998
-64.0000000	2.7800000
-63.0000000	2.7950001
-62.0000000	2.8049998
-61.0000000	2.8049998
-60.0000000	2.8000000
-59.0000000	2.7950001
-58.0000000	2.7950001
-57.0000000	2.8000000
-56.0000000	2.8000000
-55.0000000	2.7900000
-54.0000000	2.7800000
-53.0000000	2.7750001
-52.0000000	2.7550001
-51.0000000	2.7400000
-50.0000000	2.7200000
-49.0000000	2.6950002
-48.0000000	2.6700001
-47.0000000	2.6600001
-46.0000000	2.6600001
-45.0000000	2.6500001
-44.0000000	2.6550002
-43.0000000	2.6650000
-42.0000000	2.6600001
-41.0000000	2.6650000
-40.0000000	2.6750002
-39.0000000	2.6900001
-38.0000000	2.6950002
-37.0000000	2.7150002
-36.0000000	2.7233334
-35.0000000	2.7150002
-34.0000000	2.7150002
-33.0000000	2.7150002
-32.0000000	2.7300000
-31.0000000	2.7350001
-30.0000000	2.7500000
-29.0000000	2.7600000
-28.0000000	2.7750001
-27.0000000	2.7900000
-26.0000000	2.7849998
-25.0000000	2.8000000

-24.0000000	2.7950001
-23.0000000	2.8000000
-22.0000000	2.8099999
-21.0000000	2.8199999
-20.0000000	2.8299999
-19.0000000	2.8299999
-18.0000000	2.8350000
-17.0000000	2.8499999
-16.0000000	2.8699999
-15.0000000	2.8649998
-14.0000000	2.8800001
-13.0000000	2.8750000
-12.0000000	2.8850002
-11.0000000	2.8950000
-10.0000000	2.9050002
-9.0000000	2.9200001
-8.0000000	2.9250002
-7.0000000	2.9349999
-6.0000000	2.9566667
-5.0000000	2.9600000
-4.0000000	2.9700000
-3.0000000	2.9700000
-2.0000000	2.9749999
-1.0000000	2.9850001
0.0000000	2.9800000
1.0000000	2.9800000
2.0000000	2.9700000
3.0000000	2.9500000
4.0000000	2.9200001
5.0000000	2.8900001
6.0000000	2.8699999
7.0000000	2.8399999
8.0000000	2.8049998
9.0000000	2.7849998
10.0000000	2.7750001
11.0000000	2.7750001
12.0000000	2.7849998
13.0000000	2.8000000
14.0000000	2.8099999
15.0000000	2.8249998
16.0000000	2.8499999
17.0000000	2.8649998
18.0000000	2.8950000
19.0000000	2.9150000
20.0000000	2.9349999
21.0000000	2.9549999
22.0000000	2.9700000
23.0000000	2.9900000
24.0000000	3.0166664
25.0000000	3.0300000
26.0000000	3.0400000
27.0000000	3.0450001
28.0000000	3.0349998
29.0000000	3.0400000
30.0000000	3.0349998
31.0000000	3.0349998
32.0000000	3.0349998

33.0000000	3.0300000
34.0000000	3.0349998
35.0000000	3.0250001
36.0000000	3.0149999
37.0000000	3.0000000
38.0000000	2.9800000
39.0000000	2.9600000
40.0000000	2.9300001
41.0000000	2.9100001
42.0000000	2.8800001
43.0000000	2.8599999
44.0000000	2.8350000
45.0000000	2.8199999
46.0000000	2.8049998
47.0000000	2.8099999
48.0000000	2.8099999
49.0000000	2.8150001
50.0000000	2.8099999
51.0000000	2.8049998
52.0000000	2.8000000
53.0000000	2.8049998
54.0000000	2.7900000
55.0000000	2.7800000
56.0000000	2.7500000
57.0000000	2.7300000
58.0000000	2.7100000
59.0000000	2.6750002
60.0000000	2.6300001
61.0000000	2.5999999
62.0000000	2.5850000
63.0000000	2.5650001
64.0000000	2.5549998
65.0000000	2.5349998
66.0000000	2.5349998
67.0000000	2.5349998
68.0000000	2.5400000
69.0000000	2.5599999
70.0000000	2.5599999
71.0000000	2.5699999
72.0000000	2.5549998
73.0000000	2.5250001
74.0000000	2.5000000
75.0000000	2.4700000
76.0000000	2.4549999
77.0000000	2.4450002
78.0000000	2.4450002
79.0000000	2.4600000
80.0000000	2.4850001
81.0000000	2.5100000
82.0000000	2.5300000
83.0000000	2.5500000
84.0000000	2.5833333
85.0000000	2.6099999
86.0000000	2.6250000
87.0000000	2.6550002
88.0000000	2.6650000
89.0000000	2.6849999

90.0000000	2.7150002
91.0000000	2.7200000
92.0000000	2.7300000
93.0000000	2.7300000
94.0000000	2.7400000
95.0000000	2.7400000
96.0000000	2.7400000
97.0000000	2.7500000
98.0000000	2.7600000
99.0000000	2.7750001
100.0000000	2.7900000
101.0000000	2.7900000
102.0000000	2.7849998
103.0000000	2.7800000
104.0000000	2.7849998
105.0000000	2.7849998
106.0000000	2.7950001
107.0000000	2.7950001
108.0000000	2.8049998
109.0000000	2.8199999
110.0000000	2.8350000
111.0000000	2.8399999
112.0000000	2.8499999
113.0000000	2.8649998
114.0000000	2.8599999
115.0000000	2.8666666
116.0000000	2.8699999
117.0000000	2.8800001
118.0000000	2.8699999
119.0000000	2.8750000
120.0000000	2.8850002
121.0000000	2.8850002
122.0000000	2.8750000
123.0000000	2.8699999
124.0000000	2.8699999
125.0000000	2.8599999
126.0000000	2.8550000
127.0000000	2.8649998
128.0000000	2.8599999
129.0000000	2.8750000
130.0000000	2.8649998
131.0000000	2.8699999
132.0000000	2.8550000
133.0000000	2.8599999
134.0000000	2.8550000
135.0000000	2.8449998
136.0000000	2.8449998
137.0000000	2.8550000
138.0000000	2.8550000
139.0000000	2.8550000
140.0000000	2.8750000
141.0000000	2.8699999
142.0000000	2.8550000
143.0000000	2.8449998
144.0000000	2.8249998
145.0000000	2.7933333
146.0000000	2.7800000

147.0000000	2.7600000
148.0000000	2.7600000
149.0000000	2.7649999
150.0000000	2.7649999
151.0000000	2.7649999
152.0000000	2.7350001
153.0000000	2.7000000
154.0000000	2.6750002
155.0000000	2.6750002
156.0000000	2.6849999
157.0000000	2.6900001
158.0000000	2.6800001
159.0000000	2.6849999
160.0000000	2.6800001
161.0000000	2.6849999
162.0000000	2.6849999
163.0000000	2.7000000
164.0000000	2.6900001
165.0000000	2.6650000
166.0000000	2.6450000
167.0000000	2.6300001
168.0000000	2.6250000
169.0000000	2.6149998
170.0000000	2.6050000
171.0000000	2.6099999
172.0000000	2.6099999
173.0000000	2.6250000
174.0000000	2.6300001
175.0000000	2.6466668
176.0000000	2.6750002
177.0000000	2.6950002
178.0000000	2.7200000
179.0000000	2.7300000
180.0000000	2.7500000
181.0000000	2.7500000
182.0000000	2.7750001
183.0000000	2.7849998
184.0000000	2.8000000
185.0000000	2.8000000
186.0000000	2.8150001
187.0000000	2.8150001
188.0000000	2.8350000
189.0000000	2.8399999
190.0000000	2.8399999
191.0000000	2.8399999
192.0000000	2.8449998
193.0000000	2.8449998
194.0000000	2.8399999
195.0000000	2.8399999
196.0000000	2.8350000
197.0000000	2.8299999
198.0000000	2.8099999
199.0000000	2.7800000
200.0000000	2.7649999
201.0000000	2.7449999
202.0000000	2.7449999
203.0000000	2.7350001

204.0000000	2.7200000
205.0000000	2.7133334
206.0000000	2.7150002
207.0000000	2.7200000
208.0000000	2.7249999
209.0000000	2.7200000
210.0000000	2.7300000
211.0000000	2.7249999
212.0000000	2.7200000
213.0000000	2.7249999
214.0000000	2.7300000
215.0000000	2.7300000
216.0000000	2.7300000
217.0000000	2.7300000
218.0000000	2.7200000
219.0000000	2.7100000
220.0000000	2.7049999
221.0000000	2.6900001
222.0000000	2.6900001
223.0000000	2.6849999
224.0000000	2.6800001
225.0000000	2.6800001
226.0000000	2.6800001
227.0000000	2.6800001
228.0000000	2.6700001
229.0000000	2.6650000
230.0000000	2.6600001
231.0000000	2.6600001
232.0000000	2.6650000
233.0000000	2.6650000
234.0000000	2.6866667
235.0000000	2.7100000
236.0000000	2.7150002
237.0000000	2.7300000
238.0000000	2.7400000
239.0000000	2.7449999
240.0000000	2.7550001
241.0000000	2.7550001
242.0000000	2.7550001
243.0000000	2.7550001
244.0000000	2.7550001
245.0000000	2.7449999
246.0000000	2.7350001
247.0000000	2.7300000
248.0000000	2.7150002
249.0000000	2.7000000
250.0000000	2.6900001
251.0000000	2.6800001
252.0000000	2.6600001
253.0000000	2.6300001
254.0000000	2.6250000
255.0000000	2.6149998
256.0000000	2.6050000
257.0000000	2.5999999
258.0000000	2.5899999
259.0000000	2.5749998
260.0000000	2.5599999

261.0000000	2.5450001
262.0000000	2.5400000
263.0000000	2.5250001
264.0000000	2.5149999
265.0000000	2.5100000
266.0000000	2.4949999
267.0000000	2.4800003
268.0000000	2.4749999
269.0000000	2.4650002
270.0000000	2.4450002
271.0000000	2.4400001
272.0000000	2.4100001
273.0000000	2.3649998
274.0000000	2.3150001
275.0000000	2.2700000
276.0000000	2.2300000
277.0000000	2.2200000
278.0000000	2.2249999
279.0000000	2.2400000
280.0000000	2.2449999
281.0000000	2.2600000
282.0000000	2.2900000
283.0000000	2.2950001
284.0000000	2.3049998
285.0000000	2.3150001
286.0000000	2.3099999
287.0000000	2.3249998
288.0000000	2.3099999
289.0000000	2.3049998
290.0000000	2.3000000
291.0000000	2.3150001
292.0000000	2.3299999
293.0000000	2.3399999
294.0000000	2.3649998
295.0000000	2.3800001
296.0000000	2.4066668
297.0000000	2.4300001
298.0000000	2.4349999
299.0000000	2.4549999
300.0000000	2.4549999
301.0000000	2.4749999
302.0000000	2.4600000
303.0000000	2.4450002
304.0000000	2.4100001
305.0000000	2.3850002
306.0000000	2.3599999
307.0000000	2.3299999
308.0000000	2.2950001
309.0000000	2.2849998
310.0000000	2.2750001
311.0000000	2.2700000
312.0000000	2.2750001
313.0000000	2.2800000
314.0000000	2.2950001
315.0000000	2.3150001
316.0000000	2.3399999
317.0000000	2.3649998

318.0000000	2.3850002
319.0000000	2.4150000
320.0000000	2.4150000
321.0000000	2.4200001
322.0000000	2.4349999
323.0000000	2.4500000
324.0000000	2.4600000
325.0000000	2.4700000
326.0000000	2.4850001
327.0000000	2.5000000
328.0000000	2.5200000
329.0000000	2.5200000
330.0000000	2.5250001
331.0000000	2.5250001
332.0000000	2.5300000
333.0000000	2.5250001
334.0000000	2.5149999
335.0000000	2.4949999
336.0000000	2.4850001
337.0000000	2.4700000
338.0000000	2.4500000
339.0000000	2.4500000
340.0000000	2.4400001
341.0000000	2.4500000
342.0000000	2.4549999
343.0000000	2.4600000
344.0000000	2.4600000
345.0000000	2.4600000
346.0000000	2.4450002
347.0000000	2.4250002
348.0000000	2.4150000
349.0000000	2.3900001
350.0000000	2.3900001
351.0000000	2.3599999
352.0000000	2.3449998
353.0000000	2.3350000
354.0000000	2.3499999
355.0000000	2.3666666
356.0000000	2.3800001
357.0000000	2.3900001
358.0000000	2.4100001
359.0000000	2.4100001
360.0000000	2.4200001
361.0000000	2.4100001
362.0000000	2.3850002
363.0000000	2.3699999
364.0000000	2.3750000
365.0000000	2.3900001
366.0000000	2.4050002
367.0000000	2.4250002
368.0000000	2.4250002
369.0000000	2.4100001
370.0000000	2.4050002
371.0000000	2.3950000
372.0000000	2.3950000
373.0000000	2.3900001
374.0000000	2.4000001

375.0000000	2.4000001
376.0000000	2.4100001
377.0000000	2.3850002
378.0000000	2.3699999
379.0000000	2.3699999
380.0000000	2.3550000
381.0000000	2.3350000
382.0000000	2.3350000
383.0000000	2.3399999
384.0000000	2.3449998
385.0000000	2.3599999
386.0000000	2.3800001
387.0000000	2.3950000
388.0000000	2.4150000
389.0000000	2.4300001
390.0000000	2.4400001
391.0000000	2.4549999
392.0000000	2.4600000
393.0000000	2.4600000
394.0000000	2.4650002
395.0000000	2.4450002
396.0000000	2.4450002
397.0000000	2.4400001
398.0000000	2.4250002
399.0000000	2.4050002
400.0000000	2.4050002
401.0000000	2.4050002
402.0000000	2.4000001
403.0000000	2.3850002
404.0000000	2.3750000
405.0000000	2.3850002
406.0000000	2.3900001
407.0000000	2.3900001
408.0000000	2.3950000
409.0000000	2.4000001
410.0000000	2.4100001
411.0000000	2.4100001
412.0000000	2.4100001
413.0000000	2.4100001
414.0000000	2.4050002
415.0000000	2.3900001
416.0000000	2.3699999
417.0000000	2.3533332
418.0000000	2.3399999
419.0000000	2.3449998
420.0000000	2.3499999
421.0000000	2.3499999
422.0000000	2.3449998
423.0000000	2.3449998
424.0000000	2.3449998
425.0000000	2.3399999
426.0000000	2.3150001
427.0000000	2.3000000
428.0000000	2.3049998
429.0000000	2.3049998
430.0000000	2.2950001
431.0000000	2.2849998

432.0000000	2.2800000
433.0000000	2.2900000
434.0000000	2.2950001
435.0000000	2.3099999
436.0000000	2.3350000
437.0000000	2.3599999
438.0000000	2.3800001
439.0000000	2.3850002
440.0000000	2.3750000
441.0000000	2.3649998
442.0000000	2.3449998
443.0000000	2.3299999
444.0000000	2.3150001
445.0000000	2.3033333
446.0000000	2.3099999
447.0000000	2.3049998
448.0000000	2.2900000
449.0000000	2.2800000
450.0000000	2.2649999
451.0000000	2.2600000
452.0000000	2.2649999
453.0000000	2.2600000
454.0000000	2.2400000
455.0000000	2.2449999
456.0000000	2.2350001
457.0000000	2.2400000
458.0000000	2.2550001
459.0000000	2.2750001
460.0000000	2.2950001
461.0000000	2.3249998
462.0000000	2.3650000
463.0000000	2.3900001
464.0000000	2.4050002
465.0000000	2.4150000
466.0000000	2.4349999
467.0000000	2.4500000
468.0000000	2.4650002
469.0000000	2.4850001
470.0000000	2.5149999
471.0000000	2.5300000
472.0000000	2.5450001
473.0000000	2.5650001
474.0000000	2.5749998
475.0000000	2.5799999
476.0000000	2.5933332
477.0000000	2.5899999
478.0000000	2.5899999
479.0000000	2.5899999
480.0000000	2.5949998
481.0000000	2.5999999
482.0000000	2.6149998
483.0000000	2.6050000
484.0000000	2.5999999
485.0000000	2.6050000
486.0000000	2.6149998
487.0000000	2.6500001
488.0000000	2.6550002

489.0000000	2.6700001
490.0000000	2.6800001
491.0000000	2.7000000
492.0000000	2.7150002
493.0000000	2.7200000
494.0000000	2.7200000
495.0000000	2.7300000
496.0000000	2.7200000
497.0000000	2.7100000
498.0000000	2.7000000
499.0000000	2.6900001
500.0000000	2.6900001
501.0000000	2.6800001
502.0000000	2.6849999
503.0000000	2.6800001
504.0000000	2.6750002
505.0000000	2.6800001
506.0000000	2.6800001
507.0000000	2.6750002
508.0000000	2.6750002
509.0000000	2.6600001
510.0000000	2.6450000
511.0000000	2.6500001
512.0000000	2.6650000
513.0000000	2.6750002
514.0000000	2.6849999
515.0000000	2.7100000
516.0000000	2.7249999
517.0000000	2.7400000
518.0000000	2.7500000
519.0000000	2.7750001
520.0000000	2.7900000
521.0000000	2.7950001
522.0000000	2.8049998
523.0000000	2.8249998
524.0000000	2.8449998
525.0000000	2.8550000
526.0000000	2.8599999
527.0000000	2.8599999
528.0000000	2.8699999
529.0000000	2.8750000
530.0000000	2.8649998
531.0000000	2.8750000
532.0000000	2.8800001
533.0000000	2.8900001
534.0000000	2.8850002
535.0000000	2.8850002
536.0000000	2.8699999
537.0000000	2.8733332
538.0000000	2.8750000
539.0000000	2.8699999
540.0000000	2.8599999
541.0000000	2.8599999
542.0000000	2.8550000
543.0000000	2.8550000
544.0000000	2.8449998
545.0000000	2.8399999

546.0000000	2.8399999
547.0000000	2.8399999
548.0000000	2.8350000
549.0000000	2.8399999
550.0000000	2.8449998
551.0000000	2.8449998
552.0000000	2.8649998
553.0000000	2.8649998
554.0000000	2.8699999
555.0000000	2.8800001
556.0000000	2.8850002
557.0000000	2.8950000
558.0000000	2.8950000
559.0000000	2.9050002
560.0000000	2.9250002
561.0000000	2.9250002
562.0000000	2.9349999
563.0000000	2.9450002
564.0000000	2.9650002
565.0000000	2.9700000
566.0000000	2.9833333
567.0000000	2.9900000
568.0000000	3.0149999
569.0000000	3.0200000
570.0000000	3.0250001
571.0000000	3.0100000
572.0000000	3.0100000
573.0000000	3.0100000
574.0000000	3.0200000
575.0000000	3.0250001
576.0000000	3.0400000
577.0000000	3.0450001
578.0000000	3.0450001
579.0000000	3.0450001
580.0000000	3.0400000
581.0000000	3.0250001
582.0000000	3.0349998
583.0000000	3.0400000
584.0000000	3.0450001
585.0000000	3.0450001
586.0000000	3.0349998
587.0000000	3.0400000
588.0000000	3.0349998
589.0000000	3.0349998
590.0000000	3.0500000
591.0000000	3.0650001
592.0000000	3.0699999
593.0000000	3.0599999
594.0000000	3.0650001
595.0000000	3.0450001
596.0000000	3.0250001
597.0000000	3.0433333
598.0000000	3.0650001
599.0000000	3.0850000
600.0000000	3.1099999
601.0000000	3.1199999
602.0000000	3.1250000

603.0000000	3.1400001
604.0000000	3.1700001
605.0000000	3.1750002
606.0000000	3.1750002
607.0000000	3.1800001
608.0000000	3.1700001
609.0000000	3.1800001
610.0000000	3.1849999
611.0000000	3.1800001
612.0000000	3.1900001
613.0000000	3.1750002
614.0000000	3.1600001
615.0000000	3.1550002
616.0000000	3.1500001
617.0000000	3.1300001
618.0000000	3.1099999
619.0000000	3.0899999
620.0000000	3.0599999
621.0000000	3.0200000
622.0000000	2.9850001
623.0000000	2.9600000
624.0000000	2.9200001
625.0000000	2.9000001
626.0000000	2.8800001
627.0000000	2.8566666
628.0000000	2.8399999
629.0000000	2.8249998
630.0000000	2.8049998
631.0000000	2.7750001
632.0000000	2.7449999
633.0000000	2.7200000
634.0000000	2.6900001
635.0000000	2.6500001
636.0000000	2.5899999
637.0000000	2.5799999
638.0000000	2.5949998
639.0000000	2.6149998
640.0000000	2.6350002
641.0000000	2.6350002
642.0000000	2.6600001
643.0000000	2.6650000
644.0000000	2.6750002
645.0000000	2.6800001
646.0000000	2.6900001
647.0000000	2.7000000
648.0000000	2.7150002
649.0000000	2.7249999
650.0000000	2.7400000
651.0000000	2.7649999
652.0000000	2.7900000
653.0000000	2.8199999
654.0000000	2.8449998
655.0000000	2.8649998
656.0000000	2.8800001
657.0000000	2.9000001
658.0000000	2.9150000
659.0000000	2.9300001

660.0000000	2.9466667
661.0000000	2.9549999
662.0000000	2.9700000
663.0000000	2.9600000
664.0000000	2.9650002
665.0000000	2.9850001
666.0000000	2.9949999
667.0000000	3.0100000
668.0000000	3.0250001
669.0000000	3.0450001
670.0000000	3.0650001
671.0000000	3.0850000
672.0000000	3.1050000
673.0000000	3.1199999
674.0000000	3.1350002
675.0000000	3.1550002
676.0000000	3.1849999
677.0000000	3.1950002
678.0000000	3.2100000
679.0000000	3.2200000
680.0000000	3.2200000
681.0000000	3.2249999
682.0000000	3.2150002
683.0000000	3.2100000
684.0000000	3.2249999
685.0000000	3.2150002
686.0000000	3.1950002
687.0000000	3.2000000
688.0000000	3.1950002
689.0000000	3.1849999
690.0000000	3.1800003
691.0000000	3.1849999
692.0000000	3.1800001
693.0000000	3.1800001
694.0000000	3.1800001
695.0000000	3.1700001
696.0000000	3.1500001
697.0000000	3.1500001
698.0000000	3.1400001
699.0000000	3.1350002
700.0000000	3.1300001
701.0000000	3.1199999
702.0000000	3.1199999
703.0000000	3.1149998
704.0000000	3.1250000
705.0000000	3.1450000
706.0000000	3.1650000
707.0000000	3.1849999
708.0000000	3.2049999
709.0000000	3.2150002
710.0000000	3.2350001
711.0000000	3.2300000
712.0000000	3.2400000
713.0000000	3.2400000
714.0000000	3.2500000
715.0000000	3.2600000
716.0000000	3.2700000

717.0000000	3.2700000
718.0000000	3.2600000
719.0000000	3.2550001
720.0000000	3.2500000
721.0000000	3.2249999
722.0000000	3.2150002
723.0000000	3.2049999
724.0000000	3.1950002
725.0000000	3.1600001
726.0000000	3.1300001
727.0000000	3.0949998
728.0000000	3.0500000
729.0000000	3.0100000
730.0000000	2.9749999
731.0000000	2.9450002
732.0000000	2.9349999
733.0000000	2.9450002
734.0000000	2.9650002
735.0000000	2.9700000
736.0000000	2.9800000
737.0000000	2.9850001
738.0000000	2.9749999
739.0000000	2.9700000
740.0000000	2.9850001
741.0000000	3.0050001
742.0000000	3.0300000
743.0000000	3.0349998
744.0000000	3.0450001
745.0000000	3.0599999
746.0000000	3.0749998
747.0000000	3.0749998
748.0000000	3.0699999
749.0000000	3.0749998
750.0000000	3.0633333
751.0000000	3.0899999
752.0000000	3.0899999
753.0000000	3.0899999
754.0000000	3.0799999
755.0000000	3.0749998
756.0000000	3.0549998
757.0000000	3.0549998
758.0000000	3.0349998
759.0000000	3.0149999
760.0000000	3.0100000
761.0000000	3.0100000
762.0000000	3.0100000
763.0000000	3.0100000
764.0000000	3.0100000
765.0000000	2.9949999
766.0000000	2.9800000
767.0000000	2.9800000
768.0000000	2.9700000
769.0000000	2.9700000
770.0000000	2.9650002
771.0000000	2.9650002
772.0000000	2.9549999
773.0000000	2.9549999

774.0000000	2.9500000
775.0000000	2.9300001
776.0000000	2.9250002
777.0000000	2.9349999
778.0000000	2.9500000
779.0000000	2.9650002
780.0000000	2.9949999
781.0000000	3.0000000
782.0000000	3.0050001
783.0000000	3.0000000
784.0000000	2.9749999
785.0000000	2.9700000
786.0000000	2.9800000
787.0000000	2.9749999
788.0000000	2.9800000
789.0000000	2.9850001
790.0000000	2.9900000
791.0000000	2.9900000
792.0000000	2.9900000
793.0000000	2.9949999
794.0000000	3.0000000
795.0000000	2.9949999
796.0000000	2.9949999
797.0000000	2.9850001
798.0000000	2.9800000
799.0000000	2.9700000
800.0000000	2.9600000
801.0000000	2.9349999
802.0000000	2.9100001
803.0000000	2.8800001
804.0000000	2.8550000
805.0000000	2.8199999
806.0000000	2.7800000
807.0000000	2.7449999
808.0000000	2.6933334
809.0000000	2.6700001
810.0000000	2.6500001
811.0000000	2.6300001
812.0000000	2.6199999
813.0000000	2.6050000
814.0000000	2.5999999
815.0000000	2.5949998
816.0000000	2.5999999
817.0000000	2.6050000
818.0000000	2.5850000
819.0000000	2.5799999
820.0000000	2.5749998
821.0000000	2.5699999
822.0000000	2.5799999
823.0000000	2.5799999
824.0000000	2.5850000
825.0000000	2.5999999
826.0000000	2.6099999
827.0000000	2.6199999
828.0000000	2.6250000
829.0000000	2.5999999
830.0000000	2.5549998

831.0000000	2.5349998
832.0000000	2.5349998
833.0000000	2.5349998
834.0000000	2.5500000
835.0000000	2.5549998
836.0000000	2.5650001
837.0000000	2.5899999
838.0000000	2.6149998
839.0000000	2.6350002
840.0000000	2.6650000
841.0000000	2.6933334
842.0000000	2.7400000
843.0000000	2.7800000
844.0000000	2.7849998
845.0000000	2.8000000
846.0000000	2.8049998
847.0000000	2.8150001
848.0000000	2.8150001
849.0000000	2.8399999
850.0000000	2.8499999
851.0000000	2.8699999
852.0000000	2.8800001
853.0000000	2.8850002
854.0000000	2.8900001
855.0000000	2.9150000
856.0000000	2.9300001
857.0000000	2.9400001
858.0000000	2.9450002
859.0000000	2.9549999
860.0000000	2.9700000
861.0000000	2.9749999
862.0000000	2.9949999
863.0000000	3.0000000
864.0000000	3.0000000
865.0000000	3.0050001
866.0000000	3.0050001
867.0000000	3.0149999
868.0000000	3.0100000
869.0000000	3.0200000
870.0000000	2.9933331
871.0000000	2.9850001
872.0000000	3.0000000
873.0000000	3.0100000
874.0000000	3.0200000
875.0000000	3.0450001
876.0000000	3.0549998
877.0000000	3.0650001
878.0000000	3.0749998
879.0000000	3.0799999
880.0000000	3.0799999
881.0000000	3.0850000
882.0000000	3.0999999
883.0000000	3.0999999
884.0000000	3.1050000
885.0000000	3.1250000
886.0000000	3.1350002
887.0000000	3.1450000

888.0000000	3.1350002
889.0000000	3.1450000
890.0000000	3.1300001
891.0000000	3.1250000
892.0000000	3.1149998
893.0000000	3.1149998
894.0000000	3.1099999
895.0000000	3.1149998
896.0000000	3.1199999
897.0000000	3.1149998
898.0000000	3.1099999
899.0000000	3.0966663
900.0000000	3.0949998
901.0000000	3.1050000
902.0000000	3.0999999
903.0000000	3.1050000
904.0000000	3.1199999
905.0000000	3.1400001
906.0000000	3.1350002
907.0000000	3.1500001
908.0000000	3.1500001
909.0000000	3.1650000
910.0000000	3.1900001
911.0000000	3.2000000
912.0000000	3.2150002
913.0000000	3.2150002
914.0000000	3.2400000
915.0000000	3.2449999
916.0000000	3.2500000
917.0000000	3.2600000
918.0000000	3.2700000
919.0000000	3.2849998
920.0000000	3.3000000
921.0000000	3.3049998
922.0000000	3.3049998
923.0000000	3.3049998
924.0000000	3.3099999
925.0000000	3.3049998
926.0000000	3.3099999
927.0000000	3.3150001
928.0000000	3.3399999
929.0000000	3.3499997
930.0000000	3.3800001
931.0000000	3.4000001
932.0000000	3.4150000
933.0000000	3.4300001
934.0000000	3.4349999
935.0000000	3.4600000
936.0000000	3.4850001
937.0000000	3.5000000
938.0000000	3.5300000
939.0000000	3.5599999
940.0000000	3.5699999
941.0000000	3.5799999
942.0000000	3.6050000
943.0000000	3.6199999
944.0000000	3.6500001

945.0000000	3.6650000
946.0000000	3.6800001
947.0000000	3.7000000
948.0000000	3.7150002
949.0000000	3.7350001
950.0000000	3.7649999
951.0000000	3.7849998
952.0000000	3.8099999
953.0000000	3.8299999
954.0000000	3.8550000
955.0000000	3.8699999
956.0000000	3.8800001
957.0000000	3.8900001
958.0000000	3.9000001
959.0000000	3.9233334
960.0000000	3.9450002
961.0000000	3.9650002
962.0000000	3.9900000
963.0000000	4.0100002
964.0000000	4.0150003
965.0000000	4.0300002
966.0000000	4.0299997
967.0000000	4.0349998
968.0000000	4.0200000
969.0000000	4.0200000
970.0000000	4.0150003
971.0000000	4.0200000
972.0000000	4.0050001
973.0000000	3.9949999
974.0000000	3.9900000
975.0000000	3.9900000
976.0000000	3.9800000
977.0000000	3.9850001
978.0000000	3.9800000
979.0000000	3.9800000
980.0000000	3.9800000
981.0000000	3.9900000
982.0000000	3.9800000
983.0000000	3.9800000
984.0000000	3.9749999
985.0000000	3.9749999
986.0000000	3.9700000
987.0000000	3.9700000
988.0000000	3.9600000
989.0000000	3.9400001
990.0000000	3.9133332
991.0000000	3.8750000
992.0000000	3.8499999
993.0000000	3.8150001
994.0000000	3.7849998
995.0000000	3.7400000
996.0000000	3.7000000
997.0000000	3.6750002
998.0000000	3.6500001
999.0000000	3.6350002
1000.0000000	3.6350002
1001.0000000	3.6450000

1002.0000000	3.6400001
1003.0000000	3.6550002
1004.0000000	3.6450000
1005.0000000	3.6050000
1006.0000000	3.5850000
1007.0000000	3.5799999
1008.0000000	3.5599999
1009.0000000	3.5699999
1010.0000000	3.5599999
1011.0000000	3.5749998
1012.0000000	3.5699999
1013.0000000	3.5699999
1014.0000000	3.5500000
1015.0000000	3.5349998
1016.0000000	3.5200000
1017.0000000	3.4800000
1018.0000000	3.4349999
1019.0000000	3.3950000
1020.0000000	3.3433332
1021.0000000	3.3099999
1022.0000000	3.3099999
1023.0000000	3.3150001
1024.0000000	3.3099999
1025.0000000	3.2950001
1026.0000000	3.2750001
1027.0000000	3.2500000
1028.0000000	3.2350001
1029.0000000	3.2300000
1030.0000000	3.2500000
1031.0000000	3.2800000
1032.0000000	3.2900000
1033.0000000	3.3150001
1034.0000000	3.3299999
1035.0000000	3.3550000
1036.0000000	3.3950000
1037.0000000	3.4200001
1038.0000000	3.4349999
1039.0000000	3.4549999
1040.0000000	3.4600000
1041.0000000	3.4749999
1042.0000000	3.4700000
1043.0000000	3.4800000
1044.0000000	3.4749999
1045.0000000	3.5100000
1046.0000000	3.5100000
1047.0000000	3.5149999
1048.0000000	3.5250001
1049.0000000	3.5300000
1050.0000000	3.5549998
1051.0000000	3.5566666
1052.0000000	3.5500000
1053.0000000	3.5450001
1054.0000000	3.5450001
1055.0000000	3.5400000
1056.0000000	3.5200000
1057.0000000	3.4900000
1058.0000000	3.4650002

1059.0000000	3.4400001
1060.0000000	3.4250002
1061.0000000	3.4050002
1062.0000000	3.3900001
1063.0000000	3.3699999
1064.0000000	3.3649998
1065.0000000	3.3550000
1066.0000000	3.3649998
1067.0000000	3.3599999
1068.0000000	3.3599999
1069.0000000	3.3499999
1070.0000000	3.3599999
1071.0000000	3.3750000
1072.0000000	3.3800001
1073.0000000	3.3850002
1074.0000000	3.3900001
1075.0000000	3.4000001
1076.0000000	3.3950000
1077.0000000	3.3850002
1078.0000000	3.3850002
1079.0000000	3.4050002
1080.0000000	3.4233334
1081.0000000	3.4400001
1082.0000000	3.4600000
1083.0000000	3.4700000
1084.0000000	3.4900000
1085.0000000	3.5149999
1086.0000000	3.5300000
1087.0000000	3.5500000
1088.0000000	3.5650001
1089.0000000	3.5650001
1090.0000000	3.5699999
1091.0000000	3.5699999
1092.0000000	3.5599999
1093.0000000	3.5549998
1094.0000000	3.5400000
1095.0000000	3.5349998
1096.0000000	3.5200000
1097.0000000	3.5000000
1098.0000000	3.5000000
1099.0000000	3.4900000
1100.0000000	3.4949999
1101.0000000	3.4850001
1102.0000000	3.4700000
1103.0000000	3.4549999
1104.0000000	3.4349999
1105.0000000	3.4100001
1106.0000000	3.3850000
1107.0000000	3.3499999
1108.0000000	3.3199999
1109.0000000	3.3000000
1110.0000000	3.2766666
1111.0000000	3.2550001
1112.0000000	3.2500000
1113.0000000	3.2350001
1114.0000000	3.2049999
1115.0000000	3.1849999

1116.0000000	3.1500001
1117.0000000	3.1250000
1118.0000000	3.1050000
1119.0000000	3.0899999
1120.0000000	3.0699999
1121.0000000	3.0549998
1122.0000000	3.0450001
1123.0000000	3.0400000
1124.0000000	3.0349998
1125.0000000	3.0400000
1126.0000000	3.0500000
1127.0000000	3.0599999
1128.0000000	3.0650001
1129.0000000	3.0549998
1130.0000000	3.0500000
1131.0000000	3.0349998
1132.0000000	3.0200000
1133.0000000	2.9949999
1134.0000000	2.9700000
1135.0000000	2.9600000
1136.0000000	2.9549999
1137.0000000	2.9549999
1138.0000000	2.9500000
1139.0000000	2.9450002
1140.0000000	2.9333334
1141.0000000	2.9050002
1142.0000000	2.8800001
1143.0000000	2.8649998
1144.0000000	2.8750000
1145.0000000	2.8650000
1146.0000000	2.8449998
1147.0000000	2.8299999
1148.0000000	2.8550000
1149.0000000	2.8850000
1150.0000000	2.9000001
1151.0000000	2.9050002
1152.0000000	2.8950000
1153.0000000	2.8599999
1154.0000000	2.8750000
1155.0000000	2.9150000
1156.0000000	2.9400001
1157.0000000	2.9549999
1158.0000000	2.9600000
1159.0000000	2.9850001
1160.0000000	3.0050001
1161.0000000	2.9949999
1162.0000000	3.0050001
1163.0000000	3.0100000
1164.0000000	3.0200000
1165.0000000	3.0300000
1166.0000000	3.0450001
1167.0000000	3.0699999
1168.0000000	3.0799999
1169.0000000	3.0999999
1170.0000000	3.1149998
1171.0000000	3.1400001
1172.0000000	3.1550002

1173.0000000	3.1750002
1174.0000000	3.1800001
1175.0000000	3.2000000
1176.0000000	3.2200000
1177.0000000	3.2350001
1178.0000000	3.2550001
1179.0000000	3.2649999
1180.0000000	3.2600000
1181.0000000	3.2449999
1182.0000000	3.2649999
1183.0000000	3.2649999
1184.0000000	3.2649999
1185.0000000	3.2649999
1186.0000000	3.2750001
1187.0000000	3.2800000
1188.0000000	3.2649999
1189.0000000	3.2600000
1190.0000000	3.2249999
1191.0000000	3.2049999
1192.0000000	3.1750002
1193.0000000	3.1250000
1194.0000000	3.0650001
1195.0000000	2.9900000
1196.0000000	2.9300001
1197.0000000	2.8750000
1198.0000000	2.8199999
1199.0000000	2.7449999
1200.0000000	2.6900001
1201.0000000	2.6300001
1202.0000000	2.5899999
1203.0000000	2.5850000
1204.0000000	2.5850000
1205.0000000	2.5999999
1206.0000000	2.6099999
1207.0000000	2.5949998
1208.0000000	2.5999999
1209.0000000	2.6250000
1210.0000000	2.6650000
1211.0000000	2.7100000
1212.0000000	2.7550001
1213.0000000	2.7950001
1214.0000000	2.8449998
1215.0000000	2.8900001
1216.0000000	2.9150000
1217.0000000	2.9349999
1218.0000000	2.9500000
1219.0000000	2.9400001
1220.0000000	2.9349999
1221.0000000	2.9100001
1222.0000000	2.8800001
1223.0000000	2.8399999
1224.0000000	2.7900000
1225.0000000	2.7449999
1226.0000000	2.7200000
1227.0000000	2.6800001
1228.0000000	2.6550002
1229.0000000	2.6300001

1230.0000000	2.6149998
1231.0000000	2.6033332
1232.0000000	2.5899999
1233.0000000	2.5850000
1234.0000000	2.5899999
1235.0000000	2.5949998
1236.0000000	2.5899999
1237.0000000	2.5899999
1238.0000000	2.5799999
1239.0000000	2.5650001
1240.0000000	2.5599999
1241.0000000	2.5450001
1242.0000000	2.5400000
1243.0000000	2.5400000
1244.0000000	2.5400000
1245.0000000	2.5349998
1246.0000000	2.5250001
1247.0000000	2.5200000
1248.0000000	2.5250001
1249.0000000	2.5149999
1250.0000000	2.5000000
1251.0000000	2.5000000
1252.0000000	2.5100000
1253.0000000	2.5149999
1254.0000000	2.5300000
1255.0000000	2.5400000
1256.0000000	2.5400000
1257.0000000	2.5450001
1258.0000000	2.5549998
1259.0000000	2.5450001
1260.0000000	2.5400000
1261.0000000	2.5333331
1262.0000000	2.5250001
1263.0000000	2.5100000
1264.0000000	2.5050001
1265.0000000	2.5000000
1266.0000000	2.4949999
1267.0000000	2.5000000
1268.0000000	2.4900000
1269.0000000	2.5000000
1270.0000000	2.5050001
1271.0000000	2.5149999
1272.0000000	2.5200000
1273.0000000	2.5349998
1274.0000000	2.5450001
1275.0000000	2.5599999
1276.0000000	2.5850000
1277.0000000	2.5799999
1278.0000000	2.5699999
1279.0000000	2.5650001
1280.0000000	2.5799999
1281.0000000	2.5850000
1282.0000000	2.5899999
1283.0000000	2.5799999
1284.0000000	2.5850000
1285.0000000	2.5899999
1286.0000000	2.5999999

1287.0000000	2.5999999
1288.0000000	2.5949998
1289.0000000	2.5899999
1290.0000000	2.5899999
1291.0000000	2.5699999
1292.0000000	2.5650001
1293.0000000	2.5599999
1294.0000000	2.5566666
1295.0000000	2.5650001
1296.0000000	2.5599999
1297.0000000	2.5450001
1298.0000000	2.5500000
1299.0000000	2.5450001
1300.0000000	2.5450001
1301.0000000	2.5400000
1302.0000000	2.5200000
1303.0000000	2.4949999
1304.0000000	2.4650002
1305.0000000	2.4349999
1306.0000000	2.3800001
1307.0000000	2.3399999
1308.0000000	2.3249998
1309.0000000	2.3350000
1310.0000000	2.3650000
1311.0000000	2.3950000
1312.0000000	2.4200001
1313.0000000	2.4549999
1314.0000000	2.4700000
1315.0000000	2.4800000
1316.0000000	2.4949999
1317.0000000	2.4800000
1318.0000000	2.4749999
1319.0000000	2.4949999
1320.0000000	2.5050001
1321.0000000	2.5050001
1322.0000000	2.5149999
1323.0000000	2.5200000
1324.0000000	2.5300000
1325.0000000	2.5549998
1326.0000000	2.5799999
1327.0000000	2.5999999
1328.0000000	2.6250000
1329.0000000	2.6350002
1330.0000000	2.6450000
1331.0000000	2.6350002
1332.0000000	2.6149998
1333.0000000	2.5899999
1334.0000000	2.5850000
1335.0000000	2.5650001
1336.0000000	2.5549998
1337.0000000	2.5450001
1338.0000000	2.5250001
1339.0000000	2.5100000
1340.0000000	2.4900000
1341.0000000	2.4949999
1342.0000000	2.4949999
1343.0000000	2.5100000

1344.0000000	2.5200000
1345.0000000	2.5250001
1346.0000000	2.5200000
1347.0000000	2.5200000
1348.0000000	2.5250001
1349.0000000	2.5250001
1350.0000000	2.5250001
1351.0000000	2.5250001
1352.0000000	2.5349998
1353.0000000	2.5266666
1354.0000000	2.5349998
1355.0000000	2.5300000
1356.0000000	2.5349998
1357.0000000	2.5400000
1358.0000000	2.5450001
1359.0000000	2.5650001
1360.0000000	2.5549998
1361.0000000	2.5699999
1362.0000000	2.5749998
1363.0000000	2.5850000
1364.0000000	2.5949998
1365.0000000	2.5999999
1366.0000000	2.5999999
1367.0000000	2.5949998
1368.0000000	2.5999999
1369.0000000	2.5899999
1370.0000000	2.5699999
1371.0000000	2.5549998
1372.0000000	2.5650001
1373.0000000	2.5599999
1374.0000000	2.5699999
1375.0000000	2.5799999
1376.0000000	2.5650001
1377.0000000	2.5500000
1378.0000000	2.5500000
1379.0000000	2.5450001
1380.0000000	2.5300000
1381.0000000	2.5100000
1382.0000000	2.5000000
1383.0000000	2.4700000
1384.0000000	2.4450002
1385.0000000	2.4300001
1386.0000000	2.4050002
1387.0000000	2.3699999
1388.0000000	2.3449998
1389.0000000	2.3399999
1390.0000000	2.3249998
1391.0000000	2.3049998
1392.0000000	2.3000000
1393.0000000	2.3000000
1394.0000000	2.2600000
1395.0000000	2.2249999
1396.0000000	2.2000000
1397.0000000	2.1849999
1398.0000000	2.1950002
1399.0000000	2.1950002
1400.0000000	2.1900001

1401.0000000	2.1750002
1402.0000000	2.1650000
1403.0000000	2.1700001
1404.0000000	2.1700001
1405.0000000	2.1750002
1406.0000000	2.1900001
1407.0000000	2.1950002
1408.0000000	2.1950002
1409.0000000	2.1900001
1410.0000000	2.2100000
1411.0000000	2.2249999
1412.0000000	2.2400000
1413.0000000	2.2550001
1414.0000000	2.2600000
1415.0000000	2.2666667
1416.0000000	2.2649999
1417.0000000	2.2700000
1418.0000000	2.2849998
1419.0000000	2.2849998
1420.0000000	2.2900000
1421.0000000	2.3049998
1422.0000000	2.3099999
1423.0000000	2.3249998
1424.0000000	2.3150001
1425.0000000	2.3150001
1426.0000000	2.3199999
1427.0000000	2.3249998
1428.0000000	2.3299999
1429.0000000	2.3150001
1430.0000000	2.2849998
1431.0000000	2.2700000
1432.0000000	2.2550001
1433.0000000	2.2350001
1434.0000000	2.2150002
1435.0000000	2.1750002
1436.0000000	2.1700001
1437.0000000	2.1350002
1438.0000000	2.1250000
1439.0000000	2.1450000
1440.0000000	2.1700001
1441.0000000	2.1900001
1442.0000000	2.2200000
1443.0000000	2.2350001
1444.0000000	2.2600000
1445.0000000	2.2933333
1446.0000000	2.3049998
1447.0000000	2.3299999
1448.0000000	2.3299999
1449.0000000	2.3350000
1450.0000000	2.3299999
1451.0000000	2.3199999
1452.0000000	2.3049998
1453.0000000	2.2849998
1454.0000000	2.2600000
1455.0000000	2.2400000
1456.0000000	2.2249999
1457.0000000	2.2049999

1458.0000000	2.1849999
1459.0000000	2.1550002
1460.0000000	2.1099999
1461.0000000	2.0500000
1462.0000000	1.9850000
1463.0000000	1.9450001
1464.0000000	1.9200000
1465.0000000	1.9300001
1466.0000000	1.9450001
1467.0000000	1.9650000
1468.0000000	1.9900000
1469.0000000	2.0250001
1470.0000000	2.0549998
1471.0000000	2.0850000
1472.0000000	2.1199999
1473.0000000	2.1450000
1474.0000000	2.1966667
1475.0000000	2.2249999
1476.0000000	2.2700000
1477.0000000	2.3049998
1478.0000000	2.3350000
1479.0000000	2.3649998
1480.0000000	2.4000001
1481.0000000	2.4250002
1482.0000000	2.4500000
1483.0000000	2.4800000
1484.0000000	2.5149999
1485.0000000	2.5500000
1486.0000000	2.5949998
1487.0000000	2.6400001
1488.0000000	2.6750002
1489.0000000	2.7150002
1490.0000000	2.7600000
1491.0000000	2.7800000
1492.0000000	2.8199999
1493.0000000	2.8399999
1494.0000000	2.8599999
1495.0000000	2.8850002
1496.0000000	2.9050002
1497.0000000	2.9200001
1498.0000000	2.9300001
1499.0000000	2.9150000
1500.0000000	2.9250002
1501.0000000	2.9250002
1502.0000000	2.9450002
1503.0000000	2.9600000
1504.0000000	2.9900000
1505.0000000	3.0050001
1506.0000000	3.0200000
1507.0000000	3.0349998
1508.0000000	3.0400000
1509.0000000	3.0650001
1510.0000000	3.0699999
1511.0000000	3.0949998
1512.0000000	3.1300001
1513.0000000	3.1450000
1514.0000000	3.1600001

1515.0000000	3.1750002
1516.0000000	3.1849999
1517.0000000	3.2000000
1518.0000000	3.2100000
1519.0000000	3.2249999
1520.0000000	3.2300000
1521.0000000	3.2350001
1522.0000000	3.2449999
1523.0000000	3.2600000
1524.0000000	3.2700000
1525.0000000	3.2750001
1526.0000000	3.2849998
1527.0000000	3.3099999
1528.0000000	3.3099999
1529.0000000	3.3299999
1530.0000000	3.3599999
1531.0000000	3.3699999
1532.0000000	3.3850002
1533.0000000	3.4000001
1534.0000000	3.4200001
1535.0000000	3.4433334
1536.0000000	3.4650002
1537.0000000	3.4800000
1538.0000000	3.4949999
1539.0000000	3.5149999
1540.0000000	3.5250001
1541.0000000	3.5450001
1542.0000000	3.5450001
1543.0000000	3.5549998
1544.0000000	3.5549998
1545.0000000	3.5450001
1546.0000000	3.5349998
1547.0000000	3.5200000
1548.0000000	3.5050001
1549.0000000	3.4850001
1550.0000000	3.4650002
1551.0000000	3.4450002
1552.0000000	3.4200001
1553.0000000	3.3900001
1554.0000000	3.3750000
1555.0000000	3.3449998
1556.0000000	3.3350000
1557.0000000	3.3199999
1558.0000000	3.2849998
1559.0000000	3.2600000
1560.0000000	3.2550001
1561.0000000	3.2449999
1562.0000000	3.2550001
1563.0000000	3.2633333
1564.0000000	3.2449999
1565.0000000	3.2049999
1566.0000000	3.1650000
1567.0000000	3.1450000
1568.0000000	3.1650000
1569.0000000	3.1750002
1570.0000000	3.1800001
1571.0000000	3.1750002

1572.0000000	3.1800001
1573.0000000	3.1800001
1574.0000000	3.1950002
1575.0000000	3.2000000
1576.0000000	3.1950002
1577.0000000	3.1900001
1578.0000000	3.2000000
1579.0000000	3.1900001
1580.0000000	3.2000000
1581.0000000	3.1950002
1582.0000000	3.1700001
1583.0000000	3.1600001
1584.0000000	3.1450000
1585.0000000	3.1450000
1586.0000000	3.1150000
1587.0000000	3.0650001
1588.0000000	3.0400000
1589.0000000	3.0100000
1590.0000000	3.0000000
1591.0000000	2.9900000
1592.0000000	3.0050001
1593.0000000	2.9900000
1594.0000000	2.9749999
1595.0000000	2.9450002
1596.0000000	2.9399998
1597.0000000	2.9500000
1598.0000000	2.9600000
1599.0000000	2.9600000
1600.0000000	2.9549999
1601.0000000	2.9500000
1602.0000000	2.9400001
1603.0000000	2.9300001
1604.0000000	2.9150000
1605.0000000	2.8800001
1606.0000000	2.8599999
1607.0000000	2.8199999
1608.0000000	2.7750001
1609.0000000	2.7100000
1610.0000000	2.6650000
1611.0000000	2.6400001
1612.0000000	2.6400001
1613.0000000	2.6500001
1614.0000000	2.6500001
1615.0000000	2.6500001
1616.0000000	2.6450000
1617.0000000	2.6700001
1618.0000000	2.6750002
1619.0000000	2.6950002
1620.0000000	2.7000000
1621.0000000	2.7049999
1622.0000000	2.7350001
1623.0000000	2.7649999
1624.0000000	2.8000000
1625.0000000	2.8599999
1626.0000000	2.9100001
1627.0000000	2.9650002
1628.0000000	2.9900000

1629.0000000	3.0300000
1630.0000000	3.0549998
1631.0000000	3.0749998
1632.0000000	3.0850000
1633.0000000	3.0899999
1634.0000000	3.0899999
1635.0000000	3.0899999
1636.0000000	3.0650001
1637.0000000	3.0549998
1638.0000000	3.0200000
1639.0000000	2.9650002
1640.0000000	2.9400001
1641.0000000	2.9000001
1642.0000000	2.8800001
1643.0000000	2.8699999
1644.0000000	2.8649998
1645.0000000	2.8649998
1646.0000000	2.8599999
1647.0000000	2.8649998
1648.0000000	2.8800001
1649.0000000	2.8950000
1650.0000000	2.9000001
1651.0000000	2.9250002
1652.0000000	2.9600000
1653.0000000	2.9850001
1654.0000000	3.0100000
1655.0000000	3.0266666
1656.0000000	3.0599999
1657.0000000	3.0799999
1658.0000000	3.0999999
1659.0000000	3.1149998
1660.0000000	3.1400001
1661.0000000	3.1400001
1662.0000000	3.1500001
1663.0000000	3.1550002
1664.0000000	3.1550002
1665.0000000	3.1500001
1666.0000000	3.1400001
1667.0000000	3.1500001
1668.0000000	3.1300001
1669.0000000	3.1149998
1670.0000000	3.1050000
1671.0000000	3.0850000
1672.0000000	3.0699999
1673.0000000	3.0500000
1674.0000000	3.0400000
1675.0000000	3.0400000
1676.0000000	3.0500000
1677.0000000	3.0450001
1678.0000000	3.0549998
1679.0000000	3.0699999
1680.0000000	3.0549998
1681.0000000	3.0549998
1682.0000000	3.0599999
1683.0000000	3.0749998
1684.0000000	3.0850000
1685.0000000	3.1066666

1686.0000000	3.1199999
1687.0000000	3.1300001
1688.0000000	3.1350002
1689.0000000	3.1500001
1690.0000000	3.1750002
1691.0000000	3.1849999
1692.0000000	3.2150002
1693.0000000	3.2350001
1694.0000000	3.2649999
1695.0000000	3.2950001
1696.0000000	3.3049998
1697.0000000	3.3299999
1698.0000000	3.3550000
1699.0000000	3.3750000
1700.0000000	3.3900001
1701.0000000	3.4050002
1702.0000000	3.4200001
1703.0000000	3.4300001
1704.0000000	3.4549999
1705.0000000	3.4650002
1706.0000000	3.4749999
1707.0000000	3.4900000
1708.0000000	3.5050001
1709.0000000	3.5300000
1710.0000000	3.5400000
1711.0000000	3.5549998
1712.0000000	3.5599999
1713.0000000	3.5699999
1714.0000000	3.5650001
1715.0000000	3.5799999
1716.0000000	3.5799999
1717.0000000	3.5749998
1718.0000000	3.5899999
1719.0000000	3.5999999
1720.0000000	3.6099999
1721.0000000	3.6149998
1722.0000000	3.6199999
1723.0000000	3.6250000
1724.0000000	3.6149998
1725.0000000	3.5999999
1726.0000000	3.5999999
1727.0000000	3.5999999
1728.0000000	3.6050000
1729.0000000	3.6149998
1730.0000000	3.6199999
1731.0000000	3.6350002
1732.0000000	3.6450000
1733.0000000	3.6400001
1734.0000000	3.6450000
1735.0000000	3.6650000
1736.0000000	3.6700001
1737.0000000	3.6900001
1738.0000000	3.6900001
1739.0000000	3.7049999
1740.0000000	3.7200000
1741.0000000	3.7249999
1742.0000000	3.7200000

1743.0000000	3.7049999
1744.0000000	3.6750002
1745.0000000	3.6400001
1746.0000000	3.6250000
1747.0000000	3.5949998
1748.0000000	3.5799999
1749.0000000	3.5549998
1750.0000000	3.5450001
1751.0000000	3.5450001
1752.0000000	3.5400000
1753.0000000	3.5300000
1754.0000000	3.5200000
1755.0000000	3.5100000
1756.0000000	3.5000000
1757.0000000	3.4749999
1758.0000000	3.4650002
1759.0000000	3.4500000
1760.0000000	3.4300001
1761.0000000	3.4250002
1762.0000000	3.4000001
1763.0000000	3.3800001
1764.0000000	3.3550000
1765.0000000	3.3199999
1766.0000000	3.2900000
1767.0000000	3.2649999
1768.0000000	3.2249999
1769.0000000	3.1950002
1770.0000000	3.1450000
1771.0000000	3.0999999
1772.0000000	3.0749998
1773.0000000	3.0400000
1774.0000000	3.0250001
1775.0000000	3.0100000
1776.0000000	3.0050001
1777.0000000	2.9949999
1778.0000000	2.9800000
1779.0000000	2.9650002
1780.0000000	2.9300001
1781.0000000	2.9150000
1782.0000000	2.8950000
1783.0000000	2.8850002
1784.0000000	2.8900001
1785.0000000	2.9100001
1786.0000000	2.9400001
1787.0000000	2.9650002
1788.0000000	2.9749999
1789.0000000	2.9800000
1790.0000000	2.9749999
1791.0000000	2.9800000
1792.0000000	2.9650002
1793.0000000	2.9600000
1794.0000000	2.9450002
1795.0000000	2.9349999
1796.0000000	2.9150000
1797.0000000	2.9250002
1798.0000000	2.9650002
1799.0000000	3.0000000

1800.0000000	3.0250001
1801.0000000	3.0500000
1802.0000000	3.0699999
1803.0000000	3.0999999
1804.0000000	3.1149998
1805.0000000	3.1633332
1806.0000000	3.2150002
1807.0000000	3.2249999
1808.0000000	3.2500000
1809.0000000	3.2700000
1810.0000000	3.2849998
1811.0000000	3.3000000
1812.0000000	3.3099999
1813.0000000	3.3249998
1814.0000000	3.3299999
1815.0000000	3.3000000
1816.0000000	3.2900000
1817.0000000	3.2700000
1818.0000000	3.2400000
1819.0000000	3.2200000
1820.0000000	3.2100000
1821.0000000	3.1849999
1822.0000000	3.1550002
1823.0000000	3.1300001
1824.0000000	3.1099999
1825.0000000	3.1050000
1826.0000000	3.1050000
1827.0000000	3.1149998
1828.0000000	3.1149998
1829.0000000	3.1149998
1830.0000000	3.1250000
1831.0000000	3.1450000
1832.0000000	3.1900001
1833.0000000	3.2249999
1834.0000000	3.2649999
1835.0000000	3.2950001
1836.0000000	3.3449998
1837.0000000	3.3733332
1838.0000000	3.4050002
1839.0000000	3.4300001
1840.0000000	3.4500000
1841.0000000	3.4650002
1842.0000000	3.4900000
1843.0000000	3.5200000
1844.0000000	3.5250001
1845.0000000	3.5450001
1846.0000000	3.5500000
1847.0000000	3.5650001
1848.0000000	3.5599999
1849.0000000	3.5549998
1850.0000000	3.5400000
1851.0000000	3.5450001
1852.0000000	3.5500000
1853.0000000	3.5450001
1854.0000000	3.5500000
1855.0000000	3.5500000
1856.0000000	3.5699999

1857.0000000	3.5949998
1858.0000000	3.6250000
1859.0000000	3.6600001
1860.0000000	3.6849999
1861.0000000	3.7249999
1862.0000000	3.7800000
1863.0000000	3.8150001
1864.0000000	3.8649998
1865.0000000	3.9200001
1866.0000000	3.9450002
1867.0000000	3.9666665
1868.0000000	3.9949999
1869.0000000	3.9800000
1870.0000000	3.9549999
1871.0000000	3.9349999
1872.0000000	3.9250002
1873.0000000	3.9400001
1874.0000000	3.9500000
1875.0000000	3.9500000
1876.0000000	3.9700000
1877.0000000	3.9800000
1878.0000000	3.9900000
1879.0000000	4.0050001
1880.0000000	4.0050001
1881.0000000	4.0300002
1882.0000000	4.0200005
1883.0000000	4.0200000
1884.0000000	4.0100002
1885.0000000	3.9949999
1886.0000000	3.9800000
1887.0000000	3.9700000
1888.0000000	3.9650002
1889.0000000	3.9700000
1890.0000000	3.9700000
1891.0000000	3.9600000
1892.0000000	3.9450002
1893.0000000	3.9400001
1894.0000000	3.9300001
1895.0000000	3.9400001
1896.0000000	3.9600000
1897.0000000	3.9749999
1898.0000000	3.9633334
1899.0000000	3.9650002
1900.0000000	3.9700000
1901.0000000	3.9850001
1902.0000000	4.0150003
1903.0000000	4.0299997
1904.0000000	4.0250001
1905.0000000	4.0300002
1906.0000000	4.0299997
1907.0000000	4.0300002
1908.0000000	4.0150003
1909.0000000	4.0150003
1910.0000000	4.0200005
1911.0000000	4.0150003
1912.0000000	4.0250001
1913.0000000	4.0200000

1914.0000000	4.0150003
1915.0000000	3.9900000
1916.0000000	3.9850001
1917.0000000	3.9650002
1918.0000000	3.9450002
1919.0000000	3.9250002
1920.0000000	3.9000001
1921.0000000	3.8900001
1922.0000000	3.8649998
1923.0000000	3.8249998
1924.0000000	3.8000000
1925.0000000	3.7600000
1926.0000000	3.7100000
1927.0000000	3.6700001
1928.0000000	3.6233332
1929.0000000	3.5949998
1930.0000000	3.5899999
1931.0000000	3.6050000
1932.0000000	3.6149998
1933.0000000	3.6300001
1934.0000000	3.6450000
1935.0000000	3.6650000
1936.0000000	3.6800001
1937.0000000	3.7000000
1938.0000000	3.7200000
1939.0000000	3.7400000
1940.0000000	3.7550001
1941.0000000	3.7649999
1942.0000000	3.7700000
1943.0000000	3.7950001
1944.0000000	3.8049998
1945.0000000	3.8099999
1946.0000000	3.8099999
1947.0000000	3.8099999
1948.0000000	3.8199999
1949.0000000	3.8150001
1950.0000000	3.8150001
1951.0000000	3.8150001
1952.0000000	3.7950001
1953.0000000	3.8000000
1954.0000000	3.7950001
1955.0000000	3.7950001
1956.0000000	3.7950001
1957.0000000	3.7849998
1958.0000000	3.7700002
1959.0000000	3.7550001
1960.0000000	3.7200000
1961.0000000	3.6950002
1962.0000000	3.6900001
1963.0000000	3.6900001
1964.0000000	3.7100000
1965.0000000	3.7049999
1966.0000000	3.7150002
1967.0000000	3.7249999
1968.0000000	3.7200000
1969.0000000	3.7200000
1970.0000000	3.7150002

1971.0000000	3.6800001
1972.0000000	3.6650000
1973.0000000	3.6550002
1974.0000000	3.6350002
1975.0000000	3.6350002
1976.0000000	3.6250000
1977.0000000	3.6250000
1978.0000000	3.6300001
1979.0000000	3.6149998
1980.0000000	3.6149998
1981.0000000	3.6050000
1982.0000000	3.6050000
1983.0000000	3.5999999
1984.0000000	3.5899999
1985.0000000	3.5899999
1986.0000000	3.5850000
1987.0000000	3.5749998
1988.0000000	3.5833333
1989.0000000	3.5899999
1990.0000000	3.5899999
1991.0000000	3.5949998
1992.0000000	3.6149998
1993.0000000	3.6350002
1994.0000000	3.6650000
1995.0000000	3.6800001
1996.0000000	3.7100000
1997.0000000	3.7150002
1998.0000000	3.7249999
1999.0000000	3.7300000
2000.0000000	3.7449999
2001.0000000	3.7600000
2002.0000000	3.7550001
2003.0000000	3.7750001
2004.0000000	3.7700000
2005.0000000	3.7600000
2006.0000000	3.7500000
2007.0000000	3.7350001
2008.0000000	3.7249999
2009.0000000	3.7249999
2010.0000000	3.7200000
2011.0000000	3.7100000
2012.0000000	3.7200000
2013.0000000	3.7100000
2014.0000000	3.6950002
2015.0000000	3.6900001
2016.0000000	3.6600001
2017.0000000	3.6300001
2018.0000000	3.6050000
2019.0000000	3.5833333
2020.0000000	3.5599999
2021.0000000	3.5549998
2022.0000000	3.5599999
2023.0000000	3.5599999
2024.0000000	3.5850000
2025.0000000	3.5799999
2026.0000000	3.5749998
2027.0000000	3.5650001

2028.0000000	3.5400000
2029.0000000	3.5349998
2030.0000000	3.5250001
2031.0000000	3.5000000
2032.0000000	3.4600000
2033.0000000	3.4549999
2034.0000000	3.4650002
2035.0000000	3.4749999
2036.0000000	3.4850001
2037.0000000	3.4949999
2038.0000000	3.5149999
2039.0000000	3.5200000
2040.0000000	3.5349998
2041.0000000	3.5400000
2042.0000000	3.5450001
2043.0000000	3.5400000
2044.0000000	3.5400000
2045.0000000	3.5349998
2046.0000000	3.5000000
2047.0000000	3.4800000
2048.0000000	3.4650002
2049.0000000	3.4566667
2050.0000000	3.4700000
2051.0000000	3.4800000
2052.0000000	3.4949999
2053.0000000	3.5000000
2054.0000000	3.4850001
2055.0000000	3.4850001
2056.0000000	3.4949999
2057.0000000	3.4900000
2058.0000000	3.4850001
2059.0000000	3.4650002
2060.0000000	3.4349999
2061.0000000	3.4100001
2062.0000000	3.3800001
2063.0000000	3.3649998
2064.0000000	3.3350000
2065.0000000	3.3049998
2066.0000000	3.2800000
2067.0000000	3.2800000
2068.0000000	3.2600000
2069.0000000	3.2400000
2070.0000000	3.2400000
2071.0000000	3.2400000
2072.0000000	3.2500000
2073.0000000	3.2750001
2074.0000000	3.2900000
2075.0000000	3.3049998
2076.0000000	3.3350000
2077.0000000	3.3599999
2078.0000000	3.3699999
2079.0000000	3.3800001
2080.0000000	3.3866670
2081.0000000	3.3850002
2082.0000000	3.3699999
2083.0000000	3.3550000
2084.0000000	3.3449998

2085.0000000	3.3399999
2086.0000000	3.3199999
2087.0000000	3.3150001
2088.0000000	3.2950001
2089.0000000	3.2649999
2090.0000000	3.2400000
2091.0000000	3.2150002
2092.0000000	3.2000000
2093.0000000	3.1849999
2094.0000000	3.1849999
2095.0000000	3.1650000
2096.0000000	3.1450000
2097.0000000	3.1350002
2098.0000000	3.1199999
2099.0000000	3.0850000
2100.0000000	3.0400000
2101.0000000	2.9900000
2102.0000000	2.9549999
2103.0000000	2.9650002
2104.0000000	2.9850001
2105.0000000	3.0250001
2106.0000000	3.0549998
2107.0000000	3.0549998
2108.0000000	3.0349998
2109.0000000	3.0400000
2110.0000000	3.0699999
2111.0000000	3.1150000
2112.0000000	3.1550002
2113.0000000	3.2200000
2114.0000000	3.2750001
2115.0000000	3.3449998
2116.0000000	3.4100001
2117.0000000	3.4700000
2118.0000000	3.5050001
2119.0000000	3.5349998
2120.0000000	3.5500000
2121.0000000	3.5699999
2122.0000000	3.5850000
2123.0000000	3.6150000
2124.0000000	3.6300001
2125.0000000	3.6650000
2126.0000000	3.6849999
2127.0000000	3.7100000
2128.0000000	3.7500000
2129.0000000	3.7700000
2130.0000000	3.7900000
2131.0000000	3.8199999
2132.0000000	3.8350000
2133.0000000	3.8550000
2134.0000000	3.8499999
2135.0000000	3.8550000
2136.0000000	3.8699999
2137.0000000	3.8750000
2138.0000000	3.8850002
2139.0000000	3.8999999
2140.0000000	3.9100001
2141.0000000	3.9200001

2142.0000000	3.9100001
2143.0000000	3.9150000
2144.0000000	3.9300001
2145.0000000	3.9349999
2146.0000000	3.9250002
2147.0000000	3.9200001
2148.0000000	3.9150000
2149.0000000	3.9100001
2150.0000000	3.9000001
2151.0000000	3.8900001
2152.0000000	3.8699999
2153.0000000	3.8399999
2154.0000000	3.7950001
2155.0000000	3.7600000
2156.0000000	3.7249999
2157.0000000	3.6800001
2158.0000000	3.6550002
2159.0000000	3.6250000
2160.0000000	3.5850000
2161.0000000	3.5599999
2162.0000000	3.5250001
2163.0000000	3.5050001
2164.0000000	3.4850001
2165.0000000	3.4600000
2166.0000000	3.4300001
2167.0000000	3.4150000
2168.0000000	3.3699999
2169.0000000	3.3633330
2170.0000000	3.3449998
2171.0000000	3.3350000
2172.0000000	3.3350000
2173.0000000	3.3150001
2174.0000000	3.3049998
2175.0000000	3.2849998
2176.0000000	3.2600000
2177.0000000	3.2400000
2178.0000000	3.2150002
2179.0000000	3.1950002
2180.0000000	3.1800001
2181.0000000	3.1700001
2182.0000000	3.1650000
2183.0000000	3.1600001
2184.0000000	3.1450000
2185.0000000	3.1099999
2186.0000000	3.0949998
2187.0000000	3.0899999
2188.0000000	3.0749998
2189.0000000	3.0650001
2190.0000000	3.0699999
2191.0000000	3.0799999
2192.0000000	3.0749998
2193.0000000	3.0599999
2194.0000000	3.0400000
2195.0000000	3.0149999
2196.0000000	2.9900000
2197.0000000	2.9549999
2198.0000000	2.9349999

2199.0000000	2.9100001
2200.0000000	2.8900001
2201.0000000	2.8750000
2202.0000000	2.8499999
2203.0000000	2.8399999
2204.0000000	2.8650000
2205.0000000	2.8850000
2206.0000000	2.9050002
2207.0000000	2.9400001
2208.0000000	2.9700000
2209.0000000	2.9900000
2210.0000000	3.0050001
2211.0000000	3.0250001
2212.0000000	3.0349998
2213.0000000	3.0349998
2214.0000000	3.0500000
2215.0000000	3.0400000
2216.0000000	3.0500000
2217.0000000	3.0549998
2218.0000000	3.0650001
2219.0000000	3.0650001
2220.0000000	3.0749998
2221.0000000	3.0949998
2222.0000000	3.1099999
2223.0000000	3.1350002
2224.0000000	3.1450000
2225.0000000	3.1550002
2226.0000000	3.1450000
2227.0000000	3.1500001
2228.0000000	3.1400001
2229.0000000	3.1350002
2230.0000000	3.1500003
2231.0000000	3.1650000
2232.0000000	3.1600001
2233.0000000	3.1700001
2234.0000000	3.1600001
2235.0000000	3.1450000
2236.0000000	3.1250000
2237.0000000	3.1250000
2238.0000000	3.1199999
2239.0000000	3.0999999
2240.0000000	3.0999999
2241.0000000	3.0899999
2242.0000000	3.0899999
2243.0000000	3.0949998
2244.0000000	3.0949998
2245.0000000	3.0899999
2246.0000000	3.0999999
2247.0000000	3.0949998
2248.0000000	3.0999999
2249.0000000	3.0699999
2250.0000000	3.0549998
2251.0000000	3.0450001
2252.0000000	3.0300000
2253.0000000	3.0200000
2254.0000000	3.0100000
2255.0000000	3.0100000

2256.0000000	3.0100000
2257.0000000	3.0050001
2258.0000000	3.0050001
2259.0000000	3.0200000
2260.0000000	3.0450001
2261.0000000	3.0799999
2262.0000000	3.0899999
2263.0000000	3.0999999
2264.0000000	3.1050000
2265.0000000	3.1099999
2266.0000000	3.1149998
2267.0000000	3.1250000
2268.0000000	3.1300001
2269.0000000	3.1500001
2270.0000000	3.1550002
2271.0000000	3.1800001
2272.0000000	3.1849999
2273.0000000	3.2000000
2274.0000000	3.2049999
2275.0000000	3.2150002
2276.0000000	3.2249999
2277.0000000	3.2449999
2278.0000000	3.2449999
2279.0000000	3.2550001
2280.0000000	3.2700000
2281.0000000	3.2800000
2282.0000000	3.3049998
2283.0000000	3.3150001
2284.0000000	3.3350000
2285.0000000	3.3449998
2286.0000000	3.3699999
2287.0000000	3.3950000
2288.0000000	3.4050002
2289.0000000	3.4233334
2290.0000000	3.4400001
2291.0000000	3.4466667
2292.0000000	3.4500000
2293.0000000	3.4549999
2294.0000000	3.4400001
2295.0000000	3.4450002
2296.0000000	3.4250002
2297.0000000	3.4150000
2298.0000000	3.4050002
2299.0000000	3.3950000
2300.0000000	3.3850002
2301.0000000	3.3699999
2302.0000000	3.3499999
2303.0000000	3.3350000
2304.0000000	3.3000000
2305.0000000	3.2900000
2306.0000000	3.2500000
2307.0000000	3.2200000
2308.0000000	3.1900001
2309.0000000	3.1700001
2310.0000000	3.1650000
2311.0000000	3.1400001
2312.0000000	3.1350002

2313.0000000	3.1400001
2314.0000000	3.1450000
2315.0000000	3.1500001
2316.0000000	3.1550002
2317.0000000	3.1700001
2318.0000000	3.1800001
2319.0000000	3.1849999
2320.0000000	3.1833334
2321.0000000	3.1750002
2322.0000000	3.1600001
2323.0000000	3.1350002
2324.0000000	3.1400001
2325.0000000	3.1550002
2326.0000000	3.1650000
2327.0000000	3.1800001
2328.0000000	3.1849999
2329.0000000	3.2100000
2330.0000000	3.2049999
2331.0000000	3.2150002
2332.0000000	3.2300000
2333.0000000	3.2550001
2334.0000000	3.2700000
2335.0000000	3.3000000
2336.0000000	3.3299999
2337.0000000	3.3599999
2338.0000000	3.3900001
2339.0000000	3.4250002
2340.0000000	3.4400001
2341.0000000	3.4450002
2342.0000000	3.4549999
2343.0000000	3.4749999
2344.0000000	3.4850001
2345.0000000	3.5000000
2346.0000000	3.5200000
2347.0000000	3.5450001
2348.0000000	3.5549998
2349.0000000	3.5799999
2350.0000000	3.5966663
2351.0000000	3.6149998
2352.0000000	3.6350002
2353.0000000	3.6450000
2354.0000000	3.6600001
2355.0000000	3.6600001
2356.0000000	3.6650000
2357.0000000	3.6550002
2358.0000000	3.6700001
2359.0000000	3.6800001
2360.0000000	3.6849999
2361.0000000	3.6800001
2362.0000000	3.6750002
2363.0000000	3.6800001
2364.0000000	3.6700001
2365.0000000	3.6700001
2366.0000000	3.6550002
2367.0000000	3.6700001
2368.0000000	3.6600001
2369.0000000	3.6650000

2370.0000000	3.6600001
2371.0000000	3.6550002
2372.0000000	3.6199999
2373.0000000	3.6250000
2374.0000000	3.6300001
2375.0000000	3.6050000
2376.0000000	3.6099999
2377.0000000	3.6050000
2378.0000000	3.6050000
2379.0000000	3.6199999
2380.0000000	3.6300001
2381.0000000	3.6400001
2382.0000000	3.6600001
2383.0000000	3.6600001
2384.0000000	3.6550002
2385.0000000	3.6550002
2386.0000000	3.6500001
2387.0000000	3.6400001
2388.0000000	3.6500001
2389.0000000	3.6250000
2390.0000000	3.6250000
2391.0000000	3.6050000
2392.0000000	3.5949998
2393.0000000	3.5850000
2394.0000000	3.5650001
2395.0000000	3.5650001
2396.0000000	3.5450001
2397.0000000	3.5400000
2398.0000000	3.5300000
2399.0000000	3.5400000
2400.0000000	3.5349998
2401.0000000	3.5599999
2402.0000000	3.5500000
2403.0000000	3.5699999
2404.0000000	3.5799999
2405.0000000	3.5949998
2406.0000000	3.5899999
2407.0000000	3.5899999
2408.0000000	3.5949998
2409.0000000	3.6099999
2410.0000000	3.5999997
2411.0000000	3.5899999
2412.0000000	3.5599999
2413.0000000	3.5349998
2414.0000000	3.5000000
2415.0000000	3.4850001
2416.0000000	3.4749999
2417.0000000	3.4700000
2418.0000000	3.4549999
2419.0000000	3.4450002
2420.0000000	3.4450002
2421.0000000	3.4400001
2422.0000000	3.4349999
2423.0000000	3.4400001
2424.0000000	3.4349999
2425.0000000	3.4349999
2426.0000000	3.4549999

2427.0000000	3.4450002
2428.0000000	3.4500000
2429.0000000	3.4500000
2430.0000000	3.4549999
2431.0000000	3.4549999
2432.0000000	3.4650002
2433.0000000	3.4800000
2434.0000000	3.4850001
2435.0000000	3.4949999
2436.0000000	3.5000000
2437.0000000	3.4949999
2438.0000000	3.4700000
2439.0000000	3.4549999
2440.0000000	3.4333334
2441.0000000	3.4200001
2442.0000000	3.4033337
2443.0000000	3.4000001
2444.0000000	3.4000001
2445.0000000	3.4000001
2446.0000000	3.3900001
2447.0000000	3.3950000
2448.0000000	3.3850002
2449.0000000	3.3950000
2450.0000000	3.3950000
2451.0000000	3.3699999
2452.0000000	3.3499999
2453.0000000	3.3299999
2454.0000000	3.3150001
2455.0000000	3.2900000
2456.0000000	3.2500000
2457.0000000	3.2100000
2458.0000000	3.1750002
2459.0000000	3.1099999
2460.0000000	3.0650001
2461.0000000	3.0250001
2462.0000000	3.0000000
2463.0000000	2.9749999
2464.0000000	2.9549999
2465.0000000	2.9549999
2466.0000000	2.9800000
2467.0000000	3.0100000
2468.0000000	3.0450001
2469.0000000	3.0799999
2470.0000000	3.0949998
2471.0000000	3.1250000
2472.0000000	3.1433334
2473.0000000	3.1550002
2474.0000000	3.1700001
2475.0000000	3.1849999
2476.0000000	3.1900001
2477.0000000	3.1950002
2478.0000000	3.2150002
2479.0000000	3.2249999
2480.0000000	3.2400000
2481.0000000	3.2700000
2482.0000000	3.2800000
2483.0000000	3.2849998

2484.0000000	3.2900000
2485.0000000	3.2800000
2486.0000000	3.2750001
2487.0000000	3.2700000
2488.0000000	3.2700000
2489.0000000	3.2800000
2490.0000000	3.2700000
2491.0000000	3.2550001
2492.0000000	3.2600000
2493.0000000	3.2849998
2494.0000000	3.3150001
2495.0000000	3.3299999
2496.0000000	3.3550000
2497.0000000	3.3699999
2498.0000000	3.3800001
2499.0000000	3.4000001
2500.0000000	3.3950000
2501.0000000	3.3966668
2502.0000000	3.4349999
2503.0000000	3.4250002
2504.0000000	3.4400001
2505.0000000	3.4500000
2506.0000000	3.4500000
2507.0000000	3.4549999
2508.0000000	3.4500000
2509.0000000	3.4450002
2510.0000000	3.4600000
2511.0000000	3.4549999
2512.0000000	3.4600000
2513.0000000	3.4700000
2514.0000000	3.4749999
2515.0000000	3.4800000
2516.0000000	3.4850001
2517.0000000	3.4850001
2518.0000000	3.4800000
2519.0000000	3.4600000
2520.0000000	3.4600000
2521.0000000	3.4549999
2522.0000000	3.4600000
2523.0000000	3.4549999
2524.0000000	3.4549999
2525.0000000	3.4549999
2526.0000000	3.4600000
2527.0000000	3.4900000
2528.0000000	3.5149999
2529.0000000	3.5300000
2530.0000000	3.5549998
2531.0000000	3.5900002
2532.0000000	3.6050000
2533.0000000	3.6199999
2534.0000000	3.6350002
2535.0000000	3.6400001
2536.0000000	3.6600001
2537.0000000	3.6700001
2538.0000000	3.6849999
2539.0000000	3.7150002
2540.0000000	3.7249999

2541.0000000	3.7400000
2542.0000000	3.7449999
2543.0000000	3.7700000
2544.0000000	3.7900000
2545.0000000	3.7950001
2546.0000000	3.8249998
2547.0000000	3.8350000
2548.0000000	3.8449998
2549.0000000	3.8649998
2550.0000000	3.8900001
2551.0000000	3.9050002
2552.0000000	3.9100001
2553.0000000	3.9150000
2554.0000000	3.9150000
2555.0000000	3.9150000
2556.0000000	3.9150000
2557.0000000	3.9150000
2558.0000000	3.9050002
2559.0000000	3.8950000
2560.0000000	3.8800001
2561.0000000	3.8699999
2562.0000000	3.8649998
2563.0000000	3.8566666
2564.0000000	3.8449998
2565.0000000	3.8499999
2566.0000000	3.8499999
2567.0000000	3.8449998
2568.0000000	3.8449998
2569.0000000	3.8449998
2570.0000000	3.8249998
2571.0000000	3.8199999
2572.0000000	3.8199999
2573.0000000	3.8249998
2574.0000000	3.8199999
2575.0000000	3.8299999
2576.0000000	3.8099999
2577.0000000	3.8000000
2578.0000000	3.7950001
2579.0000000	3.7800000
2580.0000000	3.7649999
2581.0000000	3.7249999
2582.0000000	3.7000000
2583.0000000	3.6800001
2584.0000000	3.6650000
2585.0000000	3.6550002
2586.0000000	3.6500001
2587.0000000	3.6500001
2588.0000000	3.6550002
2589.0000000	3.6600001
2590.0000000	3.6800001
2591.0000000	3.6750002
2592.0000000	3.6966667
2593.0000000	3.6950002
2594.0000000	3.7049999
2595.0000000	3.7150002
2596.0000000	3.7200000
2597.0000000	3.7100000

2598.0000000	3.7150002
2599.0000000	3.7000000
2600.0000000	3.6950002
2601.0000000	3.7049999
2602.0000000	3.7000000
2603.0000000	3.7100000
2604.0000000	3.7100000
2605.0000000	3.7200000
2606.0000000	3.7350001
2607.0000000	3.7550001
2608.0000000	3.7950001
2609.0000000	3.8150001
2610.0000000	3.8499999
2611.0000000	3.8649998
2612.0000000	3.8800001
2613.0000000	3.9050002
2614.0000000	3.8950000
2615.0000000	3.9100001
2616.0000000	3.9200001
2617.0000000	3.9450002
2618.0000000	3.9450002
2619.0000000	3.9600000
2620.0000000	3.9549999
2621.0000000	3.9600000
2622.0000000	3.9500000
2623.0000000	3.9400001
2624.0000000	3.9500000
2625.0000000	3.9500000
2626.0000000	3.9600000
2627.0000000	3.9500000
2628.0000000	3.9500000
2629.0000000	3.9450002
2630.0000000	3.9349999
2631.0000000	3.9200001
2632.0000000	3.9150000
2633.0000000	3.9250002
2634.0000000	3.9250002
2635.0000000	3.9150000
2636.0000000	3.9150000
2637.0000000	3.9000001
2638.0000000	3.8900001
2639.0000000	3.8699999
2640.0000000	3.8550000
2641.0000000	3.8399999
2642.0000000	3.8099999
2643.0000000	3.7950001
2644.0000000	3.7649999
2645.0000000	3.7350001
2646.0000000	3.7000000
2647.0000000	3.6700001
2648.0000000	3.6400001
2649.0000000	3.6050000
2650.0000000	3.5650001
2651.0000000	3.5349998
2652.0000000	3.5133333
2653.0000000	3.4949999
2654.0000000	3.4800000

2655.0000000	3.4749999
2656.0000000	3.4600000
2657.0000000	3.4450002
2658.0000000	3.4349999
2659.0000000	3.4100001
2660.0000000	3.3699999
2661.0000000	3.3449998
2662.0000000	3.3350000
2663.0000000	3.3399999
2664.0000000	3.3499999
2665.0000000	3.3199999
2666.0000000	3.3000000
2667.0000000	3.2600000
2668.0000000	3.2400000
2669.0000000	3.2150002
2670.0000000	3.1950002
2671.0000000	3.1550002
2672.0000000	3.1300001
2673.0000000	3.0999999
2674.0000000	3.1099999
2675.0000000	3.1199999
2676.0000000	3.0999999
2677.0000000	3.1099999
2678.0000000	3.1149998
2679.0000000	3.1300001
2680.0000000	3.1550002
2681.0000000	3.1700001
2682.0000000	3.2049999
2683.0000000	3.2299998
2684.0000000	3.2700000
2685.0000000	3.2950001
2686.0000000	3.3249998
2687.0000000	3.3499999
2688.0000000	3.3800001
2689.0000000	3.4150000
2690.0000000	3.4450002
2691.0000000	3.4650002
2692.0000000	3.4850001
2693.0000000	3.5000000
2694.0000000	3.5149999
2695.0000000	3.5250001
2696.0000000	3.5450001
2697.0000000	3.5500000
2698.0000000	3.5400000
2699.0000000	3.5400000
2700.0000000	3.5349998
2701.0000000	3.5200000
2702.0000000	3.5050001
2703.0000000	3.5050001
2704.0000000	3.4850001
2705.0000000	3.4800000
2706.0000000	3.4400001
2707.0000000	3.4250002
2708.0000000	3.3900001
2709.0000000	3.3750000
2710.0000000	3.3499999
2711.0000000	3.3299999

2712.0000000	3.2950001
2713.0000000	3.2600000
2714.0000000	3.2733333
2715.0000000	3.2800000
2716.0000000	3.2900000
2717.0000000	3.2950001
2718.0000000	3.3099999
2719.0000000	3.3249998
2720.0000000	3.3499999
2721.0000000	3.3649998
2722.0000000	3.3950000
2723.0000000	3.4250002
2724.0000000	3.4450002
2725.0000000	3.4749999
2726.0000000	3.4850001
2727.0000000	3.5050001
2728.0000000	3.5200000
2729.0000000	3.5349998
2730.0000000	3.5500000
2731.0000000	3.5650001
2732.0000000	3.5749998
2733.0000000	3.5850000
2734.0000000	3.5899999
2735.0000000	3.6099999
2736.0000000	3.6199999
2737.0000000	3.6400001
2738.0000000	3.6650000
2739.0000000	3.6650000
2740.0000000	3.6600001
2741.0000000	3.6750002
2742.0000000	3.6650000
2743.0000000	3.6700001
2744.0000000	3.6700001
2745.0000000	3.6849999
2746.0000000	3.6849999
2747.0000000	3.6800001
2748.0000000	3.6900001
2749.0000000	3.7000000
2750.0000000	3.7100000
2751.0000000	3.7200000
2752.0000000	3.7200000
2753.0000000	3.7449999
2754.0000000	3.7600000
2755.0000000	3.7849998
2756.0000000	3.8150001
2757.0000000	3.8199999
2758.0000000	3.8299999
2759.0000000	3.8499999
2760.0000000	3.8399999
2761.0000000	3.8550000
2762.0000000	3.8550000
2763.0000000	3.8699999
2764.0000000	3.9050002
2765.0000000	3.8950000
2766.0000000	3.9000001
2767.0000000	3.9100001
2768.0000000	3.9150000

2769.0000000	3.9250002
2770.0000000	3.9200001
2771.0000000	3.9300001
2772.0000000	3.9200001
2773.0000000	3.9399998
2774.0000000	3.9400001
2775.0000000	3.9500000
2776.0000000	3.9549999
2777.0000000	3.9700000
2778.0000000	3.9700000
2779.0000000	3.9700000
2780.0000000	3.9700000
2781.0000000	3.9800000
2782.0000000	3.9850001
2783.0000000	3.9850001
2784.0000000	3.9850001
2785.0000000	3.9900000
2786.0000000	4.0000000
2787.0000000	4.0000000
2788.0000000	4.0150003
2789.0000000	4.0150003
2790.0000000	3.9949999
2791.0000000	3.9900000
2792.0000000	3.9800000
2793.0000000	3.9800000
2794.0000000	3.9900000
2795.0000000	3.9850001
2796.0000000	3.9800000
2797.0000000	3.9850001
2798.0000000	3.9850001
2799.0000000	4.0050001
2800.0000000	4.0500002
2801.0000000	4.0900002
2802.0000000	4.1199999
2803.0000000	4.1833334
2804.0000000	4.2449999
2805.0000000	4.3050003
2806.0000000	4.3400002
2807.0000000	4.3750000
2808.0000000	4.4250002
2809.0000000	4.4700003
2810.0000000	4.5000000
2811.0000000	4.5150003
2812.0000000	4.5600004
2813.0000000	4.5700002
2814.0000000	4.5999999
2815.0000000	4.6250000
2816.0000000	4.6400003
2817.0000000	4.6599998
2818.0000000	4.6900001
2819.0000000	4.7100000
2820.0000000	4.7100000
2821.0000000	4.7399998
2822.0000000	4.7500000
2823.0000000	4.7800002
2824.0000000	4.7799997
2825.0000000	4.7950001

2826.0000000	4.8100004
2827.0000000	4.8099999
2828.0000000	4.8199997
2829.0000000	4.8450003
2830.0000000	4.8450003
2831.0000000	4.8400002
2832.0000000	4.8750000
2833.0000000	4.8833337
2834.0000000	4.8699999
2835.0000000	4.8499999
2836.0000000	4.8450003
2837.0000000	4.8199997
2838.0000000	4.8150001
2839.0000000	4.7900000
2840.0000000	4.7750001
2841.0000000	4.7650003
2842.0000000	4.7349997
2843.0000000	4.7249999
2844.0000000	4.7100000
2845.0000000	4.6849999
2846.0000000	4.6450000
2847.0000000	4.6250000
2848.0000000	4.6149998
2849.0000000	4.5950003
2850.0000000	4.5650001
2851.0000000	4.5400000
2852.0000000	4.5200005
2853.0000000	4.4699998
2854.0000000	4.4400001
2855.0000000	4.3950000
2856.0000000	4.3800001
2857.0000000	4.3649998
2858.0000000	4.3350000
2859.0000000	4.3050003
2860.0000000	4.2700000
2861.0000000	4.2349997
2862.0000000	4.2199998
2863.0000000	4.2049999
2864.0000000	4.2150002
2865.0000000	4.2133331
2866.0000000	4.2249999
2867.0000000	4.2249999
2868.0000000	4.2249999
2869.0000000	4.2100000
2870.0000000	4.2100000
2871.0000000	4.1900001
2872.0000000	4.1949997
2873.0000000	4.1999998
2874.0000000	4.1999998
2875.0000000	4.1949997
2876.0000000	4.1849999
2877.0000000	4.1799998
2878.0000000	4.1650000
2879.0000000	4.1599998
2880.0000000	4.1499996
2881.0000000	4.1199999
2882.0000000	4.1099997

2883.0000000	4.1000004
2884.0000000	4.0900002
2885.0000000	4.0799999
2886.0000000	4.0950003
2887.0000000	4.1050000
2888.0000000	4.1050000
2889.0000000	4.1149998
2890.0000000	4.1149998
2891.0000000	4.1199999
2892.0000000	4.1250000
2893.0000000	4.1050000
2894.0000000	4.1100001
2895.0000000	4.1033340
2896.0000000	4.0900002
2897.0000000	4.0850000
2898.0000000	4.0850000
2899.0000000	4.0900002
2900.0000000	4.0749998
2901.0000000	4.0799999
2902.0000000	4.0650001
2903.0000000	4.0650001
2904.0000000	4.0450001
2905.0000000	4.0349998
2906.0000000	4.0250001
2907.0000000	4.0100002
2908.0000000	4.0000000
2909.0000000	4.0100002
2910.0000000	3.9900000
2911.0000000	4.0000000
2912.0000000	3.9600000
2913.0000000	3.9400001
2914.0000000	3.9150000
2915.0000000	3.9000001
2916.0000000	3.8950000
2917.0000000	3.9000001
2918.0000000	3.8950000
2919.0000000	3.9250002
2920.0000000	3.9250002
2921.0000000	3.9450002
2922.0000000	3.9600000
2923.0000000	3.9700000
2924.0000000	3.9850001
2925.0000000	3.9933331
2926.0000000	3.9949999
2927.0000000	4.0050001
2928.0000000	4.0150003
2929.0000000	4.0200005
2930.0000000	4.0200005
2931.0000000	4.0200005
2932.0000000	4.0250001
2933.0000000	4.0200000
2934.0000000	4.0150003
2935.0000000	3.9950001
2936.0000000	3.9749999
2937.0000000	3.9500000
2938.0000000	3.9150000
2939.0000000	3.8750000

2940.0000000	3.8449998
2941.0000000	3.8099999
2942.0000000	3.7849998
2943.0000000	3.7800000
2944.0000000	3.7750001
2945.0000000	3.7700000
2946.0000000	3.7750001
2947.0000000	3.7649999
2948.0000000	3.7400000
2949.0000000	3.7150002
2950.0000000	3.7100000
2951.0000000	3.7100000
2952.0000000	3.7100000
2953.0000000	3.7550001
2954.0000000	3.7966664
2955.0000000	3.8249998
2956.0000000	3.8650000
2957.0000000	3.8950000
2958.0000000	3.9250002
2959.0000000	3.9349999
2960.0000000	3.9549999
2961.0000000	3.9650002
2962.0000000	3.9800000
2963.0000000	4.0100002
2964.0000000	4.0100002
2965.0000000	4.0300002
2966.0000000	4.0299997
2967.0000000	4.0500002
2968.0000000	4.0799999
2969.0000000	4.0999999
2970.0000000	4.1350002
2971.0000000	4.1800003
2972.0000000	4.2249999
2973.0000000	4.2700000
2974.0000000	4.3150001
2975.0000000	4.3500004
2976.0000000	4.3900003
2977.0000000	4.4300003
2978.0000000	4.4700003
2979.0000000	4.5000000
2980.0000000	4.5250001
2981.0000000	4.5300002
2982.0000000	4.5599999
2983.0000000	4.6066670
2984.0000000	4.6450000
2985.0000000	4.6549997
2986.0000000	4.6900001
2987.0000000	4.7150002
2988.0000000	4.7249999
2989.0000000	4.7449999
2990.0000000	4.7650003
2991.0000000	4.7900000
2992.0000000	4.8150001
2993.0000000	4.8450003
2994.0000000	4.8649998
2995.0000000	4.8850002
2996.0000000	4.9150000

2997.0000000	4.9200001
2998.0000000	4.9200001
2999.0000000	4.9250002
3000.0000000	4.9399996
3001.0000000	4.9499998
3002.0000000	4.9600000
3003.0000000	4.9499998
3004.0000000	4.9349999
3005.0000000	4.9349999
3006.0000000	4.9250002
3007.0000000	4.8900003
3008.0000000	4.8649998
3009.0000000	4.8400002
3010.0000000	4.8199997
3011.0000000	4.8150001
3012.0000000	4.7849998
3013.0000000	4.7666669
3014.0000000	4.7349997
3015.0000000	4.7100000
3016.0000000	4.7049999
3017.0000000	4.6849999
3018.0000000	4.6900001
3019.0000000	4.6949997
3020.0000000	4.6949997
3021.0000000	4.7150002
3022.0000000	4.7150002
3023.0000000	4.7500000
3024.0000000	4.7550001
3025.0000000	4.7900000
3026.0000000	4.8100004
3027.0000000	4.8199997
3028.0000000	4.8249998
3029.0000000	4.8400002
3030.0000000	4.8299999
3031.0000000	4.8299999
3032.0000000	4.8400002
3033.0000000	4.8450003
3034.0000000	4.8599997
3035.0000000	4.8550000
3036.0000000	4.8550000
3037.0000000	4.8550000
3038.0000000	4.8550000
3039.0000000	4.8450003
3040.0000000	4.8350000
3041.0000000	4.8299999
3042.0000000	4.8050003
3043.0000000	4.7950001
3044.0000000	4.7733331
3045.0000000	4.7550001
3046.0000000	4.7300000
3047.0000000	4.6849999
3048.0000000	4.6450000
3049.0000000	4.6050000
3050.0000000	4.5500002
3051.0000000	4.5100002
3052.0000000	4.4650002
3053.0000000	4.4000001

3054.0000000	4.3750000
3055.0000000	4.3200002
3056.0000000	4.2750001
3057.0000000	4.2349997
3058.0000000	4.1999998
3059.0000000	4.1549997
3060.0000000	4.1199999
3061.0000000	4.0599999
3062.0000000	3.9950001
3063.0000000	3.9349999
3064.0000000	3.9050002
3065.0000000	3.8850002
3066.0000000	3.8599999
3067.0000000	3.8499999
3068.0000000	3.8350000
3069.0000000	3.8099999
3070.0000000	3.8000000
3071.0000000	3.7950001
3072.0000000	3.7900000
3073.0000000	3.7950001
3074.0000000	3.7533333
3075.0000000	3.7000000
3076.0000000	3.6900001
3077.0000000	3.7000000
3078.0000000	3.7249999
3079.0000000	3.7200000
3080.0000000	3.7350001
3081.0000000	3.7400000
3082.0000000	3.7500000
3083.0000000	3.7500000
3084.0000000	3.7550001
3085.0000000	3.7550001
3086.0000000	3.7500000
3087.0000000	3.7649999
3088.0000000	3.7750001
3089.0000000	3.8049998
3090.0000000	3.8249998
3091.0000000	3.8399999
3092.0000000	3.8599999
3093.0000000	3.8599999
3094.0000000	3.8699999
3095.0000000	3.8750000
3096.0000000	3.8800001
3097.0000000	3.8800001
3098.0000000	3.8800001
3099.0000000	3.8850002
3100.0000000	3.8950000
3101.0000000	3.8900001
3102.0000000	3.8950000
3103.0000000	3.9150000
3104.0000000	3.9250002
3105.0000000	3.9400001
3106.0000000	3.9633334
3107.0000000	3.9949999
3108.0000000	4.0200000
3109.0000000	4.0349998
3110.0000000	4.0500002

3111.0000000	4.0650001
3112.0000000	4.0700002
3113.0000000	4.0900002
3114.0000000	4.0950003
3115.0000000	4.0850000
3116.0000000	4.0699997
3117.0000000	4.0550003
3118.0000000	4.0400000
3119.0000000	4.0150003
3120.0000000	3.9850001
3121.0000000	3.9450002
3122.0000000	3.9349999
3123.0000000	3.9000001
3124.0000000	3.9000001
3125.0000000	3.8900001
3126.0000000	3.8699999
3127.0000000	3.8350000
3128.0000000	3.7900000
3129.0000000	3.7449999
3130.0000000	3.7400000
3131.0000000	3.6900001
3132.0000000	3.6600001
3133.0000000	3.6149998
3134.0000000	3.5749998
3135.0000000	3.5200000
3136.0000000	3.4949999
3137.0000000	3.4400001
3138.0000000	3.4133332
3139.0000000	3.3750000
3140.0000000	3.3649998
3141.0000000	3.3800001
3142.0000000	3.3850002
3143.0000000	3.3750000
3144.0000000	3.3950000
3145.0000000	3.4000001
3146.0000000	3.4150000
3147.0000000	3.4349999
3148.0000000	3.4600000
3149.0000000	3.4900000
3150.0000000	3.5250001
3151.0000000	3.5500000
3152.0000000	3.5850000
3153.0000000	3.6250000
3154.0000000	3.6350000
3155.0000000	3.6400001
3156.0000000	3.6400001
3157.0000000	3.6450000
3158.0000000	3.6550002
3159.0000000	3.6500001
3160.0000000	3.6750002
3161.0000000	3.6800001
3162.0000000	3.7100000
3163.0000000	3.7500000
3164.0000000	3.7900000
3165.0000000	3.8099999
3166.0000000	3.8299999
3167.0000000	3.8699999

3168.0000000	3.8850002
3169.0000000	3.9050002
3170.0000000	3.9250002
3171.0000000	3.9500000
3172.0000000	3.9549999
3173.0000000	3.9400001
3174.0000000	3.9250002
3175.0000000	3.9300001
3176.0000000	3.9500000
3177.0000000	3.9600000
3178.0000000	3.9800000
3179.0000000	4.0000000
3180.0000000	4.0050001
3181.0000000	4.0300002
3182.0000000	4.0300002
3183.0000000	4.0349998
3184.0000000	4.0250001
3185.0000000	4.0250001
3186.0000000	4.0349998
3187.0000000	4.0150003
3188.0000000	4.0050001
3189.0000000	3.9900000
3190.0000000	3.9800000
3191.0000000	3.9800000
3192.0000000	3.9749999
3193.0000000	3.9900000
3194.0000000	4.0150003
3195.0000000	4.0200000
3196.0000000	4.0333333
3197.0000000	4.0749998
3198.0000000	4.0700002
3199.0000000	4.0749998
3200.0000000	4.0799999
3201.0000000	4.0850000
3202.0000000	4.0950003
3203.0000000	4.1099997
3204.0000000	4.1399999
3205.0000000	4.1549997
3206.0000000	4.1750002
3207.0000000	4.1949997
3208.0000000	4.2049999
3209.0000000	4.2100000
3210.0000000	4.2249999
3211.0000000	4.2299995
3212.0000000	4.2550001
3213.0000000	4.2800002
3214.0000000	4.3099999
3215.0000000	4.3100004
3216.0000000	4.3200002
3217.0000000	4.3299999
3218.0000000	4.3350000
3219.0000000	4.3500004
3220.0000000	4.3500004
3221.0000000	4.3750000
3222.0000000	4.3600001
3223.0000000	4.3750000
3224.0000000	4.3699999

3225.0000000	4.3550000
3226.0000000	4.3350000
3227.0000000	4.3099999
3228.0000000	4.2700005
3229.0000000	4.2249999
3230.0000000	4.1799998
3231.0000000	4.1549997
3232.0000000	4.1199999
3233.0000000	4.0850000
3234.0000000	4.0550003
3235.0000000	4.0250001
3236.0000000	4.0050001
3237.0000000	3.9949999
3238.0000000	3.9600000
3239.0000000	3.9549999
3240.0000000	3.9500000
3241.0000000	3.9300001
3242.0000000	3.9050002
3243.0000000	3.8900001
3244.0000000	3.8550000
3245.0000000	3.8350000
3246.0000000	3.8199999
3247.0000000	3.7900000
3248.0000000	3.7449999
3249.0000000	3.6750002
3250.0000000	3.6050000
3251.0000000	3.5450001
3252.0000000	3.4949999
3253.0000000	3.4400001
3254.0000000	3.3850000
3255.0000000	3.3449998
3256.0000000	3.3350000
3257.0000000	3.3249998
3258.0000000	3.3100002
3259.0000000	3.2849998
3260.0000000	3.3049998
3261.0000000	3.3150001
3262.0000000	3.3449998
3263.0000000	3.3499999
3264.0000000	3.3499999
3265.0000000	3.3249998
3266.0000000	3.3299999
3267.0000000	3.3199999
3268.0000000	3.3199999
3269.0000000	3.3000000
3270.0000000	3.2900000
3271.0000000	3.2600000
3272.0000000	3.2449999
3273.0000000	3.2249999
3274.0000000	3.2200000
3275.0000000	3.2049999
3276.0000000	3.1900001
3277.0000000	3.1700001
3278.0000000	3.1500001
3279.0000000	3.1250000
3280.0000000	3.0949998
3281.0000000	3.0500000

3282.0000000	3.0149999
3283.0000000	2.9500000
3284.0000000	2.9050002
3285.0000000	2.8699999
3286.0000000	2.8399999
3287.0000000	2.7966664
3288.0000000	2.7900000
3289.0000000	2.8000000
3290.0000000	2.8249998
3291.0000000	2.8550000
3292.0000000	2.8699999
3293.0000000	2.8950000
3294.0000000	2.9150000
3295.0000000	2.9150000
3296.0000000	2.9000001
3297.0000000	2.8600001
3298.0000000	2.8000000
3299.0000000	2.7950001
3300.0000000	2.7700000
3301.0000000	2.7449999
3302.0000000	2.7249999
3303.0000000	2.7249999
3304.0000000	2.7350001
3305.0000000	2.7400000
3306.0000000	2.7350001
3307.0000000	2.7600000
3308.0000000	2.7900000
3309.0000000	2.8150001
3310.0000000	2.8299999
3311.0000000	2.8550000
3312.0000000	2.8699999
3313.0000000	2.8800001
3314.0000000	2.8950000
3315.0000000	2.9300001
3316.0000000	2.9233334
3317.0000000	2.9300001
3318.0000000	2.9400001
3319.0000000	2.9400001
3320.0000000	2.9349999
3321.0000000	2.9100001
3322.0000000	2.9100001
3323.0000000	2.9300001
3324.0000000	2.9549999
3325.0000000	2.9700000
3326.0000000	2.9949999
3327.0000000	3.0300000
3328.0000000	3.0250001
3329.0000000	3.0500000
3330.0000000	3.0699999
3331.0000000	3.1050000
3332.0000000	3.1199999
3333.0000000	3.1400001
3334.0000000	3.1600001
3335.0000000	3.1800001
3336.0000000	3.1900001
3337.0000000	3.1800001
3338.0000000	3.2000000

3339.0000000	3.1950002
3340.0000000	3.2150002
3341.0000000	3.2300000
3342.0000000	3.2550001
3343.0000000	3.2750001
3344.0000000	3.3049998
3345.0000000	3.3150001
3346.0000000	3.3350000
3347.0000000	3.3699999
3348.0000000	3.4000003
3349.0000000	3.4300001
3350.0000000	3.4549999
3351.0000000	3.4850001
3352.0000000	3.5050001
3353.0000000	3.5100000
3354.0000000	3.5300000
3355.0000000	3.5500000
3356.0000000	3.5699999
3357.0000000	3.5850000
3358.0000000	3.5999999
3359.0000000	3.5999999
3360.0000000	3.6199999
3361.0000000	3.6300001
3362.0000000	3.6300001
3363.0000000	3.6350002
3364.0000000	3.6400001
3365.0000000	3.6500001
3366.0000000	3.6700001
3367.0000000	3.6900001
3368.0000000	3.7000000
3369.0000000	3.7150002
3370.0000000	3.7350001
3371.0000000	3.7500000
3372.0000000	3.7649999
3373.0000000	3.7750001
3374.0000000	3.7849998
3375.0000000	3.7750001
3376.0000000	3.7900000
3377.0000000	3.7900000
3378.0000000	3.8049998
3379.0000000	3.8100002
3380.0000000	3.8049998
3381.0000000	3.8049998
3382.0000000	3.8049998
3383.0000000	3.8249998
3384.0000000	3.8199999
3385.0000000	3.8150001
3386.0000000	3.8099999
3387.0000000	3.8150001
3388.0000000	3.8049998
3389.0000000	3.7950001
3390.0000000	3.8099999
3391.0000000	3.8350000
3392.0000000	3.8350000
3393.0000000	3.8599999
3394.0000000	3.8850002
3395.0000000	3.9000001

3396.0000000	3.9200001
3397.0000000	3.9400001
3398.0000000	3.9500000
3399.0000000	3.9549999
3400.0000000	3.9700000
3401.0000000	3.9800000
3402.0000000	3.9800000
3403.0000000	4.0050001
3404.0000000	3.9949999
3405.0000000	4.0050001
3406.0000000	4.0300002
3407.0000000	4.0300002
3408.0000000	4.0500002
3409.0000000	4.0299997
3410.0000000	4.0150003
3411.0000000	4.0100002
3412.0000000	4.0000000
3413.0000000	3.9749999
3414.0000000	3.9300001
3415.0000000	3.8950000
3416.0000000	3.8599999
3417.0000000	3.8499999
3418.0000000	3.8299999
3419.0000000	3.8199999
3420.0000000	3.8249998
3421.0000000	3.8499999
3422.0000000	3.8750000
3423.0000000	3.8950000
3424.0000000	3.9050002
3425.0000000	3.9200001
3426.0000000	3.9250002
3427.0000000	3.8950000
3428.0000000	3.8750000
3429.0000000	3.8850002
3430.0000000	3.9100001
3431.0000000	3.9300001
3432.0000000	3.9450002
3433.0000000	3.9450002
3434.0000000	3.9600000
3435.0000000	3.9949999
3436.0000000	4.0050001
3437.0000000	4.0200005
3438.0000000	4.0250001
3439.0000000	4.0233331
3440.0000000	4.0150003
3441.0000000	4.0050001
3442.0000000	4.0100002
3443.0000000	3.9900000
3444.0000000	3.9700000
3445.0000000	3.9700000
3446.0000000	3.9650002
3447.0000000	3.9450002
3448.0000000	3.9250002
3449.0000000	3.9150000
3450.0000000	3.8950000
3451.0000000	3.8900001
3452.0000000	3.8599999

3453.0000000	3.8449998
3454.0000000	3.8000000
3455.0000000	3.7849998
3456.0000000	3.7550001
3457.0000000	3.7449999
3458.0000000	3.7150002
3459.0000000	3.7049999
3460.0000000	3.6700001
3461.0000000	3.6600001
3462.0000000	3.6450000
3463.0000000	3.6300001
3464.0000000	3.6300001
3465.0000000	3.5999999
3466.0000000	3.5999999
3467.0000000	3.5899999
3468.0000000	3.5933332
3469.0000000	3.5850000
3470.0000000	3.5799999
3471.0000000	3.5699999
3472.0000000	3.5549998
3473.0000000	3.5450001
3474.0000000	3.5400000
3475.0000000	3.5400000
3476.0000000	3.5300000
3477.0000000	3.5300000
3478.0000000	3.5400000
3479.0000000	3.5500000
3480.0000000	3.5549998
3481.0000000	3.5699999
3482.0000000	3.5949998
3483.0000000	3.6099999
3484.0000000	3.6400001
3485.0000000	3.6550002
3486.0000000	3.6650000
3487.0000000	3.6650000
3488.0000000	3.6450000
3489.0000000	3.6400001
3490.0000000	3.6300001
3491.0000000	3.6350002
3492.0000000	3.6350002
3493.0000000	3.6550002
3494.0000000	3.6450000
3495.0000000	3.6400001
3496.0000000	3.6350002
3497.0000000	3.6400001
3498.0000000	3.6600001
3499.0000000	3.7000000
3500.0000000	3.7400000
3501.0000000	3.7800000
3502.0000000	3.8249998
3503.0000000	3.8599999
3504.0000000	3.8900001
3505.0000000	3.9300001
3506.0000000	3.9749999
3507.0000000	4.0000000
3508.0000000	4.0299997
3509.0000000	4.0599999

3510.0000000	4.1050000
3511.0000000	4.1199999
3512.0000000	4.1549997
3513.0000000	4.1799998
3514.0000000	4.2199998
3515.0000000	4.2299995
3516.0000000	4.2299995
3517.0000000	4.2299995
3518.0000000	4.2399998
3519.0000000	4.2399998
3520.0000000	4.2600002
3521.0000000	4.2799997
3522.0000000	4.2900000
3523.0000000	4.2950001
3524.0000000	4.3200002
3525.0000000	4.3299999
3526.0000000	4.3299999
3527.0000000	4.3400002
3528.0000000	4.3599997
3529.0000000	4.3800001
3530.0000000	4.3950000
3531.0000000	4.4200001
3532.0000000	4.4300003
3533.0000000	4.4349999
3534.0000000	4.4549999
3535.0000000	4.4499998
3536.0000000	4.4499998
3537.0000000	4.4499998
3538.0000000	4.4499998
3539.0000000	4.4549999
3540.0000000	4.4600000
3541.0000000	4.4650002
3542.0000000	4.4650002
3543.0000000	4.4749999
3544.0000000	4.4700003
3545.0000000	4.4549999
3546.0000000	4.4549999
3547.0000000	4.4499998
3548.0000000	4.4250002
3549.0000000	4.4299998
3550.0000000	4.4099998
3551.0000000	4.4099998
3552.0000000	4.3999996
3553.0000000	4.4049997
3554.0000000	4.3999996
3555.0000000	4.3850002
3556.0000000	4.3800001
3557.0000000	4.3800001
3558.0000000	4.3750000
3559.0000000	4.3850002
3560.0000000	4.3766665
3561.0000000	4.3800001
3562.0000000	4.3699999
3563.0000000	4.3850002
3564.0000000	4.3899999
3565.0000000	4.3950000
3566.0000000	4.3850002

3567.0000000	4.3750000
3568.0000000	4.3699999
3569.0000000	4.3600001
3570.0000000	4.3649998
3571.0000000	4.3400002
3572.0000000	4.3450003
3573.0000000	4.3450003
3574.0000000	4.3450003
3575.0000000	4.3600001
3576.0000000	4.3649998
3577.0000000	4.3550000
3578.0000000	4.3649998
3579.0000000	4.3800001
3580.0000000	4.3750000
3581.0000000	4.3800001
3582.0000000	4.3649998
3583.0000000	4.3699999
3584.0000000	4.3500004
3585.0000000	4.3200002
3586.0000000	4.3150001
3587.0000000	4.2700000
3588.0000000	4.2349997
3589.0000000	4.1933331
3590.0000000	4.1650000
3591.0000000	4.1300001
3592.0000000	4.1149998
3593.0000000	4.0900002
3594.0000000	4.0699997
3595.0000000	4.0600004
3596.0000000	4.0349998
3597.0000000	4.0050001
3598.0000000	4.0150003
3599.0000000	4.0050001
3600.0000000	3.9949999
3601.0000000	3.9900000
3602.0000000	3.9900000
3603.0000000	4.0050001
3604.0000000	4.0150003
3605.0000000	4.0200005
3606.0000000	4.0349998
3607.0000000	4.0450001
3608.0000000	4.0749998
3609.0000000	4.0749998
3610.0000000	4.0699997
3611.0000000	4.0749998
3612.0000000	4.0799999
3613.0000000	4.0749998
3614.0000000	4.0900002
3615.0000000	4.0700002
3616.0000000	4.0650001
3617.0000000	4.0600004
3618.0000000	4.0850000
3619.0000000	4.0699997
3620.0000000	4.0466666
3621.0000000	4.0450001
3622.0000000	4.0550003
3623.0000000	4.0799999

3624.0000000	4.0900002
3625.0000000	4.0950003
3626.0000000	4.1199999
3627.0000000	4.1149998
3628.0000000	4.0950003
3629.0000000	4.0950003
3630.0000000	4.1099997
3631.0000000	4.0999999
3632.0000000	4.0950003
3633.0000000	4.0850000
3634.0000000	4.0650001
3635.0000000	4.0500002
3636.0000000	4.0599999
3637.0000000	4.0650001
3638.0000000	4.0749998
3639.0000000	4.0749998
3640.0000000	4.0900002
3641.0000000	4.1050000
3642.0000000	4.1050000
3643.0000000	4.0900002
3644.0000000	4.0749998
3645.0000000	4.0749998
3646.0000000	4.0799999
3647.0000000	4.0650001
3648.0000000	4.0599999
3649.0000000	4.0550003
3650.0000000	4.0400000
3651.0000000	4.0299997
3652.0000000	4.0400000
3653.0000000	4.0450001
3654.0000000	4.0450001
3655.0000000	4.0400000
3656.0000000	4.0700002
3657.0000000	4.0699997
3658.0000000	4.0950003
3659.0000000	4.1000004
3660.0000000	4.1050000
3661.0000000	4.1100001
3662.0000000	4.1050000
3663.0000000	4.1099997
3664.0000000	4.1199999
3665.0000000	4.1350002
3666.0000000	4.1199999
3667.0000000	4.1100001
3668.0000000	4.0950003
3669.0000000	4.0950003
3670.0000000	4.0850000
3671.0000000	4.0799999
3672.0000000	4.0799999
3673.0000000	4.0799999
3674.0000000	4.0749998
3675.0000000	4.0650001
3676.0000000	4.0400000
3677.0000000	4.0250001
3678.0000000	4.0050001
3679.0000000	3.9800000
3680.0000000	3.9766667

3681.0000000	3.9700000
3682.0000000	3.9600000
3683.0000000	3.9349999
3684.0000000	3.9300001
3685.0000000	3.9200001
3686.0000000	3.9300001
3687.0000000	3.9500000
3688.0000000	3.9700000
3689.0000000	3.9650002
3690.0000000	3.9850001
3691.0000000	3.9900000
3692.0000000	4.0200000
3693.0000000	4.0349998
3694.0000000	4.0599999
3695.0000000	4.0700002
3696.0000000	4.0900002
3697.0000000	4.0900002
3698.0000000	4.0950003
3699.0000000	4.1099997
3700.0000000	4.1199999
3701.0000000	4.1300001
3702.0000000	4.1149998
3703.0000000	4.1199999
3704.0000000	4.1099997
3705.0000000	4.1250000
3706.0000000	4.1250000
3707.0000000	4.1100001
3708.0000000	4.1100001
3709.0000000	4.1100001
3710.0000000	4.0850000
3711.0000000	4.0550003
3712.0000000	4.0400000
3713.0000000	4.0450001
3714.0000000	4.0200000
3715.0000000	3.9800000
3716.0000000	3.9850001
3717.0000000	3.9749999
3718.0000000	3.9700000
3719.0000000	3.9549999
3720.0000000	3.9349999
3721.0000000	3.9150000
3722.0000000	3.8850002
3723.0000000	3.8599999
3724.0000000	3.8399999
3725.0000000	3.8000000
3726.0000000	3.7800000
3727.0000000	3.7600000
3728.0000000	3.7150002
3729.0000000	3.7049999
3730.0000000	3.6849999
3731.0000000	3.6750002
3732.0000000	3.6550002
3733.0000000	3.6550002
3734.0000000	3.6199999
3735.0000000	3.5899999
3736.0000000	3.5450001
3737.0000000	3.5200000

3738.0000000	3.4800000
3739.0000000	3.4800000
3740.0000000	3.4450002
3741.0000000	3.4200001
3742.0000000	3.3800001
3743.0000000	3.3599999
3744.0000000	3.3249998
3745.0000000	3.2950001
3746.0000000	3.2700000
3747.0000000	3.2200000
3748.0000000	3.1849999
3749.0000000	3.1500001
3750.0000000	3.1199999
3751.0000000	3.1099999
3752.0000000	3.0850000
3753.0000000	3.0949998
3754.0000000	3.1300001
3755.0000000	3.1650000
3756.0000000	3.1750002
3757.0000000	3.2000000
3758.0000000	3.2150002
3759.0000000	3.2350001
3760.0000000	3.2449999
3761.0000000	3.2649999
3762.0000000	3.2800000
3763.0000000	3.3150001
3764.0000000	3.3249998
3765.0000000	3.3599999
3766.0000000	3.3750000
3767.0000000	3.3699999
3768.0000000	3.3649998
3769.0000000	3.3333333
3770.0000000	3.3299999
3771.0000000	3.3299999
3772.0000000	3.3449998
3773.0000000	3.3449998
3774.0000000	3.3499999
3775.0000000	3.3750000
3776.0000000	3.3550000
3777.0000000	3.3350000
3778.0000000	3.3350000
3779.0000000	3.3449998
3780.0000000	3.3350000
3781.0000000	3.3449998
3782.0000000	3.3499999
3783.0000000	3.3499999
3784.0000000	3.3399999
3785.0000000	3.2950001
3786.0000000	3.2649999
3787.0000000	3.2700000
3788.0000000	3.2900000
3789.0000000	3.3350000
3790.0000000	3.3550000
3791.0000000	3.3800001
3792.0000000	3.4100001
3793.0000000	3.4250002
3794.0000000	3.4600000

3795.0000000	3.4700000
3796.0000000	3.4850001
3797.0000000	3.4949999
3798.0000000	3.4949999
3799.0000000	3.5050001
3800.0000000	3.5100000
3801.0000000	3.5100000
3802.0000000	3.5033333
3803.0000000	3.4850001
3804.0000000	3.4949999
3805.0000000	3.4800000
3806.0000000	3.4800000
3807.0000000	3.4749999
3808.0000000	3.4700000
3809.0000000	3.4800000
3810.0000000	3.4800000
3811.0000000	3.4949999
3812.0000000	3.5000000
3813.0000000	3.5050001
3814.0000000	3.5050001
3815.0000000	3.5200000
3816.0000000	3.5100000
3817.0000000	3.5050001
3818.0000000	3.5000000
3819.0000000	3.4900000
3820.0000000	3.4850001
3821.0000000	3.4650002
3822.0000000	3.4600000
3823.0000000	3.4349999
3824.0000000	3.4150000
3825.0000000	3.4050002
3826.0000000	3.3950000
3827.0000000	3.4250002
3828.0000000	3.4549999
3829.0000000	3.4933336
3830.0000000	3.5400000
3831.0000000	3.5749998
3832.0000000	3.5999999
3833.0000000	3.6300001
3834.0000000	3.6500001
3835.0000000	3.6700001
3836.0000000	3.6849999
3837.0000000	3.7100000
3838.0000000	3.7249999
3839.0000000	3.7400000
3840.0000000	3.7249999
3841.0000000	3.7200000
3842.0000000	3.7100000
3843.0000000	3.7000000
3844.0000000	3.6849999
3845.0000000	3.6800001
3846.0000000	3.6550002
3847.0000000	3.6600001
3848.0000000	3.6550002
3849.0000000	3.6400001
3850.0000000	3.6350002
3851.0000000	3.6400001

3852.0000000	3.6149998
3853.0000000	3.6099999
3854.0000000	3.5999999
3855.0000000	3.5999999
3856.0000000	3.6149998
3857.0000000	3.6250000
3858.0000000	3.6149998
3859.0000000	3.6149998
3860.0000000	3.6099999
3861.0000000	3.5899999
3862.0000000	3.5899999
3863.0000000	3.5749998
3864.0000000	3.5699999
3865.0000000	3.5650001
3866.0000000	3.5650001
3867.0000000	3.5699999
3868.0000000	3.5500000
3869.0000000	3.5500000
3870.0000000	3.5300000
3871.0000000	3.5250001
3872.0000000	3.5200000
3873.0000000	3.5149999
3874.0000000	3.5250001
3875.0000000	3.5149999
3876.0000000	3.5000000
3877.0000000	3.4749999
3878.0000000	3.4500000
3879.0000000	3.4250002
3880.0000000	3.4100001
3881.0000000	3.3950000
3882.0000000	3.3699999
3883.0000000	3.3750000
3884.0000000	3.3550000
3885.0000000	3.3550000
3886.0000000	3.3699999
3887.0000000	3.3900001
3888.0000000	3.4100001
3889.0000000	3.4150000
3890.0000000	3.4600000
3891.0000000	3.4800000
3892.0000000	3.5000000
3893.0000000	3.5149999
3894.0000000	3.5300000
3895.0000000	3.5300000
3896.0000000	3.5200000
3897.0000000	3.5149999
3898.0000000	3.5250001
3899.0000000	3.5500000
3900.0000000	3.5699999
3901.0000000	3.5850000
3902.0000000	3.6099999
3903.0000000	3.6350002
3904.0000000	3.6600001
3905.0000000	3.6950002
3906.0000000	3.7000000
3907.0000000	3.7350001
3908.0000000	3.7449999

3909.0000000	3.7550001
3910.0000000	3.7800000
3911.0000000	3.7849998
3912.0000000	3.7849998
3913.0000000	3.7800000
3914.0000000	3.7700000
3915.0000000	3.7700000
3916.0000000	3.7849998
3917.0000000	3.7900000
3918.0000000	3.8049998
3919.0000000	3.7950001
3920.0000000	3.8000000
3921.0000000	3.8099999
3922.0000000	3.8133333
3923.0000000	3.8049998
3924.0000000	3.8099999
3925.0000000	3.7849998
3926.0000000	3.7700000
3927.0000000	3.7350001
3928.0000000	3.7400000
3929.0000000	3.7449999
3930.0000000	3.7200000
3931.0000000	3.7049999
3932.0000000	3.6800001
3933.0000000	3.6400001
3934.0000000	3.6050000
3935.0000000	3.5799999
3936.0000000	3.5549998
3937.0000000	3.5349998
3938.0000000	3.5200000
3939.0000000	3.5100000
3940.0000000	3.5200000
3941.0000000	3.5250001
3942.0000000	3.5300000
3943.0000000	3.5400000
3944.0000000	3.5400000
3945.0000000	3.5349998
3946.0000000	3.5400000
3947.0000000	3.5400000
3948.0000000	3.5250001
3949.0000000	3.5149999
3950.0000000	3.4850001
3951.0000000	3.4600000
3952.0000000	3.4466667
3953.0000000	3.4300001
3954.0000000	3.4349999
3955.0000000	3.4300001
3956.0000000	3.4300001
3957.0000000	3.4250002
3958.0000000	3.4300001
3959.0000000	3.4349999
3960.0000000	3.4549999
3961.0000000	3.4600000
3962.0000000	3.4749999
3963.0000000	3.4850001
3964.0000000	3.5050001
3965.0000000	3.5050001

3966.0000000	3.5200000
3967.0000000	3.5300000
3968.0000000	3.5450001
3969.0000000	3.5549998
3970.0000000	3.5500000
3971.0000000	3.5500000
3972.0000000	3.5349998
3973.0000000	3.5300000
3974.0000000	3.5200000
3975.0000000	3.5000000
3976.0000000	3.4850001
3977.0000000	3.4549999
3978.0000000	3.4250002
3979.0000000	3.3850000
3980.0000000	3.3499999
3981.0000000	3.2950001
3982.0000000	3.2733333
3983.0000000	3.2500000
3984.0000000	3.2249999
3985.0000000	3.2049999
3986.0000000	3.1900001
3987.0000000	3.1950002
3988.0000000	3.1849999
3989.0000000	3.1750002
3990.0000000	3.1450000
3991.0000000	3.1199999
3992.0000000	3.0999999
3993.0000000	3.0699999
3994.0000000	3.0400000
3995.0000000	3.0149999
3996.0000000	3.0050001
3997.0000000	2.9900000
3998.0000000	2.9850001
3999.0000000	2.9800000
4000.0000000	2.9800000
4001.0000000	2.9850001
4002.0000000	2.9900000
4003.0000000	2.9800000
4004.0000000	2.9800000
4005.0000000	2.9749999
4006.0000000	2.9700000
4007.0000000	2.9700000
4008.0000000	2.9900000
4009.0000000	2.9900000
4010.0000000	3.0000000
4011.0000000	3.0000000
4012.0000000	3.0200000
4013.0000000	3.0300000
4014.0000000	3.0349998
4015.0000000	3.0650001
4016.0000000	3.0699999
4017.0000000	3.0799999
4018.0000000	3.0949998
4019.0000000	3.1149998
4020.0000000	3.1400001
4021.0000000	3.1650000
4022.0000000	3.1750002

4023.0000000	3.1900001
4024.0000000	3.1750002
4025.0000000	3.1950002
4026.0000000	3.1950002
4027.0000000	3.1950002
4028.0000000	3.1950002
4029.0000000	3.1800001
4030.0000000	3.1800001
4031.0000000	3.1900001
4032.0000000	3.2100000
4033.0000000	3.2000000
4034.0000000	3.1950002
4035.0000000	3.1849999
4036.0000000	3.1800001
4037.0000000	3.1800001
4038.0000000	3.1700001
4039.0000000	3.1750002
4040.0000000	3.1750002
4041.0000000	3.1750002
4042.0000000	3.1700001
4043.0000000	3.1800001
4044.0000000	3.1933334
4045.0000000	3.2150002
4046.0000000	3.2400000
4047.0000000	3.2500000
4048.0000000	3.2500000
4049.0000000	3.2500000
4050.0000000	3.2049999
4051.0000000	3.1600001
4052.0000000	3.1350000
4053.0000000	3.1050000
4054.0000000	3.0699999
4055.0000000	3.0699999
4056.0000000	3.0699999
4057.0000000	3.0749998
4058.0000000	3.0749998
4059.0000000	3.0949998
4060.0000000	3.0999999
4061.0000000	3.0999999
4062.0000000	3.1149998
4063.0000000	3.1450000
4064.0000000	3.1650000
4065.0000000	3.1750002
4066.0000000	3.1900001
4067.0000000	3.2000000
4068.0000000	3.2000000
4069.0000000	3.2000000
4070.0000000	3.2150002
4071.0000000	3.2350001
4072.0000000	3.2699997
4073.0000000	3.2849998
4074.0000000	3.3000000
4075.0000000	3.3199999
4076.0000000	3.3249998
4077.0000000	3.3249998
4078.0000000	3.3249998
4079.0000000	3.3299999

4080.0000000	3.3199999
4081.0000000	3.3099999
4082.0000000	3.2950001
4083.0000000	3.2950001
4084.0000000	3.2900000
4085.0000000	3.2700000
4086.0000000	3.2649999
4087.0000000	3.2500000
4088.0000000	3.2400000
4089.0000000	3.2400000
4090.0000000	3.2249999
4091.0000000	3.2249999
4092.0000000	3.2150002
4093.0000000	3.2150002
4094.0000000	3.2049999
4095.0000000	3.2000000
4096.0000000	3.2000000
4097.0000000	3.2000000
4098.0000000	3.2049999
4099.0000000	3.2049999
4100.0000000	3.2100000
4101.0000000	3.1950002
4102.0000000	3.1849999
4103.0000000	3.1766665
4104.0000000	3.1650000
4105.0000000	3.1650000
4106.0000000	3.1900001
4107.0000000	3.1700001
4108.0000000	3.1550002
4109.0000000	3.1650000
4110.0000000	3.1500001
4111.0000000	3.1600001
4112.0000000	3.1700001
4113.0000000	3.1750002
4114.0000000	3.1849999
4115.0000000	3.1900001
4116.0000000	3.1950002
4117.0000000	3.2049999
4118.0000000	3.2049999
4119.0000000	3.2100000
4120.0000000	3.2200000
4121.0000000	3.2049999
4122.0000000	3.2100000
4123.0000000	3.2200000
4124.0000000	3.2300000
4125.0000000	3.2550001
4126.0000000	3.2649999
4127.0000000	3.2800000
4128.0000000	3.2900000
4129.0000000	3.3000000
4130.0000000	3.3150001
4131.0000000	3.2866666
4132.0000000	3.3049998
4133.0000000	3.3150001
4134.0000000	3.3199999
4135.0000000	3.3299999
4136.0000000	3.3449998

4137.0000000	3.4100001
4138.0000000	3.4349999
4139.0000000	3.4650002
4140.0000000	3.4900000
4141.0000000	3.5200000
4142.0000000	3.5250001
4143.0000000	3.5450001
4144.0000000	3.5549998
4145.0000000	3.5749998
4146.0000000	3.5749998
4147.0000000	3.5899999
4148.0000000	3.6050000
4149.0000000	3.6099999
4150.0000000	3.6199999
4151.0000000	3.6300001
4152.0000000	3.6350002
4153.0000000	3.6400001
4154.0000000	3.6450000
4155.0000000	3.6500001
4156.0000000	3.6600001
4157.0000000	3.6650000
4158.0000000	3.6800001
4159.0000000	3.6800001
4160.0000000	3.6849999
4161.0000000	3.6900001
4162.0000000	3.6849999
4163.0000000	3.6849999
4164.0000000	3.6750002
4165.0000000	3.6600001
4166.0000000	3.6300001
4167.0000000	3.6300001
4168.0000000	3.6099999
4169.0000000	3.6149998
4170.0000000	3.6050000
4171.0000000	3.6050000
4172.0000000	3.6149998
4173.0000000	3.6050000
4174.0000000	3.6050000
4175.0000000	3.6300001
4176.0000000	3.6350000
4177.0000000	3.6650000
4178.0000000	3.6900001
4179.0000000	3.7200000
4180.0000000	3.7150002
4181.0000000	3.7449999
4182.0000000	3.7350001
4183.0000000	3.7400000
4184.0000000	3.7400000
4185.0000000	3.7449999
4186.0000000	3.7449999
4187.0000000	3.7400000
4188.0000000	3.7500000
4189.0000000	3.7649999
4190.0000000	3.7400000
4191.0000000	3.7449999
4192.0000000	3.7600000
4193.0000000	3.7649999

4194.0000000	3.7666664
4195.0000000	3.7849998
4196.0000000	3.7800000
4197.0000000	3.7950001
4198.0000000	3.8099999
4199.0000000	3.8199999
4200.0000000	3.8199999
4201.0000000	3.8299999
4202.0000000	3.8249998
4203.0000000	3.8199999
4204.0000000	3.8099999
4205.0000000	3.8099999
4206.0000000	3.8249998
4207.0000000	3.8299999
4208.0000000	3.8399999
4209.0000000	3.8449998
4210.0000000	3.8499999
4211.0000000	3.8599999
4212.0000000	3.8499999
4213.0000000	3.8350000
4214.0000000	3.8499999
4215.0000000	3.8449998
4216.0000000	3.8249998
4217.0000000	3.8150001
4218.0000000	3.7950001
4219.0000000	3.7800000
4220.0000000	3.7649999
4221.0000000	3.7500000
4222.0000000	3.7233334
4223.0000000	3.6950002
4224.0000000	3.6650000
4225.0000000	3.6400001
4226.0000000	3.6250000
4227.0000000	3.5899999
4228.0000000	3.5650001
4229.0000000	3.5349998
4230.0000000	3.5300000
4231.0000000	3.5300000
4232.0000000	3.5250001
4233.0000000	3.5450001
4234.0000000	3.5650001
4235.0000000	3.5850000
4236.0000000	3.5999999
4237.0000000	3.6400001
4238.0000000	3.6600001
4239.0000000	3.6849999
4240.0000000	3.7000000
4241.0000000	3.7100000
4242.0000000	3.7049999
4243.0000000	3.7200000
4244.0000000	3.7249999
4245.0000000	3.7249999
4246.0000000	3.7249999
4247.0000000	3.7350001
4248.0000000	3.7350001
4249.0000000	3.7550001
4250.0000000	3.7649999

4251.0000000	3.7649999
4252.0000000	3.7550001
4253.0000000	3.7550001
4254.0000000	3.7649999
4255.0000000	3.7766666
4256.0000000	3.8049998
4257.0000000	3.8199999
4258.0000000	3.8299999
4259.0000000	3.8399999
4260.0000000	3.8550000
4261.0000000	3.8699999
4262.0000000	3.8900001
4263.0000000	3.9100001
4264.0000000	3.9150000
4265.0000000	3.9250002
4266.0000000	3.9400001
4267.0000000	3.9650002
4268.0000000	3.9749999
4269.0000000	3.9749999
4270.0000000	3.9850001
4271.0000000	3.9850001
4272.0000000	3.9850001
4273.0000000	3.9850001
4274.0000000	3.9800000
4275.0000000	3.9900000
4276.0000000	3.9749999
4277.0000000	3.9900000
4278.0000000	4.0000000
4279.0000000	3.9949999
4280.0000000	4.0050001
4281.0000000	4.0300002
4282.0000000	4.0100002
4283.0000000	4.0300002
4284.0000000	4.0266666
4285.0000000	4.0299997
4286.0000000	4.0100002
4287.0000000	4.0000000
4288.0000000	3.9949999
4289.0000000	3.9749999
4290.0000000	3.9700000
4291.0000000	3.9600000
4292.0000000	3.9600000
4293.0000000	3.9400001
4294.0000000	3.9300001
4295.0000000	3.9150000
4296.0000000	3.8950000
4297.0000000	3.8850002
4298.0000000	3.8649998
4299.0000000	3.8599999
4300.0000000	3.8499999
4301.0000000	3.8249998
4302.0000000	3.8299999
4303.0000000	3.8000000
4304.0000000	3.7849998
4305.0000000	3.7649999
4306.0000000	3.7500000
4307.0000000	3.7200000

4308.0000000	3.6900001
4309.0000000	3.6650000
4310.0000000	3.6350002
4311.0000000	3.5999999
4312.0000000	3.5749998
4313.0000000	3.5400000
4314.0000000	3.5066664
4315.0000000	3.4450002
4316.0000000	3.4100001
4317.0000000	3.3699999
4318.0000000	3.3249998
4319.0000000	3.3049998
4320.0000000	3.2800000
4321.0000000	3.2550001
4322.0000000	3.2249999
4323.0000000	3.1950002
4324.0000000	3.1600001
4325.0000000	3.1350002
4326.0000000	3.0999999
4327.0000000	3.0699999
4328.0000000	3.0349998
4329.0000000	2.9600000
4330.0000000	2.8800001
4331.0000000	2.7950001
4332.0000000	2.7150002
4333.0000000	2.6700001
4334.0000000	2.6399999
4335.0000000	2.6050000
4336.0000000	2.5749998
4337.0000000	2.5749998
4338.0000000	2.5799999
4339.0000000	2.5699999
4340.0000000	2.5799999
4341.0000000	2.5999999
4342.0000000	2.6199999
4343.0000000	2.6450000
4344.0000000	2.6400001
4345.0000000	2.6633334
4346.0000000	2.6750002
4347.0000000	2.7049999
4348.0000000	2.7200000
4349.0000000	2.7200000
4350.0000000	2.7150002
4351.0000000	2.7049999
4352.0000000	2.7150002
4353.0000000	2.7100000
4354.0000000	2.7049999
4355.0000000	2.7000000
4356.0000000	2.6950002
4357.0000000	2.6849999
4358.0000000	2.6800001
4359.0000000	2.6550002
4360.0000000	2.6250000
4361.0000000	2.6099999
4362.0000000	2.5999999
4363.0000000	2.5799999
4364.0000000	2.5749998

4365.0000000	2.5699999
4366.0000000	2.5949998
4367.0000000	2.6300001
4368.0000000	2.6600001
4369.0000000	2.6700001
4370.0000000	2.6750002
4371.0000000	2.7049999
4372.0000000	2.7200000
4373.0000000	2.7566669
4374.0000000	2.7950001
4375.0000000	2.8199999
4376.0000000	2.8449998
4377.0000000	2.8599999
4378.0000000	2.8800001
4379.0000000	2.8950000
4380.0000000	2.9250002
4381.0000000	2.9300001
4382.0000000	2.9450002
4383.0000000	2.9549999
4384.0000000	2.9600000
4385.0000000	2.9549999
4386.0000000	2.9500000
4387.0000000	2.9300001
4388.0000000	2.9200001
4389.0000000	2.9300001
4390.0000000	2.9250002
4391.0000000	2.9100001
4392.0000000	2.9050002
4393.0000000	2.9100001
4394.0000000	2.9150000
4395.0000000	2.9150000
4396.0000000	2.9300001
4397.0000000	2.9250002
4398.0000000	2.9200001
4399.0000000	2.9200001
4400.0000000	2.9450002
4401.0000000	2.9549999
4402.0000000	2.9800000
4403.0000000	2.9900000
4404.0000000	3.0050001
4405.0000000	3.0149999
4406.0000000	3.0166667
4407.0000000	3.0300000
4408.0000000	3.0300000
4409.0000000	3.0200000
4410.0000000	3.0149999
4411.0000000	3.0149999
4412.0000000	2.9949999
4413.0000000	2.9900000
4414.0000000	2.9650002
4415.0000000	2.9700000
4416.0000000	2.9500000
4417.0000000	2.9500000
4418.0000000	2.9500000
4419.0000000	2.9500000
4420.0000000	2.9800000
4421.0000000	3.0100000

4422.0000000	3.0500000
4423.0000000	3.0749998
4424.0000000	3.0799999
4425.0000000	3.0850000
4426.0000000	3.0949998
4427.0000000	3.0999999
4428.0000000	3.1199999
4429.0000000	3.1650000
4430.0000000	3.1950002
4431.0000000	3.2150002
4432.0000000	3.2500000
4433.0000000	3.2700000
4434.0000000	3.3033333
4435.0000000	3.3199999
4436.0000000	3.3249998
4437.0000000	3.3099999
4438.0000000	3.2900000
4439.0000000	3.2800000
4440.0000000	3.2500000
4441.0000000	3.2150002
4442.0000000	3.1950002
4443.0000000	3.1900001
4444.0000000	3.2049999
4445.0000000	3.2000000
4446.0000000	3.2000000
4447.0000000	3.2100000
4448.0000000	3.2200000
4449.0000000	3.2350001
4450.0000000	3.2500000
4451.0000000	3.2600000
4452.0000000	3.2800000
4453.0000000	3.2800000
4454.0000000	3.2800000
4455.0000000	3.2849998
4456.0000000	3.2800000
4457.0000000	3.2750001
4458.0000000	3.2800000
4459.0000000	3.2800000
4460.0000000	3.2800000
4461.0000000	3.2649999
4462.0000000	3.2700000
4463.0000000	3.2800000
4464.0000000	3.3049998
4465.0000000	3.3299999
4466.0000000	3.3550000
4467.0000000	3.3750000
4468.0000000	3.4050002
4469.0000000	3.4150000
4470.0000000	3.4549999
4471.0000000	3.4850001
4472.0000000	3.5000000
4473.0000000	3.5250001
4474.0000000	3.5250001
4475.0000000	3.5400000
4476.0000000	3.5500000
4477.0000000	3.5599999
4478.0000000	3.5599999

4479.0000000	3.5749998
4480.0000000	3.5650001
4481.0000000	3.5699999
4482.0000000	3.5450001
4483.0000000	3.5450001
4484.0000000	3.5599999
4485.0000000	3.5749998
4486.0000000	3.5799999
4487.0000000	3.5749998
4488.0000000	3.5650001
4489.0000000	3.5549998
4490.0000000	3.5400000
4491.0000000	3.5200000
4492.0000000	3.5050001
4493.0000000	3.5000000
4494.0000000	3.4700000
4495.0000000	3.4549999
4496.0000000	3.4300001
4497.0000000	3.4250002
4498.0000000	3.3900001
4499.0000000	3.3699999
4500.0000000	3.3550000
4501.0000000	3.3399999
4502.0000000	3.3150001
4503.0000000	3.3000000
4504.0000000	3.2849998
4505.0000000	3.2700000
4506.0000000	3.2350001
4507.0000000	3.2200000
4508.0000000	3.2000000
4509.0000000	3.1550002
4510.0000000	3.1300001
4511.0000000	3.1050000
4512.0000000	3.0999999
4513.0000000	3.0749998
4514.0000000	3.0549998
4515.0000000	3.0549998
4516.0000000	3.0699999
4517.0000000	3.0650001
4518.0000000	3.0500000
4519.0000000	3.0500000
4520.0000000	3.0450001
4521.0000000	3.0450001
4522.0000000	3.0400000
4523.0000000	3.0549998
4524.0000000	3.0549998
4525.0000000	3.0666666
4526.0000000	3.0650001
4527.0000000	3.0500000
4528.0000000	3.0349998
4529.0000000	3.0100000
4530.0000000	3.0149999
4531.0000000	3.0200000
4532.0000000	2.9949999
4533.0000000	3.0000000
4534.0000000	3.0100000
4535.0000000	3.0250001

4536.0000000	3.0349998
4537.0000000	3.0450001
4538.0000000	3.0650001
4539.0000000	3.0749998
4540.0000000	3.0799999
4541.0000000	3.0949998
4542.0000000	3.0799999
4543.0000000	3.0699999
4544.0000000	3.0400000
4545.0000000	3.0250001
4546.0000000	3.0200000
4547.0000000	3.0149999
4548.0000000	3.0100000
4549.0000000	3.0000000
4550.0000000	2.9749999
4551.0000000	2.9549999
4552.0000000	2.9400001
4553.0000000	2.9250002
4554.0000000	2.9050002
4555.0000000	2.9000003
4556.0000000	2.8950000
4557.0000000	2.8800001
4558.0000000	2.8800001
4559.0000000	2.8800001
4560.0000000	2.8699999
4561.0000000	2.8750000
4562.0000000	2.8599999
4563.0000000	2.8399999
4564.0000000	2.8299999
4565.0000000	2.8049998
4566.0000000	2.7950001
4567.0000000	2.8150001
4568.0000000	2.8199999
4569.0000000	2.8150001
4570.0000000	2.8249998
4571.0000000	2.8199999
4572.0000000	2.8249998
4573.0000000	2.8249998
4574.0000000	2.8399999
4575.0000000	2.8550000
4576.0000000	2.8800001
4577.0000000	2.9000001
4578.0000000	2.9050002
4579.0000000	2.9300001
4580.0000000	2.9450002
4581.0000000	2.9600000
4582.0000000	2.9850001
4583.0000000	3.0000000
4584.0000000	3.0149999
4585.0000000	3.0300000
4586.0000000	3.0433333
4587.0000000	3.0450001
4588.0000000	3.0450001
4589.0000000	3.0500000
4590.0000000	3.0450001
4591.0000000	3.0500000
4592.0000000	3.0500000

4593.0000000	3.0599999
4594.0000000	3.0549998
4595.0000000	3.0400000
4596.0000000	3.0149999
4597.0000000	3.0050001
4598.0000000	3.0149999
4599.0000000	3.0100000
4600.0000000	3.0050001
4601.0000000	3.0149999
4602.0000000	3.0300000
4603.0000000	3.0450001
4604.0000000	3.0699999
4605.0000000	3.0749998
4606.0000000	3.0850000
4607.0000000	3.1050000
4608.0000000	3.1199999
4609.0000000	3.1300001
4610.0000000	3.1300001
4611.0000000	3.1400001
4612.0000000	3.1300001
4613.0000000	3.1300001
4614.0000000	3.1099999
4615.0000000	3.0949998
4616.0000000	3.0233333
4617.0000000	2.9749999
4618.0000000	2.9600000
4619.0000000	2.9650002
4620.0000000	2.9600000
4621.0000000	2.9850001
4622.0000000	3.0149999
4623.0000000	3.0450001
4624.0000000	3.0699999
4625.0000000	3.0949998
4626.0000000	3.1100001
4627.0000000	3.1450000
4628.0000000	3.1750002
4629.0000000	3.1800001
4630.0000000	3.1750002
4631.0000000	3.1800001
4632.0000000	3.1750002
4633.0000000	3.1700001
4634.0000000	3.1750002
4635.0000000	3.1849999
4636.0000000	3.2150002
4637.0000000	3.2350001
4638.0000000	3.2600000
4639.0000000	3.2700000
4640.0000000	3.2750001
4641.0000000	3.2800000
4642.0000000	3.2750001
4643.0000000	3.2700000
4644.0000000	3.2800000
4645.0000000	3.3000000
4646.0000000	3.3099997
4647.0000000	3.3499999
4648.0000000	3.3499999
4649.0000000	3.3750000

4650.0000000	3.3800001
4651.0000000	3.4000001
4652.0000000	3.4200001
4653.0000000	3.4450002
4654.0000000	3.4650002
4655.0000000	3.4800000
4656.0000000	3.4900000
4657.0000000	3.5100000
4658.0000000	3.5250001
4659.0000000	3.5650001
4660.0000000	3.5799999
4661.0000000	3.6050000
4662.0000000	3.6149998
4663.0000000	3.6400001
4664.0000000	3.6700001
4665.0000000	3.6700001
4666.0000000	3.6849999
4667.0000000	3.6750002
4668.0000000	3.6849999
4669.0000000	3.6950002
4670.0000000	3.7100000
4671.0000000	3.7449999
4672.0000000	3.7449999
4673.0000000	3.7249999
4674.0000000	3.7049999
4675.0000000	3.7150002
4676.0000000	3.7133334
4677.0000000	3.7500000
4678.0000000	3.7649999
4679.0000000	3.7750001
4680.0000000	3.7700000
4681.0000000	3.7800000
4682.0000000	3.7900000
4683.0000000	3.7849998
4684.0000000	3.7900000
4685.0000000	3.7849998
4686.0000000	3.7900000
4687.0000000	3.7750001
4688.0000000	3.7800000
4689.0000000	3.7600000
4690.0000000	3.7300000
4691.0000000	3.6900001
4692.0000000	3.6650000
4693.0000000	3.6050000
4694.0000000	3.5749998
4695.0000000	3.5500000
4696.0000000	3.5250001
4697.0000000	3.4949999
4698.0000000	3.4800000
4699.0000000	3.4749999
4700.0000000	3.4650002
4701.0000000	3.4500000
4702.0000000	3.4400001
4703.0000000	3.4349999
4704.0000000	3.4200001
4705.0000000	3.4266670
4706.0000000	3.4400001

4707.0000000	3.4600000
4708.0000000	3.4800000
4709.0000000	3.4800000
4710.0000000	3.4949999
4711.0000000	3.5000000
4712.0000000	3.5000000
4713.0000000	3.4949999
4714.0000000	3.5000000
4715.0000000	3.5149999
4716.0000000	3.5450001
4717.0000000	3.5650001
4718.0000000	3.5749998
4719.0000000	3.6050000
4720.0000000	3.6149998
4721.0000000	3.6199999
4722.0000000	3.6300001
4723.0000000	3.6550002
4724.0000000	3.6750002
4725.0000000	3.7049999
4726.0000000	3.7150002
4727.0000000	3.7300000
4728.0000000	3.7550001
4729.0000000	3.7550001
4730.0000000	3.7700000
4731.0000000	3.7849998
4732.0000000	3.8000000
4733.0000000	3.8150001
4734.0000000	3.8199999
4735.0000000	3.8150001
4736.0000000	3.8150001
4737.0000000	3.8266666
4738.0000000	3.8499999
4739.0000000	3.8499999
4740.0000000	3.8699999
4741.0000000	3.8750000
4742.0000000	3.8750000
4743.0000000	3.8750000
4744.0000000	3.8750000
4745.0000000	3.8800001
4746.0000000	3.8850002
4747.0000000	3.8950000
4748.0000000	3.8900001
4749.0000000	3.8800001
4750.0000000	3.8900001
4751.0000000	3.8900001
4752.0000000	3.8850002
4753.0000000	3.8900001
4754.0000000	3.8800001
4755.0000000	3.8800001
4756.0000000	3.8900001
4757.0000000	3.8850002
4758.0000000	3.8750000
4759.0000000	3.8800001
4760.0000000	3.8800001
4761.0000000	3.8649998
4762.0000000	3.8649998
4763.0000000	3.8599999

4764.0000000	3.8399999
4765.0000000	3.8099999
4766.0000000	3.7950001
4767.0000000	3.7800000
4768.0000000	3.7600000
4769.0000000	3.7500000
4770.0000000	3.7300000
4771.0000000	3.7150002
4772.0000000	3.6849999
4773.0000000	3.6650000
4774.0000000	3.6450000
4775.0000000	3.6250000
4776.0000000	3.5949998
4777.0000000	3.5549998
4778.0000000	3.5149999
4779.0000000	3.4800000
4780.0000000	3.4150000
4781.0000000	3.3600001
4782.0000000	3.2800000
4783.0000000	3.1950002
4784.0000000	3.1350002
4785.0000000	3.0899999
4786.0000000	3.0549998
4787.0000000	3.0149999
4788.0000000	3.0000000
4789.0000000	2.9800000
4790.0000000	2.9650002
4791.0000000	2.9549999
4792.0000000	2.9600000
4793.0000000	2.9600000
4794.0000000	2.9650002
4795.0000000	2.9700000
4796.0000000	2.9833336
4797.0000000	2.9949999
4798.0000000	3.0149999
4799.0000000	2.9949999
4800.0000000	3.0050001
4801.0000000	3.0149999
4802.0000000	3.0200000
4803.0000000	3.0400000
4804.0000000	3.0200000
4805.0000000	3.0000000
4806.0000000	2.9250002
4807.0000000	2.8699999
4808.0000000	2.8599999
4809.0000000	2.8950000
4810.0000000	2.9600000
4811.0000000	2.9949999
4812.0000000	3.0149999
4813.0000000	3.0599999
4814.0000000	3.1300001
4815.0000000	3.1900001
4816.0000000	3.2350001
4817.0000000	3.2800000
4818.0000000	3.3199999
4819.0000000	3.3449998
4820.0000000	3.3699999

4821.0000000	3.4100001
4822.0000000	3.4000001
4823.0000000	3.4200001
4824.0000000	3.4200001
4825.0000000	3.4150000
4826.0000000	3.4250002
4827.0000000	3.4200001
4828.0000000	3.4300001
4829.0000000	3.4300001
4830.0000000	3.4250002
4831.0000000	3.4150000
4832.0000000	3.4200001
4833.0000000	3.4150000
4834.0000000	3.4100001
4835.0000000	3.4000001
4836.0000000	3.3850002
4837.0000000	3.3950000
4838.0000000	3.3750000
4839.0000000	3.3800001
4840.0000000	3.3800001
4841.0000000	3.3850002
4842.0000000	3.4000001
4843.0000000	3.3950000
4844.0000000	3.3950000
4845.0000000	3.4000001
4846.0000000	3.4050002
4847.0000000	3.3950000
4848.0000000	3.4000001
4849.0000000	3.4050002
4850.0000000	3.4200001
4851.0000000	3.4250002
4852.0000000	3.4400001
4853.0000000	3.4450002
4854.0000000	3.4749999
4855.0000000	3.4900000
4856.0000000	3.5000000
4857.0000000	3.5200000
4858.0000000	3.5366666
4859.0000000	3.5699999
4860.0000000	3.5749998
4861.0000000	3.5949998
4862.0000000	3.6149998
4863.0000000	3.6300001
4864.0000000	3.6550002
4865.0000000	3.6950002
4866.0000000	3.7300000
4867.0000000	3.7750001
4868.0000000	3.7849998
4869.0000000	3.8199999
4870.0000000	3.8499999
4871.0000000	3.8649998
4872.0000000	3.8850002
4873.0000000	3.8900001
4874.0000000	3.9100001
4875.0000000	3.9100001
4876.0000000	3.9250002
4877.0000000	3.9300001

4878.0000000	3.9450002
4879.0000000	3.9650002
4880.0000000	3.9749999
4881.0000000	3.9800000
4882.0000000	3.9900000
4883.0000000	4.0000000
4884.0000000	4.0050001
4885.0000000	3.9800000
4886.0000000	3.9549999
4887.0000000	3.9500000
4888.0000000	3.9466667
4889.0000000	3.9500000
4890.0000000	3.9500000
4891.0000000	3.9549999
4892.0000000	3.9749999
4893.0000000	3.9749999
4894.0000000	3.9949999
4895.0000000	3.9949999
4896.0000000	4.0000000
4897.0000000	3.9949999
4898.0000000	4.0050001
4899.0000000	4.0100002
4900.0000000	3.9950001
4901.0000000	4.0050001
4902.0000000	4.0250001
4903.0000000	4.0349998
4904.0000000	4.0450001
4905.0000000	4.0749998
4906.0000000	4.0799999
4907.0000000	4.0999999
4908.0000000	4.1250000
4909.0000000	4.1500001
4910.0000000	4.1700001
4911.0000000	4.1799998
4912.0000000	4.1949997
4913.0000000	4.1849999
4914.0000000	4.1899996
4915.0000000	4.1999998
4916.0000000	4.2150002
4917.0000000	4.2100000
4918.0000000	4.2100000
4919.0000000	4.2133336
4920.0000000	4.2399998
4921.0000000	4.2299995
4922.0000000	4.2300000
4923.0000000	4.2300000
4924.0000000	4.2200003
4925.0000000	4.2300000
4926.0000000	4.2150002
4927.0000000	4.2199998
4928.0000000	4.2249999
4929.0000000	4.2349997
4930.0000000	4.2349997
4931.0000000	4.2500000
4932.0000000	4.2449999
4933.0000000	4.2300000
4934.0000000	4.2249999

4935.0000000	4.2049999
4936.0000000	4.2100000
4937.0000000	4.2049999
4938.0000000	4.1800003
4939.0000000	4.1599998
4940.0000000	4.1450000
4941.0000000	4.0999999
4942.0000000	4.0550003
4943.0000000	4.0200005
4944.0000000	3.9700000
4945.0000000	3.9400001
4946.0000000	3.9150000
4947.0000000	3.9000001
4948.0000000	3.8866665
4949.0000000	3.8850002
4950.0000000	3.8750000
4951.0000000	3.8649998
4952.0000000	3.8499999
4953.0000000	3.8199999
4954.0000000	3.7950001
4955.0000000	3.8099999
4956.0000000	3.8199999
4957.0000000	3.8299999
4958.0000000	3.8499999
4959.0000000	3.8449998
4960.0000000	3.8199999
4961.0000000	3.8150001
4962.0000000	3.8299999
4963.0000000	3.8099999
4964.0000000	3.7950001
4965.0000000	3.7800000
4966.0000000	3.7550001
4967.0000000	3.7350001
4968.0000000	3.6900001
4969.0000000	3.6849999
4970.0000000	3.6250000
4971.0000000	3.6350002
4972.0000000	3.6450000
4973.0000000	3.6450000
4974.0000000	3.6700001
4975.0000000	3.6650000
4976.0000000	3.6350000
4977.0000000	3.5850000
4978.0000000	3.5500000
4979.0000000	3.5400000
4980.0000000	3.5166667
4981.0000000	3.4900000
4982.0000000	3.4800000
4983.0000000	3.4549999
4984.0000000	3.4150000
4985.0000000	3.3850002
4986.0000000	3.3699999
4987.0000000	3.3449998
4988.0000000	3.2950001
4989.0000000	3.2449999
4990.0000000	3.2150002
4991.0000000	3.2300000

4992.0000000	3.2649999
4993.0000000	3.2700000
4994.0000000	3.2750001
4995.0000000	3.2950001
4996.0000000	3.2950001
4997.0000000	3.2950001
4998.0000000	3.3099999
4999.0000000	3.3099999
5000.0000000	3.3449998
5001.0000000	3.3650000
5002.0000000	3.3800001
5003.0000000	3.3950000
5004.0000000	3.4050002
5005.0000000	3.4050002
5006.0000000	3.4050002
5007.0000000	3.3833332
5008.0000000	3.3800001
5009.0000000	3.3550000
5010.0000000	3.3599999
5011.0000000	3.3299999
5012.0000000	3.3449998
5013.0000000	3.3399999
5014.0000000	3.3699999
5015.0000000	3.3599999
5016.0000000	3.3599999
5017.0000000	3.3499999
5018.0000000	3.3299999
5019.0000000	3.3249998
5020.0000000	3.3049998
5021.0000000	3.2600000
5022.0000000	3.2249999
5023.0000000	3.1950002
5024.0000000	3.1550002
5025.0000000	3.1350002
5026.0000000	3.1300001
5027.0000000	3.1350002
5028.0000000	3.1300001
5029.0000000	3.1300001
5030.0000000	3.1250000
5031.0000000	3.1250000
5032.0000000	3.1199999
5033.0000000	3.1199999
5034.0000000	3.1250000
5035.0000000	3.1350002
5036.0000000	3.1250000
5037.0000000	3.1400001
5038.0000000	3.1466668
5039.0000000	3.1700001
5040.0000000	3.1900001
5041.0000000	3.2150002
5042.0000000	3.2449999
5043.0000000	3.2649999
5044.0000000	3.2700000
5045.0000000	3.2750001
5046.0000000	3.2750001
5047.0000000	3.2849998
5048.0000000	3.2849998

5049.0000000	3.2900000
5050.0000000	3.3099999
5051.0000000	3.3249998
5052.0000000	3.3199999
5053.0000000	3.3449998
5054.0000000	3.3249998
5055.0000000	3.3249998
5056.0000000	3.3099999
5057.0000000	3.2950001
5058.0000000	3.2849998
5059.0000000	3.2649999
5060.0000000	3.2750001
5061.0000000	3.2700000
5062.0000000	3.2700000
5063.0000000	3.2600000
5064.0000000	3.2300000
5065.0000000	3.2200000
5066.0000000	3.2000000
5067.0000000	3.1900001
5068.0000000	3.1833334
5069.0000000	3.1849999
5070.0000000	3.1750002
5071.0000000	3.1700001
5072.0000000	3.1849999
5073.0000000	3.1900001
5074.0000000	3.1750002
5075.0000000	3.2000000
5076.0000000	3.2100000
5077.0000000	3.2249999
5078.0000000	3.2300000
5079.0000000	3.2400000
5080.0000000	3.2350001
5081.0000000	3.2449999
5082.0000000	3.2500000
5083.0000000	3.2700000
5084.0000000	3.2849998
5085.0000000	3.2800000
5086.0000000	3.2950001
5087.0000000	3.2950001
5088.0000000	3.3049998
5089.0000000	3.3000000
5090.0000000	3.3150001
5091.0000000	3.3199999
5092.0000000	3.3099999
5093.0000000	3.3299999
5094.0000000	3.3399999
5095.0000000	3.3550000
5096.0000000	3.3850002
5097.0000000	3.4100001
5098.0000000	3.4399998
5099.0000000	3.4549999
5100.0000000	3.4800000
5101.0000000	3.5050001
5102.0000000	3.5250001
5103.0000000	3.5599999
5104.0000000	3.5749998
5105.0000000	3.5999999

5106.0000000	3.6300001
5107.0000000	3.6550002
5108.0000000	3.6650000
5109.0000000	3.7049999
5110.0000000	3.7249999
5111.0000000	3.7300000
5112.0000000	3.7500000
5113.0000000	3.7550001
5114.0000000	3.7700000
5115.0000000	3.7750001
5116.0000000	3.7950001
5117.0000000	3.7950001
5118.0000000	3.8199999
5119.0000000	3.8199999
5120.0000000	3.8099999
5121.0000000	3.8249998
5122.0000000	3.8249998
5123.0000000	3.8249998
5124.0000000	3.8499999
5125.0000000	3.8649998
5126.0000000	3.8750000
5127.0000000	3.8950000
5128.0000000	3.9050002
5129.0000000	3.9300001
5130.0000000	3.9633334
5131.0000000	3.9700000
5132.0000000	3.9850001
5133.0000000	4.0100002
5134.0000000	4.0000000
5135.0000000	4.0250001
5136.0000000	4.0299997
5137.0000000	4.0200005
5138.0000000	4.0250001
5139.0000000	4.0100002
5140.0000000	4.0050001
5141.0000000	3.9949999
5142.0000000	3.9749999
5143.0000000	3.9749999
5144.0000000	3.9500000
5145.0000000	3.9600000
5146.0000000	3.9450002
5147.0000000	3.9349999
5148.0000000	3.9400001
5149.0000000	3.9400001
5150.0000000	3.9300001
5151.0000000	3.9450002
5152.0000000	3.9500000
5153.0000000	3.9600000
5154.0000000	3.9800000
5155.0000000	3.9800000
5156.0000000	3.9850001
5157.0000000	3.9800000
5158.0000000	4.0000000
5159.0000000	4.0000000
5160.0000000	4.0000000
5161.0000000	4.0050001
5162.0000000	4.0050001

5163.0000000	3.9949999
5164.0000000	3.9800000
5165.0000000	3.9700000
5166.0000000	3.9549999
5167.0000000	3.9349999
5168.0000000	3.9200001
5169.0000000	3.8800001
5170.0000000	3.8550000
5171.0000000	3.8550000
5172.0000000	3.8350000
5173.0000000	3.8199999
5174.0000000	3.8199999
5175.0000000	3.8350000
5176.0000000	3.8499999
5177.0000000	3.8699999
5178.0000000	3.8850000
5179.0000000	3.8850002
5180.0000000	3.9100001
5181.0000000	3.9250002
5182.0000000	3.9450002
5183.0000000	3.9600000
5184.0000000	3.9700000
5185.0000000	3.9850001
5186.0000000	4.0100002
5187.0000000	4.0250001
5188.0000000	4.0250001
5189.0000000	4.0349998
5190.0000000	4.0533333
5191.0000000	4.0599999
5192.0000000	4.0700002
5193.0000000	4.0700002
5194.0000000	4.0799999
5195.0000000	4.0900002
5196.0000000	4.0850000
5197.0000000	4.0900002
5198.0000000	4.0799999
5199.0000000	4.0749998
5200.0000000	4.0500002
5201.0000000	4.0450001
5202.0000000	4.0300002
5203.0000000	4.0200000
5204.0000000	4.0000000
5205.0000000	3.9850001
5206.0000000	3.9749999
5207.0000000	3.9549999
5208.0000000	3.9349999
5209.0000000	3.9250002
5210.0000000	3.9250002
5211.0000000	3.8950000
5212.0000000	3.8900001
5213.0000000	3.8850002
5214.0000000	3.8699999
5215.0000000	3.8550000
5216.0000000	3.8249998
5217.0000000	3.7950001
5218.0000000	3.7500000
5219.0000000	3.6950002

5220.0000000	3.6300001
5221.0000000	3.5999999
5222.0000000	3.5599999
5223.0000000	3.5450001
5224.0000000	3.5450001
5225.0000000	3.5400000
5226.0000000	3.5300000
5227.0000000	3.5349998
5228.0000000	3.5549998
5229.0000000	3.5500000
5230.0000000	3.5549998
5231.0000000	3.5650001
5232.0000000	3.5599999
5233.0000000	3.5599999
5234.0000000	3.5699999
5235.0000000	3.5599999
5236.0000000	3.5549998
5237.0000000	3.5599999
5238.0000000	3.5450001
5239.0000000	3.5300000
5240.0000000	3.5100000
5241.0000000	3.4850001
5242.0000000	3.4450002
5243.0000000	3.4000001
5244.0000000	3.3650000
5245.0000000	3.3099999
5246.0000000	3.2600000
5247.0000000	3.2000000
5248.0000000	3.1300001
5249.0000000	3.0699999
5250.0000000	3.0250001
5251.0000000	2.9850001
5252.0000000	2.9366667
5253.0000000	2.9100001
5254.0000000	2.9050002
5255.0000000	2.9100001
5256.0000000	2.9300001
5257.0000000	2.9450002
5258.0000000	2.9549999
5259.0000000	2.9900000
5260.0000000	3.0149999
5261.0000000	3.0400000
5262.0000000	3.0650001
5263.0000000	3.0949998
5264.0000000	3.1050000
5265.0000000	3.1149998
5266.0000000	3.1149998
5267.0000000	3.1199999
5268.0000000	3.1350002
5269.0000000	3.1400001
5270.0000000	3.1450000
5271.0000000	3.1450000
5272.0000000	3.1450000
5273.0000000	3.1300001
5274.0000000	3.1300001
5275.0000000	3.0999999
5276.0000000	3.0749998

5277.0000000	3.0500000
5278.0000000	3.0200000
5279.0000000	3.0100000
5280.0000000	3.0166664
5281.0000000	3.0300000
5282.0000000	3.0349998
5283.0000000	3.0400000
5284.0000000	3.0650001
5285.0000000	3.0850000
5286.0000000	3.0999999
5287.0000000	3.1250000
5288.0000000	3.1300001
5289.0000000	3.1450000
5290.0000000	3.1600001
5291.0000000	3.1600001
5292.0000000	3.1700001
5293.0000000	3.1750002
5294.0000000	3.1800001
5295.0000000	3.1750002
5296.0000000	3.2000000
5297.0000000	3.2300000
5298.0000000	3.2600000
5299.0000000	3.2750001
5300.0000000	3.2900000
5301.0000000	3.2900000
5302.0000000	3.2800000
5303.0000000	3.2800000
5304.0000000	3.3000000
5305.0000000	3.3199999
5306.0000000	3.3249998
5307.0000000	3.3599999
5308.0000000	3.3800001
5309.0000000	3.4100001
5310.0000000	3.4399998
5311.0000000	3.4600000
5312.0000000	3.4900000
5313.0000000	3.5050001
5314.0000000	3.5100000
5315.0000000	3.5250001
5316.0000000	3.5300000
5317.0000000	3.5400000
5318.0000000	3.5549998
5319.0000000	3.5599999
5320.0000000	3.5799999
5321.0000000	3.5699999
5322.0000000	3.5799999
5323.0000000	3.5850000
5324.0000000	3.5850000
5325.0000000	3.5999999
5326.0000000	3.5999999
5327.0000000	3.6050000
5328.0000000	3.6050000
5329.0000000	3.6050000
5330.0000000	3.6099999
5331.0000000	3.6050000
5332.0000000	3.6149998
5333.0000000	3.6099999

5334.0000000	3.6149998
5335.0000000	3.6149998
5336.0000000	3.6150000
5337.0000000	3.5999999
5338.0000000	3.5949998
5339.0000000	3.5749998
5340.0000000	3.5699999
5341.0000000	3.5650001
5342.0000000	3.5533333
5343.0000000	3.5349998
5344.0000000	3.5250001
5345.0000000	3.4900000
5346.0000000	3.4500000
5347.0000000	3.4300001
5348.0000000	3.4150000
5349.0000000	3.4150000
5350.0000000	3.4100001
5351.0000000	3.4349999
5352.0000000	3.4300001
5353.0000000	3.4500000
5354.0000000	3.4549999
5355.0000000	3.4700000
5356.0000000	3.4749999
5357.0000000	3.4749999
5358.0000000	3.5000000
5359.0000000	3.5000000
5360.0000000	3.5149999
5361.0000000	3.5200000
5362.0000000	3.5200000
5363.0000000	3.5200000
5364.0000000	3.5300000
5365.0000000	3.5250001
5366.0000000	3.5100000
5367.0000000	3.5000000
5368.0000000	3.4949999
5369.0000000	3.4700000
5370.0000000	3.4500000
5371.0000000	3.4300001
5372.0000000	3.3966668
5373.0000000	3.3550000
5374.0000000	3.3249998
5375.0000000	3.3000000
5376.0000000	3.2700000
5377.0000000	3.2600000
5378.0000000	3.2449999
5379.0000000	3.2400000
5380.0000000	3.2249999
5381.0000000	3.2449999
5382.0000000	3.2400000
5383.0000000	3.2350001
5384.0000000	3.2449999
5385.0000000	3.2400000
5386.0000000	3.2449999
5387.0000000	3.2400000
5388.0000000	3.2500000
5389.0000000	3.2300000
5390.0000000	3.2300000

5391.0000000	3.2100000
5392.0000000	3.2049999
5393.0000000	3.1849999
5394.0000000	3.1849999
5395.0000000	3.1950002
5396.0000000	3.2100000
5397.0000000	3.2350001
5398.0000000	3.2400000
5399.0000000	3.2649999
5400.0000000	3.2666664
5401.0000000	3.2950001
5402.0000000	3.3049998
5403.0000000	3.3299999
5404.0000000	3.3499999
5405.0000000	3.3699999
5406.0000000	3.3950000
5407.0000000	3.4050002
5408.0000000	3.4300001
5409.0000000	3.4450002
5410.0000000	3.4549999
5411.0000000	3.4600000
5412.0000000	3.4700000
5413.0000000	3.4700000
5414.0000000	3.4800000
5415.0000000	3.4800000
5416.0000000	3.5000000
5417.0000000	3.4850001
5418.0000000	3.5000000
5419.0000000	3.5200000
5420.0000000	3.5450001
5421.0000000	3.5549998
5422.0000000	3.5699999
5423.0000000	3.5850000
5424.0000000	3.5850000
5425.0000000	3.5850000
5426.0000000	3.5899999
5427.0000000	3.5850000
5428.0000000	3.5949998
5429.0000000	3.6050000
5430.0000000	3.6099999
5431.0000000	3.6199999
5432.0000000	3.6199999
5433.0000000	3.6199999
5434.0000000	3.6250000
5435.0000000	3.5999999
5436.0000000	3.5999999
5437.0000000	3.5949998
5438.0000000	3.5850000
5439.0000000	3.5850000
5440.0000000	3.5749998
5441.0000000	3.5599999
5442.0000000	3.5300000
5443.0000000	3.5050001
5444.0000000	3.4850001
5445.0000000	3.4549999
5446.0000000	3.4300001
5447.0000000	3.3950000

5448.0000000	3.3699999
5449.0000000	3.3299999
5450.0000000	3.2800000
5451.0000000	3.2550001
5452.0000000	3.2200000
5453.0000000	3.2000000
5454.0000000	3.1700001
5455.0000000	3.1400001
5456.0000000	3.1199999
5457.0000000	3.1099999
5458.0000000	3.0949998
5459.0000000	3.0799999
5460.0000000	3.0749998
5461.0000000	3.0599999
5462.0000000	3.0633335
5463.0000000	3.0699999
5464.0000000	3.0650001
5465.0000000	3.0749998
5466.0000000	3.0850000
5467.0000000	3.0850000
5468.0000000	3.0799999
5469.0000000	3.0899999
5470.0000000	3.0949998
5471.0000000	3.0799999
5472.0000000	3.0650001
5473.0000000	3.0650001
5474.0000000	3.0699999
5475.0000000	3.0599999
5476.0000000	3.0699999
5477.0000000	3.0650001
5478.0000000	3.0749998
5479.0000000	3.0699999
5480.0000000	3.0650001
5481.0000000	3.0599999
5482.0000000	3.0450001
5483.0000000	3.0349998
5484.0000000	3.0349998
5485.0000000	3.0349998
5486.0000000	3.0349998
5487.0000000	3.0549998
5488.0000000	3.0699999
5489.0000000	3.0599999
5490.0000000	3.0349998
5491.0000000	3.0149999
5492.0000000	2.9900000
5493.0000000	2.9466667
5494.0000000	2.9349999
5495.0000000	2.9250002
5496.0000000	2.9150000
5497.0000000	2.9300001
5498.0000000	2.9300001
5499.0000000	2.9349999
5500.0000000	2.9150000
5501.0000000	2.9150000
5502.0000000	2.9050002
5503.0000000	2.8900001
5504.0000000	2.8850002

5505.0000000	2.8900001
5506.0000000	2.8900001
5507.0000000	2.8950000
5508.0000000	2.8850002
5509.0000000	2.8950000
5510.0000000	2.8850002
5511.0000000	2.8750000
5512.0000000	2.8699999
5513.0000000	2.8550000
5514.0000000	2.8599999
5515.0000000	2.8550000
5516.0000000	2.8550000
5517.0000000	2.8399999
5518.0000000	2.8249998
5519.0000000	2.8299999
5520.0000000	2.8299999
5521.0000000	2.8399999
5522.0000000	2.8350000
5523.0000000	2.8533332
5524.0000000	2.8750000
5525.0000000	2.8850002
5526.0000000	2.8950000
5527.0000000	2.9150000
5528.0000000	2.9300001
5529.0000000	2.9549999
5530.0000000	2.9749999
5531.0000000	2.9900000
5532.0000000	3.0200000
5533.0000000	3.0300000
5534.0000000	3.0500000
5535.0000000	3.0599999
5536.0000000	3.0599999
5537.0000000	3.0549998
5538.0000000	3.0450001
5539.0000000	3.0300000
5540.0000000	3.0450001
5541.0000000	3.0599999
5542.0000000	3.0650001
5543.0000000	3.0699999
5544.0000000	3.0699999
5545.0000000	3.0850000
5546.0000000	3.0850000
5547.0000000	3.0799999
5548.0000000	3.0749998
5549.0000000	3.0799999
5550.0000000	3.0899999
5551.0000000	3.0850000
5552.0000000	3.0699999
5553.0000000	3.0650001
5554.0000000	3.0599999
5555.0000000	3.0450001
5556.0000000	3.0250001
5557.0000000	3.0149999
5558.0000000	3.0050001
5559.0000000	3.0000000
5560.0000000	3.0000000
5561.0000000	2.9900000

5562.0000000	2.9949999
5563.0000000	2.9900000
5564.0000000	2.9850001
5565.0000000	2.9700000
5566.0000000	2.9549999
5567.0000000	2.9300001
5568.0000000	2.9000001
5569.0000000	2.8750000
5570.0000000	2.8449998
5571.0000000	2.8299999
5572.0000000	2.7950001
5573.0000000	2.7849998
5574.0000000	2.7750001
5575.0000000	2.7600000
5576.0000000	2.7500000
5577.0000000	2.7500000
5578.0000000	2.7500000
5579.0000000	2.7600000
5580.0000000	2.7750001
5581.0000000	2.7849998
5582.0000000	2.7950001
5583.0000000	2.8033333
5584.0000000	2.8099999
5585.0000000	2.8000000
5586.0000000	2.7900000
5587.0000000	2.7300000
5588.0000000	2.7000000
5589.0000000	2.7049999
5590.0000000	2.7150002
5591.0000000	2.7350001
5592.0000000	2.7500000
5593.0000000	2.7700000
5594.0000000	2.7800000
5595.0000000	2.8000000
5596.0000000	2.8150001
5597.0000000	2.8199999
5598.0000000	2.8249998
5599.0000000	2.8299999
5600.0000000	2.8299999
5601.0000000	2.8249998
5602.0000000	2.8150001
5603.0000000	2.8150001
5604.0000000	2.8049998
5605.0000000	2.7900000
5606.0000000	2.7800000
5607.0000000	2.7900000
5608.0000000	2.8049998
5609.0000000	2.8049998
5610.0000000	2.8249998
5611.0000000	2.8299999
5612.0000000	2.8499997
5613.0000000	2.8699999
5614.0000000	2.8900001
5615.0000000	2.9050002
5616.0000000	2.9200001
5617.0000000	2.9349999
5618.0000000	2.9300001

5619.0000000	2.9349999
5620.0000000	2.9300001
5621.0000000	2.9300001
5622.0000000	2.9250002
5623.0000000	2.9300001
5624.0000000	2.9250002
5625.0000000	2.9200001
5626.0000000	2.9250002
5627.0000000	2.9250002
5628.0000000	2.9200001
5629.0000000	2.9250002
5630.0000000	2.9150000
5631.0000000	2.9250002
5632.0000000	2.9450002
5633.0000000	2.9500000
5634.0000000	2.9650002
5635.0000000	2.9800000
5636.0000000	2.9800000
5637.0000000	2.9850001
5638.0000000	2.9850001
5639.0000000	2.9900000
5640.0000000	2.9949999
5641.0000000	2.9900000
5642.0000000	2.9800000
5643.0000000	2.9700000
5644.0000000	2.9500000
5645.0000000	2.9349999
5646.0000000	2.9250002
5647.0000000	2.9050002
5648.0000000	2.8950000
5649.0000000	2.8699999
5650.0000000	2.8499999
5651.0000000	2.8199999
5652.0000000	2.7900000
5653.0000000	2.7449999
5654.0000000	2.7100000
5655.0000000	2.6650000
5656.0000000	2.6199999
5657.0000000	2.5799999
5658.0000000	2.5549998
5659.0000000	2.5500000
5660.0000000	2.5450001
5661.0000000	2.5250001
5662.0000000	2.5100000
5663.0000000	2.5000000
5664.0000000	2.4949999
5665.0000000	2.4900000
5666.0000000	2.4850001
5667.0000000	2.4850001
5668.0000000	2.4900000
5669.0000000	2.5100000
5670.0000000	2.5250001
5671.0000000	2.5450001
5672.0000000	2.5549998
5673.0000000	2.5899999
5674.0000000	2.6099999
5675.0000000	2.6266668

5676.0000000	2.6450000
5677.0000000	2.6700001
5678.0000000	2.6700001
5679.0000000	2.6750002
5680.0000000	2.6500001
5681.0000000	2.6500001
5682.0000000	2.6500001
5683.0000000	2.6450000
5684.0000000	2.6500001
5685.0000000	2.6450000
5686.0000000	2.6350002
5687.0000000	2.6300001
5688.0000000	2.6400001
5689.0000000	2.6450000
5690.0000000	2.6550002
5691.0000000	2.6650000
5692.0000000	2.6600001
5693.0000000	2.6550002
5694.0000000	2.6450000
5695.0000000	2.6300001
5696.0000000	2.6199999
5697.0000000	2.5899999
5698.0000000	2.5749998
5699.0000000	2.5650001
5700.0000000	2.5549998
5701.0000000	2.5500000
5702.0000000	2.5400000
5703.0000000	2.5300000
5704.0000000	2.5250001
5705.0000000	2.5100000
5706.0000000	2.4900000
5707.0000000	2.4800000
5708.0000000	2.4700000
5709.0000000	2.4650002
5710.0000000	2.4700000
5711.0000000	2.4549999
5712.0000000	2.4450002
5713.0000000	2.4150000
5714.0000000	2.4000001
5715.0000000	2.3950000
5716.0000000	2.4050002
5717.0000000	2.4100001
5718.0000000	2.4100001
5719.0000000	2.4200001
5720.0000000	2.4150000
5721.0000000	2.4100001
5722.0000000	2.4100001
5723.0000000	2.3950000
5724.0000000	2.3900001
5725.0000000	2.3900001
5726.0000000	2.3850002
5727.0000000	2.3649998
5728.0000000	2.3649998
5729.0000000	2.3550000
5730.0000000	2.3449998
5731.0000000	2.3249998
5732.0000000	2.3099999

5733.0000000	2.3099999
5734.0000000	2.3249998
5735.0000000	2.3366668
5736.0000000	2.3550000
5737.0000000	2.3800001
5738.0000000	2.3900001
5739.0000000	2.4200001
5740.0000000	2.4450002
5741.0000000	2.4500000
5742.0000000	2.4650002
5743.0000000	2.4650002
5744.0000000	2.4800000
5745.0000000	2.4850001
5746.0000000	2.4900000
5747.0000000	2.4949999
5748.0000000	2.5000000
5749.0000000	2.5149999
5750.0000000	2.5200000
5751.0000000	2.5349998
5752.0000000	2.5349998
5753.0000000	2.5349998
5754.0000000	2.5400000
5755.0000000	2.5400000
5756.0000000	2.5400000
5757.0000000	2.5500000
5758.0000000	2.5549998
5759.0000000	2.5500000
5760.0000000	2.5450001
5761.0000000	2.5450001
5762.0000000	2.5500000
5763.0000000	2.5500000
5764.0000000	2.5549998
5765.0000000	2.5400000
5766.0000000	2.5450001
5767.0000000	2.5450001
5768.0000000	2.5450001
5769.0000000	2.5450001
5770.0000000	2.5349998
5771.0000000	2.5349998
5772.0000000	2.5200000
5773.0000000	2.5149999
5774.0000000	2.5149999
5775.0000000	2.5149999
5776.0000000	2.5250001
5777.0000000	2.5250001
5778.0000000	2.5349998
5779.0000000	2.5300000
5780.0000000	2.5349998
5781.0000000	2.5300000
5782.0000000	2.5349998
5783.0000000	2.5400000
5784.0000000	2.5400000
5785.0000000	2.5400000
5786.0000000	2.5400000
5787.0000000	2.5500000
5788.0000000	2.5549998
5789.0000000	2.5599999

5790.0000000	2.5650001
5791.0000000	2.5650001
5792.0000000	2.5699999
5793.0000000	2.5749998
5794.0000000	2.5899999
5795.0000000	2.5699999
5796.0000000	2.5749998
5797.0000000	2.5699999
5798.0000000	2.5749998
5799.0000000	2.5749998
5800.0000000	2.5799999
5801.0000000	2.5749998
5802.0000000	2.5749998
5803.0000000	2.5699999
5804.0000000	2.5599999
5805.0000000	2.5599999
5806.0000000	2.5599999
5807.0000000	2.5400000
5808.0000000	2.5349998
5809.0000000	2.5200000
5810.0000000	2.4949999
5811.0000000	2.4749999
5812.0000000	2.4549999
5813.0000000	2.4400001
5814.0000000	2.4050002
5815.0000000	2.3699999
5816.0000000	2.3350000
5817.0000000	2.2900000
5818.0000000	2.2500000
5819.0000000	2.2300000
5820.0000000	2.2200000
5821.0000000	2.2200000
5822.0000000	2.2249999
5823.0000000	2.2150002
5824.0000000	2.2299998
5825.0000000	2.2449999
5826.0000000	2.2550001
5827.0000000	2.2800000
5828.0000000	2.2950001
5829.0000000	2.3049998
5830.0000000	2.3150001
5831.0000000	2.3350000
5832.0000000	2.3299999
5833.0000000	2.3299999
5834.0000000	2.3150001
5835.0000000	2.2950001
5836.0000000	2.2950001
5837.0000000	2.3000000
5838.0000000	2.3249998
5839.0000000	2.3399999
5840.0000000	2.3499999
5841.0000000	2.3550000
5842.0000000	2.3599999
5843.0000000	2.3550000
5844.0000000	2.3449998
5845.0000000	2.3299999
5846.0000000	2.3150001

5847.0000000	2.3150001
5848.0000000	2.3049998
5849.0000000	2.3099999
5850.0000000	2.3199999
5851.0000000	2.3249998
5852.0000000	2.3249998
5853.0000000	2.3399999
5854.0000000	2.3299999
5855.0000000	2.3249998
5856.0000000	2.3266666
5857.0000000	2.3099999
5858.0000000	2.3000000
5859.0000000	2.2750001
5860.0000000	2.2500000
5861.0000000	2.2200000
5862.0000000	2.1950002
5863.0000000	2.1750002
5864.0000000	2.1600001
5865.0000000	2.1600001
5866.0000000	2.1600001
5867.0000000	2.1400001
5868.0000000	2.1099999
5869.0000000	2.1099999
5870.0000000	2.0999999
5871.0000000	2.0749998
5872.0000000	2.0850000
5873.0000000	2.0899999
5874.0000000	2.1050000
5875.0000000	2.1199999
5876.0000000	2.1300001
5877.0000000	2.1500001
5878.0000000	2.1600001
5879.0000000	2.1849999
5880.0000000	2.2049999
5881.0000000	2.2150002
5882.0000000	2.2400000
5883.0000000	2.2500000
5884.0000000	2.2600000
5885.0000000	2.2766666
5886.0000000	2.2900000
5887.0000000	2.2950001
5888.0000000	2.2950001
5889.0000000	2.3000000
5890.0000000	2.3049998
5891.0000000	2.3049998
5892.0000000	2.2950001
5893.0000000	2.2849998
5894.0000000	2.2800000
5895.0000000	2.2700000
5896.0000000	2.2600000
5897.0000000	2.2550001
5898.0000000	2.2300000
5899.0000000	2.2150002
5900.0000000	2.1950002
5901.0000000	2.1849999
5902.0000000	2.1650000
5903.0000000	2.1500001

5904.0000000	2.1250000
5905.0000000	2.1050000
5906.0000000	2.0850000
5907.0000000	2.0749998
5908.0000000	2.0599999
5909.0000000	2.0599999
5910.0000000	2.0400000
5911.0000000	2.0250001
5912.0000000	2.0050001
5913.0000000	1.9800000
5914.0000000	1.9550000
5915.0000000	1.9250000
5916.0000000	1.9033333
5917.0000000	1.8900000
5918.0000000	1.8650000
5919.0000000	1.8550000
5920.0000000	1.8450000
5921.0000000	1.8500000
5922.0000000	1.8650000
5923.0000000	1.8800000
5924.0000000	1.8800000
5925.0000000	1.8950000
5926.0000000	1.9100000
5927.0000000	1.9000000
5928.0000000	1.9150000
5929.0000000	1.9300001
5930.0000000	1.9500000
5931.0000000	1.9600000
5932.0000000	1.9700000
5933.0000000	1.9700000
5934.0000000	1.9650000
5935.0000000	1.9800000
5936.0000000	1.9800000
5937.0000000	1.9850000
5938.0000000	1.9700000
5939.0000000	1.9600000
5940.0000000	1.9600000
5941.0000000	1.9500000
5942.0000000	1.9450001
5943.0000000	1.9349999
5944.0000000	1.9250000
5945.0000000	1.9100000
5946.0000000	1.9066666
5947.0000000	1.9100000
5948.0000000	1.9200000
5949.0000000	1.9299999
5950.0000000	1.9400001
5951.0000000	1.9450001
5952.0000000	1.9450001
5953.0000000	1.9650000
5954.0000000	1.9650000
5955.0000000	1.9800000
5956.0000000	1.9800000
5957.0000000	1.9800000
5958.0000000	1.9900000
5959.0000000	1.9950000
5960.0000000	2.0100000

5961.0000000	2.0250001
5962.0000000	2.0300000
5963.0000000	2.0349998
5964.0000000	2.0349998
5965.0000000	2.0250001
5966.0000000	2.0300000
5967.0000000	2.0349998
5968.0000000	2.0300000
5969.0000000	2.0349998
5970.0000000	2.0349998
5971.0000000	2.0450001
5972.0000000	2.0549998
5973.0000000	2.0850000
5974.0000000	2.1050000
5975.0000000	2.1300001
5976.0000000	2.1566668
5977.0000000	2.1800001
5978.0000000	2.2000000
5979.0000000	2.2100000
5980.0000000	2.2100000
5981.0000000	2.2200000
5982.0000000	2.2249999
5983.0000000	2.2350001
5984.0000000	2.2400000
5985.0000000	2.2449999
5986.0000000	2.2700000
5987.0000000	2.2750001
5988.0000000	2.2750001
5989.0000000	2.2900000
5990.0000000	2.3000000
5991.0000000	2.3199999
5992.0000000	2.3399999
5993.0000000	2.3499999
5994.0000000	2.3800001
5995.0000000	2.3900001
5996.0000000	2.4050002
5997.0000000	2.4150000
5998.0000000	2.4200001
5999.0000000	2.4450002
6000.0000000	2.4549999
6001.0000000	2.4600000
6002.0000000	2.4549999
6003.0000000	2.4549999
6004.0000000	2.4500000
6005.0000000	2.4450002
6006.0000000	2.4466667
6007.0000000	2.4500000
6008.0000000	2.4600000
6009.0000000	2.4450002
6010.0000000	2.4600000
6011.0000000	2.4650002
6012.0000000	2.4700000
6013.0000000	2.4650002
6014.0000000	2.4749999
6015.0000000	2.4850001
6016.0000000	2.4949999
6017.0000000	2.4900000

6018.0000000	2.5000000
6019.0000000	2.5000000
6020.0000000	2.5149999
6021.0000000	2.5149999
6022.0000000	2.5200000
6023.0000000	2.5300000
6024.0000000	2.5300000
6025.0000000	2.5400000
6026.0000000	2.5549998
6027.0000000	2.5599999
6028.0000000	2.5749998
6029.0000000	2.5850000
6030.0000000	2.6099999
6031.0000000	2.6300001
6032.0000000	2.6350002
6033.0000000	2.6500001
6034.0000000	2.6650000
6035.0000000	2.6600001
6036.0000000	2.6700001
6037.0000000	2.6750002
6038.0000000	2.6800001
6039.0000000	2.6700001
6040.0000000	2.6700001
6041.0000000	2.6750002
6042.0000000	2.6750002
6043.0000000	2.6700001
6044.0000000	2.6750002
6045.0000000	2.6800001
6046.0000000	2.6900001
6047.0000000	2.6849999
6048.0000000	2.6750002
6049.0000000	2.6600001
6050.0000000	2.6550002
6051.0000000	2.6450000
6052.0000000	2.6300001
6053.0000000	2.6250000
6054.0000000	2.6250000
6055.0000000	2.6350002
6056.0000000	2.6400001
6057.0000000	2.6450000
6058.0000000	2.6600001
6059.0000000	2.6650000
6060.0000000	2.6800001
6061.0000000	2.6849999
6062.0000000	2.7000000
6063.0000000	2.7049999
6064.0000000	2.7100000
6065.0000000	2.7333333
6066.0000000	2.7400000
6067.0000000	2.7500000
6068.0000000	2.7550001
6069.0000000	2.7649999
6070.0000000	2.7649999
6071.0000000	2.7649999
6072.0000000	2.7649999
6073.0000000	2.7600000
6074.0000000	2.7400000

6075.0000000	2.7350001
6076.0000000	2.7200000
6077.0000000	2.6950002
6078.0000000	2.6750002
6079.0000000	2.6450000
6080.0000000	2.6149998
6081.0000000	2.5650001
6082.0000000	2.5349998
6083.0000000	2.4949999
6084.0000000	2.4450002
6085.0000000	2.3950000
6086.0000000	2.3449998
6087.0000000	2.2950001
6088.0000000	2.2649999
6089.0000000	2.2500000
6090.0000000	2.2500000
6091.0000000	2.2649999
6092.0000000	2.2750001
6093.0000000	2.3000000
6094.0000000	2.3099999
6095.0000000	2.3299999
6096.0000000	2.3399999
6097.0000000	2.3399999
6098.0000000	2.3449998
6099.0000000	2.3550000
6100.0000000	2.3699999
6101.0000000	2.3750000
6102.0000000	2.3800001
6103.0000000	2.3750000
6104.0000000	2.3750000
6105.0000000	2.3900001
6106.0000000	2.3950000
6107.0000000	2.4000001
6108.0000000	2.4000001
6109.0000000	2.4250002
6110.0000000	2.4400001
6111.0000000	2.4450002
6112.0000000	2.4450002
6113.0000000	2.4500000
6114.0000000	2.4400001
6115.0000000	2.4349999
6116.0000000	2.4200001
6117.0000000	2.4200001
6118.0000000	2.4050002
6119.0000000	2.4000001
6120.0000000	2.3950000
6121.0000000	2.3950000
6122.0000000	2.4000001
6123.0000000	2.4050002
6124.0000000	2.4150000
6125.0000000	2.4100001
6126.0000000	2.4200001
6127.0000000	2.4150000
6128.0000000	2.4250002
6129.0000000	2.4300001
6130.0000000	2.4300001
6131.0000000	2.4250002

6132.0000000	2.4150000
6133.0000000	2.3950000
6134.0000000	2.3950000
6135.0000000	2.3850002
6136.0000000	2.3599999
6137.0000000	2.3399999
6138.0000000	2.3150001
6139.0000000	2.2900000
6140.0000000	2.2649999
6141.0000000	2.2350001
6142.0000000	2.1900001
6143.0000000	2.1500001
6144.0000000	2.1149998
6145.0000000	2.0949998
6146.0000000	2.0899999
6147.0000000	2.0699999
6148.0000000	2.0599999
6149.0000000	2.0599999
6150.0000000	2.0899999
6151.0000000	2.1050000
6152.0000000	2.1149998
6153.0000000	2.1300001
6154.0000000	2.1550002
6155.0000000	2.1750002
6156.0000000	2.1933334
6157.0000000	2.2100000
6158.0000000	2.2049999
6159.0000000	2.2200000
6160.0000000	2.2200000
6161.0000000	2.2200000
6162.0000000	2.2350001
6163.0000000	2.2649999
6164.0000000	2.3000000
6165.0000000	2.3299999
6166.0000000	2.3599999
6167.0000000	2.3900001
6168.0000000	2.4250002
6169.0000000	2.4549999
6170.0000000	2.4900000
6171.0000000	2.5100000
6172.0000000	2.5450001
6173.0000000	2.5650001
6174.0000000	2.6099999
6175.0000000	2.6350002
6176.0000000	2.6700001
6177.0000000	2.6950002
6178.0000000	2.7150002
6179.0000000	2.7300000
6180.0000000	2.7700000
6181.0000000	2.7700000
6182.0000000	2.7849998
6183.0000000	2.7849998
6184.0000000	2.7750001
6185.0000000	2.7649999
6186.0000000	2.7500000
6187.0000000	2.7399998
6188.0000000	2.7400000

6189.0000000	2.7500000
6190.0000000	2.7550001
6191.0000000	2.7550001
6192.0000000	2.7550001
6193.0000000	2.7550001
6194.0000000	2.7600000
6195.0000000	2.7700000
6196.0000000	2.7600000
6197.0000000	2.7600000
6198.0000000	2.7750001
6199.0000000	2.7649999
6200.0000000	2.7649999
6201.0000000	2.7750001
6202.0000000	2.7800000
6203.0000000	2.7849998
6204.0000000	2.7700000
6205.0000000	2.7649999
6206.0000000	2.7500000
6207.0000000	2.7350001
6208.0000000	2.7300000
6209.0000000	2.7300000
6210.0000000	2.7249999
6211.0000000	2.7150002
6212.0000000	2.7150002
6213.0000000	2.7100000
6214.0000000	2.7100000
6215.0000000	2.7000000
6216.0000000	2.7000000
6217.0000000	2.6933334
6218.0000000	2.6950002
6219.0000000	2.7000000
6220.0000000	2.7000000
6221.0000000	2.7000000
6222.0000000	2.7249999
6223.0000000	2.7350001
6224.0000000	2.7550001
6225.0000000	2.7849998
6226.0000000	2.7900000
6227.0000000	2.8099999
6228.0000000	2.8299999
6229.0000000	2.8350000
6230.0000000	2.8399999
6231.0000000	2.8249998
6232.0000000	2.8350000
6233.0000000	2.8350000
6234.0000000	2.8350000
6235.0000000	2.8350000
6236.0000000	2.8399999
6237.0000000	2.8249998
6238.0000000	2.8199999
6239.0000000	2.8249998
6240.0000000	2.8099999
6241.0000000	2.7950001
6242.0000000	2.7849998
6243.0000000	2.7750001
6244.0000000	2.7649999
6245.0000000	2.7500000

6246.0000000	2.7400000
6247.0000000	2.7399998
6248.0000000	2.7200000
6249.0000000	2.7200000
6250.0000000	2.7100000
6251.0000000	2.7000000
6252.0000000	2.7000000
6253.0000000	2.6950002
6254.0000000	2.7150002
6255.0000000	2.7200000
6256.0000000	2.7300000
6257.0000000	2.7449999
6258.0000000	2.7600000
6259.0000000	2.7750001
6260.0000000	2.7900000
6261.0000000	2.8150001
6262.0000000	2.8350000
6263.0000000	2.8599999
6264.0000000	2.8850002
6265.0000000	2.8900001
6266.0000000	2.9000001
6267.0000000	2.8950000
6268.0000000	2.9000001
6269.0000000	2.9050002
6270.0000000	2.9300001
6271.0000000	2.9450002
6272.0000000	2.9549999
6273.0000000	2.9600000
6274.0000000	2.9600000
6275.0000000	2.9700000
6276.0000000	2.9650002
6277.0000000	2.9749999
6278.0000000	2.9800003
6279.0000000	2.9800000
6280.0000000	2.9900000
6281.0000000	3.0149999
6282.0000000	3.0100000
6283.0000000	3.0050001
6284.0000000	2.9949999
6285.0000000	2.9700000
6286.0000000	2.9500000
6287.0000000	2.9400001
6288.0000000	2.9250002
6289.0000000	2.9349999
6290.0000000	2.9400001
6291.0000000	2.9400001
6292.0000000	2.9400001
6293.0000000	2.9500000
6294.0000000	2.9600000
6295.0000000	2.9700000
6296.0000000	2.9749999
6297.0000000	2.9850001
6298.0000000	2.9850001
6299.0000000	2.9850001
6300.0000000	2.9850001
6301.0000000	2.9850001
6302.0000000	2.9700000

6303.0000000	2.9600000
6304.0000000	2.9400001
6305.0000000	2.9200001
6306.0000000	2.9200001
6307.0000000	2.9200001
6308.0000000	2.9050002
6309.0000000	2.9066668
6310.0000000	2.9150000
6311.0000000	2.8850002
6312.0000000	2.8950000
6313.0000000	2.8950000
6314.0000000	2.9050002
6315.0000000	2.9150000
6316.0000000	2.9250002
6317.0000000	2.9400001
6318.0000000	2.9450002
6319.0000000	2.9600000
6320.0000000	2.9700000
6321.0000000	2.9850001
6322.0000000	3.0000000
6323.0000000	3.0149999
6324.0000000	3.0200000
6325.0000000	3.0400000
6326.0000000	3.0300000
6327.0000000	3.0400000
6328.0000000	3.0400000
6329.0000000	3.0450001
6330.0000000	3.0599999
6331.0000000	3.0500000
6332.0000000	3.0500000
6333.0000000	3.0349998
6334.0000000	3.0349998
6335.0000000	3.0400000
6336.0000000	3.0200000
6337.0000000	3.0050001
6338.0000000	2.9949999
6339.0000000	2.9833333
6340.0000000	2.9500000
6341.0000000	2.9200001
6342.0000000	2.8950000
6343.0000000	2.9000001
6344.0000000	2.8900001
6345.0000000	2.8850002
6346.0000000	2.8800001
6347.0000000	2.8850002
6348.0000000	2.8850002
6349.0000000	2.8649998
6350.0000000	2.8649998
6351.0000000	2.8449998
6352.0000000	2.8449998
6353.0000000	2.8449998
6354.0000000	2.8399999
6355.0000000	2.8399999
6356.0000000	2.8449998
6357.0000000	2.8499999
6358.0000000	2.8499999
6359.0000000	2.8550000

6360.0000000	2.8649998
6361.0000000	2.8550000
6362.0000000	2.8550000
6363.0000000	2.8499999
6364.0000000	2.8550000
6365.0000000	2.8499999
6366.0000000	2.8550000
6367.0000000	2.8449998
6368.0000000	2.8499999
6369.0000000	2.8199999
6370.0000000	2.7800000
6371.0000000	2.7649999
6372.0000000	2.7600000
6373.0000000	2.7649999
6374.0000000	2.7550001
6375.0000000	2.7500000
6376.0000000	2.7500000
6377.0000000	2.7600000
6378.0000000	2.7500000
6379.0000000	2.7649999
6380.0000000	2.7800000
6381.0000000	2.7750001
6382.0000000	2.7950001
6383.0000000	2.8150001
6384.0000000	2.8299999
6385.0000000	2.8399999
6386.0000000	2.8599999
6387.0000000	2.8599999
6388.0000000	2.8800001
6389.0000000	2.9000001
6390.0000000	2.9250002
6391.0000000	2.9400001
6392.0000000	2.9500000
6393.0000000	2.9600000
6394.0000000	2.9749999
6395.0000000	2.9700000
6396.0000000	2.9800000
6397.0000000	2.9800000
6398.0000000	2.9900000
6399.0000000	3.0100002
6400.0000000	3.0100000
6401.0000000	3.0000000
6402.0000000	3.0000000
6403.0000000	2.9900000
6404.0000000	3.0000000
6405.0000000	2.9949999
6406.0000000	2.9949999
6407.0000000	2.9949999
6408.0000000	3.0000000
6409.0000000	3.0050001
6410.0000000	3.0100000
6411.0000000	3.0100000
6412.0000000	3.0050001
6413.0000000	3.0050001
6414.0000000	3.0050001
6415.0000000	3.0050001
6416.0000000	3.0250001

6417.0000000	3.0200000
6418.0000000	3.0100000
6419.0000000	3.0149999
6420.0000000	3.0300000
6421.0000000	3.0250001
6422.0000000	3.0349998
6423.0000000	3.0400000
6424.0000000	3.0549998
6425.0000000	3.0650001
6426.0000000	3.0749998
6427.0000000	3.0949998
6428.0000000	3.1149998
6429.0000000	3.1166666
6430.0000000	3.1300001
6431.0000000	3.1450000
6432.0000000	3.1550002
6433.0000000	3.1650000
6434.0000000	3.1750002
6435.0000000	3.1800001
6436.0000000	3.1900001
6437.0000000	3.2049999
6438.0000000	3.2249999
6439.0000000	3.2200000
6440.0000000	3.2249999
6441.0000000	3.2400000
6442.0000000	3.2449999
6443.0000000	3.2600000
6444.0000000	3.2700000
6445.0000000	3.2800000
6446.0000000	3.2800000
6447.0000000	3.2700000
6448.0000000	3.2400000
6449.0000000	3.2249999
6450.0000000	3.2100000
6451.0000000	3.2049999
6452.0000000	3.1900001
6453.0000000	3.1700001
6454.0000000	3.1650000
6455.0000000	3.1500001
6456.0000000	3.1450000
6457.0000000	3.1400001
6458.0000000	3.1500001
6459.0000000	3.1533334
6460.0000000	3.1750002
6461.0000000	3.1700001
6462.0000000	3.1900001
6463.0000000	3.2000000
6464.0000000	3.2000000
6465.0000000	3.2100000
6466.0000000	3.2249999
6467.0000000	3.2300000
6468.0000000	3.2300000
6469.0000000	3.2049999
6470.0000000	3.2150002
6471.0000000	3.2200000
6472.0000000	3.2200000
6473.0000000	3.2300000

6474.0000000	3.2500000
6475.0000000	3.2600000
6476.0000000	3.2700000
6477.0000000	3.2700000
6478.0000000	3.2800000
6479.0000000	3.2849998
6480.0000000	3.2649999
6481.0000000	3.2700000
6482.0000000	3.2600000
6483.0000000	3.2600000
6484.0000000	3.2649999
6485.0000000	3.2600000
6486.0000000	3.2550001
6487.0000000	3.2649999
6488.0000000	3.2600000
6489.0000000	3.2500000
6490.0000000	3.2600000
6491.0000000	3.2700000
6492.0000000	3.2600000
6493.0000000	3.2750001
6494.0000000	3.2900000
6495.0000000	3.2950001
6496.0000000	3.3049998
6497.0000000	3.3150001
6498.0000000	3.3199999
6499.0000000	3.3199999
6500.0000000	3.3199999
6501.0000000	3.3099999
6502.0000000	3.3249998
6503.0000000	3.3350000
6504.0000000	3.3299999
6505.0000000	3.3350000
6506.0000000	3.3350000
6507.0000000	3.3299999
6508.0000000	3.3299999
6509.0000000	3.3199999
6510.0000000	3.3099999
6511.0000000	3.3099999
6512.0000000	3.3000000
6513.0000000	3.2900000
6514.0000000	3.2849998
6515.0000000	3.2750001
6516.0000000	3.2849998
6517.0000000	3.2849998
6518.0000000	3.2800000
6519.0000000	3.2900000
6520.0000000	3.3133333
6521.0000000	3.3199999
6522.0000000	3.3299999
6523.0000000	3.3350000
6524.0000000	3.3249998
6525.0000000	3.3199999
6526.0000000	3.3099999
6527.0000000	3.3049998
6528.0000000	3.2950001
6529.0000000	3.2800000
6530.0000000	3.2900000

6531.0000000	3.2900000
6532.0000000	3.3099999
6533.0000000	3.3199999
6534.0000000	3.3150001
6535.0000000	3.3049998
6536.0000000	3.3000000
6537.0000000	3.3249998
6538.0000000	3.3299999
6539.0000000	3.3399999
6540.0000000	3.3449998
6541.0000000	3.3350000
6542.0000000	3.3399999
6543.0000000	3.3399999
6544.0000000	3.3449998
6545.0000000	3.3499999
6546.0000000	3.3449998
6547.0000000	3.3499999
6548.0000000	3.3599999
6549.0000000	3.3699999
6550.0000000	3.3750000
6551.0000000	3.3850002
6552.0000000	3.4000001
6553.0000000	3.4000001
6554.0000000	3.4150000
6555.0000000	3.4250002
6556.0000000	3.4349999
6557.0000000	3.4349999
6558.0000000	3.4400001
6559.0000000	3.4250002
6560.0000000	3.4150000
6561.0000000	3.4150000
6562.0000000	3.4050002
6563.0000000	3.3950000
6564.0000000	3.3850002
6565.0000000	3.3699999
6566.0000000	3.3649998
6567.0000000	3.3599999
6568.0000000	3.3499999
6569.0000000	3.3499999
6570.0000000	3.3599999
6571.0000000	3.3750000
6572.0000000	3.3750000
6573.0000000	3.3800001
6574.0000000	3.3750000
6575.0000000	3.3750000
6576.0000000	3.3900001
6577.0000000	3.4000001
6578.0000000	3.4200001
6579.0000000	3.4200001
6580.0000000	3.4266670
6581.0000000	3.4400001
6582.0000000	3.4200001
6583.0000000	3.4150000
6584.0000000	3.4000001
6585.0000000	3.3800001
6586.0000000	3.3599999
6587.0000000	3.3399999

6588.0000000	3.3000000
6589.0000000	3.2800000
6590.0000000	3.2649999
6591.0000000	3.2350001
6592.0000000	3.1950002
6593.0000000	3.1700001
6594.0000000	3.1350002
6595.0000000	3.1099999
6596.0000000	3.0749998
6597.0000000	3.0450001
6598.0000000	3.0200000
6599.0000000	2.9900000
6600.0000000	2.9650002
6601.0000000	2.9549999
6602.0000000	2.9549999
6603.0000000	2.9349999
6604.0000000	2.9349999
6605.0000000	2.9400001
6606.0000000	2.9400001
6607.0000000	2.9500000
6608.0000000	2.9650002
6609.0000000	2.9749999
6610.0000000	2.9900000
6611.0000000	3.0166664
6612.0000000	3.0400000
6613.0000000	3.0599999
6614.0000000	3.0749998
6615.0000000	3.0999999
6616.0000000	3.1300001
6617.0000000	3.1450000
6618.0000000	3.1700001
6619.0000000	3.1900001
6620.0000000	3.2049999
6621.0000000	3.2350001
6622.0000000	3.2550001
6623.0000000	3.2600000
6624.0000000	3.2849998
6625.0000000	3.3000000
6626.0000000	3.3150001
6627.0000000	3.3399999
6628.0000000	3.3499999
6629.0000000	3.3649998
6630.0000000	3.3900001
6631.0000000	3.3850002
6632.0000000	3.3950000
6633.0000000	3.3950000
6634.0000000	3.4000001
6635.0000000	3.3900001
6636.0000000	3.3699999
6637.0000000	3.3649998
6638.0000000	3.3499999
6639.0000000	3.3266666
6640.0000000	3.3350000
6641.0000000	3.3299999
6642.0000000	3.3499999
6643.0000000	3.3649998
6644.0000000	3.3900001

6645.0000000	3.4000001
6646.0000000	3.4050002
6647.0000000	3.4300001
6648.0000000	3.4450002
6649.0000000	3.4450002
6650.0000000	3.4549999
6651.0000000	3.4650002
6652.0000000	3.4700000
6653.0000000	3.4749999
6654.0000000	3.4600000
6655.0000000	3.4500000
6656.0000000	3.4549999
6657.0000000	3.4650002
6658.0000000	3.4800000
6659.0000000	3.5000000
6660.0000000	3.5100000
6661.0000000	3.5250001
6662.0000000	3.5400000
6663.0000000	3.5549998
6664.0000000	3.5699999
6665.0000000	3.5699999
6666.0000000	3.5699999
6667.0000000	3.5949998
6668.0000000	3.5899999
6669.0000000	3.5899999
6670.0000000	3.5949998
6671.0000000	3.6050000
6672.0000000	3.6149998
6673.0000000	3.6300001
6674.0000000	3.6400001
6675.0000000	3.6400001
6676.0000000	3.6400001
6677.0000000	3.6550002
6678.0000000	3.6500001
6679.0000000	3.6500001
6680.0000000	3.6650000
6681.0000000	3.6750002
6682.0000000	3.6800001
6683.0000000	3.6950002
6684.0000000	3.7100000
6685.0000000	3.7200000
6686.0000000	3.7350001
6687.0000000	3.7550001
6688.0000000	3.7600000
6689.0000000	3.7400000
6690.0000000	3.7449999
6691.0000000	3.7200000
6692.0000000	3.7100000
6693.0000000	3.7049999
6694.0000000	3.6800001
6695.0000000	3.6750002
6696.0000000	3.6450000
6697.0000000	3.6350002
6698.0000000	3.6099999
6699.0000000	3.5850000
6700.0000000	3.5749998
6701.0000000	3.5450001

6702.0000000	3.5233333
6703.0000000	3.5000000
6704.0000000	3.4850001
6705.0000000	3.4450002
6706.0000000	3.4300001
6707.0000000	3.4050002
6708.0000000	3.3900001
6709.0000000	3.3650000
6710.0000000	3.3449998
6711.0000000	3.3249998
6712.0000000	3.3049998
6713.0000000	3.2750001
6714.0000000	3.2700000
6715.0000000	3.2550001
6716.0000000	3.2400000
6717.0000000	3.2100000
6718.0000000	3.2000000
6719.0000000	3.1849999
6720.0000000	3.1650000
6721.0000000	3.1650000
6722.0000000	3.1650000
6723.0000000	3.1700001
6724.0000000	3.1800001
6725.0000000	3.1849999
6726.0000000	3.1800001
6727.0000000	3.1750002
6728.0000000	3.1700001
6729.0000000	3.1600001
6730.0000000	3.1500001
6731.0000000	3.1550002
6732.0000000	3.1400001
6733.0000000	3.1400001
6734.0000000	3.1350002
6735.0000000	3.1300001
6736.0000000	3.1250000
6737.0000000	3.1199999
6738.0000000	3.1099999
6739.0000000	3.0850000
6740.0000000	3.0549998
6741.0000000	3.0400000
6742.0000000	3.0300000
6743.0000000	3.0050001
6744.0000000	2.9900000
6745.0000000	2.9800000
6746.0000000	2.9400001
6747.0000000	2.9200001
6748.0000000	2.9050002
6749.0000000	2.8950000
6750.0000000	2.8800001
6751.0000000	2.8699999
6752.0000000	2.8649998
6753.0000000	2.8599999
6754.0000000	2.8399999
6755.0000000	2.8399999
6756.0000000	2.8299999
6757.0000000	2.8099999
6758.0000000	2.8150001

6759.0000000	2.8049998
6760.0000000	2.8000000
6761.0000000	2.7900000
6762.0000000	2.7750001
6763.0000000	2.7666667
6764.0000000	2.7449999
6765.0000000	2.7300000
6766.0000000	2.7100000
6767.0000000	2.6900001
6768.0000000	2.6750002
6769.0000000	2.6400001
6770.0000000	2.6199999
6771.0000000	2.5949998
6772.0000000	2.5549998
6773.0000000	2.5300000
6774.0000000	2.5000000
6775.0000000	2.4650002
6776.0000000	2.4349999
6777.0000000	2.4100001
6778.0000000	2.3850002
6779.0000000	2.3449998
6780.0000000	2.3350000
6781.0000000	2.3399999
6782.0000000	2.3449998
6783.0000000	2.3550000
6784.0000000	2.3750000
6785.0000000	2.3900001
6786.0000000	2.4050002
6787.0000000	2.4250002
6788.0000000	2.4450002
6789.0000000	2.4549999
6790.0000000	2.4700000
6791.0000000	2.4749999
6792.0000000	2.4900000
6793.0000000	2.5066669
6794.0000000	2.5200000
6795.0000000	2.5349998
6796.0000000	2.5450001
6797.0000000	2.5599999
6798.0000000	2.5549998
6799.0000000	2.5549998
6800.0000000	2.5549998
6801.0000000	2.5549998
6802.0000000	2.5599999
6803.0000000	2.5599999
6804.0000000	2.5500000
6805.0000000	2.5549998
6806.0000000	2.5450001
6807.0000000	2.5400000
6808.0000000	2.5149999
6809.0000000	2.5000000
6810.0000000	2.4949999
6811.0000000	2.4850001
6812.0000000	2.4749999
6813.0000000	2.4749999
6814.0000000	2.4749999
6815.0000000	2.4749999

6816.0000000	2.4800000
6817.0000000	2.4800000
6818.0000000	2.4949999
6819.0000000	2.4949999
6820.0000000	2.5050001
6821.0000000	2.5100000
6822.0000000	2.5100000
6823.0000000	2.5100000
6824.0000000	2.5050001
6825.0000000	2.5050001
6826.0000000	2.5050001
6827.0000000	2.5050001
6828.0000000	2.5149999
6829.0000000	2.5100000
6830.0000000	2.5100000
6831.0000000	2.5100000
6832.0000000	2.4949999
6833.0000000	2.4800000
6834.0000000	2.4600000
6835.0000000	2.4400001
6836.0000000	2.4200001
6837.0000000	2.4050002
6838.0000000	2.3850002
6839.0000000	2.3750000
6840.0000000	2.3550000
6841.0000000	2.3499999
6842.0000000	2.3299999
6843.0000000	2.3150001
6844.0000000	2.3049998
6845.0000000	2.2950001
6846.0000000	2.2950001
6847.0000000	2.2950001
6848.0000000	2.3150001
6849.0000000	2.3049998
6850.0000000	2.2849998
6851.0000000	2.2849998
6852.0000000	2.2750001
6853.0000000	2.2500000
6854.0000000	2.2100000
6855.0000000	2.2200000
6856.0000000	2.2100000
6857.0000000	2.2249999
6858.0000000	2.2400000
6859.0000000	2.2500000
6860.0000000	2.2600000
6861.0000000	2.2500000
6862.0000000	2.2700000
6863.0000000	2.2649999
6864.0000000	2.2750001
6865.0000000	2.2950001
6866.0000000	2.2900000
6867.0000000	2.3000000
6868.0000000	2.3099999
6869.0000000	2.3150001
6870.0000000	2.3249998
6871.0000000	2.3299999
6872.0000000	2.3449998

6873.0000000	2.3599999
6874.0000000	2.3750000
6875.0000000	2.3900001
6876.0000000	2.4100001
6877.0000000	2.4300001
6878.0000000	2.4450002
6879.0000000	2.4600000
6880.0000000	2.4850001
6881.0000000	2.4949999
6882.0000000	2.5066669
6883.0000000	2.5250001
6884.0000000	2.5300000
6885.0000000	2.5200000
6886.0000000	2.5300000
6887.0000000	2.5400000
6888.0000000	2.5349998
6889.0000000	2.5400000
6890.0000000	2.5450001
6891.0000000	2.5599999
6892.0000000	2.5799999
6893.0000000	2.5899999
6894.0000000	2.6050000
6895.0000000	2.6300001
6896.0000000	2.6350002
6897.0000000	2.6400001
6898.0000000	2.6550002
6899.0000000	2.6600001
6900.0000000	2.6750002
6901.0000000	2.6800001
6902.0000000	2.6900001
6903.0000000	2.6700001
6904.0000000	2.6700001
6905.0000000	2.6750002
6906.0000000	2.6750002
6907.0000000	2.6650000
6908.0000000	2.6600001
6909.0000000	2.6400001
6910.0000000	2.6199999
6911.0000000	2.6099999
6912.0000000	2.6033332
6913.0000000	2.5899999
6914.0000000	2.5850000
6915.0000000	2.5799999
6916.0000000	2.5749998
6917.0000000	2.5699999
6918.0000000	2.5599999
6919.0000000	2.5500000
6920.0000000	2.5300000
6921.0000000	2.5300000
6922.0000000	2.5300000
6923.0000000	2.5250001
6924.0000000	2.5400000
6925.0000000	2.5400000
6926.0000000	2.5450001
6927.0000000	2.5450001
6928.0000000	2.5549998
6929.0000000	2.5450001

6930.0000000	2.5599999
6931.0000000	2.5650001
6932.0000000	2.5799999
6933.0000000	2.5850000
6934.0000000	2.5899999
6935.0000000	2.5999999
6936.0000000	2.6099999
6937.0000000	2.6149998
6938.0000000	2.6250000
6939.0000000	2.6350002
6940.0000000	2.6300001
6941.0000000	2.6250000
6942.0000000	2.6133330
6943.0000000	2.6050000
6944.0000000	2.5949998
6945.0000000	2.5899999
6946.0000000	2.5949998
6947.0000000	2.5949998
6948.0000000	2.6099999
6949.0000000	2.6199999
6950.0000000	2.6149998
6951.0000000	2.6099999
6952.0000000	2.6099999
6953.0000000	2.5949998
6954.0000000	2.5899999
6955.0000000	2.5749998
6956.0000000	2.5650001
6957.0000000	2.5650001
6958.0000000	2.5699999
6959.0000000	2.5850000
6960.0000000	2.5850000
6961.0000000	2.5749998
6962.0000000	2.5749998
6963.0000000	2.5799999
6964.0000000	2.5899999
6965.0000000	2.6099999
6966.0000000	2.6099999
6967.0000000	2.6199999
6968.0000000	2.6300001
6969.0000000	2.6350002
6970.0000000	2.6400001
6971.0000000	2.6450000
6972.0000000	2.6566668
6973.0000000	2.6800001
6974.0000000	2.6849999
6975.0000000	2.6950002
6976.0000000	2.7100000
6977.0000000	2.7249999
6978.0000000	2.7500000
6979.0000000	2.7550001
6980.0000000	2.7649999
6981.0000000	2.7550001
6982.0000000	2.7600000
6983.0000000	2.7750001
6984.0000000	2.7800000
6985.0000000	2.7900000
6986.0000000	2.8049998

6987.0000000	2.8150001
6988.0000000	2.8150001
6989.0000000	2.8299999
6990.0000000	2.8249998
6991.0000000	2.8399999
6992.0000000	2.8299999
6993.0000000	2.8299999
6994.0000000	2.8350000
6995.0000000	2.8299999
6996.0000000	2.8249998
6997.0000000	2.8150001
6998.0000000	2.8150001
6999.0000000	2.7849998
7000.0000000	2.7750001
7001.0000000	2.7800000
7002.0000000	2.7649999
7003.0000000	2.7433331
7004.0000000	2.7350001
7005.0000000	2.7300000
7006.0000000	2.7249999
7007.0000000	2.7150002
7008.0000000	2.7150002
7009.0000000	2.7000000
7010.0000000	2.6950002
7011.0000000	2.6849999
7012.0000000	2.6950002
7013.0000000	2.6849999
7014.0000000	2.6700001
7015.0000000	2.6650000
7016.0000000	2.6650000
7017.0000000	2.6600001
7018.0000000	2.6500001
7019.0000000	2.6400001
7020.0000000	2.6300001
7021.0000000	2.6250000
7022.0000000	2.6099999
7023.0000000	2.5999999
7024.0000000	2.5899999
7025.0000000	2.5799999
7026.0000000	2.5650001
7027.0000000	2.5500000
7028.0000000	2.5450001
7029.0000000	2.5300000
7030.0000000	2.5149999
7031.0000000	2.5149999
7032.0000000	2.5100000
7033.0000000	2.5050001
7034.0000000	2.5066669
7035.0000000	2.4900000
7036.0000000	2.4800000
7037.0000000	2.4650002
7038.0000000	2.4400001
7039.0000000	2.4100001
7040.0000000	2.3599999
7041.0000000	2.3199999
7042.0000000	2.2550001
7043.0000000	2.1950002

7044.0000000	2.1300001
7045.0000000	2.0999999
7046.0000000	2.1050000
7047.0000000	2.1050000
7048.0000000	2.1199999
7049.0000000	2.1300001
7050.0000000	2.1350002
7051.0000000	2.1400001
7052.0000000	2.1600001
7053.0000000	2.1750002
7054.0000000	2.1800001
7055.0000000	2.1849999
7056.0000000	2.1800001
7057.0000000	2.1800001
7058.0000000	2.1800001
7059.0000000	2.1750002
7060.0000000	2.1650000
7061.0000000	2.1550002
7062.0000000	2.1500001
7063.0000000	2.1450000
7064.0000000	2.1433334
7065.0000000	2.1199999
7066.0000000	2.0899999
7067.0000000	2.0599999
7068.0000000	2.0549998
7069.0000000	2.0500000
7070.0000000	2.0450001
7071.0000000	2.0599999
7072.0000000	2.0850000
7073.0000000	2.0999999
7074.0000000	2.1050000
7075.0000000	2.1050000
7076.0000000	2.0949998
7077.0000000	2.0899999
7078.0000000	2.0799999
7079.0000000	2.0850000
7080.0000000	2.0850000
7081.0000000	2.0799999
7082.0000000	2.0749998
7083.0000000	2.0749998
7084.0000000	2.0799999
7085.0000000	2.0899999
7086.0000000	2.1099999
7087.0000000	2.1300001
7088.0000000	2.1450000
7089.0000000	2.1550002
7090.0000000	2.1600001
7091.0000000	2.1750002
7092.0000000	2.1866667
7093.0000000	2.2049999
7094.0000000	2.2100000
7095.0000000	2.2200000
7096.0000000	2.2400000
7097.0000000	2.2449999
7098.0000000	2.2649999
7099.0000000	2.2649999
7100.0000000	2.2700000

7101.0000000	2.2700000
7102.0000000	2.2750001
7103.0000000	2.2750001
7104.0000000	2.2800000
7105.0000000	2.2849998
7106.0000000	2.3000000
7107.0000000	2.3000000
7108.0000000	2.3099999
7109.0000000	2.3150001
7110.0000000	2.3049998
7111.0000000	2.3049998
7112.0000000	2.3049998
7113.0000000	2.2950001
7114.0000000	2.2849998
7115.0000000	2.3049998
7116.0000000	2.3000000
7117.0000000	2.3000000
7118.0000000	2.3049998
7119.0000000	2.3049998
7120.0000000	2.3099999
7121.0000000	2.3099999
7122.0000000	2.3033333
7123.0000000	2.3000000
7124.0000000	2.2849998
7125.0000000	2.2849998
7126.0000000	2.2649999
7127.0000000	2.2500000
7128.0000000	2.2500000
7129.0000000	2.2300000
7130.0000000	2.2300000
7131.0000000	2.2300000
7132.0000000	2.2249999
7133.0000000	2.2200000
7134.0000000	2.2249999
7135.0000000	2.2249999
7136.0000000	2.2200000
7137.0000000	2.2249999
7138.0000000	2.2249999
7139.0000000	2.2100000
7140.0000000	2.2049999
7141.0000000	2.1900001
7142.0000000	2.1849999
7143.0000000	2.1800001
7144.0000000	2.1650000
7145.0000000	2.1650000
7146.0000000	2.1550002
7147.0000000	2.1600001
7148.0000000	2.1600001
7149.0000000	2.1500001
7150.0000000	2.1500001
7151.0000000	2.1500001
7152.0000000	2.1500001
7153.0000000	2.1533334
7154.0000000	2.1600001
7155.0000000	2.1550002
7156.0000000	2.1700001
7157.0000000	2.1600001

7158.0000000	2.1650000
7159.0000000	2.1650000
7160.0000000	2.1700001
7161.0000000	2.1650000
7162.0000000	2.1650000
7163.0000000	2.1550002
7164.0000000	2.1600001
7165.0000000	2.1650000
7166.0000000	2.1650000
7167.0000000	2.1650000
7168.0000000	2.1750002
7169.0000000	2.1750002
7170.0000000	2.1750002
7171.0000000	2.1700001
7172.0000000	2.1750002
7173.0000000	2.1800001
7174.0000000	2.1700001
7175.0000000	2.1750002
7176.0000000	2.1800001
7177.0000000	2.1800001
7178.0000000	2.1849999
7179.0000000	2.1950002
7180.0000000	2.1950002
7181.0000000	2.2000000
7182.0000000	2.2049999
7183.0000000	2.2100000
7184.0000000	2.2200000
7185.0000000	2.2249999
7186.0000000	2.2350001
7187.0000000	2.2350001
7188.0000000	2.2400000
7189.0000000	2.2350001
7190.0000000	2.2400000
7191.0000000	2.2400000
7192.0000000	2.2500000
7193.0000000	2.2600000
7194.0000000	2.2849998
7195.0000000	2.2900000
7196.0000000	2.2900000
7197.0000000	2.2800000
7198.0000000	2.2950001
7199.0000000	2.3099999
7200.0000000	2.3099999
7201.0000000	2.3099999
7202.0000000	2.3150001
7203.0000000	2.3000000
7204.0000000	2.2900000
7205.0000000	2.2900000
7206.0000000	2.2950001
7207.0000000	2.2750001
7208.0000000	2.2449999
7209.0000000	2.2150002
7210.0000000	2.1800001
7211.0000000	2.1550002
7212.0000000	2.1250000
7213.0000000	2.0933332
7214.0000000	2.0799999

7215.0000000	2.0899999
7216.0000000	2.0899999
7217.0000000	2.0999999
7218.0000000	2.0999999
7219.0000000	2.1099999
7220.0000000	2.1099999
7221.0000000	2.1149998
7222.0000000	2.1149998
7223.0000000	2.1199999
7224.0000000	2.1199999
7225.0000000	2.1300001
7226.0000000	2.1350002
7227.0000000	2.1400001
7228.0000000	2.1500001
7229.0000000	2.1550002
7230.0000000	2.1600001
7231.0000000	2.1550002
7232.0000000	2.1600001
7233.0000000	2.1700001
7234.0000000	2.1700001
7235.0000000	2.1700001
7236.0000000	2.1550002
7237.0000000	2.1500001
7238.0000000	2.1400001
7239.0000000	2.1500001
7240.0000000	2.1500001
7241.0000000	2.1500001
7242.0000000	2.1550002
7243.0000000	2.1600001
7244.0000000	2.1366668
7245.0000000	2.1300001
7246.0000000	2.1300001
7247.0000000	2.1149998
7248.0000000	2.1149998
7249.0000000	2.0999999
7250.0000000	2.1050000
7251.0000000	2.1050000
7252.0000000	2.1149998
7253.0000000	2.1250000
7254.0000000	2.1350002
7255.0000000	2.1400001
7256.0000000	2.1500001
7257.0000000	2.1500001
7258.0000000	2.1600001
7259.0000000	2.1700001
7260.0000000	2.1700001
7261.0000000	2.1800001
7262.0000000	2.1849999
7263.0000000	2.2000000
7264.0000000	2.2000000
7265.0000000	2.2100000
7266.0000000	2.2100000
7267.0000000	2.2049999
7268.0000000	2.2049999
7269.0000000	2.2100000
7270.0000000	2.2150002
7271.0000000	2.2200000

7272.0000000	2.2150002
7273.0000000	2.2200000
7274.0000000	2.2299998
7275.0000000	2.2300000
7276.0000000	2.2350001
7277.0000000	2.2300000
7278.0000000	2.2350001
7279.0000000	2.2249999
7280.0000000	2.2300000
7281.0000000	2.2400000
7282.0000000	2.2400000
7283.0000000	2.2449999
7284.0000000	2.2500000
7285.0000000	2.2550001
7286.0000000	2.2600000
7287.0000000	2.2700000
7288.0000000	2.2750001
7289.0000000	2.2750001
7290.0000000	2.2750001
7291.0000000	2.2649999
7292.0000000	2.2600000
7293.0000000	2.2400000
7294.0000000	2.2200000
7295.0000000	2.2049999
7296.0000000	2.2000000
7297.0000000	2.1900001
7298.0000000	2.2000000
7299.0000000	2.1849999
7300.0000000	2.1750002
7301.0000000	2.1800001
7302.0000000	2.1849999
7303.0000000	2.1950002
7304.0000000	2.2000000
7305.0000000	2.2166669
7306.0000000	2.2200000
7307.0000000	2.2300000
7308.0000000	2.2150002
7309.0000000	2.2100000
7310.0000000	2.2200000
7311.0000000	2.2200000
7312.0000000	2.2249999
7313.0000000	2.2350001
7314.0000000	2.2400000
7315.0000000	2.2500000
7316.0000000	2.2550001
7317.0000000	2.2649999
7318.0000000	2.2649999
7319.0000000	2.2750001
7320.0000000	2.2800000
7321.0000000	2.2849998
7322.0000000	2.2849998
7323.0000000	2.2800000
7324.0000000	2.2900000
7325.0000000	2.2900000
7326.0000000	2.2900000
7327.0000000	2.2800000
7328.0000000	2.2750001

7329.0000000	2.2700000
7330.0000000	2.2600000
7331.0000000	2.2600000
7332.0000000	2.2500000
7333.0000000	2.2449999
7334.0000000	2.2300000
7335.0000000	2.2400000
7336.0000000	2.2366667
7337.0000000	2.2300000
7338.0000000	2.2400000
7339.0000000	2.2300000
7340.0000000	2.2249999
7341.0000000	2.2200000
7342.0000000	2.2100000
7343.0000000	2.2150002
7344.0000000	2.2049999
7345.0000000	2.1950002
7346.0000000	2.1900001
7347.0000000	2.1800001
7348.0000000	2.1849999
7349.0000000	2.1900001
7350.0000000	2.1849999
7351.0000000	2.1900001
7352.0000000	2.1900001
7353.0000000	2.1950002
7354.0000000	2.1750002
7355.0000000	2.1800001
7356.0000000	2.1800001
7357.0000000	2.1849999
7358.0000000	2.1800001
7359.0000000	2.1900001
7360.0000000	2.1900001
7361.0000000	2.2000000
7362.0000000	2.2000000
7363.0000000	2.2000000
7364.0000000	2.2100000
7365.0000000	2.2000000
7366.0000000	2.2000000
7367.0000000	2.2000000
7368.0000000	2.2000000
7369.0000000	2.2000000
7370.0000000	2.2249999
7371.0000000	2.2400000
7372.0000000	2.2449999
7373.0000000	2.2600000
7374.0000000	2.2700000
7375.0000000	2.2849998
7376.0000000	2.2950001
7377.0000000	2.3049998
7378.0000000	2.3099999
7379.0000000	2.3199999
7380.0000000	2.3199999
7381.0000000	2.3150001
7382.0000000	2.3150001
7383.0000000	2.3150001
7384.0000000	2.3150001
7385.0000000	2.3099999

7386.0000000	2.3150001
7387.0000000	2.3199999
7388.0000000	2.3150001
7389.0000000	2.3249998
7390.0000000	2.3350000
7391.0000000	2.3299999
7392.0000000	2.3350000
7393.0000000	2.3399999
7394.0000000	2.3449998
7395.0000000	2.3466666
7396.0000000	2.3449998
7397.0000000	2.3299999
7398.0000000	2.3249998
7399.0000000	2.3150001
7400.0000000	2.3049998
7401.0000000	2.2950001
7402.0000000	2.3000000
7403.0000000	2.3000000
7404.0000000	2.2950001
7405.0000000	2.2900000
7406.0000000	2.2800000
7407.0000000	2.2800000
7408.0000000	2.2750001
7409.0000000	2.2649999
7410.0000000	2.2600000
7411.0000000	2.2550001
7412.0000000	2.2500000
7413.0000000	2.2350001
7414.0000000	2.2400000
7415.0000000	2.2400000
7416.0000000	2.2449999
7417.0000000	2.2400000
7418.0000000	2.2300000
7419.0000000	2.2350001
7420.0000000	2.2300000
7421.0000000	2.2249999
7422.0000000	2.2300000
7423.0000000	2.2200000
7424.0000000	2.2249999
7425.0000000	2.2200000
7426.0000000	2.2100000
7427.0000000	2.2150002
7428.0000000	2.2000000
7429.0000000	2.2000000
7430.0000000	2.2000000
7431.0000000	2.2000000
7432.0000000	2.2100000
7433.0000000	2.2049999
7434.0000000	2.2100000
7435.0000000	2.2049999
7436.0000000	2.2049999
7437.0000000	2.1950002
7438.0000000	2.1900001
7439.0000000	2.1900001
7440.0000000	2.1750002
7441.0000000	2.1650000
7442.0000000	2.1650000

7443.0000000	2.1550002
7444.0000000	2.1500001
7445.0000000	2.1550002
7446.0000000	2.1500001
7447.0000000	2.1450000
7448.0000000	2.1350002
7449.0000000	2.1300001
7450.0000000	2.1300001
7451.0000000	2.1149998
7452.0000000	2.1050000
7453.0000000	2.0999999
7454.0000000	2.0899999
7455.0000000	2.0933332
7456.0000000	2.0799999
7457.0000000	2.0749998
7458.0000000	2.0699999
7459.0000000	2.0699999
7460.0000000	2.0699999
7461.0000000	2.0699999
7462.0000000	2.0699999
7463.0000000	2.0749998
7464.0000000	2.0799999
7465.0000000	2.0850000
7466.0000000	2.0899999
7467.0000000	2.0899999
7468.0000000	2.0999999
7469.0000000	2.0999999
7470.0000000	2.0999999
7471.0000000	2.0850000
7472.0000000	2.0799999
7473.0000000	2.0850000
7474.0000000	2.0949998
7475.0000000	2.0949998
7476.0000000	2.1050000
7477.0000000	2.0999999
7478.0000000	2.1149998
7479.0000000	2.1250000
7480.0000000	2.1450000
7481.0000000	2.1450000
7482.0000000	2.1450000
7483.0000000	2.1400001
7484.0000000	2.1250000
7485.0000000	2.1233332
7486.0000000	2.1050000
7487.0000000	2.0999999
7488.0000000	2.0699999
7489.0000000	2.0699999
7490.0000000	2.0599999
7491.0000000	2.0450001
7492.0000000	2.0450001
7493.0000000	2.0400000
7494.0000000	2.0400000
7495.0000000	2.0300000
7496.0000000	2.0300000
7497.0000000	2.0300000
7498.0000000	2.0200000
7499.0000000	2.0250001

7500.0000000	2.0200000
7501.0000000	2.0149999
7502.0000000	2.0100000
7503.0000000	1.9900000
7504.0000000	1.9750000
7505.0000000	1.9600000
7506.0000000	1.9450001
7507.0000000	1.9400001
7508.0000000	1.9200000
7509.0000000	1.9150000
7510.0000000	1.9000000
7511.0000000	1.8850000
7512.0000000	1.8850000
7513.0000000	1.8750000
7514.0000000	1.8700000
7515.0000000	1.8633333
7516.0000000	1.8600000
7517.0000000	1.8550000
7518.0000000	1.8500000
7519.0000000	1.8450000
7520.0000000	1.8450000
7521.0000000	1.8350000
7522.0000000	1.8200001
7523.0000000	1.8099999
7524.0000000	1.7950000
7525.0000000	1.7850000
7526.0000000	1.7800000
7527.0000000	1.7750000
7528.0000000	1.7700000
7529.0000000	1.7650000
7530.0000000	1.7650000
7531.0000000	1.7550000
7532.0000000	1.7500000
7533.0000000	1.7500000
7534.0000000	1.7450000
7535.0000000	1.7350000
7536.0000000	1.7350000
7537.0000000	1.7300000
7538.0000000	1.7350000
7539.0000000	1.7450000
7540.0000000	1.7500000
7541.0000000	1.7550000
7542.0000000	1.7450000
7543.0000000	1.7300000
7544.0000000	1.7450000
7545.0000000	1.7466666
7546.0000000	1.7550000
7547.0000000	1.7550000
7548.0000000	1.7650000
7549.0000000	1.7650000
7550.0000000	1.7700000
7551.0000000	1.7850000
7552.0000000	1.7900000
7553.0000000	1.8000000
7554.0000000	1.8099999
7555.0000000	1.8150001
7556.0000000	1.8200001

7557.0000000	1.8200001
7558.0000000	1.8150001
7559.0000000	1.8250000
7560.0000000	1.8450000
7561.0000000	1.8550000
7562.0000000	1.8700000
7563.0000000	1.8900000
7564.0000000	1.9050000
7565.0000000	1.9250000
7566.0000000	1.9450001
7567.0000000	1.9550000
7568.0000000	1.9650000
7569.0000000	1.9600000
7570.0000000	1.9650000
7571.0000000	1.9600000
7572.0000000	1.9750000
7573.0000000	1.9850000
7574.0000000	1.9950000
7575.0000000	2.0033333
7576.0000000	1.9950000
7577.0000000	1.9900000
7578.0000000	1.9950000
7579.0000000	2.0000000
7580.0000000	2.0100000
7581.0000000	2.0250001
7582.0000000	2.0300000
7583.0000000	2.0349998
7584.0000000	2.0300000
7585.0000000	2.0300000
7586.0000000	2.0250001
7587.0000000	2.0200000
7588.0000000	2.0149999
7589.0000000	2.0250001
7590.0000000	2.0250001
7591.0000000	2.0250001
7592.0000000	2.0250001
7593.0000000	2.0300000
7594.0000000	2.0349998
7595.0000000	2.0300000
7596.0000000	2.0349998
7597.0000000	2.0300000
7598.0000000	2.0200000
7599.0000000	2.0250001
7600.0000000	2.0300000
7601.0000000	2.0300000
7602.0000000	2.0300000
7603.0000000	2.0400000
7604.0000000	2.0450001
7605.0000000	2.0450001
7606.0000000	2.0500000
7607.0000000	2.0549998
7608.0000000	2.0699999
7609.0000000	2.0650001
7610.0000000	2.0650001
7611.0000000	2.0699999
7612.0000000	2.0699999
7613.0000000	2.0749998

7614.0000000	2.0749998
7615.0000000	2.0799999
7616.0000000	2.0799999
7617.0000000	2.0799999
7618.0000000	2.0850000
7619.0000000	2.0850000
7620.0000000	2.0850000
7621.0000000	2.0899999
7622.0000000	2.0899999
7623.0000000	2.0949998
7624.0000000	2.0949998
7625.0000000	2.0999999
7626.0000000	2.1050000
7627.0000000	2.0999999
7628.0000000	2.0999999
7629.0000000	2.0899999
7630.0000000	2.0899999
7631.0000000	2.0799999
7632.0000000	2.0799999
7633.0000000	2.0749998
7634.0000000	2.0699999
7635.0000000	2.0699999
7636.0000000	2.0633333
7637.0000000	2.0699999
7638.0000000	2.0650001
7639.0000000	2.0650001
7640.0000000	2.0699999
7641.0000000	2.0749998
7642.0000000	2.0749998
7643.0000000	2.0799999
7644.0000000	2.0899999
7645.0000000	2.0949998
7646.0000000	2.0949998
7647.0000000	2.0999999
7648.0000000	2.1099999
7649.0000000	2.1199999
7650.0000000	2.1300001
7651.0000000	2.1400001
7652.0000000	2.1550002
7653.0000000	2.1600001
7654.0000000	2.1550002
7655.0000000	2.1500001
7656.0000000	2.1400001
7657.0000000	2.1150000
7658.0000000	2.1050000
7659.0000000	2.1099999
7660.0000000	2.1149998
7661.0000000	2.1250000
7662.0000000	2.1300001
7663.0000000	2.1600001
7664.0000000	2.1700001
7665.0000000	2.1900001
7666.0000000	2.2033334
7667.0000000	2.2049999
7668.0000000	2.2000000
7669.0000000	2.2049999
7670.0000000	2.2049999

7671.0000000	2.2150002
7672.0000000	2.2249999
7673.0000000	2.2350001
7674.0000000	2.2500000
7675.0000000	2.2550001
7676.0000000	2.2700000
7677.0000000	2.2800000
7678.0000000	2.2900000
7679.0000000	2.3000000
7680.0000000	2.2900000
7681.0000000	2.2750001
7682.0000000	2.2750001
7683.0000000	2.2600000
7684.0000000	2.2449999
7685.0000000	2.2400000
7686.0000000	2.2350001
7687.0000000	2.2350001
7688.0000000	2.2350001
7689.0000000	2.2500000
7690.0000000	2.2400000
7691.0000000	2.2550001
7692.0000000	2.2550001
7693.0000000	2.2550001
7694.0000000	2.2649999
7695.0000000	2.2700000
7696.0000000	2.2700000
7697.0000000	2.2766666
7698.0000000	2.2649999
7699.0000000	2.2700000
7700.0000000	2.2700000
7701.0000000	2.2700000
7702.0000000	2.2649999
7703.0000000	2.2550001
7704.0000000	2.2500000
7705.0000000	2.2400000
7706.0000000	2.2300000
7707.0000000	2.2300000
7708.0000000	2.2249999
7709.0000000	2.2200000
7710.0000000	2.2049999
7711.0000000	2.1900001
7712.0000000	2.1849999
7713.0000000	2.1600001
7714.0000000	2.1500001
7715.0000000	2.1250000
7716.0000000	2.0999999
7717.0000000	2.0799999
7718.0000000	2.0500000
7719.0000000	2.0250001
7720.0000000	2.0100000
7721.0000000	1.9850000
7722.0000000	1.9650000
7723.0000000	1.9400001
7724.0000000	1.9250000
7725.0000000	1.9050000
7726.0000000	1.8900000
7727.0000000	1.8933333

7728.0000000	1.8950000
7729.0000000	1.9050000
7730.0000000	1.9200000
7731.0000000	1.9250000
7732.0000000	1.9400001
7733.0000000	1.9500000
7734.0000000	1.9600000
7735.0000000	1.9700000
7736.0000000	1.9800000
7737.0000000	1.9950000
7738.0000000	2.0000000
7739.0000000	2.0100000
7740.0000000	2.0149999
7741.0000000	2.0250001
7742.0000000	2.0300000
7743.0000000	2.0400000
7744.0000000	2.0599999
7745.0000000	2.0599999
7746.0000000	2.0699999
7747.0000000	2.0699999
7748.0000000	2.0749998
7749.0000000	2.0699999
7750.0000000	2.0699999
7751.0000000	2.0650001
7752.0000000	2.0650001
7753.0000000	2.0650001
7754.0000000	2.0799999
7755.0000000	2.0749998
7756.0000000	2.0749998
7757.0000000	2.0850000
7758.0000000	2.0833333
7759.0000000	2.0799999
7760.0000000	2.0850000
7761.0000000	2.0850000
7762.0000000	2.0899999
7763.0000000	2.0850000
7764.0000000	2.0899999
7765.0000000	2.0899999
7766.0000000	2.0999999
7767.0000000	2.0899999
7768.0000000	2.0899999
7769.0000000	2.0850000
7770.0000000	2.0799999
7771.0000000	2.0599999
7772.0000000	2.0500000
7773.0000000	2.0349998
7774.0000000	2.0200000
7775.0000000	2.0200000
7776.0000000	2.0300000
7777.0000000	2.0450001
7778.0000000	2.0400000
7779.0000000	2.0549998
7780.0000000	2.0699999
7781.0000000	2.0799999
7782.0000000	2.0899999
7783.0000000	2.0949998
7784.0000000	2.0899999

7785.0000000	2.1050000
7786.0000000	2.0949998
7787.0000000	2.0999999
7788.0000000	2.0899999
7789.0000000	2.0833333
7790.0000000	2.0749998
7791.0000000	2.0699999
7792.0000000	2.0650001
7793.0000000	2.0650001
7794.0000000	2.0650001
7795.0000000	2.0650001
7796.0000000	2.0599999
7797.0000000	2.0549998
7798.0000000	2.0549998
7799.0000000	2.0500000
7800.0000000	2.0549998
7801.0000000	2.0500000
7802.0000000	2.0500000
7803.0000000	2.0549998
7804.0000000	2.0300000
7805.0000000	1.9950000
7806.0000000	1.9750000
7807.0000000	1.9650000
7808.0000000	1.9500000
7809.0000000	1.9500000
7810.0000000	1.9450001
7811.0000000	1.9400001
7812.0000000	1.9349999
7813.0000000	1.9349999
7814.0000000	1.9299999
7815.0000000	1.9349999
7816.0000000	1.9450001
7817.0000000	1.9400001
7818.0000000	1.9500002
7819.0000000	1.9500000
7820.0000000	1.9550000
7821.0000000	1.9500000
7822.0000000	1.9550000
7823.0000000	1.9500000
7824.0000000	1.9600000
7825.0000000	1.9700000
7826.0000000	1.9800000
7827.0000000	1.9950000
7828.0000000	2.0050001
7829.0000000	2.0100000
7830.0000000	2.0250001
7831.0000000	2.0349998
7832.0000000	2.0400000
7833.0000000	2.0549998
7834.0000000	2.0599999
7835.0000000	2.0650001
7836.0000000	2.0650001
7837.0000000	2.0599999
7838.0000000	2.0549998
7839.0000000	2.0549998
7840.0000000	2.0549998
7841.0000000	2.0549998

7842.0000000	2.0400000
7843.0000000	2.0450001
7844.0000000	2.0300000
7845.0000000	2.0300000
7846.0000000	2.0149999
7847.0000000	2.0100000
7848.0000000	2.0000000
7849.0000000	1.9850000
7850.0000000	1.9750000
7851.0000000	1.9750000
7852.0000000	1.9650000
7853.0000000	1.9349999
7854.0000000	1.9200000
7855.0000000	1.9100000
7856.0000000	1.9100000
7857.0000000	1.9000000
7858.0000000	1.9000000
7859.0000000	1.8900000
7860.0000000	1.8900000
7861.0000000	1.8800000
7862.0000000	1.8600000
7863.0000000	1.8450000
7864.0000000	1.8350000
7865.0000000	1.8300000
7866.0000000	1.8250000
7867.0000000	1.8250000
7868.0000000	1.8200001
7869.0000000	1.8049999
7870.0000000	1.8000000
7871.0000000	1.8000000
7872.0000000	1.7950000
7873.0000000	1.8049999
7874.0000000	1.8200001
7875.0000000	1.8300000
7876.0000000	1.8350000
7877.0000000	1.8350000
7878.0000000	1.8333334
7879.0000000	1.8450000
7880.0000000	1.8450000
7881.0000000	1.8550000
7882.0000000	1.8500000
7883.0000000	1.8600000
7884.0000000	1.8650000
7885.0000000	1.8650000
7886.0000000	1.8650000
7887.0000000	1.8550000
7888.0000000	1.8500000
7889.0000000	1.8450000
7890.0000000	1.8550000
7891.0000000	1.8600000
7892.0000000	1.8550000
7893.0000000	1.8600000
7894.0000000	1.8600000
7895.0000000	1.8600000
7896.0000000	1.8600000
7897.0000000	1.8600000
7898.0000000	1.8550000

7899.0000000	1.8500000
7900.0000000	1.8550000
7901.0000000	1.8350000
7902.0000000	1.8350000
7903.0000000	1.8350000
7904.0000000	1.8350000
7905.0000000	1.8400000
7906.0000000	1.8350000
7907.0000000	1.8350000
7908.0000000	1.8300000
7909.0000000	1.8300000
7910.0000000	1.8400000
7911.0000000	1.8500000
7912.0000000	1.8550000
7913.0000000	1.8600000
7914.0000000	1.8700000
7915.0000000	1.8700000
7916.0000000	1.8750000
7917.0000000	1.8800000
7918.0000000	1.8950000
7919.0000000	1.8900000
7920.0000000	1.8900000
7921.0000000	1.8950000
7922.0000000	1.8950000
7923.0000000	1.9000000
7924.0000000	1.9100000
7925.0000000	1.9250000
7926.0000000	1.9349999
7927.0000000	1.9550000
7928.0000000	1.9650000
7929.0000000	1.9700000
7930.0000000	1.9800000
7931.0000000	1.9900000
7932.0000000	2.0050001
7933.0000000	2.0000000
7934.0000000	2.0100000
7935.0000000	2.0100000
7936.0000000	2.0200000
7937.0000000	2.0200000
7938.0000000	2.0200000
7939.0000000	2.0166667
7940.0000000	2.0149999
7941.0000000	2.0100000
7942.0000000	2.0149999
7943.0000000	2.0100000
7944.0000000	2.0200000
7945.0000000	2.0149999
7946.0000000	2.0149999
7947.0000000	2.0100000
7948.0000000	2.0149999
7949.0000000	2.0100000
7950.0000000	2.0000000
7951.0000000	2.0050001
7952.0000000	2.0149999
7953.0000000	2.0100000
7954.0000000	2.0100000
7955.0000000	2.0100000

7956.0000000	2.0100000
7957.0000000	2.0100000
7958.0000000	2.0149999
7959.0000000	2.0050001
7960.0000000	2.0149999
7961.0000000	2.0200000
7962.0000000	2.0200000
7963.0000000	2.0200000
7964.0000000	2.0200000
7965.0000000	2.0300000
7966.0000000	2.0250001
7967.0000000	2.0500000
7968.0000000	2.0549998
7969.0000000	2.0599999
7970.0000000	2.0650001
7971.0000000	2.0566666
7972.0000000	2.0500000
7973.0000000	2.0500000
7974.0000000	2.0549998
7975.0000000	2.0549998
7976.0000000	2.0500000
7977.0000000	2.0599999
7978.0000000	2.0500000
7979.0000000	2.0599999
7980.0000000	2.0500000
7981.0000000	2.0500000
7982.0000000	2.0549998
7983.0000000	2.0500000
7984.0000000	2.0450001
7985.0000000	2.0549998
7986.0000000	2.0500000
7987.0000000	2.0450001
7988.0000000	2.0500000
7989.0000000	2.0349998
7990.0000000	2.0400000
7991.0000000	2.0500000
7992.0000000	2.0549998
7993.0000000	2.0549998
7994.0000000	2.0450001
7995.0000000	2.0450001
7996.0000000	2.0500000
7997.0000000	2.0400000
7998.0000000	2.0450001
7999.0000000	2.0349998
8000.0000000	2.0366666
8001.0000000	2.0300000
8002.0000000	2.0349998
8003.0000000	2.0400000
8004.0000000	2.0400000
8005.0000000	2.0400000
8006.0000000	2.0450001
8007.0000000	2.0500000
8008.0000000	2.0450001
8009.0000000	2.0450001
8010.0000000	2.0300000
8011.0000000	2.0300000
8012.0000000	2.0149999

8013.0000000	2.0149999
8014.0000000	2.0100000
8015.0000000	2.0100000
8016.0000000	2.0200000
8017.0000000	2.0200000
8018.0000000	2.0200000
8019.0000000	2.0149999
8020.0000000	2.0050001
8021.0000000	2.0000000
8022.0000000	1.9900000
8023.0000000	1.9900000
8024.0000000	1.9800000
8025.0000000	1.9800000
8026.0000000	1.9750000
8027.0000000	1.9700000
8028.0000000	1.9700000
8029.0000000	1.9750000
8030.0000000	1.9700000
8031.0000000	1.9733334
8032.0000000	1.9850000
8033.0000000	1.9750000
8034.0000000	1.9800000
8035.0000000	1.9800000
8036.0000000	1.9800000
8037.0000000	1.9850000
8038.0000000	1.9650000
8039.0000000	1.9600000
8040.0000000	1.9550000
8041.0000000	1.9450001
8042.0000000	1.9400001
8043.0000000	1.9250000
8044.0000000	1.9250000
8045.0000000	1.9200000
8046.0000000	1.9150000
8047.0000000	1.9000000
8048.0000000	1.8750000
8049.0000000	1.8550000
8050.0000000	1.8450000
8051.0000000	1.8350000
8052.0000000	1.8350000
8053.0000000	1.8350000
8054.0000000	1.8400000
8055.0000000	1.8450000
8056.0000000	1.8400000
8057.0000000	1.8500000
8058.0000000	1.8550000
8059.0000000	1.8600000
8060.0000000	1.8550000
8061.0000000	1.8666667
8062.0000000	1.8750000
8063.0000000	1.8800000
8064.0000000	1.8850000
8065.0000000	1.8900000
8066.0000000	1.8950000
8067.0000000	1.9000000
8068.0000000	1.8950000
8069.0000000	1.9050000

8070.0000000	1.9000000
8071.0000000	1.8900000
8072.0000000	1.8750000
8073.0000000	1.8700000
8074.0000000	1.8650000
8075.0000000	1.8650000
8076.0000000	1.8600000
8077.0000000	1.8450000
8078.0000000	1.8350000
8079.0000000	1.8200001
8080.0000000	1.8099999
8081.0000000	1.8049999
8082.0000000	1.8099999
8083.0000000	1.8099999
8084.0000000	1.8150001
8085.0000000	1.8200001
8086.0000000	1.8300000
8087.0000000	1.8300000
8088.0000000	1.8500000
8089.0000000	1.8600000
8090.0000000	1.8650000
8091.0000000	1.8766667
8092.0000000	1.8850000
8093.0000000	1.8950000
8094.0000000	1.9000000
8095.0000000	1.9050000
8096.0000000	1.9050000
8097.0000000	1.9150000
8098.0000000	1.9250000
8099.0000000	1.9200000
8100.0000000	1.9299999
8101.0000000	1.9500000
8102.0000000	1.9450001
8103.0000000	1.9550000
8104.0000000	1.9500000
8105.0000000	1.9650000
8106.0000000	1.9600000
8107.0000000	1.9600000
8108.0000000	1.9600000
8109.0000000	1.9550000
8110.0000000	1.9550000
8111.0000000	1.9450001
8112.0000000	1.9500000
8113.0000000	1.9349999
8114.0000000	1.9299999
8115.0000000	1.9250000
8116.0000000	1.9250000
8117.0000000	1.9200000
8118.0000000	1.9250000
8119.0000000	1.9150000
8120.0000000	1.9150000
8121.0000000	1.9100000
8122.0000000	1.9050000
8123.0000000	1.9100000
8124.0000000	1.9000000
8125.0000000	1.8950000
8126.0000000	1.8950000

8127.0000000	1.8850000
8128.0000000	1.8850000
8129.0000000	1.8800000
8130.0000000	1.8850000
8131.0000000	1.8850000
8132.0000000	1.8850000
8133.0000000	1.8850000
8134.0000000	1.8900000
8135.0000000	1.8950000
8136.0000000	1.8900000
8137.0000000	1.8850000
8138.0000000	1.8950000
8139.0000000	1.8900000
8140.0000000	1.8950000
8141.0000000	1.9000000
8142.0000000	1.9000000
8143.0000000	1.9000000
8144.0000000	1.8950000
8145.0000000	1.8900000
8146.0000000	1.8850000
8147.0000000	1.8800000
8148.0000000	1.8750000
8149.0000000	1.8850000
8150.0000000	1.8800000
8151.0000000	1.8700000
8152.0000000	1.8550000
8153.0000000	1.8550000
8154.0000000	1.8450000
8155.0000000	1.8400000
8156.0000000	1.8350000
8157.0000000	1.8350000
8158.0000000	1.8250000
8159.0000000	1.8300000
8160.0000000	1.8300000
8161.0000000	1.8200001
8162.0000000	1.8200001
8163.0000000	1.8099999
8164.0000000	1.8000000
8165.0000000	1.7900000
8166.0000000	1.7850000
8167.0000000	1.7750000
8168.0000000	1.7650000
8169.0000000	1.7700000
8170.0000000	1.7650000
8171.0000000	1.7600000
8172.0000000	1.7500000
8173.0000000	1.7600000
8174.0000000	1.7550000
8175.0000000	1.7450000
8176.0000000	1.7450000
8177.0000000	1.7400000
8178.0000000	1.7400000
8179.0000000	1.7350000
8180.0000000	1.7333332
8181.0000000	1.7300000
8182.0000000	1.7200000
8183.0000000	1.7200000

8184.0000000	1.7100000
8185.0000000	1.7100000
8186.0000000	1.7050000
8187.0000000	1.7050000
8188.0000000	1.7000000
8189.0000000	1.7000000
8190.0000000	1.7000000
8191.0000000	1.7000000
8192.0000000	1.6950001
8193.0000000	1.6849999
8194.0000000	1.6650000
8195.0000000	1.6600000
8196.0000000	1.6550000
8197.0000000	1.6550000
8198.0000000	1.6550000
8199.0000000	1.6550000
8200.0000000	1.6550000
8201.0000000	1.6550000
8202.0000000	1.6600000
8203.0000000	1.6600000
8204.0000000	1.6600000
8205.0000000	1.6650000
8206.0000000	1.6750000
8207.0000000	1.6750000
8208.0000000	1.6799999
8209.0000000	1.6700000
8210.0000000	1.6700000
8211.0000000	1.6700000
8212.0000000	1.6700000
8213.0000000	1.6650000
8214.0000000	1.6600000
8215.0000000	1.6500000
8216.0000000	1.6450000
8217.0000000	1.6250000
8218.0000000	1.6250000
8219.0000000	1.6250000
8220.0000000	1.6150000
8221.0000000	1.6300000
8222.0000000	1.6300000
8223.0000000	1.6300000
8224.0000000	1.6250000
8225.0000000	1.6200000
8226.0000000	1.6050000
8227.0000000	1.6000000
8228.0000000	1.6000000
8229.0000000	1.6000000
8230.0000000	1.6000000
8231.0000000	1.5900000
8232.0000000	1.5900000
8233.0000000	1.5800000
8234.0000000	1.5700001
8235.0000000	1.5750000
8236.0000000	1.5700001
8237.0000000	1.5700001
8238.0000000	1.5549999
8239.0000000	1.5450000
8240.0000000	1.5300001

8241.0000000	1.5100000
8242.0000000	1.4950000
8243.0000000	1.4700000
8244.0000000	1.4550000
8245.0000000	1.4349999
8246.0000000	1.4200000
8247.0000000	1.3950000
8248.0000000	1.3700000
8249.0000000	1.3450000
8250.0000000	1.3250000
8251.0000000	1.3049999
8252.0000000	1.3000000
8253.0000000	1.2950000
8254.0000000	1.2900000
8255.0000000	1.2950000
8256.0000000	1.3000000
8257.0000000	1.3049999
8258.0000000	1.3150001
8259.0000000	1.3300000
8260.0000000	1.3400000
8261.0000000	1.3450000
8262.0000000	1.3500000
8263.0000000	1.3600000
8264.0000000	1.3600000
8265.0000000	1.3750000
8266.0000000	1.3800000
8267.0000000	1.3850000
8268.0000000	1.3900000
8269.0000000	1.4000000
8270.0000000	1.3950000
8271.0000000	1.3966666
8272.0000000	1.3850000
8273.0000000	1.3850000
8274.0000000	1.3800000
8275.0000000	1.3750000
8276.0000000	1.3700000
8277.0000000	1.3600000
8278.0000000	1.3500000
8279.0000000	1.3350000
8280.0000000	1.3250000
8281.0000000	1.3150001
8282.0000000	1.3150001
8283.0000000	1.3099999
8284.0000000	1.3099999
8285.0000000	1.3000000
8286.0000000	1.3049999
8287.0000000	1.2950000
8288.0000000	1.3000000
8289.0000000	1.2950000
8290.0000000	1.3000000
8291.0000000	1.3000000
8292.0000000	1.3099999
8293.0000000	1.3150001
8294.0000000	1.3150001
8295.0000000	1.3250000
8296.0000000	1.3250000
8297.0000000	1.3300000

8298.0000000	1.3300000
8299.0000000	1.3450000
8300.0000000	1.3500000
8301.0000000	1.3500000
8302.0000000	1.3500000
8303.0000000	1.3550000
8304.0000000	1.3600000
8305.0000000	1.3500000
8306.0000000	1.3650000
8307.0000000	1.3600000
8308.0000000	1.3550000
8309.0000000	1.3600000
8310.0000000	1.3550000
8311.0000000	1.3650000
8312.0000000	1.3600000
8313.0000000	1.3600000
8314.0000000	1.3650000
8315.0000000	1.3600000
8316.0000000	1.3700000
8317.0000000	1.3600000
8318.0000000	1.3650000
8319.0000000	1.3600000
8320.0000000	1.3500000
8321.0000000	1.3550000
8322.0000000	1.3500000
8323.0000000	1.3500000
8324.0000000	1.3500000
8325.0000000	1.3500000
8326.0000000	1.3450000
8327.0000000	1.3400000
8328.0000000	1.3350000
8329.0000000	1.3200001
8330.0000000	1.3099999
8331.0000000	1.2950000
8332.0000000	1.2900000
8333.0000000	1.2850000
8334.0000000	1.2850000
8335.0000000	1.2900000
8336.0000000	1.3000000
8337.0000000	1.3000000
8338.0000000	1.3000000
8339.0000000	1.3000000
8340.0000000	1.3000000
8341.0000000	1.3049999
8342.0000000	1.3049999
8343.0000000	1.3099999
8344.0000000	1.3099999
8345.0000000	1.3049999
8346.0000000	1.3000000
8347.0000000	1.3049999
8348.0000000	1.2950000
8349.0000000	1.2900000
8350.0000000	1.2900000
8351.0000000	1.2950000
8352.0000000	1.2900000
8353.0000000	1.2900000
8354.0000000	1.2950000

8355.0000000	1.2900000
8356.0000000	1.2950000
8357.0000000	1.3000000
8358.0000000	1.3000000
8359.0000000	1.3049999
8360.0000000	1.3099999
8361.0000000	1.3099999
8362.0000000	1.3099999
8363.0000000	1.3000000
8364.0000000	1.3049999
8365.0000000	1.3049999
8366.0000000	1.2950000
8367.0000000	1.3000000
8368.0000000	1.3000000
8369.0000000	1.3000000
8370.0000000	1.2950000
8371.0000000	1.2950000
8372.0000000	1.3000000
8373.0000000	1.2950000
8374.0000000	1.3000000
8375.0000000	1.3000000
8376.0000000	1.3049999
8377.0000000	1.3000000
8378.0000000	1.2950000
8379.0000000	1.2950000
8380.0000000	1.2900000
8381.0000000	1.2850000
8382.0000000	1.2750000
8383.0000000	1.2700000
8384.0000000	1.2600000
8385.0000000	1.2650000
8386.0000000	1.2500000
8387.0000000	1.2450000
8388.0000000	1.2300000
8389.0000000	1.2150000
8390.0000000	1.1900001
8391.0000000	1.1799999
8392.0000000	1.1666666
8393.0000000	1.1600000
8394.0000000	1.1600000
8395.0000000	1.1650000
8396.0000000	1.1600000
8397.0000000	1.1700000
8398.0000000	1.1700000
8399.0000000	1.1750000
8400.0000000	1.1799999
8401.0000000	1.1799999
8402.0000000	1.1799999
8403.0000000	1.1900001
8404.0000000	1.1900001
8405.0000000	1.1950001
8406.0000000	1.2100000
8407.0000000	1.2050000
8408.0000000	1.2100000
8409.0000000	1.2100000
8410.0000000	1.2100000
8411.0000000	1.2150000

8412.0000000	1.2150000
8413.0000000	1.2200000
8414.0000000	1.2250000
8415.0000000	1.2250000
8416.0000000	1.2300000
8417.0000000	1.2250000
8418.0000000	1.2300000
8419.0000000	1.2300000
8420.0000000	1.2200000
8421.0000000	1.2150000
8422.0000000	1.2133334
8423.0000000	1.2200000
8424.0000000	1.2150000
8425.0000000	1.2100000
8426.0000000	1.2100000
8427.0000000	1.2150000
8428.0000000	1.2100000
8429.0000000	1.2100000
8430.0000000	1.2050000
8431.0000000	1.2100000
8432.0000000	1.2050000
8433.0000000	1.2050000
8434.0000000	1.2050000
8435.0000000	1.1950001
8436.0000000	1.1950001
8437.0000000	1.1900001
8438.0000000	1.1799999
8439.0000000	1.1750000
8440.0000000	1.1700000
8441.0000000	1.1650000
8442.0000000	1.1600000
8443.0000000	1.1600000
8444.0000000	1.1600000
8445.0000000	1.1500000
8446.0000000	1.1500000
8447.0000000	1.1400000
8448.0000000	1.1350000
8449.0000000	1.1350000
8450.0000000	1.1300000
8451.0000000	1.1300000
8452.0000000	1.1250000
8453.0000000	1.1233333
8454.0000000	1.1200000
8455.0000000	1.1150000
8456.0000000	1.1050000
8457.0000000	1.1100000
8458.0000000	1.1100000
8459.0000000	1.1100000
8460.0000000	1.1000000
8461.0000000	1.1050000
8462.0000000	1.0950000
8463.0000000	1.1050000
8464.0000000	1.1000000
8465.0000000	1.1000000
8466.0000000	1.1150000
8467.0000000	1.1200000
8468.0000000	1.1300000

8469.0000000	1.1300000
8470.0000000	1.1300000
8471.0000000	1.1300000
8472.0000000	1.1200000
8473.0000000	1.1150000
8474.0000000	1.1100000
8475.0000000	1.1100000
8476.0000000	1.1100000
8477.0000000	1.1200000
8478.0000000	1.1200000
8479.0000000	1.1250000
8480.0000000	1.1350000
8481.0000000	1.1350000
8482.0000000	1.1400000
8483.0000000	1.1466666
8484.0000000	1.1550000
8485.0000000	1.1600000
8486.0000000	1.1600000
8487.0000000	1.1650000
8488.0000000	1.1650000
8489.0000000	1.1700000
8490.0000000	1.1700000
8491.0000000	1.1700000
8492.0000000	1.1700000
8493.0000000	1.1799999
8494.0000000	1.1849999
8495.0000000	1.1849999
8496.0000000	1.1900001
8497.0000000	1.1799999
8498.0000000	1.1799999
8499.0000000	1.1799999
8500.0000000	1.1799999
8501.0000000	1.1799999
8502.0000000	1.1849999
8503.0000000	1.1799999
8504.0000000	1.1849999
8505.0000000	1.1849999
8506.0000000	1.1799999
8507.0000000	1.1950001
8508.0000000	1.1950001
8509.0000000	1.1950001
8510.0000000	1.2000000
8511.0000000	1.2050000
8512.0000000	1.2100000
8513.0000000	1.2100000
8514.0000000	1.2000000
8515.0000000	1.2000000
8516.0000000	1.1950001
8517.0000000	1.1950001
8518.0000000	1.1900001
8519.0000000	1.1849999
8520.0000000	1.1799999
8521.0000000	1.1700000
8522.0000000	1.1550000
8523.0000000	1.1400000
8524.0000000	1.1400000
8525.0000000	1.1400000

8526.0000000	1.1450000
8527.0000000	1.1500000
8528.0000000	1.1550000
8529.0000000	1.1650000
8530.0000000	1.1700000
8531.0000000	1.1700000
8532.0000000	1.1750000
8533.0000000	1.1700000
8534.0000000	1.1799999
8535.0000000	1.1849999
8536.0000000	1.1799999
8537.0000000	1.1950001
8538.0000000	1.2000000
8539.0000000	1.1950001
8540.0000000	1.2000000
8541.0000000	1.2000000
8542.0000000	1.2050000
8543.0000000	1.2033334
8544.0000000	1.2100000
8545.0000000	1.2050000
8546.0000000	1.2000000
8547.0000000	1.2000000
8548.0000000	1.2000000
8549.0000000	1.2000000
8550.0000000	1.2100000
8551.0000000	1.2100000
8552.0000000	1.2100000
8553.0000000	1.2100000
8554.0000000	1.2200000
8555.0000000	1.2200000
8556.0000000	1.2150000
8557.0000000	1.2200000
8558.0000000	1.2100000
8559.0000000	1.2100000
8560.0000000	1.2100000
8561.0000000	1.2050000
8562.0000000	1.1950001
8563.0000000	1.1950001
8564.0000000	1.1799999
8565.0000000	1.1750000
8566.0000000	1.1600000
8567.0000000	1.1400000
8568.0000000	1.1350000
8569.0000000	1.1200000
8570.0000000	1.1050000
8571.0000000	1.0850000
8572.0000000	1.0800000
8573.0000000	1.0650001
8574.0000000	1.0650001
8575.0000000	1.0633334
8576.0000000	1.0700001
8577.0000000	1.0800000
8578.0000000	1.0850000
8579.0000000	1.0800000
8580.0000000	1.0950000
8581.0000000	1.1050000
8582.0000000	1.1150000

8583.0000000	1.1250000
8584.0000000	1.1350000
8585.0000000	1.1550000
8586.0000000	1.1550000
8587.0000000	1.1750000
8588.0000000	1.1799999
8589.0000000	1.1900001
8590.0000000	1.1950001
8591.0000000	1.2050000
8592.0000000	1.2100000
8593.0000000	1.2100000
8594.0000000	1.2200000
8595.0000000	1.2250000
8596.0000000	1.2350000
8597.0000000	1.2400000
8598.0000000	1.2500000
8599.0000000	1.2600000
8600.0000000	1.2650000
8601.0000000	1.2650000
8602.0000000	1.2833333
8603.0000000	1.2850000
8604.0000000	1.3000000
8605.0000000	1.3049999
8606.0000000	1.3099999
8607.0000000	1.3150001
8608.0000000	1.3300000
8609.0000000	1.3300000
8610.0000000	1.3400000
8611.0000000	1.3400000
8612.0000000	1.3500000
8613.0000000	1.3600000
8614.0000000	1.3600000
8615.0000000	1.3700000
8616.0000000	1.3700000
8617.0000000	1.3750000
8618.0000000	1.3850000
8619.0000000	1.3900000
8620.0000000	1.3950000
8621.0000000	1.4000000
8622.0000000	1.4100000
8623.0000000	1.4100000
8624.0000000	1.4200000
8625.0000000	1.4299999
8626.0000000	1.4349999
8627.0000000	1.4400001
8628.0000000	1.4400001
8629.0000000	1.4550000
8630.0000000	1.4550000
8631.0000000	1.4600000
8632.0000000	1.4700000
8633.0000000	1.4766668
8634.0000000	1.4850000
8635.0000000	1.4800000
8636.0000000	1.4850000
8637.0000000	1.4850000
8638.0000000	1.4850000
8639.0000000	1.4900000

8640.0000000	1.4900000
8641.0000000	1.4900000
8642.0000000	1.5000000
8643.0000000	1.4950000
8644.0000000	1.4900000
8645.0000000	1.4900000
8646.0000000	1.4850000
8647.0000000	1.4800000
8648.0000000	1.4800000
8649.0000000	1.4850000
8650.0000000	1.4750000
8651.0000000	1.4750000
8652.0000000	1.4650000
8653.0000000	1.4650000
8654.0000000	1.4550000
8655.0000000	1.4500000
8656.0000000	1.4400001
8657.0000000	1.4349999
8658.0000000	1.4349999
8659.0000000	1.4250000
8660.0000000	1.4200000
8661.0000000	1.4200000
8662.0000000	1.4100000
8663.0000000	1.3900000
8664.0000000	1.3800000
8665.0000000	1.3700000
8666.0000000	1.3533334
8667.0000000	1.3400000
8668.0000000	1.3150001
8669.0000000	1.2900000
8670.0000000	1.2650000
8671.0000000	1.2500000
8672.0000000	1.2300000
8673.0000000	1.2150000
8674.0000000	1.2050000
8675.0000000	1.1849999
8676.0000000	1.1799999
8677.0000000	1.1799999
8678.0000000	1.1700000
8679.0000000	1.1750000
8680.0000000	1.1700000
8681.0000000	1.1650000
8682.0000000	1.1600000
8683.0000000	1.1600000
8684.0000000	1.1650000
8685.0000000	1.1700000
8686.0000000	1.1650000
8687.0000000	1.1700000
8688.0000000	1.1700000
8689.0000000	1.1750000
8690.0000000	1.1700000
8691.0000000	1.1799999
8692.0000000	1.1799999
8693.0000000	1.1750000
8694.0000000	1.1799999
8695.0000000	1.1766666
8696.0000000	1.1650000

8697.0000000	1.1600000
8698.0000000	1.1650000
8699.0000000	1.1500000
8700.0000000	1.1500000
8701.0000000	1.1450000
8702.0000000	1.1350000
8703.0000000	1.1300000
8704.0000000	1.1350000
8705.0000000	1.1400000
8706.0000000	1.1400000
8707.0000000	1.1400000
8708.0000000	1.1400000
8709.0000000	1.1550000
8710.0000000	1.1600000
8711.0000000	1.1600000
8712.0000000	1.1650000
8713.0000000	1.1650000
8714.0000000	1.1750000
8715.0000000	1.1700000
8716.0000000	1.1799999
8717.0000000	1.1799999
8718.0000000	1.1849999
8719.0000000	1.1900001
8720.0000000	1.1900001
8721.0000000	1.1849999
8722.0000000	1.1900001
8723.0000000	1.1866666
8724.0000000	1.1900001
8725.0000000	1.1950001
8726.0000000	1.1950001
8727.0000000	1.2000000
8728.0000000	1.2050000
8729.0000000	1.2100000
8730.0000000	1.2150000
8731.0000000	1.2150000
8732.0000000	1.2200000
8733.0000000	1.2200000
8734.0000000	1.2250000
8735.0000000	1.2250000
8736.0000000	1.2250000
8737.0000000	1.2200000
8738.0000000	1.2300000
8739.0000000	1.2300000
8740.0000000	1.2300000
8741.0000000	1.2300000
8742.0000000	1.2350000
8743.0000000	1.2300000
8744.0000000	1.2250000
8745.0000000	1.2300000
8746.0000000	1.2250000
8747.0000000	1.2200000
8748.0000000	1.2250000
8749.0000000	1.2200000
8750.0000000	1.2200000
8751.0000000	1.2100000
8752.0000000	1.2050000
8753.0000000	1.2050000

8754.0000000	1.1849999
8755.0000000	1.1849999
8756.0000000	1.1799999
8757.0000000	1.1799999
8758.0000000	1.1849999
8759.0000000	1.1900001
8760.0000000	1.1849999
8761.0000000	1.1849999
8762.0000000	1.1900001
8763.0000000	1.1900001
8764.0000000	1.1799999
8765.0000000	1.1750000
8766.0000000	1.1700000
8767.0000000	1.1600000
8768.0000000	1.1450000
8769.0000000	1.1400000
8770.0000000	1.1400000
8771.0000000	1.1350000
8772.0000000	1.1450000
8773.0000000	1.1400000
8774.0000000	1.1450000
8775.0000000	1.1500000
8776.0000000	1.1500000
8777.0000000	1.1600000
8778.0000000	1.1550000
8779.0000000	1.1600000
8780.0000000	1.1600000
8781.0000000	1.1650000
8782.0000000	1.1700000
8783.0000000	1.1550000
8784.0000000	1.1550000
8785.0000000	1.1550000
8786.0000000	1.1550000
8787.0000000	1.1433333
8788.0000000	1.1350000
8789.0000000	1.1300000
8790.0000000	1.1200000
8791.0000000	1.1150000
8792.0000000	1.1150000
8793.0000000	1.1100000
8794.0000000	1.1000000
8795.0000000	1.0900000
8796.0000000	1.0850000
8797.0000000	1.0800000
8798.0000000	1.0750000
8799.0000000	1.0599999
8800.0000000	1.0700001
8801.0000000	1.0599999
8802.0000000	1.0599999
8803.0000000	1.0549999
8804.0000000	1.0599999
8805.0000000	1.0599999
8806.0000000	1.0599999
8807.0000000	1.0599999
8808.0000000	1.0650001
8809.0000000	1.0700001
8810.0000000	1.0700001

8811.0000000	1.0750000
8812.0000000	1.0700001
8813.0000000	1.0800000
8814.0000000	1.0800000
8815.0000000	1.0750000
8816.0000000	1.0800000
8817.0000000	1.0800000
8818.0000000	1.0800000
8819.0000000	1.0900000
8820.0000000	1.0950000
8821.0000000	1.0900000
8822.0000000	1.1050000
8823.0000000	1.1000000
8824.0000000	1.1050000
8825.0000000	1.1100000
8826.0000000	1.1050000
8827.0000000	1.1150000
8828.0000000	1.1100000
8829.0000000	1.1150000
8830.0000000	1.1150000
8831.0000000	1.1200000
8832.0000000	1.1250000
8833.0000000	1.1250000
8834.0000000	1.1300000
8835.0000000	1.1350000
8836.0000000	1.1300000
8837.0000000	1.1350000
8838.0000000	1.1400000
8839.0000000	1.1300000
8840.0000000	1.1350000
8841.0000000	1.1300000
8842.0000000	1.1350000
8843.0000000	1.1300000
8844.0000000	1.1300000
8845.0000000	1.1350000
8846.0000000	1.1350000
8847.0000000	1.1433333
8848.0000000	1.1400000
8849.0000000	1.1500000
8850.0000000	1.1400000
8851.0000000	1.1500000
8852.0000000	1.1550000
8853.0000000	1.1550000
8854.0000000	1.1500000
8855.0000000	1.1500000
8856.0000000	1.1500000
8857.0000000	1.1450000
8858.0000000	1.1400000
8859.0000000	1.1300000
8860.0000000	1.1250000
8861.0000000	1.1250000
8862.0000000	1.1250000
8863.0000000	1.1150000
8864.0000000	1.1100000
8865.0000000	1.1000000
8866.0000000	1.1050000
8867.0000000	1.1050000

8868.0000000	1.1100000
8869.0000000	1.1100000
8870.0000000	1.1100000
8871.0000000	1.1150000
8872.0000000	1.1200000
8873.0000000	1.1200000
8874.0000000	1.1300000
8875.0000000	1.1250000
8876.0000000	1.1300000
8877.0000000	1.1300000
8878.0000000	1.1350000
8879.0000000	1.1350000
8880.0000000	1.1450000
8881.0000000	1.1450000
8882.0000000	1.1500000
8883.0000000	1.1550000
8884.0000000	1.1550000
8885.0000000	1.1600000
8886.0000000	1.1650000
8887.0000000	1.1700000
8888.0000000	1.1700000
8889.0000000	1.1700000
8890.0000000	1.1799999
8891.0000000	1.1750000
8892.0000000	1.1750000
8893.0000000	1.1700000
8894.0000000	1.1750000
8895.0000000	1.1700000
8896.0000000	1.1799999
8897.0000000	1.1799999
8898.0000000	1.1750000
8899.0000000	1.1849999
8900.0000000	1.1799999
8901.0000000	1.1849999
8902.0000000	1.1950001
8903.0000000	1.1900001
8904.0000000	1.1950001
8905.0000000	1.2000000
8906.0000000	1.2000000
8907.0000000	1.2066667
8908.0000000	1.2100000
8909.0000000	1.2100000
8910.0000000	1.2100000
8911.0000000	1.2150000
8912.0000000	1.2100000
8913.0000000	1.2100000
8914.0000000	1.2150000
8915.0000000	1.2050000
8916.0000000	1.2050000
8917.0000000	1.2000000
8918.0000000	1.2000000
8919.0000000	1.1950001
8920.0000000	1.1900001
8921.0000000	1.1900001
8922.0000000	1.1849999
8923.0000000	1.1799999
8924.0000000	1.1750000

8925.0000000	1.1750000
8926.0000000	1.1700000
8927.0000000	1.1650000
8928.0000000	1.1600000
8929.0000000	1.1600000
8930.0000000	1.1500000
8931.0000000	1.1550000
8932.0000000	1.1400000
8933.0000000	1.1350000
8934.0000000	1.1300000
8935.0000000	1.1200000
8936.0000000	1.1100000
8937.0000000	1.0966667
8938.0000000	1.0900000
8939.0000000	1.0800000
8940.0000000	1.0700001
8941.0000000	1.0599999
8942.0000000	1.0549999
8943.0000000	1.0549999
8944.0000000	1.0450000
8945.0000000	1.0500000
8946.0000000	1.0500000
8947.0000000	1.0400000
8948.0000000	1.0500000
8949.0000000	1.0549999
8950.0000000	1.0500000
8951.0000000	1.0549999
8952.0000000	1.0500000
8953.0000000	1.0500000
8954.0000000	1.0500000
8955.0000000	1.0599999
8956.0000000	1.0650001
8957.0000000	1.0599999
8958.0000000	1.0650001
8959.0000000	1.0699999
8960.0000000	1.0750000
8961.0000000	1.0850000
8962.0000000	1.0900000
8963.0000000	1.0950000
8964.0000000	1.1000000
8965.0000000	1.1100000
8966.0000000	1.1200000
8967.0000000	1.1300000
8968.0000000	1.1300000
8969.0000000	1.1400000
8970.0000000	1.1500000
8971.0000000	1.1450000
8972.0000000	1.1600000
8973.0000000	1.1600000
8974.0000000	1.1650000
8975.0000000	1.1750000
8976.0000000	1.1700000
8977.0000000	1.1799999
8978.0000000	1.1799999
8979.0000000	1.1799999
8980.0000000	1.1900001
8981.0000000	1.1900001

8982.0000000	1.1950001
8983.0000000	1.2000000
8984.0000000	1.1950001
8985.0000000	1.2050000
8986.0000000	1.2050000
8987.0000000	1.2100000
8988.0000000	1.2150000
8989.0000000	1.2150000
8990.0000000	1.2200000
8991.0000000	1.2200000
8992.0000000	1.2200000
8993.0000000	1.2150000
8994.0000000	1.2200000
8995.0000000	1.2233334
8996.0000000	1.2200000
8997.0000000	1.2200000
8998.0000000	1.2250000
8999.0000000	1.2200000
9000.0000000	1.2200000
9001.0000000	1.2200000
9002.0000000	1.2150000
9003.0000000	1.2150000
9004.0000000	1.2100000
9005.0000000	1.2200000
9006.0000000	1.2150000
9007.0000000	1.2250000
9008.0000000	1.2250000
9009.0000000	1.2250000
9010.0000000	1.2350000
9011.0000000	1.2350000
9012.0000000	1.2350000
9013.0000000	1.2300000
9014.0000000	1.2350000
9015.0000000	1.2350000
9016.0000000	1.2350000
9017.0000000	1.2400000
9018.0000000	1.2450000
9019.0000000	1.2400000
9020.0000000	1.2450000
9021.0000000	1.2450000
9022.0000000	1.2350000
9023.0000000	1.2450000
9024.0000000	1.2550000
9025.0000000	1.2500000
9026.0000000	1.2566667
9027.0000000	1.2550000
9028.0000000	1.2650000
9029.0000000	1.2650000
9030.0000000	1.2700000
9031.0000000	1.2800000
9032.0000000	1.2700000
9033.0000000	1.2850000
9034.0000000	1.2800000
9035.0000000	1.2900000
9036.0000000	1.2900000
9037.0000000	1.2950000
9038.0000000	1.2950000

9039.0000000	1.2950000
9040.0000000	1.3000000
9041.0000000	1.3000000
9042.0000000	1.3049999
9043.0000000	1.3049999
9044.0000000	1.3000000
9045.0000000	1.3150001
9046.0000000	1.3049999
9047.0000000	1.3099999
9048.0000000	1.3099999
9049.0000000	1.3150001
9050.0000000	1.3200001
9051.0000000	1.3200001
9052.0000000	1.3250000
9053.0000000	1.3199999
9054.0000000	1.3300000
9055.0000000	1.3350000
9056.0000000	1.3366667
9057.0000000	1.3450000
9058.0000000	1.3500000
9059.0000000	1.3500000
9060.0000000	1.3550000
9061.0000000	1.3600000
9062.0000000	1.3600000
9063.0000000	1.3600000
9064.0000000	1.3650000
9065.0000000	1.3650000
9066.0000000	1.3700000
9067.0000000	1.3750000
9068.0000000	1.3700000
9069.0000000	1.3800000
9070.0000000	1.3850000
9071.0000000	1.3800000
9072.0000000	1.3800000
9073.0000000	1.3800000
9074.0000000	1.3800000
9075.0000000	1.3800000
9076.0000000	1.3850000
9077.0000000	1.3850000
9078.0000000	1.3850000
9079.0000000	1.3850000
9080.0000000	1.3800000
9081.0000000	1.3800000
9082.0000000	1.3800000
9083.0000000	1.3750000
9084.0000000	1.3650000
9085.0000000	1.3600000
9086.0000000	1.3600000
9087.0000000	1.3550000
9088.0000000	1.3550000
9089.0000000	1.3450000
9090.0000000	1.3300000
9091.0000000	1.3150001
9092.0000000	1.3000000
9093.0000000	1.2750000
9094.0000000	1.2700000
9095.0000000	1.2550000

9096.0000000	1.2500000
9097.0000000	1.2500000
9098.0000000	1.2550000
9099.0000000	1.2450000
9100.0000000	1.2450000
9101.0000000	1.2500000
9102.0000000	1.2450000
9103.0000000	1.2550000
9104.0000000	1.2600000
9105.0000000	1.2700000
9106.0000000	1.2800000
9107.0000000	1.2700000
9108.0000000	1.2750000
9109.0000000	1.2750000
9110.0000000	1.2750000
9111.0000000	1.2800000
9112.0000000	1.2800000
9113.0000000	1.2900000
9114.0000000	1.2950000
9115.0000000	1.3049999
9116.0000000	1.3166667
9117.0000000	1.3150001
9118.0000000	1.3200001
9119.0000000	1.3300000
9120.0000000	1.3300000
9121.0000000	1.3450000
9122.0000000	1.3500000
9123.0000000	1.3500000
9124.0000000	1.3550000
9125.0000000	1.3600000
9126.0000000	1.3600000
9127.0000000	1.3650000
9128.0000000	1.3700000
9129.0000000	1.3650000
9130.0000000	1.3700000
9131.0000000	1.3850000
9132.0000000	1.3800000
9133.0000000	1.3850000
9134.0000000	1.3850000
9135.0000000	1.3850000
9136.0000000	1.3950000
9137.0000000	1.3950000
9138.0000000	1.3950000
9139.0000000	1.4050000
9140.0000000	1.4100000
9141.0000000	1.4100000
9142.0000000	1.4100000
9143.0000000	1.4150000
9144.0000000	1.4000000
9145.0000000	1.4050000
9146.0000000	1.4000000
9147.0000000	1.4000000
9148.0000000	1.4100000
9149.0000000	1.4050000
9150.0000000	1.4000000
9151.0000000	1.4050000
9152.0000000	1.3950000

9153.0000000	1.3900000
9154.0000000	1.3800000
9155.0000000	1.3750000
9156.0000000	1.3700000
9157.0000000	1.3700000
9158.0000000	1.3600000
9159.0000000	1.3600000
9160.0000000	1.3550000
9161.0000000	1.3550000
9162.0000000	1.3450000
9163.0000000	1.3350000
9164.0000000	1.3200001
9165.0000000	1.3099999
9166.0000000	1.3049999
9167.0000000	1.3049999
9168.0000000	1.2900000
9169.0000000	1.2800000
9170.0000000	1.2700000
9171.0000000	1.2550000
9172.0000000	1.2400000
9173.0000000	1.2400000
9174.0000000	1.2250000
9175.0000000	1.2200000
9176.0000000	1.2100000
9177.0000000	1.2100000
9178.0000000	1.2033334
9179.0000000	1.2050000
9180.0000000	1.2000000
9181.0000000	1.2100000
9182.0000000	1.2050000
9183.0000000	1.2100000
9184.0000000	1.2100000
9185.0000000	1.2100000
9186.0000000	1.2150000
9187.0000000	1.2100000
9188.0000000	1.2100000
9189.0000000	1.2000000
9190.0000000	1.2000000
9191.0000000	1.1950001
9192.0000000	1.1849999
9193.0000000	1.1799999
9194.0000000	1.1799999
9195.0000000	1.1750000
9196.0000000	1.1650000
9197.0000000	1.1600000
9198.0000000	1.1550000
9199.0000000	1.1400000
9200.0000000	1.1400000
9201.0000000	1.1350000
9202.0000000	1.1300000
9203.0000000	1.1200000
9204.0000000	1.1300000
9205.0000000	1.1250000
9206.0000000	1.1200000
9207.0000000	1.1300000
9208.0000000	1.1166667
9209.0000000	1.1150000

9210.0000000	1.1100000
9211.0000000	1.1100000
9212.0000000	1.1100000
9213.0000000	1.1050000
9214.0000000	1.1050000
9215.0000000	1.1000000
9216.0000000	1.1000000
9217.0000000	1.0950000
9218.0000000	1.0900000
9219.0000000	1.0950000
9220.0000000	1.0900000
9221.0000000	1.0850000
9222.0000000	1.0850000
9223.0000000	1.0800000
9224.0000000	1.0750000
9225.0000000	1.0750000
9226.0000000	1.0699999
9227.0000000	1.0599999
9228.0000000	1.0549999
9229.0000000	1.0500000
9230.0000000	1.0400000
9231.0000000	1.0400000
9232.0000000	1.0300000
9233.0000000	1.0200000
9234.0000000	1.0200000
9235.0000000	1.0150000
9236.0000000	1.0100000
9237.0000000	1.0050000
9238.0000000	0.9933333
9239.0000000	0.9900000
9240.0000000	0.9800000
9241.0000000	0.9800000
9242.0000000	0.9750000
9243.0000000	0.9600000
9244.0000000	0.9600000
9245.0000000	0.9600000
9246.0000000	0.9500000
9247.0000000	0.9500000
9248.0000000	0.9400000
9249.0000000	0.9350000
9250.0000000	0.9350000
9251.0000000	0.9350000
9252.0000000	0.9350000
9253.0000000	0.9300000
9254.0000000	0.9350000
9255.0000000	0.9300000
9256.0000000	0.9300000
9257.0000000	0.9300000
9258.0000000	0.9300000
9259.0000000	0.9200000
9260.0000000	0.9200000
9261.0000000	0.9150000
9262.0000000	0.9050000
9263.0000000	0.9000000
9264.0000000	0.9050000
9265.0000000	0.9000000
9266.0000000	0.9000000

9267.0000000	0.8999999
9268.0000000	0.8950000
9269.0000000	0.8950000
9270.0000000	0.9000000
9271.0000000	0.8950000
9272.0000000	0.9000000
9273.0000000	0.9050000
9274.0000000	0.9000000
9275.0000000	0.9100000
9276.0000000	0.9050000
9277.0000000	0.9050000
9278.0000000	0.9050000
9279.0000000	0.9000000
9280.0000000	0.9000000
9281.0000000	0.8950000
9282.0000000	0.8900000
9283.0000000	0.8800000
9284.0000000	0.8800000
9285.0000000	0.8800000
9286.0000000	0.8800000
9287.0000000	0.8850000
9288.0000000	0.8800000
9289.0000000	0.8800000
9290.0000000	0.8850000
9291.0000000	0.8900000
9292.0000000	0.8900000
9293.0000000	0.8900000
9294.0000000	0.9000000
9295.0000000	0.8950000
9296.0000000	0.9000000
9297.0000000	0.8966667
9298.0000000	0.8950000
9299.0000000	0.8850000
9300.0000000	0.8850000
9301.0000000	0.8850000
9302.0000000	0.8800000
9303.0000000	0.8850000
9304.0000000	0.8800000
9305.0000000	0.8800000
9306.0000000	0.8850000
9307.0000000	0.8800000
9308.0000000	0.8800000
9309.0000000	0.8800000
9310.0000000	0.8700000
9311.0000000	0.8650000
9312.0000000	0.8650000
9313.0000000	0.8600000
9314.0000000	0.8600000
9315.0000000	0.8600000
9316.0000000	0.8550000
9317.0000000	0.8550000
9318.0000000	0.8650000
9319.0000000	0.8650000
9320.0000000	0.8700000
9321.0000000	0.8750000
9322.0000000	0.8750000
9323.0000000	0.8800000

9324.0000000	0.8900000
9325.0000000	0.8850000
9326.0000000	0.8950000
9327.0000000	0.9000000
9328.0000000	0.9000000
9329.0000000	0.9050000
9330.0000000	0.9066667
9331.0000000	0.9050000
9332.0000000	0.9150000
9333.0000000	0.9150000
9334.0000000	0.9200000
9335.0000000	0.9200000
9336.0000000	0.9200000
9337.0000000	0.9200000
9338.0000000	0.9200000
9339.0000000	0.9200000
9340.0000000	0.9200000
9341.0000000	0.9200000
9342.0000000	0.9200000
9343.0000000	0.9250000
9344.0000000	0.9200000
9345.0000000	0.9150000
9346.0000000	0.9150000
9347.0000000	0.9200000
9348.0000000	0.9150000
9349.0000000	0.9150000
9350.0000000	0.9150000
9351.0000000	0.9150000
9352.0000000	0.9150000
9353.0000000	0.9100000
9354.0000000	0.9150000
9355.0000000	0.9100000
9356.0000000	0.9100000
9357.0000000	0.9100000
9358.0000000	0.9050000
9359.0000000	0.9100000
9360.0000000	0.9050000
9361.0000000	0.9100000
9362.0000000	0.9000000
9363.0000000	0.9050000
9364.0000000	0.9050000
9365.0000000	0.9000000
9366.0000000	0.9000000
9367.0000000	0.9000000
9368.0000000	0.9000000
9369.0000000	0.8950000
9370.0000000	0.9000000
9371.0000000	0.8950000
9372.0000000	0.8900000
9373.0000000	0.8850000
9374.0000000	0.8900000
9375.0000000	0.8900000
9376.0000000	0.8850000
9377.0000000	0.8800000
9378.0000000	0.8800000
9379.0000000	0.8700000
9380.0000000	0.8700000

9381.0000000	0.8550000
9382.0000000	0.8550000
9383.0000000	0.8550000
9384.0000000	0.8550000
9385.0000000	0.8650000
9386.0000000	0.8600000
9387.0000000	0.8650000
9388.0000000	0.8700001
9389.0000000	0.8800000
9390.0000000	0.8800000
9391.0000000	0.8766667
9392.0000000	0.8750000
9393.0000000	0.8750000
9394.0000000	0.8850000
9395.0000000	0.8850000
9396.0000000	0.8850000
9397.0000000	0.8850000
9398.0000000	0.8850000
9399.0000000	0.8850000
9400.0000000	0.8850000
9401.0000000	0.8800000
9402.0000000	0.8850000
9403.0000000	0.8900000
9404.0000000	0.8950000
9405.0000000	0.8950000
9406.0000000	0.9000000
9407.0000000	0.9000000
9408.0000000	0.9050000
9409.0000000	0.9050000
9410.0000000	0.9050000
9411.0000000	0.8950000
9412.0000000	0.9000000
9413.0000000	0.8950000
9414.0000000	0.8900000
9415.0000000	0.8900000
9416.0000000	0.8900000
9417.0000000	0.8900000
9418.0000000	0.8950000
9419.0000000	0.8900000
9420.0000000	0.8966667
9421.0000000	0.9000000
9422.0000000	0.9050000
9423.0000000	0.9050000
9424.0000000	0.9000000
9425.0000000	0.9000000
9426.0000000	0.8950000
9427.0000000	0.8950000
9428.0000000	0.8900000
9429.0000000	0.8900000
9430.0000000	0.8900000
9431.0000000	0.8800000
9432.0000000	0.8800000
9433.0000000	0.8750000
9434.0000000	0.8750000
9435.0000000	0.8750000
9436.0000000	0.8700000
9437.0000000	0.8700000

9438.0000000	0.8700000
9439.0000000	0.8700000
9440.0000000	0.8700000
9441.0000000	0.8700000
9442.0000000	0.8700000
9443.0000000	0.8700000
9444.0000000	0.8600000
9445.0000000	0.8600000
9446.0000000	0.8550000
9447.0000000	0.8500000
9448.0000000	0.8500000
9449.0000000	0.8450000
9450.0000000	0.8333333
9451.0000000	0.8350000
9452.0000000	0.8350000
9453.0000000	0.8300000
9454.0000000	0.8250000
9455.0000000	0.8200000
9456.0000000	0.8150000
9457.0000000	0.8100000
9458.0000000	0.8100000
9459.0000000	0.8100000
9460.0000000	0.8100000
9461.0000000	0.8100000
9462.0000000	0.8100000
9463.0000000	0.8100000
9464.0000000	0.8050000
9465.0000000	0.8000000
9466.0000000	0.8050000
9467.0000000	0.7950000
9468.0000000	0.7950000
9469.0000000	0.7900000
9470.0000000	0.7900000
9471.0000000	0.7850000
9472.0000000	0.7800000
9473.0000000	0.7800000
9474.0000000	0.7800000
9475.0000000	0.7750000
9476.0000000	0.7800000
9477.0000000	0.7800000
9478.0000000	0.7800000
9479.0000000	0.7833333
9480.0000000	0.7800000
9481.0000000	0.7800000
9482.0000000	0.7850000
9483.0000000	0.7800000
9484.0000000	0.7800000
9485.0000000	0.7850000
9486.0000000	0.7750000
9487.0000000	0.7800000
9488.0000000	0.7700000
9489.0000000	0.7700000
9490.0000000	0.7750000
9491.0000000	0.7750000
9492.0000000	0.7750000
9493.0000000	0.7850000
9494.0000000	0.7850000

9495.0000000	0.7900000
9496.0000000	0.7900000
9497.0000000	0.7850000
9498.0000000	0.7900000
9499.0000000	0.7900000
9500.0000000	0.7950000
9501.0000000	0.7950000
9502.0000000	0.8000000
9503.0000000	0.8050000
9504.0000000	0.8100000
9505.0000000	0.8150000
9506.0000000	0.8150000
9507.0000000	0.8250000
9508.0000000	0.8300000
9509.0000000	0.8300000
9510.0000000	0.8350000
9511.0000000	0.8400000
9512.0000000	0.8400000
9513.0000000	0.8400000
9514.0000000	0.8400000
9515.0000000	0.8400000
9516.0000000	0.8400000
9517.0000000	0.8400000
9518.0000000	0.8400000
9519.0000000	0.8350000
9520.0000000	0.8300000
9521.0000000	0.8300000
9522.0000000	0.8350000
9523.0000000	0.8400000
9524.0000000	0.8400000
9525.0000000	0.8450000
9526.0000000	0.8450000
9527.0000000	0.8500000
9528.0000000	0.8600000
9529.0000000	0.8600000
9530.0000000	0.8600000
9531.0000000	0.8550000
9532.0000000	0.8600000
9533.0000000	0.8600000
9534.0000000	0.8600000
9535.0000000	0.8650000
9536.0000000	0.8700000
9537.0000000	0.8650000
9538.0000000	0.8650000
9539.0000000	0.8633334
9540.0000000	0.8600000
9541.0000000	0.8650000
9542.0000000	0.8700000
9543.0000000	0.8700000
9544.0000000	0.8700000
9545.0000000	0.8700000
9546.0000000	0.8650000
9547.0000000	0.8600000
9548.0000000	0.8600000
9549.0000000	0.8600000
9550.0000000	0.8550000
9551.0000000	0.8500000

9552.0000000	0.8600000
9553.0000000	0.8600000
9554.0000000	0.8650000
9555.0000000	0.8700000
9556.0000000	0.8700000
9557.0000000	0.8700000
9558.0000000	0.8750000
9559.0000000	0.8700000
9560.0000000	0.8750000
9561.0000000	0.8750000
9562.0000000	0.8800000
9563.0000000	0.8800000
9564.0000000	0.8850000
9565.0000000	0.8900000
9566.0000000	0.8900000
9567.0000000	0.8900000
9568.0000000	0.8850000
9569.0000000	0.8900000
9570.0000000	0.8933333
9571.0000000	0.8900000
9572.0000000	0.9000000
9573.0000000	0.9000000
9574.0000000	0.9100000
9575.0000000	0.9100000
9576.0000000	0.9150000
9577.0000000	0.9200000
9578.0000000	0.9200000
9579.0000000	0.9250000
9580.0000000	0.9300000
9581.0000000	0.9300000
9582.0000000	0.9300000
9583.0000000	0.9400000
9584.0000000	0.9400000
9585.0000000	0.9450000
9586.0000000	0.9500000
9587.0000000	0.9500000
9588.0000000	0.9550000
9589.0000000	0.9550000
9590.0000000	0.9600000
9591.0000000	0.9600000
9592.0000000	0.9700000
9593.0000000	0.9700000
9594.0000000	0.9700000
9595.0000000	0.9750000
9596.0000000	0.9850000
9597.0000000	0.9850000
9598.0000000	0.9850000
9599.0000000	0.9850000
9600.0000000	0.9933333
9601.0000000	1.0000000
9602.0000000	1.0100000
9603.0000000	1.0050000
9604.0000000	1.0100000
9605.0000000	1.0100000
9606.0000000	1.0150000
9607.0000000	1.0150000
9608.0000000	1.0100000

9609.0000000	1.0200000
9610.0000000	1.0150000
9611.0000000	1.0200000
9612.0000000	1.0100000
9613.0000000	1.0150000
9614.0000000	1.0200000
9615.0000000	1.0200000
9616.0000000	1.0300000
9617.0000000	1.0250000
9618.0000000	1.0250000
9619.0000000	1.0250000
9620.0000000	1.0300000
9621.0000000	1.0300000
9622.0000000	1.0300000
9623.0000000	1.0250000
9624.0000000	1.0200000
9625.0000000	1.0300000
9626.0000000	1.0300000
9627.0000000	1.0300000
9628.0000000	1.0300000
9629.0000000	1.0300000
9630.0000000	1.0433333
9631.0000000	1.0450000
9632.0000000	1.0450000
9633.0000000	1.0500000
9634.0000000	1.0500000
9635.0000000	1.0549999
9636.0000000	1.0599999
9637.0000000	1.0549999
9638.0000000	1.0549999
9639.0000000	1.0599999
9640.0000000	1.0599999
9641.0000000	1.0599999
9642.0000000	1.0599999
9643.0000000	1.0599999
9644.0000000	1.0599999
9645.0000000	1.0549999
9646.0000000	1.0599999
9647.0000000	1.0549999
9648.0000000	1.0500000
9649.0000000	1.0500000
9650.0000000	1.0549999
9651.0000000	1.0500000
9652.0000000	1.0450000
9653.0000000	1.0450000
9654.0000000	1.0450000
9655.0000000	1.0400000
9656.0000000	1.0400000
9657.0000000	1.0350000
9658.0000000	1.0400000
9659.0000000	1.0400000
9660.0000000	1.0400000
9661.0000000	1.0400000
9662.0000000	1.0400000
9663.0000000	1.0450000
9664.0000000	1.0400000
9665.0000000	1.0400000

9666.0000000	1.0400000
9667.0000000	1.0400000
9668.0000000	1.0400000
9669.0000000	1.0350000
9670.0000000	1.0400000
9671.0000000	1.0400000
9672.0000000	1.0400000
9673.0000000	1.0400000
9674.0000000	1.0350000
9675.0000000	1.0350000
9676.0000000	1.0350000
9677.0000000	1.0350000
9678.0000000	1.0400000
9679.0000000	1.0300000
9680.0000000	1.0400000
9681.0000000	1.0400000
9682.0000000	1.0350000
9683.0000000	1.0350000
9684.0000000	1.0300000
9685.0000000	1.0300000
9686.0000000	1.0250000
9687.0000000	1.0250000
9688.0000000	1.0250000
9689.0000000	1.0200000
9690.0000000	1.0200000
9691.0000000	1.0100000
9692.0000000	1.0100000
9693.0000000	1.0100000
9694.0000000	1.0150000
9695.0000000	1.0050000
9696.0000000	1.0100000
9697.0000000	1.0000000
9698.0000000	1.0000000
9699.0000000	1.0000000
9700.0000000	0.9900000
9701.0000000	0.9850000
9702.0000000	0.9800000
9703.0000000	0.9800000
9704.0000000	0.9750000
9705.0000000	0.9800000
9706.0000000	0.9750000
9707.0000000	0.9700000
9708.0000000	0.9700000
9709.0000000	0.9650000
9710.0000000	0.9650000
9711.0000000	0.9600000
9712.0000000	0.9600000
9713.0000000	0.9600000
9714.0000000	0.9650000
9715.0000000	0.9600000
9716.0000000	0.9500000
9717.0000000	0.9500000
9718.0000000	0.9350000
9719.0000000	0.9350000
9720.0000000	0.9300000
9721.0000000	0.9300000
9722.0000000	0.9300000

9723.0000000	0.9350000
9724.0000000	0.9350000
9725.0000000	0.9450000
9726.0000000	0.9500000
9727.0000000	0.9550000
9728.0000000	0.9600000
9729.0000000	0.9700000
9730.0000000	0.9700000
9731.0000000	0.9750000
9732.0000000	0.9800000
9733.0000000	0.9750000
9734.0000000	0.9800000
9735.0000000	0.9750000
9736.0000000	0.9800000
9737.0000000	0.9800000
9738.0000000	0.9800000
9739.0000000	0.9850000
9740.0000000	0.9800000
9741.0000000	0.9850000
9742.0000000	0.9850000
9743.0000000	0.9800000
9744.0000000	0.9850000
9745.0000000	0.9900000
9746.0000000	0.9850000
9747.0000000	0.9900000
9748.0000000	0.9900000
9749.0000000	0.9850000
9750.0000000	0.9900000
9751.0000000	0.9850000
9752.0000000	0.9900000
9753.0000000	0.9800000
9754.0000000	0.9850000
9755.0000000	0.9800000
9756.0000000	0.9800000
9757.0000000	0.9850000
9758.0000000	0.9850000
9759.0000000	0.9800000
9760.0000000	0.9800000
9761.0000000	0.9850000
9762.0000000	0.9800000
9763.0000000	0.9900000
9764.0000000	0.9900000
9765.0000000	0.9850000
9766.0000000	0.9900000
9767.0000000	0.9900000
9768.0000000	0.9900000
9769.0000000	0.9900000
9770.0000000	0.9850000
9771.0000000	0.9900000
9772.0000000	0.9900000
9773.0000000	0.9950000
9774.0000000	0.9950000
9775.0000000	1.0000000
9776.0000000	1.0050000
9777.0000000	1.0100000
9778.0000000	1.0150000
9779.0000000	1.0200000

9780.0000000	1.0200000
9781.0000000	1.0200000
9782.0000000	1.0250000
9783.0000000	1.0300000
9784.0000000	1.0300000
9785.0000000	1.0300000
9786.0000000	1.0450000
9787.0000000	1.0450000
9788.0000000	1.0500000
9789.0000000	1.0500000
9790.0000000	1.0500000
9791.0000000	1.0599999
9792.0000000	1.0500000
9793.0000000	1.0549999
9794.0000000	1.0549999
9795.0000000	1.0599999
9796.0000000	1.0549999
9797.0000000	1.0500000
9798.0000000	1.0500000
9799.0000000	1.0500000
9800.0000000	1.0549999
9801.0000000	1.0500000
9802.0000000	1.0549999
9803.0000000	1.0599999
9804.0000000	1.0500000
9805.0000000	1.0500000
9806.0000000	1.0549999
9807.0000000	1.0500000
9808.0000000	1.0549999
9809.0000000	1.0549999
9810.0000000	1.0549999
9811.0000000	1.0549999
9812.0000000	1.0599999
9813.0000000	1.0500000
9814.0000000	1.0599999
9815.0000000	1.0599999
9816.0000000	1.0549999
9817.0000000	1.0599999
9818.0000000	1.0650001
9819.0000000	1.0650001
9820.0000000	1.0599999
9821.0000000	1.0599999
9822.0000000	1.0599999
9823.0000000	1.0599999
9824.0000000	1.0599999
9825.0000000	1.0599999
9826.0000000	1.0650001
9827.0000000	1.0599999
9828.0000000	1.0599999
9829.0000000	1.0549999
9830.0000000	1.0650001
9831.0000000	1.0650001
9832.0000000	1.0700001
9833.0000000	1.0650001
9834.0000000	1.0599999
9835.0000000	1.0599999
9836.0000000	1.0599999

9837.0000000	1.0549999
9838.0000000	1.0549999
9839.0000000	1.0549999
9840.0000000	1.0500000
9841.0000000	1.0500000
9842.0000000	1.0450000
9843.0000000	1.0400000
9844.0000000	1.0300000
9845.0000000	1.0300000
9846.0000000	1.0300000
9847.0000000	1.0300000
9848.0000000	1.0300000
9849.0000000	1.0200000
9850.0000000	1.0150000
9851.0000000	1.0100000
9852.0000000	1.0050000
9853.0000000	0.9900000
9854.0000000	0.9950000
9855.0000000	0.9850000
9856.0000000	0.9850000
9857.0000000	0.9800000
9858.0000000	0.9750000
9859.0000000	0.9800000
9860.0000000	0.9750000
9861.0000000	0.9700000
9862.0000000	0.9700000
9863.0000000	0.9750000
9864.0000000	0.9700000
9865.0000000	0.9650000
9866.0000000	0.9650000
9867.0000000	0.9600000
9868.0000000	0.9600000
9869.0000000	0.9600000
9870.0000000	0.9600000
9871.0000000	0.9500000
9872.0000000	0.9500000
9873.0000000	0.9500000
9874.0000000	0.9500000
9875.0000000	0.9500000
9876.0000000	0.9450000
9877.0000000	0.9400000
9878.0000000	0.9400000
9879.0000000	0.9350000
9880.0000000	0.9400000
9881.0000000	0.9400000
9882.0000000	0.9400000
9883.0000000	0.9450000
9884.0000000	0.9400000
9885.0000000	0.9500000
9886.0000000	0.9500000
9887.0000000	0.9450000
9888.0000000	0.9600000
9889.0000000	0.9600000
9890.0000000	0.9600000
9891.0000000	0.9600000
9892.0000000	0.9650000
9893.0000000	0.9650000

9894.0000000	0.9700000
9895.0000000	0.9650000
9896.0000000	0.9700000
9897.0000000	0.9650000
9898.0000000	0.9700000
9899.0000000	0.9750000
9900.0000000	0.9700000
9901.0000000	0.9750000
9902.0000000	0.9700000
9903.0000000	0.9700000
9904.0000000	0.9700000
9905.0000000	0.9700000
9906.0000000	0.9750000
9907.0000000	0.9700000
9908.0000000	0.9800000
9909.0000000	0.9800000
9910.0000000	0.9750000
9911.0000000	0.9700000
9912.0000000	0.9700000
9913.0000000	0.9750000
9914.0000000	0.9700000
9915.0000000	0.9600000
9916.0000000	0.9700000
9917.0000000	0.9650000
9918.0000000	0.9600000
9919.0000000	0.9600000
9920.0000000	0.9550000
9921.0000000	0.9500000
9922.0000000	0.9450000
9923.0000000	0.9450000
9924.0000000	0.9300000
9925.0000000	0.9300000
9926.0000000	0.9250000
9927.0000000	0.9100000
9928.0000000	0.9150000
9929.0000000	0.9050000
9930.0000000	0.8900000
9931.0000000	0.8850000
9932.0000000	0.8800000
9933.0000000	0.8800000
9934.0000000	0.8766667
9935.0000000	0.8750000
9936.0000000	0.8750000
9937.0000000	0.8750000
9938.0000000	0.8750000
9939.0000000	0.8700000
9940.0000000	0.8700000
9941.0000000	0.8800000
9942.0000000	0.8800000
9943.0000000	0.8700000
9944.0000000	0.8750000
9945.0000000	0.8700000
9946.0000000	0.8800000
9947.0000000	0.8850000
9948.0000000	0.8750000
9949.0000000	0.8800000
9950.0000000	0.8800000

9951.0000000	0.8800000
9952.0000000	0.8950000
9953.0000000	0.8900000
9954.0000000	0.8950000
9955.0000000	0.9000000
9956.0000000	0.9050000
9957.0000000	0.9100000
9958.0000000	0.9150000
9959.0000000	0.9200000
9960.0000000	0.9250000
9961.0000000	0.9350000
9962.0000000	0.9500000
9963.0000000	0.9400000
9964.0000000	0.9500000
9965.0000000	0.9550000
9966.0000000	0.9600000
9967.0000000	0.9600000
9968.0000000	0.9700000
9969.0000000	0.9700000
9970.0000000	0.9750000
9971.0000000	0.9750000
9972.0000000	0.9800000
9973.0000000	0.9850000
9974.0000000	0.9800000
9975.0000000	0.9800000
9976.0000000	0.9850000
9977.0000000	0.9850000
9978.0000000	0.9900000
9979.0000000	0.9850000
9980.0000000	0.9900000
9981.0000000	0.9900000
9982.0000000	0.9900000
9983.0000000	0.9950000
9984.0000000	0.9850000
9985.0000000	0.9900000
9986.0000000	0.9900000
9987.0000000	0.9950000
9988.0000000	0.9900000
9989.0000000	0.9950000
9990.0000000	0.9900000
9991.0000000	0.9950000
9992.0000000	1.0000000
9993.0000000	1.0050000
9994.0000000	1.0050000
9995.0000000	1.0050000
9996.0000000	1.0050000
9997.0000000	1.0000000
9998.0000000	1.0000000
9999.0000000	0.9950000
10000.0000000	1.0000000
10001.0000000	0.9900000
10002.0000000	0.9900000
10003.0000000	0.9900000
10004.0000000	0.9850000
10005.0000000	0.9850000
10006.0000000	0.9850000
10007.0000000	0.9850000

10008.0000000	0.9800000
10009.0000000	0.9900000
10010.0000000	0.9950000
10011.0000000	0.9850000
10012.0000000	0.9800000
10013.0000000	0.9800000
10014.0000000	0.9800000
10015.0000000	0.9700000
10016.0000000	0.9750000
10017.0000000	0.9750000
10018.0000000	0.9700000
10019.0000000	0.9700000
10020.0000000	0.9700000
10021.0000000	0.9750000
10022.0000000	0.9800000
10023.0000000	0.9800000
10024.0000000	0.9850000
10025.0000000	0.9766667
10026.0000000	0.9800000
10027.0000000	0.9800000
10028.0000000	0.9800000
10029.0000000	0.9800000
10030.0000000	0.9750000
10031.0000000	0.9700000
10032.0000000	0.9650000
10033.0000000	0.9600000
10034.0000000	0.9550000
10035.0000000	0.9400000
10036.0000000	0.9400000
10037.0000000	0.9300000
10038.0000000	0.9350000
10039.0000000	0.9300000
10040.0000000	0.9350000
10041.0000000	0.9350000
10042.0000000	0.9450000
10043.0000000	0.9500000
10044.0000000	0.9600000
10045.0000000	0.9550000
10046.0000000	0.9600000
10047.0000000	0.9700000
10048.0000000	0.9700000
10049.0000000	0.9750000
10050.0000000	0.9700000
10051.0000000	0.9750000
10052.0000000	0.9800000
10053.0000000	0.9850000
10054.0000000	0.9933333
10055.0000000	0.9950000
10056.0000000	1.0000000
10057.0000000	1.0050000
10058.0000000	1.0150000
10059.0000000	1.0200000
10060.0000000	1.0150000
10061.0000000	1.0200000
10062.0000000	1.0200000
10063.0000000	1.0150000
10064.0000000	1.0200000

10065.0000000	1.0100000
10066.0000000	1.0200000
10067.0000000	1.0200000
10068.0000000	1.0200000
10069.0000000	1.0200000
10070.0000000	1.0200000
10071.0000000	1.0250000
10072.0000000	1.0250000
10073.0000000	1.0200000
10074.0000000	1.0200000
10075.0000000	1.0100000
10076.0000000	1.0000000
10077.0000000	0.9950000
10078.0000000	0.9900000
10079.0000000	0.9850000
10080.0000000	0.9700000
10081.0000000	0.9650000
10082.0000000	0.9500000
10083.0000000	0.9400000
10084.0000000	0.9200000
10085.0000000	0.9050000
10086.0000000	0.9050000
10087.0000000	0.8950000
10088.0000000	0.8900000
10089.0000000	0.8850000
10090.0000000	0.8900000
10091.0000000	0.8850000
10092.0000000	0.8850000
10093.0000000	0.8800000
10094.0000000	0.8900000
10095.0000000	0.8900000
10096.0000000	0.8850000
10097.0000000	0.8850000
10098.0000000	0.8850000
10099.0000000	0.8850000
10100.0000000	0.8900000
10101.0000000	0.8950000
10102.0000000	0.8900000
10103.0000000	0.9000000
10104.0000000	0.9000000
10105.0000000	0.9050000
10106.0000000	0.9100000
10107.0000000	0.9100000
10108.0000000	0.9200000
10109.0000000	0.9250000
10110.0000000	0.9350000
10111.0000000	0.9300000
10112.0000000	0.9400000
10113.0000000	0.9450000
10114.0000000	0.9466667
10115.0000000	0.9500000
10116.0000000	0.9550000
10117.0000000	0.9550000
10118.0000000	0.9550000
10119.0000000	0.9650000
10120.0000000	0.9600000
10121.0000000	0.9600000

10122.0000000	0.9600000
10123.0000000	0.9550000
10124.0000000	0.9550000
10125.0000000	0.9500000
10126.0000000	0.9500000
10127.0000000	0.9500000
10128.0000000	0.9500000
10129.0000000	0.9550000
10130.0000000	0.9600000
10131.0000000	0.9600000
10132.0000000	0.9600000
10133.0000000	0.9600000
10134.0000000	0.9700000
10135.0000000	0.9700000
10136.0000000	0.9800000
10137.0000000	0.9750000
10138.0000000	0.9850000
10139.0000000	0.9900000
10140.0000000	0.9950000
10141.0000000	1.0000000
10142.0000000	1.0000000
10143.0000000	1.0100000
10144.0000000	1.0133333
10145.0000000	1.0150000
10146.0000000	1.0200000
10147.0000000	1.0200000
10148.0000000	1.0250000
10149.0000000	1.0200000
10150.0000000	1.0200000
10151.0000000	1.0250000
10152.0000000	1.0200000
10153.0000000	1.0200000
10154.0000000	1.0200000
10155.0000000	1.0150000
10156.0000000	1.0250000
10157.0000000	1.0200000
10158.0000000	1.0200000
10159.0000000	1.0250000
10160.0000000	1.0200000
10161.0000000	1.0300000
10162.0000000	1.0300000
10163.0000000	1.0300000
10164.0000000	1.0300000
10165.0000000	1.0200000
10166.0000000	1.0300000
10167.0000000	1.0300000
10168.0000000	1.0300000
10169.0000000	1.0300000
10170.0000000	1.0350000
10171.0000000	1.0400000
10172.0000000	1.0400000
10173.0000000	1.0400000
10174.0000000	1.0366666
10175.0000000	1.0400000
10176.0000000	1.0400000
10177.0000000	1.0400000
10178.0000000	1.0400000

10179.0000000	1.0400000
10180.0000000	1.0400000
10181.0000000	1.0400000
10182.0000000	1.0500000
10183.0000000	1.0400000
10184.0000000	1.0450000
10185.0000000	1.0450000
10186.0000000	1.0450000
10187.0000000	1.0500000
10188.0000000	1.0450000
10189.0000000	1.0400000
10190.0000000	1.0400000
10191.0000000	1.0500000
10192.0000000	1.0400000
10193.0000000	1.0500000
10194.0000000	1.0500000
10195.0000000	1.0500000
10196.0000000	1.0450000
10197.0000000	1.0450000
10198.0000000	1.0500000
10199.0000000	1.0400000
10200.0000000	1.0400000
10201.0000000	1.0400000
10202.0000000	1.0350000
10203.0000000	1.0400000
10204.0000000	1.0400000
10205.0000000	1.0400000
10206.0000000	1.0350000
10207.0000000	1.0400000
10208.0000000	1.0400000
10209.0000000	1.0350000
10210.0000000	1.0300000
10211.0000000	1.0250000
10212.0000000	1.0200000
10213.0000000	1.0200000
10214.0000000	1.0100000
10215.0000000	1.0000000
10216.0000000	0.9900000
10217.0000000	0.9800000
10218.0000000	0.9800000
10219.0000000	0.9750000
10220.0000000	0.9700000
10221.0000000	0.9650000
10222.0000000	0.9700000
10223.0000000	0.9600000
10224.0000000	0.9550000
10225.0000000	0.9550000
10226.0000000	0.9450000
10227.0000000	0.9500000
10228.0000000	0.9450000
10229.0000000	0.9450000
10230.0000000	0.9500000
10231.0000000	0.9400000
10232.0000000	0.9400000
10233.0000000	0.9400000
10234.0000000	0.9400000
10235.0000000	0.9466667

10236.0000000	0.9400000
10237.0000000	0.9400000
10238.0000000	0.9400000
10239.0000000	0.9300000
10240.0000000	0.9300000
10241.0000000	0.9350000
10242.0000000	0.9300000
10243.0000000	0.9300000
10244.0000000	0.9400000
10245.0000000	0.9400000
10246.0000000	0.9350000
10247.0000000	0.9300000
10248.0000000	0.9400000
10249.0000000	0.9400000
10250.0000000	0.9400000
10251.0000000	0.9350000
10252.0000000	0.9350000
10253.0000000	0.9350000
10254.0000000	0.9350000
10255.0000000	0.9350000
10256.0000000	0.9400000
10257.0000000	0.9350000
10258.0000000	0.9400000
10259.0000000	0.9400000
10260.0000000	0.9350000
10261.0000000	0.9300000
10262.0000000	0.9350000
10263.0000000	0.9300000
10264.0000000	0.9200000
10265.0000000	0.9233333
10266.0000000	0.9200000
10267.0000000	0.9100000
10268.0000000	0.9150000
10269.0000000	0.9100000
10270.0000000	0.9050000
10271.0000000	0.9000000
10272.0000000	0.9050000
10273.0000000	0.9000000
10274.0000000	0.9000000
10275.0000000	0.9000000
10276.0000000	0.8900000
10277.0000000	0.8800000
10278.0000000	0.8850000
10279.0000000	0.8800000
10280.0000000	0.8750000
10281.0000000	0.8750000
10282.0000000	0.8800000
10283.0000000	0.8700000
10284.0000000	0.8700000
10285.0000000	0.8750000
10286.0000000	0.8800000
10287.0000000	0.8750000
10288.0000000	0.8800000
10289.0000000	0.8850000
10290.0000000	0.8850000
10291.0000000	0.8900000
10292.0000000	0.8900000

10293.0000000	0.8950000
10294.0000000	0.8999999
10295.0000000	0.9000000
10296.0000000	0.9050000
10297.0000000	0.9050000
10298.0000000	0.9050000
10299.0000000	0.9000000
10300.0000000	0.9000000
10301.0000000	0.8950000
10302.0000000	0.8900000
10303.0000000	0.8950000
10304.0000000	0.8850000
10305.0000000	0.8850000
10306.0000000	0.8800000
10307.0000000	0.8850000
10308.0000000	0.8900000
10309.0000000	0.8900000
10310.0000000	0.8900000
10311.0000000	0.8900000
10312.0000000	0.8900000
10313.0000000	0.8900000
10314.0000000	0.8950000
10315.0000000	0.9000000
10316.0000000	0.9000000
10317.0000000	0.8950000
10318.0000000	0.9000000
10319.0000000	0.9100000
10320.0000000	0.9000000
10321.0000000	0.9050000
10322.0000000	0.9050000
10323.0000000	0.9100000
10324.0000000	0.9100000
10325.0000000	0.9100000
10326.0000000	0.9200000
10327.0000000	0.9200000
10328.0000000	0.9250000
10329.0000000	0.9150000
10330.0000000	0.9200000
10331.0000000	0.9200000
10332.0000000	0.9200000
10333.0000000	0.9250000
10334.0000000	0.9250000
10335.0000000	0.9200000
10336.0000000	0.9250000
10337.0000000	0.9250000
10338.0000000	0.9250000
10339.0000000	0.9250000
10340.0000000	0.9350000
10341.0000000	0.9300000
10342.0000000	0.9300000
10343.0000000	0.9250000
10344.0000000	0.9300000
10345.0000000	0.9300000
10346.0000000	0.9300000
10347.0000000	0.9300000
10348.0000000	0.9300000
10349.0000000	0.9400000

10350.0000000	0.9400000
10351.0000000	0.9450000
10352.0000000	0.9400000
10353.0000000	0.9450000
10354.0000000	0.9450000
10355.0000000	0.9366667
10356.0000000	0.9350000
10357.0000000	0.9450000
10358.0000000	0.9400000
10359.0000000	0.9500000
10360.0000000	0.9500000
10361.0000000	0.9600000
10362.0000000	0.9600000
10363.0000000	0.9650000
10364.0000000	0.9650000
10365.0000000	0.9700000
10366.0000000	0.9750000
10367.0000000	0.9700000
10368.0000000	0.9800000
10369.0000000	0.9800000
10370.0000000	0.9800000
10371.0000000	0.9800000
10372.0000000	0.9800000
10373.0000000	0.9800000
10374.0000000	0.9750000
10375.0000000	0.9750000
10376.0000000	0.9800000
10377.0000000	0.9800000
10378.0000000	0.9750000
10379.0000000	0.9750000
10380.0000000	0.9750000
10381.0000000	0.9800000
10382.0000000	0.9800000
10383.0000000	0.9800000
10384.0000000	0.9800000
10385.0000000	0.9750000
10386.0000000	0.9800000
10387.0000000	0.9800000
10388.0000000	0.9800000
10389.0000000	0.9800000
10390.0000000	0.9750000
10391.0000000	0.9700000
10392.0000000	0.9700000
10393.0000000	0.9700000
10394.0000000	0.9750000
10395.0000000	0.9700000
10396.0000000	0.9800000
10397.0000000	0.9700000
10398.0000000	0.9700000
10399.0000000	0.9650000
10400.0000000	0.9700000
10401.0000000	0.9600000
10402.0000000	0.9600000
10403.0000000	0.9550000
10404.0000000	0.9600000
10405.0000000	0.9600000
10406.0000000	0.9600000

10407.0000000	0.9600000
10408.0000000	0.9600000
10409.0000000	0.9600000
10410.0000000	0.9650000
10411.0000000	0.9600000
10412.0000000	0.9600000
10413.0000000	0.9600000
10414.0000000	0.9600000
10415.0000000	0.9500000
10416.0000000	0.9533333
10417.0000000	0.9450000
10418.0000000	0.9550000
10419.0000000	0.9550000
10420.0000000	0.9600000
10421.0000000	0.9600000
10422.0000000	0.9650000
10423.0000000	0.9650000
10424.0000000	0.9700000
10425.0000000	0.9750000
10426.0000000	0.9700000
10427.0000000	0.9800000
10428.0000000	0.9800000
10429.0000000	0.9850000
10430.0000000	0.9800000
10431.0000000	0.9850000
10432.0000000	0.9950000
10433.0000000	0.9900000
10434.0000000	0.9900000
10435.0000000	0.9900000
10436.0000000	0.9950000
10437.0000000	0.9900000
10438.0000000	0.9900000
10439.0000000	1.0000000
10440.0000000	1.0000000
10441.0000000	1.0000000
10442.0000000	1.0000000
10443.0000000	1.0000000
10444.0000000	0.9900000
10445.0000000	0.9950000
10446.0000000	1.0050000
10447.0000000	1.0000000
10448.0000000	0.9966667
10449.0000000	1.0000000
10450.0000000	0.9950000
10451.0000000	0.9950000
10452.0000000	0.9900000
10453.0000000	0.9850000
10454.0000000	0.9850000
10455.0000000	0.9850000
10456.0000000	0.9850000
10457.0000000	0.9800000
10458.0000000	0.9800000
10459.0000000	0.9750000
10460.0000000	0.9700000
10461.0000000	0.9700000
10462.0000000	0.9750000
10463.0000000	0.9700000

10464.0000000	0.9750000
10465.0000000	0.9700000
10466.0000000	0.9800000
10467.0000000	0.9900000
10468.0000000	0.9900000
10469.0000000	0.9950000
10470.0000000	0.9950000
10471.0000000	1.0000000
10472.0000000	1.0050000
10473.0000000	1.0000000
10474.0000000	1.0100000
10475.0000000	1.0100000
10476.0000000	1.0200000
10477.0000000	1.0250000
10478.0000000	1.0200000
10479.0000000	1.0250000
10480.0000000	1.0300000
10481.0000000	1.0400000
10482.0000000	1.0400000
10483.0000000	1.0400000
10484.0000000	1.0450000
10485.0000000	1.0500000
10486.0000000	1.0549999
10487.0000000	1.0599999
10488.0000000	1.0549999
10489.0000000	1.0599999
10490.0000000	1.0599999
10491.0000000	1.0700001
10492.0000000	1.0700001
10493.0000000	1.0800000
10494.0000000	1.0850000
10495.0000000	1.0850000
10496.0000000	1.0900000
10497.0000000	1.0900000
10498.0000000	1.0900000
10499.0000000	1.1000000
10500.0000000	1.0950000
10501.0000000	1.1000000
10502.0000000	1.1100000
10503.0000000	1.1150000
10504.0000000	1.1100000
10505.0000000	1.1150000
10506.0000000	1.1166667
10507.0000000	1.1150000
10508.0000000	1.1200000
10509.0000000	1.1200000
10510.0000000	1.1150000
10511.0000000	1.1150000
10512.0000000	1.1150000
10513.0000000	1.1150000
10514.0000000	1.1200000
10515.0000000	1.1250000
10516.0000000	1.1200000
10517.0000000	1.1200000
10518.0000000	1.1200000
10519.0000000	1.1200000
10520.0000000	1.1250000

10521.0000000	1.1200000
10522.0000000	1.1200000
10523.0000000	1.1200000
10524.0000000	1.1150000
10525.0000000	1.1150000
10526.0000000	1.1100000
10527.0000000	1.1100000
10528.0000000	1.1150000
10529.0000000	1.1150000
10530.0000000	1.1150000
10531.0000000	1.1100000
10532.0000000	1.1150000
10533.0000000	1.1100000
10534.0000000	1.1100000
10535.0000000	1.1150000
10536.0000000	1.1150000
10537.0000000	1.1100000
10538.0000000	1.1100000
10539.0000000	1.1100000
10540.0000000	1.1150000
10541.0000000	1.1050000
10542.0000000	1.1000000
10543.0000000	1.1100000
10544.0000000	1.1050000
10545.0000000	1.1100000
10546.0000000	1.1100000
10547.0000000	1.1100000
10548.0000000	1.1100000
10549.0000000	1.1100000
10550.0000000	1.1100000
10551.0000000	1.1100000
10552.0000000	1.1200000
10553.0000000	1.1150000
10554.0000000	1.1100000
10555.0000000	1.1150000
10556.0000000	1.1150000
10557.0000000	1.1250000
10558.0000000	1.1300000
10559.0000000	1.1250000
10560.0000000	1.1250000
10561.0000000	1.1250000
10562.0000000	1.1350000
10563.0000000	1.1300000
10564.0000000	1.1300000
10565.0000000	1.1350000
10566.0000000	1.1400000
10567.0000000	1.1400000
10568.0000000	1.1400000
10569.0000000	1.1450000
10570.0000000	1.1450000
10571.0000000	1.1500000
10572.0000000	1.1550000
10573.0000000	1.1600000
10574.0000000	1.1600000
10575.0000000	1.1600000
10576.0000000	1.1650000
10577.0000000	1.1700000

10578.0000000	1.1600000
10579.0000000	1.1650000
10580.0000000	1.1700000
10581.0000000	1.1650000
10582.0000000	1.1750000
10583.0000000	1.1799999
10584.0000000	1.1799999
10585.0000000	1.1799999
10586.0000000	1.1849999
10587.0000000	1.1799999
10588.0000000	1.1799999
10589.0000000	1.1799999
10590.0000000	1.1849999
10591.0000000	1.1849999
10592.0000000	1.1849999
10593.0000000	1.1849999
10594.0000000	1.1849999
10595.0000000	1.1849999
10596.0000000	1.1849999
10597.0000000	1.1799999
10598.0000000	1.1900001
10599.0000000	1.1799999
10600.0000000	1.1900001
10601.0000000	1.1849999
10602.0000000	1.1900001
10603.0000000	1.1900001
10604.0000000	1.1900001
10605.0000000	1.1900001
10606.0000000	1.1849999
10607.0000000	1.1900001
10608.0000000	1.1900001
10609.0000000	1.1900001
10610.0000000	1.1849999
10611.0000000	1.1849999
10612.0000000	1.1849999
10613.0000000	1.1799999
10614.0000000	1.1849999
10615.0000000	1.1849999
10616.0000000	1.1799999
10617.0000000	1.1799999
10618.0000000	1.1799999
10619.0000000	1.1799999
10620.0000000	1.1849999
10621.0000000	1.1750000
10622.0000000	1.1849999
10623.0000000	1.1799999
10624.0000000	1.1799999
10625.0000000	1.1849999
10626.0000000	1.1799999
10627.0000000	1.1733333
10628.0000000	1.1799999
10629.0000000	1.1700000
10630.0000000	1.1700000
10631.0000000	1.1650000
10632.0000000	1.1700000
10633.0000000	1.1500000
10634.0000000	1.1450000

10635.0000000	1.1500000
10636.0000000	1.1500000
10637.0000000	1.1450000
10638.0000000	1.1450000
10639.0000000	1.1400000
10640.0000000	1.1450000
10641.0000000	1.1400000
10642.0000000	1.1350000
10643.0000000	1.1350000
10644.0000000	1.1300000
10645.0000000	1.1200000
10646.0000000	1.1100000
10647.0000000	1.1150000
10648.0000000	1.1050000
10649.0000000	1.1000000
10650.0000000	1.1000000
10651.0000000	1.0900000
10652.0000000	1.0900000
10653.0000000	1.0850000
10654.0000000	1.0800000
10655.0000000	1.0750000
10656.0000000	1.0750000
10657.0000000	1.0700001
10658.0000000	1.0733334
10659.0000000	1.0700001
10660.0000000	1.0700001
10661.0000000	1.0700001
10662.0000000	1.0700001
10663.0000000	1.0700001
10664.0000000	1.0700001
10665.0000000	1.0700001
10666.0000000	1.0700001
10667.0000000	1.0750000
10668.0000000	1.0700001
10669.0000000	1.0750000
10670.0000000	1.0750000
10671.0000000	1.0700001
10672.0000000	1.0800000
10673.0000000	1.0800000
10674.0000000	1.0800000
10675.0000000	1.0800000
10676.0000000	1.0800000
10677.0000000	1.0800000
10678.0000000	1.0800000
10679.0000000	1.0800000
10680.0000000	1.0800000
10681.0000000	1.0750000
10682.0000000	1.0750000
10683.0000000	1.0700001
10684.0000000	1.0700001
10685.0000000	1.0650001
10686.0000000	1.0599999
10687.0000000	1.0500000
10688.0000000	1.0533333
10689.0000000	1.0450000
10690.0000000	1.0400000
10691.0000000	1.0400000

10692.0000000	1.0350000
10693.0000000	1.0300000
10694.0000000	1.0200000
10695.0000000	1.0150000
10696.0000000	1.0100000
10697.0000000	1.0050000
10698.0000000	1.0000000
10699.0000000	0.9900000
10700.0000000	0.9900000
10701.0000000	0.9850000
10702.0000000	0.9800000
10703.0000000	0.9750000
10704.0000000	0.9750000
10705.0000000	0.9800000
10706.0000000	0.9750000
10707.0000000	0.9750000
10708.0000000	0.9700000
10709.0000000	0.9800000
10710.0000000	0.9800000
10711.0000000	0.9750000
10712.0000000	0.9800000
10713.0000000	0.9800000
10714.0000000	0.9850000
10715.0000000	0.9800000
10716.0000000	0.9900000
10717.0000000	0.9900000
10718.0000000	0.9966667
10719.0000000	1.0000000
10720.0000000	1.0050000
10721.0000000	1.0050000
10722.0000000	1.0000000
10723.0000000	1.0050000
10724.0000000	1.0000000
10725.0000000	1.0000000
10726.0000000	1.0000000
10727.0000000	1.0000000
10728.0000000	1.0000000
10729.0000000	1.0050000
10730.0000000	1.0100000
10731.0000000	1.0100000
10732.0000000	1.0150000
10733.0000000	1.0150000
10734.0000000	1.0100000
10735.0000000	1.0100000
10736.0000000	1.0100000
10737.0000000	1.0000000
10738.0000000	1.0050000
10739.0000000	1.0000000
10740.0000000	0.9950000
10741.0000000	0.9900000
10742.0000000	0.9850000
10743.0000000	0.9800000
10744.0000000	0.9750000
10745.0000000	0.9700000
10746.0000000	0.9600000
10747.0000000	0.9600000
10748.0000000	0.9500000

10749.0000000	0.9350000
10750.0000000	0.9300000
10751.0000000	0.9300000
10752.0000000	0.9200000
10753.0000000	0.9200000
10754.0000000	0.9200000
10755.0000000	0.9200000
10756.0000000	0.9150000
10757.0000000	0.9200000
10758.0000000	0.9250000
10759.0000000	0.9200000
10760.0000000	0.9200000
10761.0000000	0.9200000
10762.0000000	0.9200000
10763.0000000	0.9200000
10764.0000000	0.9200000
10765.0000000	0.9150000
10766.0000000	0.9100000
10767.0000000	0.9000000
10768.0000000	0.9000000
10769.0000000	0.8900000
10770.0000000	0.9000000
10771.0000000	0.8950000
10772.0000000	0.8950000
10773.0000000	0.8900000
10774.0000000	0.8900000
10775.0000000	0.8900000
10776.0000000	0.8900000
10777.0000000	0.9000000
10778.0000000	0.8900000
10779.0000000	0.8950000
10780.0000000	0.8933333
10781.0000000	0.8950000
10782.0000000	0.9000000
10783.0000000	0.9000000
10784.0000000	0.9000000
10785.0000000	0.9100000
10786.0000000	0.9100000
10787.0000000	0.9100000
10788.0000000	0.9100000
10789.0000000	0.9050000
10790.0000000	0.9100000
10791.0000000	0.9100000
10792.0000000	0.9100000
10793.0000000	0.9100000
10794.0000000	0.9200000
10795.0000000	0.9200000
10796.0000000	0.9200000
10797.0000000	0.9200000
10798.0000000	0.9200000
10799.0000000	0.9200000
10800.0000000	0.9200000
10801.0000000	0.9200000
10802.0000000	0.9150000
10803.0000000	0.9150000
10804.0000000	0.9150000
10805.0000000	0.9100000

10806.0000000	0.9100000
10807.0000000	0.9100000
10808.0000000	0.9100000
10809.0000000	0.9100000
10810.0000000	0.9033334
10811.0000000	0.9000000
10812.0000000	0.9050000
10813.0000000	0.9050000
10814.0000000	0.9100000
10815.0000000	0.9000000
10816.0000000	0.9000000
10817.0000000	0.9000000
10818.0000000	0.9000000
10819.0000000	0.9000000
10820.0000000	0.9000000
10821.0000000	0.9000000
10822.0000000	0.9000000
10823.0000000	0.8950000
10824.0000000	0.9000000
10825.0000000	0.9000000
10826.0000000	0.8950000
10827.0000000	0.9000000
10828.0000000	0.9000000
10829.0000000	0.9000000
10830.0000000	0.9000000
10831.0000000	0.9000000
10832.0000000	0.9000000
10833.0000000	0.9000000
10834.0000000	0.9000000
10835.0000000	0.9000000
10836.0000000	0.9000000
10837.0000000	0.9050000
10838.0000000	0.9050000
10839.0000000	0.9100000
10840.0000000	0.9100000
10841.0000000	0.9100000
10842.0000000	0.9100000
10843.0000000	0.9100000
10844.0000000	0.9150000
10845.0000000	0.9200000
10846.0000000	0.9300000
10847.0000000	0.9400000
10848.0000000	0.9400000
10849.0000000	0.9200000
10850.0000000	0.9200000
10851.0000000	0.9150000
10852.0000000	0.9150000
10853.0000000	0.9050000
10854.0000000	0.9100000
10855.0000000	0.9050000
10856.0000000	0.9100000
10857.0000000	0.9050000
10858.0000000	0.9100000
10859.0000000	0.9150000
10860.0000000	0.9200000
10861.0000000	0.9350000
10862.0000000	0.9400000

10863.0000000	0.9300000
10864.0000000	0.9300000
10865.0000000	0.9150000
10866.0000000	0.9100000
10867.0000000	0.9100000
10868.0000000	0.9050000
10869.0000000	0.9000000
10870.0000000	0.8999999
10871.0000000	0.8950000
10872.0000000	0.8950000
10873.0000000	0.9000000
10874.0000000	0.9100000
10875.0000000	0.9150000
10876.0000000	0.9100000
10877.0000000	0.9000000
10878.0000000	0.8900000
10879.0000000	0.8800000
10880.0000000	0.8650000
10881.0000000	0.8600000
10882.0000000	0.8600000
10883.0000000	0.8600000
10884.0000000	0.8550000
10885.0000000	0.8500000
10886.0000000	0.8500000
10887.0000000	0.8500000
10888.0000000	0.8400000
10889.0000000	0.8400000
10890.0000000	0.8300000
10891.0000000	0.8300000
10892.0000000	0.8350000
10893.0000000	0.8400000
10894.0000000	0.8400000
10895.0000000	0.8350000
10896.0000000	0.8400000
10897.0000000	0.8400000
10898.0000000	0.8350000
10899.0000000	0.8400000
10900.0000000	0.8400000
10901.0000000	0.8400000
10902.0000000	0.8450000
10903.0000000	0.8400000
10904.0000000	0.8300000
10905.0000000	0.8300000
10906.0000000	0.8350000
10907.0000000	0.8350000
10908.0000000	0.8300000
10909.0000000	0.8300000
10910.0000000	0.8300000
10911.0000000	0.8200000
10912.0000000	0.8250000
10913.0000000	0.8200000
10914.0000000	0.8250000
10915.0000000	0.8150000
10916.0000000	0.8100000
10917.0000000	0.8100000
10918.0000000	0.8100000
10919.0000000	0.8000000

10920.0000000	0.8050000
10921.0000000	0.8000000
10922.0000000	0.8000000
10923.0000000	0.7900000
10924.0000000	0.7950000
10925.0000000	0.7900000
10926.0000000	0.7900000
10927.0000000	0.7850000
10928.0000000	0.7900000
10929.0000000	0.7900000
10930.0000000	0.7900000
10931.0000000	0.7866666
10932.0000000	0.7850000
10933.0000000	0.7850000
10934.0000000	0.7850000
10935.0000000	0.7800000
10936.0000000	0.7850000
10937.0000000	0.7850000
10938.0000000	0.7900000
10939.0000000	0.7950000
10940.0000000	0.7900000
10941.0000000	0.7900000
10942.0000000	0.7950000
10943.0000000	0.7900000
10944.0000000	0.7950000
10945.0000000	0.7900000
10946.0000000	0.7900000
10947.0000000	0.8000000
10948.0000000	0.7900000
10949.0000000	0.7900000
10950.0000000	0.7800000
10951.0000000	0.7850000
10952.0000000	0.7800000
10953.0000000	0.7800000
10954.0000000	0.7800000
10955.0000000	0.7800000
10956.0000000	0.7800000
10957.0000000	0.7800000
10958.0000000	0.7750000
10959.0000000	0.7800000
10960.0000000	0.7750000
10961.0000000	0.7800000
10962.0000000	0.7750000
10963.0000000	0.7800000
10964.0000000	0.7800000
10965.0000000	0.7700000
10966.0000000	0.7750000
10967.0000000	0.7700000
10968.0000000	0.7650000
10969.0000000	0.7600000
10970.0000000	0.7550000
10971.0000000	0.7550000
10972.0000000	0.7500000
10973.0000000	0.7450000
10974.0000000	0.7450000
10975.0000000	0.7350000
10976.0000000	0.7350000

10977.0000000	0.7350000
10978.0000000	0.7350000
10979.0000000	0.7350000
10980.0000000	0.7350000
10981.0000000	0.7300000
10982.0000000	0.7350000
10983.0000000	0.7300000
10984.0000000	0.7300000
10985.0000000	0.7300000
10986.0000000	0.7300000
10987.0000000	0.7250000
10988.0000000	0.7250000
10989.0000000	0.7300000
10990.0000000	0.7250000
10991.0000000	0.7250000
10992.0000000	0.7233334
10993.0000000	0.7250000
10994.0000000	0.7300000
10995.0000000	0.7250000
10996.0000000	0.7300000
10997.0000000	0.7250000
10998.0000000	0.7250000
10999.0000000	0.7300000
11000.0000000	0.7300000
11001.0000000	0.7250000
11002.0000000	0.7250000
11003.0000000	0.7200000
11004.0000000	0.7200000
11005.0000000	0.7200000
11006.0000000	0.7150000
11007.0000000	0.7150000
11008.0000000	0.7050000
11009.0000000	0.7000000
11010.0000000	0.7050000
11011.0000000	0.7000000
11012.0000000	0.6950000
11013.0000000	0.6950000
11014.0000000	0.6900000
11015.0000000	0.6900000
11016.0000000	0.6900000
11017.0000000	0.6850000
11018.0000000	0.6900000
11019.0000000	0.6900000
11020.0000000	0.6833333
11021.0000000	0.6800000
11022.0000000	0.6800000
11023.0000000	0.6750000
11024.0000000	0.6750000
11025.0000000	0.6750000
11026.0000000	0.6700000
11027.0000000	0.6650000
11028.0000000	0.6600000
11029.0000000	0.6600000
11030.0000000	0.6550000
11031.0000000	0.6550000
11032.0000000	0.6500000
11033.0000000	0.6550000

11034.0000000	0.6600000
11035.0000000	0.6600000
11036.0000000	0.6600000
11037.0000000	0.6700000
11038.0000000	0.6650000
11039.0000000	0.6750000
11040.0000000	0.6700000
11041.0000000	0.6700000
11042.0000000	0.6700000
11043.0000000	0.6700000
11044.0000000	0.6650000
11045.0000000	0.6700000
11046.0000000	0.6600000
11047.0000000	0.6600000
11048.0000000	0.6600000
11049.0000000	0.6500000
11050.0000000	0.6550000
11051.0000000	0.6500000
11052.0000000	0.6550000
11053.0000000	0.6500000
11054.0000000	0.6500000
11055.0000000	0.6500000
11056.0000000	0.6500000
11057.0000000	0.6500000
11058.0000000	0.6450000
11059.0000000	0.6500000
11060.0000000	0.6450000
11061.0000000	0.6500000
11062.0000000	0.6600000
11063.0000000	0.6500000
11064.0000000	0.6550000
11065.0000000	0.6550000
11066.0000000	0.6600000
11067.0000000	0.6650000
11068.0000000	0.6600000
11069.0000000	0.6600000
11070.0000000	0.6650000
11071.0000000	0.6700000
11072.0000000	0.6750000
11073.0000000	0.6700000
11074.0000000	0.6700000
11075.0000000	0.6750000
11076.0000000	0.6750000
11077.0000000	0.6800000
11078.0000000	0.6800000
11079.0000000	0.6800000
11080.0000000	0.6850000
11081.0000000	0.6850000
11082.0000000	0.6966667
11083.0000000	0.7000000
11084.0000000	0.7000000
11085.0000000	0.7000000
11086.0000000	0.7100000
11087.0000000	0.7050000
11088.0000000	0.7100000
11089.0000000	0.7150000
11090.0000000	0.7200000

11091.0000000	0.7150000
11092.0000000	0.7150000
11093.0000000	0.7100000
11094.0000000	0.7100000
11095.0000000	0.7100000
11096.0000000	0.7050000
11097.0000000	0.7100000
11098.0000000	0.7100000
11099.0000000	0.7100000
11100.0000000	0.7200000
11101.0000000	0.7200000
11102.0000000	0.7200000
11103.0000000	0.7250000
11104.0000000	0.7300000
11105.0000000	0.7300000
11106.0000000	0.7300000
11107.0000000	0.7400000
11108.0000000	0.7450000
11109.0000000	0.7500000
11110.0000000	0.7500000
11111.0000000	0.7550000
11112.0000000	0.7633333
11113.0000000	0.7650000
11114.0000000	0.7650000
11115.0000000	0.7800000
11116.0000000	0.7750000
11117.0000000	0.7750000
11118.0000000	0.7850000
11119.0000000	0.7850000
11120.0000000	0.7850000
11121.0000000	0.7900000
11122.0000000	0.7900000
11123.0000000	0.7900000
11124.0000000	0.8000000
11125.0000000	0.8000000
11126.0000000	0.7950000
11127.0000000	0.8050000
11128.0000000	0.8000000
11129.0000000	0.8050000
11130.0000000	0.8100000
11131.0000000	0.8100000
11132.0000000	0.8100000
11133.0000000	0.8100000
11134.0000000	0.8100000
11135.0000000	0.8150000
11136.0000000	0.8250000
11137.0000000	0.8250000
11138.0000000	0.8250000
11139.0000000	0.8300000
11140.0000000	0.8366666
11141.0000000	0.8350000
11142.0000000	0.8350000
11143.0000000	0.8300000
11144.0000000	0.8350000
11145.0000000	0.8400000
11146.0000000	0.8400000
11147.0000000	0.8400000

11148.0000000	0.8450000
11149.0000000	0.8500000
11150.0000000	0.8500000
11151.0000000	0.8550000
11152.0000000	0.8500000
11153.0000000	0.8500000
11154.0000000	0.8550000
11155.0000000	0.8500000
11156.0000000	0.8500000
11157.0000000	0.8500000
11158.0000000	0.8450000
11159.0000000	0.8450000
11160.0000000	0.8450000
11161.0000000	0.8500000
11162.0000000	0.8500000
11163.0000000	0.8500000
11164.0000000	0.8550000
11165.0000000	0.8500000
11166.0000000	0.8500000
11167.0000000	0.8550000
11168.0000000	0.8600000
11169.0000000	0.8500000
11170.0000000	0.8500000
11171.0000000	0.8450000
11172.0000000	0.8500001
11173.0000000	0.8450000
11174.0000000	0.8500000
11175.0000000	0.8500000
11176.0000000	0.8450000
11177.0000000	0.8400000
11178.0000000	0.8400000
11179.0000000	0.8450000
11180.0000000	0.8450000
11181.0000000	0.8400000
11182.0000000	0.8400000
11183.0000000	0.8300000
11184.0000000	0.8300000
11185.0000000	0.8250000
11186.0000000	0.8250000
11187.0000000	0.8300000
11188.0000000	0.8200000
11189.0000000	0.8200000
11190.0000000	0.8250000
11191.0000000	0.8200000
11192.0000000	0.8200000
11193.0000000	0.8250000
11194.0000000	0.8250000
11195.0000000	0.8200000
11196.0000000	0.8250000
11197.0000000	0.8300000
11198.0000000	0.8300000
11199.0000000	0.8300000
11200.0000000	0.8350000
11201.0000000	0.8400000
11202.0000000	0.8450000
11203.0000000	0.8500000
11204.0000000	0.8450000

11205.0000000	0.8500000
11206.0000000	0.8500000
11207.0000000	0.8550000
11208.0000000	0.8600000
11209.0000000	0.8600000
11210.0000000	0.8550000
11211.0000000	0.8600000
11212.0000000	0.8600000
11213.0000000	0.8550000
11214.0000000	0.8550000
11215.0000000	0.8500000
11216.0000000	0.8450000
11217.0000000	0.8500000
11218.0000000	0.8500000
11219.0000000	0.8400000
11220.0000000	0.8400000
11221.0000000	0.8400000
11222.0000000	0.8450000
11223.0000000	0.8400000
11224.0000000	0.8450000
11225.0000000	0.8550000
11226.0000000	0.8550000
11227.0000000	0.8550000
11228.0000000	0.8600000
11229.0000000	0.8650000
11230.0000000	0.8600000
11231.0000000	0.8733333
11232.0000000	0.8750000
11233.0000000	0.8800000
11234.0000000	0.8850000
11235.0000000	0.8850000
11236.0000000	0.8900000
11237.0000000	0.8950000
11238.0000000	0.9000000
11239.0000000	0.9100000
11240.0000000	0.9050000
11241.0000000	0.9150000
11242.0000000	0.9150000
11243.0000000	0.9200000
11244.0000000	0.9250000
11245.0000000	0.9300000
11246.0000000	0.9300000
11247.0000000	0.9350000
11248.0000000	0.9300000
11249.0000000	0.9400000
11250.0000000	0.9400000
11251.0000000	0.9400000
11252.0000000	0.9450000
11253.0000000	0.9350000
11254.0000000	0.9350000
11255.0000000	0.9300000
11256.0000000	0.9350000
11257.0000000	0.9300000
11258.0000000	0.9350000
11259.0000000	0.9300000
11260.0000000	0.9300000
11261.0000000	0.9300000

11262.0000000	0.9333333
11263.0000000	0.9350000
11264.0000000	0.9350000
11265.0000000	0.9350000
11266.0000000	0.9450000
11267.0000000	0.9400000
11268.0000000	0.9500000
11269.0000000	0.9450000
11270.0000000	0.9500000
11271.0000000	0.9500000
11272.0000000	0.9600000
11273.0000000	0.9550000
11274.0000000	0.9600000
11275.0000000	0.9600000
11276.0000000	0.9600000
11277.0000000	0.9650000
11278.0000000	0.9600000
11279.0000000	0.9600000
11280.0000000	0.9600000
11281.0000000	0.9600000
11282.0000000	0.9650000
11283.0000000	0.9650000
11284.0000000	0.9600000
11285.0000000	0.9700000
11286.0000000	0.9600000
11287.0000000	0.9600000
11288.0000000	0.9600000
11289.0000000	0.9600000
11290.0000000	0.9600000
11291.0000000	0.9500000
11292.0000000	0.9400000
11293.0000000	0.9400000
11294.0000000	0.9400000
11295.0000000	0.9400000
11296.0000000	0.9350000
11297.0000000	0.9300000
11298.0000000	0.9250000
11299.0000000	0.9200000
11300.0000000	0.9150000
11301.0000000	0.9100000
11302.0000000	0.9100000
11303.0000000	0.9100000
11304.0000000	0.9150000
11305.0000000	0.9200000
11306.0000000	0.9100000
11307.0000000	0.9100000
11308.0000000	0.9100000
11309.0000000	0.9000000
11310.0000000	0.9000000
11311.0000000	0.9000000
11312.0000000	0.9000000
11313.0000000	0.9000000
11314.0000000	0.8950000
11315.0000000	0.9000000
11316.0000000	0.8900000
11317.0000000	0.8950000
11318.0000000	0.9000000

11319.0000000	0.8950000
11320.0000000	0.8950000
11321.0000000	0.8933333
11322.0000000	0.8900000
11323.0000000	0.8900000
11324.0000000	0.8900000
11325.0000000	0.8900000
11326.0000000	0.8900000
11327.0000000	0.8850000
11328.0000000	0.8900000
11329.0000000	0.8800000
11330.0000000	0.8850000
11331.0000000	0.8850000
11332.0000000	0.8900000
11333.0000000	0.8900000
11334.0000000	0.8900000
11335.0000000	0.8950000
11336.0000000	0.8900000
11337.0000000	0.9000000
11338.0000000	0.9000000
11339.0000000	0.9000000
11340.0000000	0.9100000
11341.0000000	0.9100000
11342.0000000	0.9100000
11343.0000000	0.9100000
11344.0000000	0.9050000
11345.0000000	0.9100000
11346.0000000	0.9100000
11347.0000000	0.9150000
11348.0000000	0.9150000
11349.0000000	0.9100000
11350.0000000	0.9150000
11351.0000000	0.9100000
11352.0000000	0.9100000
11353.0000000	0.9050000
11354.0000000	0.9000000
11355.0000000	0.8900000
11356.0000000	0.8950000
11357.0000000	0.8900000
11358.0000000	0.8900000
11359.0000000	0.8900000
11360.0000000	0.8850000
11361.0000000	0.8700000
11362.0000000	0.8700000
11363.0000000	0.8600000
11364.0000000	0.8650000
11365.0000000	0.8600000
11366.0000000	0.8600000
11367.0000000	0.8600000
11368.0000000	0.8600000
11369.0000000	0.8600000
11370.0000000	0.8600000
11371.0000000	0.8700000
11372.0000000	0.8600000
11373.0000000	0.8650000
11374.0000000	0.8700000
11375.0000000	0.8700000

11376.0000000	0.8700000
11377.0000000	0.8750000
11378.0000000	0.8800000
11379.0000000	0.8800000
11380.0000000	0.8850000
11381.0000000	0.8900000
11382.0000000	0.8866666
11383.0000000	0.8900000
11384.0000000	0.8950000
11385.0000000	0.8850000
11386.0000000	0.8900000
11387.0000000	0.8800000
11388.0000000	0.8750000
11389.0000000	0.8750000
11390.0000000	0.8600000
11391.0000000	0.8600000
11392.0000000	0.8500000
11393.0000000	0.8400000
11394.0000000	0.8250000
11395.0000000	0.8200000
11396.0000000	0.8150000
11397.0000000	0.8100000
11398.0000000	0.8100000
11399.0000000	0.8100000
11400.0000000	0.8050000
11401.0000000	0.8100000
11402.0000000	0.8050000
11403.0000000	0.8100000
11404.0000000	0.8100000
11405.0000000	0.8050000
11406.0000000	0.8000000
11407.0000000	0.8050000
11408.0000000	0.8050000
11409.0000000	0.8100000
11410.0000000	0.8100000
11411.0000000	0.8150000
11412.0000000	0.8100000
11413.0000000	0.8066667
11414.0000000	0.8050000
11415.0000000	0.7900000
11416.0000000	0.7850000
11417.0000000	0.7800000
11418.0000000	0.7800000
11419.0000000	0.7800000
11420.0000000	0.7800000
11421.0000000	0.7800000
11422.0000000	0.7800000
11423.0000000	0.7850000
11424.0000000	0.7850000
11425.0000000	0.7900000
11426.0000000	0.7900000
11427.0000000	0.7850000
11428.0000000	0.7900000
11429.0000000	0.7900000
11430.0000000	0.7900000
11431.0000000	0.7950000
11432.0000000	0.8000000

11433.0000000	0.8000000
11434.0000000	0.8000000
11435.0000000	0.8050000
11436.0000000	0.8050000
11437.0000000	0.8050000
11438.0000000	0.8100000
11439.0000000	0.8050000
11440.0000000	0.8050000
11441.0000000	0.8050000
11442.0000000	0.8050000
11443.0000000	0.8066667
11444.0000000	0.8050000
11445.0000000	0.8050000
11446.0000000	0.8150000
11447.0000000	0.8100000
11448.0000000	0.8100000
11449.0000000	0.8150000
11450.0000000	0.8200000
11451.0000000	0.8200000
11452.0000000	0.8200000
11453.0000000	0.8250000
11454.0000000	0.8300000
11455.0000000	0.8200000
11456.0000000	0.8250000
11457.0000000	0.8200000
11458.0000000	0.8150000
11459.0000000	0.8150000
11460.0000000	0.8150000
11461.0000000	0.8150000
11462.0000000	0.8200000
11463.0000000	0.8200000
11464.0000000	0.8150000
11465.0000000	0.8150000
11466.0000000	0.8200000
11467.0000000	0.8200000
11468.0000000	0.8300000
11469.0000000	0.8400000
11470.0000000	0.8400000
11471.0000000	0.8550000
11472.0000000	0.8950000
11473.0000000	1.0100000
11474.0000000	1.0150000
11475.0000000	0.9500000
11476.0000000	0.8950000
11477.0000000	0.8550000
11478.0000000	0.8450000
11479.0000000	0.8400000
11480.0000000	0.8300000
11481.0000000	0.8200000
11482.0000000	0.8100000
11483.0000000	0.8100000
11484.0000000	0.8050000
11485.0000000	0.7950000
11486.0000000	0.7900000
11487.0000000	0.7850000
11488.0000000	0.7900000
11489.0000000	0.7850000

11490.0000000	0.7800000
11491.0000000	0.7800000
11492.0000000	0.7750000
11493.0000000	0.7750000
11494.0000000	0.7800000
11495.0000000	0.7800000
11496.0000000	0.7800000
11497.0000000	0.7850000
11498.0000000	0.7800000
11499.0000000	0.7900000
11500.0000000	0.7900000
11501.0000000	0.7800000
11502.0000000	0.7850000
11503.0000000	0.7800000
11504.0000000	0.7733333
11505.0000000	0.7800000
11506.0000000	0.7800000
11507.0000000	0.7750000
11508.0000000	0.7800000
11509.0000000	0.7800000
11510.0000000	0.7800000
11511.0000000	0.7800000
11512.0000000	0.7850000
11513.0000000	0.7800000
11514.0000000	0.7750000
11515.0000000	0.7800000
11516.0000000	0.7700000
11517.0000000	0.7750000
11518.0000000	0.7700000
11519.0000000	0.7700000
11520.0000000	0.7700000
11521.0000000	0.7600000
11522.0000000	0.7650000
11523.0000000	0.7600000
11524.0000000	0.7700000
11525.0000000	0.7700000
11526.0000000	0.7700000
11527.0000000	0.7700000
11528.0000000	0.7750000
11529.0000000	0.7700000
11530.0000000	0.7750000
11531.0000000	0.7700000
11532.0000000	0.7650000
11533.0000000	0.7650000
11534.0000000	0.7633333
11535.0000000	0.7650000
11536.0000000	0.7600000
11537.0000000	0.7550000
11538.0000000	0.7500000
11539.0000000	0.7400000
11540.0000000	0.7350000
11541.0000000	0.7350000
11542.0000000	0.7300000
11543.0000000	0.7250000
11544.0000000	0.7200000
11545.0000000	0.7200000
11546.0000000	0.7200000

11547.0000000	0.7200000
11548.0000000	0.7250000
11549.0000000	0.7200000
11550.0000000	0.7250000
11551.0000000	0.7300000
11552.0000000	0.7200000
11553.0000000	0.7250000
11554.0000000	0.7250000
11555.0000000	0.7250000
11556.0000000	0.7350000
11557.0000000	0.7300000
11558.0000000	0.7350000
11559.0000000	0.7300000
11560.0000000	0.7300000
11561.0000000	0.7350000
11562.0000000	0.7350000
11563.0000000	0.7433333
11564.0000000	0.7450000
11565.0000000	0.7450000
11566.0000000	0.7500000
11567.0000000	0.7500000
11568.0000000	0.7450000
11569.0000000	0.7500000
11570.0000000	0.7500000
11571.0000000	0.7550000
11572.0000000	0.7550000
11573.0000000	0.7600000
11574.0000000	0.7550000
11575.0000000	0.7600000
11576.0000000	0.7600000
11577.0000000	0.7700000
11578.0000000	0.7600000
11579.0000000	0.7700000
11580.0000000	0.7700000
11581.0000000	0.7650000
11582.0000000	0.7700000
11583.0000000	0.7700000
11584.0000000	0.7650000
11585.0000000	0.7700000
11586.0000000	0.7750000
11587.0000000	0.7700000
11588.0000000	0.7800000
11589.0000000	0.7800000
11590.0000000	0.7800000
11591.0000000	0.7850000
11592.0000000	0.7800000
11593.0000000	0.7900000
11594.0000000	0.7850000
11595.0000000	0.7900000
11596.0000000	0.7850000
11597.0000000	0.7850000
11598.0000000	0.7850000
11599.0000000	0.7900000
11600.0000000	0.7850000
11601.0000000	0.7900000
11602.0000000	0.7950000
11603.0000000	0.7950000

11604.0000000	0.7900000
11605.0000000	0.7900000
11606.0000000	0.7900000
11607.0000000	0.7900000
11608.0000000	0.7900000
11609.0000000	0.7850000
11610.0000000	0.7900000
11611.0000000	0.7850000
11612.0000000	0.7850000
11613.0000000	0.7850000
11614.0000000	0.7850000
11615.0000000	0.7800000
11616.0000000	0.7800000
11617.0000000	0.7750000
11618.0000000	0.7750000
11619.0000000	0.7750000
11620.0000000	0.7700000
11621.0000000	0.7700000
11622.0000000	0.7600000
11623.0000000	0.7550000
11624.0000000	0.7500000
11625.0000000	0.7400000
11626.0000000	0.7350000
11627.0000000	0.7350000
11628.0000000	0.7350000
11629.0000000	0.7350000
11630.0000000	0.7300000
11631.0000000	0.7250000
11632.0000000	0.7250000
11633.0000000	0.7250000
11634.0000000	0.7300000
11635.0000000	0.7250000
11636.0000000	0.7250000
11637.0000000	0.7300000
11638.0000000	0.7300000
11639.0000000	0.7300000
11640.0000000	0.7300000
11641.0000000	0.7300000
11642.0000000	0.7350000
11643.0000000	0.7400000
11644.0000000	0.7300000
11645.0000000	0.7300000
11646.0000000	0.7350000
11647.0000000	0.7350000
11648.0000000	0.7300000
11649.0000000	0.7400000
11650.0000000	0.7300000
11651.0000000	0.7400000
11652.0000000	0.7350000
11653.0000000	0.7350000
11654.0000000	0.7350000
11655.0000000	0.7300000
11656.0000000	0.7300000
11657.0000000	0.7400000
11658.0000000	0.7450000
11659.0000000	0.7400000
11660.0000000	0.7400000

11661.0000000	0.7500000
11662.0000000	0.7400000
11663.0000000	0.7500000
11664.0000000	0.7500000
11665.0000000	0.7450000
11666.0000000	0.7400000
11667.0000000	0.7400000
11668.0000000	0.7450000
11669.0000000	0.7400000
11670.0000000	0.7400000
11671.0000000	0.7450000
11672.0000000	0.7400000
11673.0000000	0.7450000
11674.0000000	0.7400000
11675.0000000	0.7350000
11676.0000000	0.7400000
11677.0000000	0.7400000
11678.0000000	0.7450000
11679.0000000	0.7450000
11680.0000000	0.7500000
11681.0000000	0.7500000
11682.0000000	0.7500000
11683.0000000	0.7500000
11684.0000000	0.7550000
11685.0000000	0.7500000
11686.0000000	0.7500000
11687.0000000	0.7500000
11688.0000000	0.7500000
11689.0000000	0.7500000
11690.0000000	0.7550000
11691.0000000	0.7500000
11692.0000000	0.7500000
11693.0000000	0.7450000
11694.0000000	0.7500000
11695.0000000	0.7400000
11696.0000000	0.7400000
11697.0000000	0.7400000
11698.0000000	0.7450000
11699.0000000	0.7450000
11700.0000000	0.7450000
11701.0000000	0.7450000
11702.0000000	0.7450000
11703.0000000	0.7500000
11704.0000000	0.7500000
11705.0000000	0.7500000
11706.0000000	0.7500000
11707.0000000	0.7550000
11708.0000000	0.7600000
11709.0000000	0.7550000
11710.0000000	0.7600000
11711.0000000	0.7600000
11712.0000000	0.7700000
11713.0000000	0.7600000
11714.0000000	0.7650000
11715.0000000	0.7600000
11716.0000000	0.7600000
11717.0000000	0.7600000

11718.0000000	0.7600000
11719.0000000	0.7600000
11720.0000000	0.7550000
11721.0000000	0.7500000
11722.0000000	0.7500000
11723.0000000	0.7550000
11724.0000000	0.7550000
11725.0000000	0.7600000
11726.0000000	0.7550000
11727.0000000	0.7500000
11728.0000000	0.7400000
11729.0000000	0.7450000
11730.0000000	0.7400000
11731.0000000	0.7350000
11732.0000000	0.7300000
11733.0000000	0.7250000
11734.0000000	0.7200000
11735.0000000	0.7200000
11736.0000000	0.7200000
11737.0000000	0.7150000
11738.0000000	0.7150000
11739.0000000	0.7100000
11740.0000000	0.7100000
11741.0000000	0.7100000
11742.0000000	0.7100000
11743.0000000	0.7100000
11744.0000000	0.7100000
11745.0000000	0.7100000
11746.0000000	0.7100000
11747.0000000	0.7200000
11748.0000000	0.7100000
11749.0000000	0.7100000
11750.0000000	0.7100000
11751.0000000	0.7100000
11752.0000000	0.7100000
11753.0000000	0.7050000
11754.0000000	0.7050000
11755.0000000	0.7050000
11756.0000000	0.7000000
11757.0000000	0.7050000
11758.0000000	0.7000000
11759.0000000	0.7050000
11760.0000000	0.7000000
11761.0000000	0.7000000
11762.0000000	0.7050000
11763.0000000	0.7050000
11764.0000000	0.7100000
11765.0000000	0.7100000
11766.0000000	0.7000000
11767.0000000	0.7100000
11768.0000000	0.7050000
11769.0000000	0.7050000
11770.0000000	0.7100000
11771.0000000	0.7000000
11772.0000000	0.7050000
11773.0000000	0.7100000
11774.0000000	0.7050000

11775.0000000	0.7033333
11776.0000000	0.6950000
11777.0000000	0.7000000
11778.0000000	0.6950000
11779.0000000	0.6950000
11780.0000000	0.7000000
11781.0000000	0.7000000
11782.0000000	0.7000000
11783.0000000	0.7000000
11784.0000000	0.7000000
11785.0000000	0.7000000
11786.0000000	0.6950000
11787.0000000	0.6950000
11788.0000000	0.7000000
11789.0000000	0.7000000
11790.0000000	0.7000000
11791.0000000	0.7000000
11792.0000000	0.6950000
11793.0000000	0.6900000
11794.0000000	0.6900000
11795.0000000	0.6900000
11796.0000000	0.6950000
11797.0000000	0.6900000
11798.0000000	0.6900000
11799.0000000	0.6950000
11800.0000000	0.6950000
11801.0000000	0.6950000
11802.0000000	0.6950000
11803.0000000	0.7000000
11804.0000000	0.7000000
11805.0000000	0.7050000
11806.0000000	0.7100000
11807.0000000	0.7100000
11808.0000000	0.7100000
11809.0000000	0.7150000
11810.0000000	0.7150000
11811.0000000	0.7200000
11812.0000000	0.7250000
11813.0000000	0.7200000
11814.0000000	0.7250000
11815.0000000	0.7300000
11816.0000000	0.7350000
11817.0000000	0.7300000
11818.0000000	0.7350000
11819.0000000	0.7300000
11820.0000000	0.7300000
11821.0000000	0.7200000
11822.0000000	0.7300000
11823.0000000	0.7250000
11824.0000000	0.7200000
11825.0000000	0.7200000
11826.0000000	0.7200000
11827.0000000	0.7150000
11828.0000000	0.7200000
11829.0000000	0.7150000
11830.0000000	0.7200000
11831.0000000	0.7150000

11832.0000000	0.7150000
11833.0000000	0.7200000
11834.0000000	0.7200000
11835.0000000	0.7200000
11836.0000000	0.7200000
11837.0000000	0.7333333
11838.0000000	0.7300000
11839.0000000	0.7400000
11840.0000000	0.7400000
11841.0000000	0.7450000
11842.0000000	0.7500000
11843.0000000	0.7500000
11844.0000000	0.7550000
11845.0000000	0.7500000
11846.0000000	0.7600000
11847.0000000	0.7650000
11848.0000000	0.7650000
11849.0000000	0.7700000
11850.0000000	0.7700000
11851.0000000	0.7850000
11852.0000000	0.7800000
11853.0000000	0.7900000
11854.0000000	0.7850000
11855.0000000	0.7900000
11856.0000000	0.7900000
11857.0000000	0.7900000
11858.0000000	0.7950000
11859.0000000	0.8000000
11860.0000000	0.8050000
11861.0000000	0.8100000
11862.0000000	0.8100000
11863.0000000	0.8200000
11864.0000000	0.8200000
11865.0000000	0.8300000
11866.0000000	0.8300000
11867.0000000	0.8350000
11868.0000000	0.8333333
11869.0000000	0.8400000
11870.0000000	0.8400000
11871.0000000	0.8400000
11872.0000000	0.8400000
11873.0000000	0.8450000
11874.0000000	0.8500000
11875.0000000	0.8450000
11876.0000000	0.8500000
11877.0000000	0.8450000
11878.0000000	0.8450000
11879.0000000	0.8450000
11880.0000000	0.8500000
11881.0000000	0.8500000
11882.0000000	0.8550000
11883.0000000	0.8500000
11884.0000000	0.8500000
11885.0000000	0.8550000
11886.0000000	0.8550000
11887.0000000	0.8600000
11888.0000000	0.8500000

11889.0000000	0.8600000
11890.0000000	0.8600000
11891.0000000	0.8650000
11892.0000000	0.8600000
11893.0000000	0.8700000
11894.0000000	0.8700000
11895.0000000	0.8750000
11896.0000000	0.8700000
11897.0000000	0.8799999
11898.0000000	0.8800000
11899.0000000	0.8800000
11900.0000000	0.8850000
11901.0000000	0.8900000
11902.0000000	0.8900000
11903.0000000	0.8950000
11904.0000000	0.9000000
11905.0000000	0.9000000
11906.0000000	0.9050000
11907.0000000	0.9150000
11908.0000000	0.9200000
11909.0000000	0.9250000
11910.0000000	0.9300000
11911.0000000	0.9350000
11912.0000000	0.9350000
11913.0000000	0.9450000
11914.0000000	0.9400000
11915.0000000	0.9500000
11916.0000000	0.9550000
11917.0000000	0.9600000
11918.0000000	0.9600000
11919.0000000	0.9700000
11920.0000000	0.9700000
11921.0000000	0.9800000
11922.0000000	0.9800000
11923.0000000	0.9850000
11924.0000000	0.9900000
11925.0000000	0.9900000
11926.0000000	0.9950000
11927.0000000	0.9933333
11928.0000000	0.9950000
11929.0000000	1.0000000
11930.0000000	0.9900000
11931.0000000	0.9900000
11932.0000000	0.9900000
11933.0000000	0.9950000
11934.0000000	0.9900000
11935.0000000	0.9950000
11936.0000000	1.0000000
11937.0000000	1.0000000
11938.0000000	1.0000000
11939.0000000	1.0000000
11940.0000000	1.0050000
11941.0000000	1.0050000
11942.0000000	1.0150000
11943.0000000	1.0150000
11944.0000000	1.0100000
11945.0000000	1.0100000

11946.0000000	1.0250000
11947.0000000	1.0200000
11948.0000000	1.0250000
11949.0000000	1.0300000
11950.0000000	1.0300000
11951.0000000	1.0400000
11952.0000000	1.0350000
11953.0000000	1.0300000
11954.0000000	1.0300000
11955.0000000	1.0350000
11956.0000000	1.0400000
11957.0000000	1.0333333
11958.0000000	1.0350000
11959.0000000	1.0400000
11960.0000000	1.0400000
11961.0000000	1.0300000
11962.0000000	1.0300000
11963.0000000	1.0300000
11964.0000000	1.0250000
11965.0000000	1.0300000
11966.0000000	1.0300000
11967.0000000	1.0300000
11968.0000000	1.0300000
11969.0000000	1.0350000
11970.0000000	1.0300000
11971.0000000	1.0300000
11972.0000000	1.0300000
11973.0000000	1.0300000
11974.0000000	1.0350000
11975.0000000	1.0350000
11976.0000000	1.0300000
11977.0000000	1.0400000
11978.0000000	1.0400000
11979.0000000	1.0450000
11980.0000000	1.0450000
11981.0000000	1.0400000
11982.0000000	1.0450000
11983.0000000	1.0400000
11984.0000000	1.0400000
11985.0000000	1.0400000
11986.0000000	1.0466666
11987.0000000	1.0400000
11988.0000000	1.0500000
11989.0000000	1.0500000
11990.0000000	1.0450000
11991.0000000	1.0500000
11992.0000000	1.0500000
11993.0000000	1.0450000
11994.0000000	1.0500000
11995.0000000	1.0549999
11996.0000000	1.0549999
11997.0000000	1.0599999
11998.0000000	1.0500000
11999.0000000	1.0549999
12000.0000000	1.0599999
12001.0000000	1.0599999
12002.0000000	1.0599999

12003.0000000	1.0599999
12004.0000000	1.0700001
12005.0000000	1.0650001
12006.0000000	1.0650001
12007.0000000	1.0700001
12008.0000000	1.0700001
12009.0000000	1.0750000
12010.0000000	1.0750000
12011.0000000	1.0750000
12012.0000000	1.0800000
12013.0000000	1.0800000
12014.0000000	1.0800000
12015.0000000	1.0900000
12016.0000000	1.0800000
12017.0000000	1.0900000
12018.0000000	1.0900000
12019.0000000	1.0900000
12020.0000000	1.0950000
12021.0000000	1.1000000
12022.0000000	1.1000000
12023.0000000	1.1000000
12024.0000000	1.1000000
12025.0000000	1.0950000
12026.0000000	1.1000000
12027.0000000	1.0950000
12028.0000000	1.0950000
12029.0000000	1.1000000
12030.0000000	1.1000000
12031.0000000	1.1000000
12032.0000000	1.1000000
12033.0000000	1.0950000
12034.0000000	1.0950000
12035.0000000	1.0950000
12036.0000000	1.1000000
12037.0000000	1.0950000
12038.0000000	1.1000000
12039.0000000	1.0950000
12040.0000000	1.1000000
12041.0000000	1.1050000
12042.0000000	1.0950000
12043.0000000	1.1000000
12044.0000000	1.1050000
12045.0000000	1.1100000
12046.0000000	1.1100000
12047.0000000	1.1133333
12048.0000000	1.1200000
12049.0000000	1.1150000
12050.0000000	1.1200000
12051.0000000	1.1200000
12052.0000000	1.1200000
12053.0000000	1.1200000
12054.0000000	1.1100000
12055.0000000	1.1050000
12056.0000000	1.1100000
12057.0000000	1.1000000
12058.0000000	1.1000000
12059.0000000	1.1000000

12060.0000000	1.1050000
12061.0000000	1.1050000
12062.0000000	1.1000000
12063.0000000	1.1050000
12064.0000000	1.1100000
12065.0000000	1.1050000
12066.0000000	1.1100000
12067.0000000	1.1100000
12068.0000000	1.1100000
12069.0000000	1.1200000
12070.0000000	1.1300000
12071.0000000	1.1300000
12072.0000000	1.1300000
12073.0000000	1.1350000
12074.0000000	1.1350000
12075.0000000	1.1350000
12076.0000000	1.1300000
12077.0000000	1.1300000
12078.0000000	1.1400000
12079.0000000	1.1400000
12080.0000000	1.1400000
12081.0000000	1.1450000
12082.0000000	1.1350000
12083.0000000	1.1400000
12084.0000000	1.1400000
12085.0000000	1.1350000
12086.0000000	1.1400000
12087.0000000	1.1400000
12088.0000000	1.1400000
12089.0000000	1.1400000
12090.0000000	1.1400000
12091.0000000	1.1400000
12092.0000000	1.1400000
12093.0000000	1.1500000
12094.0000000	1.1500000
12095.0000000	1.1550000
12096.0000000	1.1600000
12097.0000000	1.1600000
12098.0000000	1.1600000
12099.0000000	1.1600000
12100.0000000	1.1650000
12101.0000000	1.1650000
12102.0000000	1.1700000
12103.0000000	1.1650000
12104.0000000	1.1700000
12105.0000000	1.1650000
12106.0000000	1.1600000
12107.0000000	1.1700000
12108.0000000	1.1700000
12109.0000000	1.1600000
12110.0000000	1.1600000
12111.0000000	1.1600000
12112.0000000	1.1600000
12113.0000000	1.1500000
12114.0000000	1.1500000
12115.0000000	1.1450000
12116.0000000	1.1350000

12117.0000000	1.1300000
12118.0000000	1.1300000
12119.0000000	1.1350000
12120.0000000	1.1400000
12121.0000000	1.1400000
12122.0000000	1.1450000
12123.0000000	1.1400000
12124.0000000	1.1400000
12125.0000000	1.1450000
12126.0000000	1.1550000
12127.0000000	1.1500000
12128.0000000	1.1500000
12129.0000000	1.1500000
12130.0000000	1.1550000
12131.0000000	1.1650000
12132.0000000	1.1650000
12133.0000000	1.1650000
12134.0000000	1.1700000
12135.0000000	1.1750000
12136.0000000	1.1799999
12137.0000000	1.1849999
12138.0000000	1.1966667
12139.0000000	1.2000000
12140.0000000	1.2050000
12141.0000000	1.2050000
12142.0000000	1.2000000
12143.0000000	1.1950001
12144.0000000	1.1950001
12145.0000000	1.1950001
12146.0000000	1.1900001
12147.0000000	1.1900001
12148.0000000	1.1900001
12149.0000000	1.1849999
12150.0000000	1.1799999
12151.0000000	1.1849999
12152.0000000	1.1799999
12153.0000000	1.1849999
12154.0000000	1.1900001
12155.0000000	1.2000000
12156.0000000	1.1950001
12157.0000000	1.2000000
12158.0000000	1.1950001
12159.0000000	1.2000000
12160.0000000	1.1950001
12161.0000000	1.2000000
12162.0000000	1.2000000
12163.0000000	1.2100000
12164.0000000	1.2050000
12165.0000000	1.2100000
12166.0000000	1.2100000
12167.0000000	1.2033334
12168.0000000	1.2100000
12169.0000000	1.2050000
12170.0000000	1.2050000
12171.0000000	1.2000000
12172.0000000	1.2100000
12173.0000000	1.2050000

12174.0000000	1.2100000
12175.0000000	1.2100000
12176.0000000	1.2100000
12177.0000000	1.2150000
12178.0000000	1.2100000
12179.0000000	1.2100000
12180.0000000	1.2100000
12181.0000000	1.2100000
12182.0000000	1.2100000
12183.0000000	1.2150000
12184.0000000	1.2050000
12185.0000000	1.2100000
12186.0000000	1.2100000
12187.0000000	1.2050000
12188.0000000	1.2050000
12189.0000000	1.2050000
12190.0000000	1.2000000
12191.0000000	1.2000000
12192.0000000	1.1950001
12193.0000000	1.1849999
12194.0000000	1.1900001
12195.0000000	1.1799999
12196.0000000	1.1750000
12197.0000000	1.1650000
12198.0000000	1.1550000
12199.0000000	1.1566666
12200.0000000	1.1600000
12201.0000000	1.1500000
12202.0000000	1.1600000
12203.0000000	1.1650000
12204.0000000	1.1550000
12205.0000000	1.1650000
12206.0000000	1.1750000
12207.0000000	1.1750000
12208.0000000	1.1799999
12209.0000000	1.1900001
12210.0000000	1.1849999
12211.0000000	1.1900001
12212.0000000	1.1900001
12213.0000000	1.1900001
12214.0000000	1.1950001
12215.0000000	1.1900001
12216.0000000	1.1950001
12217.0000000	1.1950001
12218.0000000	1.2000000
12219.0000000	1.2000000
12220.0000000	1.2050000
12221.0000000	1.2000000
12222.0000000	1.2100000
12223.0000000	1.2200000
12224.0000000	1.2200000
12225.0000000	1.2350000
12226.0000000	1.2350000
12227.0000000	1.2450000
12228.0000000	1.2466667
12229.0000000	1.2550000
12230.0000000	1.2600000

12231.0000000	1.2600000
12232.0000000	1.2650000
12233.0000000	1.2650000
12234.0000000	1.2700000
12235.0000000	1.2650000
12236.0000000	1.2700000
12237.0000000	1.2700000
12238.0000000	1.2750000
12239.0000000	1.2750000
12240.0000000	1.2700000
12241.0000000	1.2800000
12242.0000000	1.2750000
12243.0000000	1.2750000
12244.0000000	1.2800000
12245.0000000	1.2850000
12246.0000000	1.2800000
12247.0000000	1.2850000
12248.0000000	1.2850000
12249.0000000	1.2850000
12250.0000000	1.2900000
12251.0000000	1.2950000
12252.0000000	1.2900000
12253.0000000	1.3000000
12254.0000000	1.2900000
12255.0000000	1.2850000
12256.0000000	1.2900000
12257.0000000	1.2950000
12258.0000000	1.3000000
12259.0000000	1.3049999
12260.0000000	1.3000000
12261.0000000	1.3049999
12262.0000000	1.3049999
12263.0000000	1.3000000
12264.0000000	1.3049999
12265.0000000	1.3099999
12266.0000000	1.3099999
12267.0000000	1.3049999
12268.0000000	1.3049999
12269.0000000	1.3049999
12270.0000000	1.3049999
12271.0000000	1.3099999
12272.0000000	1.3049999
12273.0000000	1.3099999
12274.0000000	1.3049999
12275.0000000	1.3049999
12276.0000000	1.2950000
12277.0000000	1.2900000
12278.0000000	1.2900000
12279.0000000	1.2800000
12280.0000000	1.2750000
12281.0000000	1.2700000
12282.0000000	1.2600000
12283.0000000	1.2600000
12284.0000000	1.2450000
12285.0000000	1.2400000
12286.0000000	1.2350000
12287.0000000	1.2250000

12288.0000000	1.2300000
12289.0000000	1.2200000
12290.0000000	1.2133334
12291.0000000	1.2100000
12292.0000000	1.2200000
12293.0000000	1.2200000
12294.0000000	1.2200000
12295.0000000	1.2200000
12296.0000000	1.2150000
12297.0000000	1.2150000
12298.0000000	1.2150000
12299.0000000	1.2100000
12300.0000000	1.2100000
12301.0000000	1.2050000
12302.0000000	1.2000000
12303.0000000	1.2000000
12304.0000000	1.2000000
12305.0000000	1.1900001
12306.0000000	1.1950001
12307.0000000	1.1900001
12308.0000000	1.1849999
12309.0000000	1.1849999
12310.0000000	1.1799999
12311.0000000	1.1750000
12312.0000000	1.1799999
12313.0000000	1.1799999
12314.0000000	1.1750000
12315.0000000	1.1849999
12316.0000000	1.1849999
12317.0000000	1.1849999
12318.0000000	1.1799999
12319.0000000	1.1900001
12320.0000000	1.1950001
12321.0000000	1.1966667
12322.0000000	1.1900001
12323.0000000	1.2000000
12324.0000000	1.1950001
12325.0000000	1.2000000
12326.0000000	1.1900001
12327.0000000	1.2000000
12328.0000000	1.2050000
12329.0000000	1.2050000
12330.0000000	1.2000000
12331.0000000	1.2150000
12332.0000000	1.2150000
12333.0000000	1.2150000
12334.0000000	1.2200000
12335.0000000	1.2200000
12336.0000000	1.2200000
12337.0000000	1.2200000
12338.0000000	1.2250000
12339.0000000	1.2250000
12340.0000000	1.2350000
12341.0000000	1.2350000
12342.0000000	1.2400000
12343.0000000	1.2400000
12344.0000000	1.2500000

12345.0000000	1.2550000
12346.0000000	1.2600000
12347.0000000	1.2650000
12348.0000000	1.2700000
12349.0000000	1.2666667
12350.0000000	1.2750000
12351.0000000	1.2700000
12352.0000000	1.2800000
12353.0000000	1.2850000
12354.0000000	1.2800000
12355.0000000	1.2800000
12356.0000000	1.2850000
12357.0000000	1.2750000
12358.0000000	1.2800000
12359.0000000	1.2850000
12360.0000000	1.2850000
12361.0000000	1.2850000
12362.0000000	1.2900000
12363.0000000	1.2900000
12364.0000000	1.2850000
12365.0000000	1.2800000
12366.0000000	1.2800000
12367.0000000	1.2800000
12368.0000000	1.2700000
12369.0000000	1.2750000
12370.0000000	1.2800000
12371.0000000	1.2800000
12372.0000000	1.2850000
12373.0000000	1.2900000
12374.0000000	1.3000000
12375.0000000	1.3000000
12376.0000000	1.3099999
12377.0000000	1.3049999
12378.0000000	1.3099999
12379.0000000	1.3033333
12380.0000000	1.3099999
12381.0000000	1.3000000
12382.0000000	1.3000000
12383.0000000	1.3049999
12384.0000000	1.3099999
12385.0000000	1.3049999
12386.0000000	1.3049999
12387.0000000	1.3049999
12388.0000000	1.2950000
12389.0000000	1.2900000
12390.0000000	1.2900000
12391.0000000	1.2850000
12392.0000000	1.2850000
12393.0000000	1.2900000
12394.0000000	1.2900000
12395.0000000	1.2900000
12396.0000000	1.3000000
12397.0000000	1.3049999
12398.0000000	1.3049999
12399.0000000	1.3099999
12400.0000000	1.3099999
12401.0000000	1.3150001

12402.0000000	1.3099999
12403.0000000	1.3150001
12404.0000000	1.3099999
12405.0000000	1.3099999
12406.0000000	1.3099999
12407.0000000	1.3150001
12408.0000000	1.3150001
12409.0000000	1.3199999
12410.0000000	1.3133334
12411.0000000	1.3200001
12412.0000000	1.3200001
12413.0000000	1.3250000
12414.0000000	1.3200001
12415.0000000	1.3200001
12416.0000000	1.3250000
12417.0000000	1.3250000
12418.0000000	1.3250000
12419.0000000	1.3250000
12420.0000000	1.3250000
12421.0000000	1.3300000
12422.0000000	1.3300000
12423.0000000	1.3300000
12424.0000000	1.3350000
12425.0000000	1.3400000
12426.0000000	1.3400000
12427.0000000	1.3500000
12428.0000000	1.3500000
12429.0000000	1.3550000
12430.0000000	1.3500000
12431.0000000	1.3600000
12432.0000000	1.3600000
12433.0000000	1.3600000
12434.0000000	1.3650000
12435.0000000	1.3600000
12436.0000000	1.3600000
12437.0000000	1.3550000
12438.0000000	1.3600000
12439.0000000	1.3600000
12440.0000000	1.3600000
12441.0000000	1.3550000
12442.0000000	1.3600000
12443.0000000	1.3600000
12444.0000000	1.3600000
12445.0000000	1.3650000
12446.0000000	1.3700000
12447.0000000	1.3650000
12448.0000000	1.3600000
12449.0000000	1.3600000
12450.0000000	1.3650000
12451.0000000	1.3600000
12452.0000000	1.3600000
12453.0000000	1.3600000
12454.0000000	1.3600000
12455.0000000	1.3700000
12456.0000000	1.3700000
12457.0000000	1.3700000
12458.0000000	1.3700000

12459.0000000	1.3750000
12460.0000000	1.3800000
12461.0000000	1.3850000
12462.0000000	1.3900000
12463.0000000	1.3950000
12464.0000000	1.3950000
12465.0000000	1.3950000
12466.0000000	1.4000000
12467.0000000	1.3950000
12468.0000000	1.4000000
12469.0000000	1.4050000
12470.0000000	1.4150000
12471.0000000	1.4200000
12472.0000000	1.4200000
12473.0000000	1.4200000
12474.0000000	1.4200000
12475.0000000	1.4200000
12476.0000000	1.4299999
12477.0000000	1.4299999
12478.0000000	1.4349999
12479.0000000	1.4299999
12480.0000000	1.4349999
12481.0000000	1.4299999
12482.0000000	1.4299999
12483.0000000	1.4299999
12484.0000000	1.4299999
12485.0000000	1.4250000
12486.0000000	1.4299999
12487.0000000	1.4349999
12488.0000000	1.4250000
12489.0000000	1.4299999
12490.0000000	1.4299999
12491.0000000	1.4299999
12492.0000000	1.4299999
12493.0000000	1.4299999
12494.0000000	1.4299999
12495.0000000	1.4250000
12496.0000000	1.4200000
12497.0000000	1.4250000
12498.0000000	1.4100000
12499.0000000	1.4100000
12500.0000000	1.4133333
12501.0000000	1.4150000
12502.0000000	1.4100000
12503.0000000	1.4050000
12504.0000000	1.4100000
12505.0000000	1.4150000
12506.0000000	1.4150000
12507.0000000	1.4200000
12508.0000000	1.4349999
12509.0000000	1.4400001
12510.0000000	1.4500000
12511.0000000	1.4600000
12512.0000000	1.4600000
12513.0000000	1.4600000
12514.0000000	1.4700000
12515.0000000	1.4700000

12516.0000000	1.4750000
12517.0000000	1.4800000
12518.0000000	1.4800000
12519.0000000	1.4850000
12520.0000000	1.4850000
12521.0000000	1.4850000
12522.0000000	1.4900000
12523.0000000	1.4950000
12524.0000000	1.5050000
12525.0000000	1.5050000
12526.0000000	1.5100000
12527.0000000	1.5150000
12528.0000000	1.5100000
12529.0000000	1.5150000
12530.0000000	1.5200000
12531.0000000	1.5233332
12532.0000000	1.5350000
12533.0000000	1.5300000
12534.0000000	1.5400000
12535.0000000	1.5500000
12536.0000000	1.5500000
12537.0000000	1.5549999
12538.0000000	1.5599999
12539.0000000	1.5599999
12540.0000000	1.5650001
12541.0000000	1.5700001
12542.0000000	1.5700001
12543.0000000	1.5750000
12544.0000000	1.5750000
12545.0000000	1.5750000
12546.0000000	1.5750000
12547.0000000	1.5800000
12548.0000000	1.5800000
12549.0000000	1.5800000
12550.0000000	1.5900000
12551.0000000	1.5900000
12552.0000000	1.5900000
12553.0000000	1.5900000
12554.0000000	1.5800000
12555.0000000	1.5850000
12556.0000000	1.5800000
12557.0000000	1.5850000
12558.0000000	1.5800000
12559.0000000	1.5850000
12560.0000000	1.5750000
12561.0000000	1.5700001
12562.0000000	1.5733334
12563.0000000	1.5700001
12564.0000000	1.5750000
12565.0000000	1.5750000
12566.0000000	1.5700001
12567.0000000	1.5650001
12568.0000000	1.5750000
12569.0000000	1.5700001
12570.0000000	1.5700001
12571.0000000	1.5700001
12572.0000000	1.5750000

12573.0000000	1.5700001
12574.0000000	1.5750000
12575.0000000	1.5800000
12576.0000000	1.5850000
12577.0000000	1.5900000
12578.0000000	1.5850000
12579.0000000	1.5850000
12580.0000000	1.5900000
12581.0000000	1.5900000
12582.0000000	1.5950000
12583.0000000	1.5950000
12584.0000000	1.5900000
12585.0000000	1.5950000
12586.0000000	1.5900000
12587.0000000	1.5900000
12588.0000000	1.6000000
12589.0000000	1.6000000
12590.0000000	1.6000000
12591.0000000	1.5950000
12592.0000000	1.6033334
12593.0000000	1.6100000
12594.0000000	1.6000000
12595.0000000	1.6000000
12596.0000000	1.6000000
12597.0000000	1.5900000
12598.0000000	1.5950000
12599.0000000	1.5950000
12600.0000000	1.5900000
12601.0000000	1.5900000
12602.0000000	1.5900000
12603.0000000	1.5850000
12604.0000000	1.5800000
12605.0000000	1.5750000
12606.0000000	1.5750000
12607.0000000	1.5699999
12608.0000000	1.5700001
12609.0000000	1.5699999
12610.0000000	1.5650001
12611.0000000	1.5599999
12612.0000000	1.5549999
12613.0000000	1.5549999
12614.0000000	1.5500000
12615.0000000	1.5450000
12616.0000000	1.5450000
12617.0000000	1.5350000
12618.0000000	1.5350000
12619.0000000	1.5250000
12620.0000000	1.5150000
12621.0000000	1.5150000
12622.0000000	1.5033334
12623.0000000	1.5000000
12624.0000000	1.4850000
12625.0000000	1.4750000
12626.0000000	1.4650000
12627.0000000	1.4450001
12628.0000000	1.4400001
12629.0000000	1.4250000

12630.0000000	1.4150000
12631.0000000	1.4050000
12632.0000000	1.3950000
12633.0000000	1.3900000
12634.0000000	1.3950000
12635.0000000	1.3900000
12636.0000000	1.3850000
12637.0000000	1.3900000
12638.0000000	1.3900000
12639.0000000	1.3900000
12640.0000000	1.4000000
12641.0000000	1.4050000
12642.0000000	1.4100000
12643.0000000	1.4150000
12644.0000000	1.4200000
12645.0000000	1.4299999
12646.0000000	1.4400001
12647.0000000	1.4450001
12648.0000000	1.4600000
12649.0000000	1.4650000
12650.0000000	1.4750000
12651.0000000	1.4750000
12652.0000000	1.4833332
12653.0000000	1.4950000
12654.0000000	1.4950000
12655.0000000	1.5050000
12656.0000000	1.5100000
12657.0000000	1.5150000
12658.0000000	1.5100000
12659.0000000	1.5150000
12660.0000000	1.5250000
12661.0000000	1.5250000
12662.0000000	1.5300000
12663.0000000	1.5450000
12664.0000000	1.5500000
12665.0000000	1.5599999
12666.0000000	1.5700001
12667.0000000	1.5750000
12668.0000000	1.5900000
12669.0000000	1.5900000
12670.0000000	1.6000000
12671.0000000	1.6100000
12672.0000000	1.6100000
12673.0000000	1.6250000
12674.0000000	1.6200000
12675.0000000	1.6350000
12676.0000000	1.6350000
12677.0000000	1.6400000
12678.0000000	1.6400000
12679.0000000	1.6450000
12680.0000000	1.6500000
12681.0000000	1.6550000
12682.0000000	1.6600000
12683.0000000	1.6600000
12684.0000000	1.6733333
12685.0000000	1.6849999
12686.0000000	1.6950001

12687.0000000	1.7050000
12688.0000000	1.7100000
12689.0000000	1.7100000
12690.0000000	1.7150000
12691.0000000	1.7100000
12692.0000000	1.7150000
12693.0000000	1.7250000
12694.0000000	1.7250000
12695.0000000	1.7250000
12696.0000000	1.7300000
12697.0000000	1.7300000
12698.0000000	1.7400000
12699.0000000	1.7400000
12700.0000000	1.7450000
12701.0000000	1.7450000
12702.0000000	1.7450000
12703.0000000	1.7550000
12704.0000000	1.7500000
12705.0000000	1.7500000
12706.0000000	1.7600000
12707.0000000	1.7650000
12708.0000000	1.7650000
12709.0000000	1.7700000
12710.0000000	1.7600000
12711.0000000	1.7600000
12712.0000000	1.7600000
12713.0000000	1.7550000
12714.0000000	1.7533334
12715.0000000	1.7550000
12716.0000000	1.7550000
12717.0000000	1.7550000
12718.0000000	1.7600000
12719.0000000	1.7600000
12720.0000000	1.7600000
12721.0000000	1.7650000
12722.0000000	1.7650000
12723.0000000	1.7650000
12724.0000000	1.7700000
12725.0000000	1.7750000
12726.0000000	1.7700000
12727.0000000	1.7750000
12728.0000000	1.7850000
12729.0000000	1.7800000
12730.0000000	1.7850000
12731.0000000	1.7950000
12732.0000000	1.7900000
12733.0000000	1.7950000
12734.0000000	1.7950000
12735.0000000	1.8000000
12736.0000000	1.8049999
12737.0000000	1.8099999
12738.0000000	1.8250000
12739.0000000	1.8300000
12740.0000000	1.8300000
12741.0000000	1.8350000
12742.0000000	1.8300000
12743.0000000	1.8450000

12744.0000000	1.8500000
12745.0000000	1.8600000
12746.0000000	1.8750000
12747.0000000	1.8800000
12748.0000000	1.8750000
12749.0000000	1.8850000
12750.0000000	1.8850000
12751.0000000	1.8950000
12752.0000000	1.8850000
12753.0000000	1.8950000
12754.0000000	1.8950000
12755.0000000	1.9000000
12756.0000000	1.9100000
12757.0000000	1.9100000
12758.0000000	1.9100000
12759.0000000	1.9200000
12760.0000000	1.9299999
12761.0000000	1.9250000
12762.0000000	1.9300001
12763.0000000	1.9250000
12764.0000000	1.9299999
12765.0000000	1.9299999
12766.0000000	1.9299999
12767.0000000	1.9299999
12768.0000000	1.9349999
12769.0000000	1.9349999
12770.0000000	1.9349999
12771.0000000	1.9400001
12772.0000000	1.9450001
12773.0000000	1.9450001
12774.0000000	1.9433333
12775.0000000	1.9550000
12776.0000000	1.9550000
12777.0000000	1.9600000
12778.0000000	1.9700000
12779.0000000	1.9700000
12780.0000000	1.9800000
12781.0000000	1.9800000
12782.0000000	1.9850000
12783.0000000	1.9800000
12784.0000000	1.9800000
12785.0000000	1.9800000
12786.0000000	1.9800000
12787.0000000	1.9800000
12788.0000000	1.9800000
12789.0000000	1.9800000
12790.0000000	1.9850000
12791.0000000	1.9900000
12792.0000000	1.9800000
12793.0000000	1.9850000
12794.0000000	1.9900000
12795.0000000	1.9900000
12796.0000000	1.9900000
12797.0000000	1.9900000
12798.0000000	1.9950000
12799.0000000	1.9900000
12800.0000000	1.9900000

12801.0000000	1.9900000
12802.0000000	1.9950000
12803.0000000	1.9900000
12804.0000000	2.0000000
12805.0000000	2.0000000
12806.0000000	1.9950000
12807.0000000	1.9900000
12808.0000000	1.9900000
12809.0000000	1.9850000
12810.0000000	1.9850000
12811.0000000	1.9800000
12812.0000000	1.9700000
12813.0000000	1.9500000
12814.0000000	1.9400001
12815.0000000	1.9400001
12816.0000000	1.9349999
12817.0000000	1.9250000
12818.0000000	1.9200000
12819.0000000	1.9200000
12820.0000000	1.9100000
12821.0000000	1.9100000
12822.0000000	1.9100000
12823.0000000	1.9150000
12824.0000000	1.9200000
12825.0000000	1.9150000
12826.0000000	1.9200000
12827.0000000	1.9250000
12828.0000000	1.9250000
12829.0000000	1.9349999
12830.0000000	1.9299999
12831.0000000	1.9450001
12832.0000000	1.9450001
12833.0000000	1.9450001
12834.0000000	1.9666667
12835.0000000	1.9700000
12836.0000000	1.9800000
12837.0000000	1.9800000
12838.0000000	1.9850000
12839.0000000	1.9950000
12840.0000000	2.0050001
12841.0000000	2.0100000
12842.0000000	2.0250001
12843.0000000	2.0250001
12844.0000000	2.0400000
12845.0000000	2.0450001
12846.0000000	2.0599999
12847.0000000	2.0650001
12848.0000000	2.0699999
12849.0000000	2.0850000
12850.0000000	2.0999999
12851.0000000	2.0999999
12852.0000000	2.1199999
12853.0000000	2.1149998
12854.0000000	2.1250000
12855.0000000	2.1250000
12856.0000000	2.1350002
12857.0000000	2.1500001

12858.0000000	2.1500001
12859.0000000	2.1600001
12860.0000000	2.1650000
12861.0000000	2.1750002
12862.0000000	2.1750002
12863.0000000	2.1950002
12864.0000000	2.1900001
12865.0000000	2.1966667
12866.0000000	2.2100000
12867.0000000	2.2049999
12868.0000000	2.2150002
12869.0000000	2.2150002
12870.0000000	2.2200000
12871.0000000	2.2249999
12872.0000000	2.2150002
12873.0000000	2.2200000
12874.0000000	2.2200000
12875.0000000	2.2350001
12876.0000000	2.2300000
12877.0000000	2.2300000
12878.0000000	2.2350001
12879.0000000	2.2350001
12880.0000000	2.2350001
12881.0000000	2.2300000
12882.0000000	2.2249999
12883.0000000	2.2249999
12884.0000000	2.2150002
12885.0000000	2.2249999
12886.0000000	2.2249999
12887.0000000	2.2350001
12888.0000000	2.2350001
12889.0000000	2.2400000
12890.0000000	2.2449999
12891.0000000	2.2449999
12892.0000000	2.2500000
12893.0000000	2.2550001
12894.0000000	2.2600000
12895.0000000	2.2666667
12896.0000000	2.2649999
12897.0000000	2.2700000
12898.0000000	2.2700000
12899.0000000	2.2800000
12900.0000000	2.2800000
12901.0000000	2.2750001
12902.0000000	2.2750001
12903.0000000	2.2750001
12904.0000000	2.2750001
12905.0000000	2.2849998
12906.0000000	2.2800000
12907.0000000	2.2900000
12908.0000000	2.2950001
12909.0000000	2.2900000
12910.0000000	2.3000000
12911.0000000	2.2950001
12912.0000000	2.3000000
12913.0000000	2.3049998
12914.0000000	2.3049998

12915.0000000	2.3150001
12916.0000000	2.3099999
12917.0000000	2.3049998
12918.0000000	2.3049998
12919.0000000	2.3099999
12920.0000000	2.3099999
12921.0000000	2.3150001
12922.0000000	2.3150001
12923.0000000	2.3199999
12924.0000000	2.3199999
12925.0000000	2.3199999
12926.0000000	2.3199999
12927.0000000	2.3199999
12928.0000000	2.3299999
12929.0000000	2.3199999
12930.0000000	2.3099999
12931.0000000	2.3199999
12932.0000000	2.3099999
12933.0000000	2.3049998
12934.0000000	2.2900000
12935.0000000	2.2800000
12936.0000000	2.2649999
12937.0000000	2.2750001
12938.0000000	2.2750001
12939.0000000	2.2700000
12940.0000000	2.2750001
12941.0000000	2.2750001
12942.0000000	2.2849998
12943.0000000	2.2849998
12944.0000000	2.2950001
12945.0000000	2.3000000
12946.0000000	2.2950001
12947.0000000	2.3049998
12948.0000000	2.3000000
12949.0000000	2.3099999
12950.0000000	2.3150001
12951.0000000	2.3249998
12952.0000000	2.3449998
12953.0000000	2.3550000
12954.0000000	2.3599999
12955.0000000	2.3750000
12956.0000000	2.3833334
12957.0000000	2.3900001
12958.0000000	2.3950000
12959.0000000	2.3950000
12960.0000000	2.4050002
12961.0000000	2.4200001
12962.0000000	2.4349999
12963.0000000	2.4450002
12964.0000000	2.4349999
12965.0000000	2.4400001
12966.0000000	2.4400001
12967.0000000	2.4349999
12968.0000000	2.4349999
12969.0000000	2.4349999
12970.0000000	2.4250002
12971.0000000	2.4250002

12972.0000000	2.4200001
12973.0000000	2.4300001
12974.0000000	2.4250002
12975.0000000	2.4300001
12976.0000000	2.4250002
12977.0000000	2.4250002
12978.0000000	2.4150000
12979.0000000	2.4200001
12980.0000000	2.4100001
12981.0000000	2.4150000
12982.0000000	2.4150000
12983.0000000	2.4000001
12984.0000000	2.4000001
12985.0000000	2.3950000
12986.0000000	2.3933334
12987.0000000	2.3950000
12988.0000000	2.3950000
12989.0000000	2.3900001
12990.0000000	2.3900001
12991.0000000	2.3900001
12992.0000000	2.3850002
12993.0000000	2.3850002
12994.0000000	2.3850002
12995.0000000	2.3800001
12996.0000000	2.3800001
12997.0000000	2.3750000
12998.0000000	2.3649998
12999.0000000	2.3649998
13000.0000000	2.3499999
13001.0000000	2.3449998
13002.0000000	2.3399999
13003.0000000	2.3399999
13004.0000000	2.3299999
13005.0000000	2.3299999
13006.0000000	2.3199999
13007.0000000	2.3199999
13008.0000000	2.3099999
13009.0000000	2.3099999
13010.0000000	2.3150001
13011.0000000	2.3049998
13012.0000000	2.3049998
13013.0000000	2.3099999
13014.0000000	2.3049998
13015.0000000	2.2950001
13016.0000000	2.2733333
13017.0000000	2.2500000
13018.0000000	2.2449999
13019.0000000	2.2400000
13020.0000000	2.2350001
13021.0000000	2.2100000
13022.0000000	2.2200000
13023.0000000	2.2049999
13024.0000000	2.2000000
13025.0000000	2.1900001
13026.0000000	2.1900001
13027.0000000	2.1849999
13028.0000000	2.1800001

13029.0000000	2.1800001
13030.0000000	2.1800001
13031.0000000	2.1900001
13032.0000000	2.1849999
13033.0000000	2.1950002
13034.0000000	2.2000000
13035.0000000	2.2049999
13036.0000000	2.2049999
13037.0000000	2.2100000
13038.0000000	2.2200000
13039.0000000	2.2249999
13040.0000000	2.2350001
13041.0000000	2.2300000
13042.0000000	2.2300000
13043.0000000	2.2249999
13044.0000000	2.2249999
13045.0000000	2.2400000
13046.0000000	2.2566669
13047.0000000	2.2649999
13048.0000000	2.2750001
13049.0000000	2.2849998
13050.0000000	2.3000000
13051.0000000	2.3049998
13052.0000000	2.3099999
13053.0000000	2.3150001
13054.0000000	2.3249998
13055.0000000	2.3249998
13056.0000000	2.3350000
13057.0000000	2.3449998
13058.0000000	2.3550000
13059.0000000	2.3599999
13060.0000000	2.3599999
13061.0000000	2.3599999
13062.0000000	2.3649998
13063.0000000	2.3649998
13064.0000000	2.3649998
13065.0000000	2.3800001
13066.0000000	2.3800001
13067.0000000	2.3900001
13068.0000000	2.3800001
13069.0000000	2.3850002
13070.0000000	2.3900001
13071.0000000	2.3900001
13072.0000000	2.4050002
13073.0000000	2.4150000
13074.0000000	2.4050002
13075.0000000	2.4200001
13076.0000000	2.4200001
13077.0000000	2.4150000
13078.0000000	2.4250002
13079.0000000	2.4250002
13080.0000000	2.4150000
13081.0000000	2.4150000
13082.0000000	2.4150000
13083.0000000	2.4150000
13084.0000000	2.4300001
13085.0000000	2.4300001

13086.0000000	2.4400001
13087.0000000	2.4500000
13088.0000000	2.4500000
13089.0000000	2.4700000
13090.0000000	2.4749999
13091.0000000	2.4800000
13092.0000000	2.4949999
13093.0000000	2.4949999
13094.0000000	2.5100000
13095.0000000	2.5149999
13096.0000000	2.5300000
13097.0000000	2.5450001
13098.0000000	2.5549998
13099.0000000	2.5599999
13100.0000000	2.5749998
13101.0000000	2.5749998
13102.0000000	2.5899999
13103.0000000	2.5850000
13104.0000000	2.5899999
13105.0000000	2.5933332
13106.0000000	2.5899999
13107.0000000	2.5949998
13108.0000000	2.6050000
13109.0000000	2.6149998
13110.0000000	2.6149998
13111.0000000	2.6250000
13112.0000000	2.6350002
13113.0000000	2.6300001
13114.0000000	2.6350002
13115.0000000	2.6400001
13116.0000000	2.6400001
13117.0000000	2.6300001
13118.0000000	2.6350002
13119.0000000	2.6350002
13120.0000000	2.6300001
13121.0000000	2.6350002
13122.0000000	2.6350002
13123.0000000	2.6400001
13124.0000000	2.6350002
13125.0000000	2.6500001
13126.0000000	2.6500001
13127.0000000	2.6400001
13128.0000000	2.6500001
13129.0000000	2.6450000
13130.0000000	2.6450000
13131.0000000	2.6450000
13132.0000000	2.6400001
13133.0000000	2.6550002
13134.0000000	2.6500001
13135.0000000	2.6466668
13136.0000000	2.6300001
13137.0000000	2.6300001
13138.0000000	2.6250000
13139.0000000	2.6099999
13140.0000000	2.6099999
13141.0000000	2.6050000
13142.0000000	2.5999999

13143.0000000	2.5949998
13144.0000000	2.5949998
13145.0000000	2.5949998
13146.0000000	2.5999999
13147.0000000	2.5850000
13148.0000000	2.5799999
13149.0000000	2.5749998
13150.0000000	2.5799999
13151.0000000	2.5650001
13152.0000000	2.5799999
13153.0000000	2.5749998
13154.0000000	2.5699999
13155.0000000	2.5650001
13156.0000000	2.5599999
13157.0000000	2.5500000
13158.0000000	2.5500000
13159.0000000	2.5450001
13160.0000000	2.5400000
13161.0000000	2.5300000
13162.0000000	2.5250001
13163.0000000	2.5200000
13164.0000000	2.5100000
13165.0000000	2.5000000
13166.0000000	2.4850001
13167.0000000	2.4800000
13168.0000000	2.4749999
13169.0000000	2.4700000
13170.0000000	2.4650002
13171.0000000	2.4500000
13172.0000000	2.4450002
13173.0000000	2.4400001
13174.0000000	2.4300001
13175.0000000	2.4300001
13176.0000000	2.4349999
13177.0000000	2.4349999
13178.0000000	2.4250002
13179.0000000	2.4250002
13180.0000000	2.4200001
13181.0000000	2.4200001
13182.0000000	2.4150000
13183.0000000	2.4200001
13184.0000000	2.4150000
13185.0000000	2.4150000
13186.0000000	2.4050002
13187.0000000	2.4000001
13188.0000000	2.4000001
13189.0000000	2.3850002
13190.0000000	2.3900001
13191.0000000	2.3750000
13192.0000000	2.3800001
13193.0000000	2.3699999
13194.0000000	2.3633330
13195.0000000	2.3550000
13196.0000000	2.3499999
13197.0000000	2.3399999
13198.0000000	2.3350000
13199.0000000	2.3199999

13200.0000000	2.3199999
13201.0000000	2.3199999
13202.0000000	2.3199999
13203.0000000	2.3199999
13204.0000000	2.3049998
13205.0000000	2.3000000
13206.0000000	2.2950001
13207.0000000	2.2800000
13208.0000000	2.2800000
13209.0000000	2.2649999
13210.0000000	2.2700000
13211.0000000	2.2550001
13212.0000000	2.2600000
13213.0000000	2.2500000
13214.0000000	2.2500000
13215.0000000	2.2500000
13216.0000000	2.2449999
13217.0000000	2.2300000
13218.0000000	2.2300000
13219.0000000	2.2150002
13220.0000000	2.2049999
13221.0000000	2.2000000
13222.0000000	2.1849999
13223.0000000	2.1750002
13224.0000000	2.1750002
13225.0000000	2.1666667
13226.0000000	2.1550002
13227.0000000	2.1500001
13228.0000000	2.1400001
13229.0000000	2.1350002
13230.0000000	2.1400001
13231.0000000	2.1350002
13232.0000000	2.1350002
13233.0000000	2.1250000
13234.0000000	2.1250000
13235.0000000	2.1199999
13236.0000000	2.1050000
13237.0000000	2.1050000
13238.0000000	2.0949998
13239.0000000	2.0899999
13240.0000000	2.0799999
13241.0000000	2.0749998
13242.0000000	2.0699999
13243.0000000	2.0650001
13244.0000000	2.0400000
13245.0000000	2.0300000
13246.0000000	2.0250001
13247.0000000	2.0200000
13248.0000000	2.0050001
13249.0000000	2.0050001
13250.0000000	1.9950000
13251.0000000	1.9850000
13252.0000000	1.9850000
13253.0000000	1.9700000
13254.0000000	1.9600000
13255.0000000	1.9433333
13256.0000000	1.9250000

13257.0000000	1.9150000
13258.0000000	1.9000000
13259.0000000	1.8950000
13260.0000000	1.8800000
13261.0000000	1.8750000
13262.0000000	1.8700000
13263.0000000	1.8550000
13264.0000000	1.8500000
13265.0000000	1.8450000
13266.0000000	1.8400000
13267.0000000	1.8400000
13268.0000000	1.8450000
13269.0000000	1.8550000
13270.0000000	1.8650000
13271.0000000	1.8750000
13272.0000000	1.8750000
13273.0000000	1.8800000
13274.0000000	1.8900000
13275.0000000	1.9000000
13276.0000000	1.9150000
13277.0000000	1.9100000
13278.0000000	1.9050000
13279.0000000	1.9050000
13280.0000000	1.9150000
13281.0000000	1.9000000
13282.0000000	1.9050000
13283.0000000	1.9100000
13284.0000000	1.9100000
13285.0000000	1.9299999
13286.0000000	1.9299999
13287.0000000	1.9450001
13288.0000000	1.9450001
13289.0000000	1.9400001
13290.0000000	1.9400001
13291.0000000	1.9400001
13292.0000000	1.9500000
13293.0000000	1.9500000
13294.0000000	1.9400001
13295.0000000	1.9400001
13296.0000000	1.9450001
13297.0000000	1.9500000
13298.0000000	1.9400001
13299.0000000	1.9349999
13300.0000000	1.9299999
13301.0000000	1.9250000
13302.0000000	1.9200000
13303.0000000	1.9150000
13304.0000000	1.9100000
13305.0000000	1.9000000
13306.0000000	1.8950000
13307.0000000	1.8950000
13308.0000000	1.8850000
13309.0000000	1.8800000
13310.0000000	1.8700000
13311.0000000	1.8650000
13312.0000000	1.8700000
13313.0000000	1.8600000

13314.0000000	1.8550000
13315.0000000	1.8400000
13316.0000000	1.8266667
13317.0000000	1.8150001
13318.0000000	1.8000000
13319.0000000	1.7950000
13320.0000000	1.7850000
13321.0000000	1.7800000
13322.0000000	1.7600000
13323.0000000	1.7650000
13324.0000000	1.7500000
13325.0000000	1.7450000
13326.0000000	1.7250000
13327.0000000	1.7100000
13328.0000000	1.7100000
13329.0000000	1.7150000
13330.0000000	1.7250000
13331.0000000	1.7350000
13332.0000000	1.7450000
13333.0000000	1.7500000
13334.0000000	1.7550000
13335.0000000	1.7550000
13336.0000000	1.7600000
13337.0000000	1.7600000
13338.0000000	1.7650000
13339.0000000	1.7650000
13340.0000000	1.7700000
13341.0000000	1.7650000
13342.0000000	1.7500000
13343.0000000	1.7200000
13344.0000000	1.6950001
13345.0000000	1.6750000
13346.0000000	1.6600000
13347.0000000	1.6700000
13348.0000000	1.6600000
13349.0000000	1.6500000
13350.0000000	1.6500000
13351.0000000	1.6300000
13352.0000000	1.6300000
13353.0000000	1.6100000
13354.0000000	1.6150000
13355.0000000	1.6050000
13356.0000000	1.6000000
13357.0000000	1.5950000
13358.0000000	1.5900000
13359.0000000	1.5850000
13360.0000000	1.5950000
13361.0000000	1.5850000
13362.0000000	1.5900000
13363.0000000	1.5900000
13364.0000000	1.5900000
13365.0000000	1.5950000
13366.0000000	1.5900000
13367.0000000	1.5900000
13368.0000000	1.5950000
13369.0000000	1.5900000
13370.0000000	1.5850000

13371.0000000	1.5850000
13372.0000000	1.5900000
13373.0000000	1.6000000
13374.0000000	1.6050000
13375.0000000	1.6000000
13376.0000000	1.6033334
13377.0000000	1.6000000
13378.0000000	1.5950000
13379.0000000	1.5950000
13380.0000000	1.5850000
13381.0000000	1.5750000
13382.0000000	1.5650001
13383.0000000	1.5599999
13384.0000000	1.5500000
13385.0000000	1.5400000
13386.0000000	1.5200000
13387.0000000	1.5150000
13388.0000000	1.5150000
13389.0000000	1.5000000
13390.0000000	1.4850000
13391.0000000	1.4750000
13392.0000000	1.4650000
13393.0000000	1.4550000
13394.0000000	1.4400001
13395.0000000	1.4300001
13396.0000000	1.4100000
13397.0000000	1.4000000
13398.0000000	1.4000000
13399.0000000	1.4000000
13400.0000000	1.4100000
13401.0000000	1.4150000
13402.0000000	1.4250000
13403.0000000	1.4299999
13404.0000000	1.4400001
13405.0000000	1.4500000
13406.0000000	1.4650000
13407.0000000	1.4699999
13408.0000000	1.4800000
13409.0000000	1.4800000
13410.0000000	1.4850000
13411.0000000	1.4900000
13412.0000000	1.4950000
13413.0000000	1.4950000
13414.0000000	1.5000000
13415.0000000	1.5050000
13416.0000000	1.5100000
13417.0000000	1.5150000
13418.0000000	1.5100000
13419.0000000	1.5100000
13420.0000000	1.5150000
13421.0000000	1.5150000
13422.0000000	1.5200000
13423.0000000	1.5200000
13424.0000000	1.5250000
13425.0000000	1.5300000
13426.0000000	1.5350000
13427.0000000	1.5350000

13428.0000000	1.5450000
13429.0000000	1.5549999
13430.0000000	1.5599999
13431.0000000	1.5599999
13432.0000000	1.5750000
13433.0000000	1.5800000
13434.0000000	1.5750000
13435.0000000	1.5900000
13436.0000000	1.5950000
13437.0000000	1.5900000
13438.0000000	1.6000000
13439.0000000	1.6050000
13440.0000000	1.6050000
13441.0000000	1.6100000
13442.0000000	1.6150000
13443.0000000	1.6100000
13444.0000000	1.6100000
13445.0000000	1.6200000
13446.0000000	1.6200000
13447.0000000	1.6200000
13448.0000000	1.6350000
13449.0000000	1.6300000
13450.0000000	1.6350000
13451.0000000	1.6350000
13452.0000000	1.6400000
13453.0000000	1.6400000
13454.0000000	1.6450000
13455.0000000	1.6500000
13456.0000000	1.6500000
13457.0000000	1.6550000
13458.0000000	1.6650000
13459.0000000	1.6650000
13460.0000000	1.6600000
13461.0000000	1.6700000
13462.0000000	1.6700000
13463.0000000	1.6700000
13464.0000000	1.6750000
13465.0000000	1.6700000
13466.0000000	1.6766666
13467.0000000	1.6900001
13468.0000000	1.6900001
13469.0000000	1.6950001
13470.0000000	1.6950001
13471.0000000	1.6900001
13472.0000000	1.6900001
13473.0000000	1.6950001
13474.0000000	1.6950001
13475.0000000	1.6799999
13476.0000000	1.6799999
13477.0000000	1.6849999
13478.0000000	1.6750000
13479.0000000	1.6849999
13480.0000000	1.6799999
13481.0000000	1.6900001
13482.0000000	1.6799999
13483.0000000	1.6849999
13484.0000000	1.6800001

13485.0000000	1.6750000
13486.0000000	1.6750000
13487.0000000	1.6700000
13488.0000000	1.6650000
13489.0000000	1.6600000
13490.0000000	1.6550000
13491.0000000	1.6550000
13492.0000000	1.6450000
13493.0000000	1.6450000
13494.0000000	1.6300000
13495.0000000	1.6250000
13496.0000000	1.6250000
13497.0000000	1.6100000
13498.0000000	1.5950000
13499.0000000	1.5850000
13500.0000000	1.5650001
13501.0000000	1.5599999
13502.0000000	1.5450000
13503.0000000	1.5350000
13504.0000000	1.5300000
13505.0000000	1.5200000
13506.0000000	1.5200000
13507.0000000	1.5100000
13508.0000000	1.5050000
13509.0000000	1.5050000
13510.0000000	1.5000000
13511.0000000	1.5000000
13512.0000000	1.4950000
13513.0000000	1.4900000
13514.0000000	1.4900000
13515.0000000	1.4900000
13516.0000000	1.4850000
13517.0000000	1.4900000
13518.0000000	1.4850000
13519.0000000	1.4700000
13520.0000000	1.4750000
13521.0000000	1.4750000
13522.0000000	1.4700000
13523.0000000	1.4700000
13524.0000000	1.4650000
13525.0000000	1.4700000
13526.0000000	1.4800000
13527.0000000	1.4700000
13528.0000000	1.4766668
13529.0000000	1.4800000
13530.0000000	1.4750000
13531.0000000	1.4750000
13532.0000000	1.4800000
13533.0000000	1.4750000
13534.0000000	1.4800000
13535.0000000	1.4650000
13536.0000000	1.4600000
13537.0000000	1.4550000
13538.0000000	1.4500000
13539.0000000	1.4349999
13540.0000000	1.4299999
13541.0000000	1.4200000

13542.0000000	1.4050000
13543.0000000	1.3850000
13544.0000000	1.3750000
13545.0000000	1.3600000
13546.0000000	1.3400000
13547.0000000	1.3199999
13548.0000000	1.3049999
13549.0000000	1.2950000
13550.0000000	1.2850000
13551.0000000	1.2900000
13552.0000000	1.2900000
13553.0000000	1.2800000
13554.0000000	1.2800000
13555.0000000	1.2700000
13556.0000000	1.2750000
13557.0000000	1.2700000
13558.0000000	1.2650000
13559.0000000	1.2600000
13560.0000000	1.2550000
13561.0000000	1.2500000
13562.0000000	1.2600000
13563.0000000	1.2600000
13564.0000000	1.2650000
13565.0000000	1.2750000
13566.0000000	1.2700000
13567.0000000	1.2800000
13568.0000000	1.2850000
13569.0000000	1.3000000
13570.0000000	1.3099999
13571.0000000	1.3099999
13572.0000000	1.3200001
13573.0000000	1.3200001
13574.0000000	1.3250000
13575.0000000	1.3199999
13576.0000000	1.3200001
13577.0000000	1.3200001
13578.0000000	1.3200001
13579.0000000	1.3099999
13580.0000000	1.3099999
13581.0000000	1.3049999
13582.0000000	1.2950000
13583.0000000	1.2900000
13584.0000000	1.2850000
13585.0000000	1.2850000
13586.0000000	1.2800000
13587.0000000	1.2750000
13588.0000000	1.2600000
13589.0000000	1.2633333
13590.0000000	1.2550000
13591.0000000	1.2550000
13592.0000000	1.2600000
13593.0000000	1.2500000
13594.0000000	1.2400000
13595.0000000	1.2450000
13596.0000000	1.2450000
13597.0000000	1.2550000
13598.0000000	1.2650000

13599.0000000	1.2650000
13600.0000000	1.2800000
13601.0000000	1.2900000
13602.0000000	1.3000000
13603.0000000	1.3150001
13604.0000000	1.3150001
13605.0000000	1.3200001
13606.0000000	1.3300000
13607.0000000	1.3300000
13608.0000000	1.3350000
13609.0000000	1.3400000
13610.0000000	1.3400000
13611.0000000	1.3300000
13612.0000000	1.3200001
13613.0000000	1.3199999
13614.0000000	1.2950000
13615.0000000	1.2750000
13616.0000000	1.2600000
13617.0000000	1.2550000
13618.0000000	1.2566667
13619.0000000	1.2650000
13620.0000000	1.2600000
13621.0000000	1.2600000
13622.0000000	1.2600000
13623.0000000	1.2600000
13624.0000000	1.2600000
13625.0000000	1.2650000
13626.0000000	1.2700000
13627.0000000	1.2700000
13628.0000000	1.2700000
13629.0000000	1.2700000
13630.0000000	1.2750000
13631.0000000	1.2750000
13632.0000000	1.2750000
13633.0000000	1.2750000
13634.0000000	1.2700000
13635.0000000	1.2700000
13636.0000000	1.2700000
13637.0000000	1.2700000
13638.0000000	1.2750000
13639.0000000	1.2750000
13640.0000000	1.2750000
13641.0000000	1.2750000
13642.0000000	1.2800000
13643.0000000	1.2800000
13644.0000000	1.2800000
13645.0000000	1.2800000
13646.0000000	1.2700000
13647.0000000	1.2750000
13648.0000000	1.2800000
13649.0000000	1.2850000
13650.0000000	1.2900000
13651.0000000	1.2950000
13652.0000000	1.3000000
13653.0000000	1.3099999
13654.0000000	1.3250000
13655.0000000	1.3200001

13656.0000000	1.3350000
13657.0000000	1.3350000
13658.0000000	1.3400000
13659.0000000	1.3500000
13660.0000000	1.3400000
13661.0000000	1.3550000
13662.0000000	1.3550000
13663.0000000	1.3550000
13664.0000000	1.3600000
13665.0000000	1.3550000
13666.0000000	1.3550000
13667.0000000	1.3500000
13668.0000000	1.3400000
13669.0000000	1.3400000
13670.0000000	1.3300000
13671.0000000	1.3250000
13672.0000000	1.3300000
13673.0000000	1.3350000
13674.0000000	1.3350000
13675.0000000	1.3250000
13676.0000000	1.3250000
13677.0000000	1.3250000
13678.0000000	1.3200001
13679.0000000	1.3200001
13680.0000000	1.3066666
13681.0000000	1.3000000
13682.0000000	1.3000000
13683.0000000	1.3000000
13684.0000000	1.2950000
13685.0000000	1.2900000
13686.0000000	1.2950000
13687.0000000	1.2900000
13688.0000000	1.2950000
13689.0000000	1.2950000
13690.0000000	1.2900000
13691.0000000	1.2900000
13692.0000000	1.2900000
13693.0000000	1.2850000
13694.0000000	1.2800000
13695.0000000	1.2700000
13696.0000000	1.2700000
13697.0000000	1.2650000
13698.0000000	1.2700000
13699.0000000	1.2650000
13700.0000000	1.2650000
13701.0000000	1.2600000
13702.0000000	1.2550000
13703.0000000	1.2550000
13704.0000000	1.2450000
13705.0000000	1.2400000
13706.0000000	1.2300000
13707.0000000	1.2300000
13708.0000000	1.2300000
13709.0000000	1.2200000
13710.0000000	1.2200000
13711.0000000	1.2100000
13712.0000000	1.2150000

13713.0000000	1.2100000
13714.0000000	1.2100000
13715.0000000	1.2000000
13716.0000000	1.2050000
13717.0000000	1.2050000
13718.0000000	1.2050000
13719.0000000	1.2050000
13720.0000000	1.2100000
13721.0000000	1.2150000
13722.0000000	1.2100000
13723.0000000	1.2200000
13724.0000000	1.2200000
13725.0000000	1.2200000
13726.0000000	1.2200000
13727.0000000	1.2300000
13728.0000000	1.2300000
13729.0000000	1.2300000
13730.0000000	1.2300000
13731.0000000	1.2300000
13732.0000000	1.2300000
13733.0000000	1.2300000
13734.0000000	1.2400000
13735.0000000	1.2350000
13736.0000000	1.2400000
13737.0000000	1.2400000
13738.0000000	1.2450000
13739.0000000	1.2600000
13740.0000000	1.2650000
13741.0000000	1.2650000
13742.0000000	1.2700000
13743.0000000	1.2700000
13744.0000000	1.2750000
13745.0000000	1.2850000
13746.0000000	1.2800000
13747.0000000	1.2900000
13748.0000000	1.2850000
13749.0000000	1.3000000
13750.0000000	1.3000000
13751.0000000	1.3000000
13752.0000000	1.3099999
13753.0000000	1.3150001
13754.0000000	1.3200001
13755.0000000	1.3300000
13756.0000000	1.3400000
13757.0000000	1.3450000
13758.0000000	1.3550000
13759.0000000	1.3550000
13760.0000000	1.3700000
13761.0000000	1.3750000
13762.0000000	1.3750000
13763.0000000	1.3850000
13764.0000000	1.3950000
13765.0000000	1.3950000
13766.0000000	1.4000000
13767.0000000	1.4100000
13768.0000000	1.4150000
13769.0000000	1.4166666

13770.0000000	1.4200000
13771.0000000	1.4299999
13772.0000000	1.4299999
13773.0000000	1.4349999
13774.0000000	1.4349999
13775.0000000	1.4400001
13776.0000000	1.4450001
13777.0000000	1.4400001
13778.0000000	1.4349999
13779.0000000	1.4299999
13780.0000000	1.4400001
13781.0000000	1.4299999
13782.0000000	1.4400001
13783.0000000	1.4299999
13784.0000000	1.4349999
13785.0000000	1.4299999
13786.0000000	1.4250000
13787.0000000	1.4200000
13788.0000000	1.4300001
13789.0000000	1.4250000
13790.0000000	1.4299999
13791.0000000	1.4349999
13792.0000000	1.4400001
13793.0000000	1.4500000
13794.0000000	1.4500000
13795.0000000	1.4550000
13796.0000000	1.4700000
13797.0000000	1.4650000
13798.0000000	1.4700000
13799.0000000	1.4800000
13800.0000000	1.4900000
13801.0000000	1.4950000
13802.0000000	1.5000000
13803.0000000	1.5050000
13804.0000000	1.5100000
13805.0000000	1.5050000
13806.0000000	1.5100000
13807.0000000	1.5050000
13808.0000000	1.5000000
13809.0000000	1.5000000
13810.0000000	1.4900000
13811.0000000	1.4900000
13812.0000000	1.4700000
13813.0000000	1.4800000
13814.0000000	1.4750000
13815.0000000	1.4800000
13816.0000000	1.4800000
13817.0000000	1.4800000
13818.0000000	1.4850000
13819.0000000	1.4800000
13820.0000000	1.4850000
13821.0000000	1.4850000
13822.0000000	1.4800000
13823.0000000	1.4750000
13824.0000000	1.4750000
13825.0000000	1.4600000
13826.0000000	1.4550000

13827.0000000	1.4550000
13828.0000000	1.4500000
13829.0000000	1.4533334
13830.0000000	1.4600000
13831.0000000	1.4650000
13832.0000000	1.4650000
13833.0000000	1.4700000
13834.0000000	1.4700000
13835.0000000	1.4750000
13836.0000000	1.4800000
13837.0000000	1.4750000
13838.0000000	1.4850000
13839.0000000	1.4800000
13840.0000000	1.4850000
13841.0000000	1.4850000
13842.0000000	1.4800000
13843.0000000	1.4800000
13844.0000000	1.4750000
13845.0000000	1.4750000
13846.0000000	1.4750000
13847.0000000	1.4750000
13848.0000000	1.4650000
13849.0000000	1.4700000
13850.0000000	1.4650000
13851.0000000	1.4600000
13852.0000000	1.4600000
13853.0000000	1.4600000
13854.0000000	1.4600000
13855.0000000	1.4600000
13856.0000000	1.4600000
13857.0000000	1.4600000
13858.0000000	1.4500000
13859.0000000	1.4466667
13860.0000000	1.4500000
13861.0000000	1.4450001
13862.0000000	1.4500000
13863.0000000	1.4500000
13864.0000000	1.4500000
13865.0000000	1.4500000
13866.0000000	1.4500000
13867.0000000	1.4550000
13868.0000000	1.4550000
13869.0000000	1.4600000
13870.0000000	1.4600000
13871.0000000	1.4600000
13872.0000000	1.4600000
13873.0000000	1.4650000
13874.0000000	1.4650000
13875.0000000	1.4600000
13876.0000000	1.4600000
13877.0000000	1.4650000
13878.0000000	1.4650000
13879.0000000	1.4550000
13880.0000000	1.4700000
13881.0000000	1.4600000
13882.0000000	1.4650000
13883.0000000	1.4600000

13884.0000000	1.4600000
13885.0000000	1.4600000
13886.0000000	1.4650000
13887.0000000	1.4650000
13888.0000000	1.4700000
13889.0000000	1.4750000
13890.0000000	1.4850000
13891.0000000	1.4933333
13892.0000000	1.4900000
13893.0000000	1.5050000
13894.0000000	1.5050000
13895.0000000	1.5100000
13896.0000000	1.5050000
13897.0000000	1.5100000
13898.0000000	1.5100000
13899.0000000	1.5100000
13900.0000000	1.5050000
13901.0000000	1.5050000
13902.0000000	1.5050000
13903.0000000	1.5100000
13904.0000000	1.5150000
13905.0000000	1.5150000
13906.0000000	1.5200000
13907.0000000	1.5100000
13908.0000000	1.5150000
13909.0000000	1.5200000
13910.0000000	1.5250000
13911.0000000	1.5200000
13912.0000000	1.5250000
13913.0000000	1.5200000
13914.0000000	1.5250000
13915.0000000	1.5300000
13916.0000000	1.5300000
13917.0000000	1.5300000
13918.0000000	1.5300000
13919.0000000	1.5300000
13920.0000000	1.5450000
13921.0000000	1.5400000
13922.0000000	1.5400000
13923.0000000	1.5400000
13924.0000000	1.5500000
13925.0000000	1.5400000
13926.0000000	1.5450000
13927.0000000	1.5450000
13928.0000000	1.5350000
13929.0000000	1.5400000
13930.0000000	1.5400000
13931.0000000	1.5400000
13932.0000000	1.5450000
13933.0000000	1.5350000
13934.0000000	1.5400000
13935.0000000	1.5400000
13936.0000000	1.5400000
13937.0000000	1.5400000
13938.0000000	1.5300000
13939.0000000	1.5300000
13940.0000000	1.5300000

13941.0000000	1.5300000
13942.0000000	1.5300000
13943.0000000	1.5300000
13944.0000000	1.5300000
13945.0000000	1.5300000
13946.0000000	1.5250000
13947.0000000	1.5250000
13948.0000000	1.5350000
13949.0000000	1.5300000
13950.0000000	1.5350000
13951.0000000	1.5350000
13952.0000000	1.5300001
13953.0000000	1.5200000
13954.0000000	1.5300000
13955.0000000	1.5300000
13956.0000000	1.5300000
13957.0000000	1.5350000
13958.0000000	1.5400000
13959.0000000	1.5400000
13960.0000000	1.5400000
13961.0000000	1.5500000
13962.0000000	1.5500000
13963.0000000	1.5450000
13964.0000000	1.5400000
13965.0000000	1.5500000
13966.0000000	1.5500000
13967.0000000	1.5450000
13968.0000000	1.5400000
13969.0000000	1.5400000
13970.0000000	1.5400000
13971.0000000	1.5400000
13972.0000000	1.5400000
13973.0000000	1.5250000
13974.0000000	1.5250000
13975.0000000	1.5300000
13976.0000000	1.5200000
13977.0000000	1.5200000
13978.0000000	1.5100000
13979.0000000	1.5100000
13980.0000000	1.5100000
13981.0000000	1.5000000
13982.0000000	1.4933333
13983.0000000	1.4850000
13984.0000000	1.4850000
13985.0000000	1.4900000
13986.0000000	1.4800000
13987.0000000	1.4750000
13988.0000000	1.4800000
13989.0000000	1.4650000
13990.0000000	1.4650000
13991.0000000	1.4600000
13992.0000000	1.4650000
13993.0000000	1.4600000
13994.0000000	1.4650000
13995.0000000	1.4600000
13996.0000000	1.4600000
13997.0000000	1.4600000

13998.0000000	1.4600000
13999.0000000	1.4550000
14000.0000000	1.4550000
14001.0000000	1.4500000
14002.0000000	1.4500000
14003.0000000	1.4550000
14004.0000000	1.4600000
14005.0000000	1.4600000
14006.0000000	1.4650000
14007.0000000	1.4600000
14008.0000000	1.4700000
14009.0000000	1.4650000
14010.0000000	1.4600000
14011.0000000	1.4550000
14012.0000000	1.4533334
14013.0000000	1.4400001
14014.0000000	1.4349999
14015.0000000	1.4300001
14016.0000000	1.4299999
14017.0000000	1.4250000
14018.0000000	1.4299999
14019.0000000	1.4250000
14020.0000000	1.4250000
14021.0000000	1.4250000
14022.0000000	1.4300001
14023.0000000	1.4250000
14024.0000000	1.4299999
14025.0000000	1.4299999
14026.0000000	1.4299999
14027.0000000	1.4299999
14028.0000000	1.4299999
14029.0000000	1.4299999
14030.0000000	1.4349999
14031.0000000	1.4299999
14032.0000000	1.4349999
14033.0000000	1.4349999
14034.0000000	1.4349999
14035.0000000	1.4349999
14036.0000000	1.4400001
14037.0000000	1.4400001
14038.0000000	1.4400001
14039.0000000	1.4400001
14040.0000000	1.4400001
14041.0000000	1.4333333
14042.0000000	1.4299999
14043.0000000	1.4299999
14044.0000000	1.4349999
14045.0000000	1.4349999
14046.0000000	1.4299999
14047.0000000	1.4299999
14048.0000000	1.4250000
14049.0000000	1.4250000
14050.0000000	1.4349999
14051.0000000	1.4299999
14052.0000000	1.4200000
14053.0000000	1.4150000
14054.0000000	1.4050000

14055.0000000	1.4000000
14056.0000000	1.3850000
14057.0000000	1.3850000
14058.0000000	1.3700000
14059.0000000	1.3700000
14060.0000000	1.3700000
14061.0000000	1.3650000
14062.0000000	1.3600000
14063.0000000	1.3600000
14064.0000000	1.3550000
14065.0000000	1.3600000
14066.0000000	1.3500000
14067.0000000	1.3500000
14068.0000000	1.3550000
14069.0000000	1.3400000
14070.0000000	1.3400000
14071.0000000	1.3300000
14072.0000000	1.3250000
14073.0000000	1.3233334
14074.0000000	1.3200001
14075.0000000	1.3200001
14076.0000000	1.3200001
14077.0000000	1.3099999
14078.0000000	1.3099999
14079.0000000	1.3099999
14080.0000000	1.3099999
14081.0000000	1.3049999
14082.0000000	1.3049999
14083.0000000	1.3099999
14084.0000000	1.3000000
14085.0000000	1.2950000
14086.0000000	1.2900000
14087.0000000	1.2900000
14088.0000000	1.2800000
14089.0000000	1.2750000
14090.0000000	1.2850000
14091.0000000	1.2850000
14092.0000000	1.2900000
14093.0000000	1.2900000
14094.0000000	1.2950000
14095.0000000	1.2900000
14096.0000000	1.2900000
14097.0000000	1.3000000
14098.0000000	1.3099999
14099.0000000	1.3099999
14100.0000000	1.3099999
14101.0000000	1.3200001
14102.0000000	1.3200001
14103.0000000	1.3200001
14104.0000000	1.3250000
14105.0000000	1.3300000
14106.0000000	1.3350000
14107.0000000	1.3300000
14108.0000000	1.3350000
14109.0000000	1.3250000
14110.0000000	1.3300000
14111.0000000	1.3300000

14112.0000000	1.3350000
14113.0000000	1.3350000
14114.0000000	1.3400000
14115.0000000	1.3350000
14116.0000000	1.3400000
14117.0000000	1.3450000
14118.0000000	1.3500000
14119.0000000	1.3550000
14120.0000000	1.3500000
14121.0000000	1.3550000
14122.0000000	1.3500000
14123.0000000	1.3550000
14124.0000000	1.3400000
14125.0000000	1.3350000
14126.0000000	1.3350000
14127.0000000	1.3300000
14128.0000000	1.3350000
14129.0000000	1.3250000
14130.0000000	1.3300000
14131.0000000	1.3250000
14132.0000000	1.3266667
14133.0000000	1.3200001
14134.0000000	1.3250000
14135.0000000	1.3200001
14136.0000000	1.3200001
14137.0000000	1.3200001
14138.0000000	1.3150001
14139.0000000	1.3150001
14140.0000000	1.3150001
14141.0000000	1.3150001
14142.0000000	1.3150001
14143.0000000	1.3150001
14144.0000000	1.3099999
14145.0000000	1.3099999
14146.0000000	1.3150001
14147.0000000	1.3000000
14148.0000000	1.3099999
14149.0000000	1.3049999
14150.0000000	1.3000000
14151.0000000	1.3049999
14152.0000000	1.3049999
14153.0000000	1.3049999
14154.0000000	1.2900000
14155.0000000	1.2950000
14156.0000000	1.2900000
14157.0000000	1.2800000
14158.0000000	1.2700000
14159.0000000	1.2700000
14160.0000000	1.2650000
14161.0000000	1.2600000
14162.0000000	1.2600000
14163.0000000	1.2500000
14164.0000000	1.2450000
14165.0000000	1.2450000
14166.0000000	1.2450000
14167.0000000	1.2400000
14168.0000000	1.2400000

14169.0000000	1.2400000
14170.0000000	1.2400000
14171.0000000	1.2400000
14172.0000000	1.2350000
14173.0000000	1.2400000
14174.0000000	1.2400000
14175.0000000	1.2300000
14176.0000000	1.2250000
14177.0000000	1.2200000
14178.0000000	1.2150000
14179.0000000	1.2150000
14180.0000000	1.2150000
14181.0000000	1.2100000
14182.0000000	1.2050000
14183.0000000	1.2100000
14184.0000000	1.2000000
14185.0000000	1.2000000
14186.0000000	1.2050000
14187.0000000	1.2050000
14188.0000000	1.2100000
14189.0000000	1.2100000
14190.0000000	1.2150000
14191.0000000	1.2200000
14192.0000000	1.2200000
14193.0000000	1.2150000
14194.0000000	1.2200000
14195.0000000	1.2200000
14196.0000000	1.2150000
14197.0000000	1.2200000
14198.0000000	1.2200000
14199.0000000	1.2250000
14200.0000000	1.2300000
14201.0000000	1.2200000
14202.0000000	1.2250000
14203.0000000	1.2300000
14204.0000000	1.2250000
14205.0000000	1.2200000
14206.0000000	1.2200000
14207.0000000	1.2200000
14208.0000000	1.2150000
14209.0000000	1.2100000
14210.0000000	1.2100000
14211.0000000	1.2100000
14212.0000000	1.2100000
14213.0000000	1.2000000
14214.0000000	1.2050000
14215.0000000	1.1950001
14216.0000000	1.1900001
14217.0000000	1.1849999
14218.0000000	1.1799999
14219.0000000	1.1799999
14220.0000000	1.1700000
14221.0000000	1.1700000
14222.0000000	1.1650000
14223.0000000	1.1650000
14224.0000000	1.1633333
14225.0000000	1.1600000

14226.0000000	1.1550000
14227.0000000	1.1550000
14228.0000000	1.1450000
14229.0000000	1.1450000
14230.0000000	1.1400000
14231.0000000	1.1300000
14232.0000000	1.1350000
14233.0000000	1.1350000
14234.0000000	1.1350000
14235.0000000	1.1350000
14236.0000000	1.1150000
14237.0000000	1.1150000
14238.0000000	1.1050000
14239.0000000	1.1050000
14240.0000000	1.0950000
14241.0000000	1.0950000
14242.0000000	1.0850000
14243.0000000	1.0850000
14244.0000000	1.0850000
14245.0000000	1.0800000
14246.0000000	1.0800000
14247.0000000	1.0700001
14248.0000000	1.0700001
14249.0000000	1.0700001
14250.0000000	1.0599999
14251.0000000	1.0549999
14252.0000000	1.0549999
14253.0000000	1.0500000
14254.0000000	1.0400000
14255.0000000	1.0366666
14256.0000000	1.0350000
14257.0000000	1.0300000
14258.0000000	1.0300000
14259.0000000	1.0250000
14260.0000000	1.0300000
14261.0000000	1.0250000
14262.0000000	1.0300000
14263.0000000	1.0250000
14264.0000000	1.0250000
14265.0000000	1.0300000
14266.0000000	1.0250000
14267.0000000	1.0300000
14268.0000000	1.0400000
14269.0000000	1.0300000
14270.0000000	1.0300000
14271.0000000	1.0300000
14272.0000000	1.0300000
14273.0000000	1.0300000
14274.0000000	1.0350000
14275.0000000	1.0350000
14276.0000000	1.0400000
14277.0000000	1.0400000
14278.0000000	1.0400000
14279.0000000	1.0500000
14280.0000000	1.0500000
14281.0000000	1.0549999
14282.0000000	1.0549999

14283.0000000	1.0599999
14284.0000000	1.0599999
14285.0000000	1.0633334
14286.0000000	1.0700001
14287.0000000	1.0650001
14288.0000000	1.0650001
14289.0000000	1.0700001
14290.0000000	1.0650001
14291.0000000	1.0700001
14292.0000000	1.0700001
14293.0000000	1.0650001
14294.0000000	1.0599999
14295.0000000	1.0599999
14296.0000000	1.0650001
14297.0000000	1.0500000
14298.0000000	1.0450000
14299.0000000	1.0500000
14300.0000000	1.0450000
14301.0000000	1.0400000
14302.0000000	1.0450000
14303.0000000	1.0450000
14304.0000000	1.0400000
14305.0000000	1.0400000
14306.0000000	1.0300000
14307.0000000	1.0300000
14308.0000000	1.0250000
14309.0000000	1.0200000
14310.0000000	1.0150000
14311.0000000	1.0100000
14312.0000000	1.0050000
14313.0000000	0.9900000
14314.0000000	0.9833333
14315.0000000	0.9650000
14316.0000000	0.9600000
14317.0000000	0.9550000
14318.0000000	0.9450000
14319.0000000	0.9350000
14320.0000000	0.9300000
14321.0000000	0.9200000
14322.0000000	0.9200000
14323.0000000	0.9100000
14324.0000000	0.9150000
14325.0000000	0.9200000
14326.0000000	0.9100000
14327.0000000	0.9050000
14328.0000000	0.9050000
14329.0000000	0.8850000
14330.0000000	0.8800000
14331.0000000	0.8650000
14332.0000000	0.8500000
14333.0000000	0.8450000
14334.0000000	0.8500000
14335.0000000	0.8450000
14336.0000000	0.8400000
14337.0000000	0.8450000
14338.0000000	0.8350000
14339.0000000	0.8250000

14340.0000000	0.8300000
14341.0000000	0.8250000
14342.0000000	0.8250000
14343.0000000	0.8200000
14344.0000000	0.8250000
14345.0000000	0.8100000
14346.0000000	0.8000000
14347.0000000	0.7950000
14348.0000000	0.7900000
14349.0000000	0.7900000
14350.0000000	0.7950000
14351.0000000	0.8000000
14352.0000000	0.8000000
14353.0000000	0.8000000
14354.0000000	0.8000000
14355.0000000	0.7950000
14356.0000000	0.8000000
14357.0000000	0.8000000
14358.0000000	0.8050000
14359.0000000	0.7950000
14360.0000000	0.8000000
14361.0000000	0.7900000
14362.0000000	0.7900000
14363.0000000	0.7900000
14364.0000000	0.7950000
14365.0000000	0.7950000
14366.0000000	0.7950000
14367.0000000	0.8000000
14368.0000000	0.8000000
14369.0000000	0.8050000
14370.0000000	0.8000000
14371.0000000	0.8000000
14372.0000000	0.8050000
14373.0000000	0.8000000
14374.0000000	0.7966667
14375.0000000	0.7900000
14376.0000000	0.7900000
14377.0000000	0.7900000
14378.0000000	0.7850000
14379.0000000	0.7750000
14380.0000000	0.7750000
14381.0000000	0.7600000
14382.0000000	0.7600000
14383.0000000	0.7500000
14384.0000000	0.7450000
14385.0000000	0.7400000
14386.0000000	0.7450000
14387.0000000	0.7450000
14388.0000000	0.7450000
14389.0000000	0.7500000
14390.0000000	0.7500000
14391.0000000	0.7500000
14392.0000000	0.7500000
14393.0000000	0.7550000
14394.0000000	0.7550000
14395.0000000	0.7600000
14396.0000000	0.7550000

14397.0000000	0.7600000
14398.0000000	0.7600000
14399.0000000	0.7650000
14400.0000000	0.7600000
14401.0000000	0.7650000
14402.0000000	0.7650000
14403.0000000	0.7600000
14404.0000000	0.7600000
14405.0000000	0.7600000
14406.0000000	0.7550000
14407.0000000	0.7550000
14408.0000000	0.7550000
14409.0000000	0.7600000
14410.0000000	0.7550000
14411.0000000	0.7550000
14412.0000000	0.7550000
14413.0000000	0.7600000
14414.0000000	0.7600000
14415.0000000	0.7550000
14416.0000000	0.7550000
14417.0000000	0.7500000
14418.0000000	0.7500000
14419.0000000	0.7400000
14420.0000000	0.7400000
14421.0000000	0.7400000
14422.0000000	0.7350000
14423.0000000	0.7300000
14424.0000000	0.7250000
14425.0000000	0.7250000
14426.0000000	0.7200000
14427.0000000	0.7200000
14428.0000000	0.7100000
14429.0000000	0.7100000
14430.0000000	0.7100000
14431.0000000	0.7050000
14432.0000000	0.7100000
14433.0000000	0.7000000
14434.0000000	0.7050000
14435.0000000	0.6966667
14436.0000000	0.6950000
14437.0000000	0.6900000
14438.0000000	0.6900000
14439.0000000	0.6900000
14440.0000000	0.6900000
14441.0000000	0.6800000
14442.0000000	0.6850000
14443.0000000	0.6800000
14444.0000000	0.6800000
14445.0000000	0.6800000
14446.0000000	0.6700000
14447.0000000	0.6750000
14448.0000000	0.6700000
14449.0000000	0.6650000
14450.0000000	0.6600000
14451.0000000	0.6550000
14452.0000000	0.6600000
14453.0000000	0.6600000

14454.0000000	0.6550000
14455.0000000	0.6550000
14456.0000000	0.6500000
14457.0000000	0.6500000
14458.0000000	0.6450000
14459.0000000	0.6350000
14460.0000000	0.6300000
14461.0000000	0.6350000
14462.0000000	0.6350000
14463.0000000	0.6300000
14464.0000000	0.6300000
14465.0000000	0.6333333
14466.0000000	0.6300000
14467.0000000	0.6300000
14468.0000000	0.6300000
14469.0000000	0.6350000
14470.0000000	0.6300000
14471.0000000	0.6300000
14472.0000000	0.6250000
14473.0000000	0.6200000
14474.0000000	0.6200000
14475.0000000	0.6200000
14476.0000000	0.6250000
14477.0000000	0.6150000
14478.0000000	0.6150000
14479.0000000	0.6150000
14480.0000000	0.6100000
14481.0000000	0.6100000
14482.0000000	0.6050000
14483.0000000	0.6100000
14484.0000000	0.6100000
14485.0000000	0.6050000
14486.0000000	0.6050000
14487.0000000	0.6000000
14488.0000000	0.5950000
14489.0000000	0.6000000
14490.0000000	0.6000000
14491.0000000	0.6000000
14492.0000000	0.6000000
14493.0000000	0.6000000
14494.0000000	0.5950000
14495.0000000	0.5900000
14496.0000000	0.5900000
14497.0000000	0.5900000
14498.0000000	0.5950000
14499.0000000	0.6000000
14500.0000000	0.6000000
14501.0000000	0.6000000
14502.0000000	0.6050000
14503.0000000	0.6000000
14504.0000000	0.6000000
14505.0000000	0.6000000
14506.0000000	0.6000000
14507.0000000	0.6050000
14508.0000000	0.6050000
14509.0000000	0.6050000
14510.0000000	0.6050000

14511.0000000	0.6100000
14512.0000000	0.6200000
14513.0000000	0.6100000
14514.0000000	0.6150000
14515.0000000	0.6200000
14516.0000000	0.6250000
14517.0000000	0.6250000
14518.0000000	0.6250000
14519.0000000	0.6250000
14520.0000000	0.6300000
14521.0000000	0.6300000
14522.0000000	0.6350000
14523.0000000	0.6350000
14524.0000000	0.6300000
14525.0000000	0.6400000
14526.0000000	0.6400000
14527.0000000	0.6400000
14528.0000000	0.6450000
14529.0000000	0.6450000
14530.0000000	0.6450000
14531.0000000	0.6450000
14532.0000000	0.6450000
14533.0000000	0.6450000
14534.0000000	0.6400000
14535.0000000	0.6450000
14536.0000000	0.6500000
14537.0000000	0.6450000
14538.0000000	0.6400000
14539.0000000	0.6400000
14540.0000000	0.6450000
14541.0000000	0.6400000
14542.0000000	0.6450000
14543.0000000	0.6400000
14544.0000000	0.6400000
14545.0000000	0.6300000
14546.0000000	0.6300000
14547.0000000	0.6300000
14548.0000000	0.6350000
14549.0000000	0.6300000
14550.0000000	0.6300000
14551.0000000	0.6400000
14552.0000000	0.6300000
14553.0000000	0.6300000
14554.0000000	0.6350000
14555.0000000	0.6300000
14556.0000000	0.6200000
14557.0000000	0.6200000
14558.0000000	0.6200000
14559.0000000	0.6150000
14560.0000000	0.6150000
14561.0000000	0.6150000
14562.0000000	0.6100000
14563.0000000	0.6150000
14564.0000000	0.6200000
14565.0000000	0.6100000
14566.0000000	0.6150000
14567.0000000	0.6150000

14568.0000000	0.6150000
14569.0000000	0.6200000
14570.0000000	0.6200000
14571.0000000	0.6250000
14572.0000000	0.6200000
14573.0000000	0.6300000
14574.0000000	0.6300000
14575.0000000	0.6300000
14576.0000000	0.6300000
14577.0000000	0.6350000
14578.0000000	0.6350000
14579.0000000	0.6350000
14580.0000000	0.6400000
14581.0000000	0.6400000
14582.0000000	0.6450000
14583.0000000	0.6400000
14584.0000000	0.6550000
14585.0000000	0.6533333
14586.0000000	0.6500000
14587.0000000	0.6600000
14588.0000000	0.6550000
14589.0000000	0.6600000
14590.0000000	0.6600000
14591.0000000	0.6600000
14592.0000000	0.6650000
14593.0000000	0.6650000
14594.0000000	0.6650000
14595.0000000	0.6650000
14596.0000000	0.6600000
14597.0000000	0.6700000
14598.0000000	0.6650000
14599.0000000	0.6700000
14600.0000000	0.6700000
14601.0000000	0.6700000
14602.0000000	0.6700000
14603.0000000	0.6700000
14604.0000000	0.6700000
14605.0000000	0.6700000
14606.0000000	0.6750000
14607.0000000	0.6950000
14608.0000000	0.7500000
14609.0000000	0.6650000
14610.0000000	0.6650000
14611.0000000	0.6600000
14612.0000000	0.6600000
14613.0000000	0.6600000
14614.0000000	0.6550000
14615.0000000	0.6450000
14616.0000000	0.6366667
14617.0000000	0.6250000
14618.0000000	0.6200000
14619.0000000	0.6100000
14620.0000000	0.6050000
14621.0000000	0.6100000
14622.0000000	0.6000000
14623.0000000	0.6000000
14624.0000000	0.6000000

14625.0000000	0.5950000
14626.0000000	0.5950000
14627.0000000	0.6000000
14628.0000000	0.6000000
14629.0000000	0.5950000
14630.0000000	0.6000000
14631.0000000	0.6050000
14632.0000000	0.6000000
14633.0000000	0.6000000
14634.0000000	0.6000000
14635.0000000	0.6000000
14636.0000000	0.6000000
14637.0000000	0.6000000
14638.0000000	0.6000000
14639.0000000	0.6050000
14640.0000000	0.5950000
14641.0000000	0.6000000
14642.0000000	0.5950000
14643.0000000	0.6000000
14644.0000000	0.6000000
14645.0000000	0.6000000
14646.0000000	0.5966667
14647.0000000	0.5950000
14648.0000000	0.5900000
14649.0000000	0.5900000
14650.0000000	0.5900000
14651.0000000	0.5900000
14652.0000000	0.5850000
14653.0000000	0.5800000
14654.0000000	0.5700000
14655.0000000	0.5700000
14656.0000000	0.5650000
14657.0000000	0.5650000
14658.0000000	0.5600000
14659.0000000	0.5550000
14660.0000000	0.5500000
14661.0000000	0.5450000
14662.0000000	0.5450000
14663.0000000	0.5450000
14664.0000000	0.5350000
14665.0000000	0.5350000
14666.0000000	0.5250000
14667.0000000	0.5200000
14668.0000000	0.5150000
14669.0000000	0.5100000
14670.0000000	0.5050000
14671.0000000	0.5000000
14672.0000000	0.4950000
14673.0000000	0.4900000
14674.0000000	0.4900000
14675.0000000	0.4850000
14676.0000000	0.4766667
14677.0000000	0.4700000
14678.0000000	0.4700000
14679.0000000	0.4700000
14680.0000000	0.4600000
14681.0000000	0.4650000

14682.0000000	0.4600000
14683.0000000	0.4550000
14684.0000000	0.4500000
14685.0000000	0.4450000
14686.0000000	0.4450000
14687.0000000	0.4400000
14688.0000000	0.4400000
14689.0000000	0.4400000
14690.0000000	0.4300000
14691.0000000	0.4350000
14692.0000000	0.4300000
14693.0000000	0.4300000
14694.0000000	0.4300000
14695.0000000	0.4300000
14696.0000000	0.4350000
14697.0000000	0.4250000
14698.0000000	0.4200000
14699.0000000	0.4200000
14700.0000000	0.4200000
14701.0000000	0.4050000
14702.0000000	0.4050000
14703.0000000	0.4000000
14704.0000000	0.4000000
14705.0000000	0.4000000
14706.0000000	0.4000000
14707.0000000	0.3933333
14708.0000000	0.3900000
14709.0000000	0.3850000
14710.0000000	0.3850000
14711.0000000	0.3850000
14712.0000000	0.3750000
14713.0000000	0.3700000
14714.0000000	0.3600000
14715.0000000	0.3600000
14716.0000000	0.3600000
14717.0000000	0.3550000
14718.0000000	0.3500000
14719.0000000	0.3500000
14720.0000000	0.3450000
14721.0000000	0.3400000
14722.0000000	0.3350000
14723.0000000	0.3400000
14724.0000000	0.3400000
14725.0000000	0.3300000
14726.0000000	0.3350000
14727.0000000	0.3300000
14728.0000000	0.3200000
14729.0000000	0.3250000
14730.0000000	0.3250000
14731.0000000	0.3200000
14732.0000000	0.3200000
14733.0000000	0.3250000
14734.0000000	0.3200000
14735.0000000	0.3200000
14736.0000000	0.3200000
14737.0000000	0.3133333
14738.0000000	0.3100000

14739.0000000	0.3100000
14740.0000000	0.3150000
14741.0000000	0.3100000
14742.0000000	0.3100000
14743.0000000	0.3100000
14744.0000000	0.3100000
14745.0000000	0.3100000
14746.0000000	0.3100000
14747.0000000	0.3050000
14748.0000000	0.3000000
14749.0000000	0.3000000
14750.0000000	0.3000000
14751.0000000	0.2900000
14752.0000000	0.2950000
14753.0000000	0.2950000
14754.0000000	0.2900000
14755.0000000	0.2950000
14756.0000000	0.2950000
14757.0000000	0.2900000
14758.0000000	0.2900000
14759.0000000	0.2900000
14760.0000000	0.2800000
14761.0000000	0.2750000
14762.0000000	0.2750000
14763.0000000	0.2700000
14764.0000000	0.2700000
14765.0000000	0.2600000
14766.0000000	0.2633333
14767.0000000	0.2500000
14768.0000000	0.2450000
14769.0000000	0.2400000
14770.0000000	0.2350000
14771.0000000	0.2300000
14772.0000000	0.2300000
14773.0000000	0.2200000
14774.0000000	0.2150000
14775.0000000	0.2100000
14776.0000000	0.2100000
14777.0000000	0.2000000
14778.0000000	0.1950000
14779.0000000	0.1900000
14780.0000000	0.1850000
14781.0000000	0.1800000
14782.0000000	0.1800000
14783.0000000	0.1750000
14784.0000000	0.1700000
14785.0000000	0.1650000
14786.0000000	0.1600000
14787.0000000	0.1500000
14788.0000000	0.1500000
14789.0000000	0.1450000
14790.0000000	0.1400000
14791.0000000	0.1450000
14792.0000000	0.1400000
14793.0000000	0.1400000
14794.0000000	0.1400000
14795.0000000	0.1400000

14796.0000000	0.1400000
14797.0000000	0.1350000
14798.0000000	0.1366667
14799.0000000	0.1400000
14800.0000000	0.1400000
14801.0000000	0.1400000
14802.0000000	0.1400000
14803.0000000	0.1400000
14804.0000000	0.1400000
14805.0000000	0.1450000
14806.0000000	0.1400000
14807.0000000	0.1400000
14808.0000000	0.1400000
14809.0000000	0.1450000
14810.0000000	0.1450000
14811.0000000	0.1500000
14812.0000000	0.1450000
14813.0000000	0.1450000
14814.0000000	0.1500000
14815.0000000	0.1400000
14816.0000000	0.1450000
14817.0000000	0.1450000
14818.0000000	0.1400000
14819.0000000	0.1400000
14820.0000000	0.1450000
14821.0000000	0.1400000
14822.0000000	0.1400000
14823.0000000	0.1350000
14824.0000000	0.1350000
14825.0000000	0.1300000
14826.0000000	0.1300000
14827.0000000	0.1333333
14828.0000000	0.1300000
14829.0000000	0.1300000
14830.0000000	0.1200000
14831.0000000	0.1200000
14832.0000000	0.1150000
14833.0000000	0.1150000
14834.0000000	0.1100000
14835.0000000	0.1100000
14836.0000000	0.1050000
14837.0000000	0.1000000
14838.0000000	0.0950000
14839.0000000	0.0900000
14840.0000000	0.0800000
14841.0000000	0.0750000
14842.0000000	0.0750000
14843.0000000	0.0600000
14844.0000000	0.0600000
14845.0000000	0.0500000
14846.0000000	0.0450000
14847.0000000	0.0400000
14848.0000000	0.0350000
14849.0000000	0.0300000
14850.0000000	0.0200000
14851.0000000	0.0150000
14852.0000000	0.0050000

14853.0000000	0.0000000
14854.0000000	-0.0050000
14855.0000000	-0.0100000
14856.0000000	-0.0200000
14857.0000000	-0.0300000
14858.0000000	-0.0333333
14859.0000000	-0.0350000
14860.0000000	-0.0450000
14861.0000000	-0.0500000
14862.0000000	-0.0550000
14863.0000000	-0.0600000
14864.0000000	-0.0650000
14865.0000000	-0.0700000
14866.0000000	-0.0800000
14867.0000000	-0.0800000
14868.0000000	-0.0900000
14869.0000000	-0.0950000
14870.0000000	-0.0950000
14871.0000000	-0.1000000
14872.0000000	-0.1100000
14873.0000000	-0.1150000
14874.0000000	-0.1200000
14875.0000000	-0.1200000
14876.0000000	-0.1200000
14877.0000000	-0.1250000
14878.0000000	-0.1350000
14879.0000000	-0.1350000
14880.0000000	-0.1400000
14881.0000000	-0.1400000
14882.0000000	-0.1400000
14883.0000000	-0.1400000
14884.0000000	-0.1400000
14885.0000000	-0.1500000
14886.0000000	-0.1600000
14887.0000000	-0.1600000
14888.0000000	-0.1650000
14889.0000000	-0.1650000
14890.0000000	-0.1650000
14891.0000000	-0.1700000
14892.0000000	-0.1700000
14893.0000000	-0.1700000
14894.0000000	-0.1750000
14895.0000000	-0.1800000
14896.0000000	-0.1800000
14897.0000000	-0.1800000
14898.0000000	-0.1850000
14899.0000000	-0.1900000
14900.0000000	-0.1900000
14901.0000000	-0.1900000
14902.0000000	-0.1950000
14903.0000000	-0.1950000
14904.0000000	-0.1950000
14905.0000000	-0.2000000
14906.0000000	-0.2000000
14907.0000000	-0.2000000
14908.0000000	-0.2100000
14909.0000000	-0.2150000

14910.0000000	-0.2150000
14911.0000000	-0.2200000
14912.0000000	-0.2250000
14913.0000000	-0.2200000
14914.0000000	-0.2200000
14915.0000000	-0.2250000
14916.0000000	-0.2250000
14917.0000000	-0.2250000
14918.0000000	-0.2200000
14919.0000000	-0.2266667
14920.0000000	-0.2250000
14921.0000000	-0.2200000
14922.0000000	-0.2300000
14923.0000000	-0.2300000
14924.0000000	-0.2250000
14925.0000000	-0.2300000
14926.0000000	-0.2300000
14927.0000000	-0.2300000
14928.0000000	-0.2350000
14929.0000000	-0.2400000
14930.0000000	-0.2450000
14931.0000000	-0.2450000
14932.0000000	-0.2500000
14933.0000000	-0.2500000
14934.0000000	-0.2550000
14935.0000000	-0.2600000
14936.0000000	-0.2600000
14937.0000000	-0.2600000
14938.0000000	-0.2600000
14939.0000000	-0.2600000
14940.0000000	-0.2600000
14941.0000000	-0.2650000
14942.0000000	-0.2650000
14943.0000000	-0.2700000
14944.0000000	-0.2750000
14945.0000000	-0.2750000
14946.0000000	-0.2750000
14947.0000000	-0.2766667
14948.0000000	-0.2800000
14949.0000000	-0.2850000
14950.0000000	-0.2850000
14951.0000000	-0.2850000
14952.0000000	-0.2900000
14953.0000000	-0.2900000
14954.0000000	-0.2950000
14955.0000000	-0.3000000
14956.0000000	-0.2950000
14957.0000000	-0.3100000
14958.0000000	-0.3000000
14959.0000000	-0.3050000
14960.0000000	-0.3050000
14961.0000000	-0.3100000
14962.0000000	-0.3050000
14963.0000000	-0.3050000
14964.0000000	-0.3100000
14965.0000000	-0.3100000
14966.0000000	-0.3100000

14967.0000000	-0.3100000
14968.0000000	-0.3150000
14969.0000000	-0.3200000
14970.0000000	-0.3200000
14971.0000000	-0.3250000
14972.0000000	-0.3200000
14973.0000000	-0.3200000
14974.0000000	-0.3200000
14975.0000000	-0.3250000
14976.0000000	-0.3250000
14977.0000000	-0.3300000
14978.0000000	-0.3300000
14979.0000000	-0.3333333
14980.0000000	-0.3300000
14981.0000000	-0.3350000
14982.0000000	-0.3350000
14983.0000000	-0.3350000
14984.0000000	-0.3350000
14985.0000000	-0.3400000
14986.0000000	-0.3400000
14987.0000000	-0.3400000
14988.0000000	-0.3400000
14989.0000000	-0.3450000
14990.0000000	-0.3450000
14991.0000000	-0.3400000
14992.0000000	-0.3350000
14993.0000000	-0.3400000
14994.0000000	-0.3400000
14995.0000000	-0.3350000
14996.0000000	-0.3350000
14997.0000000	-0.3350000
14998.0000000	-0.3400000
14999.0000000	-0.3400000
15000.0000000	-0.3400000
15001.0000000	-0.3400000
15002.0000000	-0.3400000
15003.0000000	-0.3450000
15004.0000000	-0.3400000
15005.0000000	-0.3500000
15006.0000000	-0.3500000
15007.0000000	-0.3450000
15008.0000000	-0.3550000
15009.0000000	-0.3566667
15010.0000000	-0.3500000
15011.0000000	-0.3550000
15012.0000000	-0.3550000
15013.0000000	-0.3600000
15014.0000000	-0.3500000
15015.0000000	-0.3600000
15016.0000000	-0.3550000
15017.0000000	-0.3550000
15018.0000000	-0.3600000
15019.0000000	-0.3550000
15020.0000000	-0.3500000
15021.0000000	-0.3600000
15022.0000000	-0.3550000
15023.0000000	-0.3550000

15024.0000000	-0.3600000
15025.0000000	-0.3600000
15026.0000000	-0.3600000
15027.0000000	-0.3650000
15028.0000000	-0.3550000
15029.0000000	-0.3650000
15030.0000000	-0.3600000
15031.0000000	-0.3600000
15032.0000000	-0.3650000
15033.0000000	-0.3700000
15034.0000000	-0.3650000
15035.0000000	-0.3700000
15036.0000000	-0.3650000
15037.0000000	-0.3700000
15038.0000000	-0.3650000
15039.0000000	-0.3700000
15040.0000000	-0.3700000
15041.0000000	-0.3700000
15042.0000000	-0.3700000
15043.0000000	-0.3650000
15044.0000000	-0.3700000
15045.0000000	-0.3700000
15046.0000000	-0.3650000
15047.0000000	-0.3650000
15048.0000000	-0.3700000
15049.0000000	-0.3650000
15050.0000000	-0.3700000
15051.0000000	-0.3700000
15052.0000000	-0.3750000
15053.0000000	-0.3750000
15054.0000000	-0.3700000
15055.0000000	-0.3800000
15056.0000000	-0.3750000
15057.0000000	-0.3750000
15058.0000000	-0.3750000
15059.0000000	-0.3800000
15060.0000000	-0.3750000
15061.0000000	-0.3750000
15062.0000000	-0.3750000
15063.0000000	-0.3750000
15064.0000000	-0.3750000
15065.0000000	-0.3700000
15066.0000000	-0.3700000
15067.0000000	-0.3700000
15068.0000000	-0.3750000
15069.0000000	-0.3700000
15070.0000000	-0.3733333
15071.0000000	-0.3700000
15072.0000000	-0.3700000
15073.0000000	-0.3650000
15074.0000000	-0.3700000
15075.0000000	-0.3750000
15076.0000000	-0.3700000
15077.0000000	-0.3650000
15078.0000000	-0.3750000
15079.0000000	-0.3700000
15080.0000000	-0.3700000

15081.0000000	-0.3700000
15082.0000000	-0.3650000
15083.0000000	-0.3700000
15084.0000000	-0.3700000
15085.0000000	-0.3700000
15086.0000000	-0.3700000
15087.0000000	-0.3650000
15088.0000000	-0.3650000
15089.0000000	-0.3700000
15090.0000000	-0.3650000
15091.0000000	-0.3600000
15092.0000000	-0.3700000
15093.0000000	-0.3650000
15094.0000000	-0.3700000
15095.0000000	-0.3650000
15096.0000000	-0.3700000
15097.0000000	-0.3750000
15098.0000000	-0.3700000
15099.0000000	-0.3750000
15100.0000000	-0.3766667
15101.0000000	-0.3750000
15102.0000000	-0.3750000
15103.0000000	-0.3750000
15104.0000000	-0.3800000
15105.0000000	-0.3700000
15106.0000000	-0.3750000
15107.0000000	-0.3800000
15108.0000000	-0.3750000
15109.0000000	-0.3800000
15110.0000000	-0.3750000
15111.0000000	-0.3800000
15112.0000000	-0.3750000
15113.0000000	-0.3800000
15114.0000000	-0.3800000
15115.0000000	-0.3800000
15116.0000000	-0.3850000
15117.0000000	-0.3800000
15118.0000000	-0.3850000
15119.0000000	-0.3900000
15120.0000000	-0.3850000
15121.0000000	-0.3850000
15122.0000000	-0.3850000
15123.0000000	-0.3900000
15124.0000000	-0.3850000
15125.0000000	-0.3850000
15126.0000000	-0.3900000
15127.0000000	-0.3900000
15128.0000000	-0.3900000
15129.0000000	-0.3850000
15130.0000000	-0.3900000
15131.0000000	-0.3866667
15132.0000000	-0.3800000
15133.0000000	-0.3900000
15134.0000000	-0.3900000
15135.0000000	-0.3900000
15136.0000000	-0.3800000
15137.0000000	-0.3850000

15138.0000000	-0.3850000
15139.0000000	-0.3850000
15140.0000000	-0.3850000
15141.0000000	-0.3900000
15142.0000000	-0.3900000
15143.0000000	-0.3850000
15144.0000000	-0.3950000
15145.0000000	-0.3950000
15146.0000000	-0.3900000
15147.0000000	-0.3950000
15148.0000000	-0.3950000
15149.0000000	-0.3950000
15150.0000000	-0.3950000
15151.0000000	-0.3950000
15152.0000000	-0.3900000
15153.0000000	-0.3950000
15154.0000000	-0.3900000
15155.0000000	-0.3900000
15156.0000000	-0.3950000
15157.0000000	-0.3900000
15158.0000000	-0.3900000
15159.0000000	-0.3850000
15160.0000000	-0.3900000
15161.0000000	-0.3900000
15162.0000000	-0.3900000
15163.0000000	-0.3850000
15164.0000000	-0.3800000
15165.0000000	-0.3850000
15166.0000000	-0.3850000
15167.0000000	-0.3900000
15168.0000000	-0.3850000
15169.0000000	-0.3850000
15170.0000000	-0.3850000
15171.0000000	-0.3850000
15172.0000000	-0.3850000
15173.0000000	-0.3800000
15174.0000000	-0.3850000
15175.0000000	-0.3850000
15176.0000000	-0.3800000
15177.0000000	-0.3800000
15178.0000000	-0.3800000
15179.0000000	-0.3850000
15180.0000000	-0.3850000
15181.0000000	-0.3800000
15182.0000000	-0.3800000
15183.0000000	-0.3750000
15184.0000000	-0.3750000
15185.0000000	-0.3800000
15186.0000000	-0.3800000
15187.0000000	-0.3850000
15188.0000000	-0.3800000
15189.0000000	-0.3850000
15190.0000000	-0.3800000
15191.0000000	-0.3833333
15192.0000000	-0.3750000
15193.0000000	-0.3850000
15194.0000000	-0.3800000

15195.0000000	-0.3800000
15196.0000000	-0.3800000
15197.0000000	-0.3800000
15198.0000000	-0.3800000
15199.0000000	-0.3850000
15200.0000000	-0.3750000
15201.0000000	-0.3800000
15202.0000000	-0.3800000
15203.0000000	-0.3750000
15204.0000000	-0.3800000
15205.0000000	-0.3750000
15206.0000000	-0.3800000
15207.0000000	-0.3700000
15208.0000000	-0.3850000
15209.0000000	-0.3750000
15210.0000000	-0.3800000
15211.0000000	-0.3800000
15212.0000000	-0.3750000
15213.0000000	-0.3750000
15214.0000000	-0.3800000
15215.0000000	-0.3700000
15216.0000000	-0.3800000
15217.0000000	-0.3750000
15218.0000000	-0.3800000
15219.0000000	-0.3750000
15220.0000000	-0.3800000
15221.0000000	-0.3733333
15222.0000000	-0.3800000
15223.0000000	-0.3750000
15224.0000000	-0.3750000
15225.0000000	-0.3750000
15226.0000000	-0.3800000
15227.0000000	-0.3800000
15228.0000000	-0.3850000
15229.0000000	-0.3800000
15230.0000000	-0.3850000
15231.0000000	-0.3850000
15232.0000000	-0.3850000
15233.0000000	-0.3850000
15234.0000000	-0.3900000
15235.0000000	-0.3900000
15236.0000000	-0.3900000
15237.0000000	-0.3850000
15238.0000000	-0.3950000
15239.0000000	-0.3950000
15240.0000000	-0.3950000
15241.0000000	-0.3950000
15242.0000000	-0.4000000
15243.0000000	-0.3950000
15244.0000000	-0.3950000
15245.0000000	-0.4000000
15246.0000000	-0.4000000
15247.0000000	-0.3950000
15248.0000000	-0.3950000
15249.0000000	-0.4000000
15250.0000000	-0.4050000
15251.0000000	-0.4000000

15252.0000000	-0.4050000
15253.0000000	-0.4050000
15254.0000000	-0.4000000
15255.0000000	-0.4050000
15256.0000000	-0.4050000
15257.0000000	-0.4050000
15258.0000000	-0.4150000
15259.0000000	-0.4100000
15260.0000000	-0.4150000
15261.0000000	-0.4150000
15262.0000000	-0.4150000
15263.0000000	-0.4150000
15264.0000000	-0.4150000
15265.0000000	-0.4200000
15266.0000000	-0.4200000
15267.0000000	-0.4200000
15268.0000000	-0.4250000
15269.0000000	-0.4200000
15270.0000000	-0.4200000
15271.0000000	-0.4250000
15272.0000000	-0.4250000
15273.0000000	-0.4300000
15274.0000000	-0.4250000
15275.0000000	-0.4250000
15276.0000000	-0.4300000
15277.0000000	-0.4300000
15278.0000000	-0.4350000
15279.0000000	-0.4350000
15280.0000000	-0.4400000
15281.0000000	-0.4400000
15282.0000000	-0.4400000
15283.0000000	-0.4400000
15284.0000000	-0.4450000
15285.0000000	-0.4500000
15286.0000000	-0.4500000
15287.0000000	-0.4500000
15288.0000000	-0.4500000
15289.0000000	-0.4500000
15290.0000000	-0.4600000
15291.0000000	-0.4550000
15292.0000000	-0.4600000
15293.0000000	-0.4600000
15294.0000000	-0.4550000
15295.0000000	-0.4600000
15296.0000000	-0.4650000
15297.0000000	-0.4600000
15298.0000000	-0.4750000
15299.0000000	-0.4700000
15300.0000000	-0.4750000
15301.0000000	-0.4750000
15302.0000000	-0.4800000
15303.0000000	-0.4750000
15304.0000000	-0.4850000
15305.0000000	-0.4800000
15306.0000000	-0.4850000
15307.0000000	-0.4850000
15308.0000000	-0.4850000

15309.0000000	-0.4850000
15310.0000000	-0.4850000
15311.0000000	-0.4866667
15312.0000000	-0.4850000
15313.0000000	-0.4900000
15314.0000000	-0.4950000
15315.0000000	-0.5000000
15316.0000000	-0.5000000
15317.0000000	-0.5050000
15318.0000000	-0.5050000
15319.0000000	-0.5050000
15320.0000000	-0.5100000
15321.0000000	-0.5000000
15322.0000000	-0.5050000
15323.0000000	-0.5100000
15324.0000000	-0.5100000
15325.0000000	-0.5200000
15326.0000000	-0.5200000
15327.0000000	-0.5200000
15328.0000000	-0.5200000
15329.0000000	-0.5200000
15330.0000000	-0.5200000
15331.0000000	-0.5200000
15332.0000000	-0.5250000
15333.0000000	-0.5300000
15334.0000000	-0.5300000
15335.0000000	-0.5300000
15336.0000000	-0.5300000
15337.0000000	-0.5350000
15338.0000000	-0.5300000
15339.0000000	-0.5350000
15340.0000000	-0.5350000
15341.0000000	-0.5400000
15342.0000000	-0.5433334
15343.0000000	-0.5400000
15344.0000000	-0.5400000
15345.0000000	-0.5400000
15346.0000000	-0.5400000
15347.0000000	-0.5450000
15348.0000000	-0.5450000
15349.0000000	-0.5500000
15350.0000000	-0.5550000
15351.0000000	-0.5500000
15352.0000000	-0.5550000
15353.0000000	-0.5550000
15354.0000000	-0.5550000
15355.0000000	-0.5650000
15356.0000000	-0.5600000
15357.0000000	-0.5700000
15358.0000000	-0.5700000
15359.0000000	-0.5700000
15360.0000000	-0.5700000
15361.0000000	-0.5750000
15362.0000000	-0.5800000
15363.0000000	-0.5750000
15364.0000000	-0.5800000
15365.0000000	-0.5800000

15366.0000000	-0.5800000
15367.0000000	-0.5900000
15368.0000000	-0.5950000
15369.0000000	-0.5950000
15370.0000000	-0.5950000
15371.0000000	-0.5966666
15372.0000000	-0.6050000
15373.0000000	-0.6000000
15374.0000000	-0.6100000
15375.0000000	-0.6100000
15376.0000000	-0.6050000
15377.0000000	-0.6150000
15378.0000000	-0.6100000
15379.0000000	-0.6150000
15380.0000000	-0.6150000
15381.0000000	-0.6150000
15382.0000000	-0.6150000
15383.0000000	-0.6200000
15384.0000000	-0.6250000
15385.0000000	-0.6200000
15386.0000000	-0.6250000
15387.0000000	-0.6250000
15388.0000000	-0.6250000
15389.0000000	-0.6300000
15390.0000000	-0.6300000
15391.0000000	-0.6300000
15392.0000000	-0.6350000
15393.0000000	-0.6400000
15394.0000000	-0.6450000
15395.0000000	-0.6400000
15396.0000000	-0.6400000
15397.0000000	-0.6450000
15398.0000000	-0.6450000
15399.0000000	-0.6500000
15400.0000000	-0.6600000
15401.0000000	-0.6600000
15402.0000000	-0.6650000
15403.0000000	-0.6600000
15404.0000000	-0.6600000
15405.0000000	-0.6550000
15406.0000000	-0.6550000
15407.0000000	-0.6600000
15408.0000000	-0.6700000
15409.0000000	-0.6600000
15410.0000000	-0.6650000
15411.0000000	-0.6650000
15412.0000000	-0.6600000
15413.0000000	-0.6600000
15414.0000000	-0.6600000
15415.0000000	-0.6650000
15416.0000000	-0.6600000
15417.0000000	-0.6650000
15418.0000000	-0.6600000
15419.0000000	-0.6600000
15420.0000000	-0.6650000
15421.0000000	-0.6600000
15422.0000000	-0.6650000

15423.0000000	-0.6600000
15424.0000000	-0.6600000
15425.0000000	-0.6600000
15426.0000000	-0.6600000
15427.0000000	-0.6650000
15428.0000000	-0.6600000
15429.0000000	-0.6600000
15430.0000000	-0.6650000
15431.0000000	-0.6600000
15432.0000000	-0.6600000
15433.0000000	-0.6600000
15434.0000000	-0.6700000
15435.0000000	-0.6650000
15436.0000000	-0.6650000
15437.0000000	-0.6650000
15438.0000000	-0.6650000
15439.0000000	-0.6700000
15440.0000000	-0.6600000
15441.0000000	-0.6650000
15442.0000000	-0.6650000
15443.0000000	-0.6650000
15444.0000000	-0.6650000
15445.0000000	-0.6650000
15446.0000000	-0.6600000
15447.0000000	-0.6600000
15448.0000000	-0.6550000
15449.0000000	-0.6500000
15450.0000000	-0.6500000
15451.0000000	-0.6500000
15452.0000000	-0.6450000
15453.0000000	-0.6450000
15454.0000000	-0.6400000
15455.0000000	-0.6400000
15456.0000000	-0.6350000
15457.0000000	-0.6400000
15458.0000000	-0.6300000
15459.0000000	-0.6300000
15460.0000000	-0.6300000
15461.0000000	-0.6250000
15462.0000000	-0.6300000
15463.0000000	-0.6266667
15464.0000000	-0.6250000
15465.0000000	-0.6300000
15466.0000000	-0.6300000
15467.0000000	-0.6350000
15468.0000000	-0.6250000
15469.0000000	-0.6300000
15470.0000000	-0.6300000
15471.0000000	-0.6350000
15472.0000000	-0.6350000
15473.0000000	-0.6350000
15474.0000000	-0.6300000
15475.0000000	-0.6300000
15476.0000000	-0.6250000
15477.0000000	-0.6300000
15478.0000000	-0.6250000
15479.0000000	-0.6250000

15480.0000000	-0.6250000
15481.0000000	-0.6300000
15482.0000000	-0.6250000
15483.0000000	-0.6250000
15484.0000000	-0.6200000
15485.0000000	-0.6250000
15486.0000000	-0.6200000
15487.0000000	-0.6200000
15488.0000000	-0.6200000
15489.0000000	-0.6200000
15490.0000000	-0.6250000
15491.0000000	-0.6200000
15492.0000000	-0.6200000
15493.0000000	-0.6233333
15494.0000000	-0.6250000
15495.0000000	-0.6200000
15496.0000000	-0.6250000
15497.0000000	-0.6300000
15498.0000000	-0.6250000
15499.0000000	-0.6200000
15500.0000000	-0.6200000
15501.0000000	-0.6200000
15502.0000000	-0.6150000
15503.0000000	-0.6150000
15504.0000000	-0.6200000
15505.0000000	-0.6150000
15506.0000000	-0.6200000
15507.0000000	-0.6200000
15508.0000000	-0.6200000
15509.0000000	-0.6200000
15510.0000000	-0.6150000
15511.0000000	-0.6200000
15512.0000000	-0.6200000
15513.0000000	-0.6200000
15514.0000000	-0.6250000
15515.0000000	-0.6150000
15516.0000000	-0.6200000
15517.0000000	-0.6200000
15518.0000000	-0.6200000
15519.0000000	-0.6150000
15520.0000000	-0.6150000
15521.0000000	-0.6100000
15522.0000000	-0.6100000
15523.0000000	-0.6200000
15524.0000000	-0.6200000
15525.0000000	-0.6150000
15526.0000000	-0.6200000
15527.0000000	-0.6200000
15528.0000000	-0.6150000
15529.0000000	-0.6200000
15530.0000000	-0.6250000
15531.0000000	-0.6200000
15532.0000000	-0.6200000
15533.0000000	-0.6250000
15534.0000000	-0.6150000
15535.0000000	-0.6200000
15536.0000000	-0.6200000

15537.0000000	-0.6150000
15538.0000000	-0.6200000
15539.0000000	-0.6150000
15540.0000000	-0.6200000
15541.0000000	-0.6100000
15542.0000000	-0.6200000
15543.0000000	-0.6150000
15544.0000000	-0.6150000
15545.0000000	-0.6250000
15546.0000000	-0.6200000
15547.0000000	-0.6200000
15548.0000000	-0.6200000
15549.0000000	-0.6150000
15550.0000000	-0.6150000
15551.0000000	-0.6150000
15552.0000000	-0.6150000
15553.0000000	-0.6066667
15554.0000000	-0.6150000
15555.0000000	-0.6150000
15556.0000000	-0.6100000
15557.0000000	-0.6200000
15558.0000000	-0.6150000
15559.0000000	-0.6100000
15560.0000000	-0.6150000
15561.0000000	-0.6100000
15562.0000000	-0.6100000
15563.0000000	-0.6100000
15564.0000000	-0.6050000
15565.0000000	-0.6100000
15566.0000000	-0.6100000
15567.0000000	-0.6050000
15568.0000000	-0.6050000
15569.0000000	-0.6100000
15570.0000000	-0.6100000
15571.0000000	-0.6050000
15572.0000000	-0.6100000
15573.0000000	-0.6050000
15574.0000000	-0.6050000
15575.0000000	-0.6100000
15576.0000000	-0.6100000
15577.0000000	-0.6100000
15578.0000000	-0.6150000
15579.0000000	-0.6150000
15580.0000000	-0.6050000
15581.0000000	-0.6050000
15582.0000000	-0.6050000
15583.0000000	-0.6000000
15584.0000000	-0.6066667
15585.0000000	-0.6050000
15586.0000000	-0.6050000
15587.0000000	-0.6050000
15588.0000000	-0.6050000
15589.0000000	-0.6050000
15590.0000000	-0.6050000
15591.0000000	-0.6000000
15592.0000000	-0.6000000
15593.0000000	-0.5950000

15594.0000000	-0.5950000
15595.0000000	-0.5950000
15596.0000000	-0.5900000
15597.0000000	-0.5900000
15598.0000000	-0.5900000
15599.0000000	-0.5950000
15600.0000000	-0.5950000
15601.0000000	-0.5950000
15602.0000000	-0.5950000
15603.0000000	-0.6000000
15604.0000000	-0.6000000
15605.0000000	-0.5950000
15606.0000000	-0.6050000
15607.0000000	-0.6000000
15608.0000000	-0.6000000
15609.0000000	-0.6000000
15610.0000000	-0.6050000
15611.0000000	-0.6000000
15612.0000000	-0.6000000
15613.0000000	-0.6033334
15614.0000000	-0.6000000
15615.0000000	-0.5950000
15616.0000000	-0.6000000
15617.0000000	-0.6000000
15618.0000000	-0.6000000
15619.0000000	-0.6050000
15620.0000000	-0.6050000
15621.0000000	-0.6000000
15622.0000000	-0.6000000
15623.0000000	-0.5950000
15624.0000000	-0.5950000
15625.0000000	-0.5950000
15626.0000000	-0.6000000
15627.0000000	-0.6000000
15628.0000000	-0.5950000
15629.0000000	-0.6000000
15630.0000000	-0.6050000
15631.0000000	-0.6000000
15632.0000000	-0.6000000
15633.0000000	-0.6000000
15634.0000000	-0.5950000
15635.0000000	-0.6000000
15636.0000000	-0.6000000
15637.0000000	-0.5950000
15638.0000000	-0.5950000
15639.0000000	-0.6000000
15640.0000000	-0.5950000
15641.0000000	-0.5950000
15642.0000000	-0.5900000
15643.0000000	-0.5900000
15644.0000000	-0.5933333
15645.0000000	-0.5900000
15646.0000000	-0.5900000
15647.0000000	-0.5800000
15648.0000000	-0.5900000
15649.0000000	-0.5850000
15650.0000000	-0.5850000

15651.0000000	-0.5800000
15652.0000000	-0.5800000
15653.0000000	-0.5800000
15654.0000000	-0.5750000
15655.0000000	-0.5750000
15656.0000000	-0.5750000
15657.0000000	-0.5750000
15658.0000000	-0.5650000
15659.0000000	-0.5650000
15660.0000000	-0.5650000
15661.0000000	-0.5600000
15662.0000000	-0.5600000
15663.0000000	-0.5600000
15664.0000000	-0.5550000
15665.0000000	-0.5550000
15666.0000000	-0.5600000
15667.0000000	-0.5550000
15668.0000000	-0.5550000
15669.0000000	-0.5500000
15670.0000000	-0.5500000
15671.0000000	-0.5500000
15672.0000000	-0.5566667
15673.0000000	-0.5550000
15674.0000000	-0.5550000
15675.0000000	-0.5600000
15676.0000000	-0.5600000
15677.0000000	-0.5550000
15678.0000000	-0.5550000
15679.0000000	-0.5550000
15680.0000000	-0.5600000
15681.0000000	-0.5600000
15682.0000000	-0.5550000
15683.0000000	-0.5550000
15684.0000000	-0.5550000
15685.0000000	-0.5550000
15686.0000000	-0.5550000
15687.0000000	-0.5550000
15688.0000000	-0.5550000
15689.0000000	-0.5600000
15690.0000000	-0.5600000
15691.0000000	-0.5600000
15692.0000000	-0.5600000
15693.0000000	-0.5600000
15694.0000000	-0.5600000
15695.0000000	-0.5600000
15696.0000000	-0.5550000
15697.0000000	-0.5550000
15698.0000000	-0.5600000
15699.0000000	-0.5550000
15700.0000000	-0.5500000
15701.0000000	-0.5600000
15702.0000000	-0.5566667
15703.0000000	-0.5550000
15704.0000000	-0.5550000
15705.0000000	-0.5550000
15706.0000000	-0.5500000
15707.0000000	-0.5500000

15708.0000000	-0.5550000
15709.0000000	-0.5500000
15710.0000000	-0.5550000
15711.0000000	-0.5500000
15712.0000000	-0.5600000
15713.0000000	-0.5550000
15714.0000000	-0.5550000
15715.0000000	-0.5500000
15716.0000000	-0.5500000
15717.0000000	-0.5500000
15718.0000000	-0.5500000
15719.0000000	-0.5550000
15720.0000000	-0.5550000
15721.0000000	-0.5500000
15722.0000000	-0.5600000
15723.0000000	-0.5550000
15724.0000000	-0.5500000
15725.0000000	-0.5600000
15726.0000000	-0.5550000
15727.0000000	-0.5600000
15728.0000000	-0.5600000
15729.0000000	-0.5550000
15730.0000000	-0.5650000
15731.0000000	-0.5650000
15732.0000000	-0.5700000
15733.0000000	-0.5700000
15734.0000000	-0.5750000
15735.0000000	-0.5750000
15736.0000000	-0.5700000
15737.0000000	-0.5750000
15738.0000000	-0.5800000
15739.0000000	-0.5800000
15740.0000000	-0.5750000
15741.0000000	-0.5800000
15742.0000000	-0.5800000
15743.0000000	-0.5750000
15744.0000000	-0.5800000
15745.0000000	-0.5850000
15746.0000000	-0.5800000
15747.0000000	-0.5850000
15748.0000000	-0.5900000
15749.0000000	-0.5850000
15750.0000000	-0.5850000
15751.0000000	-0.5900000
15752.0000000	-0.5900000
15753.0000000	-0.5900000
15754.0000000	-0.5900000
15755.0000000	-0.5950000
15756.0000000	-0.5950000
15757.0000000	-0.6000000
15758.0000000	-0.6000000
15759.0000000	-0.6050000
15760.0000000	-0.6050000
15761.0000000	-0.6000000
15762.0000000	-0.6000000
15763.0000000	-0.6050000
15764.0000000	-0.6050000

15765.0000000	-0.6100000
15766.0000000	-0.6150000
15767.0000000	-0.6150000
15768.0000000	-0.6150000
15769.0000000	-0.6150000
15770.0000000	-0.6200000
15771.0000000	-0.6200000
15772.0000000	-0.6250000
15773.0000000	-0.6250000
15774.0000000	-0.6250000
15775.0000000	-0.6200000
15776.0000000	-0.6250000
15777.0000000	-0.6300000
15778.0000000	-0.6250000
15779.0000000	-0.6300000
15780.0000000	-0.6300000
15781.0000000	-0.6250000
15782.0000000	-0.6350000
15783.0000000	-0.6350000
15784.0000000	-0.6350000
15785.0000000	-0.6400000
15786.0000000	-0.6350000
15787.0000000	-0.6350000
15788.0000000	-0.6350000
15789.0000000	-0.6400000
15790.0000000	-0.6400000
15791.0000000	-0.6400000
15792.0000000	-0.6450000
15793.0000000	-0.6450000
15794.0000000	-0.6400000
15795.0000000	-0.6500000
15796.0000000	-0.6500000
15797.0000000	-0.6550000
15798.0000000	-0.6550000
15799.0000000	-0.6500000
15800.0000000	-0.6600000
15801.0000000	-0.6600000
15802.0000000	-0.6600000
15803.0000000	-0.6550000
15804.0000000	-0.6600000
15805.0000000	-0.6600000
15806.0000000	-0.6600000
15807.0000000	-0.6600000
15808.0000000	-0.6600000
15809.0000000	-0.6600000
15810.0000000	-0.6650000
15811.0000000	-0.6650000
15812.0000000	-0.6650000
15813.0000000	-0.6650000
15814.0000000	-0.6700000
15815.0000000	-0.6700000
15816.0000000	-0.6650000
15817.0000000	-0.6650000
15818.0000000	-0.6700000
15819.0000000	-0.6700000
15820.0000000	-0.6700000
15821.0000000	-0.6750000

15822.0000000	-0.6750000
15823.0000000	-0.6750000
15824.0000000	-0.6800000
15825.0000000	-0.6800000
15826.0000000	-0.6766667
15827.0000000	-0.6800000
15828.0000000	-0.6800000
15829.0000000	-0.6800000
15830.0000000	-0.6850000
15831.0000000	-0.6850000
15832.0000000	-0.6900000
15833.0000000	-0.6900000
15834.0000000	-0.7000000
15835.0000000	-0.7000000
15836.0000000	-0.7050000
15837.0000000	-0.7050000
15838.0000000	-0.7100000
15839.0000000	-0.7000000
15840.0000000	-0.7000000
15841.0000000	-0.7000000
15842.0000000	-0.7000000
15843.0000000	-0.6950000
15844.0000000	-0.7000000
15845.0000000	-0.7000000
15846.0000000	-0.7050000
15847.0000000	-0.7050000
15848.0000000	-0.7050000
15849.0000000	-0.7100000
15850.0000000	-0.7050000
15851.0000000	-0.7100000
15852.0000000	-0.7100000
15853.0000000	-0.7100000
15854.0000000	-0.7100000
15855.0000000	-0.7100000
15856.0000000	-0.7133334
15857.0000000	-0.7150000
15858.0000000	-0.7150000
15859.0000000	-0.7200000
15860.0000000	-0.7200000
15861.0000000	-0.7250000
15862.0000000	-0.7250000
15863.0000000	-0.7300000
15864.0000000	-0.7250000
15865.0000000	-0.7250000
15866.0000000	-0.7250000
15867.0000000	-0.7300000
15868.0000000	-0.7300000
15869.0000000	-0.7300000
15870.0000000	-0.7300000
15871.0000000	-0.7350000
15872.0000000	-0.7350000
15873.0000000	-0.7400000
15874.0000000	-0.7350000
15875.0000000	-0.7400000
15876.0000000	-0.7350000
15877.0000000	-0.7400000
15878.0000000	-0.7350000

15879.0000000	-0.7350000
15880.0000000	-0.7350000
15881.0000000	-0.7350000
15882.0000000	-0.7400000
15883.0000000	-0.7400000
15884.0000000	-0.7450000
15885.0000000	-0.7450000
15886.0000000	-0.7366667
15887.0000000	-0.7450000
15888.0000000	-0.7450000
15889.0000000	-0.7450000
15890.0000000	-0.7450000
15891.0000000	-0.7450000
15892.0000000	-0.7500000
15893.0000000	-0.7500000
15894.0000000	-0.7450000
15895.0000000	-0.7550000
15896.0000000	-0.7550000
15897.0000000	-0.7600000
15898.0000000	-0.7600000
15899.0000000	-0.7600000
15900.0000000	-0.7650000
15901.0000000	-0.7600000
15902.0000000	-0.7600000
15903.0000000	-0.7700000
15904.0000000	-0.7650000
15905.0000000	-0.7650000
15906.0000000	-0.7700000
15907.0000000	-0.7750000
15908.0000000	-0.7750000
15909.0000000	-0.7850000
15910.0000000	-0.7800000
15911.0000000	-0.7800000
15912.0000000	-0.7800000
15913.0000000	-0.7800000
15914.0000000	-0.7850000
15915.0000000	-0.7850000
15916.0000000	-0.7900000
15917.0000000	-0.7900000
15918.0000000	-0.7850000
15919.0000000	-0.7900000
15920.0000000	-0.7900000
15921.0000000	-0.8000000
15922.0000000	-0.8000000
15923.0000000	-0.8000000
15924.0000000	-0.7950000
15925.0000000	-0.8000000
15926.0000000	-0.8050000
15927.0000000	-0.8050000
15928.0000000	-0.8050000
15929.0000000	-0.8100000
15930.0000000	-0.8100000
15931.0000000	-0.8100000
15932.0000000	-0.8100000
15933.0000000	-0.8050000
15934.0000000	-0.8150000
15935.0000000	-0.8150000

15936.0000000	-0.8150000
15937.0000000	-0.8100000
15938.0000000	-0.8100000
15939.0000000	-0.8100000
15940.0000000	-0.8150000
15941.0000000	-0.8150000
15942.0000000	-0.8100000
15943.0000000	-0.8100000
15944.0000000	-0.8150000
15945.0000000	-0.8150000
15946.0000000	-0.8133333
15947.0000000	-0.8150000
15948.0000000	-0.8100000
15949.0000000	-0.8150000
15950.0000000	-0.8150000
15951.0000000	-0.8150000
15952.0000000	-0.8250000
15953.0000000	-0.8250000
15954.0000000	-0.8250000
15955.0000000	-0.8300000
15956.0000000	-0.8300000
15957.0000000	-0.8250000
15958.0000000	-0.8300000
15959.0000000	-0.8300000
15960.0000000	-0.8300000
15961.0000000	-0.8350000
15962.0000000	-0.8350000
15963.0000000	-0.8300000
15964.0000000	-0.8350000
15965.0000000	-0.8400000
15966.0000000	-0.8400000
15967.0000000	-0.8350000
15968.0000000	-0.8400000
15969.0000000	-0.8350000
15970.0000000	-0.8400000
15971.0000000	-0.8400000
15972.0000000	-0.8450000
15973.0000000	-0.8400000
15974.0000000	-0.8433333
15975.0000000	-0.8400000
15976.0000000	-0.8450000
15977.0000000	-0.8450000
15978.0000000	-0.8500000
15979.0000000	-0.8400000
15980.0000000	-0.8450000
15981.0000000	-0.8450000
15982.0000000	-0.8450000
15983.0000000	-0.8450000
15984.0000000	-0.8500000
15985.0000000	-0.8500000
15986.0000000	-0.8450000
15987.0000000	-0.8450000
15988.0000000	-0.8400000
15989.0000000	-0.8400000
15990.0000000	-0.8350000
15991.0000000	-0.8400000
15992.0000000	-0.8350000

15993.0000000	-0.8300000
15994.0000000	-0.8300000
15995.0000000	-0.8300000
15996.0000000	-0.8250000
15997.0000000	-0.8250000
15998.0000000	-0.8200000
15999.0000000	-0.8200000
16000.0000000	-0.8250000
16001.0000000	-0.8250000
16002.0000000	-0.8300000
16003.0000000	-0.8150000
16004.0000000	-0.8200000
16005.0000000	-0.8200000
16006.0000000	-0.8250000
16007.0000000	-0.8150000
16008.0000000	-0.8150000
16009.0000000	-0.8100000
16010.0000000	-0.8150000
16011.0000000	-0.8150000
16012.0000000	-0.8050000
16013.0000000	-0.8100000
16014.0000000	-0.8100000
16015.0000000	-0.8100000
16016.0000000	-0.8050000
16017.0000000	-0.8100000
16018.0000000	-0.8000000
16019.0000000	-0.8000000
16020.0000000	-0.8050000
16021.0000000	-0.8000000
16022.0000000	-0.8050000
16023.0000000	-0.8000000
16024.0000000	-0.8000000
16025.0000000	-0.7950000
16026.0000000	-0.8000000
16027.0000000	-0.8050000
16028.0000000	-0.8000000
16029.0000000	-0.8000000
16030.0000000	-0.7950000
16031.0000000	-0.8050000
16032.0000000	-0.8000000
16033.0000000	-0.8000000
16034.0000000	-0.7950000
16035.0000000	-0.7966667
16036.0000000	-0.7950000
16037.0000000	-0.8000000
16038.0000000	-0.7950000
16039.0000000	-0.8050000
16040.0000000	-0.8050000
16041.0000000	-0.8000000
16042.0000000	-0.8000000
16043.0000000	-0.8100000
16044.0000000	-0.8100000
16045.0000000	-0.8050000
16046.0000000	-0.8100000
16047.0000000	-0.8100000
16048.0000000	-0.8100000
16049.0000000	-0.8100000

16050.0000000	-0.8150000
16051.0000000	-0.8100000
16052.0000000	-0.8150000
16053.0000000	-0.8200000
16054.0000000	-0.8150000
16055.0000000	-0.8200000
16056.0000000	-0.8150000
16057.0000000	-0.8150000
16058.0000000	-0.8150000
16059.0000000	-0.8150000
16060.0000000	-0.8100000
16061.0000000	-0.8150000
16062.0000000	-0.8150000
16063.0000000	-0.8150000
16064.0000000	-0.8150000
16065.0000000	-0.8133333
16066.0000000	-0.8150000
16067.0000000	-0.8050000
16068.0000000	-0.8150000
16069.0000000	-0.8150000
16070.0000000	-0.8200000
16071.0000000	-0.8200000
16072.0000000	-0.8150000
16073.0000000	-0.8150000
16074.0000000	-0.8150000
16075.0000000	-0.8150000
16076.0000000	-0.8150000
16077.0000000	-0.8100000
16078.0000000	-0.8100000
16079.0000000	-0.8150000
16080.0000000	-0.8100000
16081.0000000	-0.8100000
16082.0000000	-0.8100000
16083.0000000	-0.8150000
16084.0000000	-0.8150000
16085.0000000	-0.8100000
16086.0000000	-0.8100000
16087.0000000	-0.8050000
16088.0000000	-0.8050000
16089.0000000	-0.8100000
16090.0000000	-0.8150000
16091.0000000	-0.8100000
16092.0000000	-0.8100000
16093.0000000	-0.8100000
16094.0000000	-0.8050000
16095.0000000	-0.8050000
16096.0000000	-0.8133333
16097.0000000	-0.8150000
16098.0000000	-0.8150000
16099.0000000	-0.8150000
16100.0000000	-0.8200000
16101.0000000	-0.8100000
16102.0000000	-0.8050000
16103.0000000	-0.8050000
16104.0000000	-0.8150000
16105.0000000	-0.8150000
16106.0000000	-0.8150000

16107.0000000	-0.8150000
16108.0000000	-0.8150000
16109.0000000	-0.8150000
16110.0000000	-0.8100000
16111.0000000	-0.8150000
16112.0000000	-0.8150000
16113.0000000	-0.8150000
16114.0000000	-0.8100000
16115.0000000	-0.8100000
16116.0000000	-0.8050000
16117.0000000	-0.8100000
16118.0000000	-0.8050000
16119.0000000	-0.8050000
16120.0000000	-0.8050000
16121.0000000	-0.8050000
16122.0000000	-0.8050000
16123.0000000	-0.8050000
16124.0000000	-0.8050000
16125.0000000	-0.8100000
16126.0000000	-0.8050000
16127.0000000	-0.8066667
16128.0000000	-0.8050000
16129.0000000	-0.8000000
16130.0000000	-0.8050000
16131.0000000	-0.7950000
16132.0000000	-0.8050000
16133.0000000	-0.8000000
16134.0000000	-0.8000000
16135.0000000	-0.8000000
16136.0000000	-0.8050000
16137.0000000	-0.8000000
16138.0000000	-0.8050000
16139.0000000	-0.8050000
16140.0000000	-0.8000000
16141.0000000	-0.8050000
16142.0000000	-0.7950000
16143.0000000	-0.8000000
16144.0000000	-0.7950000
16145.0000000	-0.7950000
16146.0000000	-0.8000000
16147.0000000	-0.7950000
16148.0000000	-0.7900000
16149.0000000	-0.8000000
16150.0000000	-0.7950000
16151.0000000	-0.7850000
16152.0000000	-0.7850000
16153.0000000	-0.7900000
16154.0000000	-0.7800000
16155.0000000	-0.7900000
16156.0000000	-0.7850000
16157.0000000	-0.7900000
16158.0000000	-0.7900000
16159.0000000	-0.7900000
16160.0000000	-0.7900000
16161.0000000	-0.7950000
16162.0000000	-0.7950000
16163.0000000	-0.8000000

16164.0000000	-0.8000000
16165.0000000	-0.8000000
16166.0000000	-0.7950000
16167.0000000	-0.8000000
16168.0000000	-0.8000000
16169.0000000	-0.8050000
16170.0000000	-0.8050000
16171.0000000	-0.8050000
16172.0000000	-0.8150000
16173.0000000	-0.8100000
16174.0000000	-0.8150000
16175.0000000	-0.8150000
16176.0000000	-0.8200000
16177.0000000	-0.8150000
16178.0000000	-0.8150000
16179.0000000	-0.8150000
16180.0000000	-0.8150000
16181.0000000	-0.8150000
16182.0000000	-0.8150000
16183.0000000	-0.8200000
16184.0000000	-0.8250000
16185.0000000	-0.8250000
16186.0000000	-0.8200000
16187.0000000	-0.8250000
16188.0000000	-0.8233333
16189.0000000	-0.8200000
16190.0000000	-0.8250000
16191.0000000	-0.8250000
16192.0000000	-0.8300000
16193.0000000	-0.8250000
16194.0000000	-0.8250000
16195.0000000	-0.8300000
16196.0000000	-0.8300000
16197.0000000	-0.8250000
16198.0000000	-0.8300000
16199.0000000	-0.8250000
16200.0000000	-0.8150000
16201.0000000	-0.8150000
16202.0000000	-0.8150000
16203.0000000	-0.8100000
16204.0000000	-0.8050000
16205.0000000	-0.8100000
16206.0000000	-0.8050000
16207.0000000	-0.8000000
16208.0000000	-0.7950000
16209.0000000	-0.7950000
16210.0000000	-0.7950000
16211.0000000	-0.7950000
16212.0000000	-0.7900000
16213.0000000	-0.7900000
16214.0000000	-0.7850000
16215.0000000	-0.7850000
16216.0000000	-0.7866666
16217.0000000	-0.7800000
16218.0000000	-0.7850000
16219.0000000	-0.7850000
16220.0000000	-0.7800000

16221.0000000	-0.7800000
16222.0000000	-0.7800000
16223.0000000	-0.7700000
16224.0000000	-0.7700000
16225.0000000	-0.7700000
16226.0000000	-0.7600000
16227.0000000	-0.7550000
16228.0000000	-0.7550000
16229.0000000	-0.7500000
16230.0000000	-0.7450000
16231.0000000	-0.7500000
16232.0000000	-0.7400000
16233.0000000	-0.7400000
16234.0000000	-0.7350000
16235.0000000	-0.7350000
16236.0000000	-0.7250000
16237.0000000	-0.7250000
16238.0000000	-0.7250000
16239.0000000	-0.7250000
16240.0000000	-0.7200000
16241.0000000	-0.7250000
16242.0000000	-0.7200000
16243.0000000	-0.7200000
16244.0000000	-0.7200000
16245.0000000	-0.7150000
16246.0000000	-0.7133334
16247.0000000	-0.7200000
16248.0000000	-0.7200000
16249.0000000	-0.7150000
16250.0000000	-0.7100000
16251.0000000	-0.7200000
16252.0000000	-0.7100000
16253.0000000	-0.7050000
16254.0000000	-0.7150000
16255.0000000	-0.7150000
16256.0000000	-0.7050000
16257.0000000	-0.7100000
16258.0000000	-0.7100000
16259.0000000	-0.7100000
16260.0000000	-0.7100000
16261.0000000	-0.7050000
16262.0000000	-0.7100000
16263.0000000	-0.7100000
16264.0000000	-0.7050000
16265.0000000	-0.7050000
16266.0000000	-0.7050000
16267.0000000	-0.7000000
16268.0000000	-0.6950000
16269.0000000	-0.7000000
16270.0000000	-0.7000000
16271.0000000	-0.6950000
16272.0000000	-0.7050000
16273.0000000	-0.7000000
16274.0000000	-0.7050000
16275.0000000	-0.7000000
16276.0000000	-0.6966667
16277.0000000	-0.7000000

16278.0000000	-0.6950000
16279.0000000	-0.6950000
16280.0000000	-0.6950000
16281.0000000	-0.6950000
16282.0000000	-0.6900000
16283.0000000	-0.6900000
16284.0000000	-0.6950000
16285.0000000	-0.6900000
16286.0000000	-0.6900000
16287.0000000	-0.6900000
16288.0000000	-0.6900000
16289.0000000	-0.6900000
16290.0000000	-0.6850000
16291.0000000	-0.6850000
16292.0000000	-0.6900000
16293.0000000	-0.6850000
16294.0000000	-0.6900000
16295.0000000	-0.6900000
16296.0000000	-0.6850000
16297.0000000	-0.6950000
16298.0000000	-0.6950000
16299.0000000	-0.6900000
16300.0000000	-0.6950000
16301.0000000	-0.7000000
16302.0000000	-0.6950000
16303.0000000	-0.7000000
16304.0000000	-0.6950000
16305.0000000	-0.7000000
16306.0000000	-0.7000000
16307.0000000	-0.6933333
16308.0000000	-0.7000000
16309.0000000	-0.7000000
16310.0000000	-0.6950000
16311.0000000	-0.7000000
16312.0000000	-0.7050000
16313.0000000	-0.7000000
16314.0000000	-0.7000000
16315.0000000	-0.6950000
16316.0000000	-0.7000000
16317.0000000	-0.6950000
16318.0000000	-0.7000000
16319.0000000	-0.7000000
16320.0000000	-0.7050000
16321.0000000	-0.7050000
16322.0000000	-0.7050000
16323.0000000	-0.7050000
16324.0000000	-0.7100000
16325.0000000	-0.7100000
16326.0000000	-0.7050000
16327.0000000	-0.7050000
16328.0000000	-0.7050000
16329.0000000	-0.7100000
16330.0000000	-0.7100000
16331.0000000	-0.7100000
16332.0000000	-0.7050000
16333.0000000	-0.7050000
16334.0000000	-0.7100000

16335.0000000	-0.7100000
16336.0000000	-0.7050000
16337.0000000	-0.7100000
16338.0000000	-0.7050000
16339.0000000	-0.7150000
16340.0000000	-0.7200000
16341.0000000	-0.7150000
16342.0000000	-0.7150000
16343.0000000	-0.7150000
16344.0000000	-0.7100000
16345.0000000	-0.7200000
16346.0000000	-0.7150000
16347.0000000	-0.7150000
16348.0000000	-0.7150000
16349.0000000	-0.7200000
16350.0000000	-0.7150000
16351.0000000	-0.7200000
16352.0000000	-0.7150000
16353.0000000	-0.7150000
16354.0000000	-0.7200000
16355.0000000	-0.7150000
16356.0000000	-0.7150000
16357.0000000	-0.7150000
16358.0000000	-0.7200000
16359.0000000	-0.7150000
16360.0000000	-0.7200000
16361.0000000	-0.7150000
16362.0000000	-0.7150000
16363.0000000	-0.7150000
16364.0000000	-0.7200000
16365.0000000	-0.7200000
16366.0000000	-0.7200000
16367.0000000	-0.7150000
16368.0000000	-0.7050000
16369.0000000	-0.7133334
16370.0000000	-0.7100000
16371.0000000	-0.7050000
16372.0000000	-0.7150000
16373.0000000	-0.7150000
16374.0000000	-0.7050000
16375.0000000	-0.7100000
16376.0000000	-0.7150000
16377.0000000	-0.7150000
16378.0000000	-0.7150000
16379.0000000	-0.7100000
16380.0000000	-0.7150000
16381.0000000	-0.7150000
16382.0000000	-0.7100000
16383.0000000	-0.7100000
16384.0000000	-0.7050000
16385.0000000	-0.7150000
16386.0000000	-0.7050000
16387.0000000	-0.7050000
16388.0000000	-0.7050000
16389.0000000	-0.7100000
16390.0000000	-0.7100000
16391.0000000	-0.7000000

16392.0000000	-0.7050000
16393.0000000	-0.7000000
16394.0000000	-0.7050000
16395.0000000	-0.7000000
16396.0000000	-0.6950000
16397.0000000	-0.6950000
16398.0000000	-0.6950000
16399.0000000	-0.6966667
16400.0000000	-0.6950000
16401.0000000	-0.6950000
16402.0000000	-0.6950000
16403.0000000	-0.6950000
16404.0000000	-0.6950000
16405.0000000	-0.6950000
16406.0000000	-0.6850000
16407.0000000	-0.6850000
16408.0000000	-0.6800000
16409.0000000	-0.6800000
16410.0000000	-0.6800000
16411.0000000	-0.6750000
16412.0000000	-0.6800000
16413.0000000	-0.6750000
16414.0000000	-0.6800000
16415.0000000	-0.6800000
16416.0000000	-0.6850000
16417.0000000	-0.6750000
16418.0000000	-0.6850000
16419.0000000	-0.6800000
16420.0000000	-0.6850000
16421.0000000	-0.6850000
16422.0000000	-0.6800000
16423.0000000	-0.6850000
16424.0000000	-0.6800000
16425.0000000	-0.6850000
16426.0000000	-0.6850000
16427.0000000	-0.6850000
16428.0000000	-0.6833333
16429.0000000	-0.6800000
16430.0000000	-0.6850000
16431.0000000	-0.6850000
16432.0000000	-0.6850000
16433.0000000	-0.6850000
16434.0000000	-0.6850000
16435.0000000	-0.6800000
16436.0000000	-0.6800000
16437.0000000	-0.6750000
16438.0000000	-0.6800000
16439.0000000	-0.6750000
16440.0000000	-0.6800000
16441.0000000	-0.6750000
16442.0000000	-0.6750000
16443.0000000	-0.6800000
16444.0000000	-0.6700000
16445.0000000	-0.6750000
16446.0000000	-0.6750000
16447.0000000	-0.6750000
16448.0000000	-0.6700000

16449.0000000	-0.6750000
16450.0000000	-0.6750000
16451.0000000	-0.6650000
16452.0000000	-0.6750000
16453.0000000	-0.6700000
16454.0000000	-0.6700000
16455.0000000	-0.6800000
16456.0000000	-0.6800000
16457.0000000	-0.6800000
16458.0000000	-0.6800000
16459.0000000	-0.6800000
16460.0000000	-0.6800000
16461.0000000	-0.6800000
16462.0000000	-0.6850000
16463.0000000	-0.6800000
16464.0000000	-0.6900000
16465.0000000	-0.6900000
16466.0000000	-0.6850000
16467.0000000	-0.6900000
16468.0000000	-0.6900000
16469.0000000	-0.6850000
16470.0000000	-0.6900000
16471.0000000	-0.6950000
16472.0000000	-0.6950000
16473.0000000	-0.6950000
16474.0000000	-0.7000000
16475.0000000	-0.6900000
16476.0000000	-0.6900000
16477.0000000	-0.6950000
16478.0000000	-0.7000000
16479.0000000	-0.6950000
16480.0000000	-0.6950000
16481.0000000	-0.6950000
16482.0000000	-0.7000000
16483.0000000	-0.6950000
16484.0000000	-0.6950000
16485.0000000	-0.6950000
16486.0000000	-0.6950000
16487.0000000	-0.6950000
16488.0000000	-0.6950000
16489.0000000	-0.6950000
16490.0000000	-0.6966667
16491.0000000	-0.7050000
16492.0000000	-0.7000000
16493.0000000	-0.7000000
16494.0000000	-0.7000000
16495.0000000	-0.7050000
16496.0000000	-0.7050000
16497.0000000	-0.7050000
16498.0000000	-0.7100000
16499.0000000	-0.7150000
16500.0000000	-0.7150000
16501.0000000	-0.7100000
16502.0000000	-0.7150000
16503.0000000	-0.7150000
16504.0000000	-0.7200000
16505.0000000	-0.7150000

16506.0000000	-0.7200000
16507.0000000	-0.7250000
16508.0000000	-0.7200000
16509.0000000	-0.7150000
16510.0000000	-0.7200000
16511.0000000	-0.7250000
16512.0000000	-0.7200000
16513.0000000	-0.7200000
16514.0000000	-0.7200000
16515.0000000	-0.7200000
16516.0000000	-0.7250000
16517.0000000	-0.7250000
16518.0000000	-0.7300000
16519.0000000	-0.7200000
16520.0000000	-0.7233334
16521.0000000	-0.7200000
16522.0000000	-0.7200000
16523.0000000	-0.7200000
16524.0000000	-0.7250000
16525.0000000	-0.7250000
16526.0000000	-0.7200000
16527.0000000	-0.7200000
16528.0000000	-0.7200000
16529.0000000	-0.7200000
16530.0000000	-0.7250000
16531.0000000	-0.7250000
16532.0000000	-0.7300000
16533.0000000	-0.7300000
16534.0000000	-0.7250000
16535.0000000	-0.7250000
16536.0000000	-0.7250000
16537.0000000	-0.7300000
16538.0000000	-0.7300000
16539.0000000	-0.7250000
16540.0000000	-0.7300000
16541.0000000	-0.7350000
16542.0000000	-0.7300000
16543.0000000	-0.7300000
16544.0000000	-0.7350000
16545.0000000	-0.7350000
16546.0000000	-0.7350000
16547.0000000	-0.7400000
16548.0000000	-0.7300000
16549.0000000	-0.7300000
16550.0000000	-0.7333333
16551.0000000	-0.7350000
16552.0000000	-0.7300000
16553.0000000	-0.7300000
16554.0000000	-0.7350000
16555.0000000	-0.7350000
16556.0000000	-0.7300000
16557.0000000	-0.7400000
16558.0000000	-0.7300000
16559.0000000	-0.7250000
16560.0000000	-0.7300000
16561.0000000	-0.7350000
16562.0000000	-0.7300000

16563.0000000	-0.7300000
16564.0000000	-0.7300000
16565.0000000	-0.7350000
16566.0000000	-0.7300000
16567.0000000	-0.7300000
16568.0000000	-0.7350000
16569.0000000	-0.7250000
16570.0000000	-0.7350000
16571.0000000	-0.7300000
16572.0000000	-0.7300000
16573.0000000	-0.7350000
16574.0000000	-0.7300000
16575.0000000	-0.7350000
16576.0000000	-0.7300000
16577.0000000	-0.7300000
16578.0000000	-0.7400000
16579.0000000	-0.7300000
16580.0000000	-0.7366667
16581.0000000	-0.7400000
16582.0000000	-0.7400000
16583.0000000	-0.7400000
16584.0000000	-0.7450000
16585.0000000	-0.7400000
16586.0000000	-0.7450000
16587.0000000	-0.7450000
16588.0000000	-0.7450000
16589.0000000	-0.7500000
16590.0000000	-0.7500000
16591.0000000	-0.7450000
16592.0000000	-0.7500000
16593.0000000	-0.7550000
16594.0000000	-0.7500000
16595.0000000	-0.7550000
16596.0000000	-0.7550000
16597.0000000	-0.7550000
16598.0000000	-0.7600000
16599.0000000	-0.7550000
16600.0000000	-0.7650000
16601.0000000	-0.7600000
16602.0000000	-0.7700000
16603.0000000	-0.7650000
16604.0000000	-0.7700000
16605.0000000	-0.7700000
16606.0000000	-0.7650000
16607.0000000	-0.7650000
16608.0000000	-0.7700000
16609.0000000	-0.7750000
16610.0000000	-0.7700000
16611.0000000	-0.7733333
16612.0000000	-0.7750000
16613.0000000	-0.7750000
16614.0000000	-0.7750000
16615.0000000	-0.7750000
16616.0000000	-0.7750000
16617.0000000	-0.7800000
16618.0000000	-0.7750000
16619.0000000	-0.7750000

16620.0000000	-0.7750000
16621.0000000	-0.7750000
16622.0000000	-0.7750000
16623.0000000	-0.7850000
16624.0000000	-0.7800000
16625.0000000	-0.7850000
16626.0000000	-0.7850000
16627.0000000	-0.7800000
16628.0000000	-0.7800000
16629.0000000	-0.7800000
16630.0000000	-0.7850000
16631.0000000	-0.7800000
16632.0000000	-0.7800000
16633.0000000	-0.7750000
16634.0000000	-0.7850000
16635.0000000	-0.7800000
16636.0000000	-0.7850000
16637.0000000	-0.7800000
16638.0000000	-0.7850000
16639.0000000	-0.7850000
16640.0000000	-0.7850000
16641.0000000	-0.7900000
16642.0000000	-0.7950000
16643.0000000	-0.7900000
16644.0000000	-0.8000000
16645.0000000	-0.7950000
16646.0000000	-0.8000000
16647.0000000	-0.8000000
16648.0000000	-0.8050000
16649.0000000	-0.8050000
16650.0000000	-0.8050000
16651.0000000	-0.8100000
16652.0000000	-0.8200000
16653.0000000	-0.8150000
16654.0000000	-0.8250000
16655.0000000	-0.8200000
16656.0000000	-0.8250000
16657.0000000	-0.8250000
16658.0000000	-0.8250000
16659.0000000	-0.8300000
16660.0000000	-0.8300000
16661.0000000	-0.8300000
16662.0000000	-0.8350000
16663.0000000	-0.8250000
16664.0000000	-0.8350000
16665.0000000	-0.8300000
16666.0000000	-0.8300000
16667.0000000	-0.8350000
16668.0000000	-0.8350000
16669.0000000	-0.8350000
16670.0000000	-0.8350000
16671.0000000	-0.8400000
16672.0000000	-0.8400000
16673.0000000	-0.8450000
16674.0000000	-0.8450000
16675.0000000	-0.8500000
16676.0000000	-0.8450000

16677.0000000	-0.8450000
16678.0000000	-0.8500000
16679.0000000	-0.8500000
16680.0000000	-0.8500000
16681.0000000	-0.8550000
16682.0000000	-0.8500000
16683.0000000	-0.8550000
16684.0000000	-0.8550000
16685.0000000	-0.8550000
16686.0000000	-0.8550000
16687.0000000	-0.8550000
16688.0000000	-0.8600000
16689.0000000	-0.8550000
16690.0000000	-0.8550000
16691.0000000	-0.8600000
16692.0000000	-0.8500000
16693.0000000	-0.8600000
16694.0000000	-0.8550000
16695.0000000	-0.8600000
16696.0000000	-0.8650000
16697.0000000	-0.8550000
16698.0000000	-0.8650000
16699.0000000	-0.8650000
16700.0000000	-0.8700001
16701.0000000	-0.8650000
16702.0000000	-0.8650000
16703.0000000	-0.8650000
16704.0000000	-0.8650000
16705.0000000	-0.8650000
16706.0000000	-0.8600000
16707.0000000	-0.8700000
16708.0000000	-0.8650000
16709.0000000	-0.8700000
16710.0000000	-0.8700000
16711.0000000	-0.8700000
16712.0000000	-0.8750000
16713.0000000	-0.8750000
16714.0000000	-0.8750000
16715.0000000	-0.8750000
16716.0000000	-0.8700000
16717.0000000	-0.8750000
16718.0000000	-0.8750000
16719.0000000	-0.8750000
16720.0000000	-0.8800000
16721.0000000	-0.8800000
16722.0000000	-0.8800000
16723.0000000	-0.8750000
16724.0000000	-0.8800000
16725.0000000	-0.8850000
16726.0000000	-0.8800000
16727.0000000	-0.8800000
16728.0000000	-0.8850000
16729.0000000	-0.8800000
16730.0000000	-0.8850000
16731.0000000	-0.8799999
16732.0000000	-0.8800000
16733.0000000	-0.8800000

16734.0000000	-0.8700000
16735.0000000	-0.8750000
16736.0000000	-0.8650000
16737.0000000	-0.8650000
16738.0000000	-0.8700000
16739.0000000	-0.8600000
16740.0000000	-0.8650000
16741.0000000	-0.8600000
16742.0000000	-0.8600000
16743.0000000	-0.8600000
16744.0000000	-0.8550000
16745.0000000	-0.8550000
16746.0000000	-0.8550000
16747.0000000	-0.8500000
16748.0000000	-0.8550000
16749.0000000	-0.8550000
16750.0000000	-0.8550000
16751.0000000	-0.8550000
16752.0000000	-0.8500000
16753.0000000	-0.8500000
16754.0000000	-0.8600000
16755.0000000	-0.8550000
16756.0000000	-0.8550000
16757.0000000	-0.8500000
16758.0000000	-0.8550000
16759.0000000	-0.8550000
16760.0000000	-0.8550000
16761.0000000	-0.8600000
16762.0000000	-0.8600000
16763.0000000	-0.8600000
16764.0000000	-0.8600000
16765.0000000	-0.8600000
16766.0000000	-0.8600000
16767.0000000	-0.8550000
16768.0000000	-0.8550000
16769.0000000	-0.8600000
16770.0000000	-0.8550000
16771.0000000	-0.8550000
16772.0000000	-0.8500000
16773.0000000	-0.8500000
16774.0000000	-0.8500000
16775.0000000	-0.8500000
16776.0000000	-0.8450000
16777.0000000	-0.8450000
16778.0000000	-0.8400000
16779.0000000	-0.8400000
16780.0000000	-0.8400000
16781.0000000	-0.8450000
16782.0000000	-0.8350000
16783.0000000	-0.8300000
16784.0000000	-0.8350000
16785.0000000	-0.8350000
16786.0000000	-0.8350000
16787.0000000	-0.8300000
16788.0000000	-0.8250000
16789.0000000	-0.8200000
16790.0000000	-0.8250000

16791.0000000	-0.8250000
16792.0000000	-0.8266667
16793.0000000	-0.8300000
16794.0000000	-0.8250000
16795.0000000	-0.8300000
16796.0000000	-0.8300000
16797.0000000	-0.8250000
16798.0000000	-0.8300000
16799.0000000	-0.8250000
16800.0000000	-0.8300000
16801.0000000	-0.8200000
16802.0000000	-0.8250000
16803.0000000	-0.8200000
16804.0000000	-0.8200000
16805.0000000	-0.8200000
16806.0000000	-0.8150000
16807.0000000	-0.8200000
16808.0000000	-0.8150000
16809.0000000	-0.8100000
16810.0000000	-0.8050000
16811.0000000	-0.8050000
16812.0000000	-0.8000000
16813.0000000	-0.7950000
16814.0000000	-0.8000000
16815.0000000	-0.7950000
16816.0000000	-0.7950000
16817.0000000	-0.8000000
16818.0000000	-0.7950000
16819.0000000	-0.7950000
16820.0000000	-0.7950000
16821.0000000	-0.7900000
16822.0000000	-0.7933334
16823.0000000	-0.8000000
16824.0000000	-0.7900000
16825.0000000	-0.7950000
16826.0000000	-0.7950000
16827.0000000	-0.7900000
16828.0000000	-0.7900000
16829.0000000	-0.7850000
16830.0000000	-0.7800000
16831.0000000	-0.7750000
16832.0000000	-0.7750000
16833.0000000	-0.7700000
16834.0000000	-0.7750000
16835.0000000	-0.7700000
16836.0000000	-0.7650000
16837.0000000	-0.7650000
16838.0000000	-0.7650000
16839.0000000	-0.7700000
16840.0000000	-0.7600000
16841.0000000	-0.7600000
16842.0000000	-0.7600000
16843.0000000	-0.7500000
16844.0000000	-0.7500000
16845.0000000	-0.7450000
16846.0000000	-0.7450000
16847.0000000	-0.7500000

16848.0000000	-0.7450000
16849.0000000	-0.7450000
16850.0000000	-0.7450000
16851.0000000	-0.7400000
16852.0000000	-0.7400000
16853.0000000	-0.7350000
16854.0000000	-0.7350000
16855.0000000	-0.7400000
16856.0000000	-0.7400000
16857.0000000	-0.7400000
16858.0000000	-0.7350000
16859.0000000	-0.7350000
16860.0000000	-0.7300000
16861.0000000	-0.7300000
16862.0000000	-0.7300000
16863.0000000	-0.7200000
16864.0000000	-0.7200000
16865.0000000	-0.7200000
16866.0000000	-0.7200000
16867.0000000	-0.7200000
16868.0000000	-0.7250000
16869.0000000	-0.7200000
16870.0000000	-0.7200000
16871.0000000	-0.7250000
16872.0000000	-0.7200000
16873.0000000	-0.7150000
16874.0000000	-0.7150000
16875.0000000	-0.7150000
16876.0000000	-0.7100000
16877.0000000	-0.7150000
16878.0000000	-0.7150000
16879.0000000	-0.7150000
16880.0000000	-0.7150000
16881.0000000	-0.7100000
16882.0000000	-0.7066666
16883.0000000	-0.7050000
16884.0000000	-0.7000000
16885.0000000	-0.7000000
16886.0000000	-0.7000000
16887.0000000	-0.6950000
16888.0000000	-0.7000000
16889.0000000	-0.7050000
16890.0000000	-0.7000000
16891.0000000	-0.7000000
16892.0000000	-0.7050000
16893.0000000	-0.7050000
16894.0000000	-0.7050000
16895.0000000	-0.7050000
16896.0000000	-0.7050000
16897.0000000	-0.7100000
16898.0000000	-0.7050000
16899.0000000	-0.7100000
16900.0000000	-0.7100000
16901.0000000	-0.7050000
16902.0000000	-0.7050000
16903.0000000	-0.7150000
16904.0000000	-0.7100000

16905.0000000	-0.7150000
16906.0000000	-0.7150000
16907.0000000	-0.7150000
16908.0000000	-0.7200000
16909.0000000	-0.7200000
16910.0000000	-0.7150000
16911.0000000	-0.7200000
16912.0000000	-0.7150000
16913.0000000	-0.7200000
16914.0000000	-0.7200000
16915.0000000	-0.7150000
16916.0000000	-0.7300000
16917.0000000	-0.7250000
16918.0000000	-0.7200000
16919.0000000	-0.7200000
16920.0000000	-0.7150000
16921.0000000	-0.7150000
16922.0000000	-0.7150000
16923.0000000	-0.7200000
16924.0000000	-0.7300000
16925.0000000	-0.7300000
16926.0000000	-0.7300000
16927.0000000	-0.7300000
16928.0000000	-0.7300000
16929.0000000	-0.7200000
16930.0000000	-0.7250000
16931.0000000	-0.7250000
16932.0000000	-0.7250000
16933.0000000	-0.7250000
16934.0000000	-0.7200000
16935.0000000	-0.7200000
16936.0000000	-0.7150000
16937.0000000	-0.7150000
16938.0000000	-0.7100000
16939.0000000	-0.7100000
16940.0000000	-0.7150000
16941.0000000	-0.7200000
16942.0000000	-0.7166667
16943.0000000	-0.7150000
16944.0000000	-0.7200000
16945.0000000	-0.7150000
16946.0000000	-0.7150000
16947.0000000	-0.7150000
16948.0000000	-0.7100000
16949.0000000	-0.7100000
16950.0000000	-0.7000000
16951.0000000	-0.6950000
16952.0000000	-0.6950000
16953.0000000	-0.6950000
16954.0000000	-0.6900000
16955.0000000	-0.6900000
16956.0000000	-0.6950000
16957.0000000	-0.6900000
16958.0000000	-0.6950000
16959.0000000	-0.7000000
16960.0000000	-0.6900000
16961.0000000	-0.7000000

16962.0000000	-0.6950000
16963.0000000	-0.6800000
16964.0000000	-0.6850000
16965.0000000	-0.6850000
16966.0000000	-0.6850000
16967.0000000	-0.6850000
16968.0000000	-0.6850000
16969.0000000	-0.6850000
16970.0000000	-0.6900000
16971.0000000	-0.6850000
16972.0000000	-0.6800000
16973.0000000	-0.6850000
16974.0000000	-0.6700000
16975.0000000	-0.6700000
16976.0000000	-0.6700000
16977.0000000	-0.6750000
16978.0000000	-0.6750000
16979.0000000	-0.6750000
16980.0000000	-0.6750000
16981.0000000	-0.6700000
16982.0000000	-0.6750000
16983.0000000	-0.6750000
16984.0000000	-0.6750000
16985.0000000	-0.6700000
16986.0000000	-0.6750000
16987.0000000	-0.6800000
16988.0000000	-0.6750000
16989.0000000	-0.6750000
16990.0000000	-0.6700000
16991.0000000	-0.6700000
16992.0000000	-0.6600000
16993.0000000	-0.6650000
16994.0000000	-0.6700000
16995.0000000	-0.6650000
16996.0000000	-0.6650000
16997.0000000	-0.6700000
16998.0000000	-0.6800000
16999.0000000	-0.6750000
17000.0000000	-0.6750000
17001.0000000	-0.6650000
17002.0000000	-0.6700000
17003.0000000	-0.6733333
17004.0000000	-0.6750000
17005.0000000	-0.6700000
17006.0000000	-0.6700000
17007.0000000	-0.6700000
17008.0000000	-0.6700000
17009.0000000	-0.6750000
17010.0000000	-0.6750000
17011.0000000	-0.6800000
17012.0000000	-0.6800000
17013.0000000	-0.6750000
17014.0000000	-0.6700000
17015.0000000	-0.6700000
17016.0000000	-0.6700000
17017.0000000	-0.6700000
17018.0000000	-0.6700000

17019.0000000	-0.6650000
17020.0000000	-0.6700000
17021.0000000	-0.6600000
17022.0000000	-0.6600000
17023.0000000	-0.6600000
17024.0000000	-0.6550000
17025.0000000	-0.6650000
17026.0000000	-0.6600000
17027.0000000	-0.6600000
17028.0000000	-0.6650000
17029.0000000	-0.6600000
17030.0000000	-0.6600000
17031.0000000	-0.6600000
17032.0000000	-0.6600000
17033.0000000	-0.6566667
17034.0000000	-0.6550000
17035.0000000	-0.6550000
17036.0000000	-0.6650000
17037.0000000	-0.6600000
17038.0000000	-0.6550000
17039.0000000	-0.6600000
17040.0000000	-0.6600000
17041.0000000	-0.6650000
17042.0000000	-0.6650000
17043.0000000	-0.6700000
17044.0000000	-0.6650000
17045.0000000	-0.6650000
17046.0000000	-0.6650000
17047.0000000	-0.6600000
17048.0000000	-0.6550000
17049.0000000	-0.6600000
17050.0000000	-0.6600000
17051.0000000	-0.6550000
17052.0000000	-0.6600000
17053.0000000	-0.6550000
17054.0000000	-0.6600000
17055.0000000	-0.6550000
17056.0000000	-0.6600000
17057.0000000	-0.6550000
17058.0000000	-0.6550000
17059.0000000	-0.6550000
17060.0000000	-0.6550000
17061.0000000	-0.6550000
17062.0000000	-0.6550000
17063.0000000	-0.6550000
17064.0000000	-0.6566667
17065.0000000	-0.6600000
17066.0000000	-0.6600000
17067.0000000	-0.6550000
17068.0000000	-0.6550000
17069.0000000	-0.6550000
17070.0000000	-0.6500000
17071.0000000	-0.6550000
17072.0000000	-0.6600000
17073.0000000	-0.6550000
17074.0000000	-0.6600000
17075.0000000	-0.6600000

17076.0000000	-0.6600000
17077.0000000	-0.6600000
17078.0000000	-0.6650000
17079.0000000	-0.6650000
17080.0000000	-0.6600000
17081.0000000	-0.6650000
17082.0000000	-0.6650000
17083.0000000	-0.6650000
17084.0000000	-0.6650000
17085.0000000	-0.6700000
17086.0000000	-0.6650000
17087.0000000	-0.6650000
17088.0000000	-0.6650000
17089.0000000	-0.6650000
17090.0000000	-0.6600000
17091.0000000	-0.6650000
17092.0000000	-0.6600000
17093.0000000	-0.6550000
17094.0000000	-0.6633334
17095.0000000	-0.6600000
17096.0000000	-0.6600000
17097.0000000	-0.6600000
17098.0000000	-0.6600000
17099.0000000	-0.6600000
17100.0000000	-0.6600000
17101.0000000	-0.6600000
17102.0000000	-0.6600000
17103.0000000	-0.6650000
17104.0000000	-0.6550000
17105.0000000	-0.6550000
17106.0000000	-0.6500000
17107.0000000	-0.6550000
17108.0000000	-0.6550000
17109.0000000	-0.6550000
17110.0000000	-0.6600000
17111.0000000	-0.6600000
17112.0000000	-0.6550000
17113.0000000	-0.6550000
17114.0000000	-0.6450000
17115.0000000	-0.6500000
17116.0000000	-0.6500000
17117.0000000	-0.6550000
17118.0000000	-0.6550000
17119.0000000	-0.6600000
17120.0000000	-0.6550000
17121.0000000	-0.6600000
17122.0000000	-0.6550000
17123.0000000	-0.6500000
17124.0000000	-0.6533333
17125.0000000	-0.6550000
17126.0000000	-0.6600000
17127.0000000	-0.6600000
17128.0000000	-0.6600000
17129.0000000	-0.6600000
17130.0000000	-0.6600000
17131.0000000	-0.6550000
17132.0000000	-0.6600000

17133.0000000	-0.6550000
17134.0000000	-0.6600000
17135.0000000	-0.6600000
17136.0000000	-0.6600000
17137.0000000	-0.6600000
17138.0000000	-0.6600000
17139.0000000	-0.6600000
17140.0000000	-0.6650000
17141.0000000	-0.6650000
17142.0000000	-0.6650000
17143.0000000	-0.6700000
17144.0000000	-0.6700000
17145.0000000	-0.6650000
17146.0000000	-0.6650000
17147.0000000	-0.6750000
17148.0000000	-0.6650000
17149.0000000	-0.6700000
17150.0000000	-0.6700000
17151.0000000	-0.6750000
17152.0000000	-0.6750000
17153.0000000	-0.6666667
17154.0000000	-0.6700000
17155.0000000	-0.6650000
17156.0000000	-0.6700000
17157.0000000	-0.6650000
17158.0000000	-0.6700000
17159.0000000	-0.6650000
17160.0000000	-0.6700000
17161.0000000	-0.6700000
17162.0000000	-0.6650000
17163.0000000	-0.6700000
17164.0000000	-0.6750000
17165.0000000	-0.6700000
17166.0000000	-0.6700000
17167.0000000	-0.6650000
17168.0000000	-0.6700000
17169.0000000	-0.6700000
17170.0000000	-0.6700000
17171.0000000	-0.6700000
17172.0000000	-0.6650000
17173.0000000	-0.6650000
17174.0000000	-0.6600000
17175.0000000	-0.6600000
17176.0000000	-0.6650000
17177.0000000	-0.6600000
17178.0000000	-0.6550000
17179.0000000	-0.6550000
17180.0000000	-0.6600000
17181.0000000	-0.6550000
17182.0000000	-0.6550000
17183.0000000	-0.6550000
17184.0000000	-0.6566667
17185.0000000	-0.6600000
17186.0000000	-0.6600000
17187.0000000	-0.6550000
17188.0000000	-0.6550000
17189.0000000	-0.6500000

17190.0000000	-0.6550000
17191.0000000	-0.6550000
17192.0000000	-0.6550000
17193.0000000	-0.6550000
17194.0000000	-0.6550000
17195.0000000	-0.6500000
17196.0000000	-0.6550000
17197.0000000	-0.6500000
17198.0000000	-0.6500000
17199.0000000	-0.6500000
17200.0000000	-0.6500000
17201.0000000	-0.6450000
17202.0000000	-0.6550000
17203.0000000	-0.6550000
17204.0000000	-0.6500000
17205.0000000	-0.6600000
17206.0000000	-0.6500000
17207.0000000	-0.6550000
17208.0000000	-0.6550000
17209.0000000	-0.6550000
17210.0000000	-0.6500000
17211.0000000	-0.6550000
17212.0000000	-0.6550000
17213.0000000	-0.6500000
17214.0000000	-0.6500000
17215.0000000	-0.6500000
17216.0000000	-0.6450000
17217.0000000	-0.6500000
17218.0000000	-0.6500000
17219.0000000	-0.6450000
17220.0000000	-0.6450000
17221.0000000	-0.6500000
17222.0000000	-0.6400000
17223.0000000	-0.6450000
17224.0000000	-0.6400000
17225.0000000	-0.6400000
17226.0000000	-0.6400000
17227.0000000	-0.6400000
17228.0000000	-0.6400000
17229.0000000	-0.6350000
17230.0000000	-0.6350000
17231.0000000	-0.6350000
17232.0000000	-0.6350000
17233.0000000	-0.6300000
17234.0000000	-0.6350000
17235.0000000	-0.6400000
17236.0000000	-0.6350000
17237.0000000	-0.6400000
17238.0000000	-0.6350000
17239.0000000	-0.6350000
17240.0000000	-0.6350000
17241.0000000	-0.6300000
17242.0000000	-0.6300000
17243.0000000	-0.6300000
17244.0000000	-0.6333333
17245.0000000	-0.6300000
17246.0000000	-0.6300000

17247.0000000	-0.6300000
17248.0000000	-0.6250000
17249.0000000	-0.6250000
17250.0000000	-0.6250000
17251.0000000	-0.6300000
17252.0000000	-0.6250000
17253.0000000	-0.6200000
17254.0000000	-0.6200000
17255.0000000	-0.6200000
17256.0000000	-0.6200000
17257.0000000	-0.6250000
17258.0000000	-0.6250000
17259.0000000	-0.6250000
17260.0000000	-0.6200000
17261.0000000	-0.6250000
17262.0000000	-0.6200000
17263.0000000	-0.6150000
17264.0000000	-0.6150000
17265.0000000	-0.6200000
17266.0000000	-0.6150000
17267.0000000	-0.6200000
17268.0000000	-0.6200000
17269.0000000	-0.6200000
17270.0000000	-0.6200000
17271.0000000	-0.6150000
17272.0000000	-0.6200000
17273.0000000	-0.6150000
17274.0000000	-0.6166667
17275.0000000	-0.6150000
17276.0000000	-0.6200000
17277.0000000	-0.6100000
17278.0000000	-0.6100000
17279.0000000	-0.6100000
17280.0000000	-0.6100000
17281.0000000	-0.6050000
17282.0000000	-0.6100000
17283.0000000	-0.6050000
17284.0000000	-0.6050000
17285.0000000	-0.6050000
17286.0000000	-0.6050000
17287.0000000	-0.6100000
17288.0000000	-0.6000000
17289.0000000	-0.6050000
17290.0000000	-0.6000000
17291.0000000	-0.6000000
17292.0000000	-0.6050000
17293.0000000	-0.6000000
17294.0000000	-0.6050000
17295.0000000	-0.6000000
17296.0000000	-0.6000000
17297.0000000	-0.6050000
17298.0000000	-0.6000000
17299.0000000	-0.6000000
17300.0000000	-0.6000000
17301.0000000	-0.6000000
17302.0000000	-0.6000000
17303.0000000	-0.6050000

17304.0000000	-0.6033334
17305.0000000	-0.6000000
17306.0000000	-0.6050000
17307.0000000	-0.6050000
17308.0000000	-0.6050000
17309.0000000	-0.6050000
17310.0000000	-0.6100000
17311.0000000	-0.6100000
17312.0000000	-0.6050000
17313.0000000	-0.5950000
17314.0000000	-0.6000000
17315.0000000	-0.6000000
17316.0000000	-0.5950000
17317.0000000	-0.6000000
17318.0000000	-0.6000000
17319.0000000	-0.6050000
17320.0000000	-0.6000000
17321.0000000	-0.6000000
17322.0000000	-0.6000000
17323.0000000	-0.5950000
17324.0000000	-0.6000000
17325.0000000	-0.5950000
17326.0000000	-0.5950000
17327.0000000	-0.5950000
17328.0000000	-0.6000000
17329.0000000	-0.6050000
17330.0000000	-0.6000000
17331.0000000	-0.6000000
17332.0000000	-0.6050000
17333.0000000	-0.6050000
17334.0000000	-0.6000000
17335.0000000	-0.6050000
17336.0000000	-0.6000000
17337.0000000	-0.6050000
17338.0000000	-0.6050000
17339.0000000	-0.6000000
17340.0000000	-0.6050000
17341.0000000	-0.6000000
17342.0000000	-0.6050000
17343.0000000	-0.6050000
17344.0000000	-0.6000000
17345.0000000	-0.6000000
17346.0000000	-0.6000000
17347.0000000	-0.5950000
17348.0000000	-0.5900000
17349.0000000	-0.5950000
17350.0000000	-0.6000000
17351.0000000	-0.6000000
17352.0000000	-0.6000000
17353.0000000	-0.6050000
17354.0000000	-0.6000000
17355.0000000	-0.6000000
17356.0000000	-0.6000000
17357.0000000	-0.6000000
17358.0000000	-0.6050000
17359.0000000	-0.6050000
17360.0000000	-0.6050000

17361.0000000	-0.6100000
17362.0000000	-0.6100000
17363.0000000	-0.6050000
17364.0000000	-0.6133333
17365.0000000	-0.6100000
17366.0000000	-0.6150000
17367.0000000	-0.6150000
17368.0000000	-0.6150000
17369.0000000	-0.6150000
17370.0000000	-0.6200000
17371.0000000	-0.6200000
17372.0000000	-0.6250000
17373.0000000	-0.6250000
17374.0000000	-0.6250000
17375.0000000	-0.6300000
17376.0000000	-0.6200000
17377.0000000	-0.6200000
17378.0000000	-0.6300000
17379.0000000	-0.6250000
17380.0000000	-0.6300000
17381.0000000	-0.6300000
17382.0000000	-0.6350000
17383.0000000	-0.6350000
17384.0000000	-0.6350000
17385.0000000	-0.6400000
17386.0000000	-0.6350000
17387.0000000	-0.6400000
17388.0000000	-0.6400000
17389.0000000	-0.6450000
17390.0000000	-0.6400000
17391.0000000	-0.6400000
17392.0000000	-0.6400000
17393.0000000	-0.6400000
17394.0000000	-0.6400000
17395.0000000	-0.6400000
17396.0000000	-0.6400000
17397.0000000	-0.6450000
17398.0000000	-0.6350000
17399.0000000	-0.6450000
17400.0000000	-0.6400000
17401.0000000	-0.6400000
17402.0000000	-0.6500000
17403.0000000	-0.6450000
17404.0000000	-0.6500000
17405.0000000	-0.6500000
17406.0000000	-0.6500000
17407.0000000	-0.6500000
17408.0000000	-0.6450000
17409.0000000	-0.6500000
17410.0000000	-0.6550000
17411.0000000	-0.6550000
17412.0000000	-0.6550000
17413.0000000	-0.6550000
17414.0000000	-0.6550000
17415.0000000	-0.6500000
17416.0000000	-0.6550000
17417.0000000	-0.6550000

17418.0000000	-0.6450000
17419.0000000	-0.6550000
17420.0000000	-0.6450000
17421.0000000	-0.6500000
17422.0000000	-0.6450000
17423.0000000	-0.6400000
17424.0000000	-0.6433333
17425.0000000	-0.6400000
17426.0000000	-0.6350000
17427.0000000	-0.6350000
17428.0000000	-0.6400000
17429.0000000	-0.6350000
17430.0000000	-0.6350000
17431.0000000	-0.6400000
17432.0000000	-0.6400000
17433.0000000	-0.6350000
17434.0000000	-0.6350000
17435.0000000	-0.6400000
17436.0000000	-0.6300000
17437.0000000	-0.6400000
17438.0000000	-0.6400000
17439.0000000	-0.6450000
17440.0000000	-0.6450000
17441.0000000	-0.6400000
17442.0000000	-0.6400000
17443.0000000	-0.6400000
17444.0000000	-0.6450000
17445.0000000	-0.6450000
17446.0000000	-0.6450000
17447.0000000	-0.6450000
17448.0000000	-0.6450000
17449.0000000	-0.6500000
17450.0000000	-0.6550000
17451.0000000	-0.6500000
17452.0000000	-0.6500000
17453.0000000	-0.6500000
17454.0000000	-0.6466666
17455.0000000	-0.6550000
17456.0000000	-0.6500000
17457.0000000	-0.6500000
17458.0000000	-0.6500000
17459.0000000	-0.6550000
17460.0000000	-0.6450000
17461.0000000	-0.6550000
17462.0000000	-0.6550000
17463.0000000	-0.6500000
17464.0000000	-0.6600000
17465.0000000	-0.6550000
17466.0000000	-0.6550000
17467.0000000	-0.6500000
17468.0000000	-0.6550000
17469.0000000	-0.6550000
17470.0000000	-0.6550000
17471.0000000	-0.6550000
17472.0000000	-0.6550000
17473.0000000	-0.6600000
17474.0000000	-0.6500000

17475.0000000	-0.6550000
17476.0000000	-0.6550000
17477.0000000	-0.6550000
17478.0000000	-0.6550000
17479.0000000	-0.6600000
17480.0000000	-0.6550000
17481.0000000	-0.6550000
17482.0000000	-0.6550000
17483.0000000	-0.6550000
17484.0000000	-0.6600000
17485.0000000	-0.6566667
17486.0000000	-0.6550000
17487.0000000	-0.6550000
17488.0000000	-0.6550000
17489.0000000	-0.6550000
17490.0000000	-0.6600000
17491.0000000	-0.6550000
17492.0000000	-0.6550000
17493.0000000	-0.6650000
17494.0000000	-0.6650000
17495.0000000	-0.6650000
17496.0000000	-0.6650000
17497.0000000	-0.6700000
17498.0000000	-0.6600000
17499.0000000	-0.6600000
17500.0000000	-0.6650000
17501.0000000	-0.6650000
17502.0000000	-0.6700000
17503.0000000	-0.6700000
17504.0000000	-0.6700000
17505.0000000	-0.6750000
17506.0000000	-0.6750000
17507.0000000	-0.6700000
17508.0000000	-0.6750000
17509.0000000	-0.6700000
17510.0000000	-0.6750000
17511.0000000	-0.6750000
17512.0000000	-0.6750000
17513.0000000	-0.6700000
17514.0000000	-0.6750000
17515.0000000	-0.6800000
17516.0000000	-0.6750000
17517.0000000	-0.6733333
17518.0000000	-0.6800000
17519.0000000	-0.6800000
17520.0000000	-0.6800000
17521.0000000	-0.6800000
17522.0000000	-0.6800000
17523.0000000	-0.6800000
17524.0000000	-0.6800000
17525.0000000	-0.6750000
17526.0000000	-0.6800000
17527.0000000	-0.6800000
17528.0000000	-0.6800000
17529.0000000	-0.6800000
17530.0000000	-0.6800000
17531.0000000	-0.6850000

17532.0000000	-0.6850000
17533.0000000	-0.6850000
17534.0000000	-0.6850000
17535.0000000	-0.6850000
17536.0000000	-0.6850000
17537.0000000	-0.6900000
17538.0000000	-0.6850000
17539.0000000	-0.6900000
17540.0000000	-0.6850000
17541.0000000	-0.6850000
17542.0000000	-0.6850000
17543.0000000	-0.6800000
17544.0000000	-0.6800000
17545.0000000	-0.6800000
17546.0000000	-0.6750000
17547.0000000	-0.6800000
17548.0000000	-0.6700000
17549.0000000	-0.6800000
17550.0000000	-0.6750000
17551.0000000	-0.6800000
17552.0000000	-0.6750000
17553.0000000	-0.6750000
17554.0000000	-0.6800000
17555.0000000	-0.6750000
17556.0000000	-0.6750000
17557.0000000	-0.6750000
17558.0000000	-0.6750000
17559.0000000	-0.6700000
17560.0000000	-0.6800000
17561.0000000	-0.6750000
17562.0000000	-0.6750000
17563.0000000	-0.6800000
17564.0000000	-0.6800000
17565.0000000	-0.6700000
17566.0000000	-0.6850000
17567.0000000	-0.6700000
17568.0000000	-0.6700000
17569.0000000	-0.6750000
17570.0000000	-0.6700000
17571.0000000	-0.6750000
17572.0000000	-0.6750000
17573.0000000	-0.6750000
17574.0000000	-0.6750000
17575.0000000	-0.6650000
17576.0000000	-0.6700000
17577.0000000	-0.6750000
17578.0000000	-0.6700000
17579.0000000	-0.6750000
17580.0000000	-0.6700000
17581.0000000	-0.6750000
17582.0000000	-0.6750000
17583.0000000	-0.6700000
17584.0000000	-0.6700000
17585.0000000	-0.6700000
17586.0000000	-0.6650000
17587.0000000	-0.6700000
17588.0000000	-0.6650000

17589.0000000	-0.6650000
17590.0000000	-0.6700000
17591.0000000	-0.6700000
17592.0000000	-0.6650000
17593.0000000	-0.6650000
17594.0000000	-0.6700000
17595.0000000	-0.6600000
17596.0000000	-0.6650000
17597.0000000	-0.6650000
17598.0000000	-0.6650000
17599.0000000	-0.6600000
17600.0000000	-0.6600000
17601.0000000	-0.6600000
17602.0000000	-0.6600000
17603.0000000	-0.6550000
17604.0000000	-0.6550000
17605.0000000	-0.6550000
17606.0000000	-0.6500000
17607.0000000	-0.6500000
17608.0000000	-0.6450000
17609.0000000	-0.6450000
17610.0000000	-0.6450000
17611.0000000	-0.6500000
17612.0000000	-0.6450000
17613.0000000	-0.6500000
17614.0000000	-0.6500000
17615.0000000	-0.6450000
17616.0000000	-0.6500000
17617.0000000	-0.6400000
17618.0000000	-0.6400000
17619.0000000	-0.6400000
17620.0000000	-0.6350000
17621.0000000	-0.6400000
17622.0000000	-0.6400000
17623.0000000	-0.6450000
17624.0000000	-0.6400000
17625.0000000	-0.6400000
17626.0000000	-0.6450000
17627.0000000	-0.6450000
17628.0000000	-0.6450000
17629.0000000	-0.6500000
17630.0000000	-0.6350000
17631.0000000	-0.6500000
17632.0000000	-0.6450000
17633.0000000	-0.6450000
17634.0000000	-0.6500000
17635.0000000	-0.6450000
17636.0000000	-0.6450000
17637.0000000	-0.6466666
17638.0000000	-0.6450000
17639.0000000	-0.6400000
17640.0000000	-0.6450000
17641.0000000	-0.6400000
17642.0000000	-0.6450000
17643.0000000	-0.6450000
17644.0000000	-0.6450000
17645.0000000	-0.6500000

17646.0000000	-0.6450000
17647.0000000	-0.6400000
17648.0000000	-0.6450000
17649.0000000	-0.6400000
17650.0000000	-0.6450000
17651.0000000	-0.6400000
17652.0000000	-0.6400000
17653.0000000	-0.6400000
17654.0000000	-0.6300000
17655.0000000	-0.6300000
17656.0000000	-0.6300000
17657.0000000	-0.6250000
17658.0000000	-0.6300000
17659.0000000	-0.6250000
17660.0000000	-0.6300000
17661.0000000	-0.6350000
17662.0000000	-0.6350000
17663.0000000	-0.6350000
17664.0000000	-0.6300000
17665.0000000	-0.6300000
17666.0000000	-0.6350000
17667.0000000	-0.6266667
17668.0000000	-0.6350000
17669.0000000	-0.6300000
17670.0000000	-0.6300000
17671.0000000	-0.6300000
17672.0000000	-0.6350000
17673.0000000	-0.6350000
17674.0000000	-0.6300000
17675.0000000	-0.6300000
17676.0000000	-0.6300000
17677.0000000	-0.6250000
17678.0000000	-0.6350000
17679.0000000	-0.6300000
17680.0000000	-0.6250000
17681.0000000	-0.6300000
17682.0000000	-0.6250000
17683.0000000	-0.6250000
17684.0000000	-0.6250000
17685.0000000	-0.6300000
17686.0000000	-0.6300000
17687.0000000	-0.6300000
17688.0000000	-0.6250000
17689.0000000	-0.6250000
17690.0000000	-0.6200000
17691.0000000	-0.6200000
17692.0000000	-0.6250000
17693.0000000	-0.6200000
17694.0000000	-0.6250000
17695.0000000	-0.6200000
17696.0000000	-0.6150000
17697.0000000	-0.6066667
17698.0000000	-0.6100000
17699.0000000	-0.6050000
17700.0000000	-0.6000000
17701.0000000	-0.6000000
17702.0000000	-0.6000000

17703.0000000	-0.5950000
17704.0000000	-0.5950000
17705.0000000	-0.6000000
17706.0000000	-0.5900000
17707.0000000	-0.5950000
17708.0000000	-0.5950000
17709.0000000	-0.5950000
17710.0000000	-0.5850000
17711.0000000	-0.5900000
17712.0000000	-0.5850000
17713.0000000	-0.5900000
17714.0000000	-0.5900000
17715.0000000	-0.5900000
17716.0000000	-0.5850000
17717.0000000	-0.5900000
17718.0000000	-0.5850000
17719.0000000	-0.5850000
17720.0000000	-0.5800000
17721.0000000	-0.5800000
17722.0000000	-0.5750000
17723.0000000	-0.5750000
17724.0000000	-0.5750000
17725.0000000	-0.5750000
17726.0000000	-0.5766667
17727.0000000	-0.5750000
17728.0000000	-0.5750000
17729.0000000	-0.5750000
17730.0000000	-0.5700000
17731.0000000	-0.5700000
17732.0000000	-0.5700000
17733.0000000	-0.5700000
17734.0000000	-0.5600000
17735.0000000	-0.5650000
17736.0000000	-0.5600000
17737.0000000	-0.5500000
17738.0000000	-0.5550000
17739.0000000	-0.5550000
17740.0000000	-0.5500000
17741.0000000	-0.5500000
17742.0000000	-0.5450000
17743.0000000	-0.5450000
17744.0000000	-0.5450000
17745.0000000	-0.5500000
17746.0000000	-0.5450000
17747.0000000	-0.5450000
17748.0000000	-0.5450000
17749.0000000	-0.5400000
17750.0000000	-0.5350000
17751.0000000	-0.5400000
17752.0000000	-0.5350000
17753.0000000	-0.5350000
17754.0000000	-0.5300000
17755.0000000	-0.5300000
17756.0000000	-0.5300000
17757.0000000	-0.5250000
17758.0000000	-0.5250000
17759.0000000	-0.5233333

17760.0000000	-0.5200000
17761.0000000	-0.5150000
17762.0000000	-0.5150000
17763.0000000	-0.5200000
17764.0000000	-0.5150000
17765.0000000	-0.5200000
17766.0000000	-0.5150000
17767.0000000	-0.5150000
17768.0000000	-0.5100000
17769.0000000	-0.5150000
17770.0000000	-0.5150000
17771.0000000	-0.5100000
17772.0000000	-0.5150000
17773.0000000	-0.5200000
17774.0000000	-0.5100000
17775.0000000	-0.5150000
17776.0000000	-0.5150000
17777.0000000	-0.5100000
17778.0000000	-0.5100000
17779.0000000	-0.5150000
17780.0000000	-0.5100000
17781.0000000	-0.5050000
17782.0000000	-0.5050000
17783.0000000	-0.5050000
17784.0000000	-0.5050000
17785.0000000	-0.5000000
17786.0000000	-0.5000000
17787.0000000	-0.5000000
17788.0000000	-0.5000000
17789.0000000	-0.4950000
17790.0000000	-0.4950000
17791.0000000	-0.4950000
17792.0000000	-0.4900000
17793.0000000	-0.4950000
17794.0000000	-0.4900000
17795.0000000	-0.4900000
17796.0000000	-0.4900000
17797.0000000	-0.4950000
17798.0000000	-0.4900000
17799.0000000	-0.4950000
17800.0000000	-0.4900000
17801.0000000	-0.4900000
17802.0000000	-0.4950000
17803.0000000	-0.4950000
17804.0000000	-0.4900000
17805.0000000	-0.4900000
17806.0000000	-0.4950000
17807.0000000	-0.4950000
17808.0000000	-0.4850000
17809.0000000	-0.4900000
17810.0000000	-0.4900000
17811.0000000	-0.4950000
17812.0000000	-0.4950000
17813.0000000	-0.5000000
17814.0000000	-0.4950000
17815.0000000	-0.5000000
17816.0000000	-0.5000000

17817.0000000	-0.5050000
17818.0000000	-0.5033333
17819.0000000	-0.5000000
17820.0000000	-0.5000000
17821.0000000	-0.5000000
17822.0000000	-0.5050000
17823.0000000	-0.5000000
17824.0000000	-0.5000000
17825.0000000	-0.4950000
17826.0000000	-0.5000000
17827.0000000	-0.4950000
17828.0000000	-0.5000000
17829.0000000	-0.4950000
17830.0000000	-0.5000000
17831.0000000	-0.4950000
17832.0000000	-0.5000000
17833.0000000	-0.5050000
17834.0000000	-0.5000000
17835.0000000	-0.5000000
17836.0000000	-0.5000000
17837.0000000	-0.5000000
17838.0000000	-0.5000000
17839.0000000	-0.4950000
17840.0000000	-0.4950000
17841.0000000	-0.5000000
17842.0000000	-0.4950000
17843.0000000	-0.5000000
17844.0000000	-0.4950000
17845.0000000	-0.4900000
17846.0000000	-0.5000000
17847.0000000	-0.4966667
17848.0000000	-0.4950000
17849.0000000	-0.4900000
17850.0000000	-0.4900000
17851.0000000	-0.5000000
17852.0000000	-0.4950000
17853.0000000	-0.4950000
17854.0000000	-0.4950000
17855.0000000	-0.4900000
17856.0000000	-0.4950000
17857.0000000	-0.4950000
17858.0000000	-0.4900000
17859.0000000	-0.4900000
17860.0000000	-0.4850000
17861.0000000	-0.4850000
17862.0000000	-0.4850000
17863.0000000	-0.4850000
17864.0000000	-0.4850000
17865.0000000	-0.4800000
17866.0000000	-0.4800000
17867.0000000	-0.4750000
17868.0000000	-0.4750000
17869.0000000	-0.4850000
17870.0000000	-0.4750000
17871.0000000	-0.4800000
17872.0000000	-0.4750000
17873.0000000	-0.4750000

17874.0000000	-0.4800000
17875.0000000	-0.4750000
17876.0000000	-0.4800000
17877.0000000	-0.4750000
17878.0000000	-0.4750000
17879.0000000	-0.4733333
17880.0000000	-0.4700000
17881.0000000	-0.4600000
17882.0000000	-0.4650000
17883.0000000	-0.4650000
17884.0000000	-0.4600000
17885.0000000	-0.4600000
17886.0000000	-0.4600000
17887.0000000	-0.4600000
17888.0000000	-0.4550000
17889.0000000	-0.4600000
17890.0000000	-0.4550000
17891.0000000	-0.4550000
17892.0000000	-0.4500000
17893.0000000	-0.4550000
17894.0000000	-0.4550000
17895.0000000	-0.4500000
17896.0000000	-0.4500000
17897.0000000	-0.4500000
17898.0000000	-0.4500000
17899.0000000	-0.4450000
17900.0000000	-0.4500000
17901.0000000	-0.4500000
17902.0000000	-0.4500000
17903.0000000	-0.4500000
17904.0000000	-0.4450000
17905.0000000	-0.4450000
17906.0000000	-0.4500000
17907.0000000	-0.4500000
17908.0000000	-0.4450000
17909.0000000	-0.4500000
17910.0000000	-0.4533333
17911.0000000	-0.4500000
17912.0000000	-0.4500000
17913.0000000	-0.4450000
17914.0000000	-0.4400000
17915.0000000	-0.4450000
17916.0000000	-0.4500000
17917.0000000	-0.4450000
17918.0000000	-0.4450000
17919.0000000	-0.4450000
17920.0000000	-0.4450000
17921.0000000	-0.4400000
17922.0000000	-0.4450000
17923.0000000	-0.4400000
17924.0000000	-0.4450000
17925.0000000	-0.4450000
17926.0000000	-0.4400000
17927.0000000	-0.4400000
17928.0000000	-0.4450000
17929.0000000	-0.4400000
17930.0000000	-0.4400000

17931.0000000	-0.4400000
17932.0000000	-0.4400000
17933.0000000	-0.4400000
17934.0000000	-0.4350000
17935.0000000	-0.4350000
17936.0000000	-0.4400000
17937.0000000	-0.4350000
17938.0000000	-0.4400000
17939.0000000	-0.4350000
17940.0000000	-0.4333333
17941.0000000	-0.4300000
17942.0000000	-0.4250000
17943.0000000	-0.4350000
17944.0000000	-0.4300000
17945.0000000	-0.4250000
17946.0000000	-0.4300000
17947.0000000	-0.4250000
17948.0000000	-0.4250000
17949.0000000	-0.4250000
17950.0000000	-0.4300000
17951.0000000	-0.4300000
17952.0000000	-0.4300000
17953.0000000	-0.4250000
17954.0000000	-0.4300000
17955.0000000	-0.4250000
17956.0000000	-0.4200000
17957.0000000	-0.4300000
17958.0000000	-0.4250000
17959.0000000	-0.4250000
17960.0000000	-0.4300000
17961.0000000	-0.4300000
17962.0000000	-0.4250000
17963.0000000	-0.4250000
17964.0000000	-0.4300000
17965.0000000	-0.4200000
17966.0000000	-0.4250000
17967.0000000	-0.4200000
17968.0000000	-0.4250000
17969.0000000	-0.4300000
17970.0000000	-0.4266666
17971.0000000	-0.4200000
17972.0000000	-0.4200000
17973.0000000	-0.4200000
17974.0000000	-0.4250000
17975.0000000	-0.4200000
17976.0000000	-0.4200000
17977.0000000	-0.4200000
17978.0000000	-0.4150000
17979.0000000	-0.4200000
17980.0000000	-0.4200000
17981.0000000	-0.4200000
17982.0000000	-0.4150000
17983.0000000	-0.4200000
17984.0000000	-0.4200000
17985.0000000	-0.4200000
17986.0000000	-0.4200000
17987.0000000	-0.4200000

17988.0000000	-0.4150000
17989.0000000	-0.4200000
17990.0000000	-0.4150000
17991.0000000	-0.4200000
17992.0000000	-0.4150000
17993.0000000	-0.4200000
17994.0000000	-0.4150000
17995.0000000	-0.4150000
17996.0000000	-0.4200000
17997.0000000	-0.4150000
17998.0000000	-0.4150000
17999.0000000	-0.4150000
18000.0000000	-0.4133333
18001.0000000	-0.4150000
18002.0000000	-0.4100000
18003.0000000	-0.4150000
18004.0000000	-0.4200000
18005.0000000	-0.4200000
18006.0000000	-0.4200000
18007.0000000	-0.4150000
18008.0000000	-0.4150000
18009.0000000	-0.4150000
18010.0000000	-0.4150000
18011.0000000	-0.4150000
18012.0000000	-0.4200000
18013.0000000	-0.4200000
18014.0000000	-0.4200000
18015.0000000	-0.4200000
18016.0000000	-0.4250000
18017.0000000	-0.4250000
18018.0000000	-0.4200000
18019.0000000	-0.4200000
18020.0000000	-0.4150000
18021.0000000	-0.4200000
18022.0000000	-0.4250000
18023.0000000	-0.4150000
18024.0000000	-0.4200000
18025.0000000	-0.4150000
18026.0000000	-0.4150000
18027.0000000	-0.4200000
18028.0000000	-0.4150000
18029.0000000	-0.4200000
18030.0000000	-0.4166667
18031.0000000	-0.4150000
18032.0000000	-0.4150000
18033.0000000	-0.4100000
18034.0000000	-0.4100000
18035.0000000	-0.4150000
18036.0000000	-0.4150000
18037.0000000	-0.4200000
18038.0000000	-0.4150000
18039.0000000	-0.4150000
18040.0000000	-0.4150000
18041.0000000	-0.4100000
18042.0000000	-0.4150000
18043.0000000	-0.4150000
18044.0000000	-0.4200000

18045.0000000	-0.4150000
18046.0000000	-0.4100000
18047.0000000	-0.4150000
18048.0000000	-0.4100000
18049.0000000	-0.4100000
18050.0000000	-0.4200000
18051.0000000	-0.4100000
18052.0000000	-0.4150000
18053.0000000	-0.4150000
18054.0000000	-0.4200000
18055.0000000	-0.4150000
18056.0000000	-0.4200000
18057.0000000	-0.4150000
18058.0000000	-0.4200000
18059.0000000	-0.4200000
18060.0000000	-0.4150000
18061.0000000	-0.4200000
18062.0000000	-0.4200000
18063.0000000	-0.4150000
18064.0000000	-0.4200000
18065.0000000	-0.4150000
18066.0000000	-0.4150000
18067.0000000	-0.4150000
18068.0000000	-0.4200000
18069.0000000	-0.4150000
18070.0000000	-0.4150000
18071.0000000	-0.4200000
18072.0000000	-0.4200000
18073.0000000	-0.4200000
18074.0000000	-0.4200000
18075.0000000	-0.4250000
18076.0000000	-0.4200000
18077.0000000	-0.4250000
18078.0000000	-0.4200000
18079.0000000	-0.4200000
18080.0000000	-0.4250000
18081.0000000	-0.4200000
18082.0000000	-0.4250000
18083.0000000	-0.4250000
18084.0000000	-0.4200000
18085.0000000	-0.4200000
18086.0000000	-0.4250000
18087.0000000	-0.4250000
18088.0000000	-0.4250000
18089.0000000	-0.4250000
18090.0000000	-0.4250000
18091.0000000	-0.4266666
18092.0000000	-0.4250000
18093.0000000	-0.4300000
18094.0000000	-0.4250000
18095.0000000	-0.4250000
18096.0000000	-0.4200000
18097.0000000	-0.4250000
18098.0000000	-0.4200000
18099.0000000	-0.4200000
18100.0000000	-0.4250000
18101.0000000	-0.4250000

18102.0000000	-0.4250000
18103.0000000	-0.4250000
18104.0000000	-0.4250000
18105.0000000	-0.4300000
18106.0000000	-0.4300000
18107.0000000	-0.4250000
18108.0000000	-0.4250000
18109.0000000	-0.4300000
18110.0000000	-0.4300000
18111.0000000	-0.4350000
18112.0000000	-0.4250000
18113.0000000	-0.4300000
18114.0000000	-0.4300000
18115.0000000	-0.4250000
18116.0000000	-0.4300000
18117.0000000	-0.4350000
18118.0000000	-0.4250000
18119.0000000	-0.4250000
18120.0000000	-0.4300000
18121.0000000	-0.4250000
18122.0000000	-0.4333333
18123.0000000	-0.4350000
18124.0000000	-0.4300000
18125.0000000	-0.4300000
18126.0000000	-0.4350000
18127.0000000	-0.4350000
18128.0000000	-0.4300000
18129.0000000	-0.4300000
18130.0000000	-0.4350000
18131.0000000	-0.4400000
18132.0000000	-0.4350000
18133.0000000	-0.4400000
18134.0000000	-0.4400000
18135.0000000	-0.4400000
18136.0000000	-0.4400000
18137.0000000	-0.4400000
18138.0000000	-0.4400000
18139.0000000	-0.4450000
18140.0000000	-0.4500000
18141.0000000	-0.4450000
18142.0000000	-0.4450000
18143.0000000	-0.4500000
18144.0000000	-0.4500000
18145.0000000	-0.4500000
18146.0000000	-0.4500000
18147.0000000	-0.4500000
18148.0000000	-0.4550000
18149.0000000	-0.4500000
18150.0000000	-0.4550000
18151.0000000	-0.4533333
18152.0000000	-0.4550000
18153.0000000	-0.4600000
18154.0000000	-0.4600000
18155.0000000	-0.4550000
18156.0000000	-0.4550000
18157.0000000	-0.4600000
18158.0000000	-0.4600000

18159.0000000	-0.4600000
18160.0000000	-0.4550000
18161.0000000	-0.4550000
18162.0000000	-0.4600000
18163.0000000	-0.4550000
18164.0000000	-0.4550000
18165.0000000	-0.4600000
18166.0000000	-0.4550000
18167.0000000	-0.4550000
18168.0000000	-0.4550000
18169.0000000	-0.4500000
18170.0000000	-0.4500000
18171.0000000	-0.4550000
18172.0000000	-0.4450000
18173.0000000	-0.4500000
18174.0000000	-0.4500000
18175.0000000	-0.4500000
18176.0000000	-0.4500000
18177.0000000	-0.4450000
18178.0000000	-0.4450000
18179.0000000	-0.4500000
18180.0000000	-0.4500000
18181.0000000	-0.4500000
18182.0000000	-0.4400000
18183.0000000	-0.4500000
18184.0000000	-0.4450000
18185.0000000	-0.4400000
18186.0000000	-0.4450000
18187.0000000	-0.4400000
18188.0000000	-0.4400000
18189.0000000	-0.4400000
18190.0000000	-0.4350000
18191.0000000	-0.4400000
18192.0000000	-0.4350000
18193.0000000	-0.4350000
18194.0000000	-0.4400000
18195.0000000	-0.4350000
18196.0000000	-0.4350000
18197.0000000	-0.4350000
18198.0000000	-0.4400000
18199.0000000	-0.4300000
18200.0000000	-0.4350000
18201.0000000	-0.4350000
18202.0000000	-0.4350000
18203.0000000	-0.4300000
18204.0000000	-0.4300000
18205.0000000	-0.4350000
18206.0000000	-0.4350000
18207.0000000	-0.4350000
18208.0000000	-0.4300000
18209.0000000	-0.4300000
18210.0000000	-0.4333333
18211.0000000	-0.4350000
18212.0000000	-0.4350000
18213.0000000	-0.4400000
18214.0000000	-0.4300000
18215.0000000	-0.4350000

18216.0000000	-0.4250000
18217.0000000	-0.4300000
18218.0000000	-0.4300000
18219.0000000	-0.4300000
18220.0000000	-0.4350000
18221.0000000	-0.4300000
18222.0000000	-0.4350000
18223.0000000	-0.4300000
18224.0000000	-0.4300000
18225.0000000	-0.4300000
18226.0000000	-0.4250000
18227.0000000	-0.4300000
18228.0000000	-0.4250000
18229.0000000	-0.4300000
18230.0000000	-0.4350000
18231.0000000	-0.4300000
18232.0000000	-0.4350000
18233.0000000	-0.4300000
18234.0000000	-0.4300000
18235.0000000	-0.4400000
18236.0000000	-0.4250000
18237.0000000	-0.4250000
18238.0000000	-0.4300000
18239.0000000	-0.4300000
18240.0000000	-0.4300000
18241.0000000	-0.4300000
18242.0000000	-0.4300000
18243.0000000	-0.4350000
18244.0000000	-0.4300000
18245.0000000	-0.4300000
18246.0000000	-0.4350000
18247.0000000	-0.4350000
18248.0000000	-0.4350000
18249.0000000	-0.4300000
18250.0000000	-0.4350000
18251.0000000	-0.4350000
18252.0000000	-0.4350000
18253.0000000	-0.4400000
18254.0000000	-0.4300000
18255.0000000	-0.4300000
18256.0000000	-0.4350000
18257.0000000	-0.4400000
18258.0000000	-0.4350000
18259.0000000	-0.4350000
18260.0000000	-0.4350000
18261.0000000	-0.4350000
18262.0000000	-0.4350000
18263.0000000	-0.4300000
18264.0000000	-0.4300000
18265.0000000	-0.4300000
18266.0000000	-0.4300000
18267.0000000	-0.4350000
18268.0000000	-0.4300000
18269.0000000	-0.4300000
18270.0000000	-0.4400000
18271.0000000	-0.4266666
18272.0000000	-0.4300000

18273.0000000	-0.4350000
18274.0000000	-0.4350000
18275.0000000	-0.4350000
18276.0000000	-0.4350000
18277.0000000	-0.4350000
18278.0000000	-0.4300000
18279.0000000	-0.4300000
18280.0000000	-0.4300000
18281.0000000	-0.4300000
18282.0000000	-0.4350000
18283.0000000	-0.4350000
18284.0000000	-0.4200000
18285.0000000	-0.4300000
18286.0000000	-0.4250000
18287.0000000	-0.4250000
18288.0000000	-0.4300000
18289.0000000	-0.4200000
18290.0000000	-0.4250000
18291.0000000	-0.4300000
18292.0000000	-0.4350000
18293.0000000	-0.4250000
18294.0000000	-0.4300000
18295.0000000	-0.4300000
18296.0000000	-0.4250000
18297.0000000	-0.4300000
18298.0000000	-0.4350000
18299.0000000	-0.4300000
18300.0000000	-0.4300000
18301.0000000	-0.4333333
18302.0000000	-0.4300000
18303.0000000	-0.4300000
18304.0000000	-0.4300000
18305.0000000	-0.4250000
18306.0000000	-0.4350000
18307.0000000	-0.4250000
18308.0000000	-0.4250000
18309.0000000	-0.4300000
18310.0000000	-0.4250000
18311.0000000	-0.4250000
18312.0000000	-0.4250000
18313.0000000	-0.4250000
18314.0000000	-0.4300000
18315.0000000	-0.4300000
18316.0000000	-0.4250000
18317.0000000	-0.4300000
18318.0000000	-0.4250000
18319.0000000	-0.4200000
18320.0000000	-0.4200000
18321.0000000	-0.4250000
18322.0000000	-0.4200000
18323.0000000	-0.4250000
18324.0000000	-0.4200000
18325.0000000	-0.4200000
18326.0000000	-0.4150000
18327.0000000	-0.4200000
18328.0000000	-0.4150000
18329.0000000	-0.4150000

18330.0000000	-0.4150000
18331.0000000	-0.4150000
18332.0000000	-0.4133333
18333.0000000	-0.4100000
18334.0000000	-0.4100000
18335.0000000	-0.4100000
18336.0000000	-0.4200000
18337.0000000	-0.4150000
18338.0000000	-0.4100000
18339.0000000	-0.4100000
18340.0000000	-0.4100000
18341.0000000	-0.4150000
18342.0000000	-0.4150000
18343.0000000	-0.4050000
18344.0000000	-0.4100000
18345.0000000	-0.4100000
18346.0000000	-0.4150000
18347.0000000	-0.4150000
18348.0000000	-0.4100000
18349.0000000	-0.4100000
18350.0000000	-0.4000000
18351.0000000	-0.4050000
18352.0000000	-0.4050000
18353.0000000	-0.4100000
18354.0000000	-0.4100000
18355.0000000	-0.4100000
18356.0000000	-0.4050000
18357.0000000	-0.4050000
18358.0000000	-0.4100000
18359.0000000	-0.4100000
18360.0000000	-0.4100000
18361.0000000	-0.4150000
18362.0000000	-0.4133333
18363.0000000	-0.4150000
18364.0000000	-0.4100000
18365.0000000	-0.4100000
18366.0000000	-0.4150000
18367.0000000	-0.4150000
18368.0000000	-0.4200000
18369.0000000	-0.4200000
18370.0000000	-0.4200000
18371.0000000	-0.4200000
18372.0000000	-0.4250000
18373.0000000	-0.4200000
18374.0000000	-0.4200000
18375.0000000	-0.4150000
18376.0000000	-0.4150000
18377.0000000	-0.4200000
18378.0000000	-0.4250000
18379.0000000	-0.4200000
18380.0000000	-0.4200000
18381.0000000	-0.4200000
18382.0000000	-0.4150000
18383.0000000	-0.4200000
18384.0000000	-0.4200000
18385.0000000	-0.4200000
18386.0000000	-0.4250000

18387.0000000	-0.4200000
18388.0000000	-0.4250000
18389.0000000	-0.4200000
18390.0000000	-0.4200000
18391.0000000	-0.4200000
18392.0000000	-0.4133333
18393.0000000	-0.4100000
18394.0000000	-0.4150000
18395.0000000	-0.4100000
18396.0000000	-0.4050000
18397.0000000	-0.4150000
18398.0000000	-0.4150000
18399.0000000	-0.4150000
18400.0000000	-0.4100000
18401.0000000	-0.4050000
18402.0000000	-0.4100000
18403.0000000	-0.4050000
18404.0000000	-0.4050000
18405.0000000	-0.4050000
18406.0000000	-0.4000000
18407.0000000	-0.4050000
18408.0000000	-0.4050000
18409.0000000	-0.4000000
18410.0000000	-0.3950000
18411.0000000	-0.4000000
18412.0000000	-0.3950000
18413.0000000	-0.3950000
18414.0000000	-0.3900000
18415.0000000	-0.3900000
18416.0000000	-0.3900000
18417.0000000	-0.3850000
18418.0000000	-0.3950000
18419.0000000	-0.3900000
18420.0000000	-0.3850000
18421.0000000	-0.3833333
18422.0000000	-0.3800000
18423.0000000	-0.3800000
18424.0000000	-0.3800000
18425.0000000	-0.3800000
18426.0000000	-0.3750000
18427.0000000	-0.3700000
18428.0000000	-0.3750000
18429.0000000	-0.3650000
18430.0000000	-0.3700000
18431.0000000	-0.3700000
18432.0000000	-0.3700000
18433.0000000	-0.3600000
18434.0000000	-0.3650000
18435.0000000	-0.3650000
18436.0000000	-0.3650000
18437.0000000	-0.3600000
18438.0000000	-0.3650000
18439.0000000	-0.3550000
18440.0000000	-0.3600000
18441.0000000	-0.3550000
18442.0000000	-0.3550000
18443.0000000	-0.3550000

18444.0000000	-0.3600000
18445.0000000	-0.3550000
18446.0000000	-0.3450000
18447.0000000	-0.3600000
18448.0000000	-0.3450000
18449.0000000	-0.3500000
18450.0000000	-0.3450000
18451.0000000	-0.3450000
18452.0000000	-0.3450000
18453.0000000	-0.3333333
18454.0000000	-0.3400000
18455.0000000	-0.3350000
18456.0000000	-0.3400000
18457.0000000	-0.3400000
18458.0000000	-0.3350000
18459.0000000	-0.3350000
18460.0000000	-0.3400000
18461.0000000	-0.3300000
18462.0000000	-0.3300000
18463.0000000	-0.3300000
18464.0000000	-0.3250000
18465.0000000	-0.3300000
18466.0000000	-0.3300000
18467.0000000	-0.3300000
18468.0000000	-0.3300000
18469.0000000	-0.3300000
18470.0000000	-0.3250000
18471.0000000	-0.3200000
18472.0000000	-0.3200000
18473.0000000	-0.3200000
18474.0000000	-0.3200000
18475.0000000	-0.3200000
18476.0000000	-0.3200000
18477.0000000	-0.3150000
18478.0000000	-0.3150000
18479.0000000	-0.3200000
18480.0000000	-0.3200000
18481.0000000	-0.3150000
18482.0000000	-0.3100000
18483.0000000	-0.3100000
18484.0000000	-0.3050000
18485.0000000	-0.3100000
18486.0000000	-0.3150000
18487.0000000	-0.3100000
18488.0000000	-0.3100000
18489.0000000	-0.3150000
18490.0000000	-0.3050000
18491.0000000	-0.3000000
18492.0000000	-0.3000000
18493.0000000	-0.3000000
18494.0000000	-0.2950000
18495.0000000	-0.3050000
18496.0000000	-0.3000000
18497.0000000	-0.3000000
18498.0000000	-0.3000000
18499.0000000	-0.2950000
18500.0000000	-0.2950000

18501.0000000	-0.3000000
18502.0000000	-0.3000000
18503.0000000	-0.2950000
18504.0000000	-0.3000000
18505.0000000	-0.2950000
18506.0000000	-0.2950000
18507.0000000	-0.2900000
18508.0000000	-0.2900000
18509.0000000	-0.2950000
18510.0000000	-0.2900000
18511.0000000	-0.2950000
18512.0000000	-0.2900000
18513.0000000	-0.2900000
18514.0000000	-0.2950000
18515.0000000	-0.2950000
18516.0000000	-0.2950000
18517.0000000	-0.3000000
18518.0000000	-0.2950000
18519.0000000	-0.2900000
18520.0000000	-0.2850000
18521.0000000	-0.2900000
18522.0000000	-0.2850000
18523.0000000	-0.2850000
18524.0000000	-0.2900000
18525.0000000	-0.2900000
18526.0000000	-0.2900000
18527.0000000	-0.2900000
18528.0000000	-0.2900000
18529.0000000	-0.2900000
18530.0000000	-0.2900000
18531.0000000	-0.2900000
18532.0000000	-0.2800000
18533.0000000	-0.2800000
18534.0000000	-0.2750000
18535.0000000	-0.2900000
18536.0000000	-0.2950000
18537.0000000	-0.2900000
18538.0000000	-0.2900000
18539.0000000	-0.2950000
18540.0000000	-0.2900000
18541.0000000	-0.2950000
18542.0000000	-0.2900000
18543.0000000	-0.2900000
18544.0000000	-0.2950000
18545.0000000	-0.2950000
18546.0000000	-0.3000000
18547.0000000	-0.2900000
18548.0000000	-0.2900000
18549.0000000	-0.2950000
18550.0000000	-0.2950000
18551.0000000	-0.2950000
18552.0000000	-0.2950000
18553.0000000	-0.2950000
18554.0000000	-0.2900000
18555.0000000	-0.2950000
18556.0000000	-0.2900000
18557.0000000	-0.2900000

18558.0000000	-0.2850000
18559.0000000	-0.2850000
18560.0000000	-0.2850000
18561.0000000	-0.2850000
18562.0000000	-0.2900000
18563.0000000	-0.2900000
18564.0000000	-0.2850000
18565.0000000	-0.2850000
18566.0000000	-0.2800000
18567.0000000	-0.2850000
18568.0000000	-0.2800000
18569.0000000	-0.2750000
18570.0000000	-0.2800000
18571.0000000	-0.2850000
18572.0000000	-0.2800000
18573.0000000	-0.2800000
18574.0000000	-0.2800000
18575.0000000	-0.2850000
18576.0000000	-0.2800000
18577.0000000	-0.2800000
18578.0000000	-0.2750000
18579.0000000	-0.2750000
18580.0000000	-0.2800000
18581.0000000	-0.2750000
18582.0000000	-0.2800000
18583.0000000	-0.2800000
18584.0000000	-0.2750000
18585.0000000	-0.2750000
18586.0000000	-0.2800000
18587.0000000	-0.2750000
18588.0000000	-0.2750000
18589.0000000	-0.2750000
18590.0000000	-0.2750000
18591.0000000	-0.2700000
18592.0000000	-0.2700000
18593.0000000	-0.2750000
18594.0000000	-0.2750000
18595.0000000	-0.2750000
18596.0000000	-0.2750000
18597.0000000	-0.2700000
18598.0000000	-0.2700000
18599.0000000	-0.2750000
18600.0000000	-0.2700000
18601.0000000	-0.2700000
18602.0000000	-0.2750000
18603.0000000	-0.2700000
18604.0000000	-0.2666667
18605.0000000	-0.2650000
18606.0000000	-0.2600000
18607.0000000	-0.2700000
18608.0000000	-0.2650000
18609.0000000	-0.2700000
18610.0000000	-0.2650000
18611.0000000	-0.2700000
18612.0000000	-0.2650000
18613.0000000	-0.2650000
18614.0000000	-0.2700000

18615.0000000	-0.2650000
18616.0000000	-0.2600000
18617.0000000	-0.2600000
18618.0000000	-0.2600000
18619.0000000	-0.2550000
18620.0000000	-0.2600000
18621.0000000	-0.2550000
18622.0000000	-0.2600000
18623.0000000	-0.2600000
18624.0000000	-0.2600000
18625.0000000	-0.2600000
18626.0000000	-0.2600000
18627.0000000	-0.2550000
18628.0000000	-0.2600000
18629.0000000	-0.2550000
18630.0000000	-0.2550000
18631.0000000	-0.2550000
18632.0000000	-0.2500000
18633.0000000	-0.2500000
18634.0000000	-0.2450000
18635.0000000	-0.2466667
18636.0000000	-0.2450000
18637.0000000	-0.2450000
18638.0000000	-0.2400000
18639.0000000	-0.2450000
18640.0000000	-0.2400000
18641.0000000	-0.2350000
18642.0000000	-0.2350000
18643.0000000	-0.2400000
18644.0000000	-0.2350000
18645.0000000	-0.2350000
18646.0000000	-0.2300000
18647.0000000	-0.2300000
18648.0000000	-0.2300000
18649.0000000	-0.2250000
18650.0000000	-0.2300000
18651.0000000	-0.2250000
18652.0000000	-0.2250000
18653.0000000	-0.2200000
18654.0000000	-0.2200000
18655.0000000	-0.2250000
18656.0000000	-0.2200000
18657.0000000	-0.2250000
18658.0000000	-0.2200000
18659.0000000	-0.2200000
18660.0000000	-0.2200000
18661.0000000	-0.2150000
18662.0000000	-0.2150000
18663.0000000	-0.2200000
18664.0000000	-0.2100000
18665.0000000	-0.2133333
18666.0000000	-0.2200000
18667.0000000	-0.2150000
18668.0000000	-0.2200000
18669.0000000	-0.2100000
18670.0000000	-0.2150000
18671.0000000	-0.2150000

18672.0000000	-0.2150000
18673.0000000	-0.2100000
18674.0000000	-0.2150000
18675.0000000	-0.2050000
18676.0000000	-0.2100000
18677.0000000	-0.2100000
18678.0000000	-0.2000000
18679.0000000	-0.2100000
18680.0000000	-0.2050000
18681.0000000	-0.2050000
18682.0000000	-0.2050000
18683.0000000	-0.2050000
18684.0000000	-0.2000000
18685.0000000	-0.2000000
18686.0000000	-0.2050000
18687.0000000	-0.2000000
18688.0000000	-0.1950000
18689.0000000	-0.1900000
18690.0000000	-0.1950000
18691.0000000	-0.1950000
18692.0000000	-0.1950000
18693.0000000	-0.1900000
18694.0000000	-0.1900000
18695.0000000	-0.1900000
18696.0000000	-0.1866667
18697.0000000	-0.1850000
18698.0000000	-0.1800000
18699.0000000	-0.1800000
18700.0000000	-0.1750000
18701.0000000	-0.1750000
18702.0000000	-0.1750000
18703.0000000	-0.1750000
18704.0000000	-0.1750000
18705.0000000	-0.1750000
18706.0000000	-0.1750000
18707.0000000	-0.1750000
18708.0000000	-0.1750000
18709.0000000	-0.1700000
18710.0000000	-0.1750000
18711.0000000	-0.1700000
18712.0000000	-0.1700000
18713.0000000	-0.1700000
18714.0000000	-0.1650000
18715.0000000	-0.1700000
18716.0000000	-0.1650000
18717.0000000	-0.1650000
18718.0000000	-0.1650000
18719.0000000	-0.1650000
18720.0000000	-0.1600000
18721.0000000	-0.1600000
18722.0000000	-0.1600000
18723.0000000	-0.1550000
18724.0000000	-0.1550000
18725.0000000	-0.1550000
18726.0000000	-0.1533333
18727.0000000	-0.1500000
18728.0000000	-0.1550000

18729.0000000	-0.1500000
18730.0000000	-0.1500000
18731.0000000	-0.1500000
18732.0000000	-0.1500000
18733.0000000	-0.1400000
18734.0000000	-0.1400000
18735.0000000	-0.1450000
18736.0000000	-0.1450000
18737.0000000	-0.1450000
18738.0000000	-0.1400000
18739.0000000	-0.1450000
18740.0000000	-0.1400000
18741.0000000	-0.1350000
18742.0000000	-0.1400000
18743.0000000	-0.1400000
18744.0000000	-0.1400000
18745.0000000	-0.1400000
18746.0000000	-0.1400000
18747.0000000	-0.1350000
18748.0000000	-0.1300000
18749.0000000	-0.1400000
18750.0000000	-0.1400000
18751.0000000	-0.1350000
18752.0000000	-0.1350000
18753.0000000	-0.1350000
18754.0000000	-0.1300000
18755.0000000	-0.1300000
18756.0000000	-0.1300000
18757.0000000	-0.1300000
18758.0000000	-0.1250000
18759.0000000	-0.1300000
18760.0000000	-0.1250000
18761.0000000	-0.1250000
18762.0000000	-0.1200000
18763.0000000	-0.1200000
18764.0000000	-0.1150000
18765.0000000	-0.1200000
18766.0000000	-0.1200000
18767.0000000	-0.1200000
18768.0000000	-0.1200000
18769.0000000	-0.1200000
18770.0000000	-0.1150000
18771.0000000	-0.1100000
18772.0000000	-0.1100000
18773.0000000	-0.1050000
18774.0000000	-0.1100000
18775.0000000	-0.1100000
18776.0000000	-0.1050000
18777.0000000	-0.1050000
18778.0000000	-0.1000000
18779.0000000	-0.1000000
18780.0000000	-0.1000000
18781.0000000	-0.0950000
18782.0000000	-0.0950000
18783.0000000	-0.0950000
18784.0000000	-0.0950000
18785.0000000	-0.0950000

18786.0000000	-0.0900000
18787.0000000	-0.0900000
18788.0000000	-0.0900000
18789.0000000	-0.0850000
18790.0000000	-0.0900000
18791.0000000	-0.0800000
18792.0000000	-0.0800000
18793.0000000	-0.0850000
18794.0000000	-0.0800000
18795.0000000	-0.0800000
18796.0000000	-0.0750000
18797.0000000	-0.0800000
18798.0000000	-0.0700000
18799.0000000	-0.0700000
18800.0000000	-0.0750000
18801.0000000	-0.0700000
18802.0000000	-0.0650000
18803.0000000	-0.0700000
18804.0000000	-0.0650000
18805.0000000	-0.0650000
18806.0000000	-0.0650000
18807.0000000	-0.0600000
18808.0000000	-0.0550000
18809.0000000	-0.0600000
18810.0000000	-0.0550000
18811.0000000	-0.0600000
18812.0000000	-0.0600000
18813.0000000	-0.0550000
18814.0000000	-0.0550000
18815.0000000	-0.0550000
18816.0000000	-0.0533333
18817.0000000	-0.0550000
18818.0000000	-0.0500000
18819.0000000	-0.0500000
18820.0000000	-0.0500000
18821.0000000	-0.0450000
18822.0000000	-0.0500000
18823.0000000	-0.0400000
18824.0000000	-0.0450000
18825.0000000	-0.0400000
18826.0000000	-0.0400000
18827.0000000	-0.0350000
18828.0000000	-0.0350000
18829.0000000	-0.0300000
18830.0000000	-0.0350000
18831.0000000	-0.0300000
18832.0000000	-0.0300000
18833.0000000	-0.0300000
18834.0000000	-0.0350000
18835.0000000	-0.0250000
18836.0000000	-0.0300000
18837.0000000	-0.0300000
18838.0000000	-0.0250000
18839.0000000	-0.0300000
18840.0000000	-0.0350000
18841.0000000	-0.0350000
18842.0000000	-0.0250000

18843.0000000	-0.0300000
18844.0000000	-0.0300000
18845.0000000	-0.0300000
18846.0000000	-0.0300000
18847.0000000	-0.0200000
18848.0000000	-0.0200000
18849.0000000	-0.0200000
18850.0000000	-0.0200000
18851.0000000	-0.0200000
18852.0000000	-0.0150000
18853.0000000	-0.0200000
18854.0000000	-0.0200000
18855.0000000	-0.0150000
18856.0000000	-0.0100000
18857.0000000	-0.0100000
18858.0000000	-0.0050000
18859.0000000	-0.0100000
18860.0000000	-0.0100000
18861.0000000	-0.0050000
18862.0000000	-0.0050000
18863.0000000	-0.0100000
18864.0000000	-0.0050000
18865.0000000	-0.0050000
18866.0000000	-0.0050000
18867.0000000	0.0000000
18868.0000000	0.0000000
18869.0000000	-0.0050000
18870.0000000	0.0050000
18871.0000000	0.0000000
18872.0000000	0.0000000
18873.0000000	0.0000000
18874.0000000	0.0150000
18875.0000000	0.0050000
18876.0000000	0.0100000
18877.0000000	0.0100000
18878.0000000	0.0100000
18879.0000000	0.0100000
18880.0000000	0.0200000
18881.0000000	0.0200000
18882.0000000	0.0200000
18883.0000000	0.0150000
18884.0000000	0.0200000
18885.0000000	0.0250000
18886.0000000	0.0300000
18887.0000000	0.0300000
18888.0000000	0.0250000
18889.0000000	0.0300000
18890.0000000	0.0300000
18891.0000000	0.0350000
18892.0000000	0.0350000
18893.0000000	0.0400000
18894.0000000	0.0350000
18895.0000000	0.0350000
18896.0000000	0.0400000
18897.0000000	0.0400000
18898.0000000	0.0450000
18899.0000000	0.0450000

18900.0000000	0.0400000
18901.0000000	0.0500000
18902.0000000	0.0450000
18903.0000000	0.0500000
18904.0000000	0.0500000
18905.0000000	0.0550000
18906.0000000	0.0500000
18907.0000000	0.0633333
18908.0000000	0.0550000
18909.0000000	0.0600000
18910.0000000	0.0600000
18911.0000000	0.0600000
18912.0000000	0.0600000
18913.0000000	0.0700000
18914.0000000	0.0700000
18915.0000000	0.0750000
18916.0000000	0.0750000
18917.0000000	0.0700000
18918.0000000	0.0750000
18919.0000000	0.0800000
18920.0000000	0.0750000
18921.0000000	0.0800000
18922.0000000	0.0850000
18923.0000000	0.0850000
18924.0000000	0.0900000
18925.0000000	0.0850000
18926.0000000	0.0850000
18927.0000000	0.0900000
18928.0000000	0.0900000
18929.0000000	0.0900000
18930.0000000	0.0950000
18931.0000000	0.1000000
18932.0000000	0.1000000
18933.0000000	0.1050000
18934.0000000	0.1000000
18935.0000000	0.1050000
18936.0000000	0.1000000
18937.0000000	0.1066667
18938.0000000	0.1100000
18939.0000000	0.1100000
18940.0000000	0.1100000
18941.0000000	0.1100000
18942.0000000	0.1150000
18943.0000000	0.1200000
18944.0000000	0.1200000
18945.0000000	0.1200000
18946.0000000	0.1250000
18947.0000000	0.1200000
18948.0000000	0.1250000
18949.0000000	0.1300000
18950.0000000	0.1300000
18951.0000000	0.1300000
18952.0000000	0.1250000
18953.0000000	0.1350000
18954.0000000	0.1300000
18955.0000000	0.1300000
18956.0000000	0.1300000

18957.0000000	0.1350000
18958.0000000	0.1400000
18959.0000000	0.1400000
18960.0000000	0.1400000
18961.0000000	0.1400000
18962.0000000	0.1400000
18963.0000000	0.1400000
18964.0000000	0.1400000
18965.0000000	0.1400000
18966.0000000	0.1450000
18967.0000000	0.1433333
18968.0000000	0.1400000
18969.0000000	0.1400000
18970.0000000	0.1400000
18971.0000000	0.1450000
18972.0000000	0.1450000
18973.0000000	0.1500000
18974.0000000	0.1500000
18975.0000000	0.1450000
18976.0000000	0.1450000
18977.0000000	0.1550000
18978.0000000	0.1500000
18979.0000000	0.1500000
18980.0000000	0.1550000
18981.0000000	0.1550000
18982.0000000	0.1500000
18983.0000000	0.1500000
18984.0000000	0.1550000
18985.0000000	0.1600000
18986.0000000	0.1550000
18987.0000000	0.1600000
18988.0000000	0.1600000
18989.0000000	0.1500000
18990.0000000	0.1600000
18991.0000000	0.1550000
18992.0000000	0.1550000
18993.0000000	0.1600000
18994.0000000	0.1600000
18995.0000000	0.1600000
18996.0000000	0.1600000
18997.0000000	0.1633333
18998.0000000	0.1600000
18999.0000000	0.1600000
19000.0000000	0.1650000
19001.0000000	0.1650000
19002.0000000	0.1600000
19003.0000000	0.1600000
19004.0000000	0.1600000
19005.0000000	0.1600000
19006.0000000	0.1600000
19007.0000000	0.1600000
19008.0000000	0.1600000
19009.0000000	0.1650000
19010.0000000	0.1600000
19011.0000000	0.1650000
19012.0000000	0.1600000
19013.0000000	0.1600000

19014.0000000	0.1600000
19015.0000000	0.1650000
19016.0000000	0.1650000
19017.0000000	0.1550000
19018.0000000	0.1600000
19019.0000000	0.1600000
19020.0000000	0.1600000
19021.0000000	0.1550000
19022.0000000	0.1600000
19023.0000000	0.1600000
19024.0000000	0.1600000
19025.0000000	0.1600000
19026.0000000	0.1600000
19027.0000000	0.1633333
19028.0000000	0.1650000
19029.0000000	0.1650000
19030.0000000	0.1650000
19031.0000000	0.1700000
19032.0000000	0.1700000
19033.0000000	0.1700000
19034.0000000	0.1700000
19035.0000000	0.1750000
19036.0000000	0.1700000
19037.0000000	0.1700000
19038.0000000	0.1750000
19039.0000000	0.1750000
19040.0000000	0.1750000
19041.0000000	0.1750000
19042.0000000	0.1800000
19043.0000000	0.1800000
19044.0000000	0.1850000
19045.0000000	0.1800000
19046.0000000	0.1900000
19047.0000000	0.1800000
19048.0000000	0.1850000
19049.0000000	0.1850000
19050.0000000	0.1850000
19051.0000000	0.1900000
19052.0000000	0.1900000
19053.0000000	0.1900000
19054.0000000	0.1950000
19055.0000000	0.1900000
19056.0000000	0.1866667
19057.0000000	0.1900000
19058.0000000	0.1950000
19059.0000000	0.1950000
19060.0000000	0.1950000
19061.0000000	0.2000000
19062.0000000	0.2000000
19063.0000000	0.2050000
19064.0000000	0.2050000
19065.0000000	0.2000000
19066.0000000	0.2050000
19067.0000000	0.2000000
19068.0000000	0.2050000
19069.0000000	0.2050000
19070.0000000	0.2050000

19071.0000000	0.2000000
19072.0000000	0.2050000
19073.0000000	0.2100000
19074.0000000	0.2050000
19075.0000000	0.2000000
19076.0000000	0.2050000
19077.0000000	0.2100000
19078.0000000	0.2050000
19079.0000000	0.2000000
19080.0000000	0.2050000
19081.0000000	0.2050000
19082.0000000	0.2000000
19083.0000000	0.2050000
19084.0000000	0.2050000
19085.0000000	0.2000000
19086.0000000	0.2000000
19087.0000000	0.2000000
19088.0000000	0.2000000
19089.0000000	0.1950000
19090.0000000	0.2000000
19091.0000000	0.2000000
19092.0000000	0.2000000
19093.0000000	0.2000000
19094.0000000	0.2000000
19095.0000000	0.1950000
19096.0000000	0.2000000
19097.0000000	0.2000000
19098.0000000	0.1950000
19099.0000000	0.1950000
19100.0000000	0.1950000
19101.0000000	0.1950000
19102.0000000	0.1900000
19103.0000000	0.1900000
19104.0000000	0.1950000
19105.0000000	0.1900000
19106.0000000	0.1900000
19107.0000000	0.1900000
19108.0000000	0.1850000
19109.0000000	0.1900000
19110.0000000	0.1900000
19111.0000000	0.1900000
19112.0000000	0.1900000
19113.0000000	0.1950000
19114.0000000	0.2000000
19115.0000000	0.1900000
19116.0000000	0.1933333
19117.0000000	0.2000000
19118.0000000	0.1950000
19119.0000000	0.1950000
19120.0000000	0.2000000
19121.0000000	0.2000000
19122.0000000	0.2000000
19123.0000000	0.2000000
19124.0000000	0.2000000
19125.0000000	0.2050000
19126.0000000	0.2000000
19127.0000000	0.2000000

19128.0000000	0.1950000
19129.0000000	0.2000000
19130.0000000	0.1900000
19131.0000000	0.1900000
19132.0000000	0.1950000
19133.0000000	0.2000000
19134.0000000	0.2000000
19135.0000000	0.1950000
19136.0000000	0.2000000
19137.0000000	0.2000000
19138.0000000	0.2050000
19139.0000000	0.2050000
19140.0000000	0.2050000
19141.0000000	0.2100000
19142.0000000	0.2100000
19143.0000000	0.2100000
19144.0000000	0.2150000
19145.0000000	0.2200000
19146.0000000	0.2200000
19147.0000000	0.2200000
19148.0000000	0.2200000
19149.0000000	0.2250000
19150.0000000	0.2300000
19151.0000000	0.2250000
19152.0000000	0.2300000
19153.0000000	0.2300000
19154.0000000	0.2300000
19155.0000000	0.2300000
19156.0000000	0.2300000
19157.0000000	0.2300000
19158.0000000	0.2350000
19159.0000000	0.2350000
19160.0000000	0.2300000
19161.0000000	0.2350000
19162.0000000	0.2400000
19163.0000000	0.2350000
19164.0000000	0.2350000
19165.0000000	0.2400000
19166.0000000	0.2400000
19167.0000000	0.2400000
19168.0000000	0.2450000
19169.0000000	0.2350000
19170.0000000	0.2400000
19171.0000000	0.2400000
19172.0000000	0.2350000
19173.0000000	0.2350000
19174.0000000	0.2400000
19175.0000000	0.2400000
19176.0000000	0.2433333
19177.0000000	0.2350000
19178.0000000	0.2400000
19179.0000000	0.2350000
19180.0000000	0.2400000
19181.0000000	0.2400000
19182.0000000	0.2500000
19183.0000000	0.2500000
19184.0000000	0.2500000

19185.0000000	0.2550000
19186.0000000	0.2600000
19187.0000000	0.2550000
19188.0000000	0.2650000
19189.0000000	0.2600000
19190.0000000	0.2600000
19191.0000000	0.2600000
19192.0000000	0.2650000
19193.0000000	0.2550000
19194.0000000	0.2600000
19195.0000000	0.2600000
19196.0000000	0.2550000
19197.0000000	0.2550000
19198.0000000	0.2600000
19199.0000000	0.2600000
19200.0000000	0.2600000
19201.0000000	0.2600000
19202.0000000	0.2550000
19203.0000000	0.2600000
19204.0000000	0.2650000
19205.0000000	0.2600000
19206.0000000	0.2600000
19207.0000000	0.2633333
19208.0000000	0.2600000
19209.0000000	0.2600000
19210.0000000	0.2650000
19211.0000000	0.2650000
19212.0000000	0.2700000
19213.0000000	0.2700000
19214.0000000	0.2700000
19215.0000000	0.2700000
19216.0000000	0.2650000
19217.0000000	0.2700000
19218.0000000	0.2700000
19219.0000000	0.2750000
19220.0000000	0.2750000
19221.0000000	0.2750000
19222.0000000	0.2750000
19223.0000000	0.2750000
19224.0000000	0.2800000
19225.0000000	0.2800000
19226.0000000	0.2800000
19227.0000000	0.2850000
19228.0000000	0.2850000
19229.0000000	0.2850000
19230.0000000	0.2850000
19231.0000000	0.2850000
19232.0000000	0.2850000
19233.0000000	0.2850000
19234.0000000	0.2850000
19235.0000000	0.2850000
19236.0000000	0.2900000
19237.0000000	0.2900000
19238.0000000	0.2900000
19239.0000000	0.2900000
19240.0000000	0.2850000
19241.0000000	0.2850000

19242.0000000	0.2850000
19243.0000000	0.2850000
19244.0000000	0.2850000
19245.0000000	0.2800000
19246.0000000	0.2800000
19247.0000000	0.2700000
19248.0000000	0.2700000
19249.0000000	0.2600000
19250.0000000	0.2600000
19251.0000000	0.2600000
19252.0000000	0.2650000
19253.0000000	0.2650000
19254.0000000	0.2600000
19255.0000000	0.2700000
19256.0000000	0.2650000
19257.0000000	0.2700000
19258.0000000	0.2700000
19259.0000000	0.2750000
19260.0000000	0.2750000
19261.0000000	0.2700000
19262.0000000	0.2700000
19263.0000000	0.2700000
19264.0000000	0.2750000
19265.0000000	0.2800000
19266.0000000	0.2800000
19267.0000000	0.2850000
19268.0000000	0.2866667
19269.0000000	0.3000000
19270.0000000	0.2900000
19271.0000000	0.2800000
19272.0000000	0.2800000
19273.0000000	0.2750000
19274.0000000	0.2750000
19275.0000000	0.2700000
19276.0000000	0.2700000
19277.0000000	0.2700000
19278.0000000	0.2750000
19279.0000000	0.2750000
19280.0000000	0.2750000
19281.0000000	0.2700000
19282.0000000	0.2750000
19283.0000000	0.2800000
19284.0000000	0.2800000
19285.0000000	0.2800000
19286.0000000	0.2800000
19287.0000000	0.2800000
19288.0000000	0.2800000
19289.0000000	0.2750000
19290.0000000	0.2800000
19291.0000000	0.2850000
19292.0000000	0.2900000
19293.0000000	0.2850000
19294.0000000	0.2850000
19295.0000000	0.2950000
19296.0000000	0.2900000
19297.0000000	0.2950000
19298.0000000	0.3033333

19299.0000000	0.3000000
19300.0000000	0.3050000
19301.0000000	0.3050000
19302.0000000	0.3000000
19303.0000000	0.3050000
19304.0000000	0.3050000
19305.0000000	0.3100000
19306.0000000	0.3100000
19307.0000000	0.3100000
19308.0000000	0.3200000
19309.0000000	0.3200000
19310.0000000	0.3150000
19311.0000000	0.3200000
19312.0000000	0.3150000
19313.0000000	0.3200000
19314.0000000	0.3200000
19315.0000000	0.3200000
19316.0000000	0.3250000
19317.0000000	0.3200000
19318.0000000	0.3250000
19319.0000000	0.3200000
19320.0000000	0.3250000
19321.0000000	0.3250000
19322.0000000	0.3250000
19323.0000000	0.3300000
19324.0000000	0.3250000
19325.0000000	0.3300000
19326.0000000	0.3300000
19327.0000000	0.3300000
19328.0000000	0.3333333
19329.0000000	0.3300000
19330.0000000	0.3350000
19331.0000000	0.3400000
19332.0000000	0.3400000
19333.0000000	0.3400000
19334.0000000	0.3450000
19335.0000000	0.3450000
19336.0000000	0.3550000
19337.0000000	0.3500000
19338.0000000	0.3600000
19339.0000000	0.3550000
19340.0000000	0.3550000
19341.0000000	0.3600000
19342.0000000	0.3600000
19343.0000000	0.3600000
19344.0000000	0.3550000
19345.0000000	0.3600000
19346.0000000	0.3600000
19347.0000000	0.3550000
19348.0000000	0.3600000
19349.0000000	0.3600000
19350.0000000	0.3650000
19351.0000000	0.3600000
19352.0000000	0.3600000
19353.0000000	0.3600000
19354.0000000	0.3600000
19355.0000000	0.3650000

19356.0000000	0.3700000
19357.0000000	0.3700000
19358.0000000	0.3700000
19359.0000000	0.3700000
19360.0000000	0.3650000
19361.0000000	0.3750000
19362.0000000	0.3800000
19363.0000000	0.3800000
19364.0000000	0.3800000
19365.0000000	0.3800000
19366.0000000	0.3850000
19367.0000000	0.3800000
19368.0000000	0.3800000
19369.0000000	0.3800000
19370.0000000	0.3750000
19371.0000000	0.3800000
19372.0000000	0.3750000
19373.0000000	0.3800000
19374.0000000	0.3850000
19375.0000000	0.3800000
19376.0000000	0.3850000
19377.0000000	0.3900000
19378.0000000	0.3900000
19379.0000000	0.3950000
19380.0000000	0.4000000
19381.0000000	0.4000000
19382.0000000	0.4000000
19383.0000000	0.4000000
19384.0000000	0.4000000
19385.0000000	0.4050000
19386.0000000	0.4000000
19387.0000000	0.4000000
19388.0000000	0.4050000
19389.0000000	0.4000000
19390.0000000	0.4100000
19391.0000000	0.4100000
19392.0000000	0.4150000
19393.0000000	0.4100000
19394.0000000	0.4150000
19395.0000000	0.4150000
19396.0000000	0.4200000
19397.0000000	0.4200000
19398.0000000	0.4200000
19399.0000000	0.4250000
19400.0000000	0.4200000
19401.0000000	0.4150000
19402.0000000	0.4200000
19403.0000000	0.4200000
19404.0000000	0.4200000
19405.0000000	0.4150000
19406.0000000	0.4200000
19407.0000000	0.4200000
19408.0000000	0.4200000
19409.0000000	0.4250000
19410.0000000	0.4150000
19411.0000000	0.4200000
19412.0000000	0.4250000

19413.0000000	0.4300000
19414.0000000	0.4300000
19415.0000000	0.4300000
19416.0000000	0.4300000
19417.0000000	0.4300000
19418.0000000	0.4300000
19419.0000000	0.4300000
19420.0000000	0.4300000
19421.0000000	0.4350000
19422.0000000	0.4350000
19423.0000000	0.4300000
19424.0000000	0.4300000
19425.0000000	0.4300000
19426.0000000	0.4300000
19427.0000000	0.4350000
19428.0000000	0.4350000
19429.0000000	0.4300000
19430.0000000	0.4400000
19431.0000000	0.4300000
19432.0000000	0.4350000
19433.0000000	0.4400000
19434.0000000	0.4350000
19435.0000000	0.4350000
19436.0000000	0.4400000
19437.0000000	0.4450000
19438.0000000	0.4300000
19439.0000000	0.4350000
19440.0000000	0.4350000
19441.0000000	0.4300000
19442.0000000	0.4250000
19443.0000000	0.4300000
19444.0000000	0.4350000
19445.0000000	0.4300000
19446.0000000	0.4400000
19447.0000000	0.4400000
19448.0000000	0.4400000
19449.0000000	0.4400000
19450.0000000	0.4433333
19451.0000000	0.4450000
19452.0000000	0.4450000
19453.0000000	0.4500000
19454.0000000	0.4500000
19455.0000000	0.4500000
19456.0000000	0.4450000
19457.0000000	0.4400000
19458.0000000	0.4500000
19459.0000000	0.4450000
19460.0000000	0.4450000
19461.0000000	0.4350000
19462.0000000	0.4350000
19463.0000000	0.4300000
19464.0000000	0.4300000
19465.0000000	0.4200000
19466.0000000	0.4200000
19467.0000000	0.4200000
19468.0000000	0.4200000
19469.0000000	0.4150000

19470.0000000	0.4150000
19471.0000000	0.4200000
19472.0000000	0.4300000
19473.0000000	0.4300000
19474.0000000	0.4300000
19475.0000000	0.4300000
19476.0000000	0.4300000
19477.0000000	0.4300000
19478.0000000	0.4300000
19479.0000000	0.4350000
19480.0000000	0.4333333
19481.0000000	0.4350000
19482.0000000	0.4400000
19483.0000000	0.4400000
19484.0000000	0.4450000
19485.0000000	0.4500000
19486.0000000	0.4550000
19487.0000000	0.4600000
19488.0000000	0.4600000
19489.0000000	0.4650000
19490.0000000	0.4700000
19491.0000000	0.4750000
19492.0000000	0.4750000
19493.0000000	0.4700000
19494.0000000	0.4750000
19495.0000000	0.4800000
19496.0000000	0.4800000
19497.0000000	0.4750000
19498.0000000	0.4800000
19499.0000000	0.4850000
19500.0000000	0.4850000
19501.0000000	0.4800000
19502.0000000	0.4900000
19503.0000000	0.4900000
19504.0000000	0.4850000
19505.0000000	0.4800000
19506.0000000	0.4850000
19507.0000000	0.4850000
19508.0000000	0.4800000
19509.0000000	0.4850000
19510.0000000	0.4850000
19511.0000000	0.4800000
19512.0000000	0.4800000
19513.0000000	0.4700000
19514.0000000	0.4650000
19515.0000000	0.4650000
19516.0000000	0.4600000
19517.0000000	0.4550000
19518.0000000	0.4500000
19519.0000000	0.4450000
19520.0000000	0.4400000
19521.0000000	0.4400000
19522.0000000	0.4400000
19523.0000000	0.4400000
19524.0000000	0.4350000
19525.0000000	0.4300000
19526.0000000	0.4250000

19527.0000000	0.4200000
19528.0000000	0.4200000
19529.0000000	0.4200000
19530.0000000	0.4100000
19531.0000000	0.4150000
19532.0000000	0.4100000
19533.0000000	0.4100000
19534.0000000	0.4200000
19535.0000000	0.4100000
19536.0000000	0.4150000
19537.0000000	0.4150000
19538.0000000	0.4150000
19539.0000000	0.4200000
19540.0000000	0.4200000
19541.0000000	0.4233333
19542.0000000	0.4300000
19543.0000000	0.4300000
19544.0000000	0.4350000
19545.0000000	0.4400000
19546.0000000	0.4450000
19547.0000000	0.4500000
19548.0000000	0.4500000
19549.0000000	0.4550000
19550.0000000	0.4550000
19551.0000000	0.4600000
19552.0000000	0.4550000
19553.0000000	0.4600000
19554.0000000	0.4650000
19555.0000000	0.4700000
19556.0000000	0.4700000
19557.0000000	0.4700000
19558.0000000	0.4700000
19559.0000000	0.4700000
19560.0000000	0.4600000
19561.0000000	0.4650000
19562.0000000	0.4700000
19563.0000000	0.4750000
19564.0000000	0.4800000
19565.0000000	0.4800000
19566.0000000	0.4900000
19567.0000000	0.4900000
19568.0000000	0.4900000
19569.0000000	0.4950000
19570.0000000	0.4900000
19571.0000000	0.4900000
19572.0000000	0.4900000
19573.0000000	0.4950000
19574.0000000	0.4900000
19575.0000000	0.4950000
19576.0000000	0.4900000
19577.0000000	0.4950000
19578.0000000	0.4950000
19579.0000000	0.4950000
19580.0000000	0.5000000
19581.0000000	0.5000000
19582.0000000	0.5050000
19583.0000000	0.5050000

19584.0000000	0.5050000
19585.0000000	0.5050000
19586.0000000	0.5150000
19587.0000000	0.5100000
19588.0000000	0.5200000
19589.0000000	0.5200000
19590.0000000	0.5200000
19591.0000000	0.5200000
19592.0000000	0.5250000
19593.0000000	0.5200000
19594.0000000	0.5250000
19595.0000000	0.5200000
19596.0000000	0.5250000
19597.0000000	0.5250000
19598.0000000	0.5200000
19599.0000000	0.5250000
19600.0000000	0.5250000
19601.0000000	0.5300000
19602.0000000	0.5300000
19603.0000000	0.5300000
19604.0000000	0.5350000
19605.0000000	0.5350000
19606.0000000	0.5400000
19607.0000000	0.5400000
19608.0000000	0.5400000
19609.0000000	0.5400000
19610.0000000	0.5450000
19611.0000000	0.5400000
19612.0000000	0.5450000
19613.0000000	0.5500000
19614.0000000	0.5500000
19615.0000000	0.5550000
19616.0000000	0.5550000
19617.0000000	0.5550000
19618.0000000	0.5550000
19619.0000000	0.5550000
19620.0000000	0.5550000
19621.0000000	0.5550000
19622.0000000	0.5550000
19623.0000000	0.5600000
19624.0000000	0.5550000
19625.0000000	0.5550000
19626.0000000	0.5550000
19627.0000000	0.5550000
19628.0000000	0.5500000
19629.0000000	0.5600000
19630.0000000	0.5550000
19631.0000000	0.5500000
19632.0000000	0.5500000
19633.0000000	0.5500000
19634.0000000	0.5550000
19635.0000000	0.5500000
19636.0000000	0.5500000
19637.0000000	0.5500000
19638.0000000	0.5500000
19639.0000000	0.5500000
19640.0000000	0.5500000

19641.0000000	0.5500000
19642.0000000	0.5450000
19643.0000000	0.5450000
19644.0000000	0.5400000
19645.0000000	0.5350000
19646.0000000	0.5400000
19647.0000000	0.5400000
19648.0000000	0.5400000
19649.0000000	0.5300000
19650.0000000	0.5300000
19651.0000000	0.5300000
19652.0000000	0.5300000
19653.0000000	0.5300000
19654.0000000	0.5150000
19655.0000000	0.5200000
19656.0000000	0.5150000
19657.0000000	0.5150000
19658.0000000	0.5200000
19659.0000000	0.5150000
19660.0000000	0.5150000
19661.0000000	0.5200000
19662.0000000	0.5166667
19663.0000000	0.5200000
19664.0000000	0.5200000
19665.0000000	0.5250000
19666.0000000	0.5250000
19667.0000000	0.5300000
19668.0000000	0.5300000
19669.0000000	0.5250000
19670.0000000	0.5300000
19671.0000000	0.5400000
19672.0000000	0.5300000
19673.0000000	0.5400000
19674.0000000	0.5400000
19675.0000000	0.5400000
19676.0000000	0.5450000
19677.0000000	0.5400000
19678.0000000	0.5300000
19679.0000000	0.5400000
19680.0000000	0.5350000
19681.0000000	0.5300000
19682.0000000	0.5350000
19683.0000000	0.5300000
19684.0000000	0.5300000
19685.0000000	0.5300000
19686.0000000	0.5250000
19687.0000000	0.5200000
19688.0000000	0.5300000
19689.0000000	0.5250000
19690.0000000	0.5200000
19691.0000000	0.5200000
19692.0000000	0.5200000
19693.0000000	0.5200000
19694.0000000	0.5200000
19695.0000000	0.5250000
19696.0000000	0.5250000
19697.0000000	0.5300000

19698.0000000	0.5300000
19699.0000000	0.5400000
19700.0000000	0.5450000
19701.0000000	0.5400000
19702.0000000	0.5500000
19703.0000000	0.5500000
19704.0000000	0.5450000
19705.0000000	0.5550000
19706.0000000	0.5550000
19707.0000000	0.5600000
19708.0000000	0.5550000
19709.0000000	0.5650000
19710.0000000	0.5650000
19711.0000000	0.5600000
19712.0000000	0.5700000
19713.0000000	0.5700000
19714.0000000	0.5700000
19715.0000000	0.5650000
19716.0000000	0.5700000
19717.0000000	0.5700000
19718.0000000	0.5700000
19719.0000000	0.5700000
19720.0000000	0.5700000
19721.0000000	0.5750000
19722.0000000	0.5633333
19723.0000000	0.5550000
19724.0000000	0.5550000
19725.0000000	0.5500000
19726.0000000	0.5500000
19727.0000000	0.5500000
19728.0000000	0.5500000
19729.0000000	0.5500000
19730.0000000	0.5550000
19731.0000000	0.5550000
19732.0000000	0.5500000
19733.0000000	0.5500000
19734.0000000	0.5500000
19735.0000000	0.5550000
19736.0000000	0.5550000
19737.0000000	0.5500000
19738.0000000	0.5600000
19739.0000000	0.5600000
19740.0000000	0.5650000
19741.0000000	0.5600000
19742.0000000	0.5700000
19743.0000000	0.5700000
19744.0000000	0.5700000
19745.0000000	0.5750000
19746.0000000	0.5800000
19747.0000000	0.5800000
19748.0000000	0.5800000
19749.0000000	0.5700000
19750.0000000	0.5700000
19751.0000000	0.5700000
19752.0000000	0.5600000
19753.0000000	0.5600000
19754.0000000	0.5650000

19755.0000000	0.5600000
19756.0000000	0.5650000
19757.0000000	0.5650000
19758.0000000	0.5700000
19759.0000000	0.5700000
19760.0000000	0.5650000
19761.0000000	0.5700000
19762.0000000	0.5700000
19763.0000000	0.5700000
19764.0000000	0.5650000
19765.0000000	0.5750000
19766.0000000	0.5700000
19767.0000000	0.5700000
19768.0000000	0.5700000
19769.0000000	0.5700000
19770.0000000	0.5750000
19771.0000000	0.5700000
19772.0000000	0.5750000
19773.0000000	0.5750000
19774.0000000	0.5800000
19775.0000000	0.5800000
19776.0000000	0.5700000
19777.0000000	0.5800000
19778.0000000	0.5800000
19779.0000000	0.5800000
19780.0000000	0.5800000
19781.0000000	0.5866666
19782.0000000	0.5850000
19783.0000000	0.5800000
19784.0000000	0.5900000
19785.0000000	0.5900000
19786.0000000	0.5900000
19787.0000000	0.5950000
19788.0000000	0.5900000
19789.0000000	0.5950000
19790.0000000	0.6000000
19791.0000000	0.5900000
19792.0000000	0.5900000
19793.0000000	0.5900000
19794.0000000	0.5950000
19795.0000000	0.6000000
19796.0000000	0.5900000
19797.0000000	0.6000000
19798.0000000	0.6000000
19799.0000000	0.6000000
19800.0000000	0.6050000
19801.0000000	0.6050000
19802.0000000	0.6000000
19803.0000000	0.6000000
19804.0000000	0.6000000
19805.0000000	0.6000000
19806.0000000	0.6000000
19807.0000000	0.6050000
19808.0000000	0.6000000
19809.0000000	0.6050000
19810.0000000	0.6000000
19811.0000000	0.6033334

19812.0000000	0.6000000
19813.0000000	0.6000000
19814.0000000	0.6000000
19815.0000000	0.5900000
19816.0000000	0.5900000
19817.0000000	0.5850000
19818.0000000	0.5850000
19819.0000000	0.5850000
19820.0000000	0.5850000
19821.0000000	0.5800000
19822.0000000	0.5800000
19823.0000000	0.5850000
19824.0000000	0.5850000
19825.0000000	0.5900000
19826.0000000	0.5950000
19827.0000000	0.6000000
19828.0000000	0.6000000
19829.0000000	0.6000000
19830.0000000	0.6000000
19831.0000000	0.6050000
19832.0000000	0.6000000
19833.0000000	0.6100000
19834.0000000	0.6100000
19835.0000000	0.6100000
19836.0000000	0.6150000
19837.0000000	0.6200000
19838.0000000	0.6150000
19839.0000000	0.6300000
19840.0000000	0.6200000
19841.0000000	0.6266667
19842.0000000	0.6300000
19843.0000000	0.6300000
19844.0000000	0.6300000
19845.0000000	0.6350000
19846.0000000	0.6300000
19847.0000000	0.6300000
19848.0000000	0.6300000
19849.0000000	0.6200000
19850.0000000	0.6100000
19851.0000000	0.6150000
19852.0000000	0.6100000
19853.0000000	0.6000000
19854.0000000	0.6000000
19855.0000000	0.6000000
19856.0000000	0.5950000
19857.0000000	0.5900000
19858.0000000	0.5900000
19859.0000000	0.5900000
19860.0000000	0.5900000
19861.0000000	0.5900000
19862.0000000	0.5850000
19863.0000000	0.5900000
19864.0000000	0.5850000
19865.0000000	0.5800000
19866.0000000	0.5850000
19867.0000000	0.5750000
19868.0000000	0.5750000

19869.0000000	0.5700000
19870.0000000	0.5800000
19871.0000000	0.5800000
19872.0000000	0.5800000
19873.0000000	0.5800000
19874.0000000	0.5850000
19875.0000000	0.5800000
19876.0000000	0.5850000
19877.0000000	0.5900000
19878.0000000	0.5900000
19879.0000000	0.5900000
19880.0000000	0.5900000
19881.0000000	0.5950000
19882.0000000	0.5950000
19883.0000000	0.5950000
19884.0000000	0.6000000
19885.0000000	0.6000000
19886.0000000	0.5950000
19887.0000000	0.6000000
19888.0000000	0.6000000
19889.0000000	0.6000000
19890.0000000	0.6000000
19891.0000000	0.5950000
19892.0000000	0.6000000
19893.0000000	0.6000000
19894.0000000	0.5950000
19895.0000000	0.5950000
19896.0000000	0.5950000
19897.0000000	0.5950000
19898.0000000	0.5900000
19899.0000000	0.5900000
19900.0000000	0.5900000
19901.0000000	0.5850000
19902.0000000	0.5800000
19903.0000000	0.5800000
19904.0000000	0.5850000
19905.0000000	0.5800000
19906.0000000	0.5750000
19907.0000000	0.5800000
19908.0000000	0.5700000
19909.0000000	0.5700000
19910.0000000	0.5700000
19911.0000000	0.5650000
19912.0000000	0.5700000
19913.0000000	0.5650000
19914.0000000	0.5600000
19915.0000000	0.5650000
19916.0000000	0.5650000
19917.0000000	0.5650000
19918.0000000	0.5600000
19919.0000000	0.5650000
19920.0000000	0.5700000
19921.0000000	0.5650000
19922.0000000	0.5700000
19923.0000000	0.5650000
19924.0000000	0.5700000
19925.0000000	0.5750000

19926.0000000	0.5700000
19927.0000000	0.5750000
19928.0000000	0.5700000
19929.0000000	0.5700000
19930.0000000	0.5700000
19931.0000000	0.5650000
19932.0000000	0.5533333
19933.0000000	0.5600000
19934.0000000	0.5500000
19935.0000000	0.5500000
19936.0000000	0.5450000
19937.0000000	0.5550000
19938.0000000	0.5500000
19939.0000000	0.5500000
19940.0000000	0.5500000
19941.0000000	0.5550000
19942.0000000	0.5550000
19943.0000000	0.5600000
19944.0000000	0.5550000
19945.0000000	0.5550000
19946.0000000	0.5500000
19947.0000000	0.5500000
19948.0000000	0.5500000
19949.0000000	0.5550000
19950.0000000	0.5500000
19951.0000000	0.5550000
19952.0000000	0.5500000
19953.0000000	0.5550000
19954.0000000	0.5500000
19955.0000000	0.5600000
19956.0000000	0.5550000
19957.0000000	0.5500000
19958.0000000	0.5500000
19959.0000000	0.5500000
19960.0000000	0.5350000
19961.0000000	0.5450000
19962.0000000	0.5350000
19963.0000000	0.5266666
19964.0000000	0.5250000
19965.0000000	0.5300000
19966.0000000	0.5350000
19967.0000000	0.5300000
19968.0000000	0.5300000
19969.0000000	0.5300000
19970.0000000	0.5350000
19971.0000000	0.5400000
19972.0000000	0.5350000
19973.0000000	0.5400000
19974.0000000	0.5400000
19975.0000000	0.5500000
19976.0000000	0.5500000
19977.0000000	0.5500000
19978.0000000	0.5500000
19979.0000000	0.5500000
19980.0000000	0.5550000
19981.0000000	0.5500000
19982.0000000	0.5550000

19983.0000000	0.5600000
19984.0000000	0.5600000
19985.0000000	0.5700000
19986.0000000	0.5600000
19987.0000000	0.5700000
19988.0000000	0.5650000
19989.0000000	0.5700000
19990.0000000	0.5700000
19991.0000000	0.5600000
19992.0000000	0.5650000
19993.0000000	0.5600000
19994.0000000	0.5550000
19995.0000000	0.5500000
19996.0000000	0.5500000
19997.0000000	0.5400000
19998.0000000	0.5350000
19999.0000000	0.5400000
20000.0000000	0.5350000
20001.0000000	0.5250000
20002.0000000	0.5300000
20003.0000000	0.5200000
20004.0000000	0.5300000
20005.0000000	0.5200000
20006.0000000	0.5300000
20007.0000000	0.5250000
20008.0000000	0.5300000
20009.0000000	0.5300000
20010.0000000	0.5300000
20011.0000000	0.5350000
20012.0000000	0.5300000
20013.0000000	0.5350000
20014.0000000	0.5400000
20015.0000000	0.5350000
20016.0000000	0.5400000
20017.0000000	0.5350000
20018.0000000	0.5400000
20019.0000000	0.5400000
20020.0000000	0.5400000
20021.0000000	0.5450000
20022.0000000	0.5400000
20023.0000000	0.5400000
20024.0000000	0.5400000
20025.0000000	0.5400000
20026.0000000	0.5450000
20027.0000000	0.5450000
20028.0000000	0.5450000
20029.0000000	0.5550000
20030.0000000	0.5500000
20031.0000000	0.5450000
20032.0000000	0.5550000
20033.0000000	0.5550000
20034.0000000	0.5600000
20035.0000000	0.5600000
20036.0000000	0.5600000
20037.0000000	0.5600000
20038.0000000	0.5600000
20039.0000000	0.5600000

20040.0000000	0.5650000
20041.0000000	0.5700000
20042.0000000	0.5700000
20043.0000000	0.5700000
20044.0000000	0.5700000
20045.0000000	0.5750000
20046.0000000	0.5800000
20047.0000000	0.5850000
20048.0000000	0.5800000
20049.0000000	0.5850000
20050.0000000	0.5800000
20051.0000000	0.5800000
20052.0000000	0.5800000
20053.0000000	0.5750000
20054.0000000	0.5800000
20055.0000000	0.5800000
20056.0000000	0.5750000
20057.0000000	0.5750000
20058.0000000	0.5700000
20059.0000000	0.5800000
20060.0000000	0.5750000
20061.0000000	0.5750000
20062.0000000	0.5750000
20063.0000000	0.5750000
20064.0000000	0.5800000
20065.0000000	0.5700000
20066.0000000	0.5700000
20067.0000000	0.5700000
20068.0000000	0.5700000
20069.0000000	0.5700000
20070.0000000	0.5700000
20071.0000000	0.5650000
20072.0000000	0.5700000
20073.0000000	0.5700000
20074.0000000	0.5650000
20075.0000000	0.5650000
20076.0000000	0.5650000
20077.0000000	0.5650000
20078.0000000	0.5650000
20079.0000000	0.5650000
20080.0000000	0.5700000
20081.0000000	0.5600000
20082.0000000	0.5650000
20083.0000000	0.5600000
20084.0000000	0.5600000
20085.0000000	0.5550000
20086.0000000	0.5600000
20087.0000000	0.5500000
20088.0000000	0.5600000
20089.0000000	0.5550000
20090.0000000	0.5600000
20091.0000000	0.5600000
20092.0000000	0.5600000
20093.0000000	0.5650000
20094.0000000	0.5600000
20095.0000000	0.5650000
20096.0000000	0.5650000

20097.0000000	0.5650000
20098.0000000	0.5600000
20099.0000000	0.5650000
20100.0000000	0.5600000
20101.0000000	0.5600000
20102.0000000	0.5600000
20103.0000000	0.5550000
20104.0000000	0.5550000
20105.0000000	0.5550000
20106.0000000	0.5500000
20107.0000000	0.5550000
20108.0000000	0.5550000
20109.0000000	0.5450000
20110.0000000	0.5450000
20111.0000000	0.5450000
20112.0000000	0.5500000
20113.0000000	0.5500000
20114.0000000	0.5500000
20115.0000000	0.5600000
20116.0000000	0.5550000
20117.0000000	0.5600000
20118.0000000	0.5600000
20119.0000000	0.5600000
20120.0000000	0.5650000
20121.0000000	0.5650000
20122.0000000	0.5600000
20123.0000000	0.5700000
20124.0000000	0.5600000
20125.0000000	0.5600000
20126.0000000	0.5600000
20127.0000000	0.5550000
20128.0000000	0.5500000
20129.0000000	0.5500000
20130.0000000	0.5450000
20131.0000000	0.5450000
20132.0000000	0.5350000
20133.0000000	0.5300000
20134.0000000	0.5300000
20135.0000000	0.5250000
20136.0000000	0.5250000
20137.0000000	0.5200000
20138.0000000	0.5200000
20139.0000000	0.5200000
20140.0000000	0.5150000
20141.0000000	0.5200000
20142.0000000	0.5150000
20143.0000000	0.5150000
20144.0000000	0.5150000
20145.0000000	0.5133333
20146.0000000	0.5200000
20147.0000000	0.5200000
20148.0000000	0.5200000
20149.0000000	0.5200000
20150.0000000	0.5200000
20151.0000000	0.5200000
20152.0000000	0.5150000
20153.0000000	0.5100000

20154.0000000	0.5100000
20155.0000000	0.5100000
20156.0000000	0.5100000
20157.0000000	0.5100000
20158.0000000	0.5150000
20159.0000000	0.5200000
20160.0000000	0.5200000
20161.0000000	0.5250000
20162.0000000	0.5250000
20163.0000000	0.5350000
20164.0000000	0.5400000
20165.0000000	0.5400000
20166.0000000	0.5500000
20167.0000000	0.5500000
20168.0000000	0.5550000
20169.0000000	0.5600000
20170.0000000	0.5600000
20171.0000000	0.5700000
20172.0000000	0.5700000
20173.0000000	0.5750000
20174.0000000	0.5700000
20175.0000000	0.5700000
20176.0000000	0.5750000
20177.0000000	0.5700000
20178.0000000	0.5700000
20179.0000000	0.5700000
20180.0000000	0.5750000
20181.0000000	0.5700000
20182.0000000	0.5800000
20183.0000000	0.5800000
20184.0000000	0.5750000
20185.0000000	0.5850000
20186.0000000	0.5850000
20187.0000000	0.5800000
20188.0000000	0.5800000
20189.0000000	0.5900000
20190.0000000	0.5850000
20191.0000000	0.5850000
20192.0000000	0.5900000
20193.0000000	0.5900000
20194.0000000	0.5950000
20195.0000000	0.5950000
20196.0000000	0.5900000
20197.0000000	0.5900000
20198.0000000	0.5950000
20199.0000000	0.5950000
20200.0000000	0.5850000
20201.0000000	0.5900000
20202.0000000	0.5900000
20203.0000000	0.5800000
20204.0000000	0.5800000
20205.0000000	0.5800000
20206.0000000	0.5733333
20207.0000000	0.5800000
20208.0000000	0.5750000
20209.0000000	0.5800000
20210.0000000	0.5850000

20211.0000000	0.5800000
20212.0000000	0.5900000
20213.0000000	0.5850000
20214.0000000	0.5900000
20215.0000000	0.5900000
20216.0000000	0.5900000
20217.0000000	0.5950000
20218.0000000	0.5950000
20219.0000000	0.5950000
20220.0000000	0.5900000
20221.0000000	0.5950000
20222.0000000	0.5950000
20223.0000000	0.5950000
20224.0000000	0.5950000
20225.0000000	0.5900000
20226.0000000	0.6000000
20227.0000000	0.6000000
20228.0000000	0.6000000
20229.0000000	0.6000000
20230.0000000	0.6100000
20231.0000000	0.6100000
20232.0000000	0.6150000
20233.0000000	0.6100000
20234.0000000	0.6150000
20235.0000000	0.6150000
20236.0000000	0.6200000
20237.0000000	0.6250000
20238.0000000	0.6200000
20239.0000000	0.6200000
20240.0000000	0.6250000
20241.0000000	0.6300000
20242.0000000	0.6300000
20243.0000000	0.6300000
20244.0000000	0.6300000
20245.0000000	0.6300000
20246.0000000	0.6350000
20247.0000000	0.6350000
20248.0000000	0.6400000
20249.0000000	0.6400000
20250.0000000	0.6400000
20251.0000000	0.6400000
20252.0000000	0.6450000
20253.0000000	0.6450000
20254.0000000	0.6500000
20255.0000000	0.6500000
20256.0000000	0.6500000
20257.0000000	0.6450000
20258.0000000	0.6500000
20259.0000000	0.6500000
20260.0000000	0.6500000
20261.0000000	0.6500000
20262.0000000	0.6500000
20263.0000000	0.6500000
20264.0000000	0.6500000
20265.0000000	0.6600000
20266.0000000	0.6500000
20267.0000000	0.6533333

20268.0000000	0.6600000
20269.0000000	0.6500000
20270.0000000	0.6550000
20271.0000000	0.6550000
20272.0000000	0.6500000
20273.0000000	0.6500000
20274.0000000	0.6450000
20275.0000000	0.6450000
20276.0000000	0.6450000
20277.0000000	0.6350000
20278.0000000	0.6350000
20279.0000000	0.6350000
20280.0000000	0.6300000
20281.0000000	0.6250000
20282.0000000	0.6350000
20283.0000000	0.6200000
20284.0000000	0.6300000
20285.0000000	0.6300000
20286.0000000	0.6300000
20287.0000000	0.6350000
20288.0000000	0.6300000
20289.0000000	0.6400000
20290.0000000	0.6350000
20291.0000000	0.6350000
20292.0000000	0.6400000
20293.0000000	0.6400000
20294.0000000	0.6400000
20295.0000000	0.6400000
20296.0000000	0.6450000
20297.0000000	0.6400000
20298.0000000	0.6400000
20299.0000000	0.6450000
20300.0000000	0.6450000
20301.0000000	0.6400000
20302.0000000	0.6400000
20303.0000000	0.6350000
20304.0000000	0.6400000
20305.0000000	0.6300000
20306.0000000	0.6350000
20307.0000000	0.6350000
20308.0000000	0.6350000
20309.0000000	0.6350000
20310.0000000	0.6350000
20311.0000000	0.6350000
20312.0000000	0.6350000
20313.0000000	0.6400000
20314.0000000	0.6350000
20315.0000000	0.6350000
20316.0000000	0.6350000
20317.0000000	0.6350000
20318.0000000	0.6350000
20319.0000000	0.6300000
20320.0000000	0.6400000
20321.0000000	0.6400000
20322.0000000	0.6450000
20323.0000000	0.6450000
20324.0000000	0.6550000

20325.0000000	0.6500000
20326.0000000	0.6500000
20327.0000000	0.6600000
20328.0000000	0.6600000
20329.0000000	0.6600000
20330.0000000	0.6600000
20331.0000000	0.6600000
20332.0000000	0.6700000
20333.0000000	0.6650000
20334.0000000	0.6700000
20335.0000000	0.6800000
20336.0000000	0.6750000
20337.0000000	0.6700000
20338.0000000	0.6750000
20339.0000000	0.6800000
20340.0000000	0.6700000
20341.0000000	0.6750000
20342.0000000	0.6800000
20343.0000000	0.6800000
20344.0000000	0.6800000
20345.0000000	0.6750000
20346.0000000	0.6700000
20347.0000000	0.6750000
20348.0000000	0.6700000
20349.0000000	0.6700000
20350.0000000	0.6700000
20351.0000000	0.6700000
20352.0000000	0.6700000
20353.0000000	0.6700000
20354.0000000	0.6666667
20355.0000000	0.6600000
20356.0000000	0.6600000
20357.0000000	0.6600000
20358.0000000	0.6500000
20359.0000000	0.6450000
20360.0000000	0.6450000
20361.0000000	0.6450000
20362.0000000	0.6400000
20363.0000000	0.6350000
20364.0000000	0.6300000
20365.0000000	0.6250000
20366.0000000	0.6200000
20367.0000000	0.6200000
20368.0000000	0.6200000
20369.0000000	0.6150000
20370.0000000	0.6200000
20371.0000000	0.6200000
20372.0000000	0.6200000
20373.0000000	0.6200000
20374.0000000	0.6200000
20375.0000000	0.6200000
20376.0000000	0.6150000
20377.0000000	0.6200000
20378.0000000	0.6200000
20379.0000000	0.6100000
20380.0000000	0.6150000
20381.0000000	0.6050000

20382.0000000	0.6000000
20383.0000000	0.6000000
20384.0000000	0.5850000
20385.0000000	0.5766667
20386.0000000	0.5700000
20387.0000000	0.5650000
20388.0000000	0.5600000
20389.0000000	0.5600000
20390.0000000	0.5650000
20391.0000000	0.5600000
20392.0000000	0.5600000
20393.0000000	0.5600000
20394.0000000	0.5500000
20395.0000000	0.5550000
20396.0000000	0.5500000
20397.0000000	0.5450000
20398.0000000	0.5450000
20399.0000000	0.5400000
20400.0000000	0.5300000
20401.0000000	0.5250000
20402.0000000	0.5100000
20403.0000000	0.5100000
20404.0000000	0.5100000
20405.0000000	0.5100000
20406.0000000	0.5100000
20407.0000000	0.5100000
20408.0000000	0.5000000
20409.0000000	0.5100000
20410.0000000	0.5100000
20411.0000000	0.5050000
20412.0000000	0.5100000
20413.0000000	0.5100000
20414.0000000	0.5100000
20415.0000000	0.5200000
20416.0000000	0.5150000
20417.0000000	0.5250000
20418.0000000	0.5250000
20419.0000000	0.5300000
20420.0000000	0.5400000
20421.0000000	0.5400000
20422.0000000	0.5400000
20423.0000000	0.5500000
20424.0000000	0.5450000
20425.0000000	0.5500000
20426.0000000	0.5550000
20427.0000000	0.5550000
20428.0000000	0.5600000
20429.0000000	0.5600000
20430.0000000	0.5650000
20431.0000000	0.5650000
20432.0000000	0.5600000
20433.0000000	0.5700000
20434.0000000	0.5650000
20435.0000000	0.5750000
20436.0000000	0.5750000
20437.0000000	0.5800000
20438.0000000	0.5800000

20439.0000000	0.5800000
20440.0000000	0.5800000
20441.0000000	0.5800000
20442.0000000	0.5750000
20443.0000000	0.5850000
20444.0000000	0.5800000
20445.0000000	0.5850000
20446.0000000	0.5850000
20447.0000000	0.5850000
20448.0000000	0.5866666
20449.0000000	0.5850000
20450.0000000	0.5900000
20451.0000000	0.5850000
20452.0000000	0.5850000
20453.0000000	0.5850000
20454.0000000	0.5800000
20455.0000000	0.5850000
20456.0000000	0.5750000
20457.0000000	0.5700000
20458.0000000	0.5750000
20459.0000000	0.5700000
20460.0000000	0.5700000
20461.0000000	0.5700000
20462.0000000	0.5650000
20463.0000000	0.5700000
20464.0000000	0.5750000
20465.0000000	0.5750000
20466.0000000	0.5800000
20467.0000000	0.5800000
20468.0000000	0.5850000
20469.0000000	0.5850000
20470.0000000	0.5800000
20471.0000000	0.5850000
20472.0000000	0.5850000
20473.0000000	0.5800000
20474.0000000	0.5750000
20475.0000000	0.5800000
20476.0000000	0.5750000
20477.0000000	0.5766667
20478.0000000	0.5800000
20479.0000000	0.5700000
20480.0000000	0.5750000
20481.0000000	0.5750000
20482.0000000	0.5700000
20483.0000000	0.5700000
20484.0000000	0.5650000
20485.0000000	0.5600000
20486.0000000	0.5550000
20487.0000000	0.5550000
20488.0000000	0.5500000
20489.0000000	0.5500000
20490.0000000	0.5500000
20491.0000000	0.5400000
20492.0000000	0.5350000
20493.0000000	0.5300000
20494.0000000	0.5200000
20495.0000000	0.5150000

20496.0000000	0.5050000
20497.0000000	0.5000000
20498.0000000	0.4900000
20499.0000000	0.4900000
20500.0000000	0.4850000
20501.0000000	0.4850000
20502.0000000	0.4950000
20503.0000000	0.4900000
20504.0000000	0.5000000
20505.0000000	0.5100000
20506.0000000	0.5000000
20507.0000000	0.5000000
20508.0000000	0.5033333
20509.0000000	0.5050000
20510.0000000	0.5100000
20511.0000000	0.5100000
20512.0000000	0.5150000
20513.0000000	0.5200000
20514.0000000	0.5200000
20515.0000000	0.5200000
20516.0000000	0.5200000
20517.0000000	0.5300000
20518.0000000	0.5300000
20519.0000000	0.5300000
20520.0000000	0.5300000
20521.0000000	0.5300000
20522.0000000	0.5300000
20523.0000000	0.5300000
20524.0000000	0.5250000
20525.0000000	0.5250000
20526.0000000	0.5250000
20527.0000000	0.5200000
20528.0000000	0.5250000
20529.0000000	0.5200000
20530.0000000	0.5200000
20531.0000000	0.5300000
20532.0000000	0.5200000
20533.0000000	0.5200000
20534.0000000	0.5150000
20535.0000000	0.5100000
20536.0000000	0.5200000
20537.0000000	0.5150000
20538.0000000	0.5200000
20539.0000000	0.5200000
20540.0000000	0.5200000
20541.0000000	0.5150000
20542.0000000	0.5150000
20543.0000000	0.5150000
20544.0000000	0.5200000
20545.0000000	0.5200000
20546.0000000	0.5200000
20547.0000000	0.5150000
20548.0000000	0.5150000
20549.0000000	0.5100000
20550.0000000	0.5100000
20551.0000000	0.5100000
20552.0000000	0.5050000

20553.0000000	0.5150000
20554.0000000	0.5100000
20555.0000000	0.5100000
20556.0000000	0.5100000
20557.0000000	0.5000000
20558.0000000	0.5000000
20559.0000000	0.5000000
20560.0000000	0.5000000
20561.0000000	0.5000000
20562.0000000	0.5000000
20563.0000000	0.5000000
20564.0000000	0.5000000
20565.0000000	0.5000000
20566.0000000	0.5050000
20567.0000000	0.5033333
20568.0000000	0.4950000
20569.0000000	0.4950000
20570.0000000	0.5000000
20571.0000000	0.4900000
20572.0000000	0.4900000
20573.0000000	0.4900000
20574.0000000	0.4850000
20575.0000000	0.4800000
20576.0000000	0.4700000
20577.0000000	0.4750000
20578.0000000	0.4700000
20579.0000000	0.4700000
20580.0000000	0.4700000
20581.0000000	0.4750000
20582.0000000	0.4700000
20583.0000000	0.4750000
20584.0000000	0.4700000
20585.0000000	0.4750000
20586.0000000	0.4700000
20587.0000000	0.4700000
20588.0000000	0.4750000
20589.0000000	0.4700000
20590.0000000	0.4700000
20591.0000000	0.4650000
20592.0000000	0.4700000
20593.0000000	0.4600000
20594.0000000	0.4650000
20595.0000000	0.4650000
20596.0000000	0.4650000
20597.0000000	0.4700000
20598.0000000	0.4733333
20599.0000000	0.4750000
20600.0000000	0.4700000
20601.0000000	0.4750000
20602.0000000	0.4750000
20603.0000000	0.4750000
20604.0000000	0.4800000
20605.0000000	0.4800000
20606.0000000	0.4800000
20607.0000000	0.4950000
20608.0000000	0.4900000
20609.0000000	0.4950000

20610.0000000	0.4900000
20611.0000000	0.4950000
20612.0000000	0.5000000
20613.0000000	0.5000000
20614.0000000	0.5000000
20615.0000000	0.5000000
20616.0000000	0.4900000
20617.0000000	0.4950000
20618.0000000	0.4950000
20619.0000000	0.4800000
20620.0000000	0.4850000
20621.0000000	0.4800000
20622.0000000	0.4850000
20623.0000000	0.4850000
20624.0000000	0.4850000
20625.0000000	0.4850000
20626.0000000	0.4900000
20627.0000000	0.4900000
20628.0000000	0.4900000
20629.0000000	0.4850000
20630.0000000	0.4850000
20631.0000000	0.4900000
20632.0000000	0.4800000
20633.0000000	0.4900000
20634.0000000	0.4850000
20635.0000000	0.4850000
20636.0000000	0.4900000
20637.0000000	0.4850000
20638.0000000	0.4900000
20639.0000000	0.4900000
20640.0000000	0.4900000
20641.0000000	0.4850000
20642.0000000	0.4900000
20643.0000000	0.4900000
20644.0000000	0.4900000
20645.0000000	0.4850000
20646.0000000	0.4700000
20647.0000000	0.4650000
20648.0000000	0.4600000
20649.0000000	0.4600000
20650.0000000	0.4650000
20651.0000000	0.4600000
20652.0000000	0.4600000
20653.0000000	0.4600000
20654.0000000	0.4650000
20655.0000000	0.4600000
20656.0000000	0.4550000
20657.0000000	0.4600000
20658.0000000	0.4600000
20659.0000000	0.4550000
20660.0000000	0.4550000
20661.0000000	0.4550000
20662.0000000	0.4500000
20663.0000000	0.4550000
20664.0000000	0.4550000
20665.0000000	0.4450000
20666.0000000	0.4400000

20667.0000000	0.4400000
20668.0000000	0.4350000
20669.0000000	0.4400000
20670.0000000	0.4350000
20671.0000000	0.4400000
20672.0000000	0.4400000
20673.0000000	0.4350000
20674.0000000	0.4300000
20675.0000000	0.4300000
20676.0000000	0.4350000
20677.0000000	0.4300000
20678.0000000	0.4300000
20679.0000000	0.4250000
20680.0000000	0.4250000
20681.0000000	0.4250000
20682.0000000	0.4200000
20683.0000000	0.4150000
20684.0000000	0.4100000
20685.0000000	0.4100000
20686.0000000	0.4050000
20687.0000000	0.4000000
20688.0000000	0.3900000
20689.0000000	0.3850000
20690.0000000	0.3800000
20691.0000000	0.3850000
20692.0000000	0.3850000
20693.0000000	0.3850000
20694.0000000	0.3900000
20695.0000000	0.3950000
20696.0000000	0.3900000
20697.0000000	0.3950000
20698.0000000	0.3950000
20699.0000000	0.3950000
20700.0000000	0.4000000
20701.0000000	0.4000000
20702.0000000	0.4000000
20703.0000000	0.4000000
20704.0000000	0.3950000
20705.0000000	0.4000000
20706.0000000	0.3950000
20707.0000000	0.4000000
20708.0000000	0.4000000
20709.0000000	0.4050000
20710.0000000	0.4050000
20711.0000000	0.4050000
20712.0000000	0.4050000
20713.0000000	0.4000000
20714.0000000	0.4000000
20715.0000000	0.3900000
20716.0000000	0.3950000
20717.0000000	0.3966666
20718.0000000	0.3900000
20719.0000000	0.3900000
20720.0000000	0.3950000
20721.0000000	0.3900000
20722.0000000	0.3900000
20723.0000000	0.3900000

20724.0000000	0.3950000
20725.0000000	0.3950000
20726.0000000	0.3900000
20727.0000000	0.3950000
20728.0000000	0.3950000
20729.0000000	0.3950000
20730.0000000	0.4000000
20731.0000000	0.4050000
20732.0000000	0.4000000
20733.0000000	0.4000000
20734.0000000	0.4000000
20735.0000000	0.4000000
20736.0000000	0.4000000
20737.0000000	0.4000000
20738.0000000	0.4050000
20739.0000000	0.4000000
20740.0000000	0.4000000
20741.0000000	0.4000000
20742.0000000	0.4000000
20743.0000000	0.4000000
20744.0000000	0.4000000
20745.0000000	0.4050000
20746.0000000	0.4000000
20747.0000000	0.4066667
20748.0000000	0.4000000
20749.0000000	0.4050000
20750.0000000	0.4050000
20751.0000000	0.4050000
20752.0000000	0.4100000
20753.0000000	0.4100000
20754.0000000	0.4050000
20755.0000000	0.4150000
20756.0000000	0.4100000
20757.0000000	0.4150000
20758.0000000	0.4200000
20759.0000000	0.4200000
20760.0000000	0.4200000
20761.0000000	0.4250000
20762.0000000	0.4200000
20763.0000000	0.4300000
20764.0000000	0.4250000
20765.0000000	0.4250000
20766.0000000	0.4300000
20767.0000000	0.4350000
20768.0000000	0.4300000
20769.0000000	0.4350000
20770.0000000	0.4350000
20771.0000000	0.4400000
20772.0000000	0.4400000
20773.0000000	0.4400000
20774.0000000	0.4450000
20775.0000000	0.4350000
20776.0000000	0.4400000
20777.0000000	0.4350000
20778.0000000	0.4366666
20779.0000000	0.4300000
20780.0000000	0.4350000

20781.0000000	0.4300000
20782.0000000	0.4300000
20783.0000000	0.4250000
20784.0000000	0.4250000
20785.0000000	0.4250000
20786.0000000	0.4200000
20787.0000000	0.4200000
20788.0000000	0.4200000
20789.0000000	0.4200000
20790.0000000	0.4150000
20791.0000000	0.4200000
20792.0000000	0.4100000
20793.0000000	0.4200000
20794.0000000	0.4100000
20795.0000000	0.4100000
20796.0000000	0.4100000
20797.0000000	0.4100000
20798.0000000	0.4000000
20799.0000000	0.4050000
20800.0000000	0.3900000
20801.0000000	0.3900000
20802.0000000	0.3850000
20803.0000000	0.3850000
20804.0000000	0.3800000
20805.0000000	0.3850000
20806.0000000	0.3800000
20807.0000000	0.3850000
20808.0000000	0.3833333
20809.0000000	0.3850000
20810.0000000	0.3900000
20811.0000000	0.3900000
20812.0000000	0.3900000
20813.0000000	0.3950000
20814.0000000	0.3900000
20815.0000000	0.3900000
20816.0000000	0.3900000
20817.0000000	0.3900000
20818.0000000	0.3850000
20819.0000000	0.3800000
20820.0000000	0.3750000
20821.0000000	0.3700000
20822.0000000	0.3600000
20823.0000000	0.3600000
20824.0000000	0.3600000
20825.0000000	0.3550000
20826.0000000	0.3550000
20827.0000000	0.3550000
20828.0000000	0.3650000
20829.0000000	0.3600000
20830.0000000	0.3700000
20831.0000000	0.3750000
20832.0000000	0.3650000
20833.0000000	0.3700000
20834.0000000	0.3700000
20835.0000000	0.3750000
20836.0000000	0.3750000
20837.0000000	0.3800000

20838.0000000	0.3800000
20839.0000000	0.3800000
20840.0000000	0.3833333
20841.0000000	0.3850000
20842.0000000	0.3800000
20843.0000000	0.3800000
20844.0000000	0.3900000
20845.0000000	0.3850000
20846.0000000	0.3950000
20847.0000000	0.3950000
20848.0000000	0.4000000
20849.0000000	0.4050000
20850.0000000	0.4050000
20851.0000000	0.4100000
20852.0000000	0.4150000
20853.0000000	0.4100000
20854.0000000	0.4150000
20855.0000000	0.4200000
20856.0000000	0.4300000
20857.0000000	0.4300000
20858.0000000	0.4300000
20859.0000000	0.4350000
20860.0000000	0.4350000
20861.0000000	0.4400000
20862.0000000	0.4350000
20863.0000000	0.4400000
20864.0000000	0.4450000
20865.0000000	0.4400000
20866.0000000	0.4450000
20867.0000000	0.4450000
20868.0000000	0.4450000
20869.0000000	0.4400000
20870.0000000	0.4400000
20871.0000000	0.4366666
20872.0000000	0.4350000
20873.0000000	0.4400000
20874.0000000	0.4350000
20875.0000000	0.4350000
20876.0000000	0.4300000
20877.0000000	0.4350000
20878.0000000	0.4350000
20879.0000000	0.4400000
20880.0000000	0.4300000
20881.0000000	0.4400000
20882.0000000	0.4400000
20883.0000000	0.4400000
20884.0000000	0.4400000
20885.0000000	0.4500000
20886.0000000	0.4450000
20887.0000000	0.4450000
20888.0000000	0.4500000
20889.0000000	0.4450000
20890.0000000	0.4450000
20891.0000000	0.4450000
20892.0000000	0.4450000
20893.0000000	0.4450000
20894.0000000	0.4500000

20895.0000000	0.4450000
20896.0000000	0.4500000
20897.0000000	0.4500000
20898.0000000	0.4450000
20899.0000000	0.4450000
20900.0000000	0.4350000
20901.0000000	0.4433333
20902.0000000	0.4350000
20903.0000000	0.4350000
20904.0000000	0.4400000
20905.0000000	0.4400000
20906.0000000	0.4400000
20907.0000000	0.4350000
20908.0000000	0.4400000
20909.0000000	0.4350000
20910.0000000	0.4350000
20911.0000000	0.4350000
20912.0000000	0.4350000
20913.0000000	0.4400000
20914.0000000	0.4350000
20915.0000000	0.4350000
20916.0000000	0.4350000
20917.0000000	0.4400000
20918.0000000	0.4300000
20919.0000000	0.4350000
20920.0000000	0.4350000
20921.0000000	0.4400000
20922.0000000	0.4400000
20923.0000000	0.4400000
20924.0000000	0.4400000
20925.0000000	0.4400000
20926.0000000	0.4500000
20927.0000000	0.4500000
20928.0000000	0.4500000
20929.0000000	0.4500000
20930.0000000	0.4550000
20931.0000000	0.4500000
20932.0000000	0.4500000
20933.0000000	0.4500000
20934.0000000	0.4450000
20935.0000000	0.4500000
20936.0000000	0.4450000
20937.0000000	0.4400000
20938.0000000	0.4400000
20939.0000000	0.4400000
20940.0000000	0.4500000
20941.0000000	0.4450000
20942.0000000	0.4400000
20943.0000000	0.4500000
20944.0000000	0.4500000
20945.0000000	0.4550000
20946.0000000	0.4500000
20947.0000000	0.4500000
20948.0000000	0.4500000
20949.0000000	0.4550000
20950.0000000	0.4550000
20951.0000000	0.4600000

20952.0000000	0.4650000
20953.0000000	0.4600000
20954.0000000	0.4600000
20955.0000000	0.4650000
20956.0000000	0.4650000
20957.0000000	0.4650000
20958.0000000	0.4700000
20959.0000000	0.4650000
20960.0000000	0.4700000
20961.0000000	0.4700000
20962.0000000	0.4733333
20963.0000000	0.4750000
20964.0000000	0.4800000
20965.0000000	0.4800000
20966.0000000	0.4800000
20967.0000000	0.4750000
20968.0000000	0.4800000
20969.0000000	0.4800000
20970.0000000	0.4700000
20971.0000000	0.4800000
20972.0000000	0.4750000
20973.0000000	0.4750000
20974.0000000	0.4750000
20975.0000000	0.4800000
20976.0000000	0.4750000
20977.0000000	0.4700000
20978.0000000	0.4800000
20979.0000000	0.4750000
20980.0000000	0.4750000
20981.0000000	0.4750000
20982.0000000	0.4800000
20983.0000000	0.4750000
20984.0000000	0.4750000
20985.0000000	0.4800000
20986.0000000	0.4850000
20987.0000000	0.4850000
20988.0000000	0.4900000
20989.0000000	0.4900000
20990.0000000	0.4850000
20991.0000000	0.4833333
20992.0000000	0.4900000
20993.0000000	0.4900000
20994.0000000	0.4900000
20995.0000000	0.4950000
20996.0000000	0.4950000
20997.0000000	0.4950000
20998.0000000	0.4950000
20999.0000000	0.4950000
21000.0000000	0.4900000
21001.0000000	0.4900000
21002.0000000	0.4900000
21003.0000000	0.4800000
21004.0000000	0.4800000
21005.0000000	0.4750000
21006.0000000	0.4700000
21007.0000000	0.4700000
21008.0000000	0.4700000

21009.0000000	0.4700000
21010.0000000	0.4750000
21011.0000000	0.4750000
21012.0000000	0.4750000
21013.0000000	0.4800000
21014.0000000	0.4750000
21015.0000000	0.4800000
21016.0000000	0.4750000
21017.0000000	0.4850000
21018.0000000	0.4850000
21019.0000000	0.4850000
21020.0000000	0.4900000
21021.0000000	0.4966667
21022.0000000	0.5000000
21023.0000000	0.5000000
21024.0000000	0.5050000
21025.0000000	0.5050000
21026.0000000	0.5050000
21027.0000000	0.5100000
21028.0000000	0.5150000
21029.0000000	0.5200000
21030.0000000	0.5250000
21031.0000000	0.5250000
21032.0000000	0.5300000
21033.0000000	0.5350000
21034.0000000	0.5350000
21035.0000000	0.5400000
21036.0000000	0.5400000
21037.0000000	0.5400000
21038.0000000	0.5450000
21039.0000000	0.5450000
21040.0000000	0.5550000
21041.0000000	0.5550000
21042.0000000	0.5600000
21043.0000000	0.5600000
21044.0000000	0.5600000
21045.0000000	0.5600000
21046.0000000	0.5600000
21047.0000000	0.5650000
21048.0000000	0.5700000
21049.0000000	0.5700000
21050.0000000	0.5700000
21051.0000000	0.5766667
21052.0000000	0.5700000
21053.0000000	0.5700000
21054.0000000	0.5700000
21055.0000000	0.5700000
21056.0000000	0.5750000
21057.0000000	0.5800000
21058.0000000	0.5800000
21059.0000000	0.5800000
21060.0000000	0.5850000
21061.0000000	0.5800000
21062.0000000	0.5800000
21063.0000000	0.5850000
21064.0000000	0.5800000
21065.0000000	0.5850000

21066.0000000	0.5850000
21067.0000000	0.5800000
21068.0000000	0.5800000
21069.0000000	0.5800000
21070.0000000	0.5850000
21071.0000000	0.5800000
21072.0000000	0.5800000
21073.0000000	0.5800000
21074.0000000	0.5850000
21075.0000000	0.5950000
21076.0000000	0.5900000
21077.0000000	0.5950000
21078.0000000	0.6000000
21079.0000000	0.6000000
21080.0000000	0.6033334
21081.0000000	0.6050000
21082.0000000	0.6100000
21083.0000000	0.6100000
21084.0000000	0.6150000
21085.0000000	0.6100000
21086.0000000	0.6100000
21087.0000000	0.6150000
21088.0000000	0.6150000
21089.0000000	0.6150000
21090.0000000	0.6100000
21091.0000000	0.6150000
21092.0000000	0.6100000
21093.0000000	0.6100000
21094.0000000	0.6100000
21095.0000000	0.6100000
21096.0000000	0.6200000
21097.0000000	0.6200000
21098.0000000	0.6200000
21099.0000000	0.6200000
21100.0000000	0.6200000
21101.0000000	0.6250000
21102.0000000	0.6250000
21103.0000000	0.6250000
21104.0000000	0.6300000
21105.0000000	0.6300000
21106.0000000	0.6350000
21107.0000000	0.6350000
21108.0000000	0.6400000
21109.0000000	0.6350000
21110.0000000	0.6366667
21111.0000000	0.6400000
21112.0000000	0.6400000
21113.0000000	0.6500000
21114.0000000	0.6400000
21115.0000000	0.6400000
21116.0000000	0.6350000
21117.0000000	0.6300000
21118.0000000	0.6250000
21119.0000000	0.6250000
21120.0000000	0.6250000
21121.0000000	0.6150000
21122.0000000	0.6150000

21123.0000000	0.6000000
21124.0000000	0.6000000
21125.0000000	0.5900000
21126.0000000	0.5850000
21127.0000000	0.5800000
21128.0000000	0.5800000
21129.0000000	0.5750000
21130.0000000	0.5750000
21131.0000000	0.5700000
21132.0000000	0.5650000
21133.0000000	0.5600000
21134.0000000	0.5600000
21135.0000000	0.5500000
21136.0000000	0.5500000
21137.0000000	0.5450000
21138.0000000	0.5550000
21139.0000000	0.5500000
21140.0000000	0.5600000
21141.0000000	0.5600000
21142.0000000	0.5550000
21143.0000000	0.5600000
21144.0000000	0.5500000
21145.0000000	0.5500000
21146.0000000	0.5550000
21147.0000000	0.5600000
21148.0000000	0.5650000
21149.0000000	0.5700000
21150.0000000	0.5700000
21151.0000000	0.5700000
21152.0000000	0.5700000
21153.0000000	0.5700000
21154.0000000	0.5800000
21155.0000000	0.5800000
21156.0000000	0.5850000
21157.0000000	0.5850000
21158.0000000	0.5900000
21159.0000000	0.5850000
21160.0000000	0.5800000
21161.0000000	0.5800000
21162.0000000	0.5750000
21163.0000000	0.5800000
21164.0000000	0.5850000
21165.0000000	0.5800000
21166.0000000	0.5850000
21167.0000000	0.5800000
21168.0000000	0.5900000
21169.0000000	0.5900000
21170.0000000	0.5900000
21171.0000000	0.5900000
21172.0000000	0.5900000
21173.0000000	0.5950000
21174.0000000	0.5900000
21175.0000000	0.5850000
21176.0000000	0.5850000
21177.0000000	0.5800000
21178.0000000	0.5900000
21179.0000000	0.5900000

21180.0000000	0.5900000
21181.0000000	0.5900000
21182.0000000	0.6000000
21183.0000000	0.6000000
21184.0000000	0.6050000
21185.0000000	0.6100000
21186.0000000	0.6150000
21187.0000000	0.6100000
21188.0000000	0.6100000
21189.0000000	0.6200000
21190.0000000	0.6200000
21191.0000000	0.6300000
21192.0000000	0.6300000
21193.0000000	0.6350000
21194.0000000	0.6400000
21195.0000000	0.6450000
21196.0000000	0.6400000
21197.0000000	0.6400000
21198.0000000	0.6400000
21199.0000000	0.6450000
21200.0000000	0.6450000
21201.0000000	0.6400000
21202.0000000	0.6433333
21203.0000000	0.6400000
21204.0000000	0.6400000
21205.0000000	0.6400000
21206.0000000	0.6400000
21207.0000000	0.6400000
21208.0000000	0.6400000
21209.0000000	0.6350000
21210.0000000	0.6400000
21211.0000000	0.6350000
21212.0000000	0.6350000
21213.0000000	0.6400000
21214.0000000	0.6350000
21215.0000000	0.6300000
21216.0000000	0.6300000
21217.0000000	0.6300000
21218.0000000	0.6300000
21219.0000000	0.6350000
21220.0000000	0.6350000
21221.0000000	0.6400000
21222.0000000	0.6450000
21223.0000000	0.6500000
21224.0000000	0.6450000
21225.0000000	0.6500000
21226.0000000	0.6450000
21227.0000000	0.6450000
21228.0000000	0.6500000
21229.0000000	0.6450000
21230.0000000	0.6450000
21231.0000000	0.6400000
21232.0000000	0.6400000
21233.0000000	0.6400000
21234.0000000	0.6500000
21235.0000000	0.6400000
21236.0000000	0.6500000

21237.0000000	0.6450000
21238.0000000	0.6500000
21239.0000000	0.6500000
21240.0000000	0.6500000
21241.0000000	0.6500000
21242.0000000	0.6400000
21243.0000000	0.6450000
21244.0000000	0.6450000
21245.0000000	0.6400000
21246.0000000	0.6400000
21247.0000000	0.6450000
21248.0000000	0.6300000
21249.0000000	0.6350000
21250.0000000	0.6350000
21251.0000000	0.6400000
21252.0000000	0.6350000
21253.0000000	0.6300000
21254.0000000	0.6350000
21255.0000000	0.6350000
21256.0000000	0.6300000
21257.0000000	0.6250000
21258.0000000	0.6200000
21259.0000000	0.6300000
21260.0000000	0.6200000
21261.0000000	0.6266667
21262.0000000	0.6200000
21263.0000000	0.6150000
21264.0000000	0.6200000
21265.0000000	0.6200000
21266.0000000	0.6200000
21267.0000000	0.6150000
21268.0000000	0.6200000
21269.0000000	0.6200000
21270.0000000	0.6200000
21271.0000000	0.6200000
21272.0000000	0.6200000
21273.0000000	0.6150000
21274.0000000	0.6150000
21275.0000000	0.6250000
21276.0000000	0.6200000
21277.0000000	0.6200000
21278.0000000	0.6200000
21279.0000000	0.6200000
21280.0000000	0.6250000
21281.0000000	0.6300000
21282.0000000	0.6250000
21283.0000000	0.6300000
21284.0000000	0.6400000
21285.0000000	0.6300000
21286.0000000	0.6400000
21287.0000000	0.6450000
21288.0000000	0.6350000
21289.0000000	0.6450000
21290.0000000	0.6450000
21291.0000000	0.6466666
21292.0000000	0.6450000
21293.0000000	0.6400000

21294.0000000	0.6450000
21295.0000000	0.6400000
21296.0000000	0.6350000
21297.0000000	0.6400000
21298.0000000	0.6400000
21299.0000000	0.6350000
21300.0000000	0.6350000
21301.0000000	0.6350000
21302.0000000	0.6400000
21303.0000000	0.6400000
21304.0000000	0.6450000
21305.0000000	0.6450000
21306.0000000	0.6400000
21307.0000000	0.6450000
21308.0000000	0.6450000
21309.0000000	0.6400000
21310.0000000	0.6450000
21311.0000000	0.6550000
21312.0000000	0.6550000
21313.0000000	0.6600000
21314.0000000	0.6650000
21315.0000000	0.6650000
21316.0000000	0.6750000
21317.0000000	0.6800000
21318.0000000	0.6800000
21319.0000000	0.6850000
21320.0000000	0.7000000
21321.0000000	0.7000000
21322.0000000	0.7050000
21323.0000000	0.7100000
21324.0000000	0.7200000
21325.0000000	0.7300000
21326.0000000	0.7300000
21327.0000000	0.7400000
21328.0000000	0.7500000
21329.0000000	0.7500000
21330.0000000	0.7550000
21331.0000000	0.7600000
21332.0000000	0.7650000
21333.0000000	0.7750000
21334.0000000	0.7700000
21335.0000000	0.7700000
21336.0000000	0.7850000
21337.0000000	0.7850000
21338.0000000	0.7900000
21339.0000000	0.7950000
21340.0000000	0.8000000
21341.0000000	0.8000000
21342.0000000	0.7900000
21343.0000000	0.8050000
21344.0000000	0.8000000
21345.0000000	0.8050000
21346.0000000	0.8050000
21347.0000000	0.8100000
21348.0000000	0.8150000
21349.0000000	0.8100000
21350.0000000	0.8200000

21351.0000000	0.8200000
21352.0000000	0.8200000
21353.0000000	0.8250000
21354.0000000	0.8200000
21355.0000000	0.8200000
21356.0000000	0.8200000
21357.0000000	0.8200000
21358.0000000	0.8200000
21359.0000000	0.8200000
21360.0000000	0.8200000
21361.0000000	0.8150000
21362.0000000	0.8150000
21363.0000000	0.8200000
21364.0000000	0.8200000
21365.0000000	0.8200000
21366.0000000	0.8200000
21367.0000000	0.8200000
21368.0000000	0.8100000
21369.0000000	0.8150000
21370.0000000	0.8200000
21371.0000000	0.8150000
21372.0000000	0.8200000
21373.0000000	0.8150000
21374.0000000	0.8150000
21375.0000000	0.8150000
21376.0000000	0.8100000
21377.0000000	0.8100000
21378.0000000	0.8050000
21379.0000000	0.8000000
21380.0000000	0.8050000
21381.0000000	0.8000000
21382.0000000	0.8000000
21383.0000000	0.8050000
21384.0000000	0.8000000
21385.0000000	0.8000000
21386.0000000	0.8000000
21387.0000000	0.7950000
21388.0000000	0.7900000
21389.0000000	0.7900000
21390.0000000	0.7900000
21391.0000000	0.7900000
21392.0000000	0.7850000
21393.0000000	0.7800000
21394.0000000	0.7850000
21395.0000000	0.7750000
21396.0000000	0.7800000
21397.0000000	0.7800000
21398.0000000	0.7750000
21399.0000000	0.7700000
21400.0000000	0.7700000
21401.0000000	0.7650000
21402.0000000	0.7550000
21403.0000000	0.7600000
21404.0000000	0.7500000
21405.0000000	0.7600000
21406.0000000	0.7500000
21407.0000000	0.7450000

21408.0000000	0.7400000
21409.0000000	0.7400000
21410.0000000	0.7350000
21411.0000000	0.7300000
21412.0000000	0.7266667
21413.0000000	0.7200000
21414.0000000	0.7150000
21415.0000000	0.7150000
21416.0000000	0.7100000
21417.0000000	0.7050000
21418.0000000	0.7100000
21419.0000000	0.7000000
21420.0000000	0.7100000
21421.0000000	0.7050000
21422.0000000	0.7050000
21423.0000000	0.7050000
21424.0000000	0.7050000
21425.0000000	0.7100000
21426.0000000	0.7200000
21427.0000000	0.7200000
21428.0000000	0.7250000
21429.0000000	0.7300000
21430.0000000	0.7350000
21431.0000000	0.7350000
21432.0000000	0.7400000
21433.0000000	0.7400000
21434.0000000	0.7400000
21435.0000000	0.7500000
21436.0000000	0.7500000
21437.0000000	0.7550000
21438.0000000	0.7650000
21439.0000000	0.7700000
21440.0000000	0.7700000
21441.0000000	0.7750000
21442.0000000	0.7800000
21443.0000000	0.7850000
21444.0000000	0.7900000
21445.0000000	0.7900000
21446.0000000	0.7900000
21447.0000000	0.7950000
21448.0000000	0.7900000
21449.0000000	0.7900000
21450.0000000	0.7900000
21451.0000000	0.7950000
21452.0000000	0.7900000
21453.0000000	0.7900000
21454.0000000	0.7850000
21455.0000000	0.7800000
21456.0000000	0.7800000
21457.0000000	0.7750000
21458.0000000	0.7750000
21459.0000000	0.7800000
21460.0000000	0.7750000
21461.0000000	0.7750000
21462.0000000	0.7700000
21463.0000000	0.7750000
21464.0000000	0.7700000

21465.0000000	0.7700000
21466.0000000	0.7700000
21467.0000000	0.7600000
21468.0000000	0.7650000
21469.0000000	0.7550000
21470.0000000	0.7550000
21471.0000000	0.7600000
21472.0000000	0.7550000
21473.0000000	0.7600000
21474.0000000	0.7533333
21475.0000000	0.7450000
21476.0000000	0.7450000
21477.0000000	0.7500000
21478.0000000	0.7500000
21479.0000000	0.7450000
21480.0000000	0.7400000
21481.0000000	0.7400000
21482.0000000	0.7300000
21483.0000000	0.7300000
21484.0000000	0.7250000
21485.0000000	0.7200000
21486.0000000	0.7200000
21487.0000000	0.7200000
21488.0000000	0.7200000
21489.0000000	0.7150000
21490.0000000	0.7150000
21491.0000000	0.7200000
21492.0000000	0.7250000
21493.0000000	0.7200000
21494.0000000	0.7300000
21495.0000000	0.7300000
21496.0000000	0.7350000
21497.0000000	0.7300000
21498.0000000	0.7300000
21499.0000000	0.7250000
21500.0000000	0.7200000
21501.0000000	0.7100000
21502.0000000	0.7050000
21503.0000000	0.6900000
21504.0000000	0.6800000
21505.0000000	0.6800000
21506.0000000	0.6750000
21507.0000000	0.6750000
21508.0000000	0.6800000
21509.0000000	0.6800000
21510.0000000	0.6800000
21511.0000000	0.6850000
21512.0000000	0.6900000
21513.0000000	0.6950000
21514.0000000	0.6950000
21515.0000000	0.7000000
21516.0000000	0.7050000
21517.0000000	0.7100000
21518.0000000	0.7050000
21519.0000000	0.7000000
21520.0000000	0.7100000
21521.0000000	0.7050000

21522.0000000	0.7000000
21523.0000000	0.6950000
21524.0000000	0.6950000
21525.0000000	0.7000000
21526.0000000	0.6950000
21527.0000000	0.7000000
21528.0000000	0.7000000
21529.0000000	0.7000000
21530.0000000	0.6900000
21531.0000000	0.6900000
21532.0000000	0.6900000
21533.0000000	0.6900000
21534.0000000	0.6966667
21535.0000000	0.7000000
21536.0000000	0.7000000
21537.0000000	0.7000000
21538.0000000	0.7100000
21539.0000000	0.7100000
21540.0000000	0.7150000
21541.0000000	0.7200000
21542.0000000	0.7150000
21543.0000000	0.7200000
21544.0000000	0.7200000
21545.0000000	0.7200000
21546.0000000	0.7300000
21547.0000000	0.7300000
21548.0000000	0.7250000
21549.0000000	0.7300000
21550.0000000	0.7250000
21551.0000000	0.7300000
21552.0000000	0.7250000
21553.0000000	0.7200000
21554.0000000	0.7200000
21555.0000000	0.7300000
21556.0000000	0.7250000
21557.0000000	0.7200000
21558.0000000	0.7250000
21559.0000000	0.7200000
21560.0000000	0.7200000
21561.0000000	0.7200000
21562.0000000	0.7200000
21563.0000000	0.7150000
21564.0000000	0.7100000
21565.0000000	0.7100000
21566.0000000	0.7000000
21567.0000000	0.7000000
21568.0000000	0.6900000
21569.0000000	0.6900000
21570.0000000	0.6950000
21571.0000000	0.6900000
21572.0000000	0.6900000
21573.0000000	0.6850000
21574.0000000	0.6800000
21575.0000000	0.6900000
21576.0000000	0.6900000
21577.0000000	0.6850000
21578.0000000	0.6800000

21579.0000000	0.6700000
21580.0000000	0.6700000
21581.0000000	0.6650000
21582.0000000	0.6700000
21583.0000000	0.6750000
21584.0000000	0.6750000
21585.0000000	0.6850000
21586.0000000	0.6800000
21587.0000000	0.6850000
21588.0000000	0.6950000
21589.0000000	0.6950000
21590.0000000	0.7000000
21591.0000000	0.7000000
21592.0000000	0.7100000
21593.0000000	0.7100000
21594.0000000	0.7200000
21595.0000000	0.7200000
21596.0000000	0.7250000
21597.0000000	0.7250000
21598.0000000	0.7300000
21599.0000000	0.7300000
21600.0000000	0.7350000
21601.0000000	0.7400000
21602.0000000	0.7350000
21603.0000000	0.7400000
21604.0000000	0.7400000
21605.0000000	0.7400000
21606.0000000	0.7400000
21607.0000000	0.7500000
21608.0000000	0.7450000
21609.0000000	0.7450000
21610.0000000	0.7400000
21611.0000000	0.7450000
21612.0000000	0.7400000
21613.0000000	0.7400000
21614.0000000	0.7400000
21615.0000000	0.7400000
21616.0000000	0.7400000
21617.0000000	0.7400000
21618.0000000	0.7400000
21619.0000000	0.7400000
21620.0000000	0.7450000
21621.0000000	0.7400000
21622.0000000	0.7400000
21623.0000000	0.7450000
21624.0000000	0.7450000
21625.0000000	0.7400000
21626.0000000	0.7366667
21627.0000000	0.7350000
21628.0000000	0.7350000
21629.0000000	0.7300000
21630.0000000	0.7300000
21631.0000000	0.7300000
21632.0000000	0.7250000
21633.0000000	0.7250000
21634.0000000	0.7300000
21635.0000000	0.7300000

21636.0000000	0.7300000
21637.0000000	0.7350000
21638.0000000	0.7300000
21639.0000000	0.7350000
21640.0000000	0.7400000
21641.0000000	0.7400000
21642.0000000	0.7350000
21643.0000000	0.7200000
21644.0000000	0.7050000
21645.0000000	0.7050000
21646.0000000	0.7000000
21647.0000000	0.7000000
21648.0000000	0.7050000
21649.0000000	0.7050000
21650.0000000	0.7050000
21651.0000000	0.7100000
21652.0000000	0.7100000
21653.0000000	0.7200000
21654.0000000	0.7200000
21655.0000000	0.7266667
21656.0000000	0.7300000
21657.0000000	0.7300000
21658.0000000	0.7350000
21659.0000000	0.7350000
21660.0000000	0.7350000
21661.0000000	0.7300000
21662.0000000	0.7350000
21663.0000000	0.7300000
21664.0000000	0.7300000
21665.0000000	0.7350000
21666.0000000	0.7350000
21667.0000000	0.7350000
21668.0000000	0.7400000
21669.0000000	0.7400000
21670.0000000	0.7400000
21671.0000000	0.7450000
21672.0000000	0.7500000
21673.0000000	0.7550000
21674.0000000	0.7650000
21675.0000000	0.7700000
21676.0000000	0.7750000
21677.0000000	0.7750000
21678.0000000	0.7800000
21679.0000000	0.7750000
21680.0000000	0.7800000
21681.0000000	0.7750000
21682.0000000	0.7800000
21683.0000000	0.7800000
21684.0000000	0.7850000
21685.0000000	0.7900000
21686.0000000	0.8000000
21687.0000000	0.8050000
21688.0000000	0.8100000
21689.0000000	0.8100000
21690.0000000	0.8100000
21691.0000000	0.8100000
21692.0000000	0.8100000

21693.0000000	0.8100000
21694.0000000	0.8100000
21695.0000000	0.8100000
21696.0000000	0.8050000
21697.0000000	0.8000000
21698.0000000	0.8000000
21699.0000000	0.8000000
21700.0000000	0.7950000
21701.0000000	0.7900000
21702.0000000	0.7950000
21703.0000000	0.7900000
21704.0000000	0.7850000
21705.0000000	0.7800000
21706.0000000	0.7750000
21707.0000000	0.7700000
21708.0000000	0.7600000
21709.0000000	0.7650000
21710.0000000	0.7700000
21711.0000000	0.7650000
21712.0000000	0.7600000
21713.0000000	0.7600000
21714.0000000	0.7600000
21715.0000000	0.7533333
21716.0000000	0.7500000
21717.0000000	0.7500000
21718.0000000	0.7450000
21719.0000000	0.7500000
21720.0000000	0.7500000
21721.0000000	0.7500000
21722.0000000	0.7500000
21723.0000000	0.7500000
21724.0000000	0.7500000
21725.0000000	0.7450000
21726.0000000	0.7400000
21727.0000000	0.7400000
21728.0000000	0.7350000
21729.0000000	0.7300000
21730.0000000	0.7200000
21731.0000000	0.7100000
21732.0000000	0.7150000
21733.0000000	0.7050000
21734.0000000	0.7200000
21735.0000000	0.7150000
21736.0000000	0.7050000
21737.0000000	0.7050000
21738.0000000	0.7050000
21739.0000000	0.7100000
21740.0000000	0.7100000
21741.0000000	0.7350000
21742.0000000	0.7850000
21743.0000000	0.8500000
21744.0000000	0.8200000
21745.0000000	0.7500000
21746.0000000	0.7300000
21747.0000000	0.7233334
21748.0000000	0.7250000
21749.0000000	0.7250000

21750.0000000	0.7300000
21751.0000000	0.7250000
21752.0000000	0.7350000
21753.0000000	0.7250000
21754.0000000	0.7250000
21755.0000000	0.7200000
21756.0000000	0.7200000
21757.0000000	0.7150000
21758.0000000	0.7100000
21759.0000000	0.7000000
21760.0000000	0.7000000
21761.0000000	0.7100000
21762.0000000	0.7100000
21763.0000000	0.7050000
21764.0000000	0.7200000
21765.0000000	0.7500000
21766.0000000	0.7550000
21767.0000000	0.7300000
21768.0000000	0.7150000
21769.0000000	0.7100000
21770.0000000	0.7200000
21771.0000000	0.7300000
21772.0000000	0.7450000
21773.0000000	0.7700000
21774.0000000	0.8200000
21775.0000000	0.8333333
21776.0000000	0.7900000
21777.0000000	0.7550000
21778.0000000	0.7400000
21779.0000000	0.7400000
21780.0000000	0.7450000
21781.0000000	0.7450000
21782.0000000	0.7500000
21783.0000000	0.7500000
21784.0000000	0.7550000
21785.0000000	0.7500000
21786.0000000	0.7450000
21787.0000000	0.7450000
21788.0000000	0.7300000
21789.0000000	0.7300000
21790.0000000	0.7200000
21791.0000000	0.7200000
21792.0000000	0.7150000
21793.0000000	0.7250000
21794.0000000	0.7250000
21795.0000000	0.7250000
21796.0000000	0.7300000
21797.0000000	0.7300000
21798.0000000	0.7250000
21799.0000000	0.7200000
21800.0000000	0.7100000
21801.0000000	0.7100000
21802.0000000	0.7050000
21803.0000000	0.7050000
21804.0000000	0.7100000
21805.0000000	0.7000000
21806.0000000	0.7000000

21807.0000000	0.7000000
21808.0000000	0.7000000
21809.0000000	0.6950000
21810.0000000	0.6900000
21811.0000000	0.6900000
21812.0000000	0.6900000
21813.0000000	0.6850000
21814.0000000	0.6800000
21815.0000000	0.6800000
21816.0000000	0.6700000
21817.0000000	0.6800000
21818.0000000	0.6750000
21819.0000000	0.6800000
21820.0000000	0.6750000
21821.0000000	0.6750000
21822.0000000	0.6750000
21823.0000000	0.6700000
21824.0000000	0.6750000
21825.0000000	0.6750000
21826.0000000	0.6800000
21827.0000000	0.6800000
21828.0000000	0.6850000
21829.0000000	0.6850000
21830.0000000	0.6900000
21831.0000000	0.6850000
21832.0000000	0.6900000
21833.0000000	0.6950000
21834.0000000	0.6950000
21835.0000000	0.6900000
21836.0000000	0.6900000
21837.0000000	0.6800000
21838.0000000	0.6800000
21839.0000000	0.6800000
21840.0000000	0.6800000
21841.0000000	0.6850000
21842.0000000	0.6800000
21843.0000000	0.6800000
21844.0000000	0.6750000
21845.0000000	0.6750000
21846.0000000	0.6700000
21847.0000000	0.6750000
21848.0000000	0.6700000
21849.0000000	0.6750000
21850.0000000	0.6700000
21851.0000000	0.6650000
21852.0000000	0.6700000
21853.0000000	0.6650000
21854.0000000	0.6700000
21855.0000000	0.6650000
21856.0000000	0.6700000
21857.0000000	0.6700000
21858.0000000	0.6700000
21859.0000000	0.6650000
21860.0000000	0.6700000
21861.0000000	0.6750000
21862.0000000	0.6800000
21863.0000000	0.6800000

21864.0000000	0.6850000
21865.0000000	0.6950000
21866.0000000	0.6966667
21867.0000000	0.7000000
21868.0000000	0.7050000
21869.0000000	0.7150000
21870.0000000	0.7100000
21871.0000000	0.7150000
21872.0000000	0.7100000
21873.0000000	0.7100000
21874.0000000	0.7100000
21875.0000000	0.7150000
21876.0000000	0.7100000
21877.0000000	0.7100000
21878.0000000	0.7100000
21879.0000000	0.7050000
21880.0000000	0.7050000
21881.0000000	0.7100000
21882.0000000	0.7000000
21883.0000000	0.7050000
21884.0000000	0.7000000
21885.0000000	0.7050000
21886.0000000	0.7050000
21887.0000000	0.6950000
21888.0000000	0.7000000
21889.0000000	0.6950000
21890.0000000	0.6950000
21891.0000000	0.6950000
21892.0000000	0.6900000
21893.0000000	0.6900000
21894.0000000	0.6900000
21895.0000000	0.6950000
21896.0000000	0.7000000
21897.0000000	0.6900000
21898.0000000	0.6900000
21899.0000000	0.6900000
21900.0000000	0.6850000
21901.0000000	0.6850000
21902.0000000	0.6900000
21903.0000000	0.6900000
21904.0000000	0.6850000
21905.0000000	0.6850000
21906.0000000	0.6800000
21907.0000000	0.6850000
21908.0000000	0.6900000
21909.0000000	0.7000000
21910.0000000	0.7000000
21911.0000000	0.7100000
21912.0000000	0.7100000
21913.0000000	0.7100000
21914.0000000	0.7150000
21915.0000000	0.7150000
21916.0000000	0.7200000
21917.0000000	0.7200000
21918.0000000	0.7200000
21919.0000000	0.7250000
21920.0000000	0.7300000

21921.0000000	0.7300000
21922.0000000	0.7350000
21923.0000000	0.7350000
21924.0000000	0.7400000
21925.0000000	0.7350000
21926.0000000	0.7350000
21927.0000000	0.7350000
21928.0000000	0.7400000
21929.0000000	0.7450000
21930.0000000	0.7500000
21931.0000000	0.7500000
21932.0000000	0.7550000
21933.0000000	0.7450000
21934.0000000	0.7500000
21935.0000000	0.7500000
21936.0000000	0.7500000
21937.0000000	0.7600000
21938.0000000	0.7550000
21939.0000000	0.7650000
21940.0000000	0.7650000
21941.0000000	0.7600000
21942.0000000	0.7600000
21943.0000000	0.7650000
21944.0000000	0.7600000
21945.0000000	0.7600000
21946.0000000	0.7700000
21947.0000000	0.7650000
21948.0000000	0.7700000
21949.0000000	0.7650000
21950.0000000	0.7600000
21951.0000000	0.7600000
21952.0000000	0.7500000
21953.0000000	0.7500000
21954.0000000	0.7450000
21955.0000000	0.7400000
21956.0000000	0.7400000
21957.0000000	0.7350000
21958.0000000	0.7400000
21959.0000000	0.7400000
21960.0000000	0.7450000
21961.0000000	0.7500000
21962.0000000	0.7500000
21963.0000000	0.7500000
21964.0000000	0.7500000
21965.0000000	0.7450000
21966.0000000	0.7500000
21967.0000000	0.7500000
21968.0000000	0.7500000
21969.0000000	0.7500000
21970.0000000	0.7450000
21971.0000000	0.7500000
21972.0000000	0.7400000
21973.0000000	0.7500000
21974.0000000	0.7450000
21975.0000000	0.7500000
21976.0000000	0.7500000
21977.0000000	0.7400000

21978.0000000	0.7500000
21979.0000000	0.7450000
21980.0000000	0.7500000
21981.0000000	0.7500000
21982.0000000	0.7400000
21983.0000000	0.7500000
21984.0000000	0.7450000
21985.0000000	0.7400000
21986.0000000	0.7400000
21987.0000000	0.7400000
21988.0000000	0.7466667
21989.0000000	0.7450000
21990.0000000	0.7400000
21991.0000000	0.7400000
21992.0000000	0.7300000
21993.0000000	0.7200000
21994.0000000	0.7200000
21995.0000000	0.7100000
21996.0000000	0.7100000
21997.0000000	0.7100000
21998.0000000	0.7000000
21999.0000000	0.6900000
22000.0000000	0.6950000
22001.0000000	0.6900000
22002.0000000	0.6850000
22003.0000000	0.6850000
22004.0000000	0.6900000
22005.0000000	0.6900000
22006.0000000	0.6950000
22007.0000000	0.6800000
22008.0000000	0.6850000
22009.0000000	0.6800000
22010.0000000	0.6800000
22011.0000000	0.6800000
22012.0000000	0.6850000
22013.0000000	0.6850000
22014.0000000	0.6900000
22015.0000000	0.6950000
22016.0000000	0.7000000
22017.0000000	0.7000000
22018.0000000	0.7000000
22019.0000000	0.7050000
22020.0000000	0.7050000
22021.0000000	0.7100000
22022.0000000	0.7200000
22023.0000000	0.7250000
22024.0000000	0.7350000
22025.0000000	0.7400000
22026.0000000	0.7450000
22027.0000000	0.7500000
22028.0000000	0.7500000
22029.0000000	0.7500000
22030.0000000	0.7550000
22031.0000000	0.7600000
22032.0000000	0.7600000
22033.0000000	0.7700000
22034.0000000	0.7750000

22035.0000000	0.7700000
22036.0000000	0.7800000
22037.0000000	0.7750000
22038.0000000	0.7800000
22039.0000000	0.7800000
22040.0000000	0.7800000
22041.0000000	0.7800000
22042.0000000	0.7800000
22043.0000000	0.7900000
22044.0000000	0.7900000
22045.0000000	0.7850000
22046.0000000	0.7900000
22047.0000000	0.7850000
22048.0000000	0.7900000
22049.0000000	0.7900000
22050.0000000	0.7900000
22051.0000000	0.7950000
22052.0000000	0.8000000
22053.0000000	0.8000000
22054.0000000	0.8000000
22055.0000000	0.8100000
22056.0000000	0.8100000
22057.0000000	0.8150000
22058.0000000	0.8150000
22059.0000000	0.8150000
22060.0000000	0.8150000
22061.0000000	0.8150000
22062.0000000	0.8100000
22063.0000000	0.8150000
22064.0000000	0.8100000
22065.0000000	0.8100000
22066.0000000	0.8150000
22067.0000000	0.8100000
22068.0000000	0.8100000
22069.0000000	0.8100000
22070.0000000	0.8050000
22071.0000000	0.8100000
22072.0000000	0.8100000
22073.0000000	0.8100000
22074.0000000	0.8100000
22075.0000000	0.8100000
22076.0000000	0.8150000
22077.0000000	0.8100000
22078.0000000	0.8200000
22079.0000000	0.8166667
22080.0000000	0.8200000
22081.0000000	0.8250000
22082.0000000	0.8300000
22083.0000000	0.8350000
22084.0000000	0.8300000
22085.0000000	0.8350000
22086.0000000	0.8350000
22087.0000000	0.8450000
22088.0000000	0.8400000
22089.0000000	0.8450000
22090.0000000	0.8500000
22091.0000000	0.8500000

22092.0000000	0.8550000
22093.0000000	0.8500000
22094.0000000	0.8550000
22095.0000000	0.8450000
22096.0000000	0.8450000
22097.0000000	0.8450000
22098.0000000	0.8400000
22099.0000000	0.8400000
22100.0000000	0.8400000
22101.0000000	0.8300000
22102.0000000	0.8400000
22103.0000000	0.8300000
22104.0000000	0.8350000
22105.0000000	0.8350000
22106.0000000	0.8300000
22107.0000000	0.8300000
22108.0000000	0.8300000
22109.0000000	0.8300000
22110.0000000	0.8300000
22111.0000000	0.8350000
22112.0000000	0.8300000
22113.0000000	0.8350000
22114.0000000	0.8400000
22115.0000000	0.8400000
22116.0000000	0.8400000
22117.0000000	0.8400000
22118.0000000	0.8400000
22119.0000000	0.8400000
22120.0000000	0.8400000
22121.0000000	0.8400000
22122.0000000	0.8450000
22123.0000000	0.8350000
22124.0000000	0.8350000
22125.0000000	0.8350000
22126.0000000	0.8250000
22127.0000000	0.8300000
22128.0000000	0.8150000
22129.0000000	0.8100000
22130.0000000	0.8100000
22131.0000000	0.8000000
22132.0000000	0.8050000
22133.0000000	0.8100000
22134.0000000	0.8150000
22135.0000000	0.8100000
22136.0000000	0.8250000
22137.0000000	0.8200000
22138.0000000	0.8200000
22139.0000000	0.8233333
22140.0000000	0.8100000
22141.0000000	0.8100000
22142.0000000	0.8150000
22143.0000000	0.8100000
22144.0000000	0.8250000
22145.0000000	0.8250000
22146.0000000	0.8250000
22147.0000000	0.8250000
22148.0000000	0.8250000

22149.0000000	0.8250000
22150.0000000	0.8250000
22151.0000000	0.8350000
22152.0000000	0.8300000
22153.0000000	0.8300000
22154.0000000	0.8300000
22155.0000000	0.8250000
22156.0000000	0.8250000
22157.0000000	0.8250000
22158.0000000	0.8200000
22159.0000000	0.8200000
22160.0000000	0.8150000
22161.0000000	0.8050000
22162.0000000	0.8050000
22163.0000000	0.8000000
22164.0000000	0.8050000
22165.0000000	0.8050000
22166.0000000	0.8000000
22167.0000000	0.8000000
22168.0000000	0.8000000
22169.0000000	0.7950000
22170.0000000	0.8000000
22171.0000000	0.7900000
22172.0000000	0.7950000
22173.0000000	0.7950000
22174.0000000	0.7900000
22175.0000000	0.8000000
22176.0000000	0.7950000
22177.0000000	0.7950000
22178.0000000	0.8000000
22179.0000000	0.7900000
22180.0000000	0.7900000
22181.0000000	0.7900000
22182.0000000	0.7900000
22183.0000000	0.7950000
22184.0000000	0.8000000
22185.0000000	0.8000000
22186.0000000	0.8100000
22187.0000000	0.8150000
22188.0000000	0.8150000
22189.0000000	0.8200000
22190.0000000	0.8100000
22191.0000000	0.8150000
22192.0000000	0.8100000
22193.0000000	0.8000000
22194.0000000	0.8000000
22195.0000000	0.7950000
22196.0000000	0.7950000
22197.0000000	0.8000000
22198.0000000	0.7900000
22199.0000000	0.7950000
22200.0000000	0.7966667
22201.0000000	0.8050000
22202.0000000	0.8200000
22203.0000000	0.8200000
22204.0000000	0.8250000
22205.0000000	0.8250000

22206.0000000	0.8350000
22207.0000000	0.8400000
22208.0000000	0.8400000
22209.0000000	0.8450000
22210.0000000	0.8500000
22211.0000000	0.8500000
22212.0000000	0.8500000
22213.0000000	0.8450000
22214.0000000	0.8500000
22215.0000000	0.8500000
22216.0000000	0.8550000
22217.0000000	0.8550000
22218.0000000	0.8550000
22219.0000000	0.8550000
22220.0000000	0.8600000
22221.0000000	0.8600000
22222.0000000	0.8500000
22223.0000000	0.8550000
22224.0000000	0.8550000
22225.0000000	0.8550000
22226.0000000	0.8500000
22227.0000000	0.8600000
22228.0000000	0.8633334
22229.0000000	0.8650000
22230.0000000	0.8650000
22231.0000000	0.8700000
22232.0000000	0.8700000
22233.0000000	0.8700000
22234.0000000	0.8800000
22235.0000000	0.8800000
22236.0000000	0.8800000
22237.0000000	0.8850000
22238.0000000	0.8950000
22239.0000000	0.9000000
22240.0000000	0.9050000
22241.0000000	0.9150000
22242.0000000	0.9200000
22243.0000000	0.9200000
22244.0000000	0.9250000
22245.0000000	0.9300000
22246.0000000	0.9500000
22247.0000000	0.9600000
22248.0000000	0.9350000
22249.0000000	0.9200000
22250.0000000	0.9100000
22251.0000000	0.9000000
22252.0000000	0.8950000
22253.0000000	0.8800000
22254.0000000	0.8800000
22255.0000000	0.8750000
22256.0000000	0.8700000
22257.0000000	0.8650000
22258.0000000	0.8550000
22259.0000000	0.8500000
22260.0000000	0.8400000
22261.0000000	0.8466666
22262.0000000	0.8400000

22263.0000000	0.8450000
22264.0000000	0.8350000
22265.0000000	0.8250000
22266.0000000	0.8200000
22267.0000000	0.8050000
22268.0000000	0.7950000
22269.0000000	0.7950000
22270.0000000	0.7950000
22271.0000000	0.7950000
22272.0000000	0.8000000
22273.0000000	0.8050000
22274.0000000	0.8050000
22275.0000000	0.8100000
22276.0000000	0.8100000
22277.0000000	0.8200000
22278.0000000	0.8100000
22279.0000000	0.8150000
22280.0000000	0.8100000
22281.0000000	0.8100000
22282.0000000	0.8200000
22283.0000000	0.8100000
22284.0000000	0.8100000
22285.0000000	0.8100000
22286.0000000	0.8050000
22287.0000000	0.8000000
22288.0000000	0.8000000
22289.0000000	0.7950000
22290.0000000	0.7900000
22291.0000000	0.7800000
22292.0000000	0.7750000
22293.0000000	0.7750000
22294.0000000	0.7650000
22295.0000000	0.7650000
22296.0000000	0.7700000
22297.0000000	0.7600000
22298.0000000	0.7600000
22299.0000000	0.7650000
22300.0000000	0.7600000
22301.0000000	0.7600000
22302.0000000	0.7600000
22303.0000000	0.7600000
22304.0000000	0.7600000
22305.0000000	0.7600000
22306.0000000	0.7600000
22307.0000000	0.7600000
22308.0000000	0.7500000
22309.0000000	0.7450000
22310.0000000	0.7450000
22311.0000000	0.7400000
22312.0000000	0.7500000
22313.0000000	0.7450000
22314.0000000	0.7500000
22315.0000000	0.7500000
22316.0000000	0.7550000
22317.0000000	0.7500000
22318.0000000	0.7500000
22319.0000000	0.7400000

22320.0000000	0.7450000
22321.0000000	0.7450000
22322.0000000	0.7500000
22323.0000000	0.7600000
22324.0000000	0.7650000
22325.0000000	0.7700000
22326.0000000	0.7700000
22327.0000000	0.7750000
22328.0000000	0.7750000
22329.0000000	0.7800000
22330.0000000	0.7850000
22331.0000000	0.7950000
22332.0000000	0.8000000
22333.0000000	0.8000000
22334.0000000	0.8050000
22335.0000000	0.8000000
22336.0000000	0.8050000
22337.0000000	0.8000000
22338.0000000	0.8100000
22339.0000000	0.8100000
22340.0000000	0.8050000
22341.0000000	0.8150000
22342.0000000	0.8150000
22343.0000000	0.8150000
22344.0000000	0.8100000
22345.0000000	0.8100000
22346.0000000	0.8100000
22347.0000000	0.8100000
22348.0000000	0.8100000
22349.0000000	0.8133333
22350.0000000	0.8250000
22351.0000000	0.8200000
22352.0000000	0.8200000
22353.0000000	0.8200000
22354.0000000	0.8300000
22355.0000000	0.8250000
22356.0000000	0.8200000
22357.0000000	0.8150000
22358.0000000	0.8100000
22359.0000000	0.8050000
22360.0000000	0.7950000
22361.0000000	0.8000000
22362.0000000	0.7900000
22363.0000000	0.7900000
22364.0000000	0.7900000
22365.0000000	0.7950000
22366.0000000	0.7900000
22367.0000000	0.7800000
22368.0000000	0.7900000
22369.0000000	0.7850000
22370.0000000	0.7900000
22371.0000000	0.8000000
22372.0000000	0.8000000
22373.0000000	0.8150000
22374.0000000	0.8150000
22375.0000000	0.8250000
22376.0000000	0.8300000

22377.0000000	0.8300000
22378.0000000	0.8400000
22379.0000000	0.8350000
22380.0000000	0.8400000
22381.0000000	0.8500000
22382.0000000	0.8500000
22383.0000000	0.8550000
22384.0000000	0.8550000
22385.0000000	0.8550000
22386.0000000	0.8600000
22387.0000000	0.8600000
22388.0000000	0.8600000
22389.0000000	0.8600000
22390.0000000	0.8600000
22391.0000000	0.8600000
22392.0000000	0.8600000
22393.0000000	0.8650000
22394.0000000	0.8650000
22395.0000000	0.8600000
22396.0000000	0.8600000
22397.0000000	0.8600000
22398.0000000	0.8600000
22399.0000000	0.8500000
22400.0000000	0.8500000
22401.0000000	0.8400000
22402.0000000	0.8350000
22403.0000000	0.8400000
22404.0000000	0.8450000
22405.0000000	0.8400000
22406.0000000	0.8500000
22407.0000000	0.8500000
22408.0000000	0.8450000
22409.0000000	0.8550000
22410.0000000	0.8500000
22411.0000000	0.8500001
22412.0000000	0.8500000
22413.0000000	0.8450000
22414.0000000	0.8400000
22415.0000000	0.8450000
22416.0000000	0.8400000
22417.0000000	0.8350000
22418.0000000	0.8250000
22419.0000000	0.8250000
22420.0000000	0.8150000
22421.0000000	0.8200000
22422.0000000	0.8250000
22423.0000000	0.8250000
22424.0000000	0.8250000
22425.0000000	0.8300000
22426.0000000	0.8300000
22427.0000000	0.8400000
22428.0000000	0.8400000
22429.0000000	0.8400000
22430.0000000	0.8400000
22431.0000000	0.8400000
22432.0000000	0.8400000
22433.0000000	0.8450000

22434.0000000	0.8450000
22435.0000000	0.8500000
22436.0000000	0.8550000
22437.0000000	0.8600000
22438.0000000	0.8600000
22439.0000000	0.8600000
22440.0000000	0.8550000
22441.0000000	0.8533333
22442.0000000	0.8550000
22443.0000000	0.8500000
22444.0000000	0.8500000
22445.0000000	0.8550000
22446.0000000	0.8600000
22447.0000000	0.8600000
22448.0000000	0.8600000
22449.0000000	0.8600000
22450.0000000	0.8550000
22451.0000000	0.8600000
22452.0000000	0.8550000
22453.0000000	0.8600000
22454.0000000	0.8550000
22455.0000000	0.8600000
22456.0000000	0.8650000
22457.0000000	0.8650000
22458.0000000	0.8700000
22459.0000000	0.8750000
22460.0000000	0.8800000
22461.0000000	0.8750000
22462.0000000	0.8750000
22463.0000000	0.8800000
22464.0000000	0.8900000
22465.0000000	0.8900000
22466.0000000	0.8950000
22467.0000000	0.9100000
22468.0000000	0.9100000
22469.0000000	0.9100000
22470.0000000	0.9100000
22471.0000000	0.9166667
22472.0000000	0.9200000
22473.0000000	0.9200000
22474.0000000	0.9250000
22475.0000000	0.9200000
22476.0000000	0.9250000
22477.0000000	0.9250000
22478.0000000	0.9250000
22479.0000000	0.9250000
22480.0000000	0.9300000
22481.0000000	0.9350000
22482.0000000	0.9350000
22483.0000000	0.9350000
22484.0000000	0.9350000
22485.0000000	0.9350000
22486.0000000	0.9400000
22487.0000000	0.9400000
22488.0000000	0.9450000
22489.0000000	0.9450000
22490.0000000	0.9500000

22491.0000000	0.9500000
22492.0000000	0.9600000
22493.0000000	0.9500000
22494.0000000	0.9600000
22495.0000000	0.9700000
22496.0000000	0.9600000
22497.0000000	0.9650000
22498.0000000	0.9650000
22499.0000000	0.9600000
22500.0000000	0.9600000
22501.0000000	0.9600000
22502.0000000	0.9650000
22503.0000000	0.9600000
22504.0000000	0.9600000
22505.0000000	0.9500000
22506.0000000	0.9500000
22507.0000000	0.9350000
22508.0000000	0.9300000
22509.0000000	0.9200000
22510.0000000	0.9150000
22511.0000000	0.9000000
22512.0000000	0.9000000
22513.0000000	0.9000000
22514.0000000	0.8950000
22515.0000000	0.8900000
22516.0000000	0.8900000
22517.0000000	0.8900000
22518.0000000	0.8900000
22519.0000000	0.9000000
22520.0000000	0.9000000
22521.0000000	0.9000000
22522.0000000	0.9050000
22523.0000000	0.9050000
22524.0000000	0.9100000
22525.0000000	0.9100000
22526.0000000	0.9150000
22527.0000000	0.9100000
22528.0000000	0.9150000
22529.0000000	0.9200000
22530.0000000	0.9200000
22531.0000000	0.9266667
22532.0000000	0.9250000
22533.0000000	0.9250000
22534.0000000	0.9250000
22535.0000000	0.9300000
22536.0000000	0.9300000
22537.0000000	0.9350000
22538.0000000	0.9400000
22539.0000000	0.9450000
22540.0000000	0.9450000
22541.0000000	0.9500000
22542.0000000	0.9500000
22543.0000000	0.9500000
22544.0000000	0.9550000
22545.0000000	0.9500000
22546.0000000	0.9550000
22547.0000000	0.9550000

22548.0000000	0.9500000
22549.0000000	0.9550000
22550.0000000	0.9600000
22551.0000000	0.9500000
22552.0000000	0.9500000
22553.0000000	0.9500000
22554.0000000	0.9400000
22555.0000000	0.9500000
22556.0000000	0.9450000
22557.0000000	0.9450000
22558.0000000	0.9450000
22559.0000000	0.9350000
22560.0000000	0.9400000
22561.0000000	0.9333333
22562.0000000	0.9300000
22563.0000000	0.9300000
22564.0000000	0.9200000
22565.0000000	0.9200000
22566.0000000	0.9100000
22567.0000000	0.8950000
22568.0000000	0.8750000
22569.0000000	0.8550000
22570.0000000	0.8400000
22571.0000000	0.8400000
22572.0000000	0.8200000
22573.0000000	0.8100000
22574.0000000	0.8100000
22575.0000000	0.8100000
22576.0000000	0.8150000
22577.0000000	0.8100000
22578.0000000	0.8100000
22579.0000000	0.8050000
22580.0000000	0.8000000
22581.0000000	0.8000000
22582.0000000	0.7950000
22583.0000000	0.7900000
22584.0000000	0.7950000
22585.0000000	0.7950000
22586.0000000	0.7900000
22587.0000000	0.7900000
22588.0000000	0.7850000
22589.0000000	0.7800000
22590.0000000	0.7850000
22591.0000000	0.7800000
22592.0000000	0.7733333
22593.0000000	0.7800000
22594.0000000	0.7850000
22595.0000000	0.7800000
22596.0000000	0.7850000
22597.0000000	0.7900000
22598.0000000	0.7900000
22599.0000000	0.7900000
22600.0000000	0.8000000
22601.0000000	0.8000000
22602.0000000	0.8150000
22603.0000000	0.8250000
22604.0000000	0.8300000

22605.0000000	0.8400000
22606.0000000	0.8400000
22607.0000000	0.8450000
22608.0000000	0.8550000
22609.0000000	0.8650000
22610.0000000	0.8950000
22611.0000000	0.9600000
22612.0000000	0.9200000
22613.0000000	0.8750000
22614.0000000	0.8750000
22615.0000000	0.8650000
22616.0000000	0.8700000
22617.0000000	0.8700000
22618.0000000	0.8600000
22619.0000000	0.8650000
22620.0000000	0.8700000
22621.0000000	0.8700001
22622.0000000	0.8700000
22623.0000000	0.8700000
22624.0000000	0.8700000
22625.0000000	0.8650000
22626.0000000	0.8750000
22627.0000000	0.8800000
22628.0000000	0.8750000
22629.0000000	0.8700000
22630.0000000	0.8750000
22631.0000000	0.8800000
22632.0000000	0.8750000
22633.0000000	0.8850000
22634.0000000	0.8850000
22635.0000000	0.8900000
22636.0000000	0.8900000
22637.0000000	0.8750000
22638.0000000	0.8750000
22639.0000000	0.8700000
22640.0000000	0.8650000
22641.0000000	0.8750000
22642.0000000	0.8800000
22643.0000000	0.8900000
22644.0000000	0.8900000
22645.0000000	0.9000000
22646.0000000	0.8800000
22647.0000000	0.8650000
22648.0000000	0.8650000
22649.0000000	0.8600000
22650.0000000	0.8550000
22651.0000000	0.8450000
22652.0000000	0.8433333
22653.0000000	0.8350000
22654.0000000	0.8300000
22655.0000000	0.8350000
22656.0000000	0.8300000
22657.0000000	0.8400000
22658.0000000	0.8450000
22659.0000000	0.8400000
22660.0000000	0.8400000
22661.0000000	0.8500000

22662.0000000	0.8500000
22663.0000000	0.8500000
22664.0000000	0.8600000
22665.0000000	0.8600000
22666.0000000	0.8600000
22667.0000000	0.8700000
22668.0000000	0.8650000
22669.0000000	0.8700000
22670.0000000	0.8750000
22671.0000000	0.8800000
22672.0000000	0.8700000
22673.0000000	0.8750000
22674.0000000	0.8650000
22675.0000000	0.8600000
22676.0000000	0.8600000
22677.0000000	0.8600000
22678.0000000	0.8600000
22679.0000000	0.8700000
22680.0000000	0.8650000
22681.0000000	0.8750000
22682.0000000	0.8766667
22683.0000000	0.8900000
22684.0000000	0.8850000
22685.0000000	0.8950000
22686.0000000	0.8950000
22687.0000000	0.9000000
22688.0000000	0.9100000
22689.0000000	0.9150000
22690.0000000	0.9200000
22691.0000000	0.9250000
22692.0000000	0.9300000
22693.0000000	0.9250000
22694.0000000	0.9350000
22695.0000000	0.9300000
22696.0000000	0.9300000
22697.0000000	0.9350000
22698.0000000	0.9250000
22699.0000000	0.9250000
22700.0000000	0.9250000
22701.0000000	0.9100000
22702.0000000	0.9150000
22703.0000000	0.9150000
22704.0000000	0.9100000
22705.0000000	0.9100000
22706.0000000	0.9100000
22707.0000000	0.9200000
22708.0000000	0.9150000
22709.0000000	0.9200000
22710.0000000	0.9250000
22711.0000000	0.9250000
22712.0000000	0.9366667
22713.0000000	0.9350000
22714.0000000	0.9400000
22715.0000000	0.9350000
22716.0000000	0.9400000
22717.0000000	0.9300000
22718.0000000	0.9300000

22719.0000000	0.9350000
22720.0000000	0.9400000
22721.0000000	0.9350000
22722.0000000	0.9300000
22723.0000000	0.9400000
22724.0000000	0.9300000
22725.0000000	0.9350000
22726.0000000	0.9450000
22727.0000000	0.9400000
22728.0000000	0.9550000
22729.0000000	0.9550000
22730.0000000	0.9600000
22731.0000000	0.9600000
22732.0000000	0.9650000
22733.0000000	0.9700000
22734.0000000	0.9700000
22735.0000000	0.9850000
22736.0000000	0.9850000
22737.0000000	0.9800000
22738.0000000	0.9800000
22739.0000000	0.9800000
22740.0000000	0.9700000
22741.0000000	0.9700000
22742.0000000	0.9700000
22743.0000000	0.9633333
22744.0000000	0.9600000
22745.0000000	0.9600000
22746.0000000	0.9600000
22747.0000000	0.9600000
22748.0000000	0.9550000
22749.0000000	0.9550000
22750.0000000	0.9600000
22751.0000000	0.9600000
22752.0000000	0.9650000
22753.0000000	0.9700000
22754.0000000	0.9700000
22755.0000000	0.9700000
22756.0000000	0.9750000
22757.0000000	0.9800000
22758.0000000	0.9750000
22759.0000000	0.9800000
22760.0000000	0.9700000
22761.0000000	0.9650000
22762.0000000	0.9600000
22763.0000000	0.9600000
22764.0000000	0.9600000
22765.0000000	0.9500000
22766.0000000	0.9600000
22767.0000000	0.9500000
22768.0000000	0.9500000
22769.0000000	0.9550000
22770.0000000	0.9550000
22771.0000000	0.9550000
22772.0000000	0.9650000
22773.0000000	0.9633334
22774.0000000	0.9650000
22775.0000000	0.9700000

22776.0000000	0.9700000
22777.0000000	0.9750000
22778.0000000	0.9800000
22779.0000000	0.9800000
22780.0000000	0.9800000
22781.0000000	0.9900000
22782.0000000	0.9900000
22783.0000000	0.9950000
22784.0000000	0.9950000
22785.0000000	1.0050000
22786.0000000	1.0050000
22787.0000000	1.0100000
22788.0000000	1.0150000
22789.0000000	1.0200000
22790.0000000	1.0150000
22791.0000000	1.0200000
22792.0000000	1.0250000
22793.0000000	1.0200000
22794.0000000	1.0300000
22795.0000000	1.0300000
22796.0000000	1.0300000
22797.0000000	1.0350000
22798.0000000	1.0350000
22799.0000000	1.0250000
22800.0000000	1.0250000
22801.0000000	1.0200000
22802.0000000	1.0200000
22803.0000000	1.0200000
22804.0000000	1.0133333
22805.0000000	1.0000000
22806.0000000	0.9950000
22807.0000000	0.9950000
22808.0000000	0.9900000
22809.0000000	0.9750000
22810.0000000	0.9750000
22811.0000000	0.9700000
22812.0000000	0.9650000
22813.0000000	0.9650000
22814.0000000	0.9600000
22815.0000000	0.9600000
22816.0000000	0.9550000
22817.0000000	0.9550000
22818.0000000	0.9600000
22819.0000000	0.9450000
22820.0000000	0.9500000
22821.0000000	0.9400000
22822.0000000	0.9400000
22823.0000000	0.9450000
22824.0000000	0.9400000
22825.0000000	0.9500000
22826.0000000	0.9500000
22827.0000000	0.9500000
22828.0000000	0.9500000
22829.0000000	0.9550000
22830.0000000	0.9600000
22831.0000000	0.9650000
22832.0000000	0.9700000

22833.0000000	0.9700000
22834.0000000	0.9733334
22835.0000000	0.9800000
22836.0000000	0.9850000
22837.0000000	0.9950000
22838.0000000	0.9900000
22839.0000000	1.0000000
22840.0000000	1.0000000
22841.0000000	1.0050000
22842.0000000	1.0000000
22843.0000000	1.0000000
22844.0000000	1.0000000
22845.0000000	1.0000000
22846.0000000	1.0000000
22847.0000000	1.0050000
22848.0000000	1.0000000
22849.0000000	1.0000000
22850.0000000	1.0050000
22851.0000000	1.0000000
22852.0000000	1.0000000
22853.0000000	1.0100000
22854.0000000	1.0100000
22855.0000000	1.0150000
22856.0000000	1.0150000
22857.0000000	1.0100000
22858.0000000	1.0200000
22859.0000000	1.0200000
22860.0000000	1.0150000
22861.0000000	1.0200000
22862.0000000	1.0250000
22863.0000000	1.0300000
22864.0000000	1.0300000
22865.0000000	1.0266666
22866.0000000	1.0300000
22867.0000000	1.0300000
22868.0000000	1.0250000
22869.0000000	1.0300000
22870.0000000	1.0300000
22871.0000000	1.0250000
22872.0000000	1.0200000
22873.0000000	1.0300000
22874.0000000	1.0150000
22875.0000000	1.0150000
22876.0000000	1.0200000
22877.0000000	1.0100000
22878.0000000	1.0200000
22879.0000000	1.0150000
22880.0000000	1.0150000
22881.0000000	1.0150000
22882.0000000	1.0150000
22883.0000000	1.0100000
22884.0000000	1.0100000
22885.0000000	1.0150000
22886.0000000	1.0100000
22887.0000000	1.0100000
22888.0000000	1.0000000
22889.0000000	1.0000000

22890.0000000	0.9900000
22891.0000000	0.9900000
22892.0000000	0.9900000
22893.0000000	0.9800000
22894.0000000	0.9850000
22895.0000000	0.9833333
22896.0000000	0.9800000
22897.0000000	0.9850000
22898.0000000	0.9800000
22899.0000000	0.9850000
22900.0000000	0.9900000
22901.0000000	0.9950000
22902.0000000	0.9900000
22903.0000000	0.9900000
22904.0000000	0.9950000
22905.0000000	0.9850000
22906.0000000	0.9850000
22907.0000000	0.9850000
22908.0000000	0.9850000
22909.0000000	0.9850000
22910.0000000	0.9850000
22911.0000000	0.9750000
22912.0000000	0.9700000
22913.0000000	0.9700000
22914.0000000	0.9800000
22915.0000000	0.9750000
22916.0000000	0.9750000
22917.0000000	0.9700000
22918.0000000	0.9650000
22919.0000000	0.9650000
22920.0000000	0.9700000
22921.0000000	0.9800000
22922.0000000	0.9800000
22923.0000000	0.9850000
22924.0000000	0.9900000
22925.0000000	0.9966667
22926.0000000	1.0100000
22927.0000000	1.0200000
22928.0000000	1.0200000
22929.0000000	1.0200000
22930.0000000	1.0350000
22931.0000000	1.0300000
22932.0000000	1.0350000
22933.0000000	1.0350000
22934.0000000	1.0300000
22935.0000000	1.0200000
22936.0000000	1.0200000
22937.0000000	1.0200000
22938.0000000	1.0200000
22939.0000000	1.0250000
22940.0000000	1.0300000
22941.0000000	1.0300000
22942.0000000	1.0250000
22943.0000000	1.0250000
22944.0000000	1.0300000
22945.0000000	1.0300000
22946.0000000	1.0200000

22947.0000000	1.0200000
22948.0000000	1.0200000
22949.0000000	1.0150000
22950.0000000	1.0200000
22951.0000000	1.0150000
22952.0000000	1.0100000
22953.0000000	1.0050000
22954.0000000	1.0050000
22955.0000000	1.0100000
22956.0000000	1.0066667
22957.0000000	1.0050000
22958.0000000	1.0100000
22959.0000000	1.0050000
22960.0000000	1.0050000
22961.0000000	1.0100000
22962.0000000	1.0050000
22963.0000000	1.0050000
22964.0000000	1.0000000
22965.0000000	1.0050000
22966.0000000	1.0100000
22967.0000000	1.0100000
22968.0000000	1.0050000
22969.0000000	1.0050000
22970.0000000	1.0100000
22971.0000000	1.0150000
22972.0000000	1.0150000
22973.0000000	1.0150000
22974.0000000	1.0200000
22975.0000000	1.0250000
22976.0000000	1.0550001
22977.0000000	1.0500000
22978.0000000	1.0350000
22979.0000000	1.0300000
22980.0000000	1.0350000
22981.0000000	1.0350000
22982.0000000	1.0400000
22983.0000000	1.0450000
22984.0000000	1.0500000
22985.0000000	1.0533333
22986.0000000	1.0549999
22987.0000000	1.0500000
22988.0000000	1.0549999
22989.0000000	1.0500000
22990.0000000	1.0500000
22991.0000000	1.0500000
22992.0000000	1.0450000
22993.0000000	1.0400000
22994.0000000	1.0350000
22995.0000000	1.0300000
22996.0000000	1.0300000
22997.0000000	1.0250000
22998.0000000	1.0250000
22999.0000000	1.0100000
23000.0000000	1.0000000
23001.0000000	0.9850000
23002.0000000	0.9750000
23003.0000000	0.9700000

23004.0000000	0.9600000
23005.0000000	0.9650000
23006.0000000	0.9550000
23007.0000000	0.9550000
23008.0000000	0.9500000
23009.0000000	0.9500000
23010.0000000	0.9500000
23011.0000000	0.9500000
23012.0000000	0.9550000
23013.0000000	0.9550000
23014.0000000	0.9600000
23015.0000000	0.9600000
23016.0000000	0.9600000
23017.0000000	0.9700000
23018.0000000	0.9750000
23019.0000000	0.9700000
23020.0000000	0.9850000
23021.0000000	0.9800000
23022.0000000	0.9950000
23023.0000000	1.0000000
23024.0000000	1.0000000
23025.0000000	1.0100000
23026.0000000	1.0150000
23027.0000000	1.0200000
23028.0000000	1.0200000
23029.0000000	1.0200000
23030.0000000	1.0100000
23031.0000000	1.0100000
23032.0000000	1.0050000
23033.0000000	1.0000000
23034.0000000	1.0050000
23035.0000000	1.0050000
23036.0000000	1.0000000
23037.0000000	1.0000000
23038.0000000	0.9950000
23039.0000000	1.0000000
23040.0000000	0.9950000
23041.0000000	0.9950000
23042.0000000	0.9950000
23043.0000000	1.0000000
23044.0000000	1.0000000
23045.0000000	1.0000000
23046.0000000	1.0000000
23047.0000000	1.0000000
23048.0000000	1.0000000
23049.0000000	1.0050000
23050.0000000	1.0050000
23051.0000000	1.0100000
23052.0000000	1.0100000
23053.0000000	1.0150000
23054.0000000	1.0200000
23055.0000000	1.0150000
23056.0000000	1.0200000
23057.0000000	1.0250000
23058.0000000	1.0200000
23059.0000000	1.0200000
23060.0000000	1.0300000

23061.0000000	1.0250000
23062.0000000	1.0300000
23063.0000000	1.0400000
23064.0000000	1.0400000
23065.0000000	1.0450000
23066.0000000	1.0450000
23067.0000000	1.0450000
23068.0000000	1.0450000
23069.0000000	1.0400000
23070.0000000	1.0400000
23071.0000000	1.0400000
23072.0000000	1.0300000
23073.0000000	1.0350000
23074.0000000	1.0233333
23075.0000000	1.0250000
23076.0000000	1.0250000
23077.0000000	1.0200000
23078.0000000	1.0200000
23079.0000000	1.0150000
23080.0000000	1.0100000
23081.0000000	1.0000000
23082.0000000	0.9950000
23083.0000000	0.9850000
23084.0000000	0.9750000
23085.0000000	0.9650000
23086.0000000	0.9600000
23087.0000000	0.9500000
23088.0000000	0.9500000
23089.0000000	0.9500000
23090.0000000	0.9500000
23091.0000000	0.9450000
23092.0000000	0.9500000
23093.0000000	0.9500000
23094.0000000	0.9500000
23095.0000000	0.9600000
23096.0000000	0.9500000
23097.0000000	0.9600000
23098.0000000	0.9650000
23099.0000000	0.9650000
23100.0000000	0.9650000
23101.0000000	0.9700000
23102.0000000	0.9800000
23103.0000000	0.9750000
23104.0000000	0.9800000
23105.0000000	0.9800000
23106.0000000	0.9700000
23107.0000000	0.9700000
23108.0000000	0.9600000
23109.0000000	0.9550000
23110.0000000	0.9400000
23111.0000000	0.9450000
23112.0000000	0.9400000
23113.0000000	0.9400000
23114.0000000	0.9350000
23115.0000000	0.9350000
23116.0000000	0.9350000
23117.0000000	0.9350000

23118.0000000	0.9400000
23119.0000000	0.9450000
23120.0000000	0.9600000
23121.0000000	0.9650000
23122.0000000	0.9650000
23123.0000000	0.9800000
23124.0000000	0.9850000
23125.0000000	0.9900000
23126.0000000	0.9950000
23127.0000000	1.0000000
23128.0000000	1.0000000
23129.0000000	1.0050000
23130.0000000	1.0100000
23131.0000000	1.0150000
23132.0000000	1.0200000
23133.0000000	1.0200000
23134.0000000	1.0233333
23135.0000000	1.0300000
23136.0000000	1.0300000
23137.0000000	1.0400000
23138.0000000	1.0350000
23139.0000000	1.0350000
23140.0000000	1.0400000
23141.0000000	1.0350000
23142.0000000	1.0400000
23143.0000000	1.0450000
23144.0000000	1.0400000
23145.0000000	1.0350000
23146.0000000	1.0350000
23147.0000000	1.0300000
23148.0000000	1.0250000
23149.0000000	1.0200000
23150.0000000	1.0150000
23151.0000000	1.0150000
23152.0000000	1.0100000
23153.0000000	1.0100000
23154.0000000	1.0050000
23155.0000000	1.0100000
23156.0000000	1.0150000
23157.0000000	1.0050000
23158.0000000	1.0100000
23159.0000000	1.0100000
23160.0000000	1.0000000
23161.0000000	1.0100000
23162.0000000	1.0300000
23163.0000000	1.0000000
23164.0000000	1.0000000
23165.0000000	1.0000000
23166.0000000	1.0000000
23167.0000000	1.0000000
23168.0000000	1.0050000
23169.0000000	1.0000000
23170.0000000	1.0050000
23171.0000000	1.0100000
23172.0000000	1.0200000
23173.0000000	1.0250000
23174.0000000	1.0200000

23175.0000000	1.0300000
23176.0000000	1.0300000
23177.0000000	1.0300000
23178.0000000	1.0350000
23179.0000000	1.0350000
23180.0000000	1.0350000
23181.0000000	1.0350000
23182.0000000	1.0400000
23183.0000000	1.0450000
23184.0000000	1.0400000
23185.0000000	1.0350000
23186.0000000	1.0400000
23187.0000000	1.0450000
23188.0000000	1.0400000
23189.0000000	1.0400000
23190.0000000	1.0450000
23191.0000000	1.0350000
23192.0000000	1.0350000
23193.0000000	1.0300000
23194.0000000	1.0200000
23195.0000000	1.0133333
23196.0000000	1.0100000
23197.0000000	1.0050000
23198.0000000	0.9950000
23199.0000000	0.9900000
23200.0000000	0.9800000
23201.0000000	0.9700000
23202.0000000	0.9650000
23203.0000000	0.9550000
23204.0000000	0.9600000
23205.0000000	0.9600000
23206.0000000	0.9550000
23207.0000000	0.9700000
23208.0000000	0.9750000
23209.0000000	0.9850000
23210.0000000	1.0050000
23211.0000000	1.0100000
23212.0000000	1.0200000
23213.0000000	1.0200000
23214.0000000	1.0300000
23215.0000000	1.0350000
23216.0000000	1.0350000
23217.0000000	1.0350000
23218.0000000	1.0350000
23219.0000000	1.0400000
23220.0000000	1.0500000
23221.0000000	1.0400000
23222.0000000	1.0450000
23223.0000000	1.0350000
23224.0000000	1.0350000
23225.0000000	1.0400000
23226.0000000	1.0300000
23227.0000000	1.0350000
23228.0000000	1.0200000
23229.0000000	1.0200000
23230.0000000	1.0150000
23231.0000000	1.0100000

23232.0000000	1.0100000
23233.0000000	1.0100000
23234.0000000	1.0050000
23235.0000000	1.0050000
23236.0000000	0.9950000
23237.0000000	0.9950000
23238.0000000	0.9850000
23239.0000000	0.9850000
23240.0000000	0.9850000
23241.0000000	0.9800000
23242.0000000	0.9800000
23243.0000000	0.9750000
23244.0000000	0.9750000
23245.0000000	0.9800000
23246.0000000	0.9800000
23247.0000000	0.9850000
23248.0000000	0.9800000
23249.0000000	0.9800000
23250.0000000	0.9850000
23251.0000000	0.9950000
23252.0000000	0.9900000
23253.0000000	0.9850000
23254.0000000	0.9800000
23255.0000000	0.9766667
23256.0000000	0.9700000
23257.0000000	0.9650000
23258.0000000	0.9600000
23259.0000000	0.9700000
23260.0000000	0.9700000
23261.0000000	0.9600000
23262.0000000	0.9600000
23263.0000000	0.9550000
23264.0000000	0.9550000
23265.0000000	0.9550000
23266.0000000	0.9500000
23267.0000000	0.9550000
23268.0000000	0.9600000
23269.0000000	0.9550000
23270.0000000	0.9650000
23271.0000000	0.9650000
23272.0000000	0.9600000
23273.0000000	0.9650000
23274.0000000	0.9650000
23275.0000000	0.9600000
23276.0000000	0.9650000
23277.0000000	0.9650000
23278.0000000	0.9700000
23279.0000000	0.9700000
23280.0000000	0.9700000
23281.0000000	0.9700000
23282.0000000	0.9700000
23283.0000000	0.9800000
23284.0000000	0.9750000
23285.0000000	0.9766667
23286.0000000	0.9800000
23287.0000000	0.9800000
23288.0000000	0.9900000

23289.0000000	0.9900000
23290.0000000	0.9900000
23291.0000000	0.9950000
23292.0000000	0.9950000
23293.0000000	1.0000000
23294.0000000	1.0000000
23295.0000000	1.0000000
23296.0000000	1.0100000
23297.0000000	1.0150000
23298.0000000	1.0200000
23299.0000000	1.0300000
23300.0000000	1.0300000
23301.0000000	1.0350000
23302.0000000	1.0300000
23303.0000000	1.0350000
23304.0000000	1.0400000
23305.0000000	1.0400000
23306.0000000	1.0400000
23307.0000000	1.0500000
23308.0000000	1.0599999
23309.0000000	1.0549999
23310.0000000	1.0400000
23311.0000000	1.0300000
23312.0000000	1.0300000
23313.0000000	1.0300000
23314.0000000	1.0300000
23315.0000000	1.0300000
23316.0000000	1.0200000
23317.0000000	1.0250000
23318.0000000	1.0300000
23319.0000000	1.0250000
23320.0000000	1.0300000
23321.0000000	1.0300000
23322.0000000	1.0300000
23323.0000000	1.0300000
23324.0000000	1.0200000
23325.0000000	1.0200000
23326.0000000	1.0200000
23327.0000000	1.0200000
23328.0000000	1.0150000
23329.0000000	1.0050000
23330.0000000	1.0000000
23331.0000000	0.9900000
23332.0000000	0.9900000
23333.0000000	0.9850000
23334.0000000	0.9850000
23335.0000000	0.9800000
23336.0000000	0.9800000
23337.0000000	0.9700000
23338.0000000	0.9700000
23339.0000000	0.9750000
23340.0000000	0.9650000
23341.0000000	0.9750000
23342.0000000	0.9700000
23343.0000000	0.9700000
23344.0000000	0.9750000
23345.0000000	0.9700000

23346.0000000	0.9766667
23347.0000000	0.9850000
23348.0000000	0.9900000
23349.0000000	0.9950000
23350.0000000	0.9950000
23351.0000000	1.0000000
23352.0000000	1.0000000
23353.0000000	1.0000000
23354.0000000	1.0100000
23355.0000000	1.0100000
23356.0000000	1.0100000
23357.0000000	1.0200000
23358.0000000	1.0200000
23359.0000000	1.0200000
23360.0000000	1.0250000
23361.0000000	1.0300000
23362.0000000	1.0300000
23363.0000000	1.0300000
23364.0000000	1.0300000
23365.0000000	1.0250000
23366.0000000	1.0200000
23367.0000000	1.0200000
23368.0000000	1.0200000
23369.0000000	1.0150000
23370.0000000	1.0150000
23371.0000000	1.0100000
23372.0000000	1.0100000
23373.0000000	1.0000000
23374.0000000	0.9950000
23375.0000000	1.0000000
23376.0000000	0.9966667
23377.0000000	0.9950000
23378.0000000	0.9950000
23379.0000000	0.9850000
23380.0000000	0.9850000
23381.0000000	0.9800000
23382.0000000	0.9800000
23383.0000000	0.9850000
23384.0000000	0.9800000
23385.0000000	0.9850000
23386.0000000	0.9900000
23387.0000000	0.9900000
23388.0000000	0.9850000
23389.0000000	0.9900000
23390.0000000	0.9800000
23391.0000000	0.9800000
23392.0000000	0.9750000
23393.0000000	0.9650000
23394.0000000	0.9650000
23395.0000000	0.9700000
23396.0000000	0.9650000
23397.0000000	0.9650000
23398.0000000	0.9650000
23399.0000000	0.9550000
23400.0000000	0.9600000
23401.0000000	0.9600000
23402.0000000	0.9650000

23403.0000000	0.9600000
23404.0000000	0.9600000
23405.0000000	0.9650000
23406.0000000	0.9700000
23407.0000000	0.9700000
23408.0000000	0.9800000
23409.0000000	0.9850000
23410.0000000	0.9900000
23411.0000000	0.9900000
23412.0000000	0.9950000
23413.0000000	0.9900000
23414.0000000	0.9950000
23415.0000000	1.0000000
23416.0000000	1.0000000
23417.0000000	1.0000000
23418.0000000	0.9950000
23419.0000000	1.0000000
23420.0000000	0.9900000
23421.0000000	0.9950000
23422.0000000	0.9950000
23423.0000000	1.0000000
23424.0000000	1.0000000
23425.0000000	1.0100000
23426.0000000	1.0000000
23427.0000000	1.0050000
23428.0000000	1.0050000
23429.0000000	1.0100000
23430.0000000	1.0100000
23431.0000000	1.0050000
23432.0000000	1.0100000
23433.0000000	1.0100000
23434.0000000	1.0150000
23435.0000000	1.0250000
23436.0000000	1.0250000
23437.0000000	1.0266666
23438.0000000	1.0300000
23439.0000000	1.0300000
23440.0000000	1.0250000
23441.0000000	1.0250000
23442.0000000	1.0200000
23443.0000000	1.0200000
23444.0000000	1.0250000
23445.0000000	1.0150000
23446.0000000	1.0050000
23447.0000000	0.9950000
23448.0000000	0.9850000
23449.0000000	0.9800000
23450.0000000	0.9750000
23451.0000000	0.9650000
23452.0000000	0.9500000
23453.0000000	0.9400000
23454.0000000	0.9400000
23455.0000000	0.9350000
23456.0000000	0.9250000
23457.0000000	0.9250000
23458.0000000	0.9250000
23459.0000000	0.9200000

23460.0000000	0.9200000
23461.0000000	0.9250000
23462.0000000	0.9250000
23463.0000000	0.9300000
23464.0000000	0.9350000
23465.0000000	0.9450000
23466.0000000	0.9500000
23467.0000000	0.9533333
23468.0000000	0.9700000
23469.0000000	0.9700000
23470.0000000	0.9700000
23471.0000000	0.9700000
23472.0000000	0.9700000
23473.0000000	0.9650000
23474.0000000	0.9600000
23475.0000000	0.9700000
23476.0000000	0.9650000
23477.0000000	0.9700000
23478.0000000	0.9750000
23479.0000000	0.9700000
23480.0000000	0.9800000
23481.0000000	0.9750000
23482.0000000	0.9750000
23483.0000000	0.9800000
23484.0000000	0.9800000
23485.0000000	0.9800000
23486.0000000	0.9800000
23487.0000000	0.9800000
23488.0000000	0.9750000
23489.0000000	0.9750000
23490.0000000	0.9650000
23491.0000000	0.9600000
23492.0000000	0.9500000
23493.0000000	0.9450000
23494.0000000	0.9350000
23495.0000000	0.9200000
23496.0000000	0.9100000
23497.0000000	0.8966667
23498.0000000	0.8850000
23499.0000000	0.8850000
23500.0000000	0.8800000
23501.0000000	0.8800000
23502.0000000	0.8750000
23503.0000000	0.8700000
23504.0000000	0.8650000
23505.0000000	0.8650000
23506.0000000	0.8700000
23507.0000000	0.8850000
23508.0000000	0.8900000
23509.0000000	0.8950000
23510.0000000	0.8950000
23511.0000000	0.8950000
23512.0000000	0.8900000
23513.0000000	0.8900000
23514.0000000	0.8900000
23515.0000000	0.8850000
23516.0000000	0.8850000

23517.0000000	0.8850000
23518.0000000	0.9000000
23519.0000000	0.9000000
23520.0000000	0.9150000
23521.0000000	0.9200000
23522.0000000	0.9200000
23523.0000000	0.9250000
23524.0000000	0.9200000
23525.0000000	0.9150000
23526.0000000	0.9250000
23527.0000000	0.9300000
23528.0000000	0.9366667
23529.0000000	0.9500000
23530.0000000	0.9500000
23531.0000000	0.9600000
23532.0000000	0.9600000
23533.0000000	0.9650000
23534.0000000	0.9700000
23535.0000000	0.9700000
23536.0000000	0.9700000
23537.0000000	0.9600000
23538.0000000	0.9600000
23539.0000000	0.9650000
23540.0000000	0.9600000
23541.0000000	0.9750000
23542.0000000	0.9700000
23543.0000000	0.9800000
23544.0000000	0.9800000
23545.0000000	0.9750000
23546.0000000	0.9750000
23547.0000000	0.9700000
23548.0000000	0.9750000
23549.0000000	0.9750000
23550.0000000	0.9800000
23551.0000000	0.9800000
23552.0000000	0.9900000
23553.0000000	1.0000000
23554.0000000	1.0000000
23555.0000000	1.0050000
23556.0000000	1.0150000
23557.0000000	1.0200000
23558.0000000	1.0200000
23559.0000000	1.0300000
23560.0000000	1.0350000
23561.0000000	1.0350000
23562.0000000	1.0350000
23563.0000000	1.0450000
23564.0000000	1.0450000
23565.0000000	1.0500000
23566.0000000	1.0450000
23567.0000000	1.0500000
23568.0000000	1.0549999
23569.0000000	1.0599999
23570.0000000	1.0599999
23571.0000000	1.0700001
23572.0000000	1.0700001
23573.0000000	1.0700001

23574.0000000	1.0700001
23575.0000000	1.0650001
23576.0000000	1.0750000
23577.0000000	1.0800000
23578.0000000	1.0900000
23579.0000000	1.0850000
23580.0000000	1.0900000
23581.0000000	1.0950000
23582.0000000	1.0900000
23583.0000000	1.0850000
23584.0000000	1.0800000
23585.0000000	1.0800000
23586.0000000	1.0850000
23587.0000000	1.0800000
23588.0000000	1.0833334
23589.0000000	1.0850000
23590.0000000	1.0900000
23591.0000000	1.0900000
23592.0000000	1.0900000
23593.0000000	1.0850000
23594.0000000	1.0900000
23595.0000000	1.0950000
23596.0000000	1.1000000
23597.0000000	1.1000000
23598.0000000	1.1000000
23599.0000000	1.1100000
23600.0000000	1.1050000
23601.0000000	1.0950000
23602.0000000	1.0950000
23603.0000000	1.0950000
23604.0000000	1.0900000
23605.0000000	1.0950000
23606.0000000	1.0950000
23607.0000000	1.0900000
23608.0000000	1.0950000
23609.0000000	1.0950000
23610.0000000	1.0950000
23611.0000000	1.0950000
23612.0000000	1.0950000
23613.0000000	1.0900000
23614.0000000	1.0900000
23615.0000000	1.0950000
23616.0000000	1.1000000
23617.0000000	1.0933334
23618.0000000	1.1000000
23619.0000000	1.1000000
23620.0000000	1.1000000
23621.0000000	1.1000000
23622.0000000	1.0850000
23623.0000000	1.0800000
23624.0000000	1.0750000
23625.0000000	1.0650001
23626.0000000	1.0549999
23627.0000000	1.0350000
23628.0000000	1.0400000
23629.0000000	1.0300000
23630.0000000	1.0300000

23631.0000000	1.0200000
23632.0000000	1.0200000
23633.0000000	1.0200000
23634.0000000	1.0150000
23635.0000000	1.0150000
23636.0000000	1.0100000
23637.0000000	1.0050000
23638.0000000	1.0100000
23639.0000000	1.0100000
23640.0000000	1.0000000
23641.0000000	1.0050000
23642.0000000	1.0000000
23643.0000000	1.0050000
23644.0000000	1.0150000
23645.0000000	1.0200000
23646.0000000	1.0300000
23647.0000000	1.0300000
23648.0000000	1.0466666
23649.0000000	1.0599999
23650.0000000	1.0599999
23651.0000000	1.0700001
23652.0000000	1.0700001
23653.0000000	1.0800000
23654.0000000	1.0700001
23655.0000000	1.0700001
23656.0000000	1.0800000
23657.0000000	1.0700001
23658.0000000	1.0650001
23659.0000000	1.0650001
23660.0000000	1.0599999
23661.0000000	1.0650001
23662.0000000	1.0599999
23663.0000000	1.0599999
23664.0000000	1.0500000
23665.0000000	1.0400000
23666.0000000	1.0500000
23667.0000000	1.0450000
23668.0000000	1.0400000
23669.0000000	1.0350000
23670.0000000	1.0400000
23671.0000000	1.0450000
23672.0000000	1.0300000
23673.0000000	1.0400000
23674.0000000	1.0400000
23675.0000000	1.0400000
23676.0000000	1.0450000
23677.0000000	1.0450000
23678.0000000	1.0450000
23679.0000000	1.0400000
23680.0000000	1.0466666
23681.0000000	1.0450000
23682.0000000	1.0450000
23683.0000000	1.0500000
23684.0000000	1.0500000
23685.0000000	1.0450000
23686.0000000	1.0500000
23687.0000000	1.0500000

23688.0000000	1.0549999
23689.0000000	1.0599999
23690.0000000	1.0650001
23691.0000000	1.0599999
23692.0000000	1.0700001
23693.0000000	1.0700001
23694.0000000	1.0700001
23695.0000000	1.0650001
23696.0000000	1.0700001
23697.0000000	1.0549999
23698.0000000	1.0549999
23699.0000000	1.0549999
23700.0000000	1.0500000
23701.0000000	1.0549999
23702.0000000	1.0549999
23703.0000000	1.0599999
23704.0000000	1.0599999
23705.0000000	1.0650001
23706.0000000	1.0599999
23707.0000000	1.0599999
23708.0000000	1.0500000
23709.0000000	1.0566666
23710.0000000	1.0500000
23711.0000000	1.0549999
23712.0000000	1.0549999
23713.0000000	1.0650001
23714.0000000	1.0549999
23715.0000000	1.0549999
23716.0000000	1.0599999
23717.0000000	1.0599999
23718.0000000	1.0599999
23719.0000000	1.0599999
23720.0000000	1.0599999
23721.0000000	1.0549999
23722.0000000	1.0599999
23723.0000000	1.0599999
23724.0000000	1.0650001
23725.0000000	1.0650001
23726.0000000	1.0650001
23727.0000000	1.0700001
23728.0000000	1.0800000
23729.0000000	1.0800000
23730.0000000	1.0850000
23731.0000000	1.0850000
23732.0000000	1.0950000
23733.0000000	1.0950000
23734.0000000	1.0900000
23735.0000000	1.1000000
23736.0000000	1.0950000
23737.0000000	1.1000000
23738.0000000	1.1000000
23739.0000000	1.0900000
23740.0000000	1.0900000
23741.0000000	1.0900000
23742.0000000	1.0850000
23743.0000000	1.0800000
23744.0000000	1.0900000

23745.0000000	1.0800000
23746.0000000	1.0800000
23747.0000000	1.0850000
23748.0000000	1.0800000
23749.0000000	1.0850000
23750.0000000	1.0850000
23751.0000000	1.0900000
23752.0000000	1.0850000
23753.0000000	1.0900000
23754.0000000	1.1000000
23755.0000000	1.0950000
23756.0000000	1.0950000
23757.0000000	1.1050000
23758.0000000	1.1100000
23759.0000000	1.1100000
23760.0000000	1.1100000
23761.0000000	1.1100000
23762.0000000	1.1150000
23763.0000000	1.1150000
23764.0000000	1.1250000
23765.0000000	1.1250000
23766.0000000	1.1300000
23767.0000000	1.1300000
23768.0000000	1.1233333
23769.0000000	1.1250000
23770.0000000	1.1250000
23771.0000000	1.1350000
23772.0000000	1.1300000
23773.0000000	1.1300000
23774.0000000	1.1300000
23775.0000000	1.1350000
23776.0000000	1.1350000
23777.0000000	1.1300000
23778.0000000	1.1350000
23779.0000000	1.1400000
23780.0000000	1.1400000
23781.0000000	1.1400000
23782.0000000	1.1350000
23783.0000000	1.1300000
23784.0000000	1.1300000
23785.0000000	1.1400000
23786.0000000	1.1400000
23787.0000000	1.1450000
23788.0000000	1.1400000
23789.0000000	1.1450000
23790.0000000	1.1400000
23791.0000000	1.1500000
23792.0000000	1.1550000
23793.0000000	1.1600000
23794.0000000	1.1650000
23795.0000000	1.2000000
23796.0000000	1.1949999
23797.0000000	1.1600000
23798.0000000	1.1600000
23799.0000000	1.1566666
23800.0000000	1.1550000
23801.0000000	1.1600000

23802.0000000	1.1500000
23803.0000000	1.1500000
23804.0000000	1.1400000
23805.0000000	1.1300000
23806.0000000	1.1200000
23807.0000000	1.1150000
23808.0000000	1.1100000
23809.0000000	1.1050000
23810.0000000	1.1000000
23811.0000000	1.1000000
23812.0000000	1.1100000
23813.0000000	1.1150000
23814.0000000	1.1200000
23815.0000000	1.1350000
23816.0000000	1.1450000
23817.0000000	1.1400000
23818.0000000	1.1450000
23819.0000000	1.1500000
23820.0000000	1.1450000
23821.0000000	1.1400000
23822.0000000	1.1450000
23823.0000000	1.1500000
23824.0000000	1.1400000
23825.0000000	1.1300000
23826.0000000	1.1300000
23827.0000000	1.1300000
23828.0000000	1.1250000
23829.0000000	1.1200000
23830.0000000	1.1150000
23831.0000000	1.1050000
23832.0000000	1.1100000
23833.0000000	1.1000000
23834.0000000	1.1000000
23835.0000000	1.0950000
23836.0000000	1.0950000
23837.0000000	1.0850000
23838.0000000	1.0800000
23839.0000000	1.0700001
23840.0000000	1.0599999
23841.0000000	1.0549999
23842.0000000	1.0599999
23843.0000000	1.0549999
23844.0000000	1.0650001
23845.0000000	1.0599999
23846.0000000	1.0650001
23847.0000000	1.0700001
23848.0000000	1.0700001
23849.0000000	1.0700001
23850.0000000	1.0500000
23851.0000000	1.0500000
23852.0000000	1.0450000
23853.0000000	1.0400000
23854.0000000	1.0350000
23855.0000000	1.0350000
23856.0000000	1.0300000
23857.0000000	1.0400000
23858.0000000	1.0350000

23859.0000000	1.0433333
23860.0000000	1.0500000
23861.0000000	1.0599999
23862.0000000	1.0650001
23863.0000000	1.0700001
23864.0000000	1.0750000
23865.0000000	1.0700001
23866.0000000	1.0750000
23867.0000000	1.0800000
23868.0000000	1.0800000
23869.0000000	1.0850000
23870.0000000	1.0850000
23871.0000000	1.0900000
23872.0000000	1.0950000
23873.0000000	1.0950000
23874.0000000	1.0800000
23875.0000000	1.0850000
23876.0000000	1.0900000
23877.0000000	1.0850000
23878.0000000	1.0850000
23879.0000000	1.0850000
23880.0000000	1.0900000
23881.0000000	1.0950000
23882.0000000	1.1000000
23883.0000000	1.0900000
23884.0000000	1.1050000
23885.0000000	1.1000000
23886.0000000	1.1100000
23887.0000000	1.1050000
23888.0000000	1.1000000
23889.0000000	1.1100000
23890.0000000	1.1100000
23891.0000000	1.1050000
23892.0000000	1.1050000
23893.0000000	1.1000000
23894.0000000	1.0900000
23895.0000000	1.0750000
23896.0000000	1.0650001
23897.0000000	1.0599999
23898.0000000	1.0549999
23899.0000000	1.0500000
23900.0000000	1.0549999
23901.0000000	1.0549999
23902.0000000	1.0549999
23903.0000000	1.0500000
23904.0000000	1.0400000
23905.0000000	1.0450000
23906.0000000	1.0350000
23907.0000000	1.0300000
23908.0000000	1.0300000
23909.0000000	1.0300000
23910.0000000	1.0300000
23911.0000000	1.0300000
23912.0000000	1.0350000
23913.0000000	1.0350000
23914.0000000	1.0300000
23915.0000000	1.0400000

23916.0000000	1.0400000
23917.0000000	1.0400000
23918.0000000	1.0400000
23919.0000000	1.0400000
23920.0000000	1.0466666
23921.0000000	1.0400000
23922.0000000	1.0400000
23923.0000000	1.0500000
23924.0000000	1.0450000
23925.0000000	1.0400000
23926.0000000	1.0450000
23927.0000000	1.0400000
23928.0000000	1.0400000
23929.0000000	1.0350000
23930.0000000	1.0300000
23931.0000000	1.0300000
23932.0000000	1.0150000
23933.0000000	1.0100000
23934.0000000	1.0050000
23935.0000000	1.0000000
23936.0000000	1.0050000
23937.0000000	1.0050000
23938.0000000	1.0000000
23939.0000000	0.9950000
23940.0000000	0.9900000
23941.0000000	0.9950000
23942.0000000	0.9900000
23943.0000000	0.9850000
23944.0000000	0.9850000
23945.0000000	0.9900000
23946.0000000	0.9850000
23947.0000000	0.9900000
23948.0000000	0.9950000
23949.0000000	0.9900000
23950.0000000	1.0000000
23951.0000000	0.9966667
23952.0000000	1.0000000
23953.0000000	1.0050000
23954.0000000	1.0100000
23955.0000000	1.0150000
23956.0000000	1.0100000
23957.0000000	1.0200000
23958.0000000	1.0100000
23959.0000000	1.0200000
23960.0000000	1.0250000
23961.0000000	1.0200000
23962.0000000	1.0200000
23963.0000000	1.0200000
23964.0000000	1.0200000
23965.0000000	1.0200000
23966.0000000	1.0150000
23967.0000000	1.0200000
23968.0000000	1.0150000
23969.0000000	1.0150000
23970.0000000	1.0200000
23971.0000000	1.0150000
23972.0000000	1.0200000

23973.0000000	1.0200000
23974.0000000	1.0200000
23975.0000000	1.0250000
23976.0000000	1.0200000
23977.0000000	1.0250000
23978.0000000	1.0150000
23979.0000000	1.0200000
23980.0000000	1.0300000
23981.0000000	1.0300000
23982.0000000	1.0350000
23983.0000000	1.0400000
23984.0000000	1.0400000
23985.0000000	1.0400000
23986.0000000	1.0400000
23987.0000000	1.0400000
23988.0000000	1.0450000
23989.0000000	1.0450000
23990.0000000	1.0400000
23991.0000000	1.0400000
23992.0000000	1.0400000
23993.0000000	1.0400000
23994.0000000	1.0450000
23995.0000000	1.0450000
23996.0000000	1.0450000
23997.0000000	1.0549999
23998.0000000	1.0599999
23999.0000000	1.0599999
24000.0000000	1.0599999
24001.0000000	1.0650001
24002.0000000	1.0650001
24003.0000000	1.0650001
24004.0000000	1.0750000
24005.0000000	1.0750000
24006.0000000	1.0800000
24007.0000000	1.0900000
24008.0000000	1.0850000
24009.0000000	1.0900000
24010.0000000	1.1000000
24011.0000000	1.1000000
24012.0000000	1.1000000
24013.0000000	1.1100000
24014.0000000	1.1150000
24015.0000000	1.1150000
24016.0000000	1.1200000
24017.0000000	1.1200000
24018.0000000	1.1300000
24019.0000000	1.1300000
24020.0000000	1.1300000
24021.0000000	1.1350000
24022.0000000	1.1400000
24023.0000000	1.1400000
24024.0000000	1.1400000
24025.0000000	1.1400000
24026.0000000	1.1400000
24027.0000000	1.1400000
24028.0000000	1.1400000
24029.0000000	1.1300000

24030.0000000	1.1300000
24031.0000000	1.1250000
24032.0000000	1.1300000
24033.0000000	1.1400000
24034.0000000	1.1400000
24035.0000000	1.1500000
24036.0000000	1.1500000
24037.0000000	1.1550000
24038.0000000	1.1600000
24039.0000000	1.1500000
24040.0000000	1.1550000
24041.0000000	1.1550000
24042.0000000	1.1533333
24043.0000000	1.1500000
24044.0000000	1.1450000
24045.0000000	1.1400000
24046.0000000	1.1300000
24047.0000000	1.1350000
24048.0000000	1.1250000
24049.0000000	1.1150000
24050.0000000	1.1150000
24051.0000000	1.1200000
24052.0000000	1.1150000
24053.0000000	1.1200000
24054.0000000	1.1200000
24055.0000000	1.1100000
24056.0000000	1.1150000
24057.0000000	1.1150000
24058.0000000	1.1150000
24059.0000000	1.1150000
24060.0000000	1.1200000
24061.0000000	1.1200000
24062.0000000	1.1250000
24063.0000000	1.1350000
24064.0000000	1.1300000
24065.0000000	1.1300000
24066.0000000	1.1300000
24067.0000000	1.1300000
24068.0000000	1.1300000
24069.0000000	1.1250000
24070.0000000	1.1100000
24071.0000000	1.0950000
24072.0000000	1.0833334
24073.0000000	1.0800000
24074.0000000	1.0750000
24075.0000000	1.0700001
24076.0000000	1.0699999
24077.0000000	1.0700001
24078.0000000	1.0700001
24079.0000000	1.0700001
24080.0000000	1.0700001
24081.0000000	1.0700001
24082.0000000	1.0700001
24083.0000000	1.0800000
24084.0000000	1.0750000
24085.0000000	1.0800000
24086.0000000	1.0850000

24087.0000000	1.0950000
24088.0000000	1.0950000
24089.0000000	1.1050000
24090.0000000	1.1100000
24091.0000000	1.1200000
24092.0000000	1.1200000
24093.0000000	1.1200000
24094.0000000	1.1200000
24095.0000000	1.1200000
24096.0000000	1.1250000
24097.0000000	1.1300000
24098.0000000	1.1350000
24099.0000000	1.1350000
24100.0000000	1.1400000
24101.0000000	1.1450000
24102.0000000	1.1400000
24103.0000000	1.1533333
24104.0000000	1.1550000
24105.0000000	1.1600000
24106.0000000	1.1600000
24107.0000000	1.1650000
24108.0000000	1.1750000
24109.0000000	1.1849999
24110.0000000	1.1799999
24111.0000000	1.1849999
24112.0000000	1.1799999
24113.0000000	1.1799999
24114.0000000	1.1849999
24115.0000000	1.1849999
24116.0000000	1.1950001
24117.0000000	1.1900001
24118.0000000	1.1950001
24119.0000000	1.1900001
24120.0000000	1.1900001
24121.0000000	1.1900001
24122.0000000	1.1900001
24123.0000000	1.1799999
24124.0000000	1.1900001
24125.0000000	1.1900001
24126.0000000	1.1900001
24127.0000000	1.2000000
24128.0000000	1.2000000
24129.0000000	1.2050000
24130.0000000	1.2000000
24131.0000000	1.2050000
24132.0000000	1.2100000
24133.0000000	1.2100000
24134.0000000	1.2100000
24135.0000000	1.2100000
24136.0000000	1.2200000
24137.0000000	1.2200000
24138.0000000	1.2200000
24139.0000000	1.2200000
24140.0000000	1.2300000
24141.0000000	1.2300000
24142.0000000	1.2300000
24143.0000000	1.2300000

24144.0000000	1.2200000
24145.0000000	1.2200000
24146.0000000	1.2250000
24147.0000000	1.2250000
24148.0000000	1.2250000
24149.0000000	1.2200000
24150.0000000	1.2200000
24151.0000000	1.2200000
24152.0000000	1.2150000
24153.0000000	1.2200000
24154.0000000	1.2100000
24155.0000000	1.2200000
24156.0000000	1.2250000
24157.0000000	1.2200000
24158.0000000	1.2250000
24159.0000000	1.2300000
24160.0000000	1.2300000
24161.0000000	1.2400000
24162.0000000	1.2400000
24163.0000000	1.2400000
24164.0000000	1.2450000
24165.0000000	1.2450000
24166.0000000	1.2500000
24167.0000000	1.2500000
24168.0000000	1.2500000
24169.0000000	1.2500000
24170.0000000	1.2550000
24171.0000000	1.2550000
24172.0000000	1.2600000
24173.0000000	1.2600000
24174.0000000	1.2600000
24175.0000000	1.2600000
24176.0000000	1.2600000
24177.0000000	1.2600000
24178.0000000	1.2600000
24179.0000000	1.2550000
24180.0000000	1.2550000
24181.0000000	1.2550000
24182.0000000	1.2400000
24183.0000000	1.2400000
24184.0000000	1.2450000
24185.0000000	1.2400000
24186.0000000	1.2400000
24187.0000000	1.2400000
24188.0000000	1.2300000
24189.0000000	1.2300000
24190.0000000	1.2250000
24191.0000000	1.2200000
24192.0000000	1.2150000
24193.0000000	1.2050000
24194.0000000	1.2100000
24195.0000000	1.1950001
24196.0000000	1.1900001
24197.0000000	1.1900001
24198.0000000	1.1950001
24199.0000000	1.2000000
24200.0000000	1.1950001

24201.0000000	1.2050000
24202.0000000	1.2000000
24203.0000000	1.1950001
24204.0000000	1.1900001
24205.0000000	1.1799999
24206.0000000	1.1700000
24207.0000000	1.1700000
24208.0000000	1.1650000
24209.0000000	1.1550000
24210.0000000	1.1550000
24211.0000000	1.1500000
24212.0000000	1.1400000
24213.0000000	1.1400000
24214.0000000	1.1300000
24215.0000000	1.1350000
24216.0000000	1.1300000
24217.0000000	1.1300000
24218.0000000	1.1300000
24219.0000000	1.1300000
24220.0000000	1.1300000
24221.0000000	1.1300000
24222.0000000	1.1350000
24223.0000000	1.1400000
24224.0000000	1.1400000
24225.0000000	1.1450000
24226.0000000	1.1400000
24227.0000000	1.1500000
24228.0000000	1.1500000
24229.0000000	1.1600000
24230.0000000	1.1600000
24231.0000000	1.1550000
24232.0000000	1.1650000
24233.0000000	1.1650000
24234.0000000	1.1700000
24235.0000000	1.1700000
24236.0000000	1.1799999
24237.0000000	1.1900001
24238.0000000	1.1900001
24239.0000000	1.1900001
24240.0000000	1.1900001
24241.0000000	1.1900001
24242.0000000	1.1900001
24243.0000000	1.1950001
24244.0000000	1.1950001
24245.0000000	1.2100000
24246.0000000	1.2050000
24247.0000000	1.2150000
24248.0000000	1.2250000
24249.0000000	1.2250000
24250.0000000	1.2300000
24251.0000000	1.2300000
24252.0000000	1.2250000
24253.0000000	1.2200000
24254.0000000	1.2200000
24255.0000000	1.2166667
24256.0000000	1.2100000
24257.0000000	1.2050000

24258.0000000	1.2000000
24259.0000000	1.1950001
24260.0000000	1.1900001
24261.0000000	1.1799999
24262.0000000	1.1700000
24263.0000000	1.1700000
24264.0000000	1.1650000
24265.0000000	1.1600000
24266.0000000	1.1600000
24267.0000000	1.1600000
24268.0000000	1.1500000
24269.0000000	1.1550000
24270.0000000	1.1400000
24271.0000000	1.1450000
24272.0000000	1.1450000
24273.0000000	1.1400000
24274.0000000	1.1400000
24275.0000000	1.1500000
24276.0000000	1.1550000
24277.0000000	1.1650000
24278.0000000	1.1650000
24279.0000000	1.1650000
24280.0000000	1.1600000
24281.0000000	1.1600000
24282.0000000	1.1550000
24283.0000000	1.1600000
24284.0000000	1.1500000
24285.0000000	1.1600000
24286.0000000	1.1650000
24287.0000000	1.1700000
24288.0000000	1.1700000
24289.0000000	1.1750000
24290.0000000	1.1799999
24291.0000000	1.1750000
24292.0000000	1.1799999
24293.0000000	1.1900001
24294.0000000	1.1900001
24295.0000000	1.1950001
24296.0000000	1.2000000
24297.0000000	1.2000000
24298.0000000	1.2000000
24299.0000000	1.2000000
24300.0000000	1.2050000
24301.0000000	1.2050000
24302.0000000	1.2000000
24303.0000000	1.2000000
24304.0000000	1.2000000
24305.0000000	1.2050000
24306.0000000	1.2000000
24307.0000000	1.2100000
24308.0000000	1.2050000
24309.0000000	1.2050000
24310.0000000	1.2000000
24311.0000000	1.2000000
24312.0000000	1.2100000
24313.0000000	1.2000000
24314.0000000	1.2100000

24315.0000000	1.2100000
24316.0000000	1.2200000
24317.0000000	1.2200000
24318.0000000	1.2200000
24319.0000000	1.2300000
24320.0000000	1.2300000
24321.0000000	1.2350000
24322.0000000	1.2300000
24323.0000000	1.2300000
24324.0000000	1.2250000
24325.0000000	1.2250000
24326.0000000	1.2350000
24327.0000000	1.2350000
24328.0000000	1.2300000
24329.0000000	1.2350000
24330.0000000	1.2400000
24331.0000000	1.2400000
24332.0000000	1.2400000
24333.0000000	1.2400000
24334.0000000	1.2350000
24335.0000000	1.2350000
24336.0000000	1.2300000
24337.0000000	1.2350000
24338.0000000	1.2300000
24339.0000000	1.2200000
24340.0000000	1.2200000
24341.0000000	1.2200000
24342.0000000	1.2100000
24343.0000000	1.2150000
24344.0000000	1.2166667
24345.0000000	1.2200000
24346.0000000	1.2150000
24347.0000000	1.2150000
24348.0000000	1.2050000
24349.0000000	1.2000000
24350.0000000	1.1950001
24351.0000000	1.1900001
24352.0000000	1.1849999
24353.0000000	1.1849999
24354.0000000	1.1799999
24355.0000000	1.1700000
24356.0000000	1.1600000
24357.0000000	1.1350000
24358.0000000	1.1350000
24359.0000000	1.1300000
24360.0000000	1.1250000
24361.0000000	1.1350000
24362.0000000	1.1350000
24363.0000000	1.1400000
24364.0000000	1.1400000
24365.0000000	1.1500000
24366.0000000	1.1550000
24367.0000000	1.1550000
24368.0000000	1.1600000
24369.0000000	1.1600000
24370.0000000	1.1600000
24371.0000000	1.1700000

24372.0000000	1.1700000
24373.0000000	1.1799999
24374.0000000	1.1849999
24375.0000000	1.1933334
24376.0000000	1.2050000
24377.0000000	1.2050000
24378.0000000	1.2100000
24379.0000000	1.2100000
24380.0000000	1.2150000
24381.0000000	1.2150000
24382.0000000	1.2150000
24383.0000000	1.2200000
24384.0000000	1.2200000
24385.0000000	1.2200000
24386.0000000	1.2250000
24387.0000000	1.2250000
24388.0000000	1.2200000
24389.0000000	1.2200000
24390.0000000	1.2100000
24391.0000000	1.2100000
24392.0000000	1.2050000
24393.0000000	1.1950001
24394.0000000	1.1950001
24395.0000000	1.1849999
24396.0000000	1.1799999
24397.0000000	1.1700000
24398.0000000	1.1600000
24399.0000000	1.1600000
24400.0000000	1.1550000
24401.0000000	1.1500000
24402.0000000	1.1400000
24403.0000000	1.1333333
24404.0000000	1.1300000
24405.0000000	1.1300000
24406.0000000	1.1200000
24407.0000000	1.1250000
24408.0000000	1.1300000
24409.0000000	1.1350000
24410.0000000	1.1300000
24411.0000000	1.1450000
24412.0000000	1.1500000
24413.0000000	1.1550000
24414.0000000	1.1650000
24415.0000000	1.1600000
24416.0000000	1.1600000
24417.0000000	1.1550000
24418.0000000	1.1550000
24419.0000000	1.1600000
24420.0000000	1.1600000
24421.0000000	1.1600000
24422.0000000	1.1650000
24423.0000000	1.1700000
24424.0000000	1.1650000
24425.0000000	1.1700000
24426.0000000	1.1750000
24427.0000000	1.1849999
24428.0000000	1.1750000

24429.0000000	1.1849999
24430.0000000	1.1900001
24431.0000000	1.2000000
24432.0000000	1.1950001
24433.0000000	1.2000000
24434.0000000	1.2050000
24435.0000000	1.2000000
24436.0000000	1.2000000
24437.0000000	1.2050000
24438.0000000	1.2150000
24439.0000000	1.2200000
24440.0000000	1.2200000
24441.0000000	1.2200000
24442.0000000	1.2300000
24443.0000000	1.2350000
24444.0000000	1.2400000
24445.0000000	1.2500000
24446.0000000	1.2450000
24447.0000000	1.2450000
24448.0000000	1.2400000
24449.0000000	1.2400000
24450.0000000	1.2400000
24451.0000000	1.2400000
24452.0000000	1.2300000
24453.0000000	1.2250000
24454.0000000	1.2250000
24455.0000000	1.2200000
24456.0000000	1.2150000
24457.0000000	1.2100000
24458.0000000	1.2100000
24459.0000000	1.2100000
24460.0000000	1.1950001
24461.0000000	1.1950001
24462.0000000	1.1900001
24463.0000000	1.1799999
24464.0000000	1.1650000
24465.0000000	1.1600000
24466.0000000	1.1550000
24467.0000000	1.1600000
24468.0000000	1.1700000
24469.0000000	1.1750000
24470.0000000	1.1849999
24471.0000000	1.1799999
24472.0000000	1.1849999
24473.0000000	1.1849999
24474.0000000	1.1799999
24475.0000000	1.1750000
24476.0000000	1.1750000
24477.0000000	1.1700000
24478.0000000	1.1700000
24479.0000000	1.1799999
24480.0000000	1.1799999
24481.0000000	1.1849999
24482.0000000	1.1950001
24483.0000000	1.1799999
24484.0000000	1.1799999
24485.0000000	1.1700000

24486.0000000	1.1600000
24487.0000000	1.1700000
24488.0000000	1.1750000
24489.0000000	1.1700000
24490.0000000	1.1799999
24491.0000000	1.1750000
24492.0000000	1.1849999
24493.0000000	1.1799999
24494.0000000	1.1833333
24495.0000000	1.1900001
24496.0000000	1.1849999
24497.0000000	1.1849999
24498.0000000	1.1849999
24499.0000000	1.1849999
24500.0000000	1.1700000
24501.0000000	1.1700000
24502.0000000	1.1700000
24503.0000000	1.1600000
24504.0000000	1.1700000
24505.0000000	1.1700000
24506.0000000	1.1650000
24507.0000000	1.1700000
24508.0000000	1.1700000
24509.0000000	1.1750000
24510.0000000	1.1650000
24511.0000000	1.1650000
24512.0000000	1.1650000
24513.0000000	1.1650000
24514.0000000	1.1650000
24515.0000000	1.1650000
24516.0000000	1.1650000
24517.0000000	1.1700000
24518.0000000	1.1700000
24519.0000000	1.1700000
24520.0000000	1.1700000
24521.0000000	1.1650000
24522.0000000	1.1700000
24523.0000000	1.1700000
24524.0000000	1.1733333
24525.0000000	1.1750000
24526.0000000	1.1849999
24527.0000000	1.1849999
24528.0000000	1.1799999
24529.0000000	1.1799999
24530.0000000	1.1799999
24531.0000000	1.1799999
24532.0000000	1.1750000
24533.0000000	1.1750000
24534.0000000	1.1700000
24535.0000000	1.1700000
24536.0000000	1.1650000
24537.0000000	1.1650000
24538.0000000	1.1700000
24539.0000000	1.1600000
24540.0000000	1.1650000
24541.0000000	1.1650000
24542.0000000	1.1650000

24543.0000000	1.1600000
24544.0000000	1.1600000
24545.0000000	1.1600000
24546.0000000	1.1600000
24547.0000000	1.1600000
24548.0000000	1.1600000
24549.0000000	1.1550000
24550.0000000	1.1600000
24551.0000000	1.1550000
24552.0000000	1.1450000
24553.0000000	1.1500000
24554.0000000	1.1500000
24555.0000000	1.1466666
24556.0000000	1.1400000
24557.0000000	1.1400000
24558.0000000	1.1400000
24559.0000000	1.1350000
24560.0000000	1.1300000
24561.0000000	1.1250000
24562.0000000	1.1300000
24563.0000000	1.1250000
24564.0000000	1.1150000
24565.0000000	1.1150000
24566.0000000	1.1100000
24567.0000000	1.1100000
24568.0000000	1.1000000
24569.0000000	1.1050000
24570.0000000	1.1000000
24571.0000000	1.1000000
24572.0000000	1.1000000
24573.0000000	1.1000000
24574.0000000	1.1050000
24575.0000000	1.1100000
24576.0000000	1.1100000
24577.0000000	1.1050000
24578.0000000	1.1100000
24579.0000000	1.1100000
24580.0000000	1.1150000
24581.0000000	1.1200000
24582.0000000	1.1200000
24583.0000000	1.1200000
24584.0000000	1.1250000
24585.0000000	1.1233333
24586.0000000	1.1300000
24587.0000000	1.1200000
24588.0000000	1.1200000
24589.0000000	1.1200000
24590.0000000	1.1100000
24591.0000000	1.1200000
24592.0000000	1.1100000
24593.0000000	1.1100000
24594.0000000	1.1050000
24595.0000000	1.1000000
24596.0000000	1.1050000
24597.0000000	1.1000000
24598.0000000	1.0950000
24599.0000000	1.0950000

24600.0000000	1.0950000
24601.0000000	1.0900000
24602.0000000	1.0900000
24603.0000000	1.0850000
24604.0000000	1.0850000
24605.0000000	1.0850000
24606.0000000	1.0900000
24607.0000000	1.0800000
24608.0000000	1.0800000
24609.0000000	1.0700001
24610.0000000	1.0500000
24611.0000000	1.0500000
24612.0000000	1.0500000
24613.0000000	1.0500000
24614.0000000	1.0549999
24615.0000000	1.0666667
24616.0000000	1.0650001
24617.0000000	1.0700001
24618.0000000	1.0700001
24619.0000000	1.0700001
24620.0000000	1.0750000
24621.0000000	1.0800000
24622.0000000	1.0800000
24623.0000000	1.0800000
24624.0000000	1.0750000
24625.0000000	1.0750000
24626.0000000	1.0800000
24627.0000000	1.0850000
24628.0000000	1.0850000
24629.0000000	1.0800000
24630.0000000	1.0900000
24631.0000000	1.0900000
24632.0000000	1.0900000
24633.0000000	1.0950000
24634.0000000	1.1000000
24635.0000000	1.1000000
24636.0000000	1.1050000
24637.0000000	1.1000000
24638.0000000	1.1100000
24639.0000000	1.1100000
24640.0000000	1.1150000
24641.0000000	1.1200000
24642.0000000	1.1100000
24643.0000000	1.1200000
24644.0000000	1.1250000
24645.0000000	1.1250000
24646.0000000	1.1200000
24647.0000000	1.1250000
24648.0000000	1.1300000
24649.0000000	1.1200000
24650.0000000	1.1150000
24651.0000000	1.1150000
24652.0000000	1.1100000
24653.0000000	1.1050000
24654.0000000	1.0800000
24655.0000000	1.0750000
24656.0000000	1.0549999

24657.0000000	1.0350000
24658.0000000	1.0250000
24659.0000000	1.0150000
24660.0000000	1.0100000
24661.0000000	1.0050000
24662.0000000	1.0150000
24663.0000000	1.0050000
24664.0000000	1.0100000
24665.0000000	1.0250000
24666.0000000	1.0200000
24667.0000000	1.0250000
24668.0000000	1.0200000
24669.0000000	1.0150000
24670.0000000	1.0150000
24671.0000000	1.0250000
24672.0000000	1.0300000
24673.0000000	1.0400000
24674.0000000	1.0599999
24675.0000000	1.0650001
24676.0000000	1.0766667
24677.0000000	1.0750000
24678.0000000	1.0800000
24679.0000000	1.0850000
24680.0000000	1.0900000
24681.0000000	1.0900000
24682.0000000	1.0850000
24683.0000000	1.0900000
24684.0000000	1.0900000
24685.0000000	1.0950000
24686.0000000	1.1000000
24687.0000000	1.1000000
24688.0000000	1.0900000
24689.0000000	1.0950000
24690.0000000	1.0900000
24691.0000000	1.0900000
24692.0000000	1.0950000
24693.0000000	1.0900000
24694.0000000	1.0900000
24695.0000000	1.0900000
24696.0000000	1.0900000
24697.0000000	1.0950000
24698.0000000	1.0900000
24699.0000000	1.0900000
24700.0000000	1.0800000
24701.0000000	1.0800000
24702.0000000	1.0800000
24703.0000000	1.0700001
24704.0000000	1.0700001
24705.0000000	1.0700001
24706.0000000	1.0750000
24707.0000000	1.0633334
24708.0000000	1.0599999
24709.0000000	1.0549999
24710.0000000	1.0500000
24711.0000000	1.0500000
24712.0000000	1.0549999
24713.0000000	1.0500000

24714.0000000	1.0500000
24715.0000000	1.0500000
24716.0000000	1.0500000
24717.0000000	1.0500000
24718.0000000	1.0500000
24719.0000000	1.0500000
24720.0000000	1.0500000
24721.0000000	1.0549999
24722.0000000	1.0599999
24723.0000000	1.0549999
24724.0000000	1.0650001
24725.0000000	1.0549999
24726.0000000	1.0549999
24727.0000000	1.0599999
24728.0000000	1.0500000
24729.0000000	1.0500000
24730.0000000	1.0400000
24731.0000000	1.0350000
24732.0000000	1.0400000
24733.0000000	1.0350000
24734.0000000	1.0400000
24735.0000000	1.0400000
24736.0000000	1.0450000
24737.0000000	1.0549999
24738.0000000	1.0533333
24739.0000000	1.0549999
24740.0000000	1.0549999
24741.0000000	1.0500000
24742.0000000	1.0400000
24743.0000000	1.0350000
24744.0000000	1.0300000
24745.0000000	1.0250000
24746.0000000	1.0150000
24747.0000000	1.0200000
24748.0000000	1.0100000
24749.0000000	1.0200000
24750.0000000	1.0150000
24751.0000000	1.0200000
24752.0000000	1.0200000
24753.0000000	1.0200000
24754.0000000	1.0250000
24755.0000000	1.0250000
24756.0000000	1.0300000
24757.0000000	1.0300000
24758.0000000	1.0300000
24759.0000000	1.0400000
24760.0000000	1.0400000
24761.0000000	1.0400000
24762.0000000	1.0350000
24763.0000000	1.0350000
24764.0000000	1.0350000
24765.0000000	1.0200000
24766.0000000	1.0150000
24767.0000000	1.0066667
24768.0000000	1.0050000
24769.0000000	0.9950000
24770.0000000	0.9950000

24771.0000000	0.9850000
24772.0000000	0.9850000
24773.0000000	0.9750000
24774.0000000	0.9700000
24775.0000000	0.9650000
24776.0000000	0.9550000
24777.0000000	0.9550000
24778.0000000	0.9500000
24779.0000000	0.9450000
24780.0000000	0.9500000
24781.0000000	0.9400000
24782.0000000	0.9450000
24783.0000000	0.9400000
24784.0000000	0.9400000
24785.0000000	0.9400000
24786.0000000	0.9400000
24787.0000000	0.9450000
24788.0000000	0.9400000
24789.0000000	0.9450000
24790.0000000	0.9400000
24791.0000000	0.9350000
24792.0000000	0.9400000
24793.0000000	0.9400000
24794.0000000	0.9400000
24795.0000000	0.9400000
24796.0000000	0.9400000
24797.0000000	0.9400000
24798.0000000	0.9433333
24799.0000000	0.9400000
24800.0000000	0.9400000
24801.0000000	0.9450000
24802.0000000	0.9500000
24803.0000000	0.9500000
24804.0000000	0.9600000
24805.0000000	0.9600000
24806.0000000	0.9700000
24807.0000000	0.9750000
24808.0000000	0.9750000
24809.0000000	0.9850000
24810.0000000	0.9800000
24811.0000000	0.9800000
24812.0000000	0.9800000
24813.0000000	0.9800000
24814.0000000	0.9850000
24815.0000000	0.9750000
24816.0000000	0.9750000
24817.0000000	0.9700000
24818.0000000	0.9700000
24819.0000000	0.9700000
24820.0000000	0.9700000
24821.0000000	0.9700000
24822.0000000	0.9650000
24823.0000000	0.9700000
24824.0000000	0.9700000
24825.0000000	0.9700000
24826.0000000	0.9600000
24827.0000000	0.9650000

24828.0000000	0.9700000
24829.0000000	0.9700000
24830.0000000	0.9700000
24831.0000000	0.9750000
24832.0000000	0.9750000
24833.0000000	0.9700000
24834.0000000	0.9700000
24835.0000000	0.9650000
24836.0000000	0.9650000
24837.0000000	0.9550000
24838.0000000	0.9400000
24839.0000000	0.9300000
24840.0000000	0.9150000
24841.0000000	0.8950000
24842.0000000	0.8800000
24843.0000000	0.8750000
24844.0000000	0.8650000
24845.0000000	0.8650000
24846.0000000	0.8600000
24847.0000000	0.8700000
24848.0000000	0.8750000
24849.0000000	0.8750000
24850.0000000	0.8750000
24851.0000000	0.8800000
24852.0000000	0.8850000
24853.0000000	0.8900000
24854.0000000	0.8900000
24855.0000000	0.9000000
24856.0000000	0.9100000
24857.0000000	0.9050000
24858.0000000	0.9133334
24859.0000000	0.9200000
24860.0000000	0.9200000
24861.0000000	0.9200000
24862.0000000	0.9150000
24863.0000000	0.9200000
24864.0000000	0.9200000
24865.0000000	0.9150000
24866.0000000	0.9100000
24867.0000000	0.9100000
24868.0000000	0.8950000
24869.0000000	0.8900000
24870.0000000	0.8800000
24871.0000000	0.8900000
24872.0000000	0.8900000
24873.0000000	0.8850000
24874.0000000	0.8800000
24875.0000000	0.8750000
24876.0000000	0.8750000
24877.0000000	0.8850000
24878.0000000	0.9000000
24879.0000000	0.9050000
24880.0000000	0.9000000
24881.0000000	0.9050000
24882.0000000	0.9100000
24883.0000000	0.9200000
24884.0000000	0.9200000

24885.0000000	0.9150000
24886.0000000	0.9200000
24887.0000000	0.9200000
24888.0000000	0.9200000
24889.0000000	0.9233333
24890.0000000	0.9300000
24891.0000000	0.9300000
24892.0000000	0.9300000
24893.0000000	0.9300000
24894.0000000	0.9400000
24895.0000000	0.9400000
24896.0000000	0.9400000
24897.0000000	0.9300000
24898.0000000	0.9350000
24899.0000000	0.9400000
24900.0000000	0.9400000
24901.0000000	0.9400000
24902.0000000	0.9350000
24903.0000000	0.9350000
24904.0000000	0.9300000
24905.0000000	0.9250000
24906.0000000	0.9200000
24907.0000000	0.9200000
24908.0000000	0.9250000
24909.0000000	0.9200000
24910.0000000	0.9200000
24911.0000000	0.9250000
24912.0000000	0.9300000
24913.0000000	0.9300000
24914.0000000	0.9300000
24915.0000000	0.9350000
24916.0000000	0.9350000
24917.0000000	0.9350000
24918.0000000	0.9350000
24919.0000000	0.9400000
24920.0000000	0.9400000
24921.0000000	0.9400000
24922.0000000	0.9350000
24923.0000000	0.9300000
24924.0000000	0.9300000
24925.0000000	0.9200000
24926.0000000	0.9150000
24927.0000000	0.9100000
24928.0000000	0.9150000
24929.0000000	0.9100000
24930.0000000	0.9000000
24931.0000000	0.9000000
24932.0000000	0.8900000
24933.0000000	0.8950000
24934.0000000	0.8900000
24935.0000000	0.8900000
24936.0000000	0.8850000
24937.0000000	0.8850000
24938.0000000	0.8750000
24939.0000000	0.8800000
24940.0000000	0.8650000
24941.0000000	0.8700000

24942.0000000	0.8650000
24943.0000000	0.8700000
24944.0000000	0.8700000
24945.0000000	0.8750000
24946.0000000	0.8800000
24947.0000000	0.8900000
24948.0000000	0.8950000
24949.0000000	0.8999999
24950.0000000	0.9000000
24951.0000000	0.8950000
24952.0000000	0.9000000
24953.0000000	0.8900000
24954.0000000	0.8900000
24955.0000000	0.8850000
24956.0000000	0.8800000
24957.0000000	0.8850000
24958.0000000	0.8900000
24959.0000000	0.8800000
24960.0000000	0.8850000
24961.0000000	0.8800000
24962.0000000	0.8700000
24963.0000000	0.8700000
24964.0000000	0.8700000
24965.0000000	0.8700000
24966.0000000	0.8750000
24967.0000000	0.8700000
24968.0000000	0.8700000
24969.0000000	0.8700000
24970.0000000	0.8700000
24971.0000000	0.8700000
24972.0000000	0.8700000
24973.0000000	0.8700000
24974.0000000	0.8700000
24975.0000000	0.8650000
24976.0000000	0.8650000
24977.0000000	0.8700000
24978.0000000	0.8700000
24979.0000000	0.8600000
24980.0000000	0.8533333
24981.0000000	0.8550000
24982.0000000	0.8500000
24983.0000000	0.8500000
24984.0000000	0.8500000
24985.0000000	0.8500000
24986.0000000	0.8500000
24987.0000000	0.8500000
24988.0000000	0.8500000
24989.0000000	0.8500000
24990.0000000	0.8550000
24991.0000000	0.8500000
24992.0000000	0.8400000
24993.0000000	0.8400000
24994.0000000	0.8350000
24995.0000000	0.8350000
24996.0000000	0.8350000
24997.0000000	0.8300000
24998.0000000	0.8300000

24999.0000000	0.8250000
25000.0000000	0.8250000
25001.0000000	0.8300000
25002.0000000	0.8300000
25003.0000000	0.8300000
25004.0000000	0.8350000
25005.0000000	0.8400000
25006.0000000	0.8450000
25007.0000000	0.8500000
25008.0000000	0.8450000
25009.0000000	0.8450000
25010.0000000	0.8500001
25011.0000000	0.8450000
25012.0000000	0.8550000
25013.0000000	0.8550000
25014.0000000	0.8550000
25015.0000000	0.8600000
25016.0000000	0.8600000
25017.0000000	0.8600000
25018.0000000	0.8600000
25019.0000000	0.8600000
25020.0000000	0.8700000
25021.0000000	0.8650000
25022.0000000	0.8600000
25023.0000000	0.8600000
25024.0000000	0.8500000
25025.0000000	0.8450000
25026.0000000	0.8400000
25027.0000000	0.8450000
25028.0000000	0.8400000
25029.0000000	0.8400000
25030.0000000	0.8350000
25031.0000000	0.8250000
25032.0000000	0.8250000
25033.0000000	0.8250000
25034.0000000	0.8250000
25035.0000000	0.8300000
25036.0000000	0.8300000
25037.0000000	0.8250000
25038.0000000	0.8350000
25039.0000000	0.8366666
25040.0000000	0.8400000
25041.0000000	0.8400000
25042.0000000	0.8400000
25043.0000000	0.8450000
25044.0000000	0.8500000
25045.0000000	0.8550000
25046.0000000	0.8550000
25047.0000000	0.8600000
25048.0000000	0.8650000
25049.0000000	0.8600000
25050.0000000	0.8650000
25051.0000000	0.8600000
25052.0000000	0.8650000
25053.0000000	0.8600000
25054.0000000	0.8650000
25055.0000000	0.8550000

25056.0000000	0.8500000
25057.0000000	0.8500000
25058.0000000	0.8500000
25059.0000000	0.8500000
25060.0000000	0.8500000
25061.0000000	0.8550000
25062.0000000	0.8550000
25063.0000000	0.8700000
25064.0000000	0.8750000
25065.0000000	0.8850000
25066.0000000	0.8950000
25067.0000000	0.9000000
25068.0000000	0.9050000
25069.0000000	0.9100000
25070.0000000	0.9150000
25071.0000000	0.9100000
25072.0000000	0.9100000
25073.0000000	0.9050000
25074.0000000	0.9000000
25075.0000000	0.9050000
25076.0000000	0.9100000
25077.0000000	0.9100000
25078.0000000	0.9100000
25079.0000000	0.9100000
25080.0000000	0.9200000
25081.0000000	0.9150000
25082.0000000	0.9200000
25083.0000000	0.9200000
25084.0000000	0.9250000
25085.0000000	0.9250000
25086.0000000	0.9200000
25087.0000000	0.9300000
25088.0000000	0.9250000
25089.0000000	0.9200000
25090.0000000	0.9200000
25091.0000000	0.9100000
25092.0000000	0.9000000
25093.0000000	0.9000000
25094.0000000	0.8900000
25095.0000000	0.8900000
25096.0000000	0.8850000
25097.0000000	0.8850000
25098.0000000	0.8800000
25099.0000000	0.8800000
25100.0000000	0.8833334
25101.0000000	0.8850000
25102.0000000	0.8800000
25103.0000000	0.8900000
25104.0000000	0.8800000
25105.0000000	0.8750000
25106.0000000	0.8650000
25107.0000000	0.8600000
25108.0000000	0.8600000
25109.0000000	0.8450000
25110.0000000	0.8400000
25111.0000000	0.8450000
25112.0000000	0.8400000

25113.0000000	0.8400000
25114.0000000	0.8400000
25115.0000000	0.8450000
25116.0000000	0.8450000
25117.0000000	0.8500000
25118.0000000	0.8550000
25119.0000000	0.8600000
25120.0000000	0.8650000
25121.0000000	0.8600000
25122.0000000	0.8700000
25123.0000000	0.8650000
25124.0000000	0.8600000
25125.0000000	0.8600000
25126.0000000	0.8500000
25127.0000000	0.8450000
25128.0000000	0.8400000
25129.0000000	0.8250000
25130.0000000	0.8200000
25131.0000000	0.8166667
25132.0000000	0.8100000
25133.0000000	0.8200000
25134.0000000	0.8150000
25135.0000000	0.8200000
25136.0000000	0.8100000
25137.0000000	0.8150000
25138.0000000	0.8050000
25139.0000000	0.8050000
25140.0000000	0.8050000
25141.0000000	0.8000000
25142.0000000	0.8000000
25143.0000000	0.7850000
25144.0000000	0.7850000
25145.0000000	0.7850000
25146.0000000	0.7800000
25147.0000000	0.7900000
25148.0000000	0.7900000
25149.0000000	0.7900000
25150.0000000	0.7900000
25151.0000000	0.7900000
25152.0000000	0.7850000
25153.0000000	0.7800000
25154.0000000	0.7900000
25155.0000000	0.7900000
25156.0000000	0.7950000
25157.0000000	0.8050000
25158.0000000	0.8100000
25159.0000000	0.8200000
25160.0000000	0.8200000
25161.0000000	0.8233333
25162.0000000	0.8200000
25163.0000000	0.8250000
25164.0000000	0.8250000
25165.0000000	0.8300000
25166.0000000	0.8400000
25167.0000000	0.8450000
25168.0000000	0.8450000
25169.0000000	0.8500000

25170.0000000	0.8500000
25171.0000000	0.8500000
25172.0000000	0.8500000
25173.0000000	0.8600000
25174.0000000	0.8600000
25175.0000000	0.8650000
25176.0000000	0.8700000
25177.0000000	0.8700000
25178.0000000	0.8650000
25179.0000000	0.8600000
25180.0000000	0.8700000
25181.0000000	0.8600000
25182.0000000	0.8650000
25183.0000000	0.8650000
25184.0000000	0.8600000
25185.0000000	0.8600000
25186.0000000	0.8600000
25187.0000000	0.8650000
25188.0000000	0.8550000
25189.0000000	0.8550000
25190.0000000	0.8533333
25191.0000000	0.8450000
25192.0000000	0.8450000
25193.0000000	0.8400000
25194.0000000	0.8300000
25195.0000000	0.8200000
25196.0000000	0.8200000
25197.0000000	0.8100000
25198.0000000	0.8000000
25199.0000000	0.8000000
25200.0000000	0.8000000
25201.0000000	0.8000000
25202.0000000	0.8000000
25203.0000000	0.8100000
25204.0000000	0.8200000
25205.0000000	0.8250000
25206.0000000	0.8250000
25207.0000000	0.8300000
25208.0000000	0.8350000
25209.0000000	0.8400000
25210.0000000	0.8350000
25211.0000000	0.8300000
25212.0000000	0.8200000
25213.0000000	0.8200000
25214.0000000	0.8200000
25215.0000000	0.8200000
25216.0000000	0.8100000
25217.0000000	0.8100000
25218.0000000	0.8150000
25219.0000000	0.8200000
25220.0000000	0.8300000
25221.0000000	0.8300000
25222.0000000	0.8300000
25223.0000000	0.8300000
25224.0000000	0.8400000
25225.0000000	0.8400000
25226.0000000	0.8400000

25227.0000000	0.8500000
25228.0000000	0.8550000
25229.0000000	0.8600000
25230.0000000	0.8600000
25231.0000000	0.8700000
25232.0000000	0.8700000
25233.0000000	0.8750000
25234.0000000	0.8800000
25235.0000000	0.8850000
25236.0000000	0.8850000
25237.0000000	0.8800000
25238.0000000	0.8800000
25239.0000000	0.8800000
25240.0000000	0.8800000
25241.0000000	0.8850000
25242.0000000	0.8900000
25243.0000000	0.8850000
25244.0000000	0.8750000
25245.0000000	0.8750000
25246.0000000	0.8750000
25247.0000000	0.8650000
25248.0000000	0.8600000
25249.0000000	0.8700000
25250.0000000	0.8600000
25251.0000000	0.8600000
25252.0000000	0.8500001
25253.0000000	0.8400000
25254.0000000	0.8400000
25255.0000000	0.8400000
25256.0000000	0.8300000
25257.0000000	0.8400000
25258.0000000	0.8350000
25259.0000000	0.8300000
25260.0000000	0.8300000
25261.0000000	0.8300000
25262.0000000	0.8300000
25263.0000000	0.8300000
25264.0000000	0.8300000
25265.0000000	0.8350000
25266.0000000	0.8300000
25267.0000000	0.8300000
25268.0000000	0.8350000
25269.0000000	0.8300000
25270.0000000	0.8250000
25271.0000000	0.8350000
25272.0000000	0.8300000
25273.0000000	0.8250000
25274.0000000	0.8250000
25275.0000000	0.8200000
25276.0000000	0.8150000
25277.0000000	0.8050000
25278.0000000	0.8000000
25279.0000000	0.7950000
25280.0000000	0.7950000
25281.0000000	0.7900000
25282.0000000	0.7900000
25283.0000000	0.7900000

25284.0000000	0.7850000
25285.0000000	0.7800000
25286.0000000	0.7800000
25287.0000000	0.7800000
25288.0000000	0.7700000
25289.0000000	0.7650000
25290.0000000	0.7600000
25291.0000000	0.7500000
25292.0000000	0.7450000
25293.0000000	0.7350000
25294.0000000	0.7300000
25295.0000000	0.7200000
25296.0000000	0.7200000
25297.0000000	0.7200000
25298.0000000	0.7250000
25299.0000000	0.7350000
25300.0000000	0.7350000
25301.0000000	0.7300000
25302.0000000	0.7300000
25303.0000000	0.7200000
25304.0000000	0.7300000
25305.0000000	0.7450000
25306.0000000	0.7550000
25307.0000000	0.7600000
25308.0000000	0.7600000
25309.0000000	0.7650000
25310.0000000	0.7650000
25311.0000000	0.7600000
25312.0000000	0.7700000
25313.0000000	0.7750000
25314.0000000	0.7750000
25315.0000000	0.7800000
25316.0000000	0.7900000
25317.0000000	0.7850000
25318.0000000	0.7900000
25319.0000000	0.8000000
25320.0000000	0.7950000
25321.0000000	0.8000000
25322.0000000	0.8000000
25323.0000000	0.8050000
25324.0000000	0.8100000
25325.0000000	0.8150000
25326.0000000	0.8200000
25327.0000000	0.8250000
25328.0000000	0.8300000
25329.0000000	0.8300000
25330.0000000	0.8350000
25331.0000000	0.8400000
25332.0000000	0.8400000
25333.0000000	0.8400000
25334.0000000	0.8400000
25335.0000000	0.8500000
25336.0000000	0.8450000
25337.0000000	0.8450000
25338.0000000	0.8350000
25339.0000000	0.8300000
25340.0000000	0.8300000

25341.0000000	0.8266667
25342.0000000	0.8250000
25343.0000000	0.8250000
25344.0000000	0.8200000
25345.0000000	0.8200000
25346.0000000	0.8150000
25347.0000000	0.8100000
25348.0000000	0.8150000
25349.0000000	0.8050000
25350.0000000	0.8000000
25351.0000000	0.8000000
25352.0000000	0.7950000
25353.0000000	0.7800000
25354.0000000	0.7800000
25355.0000000	0.7800000
25356.0000000	0.7700000
25357.0000000	0.7700000
25358.0000000	0.7600000
25359.0000000	0.7500000
25360.0000000	0.7650000
25361.0000000	0.7650000
25362.0000000	0.7700000
25363.0000000	0.7700000
25364.0000000	0.7750000
25365.0000000	0.7700000
25366.0000000	0.7700000
25367.0000000	0.7750000
25368.0000000	0.7700000
25369.0000000	0.7800000
25370.0000000	0.7800000
25371.0000000	0.7900000
25372.0000000	0.7900000
25373.0000000	0.7900000
25374.0000000	0.7950000
25375.0000000	0.7950000
25376.0000000	0.7900000
25377.0000000	0.8000000
25378.0000000	0.8000000
25379.0000000	0.8000000
25380.0000000	0.8000000
25381.0000000	0.7900000
25382.0000000	0.8000000
25383.0000000	0.7950000
25384.0000000	0.7950000
25385.0000000	0.7950000
25386.0000000	0.8000000
25387.0000000	0.8050000
25388.0000000	0.7950000
25389.0000000	0.8000000
25390.0000000	0.8050000
25391.0000000	0.8100000
25392.0000000	0.8200000
25393.0000000	0.8100000
25394.0000000	0.8200000
25395.0000000	0.8200000
25396.0000000	0.8250000
25397.0000000	0.8350000

25398.0000000	0.8300000
25399.0000000	0.8300000
25400.0000000	0.8300000
25401.0000000	0.8250000
25402.0000000	0.8333333
25403.0000000	0.8400000
25404.0000000	0.8450000
25405.0000000	0.8450000
25406.0000000	0.8450000
25407.0000000	0.8450000
25408.0000000	0.8400000
25409.0000000	0.8400000
25410.0000000	0.8450000
25411.0000000	0.8400000
25412.0000000	0.8400000
25413.0000000	0.8400000
25414.0000000	0.8300000
25415.0000000	0.8350000
25416.0000000	0.8350000
25417.0000000	0.8300000
25418.0000000	0.8300000
25419.0000000	0.8300000
25420.0000000	0.8300000
25421.0000000	0.8300000
25422.0000000	0.8400000
25423.0000000	0.8300000
25424.0000000	0.8350000
25425.0000000	0.8350000
25426.0000000	0.8300000
25427.0000000	0.8300000
25428.0000000	0.8300000
25429.0000000	0.8300000
25430.0000000	0.8300000
25431.0000000	0.8400000
25432.0000000	0.8400000
25433.0000000	0.8400000
25434.0000000	0.8400000
25435.0000000	0.8350000
25436.0000000	0.8400000
25437.0000000	0.8450000
25438.0000000	0.8400000
25439.0000000	0.8450000
25440.0000000	0.8400000
25441.0000000	0.8450000
25442.0000000	0.8400000
25443.0000000	0.8350000
25444.0000000	0.8400000
25445.0000000	0.8400000
25446.0000000	0.8350000
25447.0000000	0.8400000
25448.0000000	0.8400000
25449.0000000	0.8300000
25450.0000000	0.8300000
25451.0000000	0.8300000
25452.0000000	0.8250000
25453.0000000	0.8200000
25454.0000000	0.8200000

25455.0000000	0.8150000
25456.0000000	0.8100000
25457.0000000	0.8050000
25458.0000000	0.8000000
25459.0000000	0.7900000
25460.0000000	0.7900000
25461.0000000	0.7950000
25462.0000000	0.8000000
25463.0000000	0.8033333
25464.0000000	0.8100000
25465.0000000	0.8150000
25466.0000000	0.8100000
25467.0000000	0.8100000
25468.0000000	0.8000000
25469.0000000	0.8000000
25470.0000000	0.7950000
25471.0000000	0.7950000
25472.0000000	0.7950000
25473.0000000	0.7850000
25474.0000000	0.7900000
25475.0000000	0.7900000
25476.0000000	0.7900000
25477.0000000	0.8000000
25478.0000000	0.7950000
25479.0000000	0.7900000
25480.0000000	0.7900000
25481.0000000	0.7850000
25482.0000000	0.7750000
25483.0000000	0.7700000
25484.0000000	0.7700000
25485.0000000	0.7700000
25486.0000000	0.7750000
25487.0000000	0.7700000
25488.0000000	0.7700000
25489.0000000	0.7650000
25490.0000000	0.7600000
25491.0000000	0.7700000
25492.0000000	0.7650000
25493.0000000	0.7666667
25494.0000000	0.7650000
25495.0000000	0.7700000
25496.0000000	0.7700000
25497.0000000	0.7750000
25498.0000000	0.7800000
25499.0000000	0.7800000
25500.0000000	0.7800000
25501.0000000	0.7850000
25502.0000000	0.7900000
25503.0000000	0.7900000
25504.0000000	0.8000000
25505.0000000	0.8050000
25506.0000000	0.8050000
25507.0000000	0.8150000
25508.0000000	0.8150000
25509.0000000	0.8200000
25510.0000000	0.8200000
25511.0000000	0.8200000

25512.0000000	0.8300000
25513.0000000	0.8250000
25514.0000000	0.8300000
25515.0000000	0.8300000
25516.0000000	0.8300000
25517.0000000	0.8350000
25518.0000000	0.8400000
25519.0000000	0.8400000
25520.0000000	0.8300000
25521.0000000	0.8333333
25522.0000000	0.8450000
25523.0000000	0.8400000
25524.0000000	0.8400000
25525.0000000	0.8500000
25526.0000000	0.8500000
25527.0000000	0.8600000
25528.0000000	0.8700000
25529.0000000	0.8700000
25530.0000000	0.8750000
25531.0000000	0.8750000
25532.0000000	0.8800000
25533.0000000	0.8800000
25534.0000000	0.8900000
25535.0000000	0.8900000
25536.0000000	0.8950000
25537.0000000	0.9000000
25538.0000000	0.9000000
25539.0000000	0.9000000
25540.0000000	0.9050000
25541.0000000	0.9050000
25542.0000000	0.9000000
25543.0000000	0.9100000
25544.0000000	0.9100000
25545.0000000	0.9150000
25546.0000000	0.9100000
25547.0000000	0.9150000
25548.0000000	0.9200000
25549.0000000	0.9200000
25550.0000000	0.9250000
25551.0000000	0.9233333
25552.0000000	0.9300000
25553.0000000	0.9300000
25554.0000000	0.9300000
25555.0000000	0.9250000
25556.0000000	0.9300000
25557.0000000	0.9300000
25558.0000000	0.9300000
25559.0000000	0.9250000
25560.0000000	0.9300000
25561.0000000	0.9250000
25562.0000000	0.9200000
25563.0000000	0.9200000
25564.0000000	0.9200000
25565.0000000	0.9150000
25566.0000000	0.9100000
25567.0000000	0.9000000
25568.0000000	0.9050000

25569.0000000	0.8950000
25570.0000000	0.8950000
25571.0000000	0.8900000
25572.0000000	0.8950000
25573.0000000	0.8850000
25574.0000000	0.8800000
25575.0000000	0.8800000
25576.0000000	0.8750000
25577.0000000	0.8700000
25578.0000000	0.8700000
25579.0000000	0.8650000
25580.0000000	0.8650000
25581.0000000	0.8600000
25582.0000000	0.8700000
25583.0000000	0.8600000
25584.0000000	0.8633334
25585.0000000	0.8700000
25586.0000000	0.8800000
25587.0000000	0.8800000
25588.0000000	0.8800000
25589.0000000	0.8900000
25590.0000000	0.8950000
25591.0000000	0.9050000
25592.0000000	0.9000000
25593.0000000	0.9050000
25594.0000000	0.9100000
25595.0000000	0.9100000
25596.0000000	0.9100000
25597.0000000	0.9100000
25598.0000000	0.9150000
25599.0000000	0.9200000
25600.0000000	0.9200000
25601.0000000	0.9150000
25602.0000000	0.9100000
25603.0000000	0.9150000
25604.0000000	0.9150000
25605.0000000	0.9200000
25606.0000000	0.9150000
25607.0000000	0.9100000
25608.0000000	0.9100000
25609.0000000	0.9100000
25610.0000000	0.9200000
25611.0000000	0.9150000
25612.0000000	0.9100000
25613.0000000	0.9150000
25614.0000000	0.9133334
25615.0000000	0.9050000
25616.0000000	0.9000000
25617.0000000	0.9100000
25618.0000000	0.9000000
25619.0000000	0.9000000
25620.0000000	0.9050000
25621.0000000	0.9050000
25622.0000000	0.9100000
25623.0000000	0.9150000
25624.0000000	0.9100000
25625.0000000	0.9100000

25626.0000000	0.9150000
25627.0000000	0.9150000
25628.0000000	0.9100000
25629.0000000	0.9100000
25630.0000000	0.9100000
25631.0000000	0.9050000
25632.0000000	0.9050000
25633.0000000	0.9100000
25634.0000000	0.9100000
25635.0000000	0.9150000
25636.0000000	0.9150000
25637.0000000	0.9050000
25638.0000000	0.9050000
25639.0000000	0.9050000
25640.0000000	0.9000000
25641.0000000	0.9000000
25642.0000000	0.8950000
25643.0000000	0.9000000
25644.0000000	0.9033334
25645.0000000	0.9000000
25646.0000000	0.9000000
25647.0000000	0.9000000
25648.0000000	0.9000000
25649.0000000	0.9000000
25650.0000000	0.9000000
25651.0000000	0.9000000
25652.0000000	0.8950000
25653.0000000	0.8950000
25654.0000000	0.8950000
25655.0000000	0.8950000
25656.0000000	0.8900000
25657.0000000	0.8950000
25658.0000000	0.8900000
25659.0000000	0.8950000
25660.0000000	0.8900000
25661.0000000	0.8950000
25662.0000000	0.9000000
25663.0000000	0.8900000
25664.0000000	0.8900000
25665.0000000	0.8900000
25666.0000000	0.8900000
25667.0000000	0.8800000
25668.0000000	0.8850000
25669.0000000	0.8800000
25670.0000000	0.8750000
25671.0000000	0.8800000
25672.0000000	0.8750000
25673.0000000	0.8750000
25674.0000000	0.8750000
25675.0000000	0.8799999
25676.0000000	0.8850000
25677.0000000	0.8850000
25678.0000000	0.8750000
25679.0000000	0.8800000
25680.0000000	0.8800000
25681.0000000	0.8800000
25682.0000000	0.8800000

25683.0000000	0.8850000
25684.0000000	0.8800000
25685.0000000	0.8800000
25686.0000000	0.8900000
25687.0000000	0.8800000
25688.0000000	0.8800000
25689.0000000	0.8800000
25690.0000000	0.8800000
25691.0000000	0.8800000
25692.0000000	0.8750000
25693.0000000	0.8750000
25694.0000000	0.8700000
25695.0000000	0.8650000
25696.0000000	0.8650000
25697.0000000	0.8600000
25698.0000000	0.8500000
25699.0000000	0.8500000
25700.0000000	0.8500000
25701.0000000	0.8400000
25702.0000000	0.8400000
25703.0000000	0.8400000
25704.0000000	0.8400000
25705.0000000	0.8350000
25706.0000000	0.8300000
25707.0000000	0.8350000
25708.0000000	0.8350000
25709.0000000	0.8400000
25710.0000000	0.8350000
25711.0000000	0.8350000
25712.0000000	0.8350000
25713.0000000	0.8400000
25714.0000000	0.8400000
25715.0000000	0.8450000
25716.0000000	0.8550000
25717.0000000	0.8500000
25718.0000000	0.8550000
25719.0000000	0.8550000
25720.0000000	0.8500000
25721.0000000	0.8450000
25722.0000000	0.8400000
25723.0000000	0.8350000
25724.0000000	0.8400000
25725.0000000	0.8350000
25726.0000000	0.8400000
25727.0000000	0.8400000
25728.0000000	0.8400000
25729.0000000	0.8350000
25730.0000000	0.8350000
25731.0000000	0.8300000
25732.0000000	0.8400000
25733.0000000	0.8300000
25734.0000000	0.8400000
25735.0000000	0.8400000
25736.0000000	0.8400000
25737.0000000	0.8350000
25738.0000000	0.8400000
25739.0000000	0.8350000

25740.0000000	0.8350000
25741.0000000	0.8300000
25742.0000000	0.8300000
25743.0000000	0.8400000
25744.0000000	0.8300000
25745.0000000	0.8350000
25746.0000000	0.8300000
25747.0000000	0.8300000
25748.0000000	0.8300000
25749.0000000	0.8200000
25750.0000000	0.8250000
25751.0000000	0.8200000
25752.0000000	0.8200000
25753.0000000	0.8250000
25754.0000000	0.8200000
25755.0000000	0.8200000
25756.0000000	0.8200000
25757.0000000	0.8200000
25758.0000000	0.8250000
25759.0000000	0.8250000
25760.0000000	0.8200000
25761.0000000	0.8250000
25762.0000000	0.8200000
25763.0000000	0.8150000
25764.0000000	0.8250000
25765.0000000	0.8166667
25766.0000000	0.8200000
25767.0000000	0.8250000
25768.0000000	0.8350000
25769.0000000	0.8300000
25770.0000000	0.8300000
25771.0000000	0.8400000
25772.0000000	0.8400000
25773.0000000	0.8450000
25774.0000000	0.8500000
25775.0000000	0.8550000
25776.0000000	0.8600000
25777.0000000	0.8650000
25778.0000000	0.8700000
25779.0000000	0.8750000
25780.0000000	0.8850000
25781.0000000	0.8900000
25782.0000000	0.8850000
25783.0000000	0.8850000
25784.0000000	0.8750000
25785.0000000	0.8700000
25786.0000000	0.8650000
25787.0000000	0.8550000
25788.0000000	0.8550000
25789.0000000	0.8400000
25790.0000000	0.8500000
25791.0000000	0.8350000
25792.0000000	0.8350000
25793.0000000	0.8300000
25794.0000000	0.8350000
25795.0000000	0.8400000
25796.0000000	0.8450000

25797.0000000	0.8450000
25798.0000000	0.8500000
25799.0000000	0.8500000
25800.0000000	0.8500000
25801.0000000	0.8600000
25802.0000000	0.8600000
25803.0000000	0.8650000
25804.0000000	0.8700000
25805.0000000	0.8750000
25806.0000000	0.8800000
25807.0000000	0.8850000
25808.0000000	0.8800000
25809.0000000	0.8900000
25810.0000000	0.8900000
25811.0000000	0.8850000
25812.0000000	0.8900000
25813.0000000	0.8850000
25814.0000000	0.8850000
25815.0000000	0.8900000
25816.0000000	0.8850000
25817.0000000	0.8800000
25818.0000000	0.8700000
25819.0000000	0.8700000
25820.0000000	0.8600000
25821.0000000	0.8700000
25822.0000000	0.8650000
25823.0000000	0.8600000
25824.0000000	0.8650000
25825.0000000	0.8650000
25826.0000000	0.8666666
25827.0000000	0.8650000
25828.0000000	0.8700000
25829.0000000	0.8600000
25830.0000000	0.8650000
25831.0000000	0.8700000
25832.0000000	0.8650000
25833.0000000	0.8650000
25834.0000000	0.8700000
25835.0000000	0.8600000
25836.0000000	0.8650000
25837.0000000	0.8700000
25838.0000000	0.8700000
25839.0000000	0.8650000
25840.0000000	0.8750000
25841.0000000	0.8700000
25842.0000000	0.8800000
25843.0000000	0.8800000
25844.0000000	0.8900000
25845.0000000	0.8850000
25846.0000000	0.8800000
25847.0000000	0.8850000
25848.0000000	0.8900000
25849.0000000	0.8850000
25850.0000000	0.8850000
25851.0000000	0.8950000
25852.0000000	0.8950000
25853.0000000	0.8900000

25854.0000000	0.9000000
25855.0000000	0.8900000
25856.0000000	0.8999999
25857.0000000	0.9050000
25858.0000000	0.9000000
25859.0000000	0.9000000
25860.0000000	0.9050000
25861.0000000	0.9050000
25862.0000000	0.9100000
25863.0000000	0.9100000
25864.0000000	0.9100000
25865.0000000	0.9200000
25866.0000000	0.9250000
25867.0000000	0.9200000
25868.0000000	0.9300000
25869.0000000	0.9350000
25870.0000000	0.9350000
25871.0000000	0.9350000
25872.0000000	0.9400000
25873.0000000	0.9400000
25874.0000000	0.9400000
25875.0000000	0.9450000
25876.0000000	0.9500000
25877.0000000	0.9500000
25878.0000000	0.9450000
25879.0000000	0.9450000
25880.0000000	0.9450000
25881.0000000	0.9500000
25882.0000000	0.9500000
25883.0000000	0.9500000
25884.0000000	0.9500000
25885.0000000	0.9500000
25886.0000000	0.9566666
25887.0000000	0.9600000
25888.0000000	0.9600000
25889.0000000	0.9700000
25890.0000000	0.9650000
25891.0000000	0.9700000
25892.0000000	0.9800000
25893.0000000	0.9800000
25894.0000000	0.9750000
25895.0000000	0.9750000
25896.0000000	0.9750000
25897.0000000	0.9800000
25898.0000000	0.9900000
25899.0000000	0.9950000
25900.0000000	0.9900000
25901.0000000	0.9950000
25902.0000000	0.9950000
25903.0000000	0.9950000
25904.0000000	0.9950000
25905.0000000	1.0000000
25906.0000000	1.0000000
25907.0000000	1.0100000
25908.0000000	1.0100000
25909.0000000	1.0100000
25910.0000000	1.0100000

25911.0000000	1.0100000
25912.0000000	1.0200000
25913.0000000	1.0250000
25914.0000000	1.0200000
25915.0000000	1.0200000
25916.0000000	1.0200000
25917.0000000	1.0250000
25918.0000000	1.0200000
25919.0000000	1.0250000
25920.0000000	1.0200000
25921.0000000	1.0200000
25922.0000000	1.0200000
25923.0000000	1.0200000
25924.0000000	1.0200000
25925.0000000	1.0100000
25926.0000000	1.0100000
25927.0000000	1.0050000
25928.0000000	0.9950000
25929.0000000	0.9850000
25930.0000000	0.9800000
25931.0000000	0.9750000
25932.0000000	0.9750000
25933.0000000	0.9650000
25934.0000000	0.9550000
25935.0000000	0.9400000
25936.0000000	0.9350000
25937.0000000	0.9300000
25938.0000000	0.9150000
25939.0000000	0.9050000
25940.0000000	0.8950000
25941.0000000	0.8950000
25942.0000000	0.8800000
25943.0000000	0.8700000
25944.0000000	0.8633334
25945.0000000	0.8550000
25946.0000000	0.8550000
25947.0000000	0.8600000
25948.0000000	0.8550000
25949.0000000	0.8450000
25950.0000000	0.8450000
25951.0000000	0.8450000
25952.0000000	0.8450000
25953.0000000	0.8450000
25954.0000000	0.8400000
25955.0000000	0.8450000
25956.0000000	0.8400000
25957.0000000	0.8450000
25958.0000000	0.8450000
25959.0000000	0.8400000
25960.0000000	0.8400000
25961.0000000	0.8400000
25962.0000000	0.8450000
25963.0000000	0.8500000
25964.0000000	0.8500000
25965.0000000	0.8500000
25966.0000000	0.8500000
25967.0000000	0.8550000

25968.0000000	0.8600000
25969.0000000	0.8650000
25970.0000000	0.8750000
25971.0000000	0.8800000
25972.0000000	0.8850000
25973.0000000	0.8800000
25974.0000000	0.8850000
25975.0000000	0.8866666
25976.0000000	0.8900000
25977.0000000	0.8850000
25978.0000000	0.8800000
25979.0000000	0.8800000
25980.0000000	0.8800000
25981.0000000	0.8800000
25982.0000000	0.8800000
25983.0000000	0.8800000
25984.0000000	0.8850000
25985.0000000	0.8800000
25986.0000000	0.8800000
25987.0000000	0.8800000
25988.0000000	0.8800000
25989.0000000	0.8800000
25990.0000000	0.8800000
25991.0000000	0.8750000
25992.0000000	0.8650000
25993.0000000	0.8600000
25994.0000000	0.8400000
25995.0000000	0.8400000
25996.0000000	0.8300000
25997.0000000	0.8250000
25998.0000000	0.8250000
25999.0000000	0.8250000
26000.0000000	0.8200000
26001.0000000	0.8300000
26002.0000000	0.8250000
26003.0000000	0.8300000
26004.0000000	0.8300000
26005.0000000	0.8300000
26006.0000000	0.8333333
26007.0000000	0.8350000
26008.0000000	0.8400000
26009.0000000	0.8400000
26010.0000000	0.8400000
26011.0000000	0.8400000
26012.0000000	0.8400000
26013.0000000	0.8450000
26014.0000000	0.8350000
26015.0000000	0.8300000
26016.0000000	0.8300000
26017.0000000	0.8250000
26018.0000000	0.8250000
26019.0000000	0.8150000
26020.0000000	0.8150000
26021.0000000	0.8000000
26022.0000000	0.8100000
26023.0000000	0.8100000
26024.0000000	0.8100000

26025.0000000	0.8100000
26026.0000000	0.8100000
26027.0000000	0.8050000
26028.0000000	0.8050000
26029.0000000	0.8150000
26030.0000000	0.8100000
26031.0000000	0.8100000
26032.0000000	0.8100000
26033.0000000	0.8150000
26034.0000000	0.8150000
26035.0000000	0.8200000
26036.0000000	0.8200000
26037.0000000	0.8200000
26038.0000000	0.8300000
26039.0000000	0.8250000
26040.0000000	0.8250000
26041.0000000	0.8300000
26042.0000000	0.8350000
26043.0000000	0.8300000
26044.0000000	0.8300000
26045.0000000	0.8300000
26046.0000000	0.8300000
26047.0000000	0.8300000
26048.0000000	0.8300000
26049.0000000	0.8400000
26050.0000000	0.8400000
26051.0000000	0.8400000
26052.0000000	0.8450000
26053.0000000	0.8400000
26054.0000000	0.8450000
26055.0000000	0.8500000
26056.0000000	0.8500000
26057.0000000	0.8500000
26058.0000000	0.8450000
26059.0000000	0.8450000
26060.0000000	0.8400000
26061.0000000	0.8400000
26062.0000000	0.8400000
26063.0000000	0.8400000
26064.0000000	0.8350000
26065.0000000	0.8300000
26066.0000000	0.8366666
26067.0000000	0.8300000
26068.0000000	0.8150000
26069.0000000	0.8050000
26070.0000000	0.7950000
26071.0000000	0.7700000
26072.0000000	0.7650000
26073.0000000	0.7600000
26074.0000000	0.7600000
26075.0000000	0.7500000
26076.0000000	0.7500000
26077.0000000	0.7600000
26078.0000000	0.7550000
26079.0000000	0.7550000
26080.0000000	0.7500000
26081.0000000	0.7450000

26082.0000000	0.7500000
26083.0000000	0.7450000
26084.0000000	0.7550000
26085.0000000	0.7500000
26086.0000000	0.7550000
26087.0000000	0.7600000
26088.0000000	0.7600000
26089.0000000	0.7600000
26090.0000000	0.7700000
26091.0000000	0.7700000
26092.0000000	0.7700000
26093.0000000	0.7850000
26094.0000000	0.7800000
26095.0000000	0.7900000
26096.0000000	0.7933334
26097.0000000	0.7950000
26098.0000000	0.8000000
26099.0000000	0.8000000
26100.0000000	0.8000000
26101.0000000	0.7950000
26102.0000000	0.7950000
26103.0000000	0.7950000
26104.0000000	0.8050000
26105.0000000	0.8100000
26106.0000000	0.8100000
26107.0000000	0.8100000
26108.0000000	0.8100000
26109.0000000	0.8050000
26110.0000000	0.8050000
26111.0000000	0.8050000
26112.0000000	0.8000000
26113.0000000	0.8000000
26114.0000000	0.7950000
26115.0000000	0.7950000
26116.0000000	0.7900000
26117.0000000	0.7900000
26118.0000000	0.7900000
26119.0000000	0.7900000
26120.0000000	0.7950000
26121.0000000	0.7950000
26122.0000000	0.7900000
26123.0000000	0.7900000
26124.0000000	0.7850000
26125.0000000	0.7800000
26126.0000000	0.7800000
26127.0000000	0.7900000
26128.0000000	0.7950000
26129.0000000	0.8000000
26130.0000000	0.7900000
26131.0000000	0.8050000
26132.0000000	0.8100000
26133.0000000	0.8150000
26134.0000000	0.8150000
26135.0000000	0.8200000
26136.0000000	0.8200000
26137.0000000	0.8100000
26138.0000000	0.8100000

26139.0000000	0.8100000
26140.0000000	0.8050000
26141.0000000	0.8000000
26142.0000000	0.8000000
26143.0000000	0.8000000
26144.0000000	0.7850000
26145.0000000	0.7900000
26146.0000000	0.7850000
26147.0000000	0.7800000
26148.0000000	0.7750000
26149.0000000	0.7750000
26150.0000000	0.7750000
26151.0000000	0.7700000
26152.0000000	0.7800000
26153.0000000	0.7700000
26154.0000000	0.7700000
26155.0000000	0.7650000
26156.0000000	0.7600000
26157.0000000	0.7600000
26158.0000000	0.7600000
26159.0000000	0.7600000
26160.0000000	0.7600000
26161.0000000	0.7550000
26162.0000000	0.7550000
26163.0000000	0.7550000
26164.0000000	0.7450000
26165.0000000	0.7450000
26166.0000000	0.7450000
26167.0000000	0.7400000
26168.0000000	0.7300000
26169.0000000	0.7300000
26170.0000000	0.7250000
26171.0000000	0.7200000
26172.0000000	0.7000000
26173.0000000	0.6950000
26174.0000000	0.6950000
26175.0000000	0.6800000
26176.0000000	0.6800000
26177.0000000	0.6750000
26178.0000000	0.6800000
26179.0000000	0.6700000
26180.0000000	0.6700000
26181.0000000	0.6750000
26182.0000000	0.6750000
26183.0000000	0.6700000
26184.0000000	0.6750000
26185.0000000	0.6700000
26186.0000000	0.6700000
26187.0000000	0.6733333
26188.0000000	0.6800000
26189.0000000	0.6700000
26190.0000000	0.6800000
26191.0000000	0.6700000
26192.0000000	0.6750000
26193.0000000	0.6700000
26194.0000000	0.6700000
26195.0000000	0.6700000

26196.0000000	0.6800000
26197.0000000	0.6800000
26198.0000000	0.6700000
26199.0000000	0.6750000
26200.0000000	0.6700000
26201.0000000	0.6700000
26202.0000000	0.6700000
26203.0000000	0.6700000
26204.0000000	0.6800000
26205.0000000	0.6800000
26206.0000000	0.6800000
26207.0000000	0.6950000
26208.0000000	0.6900000
26209.0000000	0.6900000
26210.0000000	0.6950000
26211.0000000	0.7000000
26212.0000000	0.7000000
26213.0000000	0.7050000
26214.0000000	0.7100000
26215.0000000	0.7200000
26216.0000000	0.7200000
26217.0000000	0.7200000
26218.0000000	0.7266667
26219.0000000	0.7250000
26220.0000000	0.7300000
26221.0000000	0.7400000
26222.0000000	0.7400000
26223.0000000	0.7400000
26224.0000000	0.7500000
26225.0000000	0.7400000
26226.0000000	0.7500000
26227.0000000	0.7500000
26228.0000000	0.7550000
26229.0000000	0.7600000
26230.0000000	0.7600000
26231.0000000	0.7600000
26232.0000000	0.7550000
26233.0000000	0.7450000
26234.0000000	0.7450000
26235.0000000	0.7300000
26236.0000000	0.7200000
26237.0000000	0.7200000
26238.0000000	0.7250000
26239.0000000	0.7250000
26240.0000000	0.7300000
26241.0000000	0.7250000
26242.0000000	0.7200000
26243.0000000	0.7200000
26244.0000000	0.7200000
26245.0000000	0.7200000
26246.0000000	0.7200000
26247.0000000	0.7200000
26248.0000000	0.7166667
26249.0000000	0.7250000
26250.0000000	0.7200000
26251.0000000	0.7250000
26252.0000000	0.7250000

26253.0000000	0.7300000
26254.0000000	0.7300000
26255.0000000	0.7300000
26256.0000000	0.7400000
26257.0000000	0.7400000
26258.0000000	0.7450000
26259.0000000	0.7450000
26260.0000000	0.7500000
26261.0000000	0.7600000
26262.0000000	0.7600000
26263.0000000	0.7600000
26264.0000000	0.7600000
26265.0000000	0.7650000
26266.0000000	0.7600000
26267.0000000	0.7600000
26268.0000000	0.7600000
26269.0000000	0.7500000
26270.0000000	0.7500000
26271.0000000	0.7400000
26272.0000000	0.7300000
26273.0000000	0.7300000
26274.0000000	0.7200000
26275.0000000	0.7350000
26276.0000000	0.7233334
26277.0000000	0.7200000
26278.0000000	0.7233334
26279.0000000	0.7300000
26280.0000000	0.7200000
26281.0000000	0.7250000
26282.0000000	0.7200000
26283.0000000	0.7200000
26284.0000000	0.7200000
26285.0000000	0.7100000
26286.0000000	0.7150000
26287.0000000	0.7150000
26288.0000000	0.7200000
26289.0000000	0.7100000
26290.0000000	0.7000000
26291.0000000	0.7000000
26292.0000000	0.7000000
26293.0000000	0.7000000
26294.0000000	0.7000000
26295.0000000	0.6950000
26296.0000000	0.7000000
26297.0000000	0.6900000
26298.0000000	0.6950000
26299.0000000	0.6950000
26300.0000000	0.6850000
26301.0000000	0.7000000
26302.0000000	0.7000000
26303.0000000	0.7050000
26304.0000000	0.7100000
26305.0000000	0.7150000
26306.0000000	0.7100000
26307.0000000	0.7200000
26308.0000000	0.7233334
26309.0000000	0.7250000

26310.0000000	0.7250000
26311.0000000	0.7350000
26312.0000000	0.7400000
26313.0000000	0.7400000
26314.0000000	0.7400000
26315.0000000	0.7400000
26316.0000000	0.7400000
26317.0000000	0.7450000
26318.0000000	0.7450000
26319.0000000	0.7400000
26320.0000000	0.7450000
26321.0000000	0.7450000
26322.0000000	0.7400000
26323.0000000	0.7450000
26324.0000000	0.7400000
26325.0000000	0.7450000
26326.0000000	0.7400000
26327.0000000	0.7400000
26328.0000000	0.7400000
26329.0000000	0.7450000
26330.0000000	0.7350000
26331.0000000	0.7450000
26332.0000000	0.7400000
26333.0000000	0.7450000
26334.0000000	0.7450000
26335.0000000	0.7500000
26336.0000000	0.7550000
26337.0000000	0.7533333
26338.0000000	0.7600000
26339.0000000	0.7566667
26340.0000000	0.7600000
26341.0000000	0.7600000
26342.0000000	0.7700000
26343.0000000	0.7650000
26344.0000000	0.7700000
26345.0000000	0.7700000
26346.0000000	0.7700000
26347.0000000	0.7650000
26348.0000000	0.7600000
26349.0000000	0.7600000
26350.0000000	0.7600000
26351.0000000	0.7550000
26352.0000000	0.7600000
26353.0000000	0.7650000
26354.0000000	0.7650000
26355.0000000	0.7700000
26356.0000000	0.7700000
26357.0000000	0.7750000
26358.0000000	0.7800000
26359.0000000	0.7850000
26360.0000000	0.7800000
26361.0000000	0.7850000
26362.0000000	0.7800000
26363.0000000	0.7850000
26364.0000000	0.7750000
26365.0000000	0.7700000
26366.0000000	0.7600000

26367.0000000	0.7600000
26368.0000000	0.7666667
26369.0000000	0.7700000
26370.0000000	0.7700000
26371.0000000	0.7700000
26372.0000000	0.7750000
26373.0000000	0.7800000
26374.0000000	0.7800000
26375.0000000	0.7800000
26376.0000000	0.7800000
26377.0000000	0.7800000
26378.0000000	0.7850000
26379.0000000	0.7900000
26380.0000000	0.7950000
26381.0000000	0.7900000
26382.0000000	0.7950000
26383.0000000	0.7950000
26384.0000000	0.8000000
26385.0000000	0.8000000
26386.0000000	0.8100000
26387.0000000	0.8150000
26388.0000000	0.8150000
26389.0000000	0.8200000
26390.0000000	0.8300000
26391.0000000	0.8250000
26392.0000000	0.8300000
26393.0000000	0.8300000
26394.0000000	0.8300000
26395.0000000	0.8350000
26396.0000000	0.8350000
26397.0000000	0.8350000
26398.0000000	0.8333333
26399.0000000	0.8300000
26400.0000000	0.8250000
26401.0000000	0.8300000
26402.0000000	0.8350000
26403.0000000	0.8300000
26404.0000000	0.8350000
26405.0000000	0.8400000
26406.0000000	0.8400000
26407.0000000	0.8400000
26408.0000000	0.8450000
26409.0000000	0.8500000
26410.0000000	0.8550000
26411.0000000	0.8600000
26412.0000000	0.8550000
26413.0000000	0.8600000
26414.0000000	0.8600000
26415.0000000	0.8550000
26416.0000000	0.8600000
26417.0000000	0.8550000
26418.0000000	0.8600000
26419.0000000	0.8550000
26420.0000000	0.8550000
26421.0000000	0.8550000
26422.0000000	0.8600000
26423.0000000	0.8600000

26424.0000000	0.8600000
26425.0000000	0.8550000
26426.0000000	0.8500000
26427.0000000	0.8500000
26428.0000000	0.8466666
26429.0000000	0.8500000
26430.0000000	0.8450000
26431.0000000	0.8450000
26432.0000000	0.8450000
26433.0000000	0.8500000
26434.0000000	0.8500000
26435.0000000	0.8400000
26436.0000000	0.8400000
26437.0000000	0.8350000
26438.0000000	0.8300000
26439.0000000	0.8250000
26440.0000000	0.8100000
26441.0000000	0.8100000
26442.0000000	0.8000000
26443.0000000	0.8000000
26444.0000000	0.8000000
26445.0000000	0.7950000
26446.0000000	0.7900000
26447.0000000	0.7900000
26448.0000000	0.7900000
26449.0000000	0.7800000
26450.0000000	0.7750000
26451.0000000	0.7700000
26452.0000000	0.7600000
26453.0000000	0.7550000
26454.0000000	0.7550000
26455.0000000	0.7550000
26456.0000000	0.7500000
26457.0000000	0.7400000
26458.0000000	0.7400000
26459.0000000	0.7400000
26460.0000000	0.7450000
26461.0000000	0.7400000
26462.0000000	0.7400000
26463.0000000	0.7400000
26464.0000000	0.7350000
26465.0000000	0.7300000
26466.0000000	0.7300000
26467.0000000	0.7300000
26468.0000000	0.7350000
26469.0000000	0.7350000
26470.0000000	0.7350000
26471.0000000	0.7450000
26472.0000000	0.7500000
26473.0000000	0.7500000
26474.0000000	0.7550000
26475.0000000	0.7600000
26476.0000000	0.7700000
26477.0000000	0.7750000
26478.0000000	0.7800000
26479.0000000	0.7850000
26480.0000000	0.7850000

26481.0000000	0.7900000
26482.0000000	0.7900000
26483.0000000	0.7900000
26484.0000000	0.7900000
26485.0000000	0.7950000
26486.0000000	0.7900000
26487.0000000	0.8000000
26488.0000000	0.8000000
26489.0000000	0.8066667
26490.0000000	0.8150000
26491.0000000	0.8150000
26492.0000000	0.8100000
26493.0000000	0.8200000
26494.0000000	0.8200000
26495.0000000	0.8200000
26496.0000000	0.8300000
26497.0000000	0.8300000
26498.0000000	0.8250000
26499.0000000	0.8300000
26500.0000000	0.8250000
26501.0000000	0.8250000
26502.0000000	0.8200000
26503.0000000	0.8200000
26504.0000000	0.8200000
26505.0000000	0.8100000
26506.0000000	0.8150000
26507.0000000	0.8150000
26508.0000000	0.8200000
26509.0000000	0.8300000
26510.0000000	0.8300000
26511.0000000	0.8350000
26512.0000000	0.8350000
26513.0000000	0.8350000
26514.0000000	0.8400000
26515.0000000	0.8450000
26516.0000000	0.8450000
26517.0000000	0.8500000
26518.0000000	0.8600000
26519.0000000	0.8600000
26520.0000000	0.8650000
26521.0000000	0.8650000
26522.0000000	0.8750000
26523.0000000	0.8700000
26524.0000000	0.8700000
26525.0000000	0.8750000
26526.0000000	0.8750000
26527.0000000	0.8750000
26528.0000000	0.8800000
26529.0000000	0.8850000
26530.0000000	0.8800000
26531.0000000	0.8850000
26532.0000000	0.8900000
26533.0000000	0.8900000
26534.0000000	0.8950000
26535.0000000	0.8900000
26536.0000000	0.8950000
26537.0000000	0.8950000

26538.0000000	0.9000000
26539.0000000	0.8950000
26540.0000000	0.9050000
26541.0000000	0.9050000
26542.0000000	0.9000000
26543.0000000	0.9000000
26544.0000000	0.9000000
26545.0000000	0.9000000
26546.0000000	0.8900000
26547.0000000	0.8950000
26548.0000000	0.8950000
26549.0000000	0.8950000
26550.0000000	0.8900000
26551.0000000	0.8900000
26552.0000000	0.8900000
26553.0000000	0.8950000
26554.0000000	0.8900000
26555.0000000	0.8900000
26556.0000000	0.8900000
26557.0000000	0.8800000
26558.0000000	0.8800000
26559.0000000	0.8800000
26560.0000000	0.8800000
26561.0000000	0.8800000
26562.0000000	0.8750000
26563.0000000	0.8750000
26564.0000000	0.8750000
26565.0000000	0.8800000
26566.0000000	0.8800000
26567.0000000	0.8850000
26568.0000000	0.8900000
26569.0000000	0.8900000
26570.0000000	0.8900000
26571.0000000	0.8950000
26572.0000000	0.8950000
26573.0000000	0.9000000
26574.0000000	0.9000000
26575.0000000	0.9050000
26576.0000000	0.9050000
26577.0000000	0.9050000
26578.0000000	0.9050000
26579.0000000	0.9100000
26580.0000000	0.9150000
26581.0000000	0.9100000
26582.0000000	0.9150000
26583.0000000	0.9150000
26584.0000000	0.9100000
26585.0000000	0.9050000
26586.0000000	0.9050000
26587.0000000	0.9000000
26588.0000000	0.9000000
26589.0000000	0.8900000
26590.0000000	0.8900000
26591.0000000	0.8950000
26592.0000000	0.8850000
26593.0000000	0.8850000
26594.0000000	0.8900000

26595.0000000	0.8950000
26596.0000000	0.8800000
26597.0000000	0.8800000
26598.0000000	0.8850000
26599.0000000	0.8850000
26600.0000000	0.8800000
26601.0000000	0.8800000
26602.0000000	0.8750000
26603.0000000	0.8700000
26604.0000000	0.8650000
26605.0000000	0.8600000
26606.0000000	0.8650000
26607.0000000	0.8600000
26608.0000000	0.8600000
26609.0000000	0.8633334
26610.0000000	0.8500000
26611.0000000	0.8500000
26612.0000000	0.8550000
26613.0000000	0.8450000
26614.0000000	0.8500000
26615.0000000	0.8500000
26616.0000000	0.8400000
26617.0000000	0.8450000
26618.0000000	0.8450000
26619.0000000	0.8400000
26620.0000000	0.8400000
26621.0000000	0.8350000
26622.0000000	0.8250000
26623.0000000	0.8350000
26624.0000000	0.8350000
26625.0000000	0.8400000
26626.0000000	0.8550000
26627.0000000	0.8450000
26628.0000000	0.8450000
26629.0000000	0.8450000
26630.0000000	0.8500000
26631.0000000	0.8600000
26632.0000000	0.8500000
26633.0000000	0.8600000
26634.0000000	0.8600000
26635.0000000	0.8650000
26636.0000000	0.8700000
26637.0000000	0.8600000
26638.0000000	0.8650000
26639.0000000	0.8666666
26640.0000000	0.8750000
26641.0000000	0.8700000
26642.0000000	0.8700000
26643.0000000	0.8750000
26644.0000000	0.8750000
26645.0000000	0.8800000
26646.0000000	0.8750000
26647.0000000	0.8700000
26648.0000000	0.8600000
26649.0000000	0.8600000
26650.0000000	0.8600000
26651.0000000	0.8600000

26652.0000000	0.8500000
26653.0000000	0.8500000
26654.0000000	0.8450000
26655.0000000	0.8350000
26656.0000000	0.8350000
26657.0000000	0.8250000
26658.0000000	0.8200000
26659.0000000	0.8200000
26660.0000000	0.8100000
26661.0000000	0.8050000
26662.0000000	0.8050000
26663.0000000	0.8050000
26664.0000000	0.7950000
26665.0000000	0.7950000
26666.0000000	0.7850000
26667.0000000	0.7700000
26668.0000000	0.7700000
26669.0000000	0.7600000
26670.0000000	0.7600000
26671.0000000	0.7533333
26672.0000000	0.7450000
26673.0000000	0.7550000
26674.0000000	0.7600000
26675.0000000	0.7700000
26676.0000000	0.7750000
26677.0000000	0.7800000
26678.0000000	0.7800000
26679.0000000	0.7850000
26680.0000000	0.7850000
26681.0000000	0.7950000
26682.0000000	0.7900000
26683.0000000	0.8000000
26684.0000000	0.8050000
26685.0000000	0.8050000
26686.0000000	0.8100000
26687.0000000	0.8200000
26688.0000000	0.8250000
26689.0000000	0.8300000
26690.0000000	0.8400000
26691.0000000	0.8400000
26692.0000000	0.8500000
26693.0000000	0.8500000
26694.0000000	0.8550000
26695.0000000	0.8650000
26696.0000000	0.8650000
26697.0000000	0.8600000
26698.0000000	0.8600000
26699.0000000	0.8600000
26700.0000000	0.8650000
26701.0000000	0.8650000
26702.0000000	0.8700000
26703.0000000	0.8700000
26704.0000000	0.8700000
26705.0000000	0.8850000
26706.0000000	0.8800000
26707.0000000	0.8800000
26708.0000000	0.8900000

26709.0000000	0.8900000
26710.0000000	0.9050000
26711.0000000	0.9000000
26712.0000000	0.9000000
26713.0000000	0.8950000
26714.0000000	0.8950000
26715.0000000	0.8950000
26716.0000000	0.8950000
26717.0000000	0.8900000
26718.0000000	0.8800000
26719.0000000	0.8800000
26720.0000000	0.8850000
26721.0000000	0.8700000
26722.0000000	0.8650000
26723.0000000	0.8650000
26724.0000000	0.8600000
26725.0000000	0.8600000
26726.0000000	0.8550000
26727.0000000	0.8500000
26728.0000000	0.8500000
26729.0000000	0.8500000
26730.0000000	0.8500000
26731.0000000	0.8450000
26732.0000000	0.8433333
26733.0000000	0.8500000
26734.0000000	0.8450000
26735.0000000	0.8500000
26736.0000000	0.8550000
26737.0000000	0.8500000
26738.0000000	0.8600000
26739.0000000	0.8600000
26740.0000000	0.8600000
26741.0000000	0.8700000
26742.0000000	0.8650000
26743.0000000	0.8700000
26744.0000000	0.8700000
26745.0000000	0.8700000
26746.0000000	0.8700000
26747.0000000	0.8650000
26748.0000000	0.8750000
26749.0000000	0.8750000
26750.0000000	0.8850000
26751.0000000	0.8800000
26752.0000000	0.8850000
26753.0000000	0.8900000
26754.0000000	0.8950000
26755.0000000	0.8950000
26756.0000000	0.9000000
26757.0000000	0.9050000
26758.0000000	0.9100000
26759.0000000	0.9050000
26760.0000000	0.9150000
26761.0000000	0.9100000
26762.0000000	0.9100000
26763.0000000	0.9150000
26764.0000000	0.9200000
26765.0000000	0.9100000

26766.0000000	0.9150000
26767.0000000	0.9200000
26768.0000000	0.9150000
26769.0000000	0.9200000
26770.0000000	0.9150000
26771.0000000	0.9150000
26772.0000000	0.9150000
26773.0000000	0.9150000
26774.0000000	0.9100000
26775.0000000	0.9100000
26776.0000000	0.9050000
26777.0000000	0.9000000
26778.0000000	0.8950000
26779.0000000	0.8850000
26780.0000000	0.8850000
26781.0000000	0.8800000
26782.0000000	0.8800000
26783.0000000	0.8700000
26784.0000000	0.8700000
26785.0000000	0.8600000
26786.0000000	0.8550000
26787.0000000	0.8450000
26788.0000000	0.8500000
26789.0000000	0.8450000
26790.0000000	0.8400000
26791.0000000	0.8400000
26792.0000000	0.8400000
26793.0000000	0.8400000
26794.0000000	0.8400000
26795.0000000	0.8400000
26796.0000000	0.8400000
26797.0000000	0.8400000
26798.0000000	0.8400000
26799.0000000	0.8400000
26800.0000000	0.8500000
26801.0000000	0.8450000
26802.0000000	0.8550000
26803.0000000	0.8500000
26804.0000000	0.8550000
26805.0000000	0.8550000
26806.0000000	0.8600000
26807.0000000	0.8600000
26808.0000000	0.8650000
26809.0000000	0.8550000
26810.0000000	0.8550000
26811.0000000	0.8500000
26812.0000000	0.8450000
26813.0000000	0.8350000
26814.0000000	0.8200000
26815.0000000	0.8100000
26816.0000000	0.7900000
26817.0000000	0.7900000
26818.0000000	0.7750000
26819.0000000	0.7700000
26820.0000000	0.7650000
26821.0000000	0.7633333
26822.0000000	0.7500000

26823.0000000	0.7500000
26824.0000000	0.7500000
26825.0000000	0.7500000
26826.0000000	0.7500000
26827.0000000	0.7550000
26828.0000000	0.7550000
26829.0000000	0.7600000
26830.0000000	0.7600000
26831.0000000	0.7650000
26832.0000000	0.7600000
26833.0000000	0.7700000
26834.0000000	0.7600000
26835.0000000	0.7700000
26836.0000000	0.7700000
26837.0000000	0.7650000
26838.0000000	0.7700000
26839.0000000	0.7600000
26840.0000000	0.7700000
26841.0000000	0.7700000
26842.0000000	0.7650000
26843.0000000	0.7600000
26844.0000000	0.7600000
26845.0000000	0.7650000
26846.0000000	0.7600000
26847.0000000	0.7600000
26848.0000000	0.7650000
26849.0000000	0.7650000
26850.0000000	0.7600000
26851.0000000	0.7600000
26852.0000000	0.7600000
26853.0000000	0.7600000
26854.0000000	0.7600000
26855.0000000	0.7600000
26856.0000000	0.7500000
26857.0000000	0.7500000
26858.0000000	0.7400000
26859.0000000	0.7400000
26860.0000000	0.7400000
26861.0000000	0.7400000
26862.0000000	0.7400000
26863.0000000	0.7300000
26864.0000000	0.7300000
26865.0000000	0.7350000
26866.0000000	0.7300000
26867.0000000	0.7250000
26868.0000000	0.7350000
26869.0000000	0.7300000
26870.0000000	0.7250000
26871.0000000	0.7200000
26872.0000000	0.7100000
26873.0000000	0.7000000
26874.0000000	0.6900000
26875.0000000	0.6800000
26876.0000000	0.6800000
26877.0000000	0.6850000
26878.0000000	0.6850000
26879.0000000	0.7000000

26880.0000000	0.7000000
26881.0000000	0.7000000
26882.0000000	0.7033333
26883.0000000	0.7050000
26884.0000000	0.7050000
26885.0000000	0.7150000
26886.0000000	0.7150000
26887.0000000	0.7250000
26888.0000000	0.7200000
26889.0000000	0.7200000
26890.0000000	0.7200000
26891.0000000	0.7250000
26892.0000000	0.7200000
26893.0000000	0.7300000
26894.0000000	0.7350000
26895.0000000	0.7300000
26896.0000000	0.7300000
26897.0000000	0.7250000
26898.0000000	0.7200000
26899.0000000	0.7250000
26900.0000000	0.7200000
26901.0000000	0.7200000
26902.0000000	0.7150000
26903.0000000	0.7100000
26904.0000000	0.7100000
26905.0000000	0.7000000
26906.0000000	0.6800000
26907.0000000	0.6800000
26908.0000000	0.6750000
26909.0000000	0.6700000
26910.0000000	0.6750000
26911.0000000	0.6800000
26912.0000000	0.6833333
26913.0000000	0.6900000
26914.0000000	0.6900000
26915.0000000	0.6950000
26916.0000000	0.7000000
26917.0000000	0.7000000
26918.0000000	0.6950000
26919.0000000	0.6900000
26920.0000000	0.6950000
26921.0000000	0.6900000
26922.0000000	0.6950000
26923.0000000	0.7000000
26924.0000000	0.7000000
26925.0000000	0.6950000
26926.0000000	0.6850000
26927.0000000	0.6800000
26928.0000000	0.6750000
26929.0000000	0.6700000
26930.0000000	0.6650000
26931.0000000	0.6650000
26932.0000000	0.6500000
26933.0000000	0.6500000
26934.0000000	0.6450000
26935.0000000	0.6500000
26936.0000000	0.6500000

26937.0000000	0.6650000
26938.0000000	0.6600000
26939.0000000	0.6600000
26940.0000000	0.6650000
26941.0000000	0.6650000
26942.0000000	0.6733333
26943.0000000	0.6800000
26944.0000000	0.6900000
26945.0000000	0.6800000
26946.0000000	0.6900000
26947.0000000	0.6900000
26948.0000000	0.6900000
26949.0000000	0.6900000
26950.0000000	0.6850000
26951.0000000	0.6850000
26952.0000000	0.6800000
26953.0000000	0.6800000
26954.0000000	0.6800000
26955.0000000	0.6850000
26956.0000000	0.6800000
26957.0000000	0.6850000
26958.0000000	0.6800000
26959.0000000	0.6800000
26960.0000000	0.6800000
26961.0000000	0.6800000
26962.0000000	0.6800000
26963.0000000	0.6850000
26964.0000000	0.6850000
26965.0000000	0.6800000
26966.0000000	0.6850000
26967.0000000	0.6900000
26968.0000000	0.6900000
26969.0000000	0.6900000
26970.0000000	0.6900000
26971.0000000	0.6950000
26972.0000000	0.6933333
26973.0000000	0.7000000
26974.0000000	0.7000000
26975.0000000	0.6950000
26976.0000000	0.6900000
26977.0000000	0.6900000
26978.0000000	0.6950000
26979.0000000	0.6900000
26980.0000000	0.6950000
26981.0000000	0.6850000
26982.0000000	0.6900000
26983.0000000	0.6900000
26984.0000000	0.6950000
26985.0000000	0.6900000
26986.0000000	0.6900000
26987.0000000	0.6950000
26988.0000000	0.6850000
26989.0000000	0.6800000
26990.0000000	0.6800000
26991.0000000	0.6800000
26992.0000000	0.6750000
26993.0000000	0.6750000

26994.0000000	0.6700000
26995.0000000	0.6700000
26996.0000000	0.6700000
26997.0000000	0.6650000
26998.0000000	0.6650000
26999.0000000	0.6550000
27000.0000000	0.6500000
27001.0000000	0.6466666
27002.0000000	0.6300000
27003.0000000	0.6200000
27004.0000000	0.6200000
27005.0000000	0.6200000
27006.0000000	0.6200000
27007.0000000	0.6150000
27008.0000000	0.6150000
27009.0000000	0.6200000
27010.0000000	0.6200000
27011.0000000	0.6300000
27012.0000000	0.6300000
27013.0000000	0.6350000
27014.0000000	0.6300000
27015.0000000	0.6400000
27016.0000000	0.6350000
27017.0000000	0.6300000
27018.0000000	0.6400000
27019.0000000	0.6450000
27020.0000000	0.6500000
27021.0000000	0.6650000
27022.0000000	0.6650000
27023.0000000	0.6650000
27024.0000000	0.6750000
27025.0000000	0.6750000
27026.0000000	0.6850000
27027.0000000	0.6800000
27028.0000000	0.6850000
27029.0000000	0.6850000
27030.0000000	0.6800000
27031.0000000	0.6800000
27032.0000000	0.6850000
27033.0000000	0.6800000
27034.0000000	0.6800000
27035.0000000	0.6850000
27036.0000000	0.6800000
27037.0000000	0.6800000
27038.0000000	0.6700000
27039.0000000	0.6650000
27040.0000000	0.6600000
27041.0000000	0.6350000
27042.0000000	0.6300000
27043.0000000	0.6300000
27044.0000000	0.6300000
27045.0000000	0.6250000
27046.0000000	0.6250000
27047.0000000	0.6200000
27048.0000000	0.6300000
27049.0000000	0.6350000
27050.0000000	0.6400000

27051.0000000	0.6400000
27052.0000000	0.6550000
27053.0000000	0.6600000
27054.0000000	0.6750000
27055.0000000	0.6750000
27056.0000000	0.6850000
27057.0000000	0.6950000
27058.0000000	0.6950000
27059.0000000	0.7050000
27060.0000000	0.7100000
27061.0000000	0.7200000
27062.0000000	0.7200000
27063.0000000	0.7300000
27064.0000000	0.7300000
27065.0000000	0.7400000
27066.0000000	0.7450000
27067.0000000	0.7500000
27068.0000000	0.7500000
27069.0000000	0.7500000
27070.0000000	0.7450000
27071.0000000	0.7450000
27072.0000000	0.7400000
27073.0000000	0.7450000
27074.0000000	0.7350000
27075.0000000	0.7400000
27076.0000000	0.7400000
27077.0000000	0.7400000
27078.0000000	0.7400000
27079.0000000	0.7400000
27080.0000000	0.7400000
27081.0000000	0.7300000
27082.0000000	0.7400000
27083.0000000	0.7350000
27084.0000000	0.7350000
27085.0000000	0.7250000
27086.0000000	0.7300000
27087.0000000	0.7250000
27088.0000000	0.7200000
27089.0000000	0.7200000
27090.0000000	0.7200000
27091.0000000	0.7200000
27092.0000000	0.7150000
27093.0000000	0.7100000
27094.0000000	0.7100000
27095.0000000	0.7100000
27096.0000000	0.7100000
27097.0000000	0.7050000
27098.0000000	0.7000000
27099.0000000	0.7050000
27100.0000000	0.7050000
27101.0000000	0.7100000
27102.0000000	0.7100000
27103.0000000	0.7100000
27104.0000000	0.7100000
27105.0000000	0.7050000
27106.0000000	0.7000000
27107.0000000	0.7000000

27108.0000000	0.6950000
27109.0000000	0.6950000
27110.0000000	0.6900000
27111.0000000	0.6850000
27112.0000000	0.6700000
27113.0000000	0.6650000
27114.0000000	0.6600000
27115.0000000	0.6550000
27116.0000000	0.6500000
27117.0000000	0.6500000
27118.0000000	0.6500000
27119.0000000	0.6400000
27120.0000000	0.6350000
27121.0000000	0.6250000
27122.0000000	0.6200000
27123.0000000	0.6100000
27124.0000000	0.6066667
27125.0000000	0.6250000
27126.0000000	0.6250000
27127.0000000	0.6200000
27128.0000000	0.6200000
27129.0000000	0.6250000
27130.0000000	0.6250000
27131.0000000	0.6250000
27132.0000000	0.6300000
27133.0000000	0.6350000
27134.0000000	0.6400000
27135.0000000	0.6450000
27136.0000000	0.6450000
27137.0000000	0.6450000
27138.0000000	0.6400000
27139.0000000	0.6400000
27140.0000000	0.6450000
27141.0000000	0.6350000
27142.0000000	0.6350000
27143.0000000	0.6250000
27144.0000000	0.6200000
27145.0000000	0.6250000
27146.0000000	0.6300000
27147.0000000	0.6300000
27148.0000000	0.6300000
27149.0000000	0.6400000
27150.0000000	0.6450000
27151.0000000	0.6400000
27152.0000000	0.6400000
27153.0000000	0.6400000
27154.0000000	0.6450000
27155.0000000	0.6533333
27156.0000000	0.6600000
27157.0000000	0.6600000
27158.0000000	0.6600000
27159.0000000	0.6650000
27160.0000000	0.6650000
27161.0000000	0.6650000
27162.0000000	0.6700000
27163.0000000	0.6650000
27164.0000000	0.6650000

27165.0000000	0.6650000
27166.0000000	0.6700000
27167.0000000	0.6700000
27168.0000000	0.6750000
27169.0000000	0.6700000
27170.0000000	0.6800000
27171.0000000	0.6800000
27172.0000000	0.6800000
27173.0000000	0.6800000
27174.0000000	0.6750000
27175.0000000	0.6750000
27176.0000000	0.6650000
27177.0000000	0.6700000
27178.0000000	0.6650000
27179.0000000	0.6600000
27180.0000000	0.6650000
27181.0000000	0.6650000
27182.0000000	0.6600000
27183.0000000	0.6650000
27184.0000000	0.6633334
27185.0000000	0.6600000
27186.0000000	0.6500000
27187.0000000	0.6500000
27188.0000000	0.6400000
27189.0000000	0.6500000
27190.0000000	0.6400000
27191.0000000	0.6400000
27192.0000000	0.6400000
27193.0000000	0.6200000
27194.0000000	0.6100000
27195.0000000	0.6000000
27196.0000000	0.5950000
27197.0000000	0.5950000
27198.0000000	0.5950000
27199.0000000	0.6000000
27200.0000000	0.6000000
27201.0000000	0.6000000
27202.0000000	0.6050000
27203.0000000	0.6000000
27204.0000000	0.6000000
27205.0000000	0.5950000
27206.0000000	0.5950000
27207.0000000	0.5900000
27208.0000000	0.5850000
27209.0000000	0.5900000
27210.0000000	0.5900000
27211.0000000	0.5950000
27212.0000000	0.5900000
27213.0000000	0.5933333
27214.0000000	0.5950000
27215.0000000	0.5900000
27216.0000000	0.6050000
27217.0000000	0.6050000
27218.0000000	0.6150000
27219.0000000	0.6200000
27220.0000000	0.6200000
27221.0000000	0.6200000

27222.0000000	0.6300000
27223.0000000	0.6350000
27224.0000000	0.6400000
27225.0000000	0.6400000
27226.0000000	0.6400000
27227.0000000	0.6450000
27228.0000000	0.6500000
27229.0000000	0.6600000
27230.0000000	0.6500000
27231.0000000	0.6500000
27232.0000000	0.6600000
27233.0000000	0.6550000
27234.0000000	0.6600000
27235.0000000	0.6500000
27236.0000000	0.6550000
27237.0000000	0.6550000
27238.0000000	0.6550000
27239.0000000	0.6600000
27240.0000000	0.6600000
27241.0000000	0.6600000
27242.0000000	0.6650000
27243.0000000	0.6700000
27244.0000000	0.6733333
27245.0000000	0.6700000
27246.0000000	0.6800000
27247.0000000	0.6850000
27248.0000000	0.6900000
27249.0000000	0.6950000
27250.0000000	0.6900000
27251.0000000	0.7000000
27252.0000000	0.7000000
27253.0000000	0.7000000
27254.0000000	0.7000000
27255.0000000	0.7000000
27256.0000000	0.7000000
27257.0000000	0.7000000
27258.0000000	0.7100000
27259.0000000	0.7100000
27260.0000000	0.7000000
27261.0000000	0.7000000
27262.0000000	0.7000000
27263.0000000	0.7000000
27264.0000000	0.6950000
27265.0000000	0.6950000
27266.0000000	0.6850000
27267.0000000	0.6800000
27268.0000000	0.6850000
27269.0000000	0.6850000
27270.0000000	0.6850000
27271.0000000	0.6850000
27272.0000000	0.6900000
27273.0000000	0.6900000
27274.0000000	0.6950000
27275.0000000	0.7000000
27276.0000000	0.7000000
27277.0000000	0.7000000
27278.0000000	0.7000000

27279.0000000	0.7100000
27280.0000000	0.7100000
27281.0000000	0.7100000
27282.0000000	0.7150000
27283.0000000	0.7100000
27284.0000000	0.7200000
27285.0000000	0.7150000
27286.0000000	0.7250000
27287.0000000	0.7250000
27288.0000000	0.7300000
27289.0000000	0.7300000
27290.0000000	0.7400000
27291.0000000	0.7400000
27292.0000000	0.7500000
27293.0000000	0.7500000
27294.0000000	0.7550000
27295.0000000	0.7550000
27296.0000000	0.7550000
27297.0000000	0.7600000
27298.0000000	0.7500000
27299.0000000	0.7600000
27300.0000000	0.7650000
27301.0000000	0.7700000
27302.0000000	0.7600000
27303.0000000	0.7600000
27304.0000000	0.7600000
27305.0000000	0.7600000
27306.0000000	0.7650000
27307.0000000	0.7700000
27308.0000000	0.7600000
27309.0000000	0.7600000
27310.0000000	0.7600000
27311.0000000	0.7650000
27312.0000000	0.7700000
27313.0000000	0.7650000
27314.0000000	0.7600000
27315.0000000	0.7600000
27316.0000000	0.7650000
27317.0000000	0.7600000
27318.0000000	0.7600000
27319.0000000	0.7550000
27320.0000000	0.7500000
27321.0000000	0.7450000
27322.0000000	0.7400000
27323.0000000	0.7400000
27324.0000000	0.7300000
27325.0000000	0.7250000
27326.0000000	0.7200000
27327.0000000	0.7100000
27328.0000000	0.7050000
27329.0000000	0.7050000
27330.0000000	0.7050000
27331.0000000	0.7000000
27332.0000000	0.7000000
27333.0000000	0.7033333
27334.0000000	0.7000000
27335.0000000	0.7050000

27336.0000000	0.7050000
27337.0000000	0.7100000
27338.0000000	0.7150000
27339.0000000	0.7200000
27340.0000000	0.7150000
27341.0000000	0.7200000
27342.0000000	0.7200000
27343.0000000	0.7250000
27344.0000000	0.7250000
27345.0000000	0.7250000
27346.0000000	0.7250000
27347.0000000	0.7200000
27348.0000000	0.7150000
27349.0000000	0.7100000
27350.0000000	0.7100000
27351.0000000	0.7100000
27352.0000000	0.7100000
27353.0000000	0.7150000
27354.0000000	0.7150000
27355.0000000	0.7100000
27356.0000000	0.7150000
27357.0000000	0.7150000
27358.0000000	0.7150000
27359.0000000	0.7100000
27360.0000000	0.7100000
27361.0000000	0.7150000
27362.0000000	0.7250000
27363.0000000	0.7300000
27364.0000000	0.7300000
27365.0000000	0.7266667
27366.0000000	0.7200000
27367.0000000	0.7300000
27368.0000000	0.7300000
27369.0000000	0.7300000
27370.0000000	0.7300000
27371.0000000	0.7300000
27372.0000000	0.7350000
27373.0000000	0.7350000
27374.0000000	0.7350000
27375.0000000	0.7350000
27376.0000000	0.7300000
27377.0000000	0.7350000
27378.0000000	0.7300000
27379.0000000	0.7400000
27380.0000000	0.7350000
27381.0000000	0.7300000
27382.0000000	0.7250000
27383.0000000	0.7250000
27384.0000000	0.7200000
27385.0000000	0.7250000
27386.0000000	0.7100000
27387.0000000	0.7100000
27388.0000000	0.7000000
27389.0000000	0.7000000
27390.0000000	0.6950000
27391.0000000	0.6900000
27392.0000000	0.6900000

27393.0000000	0.6900000
27394.0000000	0.6850000
27395.0000000	0.6850000
27396.0000000	0.6900000
27397.0000000	0.6950000
27398.0000000	0.6950000
27399.0000000	0.6900000
27400.0000000	0.6850000
27401.0000000	0.6800000
27402.0000000	0.6850000
27403.0000000	0.6850000
27404.0000000	0.6800000
27405.0000000	0.6850000
27406.0000000	0.6850000
27407.0000000	0.6800000
27408.0000000	0.6750000
27409.0000000	0.6800000
27410.0000000	0.6800000
27411.0000000	0.6800000
27412.0000000	0.6850000
27413.0000000	0.6850000
27414.0000000	0.6850000
27415.0000000	0.6950000
27416.0000000	0.6900000
27417.0000000	0.6950000
27418.0000000	0.7000000
27419.0000000	0.7000000
27420.0000000	0.7000000
27421.0000000	0.7100000
27422.0000000	0.7100000
27423.0000000	0.7050000
27424.0000000	0.7000000
27425.0000000	0.7000000
27426.0000000	0.7100000
27427.0000000	0.7050000
27428.0000000	0.7100000
27429.0000000	0.7100000
27430.0000000	0.7100000
27431.0000000	0.7100000
27432.0000000	0.7150000
27433.0000000	0.7050000
27434.0000000	0.7100000
27435.0000000	0.7050000
27436.0000000	0.7000000
27437.0000000	0.7000000
27438.0000000	0.7000000
27439.0000000	0.7050000
27440.0000000	0.7050000
27441.0000000	0.7050000
27442.0000000	0.7100000
27443.0000000	0.7100000
27444.0000000	0.7100000
27445.0000000	0.7100000
27446.0000000	0.7150000
27447.0000000	0.7100000
27448.0000000	0.7150000
27449.0000000	0.7150000

27450.0000000	0.7100000
27451.0000000	0.7150000
27452.0000000	0.7200000
27453.0000000	0.7200000
27454.0000000	0.7250000
27455.0000000	0.7350000
27456.0000000	0.7300000
27457.0000000	0.7366667
27458.0000000	0.7400000
27459.0000000	0.7350000
27460.0000000	0.7400000
27461.0000000	0.7400000
27462.0000000	0.7350000
27463.0000000	0.7350000
27464.0000000	0.7300000
27465.0000000	0.7250000
27466.0000000	0.7200000
27467.0000000	0.7100000
27468.0000000	0.7000000
27469.0000000	0.7000000
27470.0000000	0.6900000
27471.0000000	0.6850000
27472.0000000	0.6750000
27473.0000000	0.6650000
27474.0000000	0.6550000
27475.0000000	0.6400000
27476.0000000	0.6400000
27477.0000000	0.6250000
27478.0000000	0.6150000
27479.0000000	0.6100000
27480.0000000	0.6000000
27481.0000000	0.5950000
27482.0000000	0.5850000
27483.0000000	0.5850000
27484.0000000	0.5800000
27485.0000000	0.5800000
27486.0000000	0.5750000
27487.0000000	0.5766667
27488.0000000	0.5750000
27489.0000000	0.5750000
27490.0000000	0.5700000
27491.0000000	0.5650000
27492.0000000	0.5700000
27493.0000000	0.5600000
27494.0000000	0.5600000
27495.0000000	0.5600000
27496.0000000	0.5700000
27497.0000000	0.5750000
27498.0000000	0.5800000
27499.0000000	0.5900000
27500.0000000	0.5900000
27501.0000000	0.6000000
27502.0000000	0.6000000
27503.0000000	0.6000000
27504.0000000	0.6100000
27505.0000000	0.6050000
27506.0000000	0.6100000

27507.0000000	0.6100000
27508.0000000	0.6100000
27509.0000000	0.6000000
27510.0000000	0.6000000
27511.0000000	0.6000000
27512.0000000	0.5900000
27513.0000000	0.5950000
27514.0000000	0.5950000
27515.0000000	0.5866666
27516.0000000	0.5750000
27517.0000000	0.5550000
27518.0000000	0.5500000
27519.0000000	0.5450000
27520.0000000	0.5450000
27521.0000000	0.5350000
27522.0000000	0.5300000
27523.0000000	0.5300000
27524.0000000	0.5250000
27525.0000000	0.5300000
27526.0000000	0.5250000
27527.0000000	0.5300000
27528.0000000	0.5300000
27529.0000000	0.5400000
27530.0000000	0.5400000
27531.0000000	0.5450000
27532.0000000	0.5500000
27533.0000000	0.5500000
27534.0000000	0.5550000
27535.0000000	0.5600000
27536.0000000	0.5650000
27537.0000000	0.5750000
27538.0000000	0.5800000
27539.0000000	0.5850000
27540.0000000	0.5850000
27541.0000000	0.6000000
27542.0000000	0.6000000
27543.0000000	0.6000000
27544.0000000	0.6000000
27545.0000000	0.6100000
27546.0000000	0.6100000
27547.0000000	0.6050000
27548.0000000	0.6100000
27549.0000000	0.6150000
27550.0000000	0.6200000
27551.0000000	0.6200000
27552.0000000	0.6250000
27553.0000000	0.6200000
27554.0000000	0.6150000
27555.0000000	0.6000000
27556.0000000	0.6000000
27557.0000000	0.5900000
27558.0000000	0.5800000
27559.0000000	0.5800000
27560.0000000	0.5750000
27561.0000000	0.5650000
27562.0000000	0.5600000
27563.0000000	0.5550000

27564.0000000	0.5550000
27565.0000000	0.5400000
27566.0000000	0.5500000
27567.0000000	0.5500000
27568.0000000	0.5400000
27569.0000000	0.5400000
27570.0000000	0.5450000
27571.0000000	0.5400000
27572.0000000	0.5500000
27573.0000000	0.5500000
27574.0000000	0.5600000
27575.0000000	0.5650000
27576.0000000	0.5600000
27577.0000000	0.5566667
27578.0000000	0.5600000
27579.0000000	0.5650000
27580.0000000	0.5650000
27581.0000000	0.5600000
27582.0000000	0.5600000
27583.0000000	0.5600000
27584.0000000	0.5600000
27585.0000000	0.5550000
27586.0000000	0.5500000
27587.0000000	0.5500000
27588.0000000	0.5600000
27589.0000000	0.5500000
27590.0000000	0.5500000
27591.0000000	0.5550000
27592.0000000	0.5600000
27593.0000000	0.5600000
27594.0000000	0.5550000
27595.0000000	0.5550000
27596.0000000	0.5550000
27597.0000000	0.5600000
27598.0000000	0.5500000
27599.0000000	0.5550000
27600.0000000	0.5550000
27601.0000000	0.5600000
27602.0000000	0.5600000
27603.0000000	0.5650000
27604.0000000	0.5700000
27605.0000000	0.5800000
27606.0000000	0.5850000
27607.0000000	0.5900000
27608.0000000	0.6000000
27609.0000000	0.6050000
27610.0000000	0.6100000
27611.0000000	0.6100000
27612.0000000	0.6150000
27613.0000000	0.6250000
27614.0000000	0.6250000
27615.0000000	0.6250000
27616.0000000	0.6300000
27617.0000000	0.6300000
27618.0000000	0.6300000
27619.0000000	0.6300000
27620.0000000	0.6300000

27621.0000000	0.6300000
27622.0000000	0.6300000
27623.0000000	0.6250000
27624.0000000	0.6250000
27625.0000000	0.6250000
27626.0000000	0.6250000
27627.0000000	0.6150000
27628.0000000	0.6100000
27629.0000000	0.6150000
27630.0000000	0.6000000
27631.0000000	0.5900000
27632.0000000	0.5950000
27633.0000000	0.6000000
27634.0000000	0.5900000
27635.0000000	0.5800000
27636.0000000	0.5800000
27637.0000000	0.5800000
27638.0000000	0.5733333
27639.0000000	0.5750000
27640.0000000	0.5750000
27641.0000000	0.5800000
27642.0000000	0.5800000
27643.0000000	0.5800000
27644.0000000	0.5800000
27645.0000000	0.5850000
27646.0000000	0.5850000
27647.0000000	0.5900000
27648.0000000	0.5900000
27649.0000000	0.5900000
27650.0000000	0.5950000
27651.0000000	0.6000000
27652.0000000	0.6000000
27653.0000000	0.6050000
27654.0000000	0.6050000
27655.0000000	0.6000000
27656.0000000	0.6000000
27657.0000000	0.6000000
27658.0000000	0.6000000
27659.0000000	0.6050000
27660.0000000	0.5950000
27661.0000000	0.5950000
27662.0000000	0.5900000
27663.0000000	0.5900000
27664.0000000	0.5800000
27665.0000000	0.5850000
27666.0000000	0.5900000
27667.0000000	0.5833333
27668.0000000	0.5800000
27669.0000000	0.5750000
27670.0000000	0.5700000
27671.0000000	0.5700000
27672.0000000	0.5650000
27673.0000000	0.5650000
27674.0000000	0.5600000
27675.0000000	0.5600000
27676.0000000	0.5600000
27677.0000000	0.5550000

27678.0000000	0.5500000
27679.0000000	0.5500000
27680.0000000	0.5600000
27681.0000000	0.5600000
27682.0000000	0.5600000
27683.0000000	0.5600000
27684.0000000	0.5600000
27685.0000000	0.5600000
27686.0000000	0.5600000
27687.0000000	0.5700000
27688.0000000	0.5700000
27689.0000000	0.5700000
27690.0000000	0.5700000
27691.0000000	0.5750000
27692.0000000	0.5600000
27693.0000000	0.5600000
27694.0000000	0.5600000
27695.0000000	0.5500000
27696.0000000	0.5600000
27697.0000000	0.5600000
27698.0000000	0.5500000
27699.0000000	0.5600000
27700.0000000	0.5500000
27701.0000000	0.5500000
27702.0000000	0.5500000
27703.0000000	0.5500000
27704.0000000	0.5500000
27705.0000000	0.5550000
27706.0000000	0.5500000
27707.0000000	0.5600000
27708.0000000	0.5550000
27709.0000000	0.5600000
27710.0000000	0.5550000
27711.0000000	0.5600000
27712.0000000	0.5600000
27713.0000000	0.5600000
27714.0000000	0.5500000
27715.0000000	0.5450000
27716.0000000	0.5500000
27717.0000000	0.5450000
27718.0000000	0.5350000
27719.0000000	0.5300000
27720.0000000	0.5300000
27721.0000000	0.5200000
27722.0000000	0.5200000
27723.0000000	0.5200000
27724.0000000	0.5250000
27725.0000000	0.5300000
27726.0000000	0.5300000
27727.0000000	0.5300000
27728.0000000	0.5333333
27729.0000000	0.5400000
27730.0000000	0.5300000
27731.0000000	0.5300000
27732.0000000	0.5300000
27733.0000000	0.5250000
27734.0000000	0.5200000

27735.0000000	0.5200000
27736.0000000	0.5200000
27737.0000000	0.5200000
27738.0000000	0.5250000
27739.0000000	0.5250000
27740.0000000	0.5250000
27741.0000000	0.5250000
27742.0000000	0.5350000
27743.0000000	0.5300000
27744.0000000	0.5350000
27745.0000000	0.5350000
27746.0000000	0.5400000
27747.0000000	0.5350000
27748.0000000	0.5350000
27749.0000000	0.5400000
27750.0000000	0.5400000
27751.0000000	0.5400000
27752.0000000	0.5400000
27753.0000000	0.5450000
27754.0000000	0.5400000
27755.0000000	0.5400000
27756.0000000	0.5350000
27757.0000000	0.5450000
27758.0000000	0.5300000
27759.0000000	0.5300000
27760.0000000	0.5300000
27761.0000000	0.5250000
27762.0000000	0.5300000
27763.0000000	0.5300000
27764.0000000	0.5300000
27765.0000000	0.5250000
27766.0000000	0.5300000
27767.0000000	0.5200000
27768.0000000	0.5200000
27769.0000000	0.5250000
27770.0000000	0.5250000
27771.0000000	0.5250000
27772.0000000	0.5200000
27773.0000000	0.5200000
27774.0000000	0.5250000
27775.0000000	0.5200000
27776.0000000	0.5200000
27777.0000000	0.5200000
27778.0000000	0.5250000
27779.0000000	0.5300000
27780.0000000	0.5250000
27781.0000000	0.5300000
27782.0000000	0.5300000
27783.0000000	0.5350000
27784.0000000	0.5450000
27785.0000000	0.5450000
27786.0000000	0.5400000
27787.0000000	0.5450000
27788.0000000	0.5500000
27789.0000000	0.5500000
27790.0000000	0.5550000
27791.0000000	0.5600000

27792.0000000	0.5600000
27793.0000000	0.5600000
27794.0000000	0.5700000
27795.0000000	0.5600000
27796.0000000	0.5700000
27797.0000000	0.5600000
27798.0000000	0.5550000
27799.0000000	0.5500000
27800.0000000	0.5500000
27801.0000000	0.5450000
27802.0000000	0.5450000
27803.0000000	0.5300000
27804.0000000	0.5300000
27805.0000000	0.5300000
27806.0000000	0.5250000
27807.0000000	0.5200000
27808.0000000	0.5250000
27809.0000000	0.5250000
27810.0000000	0.5250000
27811.0000000	0.5300000
27812.0000000	0.5300000
27813.0000000	0.5300000
27814.0000000	0.5300000
27815.0000000	0.5400000
27816.0000000	0.5300000
27817.0000000	0.5266666
27818.0000000	0.5300000
27819.0000000	0.5300000
27820.0000000	0.5300000
27821.0000000	0.5250000
27822.0000000	0.5250000
27823.0000000	0.5300000
27824.0000000	0.5250000
27825.0000000	0.5200000
27826.0000000	0.5200000
27827.0000000	0.5150000
27828.0000000	0.5000000
27829.0000000	0.5050000
27830.0000000	0.5000000
27831.0000000	0.4950000
27832.0000000	0.4900000
27833.0000000	0.4950000
27834.0000000	0.4900000
27835.0000000	0.4850000
27836.0000000	0.4800000
27837.0000000	0.4750000
27838.0000000	0.4750000
27839.0000000	0.4800000
27840.0000000	0.4800000
27841.0000000	0.4800000
27842.0000000	0.4800000
27843.0000000	0.4850000
27844.0000000	0.4900000
27845.0000000	0.4900000
27846.0000000	0.4950000
27847.0000000	0.5050000
27848.0000000	0.5000000

27849.0000000	0.5000000
27850.0000000	0.5050000
27851.0000000	0.5000000
27852.0000000	0.5050000
27853.0000000	0.5100000
27854.0000000	0.5100000
27855.0000000	0.5150000
27856.0000000	0.5150000
27857.0000000	0.5100000
27858.0000000	0.5150000
27859.0000000	0.5200000
27860.0000000	0.5200000
27861.0000000	0.5250000
27862.0000000	0.5200000
27863.0000000	0.5200000
27864.0000000	0.5200000
27865.0000000	0.5200000
27866.0000000	0.5100000
27867.0000000	0.5150000
27868.0000000	0.5150000
27869.0000000	0.5050000
27870.0000000	0.5100000
27871.0000000	0.5100000
27872.0000000	0.5050000
27873.0000000	0.5050000
27874.0000000	0.5050000
27875.0000000	0.5000000
27876.0000000	0.5000000
27877.0000000	0.5000000
27878.0000000	0.5000000
27879.0000000	0.5000000
27880.0000000	0.4950000
27881.0000000	0.4950000
27882.0000000	0.4850000
27883.0000000	0.4900000
27884.0000000	0.4850000
27885.0000000	0.4800000
27886.0000000	0.4800000
27887.0000000	0.4850000
27888.0000000	0.4800000
27889.0000000	0.4750000
27890.0000000	0.4800000
27891.0000000	0.4750000
27892.0000000	0.4700000
27893.0000000	0.4600000
27894.0000000	0.4550000
27895.0000000	0.4600000
27896.0000000	0.4450000
27897.0000000	0.4500000
27898.0000000	0.4400000
27899.0000000	0.4400000
27900.0000000	0.4400000
27901.0000000	0.4300000
27902.0000000	0.4350000
27903.0000000	0.4300000
27904.0000000	0.4350000
27905.0000000	0.4300000

27906.0000000	0.4300000
27907.0000000	0.4300000
27908.0000000	0.4300000
27909.0000000	0.4333333
27910.0000000	0.4350000
27911.0000000	0.4350000
27912.0000000	0.4350000
27913.0000000	0.4350000
27914.0000000	0.4300000
27915.0000000	0.4300000
27916.0000000	0.4250000
27917.0000000	0.4400000
27918.0000000	0.4400000
27919.0000000	0.4350000
27920.0000000	0.4400000
27921.0000000	0.4400000
27922.0000000	0.4400000
27923.0000000	0.4400000
27924.0000000	0.4400000
27925.0000000	0.4500000
27926.0000000	0.4550000
27927.0000000	0.4500000
27928.0000000	0.4500000
27929.0000000	0.4500000
27930.0000000	0.4550000
27931.0000000	0.4600000
27932.0000000	0.4650000
27933.0000000	0.4600000
27934.0000000	0.4650000
27935.0000000	0.4650000
27936.0000000	0.4700000
27937.0000000	0.4750000
27938.0000000	0.4700000
27939.0000000	0.4733333
27940.0000000	0.4750000
27941.0000000	0.4750000
27942.0000000	0.4800000
27943.0000000	0.4700000
27944.0000000	0.4750000
27945.0000000	0.4700000
27946.0000000	0.4800000
27947.0000000	0.4800000
27948.0000000	0.4700000
27949.0000000	0.4700000
27950.0000000	0.4700000
27951.0000000	0.4700000
27952.0000000	0.4700000
27953.0000000	0.4650000
27954.0000000	0.4700000
27955.0000000	0.4700000
27956.0000000	0.4700000
27957.0000000	0.4800000
27958.0000000	0.4800000
27959.0000000	0.4800000
27960.0000000	0.4800000
27961.0000000	0.4800000
27962.0000000	0.4800000

27963.0000000	0.4850000
27964.0000000	0.4750000
27965.0000000	0.4800000
27966.0000000	0.4800000
27967.0000000	0.4800000
27968.0000000	0.4833333
27969.0000000	0.4800000
27970.0000000	0.4900000
27971.0000000	0.4866667
27972.0000000	0.4900000
27973.0000000	0.4900000
27974.0000000	0.4950000
27975.0000000	0.5000000
27976.0000000	0.5000000
27977.0000000	0.4950000
27978.0000000	0.5000000
27979.0000000	0.5000000
27980.0000000	0.5000000
27981.0000000	0.5000000
27982.0000000	0.5000000
27983.0000000	0.5000000
27984.0000000	0.5000000
27985.0000000	0.5050000
27986.0000000	0.5000000
27987.0000000	0.5100000
27988.0000000	0.5100000
27989.0000000	0.5200000
27990.0000000	0.5150000
27991.0000000	0.5200000
27992.0000000	0.5200000
27993.0000000	0.5250000
27994.0000000	0.5200000
27995.0000000	0.5300000
27996.0000000	0.5300000
27997.0000000	0.5300000
27998.0000000	0.5400000
27999.0000000	0.5400000
28000.0000000	0.5466667
28001.0000000	0.5550000
28002.0000000	0.5500000
28003.0000000	0.5550000
28004.0000000	0.5500000
28005.0000000	0.5550000
28006.0000000	0.5500000
28007.0000000	0.5500000
28008.0000000	0.5500000
28009.0000000	0.5500000
28010.0000000	0.5500000
28011.0000000	0.5600000
28012.0000000	0.5500000
28013.0000000	0.5550000
28014.0000000	0.5550000
28015.0000000	0.5600000
28016.0000000	0.5550000
28017.0000000	0.5600000
28018.0000000	0.5550000
28019.0000000	0.5600000

28020.0000000	0.5500000
28021.0000000	0.5600000
28022.0000000	0.5600000
28023.0000000	0.5650000
28024.0000000	0.5650000
28025.0000000	0.5600000
28026.0000000	0.5600000
28027.0000000	0.5700000
28028.0000000	0.5650000
28029.0000000	0.5650000
28030.0000000	0.5600000
28031.0000000	0.5650000
28032.0000000	0.5600000
28033.0000000	0.5600000
28034.0000000	0.5600000
28035.0000000	0.5600000
28036.0000000	0.5600000
28037.0000000	0.5450000
28038.0000000	0.5450000
28039.0000000	0.5400000
28040.0000000	0.5400000
28041.0000000	0.5300000
28042.0000000	0.5200000
28043.0000000	0.5150000
28044.0000000	0.5100000
28045.0000000	0.5100000
28046.0000000	0.5100000
28047.0000000	0.5050000
28048.0000000	0.5000000
28049.0000000	0.5000000
28050.0000000	0.5000000
28051.0000000	0.5000000
28052.0000000	0.5000000
28053.0000000	0.5000000
28054.0000000	0.5050000
28055.0000000	0.5050000
28056.0000000	0.5000000
28057.0000000	0.5000000
28058.0000000	0.5000000
28059.0000000	0.5033333
28060.0000000	0.5050000
28061.0000000	0.5100000
28062.0000000	0.5100000
28063.0000000	0.5100000
28064.0000000	0.5100000
28065.0000000	0.5250000
28066.0000000	0.5200000
28067.0000000	0.5200000
28068.0000000	0.5200000
28069.0000000	0.5100000
28070.0000000	0.5050000
28071.0000000	0.5000000
28072.0000000	0.4900000
28073.0000000	0.4800000
28074.0000000	0.4850000
28075.0000000	0.4750000
28076.0000000	0.4700000

28077.0000000	0.4700000
28078.0000000	0.4600000
28079.0000000	0.4650000
28080.0000000	0.4650000
28081.0000000	0.4700000
28082.0000000	0.4700000
28083.0000000	0.4700000
28084.0000000	0.4800000
28085.0000000	0.4750000
28086.0000000	0.4800000
28087.0000000	0.4800000
28088.0000000	0.4800000
28089.0000000	0.4850000
28090.0000000	0.4900000
28091.0000000	0.4900000
28092.0000000	0.4850000
28093.0000000	0.4900000
28094.0000000	0.4950000
28095.0000000	0.5000000
28096.0000000	0.5000000
28097.0000000	0.5000000
28098.0000000	0.5000000
28099.0000000	0.5000000
28100.0000000	0.5000000
28101.0000000	0.4950000
28102.0000000	0.4950000
28103.0000000	0.5000000
28104.0000000	0.5000000
28105.0000000	0.4950000
28106.0000000	0.4950000
28107.0000000	0.4900000
28108.0000000	0.4900000
28109.0000000	0.4900000
28110.0000000	0.4800000
28111.0000000	0.4800000
28112.0000000	0.4750000
28113.0000000	0.4750000
28114.0000000	0.4700000
28115.0000000	0.4700000
28116.0000000	0.4750000
28117.0000000	0.4800000
28118.0000000	0.4800000
28119.0000000	0.4850000
28120.0000000	0.4866667
28121.0000000	0.4900000
28122.0000000	0.4950000
28123.0000000	0.4900000
28124.0000000	0.4900000
28125.0000000	0.4900000
28126.0000000	0.4950000
28127.0000000	0.4950000
28128.0000000	0.4950000
28129.0000000	0.5050000
28130.0000000	0.5000000
28131.0000000	0.5050000
28132.0000000	0.5050000
28133.0000000	0.4950000

28134.0000000	0.5000000
28135.0000000	0.4950000
28136.0000000	0.5000000
28137.0000000	0.4950000
28138.0000000	0.4850000
28139.0000000	0.4900000
28140.0000000	0.4900000
28141.0000000	0.4900000
28142.0000000	0.4900000
28143.0000000	0.4900000
28144.0000000	0.4900000
28145.0000000	0.4800000
28146.0000000	0.4850000
28147.0000000	0.4800000
28148.0000000	0.4800000
28149.0000000	0.4850000
28150.0000000	0.4800000
28151.0000000	0.4766667
28152.0000000	0.4750000
28153.0000000	0.4750000
28154.0000000	0.4750000
28155.0000000	0.4800000
28156.0000000	0.4850000
28157.0000000	0.4850000
28158.0000000	0.4900000
28159.0000000	0.4900000
28160.0000000	0.4950000
28161.0000000	0.4900000
28162.0000000	0.4900000
28163.0000000	0.4950000
28164.0000000	0.5000000
28165.0000000	0.4950000
28166.0000000	0.4900000
28167.0000000	0.4900000
28168.0000000	0.4850000
28169.0000000	0.4800000
28170.0000000	0.4750000
28171.0000000	0.4750000
28172.0000000	0.4600000
28173.0000000	0.4600000
28174.0000000	0.4550000
28175.0000000	0.4550000
28176.0000000	0.4500000
28177.0000000	0.4450000
28178.0000000	0.4450000
28179.0000000	0.4450000
28180.0000000	0.4400000
28181.0000000	0.4400000
28182.0000000	0.4500000
28183.0000000	0.4500000
28184.0000000	0.4550000
28185.0000000	0.4600000
28186.0000000	0.4650000
28187.0000000	0.4600000
28188.0000000	0.4600000
28189.0000000	0.4650000
28190.0000000	0.4600000

28191.0000000	0.4600000
28192.0000000	0.4550000
28193.0000000	0.4600000
28194.0000000	0.4600000
28195.0000000	0.4550000
28196.0000000	0.4500000
28197.0000000	0.4500000
28198.0000000	0.4500000
28199.0000000	0.4400000
28200.0000000	0.4350000
28201.0000000	0.4300000
28202.0000000	0.4150000
28203.0000000	0.4100000
28204.0000000	0.4050000
28205.0000000	0.3950000
28206.0000000	0.3850000
28207.0000000	0.3900000
28208.0000000	0.3850000
28209.0000000	0.3850000
28210.0000000	0.3950000
28211.0000000	0.3950000
28212.0000000	0.3966666
28213.0000000	0.4000000
28214.0000000	0.3900000
28215.0000000	0.4050000
28216.0000000	0.4100000
28217.0000000	0.4100000
28218.0000000	0.4150000
28219.0000000	0.4150000
28220.0000000	0.4200000
28221.0000000	0.4250000
28222.0000000	0.4250000
28223.0000000	0.4300000
28224.0000000	0.4400000
28225.0000000	0.4350000
28226.0000000	0.4400000
28227.0000000	0.4400000
28228.0000000	0.4400000
28229.0000000	0.4500000
28230.0000000	0.4500000
28231.0000000	0.4550000
28232.0000000	0.4550000
28233.0000000	0.4600000
28234.0000000	0.4650000
28235.0000000	0.4650000
28236.0000000	0.4600000
28237.0000000	0.4650000
28238.0000000	0.4700000
28239.0000000	0.4800000
28240.0000000	0.4700000
28241.0000000	0.4750000
28242.0000000	0.4700000
28243.0000000	0.4700000
28244.0000000	0.4700000
28245.0000000	0.4700000
28246.0000000	0.4750000
28247.0000000	0.4700000

28248.0000000	0.4700000
28249.0000000	0.4700000
28250.0000000	0.4800000
28251.0000000	0.4850000
28252.0000000	0.4750000
28253.0000000	0.4700000
28254.0000000	0.4700000
28255.0000000	0.4650000
28256.0000000	0.4600000
28257.0000000	0.4500000
28258.0000000	0.4450000
28259.0000000	0.4400000
28260.0000000	0.4400000
28261.0000000	0.4450000
28262.0000000	0.4400000
28263.0000000	0.4300000
28264.0000000	0.4300000
28265.0000000	0.4150000
28266.0000000	0.4150000
28267.0000000	0.4150000
28268.0000000	0.4100000
28269.0000000	0.4150000
28270.0000000	0.4100000
28271.0000000	0.4100000
28272.0000000	0.4200000
28273.0000000	0.4200000
28274.0000000	0.4250000
28275.0000000	0.4250000
28276.0000000	0.4350000
28277.0000000	0.4350000
28278.0000000	0.4350000
28279.0000000	0.4450000
28280.0000000	0.4400000
28281.0000000	0.4400000
28282.0000000	0.4500000
28283.0000000	0.4400000
28284.0000000	0.4400000
28285.0000000	0.4400000
28286.0000000	0.4400000
28287.0000000	0.4400000
28288.0000000	0.4500000
28289.0000000	0.4500000
28290.0000000	0.4550000
28291.0000000	0.4600000
28292.0000000	0.4600000
28293.0000000	0.4600000
28294.0000000	0.4650000
28295.0000000	0.4700000
28296.0000000	0.4700000
28297.0000000	0.4850000
28298.0000000	0.4800000
28299.0000000	0.4850000
28300.0000000	0.4850000
28301.0000000	0.4800000
28302.0000000	0.4866667
28303.0000000	0.4800000
28304.0000000	0.4900000

28305.0000000	0.4900000
28306.0000000	0.4850000
28307.0000000	0.4800000
28308.0000000	0.4800000
28309.0000000	0.4850000
28310.0000000	0.4800000
28311.0000000	0.4900000
28312.0000000	0.4900000
28313.0000000	0.4900000
28314.0000000	0.4850000
28315.0000000	0.4850000
28316.0000000	0.4850000
28317.0000000	0.4900000
28318.0000000	0.4850000
28319.0000000	0.4900000
28320.0000000	0.4900000
28321.0000000	0.4800000
28322.0000000	0.4800000
28323.0000000	0.4800000
28324.0000000	0.4800000
28325.0000000	0.4750000
28326.0000000	0.4800000
28327.0000000	0.4750000
28328.0000000	0.4800000
28329.0000000	0.4700000
28330.0000000	0.4700000
28331.0000000	0.4733333
28332.0000000	0.4700000
28333.0000000	0.4700000
28334.0000000	0.4650000
28335.0000000	0.4600000
28336.0000000	0.4650000
28337.0000000	0.4600000
28338.0000000	0.4600000
28339.0000000	0.4600000
28340.0000000	0.4600000
28341.0000000	0.4600000
28342.0000000	0.4650000
28343.0000000	0.4600000
28344.0000000	0.4600000
28345.0000000	0.4650000
28346.0000000	0.4700000
28347.0000000	0.4700000
28348.0000000	0.4650000
28349.0000000	0.4600000
28350.0000000	0.4650000
28351.0000000	0.4700000
28352.0000000	0.4700000
28353.0000000	0.4700000
28354.0000000	0.4650000
28355.0000000	0.4700000
28356.0000000	0.4700000
28357.0000000	0.4700000
28358.0000000	0.4750000
28359.0000000	0.4850000
28360.0000000	0.4800000
28361.0000000	0.4800000

28362.0000000	0.4833333
28363.0000000	0.4800000
28364.0000000	0.4850000
28365.0000000	0.4800000
28366.0000000	0.4850000
28367.0000000	0.4800000
28368.0000000	0.4700000
28369.0000000	0.4700000
28370.0000000	0.4650000
28371.0000000	0.4650000
28372.0000000	0.4650000
28373.0000000	0.4750000
28374.0000000	0.4700000
28375.0000000	0.4800000
28376.0000000	0.4750000
28377.0000000	0.4700000
28378.0000000	0.4750000
28379.0000000	0.4650000
28380.0000000	0.4600000
28381.0000000	0.4550000
28382.0000000	0.4600000
28383.0000000	0.4600000
28384.0000000	0.4650000
28385.0000000	0.4650000
28386.0000000	0.4750000
28387.0000000	0.4700000
28388.0000000	0.4750000
28389.0000000	0.4700000
28390.0000000	0.4700000
28391.0000000	0.4766667
28392.0000000	0.4700000
28393.0000000	0.4600000
28394.0000000	0.4550000
28395.0000000	0.4550000
28396.0000000	0.4500000
28397.0000000	0.4400000
28398.0000000	0.4400000
28399.0000000	0.4400000
28400.0000000	0.4400000
28401.0000000	0.4300000
28402.0000000	0.4250000
28403.0000000	0.4300000
28404.0000000	0.4200000
28405.0000000	0.4300000
28406.0000000	0.4300000
28407.0000000	0.4300000
28408.0000000	0.4400000
28409.0000000	0.4400000
28410.0000000	0.4450000
28411.0000000	0.4450000
28412.0000000	0.4500000
28413.0000000	0.4550000
28414.0000000	0.4500000
28415.0000000	0.4600000
28416.0000000	0.4600000
28417.0000000	0.4600000
28418.0000000	0.4600000

28419.0000000	0.4550000
28420.0000000	0.4600000
28421.0000000	0.4600000
28422.0000000	0.4600000
28423.0000000	0.4600000
28424.0000000	0.4650000
28425.0000000	0.4700000
28426.0000000	0.4750000
28427.0000000	0.4800000
28428.0000000	0.4800000
28429.0000000	0.4700000
28430.0000000	0.4800000
28431.0000000	0.4700000
28432.0000000	0.4750000
28433.0000000	0.4750000
28434.0000000	0.4750000
28435.0000000	0.4800000
28436.0000000	0.4800000
28437.0000000	0.4800000
28438.0000000	0.4800000
28439.0000000	0.4750000
28440.0000000	0.4750000
28441.0000000	0.4700000
28442.0000000	0.4700000
28443.0000000	0.4750000
28444.0000000	0.4700000
28445.0000000	0.4700000
28446.0000000	0.4650000
28447.0000000	0.4600000
28448.0000000	0.4550000
28449.0000000	0.4500000
28450.0000000	0.4400000
28451.0000000	0.4300000
28452.0000000	0.4250000
28453.0000000	0.4166667
28454.0000000	0.4150000
28455.0000000	0.4150000
28456.0000000	0.4000000
28457.0000000	0.4000000
28458.0000000	0.3900000
28459.0000000	0.3800000
28460.0000000	0.3800000
28461.0000000	0.3800000
28462.0000000	0.3750000
28463.0000000	0.3750000
28464.0000000	0.3800000
28465.0000000	0.3800000
28466.0000000	0.3850000
28467.0000000	0.3950000
28468.0000000	0.4000000
28469.0000000	0.4050000
28470.0000000	0.4100000
28471.0000000	0.4200000
28472.0000000	0.4200000
28473.0000000	0.4300000
28474.0000000	0.4300000
28475.0000000	0.4300000

28476.0000000	0.4300000
28477.0000000	0.4300000
28478.0000000	0.4300000
28479.0000000	0.4250000
28480.0000000	0.4200000
28481.0000000	0.4150000
28482.0000000	0.4200000
28483.0000000	0.4200000
28484.0000000	0.4100000
28485.0000000	0.4100000
28486.0000000	0.4150000
28487.0000000	0.4100000
28488.0000000	0.4100000
28489.0000000	0.4150000
28490.0000000	0.4100000
28491.0000000	0.4100000
28492.0000000	0.4050000
28493.0000000	0.4100000
28494.0000000	0.4100000
28495.0000000	0.4050000
28496.0000000	0.4050000
28497.0000000	0.4000000
28498.0000000	0.4000000
28499.0000000	0.3900000
28500.0000000	0.3900000
28501.0000000	0.3900000
28502.0000000	0.3900000
28503.0000000	0.3900000
28504.0000000	0.3950000
28505.0000000	0.4000000
28506.0000000	0.4000000
28507.0000000	0.4100000
28508.0000000	0.4000000
28509.0000000	0.4050000
28510.0000000	0.4050000
28511.0000000	0.4000000
28512.0000000	0.3900000
28513.0000000	0.3833333
28514.0000000	0.3850000
28515.0000000	0.3850000
28516.0000000	0.3950000
28517.0000000	0.3900000
28518.0000000	0.3950000
28519.0000000	0.3900000
28520.0000000	0.3950000
28521.0000000	0.4000000
28522.0000000	0.3950000
28523.0000000	0.4000000
28524.0000000	0.4000000
28525.0000000	0.4000000
28526.0000000	0.4050000
28527.0000000	0.4000000
28528.0000000	0.4000000
28529.0000000	0.4000000
28530.0000000	0.4100000
28531.0000000	0.4100000
28532.0000000	0.4100000

28533.0000000	0.4100000
28534.0000000	0.4100000
28535.0000000	0.4050000
28536.0000000	0.4100000
28537.0000000	0.4150000
28538.0000000	0.4100000
28539.0000000	0.4150000
28540.0000000	0.4150000
28541.0000000	0.4200000
28542.0000000	0.4100000
28543.0000000	0.4150000
28544.0000000	0.4200000
28545.0000000	0.4200000
28546.0000000	0.4200000
28547.0000000	0.4200000
28548.0000000	0.4200000
28549.0000000	0.4200000
28550.0000000	0.4200000
28551.0000000	0.4200000
28552.0000000	0.4250000
28553.0000000	0.4200000
28554.0000000	0.4200000
28555.0000000	0.4200000
28556.0000000	0.4200000
28557.0000000	0.4200000
28558.0000000	0.4200000
28559.0000000	0.4250000
28560.0000000	0.4200000
28561.0000000	0.4150000
28562.0000000	0.4200000
28563.0000000	0.4150000
28564.0000000	0.4100000
28565.0000000	0.4150000
28566.0000000	0.4100000
28567.0000000	0.4150000
28568.0000000	0.4100000
28569.0000000	0.4200000
28570.0000000	0.4200000
28571.0000000	0.4300000
28572.0000000	0.4250000
28573.0000000	0.4333333
28574.0000000	0.4350000
28575.0000000	0.4400000
28576.0000000	0.4400000
28577.0000000	0.4400000
28578.0000000	0.4500000
28579.0000000	0.4500000
28580.0000000	0.4550000
28581.0000000	0.4600000
28582.0000000	0.4700000
28583.0000000	0.4700000
28584.0000000	0.4750000
28585.0000000	0.4800000
28586.0000000	0.4750000
28587.0000000	0.4750000
28588.0000000	0.4700000
28589.0000000	0.4750000

28590.0000000	0.4750000
28591.0000000	0.4700000
28592.0000000	0.4700000
28593.0000000	0.4750000
28594.0000000	0.4750000
28595.0000000	0.4700000
28596.0000000	0.4700000
28597.0000000	0.4700000
28598.0000000	0.4750000
28599.0000000	0.4750000
28600.0000000	0.4700000
28601.0000000	0.4750000
28602.0000000	0.4700000
28603.0000000	0.4766667
28604.0000000	0.4750000
28605.0000000	0.4750000
28606.0000000	0.4750000
28607.0000000	0.4800000
28608.0000000	0.4850000
28609.0000000	0.4900000
28610.0000000	0.4850000
28611.0000000	0.4850000
28612.0000000	0.4900000
28613.0000000	0.4900000
28614.0000000	0.4900000
28615.0000000	0.4900000
28616.0000000	0.4850000
28617.0000000	0.4750000
28618.0000000	0.4850000
28619.0000000	0.4750000
28620.0000000	0.4750000
28621.0000000	0.4850000
28622.0000000	0.4900000
28623.0000000	0.4900000
28624.0000000	0.5000000
28625.0000000	0.5000000
28626.0000000	0.5050000
28627.0000000	0.5000000
28628.0000000	0.5050000
28629.0000000	0.5050000
28630.0000000	0.5050000
28631.0000000	0.5150000
28632.0000000	0.5100000
28633.0000000	0.5100000
28634.0000000	0.5100000
28635.0000000	0.5050000
28636.0000000	0.5100000
28637.0000000	0.5050000
28638.0000000	0.5000000
28639.0000000	0.4950000
28640.0000000	0.5000000
28641.0000000	0.5050000
28642.0000000	0.5000000
28643.0000000	0.5000000
28644.0000000	0.5000000
28645.0000000	0.5000000
28646.0000000	0.4950000

28647.0000000	0.5000000
28648.0000000	0.5000000
28649.0000000	0.4950000
28650.0000000	0.4900000
28651.0000000	0.4900000
28652.0000000	0.4800000
28653.0000000	0.4850000
28654.0000000	0.4850000
28655.0000000	0.4800000
28656.0000000	0.4800000
28657.0000000	0.4800000
28658.0000000	0.4800000
28659.0000000	0.4750000
28660.0000000	0.4750000
28661.0000000	0.4700000
28662.0000000	0.4700000
28663.0000000	0.4666667
28664.0000000	0.4600000
28665.0000000	0.4600000
28666.0000000	0.4500000
28667.0000000	0.4450000
28668.0000000	0.4550000
28669.0000000	0.4600000
28670.0000000	0.4600000
28671.0000000	0.4600000
28672.0000000	0.4650000
28673.0000000	0.4600000
28674.0000000	0.4600000
28675.0000000	0.4600000
28676.0000000	0.4650000
28677.0000000	0.4550000
28678.0000000	0.4500000
28679.0000000	0.4500000
28680.0000000	0.4450000
28681.0000000	0.4350000
28682.0000000	0.4350000
28683.0000000	0.4400000
28684.0000000	0.4300000
28685.0000000	0.4300000
28686.0000000	0.4200000
28687.0000000	0.4150000
28688.0000000	0.4200000
28689.0000000	0.4200000
28690.0000000	0.4150000
28691.0000000	0.4200000
28692.0000000	0.4200000
28693.0000000	0.4266666
28694.0000000	0.4300000
28695.0000000	0.4300000
28696.0000000	0.4350000
28697.0000000	0.4350000
28698.0000000	0.4350000
28699.0000000	0.4350000
28700.0000000	0.4300000
28701.0000000	0.4350000
28702.0000000	0.4400000
28703.0000000	0.4400000

28704.0000000	0.4350000
28705.0000000	0.4350000
28706.0000000	0.4250000
28707.0000000	0.4200000
28708.0000000	0.4100000
28709.0000000	0.4100000
28710.0000000	0.4050000
28711.0000000	0.4050000
28712.0000000	0.4100000
28713.0000000	0.4100000
28714.0000000	0.4150000
28715.0000000	0.4150000
28716.0000000	0.4200000
28717.0000000	0.4300000
28718.0000000	0.4200000
28719.0000000	0.4200000
28720.0000000	0.4200000
28721.0000000	0.4200000
28722.0000000	0.4150000
28723.0000000	0.4150000
28724.0000000	0.4150000
28725.0000000	0.4100000
28726.0000000	0.4100000
28727.0000000	0.4200000
28728.0000000	0.4150000
28729.0000000	0.4200000
28730.0000000	0.4300000
28731.0000000	0.4200000
28732.0000000	0.4300000
28733.0000000	0.4350000
28734.0000000	0.4300000
28735.0000000	0.4300000
28736.0000000	0.4350000
28737.0000000	0.4400000
28738.0000000	0.4350000
28739.0000000	0.4400000
28740.0000000	0.4400000
28741.0000000	0.4450000
28742.0000000	0.4500000
28743.0000000	0.4550000
28744.0000000	0.4550000
28745.0000000	0.4500000
28746.0000000	0.4600000
28747.0000000	0.4700000
28748.0000000	0.4700000
28749.0000000	0.4700000
28750.0000000	0.4800000
28751.0000000	0.4800000
28752.0000000	0.4800000
28753.0000000	0.4800000
28754.0000000	0.4800000
28755.0000000	0.4866667
28756.0000000	0.4800000
28757.0000000	0.4800000
28758.0000000	0.4800000
28759.0000000	0.4800000
28760.0000000	0.4750000

28761.0000000	0.4800000
28762.0000000	0.4800000
28763.0000000	0.4800000
28764.0000000	0.4850000
28765.0000000	0.4800000
28766.0000000	0.4800000
28767.0000000	0.4800000
28768.0000000	0.4800000
28769.0000000	0.4800000
28770.0000000	0.4850000
28771.0000000	0.4900000
28772.0000000	0.4900000
28773.0000000	0.4900000
28774.0000000	0.4900000
28775.0000000	0.4900000
28776.0000000	0.4900000
28777.0000000	0.4950000
28778.0000000	0.4900000
28779.0000000	0.5000000
28780.0000000	0.5000000
28781.0000000	0.5000000
28782.0000000	0.5000000
28783.0000000	0.4950000
28784.0000000	0.5000000
28785.0000000	0.4900000
28786.0000000	0.5000000
28787.0000000	0.4950000
28788.0000000	0.4900000
28789.0000000	0.4900000
28790.0000000	0.4850000
28791.0000000	0.4750000
28792.0000000	0.4700000
28793.0000000	0.4700000
28794.0000000	0.4650000
28795.0000000	0.4700000
28796.0000000	0.4650000
28797.0000000	0.4600000
28798.0000000	0.4600000
28799.0000000	0.4500000
28800.0000000	0.4500000
28801.0000000	0.4450000
28802.0000000	0.4450000
28803.0000000	0.4550000
28804.0000000	0.4450000
28805.0000000	0.4550000
28806.0000000	0.4500000
28807.0000000	0.4550000
28808.0000000	0.4600000
28809.0000000	0.4550000
28810.0000000	0.4550000
28811.0000000	0.4600000
28812.0000000	0.4600000
28813.0000000	0.4600000
28814.0000000	0.4700000
28815.0000000	0.4650000
28816.0000000	0.4700000
28817.0000000	0.4800000

28818.0000000	0.4800000
28819.0000000	0.4750000
28820.0000000	0.4750000
28821.0000000	0.4750000
28822.0000000	0.4750000
28823.0000000	0.4750000
28824.0000000	0.4650000
28825.0000000	0.4750000
28826.0000000	0.4700000
28827.0000000	0.4700000
28828.0000000	0.4750000
28829.0000000	0.4700000
28830.0000000	0.4700000
28831.0000000	0.4700000
28832.0000000	0.4700000
28833.0000000	0.4600000
28834.0000000	0.4650000
28835.0000000	0.4650000
28836.0000000	0.4600000
28837.0000000	0.4600000
28838.0000000	0.4550000
28839.0000000	0.4600000
28840.0000000	0.4600000
28841.0000000	0.4600000
28842.0000000	0.4650000
28843.0000000	0.4650000
28844.0000000	0.4700000
28845.0000000	0.4700000
28846.0000000	0.4700000
28847.0000000	0.4750000
28848.0000000	0.4800000
28849.0000000	0.4800000
28850.0000000	0.4800000
28851.0000000	0.4800000
28852.0000000	0.4800000
28853.0000000	0.4850000
28854.0000000	0.4850000
28855.0000000	0.4900000
28856.0000000	0.5000000
28857.0000000	0.5000000
28858.0000000	0.4950000
28859.0000000	0.5000000
28860.0000000	0.5000000
28861.0000000	0.5050000
28862.0000000	0.5000000
28863.0000000	0.5000000
28864.0000000	0.5050000
28865.0000000	0.5050000
28866.0000000	0.4950000
28867.0000000	0.4950000
28868.0000000	0.5000000
28869.0000000	0.4950000
28870.0000000	0.4950000
28871.0000000	0.4900000
28872.0000000	0.4900000
28873.0000000	0.4900000
28874.0000000	0.4933333

28875.0000000	0.4900000
28876.0000000	0.4933333
28877.0000000	0.4900000
28878.0000000	0.4900000
28879.0000000	0.4950000
28880.0000000	0.5000000
28881.0000000	0.5000000
28882.0000000	0.5000000
28883.0000000	0.4900000
28884.0000000	0.4950000
28885.0000000	0.5000000
28886.0000000	0.5000000
28887.0000000	0.4900000
28888.0000000	0.4900000
28889.0000000	0.4900000
28890.0000000	0.4950000
28891.0000000	0.4900000
28892.0000000	0.4900000
28893.0000000	0.4900000
28894.0000000	0.4900000
28895.0000000	0.4900000
28896.0000000	0.5000000
28897.0000000	0.4950000
28898.0000000	0.4950000
28899.0000000	0.4900000
28900.0000000	0.5000000
28901.0000000	0.4950000
28902.0000000	0.4950000
28903.0000000	0.4900000
28904.0000000	0.4900000
28905.0000000	0.4800000
28906.0000000	0.4800000
28907.0000000	0.4700000
28908.0000000	0.4700000
28909.0000000	0.4750000
28910.0000000	0.4800000
28911.0000000	0.4800000
28912.0000000	0.4750000
28913.0000000	0.4800000
28914.0000000	0.4850000
28915.0000000	0.4850000
28916.0000000	0.4850000
28917.0000000	0.4800000
28918.0000000	0.4800000
28919.0000000	0.4800000
28920.0000000	0.4800000
28921.0000000	0.4750000
28922.0000000	0.4800000
28923.0000000	0.4800000
28924.0000000	0.4850000
28925.0000000	0.4800000
28926.0000000	0.4800000
28927.0000000	0.4800000
28928.0000000	0.4750000
28929.0000000	0.4700000
28930.0000000	0.4650000
28931.0000000	0.4550000

28932.0000000	0.4600000
28933.0000000	0.4500000
28934.0000000	0.4450000
28935.0000000	0.4433333
28936.0000000	0.4400000
28937.0000000	0.4400000
28938.0000000	0.4450000
28939.0000000	0.4450000
28940.0000000	0.4450000
28941.0000000	0.4500000
28942.0000000	0.4550000
28943.0000000	0.4600000
28944.0000000	0.4600000
28945.0000000	0.4600000
28946.0000000	0.4700000
28947.0000000	0.4700000
28948.0000000	0.4750000
28949.0000000	0.4750000
28950.0000000	0.4900000
28951.0000000	0.4900000
28952.0000000	0.4900000
28953.0000000	0.4900000
28954.0000000	0.4950000
28955.0000000	0.4900000
28956.0000000	0.4900000
28957.0000000	0.4950000
28958.0000000	0.4900000
28959.0000000	0.4900000
28960.0000000	0.4900000
28961.0000000	0.4950000
28962.0000000	0.4800000
28963.0000000	0.4900000
28964.0000000	0.4950000
28965.0000000	0.4900000
28966.0000000	0.5000000
28967.0000000	0.5000000
28968.0000000	0.4950000
28969.0000000	0.4900000
28970.0000000	0.4950000
28971.0000000	0.4950000
28972.0000000	0.4900000
28973.0000000	0.4850000
28974.0000000	0.4800000
28975.0000000	0.4850000
28976.0000000	0.4700000
28977.0000000	0.4700000
28978.0000000	0.4700000
28979.0000000	0.4750000
28980.0000000	0.4700000
28981.0000000	0.4600000
28982.0000000	0.4600000
28983.0000000	0.4550000
28984.0000000	0.4500000
28985.0000000	0.4500000
28986.0000000	0.4450000
28987.0000000	0.4400000
28988.0000000	0.4450000

28989.0000000	0.4450000
28990.0000000	0.4450000
28991.0000000	0.4400000
28992.0000000	0.4450000
28993.0000000	0.4500000
28994.0000000	0.4600000
28995.0000000	0.4600000
28996.0000000	0.4650000
28997.0000000	0.4700000
28998.0000000	0.4750000
28999.0000000	0.4800000
29000.0000000	0.4800000
29001.0000000	0.4850000
29002.0000000	0.4900000
29003.0000000	0.4900000
29004.0000000	0.4900000
29005.0000000	0.4950000
29006.0000000	0.4900000
29007.0000000	0.4850000
29008.0000000	0.4800000
29009.0000000	0.4800000
29010.0000000	0.4750000
29011.0000000	0.4800000
29012.0000000	0.4700000
29013.0000000	0.4600000
29014.0000000	0.4600000
29015.0000000	0.4600000
29016.0000000	0.4600000
29017.0000000	0.4600000
29018.0000000	0.4650000
29019.0000000	0.4750000
29020.0000000	0.4750000
29021.0000000	0.4750000
29022.0000000	0.4850000
29023.0000000	0.4850000
29024.0000000	0.4900000
29025.0000000	0.4900000
29026.0000000	0.4933333
29027.0000000	0.4900000
29028.0000000	0.5000000
29029.0000000	0.4950000
29030.0000000	0.4900000
29031.0000000	0.4950000
29032.0000000	0.4900000
29033.0000000	0.4900000
29034.0000000	0.4950000
29035.0000000	0.4950000
29036.0000000	0.4950000
29037.0000000	0.5000000
29038.0000000	0.4950000
29039.0000000	0.4950000
29040.0000000	0.4950000
29041.0000000	0.4900000
29042.0000000	0.4900000
29043.0000000	0.4950000
29044.0000000	0.4900000
29045.0000000	0.4950000

29046.0000000	0.4900000
29047.0000000	0.5000000
29048.0000000	0.5000000
29049.0000000	0.5000000
29050.0000000	0.5050000
29051.0000000	0.5100000
29052.0000000	0.5150000
29053.0000000	0.5250000
29054.0000000	0.5200000
29055.0000000	0.5250000
29056.0000000	0.5266666
29057.0000000	0.5250000
29058.0000000	0.5300000
29059.0000000	0.5350000
29060.0000000	0.5400000
29061.0000000	0.5400000
29062.0000000	0.5400000
29063.0000000	0.5500000
29064.0000000	0.5500000
29065.0000000	0.5500000
29066.0000000	0.5500000
29067.0000000	0.5400000
29068.0000000	0.5400000
29069.0000000	0.5400000
29070.0000000	0.5400000
29071.0000000	0.5400000
29072.0000000	0.5450000
29073.0000000	0.5400000
29074.0000000	0.5400000
29075.0000000	0.5400000
29076.0000000	0.5350000
29077.0000000	0.5450000
29078.0000000	0.5400000
29079.0000000	0.5450000
29080.0000000	0.5550000
29081.0000000	0.5550000
29082.0000000	0.5600000
29083.0000000	0.5650000
29084.0000000	0.5700000
29085.0000000	0.5750000
29086.0000000	0.5800000
29087.0000000	0.5866666
29088.0000000	0.5900000
29089.0000000	0.6000000
29090.0000000	0.6050000
29091.0000000	0.6050000
29092.0000000	0.6100000
29093.0000000	0.6100000
29094.0000000	0.6150000
29095.0000000	0.6200000
29096.0000000	0.6200000
29097.0000000	0.6200000
29098.0000000	0.6300000
29099.0000000	0.6350000
29100.0000000	0.6350000
29101.0000000	0.6450000
29102.0000000	0.6500000

29103.0000000	0.6550000
29104.0000000	0.6600000
29105.0000000	0.6700000
29106.0000000	0.6750000
29107.0000000	0.6850000
29108.0000000	0.6950000
29109.0000000	0.6900000
29110.0000000	0.7000000
29111.0000000	0.7000000
29112.0000000	0.7100000
29113.0000000	0.7150000
29114.0000000	0.7250000
29115.0000000	0.7200000
29116.0000000	0.7300000
29117.0000000	0.7366667
29118.0000000	0.7450000
29119.0000000	0.7500000
29120.0000000	0.7550000
29121.0000000	0.7700000
29122.0000000	0.7700000
29123.0000000	0.7700000
29124.0000000	0.7800000
29125.0000000	0.7900000
29126.0000000	0.7900000
29127.0000000	0.8000000
29128.0000000	0.8000000
29129.0000000	0.8100000
29130.0000000	0.8150000
29131.0000000	0.8200000
29132.0000000	0.8250000
29133.0000000	0.8300000
29134.0000000	0.8350000
29135.0000000	0.8350000
29136.0000000	0.8350000
29137.0000000	0.8400000
29138.0000000	0.8450000
29139.0000000	0.8450000
29140.0000000	0.8550000
29141.0000000	0.8550000
29142.0000000	0.8550000
29143.0000000	0.8600000
29144.0000000	0.8600000
29145.0000000	0.8650000
29146.0000000	0.8700000
29147.0000000	0.8666666
29148.0000000	0.8700000
29149.0000000	0.8800000
29150.0000000	0.8800000
29151.0000000	0.8800000
29152.0000000	0.8750000
29153.0000000	0.8850000
29154.0000000	0.8950000
29155.0000000	0.9000000
29156.0000000	0.9000000
29157.0000000	0.9000000
29158.0000000	0.9050000
29159.0000000	0.9200000

29160.0000000	0.9200000
29161.0000000	0.9250000
29162.0000000	0.9250000
29163.0000000	0.9300000
29164.0000000	0.9450000
29165.0000000	0.9500000
29166.0000000	0.9500000
29167.0000000	0.9550000
29168.0000000	0.9500000
29169.0000000	0.9550000
29170.0000000	0.9500000
29171.0000000	0.9450000
29172.0000000	0.9550000
29173.0000000	0.9550000
29174.0000000	0.9600000
29175.0000000	0.9550000
29176.0000000	0.9550000
29177.0000000	0.9500000
29178.0000000	0.9566666
29179.0000000	0.9500000
29180.0000000	0.9550000
29181.0000000	0.9400000
29182.0000000	0.9350000
29183.0000000	0.9300000
29184.0000000	0.9300000
29185.0000000	0.9300000
29186.0000000	0.9350000
29187.0000000	0.9400000
29188.0000000	0.9400000
29189.0000000	0.9450000
29190.0000000	0.9500000
29191.0000000	0.9500000
29192.0000000	0.9550000
29193.0000000	0.9650000
29194.0000000	0.9750000
29195.0000000	0.9950000
29196.0000000	0.9900000
29197.0000000	0.9950000
29198.0000000	1.0000000
29199.0000000	1.0050000
29200.0000000	1.0050000
29201.0000000	1.0100000
29202.0000000	1.0100000
29203.0000000	1.0250000
29204.0000000	1.0300000
29205.0000000	1.0400000
29206.0000000	1.0400000
29207.0000000	1.0500000
29208.0000000	1.0549999
29209.0000000	1.0633334
29210.0000000	1.0700001
29211.0000000	1.0800000
29212.0000000	1.0900000
29213.0000000	1.0900000
29214.0000000	1.1000000
29215.0000000	1.1100000
29216.0000000	1.1250000

29217.0000000	1.1250000
29218.0000000	1.1400000
29219.0000000	1.1400000
29220.0000000	1.1400000
29221.0000000	1.1450000
29222.0000000	1.1500000
29223.0000000	1.1600000
29224.0000000	1.1650000
29225.0000000	1.1600000
29226.0000000	1.1750000
29227.0000000	1.1799999
29228.0000000	1.1900001
29229.0000000	1.2000000
29230.0000000	1.2000000
29231.0000000	1.2050000
29232.0000000	1.2100000
29233.0000000	1.2200000
29234.0000000	1.2300000
29235.0000000	1.2450000
29236.0000000	1.2450000
29237.0000000	1.2600000
29238.0000000	1.2700000
29239.0000000	1.2750000
29240.0000000	1.2800000
29241.0000000	1.2900000
29242.0000000	1.2950000
29243.0000000	1.3049999
29244.0000000	1.3200001
29245.0000000	1.3250000
29246.0000000	1.3250000
29247.0000000	1.3250000
29248.0000000	1.3300000
29249.0000000	1.3350000
29250.0000000	1.3400000
29251.0000000	1.3300000
29252.0000000	1.3350000
29253.0000000	1.3450000
29254.0000000	1.3400000
29255.0000000	1.3450000
29256.0000000	1.3550000
29257.0000000	1.3650000
29258.0000000	1.3650000
29259.0000000	1.3800000
29260.0000000	1.3900000
29261.0000000	1.3900000
29262.0000000	1.4000000
29263.0000000	1.4050000
29264.0000000	1.4100000
29265.0000000	1.4250000
29266.0000000	1.4349999
29267.0000000	1.4400001
29268.0000000	1.4433333
29269.0000000	1.4550000
29270.0000000	1.4600000
29271.0000000	1.4550000
29272.0000000	1.4500000
29273.0000000	1.4600000

29274.0000000	1.4550000
29275.0000000	1.4500000
29276.0000000	1.4400001
29277.0000000	1.4400001
29278.0000000	1.4299999
29279.0000000	1.4299999
29280.0000000	1.4200000
29281.0000000	1.4200000
29282.0000000	1.4200000
29283.0000000	1.4200000
29284.0000000	1.4100000
29285.0000000	1.4200000
29286.0000000	1.4200000
29287.0000000	1.4200000
29288.0000000	1.4100000
29289.0000000	1.4100000
29290.0000000	1.4100000
29291.0000000	1.4050000
29292.0000000	1.4100000
29293.0000000	1.4050000
29294.0000000	1.4100000
29295.0000000	1.4000000
29296.0000000	1.4000000
29297.0000000	1.4000000
29298.0000000	1.4033333
29299.0000000	1.4000000
29300.0000000	1.3900000
29301.0000000	1.3950000
29302.0000000	1.3950000
29303.0000000	1.3950000
29304.0000000	1.3900000
29305.0000000	1.3950000
29306.0000000	1.4000000
29307.0000000	1.3950000
29308.0000000	1.3850000
29309.0000000	1.3800000
29310.0000000	1.3800000
29311.0000000	1.3700000
29312.0000000	1.3600000
29313.0000000	1.3500000
29314.0000000	1.3600000
29315.0000000	1.3600000
29316.0000000	1.3650000
29317.0000000	1.3700000
29318.0000000	1.3800000
29319.0000000	1.3850000
29320.0000000	1.4000000
29321.0000000	1.4000000
29322.0000000	1.4000000
29323.0000000	1.4000000
29324.0000000	1.4000000
29325.0000000	1.4000000
29326.0000000	1.4100000
29327.0000000	1.4050000
29328.0000000	1.4200000
29329.0000000	1.4200000
29330.0000000	1.4299999

29331.0000000	1.4400001
29332.0000000	1.4450001
29333.0000000	1.4600000
29334.0000000	1.4600000
29335.0000000	1.4650000
29336.0000000	1.4750000
29337.0000000	1.4750000
29338.0000000	1.4900000
29339.0000000	1.4950000
29340.0000000	1.5000000
29341.0000000	1.5000000
29342.0000000	1.5000000
29343.0000000	1.4950000
29344.0000000	1.5000000
29345.0000000	1.5050000
29346.0000000	1.5100000
29347.0000000	1.5100000
29348.0000000	1.5150000
29349.0000000	1.5050000
29350.0000000	1.5050000
29351.0000000	1.5150000
29352.0000000	1.5200000
29353.0000000	1.5300000
29354.0000000	1.5350000
29355.0000000	1.5400000
29356.0000000	1.5400000
29357.0000000	1.5500000
29358.0000000	1.5650001
29359.0000000	1.5650001
29360.0000000	1.5700001
29361.0000000	1.5750000
29362.0000000	1.5700001
29363.0000000	1.5800000
29364.0000000	1.5700001
29365.0000000	1.5700001
29366.0000000	1.5750000
29367.0000000	1.5750000
29368.0000000	1.5750000
29369.0000000	1.5850000
29370.0000000	1.5850000
29371.0000000	1.5900000
29372.0000000	1.6000000
29373.0000000	1.6100000
29374.0000000	1.6100000
29375.0000000	1.6150000
29376.0000000	1.6100000
29377.0000000	1.6150000
29378.0000000	1.6100000
29379.0000000	1.6100000
29380.0000000	1.6150000
29381.0000000	1.6300000
29382.0000000	1.6350000
29383.0000000	1.6450000
29384.0000000	1.6550000
29385.0000000	1.6600000
29386.0000000	1.6650000
29387.0000000	1.6750000

29388.0000000	1.6700000
29389.0000000	1.6700000
29390.0000000	1.6600000
29391.0000000	1.6600000
29392.0000000	1.6700000
29393.0000000	1.6650000
29394.0000000	1.6700000
29395.0000000	1.6700000
29396.0000000	1.6750000
29397.0000000	1.6700000
29398.0000000	1.6799999
29399.0000000	1.6799999
29400.0000000	1.6849999
29401.0000000	1.6900001
29402.0000000	1.6900001
29403.0000000	1.6950001
29404.0000000	1.7000000
29405.0000000	1.6950001
29406.0000000	1.6950001
29407.0000000	1.7000000
29408.0000000	1.7050000
29409.0000000	1.7000000
29410.0000000	1.7150000
29411.0000000	1.7200000
29412.0000000	1.7200000
29413.0000000	1.7150000
29414.0000000	1.7300000
29415.0000000	1.7300000
29416.0000000	1.7300000
29417.0000000	1.7250000
29418.0000000	1.7300000
29419.0000000	1.7366667
29420.0000000	1.7300000
29421.0000000	1.7300000
29422.0000000	1.7200000
29423.0000000	1.7050000
29424.0000000	1.7200000
29425.0000000	1.7150000
29426.0000000	1.7250000
29427.0000000	1.7200000
29428.0000000	1.7400000
29429.0000000	1.7300000
29430.0000000	1.7350000
29431.0000000	1.7350000
29432.0000000	1.7300000
29433.0000000	1.7300000
29434.0000000	1.7300000
29435.0000000	1.7350000
29436.0000000	1.7400000
29437.0000000	1.7450000
29438.0000000	1.7500000
29439.0000000	1.7500000
29440.0000000	1.7650000
29441.0000000	1.7600000
29442.0000000	1.7700000
29443.0000000	1.7600000
29444.0000000	1.7700000

29445.0000000	1.7700000
29446.0000000	1.7750000
29447.0000000	1.7600000
29448.0000000	1.7700000
29449.0000000	1.7600000
29450.0000000	1.7633333
29451.0000000	1.7700000
29452.0000000	1.7700000
29453.0000000	1.7600000
29454.0000000	1.7500000
29455.0000000	1.7550000
29456.0000000	1.7550000
29457.0000000	1.7500000
29458.0000000	1.7500000
29459.0000000	1.7500000
29460.0000000	1.7500000
29461.0000000	1.7550000
29462.0000000	1.7650000
29463.0000000	1.7600000
29464.0000000	1.7700000
29465.0000000	1.7800000
29466.0000000	1.7800000
29467.0000000	1.7800000
29468.0000000	1.7900000
29469.0000000	1.7900000
29470.0000000	1.7850000
29471.0000000	1.7900000
29472.0000000	1.7900000
29473.0000000	1.7850000
29474.0000000	1.7800000
29475.0000000	1.7900000
29476.0000000	1.7800000
29477.0000000	1.7800000
29478.0000000	1.7550000
29479.0000000	1.7250000
29480.0000000	1.7100000
29481.0000000	1.6966667
29482.0000000	1.6800001
29483.0000000	1.6750000
29484.0000000	1.6600000
29485.0000000	1.6600000
29486.0000000	1.6500000
29487.0000000	1.6500000
29488.0000000	1.6500000
29489.0000000	1.6550000
29490.0000000	1.6500000
29491.0000000	1.6400000
29492.0000000	1.6300000
29493.0000000	1.6200000
29494.0000000	1.6250000
29495.0000000	1.6300000
29496.0000000	1.6250000
29497.0000000	1.6300000
29498.0000000	1.6400000
29499.0000000	1.6400000
29500.0000000	1.6400000
29501.0000000	1.6500000

29502.0000000	1.6600000
29503.0000000	1.6700000
29504.0000000	1.6900001
29505.0000000	1.7000000
29506.0000000	1.7150000
29507.0000000	1.7250000
29508.0000000	1.7450000
29509.0000000	1.7500000
29510.0000000	1.7600000
29511.0000000	1.7600000
29512.0000000	1.7766666
29513.0000000	1.7900000
29514.0000000	1.7900000
29515.0000000	1.7950000
29516.0000000	1.7950000
29517.0000000	1.7950000
29518.0000000	1.7850000
29519.0000000	1.7900000
29520.0000000	1.7950000
29521.0000000	1.7950000
29522.0000000	1.8000000
29523.0000000	1.8099999
29524.0000000	1.8099999
29525.0000000	1.8099999
29526.0000000	1.8250000
29527.0000000	1.8250000
29528.0000000	1.8350000
29529.0000000	1.8300000
29530.0000000	1.8350000
29531.0000000	1.8400000
29532.0000000	1.8450000
29533.0000000	1.8400000
29534.0000000	1.8450000
29535.0000000	1.8550000
29536.0000000	1.8600000
29537.0000000	1.8600000
29538.0000000	1.8600000
29539.0000000	1.8550000
29540.0000000	1.8466667
29541.0000000	1.8600000
29542.0000000	1.8650000
29543.0000000	1.8800000
29544.0000000	1.8700000
29545.0000000	1.8700000
29546.0000000	1.8700000
29547.0000000	1.8750000
29548.0000000	1.8700000
29549.0000000	1.8700000
29550.0000000	1.8750000
29551.0000000	1.8800000
29552.0000000	1.8800000
29553.0000000	1.8800000
29554.0000000	1.8750000
29555.0000000	1.8600000
29556.0000000	1.8550000
29557.0000000	1.8500000
29558.0000000	1.8500000

29559.0000000	1.8500000
29560.0000000	1.8450000
29561.0000000	1.8400000
29562.0000000	1.8400000
29563.0000000	1.8450000
29564.0000000	1.8400000
29565.0000000	1.8350000
29566.0000000	1.8350000
29567.0000000	1.8300000
29568.0000000	1.8200001
29569.0000000	1.8150001
29570.0000000	1.8150001
29571.0000000	1.8200001
29572.0000000	1.8300000
29573.0000000	1.8350000
29574.0000000	1.8400000
29575.0000000	1.8500000
29576.0000000	1.8600000
29577.0000000	1.8700000
29578.0000000	1.8750000
29579.0000000	1.8800000
29580.0000000	1.8900000
29581.0000000	1.8950000
29582.0000000	1.8900000
29583.0000000	1.8850000
29584.0000000	1.8800000
29585.0000000	1.8800000
29586.0000000	1.8750000
29587.0000000	1.8650000
29588.0000000	1.8650000
29589.0000000	1.8700000
29590.0000000	1.8600000
29591.0000000	1.8500000
29592.0000000	1.8550000
29593.0000000	1.8500000
29594.0000000	1.8600000
29595.0000000	1.8550000
29596.0000000	1.8750000
29597.0000000	1.8850000
29598.0000000	1.8950000
29599.0000000	1.9150000
29600.0000000	1.9233333
29601.0000000	1.9250000
29602.0000000	1.9349999
29603.0000000	1.9500000
29604.0000000	1.9500000
29605.0000000	1.9500000
29606.0000000	1.9450001
29607.0000000	1.9550000
29608.0000000	1.9500000
29609.0000000	1.9550000
29610.0000000	1.9500000
29611.0000000	1.9600000
29612.0000000	1.9650000
29613.0000000	1.9650000
29614.0000000	1.9600000
29615.0000000	1.9500000

29616.0000000	1.9600000
29617.0000000	1.9600000
29618.0000000	1.9600000
29619.0000000	1.9600000
29620.0000000	1.9600000
29621.0000000	1.9650000
29622.0000000	1.9600000
29623.0000000	1.9600000
29624.0000000	1.9650000
29625.0000000	1.9600000
29626.0000000	1.9600000
29627.0000000	1.9600000
29628.0000000	1.9500000
29629.0000000	1.9533334
29630.0000000	1.9600000
29631.0000000	1.9433333
29632.0000000	1.9450001
29633.0000000	1.9450001
29634.0000000	1.9349999
29635.0000000	1.9349999
29636.0000000	1.9299999
29637.0000000	1.9400001
29638.0000000	1.9400001
29639.0000000	1.9500000
29640.0000000	1.9550000
29641.0000000	1.9550000
29642.0000000	1.9600000
29643.0000000	1.9650000
29644.0000000	1.9750000
29645.0000000	1.9900000
29646.0000000	1.9900000
29647.0000000	1.9900000
29648.0000000	2.0000000
29649.0000000	2.0050001
29650.0000000	2.0000000
29651.0000000	2.0100000
29652.0000000	2.0149999
29653.0000000	2.0100000
29654.0000000	2.0100000
29655.0000000	2.0100000
29656.0000000	2.0250001
29657.0000000	2.0200000
29658.0000000	2.0300000
29659.0000000	2.0349998
29660.0000000	2.0349998
29661.0000000	2.0300000
29662.0000000	2.0333331
29663.0000000	2.0300000
29664.0000000	2.0250001
29665.0000000	2.0300000
29666.0000000	2.0250001
29667.0000000	2.0250001
29668.0000000	2.0300000
29669.0000000	2.0250001
29670.0000000	2.0250001
29671.0000000	2.0349998
29672.0000000	2.0300000

29673.0000000	2.0250001
29674.0000000	2.0250001
29675.0000000	2.0349998
29676.0000000	2.0349998
29677.0000000	2.0349998
29678.0000000	2.0500000
29679.0000000	2.0599999
29680.0000000	2.0599999
29681.0000000	2.0599999
29682.0000000	2.0650001
29683.0000000	2.0699999
29684.0000000	2.0699999
29685.0000000	2.0699999
29686.0000000	2.0749998
29687.0000000	2.0699999
29688.0000000	2.0749998
29689.0000000	2.0650001
29690.0000000	2.0699999
29691.0000000	2.0599999
29692.0000000	2.0699999
29693.0000000	2.0650001
29694.0000000	2.0749998
29695.0000000	2.0799999
29696.0000000	2.0799999
29697.0000000	2.0850000
29698.0000000	2.0850000
29699.0000000	2.0850000
29700.0000000	2.0799999
29701.0000000	2.0749998
29702.0000000	2.0650001
29703.0000000	2.0650001
29704.0000000	2.0650001
29705.0000000	2.0699999
29706.0000000	2.0650001
29707.0000000	2.0749998
29708.0000000	2.0699999
29709.0000000	2.0749998
29710.0000000	2.0799999
29711.0000000	2.0899999
29712.0000000	2.0899999
29713.0000000	2.0999999
29714.0000000	2.0949998
29715.0000000	2.0999999
29716.0000000	2.1050000
29717.0000000	2.1099999
29718.0000000	2.1099999
29719.0000000	2.1099999
29720.0000000	2.1050000
29721.0000000	2.1099999
29722.0000000	2.0999999
29723.0000000	2.1050000
29724.0000000	2.1050000
29725.0000000	2.1050000
29726.0000000	2.1149998
29727.0000000	2.1050000
29728.0000000	2.0949998
29729.0000000	2.0949998

29730.0000000	2.0899999
29731.0000000	2.0899999
29732.0000000	2.0899999
29733.0000000	2.0850000
29734.0000000	2.0949998
29735.0000000	2.0949998
29736.0000000	2.0949998
29737.0000000	2.0899999
29738.0000000	2.0949998
29739.0000000	2.0899999
29740.0000000	2.0850000
29741.0000000	2.0749998
29742.0000000	2.0749998
29743.0000000	2.0699999
29744.0000000	2.0749998
29745.0000000	2.0699999
29746.0000000	2.0799999
29747.0000000	2.0749998
29748.0000000	2.0850000
29749.0000000	2.0850000
29750.0000000	2.0899999
29751.0000000	2.0933332
29752.0000000	2.0999999
29753.0000000	2.0899999
29754.0000000	2.0699999
29755.0000000	2.0699999
29756.0000000	2.0699999
29757.0000000	2.0699999
29758.0000000	2.0799999
29759.0000000	2.0899999
29760.0000000	2.0850000
29761.0000000	2.0799999
29762.0000000	2.0799999
29763.0000000	2.0850000
29764.0000000	2.0850000
29765.0000000	2.0799999
29766.0000000	2.0899999
29767.0000000	2.0949998
29768.0000000	2.0949998
29769.0000000	2.0949998
29770.0000000	2.0899999
29771.0000000	2.0899999
29772.0000000	2.0799999
29773.0000000	2.0799999
29774.0000000	2.0899999
29775.0000000	2.0899999
29776.0000000	2.0949998
29777.0000000	2.1050000
29778.0000000	2.0999999
29779.0000000	2.1050000
29780.0000000	2.0999999
29781.0000000	2.0966666
29782.0000000	2.0999999
29783.0000000	2.0949998
29784.0000000	2.0899999
29785.0000000	2.0899999
29786.0000000	2.0899999

29787.0000000	2.0850000
29788.0000000	2.0850000
29789.0000000	2.0749998
29790.0000000	2.0699999
29791.0000000	2.0650001
29792.0000000	2.0699999
29793.0000000	2.0500000
29794.0000000	2.0549998
29795.0000000	2.0549998
29796.0000000	2.0599999
29797.0000000	2.0599999
29798.0000000	2.0749998
29799.0000000	2.0799999
29800.0000000	2.0749998
29801.0000000	2.0699999
29802.0000000	2.0699999
29803.0000000	2.0650001
29804.0000000	2.0549998
29805.0000000	2.0500000
29806.0000000	2.0500000
29807.0000000	2.0400000
29808.0000000	2.0400000
29809.0000000	2.0400000
29810.0000000	2.0300000
29811.0000000	2.0066667
29812.0000000	2.0100000
29813.0000000	2.0050001
29814.0000000	2.0033333
29815.0000000	1.9950000
29816.0000000	1.9900000
29817.0000000	1.9850000
29818.0000000	1.9800000
29819.0000000	1.9800000
29820.0000000	1.9750000
29821.0000000	1.9700000
29822.0000000	1.9750000
29823.0000000	1.9850000
29824.0000000	1.9800000
29825.0000000	1.9850000
29826.0000000	1.9900000
29827.0000000	1.9850000
29828.0000000	1.9900000
29829.0000000	1.9950000
29830.0000000	2.0000000
29831.0000000	2.0000000
29832.0000000	2.0050001
29833.0000000	2.0100000
29834.0000000	2.0200000
29835.0000000	2.0100000
29836.0000000	2.0100000
29837.0000000	2.0100000
29838.0000000	2.0050001
29839.0000000	2.0100000
29840.0000000	2.0250001
29841.0000000	2.0300000
29842.0000000	2.0300000
29843.0000000	2.0366666

29844.0000000	2.0400000
29845.0000000	2.0450001
29846.0000000	2.0500000
29847.0000000	2.0450001
29848.0000000	2.0500000
29849.0000000	2.0549998
29850.0000000	2.0549998
29851.0000000	2.0599999
29852.0000000	2.0599999
29853.0000000	2.0650001
29854.0000000	2.0599999
29855.0000000	2.0549998
29856.0000000	2.0599999
29857.0000000	2.0500000
29858.0000000	2.0549998
29859.0000000	2.0549998
29860.0000000	2.0549998
29861.0000000	2.0599999
29862.0000000	2.0599999
29863.0000000	2.0599999
29864.0000000	2.0599999
29865.0000000	2.0500000
29866.0000000	2.0400000
29867.0000000	2.0300000
29868.0000000	2.0250001
29869.0000000	2.0149999
29870.0000000	1.9950000
29871.0000000	1.9866667
29872.0000000	1.9750000
29873.0000000	1.9700000
29874.0000000	1.9700000
29875.0000000	1.9750000
29876.0000000	1.9800000
29877.0000000	1.9800000
29878.0000000	1.9650000
29879.0000000	1.9550000
29880.0000000	1.9550000
29881.0000000	1.9450001
29882.0000000	1.9450001
29883.0000000	1.9600000
29884.0000000	1.9650000
29885.0000000	1.9800000
29886.0000000	1.9950000
29887.0000000	2.0050001
29888.0000000	2.0250001
29889.0000000	2.0250001
29890.0000000	2.0349998
29891.0000000	2.0400000
29892.0000000	2.0500000
29893.0000000	2.0500000
29894.0000000	2.0549998
29895.0000000	2.0500000
29896.0000000	2.0450001
29897.0000000	2.0300000
29898.0000000	2.0250001
29899.0000000	2.0100000
29900.0000000	2.0149999

29901.0000000	2.0100000
29902.0000000	2.0200000
29903.0000000	2.0266666
29904.0000000	2.0300000
29905.0000000	2.0300000
29906.0000000	2.0250001
29907.0000000	2.0250001
29908.0000000	2.0349998
29909.0000000	2.0400000
29910.0000000	2.0500000
29911.0000000	2.0599999
29912.0000000	2.0599999
29913.0000000	2.0650001
29914.0000000	2.0699999
29915.0000000	2.0749998
29916.0000000	2.0799999
29917.0000000	2.0899999
29918.0000000	2.0850000
29919.0000000	2.0999999
29920.0000000	2.0999999
29921.0000000	2.0999999
29922.0000000	2.0949998
29923.0000000	2.0850000
29924.0000000	2.0850000
29925.0000000	2.0799999
29926.0000000	2.0599999
29927.0000000	2.0450001
29928.0000000	2.0349998
29929.0000000	2.0200000
29930.0000000	2.0050001
29931.0000000	1.9850000
29932.0000000	1.9766668
29933.0000000	1.9800000
29934.0000000	1.9766668
29935.0000000	1.9750000
29936.0000000	1.9800000
29937.0000000	1.9900000
29938.0000000	2.0000000
29939.0000000	2.0149999
29940.0000000	2.0300000
29941.0000000	2.0549998
29942.0000000	2.0650001
29943.0000000	2.0749998
29944.0000000	2.0949998
29945.0000000	2.0999999
29946.0000000	2.1050000
29947.0000000	2.1149998
29948.0000000	2.1300001
29949.0000000	2.1400001
29950.0000000	2.1450000
29951.0000000	2.1550002
29952.0000000	2.1600001
29953.0000000	2.1750002
29954.0000000	2.1800001
29955.0000000	2.1750002
29956.0000000	2.1700001
29957.0000000	2.1700001

29958.0000000	2.1800001
29959.0000000	2.1849999
29960.0000000	2.2049999
29961.0000000	2.2049999
29962.0000000	2.2150002
29963.0000000	2.2233334
29964.0000000	2.2249999
29965.0000000	2.2300000
29966.0000000	2.2249999
29967.0000000	2.2300000
29968.0000000	2.2150002
29969.0000000	2.2100000
29970.0000000	2.2100000
29971.0000000	2.2100000
29972.0000000	2.2300000
29973.0000000	2.2249999
29974.0000000	2.2449999
29975.0000000	2.2649999
29976.0000000	2.2800000
29977.0000000	2.2849998
29978.0000000	2.3000000
29979.0000000	2.3099999
29980.0000000	2.3150001
29981.0000000	2.3299999
29982.0000000	2.3350000
29983.0000000	2.3399999
29984.0000000	2.3399999
29985.0000000	2.3350000
29986.0000000	2.3350000
29987.0000000	2.3249998
29988.0000000	2.3299999
29989.0000000	2.3249998
29990.0000000	2.3150001
29991.0000000	2.3099999
29992.0000000	2.3000000
29993.0000000	2.3000000
29994.0000000	2.3000000
29995.0000000	2.3066666
29996.0000000	2.3049998
29997.0000000	2.3000000
29998.0000000	2.2950001
29999.0000000	2.2849998
30000.0000000	2.2800000
30001.0000000	2.2800000
30002.0000000	2.2750001
30003.0000000	2.2700000
30004.0000000	2.2750001
30005.0000000	2.2700000
30006.0000000	2.2750001
30007.0000000	2.2800000
30008.0000000	2.2700000
30009.0000000	2.2700000
30010.0000000	2.2750001
30011.0000000	2.2649999
30012.0000000	2.2750001
30013.0000000	2.2849998
30014.0000000	2.2800000

30015.0000000	2.2900000
30016.0000000	2.3049998
30017.0000000	2.3199999
30018.0000000	2.3449998
30019.0000000	2.3599999
30020.0000000	2.3800001
30021.0000000	2.3900001
30022.0000000	2.3950000
30023.0000000	2.4100001
30024.0000000	2.4300001
30025.0000000	2.4400001
30026.0000000	2.4600000
30027.0000000	2.4749999
30028.0000000	2.4900000
30029.0000000	2.4900000
30030.0000000	2.5050001
30031.0000000	2.5100000
30032.0000000	2.5149999
30033.0000000	2.5250001
30034.0000000	2.5300000
30035.0000000	2.5349998
30036.0000000	2.5450001
30037.0000000	2.5450001
30038.0000000	2.5500000
30039.0000000	2.5450001
30040.0000000	2.5500000
30041.0000000	2.5400000
30042.0000000	2.5450001
30043.0000000	2.5450001
30044.0000000	2.5400000
30045.0000000	2.5450001
30046.0000000	2.5400000
30047.0000000	2.5400000
30048.0000000	2.5349998
30049.0000000	2.5200000
30050.0000000	2.5200000
30051.0000000	2.5149999
30052.0000000	2.5149999
30053.0000000	2.5100000
30054.0000000	2.5100000
30055.0000000	2.4949999
30056.0000000	2.5000000
30057.0000000	2.4900000
30058.0000000	2.4949999
30059.0000000	2.4850001
30060.0000000	2.5050001
30061.0000000	2.5100000
30062.0000000	2.5250001
30063.0000000	2.5500000
30064.0000000	2.5500000
30065.0000000	2.5650001
30066.0000000	2.5799999
30067.0000000	2.5899999
30068.0000000	2.6099999
30069.0000000	2.6250000
30070.0000000	2.6300001
30071.0000000	2.6450000

30072.0000000	2.6550002
30073.0000000	2.6600001
30074.0000000	2.6700001
30075.0000000	2.6750002
30076.0000000	2.6800001
30077.0000000	2.6900001
30078.0000000	2.6900001
30079.0000000	2.6950002
30080.0000000	2.6950002
30081.0000000	2.7049999
30082.0000000	2.7150002
30083.0000000	2.7266667
30084.0000000	2.7449999
30085.0000000	2.7449999
30086.0000000	2.7500000
30087.0000000	2.7649999
30088.0000000	2.7700000
30089.0000000	2.7900000
30090.0000000	2.8000000
30091.0000000	2.8000000
30092.0000000	2.8049998
30093.0000000	2.8150001
30094.0000000	2.8150001
30095.0000000	2.8350000
30096.0000000	2.8399999
30097.0000000	2.8499999
30098.0000000	2.8599999
30099.0000000	2.8699999
30100.0000000	2.8850002
30101.0000000	2.9000001
30102.0000000	2.9100001
30103.0000000	2.9100001
30104.0000000	2.9349999
30105.0000000	2.9450002
30106.0000000	2.9700000
30107.0000000	2.9749999
30108.0000000	2.9900000
30109.0000000	3.0050001
30110.0000000	3.0149999
30111.0000000	3.0250001
30112.0000000	3.0250001
30113.0000000	3.0233333
30114.0000000	3.0250001
30115.0000000	3.0250001
30116.0000000	3.0200000
30117.0000000	3.0200000
30118.0000000	3.0200000
30119.0000000	3.0250001
30120.0000000	3.0250001
30121.0000000	3.0250001
30122.0000000	3.0250001
30123.0000000	3.0300000
30124.0000000	3.0149999
30125.0000000	3.0250001
30126.0000000	3.0250001
30127.0000000	3.0349998
30128.0000000	3.0250001

30129.0000000	3.0349998
30130.0000000	3.0300000
30131.0000000	3.0300000
30132.0000000	3.0300000
30133.0000000	3.0250001
30134.0000000	3.0250001
30135.0000000	3.0250001
30136.0000000	3.0250001
30137.0000000	3.0300000
30138.0000000	3.0349998
30139.0000000	3.0300000
30140.0000000	3.0349998
30141.0000000	3.0450001
30142.0000000	3.0549998
30143.0000000	3.0549998
30144.0000000	3.0633335
30145.0000000	3.0650001
30146.0000000	3.0599999
30147.0000000	3.0599999
30148.0000000	3.0500000
30149.0000000	3.0400000
30150.0000000	3.0400000
30151.0000000	3.0400000
30152.0000000	3.0450001
30153.0000000	3.0349998
30154.0000000	3.0400000
30155.0000000	3.0300000
30156.0000000	3.0300000
30157.0000000	3.0300000
30158.0000000	3.0400000
30159.0000000	3.0300000
30160.0000000	3.0400000
30161.0000000	3.0300000
30162.0000000	3.0349998
30163.0000000	3.0300000
30164.0000000	3.0300000
30165.0000000	3.0050001
30166.0000000	3.0050001
30167.0000000	2.9949999
30168.0000000	2.9800000
30169.0000000	2.9850001
30170.0000000	2.9800000
30171.0000000	2.9949999
30172.0000000	3.0100000
30173.0000000	3.0200000
30174.0000000	3.0433333
30175.0000000	3.0650001
30176.0000000	3.0850000
30177.0000000	3.1099999
30178.0000000	3.1300001
30179.0000000	3.1300001
30180.0000000	3.1400001
30181.0000000	3.1450000
30182.0000000	3.1500001
30183.0000000	3.1450000
30184.0000000	3.1300001
30185.0000000	3.1400001

30186.0000000	3.1350002
30187.0000000	3.1350002
30188.0000000	3.1350002
30189.0000000	3.1300001
30190.0000000	3.1149998
30191.0000000	3.1149998
30192.0000000	3.1149998
30193.0000000	3.1250000
30194.0000000	3.1350002
30195.0000000	3.1500001
30196.0000000	3.1600001
30197.0000000	3.1600001
30198.0000000	3.1550002
30199.0000000	3.1600001
30200.0000000	3.1750002
30201.0000000	3.1800001
30202.0000000	3.1950002
30203.0000000	3.2100000
30204.0000000	3.2150002
30205.0000000	3.2350001
30206.0000000	3.2400000
30207.0000000	3.2500000
30208.0000000	3.2550001
30209.0000000	3.2649999
30210.0000000	3.2700000
30211.0000000	3.2750001
30212.0000000	3.2900000
30213.0000000	3.3000000
30214.0000000	3.3099999
30215.0000000	3.3299999
30216.0000000	3.3449998
30217.0000000	3.3499999
30218.0000000	3.3649998
30219.0000000	3.3649998
30220.0000000	3.3649998
30221.0000000	3.3649998
30222.0000000	3.3550000
30223.0000000	3.3649998
30224.0000000	3.3599999
30225.0000000	3.3599999
30226.0000000	3.3750000
30227.0000000	3.3800001
30228.0000000	3.3900001
30229.0000000	3.4000001
30230.0000000	3.4100001
30231.0000000	3.4200001
30232.0000000	3.4300001
30233.0000000	3.4400001
30234.0000000	3.4450002
30235.0000000	3.4500000
30236.0000000	3.4400001
30237.0000000	3.4333334
30238.0000000	3.4200001
30239.0000000	3.4050002
30240.0000000	3.4100001
30241.0000000	3.4050002
30242.0000000	3.3950000

30243.0000000	3.3750000
30244.0000000	3.3699999
30245.0000000	3.3649998
30246.0000000	3.3699999
30247.0000000	3.3699999
30248.0000000	3.3699999
30249.0000000	3.3750000
30250.0000000	3.3850002
30251.0000000	3.3850002
30252.0000000	3.3800001
30253.0000000	3.3850002
30254.0000000	3.3850002
30255.0000000	3.3950000
30256.0000000	3.3950000
30257.0000000	3.4050002
30258.0000000	3.4050002
30259.0000000	3.3950000
30260.0000000	3.3900001
30261.0000000	3.3850002
30262.0000000	3.4000001
30263.0000000	3.4050002
30264.0000000	3.4266670
30265.0000000	3.4200001
30266.0000000	3.4300001
30267.0000000	3.4500000
30268.0000000	3.4600000
30269.0000000	3.4700000
30270.0000000	3.4800000
30271.0000000	3.4900000
30272.0000000	3.4949999
30273.0000000	3.5149999
30274.0000000	3.5200000
30275.0000000	3.5450001
30276.0000000	3.5549998
30277.0000000	3.5699999
30278.0000000	3.5799999
30279.0000000	3.5699999
30280.0000000	3.5699999
30281.0000000	3.5749998
30282.0000000	3.5799999
30283.0000000	3.5749998
30284.0000000	3.5850000
30285.0000000	3.5899999
30286.0000000	3.5799999
30287.0000000	3.5850000
30288.0000000	3.5850000
30289.0000000	3.5749998
30290.0000000	3.5749998
30291.0000000	3.5599999
30292.0000000	3.5500000
30293.0000000	3.5549998
30294.0000000	3.5500000
30295.0000000	3.5549998
30296.0000000	3.5400000
30297.0000000	3.5250001
30298.0000000	3.5100000
30299.0000000	3.5000000

30300.0000000	3.4900000
30301.0000000	3.4800000
30302.0000000	3.4749999
30303.0000000	3.4749999
30304.0000000	3.4549999
30305.0000000	3.4450002
30306.0000000	3.4349999
30307.0000000	3.4300001
30308.0000000	3.4349999
30309.0000000	3.4349999
30310.0000000	3.4400001
30311.0000000	3.4500000
30312.0000000	3.4700000
30313.0000000	3.4749999
30314.0000000	3.5000000
30315.0000000	3.5300000
30316.0000000	3.5349998
30317.0000000	3.5599999
30318.0000000	3.5799999
30319.0000000	3.5899999
30320.0000000	3.5749998
30321.0000000	3.5699999
30322.0000000	3.5549998
30323.0000000	3.5599999
30324.0000000	3.5549998
30325.0000000	3.5599997
30326.0000000	3.5500000
30327.0000000	3.5699999
30328.0000000	3.5549998
30329.0000000	3.5799999
30330.0000000	3.5850000
30331.0000000	3.5850000
30332.0000000	3.5850000
30333.0000000	3.5899999
30334.0000000	3.5749998
30335.0000000	3.5799999
30336.0000000	3.5749998
30337.0000000	3.5699999
30338.0000000	3.5749998
30339.0000000	3.5850000
30340.0000000	3.5749998
30341.0000000	3.5650001
30342.0000000	3.5650001
30343.0000000	3.5549998
30344.0000000	3.5549998
30345.0000000	3.5500000
30346.0000000	3.5599999
30347.0000000	3.5749998
30348.0000000	3.5799999
30349.0000000	3.5850000
30350.0000000	3.5899999
30351.0000000	3.6050000
30352.0000000	3.6149998
30353.0000000	3.6350002
30354.0000000	3.6350002
30355.0000000	3.6550002
30356.0000000	3.6800003

30357.0000000	3.6900001
30358.0000000	3.7000000
30359.0000000	3.7150002
30360.0000000	3.7350001
30361.0000000	3.7449999
30362.0000000	3.7550001
30363.0000000	3.7550001
30364.0000000	3.7649999
30365.0000000	3.7649999
30366.0000000	3.7750001
30367.0000000	3.7950001
30368.0000000	3.8049998
30369.0000000	3.8000000
30370.0000000	3.8049998
30371.0000000	3.8049998
30372.0000000	3.8049998
30373.0000000	3.8099999
30374.0000000	3.8199999
30375.0000000	3.8249998
30376.0000000	3.8249998
30377.0000000	3.8299999
30378.0000000	3.8350000
30379.0000000	3.8399999
30380.0000000	3.8499999
30381.0000000	3.8499999
30382.0000000	3.8350000
30383.0000000	3.8249998
30384.0000000	3.8350000
30385.0000000	3.8399999
30386.0000000	3.8649998
30387.0000000	3.8699999
30388.0000000	3.9033334
30389.0000000	3.9300001
30390.0000000	3.9549999
30391.0000000	3.9700000
30392.0000000	3.9850001
30393.0000000	4.0000000
30394.0000000	4.0100002
30395.0000000	4.0200000
30396.0000000	4.0150003
30397.0000000	4.0299997
30398.0000000	4.0349998
30399.0000000	4.0450001
30400.0000000	4.0550003
30401.0000000	4.0799999
30402.0000000	4.0950003
30403.0000000	4.1050000
30404.0000000	4.1199999
30405.0000000	4.1399999
30406.0000000	4.1450000
30407.0000000	4.1599998
30408.0000000	4.1650000
30409.0000000	4.1599998
30410.0000000	4.1750002
30411.0000000	4.1849999
30412.0000000	4.1999998
30413.0000000	4.2100000

30414.0000000	4.2249999
30415.0000000	4.2249999
30416.0000000	4.2366662
30417.0000000	4.2500000
30418.0000000	4.2533336
30419.0000000	4.2600002
30420.0000000	4.2600002
30421.0000000	4.2700000
30422.0000000	4.2700000
30423.0000000	4.2849998
30424.0000000	4.2800002
30425.0000000	4.2849998
30426.0000000	4.2950001
30427.0000000	4.3000002
30428.0000000	4.3050003
30429.0000000	4.3050003
30430.0000000	4.3100004
30431.0000000	4.3150001
30432.0000000	4.3299999
30433.0000000	4.3249998
30434.0000000	4.3199997
30435.0000000	4.3200002
30436.0000000	4.3299999
30437.0000000	4.3299999
30438.0000000	4.3199997
30439.0000000	4.3299999
30440.0000000	4.3199997
30441.0000000	4.3199997
30442.0000000	4.3249998
30443.0000000	4.3400002
30444.0000000	4.3400002
30445.0000000	4.3450003
30446.0000000	4.3433337
30447.0000000	4.3450003
30448.0000000	4.3450003
30449.0000000	4.3450003
30450.0000000	4.3400002
30451.0000000	4.3299999
30452.0000000	4.3100004
30453.0000000	4.3050003
30454.0000000	4.3050003
30455.0000000	4.3050003
30456.0000000	4.2950001
30457.0000000	4.3050003
30458.0000000	4.2950001
30459.0000000	4.2950001
30460.0000000	4.2799997
30461.0000000	4.2500000
30462.0000000	4.2100000
30463.0000000	4.1899996
30464.0000000	4.1650000
30465.0000000	4.1499996
30466.0000000	4.1549997
30467.0000000	4.1599998
30468.0000000	4.1650000
30469.0000000	4.1849999
30470.0000000	4.1949997

30471.0000000	4.2100000
30472.0000000	4.2150002
30473.0000000	4.2399998
30474.0000000	4.2500000
30475.0000000	4.2449999
30476.0000000	4.2600002
30477.0000000	4.2750001
30478.0000000	4.2800002
30479.0000000	4.2800002
30480.0000000	4.2900000
30481.0000000	4.2950001
30482.0000000	4.2900000
30483.0000000	4.2950001
30484.0000000	4.3099999
30485.0000000	4.3200002
30486.0000000	4.3199997
30487.0000000	4.3249998
30488.0000000	4.3299999
30489.0000000	4.3350000
30490.0000000	4.3450003
30491.0000000	4.3450003
30492.0000000	4.3550000
30493.0000000	4.3600001
30494.0000000	4.3600001
30495.0000000	4.3750000
30496.0000000	4.3850002
30497.0000000	4.4000001
30498.0000000	4.4000001
30499.0000000	4.4049997
30500.0000000	4.3999996
30501.0000000	4.4099998
30502.0000000	4.4049997
30503.0000000	4.4150000
30504.0000000	4.4300003
30505.0000000	4.4499998
30506.0000000	4.4499998
30507.0000000	4.4549999
30508.0000000	4.4633331
30509.0000000	4.4700003
30510.0000000	4.4800000
30511.0000000	4.4800000
30512.0000000	4.4899998
30513.0000000	4.4899998
30514.0000000	4.4899998
30515.0000000	4.5050001
30516.0000000	4.4949999
30517.0000000	4.5150003
30518.0000000	4.5250001
30519.0000000	4.5400000
30520.0000000	4.5500002
30521.0000000	4.5650001
30522.0000000	4.5699997
30523.0000000	4.5799999
30524.0000000	4.5900002
30525.0000000	4.5900002
30526.0000000	4.5900002
30527.0000000	4.5950003

30528.0000000	4.5999999
30529.0000000	4.5950003
30530.0000000	4.6050000
30531.0000000	4.6050000
30532.0000000	4.5850000
30533.0000000	4.5850000
30534.0000000	4.5850000
30535.0000000	4.6050000
30536.0000000	4.6099997
30537.0000000	4.6266665
30538.0000000	4.6500001
30539.0000000	4.6650000
30540.0000000	4.6849999
30541.0000000	4.6849999
30542.0000000	4.7049999
30543.0000000	4.7049999
30544.0000000	4.6999998
30545.0000000	4.7200003
30546.0000000	4.6999998
30547.0000000	4.7100000
30548.0000000	4.7150002
30549.0000000	4.7100000
30550.0000000	4.7150002
30551.0000000	4.6999998
30552.0000000	4.6900001
30553.0000000	4.6700001
30554.0000000	4.6499996
30555.0000000	4.6399999
30556.0000000	4.6350002
30557.0000000	4.6300001
30558.0000000	4.6399999
30559.0000000	4.6500001
30560.0000000	4.6500001
30561.0000000	4.6500001
30562.0000000	4.6399999
30563.0000000	4.6350002
30564.0000000	4.6250000
30565.0000000	4.6199999
30566.0000000	4.6199999
30567.0000000	4.6000004
30568.0000000	4.5900002
30569.0000000	4.5749998
30570.0000000	4.5799999
30571.0000000	4.5699997
30572.0000000	4.5749998
30573.0000000	4.5700002
30574.0000000	4.5700002
30575.0000000	4.5699997
30576.0000000	4.5749998
30577.0000000	4.5650001
30578.0000000	4.5599999
30579.0000000	4.5650001
30580.0000000	4.5749998
30581.0000000	4.5550003
30582.0000000	4.5450001
30583.0000000	4.5400000
30584.0000000	4.5349998

30585.0000000	4.5299997
30586.0000000	4.5250001
30587.0000000	4.5349998
30588.0000000	4.5450001
30589.0000000	4.5550003
30590.0000000	4.5650001
30591.0000000	4.5650001
30592.0000000	4.5550003
30593.0000000	4.5550003
30594.0000000	4.5550003
30595.0000000	4.5349998
30596.0000000	4.5400000
30597.0000000	4.5450001
30598.0000000	4.5599999
30599.0000000	4.5650001
30600.0000000	4.5749998
30601.0000000	4.5950003
30602.0000000	4.6050000
30603.0000000	4.6149998
30604.0000000	4.6199999
30605.0000000	4.6199999
30606.0000000	4.6350002
30607.0000000	4.6450000
30608.0000000	4.6549997
30609.0000000	4.6549997
30610.0000000	4.6599998
30611.0000000	4.6650000
30612.0000000	4.6650000
30613.0000000	4.6849999
30614.0000000	4.6750002
30615.0000000	4.6949997
30616.0000000	4.7100000
30617.0000000	4.7199998
30618.0000000	4.7249999
30619.0000000	4.7299995
30620.0000000	4.7299995
30621.0000000	4.7100000
30622.0000000	4.6949997
30623.0000000	4.6849999
30624.0000000	4.6949997
30625.0000000	4.6849999
30626.0000000	4.6849999
30627.0000000	4.6900001
30628.0000000	4.6949997
30629.0000000	4.7049999
30630.0000000	4.7199998
30631.0000000	4.7349997
30632.0000000	4.7399998
30633.0000000	4.7500000
30634.0000000	4.7550001
30635.0000000	4.7600002
30636.0000000	4.7700005
30637.0000000	4.7799997
30638.0000000	4.7900000
30639.0000000	4.8000002
30640.0000000	4.8150001
30641.0000000	4.8249998

30642.0000000	4.8350000
30643.0000000	4.8499999
30644.0000000	4.8600001
30645.0000000	4.8750000
30646.0000000	4.8850002
30647.0000000	4.9150000
30648.0000000	4.9200001
30649.0000000	4.9400001
30650.0000000	4.9499998
30651.0000000	4.9699998
30652.0000000	4.9749999
30653.0000000	4.9800000
30654.0000000	5.0050001
30655.0000000	5.0100002
30656.0000000	5.0299997
30657.0000000	5.0400000
30658.0000000	5.0599999
30659.0000000	5.0833335
30660.0000000	5.1050000
30661.0000000	5.1099997
30662.0000000	5.1250000
30663.0000000	5.1149998
30664.0000000	5.1199999
30665.0000000	5.1250000
30666.0000000	5.1199999
30667.0000000	5.1300001
30668.0000000	5.1250000
30669.0000000	5.1250000
30670.0000000	5.1350002
30671.0000000	5.1300001
30672.0000000	5.1400003
30673.0000000	5.1599998
30674.0000000	5.1650000
30675.0000000	5.1750002
30676.0000000	5.1949997
30677.0000000	5.1949997
30678.0000000	5.2100000
30679.0000000	5.2049999
30680.0000000	5.2100000
30681.0000000	5.2100000
30682.0000000	5.2100000
30683.0000000	5.2049999
30684.0000000	5.1949997
30685.0000000	5.1949997
30686.0000000	5.2049999
30687.0000000	5.2150002
30688.0000000	5.2100000
30689.0000000	5.2199998
30690.0000000	5.2100000
30691.0000000	5.1999998
30692.0000000	5.1700001
30693.0000000	5.1450000
30694.0000000	5.1400003
30695.0000000	5.1199999
30696.0000000	5.1050000
30697.0000000	5.0699997
30698.0000000	5.0500002

30699.0000000	5.0450001
30700.0000000	5.0599999
30701.0000000	5.0599999
30702.0000000	5.0400000
30703.0000000	5.0450001
30704.0000000	5.0400000
30705.0000000	5.0250001
30706.0000000	5.0200000
30707.0000000	5.0300002
30708.0000000	5.0200005
30709.0000000	5.0200000
30710.0000000	5.0450001
30711.0000000	5.0900002
30712.0000000	5.1199999
30713.0000000	5.1400003
30714.0000000	5.1849999
30715.0000000	5.2249999
30716.0000000	5.2449999
30717.0000000	5.2900000
30718.0000000	5.3299999
30719.0000000	5.3599997
30720.0000000	5.4000001
30721.0000000	5.4499998
30722.0000000	5.4699998
30723.0000000	5.5100002
30724.0000000	5.5349998
30725.0000000	5.5550003
30726.0000000	5.5650001
30727.0000000	5.5850000
30728.0000000	5.6000004
30729.0000000	5.5950003
30730.0000000	5.5950003
30731.0000000	5.6050000
30732.0000000	5.6000004
30733.0000000	5.6100001
30734.0000000	5.6099997
30735.0000000	5.6000004
30736.0000000	5.6050000
30737.0000000	5.6099997
30738.0000000	5.6050000
30739.0000000	5.6100001
30740.0000000	5.6149998
30741.0000000	5.6149998
30742.0000000	5.6149998
30743.0000000	5.5850000
30744.0000000	5.5900002
30745.0000000	5.5850000
30746.0000000	5.5700002
30747.0000000	5.5500002
30748.0000000	5.5400000
30749.0000000	5.5300002
30750.0000000	5.5166669
30751.0000000	5.5000000
30752.0000000	5.4899998
30753.0000000	5.4749999
30754.0000000	5.4749999
30755.0000000	5.4899998

30756.0000000	5.4799995
30757.0000000	5.4749999
30758.0000000	5.4800000
30759.0000000	5.4899998
30760.0000000	5.4799995
30761.0000000	5.4700003
30762.0000000	5.4600000
30763.0000000	5.4499998
30764.0000000	5.4399996
30765.0000000	5.4349999
30766.0000000	5.4250002
30767.0000000	5.4200001
30768.0000000	5.4250002
30769.0000000	5.4200001
30770.0000000	5.4349999
30771.0000000	5.4399996
30772.0000000	5.4449997
30773.0000000	5.4549999
30774.0000000	5.4600000
30775.0000000	5.4600000
30776.0000000	5.4499998
30777.0000000	5.4499998
30778.0000000	5.4650002
30779.0000000	5.4733334
30780.0000000	5.4849997
30781.0000000	5.4849997
30782.0000000	5.4749999
30783.0000000	5.4749999
30784.0000000	5.4849997
30785.0000000	5.5000000
30786.0000000	5.5050001
30787.0000000	5.5000000
30788.0000000	5.4749999
30789.0000000	5.4700003
30790.0000000	5.4549999
30791.0000000	5.4499998
30792.0000000	5.4250002
30793.0000000	5.4049997
30794.0000000	5.3900003
30795.0000000	5.4049997
30796.0000000	5.3999996
30797.0000000	5.4000001
30798.0000000	5.3950000
30799.0000000	5.3800001
30800.0000000	5.3500004
30801.0000000	5.3249998
30802.0000000	5.3099999
30803.0000000	5.3099999
30804.0000000	5.3199997
30805.0000000	5.3249998
30806.0000000	5.3350000
30807.0000000	5.3299999
30808.0000000	5.3400002
30809.0000000	5.3599997
30810.0000000	5.3750000
30811.0000000	5.3700004
30812.0000000	5.3899999

30813.0000000	5.3850002
30814.0000000	5.3900003
30815.0000000	5.3699999
30816.0000000	5.3800001
30817.0000000	5.3750000
30818.0000000	5.3800001
30819.0000000	5.3800001
30820.0000000	5.3750000
30821.0000000	5.3599997
30822.0000000	5.3599997
30823.0000000	5.3400002
30824.0000000	5.3249998
30825.0000000	5.3150001
30826.0000000	5.2950001
30827.0000000	5.2849998
30828.0000000	5.2799997
30829.0000000	5.2600002
30830.0000000	5.2399998
30831.0000000	5.2199998
30832.0000000	5.1949997
30833.0000000	5.1499996
30834.0000000	5.1149998
30835.0000000	5.0850000
30836.0000000	5.0650001
30837.0000000	5.0450001
30838.0000000	5.0349998
30839.0000000	5.0150003
30840.0000000	5.0033336
30841.0000000	5.0100002
30842.0000000	4.9849997
30843.0000000	4.9650002
30844.0000000	4.9650002
30845.0000000	4.9499998
30846.0000000	4.9399996
30847.0000000	4.9549999
30848.0000000	4.9699998
30849.0000000	4.9600000
30850.0000000	4.9549999
30851.0000000	4.9600000
30852.0000000	4.9700003
30853.0000000	4.9749999
30854.0000000	4.9800000
30855.0000000	4.9699998
30856.0000000	4.9699998
30857.0000000	4.9549999
30858.0000000	4.9349999
30859.0000000	4.9250002
30860.0000000	4.9250002
30861.0000000	4.9250002
30862.0000000	4.9250002
30863.0000000	4.9449997
30864.0000000	4.9650002
30865.0000000	4.9699998
30866.0000000	4.9650002
30867.0000000	4.9700003
30868.0000000	4.9700003
30869.0000000	4.9899998

30870.0000000	5.0200000
30871.0000000	5.0700002
30872.0000000	5.1050000
30873.0000000	5.1350002
30874.0000000	5.1750002
30875.0000000	5.1899996
30876.0000000	5.2100000
30877.0000000	5.2100000
30878.0000000	5.2349997
30879.0000000	5.2500000
30880.0000000	5.2650003
30881.0000000	5.2849998
30882.0000000	5.2849998
30883.0000000	5.2950001
30884.0000000	5.3100004
30885.0000000	5.3150001
30886.0000000	5.3249998
30887.0000000	5.3350000
30888.0000000	5.3400002
30889.0000000	5.3500004
30890.0000000	5.3699999
30891.0000000	5.3800001
30892.0000000	5.3900003
30893.0000000	5.4200001
30894.0000000	5.4499998
30895.0000000	5.4749999
30896.0000000	5.5000000
30897.0000000	5.5300002
30898.0000000	5.5700002
30899.0000000	5.5950003
30900.0000000	5.6133332
30901.0000000	5.6450000
30902.0000000	5.6650000
30903.0000000	5.6800003
30904.0000000	5.6849999
30905.0000000	5.6849999
30906.0000000	5.6849999
30907.0000000	5.6849999
30908.0000000	5.6800003
30909.0000000	5.6949997
30910.0000000	5.6849999
30911.0000000	5.6999998
30912.0000000	5.7100000
30913.0000000	5.7299995
30914.0000000	5.7249999
30915.0000000	5.7299995
30916.0000000	5.7249999
30917.0000000	5.7150002
30918.0000000	5.7199998
30919.0000000	5.7150002
30920.0000000	5.7249999
30921.0000000	5.7249999
30922.0000000	5.7150002
30923.0000000	5.6999998
30924.0000000	5.6949997
30925.0000000	5.6599998
30926.0000000	5.6499996

30927.0000000	5.6350002
30928.0000000	5.6050000
30929.0000000	5.6050000
30930.0000000	5.6000004
30931.0000000	5.5900002
30932.0000000	5.5699997
30933.0000000	5.5450001
30934.0000000	5.5450001
30935.0000000	5.5349998
30936.0000000	5.5400000
30937.0000000	5.5450001
30938.0000000	5.5450001
30939.0000000	5.5600004
30940.0000000	5.5600004
30941.0000000	5.5600004
30942.0000000	5.5600004
30943.0000000	5.5600004
30944.0000000	5.5599999
30945.0000000	5.5500002
30946.0000000	5.5500002
30947.0000000	5.5450001
30948.0000000	5.5500002
30949.0000000	5.5500002
30950.0000000	5.5349998
30951.0000000	5.5400000
30952.0000000	5.5349998
30953.0000000	5.5200000
30954.0000000	5.5050001
30955.0000000	5.5000000
30956.0000000	5.5000000
30957.0000000	5.4849997
30958.0000000	5.4849997
30959.0000000	5.5000000
30960.0000000	5.5299997
30961.0000000	5.5400000
30962.0000000	5.5550003
30963.0000000	5.5850000
30964.0000000	5.5749998
30965.0000000	5.5850000
30966.0000000	5.5950003
30967.0000000	5.5950003
30968.0000000	5.5900002
30969.0000000	5.5950003
30970.0000000	5.5900002
30971.0000000	5.5900002
30972.0000000	5.5999999
30973.0000000	5.6199999
30974.0000000	5.6350002
30975.0000000	5.6650000
30976.0000000	5.6750002
30977.0000000	5.6949997
30978.0000000	5.7200003
30979.0000000	5.7349997
30980.0000000	5.7600002
30981.0000000	5.7750001
30982.0000000	5.7800002
30983.0000000	5.8050003

30984.0000000	5.8199997
30985.0000000	5.8299999
30986.0000000	5.8599997
30987.0000000	5.8699999
30988.0000000	5.8999996
30989.0000000	5.9150000
30990.0000000	5.9200001
30991.0000000	5.9166665
30992.0000000	5.9049997
30993.0000000	5.9049997
30994.0000000	5.9200001
30995.0000000	5.9200001
30996.0000000	5.9200001
30997.0000000	5.9300003
30998.0000000	5.9349999
30999.0000000	5.9299998
31000.0000000	5.9399996
31001.0000000	5.9499998
31002.0000000	5.9749999
31003.0000000	5.9749999
31004.0000000	6.0200000
31005.0000000	6.0299997
31006.0000000	6.0450001
31007.0000000	6.0599999
31008.0000000	6.0550003
31009.0000000	6.0550003
31010.0000000	6.0500002
31011.0000000	6.0500002
31012.0000000	6.0550003
31013.0000000	6.0650001
31014.0000000	6.0599999
31015.0000000	6.0500002
31016.0000000	6.0500002
31017.0000000	6.0550003
31018.0000000	6.0550003
31019.0000000	6.0349998
31020.0000000	6.0233331
31021.0000000	6.0300002
31022.0000000	6.0200005
31023.0000000	6.0150003
31024.0000000	6.0100002
31025.0000000	5.9949999
31026.0000000	5.9849997
31027.0000000	5.9650002
31028.0000000	5.9449997
31029.0000000	5.9200001
31030.0000000	5.9049997
31031.0000000	5.8599997
31032.0000000	5.8299999
31033.0000000	5.7950001
31034.0000000	5.7849998
31035.0000000	5.7800002
31036.0000000	5.7849998
31037.0000000	5.7900000
31038.0000000	5.8099999
31039.0000000	5.8150001
31040.0000000	5.8249998

31041.0000000	5.8499999
31042.0000000	5.8800001
31043.0000000	5.8899999
31044.0000000	5.9099998
31045.0000000	5.9099998
31046.0000000	5.9099998
31047.0000000	5.9150000
31048.0000000	5.9150000
31049.0000000	5.9200001
31050.0000000	5.9300003
31051.0000000	5.9400001
31052.0000000	5.9300003
31053.0000000	5.9150000
31054.0000000	5.8999996
31055.0000000	5.8850002
31056.0000000	5.8649998
31057.0000000	5.8350000
31058.0000000	5.8050003
31059.0000000	5.8000002
31060.0000000	5.7950001
31061.0000000	5.7849998
31062.0000000	5.7550001
31063.0000000	5.7299995
31064.0000000	5.7199998
31065.0000000	5.7049999
31066.0000000	5.6750002
31067.0000000	5.6450000
31068.0000000	5.6050000
31069.0000000	5.5999999
31070.0000000	5.5850000
31071.0000000	5.5749998
31072.0000000	5.5749998
31073.0000000	5.5550003
31074.0000000	5.5500002
31075.0000000	5.5749998
31076.0000000	5.5850000
31077.0000000	5.6250000
31078.0000000	5.6599998
31079.0000000	5.6949997
31080.0000000	5.7249999
31081.0000000	5.7800002
31082.0000000	5.8100004
31083.0000000	5.8400002
31084.0000000	5.8600001
31085.0000000	5.8800001
31086.0000000	5.8899999
31087.0000000	5.8750000
31088.0000000	5.8900003
31089.0000000	5.9000001
31090.0000000	5.8750000
31091.0000000	5.8699999
31092.0000000	5.8350000
31093.0000000	5.8249998
31094.0000000	5.7849998
31095.0000000	5.7600002
31096.0000000	5.7049999
31097.0000000	5.6500001

31098.0000000	5.6100001
31099.0000000	5.5599999
31100.0000000	5.5299997
31101.0000000	5.4949999
31102.0000000	5.4549999
31103.0000000	5.4250002
31104.0000000	5.4049997
31105.0000000	5.4000001
31106.0000000	5.3850002
31107.0000000	5.3649998
31108.0000000	5.3249998
31109.0000000	5.3249998
31110.0000000	5.3450003
31111.0000000	5.3550000
31112.0000000	5.3799996
31113.0000000	5.4349999
31114.0000000	5.4549999
31115.0000000	5.4849997
31116.0000000	5.4899998
31117.0000000	5.4800000
31118.0000000	5.5050001
31119.0000000	5.5349998
31120.0000000	5.5299997
31121.0000000	5.5400000
31122.0000000	5.5650001
31123.0000000	5.5699997
31124.0000000	5.5850000
31125.0000000	5.6100001
31126.0000000	5.6300001
31127.0000000	5.6350002
31128.0000000	5.6399999
31129.0000000	5.6499996
31130.0000000	5.6750002
31131.0000000	5.6900001
31132.0000000	5.7049999
31133.0000000	5.7399998
31134.0000000	5.7600002
31135.0000000	5.7500000
31136.0000000	5.7299995
31137.0000000	5.7150002
31138.0000000	5.6999998
31139.0000000	5.6700001
31140.0000000	5.6450000
31141.0000000	5.6350002
31142.0000000	5.6266670
31143.0000000	5.6100001
31144.0000000	5.5799999
31145.0000000	5.5749998
31146.0000000	5.5999999
31147.0000000	5.5950003
31148.0000000	5.5999999
31149.0000000	5.5999999
31150.0000000	5.5850000
31151.0000000	5.5650001
31152.0000000	5.5450001
31153.0000000	5.5349998
31154.0000000	5.5250001

31155.0000000	5.5400000
31156.0000000	5.5200005
31157.0000000	5.5349998
31158.0000000	5.5400000
31159.0000000	5.5699997
31160.0000000	5.5850000
31161.0000000	5.6050000
31162.0000000	5.6149998
31163.0000000	5.6350002
31164.0000000	5.6400003
31165.0000000	5.6399999
31166.0000000	5.6300001
31167.0000000	5.6300001
31168.0000000	5.6199999
31169.0000000	5.6149998
31170.0000000	5.6050000
31171.0000000	5.6149998
31172.0000000	5.6199999
31173.0000000	5.6299996
31174.0000000	5.6450000
31175.0000000	5.6750002
31176.0000000	5.6799998
31177.0000000	5.6999998
31178.0000000	5.7100000
31179.0000000	5.6899996
31180.0000000	5.6999998
31181.0000000	5.7150002
31182.0000000	5.7349997
31183.0000000	5.7500000
31184.0000000	5.7500000
31185.0000000	5.7550001
31186.0000000	5.7700000
31187.0000000	5.7849998
31188.0000000	5.7900000
31189.0000000	5.8000002
31190.0000000	5.8100004
31191.0000000	5.8150001
31192.0000000	5.8150001
31193.0000000	5.8249998
31194.0000000	5.8350000
31195.0000000	5.8550000
31196.0000000	5.8550000
31197.0000000	5.8649998
31198.0000000	5.8699999
31199.0000000	5.8699999
31200.0000000	5.8500004
31201.0000000	5.8100004
31202.0000000	5.7600002
31203.0000000	5.7666664
31204.0000000	5.7299995
31205.0000000	5.7150002
31206.0000000	5.6999998
31207.0000000	5.6700001
31208.0000000	5.6499996
31209.0000000	5.6149998
31210.0000000	5.5900002
31211.0000000	5.5500002

31212.0000000	5.5100002
31213.0000000	5.4650002
31214.0000000	5.4549999
31215.0000000	5.4349999
31216.0000000	5.4049997
31217.0000000	5.3850002
31218.0000000	5.3550000
31219.0000000	5.3400002
31220.0000000	5.3050003
31221.0000000	5.2900000
31222.0000000	5.2800002
31223.0000000	5.2799997
31224.0000000	5.3050003
31225.0000000	5.3200002
31226.0000000	5.3299999
31227.0000000	5.3450003
31228.0000000	5.3350000
31229.0000000	5.3249998
31230.0000000	5.3150001
31231.0000000	5.3099999
31232.0000000	5.3266664
31233.0000000	5.3450003
31234.0000000	5.3550000
31235.0000000	5.3649998
31236.0000000	5.3550000
31237.0000000	5.3350000
31238.0000000	5.3199997
31239.0000000	5.2849998
31240.0000000	5.2700000
31241.0000000	5.2449999
31242.0000000	5.2249999
31243.0000000	5.1900001
31244.0000000	5.1700001
31245.0000000	5.1499996
31246.0000000	5.1499996
31247.0000000	5.1450000
31248.0000000	5.1450000
31249.0000000	5.1450000
31250.0000000	5.1599998
31251.0000000	5.1549997
31252.0000000	5.1700001
31253.0000000	5.1599998
31254.0000000	5.1650000
31255.0000000	5.1949997
31256.0000000	5.2200003
31257.0000000	5.2349997
31258.0000000	5.2650003
31259.0000000	5.2849998
31260.0000000	5.3000002
31261.0000000	5.3000002
31262.0000000	5.2633338
31263.0000000	5.2500000
31264.0000000	5.2399998
31265.0000000	5.2150002
31266.0000000	5.1949997
31267.0000000	5.1700001
31268.0000000	5.1549997

31269.0000000	5.1350002
31270.0000000	5.1149998
31271.0000000	5.0950003
31272.0000000	5.0850000
31273.0000000	5.0799999
31274.0000000	5.0650001
31275.0000000	5.0700002
31276.0000000	5.0799999
31277.0000000	5.0749998
31278.0000000	5.0699997
31279.0000000	5.0550003
31280.0000000	5.0400000
31281.0000000	5.0400000
31282.0000000	5.0349998
31283.0000000	5.0450001
31284.0000000	5.0550003
31285.0000000	5.0450001
31286.0000000	5.0550003
31287.0000000	5.0799999
31288.0000000	5.0850000
31289.0000000	5.0999999
31290.0000000	5.1000004
31291.0000000	5.1050000
31292.0000000	5.1099997
31293.0000000	5.1250000
31294.0000000	5.1266665
31295.0000000	5.1499996
31296.0000000	5.1549997
31297.0000000	5.1650000
31298.0000000	5.1799998
31299.0000000	5.1849999
31300.0000000	5.2100000
31301.0000000	5.2399998
31302.0000000	5.2500000
31303.0000000	5.2650003
31304.0000000	5.2750001
31305.0000000	5.2750001
31306.0000000	5.2650003
31307.0000000	5.2650003
31308.0000000	5.2600002
31309.0000000	5.2700005
31310.0000000	5.2800002
31311.0000000	5.2950001
31312.0000000	5.2950001
31313.0000000	5.3100004
31314.0000000	5.3150001
31315.0000000	5.3200002
31316.0000000	5.3000002
31317.0000000	5.2650003
31318.0000000	5.2199998
31319.0000000	5.1750002
31320.0000000	5.1300001
31321.0000000	5.0950003
31322.0000000	5.0700002
31323.0000000	5.0500002
31324.0000000	5.0400000
31325.0000000	5.0400000

31326.0000000	5.0250001
31327.0000000	5.0000000
31328.0000000	4.9699998
31329.0000000	4.9600000
31330.0000000	4.9600000
31331.0000000	4.9650002
31332.0000000	4.9849997
31333.0000000	5.0100002
31334.0000000	4.9899998
31335.0000000	5.0000000
31336.0000000	5.0000000
31337.0000000	5.0150003
31338.0000000	5.0250001
31339.0000000	5.0200005
31340.0000000	5.0100002
31341.0000000	5.0100002
31342.0000000	5.0100002
31343.0000000	5.0100002
31344.0000000	5.0050001
31345.0000000	4.9949999
31346.0000000	4.9800000
31347.0000000	4.9650002
31348.0000000	4.9549999
31349.0000000	4.9449997
31350.0000000	4.9449997
31351.0000000	4.9300003
31352.0000000	4.9349999
31353.0000000	4.9466662
31354.0000000	4.9549999
31355.0000000	4.9549999
31356.0000000	4.9499998
31357.0000000	4.9499998
31358.0000000	4.9299998
31359.0000000	4.9250002
31360.0000000	4.9099998
31361.0000000	4.8900003
31362.0000000	4.8649998
31363.0000000	4.8299999
31364.0000000	4.8000002
31365.0000000	4.7650003
31366.0000000	4.7500000
31367.0000000	4.7449999
31368.0000000	4.7349997
31369.0000000	4.7500000
31370.0000000	4.7449999
31371.0000000	4.7249999
31372.0000000	4.7300000
31373.0000000	4.7249999
31374.0000000	4.6949997
31375.0000000	4.6700001
31376.0000000	4.6549997
31377.0000000	4.6300001
31378.0000000	4.6300001
31379.0000000	4.6350002
31380.0000000	4.6399999
31381.0000000	4.6450000
31382.0000000	4.6499996

31383.0000000	4.6599998
31384.0000000	4.6366668
31385.0000000	4.6199999
31386.0000000	4.6149998
31387.0000000	4.5850000
31388.0000000	4.5650001
31389.0000000	4.5450001
31390.0000000	4.5349998
31391.0000000	4.5050001
31392.0000000	4.4699998
31393.0000000	4.4049997
31394.0000000	4.3200002
31395.0000000	4.2500000
31396.0000000	4.1650000
31397.0000000	4.0749998
31398.0000000	3.9800000
31399.0000000	3.9400001
31400.0000000	3.9150000
31401.0000000	3.9100001
31402.0000000	3.9100001
31403.0000000	3.9000001
31404.0000000	3.9000001
31405.0000000	3.9050002
31406.0000000	3.9100001
31407.0000000	3.9000001
31408.0000000	3.9250002
31409.0000000	3.9349999
31410.0000000	3.9650002
31411.0000000	3.9850001
31412.0000000	3.9800000
31413.0000000	3.9949999
31414.0000000	4.0066667
31415.0000000	4.0150003
31416.0000000	4.0200000
31417.0000000	4.0250001
31418.0000000	4.0299997
31419.0000000	4.0349998
31420.0000000	4.0450001
31421.0000000	4.0550003
31422.0000000	4.0650001
31423.0000000	4.0799999
31424.0000000	4.0850000
31425.0000000	4.0900002
31426.0000000	4.0900002
31427.0000000	4.0850000
31428.0000000	4.0950003
31429.0000000	4.0799999
31430.0000000	4.0799999
31431.0000000	4.0699997
31432.0000000	4.0699997
31433.0000000	4.0699997
31434.0000000	4.0749998
31435.0000000	4.0599999
31436.0000000	4.0599999
31437.0000000	4.0450001
31438.0000000	4.0299997
31439.0000000	4.0299997

31440.0000000	4.0450001
31441.0000000	4.0749998
31442.0000000	4.0950003
31443.0000000	4.1050000
31444.0000000	4.1233335
31445.0000000	4.1400003
31446.0000000	4.1399999
31447.0000000	4.1350002
31448.0000000	4.1350002
31449.0000000	4.1199999
31450.0000000	4.0900002
31451.0000000	4.0450001
31452.0000000	4.0100002
31453.0000000	3.9949999
31454.0000000	3.9949999
31455.0000000	3.9949999
31456.0000000	4.0000000
31457.0000000	3.9900000
31458.0000000	3.9900000
31459.0000000	3.9850001
31460.0000000	3.9900000
31461.0000000	3.9900000
31462.0000000	4.0050001
31463.0000000	4.0200000
31464.0000000	4.0150003
31465.0000000	4.0200000
31466.0000000	4.0299997
31467.0000000	4.0349998
31468.0000000	4.0349998
31469.0000000	4.0450001
31470.0000000	4.0600004
31471.0000000	4.0749998
31472.0000000	4.0749998
31473.0000000	4.0799999
31474.0000000	4.1000004
31475.0000000	4.0900002
31476.0000000	4.1050000
31477.0000000	4.1100001
31478.0000000	4.1149998
31479.0000000	4.1149998
31480.0000000	4.1300001
31481.0000000	4.1350002
31482.0000000	4.1499996
31483.0000000	4.1500001
31484.0000000	4.1650000
31485.0000000	4.1700001
31486.0000000	4.1800003
31487.0000000	4.1849999
31488.0000000	4.1900001
31489.0000000	4.1899996
31490.0000000	4.1999998
31491.0000000	4.2100000
31492.0000000	4.2299995
31493.0000000	4.2399998
31494.0000000	4.2550001
31495.0000000	4.2750001
31496.0000000	4.2849998

31497.0000000	4.3150001
31498.0000000	4.3299999
31499.0000000	4.3450003
31500.0000000	4.3699999
31501.0000000	4.3899999
31502.0000000	4.4099998
31503.0000000	4.4366665
31504.0000000	4.4400001
31505.0000000	4.4566665
31506.0000000	4.4600000
31507.0000000	4.4549999
31508.0000000	4.4549999
31509.0000000	4.4499998
31510.0000000	4.4449997
31511.0000000	4.4449997
31512.0000000	4.4449997
31513.0000000	4.4399996
31514.0000000	4.4499998
31515.0000000	4.4499998
31516.0000000	4.4600000
31517.0000000	4.4699998
31518.0000000	4.4800000
31519.0000000	4.4899998
31520.0000000	4.4899998
31521.0000000	4.5000000
31522.0000000	4.5050001
31523.0000000	4.5200000
31524.0000000	4.5250001
31525.0000000	4.5250001
31526.0000000	4.5200000
31527.0000000	4.5150003
31528.0000000	4.5100002
31529.0000000	4.5000000
31530.0000000	4.4800000
31531.0000000	4.4650002
31532.0000000	4.4449997
31533.0000000	4.4366665
31534.0000000	4.4099998
31535.0000000	4.4200001
31536.0000000	4.4150000
31537.0000000	4.4099998
31538.0000000	4.4049997
31539.0000000	4.3950000
31540.0000000	4.3899999
31541.0000000	4.3900003
31542.0000000	4.3900003
31543.0000000	4.3850002
31544.0000000	4.3899999
31545.0000000	4.3850002
31546.0000000	4.3800001
31547.0000000	4.3699999
31548.0000000	4.3600001
31549.0000000	4.3299999
31550.0000000	4.2900000
31551.0000000	4.2449999
31552.0000000	4.2150002
31553.0000000	4.1900001

31554.0000000	4.1800003
31555.0000000	4.1900001
31556.0000000	4.1949997
31557.0000000	4.1849999
31558.0000000	4.1650000
31559.0000000	4.1599998
31560.0000000	4.1350002
31561.0000000	4.1050000
31562.0000000	4.0999999
31563.0000000	4.0900002
31564.0000000	4.0933337
31565.0000000	4.0950003
31566.0000000	4.0999999
31567.0000000	4.1199999
31568.0000000	4.1450000
31569.0000000	4.1599998
31570.0000000	4.1799998
31571.0000000	4.1999998
31572.0000000	4.2399998
31573.0000000	4.2750001
31574.0000000	4.2950001
31575.0000000	4.3099999
31576.0000000	4.3050003
31577.0000000	4.2950001
31578.0000000	4.2799997
31579.0000000	4.2650003
31580.0000000	4.2399998
31581.0000000	4.2100000
31582.0000000	4.1700001
31583.0000000	4.1599998
31584.0000000	4.1450000
31585.0000000	4.1250000
31586.0000000	4.1250000
31587.0000000	4.1350002
31588.0000000	4.1450000
31589.0000000	4.1399999
31590.0000000	4.1500001
31591.0000000	4.1549997
31592.0000000	4.1700001
31593.0000000	4.1900001
31594.0000000	4.2249999
31595.0000000	4.2533336
31596.0000000	4.2799997
31597.0000000	4.2849998
31598.0000000	4.2900000
31599.0000000	4.3050003
31600.0000000	4.3099999
31601.0000000	4.3200002
31602.0000000	4.3450003
31603.0000000	4.3649998
31604.0000000	4.3699999
31605.0000000	4.3649998
31606.0000000	4.3699999
31607.0000000	4.3850002
31608.0000000	4.3750000
31609.0000000	4.3800001
31610.0000000	4.3950000

31611.0000000	4.3950000
31612.0000000	4.4049997
31613.0000000	4.4250002
31614.0000000	4.4300003
31615.0000000	4.4399996
31616.0000000	4.4600000
31617.0000000	4.4749999
31618.0000000	4.4849997
31619.0000000	4.5000000
31620.0000000	4.5100002
31621.0000000	4.5250001
31622.0000000	4.5349998
31623.0000000	4.5600004
31624.0000000	4.5866666
31625.0000000	4.6100001
31626.0000000	4.6199999
31627.0000000	4.6350002
31628.0000000	4.6499996
31629.0000000	4.6650000
31630.0000000	4.6900001
31631.0000000	4.7199998
31632.0000000	4.7349997
31633.0000000	4.7500000
31634.0000000	4.7449999
31635.0000000	4.7550001
31636.0000000	4.7550001
31637.0000000	4.7500000
31638.0000000	4.7600002
31639.0000000	4.7849998
31640.0000000	4.8200002
31641.0000000	4.8099999
31642.0000000	4.8249998
31643.0000000	4.8200002
31644.0000000	4.8299999
31645.0000000	4.8199997
31646.0000000	4.8249998
31647.0000000	4.8350000
31648.0000000	4.8150001
31649.0000000	4.8249998
31650.0000000	4.8299999
31651.0000000	4.8350000
31652.0000000	4.8249998
31653.0000000	4.8249998
31654.0000000	4.8199997
31655.0000000	4.8499999
31656.0000000	4.8649998
31657.0000000	4.8900003
31658.0000000	4.9200001
31659.0000000	4.9299998
31660.0000000	4.9600000
31661.0000000	4.9749999
31662.0000000	5.0000000
31663.0000000	5.0150003
31664.0000000	5.0250001
31665.0000000	5.0250001
31666.0000000	5.0299997
31667.0000000	5.0349998

31668.0000000	5.0400000
31669.0000000	5.0299997
31670.0000000	5.0400000
31671.0000000	5.0250001
31672.0000000	5.0250001
31673.0000000	5.0250001
31674.0000000	5.0300002
31675.0000000	5.0299997
31676.0000000	5.0349998
31677.0000000	5.0349998
31678.0000000	5.0349998
31679.0000000	5.0349998
31680.0000000	5.0349998
31681.0000000	5.0349998
31682.0000000	5.0250001
31683.0000000	5.0250001
31684.0000000	5.0066667
31685.0000000	5.0100002
31686.0000000	5.0033336
31687.0000000	5.0050001
31688.0000000	4.9949999
31689.0000000	4.9799995
31690.0000000	4.9650002
31691.0000000	4.9549999
31692.0000000	4.9299998
31693.0000000	4.9300003
31694.0000000	4.9150000
31695.0000000	4.8999996
31696.0000000	4.8850002
31697.0000000	4.8899999
31698.0000000	4.8699999
31699.0000000	4.8649998
31700.0000000	4.8450003
31701.0000000	4.8400002
31702.0000000	4.8299999
31703.0000000	4.8400002
31704.0000000	4.8350000
31705.0000000	4.8350000
31706.0000000	4.8299999
31707.0000000	4.8450003
31708.0000000	4.8450003
31709.0000000	4.8550000
31710.0000000	4.8550000
31711.0000000	4.8550000
31712.0000000	4.8800001
31713.0000000	4.9049997
31714.0000000	4.9200001
31715.0000000	4.9250002
31716.0000000	4.9400001
31717.0000000	4.9650002
31718.0000000	4.9899998
31719.0000000	5.0000000
31720.0000000	5.0349998
31721.0000000	5.0450001
31722.0000000	5.0799999
31723.0000000	5.0950003
31724.0000000	5.1199999

31725.0000000	5.1350002
31726.0000000	5.1399999
31727.0000000	5.1450000
31728.0000000	5.1499996
31729.0000000	5.1450000
31730.0000000	5.1549997
31731.0000000	5.1599998
31732.0000000	5.1750002
31733.0000000	5.1849999
31734.0000000	5.1949997
31735.0000000	5.1999998
31736.0000000	5.2100000
31737.0000000	5.2200003
31738.0000000	5.2249999
31739.0000000	5.2199998
31740.0000000	5.2300000
31741.0000000	5.2299995
31742.0000000	5.2349997
31743.0000000	5.2500000
31744.0000000	5.2550001
31745.0000000	5.2666669
31746.0000000	5.2800002
31747.0000000	5.2733331
31748.0000000	5.2750001
31749.0000000	5.2750001
31750.0000000	5.2650003
31751.0000000	5.2500000
31752.0000000	5.2399998
31753.0000000	5.2399998
31754.0000000	5.2249999
31755.0000000	5.2100000
31756.0000000	5.1949997
31757.0000000	5.1750002
31758.0000000	5.1499996
31759.0000000	5.1350002
31760.0000000	5.1149998
31761.0000000	5.0900002
31762.0000000	5.0749998
31763.0000000	5.0500002
31764.0000000	5.0349998
31765.0000000	5.0050001
31766.0000000	4.9899998
31767.0000000	4.9700003
31768.0000000	4.9499998
31769.0000000	4.9150000
31770.0000000	4.8950000
31771.0000000	4.8850002
31772.0000000	4.8850002
31773.0000000	4.8649998
31774.0000000	4.8500004
31775.0000000	4.8450003
31776.0000000	4.8233333
31777.0000000	4.8199997
31778.0000000	4.8099999
31779.0000000	4.7950001
31780.0000000	4.7900000
31781.0000000	4.8050003

31782.0000000	4.8249998
31783.0000000	4.8400002
31784.0000000	4.8550000
31785.0000000	4.8800001
31786.0000000	4.9049997
31787.0000000	4.9250002
31788.0000000	4.9499998
31789.0000000	4.9699998
31790.0000000	4.9899998
31791.0000000	5.0000000
31792.0000000	5.0150003
31793.0000000	5.0200005
31794.0000000	5.0250001
31795.0000000	5.0200005
31796.0000000	5.0150003
31797.0000000	5.0150003
31798.0000000	5.0200005
31799.0000000	5.0250001
31800.0000000	5.0250001
31801.0000000	5.0300002
31802.0000000	5.0299997
31803.0000000	5.0250001
31804.0000000	5.0349998
31805.0000000	5.0349998
31806.0000000	5.0400000
31807.0000000	5.0200000
31808.0000000	5.0050001
31809.0000000	4.9749999
31810.0000000	4.9499998
31811.0000000	4.9299998
31812.0000000	4.9049997
31813.0000000	4.8950000
31814.0000000	4.8649998
31815.0000000	4.8500004
31816.0000000	4.8200002
31817.0000000	4.7700000
31818.0000000	4.7299995
31819.0000000	4.6700001
31820.0000000	4.6199999
31821.0000000	4.5500002
31822.0000000	4.5349998
31823.0000000	4.5349998
31824.0000000	4.5500002
31825.0000000	4.5450001
31826.0000000	4.5450001
31827.0000000	4.5749998
31828.0000000	4.6050000
31829.0000000	4.6350002
31830.0000000	4.6700001
31831.0000000	4.6999998
31832.0000000	4.7299995
31833.0000000	4.7550001
31834.0000000	4.7750001
31835.0000000	4.7799997
31836.0000000	4.7849998
31837.0000000	4.7866664
31838.0000000	4.8050003

31839.0000000	4.8150001
31840.0000000	4.8400002
31841.0000000	4.8500004
31842.0000000	4.8800001
31843.0000000	4.8950000
31844.0000000	4.8999996
31845.0000000	4.9049997
31846.0000000	4.9099998
31847.0000000	4.8800001
31848.0000000	4.8599997
31849.0000000	4.8400002
31850.0000000	4.8000002
31851.0000000	4.7650003
31852.0000000	4.7349997
31853.0000000	4.6849999
31854.0000000	4.6450000
31855.0000000	4.5799999
31856.0000000	4.5300002
31857.0000000	4.4899998
31858.0000000	4.4699998
31859.0000000	4.4600000
31860.0000000	4.4200001
31861.0000000	4.4400001
31862.0000000	4.4400001
31863.0000000	4.4549999
31864.0000000	4.4400001
31865.0000000	4.4399996
31866.0000000	4.4399996
31867.0000000	4.4400001
31868.0000000	4.4299998
31869.0000000	4.4349999
31870.0000000	4.4349999
31871.0000000	4.4499998
31872.0000000	4.4449997
31873.0000000	4.4600000
31874.0000000	4.4650002
31875.0000000	4.4749999
31876.0000000	4.4499998
31877.0000000	4.4349999
31878.0000000	4.4150000
31879.0000000	4.4150000
31880.0000000	4.4099998
31881.0000000	4.4150000
31882.0000000	4.4349999
31883.0000000	4.4549999
31884.0000000	4.5000000
31885.0000000	4.5250001
31886.0000000	4.5450001
31887.0000000	4.5700002
31888.0000000	4.5799999
31889.0000000	4.5900002
31890.0000000	4.6099997
31891.0000000	4.6050000
31892.0000000	4.6199999
31893.0000000	4.6199999
31894.0000000	4.6250000
31895.0000000	4.6350002

31896.0000000	4.6300001
31897.0000000	4.6599998
31898.0000000	4.6700001
31899.0000000	4.6750002
31900.0000000	4.6849999
31901.0000000	4.6899996
31902.0000000	4.7049999
31903.0000000	4.7100000
31904.0000000	4.7100000
31905.0000000	4.7200003
31906.0000000	4.7299995
31907.0000000	4.7199998
31908.0000000	4.7150002
31909.0000000	4.6949997
31910.0000000	4.6599998
31911.0000000	4.5950003
31912.0000000	4.5200000
31913.0000000	4.4749999
31914.0000000	4.4400001
31915.0000000	4.4150000
31916.0000000	4.3800001
31917.0000000	4.3550000
31918.0000000	4.3499999
31919.0000000	4.3600001
31920.0000000	4.3800001
31921.0000000	4.4000001
31922.0000000	4.4250002
31923.0000000	4.4650002
31924.0000000	4.4949999
31925.0000000	4.5299997
31926.0000000	4.5633335
31927.0000000	4.5999999
31928.0000000	4.6299996
31929.0000000	4.6549997
31930.0000000	4.6750002
31931.0000000	4.6799998
31932.0000000	4.6949997
31933.0000000	4.7100000
31934.0000000	4.7199998
31935.0000000	4.7300000
31936.0000000	4.7199998
31937.0000000	4.6949997
31938.0000000	4.6949997
31939.0000000	4.6900001
31940.0000000	4.6800003
31941.0000000	4.6750002
31942.0000000	4.6800003
31943.0000000	4.6849999
31944.0000000	4.6999998
31945.0000000	4.6999998
31946.0000000	4.6999998
31947.0000000	4.6999998
31948.0000000	4.6949997
31949.0000000	4.6900001
31950.0000000	4.6650000
31951.0000000	4.6750002
31952.0000000	4.6849999

31953.0000000	4.6849999
31954.0000000	4.6799998
31955.0000000	4.6899996
31956.0000000	4.6866665
31957.0000000	4.6999998
31958.0000000	4.6949997
31959.0000000	4.6849999
31960.0000000	4.6949997
31961.0000000	4.6949997
31962.0000000	4.6849999
31963.0000000	4.6849999
31964.0000000	4.6800003
31965.0000000	4.6650000
31966.0000000	4.6549997
31967.0000000	4.6300001
31968.0000000	4.6149998
31969.0000000	4.6000004
31970.0000000	4.5999999
31971.0000000	4.5799999
31972.0000000	4.5699997
31973.0000000	4.5650001
31974.0000000	4.5599999
31975.0000000	4.5349998
31976.0000000	4.5100002
31977.0000000	4.4700003
31978.0000000	4.4250002
31979.0000000	4.4200001
31980.0000000	4.4049997
31981.0000000	4.4049997
31982.0000000	4.3950000
31983.0000000	4.3999996
31984.0000000	4.4099998
31985.0000000	4.4349999
31986.0000000	4.4449997
31987.0000000	4.4600000
31988.0000000	4.4849997
31989.0000000	4.4749999
31990.0000000	4.4949999
31991.0000000	4.5000000
31992.0000000	4.5000000
31993.0000000	4.4899998
31994.0000000	4.4899998
31995.0000000	4.4949999
31996.0000000	4.5100002
31997.0000000	4.5200000
31998.0000000	4.5200000
31999.0000000	4.5400000
32000.0000000	4.5400000
32001.0000000	4.5349998
32002.0000000	4.5650001
32003.0000000	4.5650001
32004.0000000	4.5650001
32005.0000000	4.5650001
32006.0000000	4.5550003
32007.0000000	4.5400000
32008.0000000	4.5200005
32009.0000000	4.5000000

32010.0000000	4.4799995
32011.0000000	4.4949999
32012.0000000	4.5000000
32013.0000000	4.5300002
32014.0000000	4.5450001
32015.0000000	4.5550003
32016.0000000	4.5799999
32017.0000000	4.5749998
32018.0000000	4.5966668
32019.0000000	4.6300001
32020.0000000	4.6250000
32021.0000000	4.6300001
32022.0000000	4.6250000
32023.0000000	4.6199999
32024.0000000	4.6100001
32025.0000000	4.6000004
32026.0000000	4.6250000
32027.0000000	4.6399999
32028.0000000	4.6549997
32029.0000000	4.6599998
32030.0000000	4.6650000
32031.0000000	4.6750002
32032.0000000	4.6849999
32033.0000000	4.7100000
32034.0000000	4.7399998
32035.0000000	4.7449999
32036.0000000	4.7700000
32037.0000000	4.7950001
32038.0000000	4.8050003
32039.0000000	4.8100004
32040.0000000	4.8299999
32041.0000000	4.8350000
32042.0000000	4.8249998
32043.0000000	4.8450003
32044.0000000	4.8599997
32045.0000000	4.8649998
32046.0000000	4.8750000
32047.0000000	4.8850002
32048.0000000	4.8750000
32049.0000000	4.8733335
32050.0000000	4.8800001
32051.0000000	4.8750000
32052.0000000	4.8750000
32053.0000000	4.8599997
32054.0000000	4.8550000
32055.0000000	4.8499999
32056.0000000	4.8649998
32057.0000000	4.8750000
32058.0000000	4.8850002
32059.0000000	4.8950000
32060.0000000	4.8999996
32061.0000000	4.9049997
32062.0000000	4.8899999
32063.0000000	4.9099998
32064.0000000	4.8900003
32065.0000000	4.8900003
32066.0000000	4.8850002

32067.0000000	4.8800001
32068.0000000	4.8699999
32069.0000000	4.8850002
32070.0000000	4.8899999
32071.0000000	4.8850002
32072.0000000	4.8750000
32073.0000000	4.8550000
32074.0000000	4.8299999
32075.0000000	4.8050003
32076.0000000	4.7950001
32077.0000000	4.7650003
32078.0000000	4.7466664
32079.0000000	4.7199998
32080.0000000	4.7049999
32081.0000000	4.6949997
32082.0000000	4.6800003
32083.0000000	4.6800003
32084.0000000	4.6750002
32085.0000000	4.6700001
32086.0000000	4.6549997
32087.0000000	4.6400003
32088.0000000	4.6149998
32089.0000000	4.6000004
32090.0000000	4.5749998
32091.0000000	4.5550003
32092.0000000	4.5299997
32093.0000000	4.5100002
32094.0000000	4.5000000
32095.0000000	4.4899998
32096.0000000	4.5000000
32097.0000000	4.5050001
32098.0000000	4.5050001
32099.0000000	4.4899998
32100.0000000	4.4949999
32101.0000000	4.4800000
32102.0000000	4.4549999
32103.0000000	4.4099998
32104.0000000	4.3699999
32105.0000000	4.3150001
32106.0000000	4.2449999
32107.0000000	4.1800003
32108.0000000	4.1566663
32109.0000000	4.1500001
32110.0000000	4.1500001
32111.0000000	4.1599998
32112.0000000	4.1849999
32113.0000000	4.2150002
32114.0000000	4.2349997
32115.0000000	4.2500000
32116.0000000	4.2600002
32117.0000000	4.2550001
32118.0000000	4.2700000
32119.0000000	4.2900000
32120.0000000	4.2900000
32121.0000000	4.2900000
32122.0000000	4.3000002
32123.0000000	4.3050003

32124.0000000	4.3299999
32125.0000000	4.3450003
32126.0000000	4.3450003
32127.0000000	4.3550000
32128.0000000	4.3699999
32129.0000000	4.3900003
32130.0000000	4.3950000
32131.0000000	4.3950000
32132.0000000	4.3750000
32133.0000000	4.3800001
32134.0000000	4.3800001
32135.0000000	4.3850002
32136.0000000	4.3750000
32137.0000000	4.3733335
32138.0000000	4.3800001
32139.0000000	4.3699999
32140.0000000	4.3899999
32141.0000000	4.3950000
32142.0000000	4.4150000
32143.0000000	4.4200001
32144.0000000	4.4349999
32145.0000000	4.4399996
32146.0000000	4.4650002
32147.0000000	4.4749999
32148.0000000	4.4849997
32149.0000000	4.5000000
32150.0000000	4.5150003
32151.0000000	4.5200005
32152.0000000	4.5100002
32153.0000000	4.5150003
32154.0000000	4.5200005
32155.0000000	4.5299997
32156.0000000	4.5450001
32157.0000000	4.5500002
32158.0000000	4.5500002
32159.0000000	4.5550003
32160.0000000	4.5550003
32161.0000000	4.5850000
32162.0000000	4.5900002
32163.0000000	4.5950003
32164.0000000	4.5950003
32165.0000000	4.5850000
32166.0000000	4.5850000
32167.0000000	4.5850000
32168.0000000	4.5900002
32169.0000000	4.5933337
32170.0000000	4.5950003
32171.0000000	4.5749998
32172.0000000	4.5650001
32173.0000000	4.5600004
32174.0000000	4.5550003
32175.0000000	4.5500002
32176.0000000	4.5500002
32177.0000000	4.5349998
32178.0000000	4.5349998
32179.0000000	4.5349998
32180.0000000	4.5300002

32181.0000000	4.5250001
32182.0000000	4.5349998
32183.0000000	4.5299997
32184.0000000	4.5250001
32185.0000000	4.5299997
32186.0000000	4.5300002
32187.0000000	4.5450001
32188.0000000	4.5650001
32189.0000000	4.5700002
32190.0000000	4.5699997
32191.0000000	4.5749998
32192.0000000	4.5699997
32193.0000000	4.5749998
32194.0000000	4.5599999
32195.0000000	4.5450001
32196.0000000	4.5250001
32197.0000000	4.5100002
32198.0000000	4.4899998
32199.0000000	4.4799995
32200.0000000	4.4600000
32201.0000000	4.4399996
32202.0000000	4.4150000
32203.0000000	4.3900003
32204.0000000	4.3750000
32205.0000000	4.3699999
32206.0000000	4.3649998
32207.0000000	4.3550000
32208.0000000	4.3500004
32209.0000000	4.3500004
32210.0000000	4.3550000
32211.0000000	4.3600001
32212.0000000	4.3550000
32213.0000000	4.3500004
32214.0000000	4.3350000
32215.0000000	4.3150001
32216.0000000	4.3100004
32217.0000000	4.3050003
32218.0000000	4.2849998
32219.0000000	4.2750001
32220.0000000	4.2600002
32221.0000000	4.2449999
32222.0000000	4.2200003
32223.0000000	4.1949997
32224.0000000	4.1549997
32225.0000000	4.1399999
32226.0000000	4.1199999
32227.0000000	4.1000004
32228.0000000	4.1050000
32229.0000000	4.1033330
32230.0000000	4.0950003
32231.0000000	4.0900002
32232.0000000	4.0950003
32233.0000000	4.0999999
32234.0000000	4.1050000
32235.0000000	4.1000004
32236.0000000	4.0900002
32237.0000000	4.0850000

32238.0000000	4.0950003
32239.0000000	4.0749998
32240.0000000	4.0850000
32241.0000000	4.0900002
32242.0000000	4.0950003
32243.0000000	4.1050000
32244.0000000	4.1050000
32245.0000000	4.1149998
32246.0000000	4.1199999
32247.0000000	4.1149998
32248.0000000	4.1099997
32249.0000000	4.0999999
32250.0000000	4.0799999
32251.0000000	4.0850000
32252.0000000	4.0799999
32253.0000000	4.0749998
32254.0000000	4.0650001
32255.0000000	4.0500002
32256.0000000	4.0550003
32257.0000000	4.0400000
32258.0000000	4.0300002
32259.0000000	4.0200000
32260.0000000	3.9800000
32261.0000000	3.9600000
32262.0000000	3.9200001
32263.0000000	3.8499999
32264.0000000	3.7950001
32265.0000000	3.7550001
32266.0000000	3.7449999
32267.0000000	3.7600000
32268.0000000	3.7200000
32269.0000000	3.7249999
32270.0000000	3.7500000
32271.0000000	3.7550001
32272.0000000	3.7500000
32273.0000000	3.7350001
32274.0000000	3.7100000
32275.0000000	3.6950002
32276.0000000	3.7100000
32277.0000000	3.7150002
32278.0000000	3.6950002
32279.0000000	3.6900001
32280.0000000	3.7100000
32281.0000000	3.7200000
32282.0000000	3.7100000
32283.0000000	3.7200000
32284.0000000	3.7449999
32285.0000000	3.7550001
32286.0000000	3.8000000
32287.0000000	3.8049998
32288.0000000	3.8249998
32289.0000000	3.8333333
32290.0000000	3.8550000
32291.0000000	3.8850000
32292.0000000	3.9000001
32293.0000000	3.9200001
32294.0000000	3.9349999

32295.0000000	3.9600000
32296.0000000	3.9850001
32297.0000000	4.0050001
32298.0000000	4.0150003
32299.0000000	4.0200000
32300.0000000	4.0299997
32301.0000000	4.0450001
32302.0000000	4.0349998
32303.0000000	4.0500002
32304.0000000	4.0600004
32305.0000000	4.0700002
32306.0000000	4.0999999
32307.0000000	4.1199999
32308.0000000	4.1400003
32309.0000000	4.1750002
32310.0000000	4.2049999
32311.0000000	4.2150002
32312.0000000	4.2349997
32313.0000000	4.2449999
32314.0000000	4.2600002
32315.0000000	4.2750001
32316.0000000	4.2700000
32317.0000000	4.3000002
32318.0000000	4.3150001
32319.0000000	4.3350000
32320.0000000	4.3400002
32321.0000000	4.3699999
32322.0000000	4.3800001
32323.0000000	4.3999996
32324.0000000	4.4099998
32325.0000000	4.4150000
32326.0000000	4.4099998
32327.0000000	4.4200001
32328.0000000	4.4399996
32329.0000000	4.4349999
32330.0000000	4.4449997
32331.0000000	4.4400001
32332.0000000	4.4299998
32333.0000000	4.4000001
32334.0000000	4.3899999
32335.0000000	4.3500004
32336.0000000	4.3350000
32337.0000000	4.3050003
32338.0000000	4.2849998
32339.0000000	4.2800002
32340.0000000	4.2800002
32341.0000000	4.2799997
32342.0000000	4.2849998
32343.0000000	4.2750001
32344.0000000	4.2600002
32345.0000000	4.2650003
32346.0000000	4.2600002
32347.0000000	4.2799997
32348.0000000	4.2849998
32349.0000000	4.2950001
32350.0000000	4.3099999
32351.0000000	4.3150001

32352.0000000	4.3350000
32353.0000000	4.3550000
32354.0000000	4.3750000
32355.0000000	4.3850002
32356.0000000	4.4099998
32357.0000000	4.4399996
32358.0000000	4.4549999
32359.0000000	4.4849997
32360.0000000	4.5300002
32361.0000000	4.5550003
32362.0000000	4.5749998
32363.0000000	4.6099997
32364.0000000	4.6099997
32365.0000000	4.6250000
32366.0000000	4.6350002
32367.0000000	4.6500001
32368.0000000	4.6450000
32369.0000000	4.6499996
32370.0000000	4.6650000
32371.0000000	4.6700001
32372.0000000	4.6899996
32373.0000000	4.6949997
32374.0000000	4.6999998
32375.0000000	4.7049999
32376.0000000	4.7199998
32377.0000000	4.7249999
32378.0000000	4.7399998
32379.0000000	4.7533336
32380.0000000	4.7700000
32381.0000000	4.7633338
32382.0000000	4.7449999
32383.0000000	4.7600002
32384.0000000	4.7700005
32385.0000000	4.7799997
32386.0000000	4.7900000
32387.0000000	4.7950001
32388.0000000	4.8000002
32389.0000000	4.8150001
32390.0000000	4.8150001
32391.0000000	4.8100004
32392.0000000	4.8150001
32393.0000000	4.8249998
32394.0000000	4.8150001
32395.0000000	4.8150001
32396.0000000	4.8099999
32397.0000000	4.8099999
32398.0000000	4.8100004
32399.0000000	4.8099999
32400.0000000	4.8199997
32401.0000000	4.8150001
32402.0000000	4.8200002
32403.0000000	4.8299999
32404.0000000	4.8550000
32405.0000000	4.8750000
32406.0000000	4.8699999
32407.0000000	4.8649998
32408.0000000	4.8750000

32409.0000000	4.8899999
32410.0000000	4.9033332
32411.0000000	4.9250002
32412.0000000	4.9499998
32413.0000000	4.9699998
32414.0000000	4.9849997
32415.0000000	4.9799995
32416.0000000	5.0100002
32417.0000000	5.0050001
32418.0000000	5.0299997
32419.0000000	5.0349998
32420.0000000	5.0400000
32421.0000000	5.0550003
32422.0000000	5.0600004
32423.0000000	5.0749998
32424.0000000	5.0850000
32425.0000000	5.1000004
32426.0000000	5.0950003
32427.0000000	5.0999999
32428.0000000	5.1050000
32429.0000000	5.0999999
32430.0000000	5.1099997
32431.0000000	5.1050000
32432.0000000	5.0950003
32433.0000000	5.0799999
32434.0000000	5.0749998
32435.0000000	5.0699997
32436.0000000	5.0700002
32437.0000000	5.0500002
32438.0000000	5.0349998
32439.0000000	5.0050001
32440.0000000	4.9600000
32441.0000000	4.9466667
32442.0000000	4.9000001
32443.0000000	4.8850002
32444.0000000	4.8599997
32445.0000000	4.8350000
32446.0000000	4.8350000
32447.0000000	4.8400002
32448.0000000	4.8400002
32449.0000000	4.8400002
32450.0000000	4.8350000
32451.0000000	4.8649998
32452.0000000	4.8750000
32453.0000000	4.9099998
32454.0000000	4.9349999
32455.0000000	4.9549999
32456.0000000	4.9600000
32457.0000000	4.9549999
32458.0000000	4.9549999
32459.0000000	4.9650002
32460.0000000	4.9749999
32461.0000000	4.9899998
32462.0000000	4.9849997
32463.0000000	4.9949999
32464.0000000	5.0000000
32465.0000000	5.0150003

32466.0000000	5.0100002
32467.0000000	5.0150003
32468.0000000	5.0100002
32469.0000000	5.0250001
32470.0000000	5.0150003
32471.0000000	5.0166669
32472.0000000	5.0400000
32473.0000000	5.0200005
32474.0000000	5.0200005
32475.0000000	5.0100002
32476.0000000	5.0100002
32477.0000000	5.0050001
32478.0000000	4.9899998
32479.0000000	4.9800000
32480.0000000	4.9650002
32481.0000000	4.9549999
32482.0000000	4.9549999
32483.0000000	4.9349999
32484.0000000	4.9250002
32485.0000000	4.9200001
32486.0000000	4.9300003
32487.0000000	4.9250002
32488.0000000	4.9150000
32489.0000000	4.8699999
32490.0000000	4.8099999
32491.0000000	4.7700000
32492.0000000	4.7550001
32493.0000000	4.7550001
32494.0000000	4.7600002
32495.0000000	4.7650003
32496.0000000	4.7700000
32497.0000000	4.7650003
32498.0000000	4.7600002
32499.0000000	4.7600002
32500.0000000	4.7449999
32501.0000000	4.7349997
32502.0000000	4.7033334
32503.0000000	4.6750002
32504.0000000	4.6499996
32505.0000000	4.6700001
32506.0000000	4.6700001
32507.0000000	4.6500001
32508.0000000	4.6350002
32509.0000000	4.6350002
32510.0000000	4.6599998
32511.0000000	4.6949997
32512.0000000	4.7199998
32513.0000000	4.7449999
32514.0000000	4.7550001
32515.0000000	4.7750001
32516.0000000	4.7799997
32517.0000000	4.7799997
32518.0000000	4.7849998
32519.0000000	4.8050003
32520.0000000	4.8100004
32521.0000000	4.8000002
32522.0000000	4.7950001

32523.0000000	4.7950001
32524.0000000	4.7849998
32525.0000000	4.7750001
32526.0000000	4.7650003
32527.0000000	4.7500000
32528.0000000	4.7300000
32529.0000000	4.7299995
32530.0000000	4.7133336
32531.0000000	4.6849999
32532.0000000	4.6500001
32533.0000000	4.6350002
32534.0000000	4.6149998
32535.0000000	4.5799999
32536.0000000	4.5650001
32537.0000000	4.5550003
32538.0000000	4.5450001
32539.0000000	4.5349998
32540.0000000	4.5250001
32541.0000000	4.5300002
32542.0000000	4.5299997
32543.0000000	4.5200005
32544.0000000	4.5100002
32545.0000000	4.4899998
32546.0000000	4.4899998
32547.0000000	4.4849997
32548.0000000	4.4749999
32549.0000000	4.4549999
32550.0000000	4.4250002
32551.0000000	4.3900003
32552.0000000	4.3450003
32553.0000000	4.3000002
32554.0000000	4.2800002
32555.0000000	4.2849998
32556.0000000	4.2750001
32557.0000000	4.2750001
32558.0000000	4.2700000
32559.0000000	4.2900000
32560.0000000	4.2950001
32561.0000000	4.2900000
32562.0000000	4.3000002
32563.0000000	4.3100004
32564.0000000	4.3299999
32565.0000000	4.3199997
32566.0000000	4.3050003
32567.0000000	4.3000002
32568.0000000	4.2950001
32569.0000000	4.2849998
32570.0000000	4.2650003
32571.0000000	4.2650003
32572.0000000	4.2750001
32573.0000000	4.2849998
32574.0000000	4.2900000
32575.0000000	4.2900000
32576.0000000	4.2849998
32577.0000000	4.2750001
32578.0000000	4.2800002
32579.0000000	4.2600002

32580.0000000	4.2449999
32581.0000000	4.2349997
32582.0000000	4.2150002
32583.0000000	4.2100000
32584.0000000	4.1949997
32585.0000000	4.1750002
32586.0000000	4.1750002
32587.0000000	4.1949997
32588.0000000	4.2200003
32589.0000000	4.2500000
32590.0000000	4.2650003
32591.0000000	4.2999997
32592.0000000	4.3200002
32593.0000000	4.3150001
32594.0000000	4.3199997
32595.0000000	4.3150001
32596.0000000	4.3050003
32597.0000000	4.3050003
32598.0000000	4.3100004
32599.0000000	4.3249998
32600.0000000	4.3200002
32601.0000000	4.3050003
32602.0000000	4.2900000
32603.0000000	4.2650003
32604.0000000	4.2449999
32605.0000000	4.2500000
32606.0000000	4.2900000
32607.0000000	4.3199997
32608.0000000	4.3500004
32609.0000000	4.3500004
32610.0000000	4.3750000
32611.0000000	4.3800001
32612.0000000	4.3950000
32613.0000000	4.4099998
32614.0000000	4.3950000
32615.0000000	4.3500004
32616.0000000	4.3050003
32617.0000000	4.2950001
32618.0000000	4.3000002
32619.0000000	4.3150001
32620.0000000	4.3299999
32621.0000000	4.3633332
32622.0000000	4.3800001
32623.0000000	4.4033332
32624.0000000	4.4250002
32625.0000000	4.4400001
32626.0000000	4.4449997
32627.0000000	4.4600000
32628.0000000	4.4849997
32629.0000000	4.5100002
32630.0000000	4.5400000
32631.0000000	4.5850000
32632.0000000	4.6050000
32633.0000000	4.6250000
32634.0000000	4.6399999
32635.0000000	4.6599998
32636.0000000	4.6549997

32637.0000000	4.6400003
32638.0000000	4.6199999
32639.0000000	4.5900002
32640.0000000	4.5599999
32641.0000000	4.5550003
32642.0000000	4.5400000
32643.0000000	4.5150003
32644.0000000	4.5000000
32645.0000000	4.5050001
32646.0000000	4.5050001
32647.0000000	4.5200005
32648.0000000	4.5400000
32649.0000000	4.5550003
32650.0000000	4.5699997
32651.0000000	4.5833335
32652.0000000	4.5749998
32653.0000000	4.5799999
32654.0000000	4.5999999
32655.0000000	4.5999999
32656.0000000	4.6050000
32657.0000000	4.5900002
32658.0000000	4.5850000
32659.0000000	4.5700002
32660.0000000	4.5900002
32661.0000000	4.5850000
32662.0000000	4.5950003
32663.0000000	4.5850000
32664.0000000	4.5950003
32665.0000000	4.5999999
32666.0000000	4.5799999
32667.0000000	4.5749998
32668.0000000	4.5700002
32669.0000000	4.5650001
32670.0000000	4.5550003
32671.0000000	4.5650001
32672.0000000	4.5650001
32673.0000000	4.5500002
32674.0000000	4.5400000
32675.0000000	4.5050001
32676.0000000	4.4749999
32677.0000000	4.4499998
32678.0000000	4.4400001
32679.0000000	4.4200001
32680.0000000	4.4300003
32681.0000000	4.4466667
32682.0000000	4.4499998
32683.0000000	4.4600000
32684.0000000	4.4749999
32685.0000000	4.4949999
32686.0000000	4.5050001
32687.0000000	4.5100002
32688.0000000	4.5250001
32689.0000000	4.5349998
32690.0000000	4.5650001
32691.0000000	4.5799999
32692.0000000	4.5850000
32693.0000000	4.6149998

32694.0000000	4.6300001
32695.0000000	4.6500001
32696.0000000	4.6799998
32697.0000000	4.7150002
32698.0000000	4.7300000
32699.0000000	4.7500000
32700.0000000	4.7550001
32701.0000000	4.7950001
32702.0000000	4.8249998
32703.0000000	4.8550000
32704.0000000	4.8750000
32705.0000000	4.9000001
32706.0000000	4.9200001
32707.0000000	4.9299998
32708.0000000	4.9250002
32709.0000000	4.9300003
32710.0000000	4.9349999
32711.0000000	4.9600000
32712.0000000	4.9800000
32713.0000000	4.9749999
32714.0000000	4.9700003
32715.0000000	4.9700003
32716.0000000	4.9800000
32717.0000000	4.9849997
32718.0000000	4.9899998
32719.0000000	4.9899998
32720.0000000	4.9899998
32721.0000000	4.9949999
32722.0000000	5.0050001
32723.0000000	5.0100002
32724.0000000	5.0100002
32725.0000000	5.0100002
32726.0000000	5.0050001
32727.0000000	5.0050001
32728.0000000	5.0250001
32729.0000000	5.0450001
32730.0000000	5.0550003
32731.0000000	5.0550003
32732.0000000	5.0600004
32733.0000000	5.0600004
32734.0000000	5.0550003
32735.0000000	5.0650001
32736.0000000	5.0450001
32737.0000000	5.0550003
32738.0000000	5.0450001
32739.0000000	5.0349998
32740.0000000	5.0550003
32741.0000000	5.0500002
32742.0000000	5.0366664
32743.0000000	5.0450001
32744.0000000	5.0550003
32745.0000000	5.0550003
32746.0000000	5.0650001
32747.0000000	5.0799999
32748.0000000	5.0950003
32749.0000000	5.1100001
32750.0000000	5.1199999

32751.0000000	5.1450000
32752.0000000	5.1599998
32753.0000000	5.1899996
32754.0000000	5.2049999
32755.0000000	5.2199998
32756.0000000	5.2249999
32757.0000000	5.2299995
32758.0000000	5.2349997
32759.0000000	5.2399998
32760.0000000	5.2449999
32761.0000000	5.2500000
32762.0000000	5.2449999
32763.0000000	5.2449999
32764.0000000	5.2399998
32765.0000000	5.2299995
32766.0000000	5.2100000
32767.0000000	5.2100000
32768.0000000	5.1999998
32769.0000000	5.1900001
32770.0000000	5.1750002
32771.0000000	5.1700001
32772.0000000	5.1549997
32773.0000000	5.1533332
32774.0000000	5.1499996
32775.0000000	5.1549997
32776.0000000	5.1500001
32777.0000000	5.1450000
32778.0000000	5.1599998
32779.0000000	5.1750002
32780.0000000	5.1750002
32781.0000000	5.1900001
32782.0000000	5.1999998
32783.0000000	5.1999998
32784.0000000	5.1999998
32785.0000000	5.2150002
32786.0000000	5.2200003
32787.0000000	5.2200003
32788.0000000	5.2100000
32789.0000000	5.1949997
32790.0000000	5.1899996
32791.0000000	5.1849999
32792.0000000	5.1650000
32793.0000000	5.1549997
32794.0000000	5.1300001
32795.0000000	5.1050000
32796.0000000	5.0850000
32797.0000000	5.0599999
32798.0000000	5.0400000
32799.0000000	5.0400000
32800.0000000	5.0450001
32801.0000000	5.0400000
32802.0000000	5.0499997
32803.0000000	5.0699997
32804.0000000	5.0900002
32805.0000000	5.1250000
32806.0000000	5.1549997
32807.0000000	5.1700001

32808.0000000	5.1650000
32809.0000000	5.1800003
32810.0000000	5.1949997
32811.0000000	5.2100000
32812.0000000	5.2249999
32813.0000000	5.2449999
32814.0000000	5.2550001
32815.0000000	5.2650003
32816.0000000	5.2849998
32817.0000000	5.2849998
32818.0000000	5.2650003
32819.0000000	5.2550001
32820.0000000	5.2449999
32821.0000000	5.2150002
32822.0000000	5.2049999
32823.0000000	5.1849999
32824.0000000	5.1750002
32825.0000000	5.1599998
32826.0000000	5.1499996
32827.0000000	5.1300001
32828.0000000	5.1199999
32829.0000000	5.1149998
32830.0000000	5.1050000
32831.0000000	5.1149998
32832.0000000	5.1199999
32833.0000000	5.1100001
32834.0000000	5.0933337
32835.0000000	5.1000004
32836.0000000	5.0950003
32837.0000000	5.1050000
32838.0000000	5.1050000
32839.0000000	5.1350002
32840.0000000	5.1300001
32841.0000000	5.1300001
32842.0000000	5.1350002
32843.0000000	5.1199999
32844.0000000	5.1300001
32845.0000000	5.1250000
32846.0000000	5.1149998
32847.0000000	5.0900002
32848.0000000	5.0600004
32849.0000000	5.0349998
32850.0000000	5.0050001
32851.0000000	4.9699998
32852.0000000	4.9499998
32853.0000000	4.9349999
32854.0000000	4.9200001
32855.0000000	4.9049997
32856.0000000	4.9049997
32857.0000000	4.8900003
32858.0000000	4.8850002
32859.0000000	4.8750000
32860.0000000	4.8550000
32861.0000000	4.8550000
32862.0000000	4.8450003
32863.0000000	4.8533335
32864.0000000	4.8499999

32865.0000000	4.8866668
32866.0000000	4.8900003
32867.0000000	4.9150000
32868.0000000	4.9250002
32869.0000000	4.9399996
32870.0000000	4.9650002
32871.0000000	4.9849997
32872.0000000	5.0100002
32873.0000000	5.0200005
32874.0000000	5.0200005
32875.0000000	5.0100002
32876.0000000	5.0200005
32877.0000000	5.0250001
32878.0000000	5.0450001
32879.0000000	5.0550003
32880.0000000	5.0599999
32881.0000000	5.0699997
32882.0000000	5.0950003
32883.0000000	5.0950003
32884.0000000	5.1000004
32885.0000000	5.1050000
32886.0000000	5.1000004
32887.0000000	5.1050000
32888.0000000	5.1099997
32889.0000000	5.1149998
32890.0000000	5.1149998
32891.0000000	5.1149998
32892.0000000	5.1000004
32893.0000000	5.0950003
32894.0000000	5.0833335
32895.0000000	5.0599999
32896.0000000	5.0450001
32897.0000000	5.0400000
32898.0000000	5.0300002
32899.0000000	5.0050001
32900.0000000	4.9849997
32901.0000000	4.9700003
32902.0000000	4.9499998
32903.0000000	4.9400001
32904.0000000	4.9250002
32905.0000000	4.9200001
32906.0000000	4.9200001
32907.0000000	4.9049997
32908.0000000	4.8999996
32909.0000000	4.8900003
32910.0000000	4.8900003
32911.0000000	4.8800001
32912.0000000	4.8850002
32913.0000000	4.8750000
32914.0000000	4.8900003
32915.0000000	4.8999996
32916.0000000	4.8950000
32917.0000000	4.9349999
32918.0000000	4.9699998
32919.0000000	4.9949999
32920.0000000	5.0250001
32921.0000000	5.0450001

32922.0000000	5.0699997
32923.0000000	5.0900002
32924.0000000	5.1066666
32925.0000000	5.1149998
32926.0000000	5.1149998
32927.0000000	5.1149998
32928.0000000	5.1350002
32929.0000000	5.1499996
32930.0000000	5.1750002
32931.0000000	5.1949997
32932.0000000	5.1949997
32933.0000000	5.1899996
32934.0000000	5.1899996
32935.0000000	5.1800003
32936.0000000	5.1949997
32937.0000000	5.1849999
32938.0000000	5.2049999
32939.0000000	5.2100000
32940.0000000	5.2150002
32941.0000000	5.2249999
32942.0000000	5.2150002
32943.0000000	5.2249999
32944.0000000	5.2150002
32945.0000000	5.2349997
32946.0000000	5.2349997
32947.0000000	5.2249999
32948.0000000	5.1999998
32949.0000000	5.2200003
32950.0000000	5.2049999
32951.0000000	5.2049999
32952.0000000	5.2049999
32953.0000000	5.1949997
32954.0000000	5.1833334
32955.0000000	5.1399999
32956.0000000	5.1266665
32957.0000000	5.0900002
32958.0000000	5.0600004
32959.0000000	5.0200005
32960.0000000	4.9699998
32961.0000000	4.9250002
32962.0000000	4.8800001
32963.0000000	4.8450003
32964.0000000	4.8050003
32965.0000000	4.7600002
32966.0000000	4.7300000
32967.0000000	4.6999998
32968.0000000	4.6599998
32969.0000000	4.6350002
32970.0000000	4.6100001
32971.0000000	4.5950003
32972.0000000	4.5850000
32973.0000000	4.5850000
32974.0000000	4.5850000
32975.0000000	4.5749998
32976.0000000	4.5450001
32977.0000000	4.5200000
32978.0000000	4.5200000

32979.0000000	4.5400000
32980.0000000	4.5349998
32981.0000000	4.5450001
32982.0000000	4.5650001
32983.0000000	4.5900002
32984.0000000	4.5900002
32985.0000000	4.6100001
32986.0000000	4.6100001
32987.0000000	4.6350002
32988.0000000	4.6450000
32989.0000000	4.6650000
32990.0000000	4.6750002
32991.0000000	4.6849999
32992.0000000	4.7100000
32993.0000000	4.7199998
32994.0000000	4.7399998
32995.0000000	4.7449999
32996.0000000	4.7500000
32997.0000000	4.7500000
32998.0000000	4.7550001
32999.0000000	4.7750001
33000.0000000	4.7750001
33001.0000000	4.7750001
33002.0000000	4.7650003
33003.0000000	4.7750001
33004.0000000	4.7799997
33005.0000000	4.7849998
33006.0000000	4.7900000
33007.0000000	4.7900000
33008.0000000	4.8099999
33009.0000000	4.8299999
33010.0000000	4.8249998
33011.0000000	4.8350000
33012.0000000	4.8400002
33013.0000000	4.8450003
33014.0000000	4.8400002
33015.0000000	4.8249998
33016.0000000	4.8166671
33017.0000000	4.8000002
33018.0000000	4.7849998
33019.0000000	4.7600002
33020.0000000	4.7199998
33021.0000000	4.6849999
33022.0000000	4.6400003
33023.0000000	4.6000004
33024.0000000	4.5650001
33025.0000000	4.5300002
33026.0000000	4.5050001
33027.0000000	4.4499998
33028.0000000	4.3899999
33029.0000000	4.3450003
33030.0000000	4.3050003
33031.0000000	4.2650003
33032.0000000	4.2500000
33033.0000000	4.2500000
33034.0000000	4.2550001
33035.0000000	4.2650003

33036.0000000	4.2650003
33037.0000000	4.2950001
33038.0000000	4.3150001
33039.0000000	4.3450003
33040.0000000	4.3649998
33041.0000000	4.3900003
33042.0000000	4.3999996
33043.0000000	4.4150000
33044.0000000	4.4250002
33045.0000000	4.4200001
33046.0000000	4.4099998
33047.0000000	4.3899999
33048.0000000	4.3699999
33049.0000000	4.3249998
33050.0000000	4.2900000
33051.0000000	4.2399998
33052.0000000	4.1949997
33053.0000000	4.1799998
33054.0000000	4.1750002
33055.0000000	4.1949997
33056.0000000	4.2150002
33057.0000000	4.2249999
33058.0000000	4.2399998
33059.0000000	4.2399998
33060.0000000	4.2100000
33061.0000000	4.1800003
33062.0000000	4.0950003
33063.0000000	4.0349998
33064.0000000	3.9949999
33065.0000000	3.9900000
33066.0000000	3.9800000
33067.0000000	3.9850001
33068.0000000	3.9900000
33069.0000000	3.9950001
33070.0000000	3.9949999
33071.0000000	4.0100002
33072.0000000	4.0500002
33073.0000000	4.0749998
33074.0000000	4.0900002
33075.0000000	4.1066670
33076.0000000	4.1199999
33077.0000000	4.1400003
33078.0000000	4.1450000
33079.0000000	4.1650000
33080.0000000	4.1900001
33081.0000000	4.1899996
33082.0000000	4.1999998
33083.0000000	4.2150002
33084.0000000	4.2249999
33085.0000000	4.2399998
33086.0000000	4.2449999
33087.0000000	4.2449999
33088.0000000	4.2349997
33089.0000000	4.2449999
33090.0000000	4.2449999
33091.0000000	4.2500000
33092.0000000	4.2500000

33093.0000000	4.2600002
33094.0000000	4.2550001
33095.0000000	4.2550001
33096.0000000	4.2550001
33097.0000000	4.2349997
33098.0000000	4.2500000
33099.0000000	4.2449999
33100.0000000	4.2200003
33101.0000000	4.1999998
33102.0000000	4.1750002
33103.0000000	4.1700001
33104.0000000	4.1750002
33105.0000000	4.1849999
33106.0000000	4.1666665
33107.0000000	4.1250000
33108.0000000	4.0799999
33109.0000000	4.0450001
33110.0000000	4.0200005
33111.0000000	4.0000000
33112.0000000	3.9900000
33113.0000000	3.9850001
33114.0000000	3.9850001
33115.0000000	3.9949999
33116.0000000	4.0000000
33117.0000000	4.0000000
33118.0000000	4.0000000
33119.0000000	4.0000000
33120.0000000	4.0000000
33121.0000000	4.0050001
33122.0000000	4.0349998
33123.0000000	4.0450001
33124.0000000	4.0600004
[truncated: 607,051 more chars]
