# Supplementary material for: Periodic oscillations of atmospheric electric field during snowfall in the Tokyo metropolitan area
Source: Sci Rep. 2021 Jan 26;11:2280. doi: 10.1038/s41598-021-82091-w (PMC7838252; doi:10.1038/s41598-021-82091-w)
Supplement: Supplementary file 4 — Supplementary Information 4. [file 41598_2021_82091_MOESM4_ESM.pdf]

-32400.0000000	0.2515000
-32399.0000000	0.2514285
-32398.0000000	0.2500000
-32397.0000000	0.2515000
-32396.0000000	0.2514285
-32395.0000000	0.2510000
-32394.0000000	0.2510000
-32393.0000000	0.2505000
-32392.0000000	0.2509524
-32391.0000000	0.2500000
-32390.0000000	0.2509524
-32389.0000000	0.2510000
-32388.0000000	0.2505000
-32387.0000000	0.2504762
-32386.0000000	0.2510000
-32385.0000000	0.2500000
-32384.0000000	0.2510000
-32383.0000000	0.2509524
-32382.0000000	0.2504762
-32381.0000000	0.2505000
-32380.0000000	0.2500000
-32379.0000000	0.2510000
-32378.0000000	0.2509524
-32377.0000000	0.2510000
-32376.0000000	0.2505000
-32375.0000000	0.2510000
-32374.0000000	0.2514285
-32373.0000000	0.2510000
-32372.0000000	0.2505000
-32371.0000000	0.2504762
-32370.0000000	0.2500000
-32369.0000000	0.2509524
-32368.0000000	0.2510000
-32367.0000000	0.2505000
-32366.0000000	0.2504762
-32365.0000000	0.2505000
-32364.0000000	0.2500000
-32363.0000000	0.2505000
-32362.0000000	0.2504762
-32361.0000000	0.2500000
-32360.0000000	0.2500000
-32359.0000000	0.2500000
-32358.0000000	0.2505000
-32357.0000000	0.2500000
-32356.0000000	0.2504762
-32355.0000000	0.2510000
-32354.0000000	0.2505000
-32353.0000000	0.2504762
-32352.0000000	0.2505000
-32351.0000000	0.2500000
-32350.0000000	0.2505000
-32349.0000000	0.2500000
-32348.0000000	0.2500000
-32347.0000000	0.2500000
-32346.0000000	0.2500000
-32345.0000000	0.2500000
-32344.0000000	0.2500000

-32343.0000000	0.2500000
-32342.0000000	0.2500000
-32341.0000000	0.2500000
-32340.0000000	0.2500000
-32339.0000000	0.2500000
-32338.0000000	0.2500000
-32337.0000000	0.2495000
-32336.0000000	0.2500000
-32335.0000000	0.2500000
-32334.0000000	0.2500000
-32333.0000000	0.2500000
-32332.0000000	0.2500000
-32331.0000000	0.2500000
-32330.0000000	0.2500000
-32329.0000000	0.2500000
-32328.0000000	0.2500000
-32327.0000000	0.2500000
-32326.0000000	0.2500000
-32325.0000000	0.2500000
-32324.0000000	0.2490000
-32323.0000000	0.2500000
-32322.0000000	0.2495000
-32321.0000000	0.2485714
-32320.0000000	0.2495000
-32319.0000000	0.2485000
-32318.0000000	0.2490000
-32317.0000000	0.2490476
-32316.0000000	0.2490000
-32315.0000000	0.2480000
-32314.0000000	0.2485714
-32313.0000000	0.2490000
-32312.0000000	0.2495000
-32311.0000000	0.2490000
-32310.0000000	0.2466667
-32309.0000000	0.2480952
-32308.0000000	0.2475000
-32307.0000000	0.2460000
-32306.0000000	0.2465000
-32305.0000000	0.2461905
-32304.0000000	0.2455000
-32303.0000000	0.2445000
-32302.0000000	0.2447619
-32301.0000000	0.2420000
-32300.0000000	0.2433333
-32299.0000000	0.2435000
-32298.0000000	0.2404999
-32297.0000000	0.2435000
-32296.0000000	0.2423809
-32295.0000000	0.2435000
-32294.0000000	0.2404999
-32293.0000000	0.2414285
-32292.0000000	0.2415000
-32291.0000000	0.2428571
-32290.0000000	0.2405000
-32289.0000000	0.2400000
-32288.0000000	0.2400000
-32287.0000000	0.2414285

-32286.0000000	0.2405000
-32285.0000000	0.2415000
-32284.0000000	0.2409523
-32283.0000000	0.2404999
-32282.0000000	0.2400000
-32281.0000000	0.2400000
-32280.0000000	0.2399999
-32279.0000000	0.2400000
-32278.0000000	0.2399999
-32277.0000000	0.2400000
-32276.0000000	0.2400000
-32275.0000000	0.2399999
-32274.0000000	0.2400000
-32273.0000000	0.2400000
-32272.0000000	0.2400000
-32271.0000000	0.2399999
-32270.0000000	0.2400000
-32269.0000000	0.2399999
-32268.0000000	0.2400000
-32267.0000000	0.2400000
-32266.0000000	0.2399999
-32265.0000000	0.2400000
-32264.0000000	0.2400000
-32263.0000000	0.2399999
-32262.0000000	0.2400000
-32261.0000000	0.2399999
-32260.0000000	0.2395000
-32259.0000000	0.2400000
-32258.0000000	0.2400000
-32257.0000000	0.2399999
-32256.0000000	0.2400000
-32255.0000000	0.2395000
-32254.0000000	0.2399999
-32253.0000000	0.2395000
-32252.0000000	0.2395000
-32251.0000000	0.2390000
-32250.0000000	0.2395238
-32249.0000000	0.2395000
-32248.0000000	0.2399999
-32247.0000000	0.2385000
-32246.0000000	0.2390000
-32245.0000000	0.2390476
-32244.0000000	0.2370000
-32243.0000000	0.2375000
-32242.0000000	0.2350000
-32241.0000000	0.2376190
-32240.0000000	0.2375000
-32239.0000000	0.2366666
-32238.0000000	0.2355000
-32237.0000000	0.2345000
-32236.0000000	0.2371428
-32235.0000000	0.2355000
-32234.0000000	0.2330000
-32233.0000000	0.2328571
-32232.0000000	0.2345000
-32231.0000000	0.2345000
-32230.0000000	0.2333333

-32229.0000000	0.2355000
-32228.0000000	0.2330000
-32227.0000000	0.2338095
-32226.0000000	0.2345000
-32225.0000000	0.2360000
-32224.0000000	0.2366666
-32223.0000000	0.2335000
-32222.0000000	0.2355000
-32221.0000000	0.2350000
-32220.0000000	0.2319048
-32219.0000000	0.2330000
-32218.0000000	0.2328571
-32217.0000000	0.2320000
-32216.0000000	0.2330000
-32215.0000000	0.2333333
-32214.0000000	0.2330000
-32213.0000000	0.2325000
-32212.0000000	0.2335000
-32211.0000000	0.2319048
-32210.0000000	0.2300000
-32209.0000000	0.2323809
-32208.0000000	0.2330000
-32207.0000000	0.2335000
-32206.0000000	0.2315000
-32205.0000000	0.2314286
-32204.0000000	0.2315000
-32203.0000000	0.2309524
-32202.0000000	0.2310000
-32201.0000000	0.2315000
-32200.0000000	0.2314286
-32199.0000000	0.2310000
-32198.0000000	0.2305000
-32197.0000000	0.2310000
-32196.0000000	0.2319048
-32195.0000000	0.2315000
-32194.0000000	0.2309524
-32193.0000000	0.2305000
-32192.0000000	0.2310000
-32191.0000000	0.2305000
-32190.0000000	0.2314286
-32189.0000000	0.2315000
-32188.0000000	0.2300000
-32187.0000000	0.2314286
-32186.0000000	0.2315000
-32185.0000000	0.2314286
-32184.0000000	0.2310000
-32183.0000000	0.2320000
-32182.0000000	0.2310000
-32181.0000000	0.2314285
-32180.0000000	0.2305000
-32179.0000000	0.2315000
-32178.0000000	0.2309524
-32177.0000000	0.2315000
-32176.0000000	0.2335000
-32175.0000000	0.2342857
-32174.0000000	0.2340000
-32173.0000000	0.2325000

-32172.0000000	0.2328571
-32171.0000000	0.2345000
-32170.0000000	0.2347619
-32169.0000000	0.2355000
-32168.0000000	0.2350000
-32167.0000000	0.2370000
-32166.0000000	0.2357143
-32165.0000000	0.2355000
-32164.0000000	0.2357143
-32163.0000000	0.2350000
-32162.0000000	0.2365000
-32161.0000000	0.2360000
-32160.0000000	0.2361904
-32159.0000000	0.2360000
-32158.0000000	0.2350000
-32157.0000000	0.2352381
-32156.0000000	0.2350000
-32155.0000000	0.2342857
-32154.0000000	0.2340000
-32153.0000000	0.2360000
-32152.0000000	0.2330000
-32151.0000000	0.2328571
-32150.0000000	0.2330000
-32149.0000000	0.2355000
-32148.0000000	0.2319048
-32147.0000000	0.2315000
-32146.0000000	0.2305000
-32145.0000000	0.2304762
-32144.0000000	0.2315000
-32143.0000000	0.2305000
-32142.0000000	0.2300000
-32141.0000000	0.2300000
-32140.0000000	0.2300000
-32139.0000000	0.2300000
-32138.0000000	0.2300000
-32137.0000000	0.2300000
-32136.0000000	0.2300000
-32135.0000000	0.2300000
-32134.0000000	0.2300000
-32133.0000000	0.2300000
-32132.0000000	0.2285000
-32131.0000000	0.2280000
-32130.0000000	0.2252381
-32129.0000000	0.2235000
-32128.0000000	0.2235000
-32127.0000000	0.2238095
-32126.0000000	0.2215000
-32125.0000000	0.2204762
-32124.0000000	0.2200000
-32123.0000000	0.2200000
-32122.0000000	0.2200000
-32121.0000000	0.2200000
-32120.0000000	0.2200000
-32119.0000000	0.2200000
-32118.0000000	0.2200000
-32117.0000000	0.2200000
-32116.0000000	0.2190000

-32115.0000000	0.2170000
-32114.0000000	0.2176190
-32113.0000000	0.2160000
-32112.0000000	0.2142857
-32111.0000000	0.2155000
-32110.0000000	0.2115000
-32109.0000000	0.2100000
-32108.0000000	0.2110000
-32107.0000000	0.2115000
-32106.0000000	0.2100000
-32105.0000000	0.2100000
-32104.0000000	0.2104762
-32103.0000000	0.2100000
-32102.0000000	0.2100000
-32101.0000000	0.2095000
-32100.0000000	0.2100000
-32099.0000000	0.2100000
-32098.0000000	0.2095000
-32097.0000000	0.2100000
-32096.0000000	0.2095000
-32095.0000000	0.2100000
-32094.0000000	0.2100000
-32093.0000000	0.2075000
-32092.0000000	0.2080000
-32091.0000000	0.2076191
-32090.0000000	0.2070000
-32089.0000000	0.2075000
-32088.0000000	0.2042857
-32087.0000000	0.2050000
-32086.0000000	0.2035000
-32085.0000000	0.2010000
-32084.0000000	0.2028572
-32083.0000000	0.2005000
-32082.0000000	0.2004762
-32081.0000000	0.2000000
-32080.0000000	0.2010000
-32079.0000000	0.2009524
-32078.0000000	0.2000000
-32077.0000000	0.2000000
-32076.0000000	0.2000000
-32075.0000000	0.2000000
-32074.0000000	0.2000000
-32073.0000000	0.2000000
-32072.0000000	0.2000000
-32071.0000000	0.2000000
-32070.0000000	0.1995238
-32069.0000000	0.2000000
-32068.0000000	0.1995000
-32067.0000000	0.1990000
-32066.0000000	0.1980953
-32065.0000000	0.1990000
-32064.0000000	0.1957143
-32063.0000000	0.1960000
-32062.0000000	0.1945000
-32061.0000000	0.1947619
-32060.0000000	0.1940000
-32059.0000000	0.1930000

-32058.0000000	0.1900000
-32057.0000000	0.1914286
-32056.0000000	0.1905000
-32055.0000000	0.1900000
-32054.0000000	0.1900000
-32053.0000000	0.1900000
-32052.0000000	0.1900000
-32051.0000000	0.1900000
-32050.0000000	0.1900000
-32049.0000000	0.1900000
-32048.0000000	0.1900000
-32047.0000000	0.1895000
-32046.0000000	0.1870000
-32045.0000000	0.1880953
-32044.0000000	0.1865000
-32043.0000000	0.1842858
-32042.0000000	0.1840000
-32041.0000000	0.1815000
-32040.0000000	0.1810000
-32039.0000000	0.1800000
-32038.0000000	0.1800000
-32037.0000000	0.1800000
-32036.0000000	0.1800000
-32035.0000000	0.1800000
-32034.0000000	0.1800000
-32033.0000000	0.1800000
-32032.0000000	0.1795000
-32031.0000000	0.1795239
-32030.0000000	0.1800000
-32029.0000000	0.1790000
-32028.0000000	0.1790000
-32027.0000000	0.1766667
-32026.0000000	0.1765000
-32025.0000000	0.1745000
-32024.0000000	0.1735000
-32023.0000000	0.1733334
-32022.0000000	0.1719048
-32021.0000000	0.1700000
-32020.0000000	0.1705000
-32019.0000000	0.1709524
-32018.0000000	0.1700000
-32017.0000000	0.1700000
-32016.0000000	0.1700000
-32015.0000000	0.1704762
-32014.0000000	0.1700000
-32013.0000000	0.1700000
-32012.0000000	0.1700000
-32011.0000000	0.1695000
-32010.0000000	0.1690000
-32009.0000000	0.1700000
-32008.0000000	0.1700000
-32007.0000000	0.1685000
-32006.0000000	0.1690476
-32005.0000000	0.1690000
-32004.0000000	0.1700000
-32003.0000000	0.1675000
-32002.0000000	0.1685000

-32001.0000000	0.1695000
-32000.0000000	0.1690476
-31999.0000000	0.1675000
-31998.0000000	0.1675000
-31997.0000000	0.1671429
-31996.0000000	0.1675000
-31995.0000000	0.1655000
-31994.0000000	0.1655000
-31993.0000000	0.1680953
-31992.0000000	0.1670000
-31991.0000000	0.1671429
-31990.0000000	0.1665000
-31989.0000000	0.1625000
-31988.0000000	0.1657143
-31987.0000000	0.1660000
-31986.0000000	0.1640000
-31985.0000000	0.1666667
-31984.0000000	0.1645000
-31983.0000000	0.1630000
-31982.0000000	0.1633334
-31981.0000000	0.1605000
-31980.0000000	0.1615000
-31979.0000000	0.1633334
-31978.0000000	0.1605000
-31977.0000000	0.1620000
-31976.0000000	0.1600000
-31975.0000000	0.1600000
-31974.0000000	0.1604762
-31973.0000000	0.1605000
-31972.0000000	0.1600000
-31971.0000000	0.1600000
-31970.0000000	0.1600000
-31969.0000000	0.1600000
-31968.0000000	0.1605000
-31967.0000000	0.1595238
-31966.0000000	0.1595000
-31965.0000000	0.1600000
-31964.0000000	0.1600000
-31963.0000000	0.1600000
-31962.0000000	0.1600000
-31961.0000000	0.1595238
-31960.0000000	0.1590000
-31959.0000000	0.1600000
-31958.0000000	0.1600000
-31957.0000000	0.1600000
-31956.0000000	0.1590000
-31955.0000000	0.1595238
-31954.0000000	0.1600000
-31953.0000000	0.1595000
-31952.0000000	0.1571429
-31951.0000000	0.1595000
-31950.0000000	0.1600000
-31949.0000000	0.1590476
-31948.0000000	0.1590000
-31947.0000000	0.1590000
-31946.0000000	0.1590476
-31945.0000000	0.1600000

-31944.0000000	0.1590000
-31943.0000000	0.1580953
-31942.0000000	0.1575000
-31941.0000000	0.1560000
-31940.0000000	0.1561905
-31939.0000000	0.1555000
-31938.0000000	0.1565000
-31937.0000000	0.1571429
-31936.0000000	0.1550000
-31935.0000000	0.1570000
-31934.0000000	0.1555000
-31933.0000000	0.1552381
-31932.0000000	0.1535000
-31931.0000000	0.1552381
-31930.0000000	0.1545000
-31929.0000000	0.1535000
-31928.0000000	0.1557143
-31927.0000000	0.1530000
-31926.0000000	0.1535000
-31925.0000000	0.1520000
-31924.0000000	0.1528572
-31923.0000000	0.1525000
-31922.0000000	0.1514286
-31921.0000000	0.1525000
-31920.0000000	0.1520000
-31919.0000000	0.1505000
-31918.0000000	0.1514286
-31917.0000000	0.1510000
-31916.0000000	0.1509524
-31915.0000000	0.1510000
-31914.0000000	0.1515000
-31913.0000000	0.1504762
-31912.0000000	0.1510000
-31911.0000000	0.1500000
-31910.0000000	0.1510000
-31909.0000000	0.1509524
-31908.0000000	0.1500000
-31907.0000000	0.1514286
-31906.0000000	0.1505000
-31905.0000000	0.1510000
-31904.0000000	0.1500000
-31903.0000000	0.1505000
-31902.0000000	0.1504762
-31901.0000000	0.1500000
-31900.0000000	0.1504762
-31899.0000000	0.1500000
-31898.0000000	0.1500000
-31897.0000000	0.1500000
-31896.0000000	0.1500000
-31895.0000000	0.1500000
-31894.0000000	0.1500000
-31893.0000000	0.1500000
-31892.0000000	0.1500000
-31891.0000000	0.1500000
-31890.0000000	0.1500000
-31889.0000000	0.1495000
-31888.0000000	0.1495238

-31887.0000000	0.1500000
-31886.0000000	0.1490476
-31885.0000000	0.1500000
-31884.0000000	0.1500000
-31883.0000000	0.1495238
-31882.0000000	0.1495000
-31881.0000000	0.1495000
-31880.0000000	0.1495000
-31879.0000000	0.1495238
-31878.0000000	0.1500000
-31877.0000000	0.1500000
-31876.0000000	0.1485000
-31875.0000000	0.1490000
-31874.0000000	0.1490000
-31873.0000000	0.1480953
-31872.0000000	0.1495000
-31871.0000000	0.1480000
-31870.0000000	0.1485715
-31869.0000000	0.1475000
-31868.0000000	0.1471429
-31867.0000000	0.1470000
-31866.0000000	0.1485000
-31865.0000000	0.1485000
-31864.0000000	0.1476191
-31863.0000000	0.1450000
-31862.0000000	0.1490000
-31861.0000000	0.1490476
-31860.0000000	0.1475000
-31859.0000000	0.1485000
-31858.0000000	0.1471429
-31857.0000000	0.1480000
-31856.0000000	0.1442858
-31855.0000000	0.1475000
-31854.0000000	0.1460000
-31853.0000000	0.1475000
-31852.0000000	0.1476191
-31851.0000000	0.1485000
-31850.0000000	0.1470000
-31849.0000000	0.1476191
-31848.0000000	0.1485000
-31847.0000000	0.1476191
-31846.0000000	0.1490000
-31845.0000000	0.1465000
-31844.0000000	0.1485000
-31843.0000000	0.1480953
-31842.0000000	0.1485000
-31841.0000000	0.1480000
-31840.0000000	0.1480953
-31839.0000000	0.1485000
-31838.0000000	0.1485715
-31837.0000000	0.1495000
-31836.0000000	0.1495000
-31835.0000000	0.1500000
-31834.0000000	0.1490476
-31833.0000000	0.1500000
-31832.0000000	0.1500000
-31831.0000000	0.1500000

-31830.0000000	0.1500000
-31829.0000000	0.1500000
-31828.0000000	0.1500000
-31827.0000000	0.1490476
-31826.0000000	0.1500000
-31825.0000000	0.1500000
-31824.0000000	0.1495000
-31823.0000000	0.1500000
-31822.0000000	0.1500000
-31821.0000000	0.1500000
-31820.0000000	0.1495000
-31819.0000000	0.1500000
-31818.0000000	0.1500000
-31817.0000000	0.1500000
-31816.0000000	0.1500000
-31815.0000000	0.1500000
-31814.0000000	0.1500000
-31813.0000000	0.1500000
-31812.0000000	0.1500000
-31811.0000000	0.1500000
-31810.0000000	0.1500000
-31809.0000000	0.1495000
-31808.0000000	0.1500000
-31807.0000000	0.1500000
-31806.0000000	0.1500000
-31805.0000000	0.1500000
-31804.0000000	0.1500000
-31803.0000000	0.1500000
-31802.0000000	0.1500000
-31801.0000000	0.1495238
-31800.0000000	0.1500000
-31799.0000000	0.1500000
-31798.0000000	0.1500000
-31797.0000000	0.1500000
-31796.0000000	0.1500000
-31795.0000000	0.1500000
-31794.0000000	0.1500000
-31793.0000000	0.1500000
-31792.0000000	0.1500000
-31791.0000000	0.1500000
-31790.0000000	0.1505000
-31789.0000000	0.1505000
-31788.0000000	0.1500000
-31787.0000000	0.1500000
-31786.0000000	0.1505000
-31785.0000000	0.1500000
-31784.0000000	0.1505000
-31783.0000000	0.1500000
-31782.0000000	0.1500000
-31781.0000000	0.1500000
-31780.0000000	0.1505000
-31779.0000000	0.1504762
-31778.0000000	0.1504762
-31777.0000000	0.1505000
-31776.0000000	0.1515000
-31775.0000000	0.1520000
-31774.0000000	0.1519048

-31773.0000000	0.1510000
-31772.0000000	0.1520000
-31771.0000000	0.1525000
-31770.0000000	0.1523810
-31769.0000000	0.1520000
-31768.0000000	0.1530000
-31767.0000000	0.1533334
-31766.0000000	0.1530000
-31765.0000000	0.1528572
-31764.0000000	0.1560000
-31763.0000000	0.1560000
-31762.0000000	0.1542857
-31761.0000000	0.1540000
-31760.0000000	0.1555000
-31759.0000000	0.1575000
-31758.0000000	0.1571429
-31757.0000000	0.1550000
-31756.0000000	0.1571429
-31755.0000000	0.1585000
-31754.0000000	0.1595000
-31753.0000000	0.1575000
-31752.0000000	0.1595238
-31751.0000000	0.1585000
-31750.0000000	0.1595000
-31749.0000000	0.1576191
-31748.0000000	0.1600000
-31747.0000000	0.1600000
-31746.0000000	0.1600000
-31745.0000000	0.1590000
-31744.0000000	0.1595238
-31743.0000000	0.1595000
-31742.0000000	0.1600000
-31741.0000000	0.1600000
-31740.0000000	0.1595238
-31739.0000000	0.1595000
-31738.0000000	0.1600000
-31737.0000000	0.1600000
-31736.0000000	0.1600000
-31735.0000000	0.1600000
-31734.0000000	0.1600000
-31733.0000000	0.1600000
-31732.0000000	0.1600000
-31731.0000000	0.1600000
-31730.0000000	0.1600000
-31729.0000000	0.1600000
-31728.0000000	0.1600000
-31727.0000000	0.1600000
-31726.0000000	0.1600000
-31725.0000000	0.1600000
-31724.0000000	0.1600000
-31723.0000000	0.1600000
-31722.0000000	0.1604762
-31721.0000000	0.1600000
-31720.0000000	0.1605000
-31719.0000000	0.1600000
-31718.0000000	0.1605000
-31717.0000000	0.1600000

-31716.0000000	0.1600000
-31715.0000000	0.1605000
-31714.0000000	0.1604762
-31713.0000000	0.1600000
-31712.0000000	0.1610000
-31711.0000000	0.1600000
-31710.0000000	0.1604762
-31709.0000000	0.1605000
-31708.0000000	0.1605000
-31707.0000000	0.1610000
-31706.0000000	0.1619048
-31705.0000000	0.1619048
-31704.0000000	0.1630000
-31703.0000000	0.1625000
-31702.0000000	0.1610000
-31701.0000000	0.1609524
-31700.0000000	0.1605000
-31699.0000000	0.1620000
-31698.0000000	0.1619048
-31697.0000000	0.1610000
-31696.0000000	0.1633334
-31695.0000000	0.1610000
-31694.0000000	0.1640000
-31693.0000000	0.1620000
-31692.0000000	0.1633334
-31691.0000000	0.1620000
-31690.0000000	0.1640000
-31689.0000000	0.1623810
-31688.0000000	0.1630000
-31687.0000000	0.1647619
-31686.0000000	0.1640000
-31685.0000000	0.1635000
-31684.0000000	0.1610000
-31683.0000000	0.1619048
-31682.0000000	0.1630000
-31681.0000000	0.1620000
-31680.0000000	0.1614286
-31679.0000000	0.1620000
-31678.0000000	0.1615000
-31677.0000000	0.1615000
-31676.0000000	0.1619048
-31675.0000000	0.1620000
-31674.0000000	0.1609524
-31673.0000000	0.1605000
-31672.0000000	0.1610000
-31671.0000000	0.1623810
-31670.0000000	0.1630000
-31669.0000000	0.1605000
-31668.0000000	0.1614286
-31667.0000000	0.1610000
-31666.0000000	0.1605000
-31665.0000000	0.1614286
-31664.0000000	0.1605000
-31663.0000000	0.1620000
-31662.0000000	0.1609524
-31661.0000000	0.1605000
-31660.0000000	0.1610000

-31659.0000000	0.1614286
-31658.0000000	0.1615000
-31657.0000000	0.1610000
-31656.0000000	0.1619048
-31655.0000000	0.1615000
-31654.0000000	0.1610000
-31653.0000000	0.1609524
-31652.0000000	0.1615000
-31651.0000000	0.1615000
-31650.0000000	0.1600000
-31649.0000000	0.1615000
-31648.0000000	0.1615000
-31647.0000000	0.1625000
-31646.0000000	0.1604762
-31645.0000000	0.1605000
-31644.0000000	0.1614286
-31643.0000000	0.1625000
-31642.0000000	0.1615000
-31641.0000000	0.1600000
-31640.0000000	0.1605000
-31639.0000000	0.1630000
-31638.0000000	0.1615000
-31637.0000000	0.1628572
-31636.0000000	0.1605000
-31635.0000000	0.1623810
-31634.0000000	0.1615000
-31633.0000000	0.1630000
-31632.0000000	0.1614286
-31631.0000000	0.1600000
-31630.0000000	0.1630000
-31629.0000000	0.1614286
-31628.0000000	0.1630000
-31627.0000000	0.1620000
-31626.0000000	0.1623810
-31625.0000000	0.1615000
-31624.0000000	0.1625000
-31623.0000000	0.1619048
-31622.0000000	0.1615000
-31621.0000000	0.1610000
-31620.0000000	0.1604762
-31619.0000000	0.1630000
-31618.0000000	0.1610000
-31617.0000000	0.1610000
-31616.0000000	0.1609524
-31615.0000000	0.1605000
-31614.0000000	0.1609524
-31613.0000000	0.1600000
-31612.0000000	0.1605000
-31611.0000000	0.1600000
-31610.0000000	0.1600000
-31609.0000000	0.1605000
-31608.0000000	0.1610000
-31607.0000000	0.1600000
-31606.0000000	0.1600000
-31605.0000000	0.1600000
-31604.0000000	0.1600000
-31603.0000000	0.1600000

-31602.0000000	0.1600000
-31601.0000000	0.1600000
-31600.0000000	0.1600000
-31599.0000000	0.1600000
-31598.0000000	0.1600000
-31597.0000000	0.1600000
-31596.0000000	0.1600000
-31595.0000000	0.1600000
-31594.0000000	0.1600000
-31593.0000000	0.1595000
-31592.0000000	0.1600000
-31591.0000000	0.1600000
-31590.0000000	0.1600000
-31589.0000000	0.1600000
-31588.0000000	0.1600000
-31587.0000000	0.1595000
-31586.0000000	0.1580953
-31585.0000000	0.1585000
-31584.0000000	0.1595000
-31583.0000000	0.1576191
-31582.0000000	0.1585000
-31581.0000000	0.1595238
-31580.0000000	0.1585000
-31579.0000000	0.1580000
-31578.0000000	0.1575000
-31577.0000000	0.1585715
-31576.0000000	0.1570000
-31575.0000000	0.1590476
-31574.0000000	0.1555000
-31573.0000000	0.1555000
-31572.0000000	0.1565000
-31571.0000000	0.1552381
-31570.0000000	0.1575000
-31569.0000000	0.1557143
-31568.0000000	0.1555000
-31567.0000000	0.1550000
-31566.0000000	0.1566667
-31565.0000000	0.1535000
-31564.0000000	0.1540000
-31563.0000000	0.1545000
-31562.0000000	0.1542857
-31561.0000000	0.1525000
-31560.0000000	0.1533334
-31559.0000000	0.1510000
-31558.0000000	0.1540000
-31557.0000000	0.1535000
-31556.0000000	0.1519048
-31555.0000000	0.1525000
-31554.0000000	0.1520000
-31553.0000000	0.1523810
-31552.0000000	0.1515000
-31551.0000000	0.1514286
-31550.0000000	0.1525000
-31549.0000000	0.1515000
-31548.0000000	0.1505000
-31547.0000000	0.1500000
-31546.0000000	0.1510000

-31545.0000000	0.1505000
-31544.0000000	0.1504762
-31543.0000000	0.1505000
-31542.0000000	0.1505000
-31541.0000000	0.1500000
-31540.0000000	0.1500000
-31539.0000000	0.1505000
-31538.0000000	0.1504762
-31537.0000000	0.1500000
-31536.0000000	0.1505000
-31535.0000000	0.1504762
-31534.0000000	0.1510000
-31533.0000000	0.1515000
-31532.0000000	0.1514286
-31531.0000000	0.1515000
-31530.0000000	0.1514286
-31529.0000000	0.1505000
-31528.0000000	0.1510000
-31527.0000000	0.1515000
-31526.0000000	0.1500000
-31525.0000000	0.1505000
-31524.0000000	0.1505000
-31523.0000000	0.1509524
-31522.0000000	0.1505000
-31521.0000000	0.1500000
-31520.0000000	0.1500000
-31519.0000000	0.1510000
-31518.0000000	0.1520000
-31517.0000000	0.1519048
-31516.0000000	0.1515000
-31515.0000000	0.1510000
-31514.0000000	0.1504762
-31513.0000000	0.1505000
-31512.0000000	0.1505000
-31511.0000000	0.1505000
-31510.0000000	0.1504762
-31509.0000000	0.1500000
-31508.0000000	0.1500000
-31507.0000000	0.1505000
-31506.0000000	0.1505000
-31505.0000000	0.1500000
-31504.0000000	0.1500000
-31503.0000000	0.1505000
-31502.0000000	0.1505000
-31501.0000000	0.1500000
-31500.0000000	0.1500000
-31499.0000000	0.1500000
-31498.0000000	0.1505000
-31497.0000000	0.1500000
-31496.0000000	0.1504762
-31495.0000000	0.1500000
-31494.0000000	0.1500000
-31493.0000000	0.1500000
-31492.0000000	0.1500000
-31491.0000000	0.1504762
-31490.0000000	0.1505000
-31489.0000000	0.1500000

-31488.0000000	0.1505000
-31487.0000000	0.1500000
-31486.0000000	0.1505000
-31485.0000000	0.1515000
-31484.0000000	0.1500000
-31483.0000000	0.1510000
-31482.0000000	0.1520000
-31481.0000000	0.1510000
-31480.0000000	0.1519048
-31479.0000000	0.1510000
-31478.0000000	0.1528572
-31477.0000000	0.1525000
-31476.0000000	0.1540000
-31475.0000000	0.1533334
-31474.0000000	0.1555000
-31473.0000000	0.1540000
-31472.0000000	0.1525000
-31471.0000000	0.1552381
-31470.0000000	0.1540000
-31469.0000000	0.1552381
-31468.0000000	0.1540000
-31467.0000000	0.1535000
-31466.0000000	0.1533334
-31465.0000000	0.1540000
-31464.0000000	0.1555000
-31463.0000000	0.1550000
-31462.0000000	0.1533334
-31461.0000000	0.1545000
-31460.0000000	0.1552381
-31459.0000000	0.1560000
-31458.0000000	0.1575000
-31457.0000000	0.1576191
-31456.0000000	0.1540000
-31455.0000000	0.1560000
-31454.0000000	0.1575000
-31453.0000000	0.1547619
-31452.0000000	0.1550000
-31451.0000000	0.1565000
-31450.0000000	0.1590476
-31449.0000000	0.1590000
-31448.0000000	0.1580953
-31447.0000000	0.1565000
-31446.0000000	0.1580000
-31445.0000000	0.1585715
-31444.0000000	0.1585000
-31443.0000000	0.1575000
-31442.0000000	0.1580000
-31441.0000000	0.1590476
-31440.0000000	0.1580000
-31439.0000000	0.1590476
-31438.0000000	0.1590000
-31437.0000000	0.1585000
-31436.0000000	0.1585715
-31435.0000000	0.1595000
-31434.0000000	0.1595000
-31433.0000000	0.1600000
-31432.0000000	0.1590476

-31431.0000000	0.1595000
-31430.0000000	0.1590476
-31429.0000000	0.1595000
-31428.0000000	0.1585000
-31427.0000000	0.1595000
-31426.0000000	0.1585715
-31425.0000000	0.1590000
-31424.0000000	0.1595000
-31423.0000000	0.1590476
-31422.0000000	0.1600000
-31421.0000000	0.1600000
-31420.0000000	0.1590476
-31419.0000000	0.1590000
-31418.0000000	0.1595000
-31417.0000000	0.1595238
-31416.0000000	0.1595000
-31415.0000000	0.1590476
-31414.0000000	0.1600000
-31413.0000000	0.1600000
-31412.0000000	0.1595000
-31411.0000000	0.1600000
-31410.0000000	0.1600000
-31409.0000000	0.1595238
-31408.0000000	0.1600000
-31407.0000000	0.1600000
-31406.0000000	0.1600000
-31405.0000000	0.1595238
-31404.0000000	0.1600000
-31403.0000000	0.1595000
-31402.0000000	0.1595238
-31401.0000000	0.1600000
-31400.0000000	0.1600000
-31399.0000000	0.1600000
-31398.0000000	0.1600000
-31397.0000000	0.1600000
-31396.0000000	0.1600000
-31395.0000000	0.1600000
-31394.0000000	0.1600000
-31393.0000000	0.1600000
-31392.0000000	0.1600000
-31391.0000000	0.1600000
-31390.0000000	0.1600000
-31389.0000000	0.1600000
-31388.0000000	0.1600000
-31387.0000000	0.1600000
-31386.0000000	0.1600000
-31385.0000000	0.1600000
-31384.0000000	0.1600000
-31383.0000000	0.1600000
-31382.0000000	0.1600000
-31381.0000000	0.1600000
-31380.0000000	0.1600000
-31379.0000000	0.1600000
-31378.0000000	0.1600000
-31377.0000000	0.1600000
-31376.0000000	0.1600000
-31375.0000000	0.1604762

-31374.0000000	0.1600000
-31373.0000000	0.1600000
-31372.0000000	0.1600000
-31371.0000000	0.1600000
-31370.0000000	0.1600000
-31369.0000000	0.1600000
-31368.0000000	0.1600000
-31367.0000000	0.1600000
-31366.0000000	0.1600000
-31365.0000000	0.1600000
-31364.0000000	0.1600000
-31363.0000000	0.1600000
-31362.0000000	0.1600000
-31361.0000000	0.1600000
-31360.0000000	0.1600000
-31359.0000000	0.1600000
-31358.0000000	0.1600000
-31357.0000000	0.1600000
-31356.0000000	0.1600000
-31355.0000000	0.1600000
-31354.0000000	0.1600000
-31353.0000000	0.1600000
-31352.0000000	0.1600000
-31351.0000000	0.1600000
-31350.0000000	0.1600000
-31349.0000000	0.1600000
-31348.0000000	0.1600000
-31347.0000000	0.1600000
-31346.0000000	0.1600000
-31345.0000000	0.1600000
-31344.0000000	0.1600000
-31343.0000000	0.1600000
-31342.0000000	0.1600000
-31341.0000000	0.1600000
-31340.0000000	0.1600000
-31339.0000000	0.1600000
-31338.0000000	0.1600000
-31337.0000000	0.1600000
-31336.0000000	0.1600000
-31335.0000000	0.1600000
-31334.0000000	0.1600000
-31333.0000000	0.1600000
-31332.0000000	0.1600000
-31331.0000000	0.1600000
-31330.0000000	0.1600000
-31329.0000000	0.1600000
-31328.0000000	0.1605000
-31327.0000000	0.1604762
-31326.0000000	0.1600000
-31325.0000000	0.1605000
-31324.0000000	0.1600000
-31323.0000000	0.1600000
-31322.0000000	0.1600000
-31321.0000000	0.1604762
-31320.0000000	0.1600000
-31319.0000000	0.1600000
-31318.0000000	0.1600000

-31317.0000000	0.1600000
-31316.0000000	0.1600000
-31315.0000000	0.1600000
-31314.0000000	0.1600000
-31313.0000000	0.1600000
-31312.0000000	0.1600000
-31311.0000000	0.1605000
-31310.0000000	0.1600000
-31309.0000000	0.1600000
-31308.0000000	0.1600000
-31307.0000000	0.1600000
-31306.0000000	0.1600000
-31305.0000000	0.1600000
-31304.0000000	0.1600000
-31303.0000000	0.1600000
-31302.0000000	0.1600000
-31301.0000000	0.1600000
-31300.0000000	0.1600000
-31299.0000000	0.1600000
-31298.0000000	0.1600000
-31297.0000000	0.1600000
-31296.0000000	0.1600000
-31295.0000000	0.1600000
-31294.0000000	0.1600000
-31293.0000000	0.1595000
-31292.0000000	0.1600000
-31291.0000000	0.1600000
-31290.0000000	0.1595000
-31289.0000000	0.1595000
-31288.0000000	0.1595238
-31287.0000000	0.1600000
-31286.0000000	0.1600000
-31285.0000000	0.1595000
-31284.0000000	0.1595238
-31283.0000000	0.1595000
-31282.0000000	0.1595238
-31281.0000000	0.1585000
-31280.0000000	0.1595000
-31279.0000000	0.1595238
-31278.0000000	0.1590000
-31277.0000000	0.1590000
-31276.0000000	0.1595000
-31275.0000000	0.1585715
-31274.0000000	0.1575000
-31273.0000000	0.1585715
-31272.0000000	0.1600000
-31271.0000000	0.1585000
-31270.0000000	0.1570000
-31269.0000000	0.1580953
-31268.0000000	0.1570000
-31267.0000000	0.1565000
-31266.0000000	0.1585715
-31265.0000000	0.1570000
-31264.0000000	0.1576191
-31263.0000000	0.1565000
-31262.0000000	0.1585000
-31261.0000000	0.1570000

-31260.0000000	0.1561905
-31259.0000000	0.1555000
-31258.0000000	0.1570000
-31257.0000000	0.1566667
-31256.0000000	0.1545000
-31255.0000000	0.1550000
-31254.0000000	0.1561905
-31253.0000000	0.1550000
-31252.0000000	0.1557143
-31251.0000000	0.1565000
-31250.0000000	0.1550000
-31249.0000000	0.1555000
-31248.0000000	0.1566667
-31247.0000000	0.1560000
-31246.0000000	0.1545000
-31245.0000000	0.1552381
-31244.0000000	0.1555000
-31243.0000000	0.1547619
-31242.0000000	0.1540000
-31241.0000000	0.1555000
-31240.0000000	0.1550000
-31239.0000000	0.1547619
-31238.0000000	0.1540000
-31237.0000000	0.1550000
-31236.0000000	0.1533334
-31235.0000000	0.1525000
-31234.0000000	0.1542857
-31233.0000000	0.1545000
-31232.0000000	0.1525000
-31231.0000000	0.1525000
-31230.0000000	0.1528572
-31229.0000000	0.1525000
-31228.0000000	0.1545000
-31227.0000000	0.1528572
-31226.0000000	0.1515000
-31225.0000000	0.1515000
-31224.0000000	0.1530000
-31223.0000000	0.1523810
-31222.0000000	0.1514286
-31221.0000000	0.1510000
-31220.0000000	0.1525000
-31219.0000000	0.1515000
-31218.0000000	0.1500000
-31217.0000000	0.1500000
-31216.0000000	0.1505000
-31215.0000000	0.1500000
-31214.0000000	0.1515000
-31213.0000000	0.1500000
-31212.0000000	0.1500000
-31211.0000000	0.1505000
-31210.0000000	0.1500000
-31209.0000000	0.1500000
-31208.0000000	0.1505000
-31207.0000000	0.1500000
-31206.0000000	0.1500000
-31205.0000000	0.1500000
-31204.0000000	0.1500000

-31203.0000000	0.1500000
-31202.0000000	0.1500000
-31201.0000000	0.1500000
-31200.0000000	0.1500000
-31199.0000000	0.1500000
-31198.0000000	0.1500000
-31197.0000000	0.1500000
-31196.0000000	0.1495000
-31195.0000000	0.1500000
-31194.0000000	0.1495000
-31193.0000000	0.1500000
-31192.0000000	0.1500000
-31191.0000000	0.1500000
-31190.0000000	0.1495000
-31189.0000000	0.1495000
-31188.0000000	0.1490476
-31187.0000000	0.1495000
-31186.0000000	0.1495000
-31185.0000000	0.1500000
-31184.0000000	0.1490476
-31183.0000000	0.1485000
-31182.0000000	0.1490476
-31181.0000000	0.1500000
-31180.0000000	0.1500000
-31179.0000000	0.1495238
-31178.0000000	0.1495000
-31177.0000000	0.1500000
-31176.0000000	0.1500000
-31175.0000000	0.1495238
-31174.0000000	0.1495238
-31173.0000000	0.1495000
-31172.0000000	0.1490000
-31171.0000000	0.1500000
-31170.0000000	0.1476191
-31169.0000000	0.1480000
-31168.0000000	0.1480000
-31167.0000000	0.1457143
-31166.0000000	0.1445000
-31165.0000000	0.1435000
-31164.0000000	0.1425000
-31163.0000000	0.1433334
-31162.0000000	0.1415000
-31161.0000000	0.1414286
-31160.0000000	0.1410000
-31159.0000000	0.1400000
-31158.0000000	0.1409524
-31157.0000000	0.1405000
-31156.0000000	0.1410000
-31155.0000000	0.1405000
-31154.0000000	0.1423810
-31153.0000000	0.1405000
-31152.0000000	0.1409524
-31151.0000000	0.1405000
-31150.0000000	0.1405000
-31149.0000000	0.1409524
-31148.0000000	0.1400000
-31147.0000000	0.1400000

-31146.0000000	0.1410000
-31145.0000000	0.1400000
-31144.0000000	0.1400000
-31143.0000000	0.1400000
-31142.0000000	0.1400000
-31141.0000000	0.1400000
-31140.0000000	0.1400000
-31139.0000000	0.1405000
-31138.0000000	0.1400000
-31137.0000000	0.1405000
-31136.0000000	0.1400000
-31135.0000000	0.1405000
-31134.0000000	0.1400000
-31133.0000000	0.1409524
-31132.0000000	0.1405000
-31131.0000000	0.1400000
-31130.0000000	0.1405000
-31129.0000000	0.1405000
-31128.0000000	0.1423810
-31127.0000000	0.1400000
-31126.0000000	0.1410000
-31125.0000000	0.1410000
-31124.0000000	0.1409524
-31123.0000000	0.1405000
-31122.0000000	0.1419048
-31121.0000000	0.1420000
-31120.0000000	0.1410000
-31119.0000000	0.1415000
-31118.0000000	0.1419048
-31117.0000000	0.1425000
-31116.0000000	0.1410000
-31115.0000000	0.1414286
-31114.0000000	0.1410000
-31113.0000000	0.1428572
-31112.0000000	0.1440000
-31111.0000000	0.1435000
-31110.0000000	0.1425000
-31109.0000000	0.1447619
-31108.0000000	0.1445000
-31107.0000000	0.1490000
-31106.0000000	0.1480953
-31105.0000000	0.1480000
-31104.0000000	0.1485000
-31103.0000000	0.1480953
-31102.0000000	0.1500000
-31101.0000000	0.1485715
-31100.0000000	0.1495000
-31099.0000000	0.1490000
-31098.0000000	0.1452381
-31097.0000000	0.1450000
-31096.0000000	0.1455000
-31095.0000000	0.1470000
-31094.0000000	0.1447619
-31093.0000000	0.1465000
-31092.0000000	0.1476191
-31091.0000000	0.1485000
-31090.0000000	0.1460000

-31089.0000000	0.1460000
-31088.0000000	0.1466667
-31087.0000000	0.1470000
-31086.0000000	0.1480000
-31085.0000000	0.1485715
-31084.0000000	0.1490000
-31083.0000000	0.1500000
-31082.0000000	0.1495000
-31081.0000000	0.1500000
-31080.0000000	0.1500000
-31079.0000000	0.1500000
-31078.0000000	0.1500000
-31077.0000000	0.1500000
-31076.0000000	0.1500000
-31075.0000000	0.1500000
-31074.0000000	0.1500000
-31073.0000000	0.1500000
-31072.0000000	0.1500000
-31071.0000000	0.1500000
-31070.0000000	0.1500000
-31069.0000000	0.1500000
-31068.0000000	0.1500000
-31067.0000000	0.1505000
-31066.0000000	0.1500000
-31065.0000000	0.1500000
-31064.0000000	0.1500000
-31063.0000000	0.1500000
-31062.0000000	0.1505000
-31061.0000000	0.1500000
-31060.0000000	0.1500000
-31059.0000000	0.1500000
-31058.0000000	0.1509524
-31057.0000000	0.1500000
-31056.0000000	0.1500000
-31055.0000000	0.1500000
-31054.0000000	0.1505000
-31053.0000000	0.1500000
-31052.0000000	0.1509524
-31051.0000000	0.1505000
-31050.0000000	0.1510000
-31049.0000000	0.1504762
-31048.0000000	0.1500000
-31047.0000000	0.1510000
-31046.0000000	0.1504762
-31045.0000000	0.1510000
-31044.0000000	0.1505000
-31043.0000000	0.1505000
-31042.0000000	0.1504762
-31041.0000000	0.1500000
-31040.0000000	0.1514286
-31039.0000000	0.1515000
-31038.0000000	0.1515000
-31037.0000000	0.1514286
-31036.0000000	0.1515000
-31035.0000000	0.1530000
-31034.0000000	0.1542857
-31033.0000000	0.1545000

-31032.0000000	0.1530000
-31031.0000000	0.1509524
-31030.0000000	0.1530000
-31029.0000000	0.1525000
-31028.0000000	0.1535000
-31027.0000000	0.1528572
-31026.0000000	0.1525000
-31025.0000000	0.1523810
-31024.0000000	0.1520000
-31023.0000000	0.1545000
-31022.0000000	0.1542857
-31021.0000000	0.1550000
-31020.0000000	0.1565000
-31019.0000000	0.1576191
-31018.0000000	0.1585000
-31017.0000000	0.1555000
-31016.0000000	0.1566667
-31015.0000000	0.1565000
-31014.0000000	0.1575000
-31013.0000000	0.1580000
-31012.0000000	0.1580953
-31011.0000000	0.1590000
-31010.0000000	0.1585715
-31009.0000000	0.1600000
-31008.0000000	0.1585000
-31007.0000000	0.1580953
-31006.0000000	0.1585000
-31005.0000000	0.1595000
-31004.0000000	0.1595238
-31003.0000000	0.1590000
-31002.0000000	0.1600000
-31001.0000000	0.1595238
-31000.0000000	0.1600000
-30999.0000000	0.1600000
-30998.0000000	0.1595000
-30997.0000000	0.1595238
-30996.0000000	0.1595000
-30995.0000000	0.1595238
-30994.0000000	0.1600000
-30993.0000000	0.1600000
-30992.0000000	0.1600000
-30991.0000000	0.1600000
-30990.0000000	0.1600000
-30989.0000000	0.1600000
-30988.0000000	0.1600000
-30987.0000000	0.1600000
-30986.0000000	0.1600000
-30985.0000000	0.1600000
-30984.0000000	0.1600000
-30983.0000000	0.1600000
-30982.0000000	0.1595238
-30981.0000000	0.1600000
-30980.0000000	0.1595000
-30979.0000000	0.1600000
-30978.0000000	0.1600000
-30977.0000000	0.1600000
-30976.0000000	0.1600000

-30975.0000000	0.1600000
-30974.0000000	0.1600000
-30973.0000000	0.1600000
-30972.0000000	0.1600000
-30971.0000000	0.1600000
-30970.0000000	0.1600000
-30969.0000000	0.1600000
-30968.0000000	0.1600000
-30967.0000000	0.1600000
-30966.0000000	0.1600000
-30965.0000000	0.1600000
-30964.0000000	0.1600000
-30963.0000000	0.1600000
-30962.0000000	0.1600000
-30961.0000000	0.1600000
-30960.0000000	0.1600000
-30959.0000000	0.1600000
-30958.0000000	0.1600000
-30957.0000000	0.1600000
-30956.0000000	0.1600000
-30955.0000000	0.1600000
-30954.0000000	0.1600000
-30953.0000000	0.1600000
-30952.0000000	0.1600000
-30951.0000000	0.1600000
-30950.0000000	0.1595000
-30949.0000000	0.1600000
-30948.0000000	0.1600000
-30947.0000000	0.1600000
-30946.0000000	0.1595000
-30945.0000000	0.1600000
-30944.0000000	0.1600000
-30943.0000000	0.1600000
-30942.0000000	0.1600000
-30941.0000000	0.1600000
-30940.0000000	0.1600000
-30939.0000000	0.1600000
-30938.0000000	0.1600000
-30937.0000000	0.1600000
-30936.0000000	0.1600000
-30935.0000000	0.1600000
-30934.0000000	0.1600000
-30933.0000000	0.1600000
-30932.0000000	0.1600000
-30931.0000000	0.1600000
-30930.0000000	0.1600000
-30929.0000000	0.1600000
-30928.0000000	0.1600000
-30927.0000000	0.1600000
-30926.0000000	0.1604762
-30925.0000000	0.1600000
-30924.0000000	0.1605000
-30923.0000000	0.1600000
-30922.0000000	0.1600000
-30921.0000000	0.1600000
-30920.0000000	0.1600000
-30919.0000000	0.1605000

-30918.0000000	0.1600000
-30917.0000000	0.1600000
-30916.0000000	0.1605000
-30915.0000000	0.1615000
-30914.0000000	0.1610000
-30913.0000000	0.1609524
-30912.0000000	0.1610000
-30911.0000000	0.1605000
-30910.0000000	0.1619048
-30909.0000000	0.1600000
-30908.0000000	0.1610000
-30907.0000000	0.1630000
-30906.0000000	0.1628572
-30905.0000000	0.1642857
-30904.0000000	0.1645000
-30903.0000000	0.1650000
-30902.0000000	0.1630000
-30901.0000000	0.1657143
-30900.0000000	0.1630000
-30899.0000000	0.1660000
-30898.0000000	0.1645000
-30897.0000000	0.1661905
-30896.0000000	0.1671429
-30895.0000000	0.1665000
-30894.0000000	0.1660000
-30893.0000000	0.1685000
-30892.0000000	0.1685714
-30891.0000000	0.1685000
-30890.0000000	0.1695000
-30889.0000000	0.1680000
-30888.0000000	0.1695238
-30887.0000000	0.1700000
-30886.0000000	0.1695000
-30885.0000000	0.1700000
-30884.0000000	0.1700000
-30883.0000000	0.1700000
-30882.0000000	0.1700000
-30881.0000000	0.1700000
-30880.0000000	0.1700000
-30879.0000000	0.1700000
-30878.0000000	0.1700000
-30877.0000000	0.1700000
-30876.0000000	0.1700000
-30875.0000000	0.1700000
-30874.0000000	0.1704762
-30873.0000000	0.1705000
-30872.0000000	0.1725000
-30871.0000000	0.1723810
-30870.0000000	0.1720000
-30869.0000000	0.1735000
-30868.0000000	0.1735000
-30867.0000000	0.1757143
-30866.0000000	0.1775000
-30865.0000000	0.1752381
-30864.0000000	0.1775000
-30863.0000000	0.1780000
-30862.0000000	0.1785715

-30861.0000000	0.1790000
-30860.0000000	0.1795000
-30859.0000000	0.1785000
-30858.0000000	0.1795239
-30857.0000000	0.1795238
-30856.0000000	0.1795000
-30855.0000000	0.1800000
-30854.0000000	0.1800000
-30853.0000000	0.1800000
-30852.0000000	0.1800000
-30851.0000000	0.1800000
-30850.0000000	0.1800000
-30849.0000000	0.1800000
-30848.0000000	0.1800000
-30847.0000000	0.1800000
-30846.0000000	0.1800000
-30845.0000000	0.1800000
-30844.0000000	0.1800000
-30843.0000000	0.1800000
-30842.0000000	0.1800000
-30841.0000000	0.1800000
-30840.0000000	0.1800000
-30839.0000000	0.1800000
-30838.0000000	0.1800000
-30837.0000000	0.1804762
-30836.0000000	0.1800000
-30835.0000000	0.1800000
-30834.0000000	0.1800000
-30833.0000000	0.1800000
-30832.0000000	0.1800000
-30831.0000000	0.1800000
-30830.0000000	0.1805000
-30829.0000000	0.1800000
-30828.0000000	0.1800000
-30827.0000000	0.1800000
-30826.0000000	0.1800000
-30825.0000000	0.1805000
-30824.0000000	0.1805000
-30823.0000000	0.1804762
-30822.0000000	0.1800000
-30821.0000000	0.1815000
-30820.0000000	0.1805000
-30819.0000000	0.1800000
-30818.0000000	0.1815000
-30817.0000000	0.1815000
-30816.0000000	0.1804762
-30815.0000000	0.1800000
-30814.0000000	0.1804762
-30813.0000000	0.1800000
-30812.0000000	0.1800000
-30811.0000000	0.1804762
-30810.0000000	0.1805000
-30809.0000000	0.1810000
-30808.0000000	0.1810000
-30807.0000000	0.1809524
-30806.0000000	0.1830000
-30805.0000000	0.1828572

-30804.0000000	0.1840000
-30803.0000000	0.1850000
-30802.0000000	0.1845000
-30801.0000000	0.1876191
-30800.0000000	0.1860000
-30799.0000000	0.1880000
-30798.0000000	0.1871429
-30797.0000000	0.1865000
-30796.0000000	0.1871429
-30795.0000000	0.1885000
-30794.0000000	0.1875000
-30793.0000000	0.1885000
-30792.0000000	0.1885715
-30791.0000000	0.1890000
-30790.0000000	0.1900000
-30789.0000000	0.1895238
-30788.0000000	0.1890000
-30787.0000000	0.1900000
-30786.0000000	0.1900000
-30785.0000000	0.1895000
-30784.0000000	0.1900000
-30783.0000000	0.1895238
-30782.0000000	0.1900000
-30781.0000000	0.1900000
-30780.0000000	0.1900000
-30779.0000000	0.1900000
-30778.0000000	0.1895000
-30777.0000000	0.1900000
-30776.0000000	0.1900000
-30775.0000000	0.1900000
-30774.0000000	0.1900000
-30773.0000000	0.1900000
-30772.0000000	0.1900000
-30771.0000000	0.1900000
-30770.0000000	0.1900000
-30769.0000000	0.1900000
-30768.0000000	0.1900000
-30767.0000000	0.1900000
-30766.0000000	0.1904762
-30765.0000000	0.1900000
-30764.0000000	0.1900000
-30763.0000000	0.1905000
-30762.0000000	0.1909524
-30761.0000000	0.1910000
-30760.0000000	0.1905000
-30759.0000000	0.1909524
-30758.0000000	0.1905000
-30757.0000000	0.1910000
-30756.0000000	0.1904762
-30755.0000000	0.1910000
-30754.0000000	0.1935000
-30753.0000000	0.1914286
-30752.0000000	0.1920000
-30751.0000000	0.1919048
-30750.0000000	0.1930000
-30749.0000000	0.1960000
-30748.0000000	0.1925000

-30747.0000000	0.1957143
-30746.0000000	0.1960000
-30745.0000000	0.1975000
-30744.0000000	0.1985715
-30743.0000000	0.1980000
-30742.0000000	0.1995000
-30741.0000000	0.2000000
-30740.0000000	0.2000000
-30739.0000000	0.2000000
-30738.0000000	0.2000000
-30737.0000000	0.2000000
-30736.0000000	0.2000000
-30735.0000000	0.2000000
-30734.0000000	0.2000000
-30733.0000000	0.2000000
-30732.0000000	0.2000000
-30731.0000000	0.2000000
-30730.0000000	0.2000000
-30729.0000000	0.2009524
-30728.0000000	0.2020000
-30727.0000000	0.2015000
-30726.0000000	0.2035000
-30725.0000000	0.2047619
-30724.0000000	0.2050000
-30723.0000000	0.2076191
-30722.0000000	0.2075000
-30721.0000000	0.2090000
-30720.0000000	0.2095238
-30719.0000000	0.2100000
-30718.0000000	0.2095000
-30717.0000000	0.2100000
-30716.0000000	0.2100000
-30715.0000000	0.2100000
-30714.0000000	0.2100000
-30713.0000000	0.2105000
-30712.0000000	0.2100000
-30711.0000000	0.2105000
-30710.0000000	0.2119048
-30709.0000000	0.2105000
-30708.0000000	0.2109524
-30707.0000000	0.2130000
-30706.0000000	0.2120000
-30705.0000000	0.2138095
-30704.0000000	0.2120000
-30703.0000000	0.2145000
-30702.0000000	0.2130000
-30701.0000000	0.2166667
-30700.0000000	0.2165000
-30699.0000000	0.2166667
-30698.0000000	0.2155000
-30697.0000000	0.2160000
-30696.0000000	0.2145000
-30695.0000000	0.2166667
-30694.0000000	0.2160000
-30693.0000000	0.2180000
-30692.0000000	0.2176190
-30691.0000000	0.2175000

-30690.0000000	0.2180952
-30689.0000000	0.2180000
-30688.0000000	0.2185000
-30687.0000000	0.2185714
-30686.0000000	0.2195000
-30685.0000000	0.2190000
-30684.0000000	0.2195238
-30683.0000000	0.2200000
-30682.0000000	0.2200000
-30681.0000000	0.2190000
-30680.0000000	0.2200000
-30679.0000000	0.2200000
-30678.0000000	0.2200000
-30677.0000000	0.2200000
-30676.0000000	0.2200000
-30675.0000000	0.2200000
-30674.0000000	0.2200000
-30673.0000000	0.2200000
-30672.0000000	0.2200000
-30671.0000000	0.2200000
-30670.0000000	0.2200000
-30669.0000000	0.2200000
-30668.0000000	0.2200000
-30667.0000000	0.2200000
-30666.0000000	0.2200000
-30665.0000000	0.2200000
-30664.0000000	0.2200000
-30663.0000000	0.2200000
-30662.0000000	0.2200000
-30661.0000000	0.2200000
-30660.0000000	0.2200000
-30659.0000000	0.2200000
-30658.0000000	0.2200000
-30657.0000000	0.2200000
-30656.0000000	0.2200000
-30655.0000000	0.2200000
-30654.0000000	0.2200000
-30653.0000000	0.2200000
-30652.0000000	0.2200000
-30651.0000000	0.2205000
-30650.0000000	0.2200000
-30649.0000000	0.2205000
-30648.0000000	0.2204762
-30647.0000000	0.2210000
-30646.0000000	0.2205000
-30645.0000000	0.2205000
-30644.0000000	0.2204762
-30643.0000000	0.2200000
-30642.0000000	0.2200000
-30641.0000000	0.2200000
-30640.0000000	0.2205000
-30639.0000000	0.2209524
-30638.0000000	0.2210000
-30637.0000000	0.2200000
-30636.0000000	0.2210000
-30635.0000000	0.2204762
-30634.0000000	0.2220000

-30633.0000000	0.2220000
-30632.0000000	0.2214286
-30631.0000000	0.2220000
-30630.0000000	0.2214286
-30629.0000000	0.2220000
-30628.0000000	0.2215000
-30627.0000000	0.2215000
-30626.0000000	0.2214286
-30625.0000000	0.2230000
-30624.0000000	0.2220000
-30623.0000000	0.2228571
-30622.0000000	0.2245000
-30621.0000000	0.2235000
-30620.0000000	0.2245000
-30619.0000000	0.2233333
-30618.0000000	0.2238095
-30617.0000000	0.2235000
-30616.0000000	0.2235000
-30615.0000000	0.2245000
-30614.0000000	0.2242857
-30613.0000000	0.2235000
-30612.0000000	0.2220000
-30611.0000000	0.2238095
-30610.0000000	0.2250000
-30609.0000000	0.2261905
-30608.0000000	0.2250000
-30607.0000000	0.2270000
-30606.0000000	0.2260000
-30605.0000000	0.2261905
-30604.0000000	0.2285000
-30603.0000000	0.2285000
-30602.0000000	0.2285714
-30601.0000000	0.2295000
-30600.0000000	0.2261905
-30599.0000000	0.2260000
-30598.0000000	0.2270000
-30597.0000000	0.2280000
-30596.0000000	0.2285714
-30595.0000000	0.2280000
-30594.0000000	0.2285000
-30593.0000000	0.2285714
-30592.0000000	0.2295000
-30591.0000000	0.2290000
-30590.0000000	0.2290000
-30589.0000000	0.2290476
-30588.0000000	0.2300000
-30587.0000000	0.2290476
-30586.0000000	0.2300000
-30585.0000000	0.2295000
-30584.0000000	0.2290476
-30583.0000000	0.2290000
-30582.0000000	0.2295000
-30581.0000000	0.2295000
-30580.0000000	0.2295238
-30579.0000000	0.2290000
-30578.0000000	0.2295238
-30577.0000000	0.2290000

-30576.0000000	0.2285000
-30575.0000000	0.2295238
-30574.0000000	0.2280000
-30573.0000000	0.2295000
-30572.0000000	0.2290000
-30571.0000000	0.2295238
-30570.0000000	0.2290476
-30569.0000000	0.2265000
-30568.0000000	0.2285000
-30567.0000000	0.2280000
-30566.0000000	0.2290476
-30565.0000000	0.2290000
-30564.0000000	0.2285000
-30563.0000000	0.2285714
-30562.0000000	0.2295000
-30561.0000000	0.2295000
-30560.0000000	0.2285000
-30559.0000000	0.2295238
-30558.0000000	0.2285000
-30557.0000000	0.2295238
-30556.0000000	0.2300000
-30555.0000000	0.2275000
-30554.0000000	0.2266667
-30553.0000000	0.2290000
-30552.0000000	0.2285000
-30551.0000000	0.2290000
-30550.0000000	0.2280952
-30549.0000000	0.2275000
-30548.0000000	0.2271429
-30547.0000000	0.2285000
-30546.0000000	0.2275000
-30545.0000000	0.2266667
-30544.0000000	0.2270000
-30543.0000000	0.2270000
-30542.0000000	0.2250000
-30541.0000000	0.2242857
-30540.0000000	0.2245000
-30539.0000000	0.2252381
-30538.0000000	0.2255000
-30537.0000000	0.2260000
-30536.0000000	0.2257143
-30535.0000000	0.2240000
-30534.0000000	0.2245000
-30533.0000000	0.2228571
-30532.0000000	0.2250000
-30531.0000000	0.2250000
-30530.0000000	0.2260000
-30529.0000000	0.2238095
-30528.0000000	0.2225000
-30527.0000000	0.2228571
-30526.0000000	0.2235000
-30525.0000000	0.2250000
-30524.0000000	0.2228571
-30523.0000000	0.2235000
-30522.0000000	0.2225000
-30521.0000000	0.2245000
-30520.0000000	0.2247619

-30519.0000000	0.2255000
-30518.0000000	0.2209524
-30517.0000000	0.2230000
-30516.0000000	0.2225000
-30515.0000000	0.2245000
-30514.0000000	0.2223809
-30513.0000000	0.2220000
-30512.0000000	0.2220000
-30511.0000000	0.2219048
-30510.0000000	0.2210000
-30509.0000000	0.2204762
-30508.0000000	0.2210000
-30507.0000000	0.2210000
-30506.0000000	0.2215000
-30505.0000000	0.2200000
-30504.0000000	0.2200000
-30503.0000000	0.2200000
-30502.0000000	0.2205000
-30501.0000000	0.2205000
-30500.0000000	0.2200000
-30499.0000000	0.2200000
-30498.0000000	0.2205000
-30497.0000000	0.2200000
-30496.0000000	0.2200000
-30495.0000000	0.2200000
-30494.0000000	0.2200000
-30493.0000000	0.2200000
-30492.0000000	0.2200000
-30491.0000000	0.2200000
-30490.0000000	0.2200000
-30489.0000000	0.2200000
-30488.0000000	0.2195238
-30487.0000000	0.2200000
-30486.0000000	0.2195000
-30485.0000000	0.2200000
-30484.0000000	0.2200000
-30483.0000000	0.2190000
-30482.0000000	0.2195000
-30481.0000000	0.2185714
-30480.0000000	0.2190000
-30479.0000000	0.2190476
-30478.0000000	0.2180000
-30477.0000000	0.2185000
-30476.0000000	0.2180000
-30475.0000000	0.2190476
-30474.0000000	0.2190000
-30473.0000000	0.2195000
-30472.0000000	0.2171429
-30471.0000000	0.2185000
-30470.0000000	0.2170000
-30469.0000000	0.2176190
-30468.0000000	0.2170000
-30467.0000000	0.2165000
-30466.0000000	0.2157143
-30465.0000000	0.2170000
-30464.0000000	0.2157143
-30463.0000000	0.2140000

-30462.0000000	0.2165000
-30461.0000000	0.2140000
-30460.0000000	0.2138095
-30459.0000000	0.2130000
-30458.0000000	0.2125000
-30457.0000000	0.2114286
-30456.0000000	0.2120000
-30455.0000000	0.2130000
-30454.0000000	0.2138095
-30453.0000000	0.2135000
-30452.0000000	0.2120000
-30451.0000000	0.2128572
-30450.0000000	0.2135000
-30449.0000000	0.2120000
-30448.0000000	0.2123810
-30447.0000000	0.2135000
-30446.0000000	0.2125000
-30445.0000000	0.2114286
-30444.0000000	0.2110000
-30443.0000000	0.2115000
-30442.0000000	0.2119048
-30441.0000000	0.2115000
-30440.0000000	0.2110000
-30439.0000000	0.2114286
-30438.0000000	0.2120000
-30437.0000000	0.2110000
-30436.0000000	0.2109524
-30435.0000000	0.2130000
-30434.0000000	0.2119048
-30433.0000000	0.2115000
-30432.0000000	0.2110000
-30431.0000000	0.2100000
-30430.0000000	0.2109524
-30429.0000000	0.2115000
-30428.0000000	0.2105000
-30427.0000000	0.2104762
-30426.0000000	0.2100000
-30425.0000000	0.2105000
-30424.0000000	0.2100000
-30423.0000000	0.2104762
-30422.0000000	0.2105000
-30421.0000000	0.2100000
-30420.0000000	0.2100000
-30419.0000000	0.2100000
-30418.0000000	0.2104762
-30417.0000000	0.2100000
-30416.0000000	0.2100000
-30415.0000000	0.2100000
-30414.0000000	0.2100000
-30413.0000000	0.2100000
-30412.0000000	0.2100000
-30411.0000000	0.2100000
-30410.0000000	0.2100000
-30409.0000000	0.2100000
-30408.0000000	0.2100000
-30407.0000000	0.2100000
-30406.0000000	0.2100000

-30405.0000000	0.2100000
-30404.0000000	0.2100000
-30403.0000000	0.2100000
-30402.0000000	0.2100000
-30401.0000000	0.2100000
-30400.0000000	0.2100000
-30399.0000000	0.2100000
-30398.0000000	0.2095000
-30397.0000000	0.2100000
-30396.0000000	0.2100000
-30395.0000000	0.2100000
-30394.0000000	0.2100000
-30393.0000000	0.2100000
-30392.0000000	0.2100000
-30391.0000000	0.2100000
-30390.0000000	0.2100000
-30389.0000000	0.2100000
-30388.0000000	0.2100000
-30387.0000000	0.2095000
-30386.0000000	0.2095000
-30385.0000000	0.2100000
-30384.0000000	0.2100000
-30383.0000000	0.2100000
-30382.0000000	0.2095238
-30381.0000000	0.2090000
-30380.0000000	0.2100000
-30379.0000000	0.2100000
-30378.0000000	0.2095238
-30377.0000000	0.2100000
-30376.0000000	0.2090000
-30375.0000000	0.2085715
-30374.0000000	0.2090000
-30373.0000000	0.2095238
-30372.0000000	0.2075000
-30371.0000000	0.2080000
-30370.0000000	0.2085715
-30369.0000000	0.2085000
-30368.0000000	0.2090000
-30367.0000000	0.2066667
-30366.0000000	0.2080000
-30365.0000000	0.2070000
-30364.0000000	0.2050000
-30363.0000000	0.2047619
-30362.0000000	0.2055000
-30361.0000000	0.2052381
-30360.0000000	0.2025000
-30359.0000000	0.2050000
-30358.0000000	0.2038095
-30357.0000000	0.2045000
-30356.0000000	0.2045000
-30355.0000000	0.2030000
-30354.0000000	0.2033333
-30353.0000000	0.2015000
-30352.0000000	0.2023810
-30351.0000000	0.2010000
-30350.0000000	0.2010000
-30349.0000000	0.2015000

-30348.0000000	0.2004762
-30347.0000000	0.2015000
-30346.0000000	0.2015000
-30345.0000000	0.2019048
-30344.0000000	0.2005000
-30343.0000000	0.2004762
-30342.0000000	0.2000000
-30341.0000000	0.2000000
-30340.0000000	0.2005000
-30339.0000000	0.2000000
-30338.0000000	0.2000000
-30337.0000000	0.2005000
-30336.0000000	0.2000000
-30335.0000000	0.2000000
-30334.0000000	0.2000000
-30333.0000000	0.2000000
-30332.0000000	0.2000000
-30331.0000000	0.2000000
-30330.0000000	0.2000000
-30329.0000000	0.2005000
-30328.0000000	0.2000000
-30327.0000000	0.2000000
-30326.0000000	0.2000000
-30325.0000000	0.2000000
-30324.0000000	0.2000000
-30323.0000000	0.2000000
-30322.0000000	0.2000000
-30321.0000000	0.2000000
-30320.0000000	0.2000000
-30319.0000000	0.2000000
-30318.0000000	0.2000000
-30317.0000000	0.2000000
-30316.0000000	0.2000000
-30315.0000000	0.1995238
-30314.0000000	0.2000000
-30313.0000000	0.2000000
-30312.0000000	0.2000000
-30311.0000000	0.2000000
-30310.0000000	0.2000000
-30309.0000000	0.1995238
-30308.0000000	0.2000000
-30307.0000000	0.2000000
-30306.0000000	0.2000000
-30305.0000000	0.1995000
-30304.0000000	0.2000000
-30303.0000000	0.1995000
-30302.0000000	0.1995238
-30301.0000000	0.2000000
-30300.0000000	0.2000000
-30299.0000000	0.1995000
-30298.0000000	0.2000000
-30297.0000000	0.1995238
-30296.0000000	0.2000000
-30295.0000000	0.1995000
-30294.0000000	0.1995000
-30293.0000000	0.2000000
-30292.0000000	0.2000000

-30291.0000000	0.2000000
-30290.0000000	0.2000000
-30289.0000000	0.1995000
-30288.0000000	0.2000000
-30287.0000000	0.2000000
-30286.0000000	0.1995000
-30285.0000000	0.1995238
-30284.0000000	0.1995000
-30283.0000000	0.2000000
-30282.0000000	0.2000000
-30281.0000000	0.2000000
-30280.0000000	0.2000000
-30279.0000000	0.2000000
-30278.0000000	0.2000000
-30277.0000000	0.2000000
-30276.0000000	0.1995238
-30275.0000000	0.1995000
-30274.0000000	0.2000000
-30273.0000000	0.2000000
-30272.0000000	0.2000000
-30271.0000000	0.2000000
-30270.0000000	0.2000000
-30269.0000000	0.1995000
-30268.0000000	0.1995000
-30267.0000000	0.1995238
-30266.0000000	0.2000000
-30265.0000000	0.2000000
-30264.0000000	0.2000000
-30263.0000000	0.1995238
-30262.0000000	0.1995000
-30261.0000000	0.1995238
-30260.0000000	0.1990000
-30259.0000000	0.1990000
-30258.0000000	0.1990477
-30257.0000000	0.2000000
-30256.0000000	0.1990000
-30255.0000000	0.1985000
-30254.0000000	0.1990476
-30253.0000000	0.1990000
-30252.0000000	0.1985715
-30251.0000000	0.1985000
-30250.0000000	0.1985000
-30249.0000000	0.1995238
-30248.0000000	0.1995000
-30247.0000000	0.1995000
-30246.0000000	0.1980953
-30245.0000000	0.1970000
-30244.0000000	0.1980000
-30243.0000000	0.1985000
-30242.0000000	0.1990477
-30241.0000000	0.1990000
-30240.0000000	0.1976191
-30239.0000000	0.1985000
-30238.0000000	0.1995000
-30237.0000000	0.1990477
-30236.0000000	0.1980000
-30235.0000000	0.1995000

-30234.0000000	0.1990000
-30233.0000000	0.1985715
-30232.0000000	0.1995000
-30231.0000000	0.1985715
-30230.0000000	0.1985000
-30229.0000000	0.1970000
-30228.0000000	0.1995238
-30227.0000000	0.1970000
-30226.0000000	0.1980000
-30225.0000000	0.1980000
-30224.0000000	0.1976191
-30223.0000000	0.1985000
-30222.0000000	0.1976191
-30221.0000000	0.1965000
-30220.0000000	0.1960000
-30219.0000000	0.1971429
-30218.0000000	0.1975000
-30217.0000000	0.1955000
-30216.0000000	0.1966667
-30215.0000000	0.1970000
-30214.0000000	0.1980000
-30213.0000000	0.1950000
-30212.0000000	0.1966667
-30211.0000000	0.1955000
-30210.0000000	0.1942857
-30209.0000000	0.1950000
-30208.0000000	0.1970000
-30207.0000000	0.1942857
-30206.0000000	0.1955000
-30205.0000000	0.1950000
-30204.0000000	0.1925000
-30203.0000000	0.1942857
-30202.0000000	0.1950000
-30201.0000000	0.1947619
-30200.0000000	0.1925000
-30199.0000000	0.1945000
-30198.0000000	0.1915000
-30197.0000000	0.1938096
-30196.0000000	0.1930000
-30195.0000000	0.1925000
-30194.0000000	0.1914286
-30193.0000000	0.1930000
-30192.0000000	0.1900000
-30191.0000000	0.1920000
-30190.0000000	0.1915000
-30189.0000000	0.1910000
-30188.0000000	0.1904762
-30187.0000000	0.1910000
-30186.0000000	0.1915000
-30185.0000000	0.1904762
-30184.0000000	0.1925000
-30183.0000000	0.1915000
-30182.0000000	0.1904762
-30181.0000000	0.1900000
-30180.0000000	0.1905000
-30179.0000000	0.1904762
-30178.0000000	0.1910000

-30177.0000000	0.1900000
-30176.0000000	0.1900000
-30175.0000000	0.1900000
-30174.0000000	0.1905000
-30173.0000000	0.1904762
-30172.0000000	0.1900000
-30171.0000000	0.1900000
-30170.0000000	0.1900000
-30169.0000000	0.1905000
-30168.0000000	0.1900000
-30167.0000000	0.1900000
-30166.0000000	0.1900000
-30165.0000000	0.1900000
-30164.0000000	0.1900000
-30163.0000000	0.1900000
-30162.0000000	0.1900000
-30161.0000000	0.1900000
-30160.0000000	0.1900000
-30159.0000000	0.1900000
-30158.0000000	0.1900000
-30157.0000000	0.1900000
-30156.0000000	0.1900000
-30155.0000000	0.1900000
-30154.0000000	0.1900000
-30153.0000000	0.1900000
-30152.0000000	0.1900000
-30151.0000000	0.1900000
-30150.0000000	0.1900000
-30149.0000000	0.1900000
-30148.0000000	0.1900000
-30147.0000000	0.1900000
-30146.0000000	0.1900000
-30145.0000000	0.1900000
-30144.0000000	0.1900000
-30143.0000000	0.1900000
-30142.0000000	0.1900000
-30141.0000000	0.1900000
-30140.0000000	0.1900000
-30139.0000000	0.1900000
-30138.0000000	0.1900000
-30137.0000000	0.1900000
-30136.0000000	0.1900000
-30135.0000000	0.1900000
-30134.0000000	0.1900000
-30133.0000000	0.1900000
-30132.0000000	0.1900000
-30131.0000000	0.1900000
-30130.0000000	0.1900000
-30129.0000000	0.1905000
-30128.0000000	0.1900000
-30127.0000000	0.1900000
-30126.0000000	0.1900000
-30125.0000000	0.1900000
-30124.0000000	0.1900000
-30123.0000000	0.1900000
-30122.0000000	0.1900000
-30121.0000000	0.1900000

-30120.0000000	0.1900000
-30119.0000000	0.1900000
-30118.0000000	0.1900000
-30117.0000000	0.1895238
-30116.0000000	0.1895000
-30115.0000000	0.1900000
-30114.0000000	0.1900000
-30113.0000000	0.1900000
-30112.0000000	0.1900000
-30111.0000000	0.1895000
-30110.0000000	0.1900000
-30109.0000000	0.1900000
-30108.0000000	0.1900000
-30107.0000000	0.1900000
-30106.0000000	0.1900000
-30105.0000000	0.1900000
-30104.0000000	0.1900000
-30103.0000000	0.1900000
-30102.0000000	0.1900000
-30101.0000000	0.1900000
-30100.0000000	0.1900000
-30099.0000000	0.1900000
-30098.0000000	0.1895000
-30097.0000000	0.1900000
-30096.0000000	0.1900000
-30095.0000000	0.1900000
-30094.0000000	0.1900000
-30093.0000000	0.1895000
-30092.0000000	0.1900000
-30091.0000000	0.1900000
-30090.0000000	0.1900000
-30089.0000000	0.1900000
-30088.0000000	0.1900000
-30087.0000000	0.1900000
-30086.0000000	0.1900000
-30085.0000000	0.1900000
-30084.0000000	0.1900000
-30083.0000000	0.1900000
-30082.0000000	0.1900000
-30081.0000000	0.1900000
-30080.0000000	0.1900000
-30079.0000000	0.1900000
-30078.0000000	0.1900000
-30077.0000000	0.1900000
-30076.0000000	0.1900000
-30075.0000000	0.1900000
-30074.0000000	0.1895238
-30073.0000000	0.1900000
-30072.0000000	0.1900000
-30071.0000000	0.1900000
-30070.0000000	0.1900000
-30069.0000000	0.1900000
-30068.0000000	0.1900000
-30067.0000000	0.1900000
-30066.0000000	0.1900000
-30065.0000000	0.1900000
-30064.0000000	0.1900000

-30063.0000000	0.1900000
-30062.0000000	0.1900000
-30061.0000000	0.1900000
-30060.0000000	0.1900000
-30059.0000000	0.1900000
-30058.0000000	0.1890476
-30057.0000000	0.1895000
-30056.0000000	0.1895238
-30055.0000000	0.1900000
-30054.0000000	0.1895000
-30053.0000000	0.1900000
-30052.0000000	0.1900000
-30051.0000000	0.1900000
-30050.0000000	0.1900000
-30049.0000000	0.1900000
-30048.0000000	0.1900000
-30047.0000000	0.1900000
-30046.0000000	0.1900000
-30045.0000000	0.1900000
-30044.0000000	0.1900000
-30043.0000000	0.1900000
-30042.0000000	0.1900000
-30041.0000000	0.1900000
-30040.0000000	0.1900000
-30039.0000000	0.1900000
-30038.0000000	0.1900000
-30037.0000000	0.1900000
-30036.0000000	0.1900000
-30035.0000000	0.1904762
-30034.0000000	0.1900000
-30033.0000000	0.1900000
-30032.0000000	0.1900000
-30031.0000000	0.1900000
-30030.0000000	0.1900000
-30029.0000000	0.1900000
-30028.0000000	0.1900000
-30027.0000000	0.1900000
-30026.0000000	0.1900000
-30025.0000000	0.1900000
-30024.0000000	0.1905000
-30023.0000000	0.1900000
-30022.0000000	0.1904762
-30021.0000000	0.1900000
-30020.0000000	0.1900000
-30019.0000000	0.1900000
-30018.0000000	0.1905000
-30017.0000000	0.1900000
-30016.0000000	0.1900000
-30015.0000000	0.1909524
-30014.0000000	0.1900000
-30013.0000000	0.1900000
-30012.0000000	0.1900000
-30011.0000000	0.1900000
-30010.0000000	0.1900000
-30009.0000000	0.1905000
-30008.0000000	0.1900000
-30007.0000000	0.1904762

-30006.0000000	0.1900000
-30005.0000000	0.1909524
-30004.0000000	0.1910000
-30003.0000000	0.1905000
-30002.0000000	0.1905000
-30001.0000000	0.1909524
-30000.0000000	0.1910000
-29999.0000000	0.1910000
-29998.0000000	0.1914286
-29997.0000000	0.1910000
-29996.0000000	0.1904762
-29995.0000000	0.1905000
-29994.0000000	0.1915000
-29993.0000000	0.1900000
-29992.0000000	0.1909524
-29991.0000000	0.1910000
-29990.0000000	0.1905000
-29989.0000000	0.1909524
-29988.0000000	0.1900000
-29987.0000000	0.1900000
-29986.0000000	0.1925000
-29985.0000000	0.1904762
-29984.0000000	0.1905000
-29983.0000000	0.1900000
-29982.0000000	0.1905000
-29981.0000000	0.1910000
-29980.0000000	0.1914286
-29979.0000000	0.1910000
-29978.0000000	0.1905000
-29977.0000000	0.1905000
-29976.0000000	0.1909524
-29975.0000000	0.1905000
-29974.0000000	0.1904762
-29973.0000000	0.1915000
-29972.0000000	0.1900000
-29971.0000000	0.1904762
-29970.0000000	0.1900000
-29969.0000000	0.1910000
-29968.0000000	0.1904762
-29967.0000000	0.1920000
-29966.0000000	0.1900000
-29965.0000000	0.1905000
-29964.0000000	0.1905000
-29963.0000000	0.1905000
-29962.0000000	0.1904762
-29961.0000000	0.1915000
-29960.0000000	0.1900000
-29959.0000000	0.1904762
-29958.0000000	0.1920000
-29957.0000000	0.1915000
-29956.0000000	0.1905000
-29955.0000000	0.1914286
-29954.0000000	0.1925000
-29953.0000000	0.1933334
-29952.0000000	0.1920000
-29951.0000000	0.1930000
-29950.0000000	0.1933334

-29949.0000000	0.1940000
-29948.0000000	0.1940000
-29947.0000000	0.1925000
-29946.0000000	0.1919048
-29945.0000000	0.1935000
-29944.0000000	0.1938096
-29943.0000000	0.1930000
-29942.0000000	0.1930000
-29941.0000000	0.1933334
-29940.0000000	0.1930000
-29939.0000000	0.1935000
-29938.0000000	0.1960000
-29937.0000000	0.1952381
-29936.0000000	0.1965000
-29935.0000000	0.1961905
-29934.0000000	0.1965000
-29933.0000000	0.1985000
-29932.0000000	0.1976191
-29931.0000000	0.1970000
-29930.0000000	0.1975000
-29929.0000000	0.1980953
-29928.0000000	0.1980000
-29927.0000000	0.1990000
-29926.0000000	0.1985000
-29925.0000000	0.1971429
-29924.0000000	0.1980000
-29923.0000000	0.1990477
-29922.0000000	0.1990000
-29921.0000000	0.1985000
-29920.0000000	0.1985715
-29919.0000000	0.1990000
-29918.0000000	0.1995000
-29917.0000000	0.1990000
-29916.0000000	0.1980953
-29915.0000000	0.2000000
-29914.0000000	0.1995238
-29913.0000000	0.1985000
-29912.0000000	0.2000000
-29911.0000000	0.1995000
-29910.0000000	0.1990477
-29909.0000000	0.1995000
-29908.0000000	0.1990000
-29907.0000000	0.1985715
-29906.0000000	0.1990000
-29905.0000000	0.2000000
-29904.0000000	0.1990000
-29903.0000000	0.1995000
-29902.0000000	0.1995000
-29901.0000000	0.1985715
-29900.0000000	0.1980000
-29899.0000000	0.1990477
-29898.0000000	0.1985000
-29897.0000000	0.1995000
-29896.0000000	0.1995000
-29895.0000000	0.1980952
-29894.0000000	0.1990000
-29893.0000000	0.1995238

-29892.0000000	0.1965000
-29891.0000000	0.1985000
-29890.0000000	0.1980953
-29889.0000000	0.1985000
-29888.0000000	0.1990000
-29887.0000000	0.1970000
-29886.0000000	0.1990477
-29885.0000000	0.1990000
-29884.0000000	0.1980000
-29883.0000000	0.1990477
-29882.0000000	0.1990000
-29881.0000000	0.1990000
-29880.0000000	0.2000000
-29879.0000000	0.1985000
-29878.0000000	0.1995000
-29877.0000000	0.2000000
-29876.0000000	0.2000000
-29875.0000000	0.2000000
-29874.0000000	0.2000000
-29873.0000000	0.2000000
-29872.0000000	0.2000000
-29871.0000000	0.2000000
-29870.0000000	0.2000000
-29869.0000000	0.2000000
-29868.0000000	0.2000000
-29867.0000000	0.2000000
-29866.0000000	0.2000000
-29865.0000000	0.2000000
-29864.0000000	0.2000000
-29863.0000000	0.2000000
-29862.0000000	0.2000000
-29861.0000000	0.2005000
-29860.0000000	0.2000000
-29859.0000000	0.2000000
-29858.0000000	0.2000000
-29857.0000000	0.2000000
-29856.0000000	0.2000000
-29855.0000000	0.2005000
-29854.0000000	0.2000000
-29853.0000000	0.2000000
-29852.0000000	0.2000000
-29851.0000000	0.2000000
-29850.0000000	0.2000000
-29849.0000000	0.2005000
-29848.0000000	0.2005000
-29847.0000000	0.2004762
-29846.0000000	0.2010000
-29845.0000000	0.2005000
-29844.0000000	0.2004762
-29843.0000000	0.2010000
-29842.0000000	0.2005000
-29841.0000000	0.2009524
-29840.0000000	0.2015000
-29839.0000000	0.2010000
-29838.0000000	0.2019048
-29837.0000000	0.2010000
-29836.0000000	0.2000000

-29835.0000000	0.2014286
-29834.0000000	0.2005000
-29833.0000000	0.2025000
-29832.0000000	0.2014286
-29831.0000000	0.2025000
-29830.0000000	0.2009524
-29829.0000000	0.2025000
-29828.0000000	0.2015000
-29827.0000000	0.2020000
-29826.0000000	0.2028572
-29825.0000000	0.2020000
-29824.0000000	0.2025000
-29823.0000000	0.2019048
-29822.0000000	0.2040000
-29821.0000000	0.2035000
-29820.0000000	0.2025000
-29819.0000000	0.2033333
-29818.0000000	0.2040000
-29817.0000000	0.2028572
-29816.0000000	0.2050000
-29815.0000000	0.2035000
-29814.0000000	0.2047619
-29813.0000000	0.2060000
-29812.0000000	0.2090000
-29811.0000000	0.2080953
-29810.0000000	0.2080000
-29809.0000000	0.2090000
-29808.0000000	0.2100000
-29807.0000000	0.2095000
-29806.0000000	0.2080000
-29805.0000000	0.2080953
-29804.0000000	0.2090000
-29803.0000000	0.2095000
-29802.0000000	0.2100000
-29801.0000000	0.2090000
-29800.0000000	0.2095238
-29799.0000000	0.2100000
-29798.0000000	0.2100000
-29797.0000000	0.2100000
-29796.0000000	0.2100000
-29795.0000000	0.2095000
-29794.0000000	0.2095000
-29793.0000000	0.2100000
-29792.0000000	0.2100000
-29791.0000000	0.2100000
-29790.0000000	0.2100000
-29789.0000000	0.2100000
-29788.0000000	0.2095000
-29787.0000000	0.2095238
-29786.0000000	0.2100000
-29785.0000000	0.2100000
-29784.0000000	0.2100000
-29783.0000000	0.2100000
-29782.0000000	0.2100000
-29781.0000000	0.2100000
-29780.0000000	0.2100000
-29779.0000000	0.2100000

-29778.0000000	0.2100000
-29777.0000000	0.2100000
-29776.0000000	0.2100000
-29775.0000000	0.2100000
-29774.0000000	0.2100000
-29773.0000000	0.2100000
-29772.0000000	0.2105000
-29771.0000000	0.2100000
-29770.0000000	0.2105000
-29769.0000000	0.2100000
-29768.0000000	0.2100000
-29767.0000000	0.2105000
-29766.0000000	0.2100000
-29765.0000000	0.2100000
-29764.0000000	0.2105000
-29763.0000000	0.2110000
-29762.0000000	0.2100000
-29761.0000000	0.2105000
-29760.0000000	0.2100000
-29759.0000000	0.2109524
-29758.0000000	0.2110000
-29757.0000000	0.2100000
-29756.0000000	0.2120000
-29755.0000000	0.2115000
-29754.0000000	0.2128572
-29753.0000000	0.2120000
-29752.0000000	0.2120000
-29751.0000000	0.2125000
-29750.0000000	0.2133333
-29749.0000000	0.2140000
-29748.0000000	0.2123810
-29747.0000000	0.2150000
-29746.0000000	0.2160000
-29745.0000000	0.2140000
-29744.0000000	0.2161905
-29743.0000000	0.2170000
-29742.0000000	0.2180000
-29741.0000000	0.2190476
-29740.0000000	0.2165000
-29739.0000000	0.2176191
-29738.0000000	0.2180000
-29737.0000000	0.2185000
-29736.0000000	0.2200000
-29735.0000000	0.2185000
-29734.0000000	0.2190000
-29733.0000000	0.2200000
-29732.0000000	0.2190476
-29731.0000000	0.2200000
-29730.0000000	0.2200000
-29729.0000000	0.2195238
-29728.0000000	0.2200000
-29727.0000000	0.2200000
-29726.0000000	0.2195000
-29725.0000000	0.2200000
-29724.0000000	0.2195000
-29723.0000000	0.2200000
-29722.0000000	0.2195000

-29721.0000000	0.2195000
-29720.0000000	0.2190476
-29719.0000000	0.2195000
-29718.0000000	0.2200000
-29717.0000000	0.2200000
-29716.0000000	0.2200000
-29715.0000000	0.2200000
-29714.0000000	0.2200000
-29713.0000000	0.2200000
-29712.0000000	0.2200000
-29711.0000000	0.2195238
-29710.0000000	0.2200000
-29709.0000000	0.2200000
-29708.0000000	0.2195000
-29707.0000000	0.2200000
-29706.0000000	0.2195000
-29705.0000000	0.2200000
-29704.0000000	0.2200000
-29703.0000000	0.2200000
-29702.0000000	0.2200000
-29701.0000000	0.2200000
-29700.0000000	0.2200000
-29699.0000000	0.2195000
-29698.0000000	0.2195238
-29697.0000000	0.2200000
-29696.0000000	0.2195000
-29695.0000000	0.2200000
-29694.0000000	0.2200000
-29693.0000000	0.2195238
-29692.0000000	0.2200000
-29691.0000000	0.2195000
-29690.0000000	0.2195000
-29689.0000000	0.2200000
-29688.0000000	0.2185000
-29687.0000000	0.2200000
-29686.0000000	0.2200000
-29685.0000000	0.2195000
-29684.0000000	0.2200000
-29683.0000000	0.2200000
-29682.0000000	0.2190000
-29681.0000000	0.2200000
-29680.0000000	0.2200000
-29679.0000000	0.2195238
-29678.0000000	0.2200000
-29677.0000000	0.2200000
-29676.0000000	0.2200000
-29675.0000000	0.2200000
-29674.0000000	0.2200000
-29673.0000000	0.2200000
-29672.0000000	0.2200000
-29671.0000000	0.2200000
-29670.0000000	0.2200000
-29669.0000000	0.2200000
-29668.0000000	0.2200000
-29667.0000000	0.2200000
-29666.0000000	0.2200000
-29665.0000000	0.2200000

-29664.0000000	0.2210000
-29663.0000000	0.2204762
-29662.0000000	0.2200000
-29661.0000000	0.2200000
-29660.0000000	0.2210000
-29659.0000000	0.2204762
-29658.0000000	0.2205000
-29657.0000000	0.2204762
-29656.0000000	0.2215000
-29655.0000000	0.2220000
-29654.0000000	0.2204762
-29653.0000000	0.2220000
-29652.0000000	0.2210000
-29651.0000000	0.2220000
-29650.0000000	0.2214286
-29649.0000000	0.2223810
-29648.0000000	0.2205000
-29647.0000000	0.2245000
-29646.0000000	0.2225000
-29645.0000000	0.2247619
-29644.0000000	0.2240000
-29643.0000000	0.2220000
-29642.0000000	0.2238095
-29641.0000000	0.2215000
-29640.0000000	0.2230000
-29639.0000000	0.2230000
-29638.0000000	0.2228571
-29637.0000000	0.2230000
-29636.0000000	0.2247619
-29635.0000000	0.2235000
-29634.0000000	0.2230000
-29633.0000000	0.2261904
-29632.0000000	0.2270000
-29631.0000000	0.2245000
-29630.0000000	0.2225000
-29629.0000000	0.2261904
-29628.0000000	0.2245000
-29627.0000000	0.2242857
-29626.0000000	0.2245000
-29625.0000000	0.2225000
-29624.0000000	0.2242857
-29623.0000000	0.2225000
-29622.0000000	0.2215000
-29621.0000000	0.2205000
-29620.0000000	0.2214286
-29619.0000000	0.2210000
-29618.0000000	0.2219047
-29617.0000000	0.2215000
-29616.0000000	0.2210000
-29615.0000000	0.2209524
-29614.0000000	0.2225000
-29613.0000000	0.2215000
-29612.0000000	0.2204762
-29611.0000000	0.2205000
-29610.0000000	0.2205000
-29609.0000000	0.2205000
-29608.0000000	0.2200000

-29607.0000000	0.2205000
-29606.0000000	0.2204762
-29605.0000000	0.2205000
-29604.0000000	0.2210000
-29603.0000000	0.2209524
-29602.0000000	0.2200000
-29601.0000000	0.2200000
-29600.0000000	0.2200000
-29599.0000000	0.2200000
-29598.0000000	0.2200000
-29597.0000000	0.2200000
-29596.0000000	0.2200000
-29595.0000000	0.2200000
-29594.0000000	0.2200000
-29593.0000000	0.2200000
-29592.0000000	0.2200000
-29591.0000000	0.2200000
-29590.0000000	0.2200000
-29589.0000000	0.2200000
-29588.0000000	0.2200000
-29587.0000000	0.2200000
-29586.0000000	0.2200000
-29585.0000000	0.2200000
-29584.0000000	0.2200000
-29583.0000000	0.2200000
-29582.0000000	0.2195238
-29581.0000000	0.2200000
-29580.0000000	0.2200000
-29579.0000000	0.2200000
-29578.0000000	0.2195238
-29577.0000000	0.2200000
-29576.0000000	0.2200000
-29575.0000000	0.2195238
-29574.0000000	0.2200000
-29573.0000000	0.2200000
-29572.0000000	0.2200000
-29571.0000000	0.2195000
-29570.0000000	0.2195000
-29569.0000000	0.2200000
-29568.0000000	0.2195000
-29567.0000000	0.2200000
-29566.0000000	0.2195000
-29565.0000000	0.2190000
-29564.0000000	0.2190000
-29563.0000000	0.2195238
-29562.0000000	0.2200000
-29561.0000000	0.2190000
-29560.0000000	0.2190476
-29559.0000000	0.2200000
-29558.0000000	0.2200000
-29557.0000000	0.2200000
-29556.0000000	0.2200000
-29555.0000000	0.2200000
-29554.0000000	0.2200000
-29553.0000000	0.2195000
-29552.0000000	0.2200000
-29551.0000000	0.2200000

-29550.0000000	0.2200000
-29549.0000000	0.2195000
-29548.0000000	0.2185714
-29547.0000000	0.2200000
-29546.0000000	0.2190000
-29545.0000000	0.2200000
-29544.0000000	0.2200000
-29543.0000000	0.2200000
-29542.0000000	0.2185000
-29541.0000000	0.2190000
-29540.0000000	0.2185000
-29539.0000000	0.2180952
-29538.0000000	0.2190000
-29537.0000000	0.2180000
-29536.0000000	0.2200000
-29535.0000000	0.2180000
-29534.0000000	0.2165000
-29533.0000000	0.2161905
-29532.0000000	0.2135000
-29531.0000000	0.2125000
-29530.0000000	0.2133334
-29529.0000000	0.2115000
-29528.0000000	0.2115000
-29527.0000000	0.2109524
-29526.0000000	0.2115000
-29525.0000000	0.2115000
-29524.0000000	0.2119048
-29523.0000000	0.2120000
-29522.0000000	0.2125000
-29521.0000000	0.2123810
-29520.0000000	0.2105000
-29519.0000000	0.2110000
-29518.0000000	0.2104762
-29517.0000000	0.2120000
-29516.0000000	0.2100000
-29515.0000000	0.2109524
-29514.0000000	0.2110000
-29513.0000000	0.2119048
-29512.0000000	0.2120000
-29511.0000000	0.2120000
-29510.0000000	0.2115000
-29509.0000000	0.2100000
-29508.0000000	0.2115000
-29507.0000000	0.2110000
-29506.0000000	0.2109524
-29505.0000000	0.2115000
-29504.0000000	0.2110000
-29503.0000000	0.2105000
-29502.0000000	0.2123810
-29501.0000000	0.2125000
-29500.0000000	0.2128572
-29499.0000000	0.2145000
-29498.0000000	0.2135000
-29497.0000000	0.2138095
-29496.0000000	0.2130000
-29495.0000000	0.2125000
-29494.0000000	0.2147619

-29493.0000000	0.2160000
-29492.0000000	0.2130000
-29491.0000000	0.2166667
-29490.0000000	0.2155000
-29489.0000000	0.2145000
-29488.0000000	0.2180000
-29487.0000000	0.2166667
-29486.0000000	0.2165000
-29485.0000000	0.2133333
-29484.0000000	0.2155000
-29483.0000000	0.2150000
-29482.0000000	0.2161905
-29481.0000000	0.2170000
-29480.0000000	0.2160000
-29479.0000000	0.2147619
-29478.0000000	0.2135000
-29477.0000000	0.2135000
-29476.0000000	0.2138095
-29475.0000000	0.2150000
-29474.0000000	0.2135000
-29473.0000000	0.2140000
-29472.0000000	0.2138095
-29471.0000000	0.2135000
-29470.0000000	0.2142857
-29469.0000000	0.2110000
-29468.0000000	0.2135000
-29467.0000000	0.2123810
-29466.0000000	0.2140000
-29465.0000000	0.2140000
-29464.0000000	0.2120000
-29463.0000000	0.2128572
-29462.0000000	0.2150000
-29461.0000000	0.2138095
-29460.0000000	0.2140000
-29459.0000000	0.2110000
-29458.0000000	0.2135000
-29457.0000000	0.2119048
-29456.0000000	0.2125000
-29455.0000000	0.2105000
-29454.0000000	0.2119048
-29453.0000000	0.2120000
-29452.0000000	0.2128572
-29451.0000000	0.2140000
-29450.0000000	0.2145000
-29449.0000000	0.2128572
-29448.0000000	0.2150000
-29447.0000000	0.2135000
-29446.0000000	0.2120000
-29445.0000000	0.2133334
-29444.0000000	0.2130000
-29443.0000000	0.2120000
-29442.0000000	0.2123810
-29441.0000000	0.2130000
-29440.0000000	0.2109524
-29439.0000000	0.2110000
-29438.0000000	0.2105000
-29437.0000000	0.2125000

-29436.0000000	0.2119048
-29435.0000000	0.2110000
-29434.0000000	0.2115000
-29433.0000000	0.2109524
-29432.0000000	0.2120000
-29431.0000000	0.2119048
-29430.0000000	0.2130000
-29429.0000000	0.2135000
-29428.0000000	0.2105000
-29427.0000000	0.2133334
-29426.0000000	0.2125000
-29425.0000000	0.2125000
-29424.0000000	0.2128572
-29423.0000000	0.2140000
-29422.0000000	0.2147619
-29421.0000000	0.2165000
-29420.0000000	0.2155000
-29419.0000000	0.2140000
-29418.0000000	0.2147619
-29417.0000000	0.2155000
-29416.0000000	0.2140000
-29415.0000000	0.2152381
-29414.0000000	0.2165000
-29413.0000000	0.2150000
-29412.0000000	0.2176191
-29411.0000000	0.2170000
-29410.0000000	0.2152381
-29409.0000000	0.2150000
-29408.0000000	0.2150000
-29407.0000000	0.2160000
-29406.0000000	0.2171429
-29405.0000000	0.2150000
-29404.0000000	0.2170000
-29403.0000000	0.2140000
-29402.0000000	0.2160000
-29401.0000000	0.2155000
-29400.0000000	0.2152381
-29399.0000000	0.2145000
-29398.0000000	0.2130000
-29397.0000000	0.2128572
-29396.0000000	0.2120000
-29395.0000000	0.2140000
-29394.0000000	0.2115000
-29393.0000000	0.2138095
-29392.0000000	0.2130000
-29391.0000000	0.2128572
-29390.0000000	0.2120000
-29389.0000000	0.2130000
-29388.0000000	0.2119048
-29387.0000000	0.2120000
-29386.0000000	0.2110000
-29385.0000000	0.2115000
-29384.0000000	0.2109524
-29383.0000000	0.2110000
-29382.0000000	0.2114286
-29381.0000000	0.2115000
-29380.0000000	0.2105000

-29379.0000000	0.2104762
-29378.0000000	0.2110000
-29377.0000000	0.2120000
-29376.0000000	0.2105000
-29375.0000000	0.2104762
-29374.0000000	0.2110000
-29373.0000000	0.2109524
-29372.0000000	0.2105000
-29371.0000000	0.2110000
-29370.0000000	0.2104762
-29369.0000000	0.2125000
-29368.0000000	0.2100000
-29367.0000000	0.2123810
-29366.0000000	0.2105000
-29365.0000000	0.2110000
-29364.0000000	0.2120000
-29363.0000000	0.2119048
-29362.0000000	0.2125000
-29361.0000000	0.2109524
-29360.0000000	0.2115000
-29359.0000000	0.2120000
-29358.0000000	0.2104762
-29357.0000000	0.2125000
-29356.0000000	0.2110000
-29355.0000000	0.2125000
-29354.0000000	0.2104762
-29353.0000000	0.2120000
-29352.0000000	0.2119048
-29351.0000000	0.2130000
-29350.0000000	0.2100000
-29349.0000000	0.2123810
-29348.0000000	0.2110000
-29347.0000000	0.2105000
-29346.0000000	0.2110000
-29345.0000000	0.2123810
-29344.0000000	0.2115000
-29343.0000000	0.2114286
-29342.0000000	0.2110000
-29341.0000000	0.2115000
-29340.0000000	0.2119048
-29339.0000000	0.2115000
-29338.0000000	0.2120000
-29337.0000000	0.2110000
-29336.0000000	0.2123810
-29335.0000000	0.2115000
-29334.0000000	0.2115000
-29333.0000000	0.2119048
-29332.0000000	0.2130000
-29331.0000000	0.2128572
-29330.0000000	0.2110000
-29329.0000000	0.2115000
-29328.0000000	0.2123810
-29327.0000000	0.2130000
-29326.0000000	0.2105000
-29325.0000000	0.2110000
-29324.0000000	0.2133333
-29323.0000000	0.2105000

-29322.0000000	0.2128572
-29321.0000000	0.2115000
-29320.0000000	0.2125000
-29319.0000000	0.2125000
-29318.0000000	0.2128572
-29317.0000000	0.2130000
-29316.0000000	0.2140000
-29315.0000000	0.2123810
-29314.0000000	0.2135000
-29313.0000000	0.2147619
-29312.0000000	0.2165000
-29311.0000000	0.2135000
-29310.0000000	0.2140000
-29309.0000000	0.2133333
-29308.0000000	0.2135000
-29307.0000000	0.2130000
-29306.0000000	0.2138095
-29305.0000000	0.2140000
-29304.0000000	0.2130000
-29303.0000000	0.2161905
-29302.0000000	0.2178947
-29301.0000000	0.2147619
-29300.0000000	0.2142857
-29299.0000000	0.2165000
-29298.0000000	0.2161905
-29297.0000000	0.2170000
-29296.0000000	0.2160000
-29295.0000000	0.2155000
-29294.0000000	0.2157143
-29293.0000000	0.2175000
-29292.0000000	0.2165000
-29291.0000000	0.2161905
-29290.0000000	0.2165000
-29289.0000000	0.2175000
-29288.0000000	0.2147619
-29287.0000000	0.2190000
-29286.0000000	0.2165000
-29285.0000000	0.2157143
-29284.0000000	0.2185000
-29283.0000000	0.2171429
-29282.0000000	0.2175000
-29281.0000000	0.2145000
-29280.0000000	0.2160000
-29279.0000000	0.2152381
-29278.0000000	0.2135000
-29277.0000000	0.2150000
-29276.0000000	0.2147619
-29275.0000000	0.2150000
-29274.0000000	0.2130000
-29273.0000000	0.2135000
-29272.0000000	0.2152381
-29271.0000000	0.2150000
-29270.0000000	0.2147619
-29269.0000000	0.2140000
-29268.0000000	0.2152381
-29267.0000000	0.2155000
-29266.0000000	0.2160000

-29265.0000000	0.2125000
-29264.0000000	0.2133334
-29263.0000000	0.2155000
-29262.0000000	0.2160000
-29261.0000000	0.2161905
-29260.0000000	0.2160000
-29259.0000000	0.2165000
-29258.0000000	0.2166667
-29257.0000000	0.2160000
-29256.0000000	0.2185000
-29255.0000000	0.2180952
-29254.0000000	0.2160000
-29253.0000000	0.2160000
-29252.0000000	0.2185714
-29251.0000000	0.2185000
-29250.0000000	0.2180000
-29249.0000000	0.2176191
-29248.0000000	0.2195000
-29247.0000000	0.2190000
-29246.0000000	0.2195238
-29245.0000000	0.2185000
-29244.0000000	0.2200000
-29243.0000000	0.2200000
-29242.0000000	0.2185714
-29241.0000000	0.2180000
-29240.0000000	0.2190476
-29239.0000000	0.2190000
-29238.0000000	0.2180000
-29237.0000000	0.2190476
-29236.0000000	0.2190000
-29235.0000000	0.2180000
-29234.0000000	0.2176191
-29233.0000000	0.2190000
-29232.0000000	0.2195000
-29231.0000000	0.2180952
-29230.0000000	0.2190000
-29229.0000000	0.2175000
-29228.0000000	0.2190000
-29227.0000000	0.2185714
-29226.0000000	0.2190000
-29225.0000000	0.2190476
-29224.0000000	0.2190000
-29223.0000000	0.2185000
-29222.0000000	0.2195238
-29221.0000000	0.2185000
-29220.0000000	0.2170000
-29219.0000000	0.2180000
-29218.0000000	0.2190476
-29217.0000000	0.2185000
-29216.0000000	0.2185714
-29215.0000000	0.2195000
-29214.0000000	0.2190000
-29213.0000000	0.2200000
-29212.0000000	0.2185714
-29211.0000000	0.2185000
-29210.0000000	0.2175000
-29209.0000000	0.2190476

-29208.0000000	0.2170000
-29207.0000000	0.2185714
-29206.0000000	0.2175000
-29205.0000000	0.2165000
-29204.0000000	0.2195238
-29203.0000000	0.2190000
-29202.0000000	0.2170000
-29201.0000000	0.2185714
-29200.0000000	0.2190000
-29199.0000000	0.2175000
-29198.0000000	0.2185000
-29197.0000000	0.2166667
-29196.0000000	0.2190000
-29195.0000000	0.2180952
-29194.0000000	0.2175000
-29193.0000000	0.2180000
-29192.0000000	0.2190476
-29191.0000000	0.2195000
-29190.0000000	0.2195000
-29189.0000000	0.2190000
-29188.0000000	0.2185714
-29187.0000000	0.2185000
-29186.0000000	0.2176190
-29185.0000000	0.2185000
-29184.0000000	0.2185000
-29183.0000000	0.2185000
-29182.0000000	0.2190476
-29181.0000000	0.2160000
-29180.0000000	0.2180000
-29179.0000000	0.2152381
-29178.0000000	0.2165000
-29177.0000000	0.2180952
-29176.0000000	0.2165000
-29175.0000000	0.2160000
-29174.0000000	0.2171429
-29173.0000000	0.2155000
-29172.0000000	0.2160000
-29171.0000000	0.2155000
-29170.0000000	0.2157143
-29169.0000000	0.2165000
-29168.0000000	0.2165000
-29167.0000000	0.2171429
-29166.0000000	0.2165000
-29165.0000000	0.2161905
-29164.0000000	0.2165000
-29163.0000000	0.2155000
-29162.0000000	0.2180000
-29161.0000000	0.2157143
-29160.0000000	0.2160000
-29159.0000000	0.2160000
-29158.0000000	0.2133333
-29157.0000000	0.2150000
-29156.0000000	0.2145000
-29155.0000000	0.2147619
-29154.0000000	0.2150000
-29153.0000000	0.2130000
-29152.0000000	0.2123810

-29151.0000000	0.2115000
-29150.0000000	0.2130000
-29149.0000000	0.2123810
-29148.0000000	0.2115000
-29147.0000000	0.2119048
-29146.0000000	0.2105000
-29145.0000000	0.2115000
-29144.0000000	0.2100000
-29143.0000000	0.2100000
-29142.0000000	0.2100000
-29141.0000000	0.2100000
-29140.0000000	0.2100000
-29139.0000000	0.2100000
-29138.0000000	0.2105000
-29137.0000000	0.2100000
-29136.0000000	0.2100000
-29135.0000000	0.2100000
-29134.0000000	0.2100000
-29133.0000000	0.2100000
-29132.0000000	0.2100000
-29131.0000000	0.2100000
-29130.0000000	0.2100000
-29129.0000000	0.2100000
-29128.0000000	0.2090476
-29127.0000000	0.2095000
-29126.0000000	0.2100000
-29125.0000000	0.2100000
-29124.0000000	0.2100000
-29123.0000000	0.2100000
-29122.0000000	0.2100000
-29121.0000000	0.2090000
-29120.0000000	0.2100000
-29119.0000000	0.2100000
-29118.0000000	0.2100000
-29117.0000000	0.2100000
-29116.0000000	0.2095000
-29115.0000000	0.2100000
-29114.0000000	0.2100000
-29113.0000000	0.2095238
-29112.0000000	0.2095000
-29111.0000000	0.2095000
-29110.0000000	0.2100000
-29109.0000000	0.2100000
-29108.0000000	0.2090000
-29107.0000000	0.2080000
-29106.0000000	0.2090476
-29105.0000000	0.2085000
-29104.0000000	0.2095238
-29103.0000000	0.2085000
-29102.0000000	0.2060000
-29101.0000000	0.2066667
-29100.0000000	0.2065000
-29099.0000000	0.2085000
-29098.0000000	0.2075000
-29097.0000000	0.2080953
-29096.0000000	0.2066667
-29095.0000000	0.2070000

-29094.0000000	0.2075000
-29093.0000000	0.2055000
-29092.0000000	0.2061905
-29091.0000000	0.2050000
-29090.0000000	0.2040000
-29089.0000000	0.2035000
-29088.0000000	0.2038096
-29087.0000000	0.2038096
-29086.0000000	0.2025000
-29085.0000000	0.2040000
-29084.0000000	0.2020000
-29083.0000000	0.2028572
-29082.0000000	0.2020000
-29081.0000000	0.2015000
-29080.0000000	0.2014286
-29079.0000000	0.2000000
-29078.0000000	0.2000000
-29077.0000000	0.2010000
-29076.0000000	0.2000000
-29075.0000000	0.2000000
-29074.0000000	0.2000000
-29073.0000000	0.2000000
-29072.0000000	0.2000000
-29071.0000000	0.2000000
-29070.0000000	0.2000000
-29069.0000000	0.2000000
-29068.0000000	0.2000000
-29067.0000000	0.2000000
-29066.0000000	0.2000000
-29065.0000000	0.2000000
-29064.0000000	0.2000000
-29063.0000000	0.2000000
-29062.0000000	0.2000000
-29061.0000000	0.2000000
-29060.0000000	0.1995000
-29059.0000000	0.1995000
-29058.0000000	0.2000000
-29057.0000000	0.1995000
-29056.0000000	0.1990477
-29055.0000000	0.2000000
-29054.0000000	0.2000000
-29053.0000000	0.1995238
-29052.0000000	0.1980000
-29051.0000000	0.1995000
-29050.0000000	0.1990476
-29049.0000000	0.1980000
-29048.0000000	0.1975000
-29047.0000000	0.1970000
-29046.0000000	0.1957143
-29045.0000000	0.1960000
-29044.0000000	0.1950000
-29043.0000000	0.1933334
-29042.0000000	0.1950000
-29041.0000000	0.1952381
-29040.0000000	0.1915000
-29039.0000000	0.1910000
-29038.0000000	0.1905000

-29037.0000000	0.1904762
-29036.0000000	0.1900000
-29035.0000000	0.1900000
-29034.0000000	0.1900000
-29033.0000000	0.1900000
-29032.0000000	0.1900000
-29031.0000000	0.1900000
-29030.0000000	0.1900000
-29029.0000000	0.1900000
-29028.0000000	0.1900000
-29027.0000000	0.1900000
-29026.0000000	0.1900000
-29025.0000000	0.1895000
-29024.0000000	0.1900000
-29023.0000000	0.1900000
-29022.0000000	0.1900000
-29021.0000000	0.1895000
-29020.0000000	0.1900000
-29019.0000000	0.1890476
-29018.0000000	0.1885000
-29017.0000000	0.1885000
-29016.0000000	0.1880953
-29015.0000000	0.1880000
-29014.0000000	0.1861905
-29013.0000000	0.1860000
-29012.0000000	0.1860000
-29011.0000000	0.1852381
-29010.0000000	0.1850000
-29009.0000000	0.1830000
-29008.0000000	0.1840000
-29007.0000000	0.1828572
-29006.0000000	0.1830000
-29005.0000000	0.1819048
-29004.0000000	0.1815000
-29003.0000000	0.1805000
-29002.0000000	0.1800000
-29001.0000000	0.1804762
-29000.0000000	0.1800000
-28999.0000000	0.1800000
-28998.0000000	0.1800000
-28997.0000000	0.1800000
-28996.0000000	0.1800000
-28995.0000000	0.1800000
-28994.0000000	0.1800000
-28993.0000000	0.1800000
-28992.0000000	0.1800000
-28991.0000000	0.1795000
-28990.0000000	0.1785000
-28989.0000000	0.1800000
-28988.0000000	0.1785000
-28987.0000000	0.1780000
-28986.0000000	0.1733334
-28985.0000000	0.1755000
-28984.0000000	0.1725000
-28983.0000000	0.1714286
-28982.0000000	0.1715000
-28981.0000000	0.1704762

-28980.0000000	0.1700000
-28979.0000000	0.1705000
-28978.0000000	0.1700000
-28977.0000000	0.1714286
-28976.0000000	0.1700000
-28975.0000000	0.1700000
-28974.0000000	0.1700000
-28973.0000000	0.1700000
-28972.0000000	0.1700000
-28971.0000000	0.1700000
-28970.0000000	0.1700000
-28969.0000000	0.1700000
-28968.0000000	0.1700000
-28967.0000000	0.1695000
-28966.0000000	0.1700000
-28965.0000000	0.1695238
-28964.0000000	0.1695000
-28963.0000000	0.1700000
-28962.0000000	0.1700000
-28961.0000000	0.1700000
-28960.0000000	0.1700000
-28959.0000000	0.1700000
-28958.0000000	0.1705000
-28957.0000000	0.1700000
-28956.0000000	0.1700000
-28955.0000000	0.1700000
-28954.0000000	0.1705000
-28953.0000000	0.1704762
-28952.0000000	0.1700000
-28951.0000000	0.1705000
-28950.0000000	0.1700000
-28949.0000000	0.1705000
-28948.0000000	0.1705000
-28947.0000000	0.1709524
-28946.0000000	0.1710000
-28945.0000000	0.1700000
-28944.0000000	0.1714286
-28943.0000000	0.1700000
-28942.0000000	0.1710000
-28941.0000000	0.1704762
-28940.0000000	0.1705000
-28939.0000000	0.1700000
-28938.0000000	0.1700000
-28937.0000000	0.1700000
-28936.0000000	0.1700000
-28935.0000000	0.1700000
-28934.0000000	0.1700000
-28933.0000000	0.1700000
-28932.0000000	0.1695238
-28931.0000000	0.1700000
-28930.0000000	0.1680000
-28929.0000000	0.1690476
-28928.0000000	0.1670000
-28927.0000000	0.1655000
-28926.0000000	0.1645000
-28925.0000000	0.1652381
-28924.0000000	0.1615000

-28923.0000000	0.1614286
-28922.0000000	0.1600000
-28921.0000000	0.1605000
-28920.0000000	0.1600000
-28919.0000000	0.1605000
-28918.0000000	0.1600000
-28917.0000000	0.1600000
-28916.0000000	0.1600000
-28915.0000000	0.1600000
-28914.0000000	0.1600000
-28913.0000000	0.1600000
-28912.0000000	0.1600000
-28911.0000000	0.1600000
-28910.0000000	0.1600000
-28909.0000000	0.1600000
-28908.0000000	0.1600000
-28907.0000000	0.1600000
-28906.0000000	0.1600000
-28905.0000000	0.1604762
-28904.0000000	0.1600000
-28903.0000000	0.1600000
-28902.0000000	0.1600000
-28901.0000000	0.1614286
-28900.0000000	0.1600000
-28899.0000000	0.1614286
-28898.0000000	0.1605000
-28897.0000000	0.1610000
-28896.0000000	0.1635000
-28895.0000000	0.1628572
-28894.0000000	0.1645000
-28893.0000000	0.1625000
-28892.0000000	0.1642857
-28891.0000000	0.1665000
-28890.0000000	0.1661905
-28889.0000000	0.1630000
-28888.0000000	0.1625000
-28887.0000000	0.1680000
-28886.0000000	0.1671429
-28885.0000000	0.1685000
-28884.0000000	0.1680953
-28883.0000000	0.1700000
-28882.0000000	0.1685000
-28881.0000000	0.1685000
-28880.0000000	0.1685714
-28879.0000000	0.1690000
-28878.0000000	0.1700000
-28877.0000000	0.1700000
-28876.0000000	0.1700000
-28875.0000000	0.1695238
-28874.0000000	0.1700000
-28873.0000000	0.1700000
-28872.0000000	0.1700000
-28871.0000000	0.1695238
-28870.0000000	0.1700000
-28869.0000000	0.1700000
-28868.0000000	0.1700000
-28867.0000000	0.1700000

-28866.0000000	0.1700000
-28865.0000000	0.1700000
-28864.0000000	0.1705000
-28863.0000000	0.1700000
-28862.0000000	0.1700000
-28861.0000000	0.1705000
-28860.0000000	0.1700000
-28859.0000000	0.1700000
-28858.0000000	0.1705000
-28857.0000000	0.1705000
-28856.0000000	0.1704762
-28855.0000000	0.1705000
-28854.0000000	0.1700000
-28853.0000000	0.1723810
-28852.0000000	0.1715000
-28851.0000000	0.1700000
-28850.0000000	0.1704762
-28849.0000000	0.1720000
-28848.0000000	0.1700000
-28847.0000000	0.1700000
-28846.0000000	0.1715000
-28845.0000000	0.1700000
-28844.0000000	0.1719048
-28843.0000000	0.1715000
-28842.0000000	0.1710000
-28841.0000000	0.1719048
-28840.0000000	0.1710000
-28839.0000000	0.1723810
-28838.0000000	0.1715000
-28837.0000000	0.1720000
-28836.0000000	0.1750000
-28835.0000000	0.1733334
-28834.0000000	0.1730000
-28833.0000000	0.1735000
-28832.0000000	0.1738096
-28831.0000000	0.1730000
-28830.0000000	0.1728572
-28829.0000000	0.1740000
-28828.0000000	0.1720000
-28827.0000000	0.1720000
-28826.0000000	0.1723810
-28825.0000000	0.1720000
-28824.0000000	0.1720000
-28823.0000000	0.1719048
-28822.0000000	0.1725000
-28821.0000000	0.1730000
-28820.0000000	0.1725000
-28819.0000000	0.1714286
-28818.0000000	0.1700000
-28817.0000000	0.1723810
-28816.0000000	0.1735000
-28815.0000000	0.1735000
-28814.0000000	0.1723810
-28813.0000000	0.1725000
-28812.0000000	0.1730000
-28811.0000000	0.1723810
-28810.0000000	0.1710000

-28809.0000000	0.1720000
-28808.0000000	0.1723810
-28807.0000000	0.1715000
-28806.0000000	0.1715000
-28805.0000000	0.1733334
-28804.0000000	0.1740000
-28803.0000000	0.1740000
-28802.0000000	0.1728572
-28801.0000000	0.1720000
-28800.0000000	0.1742857
-28799.0000000	0.1720000
-28798.0000000	0.1740000
-28797.0000000	0.1750000
-28796.0000000	0.1733334
-28795.0000000	0.1745000
-28794.0000000	0.1760000
-28793.0000000	0.1738096
-28792.0000000	0.1755000
-28791.0000000	0.1750000
-28790.0000000	0.1755000
-28789.0000000	0.1742857
-28788.0000000	0.1775000
-28787.0000000	0.1776191
-28786.0000000	0.1770000
-28785.0000000	0.1770000
-28784.0000000	0.1771429
-28783.0000000	0.1780000
-28782.0000000	0.1770000
-28781.0000000	0.1775000
-28780.0000000	0.1776191
-28779.0000000	0.1771429
-28778.0000000	0.1765000
-28777.0000000	0.1755000
-28776.0000000	0.1765000
-28775.0000000	0.1766667
-28774.0000000	0.1775000
-28773.0000000	0.1770000
-28772.0000000	0.1770000
-28771.0000000	0.1747619
-28770.0000000	0.1766667
-28769.0000000	0.1760000
-28768.0000000	0.1745000
-28767.0000000	0.1750000
-28766.0000000	0.1742857
-28765.0000000	0.1780000
-28764.0000000	0.1770000
-28763.0000000	0.1757143
-28762.0000000	0.1790000
-28761.0000000	0.1775000
-28760.0000000	0.1780000
-28759.0000000	0.1800000
-28758.0000000	0.1800000
-28757.0000000	0.1790477
-28756.0000000	0.1795000
-28755.0000000	0.1800000
-28754.0000000	0.1800000
-28753.0000000	0.1795000

-28752.0000000	0.1800000
-28751.0000000	0.1800000
-28750.0000000	0.1800000
-28749.0000000	0.1800000
-28748.0000000	0.1800000
-28747.0000000	0.1800000
-28746.0000000	0.1800000
-28745.0000000	0.1800000
-28744.0000000	0.1800000
-28743.0000000	0.1800000
-28742.0000000	0.1800000
-28741.0000000	0.1800000
-28740.0000000	0.1800000
-28739.0000000	0.1800000
-28738.0000000	0.1800000
-28737.0000000	0.1800000
-28736.0000000	0.1800000
-28735.0000000	0.1800000
-28734.0000000	0.1800000
-28733.0000000	0.1800000
-28732.0000000	0.1800000
-28731.0000000	0.1800000
-28730.0000000	0.1805000
-28729.0000000	0.1800000
-28728.0000000	0.1805000
-28727.0000000	0.1800000
-28726.0000000	0.1805000
-28725.0000000	0.1805000
-28724.0000000	0.1804762
-28723.0000000	0.1800000
-28722.0000000	0.1805000
-28721.0000000	0.1805000
-28720.0000000	0.1800000
-28719.0000000	0.1800000
-28718.0000000	0.1809524
-28717.0000000	0.1810000
-28716.0000000	0.1805000
-28715.0000000	0.1800000
-28714.0000000	0.1804762
-28713.0000000	0.1800000
-28712.0000000	0.1810000
-28711.0000000	0.1800000
-28710.0000000	0.1820000
-28709.0000000	0.1814286
-28708.0000000	0.1805000
-28707.0000000	0.1805000
-28706.0000000	0.1800000
-28705.0000000	0.1814286
-28704.0000000	0.1815000
-28703.0000000	0.1820000
-28702.0000000	0.1819048
-28701.0000000	0.1815000
-28700.0000000	0.1825000
-28699.0000000	0.1828572
-28698.0000000	0.1830000
-28697.0000000	0.1828572
-28696.0000000	0.1860000

-28695.0000000	0.1855000
-28694.0000000	0.1833334
-28693.0000000	0.1825000
-28692.0000000	0.1820000
-28691.0000000	0.1840000
-28690.0000000	0.1833334
-28689.0000000	0.1835000
-28688.0000000	0.1847619
-28687.0000000	0.1840000
-28686.0000000	0.1850000
-28685.0000000	0.1845000
-28684.0000000	0.1857143
-28683.0000000	0.1860000
-28682.0000000	0.1875000
-28681.0000000	0.1871429
-28680.0000000	0.1880000
-28679.0000000	0.1871429
-28678.0000000	0.1885000
-28677.0000000	0.1890000
-28676.0000000	0.1880000
-28675.0000000	0.1880953
-28674.0000000	0.1890000
-28673.0000000	0.1895000
-28672.0000000	0.1895238
-28671.0000000	0.1900000
-28670.0000000	0.1900000
-28669.0000000	0.1895000
-28668.0000000	0.1900000
-28667.0000000	0.1900000
-28666.0000000	0.1900000
-28665.0000000	0.1900000
-28664.0000000	0.1900000
-28663.0000000	0.1900000
-28662.0000000	0.1900000
-28661.0000000	0.1900000
-28660.0000000	0.1900000
-28659.0000000	0.1895000
-28658.0000000	0.1900000
-28657.0000000	0.1900000
-28656.0000000	0.1900000
-28655.0000000	0.1900000
-28654.0000000	0.1900000
-28653.0000000	0.1905000
-28652.0000000	0.1900000
-28651.0000000	0.1900000
-28650.0000000	0.1900000
-28649.0000000	0.1900000
-28648.0000000	0.1900000
-28647.0000000	0.1900000
-28646.0000000	0.1910000
-28645.0000000	0.1914286
-28644.0000000	0.1900000
-28643.0000000	0.1900000
-28642.0000000	0.1904762
-28641.0000000	0.1905000
-28640.0000000	0.1900000
-28639.0000000	0.1900000

-28638.0000000	0.1900000
-28637.0000000	0.1900000
-28636.0000000	0.1900000
-28635.0000000	0.1900000
-28634.0000000	0.1905000
-28633.0000000	0.1900000
-28632.0000000	0.1905000
-28631.0000000	0.1915000
-28630.0000000	0.1904762
-28629.0000000	0.1905000
-28628.0000000	0.1905000
-28627.0000000	0.1904762
-28626.0000000	0.1910000
-28625.0000000	0.1910000
-28624.0000000	0.1910000
-28623.0000000	0.1904762
-28622.0000000	0.1900000
-28621.0000000	0.1904762
-28620.0000000	0.1900000
-28619.0000000	0.1905000
-28618.0000000	0.1904762
-28617.0000000	0.1905000
-28616.0000000	0.1900000
-28615.0000000	0.1900000
-28614.0000000	0.1900000
-28613.0000000	0.1900000
-28612.0000000	0.1904762
-28611.0000000	0.1900000
-28610.0000000	0.1900000
-28609.0000000	0.1900000
-28608.0000000	0.1909524
-28607.0000000	0.1900000
-28606.0000000	0.1900000
-28605.0000000	0.1905000
-28604.0000000	0.1905000
-28603.0000000	0.1904762
-28602.0000000	0.1910000
-28601.0000000	0.1910000
-28600.0000000	0.1914286
-28599.0000000	0.1905000
-28598.0000000	0.1915000
-28597.0000000	0.1914286
-28596.0000000	0.1905000
-28595.0000000	0.1905000
-28594.0000000	0.1900000
-28593.0000000	0.1904762
-28592.0000000	0.1900000
-28591.0000000	0.1900000
-28590.0000000	0.1910000
-28589.0000000	0.1905000
-28588.0000000	0.1909524
-28587.0000000	0.1905000
-28586.0000000	0.1905000
-28585.0000000	0.1920000
-28584.0000000	0.1904762
-28583.0000000	0.1905000
-28582.0000000	0.1904762

-28581.0000000	0.1900000
-28580.0000000	0.1900000
-28579.0000000	0.1900000
-28578.0000000	0.1900000
-28577.0000000	0.1900000
-28576.0000000	0.1900000
-28575.0000000	0.1904762
-28574.0000000	0.1900000
-28573.0000000	0.1900000
-28572.0000000	0.1900000
-28571.0000000	0.1900000
-28570.0000000	0.1900000
-28569.0000000	0.1900000
-28568.0000000	0.1900000
-28567.0000000	0.1900000
-28566.0000000	0.1900000
-28565.0000000	0.1900000
-28564.0000000	0.1900000
-28563.0000000	0.1900000
-28562.0000000	0.1900000
-28561.0000000	0.1890476
-28560.0000000	0.1900000
-28559.0000000	0.1900000
-28558.0000000	0.1895000
-28557.0000000	0.1895238
-28556.0000000	0.1900000
-28555.0000000	0.1890000
-28554.0000000	0.1895238
-28553.0000000	0.1890000
-28552.0000000	0.1890476
-28551.0000000	0.1895000
-28550.0000000	0.1875000
-28549.0000000	0.1900000
-28548.0000000	0.1885715
-28547.0000000	0.1890000
-28546.0000000	0.1895000
-28545.0000000	0.1871429
-28544.0000000	0.1885000
-28543.0000000	0.1885715
-28542.0000000	0.1875000
-28541.0000000	0.1850000
-28540.0000000	0.1850000
-28539.0000000	0.1847619
-28538.0000000	0.1850000
-28537.0000000	0.1830000
-28536.0000000	0.1838096
-28535.0000000	0.1835000
-28534.0000000	0.1820000
-28533.0000000	0.1809524
-28532.0000000	0.1820000
-28531.0000000	0.1814286
-28530.0000000	0.1810000
-28529.0000000	0.1815000
-28528.0000000	0.1810000
-28527.0000000	0.1800000
-28526.0000000	0.1805000
-28525.0000000	0.1810000

-28524.0000000	0.1800000
-28523.0000000	0.1800000
-28522.0000000	0.1804762
-28521.0000000	0.1800000
-28520.0000000	0.1800000
-28519.0000000	0.1800000
-28518.0000000	0.1800000
-28517.0000000	0.1800000
-28516.0000000	0.1800000
-28515.0000000	0.1800000
-28514.0000000	0.1800000
-28513.0000000	0.1800000
-28512.0000000	0.1800000
-28511.0000000	0.1800000
-28510.0000000	0.1800000
-28509.0000000	0.1800000
-28508.0000000	0.1800000
-28507.0000000	0.1800000
-28506.0000000	0.1800000
-28505.0000000	0.1800000
-28504.0000000	0.1800000
-28503.0000000	0.1800000
-28502.0000000	0.1800000
-28501.0000000	0.1800000
-28500.0000000	0.1800000
-28499.0000000	0.1800000
-28498.0000000	0.1800000
-28497.0000000	0.1795239
-28496.0000000	0.1795000
-28495.0000000	0.1795000
-28494.0000000	0.1795000
-28493.0000000	0.1800000
-28492.0000000	0.1800000
-28491.0000000	0.1795000
-28490.0000000	0.1785000
-28489.0000000	0.1790000
-28488.0000000	0.1795238
-28487.0000000	0.1795000
-28486.0000000	0.1780000
-28485.0000000	0.1800000
-28484.0000000	0.1780953
-28483.0000000	0.1795239
-28482.0000000	0.1790000
-28481.0000000	0.1795000
-28480.0000000	0.1795000
-28479.0000000	0.1800000
-28478.0000000	0.1775000
-28477.0000000	0.1785000
-28476.0000000	0.1790477
-28475.0000000	0.1795000
-28474.0000000	0.1795000
-28473.0000000	0.1785000
-28472.0000000	0.1785715
-28471.0000000	0.1785000
-28470.0000000	0.1776191
-28469.0000000	0.1785000
-28468.0000000	0.1780000

-28467.0000000	0.1780953
-28466.0000000	0.1775000
-28465.0000000	0.1765000
-28464.0000000	0.1760000
-28463.0000000	0.1771429
-28462.0000000	0.1765000
-28461.0000000	0.1771429
-28460.0000000	0.1750000
-28459.0000000	0.1730000
-28458.0000000	0.1747619
-28457.0000000	0.1735000
-28456.0000000	0.1765000
-28455.0000000	0.1740000
-28454.0000000	0.1728572
-28453.0000000	0.1755000
-28452.0000000	0.1714286
-28451.0000000	0.1740000
-28450.0000000	0.1720000
-28449.0000000	0.1733334
-28448.0000000	0.1750000
-28447.0000000	0.1720000
-28446.0000000	0.1723810
-28445.0000000	0.1740000
-28444.0000000	0.1725000
-28443.0000000	0.1725000
-28442.0000000	0.1723810
-28441.0000000	0.1735000
-28440.0000000	0.1745000
-28439.0000000	0.1719048
-28438.0000000	0.1730000
-28437.0000000	0.1733334
-28436.0000000	0.1735000
-28435.0000000	0.1730000
-28434.0000000	0.1730000
-28433.0000000	0.1752381
-28432.0000000	0.1725000
-28431.0000000	0.1733334
-28430.0000000	0.1730000
-28429.0000000	0.1735000
-28428.0000000	0.1715000
-28427.0000000	0.1723810
-28426.0000000	0.1710000
-28425.0000000	0.1730000
-28424.0000000	0.1719048
-28423.0000000	0.1700000
-28422.0000000	0.1709524
-28421.0000000	0.1730000
-28420.0000000	0.1725000
-28419.0000000	0.1710000
-28418.0000000	0.1709524
-28417.0000000	0.1725000
-28416.0000000	0.1709524
-28415.0000000	0.1700000
-28414.0000000	0.1705000
-28413.0000000	0.1705000
-28412.0000000	0.1719048
-28411.0000000	0.1700000

-28410.0000000	0.1719048
-28409.0000000	0.1705000
-28408.0000000	0.1710000
-28407.0000000	0.1704762
-28406.0000000	0.1700000
-28405.0000000	0.1715000
-28404.0000000	0.1705000
-28403.0000000	0.1709524
-28402.0000000	0.1715000
-28401.0000000	0.1709524
-28400.0000000	0.1700000
-28399.0000000	0.1710000
-28398.0000000	0.1705000
-28397.0000000	0.1704762
-28396.0000000	0.1700000
-28395.0000000	0.1705000
-28394.0000000	0.1700000
-28393.0000000	0.1705000
-28392.0000000	0.1704762
-28391.0000000	0.1710000
-28390.0000000	0.1705000
-28389.0000000	0.1700000
-28388.0000000	0.1700000
-28387.0000000	0.1710000
-28386.0000000	0.1700000
-28385.0000000	0.1709524
-28384.0000000	0.1705000
-28383.0000000	0.1705000
-28382.0000000	0.1714286
-28381.0000000	0.1710000
-28380.0000000	0.1700000
-28379.0000000	0.1700000
-28378.0000000	0.1710000
-28377.0000000	0.1700000
-28376.0000000	0.1705000
-28375.0000000	0.1700000
-28374.0000000	0.1700000
-28373.0000000	0.1700000
-28372.0000000	0.1700000
-28371.0000000	0.1700000
-28370.0000000	0.1704762
-28369.0000000	0.1700000
-28368.0000000	0.1700000
-28367.0000000	0.1700000
-28366.0000000	0.1705000
-28365.0000000	0.1700000
-28364.0000000	0.1709524
-28363.0000000	0.1700000
-28362.0000000	0.1704762
-28361.0000000	0.1700000
-28360.0000000	0.1700000
-28359.0000000	0.1700000
-28358.0000000	0.1700000
-28357.0000000	0.1700000
-28356.0000000	0.1700000
-28355.0000000	0.1700000
-28354.0000000	0.1700000

-28353.0000000	0.1705000
-28352.0000000	0.1700000
-28351.0000000	0.1700000
-28350.0000000	0.1700000
-28349.0000000	0.1700000
-28348.0000000	0.1700000
-28347.0000000	0.1700000
-28346.0000000	0.1700000
-28345.0000000	0.1700000
-28344.0000000	0.1700000
-28343.0000000	0.1700000
-28342.0000000	0.1700000
-28341.0000000	0.1700000
-28340.0000000	0.1700000
-28339.0000000	0.1700000
-28338.0000000	0.1700000
-28337.0000000	0.1700000
-28336.0000000	0.1700000
-28335.0000000	0.1705000
-28334.0000000	0.1700000
-28333.0000000	0.1700000
-28332.0000000	0.1695000
-28331.0000000	0.1695238
-28330.0000000	0.1700000
-28329.0000000	0.1700000
-28328.0000000	0.1695238
-28327.0000000	0.1700000
-28326.0000000	0.1695000
-28325.0000000	0.1695238
-28324.0000000	0.1705000
-28323.0000000	0.1700000
-28322.0000000	0.1700000
-28321.0000000	0.1700000
-28320.0000000	0.1700000
-28319.0000000	0.1700000
-28318.0000000	0.1700000
-28317.0000000	0.1700000
-28316.0000000	0.1700000
-28315.0000000	0.1700000
-28314.0000000	0.1700000
-28313.0000000	0.1700000
-28312.0000000	0.1700000
-28311.0000000	0.1700000
-28310.0000000	0.1700000
-28309.0000000	0.1700000
-28308.0000000	0.1700000
-28307.0000000	0.1695000
-28306.0000000	0.1695238
-28305.0000000	0.1695000
-28304.0000000	0.1700000
-28303.0000000	0.1700000
-28302.0000000	0.1700000
-28301.0000000	0.1700000
-28300.0000000	0.1700000
-28299.0000000	0.1680000
-28298.0000000	0.1700000
-28297.0000000	0.1690476

-28296.0000000	0.1700000
-28295.0000000	0.1685714
-28294.0000000	0.1685000
-28293.0000000	0.1685000
-28292.0000000	0.1675000
-28291.0000000	0.1647619
-28290.0000000	0.1650000
-28289.0000000	0.1666667
-28288.0000000	0.1665000
-28287.0000000	0.1650000
-28286.0000000	0.1652381
-28285.0000000	0.1625000
-28284.0000000	0.1650000
-28283.0000000	0.1666667
-28282.0000000	0.1640000
-28281.0000000	0.1640000
-28280.0000000	0.1652381
-28279.0000000	0.1650000
-28278.0000000	0.1635000
-28277.0000000	0.1645000
-28276.0000000	0.1666667
-28275.0000000	0.1645000
-28274.0000000	0.1638096
-28273.0000000	0.1650000
-28272.0000000	0.1650000
-28271.0000000	0.1638096
-28270.0000000	0.1630000
-28269.0000000	0.1625000
-28268.0000000	0.1620000
-28267.0000000	0.1652381
-28266.0000000	0.1640000
-28265.0000000	0.1642857
-28264.0000000	0.1635000
-28263.0000000	0.1630000
-28262.0000000	0.1630000
-28261.0000000	0.1609524
-28260.0000000	0.1630000
-28259.0000000	0.1630000
-28258.0000000	0.1609524
-28257.0000000	0.1620000
-28256.0000000	0.1614286
-28255.0000000	0.1610000
-28254.0000000	0.1620000
-28253.0000000	0.1623810
-28252.0000000	0.1615000
-28251.0000000	0.1610000
-28250.0000000	0.1610000
-28249.0000000	0.1614286
-28248.0000000	0.1605000
-28247.0000000	0.1610000
-28246.0000000	0.1614286
-28245.0000000	0.1615000
-28244.0000000	0.1609524
-28243.0000000	0.1605000
-28242.0000000	0.1605000
-28241.0000000	0.1600000
-28240.0000000	0.1614286

-28239.0000000	0.1600000
-28238.0000000	0.1600000
-28237.0000000	0.1600000
-28236.0000000	0.1605000
-28235.0000000	0.1600000
-28234.0000000	0.1605000
-28233.0000000	0.1600000
-28232.0000000	0.1605000
-28231.0000000	0.1600000
-28230.0000000	0.1595000
-28229.0000000	0.1600000
-28228.0000000	0.1600000
-28227.0000000	0.1600000
-28226.0000000	0.1600000
-28225.0000000	0.1600000
-28224.0000000	0.1600000
-28223.0000000	0.1600000
-28222.0000000	0.1600000
-28221.0000000	0.1600000
-28220.0000000	0.1600000
-28219.0000000	0.1600000
-28218.0000000	0.1600000
-28217.0000000	0.1600000
-28216.0000000	0.1600000
-28215.0000000	0.1600000
-28214.0000000	0.1595238
-28213.0000000	0.1600000
-28212.0000000	0.1600000
-28211.0000000	0.1600000
-28210.0000000	0.1600000
-28209.0000000	0.1600000
-28208.0000000	0.1600000
-28207.0000000	0.1600000
-28206.0000000	0.1600000
-28205.0000000	0.1600000
-28204.0000000	0.1595000
-28203.0000000	0.1600000
-28202.0000000	0.1600000
-28201.0000000	0.1600000
-28200.0000000	0.1600000
-28199.0000000	0.1600000
-28198.0000000	0.1590476
-28197.0000000	0.1580000
-28196.0000000	0.1580953
-28195.0000000	0.1600000
-28194.0000000	0.1570000
-28193.0000000	0.1595000
-28192.0000000	0.1576191
-28191.0000000	0.1590000
-28190.0000000	0.1585000
-28189.0000000	0.1585715
-28188.0000000	0.1575000
-28187.0000000	0.1565000
-28186.0000000	0.1585000
-28185.0000000	0.1566667
-28184.0000000	0.1575000
-28183.0000000	0.1580953

-28182.0000000	0.1575000
-28181.0000000	0.1555000
-28180.0000000	0.1566667
-28179.0000000	0.1545000
-28178.0000000	0.1555000
-28177.0000000	0.1570000
-28176.0000000	0.1566667
-28175.0000000	0.1575000
-28174.0000000	0.1566667
-28173.0000000	0.1570000
-28172.0000000	0.1550000
-28171.0000000	0.1561905
-28170.0000000	0.1560000
-28169.0000000	0.1570000
-28168.0000000	0.1555000
-28167.0000000	0.1566667
-28166.0000000	0.1523810
-28165.0000000	0.1530000
-28164.0000000	0.1530000
-28163.0000000	0.1540000
-28162.0000000	0.1547619
-28161.0000000	0.1545000
-28160.0000000	0.1535000
-28159.0000000	0.1538095
-28158.0000000	0.1555000
-28157.0000000	0.1540000
-28156.0000000	0.1515000
-28155.0000000	0.1533334
-28154.0000000	0.1525000
-28153.0000000	0.1523810
-28152.0000000	0.1520000
-28151.0000000	0.1545000
-28150.0000000	0.1523810
-28149.0000000	0.1520000
-28148.0000000	0.1525000
-28147.0000000	0.1530000
-28146.0000000	0.1514286
-28145.0000000	0.1525000
-28144.0000000	0.1504762
-28143.0000000	0.1510000
-28142.0000000	0.1515000
-28141.0000000	0.1509524
-28140.0000000	0.1500000
-28139.0000000	0.1505000
-28138.0000000	0.1505000
-28137.0000000	0.1509524
-28136.0000000	0.1500000
-28135.0000000	0.1500000
-28134.0000000	0.1505000
-28133.0000000	0.1510000
-28132.0000000	0.1500000
-28131.0000000	0.1500000
-28130.0000000	0.1500000
-28129.0000000	0.1500000
-28128.0000000	0.1505000
-28127.0000000	0.1500000
-28126.0000000	0.1500000

-28125.0000000	0.1500000
-28124.0000000	0.1500000
-28123.0000000	0.1504762
-28122.0000000	0.1500000
-28121.0000000	0.1500000
-28120.0000000	0.1500000
-28119.0000000	0.1500000
-28118.0000000	0.1500000
-28117.0000000	0.1500000
-28116.0000000	0.1500000
-28115.0000000	0.1500000
-28114.0000000	0.1500000
-28113.0000000	0.1495000
-28112.0000000	0.1500000
-28111.0000000	0.1500000
-28110.0000000	0.1500000
-28109.0000000	0.1500000
-28108.0000000	0.1500000
-28107.0000000	0.1500000
-28106.0000000	0.1500000
-28105.0000000	0.1500000
-28104.0000000	0.1495000
-28103.0000000	0.1500000
-28102.0000000	0.1495000
-28101.0000000	0.1500000
-28100.0000000	0.1495000
-28099.0000000	0.1500000
-28098.0000000	0.1500000
-28097.0000000	0.1500000
-28096.0000000	0.1500000
-28095.0000000	0.1490476
-28094.0000000	0.1500000
-28093.0000000	0.1485000
-28092.0000000	0.1495238
-28091.0000000	0.1490000
-28090.0000000	0.1495238
-28089.0000000	0.1485000
-28088.0000000	0.1480000
-28087.0000000	0.1495000
-28086.0000000	0.1471429
-28085.0000000	0.1470000
-28084.0000000	0.1485715
-28083.0000000	0.1485000
-28082.0000000	0.1495000
-28081.0000000	0.1495000
-28080.0000000	0.1495238
-28079.0000000	0.1480000
-28078.0000000	0.1490000
-28077.0000000	0.1480953
-28076.0000000	0.1490000
-28075.0000000	0.1490476
-28074.0000000	0.1490000
-28073.0000000	0.1495000
-28072.0000000	0.1490000
-28071.0000000	0.1495238
-28070.0000000	0.1490000
-28069.0000000	0.1500000

-28068.0000000	0.1500000
-28067.0000000	0.1500000
-28066.0000000	0.1495000
-28065.0000000	0.1500000
-28064.0000000	0.1490000
-28063.0000000	0.1495000
-28062.0000000	0.1495238
-28061.0000000	0.1490000
-28060.0000000	0.1485715
-28059.0000000	0.1500000
-28058.0000000	0.1500000
-28057.0000000	0.1500000
-28056.0000000	0.1500000
-28055.0000000	0.1490000
-28054.0000000	0.1500000
-28053.0000000	0.1500000
-28052.0000000	0.1485000
-28051.0000000	0.1500000
-28050.0000000	0.1495238
-28049.0000000	0.1490000
-28048.0000000	0.1490000
-28047.0000000	0.1500000
-28046.0000000	0.1490000
-28045.0000000	0.1495000
-28044.0000000	0.1495238
-28043.0000000	0.1495000
-28042.0000000	0.1490000
-28041.0000000	0.1500000
-28040.0000000	0.1500000
-28039.0000000	0.1495000
-28038.0000000	0.1500000
-28037.0000000	0.1500000
-28036.0000000	0.1500000
-28035.0000000	0.1495238
-28034.0000000	0.1500000
-28033.0000000	0.1490000
-28032.0000000	0.1490476
-28031.0000000	0.1500000
-28030.0000000	0.1500000
-28029.0000000	0.1500000
-28028.0000000	0.1500000
-28027.0000000	0.1500000
-28026.0000000	0.1495238
-28025.0000000	0.1500000
-28024.0000000	0.1495000
-28023.0000000	0.1500000
-28022.0000000	0.1500000
-28021.0000000	0.1500000
-28020.0000000	0.1500000
-28019.0000000	0.1500000
-28018.0000000	0.1500000
-28017.0000000	0.1500000
-28016.0000000	0.1505000
-28015.0000000	0.1500000
-28014.0000000	0.1500000
-28013.0000000	0.1500000
-28012.0000000	0.1500000

-28011.0000000	0.1500000
-28010.0000000	0.1500000
-28009.0000000	0.1500000
-28008.0000000	0.1500000
-28007.0000000	0.1500000
-28006.0000000	0.1505000
-28005.0000000	0.1500000
-28004.0000000	0.1509524
-28003.0000000	0.1505000
-28002.0000000	0.1509524
-28001.0000000	0.1510000
-28000.0000000	0.1510000
-27999.0000000	0.1514286
-27998.0000000	0.1510000
-27997.0000000	0.1510000
-27996.0000000	0.1514286
-27995.0000000	0.1520000
-27994.0000000	0.1525000
-27993.0000000	0.1519048
-27992.0000000	0.1545000
-27991.0000000	0.1525000
-27990.0000000	0.1525000
-27989.0000000	0.1528572
-27988.0000000	0.1500000
-27987.0000000	0.1523810
-27986.0000000	0.1525000
-27985.0000000	0.1515000
-27984.0000000	0.1514286
-27983.0000000	0.1530000
-27982.0000000	0.1535000
-27981.0000000	0.1525000
-27980.0000000	0.1514286
-27979.0000000	0.1540000
-27978.0000000	0.1542857
-27977.0000000	0.1545000
-27976.0000000	0.1540000
-27975.0000000	0.1550000
-27974.0000000	0.1552381
-27973.0000000	0.1580000
-27972.0000000	0.1585000
-27971.0000000	0.1595238
-27970.0000000	0.1570000
-27969.0000000	0.1580953
-27968.0000000	0.1580000
-27967.0000000	0.1580000
-27966.0000000	0.1580953
-27965.0000000	0.1585000
-27964.0000000	0.1590000
-27963.0000000	0.1600000
-27962.0000000	0.1585715
-27961.0000000	0.1590000
-27960.0000000	0.1600000
-27959.0000000	0.1595238
-27958.0000000	0.1600000
-27957.0000000	0.1600000
-27956.0000000	0.1600000
-27955.0000000	0.1595000

-27954.0000000	0.1595238
-27953.0000000	0.1600000
-27952.0000000	0.1600000
-27951.0000000	0.1595000
-27950.0000000	0.1600000
-27949.0000000	0.1600000
-27948.0000000	0.1590476
-27947.0000000	0.1600000
-27946.0000000	0.1600000
-27945.0000000	0.1600000
-27944.0000000	0.1600000
-27943.0000000	0.1600000
-27942.0000000	0.1600000
-27941.0000000	0.1600000
-27940.0000000	0.1600000
-27939.0000000	0.1600000
-27938.0000000	0.1600000
-27937.0000000	0.1600000
-27936.0000000	0.1600000
-27935.0000000	0.1600000
-27934.0000000	0.1600000
-27933.0000000	0.1600000
-27932.0000000	0.1600000
-27931.0000000	0.1600000
-27930.0000000	0.1600000
-27929.0000000	0.1600000
-27928.0000000	0.1600000
-27927.0000000	0.1600000
-27926.0000000	0.1600000
-27925.0000000	0.1600000
-27924.0000000	0.1600000
-27923.0000000	0.1600000
-27922.0000000	0.1595000
-27921.0000000	0.1595000
-27920.0000000	0.1600000
-27919.0000000	0.1600000
-27918.0000000	0.1600000
-27917.0000000	0.1600000
-27916.0000000	0.1600000
-27915.0000000	0.1600000
-27914.0000000	0.1600000
-27913.0000000	0.1600000
-27912.0000000	0.1600000
-27911.0000000	0.1600000
-27910.0000000	0.1600000
-27909.0000000	0.1600000
-27908.0000000	0.1600000
-27907.0000000	0.1605000
-27906.0000000	0.1600000
-27905.0000000	0.1600000
-27904.0000000	0.1605000
-27903.0000000	0.1600000
-27902.0000000	0.1600000
-27901.0000000	0.1600000
-27900.0000000	0.1600000
-27899.0000000	0.1600000
-27898.0000000	0.1600000

-27897.0000000	0.1600000
-27896.0000000	0.1600000
-27895.0000000	0.1605000
-27894.0000000	0.1605000
-27893.0000000	0.1609524
-27892.0000000	0.1605000
-27891.0000000	0.1600000
-27890.0000000	0.1600000
-27889.0000000	0.1600000
-27888.0000000	0.1609524
-27887.0000000	0.1605000
-27886.0000000	0.1610000
-27885.0000000	0.1610000
-27884.0000000	0.1619048
-27883.0000000	0.1625000
-27882.0000000	0.1615000
-27881.0000000	0.1609524
-27880.0000000	0.1625000
-27879.0000000	0.1604762
-27878.0000000	0.1610000
-27877.0000000	0.1620000
-27876.0000000	0.1625000
-27875.0000000	0.1638096
-27874.0000000	0.1640000
-27873.0000000	0.1620000
-27872.0000000	0.1633333
-27871.0000000	0.1625000
-27870.0000000	0.1650000
-27869.0000000	0.1635000
-27868.0000000	0.1633334
-27867.0000000	0.1645000
-27866.0000000	0.1647619
-27865.0000000	0.1670000
-27864.0000000	0.1670000
-27863.0000000	0.1661905
-27862.0000000	0.1670000
-27861.0000000	0.1665000
-27860.0000000	0.1680000
-27859.0000000	0.1676191
-27858.0000000	0.1690000
-27857.0000000	0.1685714
-27856.0000000	0.1695000
-27855.0000000	0.1695000
-27854.0000000	0.1676191
-27853.0000000	0.1695000
-27852.0000000	0.1685000
-27851.0000000	0.1690000
-27850.0000000	0.1700000
-27849.0000000	0.1695000
-27848.0000000	0.1690476
-27847.0000000	0.1690000
-27846.0000000	0.1685000
-27845.0000000	0.1695238
-27844.0000000	0.1700000
-27843.0000000	0.1680000
-27842.0000000	0.1700000
-27841.0000000	0.1680000

-27840.0000000	0.1700000
-27839.0000000	0.1700000
-27838.0000000	0.1700000
-27837.0000000	0.1690000
-27836.0000000	0.1695238
-27835.0000000	0.1695000
-27834.0000000	0.1695000
-27833.0000000	0.1700000
-27832.0000000	0.1700000
-27831.0000000	0.1700000
-27830.0000000	0.1695000
-27829.0000000	0.1685714
-27828.0000000	0.1700000
-27827.0000000	0.1700000
-27826.0000000	0.1695000
-27825.0000000	0.1695000
-27824.0000000	0.1690000
-27823.0000000	0.1685714
-27822.0000000	0.1700000
-27821.0000000	0.1695000
-27820.0000000	0.1695238
-27819.0000000	0.1695000
-27818.0000000	0.1700000
-27817.0000000	0.1700000
-27816.0000000	0.1700000
-27815.0000000	0.1695238
-27814.0000000	0.1700000
-27813.0000000	0.1695000
-27812.0000000	0.1700000
-27811.0000000	0.1700000
-27810.0000000	0.1700000
-27809.0000000	0.1700000
-27808.0000000	0.1700000
-27807.0000000	0.1700000
-27806.0000000	0.1700000
-27805.0000000	0.1700000
-27804.0000000	0.1700000
-27803.0000000	0.1700000
-27802.0000000	0.1700000
-27801.0000000	0.1700000
-27800.0000000	0.1700000
-27799.0000000	0.1700000
-27798.0000000	0.1700000
-27797.0000000	0.1700000
-27796.0000000	0.1700000
-27795.0000000	0.1700000
-27794.0000000	0.1700000
-27793.0000000	0.1700000
-27792.0000000	0.1700000
-27791.0000000	0.1700000
-27790.0000000	0.1700000
-27789.0000000	0.1700000
-27788.0000000	0.1700000
-27787.0000000	0.1700000
-27786.0000000	0.1700000
-27785.0000000	0.1700000
-27784.0000000	0.1700000

-27783.0000000	0.1700000
-27782.0000000	0.1700000
-27781.0000000	0.1700000
-27780.0000000	0.1695000
-27779.0000000	0.1700000
-27778.0000000	0.1700000
-27777.0000000	0.1695000
-27776.0000000	0.1700000
-27775.0000000	0.1700000
-27774.0000000	0.1700000
-27773.0000000	0.1690476
-27772.0000000	0.1695000
-27771.0000000	0.1690000
-27770.0000000	0.1690000
-27769.0000000	0.1680953
-27768.0000000	0.1695000
-27767.0000000	0.1695000
-27766.0000000	0.1690476
-27765.0000000	0.1670000
-27764.0000000	0.1670000
-27763.0000000	0.1676191
-27762.0000000	0.1655000
-27761.0000000	0.1670000
-27760.0000000	0.1661905
-27759.0000000	0.1640000
-27758.0000000	0.1645000
-27757.0000000	0.1642857
-27756.0000000	0.1635000
-27755.0000000	0.1640000
-27754.0000000	0.1628572
-27753.0000000	0.1640000
-27752.0000000	0.1666667
-27751.0000000	0.1640000
-27750.0000000	0.1655000
-27749.0000000	0.1660000
-27748.0000000	0.1652381
-27747.0000000	0.1635000
-27746.0000000	0.1650000
-27745.0000000	0.1666667
-27744.0000000	0.1660000
-27743.0000000	0.1642857
-27742.0000000	0.1665000
-27741.0000000	0.1675000
-27740.0000000	0.1650000
-27739.0000000	0.1647619
-27738.0000000	0.1645000
-27737.0000000	0.1655000
-27736.0000000	0.1642857
-27735.0000000	0.1635000
-27734.0000000	0.1645000
-27733.0000000	0.1652381
-27732.0000000	0.1660000
-27731.0000000	0.1650000
-27730.0000000	0.1642857
-27729.0000000	0.1630000
-27728.0000000	0.1645000
-27727.0000000	0.1642857

-27726.0000000	0.1645000
-27725.0000000	0.1645000
-27724.0000000	0.1661905
-27723.0000000	0.1640000
-27722.0000000	0.1620000
-27721.0000000	0.1642857
-27720.0000000	0.1610000
-27719.0000000	0.1620000
-27718.0000000	0.1609524
-27717.0000000	0.1635000
-27716.0000000	0.1615000
-27715.0000000	0.1609524
-27714.0000000	0.1605000
-27713.0000000	0.1609524
-27712.0000000	0.1605000
-27711.0000000	0.1600000
-27710.0000000	0.1615000
-27709.0000000	0.1609524
-27708.0000000	0.1600000
-27707.0000000	0.1610000
-27706.0000000	0.1614286
-27705.0000000	0.1605000
-27704.0000000	0.1615000
-27703.0000000	0.1610000
-27702.0000000	0.1609524
-27701.0000000	0.1615000
-27700.0000000	0.1604762
-27699.0000000	0.1600000
-27698.0000000	0.1605000
-27697.0000000	0.1600000
-27696.0000000	0.1605000
-27695.0000000	0.1610000
-27694.0000000	0.1600000
-27693.0000000	0.1600000
-27692.0000000	0.1600000
-27691.0000000	0.1604762
-27690.0000000	0.1600000
-27689.0000000	0.1605000
-27688.0000000	0.1604762
-27687.0000000	0.1600000
-27686.0000000	0.1600000
-27685.0000000	0.1605000
-27684.0000000	0.1600000
-27683.0000000	0.1600000
-27682.0000000	0.1600000
-27681.0000000	0.1600000
-27680.0000000	0.1600000
-27679.0000000	0.1600000
-27678.0000000	0.1600000
-27677.0000000	0.1600000
-27676.0000000	0.1600000
-27675.0000000	0.1600000
-27674.0000000	0.1600000
-27673.0000000	0.1600000
-27672.0000000	0.1600000
-27671.0000000	0.1600000
-27670.0000000	0.1600000

-27669.0000000	0.1600000
-27668.0000000	0.1600000
-27667.0000000	0.1600000
-27666.0000000	0.1600000
-27665.0000000	0.1595000
-27664.0000000	0.1600000
-27663.0000000	0.1600000
-27662.0000000	0.1600000
-27661.0000000	0.1600000
-27660.0000000	0.1600000
-27659.0000000	0.1600000
-27658.0000000	0.1600000
-27657.0000000	0.1600000
-27656.0000000	0.1600000
-27655.0000000	0.1595000
-27654.0000000	0.1595238
-27653.0000000	0.1585000
-27652.0000000	0.1590476
-27651.0000000	0.1600000
-27650.0000000	0.1590000
-27649.0000000	0.1590476
-27648.0000000	0.1595000
-27647.0000000	0.1595000
-27646.0000000	0.1580000
-27645.0000000	0.1595238
-27644.0000000	0.1580000
-27643.0000000	0.1580000
-27642.0000000	0.1590476
-27641.0000000	0.1590000
-27640.0000000	0.1585715
-27639.0000000	0.1584211
-27638.0000000	0.1580953
-27637.0000000	0.1570000
-27636.0000000	0.1566667
-27635.0000000	0.1595000
-27634.0000000	0.1580000
-27633.0000000	0.1585715
-27632.0000000	0.1585000
-27631.0000000	0.1595238
-27630.0000000	0.1590000
-27629.0000000	0.1585000
-27628.0000000	0.1595000
-27627.0000000	0.1590476
-27626.0000000	0.1590000
-27625.0000000	0.1575000
-27624.0000000	0.1600000
-27623.0000000	0.1600000
-27622.0000000	0.1590476
-27621.0000000	0.1600000
-27620.0000000	0.1585000
-27619.0000000	0.1590000
-27618.0000000	0.1590476
-27617.0000000	0.1595000
-27616.0000000	0.1585000
-27615.0000000	0.1585715
-27614.0000000	0.1585000
-27613.0000000	0.1570000

-27612.0000000	0.1595000
-27611.0000000	0.1561905
-27610.0000000	0.1576191
-27609.0000000	0.1585000
-27608.0000000	0.1580000
-27607.0000000	0.1590000
-27606.0000000	0.1571429
-27605.0000000	0.1595000
-27604.0000000	0.1560000
-27603.0000000	0.1576191
-27602.0000000	0.1580000
-27601.0000000	0.1585715
-27600.0000000	0.1580000
-27599.0000000	0.1585000
-27598.0000000	0.1585000
-27597.0000000	0.1595238
-27596.0000000	0.1585000
-27595.0000000	0.1585000
-27594.0000000	0.1580953
-27593.0000000	0.1590000
-27592.0000000	0.1590476
-27591.0000000	0.1585000
-27590.0000000	0.1595000
-27589.0000000	0.1580000
-27588.0000000	0.1580953
-27587.0000000	0.1600000
-27586.0000000	0.1585000
-27585.0000000	0.1590476
-27584.0000000	0.1600000
-27583.0000000	0.1595000
-27582.0000000	0.1590000
-27581.0000000	0.1590476
-27580.0000000	0.1575000
-27579.0000000	0.1580953
-27578.0000000	0.1575000
-27577.0000000	0.1560000
-27576.0000000	0.1566667
-27575.0000000	0.1585000
-27574.0000000	0.1555000
-27573.0000000	0.1555000
-27572.0000000	0.1561905
-27571.0000000	0.1575000
-27570.0000000	0.1566667
-27569.0000000	0.1535000
-27568.0000000	0.1555000
-27567.0000000	0.1547619
-27566.0000000	0.1530000
-27565.0000000	0.1560000
-27564.0000000	0.1561905
-27563.0000000	0.1560000
-27562.0000000	0.1565000
-27561.0000000	0.1571429
-27560.0000000	0.1545000
-27559.0000000	0.1560000
-27558.0000000	0.1576191
-27557.0000000	0.1575000
-27556.0000000	0.1580000

-27555.0000000	0.1576191
-27554.0000000	0.1560000
-27553.0000000	0.1555000
-27552.0000000	0.1560000
-27551.0000000	0.1538095
-27550.0000000	0.1560000
-27549.0000000	0.1557143
-27548.0000000	0.1555000
-27547.0000000	0.1555000
-27546.0000000	0.1557143
-27545.0000000	0.1540000
-27544.0000000	0.1565000
-27543.0000000	0.1540000
-27542.0000000	0.1552381
-27541.0000000	0.1540000
-27540.0000000	0.1538095
-27539.0000000	0.1535000
-27538.0000000	0.1545000
-27537.0000000	0.1547619
-27536.0000000	0.1540000
-27535.0000000	0.1550000
-27534.0000000	0.1557143
-27533.0000000	0.1555000
-27532.0000000	0.1545000
-27531.0000000	0.1542857
-27530.0000000	0.1530000
-27529.0000000	0.1525000
-27528.0000000	0.1514286
-27527.0000000	0.1510000
-27526.0000000	0.1520000
-27525.0000000	0.1523810
-27524.0000000	0.1515000
-27523.0000000	0.1510000
-27522.0000000	0.1515000
-27521.0000000	0.1509524
-27520.0000000	0.1510000
-27519.0000000	0.1504762
-27518.0000000	0.1510000
-27517.0000000	0.1500000
-27516.0000000	0.1504762
-27515.0000000	0.1505000
-27514.0000000	0.1510000
-27513.0000000	0.1505000
-27512.0000000	0.1509524
-27511.0000000	0.1505000
-27510.0000000	0.1504762
-27509.0000000	0.1505000
-27508.0000000	0.1505000
-27507.0000000	0.1505000
-27506.0000000	0.1504762
-27505.0000000	0.1500000
-27504.0000000	0.1510000
-27503.0000000	0.1504762
-27502.0000000	0.1500000
-27501.0000000	0.1500000
-27500.0000000	0.1500000
-27499.0000000	0.1500000

-27498.0000000	0.1500000
-27497.0000000	0.1500000
-27496.0000000	0.1500000
-27495.0000000	0.1500000
-27494.0000000	0.1500000
-27493.0000000	0.1495000
-27492.0000000	0.1500000
-27491.0000000	0.1500000
-27490.0000000	0.1500000
-27489.0000000	0.1500000
-27488.0000000	0.1500000
-27487.0000000	0.1500000
-27486.0000000	0.1500000
-27485.0000000	0.1500000
-27484.0000000	0.1500000
-27483.0000000	0.1500000
-27482.0000000	0.1500000
-27481.0000000	0.1500000
-27480.0000000	0.1500000
-27479.0000000	0.1500000
-27478.0000000	0.1500000
-27477.0000000	0.1500000
-27476.0000000	0.1500000
-27475.0000000	0.1500000
-27474.0000000	0.1500000
-27473.0000000	0.1500000
-27472.0000000	0.1500000
-27471.0000000	0.1500000
-27470.0000000	0.1500000
-27469.0000000	0.1500000
-27468.0000000	0.1500000
-27467.0000000	0.1500000
-27466.0000000	0.1500000
-27465.0000000	0.1500000
-27464.0000000	0.1500000
-27463.0000000	0.1500000
-27462.0000000	0.1500000
-27461.0000000	0.1500000
-27460.0000000	0.1500000
-27459.0000000	0.1500000
-27458.0000000	0.1500000
-27457.0000000	0.1500000
-27456.0000000	0.1500000
-27455.0000000	0.1500000
-27454.0000000	0.1500000
-27453.0000000	0.1500000
-27452.0000000	0.1500000
-27451.0000000	0.1500000
-27450.0000000	0.1505000
-27449.0000000	0.1500000
-27448.0000000	0.1500000
-27447.0000000	0.1500000
-27446.0000000	0.1500000
-27445.0000000	0.1500000
-27444.0000000	0.1500000
-27443.0000000	0.1500000
-27442.0000000	0.1500000

-27441.0000000	0.1500000
-27440.0000000	0.1500000
-27439.0000000	0.1500000
-27438.0000000	0.1500000
-27437.0000000	0.1500000
-27436.0000000	0.1500000
-27435.0000000	0.1500000
-27434.0000000	0.1500000
-27433.0000000	0.1500000
-27432.0000000	0.1500000
-27431.0000000	0.1500000
-27430.0000000	0.1500000
-27429.0000000	0.1500000
-27428.0000000	0.1500000
-27427.0000000	0.1500000
-27426.0000000	0.1500000
-27425.0000000	0.1500000
-27424.0000000	0.1500000
-27423.0000000	0.1500000
-27422.0000000	0.1500000
-27421.0000000	0.1500000
-27420.0000000	0.1500000
-27419.0000000	0.1500000
-27418.0000000	0.1500000
-27417.0000000	0.1500000
-27416.0000000	0.1495000
-27415.0000000	0.1500000
-27414.0000000	0.1500000
-27413.0000000	0.1495238
-27412.0000000	0.1495000
-27411.0000000	0.1500000
-27410.0000000	0.1495238
-27409.0000000	0.1495000
-27408.0000000	0.1500000
-27407.0000000	0.1495000
-27406.0000000	0.1500000
-27405.0000000	0.1490000
-27404.0000000	0.1500000
-27403.0000000	0.1500000
-27402.0000000	0.1500000
-27401.0000000	0.1500000
-27400.0000000	0.1500000
-27399.0000000	0.1500000
-27398.0000000	0.1500000
-27397.0000000	0.1500000
-27396.0000000	0.1495238
-27395.0000000	0.1500000
-27394.0000000	0.1500000
-27393.0000000	0.1500000
-27392.0000000	0.1500000
-27391.0000000	0.1490000
-27390.0000000	0.1500000
-27389.0000000	0.1500000
-27388.0000000	0.1500000
-27387.0000000	0.1500000
-27386.0000000	0.1500000
-27385.0000000	0.1500000

-27384.0000000	0.1495000
-27383.0000000	0.1500000
-27382.0000000	0.1500000
-27381.0000000	0.1500000
-27380.0000000	0.1500000
-27379.0000000	0.1495000
-27378.0000000	0.1500000
-27377.0000000	0.1495000
-27376.0000000	0.1500000
-27375.0000000	0.1500000
-27374.0000000	0.1500000
-27373.0000000	0.1500000
-27372.0000000	0.1500000
-27371.0000000	0.1500000
-27370.0000000	0.1500000
-27369.0000000	0.1500000
-27368.0000000	0.1500000
-27367.0000000	0.1500000
-27366.0000000	0.1500000
-27365.0000000	0.1500000
-27364.0000000	0.1500000
-27363.0000000	0.1500000
-27362.0000000	0.1500000
-27361.0000000	0.1500000
-27360.0000000	0.1500000
-27359.0000000	0.1500000
-27358.0000000	0.1500000
-27357.0000000	0.1500000
-27356.0000000	0.1500000
-27355.0000000	0.1500000
-27354.0000000	0.1500000
-27353.0000000	0.1500000
-27352.0000000	0.1500000
-27351.0000000	0.1500000
-27350.0000000	0.1500000
-27349.0000000	0.1500000
-27348.0000000	0.1500000
-27347.0000000	0.1505000
-27346.0000000	0.1500000
-27345.0000000	0.1500000
-27344.0000000	0.1500000
-27343.0000000	0.1505000
-27342.0000000	0.1500000
-27341.0000000	0.1500000
-27340.0000000	0.1500000
-27339.0000000	0.1500000
-27338.0000000	0.1505000
-27337.0000000	0.1504762
-27336.0000000	0.1500000
-27335.0000000	0.1500000
-27334.0000000	0.1500000
-27333.0000000	0.1500000
-27332.0000000	0.1500000
-27331.0000000	0.1500000
-27330.0000000	0.1500000
-27329.0000000	0.1500000
-27328.0000000	0.1500000

-27327.0000000	0.1500000
-27326.0000000	0.1500000
-27325.0000000	0.1500000
-27324.0000000	0.1495000
-27323.0000000	0.1500000
-27322.0000000	0.1500000
-27321.0000000	0.1500000
-27320.0000000	0.1495000
-27319.0000000	0.1500000
-27318.0000000	0.1490000
-27317.0000000	0.1490000
-27316.0000000	0.1471429
-27315.0000000	0.1490000
-27314.0000000	0.1471429
-27313.0000000	0.1465000
-27312.0000000	0.1480000
-27311.0000000	0.1465000
-27310.0000000	0.1461905
-27309.0000000	0.1490000
-27308.0000000	0.1500000
-27307.0000000	0.1476191
-27306.0000000	0.1485000
-27305.0000000	0.1471429
-27304.0000000	0.1470000
-27303.0000000	0.1470000
-27302.0000000	0.1475000
-27301.0000000	0.1471429
-27300.0000000	0.1475000
-27299.0000000	0.1480000
-27298.0000000	0.1490476
-27297.0000000	0.1485000
-27296.0000000	0.1475000
-27295.0000000	0.1480000
-27294.0000000	0.1480953
-27293.0000000	0.1470000
-27292.0000000	0.1466667
-27291.0000000	0.1435000
-27290.0000000	0.1425000
-27289.0000000	0.1428572
-27288.0000000	0.1410000
-27287.0000000	0.1410000
-27286.0000000	0.1420000
-27285.0000000	0.1400000
-27284.0000000	0.1400000
-27283.0000000	0.1400000
-27282.0000000	0.1410000
-27281.0000000	0.1410000
-27280.0000000	0.1404762
-27279.0000000	0.1400000
-27278.0000000	0.1400000
-27277.0000000	0.1400000
-27276.0000000	0.1400000
-27275.0000000	0.1400000
-27274.0000000	0.1400000
-27273.0000000	0.1400000
-27272.0000000	0.1400000
-27271.0000000	0.1400000

-27270.0000000	0.1400000
-27269.0000000	0.1395000
-27268.0000000	0.1395238
-27267.0000000	0.1390000
-27266.0000000	0.1385000
-27265.0000000	0.1390000
-27264.0000000	0.1385715
-27263.0000000	0.1395000
-27262.0000000	0.1361905
-27261.0000000	0.1365000
-27260.0000000	0.1395000
-27259.0000000	0.1347619
-27258.0000000	0.1350000
-27257.0000000	0.1350000
-27256.0000000	0.1320000
-27255.0000000	0.1328572
-27254.0000000	0.1335000
-27253.0000000	0.1333334
-27252.0000000	0.1330000
-27251.0000000	0.1315000
-27250.0000000	0.1323810
-27249.0000000	0.1320000
-27248.0000000	0.1305000
-27247.0000000	0.1300000
-27246.0000000	0.1305000
-27245.0000000	0.1305000
-27244.0000000	0.1304762
-27243.0000000	0.1300000
-27242.0000000	0.1300000
-27241.0000000	0.1300000
-27240.0000000	0.1300000
-27239.0000000	0.1300000
-27238.0000000	0.1300000
-27237.0000000	0.1300000
-27236.0000000	0.1300000
-27235.0000000	0.1300000
-27234.0000000	0.1300000
-27233.0000000	0.1300000
-27232.0000000	0.1300000
-27231.0000000	0.1300000
-27230.0000000	0.1295000
-27229.0000000	0.1300000
-27228.0000000	0.1300000
-27227.0000000	0.1300000
-27226.0000000	0.1295000
-27225.0000000	0.1300000
-27224.0000000	0.1300000
-27223.0000000	0.1300000
-27222.0000000	0.1300000
-27221.0000000	0.1300000
-27220.0000000	0.1300000
-27219.0000000	0.1300000
-27218.0000000	0.1290000
-27217.0000000	0.1300000
-27216.0000000	0.1290000
-27215.0000000	0.1300000
-27214.0000000	0.1300000

-27213.0000000	0.1295000
-27212.0000000	0.1290000
-27211.0000000	0.1290000
-27210.0000000	0.1276191
-27209.0000000	0.1275000
-27208.0000000	0.1271429
-27207.0000000	0.1285000
-27206.0000000	0.1270000
-27205.0000000	0.1250000
-27204.0000000	0.1261905
-27203.0000000	0.1235000
-27202.0000000	0.1240000
-27201.0000000	0.1242857
-27200.0000000	0.1220000
-27199.0000000	0.1209524
-27198.0000000	0.1210000
-27197.0000000	0.1215000
-27196.0000000	0.1220000
-27195.0000000	0.1223809
-27194.0000000	0.1205000
-27193.0000000	0.1209524
-27192.0000000	0.1205000
-27191.0000000	0.1205000
-27190.0000000	0.1205000
-27189.0000000	0.1204762
-27188.0000000	0.1215000
-27187.0000000	0.1204762
-27186.0000000	0.1205000
-27185.0000000	0.1205000
-27184.0000000	0.1200000
-27183.0000000	0.1200000
-27182.0000000	0.1205000
-27181.0000000	0.1205000
-27180.0000000	0.1214286
-27179.0000000	0.1205000
-27178.0000000	0.1200000
-27177.0000000	0.1210000
-27176.0000000	0.1205000
-27175.0000000	0.1205000
-27174.0000000	0.1200000
-27173.0000000	0.1205000
-27172.0000000	0.1200000
-27171.0000000	0.1204762
-27170.0000000	0.1200000
-27169.0000000	0.1200000
-27168.0000000	0.1200000
-27167.0000000	0.1205000
-27166.0000000	0.1200000
-27165.0000000	0.1200000
-27164.0000000	0.1200000
-27163.0000000	0.1200000
-27162.0000000	0.1204762
-27161.0000000	0.1200000
-27160.0000000	0.1200000
-27159.0000000	0.1200000
-27158.0000000	0.1200000
-27157.0000000	0.1200000

-27156.0000000	0.1200000
-27155.0000000	0.1200000
-27154.0000000	0.1200000
-27153.0000000	0.1200000
-27152.0000000	0.1200000
-27151.0000000	0.1200000
-27150.0000000	0.1200000
-27149.0000000	0.1200000
-27148.0000000	0.1195238
-27147.0000000	0.1200000
-27146.0000000	0.1195000
-27145.0000000	0.1200000
-27144.0000000	0.1200000
-27143.0000000	0.1195000
-27142.0000000	0.1200000
-27141.0000000	0.1200000
-27140.0000000	0.1195000
-27139.0000000	0.1200000
-27138.0000000	0.1200000
-27137.0000000	0.1190000
-27136.0000000	0.1200000
-27135.0000000	0.1190476
-27134.0000000	0.1195000
-27133.0000000	0.1195000
-27132.0000000	0.1200000
-27131.0000000	0.1180000
-27130.0000000	0.1195000
-27129.0000000	0.1190000
-27128.0000000	0.1180952
-27127.0000000	0.1185000
-27126.0000000	0.1185714
-27125.0000000	0.1180000
-27124.0000000	0.1180000
-27123.0000000	0.1180952
-27122.0000000	0.1170000
-27121.0000000	0.1185000
-27120.0000000	0.1180952
-27119.0000000	0.1185000
-27118.0000000	0.1185714
-27117.0000000	0.1180000
-27116.0000000	0.1190000
-27115.0000000	0.1190000
-27114.0000000	0.1180952
-27113.0000000	0.1170000
-27112.0000000	0.1180000
-27111.0000000	0.1152381
-27110.0000000	0.1165000
-27109.0000000	0.1166666
-27108.0000000	0.1150000
-27107.0000000	0.1155000
-27106.0000000	0.1145000
-27105.0000000	0.1142857
-27104.0000000	0.1140000
-27103.0000000	0.1130000
-27102.0000000	0.1133333
-27101.0000000	0.1130000
-27100.0000000	0.1130000

-27099.0000000	0.1140000
-27098.0000000	0.1133333
-27097.0000000	0.1125000
-27096.0000000	0.1114286
-27095.0000000	0.1135000
-27094.0000000	0.1130000
-27093.0000000	0.1128571
-27092.0000000	0.1115000
-27091.0000000	0.1120000
-27090.0000000	0.1130000
-27089.0000000	0.1114286
-27088.0000000	0.1110000
-27087.0000000	0.1104762
-27086.0000000	0.1110000
-27085.0000000	0.1105000
-27084.0000000	0.1104762
-27083.0000000	0.1105000
-27082.0000000	0.1110000
-27081.0000000	0.1105000
-27080.0000000	0.1100000
-27079.0000000	0.1119047
-27078.0000000	0.1110000
-27077.0000000	0.1110000
-27076.0000000	0.1105000
-27075.0000000	0.1104762
-27074.0000000	0.1100000
-27073.0000000	0.1100000
-27072.0000000	0.1100000
-27071.0000000	0.1100000
-27070.0000000	0.1100000
-27069.0000000	0.1100000
-27068.0000000	0.1100000
-27067.0000000	0.1095000
-27066.0000000	0.1100000
-27065.0000000	0.1100000
-27064.0000000	0.1105000
-27063.0000000	0.1100000
-27062.0000000	0.1100000
-27061.0000000	0.1100000
-27060.0000000	0.1100000
-27059.0000000	0.1100000
-27058.0000000	0.1100000
-27057.0000000	0.1100000
-27056.0000000	0.1100000
-27055.0000000	0.1100000
-27054.0000000	0.1100000
-27053.0000000	0.1100000
-27052.0000000	0.1100000
-27051.0000000	0.1100000
-27050.0000000	0.1100000
-27049.0000000	0.1100000
-27048.0000000	0.1100000
-27047.0000000	0.1095000
-27046.0000000	0.1100000
-27045.0000000	0.1100000
-27044.0000000	0.1100000
-27043.0000000	0.1100000

-27042.0000000	0.1095000
-27041.0000000	0.1100000
-27040.0000000	0.1100000
-27039.0000000	0.1100000
-27038.0000000	0.1100000
-27037.0000000	0.1095000
-27036.0000000	0.1100000
-27035.0000000	0.1100000
-27034.0000000	0.1100000
-27033.0000000	0.1100000
-27032.0000000	0.1100000
-27031.0000000	0.1100000
-27030.0000000	0.1100000
-27029.0000000	0.1095238
-27028.0000000	0.1095000
-27027.0000000	0.1100000
-27026.0000000	0.1090000
-27025.0000000	0.1100000
-27024.0000000	0.1090000
-27023.0000000	0.1100000
-27022.0000000	0.1100000
-27021.0000000	0.1100000
-27020.0000000	0.1100000
-27019.0000000	0.1085000
-27018.0000000	0.1076190
-27017.0000000	0.1090000
-27016.0000000	0.1100000
-27015.0000000	0.1095000
-27014.0000000	0.1100000
-27013.0000000	0.1095000
-27012.0000000	0.1095000
-27011.0000000	0.1100000
-27010.0000000	0.1095000
-27009.0000000	0.1100000
-27008.0000000	0.1100000
-27007.0000000	0.1100000
-27006.0000000	0.1100000
-27005.0000000	0.1100000
-27004.0000000	0.1100000
-27003.0000000	0.1100000
-27002.0000000	0.1100000
-27001.0000000	0.1095000
-27000.0000000	0.1100000
-26999.0000000	0.1100000
-26998.0000000	0.1100000
-26997.0000000	0.1100000
-26996.0000000	0.1100000
-26995.0000000	0.1100000
-26994.0000000	0.1100000
-26993.0000000	0.1100000
-26992.0000000	0.1100000
-26991.0000000	0.1100000
-26990.0000000	0.1095238
-26989.0000000	0.1095000
-26988.0000000	0.1100000
-26987.0000000	0.1100000
-26986.0000000	0.1090000

-26985.0000000	0.1090000
-26984.0000000	0.1095238
-26983.0000000	0.1095000
-26982.0000000	0.1095000
-26981.0000000	0.1095238
-26980.0000000	0.1100000
-26979.0000000	0.1080000
-26978.0000000	0.1095000
-26977.0000000	0.1076190
-26976.0000000	0.1100000
-26975.0000000	0.1071429
-26974.0000000	0.1075000
-26973.0000000	0.1075000
-26972.0000000	0.1085714
-26971.0000000	0.1075000
-26970.0000000	0.1090000
-26969.0000000	0.1076190
-26968.0000000	0.1060000
-26967.0000000	0.1070000
-26966.0000000	0.1057143
-26965.0000000	0.1065000
-26964.0000000	0.1070000
-26963.0000000	0.1071429
-26962.0000000	0.1080000
-26961.0000000	0.1060000
-26960.0000000	0.1071429
-26959.0000000	0.1060000
-26958.0000000	0.1060000
-26957.0000000	0.1061905
-26956.0000000	0.1070000
-26955.0000000	0.1060000
-26954.0000000	0.1033333
-26953.0000000	0.1025000
-26952.0000000	0.1030000
-26951.0000000	0.1009524
-26950.0000000	0.1020000
-26949.0000000	0.1030000
-26948.0000000	0.1015000
-26947.0000000	0.1023810
-26946.0000000	0.1020000
-26945.0000000	0.1019048
-26944.0000000	0.1015000
-26943.0000000	0.1010000
-26942.0000000	0.1019048
-26941.0000000	0.1020000
-26940.0000000	0.1025000
-26939.0000000	0.1025000
-26938.0000000	0.1033333
-26937.0000000	0.1030000
-26936.0000000	0.1019048
-26935.0000000	0.1030000
-26934.0000000	0.1040000
-26933.0000000	0.1020000
-26932.0000000	0.1019048
-26931.0000000	0.1025000
-26930.0000000	0.1023810
-26929.0000000	0.1025000

-26928.0000000	0.1065000
-26927.0000000	0.1038095
-26926.0000000	0.1055000
-26925.0000000	0.1060000
-26924.0000000	0.1033333
-26923.0000000	0.1045000
-26922.0000000	0.1035000
-26921.0000000	0.1033333
-26920.0000000	0.1045000
-26919.0000000	0.1035000
-26918.0000000	0.1060000
-26917.0000000	0.1085714
-26916.0000000	0.1060000
-26915.0000000	0.1052381
-26914.0000000	0.1035000
-26913.0000000	0.1060000
-26912.0000000	0.1057143
-26911.0000000	0.1050000
-26910.0000000	0.1050000
-26909.0000000	0.1055000
-26908.0000000	0.1061905
-26907.0000000	0.1055000
-26906.0000000	0.1061905
-26905.0000000	0.1050000
-26904.0000000	0.1050000
-26903.0000000	0.1045000
-26902.0000000	0.1076190
-26901.0000000	0.1050000
-26900.0000000	0.1061905
-26899.0000000	0.1060000
-26898.0000000	0.1070000
-26897.0000000	0.1052381
-26896.0000000	0.1065000
-26895.0000000	0.1060000
-26894.0000000	0.1080000
-26893.0000000	0.1061905
-26892.0000000	0.1070000
-26891.0000000	0.1071429
-26890.0000000	0.1060000
-26889.0000000	0.1050000
-26888.0000000	0.1055000
-26887.0000000	0.1061905
-26886.0000000	0.1050000
-26885.0000000	0.1045000
-26884.0000000	0.1042857
-26883.0000000	0.1050000
-26882.0000000	0.1042857
-26881.0000000	0.1030000
-26880.0000000	0.1050000
-26879.0000000	0.1050000
-26878.0000000	0.1061905
-26877.0000000	0.1040000
-26876.0000000	0.1040000
-26875.0000000	0.1052381
-26874.0000000	0.1050000
-26873.0000000	0.1050000
-26872.0000000	0.1038095

-26871.0000000	0.1055000
-26870.0000000	0.1052381
-26869.0000000	0.1065000
-26868.0000000	0.1040000
-26867.0000000	0.1061905
-26866.0000000	0.1065000
-26865.0000000	0.1050000
-26864.0000000	0.1070000
-26863.0000000	0.1076190
-26862.0000000	0.1070000
-26861.0000000	0.1066667
-26860.0000000	0.1070000
-26859.0000000	0.1095000
-26858.0000000	0.1085000
-26857.0000000	0.1076190
-26856.0000000	0.1095000
-26855.0000000	0.1090000
-26854.0000000	0.1076190
-26853.0000000	0.1085000
-26852.0000000	0.1095238
-26851.0000000	0.1085000
-26850.0000000	0.1085000
-26849.0000000	0.1085000
-26848.0000000	0.1076190
-26847.0000000	0.1075000
-26846.0000000	0.1085000
-26845.0000000	0.1085714
-26844.0000000	0.1090000
-26843.0000000	0.1080000
-26842.0000000	0.1076190
-26841.0000000	0.1090000
-26840.0000000	0.1090000
-26839.0000000	0.1090476
-26838.0000000	0.1090000
-26837.0000000	0.1085000
-26836.0000000	0.1085714
-26835.0000000	0.1085000
-26834.0000000	0.1075000
-26833.0000000	0.1080952
-26832.0000000	0.1095000
-26831.0000000	0.1100000
-26830.0000000	0.1085000
-26829.0000000	0.1095000
-26828.0000000	0.1095000
-26827.0000000	0.1100000
-26826.0000000	0.1095000
-26825.0000000	0.1095000
-26824.0000000	0.1100000
-26823.0000000	0.1095000
-26822.0000000	0.1100000
-26821.0000000	0.1090000
-26820.0000000	0.1100000
-26819.0000000	0.1090000
-26818.0000000	0.1100000
-26817.0000000	0.1100000
-26816.0000000	0.1095000
-26815.0000000	0.1090476

-26814.0000000	0.1095000
-26813.0000000	0.1095000
-26812.0000000	0.1100000
-26811.0000000	0.1095238
-26810.0000000	0.1085000
-26809.0000000	0.1090476
-26808.0000000	0.1090000
-26807.0000000	0.1090000
-26806.0000000	0.1095238
-26805.0000000	0.1080000
-26804.0000000	0.1100000
-26803.0000000	0.1100000
-26802.0000000	0.1100000
-26801.0000000	0.1095238
-26800.0000000	0.1100000
-26799.0000000	0.1100000
-26798.0000000	0.1100000
-26797.0000000	0.1100000
-26796.0000000	0.1100000
-26795.0000000	0.1100000
-26794.0000000	0.1100000
-26793.0000000	0.1100000
-26792.0000000	0.1100000
-26791.0000000	0.1100000
-26790.0000000	0.1100000
-26789.0000000	0.1100000
-26788.0000000	0.1100000
-26787.0000000	0.1100000
-26786.0000000	0.1100000
-26785.0000000	0.1100000
-26784.0000000	0.1100000
-26783.0000000	0.1100000
-26782.0000000	0.1100000
-26781.0000000	0.1100000
-26780.0000000	0.1100000
-26779.0000000	0.1100000
-26778.0000000	0.1100000
-26777.0000000	0.1100000
-26776.0000000	0.1100000
-26775.0000000	0.1100000
-26774.0000000	0.1100000
-26773.0000000	0.1100000
-26772.0000000	0.1100000
-26771.0000000	0.1105000
-26770.0000000	0.1100000
-26769.0000000	0.1100000
-26768.0000000	0.1100000
-26767.0000000	0.1100000
-26766.0000000	0.1100000
-26765.0000000	0.1100000
-26764.0000000	0.1100000
-26763.0000000	0.1100000
-26762.0000000	0.1100000
-26761.0000000	0.1100000
-26760.0000000	0.1100000
-26759.0000000	0.1100000
-26758.0000000	0.1100000

-26757.0000000	0.1100000
-26756.0000000	0.1100000
-26755.0000000	0.1100000
-26754.0000000	0.1100000
-26753.0000000	0.1105000
-26752.0000000	0.1100000
-26751.0000000	0.1100000
-26750.0000000	0.1100000
-26749.0000000	0.1104762
-26748.0000000	0.1100000
-26747.0000000	0.1100000
-26746.0000000	0.1109524
-26745.0000000	0.1100000
-26744.0000000	0.1100000
-26743.0000000	0.1100000
-26742.0000000	0.1100000
-26741.0000000	0.1100000
-26740.0000000	0.1100000
-26739.0000000	0.1105000
-26738.0000000	0.1105000
-26737.0000000	0.1100000
-26736.0000000	0.1100000
-26735.0000000	0.1105000
-26734.0000000	0.1100000
-26733.0000000	0.1100000
-26732.0000000	0.1100000
-26731.0000000	0.1100000
-26730.0000000	0.1100000
-26729.0000000	0.1100000
-26728.0000000	0.1100000
-26727.0000000	0.1100000
-26726.0000000	0.1105000
-26725.0000000	0.1100000
-26724.0000000	0.1100000
-26723.0000000	0.1100000
-26722.0000000	0.1100000
-26721.0000000	0.1100000
-26720.0000000	0.1100000
-26719.0000000	0.1100000
-26718.0000000	0.1100000
-26717.0000000	0.1100000
-26716.0000000	0.1100000
-26715.0000000	0.1100000
-26714.0000000	0.1100000
-26713.0000000	0.1100000
-26712.0000000	0.1100000
-26711.0000000	0.1100000
-26710.0000000	0.1100000
-26709.0000000	0.1100000
-26708.0000000	0.1100000
-26707.0000000	0.1100000
-26706.0000000	0.1090476
-26705.0000000	0.1100000
-26704.0000000	0.1100000
-26703.0000000	0.1100000
-26702.0000000	0.1095000
-26701.0000000	0.1100000

-26700.0000000	0.1100000
-26699.0000000	0.1100000
-26698.0000000	0.1100000
-26697.0000000	0.1095238
-26696.0000000	0.1090000
-26695.0000000	0.1090000
-26694.0000000	0.1100000
-26693.0000000	0.1100000
-26692.0000000	0.1090000
-26691.0000000	0.1090000
-26690.0000000	0.1090476
-26689.0000000	0.1090000
-26688.0000000	0.1085714
-26687.0000000	0.1095000
-26686.0000000	0.1100000
-26685.0000000	0.1095238
-26684.0000000	0.1095000
-26683.0000000	0.1095000
-26682.0000000	0.1095238
-26681.0000000	0.1100000
-26680.0000000	0.1100000
-26679.0000000	0.1100000
-26678.0000000	0.1085000
-26677.0000000	0.1095000
-26676.0000000	0.1100000
-26675.0000000	0.1095000
-26674.0000000	0.1100000
-26673.0000000	0.1100000
-26672.0000000	0.1090000
-26671.0000000	0.1095238
-26670.0000000	0.1095000
-26669.0000000	0.1100000
-26668.0000000	0.1100000
-26667.0000000	0.1100000
-26666.0000000	0.1095000
-26665.0000000	0.1095000
-26664.0000000	0.1090476
-26663.0000000	0.1095000
-26662.0000000	0.1085000
-26661.0000000	0.1095000
-26660.0000000	0.1100000
-26659.0000000	0.1090000
-26658.0000000	0.1095238
-26657.0000000	0.1100000
-26656.0000000	0.1100000
-26655.0000000	0.1095238
-26654.0000000	0.1095000
-26653.0000000	0.1100000
-26652.0000000	0.1100000
-26651.0000000	0.1100000
-26650.0000000	0.1100000
-26649.0000000	0.1100000
-26648.0000000	0.1095000
-26647.0000000	0.1095000
-26646.0000000	0.1100000
-26645.0000000	0.1100000
-26644.0000000	0.1100000

-26643.0000000	0.1100000
-26642.0000000	0.1100000
-26641.0000000	0.1100000
-26640.0000000	0.1100000
-26639.0000000	0.1100000
-26638.0000000	0.1095000
-26637.0000000	0.1095238
-26636.0000000	0.1100000
-26635.0000000	0.1100000
-26634.0000000	0.1100000
-26633.0000000	0.1100000
-26632.0000000	0.1095000
-26631.0000000	0.1100000
-26630.0000000	0.1100000
-26629.0000000	0.1100000
-26628.0000000	0.1100000
-26627.0000000	0.1100000
-26626.0000000	0.1100000
-26625.0000000	0.1100000
-26624.0000000	0.1100000
-26623.0000000	0.1100000
-26622.0000000	0.1100000
-26621.0000000	0.1100000
-26620.0000000	0.1100000
-26619.0000000	0.1100000
-26618.0000000	0.1100000
-26617.0000000	0.1100000
-26616.0000000	0.1100000
-26615.0000000	0.1100000
-26614.0000000	0.1100000
-26613.0000000	0.1100000
-26612.0000000	0.1100000
-26611.0000000	0.1100000
-26610.0000000	0.1100000
-26609.0000000	0.1100000
-26608.0000000	0.1100000
-26607.0000000	0.1100000
-26606.0000000	0.1100000
-26605.0000000	0.1095000
-26604.0000000	0.1100000
-26603.0000000	0.1100000
-26602.0000000	0.1100000
-26601.0000000	0.1100000
-26600.0000000	0.1100000
-26599.0000000	0.1100000
-26598.0000000	0.1100000
-26597.0000000	0.1100000
-26596.0000000	0.1100000
-26595.0000000	0.1095238
-26594.0000000	0.1100000
-26593.0000000	0.1100000
-26592.0000000	0.1100000
-26591.0000000	0.1100000
-26590.0000000	0.1100000
-26589.0000000	0.1100000
-26588.0000000	0.1100000
-26587.0000000	0.1100000

-26586.0000000	0.1100000
-26585.0000000	0.1095238
-26584.0000000	0.1100000
-26583.0000000	0.1100000
-26582.0000000	0.1100000
-26581.0000000	0.1100000
-26580.0000000	0.1100000
-26579.0000000	0.1100000
-26578.0000000	0.1100000
-26577.0000000	0.1100000
-26576.0000000	0.1100000
-26575.0000000	0.1100000
-26574.0000000	0.1100000
-26573.0000000	0.1100000
-26572.0000000	0.1095000
-26571.0000000	0.1100000
-26570.0000000	0.1100000
-26569.0000000	0.1095000
-26568.0000000	0.1100000
-26567.0000000	0.1100000
-26566.0000000	0.1100000
-26565.0000000	0.1100000
-26564.0000000	0.1105000
-26563.0000000	0.1100000
-26562.0000000	0.1105000
-26561.0000000	0.1104762
-26560.0000000	0.1100000
-26559.0000000	0.1100000
-26558.0000000	0.1104762
-26557.0000000	0.1105000
-26556.0000000	0.1100000
-26555.0000000	0.1100000
-26554.0000000	0.1100000
-26553.0000000	0.1110000
-26552.0000000	0.1104762
-26551.0000000	0.1100000
-26550.0000000	0.1100000
-26549.0000000	0.1100000
-26548.0000000	0.1100000
-26547.0000000	0.1100000
-26546.0000000	0.1100000
-26545.0000000	0.1100000
-26544.0000000	0.1100000
-26543.0000000	0.1100000
-26542.0000000	0.1100000
-26541.0000000	0.1100000
-26540.0000000	0.1100000
-26539.0000000	0.1100000
-26538.0000000	0.1100000
-26537.0000000	0.1100000
-26536.0000000	0.1100000
-26535.0000000	0.1100000
-26534.0000000	0.1100000
-26533.0000000	0.1100000
-26532.0000000	0.1100000
-26531.0000000	0.1100000
-26530.0000000	0.1100000

-26529.0000000	0.1100000
-26528.0000000	0.1100000
-26527.0000000	0.1100000
-26526.0000000	0.1100000
-26525.0000000	0.1100000
-26524.0000000	0.1105000
-26523.0000000	0.1105000
-26522.0000000	0.1100000
-26521.0000000	0.1100000
-26520.0000000	0.1100000
-26519.0000000	0.1104762
-26518.0000000	0.1105000
-26517.0000000	0.1100000
-26516.0000000	0.1109524
-26515.0000000	0.1105000
-26514.0000000	0.1104762
-26513.0000000	0.1100000
-26512.0000000	0.1105000
-26511.0000000	0.1105000
-26510.0000000	0.1100000
-26509.0000000	0.1100000
-26508.0000000	0.1110000
-26507.0000000	0.1100000
-26506.0000000	0.1100000
-26505.0000000	0.1100000
-26504.0000000	0.1100000
-26503.0000000	0.1105000
-26502.0000000	0.1100000
-26501.0000000	0.1100000
-26500.0000000	0.1100000
-26499.0000000	0.1100000
-26498.0000000	0.1100000
-26497.0000000	0.1100000
-26496.0000000	0.1100000
-26495.0000000	0.1100000
-26494.0000000	0.1100000
-26493.0000000	0.1100000
-26492.0000000	0.1100000
-26491.0000000	0.1100000
-26490.0000000	0.1105000
-26489.0000000	0.1109524
-26488.0000000	0.1105000
-26487.0000000	0.1100000
-26486.0000000	0.1100000
-26485.0000000	0.1100000
-26484.0000000	0.1100000
-26483.0000000	0.1105000
-26482.0000000	0.1100000
-26481.0000000	0.1100000
-26480.0000000	0.1100000
-26479.0000000	0.1105000
-26478.0000000	0.1100000
-26477.0000000	0.1115000
-26476.0000000	0.1109524
-26475.0000000	0.1109524
-26474.0000000	0.1110000
-26473.0000000	0.1100000

-26472.0000000	0.1105000
-26471.0000000	0.1104762
-26470.0000000	0.1110000
-26469.0000000	0.1105000
-26468.0000000	0.1109524
-26467.0000000	0.1105000
-26466.0000000	0.1105000
-26465.0000000	0.1110000
-26464.0000000	0.1109524
-26463.0000000	0.1120000
-26462.0000000	0.1109524
-26461.0000000	0.1110000
-26460.0000000	0.1100000
-26459.0000000	0.1119047
-26458.0000000	0.1120000
-26457.0000000	0.1115000
-26456.0000000	0.1130000
-26455.0000000	0.1123809
-26454.0000000	0.1130000
-26453.0000000	0.1114286
-26452.0000000	0.1130000
-26451.0000000	0.1125000
-26450.0000000	0.1114286
-26449.0000000	0.1115000
-26448.0000000	0.1125000
-26447.0000000	0.1140000
-26446.0000000	0.1114286
-26445.0000000	0.1140000
-26444.0000000	0.1123809
-26443.0000000	0.1115000
-26442.0000000	0.1130000
-26441.0000000	0.1104762
-26440.0000000	0.1125000
-26439.0000000	0.1125000
-26438.0000000	0.1115000
-26437.0000000	0.1138095
-26436.0000000	0.1125000
-26435.0000000	0.1130000
-26434.0000000	0.1142857
-26433.0000000	0.1130000
-26432.0000000	0.1119047
-26431.0000000	0.1115000
-26430.0000000	0.1115000
-26429.0000000	0.1119047
-26428.0000000	0.1115000
-26427.0000000	0.1110000
-26426.0000000	0.1120000
-26425.0000000	0.1123809
-26424.0000000	0.1125000
-26423.0000000	0.1123809
-26422.0000000	0.1115000
-26421.0000000	0.1125000
-26420.0000000	0.1115000
-26419.0000000	0.1119047
-26418.0000000	0.1130000
-26417.0000000	0.1135000
-26416.0000000	0.1152381

-26415.0000000	0.1160000
-26414.0000000	0.1152381
-26413.0000000	0.1140000
-26412.0000000	0.1135000
-26411.0000000	0.1140000
-26410.0000000	0.1157143
-26409.0000000	0.1155000
-26408.0000000	0.1145000
-26407.0000000	0.1161905
-26406.0000000	0.1150000
-26405.0000000	0.1155000
-26404.0000000	0.1155000
-26403.0000000	0.1142857
-26402.0000000	0.1133333
-26401.0000000	0.1145000
-26400.0000000	0.1155000
-26399.0000000	0.1138095
-26398.0000000	0.1145000
-26397.0000000	0.1140000
-26396.0000000	0.1155000
-26395.0000000	0.1166666
-26394.0000000	0.1150000
-26393.0000000	0.1157143
-26392.0000000	0.1140000
-26391.0000000	0.1165000
-26390.0000000	0.1185000
-26389.0000000	0.1171428
-26388.0000000	0.1165000
-26387.0000000	0.1165000
-26386.0000000	0.1157143
-26385.0000000	0.1170000
-26384.0000000	0.1142857
-26383.0000000	0.1170000
-26382.0000000	0.1175000
-26381.0000000	0.1160000
-26380.0000000	0.1185714
-26379.0000000	0.1155000
-26378.0000000	0.1175000
-26377.0000000	0.1157143
-26376.0000000	0.1145000
-26375.0000000	0.1160000
-26374.0000000	0.1130000
-26373.0000000	0.1147619
-26372.0000000	0.1150000
-26371.0000000	0.1138095
-26370.0000000	0.1130000
-26369.0000000	0.1135000
-26368.0000000	0.1128571
-26367.0000000	0.1140000
-26366.0000000	0.1135000
-26365.0000000	0.1133333
-26364.0000000	0.1125000
-26363.0000000	0.1130000
-26362.0000000	0.1114286
-26361.0000000	0.1110000
-26360.0000000	0.1115000
-26359.0000000	0.1109524

-26358.0000000	0.1125000
-26357.0000000	0.1105000
-26356.0000000	0.1104762
-26355.0000000	0.1110000
-26354.0000000	0.1110000
-26353.0000000	0.1109524
-26352.0000000	0.1105000
-26351.0000000	0.1100000
-26350.0000000	0.1100000
-26349.0000000	0.1100000
-26348.0000000	0.1100000
-26347.0000000	0.1100000
-26346.0000000	0.1105000
-26345.0000000	0.1100000
-26344.0000000	0.1100000
-26343.0000000	0.1100000
-26342.0000000	0.1100000
-26341.0000000	0.1100000
-26340.0000000	0.1095000
-26339.0000000	0.1100000
-26338.0000000	0.1100000
-26337.0000000	0.1100000
-26336.0000000	0.1095000
-26335.0000000	0.1095238
-26334.0000000	0.1100000
-26333.0000000	0.1100000
-26332.0000000	0.1100000
-26331.0000000	0.1095000
-26330.0000000	0.1100000
-26329.0000000	0.1100000
-26328.0000000	0.1100000
-26327.0000000	0.1100000
-26326.0000000	0.1100000
-26325.0000000	0.1100000
-26324.0000000	0.1100000
-26323.0000000	0.1100000
-26322.0000000	0.1100000
-26321.0000000	0.1100000
-26320.0000000	0.1100000
-26319.0000000	0.1100000
-26318.0000000	0.1100000
-26317.0000000	0.1100000
-26316.0000000	0.1100000
-26315.0000000	0.1100000
-26314.0000000	0.1100000
-26313.0000000	0.1104762
-26312.0000000	0.1100000
-26311.0000000	0.1100000
-26310.0000000	0.1100000
-26309.0000000	0.1100000
-26308.0000000	0.1100000
-26307.0000000	0.1105000
-26306.0000000	0.1100000
-26305.0000000	0.1104762
-26304.0000000	0.1105000
-26303.0000000	0.1100000
-26302.0000000	0.1104762

-26301.0000000	0.1115000
-26300.0000000	0.1110000
-26299.0000000	0.1125000
-26298.0000000	0.1123809
-26297.0000000	0.1145000
-26296.0000000	0.1119047
-26295.0000000	0.1150000
-26294.0000000	0.1115000
-26293.0000000	0.1138095
-26292.0000000	0.1140000
-26291.0000000	0.1125000
-26290.0000000	0.1160000
-26289.0000000	0.1152381
-26288.0000000	0.1140000
-26287.0000000	0.1152381
-26286.0000000	0.1145000
-26285.0000000	0.1145000
-26284.0000000	0.1170000
-26283.0000000	0.1176190
-26282.0000000	0.1135000
-26281.0000000	0.1155000
-26280.0000000	0.1166666
-26279.0000000	0.1155000
-26278.0000000	0.1166666
-26277.0000000	0.1170000
-26276.0000000	0.1145000
-26275.0000000	0.1170000
-26274.0000000	0.1166666
-26273.0000000	0.1150000
-26272.0000000	0.1170000
-26271.0000000	0.1142857
-26270.0000000	0.1150000
-26269.0000000	0.1180000
-26268.0000000	0.1161905
-26267.0000000	0.1165000
-26266.0000000	0.1161905
-26265.0000000	0.1165000
-26264.0000000	0.1175000
-26263.0000000	0.1180952
-26262.0000000	0.1180000
-26261.0000000	0.1165000
-26260.0000000	0.1185000
-26259.0000000	0.1171428
-26258.0000000	0.1185000
-26257.0000000	0.1200000
-26256.0000000	0.1190000
-26255.0000000	0.1185000
-26254.0000000	0.1180000
-26253.0000000	0.1176190
-26252.0000000	0.1190000
-26251.0000000	0.1190000
-26250.0000000	0.1190476
-26249.0000000	0.1185000
-26248.0000000	0.1195238
-26247.0000000	0.1195000
-26246.0000000	0.1175000
-26245.0000000	0.1185000

-26244.0000000	0.1185714
-26243.0000000	0.1200000
-26242.0000000	0.1195000
-26241.0000000	0.1195238
-26240.0000000	0.1190000
-26239.0000000	0.1200000
-26238.0000000	0.1200000
-26237.0000000	0.1200000
-26236.0000000	0.1200000
-26235.0000000	0.1200000
-26234.0000000	0.1200000
-26233.0000000	0.1200000
-26232.0000000	0.1200000
-26231.0000000	0.1200000
-26230.0000000	0.1200000
-26229.0000000	0.1200000
-26228.0000000	0.1200000
-26227.0000000	0.1200000
-26226.0000000	0.1200000
-26225.0000000	0.1200000
-26224.0000000	0.1200000
-26223.0000000	0.1200000
-26222.0000000	0.1200000
-26221.0000000	0.1200000
-26220.0000000	0.1204762
-26219.0000000	0.1200000
-26218.0000000	0.1204762
-26217.0000000	0.1200000
-26216.0000000	0.1205000
-26215.0000000	0.1200000
-26214.0000000	0.1204762
-26213.0000000	0.1205000
-26212.0000000	0.1200000
-26211.0000000	0.1204762
-26210.0000000	0.1205000
-26209.0000000	0.1205000
-26208.0000000	0.1205000
-26207.0000000	0.1204762
-26206.0000000	0.1200000
-26205.0000000	0.1200000
-26204.0000000	0.1210000
-26203.0000000	0.1205000
-26202.0000000	0.1219047
-26201.0000000	0.1200000
-26200.0000000	0.1220000
-26199.0000000	0.1205000
-26198.0000000	0.1200000
-26197.0000000	0.1200000
-26196.0000000	0.1205000
-26195.0000000	0.1210000
-26194.0000000	0.1215000
-26193.0000000	0.1214285
-26192.0000000	0.1215000
-26191.0000000	0.1205000
-26190.0000000	0.1209524
-26189.0000000	0.1220000
-26188.0000000	0.1200000

-26187.0000000	0.1210000
-26186.0000000	0.1205000
-26185.0000000	0.1215000
-26184.0000000	0.1219047
-26183.0000000	0.1205000
-26182.0000000	0.1200000
-26181.0000000	0.1214286
-26180.0000000	0.1220000
-26179.0000000	0.1210000
-26178.0000000	0.1215000
-26177.0000000	0.1219048
-26176.0000000	0.1215000
-26175.0000000	0.1228571
-26174.0000000	0.1220000
-26173.0000000	0.1240000
-26172.0000000	0.1228571
-26171.0000000	0.1245000
-26170.0000000	0.1235000
-26169.0000000	0.1235000
-26168.0000000	0.1223809
-26167.0000000	0.1230000
-26166.0000000	0.1238095
-26165.0000000	0.1245000
-26164.0000000	0.1230000
-26163.0000000	0.1214286
-26162.0000000	0.1230000
-26161.0000000	0.1230000
-26160.0000000	0.1240000
-26159.0000000	0.1223809
-26158.0000000	0.1240000
-26157.0000000	0.1228571
-26156.0000000	0.1250000
-26155.0000000	0.1235000
-26154.0000000	0.1238095
-26153.0000000	0.1265000
-26152.0000000	0.1270000
-26151.0000000	0.1271428
-26150.0000000	0.1245000
-26149.0000000	0.1265000
-26148.0000000	0.1240000
-26147.0000000	0.1252381
-26146.0000000	0.1265000
-26145.0000000	0.1240000
-26144.0000000	0.1257143
-26143.0000000	0.1265000
-26142.0000000	0.1266667
-26141.0000000	0.1265000
-26140.0000000	0.1265000
-26139.0000000	0.1240000
-26138.0000000	0.1261905
-26137.0000000	0.1270000
-26136.0000000	0.1261905
-26135.0000000	0.1250000
-26134.0000000	0.1240000
-26133.0000000	0.1250000
-26132.0000000	0.1252381
-26131.0000000	0.1235000

-26130.0000000	0.1260000
-26129.0000000	0.1252381
-26128.0000000	0.1230000
-26127.0000000	0.1228571
-26126.0000000	0.1245000
-26125.0000000	0.1240000
-26124.0000000	0.1266667
-26123.0000000	0.1260000
-26122.0000000	0.1245000
-26121.0000000	0.1240000
-26120.0000000	0.1271429
-26119.0000000	0.1250000
-26118.0000000	0.1260000
-26117.0000000	0.1276191
-26116.0000000	0.1270000
-26115.0000000	0.1271429
-26114.0000000	0.1280000
-26113.0000000	0.1285000
-26112.0000000	0.1280953
-26111.0000000	0.1285000
-26110.0000000	0.1290000
-26109.0000000	0.1275000
-26108.0000000	0.1290476
-26107.0000000	0.1290000
-26106.0000000	0.1280953
-26105.0000000	0.1285000
-26104.0000000	0.1285000
-26103.0000000	0.1290000
-26102.0000000	0.1285714
-26101.0000000	0.1285000
-26100.0000000	0.1295000
-26099.0000000	0.1261905
-26098.0000000	0.1270000
-26097.0000000	0.1266667
-26096.0000000	0.1275000
-26095.0000000	0.1255000
-26094.0000000	0.1280000
-26093.0000000	0.1276190
-26092.0000000	0.1280000
-26091.0000000	0.1270000
-26090.0000000	0.1280952
-26089.0000000	0.1255000
-26088.0000000	0.1255000
-26087.0000000	0.1271429
-26086.0000000	0.1275000
-26085.0000000	0.1240000
-26084.0000000	0.1276190
-26083.0000000	0.1260000
-26082.0000000	0.1247619
-26081.0000000	0.1250000
-26080.0000000	0.1250000
-26079.0000000	0.1235000
-26078.0000000	0.1223809
-26077.0000000	0.1250000
-26076.0000000	0.1220000
-26075.0000000	0.1219047
-26074.0000000	0.1225000

-26073.0000000	0.1230000
-26072.0000000	0.1223809
-26071.0000000	0.1225000
-26070.0000000	0.1240000
-26069.0000000	0.1223809
-26068.0000000	0.1235000
-26067.0000000	0.1219047
-26066.0000000	0.1205000
-26065.0000000	0.1220000
-26064.0000000	0.1200000
-26063.0000000	0.1223809
-26062.0000000	0.1200000
-26061.0000000	0.1205000
-26060.0000000	0.1219047
-26059.0000000	0.1205000
-26058.0000000	0.1210000
-26057.0000000	0.1220000
-26056.0000000	0.1223809
-26055.0000000	0.1220000
-26054.0000000	0.1214286
-26053.0000000	0.1235000
-26052.0000000	0.1220000
-26051.0000000	0.1219047
-26050.0000000	0.1215000
-26049.0000000	0.1225000
-26048.0000000	0.1219047
-26047.0000000	0.1225000
-26046.0000000	0.1215000
-26045.0000000	0.1223809
-26044.0000000	0.1205000
-26043.0000000	0.1205000
-26042.0000000	0.1204762
-26041.0000000	0.1210000
-26040.0000000	0.1200000
-26039.0000000	0.1200000
-26038.0000000	0.1205000
-26037.0000000	0.1200000
-26036.0000000	0.1200000
-26035.0000000	0.1200000
-26034.0000000	0.1205000
-26033.0000000	0.1204762
-26032.0000000	0.1200000
-26031.0000000	0.1210000
-26030.0000000	0.1204762
-26029.0000000	0.1205000
-26028.0000000	0.1200000
-26027.0000000	0.1200000
-26026.0000000	0.1200000
-26025.0000000	0.1200000
-26024.0000000	0.1200000
-26023.0000000	0.1200000
-26022.0000000	0.1205000
-26021.0000000	0.1200000
-26020.0000000	0.1200000
-26019.0000000	0.1200000
-26018.0000000	0.1200000
-26017.0000000	0.1200000

-26016.0000000	0.1200000
-26015.0000000	0.1204762
-26014.0000000	0.1205000
-26013.0000000	0.1205000
-26012.0000000	0.1215000
-26011.0000000	0.1200000
-26010.0000000	0.1200000
-26009.0000000	0.1200000
-26008.0000000	0.1200000
-26007.0000000	0.1200000
-26006.0000000	0.1204762
-26005.0000000	0.1200000
-26004.0000000	0.1200000
-26003.0000000	0.1200000
-26002.0000000	0.1200000
-26001.0000000	0.1200000
-26000.0000000	0.1200000
-25999.0000000	0.1205000
-25998.0000000	0.1205000
-25997.0000000	0.1205000
-25996.0000000	0.1204762
-25995.0000000	0.1205000
-25994.0000000	0.1204762
-25993.0000000	0.1205000
-25992.0000000	0.1205000
-25991.0000000	0.1209524
-25990.0000000	0.1205000
-25989.0000000	0.1210000
-25988.0000000	0.1205000
-25987.0000000	0.1200000
-25986.0000000	0.1210000
-25985.0000000	0.1204762
-25984.0000000	0.1205000
-25983.0000000	0.1200000
-25982.0000000	0.1200000
-25981.0000000	0.1209524
-25980.0000000	0.1200000
-25979.0000000	0.1204762
-25978.0000000	0.1200000
-25977.0000000	0.1205000
-25976.0000000	0.1209524
-25975.0000000	0.1205000
-25974.0000000	0.1210000
-25973.0000000	0.1205000
-25972.0000000	0.1200000
-25971.0000000	0.1200000
-25970.0000000	0.1200000
-25969.0000000	0.1200000
-25968.0000000	0.1200000
-25967.0000000	0.1205000
-25966.0000000	0.1200000
-25965.0000000	0.1200000
-25964.0000000	0.1205000
-25963.0000000	0.1209524
-25962.0000000	0.1205000
-25961.0000000	0.1214286
-25960.0000000	0.1220000

-25959.0000000	0.1220000
-25958.0000000	0.1205000
-25957.0000000	0.1204762
-25956.0000000	0.1200000
-25955.0000000	0.1205000
-25954.0000000	0.1209524
-25953.0000000	0.1200000
-25952.0000000	0.1200000
-25951.0000000	0.1209524
-25950.0000000	0.1210000
-25949.0000000	0.1214285
-25948.0000000	0.1220000
-25947.0000000	0.1215000
-25946.0000000	0.1204762
-25945.0000000	0.1200000
-25944.0000000	0.1215000
-25943.0000000	0.1205000
-25942.0000000	0.1204762
-25941.0000000	0.1220000
-25940.0000000	0.1209524
-25939.0000000	0.1200000
-25938.0000000	0.1205000
-25937.0000000	0.1210000
-25936.0000000	0.1223809
-25935.0000000	0.1210000
-25934.0000000	0.1225000
-25933.0000000	0.1214286
-25932.0000000	0.1215000
-25931.0000000	0.1219047
-25930.0000000	0.1220000
-25929.0000000	0.1210000
-25928.0000000	0.1225000
-25927.0000000	0.1228571
-25926.0000000	0.1225000
-25925.0000000	0.1230000
-25924.0000000	0.1233333
-25923.0000000	0.1230000
-25922.0000000	0.1235000
-25921.0000000	0.1252381
-25920.0000000	0.1235000
-25919.0000000	0.1250000
-25918.0000000	0.1233333
-25917.0000000	0.1260000
-25916.0000000	0.1275000
-25915.0000000	0.1261905
-25914.0000000	0.1270000
-25913.0000000	0.1250000
-25912.0000000	0.1261905
-25911.0000000	0.1280000
-25910.0000000	0.1276191
-25909.0000000	0.1285000
-25908.0000000	0.1275000
-25907.0000000	0.1295000
-25906.0000000	0.1290476
-25905.0000000	0.1280000
-25904.0000000	0.1280000
-25903.0000000	0.1290476

-25902.0000000	0.1300000
-25901.0000000	0.1290476
-25900.0000000	0.1280000
-25899.0000000	0.1295000
-25898.0000000	0.1285000
-25897.0000000	0.1295238
-25896.0000000	0.1295000
-25895.0000000	0.1285000
-25894.0000000	0.1290476
-25893.0000000	0.1285000
-25892.0000000	0.1300000
-25891.0000000	0.1295000
-25890.0000000	0.1300000
-25889.0000000	0.1295000
-25888.0000000	0.1295238
-25887.0000000	0.1300000
-25886.0000000	0.1300000
-25885.0000000	0.1295238
-25884.0000000	0.1300000
-25883.0000000	0.1300000
-25882.0000000	0.1295000
-25881.0000000	0.1295238
-25880.0000000	0.1290476
-25879.0000000	0.1300000
-25878.0000000	0.1300000
-25877.0000000	0.1305000
-25876.0000000	0.1295238
-25875.0000000	0.1300000
-25874.0000000	0.1300000
-25873.0000000	0.1300000
-25872.0000000	0.1295238
-25871.0000000	0.1300000
-25870.0000000	0.1300000
-25869.0000000	0.1300000
-25868.0000000	0.1290000
-25867.0000000	0.1300000
-25866.0000000	0.1295000
-25865.0000000	0.1295000
-25864.0000000	0.1295238
-25863.0000000	0.1295000
-25862.0000000	0.1295000
-25861.0000000	0.1295000
-25860.0000000	0.1300000
-25859.0000000	0.1300000
-25858.0000000	0.1300000
-25857.0000000	0.1300000
-25856.0000000	0.1300000
-25855.0000000	0.1300000
-25854.0000000	0.1305000
-25853.0000000	0.1300000
-25852.0000000	0.1300000
-25851.0000000	0.1300000
-25850.0000000	0.1300000
-25849.0000000	0.1300000
-25848.0000000	0.1300000
-25847.0000000	0.1300000
-25846.0000000	0.1300000

-25845.0000000	0.1300000
-25844.0000000	0.1300000
-25843.0000000	0.1300000
-25842.0000000	0.1300000
-25841.0000000	0.1300000
-25840.0000000	0.1300000
-25839.0000000	0.1300000
-25838.0000000	0.1300000
-25837.0000000	0.1304762
-25836.0000000	0.1300000
-25835.0000000	0.1300000
-25834.0000000	0.1300000
-25833.0000000	0.1309524
-25832.0000000	0.1310000
-25831.0000000	0.1310000
-25830.0000000	0.1304762
-25829.0000000	0.1300000
-25828.0000000	0.1304762
-25827.0000000	0.1315000
-25826.0000000	0.1325000
-25825.0000000	0.1304762
-25824.0000000	0.1310000
-25823.0000000	0.1315000
-25822.0000000	0.1310000
-25821.0000000	0.1314286
-25820.0000000	0.1310000
-25819.0000000	0.1309524
-25818.0000000	0.1305000
-25817.0000000	0.1300000
-25816.0000000	0.1325000
-25815.0000000	0.1300000
-25814.0000000	0.1305000
-25813.0000000	0.1310000
-25812.0000000	0.1304762
-25811.0000000	0.1300000
-25810.0000000	0.1300000
-25809.0000000	0.1310000
-25808.0000000	0.1315000
-25807.0000000	0.1304762
-25806.0000000	0.1300000
-25805.0000000	0.1310000
-25804.0000000	0.1300000
-25803.0000000	0.1300000
-25802.0000000	0.1300000
-25801.0000000	0.1309524
-25800.0000000	0.1300000
-25799.0000000	0.1300000
-25798.0000000	0.1305000
-25797.0000000	0.1300000
-25796.0000000	0.1300000
-25795.0000000	0.1300000
-25794.0000000	0.1300000
-25793.0000000	0.1305000
-25792.0000000	0.1300000
-25791.0000000	0.1305000
-25790.0000000	0.1300000
-25789.0000000	0.1300000

-25788.0000000	0.1300000
-25787.0000000	0.1300000
-25786.0000000	0.1300000
-25785.0000000	0.1300000
-25784.0000000	0.1300000
-25783.0000000	0.1300000
-25782.0000000	0.1295000
-25781.0000000	0.1300000
-25780.0000000	0.1300000
-25779.0000000	0.1300000
-25778.0000000	0.1300000
-25777.0000000	0.1300000
-25776.0000000	0.1305000
-25775.0000000	0.1300000
-25774.0000000	0.1300000
-25773.0000000	0.1300000
-25772.0000000	0.1300000
-25771.0000000	0.1300000
-25770.0000000	0.1300000
-25769.0000000	0.1300000
-25768.0000000	0.1300000
-25767.0000000	0.1300000
-25766.0000000	0.1300000
-25765.0000000	0.1300000
-25764.0000000	0.1300000
-25763.0000000	0.1300000
-25762.0000000	0.1300000
-25761.0000000	0.1300000
-25760.0000000	0.1300000
-25759.0000000	0.1300000
-25758.0000000	0.1304762
-25757.0000000	0.1304762
-25756.0000000	0.1310000
-25755.0000000	0.1300000
-25754.0000000	0.1300000
-25753.0000000	0.1309524
-25752.0000000	0.1300000
-25751.0000000	0.1310000
-25750.0000000	0.1304762
-25749.0000000	0.1315000
-25748.0000000	0.1319048
-25747.0000000	0.1330000
-25746.0000000	0.1315000
-25745.0000000	0.1325000
-25744.0000000	0.1342857
-25743.0000000	0.1310000
-25742.0000000	0.1330000
-25741.0000000	0.1319048
-25740.0000000	0.1335000
-25739.0000000	0.1325000
-25738.0000000	0.1330000
-25737.0000000	0.1328571
-25736.0000000	0.1315000
-25735.0000000	0.1314286
-25734.0000000	0.1310000
-25733.0000000	0.1310000
-25732.0000000	0.1314286

-25731.0000000	0.1310000
-25730.0000000	0.1305000
-25729.0000000	0.1310000
-25728.0000000	0.1304762
-25727.0000000	0.1300000
-25726.0000000	0.1309524
-25725.0000000	0.1315000
-25724.0000000	0.1305000
-25723.0000000	0.1304762
-25722.0000000	0.1310000
-25721.0000000	0.1305000
-25720.0000000	0.1300000
-25719.0000000	0.1300000
-25718.0000000	0.1300000
-25717.0000000	0.1304762
-25716.0000000	0.1300000
-25715.0000000	0.1300000
-25714.0000000	0.1295238
-25713.0000000	0.1300000
-25712.0000000	0.1300000
-25711.0000000	0.1300000
-25710.0000000	0.1300000
-25709.0000000	0.1295000
-25708.0000000	0.1295000
-25707.0000000	0.1300000
-25706.0000000	0.1300000
-25705.0000000	0.1295000
-25704.0000000	0.1295238
-25703.0000000	0.1300000
-25702.0000000	0.1300000
-25701.0000000	0.1300000
-25700.0000000	0.1295000
-25699.0000000	0.1300000
-25698.0000000	0.1285714
-25697.0000000	0.1285000
-25696.0000000	0.1295238
-25695.0000000	0.1290000
-25694.0000000	0.1285000
-25693.0000000	0.1280000
-25692.0000000	0.1276191
-25691.0000000	0.1290000
-25690.0000000	0.1295238
-25689.0000000	0.1290000
-25688.0000000	0.1300000
-25687.0000000	0.1300000
-25686.0000000	0.1300000
-25685.0000000	0.1295000
-25684.0000000	0.1295238
-25683.0000000	0.1278947
-25682.0000000	0.1276191
-25681.0000000	0.1242857
-25680.0000000	0.1255000
-25679.0000000	0.1250000
-25678.0000000	0.1245000
-25677.0000000	0.1233333
-25676.0000000	0.1235000
-25675.0000000	0.1252381

-25674.0000000	0.1250000
-25673.0000000	0.1255000
-25672.0000000	0.1261905
-25671.0000000	0.1260000
-25670.0000000	0.1265000
-25669.0000000	0.1245000
-25668.0000000	0.1242857
-25667.0000000	0.1240000
-25666.0000000	0.1228571
-25665.0000000	0.1245000
-25664.0000000	0.1235000
-25663.0000000	0.1210000
-25662.0000000	0.1233333
-25661.0000000	0.1265000
-25660.0000000	0.1240000
-25659.0000000	0.1233333
-25658.0000000	0.1245000
-25657.0000000	0.1252381
-25656.0000000	0.1255000
-25655.0000000	0.1260000
-25654.0000000	0.1230000
-25653.0000000	0.1233333
-25652.0000000	0.1240000
-25651.0000000	0.1247619
-25650.0000000	0.1235000
-25649.0000000	0.1235000
-25648.0000000	0.1220000
-25647.0000000	0.1238095
-25646.0000000	0.1235000
-25645.0000000	0.1235000
-25644.0000000	0.1219047
-25643.0000000	0.1215000
-25642.0000000	0.1209524
-25641.0000000	0.1220000
-25640.0000000	0.1210000
-25639.0000000	0.1215000
-25638.0000000	0.1204762
-25637.0000000	0.1205000
-25636.0000000	0.1205000
-25635.0000000	0.1200000
-25634.0000000	0.1205000
-25633.0000000	0.1210000
-25632.0000000	0.1200000
-25631.0000000	0.1200000
-25630.0000000	0.1200000
-25629.0000000	0.1204762
-25628.0000000	0.1205000
-25627.0000000	0.1200000
-25626.0000000	0.1204762
-25625.0000000	0.1200000
-25624.0000000	0.1205000
-25623.0000000	0.1204762
-25622.0000000	0.1205000
-25621.0000000	0.1200000
-25620.0000000	0.1205000
-25619.0000000	0.1200000
-25618.0000000	0.1200000

-25617.0000000	0.1200000
-25616.0000000	0.1200000
-25615.0000000	0.1200000
-25614.0000000	0.1200000
-25613.0000000	0.1200000
-25612.0000000	0.1200000
-25611.0000000	0.1200000
-25610.0000000	0.1200000
-25609.0000000	0.1200000
-25608.0000000	0.1200000
-25607.0000000	0.1200000
-25606.0000000	0.1200000
-25605.0000000	0.1204762
-25604.0000000	0.1205000
-25603.0000000	0.1205000
-25602.0000000	0.1209524
-25601.0000000	0.1205000
-25600.0000000	0.1205000
-25599.0000000	0.1200000
-25598.0000000	0.1225000
-25597.0000000	0.1200000
-25596.0000000	0.1219047
-25595.0000000	0.1210000
-25594.0000000	0.1200000
-25593.0000000	0.1200000
-25592.0000000	0.1200000
-25591.0000000	0.1200000
-25590.0000000	0.1200000
-25589.0000000	0.1200000
-25588.0000000	0.1200000
-25587.0000000	0.1200000
-25586.0000000	0.1200000
-25585.0000000	0.1200000
-25584.0000000	0.1200000
-25583.0000000	0.1200000
-25582.0000000	0.1200000
-25581.0000000	0.1200000
-25580.0000000	0.1200000
-25579.0000000	0.1200000
-25578.0000000	0.1200000
-25577.0000000	0.1200000
-25576.0000000	0.1200000
-25575.0000000	0.1200000
-25574.0000000	0.1205000
-25573.0000000	0.1205000
-25572.0000000	0.1205000
-25571.0000000	0.1200000
-25570.0000000	0.1200000
-25569.0000000	0.1200000
-25568.0000000	0.1205000
-25567.0000000	0.1200000
-25566.0000000	0.1200000
-25565.0000000	0.1200000
-25564.0000000	0.1220000
-25563.0000000	0.1205000
-25562.0000000	0.1214286
-25561.0000000	0.1215000

-25560.0000000	0.1209524
-25559.0000000	0.1205000
-25558.0000000	0.1210000
-25557.0000000	0.1219048
-25556.0000000	0.1220000
-25555.0000000	0.1220000
-25554.0000000	0.1225000
-25553.0000000	0.1223809
-25552.0000000	0.1238095
-25551.0000000	0.1230000
-25550.0000000	0.1220000
-25549.0000000	0.1220000
-25548.0000000	0.1223809
-25547.0000000	0.1220000
-25546.0000000	0.1215000
-25545.0000000	0.1214286
-25544.0000000	0.1215000
-25543.0000000	0.1220000
-25542.0000000	0.1220000
-25541.0000000	0.1219047
-25540.0000000	0.1230000
-25539.0000000	0.1228571
-25538.0000000	0.1235000
-25537.0000000	0.1215000
-25536.0000000	0.1223809
-25535.0000000	0.1205000
-25534.0000000	0.1200000
-25533.0000000	0.1205000
-25532.0000000	0.1204762
-25531.0000000	0.1220000
-25530.0000000	0.1204762
-25529.0000000	0.1210000
-25528.0000000	0.1215000
-25527.0000000	0.1200000
-25526.0000000	0.1215000
-25525.0000000	0.1205000
-25524.0000000	0.1210000
-25523.0000000	0.1200000
-25522.0000000	0.1200000
-25521.0000000	0.1204762
-25520.0000000	0.1200000
-25519.0000000	0.1200000
-25518.0000000	0.1200000
-25517.0000000	0.1200000
-25516.0000000	0.1200000
-25515.0000000	0.1200000
-25514.0000000	0.1200000
-25513.0000000	0.1200000
-25512.0000000	0.1200000
-25511.0000000	0.1200000
-25510.0000000	0.1200000
-25509.0000000	0.1200000
-25508.0000000	0.1200000
-25507.0000000	0.1200000
-25506.0000000	0.1200000
-25505.0000000	0.1195238
-25504.0000000	0.1190000

-25503.0000000	0.1195000
-25502.0000000	0.1185714
-25501.0000000	0.1185000
-25500.0000000	0.1166666
-25499.0000000	0.1185000
-25498.0000000	0.1195000
-25497.0000000	0.1185000
-25496.0000000	0.1185714
-25495.0000000	0.1190000
-25494.0000000	0.1190000
-25493.0000000	0.1195238
-25492.0000000	0.1200000
-25491.0000000	0.1200000
-25490.0000000	0.1190000
-25489.0000000	0.1195000
-25488.0000000	0.1195000
-25487.0000000	0.1190476
-25486.0000000	0.1200000
-25485.0000000	0.1195000
-25484.0000000	0.1200000
-25483.0000000	0.1195000
-25482.0000000	0.1200000
-25481.0000000	0.1200000
-25480.0000000	0.1200000
-25479.0000000	0.1185714
-25478.0000000	0.1195000
-25477.0000000	0.1200000
-25476.0000000	0.1195000
-25475.0000000	0.1195238
-25474.0000000	0.1190000
-25473.0000000	0.1190000
-25472.0000000	0.1171428
-25471.0000000	0.1185000
-25470.0000000	0.1185714
-25469.0000000	0.1165000
-25468.0000000	0.1140000
-25467.0000000	0.1155000
-25466.0000000	0.1142857
-25465.0000000	0.1115000
-25464.0000000	0.1110000
-25463.0000000	0.1123809
-25462.0000000	0.1120000
-25461.0000000	0.1123809
-25460.0000000	0.1120000
-25459.0000000	0.1120000
-25458.0000000	0.1150000
-25457.0000000	0.1119047
-25456.0000000	0.1125000
-25455.0000000	0.1125000
-25454.0000000	0.1123809
-25453.0000000	0.1135000
-25452.0000000	0.1120000
-25451.0000000	0.1130000
-25450.0000000	0.1123809
-25449.0000000	0.1110000
-25448.0000000	0.1109524
-25447.0000000	0.1105000

-25446.0000000	0.1120000
-25445.0000000	0.1114286
-25444.0000000	0.1115000
-25443.0000000	0.1120000
-25442.0000000	0.1120000
-25441.0000000	0.1123809
-25440.0000000	0.1100000
-25439.0000000	0.1109524
-25438.0000000	0.1120000
-25437.0000000	0.1110000
-25436.0000000	0.1109524
-25435.0000000	0.1105000
-25434.0000000	0.1110000
-25433.0000000	0.1119047
-25432.0000000	0.1125000
-25431.0000000	0.1109524
-25430.0000000	0.1115000
-25429.0000000	0.1115000
-25428.0000000	0.1125000
-25427.0000000	0.1104762
-25426.0000000	0.1110000
-25425.0000000	0.1105000
-25424.0000000	0.1123809
-25423.0000000	0.1120000
-25422.0000000	0.1110000
-25421.0000000	0.1110000
-25420.0000000	0.1119047
-25419.0000000	0.1115000
-25418.0000000	0.1114286
-25417.0000000	0.1110000
-25416.0000000	0.1130000
-25415.0000000	0.1114286
-25414.0000000	0.1130000
-25413.0000000	0.1125000
-25412.0000000	0.1125000
-25411.0000000	0.1123809
-25410.0000000	0.1135000
-25409.0000000	0.1123809
-25408.0000000	0.1145000
-25407.0000000	0.1140000
-25406.0000000	0.1133333
-25405.0000000	0.1140000
-25404.0000000	0.1150000
-25403.0000000	0.1138095
-25402.0000000	0.1140000
-25401.0000000	0.1155000
-25400.0000000	0.1123809
-25399.0000000	0.1140000
-25398.0000000	0.1140000
-25397.0000000	0.1133333
-25396.0000000	0.1155000
-25395.0000000	0.1120000
-25394.0000000	0.1138095
-25393.0000000	0.1140000
-25392.0000000	0.1125000
-25391.0000000	0.1135000
-25390.0000000	0.1119047

-25389.0000000	0.1120000
-25388.0000000	0.1123809
-25387.0000000	0.1125000
-25386.0000000	0.1120000
-25385.0000000	0.1119047
-25384.0000000	0.1120000
-25383.0000000	0.1120000
-25382.0000000	0.1115000
-25381.0000000	0.1114286
-25380.0000000	0.1105000
-25379.0000000	0.1109524
-25378.0000000	0.1115000
-25377.0000000	0.1120000
-25376.0000000	0.1130000
-25375.0000000	0.1109524
-25374.0000000	0.1110000
-25373.0000000	0.1105000
-25372.0000000	0.1119047
-25371.0000000	0.1100000
-25370.0000000	0.1100000
-25369.0000000	0.1105000
-25368.0000000	0.1100000
-25367.0000000	0.1100000
-25366.0000000	0.1100000
-25365.0000000	0.1100000
-25364.0000000	0.1100000
-25363.0000000	0.1095000
-25362.0000000	0.1100000
-25361.0000000	0.1100000
-25360.0000000	0.1095238
-25359.0000000	0.1090000
-25358.0000000	0.1100000
-25357.0000000	0.1100000
-25356.0000000	0.1085000
-25355.0000000	0.1095238
-25354.0000000	0.1095000
-25353.0000000	0.1090000
-25352.0000000	0.1075000
-25351.0000000	0.1080952
-25350.0000000	0.1075000
-25349.0000000	0.1080952
-25348.0000000	0.1085000
-25347.0000000	0.1090000
-25346.0000000	0.1080000
-25345.0000000	0.1090476
-25344.0000000	0.1070000
-25343.0000000	0.1090000
-25342.0000000	0.1071429
-25341.0000000	0.1060000
-25340.0000000	0.1085714
-25339.0000000	0.1085000
-25338.0000000	0.1075000
-25337.0000000	0.1080000
-25336.0000000	0.1076190
-25335.0000000	0.1060000
-25334.0000000	0.1085714
-25333.0000000	0.1085000

-25332.0000000	0.1085000
-25331.0000000	0.1090000
-25330.0000000	0.1080952
-25329.0000000	0.1090000
-25328.0000000	0.1085000
-25327.0000000	0.1095238
-25326.0000000	0.1090000
-25325.0000000	0.1100000
-25324.0000000	0.1090000
-25323.0000000	0.1080000
-25322.0000000	0.1095000
-25321.0000000	0.1080952
-25320.0000000	0.1090000
-25319.0000000	0.1085000
-25318.0000000	0.1090476
-25317.0000000	0.1085000
-25316.0000000	0.1085000
-25315.0000000	0.1085714
-25314.0000000	0.1080000
-25313.0000000	0.1095000
-25312.0000000	0.1080952
-25311.0000000	0.1075000
-25310.0000000	0.1080000
-25309.0000000	0.1076190
-25308.0000000	0.1090000
-25307.0000000	0.1080000
-25306.0000000	0.1071429
-25305.0000000	0.1085000
-25304.0000000	0.1066667
-25303.0000000	0.1030000
-25302.0000000	0.1045000
-25301.0000000	0.1040000
-25300.0000000	0.1028572
-25299.0000000	0.1030000
-25298.0000000	0.1020000
-25297.0000000	0.1009524
-25296.0000000	0.1015000
-25295.0000000	0.1009524
-25294.0000000	0.1010000
-25293.0000000	0.1000000
-25292.0000000	0.1010000
-25291.0000000	0.1009524
-25290.0000000	0.1010000
-25289.0000000	0.1005000
-25288.0000000	0.1019048
-25287.0000000	0.1010000
-25286.0000000	0.1005000
-25285.0000000	0.1009524
-25284.0000000	0.1000000
-25283.0000000	0.1000000
-25282.0000000	0.1019048
-25281.0000000	0.1005000
-25280.0000000	0.1015000
-25279.0000000	0.1004762
-25278.0000000	0.1005000
-25277.0000000	0.1005000
-25276.0000000	0.1000000

-25275.0000000	0.1000000
-25274.0000000	0.1000000
-25273.0000000	0.1000000
-25272.0000000	0.1000000
-25271.0000000	0.1000000
-25270.0000000	0.1000000
-25269.0000000	0.1000000
-25268.0000000	0.1000000
-25267.0000000	0.1000000
-25266.0000000	0.1000000
-25265.0000000	0.1000000
-25264.0000000	0.1000000
-25263.0000000	0.1000000
-25262.0000000	0.1000000
-25261.0000000	0.1000000
-25260.0000000	0.1005000
-25259.0000000	0.1000000
-25258.0000000	0.1000000
-25257.0000000	0.1005000
-25256.0000000	0.1000000
-25255.0000000	0.1000000
-25254.0000000	0.1000000
-25253.0000000	0.1000000
-25252.0000000	0.1000000
-25251.0000000	0.1000000
-25250.0000000	0.1000000
-25249.0000000	0.1000000
-25248.0000000	0.1000000
-25247.0000000	0.1000000
-25246.0000000	0.1000000
-25245.0000000	0.1000000
-25244.0000000	0.1000000
-25243.0000000	0.1000000
-25242.0000000	0.1000000
-25241.0000000	0.1000000
-25240.0000000	0.0995000
-25239.0000000	0.0995238
-25238.0000000	0.0995000
-25237.0000000	0.1000000
-25236.0000000	0.0990476
-25235.0000000	0.0995238
-25234.0000000	0.0995000
-25233.0000000	0.1000000
-25232.0000000	0.1000000
-25231.0000000	0.0985714
-25230.0000000	0.0995000
-25229.0000000	0.0990000
-25228.0000000	0.0995238
-25227.0000000	0.0990000
-25226.0000000	0.0960000
-25225.0000000	0.0970000
-25224.0000000	0.0980952
-25223.0000000	0.0945000
-25222.0000000	0.0957143
-25221.0000000	0.0925000
-25220.0000000	0.0945000
-25219.0000000	0.0938095

-25218.0000000	0.0945000
-25217.0000000	0.0950000
-25216.0000000	0.0930000
-25215.0000000	0.0919048
-25214.0000000	0.0935000
-25213.0000000	0.0923810
-25212.0000000	0.0900000
-25211.0000000	0.0915000
-25210.0000000	0.0915000
-25209.0000000	0.0923810
-25208.0000000	0.0915000
-25207.0000000	0.0915000
-25206.0000000	0.0923810
-25205.0000000	0.0925000
-25204.0000000	0.0923810
-25203.0000000	0.0915000
-25202.0000000	0.0925000
-25201.0000000	0.0942857
-25200.0000000	0.0930000
-25199.0000000	0.0930000
-25198.0000000	0.0920000
-25197.0000000	0.0938095
-25196.0000000	0.0960000
-25195.0000000	0.0955000
-25194.0000000	0.0961905
-25193.0000000	0.0965000
-25192.0000000	0.0957143
-25191.0000000	0.0965000
-25190.0000000	0.0965000
-25189.0000000	0.0955000
-25188.0000000	0.0961905
-25187.0000000	0.0955000
-25186.0000000	0.0940000
-25185.0000000	0.0952381
-25184.0000000	0.0955000
-25183.0000000	0.0961905
-25182.0000000	0.0970000
-25181.0000000	0.0965000
-25180.0000000	0.0960000
-25179.0000000	0.0976191
-25178.0000000	0.0945000
-25177.0000000	0.0955000
-25176.0000000	0.0938095
-25175.0000000	0.0935000
-25174.0000000	0.0952381
-25173.0000000	0.0950000
-25172.0000000	0.0935000
-25171.0000000	0.0940000
-25170.0000000	0.0942857
-25169.0000000	0.0925000
-25168.0000000	0.0915000
-25167.0000000	0.0909524
-25166.0000000	0.0910000
-25165.0000000	0.0910000
-25164.0000000	0.0900000
-25163.0000000	0.0900000
-25162.0000000	0.0909524

-25161.0000000	0.0900000
-25160.0000000	0.0905000
-25159.0000000	0.0905000
-25158.0000000	0.0900000
-25157.0000000	0.0910000
-25156.0000000	0.0905000
-25155.0000000	0.0915000
-25154.0000000	0.0923810
-25153.0000000	0.0923810
-25152.0000000	0.0940000
-25151.0000000	0.0930000
-25150.0000000	0.0920000
-25149.0000000	0.0933333
-25148.0000000	0.0930000
-25147.0000000	0.0945000
-25146.0000000	0.0933333
-25145.0000000	0.0940000
-25144.0000000	0.0933333
-25143.0000000	0.0930000
-25142.0000000	0.0930000
-25141.0000000	0.0940000
-25140.0000000	0.0919048
-25139.0000000	0.0930000
-25138.0000000	0.0930000
-25137.0000000	0.0961905
-25136.0000000	0.0930000
-25135.0000000	0.0945000
-25134.0000000	0.0960000
-25133.0000000	0.0966667
-25132.0000000	0.0935000
-25131.0000000	0.0952381
-25130.0000000	0.0960000
-25129.0000000	0.0930000
-25128.0000000	0.0961905
-25127.0000000	0.0950000
-25126.0000000	0.0965000
-25125.0000000	0.0970000
-25124.0000000	0.0966667
-25123.0000000	0.0975000
-25122.0000000	0.0990476
-25121.0000000	0.0970000
-25120.0000000	0.0985000
-25119.0000000	0.0976191
-25118.0000000	0.0985000
-25117.0000000	0.0985000
-25116.0000000	0.0980953
-25115.0000000	0.0980000
-25114.0000000	0.0971429
-25113.0000000	0.0975000
-25112.0000000	0.0975000
-25111.0000000	0.0985000
-25110.0000000	0.0990476
-25109.0000000	0.0985000
-25108.0000000	0.0975000
-25107.0000000	0.0995238
-25106.0000000	0.0995000
-25105.0000000	0.0985000

-25104.0000000	0.0985000
-25103.0000000	0.0995238
-25102.0000000	0.0985000
-25101.0000000	0.0985714
-25100.0000000	0.0985000
-25099.0000000	0.0995000
-25098.0000000	0.0985714
-25097.0000000	0.0995000
-25096.0000000	0.0990000
-25095.0000000	0.1000000
-25094.0000000	0.0990476
-25093.0000000	0.0990000
-25092.0000000	0.0980953
-25091.0000000	0.0990000
-25090.0000000	0.0985000
-25089.0000000	0.0980952
-25088.0000000	0.0990000
-25087.0000000	0.0995000
-25086.0000000	0.0985000
-25085.0000000	0.1000000
-25084.0000000	0.0990000
-25083.0000000	0.1000000
-25082.0000000	0.0985000
-25081.0000000	0.0990000
-25080.0000000	0.1000000
-25079.0000000	0.0995000
-25078.0000000	0.1000000
-25077.0000000	0.0995238
-25076.0000000	0.1000000
-25075.0000000	0.0995000
-25074.0000000	0.1000000
-25073.0000000	0.1000000
-25072.0000000	0.0995000
-25071.0000000	0.0995238
-25070.0000000	0.1000000
-25069.0000000	0.0995000
-25068.0000000	0.1000000
-25067.0000000	0.1000000
-25066.0000000	0.1000000
-25065.0000000	0.1000000
-25064.0000000	0.1000000
-25063.0000000	0.1000000
-25062.0000000	0.1000000
-25061.0000000	0.1000000
-25060.0000000	0.1000000
-25059.0000000	0.1000000
-25058.0000000	0.1000000
-25057.0000000	0.1000000
-25056.0000000	0.1000000
-25055.0000000	0.1000000
-25054.0000000	0.0995000
-25053.0000000	0.1000000
-25052.0000000	0.1000000
-25051.0000000	0.1000000
-25050.0000000	0.1000000
-25049.0000000	0.1000000
-25048.0000000	0.1000000

-25047.0000000	0.1000000
-25046.0000000	0.1000000
-25045.0000000	0.1000000
-25044.0000000	0.1000000
-25043.0000000	0.1000000
-25042.0000000	0.1000000
-25041.0000000	0.1000000
-25040.0000000	0.1000000
-25039.0000000	0.1000000
-25038.0000000	0.1000000
-25037.0000000	0.1000000
-25036.0000000	0.1000000
-25035.0000000	0.1000000
-25034.0000000	0.1000000
-25033.0000000	0.0995000
-25032.0000000	0.1000000
-25031.0000000	0.1000000
-25030.0000000	0.1000000
-25029.0000000	0.1000000
-25028.0000000	0.1000000
-25027.0000000	0.1000000
-25026.0000000	0.1000000
-25025.0000000	0.1000000
-25024.0000000	0.1010000
-25023.0000000	0.1000000
-25022.0000000	0.1000000
-25021.0000000	0.1005000
-25020.0000000	0.1015000
-25019.0000000	0.1014286
-25018.0000000	0.1010000
-25017.0000000	0.1014286
-25016.0000000	0.1030000
-25015.0000000	0.1020000
-25014.0000000	0.1035000
-25013.0000000	0.1014286
-25012.0000000	0.1035000
-25011.0000000	0.1040000
-25010.0000000	0.1028572
-25009.0000000	0.1030000
-25008.0000000	0.1023810
-25007.0000000	0.1040000
-25006.0000000	0.1045000
-25005.0000000	0.1055000
-25004.0000000	0.1038095
-25003.0000000	0.1050000
-25002.0000000	0.1045000
-25001.0000000	0.1071429
-25000.0000000	0.1080000
-24999.0000000	0.1070000
-24998.0000000	0.1085714
-24997.0000000	0.1080000
-24996.0000000	0.1085000
-24995.0000000	0.1076190
-24994.0000000	0.1095000
-24993.0000000	0.1095000
-24992.0000000	0.1100000
-24991.0000000	0.1075000

-24990.0000000	0.1095000
-24989.0000000	0.1090476
-24988.0000000	0.1095000
-24987.0000000	0.1090000
-24986.0000000	0.1100000
-24985.0000000	0.1100000
-24984.0000000	0.1100000
-24983.0000000	0.1100000
-24982.0000000	0.1095000
-24981.0000000	0.1100000
-24980.0000000	0.1100000
-24979.0000000	0.1100000
-24978.0000000	0.1100000
-24977.0000000	0.1100000
-24976.0000000	0.1100000
-24975.0000000	0.1100000
-24974.0000000	0.1100000
-24973.0000000	0.1100000
-24972.0000000	0.1100000
-24971.0000000	0.1100000
-24970.0000000	0.1090000
-24969.0000000	0.1090000
-24968.0000000	0.1100000
-24967.0000000	0.1095238
-24966.0000000	0.1100000
-24965.0000000	0.1100000
-24964.0000000	0.1090000
-24963.0000000	0.1090000
-24962.0000000	0.1100000
-24961.0000000	0.1090000
-24960.0000000	0.1100000
-24959.0000000	0.1100000
-24958.0000000	0.1095238
-24957.0000000	0.1070000
-24956.0000000	0.1100000
-24955.0000000	0.1090000
-24954.0000000	0.1085000
-24953.0000000	0.1080952
-24952.0000000	0.1095000
-24951.0000000	0.1090000
-24950.0000000	0.1095238
-24949.0000000	0.1090000
-24948.0000000	0.1100000
-24947.0000000	0.1100000
-24946.0000000	0.1100000
-24945.0000000	0.1095000
-24944.0000000	0.1100000
-24943.0000000	0.1100000
-24942.0000000	0.1100000
-24941.0000000	0.1100000
-24940.0000000	0.1100000
-24939.0000000	0.1100000
-24938.0000000	0.1100000
-24937.0000000	0.1100000
-24936.0000000	0.1100000
-24935.0000000	0.1104762
-24934.0000000	0.1105000

-24933.0000000	0.1100000
-24932.0000000	0.1104762
-24931.0000000	0.1105000
-24930.0000000	0.1105000
-24929.0000000	0.1110000
-24928.0000000	0.1109524
-24927.0000000	0.1110000
-24926.0000000	0.1100000
-24925.0000000	0.1110000
-24924.0000000	0.1105000
-24923.0000000	0.1105000
-24922.0000000	0.1104762
-24921.0000000	0.1100000
-24920.0000000	0.1100000
-24919.0000000	0.1114286
-24918.0000000	0.1105000
-24917.0000000	0.1104762
-24916.0000000	0.1100000
-24915.0000000	0.1115000
-24914.0000000	0.1114286
-24913.0000000	0.1120000
-24912.0000000	0.1130000
-24911.0000000	0.1115000
-24910.0000000	0.1128571
-24909.0000000	0.1130000
-24908.0000000	0.1110000
-24907.0000000	0.1109524
-24906.0000000	0.1120000
-24905.0000000	0.1119047
-24904.0000000	0.1120000
-24903.0000000	0.1125000
-24902.0000000	0.1130000
-24901.0000000	0.1114286
-24900.0000000	0.1140000
-24899.0000000	0.1140000
-24898.0000000	0.1142857
-24897.0000000	0.1135000
-24896.0000000	0.1147619
-24895.0000000	0.1135000
-24894.0000000	0.1140000
-24893.0000000	0.1160000
-24892.0000000	0.1161905
-24891.0000000	0.1170000
-24890.0000000	0.1175000
-24889.0000000	0.1185714
-24888.0000000	0.1165000
-24887.0000000	0.1195238
-24886.0000000	0.1180000
-24885.0000000	0.1195000
-24884.0000000	0.1195238
-24883.0000000	0.1195000
-24882.0000000	0.1200000
-24881.0000000	0.1190000
-24880.0000000	0.1190476
-24879.0000000	0.1190000
-24878.0000000	0.1195000
-24877.0000000	0.1190000

-24876.0000000	0.1195238
-24875.0000000	0.1190476
-24874.0000000	0.1180000
-24873.0000000	0.1200000
-24872.0000000	0.1185000
-24871.0000000	0.1180952
-24870.0000000	0.1155000
-24869.0000000	0.1170000
-24868.0000000	0.1142857
-24867.0000000	0.1155000
-24866.0000000	0.1128571
-24865.0000000	0.1150000
-24864.0000000	0.1125000
-24863.0000000	0.1135000
-24862.0000000	0.1166666
-24861.0000000	0.1160000
-24860.0000000	0.1180000
-24859.0000000	0.1176190
-24858.0000000	0.1190000
-24857.0000000	0.1171428
-24856.0000000	0.1190000
-24855.0000000	0.1180000
-24854.0000000	0.1185000
-24853.0000000	0.1185714
-24852.0000000	0.1185000
-24851.0000000	0.1190000
-24850.0000000	0.1195238
-24849.0000000	0.1195000
-24848.0000000	0.1200000
-24847.0000000	0.1190000
-24846.0000000	0.1200000
-24845.0000000	0.1195000
-24844.0000000	0.1195238
-24843.0000000	0.1200000
-24842.0000000	0.1200000
-24841.0000000	0.1200000
-24840.0000000	0.1200000
-24839.0000000	0.1200000
-24838.0000000	0.1200000
-24837.0000000	0.1200000
-24836.0000000	0.1200000
-24835.0000000	0.1195238
-24834.0000000	0.1200000
-24833.0000000	0.1200000
-24832.0000000	0.1200000
-24831.0000000	0.1200000
-24830.0000000	0.1200000
-24829.0000000	0.1195238
-24828.0000000	0.1200000
-24827.0000000	0.1200000
-24826.0000000	0.1200000
-24825.0000000	0.1200000
-24824.0000000	0.1200000
-24823.0000000	0.1200000
-24822.0000000	0.1200000
-24821.0000000	0.1190000
-24820.0000000	0.1200000

-24819.0000000	0.1200000
-24818.0000000	0.1200000
-24817.0000000	0.1200000
-24816.0000000	0.1200000
-24815.0000000	0.1200000
-24814.0000000	0.1200000
-24813.0000000	0.1200000
-24812.0000000	0.1200000
-24811.0000000	0.1200000
-24810.0000000	0.1200000
-24809.0000000	0.1200000
-24808.0000000	0.1200000
-24807.0000000	0.1200000
-24806.0000000	0.1200000
-24805.0000000	0.1200000
-24804.0000000	0.1200000
-24803.0000000	0.1200000
-24802.0000000	0.1200000
-24801.0000000	0.1200000
-24800.0000000	0.1200000
-24799.0000000	0.1200000
-24798.0000000	0.1200000
-24797.0000000	0.1200000
-24796.0000000	0.1200000
-24795.0000000	0.1205000
-24794.0000000	0.1210000
-24793.0000000	0.1204762
-24792.0000000	0.1210000
-24791.0000000	0.1200000
-24790.0000000	0.1200000
-24789.0000000	0.1200000
-24788.0000000	0.1200000
-24787.0000000	0.1200000
-24786.0000000	0.1200000
-24785.0000000	0.1200000
-24784.0000000	0.1200000
-24783.0000000	0.1200000
-24782.0000000	0.1200000
-24781.0000000	0.1200000
-24780.0000000	0.1195000
-24779.0000000	0.1200000
-24778.0000000	0.1200000
-24777.0000000	0.1200000
-24776.0000000	0.1200000
-24775.0000000	0.1200000
-24774.0000000	0.1200000
-24773.0000000	0.1200000
-24772.0000000	0.1200000
-24771.0000000	0.1200000
-24770.0000000	0.1200000
-24769.0000000	0.1200000
-24768.0000000	0.1200000
-24767.0000000	0.1200000
-24766.0000000	0.1200000
-24765.0000000	0.1200000
-24764.0000000	0.1200000
-24763.0000000	0.1200000

-24762.0000000	0.1200000
-24761.0000000	0.1200000
-24760.0000000	0.1204762
-24759.0000000	0.1200000
-24758.0000000	0.1200000
-24757.0000000	0.1200000
-24756.0000000	0.1200000
-24755.0000000	0.1200000
-24754.0000000	0.1200000
-24753.0000000	0.1200000
-24752.0000000	0.1195000
-24751.0000000	0.1195238
-24750.0000000	0.1190000
-24749.0000000	0.1195000
-24748.0000000	0.1200000
-24747.0000000	0.1200000
-24746.0000000	0.1200000
-24745.0000000	0.1195238
-24744.0000000	0.1200000
-24743.0000000	0.1200000
-24742.0000000	0.1195000
-24741.0000000	0.1200000
-24740.0000000	0.1200000
-24739.0000000	0.1205000
-24738.0000000	0.1200000
-24737.0000000	0.1200000
-24736.0000000	0.1200000
-24735.0000000	0.1200000
-24734.0000000	0.1200000
-24733.0000000	0.1200000
-24732.0000000	0.1200000
-24731.0000000	0.1200000
-24730.0000000	0.1195238
-24729.0000000	0.1200000
-24728.0000000	0.1200000
-24727.0000000	0.1200000
-24726.0000000	0.1200000
-24725.0000000	0.1200000
-24724.0000000	0.1200000
-24723.0000000	0.1200000
-24722.0000000	0.1200000
-24721.0000000	0.1200000
-24720.0000000	0.1200000
-24719.0000000	0.1205000
-24718.0000000	0.1200000
-24717.0000000	0.1200000
-24716.0000000	0.1205000
-24715.0000000	0.1205000
-24714.0000000	0.1214285
-24713.0000000	0.1210000
-24712.0000000	0.1210000
-24711.0000000	0.1209524
-24710.0000000	0.1215000
-24709.0000000	0.1205000
-24708.0000000	0.1223809
-24707.0000000	0.1220000
-24706.0000000	0.1219047

-24705.0000000	0.1230000
-24704.0000000	0.1225000
-24703.0000000	0.1225000
-24702.0000000	0.1223809
-24701.0000000	0.1235000
-24700.0000000	0.1230000
-24699.0000000	0.1247619
-24698.0000000	0.1245000
-24697.0000000	0.1260000
-24696.0000000	0.1252381
-24695.0000000	0.1280000
-24694.0000000	0.1295000
-24693.0000000	0.1300000
-24692.0000000	0.1290000
-24691.0000000	0.1290476
-24690.0000000	0.1300000
-24689.0000000	0.1300000
-24688.0000000	0.1290000
-24687.0000000	0.1295238
-24686.0000000	0.1295000
-24685.0000000	0.1300000
-24684.0000000	0.1300000
-24683.0000000	0.1300000
-24682.0000000	0.1300000
-24681.0000000	0.1300000
-24680.0000000	0.1305000
-24679.0000000	0.1300000
-24678.0000000	0.1300000
-24677.0000000	0.1300000
-24676.0000000	0.1300000
-24675.0000000	0.1300000
-24674.0000000	0.1300000
-24673.0000000	0.1300000
-24672.0000000	0.1295238
-24671.0000000	0.1295000
-24670.0000000	0.1295000
-24669.0000000	0.1290476
-24668.0000000	0.1280000
-24667.0000000	0.1295000
-24666.0000000	0.1285714
-24665.0000000	0.1270000
-24664.0000000	0.1290000
-24663.0000000	0.1290476
-24662.0000000	0.1275000
-24661.0000000	0.1266667
-24660.0000000	0.1250000
-24659.0000000	0.1250000
-24658.0000000	0.1250000
-24657.0000000	0.1257143
-24656.0000000	0.1255000
-24655.0000000	0.1245000
-24654.0000000	0.1247619
-24653.0000000	0.1255000
-24652.0000000	0.1235000
-24651.0000000	0.1225000
-24650.0000000	0.1233333
-24649.0000000	0.1215000

-24648.0000000	0.1238095
-24647.0000000	0.1225000
-24646.0000000	0.1245000
-24645.0000000	0.1242857
-24644.0000000	0.1235000
-24643.0000000	0.1240000
-24642.0000000	0.1265000
-24641.0000000	0.1261905
-24640.0000000	0.1255000
-24639.0000000	0.1261905
-24638.0000000	0.1275000
-24637.0000000	0.1285000
-24636.0000000	0.1280000
-24635.0000000	0.1271429
-24634.0000000	0.1290000
-24633.0000000	0.1290000
-24632.0000000	0.1295238
-24631.0000000	0.1285714
-24630.0000000	0.1295000
-24629.0000000	0.1295000
-24628.0000000	0.1295000
-24627.0000000	0.1290476
-24626.0000000	0.1300000
-24625.0000000	0.1295000
-24624.0000000	0.1300000
-24623.0000000	0.1295000
-24622.0000000	0.1300000
-24621.0000000	0.1300000
-24620.0000000	0.1290476
-24619.0000000	0.1295000
-24618.0000000	0.1290476
-24617.0000000	0.1290000
-24616.0000000	0.1295000
-24615.0000000	0.1290476
-24614.0000000	0.1290000
-24613.0000000	0.1295000
-24612.0000000	0.1295000
-24611.0000000	0.1290476
-24610.0000000	0.1295000
-24609.0000000	0.1285714
-24608.0000000	0.1280000
-24607.0000000	0.1280000
-24606.0000000	0.1280000
-24605.0000000	0.1285714
-24604.0000000	0.1275000
-24603.0000000	0.1295000
-24602.0000000	0.1285714
-24601.0000000	0.1285000
-24600.0000000	0.1290476
-24599.0000000	0.1285000
-24598.0000000	0.1285000
-24597.0000000	0.1290476
-24596.0000000	0.1285000
-24595.0000000	0.1290000
-24594.0000000	0.1295000
-24593.0000000	0.1290476
-24592.0000000	0.1295000

-24591.0000000	0.1290000
-24590.0000000	0.1295238
-24589.0000000	0.1300000
-24588.0000000	0.1300000
-24587.0000000	0.1295000
-24586.0000000	0.1300000
-24585.0000000	0.1300000
-24584.0000000	0.1285714
-24583.0000000	0.1300000
-24582.0000000	0.1300000
-24581.0000000	0.1300000
-24580.0000000	0.1300000
-24579.0000000	0.1300000
-24578.0000000	0.1300000
-24577.0000000	0.1300000
-24576.0000000	0.1300000
-24575.0000000	0.1300000
-24574.0000000	0.1300000
-24573.0000000	0.1300000
-24572.0000000	0.1300000
-24571.0000000	0.1300000
-24570.0000000	0.1300000
-24569.0000000	0.1300000
-24568.0000000	0.1300000
-24567.0000000	0.1300000
-24566.0000000	0.1300000
-24565.0000000	0.1305000
-24564.0000000	0.1300000
-24563.0000000	0.1300000
-24562.0000000	0.1305000
-24561.0000000	0.1300000
-24560.0000000	0.1305000
-24559.0000000	0.1304762
-24558.0000000	0.1304762
-24557.0000000	0.1305000
-24556.0000000	0.1325000
-24555.0000000	0.1315000
-24554.0000000	0.1328572
-24553.0000000	0.1330000
-24552.0000000	0.1320000
-24551.0000000	0.1338095
-24550.0000000	0.1335000
-24549.0000000	0.1342857
-24548.0000000	0.1330000
-24547.0000000	0.1330000
-24546.0000000	0.1330000
-24545.0000000	0.1323809
-24544.0000000	0.1330000
-24543.0000000	0.1315000
-24542.0000000	0.1338095
-24541.0000000	0.1300000
-24540.0000000	0.1314286
-24539.0000000	0.1320000
-24538.0000000	0.1315000
-24537.0000000	0.1310000
-24536.0000000	0.1309524
-24535.0000000	0.1300000

-24534.0000000	0.1310000
-24533.0000000	0.1304762
-24532.0000000	0.1300000
-24531.0000000	0.1310000
-24530.0000000	0.1300000
-24529.0000000	0.1314286
-24528.0000000	0.1305000
-24527.0000000	0.1319048
-24526.0000000	0.1335000
-24525.0000000	0.1330000
-24524.0000000	0.1319048
-24523.0000000	0.1350000
-24522.0000000	0.1345000
-24521.0000000	0.1350000
-24520.0000000	0.1361905
-24519.0000000	0.1376191
-24518.0000000	0.1385000
-24517.0000000	0.1365000
-24516.0000000	0.1370000
-24515.0000000	0.1385714
-24514.0000000	0.1385000
-24513.0000000	0.1380000
-24512.0000000	0.1390477
-24511.0000000	0.1390000
-24510.0000000	0.1385715
-24509.0000000	0.1390000
-24508.0000000	0.1385000
-24507.0000000	0.1380000
-24506.0000000	0.1380953
-24505.0000000	0.1380000
-24504.0000000	0.1390000
-24503.0000000	0.1376191
-24502.0000000	0.1360000
-24501.0000000	0.1355000
-24500.0000000	0.1350000
-24499.0000000	0.1347619
-24498.0000000	0.1345000
-24497.0000000	0.1314286
-24496.0000000	0.1305000
-24495.0000000	0.1300000
-24494.0000000	0.1300000
-24493.0000000	0.1295000
-24492.0000000	0.1295000
-24491.0000000	0.1300000
-24490.0000000	0.1295238
-24489.0000000	0.1290000
-24488.0000000	0.1295238
-24487.0000000	0.1290000
-24486.0000000	0.1285000
-24485.0000000	0.1300000
-24484.0000000	0.1295000
-24483.0000000	0.1295000
-24482.0000000	0.1285000
-24481.0000000	0.1290476
-24480.0000000	0.1300000
-24479.0000000	0.1300000
-24478.0000000	0.1300000

-24477.0000000	0.1300000
-24476.0000000	0.1300000
-24475.0000000	0.1295000
-24474.0000000	0.1300000
-24473.0000000	0.1295238
-24472.0000000	0.1300000
-24471.0000000	0.1300000
-24470.0000000	0.1300000
-24469.0000000	0.1300000
-24468.0000000	0.1300000
-24467.0000000	0.1300000
-24466.0000000	0.1300000
-24465.0000000	0.1300000
-24464.0000000	0.1300000
-24463.0000000	0.1300000
-24462.0000000	0.1300000
-24461.0000000	0.1300000
-24460.0000000	0.1300000
-24459.0000000	0.1300000
-24458.0000000	0.1300000
-24457.0000000	0.1300000
-24456.0000000	0.1300000
-24455.0000000	0.1300000
-24454.0000000	0.1300000
-24453.0000000	0.1300000
-24452.0000000	0.1300000
-24451.0000000	0.1300000
-24450.0000000	0.1300000
-24449.0000000	0.1300000
-24448.0000000	0.1300000
-24447.0000000	0.1300000
-24446.0000000	0.1295000
-24445.0000000	0.1300000
-24444.0000000	0.1300000
-24443.0000000	0.1300000
-24442.0000000	0.1300000
-24441.0000000	0.1295000
-24440.0000000	0.1300000
-24439.0000000	0.1300000
-24438.0000000	0.1300000
-24437.0000000	0.1295238
-24436.0000000	0.1290000
-24435.0000000	0.1295000
-24434.0000000	0.1300000
-24433.0000000	0.1300000
-24432.0000000	0.1295000
-24431.0000000	0.1295000
-24430.0000000	0.1285714
-24429.0000000	0.1265000
-24428.0000000	0.1285714
-24427.0000000	0.1290000
-24426.0000000	0.1290000
-24425.0000000	0.1285000
-24424.0000000	0.1280952
-24423.0000000	0.1280000
-24422.0000000	0.1265000
-24421.0000000	0.1247619

-24420.0000000	0.1265000
-24419.0000000	0.1280952
-24418.0000000	0.1265000
-24417.0000000	0.1265000
-24416.0000000	0.1270000
-24415.0000000	0.1233333
-24414.0000000	0.1240000
-24413.0000000	0.1261905
-24412.0000000	0.1230000
-24411.0000000	0.1245000
-24410.0000000	0.1245000
-24409.0000000	0.1233333
-24408.0000000	0.1240000
-24407.0000000	0.1225000
-24406.0000000	0.1233333
-24405.0000000	0.1215000
-24404.0000000	0.1219047
-24403.0000000	0.1225000
-24402.0000000	0.1220000
-24401.0000000	0.1260000
-24400.0000000	0.1247619
-24399.0000000	0.1230000
-24398.0000000	0.1245000
-24397.0000000	0.1242857
-24396.0000000	0.1250000
-24395.0000000	0.1235000
-24394.0000000	0.1233333
-24393.0000000	0.1225000
-24392.0000000	0.1235000
-24391.0000000	0.1228571
-24390.0000000	0.1220000
-24389.0000000	0.1200000
-24388.0000000	0.1223809
-24387.0000000	0.1220000
-24386.0000000	0.1220000
-24385.0000000	0.1228571
-24384.0000000	0.1225000
-24383.0000000	0.1225000
-24382.0000000	0.1214286
-24381.0000000	0.1215000
-24380.0000000	0.1220000
-24379.0000000	0.1219047
-24378.0000000	0.1205000
-24377.0000000	0.1210000
-24376.0000000	0.1204762
-24375.0000000	0.1205000
-24374.0000000	0.1204762
-24373.0000000	0.1205000
-24372.0000000	0.1210000
-24371.0000000	0.1210000
-24370.0000000	0.1200000
-24369.0000000	0.1205000
-24368.0000000	0.1215000
-24367.0000000	0.1204762
-24366.0000000	0.1200000
-24365.0000000	0.1210000
-24364.0000000	0.1210000

-24363.0000000	0.1219047
-24362.0000000	0.1225000
-24361.0000000	0.1223809
-24360.0000000	0.1210000
-24359.0000000	0.1225000
-24358.0000000	0.1219047
-24357.0000000	0.1225000
-24356.0000000	0.1230000
-24355.0000000	0.1233333
-24354.0000000	0.1210000
-24353.0000000	0.1215000
-24352.0000000	0.1219047
-24351.0000000	0.1220000
-24350.0000000	0.1220000
-24349.0000000	0.1209524
-24348.0000000	0.1215000
-24347.0000000	0.1205000
-24346.0000000	0.1214285
-24345.0000000	0.1220000
-24344.0000000	0.1219047
-24343.0000000	0.1220000
-24342.0000000	0.1205000
-24341.0000000	0.1205000
-24340.0000000	0.1200000
-24339.0000000	0.1205000
-24338.0000000	0.1200000
-24337.0000000	0.1200000
-24336.0000000	0.1205000
-24335.0000000	0.1200000
-24334.0000000	0.1200000
-24333.0000000	0.1200000
-24332.0000000	0.1200000
-24331.0000000	0.1200000
-24330.0000000	0.1200000
-24329.0000000	0.1200000
-24328.0000000	0.1200000
-24327.0000000	0.1195000
-24326.0000000	0.1190000
-24325.0000000	0.1190000
-24324.0000000	0.1190476
-24323.0000000	0.1195000
-24322.0000000	0.1185714
-24321.0000000	0.1170000
-24320.0000000	0.1180000
-24319.0000000	0.1170000
-24318.0000000	0.1195238
-24317.0000000	0.1185000
-24316.0000000	0.1190000
-24315.0000000	0.1195238
-24314.0000000	0.1200000
-24313.0000000	0.1195000
-24312.0000000	0.1195000
-24311.0000000	0.1200000
-24310.0000000	0.1200000
-24309.0000000	0.1195000
-24308.0000000	0.1200000
-24307.0000000	0.1200000

-24306.0000000	0.1190476
-24305.0000000	0.1190000
-24304.0000000	0.1180000
-24303.0000000	0.1195238
-24302.0000000	0.1195000
-24301.0000000	0.1185714
-24300.0000000	0.1195000
-24299.0000000	0.1195000
-24298.0000000	0.1195238
-24297.0000000	0.1195000
-24296.0000000	0.1190000
-24295.0000000	0.1195000
-24294.0000000	0.1190476
-24293.0000000	0.1190000
-24292.0000000	0.1180952
-24291.0000000	0.1175000
-24290.0000000	0.1170000
-24289.0000000	0.1145000
-24288.0000000	0.1147619
-24287.0000000	0.1145000
-24286.0000000	0.1145000
-24285.0000000	0.1133333
-24284.0000000	0.1150000
-24283.0000000	0.1133333
-24282.0000000	0.1140000
-24281.0000000	0.1135000
-24280.0000000	0.1128571
-24279.0000000	0.1150000
-24278.0000000	0.1165000
-24277.0000000	0.1130000
-24276.0000000	0.1157143
-24275.0000000	0.1150000
-24274.0000000	0.1135000
-24273.0000000	0.1142857
-24272.0000000	0.1110000
-24271.0000000	0.1133333
-24270.0000000	0.1130000
-24269.0000000	0.1145000
-24268.0000000	0.1120000
-24267.0000000	0.1128571
-24266.0000000	0.1130000
-24265.0000000	0.1130000
-24264.0000000	0.1147619
-24263.0000000	0.1140000
-24262.0000000	0.1119047
-24261.0000000	0.1105000
-24260.0000000	0.1110000
-24259.0000000	0.1110000
-24258.0000000	0.1119047
-24257.0000000	0.1110000
-24256.0000000	0.1120000
-24255.0000000	0.1119047
-24254.0000000	0.1105000
-24253.0000000	0.1114286
-24252.0000000	0.1115000
-24251.0000000	0.1110000
-24250.0000000	0.1110000

-24249.0000000	0.1119047
-24248.0000000	0.1110000
-24247.0000000	0.1110000
-24246.0000000	0.1100000
-24245.0000000	0.1110000
-24244.0000000	0.1105000
-24243.0000000	0.1100000
-24242.0000000	0.1100000
-24241.0000000	0.1100000
-24240.0000000	0.1100000
-24239.0000000	0.1105000
-24238.0000000	0.1100000
-24237.0000000	0.1104762
-24236.0000000	0.1100000
-24235.0000000	0.1100000
-24234.0000000	0.1100000
-24233.0000000	0.1100000
-24232.0000000	0.1100000
-24231.0000000	0.1100000
-24230.0000000	0.1100000
-24229.0000000	0.1100000
-24228.0000000	0.1095238
-24227.0000000	0.1100000
-24226.0000000	0.1100000
-24225.0000000	0.1095238
-24224.0000000	0.1085000
-24223.0000000	0.1080952
-24222.0000000	0.1085000
-24221.0000000	0.1085000
-24220.0000000	0.1085000
-24219.0000000	0.1090476
-24218.0000000	0.1070000
-24217.0000000	0.1070000
-24216.0000000	0.1095238
-24215.0000000	0.1075000
-24214.0000000	0.1075000
-24213.0000000	0.1070000
-24212.0000000	0.1076190
-24211.0000000	0.1070000
-24210.0000000	0.1080952
-24209.0000000	0.1080000
-24208.0000000	0.1080000
-24207.0000000	0.1066667
-24206.0000000	0.1070000
-24205.0000000	0.1065000
-24204.0000000	0.1065000
-24203.0000000	0.1071429
-24202.0000000	0.1090000
-24201.0000000	0.1066667
-24200.0000000	0.1075000
-24199.0000000	0.1060000
-24198.0000000	0.1080952
-24197.0000000	0.1080000
-24196.0000000	0.1060000
-24195.0000000	0.1076190
-24194.0000000	0.1060000
-24193.0000000	0.1075000

-24192.0000000	0.1076190
-24191.0000000	0.1085000
-24190.0000000	0.1090000
-24189.0000000	0.1095238
-24188.0000000	0.1095000
-24187.0000000	0.1085000
-24186.0000000	0.1100000
-24185.0000000	0.1100000
-24184.0000000	0.1095000
-24183.0000000	0.1100000
-24182.0000000	0.1090476
-24181.0000000	0.1090000
-24180.0000000	0.1095238
-24179.0000000	0.1095000
-24178.0000000	0.1100000
-24177.0000000	0.1100000
-24176.0000000	0.1100000
-24175.0000000	0.1100000
-24174.0000000	0.1100000
-24173.0000000	0.1100000
-24172.0000000	0.1100000
-24171.0000000	0.1100000
-24170.0000000	0.1090000
-24169.0000000	0.1090000
-24168.0000000	0.1085000
-24167.0000000	0.1090476
-24166.0000000	0.1085000
-24165.0000000	0.1085714
-24164.0000000	0.1095000
-24163.0000000	0.1090000
-24162.0000000	0.1090476
-24161.0000000	0.1090000
-24160.0000000	0.1085000
-24159.0000000	0.1080000
-24158.0000000	0.1100000
-24157.0000000	0.1095000
-24156.0000000	0.1090476
-24155.0000000	0.1090000
-24154.0000000	0.1090000
-24153.0000000	0.1080000
-24152.0000000	0.1080952
-24151.0000000	0.1090000
-24150.0000000	0.1095238
-24149.0000000	0.1095000
-24148.0000000	0.1100000
-24147.0000000	0.1095238
-24146.0000000	0.1100000
-24145.0000000	0.1070000
-24144.0000000	0.1085000
-24143.0000000	0.1080952
-24142.0000000	0.1095000
-24141.0000000	0.1071429
-24140.0000000	0.1065000
-24139.0000000	0.1070000
-24138.0000000	0.1085000
-24137.0000000	0.1061905
-24136.0000000	0.1095000

-24135.0000000	0.1095000
-24134.0000000	0.1080952
-24133.0000000	0.1080000
-24132.0000000	0.1066667
-24131.0000000	0.1055000
-24130.0000000	0.1060000
-24129.0000000	0.1070000
-24128.0000000	0.1071429
-24127.0000000	0.1070000
-24126.0000000	0.1080952
-24125.0000000	0.1080000
-24124.0000000	0.1065000
-24123.0000000	0.1075000
-24122.0000000	0.1076190
-24121.0000000	0.1065000
-24120.0000000	0.1075000
-24119.0000000	0.1071429
-24118.0000000	0.1075000
-24117.0000000	0.1080952
-24116.0000000	0.1085000
-24115.0000000	0.1095000
-24114.0000000	0.1095000
-24113.0000000	0.1100000
-24112.0000000	0.1090000
-24111.0000000	0.1100000
-24110.0000000	0.1100000
-24109.0000000	0.1100000
-24108.0000000	0.1100000
-24107.0000000	0.1100000
-24106.0000000	0.1100000
-24105.0000000	0.1100000
-24104.0000000	0.1100000
-24103.0000000	0.1100000
-24102.0000000	0.1100000
-24101.0000000	0.1100000
-24100.0000000	0.1100000
-24099.0000000	0.1100000
-24098.0000000	0.1100000
-24097.0000000	0.1095000
-24096.0000000	0.1100000
-24095.0000000	0.1095000
-24094.0000000	0.1100000
-24093.0000000	0.1090000
-24092.0000000	0.1095238
-24091.0000000	0.1100000
-24090.0000000	0.1090000
-24089.0000000	0.1100000
-24088.0000000	0.1100000
-24087.0000000	0.1100000
-24086.0000000	0.1100000
-24085.0000000	0.1100000
-24084.0000000	0.1100000
-24083.0000000	0.1100000
-24082.0000000	0.1100000
-24081.0000000	0.1100000
-24080.0000000	0.1100000
-24079.0000000	0.1100000

-24078.0000000	0.1100000
-24077.0000000	0.1100000
-24076.0000000	0.1100000
-24075.0000000	0.1100000
-24074.0000000	0.1100000
-24073.0000000	0.1100000
-24072.0000000	0.1100000
-24071.0000000	0.1095238
-24070.0000000	0.1100000
-24069.0000000	0.1100000
-24068.0000000	0.1095238
-24067.0000000	0.1100000
-24066.0000000	0.1100000
-24065.0000000	0.1100000
-24064.0000000	0.1095000
-24063.0000000	0.1095000
-24062.0000000	0.1095238
-24061.0000000	0.1095000
-24060.0000000	0.1100000
-24059.0000000	0.1100000
-24058.0000000	0.1100000
-24057.0000000	0.1100000
-24056.0000000	0.1100000
-24055.0000000	0.1100000
-24054.0000000	0.1100000
-24053.0000000	0.1090476
-24052.0000000	0.1100000
-24051.0000000	0.1100000
-24050.0000000	0.1100000
-24049.0000000	0.1095000
-24048.0000000	0.1095000
-24047.0000000	0.1100000
-24046.0000000	0.1095238
-24045.0000000	0.1100000
-24044.0000000	0.1100000
-24043.0000000	0.1100000
-24042.0000000	0.1100000
-24041.0000000	0.1100000
-24040.0000000	0.1100000
-24039.0000000	0.1095000
-24038.0000000	0.1100000
-24037.0000000	0.1104762
-24036.0000000	0.1100000
-24035.0000000	0.1100000
-24034.0000000	0.1100000
-24033.0000000	0.1100000
-24032.0000000	0.1100000
-24031.0000000	0.1100000
-24030.0000000	0.1100000
-24029.0000000	0.1100000
-24028.0000000	0.1100000
-24027.0000000	0.1100000
-24026.0000000	0.1100000
-24025.0000000	0.1100000
-24024.0000000	0.1100000
-24023.0000000	0.1100000
-24022.0000000	0.1100000

-24021.0000000	0.1115000
-24020.0000000	0.1100000
-24019.0000000	0.1100000
-24018.0000000	0.1100000
-24017.0000000	0.1100000
-24016.0000000	0.1104762
-24015.0000000	0.1100000
-24014.0000000	0.1109524
-24013.0000000	0.1100000
-24012.0000000	0.1110000
-24011.0000000	0.1114286
-24010.0000000	0.1120000
-24009.0000000	0.1115000
-24008.0000000	0.1115000
-24007.0000000	0.1138095
-24006.0000000	0.1135000
-24005.0000000	0.1152381
-24004.0000000	0.1125000
-24003.0000000	0.1145000
-24002.0000000	0.1147619
-24001.0000000	0.1135000
-24000.0000000	0.1145000
-23999.0000000	0.1135000
-23998.0000000	0.1142857
-23997.0000000	0.1140000
-23996.0000000	0.1138095
-23995.0000000	0.1140000
-23994.0000000	0.1135000
-23993.0000000	0.1119047
-23992.0000000	0.1110000
-23991.0000000	0.1130000
-23990.0000000	0.1115000
-23989.0000000	0.1123809
-23988.0000000	0.1115000
-23987.0000000	0.1115000
-23986.0000000	0.1104762
-23985.0000000	0.1125000
-23984.0000000	0.1133333
-23983.0000000	0.1120000
-23982.0000000	0.1140000
-23981.0000000	0.1150000
-23980.0000000	0.1138095
-23979.0000000	0.1150000
-23978.0000000	0.1150000
-23977.0000000	0.1147619
-23976.0000000	0.1160000
-23975.0000000	0.1157143
-23974.0000000	0.1145000
-23973.0000000	0.1135000
-23972.0000000	0.1140000
-23971.0000000	0.1138095
-23970.0000000	0.1150000
-23969.0000000	0.1155000
-23968.0000000	0.1138095
-23967.0000000	0.1150000
-23966.0000000	0.1133333
-23965.0000000	0.1160000

-23964.0000000	0.1140000
-23963.0000000	0.1152381
-23962.0000000	0.1155000
-23961.0000000	0.1175000
-23960.0000000	0.1140000
-23959.0000000	0.1157143
-23958.0000000	0.1160000
-23957.0000000	0.1165000
-23956.0000000	0.1140000
-23955.0000000	0.1142857
-23954.0000000	0.1119047
-23953.0000000	0.1115000
-23952.0000000	0.1120000
-23951.0000000	0.1135000
-23950.0000000	0.1123809
-23949.0000000	0.1110000
-23948.0000000	0.1130000
-23947.0000000	0.1133333
-23946.0000000	0.1125000
-23945.0000000	0.1109524
-23944.0000000	0.1115000
-23943.0000000	0.1120000
-23942.0000000	0.1105000
-23941.0000000	0.1104762
-23940.0000000	0.1105000
-23939.0000000	0.1110000
-23938.0000000	0.1104762
-23937.0000000	0.1100000
-23936.0000000	0.1104762
-23935.0000000	0.1100000
-23934.0000000	0.1115000
-23933.0000000	0.1100000
-23932.0000000	0.1100000
-23931.0000000	0.1100000
-23930.0000000	0.1100000
-23929.0000000	0.1104762
-23928.0000000	0.1100000
-23927.0000000	0.1105000
-23926.0000000	0.1105000
-23925.0000000	0.1109524
-23924.0000000	0.1100000
-23923.0000000	0.1100000
-23922.0000000	0.1105000
-23921.0000000	0.1105000
-23920.0000000	0.1100000
-23919.0000000	0.1100000
-23918.0000000	0.1100000
-23917.0000000	0.1100000
-23916.0000000	0.1104762
-23915.0000000	0.1115000
-23914.0000000	0.1100000
-23913.0000000	0.1100000
-23912.0000000	0.1100000
-23911.0000000	0.1100000
-23910.0000000	0.1100000
-23909.0000000	0.1100000
-23908.0000000	0.1100000

-23907.0000000	0.1105000
-23906.0000000	0.1100000
-23905.0000000	0.1100000
-23904.0000000	0.1100000
-23903.0000000	0.1105000
-23902.0000000	0.1100000
-23901.0000000	0.1100000
-23900.0000000	0.1100000
-23899.0000000	0.1100000
-23898.0000000	0.1120000
-23897.0000000	0.1100000
-23896.0000000	0.1120000
-23895.0000000	0.1109524
-23894.0000000	0.1105000
-23893.0000000	0.1104762
-23892.0000000	0.1110000
-23891.0000000	0.1115000
-23890.0000000	0.1114286
-23889.0000000	0.1110000
-23888.0000000	0.1120000
-23887.0000000	0.1115000
-23886.0000000	0.1109524
-23885.0000000	0.1130000
-23884.0000000	0.1123809
-23883.0000000	0.1125000
-23882.0000000	0.1150000
-23881.0000000	0.1123809
-23880.0000000	0.1115000
-23879.0000000	0.1115000
-23878.0000000	0.1133333
-23877.0000000	0.1125000
-23876.0000000	0.1155000
-23875.0000000	0.1147619
-23874.0000000	0.1150000
-23873.0000000	0.1180000
-23872.0000000	0.1161905
-23871.0000000	0.1180000
-23870.0000000	0.1195000
-23869.0000000	0.1195238
-23868.0000000	0.1200000
-23867.0000000	0.1200000
-23866.0000000	0.1200000
-23865.0000000	0.1195238
-23864.0000000	0.1200000
-23863.0000000	0.1195238
-23862.0000000	0.1200000
-23861.0000000	0.1200000
-23860.0000000	0.1200000
-23859.0000000	0.1200000
-23858.0000000	0.1200000
-23857.0000000	0.1200000
-23856.0000000	0.1200000
-23855.0000000	0.1205000
-23854.0000000	0.1200000
-23853.0000000	0.1205000
-23852.0000000	0.1205000
-23851.0000000	0.1200000

-23850.0000000	0.1204762
-23849.0000000	0.1210000
-23848.0000000	0.1215000
-23847.0000000	0.1219047
-23846.0000000	0.1220000
-23845.0000000	0.1219047
-23844.0000000	0.1215000
-23843.0000000	0.1210000
-23842.0000000	0.1205000
-23841.0000000	0.1219047
-23840.0000000	0.1230000
-23839.0000000	0.1228571
-23838.0000000	0.1245000
-23837.0000000	0.1250000
-23836.0000000	0.1245000
-23835.0000000	0.1242857
-23834.0000000	0.1235000
-23833.0000000	0.1242857
-23832.0000000	0.1240000
-23831.0000000	0.1220000
-23830.0000000	0.1223810
-23829.0000000	0.1225000
-23828.0000000	0.1230000
-23827.0000000	0.1230000
-23826.0000000	0.1209524
-23825.0000000	0.1220000
-23824.0000000	0.1214286
-23823.0000000	0.1230000
-23822.0000000	0.1245000
-23821.0000000	0.1270000
-23820.0000000	0.1247619
-23819.0000000	0.1245000
-23818.0000000	0.1265000
-23817.0000000	0.1257143
-23816.0000000	0.1260000
-23815.0000000	0.1257143
-23814.0000000	0.1260000
-23813.0000000	0.1270000
-23812.0000000	0.1285000
-23811.0000000	0.1276190
-23810.0000000	0.1280000
-23809.0000000	0.1290476
-23808.0000000	0.1285000
-23807.0000000	0.1290000
-23806.0000000	0.1270000
-23805.0000000	0.1295238
-23804.0000000	0.1280000
-23803.0000000	0.1295000
-23802.0000000	0.1290476
-23801.0000000	0.1295000
-23800.0000000	0.1300000
-23799.0000000	0.1300000
-23798.0000000	0.1295000
-23797.0000000	0.1300000
-23796.0000000	0.1300000
-23795.0000000	0.1300000
-23794.0000000	0.1300000

-23793.0000000	0.1295238
-23792.0000000	0.1290000
-23791.0000000	0.1300000
-23790.0000000	0.1300000
-23789.0000000	0.1300000
-23788.0000000	0.1300000
-23787.0000000	0.1300000
-23786.0000000	0.1300000
-23785.0000000	0.1300000
-23784.0000000	0.1300000
-23783.0000000	0.1300000
-23782.0000000	0.1300000
-23781.0000000	0.1300000
-23780.0000000	0.1305000
-23779.0000000	0.1300000
-23778.0000000	0.1300000
-23777.0000000	0.1305000
-23776.0000000	0.1305000
-23775.0000000	0.1309524
-23774.0000000	0.1300000
-23773.0000000	0.1300000
-23772.0000000	0.1300000
-23771.0000000	0.1305000
-23770.0000000	0.1300000
-23769.0000000	0.1305000
-23768.0000000	0.1305000
-23767.0000000	0.1305000
-23766.0000000	0.1314286
-23765.0000000	0.1300000
-23764.0000000	0.1305000
-23763.0000000	0.1314286
-23762.0000000	0.1310000
-23761.0000000	0.1315000
-23760.0000000	0.1314286
-23759.0000000	0.1320000
-23758.0000000	0.1315000
-23757.0000000	0.1328571
-23756.0000000	0.1330000
-23755.0000000	0.1325000
-23754.0000000	0.1314286
-23753.0000000	0.1325000
-23752.0000000	0.1320000
-23751.0000000	0.1314286
-23750.0000000	0.1305000
-23749.0000000	0.1300000
-23748.0000000	0.1300000
-23747.0000000	0.1305000
-23746.0000000	0.1315000
-23745.0000000	0.1309524
-23744.0000000	0.1310000
-23743.0000000	0.1320000
-23742.0000000	0.1319048
-23741.0000000	0.1360000
-23740.0000000	0.1352381
-23739.0000000	0.1355000
-23738.0000000	0.1375000
-23737.0000000	0.1375000

-23736.0000000	0.1395238
-23735.0000000	0.1395000
-23734.0000000	0.1390000
-23733.0000000	0.1395238
-23732.0000000	0.1395000
-23731.0000000	0.1395000
-23730.0000000	0.1395000
-23729.0000000	0.1395238
-23728.0000000	0.1395000
-23727.0000000	0.1400000
-23726.0000000	0.1400000
-23725.0000000	0.1400000
-23724.0000000	0.1400000
-23723.0000000	0.1400000
-23722.0000000	0.1400000
-23721.0000000	0.1400000
-23720.0000000	0.1400000
-23719.0000000	0.1400000
-23718.0000000	0.1400000
-23717.0000000	0.1400000
-23716.0000000	0.1400000
-23715.0000000	0.1400000
-23714.0000000	0.1400000
-23713.0000000	0.1400000
-23712.0000000	0.1400000
-23711.0000000	0.1400000
-23710.0000000	0.1400000
-23709.0000000	0.1400000
-23708.0000000	0.1400000
-23707.0000000	0.1400000
-23706.0000000	0.1400000
-23705.0000000	0.1400000
-23704.0000000	0.1400000
-23703.0000000	0.1400000
-23702.0000000	0.1395238
-23701.0000000	0.1400000
-23700.0000000	0.1400000
-23699.0000000	0.1400000
-23698.0000000	0.1400000
-23697.0000000	0.1400000
-23696.0000000	0.1405000
-23695.0000000	0.1415000
-23694.0000000	0.1433334
-23693.0000000	0.1435000
-23692.0000000	0.1440000
-23691.0000000	0.1450000
-23690.0000000	0.1457143
-23689.0000000	0.1465000
-23688.0000000	0.1476191
-23687.0000000	0.1470000
-23686.0000000	0.1495000
-23685.0000000	0.1490476
-23684.0000000	0.1485000
-23683.0000000	0.1490000
-23682.0000000	0.1485000
-23681.0000000	0.1476191
-23680.0000000	0.1485000

-23679.0000000	0.1490476
-23678.0000000	0.1480000
-23677.0000000	0.1490000
-23676.0000000	0.1500000
-23675.0000000	0.1490000
-23674.0000000	0.1495000
-23673.0000000	0.1495000
-23672.0000000	0.1500000
-23671.0000000	0.1500000
-23670.0000000	0.1500000
-23669.0000000	0.1500000
-23668.0000000	0.1500000
-23667.0000000	0.1500000
-23666.0000000	0.1500000
-23665.0000000	0.1500000
-23664.0000000	0.1500000
-23663.0000000	0.1500000
-23662.0000000	0.1500000
-23661.0000000	0.1500000
-23660.0000000	0.1500000
-23659.0000000	0.1500000
-23658.0000000	0.1504762
-23657.0000000	0.1500000
-23656.0000000	0.1505000
-23655.0000000	0.1510000
-23654.0000000	0.1500000
-23653.0000000	0.1500000
-23652.0000000	0.1500000
-23651.0000000	0.1500000
-23650.0000000	0.1500000
-23649.0000000	0.1509524
-23648.0000000	0.1500000
-23647.0000000	0.1535000
-23646.0000000	0.1515000
-23645.0000000	0.1528572
-23644.0000000	0.1515000
-23643.0000000	0.1540000
-23642.0000000	0.1538095
-23641.0000000	0.1570000
-23640.0000000	0.1555000
-23639.0000000	0.1575000
-23638.0000000	0.1566667
-23637.0000000	0.1547619
-23636.0000000	0.1545000
-23635.0000000	0.1550000
-23634.0000000	0.1560000
-23633.0000000	0.1533334
-23632.0000000	0.1530000
-23631.0000000	0.1510000
-23630.0000000	0.1519048
-23629.0000000	0.1515000
-23628.0000000	0.1528572
-23627.0000000	0.1520000
-23626.0000000	0.1500000
-23625.0000000	0.1515000
-23624.0000000	0.1504762
-23623.0000000	0.1500000

-23622.0000000	0.1500000
-23621.0000000	0.1500000
-23620.0000000	0.1505000
-23619.0000000	0.1500000
-23618.0000000	0.1510000
-23617.0000000	0.1500000
-23616.0000000	0.1500000
-23615.0000000	0.1509524
-23614.0000000	0.1510000
-23613.0000000	0.1520000
-23612.0000000	0.1519048
-23611.0000000	0.1510000
-23610.0000000	0.1520000
-23609.0000000	0.1530000
-23608.0000000	0.1528572
-23607.0000000	0.1555000
-23606.0000000	0.1542857
-23605.0000000	0.1545000
-23604.0000000	0.1570000
-23603.0000000	0.1590476
-23602.0000000	0.1580000
-23601.0000000	0.1595000
-23600.0000000	0.1575000
-23599.0000000	0.1585715
-23598.0000000	0.1595000
-23597.0000000	0.1580953
-23596.0000000	0.1585000
-23595.0000000	0.1585000
-23594.0000000	0.1590476
-23593.0000000	0.1570000
-23592.0000000	0.1585000
-23591.0000000	0.1590000
-23590.0000000	0.1585715
-23589.0000000	0.1595000
-23588.0000000	0.1595238
-23587.0000000	0.1600000
-23586.0000000	0.1595000
-23585.0000000	0.1595238
-23584.0000000	0.1600000
-23583.0000000	0.1600000
-23582.0000000	0.1595238
-23581.0000000	0.1600000
-23580.0000000	0.1595000
-23579.0000000	0.1590000
-23578.0000000	0.1595238
-23577.0000000	0.1590000
-23576.0000000	0.1600000
-23575.0000000	0.1600000
-23574.0000000	0.1600000
-23573.0000000	0.1600000
-23572.0000000	0.1595000
-23571.0000000	0.1600000
-23570.0000000	0.1600000
-23569.0000000	0.1600000
-23568.0000000	0.1600000
-23567.0000000	0.1604762
-23566.0000000	0.1610000

-23565.0000000	0.1605000
-23564.0000000	0.1620000
-23563.0000000	0.1609524
-23562.0000000	0.1640000
-23561.0000000	0.1671429
-23560.0000000	0.1675000
-23559.0000000	0.1685000
-23558.0000000	0.1695238
-23557.0000000	0.1695000
-23556.0000000	0.1695000
-23555.0000000	0.1700000
-23554.0000000	0.1700000
-23553.0000000	0.1700000
-23552.0000000	0.1695238
-23551.0000000	0.1700000
-23550.0000000	0.1700000
-23549.0000000	0.1700000
-23548.0000000	0.1700000
-23547.0000000	0.1700000
-23546.0000000	0.1700000
-23545.0000000	0.1700000
-23544.0000000	0.1705000
-23543.0000000	0.1709524
-23542.0000000	0.1700000
-23541.0000000	0.1700000
-23540.0000000	0.1705000
-23539.0000000	0.1714286
-23538.0000000	0.1715000
-23537.0000000	0.1728572
-23536.0000000	0.1750000
-23535.0000000	0.1730000
-23534.0000000	0.1740000
-23533.0000000	0.1766667
-23532.0000000	0.1770000
-23531.0000000	0.1747619
-23530.0000000	0.1765000
-23529.0000000	0.1775000
-23528.0000000	0.1776191
-23527.0000000	0.1765000
-23526.0000000	0.1775000
-23525.0000000	0.1760000
-23524.0000000	0.1761905
-23523.0000000	0.1765000
-23522.0000000	0.1790477
-23521.0000000	0.1790000
-23520.0000000	0.1785000
-23519.0000000	0.1790000
-23518.0000000	0.1790477
-23517.0000000	0.1800000
-23516.0000000	0.1800000
-23515.0000000	0.1800000
-23514.0000000	0.1800000
-23513.0000000	0.1800000
-23512.0000000	0.1800000
-23511.0000000	0.1800000
-23510.0000000	0.1805000
-23509.0000000	0.1804762

-23508.0000000	0.1810000
-23507.0000000	0.1810000
-23506.0000000	0.1814286
-23505.0000000	0.1810000
-23504.0000000	0.1830000
-23503.0000000	0.1847619
-23502.0000000	0.1830000
-23501.0000000	0.1830000
-23500.0000000	0.1838096
-23499.0000000	0.1855000
-23498.0000000	0.1852381
-23497.0000000	0.1860000
-23496.0000000	0.1870000
-23495.0000000	0.1885000
-23494.0000000	0.1890476
-23493.0000000	0.1885000
-23492.0000000	0.1890476
-23491.0000000	0.1895000
-23490.0000000	0.1895000
-23489.0000000	0.1875000
-23488.0000000	0.1890476
-23487.0000000	0.1895000
-23486.0000000	0.1900000
-23485.0000000	0.1900000
-23484.0000000	0.1895000
-23483.0000000	0.1900000
-23482.0000000	0.1895000
-23481.0000000	0.1895000
-23480.0000000	0.1900000
-23479.0000000	0.1900000
-23478.0000000	0.1900000
-23477.0000000	0.1900000
-23476.0000000	0.1900000
-23475.0000000	0.1915000
-23474.0000000	0.1910000
-23473.0000000	0.1904762
-23472.0000000	0.1900000
-23471.0000000	0.1905000
-23470.0000000	0.1900000
-23469.0000000	0.1905000
-23468.0000000	0.1900000
-23467.0000000	0.1909524
-23466.0000000	0.1900000
-23465.0000000	0.1900000
-23464.0000000	0.1900000
-23463.0000000	0.1900000
-23462.0000000	0.1900000
-23461.0000000	0.1900000
-23460.0000000	0.1900000
-23459.0000000	0.1900000
-23458.0000000	0.1900000
-23457.0000000	0.1900000
-23456.0000000	0.1900000
-23455.0000000	0.1900000
-23454.0000000	0.1900000
-23453.0000000	0.1914286
-23452.0000000	0.1910000

-23451.0000000	0.1900000
-23450.0000000	0.1910000
-23449.0000000	0.1904762
-23448.0000000	0.1900000
-23447.0000000	0.1900000
-23446.0000000	0.1900000
-23445.0000000	0.1900000
-23444.0000000	0.1900000
-23443.0000000	0.1900000
-23442.0000000	0.1900000
-23441.0000000	0.1900000
-23440.0000000	0.1900000
-23439.0000000	0.1900000
-23438.0000000	0.1900000
-23437.0000000	0.1900000
-23436.0000000	0.1900000
-23435.0000000	0.1900000
-23434.0000000	0.1900000
-23433.0000000	0.1900000
-23432.0000000	0.1900000
-23431.0000000	0.1915000
-23430.0000000	0.1910000
-23429.0000000	0.1900000
-23428.0000000	0.1909524
-23427.0000000	0.1910000
-23426.0000000	0.1915000
-23425.0000000	0.1910000
-23424.0000000	0.1923810
-23423.0000000	0.1914286
-23422.0000000	0.1910000
-23421.0000000	0.1915000
-23420.0000000	0.1920000
-23419.0000000	0.1909524
-23418.0000000	0.1925000
-23417.0000000	0.1905000
-23416.0000000	0.1914286
-23415.0000000	0.1935000
-23414.0000000	0.1945000
-23413.0000000	0.1940000
-23412.0000000	0.1928572
-23411.0000000	0.1915000
-23410.0000000	0.1938096
-23409.0000000	0.1925000
-23408.0000000	0.1920000
-23407.0000000	0.1919048
-23406.0000000	0.1900000
-23405.0000000	0.1910000
-23404.0000000	0.1905000
-23403.0000000	0.1909524
-23402.0000000	0.1900000
-23401.0000000	0.1900000
-23400.0000000	0.1905000
-23399.0000000	0.1900000
-23398.0000000	0.1904762
-23397.0000000	0.1910000
-23396.0000000	0.1910000
-23395.0000000	0.1925000

-23394.0000000	0.1928572
-23393.0000000	0.1930000
-23392.0000000	0.1909524
-23391.0000000	0.1910000
-23390.0000000	0.1905000
-23389.0000000	0.1914286
-23388.0000000	0.1905000
-23387.0000000	0.1910000
-23386.0000000	0.1910000
-23385.0000000	0.1904762
-23384.0000000	0.1910000
-23383.0000000	0.1900000
-23382.0000000	0.1900000
-23381.0000000	0.1900000
-23380.0000000	0.1900000
-23379.0000000	0.1900000
-23378.0000000	0.1900000
-23377.0000000	0.1900000
-23376.0000000	0.1900000
-23375.0000000	0.1900000
-23374.0000000	0.1900000
-23373.0000000	0.1900000
-23372.0000000	0.1895000
-23371.0000000	0.1900000
-23370.0000000	0.1900000
-23369.0000000	0.1900000
-23368.0000000	0.1900000
-23367.0000000	0.1900000
-23366.0000000	0.1905000
-23365.0000000	0.1905000
-23364.0000000	0.1904762
-23363.0000000	0.1900000
-23362.0000000	0.1904762
-23361.0000000	0.1910000
-23360.0000000	0.1920000
-23359.0000000	0.1904762
-23358.0000000	0.1905000
-23357.0000000	0.1900000
-23356.0000000	0.1900000
-23355.0000000	0.1900000
-23354.0000000	0.1900000
-23353.0000000	0.1900000
-23352.0000000	0.1900000
-23351.0000000	0.1900000
-23350.0000000	0.1900000
-23349.0000000	0.1900000
-23348.0000000	0.1910000
-23347.0000000	0.1900000
-23346.0000000	0.1904762
-23345.0000000	0.1905000
-23344.0000000	0.1900000
-23343.0000000	0.1904762
-23342.0000000	0.1900000
-23341.0000000	0.1900000
-23340.0000000	0.1900000
-23339.0000000	0.1900000
-23338.0000000	0.1895000

-23337.0000000	0.1895238
-23336.0000000	0.1885000
-23335.0000000	0.1880000
-23334.0000000	0.1857143
-23333.0000000	0.1865000
-23332.0000000	0.1857143
-23331.0000000	0.1835000
-23330.0000000	0.1825000
-23329.0000000	0.1835000
-23328.0000000	0.1861905
-23327.0000000	0.1840000
-23326.0000000	0.1850000
-23325.0000000	0.1814286
-23324.0000000	0.1850000
-23323.0000000	0.1825000
-23322.0000000	0.1850000
-23321.0000000	0.1828572
-23320.0000000	0.1845000
-23319.0000000	0.1847619
-23318.0000000	0.1890000
-23317.0000000	0.1865000
-23316.0000000	0.1895238
-23315.0000000	0.1890000
-23314.0000000	0.1900000
-23313.0000000	0.1876191
-23312.0000000	0.1885000
-23311.0000000	0.1890000
-23310.0000000	0.1895238
-23309.0000000	0.1880000
-23308.0000000	0.1880000
-23307.0000000	0.1866667
-23306.0000000	0.1830000
-23305.0000000	0.1810000
-23304.0000000	0.1804762
-23303.0000000	0.1805000
-23302.0000000	0.1800000
-23301.0000000	0.1800000
-23300.0000000	0.1800000
-23299.0000000	0.1800000
-23298.0000000	0.1800000
-23297.0000000	0.1805000
-23296.0000000	0.1800000
-23295.0000000	0.1800000
-23294.0000000	0.1800000
-23293.0000000	0.1800000
-23292.0000000	0.1805000
-23291.0000000	0.1809524
-23290.0000000	0.1815000
-23289.0000000	0.1800000
-23288.0000000	0.1825000
-23287.0000000	0.1820000
-23286.0000000	0.1828572
-23285.0000000	0.1810000
-23284.0000000	0.1810000
-23283.0000000	0.1845000
-23282.0000000	0.1828572
-23281.0000000	0.1815000

-23280.0000000	0.1809524
-23279.0000000	0.1810000
-23278.0000000	0.1815000
-23277.0000000	0.1809524
-23276.0000000	0.1805000
-23275.0000000	0.1805000
-23274.0000000	0.1800000
-23273.0000000	0.1800000
-23272.0000000	0.1805000
-23271.0000000	0.1800000
-23270.0000000	0.1800000
-23269.0000000	0.1800000
-23268.0000000	0.1800000
-23267.0000000	0.1800000
-23266.0000000	0.1800000
-23265.0000000	0.1800000
-23264.0000000	0.1800000
-23263.0000000	0.1800000
-23262.0000000	0.1800000
-23261.0000000	0.1800000
-23260.0000000	0.1800000
-23259.0000000	0.1800000
-23258.0000000	0.1800000
-23257.0000000	0.1800000
-23256.0000000	0.1800000
-23255.0000000	0.1800000
-23254.0000000	0.1800000
-23253.0000000	0.1795000
-23252.0000000	0.1795239
-23251.0000000	0.1805000
-23250.0000000	0.1800000
-23249.0000000	0.1800000
-23248.0000000	0.1800000
-23247.0000000	0.1800000
-23246.0000000	0.1800000
-23245.0000000	0.1805000
-23244.0000000	0.1800000
-23243.0000000	0.1800000
-23242.0000000	0.1800000
-23241.0000000	0.1800000
-23240.0000000	0.1790000
-23239.0000000	0.1795000
-23238.0000000	0.1790000
-23237.0000000	0.1790477
-23236.0000000	0.1780000
-23235.0000000	0.1785715
-23234.0000000	0.1780000
-23233.0000000	0.1795000
-23232.0000000	0.1800000
-23231.0000000	0.1795239
-23230.0000000	0.1790000
-23229.0000000	0.1795238
-23228.0000000	0.1800000
-23227.0000000	0.1800000
-23226.0000000	0.1795238
-23225.0000000	0.1790000
-23224.0000000	0.1800000

-23223.0000000	0.1795000
-23222.0000000	0.1800000
-23221.0000000	0.1790000
-23220.0000000	0.1795239
-23219.0000000	0.1795000
-23218.0000000	0.1790000
-23217.0000000	0.1750000
-23216.0000000	0.1757143
-23215.0000000	0.1740000
-23214.0000000	0.1752381
-23213.0000000	0.1725000
-23212.0000000	0.1720000
-23211.0000000	0.1709524
-23210.0000000	0.1715000
-23209.0000000	0.1700000
-23208.0000000	0.1705000
-23207.0000000	0.1700000
-23206.0000000	0.1700000
-23205.0000000	0.1700000
-23204.0000000	0.1700000
-23203.0000000	0.1695000
-23202.0000000	0.1670000
-23201.0000000	0.1700000
-23200.0000000	0.1670000
-23199.0000000	0.1680000
-23198.0000000	0.1690476
-23197.0000000	0.1690000
-23196.0000000	0.1685714
-23195.0000000	0.1695000
-23194.0000000	0.1695000
-23193.0000000	0.1690000
-23192.0000000	0.1690476
-23191.0000000	0.1695000
-23190.0000000	0.1700000
-23189.0000000	0.1695238
-23188.0000000	0.1690000
-23187.0000000	0.1685000
-23186.0000000	0.1700000
-23185.0000000	0.1695000
-23184.0000000	0.1690000
-23183.0000000	0.1700000
-23182.0000000	0.1695000
-23181.0000000	0.1695000
-23180.0000000	0.1685714
-23179.0000000	0.1680000
-23178.0000000	0.1680000
-23177.0000000	0.1657143
-23176.0000000	0.1650000
-23175.0000000	0.1642857
-23174.0000000	0.1630000
-23173.0000000	0.1620000
-23172.0000000	0.1615000
-23171.0000000	0.1614286
-23170.0000000	0.1615000
-23169.0000000	0.1610000
-23168.0000000	0.1604762
-23167.0000000	0.1610000

-23166.0000000	0.1600000
-23165.0000000	0.1605000
-23164.0000000	0.1605000
-23163.0000000	0.1625000
-23162.0000000	0.1604762
-23161.0000000	0.1625000
-23160.0000000	0.1615000
-23159.0000000	0.1604762
-23158.0000000	0.1615000
-23157.0000000	0.1610000
-23156.0000000	0.1610000
-23155.0000000	0.1628572
-23154.0000000	0.1610000
-23153.0000000	0.1614286
-23152.0000000	0.1620000
-23151.0000000	0.1600000
-23150.0000000	0.1600000
-23149.0000000	0.1600000
-23148.0000000	0.1600000
-23147.0000000	0.1604762
-23146.0000000	0.1600000
-23145.0000000	0.1600000
-23144.0000000	0.1600000
-23143.0000000	0.1600000
-23142.0000000	0.1600000
-23141.0000000	0.1600000
-23140.0000000	0.1600000
-23139.0000000	0.1600000
-23138.0000000	0.1590476
-23137.0000000	0.1600000
-23136.0000000	0.1590476
-23135.0000000	0.1595000
-23134.0000000	0.1595000
-23133.0000000	0.1600000
-23132.0000000	0.1595238
-23131.0000000	0.1590000
-23130.0000000	0.1590000
-23129.0000000	0.1600000
-23128.0000000	0.1595000
-23127.0000000	0.1590000
-23126.0000000	0.1590000
-23125.0000000	0.1595238
-23124.0000000	0.1600000
-23123.0000000	0.1580953
-23122.0000000	0.1585000
-23121.0000000	0.1595000
-23120.0000000	0.1571429
-23119.0000000	0.1550000
-23118.0000000	0.1560000
-23117.0000000	0.1530000
-23116.0000000	0.1523810
-23115.0000000	0.1530000
-23114.0000000	0.1538095
-23113.0000000	0.1525000
-23112.0000000	0.1545000
-23111.0000000	0.1542857
-23110.0000000	0.1540000

-23109.0000000	0.1530000
-23108.0000000	0.1525000
-23107.0000000	0.1519048
-23106.0000000	0.1523810
-23105.0000000	0.1520000
-23104.0000000	0.1520000
-23103.0000000	0.1500000
-23102.0000000	0.1519048
-23101.0000000	0.1505000
-23100.0000000	0.1510000
-23099.0000000	0.1509524
-23098.0000000	0.1505000
-23097.0000000	0.1500000
-23096.0000000	0.1500000
-23095.0000000	0.1500000
-23094.0000000	0.1495000
-23093.0000000	0.1500000
-23092.0000000	0.1500000
-23091.0000000	0.1500000
-23090.0000000	0.1500000
-23089.0000000	0.1500000
-23088.0000000	0.1500000
-23087.0000000	0.1500000
-23086.0000000	0.1500000
-23085.0000000	0.1500000
-23084.0000000	0.1500000
-23083.0000000	0.1500000
-23082.0000000	0.1500000
-23081.0000000	0.1500000
-23080.0000000	0.1500000
-23079.0000000	0.1500000
-23078.0000000	0.1500000
-23077.0000000	0.1500000
-23076.0000000	0.1500000
-23075.0000000	0.1500000
-23074.0000000	0.1500000
-23073.0000000	0.1500000
-23072.0000000	0.1500000
-23071.0000000	0.1500000
-23070.0000000	0.1500000
-23069.0000000	0.1500000
-23068.0000000	0.1495238
-23067.0000000	0.1500000
-23066.0000000	0.1500000
-23065.0000000	0.1500000
-23064.0000000	0.1495000
-23063.0000000	0.1485715
-23062.0000000	0.1490000
-23061.0000000	0.1495000
-23060.0000000	0.1495000
-23059.0000000	0.1500000
-23058.0000000	0.1500000
-23057.0000000	0.1500000
-23056.0000000	0.1490476
-23055.0000000	0.1495000
-23054.0000000	0.1490476
-23053.0000000	0.1470000

-23052.0000000	0.1465000
-23051.0000000	0.1465000
-23050.0000000	0.1476191
-23049.0000000	0.1455000
-23048.0000000	0.1460000
-23047.0000000	0.1452381
-23046.0000000	0.1425000
-23045.0000000	0.1423810
-23044.0000000	0.1425000
-23043.0000000	0.1415000
-23042.0000000	0.1425000
-23041.0000000	0.1428572
-23040.0000000	0.1415000
-23039.0000000	0.1425000
-23038.0000000	0.1428572
-23037.0000000	0.1420000
-23036.0000000	0.1435000
-23035.0000000	0.1445000
-23034.0000000	0.1423810
-23033.0000000	0.1414286
-23032.0000000	0.1425000
-23031.0000000	0.1420000
-23030.0000000	0.1415000
-23029.0000000	0.1409524
-23028.0000000	0.1405000
-23027.0000000	0.1400000
-23026.0000000	0.1400000
-23025.0000000	0.1405000
-23024.0000000	0.1400000
-23023.0000000	0.1400000
-23022.0000000	0.1400000
-23021.0000000	0.1400000
-23020.0000000	0.1400000
-23019.0000000	0.1400000
-23018.0000000	0.1400000
-23017.0000000	0.1395238
-23016.0000000	0.1400000
-23015.0000000	0.1400000
-23014.0000000	0.1400000
-23013.0000000	0.1400000
-23012.0000000	0.1400000
-23011.0000000	0.1400000
-23010.0000000	0.1400000
-23009.0000000	0.1400000
-23008.0000000	0.1404762
-23007.0000000	0.1400000
-23006.0000000	0.1405000
-23005.0000000	0.1405000
-23004.0000000	0.1404762
-23003.0000000	0.1400000
-23002.0000000	0.1400000
-23001.0000000	0.1400000
-23000.0000000	0.1405000
-22999.0000000	0.1400000
-22998.0000000	0.1405000
-22997.0000000	0.1415000
-22996.0000000	0.1410000

-22995.0000000	0.1400000
-22994.0000000	0.1400000
-22993.0000000	0.1400000
-22992.0000000	0.1405000
-22991.0000000	0.1400000
-22990.0000000	0.1400000
-22989.0000000	0.1400000
-22988.0000000	0.1400000
-22987.0000000	0.1400000
-22986.0000000	0.1400000
-22985.0000000	0.1400000
-22984.0000000	0.1404762
-22983.0000000	0.1400000
-22982.0000000	0.1400000
-22981.0000000	0.1400000
-22980.0000000	0.1410000
-22979.0000000	0.1400000
-22978.0000000	0.1404762
-22977.0000000	0.1400000
-22976.0000000	0.1405000
-22975.0000000	0.1405000
-22974.0000000	0.1409524
-22973.0000000	0.1400000
-22972.0000000	0.1400000
-22971.0000000	0.1400000
-22970.0000000	0.1400000
-22969.0000000	0.1414286
-22968.0000000	0.1400000
-22967.0000000	0.1400000
-22966.0000000	0.1400000
-22965.0000000	0.1400000
-22964.0000000	0.1400000
-22963.0000000	0.1400000
-22962.0000000	0.1400000
-22961.0000000	0.1405000
-22960.0000000	0.1400000
-22959.0000000	0.1400000
-22958.0000000	0.1400000
-22957.0000000	0.1400000
-22956.0000000	0.1400000
-22955.0000000	0.1400000
-22954.0000000	0.1400000
-22953.0000000	0.1405000
-22952.0000000	0.1400000
-22951.0000000	0.1400000
-22950.0000000	0.1400000
-22949.0000000	0.1400000
-22948.0000000	0.1400000
-22947.0000000	0.1400000
-22946.0000000	0.1405000
-22945.0000000	0.1400000
-22944.0000000	0.1400000
-22943.0000000	0.1400000
-22942.0000000	0.1400000
-22941.0000000	0.1400000
-22940.0000000	0.1395000
-22939.0000000	0.1400000

-22938.0000000	0.1400000
-22937.0000000	0.1395000
-22936.0000000	0.1390000
-22935.0000000	0.1400000
-22934.0000000	0.1395000
-22933.0000000	0.1385000
-22932.0000000	0.1385715
-22931.0000000	0.1395000
-22930.0000000	0.1385000
-22929.0000000	0.1380953
-22928.0000000	0.1395000
-22927.0000000	0.1390477
-22926.0000000	0.1395000
-22925.0000000	0.1400000
-22924.0000000	0.1390477
-22923.0000000	0.1400000
-22922.0000000	0.1400000
-22921.0000000	0.1400000
-22920.0000000	0.1400000
-22919.0000000	0.1400000
-22918.0000000	0.1395238
-22917.0000000	0.1395000
-22916.0000000	0.1395000
-22915.0000000	0.1390000
-22914.0000000	0.1371429
-22913.0000000	0.1385000
-22912.0000000	0.1380000
-22911.0000000	0.1385714
-22910.0000000	0.1380000
-22909.0000000	0.1380953
-22908.0000000	0.1390000
-22907.0000000	0.1375000
-22906.0000000	0.1390000
-22905.0000000	0.1380953
-22904.0000000	0.1370000
-22903.0000000	0.1380952
-22902.0000000	0.1370000
-22901.0000000	0.1385000
-22900.0000000	0.1390000
-22899.0000000	0.1385714
-22898.0000000	0.1375000
-22897.0000000	0.1375000
-22896.0000000	0.1380952
-22895.0000000	0.1390000
-22894.0000000	0.1380953
-22893.0000000	0.1380000
-22892.0000000	0.1375000
-22891.0000000	0.1385000
-22890.0000000	0.1385714
-22889.0000000	0.1395000
-22888.0000000	0.1395238
-22887.0000000	0.1390000
-22886.0000000	0.1385000
-22885.0000000	0.1375000
-22884.0000000	0.1380952
-22883.0000000	0.1365000
-22882.0000000	0.1375000

-22881.0000000	0.1380953
-22880.0000000	0.1360000
-22879.0000000	0.1357143
-22878.0000000	0.1340000
-22877.0000000	0.1335000
-22876.0000000	0.1345000
-22875.0000000	0.1342857
-22874.0000000	0.1330000
-22873.0000000	0.1335000
-22872.0000000	0.1319048
-22871.0000000	0.1310000
-22870.0000000	0.1315000
-22869.0000000	0.1309524
-22868.0000000	0.1310000
-22867.0000000	0.1315000
-22866.0000000	0.1309524
-22865.0000000	0.1305000
-22864.0000000	0.1315000
-22863.0000000	0.1300000
-22862.0000000	0.1305000
-22861.0000000	0.1310000
-22860.0000000	0.1323810
-22859.0000000	0.1310000
-22858.0000000	0.1314286
-22857.0000000	0.1310000
-22856.0000000	0.1305000
-22855.0000000	0.1325000
-22854.0000000	0.1309524
-22853.0000000	0.1315000
-22852.0000000	0.1300000
-22851.0000000	0.1304762
-22850.0000000	0.1315000
-22849.0000000	0.1309524
-22848.0000000	0.1315000
-22847.0000000	0.1300000
-22846.0000000	0.1300000
-22845.0000000	0.1300000
-22844.0000000	0.1305000
-22843.0000000	0.1300000
-22842.0000000	0.1304762
-22841.0000000	0.1300000
-22840.0000000	0.1300000
-22839.0000000	0.1300000
-22838.0000000	0.1295238
-22837.0000000	0.1300000
-22836.0000000	0.1290476
-22835.0000000	0.1285000
-22834.0000000	0.1285000
-22833.0000000	0.1290476
-22832.0000000	0.1275000
-22831.0000000	0.1280000
-22830.0000000	0.1280000
-22829.0000000	0.1280952
-22828.0000000	0.1266667
-22827.0000000	0.1270000
-22826.0000000	0.1270000
-22825.0000000	0.1285000

-22824.0000000	0.1276190
-22823.0000000	0.1265000
-22822.0000000	0.1270000
-22821.0000000	0.1271429
-22820.0000000	0.1290000
-22819.0000000	0.1280952
-22818.0000000	0.1280000
-22817.0000000	0.1270000
-22816.0000000	0.1295000
-22815.0000000	0.1295238
-22814.0000000	0.1300000
-22813.0000000	0.1290000
-22812.0000000	0.1295238
-22811.0000000	0.1295000
-22810.0000000	0.1295000
-22809.0000000	0.1295000
-22808.0000000	0.1285714
-22807.0000000	0.1290000
-22806.0000000	0.1300000
-22805.0000000	0.1295000
-22804.0000000	0.1290000
-22803.0000000	0.1300000
-22802.0000000	0.1295000
-22801.0000000	0.1300000
-22800.0000000	0.1295000
-22799.0000000	0.1300000
-22798.0000000	0.1300000
-22797.0000000	0.1285714
-22796.0000000	0.1280000
-22795.0000000	0.1300000
-22794.0000000	0.1300000
-22793.0000000	0.1300000
-22792.0000000	0.1295000
-22791.0000000	0.1295000
-22790.0000000	0.1300000
-22789.0000000	0.1300000
-22788.0000000	0.1300000
-22787.0000000	0.1300000
-22786.0000000	0.1300000
-22785.0000000	0.1295238
-22784.0000000	0.1295000
-22783.0000000	0.1295000
-22782.0000000	0.1285000
-22781.0000000	0.1290476
-22780.0000000	0.1285000
-22779.0000000	0.1295000
-22778.0000000	0.1295238
-22777.0000000	0.1300000
-22776.0000000	0.1295238
-22775.0000000	0.1295000
-22774.0000000	0.1300000
-22773.0000000	0.1300000
-22772.0000000	0.1300000
-22771.0000000	0.1300000
-22770.0000000	0.1290000
-22769.0000000	0.1295238
-22768.0000000	0.1295000

-22767.0000000	0.1300000
-22766.0000000	0.1300000
-22765.0000000	0.1290000
-22764.0000000	0.1295238
-22763.0000000	0.1295000
-22762.0000000	0.1300000
-22761.0000000	0.1300000
-22760.0000000	0.1295238
-22759.0000000	0.1290000
-22758.0000000	0.1300000
-22757.0000000	0.1300000
-22756.0000000	0.1290000
-22755.0000000	0.1295238
-22754.0000000	0.1290000
-22753.0000000	0.1300000
-22752.0000000	0.1295000
-22751.0000000	0.1295238
-22750.0000000	0.1280000
-22749.0000000	0.1285000
-22748.0000000	0.1290000
-22747.0000000	0.1276191
-22746.0000000	0.1271429
-22745.0000000	0.1280000
-22744.0000000	0.1285000
-22743.0000000	0.1280952
-22742.0000000	0.1260000
-22741.0000000	0.1265000
-22740.0000000	0.1240000
-22739.0000000	0.1247619
-22738.0000000	0.1245000
-22737.0000000	0.1228571
-22736.0000000	0.1230000
-22735.0000000	0.1240000
-22734.0000000	0.1240000
-22733.0000000	0.1223809
-22732.0000000	0.1230000
-22731.0000000	0.1210000
-22730.0000000	0.1219047
-22729.0000000	0.1210000
-22728.0000000	0.1219048
-22727.0000000	0.1210000
-22726.0000000	0.1210000
-22725.0000000	0.1210000
-22724.0000000	0.1204762
-22723.0000000	0.1215000
-22722.0000000	0.1215000
-22721.0000000	0.1228571
-22720.0000000	0.1225000
-22719.0000000	0.1240000
-22718.0000000	0.1215000
-22717.0000000	0.1233333
-22716.0000000	0.1225000
-22715.0000000	0.1219047
-22714.0000000	0.1230000
-22713.0000000	0.1235000
-22712.0000000	0.1233333
-22711.0000000	0.1245000

-22710.0000000	0.1215000
-22709.0000000	0.1219047
-22708.0000000	0.1215000
-22707.0000000	0.1230000
-22706.0000000	0.1233333
-22705.0000000	0.1215000
-22704.0000000	0.1210000
-22703.0000000	0.1209524
-22702.0000000	0.1215000
-22701.0000000	0.1210000
-22700.0000000	0.1214285
-22699.0000000	0.1200000
-22698.0000000	0.1214285
-22697.0000000	0.1200000
-22696.0000000	0.1210000
-22695.0000000	0.1210000
-22694.0000000	0.1204762
-22693.0000000	0.1215000
-22692.0000000	0.1200000
-22691.0000000	0.1200000
-22690.0000000	0.1200000
-22689.0000000	0.1200000
-22688.0000000	0.1205000
-22687.0000000	0.1204762
-22686.0000000	0.1200000
-22685.0000000	0.1200000
-22684.0000000	0.1205000
-22683.0000000	0.1195000
-22682.0000000	0.1204762
-22681.0000000	0.1210000
-22680.0000000	0.1200000
-22679.0000000	0.1204762
-22678.0000000	0.1205000
-22677.0000000	0.1210000
-22676.0000000	0.1200000
-22675.0000000	0.1205000
-22674.0000000	0.1215000
-22673.0000000	0.1209524
-22672.0000000	0.1200000
-22671.0000000	0.1200000
-22670.0000000	0.1209524
-22669.0000000	0.1200000
-22668.0000000	0.1200000
-22667.0000000	0.1204762
-22666.0000000	0.1200000
-22665.0000000	0.1200000
-22664.0000000	0.1200000
-22663.0000000	0.1200000
-22662.0000000	0.1200000
-22661.0000000	0.1200000
-22660.0000000	0.1200000
-22659.0000000	0.1200000
-22658.0000000	0.1200000
-22657.0000000	0.1200000
-22656.0000000	0.1200000
-22655.0000000	0.1200000
-22654.0000000	0.1200000

-22653.0000000	0.1200000
-22652.0000000	0.1200000
-22651.0000000	0.1200000
-22650.0000000	0.1200000
-22649.0000000	0.1200000
-22648.0000000	0.1200000
-22647.0000000	0.1200000
-22646.0000000	0.1200000
-22645.0000000	0.1200000
-22644.0000000	0.1200000
-22643.0000000	0.1200000
-22642.0000000	0.1200000
-22641.0000000	0.1205000
-22640.0000000	0.1204762
-22639.0000000	0.1200000
-22638.0000000	0.1210000
-22637.0000000	0.1200000
-22636.0000000	0.1200000
-22635.0000000	0.1200000
-22634.0000000	0.1200000
-22633.0000000	0.1204762
-22632.0000000	0.1200000
-22631.0000000	0.1200000
-22630.0000000	0.1200000
-22629.0000000	0.1200000
-22628.0000000	0.1200000
-22627.0000000	0.1200000
-22626.0000000	0.1200000
-22625.0000000	0.1200000
-22624.0000000	0.1195000
-22623.0000000	0.1200000
-22622.0000000	0.1200000
-22621.0000000	0.1200000
-22620.0000000	0.1180000
-22619.0000000	0.1195000
-22618.0000000	0.1195238
-22617.0000000	0.1185000
-22616.0000000	0.1200000
-22615.0000000	0.1180000
-22614.0000000	0.1185000
-22613.0000000	0.1190000
-22612.0000000	0.1185714
-22611.0000000	0.1180000
-22610.0000000	0.1190476
-22609.0000000	0.1185000
-22608.0000000	0.1180000
-22607.0000000	0.1185714
-22606.0000000	0.1195000
-22605.0000000	0.1200000
-22604.0000000	0.1190000
-22603.0000000	0.1200000
-22602.0000000	0.1195000
-22601.0000000	0.1195238
-22600.0000000	0.1200000
-22599.0000000	0.1200000
-22598.0000000	0.1200000
-22597.0000000	0.1200000

-22596.0000000	0.1195000
-22595.0000000	0.1195000
-22594.0000000	0.1200000
-22593.0000000	0.1200000
-22592.0000000	0.1200000
-22591.0000000	0.1200000
-22590.0000000	0.1200000
-22589.0000000	0.1200000
-22588.0000000	0.1200000
-22587.0000000	0.1200000
-22586.0000000	0.1200000
-22585.0000000	0.1195238
-22584.0000000	0.1200000
-22583.0000000	0.1200000
-22582.0000000	0.1200000
-22581.0000000	0.1200000
-22580.0000000	0.1200000
-22579.0000000	0.1200000
-22578.0000000	0.1200000
-22577.0000000	0.1200000
-22576.0000000	0.1200000
-22575.0000000	0.1200000
-22574.0000000	0.1200000
-22573.0000000	0.1195238
-22572.0000000	0.1200000
-22571.0000000	0.1200000
-22570.0000000	0.1195000
-22569.0000000	0.1195000
-22568.0000000	0.1180000
-22567.0000000	0.1190476
-22566.0000000	0.1185000
-22565.0000000	0.1185000
-22564.0000000	0.1185714
-22563.0000000	0.1195000
-22562.0000000	0.1180952
-22561.0000000	0.1190000
-22560.0000000	0.1185000
-22559.0000000	0.1195000
-22558.0000000	0.1200000
-22557.0000000	0.1195000
-22556.0000000	0.1190000
-22555.0000000	0.1195238
-22554.0000000	0.1200000
-22553.0000000	0.1200000
-22552.0000000	0.1200000
-22551.0000000	0.1200000
-22550.0000000	0.1195000
-22549.0000000	0.1200000
-22548.0000000	0.1200000
-22547.0000000	0.1200000
-22546.0000000	0.1200000
-22545.0000000	0.1195000
-22544.0000000	0.1200000
-22543.0000000	0.1200000
-22542.0000000	0.1200000
-22541.0000000	0.1200000
-22540.0000000	0.1200000

-22539.0000000	0.1200000
-22538.0000000	0.1200000
-22537.0000000	0.1200000
-22536.0000000	0.1200000
-22535.0000000	0.1200000
-22534.0000000	0.1200000
-22533.0000000	0.1200000
-22532.0000000	0.1200000
-22531.0000000	0.1200000
-22530.0000000	0.1200000
-22529.0000000	0.1205000
-22528.0000000	0.1200000
-22527.0000000	0.1200000
-22526.0000000	0.1200000
-22525.0000000	0.1200000
-22524.0000000	0.1200000
-22523.0000000	0.1200000
-22522.0000000	0.1200000
-22521.0000000	0.1204762
-22520.0000000	0.1200000
-22519.0000000	0.1200000
-22518.0000000	0.1200000
-22517.0000000	0.1200000
-22516.0000000	0.1200000
-22515.0000000	0.1195000
-22514.0000000	0.1200000
-22513.0000000	0.1200000
-22512.0000000	0.1200000
-22511.0000000	0.1200000
-22510.0000000	0.1200000
-22509.0000000	0.1195000
-22508.0000000	0.1200000
-22507.0000000	0.1200000
-22506.0000000	0.1195000
-22505.0000000	0.1195000
-22504.0000000	0.1200000
-22503.0000000	0.1195238
-22502.0000000	0.1200000
-22501.0000000	0.1190000
-22500.0000000	0.1200000
-22499.0000000	0.1195000
-22498.0000000	0.1200000
-22497.0000000	0.1200000
-22496.0000000	0.1200000
-22495.0000000	0.1200000
-22494.0000000	0.1200000
-22493.0000000	0.1200000
-22492.0000000	0.1200000
-22491.0000000	0.1200000
-22490.0000000	0.1200000
-22489.0000000	0.1200000
-22488.0000000	0.1200000
-22487.0000000	0.1200000
-22486.0000000	0.1200000
-22485.0000000	0.1200000
-22484.0000000	0.1200000
-22483.0000000	0.1215000

-22482.0000000	0.1209524
-22481.0000000	0.1220000
-22480.0000000	0.1209524
-22479.0000000	0.1210000
-22478.0000000	0.1215000
-22477.0000000	0.1214286
-22476.0000000	0.1215000
-22475.0000000	0.1215000
-22474.0000000	0.1220000
-22473.0000000	0.1219047
-22472.0000000	0.1220000
-22471.0000000	0.1223809
-22470.0000000	0.1230000
-22469.0000000	0.1240000
-22468.0000000	0.1271429
-22467.0000000	0.1270000
-22466.0000000	0.1270000
-22465.0000000	0.1280000
-22464.0000000	0.1290476
-22463.0000000	0.1285000
-22462.0000000	0.1270000
-22461.0000000	0.1276191
-22460.0000000	0.1285000
-22459.0000000	0.1285714
-22458.0000000	0.1290000
-22457.0000000	0.1290000
-22456.0000000	0.1285714
-22455.0000000	0.1295000
-22454.0000000	0.1255000
-22453.0000000	0.1250000
-22452.0000000	0.1280953
-22451.0000000	0.1250000
-22450.0000000	0.1266667
-22449.0000000	0.1260000
-22448.0000000	0.1275000
-22447.0000000	0.1270000
-22446.0000000	0.1280952
-22445.0000000	0.1290000
-22444.0000000	0.1290000
-22443.0000000	0.1295238
-22442.0000000	0.1290000
-22441.0000000	0.1290476
-22440.0000000	0.1300000
-22439.0000000	0.1300000
-22438.0000000	0.1300000
-22437.0000000	0.1300000
-22436.0000000	0.1300000
-22435.0000000	0.1300000
-22434.0000000	0.1300000
-22433.0000000	0.1305000
-22432.0000000	0.1300000
-22431.0000000	0.1300000
-22430.0000000	0.1300000
-22429.0000000	0.1300000
-22428.0000000	0.1300000
-22427.0000000	0.1300000
-22426.0000000	0.1300000

-22425.0000000	0.1300000
-22424.0000000	0.1300000
-22423.0000000	0.1300000
-22422.0000000	0.1300000
-22421.0000000	0.1300000
-22420.0000000	0.1300000
-22419.0000000	0.1300000
-22418.0000000	0.1300000
-22417.0000000	0.1300000
-22416.0000000	0.1290476
-22415.0000000	0.1295000
-22414.0000000	0.1300000
-22413.0000000	0.1295238
-22412.0000000	0.1290000
-22411.0000000	0.1285714
-22410.0000000	0.1300000
-22409.0000000	0.1285000
-22408.0000000	0.1295000
-22407.0000000	0.1285714
-22406.0000000	0.1275000
-22405.0000000	0.1295000
-22404.0000000	0.1271429
-22403.0000000	0.1285000
-22402.0000000	0.1285000
-22401.0000000	0.1295000
-22400.0000000	0.1285714
-22399.0000000	0.1300000
-22398.0000000	0.1290476
-22397.0000000	0.1270000
-22396.0000000	0.1260000
-22395.0000000	0.1228571
-22394.0000000	0.1255000
-22393.0000000	0.1260000
-22392.0000000	0.1276191
-22391.0000000	0.1275000
-22390.0000000	0.1270000
-22389.0000000	0.1257143
-22388.0000000	0.1285000
-22387.0000000	0.1270000
-22386.0000000	0.1276191
-22385.0000000	0.1290000
-22384.0000000	0.1295000
-22383.0000000	0.1295238
-22382.0000000	0.1295000
-22381.0000000	0.1295238
-22380.0000000	0.1285000
-22379.0000000	0.1300000
-22378.0000000	0.1295000
-22377.0000000	0.1290476
-22376.0000000	0.1300000
-22375.0000000	0.1300000
-22374.0000000	0.1300000
-22373.0000000	0.1295000
-22372.0000000	0.1300000
-22371.0000000	0.1300000
-22370.0000000	0.1295238
-22369.0000000	0.1295000

-22368.0000000	0.1300000
-22367.0000000	0.1300000
-22366.0000000	0.1295000
-22365.0000000	0.1290476
-22364.0000000	0.1300000
-22363.0000000	0.1295000
-22362.0000000	0.1295238
-22361.0000000	0.1295000
-22360.0000000	0.1295000
-22359.0000000	0.1295238
-22358.0000000	0.1285000
-22357.0000000	0.1290000
-22356.0000000	0.1300000
-22355.0000000	0.1300000
-22354.0000000	0.1295000
-22353.0000000	0.1295000
-22352.0000000	0.1271429
-22351.0000000	0.1270000
-22350.0000000	0.1276190
-22349.0000000	0.1265000
-22348.0000000	0.1265000
-22347.0000000	0.1257143
-22346.0000000	0.1260000
-22345.0000000	0.1255000
-22344.0000000	0.1271428
-22343.0000000	0.1240000
-22342.0000000	0.1245000
-22341.0000000	0.1245000
-22340.0000000	0.1247619
-22339.0000000	0.1225000
-22338.0000000	0.1238095
-22337.0000000	0.1255000
-22336.0000000	0.1275000
-22335.0000000	0.1271429
-22334.0000000	0.1270000
-22333.0000000	0.1265000
-22332.0000000	0.1290000
-22331.0000000	0.1295238
-22330.0000000	0.1290000
-22329.0000000	0.1295238
-22328.0000000	0.1285000
-22327.0000000	0.1285000
-22326.0000000	0.1285000
-22325.0000000	0.1285714
-22324.0000000	0.1290000
-22323.0000000	0.1285714
-22322.0000000	0.1290000
-22321.0000000	0.1295000
-22320.0000000	0.1285714
-22319.0000000	0.1300000
-22318.0000000	0.1285000
-22317.0000000	0.1300000
-22316.0000000	0.1290476
-22315.0000000	0.1295000
-22314.0000000	0.1300000
-22313.0000000	0.1300000
-22312.0000000	0.1300000

-22311.0000000	0.1300000
-22310.0000000	0.1304762
-22309.0000000	0.1300000
-22308.0000000	0.1300000
-22307.0000000	0.1300000
-22306.0000000	0.1300000
-22305.0000000	0.1295238
-22304.0000000	0.1285000
-22303.0000000	0.1300000
-22302.0000000	0.1300000
-22301.0000000	0.1300000
-22300.0000000	0.1300000
-22299.0000000	0.1290476
-22298.0000000	0.1300000
-22297.0000000	0.1300000
-22296.0000000	0.1300000
-22295.0000000	0.1295238
-22294.0000000	0.1300000
-22293.0000000	0.1300000
-22292.0000000	0.1300000
-22291.0000000	0.1300000
-22290.0000000	0.1300000
-22289.0000000	0.1300000
-22288.0000000	0.1300000
-22287.0000000	0.1300000
-22286.0000000	0.1300000
-22285.0000000	0.1300000
-22284.0000000	0.1304762
-22283.0000000	0.1300000
-22282.0000000	0.1300000
-22281.0000000	0.1305000
-22280.0000000	0.1300000
-22279.0000000	0.1305000
-22278.0000000	0.1300000
-22277.0000000	0.1304762
-22276.0000000	0.1300000
-22275.0000000	0.1300000
-22274.0000000	0.1300000
-22273.0000000	0.1305000
-22272.0000000	0.1300000
-22271.0000000	0.1300000
-22270.0000000	0.1305000
-22269.0000000	0.1305000
-22268.0000000	0.1304762
-22267.0000000	0.1305000
-22266.0000000	0.1300000
-22265.0000000	0.1300000
-22264.0000000	0.1300000
-22263.0000000	0.1315000
-22262.0000000	0.1304762
-22261.0000000	0.1305000
-22260.0000000	0.1315000
-22259.0000000	0.1300000
-22258.0000000	0.1305000
-22257.0000000	0.1300000
-22256.0000000	0.1314286
-22255.0000000	0.1315000

-22254.0000000	0.1314286
-22253.0000000	0.1320000
-22252.0000000	0.1310000
-22251.0000000	0.1310000
-22250.0000000	0.1319048
-22249.0000000	0.1325000
-22248.0000000	0.1325000
-22247.0000000	0.1323810
-22246.0000000	0.1320000
-22245.0000000	0.1314286
-22244.0000000	0.1320000
-22243.0000000	0.1320000
-22242.0000000	0.1335000
-22241.0000000	0.1328571
-22240.0000000	0.1330000
-22239.0000000	0.1335000
-22238.0000000	0.1338095
-22237.0000000	0.1330000
-22236.0000000	0.1355000
-22235.0000000	0.1325000
-22234.0000000	0.1328572
-22233.0000000	0.1320000
-22232.0000000	0.1328571
-22231.0000000	0.1325000
-22230.0000000	0.1320000
-22229.0000000	0.1347619
-22228.0000000	0.1360000
-22227.0000000	0.1365000
-22226.0000000	0.1357143
-22225.0000000	0.1360000
-22224.0000000	0.1390477
-22223.0000000	0.1370000
-22222.0000000	0.1385000
-22221.0000000	0.1395000
-22220.0000000	0.1385715
-22219.0000000	0.1395000
-22218.0000000	0.1400000
-22217.0000000	0.1400000
-22216.0000000	0.1395000
-22215.0000000	0.1395238
-22214.0000000	0.1400000
-22213.0000000	0.1400000
-22212.0000000	0.1400000
-22211.0000000	0.1400000
-22210.0000000	0.1400000
-22209.0000000	0.1400000
-22208.0000000	0.1395238
-22207.0000000	0.1400000
-22206.0000000	0.1400000
-22205.0000000	0.1400000
-22204.0000000	0.1400000
-22203.0000000	0.1400000
-22202.0000000	0.1400000
-22201.0000000	0.1400000
-22200.0000000	0.1400000
-22199.0000000	0.1400000
-22198.0000000	0.1400000

-22197.0000000	0.1400000
-22196.0000000	0.1400000
-22195.0000000	0.1400000
-22194.0000000	0.1400000
-22193.0000000	0.1400000
-22192.0000000	0.1400000
-22191.0000000	0.1400000
-22190.0000000	0.1400000
-22189.0000000	0.1400000
-22188.0000000	0.1400000
-22187.0000000	0.1395238
-22186.0000000	0.1395000
-22185.0000000	0.1395000
-22184.0000000	0.1400000
-22183.0000000	0.1400000
-22182.0000000	0.1400000
-22181.0000000	0.1400000
-22180.0000000	0.1400000
-22179.0000000	0.1400000
-22178.0000000	0.1400000
-22177.0000000	0.1400000
-22176.0000000	0.1400000
-22175.0000000	0.1405000
-22174.0000000	0.1400000
-22173.0000000	0.1400000
-22172.0000000	0.1400000
-22171.0000000	0.1400000
-22170.0000000	0.1395000
-22169.0000000	0.1400000
-22168.0000000	0.1400000
-22167.0000000	0.1400000
-22166.0000000	0.1395238
-22165.0000000	0.1395000
-22164.0000000	0.1400000
-22163.0000000	0.1400000
-22162.0000000	0.1400000
-22161.0000000	0.1400000
-22160.0000000	0.1400000
-22159.0000000	0.1400000
-22158.0000000	0.1400000
-22157.0000000	0.1400000
-22156.0000000	0.1400000
-22155.0000000	0.1400000
-22154.0000000	0.1415000
-22153.0000000	0.1404762
-22152.0000000	0.1410000
-22151.0000000	0.1405000
-22150.0000000	0.1419048
-22149.0000000	0.1420000
-22148.0000000	0.1425000
-22147.0000000	0.1415000
-22146.0000000	0.1414286
-22145.0000000	0.1415000
-22144.0000000	0.1433334
-22143.0000000	0.1435000
-22142.0000000	0.1420000
-22141.0000000	0.1419048

-22140.0000000	0.1420000
-22139.0000000	0.1415000
-22138.0000000	0.1405000
-22137.0000000	0.1409524
-22136.0000000	0.1414286
-22135.0000000	0.1405000
-22134.0000000	0.1400000
-22133.0000000	0.1400000
-22132.0000000	0.1404762
-22131.0000000	0.1415000
-22130.0000000	0.1405000
-22129.0000000	0.1415000
-22128.0000000	0.1428572
-22127.0000000	0.1428572
-22126.0000000	0.1425000
-22125.0000000	0.1465000
-22124.0000000	0.1450000
-22123.0000000	0.1461905
-22122.0000000	0.1450000
-22121.0000000	0.1470000
-22120.0000000	0.1485715
-22119.0000000	0.1490000
-22118.0000000	0.1490000
-22117.0000000	0.1485000
-22116.0000000	0.1495238
-22115.0000000	0.1490000
-22114.0000000	0.1490476
-22113.0000000	0.1495000
-22112.0000000	0.1490000
-22111.0000000	0.1485715
-22110.0000000	0.1490000
-22109.0000000	0.1495000
-22108.0000000	0.1495000
-22107.0000000	0.1495238
-22106.0000000	0.1500000
-22105.0000000	0.1500000
-22104.0000000	0.1500000
-22103.0000000	0.1500000
-22102.0000000	0.1500000
-22101.0000000	0.1500000
-22100.0000000	0.1500000
-22099.0000000	0.1490000
-22098.0000000	0.1500000
-22097.0000000	0.1490000
-22096.0000000	0.1500000
-22095.0000000	0.1495000
-22094.0000000	0.1500000
-22093.0000000	0.1500000
-22092.0000000	0.1500000
-22091.0000000	0.1500000
-22090.0000000	0.1500000
-22089.0000000	0.1500000
-22088.0000000	0.1500000
-22087.0000000	0.1500000
-22086.0000000	0.1495238
-22085.0000000	0.1495000
-22084.0000000	0.1485715

-22083.0000000	0.1485000
-22082.0000000	0.1490000
-22081.0000000	0.1490476
-22080.0000000	0.1495000
-22079.0000000	0.1485000
-22078.0000000	0.1495000
-22077.0000000	0.1476191
-22076.0000000	0.1475000
-22075.0000000	0.1485715
-22074.0000000	0.1485000
-22073.0000000	0.1500000
-22072.0000000	0.1485000
-22071.0000000	0.1490476
-22070.0000000	0.1490000
-22069.0000000	0.1495000
-22068.0000000	0.1500000
-22067.0000000	0.1495000
-22066.0000000	0.1500000
-22065.0000000	0.1500000
-22064.0000000	0.1500000
-22063.0000000	0.1490000
-22062.0000000	0.1495238
-22061.0000000	0.1500000
-22060.0000000	0.1480000
-22059.0000000	0.1495238
-22058.0000000	0.1495000
-22057.0000000	0.1495000
-22056.0000000	0.1485000
-22055.0000000	0.1476191
-22054.0000000	0.1476191
-22053.0000000	0.1485000
-22052.0000000	0.1485000
-22051.0000000	0.1480000
-22050.0000000	0.1480953
-22049.0000000	0.1490000
-22048.0000000	0.1490000
-22047.0000000	0.1500000
-22046.0000000	0.1485000
-22045.0000000	0.1490476
-22044.0000000	0.1495000
-22043.0000000	0.1500000
-22042.0000000	0.1490000
-22041.0000000	0.1495238
-22040.0000000	0.1500000
-22039.0000000	0.1490000
-22038.0000000	0.1480953
-22037.0000000	0.1495000
-22036.0000000	0.1485715
-22035.0000000	0.1485000
-22034.0000000	0.1485000
-22033.0000000	0.1500000
-22032.0000000	0.1500000
-22031.0000000	0.1470000
-22030.0000000	0.1490000
-22029.0000000	0.1485715
-22028.0000000	0.1495000
-22027.0000000	0.1475000

-22026.0000000	0.1490000
-22025.0000000	0.1495238
-22024.0000000	0.1490000
-22023.0000000	0.1480953
-22022.0000000	0.1490000
-22021.0000000	0.1490000
-22020.0000000	0.1495238
-22019.0000000	0.1490000
-22018.0000000	0.1480000
-22017.0000000	0.1457143
-22016.0000000	0.1460000
-22015.0000000	0.1442858
-22014.0000000	0.1425000
-22013.0000000	0.1415000
-22012.0000000	0.1415000
-22011.0000000	0.1423810
-22010.0000000	0.1405000
-22009.0000000	0.1400000
-22008.0000000	0.1404762
-22007.0000000	0.1405000
-22006.0000000	0.1400000
-22005.0000000	0.1415000
-22004.0000000	0.1400000
-22003.0000000	0.1410000
-22002.0000000	0.1400000
-22001.0000000	0.1405000
-22000.0000000	0.1405000
-21999.0000000	0.1400000
-21998.0000000	0.1405000
-21997.0000000	0.1400000
-21996.0000000	0.1400000
-21995.0000000	0.1400000
-21994.0000000	0.1400000
-21993.0000000	0.1404762
-21992.0000000	0.1400000
-21991.0000000	0.1400000
-21990.0000000	0.1400000
-21989.0000000	0.1400000
-21988.0000000	0.1405000
-21987.0000000	0.1400000
-21986.0000000	0.1400000
-21985.0000000	0.1400000
-21984.0000000	0.1400000
-21983.0000000	0.1400000
-21982.0000000	0.1400000
-21981.0000000	0.1400000
-21980.0000000	0.1400000
-21979.0000000	0.1400000
-21978.0000000	0.1400000
-21977.0000000	0.1400000
-21976.0000000	0.1390000
-21975.0000000	0.1390477
-21974.0000000	0.1395000
-21973.0000000	0.1390000
-21972.0000000	0.1380953
-21971.0000000	0.1390000
-21970.0000000	0.1395000

-21969.0000000	0.1395238
-21968.0000000	0.1390000
-21967.0000000	0.1395000
-21966.0000000	0.1390000
-21965.0000000	0.1400000
-21964.0000000	0.1400000
-21963.0000000	0.1400000
-21962.0000000	0.1400000
-21961.0000000	0.1400000
-21960.0000000	0.1395238
-21959.0000000	0.1400000
-21958.0000000	0.1400000
-21957.0000000	0.1400000
-21956.0000000	0.1400000
-21955.0000000	0.1400000
-21954.0000000	0.1400000
-21953.0000000	0.1400000
-21952.0000000	0.1400000
-21951.0000000	0.1400000
-21950.0000000	0.1400000
-21949.0000000	0.1400000
-21948.0000000	0.1400000
-21947.0000000	0.1400000
-21946.0000000	0.1400000
-21945.0000000	0.1409524
-21944.0000000	0.1405000
-21943.0000000	0.1405000
-21942.0000000	0.1400000
-21941.0000000	0.1404762
-21940.0000000	0.1405000
-21939.0000000	0.1409524
-21938.0000000	0.1405000
-21937.0000000	0.1405000
-21936.0000000	0.1400000
-21935.0000000	0.1414286
-21934.0000000	0.1410000
-21933.0000000	0.1404762
-21932.0000000	0.1410000
-21931.0000000	0.1400000
-21930.0000000	0.1404762
-21929.0000000	0.1410000
-21928.0000000	0.1400000
-21927.0000000	0.1400000
-21926.0000000	0.1400000
-21925.0000000	0.1400000
-21924.0000000	0.1409524
-21923.0000000	0.1400000
-21922.0000000	0.1405000
-21921.0000000	0.1400000
-21920.0000000	0.1404762
-21919.0000000	0.1400000
-21918.0000000	0.1414286
-21917.0000000	0.1400000
-21916.0000000	0.1400000
-21915.0000000	0.1400000
-21914.0000000	0.1395000
-21913.0000000	0.1400000

-21912.0000000	0.1400000
-21911.0000000	0.1400000
-21910.0000000	0.1400000
-21909.0000000	0.1400000
-21908.0000000	0.1400000
-21907.0000000	0.1400000
-21906.0000000	0.1400000
-21905.0000000	0.1400000
-21904.0000000	0.1400000
-21903.0000000	0.1400000
-21902.0000000	0.1400000
-21901.0000000	0.1400000
-21900.0000000	0.1400000
-21899.0000000	0.1400000
-21898.0000000	0.1400000
-21897.0000000	0.1400000
-21896.0000000	0.1400000
-21895.0000000	0.1400000
-21894.0000000	0.1400000
-21893.0000000	0.1400000
-21892.0000000	0.1400000
-21891.0000000	0.1405000
-21890.0000000	0.1400000
-21889.0000000	0.1400000
-21888.0000000	0.1400000
-21887.0000000	0.1400000
-21886.0000000	0.1400000
-21885.0000000	0.1400000
-21884.0000000	0.1400000
-21883.0000000	0.1405000
-21882.0000000	0.1400000
-21881.0000000	0.1400000
-21880.0000000	0.1400000
-21879.0000000	0.1404762
-21878.0000000	0.1400000
-21877.0000000	0.1400000
-21876.0000000	0.1400000
-21875.0000000	0.1404762
-21874.0000000	0.1405000
-21873.0000000	0.1400000
-21872.0000000	0.1409524
-21871.0000000	0.1405000
-21870.0000000	0.1400000
-21869.0000000	0.1400000
-21868.0000000	0.1405000
-21867.0000000	0.1410000
-21866.0000000	0.1400000
-21865.0000000	0.1400000
-21864.0000000	0.1405000
-21863.0000000	0.1404762
-21862.0000000	0.1400000
-21861.0000000	0.1405000
-21860.0000000	0.1404762
-21859.0000000	0.1400000
-21858.0000000	0.1400000
-21857.0000000	0.1400000
-21856.0000000	0.1400000

-21855.0000000	0.1395000
-21854.0000000	0.1400000
-21853.0000000	0.1400000
-21852.0000000	0.1400000
-21851.0000000	0.1400000
-21850.0000000	0.1400000
-21849.0000000	0.1400000
-21848.0000000	0.1400000
-21847.0000000	0.1390000
-21846.0000000	0.1385000
-21845.0000000	0.1385715
-21844.0000000	0.1368421
-21843.0000000	0.1371429
-21842.0000000	0.1352381
-21841.0000000	0.1385000
-21840.0000000	0.1366667
-21839.0000000	0.1375000
-21838.0000000	0.1365000
-21837.0000000	0.1360000
-21836.0000000	0.1366667
-21835.0000000	0.1370000
-21834.0000000	0.1355000
-21833.0000000	0.1376191
-21832.0000000	0.1370000
-21831.0000000	0.1390000
-21830.0000000	0.1380000
-21829.0000000	0.1400000
-21828.0000000	0.1390000
-21827.0000000	0.1390477
-21826.0000000	0.1395000
-21825.0000000	0.1400000
-21824.0000000	0.1395238
-21823.0000000	0.1395000
-21822.0000000	0.1395000
-21821.0000000	0.1400000
-21820.0000000	0.1400000
-21819.0000000	0.1400000
-21818.0000000	0.1400000
-21817.0000000	0.1400000
-21816.0000000	0.1390000
-21815.0000000	0.1385714
-21814.0000000	0.1400000
-21813.0000000	0.1390000
-21812.0000000	0.1380000
-21811.0000000	0.1390477
-21810.0000000	0.1400000
-21809.0000000	0.1385000
-21808.0000000	0.1395000
-21807.0000000	0.1385000
-21806.0000000	0.1385714
-21805.0000000	0.1375000
-21804.0000000	0.1375000
-21803.0000000	0.1390476
-21802.0000000	0.1360000
-21801.0000000	0.1355000
-21800.0000000	0.1360000
-21799.0000000	0.1366667

-21798.0000000	0.1365000
-21797.0000000	0.1376191
-21796.0000000	0.1375000
-21795.0000000	0.1375000
-21794.0000000	0.1376191
-21793.0000000	0.1385000
-21792.0000000	0.1390000
-21791.0000000	0.1370000
-21790.0000000	0.1376191
-21789.0000000	0.1370000
-21788.0000000	0.1371429
-21787.0000000	0.1365000
-21786.0000000	0.1380000
-21785.0000000	0.1371429
-21784.0000000	0.1385000
-21783.0000000	0.1375000
-21782.0000000	0.1370000
-21781.0000000	0.1376191
-21780.0000000	0.1380000
-21779.0000000	0.1371429
-21778.0000000	0.1375000
-21777.0000000	0.1380000
-21776.0000000	0.1395238
-21775.0000000	0.1390000
-21774.0000000	0.1390000
-21773.0000000	0.1390000
-21772.0000000	0.1395238
-21771.0000000	0.1385000
-21770.0000000	0.1385000
-21769.0000000	0.1390476
-21768.0000000	0.1390000
-21767.0000000	0.1395238
-21766.0000000	0.1365000
-21765.0000000	0.1390000
-21764.0000000	0.1400000
-21763.0000000	0.1385000
-21762.0000000	0.1385000
-21761.0000000	0.1395000
-21760.0000000	0.1395238
-21759.0000000	0.1400000
-21758.0000000	0.1400000
-21757.0000000	0.1400000
-21756.0000000	0.1400000
-21755.0000000	0.1395000
-21754.0000000	0.1400000
-21753.0000000	0.1400000
-21752.0000000	0.1390000
-21751.0000000	0.1380953
-21750.0000000	0.1380000
-21749.0000000	0.1376191
-21748.0000000	0.1355000
-21747.0000000	0.1370000
-21746.0000000	0.1355000
-21745.0000000	0.1342857
-21744.0000000	0.1330000
-21743.0000000	0.1340000
-21742.0000000	0.1342857

-21741.0000000	0.1335000
-21740.0000000	0.1325000
-21739.0000000	0.1335000
-21738.0000000	0.1314286
-21737.0000000	0.1314286
-21736.0000000	0.1315000
-21735.0000000	0.1315000
-21734.0000000	0.1315000
-21733.0000000	0.1309524
-21732.0000000	0.1305000
-21731.0000000	0.1315000
-21730.0000000	0.1323810
-21729.0000000	0.1305000
-21728.0000000	0.1314286
-21727.0000000	0.1300000
-21726.0000000	0.1300000
-21725.0000000	0.1305000
-21724.0000000	0.1314286
-21723.0000000	0.1310000
-21722.0000000	0.1310000
-21721.0000000	0.1314286
-21720.0000000	0.1310000
-21719.0000000	0.1323810
-21718.0000000	0.1345000
-21717.0000000	0.1345000
-21716.0000000	0.1345000
-21715.0000000	0.1333333
-21714.0000000	0.1335000
-21713.0000000	0.1330000
-21712.0000000	0.1328571
-21711.0000000	0.1330000
-21710.0000000	0.1330000
-21709.0000000	0.1330000
-21708.0000000	0.1333334
-21707.0000000	0.1340000
-21706.0000000	0.1314286
-21705.0000000	0.1320000
-21704.0000000	0.1320000
-21703.0000000	0.1309524
-21702.0000000	0.1315000
-21701.0000000	0.1315000
-21700.0000000	0.1314286
-21699.0000000	0.1330000
-21698.0000000	0.1315000
-21697.0000000	0.1314286
-21696.0000000	0.1305000
-21695.0000000	0.1300000
-21694.0000000	0.1304762
-21693.0000000	0.1300000
-21692.0000000	0.1300000
-21691.0000000	0.1300000
-21690.0000000	0.1300000
-21689.0000000	0.1295238
-21688.0000000	0.1300000
-21687.0000000	0.1295000
-21686.0000000	0.1300000
-21685.0000000	0.1300000

-21684.0000000	0.1295000
-21683.0000000	0.1300000
-21682.0000000	0.1300000
-21681.0000000	0.1300000
-21680.0000000	0.1300000
-21679.0000000	0.1300000
-21678.0000000	0.1300000
-21677.0000000	0.1300000
-21676.0000000	0.1300000
-21675.0000000	0.1300000
-21674.0000000	0.1305000
-21673.0000000	0.1314286
-21672.0000000	0.1305000
-21671.0000000	0.1310000
-21670.0000000	0.1305000
-21669.0000000	0.1304762
-21668.0000000	0.1300000
-21667.0000000	0.1304762
-21666.0000000	0.1300000
-21665.0000000	0.1300000
-21664.0000000	0.1300000
-21663.0000000	0.1305000
-21662.0000000	0.1305000
-21661.0000000	0.1304762
-21660.0000000	0.1305000
-21659.0000000	0.1305000
-21658.0000000	0.1323810
-21657.0000000	0.1310000
-21656.0000000	0.1330000
-21655.0000000	0.1333333
-21654.0000000	0.1340000
-21653.0000000	0.1350000
-21652.0000000	0.1347619
-21651.0000000	0.1340000
-21650.0000000	0.1355000
-21649.0000000	0.1355000
-21648.0000000	0.1366667
-21647.0000000	0.1330000
-21646.0000000	0.1338095
-21645.0000000	0.1330000
-21644.0000000	0.1325000
-21643.0000000	0.1333333
-21642.0000000	0.1315000
-21641.0000000	0.1325000
-21640.0000000	0.1315000
-21639.0000000	0.1309524
-21638.0000000	0.1325000
-21637.0000000	0.1319048
-21636.0000000	0.1305000
-21635.0000000	0.1335000
-21634.0000000	0.1330000
-21633.0000000	0.1333333
-21632.0000000	0.1325000
-21631.0000000	0.1304762
-21630.0000000	0.1315000
-21629.0000000	0.1350000
-21628.0000000	0.1319048

-21627.0000000	0.1335000
-21626.0000000	0.1345000
-21625.0000000	0.1330000
-21624.0000000	0.1338095
-21623.0000000	0.1335000
-21622.0000000	0.1352381
-21621.0000000	0.1325000
-21620.0000000	0.1315000
-21619.0000000	0.1330000
-21618.0000000	0.1314286
-21617.0000000	0.1315000
-21616.0000000	0.1300000
-21615.0000000	0.1300000
-21614.0000000	0.1300000
-21613.0000000	0.1300000
-21612.0000000	0.1300000
-21611.0000000	0.1300000
-21610.0000000	0.1300000
-21609.0000000	0.1304762
-21608.0000000	0.1305000
-21607.0000000	0.1300000
-21606.0000000	0.1305000
-21605.0000000	0.1320000
-21604.0000000	0.1305000
-21603.0000000	0.1304762
-21602.0000000	0.1315000
-21601.0000000	0.1305000
-21600.0000000	0.1323810
-21599.0000000	0.1310000
-21598.0000000	0.1314286
-21597.0000000	0.1315000
-21596.0000000	0.1305000
-21595.0000000	0.1305000
-21594.0000000	0.1304762
-21593.0000000	0.1300000
-21592.0000000	0.1300000
-21591.0000000	0.1305000
-21590.0000000	0.1300000
-21589.0000000	0.1300000
-21588.0000000	0.1300000
-21587.0000000	0.1305000
-21586.0000000	0.1300000
-21585.0000000	0.1300000
-21584.0000000	0.1305000
-21583.0000000	0.1304762
-21582.0000000	0.1300000
-21581.0000000	0.1300000
-21580.0000000	0.1300000
-21579.0000000	0.1300000
-21578.0000000	0.1300000
-21577.0000000	0.1305000
-21576.0000000	0.1300000
-21575.0000000	0.1300000
-21574.0000000	0.1315000
-21573.0000000	0.1309524
-21572.0000000	0.1305000
-21571.0000000	0.1305000

-21570.0000000	0.1300000
-21569.0000000	0.1300000
-21568.0000000	0.1305000
-21567.0000000	0.1304762
-21566.0000000	0.1315000
-21565.0000000	0.1305000
-21564.0000000	0.1304762
-21563.0000000	0.1310000
-21562.0000000	0.1300000
-21561.0000000	0.1300000
-21560.0000000	0.1300000
-21559.0000000	0.1305000
-21558.0000000	0.1300000
-21557.0000000	0.1295000
-21556.0000000	0.1300000
-21555.0000000	0.1300000
-21554.0000000	0.1300000
-21553.0000000	0.1300000
-21552.0000000	0.1300000
-21551.0000000	0.1300000
-21550.0000000	0.1300000
-21549.0000000	0.1300000
-21548.0000000	0.1300000
-21547.0000000	0.1300000
-21546.0000000	0.1300000
-21545.0000000	0.1300000
-21544.0000000	0.1300000
-21543.0000000	0.1300000
-21542.0000000	0.1300000
-21541.0000000	0.1300000
-21540.0000000	0.1300000
-21539.0000000	0.1300000
-21538.0000000	0.1300000
-21537.0000000	0.1300000
-21536.0000000	0.1295000
-21535.0000000	0.1300000
-21534.0000000	0.1300000
-21533.0000000	0.1300000
-21532.0000000	0.1300000
-21531.0000000	0.1300000
-21530.0000000	0.1300000
-21529.0000000	0.1300000
-21528.0000000	0.1295238
-21527.0000000	0.1300000
-21526.0000000	0.1300000
-21525.0000000	0.1300000
-21524.0000000	0.1295000
-21523.0000000	0.1300000
-21522.0000000	0.1300000
-21521.0000000	0.1300000
-21520.0000000	0.1300000
-21519.0000000	0.1300000
-21518.0000000	0.1300000
-21517.0000000	0.1300000
-21516.0000000	0.1300000
-21515.0000000	0.1300000
-21514.0000000	0.1300000

-21513.0000000	0.1300000
-21512.0000000	0.1300000
-21511.0000000	0.1300000
-21510.0000000	0.1300000
-21509.0000000	0.1300000
-21508.0000000	0.1300000
-21507.0000000	0.1300000
-21506.0000000	0.1300000
-21505.0000000	0.1300000
-21504.0000000	0.1300000
-21503.0000000	0.1300000
-21502.0000000	0.1300000
-21501.0000000	0.1300000
-21500.0000000	0.1300000
-21499.0000000	0.1310000
-21498.0000000	0.1315000
-21497.0000000	0.1300000
-21496.0000000	0.1310000
-21495.0000000	0.1310000
-21494.0000000	0.1314286
-21493.0000000	0.1300000
-21492.0000000	0.1320000
-21491.0000000	0.1315000
-21490.0000000	0.1330000
-21489.0000000	0.1323810
-21488.0000000	0.1310000
-21487.0000000	0.1310000
-21486.0000000	0.1320000
-21485.0000000	0.1309524
-21484.0000000	0.1305000
-21483.0000000	0.1300000
-21482.0000000	0.1300000
-21481.0000000	0.1300000
-21480.0000000	0.1300000
-21479.0000000	0.1300000
-21478.0000000	0.1300000
-21477.0000000	0.1300000
-21476.0000000	0.1290000
-21475.0000000	0.1300000
-21474.0000000	0.1295000
-21473.0000000	0.1285714
-21472.0000000	0.1280000
-21471.0000000	0.1280952
-21470.0000000	0.1285000
-21469.0000000	0.1265000
-21468.0000000	0.1280952
-21467.0000000	0.1275000
-21466.0000000	0.1285000
-21465.0000000	0.1260000
-21464.0000000	0.1271429
-21463.0000000	0.1275000
-21462.0000000	0.1271429
-21461.0000000	0.1285000
-21460.0000000	0.1295000
-21459.0000000	0.1285714
-21458.0000000	0.1295000
-21457.0000000	0.1290000

-21456.0000000	0.1300000
-21455.0000000	0.1290476
-21454.0000000	0.1285000
-21453.0000000	0.1300000
-21452.0000000	0.1280952
-21451.0000000	0.1270000
-21450.0000000	0.1300000
-21449.0000000	0.1295000
-21448.0000000	0.1300000
-21447.0000000	0.1300000
-21446.0000000	0.1300000
-21445.0000000	0.1300000
-21444.0000000	0.1300000
-21443.0000000	0.1300000
-21442.0000000	0.1300000
-21441.0000000	0.1304762
-21440.0000000	0.1300000
-21439.0000000	0.1300000
-21438.0000000	0.1300000
-21437.0000000	0.1300000
-21436.0000000	0.1300000
-21435.0000000	0.1310000
-21434.0000000	0.1304762
-21433.0000000	0.1305000
-21432.0000000	0.1300000
-21431.0000000	0.1305000
-21430.0000000	0.1300000
-21429.0000000	0.1300000
-21428.0000000	0.1304762
-21427.0000000	0.1300000
-21426.0000000	0.1310000
-21425.0000000	0.1300000
-21424.0000000	0.1300000
-21423.0000000	0.1305000
-21422.0000000	0.1300000
-21421.0000000	0.1300000
-21420.0000000	0.1300000
-21419.0000000	0.1300000
-21418.0000000	0.1300000
-21417.0000000	0.1300000
-21416.0000000	0.1300000
-21415.0000000	0.1305000
-21414.0000000	0.1305000
-21413.0000000	0.1300000
-21412.0000000	0.1300000
-21411.0000000	0.1300000
-21410.0000000	0.1300000
-21409.0000000	0.1300000
-21408.0000000	0.1300000
-21407.0000000	0.1300000
-21406.0000000	0.1305000
-21405.0000000	0.1300000
-21404.0000000	0.1300000
-21403.0000000	0.1300000
-21402.0000000	0.1300000
-21401.0000000	0.1300000
-21400.0000000	0.1300000

-21399.0000000	0.1300000
-21398.0000000	0.1300000
-21397.0000000	0.1300000
-21396.0000000	0.1300000
-21395.0000000	0.1304762
-21394.0000000	0.1300000
-21393.0000000	0.1300000
-21392.0000000	0.1300000
-21391.0000000	0.1300000
-21390.0000000	0.1295000
-21389.0000000	0.1300000
-21388.0000000	0.1295000
-21387.0000000	0.1290000
-21386.0000000	0.1290476
-21385.0000000	0.1280000
-21384.0000000	0.1290000
-21383.0000000	0.1295238
-21382.0000000	0.1300000
-21381.0000000	0.1300000
-21380.0000000	0.1300000
-21379.0000000	0.1300000
-21378.0000000	0.1295000
-21377.0000000	0.1280952
-21376.0000000	0.1295000
-21375.0000000	0.1300000
-21374.0000000	0.1295238
-21373.0000000	0.1300000
-21372.0000000	0.1290000
-21371.0000000	0.1300000
-21370.0000000	0.1295000
-21369.0000000	0.1300000
-21368.0000000	0.1295238
-21367.0000000	0.1290000
-21366.0000000	0.1300000
-21365.0000000	0.1300000
-21364.0000000	0.1290000
-21363.0000000	0.1300000
-21362.0000000	0.1300000
-21361.0000000	0.1300000
-21360.0000000	0.1300000
-21359.0000000	0.1295238
-21358.0000000	0.1295000
-21357.0000000	0.1295000
-21356.0000000	0.1295238
-21355.0000000	0.1285000
-21354.0000000	0.1265000
-21353.0000000	0.1285000
-21352.0000000	0.1276191
-21351.0000000	0.1285000
-21350.0000000	0.1271429
-21349.0000000	0.1260000
-21348.0000000	0.1250000
-21347.0000000	0.1275000
-21346.0000000	0.1242857
-21345.0000000	0.1245000
-21344.0000000	0.1228571
-21343.0000000	0.1240000

-21342.0000000	0.1230000
-21341.0000000	0.1219047
-21340.0000000	0.1225000
-21339.0000000	0.1230000
-21338.0000000	0.1225000
-21337.0000000	0.1242857
-21336.0000000	0.1220000
-21335.0000000	0.1223809
-21334.0000000	0.1230000
-21333.0000000	0.1235000
-21332.0000000	0.1225000
-21331.0000000	0.1238095
-21330.0000000	0.1230000
-21329.0000000	0.1255000
-21328.0000000	0.1261905
-21327.0000000	0.1245000
-21326.0000000	0.1242857
-21325.0000000	0.1220000
-21324.0000000	0.1240000
-21323.0000000	0.1225000
-21322.0000000	0.1238095
-21321.0000000	0.1260000
-21320.0000000	0.1266667
-21319.0000000	0.1250000
-21318.0000000	0.1255000
-21317.0000000	0.1245000
-21316.0000000	0.1247619
-21315.0000000	0.1250000
-21314.0000000	0.1242857
-21313.0000000	0.1250000
-21312.0000000	0.1245000
-21311.0000000	0.1228571
-21310.0000000	0.1235000
-21309.0000000	0.1205000
-21308.0000000	0.1260000
-21307.0000000	0.1247619
-21306.0000000	0.1255000
-21305.0000000	0.1209524
-21304.0000000	0.1225000
-21303.0000000	0.1210000
-21302.0000000	0.1200000
-21301.0000000	0.1200000
-21300.0000000	0.1200000
-21299.0000000	0.1200000
-21298.0000000	0.1204762
-21297.0000000	0.1200000
-21296.0000000	0.1200000
-21295.0000000	0.1205000
-21294.0000000	0.1200000
-21293.0000000	0.1200000
-21292.0000000	0.1200000
-21291.0000000	0.1200000
-21290.0000000	0.1200000
-21289.0000000	0.1200000
-21288.0000000	0.1200000
-21287.0000000	0.1200000
-21286.0000000	0.1200000

-21285.0000000	0.1205000
-21284.0000000	0.1219048
-21283.0000000	0.1215000
-21282.0000000	0.1220000
-21281.0000000	0.1214286
-21280.0000000	0.1255000
-21279.0000000	0.1225000
-21278.0000000	0.1245000
-21277.0000000	0.1242857
-21276.0000000	0.1240000
-21275.0000000	0.1238095
-21274.0000000	0.1260000
-21273.0000000	0.1270000
-21272.0000000	0.1250000
-21271.0000000	0.1280952
-21270.0000000	0.1265000
-21269.0000000	0.1255000
-21268.0000000	0.1257143
-21267.0000000	0.1275000
-21266.0000000	0.1271429
-21265.0000000	0.1280000
-21264.0000000	0.1280000
-21263.0000000	0.1280000
-21262.0000000	0.1295238
-21261.0000000	0.1290000
-21260.0000000	0.1290000
-21259.0000000	0.1295238
-21258.0000000	0.1300000
-21257.0000000	0.1300000
-21256.0000000	0.1280000
-21255.0000000	0.1295238
-21254.0000000	0.1300000
-21253.0000000	0.1300000
-21252.0000000	0.1300000
-21251.0000000	0.1300000
-21250.0000000	0.1300000
-21249.0000000	0.1300000
-21248.0000000	0.1300000
-21247.0000000	0.1300000
-21246.0000000	0.1300000
-21245.0000000	0.1300000
-21244.0000000	0.1300000
-21243.0000000	0.1305000
-21242.0000000	0.1305000
-21241.0000000	0.1300000
-21240.0000000	0.1300000
-21239.0000000	0.1300000
-21238.0000000	0.1300000
-21237.0000000	0.1300000
-21236.0000000	0.1300000
-21235.0000000	0.1300000
-21234.0000000	0.1300000
-21233.0000000	0.1300000
-21232.0000000	0.1300000
-21231.0000000	0.1300000
-21230.0000000	0.1295000
-21229.0000000	0.1290476

-21228.0000000	0.1295000
-21227.0000000	0.1290000
-21226.0000000	0.1270000
-21225.0000000	0.1266667
-21224.0000000	0.1260000
-21223.0000000	0.1247619
-21222.0000000	0.1240000
-21221.0000000	0.1215000
-21220.0000000	0.1209524
-21219.0000000	0.1205000
-21218.0000000	0.1210000
-21217.0000000	0.1210000
-21216.0000000	0.1200000
-21215.0000000	0.1204762
-21214.0000000	0.1200000
-21213.0000000	0.1210000
-21212.0000000	0.1205000
-21211.0000000	0.1200000
-21210.0000000	0.1200000
-21209.0000000	0.1200000
-21208.0000000	0.1210000
-21207.0000000	0.1200000
-21206.0000000	0.1200000
-21205.0000000	0.1205000
-21204.0000000	0.1215000
-21203.0000000	0.1200000
-21202.0000000	0.1204762
-21201.0000000	0.1205000
-21200.0000000	0.1225000
-21199.0000000	0.1209524
-21198.0000000	0.1210000
-21197.0000000	0.1220000
-21196.0000000	0.1205000
-21195.0000000	0.1209524
-21194.0000000	0.1235000
-21193.0000000	0.1238095
-21192.0000000	0.1235000
-21191.0000000	0.1245000
-21190.0000000	0.1257143
-21189.0000000	0.1235000
-21188.0000000	0.1260000
-21187.0000000	0.1270000
-21186.0000000	0.1271429
-21185.0000000	0.1270000
-21184.0000000	0.1276191
-21183.0000000	0.1280000
-21182.0000000	0.1295000
-21181.0000000	0.1285714
-21180.0000000	0.1280000
-21179.0000000	0.1270000
-21178.0000000	0.1270000
-21177.0000000	0.1276191
-21176.0000000	0.1270000
-21175.0000000	0.1238095
-21174.0000000	0.1250000
-21173.0000000	0.1260000
-21172.0000000	0.1252381

-21171.0000000	0.1265000
-21170.0000000	0.1260000
-21169.0000000	0.1255000
-21168.0000000	0.1238095
-21167.0000000	0.1240000
-21166.0000000	0.1235000
-21165.0000000	0.1233333
-21164.0000000	0.1240000
-21163.0000000	0.1223809
-21162.0000000	0.1205000
-21161.0000000	0.1225000
-21160.0000000	0.1209524
-21159.0000000	0.1210000
-21158.0000000	0.1205000
-21157.0000000	0.1200000
-21156.0000000	0.1209524
-21155.0000000	0.1200000
-21154.0000000	0.1200000
-21153.0000000	0.1200000
-21152.0000000	0.1200000
-21151.0000000	0.1200000
-21150.0000000	0.1200000
-21149.0000000	0.1200000
-21148.0000000	0.1200000
-21147.0000000	0.1200000
-21146.0000000	0.1200000
-21145.0000000	0.1200000
-21144.0000000	0.1200000
-21143.0000000	0.1200000
-21142.0000000	0.1200000
-21141.0000000	0.1200000
-21140.0000000	0.1200000
-21139.0000000	0.1200000
-21138.0000000	0.1200000
-21137.0000000	0.1200000
-21136.0000000	0.1200000
-21135.0000000	0.1200000
-21134.0000000	0.1200000
-21133.0000000	0.1200000
-21132.0000000	0.1200000
-21131.0000000	0.1200000
-21130.0000000	0.1195238
-21129.0000000	0.1200000
-21128.0000000	0.1195000
-21127.0000000	0.1195000
-21126.0000000	0.1190476
-21125.0000000	0.1200000
-21124.0000000	0.1200000
-21123.0000000	0.1195000
-21122.0000000	0.1195000
-21121.0000000	0.1200000
-21120.0000000	0.1200000
-21119.0000000	0.1200000
-21118.0000000	0.1200000
-21117.0000000	0.1200000
-21116.0000000	0.1200000
-21115.0000000	0.1200000

-21114.0000000	0.1200000
-21113.0000000	0.1200000
-21112.0000000	0.1200000
-21111.0000000	0.1200000
-21110.0000000	0.1200000
-21109.0000000	0.1205000
-21108.0000000	0.1200000
-21107.0000000	0.1200000
-21106.0000000	0.1200000
-21105.0000000	0.1200000
-21104.0000000	0.1200000
-21103.0000000	0.1200000
-21102.0000000	0.1200000
-21101.0000000	0.1200000
-21100.0000000	0.1200000
-21099.0000000	0.1200000
-21098.0000000	0.1200000
-21097.0000000	0.1200000
-21096.0000000	0.1200000
-21095.0000000	0.1200000
-21094.0000000	0.1200000
-21093.0000000	0.1200000
-21092.0000000	0.1200000
-21091.0000000	0.1200000
-21090.0000000	0.1204762
-21089.0000000	0.1205000
-21088.0000000	0.1215000
-21087.0000000	0.1209524
-21086.0000000	0.1205000
-21085.0000000	0.1205000
-21084.0000000	0.1200000
-21083.0000000	0.1200000
-21082.0000000	0.1200000
-21081.0000000	0.1200000
-21080.0000000	0.1200000
-21079.0000000	0.1200000
-21078.0000000	0.1200000
-21077.0000000	0.1200000
-21076.0000000	0.1200000
-21075.0000000	0.1200000
-21074.0000000	0.1195238
-21073.0000000	0.1200000
-21072.0000000	0.1200000
-21071.0000000	0.1200000
-21070.0000000	0.1200000
-21069.0000000	0.1195238
-21068.0000000	0.1200000
-21067.0000000	0.1200000
-21066.0000000	0.1195238
-21065.0000000	0.1200000
-21064.0000000	0.1200000
-21063.0000000	0.1200000
-21062.0000000	0.1200000
-21061.0000000	0.1200000
-21060.0000000	0.1200000
-21059.0000000	0.1200000
-21058.0000000	0.1195000

-21057.0000000	0.1200000
-21056.0000000	0.1200000
-21055.0000000	0.1200000
-21054.0000000	0.1200000
-21053.0000000	0.1195000
-21052.0000000	0.1200000
-21051.0000000	0.1180952
-21050.0000000	0.1165000
-21049.0000000	0.1170000
-21048.0000000	0.1166666
-21047.0000000	0.1160000
-21046.0000000	0.1185000
-21045.0000000	0.1185000
-21044.0000000	0.1166666
-21043.0000000	0.1195000
-21042.0000000	0.1195238
-21041.0000000	0.1185000
-21040.0000000	0.1195000
-21039.0000000	0.1195238
-21038.0000000	0.1185000
-21037.0000000	0.1195000
-21036.0000000	0.1195000
-21035.0000000	0.1195238
-21034.0000000	0.1195000
-21033.0000000	0.1200000
-21032.0000000	0.1195000
-21031.0000000	0.1200000
-21030.0000000	0.1195000
-21029.0000000	0.1200000
-21028.0000000	0.1200000
-21027.0000000	0.1195238
-21026.0000000	0.1200000
-21025.0000000	0.1200000
-21024.0000000	0.1200000
-21023.0000000	0.1190000
-21022.0000000	0.1195000
-21021.0000000	0.1195000
-21020.0000000	0.1190476
-21019.0000000	0.1195000
-21018.0000000	0.1200000
-21017.0000000	0.1200000
-21016.0000000	0.1200000
-21015.0000000	0.1200000
-21014.0000000	0.1190476
-21013.0000000	0.1195000
-21012.0000000	0.1195000
-21011.0000000	0.1200000
-21010.0000000	0.1195000
-21009.0000000	0.1200000
-21008.0000000	0.1200000
-21007.0000000	0.1200000
-21006.0000000	0.1200000
-21005.0000000	0.1200000
-21004.0000000	0.1200000
-21003.0000000	0.1200000
-21002.0000000	0.1200000
-21001.0000000	0.1200000

-21000.0000000	0.1195000
-20999.0000000	0.1195238
-20998.0000000	0.1180000
-20997.0000000	0.1200000
-20996.0000000	0.1165000
-20995.0000000	0.1170000
-20994.0000000	0.1190476
-20993.0000000	0.1180000
-20992.0000000	0.1190000
-20991.0000000	0.1190000
-20990.0000000	0.1200000
-20989.0000000	0.1200000
-20988.0000000	0.1200000
-20987.0000000	0.1200000
-20986.0000000	0.1200000
-20985.0000000	0.1200000
-20984.0000000	0.1200000
-20983.0000000	0.1200000
-20982.0000000	0.1200000
-20981.0000000	0.1200000
-20980.0000000	0.1195000
-20979.0000000	0.1195238
-20978.0000000	0.1195000
-20977.0000000	0.1195000
-20976.0000000	0.1195000
-20975.0000000	0.1195238
-20974.0000000	0.1185000
-20973.0000000	0.1190000
-20972.0000000	0.1200000
-20971.0000000	0.1185000
-20970.0000000	0.1195000
-20969.0000000	0.1190476
-20968.0000000	0.1165000
-20967.0000000	0.1171428
-20966.0000000	0.1150000
-20965.0000000	0.1140000
-20964.0000000	0.1110000
-20963.0000000	0.1119047
-20962.0000000	0.1110000
-20961.0000000	0.1105000
-20960.0000000	0.1109524
-20959.0000000	0.1105000
-20958.0000000	0.1100000
-20957.0000000	0.1105000
-20956.0000000	0.1100000
-20955.0000000	0.1100000
-20954.0000000	0.1100000
-20953.0000000	0.1100000
-20952.0000000	0.1100000
-20951.0000000	0.1100000
-20950.0000000	0.1100000
-20949.0000000	0.1100000
-20948.0000000	0.1100000
-20947.0000000	0.1100000
-20946.0000000	0.1100000
-20945.0000000	0.1100000
-20944.0000000	0.1100000

-20943.0000000	0.1105000
-20942.0000000	0.1100000
-20941.0000000	0.1100000
-20940.0000000	0.1100000
-20939.0000000	0.1100000
-20938.0000000	0.1100000
-20937.0000000	0.1100000
-20936.0000000	0.1104762
-20935.0000000	0.1100000
-20934.0000000	0.1105000
-20933.0000000	0.1100000
-20932.0000000	0.1100000
-20931.0000000	0.1100000
-20930.0000000	0.1100000
-20929.0000000	0.1100000
-20928.0000000	0.1100000
-20927.0000000	0.1105000
-20926.0000000	0.1105000
-20925.0000000	0.1105000
-20924.0000000	0.1100000
-20923.0000000	0.1100000
-20922.0000000	0.1100000
-20921.0000000	0.1100000
-20920.0000000	0.1100000
-20919.0000000	0.1095238
-20918.0000000	0.1095000
-20917.0000000	0.1090000
-20916.0000000	0.1095000
-20915.0000000	0.1090476
-20914.0000000	0.1090000
-20913.0000000	0.1100000
-20912.0000000	0.1095238
-20911.0000000	0.1100000
-20910.0000000	0.1100000
-20909.0000000	0.1100000
-20908.0000000	0.1095238
-20907.0000000	0.1100000
-20906.0000000	0.1100000
-20905.0000000	0.1100000
-20904.0000000	0.1100000
-20903.0000000	0.1104762
-20902.0000000	0.1100000
-20901.0000000	0.1100000
-20900.0000000	0.1100000
-20899.0000000	0.1100000
-20898.0000000	0.1100000
-20897.0000000	0.1100000
-20896.0000000	0.1110000
-20895.0000000	0.1100000
-20894.0000000	0.1100000
-20893.0000000	0.1105000
-20892.0000000	0.1100000
-20891.0000000	0.1100000
-20890.0000000	0.1100000
-20889.0000000	0.1100000
-20888.0000000	0.1100000
-20887.0000000	0.1100000

-20886.0000000	0.1105000
-20885.0000000	0.1100000
-20884.0000000	0.1100000
-20883.0000000	0.1100000
-20882.0000000	0.1100000
-20881.0000000	0.1100000
-20880.0000000	0.1100000
-20879.0000000	0.1100000
-20878.0000000	0.1100000
-20877.0000000	0.1100000
-20876.0000000	0.1090476
-20875.0000000	0.1090000
-20874.0000000	0.1100000
-20873.0000000	0.1095238
-20872.0000000	0.1095000
-20871.0000000	0.1090000
-20870.0000000	0.1085000
-20869.0000000	0.1095238
-20868.0000000	0.1100000
-20867.0000000	0.1095238
-20866.0000000	0.1095000
-20865.0000000	0.1090000
-20864.0000000	0.1090476
-20863.0000000	0.1100000
-20862.0000000	0.1100000
-20861.0000000	0.1100000
-20860.0000000	0.1100000
-20859.0000000	0.1100000
-20858.0000000	0.1100000
-20857.0000000	0.1100000
-20856.0000000	0.1095000
-20855.0000000	0.1100000
-20854.0000000	0.1100000
-20853.0000000	0.1100000
-20852.0000000	0.1095000
-20851.0000000	0.1085714
-20850.0000000	0.1100000
-20849.0000000	0.1080000
-20848.0000000	0.1090476
-20847.0000000	0.1085000
-20846.0000000	0.1085714
-20845.0000000	0.1090000
-20844.0000000	0.1090000
-20843.0000000	0.1090476
-20842.0000000	0.1080000
-20841.0000000	0.1085000
-20840.0000000	0.1065000
-20839.0000000	0.1090476
-20838.0000000	0.1095000
-20837.0000000	0.1095238
-20836.0000000	0.1085000
-20835.0000000	0.1095000
-20834.0000000	0.1090000
-20833.0000000	0.1095238
-20832.0000000	0.1100000
-20831.0000000	0.1075000
-20830.0000000	0.1080952

-20829.0000000	0.1045000
-20828.0000000	0.1042857
-20827.0000000	0.1005000
-20826.0000000	0.1000000
-20825.0000000	0.1020000
-20824.0000000	0.1004762
-20823.0000000	0.1000000
-20822.0000000	0.1005000
-20821.0000000	0.1014286
-20820.0000000	0.1005000
-20819.0000000	0.1000000
-20818.0000000	0.1020000
-20817.0000000	0.1023810
-20816.0000000	0.1030000
-20815.0000000	0.1028572
-20814.0000000	0.1060000
-20813.0000000	0.1052381
-20812.0000000	0.1035000
-20811.0000000	0.1040000
-20810.0000000	0.1070000
-20809.0000000	0.1076190
-20808.0000000	0.1035000
-20807.0000000	0.1080952
-20806.0000000	0.1055000
-20805.0000000	0.1070000
-20804.0000000	0.1075000
-20803.0000000	0.1061905
-20802.0000000	0.1090000
-20801.0000000	0.1095000
-20800.0000000	0.1090476
-20799.0000000	0.1075000
-20798.0000000	0.1080952
-20797.0000000	0.1095000
-20796.0000000	0.1060000
-20795.0000000	0.1050000
-20794.0000000	0.1038095
-20793.0000000	0.1020000
-20792.0000000	0.1025000
-20791.0000000	0.1014286
-20790.0000000	0.1025000
-20789.0000000	0.1035000
-20788.0000000	0.1030000
-20787.0000000	0.1052381
-20786.0000000	0.1040000
-20785.0000000	0.1019048
-20784.0000000	0.1035000
-20783.0000000	0.1030000
-20782.0000000	0.1033333
-20781.0000000	0.1015000
-20780.0000000	0.1020000
-20779.0000000	0.1004762
-20778.0000000	0.1015000
-20777.0000000	0.1000000
-20776.0000000	0.1019048
-20775.0000000	0.1000000
-20774.0000000	0.1000000
-20773.0000000	0.1009524

-20772.0000000	0.1010000
-20771.0000000	0.1025000
-20770.0000000	0.1019048
-20769.0000000	0.1005000
-20768.0000000	0.1010000
-20767.0000000	0.1014286
-20766.0000000	0.1015000
-20765.0000000	0.1010000
-20764.0000000	0.1033333
-20763.0000000	0.1020000
-20762.0000000	0.1040000
-20761.0000000	0.1009524
-20760.0000000	0.1025000
-20759.0000000	0.1015000
-20758.0000000	0.1020000
-20757.0000000	0.1014286
-20756.0000000	0.1010000
-20755.0000000	0.1000000
-20754.0000000	0.1005000
-20753.0000000	0.1005000
-20752.0000000	0.1009524
-20751.0000000	0.1000000
-20750.0000000	0.1005000
-20749.0000000	0.1000000
-20748.0000000	0.1000000
-20747.0000000	0.1000000
-20746.0000000	0.1004762
-20745.0000000	0.1000000
-20744.0000000	0.1005000
-20743.0000000	0.1010000
-20742.0000000	0.1000000
-20741.0000000	0.1000000
-20740.0000000	0.1009524
-20739.0000000	0.1005000
-20738.0000000	0.1030000
-20737.0000000	0.1019048
-20736.0000000	0.1055000
-20735.0000000	0.1045000
-20734.0000000	0.1070000
-20733.0000000	0.1038095
-20732.0000000	0.1045000
-20731.0000000	0.1042857
-20730.0000000	0.1030000
-20729.0000000	0.1025000
-20728.0000000	0.1035000
-20727.0000000	0.1057143
-20726.0000000	0.1035000
-20725.0000000	0.1038095
-20724.0000000	0.1050000
-20723.0000000	0.1040000
-20722.0000000	0.1047619
-20721.0000000	0.1020000
-20720.0000000	0.1025000
-20719.0000000	0.1030000
-20718.0000000	0.1023810
-20717.0000000	0.1015000
-20716.0000000	0.1009524

-20715.0000000	0.1010000
-20714.0000000	0.1030000
-20713.0000000	0.1040000
-20712.0000000	0.1057143
-20711.0000000	0.1045000
-20710.0000000	0.1052381
-20709.0000000	0.1070000
-20708.0000000	0.1055000
-20707.0000000	0.1042857
-20706.0000000	0.1060000
-20705.0000000	0.1045000
-20704.0000000	0.1080000
-20703.0000000	0.1085714
-20702.0000000	0.1075000
-20701.0000000	0.1076190
-20700.0000000	0.1090000
-20699.0000000	0.1095000
-20698.0000000	0.1095000
-20697.0000000	0.1095238
-20696.0000000	0.1100000
-20695.0000000	0.1100000
-20694.0000000	0.1095238
-20693.0000000	0.1085000
-20692.0000000	0.1100000
-20691.0000000	0.1095000
-20690.0000000	0.1100000
-20689.0000000	0.1100000
-20688.0000000	0.1090476
-20687.0000000	0.1095000
-20686.0000000	0.1100000
-20685.0000000	0.1100000
-20684.0000000	0.1100000
-20683.0000000	0.1100000
-20682.0000000	0.1100000
-20681.0000000	0.1100000
-20680.0000000	0.1100000
-20679.0000000	0.1100000
-20678.0000000	0.1100000
-20677.0000000	0.1105000
-20676.0000000	0.1100000
-20675.0000000	0.1100000
-20674.0000000	0.1100000
-20673.0000000	0.1100000
-20672.0000000	0.1100000
-20671.0000000	0.1100000
-20670.0000000	0.1100000
-20669.0000000	0.1100000
-20668.0000000	0.1100000
-20667.0000000	0.1095238
-20666.0000000	0.1095000
-20665.0000000	0.1100000
-20664.0000000	0.1100000
-20663.0000000	0.1090000
-20662.0000000	0.1100000
-20661.0000000	0.1100000
-20660.0000000	0.1080000
-20659.0000000	0.1070000

-20658.0000000	0.1071429
-20657.0000000	0.1060000
-20656.0000000	0.1070000
-20655.0000000	0.1057143
-20654.0000000	0.1045000
-20653.0000000	0.1040000
-20652.0000000	0.1042857
-20651.0000000	0.1025000
-20650.0000000	0.1023810
-20649.0000000	0.1020000
-20648.0000000	0.1030000
-20647.0000000	0.1015000
-20646.0000000	0.1028572
-20645.0000000	0.1015000
-20644.0000000	0.1005000
-20643.0000000	0.1009524
-20642.0000000	0.1005000
-20641.0000000	0.1000000
-20640.0000000	0.1010000
-20639.0000000	0.1000000
-20638.0000000	0.1000000
-20637.0000000	0.1000000
-20636.0000000	0.1000000
-20635.0000000	0.1000000
-20634.0000000	0.1000000
-20633.0000000	0.1000000
-20632.0000000	0.1000000
-20631.0000000	0.1000000
-20630.0000000	0.1005000
-20629.0000000	0.1000000
-20628.0000000	0.1000000
-20627.0000000	0.1000000
-20626.0000000	0.1000000
-20625.0000000	0.1000000
-20624.0000000	0.1000000
-20623.0000000	0.1000000
-20622.0000000	0.1000000
-20621.0000000	0.1000000
-20620.0000000	0.1000000
-20619.0000000	0.1000000
-20618.0000000	0.1000000
-20617.0000000	0.1000000
-20616.0000000	0.1000000
-20615.0000000	0.1000000
-20614.0000000	0.1000000
-20613.0000000	0.0995000
-20612.0000000	0.1000000
-20611.0000000	0.1000000
-20610.0000000	0.0995238
-20609.0000000	0.1000000
-20608.0000000	0.1000000
-20607.0000000	0.0990476
-20606.0000000	0.1000000
-20605.0000000	0.0995000
-20604.0000000	0.0990000
-20603.0000000	0.0990476
-20602.0000000	0.1000000

-20601.0000000	0.0995000
-20600.0000000	0.1000000
-20599.0000000	0.1000000
-20598.0000000	0.1000000
-20597.0000000	0.1000000
-20596.0000000	0.1000000
-20595.0000000	0.1000000
-20594.0000000	0.1000000
-20593.0000000	0.0995000
-20592.0000000	0.1000000
-20591.0000000	0.1000000
-20590.0000000	0.1000000
-20589.0000000	0.0990476
-20588.0000000	0.0995000
-20587.0000000	0.0995000
-20586.0000000	0.0995238
-20585.0000000	0.0985000
-20584.0000000	0.0990000
-20583.0000000	0.1000000
-20582.0000000	0.1000000
-20581.0000000	0.0995000
-20580.0000000	0.0995238
-20579.0000000	0.0990000
-20578.0000000	0.0990000
-20577.0000000	0.1000000
-20576.0000000	0.0990000
-20575.0000000	0.0995000
-20574.0000000	0.0995000
-20573.0000000	0.1000000
-20572.0000000	0.1000000
-20571.0000000	0.1000000
-20570.0000000	0.1000000
-20569.0000000	0.0995000
-20568.0000000	0.1000000
-20567.0000000	0.1000000
-20566.0000000	0.1000000
-20565.0000000	0.1000000
-20564.0000000	0.0995000
-20563.0000000	0.1000000
-20562.0000000	0.1000000
-20561.0000000	0.1000000
-20560.0000000	0.1000000
-20559.0000000	0.1000000
-20558.0000000	0.1000000
-20557.0000000	0.0995000
-20556.0000000	0.1000000
-20555.0000000	0.1000000
-20554.0000000	0.1000000
-20553.0000000	0.0995000
-20552.0000000	0.1000000
-20551.0000000	0.1000000
-20550.0000000	0.1000000
-20549.0000000	0.1000000
-20548.0000000	0.1000000
-20547.0000000	0.1005000
-20546.0000000	0.1000000
-20545.0000000	0.1000000

-20544.0000000	0.1000000
-20543.0000000	0.1000000
-20542.0000000	0.1000000
-20541.0000000	0.1000000
-20540.0000000	0.1005000
-20539.0000000	0.1000000
-20538.0000000	0.1000000
-20537.0000000	0.1000000
-20536.0000000	0.1000000
-20535.0000000	0.1005000
-20534.0000000	0.1000000
-20533.0000000	0.1010000
-20532.0000000	0.1005000
-20531.0000000	0.1000000
-20530.0000000	0.1005000
-20529.0000000	0.1004762
-20528.0000000	0.1005000
-20527.0000000	0.1000000
-20526.0000000	0.1019048
-20525.0000000	0.1030000
-20524.0000000	0.1015000
-20523.0000000	0.1025000
-20522.0000000	0.1009524
-20521.0000000	0.1005000
-20520.0000000	0.1028572
-20519.0000000	0.1020000
-20518.0000000	0.1030000
-20517.0000000	0.1045000
-20516.0000000	0.1028572
-20515.0000000	0.1015000
-20514.0000000	0.1010000
-20513.0000000	0.1004762
-20512.0000000	0.1005000
-20511.0000000	0.1004762
-20510.0000000	0.1000000
-20509.0000000	0.1000000
-20508.0000000	0.1000000
-20507.0000000	0.1000000
-20506.0000000	0.1000000
-20505.0000000	0.1000000
-20504.0000000	0.1000000
-20503.0000000	0.1000000
-20502.0000000	0.1000000
-20501.0000000	0.1000000
-20500.0000000	0.1000000
-20499.0000000	0.1000000
-20498.0000000	0.1000000
-20497.0000000	0.1000000
-20496.0000000	0.1000000
-20495.0000000	0.0990000
-20494.0000000	0.1000000
-20493.0000000	0.1000000
-20492.0000000	0.1000000
-20491.0000000	0.1000000
-20490.0000000	0.1000000
-20489.0000000	0.1000000
-20488.0000000	0.1000000

-20487.0000000	0.1000000
-20486.0000000	0.0995238
-20485.0000000	0.1000000
-20484.0000000	0.1000000
-20483.0000000	0.1000000
-20482.0000000	0.1000000
-20481.0000000	0.0995238
-20480.0000000	0.1000000
-20479.0000000	0.1000000
-20478.0000000	0.1000000
-20477.0000000	0.0995238
-20476.0000000	0.0985000
-20475.0000000	0.0975000
-20474.0000000	0.0980953
-20473.0000000	0.0985000
-20472.0000000	0.0985000
-20471.0000000	0.0965000
-20470.0000000	0.0980953
-20469.0000000	0.0990000
-20468.0000000	0.0947619
-20467.0000000	0.0970000
-20466.0000000	0.0971429
-20465.0000000	0.0975000
-20464.0000000	0.0960000
-20463.0000000	0.0930000
-20462.0000000	0.0971429
-20461.0000000	0.0965000
-20460.0000000	0.0970000
-20459.0000000	0.0990476
-20458.0000000	0.0990000
-20457.0000000	0.0955000
-20456.0000000	0.0957143
-20455.0000000	0.0985000
-20454.0000000	0.0990000
-20453.0000000	0.0985714
-20452.0000000	0.0990000
-20451.0000000	0.0995000
-20450.0000000	0.0985714
-20449.0000000	0.1000000
-20448.0000000	0.0990000
-20447.0000000	0.0995238
-20446.0000000	0.1000000
-20445.0000000	0.0990000
-20444.0000000	0.0995238
-20443.0000000	0.0990000
-20442.0000000	0.1000000
-20441.0000000	0.1000000
-20440.0000000	0.0995238
-20439.0000000	0.1000000
-20438.0000000	0.1000000
-20437.0000000	0.1000000
-20436.0000000	0.1000000
-20435.0000000	0.1000000
-20434.0000000	0.1000000
-20433.0000000	0.1000000
-20432.0000000	0.1000000
-20431.0000000	0.1000000

-20430.0000000	0.1000000
-20429.0000000	0.1000000
-20428.0000000	0.1000000
-20427.0000000	0.1000000
-20426.0000000	0.1000000
-20425.0000000	0.1000000
-20424.0000000	0.1000000
-20423.0000000	0.1000000
-20422.0000000	0.1000000
-20421.0000000	0.1000000
-20420.0000000	0.1000000
-20419.0000000	0.1000000
-20418.0000000	0.1005000
-20417.0000000	0.1000000
-20416.0000000	0.1000000
-20415.0000000	0.1000000
-20414.0000000	0.1000000
-20413.0000000	0.1000000
-20412.0000000	0.1000000
-20411.0000000	0.1000000
-20410.0000000	0.1000000
-20409.0000000	0.1000000
-20408.0000000	0.1000000
-20407.0000000	0.1000000
-20406.0000000	0.1000000
-20405.0000000	0.1004762
-20404.0000000	0.1000000
-20403.0000000	0.1000000
-20402.0000000	0.1000000
-20401.0000000	0.1000000
-20400.0000000	0.0995000
-20399.0000000	0.1000000
-20398.0000000	0.1000000
-20397.0000000	0.1000000
-20396.0000000	0.1000000
-20395.0000000	0.1000000
-20394.0000000	0.0995238
-20393.0000000	0.0980953
-20392.0000000	0.0995000
-20391.0000000	0.0990000
-20390.0000000	0.0990476
-20389.0000000	0.0975000
-20388.0000000	0.0970000
-20387.0000000	0.0960000
-20386.0000000	0.0966667
-20385.0000000	0.0980000
-20384.0000000	0.0942857
-20383.0000000	0.0970000
-20382.0000000	0.0965000
-20381.0000000	0.0990000
-20380.0000000	0.0995238
-20379.0000000	0.0995000
-20378.0000000	0.1000000
-20377.0000000	0.1000000
-20376.0000000	0.1000000
-20375.0000000	0.1000000
-20374.0000000	0.0990000

-20373.0000000	0.1000000
-20372.0000000	0.1000000
-20371.0000000	0.1000000
-20370.0000000	0.1000000
-20369.0000000	0.1000000
-20368.0000000	0.1000000
-20367.0000000	0.1000000
-20366.0000000	0.1000000
-20365.0000000	0.1004762
-20364.0000000	0.1000000
-20363.0000000	0.1000000
-20362.0000000	0.1000000
-20361.0000000	0.1000000
-20360.0000000	0.1000000
-20359.0000000	0.1000000
-20358.0000000	0.1000000
-20357.0000000	0.1000000
-20356.0000000	0.1000000
-20355.0000000	0.1000000
-20354.0000000	0.1000000
-20353.0000000	0.1005000
-20352.0000000	0.1000000
-20351.0000000	0.1000000
-20350.0000000	0.1000000
-20349.0000000	0.1000000
-20348.0000000	0.1000000
-20347.0000000	0.1000000
-20346.0000000	0.1000000
-20345.0000000	0.1000000
-20344.0000000	0.1000000
-20343.0000000	0.1000000
-20342.0000000	0.1000000
-20341.0000000	0.1000000
-20340.0000000	0.1000000
-20339.0000000	0.1000000
-20338.0000000	0.1000000
-20337.0000000	0.1000000
-20336.0000000	0.1000000
-20335.0000000	0.1000000
-20334.0000000	0.1004762
-20333.0000000	0.1000000
-20332.0000000	0.1000000
-20331.0000000	0.1005000
-20330.0000000	0.1000000
-20329.0000000	0.1000000
-20328.0000000	0.1005000
-20327.0000000	0.1005000
-20326.0000000	0.1000000
-20325.0000000	0.1005000
-20324.0000000	0.1009524
-20323.0000000	0.1015000
-20322.0000000	0.1010000
-20321.0000000	0.1000000
-20320.0000000	0.1000000
-20319.0000000	0.1000000
-20318.0000000	0.1000000
-20317.0000000	0.1000000

-20316.0000000	0.1000000
-20315.0000000	0.1000000
-20314.0000000	0.1000000
-20313.0000000	0.1000000
-20312.0000000	0.1000000
-20311.0000000	0.1000000
-20310.0000000	0.1000000
-20309.0000000	0.0965000
-20308.0000000	0.0966667
-20307.0000000	0.0935000
-20306.0000000	0.0960000
-20305.0000000	0.0975000
-20304.0000000	0.0971429
-20303.0000000	0.0980000
-20302.0000000	0.0980953
-20301.0000000	0.0995000
-20300.0000000	0.0995000
-20299.0000000	0.0990476
-20298.0000000	0.0995000
-20297.0000000	0.0995000
-20296.0000000	0.0995000
-20295.0000000	0.0995238
-20294.0000000	0.1000000
-20293.0000000	0.0990476
-20292.0000000	0.1000000
-20291.0000000	0.1000000
-20290.0000000	0.0995238
-20289.0000000	0.1000000
-20288.0000000	0.0985000
-20287.0000000	0.1000000
-20286.0000000	0.1000000
-20285.0000000	0.1000000
-20284.0000000	0.0995000
-20283.0000000	0.1000000
-20282.0000000	0.1000000
-20281.0000000	0.0995238
-20280.0000000	0.1000000
-20279.0000000	0.1000000
-20278.0000000	0.1000000
-20277.0000000	0.1000000
-20276.0000000	0.1000000
-20275.0000000	0.1000000
-20274.0000000	0.1000000
-20273.0000000	0.1000000
-20272.0000000	0.1000000
-20271.0000000	0.1000000
-20270.0000000	0.1000000
-20269.0000000	0.0985714
-20268.0000000	0.0985000
-20267.0000000	0.0995000
-20266.0000000	0.0975000
-20265.0000000	0.0985714
-20264.0000000	0.0995000
-20263.0000000	0.0995238
-20262.0000000	0.0985000
-20261.0000000	0.0970000
-20260.0000000	0.0971429

-20259.0000000	0.0970000
-20258.0000000	0.0980000
-20257.0000000	0.0985000
-20256.0000000	0.0976191
-20255.0000000	0.0980000
-20254.0000000	0.1000000
-20253.0000000	0.0990000
-20252.0000000	0.0980000
-20251.0000000	0.1000000
-20250.0000000	0.0985000
-20249.0000000	0.0980000
-20248.0000000	0.0985000
-20247.0000000	0.0947619
-20246.0000000	0.0940000
-20245.0000000	0.0935000
-20244.0000000	0.0947619
-20243.0000000	0.0945000
-20242.0000000	0.0966667
-20241.0000000	0.0985000
-20240.0000000	0.0970000
-20239.0000000	0.0980953
-20238.0000000	0.0985000
-20237.0000000	0.0985000
-20236.0000000	0.0985000
-20235.0000000	0.0971429
-20234.0000000	0.0990000
-20233.0000000	0.0995238
-20232.0000000	0.0995000
-20231.0000000	0.1000000
-20230.0000000	0.1000000
-20229.0000000	0.1000000
-20228.0000000	0.1000000
-20227.0000000	0.1000000
-20226.0000000	0.1000000
-20225.0000000	0.1000000
-20224.0000000	0.1000000
-20223.0000000	0.1000000
-20222.0000000	0.1000000
-20221.0000000	0.1005000
-20220.0000000	0.1004762
-20219.0000000	0.1000000
-20218.0000000	0.1000000
-20217.0000000	0.1000000
-20216.0000000	0.1000000
-20215.0000000	0.1000000
-20214.0000000	0.1000000
-20213.0000000	0.0995000
-20212.0000000	0.0995238
-20211.0000000	0.1000000
-20210.0000000	0.1000000
-20209.0000000	0.1000000
-20208.0000000	0.1000000
-20207.0000000	0.0990000
-20206.0000000	0.1000000
-20205.0000000	0.1000000
-20204.0000000	0.0995000
-20203.0000000	0.1000000

-20202.0000000	0.1000000
-20201.0000000	0.1000000
-20200.0000000	0.1000000
-20199.0000000	0.1000000
-20198.0000000	0.0990000
-20197.0000000	0.1000000
-20196.0000000	0.0985714
-20195.0000000	0.0990000
-20194.0000000	0.0990476
-20193.0000000	0.0990000
-20192.0000000	0.1000000
-20191.0000000	0.1000000
-20190.0000000	0.1000000
-20189.0000000	0.1000000
-20188.0000000	0.1000000
-20187.0000000	0.1000000
-20186.0000000	0.1005000
-20185.0000000	0.1000000
-20184.0000000	0.1000000
-20183.0000000	0.1000000
-20182.0000000	0.1000000
-20181.0000000	0.1000000
-20180.0000000	0.1000000
-20179.0000000	0.1000000
-20178.0000000	0.1000000
-20177.0000000	0.1000000
-20176.0000000	0.1000000
-20175.0000000	0.1000000
-20174.0000000	0.1000000
-20173.0000000	0.1000000
-20172.0000000	0.1000000
-20171.0000000	0.1000000
-20170.0000000	0.1000000
-20169.0000000	0.1000000
-20168.0000000	0.1000000
-20167.0000000	0.1000000
-20166.0000000	0.1000000
-20165.0000000	0.1000000
-20164.0000000	0.1000000
-20163.0000000	0.1000000
-20162.0000000	0.0990000
-20161.0000000	0.0995000
-20160.0000000	0.0995238
-20159.0000000	0.0980000
-20158.0000000	0.0985000
-20157.0000000	0.0990476
-20156.0000000	0.0995000
-20155.0000000	0.0990000
-20154.0000000	0.0985000
-20153.0000000	0.1000000
-20152.0000000	0.0995000
-20151.0000000	0.0995238
-20150.0000000	0.1000000
-20149.0000000	0.0995000
-20148.0000000	0.0990476
-20147.0000000	0.0995000
-20146.0000000	0.0995000

-20145.0000000	0.1000000
-20144.0000000	0.0995000
-20143.0000000	0.0995000
-20142.0000000	0.0995238
-20141.0000000	0.1000000
-20140.0000000	0.1000000
-20139.0000000	0.1000000
-20138.0000000	0.1000000
-20137.0000000	0.0990000
-20136.0000000	0.1000000
-20135.0000000	0.0995000
-20134.0000000	0.1000000
-20133.0000000	0.1000000
-20132.0000000	0.1000000
-20131.0000000	0.0990000
-20130.0000000	0.1000000
-20129.0000000	0.0995000
-20128.0000000	0.0995000
-20127.0000000	0.0995238
-20126.0000000	0.1000000
-20125.0000000	0.1000000
-20124.0000000	0.1000000
-20123.0000000	0.1000000
-20122.0000000	0.1000000
-20121.0000000	0.0985714
-20120.0000000	0.0990000
-20119.0000000	0.1000000
-20118.0000000	0.1000000
-20117.0000000	0.0990000
-20116.0000000	0.0975000
-20115.0000000	0.0995238
-20114.0000000	0.0995000
-20113.0000000	0.0990000
-20112.0000000	0.1000000
-20111.0000000	0.0995000
-20110.0000000	0.0995000
-20109.0000000	0.0985000
-20108.0000000	0.1000000
-20107.0000000	0.1000000
-20106.0000000	0.0990476
-20105.0000000	0.0980000
-20104.0000000	0.0995000
-20103.0000000	0.0971429
-20102.0000000	0.0985000
-20101.0000000	0.0970000
-20100.0000000	0.0955000
-20099.0000000	0.0961905
-20098.0000000	0.0935000
-20097.0000000	0.0971429
-20096.0000000	0.0980000
-20095.0000000	0.0990000
-20094.0000000	0.0960000
-20093.0000000	0.0961905
-20092.0000000	0.0985000
-20091.0000000	0.0965000
-20090.0000000	0.0961905
-20089.0000000	0.0960000

-20088.0000000	0.0971429
-20087.0000000	0.0955000
-20086.0000000	0.0955000
-20085.0000000	0.0980000
-20084.0000000	0.0966667
-20083.0000000	0.0980000
-20082.0000000	0.0985000
-20081.0000000	0.0966667
-20080.0000000	0.0975000
-20079.0000000	0.0985000
-20078.0000000	0.0985714
-20077.0000000	0.0995000
-20076.0000000	0.0995238
-20075.0000000	0.1000000
-20074.0000000	0.1000000
-20073.0000000	0.1000000
-20072.0000000	0.1000000
-20071.0000000	0.1000000
-20070.0000000	0.1000000
-20069.0000000	0.1004762
-20068.0000000	0.1000000
-20067.0000000	0.1000000
-20066.0000000	0.1000000
-20065.0000000	0.1000000
-20064.0000000	0.1000000
-20063.0000000	0.1000000
-20062.0000000	0.0995000
-20061.0000000	0.1000000
-20060.0000000	0.1000000
-20059.0000000	0.1000000
-20058.0000000	0.0995238
-20057.0000000	0.0980000
-20056.0000000	0.0975000
-20055.0000000	0.0955000
-20054.0000000	0.0971429
-20053.0000000	0.0955000
-20052.0000000	0.0965000
-20051.0000000	0.0942857
-20050.0000000	0.0960000
-20049.0000000	0.0950000
-20048.0000000	0.0933333
-20047.0000000	0.0945000
-20046.0000000	0.0923810
-20045.0000000	0.0920000
-20044.0000000	0.0925000
-20043.0000000	0.0920000
-20042.0000000	0.0933333
-20041.0000000	0.0930000
-20040.0000000	0.0925000
-20039.0000000	0.0923810
-20038.0000000	0.0940000
-20037.0000000	0.0938095
-20036.0000000	0.0935000
-20035.0000000	0.0925000
-20034.0000000	0.0930000
-20033.0000000	0.0947619
-20032.0000000	0.0955000

-20031.0000000	0.0965000
-20030.0000000	0.0947619
-20029.0000000	0.0970000
-20028.0000000	0.0957143
-20027.0000000	0.0970000
-20026.0000000	0.0970000
-20025.0000000	0.0960000
-20024.0000000	0.0947619
-20023.0000000	0.0950000
-20022.0000000	0.0945000
-20021.0000000	0.0961905
-20020.0000000	0.0945000
-20019.0000000	0.0950000
-20018.0000000	0.0960000
-20017.0000000	0.0961905
-20016.0000000	0.0965000
-20015.0000000	0.0947619
-20014.0000000	0.0945000
-20013.0000000	0.0935000
-20012.0000000	0.0928572
-20011.0000000	0.0905000
-20010.0000000	0.0915000
-20009.0000000	0.0910000
-20008.0000000	0.0914286
-20007.0000000	0.0938095
-20006.0000000	0.0935000
-20005.0000000	0.0930000
-20004.0000000	0.0915000
-20003.0000000	0.0919048
-20002.0000000	0.0910000
-20001.0000000	0.0930000
-20000.0000000	0.0920000
-19999.0000000	0.0914286
-19998.0000000	0.0914286
-19997.0000000	0.0900000
-19996.0000000	0.0900000
-19995.0000000	0.0900000
-19994.0000000	0.0909524
-19993.0000000	0.0900000
-19992.0000000	0.0900000
-19991.0000000	0.0900000
-19990.0000000	0.0900000
-19989.0000000	0.0900000
-19988.0000000	0.0910000
-19987.0000000	0.0914286
-19986.0000000	0.0925000
-19985.0000000	0.0923810
-19984.0000000	0.0935000
-19983.0000000	0.0945000
-19982.0000000	0.0933333
-19981.0000000	0.0940000
-19980.0000000	0.0950000
-19979.0000000	0.0910000
-19978.0000000	0.0914286
-19977.0000000	0.0920000
-19976.0000000	0.0904762
-19975.0000000	0.0900000

-19974.0000000	0.0900000
-19973.0000000	0.0914286
-19972.0000000	0.0905000
-19971.0000000	0.0905000
-19970.0000000	0.0900000
-19969.0000000	0.0909524
-19968.0000000	0.0905000
-19967.0000000	0.0900000
-19966.0000000	0.0905000
-19965.0000000	0.0900000
-19964.0000000	0.0900000
-19963.0000000	0.0915000
-19962.0000000	0.0900000
-19961.0000000	0.0900000
-19960.0000000	0.0900000
-19959.0000000	0.0900000
-19958.0000000	0.0900000
-19957.0000000	0.0900000
-19956.0000000	0.0900000
-19955.0000000	0.0900000
-19954.0000000	0.0900000
-19953.0000000	0.0900000
-19952.0000000	0.0900000
-19951.0000000	0.0900000
-19950.0000000	0.0905000
-19949.0000000	0.0900000
-19948.0000000	0.0900000
-19947.0000000	0.0900000
-19946.0000000	0.0904762
-19945.0000000	0.0900000
-19944.0000000	0.0900000
-19943.0000000	0.0900000
-19942.0000000	0.0900000
-19941.0000000	0.0900000
-19940.0000000	0.0900000
-19939.0000000	0.0900000
-19938.0000000	0.0900000
-19937.0000000	0.0900000
-19936.0000000	0.0900000
-19935.0000000	0.0910000
-19934.0000000	0.0900000
-19933.0000000	0.0900000
-19932.0000000	0.0915000
-19931.0000000	0.0909524
-19930.0000000	0.0925000
-19929.0000000	0.0905000
-19928.0000000	0.0920000
-19927.0000000	0.0933333
-19926.0000000	0.0920000
-19925.0000000	0.0909524
-19924.0000000	0.0920000
-19923.0000000	0.0905000
-19922.0000000	0.0914286
-19921.0000000	0.0900000
-19920.0000000	0.0900000
-19919.0000000	0.0900000
-19918.0000000	0.0904762

-19917.0000000	0.0900000
-19916.0000000	0.0900000
-19915.0000000	0.0900000
-19914.0000000	0.0900000
-19913.0000000	0.0900000
-19912.0000000	0.0909524
-19911.0000000	0.0905000
-19910.0000000	0.0910000
-19909.0000000	0.0900000
-19908.0000000	0.0910000
-19907.0000000	0.0909524
-19906.0000000	0.0900000
-19905.0000000	0.0900000
-19904.0000000	0.0900000
-19903.0000000	0.0900000
-19902.0000000	0.0900000
-19901.0000000	0.0900000
-19900.0000000	0.0900000
-19899.0000000	0.0900000
-19898.0000000	0.0895000
-19897.0000000	0.0900000
-19896.0000000	0.0900000
-19895.0000000	0.0900000
-19894.0000000	0.0900000
-19893.0000000	0.0900000
-19892.0000000	0.0900000
-19891.0000000	0.0900000
-19890.0000000	0.0900000
-19889.0000000	0.0900000
-19888.0000000	0.0900000
-19887.0000000	0.0900000
-19886.0000000	0.0895000
-19885.0000000	0.0890476
-19884.0000000	0.0895000
-19883.0000000	0.0895000
-19882.0000000	0.0900000
-19881.0000000	0.0895000
-19880.0000000	0.0890000
-19879.0000000	0.0890476
-19878.0000000	0.0900000
-19877.0000000	0.0890000
-19876.0000000	0.0900000
-19875.0000000	0.0900000
-19874.0000000	0.0900000
-19873.0000000	0.0880953
-19872.0000000	0.0900000
-19871.0000000	0.0895000
-19870.0000000	0.0900000
-19869.0000000	0.0895000
-19868.0000000	0.0895000
-19867.0000000	0.0900000
-19866.0000000	0.0890000
-19865.0000000	0.0880000
-19864.0000000	0.0880953
-19863.0000000	0.0875000
-19862.0000000	0.0857143
-19861.0000000	0.0880000

-19860.0000000	0.0880000
-19859.0000000	0.0885000
-19858.0000000	0.0866667
-19857.0000000	0.0855000
-19856.0000000	0.0860000
-19855.0000000	0.0861905
-19854.0000000	0.0855000
-19853.0000000	0.0855000
-19852.0000000	0.0876191
-19851.0000000	0.0875000
-19850.0000000	0.0860000
-19849.0000000	0.0880953
-19848.0000000	0.0855000
-19847.0000000	0.0865000
-19846.0000000	0.0885715
-19845.0000000	0.0850000
-19844.0000000	0.0895000
-19843.0000000	0.0885715
-19842.0000000	0.0875000
-19841.0000000	0.0890000
-19840.0000000	0.0885714
-19839.0000000	0.0890000
-19838.0000000	0.0875000
-19837.0000000	0.0895000
-19836.0000000	0.0895238
-19835.0000000	0.0895000
-19834.0000000	0.0900000
-19833.0000000	0.0900000
-19832.0000000	0.0900000
-19831.0000000	0.0900000
-19830.0000000	0.0900000
-19829.0000000	0.0900000
-19828.0000000	0.0900000
-19827.0000000	0.0900000
-19826.0000000	0.0900000
-19825.0000000	0.0900000
-19824.0000000	0.0900000
-19823.0000000	0.0900000
-19822.0000000	0.0900000
-19821.0000000	0.0900000
-19820.0000000	0.0900000
-19819.0000000	0.0900000
-19818.0000000	0.0900000
-19817.0000000	0.0895000
-19816.0000000	0.0895238
-19815.0000000	0.0895000
-19814.0000000	0.0890000
-19813.0000000	0.0900000
-19812.0000000	0.0895238
-19811.0000000	0.0900000
-19810.0000000	0.0895238
-19809.0000000	0.0900000
-19808.0000000	0.0900000
-19807.0000000	0.0900000
-19806.0000000	0.0900000
-19805.0000000	0.0900000
-19804.0000000	0.0900000

-19803.0000000	0.0900000
-19802.0000000	0.0900000
-19801.0000000	0.0900000
-19800.0000000	0.0900000
-19799.0000000	0.0895000
-19798.0000000	0.0900000
-19797.0000000	0.0890000
-19796.0000000	0.0890000
-19795.0000000	0.0885715
-19794.0000000	0.0895000
-19793.0000000	0.0880000
-19792.0000000	0.0900000
-19791.0000000	0.0890476
-19790.0000000	0.0895000
-19789.0000000	0.0895238
-19788.0000000	0.0895000
-19787.0000000	0.0895000
-19786.0000000	0.0900000
-19785.0000000	0.0900000
-19784.0000000	0.0900000
-19783.0000000	0.0895000
-19782.0000000	0.0900000
-19781.0000000	0.0900000
-19780.0000000	0.0900000
-19779.0000000	0.0900000
-19778.0000000	0.0900000
-19777.0000000	0.0900000
-19776.0000000	0.0900000
-19775.0000000	0.0900000
-19774.0000000	0.0900000
-19773.0000000	0.0900000
-19772.0000000	0.0900000
-19771.0000000	0.0900000
-19770.0000000	0.0900000
-19769.0000000	0.0895000
-19768.0000000	0.0900000
-19767.0000000	0.0900000
-19766.0000000	0.0895000
-19765.0000000	0.0900000
-19764.0000000	0.0900000
-19763.0000000	0.0900000
-19762.0000000	0.0900000
-19761.0000000	0.0900000
-19760.0000000	0.0900000
-19759.0000000	0.0900000
-19758.0000000	0.0900000
-19757.0000000	0.0900000
-19756.0000000	0.0900000
-19755.0000000	0.0900000
-19754.0000000	0.0900000
-19753.0000000	0.0900000
-19752.0000000	0.0900000
-19751.0000000	0.0900000
-19750.0000000	0.0900000
-19749.0000000	0.0900000
-19748.0000000	0.0900000
-19747.0000000	0.0900000

-19746.0000000	0.0900000
-19745.0000000	0.0900000
-19744.0000000	0.0900000
-19743.0000000	0.0900000
-19742.0000000	0.0900000
-19741.0000000	0.0900000
-19740.0000000	0.0900000
-19739.0000000	0.0900000
-19738.0000000	0.0900000
-19737.0000000	0.0900000
-19736.0000000	0.0900000
-19735.0000000	0.0900000
-19734.0000000	0.0900000
-19733.0000000	0.0900000
-19732.0000000	0.0900000
-19731.0000000	0.0895000
-19730.0000000	0.0900000
-19729.0000000	0.0900000
-19728.0000000	0.0900000
-19727.0000000	0.0900000
-19726.0000000	0.0905000
-19725.0000000	0.0900000
-19724.0000000	0.0900000
-19723.0000000	0.0900000
-19722.0000000	0.0904762
-19721.0000000	0.0900000
-19720.0000000	0.0900000
-19719.0000000	0.0905000
-19718.0000000	0.0910000
-19717.0000000	0.0900000
-19716.0000000	0.0900000
-19715.0000000	0.0895000
-19714.0000000	0.0900000
-19713.0000000	0.0900000
-19712.0000000	0.0900000
-19711.0000000	0.0900000
-19710.0000000	0.0900000
-19709.0000000	0.0895000
-19708.0000000	0.0900000
-19707.0000000	0.0900000
-19706.0000000	0.0900000
-19705.0000000	0.0900000
-19704.0000000	0.0900000
-19703.0000000	0.0900000
-19702.0000000	0.0900000
-19701.0000000	0.0905000
-19700.0000000	0.0900000
-19699.0000000	0.0905000
-19698.0000000	0.0900000
-19697.0000000	0.0900000
-19696.0000000	0.0900000
-19695.0000000	0.0900000
-19694.0000000	0.0900000
-19693.0000000	0.0905000
-19692.0000000	0.0905000
-19691.0000000	0.0909524
-19690.0000000	0.0900000

-19689.0000000	0.0900000
-19688.0000000	0.0900000
-19687.0000000	0.0900000
-19686.0000000	0.0900000
-19685.0000000	0.0900000
-19684.0000000	0.0900000
-19683.0000000	0.0900000
-19682.0000000	0.0900000
-19681.0000000	0.0885715
-19680.0000000	0.0895000
-19679.0000000	0.0900000
-19678.0000000	0.0900000
-19677.0000000	0.0900000
-19676.0000000	0.0900000
-19675.0000000	0.0900000
-19674.0000000	0.0900000
-19673.0000000	0.0895000
-19672.0000000	0.0900000
-19671.0000000	0.0900000
-19670.0000000	0.0900000
-19669.0000000	0.0900000
-19668.0000000	0.0904762
-19667.0000000	0.0905000
-19666.0000000	0.0910000
-19665.0000000	0.0914286
-19664.0000000	0.0910000
-19663.0000000	0.0910000
-19662.0000000	0.0920000
-19661.0000000	0.0919048
-19660.0000000	0.0905000
-19659.0000000	0.0904762
-19658.0000000	0.0915000
-19657.0000000	0.0900000
-19656.0000000	0.0904762
-19655.0000000	0.0900000
-19654.0000000	0.0900000
-19653.0000000	0.0900000
-19652.0000000	0.0900000
-19651.0000000	0.0900000
-19650.0000000	0.0900000
-19649.0000000	0.0900000
-19648.0000000	0.0900000
-19647.0000000	0.0900000
-19646.0000000	0.0900000
-19645.0000000	0.0900000
-19644.0000000	0.0900000
-19643.0000000	0.0900000
-19642.0000000	0.0900000
-19641.0000000	0.0900000
-19640.0000000	0.0895238
-19639.0000000	0.0895000
-19638.0000000	0.0900000
-19637.0000000	0.0900000
-19636.0000000	0.0895000
-19635.0000000	0.0900000
-19634.0000000	0.0900000
-19633.0000000	0.0900000

-19632.0000000	0.0900000
-19631.0000000	0.0900000
-19630.0000000	0.0900000
-19629.0000000	0.0900000
-19628.0000000	0.0900000
-19627.0000000	0.0900000
-19626.0000000	0.0900000
-19625.0000000	0.0900000
-19624.0000000	0.0900000
-19623.0000000	0.0900000
-19622.0000000	0.0900000
-19621.0000000	0.0900000
-19620.0000000	0.0900000
-19619.0000000	0.0900000
-19618.0000000	0.0900000
-19617.0000000	0.0900000
-19616.0000000	0.0900000
-19615.0000000	0.0900000
-19614.0000000	0.0885715
-19613.0000000	0.0875000
-19612.0000000	0.0895000
-19611.0000000	0.0855000
-19610.0000000	0.0876191
-19609.0000000	0.0885000
-19608.0000000	0.0890000
-19607.0000000	0.0895238
-19606.0000000	0.0900000
-19605.0000000	0.0900000
-19604.0000000	0.0900000
-19603.0000000	0.0900000
-19602.0000000	0.0900000
-19601.0000000	0.0900000
-19600.0000000	0.0900000
-19599.0000000	0.0900000
-19598.0000000	0.0900000
-19597.0000000	0.0900000
-19596.0000000	0.0900000
-19595.0000000	0.0900000
-19594.0000000	0.0900000
-19593.0000000	0.0900000
-19592.0000000	0.0900000
-19591.0000000	0.0900000
-19590.0000000	0.0900000
-19589.0000000	0.0900000
-19588.0000000	0.0900000
-19587.0000000	0.0900000
-19586.0000000	0.0900000
-19585.0000000	0.0905000
-19584.0000000	0.0905000
-19583.0000000	0.0900000
-19582.0000000	0.0900000
-19581.0000000	0.0900000
-19580.0000000	0.0900000
-19579.0000000	0.0900000
-19578.0000000	0.0905000
-19577.0000000	0.0904762
-19576.0000000	0.0900000

-19575.0000000	0.0900000
-19574.0000000	0.0915000
-19573.0000000	0.0910000
-19572.0000000	0.0910000
-19571.0000000	0.0900000
-19570.0000000	0.0900000
-19569.0000000	0.0905000
-19568.0000000	0.0904762
-19567.0000000	0.0910000
-19566.0000000	0.0910000
-19565.0000000	0.0909524
-19564.0000000	0.0915000
-19563.0000000	0.0910000
-19562.0000000	0.0900000
-19561.0000000	0.0910000
-19560.0000000	0.0920000
-19559.0000000	0.0904762
-19558.0000000	0.0925000
-19557.0000000	0.0935000
-19556.0000000	0.0942857
-19555.0000000	0.0915000
-19554.0000000	0.0915000
-19553.0000000	0.0914286
-19552.0000000	0.0920000
-19551.0000000	0.0910000
-19550.0000000	0.0919048
-19549.0000000	0.0920000
-19548.0000000	0.0945000
-19547.0000000	0.0938095
-19546.0000000	0.0925000
-19545.0000000	0.0947619
-19544.0000000	0.0935000
-19543.0000000	0.0985000
-19542.0000000	0.0940000
-19541.0000000	0.0961905
-19540.0000000	0.0960000
-19539.0000000	0.0980000
-19538.0000000	0.0985714
-19537.0000000	0.0985000
-19536.0000000	0.0985000
-19535.0000000	0.0995000
-19534.0000000	0.0966667
-19533.0000000	0.0985000
-19532.0000000	0.0980952
-19531.0000000	0.0985000
-19530.0000000	0.0985000
-19529.0000000	0.0976191
-19528.0000000	0.0980000
-19527.0000000	0.0965000
-19526.0000000	0.0955000
-19525.0000000	0.0961905
-19524.0000000	0.0955000
-19523.0000000	0.0933333
-19522.0000000	0.0930000
-19521.0000000	0.0925000
-19520.0000000	0.0905000
-19519.0000000	0.0909524

-19518.0000000	0.0920000
-19517.0000000	0.0904762
-19516.0000000	0.0915000
-19515.0000000	0.0914286
-19514.0000000	0.0900000
-19513.0000000	0.0900000
-19512.0000000	0.0905000
-19511.0000000	0.0900000
-19510.0000000	0.0905000
-19509.0000000	0.0915000
-19508.0000000	0.0919048
-19507.0000000	0.0940000
-19506.0000000	0.0930000
-19505.0000000	0.0930000
-19504.0000000	0.0942857
-19503.0000000	0.0920000
-19502.0000000	0.0923810
-19501.0000000	0.0910000
-19500.0000000	0.0925000
-19499.0000000	0.0938095
-19498.0000000	0.0910000
-19497.0000000	0.0920000
-19496.0000000	0.0930000
-19495.0000000	0.0909524
-19494.0000000	0.0920000
-19493.0000000	0.0914286
-19492.0000000	0.0910000
-19491.0000000	0.0910000
-19490.0000000	0.0945000
-19489.0000000	0.0938095
-19488.0000000	0.0945000
-19487.0000000	0.0960000
-19486.0000000	0.0952381
-19485.0000000	0.0965000
-19484.0000000	0.0961905
-19483.0000000	0.0990000
-19482.0000000	0.0965000
-19481.0000000	0.0961905
-19480.0000000	0.0965000
-19479.0000000	0.0975000
-19478.0000000	0.0965000
-19477.0000000	0.0971429
-19476.0000000	0.0980000
-19475.0000000	0.0990000
-19474.0000000	0.0966667
-19473.0000000	0.0985000
-19472.0000000	0.0985714
-19471.0000000	0.0990000
-19470.0000000	0.0980000
-19469.0000000	0.0990476
-19468.0000000	0.0990000
-19467.0000000	0.0995000
-19466.0000000	0.0990000
-19465.0000000	0.0995238
-19464.0000000	0.1000000
-19463.0000000	0.1000000
-19462.0000000	0.1000000

-19461.0000000	0.0995000
-19460.0000000	0.0995000
-19459.0000000	0.0990476
-19458.0000000	0.0975000
-19457.0000000	0.0920000
-19456.0000000	0.0909524
-19455.0000000	0.0915000
-19454.0000000	0.0920000
-19453.0000000	0.0933333
-19452.0000000	0.0940000
-19451.0000000	0.0933333
-19450.0000000	0.0925000
-19449.0000000	0.0950000
-19448.0000000	0.0925000
-19447.0000000	0.0928572
-19446.0000000	0.0910000
-19445.0000000	0.0910000
-19444.0000000	0.0904762
-19443.0000000	0.0900000
-19442.0000000	0.0900000
-19441.0000000	0.0900000
-19440.0000000	0.0900000
-19439.0000000	0.0900000
-19438.0000000	0.0900000
-19437.0000000	0.0910000
-19436.0000000	0.0920000
-19435.0000000	0.0952381
-19434.0000000	0.0965000
-19433.0000000	0.0971429
-19432.0000000	0.0995000
-19431.0000000	0.0985000
-19430.0000000	0.0995000
-19429.0000000	0.1000000
-19428.0000000	0.0995000
-19427.0000000	0.0975000
-19426.0000000	0.0966667
-19425.0000000	0.0920000
-19424.0000000	0.0900000
-19423.0000000	0.0915000
-19422.0000000	0.0915000
-19421.0000000	0.0900000
-19420.0000000	0.0914286
-19419.0000000	0.0900000
-19418.0000000	0.0910000
-19417.0000000	0.0904762
-19416.0000000	0.0920000
-19415.0000000	0.0910000
-19414.0000000	0.0915000
-19413.0000000	0.0914286
-19412.0000000	0.0905000
-19411.0000000	0.0928572
-19410.0000000	0.0900000
-19409.0000000	0.0925000
-19408.0000000	0.0900000
-19407.0000000	0.0910000
-19406.0000000	0.0905000
-19405.0000000	0.0900000

-19404.0000000	0.0900000
-19403.0000000	0.0900000
-19402.0000000	0.0900000
-19401.0000000	0.0900000
-19400.0000000	0.0900000
-19399.0000000	0.0900000
-19398.0000000	0.0905000
-19397.0000000	0.0900000
-19396.0000000	0.0900000
-19395.0000000	0.0900000
-19394.0000000	0.0900000
-19393.0000000	0.0900000
-19392.0000000	0.0900000
-19391.0000000	0.0900000
-19390.0000000	0.0900000
-19389.0000000	0.0900000
-19388.0000000	0.0900000
-19387.0000000	0.0900000
-19386.0000000	0.0900000
-19385.0000000	0.0900000
-19384.0000000	0.0905000
-19383.0000000	0.0900000
-19382.0000000	0.0900000
-19381.0000000	0.0900000
-19380.0000000	0.0900000
-19379.0000000	0.0915000
-19378.0000000	0.0904762
-19377.0000000	0.0910000
-19376.0000000	0.0910000
-19375.0000000	0.0915000
-19374.0000000	0.0904762
-19373.0000000	0.0925000
-19372.0000000	0.0914286
-19371.0000000	0.0920000
-19370.0000000	0.0950000
-19369.0000000	0.0928572
-19368.0000000	0.0935000
-19367.0000000	0.0950000
-19366.0000000	0.0961905
-19365.0000000	0.0955000
-19364.0000000	0.0965000
-19363.0000000	0.0980953
-19362.0000000	0.0975000
-19361.0000000	0.0960000
-19360.0000000	0.0976191
-19359.0000000	0.0970000
-19358.0000000	0.0980000
-19357.0000000	0.0980953
-19356.0000000	0.0995000
-19355.0000000	0.0985000
-19354.0000000	0.0975000
-19353.0000000	0.0971429
-19352.0000000	0.0980000
-19351.0000000	0.0990476
-19350.0000000	0.0980000
-19349.0000000	0.0970000
-19348.0000000	0.0952381

-19347.0000000	0.0945000
-19346.0000000	0.0935000
-19345.0000000	0.0950000
-19344.0000000	0.0961905
-19343.0000000	0.0935000
-19342.0000000	0.0952381
-19341.0000000	0.0970000
-19340.0000000	0.0985000
-19339.0000000	0.0980000
-19338.0000000	0.0980953
-19337.0000000	0.0985000
-19336.0000000	0.1000000
-19335.0000000	0.1000000
-19334.0000000	0.1000000
-19333.0000000	0.0995238
-19332.0000000	0.1000000
-19331.0000000	0.0995000
-19330.0000000	0.0985714
-19329.0000000	0.0965000
-19328.0000000	0.0960000
-19327.0000000	0.0947619
-19326.0000000	0.0935000
-19325.0000000	0.0910000
-19324.0000000	0.0905000
-19323.0000000	0.0900000
-19322.0000000	0.0905000
-19321.0000000	0.0900000
-19320.0000000	0.0900000
-19319.0000000	0.0905000
-19318.0000000	0.0900000
-19317.0000000	0.0900000
-19316.0000000	0.0900000
-19315.0000000	0.0905000
-19314.0000000	0.0900000
-19313.0000000	0.0900000
-19312.0000000	0.0900000
-19311.0000000	0.0905000
-19310.0000000	0.0900000
-19309.0000000	0.0900000
-19308.0000000	0.0900000
-19307.0000000	0.0900000
-19306.0000000	0.0905000
-19305.0000000	0.0900000
-19304.0000000	0.0900000
-19303.0000000	0.0895238
-19302.0000000	0.0900000
-19301.0000000	0.0885000
-19300.0000000	0.0885000
-19299.0000000	0.0895238
-19298.0000000	0.0890000
-19297.0000000	0.0885714
-19296.0000000	0.0895000
-19295.0000000	0.0895000
-19294.0000000	0.0900000
-19293.0000000	0.0900000
-19292.0000000	0.0895000
-19291.0000000	0.0900000

-19290.0000000	0.0900000
-19289.0000000	0.0900000
-19288.0000000	0.0900000
-19287.0000000	0.0900000
-19286.0000000	0.0900000
-19285.0000000	0.0900000
-19284.0000000	0.0900000
-19283.0000000	0.0900000
-19282.0000000	0.0900000
-19281.0000000	0.0900000
-19280.0000000	0.0900000
-19279.0000000	0.0900000
-19278.0000000	0.0904762
-19277.0000000	0.0900000
-19276.0000000	0.0900000
-19275.0000000	0.0904762
-19274.0000000	0.0905000
-19273.0000000	0.0905000
-19272.0000000	0.0909524
-19271.0000000	0.0900000
-19270.0000000	0.0900000
-19269.0000000	0.0900000
-19268.0000000	0.0900000
-19267.0000000	0.0900000
-19266.0000000	0.0900000
-19265.0000000	0.0900000
-19264.0000000	0.0900000
-19263.0000000	0.0900000
-19262.0000000	0.0900000
-19261.0000000	0.0900000
-19260.0000000	0.0900000
-19259.0000000	0.0900000
-19258.0000000	0.0904762
-19257.0000000	0.0900000
-19256.0000000	0.0910000
-19255.0000000	0.0900000
-19254.0000000	0.0909524
-19253.0000000	0.0935000
-19252.0000000	0.0930000
-19251.0000000	0.0933334
-19250.0000000	0.0935000
-19249.0000000	0.0935000
-19248.0000000	0.0930000
-19247.0000000	0.0938095
-19246.0000000	0.0945000
-19245.0000000	0.0971429
-19244.0000000	0.0990000
-19243.0000000	0.0965000
-19242.0000000	0.0980953
-19241.0000000	0.0975000
-19240.0000000	0.0985000
-19239.0000000	0.0995238
-19238.0000000	0.0995000
-19237.0000000	0.0990000
-19236.0000000	0.0985714
-19235.0000000	0.1000000
-19234.0000000	0.0990000

-19233.0000000	0.0990476
-19232.0000000	0.0975000
-19231.0000000	0.0975000
-19230.0000000	0.0976191
-19229.0000000	0.0960000
-19228.0000000	0.0952381
-19227.0000000	0.0965000
-19226.0000000	0.0925000
-19225.0000000	0.0910000
-19224.0000000	0.0914286
-19223.0000000	0.0915000
-19222.0000000	0.0915000
-19221.0000000	0.0914286
-19220.0000000	0.0900000
-19219.0000000	0.0905000
-19218.0000000	0.0900000
-19217.0000000	0.0900000
-19216.0000000	0.0900000
-19215.0000000	0.0900000
-19214.0000000	0.0900000
-19213.0000000	0.0900000
-19212.0000000	0.0904762
-19211.0000000	0.0900000
-19210.0000000	0.0910000
-19209.0000000	0.0905000
-19208.0000000	0.0900000
-19207.0000000	0.0900000
-19206.0000000	0.0900000
-19205.0000000	0.0900000
-19204.0000000	0.0900000
-19203.0000000	0.0900000
-19202.0000000	0.0900000
-19201.0000000	0.0900000
-19200.0000000	0.0900000
-19199.0000000	0.0900000
-19198.0000000	0.0900000
-19197.0000000	0.0900000
-19196.0000000	0.0900000
-19195.0000000	0.0900000
-19194.0000000	0.0900000
-19193.0000000	0.0900000
-19192.0000000	0.0900000
-19191.0000000	0.0900000
-19190.0000000	0.0900000
-19189.0000000	0.0900000
-19188.0000000	0.0915000
-19187.0000000	0.0923810
-19186.0000000	0.0925000
-19185.0000000	0.0923810
-19184.0000000	0.0950000
-19183.0000000	0.0915000
-19182.0000000	0.0914286
-19181.0000000	0.0910000
-19180.0000000	0.0905000
-19179.0000000	0.0915000
-19178.0000000	0.0923810
-19177.0000000	0.0915000

-19176.0000000	0.0904762
-19175.0000000	0.0900000
-19174.0000000	0.0900000
-19173.0000000	0.0900000
-19172.0000000	0.0900000
-19171.0000000	0.0920000
-19170.0000000	0.0900000
-19169.0000000	0.0919048
-19168.0000000	0.0905000
-19167.0000000	0.0914286
-19166.0000000	0.0940000
-19165.0000000	0.0935000
-19164.0000000	0.0925000
-19163.0000000	0.0909524
-19162.0000000	0.0905000
-19161.0000000	0.0900000
-19160.0000000	0.0900000
-19159.0000000	0.0900000
-19158.0000000	0.0900000
-19157.0000000	0.0900000
-19156.0000000	0.0900000
-19155.0000000	0.0895238
-19154.0000000	0.0895000
-19153.0000000	0.0900000
-19152.0000000	0.0900000
-19151.0000000	0.0900000
-19150.0000000	0.0900000
-19149.0000000	0.0900000
-19148.0000000	0.0904762
-19147.0000000	0.0905000
-19146.0000000	0.0923810
-19145.0000000	0.0910000
-19144.0000000	0.0910000
-19143.0000000	0.0900000
-19142.0000000	0.0904762
-19141.0000000	0.0900000
-19140.0000000	0.0900000
-19139.0000000	0.0900000
-19138.0000000	0.0900000
-19137.0000000	0.0900000
-19136.0000000	0.0905000
-19135.0000000	0.0910000
-19134.0000000	0.0900000
-19133.0000000	0.0900000
-19132.0000000	0.0905000
-19131.0000000	0.0920000
-19130.0000000	0.0928572
-19129.0000000	0.0955000
-19128.0000000	0.0970000
-19127.0000000	0.0975000
-19126.0000000	0.0966667
-19125.0000000	0.0976191
-19124.0000000	0.0985000
-19123.0000000	0.0980000
-19122.0000000	0.0955000
-19121.0000000	0.0957143
-19120.0000000	0.0975000

-19119.0000000	0.0980000
-19118.0000000	0.0947619
-19117.0000000	0.0940000
-19116.0000000	0.0947619
-19115.0000000	0.0930000
-19114.0000000	0.0910000
-19113.0000000	0.0905000
-19112.0000000	0.0900000
-19111.0000000	0.0900000
-19110.0000000	0.0900000
-19109.0000000	0.0904762
-19108.0000000	0.0900000
-19107.0000000	0.0900000
-19106.0000000	0.0900000
-19105.0000000	0.0905000
-19104.0000000	0.0910000
-19103.0000000	0.0919048
-19102.0000000	0.0910000
-19101.0000000	0.0915000
-19100.0000000	0.0933333
-19099.0000000	0.0945000
-19098.0000000	0.0925000
-19097.0000000	0.0930000
-19096.0000000	0.0919048
-19095.0000000	0.0935000
-19094.0000000	0.0942857
-19093.0000000	0.0945000
-19092.0000000	0.0940000
-19091.0000000	0.0980953
-19090.0000000	0.0965000
-19089.0000000	0.0985000
-19088.0000000	0.0980000
-19087.0000000	0.0990476
-19086.0000000	0.0990000
-19085.0000000	0.0990476
-19084.0000000	0.0995000
-19083.0000000	0.0995000
-19082.0000000	0.1000000
-19081.0000000	0.1000000
-19080.0000000	0.0990000
-19079.0000000	0.0995238
-19078.0000000	0.1000000
-19077.0000000	0.0980953
-19076.0000000	0.0980000
-19075.0000000	0.0990000
-19074.0000000	0.0985000
-19073.0000000	0.0995238
-19072.0000000	0.0990000
-19071.0000000	0.0995000
-19070.0000000	0.1000000
-19069.0000000	0.0985000
-19068.0000000	0.1000000
-19067.0000000	0.0995000
-19066.0000000	0.0990476
-19065.0000000	0.0990000
-19064.0000000	0.0990476
-19063.0000000	0.0965000

-19062.0000000	0.0940000
-19061.0000000	0.0914286
-19060.0000000	0.0900000
-19059.0000000	0.0900000
-19058.0000000	0.0900000
-19057.0000000	0.0900000
-19056.0000000	0.0900000
-19055.0000000	0.0900000
-19054.0000000	0.0900000
-19053.0000000	0.0900000
-19052.0000000	0.0900000
-19051.0000000	0.0900000
-19050.0000000	0.0895000
-19049.0000000	0.0900000
-19048.0000000	0.0895238
-19047.0000000	0.0890000
-19046.0000000	0.0900000
-19045.0000000	0.0900000
-19044.0000000	0.0900000
-19043.0000000	0.0900000
-19042.0000000	0.0900000
-19041.0000000	0.0905000
-19040.0000000	0.0909524
-19039.0000000	0.0915000
-19038.0000000	0.0915000
-19037.0000000	0.0920000
-19036.0000000	0.0923810
-19035.0000000	0.0905000
-19034.0000000	0.0904762
-19033.0000000	0.0905000
-19032.0000000	0.0910000
-19031.0000000	0.0923810
-19030.0000000	0.0935000
-19029.0000000	0.0935000
-19028.0000000	0.0930000
-19027.0000000	0.0919048
-19026.0000000	0.0910000
-19025.0000000	0.0914286
-19024.0000000	0.0925000
-19023.0000000	0.0920000
-19022.0000000	0.0920000
-19021.0000000	0.0920000
-19020.0000000	0.0966667
-19019.0000000	0.0965000
-19018.0000000	0.0961905
-19017.0000000	0.0955000
-19016.0000000	0.0990476
-19015.0000000	0.0985000
-19014.0000000	0.0975000
-19013.0000000	0.0970000
-19012.0000000	0.0980953
-19011.0000000	0.0970000
-19010.0000000	0.0966667
-19009.0000000	0.0960000
-19008.0000000	0.0940000
-19007.0000000	0.0935000
-19006.0000000	0.0928572

-19005.0000000	0.0945000
-19004.0000000	0.0920000
-19003.0000000	0.0923810
-19002.0000000	0.0915000
-19001.0000000	0.0923810
-19000.0000000	0.0910000
-18999.0000000	0.0940000
-18998.0000000	0.0935000
-18997.0000000	0.0971429
-18996.0000000	0.0970000
-18995.0000000	0.0952381
-18994.0000000	0.0950000
-18993.0000000	0.0940000
-18992.0000000	0.0925000
-18991.0000000	0.0933333
-18990.0000000	0.0930000
-18989.0000000	0.0940000
-18988.0000000	0.0914286
-18987.0000000	0.0910000
-18986.0000000	0.0900000
-18985.0000000	0.0900000
-18984.0000000	0.0905000
-18983.0000000	0.0900000
-18982.0000000	0.0909524
-18981.0000000	0.0910000
-18980.0000000	0.0909524
-18979.0000000	0.0935000
-18978.0000000	0.0950000
-18977.0000000	0.0940000
-18976.0000000	0.0976191
-18975.0000000	0.0940000
-18974.0000000	0.0950000
-18973.0000000	0.0961905
-18972.0000000	0.0955000
-18971.0000000	0.0961905
-18970.0000000	0.0950000
-18969.0000000	0.0960000
-18968.0000000	0.0960000
-18967.0000000	0.0952381
-18966.0000000	0.0960000
-18965.0000000	0.0960000
-18964.0000000	0.0952381
-18963.0000000	0.0950000
-18962.0000000	0.0945000
-18961.0000000	0.0957143
-18960.0000000	0.0970000
-18959.0000000	0.0980000
-18958.0000000	0.0985714
-18957.0000000	0.0980000
-18956.0000000	0.0980000
-18955.0000000	0.0976191
-18954.0000000	0.0985000
-18953.0000000	0.0980000
-18952.0000000	0.0980952
-18951.0000000	0.0980000
-18950.0000000	0.0966667
-18949.0000000	0.0915000

-18948.0000000	0.0900000
-18947.0000000	0.0905000
-18946.0000000	0.0900000
-18945.0000000	0.0900000
-18944.0000000	0.0900000
-18943.0000000	0.0900000
-18942.0000000	0.0900000
-18941.0000000	0.0904762
-18940.0000000	0.0905000
-18939.0000000	0.0905000
-18938.0000000	0.0915000
-18937.0000000	0.0904762
-18936.0000000	0.0910000
-18935.0000000	0.0920000
-18934.0000000	0.0923810
-18933.0000000	0.0900000
-18932.0000000	0.0915000
-18931.0000000	0.0910000
-18930.0000000	0.0900000
-18929.0000000	0.0905000
-18928.0000000	0.0900000
-18927.0000000	0.0905000
-18926.0000000	0.0910000
-18925.0000000	0.0900000
-18924.0000000	0.0900000
-18923.0000000	0.0900000
-18922.0000000	0.0900000
-18921.0000000	0.0895238
-18920.0000000	0.0900000
-18919.0000000	0.0900000
-18918.0000000	0.0900000
-18917.0000000	0.0900000
-18916.0000000	0.0900000
-18915.0000000	0.0900000
-18914.0000000	0.0905000
-18913.0000000	0.0910000
-18912.0000000	0.0904762
-18911.0000000	0.0900000
-18910.0000000	0.0910000
-18909.0000000	0.0905000
-18908.0000000	0.0905000
-18907.0000000	0.0909524
-18906.0000000	0.0900000
-18905.0000000	0.0900000
-18904.0000000	0.0900000
-18903.0000000	0.0900000
-18902.0000000	0.0900000
-18901.0000000	0.0905000
-18900.0000000	0.0904762
-18899.0000000	0.0910000
-18898.0000000	0.0914286
-18897.0000000	0.0915000
-18896.0000000	0.0920000
-18895.0000000	0.0900000
-18894.0000000	0.0905000
-18893.0000000	0.0900000
-18892.0000000	0.0905000

-18891.0000000	0.0900000
-18890.0000000	0.0905000
-18889.0000000	0.0900000
-18888.0000000	0.0910000
-18887.0000000	0.0900000
-18886.0000000	0.0909524
-18885.0000000	0.0910000
-18884.0000000	0.0910000
-18883.0000000	0.0910000
-18882.0000000	0.0909524
-18881.0000000	0.0910000
-18880.0000000	0.0909524
-18879.0000000	0.0915000
-18878.0000000	0.0920000
-18877.0000000	0.0909524
-18876.0000000	0.0920000
-18875.0000000	0.0915000
-18874.0000000	0.0920000
-18873.0000000	0.0919048
-18872.0000000	0.0910000
-18871.0000000	0.0905000
-18870.0000000	0.0900000
-18869.0000000	0.0900000
-18868.0000000	0.0900000
-18867.0000000	0.0900000
-18866.0000000	0.0900000
-18865.0000000	0.0900000
-18864.0000000	0.0905000
-18863.0000000	0.0900000
-18862.0000000	0.0905000
-18861.0000000	0.0909524
-18860.0000000	0.0905000
-18859.0000000	0.0909524
-18858.0000000	0.0900000
-18857.0000000	0.0900000
-18856.0000000	0.0900000
-18855.0000000	0.0900000
-18854.0000000	0.0900000
-18853.0000000	0.0900000
-18852.0000000	0.0890476
-18851.0000000	0.0885000
-18850.0000000	0.0885714
-18849.0000000	0.0890000
-18848.0000000	0.0885000
-18847.0000000	0.0890000
-18846.0000000	0.0871429
-18845.0000000	0.0835000
-18844.0000000	0.0825000
-18843.0000000	0.0814286
-18842.0000000	0.0810000
-18841.0000000	0.0805000
-18840.0000000	0.0800000
-18839.0000000	0.0810000
-18838.0000000	0.0800000
-18837.0000000	0.0804762
-18836.0000000	0.0800000
-18835.0000000	0.0809524

-18834.0000000	0.0820000
-18833.0000000	0.0810000
-18832.0000000	0.0845000
-18831.0000000	0.0847619
-18830.0000000	0.0865000
-18829.0000000	0.0885000
-18828.0000000	0.0876191
-18827.0000000	0.0870000
-18826.0000000	0.0875000
-18825.0000000	0.0876191
-18824.0000000	0.0850000
-18823.0000000	0.0885000
-18822.0000000	0.0866667
-18821.0000000	0.0845000
-18820.0000000	0.0847619
-18819.0000000	0.0855000
-18818.0000000	0.0850000
-18817.0000000	0.0845000
-18816.0000000	0.0833334
-18815.0000000	0.0840000
-18814.0000000	0.0825000
-18813.0000000	0.0861905
-18812.0000000	0.0865000
-18811.0000000	0.0855000
-18810.0000000	0.0819048
-18809.0000000	0.0830000
-18808.0000000	0.0830000
-18807.0000000	0.0842857
-18806.0000000	0.0845000
-18805.0000000	0.0847619
-18804.0000000	0.0840000
-18803.0000000	0.0835000
-18802.0000000	0.0815000
-18801.0000000	0.0833333
-18800.0000000	0.0840000
-18799.0000000	0.0850000
-18798.0000000	0.0842857
-18797.0000000	0.0860000
-18796.0000000	0.0870000
-18795.0000000	0.0861905
-18794.0000000	0.0875000
-18793.0000000	0.0875000
-18792.0000000	0.0871429
-18791.0000000	0.0885000
-18790.0000000	0.0855000
-18789.0000000	0.0871429
-18788.0000000	0.0860000
-18787.0000000	0.0870000
-18786.0000000	0.0880953
-18785.0000000	0.0875000
-18784.0000000	0.0865000
-18783.0000000	0.0885715
-18782.0000000	0.0890000
-18781.0000000	0.0900000
-18780.0000000	0.0900000
-18779.0000000	0.0895000
-18778.0000000	0.0890000

-18777.0000000	0.0880953
-18776.0000000	0.0895000
-18775.0000000	0.0871429
-18774.0000000	0.0840000
-18773.0000000	0.0810000
-18772.0000000	0.0800000
-18771.0000000	0.0800000
-18770.0000000	0.0800000
-18769.0000000	0.0805000
-18768.0000000	0.0804762
-18767.0000000	0.0800000
-18766.0000000	0.0800000
-18765.0000000	0.0800000
-18764.0000000	0.0800000
-18763.0000000	0.0800000
-18762.0000000	0.0800000
-18761.0000000	0.0800000
-18760.0000000	0.0800000
-18759.0000000	0.0800000
-18758.0000000	0.0800000
-18757.0000000	0.0800000
-18756.0000000	0.0805000
-18755.0000000	0.0800000
-18754.0000000	0.0800000
-18753.0000000	0.0800000
-18752.0000000	0.0800000
-18751.0000000	0.0800000
-18750.0000000	0.0800000
-18749.0000000	0.0790000
-18748.0000000	0.0795000
-18747.0000000	0.0790000
-18746.0000000	0.0800000
-18745.0000000	0.0800000
-18744.0000000	0.0795000
-18743.0000000	0.0800000
-18742.0000000	0.0800000
-18741.0000000	0.0800000
-18740.0000000	0.0800000
-18739.0000000	0.0805000
-18738.0000000	0.0800000
-18737.0000000	0.0804762
-18736.0000000	0.0810000
-18735.0000000	0.0805000
-18734.0000000	0.0809524
-18733.0000000	0.0810000
-18732.0000000	0.0810000
-18731.0000000	0.0804762
-18730.0000000	0.0800000
-18729.0000000	0.0804762
-18728.0000000	0.0805000
-18727.0000000	0.0810000
-18726.0000000	0.0800000
-18725.0000000	0.0809524
-18724.0000000	0.0825000
-18723.0000000	0.0828572
-18722.0000000	0.0820000
-18721.0000000	0.0835000

-18720.0000000	0.0838095
-18719.0000000	0.0840000
-18718.0000000	0.0860000
-18717.0000000	0.0870000
-18716.0000000	0.0871429
-18715.0000000	0.0875000
-18714.0000000	0.0847619
-18713.0000000	0.0890000
-18712.0000000	0.0885000
-18711.0000000	0.0871429
-18710.0000000	0.0860000
-18709.0000000	0.0850000
-18708.0000000	0.0840000
-18707.0000000	0.0823810
-18706.0000000	0.0830000
-18705.0000000	0.0835000
-18704.0000000	0.0838095
-18703.0000000	0.0840000
-18702.0000000	0.0828572
-18701.0000000	0.0805000
-18700.0000000	0.0815000
-18699.0000000	0.0833333
-18698.0000000	0.0820000
-18697.0000000	0.0810000
-18696.0000000	0.0810000
-18695.0000000	0.0809524
-18694.0000000	0.0810000
-18693.0000000	0.0809524
-18692.0000000	0.0805000
-18691.0000000	0.0810000
-18690.0000000	0.0810000
-18689.0000000	0.0809524
-18688.0000000	0.0805000
-18687.0000000	0.0800000
-18686.0000000	0.0800000
-18685.0000000	0.0800000
-18684.0000000	0.0800000
-18683.0000000	0.0800000
-18682.0000000	0.0805000
-18681.0000000	0.0800000
-18680.0000000	0.0804762
-18679.0000000	0.0800000
-18678.0000000	0.0800000
-18677.0000000	0.0800000
-18676.0000000	0.0800000
-18675.0000000	0.0800000
-18674.0000000	0.0800000
-18673.0000000	0.0800000
-18672.0000000	0.0800000
-18671.0000000	0.0800000
-18670.0000000	0.0800000
-18669.0000000	0.0800000
-18668.0000000	0.0800000
-18667.0000000	0.0800000
-18666.0000000	0.0800000
-18665.0000000	0.0800000
-18664.0000000	0.0800000

-18663.0000000	0.0800000
-18662.0000000	0.0795238
-18661.0000000	0.0785000
-18660.0000000	0.0770000
-18659.0000000	0.0790000
-18658.0000000	0.0750000
-18657.0000000	0.0771429
-18656.0000000	0.0785714
-18655.0000000	0.0780000
-18654.0000000	0.0776191
-18653.0000000	0.0780000
-18652.0000000	0.0750000
-18651.0000000	0.0775000
-18650.0000000	0.0790476
-18649.0000000	0.0755000
-18648.0000000	0.0760000
-18647.0000000	0.0766667
-18646.0000000	0.0760000
-18645.0000000	0.0760000
-18644.0000000	0.0742857
-18643.0000000	0.0765000
-18642.0000000	0.0760000
-18641.0000000	0.0757143
-18640.0000000	0.0775000
-18639.0000000	0.0785714
-18638.0000000	0.0760000
-18637.0000000	0.0790000
-18636.0000000	0.0795000
-18635.0000000	0.0790476
-18634.0000000	0.0800000
-18633.0000000	0.0800000
-18632.0000000	0.0800000
-18631.0000000	0.0790000
-18630.0000000	0.0800000
-18629.0000000	0.0800000
-18628.0000000	0.0800000
-18627.0000000	0.0800000
-18626.0000000	0.0800000
-18625.0000000	0.0800000
-18624.0000000	0.0800000
-18623.0000000	0.0800000
-18622.0000000	0.0800000
-18621.0000000	0.0800000
-18620.0000000	0.0800000
-18619.0000000	0.0800000
-18618.0000000	0.0800000
-18617.0000000	0.0800000
-18616.0000000	0.0800000
-18615.0000000	0.0800000
-18614.0000000	0.0800000
-18613.0000000	0.0800000
-18612.0000000	0.0800000
-18611.0000000	0.0800000
-18610.0000000	0.0800000
-18609.0000000	0.0800000
-18608.0000000	0.0800000
-18607.0000000	0.0800000

-18606.0000000	0.0800000
-18605.0000000	0.0800000
-18604.0000000	0.0800000
-18603.0000000	0.0800000
-18602.0000000	0.0800000
-18601.0000000	0.0800000
-18600.0000000	0.0800000
-18599.0000000	0.0800000
-18598.0000000	0.0800000
-18597.0000000	0.0800000
-18596.0000000	0.0800000
-18595.0000000	0.0800000
-18594.0000000	0.0800000
-18593.0000000	0.0800000
-18592.0000000	0.0795238
-18591.0000000	0.0795000
-18590.0000000	0.0800000
-18589.0000000	0.0800000
-18588.0000000	0.0795000
-18587.0000000	0.0800000
-18586.0000000	0.0800000
-18585.0000000	0.0800000
-18584.0000000	0.0795000
-18583.0000000	0.0800000
-18582.0000000	0.0800000
-18581.0000000	0.0800000
-18580.0000000	0.0795000
-18579.0000000	0.0800000
-18578.0000000	0.0795000
-18577.0000000	0.0800000
-18576.0000000	0.0800000
-18575.0000000	0.0795000
-18574.0000000	0.0795238
-18573.0000000	0.0790000
-18572.0000000	0.0795000
-18571.0000000	0.0766667
-18570.0000000	0.0785000
-18569.0000000	0.0795000
-18568.0000000	0.0790476
-18567.0000000	0.0770000
-18566.0000000	0.0775000
-18565.0000000	0.0776191
-18564.0000000	0.0755000
-18563.0000000	0.0755000
-18562.0000000	0.0757143
-18561.0000000	0.0780000
-18560.0000000	0.0761905
-18559.0000000	0.0780000
-18558.0000000	0.0775000
-18557.0000000	0.0785000
-18556.0000000	0.0776191
-18555.0000000	0.0790000
-18554.0000000	0.0800000
-18553.0000000	0.0800000
-18552.0000000	0.0800000
-18551.0000000	0.0795238
-18550.0000000	0.0800000

-18549.0000000	0.0800000
-18548.0000000	0.0800000
-18547.0000000	0.0800000
-18546.0000000	0.0800000
-18545.0000000	0.0800000
-18544.0000000	0.0800000
-18543.0000000	0.0800000
-18542.0000000	0.0800000
-18541.0000000	0.0800000
-18540.0000000	0.0800000
-18539.0000000	0.0800000
-18538.0000000	0.0800000
-18537.0000000	0.0800000
-18536.0000000	0.0800000
-18535.0000000	0.0804762
-18534.0000000	0.0800000
-18533.0000000	0.0800000
-18532.0000000	0.0800000
-18531.0000000	0.0804762
-18530.0000000	0.0800000
-18529.0000000	0.0800000
-18528.0000000	0.0805000
-18527.0000000	0.0800000
-18526.0000000	0.0800000
-18525.0000000	0.0805000
-18524.0000000	0.0800000
-18523.0000000	0.0800000
-18522.0000000	0.0800000
-18521.0000000	0.0800000
-18520.0000000	0.0805000
-18519.0000000	0.0800000
-18518.0000000	0.0800000
-18517.0000000	0.0809524
-18516.0000000	0.0815000
-18515.0000000	0.0815000
-18514.0000000	0.0830000
-18513.0000000	0.0828572
-18512.0000000	0.0840000
-18511.0000000	0.0825000
-18510.0000000	0.0833333
-18509.0000000	0.0830000
-18508.0000000	0.0842857
-18507.0000000	0.0855000
-18506.0000000	0.0845000
-18505.0000000	0.0838095
-18504.0000000	0.0845000
-18503.0000000	0.0855000
-18502.0000000	0.0840000
-18501.0000000	0.0842857
-18500.0000000	0.0820000
-18499.0000000	0.0823810
-18498.0000000	0.0805000
-18497.0000000	0.0800000
-18496.0000000	0.0800000
-18495.0000000	0.0805000
-18494.0000000	0.0805000
-18493.0000000	0.0810000

-18492.0000000	0.0800000
-18491.0000000	0.0800000
-18490.0000000	0.0804762
-18489.0000000	0.0810000
-18488.0000000	0.0800000
-18487.0000000	0.0804762
-18486.0000000	0.0810000
-18485.0000000	0.0815000
-18484.0000000	0.0825000
-18483.0000000	0.0814286
-18482.0000000	0.0815000
-18481.0000000	0.0820000
-18480.0000000	0.0828572
-18479.0000000	0.0835000
-18478.0000000	0.0819048
-18477.0000000	0.0835000
-18476.0000000	0.0855000
-18475.0000000	0.0842857
-18474.0000000	0.0850000
-18473.0000000	0.0870000
-18472.0000000	0.0855000
-18471.0000000	0.0871429
-18470.0000000	0.0865000
-18469.0000000	0.0857143
-18468.0000000	0.0850000
-18467.0000000	0.0840000
-18466.0000000	0.0835000
-18465.0000000	0.0809524
-18464.0000000	0.0815000
-18463.0000000	0.0820000
-18462.0000000	0.0828572
-18461.0000000	0.0820000
-18460.0000000	0.0819048
-18459.0000000	0.0825000
-18458.0000000	0.0805000
-18457.0000000	0.0805000
-18456.0000000	0.0828572
-18455.0000000	0.0820000
-18454.0000000	0.0810000
-18453.0000000	0.0804762
-18452.0000000	0.0800000
-18451.0000000	0.0800000
-18450.0000000	0.0804762
-18449.0000000	0.0800000
-18448.0000000	0.0800000
-18447.0000000	0.0800000
-18446.0000000	0.0800000
-18445.0000000	0.0800000
-18444.0000000	0.0800000
-18443.0000000	0.0800000
-18442.0000000	0.0800000
-18441.0000000	0.0795238
-18440.0000000	0.0800000
-18439.0000000	0.0800000
-18438.0000000	0.0800000
-18437.0000000	0.0800000
-18436.0000000	0.0800000

-18435.0000000	0.0800000
-18434.0000000	0.0800000
-18433.0000000	0.0800000
-18432.0000000	0.0800000
-18431.0000000	0.0800000
-18430.0000000	0.0800000
-18429.0000000	0.0800000
-18428.0000000	0.0800000
-18427.0000000	0.0800000
-18426.0000000	0.0800000
-18425.0000000	0.0805000
-18424.0000000	0.0810000
-18423.0000000	0.0804762
-18422.0000000	0.0805000
-18421.0000000	0.0825000
-18420.0000000	0.0809524
-18419.0000000	0.0820000
-18418.0000000	0.0815000
-18417.0000000	0.0833333
-18416.0000000	0.0840000
-18415.0000000	0.0866667
-18414.0000000	0.0850000
-18413.0000000	0.0855000
-18412.0000000	0.0850000
-18411.0000000	0.0871429
-18410.0000000	0.0875000
-18409.0000000	0.0880000
-18408.0000000	0.0866667
-18407.0000000	0.0880000
-18406.0000000	0.0880000
-18405.0000000	0.0875000
-18404.0000000	0.0876191
-18403.0000000	0.0840000
-18402.0000000	0.0847619
-18401.0000000	0.0840000
-18400.0000000	0.0845000
-18399.0000000	0.0866667
-18398.0000000	0.0840000
-18397.0000000	0.0855000
-18396.0000000	0.0855000
-18395.0000000	0.0842857
-18394.0000000	0.0835000
-18393.0000000	0.0833333
-18392.0000000	0.0835000
-18391.0000000	0.0845000
-18390.0000000	0.0847619
-18389.0000000	0.0850000
-18388.0000000	0.0875000
-18387.0000000	0.0885715
-18386.0000000	0.0865000
-18385.0000000	0.0885714
-18384.0000000	0.0890000
-18383.0000000	0.0880000
-18382.0000000	0.0870000
-18381.0000000	0.0857143
-18380.0000000	0.0875000
-18379.0000000	0.0875000

-18378.0000000	0.0876191
-18377.0000000	0.0880000
-18376.0000000	0.0840000
-18375.0000000	0.0890000
-18374.0000000	0.0876191
-18373.0000000	0.0880000
-18372.0000000	0.0885715
-18371.0000000	0.0890000
-18370.0000000	0.0885000
-18369.0000000	0.0895238
-18368.0000000	0.0895000
-18367.0000000	0.0890000
-18366.0000000	0.0900000
-18365.0000000	0.0895238
-18364.0000000	0.0900000
-18363.0000000	0.0895238
-18362.0000000	0.0895000
-18361.0000000	0.0900000
-18360.0000000	0.0895238
-18359.0000000	0.0895000
-18358.0000000	0.0895000
-18357.0000000	0.0890000
-18356.0000000	0.0895238
-18355.0000000	0.0900000
-18354.0000000	0.0885714
-18353.0000000	0.0900000
-18352.0000000	0.0890000
-18351.0000000	0.0885714
-18350.0000000	0.0895000
-18349.0000000	0.0895000
-18348.0000000	0.0890000
-18347.0000000	0.0880953
-18346.0000000	0.0895000
-18345.0000000	0.0870000
-18344.0000000	0.0880953
-18343.0000000	0.0855000
-18342.0000000	0.0842857
-18341.0000000	0.0850000
-18340.0000000	0.0845000
-18339.0000000	0.0847619
-18338.0000000	0.0870000
-18337.0000000	0.0865000
-18336.0000000	0.0875000
-18335.0000000	0.0880953
-18334.0000000	0.0895000
-18333.0000000	0.0890476
-18332.0000000	0.0895000
-18331.0000000	0.0895000
-18330.0000000	0.0890000
-18329.0000000	0.0890476
-18328.0000000	0.0895000
-18327.0000000	0.0900000
-18326.0000000	0.0900000
-18325.0000000	0.0900000
-18324.0000000	0.0900000
-18323.0000000	0.0900000
-18322.0000000	0.0900000

-18321.0000000	0.0900000
-18320.0000000	0.0900000
-18319.0000000	0.0900000
-18318.0000000	0.0900000
-18317.0000000	0.0909524
-18316.0000000	0.0900000
-18315.0000000	0.0905000
-18314.0000000	0.0900000
-18313.0000000	0.0900000
-18312.0000000	0.0900000
-18311.0000000	0.0900000
-18310.0000000	0.0900000
-18309.0000000	0.0900000
-18308.0000000	0.0900000
-18307.0000000	0.0900000
-18306.0000000	0.0900000
-18305.0000000	0.0900000
-18304.0000000	0.0900000
-18303.0000000	0.0900000
-18302.0000000	0.0895000
-18301.0000000	0.0895000
-18300.0000000	0.0890000
-18299.0000000	0.0880953
-18298.0000000	0.0865000
-18297.0000000	0.0840000
-18296.0000000	0.0819048
-18295.0000000	0.0825000
-18294.0000000	0.0833333
-18293.0000000	0.0870000
-18292.0000000	0.0885000
-18291.0000000	0.0890000
-18290.0000000	0.0880953
-18289.0000000	0.0900000
-18288.0000000	0.0900000
-18287.0000000	0.0895238
-18286.0000000	0.0900000
-18285.0000000	0.0895000
-18284.0000000	0.0895238
-18283.0000000	0.0895000
-18282.0000000	0.0895000
-18281.0000000	0.0900000
-18280.0000000	0.0880000
-18279.0000000	0.0871429
-18278.0000000	0.0855000
-18277.0000000	0.0830000
-18276.0000000	0.0845000
-18275.0000000	0.0828572
-18274.0000000	0.0830000
-18273.0000000	0.0830000
-18272.0000000	0.0809524
-18271.0000000	0.0830000
-18270.0000000	0.0830000
-18269.0000000	0.0828572
-18268.0000000	0.0830000
-18267.0000000	0.0830000
-18266.0000000	0.0823810
-18265.0000000	0.0825000

-18264.0000000	0.0845000
-18263.0000000	0.0847619
-18262.0000000	0.0820000
-18261.0000000	0.0825000
-18260.0000000	0.0804762
-18259.0000000	0.0800000
-18258.0000000	0.0820000
-18257.0000000	0.0828572
-18256.0000000	0.0815000
-18255.0000000	0.0815000
-18254.0000000	0.0814286
-18253.0000000	0.0810000
-18252.0000000	0.0815000
-18251.0000000	0.0814286
-18250.0000000	0.0850000
-18249.0000000	0.0852381
-18248.0000000	0.0860000
-18247.0000000	0.0880000
-18246.0000000	0.0875000
-18245.0000000	0.0885715
-18244.0000000	0.0895000
-18243.0000000	0.0895000
-18242.0000000	0.0900000
-18241.0000000	0.0900000
-18240.0000000	0.0900000
-18239.0000000	0.0895238
-18238.0000000	0.0900000
-18237.0000000	0.0895000
-18236.0000000	0.0900000
-18235.0000000	0.0890000
-18234.0000000	0.0895000
-18233.0000000	0.0900000
-18232.0000000	0.0890000
-18231.0000000	0.0880000
-18230.0000000	0.0876191
-18229.0000000	0.0885000
-18228.0000000	0.0880000
-18227.0000000	0.0885714
-18226.0000000	0.0890000
-18225.0000000	0.0885000
-18224.0000000	0.0890476
-18223.0000000	0.0890000
-18222.0000000	0.0885000
-18221.0000000	0.0885000
-18220.0000000	0.0895238
-18219.0000000	0.0880953
-18218.0000000	0.0890000
-18217.0000000	0.0900000
-18216.0000000	0.0900000
-18215.0000000	0.0890476
-18214.0000000	0.0900000
-18213.0000000	0.0895000
-18212.0000000	0.0900000
-18211.0000000	0.0895000
-18210.0000000	0.0900000
-18209.0000000	0.0900000
-18208.0000000	0.0900000

-18207.0000000	0.0900000
-18206.0000000	0.0900000
-18205.0000000	0.0900000
-18204.0000000	0.0900000
-18203.0000000	0.0900000
-18202.0000000	0.0900000
-18201.0000000	0.0895000
-18200.0000000	0.0890000
-18199.0000000	0.0900000
-18198.0000000	0.0900000
-18197.0000000	0.0895238
-18196.0000000	0.0900000
-18195.0000000	0.0895000
-18194.0000000	0.0900000
-18193.0000000	0.0885000
-18192.0000000	0.0880000
-18191.0000000	0.0865000
-18190.0000000	0.0861905
-18189.0000000	0.0850000
-18188.0000000	0.0828572
-18187.0000000	0.0835000
-18186.0000000	0.0820000
-18185.0000000	0.0814286
-18184.0000000	0.0805000
-18183.0000000	0.0810000
-18182.0000000	0.0810000
-18181.0000000	0.0814286
-18180.0000000	0.0805000
-18179.0000000	0.0825000
-18178.0000000	0.0814286
-18177.0000000	0.0800000
-18176.0000000	0.0804762
-18175.0000000	0.0805000
-18174.0000000	0.0820000
-18173.0000000	0.0809524
-18172.0000000	0.0815000
-18171.0000000	0.0815000
-18170.0000000	0.0825000
-18169.0000000	0.0804762
-18168.0000000	0.0815000
-18167.0000000	0.0819048
-18166.0000000	0.0815000
-18165.0000000	0.0810000
-18164.0000000	0.0810000
-18163.0000000	0.0809524
-18162.0000000	0.0815000
-18161.0000000	0.0810000
-18160.0000000	0.0814286
-18159.0000000	0.0805000
-18158.0000000	0.0823810
-18157.0000000	0.0840000
-18156.0000000	0.0820000
-18155.0000000	0.0820000
-18154.0000000	0.0833333
-18153.0000000	0.0805000
-18152.0000000	0.0810000
-18151.0000000	0.0828572

-18150.0000000	0.0810000
-18149.0000000	0.0805000
-18148.0000000	0.0810000
-18147.0000000	0.0828572
-18146.0000000	0.0819048
-18145.0000000	0.0825000
-18144.0000000	0.0840000
-18143.0000000	0.0847619
-18142.0000000	0.0820000
-18141.0000000	0.0860000
-18140.0000000	0.0850000
-18139.0000000	0.0828572
-18138.0000000	0.0835000
-18137.0000000	0.0861905
-18136.0000000	0.0855000
-18135.0000000	0.0850000
-18134.0000000	0.0865000
-18133.0000000	0.0857143
-18132.0000000	0.0840000
-18131.0000000	0.0885000
-18130.0000000	0.0885715
-18129.0000000	0.0880000
-18128.0000000	0.0900000
-18127.0000000	0.0895000
-18126.0000000	0.0900000
-18125.0000000	0.0895000
-18124.0000000	0.0900000
-18123.0000000	0.0895000
-18122.0000000	0.0900000
-18121.0000000	0.0900000
-18120.0000000	0.0895000
-18119.0000000	0.0890000
-18118.0000000	0.0890476
-18117.0000000	0.0880000
-18116.0000000	0.0890000
-18115.0000000	0.0885715
-18114.0000000	0.0885000
-18113.0000000	0.0885714
-18112.0000000	0.0890000
-18111.0000000	0.0885000
-18110.0000000	0.0895000
-18109.0000000	0.0880953
-18108.0000000	0.0900000
-18107.0000000	0.0895000
-18106.0000000	0.0885714
-18105.0000000	0.0900000
-18104.0000000	0.0900000
-18103.0000000	0.0900000
-18102.0000000	0.0900000
-18101.0000000	0.0900000
-18100.0000000	0.0900000
-18099.0000000	0.0900000
-18098.0000000	0.0900000
-18097.0000000	0.0900000
-18096.0000000	0.0895000
-18095.0000000	0.0880000
-18094.0000000	0.0866667

-18093.0000000	0.0845000
-18092.0000000	0.0830000
-18091.0000000	0.0838095
-18090.0000000	0.0825000
-18089.0000000	0.0805000
-18088.0000000	0.0815000
-18087.0000000	0.0828571
-18086.0000000	0.0810000
-18085.0000000	0.0809524
-18084.0000000	0.0810000
-18083.0000000	0.0819048
-18082.0000000	0.0810000
-18081.0000000	0.0810000
-18080.0000000	0.0805000
-18079.0000000	0.0800000
-18078.0000000	0.0810000
-18077.0000000	0.0810000
-18076.0000000	0.0809524
-18075.0000000	0.0810000
-18074.0000000	0.0805000
-18073.0000000	0.0815000
-18072.0000000	0.0804762
-18071.0000000	0.0805000
-18070.0000000	0.0800000
-18069.0000000	0.0800000
-18068.0000000	0.0800000
-18067.0000000	0.0800000
-18066.0000000	0.0800000
-18065.0000000	0.0800000
-18064.0000000	0.0800000
-18063.0000000	0.0804762
-18062.0000000	0.0800000
-18061.0000000	0.0800000
-18060.0000000	0.0800000
-18059.0000000	0.0800000
-18058.0000000	0.0800000
-18057.0000000	0.0800000
-18056.0000000	0.0800000
-18055.0000000	0.0800000
-18054.0000000	0.0800000
-18053.0000000	0.0800000
-18052.0000000	0.0800000
-18051.0000000	0.0800000
-18050.0000000	0.0800000
-18049.0000000	0.0800000
-18048.0000000	0.0800000
-18047.0000000	0.0800000
-18046.0000000	0.0800000
-18045.0000000	0.0800000
-18044.0000000	0.0800000
-18043.0000000	0.0800000
-18042.0000000	0.0800000
-18041.0000000	0.0805000
-18040.0000000	0.0804762
-18039.0000000	0.0800000
-18038.0000000	0.0800000
-18037.0000000	0.0800000

-18036.0000000	0.0800000
-18035.0000000	0.0800000
-18034.0000000	0.0800000
-18033.0000000	0.0800000
-18032.0000000	0.0800000
-18031.0000000	0.0800000
-18030.0000000	0.0800000
-18029.0000000	0.0800000
-18028.0000000	0.0800000
-18027.0000000	0.0800000
-18026.0000000	0.0800000
-18025.0000000	0.0800000
-18024.0000000	0.0800000
-18023.0000000	0.0800000
-18022.0000000	0.0800000
-18021.0000000	0.0800000
-18020.0000000	0.0800000
-18019.0000000	0.0800000
-18018.0000000	0.0800000
-18017.0000000	0.0800000
-18016.0000000	0.0800000
-18015.0000000	0.0800000
-18014.0000000	0.0800000
-18013.0000000	0.0800000
-18012.0000000	0.0800000
-18011.0000000	0.0800000
-18010.0000000	0.0800000
-18009.0000000	0.0800000
-18008.0000000	0.0800000
-18007.0000000	0.0795000
-18006.0000000	0.0800000
-18005.0000000	0.0800000
-18004.0000000	0.0800000
-18003.0000000	0.0800000
-18002.0000000	0.0800000
-18001.0000000	0.0800000
-18000.0000000	0.0800000
-17999.0000000	0.0800000
-17998.0000000	0.0800000
-17997.0000000	0.0800000
-17996.0000000	0.0800000
-17995.0000000	0.0800000
-17994.0000000	0.0800000
-17993.0000000	0.0800000
-17992.0000000	0.0809524
-17991.0000000	0.0805000
-17990.0000000	0.0810000
-17989.0000000	0.0825000
-17988.0000000	0.0823810
-17987.0000000	0.0840000
-17986.0000000	0.0830000
-17985.0000000	0.0838095
-17984.0000000	0.0815000
-17983.0000000	0.0810000
-17982.0000000	0.0830000
-17981.0000000	0.0833333
-17980.0000000	0.0825000

-17979.0000000	0.0823810
-17978.0000000	0.0840000
-17977.0000000	0.0828572
-17976.0000000	0.0845000
-17975.0000000	0.0835000
-17974.0000000	0.0825000
-17973.0000000	0.0828572
-17972.0000000	0.0860000
-17971.0000000	0.0830000
-17970.0000000	0.0823810
-17969.0000000	0.0840000
-17968.0000000	0.0820000
-17967.0000000	0.0823810
-17966.0000000	0.0820000
-17965.0000000	0.0835000
-17964.0000000	0.0833333
-17963.0000000	0.0840000
-17962.0000000	0.0842857
-17961.0000000	0.0850000
-17960.0000000	0.0860000
-17959.0000000	0.0865000
-17958.0000000	0.0871429
-17957.0000000	0.0875000
-17956.0000000	0.0850000
-17955.0000000	0.0847619
-17954.0000000	0.0845000
-17953.0000000	0.0825000
-17952.0000000	0.0810000
-17951.0000000	0.0804762
-17950.0000000	0.0800000
-17949.0000000	0.0809524
-17948.0000000	0.0805000
-17947.0000000	0.0805000
-17946.0000000	0.0800000
-17945.0000000	0.0805000
-17944.0000000	0.0805000
-17943.0000000	0.0800000
-17942.0000000	0.0810000
-17941.0000000	0.0800000
-17940.0000000	0.0804762
-17939.0000000	0.0800000
-17938.0000000	0.0805000
-17937.0000000	0.0800000
-17936.0000000	0.0835000
-17935.0000000	0.0810000
-17934.0000000	0.0819048
-17933.0000000	0.0825000
-17932.0000000	0.0845000
-17931.0000000	0.0838095
-17930.0000000	0.0840000
-17929.0000000	0.0840000
-17928.0000000	0.0842857
-17927.0000000	0.0835000
-17926.0000000	0.0835000
-17925.0000000	0.0876191
-17924.0000000	0.0835000
-17923.0000000	0.0855000

-17922.0000000	0.0860000
-17921.0000000	0.0871429
-17920.0000000	0.0865000
-17919.0000000	0.0885715
-17918.0000000	0.0890000
-17917.0000000	0.0880952
-17916.0000000	0.0890000
-17915.0000000	0.0890000
-17914.0000000	0.0900000
-17913.0000000	0.0900000
-17912.0000000	0.0895000
-17911.0000000	0.0900000
-17910.0000000	0.0900000
-17909.0000000	0.0900000
-17908.0000000	0.0895000
-17907.0000000	0.0890000
-17906.0000000	0.0900000
-17905.0000000	0.0900000
-17904.0000000	0.0895238
-17903.0000000	0.0900000
-17902.0000000	0.0900000
-17901.0000000	0.0890476
-17900.0000000	0.0900000
-17899.0000000	0.0900000
-17898.0000000	0.0895000
-17897.0000000	0.0895238
-17896.0000000	0.0890000
-17895.0000000	0.0900000
-17894.0000000	0.0890000
-17893.0000000	0.0900000
-17892.0000000	0.0900000
-17891.0000000	0.0895238
-17890.0000000	0.0900000
-17889.0000000	0.0900000
-17888.0000000	0.0895238
-17887.0000000	0.0895000
-17886.0000000	0.0900000
-17885.0000000	0.0900000
-17884.0000000	0.0900000
-17883.0000000	0.0900000
-17882.0000000	0.0900000
-17881.0000000	0.0900000
-17880.0000000	0.0900000
-17879.0000000	0.0900000
-17878.0000000	0.0900000
-17877.0000000	0.0900000
-17876.0000000	0.0900000
-17875.0000000	0.0895000
-17874.0000000	0.0900000
-17873.0000000	0.0900000
-17872.0000000	0.0900000
-17871.0000000	0.0880953
-17870.0000000	0.0895000
-17869.0000000	0.0880000
-17868.0000000	0.0865000
-17867.0000000	0.0847619
-17866.0000000	0.0850000

-17865.0000000	0.0842857
-17864.0000000	0.0855000
-17863.0000000	0.0850000
-17862.0000000	0.0855000
-17861.0000000	0.0847619
-17860.0000000	0.0855000
-17859.0000000	0.0855000
-17858.0000000	0.0866667
-17857.0000000	0.0865000
-17856.0000000	0.0871429
-17855.0000000	0.0875000
-17854.0000000	0.0875000
-17853.0000000	0.0861905
-17852.0000000	0.0845000
-17851.0000000	0.0820000
-17850.0000000	0.0825000
-17849.0000000	0.0814286
-17848.0000000	0.0810000
-17847.0000000	0.0805000
-17846.0000000	0.0805000
-17845.0000000	0.0800000
-17844.0000000	0.0800000
-17843.0000000	0.0800000
-17842.0000000	0.0800000
-17841.0000000	0.0800000
-17840.0000000	0.0800000
-17839.0000000	0.0800000
-17838.0000000	0.0800000
-17837.0000000	0.0800000
-17836.0000000	0.0800000
-17835.0000000	0.0800000
-17834.0000000	0.0800000
-17833.0000000	0.0800000
-17832.0000000	0.0805000
-17831.0000000	0.0800000
-17830.0000000	0.0805000
-17829.0000000	0.0805000
-17828.0000000	0.0800000
-17827.0000000	0.0805000
-17826.0000000	0.0823810
-17825.0000000	0.0830000
-17824.0000000	0.0830000
-17823.0000000	0.0820000
-17822.0000000	0.0828572
-17821.0000000	0.0840000
-17820.0000000	0.0840000
-17819.0000000	0.0833333
-17818.0000000	0.0835000
-17817.0000000	0.0860000
-17816.0000000	0.0860000
-17815.0000000	0.0861905
-17814.0000000	0.0845000
-17813.0000000	0.0885714
-17812.0000000	0.0880000
-17811.0000000	0.0865000
-17810.0000000	0.0895238
-17809.0000000	0.0870000

-17808.0000000	0.0890000
-17807.0000000	0.0895238
-17806.0000000	0.0895000
-17805.0000000	0.0895000
-17804.0000000	0.0895238
-17803.0000000	0.0900000
-17802.0000000	0.0900000
-17801.0000000	0.0900000
-17800.0000000	0.0900000
-17799.0000000	0.0900000
-17798.0000000	0.0900000
-17797.0000000	0.0895000
-17796.0000000	0.0900000
-17795.0000000	0.0900000
-17794.0000000	0.0900000
-17793.0000000	0.0900000
-17792.0000000	0.0900000
-17791.0000000	0.0895000
-17790.0000000	0.0900000
-17789.0000000	0.0900000
-17788.0000000	0.0900000
-17787.0000000	0.0900000
-17786.0000000	0.0900000
-17785.0000000	0.0885714
-17784.0000000	0.0890000
-17783.0000000	0.0900000
-17782.0000000	0.0885000
-17781.0000000	0.0880000
-17780.0000000	0.0866667
-17779.0000000	0.0860000
-17778.0000000	0.0880000
-17777.0000000	0.0890476
-17776.0000000	0.0870000
-17775.0000000	0.0890000
-17774.0000000	0.0895238
-17773.0000000	0.0875000
-17772.0000000	0.0900000
-17771.0000000	0.0895238
-17770.0000000	0.0895000
-17769.0000000	0.0875000
-17768.0000000	0.0900000
-17767.0000000	0.0890000
-17766.0000000	0.0885000
-17765.0000000	0.0885714
-17764.0000000	0.0885000
-17763.0000000	0.0885000
-17762.0000000	0.0900000
-17761.0000000	0.0900000
-17760.0000000	0.0900000
-17759.0000000	0.0900000
-17758.0000000	0.0900000
-17757.0000000	0.0900000
-17756.0000000	0.0900000
-17755.0000000	0.0904762
-17754.0000000	0.0900000
-17753.0000000	0.0900000
-17752.0000000	0.0900000

-17751.0000000	0.0900000
-17750.0000000	0.0900000
-17749.0000000	0.0900000
-17748.0000000	0.0900000
-17747.0000000	0.0900000
-17746.0000000	0.0900000
-17745.0000000	0.0900000
-17744.0000000	0.0900000
-17743.0000000	0.0900000
-17742.0000000	0.0905000
-17741.0000000	0.0900000
-17740.0000000	0.0904762
-17739.0000000	0.0900000
-17738.0000000	0.0900000
-17737.0000000	0.0900000
-17736.0000000	0.0905000
-17735.0000000	0.0900000
-17734.0000000	0.0900000
-17733.0000000	0.0900000
-17732.0000000	0.0905000
-17731.0000000	0.0904762
-17730.0000000	0.0900000
-17729.0000000	0.0900000
-17728.0000000	0.0900000
-17727.0000000	0.0900000
-17726.0000000	0.0910000
-17725.0000000	0.0914286
-17724.0000000	0.0920000
-17723.0000000	0.0925000
-17722.0000000	0.0923810
-17721.0000000	0.0925000
-17720.0000000	0.0938095
-17719.0000000	0.0915000
-17718.0000000	0.0930000
-17717.0000000	0.0909524
-17716.0000000	0.0900000
-17715.0000000	0.0900000
-17714.0000000	0.0900000
-17713.0000000	0.0890476
-17712.0000000	0.0890000
-17711.0000000	0.0895000
-17710.0000000	0.0890476
-17709.0000000	0.0900000
-17708.0000000	0.0895238
-17707.0000000	0.0900000
-17706.0000000	0.0900000
-17705.0000000	0.0900000
-17704.0000000	0.0900000
-17703.0000000	0.0900000
-17702.0000000	0.0900000
-17701.0000000	0.0900000
-17700.0000000	0.0895000
-17699.0000000	0.0890476
-17698.0000000	0.0895000
-17697.0000000	0.0900000
-17696.0000000	0.0900000
-17695.0000000	0.0900000

-17694.0000000	0.0900000
-17693.0000000	0.0900000
-17692.0000000	0.0900000
-17691.0000000	0.0900000
-17690.0000000	0.0900000
-17689.0000000	0.0900000
-17688.0000000	0.0900000
-17687.0000000	0.0900000
-17686.0000000	0.0900000
-17685.0000000	0.0900000
-17684.0000000	0.0905000
-17683.0000000	0.0904762
-17682.0000000	0.0915000
-17681.0000000	0.0920000
-17680.0000000	0.0920000
-17679.0000000	0.0966667
-17678.0000000	0.0947619
-17677.0000000	0.0955000
-17676.0000000	0.0975000
-17675.0000000	0.0965000
-17674.0000000	0.0976191
-17673.0000000	0.0990000
-17672.0000000	0.0965000
-17671.0000000	0.0961905
-17670.0000000	0.0975000
-17669.0000000	0.0971429
-17668.0000000	0.0975000
-17667.0000000	0.0970000
-17666.0000000	0.0960000
-17665.0000000	0.0976191
-17664.0000000	0.0970000
-17663.0000000	0.0990000
-17662.0000000	0.0985714
-17661.0000000	0.0990000
-17660.0000000	0.0985714
-17659.0000000	0.0985000
-17658.0000000	0.0990000
-17657.0000000	0.0975000
-17656.0000000	0.0985714
-17655.0000000	0.0980000
-17654.0000000	0.0975000
-17653.0000000	0.0980953
-17652.0000000	0.0975000
-17651.0000000	0.0975000
-17650.0000000	0.0940000
-17649.0000000	0.0961905
-17648.0000000	0.0930000
-17647.0000000	0.0904762
-17646.0000000	0.0910000
-17645.0000000	0.0905000
-17644.0000000	0.0900000
-17643.0000000	0.0905000
-17642.0000000	0.0900000
-17641.0000000	0.0900000
-17640.0000000	0.0905000
-17639.0000000	0.0905000
-17638.0000000	0.0923810

-17637.0000000	0.0935000
-17636.0000000	0.0935000
-17635.0000000	0.0947619
-17634.0000000	0.0965000
-17633.0000000	0.0970000
-17632.0000000	0.0980953
-17631.0000000	0.0995000
-17630.0000000	0.0985000
-17629.0000000	0.1000000
-17628.0000000	0.0990000
-17627.0000000	0.0995000
-17626.0000000	0.1000000
-17625.0000000	0.0975000
-17624.0000000	0.0980000
-17623.0000000	0.0985714
-17622.0000000	0.0980000
-17621.0000000	0.0980000
-17620.0000000	0.0980000
-17619.0000000	0.0980952
-17618.0000000	0.0980000
-17617.0000000	0.0971429
-17616.0000000	0.0975000
-17615.0000000	0.0950000
-17614.0000000	0.0952381
-17613.0000000	0.0960000
-17612.0000000	0.0940000
-17611.0000000	0.0961905
-17610.0000000	0.0955000
-17609.0000000	0.0935000
-17608.0000000	0.0952381
-17607.0000000	0.0960000
-17606.0000000	0.0940000
-17605.0000000	0.0925000
-17604.0000000	0.0938095
-17603.0000000	0.0915000
-17602.0000000	0.0904762
-17601.0000000	0.0910000
-17600.0000000	0.0915000
-17599.0000000	0.0909524
-17598.0000000	0.0900000
-17597.0000000	0.0900000
-17596.0000000	0.0900000
-17595.0000000	0.0895238
-17594.0000000	0.0900000
-17593.0000000	0.0900000
-17592.0000000	0.0890000
-17591.0000000	0.0895000
-17590.0000000	0.0875000
-17589.0000000	0.0866667
-17588.0000000	0.0845000
-17587.0000000	0.0860000
-17586.0000000	0.0852381
-17585.0000000	0.0865000
-17584.0000000	0.0861905
-17583.0000000	0.0875000
-17582.0000000	0.0890000
-17581.0000000	0.0895238

-17580.0000000	0.0880000
-17579.0000000	0.0885000
-17578.0000000	0.0876191
-17577.0000000	0.0880000
-17576.0000000	0.0845000
-17575.0000000	0.0815000
-17574.0000000	0.0819048
-17573.0000000	0.0805000
-17572.0000000	0.0828572
-17571.0000000	0.0840000
-17570.0000000	0.0835000
-17569.0000000	0.0814286
-17568.0000000	0.0865000
-17567.0000000	0.0855000
-17566.0000000	0.0870000
-17565.0000000	0.0852381
-17564.0000000	0.0870000
-17563.0000000	0.0876191
-17562.0000000	0.0885000
-17561.0000000	0.0890000
-17560.0000000	0.0895000
-17559.0000000	0.0900000
-17558.0000000	0.0900000
-17557.0000000	0.0900000
-17556.0000000	0.0900000
-17555.0000000	0.0900000
-17554.0000000	0.0900000
-17553.0000000	0.0900000
-17552.0000000	0.0900000
-17551.0000000	0.0904762
-17550.0000000	0.0900000
-17549.0000000	0.0900000
-17548.0000000	0.0910000
-17547.0000000	0.0909524
-17546.0000000	0.0910000
-17545.0000000	0.0945000
-17544.0000000	0.0923810
-17543.0000000	0.0965000
-17542.0000000	0.0985714
-17541.0000000	0.0980000
-17540.0000000	0.0975000
-17539.0000000	0.0980000
-17538.0000000	0.0985714
-17537.0000000	0.0975000
-17536.0000000	0.0970000
-17535.0000000	0.0938095
-17534.0000000	0.0935000
-17533.0000000	0.0928572
-17532.0000000	0.0905000
-17531.0000000	0.0905000
-17530.0000000	0.0900000
-17529.0000000	0.0900000
-17528.0000000	0.0905000
-17527.0000000	0.0910000
-17526.0000000	0.0909524
-17525.0000000	0.0935000
-17524.0000000	0.0928572

-17523.0000000	0.0905000
-17522.0000000	0.0900000
-17521.0000000	0.0900000
-17520.0000000	0.0900000
-17519.0000000	0.0900000
-17518.0000000	0.0900000
-17517.0000000	0.0900000
-17516.0000000	0.0885000
-17515.0000000	0.0895000
-17514.0000000	0.0880000
-17513.0000000	0.0890476
-17512.0000000	0.0885000
-17511.0000000	0.0900000
-17510.0000000	0.0895000
-17509.0000000	0.0900000
-17508.0000000	0.0900000
-17507.0000000	0.0900000
-17506.0000000	0.0900000
-17505.0000000	0.0900000
-17504.0000000	0.0900000
-17503.0000000	0.0900000
-17502.0000000	0.0900000
-17501.0000000	0.0900000
-17500.0000000	0.0900000
-17499.0000000	0.0900000
-17498.0000000	0.0900000
-17497.0000000	0.0900000
-17496.0000000	0.0900000
-17495.0000000	0.0900000
-17494.0000000	0.0904762
-17493.0000000	0.0900000
-17492.0000000	0.0900000
-17491.0000000	0.0900000
-17490.0000000	0.0900000
-17489.0000000	0.0900000
-17488.0000000	0.0900000
-17487.0000000	0.0895238
-17486.0000000	0.0900000
-17485.0000000	0.0895000
-17484.0000000	0.0900000
-17483.0000000	0.0900000
-17482.0000000	0.0895000
-17481.0000000	0.0900000
-17480.0000000	0.0900000
-17479.0000000	0.0900000
-17478.0000000	0.0900000
-17477.0000000	0.0900000
-17476.0000000	0.0900000
-17475.0000000	0.0904762
-17474.0000000	0.0900000
-17473.0000000	0.0905000
-17472.0000000	0.0900000
-17471.0000000	0.0900000
-17470.0000000	0.0905000
-17469.0000000	0.0904762
-17468.0000000	0.0905000
-17467.0000000	0.0915000

-17466.0000000	0.0904762
-17465.0000000	0.0910000
-17464.0000000	0.0905000
-17463.0000000	0.0909524
-17462.0000000	0.0905000
-17461.0000000	0.0900000
-17460.0000000	0.0914286
-17459.0000000	0.0920000
-17458.0000000	0.0915000
-17457.0000000	0.0923810
-17456.0000000	0.0920000
-17455.0000000	0.0935000
-17454.0000000	0.0940000
-17453.0000000	0.0957143
-17452.0000000	0.0975000
-17451.0000000	0.0961905
-17450.0000000	0.0965000
-17449.0000000	0.0940000
-17448.0000000	0.0923810
-17447.0000000	0.0955000
-17446.0000000	0.0950000
-17445.0000000	0.0933334
-17444.0000000	0.0925000
-17443.0000000	0.0905000
-17442.0000000	0.0928572
-17441.0000000	0.0945000
-17440.0000000	0.0930000
-17439.0000000	0.0915000
-17438.0000000	0.0919048
-17437.0000000	0.0950000
-17436.0000000	0.0938095
-17435.0000000	0.0960000
-17434.0000000	0.0955000
-17433.0000000	0.0976191
-17432.0000000	0.0990000
-17431.0000000	0.0975000
-17430.0000000	0.0975000
-17429.0000000	0.0995238
-17428.0000000	0.0990000
-17427.0000000	0.0966667
-17426.0000000	0.0970000
-17425.0000000	0.0960000
-17424.0000000	0.0950000
-17423.0000000	0.0942857
-17422.0000000	0.0940000
-17421.0000000	0.0910000
-17420.0000000	0.0923810
-17419.0000000	0.0940000
-17418.0000000	0.0938095
-17417.0000000	0.0960000
-17416.0000000	0.0960000
-17415.0000000	0.0990476
-17414.0000000	0.0985000
-17413.0000000	0.0995000
-17412.0000000	0.0995000
-17411.0000000	0.1000000
-17410.0000000	0.1000000

-17409.0000000	0.1000000
-17408.0000000	0.1000000
-17407.0000000	0.1000000
-17406.0000000	0.1000000
-17405.0000000	0.1000000
-17404.0000000	0.1000000
-17403.0000000	0.1000000
-17402.0000000	0.1000000
-17401.0000000	0.1000000
-17400.0000000	0.1000000
-17399.0000000	0.1000000
-17398.0000000	0.1000000
-17397.0000000	0.1000000
-17396.0000000	0.1000000
-17395.0000000	0.0995000
-17394.0000000	0.1000000
-17393.0000000	0.0995238
-17392.0000000	0.0965000
-17391.0000000	0.0960000
-17390.0000000	0.0957143
-17389.0000000	0.0930000
-17388.0000000	0.0952381
-17387.0000000	0.0920000
-17386.0000000	0.0915000
-17385.0000000	0.0904762
-17384.0000000	0.0905000
-17383.0000000	0.0910000
-17382.0000000	0.0905000
-17381.0000000	0.0900000
-17380.0000000	0.0900000
-17379.0000000	0.0900000
-17378.0000000	0.0900000
-17377.0000000	0.0900000
-17376.0000000	0.0900000
-17375.0000000	0.0900000
-17374.0000000	0.0900000
-17373.0000000	0.0900000
-17372.0000000	0.0900000
-17371.0000000	0.0900000
-17370.0000000	0.0900000
-17369.0000000	0.0900000
-17368.0000000	0.0900000
-17367.0000000	0.0900000
-17366.0000000	0.0900000
-17365.0000000	0.0905000
-17364.0000000	0.0900000
-17363.0000000	0.0900000
-17362.0000000	0.0905000
-17361.0000000	0.0900000
-17360.0000000	0.0900000
-17359.0000000	0.0900000
-17358.0000000	0.0910000
-17357.0000000	0.0900000
-17356.0000000	0.0900000
-17355.0000000	0.0915000
-17354.0000000	0.0928572
-17353.0000000	0.0935000

-17352.0000000	0.0905000
-17351.0000000	0.0933334
-17350.0000000	0.0920000
-17349.0000000	0.0955000
-17348.0000000	0.0930000
-17347.0000000	0.0961905
-17346.0000000	0.0935000
-17345.0000000	0.0952381
-17344.0000000	0.0965000
-17343.0000000	0.0965000
-17342.0000000	0.0980952
-17341.0000000	0.0965000
-17340.0000000	0.0965000
-17339.0000000	0.0961905
-17338.0000000	0.0945000
-17337.0000000	0.0940000
-17336.0000000	0.0942857
-17335.0000000	0.0955000
-17334.0000000	0.0945000
-17333.0000000	0.0961905
-17332.0000000	0.0955000
-17331.0000000	0.0930000
-17330.0000000	0.0952381
-17329.0000000	0.0970000
-17328.0000000	0.0965000
-17327.0000000	0.0961905
-17326.0000000	0.0975000
-17325.0000000	0.0965000
-17324.0000000	0.0995238
-17323.0000000	0.0970000
-17322.0000000	0.0975000
-17321.0000000	0.0971429
-17320.0000000	0.1000000
-17319.0000000	0.0995000
-17318.0000000	0.1000000
-17317.0000000	0.0995238
-17316.0000000	0.0995000
-17315.0000000	0.1000000
-17314.0000000	0.1000000
-17313.0000000	0.0995000
-17312.0000000	0.1000000
-17311.0000000	0.1000000
-17310.0000000	0.1000000
-17309.0000000	0.1000000
-17308.0000000	0.1000000
-17307.0000000	0.0995000
-17306.0000000	0.1000000
-17305.0000000	0.1000000
-17304.0000000	0.1000000
-17303.0000000	0.1000000
-17302.0000000	0.1000000
-17301.0000000	0.1000000
-17300.0000000	0.1000000
-17299.0000000	0.1000000
-17298.0000000	0.1000000
-17297.0000000	0.1000000
-17296.0000000	0.1000000

-17295.0000000	0.1000000
-17294.0000000	0.0995238
-17293.0000000	0.0995000
-17292.0000000	0.0995000
-17291.0000000	0.0995238
-17290.0000000	0.0990000
-17289.0000000	0.0980000
-17288.0000000	0.1000000
-17287.0000000	0.0980953
-17286.0000000	0.0990000
-17285.0000000	0.0995238
-17284.0000000	0.0995000
-17283.0000000	0.1000000
-17282.0000000	0.0995238
-17281.0000000	0.0995000
-17280.0000000	0.0970000
-17279.0000000	0.0966667
-17278.0000000	0.0970000
-17277.0000000	0.0950000
-17276.0000000	0.0952381
-17275.0000000	0.0960000
-17274.0000000	0.0980000
-17273.0000000	0.0985000
-17272.0000000	0.0980953
-17271.0000000	0.0940000
-17270.0000000	0.0919048
-17269.0000000	0.0950000
-17268.0000000	0.0960000
-17267.0000000	0.0976191
-17266.0000000	0.0965000
-17265.0000000	0.0960000
-17264.0000000	0.0970000
-17263.0000000	0.0966667
-17262.0000000	0.0975000
-17261.0000000	0.0966667
-17260.0000000	0.0975000
-17259.0000000	0.0960000
-17258.0000000	0.0965000
-17257.0000000	0.0966667
-17256.0000000	0.0945000
-17255.0000000	0.0985000
-17254.0000000	0.0976191
-17253.0000000	0.0980000
-17252.0000000	0.0985714
-17251.0000000	0.0995000
-17250.0000000	0.1000000
-17249.0000000	0.0995238
-17248.0000000	0.1000000
-17247.0000000	0.1000000
-17246.0000000	0.1000000
-17245.0000000	0.0990476
-17244.0000000	0.1000000
-17243.0000000	0.1000000
-17242.0000000	0.1000000
-17241.0000000	0.1000000
-17240.0000000	0.0995238
-17239.0000000	0.0995000

-17238.0000000	0.1000000
-17237.0000000	0.0995000
-17236.0000000	0.0995238
-17235.0000000	0.0980000
-17234.0000000	0.0990000
-17233.0000000	0.0990476
-17232.0000000	0.0995000
-17231.0000000	0.1000000
-17230.0000000	0.0995000
-17229.0000000	0.0995000
-17228.0000000	0.1000000
-17227.0000000	0.1000000
-17226.0000000	0.1000000
-17225.0000000	0.1000000
-17224.0000000	0.1000000
-17223.0000000	0.0995000
-17222.0000000	0.1000000
-17221.0000000	0.1000000
-17220.0000000	0.0990000
-17219.0000000	0.0995238
-17218.0000000	0.0985000
-17217.0000000	0.0985000
-17216.0000000	0.0985000
-17215.0000000	0.0985714
-17214.0000000	0.0995000
-17213.0000000	0.1000000
-17212.0000000	0.0995000
-17211.0000000	0.1000000
-17210.0000000	0.0995238
-17209.0000000	0.0990000
-17208.0000000	0.1000000
-17207.0000000	0.0995000
-17206.0000000	0.0990476
-17205.0000000	0.1000000
-17204.0000000	0.1000000
-17203.0000000	0.1000000
-17202.0000000	0.1000000
-17201.0000000	0.0995238
-17200.0000000	0.1000000
-17199.0000000	0.1000000
-17198.0000000	0.1000000
-17197.0000000	0.1000000
-17196.0000000	0.1000000
-17195.0000000	0.1000000
-17194.0000000	0.1000000
-17193.0000000	0.1000000
-17192.0000000	0.1000000
-17191.0000000	0.1005000
-17190.0000000	0.1000000
-17189.0000000	0.1005000
-17188.0000000	0.1000000
-17187.0000000	0.1000000
-17186.0000000	0.1005000
-17185.0000000	0.1000000
-17184.0000000	0.1000000
-17183.0000000	0.1000000
-17182.0000000	0.1000000

-17181.0000000	0.1000000
-17180.0000000	0.1000000
-17179.0000000	0.1000000
-17178.0000000	0.1000000
-17177.0000000	0.1000000
-17176.0000000	0.1004762
-17175.0000000	0.1000000
-17174.0000000	0.1010000
-17173.0000000	0.1000000
-17172.0000000	0.1014286
-17171.0000000	0.1010000
-17170.0000000	0.1009524
-17169.0000000	0.1000000
-17168.0000000	0.1000000
-17167.0000000	0.1004762
-17166.0000000	0.1005000
-17165.0000000	0.1000000
-17164.0000000	0.1000000
-17163.0000000	0.1000000
-17162.0000000	0.1000000
-17161.0000000	0.1000000
-17160.0000000	0.1000000
-17159.0000000	0.1000000
-17158.0000000	0.1000000
-17157.0000000	0.1004762
-17156.0000000	0.1010000
-17155.0000000	0.1014286
-17154.0000000	0.1015000
-17153.0000000	0.1005000
-17152.0000000	0.1010000
-17151.0000000	0.1009524
-17150.0000000	0.1005000
-17149.0000000	0.1000000
-17148.0000000	0.1005000
-17147.0000000	0.1005000
-17146.0000000	0.1000000
-17145.0000000	0.1005000
-17144.0000000	0.1000000
-17143.0000000	0.1000000
-17142.0000000	0.1000000
-17141.0000000	0.1000000
-17140.0000000	0.1004762
-17139.0000000	0.1005000
-17138.0000000	0.1000000
-17137.0000000	0.1000000
-17136.0000000	0.1004762
-17135.0000000	0.1000000
-17134.0000000	0.1000000
-17133.0000000	0.1000000
-17132.0000000	0.1000000
-17131.0000000	0.1000000
-17130.0000000	0.1010000
-17129.0000000	0.1000000
-17128.0000000	0.1000000
-17127.0000000	0.1000000
-17126.0000000	0.1000000
-17125.0000000	0.1000000

-17124.0000000	0.1015000
-17123.0000000	0.1010000
-17122.0000000	0.1010000
-17121.0000000	0.1028572
-17120.0000000	0.1025000
-17119.0000000	0.1047619
-17118.0000000	0.1045000
-17117.0000000	0.1060000
-17116.0000000	0.1047619
-17115.0000000	0.1020000
-17114.0000000	0.1045000
-17113.0000000	0.1070000
-17112.0000000	0.1033333
-17111.0000000	0.1060000
-17110.0000000	0.1023810
-17109.0000000	0.1040000
-17108.0000000	0.1015000
-17107.0000000	0.1005000
-17106.0000000	0.1000000
-17105.0000000	0.1000000
-17104.0000000	0.0995238
-17103.0000000	0.0980000
-17102.0000000	0.0980000
-17101.0000000	0.0985714
-17100.0000000	0.0990000
-17099.0000000	0.0995000
-17098.0000000	0.0995000
-17097.0000000	0.0985714
-17096.0000000	0.0995000
-17095.0000000	0.0995238
-17094.0000000	0.0990000
-17093.0000000	0.0990000
-17092.0000000	0.0995000
-17091.0000000	0.1000000
-17090.0000000	0.1000000
-17089.0000000	0.0995000
-17088.0000000	0.0990476
-17087.0000000	0.0975000
-17086.0000000	0.0966667
-17085.0000000	0.0980000
-17084.0000000	0.0970000
-17083.0000000	0.0971429
-17082.0000000	0.0985000
-17081.0000000	0.0985000
-17080.0000000	0.1000000
-17079.0000000	0.0980953
-17078.0000000	0.0975000
-17077.0000000	0.0990000
-17076.0000000	0.1000000
-17075.0000000	0.1000000
-17074.0000000	0.0995238
-17073.0000000	0.0985000
-17072.0000000	0.0975000
-17071.0000000	0.0975000
-17070.0000000	0.0980953
-17069.0000000	0.0970000
-17068.0000000	0.0970000

-17067.0000000	0.0976191
-17066.0000000	0.0980000
-17065.0000000	0.0985714
-17064.0000000	0.0995000
-17063.0000000	0.0985000
-17062.0000000	0.0980000
-17061.0000000	0.1000000
-17060.0000000	0.1000000
-17059.0000000	0.1000000
-17058.0000000	0.1000000
-17057.0000000	0.1000000
-17056.0000000	0.1000000
-17055.0000000	0.1000000
-17054.0000000	0.1000000
-17053.0000000	0.1000000
-17052.0000000	0.1000000
-17051.0000000	0.1000000
-17050.0000000	0.1000000
-17049.0000000	0.1000000
-17048.0000000	0.1000000
-17047.0000000	0.1010000
-17046.0000000	0.1000000
-17045.0000000	0.1000000
-17044.0000000	0.1005000
-17043.0000000	0.1004762
-17042.0000000	0.1000000
-17041.0000000	0.1010000
-17040.0000000	0.1004762
-17039.0000000	0.1015000
-17038.0000000	0.1020000
-17037.0000000	0.1020000
-17036.0000000	0.1019048
-17035.0000000	0.1014286
-17034.0000000	0.1010000
-17033.0000000	0.1000000
-17032.0000000	0.1005000
-17031.0000000	0.1000000
-17030.0000000	0.1005000
-17029.0000000	0.1005000
-17028.0000000	0.1004762
-17027.0000000	0.1005000
-17026.0000000	0.1000000
-17025.0000000	0.1000000
-17024.0000000	0.1000000
-17023.0000000	0.1000000
-17022.0000000	0.1000000
-17021.0000000	0.1000000
-17020.0000000	0.1000000
-17019.0000000	0.1009524
-17018.0000000	0.1005000
-17017.0000000	0.1010000
-17016.0000000	0.1025000
-17015.0000000	0.1023810
-17014.0000000	0.1025000
-17013.0000000	0.1038095
-17012.0000000	0.1025000
-17011.0000000	0.1035000

-17010.0000000	0.1061905
-17009.0000000	0.1050000
-17008.0000000	0.1035000
-17007.0000000	0.1040000
-17006.0000000	0.1019048
-17005.0000000	0.1015000
-17004.0000000	0.1014286
-17003.0000000	0.1025000
-17002.0000000	0.1020000
-17001.0000000	0.1019048
-17000.0000000	0.1025000
-16999.0000000	0.1040000
-16998.0000000	0.1042857
-16997.0000000	0.1040000
-16996.0000000	0.1045000
-16995.0000000	0.1071429
-16994.0000000	0.1060000
-16993.0000000	0.1045000
-16992.0000000	0.1038095
-16991.0000000	0.1055000
-16990.0000000	0.1050000
-16989.0000000	0.1042857
-16988.0000000	0.1060000
-16987.0000000	0.1030000
-16986.0000000	0.1030000
-16985.0000000	0.1014286
-16984.0000000	0.1010000
-16983.0000000	0.1009524
-16982.0000000	0.1000000
-16981.0000000	0.1005000
-16980.0000000	0.1014286
-16979.0000000	0.1010000
-16978.0000000	0.1035000
-16977.0000000	0.1005000
-16976.0000000	0.1023810
-16975.0000000	0.1030000
-16974.0000000	0.1038095
-16973.0000000	0.1060000
-16972.0000000	0.1065000
-16971.0000000	0.1070000
-16970.0000000	0.1066667
-16969.0000000	0.1090000
-16968.0000000	0.1085714
-16967.0000000	0.1100000
-16966.0000000	0.1095000
-16965.0000000	0.1090476
-16964.0000000	0.1090000
-16963.0000000	0.1090000
-16962.0000000	0.1090000
-16961.0000000	0.1095238
-16960.0000000	0.1100000
-16959.0000000	0.1100000
-16958.0000000	0.1095000
-16957.0000000	0.1085000
-16956.0000000	0.1100000
-16955.0000000	0.1085714
-16954.0000000	0.1090000

-16953.0000000	0.1095000
-16952.0000000	0.1080952
-16951.0000000	0.1090000
-16950.0000000	0.1090476
-16949.0000000	0.1090000
-16948.0000000	0.1095000
-16947.0000000	0.1095000
-16946.0000000	0.1090476
-16945.0000000	0.1090000
-16944.0000000	0.1095238
-16943.0000000	0.1090000
-16942.0000000	0.1095000
-16941.0000000	0.1075000
-16940.0000000	0.1038095
-16939.0000000	0.1040000
-16938.0000000	0.1019048
-16937.0000000	0.1015000
-16936.0000000	0.1010000
-16935.0000000	0.1014286
-16934.0000000	0.1015000
-16933.0000000	0.1005000
-16932.0000000	0.1010000
-16931.0000000	0.1009524
-16930.0000000	0.1010000
-16929.0000000	0.1000000
-16928.0000000	0.1005000
-16927.0000000	0.1000000
-16926.0000000	0.0995000
-16925.0000000	0.1000000
-16924.0000000	0.1000000
-16923.0000000	0.1000000
-16922.0000000	0.0995238
-16921.0000000	0.1000000
-16920.0000000	0.1000000
-16919.0000000	0.1000000
-16918.0000000	0.1000000
-16917.0000000	0.1000000
-16916.0000000	0.1000000
-16915.0000000	0.1000000
-16914.0000000	0.0995000
-16913.0000000	0.1000000
-16912.0000000	0.1000000
-16911.0000000	0.1005000
-16910.0000000	0.1000000
-16909.0000000	0.1005000
-16908.0000000	0.1000000
-16907.0000000	0.1005000
-16906.0000000	0.1000000
-16905.0000000	0.1000000
-16904.0000000	0.1004762
-16903.0000000	0.1000000
-16902.0000000	0.1000000
-16901.0000000	0.1000000
-16900.0000000	0.1000000
-16899.0000000	0.1000000
-16898.0000000	0.1000000
-16897.0000000	0.1000000

-16896.0000000	0.1000000
-16895.0000000	0.0995238
-16894.0000000	0.1000000
-16893.0000000	0.1000000
-16892.0000000	0.1000000
-16891.0000000	0.1000000
-16890.0000000	0.1000000
-16889.0000000	0.1000000
-16888.0000000	0.1010000
-16887.0000000	0.1020000
-16886.0000000	0.1019048
-16885.0000000	0.1015000
-16884.0000000	0.1030000
-16883.0000000	0.1066667
-16882.0000000	0.1055000
-16881.0000000	0.1055000
-16880.0000000	0.1060000
-16879.0000000	0.1028572
-16878.0000000	0.1040000
-16877.0000000	0.1028572
-16876.0000000	0.1030000
-16875.0000000	0.1020000
-16874.0000000	0.1023810
-16873.0000000	0.1005000
-16872.0000000	0.1000000
-16871.0000000	0.1010000
-16870.0000000	0.1023810
-16869.0000000	0.1020000
-16868.0000000	0.1004762
-16867.0000000	0.1000000
-16866.0000000	0.1005000
-16865.0000000	0.1000000
-16864.0000000	0.1005000
-16863.0000000	0.1000000
-16862.0000000	0.1004762
-16861.0000000	0.1005000
-16860.0000000	0.1009524
-16859.0000000	0.1005000
-16858.0000000	0.1005000
-16857.0000000	0.1000000
-16856.0000000	0.1000000
-16855.0000000	0.1000000
-16854.0000000	0.0985000
-16853.0000000	0.0985714
-16852.0000000	0.0975000
-16851.0000000	0.0940000
-16850.0000000	0.0930000
-16849.0000000	0.0909524
-16848.0000000	0.0915000
-16847.0000000	0.0904762
-16846.0000000	0.0935000
-16845.0000000	0.0935000
-16844.0000000	0.0957143
-16843.0000000	0.0930000
-16842.0000000	0.0920000
-16841.0000000	0.0925000
-16840.0000000	0.0919048

-16839.0000000	0.0930000
-16838.0000000	0.0909524
-16837.0000000	0.0915000
-16836.0000000	0.0915000
-16835.0000000	0.0919048
-16834.0000000	0.0910000
-16833.0000000	0.0930000
-16832.0000000	0.0904762
-16831.0000000	0.0905000
-16830.0000000	0.0915000
-16829.0000000	0.0914286
-16828.0000000	0.0905000
-16827.0000000	0.0910000
-16826.0000000	0.0914286
-16825.0000000	0.0915000
-16824.0000000	0.0910000
-16823.0000000	0.0923810
-16822.0000000	0.0935000
-16821.0000000	0.0955000
-16820.0000000	0.0930000
-16819.0000000	0.0947619
-16818.0000000	0.0925000
-16817.0000000	0.0966667
-16816.0000000	0.0980000
-16815.0000000	0.0925000
-16814.0000000	0.0947619
-16813.0000000	0.0935000
-16812.0000000	0.0930000
-16811.0000000	0.0955000
-16810.0000000	0.0947619
-16809.0000000	0.0950000
-16808.0000000	0.0961905
-16807.0000000	0.0965000
-16806.0000000	0.0950000
-16805.0000000	0.0960000
-16804.0000000	0.0966667
-16803.0000000	0.0985000
-16802.0000000	0.0995238
-16801.0000000	0.0990000
-16800.0000000	0.0995000
-16799.0000000	0.0995238
-16798.0000000	0.0985000
-16797.0000000	0.0985000
-16796.0000000	0.0985000
-16795.0000000	0.0990476
-16794.0000000	0.0975000
-16793.0000000	0.0985714
-16792.0000000	0.1000000
-16791.0000000	0.0995000
-16790.0000000	0.1000000
-16789.0000000	0.0995238
-16788.0000000	0.1000000
-16787.0000000	0.1000000
-16786.0000000	0.1000000
-16785.0000000	0.1000000
-16784.0000000	0.1000000
-16783.0000000	0.1000000

-16782.0000000	0.1000000
-16781.0000000	0.1000000
-16780.0000000	0.1000000
-16779.0000000	0.1000000
-16778.0000000	0.1000000
-16777.0000000	0.1000000
-16776.0000000	0.1000000
-16775.0000000	0.1000000
-16774.0000000	0.1000000
-16773.0000000	0.1000000
-16772.0000000	0.1000000
-16771.0000000	0.1005000
-16770.0000000	0.1000000
-16769.0000000	0.1000000
-16768.0000000	0.0995238
-16767.0000000	0.1000000
-16766.0000000	0.0980000
-16765.0000000	0.0961905
-16764.0000000	0.0980000
-16763.0000000	0.0976191
-16762.0000000	0.0975000
-16761.0000000	0.0995000
-16760.0000000	0.0960000
-16759.0000000	0.0971429
-16758.0000000	0.0970000
-16757.0000000	0.0965000
-16756.0000000	0.0980952
-16755.0000000	0.0980000
-16754.0000000	0.0985714
-16753.0000000	0.0985000
-16752.0000000	0.0985000
-16751.0000000	0.0985000
-16750.0000000	0.0961905
-16749.0000000	0.0985000
-16748.0000000	0.0970000
-16747.0000000	0.0957143
-16746.0000000	0.0950000
-16745.0000000	0.0960000
-16744.0000000	0.0965000
-16743.0000000	0.0952381
-16742.0000000	0.0952381
-16741.0000000	0.0950000
-16740.0000000	0.0945000
-16739.0000000	0.0950000
-16738.0000000	0.0985714
-16737.0000000	0.0980000
-16736.0000000	0.0970000
-16735.0000000	0.0990476
-16734.0000000	0.0985000
-16733.0000000	0.1000000
-16732.0000000	0.1000000
-16731.0000000	0.0995000
-16730.0000000	0.0990000
-16729.0000000	0.0995238
-16728.0000000	0.0995000
-16727.0000000	0.0990000
-16726.0000000	0.0990476

-16725.0000000	0.0995000
-16724.0000000	0.0970000
-16723.0000000	0.0947619
-16722.0000000	0.0945000
-16721.0000000	0.0955000
-16720.0000000	0.0957143
-16719.0000000	0.0970000
-16718.0000000	0.0965000
-16717.0000000	0.0980952
-16716.0000000	0.0995000
-16715.0000000	0.0985000
-16714.0000000	0.1000000
-16713.0000000	0.1000000
-16712.0000000	0.1000000
-16711.0000000	0.1000000
-16710.0000000	0.1000000
-16709.0000000	0.1000000
-16708.0000000	0.1000000
-16707.0000000	0.1000000
-16706.0000000	0.1000000
-16705.0000000	0.1000000
-16704.0000000	0.1000000
-16703.0000000	0.1000000
-16702.0000000	0.1000000
-16701.0000000	0.0995000
-16700.0000000	0.0995000
-16699.0000000	0.0995238
-16698.0000000	0.1000000
-16697.0000000	0.0990000
-16696.0000000	0.0995238
-16695.0000000	0.0990000
-16694.0000000	0.0995000
-16693.0000000	0.1000000
-16692.0000000	0.1000000
-16691.0000000	0.1000000
-16690.0000000	0.1000000
-16689.0000000	0.1000000
-16688.0000000	0.1000000
-16687.0000000	0.1000000
-16686.0000000	0.1000000
-16685.0000000	0.1000000
-16684.0000000	0.1000000
-16683.0000000	0.1000000
-16682.0000000	0.1000000
-16681.0000000	0.1000000
-16680.0000000	0.1000000
-16679.0000000	0.1000000
-16678.0000000	0.1000000
-16677.0000000	0.1000000
-16676.0000000	0.1000000
-16675.0000000	0.0995000
-16674.0000000	0.0995238
-16673.0000000	0.1000000
-16672.0000000	0.1000000
-16671.0000000	0.0990000
-16670.0000000	0.0985000
-16669.0000000	0.0975000

-16668.0000000	0.0976191
-16667.0000000	0.0995000
-16666.0000000	0.0971429
-16665.0000000	0.0970000
-16664.0000000	0.0980000
-16663.0000000	0.0990476
-16662.0000000	0.0990000
-16661.0000000	0.0980000
-16660.0000000	0.0995000
-16659.0000000	0.0995238
-16658.0000000	0.0995000
-16657.0000000	0.0990476
-16656.0000000	0.1000000
-16655.0000000	0.1000000
-16654.0000000	0.1000000
-16653.0000000	0.0995238
-16652.0000000	0.1000000
-16651.0000000	0.1000000
-16650.0000000	0.1000000
-16649.0000000	0.1000000
-16648.0000000	0.1000000
-16647.0000000	0.1000000
-16646.0000000	0.1000000
-16645.0000000	0.1000000
-16644.0000000	0.1000000
-16643.0000000	0.1000000
-16642.0000000	0.1000000
-16641.0000000	0.1005000
-16640.0000000	0.1005000
-16639.0000000	0.1000000
-16638.0000000	0.1000000
-16637.0000000	0.1000000
-16636.0000000	0.1004762
-16635.0000000	0.1000000
-16634.0000000	0.1000000
-16633.0000000	0.1000000
-16632.0000000	0.1000000
-16631.0000000	0.1000000
-16630.0000000	0.1000000
-16629.0000000	0.1000000
-16628.0000000	0.1000000
-16627.0000000	0.1000000
-16626.0000000	0.1000000
-16625.0000000	0.1000000
-16624.0000000	0.1000000
-16623.0000000	0.1000000
-16622.0000000	0.1000000
-16621.0000000	0.1000000
-16620.0000000	0.1000000
-16619.0000000	0.1000000
-16618.0000000	0.1000000
-16617.0000000	0.0995000
-16616.0000000	0.1000000
-16615.0000000	0.1000000
-16614.0000000	0.1000000
-16613.0000000	0.1000000
-16612.0000000	0.1000000

-16611.0000000	0.1000000
-16610.0000000	0.1000000
-16609.0000000	0.0995000
-16608.0000000	0.1000000
-16607.0000000	0.1000000
-16606.0000000	0.1000000
-16605.0000000	0.1000000
-16604.0000000	0.1000000
-16603.0000000	0.1000000
-16602.0000000	0.1000000
-16601.0000000	0.1000000
-16600.0000000	0.1005000
-16599.0000000	0.1004762
-16598.0000000	0.1000000
-16597.0000000	0.1000000
-16596.0000000	0.1000000
-16595.0000000	0.1000000
-16594.0000000	0.1000000
-16593.0000000	0.1000000
-16592.0000000	0.1000000
-16591.0000000	0.1000000
-16590.0000000	0.1000000
-16589.0000000	0.1000000
-16588.0000000	0.1000000
-16587.0000000	0.0975000
-16586.0000000	0.0995000
-16585.0000000	0.1000000
-16584.0000000	0.1000000
-16583.0000000	0.1000000
-16582.0000000	0.0990000
-16581.0000000	0.0990476
-16580.0000000	0.0985000
-16579.0000000	0.0975000
-16578.0000000	0.0965000
-16577.0000000	0.0947619
-16576.0000000	0.0952381
-16575.0000000	0.0940000
-16574.0000000	0.0965000
-16573.0000000	0.0980000
-16572.0000000	0.0952381
-16571.0000000	0.0945000
-16570.0000000	0.0955000
-16569.0000000	0.0975000
-16568.0000000	0.0952381
-16567.0000000	0.0971429
-16566.0000000	0.0995000
-16565.0000000	0.1000000
-16564.0000000	0.0990000
-16563.0000000	0.0995238
-16562.0000000	0.0995000
-16561.0000000	0.1000000
-16560.0000000	0.1000000
-16559.0000000	0.1000000
-16558.0000000	0.1000000
-16557.0000000	0.1005000
-16556.0000000	0.1010000
-16555.0000000	0.1005000

-16554.0000000	0.1023810
-16553.0000000	0.1010000
-16552.0000000	0.1005000
-16551.0000000	0.1000000
-16550.0000000	0.1000000
-16549.0000000	0.1010000
-16548.0000000	0.1010000
-16547.0000000	0.1000000
-16546.0000000	0.1010000
-16545.0000000	0.1004762
-16544.0000000	0.1005000
-16543.0000000	0.1000000
-16542.0000000	0.1023810
-16541.0000000	0.1005000
-16540.0000000	0.1015000
-16539.0000000	0.1005000
-16538.0000000	0.1009524
-16537.0000000	0.1020000
-16536.0000000	0.1019048
-16535.0000000	0.1015000
-16534.0000000	0.1040000
-16533.0000000	0.1042857
-16532.0000000	0.1045000
-16531.0000000	0.1045000
-16530.0000000	0.1045000
-16529.0000000	0.1042857
-16528.0000000	0.1050000
-16527.0000000	0.1042857
-16526.0000000	0.1025000
-16525.0000000	0.1040000
-16524.0000000	0.1028572
-16523.0000000	0.1020000
-16522.0000000	0.1015000
-16521.0000000	0.1014286
-16520.0000000	0.1005000
-16519.0000000	0.1005000
-16518.0000000	0.1005000
-16517.0000000	0.1000000
-16516.0000000	0.1000000
-16515.0000000	0.1000000
-16514.0000000	0.1000000
-16513.0000000	0.1000000
-16512.0000000	0.1000000
-16511.0000000	0.1000000
-16510.0000000	0.1010000
-16509.0000000	0.1000000
-16508.0000000	0.1000000
-16507.0000000	0.1000000
-16506.0000000	0.1023810
-16505.0000000	0.1025000
-16504.0000000	0.1000000
-16503.0000000	0.1005000
-16502.0000000	0.1019048
-16501.0000000	0.1015000
-16500.0000000	0.1015000
-16499.0000000	0.1023810
-16498.0000000	0.1045000

-16497.0000000	0.1042857
-16496.0000000	0.1045000
-16495.0000000	0.1040000
-16494.0000000	0.1070000
-16493.0000000	0.1047619
-16492.0000000	0.1090000
-16491.0000000	0.1085714
-16490.0000000	0.1080000
-16489.0000000	0.1100000
-16488.0000000	0.1095000
-16487.0000000	0.1090476
-16486.0000000	0.1095000
-16485.0000000	0.1095000
-16484.0000000	0.1100000
-16483.0000000	0.1100000
-16482.0000000	0.1095238
-16481.0000000	0.1100000
-16480.0000000	0.1100000
-16479.0000000	0.1095000
-16478.0000000	0.1085714
-16477.0000000	0.1055000
-16476.0000000	0.1047619
-16475.0000000	0.1035000
-16474.0000000	0.1030000
-16473.0000000	0.1025000
-16472.0000000	0.1047619
-16471.0000000	0.1015000
-16470.0000000	0.1010000
-16469.0000000	0.1004762
-16468.0000000	0.1005000
-16467.0000000	0.1009524
-16466.0000000	0.1005000
-16465.0000000	0.1010000
-16464.0000000	0.1000000
-16463.0000000	0.1000000
-16462.0000000	0.1000000
-16461.0000000	0.1000000
-16460.0000000	0.1000000
-16459.0000000	0.1000000
-16458.0000000	0.1000000
-16457.0000000	0.1004762
-16456.0000000	0.1000000
-16455.0000000	0.1000000
-16454.0000000	0.1004762
-16453.0000000	0.1010000
-16452.0000000	0.1004762
-16451.0000000	0.1000000
-16450.0000000	0.1010000
-16449.0000000	0.1005000
-16448.0000000	0.1000000
-16447.0000000	0.1005000
-16446.0000000	0.1000000
-16445.0000000	0.1014286
-16444.0000000	0.1000000
-16443.0000000	0.1005000
-16442.0000000	0.1000000
-16441.0000000	0.1005000

-16440.0000000	0.1000000
-16439.0000000	0.1005000
-16438.0000000	0.1000000
-16437.0000000	0.1005000
-16436.0000000	0.1000000
-16435.0000000	0.1005000
-16434.0000000	0.1000000
-16433.0000000	0.1000000
-16432.0000000	0.1010000
-16431.0000000	0.1000000
-16430.0000000	0.1000000
-16429.0000000	0.1000000
-16428.0000000	0.1000000
-16427.0000000	0.1000000
-16426.0000000	0.1000000
-16425.0000000	0.1000000
-16424.0000000	0.1000000
-16423.0000000	0.1000000
-16422.0000000	0.1000000
-16421.0000000	0.1000000
-16420.0000000	0.1000000
-16419.0000000	0.1000000
-16418.0000000	0.1000000
-16417.0000000	0.1000000
-16416.0000000	0.0995000
-16415.0000000	0.0995238
-16414.0000000	0.1000000
-16413.0000000	0.0995000
-16412.0000000	0.1000000
-16411.0000000	0.1000000
-16410.0000000	0.1000000
-16409.0000000	0.0995238
-16408.0000000	0.1000000
-16407.0000000	0.1000000
-16406.0000000	0.1000000
-16405.0000000	0.1000000
-16404.0000000	0.1000000
-16403.0000000	0.1000000
-16402.0000000	0.1000000
-16401.0000000	0.1000000
-16400.0000000	0.1000000
-16399.0000000	0.1000000
-16398.0000000	0.1000000
-16397.0000000	0.1000000
-16396.0000000	0.0995000
-16395.0000000	0.0995000
-16394.0000000	0.1000000
-16393.0000000	0.1000000
-16392.0000000	0.1000000
-16391.0000000	0.0995000
-16390.0000000	0.1000000
-16389.0000000	0.1000000
-16388.0000000	0.0995238
-16387.0000000	0.1000000
-16386.0000000	0.0995000
-16385.0000000	0.0995238
-16384.0000000	0.1000000

-16383.0000000	0.0995000
-16382.0000000	0.0990000
-16381.0000000	0.0980953
-16380.0000000	0.0990000
-16379.0000000	0.0966667
-16378.0000000	0.0990000
-16377.0000000	0.0995000
-16376.0000000	0.0985714
-16375.0000000	0.0990000
-16374.0000000	0.0985000
-16373.0000000	0.0995000
-16372.0000000	0.0980953
-16371.0000000	0.0990000
-16370.0000000	0.0990476
-16369.0000000	0.0995000
-16368.0000000	0.0980000
-16367.0000000	0.0976191
-16366.0000000	0.1000000
-16365.0000000	0.1000000
-16364.0000000	0.0995000
-16363.0000000	0.0995238
-16362.0000000	0.1000000
-16361.0000000	0.1000000
-16360.0000000	0.0995000
-16359.0000000	0.1000000
-16358.0000000	0.1000000
-16357.0000000	0.0995000
-16356.0000000	0.0985000
-16355.0000000	0.0985714
-16354.0000000	0.0990000
-16353.0000000	0.0970000
-16352.0000000	0.0955000
-16351.0000000	0.0976191
-16350.0000000	0.0970000
-16349.0000000	0.0995238
-16348.0000000	0.1000000
-16347.0000000	0.1000000
-16346.0000000	0.0995238
-16345.0000000	0.1000000
-16344.0000000	0.1000000
-16343.0000000	0.1000000
-16342.0000000	0.1000000
-16341.0000000	0.1000000
-16340.0000000	0.0995238
-16339.0000000	0.1000000
-16338.0000000	0.1000000
-16337.0000000	0.1000000
-16336.0000000	0.1000000
-16335.0000000	0.1000000
-16334.0000000	0.1000000
-16333.0000000	0.1000000
-16332.0000000	0.1000000
-16331.0000000	0.1000000
-16330.0000000	0.1000000
-16329.0000000	0.1000000
-16328.0000000	0.0995000
-16327.0000000	0.0995238

-16326.0000000	0.1000000
-16325.0000000	0.0995238
-16324.0000000	0.0995000
-16323.0000000	0.0990000
-16322.0000000	0.0975000
-16321.0000000	0.0971429
-16320.0000000	0.0940000
-16319.0000000	0.0970000
-16318.0000000	0.0952381
-16317.0000000	0.0955000
-16316.0000000	0.0966667
-16315.0000000	0.0970000
-16314.0000000	0.0980000
-16313.0000000	0.0980000
-16312.0000000	0.0980953
-16311.0000000	0.0990000
-16310.0000000	0.0990000
-16309.0000000	0.1000000
-16308.0000000	0.0995000
-16307.0000000	0.0995000
-16306.0000000	0.1000000
-16305.0000000	0.1000000
-16304.0000000	0.0990000
-16303.0000000	0.0985714
-16302.0000000	0.1000000
-16301.0000000	0.1000000
-16300.0000000	0.1000000
-16299.0000000	0.0995000
-16298.0000000	0.1000000
-16297.0000000	0.1000000
-16296.0000000	0.1000000
-16295.0000000	0.1000000
-16294.0000000	0.1000000
-16293.0000000	0.1005000
-16292.0000000	0.1000000
-16291.0000000	0.1004762
-16290.0000000	0.1000000
-16289.0000000	0.1000000
-16288.0000000	0.1004762
-16287.0000000	0.1005000
-16286.0000000	0.1000000
-16285.0000000	0.1005000
-16284.0000000	0.1005000
-16283.0000000	0.1000000
-16282.0000000	0.1000000
-16281.0000000	0.1000000
-16280.0000000	0.1000000
-16279.0000000	0.1000000
-16278.0000000	0.1005000
-16277.0000000	0.1000000
-16276.0000000	0.1000000
-16275.0000000	0.1000000
-16274.0000000	0.1000000
-16273.0000000	0.1000000
-16272.0000000	0.1000000
-16271.0000000	0.1025000
-16270.0000000	0.1014286

-16269.0000000	0.1025000
-16268.0000000	0.1010000
-16267.0000000	0.1004762
-16266.0000000	0.1000000
-16265.0000000	0.1000000
-16264.0000000	0.1005000
-16263.0000000	0.1000000
-16262.0000000	0.1005000
-16261.0000000	0.1000000
-16260.0000000	0.1000000
-16259.0000000	0.1000000
-16258.0000000	0.1000000
-16257.0000000	0.1000000
-16256.0000000	0.1000000
-16255.0000000	0.1000000
-16254.0000000	0.1000000
-16253.0000000	0.1000000
-16252.0000000	0.1000000
-16251.0000000	0.1000000
-16250.0000000	0.0995000
-16249.0000000	0.1000000
-16248.0000000	0.0990000
-16247.0000000	0.0995000
-16246.0000000	0.1000000
-16245.0000000	0.0995238
-16244.0000000	0.1000000
-16243.0000000	0.1000000
-16242.0000000	0.0995000
-16241.0000000	0.0985000
-16240.0000000	0.0995238
-16239.0000000	0.1000000
-16238.0000000	0.1000000
-16237.0000000	0.1000000
-16236.0000000	0.1000000
-16235.0000000	0.1000000
-16234.0000000	0.1000000
-16233.0000000	0.1000000
-16232.0000000	0.1000000
-16231.0000000	0.1000000
-16230.0000000	0.1000000
-16229.0000000	0.1000000
-16228.0000000	0.0995000
-16227.0000000	0.0990476
-16226.0000000	0.0990000
-16225.0000000	0.0961905
-16224.0000000	0.0975000
-16223.0000000	0.0975000
-16222.0000000	0.0980953
-16221.0000000	0.0970000
-16220.0000000	0.0965000
-16219.0000000	0.0947619
-16218.0000000	0.0940000
-16217.0000000	0.0945000
-16216.0000000	0.0930000
-16215.0000000	0.0952381
-16214.0000000	0.0965000
-16213.0000000	0.0957143

-16212.0000000	0.0955000
-16211.0000000	0.0955000
-16210.0000000	0.0976191
-16209.0000000	0.0980000
-16208.0000000	0.0985000
-16207.0000000	0.0990000
-16206.0000000	0.1000000
-16205.0000000	0.1000000
-16204.0000000	0.1000000
-16203.0000000	0.1000000
-16202.0000000	0.1000000
-16201.0000000	0.1000000
-16200.0000000	0.1004762
-16199.0000000	0.1005000
-16198.0000000	0.1020000
-16197.0000000	0.1014286
-16196.0000000	0.1035000
-16195.0000000	0.1028572
-16194.0000000	0.1015000
-16193.0000000	0.1045000
-16192.0000000	0.1028572
-16191.0000000	0.1030000
-16190.0000000	0.1040000
-16189.0000000	0.1042857
-16188.0000000	0.1040000
-16187.0000000	0.1025000
-16186.0000000	0.1035000
-16185.0000000	0.1033333
-16184.0000000	0.1025000
-16183.0000000	0.1028572
-16182.0000000	0.1035000
-16181.0000000	0.1030000
-16180.0000000	0.1023810
-16179.0000000	0.1020000
-16178.0000000	0.1005000
-16177.0000000	0.1005000
-16176.0000000	0.1004762
-16175.0000000	0.1000000
-16174.0000000	0.1000000
-16173.0000000	0.1005000
-16172.0000000	0.1000000
-16171.0000000	0.1000000
-16170.0000000	0.1004762
-16169.0000000	0.1000000
-16168.0000000	0.1000000
-16167.0000000	0.1000000
-16166.0000000	0.1000000
-16165.0000000	0.1000000
-16164.0000000	0.1000000
-16163.0000000	0.1000000
-16162.0000000	0.1005000
-16161.0000000	0.1009524
-16160.0000000	0.1000000
-16159.0000000	0.1004762
-16158.0000000	0.1000000
-16157.0000000	0.1000000
-16156.0000000	0.1000000

-16155.0000000	0.1000000
-16154.0000000	0.1000000
-16153.0000000	0.1000000
-16152.0000000	0.1004762
-16151.0000000	0.1000000
-16150.0000000	0.1000000
-16149.0000000	0.1000000
-16148.0000000	0.1000000
-16147.0000000	0.1000000
-16146.0000000	0.1004762
-16145.0000000	0.1000000
-16144.0000000	0.1000000
-16143.0000000	0.1000000
-16142.0000000	0.1000000
-16141.0000000	0.1000000
-16140.0000000	0.1004762
-16139.0000000	0.1000000
-16138.0000000	0.1000000
-16137.0000000	0.1000000
-16136.0000000	0.1005000
-16135.0000000	0.1000000
-16134.0000000	0.1000000
-16133.0000000	0.1000000
-16132.0000000	0.1000000
-16131.0000000	0.1000000
-16130.0000000	0.1005000
-16129.0000000	0.1000000
-16128.0000000	0.1000000
-16127.0000000	0.1000000
-16126.0000000	0.1000000
-16125.0000000	0.1004762
-16124.0000000	0.1000000
-16123.0000000	0.1005000
-16122.0000000	0.1004762
-16121.0000000	0.1000000
-16120.0000000	0.1004762
-16119.0000000	0.1005000
-16118.0000000	0.1000000
-16117.0000000	0.1000000
-16116.0000000	0.1004762
-16115.0000000	0.1000000
-16114.0000000	0.1000000
-16113.0000000	0.1000000
-16112.0000000	0.1000000
-16111.0000000	0.1000000
-16110.0000000	0.1000000
-16109.0000000	0.1009524
-16108.0000000	0.1000000
-16107.0000000	0.1009524
-16106.0000000	0.1010000
-16105.0000000	0.1015000
-16104.0000000	0.1014286
-16103.0000000	0.1040000
-16102.0000000	0.1040000
-16101.0000000	0.1070000
-16100.0000000	0.1076190
-16099.0000000	0.1085714

-16098.0000000	0.1085000
-16097.0000000	0.1090000
-16096.0000000	0.1085000
-16095.0000000	0.1090476
-16094.0000000	0.1090000
-16093.0000000	0.1085000
-16092.0000000	0.1095238
-16091.0000000	0.1100000
-16090.0000000	0.1095238
-16089.0000000	0.1100000
-16088.0000000	0.1095000
-16087.0000000	0.1100000
-16086.0000000	0.1100000
-16085.0000000	0.1095000
-16084.0000000	0.1095000
-16083.0000000	0.1090476
-16082.0000000	0.1075000
-16081.0000000	0.1055000
-16080.0000000	0.1050000
-16079.0000000	0.1038095
-16078.0000000	0.1020000
-16077.0000000	0.1009524
-16076.0000000	0.1000000
-16075.0000000	0.1000000
-16074.0000000	0.0995238
-16073.0000000	0.0990000
-16072.0000000	0.0970000
-16071.0000000	0.0960000
-16070.0000000	0.0942857
-16069.0000000	0.0935000
-16068.0000000	0.0957143
-16067.0000000	0.0950000
-16066.0000000	0.0970000
-16065.0000000	0.0971429
-16064.0000000	0.0990000
-16063.0000000	0.0995000
-16062.0000000	0.1000000
-16061.0000000	0.1000000
-16060.0000000	0.1005000
-16059.0000000	0.1000000
-16058.0000000	0.1000000
-16057.0000000	0.1020000
-16056.0000000	0.1014286
-16055.0000000	0.1035000
-16054.0000000	0.1065000
-16053.0000000	0.1076190
-16052.0000000	0.1085000
-16051.0000000	0.1065000
-16050.0000000	0.1085000
-16049.0000000	0.1080952
-16048.0000000	0.1070000
-16047.0000000	0.1066667
-16046.0000000	0.1035000
-16045.0000000	0.1010000
-16044.0000000	0.1009524
-16043.0000000	0.1005000
-16042.0000000	0.1005000

-16041.0000000	0.1015000
-16040.0000000	0.1004762
-16039.0000000	0.1010000
-16038.0000000	0.1004762
-16037.0000000	0.1005000
-16036.0000000	0.1000000
-16035.0000000	0.1000000
-16034.0000000	0.1000000
-16033.0000000	0.0995000
-16032.0000000	0.0980000
-16031.0000000	0.0966667
-16030.0000000	0.0985000
-16029.0000000	0.0985714
-16028.0000000	0.0990000
-16027.0000000	0.0980000
-16026.0000000	0.0990476
-16025.0000000	0.0985000
-16024.0000000	0.0990000
-16023.0000000	0.0990476
-16022.0000000	0.0995000
-16021.0000000	0.0995000
-16020.0000000	0.1000000
-16019.0000000	0.1000000
-16018.0000000	0.1000000
-16017.0000000	0.1000000
-16016.0000000	0.1000000
-16015.0000000	0.1000000
-16014.0000000	0.1000000
-16013.0000000	0.1000000
-16012.0000000	0.1000000
-16011.0000000	0.1000000
-16010.0000000	0.1000000
-16009.0000000	0.1000000
-16008.0000000	0.1000000
-16007.0000000	0.1000000
-16006.0000000	0.1000000
-16005.0000000	0.1000000
-16004.0000000	0.1000000
-16003.0000000	0.1000000
-16002.0000000	0.1000000
-16001.0000000	0.1000000
-16000.0000000	0.1000000
-15999.0000000	0.0995238
-15998.0000000	0.0995000
-15997.0000000	0.1000000
-15996.0000000	0.1000000
-15995.0000000	0.1000000
-15994.0000000	0.0995000
-15993.0000000	0.1000000
-15992.0000000	0.1000000
-15991.0000000	0.1000000
-15990.0000000	0.1000000
-15989.0000000	0.1000000
-15988.0000000	0.1000000
-15987.0000000	0.1000000
-15986.0000000	0.1000000
-15985.0000000	0.1000000

-15984.0000000	0.1000000
-15983.0000000	0.1000000
-15982.0000000	0.1000000
-15981.0000000	0.1000000
-15980.0000000	0.1000000
-15979.0000000	0.1000000
-15978.0000000	0.1000000
-15977.0000000	0.1000000
-15976.0000000	0.1005000
-15975.0000000	0.1000000
-15974.0000000	0.1000000
-15973.0000000	0.0995000
-15972.0000000	0.0960000
-15971.0000000	0.0919048
-15970.0000000	0.0900000
-15969.0000000	0.0900000
-15968.0000000	0.0900000
-15967.0000000	0.0905000
-15966.0000000	0.0900000
-15965.0000000	0.0900000
-15964.0000000	0.0900000
-15963.0000000	0.0900000
-15962.0000000	0.0900000
-15961.0000000	0.0900000
-15960.0000000	0.0900000
-15959.0000000	0.0900000
-15958.0000000	0.0900000
-15957.0000000	0.0905000
-15956.0000000	0.0910000
-15955.0000000	0.0928572
-15954.0000000	0.0919048
-15953.0000000	0.0945000
-15952.0000000	0.0915000
-15951.0000000	0.0930000
-15950.0000000	0.0971429
-15949.0000000	0.0975000
-15948.0000000	0.0990000
-15947.0000000	0.0980952
-15946.0000000	0.0995000
-15945.0000000	0.1000000
-15944.0000000	0.0995000
-15943.0000000	0.0995238
-15942.0000000	0.1000000
-15941.0000000	0.1000000
-15940.0000000	0.1000000
-15939.0000000	0.1000000
-15938.0000000	0.0995238
-15937.0000000	0.0995000
-15936.0000000	0.1000000
-15935.0000000	0.1000000
-15934.0000000	0.1000000
-15933.0000000	0.0995238
-15932.0000000	0.1000000
-15931.0000000	0.0985000
-15930.0000000	0.1000000
-15929.0000000	0.1000000
-15928.0000000	0.1000000

-15927.0000000	0.0995000
-15926.0000000	0.0990000
-15925.0000000	0.0995238
-15924.0000000	0.1000000
-15923.0000000	0.1000000
-15922.0000000	0.0995000
-15921.0000000	0.0995000
-15920.0000000	0.0985714
-15919.0000000	0.1000000
-15918.0000000	0.1000000
-15917.0000000	0.1000000
-15916.0000000	0.0990000
-15915.0000000	0.0995000
-15914.0000000	0.1000000
-15913.0000000	0.1000000
-15912.0000000	0.1000000
-15911.0000000	0.1000000
-15910.0000000	0.1000000
-15909.0000000	0.1000000
-15908.0000000	0.1000000
-15907.0000000	0.1000000
-15906.0000000	0.1000000
-15905.0000000	0.1005000
-15904.0000000	0.1009524
-15903.0000000	0.1005000
-15902.0000000	0.1019048
-15901.0000000	0.1030000
-15900.0000000	0.1005000
-15899.0000000	0.1033333
-15898.0000000	0.1050000
-15897.0000000	0.1050000
-15896.0000000	0.1060000
-15895.0000000	0.1047619
-15894.0000000	0.1075000
-15893.0000000	0.1071429
-15892.0000000	0.1080000
-15891.0000000	0.1075000
-15890.0000000	0.1076190
-15889.0000000	0.1045000
-15888.0000000	0.1065000
-15887.0000000	0.1045000
-15886.0000000	0.1038095
-15885.0000000	0.1030000
-15884.0000000	0.1015000
-15883.0000000	0.1023810
-15882.0000000	0.1010000
-15881.0000000	0.1004762
-15880.0000000	0.1000000
-15879.0000000	0.1000000
-15878.0000000	0.1009524
-15877.0000000	0.1000000
-15876.0000000	0.1000000
-15875.0000000	0.1000000
-15874.0000000	0.1000000
-15873.0000000	0.1000000
-15872.0000000	0.1000000
-15871.0000000	0.1000000

-15870.0000000	0.1000000
-15869.0000000	0.1000000
-15868.0000000	0.1000000
-15867.0000000	0.1000000
-15866.0000000	0.1000000
-15865.0000000	0.1000000
-15864.0000000	0.1000000
-15863.0000000	0.1000000
-15862.0000000	0.1000000
-15861.0000000	0.1000000
-15860.0000000	0.1000000
-15859.0000000	0.1004762
-15858.0000000	0.1000000
-15857.0000000	0.1000000
-15856.0000000	0.1000000
-15855.0000000	0.1000000
-15854.0000000	0.1000000
-15853.0000000	0.1000000
-15852.0000000	0.0980000
-15851.0000000	0.0975000
-15850.0000000	0.0947619
-15849.0000000	0.0925000
-15848.0000000	0.0933333
-15847.0000000	0.0940000
-15846.0000000	0.0945000
-15845.0000000	0.0940000
-15844.0000000	0.0919048
-15843.0000000	0.0920000
-15842.0000000	0.0923810
-15841.0000000	0.0920000
-15840.0000000	0.0915000
-15839.0000000	0.0905000
-15838.0000000	0.0904762
-15837.0000000	0.0920000
-15836.0000000	0.0935000
-15835.0000000	0.0933333
-15834.0000000	0.0980000
-15833.0000000	0.0961905
-15832.0000000	0.0970000
-15831.0000000	0.0940000
-15830.0000000	0.0945000
-15829.0000000	0.0919048
-15828.0000000	0.0915000
-15827.0000000	0.0920000
-15826.0000000	0.0923810
-15825.0000000	0.0920000
-15824.0000000	0.0905000
-15823.0000000	0.0914286
-15822.0000000	0.0930000
-15821.0000000	0.0930000
-15820.0000000	0.0942857
-15819.0000000	0.0955000
-15818.0000000	0.0938095
-15817.0000000	0.0930000
-15816.0000000	0.0935000
-15815.0000000	0.0950000
-15814.0000000	0.0938095

-15813.0000000	0.0955000
-15812.0000000	0.0975000
-15811.0000000	0.0976191
-15810.0000000	0.0960000
-15809.0000000	0.0975000
-15808.0000000	0.0985714
-15807.0000000	0.0980000
-15806.0000000	0.0980000
-15805.0000000	0.0971429
-15804.0000000	0.0970000
-15803.0000000	0.0975000
-15802.0000000	0.0947619
-15801.0000000	0.0925000
-15800.0000000	0.0950000
-15799.0000000	0.0952381
-15798.0000000	0.0950000
-15797.0000000	0.0945000
-15796.0000000	0.0947619
-15795.0000000	0.0950000
-15794.0000000	0.0965000
-15793.0000000	0.0957143
-15792.0000000	0.0955000
-15791.0000000	0.0970000
-15790.0000000	0.0980000
-15789.0000000	0.0990476
-15788.0000000	0.0980952
-15787.0000000	0.0990000
-15786.0000000	0.0995000
-15785.0000000	0.0995000
-15784.0000000	0.0995238
-15783.0000000	0.1000000
-15782.0000000	0.1000000
-15781.0000000	0.0990476
-15780.0000000	0.0975000
-15779.0000000	0.0990000
-15778.0000000	0.0960000
-15777.0000000	0.0980952
-15776.0000000	0.0975000
-15775.0000000	0.0966667
-15774.0000000	0.0960000
-15773.0000000	0.0965000
-15772.0000000	0.0971429
-15771.0000000	0.0965000
-15770.0000000	0.0990000
-15769.0000000	0.0995000
-15768.0000000	0.0995238
-15767.0000000	0.0995000
-15766.0000000	0.0990476
-15765.0000000	0.0985000
-15764.0000000	0.1000000
-15763.0000000	0.0995238
-15762.0000000	0.0990000
-15761.0000000	0.0965000
-15760.0000000	0.0950000
-15759.0000000	0.0942857
-15758.0000000	0.0938095
-15757.0000000	0.0920000

-15756.0000000	0.0920000
-15755.0000000	0.0910000
-15754.0000000	0.0914286
-15753.0000000	0.0910000
-15752.0000000	0.0900000
-15751.0000000	0.0915000
-15750.0000000	0.0923810
-15749.0000000	0.0905000
-15748.0000000	0.0920000
-15747.0000000	0.0933333
-15746.0000000	0.0925000
-15745.0000000	0.0933334
-15744.0000000	0.0935000
-15743.0000000	0.0940000
-15742.0000000	0.0933333
-15741.0000000	0.0935000
-15740.0000000	0.0930000
-15739.0000000	0.0935000
-15738.0000000	0.0976191
-15737.0000000	0.0945000
-15736.0000000	0.0938095
-15735.0000000	0.0965000
-15734.0000000	0.0960000
-15733.0000000	0.0966667
-15732.0000000	0.0970000
-15731.0000000	0.0960000
-15730.0000000	0.0955000
-15729.0000000	0.0966667
-15728.0000000	0.0960000
-15727.0000000	0.0966667
-15726.0000000	0.0960000
-15725.0000000	0.0965000
-15724.0000000	0.0942857
-15723.0000000	0.0945000
-15722.0000000	0.0940000
-15721.0000000	0.0965000
-15720.0000000	0.0980953
-15719.0000000	0.0975000
-15718.0000000	0.0975000
-15717.0000000	0.0995238
-15716.0000000	0.0990000
-15715.0000000	0.0985714
-15714.0000000	0.0995000
-15713.0000000	0.0990000
-15712.0000000	0.1000000
-15711.0000000	0.0995000
-15710.0000000	0.1000000
-15709.0000000	0.1000000
-15708.0000000	0.1000000
-15707.0000000	0.1000000
-15706.0000000	0.1000000
-15705.0000000	0.1000000
-15704.0000000	0.1000000
-15703.0000000	0.1000000
-15702.0000000	0.1004762
-15701.0000000	0.1000000
-15700.0000000	0.1005000

-15699.0000000	0.1019048
-15698.0000000	0.1020000
-15697.0000000	0.1028572
-15696.0000000	0.1030000
-15695.0000000	0.1040000
-15694.0000000	0.1020000
-15693.0000000	0.1009524
-15692.0000000	0.1000000
-15691.0000000	0.1010000
-15690.0000000	0.1000000
-15689.0000000	0.1005000
-15688.0000000	0.1005000
-15687.0000000	0.1009524
-15686.0000000	0.1010000
-15685.0000000	0.1005000
-15684.0000000	0.1004762
-15683.0000000	0.1015000
-15682.0000000	0.1009524
-15681.0000000	0.1020000
-15680.0000000	0.1015000
-15679.0000000	0.1010000
-15678.0000000	0.1004762
-15677.0000000	0.1020000
-15676.0000000	0.1025000
-15675.0000000	0.1014286
-15674.0000000	0.1000000
-15673.0000000	0.1020000
-15672.0000000	0.1009524
-15671.0000000	0.1015000
-15670.0000000	0.1000000
-15669.0000000	0.1009524
-15668.0000000	0.1010000
-15667.0000000	0.1009524
-15666.0000000	0.1005000
-15665.0000000	0.1000000
-15664.0000000	0.1000000
-15663.0000000	0.1000000
-15662.0000000	0.1000000
-15661.0000000	0.1000000
-15660.0000000	0.1000000
-15659.0000000	0.1000000
-15658.0000000	0.1000000
-15657.0000000	0.1000000
-15656.0000000	0.1000000
-15655.0000000	0.1005000
-15654.0000000	0.1000000
-15653.0000000	0.1000000
-15652.0000000	0.1000000
-15651.0000000	0.1000000
-15650.0000000	0.1005000
-15649.0000000	0.1015000
-15648.0000000	0.1023810
-15647.0000000	0.1020000
-15646.0000000	0.1045000
-15645.0000000	0.1033333
-15644.0000000	0.1020000
-15643.0000000	0.1005000

-15642.0000000	0.1004762
-15641.0000000	0.1000000
-15640.0000000	0.1000000
-15639.0000000	0.1000000
-15638.0000000	0.0995000
-15637.0000000	0.0995000
-15636.0000000	0.0980952
-15635.0000000	0.0985000
-15634.0000000	0.0955000
-15633.0000000	0.0957143
-15632.0000000	0.0945000
-15631.0000000	0.0910000
-15630.0000000	0.0919048
-15629.0000000	0.0915000
-15628.0000000	0.0910000
-15627.0000000	0.0900000
-15626.0000000	0.0905000
-15625.0000000	0.0900000
-15624.0000000	0.0914286
-15623.0000000	0.0910000
-15622.0000000	0.0928572
-15621.0000000	0.0965000
-15620.0000000	0.0980000
-15619.0000000	0.0995000
-15618.0000000	0.0995238
-15617.0000000	0.0995000
-15616.0000000	0.1000000
-15615.0000000	0.1000000
-15614.0000000	0.1000000
-15613.0000000	0.1000000
-15612.0000000	0.1005000
-15611.0000000	0.1009524
-15610.0000000	0.1010000
-15609.0000000	0.1019048
-15608.0000000	0.1005000
-15607.0000000	0.1040000
-15606.0000000	0.1028572
-15605.0000000	0.1035000
-15604.0000000	0.1035000
-15603.0000000	0.1015000
-15602.0000000	0.1004762
-15601.0000000	0.1015000
-15600.0000000	0.1004762
-15599.0000000	0.1005000
-15598.0000000	0.1000000
-15597.0000000	0.1000000
-15596.0000000	0.1000000
-15595.0000000	0.1000000
-15594.0000000	0.1000000
-15593.0000000	0.1000000
-15592.0000000	0.1000000
-15591.0000000	0.1000000
-15590.0000000	0.1000000
-15589.0000000	0.1000000
-15588.0000000	0.1000000
-15587.0000000	0.1000000
-15586.0000000	0.1000000

-15585.0000000	0.1000000
-15584.0000000	0.1000000
-15583.0000000	0.1000000
-15582.0000000	0.1000000
-15581.0000000	0.1000000
-15580.0000000	0.1000000
-15579.0000000	0.1000000
-15578.0000000	0.1000000
-15577.0000000	0.1000000
-15576.0000000	0.1000000
-15575.0000000	0.1000000
-15574.0000000	0.0985000
-15573.0000000	0.0950000
-15572.0000000	0.0976191
-15571.0000000	0.0980000
-15570.0000000	0.0990476
-15569.0000000	0.1000000
-15568.0000000	0.0995000
-15567.0000000	0.0995238
-15566.0000000	0.1000000
-15565.0000000	0.0990000
-15564.0000000	0.1000000
-15563.0000000	0.0995238
-15562.0000000	0.1000000
-15561.0000000	0.1000000
-15560.0000000	0.1000000
-15559.0000000	0.1000000
-15558.0000000	0.1000000
-15557.0000000	0.1000000
-15556.0000000	0.1000000
-15555.0000000	0.1000000
-15554.0000000	0.1000000
-15553.0000000	0.1000000
-15552.0000000	0.1000000
-15551.0000000	0.1000000
-15550.0000000	0.1000000
-15549.0000000	0.1000000
-15548.0000000	0.1000000
-15547.0000000	0.1000000
-15546.0000000	0.1000000
-15545.0000000	0.0995000
-15544.0000000	0.1000000
-15543.0000000	0.1000000
-15542.0000000	0.1000000
-15541.0000000	0.1000000
-15540.0000000	0.1000000
-15539.0000000	0.1000000
-15538.0000000	0.1000000
-15537.0000000	0.1000000
-15536.0000000	0.1000000
-15535.0000000	0.1000000
-15534.0000000	0.1000000
-15533.0000000	0.1000000
-15532.0000000	0.1000000
-15531.0000000	0.1000000
-15530.0000000	0.1000000
-15529.0000000	0.1000000

-15528.0000000	0.0995000
-15527.0000000	0.0995238
-15526.0000000	0.1000000
-15525.0000000	0.0995000
-15524.0000000	0.0995238
-15523.0000000	0.0995000
-15522.0000000	0.0985000
-15521.0000000	0.0990000
-15520.0000000	0.0985714
-15519.0000000	0.0995000
-15518.0000000	0.0985714
-15517.0000000	0.0995000
-15516.0000000	0.0990476
-15515.0000000	0.1000000
-15514.0000000	0.0980000
-15513.0000000	0.0990000
-15512.0000000	0.1000000
-15511.0000000	0.1000000
-15510.0000000	0.1000000
-15509.0000000	0.1000000
-15508.0000000	0.1000000
-15507.0000000	0.1000000
-15506.0000000	0.1000000
-15505.0000000	0.1000000
-15504.0000000	0.1000000
-15503.0000000	0.1000000
-15502.0000000	0.0990000
-15501.0000000	0.0995000
-15500.0000000	0.1000000
-15499.0000000	0.1000000
-15498.0000000	0.1000000
-15497.0000000	0.0995238
-15496.0000000	0.1000000
-15495.0000000	0.1000000
-15494.0000000	0.0980953
-15493.0000000	0.0930000
-15492.0000000	0.0920000
-15491.0000000	0.0904762
-15490.0000000	0.0900000
-15489.0000000	0.0900000
-15488.0000000	0.0900000
-15487.0000000	0.0900000
-15486.0000000	0.0900000
-15485.0000000	0.0900000
-15484.0000000	0.0900000
-15483.0000000	0.0905000
-15482.0000000	0.0914286
-15481.0000000	0.0905000
-15480.0000000	0.0905000
-15479.0000000	0.0904762
-15478.0000000	0.0900000
-15477.0000000	0.0915000
-15476.0000000	0.0909524
-15475.0000000	0.0960000
-15474.0000000	0.0960000
-15473.0000000	0.0976191
-15472.0000000	0.0975000

-15471.0000000	0.0985000
-15470.0000000	0.0995238
-15469.0000000	0.0970000
-15468.0000000	0.0945000
-15467.0000000	0.0942857
-15466.0000000	0.0920000
-15465.0000000	0.0915000
-15464.0000000	0.0900000
-15463.0000000	0.0905000
-15462.0000000	0.0900000
-15461.0000000	0.0900000
-15460.0000000	0.0900000
-15459.0000000	0.0900000
-15458.0000000	0.0900000
-15457.0000000	0.0900000
-15456.0000000	0.0900000
-15455.0000000	0.0900000
-15454.0000000	0.0900000
-15453.0000000	0.0895000
-15452.0000000	0.0900000
-15451.0000000	0.0900000
-15450.0000000	0.0900000
-15449.0000000	0.0890476
-15448.0000000	0.0895000
-15447.0000000	0.0900000
-15446.0000000	0.0890000
-15445.0000000	0.0880953
-15444.0000000	0.0885000
-15443.0000000	0.0861905
-15442.0000000	0.0890000
-15441.0000000	0.0895000
-15440.0000000	0.0900000
-15439.0000000	0.0890000
-15438.0000000	0.0900000
-15437.0000000	0.0895000
-15436.0000000	0.0900000
-15435.0000000	0.0900000
-15434.0000000	0.0900000
-15433.0000000	0.0900000
-15432.0000000	0.0895000
-15431.0000000	0.0900000
-15430.0000000	0.0900000
-15429.0000000	0.0900000
-15428.0000000	0.0900000
-15427.0000000	0.0900000
-15426.0000000	0.0900000
-15425.0000000	0.0900000
-15424.0000000	0.0900000
-15423.0000000	0.0900000
-15422.0000000	0.0900000
-15421.0000000	0.0900000
-15420.0000000	0.0900000
-15419.0000000	0.0900000
-15418.0000000	0.0890476
-15417.0000000	0.0895000
-15416.0000000	0.0890000
-15415.0000000	0.0876191

-15414.0000000	0.0885000
-15413.0000000	0.0885714
-15412.0000000	0.0890000
-15411.0000000	0.0855000
-15410.0000000	0.0855000
-15409.0000000	0.0847619
-15408.0000000	0.0845000
-15407.0000000	0.0850000
-15406.0000000	0.0876191
-15405.0000000	0.0880000
-15404.0000000	0.0871429
-15403.0000000	0.0890000
-15402.0000000	0.0865000
-15401.0000000	0.0885000
-15400.0000000	0.0866667
-15399.0000000	0.0885000
-15398.0000000	0.0865000
-15397.0000000	0.0880952
-15396.0000000	0.0895000
-15395.0000000	0.0900000
-15394.0000000	0.0895000
-15393.0000000	0.0900000
-15392.0000000	0.0895238
-15391.0000000	0.0900000
-15390.0000000	0.0900000
-15389.0000000	0.0900000
-15388.0000000	0.0900000
-15387.0000000	0.0900000
-15386.0000000	0.0900000
-15385.0000000	0.0900000
-15384.0000000	0.0895238
-15383.0000000	0.0885714
-15382.0000000	0.0900000
-15381.0000000	0.0900000
-15380.0000000	0.0895000
-15379.0000000	0.0885714
-15378.0000000	0.0890000
-15377.0000000	0.0890000
-15376.0000000	0.0900000
-15375.0000000	0.0900000
-15374.0000000	0.0895238
-15373.0000000	0.0900000
-15372.0000000	0.0895000
-15371.0000000	0.0895000
-15370.0000000	0.0900000
-15369.0000000	0.0900000
-15368.0000000	0.0900000
-15367.0000000	0.0900000
-15366.0000000	0.0900000
-15365.0000000	0.0900000
-15364.0000000	0.0900000
-15363.0000000	0.0900000
-15362.0000000	0.0900000
-15361.0000000	0.0909524
-15360.0000000	0.0910000
-15359.0000000	0.0930000
-15358.0000000	0.0923810

-15357.0000000	0.0920000
-15356.0000000	0.0945000
-15355.0000000	0.0935000
-15354.0000000	0.0947619
-15353.0000000	0.0935000
-15352.0000000	0.0933333
-15351.0000000	0.0925000
-15350.0000000	0.0935000
-15349.0000000	0.0923810
-15348.0000000	0.0945000
-15347.0000000	0.0950000
-15346.0000000	0.0957143
-15345.0000000	0.0940000
-15344.0000000	0.0940000
-15343.0000000	0.0938095
-15342.0000000	0.0965000
-15341.0000000	0.0940000
-15340.0000000	0.0933333
-15339.0000000	0.0930000
-15338.0000000	0.0915000
-15337.0000000	0.0900000
-15336.0000000	0.0910000
-15335.0000000	0.0905000
-15334.0000000	0.0900000
-15333.0000000	0.0900000
-15332.0000000	0.0900000
-15331.0000000	0.0900000
-15330.0000000	0.0900000
-15329.0000000	0.0900000
-15328.0000000	0.0900000
-15327.0000000	0.0900000
-15326.0000000	0.0900000
-15325.0000000	0.0900000
-15324.0000000	0.0900000
-15323.0000000	0.0900000
-15322.0000000	0.0900000
-15321.0000000	0.0900000
-15320.0000000	0.0900000
-15319.0000000	0.0900000
-15318.0000000	0.0900000
-15317.0000000	0.0900000
-15316.0000000	0.0900000
-15315.0000000	0.0900000
-15314.0000000	0.0900000
-15313.0000000	0.0900000
-15312.0000000	0.0905000
-15311.0000000	0.0900000
-15310.0000000	0.0905000
-15309.0000000	0.0904762
-15308.0000000	0.0900000
-15307.0000000	0.0904762
-15306.0000000	0.0910000
-15305.0000000	0.0900000
-15304.0000000	0.0900000
-15303.0000000	0.0905000
-15302.0000000	0.0900000
-15301.0000000	0.0905000

-15300.0000000	0.0900000
-15299.0000000	0.0900000
-15298.0000000	0.0904762
-15297.0000000	0.0900000
-15296.0000000	0.0905000
-15295.0000000	0.0900000
-15294.0000000	0.0904762
-15293.0000000	0.0900000
-15292.0000000	0.0900000
-15291.0000000	0.0905000
-15290.0000000	0.0900000
-15289.0000000	0.0909524
-15288.0000000	0.0905000
-15287.0000000	0.0915000
-15286.0000000	0.0900000
-15285.0000000	0.0910000
-15284.0000000	0.0935000
-15283.0000000	0.0928572
-15282.0000000	0.0920000
-15281.0000000	0.0910000
-15280.0000000	0.0915000
-15279.0000000	0.0928572
-15278.0000000	0.0905000
-15277.0000000	0.0923810
-15276.0000000	0.0915000
-15275.0000000	0.0915000
-15274.0000000	0.0900000
-15273.0000000	0.0900000
-15272.0000000	0.0905000
-15271.0000000	0.0910000
-15270.0000000	0.0904762
-15269.0000000	0.0910000
-15268.0000000	0.0909524
-15267.0000000	0.0920000
-15266.0000000	0.0935000
-15265.0000000	0.0925000
-15264.0000000	0.0909524
-15263.0000000	0.0915000
-15262.0000000	0.0935000
-15261.0000000	0.0919048
-15260.0000000	0.0910000
-15259.0000000	0.0904762
-15258.0000000	0.0905000
-15257.0000000	0.0900000
-15256.0000000	0.0900000
-15255.0000000	0.0900000
-15254.0000000	0.0895000
-15253.0000000	0.0895000
-15252.0000000	0.0876191
-15251.0000000	0.0840000
-15250.0000000	0.0850000
-15249.0000000	0.0838095
-15248.0000000	0.0835000
-15247.0000000	0.0842857
-15246.0000000	0.0815000
-15245.0000000	0.0815000
-15244.0000000	0.0810000

-15243.0000000	0.0814286
-15242.0000000	0.0810000
-15241.0000000	0.0850000
-15240.0000000	0.0842857
-15239.0000000	0.0845000
-15238.0000000	0.0876191
-15237.0000000	0.0850000
-15236.0000000	0.0875000
-15235.0000000	0.0860000
-15234.0000000	0.0880953
-15233.0000000	0.0865000
-15232.0000000	0.0865000
-15231.0000000	0.0852381
-15230.0000000	0.0830000
-15229.0000000	0.0809524
-15228.0000000	0.0810000
-15227.0000000	0.0805000
-15226.0000000	0.0815000
-15225.0000000	0.0804762
-15224.0000000	0.0815000
-15223.0000000	0.0820000
-15222.0000000	0.0804762
-15221.0000000	0.0805000
-15220.0000000	0.0815000
-15219.0000000	0.0810000
-15218.0000000	0.0814286
-15217.0000000	0.0852381
-15216.0000000	0.0855000
-15215.0000000	0.0885000
-15214.0000000	0.0865000
-15213.0000000	0.0857143
-15212.0000000	0.0865000
-15211.0000000	0.0840000
-15210.0000000	0.0838095
-15209.0000000	0.0825000
-15208.0000000	0.0814286
-15207.0000000	0.0805000
-15206.0000000	0.0820000
-15205.0000000	0.0815000
-15204.0000000	0.0814286
-15203.0000000	0.0815000
-15202.0000000	0.0815000
-15201.0000000	0.0814286
-15200.0000000	0.0830000
-15199.0000000	0.0833333
-15198.0000000	0.0835000
-15197.0000000	0.0845000
-15196.0000000	0.0845000
-15195.0000000	0.0866667
-15194.0000000	0.0865000
-15193.0000000	0.0860000
-15192.0000000	0.0890476
-15191.0000000	0.0875000
-15190.0000000	0.0890000
-15189.0000000	0.0890000
-15188.0000000	0.0895238
-15187.0000000	0.0895000

-15186.0000000	0.0885714
-15185.0000000	0.0900000
-15184.0000000	0.0890000
-15183.0000000	0.0895238
-15182.0000000	0.0885000
-15181.0000000	0.0880000
-15180.0000000	0.0890000
-15179.0000000	0.0895238
-15178.0000000	0.0900000
-15177.0000000	0.0866667
-15176.0000000	0.0865000
-15175.0000000	0.0830000
-15174.0000000	0.0814286
-15173.0000000	0.0825000
-15172.0000000	0.0825000
-15171.0000000	0.0838095
-15170.0000000	0.0815000
-15169.0000000	0.0825000
-15168.0000000	0.0823810
-15167.0000000	0.0820000
-15166.0000000	0.0815000
-15165.0000000	0.0833334
-15164.0000000	0.0845000
-15163.0000000	0.0850000
-15162.0000000	0.0852381
-15161.0000000	0.0870000
-15160.0000000	0.0875000
-15159.0000000	0.0885000
-15158.0000000	0.0900000
-15157.0000000	0.0895000
-15156.0000000	0.0885715
-15155.0000000	0.0900000
-15154.0000000	0.0880000
-15153.0000000	0.0880953
-15152.0000000	0.0900000
-15151.0000000	0.0900000
-15150.0000000	0.0895238
-15149.0000000	0.0900000
-15148.0000000	0.0900000
-15147.0000000	0.0900000
-15146.0000000	0.0900000
-15145.0000000	0.0900000
-15144.0000000	0.0900000
-15143.0000000	0.0900000
-15142.0000000	0.0900000
-15141.0000000	0.0895238
-15140.0000000	0.0900000
-15139.0000000	0.0900000
-15138.0000000	0.0900000
-15137.0000000	0.0900000
-15136.0000000	0.0900000
-15135.0000000	0.0895000
-15134.0000000	0.0895238
-15133.0000000	0.0895000
-15132.0000000	0.0900000
-15131.0000000	0.0890000
-15130.0000000	0.0875000

-15129.0000000	0.0890000
-15128.0000000	0.0895238
-15127.0000000	0.0880000
-15126.0000000	0.0865000
-15125.0000000	0.0876191
-15124.0000000	0.0895000
-15123.0000000	0.0880953
-15122.0000000	0.0895000
-15121.0000000	0.0865000
-15120.0000000	0.0876191
-15119.0000000	0.0885000
-15118.0000000	0.0885000
-15117.0000000	0.0900000
-15116.0000000	0.0890000
-15115.0000000	0.0900000
-15114.0000000	0.0895000
-15113.0000000	0.0900000
-15112.0000000	0.0900000
-15111.0000000	0.0900000
-15110.0000000	0.0900000
-15109.0000000	0.0900000
-15108.0000000	0.0900000
-15107.0000000	0.0895000
-15106.0000000	0.0855000
-15105.0000000	0.0835000
-15104.0000000	0.0828572
-15103.0000000	0.0840000
-15102.0000000	0.0823810
-15101.0000000	0.0815000
-15100.0000000	0.0815000
-15099.0000000	0.0815000
-15098.0000000	0.0804762
-15097.0000000	0.0815000
-15096.0000000	0.0800000
-15095.0000000	0.0852381
-15094.0000000	0.0830000
-15093.0000000	0.0861905
-15092.0000000	0.0865000
-15091.0000000	0.0875000
-15090.0000000	0.0861905
-15089.0000000	0.0860000
-15088.0000000	0.0860000
-15087.0000000	0.0845000
-15086.0000000	0.0833333
-15085.0000000	0.0805000
-15084.0000000	0.0805000
-15083.0000000	0.0814286
-15082.0000000	0.0810000
-15081.0000000	0.0838095
-15080.0000000	0.0815000
-15079.0000000	0.0815000
-15078.0000000	0.0815000
-15077.0000000	0.0823810
-15076.0000000	0.0810000
-15075.0000000	0.0805000
-15074.0000000	0.0800000
-15073.0000000	0.0800000

-15072.0000000	0.0800000
-15071.0000000	0.0800000
-15070.0000000	0.0800000
-15069.0000000	0.0810000
-15068.0000000	0.0804762
-15067.0000000	0.0800000
-15066.0000000	0.0815000
-15065.0000000	0.0809524
-15064.0000000	0.0825000
-15063.0000000	0.0828572
-15062.0000000	0.0860000
-15061.0000000	0.0875000
-15060.0000000	0.0865000
-15059.0000000	0.0880953
-15058.0000000	0.0880000
-15057.0000000	0.0880000
-15056.0000000	0.0895238
-15055.0000000	0.0880000
-15054.0000000	0.0880000
-15053.0000000	0.0880000
-15052.0000000	0.0880953
-15051.0000000	0.0900000
-15050.0000000	0.0890476
-15049.0000000	0.0890000
-15048.0000000	0.0895000
-15047.0000000	0.0890476
-15046.0000000	0.0875000
-15045.0000000	0.0875000
-15044.0000000	0.0870000
-15043.0000000	0.0880952
-15042.0000000	0.0895238
-15041.0000000	0.0885000
-15040.0000000	0.0870000
-15039.0000000	0.0885000
-15038.0000000	0.0876191
-15037.0000000	0.0880000
-15036.0000000	0.0870000
-15035.0000000	0.0852381
-15034.0000000	0.0840000
-15033.0000000	0.0828572
-15032.0000000	0.0835000
-15031.0000000	0.0825000
-15030.0000000	0.0855000
-15029.0000000	0.0819048
-15028.0000000	0.0860000
-15027.0000000	0.0845000
-15026.0000000	0.0852381
-15025.0000000	0.0870000
-15024.0000000	0.0850000
-15023.0000000	0.0875000
-15022.0000000	0.0871429
-15021.0000000	0.0880000
-15020.0000000	0.0885715
-15019.0000000	0.0890000
-15018.0000000	0.0880000
-15017.0000000	0.0880953
-15016.0000000	0.0895000

-15015.0000000	0.0890000
-15014.0000000	0.0880000
-15013.0000000	0.0871429
-15012.0000000	0.0870000
-15011.0000000	0.0871429
-15010.0000000	0.0855000
-15009.0000000	0.0825000
-15008.0000000	0.0823810
-15007.0000000	0.0810000
-15006.0000000	0.0825000
-15005.0000000	0.0823810
-15004.0000000	0.0831250
-15003.0000000	0.0835000
-15002.0000000	0.0861905
-15001.0000000	0.0875000
-15000.0000000	0.0875000
-14999.0000000	0.0890476
-14998.0000000	0.0895000
-14997.0000000	0.0895000
-14996.0000000	0.0880000
-14995.0000000	0.0895238
-14994.0000000	0.0885000
-14993.0000000	0.0895238
-14992.0000000	0.0895000
-14991.0000000	0.0875000
-14990.0000000	0.0876191
-14989.0000000	0.0880000
-14988.0000000	0.0895000
-14987.0000000	0.0880953
-14986.0000000	0.0880000
-14985.0000000	0.0870000
-14984.0000000	0.0857143
-14983.0000000	0.0855000
-14982.0000000	0.0885000
-14981.0000000	0.0845000
-14980.0000000	0.0857143
-14979.0000000	0.0830000
-14978.0000000	0.0809524
-14977.0000000	0.0810000
-14976.0000000	0.0810000
-14975.0000000	0.0804762
-14974.0000000	0.0830000
-14973.0000000	0.0810000
-14972.0000000	0.0819048
-14971.0000000	0.0830000
-14970.0000000	0.0860000
-14969.0000000	0.0852381
-14968.0000000	0.0885000
-14967.0000000	0.0870000
-14966.0000000	0.0880000
-14965.0000000	0.0885714
-14964.0000000	0.0880000
-14963.0000000	0.0866667
-14962.0000000	0.0870000
-14961.0000000	0.0850000
-14960.0000000	0.0828572
-14959.0000000	0.0825000

-14958.0000000	0.0855000
-14957.0000000	0.0840000
-14956.0000000	0.0871429
-14955.0000000	0.0885000
-14954.0000000	0.0857143
-14953.0000000	0.0890000
-14952.0000000	0.0890000
-14951.0000000	0.0885000
-14950.0000000	0.0900000
-14949.0000000	0.0900000
-14948.0000000	0.0900000
-14947.0000000	0.0855000
-14946.0000000	0.0860000
-14945.0000000	0.0876191
-14944.0000000	0.0875000
-14943.0000000	0.0865000
-14942.0000000	0.0850000
-14941.0000000	0.0828572
-14940.0000000	0.0825000
-14939.0000000	0.0842857
-14938.0000000	0.0825000
-14937.0000000	0.0800000
-14936.0000000	0.0805000
-14935.0000000	0.0809524
-14934.0000000	0.0810000
-14933.0000000	0.0800000
-14932.0000000	0.0800000
-14931.0000000	0.0800000
-14930.0000000	0.0809524
-14929.0000000	0.0800000
-14928.0000000	0.0805000
-14927.0000000	0.0805000
-14926.0000000	0.0800000
-14925.0000000	0.0805000
-14924.0000000	0.0820000
-14923.0000000	0.0823810
-14922.0000000	0.0820000
-14921.0000000	0.0820000
-14920.0000000	0.0809524
-14919.0000000	0.0835000
-14918.0000000	0.0847619
-14917.0000000	0.0860000
-14916.0000000	0.0860000
-14915.0000000	0.0865000
-14914.0000000	0.0866667
-14913.0000000	0.0870000
-14912.0000000	0.0865000
-14911.0000000	0.0885714
-14910.0000000	0.0875000
-14909.0000000	0.0871429
-14908.0000000	0.0825000
-14907.0000000	0.0840000
-14906.0000000	0.0860000
-14905.0000000	0.0847619
-14904.0000000	0.0865000
-14903.0000000	0.0860000
-14902.0000000	0.0833334

-14901.0000000	0.0855000
-14900.0000000	0.0842857
-14899.0000000	0.0855000
-14898.0000000	0.0835000
-14897.0000000	0.0805000
-14896.0000000	0.0819048
-14895.0000000	0.0840000
-14894.0000000	0.0820000
-14893.0000000	0.0847619
-14892.0000000	0.0810000
-14891.0000000	0.0815000
-14890.0000000	0.0810000
-14889.0000000	0.0809524
-14888.0000000	0.0800000
-14887.0000000	0.0835000
-14886.0000000	0.0830000
-14885.0000000	0.0820000
-14884.0000000	0.0819048
-14883.0000000	0.0855000
-14882.0000000	0.0860000
-14881.0000000	0.0880000
-14880.0000000	0.0876191
-14879.0000000	0.0880953
-14878.0000000	0.0895000
-14877.0000000	0.0875000
-14876.0000000	0.0870000
-14875.0000000	0.0890476
-14874.0000000	0.0865000
-14873.0000000	0.0870000
-14872.0000000	0.0857143
-14871.0000000	0.0835000
-14870.0000000	0.0833334
-14869.0000000	0.0835000
-14868.0000000	0.0810000
-14867.0000000	0.0820000
-14866.0000000	0.0804762
-14865.0000000	0.0800000
-14864.0000000	0.0800000
-14863.0000000	0.0800000
-14862.0000000	0.0800000
-14861.0000000	0.0800000
-14860.0000000	0.0800000
-14859.0000000	0.0800000
-14858.0000000	0.0800000
-14857.0000000	0.0800000
-14856.0000000	0.0805000
-14855.0000000	0.0805000
-14854.0000000	0.0833334
-14853.0000000	0.0850000
-14852.0000000	0.0880000
-14851.0000000	0.0895000
-14850.0000000	0.0871429
-14849.0000000	0.0885000
-14848.0000000	0.0880952
-14847.0000000	0.0875000
-14846.0000000	0.0880000
-14845.0000000	0.0875000

-14844.0000000	0.0900000
-14843.0000000	0.0895000
-14842.0000000	0.0890476
-14841.0000000	0.0890000
-14840.0000000	0.0895000
-14839.0000000	0.0900000
-14838.0000000	0.0900000
-14837.0000000	0.0900000
-14836.0000000	0.0900000
-14835.0000000	0.0900000
-14834.0000000	0.0900000
-14833.0000000	0.0880953
-14832.0000000	0.0880000
-14831.0000000	0.0865000
-14830.0000000	0.0865000
-14829.0000000	0.0857143
-14828.0000000	0.0880000
-14827.0000000	0.0857143
-14826.0000000	0.0875000
-14825.0000000	0.0860000
-14824.0000000	0.0895238
-14823.0000000	0.0895000
-14822.0000000	0.0875000
-14821.0000000	0.0865000
-14820.0000000	0.0880952
-14819.0000000	0.0875000
-14818.0000000	0.0857143
-14817.0000000	0.0860000
-14816.0000000	0.0855000
-14815.0000000	0.0870000
-14814.0000000	0.0866667
-14813.0000000	0.0860000
-14812.0000000	0.0885714
-14811.0000000	0.0885000
-14810.0000000	0.0875000
-14809.0000000	0.0880953
-14808.0000000	0.0895000
-14807.0000000	0.0890000
-14806.0000000	0.0890000
-14805.0000000	0.0900000
-14804.0000000	0.0895000
-14803.0000000	0.0895238
-14802.0000000	0.0895000
-14801.0000000	0.0875000
-14800.0000000	0.0890000
-14799.0000000	0.0895238
-14798.0000000	0.0895000
-14797.0000000	0.0900000
-14796.0000000	0.0895000
-14795.0000000	0.0890000
-14794.0000000	0.0880953
-14793.0000000	0.0900000
-14792.0000000	0.0900000
-14791.0000000	0.0895000
-14790.0000000	0.0890476
-14789.0000000	0.0885000
-14788.0000000	0.0900000

-14787.0000000	0.0900000
-14786.0000000	0.0895000
-14785.0000000	0.0900000
-14784.0000000	0.0895238
-14783.0000000	0.0895000
-14782.0000000	0.0890476
-14781.0000000	0.0900000
-14780.0000000	0.0895000
-14779.0000000	0.0895238
-14778.0000000	0.0885000
-14777.0000000	0.0890000
-14776.0000000	0.0880000
-14775.0000000	0.0885715
-14774.0000000	0.0880000
-14773.0000000	0.0857143
-14772.0000000	0.0845000
-14771.0000000	0.0825000
-14770.0000000	0.0815000
-14769.0000000	0.0809524
-14768.0000000	0.0815000
-14767.0000000	0.0820000
-14766.0000000	0.0833333
-14765.0000000	0.0840000
-14764.0000000	0.0833334
-14763.0000000	0.0830000
-14762.0000000	0.0855000
-14761.0000000	0.0840000
-14760.0000000	0.0852381
-14759.0000000	0.0835000
-14758.0000000	0.0835000
-14757.0000000	0.0819048
-14756.0000000	0.0825000
-14755.0000000	0.0825000
-14754.0000000	0.0804762
-14753.0000000	0.0800000
-14752.0000000	0.0800000
-14751.0000000	0.0800000
-14750.0000000	0.0800000
-14749.0000000	0.0800000
-14748.0000000	0.0809524
-14747.0000000	0.0815000
-14746.0000000	0.0830000
-14745.0000000	0.0838095
-14744.0000000	0.0830000
-14743.0000000	0.0871429
-14742.0000000	0.0885000
-14741.0000000	0.0890000
-14740.0000000	0.0900000
-14739.0000000	0.0895238
-14738.0000000	0.0900000
-14737.0000000	0.0900000
-14736.0000000	0.0900000
-14735.0000000	0.0900000
-14734.0000000	0.0900000
-14733.0000000	0.0900000
-14732.0000000	0.0900000
-14731.0000000	0.0900000

-14730.0000000	0.0904762
-14729.0000000	0.0910000
-14728.0000000	0.0900000
-14727.0000000	0.0904762
-14726.0000000	0.0900000
-14725.0000000	0.0900000
-14724.0000000	0.0900000
-14723.0000000	0.0900000
-14722.0000000	0.0900000
-14721.0000000	0.0900000
-14720.0000000	0.0895000
-14719.0000000	0.0900000
-14718.0000000	0.0900000
-14717.0000000	0.0890000
-14716.0000000	0.0885000
-14715.0000000	0.0880000
-14714.0000000	0.0890476
-14713.0000000	0.0895238
-14712.0000000	0.0865000
-14711.0000000	0.0845000
-14710.0000000	0.0845000
-14709.0000000	0.0838095
-14708.0000000	0.0825000
-14707.0000000	0.0820000
-14706.0000000	0.0833334
-14705.0000000	0.0835000
-14704.0000000	0.0828572
-14703.0000000	0.0835000
-14702.0000000	0.0845000
-14701.0000000	0.0850000
-14700.0000000	0.0838095
-14699.0000000	0.0830000
-14698.0000000	0.0835000
-14697.0000000	0.0828572
-14696.0000000	0.0830000
-14695.0000000	0.0830000
-14694.0000000	0.0820000
-14693.0000000	0.0804762
-14692.0000000	0.0800000
-14691.0000000	0.0800000
-14690.0000000	0.0800000
-14689.0000000	0.0810000
-14688.0000000	0.0800000
-14687.0000000	0.0800000
-14686.0000000	0.0820000
-14685.0000000	0.0800000
-14684.0000000	0.0800000
-14683.0000000	0.0800000
-14682.0000000	0.0800000
-14681.0000000	0.0800000
-14680.0000000	0.0795000
-14679.0000000	0.0800000
-14678.0000000	0.0800000
-14677.0000000	0.0800000
-14676.0000000	0.0800000
-14675.0000000	0.0800000
-14674.0000000	0.0800000

-14673.0000000	0.0800000
-14672.0000000	0.0800000
-14671.0000000	0.0800000
-14670.0000000	0.0800000
-14669.0000000	0.0800000
-14668.0000000	0.0815000
-14667.0000000	0.0819048
-14666.0000000	0.0825000
-14665.0000000	0.0830000
-14664.0000000	0.0865000
-14663.0000000	0.0838095
-14662.0000000	0.0835000
-14661.0000000	0.0871429
-14660.0000000	0.0890000
-14659.0000000	0.0875000
-14658.0000000	0.0895238
-14657.0000000	0.0900000
-14656.0000000	0.0900000
-14655.0000000	0.0900000
-14654.0000000	0.0900000
-14653.0000000	0.0900000
-14652.0000000	0.0900000
-14651.0000000	0.0900000
-14650.0000000	0.0895000
-14649.0000000	0.0900000
-14648.0000000	0.0900000
-14647.0000000	0.0900000
-14646.0000000	0.0895238
-14645.0000000	0.0900000
-14644.0000000	0.0900000
-14643.0000000	0.0890476
-14642.0000000	0.0895000
-14641.0000000	0.0895000
-14640.0000000	0.0885000
-14639.0000000	0.0895238
-14638.0000000	0.0900000
-14637.0000000	0.0895238
-14636.0000000	0.0900000
-14635.0000000	0.0900000
-14634.0000000	0.0895000
-14633.0000000	0.0890476
-14632.0000000	0.0900000
-14631.0000000	0.0895000
-14630.0000000	0.0900000
-14629.0000000	0.0890000
-14628.0000000	0.0900000
-14627.0000000	0.0900000
-14626.0000000	0.0900000
-14625.0000000	0.0900000
-14624.0000000	0.0900000
-14623.0000000	0.0890000
-14622.0000000	0.0871429
-14621.0000000	0.0860000
-14620.0000000	0.0865000
-14619.0000000	0.0845000
-14618.0000000	0.0810000
-14617.0000000	0.0800000

-14616.0000000	0.0828572
-14615.0000000	0.0845000
-14614.0000000	0.0820000
-14613.0000000	0.0833333
-14612.0000000	0.0815000
-14611.0000000	0.0830000
-14610.0000000	0.0820000
-14609.0000000	0.0804762
-14608.0000000	0.0805000
-14607.0000000	0.0800000
-14606.0000000	0.0800000
-14605.0000000	0.0800000
-14604.0000000	0.0800000
-14603.0000000	0.0800000
-14602.0000000	0.0800000
-14601.0000000	0.0800000
-14600.0000000	0.0800000
-14599.0000000	0.0800000
-14598.0000000	0.0800000
-14597.0000000	0.0805000
-14596.0000000	0.0800000
-14595.0000000	0.0800000
-14594.0000000	0.0800000
-14593.0000000	0.0805000
-14592.0000000	0.0805000
-14591.0000000	0.0800000
-14590.0000000	0.0810000
-14589.0000000	0.0820000
-14588.0000000	0.0814286
-14587.0000000	0.0815000
-14586.0000000	0.0800000
-14585.0000000	0.0825000
-14584.0000000	0.0840000
-14583.0000000	0.0845000
-14582.0000000	0.0861905
-14581.0000000	0.0875000
-14580.0000000	0.0875000
-14579.0000000	0.0857143
-14578.0000000	0.0875000
-14577.0000000	0.0890476
-14576.0000000	0.0885000
-14575.0000000	0.0900000
-14574.0000000	0.0895000
-14573.0000000	0.0895238
-14572.0000000	0.0895000
-14571.0000000	0.0900000
-14570.0000000	0.0900000
-14569.0000000	0.0900000
-14568.0000000	0.0900000
-14567.0000000	0.0900000
-14566.0000000	0.0900000
-14565.0000000	0.0905000
-14564.0000000	0.0900000
-14563.0000000	0.0900000
-14562.0000000	0.0900000
-14561.0000000	0.0900000
-14560.0000000	0.0905000

-14559.0000000	0.0900000
-14558.0000000	0.0900000
-14557.0000000	0.0900000
-14556.0000000	0.0900000
-14555.0000000	0.0900000
-14554.0000000	0.0900000
-14553.0000000	0.0900000
-14552.0000000	0.0900000
-14551.0000000	0.0880000
-14550.0000000	0.0885000
-14549.0000000	0.0895000
-14548.0000000	0.0900000
-14547.0000000	0.0895000
-14546.0000000	0.0895238
-14545.0000000	0.0900000
-14544.0000000	0.0900000
-14543.0000000	0.0890476
-14542.0000000	0.0895000
-14541.0000000	0.0900000
-14540.0000000	0.0900000
-14539.0000000	0.0900000
-14538.0000000	0.0895238
-14537.0000000	0.0895000
-14536.0000000	0.0900000
-14535.0000000	0.0890000
-14534.0000000	0.0890476
-14533.0000000	0.0885000
-14532.0000000	0.0885000
-14531.0000000	0.0876191
-14530.0000000	0.0870000
-14529.0000000	0.0835000
-14528.0000000	0.0825000
-14527.0000000	0.0804762
-14526.0000000	0.0805000
-14525.0000000	0.0800000
-14524.0000000	0.0800000
-14523.0000000	0.0800000
-14522.0000000	0.0800000
-14521.0000000	0.0800000
-14520.0000000	0.0800000
-14519.0000000	0.0800000
-14518.0000000	0.0800000
-14517.0000000	0.0800000
-14516.0000000	0.0800000
-14515.0000000	0.0795000
-14514.0000000	0.0800000
-14513.0000000	0.0800000
-14512.0000000	0.0800000
-14511.0000000	0.0800000
-14510.0000000	0.0800000
-14509.0000000	0.0800000
-14508.0000000	0.0800000
-14507.0000000	0.0800000
-14506.0000000	0.0800000
-14505.0000000	0.0800000
-14504.0000000	0.0800000
-14503.0000000	0.0800000

-14502.0000000	0.0800000
-14501.0000000	0.0800000
-14500.0000000	0.0800000
-14499.0000000	0.0800000
-14498.0000000	0.0800000
-14497.0000000	0.0800000
-14496.0000000	0.0815000
-14495.0000000	0.0814286
-14494.0000000	0.0825000
-14493.0000000	0.0835000
-14492.0000000	0.0814286
-14491.0000000	0.0835000
-14490.0000000	0.0830000
-14489.0000000	0.0805000
-14488.0000000	0.0828572
-14487.0000000	0.0815000
-14486.0000000	0.0804762
-14485.0000000	0.0820000
-14484.0000000	0.0815000
-14483.0000000	0.0845000
-14482.0000000	0.0833333
-14481.0000000	0.0830000
-14480.0000000	0.0820000
-14479.0000000	0.0814286
-14478.0000000	0.0800000
-14477.0000000	0.0800000
-14476.0000000	0.0825000
-14475.0000000	0.0810000
-14474.0000000	0.0810000
-14473.0000000	0.0814286
-14472.0000000	0.0825000
-14471.0000000	0.0833334
-14470.0000000	0.0820000
-14469.0000000	0.0815000
-14468.0000000	0.0820000
-14467.0000000	0.0838095
-14466.0000000	0.0835000
-14465.0000000	0.0845000
-14464.0000000	0.0852381
-14463.0000000	0.0860000
-14462.0000000	0.0876191
-14461.0000000	0.0895000
-14460.0000000	0.0890000
-14459.0000000	0.0895000
-14458.0000000	0.0900000
-14457.0000000	0.0895000
-14456.0000000	0.0900000
-14455.0000000	0.0900000
-14454.0000000	0.0900000
-14453.0000000	0.0900000
-14452.0000000	0.0900000
-14451.0000000	0.0900000
-14450.0000000	0.0904762
-14449.0000000	0.0900000
-14448.0000000	0.0900000
-14447.0000000	0.0904762
-14446.0000000	0.0900000

-14445.0000000	0.0900000
-14444.0000000	0.0905000
-14443.0000000	0.0900000
-14442.0000000	0.0900000
-14441.0000000	0.0900000
-14440.0000000	0.0895000
-14439.0000000	0.0900000
-14438.0000000	0.0900000
-14437.0000000	0.0900000
-14436.0000000	0.0900000
-14435.0000000	0.0900000
-14434.0000000	0.0900000
-14433.0000000	0.0900000
-14432.0000000	0.0900000
-14431.0000000	0.0905000
-14430.0000000	0.0900000
-14429.0000000	0.0900000
-14428.0000000	0.0900000
-14427.0000000	0.0900000
-14426.0000000	0.0900000
-14425.0000000	0.0900000
-14424.0000000	0.0900000
-14423.0000000	0.0900000
-14422.0000000	0.0900000
-14421.0000000	0.0900000
-14420.0000000	0.0900000
-14419.0000000	0.0880953
-14418.0000000	0.0860000
-14417.0000000	0.0855000
-14416.0000000	0.0866667
-14415.0000000	0.0850000
-14414.0000000	0.0840000
-14413.0000000	0.0876191
-14412.0000000	0.0875000
-14411.0000000	0.0880953
-14410.0000000	0.0870000
-14409.0000000	0.0885000
-14408.0000000	0.0875000
-14407.0000000	0.0871429
-14406.0000000	0.0895000
-14405.0000000	0.0875000
-14404.0000000	0.0885714
-14403.0000000	0.0895000
-14402.0000000	0.0880953
-14401.0000000	0.0875000
-14400.0000000	0.0875000
-14399.0000000	0.0885000
-14398.0000000	0.0895238
-14397.0000000	0.0895000
-14396.0000000	0.0900000
-14395.0000000	0.0885714
-14394.0000000	0.0880000
-14393.0000000	0.0865000
-14392.0000000	0.0855000
-14391.0000000	0.0857143
-14390.0000000	0.0860000
-14389.0000000	0.0876191

-14388.0000000	0.0870000
-14387.0000000	0.0890000
-14386.0000000	0.0895238
-14385.0000000	0.0895000
-14384.0000000	0.0895000
-14383.0000000	0.0890000
-14382.0000000	0.0890476
-14381.0000000	0.0895000
-14380.0000000	0.0895238
-14379.0000000	0.0900000
-14378.0000000	0.0890000
-14377.0000000	0.0895238
-14376.0000000	0.0880000
-14375.0000000	0.0895000
-14374.0000000	0.0895000
-14373.0000000	0.0895238
-14372.0000000	0.0895000
-14371.0000000	0.0900000
-14370.0000000	0.0895000
-14369.0000000	0.0900000
-14368.0000000	0.0900000
-14367.0000000	0.0900000
-14366.0000000	0.0900000
-14365.0000000	0.0900000
-14364.0000000	0.0900000
-14363.0000000	0.0900000
-14362.0000000	0.0900000
-14361.0000000	0.0900000
-14360.0000000	0.0900000
-14359.0000000	0.0900000
-14358.0000000	0.0895000
-14357.0000000	0.0895000
-14356.0000000	0.0890476
-14355.0000000	0.0900000
-14354.0000000	0.0895000
-14353.0000000	0.0900000
-14352.0000000	0.0885714
-14351.0000000	0.0880000
-14350.0000000	0.0880952
-14349.0000000	0.0885000
-14348.0000000	0.0875000
-14347.0000000	0.0895000
-14346.0000000	0.0880953
-14345.0000000	0.0880000
-14344.0000000	0.0885000
-14343.0000000	0.0866667
-14342.0000000	0.0860000
-14341.0000000	0.0876191
-14340.0000000	0.0880000
-14339.0000000	0.0875000
-14338.0000000	0.0871429
-14337.0000000	0.0875000
-14336.0000000	0.0870000
-14335.0000000	0.0861905
-14334.0000000	0.0845000
-14333.0000000	0.0845000
-14332.0000000	0.0835000

-14331.0000000	0.0866667
-14330.0000000	0.0850000
-14329.0000000	0.0871429
-14328.0000000	0.0865000
-14327.0000000	0.0885000
-14326.0000000	0.0876191
-14325.0000000	0.0885000
-14324.0000000	0.0870000
-14323.0000000	0.0865000
-14322.0000000	0.0871429
-14321.0000000	0.0875000
-14320.0000000	0.0871429
-14319.0000000	0.0865000
-14318.0000000	0.0895000
-14317.0000000	0.0875000
-14316.0000000	0.0880953
-14315.0000000	0.0875000
-14314.0000000	0.0890000
-14313.0000000	0.0885715
-14312.0000000	0.0890000
-14311.0000000	0.0895238
-14310.0000000	0.0890000
-14309.0000000	0.0840000
-14308.0000000	0.0855000
-14307.0000000	0.0814286
-14306.0000000	0.0805000
-14305.0000000	0.0814286
-14304.0000000	0.0815000
-14303.0000000	0.0805000
-14302.0000000	0.0805000
-14301.0000000	0.0814286
-14300.0000000	0.0835000
-14299.0000000	0.0825000
-14298.0000000	0.0847619
-14297.0000000	0.0840000
-14296.0000000	0.0842857
-14295.0000000	0.0825000
-14294.0000000	0.0800000
-14293.0000000	0.0800000
-14292.0000000	0.0800000
-14291.0000000	0.0800000
-14290.0000000	0.0800000
-14289.0000000	0.0804762
-14288.0000000	0.0805000
-14287.0000000	0.0845000
-14286.0000000	0.0840000
-14285.0000000	0.0852381
-14284.0000000	0.0845000
-14283.0000000	0.0861905
-14282.0000000	0.0850000
-14281.0000000	0.0850000
-14280.0000000	0.0828572
-14279.0000000	0.0810000
-14278.0000000	0.0805000
-14277.0000000	0.0809524
-14276.0000000	0.0820000
-14275.0000000	0.0838095

-14274.0000000	0.0850000
-14273.0000000	0.0855000
-14272.0000000	0.0870000
-14271.0000000	0.0866667
-14270.0000000	0.0880000
-14269.0000000	0.0895000
-14268.0000000	0.0900000
-14267.0000000	0.0895000
-14266.0000000	0.0885715
-14265.0000000	0.0895000
-14264.0000000	0.0895000
-14263.0000000	0.0875000
-14262.0000000	0.0871429
-14261.0000000	0.0870000
-14260.0000000	0.0860000
-14259.0000000	0.0861905
-14258.0000000	0.0830000
-14257.0000000	0.0825000
-14256.0000000	0.0810000
-14255.0000000	0.0804762
-14254.0000000	0.0805000
-14253.0000000	0.0800000
-14252.0000000	0.0800000
-14251.0000000	0.0800000
-14250.0000000	0.0800000
-14249.0000000	0.0815000
-14248.0000000	0.0815000
-14247.0000000	0.0840000
-14246.0000000	0.0847619
-14245.0000000	0.0871429
-14244.0000000	0.0870000
-14243.0000000	0.0870000
-14242.0000000	0.0875000
-14241.0000000	0.0876191
-14240.0000000	0.0875000
-14239.0000000	0.0890000
-14238.0000000	0.0890476
-14237.0000000	0.0875000
-14236.0000000	0.0885715
-14235.0000000	0.0890000
-14234.0000000	0.0885000
-14233.0000000	0.0895000
-14232.0000000	0.0900000
-14231.0000000	0.0895000
-14230.0000000	0.0895000
-14229.0000000	0.0890476
-14228.0000000	0.0885000
-14227.0000000	0.0890000
-14226.0000000	0.0895000
-14225.0000000	0.0861905
-14224.0000000	0.0860000
-14223.0000000	0.0842857
-14222.0000000	0.0855000
-14221.0000000	0.0835000
-14220.0000000	0.0819048
-14219.0000000	0.0810000
-14218.0000000	0.0805000

-14217.0000000	0.0800000
-14216.0000000	0.0800000
-14215.0000000	0.0800000
-14214.0000000	0.0800000
-14213.0000000	0.0800000
-14212.0000000	0.0805000
-14211.0000000	0.0800000
-14210.0000000	0.0810000
-14209.0000000	0.0820000
-14208.0000000	0.0820000
-14207.0000000	0.0804762
-14206.0000000	0.0830000
-14205.0000000	0.0819048
-14204.0000000	0.0820000
-14203.0000000	0.0820000
-14202.0000000	0.0828572
-14201.0000000	0.0830000
-14200.0000000	0.0830000
-14199.0000000	0.0819048
-14198.0000000	0.0815000
-14197.0000000	0.0800000
-14196.0000000	0.0800000
-14195.0000000	0.0800000
-14194.0000000	0.0805000
-14193.0000000	0.0800000
-14192.0000000	0.0800000
-14191.0000000	0.0800000
-14190.0000000	0.0804762
-14189.0000000	0.0800000
-14188.0000000	0.0805000
-14187.0000000	0.0805000
-14186.0000000	0.0800000
-14185.0000000	0.0805000
-14184.0000000	0.0804762
-14183.0000000	0.0800000
-14182.0000000	0.0805000
-14181.0000000	0.0800000
-14180.0000000	0.0809524
-14179.0000000	0.0800000
-14178.0000000	0.0800000
-14177.0000000	0.0800000
-14176.0000000	0.0800000
-14175.0000000	0.0800000
-14174.0000000	0.0800000
-14173.0000000	0.0800000
-14172.0000000	0.0800000
-14171.0000000	0.0800000
-14170.0000000	0.0800000
-14169.0000000	0.0800000
-14168.0000000	0.0800000
-14167.0000000	0.0800000
-14166.0000000	0.0800000
-14165.0000000	0.0800000
-14164.0000000	0.0800000
-14163.0000000	0.0800000
-14162.0000000	0.0800000
-14161.0000000	0.0800000

-14160.0000000	0.0800000
-14159.0000000	0.0805000
-14158.0000000	0.0800000
-14157.0000000	0.0805000
-14156.0000000	0.0804762
-14155.0000000	0.0800000
-14154.0000000	0.0800000
-14153.0000000	0.0810000
-14152.0000000	0.0810000
-14151.0000000	0.0810000
-14150.0000000	0.0823810
-14149.0000000	0.0820000
-14148.0000000	0.0820000
-14147.0000000	0.0833333
-14146.0000000	0.0815000
-14145.0000000	0.0819048
-14144.0000000	0.0805000
-14143.0000000	0.0825000
-14142.0000000	0.0815000
-14141.0000000	0.0814286
-14140.0000000	0.0820000
-14139.0000000	0.0823810
-14138.0000000	0.0815000
-14137.0000000	0.0805000
-14136.0000000	0.0810000
-14135.0000000	0.0800000
-14134.0000000	0.0810000
-14133.0000000	0.0810000
-14132.0000000	0.0814286
-14131.0000000	0.0820000
-14130.0000000	0.0809524
-14129.0000000	0.0820000
-14128.0000000	0.0810000
-14127.0000000	0.0805000
-14126.0000000	0.0823810
-14125.0000000	0.0810000
-14124.0000000	0.0805000
-14123.0000000	0.0804762
-14122.0000000	0.0805000
-14121.0000000	0.0815000
-14120.0000000	0.0804762
-14119.0000000	0.0805000
-14118.0000000	0.0805000
-14117.0000000	0.0804762
-14116.0000000	0.0805000
-14115.0000000	0.0800000
-14114.0000000	0.0800000
-14113.0000000	0.0800000
-14112.0000000	0.0800000
-14111.0000000	0.0800000
-14110.0000000	0.0800000
-14109.0000000	0.0800000
-14108.0000000	0.0800000
-14107.0000000	0.0800000
-14106.0000000	0.0800000
-14105.0000000	0.0800000
-14104.0000000	0.0800000

-14103.0000000	0.0800000
-14102.0000000	0.0800000
-14101.0000000	0.0800000
-14100.0000000	0.0800000
-14099.0000000	0.0800000
-14098.0000000	0.0800000
-14097.0000000	0.0805000
-14096.0000000	0.0800000
-14095.0000000	0.0815000
-14094.0000000	0.0815000
-14093.0000000	0.0804762
-14092.0000000	0.0805000
-14091.0000000	0.0835000
-14090.0000000	0.0815000
-14089.0000000	0.0819048
-14088.0000000	0.0825000
-14087.0000000	0.0842857
-14086.0000000	0.0810000
-14085.0000000	0.0815000
-14084.0000000	0.0804762
-14083.0000000	0.0810000
-14082.0000000	0.0815000
-14081.0000000	0.0840000
-14080.0000000	0.0847619
-14079.0000000	0.0871429
-14078.0000000	0.0870000
-14077.0000000	0.0860000
-14076.0000000	0.0870000
-14075.0000000	0.0861905
-14074.0000000	0.0870000
-14073.0000000	0.0880000
-14072.0000000	0.0845000
-14071.0000000	0.0833333
-14070.0000000	0.0823810
-14069.0000000	0.0815000
-14068.0000000	0.0815000
-14067.0000000	0.0825000
-14066.0000000	0.0814286
-14065.0000000	0.0820000
-14064.0000000	0.0805000
-14063.0000000	0.0814286
-14062.0000000	0.0805000
-14061.0000000	0.0815000
-14060.0000000	0.0805000
-14059.0000000	0.0828572
-14058.0000000	0.0810000
-14057.0000000	0.0814286
-14056.0000000	0.0810000
-14055.0000000	0.0815000
-14054.0000000	0.0804762
-14053.0000000	0.0815000
-14052.0000000	0.0810000
-14051.0000000	0.0810000
-14050.0000000	0.0842857
-14049.0000000	0.0845000
-14048.0000000	0.0828571
-14047.0000000	0.0830000

-14046.0000000	0.0810000
-14045.0000000	0.0800000
-14044.0000000	0.0800000
-14043.0000000	0.0815000
-14042.0000000	0.0805000
-14041.0000000	0.0804762
-14040.0000000	0.0820000
-14039.0000000	0.0823810
-14038.0000000	0.0815000
-14037.0000000	0.0825000
-14036.0000000	0.0809524
-14035.0000000	0.0825000
-14034.0000000	0.0830000
-14033.0000000	0.0819048
-14032.0000000	0.0810000
-14031.0000000	0.0805000
-14030.0000000	0.0815000
-14029.0000000	0.0809524
-14028.0000000	0.0815000
-14027.0000000	0.0814286
-14026.0000000	0.0800000
-14025.0000000	0.0825000
-14024.0000000	0.0852381
-14023.0000000	0.0855000
-14022.0000000	0.0880000
-14021.0000000	0.0880000
-14020.0000000	0.0876191
-14019.0000000	0.0900000
-14018.0000000	0.0895238
-14017.0000000	0.0895000
-14016.0000000	0.0885000
-14015.0000000	0.0900000
-14014.0000000	0.0900000
-14013.0000000	0.0900000
-14012.0000000	0.0900000
-14011.0000000	0.0900000
-14010.0000000	0.0900000
-14009.0000000	0.0904762
-14008.0000000	0.0900000
-14007.0000000	0.0900000
-14006.0000000	0.0900000
-14005.0000000	0.0900000
-14004.0000000	0.0900000
-14003.0000000	0.0890000
-14002.0000000	0.0890476
-14001.0000000	0.0870000
-14000.0000000	0.0850000
-13999.0000000	0.0833334
-13998.0000000	0.0835000
-13997.0000000	0.0815000
-13996.0000000	0.0800000
-13995.0000000	0.0805000
-13994.0000000	0.0800000
-13993.0000000	0.0800000
-13992.0000000	0.0800000
-13991.0000000	0.0800000
-13990.0000000	0.0800000

-13989.0000000	0.0810000
-13988.0000000	0.0804762
-13987.0000000	0.0805000
-13986.0000000	0.0800000
-13985.0000000	0.0800000
-13984.0000000	0.0800000
-13983.0000000	0.0800000
-13982.0000000	0.0805000
-13981.0000000	0.0809524
-13980.0000000	0.0805000
-13979.0000000	0.0800000
-13978.0000000	0.0800000
-13977.0000000	0.0805000
-13976.0000000	0.0805000
-13975.0000000	0.0809524
-13974.0000000	0.0820000
-13973.0000000	0.0823810
-13972.0000000	0.0825000
-13971.0000000	0.0810000
-13970.0000000	0.0820000
-13969.0000000	0.0814286
-13968.0000000	0.0825000
-13967.0000000	0.0835000
-13966.0000000	0.0823810
-13965.0000000	0.0835000
-13964.0000000	0.0828572
-13963.0000000	0.0855000
-13962.0000000	0.0840000
-13961.0000000	0.0855000
-13960.0000000	0.0842857
-13959.0000000	0.0835000
-13958.0000000	0.0825000
-13957.0000000	0.0814286
-13956.0000000	0.0820000
-13955.0000000	0.0810000
-13954.0000000	0.0804762
-13953.0000000	0.0805000
-13952.0000000	0.0800000
-13951.0000000	0.0800000
-13950.0000000	0.0800000
-13949.0000000	0.0800000
-13948.0000000	0.0800000
-13947.0000000	0.0805000
-13946.0000000	0.0800000
-13945.0000000	0.0809524
-13944.0000000	0.0800000
-13943.0000000	0.0800000
-13942.0000000	0.0800000
-13941.0000000	0.0805000
-13940.0000000	0.0810000
-13939.0000000	0.0800000
-13938.0000000	0.0810000
-13937.0000000	0.0815000
-13936.0000000	0.0809524
-13935.0000000	0.0800000
-13934.0000000	0.0800000
-13933.0000000	0.0800000

-13932.0000000	0.0800000
-13931.0000000	0.0805000
-13930.0000000	0.0823810
-13929.0000000	0.0825000
-13928.0000000	0.0815000
-13927.0000000	0.0804762
-13926.0000000	0.0820000
-13925.0000000	0.0825000
-13924.0000000	0.0815000
-13923.0000000	0.0819048
-13922.0000000	0.0820000
-13921.0000000	0.0819048
-13920.0000000	0.0835000
-13919.0000000	0.0835000
-13918.0000000	0.0833333
-13917.0000000	0.0815000
-13916.0000000	0.0835000
-13915.0000000	0.0840000
-13914.0000000	0.0819048
-13913.0000000	0.0840000
-13912.0000000	0.0847619
-13911.0000000	0.0855000
-13910.0000000	0.0830000
-13909.0000000	0.0871429
-13908.0000000	0.0885000
-13907.0000000	0.0855000
-13906.0000000	0.0875000
-13905.0000000	0.0890476
-13904.0000000	0.0895238
-13903.0000000	0.0880000
-13902.0000000	0.0890000
-13901.0000000	0.0885000
-13900.0000000	0.0876191
-13899.0000000	0.0875000
-13898.0000000	0.0890000
-13897.0000000	0.0885000
-13896.0000000	0.0876191
-13895.0000000	0.0885000
-13894.0000000	0.0890000
-13893.0000000	0.0885715
-13892.0000000	0.0895000
-13891.0000000	0.0866667
-13890.0000000	0.0860000
-13889.0000000	0.0835000
-13888.0000000	0.0833334
-13887.0000000	0.0865000
-13886.0000000	0.0870000
-13885.0000000	0.0885000
-13884.0000000	0.0890476
-13883.0000000	0.0880000
-13882.0000000	0.0895238
-13881.0000000	0.0895000
-13880.0000000	0.0895000
-13879.0000000	0.0900000
-13878.0000000	0.0900000
-13877.0000000	0.0905000
-13876.0000000	0.0900000

-13875.0000000	0.0900000
-13874.0000000	0.0900000
-13873.0000000	0.0900000
-13872.0000000	0.0900000
-13871.0000000	0.0905000
-13870.0000000	0.0900000
-13869.0000000	0.0900000
-13868.0000000	0.0895000
-13867.0000000	0.0885000
-13866.0000000	0.0895238
-13865.0000000	0.0885000
-13864.0000000	0.0900000
-13863.0000000	0.0895238
-13862.0000000	0.0875000
-13861.0000000	0.0876191
-13860.0000000	0.0880000
-13859.0000000	0.0835000
-13858.0000000	0.0833334
-13857.0000000	0.0875000
-13856.0000000	0.0860000
-13855.0000000	0.0885000
-13854.0000000	0.0871429
-13853.0000000	0.0880000
-13852.0000000	0.0890000
-13851.0000000	0.0876191
-13850.0000000	0.0885000
-13849.0000000	0.0890000
-13848.0000000	0.0885714
-13847.0000000	0.0885000
-13846.0000000	0.0885000
-13845.0000000	0.0890476
-13844.0000000	0.0900000
-13843.0000000	0.0866667
-13842.0000000	0.0845000
-13841.0000000	0.0855000
-13840.0000000	0.0835000
-13839.0000000	0.0819048
-13838.0000000	0.0830000
-13837.0000000	0.0810000
-13836.0000000	0.0804762
-13835.0000000	0.0810000
-13834.0000000	0.0810000
-13833.0000000	0.0814286
-13832.0000000	0.0810000
-13831.0000000	0.0810000
-13830.0000000	0.0819048
-13829.0000000	0.0815000
-13828.0000000	0.0847619
-13827.0000000	0.0845000
-13826.0000000	0.0850000
-13825.0000000	0.0850000
-13824.0000000	0.0852381
-13823.0000000	0.0860000
-13822.0000000	0.0820000
-13821.0000000	0.0842857
-13820.0000000	0.0835000
-13819.0000000	0.0845000

-13818.0000000	0.0819048
-13817.0000000	0.0835000
-13816.0000000	0.0820000
-13815.0000000	0.0814286
-13814.0000000	0.0810000
-13813.0000000	0.0825000
-13812.0000000	0.0819048
-13811.0000000	0.0810000
-13810.0000000	0.0810000
-13809.0000000	0.0823810
-13808.0000000	0.0825000
-13807.0000000	0.0835000
-13806.0000000	0.0842857
-13805.0000000	0.0840000
-13804.0000000	0.0865000
-13803.0000000	0.0857143
-13802.0000000	0.0860000
-13801.0000000	0.0875000
-13800.0000000	0.0876191
-13799.0000000	0.0870000
-13798.0000000	0.0880952
-13797.0000000	0.0890000
-13796.0000000	0.0890000
-13795.0000000	0.0885000
-13794.0000000	0.0890476
-13793.0000000	0.0900000
-13792.0000000	0.0900000
-13791.0000000	0.0904762
-13790.0000000	0.0900000
-13789.0000000	0.0900000
-13788.0000000	0.0900000
-13787.0000000	0.0900000
-13786.0000000	0.0900000
-13785.0000000	0.0900000
-13784.0000000	0.0910000
-13783.0000000	0.0900000
-13782.0000000	0.0900000
-13781.0000000	0.0900000
-13780.0000000	0.0900000
-13779.0000000	0.0895238
-13778.0000000	0.0900000
-13777.0000000	0.0900000
-13776.0000000	0.0895238
-13775.0000000	0.0890000
-13774.0000000	0.0895000
-13773.0000000	0.0880953
-13772.0000000	0.0895000
-13771.0000000	0.0895000
-13770.0000000	0.0880953
-13769.0000000	0.0900000
-13768.0000000	0.0900000
-13767.0000000	0.0890000
-13766.0000000	0.0885000
-13765.0000000	0.0875000
-13764.0000000	0.0871429
-13763.0000000	0.0885000
-13762.0000000	0.0880000

-13761.0000000	0.0866667
-13760.0000000	0.0875000
-13759.0000000	0.0880000
-13758.0000000	0.0880000
-13757.0000000	0.0890476
-13756.0000000	0.0885000
-13755.0000000	0.0871429
-13754.0000000	0.0855000
-13753.0000000	0.0850000
-13752.0000000	0.0871429
-13751.0000000	0.0865000
-13750.0000000	0.0875000
-13749.0000000	0.0875000
-13748.0000000	0.0876191
-13747.0000000	0.0890000
-13746.0000000	0.0895238
-13745.0000000	0.0880000
-13744.0000000	0.0890000
-13743.0000000	0.0900000
-13742.0000000	0.0890000
-13741.0000000	0.0895000
-13740.0000000	0.0890000
-13739.0000000	0.0857143
-13738.0000000	0.0835000
-13737.0000000	0.0842857
-13736.0000000	0.0825000
-13735.0000000	0.0800000
-13734.0000000	0.0804762
-13733.0000000	0.0800000
-13732.0000000	0.0805000
-13731.0000000	0.0800000
-13730.0000000	0.0800000
-13729.0000000	0.0800000
-13728.0000000	0.0800000
-13727.0000000	0.0800000
-13726.0000000	0.0805000
-13725.0000000	0.0819048
-13724.0000000	0.0825000
-13723.0000000	0.0800000
-13722.0000000	0.0809524
-13721.0000000	0.0815000
-13720.0000000	0.0815000
-13719.0000000	0.0805000
-13718.0000000	0.0804762
-13717.0000000	0.0800000
-13716.0000000	0.0800000
-13715.0000000	0.0805000
-13714.0000000	0.0820000
-13713.0000000	0.0819048
-13712.0000000	0.0820000
-13711.0000000	0.0810000
-13710.0000000	0.0810000
-13709.0000000	0.0800000
-13708.0000000	0.0800000
-13707.0000000	0.0800000
-13706.0000000	0.0800000
-13705.0000000	0.0800000

-13704.0000000	0.0800000
-13703.0000000	0.0800000
-13702.0000000	0.0800000
-13701.0000000	0.0800000
-13700.0000000	0.0800000
-13699.0000000	0.0805000
-13698.0000000	0.0810000
-13697.0000000	0.0819048
-13696.0000000	0.0820000
-13695.0000000	0.0828572
-13694.0000000	0.0835000
-13693.0000000	0.0850000
-13692.0000000	0.0814286
-13691.0000000	0.0835000
-13690.0000000	0.0815000
-13689.0000000	0.0855000
-13688.0000000	0.0833333
-13687.0000000	0.0830000
-13686.0000000	0.0833333
-13685.0000000	0.0830000
-13684.0000000	0.0835000
-13683.0000000	0.0830000
-13682.0000000	0.0861905
-13681.0000000	0.0860000
-13680.0000000	0.0890000
-13679.0000000	0.0880953
-13678.0000000	0.0895000
-13677.0000000	0.0895238
-13676.0000000	0.0900000
-13675.0000000	0.0895000
-13674.0000000	0.0895000
-13673.0000000	0.0900000
-13672.0000000	0.0895000
-13671.0000000	0.0890000
-13670.0000000	0.0880953
-13669.0000000	0.0885000
-13668.0000000	0.0895000
-13667.0000000	0.0900000
-13666.0000000	0.0895000
-13665.0000000	0.0895000
-13664.0000000	0.0890476
-13663.0000000	0.0895000
-13662.0000000	0.0900000
-13661.0000000	0.0895000
-13660.0000000	0.0900000
-13659.0000000	0.0895000
-13658.0000000	0.0895238
-13657.0000000	0.0900000
-13656.0000000	0.0895000
-13655.0000000	0.0895238
-13654.0000000	0.0900000
-13653.0000000	0.0900000
-13652.0000000	0.0900000
-13651.0000000	0.0900000
-13650.0000000	0.0900000
-13649.0000000	0.0895238
-13648.0000000	0.0895000

-13647.0000000	0.0900000
-13646.0000000	0.0904762
-13645.0000000	0.0900000
-13644.0000000	0.0900000
-13643.0000000	0.0904762
-13642.0000000	0.0900000
-13641.0000000	0.0910000
-13640.0000000	0.0923810
-13639.0000000	0.0915000
-13638.0000000	0.0930000
-13637.0000000	0.0919048
-13636.0000000	0.0920000
-13635.0000000	0.0920000
-13634.0000000	0.0909524
-13633.0000000	0.0900000
-13632.0000000	0.0904762
-13631.0000000	0.0900000
-13630.0000000	0.0900000
-13629.0000000	0.0885000
-13628.0000000	0.0890476
-13627.0000000	0.0865000
-13626.0000000	0.0875000
-13625.0000000	0.0880953
-13624.0000000	0.0885000
-13623.0000000	0.0885000
-13622.0000000	0.0895000
-13621.0000000	0.0900000
-13620.0000000	0.0900000
-13619.0000000	0.0890476
-13618.0000000	0.0895000
-13617.0000000	0.0890000
-13616.0000000	0.0885714
-13615.0000000	0.0880000
-13614.0000000	0.0875000
-13613.0000000	0.0890476
-13612.0000000	0.0865000
-13611.0000000	0.0840000
-13610.0000000	0.0828572
-13609.0000000	0.0815000
-13608.0000000	0.0825000
-13607.0000000	0.0833333
-13606.0000000	0.0840000
-13605.0000000	0.0835000
-13604.0000000	0.0838095
-13603.0000000	0.0865000
-13602.0000000	0.0876191
-13601.0000000	0.0884211
-13600.0000000	0.0885714
-13599.0000000	0.0895000
-13598.0000000	0.0895238
-13597.0000000	0.0900000
-13596.0000000	0.0900000
-13595.0000000	0.0900000
-13594.0000000	0.0895000
-13593.0000000	0.0900000
-13592.0000000	0.0900000
-13591.0000000	0.0895238

-13590.0000000	0.0885000
-13589.0000000	0.0890476
-13588.0000000	0.0890000
-13587.0000000	0.0855000
-13586.0000000	0.0857143
-13585.0000000	0.0860000
-13584.0000000	0.0860000
-13583.0000000	0.0875000
-13582.0000000	0.0880953
-13581.0000000	0.0890000
-13580.0000000	0.0895238
-13579.0000000	0.0895000
-13578.0000000	0.0890000
-13577.0000000	0.0900000
-13576.0000000	0.0890000
-13575.0000000	0.0900000
-13574.0000000	0.0900000
-13573.0000000	0.0895238
-13572.0000000	0.0895000
-13571.0000000	0.0900000
-13570.0000000	0.0900000
-13569.0000000	0.0900000
-13568.0000000	0.0900000
-13567.0000000	0.0900000
-13566.0000000	0.0900000
-13565.0000000	0.0900000
-13564.0000000	0.0900000
-13563.0000000	0.0900000
-13562.0000000	0.0900000
-13561.0000000	0.0900000
-13560.0000000	0.0900000
-13559.0000000	0.0900000
-13558.0000000	0.0900000
-13557.0000000	0.0900000
-13556.0000000	0.0900000
-13555.0000000	0.0900000
-13554.0000000	0.0900000
-13553.0000000	0.0900000
-13552.0000000	0.0900000
-13551.0000000	0.0900000
-13550.0000000	0.0900000
-13549.0000000	0.0900000
-13548.0000000	0.0900000
-13547.0000000	0.0900000
-13546.0000000	0.0900000
-13545.0000000	0.0900000
-13544.0000000	0.0900000
-13543.0000000	0.0895238
-13542.0000000	0.0900000
-13541.0000000	0.0900000
-13540.0000000	0.0900000
-13539.0000000	0.0900000
-13538.0000000	0.0900000
-13537.0000000	0.0900000
-13536.0000000	0.0900000
-13535.0000000	0.0900000
-13534.0000000	0.0900000

-13533.0000000	0.0900000
-13532.0000000	0.0900000
-13531.0000000	0.0900000
-13530.0000000	0.0900000
-13529.0000000	0.0895238
-13528.0000000	0.0900000
-13527.0000000	0.0900000
-13526.0000000	0.0900000
-13525.0000000	0.0900000
-13524.0000000	0.0900000
-13523.0000000	0.0900000
-13522.0000000	0.0900000
-13521.0000000	0.0900000
-13520.0000000	0.0914286
-13519.0000000	0.0905000
-13518.0000000	0.0900000
-13517.0000000	0.0900000
-13516.0000000	0.0904762
-13515.0000000	0.0910000
-13514.0000000	0.0900000
-13513.0000000	0.0904762
-13512.0000000	0.0905000
-13511.0000000	0.0900000
-13510.0000000	0.0905000
-13509.0000000	0.0900000
-13508.0000000	0.0905000
-13507.0000000	0.0904762
-13506.0000000	0.0900000
-13505.0000000	0.0900000
-13504.0000000	0.0900000
-13503.0000000	0.0900000
-13502.0000000	0.0895000
-13501.0000000	0.0900000
-13500.0000000	0.0900000
-13499.0000000	0.0895000
-13498.0000000	0.0900000
-13497.0000000	0.0895000
-13496.0000000	0.0900000
-13495.0000000	0.0895000
-13494.0000000	0.0900000
-13493.0000000	0.0900000
-13492.0000000	0.0900000
-13491.0000000	0.0900000
-13490.0000000	0.0895000
-13489.0000000	0.0876191
-13488.0000000	0.0860000
-13487.0000000	0.0815000
-13486.0000000	0.0800000
-13485.0000000	0.0805000
-13484.0000000	0.0800000
-13483.0000000	0.0800000
-13482.0000000	0.0805000
-13481.0000000	0.0800000
-13480.0000000	0.0800000
-13479.0000000	0.0810000
-13478.0000000	0.0815000
-13477.0000000	0.0800000

-13476.0000000	0.0810000
-13475.0000000	0.0825000
-13474.0000000	0.0828572
-13473.0000000	0.0880000
-13472.0000000	0.0850000
-13471.0000000	0.0890000
-13470.0000000	0.0876191
-13469.0000000	0.0880000
-13468.0000000	0.0866667
-13467.0000000	0.0880000
-13466.0000000	0.0850000
-13465.0000000	0.0819048
-13464.0000000	0.0800000
-13463.0000000	0.0800000
-13462.0000000	0.0800000
-13461.0000000	0.0800000
-13460.0000000	0.0800000
-13459.0000000	0.0800000
-13458.0000000	0.0800000
-13457.0000000	0.0800000
-13456.0000000	0.0800000
-13455.0000000	0.0800000
-13454.0000000	0.0800000
-13453.0000000	0.0800000
-13452.0000000	0.0800000
-13451.0000000	0.0800000
-13450.0000000	0.0800000
-13449.0000000	0.0800000
-13448.0000000	0.0805000
-13447.0000000	0.0810000
-13446.0000000	0.0819048
-13445.0000000	0.0815000
-13444.0000000	0.0819048
-13443.0000000	0.0835000
-13442.0000000	0.0850000
-13441.0000000	0.0842857
-13440.0000000	0.0840000
-13439.0000000	0.0825000
-13438.0000000	0.0845000
-13437.0000000	0.0857143
-13436.0000000	0.0842857
-13435.0000000	0.0860000
-13434.0000000	0.0830000
-13433.0000000	0.0855000
-13432.0000000	0.0866667
-13431.0000000	0.0860000
-13430.0000000	0.0890000
-13429.0000000	0.0890000
-13428.0000000	0.0890476
-13427.0000000	0.0890000
-13426.0000000	0.0900000
-13425.0000000	0.0900000
-13424.0000000	0.0900000
-13423.0000000	0.0890476
-13422.0000000	0.0890000
-13421.0000000	0.0890000
-13420.0000000	0.0895238

-13419.0000000	0.0895000
-13418.0000000	0.0895000
-13417.0000000	0.0900000
-13416.0000000	0.0900000
-13415.0000000	0.0895000
-13414.0000000	0.0900000
-13413.0000000	0.0895000
-13412.0000000	0.0895000
-13411.0000000	0.0895000
-13410.0000000	0.0895238
-13409.0000000	0.0890000
-13408.0000000	0.0895000
-13407.0000000	0.0890476
-13406.0000000	0.0900000
-13405.0000000	0.0900000
-13404.0000000	0.0900000
-13403.0000000	0.0900000
-13402.0000000	0.0890476
-13401.0000000	0.0900000
-13400.0000000	0.0895000
-13399.0000000	0.0885000
-13398.0000000	0.0900000
-13397.0000000	0.0890000
-13396.0000000	0.0895000
-13395.0000000	0.0900000
-13394.0000000	0.0895000
-13393.0000000	0.0885715
-13392.0000000	0.0900000
-13391.0000000	0.0890000
-13390.0000000	0.0880000
-13389.0000000	0.0866667
-13388.0000000	0.0880000
-13387.0000000	0.0885000
-13386.0000000	0.0880953
-13385.0000000	0.0865000
-13384.0000000	0.0847619
-13383.0000000	0.0835000
-13382.0000000	0.0845000
-13381.0000000	0.0840000
-13380.0000000	0.0828572
-13379.0000000	0.0830000
-13378.0000000	0.0840000
-13377.0000000	0.0857143
-13376.0000000	0.0870000
-13375.0000000	0.0880953
-13374.0000000	0.0875000
-13373.0000000	0.0890000
-13372.0000000	0.0890000
-13371.0000000	0.0895238
-13370.0000000	0.0885000
-13369.0000000	0.0900000
-13368.0000000	0.0900000
-13367.0000000	0.0900000
-13366.0000000	0.0900000
-13365.0000000	0.0900000
-13364.0000000	0.0900000
-13363.0000000	0.0900000

-13362.0000000	0.0900000
-13361.0000000	0.0895000
-13360.0000000	0.0890000
-13359.0000000	0.0900000
-13358.0000000	0.0895000
-13357.0000000	0.0900000
-13356.0000000	0.0900000
-13355.0000000	0.0880000
-13354.0000000	0.0850000
-13353.0000000	0.0838095
-13352.0000000	0.0850000
-13351.0000000	0.0830000
-13350.0000000	0.0861905
-13349.0000000	0.0865000
-13348.0000000	0.0865000
-13347.0000000	0.0871429
-13346.0000000	0.0870000
-13345.0000000	0.0871429
-13344.0000000	0.0870000
-13343.0000000	0.0865000
-13342.0000000	0.0855000
-13341.0000000	0.0847619
-13340.0000000	0.0870000
-13339.0000000	0.0850000
-13338.0000000	0.0842857
-13337.0000000	0.0840000
-13336.0000000	0.0850000
-13335.0000000	0.0820000
-13334.0000000	0.0833334
-13333.0000000	0.0825000
-13332.0000000	0.0828572
-13331.0000000	0.0825000
-13330.0000000	0.0830000
-13329.0000000	0.0838095
-13328.0000000	0.0845000
-13327.0000000	0.0870000
-13326.0000000	0.0857143
-13325.0000000	0.0870000
-13324.0000000	0.0850000
-13323.0000000	0.0857143
-13322.0000000	0.0855000
-13321.0000000	0.0880000
-13320.0000000	0.0871429
-13319.0000000	0.0855000
-13318.0000000	0.0855000
-13317.0000000	0.0871429
-13316.0000000	0.0850000
-13315.0000000	0.0870000
-13314.0000000	0.0833333
-13313.0000000	0.0840000
-13312.0000000	0.0825000
-13311.0000000	0.0835000
-13310.0000000	0.0828572
-13309.0000000	0.0850000
-13308.0000000	0.0809524
-13307.0000000	0.0810000
-13306.0000000	0.0800000

-13305.0000000	0.0800000
-13304.0000000	0.0800000
-13303.0000000	0.0800000
-13302.0000000	0.0800000
-13301.0000000	0.0805000
-13300.0000000	0.0800000
-13299.0000000	0.0800000
-13298.0000000	0.0800000
-13297.0000000	0.0800000
-13296.0000000	0.0804762
-13295.0000000	0.0800000
-13294.0000000	0.0800000
-13293.0000000	0.0800000
-13292.0000000	0.0805000
-13291.0000000	0.0800000
-13290.0000000	0.0800000
-13289.0000000	0.0800000
-13288.0000000	0.0805000
-13287.0000000	0.0800000
-13286.0000000	0.0800000
-13285.0000000	0.0800000
-13284.0000000	0.0800000
-13283.0000000	0.0800000
-13282.0000000	0.0800000
-13281.0000000	0.0800000
-13280.0000000	0.0800000
-13279.0000000	0.0800000
-13278.0000000	0.0804762
-13277.0000000	0.0800000
-13276.0000000	0.0805000
-13275.0000000	0.0805000
-13274.0000000	0.0804762
-13273.0000000	0.0825000
-13272.0000000	0.0825000
-13271.0000000	0.0800000
-13270.0000000	0.0810000
-13269.0000000	0.0804762
-13268.0000000	0.0810000
-13267.0000000	0.0805000
-13266.0000000	0.0833333
-13265.0000000	0.0820000
-13264.0000000	0.0845000
-13263.0000000	0.0845000
-13262.0000000	0.0885715
-13261.0000000	0.0855000
-13260.0000000	0.0855000
-13259.0000000	0.0838095
-13258.0000000	0.0830000
-13257.0000000	0.0814286
-13256.0000000	0.0800000
-13255.0000000	0.0805000
-13254.0000000	0.0800000
-13253.0000000	0.0800000
-13252.0000000	0.0800000
-13251.0000000	0.0800000
-13250.0000000	0.0800000
-13249.0000000	0.0800000

-13248.0000000	0.0814286
-13247.0000000	0.0805000
-13246.0000000	0.0810000
-13245.0000000	0.0800000
-13244.0000000	0.0814286
-13243.0000000	0.0830000
-13242.0000000	0.0835000
-13241.0000000	0.0842857
-13240.0000000	0.0835000
-13239.0000000	0.0857143
-13238.0000000	0.0875000
-13237.0000000	0.0860000
-13236.0000000	0.0880953
-13235.0000000	0.0875000
-13234.0000000	0.0890000
-13233.0000000	0.0890000
-13232.0000000	0.0890476
-13231.0000000	0.0885000
-13230.0000000	0.0885000
-13229.0000000	0.0876191
-13228.0000000	0.0875000
-13227.0000000	0.0861905
-13226.0000000	0.0840000
-13225.0000000	0.0850000
-13224.0000000	0.0855000
-13223.0000000	0.0842857
-13222.0000000	0.0815000
-13221.0000000	0.0805000
-13220.0000000	0.0804762
-13219.0000000	0.0805000
-13218.0000000	0.0800000
-13217.0000000	0.0800000
-13216.0000000	0.0805000
-13215.0000000	0.0800000
-13214.0000000	0.0800000
-13213.0000000	0.0805000
-13212.0000000	0.0800000
-13211.0000000	0.0814286
-13210.0000000	0.0810000
-13209.0000000	0.0804762
-13208.0000000	0.0800000
-13207.0000000	0.0810000
-13206.0000000	0.0800000
-13205.0000000	0.0800000
-13204.0000000	0.0800000
-13203.0000000	0.0800000
-13202.0000000	0.0800000
-13201.0000000	0.0800000
-13200.0000000	0.0800000
-13199.0000000	0.0800000
-13198.0000000	0.0800000
-13197.0000000	0.0800000
-13196.0000000	0.0800000
-13195.0000000	0.0800000
-13194.0000000	0.0800000
-13193.0000000	0.0800000
-13192.0000000	0.0800000

-13191.0000000	0.0800000
-13190.0000000	0.0800000
-13189.0000000	0.0800000
-13188.0000000	0.0800000
-13187.0000000	0.0800000
-13186.0000000	0.0800000
-13185.0000000	0.0800000
-13184.0000000	0.0800000
-13183.0000000	0.0810000
-13182.0000000	0.0805000
-13181.0000000	0.0814286
-13180.0000000	0.0835000
-13179.0000000	0.0838095
-13178.0000000	0.0860000
-13177.0000000	0.0845000
-13176.0000000	0.0855000
-13175.0000000	0.0861905
-13174.0000000	0.0875000
-13173.0000000	0.0850000
-13172.0000000	0.0857143
-13171.0000000	0.0855000
-13170.0000000	0.0840000
-13169.0000000	0.0840000
-13168.0000000	0.0810000
-13167.0000000	0.0819048
-13166.0000000	0.0814286
-13165.0000000	0.0805000
-13164.0000000	0.0810000
-13163.0000000	0.0819048
-13162.0000000	0.0805000
-13161.0000000	0.0805000
-13160.0000000	0.0809524
-13159.0000000	0.0805000
-13158.0000000	0.0800000
-13157.0000000	0.0819048
-13156.0000000	0.0815000
-13155.0000000	0.0815000
-13154.0000000	0.0828572
-13153.0000000	0.0850000
-13152.0000000	0.0865000
-13151.0000000	0.0861905
-13150.0000000	0.0830000
-13149.0000000	0.0825000
-13148.0000000	0.0833333
-13147.0000000	0.0840000
-13146.0000000	0.0840000
-13145.0000000	0.0814286
-13144.0000000	0.0850000
-13143.0000000	0.0840000
-13142.0000000	0.0823810
-13141.0000000	0.0825000
-13140.0000000	0.0845000
-13139.0000000	0.0835000
-13138.0000000	0.0838095
-13137.0000000	0.0830000
-13136.0000000	0.0842857
-13135.0000000	0.0825000

-13134.0000000	0.0830000
-13133.0000000	0.0809524
-13132.0000000	0.0820000
-13131.0000000	0.0815000
-13130.0000000	0.0819048
-13129.0000000	0.0815000
-13128.0000000	0.0815000
-13127.0000000	0.0814286
-13126.0000000	0.0810000
-13125.0000000	0.0805000
-13124.0000000	0.0820000
-13123.0000000	0.0804762
-13122.0000000	0.0820000
-13121.0000000	0.0814286
-13120.0000000	0.0845000
-13119.0000000	0.0860000
-13118.0000000	0.0875000
-13117.0000000	0.0885715
-13116.0000000	0.0895000
-13115.0000000	0.0890000
-13114.0000000	0.0895238
-13113.0000000	0.0900000
-13112.0000000	0.0900000
-13111.0000000	0.0900000
-13110.0000000	0.0900000
-13109.0000000	0.0900000
-13108.0000000	0.0900000
-13107.0000000	0.0900000
-13106.0000000	0.0900000
-13105.0000000	0.0900000
-13104.0000000	0.0900000
-13103.0000000	0.0900000
-13102.0000000	0.0900000
-13101.0000000	0.0900000
-13100.0000000	0.0900000
-13099.0000000	0.0895000
-13098.0000000	0.0880000
-13097.0000000	0.0865000
-13096.0000000	0.0847619
-13095.0000000	0.0835000
-13094.0000000	0.0855000
-13093.0000000	0.0838095
-13092.0000000	0.0860000
-13091.0000000	0.0876191
-13090.0000000	0.0875000
-13089.0000000	0.0895000
-13088.0000000	0.0870000
-13087.0000000	0.0895238
-13086.0000000	0.0870000
-13085.0000000	0.0850000
-13084.0000000	0.0838095
-13083.0000000	0.0815000
-13082.0000000	0.0814286
-13081.0000000	0.0810000
-13080.0000000	0.0800000
-13079.0000000	0.0805000
-13078.0000000	0.0800000

-13077.0000000	0.0800000
-13076.0000000	0.0810000
-13075.0000000	0.0800000
-13074.0000000	0.0800000
-13073.0000000	0.0800000
-13072.0000000	0.0800000
-13071.0000000	0.0800000
-13070.0000000	0.0800000
-13069.0000000	0.0800000
-13068.0000000	0.0800000
-13067.0000000	0.0800000
-13066.0000000	0.0800000
-13065.0000000	0.0800000
-13064.0000000	0.0800000
-13063.0000000	0.0800000
-13062.0000000	0.0814286
-13061.0000000	0.0800000
-13060.0000000	0.0800000
-13059.0000000	0.0805000
-13058.0000000	0.0805000
-13057.0000000	0.0800000
-13056.0000000	0.0800000
-13055.0000000	0.0800000
-13054.0000000	0.0800000
-13053.0000000	0.0800000
-13052.0000000	0.0800000
-13051.0000000	0.0815000
-13050.0000000	0.0820000
-13049.0000000	0.0810000
-13048.0000000	0.0800000
-13047.0000000	0.0800000
-13046.0000000	0.0800000
-13045.0000000	0.0800000
-13044.0000000	0.0800000
-13043.0000000	0.0800000
-13042.0000000	0.0800000
-13041.0000000	0.0800000
-13040.0000000	0.0800000
-13039.0000000	0.0800000
-13038.0000000	0.0800000
-13037.0000000	0.0800000
-13036.0000000	0.0800000
-13035.0000000	0.0800000
-13034.0000000	0.0800000
-13033.0000000	0.0805000
-13032.0000000	0.0800000
-13031.0000000	0.0800000
-13030.0000000	0.0804762
-13029.0000000	0.0810000
-13028.0000000	0.0815000
-13027.0000000	0.0814286
-13026.0000000	0.0820000
-13025.0000000	0.0820000
-13024.0000000	0.0810000
-13023.0000000	0.0814286
-13022.0000000	0.0840000
-13021.0000000	0.0847619

-13020.0000000	0.0860000
-13019.0000000	0.0885000
-13018.0000000	0.0880953
-13017.0000000	0.0880000
-13016.0000000	0.0890000
-13015.0000000	0.0890476
-13014.0000000	0.0890000
-13013.0000000	0.0865000
-13012.0000000	0.0876191
-13011.0000000	0.0850000
-13010.0000000	0.0865000
-13009.0000000	0.0871429
-13008.0000000	0.0890000
-13007.0000000	0.0895000
-13006.0000000	0.0900000
-13005.0000000	0.0900000
-13004.0000000	0.0900000
-13003.0000000	0.0900000
-13002.0000000	0.0900000
-13001.0000000	0.0900000
-13000.0000000	0.0900000
-12999.0000000	0.0895000
-12998.0000000	0.0900000
-12997.0000000	0.0900000
-12996.0000000	0.0900000
-12995.0000000	0.0895000
-12994.0000000	0.0895000
-12993.0000000	0.0895238
-12992.0000000	0.0895000
-12991.0000000	0.0885714
-12990.0000000	0.0885000
-12989.0000000	0.0880000
-12988.0000000	0.0870000
-12987.0000000	0.0852381
-12986.0000000	0.0860000
-12985.0000000	0.0871429
-12984.0000000	0.0885000
-12983.0000000	0.0865000
-12982.0000000	0.0885715
-12981.0000000	0.0875000
-12980.0000000	0.0865000
-12979.0000000	0.0866667
-12978.0000000	0.0865000
-12977.0000000	0.0845000
-12976.0000000	0.0809524
-12975.0000000	0.0825000
-12974.0000000	0.0840000
-12973.0000000	0.0845000
-12972.0000000	0.0838095
-12971.0000000	0.0830000
-12970.0000000	0.0823810
-12969.0000000	0.0820000
-12968.0000000	0.0805000
-12967.0000000	0.0800000
-12966.0000000	0.0800000
-12965.0000000	0.0800000
-12964.0000000	0.0795000

-12963.0000000	0.0785714
-12962.0000000	0.0785000
-12961.0000000	0.0780000
-12960.0000000	0.0800000
-12959.0000000	0.0795000
-12958.0000000	0.0790000
-12957.0000000	0.0771429
-12956.0000000	0.0795000
-12955.0000000	0.0785714
-12954.0000000	0.0795000
-12953.0000000	0.0790000
-12952.0000000	0.0800000
-12951.0000000	0.0795000
-12950.0000000	0.0795000
-12949.0000000	0.0800000
-12948.0000000	0.0800000
-12947.0000000	0.0800000
-12946.0000000	0.0800000
-12945.0000000	0.0800000
-12944.0000000	0.0800000
-12943.0000000	0.0800000
-12942.0000000	0.0800000
-12941.0000000	0.0800000
-12940.0000000	0.0795000
-12939.0000000	0.0800000
-12938.0000000	0.0800000
-12937.0000000	0.0800000
-12936.0000000	0.0800000
-12935.0000000	0.0800000
-12934.0000000	0.0800000
-12933.0000000	0.0800000
-12932.0000000	0.0800000
-12931.0000000	0.0800000
-12930.0000000	0.0800000
-12929.0000000	0.0800000
-12928.0000000	0.0805000
-12927.0000000	0.0800000
-12926.0000000	0.0800000
-12925.0000000	0.0809524
-12924.0000000	0.0800000
-12923.0000000	0.0800000
-12922.0000000	0.0810000
-12921.0000000	0.0800000
-12920.0000000	0.0805000
-12919.0000000	0.0810000
-12918.0000000	0.0823810
-12917.0000000	0.0815000
-12916.0000000	0.0814286
-12915.0000000	0.0800000
-12914.0000000	0.0810000
-12913.0000000	0.0800000
-12912.0000000	0.0804762
-12911.0000000	0.0800000
-12910.0000000	0.0800000
-12909.0000000	0.0809524
-12908.0000000	0.0805000
-12907.0000000	0.0800000

-12906.0000000	0.0800000
-12905.0000000	0.0805000
-12904.0000000	0.0805000
-12903.0000000	0.0800000
-12902.0000000	0.0815000
-12901.0000000	0.0815000
-12900.0000000	0.0823810
-12899.0000000	0.0835000
-12898.0000000	0.0845000
-12897.0000000	0.0820000
-12896.0000000	0.0833333
-12895.0000000	0.0842857
-12894.0000000	0.0860000
-12893.0000000	0.0855000
-12892.0000000	0.0850000
-12891.0000000	0.0871429
-12890.0000000	0.0885000
-12889.0000000	0.0885000
-12888.0000000	0.0890000
-12887.0000000	0.0895238
-12886.0000000	0.0890476
-12885.0000000	0.0890000
-12884.0000000	0.0895000
-12883.0000000	0.0900000
-12882.0000000	0.0900000
-12881.0000000	0.0895000
-12880.0000000	0.0890000
-12879.0000000	0.0890476
-12878.0000000	0.0885000
-12877.0000000	0.0880953
-12876.0000000	0.0870000
-12875.0000000	0.0865000
-12874.0000000	0.0865000
-12873.0000000	0.0852381
-12872.0000000	0.0855000
-12871.0000000	0.0870000
-12870.0000000	0.0842857
-12869.0000000	0.0860000
-12868.0000000	0.0835000
-12867.0000000	0.0850000
-12866.0000000	0.0866667
-12865.0000000	0.0870000
-12864.0000000	0.0880953
-12863.0000000	0.0895000
-12862.0000000	0.0900000
-12861.0000000	0.0900000
-12860.0000000	0.0900000
-12859.0000000	0.0900000
-12858.0000000	0.0895000
-12857.0000000	0.0900000
-12856.0000000	0.0900000
-12855.0000000	0.0900000
-12854.0000000	0.0900000
-12853.0000000	0.0900000
-12852.0000000	0.0900000
-12851.0000000	0.0895000
-12850.0000000	0.0905000

-12849.0000000	0.0900000
-12848.0000000	0.0900000
-12847.0000000	0.0905000
-12846.0000000	0.0900000
-12845.0000000	0.0900000
-12844.0000000	0.0900000
-12843.0000000	0.0900000
-12842.0000000	0.0900000
-12841.0000000	0.0900000
-12840.0000000	0.0895238
-12839.0000000	0.0900000
-12838.0000000	0.0900000
-12837.0000000	0.0895000
-12836.0000000	0.0895238
-12835.0000000	0.0890000
-12834.0000000	0.0900000
-12833.0000000	0.0900000
-12832.0000000	0.0895000
-12831.0000000	0.0900000
-12830.0000000	0.0900000
-12829.0000000	0.0900000
-12828.0000000	0.0900000
-12827.0000000	0.0890476
-12826.0000000	0.0895000
-12825.0000000	0.0895238
-12824.0000000	0.0895000
-12823.0000000	0.0890000
-12822.0000000	0.0890000
-12821.0000000	0.0900000
-12820.0000000	0.0900000
-12819.0000000	0.0895238
-12818.0000000	0.0900000
-12817.0000000	0.0900000
-12816.0000000	0.0900000
-12815.0000000	0.0900000
-12814.0000000	0.0900000
-12813.0000000	0.0900000
-12812.0000000	0.0909524
-12811.0000000	0.0900000
-12810.0000000	0.0904762
-12809.0000000	0.0900000
-12808.0000000	0.0900000
-12807.0000000	0.0900000
-12806.0000000	0.0900000
-12805.0000000	0.0909524
-12804.0000000	0.0904762
-12803.0000000	0.0910000
-12802.0000000	0.0915000
-12801.0000000	0.0900000
-12800.0000000	0.0900000
-12799.0000000	0.0900000
-12798.0000000	0.0900000
-12797.0000000	0.0900000
-12796.0000000	0.0900000
-12795.0000000	0.0900000
-12794.0000000	0.0895000
-12793.0000000	0.0880000

-12792.0000000	0.0890000
-12791.0000000	0.0885715
-12790.0000000	0.0895000
-12789.0000000	0.0866667
-12788.0000000	0.0870000
-12787.0000000	0.0865000
-12786.0000000	0.0847619
-12785.0000000	0.0860000
-12784.0000000	0.0835000
-12783.0000000	0.0860000
-12782.0000000	0.0847619
-12781.0000000	0.0860000
-12780.0000000	0.0852381
-12779.0000000	0.0875000
-12778.0000000	0.0865000
-12777.0000000	0.0890000
-12776.0000000	0.0876191
-12775.0000000	0.0870000
-12774.0000000	0.0865000
-12773.0000000	0.0847619
-12772.0000000	0.0855000
-12771.0000000	0.0885715
-12770.0000000	0.0880000
-12769.0000000	0.0880000
-12768.0000000	0.0850000
-12767.0000000	0.0828572
-12766.0000000	0.0830000
-12765.0000000	0.0825000
-12764.0000000	0.0838095
-12763.0000000	0.0835000
-12762.0000000	0.0840000
-12761.0000000	0.0823810
-12760.0000000	0.0810000
-12759.0000000	0.0809524
-12758.0000000	0.0810000
-12757.0000000	0.0800000
-12756.0000000	0.0800000
-12755.0000000	0.0800000
-12754.0000000	0.0805000
-12753.0000000	0.0815000
-12752.0000000	0.0809524
-12751.0000000	0.0810000
-12750.0000000	0.0809524
-12749.0000000	0.0800000
-12748.0000000	0.0815000
-12747.0000000	0.0815000
-12746.0000000	0.0828572
-12745.0000000	0.0830000
-12744.0000000	0.0805000
-12743.0000000	0.0823810
-12742.0000000	0.0820000
-12741.0000000	0.0823810
-12740.0000000	0.0820000
-12739.0000000	0.0810000
-12738.0000000	0.0805000
-12737.0000000	0.0804762
-12736.0000000	0.0830000

-12735.0000000	0.0800000
-12734.0000000	0.0800000
-12733.0000000	0.0800000
-12732.0000000	0.0800000
-12731.0000000	0.0800000
-12730.0000000	0.0804762
-12729.0000000	0.0805000
-12728.0000000	0.0800000
-12727.0000000	0.0800000
-12726.0000000	0.0800000
-12725.0000000	0.0814286
-12724.0000000	0.0805000
-12723.0000000	0.0800000
-12722.0000000	0.0805000
-12721.0000000	0.0804762
-12720.0000000	0.0800000
-12719.0000000	0.0800000
-12718.0000000	0.0800000
-12717.0000000	0.0800000
-12716.0000000	0.0800000
-12715.0000000	0.0800000
-12714.0000000	0.0800000
-12713.0000000	0.0800000
-12712.0000000	0.0800000
-12711.0000000	0.0800000
-12710.0000000	0.0800000
-12709.0000000	0.0800000
-12708.0000000	0.0800000
-12707.0000000	0.0800000
-12706.0000000	0.0795000
-12705.0000000	0.0800000
-12704.0000000	0.0800000
-12703.0000000	0.0800000
-12702.0000000	0.0800000
-12701.0000000	0.0800000
-12700.0000000	0.0809524
-12699.0000000	0.0805000
-12698.0000000	0.0800000
-12697.0000000	0.0800000
-12696.0000000	0.0815000
-12695.0000000	0.0814286
-12694.0000000	0.0855000
-12693.0000000	0.0865000
-12692.0000000	0.0870000
-12691.0000000	0.0871429
-12690.0000000	0.0865000
-12689.0000000	0.0866667
-12688.0000000	0.0875000
-12687.0000000	0.0865000
-12686.0000000	0.0835000
-12685.0000000	0.0842857
-12684.0000000	0.0825000
-12683.0000000	0.0819048
-12682.0000000	0.0830000
-12681.0000000	0.0820000
-12680.0000000	0.0809524
-12679.0000000	0.0815000

-12678.0000000	0.0805000
-12677.0000000	0.0809524
-12676.0000000	0.0800000
-12675.0000000	0.0805000
-12674.0000000	0.0800000
-12673.0000000	0.0800000
-12672.0000000	0.0800000
-12671.0000000	0.0800000
-12670.0000000	0.0800000
-12669.0000000	0.0800000
-12668.0000000	0.0800000
-12667.0000000	0.0800000
-12666.0000000	0.0800000
-12665.0000000	0.0800000
-12664.0000000	0.0800000
-12663.0000000	0.0800000
-12662.0000000	0.0800000
-12661.0000000	0.0800000
-12660.0000000	0.0800000
-12659.0000000	0.0795238
-12658.0000000	0.0800000
-12657.0000000	0.0790000
-12656.0000000	0.0795000
-12655.0000000	0.0795238
-12654.0000000	0.0800000
-12653.0000000	0.0800000
-12652.0000000	0.0800000
-12651.0000000	0.0800000
-12650.0000000	0.0800000
-12649.0000000	0.0800000
-12648.0000000	0.0800000
-12647.0000000	0.0800000
-12646.0000000	0.0800000
-12645.0000000	0.0805000
-12644.0000000	0.0804762
-12643.0000000	0.0800000
-12642.0000000	0.0800000
-12641.0000000	0.0810000
-12640.0000000	0.0814286
-12639.0000000	0.0810000
-12638.0000000	0.0845000
-12637.0000000	0.0833333
-12636.0000000	0.0850000
-12635.0000000	0.0823810
-12634.0000000	0.0845000
-12633.0000000	0.0865000
-12632.0000000	0.0895000
-12631.0000000	0.0880953
-12630.0000000	0.0885000
-12629.0000000	0.0861905
-12628.0000000	0.0850000
-12627.0000000	0.0845000
-12626.0000000	0.0810000
-12625.0000000	0.0800000
-12624.0000000	0.0800000
-12623.0000000	0.0800000
-12622.0000000	0.0800000

-12621.0000000	0.0800000
-12620.0000000	0.0800000
-12619.0000000	0.0800000
-12618.0000000	0.0800000
-12617.0000000	0.0800000
-12616.0000000	0.0800000
-12615.0000000	0.0800000
-12614.0000000	0.0800000
-12613.0000000	0.0800000
-12612.0000000	0.0800000
-12611.0000000	0.0800000
-12610.0000000	0.0800000
-12609.0000000	0.0800000
-12608.0000000	0.0800000
-12607.0000000	0.0804762
-12606.0000000	0.0800000
-12605.0000000	0.0804762
-12604.0000000	0.0805000
-12603.0000000	0.0810000
-12602.0000000	0.0805000
-12601.0000000	0.0814286
-12600.0000000	0.0840000
-12599.0000000	0.0820000
-12598.0000000	0.0847619
-12597.0000000	0.0845000
-12596.0000000	0.0845000
-12595.0000000	0.0857143
-12594.0000000	0.0855000
-12593.0000000	0.0842857
-12592.0000000	0.0855000
-12591.0000000	0.0865000
-12590.0000000	0.0865000
-12589.0000000	0.0852381
-12588.0000000	0.0850000
-12587.0000000	0.0840000
-12586.0000000	0.0852381
-12585.0000000	0.0810000
-12584.0000000	0.0809524
-12583.0000000	0.0810000
-12582.0000000	0.0800000
-12581.0000000	0.0800000
-12580.0000000	0.0800000
-12579.0000000	0.0800000
-12578.0000000	0.0800000
-12577.0000000	0.0800000
-12576.0000000	0.0800000
-12575.0000000	0.0804762
-12574.0000000	0.0800000
-12573.0000000	0.0800000
-12572.0000000	0.0800000
-12571.0000000	0.0800000
-12570.0000000	0.0800000
-12569.0000000	0.0800000
-12568.0000000	0.0800000
-12567.0000000	0.0800000
-12566.0000000	0.0800000
-12565.0000000	0.0800000

-12564.0000000	0.0800000
-12563.0000000	0.0800000
-12562.0000000	0.0795238
-12561.0000000	0.0800000
-12560.0000000	0.0800000
-12559.0000000	0.0800000
-12558.0000000	0.0790000
-12557.0000000	0.0800000
-12556.0000000	0.0800000
-12555.0000000	0.0800000
-12554.0000000	0.0800000
-12553.0000000	0.0800000
-12552.0000000	0.0800000
-12551.0000000	0.0795000
-12550.0000000	0.0800000
-12549.0000000	0.0800000
-12548.0000000	0.0800000
-12547.0000000	0.0800000
-12546.0000000	0.0800000
-12545.0000000	0.0800000
-12544.0000000	0.0795000
-12543.0000000	0.0800000
-12542.0000000	0.0800000
-12541.0000000	0.0795238
-12540.0000000	0.0800000
-12539.0000000	0.0795000
-12538.0000000	0.0800000
-12537.0000000	0.0800000
-12536.0000000	0.0805000
-12535.0000000	0.0800000
-12534.0000000	0.0809524
-12533.0000000	0.0800000
-12532.0000000	0.0828572
-12531.0000000	0.0810000
-12530.0000000	0.0810000
-12529.0000000	0.0800000
-12528.0000000	0.0810000
-12527.0000000	0.0800000
-12526.0000000	0.0800000
-12525.0000000	0.0800000
-12524.0000000	0.0800000
-12523.0000000	0.0800000
-12522.0000000	0.0800000
-12521.0000000	0.0800000
-12520.0000000	0.0800000
-12519.0000000	0.0800000
-12518.0000000	0.0800000
-12517.0000000	0.0805000
-12516.0000000	0.0800000
-12515.0000000	0.0800000
-12514.0000000	0.0800000
-12513.0000000	0.0800000
-12512.0000000	0.0800000
-12511.0000000	0.0800000
-12510.0000000	0.0805000
-12509.0000000	0.0810000
-12508.0000000	0.0833333

-12507.0000000	0.0825000
-12506.0000000	0.0835000
-12505.0000000	0.0845000
-12504.0000000	0.0876191
-12503.0000000	0.0875000
-12502.0000000	0.0895000
-12501.0000000	0.0847619
-12500.0000000	0.0820000
-12499.0000000	0.0800000
-12498.0000000	0.0810000
-12497.0000000	0.0810000
-12496.0000000	0.0805000
-12495.0000000	0.0800000
-12494.0000000	0.0800000
-12493.0000000	0.0800000
-12492.0000000	0.0800000
-12491.0000000	0.0800000
-12490.0000000	0.0800000
-12489.0000000	0.0800000
-12488.0000000	0.0805000
-12487.0000000	0.0805000
-12486.0000000	0.0804762
-12485.0000000	0.0805000
-12484.0000000	0.0823810
-12483.0000000	0.0855000
-12482.0000000	0.0840000
-12481.0000000	0.0875000
-12480.0000000	0.0871429
-12479.0000000	0.0860000
-12478.0000000	0.0895238
-12477.0000000	0.0900000
-12476.0000000	0.0895000
-12475.0000000	0.0890000
-12474.0000000	0.0895238
-12473.0000000	0.0895000
-12472.0000000	0.0865000
-12471.0000000	0.0885714
-12470.0000000	0.0895000
-12469.0000000	0.0880953
-12468.0000000	0.0895000
-12467.0000000	0.0880000
-12466.0000000	0.0870000
-12465.0000000	0.0890476
-12464.0000000	0.0885000
-12463.0000000	0.0900000
-12462.0000000	0.0890476
-12461.0000000	0.0900000
-12460.0000000	0.0895000
-12459.0000000	0.0900000
-12458.0000000	0.0890000
-12457.0000000	0.0880000
-12456.0000000	0.0900000
-12455.0000000	0.0890000
-12454.0000000	0.0871429
-12453.0000000	0.0840000
-12452.0000000	0.0810000
-12451.0000000	0.0800000

-12450.0000000	0.0800000
-12449.0000000	0.0800000
-12448.0000000	0.0800000
-12447.0000000	0.0800000
-12446.0000000	0.0800000
-12445.0000000	0.0800000
-12444.0000000	0.0800000
-12443.0000000	0.0800000
-12442.0000000	0.0800000
-12441.0000000	0.0800000
-12440.0000000	0.0800000
-12439.0000000	0.0795238
-12438.0000000	0.0800000
-12437.0000000	0.0800000
-12436.0000000	0.0800000
-12435.0000000	0.0800000
-12434.0000000	0.0800000
-12433.0000000	0.0800000
-12432.0000000	0.0800000
-12431.0000000	0.0800000
-12430.0000000	0.0800000
-12429.0000000	0.0800000
-12428.0000000	0.0800000
-12427.0000000	0.0800000
-12426.0000000	0.0800000
-12425.0000000	0.0800000
-12424.0000000	0.0805000
-12423.0000000	0.0800000
-12422.0000000	0.0820000
-12421.0000000	0.0810000
-12420.0000000	0.0819048
-12419.0000000	0.0815000
-12418.0000000	0.0823810
-12417.0000000	0.0825000
-12416.0000000	0.0830000
-12415.0000000	0.0855000
-12414.0000000	0.0885714
-12413.0000000	0.0885000
-12412.0000000	0.0880000
-12411.0000000	0.0890476
-12410.0000000	0.0890000
-12409.0000000	0.0885714
-12408.0000000	0.0890000
-12407.0000000	0.0885000
-12406.0000000	0.0875000
-12405.0000000	0.0880953
-12404.0000000	0.0895000
-12403.0000000	0.0885000
-12402.0000000	0.0890476
-12401.0000000	0.0895000
-12400.0000000	0.0885000
-12399.0000000	0.0885000
-12398.0000000	0.0885714
-12397.0000000	0.0875000
-12396.0000000	0.0895238
-12395.0000000	0.0895000
-12394.0000000	0.0890000

-12393.0000000	0.0900000
-12392.0000000	0.0895000
-12391.0000000	0.0900000
-12390.0000000	0.0895000
-12389.0000000	0.0900000
-12388.0000000	0.0895238
-12387.0000000	0.0900000
-12386.0000000	0.0900000
-12385.0000000	0.0900000
-12384.0000000	0.0900000
-12383.0000000	0.0900000
-12382.0000000	0.0900000
-12381.0000000	0.0895000
-12380.0000000	0.0895238
-12379.0000000	0.0900000
-12378.0000000	0.0900000
-12377.0000000	0.0900000
-12376.0000000	0.0900000
-12375.0000000	0.0895238
-12374.0000000	0.0900000
-12373.0000000	0.0900000
-12372.0000000	0.0895238
-12371.0000000	0.0895000
-12370.0000000	0.0900000
-12369.0000000	0.0900000
-12368.0000000	0.0895238
-12367.0000000	0.0900000
-12366.0000000	0.0876191
-12365.0000000	0.0865000
-12364.0000000	0.0835000
-12363.0000000	0.0857143
-12362.0000000	0.0865000
-12361.0000000	0.0870000
-12360.0000000	0.0880000
-12359.0000000	0.0871429
-12358.0000000	0.0855000
-12357.0000000	0.0838095
-12356.0000000	0.0815000
-12355.0000000	0.0820000
-12354.0000000	0.0828572
-12353.0000000	0.0815000
-12352.0000000	0.0825000
-12351.0000000	0.0825000
-12350.0000000	0.0814286
-12349.0000000	0.0820000
-12348.0000000	0.0800000
-12347.0000000	0.0805000
-12346.0000000	0.0800000
-12345.0000000	0.0804762
-12344.0000000	0.0805000
-12343.0000000	0.0800000
-12342.0000000	0.0800000
-12341.0000000	0.0800000
-12340.0000000	0.0800000
-12339.0000000	0.0800000
-12338.0000000	0.0800000
-12337.0000000	0.0800000

-12336.0000000	0.0800000
-12335.0000000	0.0800000
-12334.0000000	0.0800000
-12333.0000000	0.0809524
-12332.0000000	0.0840000
-12331.0000000	0.0850000
-12330.0000000	0.0845000
-12329.0000000	0.0852381
-12328.0000000	0.0860000
-12327.0000000	0.0857143
-12326.0000000	0.0870000
-12325.0000000	0.0895000
-12324.0000000	0.0885000
-12323.0000000	0.0880953
-12322.0000000	0.0885000
-12321.0000000	0.0890000
-12320.0000000	0.0880953
-12319.0000000	0.0870000
-12318.0000000	0.0890476
-12317.0000000	0.0840000
-12316.0000000	0.0870000
-12315.0000000	0.0865000
-12314.0000000	0.0833333
-12313.0000000	0.0855000
-12312.0000000	0.0847619
-12311.0000000	0.0860000
-12310.0000000	0.0875000
-12309.0000000	0.0880000
-12308.0000000	0.0880953
-12307.0000000	0.0900000
-12306.0000000	0.0900000
-12305.0000000	0.0895238
-12304.0000000	0.0900000
-12303.0000000	0.0900000
-12302.0000000	0.0890000
-12301.0000000	0.0890000
-12300.0000000	0.0885000
-12299.0000000	0.0885000
-12298.0000000	0.0876191
-12297.0000000	0.0880000
-12296.0000000	0.0871429
-12295.0000000	0.0870000
-12294.0000000	0.0855000
-12293.0000000	0.0857143
-12292.0000000	0.0835000
-12291.0000000	0.0840000
-12290.0000000	0.0842857
-12289.0000000	0.0825000
-12288.0000000	0.0825000
-12287.0000000	0.0828572
-12286.0000000	0.0810000
-12285.0000000	0.0815000
-12284.0000000	0.0828572
-12283.0000000	0.0810000
-12282.0000000	0.0814286
-12281.0000000	0.0800000
-12280.0000000	0.0810000

-12279.0000000	0.0820000
-12278.0000000	0.0814286
-12277.0000000	0.0815000
-12276.0000000	0.0815000
-12275.0000000	0.0819048
-12274.0000000	0.0815000
-12273.0000000	0.0819048
-12272.0000000	0.0830000
-12271.0000000	0.0820000
-12270.0000000	0.0840000
-12269.0000000	0.0857143
-12268.0000000	0.0860000
-12267.0000000	0.0850000
-12266.0000000	0.0866667
-12265.0000000	0.0865000
-12264.0000000	0.0870000
-12263.0000000	0.0845000
-12262.0000000	0.0847619
-12261.0000000	0.0865000
-12260.0000000	0.0857143
-12259.0000000	0.0870000
-12258.0000000	0.0875000
-12257.0000000	0.0871429
-12256.0000000	0.0900000
-12255.0000000	0.0900000
-12254.0000000	0.0890476
-12253.0000000	0.0880000
-12252.0000000	0.0875000
-12251.0000000	0.0890476
-12250.0000000	0.0900000
-12249.0000000	0.0900000
-12248.0000000	0.0895238
-12247.0000000	0.0900000
-12246.0000000	0.0885000
-12245.0000000	0.0885715
-12244.0000000	0.0890000
-12243.0000000	0.0890476
-12242.0000000	0.0855000
-12241.0000000	0.0825000
-12240.0000000	0.0825000
-12239.0000000	0.0828572
-12238.0000000	0.0845000
-12237.0000000	0.0845000
-12236.0000000	0.0842857
-12235.0000000	0.0860000
-12234.0000000	0.0860000
-12233.0000000	0.0880000
-12232.0000000	0.0876191
-12231.0000000	0.0885000
-12230.0000000	0.0895238
-12229.0000000	0.0890000
-12228.0000000	0.0880000
-12227.0000000	0.0890476
-12226.0000000	0.0890000
-12225.0000000	0.0895000
-12224.0000000	0.0900000
-12223.0000000	0.0885714

-12222.0000000	0.0885000
-12221.0000000	0.0890476
-12220.0000000	0.0890000
-12219.0000000	0.0885000
-12218.0000000	0.0885715
-12217.0000000	0.0880000
-12216.0000000	0.0870000
-12215.0000000	0.0875000
-12214.0000000	0.0880953
-12213.0000000	0.0850000
-12212.0000000	0.0861905
-12211.0000000	0.0860000
-12210.0000000	0.0855000
-12209.0000000	0.0833333
-12208.0000000	0.0835000
-12207.0000000	0.0850000
-12206.0000000	0.0842857
-12205.0000000	0.0855000
-12204.0000000	0.0850000
-12203.0000000	0.0855000
-12202.0000000	0.0857143
-12201.0000000	0.0875000
-12200.0000000	0.0852381
-12199.0000000	0.0855000
-12198.0000000	0.0845000
-12197.0000000	0.0828572
-12196.0000000	0.0830000
-12195.0000000	0.0815000
-12194.0000000	0.0800000
-12193.0000000	0.0804762
-12192.0000000	0.0800000
-12191.0000000	0.0800000
-12190.0000000	0.0810000
-12189.0000000	0.0805000
-12188.0000000	0.0805000
-12187.0000000	0.0809524
-12186.0000000	0.0815000
-12185.0000000	0.0810000
-12184.0000000	0.0800000
-12183.0000000	0.0805000
-12182.0000000	0.0800000
-12181.0000000	0.0815000
-12180.0000000	0.0810000
-12179.0000000	0.0823810
-12178.0000000	0.0815000
-12177.0000000	0.0820000
-12176.0000000	0.0815000
-12175.0000000	0.0842857
-12174.0000000	0.0815000
-12173.0000000	0.0810000
-12172.0000000	0.0814286
-12171.0000000	0.0810000
-12170.0000000	0.0809524
-12169.0000000	0.0805000
-12168.0000000	0.0805000
-12167.0000000	0.0809524
-12166.0000000	0.0805000

-12165.0000000	0.0800000
-12164.0000000	0.0800000
-12163.0000000	0.0800000
-12162.0000000	0.0800000
-12161.0000000	0.0800000
-12160.0000000	0.0800000
-12159.0000000	0.0800000
-12158.0000000	0.0800000
-12157.0000000	0.0800000
-12156.0000000	0.0800000
-12155.0000000	0.0800000
-12154.0000000	0.0800000
-12153.0000000	0.0800000
-12152.0000000	0.0800000
-12151.0000000	0.0800000
-12150.0000000	0.0805000
-12149.0000000	0.0800000
-12148.0000000	0.0800000
-12147.0000000	0.0805000
-12146.0000000	0.0800000
-12145.0000000	0.0800000
-12144.0000000	0.0800000
-12143.0000000	0.0800000
-12142.0000000	0.0804762
-12141.0000000	0.0805000
-12140.0000000	0.0800000
-12139.0000000	0.0800000
-12138.0000000	0.0810000
-12137.0000000	0.0823810
-12136.0000000	0.0810000
-12135.0000000	0.0825000
-12134.0000000	0.0815000
-12133.0000000	0.0838095
-12132.0000000	0.0835000
-12131.0000000	0.0855000
-12130.0000000	0.0857143
-12129.0000000	0.0850000
-12128.0000000	0.0850000
-12127.0000000	0.0866667
-12126.0000000	0.0855000
-12125.0000000	0.0870000
-12124.0000000	0.0866667
-12123.0000000	0.0880000
-12122.0000000	0.0895000
-12121.0000000	0.0895238
-12120.0000000	0.0890000
-12119.0000000	0.0900000
-12118.0000000	0.0895238
-12117.0000000	0.0895000
-12116.0000000	0.0890000
-12115.0000000	0.0895238
-12114.0000000	0.0890000
-12113.0000000	0.0885000
-12112.0000000	0.0876191
-12111.0000000	0.0895000
-12110.0000000	0.0895000
-12109.0000000	0.0885715

-12108.0000000	0.0890000
-12107.0000000	0.0880953
-12106.0000000	0.0885000
-12105.0000000	0.0885000
-12104.0000000	0.0895000
-12103.0000000	0.0895238
-12102.0000000	0.0880000
-12101.0000000	0.0890000
-12100.0000000	0.0876191
-12099.0000000	0.0885000
-12098.0000000	0.0890000
-12097.0000000	0.0895000
-12096.0000000	0.0890476
-12095.0000000	0.0885000
-12094.0000000	0.0890476
-12093.0000000	0.0895000
-12092.0000000	0.0880000
-12091.0000000	0.0885715
-12090.0000000	0.0870000
-12089.0000000	0.0885000
-12088.0000000	0.0885714
-12087.0000000	0.0880000
-12086.0000000	0.0865000
-12085.0000000	0.0847619
-12084.0000000	0.0860000
-12083.0000000	0.0855000
-12082.0000000	0.0833334
-12081.0000000	0.0820000
-12080.0000000	0.0820000
-12079.0000000	0.0800000
-12078.0000000	0.0805000
-12077.0000000	0.0809524
-12076.0000000	0.0805000
-12075.0000000	0.0810000
-12074.0000000	0.0815000
-12073.0000000	0.0857143
-12072.0000000	0.0855000
-12071.0000000	0.0895000
-12070.0000000	0.0890476
-12069.0000000	0.0895000
-12068.0000000	0.0895000
-12067.0000000	0.0900000
-12066.0000000	0.0900000
-12065.0000000	0.0900000
-12064.0000000	0.0900000
-12063.0000000	0.0900000
-12062.0000000	0.0900000
-12061.0000000	0.0900000
-12060.0000000	0.0900000
-12059.0000000	0.0900000
-12058.0000000	0.0900000
-12057.0000000	0.0895238
-12056.0000000	0.0875000
-12055.0000000	0.0861905
-12054.0000000	0.0860000
-12053.0000000	0.0860000
-12052.0000000	0.0871429

-12051.0000000	0.0895000
-12050.0000000	0.0890000
-12049.0000000	0.0895000
-12048.0000000	0.0900000
-12047.0000000	0.0900000
-12046.0000000	0.0890476
-12045.0000000	0.0900000
-12044.0000000	0.0895000
-12043.0000000	0.0900000
-12042.0000000	0.0900000
-12041.0000000	0.0900000
-12040.0000000	0.0900000
-12039.0000000	0.0900000
-12038.0000000	0.0895000
-12037.0000000	0.0895000
-12036.0000000	0.0880953
-12035.0000000	0.0895000
-12034.0000000	0.0900000
-12033.0000000	0.0890000
-12032.0000000	0.0880000
-12031.0000000	0.0890476
-12030.0000000	0.0895000
-12029.0000000	0.0890000
-12028.0000000	0.0870000
-12027.0000000	0.0847619
-12026.0000000	0.0865000
-12025.0000000	0.0866667
-12024.0000000	0.0880000
-12023.0000000	0.0870000
-12022.0000000	0.0890000
-12021.0000000	0.0871429
-12020.0000000	0.0875000
-12019.0000000	0.0860000
-12018.0000000	0.0861905
-12017.0000000	0.0880000
-12016.0000000	0.0876191
-12015.0000000	0.0870000
-12014.0000000	0.0875000
-12013.0000000	0.0870000
-12012.0000000	0.0880952
-12011.0000000	0.0885000
-12010.0000000	0.0860000
-12009.0000000	0.0880953
-12008.0000000	0.0875000
-12007.0000000	0.0860000
-12006.0000000	0.0857143
-12005.0000000	0.0875000
-12004.0000000	0.0890476
-12003.0000000	0.0890000
-12002.0000000	0.0890000
-12001.0000000	0.0895238
-12000.0000000	0.0895000
-11999.0000000	0.0900000
-11998.0000000	0.0895000
-11997.0000000	0.0885715
-11996.0000000	0.0885000
-11995.0000000	0.0895238

-11994.0000000	0.0885000
-11993.0000000	0.0890000
-11992.0000000	0.0885000
-11991.0000000	0.0890476
-11990.0000000	0.0880000
-11989.0000000	0.0900000
-11988.0000000	0.0900000
-11987.0000000	0.0900000
-11986.0000000	0.0900000
-11985.0000000	0.0900000
-11984.0000000	0.0900000
-11983.0000000	0.0900000
-11982.0000000	0.0900000
-11981.0000000	0.0900000
-11980.0000000	0.0905000
-11979.0000000	0.0904762
-11978.0000000	0.0900000
-11977.0000000	0.0900000
-11976.0000000	0.0904762
-11975.0000000	0.0905000
-11974.0000000	0.0900000
-11973.0000000	0.0900000
-11972.0000000	0.0900000
-11971.0000000	0.0900000
-11970.0000000	0.0900000
-11969.0000000	0.0900000
-11968.0000000	0.0900000
-11967.0000000	0.0900000
-11966.0000000	0.0900000
-11965.0000000	0.0900000
-11964.0000000	0.0895238
-11963.0000000	0.0900000
-11962.0000000	0.0900000
-11961.0000000	0.0895238
-11960.0000000	0.0885000
-11959.0000000	0.0880000
-11958.0000000	0.0880953
-11957.0000000	0.0845000
-11956.0000000	0.0820000
-11955.0000000	0.0819048
-11954.0000000	0.0805000
-11953.0000000	0.0805000
-11952.0000000	0.0800000
-11951.0000000	0.0810000
-11950.0000000	0.0840000
-11949.0000000	0.0880953
-11948.0000000	0.0875000
-11947.0000000	0.0895000
-11946.0000000	0.0890476
-11945.0000000	0.0895000
-11944.0000000	0.0900000
-11943.0000000	0.0900000
-11942.0000000	0.0900000
-11941.0000000	0.0900000
-11940.0000000	0.0900000
-11939.0000000	0.0900000
-11938.0000000	0.0900000

-11937.0000000	0.0900000
-11936.0000000	0.0905000
-11935.0000000	0.0905000
-11934.0000000	0.0909524
-11933.0000000	0.0915000
-11932.0000000	0.0920000
-11931.0000000	0.0910000
-11930.0000000	0.0914286
-11929.0000000	0.0910000
-11928.0000000	0.0904762
-11927.0000000	0.0920000
-11926.0000000	0.0915000
-11925.0000000	0.0914286
-11924.0000000	0.0905000
-11923.0000000	0.0915000
-11922.0000000	0.0915000
-11921.0000000	0.0904762
-11920.0000000	0.0920000
-11919.0000000	0.0933333
-11918.0000000	0.0940000
-11917.0000000	0.0945000
-11916.0000000	0.0914286
-11915.0000000	0.0925000
-11914.0000000	0.0910000
-11913.0000000	0.0914286
-11912.0000000	0.0920000
-11911.0000000	0.0900000
-11910.0000000	0.0900000
-11909.0000000	0.0885000
-11908.0000000	0.0870000
-11907.0000000	0.0861905
-11906.0000000	0.0820000
-11905.0000000	0.0815000
-11904.0000000	0.0814286
-11903.0000000	0.0835000
-11902.0000000	0.0850000
-11901.0000000	0.0855000
-11900.0000000	0.0857143
-11899.0000000	0.0880000
-11898.0000000	0.0857143
-11897.0000000	0.0875000
-11896.0000000	0.0865000
-11895.0000000	0.0890476
-11894.0000000	0.0880000
-11893.0000000	0.0885000
-11892.0000000	0.0875000
-11891.0000000	0.0828572
-11890.0000000	0.0845000
-11889.0000000	0.0823810
-11888.0000000	0.0820000
-11887.0000000	0.0820000
-11886.0000000	0.0805000
-11885.0000000	0.0804762
-11884.0000000	0.0820000
-11883.0000000	0.0815000
-11882.0000000	0.0800000
-11881.0000000	0.0810000

-11880.0000000	0.0819048
-11879.0000000	0.0830000
-11878.0000000	0.0820000
-11877.0000000	0.0847619
-11876.0000000	0.0830000
-11875.0000000	0.0850000
-11874.0000000	0.0835000
-11873.0000000	0.0838095
-11872.0000000	0.0855000
-11871.0000000	0.0830000
-11870.0000000	0.0842857
-11869.0000000	0.0855000
-11868.0000000	0.0857143
-11867.0000000	0.0855000
-11866.0000000	0.0840000
-11865.0000000	0.0820000
-11864.0000000	0.0842857
-11863.0000000	0.0830000
-11862.0000000	0.0820000
-11861.0000000	0.0809524
-11860.0000000	0.0805000
-11859.0000000	0.0814286
-11858.0000000	0.0800000
-11857.0000000	0.0800000
-11856.0000000	0.0805000
-11855.0000000	0.0800000
-11854.0000000	0.0805000
-11853.0000000	0.0800000
-11852.0000000	0.0804762
-11851.0000000	0.0810000
-11850.0000000	0.0800000
-11849.0000000	0.0815000
-11848.0000000	0.0800000
-11847.0000000	0.0825000
-11846.0000000	0.0804762
-11845.0000000	0.0825000
-11844.0000000	0.0835000
-11843.0000000	0.0814286
-11842.0000000	0.0835000
-11841.0000000	0.0840000
-11840.0000000	0.0835000
-11839.0000000	0.0823810
-11838.0000000	0.0823810
-11837.0000000	0.0805000
-11836.0000000	0.0815000
-11835.0000000	0.0805000
-11834.0000000	0.0804762
-11833.0000000	0.0805000
-11832.0000000	0.0805000
-11831.0000000	0.0800000
-11830.0000000	0.0805000
-11829.0000000	0.0804762
-11828.0000000	0.0810000
-11827.0000000	0.0810000
-11826.0000000	0.0815000
-11825.0000000	0.0800000
-11824.0000000	0.0810000

-11823.0000000	0.0815000
-11822.0000000	0.0838095
-11821.0000000	0.0870000
-11820.0000000	0.0876191
-11819.0000000	0.0865000
-11818.0000000	0.0885000
-11817.0000000	0.0900000
-11816.0000000	0.0880953
-11815.0000000	0.0850000
-11814.0000000	0.0855000
-11813.0000000	0.0866667
-11812.0000000	0.0855000
-11811.0000000	0.0810000
-11810.0000000	0.0825000
-11809.0000000	0.0828572
-11808.0000000	0.0865000
-11807.0000000	0.0866667
-11806.0000000	0.0855000
-11805.0000000	0.0890000
-11804.0000000	0.0885715
-11803.0000000	0.0890000
-11802.0000000	0.0895000
-11801.0000000	0.0890476
-11800.0000000	0.0890000
-11799.0000000	0.0895000
-11798.0000000	0.0871429
-11797.0000000	0.0875000
-11796.0000000	0.0860000
-11795.0000000	0.0847619
-11794.0000000	0.0830000
-11793.0000000	0.0830000
-11792.0000000	0.0842857
-11791.0000000	0.0850000
-11790.0000000	0.0825000
-11789.0000000	0.0838095
-11788.0000000	0.0805000
-11787.0000000	0.0805000
-11786.0000000	0.0800000
-11785.0000000	0.0800000
-11784.0000000	0.0800000
-11783.0000000	0.0800000
-11782.0000000	0.0800000
-11781.0000000	0.0805000
-11780.0000000	0.0825000
-11779.0000000	0.0809524
-11778.0000000	0.0820000
-11777.0000000	0.0809524
-11776.0000000	0.0810000
-11775.0000000	0.0825000
-11774.0000000	0.0809524
-11773.0000000	0.0825000
-11772.0000000	0.0830000
-11771.0000000	0.0828572
-11770.0000000	0.0825000
-11769.0000000	0.0825000
-11768.0000000	0.0857143
-11767.0000000	0.0860000

-11766.0000000	0.0835000
-11765.0000000	0.0825000
-11764.0000000	0.0804762
-11763.0000000	0.0805000
-11762.0000000	0.0804762
-11761.0000000	0.0800000
-11760.0000000	0.0800000
-11759.0000000	0.0800000
-11758.0000000	0.0800000
-11757.0000000	0.0800000
-11756.0000000	0.0800000
-11755.0000000	0.0800000
-11754.0000000	0.0800000
-11753.0000000	0.0800000
-11752.0000000	0.0800000
-11751.0000000	0.0800000
-11750.0000000	0.0800000
-11749.0000000	0.0800000
-11748.0000000	0.0800000
-11747.0000000	0.0795000
-11746.0000000	0.0800000
-11745.0000000	0.0800000
-11744.0000000	0.0800000
-11743.0000000	0.0800000
-11742.0000000	0.0800000
-11741.0000000	0.0804762
-11740.0000000	0.0810000
-11739.0000000	0.0805000
-11738.0000000	0.0809524
-11737.0000000	0.0830000
-11736.0000000	0.0840000
-11735.0000000	0.0830000
-11734.0000000	0.0847619
-11733.0000000	0.0835000
-11732.0000000	0.0847619
-11731.0000000	0.0825000
-11730.0000000	0.0830000
-11729.0000000	0.0804762
-11728.0000000	0.0810000
-11727.0000000	0.0805000
-11726.0000000	0.0815000
-11725.0000000	0.0819048
-11724.0000000	0.0830000
-11723.0000000	0.0814286
-11722.0000000	0.0805000
-11721.0000000	0.0810000
-11720.0000000	0.0815000
-11719.0000000	0.0810000
-11718.0000000	0.0809524
-11717.0000000	0.0810000
-11716.0000000	0.0804762
-11715.0000000	0.0805000
-11714.0000000	0.0800000
-11713.0000000	0.0805000
-11712.0000000	0.0800000
-11711.0000000	0.0800000
-11710.0000000	0.0800000

-11709.0000000	0.0800000
-11708.0000000	0.0800000
-11707.0000000	0.0800000
-11706.0000000	0.0800000
-11705.0000000	0.0800000
-11704.0000000	0.0800000
-11703.0000000	0.0805000
-11702.0000000	0.0809524
-11701.0000000	0.0810000
-11700.0000000	0.0800000
-11699.0000000	0.0800000
-11698.0000000	0.0809524
-11697.0000000	0.0800000
-11696.0000000	0.0800000
-11695.0000000	0.0800000
-11694.0000000	0.0800000
-11693.0000000	0.0800000
-11692.0000000	0.0805000
-11691.0000000	0.0800000
-11690.0000000	0.0800000
-11689.0000000	0.0800000
-11688.0000000	0.0800000
-11687.0000000	0.0800000
-11686.0000000	0.0800000
-11685.0000000	0.0800000
-11684.0000000	0.0800000
-11683.0000000	0.0785000
-11682.0000000	0.0780000
-11681.0000000	0.0755000
-11680.0000000	0.0780952
-11679.0000000	0.0780000
-11678.0000000	0.0795000
-11677.0000000	0.0800000
-11676.0000000	0.0800000
-11675.0000000	0.0795000
-11674.0000000	0.0800000
-11673.0000000	0.0800000
-11672.0000000	0.0800000
-11671.0000000	0.0800000
-11670.0000000	0.0795000
-11669.0000000	0.0800000
-11668.0000000	0.0800000
-11667.0000000	0.0795000
-11666.0000000	0.0800000
-11665.0000000	0.0800000
-11664.0000000	0.0800000
-11663.0000000	0.0800000
-11662.0000000	0.0800000
-11661.0000000	0.0800000
-11660.0000000	0.0800000
-11659.0000000	0.0800000
-11658.0000000	0.0800000
-11657.0000000	0.0800000
-11656.0000000	0.0800000
-11655.0000000	0.0800000
-11654.0000000	0.0800000
-11653.0000000	0.0800000

-11652.0000000	0.0800000
-11651.0000000	0.0800000
-11650.0000000	0.0800000
-11649.0000000	0.0805000
-11648.0000000	0.0800000
-11647.0000000	0.0804762
-11646.0000000	0.0800000
-11645.0000000	0.0800000
-11644.0000000	0.0800000
-11643.0000000	0.0800000
-11642.0000000	0.0800000
-11641.0000000	0.0800000
-11640.0000000	0.0800000
-11639.0000000	0.0800000
-11638.0000000	0.0804762
-11637.0000000	0.0800000
-11636.0000000	0.0800000
-11635.0000000	0.0800000
-11634.0000000	0.0805000
-11633.0000000	0.0800000
-11632.0000000	0.0800000
-11631.0000000	0.0800000
-11630.0000000	0.0800000
-11629.0000000	0.0800000
-11628.0000000	0.0800000
-11627.0000000	0.0800000
-11626.0000000	0.0800000
-11625.0000000	0.0805000
-11624.0000000	0.0800000
-11623.0000000	0.0809524
-11622.0000000	0.0815000
-11621.0000000	0.0810000
-11620.0000000	0.0809524
-11619.0000000	0.0830000
-11618.0000000	0.0810000
-11617.0000000	0.0800000
-11616.0000000	0.0800000
-11615.0000000	0.0795000
-11614.0000000	0.0800000
-11613.0000000	0.0800000
-11612.0000000	0.0800000
-11611.0000000	0.0800000
-11610.0000000	0.0800000
-11609.0000000	0.0805000
-11608.0000000	0.0800000
-11607.0000000	0.0800000
-11606.0000000	0.0800000
-11605.0000000	0.0804762
-11604.0000000	0.0800000
-11603.0000000	0.0800000
-11602.0000000	0.0800000
-11601.0000000	0.0800000
-11600.0000000	0.0800000
-11599.0000000	0.0800000
-11598.0000000	0.0800000
-11597.0000000	0.0810000
-11596.0000000	0.0819048

-11595.0000000	0.0800000
-11594.0000000	0.0815000
-11593.0000000	0.0814286
-11592.0000000	0.0810000
-11591.0000000	0.0815000
-11590.0000000	0.0805000
-11589.0000000	0.0800000
-11588.0000000	0.0810000
-11587.0000000	0.0809524
-11586.0000000	0.0815000
-11585.0000000	0.0805000
-11584.0000000	0.0810000
-11583.0000000	0.0800000
-11582.0000000	0.0800000
-11581.0000000	0.0800000
-11580.0000000	0.0800000
-11579.0000000	0.0795000
-11578.0000000	0.0800000
-11577.0000000	0.0800000
-11576.0000000	0.0800000
-11575.0000000	0.0795238
-11574.0000000	0.0795000
-11573.0000000	0.0795000
-11572.0000000	0.0800000
-11571.0000000	0.0800000
-11570.0000000	0.0800000
-11569.0000000	0.0800000
-11568.0000000	0.0800000
-11567.0000000	0.0800000
-11566.0000000	0.0800000
-11565.0000000	0.0800000
-11564.0000000	0.0800000
-11563.0000000	0.0800000
-11562.0000000	0.0800000
-11561.0000000	0.0800000
-11560.0000000	0.0800000
-11559.0000000	0.0804762
-11558.0000000	0.0800000
-11557.0000000	0.0800000
-11556.0000000	0.0800000
-11555.0000000	0.0800000
-11554.0000000	0.0800000
-11553.0000000	0.0800000
-11552.0000000	0.0800000
-11551.0000000	0.0800000
-11550.0000000	0.0800000
-11549.0000000	0.0800000
-11548.0000000	0.0800000
-11547.0000000	0.0795000
-11546.0000000	0.0800000
-11545.0000000	0.0795238
-11544.0000000	0.0800000
-11543.0000000	0.0800000
-11542.0000000	0.0800000
-11541.0000000	0.0800000
-11540.0000000	0.0800000
-11539.0000000	0.0800000

-11538.0000000	0.0800000
-11537.0000000	0.0800000
-11536.0000000	0.0800000
-11535.0000000	0.0800000
-11534.0000000	0.0800000
-11533.0000000	0.0800000
-11532.0000000	0.0800000
-11531.0000000	0.0800000
-11530.0000000	0.0800000
-11529.0000000	0.0800000
-11528.0000000	0.0800000
-11527.0000000	0.0800000
-11526.0000000	0.0800000
-11525.0000000	0.0800000
-11524.0000000	0.0800000
-11523.0000000	0.0800000
-11522.0000000	0.0800000
-11521.0000000	0.0800000
-11520.0000000	0.0800000
-11519.0000000	0.0810000
-11518.0000000	0.0800000
-11517.0000000	0.0805000
-11516.0000000	0.0800000
-11515.0000000	0.0800000
-11514.0000000	0.0800000
-11513.0000000	0.0800000
-11512.0000000	0.0810000
-11511.0000000	0.0804762
-11510.0000000	0.0810000
-11509.0000000	0.0820000
-11508.0000000	0.0840000
-11507.0000000	0.0838095
-11506.0000000	0.0850000
-11505.0000000	0.0871429
-11504.0000000	0.0890000
-11503.0000000	0.0875000
-11502.0000000	0.0895238
-11501.0000000	0.0885000
-11500.0000000	0.0885000
-11499.0000000	0.0870000
-11498.0000000	0.0866667
-11497.0000000	0.0857143
-11496.0000000	0.0835000
-11495.0000000	0.0860000
-11494.0000000	0.0870000
-11493.0000000	0.0871429
-11492.0000000	0.0875000
-11491.0000000	0.0870000
-11490.0000000	0.0871429
-11489.0000000	0.0860000
-11488.0000000	0.0852381
-11487.0000000	0.0835000
-11486.0000000	0.0840000
-11485.0000000	0.0845000
-11484.0000000	0.0814286
-11483.0000000	0.0820000
-11482.0000000	0.0815000

-11481.0000000	0.0823810
-11480.0000000	0.0805000
-11479.0000000	0.0815000
-11478.0000000	0.0805000
-11477.0000000	0.0819048
-11476.0000000	0.0815000
-11475.0000000	0.0809524
-11474.0000000	0.0815000
-11473.0000000	0.0815000
-11472.0000000	0.0800000
-11471.0000000	0.0815000
-11470.0000000	0.0805000
-11469.0000000	0.0805000
-11468.0000000	0.0804762
-11467.0000000	0.0800000
-11466.0000000	0.0800000
-11465.0000000	0.0800000
-11464.0000000	0.0800000
-11463.0000000	0.0804762
-11462.0000000	0.0800000
-11461.0000000	0.0800000
-11460.0000000	0.0800000
-11459.0000000	0.0805000
-11458.0000000	0.0800000
-11457.0000000	0.0800000
-11456.0000000	0.0800000
-11455.0000000	0.0800000
-11454.0000000	0.0790476
-11453.0000000	0.0790000
-11452.0000000	0.0795000
-11451.0000000	0.0800000
-11450.0000000	0.0800000
-11449.0000000	0.0795000
-11448.0000000	0.0790000
-11447.0000000	0.0795238
-11446.0000000	0.0785000
-11445.0000000	0.0742857
-11444.0000000	0.0725000
-11443.0000000	0.0710000
-11442.0000000	0.0752381
-11441.0000000	0.0765000
-11440.0000000	0.0790000
-11439.0000000	0.0790000
-11438.0000000	0.0795238
-11437.0000000	0.0800000
-11436.0000000	0.0795238
-11435.0000000	0.0800000
-11434.0000000	0.0800000
-11433.0000000	0.0800000
-11432.0000000	0.0795238
-11431.0000000	0.0800000
-11430.0000000	0.0800000
-11429.0000000	0.0800000
-11428.0000000	0.0800000
-11427.0000000	0.0800000
-11426.0000000	0.0800000
-11425.0000000	0.0800000

-11424.0000000	0.0800000
-11423.0000000	0.0795238
-11422.0000000	0.0800000
-11421.0000000	0.0800000
-11420.0000000	0.0800000
-11419.0000000	0.0800000
-11418.0000000	0.0800000
-11417.0000000	0.0800000
-11416.0000000	0.0800000
-11415.0000000	0.0800000
-11414.0000000	0.0800000
-11413.0000000	0.0800000
-11412.0000000	0.0800000
-11411.0000000	0.0810000
-11410.0000000	0.0800000
-11409.0000000	0.0800000
-11408.0000000	0.0809524
-11407.0000000	0.0805000
-11406.0000000	0.0800000
-11405.0000000	0.0804762
-11404.0000000	0.0810526
-11403.0000000	0.0804762
-11402.0000000	0.0800000
-11401.0000000	0.0805000
-11400.0000000	0.0814286
-11399.0000000	0.0820000
-11398.0000000	0.0809524
-11397.0000000	0.0810000
-11396.0000000	0.0815000
-11395.0000000	0.0809524
-11394.0000000	0.0810000
-11393.0000000	0.0810000
-11392.0000000	0.0835000
-11391.0000000	0.0814286
-11390.0000000	0.0825000
-11389.0000000	0.0809524
-11388.0000000	0.0805000
-11387.0000000	0.0810000
-11386.0000000	0.0800000
-11385.0000000	0.0804762
-11384.0000000	0.0805000
-11383.0000000	0.0805000
-11382.0000000	0.0804762
-11381.0000000	0.0820000
-11380.0000000	0.0809524
-11379.0000000	0.0810000
-11378.0000000	0.0800000
-11377.0000000	0.0805000
-11376.0000000	0.0814286
-11375.0000000	0.0800000
-11374.0000000	0.0805000
-11373.0000000	0.0800000
-11372.0000000	0.0800000
-11371.0000000	0.0810000
-11370.0000000	0.0800000
-11369.0000000	0.0830000
-11368.0000000	0.0833333

-11367.0000000	0.0845000
-11366.0000000	0.0860000
-11365.0000000	0.0850000
-11364.0000000	0.0842857
-11363.0000000	0.0885000
-11362.0000000	0.0865000
-11361.0000000	0.0847619
-11360.0000000	0.0860000
-11359.0000000	0.0842857
-11358.0000000	0.0835000
-11357.0000000	0.0845000
-11356.0000000	0.0840000
-11355.0000000	0.0814286
-11354.0000000	0.0820000
-11353.0000000	0.0840000
-11352.0000000	0.0825000
-11351.0000000	0.0838095
-11350.0000000	0.0861905
-11349.0000000	0.0840000
-11348.0000000	0.0845000
-11347.0000000	0.0840000
-11346.0000000	0.0838095
-11345.0000000	0.0835000
-11344.0000000	0.0820000
-11343.0000000	0.0828572
-11342.0000000	0.0830000
-11341.0000000	0.0840000
-11340.0000000	0.0840000
-11339.0000000	0.0842857
-11338.0000000	0.0845000
-11337.0000000	0.0833333
-11336.0000000	0.0865000
-11335.0000000	0.0835000
-11334.0000000	0.0838095
-11333.0000000	0.0835000
-11332.0000000	0.0840000
-11331.0000000	0.0845000
-11330.0000000	0.0871429
-11329.0000000	0.0865000
-11328.0000000	0.0876191
-11327.0000000	0.0860000
-11326.0000000	0.0880000
-11325.0000000	0.0861905
-11324.0000000	0.0870000
-11323.0000000	0.0885000
-11322.0000000	0.0875000
-11321.0000000	0.0890476
-11320.0000000	0.0895238
-11319.0000000	0.0900000
-11318.0000000	0.0900000
-11317.0000000	0.0900000
-11316.0000000	0.0895238
-11315.0000000	0.0900000
-11314.0000000	0.0900000
-11313.0000000	0.0900000
-11312.0000000	0.0900000
-11311.0000000	0.0895000

-11310.0000000	0.0900000
-11309.0000000	0.0895238
-11308.0000000	0.0895000
-11307.0000000	0.0900000
-11306.0000000	0.0900000
-11305.0000000	0.0900000
-11304.0000000	0.0900000
-11303.0000000	0.0890000
-11302.0000000	0.0900000
-11301.0000000	0.0885000
-11300.0000000	0.0880953
-11299.0000000	0.0870000
-11298.0000000	0.0857143
-11297.0000000	0.0840000
-11296.0000000	0.0850000
-11295.0000000	0.0847619
-11294.0000000	0.0845000
-11293.0000000	0.0850000
-11292.0000000	0.0880000
-11291.0000000	0.0890476
-11290.0000000	0.0900000
-11289.0000000	0.0880000
-11288.0000000	0.0900000
-11287.0000000	0.0890000
-11286.0000000	0.0885000
-11285.0000000	0.0890476
-11284.0000000	0.0875000
-11283.0000000	0.0861905
-11282.0000000	0.0825000
-11281.0000000	0.0825000
-11280.0000000	0.0815000
-11279.0000000	0.0804762
-11278.0000000	0.0805000
-11277.0000000	0.0804762
-11276.0000000	0.0800000
-11275.0000000	0.0800000
-11274.0000000	0.0804762
-11273.0000000	0.0800000
-11272.0000000	0.0800000
-11271.0000000	0.0800000
-11270.0000000	0.0800000
-11269.0000000	0.0805000
-11268.0000000	0.0800000
-11267.0000000	0.0800000
-11266.0000000	0.0800000
-11265.0000000	0.0800000
-11264.0000000	0.0800000
-11263.0000000	0.0800000
-11262.0000000	0.0800000
-11261.0000000	0.0814286
-11260.0000000	0.0820000
-11259.0000000	0.0809524
-11258.0000000	0.0820000
-11257.0000000	0.0830000
-11256.0000000	0.0835000
-11255.0000000	0.0857143
-11254.0000000	0.0855000

-11253.0000000	0.0852381
-11252.0000000	0.0860000
-11251.0000000	0.0865000
-11250.0000000	0.0830000
-11249.0000000	0.0833334
-11248.0000000	0.0855000
-11247.0000000	0.0860000
-11246.0000000	0.0842857
-11245.0000000	0.0855000
-11244.0000000	0.0866667
-11243.0000000	0.0855000
-11242.0000000	0.0850000
-11241.0000000	0.0830000
-11240.0000000	0.0823810
-11239.0000000	0.0815000
-11238.0000000	0.0830000
-11237.0000000	0.0804762
-11236.0000000	0.0800000
-11235.0000000	0.0800000
-11234.0000000	0.0800000
-11233.0000000	0.0800000
-11232.0000000	0.0795000
-11231.0000000	0.0795238
-11230.0000000	0.0790000
-11229.0000000	0.0795000
-11228.0000000	0.0795238
-11227.0000000	0.0800000
-11226.0000000	0.0795000
-11225.0000000	0.0800000
-11224.0000000	0.0800000
-11223.0000000	0.0795238
-11222.0000000	0.0795000
-11221.0000000	0.0800000
-11220.0000000	0.0800000
-11219.0000000	0.0800000
-11218.0000000	0.0800000
-11217.0000000	0.0800000
-11216.0000000	0.0800000
-11215.0000000	0.0800000
-11214.0000000	0.0800000
-11213.0000000	0.0800000
-11212.0000000	0.0795000
-11211.0000000	0.0800000
-11210.0000000	0.0800000
-11209.0000000	0.0795000
-11208.0000000	0.0800000
-11207.0000000	0.0795238
-11206.0000000	0.0795000
-11205.0000000	0.0785000
-11204.0000000	0.0790476
-11203.0000000	0.0785000
-11202.0000000	0.0785000
-11201.0000000	0.0790476
-11200.0000000	0.0790000
-11199.0000000	0.0800000
-11198.0000000	0.0795238
-11197.0000000	0.0800000

-11196.0000000	0.0800000
-11195.0000000	0.0800000
-11194.0000000	0.0800000
-11193.0000000	0.0800000
-11192.0000000	0.0800000
-11191.0000000	0.0800000
-11190.0000000	0.0805000
-11189.0000000	0.0804762
-11188.0000000	0.0800000
-11187.0000000	0.0810000
-11186.0000000	0.0800000
-11185.0000000	0.0805000
-11184.0000000	0.0814286
-11183.0000000	0.0805000
-11182.0000000	0.0805000
-11181.0000000	0.0815000
-11180.0000000	0.0804762
-11179.0000000	0.0800000
-11178.0000000	0.0800000
-11177.0000000	0.0800000
-11176.0000000	0.0800000
-11175.0000000	0.0800000
-11174.0000000	0.0800000
-11173.0000000	0.0800000
-11172.0000000	0.0800000
-11171.0000000	0.0800000
-11170.0000000	0.0805000
-11169.0000000	0.0800000
-11168.0000000	0.0800000
-11167.0000000	0.0800000
-11166.0000000	0.0800000
-11165.0000000	0.0800000
-11164.0000000	0.0800000
-11163.0000000	0.0800000
-11162.0000000	0.0804762
-11161.0000000	0.0800000
-11160.0000000	0.0800000
-11159.0000000	0.0814286
-11158.0000000	0.0800000
-11157.0000000	0.0800000
-11156.0000000	0.0805000
-11155.0000000	0.0800000
-11154.0000000	0.0800000
-11153.0000000	0.0804762
-11152.0000000	0.0810000
-11151.0000000	0.0810000
-11150.0000000	0.0800000
-11149.0000000	0.0814286
-11148.0000000	0.0820000
-11147.0000000	0.0833333
-11146.0000000	0.0835000
-11145.0000000	0.0835000
-11144.0000000	0.0840000
-11143.0000000	0.0866667
-11142.0000000	0.0875000
-11141.0000000	0.0880953
-11140.0000000	0.0870000

-11139.0000000	0.0845000
-11138.0000000	0.0861905
-11137.0000000	0.0850000
-11136.0000000	0.0880000
-11135.0000000	0.0865000
-11134.0000000	0.0847619
-11133.0000000	0.0880000
-11132.0000000	0.0876191
-11131.0000000	0.0890000
-11130.0000000	0.0885000
-11129.0000000	0.0876191
-11128.0000000	0.0880000
-11127.0000000	0.0865000
-11126.0000000	0.0855000
-11125.0000000	0.0861905
-11124.0000000	0.0880000
-11123.0000000	0.0885715
-11122.0000000	0.0870000
-11121.0000000	0.0875000
-11120.0000000	0.0871429
-11119.0000000	0.0870000
-11118.0000000	0.0855000
-11117.0000000	0.0861905
-11116.0000000	0.0865000
-11115.0000000	0.0880000
-11114.0000000	0.0870000
-11113.0000000	0.0842857
-11112.0000000	0.0865000
-11111.0000000	0.0861905
-11110.0000000	0.0865000
-11109.0000000	0.0845000
-11108.0000000	0.0861905
-11107.0000000	0.0855000
-11106.0000000	0.0880000
-11105.0000000	0.0855000
-11104.0000000	0.0861905
-11103.0000000	0.0870000
-11102.0000000	0.0857143
-11101.0000000	0.0845000
-11100.0000000	0.0850000
-11099.0000000	0.0820000
-11098.0000000	0.0828572
-11097.0000000	0.0830000
-11096.0000000	0.0845000
-11095.0000000	0.0814286
-11094.0000000	0.0810000
-11093.0000000	0.0814286
-11092.0000000	0.0820000
-11091.0000000	0.0810000
-11090.0000000	0.0810000
-11089.0000000	0.0823810
-11088.0000000	0.0830000
-11087.0000000	0.0842857
-11086.0000000	0.0845000
-11085.0000000	0.0845000
-11084.0000000	0.0830000
-11083.0000000	0.0828572

-11082.0000000	0.0830000
-11081.0000000	0.0815000
-11080.0000000	0.0823810
-11079.0000000	0.0860000
-11078.0000000	0.0828572
-11077.0000000	0.0835000
-11076.0000000	0.0845000
-11075.0000000	0.0860000
-11074.0000000	0.0833333
-11073.0000000	0.0865000
-11072.0000000	0.0855000
-11071.0000000	0.0852381
-11070.0000000	0.0865000
-11069.0000000	0.0840000
-11068.0000000	0.0814286
-11067.0000000	0.0800000
-11066.0000000	0.0810000
-11065.0000000	0.0800000
-11064.0000000	0.0800000
-11063.0000000	0.0805000
-11062.0000000	0.0800000
-11061.0000000	0.0805000
-11060.0000000	0.0815000
-11059.0000000	0.0819048
-11058.0000000	0.0825000
-11057.0000000	0.0819048
-11056.0000000	0.0835000
-11055.0000000	0.0855000
-11054.0000000	0.0835000
-11053.0000000	0.0847619
-11052.0000000	0.0840000
-11051.0000000	0.0810000
-11050.0000000	0.0814286
-11049.0000000	0.0800000
-11048.0000000	0.0804762
-11047.0000000	0.0805000
-11046.0000000	0.0825000
-11045.0000000	0.0810000
-11044.0000000	0.0809524
-11043.0000000	0.0805000
-11042.0000000	0.0810000
-11041.0000000	0.0819048
-11040.0000000	0.0820000
-11039.0000000	0.0830000
-11038.0000000	0.0840000
-11037.0000000	0.0861905
-11036.0000000	0.0865000
-11035.0000000	0.0861905
-11034.0000000	0.0825000
-11033.0000000	0.0840000
-11032.0000000	0.0814286
-11031.0000000	0.0805000
-11030.0000000	0.0800000
-11029.0000000	0.0815000
-11028.0000000	0.0804762
-11027.0000000	0.0823810
-11026.0000000	0.0835000

-11025.0000000	0.0845000
-11024.0000000	0.0870000
-11023.0000000	0.0828572
-11022.0000000	0.0850000
-11021.0000000	0.0850000
-11020.0000000	0.0880000
-11019.0000000	0.0876191
-11018.0000000	0.0866667
-11017.0000000	0.0870000
-11016.0000000	0.0850000
-11015.0000000	0.0855000
-11014.0000000	0.0852381
-11013.0000000	0.0855000
-11012.0000000	0.0840000
-11011.0000000	0.0842857
-11010.0000000	0.0830000
-11009.0000000	0.0805000
-11008.0000000	0.0800000
-11007.0000000	0.0800000
-11006.0000000	0.0800000
-11005.0000000	0.0790476
-11004.0000000	0.0795000
-11003.0000000	0.0800000
-11002.0000000	0.0800000
-11001.0000000	0.0790000
-11000.0000000	0.0785000
-10999.0000000	0.0795000
-10998.0000000	0.0795238
-10997.0000000	0.0800000
-10996.0000000	0.0800000
-10995.0000000	0.0800000
-10994.0000000	0.0800000
-10993.0000000	0.0795238
-10992.0000000	0.0800000
-10991.0000000	0.0795000
-10990.0000000	0.0800000
-10989.0000000	0.0795238
-10988.0000000	0.0800000
-10987.0000000	0.0800000
-10986.0000000	0.0800000
-10985.0000000	0.0800000
-10984.0000000	0.0790476
-10983.0000000	0.0790000
-10982.0000000	0.0765000
-10981.0000000	0.0771429
-10980.0000000	0.0765000
-10979.0000000	0.0800000
-10978.0000000	0.0800000
-10977.0000000	0.0800000
-10976.0000000	0.0795000
-10975.0000000	0.0795238
-10974.0000000	0.0800000
-10973.0000000	0.0800000
-10972.0000000	0.0800000
-10971.0000000	0.0800000
-10970.0000000	0.0800000
-10969.0000000	0.0800000

-10968.0000000	0.0800000
-10967.0000000	0.0795000
-10966.0000000	0.0800000
-10965.0000000	0.0800000
-10964.0000000	0.0800000
-10963.0000000	0.0800000
-10962.0000000	0.0800000
-10961.0000000	0.0800000
-10960.0000000	0.0800000
-10959.0000000	0.0804762
-10958.0000000	0.0800000
-10957.0000000	0.0804762
-10956.0000000	0.0805000
-10955.0000000	0.0815000
-10954.0000000	0.0820000
-10953.0000000	0.0804762
-10952.0000000	0.0830000
-10951.0000000	0.0833333
-10950.0000000	0.0825000
-10949.0000000	0.0845000
-10948.0000000	0.0845000
-10947.0000000	0.0838095
-10946.0000000	0.0835000
-10945.0000000	0.0855000
-10944.0000000	0.0847619
-10943.0000000	0.0850000
-10942.0000000	0.0828572
-10941.0000000	0.0820000
-10940.0000000	0.0845000
-10939.0000000	0.0845000
-10938.0000000	0.0842857
-10937.0000000	0.0850000
-10936.0000000	0.0876191
-10935.0000000	0.0860000
-10934.0000000	0.0865000
-10933.0000000	0.0870000
-10932.0000000	0.0866667
-10931.0000000	0.0855000
-10930.0000000	0.0840000
-10929.0000000	0.0838095
-10928.0000000	0.0845000
-10927.0000000	0.0866667
-10926.0000000	0.0880000
-10925.0000000	0.0845000
-10924.0000000	0.0865000
-10923.0000000	0.0885714
-10922.0000000	0.0885000
-10921.0000000	0.0890476
-10920.0000000	0.0890000
-10919.0000000	0.0890000
-10918.0000000	0.0890000
-10917.0000000	0.0900000
-10916.0000000	0.0900000
-10915.0000000	0.0895000
-10914.0000000	0.0900000
-10913.0000000	0.0900000
-10912.0000000	0.0900000

-10911.0000000	0.0900000
-10910.0000000	0.0900000
-10909.0000000	0.0900000
-10908.0000000	0.0900000
-10907.0000000	0.0900000
-10906.0000000	0.0900000
-10905.0000000	0.0900000
-10904.0000000	0.0900000
-10903.0000000	0.0900000
-10902.0000000	0.0900000
-10901.0000000	0.0895000
-10900.0000000	0.0870000
-10899.0000000	0.0890476
-10898.0000000	0.0845000
-10897.0000000	0.0850000
-10896.0000000	0.0852381
-10895.0000000	0.0850000
-10894.0000000	0.0855000
-10893.0000000	0.0861905
-10892.0000000	0.0870000
-10891.0000000	0.0861905
-10890.0000000	0.0865000
-10889.0000000	0.0830000
-10888.0000000	0.0825000
-10887.0000000	0.0809524
-10886.0000000	0.0825000
-10885.0000000	0.0825000
-10884.0000000	0.0847619
-10883.0000000	0.0850000
-10882.0000000	0.0838095
-10881.0000000	0.0830000
-10880.0000000	0.0845000
-10879.0000000	0.0840000
-10878.0000000	0.0838095
-10877.0000000	0.0850000
-10876.0000000	0.0805000
-10875.0000000	0.0828572
-10874.0000000	0.0815000
-10873.0000000	0.0800000
-10872.0000000	0.0800000
-10871.0000000	0.0800000
-10870.0000000	0.0800000
-10869.0000000	0.0800000
-10868.0000000	0.0800000
-10867.0000000	0.0800000
-10866.0000000	0.0800000
-10865.0000000	0.0800000
-10864.0000000	0.0800000
-10863.0000000	0.0800000
-10862.0000000	0.0800000
-10861.0000000	0.0800000
-10860.0000000	0.0800000
-10859.0000000	0.0800000
-10858.0000000	0.0800000
-10857.0000000	0.0800000
-10856.0000000	0.0800000
-10855.0000000	0.0800000

-10854.0000000	0.0800000
-10853.0000000	0.0800000
-10852.0000000	0.0800000
-10851.0000000	0.0800000
-10850.0000000	0.0800000
-10849.0000000	0.0800000
-10848.0000000	0.0800000
-10847.0000000	0.0810000
-10846.0000000	0.0800000
-10845.0000000	0.0804762
-10844.0000000	0.0805000
-10843.0000000	0.0800000
-10842.0000000	0.0800000
-10841.0000000	0.0804762
-10840.0000000	0.0810000
-10839.0000000	0.0800000
-10838.0000000	0.0805000
-10837.0000000	0.0800000
-10836.0000000	0.0814286
-10835.0000000	0.0810000
-10834.0000000	0.0825000
-10833.0000000	0.0820000
-10832.0000000	0.0833333
-10831.0000000	0.0830000
-10830.0000000	0.0809524
-10829.0000000	0.0805000
-10828.0000000	0.0810000
-10827.0000000	0.0800000
-10826.0000000	0.0810000
-10825.0000000	0.0810000
-10824.0000000	0.0815000
-10823.0000000	0.0814286
-10822.0000000	0.0825000
-10821.0000000	0.0814286
-10820.0000000	0.0815000
-10819.0000000	0.0815000
-10818.0000000	0.0804762
-10817.0000000	0.0805000
-10816.0000000	0.0815000
-10815.0000000	0.0804762
-10814.0000000	0.0820000
-10813.0000000	0.0825000
-10812.0000000	0.0825000
-10811.0000000	0.0847619
-10810.0000000	0.0840000
-10809.0000000	0.0828572
-10808.0000000	0.0850000
-10807.0000000	0.0865000
-10806.0000000	0.0833334
-10805.0000000	0.0840000
-10804.0000000	0.0840000
-10803.0000000	0.0825000
-10802.0000000	0.0814286
-10801.0000000	0.0815000
-10800.0000000	0.0814286
-10799.0000000	0.0825000
-10798.0000000	0.0800000

-10797.0000000	0.0805000
-10796.0000000	0.0804762
-10795.0000000	0.0815000
-10794.0000000	0.0814286
-10793.0000000	0.0845000
-10792.0000000	0.0823810
-10791.0000000	0.0865000
-10790.0000000	0.0840000
-10789.0000000	0.0870000
-10788.0000000	0.0875000
-10787.0000000	0.0861905
-10786.0000000	0.0825000
-10785.0000000	0.0828572
-10784.0000000	0.0845000
-10783.0000000	0.0825000
-10782.0000000	0.0828572
-10781.0000000	0.0815000
-10780.0000000	0.0805000
-10779.0000000	0.0800000
-10778.0000000	0.0800000
-10777.0000000	0.0805000
-10776.0000000	0.0819048
-10775.0000000	0.0805000
-10774.0000000	0.0815000
-10773.0000000	0.0819048
-10772.0000000	0.0820000
-10771.0000000	0.0815000
-10770.0000000	0.0804762
-10769.0000000	0.0800000
-10768.0000000	0.0805000
-10767.0000000	0.0800000
-10766.0000000	0.0800000
-10765.0000000	0.0814286
-10764.0000000	0.0820000
-10763.0000000	0.0815000
-10762.0000000	0.0815000
-10761.0000000	0.0838095
-10760.0000000	0.0810000
-10759.0000000	0.0820000
-10758.0000000	0.0814286
-10757.0000000	0.0815000
-10756.0000000	0.0820000
-10755.0000000	0.0809524
-10754.0000000	0.0820000
-10753.0000000	0.0845000
-10752.0000000	0.0825000
-10751.0000000	0.0861905
-10750.0000000	0.0850000
-10749.0000000	0.0819048
-10748.0000000	0.0840000
-10747.0000000	0.0815789
-10746.0000000	0.0831818
-10745.0000000	0.0800000
-10744.0000000	0.0800000
-10743.0000000	0.0800000
-10742.0000000	0.0800000
-10741.0000000	0.0805000

-10740.0000000	0.0804762
-10739.0000000	0.0805000
-10738.0000000	0.0805000
-10737.0000000	0.0800000
-10736.0000000	0.0800000
-10735.0000000	0.0800000
-10734.0000000	0.0800000
-10733.0000000	0.0805000
-10732.0000000	0.0805000
-10731.0000000	0.0804762
-10730.0000000	0.0800000
-10729.0000000	0.0800000
-10728.0000000	0.0800000
-10727.0000000	0.0800000
-10726.0000000	0.0805000
-10725.0000000	0.0800000
-10724.0000000	0.0800000
-10723.0000000	0.0800000
-10722.0000000	0.0800000
-10721.0000000	0.0800000
-10720.0000000	0.0800000
-10719.0000000	0.0800000
-10718.0000000	0.0800000
-10717.0000000	0.0800000
-10716.0000000	0.0800000
-10715.0000000	0.0800000
-10714.0000000	0.0800000
-10713.0000000	0.0800000
-10712.0000000	0.0800000
-10711.0000000	0.0800000
-10710.0000000	0.0800000
-10709.0000000	0.0800000
-10708.0000000	0.0800000
-10707.0000000	0.0800000
-10706.0000000	0.0800000
-10705.0000000	0.0800000
-10704.0000000	0.0800000
-10703.0000000	0.0800000
-10702.0000000	0.0800000
-10701.0000000	0.0804762
-10700.0000000	0.0800000
-10699.0000000	0.0800000
-10698.0000000	0.0800000
-10697.0000000	0.0800000
-10696.0000000	0.0800000
-10695.0000000	0.0800000
-10694.0000000	0.0800000
-10693.0000000	0.0800000
-10692.0000000	0.0800000
-10691.0000000	0.0814286
-10690.0000000	0.0815000
-10689.0000000	0.0800000
-10688.0000000	0.0805000
-10687.0000000	0.0800000
-10686.0000000	0.0804762
-10685.0000000	0.0810000
-10684.0000000	0.0815000

-10683.0000000	0.0825000
-10682.0000000	0.0833333
-10681.0000000	0.0820000
-10680.0000000	0.0840000
-10679.0000000	0.0833334
-10678.0000000	0.0840000
-10677.0000000	0.0825000
-10676.0000000	0.0819048
-10675.0000000	0.0810000
-10674.0000000	0.0804762
-10673.0000000	0.0800000
-10672.0000000	0.0820000
-10671.0000000	0.0820000
-10670.0000000	0.0814286
-10669.0000000	0.0805000
-10668.0000000	0.0820000
-10667.0000000	0.0828572
-10666.0000000	0.0815000
-10665.0000000	0.0804762
-10664.0000000	0.0810000
-10663.0000000	0.0830000
-10662.0000000	0.0825000
-10661.0000000	0.0835000
-10660.0000000	0.0828572
-10659.0000000	0.0830000
-10658.0000000	0.0861905
-10657.0000000	0.0845000
-10656.0000000	0.0852381
-10655.0000000	0.0845000
-10654.0000000	0.0830000
-10653.0000000	0.0835000
-10652.0000000	0.0835000
-10651.0000000	0.0861905
-10650.0000000	0.0825000
-10649.0000000	0.0828572
-10648.0000000	0.0825000
-10647.0000000	0.0830000
-10646.0000000	0.0842857
-10645.0000000	0.0830000
-10644.0000000	0.0865000
-10643.0000000	0.0847619
-10642.0000000	0.0850000
-10641.0000000	0.0865000
-10640.0000000	0.0842857
-10639.0000000	0.0865000
-10638.0000000	0.0861905
-10637.0000000	0.0870000
-10636.0000000	0.0875000
-10635.0000000	0.0875000
-10634.0000000	0.0880953
-10633.0000000	0.0865000
-10632.0000000	0.0865000
-10631.0000000	0.0861905
-10630.0000000	0.0840000
-10629.0000000	0.0820000
-10628.0000000	0.0819048
-10627.0000000	0.0830000

-10626.0000000	0.0814286
-10625.0000000	0.0835000
-10624.0000000	0.0810000
-10623.0000000	0.0805000
-10622.0000000	0.0800000
-10621.0000000	0.0800000
-10620.0000000	0.0805000
-10619.0000000	0.0804762
-10618.0000000	0.0800000
-10617.0000000	0.0800000
-10616.0000000	0.0800000
-10615.0000000	0.0804762
-10614.0000000	0.0810000
-10613.0000000	0.0800000
-10612.0000000	0.0800000
-10611.0000000	0.0790000
-10610.0000000	0.0790476
-10609.0000000	0.0775000
-10608.0000000	0.0800000
-10607.0000000	0.0761905
-10606.0000000	0.0775000
-10605.0000000	0.0765000
-10604.0000000	0.0770000
-10603.0000000	0.0761905
-10602.0000000	0.0765000
-10601.0000000	0.0750000
-10600.0000000	0.0780952
-10599.0000000	0.0790000
-10598.0000000	0.0795238
-10597.0000000	0.0795000
-10596.0000000	0.0800000
-10595.0000000	0.0800000
-10594.0000000	0.0800000
-10593.0000000	0.0800000
-10592.0000000	0.0800000
-10591.0000000	0.0800000
-10590.0000000	0.0800000
-10589.0000000	0.0800000
-10588.0000000	0.0805000
-10587.0000000	0.0800000
-10586.0000000	0.0800000
-10585.0000000	0.0800000
-10584.0000000	0.0815000
-10583.0000000	0.0814286
-10582.0000000	0.0835000
-10581.0000000	0.0835000
-10580.0000000	0.0835000
-10579.0000000	0.0823810
-10578.0000000	0.0830000
-10577.0000000	0.0845000
-10576.0000000	0.0852381
-10575.0000000	0.0835000
-10574.0000000	0.0847619
-10573.0000000	0.0825000
-10572.0000000	0.0815000
-10571.0000000	0.0840000
-10570.0000000	0.0809524

-10569.0000000	0.0805000
-10568.0000000	0.0800000
-10567.0000000	0.0800000
-10566.0000000	0.0810000
-10565.0000000	0.0823810
-10564.0000000	0.0800000
-10563.0000000	0.0800000
-10562.0000000	0.0805000
-10561.0000000	0.0805000
-10560.0000000	0.0800000
-10559.0000000	0.0804762
-10558.0000000	0.0800000
-10557.0000000	0.0800000
-10556.0000000	0.0800000
-10555.0000000	0.0800000
-10554.0000000	0.0810000
-10553.0000000	0.0805000
-10552.0000000	0.0814286
-10551.0000000	0.0815000
-10550.0000000	0.0819048
-10549.0000000	0.0805000
-10548.0000000	0.0820000
-10547.0000000	0.0819048
-10546.0000000	0.0820000
-10545.0000000	0.0830000
-10544.0000000	0.0830000
-10543.0000000	0.0857143
-10542.0000000	0.0870000
-10541.0000000	0.0885000
-10540.0000000	0.0895238
-10539.0000000	0.0895000
-10538.0000000	0.0895238
-10537.0000000	0.0895000
-10536.0000000	0.0900000
-10535.0000000	0.0895238
-10534.0000000	0.0895000
-10533.0000000	0.0895000
-10532.0000000	0.0875000
-10531.0000000	0.0876191
-10530.0000000	0.0880000
-10529.0000000	0.0871429
-10528.0000000	0.0855000
-10527.0000000	0.0840000
-10526.0000000	0.0860000
-10525.0000000	0.0880000
-10524.0000000	0.0880953
-10523.0000000	0.0890000
-10522.0000000	0.0900000
-10521.0000000	0.0895000
-10520.0000000	0.0900000
-10519.0000000	0.0900000
-10518.0000000	0.0900000
-10517.0000000	0.0900000
-10516.0000000	0.0900000
-10515.0000000	0.0895000
-10514.0000000	0.0895000
-10513.0000000	0.0900000

-10512.0000000	0.0900000
-10511.0000000	0.0900000
-10510.0000000	0.0900000
-10509.0000000	0.0890000
-10508.0000000	0.0890000
-10507.0000000	0.0895238
-10506.0000000	0.0890000
-10505.0000000	0.0880000
-10504.0000000	0.0885715
-10503.0000000	0.0870000
-10502.0000000	0.0850000
-10501.0000000	0.0866667
-10500.0000000	0.0870000
-10499.0000000	0.0880953
-10498.0000000	0.0850000
-10497.0000000	0.0880000
-10496.0000000	0.0875000
-10495.0000000	0.0857143
-10494.0000000	0.0835000
-10493.0000000	0.0835000
-10492.0000000	0.0814286
-10491.0000000	0.0830000
-10490.0000000	0.0850000
-10489.0000000	0.0833333
-10488.0000000	0.0825000
-10487.0000000	0.0800000
-10486.0000000	0.0805000
-10485.0000000	0.0809524
-10484.0000000	0.0800000
-10483.0000000	0.0804762
-10482.0000000	0.0805000
-10481.0000000	0.0810000
-10480.0000000	0.0804762
-10479.0000000	0.0800000
-10478.0000000	0.0810000
-10477.0000000	0.0800000
-10476.0000000	0.0804762
-10475.0000000	0.0800000
-10474.0000000	0.0800000
-10473.0000000	0.0805000
-10472.0000000	0.0800000
-10471.0000000	0.0804762
-10470.0000000	0.0800000
-10469.0000000	0.0800000
-10468.0000000	0.0800000
-10467.0000000	0.0805000
-10466.0000000	0.0810000
-10465.0000000	0.0810000
-10464.0000000	0.0833333
-10463.0000000	0.0820000
-10462.0000000	0.0814286
-10461.0000000	0.0805000
-10460.0000000	0.0820000
-10459.0000000	0.0823810
-10458.0000000	0.0840000
-10457.0000000	0.0815000
-10456.0000000	0.0838095

-10455.0000000	0.0845000
-10454.0000000	0.0865000
-10453.0000000	0.0865000
-10452.0000000	0.0885714
-10451.0000000	0.0885000
-10450.0000000	0.0885000
-10449.0000000	0.0900000
-10448.0000000	0.0900000
-10447.0000000	0.0895238
-10446.0000000	0.0890000
-10445.0000000	0.0895000
-10444.0000000	0.0895238
-10443.0000000	0.0890000
-10442.0000000	0.0885000
-10441.0000000	0.0900000
-10440.0000000	0.0880953
-10439.0000000	0.0890000
-10438.0000000	0.0890476
-10437.0000000	0.0880000
-10436.0000000	0.0860000
-10435.0000000	0.0870000
-10434.0000000	0.0830000
-10433.0000000	0.0847619
-10432.0000000	0.0814286
-10431.0000000	0.0830000
-10430.0000000	0.0810000
-10429.0000000	0.0814286
-10428.0000000	0.0820000
-10427.0000000	0.0815000
-10426.0000000	0.0830000
-10425.0000000	0.0823810
-10424.0000000	0.0805000
-10423.0000000	0.0842857
-10422.0000000	0.0815000
-10421.0000000	0.0830000
-10420.0000000	0.0852381
-10419.0000000	0.0875000
-10418.0000000	0.0860000
-10417.0000000	0.0870000
-10416.0000000	0.0880952
-10415.0000000	0.0890000
-10414.0000000	0.0890000
-10413.0000000	0.0890476
-10412.0000000	0.0890000
-10411.0000000	0.0900000
-10410.0000000	0.0895000
-10409.0000000	0.0890000
-10408.0000000	0.0900000
-10407.0000000	0.0900000
-10406.0000000	0.0900000
-10405.0000000	0.0900000
-10404.0000000	0.0900000
-10403.0000000	0.0900000
-10402.0000000	0.0900000
-10401.0000000	0.0900000
-10400.0000000	0.0895000
-10399.0000000	0.0900000

-10398.0000000	0.0900000
-10397.0000000	0.0900000
-10396.0000000	0.0885000
-10395.0000000	0.0890476
-10394.0000000	0.0870000
-10393.0000000	0.0860000
-10392.0000000	0.0847619
-10391.0000000	0.0860000
-10390.0000000	0.0815000
-10389.0000000	0.0814286
-10388.0000000	0.0800000
-10387.0000000	0.0805000
-10386.0000000	0.0814286
-10385.0000000	0.0810000
-10384.0000000	0.0842857
-10383.0000000	0.0835000
-10382.0000000	0.0825000
-10381.0000000	0.0835000
-10380.0000000	0.0842857
-10379.0000000	0.0845000
-10378.0000000	0.0860000
-10377.0000000	0.0847619
-10376.0000000	0.0850000
-10375.0000000	0.0870000
-10374.0000000	0.0866667
-10373.0000000	0.0880000
-10372.0000000	0.0861905
-10371.0000000	0.0875000
-10370.0000000	0.0895000
-10369.0000000	0.0890000
-10368.0000000	0.0895238
-10367.0000000	0.0900000
-10366.0000000	0.0900000
-10365.0000000	0.0895238
-10364.0000000	0.0900000
-10363.0000000	0.0900000
-10362.0000000	0.0900000
-10361.0000000	0.0900000
-10360.0000000	0.0895000
-10359.0000000	0.0895000
-10358.0000000	0.0900000
-10357.0000000	0.0895000
-10356.0000000	0.0900000
-10355.0000000	0.0900000
-10354.0000000	0.0900000
-10353.0000000	0.0900000
-10352.0000000	0.0900000
-10351.0000000	0.0900000
-10350.0000000	0.0900000
-10349.0000000	0.0900000
-10348.0000000	0.0900000
-10347.0000000	0.0900000
-10346.0000000	0.0900000
-10345.0000000	0.0900000
-10344.0000000	0.0900000
-10343.0000000	0.0900000
-10342.0000000	0.0900000

-10341.0000000	0.0900000
-10340.0000000	0.0900000
-10339.0000000	0.0900000
-10338.0000000	0.0900000
-10337.0000000	0.0900000
-10336.0000000	0.0900000
-10335.0000000	0.0904762
-10334.0000000	0.0900000
-10333.0000000	0.0900000
-10332.0000000	0.0900000
-10331.0000000	0.0900000
-10330.0000000	0.0900000
-10329.0000000	0.0900000
-10328.0000000	0.0890000
-10327.0000000	0.0900000
-10326.0000000	0.0890476
-10325.0000000	0.0895000
-10324.0000000	0.0885715
-10323.0000000	0.0890000
-10322.0000000	0.0890000
-10321.0000000	0.0900000
-10320.0000000	0.0885714
-10319.0000000	0.0900000
-10318.0000000	0.0900000
-10317.0000000	0.0900000
-10316.0000000	0.0900000
-10315.0000000	0.0895000
-10314.0000000	0.0900000
-10313.0000000	0.0900000
-10312.0000000	0.0900000
-10311.0000000	0.0904762
-10310.0000000	0.0900000
-10309.0000000	0.0900000
-10308.0000000	0.0914286
-10307.0000000	0.0920000
-10306.0000000	0.0925000
-10305.0000000	0.0923810
-10304.0000000	0.0930000
-10303.0000000	0.0920000
-10302.0000000	0.0957143
-10301.0000000	0.0960000
-10300.0000000	0.0945000
-10299.0000000	0.0945000
-10298.0000000	0.0933333
-10297.0000000	0.0905000
-10296.0000000	0.0904762
-10295.0000000	0.0905000
-10294.0000000	0.0900000
-10293.0000000	0.0895238
-10292.0000000	0.0900000
-10291.0000000	0.0900000
-10290.0000000	0.0900000
-10289.0000000	0.0900000
-10288.0000000	0.0900000
-10287.0000000	0.0900000
-10286.0000000	0.0900000
-10285.0000000	0.0900000

-10284.0000000	0.0905000
-10283.0000000	0.0900000
-10282.0000000	0.0900000
-10281.0000000	0.0900000
-10280.0000000	0.0900000
-10279.0000000	0.0905000
-10278.0000000	0.0910000
-10277.0000000	0.0900000
-10276.0000000	0.0900000
-10275.0000000	0.0900000
-10274.0000000	0.0914286
-10273.0000000	0.0900000
-10272.0000000	0.0900000
-10271.0000000	0.0910000
-10270.0000000	0.0905000
-10269.0000000	0.0900000
-10268.0000000	0.0900000
-10267.0000000	0.0900000
-10266.0000000	0.0900000
-10265.0000000	0.0900000
-10264.0000000	0.0900000
-10263.0000000	0.0900000
-10262.0000000	0.0900000
-10261.0000000	0.0900000
-10260.0000000	0.0895000
-10259.0000000	0.0895000
-10258.0000000	0.0895238
-10257.0000000	0.0890476
-10256.0000000	0.0880000
-10255.0000000	0.0885000
-10254.0000000	0.0855000
-10253.0000000	0.0823810
-10252.0000000	0.0830000
-10251.0000000	0.0825000
-10250.0000000	0.0819048
-10249.0000000	0.0825000
-10248.0000000	0.0850000
-10247.0000000	0.0828572
-10246.0000000	0.0825000
-10245.0000000	0.0833333
-10244.0000000	0.0855000
-10243.0000000	0.0850000
-10242.0000000	0.0840000
-10241.0000000	0.0833333
-10240.0000000	0.0830000
-10239.0000000	0.0820000
-10238.0000000	0.0828572
-10237.0000000	0.0815000
-10236.0000000	0.0819048
-10235.0000000	0.0830000
-10234.0000000	0.0840000
-10233.0000000	0.0861905
-10232.0000000	0.0855000
-10231.0000000	0.0865000
-10230.0000000	0.0860000
-10229.0000000	0.0861905
-10228.0000000	0.0890000

-10227.0000000	0.0890476
-10226.0000000	0.0895000
-10225.0000000	0.0895000
-10224.0000000	0.0900000
-10223.0000000	0.0900000
-10222.0000000	0.0900000
-10221.0000000	0.0900000
-10220.0000000	0.0900000
-10219.0000000	0.0905000
-10218.0000000	0.0900000
-10217.0000000	0.0905000
-10216.0000000	0.0900000
-10215.0000000	0.0910000
-10214.0000000	0.0900000
-10213.0000000	0.0915000
-10212.0000000	0.0905000
-10211.0000000	0.0900000
-10210.0000000	0.0900000
-10209.0000000	0.0900000
-10208.0000000	0.0900000
-10207.0000000	0.0900000
-10206.0000000	0.0900000
-10205.0000000	0.0895238
-10204.0000000	0.0900000
-10203.0000000	0.0885000
-10202.0000000	0.0871429
-10201.0000000	0.0890000
-10200.0000000	0.0825000
-10199.0000000	0.0847619
-10198.0000000	0.0850000
-10197.0000000	0.0866667
-10196.0000000	0.0875000
-10195.0000000	0.0870000
-10194.0000000	0.0875000
-10193.0000000	0.0885714
-10192.0000000	0.0880000
-10191.0000000	0.0885000
-10190.0000000	0.0895238
-10189.0000000	0.0900000
-10188.0000000	0.0900000
-10187.0000000	0.0900000
-10186.0000000	0.0900000
-10185.0000000	0.0900000
-10184.0000000	0.0900000
-10183.0000000	0.0900000
-10182.0000000	0.0900000
-10181.0000000	0.0900000
-10180.0000000	0.0900000
-10179.0000000	0.0900000
-10178.0000000	0.0900000
-10177.0000000	0.0895000
-10176.0000000	0.0900000
-10175.0000000	0.0900000
-10174.0000000	0.0885714
-10173.0000000	0.0885000
-10172.0000000	0.0842857
-10171.0000000	0.0835000

-10170.0000000	0.0825000
-10169.0000000	0.0847619
-10168.0000000	0.0825000
-10167.0000000	0.0840000
-10166.0000000	0.0842857
-10165.0000000	0.0850000
-10164.0000000	0.0830000
-10163.0000000	0.0845000
-10162.0000000	0.0828572
-10161.0000000	0.0840000
-10160.0000000	0.0847619
-10159.0000000	0.0880000
-10158.0000000	0.0876191
-10157.0000000	0.0890000
-10156.0000000	0.0900000
-10155.0000000	0.0895000
-10154.0000000	0.0890476
-10153.0000000	0.0890000
-10152.0000000	0.0900000
-10151.0000000	0.0900000
-10150.0000000	0.0900000
-10149.0000000	0.0900000
-10148.0000000	0.0885000
-10147.0000000	0.0890476
-10146.0000000	0.0890000
-10145.0000000	0.0871429
-10144.0000000	0.0885000
-10143.0000000	0.0855000
-10142.0000000	0.0871429
-10141.0000000	0.0895000
-10140.0000000	0.0890000
-10139.0000000	0.0895000
-10138.0000000	0.0890476
-10137.0000000	0.0900000
-10136.0000000	0.0900000
-10135.0000000	0.0900000
-10134.0000000	0.0900000
-10133.0000000	0.0900000
-10132.0000000	0.0900000
-10131.0000000	0.0900000
-10130.0000000	0.0900000
-10129.0000000	0.0900000
-10128.0000000	0.0905000
-10127.0000000	0.0900000
-10126.0000000	0.0900000
-10125.0000000	0.0900000
-10124.0000000	0.0900000
-10123.0000000	0.0900000
-10122.0000000	0.0900000
-10121.0000000	0.0904762
-10120.0000000	0.0900000
-10119.0000000	0.0900000
-10118.0000000	0.0890476
-10117.0000000	0.0900000
-10116.0000000	0.0900000
-10115.0000000	0.0895000
-10114.0000000	0.0900000

-10113.0000000	0.0890000
-10112.0000000	0.0885000
-10111.0000000	0.0895238
-10110.0000000	0.0895000
-10109.0000000	0.0880953
-10108.0000000	0.0895000
-10107.0000000	0.0885000
-10106.0000000	0.0861905
-10105.0000000	0.0875000
-10104.0000000	0.0860000
-10103.0000000	0.0850000
-10102.0000000	0.0861905
-10101.0000000	0.0870000
-10100.0000000	0.0865000
-10099.0000000	0.0852381
-10098.0000000	0.0820000
-10097.0000000	0.0819048
-10096.0000000	0.0805000
-10095.0000000	0.0805000
-10094.0000000	0.0805000
-10093.0000000	0.0809524
-10092.0000000	0.0805000
-10091.0000000	0.0820000
-10090.0000000	0.0838095
-10089.0000000	0.0840000
-10088.0000000	0.0865000
-10087.0000000	0.0876191
-10086.0000000	0.0885000
-10085.0000000	0.0895000
-10084.0000000	0.0900000
-10083.0000000	0.0900000
-10082.0000000	0.0900000
-10081.0000000	0.0900000
-10080.0000000	0.0900000
-10079.0000000	0.0900000
-10078.0000000	0.0895238
-10077.0000000	0.0900000
-10076.0000000	0.0900000
-10075.0000000	0.0895238
-10074.0000000	0.0900000
-10073.0000000	0.0900000
-10072.0000000	0.0890476
-10071.0000000	0.0890000
-10070.0000000	0.0866667
-10069.0000000	0.0895000
-10068.0000000	0.0885000
-10067.0000000	0.0865000
-10066.0000000	0.0857143
-10065.0000000	0.0835000
-10064.0000000	0.0845000
-10063.0000000	0.0852381
-10062.0000000	0.0850000
-10061.0000000	0.0833333
-10060.0000000	0.0840000
-10059.0000000	0.0805263
-10058.0000000	0.0809524
-10057.0000000	0.0805000

-10056.0000000	0.0804762
-10055.0000000	0.0810000
-10054.0000000	0.0809524
-10053.0000000	0.0810000
-10052.0000000	0.0823810
-10051.0000000	0.0845000
-10050.0000000	0.0835000
-10049.0000000	0.0835000
-10048.0000000	0.0845000
-10047.0000000	0.0876191
-10046.0000000	0.0870000
-10045.0000000	0.0890476
-10044.0000000	0.0890000
-10043.0000000	0.0900000
-10042.0000000	0.0890476
-10041.0000000	0.0900000
-10040.0000000	0.0900000
-10039.0000000	0.0904762
-10038.0000000	0.0900000
-10037.0000000	0.0905000
-10036.0000000	0.0900000
-10035.0000000	0.0900000
-10034.0000000	0.0900000
-10033.0000000	0.0900000
-10032.0000000	0.0905000
-10031.0000000	0.0905000
-10030.0000000	0.0904762
-10029.0000000	0.0905000
-10028.0000000	0.0900000
-10027.0000000	0.0904762
-10026.0000000	0.0900000
-10025.0000000	0.0900000
-10024.0000000	0.0900000
-10023.0000000	0.0900000
-10022.0000000	0.0900000
-10021.0000000	0.0900000
-10020.0000000	0.0900000
-10019.0000000	0.0900000
-10018.0000000	0.0900000
-10017.0000000	0.0905000
-10016.0000000	0.0900000
-10015.0000000	0.0900000
-10014.0000000	0.0900000
-10013.0000000	0.0900000
-10012.0000000	0.0900000
-10011.0000000	0.0900000
-10010.0000000	0.0905000
-10009.0000000	0.0904762
-10008.0000000	0.0900000
-10007.0000000	0.0910000
-10006.0000000	0.0914286
-10005.0000000	0.0905000
-10004.0000000	0.0915000
-10003.0000000	0.0909524
-10002.0000000	0.0910000
-10001.0000000	0.0925000
-10000.0000000	0.0904762

-9999.0000000	0.0920000
-9998.0000000	0.0910000
-9997.0000000	0.0900000
-9996.0000000	0.0900000
-9995.0000000	0.0900000
-9994.0000000	0.0900000
-9993.0000000	0.0905000
-9992.0000000	0.0900000
-9991.0000000	0.0900000
-9990.0000000	0.0895000
-9989.0000000	0.0900000
-9988.0000000	0.0895000
-9987.0000000	0.0890476
-9986.0000000	0.0900000
-9985.0000000	0.0900000
-9984.0000000	0.0900000
-9983.0000000	0.0900000
-9982.0000000	0.0904762
-9981.0000000	0.0900000
-9980.0000000	0.0900000
-9979.0000000	0.0900000
-9978.0000000	0.0900000
-9977.0000000	0.0900000
-9976.0000000	0.0900000
-9975.0000000	0.0900000
-9974.0000000	0.0900000
-9973.0000000	0.0895000
-9972.0000000	0.0890476
-9971.0000000	0.0900000
-9970.0000000	0.0900000
-9969.0000000	0.0900000
-9968.0000000	0.0895000
-9967.0000000	0.0895000
-9966.0000000	0.0895238
-9965.0000000	0.0890000
-9964.0000000	0.0895000
-9963.0000000	0.0876191
-9962.0000000	0.0865000
-9961.0000000	0.0857143
-9960.0000000	0.0855000
-9959.0000000	0.0850000
-9958.0000000	0.0835000
-9957.0000000	0.0842857
-9956.0000000	0.0860000
-9955.0000000	0.0876191
-9954.0000000	0.0895000
-9953.0000000	0.0900000
-9952.0000000	0.0885000
-9951.0000000	0.0880953
-9950.0000000	0.0885000
-9949.0000000	0.0885000
-9948.0000000	0.0857143
-9947.0000000	0.0860000
-9946.0000000	0.0850000
-9945.0000000	0.0866667
-9944.0000000	0.0865000
-9943.0000000	0.0876191

-9942.0000000	0.0875000
-9941.0000000	0.0885000
-9940.0000000	0.0890476
-9939.0000000	0.0885000
-9938.0000000	0.0885000
-9937.0000000	0.0885000
-9936.0000000	0.0895238
-9935.0000000	0.0875000
-9934.0000000	0.0880953
-9933.0000000	0.0885000
-9932.0000000	0.0900000
-9931.0000000	0.0880953
-9930.0000000	0.0890000
-9929.0000000	0.0880000
-9928.0000000	0.0875000
-9927.0000000	0.0890476
-9926.0000000	0.0835000
-9925.0000000	0.0861905
-9924.0000000	0.0860000
-9923.0000000	0.0845000
-9922.0000000	0.0850000
-9921.0000000	0.0860000
-9920.0000000	0.0876191
-9919.0000000	0.0875000
-9918.0000000	0.0871429
-9917.0000000	0.0880000
-9916.0000000	0.0861905
-9915.0000000	0.0870000
-9914.0000000	0.0875000
-9913.0000000	0.0875000
-9912.0000000	0.0835000
-9911.0000000	0.0866667
-9910.0000000	0.0815000
-9909.0000000	0.0804762
-9908.0000000	0.0805000
-9907.0000000	0.0805000
-9906.0000000	0.0804762
-9905.0000000	0.0815000
-9904.0000000	0.0845000
-9903.0000000	0.0833333
-9902.0000000	0.0855000
-9901.0000000	0.0855000
-9900.0000000	0.0861905
-9899.0000000	0.0855000
-9898.0000000	0.0870000
-9897.0000000	0.0885000
-9896.0000000	0.0880952
-9895.0000000	0.0885715
-9894.0000000	0.0860000
-9893.0000000	0.0885000
-9892.0000000	0.0880000
-9891.0000000	0.0871429
-9890.0000000	0.0870000
-9889.0000000	0.0885000
-9888.0000000	0.0847619
-9887.0000000	0.0825000
-9886.0000000	0.0819048

-9885.0000000	0.0845000
-9884.0000000	0.0825000
-9883.0000000	0.0850000
-9882.0000000	0.0842857
-9881.0000000	0.0830000
-9880.0000000	0.0875000
-9879.0000000	0.0866667
-9878.0000000	0.0875000
-9877.0000000	0.0875000
-9876.0000000	0.0885715
-9875.0000000	0.0895000
-9874.0000000	0.0880000
-9873.0000000	0.0890000
-9872.0000000	0.0895238
-9871.0000000	0.0870000
-9870.0000000	0.0900000
-9869.0000000	0.0890000
-9868.0000000	0.0900000
-9867.0000000	0.0885715
-9866.0000000	0.0880000
-9865.0000000	0.0875000
-9864.0000000	0.0852381
-9863.0000000	0.0830000
-9862.0000000	0.0810000
-9861.0000000	0.0820000
-9860.0000000	0.0809524
-9859.0000000	0.0800000
-9858.0000000	0.0820000
-9857.0000000	0.0819048
-9856.0000000	0.0800000
-9855.0000000	0.0800000
-9854.0000000	0.0800000
-9853.0000000	0.0800000
-9852.0000000	0.0800000
-9851.0000000	0.0800000
-9850.0000000	0.0800000
-9849.0000000	0.0800000
-9848.0000000	0.0800000
-9847.0000000	0.0800000
-9846.0000000	0.0800000
-9845.0000000	0.0800000
-9844.0000000	0.0800000
-9843.0000000	0.0800000
-9842.0000000	0.0800000
-9841.0000000	0.0809091
-9840.0000000	0.0809524
-9839.0000000	0.0800000
-9838.0000000	0.0805000
-9837.0000000	0.0800000
-9836.0000000	0.0800000
-9835.0000000	0.0800000
-9834.0000000	0.0804762
-9833.0000000	0.0800000
-9832.0000000	0.0810000
-9831.0000000	0.0805000
-9830.0000000	0.0825000
-9829.0000000	0.0819048

-9828.0000000	0.0819048
-9827.0000000	0.0830000
-9826.0000000	0.0815000
-9825.0000000	0.0833334
-9824.0000000	0.0815000
-9823.0000000	0.0825000
-9822.0000000	0.0805000
-9821.0000000	0.0814286
-9820.0000000	0.0800000
-9819.0000000	0.0820000
-9818.0000000	0.0828572
-9817.0000000	0.0815000
-9816.0000000	0.0838095
-9815.0000000	0.0840000
-9814.0000000	0.0845000
-9813.0000000	0.0833334
-9812.0000000	0.0805000
-9811.0000000	0.0810000
-9810.0000000	0.0815000
-9809.0000000	0.0814286
-9808.0000000	0.0835000
-9807.0000000	0.0852381
-9806.0000000	0.0865000
-9805.0000000	0.0885000
-9804.0000000	0.0885714
-9803.0000000	0.0875000
-9802.0000000	0.0890000
-9801.0000000	0.0880000
-9800.0000000	0.0895238
-9799.0000000	0.0885000
-9798.0000000	0.0895238
-9797.0000000	0.0880000
-9796.0000000	0.0875000
-9795.0000000	0.0857143
-9794.0000000	0.0845000
-9793.0000000	0.0855000
-9792.0000000	0.0865000
-9791.0000000	0.0847619
-9790.0000000	0.0850000
-9789.0000000	0.0857143
-9788.0000000	0.0850000
-9787.0000000	0.0870000
-9786.0000000	0.0860000
-9785.0000000	0.0866667
-9784.0000000	0.0885000
-9783.0000000	0.0900000
-9782.0000000	0.0900000
-9781.0000000	0.0900000
-9780.0000000	0.0895000
-9779.0000000	0.0900000
-9778.0000000	0.0900000
-9777.0000000	0.0900000
-9776.0000000	0.0900000
-9775.0000000	0.0900000
-9774.0000000	0.0900000
-9773.0000000	0.0900000
-9772.0000000	0.0895000

-9771.0000000	0.0900000
-9770.0000000	0.0890000
-9769.0000000	0.0880953
-9768.0000000	0.0890476
-9767.0000000	0.0895000
-9766.0000000	0.0885000
-9765.0000000	0.0861905
-9764.0000000	0.0820000
-9763.0000000	0.0800000
-9762.0000000	0.0810000
-9761.0000000	0.0800000
-9760.0000000	0.0820000
-9759.0000000	0.0833333
-9758.0000000	0.0820000
-9757.0000000	0.0805000
-9756.0000000	0.0815000
-9755.0000000	0.0828572
-9754.0000000	0.0840000
-9753.0000000	0.0830000
-9752.0000000	0.0847619
-9751.0000000	0.0845000
-9750.0000000	0.0819048
-9749.0000000	0.0810000
-9748.0000000	0.0815000
-9747.0000000	0.0805000
-9746.0000000	0.0800000
-9745.0000000	0.0800000
-9744.0000000	0.0805000
-9743.0000000	0.0804762
-9742.0000000	0.0800000
-9741.0000000	0.0805000
-9740.0000000	0.0800000
-9739.0000000	0.0805000
-9738.0000000	0.0800000
-9737.0000000	0.0800000
-9736.0000000	0.0800000
-9735.0000000	0.0805000
-9734.0000000	0.0804762
-9733.0000000	0.0800000
-9732.0000000	0.0800000
-9731.0000000	0.0800000
-9730.0000000	0.0804762
-9729.0000000	0.0800000
-9728.0000000	0.0800000
-9727.0000000	0.0810000
-9726.0000000	0.0800000
-9725.0000000	0.0804762
-9724.0000000	0.0800000
-9723.0000000	0.0805000
-9722.0000000	0.0800000
-9721.0000000	0.0805000
-9720.0000000	0.0804762
-9719.0000000	0.0800000
-9718.0000000	0.0800000
-9717.0000000	0.0805000
-9716.0000000	0.0804762
-9715.0000000	0.0805000

-9714.0000000	0.0820000
-9713.0000000	0.0814286
-9712.0000000	0.0815000
-9711.0000000	0.0800000
-9710.0000000	0.0825000
-9709.0000000	0.0814286
-9708.0000000	0.0800000
-9707.0000000	0.0819048
-9706.0000000	0.0800000
-9705.0000000	0.0804762
-9704.0000000	0.0805000
-9703.0000000	0.0800000
-9702.0000000	0.0800000
-9701.0000000	0.0800000
-9700.0000000	0.0800000
-9699.0000000	0.0800000
-9698.0000000	0.0800000
-9697.0000000	0.0800000
-9696.0000000	0.0800000
-9695.0000000	0.0800000
-9694.0000000	0.0795238
-9693.0000000	0.0800000
-9692.0000000	0.0800000
-9691.0000000	0.0800000
-9690.0000000	0.0800000
-9689.0000000	0.0800000
-9688.0000000	0.0800000
-9687.0000000	0.0805000
-9686.0000000	0.0800000
-9685.0000000	0.0800000
-9684.0000000	0.0800000
-9683.0000000	0.0800000
-9682.0000000	0.0800000
-9681.0000000	0.0815000
-9680.0000000	0.0804762
-9679.0000000	0.0810000
-9678.0000000	0.0800000
-9677.0000000	0.0800000
-9676.0000000	0.0830000
-9675.0000000	0.0820000
-9674.0000000	0.0804762
-9673.0000000	0.0805000
-9672.0000000	0.0800000
-9671.0000000	0.0805000
-9670.0000000	0.0800000
-9669.0000000	0.0800000
-9668.0000000	0.0809524
-9667.0000000	0.0800000
-9666.0000000	0.0810000
-9665.0000000	0.0800000
-9664.0000000	0.0804762
-9663.0000000	0.0805000
-9662.0000000	0.0814286
-9661.0000000	0.0800000
-9660.0000000	0.0800000
-9659.0000000	0.0800000
-9658.0000000	0.0800000

-9657.0000000	0.0800000
-9656.0000000	0.0800000
-9655.0000000	0.0800000
-9654.0000000	0.0800000
-9653.0000000	0.0800000
-9652.0000000	0.0800000
-9651.0000000	0.0800000
-9650.0000000	0.0800000
-9649.0000000	0.0800000
-9648.0000000	0.0800000
-9647.0000000	0.0800000
-9646.0000000	0.0800000
-9645.0000000	0.0800000
-9644.0000000	0.0800000
-9643.0000000	0.0800000
-9642.0000000	0.0800000
-9641.0000000	0.0800000
-9640.0000000	0.0800000
-9639.0000000	0.0800000
-9638.0000000	0.0800000
-9637.0000000	0.0800000
-9636.0000000	0.0800000
-9635.0000000	0.0800000
-9634.0000000	0.0800000
-9633.0000000	0.0800000
-9632.0000000	0.0800000
-9631.0000000	0.0800000
-9630.0000000	0.0800000
-9629.0000000	0.0800000
-9628.0000000	0.0800000
-9627.0000000	0.0800000
-9626.0000000	0.0800000
-9625.0000000	0.0800000
-9624.0000000	0.0800000
-9623.0000000	0.0800000
-9622.0000000	0.0800000
-9621.0000000	0.0804762
-9620.0000000	0.0800000
-9619.0000000	0.0800000
-9618.0000000	0.0800000
-9617.0000000	0.0805000
-9616.0000000	0.0800000
-9615.0000000	0.0800000
-9614.0000000	0.0800000
-9613.0000000	0.0800000
-9612.0000000	0.0800000
-9611.0000000	0.0800000
-9610.0000000	0.0800000
-9609.0000000	0.0800000
-9608.0000000	0.0800000
-9607.0000000	0.0800000
-9606.0000000	0.0800000
-9605.0000000	0.0800000
-9604.0000000	0.0800000
-9603.0000000	0.0800000
-9602.0000000	0.0800000
-9601.0000000	0.0800000

-9600.0000000	0.0800000
-9599.0000000	0.0800000
-9598.0000000	0.0800000
-9597.0000000	0.0800000
-9596.0000000	0.0810000
-9595.0000000	0.0804762
-9594.0000000	0.0805000
-9593.0000000	0.0804762
-9592.0000000	0.0800000
-9591.0000000	0.0820000
-9590.0000000	0.0805000
-9589.0000000	0.0804762
-9588.0000000	0.0805000
-9587.0000000	0.0820000
-9586.0000000	0.0833333
-9585.0000000	0.0810000
-9584.0000000	0.0833333
-9583.0000000	0.0820000
-9582.0000000	0.0815000
-9581.0000000	0.0810000
-9580.0000000	0.0804762
-9579.0000000	0.0800000
-9578.0000000	0.0805000
-9577.0000000	0.0804762
-9576.0000000	0.0800000
-9575.0000000	0.0800000
-9574.0000000	0.0800000
-9573.0000000	0.0800000
-9572.0000000	0.0800000
-9571.0000000	0.0800000
-9570.0000000	0.0800000
-9569.0000000	0.0800000
-9568.0000000	0.0800000
-9567.0000000	0.0800000
-9566.0000000	0.0800000
-9565.0000000	0.0800000
-9564.0000000	0.0800000
-9563.0000000	0.0800000
-9562.0000000	0.0800000
-9561.0000000	0.0800000
-9560.0000000	0.0800000
-9559.0000000	0.0800000
-9558.0000000	0.0805000
-9557.0000000	0.0800000
-9556.0000000	0.0800000
-9555.0000000	0.0795238
-9554.0000000	0.0795238
-9553.0000000	0.0790000
-9552.0000000	0.0784211
-9551.0000000	0.0766667
-9550.0000000	0.0775000
-9549.0000000	0.0766667
-9548.0000000	0.0785000
-9547.0000000	0.0790476
-9546.0000000	0.0795000
-9545.0000000	0.0770000
-9544.0000000	0.0765000

-9543.0000000	0.0776191
-9542.0000000	0.0785000
-9541.0000000	0.0785714
-9540.0000000	0.0785000
-9539.0000000	0.0800000
-9538.0000000	0.0800000
-9537.0000000	0.0800000
-9536.0000000	0.0795000
-9535.0000000	0.0790476
-9534.0000000	0.0790000
-9533.0000000	0.0765000
-9532.0000000	0.0785714
-9531.0000000	0.0780000
-9530.0000000	0.0780000
-9529.0000000	0.0775000
-9528.0000000	0.0795000
-9527.0000000	0.0785714
-9526.0000000	0.0800000
-9525.0000000	0.0800000
-9524.0000000	0.0790000
-9523.0000000	0.0795238
-9522.0000000	0.0800000
-9521.0000000	0.0800000
-9520.0000000	0.0800000
-9519.0000000	0.0800000
-9518.0000000	0.0800000
-9517.0000000	0.0800000
-9516.0000000	0.0804762
-9515.0000000	0.0800000
-9514.0000000	0.0804762
-9513.0000000	0.0815000
-9512.0000000	0.0810000
-9511.0000000	0.0823810
-9510.0000000	0.0850000
-9509.0000000	0.0880000
-9508.0000000	0.0880000
-9507.0000000	0.0876191
-9506.0000000	0.0870000
-9505.0000000	0.0890476
-9504.0000000	0.0895000
-9503.0000000	0.0880000
-9502.0000000	0.0870000
-9501.0000000	0.0876191
-9500.0000000	0.0860000
-9499.0000000	0.0880000
-9498.0000000	0.0885715
-9497.0000000	0.0890000
-9496.0000000	0.0890476
-9495.0000000	0.0875000
-9494.0000000	0.0895000
-9493.0000000	0.0895238
-9492.0000000	0.0870000
-9491.0000000	0.0870000
-9490.0000000	0.0880000
-9489.0000000	0.0885714
-9488.0000000	0.0880000
-9487.0000000	0.0866667

-9486.0000000	0.0875000
-9485.0000000	0.0895000
-9484.0000000	0.0880000
-9483.0000000	0.0876191
-9482.0000000	0.0855000
-9481.0000000	0.0860000
-9480.0000000	0.0838095
-9479.0000000	0.0826316
-9478.0000000	0.0859091
-9477.0000000	0.0835000
-9476.0000000	0.0840000
-9475.0000000	0.0847619
-9474.0000000	0.0885000
-9473.0000000	0.0880000
-9472.0000000	0.0890000
-9471.0000000	0.0876191
-9470.0000000	0.0900000
-9469.0000000	0.0895000
-9468.0000000	0.0900000
-9467.0000000	0.0895238
-9466.0000000	0.0900000
-9465.0000000	0.0900000
-9464.0000000	0.0895000
-9463.0000000	0.0895238
-9462.0000000	0.0900000
-9461.0000000	0.0895000
-9460.0000000	0.0900000
-9459.0000000	0.0895238
-9458.0000000	0.0900000
-9457.0000000	0.0895238
-9456.0000000	0.0895000
-9455.0000000	0.0895000
-9454.0000000	0.0885000
-9453.0000000	0.0875000
-9452.0000000	0.0842857
-9451.0000000	0.0845000
-9450.0000000	0.0838095
-9449.0000000	0.0850000
-9448.0000000	0.0865000
-9447.0000000	0.0876191
-9446.0000000	0.0865000
-9445.0000000	0.0875000
-9444.0000000	0.0866667
-9443.0000000	0.0840000
-9442.0000000	0.0800000
-9441.0000000	0.0833333
-9440.0000000	0.0815000
-9439.0000000	0.0800000
-9438.0000000	0.0810000
-9437.0000000	0.0819048
-9436.0000000	0.0810000
-9435.0000000	0.0823810
-9434.0000000	0.0825000
-9433.0000000	0.0825000
-9432.0000000	0.0852381
-9431.0000000	0.0870000
-9430.0000000	0.0840000

-9429.0000000	0.0870000
-9428.0000000	0.0866667
-9427.0000000	0.0890476
-9426.0000000	0.0875000
-9425.0000000	0.0880000
-9424.0000000	0.0895000
-9423.0000000	0.0880953
-9422.0000000	0.0875000
-9421.0000000	0.0890000
-9420.0000000	0.0866667
-9419.0000000	0.0865000
-9418.0000000	0.0847619
-9417.0000000	0.0875000
-9416.0000000	0.0825000
-9415.0000000	0.0815000
-9414.0000000	0.0800000
-9413.0000000	0.0810000
-9412.0000000	0.0805000
-9411.0000000	0.0804762
-9410.0000000	0.0810000
-9409.0000000	0.0820000
-9408.0000000	0.0804762
-9407.0000000	0.0805000
-9406.0000000	0.0805263
-9405.0000000	0.0809524
-9404.0000000	0.0819048
-9403.0000000	0.0800000
-9402.0000000	0.0805000
-9401.0000000	0.0800000
-9400.0000000	0.0800000
-9399.0000000	0.0810000
-9398.0000000	0.0809524
-9397.0000000	0.0810000
-9396.0000000	0.0804762
-9395.0000000	0.0810000
-9394.0000000	0.0805000
-9393.0000000	0.0800000
-9392.0000000	0.0800000
-9391.0000000	0.0800000
-9390.0000000	0.0800000
-9389.0000000	0.0800000
-9388.0000000	0.0800000
-9387.0000000	0.0800000
-9386.0000000	0.0800000
-9385.0000000	0.0800000
-9384.0000000	0.0800000
-9383.0000000	0.0780000
-9382.0000000	0.0800000
-9381.0000000	0.0800000
-9380.0000000	0.0795238
-9379.0000000	0.0800000
-9378.0000000	0.0800000
-9377.0000000	0.0800000
-9376.0000000	0.0800000
-9375.0000000	0.0800000
-9374.0000000	0.0805000
-9373.0000000	0.0800000

-9372.0000000	0.0800000
-9371.0000000	0.0800000
-9370.0000000	0.0800000
-9369.0000000	0.0800000
-9368.0000000	0.0800000
-9367.0000000	0.0800000
-9366.0000000	0.0800000
-9365.0000000	0.0800000
-9364.0000000	0.0800000
-9363.0000000	0.0800000
-9362.0000000	0.0800000
-9361.0000000	0.0800000
-9360.0000000	0.0804762
-9359.0000000	0.0800000
-9358.0000000	0.0810000
-9357.0000000	0.0800000
-9356.0000000	0.0810000
-9355.0000000	0.0820000
-9354.0000000	0.0825000
-9353.0000000	0.0823810
-9352.0000000	0.0835000
-9351.0000000	0.0815000
-9350.0000000	0.0819048
-9349.0000000	0.0815000
-9348.0000000	0.0800000
-9347.0000000	0.0805000
-9346.0000000	0.0805000
-9345.0000000	0.0805000
-9344.0000000	0.0800000
-9343.0000000	0.0800000
-9342.0000000	0.0800000
-9341.0000000	0.0800000
-9340.0000000	0.0800000
-9339.0000000	0.0800000
-9338.0000000	0.0800000
-9337.0000000	0.0800000
-9336.0000000	0.0800000
-9335.0000000	0.0795000
-9334.0000000	0.0800000
-9333.0000000	0.0800000
-9332.0000000	0.0800000
-9331.0000000	0.0800000
-9330.0000000	0.0800000
-9329.0000000	0.0800000
-9328.0000000	0.0800000
-9327.0000000	0.0800000
-9326.0000000	0.0800000
-9325.0000000	0.0800000
-9324.0000000	0.0805000
-9323.0000000	0.0804762
-9322.0000000	0.0825000
-9321.0000000	0.0810000
-9320.0000000	0.0814286
-9319.0000000	0.0800000
-9318.0000000	0.0805000
-9317.0000000	0.0804762
-9316.0000000	0.0805000

-9315.0000000	0.0800000
-9314.0000000	0.0800000
-9313.0000000	0.0800000
-9312.0000000	0.0800000
-9311.0000000	0.0805000
-9310.0000000	0.0800000
-9309.0000000	0.0800000
-9308.0000000	0.0800000
-9307.0000000	0.0800000
-9306.0000000	0.0800000
-9305.0000000	0.0800000
-9304.0000000	0.0800000
-9303.0000000	0.0805000
-9302.0000000	0.0800000
-9301.0000000	0.0800000
-9300.0000000	0.0800000
-9299.0000000	0.0800000
-9298.0000000	0.0800000
-9297.0000000	0.0800000
-9296.0000000	0.0800000
-9295.0000000	0.0800000
-9294.0000000	0.0800000
-9293.0000000	0.0800000
-9292.0000000	0.0800000
-9291.0000000	0.0800000
-9290.0000000	0.0790000
-9289.0000000	0.0795000
-9288.0000000	0.0790000
-9287.0000000	0.0771429
-9286.0000000	0.0780000
-9285.0000000	0.0785000
-9284.0000000	0.0790476
-9283.0000000	0.0775000
-9282.0000000	0.0785000
-9281.0000000	0.0785714
-9280.0000000	0.0775000
-9279.0000000	0.0775000
-9278.0000000	0.0776191
-9277.0000000	0.0785000
-9276.0000000	0.0790000
-9275.0000000	0.0795238
-9274.0000000	0.0800000
-9273.0000000	0.0800000
-9272.0000000	0.0800000
-9271.0000000	0.0800000
-9270.0000000	0.0800000
-9269.0000000	0.0800000
-9268.0000000	0.0800000
-9267.0000000	0.0800000
-9266.0000000	0.0804762
-9265.0000000	0.0800000
-9264.0000000	0.0800000
-9263.0000000	0.0795000
-9262.0000000	0.0800000
-9261.0000000	0.0800000
-9260.0000000	0.0795238
-9259.0000000	0.0800000

-9258.0000000	0.0800000
-9257.0000000	0.0800000
-9256.0000000	0.0800000
-9255.0000000	0.0800000
-9254.0000000	0.0800000
-9253.0000000	0.0800000
-9252.0000000	0.0800000
-9251.0000000	0.0800000
-9250.0000000	0.0800000
-9249.0000000	0.0800000
-9248.0000000	0.0800000
-9247.0000000	0.0800000
-9246.0000000	0.0800000
-9245.0000000	0.0800000
-9244.0000000	0.0800000
-9243.0000000	0.0800000
-9242.0000000	0.0800000
-9241.0000000	0.0800000
-9240.0000000	0.0800000
-9239.0000000	0.0800000
-9238.0000000	0.0800000
-9237.0000000	0.0800000
-9236.0000000	0.0800000
-9235.0000000	0.0800000
-9234.0000000	0.0800000
-9233.0000000	0.0800000
-9232.0000000	0.0800000
-9231.0000000	0.0800000
-9230.0000000	0.0800000
-9229.0000000	0.0800000
-9228.0000000	0.0805000
-9227.0000000	0.0800000
-9226.0000000	0.0800000
-9225.0000000	0.0800000
-9224.0000000	0.0800000
-9223.0000000	0.0804762
-9222.0000000	0.0830000
-9221.0000000	0.0828572
-9220.0000000	0.0820000
-9219.0000000	0.0820000
-9218.0000000	0.0835000
-9217.0000000	0.0852381
-9216.0000000	0.0855000
-9215.0000000	0.0870000
-9214.0000000	0.0866667
-9213.0000000	0.0845000
-9212.0000000	0.0866667
-9211.0000000	0.0875000
-9210.0000000	0.0850000
-9209.0000000	0.0871429
-9208.0000000	0.0835000
-9207.0000000	0.0835000
-9206.0000000	0.0815000
-9205.0000000	0.0828572
-9204.0000000	0.0805000
-9203.0000000	0.0804762
-9202.0000000	0.0805000

-9201.0000000	0.0835000
-9200.0000000	0.0814286
-9199.0000000	0.0845000
-9198.0000000	0.0845000
-9197.0000000	0.0855000
-9196.0000000	0.0833333
-9195.0000000	0.0840000
-9194.0000000	0.0835000
-9193.0000000	0.0814286
-9192.0000000	0.0815000
-9191.0000000	0.0814286
-9190.0000000	0.0805000
-9189.0000000	0.0825000
-9188.0000000	0.0810000
-9187.0000000	0.0804762
-9186.0000000	0.0805000
-9185.0000000	0.0804762
-9184.0000000	0.0800000
-9183.0000000	0.0805000
-9182.0000000	0.0800000
-9181.0000000	0.0800000
-9180.0000000	0.0805000
-9179.0000000	0.0800000
-9178.0000000	0.0800000
-9177.0000000	0.0800000
-9176.0000000	0.0800000
-9175.0000000	0.0800000
-9174.0000000	0.0810000
-9173.0000000	0.0809524
-9172.0000000	0.0820000
-9171.0000000	0.0805000
-9170.0000000	0.0805000
-9169.0000000	0.0809524
-9168.0000000	0.0800000
-9167.0000000	0.0800000
-9166.0000000	0.0800000
-9165.0000000	0.0800000
-9164.0000000	0.0800000
-9163.0000000	0.0800000
-9162.0000000	0.0800000
-9161.0000000	0.0800000
-9160.0000000	0.0800000
-9159.0000000	0.0800000
-9158.0000000	0.0800000
-9157.0000000	0.0800000
-9156.0000000	0.0800000
-9155.0000000	0.0800000
-9154.0000000	0.0804762
-9153.0000000	0.0800000
-9152.0000000	0.0800000
-9151.0000000	0.0809524
-9150.0000000	0.0805000
-9149.0000000	0.0800000
-9148.0000000	0.0814286
-9147.0000000	0.0805000
-9146.0000000	0.0810000
-9145.0000000	0.0800000

-9144.0000000	0.0800000
-9143.0000000	0.0800000
-9142.0000000	0.0800000
-9141.0000000	0.0800000
-9140.0000000	0.0800000
-9139.0000000	0.0800000
-9138.0000000	0.0800000
-9137.0000000	0.0800000
-9136.0000000	0.0800000
-9135.0000000	0.0800000
-9134.0000000	0.0800000
-9133.0000000	0.0800000
-9132.0000000	0.0805000
-9131.0000000	0.0800000
-9130.0000000	0.0800000
-9129.0000000	0.0800000
-9128.0000000	0.0800000
-9127.0000000	0.0800000
-9126.0000000	0.0800000
-9125.0000000	0.0800000
-9124.0000000	0.0800000
-9123.0000000	0.0800000
-9122.0000000	0.0800000
-9121.0000000	0.0804762
-9120.0000000	0.0800000
-9119.0000000	0.0805000
-9118.0000000	0.0800000
-9117.0000000	0.0800000
-9116.0000000	0.0800000
-9115.0000000	0.0800000
-9114.0000000	0.0800000
-9113.0000000	0.0800000
-9112.0000000	0.0785714
-9111.0000000	0.0785000
-9110.0000000	0.0790000
-9109.0000000	0.0790476
-9108.0000000	0.0780000
-9107.0000000	0.0765000
-9106.0000000	0.0795000
-9105.0000000	0.0776191
-9104.0000000	0.0750000
-9103.0000000	0.0747619
-9102.0000000	0.0745000
-9101.0000000	0.0752381
-9100.0000000	0.0750000
-9099.0000000	0.0740000
-9098.0000000	0.0725000
-9097.0000000	0.0705000
-9096.0000000	0.0704762
-9095.0000000	0.0715000
-9094.0000000	0.0700000
-9093.0000000	0.0725000
-9092.0000000	0.0745000
-9091.0000000	0.0750000
-9090.0000000	0.0776191
-9089.0000000	0.0775000
-9088.0000000	0.0770000

-9087.0000000	0.0752381
-9086.0000000	0.0750000
-9085.0000000	0.0747619
-9084.0000000	0.0735000
-9083.0000000	0.0755000
-9082.0000000	0.0771429
-9081.0000000	0.0730000
-9080.0000000	0.0745000
-9079.0000000	0.0785000
-9078.0000000	0.0785714
-9077.0000000	0.0770000
-9076.0000000	0.0780952
-9075.0000000	0.0740000
-9074.0000000	0.0730000
-9073.0000000	0.0723810
-9072.0000000	0.0715000
-9071.0000000	0.0730000
-9070.0000000	0.0728572
-9069.0000000	0.0715000
-9068.0000000	0.0730000
-9067.0000000	0.0735000
-9066.0000000	0.0757143
-9065.0000000	0.0765000
-9064.0000000	0.0771429
-9063.0000000	0.0780000
-9062.0000000	0.0775000
-9061.0000000	0.0780000
-9060.0000000	0.0785714
-9059.0000000	0.0790000
-9058.0000000	0.0785714
-9057.0000000	0.0800000
-9056.0000000	0.0800000
-9055.0000000	0.0795238
-9054.0000000	0.0800000
-9053.0000000	0.0795000
-9052.0000000	0.0795000
-9051.0000000	0.0800000
-9050.0000000	0.0795000
-9049.0000000	0.0800000
-9048.0000000	0.0795238
-9047.0000000	0.0800000
-9046.0000000	0.0795238
-9045.0000000	0.0795000
-9044.0000000	0.0795000
-9043.0000000	0.0800000
-9042.0000000	0.0800000
-9041.0000000	0.0795000
-9040.0000000	0.0800000
-9039.0000000	0.0800000
-9038.0000000	0.0800000
-9037.0000000	0.0800000
-9036.0000000	0.0800000
-9035.0000000	0.0800000
-9034.0000000	0.0800000
-9033.0000000	0.0800000
-9032.0000000	0.0800000
-9031.0000000	0.0800000

-9030.0000000	0.0800000
-9029.0000000	0.0800000
-9028.0000000	0.0800000
-9027.0000000	0.0800000
-9026.0000000	0.0790000
-9025.0000000	0.0800000
-9024.0000000	0.0800000
-9023.0000000	0.0800000
-9022.0000000	0.0790000
-9021.0000000	0.0800000
-9020.0000000	0.0795000
-9019.0000000	0.0800000
-9018.0000000	0.0795238
-9017.0000000	0.0800000
-9016.0000000	0.0800000
-9015.0000000	0.0800000
-9014.0000000	0.0800000
-9013.0000000	0.0800000
-9012.0000000	0.0800000
-9011.0000000	0.0800000
-9010.0000000	0.0800000
-9009.0000000	0.0800000
-9008.0000000	0.0800000
-9007.0000000	0.0800000
-9006.0000000	0.0800000
-9005.0000000	0.0800000
-9004.0000000	0.0800000
-9003.0000000	0.0800000
-9002.0000000	0.0800000
-9001.0000000	0.0800000
-9000.0000000	0.0800000
-8999.0000000	0.0800000
-8998.0000000	0.0800000
-8997.0000000	0.0800000
-8996.0000000	0.0800000
-8995.0000000	0.0810000
-8994.0000000	0.0800000
-8993.0000000	0.0820000
-8992.0000000	0.0825000
-8991.0000000	0.0828572
-8990.0000000	0.0865000
-8989.0000000	0.0875000
-8988.0000000	0.0866667
-8987.0000000	0.0860000
-8986.0000000	0.0855000
-8985.0000000	0.0871429
-8984.0000000	0.0860000
-8983.0000000	0.0835000
-8982.0000000	0.0814286
-8981.0000000	0.0805000
-8980.0000000	0.0840000
-8979.0000000	0.0819048
-8978.0000000	0.0850000
-8977.0000000	0.0855000
-8976.0000000	0.0852381
-8975.0000000	0.0845000
-8974.0000000	0.0876191

-8973.0000000	0.0884211
-8972.0000000	0.0855000
-8971.0000000	0.0847619
-8970.0000000	0.0840000
-8969.0000000	0.0857143
-8968.0000000	0.0840000
-8967.0000000	0.0847619
-8966.0000000	0.0870000
-8965.0000000	0.0866667
-8964.0000000	0.0875000
-8963.0000000	0.0865000
-8962.0000000	0.0870000
-8961.0000000	0.0850000
-8960.0000000	0.0857143
-8959.0000000	0.0865000
-8958.0000000	0.0857143
-8957.0000000	0.0835000
-8956.0000000	0.0830000
-8955.0000000	0.0814286
-8954.0000000	0.0825000
-8953.0000000	0.0835000
-8952.0000000	0.0815000
-8951.0000000	0.0814286
-8950.0000000	0.0805000
-8949.0000000	0.0809524
-8948.0000000	0.0805000
-8947.0000000	0.0800000
-8946.0000000	0.0800000
-8945.0000000	0.0800000
-8944.0000000	0.0795000
-8943.0000000	0.0752381
-8942.0000000	0.0740000
-8941.0000000	0.0725000
-8940.0000000	0.0714286
-8939.0000000	0.0730000
-8938.0000000	0.0720000
-8937.0000000	0.0719048
-8936.0000000	0.0705000
-8935.0000000	0.0700000
-8934.0000000	0.0700000
-8933.0000000	0.0700000
-8932.0000000	0.0700000
-8931.0000000	0.0700000
-8930.0000000	0.0700000
-8929.0000000	0.0700000
-8928.0000000	0.0700000
-8927.0000000	0.0700000
-8926.0000000	0.0700000
-8925.0000000	0.0700000
-8924.0000000	0.0704762
-8923.0000000	0.0700000
-8922.0000000	0.0710000
-8921.0000000	0.0709524
-8920.0000000	0.0700000
-8919.0000000	0.0700000
-8918.0000000	0.0710000
-8917.0000000	0.0710000

-8916.0000000	0.0715000
-8915.0000000	0.0747619
-8914.0000000	0.0745000
-8913.0000000	0.0770000
-8912.0000000	0.0761905
-8911.0000000	0.0760000
-8910.0000000	0.0776191
-8909.0000000	0.0770000
-8908.0000000	0.0775000
-8907.0000000	0.0790476
-8906.0000000	0.0795000
-8905.0000000	0.0800000
-8904.0000000	0.0795000
-8903.0000000	0.0795238
-8902.0000000	0.0795000
-8901.0000000	0.0800000
-8900.0000000	0.0800000
-8899.0000000	0.0800000
-8898.0000000	0.0804762
-8897.0000000	0.0800000
-8896.0000000	0.0800000
-8895.0000000	0.0800000
-8894.0000000	0.0795238
-8893.0000000	0.0800000
-8892.0000000	0.0800000
-8891.0000000	0.0800000
-8890.0000000	0.0800000
-8889.0000000	0.0800000
-8888.0000000	0.0795000
-8887.0000000	0.0800000
-8886.0000000	0.0800000
-8885.0000000	0.0800000
-8884.0000000	0.0800000
-8883.0000000	0.0800000
-8882.0000000	0.0800000
-8881.0000000	0.0800000
-8880.0000000	0.0800000
-8879.0000000	0.0800000
-8878.0000000	0.0800000
-8877.0000000	0.0795000
-8876.0000000	0.0800000
-8875.0000000	0.0800000
-8874.0000000	0.0800000
-8873.0000000	0.0800000
-8872.0000000	0.0800000
-8871.0000000	0.0785714
-8870.0000000	0.0800000
-8869.0000000	0.0800000
-8868.0000000	0.0800000
-8867.0000000	0.0800000
-8866.0000000	0.0795000
-8865.0000000	0.0800000
-8864.0000000	0.0790476
-8863.0000000	0.0800000
-8862.0000000	0.0795000
-8861.0000000	0.0790476
-8860.0000000	0.0790000

-8859.0000000	0.0800000
-8858.0000000	0.0790000
-8857.0000000	0.0800000
-8856.0000000	0.0785000
-8855.0000000	0.0785714
-8854.0000000	0.0780000
-8853.0000000	0.0790000
-8852.0000000	0.0790476
-8851.0000000	0.0785000
-8850.0000000	0.0795000
-8849.0000000	0.0800000
-8848.0000000	0.0800000
-8847.0000000	0.0800000
-8846.0000000	0.0800000
-8845.0000000	0.0800000
-8844.0000000	0.0800000
-8843.0000000	0.0800000
-8842.0000000	0.0795000
-8841.0000000	0.0795000
-8840.0000000	0.0795238
-8839.0000000	0.0795000
-8838.0000000	0.0785000
-8837.0000000	0.0795238
-8836.0000000	0.0800000
-8835.0000000	0.0800000
-8834.0000000	0.0800000
-8833.0000000	0.0800000
-8832.0000000	0.0800000
-8831.0000000	0.0800000
-8830.0000000	0.0800000
-8829.0000000	0.0800000
-8828.0000000	0.0800000
-8827.0000000	0.0800000
-8826.0000000	0.0800000
-8825.0000000	0.0800000
-8824.0000000	0.0800000
-8823.0000000	0.0800000
-8822.0000000	0.0800000
-8821.0000000	0.0800000
-8820.0000000	0.0795000
-8819.0000000	0.0800000
-8818.0000000	0.0800000
-8817.0000000	0.0800000
-8816.0000000	0.0800000
-8815.0000000	0.0800000
-8814.0000000	0.0800000
-8813.0000000	0.0795238
-8812.0000000	0.0800000
-8811.0000000	0.0800000
-8810.0000000	0.0800000
-8809.0000000	0.0800000
-8808.0000000	0.0800000
-8807.0000000	0.0800000
-8806.0000000	0.0800000
-8805.0000000	0.0800000
-8804.0000000	0.0790000
-8803.0000000	0.0785714

-8802.0000000	0.0760000
-8801.0000000	0.0752381
-8800.0000000	0.0740000
-8799.0000000	0.0738095
-8798.0000000	0.0730000
-8797.0000000	0.0745000
-8796.0000000	0.0745000
-8795.0000000	0.0785000
-8794.0000000	0.0761905
-8793.0000000	0.0790000
-8792.0000000	0.0780952
-8791.0000000	0.0790000
-8790.0000000	0.0795000
-8789.0000000	0.0800000
-8788.0000000	0.0800000
-8787.0000000	0.0800000
-8786.0000000	0.0800000
-8785.0000000	0.0800000
-8784.0000000	0.0800000
-8783.0000000	0.0800000
-8782.0000000	0.0800000
-8781.0000000	0.0800000
-8780.0000000	0.0800000
-8779.0000000	0.0800000
-8778.0000000	0.0800000
-8777.0000000	0.0800000
-8776.0000000	0.0795238
-8775.0000000	0.0800000
-8774.0000000	0.0800000
-8773.0000000	0.0800000
-8772.0000000	0.0800000
-8771.0000000	0.0795238
-8770.0000000	0.0800000
-8769.0000000	0.0795000
-8768.0000000	0.0795238
-8767.0000000	0.0795000
-8766.0000000	0.0800000
-8765.0000000	0.0800000
-8764.0000000	0.0795238
-8763.0000000	0.0775000
-8762.0000000	0.0780952
-8761.0000000	0.0795000
-8760.0000000	0.0795000
-8759.0000000	0.0790000
-8758.0000000	0.0785714
-8757.0000000	0.0790000
-8756.0000000	0.0794737
-8755.0000000	0.0795455
-8754.0000000	0.0795000
-8753.0000000	0.0795238
-8752.0000000	0.0800000
-8751.0000000	0.0800000
-8750.0000000	0.0800000
-8749.0000000	0.0800000
-8748.0000000	0.0800000
-8747.0000000	0.0790476
-8746.0000000	0.0790000

-8745.0000000	0.0795000
-8744.0000000	0.0790476
-8743.0000000	0.0795000
-8742.0000000	0.0795000
-8741.0000000	0.0790000
-8740.0000000	0.0800000
-8739.0000000	0.0800000
-8738.0000000	0.0800000
-8737.0000000	0.0800000
-8736.0000000	0.0800000
-8735.0000000	0.0790476
-8734.0000000	0.0790000
-8733.0000000	0.0780000
-8732.0000000	0.0795238
-8731.0000000	0.0795000
-8730.0000000	0.0800000
-8729.0000000	0.0795000
-8728.0000000	0.0800000
-8727.0000000	0.0800000
-8726.0000000	0.0785000
-8725.0000000	0.0790476
-8724.0000000	0.0790476
-8723.0000000	0.0795000
-8722.0000000	0.0795000
-8721.0000000	0.0790000
-8720.0000000	0.0800000
-8719.0000000	0.0785714
-8718.0000000	0.0795000
-8717.0000000	0.0800000
-8716.0000000	0.0776191
-8715.0000000	0.0800000
-8714.0000000	0.0795000
-8713.0000000	0.0800000
-8712.0000000	0.0800000
-8711.0000000	0.0800000
-8710.0000000	0.0800000
-8709.0000000	0.0800000
-8708.0000000	0.0800000
-8707.0000000	0.0800000
-8706.0000000	0.0800000
-8705.0000000	0.0800000
-8704.0000000	0.0800000
-8703.0000000	0.0800000
-8702.0000000	0.0800000
-8701.0000000	0.0800000
-8700.0000000	0.0785000
-8699.0000000	0.0795000
-8698.0000000	0.0780000
-8697.0000000	0.0790476
-8696.0000000	0.0795238
-8695.0000000	0.0795000
-8694.0000000	0.0800000
-8693.0000000	0.0800000
-8692.0000000	0.0800000
-8691.0000000	0.0795000
-8690.0000000	0.0790000
-8689.0000000	0.0800000

-8688.0000000	0.0800000
-8687.0000000	0.0790000
-8686.0000000	0.0785714
-8685.0000000	0.0795000
-8684.0000000	0.0795000
-8683.0000000	0.0795238
-8682.0000000	0.0785000
-8681.0000000	0.0780000
-8680.0000000	0.0771429
-8679.0000000	0.0790000
-8678.0000000	0.0795000
-8677.0000000	0.0790476
-8676.0000000	0.0770000
-8675.0000000	0.0760000
-8674.0000000	0.0757143
-8673.0000000	0.0785000
-8672.0000000	0.0780952
-8671.0000000	0.0795000
-8670.0000000	0.0800000
-8669.0000000	0.0785000
-8668.0000000	0.0800000
-8667.0000000	0.0785714
-8666.0000000	0.0795000
-8665.0000000	0.0790476
-8664.0000000	0.0765000
-8663.0000000	0.0771429
-8662.0000000	0.0780000
-8661.0000000	0.0765000
-8660.0000000	0.0775000
-8659.0000000	0.0755000
-8658.0000000	0.0738095
-8657.0000000	0.0740000
-8656.0000000	0.0738095
-8655.0000000	0.0735000
-8654.0000000	0.0705000
-8653.0000000	0.0704762
-8652.0000000	0.0700000
-8651.0000000	0.0700000
-8650.0000000	0.0700000
-8649.0000000	0.0700000
-8648.0000000	0.0700000
-8647.0000000	0.0700000
-8646.0000000	0.0710000
-8645.0000000	0.0710000
-8644.0000000	0.0709524
-8643.0000000	0.0725000
-8642.0000000	0.0740000
-8641.0000000	0.0733333
-8640.0000000	0.0740000
-8639.0000000	0.0730000
-8638.0000000	0.0745000
-8637.0000000	0.0757143
-8636.0000000	0.0745000
-8635.0000000	0.0742857
-8634.0000000	0.0740000
-8633.0000000	0.0723810
-8632.0000000	0.0725000

-8631.0000000	0.0775000
-8630.0000000	0.0775000
-8629.0000000	0.0776191
-8628.0000000	0.0770000
-8627.0000000	0.0780000
-8626.0000000	0.0771429
-8625.0000000	0.0790000
-8624.0000000	0.0785000
-8623.0000000	0.0780000
-8622.0000000	0.0766667
-8621.0000000	0.0765000
-8620.0000000	0.0765000
-8619.0000000	0.0771429
-8618.0000000	0.0730000
-8617.0000000	0.0714286
-8616.0000000	0.0705000
-8615.0000000	0.0705000
-8614.0000000	0.0700000
-8613.0000000	0.0700000
-8612.0000000	0.0700000
-8611.0000000	0.0700000
-8610.0000000	0.0704762
-8609.0000000	0.0705000
-8608.0000000	0.0710000
-8607.0000000	0.0714286
-8606.0000000	0.0705000
-8605.0000000	0.0738095
-8604.0000000	0.0730000
-8603.0000000	0.0705000
-8602.0000000	0.0742857
-8601.0000000	0.0750000
-8600.0000000	0.0775000
-8599.0000000	0.0780000
-8598.0000000	0.0790476
-8597.0000000	0.0800000
-8596.0000000	0.0785000
-8595.0000000	0.0800000
-8594.0000000	0.0790000
-8593.0000000	0.0780000
-8592.0000000	0.0795238
-8591.0000000	0.0775000
-8590.0000000	0.0780000
-8589.0000000	0.0780952
-8588.0000000	0.0776191
-8587.0000000	0.0785000
-8586.0000000	0.0785000
-8585.0000000	0.0780000
-8584.0000000	0.0755000
-8583.0000000	0.0766667
-8582.0000000	0.0750000
-8581.0000000	0.0757143
-8580.0000000	0.0720000
-8579.0000000	0.0720000
-8578.0000000	0.0735000
-8577.0000000	0.0738095
-8576.0000000	0.0735000
-8575.0000000	0.0755000

-8574.0000000	0.0776191
-8573.0000000	0.0775000
-8572.0000000	0.0795238
-8571.0000000	0.0795000
-8570.0000000	0.0800000
-8569.0000000	0.0795238
-8568.0000000	0.0800000
-8567.0000000	0.0770000
-8566.0000000	0.0761905
-8565.0000000	0.0760000
-8564.0000000	0.0720000
-8563.0000000	0.0705000
-8562.0000000	0.0704762
-8561.0000000	0.0725000
-8560.0000000	0.0715000
-8559.0000000	0.0709524
-8558.0000000	0.0705000
-8557.0000000	0.0752381
-8556.0000000	0.0760000
-8555.0000000	0.0745000
-8554.0000000	0.0755000
-8553.0000000	0.0766667
-8552.0000000	0.0770000
-8551.0000000	0.0780000
-8550.0000000	0.0785714
-8549.0000000	0.0790000
-8548.0000000	0.0780000
-8547.0000000	0.0795238
-8546.0000000	0.0790000
-8545.0000000	0.0800000
-8544.0000000	0.0800000
-8543.0000000	0.0795000
-8542.0000000	0.0800000
-8541.0000000	0.0800000
-8540.0000000	0.0800000
-8539.0000000	0.0800000
-8538.0000000	0.0800000
-8537.0000000	0.0789474
-8536.0000000	0.0800000
-8535.0000000	0.0790476
-8534.0000000	0.0785000
-8533.0000000	0.0775000
-8532.0000000	0.0785000
-8531.0000000	0.0761905
-8530.0000000	0.0735000
-8529.0000000	0.0742857
-8528.0000000	0.0700000
-8527.0000000	0.0700000
-8526.0000000	0.0700000
-8525.0000000	0.0710000
-8524.0000000	0.0715000
-8523.0000000	0.0710000
-8522.0000000	0.0704762
-8521.0000000	0.0720000
-8520.0000000	0.0714286
-8519.0000000	0.0715000
-8518.0000000	0.0720000

-8517.0000000	0.0704762
-8516.0000000	0.0705000
-8515.0000000	0.0700000
-8514.0000000	0.0700000
-8513.0000000	0.0700000
-8512.0000000	0.0705000
-8511.0000000	0.0700000
-8510.0000000	0.0710000
-8509.0000000	0.0700000
-8508.0000000	0.0704762
-8507.0000000	0.0710000
-8506.0000000	0.0709524
-8505.0000000	0.0720000
-8504.0000000	0.0705000
-8503.0000000	0.0700000
-8502.0000000	0.0700000
-8501.0000000	0.0700000
-8500.0000000	0.0700000
-8499.0000000	0.0704762
-8498.0000000	0.0705000
-8497.0000000	0.0709524
-8496.0000000	0.0700000
-8495.0000000	0.0700000
-8494.0000000	0.0700000
-8493.0000000	0.0700000
-8492.0000000	0.0700000
-8491.0000000	0.0700000
-8490.0000000	0.0700000
-8489.0000000	0.0700000
-8488.0000000	0.0700000
-8487.0000000	0.0700000
-8486.0000000	0.0704762
-8485.0000000	0.0700000
-8484.0000000	0.0705000
-8483.0000000	0.0700000
-8482.0000000	0.0700000
-8481.0000000	0.0700000
-8480.0000000	0.0700000
-8479.0000000	0.0705000
-8478.0000000	0.0700000
-8477.0000000	0.0700000
-8476.0000000	0.0715000
-8475.0000000	0.0723810
-8474.0000000	0.0705000
-8473.0000000	0.0710000
-8472.0000000	0.0705000
-8471.0000000	0.0714286
-8470.0000000	0.0714286
-8469.0000000	0.0735000
-8468.0000000	0.0720000
-8467.0000000	0.0735000
-8466.0000000	0.0742105
-8465.0000000	0.0745455
-8464.0000000	0.0750000
-8463.0000000	0.0730000
-8462.0000000	0.0747619
-8461.0000000	0.0714286

-8460.0000000	0.0710000
-8459.0000000	0.0705000
-8458.0000000	0.0705000
-8457.0000000	0.0705000
-8456.0000000	0.0714286
-8455.0000000	0.0725000
-8454.0000000	0.0733333
-8453.0000000	0.0750000
-8452.0000000	0.0735000
-8451.0000000	0.0733333
-8450.0000000	0.0750000
-8449.0000000	0.0710000
-8448.0000000	0.0720000
-8447.0000000	0.0704762
-8446.0000000	0.0710000
-8445.0000000	0.0733333
-8444.0000000	0.0735000
-8443.0000000	0.0735000
-8442.0000000	0.0752381
-8441.0000000	0.0760000
-8440.0000000	0.0755000
-8439.0000000	0.0775000
-8438.0000000	0.0752381
-8437.0000000	0.0775000
-8436.0000000	0.0780952
-8435.0000000	0.0775000
-8434.0000000	0.0785000
-8433.0000000	0.0775000
-8432.0000000	0.0757143
-8431.0000000	0.0765000
-8430.0000000	0.0747619
-8429.0000000	0.0770000
-8428.0000000	0.0785000
-8427.0000000	0.0780000
-8426.0000000	0.0771429
-8425.0000000	0.0790000
-8424.0000000	0.0795000
-8423.0000000	0.0785714
-8422.0000000	0.0785714
-8421.0000000	0.0780000
-8420.0000000	0.0785000
-8419.0000000	0.0795000
-8418.0000000	0.0790000
-8417.0000000	0.0795238
-8416.0000000	0.0800000
-8415.0000000	0.0800000
-8414.0000000	0.0800000
-8413.0000000	0.0800000
-8412.0000000	0.0800000
-8411.0000000	0.0800000
-8410.0000000	0.0800000
-8409.0000000	0.0800000
-8408.0000000	0.0800000
-8407.0000000	0.0800000
-8406.0000000	0.0800000
-8405.0000000	0.0800000
-8404.0000000	0.0800000

-8403.0000000	0.0800000
-8402.0000000	0.0800000
-8401.0000000	0.0800000
-8400.0000000	0.0800000
-8399.0000000	0.0800000
-8398.0000000	0.0800000
-8397.0000000	0.0800000
-8396.0000000	0.0800000
-8395.0000000	0.0800000
-8394.0000000	0.0800000
-8393.0000000	0.0800000
-8392.0000000	0.0800000
-8391.0000000	0.0800000
-8390.0000000	0.0800000
-8389.0000000	0.0800000
-8388.0000000	0.0800000
-8387.0000000	0.0800000
-8386.0000000	0.0800000
-8385.0000000	0.0800000
-8384.0000000	0.0800000
-8383.0000000	0.0800000
-8382.0000000	0.0800000
-8381.0000000	0.0800000
-8380.0000000	0.0800000
-8379.0000000	0.0800000
-8378.0000000	0.0800000
-8377.0000000	0.0800000
-8376.0000000	0.0800000
-8375.0000000	0.0800000
-8374.0000000	0.0800000
-8373.0000000	0.0800000
-8372.0000000	0.0800000
-8371.0000000	0.0800000
-8370.0000000	0.0795238
-8369.0000000	0.0795000
-8368.0000000	0.0800000
-8367.0000000	0.0800000
-8366.0000000	0.0800000
-8365.0000000	0.0800000
-8364.0000000	0.0800000
-8363.0000000	0.0800000
-8362.0000000	0.0805000
-8361.0000000	0.0800000
-8360.0000000	0.0800000
-8359.0000000	0.0800000
-8358.0000000	0.0800000
-8357.0000000	0.0800000
-8356.0000000	0.0800000
-8355.0000000	0.0805000
-8354.0000000	0.0800000
-8353.0000000	0.0800000
-8352.0000000	0.0800000
-8351.0000000	0.0800000
-8350.0000000	0.0800000
-8349.0000000	0.0800000
-8348.0000000	0.0804762
-8347.0000000	0.0800000

-8346.0000000	0.0800000
-8345.0000000	0.0800000
-8344.0000000	0.0800000
-8343.0000000	0.0800000
-8342.0000000	0.0800000
-8341.0000000	0.0800000
-8340.0000000	0.0800000
-8339.0000000	0.0800000
-8338.0000000	0.0800000
-8337.0000000	0.0805000
-8336.0000000	0.0805000
-8335.0000000	0.0800000
-8334.0000000	0.0800000
-8333.0000000	0.0800000
-8332.0000000	0.0810000
-8331.0000000	0.0809524
-8330.0000000	0.0800000
-8329.0000000	0.0810000
-8328.0000000	0.0800000
-8327.0000000	0.0800000
-8326.0000000	0.0800000
-8325.0000000	0.0800000
-8324.0000000	0.0800000
-8323.0000000	0.0800000
-8322.0000000	0.0800000
-8321.0000000	0.0800000
-8320.0000000	0.0800000
-8319.0000000	0.0795000
-8318.0000000	0.0795238
-8317.0000000	0.0795000
-8316.0000000	0.0795000
-8315.0000000	0.0790476
-8314.0000000	0.0780000
-8313.0000000	0.0790000
-8312.0000000	0.0795000
-8311.0000000	0.0776191
-8310.0000000	0.0795000
-8309.0000000	0.0795000
-8308.0000000	0.0790476
-8307.0000000	0.0800000
-8306.0000000	0.0795000
-8305.0000000	0.0800000
-8304.0000000	0.0800000
-8303.0000000	0.0800000
-8302.0000000	0.0800000
-8301.0000000	0.0800000
-8300.0000000	0.0800000
-8299.0000000	0.0800000
-8298.0000000	0.0810000
-8297.0000000	0.0810000
-8296.0000000	0.0800000
-8295.0000000	0.0800000
-8294.0000000	0.0800000
-8293.0000000	0.0800000
-8292.0000000	0.0800000
-8291.0000000	0.0800000
-8290.0000000	0.0804762

-8289.0000000	0.0810000
-8288.0000000	0.0800000
-8287.0000000	0.0810000
-8286.0000000	0.0800000
-8285.0000000	0.0800000
-8284.0000000	0.0805000
-8283.0000000	0.0815000
-8282.0000000	0.0800000
-8281.0000000	0.0800000
-8280.0000000	0.0800000
-8279.0000000	0.0800000
-8278.0000000	0.0800000
-8277.0000000	0.0805000
-8276.0000000	0.0800000
-8275.0000000	0.0800000
-8274.0000000	0.0800000
-8273.0000000	0.0800000
-8272.0000000	0.0800000
-8271.0000000	0.0800000
-8270.0000000	0.0800000
-8269.0000000	0.0800000
-8268.0000000	0.0800000
-8267.0000000	0.0795000
-8266.0000000	0.0800000
-8265.0000000	0.0800000
-8264.0000000	0.0795238
-8263.0000000	0.0800000
-8262.0000000	0.0800000
-8261.0000000	0.0800000
-8260.0000000	0.0800000
-8259.0000000	0.0800000
-8258.0000000	0.0800000
-8257.0000000	0.0800000
-8256.0000000	0.0800000
-8255.0000000	0.0800000
-8254.0000000	0.0800000
-8253.0000000	0.0800000
-8252.0000000	0.0800000
-8251.0000000	0.0795238
-8250.0000000	0.0795000
-8249.0000000	0.0790000
-8248.0000000	0.0800000
-8247.0000000	0.0800000
-8246.0000000	0.0795000
-8245.0000000	0.0800000
-8244.0000000	0.0800000
-8243.0000000	0.0800000
-8242.0000000	0.0800000
-8241.0000000	0.0800000
-8240.0000000	0.0795238
-8239.0000000	0.0800000
-8238.0000000	0.0795000
-8237.0000000	0.0800000
-8236.0000000	0.0795238
-8235.0000000	0.0800000
-8234.0000000	0.0800000
-8233.0000000	0.0800000

-8232.0000000	0.0795000
-8231.0000000	0.0800000
-8230.0000000	0.0800000
-8229.0000000	0.0800000
-8228.0000000	0.0800000
-8227.0000000	0.0800000
-8226.0000000	0.0800000
-8225.0000000	0.0800000
-8224.0000000	0.0790000
-8223.0000000	0.0800000
-8222.0000000	0.0800000
-8221.0000000	0.0800000
-8220.0000000	0.0776191
-8219.0000000	0.0780000
-8218.0000000	0.0790476
-8217.0000000	0.0785000
-8216.0000000	0.0775000
-8215.0000000	0.0776191
-8214.0000000	0.0760000
-8213.0000000	0.0795000
-8212.0000000	0.0776191
-8211.0000000	0.0775000
-8210.0000000	0.0790000
-8209.0000000	0.0785714
-8208.0000000	0.0790000
-8207.0000000	0.0780000
-8206.0000000	0.0795238
-8205.0000000	0.0785000
-8204.0000000	0.0757143
-8203.0000000	0.0765000
-8202.0000000	0.0790000
-8201.0000000	0.0780000
-8200.0000000	0.0780952
-8199.0000000	0.0795000
-8198.0000000	0.0795000
-8197.0000000	0.0800000
-8196.0000000	0.0785000
-8195.0000000	0.0800000
-8194.0000000	0.0795000
-8193.0000000	0.0800000
-8192.0000000	0.0800000
-8191.0000000	0.0795000
-8190.0000000	0.0780952
-8189.0000000	0.0790000
-8188.0000000	0.0780952
-8187.0000000	0.0780000
-8186.0000000	0.0780000
-8185.0000000	0.0790000
-8184.0000000	0.0780952
-8183.0000000	0.0800000
-8182.0000000	0.0790000
-8181.0000000	0.0795238
-8180.0000000	0.0800000
-8179.0000000	0.0795238
-8178.0000000	0.0800000
-8177.0000000	0.0800000
-8176.0000000	0.0795238

-8175.0000000	0.0795000
-8174.0000000	0.0795000
-8173.0000000	0.0800000
-8172.0000000	0.0795000
-8171.0000000	0.0795000
-8170.0000000	0.0800000
-8169.0000000	0.0800000
-8168.0000000	0.0800000
-8167.0000000	0.0800000
-8166.0000000	0.0795000
-8165.0000000	0.0800000
-8164.0000000	0.0800000
-8163.0000000	0.0800000
-8162.0000000	0.0800000
-8161.0000000	0.0800000
-8160.0000000	0.0800000
-8159.0000000	0.0795238
-8158.0000000	0.0800000
-8157.0000000	0.0800000
-8156.0000000	0.0795000
-8155.0000000	0.0800000
-8154.0000000	0.0800000
-8153.0000000	0.0790000
-8152.0000000	0.0800000
-8151.0000000	0.0800000
-8150.0000000	0.0800000
-8149.0000000	0.0795238
-8148.0000000	0.0800000
-8147.0000000	0.0800000
-8146.0000000	0.0795000
-8145.0000000	0.0795238
-8144.0000000	0.0795000
-8143.0000000	0.0780952
-8142.0000000	0.0775000
-8141.0000000	0.0790000
-8140.0000000	0.0775000
-8139.0000000	0.0761905
-8138.0000000	0.0750000
-8137.0000000	0.0733333
-8136.0000000	0.0715000
-8135.0000000	0.0720000
-8134.0000000	0.0704762
-8133.0000000	0.0700000
-8132.0000000	0.0710000
-8131.0000000	0.0710000
-8130.0000000	0.0715000
-8129.0000000	0.0723810
-8128.0000000	0.0709524
-8127.0000000	0.0705000
-8126.0000000	0.0725000
-8125.0000000	0.0720000
-8124.0000000	0.0723810
-8123.0000000	0.0725000
-8122.0000000	0.0740000
-8121.0000000	0.0747619
-8120.0000000	0.0738095
-8119.0000000	0.0745000

-8118.0000000	0.0745000
-8117.0000000	0.0755000
-8116.0000000	0.0757143
-8115.0000000	0.0760000
-8114.0000000	0.0745000
-8113.0000000	0.0757143
-8112.0000000	0.0720000
-8111.0000000	0.0735000
-8110.0000000	0.0760000
-8109.0000000	0.0757143
-8108.0000000	0.0770000
-8107.0000000	0.0775000
-8106.0000000	0.0776191
-8105.0000000	0.0770000
-8104.0000000	0.0761905
-8103.0000000	0.0784211
-8102.0000000	0.0795238
-8101.0000000	0.0790000
-8100.0000000	0.0785714
-8099.0000000	0.0790000
-8098.0000000	0.0795000
-8097.0000000	0.0785714
-8096.0000000	0.0785000
-8095.0000000	0.0785000
-8094.0000000	0.0780000
-8093.0000000	0.0747619
-8092.0000000	0.0745000
-8091.0000000	0.0733333
-8090.0000000	0.0725000
-8089.0000000	0.0719048
-8088.0000000	0.0720000
-8087.0000000	0.0710000
-8086.0000000	0.0715000
-8085.0000000	0.0704762
-8084.0000000	0.0705000
-8083.0000000	0.0700000
-8082.0000000	0.0709524
-8081.0000000	0.0700000
-8080.0000000	0.0700000
-8079.0000000	0.0700000
-8078.0000000	0.0700000
-8077.0000000	0.0700000
-8076.0000000	0.0700000
-8075.0000000	0.0700000
-8074.0000000	0.0700000
-8073.0000000	0.0700000
-8072.0000000	0.0700000
-8071.0000000	0.0700000
-8070.0000000	0.0700000
-8069.0000000	0.0695000
-8068.0000000	0.0695238
-8067.0000000	0.0690000
-8066.0000000	0.0700000
-8065.0000000	0.0700000
-8064.0000000	0.0700000
-8063.0000000	0.0700000
-8062.0000000	0.0700000

-8061.0000000	0.0700000
-8060.0000000	0.0700000
-8059.0000000	0.0700000
-8058.0000000	0.0700000
-8057.0000000	0.0700000
-8056.0000000	0.0700000
-8055.0000000	0.0700000
-8054.0000000	0.0704762
-8053.0000000	0.0700000
-8052.0000000	0.0700000
-8051.0000000	0.0700000
-8050.0000000	0.0700000
-8049.0000000	0.0700000
-8048.0000000	0.0700000
-8047.0000000	0.0700000
-8046.0000000	0.0704762
-8045.0000000	0.0700000
-8044.0000000	0.0700000
-8043.0000000	0.0704762
-8042.0000000	0.0700000
-8041.0000000	0.0709524
-8040.0000000	0.0710000
-8039.0000000	0.0700000
-8038.0000000	0.0705000
-8037.0000000	0.0704762
-8036.0000000	0.0710000
-8035.0000000	0.0705000
-8034.0000000	0.0700000
-8033.0000000	0.0700000
-8032.0000000	0.0700000
-8031.0000000	0.0700000
-8030.0000000	0.0700000
-8029.0000000	0.0695455
-8028.0000000	0.0685000
-8027.0000000	0.0685000
-8026.0000000	0.0675000
-8025.0000000	0.0690476
-8024.0000000	0.0690000
-8023.0000000	0.0695000
-8022.0000000	0.0700000
-8021.0000000	0.0700000
-8020.0000000	0.0700000
-8019.0000000	0.0700000
-8018.0000000	0.0700000
-8017.0000000	0.0700000
-8016.0000000	0.0700000
-8015.0000000	0.0700000
-8014.0000000	0.0695000
-8013.0000000	0.0676191
-8012.0000000	0.0695000
-8011.0000000	0.0690000
-8010.0000000	0.0685714
-8009.0000000	0.0700000
-8008.0000000	0.0700000
-8007.0000000	0.0700000
-8006.0000000	0.0695000
-8005.0000000	0.0700000

-8004.0000000	0.0700000
-8003.0000000	0.0700000
-8002.0000000	0.0700000
-8001.0000000	0.0700000
-8000.0000000	0.0700000
-7999.0000000	0.0700000
-7998.0000000	0.0700000
-7997.0000000	0.0700000
-7996.0000000	0.0700000
-7995.0000000	0.0700000
-7994.0000000	0.0700000
-7993.0000000	0.0700000
-7992.0000000	0.0700000
-7991.0000000	0.0700000
-7990.0000000	0.0700000
-7989.0000000	0.0700000
-7988.0000000	0.0700000
-7987.0000000	0.0700000
-7986.0000000	0.0700000
-7985.0000000	0.0695000
-7984.0000000	0.0700000
-7983.0000000	0.0690476
-7982.0000000	0.0695000
-7981.0000000	0.0695000
-7980.0000000	0.0690000
-7979.0000000	0.0680952
-7978.0000000	0.0695000
-7977.0000000	0.0685714
-7976.0000000	0.0700000
-7975.0000000	0.0700000
-7974.0000000	0.0700000
-7973.0000000	0.0695238
-7972.0000000	0.0700000
-7971.0000000	0.0700000
-7970.0000000	0.0700000
-7969.0000000	0.0695000
-7968.0000000	0.0695238
-7967.0000000	0.0690000
-7966.0000000	0.0695000
-7965.0000000	0.0690000
-7964.0000000	0.0695000
-7963.0000000	0.0685714
-7962.0000000	0.0695238
-7961.0000000	0.0675000
-7960.0000000	0.0689474
-7959.0000000	0.0661905
-7958.0000000	0.0671429
-7957.0000000	0.0655000
-7956.0000000	0.0670000
-7955.0000000	0.0676191
-7954.0000000	0.0670000
-7953.0000000	0.0676191
-7952.0000000	0.0645000
-7951.0000000	0.0650000
-7950.0000000	0.0666667
-7949.0000000	0.0670000
-7948.0000000	0.0660000

-7947.0000000	0.0655000
-7946.0000000	0.0661905
-7945.0000000	0.0670000
-7944.0000000	0.0675000
-7943.0000000	0.0685714
-7942.0000000	0.0685000
-7941.0000000	0.0680952
-7940.0000000	0.0695000
-7939.0000000	0.0700000
-7938.0000000	0.0700000
-7937.0000000	0.0700000
-7936.0000000	0.0700000
-7935.0000000	0.0700000
-7934.0000000	0.0700000
-7933.0000000	0.0695000
-7932.0000000	0.0700000
-7931.0000000	0.0700000
-7930.0000000	0.0700000
-7929.0000000	0.0700000
-7928.0000000	0.0700000
-7927.0000000	0.0700000
-7926.0000000	0.0700000
-7925.0000000	0.0695238
-7924.0000000	0.0695000
-7923.0000000	0.0700000
-7922.0000000	0.0690000
-7921.0000000	0.0685000
-7920.0000000	0.0685000
-7919.0000000	0.0680952
-7918.0000000	0.0680000
-7917.0000000	0.0685000
-7916.0000000	0.0671429
-7915.0000000	0.0690000
-7914.0000000	0.0690476
-7913.0000000	0.0680000
-7912.0000000	0.0670000
-7911.0000000	0.0675000
-7910.0000000	0.0685714
-7909.0000000	0.0660000
-7908.0000000	0.0650000
-7907.0000000	0.0652381
-7906.0000000	0.0645000
-7905.0000000	0.0655000
-7904.0000000	0.0647619
-7903.0000000	0.0650000
-7902.0000000	0.0642857
-7901.0000000	0.0675000
-7900.0000000	0.0685000
-7899.0000000	0.0675000
-7898.0000000	0.0680953
-7897.0000000	0.0680000
-7896.0000000	0.0695000
-7895.0000000	0.0700000
-7894.0000000	0.0695000
-7893.0000000	0.0700000
-7892.0000000	0.0700000
-7891.0000000	0.0700000

-7890.0000000	0.0700000
-7889.0000000	0.0695238
-7888.0000000	0.0695000
-7887.0000000	0.0700000
-7886.0000000	0.0700000
-7885.0000000	0.0700000
-7884.0000000	0.0695238
-7883.0000000	0.0700000
-7882.0000000	0.0700000
-7881.0000000	0.0695000
-7880.0000000	0.0700000
-7879.0000000	0.0700000
-7878.0000000	0.0700000
-7877.0000000	0.0700000
-7876.0000000	0.0705000
-7875.0000000	0.0700000
-7874.0000000	0.0700000
-7873.0000000	0.0700000
-7872.0000000	0.0700000
-7871.0000000	0.0700000
-7870.0000000	0.0700000
-7869.0000000	0.0700000
-7868.0000000	0.0700000
-7867.0000000	0.0700000
-7866.0000000	0.0700000
-7865.0000000	0.0700000
-7864.0000000	0.0700000
-7863.0000000	0.0700000
-7862.0000000	0.0704762
-7861.0000000	0.0700000
-7860.0000000	0.0705000
-7859.0000000	0.0700000
-7858.0000000	0.0700000
-7857.0000000	0.0700000
-7856.0000000	0.0700000
-7855.0000000	0.0710000
-7854.0000000	0.0700000
-7853.0000000	0.0700000
-7852.0000000	0.0700000
-7851.0000000	0.0700000
-7850.0000000	0.0700000
-7849.0000000	0.0700000
-7848.0000000	0.0705000
-7847.0000000	0.0700000
-7846.0000000	0.0700000
-7845.0000000	0.0700000
-7844.0000000	0.0700000
-7843.0000000	0.0700000
-7842.0000000	0.0700000
-7841.0000000	0.0700000
-7840.0000000	0.0700000
-7839.0000000	0.0700000
-7838.0000000	0.0700000
-7837.0000000	0.0700000
-7836.0000000	0.0700000
-7835.0000000	0.0700000
-7834.0000000	0.0710000

-7833.0000000	0.0715000
-7832.0000000	0.0719048
-7831.0000000	0.0725000
-7830.0000000	0.0730000
-7829.0000000	0.0720000
-7828.0000000	0.0709524
-7827.0000000	0.0705000
-7826.0000000	0.0709524
-7825.0000000	0.0710000
-7824.0000000	0.0710000
-7823.0000000	0.0704762
-7822.0000000	0.0705000
-7821.0000000	0.0700000
-7820.0000000	0.0715000
-7819.0000000	0.0700000
-7818.0000000	0.0710000
-7817.0000000	0.0709524
-7816.0000000	0.0700000
-7815.0000000	0.0710000
-7814.0000000	0.0709524
-7813.0000000	0.0730000
-7812.0000000	0.0715000
-7811.0000000	0.0714286
-7810.0000000	0.0730000
-7809.0000000	0.0720000
-7808.0000000	0.0730000
-7807.0000000	0.0709524
-7806.0000000	0.0725000
-7805.0000000	0.0700000
-7804.0000000	0.0700000
-7803.0000000	0.0700000
-7802.0000000	0.0685714
-7801.0000000	0.0690000
-7800.0000000	0.0666667
-7799.0000000	0.0680000
-7798.0000000	0.0680000
-7797.0000000	0.0690000
-7796.0000000	0.0700000
-7795.0000000	0.0690000
-7794.0000000	0.0700000
-7793.0000000	0.0700000
-7792.0000000	0.0700000
-7791.0000000	0.0700000
-7790.0000000	0.0700000
-7789.0000000	0.0700000
-7788.0000000	0.0700000
-7787.0000000	0.0700000
-7786.0000000	0.0700000
-7785.0000000	0.0705000
-7784.0000000	0.0700000
-7783.0000000	0.0705000
-7782.0000000	0.0705000
-7781.0000000	0.0700000
-7780.0000000	0.0700000
-7779.0000000	0.0710000
-7778.0000000	0.0700000
-7777.0000000	0.0700000

-7776.0000000	0.0700000
-7775.0000000	0.0700000
-7774.0000000	0.0700000
-7773.0000000	0.0700000
-7772.0000000	0.0700000
-7771.0000000	0.0705000
-7770.0000000	0.0700000
-7769.0000000	0.0700000
-7768.0000000	0.0710000
-7767.0000000	0.0715000
-7766.0000000	0.0719048
-7765.0000000	0.0700000
-7764.0000000	0.0710000
-7763.0000000	0.0719048
-7762.0000000	0.0710000
-7761.0000000	0.0715000
-7760.0000000	0.0705000
-7759.0000000	0.0700000
-7758.0000000	0.0700000
-7757.0000000	0.0709524
-7756.0000000	0.0705000
-7755.0000000	0.0705000
-7754.0000000	0.0700000
-7753.0000000	0.0700000
-7752.0000000	0.0700000
-7751.0000000	0.0700000
-7750.0000000	0.0700000
-7749.0000000	0.0700000
-7748.0000000	0.0700000
-7747.0000000	0.0700000
-7746.0000000	0.0700000
-7745.0000000	0.0700000
-7744.0000000	0.0700000
-7743.0000000	0.0700000
-7742.0000000	0.0700000
-7741.0000000	0.0700000
-7740.0000000	0.0700000
-7739.0000000	0.0700000
-7738.0000000	0.0700000
-7737.0000000	0.0700000
-7736.0000000	0.0700000
-7735.0000000	0.0705000
-7734.0000000	0.0700000
-7733.0000000	0.0700000
-7732.0000000	0.0700000
-7731.0000000	0.0695000
-7730.0000000	0.0695000
-7729.0000000	0.0695238
-7728.0000000	0.0695000
-7727.0000000	0.0695000
-7726.0000000	0.0695238
-7725.0000000	0.0690000
-7724.0000000	0.0695238
-7723.0000000	0.0700000
-7722.0000000	0.0675000
-7721.0000000	0.0676191
-7720.0000000	0.0685000

-7719.0000000	0.0685000
-7718.0000000	0.0700000
-7717.0000000	0.0685000
-7716.0000000	0.0700000
-7715.0000000	0.0695000
-7714.0000000	0.0695238
-7713.0000000	0.0690000
-7712.0000000	0.0680953
-7711.0000000	0.0685000
-7710.0000000	0.0685000
-7709.0000000	0.0671429
-7708.0000000	0.0680000
-7707.0000000	0.0655000
-7706.0000000	0.0680000
-7705.0000000	0.0671429
-7704.0000000	0.0665000
-7703.0000000	0.0666667
-7702.0000000	0.0650000
-7701.0000000	0.0650000
-7700.0000000	0.0635000
-7699.0000000	0.0625000
-7698.0000000	0.0642857
-7697.0000000	0.0620000
-7696.0000000	0.0638095
-7695.0000000	0.0680000
-7694.0000000	0.0657143
-7693.0000000	0.0685000
-7692.0000000	0.0660000
-7691.0000000	0.0685000
-7690.0000000	0.0685714
-7689.0000000	0.0675000
-7688.0000000	0.0660000
-7687.0000000	0.0657143
-7686.0000000	0.0650000
-7685.0000000	0.0655000
-7684.0000000	0.0661905
-7683.0000000	0.0645000
-7682.0000000	0.0642857
-7681.0000000	0.0615000
-7680.0000000	0.0605000
-7679.0000000	0.0610000
-7678.0000000	0.0609524
-7677.0000000	0.0615000
-7676.0000000	0.0620000
-7675.0000000	0.0614286
-7674.0000000	0.0615000
-7673.0000000	0.0609524
-7672.0000000	0.0640000
-7671.0000000	0.0640000
-7670.0000000	0.0640000
-7669.0000000	0.0645000
-7668.0000000	0.0661905
-7667.0000000	0.0680000
-7666.0000000	0.0666667
-7665.0000000	0.0665000
-7664.0000000	0.0690476
-7663.0000000	0.0675000

-7662.0000000	0.0675000
-7661.0000000	0.0695000
-7660.0000000	0.0690000
-7659.0000000	0.0685714
-7658.0000000	0.0695000
-7657.0000000	0.0690476
-7656.0000000	0.0695000
-7655.0000000	0.0700000
-7654.0000000	0.0700000
-7653.0000000	0.0685000
-7652.0000000	0.0695000
-7651.0000000	0.0661905
-7650.0000000	0.0675000
-7649.0000000	0.0675000
-7648.0000000	0.0657143
-7647.0000000	0.0645000
-7646.0000000	0.0657143
-7645.0000000	0.0625000
-7644.0000000	0.0615000
-7643.0000000	0.0620000
-7642.0000000	0.0628571
-7641.0000000	0.0620000
-7640.0000000	0.0640000
-7639.0000000	0.0615000
-7638.0000000	0.0609524
-7637.0000000	0.0640000
-7636.0000000	0.0638095
-7635.0000000	0.0650000
-7634.0000000	0.0666667
-7633.0000000	0.0695000
-7632.0000000	0.0685000
-7631.0000000	0.0660000
-7630.0000000	0.0685714
-7629.0000000	0.0665000
-7628.0000000	0.0635000
-7627.0000000	0.0619047
-7626.0000000	0.0600000
-7625.0000000	0.0600000
-7624.0000000	0.0600000
-7623.0000000	0.0600000
-7622.0000000	0.0600000
-7621.0000000	0.0600000
-7620.0000000	0.0600000
-7619.0000000	0.0600000
-7618.0000000	0.0600000
-7617.0000000	0.0610000
-7616.0000000	0.0600000
-7615.0000000	0.0600000
-7614.0000000	0.0600000
-7613.0000000	0.0600000
-7612.0000000	0.0600000
-7611.0000000	0.0600000
-7610.0000000	0.0600000
-7609.0000000	0.0605000
-7608.0000000	0.0604762
-7607.0000000	0.0600000
-7606.0000000	0.0604762

-7605.0000000	0.0615000
-7604.0000000	0.0630000
-7603.0000000	0.0619048
-7602.0000000	0.0655000
-7601.0000000	0.0650000
-7600.0000000	0.0665000
-7599.0000000	0.0647619
-7598.0000000	0.0670000
-7597.0000000	0.0671429
-7596.0000000	0.0675000
-7595.0000000	0.0665000
-7594.0000000	0.0690476
-7593.0000000	0.0690000
-7592.0000000	0.0685000
-7591.0000000	0.0685714
-7590.0000000	0.0700000
-7589.0000000	0.0690000
-7588.0000000	0.0690476
-7587.0000000	0.0695000
-7586.0000000	0.0700000
-7585.0000000	0.0695238
-7584.0000000	0.0700000
-7583.0000000	0.0695000
-7582.0000000	0.0700000
-7581.0000000	0.0695000
-7580.0000000	0.0695000
-7579.0000000	0.0700000
-7578.0000000	0.0700000
-7577.0000000	0.0700000
-7576.0000000	0.0700000
-7575.0000000	0.0700000
-7574.0000000	0.0700000
-7573.0000000	0.0695238
-7572.0000000	0.0695000
-7571.0000000	0.0700000
-7570.0000000	0.0700000
-7569.0000000	0.0695238
-7568.0000000	0.0695000
-7567.0000000	0.0700000
-7566.0000000	0.0700000
-7565.0000000	0.0700000
-7564.0000000	0.0700000
-7563.0000000	0.0700000
-7562.0000000	0.0700000
-7561.0000000	0.0700000
-7560.0000000	0.0700000
-7559.0000000	0.0700000
-7558.0000000	0.0700000
-7557.0000000	0.0700000
-7556.0000000	0.0700000
-7555.0000000	0.0700000
-7554.0000000	0.0700000
-7553.0000000	0.0700000
-7552.0000000	0.0700000
-7551.0000000	0.0700000
-7550.0000000	0.0700000
-7549.0000000	0.0700000

-7548.0000000	0.0700000
-7547.0000000	0.0705000
-7546.0000000	0.0700000
-7545.0000000	0.0700000
-7544.0000000	0.0700000
-7543.0000000	0.0700000
-7542.0000000	0.0700000
-7541.0000000	0.0695000
-7540.0000000	0.0695000
-7539.0000000	0.0700000
-7538.0000000	0.0690000
-7537.0000000	0.0685714
-7536.0000000	0.0680000
-7535.0000000	0.0670000
-7534.0000000	0.0670000
-7533.0000000	0.0675000
-7532.0000000	0.0652381
-7531.0000000	0.0675000
-7530.0000000	0.0671429
-7529.0000000	0.0670000
-7528.0000000	0.0685714
-7527.0000000	0.0673684
-7526.0000000	0.0666667
-7525.0000000	0.0660000
-7524.0000000	0.0642857
-7523.0000000	0.0650000
-7522.0000000	0.0685000
-7521.0000000	0.0661905
-7520.0000000	0.0670000
-7519.0000000	0.0690476
-7518.0000000	0.0680000
-7517.0000000	0.0675000
-7516.0000000	0.0675000
-7515.0000000	0.0671429
-7514.0000000	0.0675000
-7513.0000000	0.0675000
-7512.0000000	0.0685714
-7511.0000000	0.0675000
-7510.0000000	0.0690000
-7509.0000000	0.0666667
-7508.0000000	0.0645000
-7507.0000000	0.0604762
-7506.0000000	0.0605000
-7505.0000000	0.0600000
-7504.0000000	0.0605000
-7503.0000000	0.0604762
-7502.0000000	0.0630000
-7501.0000000	0.0615000
-7500.0000000	0.0614286
-7499.0000000	0.0655000
-7498.0000000	0.0647619
-7497.0000000	0.0660000
-7496.0000000	0.0675000
-7495.0000000	0.0650000
-7494.0000000	0.0647619
-7493.0000000	0.0650000
-7492.0000000	0.0660000

-7491.0000000	0.0657143
-7490.0000000	0.0650000
-7489.0000000	0.0655000
-7488.0000000	0.0666667
-7487.0000000	0.0665000
-7486.0000000	0.0650000
-7485.0000000	0.0661905
-7484.0000000	0.0645000
-7483.0000000	0.0615000
-7482.0000000	0.0600000
-7481.0000000	0.0600000
-7480.0000000	0.0600000
-7479.0000000	0.0600000
-7478.0000000	0.0600000
-7477.0000000	0.0600000
-7476.0000000	0.0600000
-7475.0000000	0.0600000
-7474.0000000	0.0600000
-7473.0000000	0.0600000
-7472.0000000	0.0600000
-7471.0000000	0.0600000
-7470.0000000	0.0600000
-7469.0000000	0.0600000
-7468.0000000	0.0600000
-7467.0000000	0.0600000
-7466.0000000	0.0600000
-7465.0000000	0.0600000
-7464.0000000	0.0600000
-7463.0000000	0.0600000
-7462.0000000	0.0600000
-7461.0000000	0.0600000
-7460.0000000	0.0600000
-7459.0000000	0.0605000
-7458.0000000	0.0600000
-7457.0000000	0.0604762
-7456.0000000	0.0610000
-7455.0000000	0.0600000
-7454.0000000	0.0600000
-7453.0000000	0.0605000
-7452.0000000	0.0600000
-7451.0000000	0.0605000
-7450.0000000	0.0600000
-7449.0000000	0.0605000
-7448.0000000	0.0604762
-7447.0000000	0.0615000
-7446.0000000	0.0619048
-7445.0000000	0.0600000
-7444.0000000	0.0605000
-7443.0000000	0.0600000
-7442.0000000	0.0600000
-7441.0000000	0.0600000
-7440.0000000	0.0600000
-7439.0000000	0.0600000
-7438.0000000	0.0605000
-7437.0000000	0.0600000
-7436.0000000	0.0600000
-7435.0000000	0.0600000

-7434.0000000	0.0600000
-7433.0000000	0.0600000
-7432.0000000	0.0600000
-7431.0000000	0.0600000
-7430.0000000	0.0600000
-7429.0000000	0.0600000
-7428.0000000	0.0600000
-7427.0000000	0.0600000
-7426.0000000	0.0600000
-7425.0000000	0.0600000
-7424.0000000	0.0600000
-7423.0000000	0.0600000
-7422.0000000	0.0600000
-7421.0000000	0.0600000
-7420.0000000	0.0600000
-7419.0000000	0.0600000
-7418.0000000	0.0600000
-7417.0000000	0.0600000
-7416.0000000	0.0600000
-7415.0000000	0.0600000
-7414.0000000	0.0600000
-7413.0000000	0.0600000
-7412.0000000	0.0600000
-7411.0000000	0.0600000
-7410.0000000	0.0600000
-7409.0000000	0.0600000
-7408.0000000	0.0600000
-7407.0000000	0.0600000
-7406.0000000	0.0595000
-7405.0000000	0.0595000
-7404.0000000	0.0595000
-7403.0000000	0.0600000
-7402.0000000	0.0600000
-7401.0000000	0.0595238
-7400.0000000	0.0600000
-7399.0000000	0.0600000
-7398.0000000	0.0600000
-7397.0000000	0.0600000
-7396.0000000	0.0600000
-7395.0000000	0.0600000
-7394.0000000	0.0600000
-7393.0000000	0.0610000
-7392.0000000	0.0609524
-7391.0000000	0.0600000
-7390.0000000	0.0605000
-7389.0000000	0.0605000
-7388.0000000	0.0604762
-7387.0000000	0.0610000
-7386.0000000	0.0600000
-7385.0000000	0.0600000
-7384.0000000	0.0600000
-7383.0000000	0.0600000
-7382.0000000	0.0604762
-7381.0000000	0.0605263
-7380.0000000	0.0600000
-7379.0000000	0.0604762
-7378.0000000	0.0615000

-7377.0000000	0.0625000
-7376.0000000	0.0604762
-7375.0000000	0.0605000
-7374.0000000	0.0615000
-7373.0000000	0.0614286
-7372.0000000	0.0605000
-7371.0000000	0.0609524
-7370.0000000	0.0600000
-7369.0000000	0.0615000
-7368.0000000	0.0620000
-7367.0000000	0.0647619
-7366.0000000	0.0640000
-7365.0000000	0.0615000
-7364.0000000	0.0652381
-7363.0000000	0.0655000
-7362.0000000	0.0638095
-7361.0000000	0.0645000
-7360.0000000	0.0635000
-7359.0000000	0.0625000
-7358.0000000	0.0666667
-7357.0000000	0.0650000
-7356.0000000	0.0640000
-7355.0000000	0.0642857
-7354.0000000	0.0645000
-7353.0000000	0.0615000
-7352.0000000	0.0652381
-7351.0000000	0.0650000
-7350.0000000	0.0665000
-7349.0000000	0.0657143
-7348.0000000	0.0650000
-7347.0000000	0.0650000
-7346.0000000	0.0619048
-7345.0000000	0.0650000
-7344.0000000	0.0640000
-7343.0000000	0.0647619
-7342.0000000	0.0675000
-7341.0000000	0.0675000
-7340.0000000	0.0652381
-7339.0000000	0.0675000
-7338.0000000	0.0660000
-7337.0000000	0.0680952
-7336.0000000	0.0685000
-7335.0000000	0.0660000
-7334.0000000	0.0680953
-7333.0000000	0.0675000
-7332.0000000	0.0685714
-7331.0000000	0.0680000
-7330.0000000	0.0690000
-7329.0000000	0.0690000
-7328.0000000	0.0695238
-7327.0000000	0.0665000
-7326.0000000	0.0685000
-7325.0000000	0.0676190
-7324.0000000	0.0665000
-7323.0000000	0.0650000
-7322.0000000	0.0655000
-7321.0000000	0.0657143

-7320.0000000	0.0655000
-7319.0000000	0.0661905
-7318.0000000	0.0625000
-7317.0000000	0.0620000
-7316.0000000	0.0619048
-7315.0000000	0.0610000
-7314.0000000	0.0625000
-7313.0000000	0.0623810
-7312.0000000	0.0610000
-7311.0000000	0.0605000
-7310.0000000	0.0619048
-7309.0000000	0.0615000
-7308.0000000	0.0615000
-7307.0000000	0.0620000
-7306.0000000	0.0628571
-7305.0000000	0.0630000
-7304.0000000	0.0614286
-7303.0000000	0.0605000
-7302.0000000	0.0600000
-7301.0000000	0.0604762
-7300.0000000	0.0610000
-7299.0000000	0.0610000
-7298.0000000	0.0605000
-7297.0000000	0.0609524
-7296.0000000	0.0610000
-7295.0000000	0.0619047
-7294.0000000	0.0600000
-7293.0000000	0.0610000
-7292.0000000	0.0604762
-7291.0000000	0.0610000
-7290.0000000	0.0620000
-7289.0000000	0.0633333
-7288.0000000	0.0650000
-7287.0000000	0.0645000
-7286.0000000	0.0655000
-7285.0000000	0.0671429
-7284.0000000	0.0670000
-7283.0000000	0.0661905
-7282.0000000	0.0675000
-7281.0000000	0.0665000
-7280.0000000	0.0680953
-7279.0000000	0.0675000
-7278.0000000	0.0680000
-7277.0000000	0.0670000
-7276.0000000	0.0671429
-7275.0000000	0.0680000
-7274.0000000	0.0685714
-7273.0000000	0.0670000
-7272.0000000	0.0680000
-7271.0000000	0.0685714
-7270.0000000	0.0695000
-7269.0000000	0.0695000
-7268.0000000	0.0690000
-7267.0000000	0.0695238
-7266.0000000	0.0695000
-7265.0000000	0.0695238
-7264.0000000	0.0700000

-7263.0000000	0.0700000
-7262.0000000	0.0695000
-7261.0000000	0.0700000
-7260.0000000	0.0690000
-7259.0000000	0.0695000
-7258.0000000	0.0690476
-7257.0000000	0.0700000
-7256.0000000	0.0700000
-7255.0000000	0.0695000
-7254.0000000	0.0695000
-7253.0000000	0.0700000
-7252.0000000	0.0695000
-7251.0000000	0.0700000
-7250.0000000	0.0685000
-7249.0000000	0.0685714
-7248.0000000	0.0695000
-7247.0000000	0.0695000
-7246.0000000	0.0685714
-7245.0000000	0.0695000
-7244.0000000	0.0695238
-7243.0000000	0.0700000
-7242.0000000	0.0690000
-7241.0000000	0.0695238
-7240.0000000	0.0695000
-7239.0000000	0.0680000
-7238.0000000	0.0680000
-7237.0000000	0.0695000
-7236.0000000	0.0690476
-7235.0000000	0.0676190
-7234.0000000	0.0680000
-7233.0000000	0.0675000
-7232.0000000	0.0665000
-7231.0000000	0.0675000
-7230.0000000	0.0671429
-7229.0000000	0.0695000
-7228.0000000	0.0685714
-7227.0000000	0.0685000
-7226.0000000	0.0695238
-7225.0000000	0.0700000
-7224.0000000	0.0695000
-7223.0000000	0.0695000
-7222.0000000	0.0695238
-7221.0000000	0.0700000
-7220.0000000	0.0695000
-7219.0000000	0.0700000
-7218.0000000	0.0700000
-7217.0000000	0.0700000
-7216.0000000	0.0700000
-7215.0000000	0.0700000
-7214.0000000	0.0700000
-7213.0000000	0.0700000
-7212.0000000	0.0700000
-7211.0000000	0.0700000
-7210.0000000	0.0700000
-7209.0000000	0.0700000
-7208.0000000	0.0700000
-7207.0000000	0.0700000

-7206.0000000	0.0700000
-7205.0000000	0.0704762
-7204.0000000	0.0705000
-7203.0000000	0.0700000
-7202.0000000	0.0715000
-7201.0000000	0.0700000
-7200.0000000	0.0740000
-7199.0000000	0.0710000
-7198.0000000	0.0723810
-7197.0000000	0.0740000
-7196.0000000	0.0742857
-7195.0000000	0.0760000
-7194.0000000	0.0770000
-7193.0000000	0.0775000
-7192.0000000	0.0790476
-7191.0000000	0.0775000
-7190.0000000	0.0790000
-7189.0000000	0.0795238
-7188.0000000	0.0790000
-7187.0000000	0.0795000
-7186.0000000	0.0795238
-7185.0000000	0.0795000
-7184.0000000	0.0795000
-7183.0000000	0.0790476
-7182.0000000	0.0765000
-7181.0000000	0.0755000
-7180.0000000	0.0709524
-7179.0000000	0.0705000
-7178.0000000	0.0700000
-7177.0000000	0.0704762
-7176.0000000	0.0705000
-7175.0000000	0.0700000
-7174.0000000	0.0700000
-7173.0000000	0.0700000
-7172.0000000	0.0700000
-7171.0000000	0.0700000
-7170.0000000	0.0700000
-7169.0000000	0.0700000
-7168.0000000	0.0700000
-7167.0000000	0.0700000
-7166.0000000	0.0700000
-7165.0000000	0.0700000
-7164.0000000	0.0700000
-7163.0000000	0.0700000
-7162.0000000	0.0700000
-7161.0000000	0.0700000
-7160.0000000	0.0700000
-7159.0000000	0.0700000
-7158.0000000	0.0700000
-7157.0000000	0.0690000
-7156.0000000	0.0700000
-7155.0000000	0.0690476
-7154.0000000	0.0700000
-7153.0000000	0.0700000
-7152.0000000	0.0700000
-7151.0000000	0.0700000
-7150.0000000	0.0695238

-7149.0000000	0.0700000
-7148.0000000	0.0700000
-7147.0000000	0.0700000
-7146.0000000	0.0700000
-7145.0000000	0.0700000
-7144.0000000	0.0700000
-7143.0000000	0.0705000
-7142.0000000	0.0700000
-7141.0000000	0.0705000
-7140.0000000	0.0700000
-7139.0000000	0.0700000
-7138.0000000	0.0700000
-7137.0000000	0.0700000
-7136.0000000	0.0700000
-7135.0000000	0.0700000
-7134.0000000	0.0700000
-7133.0000000	0.0700000
-7132.0000000	0.0700000
-7131.0000000	0.0700000
-7130.0000000	0.0700000
-7129.0000000	0.0690476
-7128.0000000	0.0695000
-7127.0000000	0.0700000
-7126.0000000	0.0652381
-7125.0000000	0.0645000
-7124.0000000	0.0635000
-7123.0000000	0.0635000
-7122.0000000	0.0652381
-7121.0000000	0.0655000
-7120.0000000	0.0680000
-7119.0000000	0.0700000
-7118.0000000	0.0700000
-7117.0000000	0.0700000
-7116.0000000	0.0695000
-7115.0000000	0.0700000
-7114.0000000	0.0700000
-7113.0000000	0.0700000
-7112.0000000	0.0700000
-7111.0000000	0.0700000
-7110.0000000	0.0695238
-7109.0000000	0.0700000
-7108.0000000	0.0695238
-7107.0000000	0.0700000
-7106.0000000	0.0700000
-7105.0000000	0.0700000
-7104.0000000	0.0700000
-7103.0000000	0.0700000
-7102.0000000	0.0700000
-7101.0000000	0.0700000
-7100.0000000	0.0700000
-7099.0000000	0.0700000
-7098.0000000	0.0700000
-7097.0000000	0.0700000
-7096.0000000	0.0700000
-7095.0000000	0.0709524
-7094.0000000	0.0700000
-7093.0000000	0.0700000

-7092.0000000	0.0700000
-7091.0000000	0.0700000
-7090.0000000	0.0700000
-7089.0000000	0.0705000
-7088.0000000	0.0700000
-7087.0000000	0.0700000
-7086.0000000	0.0700000
-7085.0000000	0.0700000
-7084.0000000	0.0700000
-7083.0000000	0.0700000
-7082.0000000	0.0700000
-7081.0000000	0.0700000
-7080.0000000	0.0700000
-7079.0000000	0.0705000
-7078.0000000	0.0700000
-7077.0000000	0.0700000
-7076.0000000	0.0700000
-7075.0000000	0.0700000
-7074.0000000	0.0700000
-7073.0000000	0.0700000
-7072.0000000	0.0700000
-7071.0000000	0.0700000
-7070.0000000	0.0700000
-7069.0000000	0.0700000
-7068.0000000	0.0700000
-7067.0000000	0.0700000
-7066.0000000	0.0700000
-7065.0000000	0.0700000
-7064.0000000	0.0700000
-7063.0000000	0.0700000
-7062.0000000	0.0700000
-7061.0000000	0.0705000
-7060.0000000	0.0700000
-7059.0000000	0.0700000
-7058.0000000	0.0700000
-7057.0000000	0.0700000
-7056.0000000	0.0719048
-7055.0000000	0.0715000
-7054.0000000	0.0700000
-7053.0000000	0.0700000
-7052.0000000	0.0700000
-7051.0000000	0.0700000
-7050.0000000	0.0704762
-7049.0000000	0.0700000
-7048.0000000	0.0700000
-7047.0000000	0.0700000
-7046.0000000	0.0700000
-7045.0000000	0.0695000
-7044.0000000	0.0700000
-7043.0000000	0.0695000
-7042.0000000	0.0700000
-7041.0000000	0.0700000
-7040.0000000	0.0700000
-7039.0000000	0.0700000
-7038.0000000	0.0700000
-7037.0000000	0.0700000
-7036.0000000	0.0700000

-7035.0000000	0.0704762
-7034.0000000	0.0700000
-7033.0000000	0.0700000
-7032.0000000	0.0700000
-7031.0000000	0.0700000
-7030.0000000	0.0700000
-7029.0000000	0.0700000
-7028.0000000	0.0700000
-7027.0000000	0.0700000
-7026.0000000	0.0700000
-7025.0000000	0.0700000
-7024.0000000	0.0700000
-7023.0000000	0.0700000
-7022.0000000	0.0700000
-7021.0000000	0.0700000
-7020.0000000	0.0700000
-7019.0000000	0.0705000
-7018.0000000	0.0700000
-7017.0000000	0.0700000
-7016.0000000	0.0705000
-7015.0000000	0.0700000
-7014.0000000	0.0704762
-7013.0000000	0.0700000
-7012.0000000	0.0700000
-7011.0000000	0.0700000
-7010.0000000	0.0700000
-7009.0000000	0.0700000
-7008.0000000	0.0700000
-7007.0000000	0.0700000
-7006.0000000	0.0705000
-7005.0000000	0.0700000
-7004.0000000	0.0700000
-7003.0000000	0.0700000
-7002.0000000	0.0700000
-7001.0000000	0.0700000
-7000.0000000	0.0700000
-6999.0000000	0.0700000
-6998.0000000	0.0700000
-6997.0000000	0.0700000
-6996.0000000	0.0700000
-6995.0000000	0.0700000
-6994.0000000	0.0700000
-6993.0000000	0.0700000
-6992.0000000	0.0700000
-6991.0000000	0.0700000
-6990.0000000	0.0700000
-6989.0000000	0.0700000
-6988.0000000	0.0700000
-6987.0000000	0.0700000
-6986.0000000	0.0700000
-6985.0000000	0.0700000
-6984.0000000	0.0700000
-6983.0000000	0.0700000
-6982.0000000	0.0700000
-6981.0000000	0.0695238
-6980.0000000	0.0700000
-6979.0000000	0.0690000

-6978.0000000	0.0700000
-6977.0000000	0.0675000
-6976.0000000	0.0695000
-6975.0000000	0.0695000
-6974.0000000	0.0700000
-6973.0000000	0.0690000
-6972.0000000	0.0700000
-6971.0000000	0.0695000
-6970.0000000	0.0700000
-6969.0000000	0.0700000
-6968.0000000	0.0700000
-6967.0000000	0.0695000
-6966.0000000	0.0690000
-6965.0000000	0.0676190
-6964.0000000	0.0665000
-6963.0000000	0.0676190
-6962.0000000	0.0685000
-6961.0000000	0.0695000
-6960.0000000	0.0695000
-6959.0000000	0.0676190
-6958.0000000	0.0680000
-6957.0000000	0.0670000
-6956.0000000	0.0666667
-6955.0000000	0.0650000
-6954.0000000	0.0635000
-6953.0000000	0.0609524
-6952.0000000	0.0605000
-6951.0000000	0.0600000
-6950.0000000	0.0610000
-6949.0000000	0.0625000
-6948.0000000	0.0615000
-6947.0000000	0.0625000
-6946.0000000	0.0633333
-6945.0000000	0.0645000
-6944.0000000	0.0666667
-6943.0000000	0.0675000
-6942.0000000	0.0661905
-6941.0000000	0.0670000
-6940.0000000	0.0695000
-6939.0000000	0.0671429
-6938.0000000	0.0665000
-6937.0000000	0.0655000
-6936.0000000	0.0665000
-6935.0000000	0.0685714
-6934.0000000	0.0690000
-6933.0000000	0.0700000
-6932.0000000	0.0700000
-6931.0000000	0.0695000
-6930.0000000	0.0700000
-6929.0000000	0.0695000
-6928.0000000	0.0700000
-6927.0000000	0.0700000
-6926.0000000	0.0700000
-6925.0000000	0.0700000
-6924.0000000	0.0695238
-6923.0000000	0.0700000
-6922.0000000	0.0700000

-6921.0000000	0.0700000
-6920.0000000	0.0700000
-6919.0000000	0.0700000
-6918.0000000	0.0700000
-6917.0000000	0.0700000
-6916.0000000	0.0700000
-6915.0000000	0.0700000
-6914.0000000	0.0700000
-6913.0000000	0.0695000
-6912.0000000	0.0700000
-6911.0000000	0.0700000
-6910.0000000	0.0700000
-6909.0000000	0.0700000
-6908.0000000	0.0700000
-6907.0000000	0.0700000
-6906.0000000	0.0700000
-6905.0000000	0.0700000
-6904.0000000	0.0700000
-6903.0000000	0.0700000
-6902.0000000	0.0715000
-6901.0000000	0.0700000
-6900.0000000	0.0700000
-6899.0000000	0.0700000
-6898.0000000	0.0700000
-6897.0000000	0.0700000
-6896.0000000	0.0700000
-6895.0000000	0.0700000
-6894.0000000	0.0700000
-6893.0000000	0.0695000
-6892.0000000	0.0690000
-6891.0000000	0.0695000
-6890.0000000	0.0700000
-6889.0000000	0.0700000
-6888.0000000	0.0695000
-6887.0000000	0.0690476
-6886.0000000	0.0690000
-6885.0000000	0.0695000
-6884.0000000	0.0690476
-6883.0000000	0.0680000
-6882.0000000	0.0675000
-6881.0000000	0.0657143
-6880.0000000	0.0655000
-6879.0000000	0.0640000
-6878.0000000	0.0628571
-6877.0000000	0.0635000
-6876.0000000	0.0655000
-6875.0000000	0.0680000
-6874.0000000	0.0680000
-6873.0000000	0.0695238
-6872.0000000	0.0695238
-6871.0000000	0.0700000
-6870.0000000	0.0700000
-6869.0000000	0.0700000
-6868.0000000	0.0700000
-6867.0000000	0.0695000
-6866.0000000	0.0700000
-6865.0000000	0.0700000

-6864.0000000	0.0700000
-6863.0000000	0.0700000
-6862.0000000	0.0700000
-6861.0000000	0.0700000
-6860.0000000	0.0700000
-6859.0000000	0.0700000
-6858.0000000	0.0700000
-6857.0000000	0.0700000
-6856.0000000	0.0700000
-6855.0000000	0.0700000
-6854.0000000	0.0700000
-6853.0000000	0.0705000
-6852.0000000	0.0700000
-6851.0000000	0.0700000
-6850.0000000	0.0700000
-6849.0000000	0.0700000
-6848.0000000	0.0700000
-6847.0000000	0.0700000
-6846.0000000	0.0695000
-6845.0000000	0.0700000
-6844.0000000	0.0700000
-6843.0000000	0.0700000
-6842.0000000	0.0704762
-6841.0000000	0.0700000
-6840.0000000	0.0700000
-6839.0000000	0.0700000
-6838.0000000	0.0700000
-6837.0000000	0.0700000
-6836.0000000	0.0695238
-6835.0000000	0.0700000
-6834.0000000	0.0700000
-6833.0000000	0.0700000
-6832.0000000	0.0700000
-6831.0000000	0.0695000
-6830.0000000	0.0695000
-6829.0000000	0.0685714
-6828.0000000	0.0685000
-6827.0000000	0.0675000
-6826.0000000	0.0690476
-6825.0000000	0.0690000
-6824.0000000	0.0676191
-6823.0000000	0.0700000
-6822.0000000	0.0695000
-6821.0000000	0.0700000
-6820.0000000	0.0695238
-6819.0000000	0.0700000
-6818.0000000	0.0700000
-6817.0000000	0.0700000
-6816.0000000	0.0700000
-6815.0000000	0.0695238
-6814.0000000	0.0700000
-6813.0000000	0.0700000
-6812.0000000	0.0700000
-6811.0000000	0.0700000
-6810.0000000	0.0695000
-6809.0000000	0.0700000
-6808.0000000	0.0695238

-6807.0000000	0.0695000
-6806.0000000	0.0680952
-6805.0000000	0.0680000
-6804.0000000	0.0685714
-6803.0000000	0.0689474
-6802.0000000	0.0685714
-6801.0000000	0.0690000
-6800.0000000	0.0695000
-6799.0000000	0.0695238
-6798.0000000	0.0700000
-6797.0000000	0.0695238
-6796.0000000	0.0685000
-6795.0000000	0.0690000
-6794.0000000	0.0690000
-6793.0000000	0.0680952
-6792.0000000	0.0700000
-6791.0000000	0.0700000
-6790.0000000	0.0695238
-6789.0000000	0.0700000
-6788.0000000	0.0700000
-6787.0000000	0.0700000
-6786.0000000	0.0700000
-6785.0000000	0.0700000
-6784.0000000	0.0700000
-6783.0000000	0.0690000
-6782.0000000	0.0700000
-6781.0000000	0.0695238
-6780.0000000	0.0700000
-6779.0000000	0.0700000
-6778.0000000	0.0675000
-6777.0000000	0.0676191
-6776.0000000	0.0671429
-6775.0000000	0.0690000
-6774.0000000	0.0690000
-6773.0000000	0.0695238
-6772.0000000	0.0695000
-6771.0000000	0.0700000
-6770.0000000	0.0695000
-6769.0000000	0.0695238
-6768.0000000	0.0685000
-6767.0000000	0.0695238
-6766.0000000	0.0700000
-6765.0000000	0.0695000
-6764.0000000	0.0700000
-6763.0000000	0.0666667
-6762.0000000	0.0645000
-6761.0000000	0.0640000
-6760.0000000	0.0638095
-6759.0000000	0.0635000
-6758.0000000	0.0642857
-6757.0000000	0.0635000
-6756.0000000	0.0665000
-6755.0000000	0.0645000
-6754.0000000	0.0642857
-6753.0000000	0.0665000
-6752.0000000	0.0645000
-6751.0000000	0.0628571

-6750.0000000	0.0670000
-6749.0000000	0.0670000
-6748.0000000	0.0666667
-6747.0000000	0.0670000
-6746.0000000	0.0650000
-6745.0000000	0.0661905
-6744.0000000	0.0675000
-6743.0000000	0.0680000
-6742.0000000	0.0671429
-6741.0000000	0.0690000
-6740.0000000	0.0700000
-6739.0000000	0.0690000
-6738.0000000	0.0690476
-6737.0000000	0.0695238
-6736.0000000	0.0700000
-6735.0000000	0.0700000
-6734.0000000	0.0700000
-6733.0000000	0.0700000
-6732.0000000	0.0700000
-6731.0000000	0.0700000
-6730.0000000	0.0700000
-6729.0000000	0.0700000
-6728.0000000	0.0695238
-6727.0000000	0.0700000
-6726.0000000	0.0695000
-6725.0000000	0.0700000
-6724.0000000	0.0700000
-6723.0000000	0.0700000
-6722.0000000	0.0700000
-6721.0000000	0.0700000
-6720.0000000	0.0700000
-6719.0000000	0.0700000
-6718.0000000	0.0700000
-6717.0000000	0.0704762
-6716.0000000	0.0725000
-6715.0000000	0.0704762
-6714.0000000	0.0725000
-6713.0000000	0.0752381
-6712.0000000	0.0765000
-6711.0000000	0.0775000
-6710.0000000	0.0775000
-6709.0000000	0.0766667
-6708.0000000	0.0775000
-6707.0000000	0.0780000
-6706.0000000	0.0728572
-6705.0000000	0.0715000
-6704.0000000	0.0705000
-6703.0000000	0.0725000
-6702.0000000	0.0723810
-6701.0000000	0.0700000
-6700.0000000	0.0725000
-6699.0000000	0.0714286
-6698.0000000	0.0725000
-6697.0000000	0.0733333
-6696.0000000	0.0725000
-6695.0000000	0.0745000
-6694.0000000	0.0730000

-6693.0000000	0.0719048
-6692.0000000	0.0700000
-6691.0000000	0.0700000
-6690.0000000	0.0700000
-6689.0000000	0.0700000
-6688.0000000	0.0700000
-6687.0000000	0.0700000
-6686.0000000	0.0700000
-6685.0000000	0.0700000
-6684.0000000	0.0700000
-6683.0000000	0.0700000
-6682.0000000	0.0685714
-6681.0000000	0.0700000
-6680.0000000	0.0700000
-6679.0000000	0.0700000
-6678.0000000	0.0700000
-6677.0000000	0.0700000
-6676.0000000	0.0700000
-6675.0000000	0.0690000
-6674.0000000	0.0700000
-6673.0000000	0.0700000
-6672.0000000	0.0700000
-6671.0000000	0.0705000
-6670.0000000	0.0700000
-6669.0000000	0.0700000
-6668.0000000	0.0700000
-6667.0000000	0.0700000
-6666.0000000	0.0700000
-6665.0000000	0.0705000
-6664.0000000	0.0710000
-6663.0000000	0.0704762
-6662.0000000	0.0700000
-6661.0000000	0.0714286
-6660.0000000	0.0700000
-6659.0000000	0.0720000
-6658.0000000	0.0740000
-6657.0000000	0.0728572
-6656.0000000	0.0715000
-6655.0000000	0.0710000
-6654.0000000	0.0704762
-6653.0000000	0.0715000
-6652.0000000	0.0704762
-6651.0000000	0.0700000
-6650.0000000	0.0700000
-6649.0000000	0.0700000
-6648.0000000	0.0700000
-6647.0000000	0.0700000
-6646.0000000	0.0700000
-6645.0000000	0.0700000
-6644.0000000	0.0700000
-6643.0000000	0.0700000
-6642.0000000	0.0695238
-6641.0000000	0.0695000
-6640.0000000	0.0700000
-6639.0000000	0.0700000
-6638.0000000	0.0700000
-6637.0000000	0.0700000

-6636.0000000	0.0705000
-6635.0000000	0.0705000
-6634.0000000	0.0720000
-6633.0000000	0.0747619
-6632.0000000	0.0770000
-6631.0000000	0.0776191
-6630.0000000	0.0770000
-6629.0000000	0.0765000
-6628.0000000	0.0780000
-6627.0000000	0.0761905
-6626.0000000	0.0760000
-6625.0000000	0.0765000
-6624.0000000	0.0757143
-6623.0000000	0.0735000
-6622.0000000	0.0714286
-6621.0000000	0.0705000
-6620.0000000	0.0705000
-6619.0000000	0.0700000
-6618.0000000	0.0700000
-6617.0000000	0.0700000
-6616.0000000	0.0700000
-6615.0000000	0.0704762
-6614.0000000	0.0700000
-6613.0000000	0.0700000
-6612.0000000	0.0720000
-6611.0000000	0.0709524
-6610.0000000	0.0725000
-6609.0000000	0.0709524
-6608.0000000	0.0700000
-6607.0000000	0.0705000
-6606.0000000	0.0704762
-6605.0000000	0.0705000
-6604.0000000	0.0700000
-6603.0000000	0.0700000
-6602.0000000	0.0700000
-6601.0000000	0.0700000
-6600.0000000	0.0700000
-6599.0000000	0.0700000
-6598.0000000	0.0700000
-6597.0000000	0.0700000
-6596.0000000	0.0695000
-6595.0000000	0.0700000
-6594.0000000	0.0700000
-6593.0000000	0.0700000
-6592.0000000	0.0700000
-6591.0000000	0.0695000
-6590.0000000	0.0695000
-6589.0000000	0.0695000
-6588.0000000	0.0695238
-6587.0000000	0.0700000
-6586.0000000	0.0704762
-6585.0000000	0.0700000
-6584.0000000	0.0700000
-6583.0000000	0.0700000
-6582.0000000	0.0700000
-6581.0000000	0.0700000
-6580.0000000	0.0710000

-6579.0000000	0.0700000
-6578.0000000	0.0700000
-6577.0000000	0.0704762
-6576.0000000	0.0700000
-6575.0000000	0.0700000
-6574.0000000	0.0700000
-6573.0000000	0.0700000
-6572.0000000	0.0700000
-6571.0000000	0.0700000
-6570.0000000	0.0700000
-6569.0000000	0.0700000
-6568.0000000	0.0700000
-6567.0000000	0.0700000
-6566.0000000	0.0700000
-6565.0000000	0.0700000
-6564.0000000	0.0700000
-6563.0000000	0.0700000
-6562.0000000	0.0700000
-6561.0000000	0.0700000
-6560.0000000	0.0700000
-6559.0000000	0.0700000
-6558.0000000	0.0700000
-6557.0000000	0.0710000
-6556.0000000	0.0705000
-6555.0000000	0.0728572
-6554.0000000	0.0710000
-6553.0000000	0.0710000
-6552.0000000	0.0709524
-6551.0000000	0.0700000
-6550.0000000	0.0725000
-6549.0000000	0.0733333
-6548.0000000	0.0720000
-6547.0000000	0.0715000
-6546.0000000	0.0742857
-6545.0000000	0.0745000
-6544.0000000	0.0765000
-6543.0000000	0.0766667
-6542.0000000	0.0790000
-6541.0000000	0.0790000
-6540.0000000	0.0790476
-6539.0000000	0.0790000
-6538.0000000	0.0785000
-6537.0000000	0.0790000
-6536.0000000	0.0780952
-6535.0000000	0.0785000
-6534.0000000	0.0795238
-6533.0000000	0.0785000
-6532.0000000	0.0790000
-6531.0000000	0.0795238
-6530.0000000	0.0795000
-6529.0000000	0.0790000
-6528.0000000	0.0795000
-6527.0000000	0.0795238
-6526.0000000	0.0800000
-6525.0000000	0.0795000
-6524.0000000	0.0790476
-6523.0000000	0.0800000

-6522.0000000	0.0795238
-6521.0000000	0.0785000
-6520.0000000	0.0775000
-6519.0000000	0.0738095
-6518.0000000	0.0700000
-6517.0000000	0.0700000
-6516.0000000	0.0700000
-6515.0000000	0.0695238
-6514.0000000	0.0700000
-6513.0000000	0.0700000
-6512.0000000	0.0700000
-6511.0000000	0.0700000
-6510.0000000	0.0700000
-6509.0000000	0.0700000
-6508.0000000	0.0700000
-6507.0000000	0.0700000
-6506.0000000	0.0700000
-6505.0000000	0.0700000
-6504.0000000	0.0700000
-6503.0000000	0.0700000
-6502.0000000	0.0700000
-6501.0000000	0.0700000
-6500.0000000	0.0700000
-6499.0000000	0.0700000
-6498.0000000	0.0705000
-6497.0000000	0.0700000
-6496.0000000	0.0700000
-6495.0000000	0.0700000
-6494.0000000	0.0700000
-6493.0000000	0.0700000
-6492.0000000	0.0700000
-6491.0000000	0.0700000
-6490.0000000	0.0700000
-6489.0000000	0.0705000
-6488.0000000	0.0728572
-6487.0000000	0.0710000
-6486.0000000	0.0723810
-6485.0000000	0.0700000
-6484.0000000	0.0705000
-6483.0000000	0.0715000
-6482.0000000	0.0714286
-6481.0000000	0.0730000
-6480.0000000	0.0715000
-6479.0000000	0.0733333
-6478.0000000	0.0720000
-6477.0000000	0.0755000
-6476.0000000	0.0740000
-6475.0000000	0.0728572
-6474.0000000	0.0742857
-6473.0000000	0.0745000
-6472.0000000	0.0725000
-6471.0000000	0.0766667
-6470.0000000	0.0775000
-6469.0000000	0.0755000
-6468.0000000	0.0780000
-6467.0000000	0.0785714
-6466.0000000	0.0770000

-6465.0000000	0.0785714
-6464.0000000	0.0795000
-6463.0000000	0.0795000
-6462.0000000	0.0800000
-6461.0000000	0.0790476
-6460.0000000	0.0800000
-6459.0000000	0.0800000
-6458.0000000	0.0790000
-6457.0000000	0.0795000
-6456.0000000	0.0790000
-6455.0000000	0.0800000
-6454.0000000	0.0795000
-6453.0000000	0.0785000
-6452.0000000	0.0771429
-6451.0000000	0.0780000
-6450.0000000	0.0755000
-6449.0000000	0.0761905
-6448.0000000	0.0745000
-6447.0000000	0.0760000
-6446.0000000	0.0733333
-6445.0000000	0.0740000
-6444.0000000	0.0750000
-6443.0000000	0.0757143
-6442.0000000	0.0735000
-6441.0000000	0.0730000
-6440.0000000	0.0742857
-6439.0000000	0.0740000
-6438.0000000	0.0735000
-6437.0000000	0.0735000
-6436.0000000	0.0757143
-6435.0000000	0.0738095
-6434.0000000	0.0725000
-6433.0000000	0.0740000
-6432.0000000	0.0730000
-6431.0000000	0.0709524
-6430.0000000	0.0720000
-6429.0000000	0.0725000
-6428.0000000	0.0733333
-6427.0000000	0.0760000
-6426.0000000	0.0771429
-6425.0000000	0.0765000
-6424.0000000	0.0770000
-6423.0000000	0.0765000
-6422.0000000	0.0771429
-6421.0000000	0.0760000
-6420.0000000	0.0765000
-6419.0000000	0.0728572
-6418.0000000	0.0705000
-6417.0000000	0.0710000
-6416.0000000	0.0700000
-6415.0000000	0.0709524
-6414.0000000	0.0710000
-6413.0000000	0.0723810
-6412.0000000	0.0715000
-6411.0000000	0.0723810
-6410.0000000	0.0755000
-6409.0000000	0.0735000

-6408.0000000	0.0765000
-6407.0000000	0.0795000
-6406.0000000	0.0761905
-6405.0000000	0.0765000
-6404.0000000	0.0780952
-6403.0000000	0.0790000
-6402.0000000	0.0780000
-6401.0000000	0.0735000
-6400.0000000	0.0752381
-6399.0000000	0.0750000
-6398.0000000	0.0760000
-6397.0000000	0.0723810
-6396.0000000	0.0720000
-6395.0000000	0.0747619
-6394.0000000	0.0735000
-6393.0000000	0.0745000
-6392.0000000	0.0755000
-6391.0000000	0.0752381
-6390.0000000	0.0770000
-6389.0000000	0.0750000
-6388.0000000	0.0771429
-6387.0000000	0.0750000
-6386.0000000	0.0766667
-6385.0000000	0.0765000
-6384.0000000	0.0770000
-6383.0000000	0.0780952
-6382.0000000	0.0770000
-6381.0000000	0.0770000
-6380.0000000	0.0761905
-6379.0000000	0.0760000
-6378.0000000	0.0760000
-6377.0000000	0.0780952
-6376.0000000	0.0795000
-6375.0000000	0.0790476
-6374.0000000	0.0790000
-6373.0000000	0.0790000
-6372.0000000	0.0795000
-6371.0000000	0.0800000
-6370.0000000	0.0800000
-6369.0000000	0.0800000
-6368.0000000	0.0795000
-6367.0000000	0.0800000
-6366.0000000	0.0795238
-6365.0000000	0.0800000
-6364.0000000	0.0800000
-6363.0000000	0.0765000
-6362.0000000	0.0795000
-6361.0000000	0.0785714
-6360.0000000	0.0785000
-6359.0000000	0.0776191
-6358.0000000	0.0775000
-6357.0000000	0.0740000
-6356.0000000	0.0742857
-6355.0000000	0.0740000
-6354.0000000	0.0755000
-6353.0000000	0.0750000
-6352.0000000	0.0733333

-6351.0000000	0.0720000
-6350.0000000	0.0733333
-6349.0000000	0.0750000
-6348.0000000	0.0740000
-6347.0000000	0.0752381
-6346.0000000	0.0780000
-6345.0000000	0.0765000
-6344.0000000	0.0766667
-6343.0000000	0.0720000
-6342.0000000	0.0710000
-6341.0000000	0.0700000
-6340.0000000	0.0700000
-6339.0000000	0.0710000
-6338.0000000	0.0700000
-6337.0000000	0.0705000
-6336.0000000	0.0720000
-6335.0000000	0.0700000
-6334.0000000	0.0700000
-6333.0000000	0.0700000
-6332.0000000	0.0705000
-6331.0000000	0.0704762
-6330.0000000	0.0715000
-6329.0000000	0.0705000
-6328.0000000	0.0742857
-6327.0000000	0.0715000
-6326.0000000	0.0735000
-6325.0000000	0.0742857
-6324.0000000	0.0740000
-6323.0000000	0.0730000
-6322.0000000	0.0738095
-6321.0000000	0.0730000
-6320.0000000	0.0714286
-6319.0000000	0.0725000
-6318.0000000	0.0700000
-6317.0000000	0.0730000
-6316.0000000	0.0719048
-6315.0000000	0.0745000
-6314.0000000	0.0750000
-6313.0000000	0.0752381
-6312.0000000	0.0755000
-6311.0000000	0.0765000
-6310.0000000	0.0765000
-6309.0000000	0.0738095
-6308.0000000	0.0719048
-6307.0000000	0.0725000
-6306.0000000	0.0740000
-6305.0000000	0.0765000
-6304.0000000	0.0723810
-6303.0000000	0.0720000
-6302.0000000	0.0735000
-6301.0000000	0.0757143
-6300.0000000	0.0735000
-6299.0000000	0.0738095
-6298.0000000	0.0720000
-6297.0000000	0.0730000
-6296.0000000	0.0725000
-6295.0000000	0.0709524

-6294.0000000	0.0705000
-6293.0000000	0.0705000
-6292.0000000	0.0700000
-6291.0000000	0.0705000
-6290.0000000	0.0700000
-6289.0000000	0.0700000
-6288.0000000	0.0705000
-6287.0000000	0.0700000
-6286.0000000	0.0700000
-6285.0000000	0.0700000
-6284.0000000	0.0700000
-6283.0000000	0.0705000
-6282.0000000	0.0705000
-6281.0000000	0.0710000
-6280.0000000	0.0700000
-6279.0000000	0.0710000
-6278.0000000	0.0710000
-6277.0000000	0.0700000
-6276.0000000	0.0700000
-6275.0000000	0.0709524
-6274.0000000	0.0720000
-6273.0000000	0.0710000
-6272.0000000	0.0710000
-6271.0000000	0.0725000
-6270.0000000	0.0719048
-6269.0000000	0.0735000
-6268.0000000	0.0733333
-6267.0000000	0.0755000
-6266.0000000	0.0750000
-6265.0000000	0.0766667
-6264.0000000	0.0755000
-6263.0000000	0.0760000
-6262.0000000	0.0790000
-6261.0000000	0.0776191
-6260.0000000	0.0785714
-6259.0000000	0.0800000
-6258.0000000	0.0780000
-6257.0000000	0.0790000
-6256.0000000	0.0800000
-6255.0000000	0.0790000
-6254.0000000	0.0800000
-6253.0000000	0.0800000
-6252.0000000	0.0800000
-6251.0000000	0.0790000
-6250.0000000	0.0771429
-6249.0000000	0.0745000
-6248.0000000	0.0720000
-6247.0000000	0.0704762
-6246.0000000	0.0710000
-6245.0000000	0.0705000
-6244.0000000	0.0700000
-6243.0000000	0.0700000
-6242.0000000	0.0700000
-6241.0000000	0.0700000
-6240.0000000	0.0700000
-6239.0000000	0.0700000
-6238.0000000	0.0700000

-6237.0000000	0.0700000
-6236.0000000	0.0700000
-6235.0000000	0.0700000
-6234.0000000	0.0709524
-6233.0000000	0.0700000
-6232.0000000	0.0733333
-6231.0000000	0.0720000
-6230.0000000	0.0715000
-6229.0000000	0.0704762
-6228.0000000	0.0700000
-6227.0000000	0.0705000
-6226.0000000	0.0705000
-6225.0000000	0.0714286
-6224.0000000	0.0705263
-6223.0000000	0.0704762
-6222.0000000	0.0709524
-6221.0000000	0.0720000
-6220.0000000	0.0714286
-6219.0000000	0.0705000
-6218.0000000	0.0710000
-6217.0000000	0.0704762
-6216.0000000	0.0705000
-6215.0000000	0.0700000
-6214.0000000	0.0700000
-6213.0000000	0.0700000
-6212.0000000	0.0700000
-6211.0000000	0.0700000
-6210.0000000	0.0700000
-6209.0000000	0.0700000
-6208.0000000	0.0700000
-6207.0000000	0.0700000
-6206.0000000	0.0700000
-6205.0000000	0.0700000
-6204.0000000	0.0700000
-6203.0000000	0.0700000
-6202.0000000	0.0705000
-6201.0000000	0.0700000
-6200.0000000	0.0704762
-6199.0000000	0.0710000
-6198.0000000	0.0715000
-6197.0000000	0.0720000
-6196.0000000	0.0730000
-6195.0000000	0.0714286
-6194.0000000	0.0705000
-6193.0000000	0.0700000
-6192.0000000	0.0700000
-6191.0000000	0.0700000
-6190.0000000	0.0700000
-6189.0000000	0.0700000
-6188.0000000	0.0700000
-6187.0000000	0.0700000
-6186.0000000	0.0700000
-6185.0000000	0.0700000
-6184.0000000	0.0700000
-6183.0000000	0.0700000
-6182.0000000	0.0700000
-6181.0000000	0.0700000

-6180.0000000	0.0700000
-6179.0000000	0.0700000
-6178.0000000	0.0700000
-6177.0000000	0.0700000
-6176.0000000	0.0700000
-6175.0000000	0.0700000
-6174.0000000	0.0700000
-6173.0000000	0.0700000
-6172.0000000	0.0700000
-6171.0000000	0.0700000
-6170.0000000	0.0700000
-6169.0000000	0.0700000
-6168.0000000	0.0705000
-6167.0000000	0.0705000
-6166.0000000	0.0725000
-6165.0000000	0.0709524
-6164.0000000	0.0730000
-6163.0000000	0.0720000
-6162.0000000	0.0714286
-6161.0000000	0.0700000
-6160.0000000	0.0710000
-6159.0000000	0.0700000
-6158.0000000	0.0700000
-6157.0000000	0.0700000
-6156.0000000	0.0705000
-6155.0000000	0.0705000
-6154.0000000	0.0700000
-6153.0000000	0.0719048
-6152.0000000	0.0735000
-6151.0000000	0.0750000
-6150.0000000	0.0738095
-6149.0000000	0.0755000
-6148.0000000	0.0771429
-6147.0000000	0.0780000
-6146.0000000	0.0785000
-6145.0000000	0.0790000
-6144.0000000	0.0770000
-6143.0000000	0.0776191
-6142.0000000	0.0780000
-6141.0000000	0.0771429
-6140.0000000	0.0770000
-6139.0000000	0.0795000
-6138.0000000	0.0790476
-6137.0000000	0.0790000
-6136.0000000	0.0795000
-6135.0000000	0.0790000
-6134.0000000	0.0800000
-6133.0000000	0.0780952
-6132.0000000	0.0760000
-6131.0000000	0.0775000
-6130.0000000	0.0765000
-6129.0000000	0.0742857
-6128.0000000	0.0730000
-6127.0000000	0.0735000
-6126.0000000	0.0714286
-6125.0000000	0.0705000
-6124.0000000	0.0700000

-6123.0000000	0.0704762
-6122.0000000	0.0700000
-6121.0000000	0.0705000
-6120.0000000	0.0700000
-6119.0000000	0.0700000
-6118.0000000	0.0700000
-6117.0000000	0.0700000
-6116.0000000	0.0700000
-6115.0000000	0.0700000
-6114.0000000	0.0704762
-6113.0000000	0.0700000
-6112.0000000	0.0700000
-6111.0000000	0.0700000
-6110.0000000	0.0700000
-6109.0000000	0.0700000
-6108.0000000	0.0700000
-6107.0000000	0.0700000
-6106.0000000	0.0700000
-6105.0000000	0.0700000
-6104.0000000	0.0700000
-6103.0000000	0.0700000
-6102.0000000	0.0704762
-6101.0000000	0.0705000
-6100.0000000	0.0710000
-6099.0000000	0.0705000
-6098.0000000	0.0704762
-6097.0000000	0.0705000
-6096.0000000	0.0710000
-6095.0000000	0.0704762
-6094.0000000	0.0730000
-6093.0000000	0.0723810
-6092.0000000	0.0730000
-6091.0000000	0.0725000
-6090.0000000	0.0719048
-6089.0000000	0.0725000
-6088.0000000	0.0715000
-6087.0000000	0.0740000
-6086.0000000	0.0733333
-6085.0000000	0.0715000
-6084.0000000	0.0709524
-6083.0000000	0.0705000
-6082.0000000	0.0700000
-6081.0000000	0.0700000
-6080.0000000	0.0710000
-6079.0000000	0.0725000
-6078.0000000	0.0719048
-6077.0000000	0.0730000
-6076.0000000	0.0720000
-6075.0000000	0.0714286
-6074.0000000	0.0710000
-6073.0000000	0.0723810
-6072.0000000	0.0725000
-6071.0000000	0.0710000
-6070.0000000	0.0720000
-6069.0000000	0.0720000
-6068.0000000	0.0719048
-6067.0000000	0.0745000

-6066.0000000	0.0728572
-6065.0000000	0.0750000
-6064.0000000	0.0747619
-6063.0000000	0.0735000
-6062.0000000	0.0760000
-6061.0000000	0.0750000
-6060.0000000	0.0750000
-6059.0000000	0.0761905
-6058.0000000	0.0760000
-6057.0000000	0.0747619
-6056.0000000	0.0760000
-6055.0000000	0.0735000
-6054.0000000	0.0752381
-6053.0000000	0.0755000
-6052.0000000	0.0755000
-6051.0000000	0.0775000
-6050.0000000	0.0761905
-6049.0000000	0.0775000
-6048.0000000	0.0776191
-6047.0000000	0.0775000
-6046.0000000	0.0790000
-6045.0000000	0.0790476
-6044.0000000	0.0775000
-6043.0000000	0.0755000
-6042.0000000	0.0742857
-6041.0000000	0.0725000
-6040.0000000	0.0730000
-6039.0000000	0.0745000
-6038.0000000	0.0757143
-6037.0000000	0.0745000
-6036.0000000	0.0766667
-6035.0000000	0.0790000
-6034.0000000	0.0795000
-6033.0000000	0.0776191
-6032.0000000	0.0740000
-6031.0000000	0.0730000
-6030.0000000	0.0728572
-6029.0000000	0.0735000
-6028.0000000	0.0730000
-6027.0000000	0.0710000
-6026.0000000	0.0719048
-6025.0000000	0.0710000
-6024.0000000	0.0725000
-6023.0000000	0.0704762
-6022.0000000	0.0705000
-6021.0000000	0.0705000
-6020.0000000	0.0719048
-6019.0000000	0.0710000
-6018.0000000	0.0704762
-6017.0000000	0.0700000
-6016.0000000	0.0700000
-6015.0000000	0.0705000
-6014.0000000	0.0700000
-6013.0000000	0.0700000
-6012.0000000	0.0705000
-6011.0000000	0.0700000
-6010.0000000	0.0715000

-6009.0000000	0.0715000
-6008.0000000	0.0760000
-6007.0000000	0.0719048
-6006.0000000	0.0755000
-6005.0000000	0.0738095
-6004.0000000	0.0755000
-6003.0000000	0.0755000
-6002.0000000	0.0757143
-6001.0000000	0.0760000
-6000.0000000	0.0760000
-5999.0000000	0.0757143
-5998.0000000	0.0770000
-5997.0000000	0.0761905
-5996.0000000	0.0775000
-5995.0000000	0.0725000
-5994.0000000	0.0745000
-5993.0000000	0.0733333
-5992.0000000	0.0700000
-5991.0000000	0.0700000
-5990.0000000	0.0700000
-5989.0000000	0.0700000
-5988.0000000	0.0700000
-5987.0000000	0.0700000
-5986.0000000	0.0705000
-5985.0000000	0.0705000
-5984.0000000	0.0714286
-5983.0000000	0.0700000
-5982.0000000	0.0719048
-5981.0000000	0.0730000
-5980.0000000	0.0745000
-5979.0000000	0.0740000
-5978.0000000	0.0720000
-5977.0000000	0.0742857
-5976.0000000	0.0740000
-5975.0000000	0.0752381
-5974.0000000	0.0755000
-5973.0000000	0.0757143
-5972.0000000	0.0740000
-5971.0000000	0.0740000
-5970.0000000	0.0770000
-5969.0000000	0.0775000
-5968.0000000	0.0766667
-5967.0000000	0.0750000
-5966.0000000	0.0776191
-5965.0000000	0.0770000
-5964.0000000	0.0765000
-5963.0000000	0.0785714
-5962.0000000	0.0765000
-5961.0000000	0.0730000
-5960.0000000	0.0740000
-5959.0000000	0.0733333
-5958.0000000	0.0710000
-5957.0000000	0.0728572
-5956.0000000	0.0745000
-5955.0000000	0.0725000
-5954.0000000	0.0709524
-5953.0000000	0.0705000

-5952.0000000	0.0715000
-5951.0000000	0.0757143
-5950.0000000	0.0745000
-5949.0000000	0.0750000
-5948.0000000	0.0766667
-5947.0000000	0.0775000
-5946.0000000	0.0740000
-5945.0000000	0.0747619
-5944.0000000	0.0775000
-5943.0000000	0.0775000
-5942.0000000	0.0790476
-5941.0000000	0.0785000
-5940.0000000	0.0795000
-5939.0000000	0.0800000
-5938.0000000	0.0795000
-5937.0000000	0.0795000
-5936.0000000	0.0790476
-5935.0000000	0.0800000
-5934.0000000	0.0790000
-5933.0000000	0.0790000
-5932.0000000	0.0800000
-5931.0000000	0.0795000
-5930.0000000	0.0800000
-5929.0000000	0.0795000
-5928.0000000	0.0800000
-5927.0000000	0.0800000
-5926.0000000	0.0800000
-5925.0000000	0.0800000
-5924.0000000	0.0800000
-5923.0000000	0.0800000
-5922.0000000	0.0805000
-5921.0000000	0.0800000
-5920.0000000	0.0800000
-5919.0000000	0.0800000
-5918.0000000	0.0800000
-5917.0000000	0.0800000
-5916.0000000	0.0800000
-5915.0000000	0.0800000
-5914.0000000	0.0800000
-5913.0000000	0.0800000
-5912.0000000	0.0785000
-5911.0000000	0.0752381
-5910.0000000	0.0735000
-5909.0000000	0.0728572
-5908.0000000	0.0730000
-5907.0000000	0.0735000
-5906.0000000	0.0738095
-5905.0000000	0.0765000
-5904.0000000	0.0760000
-5903.0000000	0.0745000
-5902.0000000	0.0766667
-5901.0000000	0.0735000
-5900.0000000	0.0760000
-5899.0000000	0.0738095
-5898.0000000	0.0738095
-5897.0000000	0.0730000
-5896.0000000	0.0725000

-5895.0000000	0.0725000
-5894.0000000	0.0740000
-5893.0000000	0.0742857
-5892.0000000	0.0745000
-5891.0000000	0.0723810
-5890.0000000	0.0750000
-5889.0000000	0.0775000
-5888.0000000	0.0750000
-5887.0000000	0.0747619
-5886.0000000	0.0740000
-5885.0000000	0.0725000
-5884.0000000	0.0738095
-5883.0000000	0.0730000
-5882.0000000	0.0728572
-5881.0000000	0.0725000
-5880.0000000	0.0710000
-5879.0000000	0.0723810
-5878.0000000	0.0735000
-5877.0000000	0.0710000
-5876.0000000	0.0725000
-5875.0000000	0.0714286
-5874.0000000	0.0705000
-5873.0000000	0.0745000
-5872.0000000	0.0733333
-5871.0000000	0.0730000
-5870.0000000	0.0728572
-5869.0000000	0.0735000
-5868.0000000	0.0730000
-5867.0000000	0.0766667
-5866.0000000	0.0780000
-5865.0000000	0.0755000
-5864.0000000	0.0750000
-5863.0000000	0.0766667
-5862.0000000	0.0775000
-5861.0000000	0.0780000
-5860.0000000	0.0752381
-5859.0000000	0.0765000
-5858.0000000	0.0795000
-5857.0000000	0.0790476
-5856.0000000	0.0770000
-5855.0000000	0.0780952
-5854.0000000	0.0775000
-5853.0000000	0.0770000
-5852.0000000	0.0790000
-5851.0000000	0.0776191
-5850.0000000	0.0780000
-5849.0000000	0.0740000
-5848.0000000	0.0738095
-5847.0000000	0.0750000
-5846.0000000	0.0728572
-5845.0000000	0.0725000
-5844.0000000	0.0715000
-5843.0000000	0.0720000
-5842.0000000	0.0730000
-5841.0000000	0.0714286
-5840.0000000	0.0730000
-5839.0000000	0.0723810

-5838.0000000	0.0735000
-5837.0000000	0.0719048
-5836.0000000	0.0740000
-5835.0000000	0.0765000
-5834.0000000	0.0760000
-5833.0000000	0.0770000
-5832.0000000	0.0766667
-5831.0000000	0.0780952
-5830.0000000	0.0775000
-5829.0000000	0.0790000
-5828.0000000	0.0785000
-5827.0000000	0.0780952
-5826.0000000	0.0775000
-5825.0000000	0.0770000
-5824.0000000	0.0771429
-5823.0000000	0.0750000
-5822.0000000	0.0745000
-5821.0000000	0.0742857
-5820.0000000	0.0730000
-5819.0000000	0.0740000
-5818.0000000	0.0766667
-5817.0000000	0.0785000
-5816.0000000	0.0790000
-5815.0000000	0.0776191
-5814.0000000	0.0775000
-5813.0000000	0.0785000
-5812.0000000	0.0790476
-5811.0000000	0.0790000
-5810.0000000	0.0795000
-5809.0000000	0.0780952
-5808.0000000	0.0760000
-5807.0000000	0.0780952
-5806.0000000	0.0780000
-5805.0000000	0.0800000
-5804.0000000	0.0790000
-5803.0000000	0.0790476
-5802.0000000	0.0780000
-5801.0000000	0.0795000
-5800.0000000	0.0795238
-5799.0000000	0.0780000
-5798.0000000	0.0765000
-5797.0000000	0.0750000
-5796.0000000	0.0771429
-5795.0000000	0.0765000
-5794.0000000	0.0750000
-5793.0000000	0.0742857
-5792.0000000	0.0740000
-5791.0000000	0.0733333
-5790.0000000	0.0735000
-5789.0000000	0.0730000
-5788.0000000	0.0714286
-5787.0000000	0.0710000
-5786.0000000	0.0715000
-5785.0000000	0.0715000
-5784.0000000	0.0714286
-5783.0000000	0.0705000
-5782.0000000	0.0700000

-5781.0000000	0.0700000
-5780.0000000	0.0705000
-5779.0000000	0.0704762
-5778.0000000	0.0700000
-5777.0000000	0.0700000
-5776.0000000	0.0704762
-5775.0000000	0.0700000
-5774.0000000	0.0715000
-5773.0000000	0.0715000
-5772.0000000	0.0733333
-5771.0000000	0.0714286
-5770.0000000	0.0710000
-5769.0000000	0.0715000
-5768.0000000	0.0725000
-5767.0000000	0.0720000
-5766.0000000	0.0704762
-5765.0000000	0.0710000
-5764.0000000	0.0704762
-5763.0000000	0.0705000
-5762.0000000	0.0700000
-5761.0000000	0.0700000
-5760.0000000	0.0715000
-5759.0000000	0.0710000
-5758.0000000	0.0710000
-5757.0000000	0.0704762
-5756.0000000	0.0710000
-5755.0000000	0.0723810
-5754.0000000	0.0705000
-5753.0000000	0.0720000
-5752.0000000	0.0700000
-5751.0000000	0.0700000
-5750.0000000	0.0700000
-5749.0000000	0.0700000
-5748.0000000	0.0700000
-5747.0000000	0.0700000
-5746.0000000	0.0700000
-5745.0000000	0.0700000
-5744.0000000	0.0700000
-5743.0000000	0.0700000
-5742.0000000	0.0700000
-5741.0000000	0.0700000
-5740.0000000	0.0700000
-5739.0000000	0.0700000
-5738.0000000	0.0700000
-5737.0000000	0.0700000
-5736.0000000	0.0700000
-5735.0000000	0.0700000
-5734.0000000	0.0700000
-5733.0000000	0.0700000
-5732.0000000	0.0700000
-5731.0000000	0.0700000
-5730.0000000	0.0715000
-5729.0000000	0.0710000
-5728.0000000	0.0700000
-5727.0000000	0.0720000
-5726.0000000	0.0725000
-5725.0000000	0.0720000

-5724.0000000	0.0704762
-5723.0000000	0.0710000
-5722.0000000	0.0705000
-5721.0000000	0.0714286
-5720.0000000	0.0720000
-5719.0000000	0.0719048
-5718.0000000	0.0740000
-5717.0000000	0.0720000
-5716.0000000	0.0700000
-5715.0000000	0.0709524
-5714.0000000	0.0705000
-5713.0000000	0.0700000
-5712.0000000	0.0728572
-5711.0000000	0.0720000
-5710.0000000	0.0725000
-5709.0000000	0.0742857
-5708.0000000	0.0715000
-5707.0000000	0.0715000
-5706.0000000	0.0705000
-5705.0000000	0.0700000
-5704.0000000	0.0714286
-5703.0000000	0.0700000
-5702.0000000	0.0705000
-5701.0000000	0.0700000
-5700.0000000	0.0700000
-5699.0000000	0.0700000
-5698.0000000	0.0700000
-5697.0000000	0.0700000
-5696.0000000	0.0700000
-5695.0000000	0.0700000
-5694.0000000	0.0700000
-5693.0000000	0.0700000
-5692.0000000	0.0700000
-5691.0000000	0.0700000
-5690.0000000	0.0700000
-5689.0000000	0.0700000
-5688.0000000	0.0700000
-5687.0000000	0.0700000
-5686.0000000	0.0700000
-5685.0000000	0.0700000
-5684.0000000	0.0710000
-5683.0000000	0.0720000
-5682.0000000	0.0714286
-5681.0000000	0.0715000
-5680.0000000	0.0704762
-5679.0000000	0.0705000
-5678.0000000	0.0700000
-5677.0000000	0.0700000
-5676.0000000	0.0700000
-5675.0000000	0.0700000
-5674.0000000	0.0700000
-5673.0000000	0.0700000
-5672.0000000	0.0700000
-5671.0000000	0.0700000
-5670.0000000	0.0700000
-5669.0000000	0.0700000
-5668.0000000	0.0700000

-5667.0000000	0.0700000
-5666.0000000	0.0704762
-5665.0000000	0.0705000
-5664.0000000	0.0704762
-5663.0000000	0.0710000
-5662.0000000	0.0715000
-5661.0000000	0.0709524
-5660.0000000	0.0705000
-5659.0000000	0.0710000
-5658.0000000	0.0700000
-5657.0000000	0.0709524
-5656.0000000	0.0705000
-5655.0000000	0.0700000
-5654.0000000	0.0700000
-5653.0000000	0.0700000
-5652.0000000	0.0700000
-5651.0000000	0.0700000
-5650.0000000	0.0695000
-5649.0000000	0.0700000
-5648.0000000	0.0700000
-5647.0000000	0.0689474
-5646.0000000	0.0700000
-5645.0000000	0.0700000
-5644.0000000	0.0690476
-5643.0000000	0.0695000
-5642.0000000	0.0690000
-5641.0000000	0.0700000
-5640.0000000	0.0690476
-5639.0000000	0.0700000
-5638.0000000	0.0700000
-5637.0000000	0.0695238
-5636.0000000	0.0695000
-5635.0000000	0.0700000
-5634.0000000	0.0700000
-5633.0000000	0.0700000
-5632.0000000	0.0700000
-5631.0000000	0.0700000
-5630.0000000	0.0700000
-5629.0000000	0.0700000
-5628.0000000	0.0700000
-5627.0000000	0.0700000
-5626.0000000	0.0700000
-5625.0000000	0.0700000
-5624.0000000	0.0700000
-5623.0000000	0.0700000
-5622.0000000	0.0695000
-5621.0000000	0.0685714
-5620.0000000	0.0700000
-5619.0000000	0.0700000
-5618.0000000	0.0700000
-5617.0000000	0.0700000
-5616.0000000	0.0700000
-5615.0000000	0.0705000
-5614.0000000	0.0705000
-5613.0000000	0.0700000
-5612.0000000	0.0705000
-5611.0000000	0.0720000

-5610.0000000	0.0738095
-5609.0000000	0.0740000
-5608.0000000	0.0740000
-5607.0000000	0.0735000
-5606.0000000	0.0723810
-5605.0000000	0.0705000
-5604.0000000	0.0709524
-5603.0000000	0.0705000
-5602.0000000	0.0700000
-5601.0000000	0.0700000
-5600.0000000	0.0700000
-5599.0000000	0.0700000
-5598.0000000	0.0700000
-5597.0000000	0.0714286
-5596.0000000	0.0723810
-5595.0000000	0.0705000
-5594.0000000	0.0745000
-5593.0000000	0.0735000
-5592.0000000	0.0745000
-5591.0000000	0.0752381
-5590.0000000	0.0740000
-5589.0000000	0.0752381
-5588.0000000	0.0720000
-5587.0000000	0.0725000
-5586.0000000	0.0750000
-5585.0000000	0.0738095
-5584.0000000	0.0720000
-5583.0000000	0.0725000
-5582.0000000	0.0719048
-5581.0000000	0.0705000
-5580.0000000	0.0709524
-5579.0000000	0.0705000
-5578.0000000	0.0710000
-5577.0000000	0.0704762
-5576.0000000	0.0700000
-5575.0000000	0.0700000
-5574.0000000	0.0700000
-5573.0000000	0.0700000
-5572.0000000	0.0700000
-5571.0000000	0.0705000
-5570.0000000	0.0704762
-5569.0000000	0.0705000
-5568.0000000	0.0700000
-5567.0000000	0.0700000
-5566.0000000	0.0700000
-5565.0000000	0.0714286
-5564.0000000	0.0715000
-5563.0000000	0.0725000
-5562.0000000	0.0715000
-5561.0000000	0.0738095
-5560.0000000	0.0750000
-5559.0000000	0.0745000
-5558.0000000	0.0747619
-5557.0000000	0.0735000
-5556.0000000	0.0750000
-5555.0000000	0.0733333
-5554.0000000	0.0745000

-5553.0000000	0.0747619
-5552.0000000	0.0745000
-5551.0000000	0.0735000
-5550.0000000	0.0745000
-5549.0000000	0.0776191
-5548.0000000	0.0750000
-5547.0000000	0.0745000
-5546.0000000	0.0766667
-5545.0000000	0.0765000
-5544.0000000	0.0747619
-5543.0000000	0.0765000
-5542.0000000	0.0770000
-5541.0000000	0.0785000
-5540.0000000	0.0770000
-5539.0000000	0.0780952
-5538.0000000	0.0785000
-5537.0000000	0.0771429
-5536.0000000	0.0785000
-5535.0000000	0.0785714
-5534.0000000	0.0760000
-5533.0000000	0.0780000
-5532.0000000	0.0775000
-5531.0000000	0.0785000
-5530.0000000	0.0780952
-5529.0000000	0.0785000
-5528.0000000	0.0780952
-5527.0000000	0.0770000
-5526.0000000	0.0760000
-5525.0000000	0.0761905
-5524.0000000	0.0750000
-5523.0000000	0.0745000
-5522.0000000	0.0747619
-5521.0000000	0.0720000
-5520.0000000	0.0735000
-5519.0000000	0.0719048
-5518.0000000	0.0700000
-5517.0000000	0.0700000
-5516.0000000	0.0700000
-5515.0000000	0.0700000
-5514.0000000	0.0700000
-5513.0000000	0.0700000
-5512.0000000	0.0700000
-5511.0000000	0.0700000
-5510.0000000	0.0700000
-5509.0000000	0.0700000
-5508.0000000	0.0700000
-5507.0000000	0.0700000
-5506.0000000	0.0700000
-5505.0000000	0.0700000
-5504.0000000	0.0695000
-5503.0000000	0.0700000
-5502.0000000	0.0700000
-5501.0000000	0.0700000
-5500.0000000	0.0689474
-5499.0000000	0.0700000
-5498.0000000	0.0685714
-5497.0000000	0.0690000

-5496.0000000	0.0685000
-5495.0000000	0.0645000
-5494.0000000	0.0657143
-5493.0000000	0.0650000
-5492.0000000	0.0671429
-5491.0000000	0.0665000
-5490.0000000	0.0670000
-5489.0000000	0.0680953
-5488.0000000	0.0675000
-5487.0000000	0.0690000
-5486.0000000	0.0680952
-5485.0000000	0.0680000
-5484.0000000	0.0675000
-5483.0000000	0.0695000
-5482.0000000	0.0671429
-5481.0000000	0.0695000
-5480.0000000	0.0685000
-5479.0000000	0.0676190
-5478.0000000	0.0675000
-5477.0000000	0.0700000
-5476.0000000	0.0680000
-5475.0000000	0.0695000
-5474.0000000	0.0700000
-5473.0000000	0.0695000
-5472.0000000	0.0695000
-5471.0000000	0.0700000
-5470.0000000	0.0695238
-5469.0000000	0.0690476
-5468.0000000	0.0695000
-5467.0000000	0.0700000
-5466.0000000	0.0700000
-5465.0000000	0.0700000
-5464.0000000	0.0695238
-5463.0000000	0.0690000
-5462.0000000	0.0695238
-5461.0000000	0.0685000
-5460.0000000	0.0700000
-5459.0000000	0.0700000
-5458.0000000	0.0700000
-5457.0000000	0.0695000
-5456.0000000	0.0695000
-5455.0000000	0.0695238
-5454.0000000	0.0695000
-5453.0000000	0.0695238
-5452.0000000	0.0700000
-5451.0000000	0.0685000
-5450.0000000	0.0685714
-5449.0000000	0.0690000
-5448.0000000	0.0700000
-5447.0000000	0.0690000
-5446.0000000	0.0695238
-5445.0000000	0.0700000
-5444.0000000	0.0700000
-5443.0000000	0.0695000
-5442.0000000	0.0685000
-5441.0000000	0.0700000
-5440.0000000	0.0690476

-5439.0000000	0.0680000
-5438.0000000	0.0680953
-5437.0000000	0.0695000
-5436.0000000	0.0695000
-5435.0000000	0.0695000
-5434.0000000	0.0695238
-5433.0000000	0.0695000
-5432.0000000	0.0695000
-5431.0000000	0.0690476
-5430.0000000	0.0700000
-5429.0000000	0.0685000
-5428.0000000	0.0700000
-5427.0000000	0.0695000
-5426.0000000	0.0690000
-5425.0000000	0.0695238
-5424.0000000	0.0695000
-5423.0000000	0.0700000
-5422.0000000	0.0695238
-5421.0000000	0.0695000
-5420.0000000	0.0695000
-5419.0000000	0.0690476
-5418.0000000	0.0700000
-5417.0000000	0.0695238
-5416.0000000	0.0700000
-5415.0000000	0.0700000
-5414.0000000	0.0700000
-5413.0000000	0.0700000
-5412.0000000	0.0700000
-5411.0000000	0.0700000
-5410.0000000	0.0695238
-5409.0000000	0.0695000
-5408.0000000	0.0695238
-5407.0000000	0.0700000
-5406.0000000	0.0690000
-5405.0000000	0.0700000
-5404.0000000	0.0700000
-5403.0000000	0.0700000
-5402.0000000	0.0700000
-5401.0000000	0.0700000
-5400.0000000	0.0700000
-5399.0000000	0.0680953
-5398.0000000	0.0670000
-5397.0000000	0.0695000
-5396.0000000	0.0690000
-5395.0000000	0.0695238
-5394.0000000	0.0680000
-5393.0000000	0.0685000
-5392.0000000	0.0676190
-5391.0000000	0.0690000
-5390.0000000	0.0690000
-5389.0000000	0.0690476
-5388.0000000	0.0695000
-5387.0000000	0.0700000
-5386.0000000	0.0704762
-5385.0000000	0.0700000
-5384.0000000	0.0700000
-5383.0000000	0.0700000

-5382.0000000	0.0700000
-5381.0000000	0.0700000
-5380.0000000	0.0704762
-5379.0000000	0.0705000
-5378.0000000	0.0704762
-5377.0000000	0.0705000
-5376.0000000	0.0700000
-5375.0000000	0.0700000
-5374.0000000	0.0700000
-5373.0000000	0.0725000
-5372.0000000	0.0765000
-5371.0000000	0.0747619
-5370.0000000	0.0750000
-5369.0000000	0.0766667
-5368.0000000	0.0750000
-5367.0000000	0.0760000
-5366.0000000	0.0735000
-5365.0000000	0.0725000
-5364.0000000	0.0700000
-5363.0000000	0.0700000
-5362.0000000	0.0700000
-5361.0000000	0.0700000
-5360.0000000	0.0700000
-5359.0000000	0.0700000
-5358.0000000	0.0700000
-5357.0000000	0.0700000
-5356.0000000	0.0700000
-5355.0000000	0.0695238
-5354.0000000	0.0695000
-5353.0000000	0.0690476
-5352.0000000	0.0680000
-5351.0000000	0.0665000
-5350.0000000	0.0680952
-5349.0000000	0.0680000
-5348.0000000	0.0640000
-5347.0000000	0.0604762
-5346.0000000	0.0610000
-5345.0000000	0.0625000
-5344.0000000	0.0610000
-5343.0000000	0.0619048
-5342.0000000	0.0642857
-5341.0000000	0.0625000
-5340.0000000	0.0625000
-5339.0000000	0.0647619
-5338.0000000	0.0610000
-5337.0000000	0.0610000
-5336.0000000	0.0620000
-5335.0000000	0.0633333
-5334.0000000	0.0660000
-5333.0000000	0.0661905
-5332.0000000	0.0670000
-5331.0000000	0.0655000
-5330.0000000	0.0660000
-5329.0000000	0.0650000
-5328.0000000	0.0676190
-5327.0000000	0.0635000
-5326.0000000	0.0633333

-5325.0000000	0.0615000
-5324.0000000	0.0625000
-5323.0000000	0.0609524
-5322.0000000	0.0620000
-5321.0000000	0.0605000
-5320.0000000	0.0615000
-5319.0000000	0.0614286
-5318.0000000	0.0600000
-5317.0000000	0.0614286
-5316.0000000	0.0615000
-5315.0000000	0.0605000
-5314.0000000	0.0610000
-5313.0000000	0.0609524
-5312.0000000	0.0610000
-5311.0000000	0.0609524
-5310.0000000	0.0600000
-5309.0000000	0.0615000
-5308.0000000	0.0609524
-5307.0000000	0.0615000
-5306.0000000	0.0645000
-5305.0000000	0.0635000
-5304.0000000	0.0652381
-5303.0000000	0.0665000
-5302.0000000	0.0661905
-5301.0000000	0.0650000
-5300.0000000	0.0655000
-5299.0000000	0.0661905
-5298.0000000	0.0660000
-5297.0000000	0.0670000
-5296.0000000	0.0660000
-5295.0000000	0.0638095
-5294.0000000	0.0623809
-5293.0000000	0.0660000
-5292.0000000	0.0665000
-5291.0000000	0.0675000
-5290.0000000	0.0680952
-5289.0000000	0.0680000
-5288.0000000	0.0680000
-5287.0000000	0.0685714
-5286.0000000	0.0690000
-5285.0000000	0.0690000
-5284.0000000	0.0700000
-5283.0000000	0.0700000
-5282.0000000	0.0700000
-5281.0000000	0.0695000
-5280.0000000	0.0700000
-5279.0000000	0.0700000
-5278.0000000	0.0709524
-5277.0000000	0.0700000
-5276.0000000	0.0705000
-5275.0000000	0.0730000
-5274.0000000	0.0709524
-5273.0000000	0.0715000
-5272.0000000	0.0719048
-5271.0000000	0.0740000
-5270.0000000	0.0715000
-5269.0000000	0.0715000

-5268.0000000	0.0714286
-5267.0000000	0.0705000
-5266.0000000	0.0715000
-5265.0000000	0.0700000
-5264.0000000	0.0710000
-5263.0000000	0.0709524
-5262.0000000	0.0710000
-5261.0000000	0.0705000
-5260.0000000	0.0715000
-5259.0000000	0.0719048
-5258.0000000	0.0700000
-5257.0000000	0.0700000
-5256.0000000	0.0700000
-5255.0000000	0.0700000
-5254.0000000	0.0700000
-5253.0000000	0.0700000
-5252.0000000	0.0700000
-5251.0000000	0.0700000
-5250.0000000	0.0700000
-5249.0000000	0.0700000
-5248.0000000	0.0695238
-5247.0000000	0.0700000
-5246.0000000	0.0700000
-5245.0000000	0.0695000
-5244.0000000	0.0700000
-5243.0000000	0.0700000
-5242.0000000	0.0700000
-5241.0000000	0.0700000
-5240.0000000	0.0700000
-5239.0000000	0.0700000
-5238.0000000	0.0700000
-5237.0000000	0.0700000
-5236.0000000	0.0700000
-5235.0000000	0.0700000
-5234.0000000	0.0700000
-5233.0000000	0.0700000
-5232.0000000	0.0700000
-5231.0000000	0.0700000
-5230.0000000	0.0700000
-5229.0000000	0.0700000
-5228.0000000	0.0700000
-5227.0000000	0.0700000
-5226.0000000	0.0700000
-5225.0000000	0.0700000
-5224.0000000	0.0700000
-5223.0000000	0.0700000
-5222.0000000	0.0700000
-5221.0000000	0.0700000
-5220.0000000	0.0700000
-5219.0000000	0.0705000
-5218.0000000	0.0705000
-5217.0000000	0.0700000
-5216.0000000	0.0700000
-5215.0000000	0.0700000
-5214.0000000	0.0700000
-5213.0000000	0.0700000
-5212.0000000	0.0704762

-5211.0000000	0.0700000
-5210.0000000	0.0705263
-5209.0000000	0.0704762
-5208.0000000	0.0700000
-5207.0000000	0.0700000
-5206.0000000	0.0700000
-5205.0000000	0.0695238
-5204.0000000	0.0685000
-5203.0000000	0.0695238
-5202.0000000	0.0700000
-5201.0000000	0.0690000
-5200.0000000	0.0695000
-5199.0000000	0.0695238
-5198.0000000	0.0685000
-5197.0000000	0.0680000
-5196.0000000	0.0661905
-5195.0000000	0.0650000
-5194.0000000	0.0655000
-5193.0000000	0.0670000
-5192.0000000	0.0657143
-5191.0000000	0.0655000
-5190.0000000	0.0652381
-5189.0000000	0.0655000
-5188.0000000	0.0635000
-5187.0000000	0.0614286
-5186.0000000	0.0600000
-5185.0000000	0.0625000
-5184.0000000	0.0661905
-5183.0000000	0.0650000
-5182.0000000	0.0650000
-5181.0000000	0.0661905
-5180.0000000	0.0675000
-5179.0000000	0.0680000
-5178.0000000	0.0685000
-5177.0000000	0.0657143
-5176.0000000	0.0676191
-5175.0000000	0.0675000
-5174.0000000	0.0655000
-5173.0000000	0.0630000
-5172.0000000	0.0619048
-5171.0000000	0.0650000
-5170.0000000	0.0675000
-5169.0000000	0.0670000
-5168.0000000	0.0676190
-5167.0000000	0.0695238
-5166.0000000	0.0680000
-5165.0000000	0.0695000
-5164.0000000	0.0695000
-5163.0000000	0.0690000
-5162.0000000	0.0690476
-5161.0000000	0.0695000
-5160.0000000	0.0690476
-5159.0000000	0.0700000
-5158.0000000	0.0700000
-5157.0000000	0.0700000
-5156.0000000	0.0700000
-5155.0000000	0.0695000

-5154.0000000	0.0695000
-5153.0000000	0.0700000
-5152.0000000	0.0700000
-5151.0000000	0.0695238
-5150.0000000	0.0700000
-5149.0000000	0.0700000
-5148.0000000	0.0700000
-5147.0000000	0.0704762
-5146.0000000	0.0700000
-5145.0000000	0.0700000
-5144.0000000	0.0700000
-5143.0000000	0.0700000
-5142.0000000	0.0709524
-5141.0000000	0.0700000
-5140.0000000	0.0705000
-5139.0000000	0.0705000
-5138.0000000	0.0710000
-5137.0000000	0.0704762
-5136.0000000	0.0700000
-5135.0000000	0.0700000
-5134.0000000	0.0700000
-5133.0000000	0.0700000
-5132.0000000	0.0700000
-5131.0000000	0.0705000
-5130.0000000	0.0700000
-5129.0000000	0.0700000
-5128.0000000	0.0700000
-5127.0000000	0.0700000
-5126.0000000	0.0700000
-5125.0000000	0.0700000
-5124.0000000	0.0700000
-5123.0000000	0.0700000
-5122.0000000	0.0700000
-5121.0000000	0.0700000
-5120.0000000	0.0700000
-5119.0000000	0.0700000
-5118.0000000	0.0700000
-5117.0000000	0.0695238
-5116.0000000	0.0700000
-5115.0000000	0.0700000
-5114.0000000	0.0700000
-5113.0000000	0.0700000
-5112.0000000	0.0700000
-5111.0000000	0.0700000
-5110.0000000	0.0700000
-5109.0000000	0.0700000
-5108.0000000	0.0700000
-5107.0000000	0.0700000
-5106.0000000	0.0700000
-5105.0000000	0.0700000
-5104.0000000	0.0700000
-5103.0000000	0.0700000
-5102.0000000	0.0700000
-5101.0000000	0.0700000
-5100.0000000	0.0705000
-5099.0000000	0.0700000
-5098.0000000	0.0700000

-5097.0000000	0.0700000
-5096.0000000	0.0700000
-5095.0000000	0.0700000
-5094.0000000	0.0700000
-5093.0000000	0.0704762
-5092.0000000	0.0700000
-5091.0000000	0.0705000
-5090.0000000	0.0704762
-5089.0000000	0.0700000
-5088.0000000	0.0705000
-5087.0000000	0.0700000
-5086.0000000	0.0700000
-5085.0000000	0.0700000
-5084.0000000	0.0700000
-5083.0000000	0.0700000
-5082.0000000	0.0700000
-5081.0000000	0.0700000
-5080.0000000	0.0700000
-5079.0000000	0.0695000
-5078.0000000	0.0700000
-5077.0000000	0.0700000
-5076.0000000	0.0700000
-5075.0000000	0.0680000
-5074.0000000	0.0680000
-5073.0000000	0.0665000
-5072.0000000	0.0685714
-5071.0000000	0.0625000
-5070.0000000	0.0650000
-5069.0000000	0.0623809
-5068.0000000	0.0650000
-5067.0000000	0.0656522
-5066.0000000	0.0645000
-5065.0000000	0.0655000
-5064.0000000	0.0645000
-5063.0000000	0.0650000
-5062.0000000	0.0695238
-5061.0000000	0.0690000
-5060.0000000	0.0685714
-5059.0000000	0.0685000
-5058.0000000	0.0690000
-5057.0000000	0.0700000
-5056.0000000	0.0700000
-5055.0000000	0.0695000
-5054.0000000	0.0700000
-5053.0000000	0.0700000
-5052.0000000	0.0700000
-5051.0000000	0.0700000
-5050.0000000	0.0700000
-5049.0000000	0.0700000
-5048.0000000	0.0700000
-5047.0000000	0.0700000
-5046.0000000	0.0700000
-5045.0000000	0.0700000
-5044.0000000	0.0700000
-5043.0000000	0.0700000
-5042.0000000	0.0700000
-5041.0000000	0.0700000

-5040.0000000	0.0700000
-5039.0000000	0.0700000
-5038.0000000	0.0700000
-5037.0000000	0.0704762
-5036.0000000	0.0700000
-5035.0000000	0.0710000
-5034.0000000	0.0710000
-5033.0000000	0.0719048
-5032.0000000	0.0710000
-5031.0000000	0.0720000
-5030.0000000	0.0738095
-5029.0000000	0.0745000
-5028.0000000	0.0745000
-5027.0000000	0.0735000
-5026.0000000	0.0728572
-5025.0000000	0.0755000
-5024.0000000	0.0738095
-5023.0000000	0.0760000
-5022.0000000	0.0780000
-5021.0000000	0.0757143
-5020.0000000	0.0745000
-5019.0000000	0.0740000
-5018.0000000	0.0750000
-5017.0000000	0.0747619
-5016.0000000	0.0755000
-5015.0000000	0.0752381
-5014.0000000	0.0735000
-5013.0000000	0.0725000
-5012.0000000	0.0715000
-5011.0000000	0.0719048
-5010.0000000	0.0715000
-5009.0000000	0.0704762
-5008.0000000	0.0700000
-5007.0000000	0.0700000
-5006.0000000	0.0704762
-5005.0000000	0.0700000
-5004.0000000	0.0700000
-5003.0000000	0.0700000
-5002.0000000	0.0700000
-5001.0000000	0.0700000
-5000.0000000	0.0700000
-4999.0000000	0.0700000
-4998.0000000	0.0700000
-4997.0000000	0.0700000
-4996.0000000	0.0694737
-4995.0000000	0.0680952
-4994.0000000	0.0676191
-4993.0000000	0.0668421
-4992.0000000	0.0661905
-4991.0000000	0.0657143
-4990.0000000	0.0650000
-4989.0000000	0.0655000
-4988.0000000	0.0633333
-4987.0000000	0.0670000
-4986.0000000	0.0685000
-4985.0000000	0.0666667
-4984.0000000	0.0660000

-4983.0000000	0.0670000
-4982.0000000	0.0670000
-4981.0000000	0.0642857
-4980.0000000	0.0650000
-4979.0000000	0.0660000
-4978.0000000	0.0638095
-4977.0000000	0.0675000
-4976.0000000	0.0666667
-4975.0000000	0.0700000
-4974.0000000	0.0700000
-4973.0000000	0.0685000
-4972.0000000	0.0685714
-4971.0000000	0.0670000
-4970.0000000	0.0685714
-4969.0000000	0.0670000
-4968.0000000	0.0665000
-4967.0000000	0.0675000
-4966.0000000	0.0666667
-4965.0000000	0.0655000
-4964.0000000	0.0675000
-4963.0000000	0.0666667
-4962.0000000	0.0655000
-4961.0000000	0.0680952
-4960.0000000	0.0675000
-4959.0000000	0.0675000
-4958.0000000	0.0666667
-4957.0000000	0.0645000
-4956.0000000	0.0615000
-4955.0000000	0.0600000
-4954.0000000	0.0614286
-4953.0000000	0.0610000
-4952.0000000	0.0610000
-4951.0000000	0.0600000
-4950.0000000	0.0610000
-4949.0000000	0.0609524
-4948.0000000	0.0600000
-4947.0000000	0.0610000
-4946.0000000	0.0614286
-4945.0000000	0.0610000
-4944.0000000	0.0610000
-4943.0000000	0.0600000
-4942.0000000	0.0609524
-4941.0000000	0.0600000
-4940.0000000	0.0609524
-4939.0000000	0.0605000
-4938.0000000	0.0625000
-4937.0000000	0.0620000
-4936.0000000	0.0625000
-4935.0000000	0.0633333
-4934.0000000	0.0640000
-4933.0000000	0.0628571
-4932.0000000	0.0605000
-4931.0000000	0.0604762
-4930.0000000	0.0605000
-4929.0000000	0.0635000
-4928.0000000	0.0635000
-4927.0000000	0.0661905

-4926.0000000	0.0690000
-4925.0000000	0.0700000
-4924.0000000	0.0690476
-4923.0000000	0.0700000
-4922.0000000	0.0700000
-4921.0000000	0.0695000
-4920.0000000	0.0700000
-4919.0000000	0.0700000
-4918.0000000	0.0700000
-4917.0000000	0.0700000
-4916.0000000	0.0705000
-4915.0000000	0.0704762
-4914.0000000	0.0705000
-4913.0000000	0.0700000
-4912.0000000	0.0700000
-4911.0000000	0.0700000
-4910.0000000	0.0700000
-4909.0000000	0.0700000
-4908.0000000	0.0700000
-4907.0000000	0.0700000
-4906.0000000	0.0700000
-4905.0000000	0.0695000
-4904.0000000	0.0700000
-4903.0000000	0.0690476
-4902.0000000	0.0680000
-4901.0000000	0.0685714
-4900.0000000	0.0660000
-4899.0000000	0.0650000
-4898.0000000	0.0660000
-4897.0000000	0.0666667
-4896.0000000	0.0685000
-4895.0000000	0.0665000
-4894.0000000	0.0671429
-4893.0000000	0.0685000
-4892.0000000	0.0685000
-4891.0000000	0.0685714
-4890.0000000	0.0675000
-4889.0000000	0.0665000
-4888.0000000	0.0680953
-4887.0000000	0.0675000
-4886.0000000	0.0680000
-4885.0000000	0.0695238
-4884.0000000	0.0700000
-4883.0000000	0.0695000
-4882.0000000	0.0700000
-4881.0000000	0.0685000
-4880.0000000	0.0700000
-4879.0000000	0.0690476
-4878.0000000	0.0690000
-4877.0000000	0.0700000
-4876.0000000	0.0680000
-4875.0000000	0.0676191
-4874.0000000	0.0690476
-4873.0000000	0.0690000
-4872.0000000	0.0695000
-4871.0000000	0.0700000
-4870.0000000	0.0700000

-4869.0000000	0.0700000
-4868.0000000	0.0700000
-4867.0000000	0.0700000
-4866.0000000	0.0700000
-4865.0000000	0.0700000
-4864.0000000	0.0704762
-4863.0000000	0.0700000
-4862.0000000	0.0700000
-4861.0000000	0.0700000
-4860.0000000	0.0700000
-4859.0000000	0.0700000
-4858.0000000	0.0700000
-4857.0000000	0.0700000
-4856.0000000	0.0695000
-4855.0000000	0.0700000
-4854.0000000	0.0695000
-4853.0000000	0.0700000
-4852.0000000	0.0700000
-4851.0000000	0.0695238
-4850.0000000	0.0700000
-4849.0000000	0.0690476
-4848.0000000	0.0694737
-4847.0000000	0.0690476
-4846.0000000	0.0700000
-4845.0000000	0.0690476
-4844.0000000	0.0700000
-4843.0000000	0.0685714
-4842.0000000	0.0680000
-4841.0000000	0.0700000
-4840.0000000	0.0690476
-4839.0000000	0.0695000
-4838.0000000	0.0695000
-4837.0000000	0.0700000
-4836.0000000	0.0704762
-4835.0000000	0.0700000
-4834.0000000	0.0700000
-4833.0000000	0.0700000
-4832.0000000	0.0700000
-4831.0000000	0.0680952
-4830.0000000	0.0695000
-4829.0000000	0.0695000
-4828.0000000	0.0690000
-4827.0000000	0.0695238
-4826.0000000	0.0700000
-4825.0000000	0.0695238
-4824.0000000	0.0695000
-4823.0000000	0.0700000
-4822.0000000	0.0700000
-4821.0000000	0.0700000
-4820.0000000	0.0700000
-4819.0000000	0.0704762
-4818.0000000	0.0700000
-4817.0000000	0.0700000
-4816.0000000	0.0700000
-4815.0000000	0.0700000
-4814.0000000	0.0705000
-4813.0000000	0.0704762

-4812.0000000	0.0705000
-4811.0000000	0.0700000
-4810.0000000	0.0700000
-4809.0000000	0.0700000
-4808.0000000	0.0700000
-4807.0000000	0.0700000
-4806.0000000	0.0704762
-4805.0000000	0.0700000
-4804.0000000	0.0700000
-4803.0000000	0.0700000
-4802.0000000	0.0700000
-4801.0000000	0.0700000
-4800.0000000	0.0700000
-4799.0000000	0.0700000
-4798.0000000	0.0700000
-4797.0000000	0.0700000
-4796.0000000	0.0705000
-4795.0000000	0.0700000
-4794.0000000	0.0705000
-4793.0000000	0.0705000
-4792.0000000	0.0700000
-4791.0000000	0.0700000
-4790.0000000	0.0705000
-4789.0000000	0.0700000
-4788.0000000	0.0700000
-4787.0000000	0.0700000
-4786.0000000	0.0695238
-4785.0000000	0.0655000
-4784.0000000	0.0650000
-4783.0000000	0.0609524
-4782.0000000	0.0610000
-4781.0000000	0.0600000
-4780.0000000	0.0605000
-4779.0000000	0.0619048
-4778.0000000	0.0610526
-4777.0000000	0.0638095
-4776.0000000	0.0652381
-4775.0000000	0.0673684
-4774.0000000	0.0690476
-4773.0000000	0.0690476
-4772.0000000	0.0695000
-4771.0000000	0.0690000
-4770.0000000	0.0695238
-4769.0000000	0.0700000
-4768.0000000	0.0695000
-4767.0000000	0.0700000
-4766.0000000	0.0700000
-4765.0000000	0.0700000
-4764.0000000	0.0700000
-4763.0000000	0.0700000
-4762.0000000	0.0700000
-4761.0000000	0.0690476
-4760.0000000	0.0685000
-4759.0000000	0.0700000
-4758.0000000	0.0690476
-4757.0000000	0.0680000
-4756.0000000	0.0685000

-4755.0000000	0.0690476
-4754.0000000	0.0700000
-4753.0000000	0.0695000
-4752.0000000	0.0685714
-4751.0000000	0.0690000
-4750.0000000	0.0700000
-4749.0000000	0.0700000
-4748.0000000	0.0700000
-4747.0000000	0.0700000
-4746.0000000	0.0700000
-4745.0000000	0.0700000
-4744.0000000	0.0700000
-4743.0000000	0.0700000
-4742.0000000	0.0700000
-4741.0000000	0.0700000
-4740.0000000	0.0700000
-4739.0000000	0.0700000
-4738.0000000	0.0700000
-4737.0000000	0.0700000
-4736.0000000	0.0700000
-4735.0000000	0.0700000
-4734.0000000	0.0700000
-4733.0000000	0.0700000
-4732.0000000	0.0695000
-4731.0000000	0.0695238
-4730.0000000	0.0695000
-4729.0000000	0.0685000
-4728.0000000	0.0690476
-4727.0000000	0.0695000
-4726.0000000	0.0700000
-4725.0000000	0.0695000
-4724.0000000	0.0695238
-4723.0000000	0.0685000
-4722.0000000	0.0695238
-4721.0000000	0.0685000
-4720.0000000	0.0690000
-4719.0000000	0.0680952
-4718.0000000	0.0680000
-4717.0000000	0.0690000
-4716.0000000	0.0700000
-4715.0000000	0.0695000
-4714.0000000	0.0685000
-4713.0000000	0.0700000
-4712.0000000	0.0700000
-4711.0000000	0.0700000
-4710.0000000	0.0700000
-4709.0000000	0.0700000
-4708.0000000	0.0700000
-4707.0000000	0.0700000
-4706.0000000	0.0700000
-4705.0000000	0.0700000
-4704.0000000	0.0700000
-4703.0000000	0.0700000
-4702.0000000	0.0690476
-4701.0000000	0.0695000
-4700.0000000	0.0685714
-4699.0000000	0.0700000

-4698.0000000	0.0700000
-4697.0000000	0.0700000
-4696.0000000	0.0700000
-4695.0000000	0.0700000
-4694.0000000	0.0700000
-4693.0000000	0.0700000
-4692.0000000	0.0700000
-4691.0000000	0.0700000
-4690.0000000	0.0700000
-4689.0000000	0.0695238
-4688.0000000	0.0695000
-4687.0000000	0.0700000
-4686.0000000	0.0700000
-4685.0000000	0.0700000
-4684.0000000	0.0695000
-4683.0000000	0.0695238
-4682.0000000	0.0700000
-4681.0000000	0.0700000
-4680.0000000	0.0700000
-4679.0000000	0.0695238
-4678.0000000	0.0695000
-4677.0000000	0.0695000
-4676.0000000	0.0695238
-4675.0000000	0.0700000
-4674.0000000	0.0690476
-4673.0000000	0.0700000
-4672.0000000	0.0700000
-4671.0000000	0.0700000
-4670.0000000	0.0695238
-4669.0000000	0.0700000
-4668.0000000	0.0685714
-4667.0000000	0.0690000
-4666.0000000	0.0695000
-4665.0000000	0.0690000
-4664.0000000	0.0700000
-4663.0000000	0.0675000
-4662.0000000	0.0695000
-4661.0000000	0.0700000
-4660.0000000	0.0695000
-4659.0000000	0.0695238
-4658.0000000	0.0695000
-4657.0000000	0.0700000
-4656.0000000	0.0700000
-4655.0000000	0.0705000
-4654.0000000	0.0710000
-4653.0000000	0.0705000
-4652.0000000	0.0704762
-4651.0000000	0.0705000
-4650.0000000	0.0715000
-4649.0000000	0.0723810
-4648.0000000	0.0710000
-4647.0000000	0.0719048
-4646.0000000	0.0705000
-4645.0000000	0.0730000
-4644.0000000	0.0719048
-4643.0000000	0.0725000
-4642.0000000	0.0735000

-4641.0000000	0.0710000
-4640.0000000	0.0709524
-4639.0000000	0.0710000
-4638.0000000	0.0700000
-4637.0000000	0.0725000
-4636.0000000	0.0715000
-4635.0000000	0.0715000
-4634.0000000	0.0750000
-4633.0000000	0.0738095
-4632.0000000	0.0750000
-4631.0000000	0.0747619
-4630.0000000	0.0735000
-4629.0000000	0.0755000
-4628.0000000	0.0766667
-4627.0000000	0.0765000
-4626.0000000	0.0760000
-4625.0000000	0.0733333
-4624.0000000	0.0725000
-4623.0000000	0.0740000
-4622.0000000	0.0780952
-4621.0000000	0.0790000
-4620.0000000	0.0790476
-4619.0000000	0.0800000
-4618.0000000	0.0800000
-4617.0000000	0.0800000
-4616.0000000	0.0800000
-4615.0000000	0.0805000
-4614.0000000	0.0800000
-4613.0000000	0.0800000
-4612.0000000	0.0800000
-4611.0000000	0.0800000
-4610.0000000	0.0800000
-4609.0000000	0.0800000
-4608.0000000	0.0800000
-4607.0000000	0.0790000
-4606.0000000	0.0775000
-4605.0000000	0.0740000
-4604.0000000	0.0709524
-4603.0000000	0.0700000
-4602.0000000	0.0700000
-4601.0000000	0.0700000
-4600.0000000	0.0690000
-4599.0000000	0.0695238
-4598.0000000	0.0700000
-4597.0000000	0.0685000
-4596.0000000	0.0685000
-4595.0000000	0.0647619
-4594.0000000	0.0655000
-4593.0000000	0.0620000
-4592.0000000	0.0647619
-4591.0000000	0.0640000
-4590.0000000	0.0655000
-4589.0000000	0.0652381
-4588.0000000	0.0685000
-4587.0000000	0.0690000
-4586.0000000	0.0685714
-4585.0000000	0.0700000

-4584.0000000	0.0695000
-4583.0000000	0.0700000
-4582.0000000	0.0700000
-4581.0000000	0.0700000
-4580.0000000	0.0700000
-4579.0000000	0.0695000
-4578.0000000	0.0690000
-4577.0000000	0.0700000
-4576.0000000	0.0685000
-4575.0000000	0.0675000
-4574.0000000	0.0650000
-4573.0000000	0.0671429
-4572.0000000	0.0615000
-4571.0000000	0.0628571
-4570.0000000	0.0615000
-4569.0000000	0.0620000
-4568.0000000	0.0628571
-4567.0000000	0.0625000
-4566.0000000	0.0625000
-4565.0000000	0.0614286
-4564.0000000	0.0615000
-4563.0000000	0.0625000
-4562.0000000	0.0638095
-4561.0000000	0.0630000
-4560.0000000	0.0625000
-4559.0000000	0.0625000
-4558.0000000	0.0600000
-4557.0000000	0.0605000
-4556.0000000	0.0604762
-4555.0000000	0.0605000
-4554.0000000	0.0605000
-4553.0000000	0.0604762
-4552.0000000	0.0600000
-4551.0000000	0.0600000
-4550.0000000	0.0600000
-4549.0000000	0.0600000
-4548.0000000	0.0600000
-4547.0000000	0.0600000
-4546.0000000	0.0600000
-4545.0000000	0.0595000
-4544.0000000	0.0595000
-4543.0000000	0.0600000
-4542.0000000	0.0600000
-4541.0000000	0.0600000
-4540.0000000	0.0600000
-4539.0000000	0.0600000
-4538.0000000	0.0600000
-4537.0000000	0.0600000
-4536.0000000	0.0600000
-4535.0000000	0.0605000
-4534.0000000	0.0600000
-4533.0000000	0.0605000
-4532.0000000	0.0600000
-4531.0000000	0.0600000
-4530.0000000	0.0600000
-4529.0000000	0.0600000
-4528.0000000	0.0600000

-4527.0000000	0.0600000
-4526.0000000	0.0595000
-4525.0000000	0.0600000
-4524.0000000	0.0595238
-4523.0000000	0.0585714
-4522.0000000	0.0590000
-4521.0000000	0.0600000
-4520.0000000	0.0600000
-4519.0000000	0.0600000
-4518.0000000	0.0600000
-4517.0000000	0.0600000
-4516.0000000	0.0595000
-4515.0000000	0.0600000
-4514.0000000	0.0600000
-4513.0000000	0.0600000
-4512.0000000	0.0595000
-4511.0000000	0.0595238
-4510.0000000	0.0595000
-4509.0000000	0.0595000
-4508.0000000	0.0590476
-4507.0000000	0.0600000
-4506.0000000	0.0600000
-4505.0000000	0.0600000
-4504.0000000	0.0595238
-4503.0000000	0.0580000
-4502.0000000	0.0595238
-4501.0000000	0.0590000
-4500.0000000	0.0595000
-4499.0000000	0.0595000
-4498.0000000	0.0600000
-4497.0000000	0.0595000
-4496.0000000	0.0590000
-4495.0000000	0.0590476
-4494.0000000	0.0600000
-4493.0000000	0.0600000
-4492.0000000	0.0600000
-4491.0000000	0.0585000
-4490.0000000	0.0595000
-4489.0000000	0.0595238
-4488.0000000	0.0600000
-4487.0000000	0.0600000
-4486.0000000	0.0600000
-4485.0000000	0.0600000
-4484.0000000	0.0600000
-4483.0000000	0.0600000
-4482.0000000	0.0600000
-4481.0000000	0.0600000
-4480.0000000	0.0600000
-4479.0000000	0.0600000
-4478.0000000	0.0595000
-4477.0000000	0.0600000
-4476.0000000	0.0595000
-4475.0000000	0.0600000
-4474.0000000	0.0600000
-4473.0000000	0.0600000
-4472.0000000	0.0600000
-4471.0000000	0.0600000

-4470.0000000	0.0600000
-4469.0000000	0.0580000
-4468.0000000	0.0595238
-4467.0000000	0.0600000
-4466.0000000	0.0600000
-4465.0000000	0.0600000
-4464.0000000	0.0600000
-4463.0000000	0.0595238
-4462.0000000	0.0600000
-4461.0000000	0.0600000
-4460.0000000	0.0600000
-4459.0000000	0.0600000
-4458.0000000	0.0605000
-4457.0000000	0.0600000
-4456.0000000	0.0600000
-4455.0000000	0.0605000
-4454.0000000	0.0605000
-4453.0000000	0.0604762
-4452.0000000	0.0600000
-4451.0000000	0.0605000
-4450.0000000	0.0609524
-4449.0000000	0.0610000
-4448.0000000	0.0600000
-4447.0000000	0.0609524
-4446.0000000	0.0610000
-4445.0000000	0.0619047
-4444.0000000	0.0610000
-4443.0000000	0.0630000
-4442.0000000	0.0615000
-4441.0000000	0.0628571
-4440.0000000	0.0620000
-4439.0000000	0.0625000
-4438.0000000	0.0638095
-4437.0000000	0.0605000
-4436.0000000	0.0610000
-4435.0000000	0.0604762
-4434.0000000	0.0605000
-4433.0000000	0.0604762
-4432.0000000	0.0610000
-4431.0000000	0.0605000
-4430.0000000	0.0605000
-4429.0000000	0.0600000
-4428.0000000	0.0600000
-4427.0000000	0.0600000
-4426.0000000	0.0600000
-4425.0000000	0.0600000
-4424.0000000	0.0600000
-4423.0000000	0.0600000
-4422.0000000	0.0600000
-4421.0000000	0.0600000
-4420.0000000	0.0604762
-4419.0000000	0.0600000
-4418.0000000	0.0600000
-4417.0000000	0.0600000
-4416.0000000	0.0600000
-4415.0000000	0.0600000
-4414.0000000	0.0600000

-4413.0000000	0.0600000
-4412.0000000	0.0600000
-4411.0000000	0.0600000
-4410.0000000	0.0600000
-4409.0000000	0.0600000
-4408.0000000	0.0600000
-4407.0000000	0.0609524
-4406.0000000	0.0610000
-4405.0000000	0.0604762
-4404.0000000	0.0600000
-4403.0000000	0.0615000
-4402.0000000	0.0619047
-4401.0000000	0.0635000
-4400.0000000	0.0610000
-4399.0000000	0.0605000
-4398.0000000	0.0619048
-4397.0000000	0.0605000
-4396.0000000	0.0604762
-4395.0000000	0.0605000
-4394.0000000	0.0605000
-4393.0000000	0.0604762
-4392.0000000	0.0610000
-4391.0000000	0.0605000
-4390.0000000	0.0609524
-4389.0000000	0.0605000
-4388.0000000	0.0605000
-4387.0000000	0.0605000
-4386.0000000	0.0604762
-4385.0000000	0.0615000
-4384.0000000	0.0619047
-4383.0000000	0.0620000
-4382.0000000	0.0605000
-4381.0000000	0.0614286
-4380.0000000	0.0600000
-4379.0000000	0.0615000
-4378.0000000	0.0605000
-4377.0000000	0.0604762
-4376.0000000	0.0605000
-4375.0000000	0.0604762
-4374.0000000	0.0600000
-4373.0000000	0.0600000
-4372.0000000	0.0600000
-4371.0000000	0.0600000
-4370.0000000	0.0595000
-4369.0000000	0.0600000
-4368.0000000	0.0600000
-4367.0000000	0.0600000
-4366.0000000	0.0590476
-4365.0000000	0.0585000
-4364.0000000	0.0570000
-4363.0000000	0.0580000
-4362.0000000	0.0600000
-4361.0000000	0.0585000
-4360.0000000	0.0595000
-4359.0000000	0.0576190
-4358.0000000	0.0585000
-4357.0000000	0.0580952

-4356.0000000	0.0590000
-4355.0000000	0.0595000
-4354.0000000	0.0585714
-4353.0000000	0.0585000
-4352.0000000	0.0595000
-4351.0000000	0.0595000
-4350.0000000	0.0600000
-4349.0000000	0.0600000
-4348.0000000	0.0600000
-4347.0000000	0.0600000
-4346.0000000	0.0600000
-4345.0000000	0.0600000
-4344.0000000	0.0600000
-4343.0000000	0.0600000
-4342.0000000	0.0600000
-4341.0000000	0.0594737
-4340.0000000	0.0595238
-4339.0000000	0.0595000
-4338.0000000	0.0590476
-4337.0000000	0.0595000
-4336.0000000	0.0600000
-4335.0000000	0.0600000
-4334.0000000	0.0600000
-4333.0000000	0.0600000
-4332.0000000	0.0600000
-4331.0000000	0.0604762
-4330.0000000	0.0600000
-4329.0000000	0.0600000
-4328.0000000	0.0600000
-4327.0000000	0.0600000
-4326.0000000	0.0600000
-4325.0000000	0.0600000
-4324.0000000	0.0610000
-4323.0000000	0.0600000
-4322.0000000	0.0600000
-4321.0000000	0.0595000
-4320.0000000	0.0600000
-4319.0000000	0.0600000
-4318.0000000	0.0600000
-4317.0000000	0.0600000
-4316.0000000	0.0600000
-4315.0000000	0.0600000
-4314.0000000	0.0600000
-4313.0000000	0.0600000
-4312.0000000	0.0600000
-4311.0000000	0.0600000
-4310.0000000	0.0600000
-4309.0000000	0.0600000
-4308.0000000	0.0604762
-4307.0000000	0.0600000
-4306.0000000	0.0600000
-4305.0000000	0.0600000
-4304.0000000	0.0600000
-4303.0000000	0.0600000
-4302.0000000	0.0600000
-4301.0000000	0.0600000
-4300.0000000	0.0595000

-4299.0000000	0.0600000
-4298.0000000	0.0600000
-4297.0000000	0.0600000
-4296.0000000	0.0600000
-4295.0000000	0.0600000
-4294.0000000	0.0600000
-4293.0000000	0.0600000
-4292.0000000	0.0600000
-4291.0000000	0.0600000
-4290.0000000	0.0600000
-4289.0000000	0.0600000
-4288.0000000	0.0605000
-4287.0000000	0.0600000
-4286.0000000	0.0605000
-4285.0000000	0.0605000
-4284.0000000	0.0609524
-4283.0000000	0.0605000
-4282.0000000	0.0604762
-4281.0000000	0.0605000
-4280.0000000	0.0605000
-4279.0000000	0.0605000
-4278.0000000	0.0604762
-4277.0000000	0.0605000
-4276.0000000	0.0610000
-4275.0000000	0.0614286
-4274.0000000	0.0600000
-4273.0000000	0.0605000
-4272.0000000	0.0600000
-4271.0000000	0.0600000
-4270.0000000	0.0600000
-4269.0000000	0.0600000
-4268.0000000	0.0600000
-4267.0000000	0.0600000
-4266.0000000	0.0600000
-4265.0000000	0.0600000
-4264.0000000	0.0600000
-4263.0000000	0.0604762
-4262.0000000	0.0600000
-4261.0000000	0.0600000
-4260.0000000	0.0600000
-4259.0000000	0.0604762
-4258.0000000	0.0600000
-4257.0000000	0.0600000
-4256.0000000	0.0600000
-4255.0000000	0.0600000
-4254.0000000	0.0600000
-4253.0000000	0.0600000
-4252.0000000	0.0600000
-4251.0000000	0.0600000
-4250.0000000	0.0600000
-4249.0000000	0.0600000
-4248.0000000	0.0600000
-4247.0000000	0.0600000
-4246.0000000	0.0600000
-4245.0000000	0.0600000
-4244.0000000	0.0600000
-4243.0000000	0.0605000

-4242.0000000	0.0600000
-4241.0000000	0.0600000
-4240.0000000	0.0600000
-4239.0000000	0.0600000
-4238.0000000	0.0600000
-4237.0000000	0.0600000
-4236.0000000	0.0600000
-4235.0000000	0.0600000
-4234.0000000	0.0600000
-4233.0000000	0.0600000
-4232.0000000	0.0600000
-4231.0000000	0.0595000
-4230.0000000	0.0600000
-4229.0000000	0.0600000
-4228.0000000	0.0600000
-4227.0000000	0.0600000
-4226.0000000	0.0600000
-4225.0000000	0.0600000
-4224.0000000	0.0600000
-4223.0000000	0.0600000
-4222.0000000	0.0600000
-4221.0000000	0.0600000
-4220.0000000	0.0600000
-4219.0000000	0.0600000
-4218.0000000	0.0600000
-4217.0000000	0.0600000
-4216.0000000	0.0595000
-4215.0000000	0.0590476
-4214.0000000	0.0585000
-4213.0000000	0.0600000
-4212.0000000	0.0600000
-4211.0000000	0.0590476
-4210.0000000	0.0600000
-4209.0000000	0.0595238
-4208.0000000	0.0600000
-4207.0000000	0.0600000
-4206.0000000	0.0600000
-4205.0000000	0.0590000
-4204.0000000	0.0600000
-4203.0000000	0.0600000
-4202.0000000	0.0585000
-4201.0000000	0.0585000
-4200.0000000	0.0600000
-4199.0000000	0.0589474
-4198.0000000	0.0580952
-4197.0000000	0.0600000
-4196.0000000	0.0595000
-4195.0000000	0.0585000
-4194.0000000	0.0585714
-4193.0000000	0.0600000
-4192.0000000	0.0600000
-4191.0000000	0.0600000
-4190.0000000	0.0600000
-4189.0000000	0.0600000
-4188.0000000	0.0600000
-4187.0000000	0.0600000
-4186.0000000	0.0600000

-4185.0000000	0.0595238
-4184.0000000	0.0600000
-4183.0000000	0.0595000
-4182.0000000	0.0600000
-4181.0000000	0.0580000
-4180.0000000	0.0600000
-4179.0000000	0.0590000
-4178.0000000	0.0595238
-4177.0000000	0.0595000
-4176.0000000	0.0600000
-4175.0000000	0.0595238
-4174.0000000	0.0595000
-4173.0000000	0.0595238
-4172.0000000	0.0600000
-4171.0000000	0.0600000
-4170.0000000	0.0595238
-4169.0000000	0.0600000
-4168.0000000	0.0600000
-4167.0000000	0.0600000
-4166.0000000	0.0600000
-4165.0000000	0.0600000
-4164.0000000	0.0604762
-4163.0000000	0.0600000
-4162.0000000	0.0600000
-4161.0000000	0.0600000
-4160.0000000	0.0600000
-4159.0000000	0.0600000
-4158.0000000	0.0600000
-4157.0000000	0.0600000
-4156.0000000	0.0600000
-4155.0000000	0.0600000
-4154.0000000	0.0600000
-4153.0000000	0.0600000
-4152.0000000	0.0600000
-4151.0000000	0.0600000
-4150.0000000	0.0600000
-4149.0000000	0.0600000
-4148.0000000	0.0600000
-4147.0000000	0.0600000
-4146.0000000	0.0600000
-4145.0000000	0.0600000
-4144.0000000	0.0600000
-4143.0000000	0.0609524
-4142.0000000	0.0605000
-4141.0000000	0.0610000
-4140.0000000	0.0610000
-4139.0000000	0.0609524
-4138.0000000	0.0600000
-4137.0000000	0.0600000
-4136.0000000	0.0604762
-4135.0000000	0.0600000
-4134.0000000	0.0600000
-4133.0000000	0.0600000
-4132.0000000	0.0600000
-4131.0000000	0.0600000
-4130.0000000	0.0600000
-4129.0000000	0.0600000

-4128.0000000	0.0600000
-4127.0000000	0.0600000
-4126.0000000	0.0600000
-4125.0000000	0.0600000
-4124.0000000	0.0600000
-4123.0000000	0.0600000
-4122.0000000	0.0600000
-4121.0000000	0.0600000
-4120.0000000	0.0600000
-4119.0000000	0.0605000
-4118.0000000	0.0600000
-4117.0000000	0.0600000
-4116.0000000	0.0600000
-4115.0000000	0.0600000
-4114.0000000	0.0600000
-4113.0000000	0.0604762
-4112.0000000	0.0600000
-4111.0000000	0.0600000
-4110.0000000	0.0600000
-4109.0000000	0.0600000
-4108.0000000	0.0600000
-4107.0000000	0.0600000
-4106.0000000	0.0600000
-4105.0000000	0.0600000
-4104.0000000	0.0600000
-4103.0000000	0.0600000
-4102.0000000	0.0600000
-4101.0000000	0.0600000
-4100.0000000	0.0600000
-4099.0000000	0.0600000
-4098.0000000	0.0604762
-4097.0000000	0.0600000
-4096.0000000	0.0605000
-4095.0000000	0.0605000
-4094.0000000	0.0619048
-4093.0000000	0.0615000
-4092.0000000	0.0620000
-4091.0000000	0.0619048
-4090.0000000	0.0640000
-4089.0000000	0.0625000
-4088.0000000	0.0640000
-4087.0000000	0.0642857
-4086.0000000	0.0642857
-4085.0000000	0.0655000
-4084.0000000	0.0650000
-4083.0000000	0.0650000
-4082.0000000	0.0666667
-4081.0000000	0.0675000
-4080.0000000	0.0660000
-4079.0000000	0.0633333
-4078.0000000	0.0625000
-4077.0000000	0.0623810
-4076.0000000	0.0645000
-4075.0000000	0.0635000
-4074.0000000	0.0625000
-4073.0000000	0.0623809
-4072.0000000	0.0610000

-4071.0000000	0.0600000
-4070.0000000	0.0609524
-4069.0000000	0.0605000
-4068.0000000	0.0619048
-4067.0000000	0.0605000
-4066.0000000	0.0600000
-4065.0000000	0.0605000
-4064.0000000	0.0604762
-4063.0000000	0.0605000
-4062.0000000	0.0600000
-4061.0000000	0.0600000
-4060.0000000	0.0600000
-4059.0000000	0.0600000
-4058.0000000	0.0600000
-4057.0000000	0.0600000
-4056.0000000	0.0600000
-4055.0000000	0.0600000
-4054.0000000	0.0600000
-4053.0000000	0.0600000
-4052.0000000	0.0600000
-4051.0000000	0.0600000
-4050.0000000	0.0600000
-4049.0000000	0.0605000
-4048.0000000	0.0609524
-4047.0000000	0.0600000
-4046.0000000	0.0600000
-4045.0000000	0.0605000
-4044.0000000	0.0620000
-4043.0000000	0.0609524
-4042.0000000	0.0605000
-4041.0000000	0.0625000
-4040.0000000	0.0635000
-4039.0000000	0.0642857
-4038.0000000	0.0642857
-4037.0000000	0.0630000
-4036.0000000	0.0640000
-4035.0000000	0.0640000
-4034.0000000	0.0619048
-4033.0000000	0.0605000
-4032.0000000	0.0605000
-4031.0000000	0.0619048
-4030.0000000	0.0610000
-4029.0000000	0.0605000
-4028.0000000	0.0600000
-4027.0000000	0.0600000
-4026.0000000	0.0600000
-4025.0000000	0.0604762
-4024.0000000	0.0600000
-4023.0000000	0.0605000
-4022.0000000	0.0609524
-4021.0000000	0.0610000
-4020.0000000	0.0615000
-4019.0000000	0.0609524
-4018.0000000	0.0610000
-4017.0000000	0.0605000
-4016.0000000	0.0609524
-4015.0000000	0.0615000

-4014.0000000	0.0605000
-4013.0000000	0.0600000
-4012.0000000	0.0600000
-4011.0000000	0.0600000
-4010.0000000	0.0600000
-4009.0000000	0.0600000
-4008.0000000	0.0600000
-4007.0000000	0.0600000
-4006.0000000	0.0600000
-4005.0000000	0.0600000
-4004.0000000	0.0605000
-4003.0000000	0.0600000
-4002.0000000	0.0600000
-4001.0000000	0.0600000
-4000.0000000	0.0600000
-3999.0000000	0.0605000
-3998.0000000	0.0604762
-3997.0000000	0.0605000
-3996.0000000	0.0605000
-3995.0000000	0.0609524
-3994.0000000	0.0610000
-3993.0000000	0.0600000
-3992.0000000	0.0600000
-3991.0000000	0.0604762
-3990.0000000	0.0600000
-3989.0000000	0.0604762
-3988.0000000	0.0605000
-3987.0000000	0.0600000
-3986.0000000	0.0600000
-3985.0000000	0.0600000
-3984.0000000	0.0600000
-3983.0000000	0.0600000
-3982.0000000	0.0600000
-3981.0000000	0.0600000
-3980.0000000	0.0600000
-3979.0000000	0.0604762
-3978.0000000	0.0600000
-3977.0000000	0.0600000
-3976.0000000	0.0600000
-3975.0000000	0.0600000
-3974.0000000	0.0600000
-3973.0000000	0.0600000
-3972.0000000	0.0600000
-3971.0000000	0.0595238
-3970.0000000	0.0585000
-3969.0000000	0.0600000
-3968.0000000	0.0600000
-3967.0000000	0.0595238
-3966.0000000	0.0590000
-3965.0000000	0.0595000
-3964.0000000	0.0600000
-3963.0000000	0.0585000
-3962.0000000	0.0590476
-3961.0000000	0.0600000
-3960.0000000	0.0600000
-3959.0000000	0.0595238
-3958.0000000	0.0600000

-3957.0000000	0.0595000
-3956.0000000	0.0600000
-3955.0000000	0.0595238
-3954.0000000	0.0600000
-3953.0000000	0.0600000
-3952.0000000	0.0600000
-3951.0000000	0.0600000
-3950.0000000	0.0600000
-3949.0000000	0.0595000
-3948.0000000	0.0600000
-3947.0000000	0.0600000
-3946.0000000	0.0600000
-3945.0000000	0.0600000
-3944.0000000	0.0600000
-3943.0000000	0.0600000
-3942.0000000	0.0600000
-3941.0000000	0.0600000
-3940.0000000	0.0600000
-3939.0000000	0.0600000
-3938.0000000	0.0595000
-3937.0000000	0.0600000
-3936.0000000	0.0600000
-3935.0000000	0.0600000
-3934.0000000	0.0600000
-3933.0000000	0.0600000
-3932.0000000	0.0600000
-3931.0000000	0.0600000
-3930.0000000	0.0600000
-3929.0000000	0.0600000
-3928.0000000	0.0600000
-3927.0000000	0.0600000
-3926.0000000	0.0595000
-3925.0000000	0.0595238
-3924.0000000	0.0590000
-3923.0000000	0.0595000
-3922.0000000	0.0600000
-3921.0000000	0.0585000
-3920.0000000	0.0585000
-3919.0000000	0.0600000
-3918.0000000	0.0600000
-3917.0000000	0.0600000
-3916.0000000	0.0595238
-3915.0000000	0.0600000
-3914.0000000	0.0600000
-3913.0000000	0.0600000
-3912.0000000	0.0600000
-3911.0000000	0.0600000
-3910.0000000	0.0600000
-3909.0000000	0.0600000
-3908.0000000	0.0605000
-3907.0000000	0.0600000
-3906.0000000	0.0600000
-3905.0000000	0.0600000
-3904.0000000	0.0600000
-3903.0000000	0.0600000
-3902.0000000	0.0595238
-3901.0000000	0.0600000

-3900.0000000	0.0600000
-3899.0000000	0.0600000
-3898.0000000	0.0600000
-3897.0000000	0.0590000
-3896.0000000	0.0585000
-3895.0000000	0.0595238
-3894.0000000	0.0595000
-3893.0000000	0.0600000
-3892.0000000	0.0600000
-3891.0000000	0.0600000
-3890.0000000	0.0600000
-3889.0000000	0.0595238
-3888.0000000	0.0600000
-3887.0000000	0.0600000
-3886.0000000	0.0595000
-3885.0000000	0.0595000
-3884.0000000	0.0600000
-3883.0000000	0.0600000
-3882.0000000	0.0600000
-3881.0000000	0.0600000
-3880.0000000	0.0600000
-3879.0000000	0.0600000
-3878.0000000	0.0600000
-3877.0000000	0.0600000
-3876.0000000	0.0600000
-3875.0000000	0.0595000
-3874.0000000	0.0600000
-3873.0000000	0.0600000
-3872.0000000	0.0595238
-3871.0000000	0.0600000
-3870.0000000	0.0595000
-3869.0000000	0.0600000
-3868.0000000	0.0600000
-3867.0000000	0.0600000
-3866.0000000	0.0600000
-3865.0000000	0.0585000
-3864.0000000	0.0600000
-3863.0000000	0.0585000
-3862.0000000	0.0580952
-3861.0000000	0.0585000
-3860.0000000	0.0575000
-3859.0000000	0.0580952
-3858.0000000	0.0575000
-3857.0000000	0.0570000
-3856.0000000	0.0557143
-3855.0000000	0.0590000
-3854.0000000	0.0560000
-3853.0000000	0.0561905
-3852.0000000	0.0570000
-3851.0000000	0.0560000
-3850.0000000	0.0561905
-3849.0000000	0.0550000
-3848.0000000	0.0535000
-3847.0000000	0.0520000
-3846.0000000	0.0519048
-3845.0000000	0.0520000
-3844.0000000	0.0514286

-3843.0000000	0.0500000
-3842.0000000	0.0505000
-3841.0000000	0.0509524
-3840.0000000	0.0505000
-3839.0000000	0.0505000
-3838.0000000	0.0500000
-3837.0000000	0.0505000
-3836.0000000	0.0500000
-3835.0000000	0.0500000
-3834.0000000	0.0504762
-3833.0000000	0.0510000
-3832.0000000	0.0504762
-3831.0000000	0.0510000
-3830.0000000	0.0520000
-3829.0000000	0.0520000
-3828.0000000	0.0538095
-3827.0000000	0.0515000
-3826.0000000	0.0535000
-3825.0000000	0.0523810
-3824.0000000	0.0550000
-3823.0000000	0.0557143
-3822.0000000	0.0555000
-3821.0000000	0.0535000
-3820.0000000	0.0538095
-3819.0000000	0.0530000
-3818.0000000	0.0525000
-3817.0000000	0.0530000
-3816.0000000	0.0533333
-3815.0000000	0.0520000
-3814.0000000	0.0519048
-3813.0000000	0.0510000
-3812.0000000	0.0500000
-3811.0000000	0.0500000
-3810.0000000	0.0510000
-3809.0000000	0.0510000
-3808.0000000	0.0500000
-3807.0000000	0.0509524
-3806.0000000	0.0505000
-3805.0000000	0.0519048
-3804.0000000	0.0510000
-3803.0000000	0.0520000
-3802.0000000	0.0510000
-3801.0000000	0.0509524
-3800.0000000	0.0505000
-3799.0000000	0.0505000
-3798.0000000	0.0504762
-3797.0000000	0.0510000
-3796.0000000	0.0514286
-3795.0000000	0.0515000
-3794.0000000	0.0515000
-3793.0000000	0.0505000
-3792.0000000	0.0500000
-3791.0000000	0.0500000
-3790.0000000	0.0490000
-3789.0000000	0.0500000
-3788.0000000	0.0500000
-3787.0000000	0.0495000

-3786.0000000	0.0500000
-3785.0000000	0.0500000
-3784.0000000	0.0500000
-3783.0000000	0.0500000
-3782.0000000	0.0500000
-3781.0000000	0.0500000
-3780.0000000	0.0500000
-3779.0000000	0.0500000
-3778.0000000	0.0500000
-3777.0000000	0.0500000
-3776.0000000	0.0500000
-3775.0000000	0.0495238
-3774.0000000	0.0500000
-3773.0000000	0.0500000
-3772.0000000	0.0495000
-3771.0000000	0.0490476
-3770.0000000	0.0500000
-3769.0000000	0.0495000
-3768.0000000	0.0500000
-3767.0000000	0.0495000
-3766.0000000	0.0500000
-3765.0000000	0.0500000
-3764.0000000	0.0500000
-3763.0000000	0.0495000
-3762.0000000	0.0504762
-3761.0000000	0.0500000
-3760.0000000	0.0500000
-3759.0000000	0.0500000
-3758.0000000	0.0500000
-3757.0000000	0.0500000
-3756.0000000	0.0500000
-3755.0000000	0.0505000
-3754.0000000	0.0505000
-3753.0000000	0.0500000
-3752.0000000	0.0505000
-3751.0000000	0.0500000
-3750.0000000	0.0500000
-3749.0000000	0.0500000
-3748.0000000	0.0500000
-3747.0000000	0.0500000
-3746.0000000	0.0500000
-3745.0000000	0.0500000
-3744.0000000	0.0500000
-3743.0000000	0.0505000
-3742.0000000	0.0505000
-3741.0000000	0.0509524
-3740.0000000	0.0505000
-3739.0000000	0.0510000
-3738.0000000	0.0510000
-3737.0000000	0.0509524
-3736.0000000	0.0514286
-3735.0000000	0.0520000
-3734.0000000	0.0505000
-3733.0000000	0.0520000
-3732.0000000	0.0533333
-3731.0000000	0.0540000
-3730.0000000	0.0535000

-3729.0000000	0.0509524
-3728.0000000	0.0530000
-3727.0000000	0.0545000
-3726.0000000	0.0545000
-3725.0000000	0.0547619
-3724.0000000	0.0545000
-3723.0000000	0.0547619
-3722.0000000	0.0535000
-3721.0000000	0.0528571
-3720.0000000	0.0560000
-3719.0000000	0.0550000
-3718.0000000	0.0560000
-3717.0000000	0.0561905
-3716.0000000	0.0565000
-3715.0000000	0.0580000
-3714.0000000	0.0571428
-3713.0000000	0.0585000
-3712.0000000	0.0575000
-3711.0000000	0.0600000
-3710.0000000	0.0580952
-3709.0000000	0.0585000
-3708.0000000	0.0590000
-3707.0000000	0.0580952
-3706.0000000	0.0600000
-3705.0000000	0.0585714
-3704.0000000	0.0595000
-3703.0000000	0.0595000
-3702.0000000	0.0600000
-3701.0000000	0.0595238
-3700.0000000	0.0595000
-3699.0000000	0.0600000
-3698.0000000	0.0600000
-3697.0000000	0.0580000
-3696.0000000	0.0580952
-3695.0000000	0.0590000
-3694.0000000	0.0563158
-3693.0000000	0.0554545
-3692.0000000	0.0545000
-3691.0000000	0.0535000
-3690.0000000	0.0533333
-3689.0000000	0.0510000
-3688.0000000	0.0505000
-3687.0000000	0.0500000
-3686.0000000	0.0500000
-3685.0000000	0.0500000
-3684.0000000	0.0505000
-3683.0000000	0.0500000
-3682.0000000	0.0500000
-3681.0000000	0.0500000
-3680.0000000	0.0500000
-3679.0000000	0.0505000
-3678.0000000	0.0509524
-3677.0000000	0.0510000
-3676.0000000	0.0504762
-3675.0000000	0.0505000
-3674.0000000	0.0520000
-3673.0000000	0.0535000

-3672.0000000	0.0520000
-3671.0000000	0.0514286
-3670.0000000	0.0500000
-3669.0000000	0.0500000
-3668.0000000	0.0500000
-3667.0000000	0.0505000
-3666.0000000	0.0500000
-3665.0000000	0.0519048
-3664.0000000	0.0500000
-3663.0000000	0.0515000
-3662.0000000	0.0514286
-3661.0000000	0.0525000
-3660.0000000	0.0519048
-3659.0000000	0.0525000
-3658.0000000	0.0530000
-3657.0000000	0.0523810
-3656.0000000	0.0525000
-3655.0000000	0.0525000
-3654.0000000	0.0509524
-3653.0000000	0.0540000
-3652.0000000	0.0515000
-3651.0000000	0.0550000
-3650.0000000	0.0542857
-3649.0000000	0.0550000
-3648.0000000	0.0542857
-3647.0000000	0.0535000
-3646.0000000	0.0550000
-3645.0000000	0.0538095
-3644.0000000	0.0535000
-3643.0000000	0.0530000
-3642.0000000	0.0515000
-3641.0000000	0.0533333
-3640.0000000	0.0550000
-3639.0000000	0.0547619
-3638.0000000	0.0535000
-3637.0000000	0.0525000
-3636.0000000	0.0530000
-3635.0000000	0.0519048
-3634.0000000	0.0505000
-3633.0000000	0.0500000
-3632.0000000	0.0504762
-3631.0000000	0.0500000
-3630.0000000	0.0504762
-3629.0000000	0.0505000
-3628.0000000	0.0510000
-3627.0000000	0.0515000
-3626.0000000	0.0509524
-3625.0000000	0.0505000
-3624.0000000	0.0505000
-3623.0000000	0.0509524
-3622.0000000	0.0500000
-3621.0000000	0.0500000
-3620.0000000	0.0500000
-3619.0000000	0.0500000
-3618.0000000	0.0500000
-3617.0000000	0.0500000
-3616.0000000	0.0500000

-3615.0000000	0.0500000
-3614.0000000	0.0504762
-3613.0000000	0.0505000
-3612.0000000	0.0500000
-3611.0000000	0.0500000
-3610.0000000	0.0514286
-3609.0000000	0.0504762
-3608.0000000	0.0505000
-3607.0000000	0.0500000
-3606.0000000	0.0505000
-3605.0000000	0.0500000
-3604.0000000	0.0510000
-3603.0000000	0.0500000
-3602.0000000	0.0504762
-3601.0000000	0.0505000
-3600.0000000	0.0505000
-3599.0000000	0.0504762
-3598.0000000	0.0505000
-3597.0000000	0.0525000
-3596.0000000	0.0519048
-3595.0000000	0.0510000
-3594.0000000	0.0514286
-3593.0000000	0.0535000
-3592.0000000	0.0530000
-3591.0000000	0.0540000
-3590.0000000	0.0523810
-3589.0000000	0.0535000
-3588.0000000	0.0510000
-3587.0000000	0.0519048
-3586.0000000	0.0545000
-3585.0000000	0.0542857
-3584.0000000	0.0520000
-3583.0000000	0.0520000
-3582.0000000	0.0515000
-3581.0000000	0.0505000
-3580.0000000	0.0509524
-3579.0000000	0.0500000
-3578.0000000	0.0509524
-3577.0000000	0.0525000
-3576.0000000	0.0515000
-3575.0000000	0.0535000
-3574.0000000	0.0571428
-3573.0000000	0.0570000
-3572.0000000	0.0565000
-3571.0000000	0.0533333
-3570.0000000	0.0580952
-3569.0000000	0.0565000
-3568.0000000	0.0550000
-3567.0000000	0.0575000
-3566.0000000	0.0571428
-3565.0000000	0.0575000
-3564.0000000	0.0570000
-3563.0000000	0.0580952
-3562.0000000	0.0580000
-3561.0000000	0.0580000
-3560.0000000	0.0580952
-3559.0000000	0.0580000

-3558.0000000	0.0552381
-3557.0000000	0.0545000
-3556.0000000	0.0565000
-3555.0000000	0.0560000
-3554.0000000	0.0576190
-3553.0000000	0.0575000
-3552.0000000	0.0565000
-3551.0000000	0.0547619
-3550.0000000	0.0560000
-3549.0000000	0.0555000
-3548.0000000	0.0571428
-3547.0000000	0.0555000
-3546.0000000	0.0560000
-3545.0000000	0.0575000
-3544.0000000	0.0561905
-3543.0000000	0.0575000
-3542.0000000	0.0576190
-3541.0000000	0.0530000
-3540.0000000	0.0550000
-3539.0000000	0.0547619
-3538.0000000	0.0515000
-3537.0000000	0.0530000
-3536.0000000	0.0520000
-3535.0000000	0.0509524
-3534.0000000	0.0515000
-3533.0000000	0.0509524
-3532.0000000	0.0505000
-3531.0000000	0.0505000
-3530.0000000	0.0500000
-3529.0000000	0.0495000
-3528.0000000	0.0500000
-3527.0000000	0.0500000
-3526.0000000	0.0500000
-3525.0000000	0.0500000
-3524.0000000	0.0500000
-3523.0000000	0.0500000
-3522.0000000	0.0500000
-3521.0000000	0.0500000
-3520.0000000	0.0505000
-3519.0000000	0.0500000
-3518.0000000	0.0509524
-3517.0000000	0.0500000
-3516.0000000	0.0505000
-3515.0000000	0.0510000
-3514.0000000	0.0504762
-3513.0000000	0.0505000
-3512.0000000	0.0519048
-3511.0000000	0.0515000
-3510.0000000	0.0514286
-3509.0000000	0.0515000
-3508.0000000	0.0525000
-3507.0000000	0.0515000
-3506.0000000	0.0515000
-3505.0000000	0.0514286
-3504.0000000	0.0525000
-3503.0000000	0.0538095
-3502.0000000	0.0515000

-3501.0000000	0.0515000
-3500.0000000	0.0505000
-3499.0000000	0.0533333
-3498.0000000	0.0525000
-3497.0000000	0.0530000
-3496.0000000	0.0514286
-3495.0000000	0.0535000
-3494.0000000	0.0528571
-3493.0000000	0.0525000
-3492.0000000	0.0520000
-3491.0000000	0.0542857
-3490.0000000	0.0520000
-3489.0000000	0.0520000
-3488.0000000	0.0545000
-3487.0000000	0.0533333
-3486.0000000	0.0555000
-3485.0000000	0.0530000
-3484.0000000	0.0510000
-3483.0000000	0.0533333
-3482.0000000	0.0533333
-3481.0000000	0.0520000
-3480.0000000	0.0540000
-3479.0000000	0.0528571
-3478.0000000	0.0520000
-3477.0000000	0.0540000
-3476.0000000	0.0530000
-3475.0000000	0.0540000
-3474.0000000	0.0547619
-3473.0000000	0.0560000
-3472.0000000	0.0566667
-3471.0000000	0.0550000
-3470.0000000	0.0565000
-3469.0000000	0.0566667
-3468.0000000	0.0570000
-3467.0000000	0.0561905
-3466.0000000	0.0550000
-3465.0000000	0.0540000
-3464.0000000	0.0530000
-3463.0000000	0.0519048
-3462.0000000	0.0515000
-3461.0000000	0.0515000
-3460.0000000	0.0514286
-3459.0000000	0.0515000
-3458.0000000	0.0504762
-3457.0000000	0.0515000
-3456.0000000	0.0515000
-3455.0000000	0.0530000
-3454.0000000	0.0550000
-3453.0000000	0.0542857
-3452.0000000	0.0540000
-3451.0000000	0.0533333
-3450.0000000	0.0510000
-3449.0000000	0.0523810
-3448.0000000	0.0515000
-3447.0000000	0.0545000
-3446.0000000	0.0525000
-3445.0000000	0.0520000

-3444.0000000	0.0519048
-3443.0000000	0.0509524
-3442.0000000	0.0515000
-3441.0000000	0.0505000
-3440.0000000	0.0525000
-3439.0000000	0.0514286
-3438.0000000	0.0515000
-3437.0000000	0.0500000
-3436.0000000	0.0504762
-3435.0000000	0.0530000
-3434.0000000	0.0509524
-3433.0000000	0.0510000
-3432.0000000	0.0510000
-3431.0000000	0.0520000
-3430.0000000	0.0514286
-3429.0000000	0.0505000
-3428.0000000	0.0520000
-3427.0000000	0.0538095
-3426.0000000	0.0530000
-3425.0000000	0.0525000
-3424.0000000	0.0514286
-3423.0000000	0.0505000
-3422.0000000	0.0515000
-3421.0000000	0.0504762
-3420.0000000	0.0505000
-3419.0000000	0.0514286
-3418.0000000	0.0505000
-3417.0000000	0.0500000
-3416.0000000	0.0505000
-3415.0000000	0.0509524
-3414.0000000	0.0505000
-3413.0000000	0.0500000
-3412.0000000	0.0500000
-3411.0000000	0.0500000
-3410.0000000	0.0500000
-3409.0000000	0.0500000
-3408.0000000	0.0500000
-3407.0000000	0.0500000
-3406.0000000	0.0500000
-3405.0000000	0.0500000
-3404.0000000	0.0500000
-3403.0000000	0.0500000
-3402.0000000	0.0500000
-3401.0000000	0.0500000
-3400.0000000	0.0500000
-3399.0000000	0.0505000
-3398.0000000	0.0505000
-3397.0000000	0.0500000
-3396.0000000	0.0500000
-3395.0000000	0.0500000
-3394.0000000	0.0500000
-3393.0000000	0.0530000
-3392.0000000	0.0505000
-3391.0000000	0.0504762
-3390.0000000	0.0505000
-3389.0000000	0.0505000
-3388.0000000	0.0519048

-3387.0000000	0.0500000
-3386.0000000	0.0500000
-3385.0000000	0.0514286
-3384.0000000	0.0525000
-3383.0000000	0.0538095
-3382.0000000	0.0550000
-3381.0000000	0.0570000
-3380.0000000	0.0575000
-3379.0000000	0.0575000
-3378.0000000	0.0585714
-3377.0000000	0.0580000
-3376.0000000	0.0585714
-3375.0000000	0.0585000
-3374.0000000	0.0585714
-3373.0000000	0.0595000
-3372.0000000	0.0600000
-3371.0000000	0.0595000
-3370.0000000	0.0600000
-3369.0000000	0.0600000
-3368.0000000	0.0600000
-3367.0000000	0.0600000
-3366.0000000	0.0600000
-3365.0000000	0.0600000
-3364.0000000	0.0600000
-3363.0000000	0.0585000
-3362.0000000	0.0590000
-3361.0000000	0.0590000
-3360.0000000	0.0585714
-3359.0000000	0.0595000
-3358.0000000	0.0585714
-3357.0000000	0.0595000
-3356.0000000	0.0600000
-3355.0000000	0.0595238
-3354.0000000	0.0600000
-3353.0000000	0.0585000
-3352.0000000	0.0595238
-3351.0000000	0.0580000
-3350.0000000	0.0590000
-3349.0000000	0.0585000
-3348.0000000	0.0571428
-3347.0000000	0.0565000
-3346.0000000	0.0566667
-3345.0000000	0.0560000
-3344.0000000	0.0550000
-3343.0000000	0.0547619
-3342.0000000	0.0550000
-3341.0000000	0.0550000
-3340.0000000	0.0547619
-3339.0000000	0.0530000
-3338.0000000	0.0525000
-3337.0000000	0.0535000
-3336.0000000	0.0528571
-3335.0000000	0.0530000
-3334.0000000	0.0545000
-3333.0000000	0.0542857
-3332.0000000	0.0550000
-3331.0000000	0.0578947

-3330.0000000	0.0536364
-3329.0000000	0.0520000
-3328.0000000	0.0504762
-3327.0000000	0.0520000
-3326.0000000	0.0530000
-3325.0000000	0.0520000
-3324.0000000	0.0533333
-3323.0000000	0.0570000
-3322.0000000	0.0570000
-3321.0000000	0.0557143
-3320.0000000	0.0570000
-3319.0000000	0.0585000
-3318.0000000	0.0595000
-3317.0000000	0.0595238
-3316.0000000	0.0600000
-3315.0000000	0.0600000
-3314.0000000	0.0600000
-3313.0000000	0.0600000
-3312.0000000	0.0600000
-3311.0000000	0.0600000
-3310.0000000	0.0600000
-3309.0000000	0.0600000
-3308.0000000	0.0600000
-3307.0000000	0.0600000
-3306.0000000	0.0600000
-3305.0000000	0.0600000
-3304.0000000	0.0600000
-3303.0000000	0.0600000
-3302.0000000	0.0600000
-3301.0000000	0.0600000
-3300.0000000	0.0600000
-3299.0000000	0.0600000
-3298.0000000	0.0595000
-3297.0000000	0.0595238
-3296.0000000	0.0600000
-3295.0000000	0.0590000
-3294.0000000	0.0600000
-3293.0000000	0.0600000
-3292.0000000	0.0600000
-3291.0000000	0.0600000
-3290.0000000	0.0600000
-3289.0000000	0.0600000
-3288.0000000	0.0600000
-3287.0000000	0.0600000
-3286.0000000	0.0600000
-3285.0000000	0.0600000
-3284.0000000	0.0595000
-3283.0000000	0.0595238
-3282.0000000	0.0600000
-3281.0000000	0.0595000
-3280.0000000	0.0600000
-3279.0000000	0.0600000
-3278.0000000	0.0595238
-3277.0000000	0.0600000
-3276.0000000	0.0600000
-3275.0000000	0.0600000
-3274.0000000	0.0600000

-3273.0000000	0.0600000
-3272.0000000	0.0600000
-3271.0000000	0.0600000
-3270.0000000	0.0600000
-3269.0000000	0.0600000
-3268.0000000	0.0600000
-3267.0000000	0.0600000
-3266.0000000	0.0600000
-3265.0000000	0.0595000
-3264.0000000	0.0595238
-3263.0000000	0.0600000
-3262.0000000	0.0585000
-3261.0000000	0.0557895
-3260.0000000	0.0550000
-3259.0000000	0.0568421
-3258.0000000	0.0531818
-3257.0000000	0.0563158
-3256.0000000	0.0518182
-3255.0000000	0.0510000
-3254.0000000	0.0525000
-3253.0000000	0.0520000
-3252.0000000	0.0533333
-3251.0000000	0.0540000
-3250.0000000	0.0550000
-3249.0000000	0.0566667
-3248.0000000	0.0580000
-3247.0000000	0.0580000
-3246.0000000	0.0580952
-3245.0000000	0.0585000
-3244.0000000	0.0585000
-3243.0000000	0.0580000
-3242.0000000	0.0590476
-3241.0000000	0.0595000
-3240.0000000	0.0600000
-3239.0000000	0.0600000
-3238.0000000	0.0600000
-3237.0000000	0.0557143
-3236.0000000	0.0555000
-3235.0000000	0.0540000
-3234.0000000	0.0540000
-3233.0000000	0.0542857
-3232.0000000	0.0520000
-3231.0000000	0.0519048
-3230.0000000	0.0545000
-3229.0000000	0.0550000
-3228.0000000	0.0538095
-3227.0000000	0.0540000
-3226.0000000	0.0550000
-3225.0000000	0.0566667
-3224.0000000	0.0560000
-3223.0000000	0.0550000
-3222.0000000	0.0575000
-3221.0000000	0.0580952
-3220.0000000	0.0590000
-3219.0000000	0.0600000
-3218.0000000	0.0595000
-3217.0000000	0.0590000

-3216.0000000	0.0595238
-3215.0000000	0.0590000
-3214.0000000	0.0600000
-3213.0000000	0.0595000
-3212.0000000	0.0600000
-3211.0000000	0.0600000
-3210.0000000	0.0600000
-3209.0000000	0.0595238
-3208.0000000	0.0600000
-3207.0000000	0.0600000
-3206.0000000	0.0600000
-3205.0000000	0.0600000
-3204.0000000	0.0600000
-3203.0000000	0.0604762
-3202.0000000	0.0600000
-3201.0000000	0.0600000
-3200.0000000	0.0600000
-3199.0000000	0.0600000
-3198.0000000	0.0605000
-3197.0000000	0.0609524
-3196.0000000	0.0605000
-3195.0000000	0.0600000
-3194.0000000	0.0609524
-3193.0000000	0.0600000
-3192.0000000	0.0604762
-3191.0000000	0.0610000
-3190.0000000	0.0615000
-3189.0000000	0.0619048
-3188.0000000	0.0605000
-3187.0000000	0.0600000
-3186.0000000	0.0605000
-3185.0000000	0.0600000
-3184.0000000	0.0600000
-3183.0000000	0.0600000
-3182.0000000	0.0600000
-3181.0000000	0.0600000
-3180.0000000	0.0595238
-3179.0000000	0.0595000
-3178.0000000	0.0595000
-3177.0000000	0.0600000
-3176.0000000	0.0585000
-3175.0000000	0.0600000
-3174.0000000	0.0600000
-3173.0000000	0.0600000
-3172.0000000	0.0600000
-3171.0000000	0.0600000
-3170.0000000	0.0595238
-3169.0000000	0.0595000
-3168.0000000	0.0595000
-3167.0000000	0.0600000
-3166.0000000	0.0600000
-3165.0000000	0.0600000
-3164.0000000	0.0600000
-3163.0000000	0.0600000
-3162.0000000	0.0600000
-3161.0000000	0.0600000
-3160.0000000	0.0600000

-3159.0000000	0.0600000
-3158.0000000	0.0604762
-3157.0000000	0.0605000
-3156.0000000	0.0604762
-3155.0000000	0.0600000
-3154.0000000	0.0600000
-3153.0000000	0.0600000
-3152.0000000	0.0600000
-3151.0000000	0.0600000
-3150.0000000	0.0600000
-3149.0000000	0.0604762
-3148.0000000	0.0605000
-3147.0000000	0.0600000
-3146.0000000	0.0600000
-3145.0000000	0.0600000
-3144.0000000	0.0600000
-3143.0000000	0.0600000
-3142.0000000	0.0604762
-3141.0000000	0.0600000
-3140.0000000	0.0595000
-3139.0000000	0.0600000
-3138.0000000	0.0595000
-3137.0000000	0.0600000
-3136.0000000	0.0590000
-3135.0000000	0.0600000
-3134.0000000	0.0600000
-3133.0000000	0.0595000
-3132.0000000	0.0585000
-3131.0000000	0.0580952
-3130.0000000	0.0580000
-3129.0000000	0.0585000
-3128.0000000	0.0585714
-3127.0000000	0.0580000
-3126.0000000	0.0595000
-3125.0000000	0.0580952
-3124.0000000	0.0595000
-3123.0000000	0.0600000
-3122.0000000	0.0600000
-3121.0000000	0.0600000
-3120.0000000	0.0595000
-3119.0000000	0.0600000
-3118.0000000	0.0600000
-3117.0000000	0.0600000
-3116.0000000	0.0600000
-3115.0000000	0.0600000
-3114.0000000	0.0600000
-3113.0000000	0.0600000
-3112.0000000	0.0605000
-3111.0000000	0.0600000
-3110.0000000	0.0604762
-3109.0000000	0.0600000
-3108.0000000	0.0600000
-3107.0000000	0.0605000
-3106.0000000	0.0609524
-3105.0000000	0.0605000
-3104.0000000	0.0609524
-3103.0000000	0.0610000

-3102.0000000	0.0605000
-3101.0000000	0.0604762
-3100.0000000	0.0600000
-3099.0000000	0.0605000
-3098.0000000	0.0600000
-3097.0000000	0.0600000
-3096.0000000	0.0600000
-3095.0000000	0.0600000
-3094.0000000	0.0600000
-3093.0000000	0.0600000
-3092.0000000	0.0600000
-3091.0000000	0.0605000
-3090.0000000	0.0605000
-3089.0000000	0.0609524
-3088.0000000	0.0605000
-3087.0000000	0.0605000
-3086.0000000	0.0604762
-3085.0000000	0.0610000
-3084.0000000	0.0600000
-3083.0000000	0.0600000
-3082.0000000	0.0600000
-3081.0000000	0.0604762
-3080.0000000	0.0605000
-3079.0000000	0.0630000
-3078.0000000	0.0610000
-3077.0000000	0.0610000
-3076.0000000	0.0623809
-3075.0000000	0.0620000
-3074.0000000	0.0623809
-3073.0000000	0.0655000
-3072.0000000	0.0647619
-3071.0000000	0.0670000
-3070.0000000	0.0635000
-3069.0000000	0.0630000
-3068.0000000	0.0635000
-3067.0000000	0.0647619
-3066.0000000	0.0645000
-3065.0000000	0.0633333
-3064.0000000	0.0630000
-3063.0000000	0.0630000
-3062.0000000	0.0633333
-3061.0000000	0.0650000
-3060.0000000	0.0645000
-3059.0000000	0.0635000
-3058.0000000	0.0619048
-3057.0000000	0.0650000
-3056.0000000	0.0638095
-3055.0000000	0.0635000
-3054.0000000	0.0625000
-3053.0000000	0.0652381
-3052.0000000	0.0640000
-3051.0000000	0.0625000
-3050.0000000	0.0647619
-3049.0000000	0.0640000
-3048.0000000	0.0630000
-3047.0000000	0.0655000
-3046.0000000	0.0652381

-3045.0000000	0.0640000
-3044.0000000	0.0630000
-3043.0000000	0.0638095
-3042.0000000	0.0635000
-3041.0000000	0.0647619
-3040.0000000	0.0642105
-3039.0000000	0.0633333
-3038.0000000	0.0623809
-3037.0000000	0.0640000
-3036.0000000	0.0650000
-3035.0000000	0.0620000
-3034.0000000	0.0633333
-3033.0000000	0.0630000
-3032.0000000	0.0645000
-3031.0000000	0.0614286
-3030.0000000	0.0620000
-3029.0000000	0.0609524
-3028.0000000	0.0625000
-3027.0000000	0.0630000
-3026.0000000	0.0642857
-3025.0000000	0.0645000
-3024.0000000	0.0665000
-3023.0000000	0.0635000
-3022.0000000	0.0661905
-3021.0000000	0.0675000
-3020.0000000	0.0645000
-3019.0000000	0.0652381
-3018.0000000	0.0680000
-3017.0000000	0.0680000
-3016.0000000	0.0670000
-3015.0000000	0.0638095
-3014.0000000	0.0647619
-3013.0000000	0.0660000
-3012.0000000	0.0630000
-3011.0000000	0.0630000
-3010.0000000	0.0623809
-3009.0000000	0.0625000
-3008.0000000	0.0645000
-3007.0000000	0.0628571
-3006.0000000	0.0650000
-3005.0000000	0.0620000
-3004.0000000	0.0628571
-3003.0000000	0.0645000
-3002.0000000	0.0635000
-3001.0000000	0.0671429
-3000.0000000	0.0670000
-2999.0000000	0.0675000
-2998.0000000	0.0676190
-2997.0000000	0.0690000
-2996.0000000	0.0695000
-2995.0000000	0.0695238
-2994.0000000	0.0690000
-2993.0000000	0.0695000
-2992.0000000	0.0666667
-2991.0000000	0.0685000
-2990.0000000	0.0680953
-2989.0000000	0.0655000

-2988.0000000	0.0670000
-2987.0000000	0.0670000
-2986.0000000	0.0645000
-2985.0000000	0.0671429
-2984.0000000	0.0665000
-2983.0000000	0.0666667
-2982.0000000	0.0645000
-2981.0000000	0.0642857
-2980.0000000	0.0615000
-2979.0000000	0.0615000
-2978.0000000	0.0620000
-2977.0000000	0.0610000
-2976.0000000	0.0614286
-2975.0000000	0.0615000
-2974.0000000	0.0604762
-2973.0000000	0.0600000
-2972.0000000	0.0610000
-2971.0000000	0.0600000
-2970.0000000	0.0605000
-2969.0000000	0.0610000
-2968.0000000	0.0605000
-2967.0000000	0.0604762
-2966.0000000	0.0600000
-2965.0000000	0.0600000
-2964.0000000	0.0600000
-2963.0000000	0.0605000
-2962.0000000	0.0614286
-2961.0000000	0.0615000
-2960.0000000	0.0610000
-2959.0000000	0.0619048
-2958.0000000	0.0620000
-2957.0000000	0.0610000
-2956.0000000	0.0610000
-2955.0000000	0.0604762
-2954.0000000	0.0619048
-2953.0000000	0.0625000
-2952.0000000	0.0610000
-2951.0000000	0.0625000
-2950.0000000	0.0647619
-2949.0000000	0.0655000
-2948.0000000	0.0680000
-2947.0000000	0.0680952
-2946.0000000	0.0690000
-2945.0000000	0.0695238
-2944.0000000	0.0700000
-2943.0000000	0.0695000
-2942.0000000	0.0700000
-2941.0000000	0.0700000
-2940.0000000	0.0695238
-2939.0000000	0.0700000
-2938.0000000	0.0700000
-2937.0000000	0.0700000
-2936.0000000	0.0700000
-2935.0000000	0.0700000
-2934.0000000	0.0700000
-2933.0000000	0.0700000
-2932.0000000	0.0695000

-2931.0000000	0.0700000
-2930.0000000	0.0695000
-2929.0000000	0.0690476
-2928.0000000	0.0700000
-2927.0000000	0.0695000
-2926.0000000	0.0695238
-2925.0000000	0.0700000
-2924.0000000	0.0700000
-2923.0000000	0.0700000
-2922.0000000	0.0700000
-2921.0000000	0.0715000
-2920.0000000	0.0704762
-2919.0000000	0.0700000
-2918.0000000	0.0700000
-2917.0000000	0.0700000
-2916.0000000	0.0700000
-2915.0000000	0.0700000
-2914.0000000	0.0700000
-2913.0000000	0.0700000
-2912.0000000	0.0695000
-2911.0000000	0.0690476
-2910.0000000	0.0695000
-2909.0000000	0.0700000
-2908.0000000	0.0690000
-2907.0000000	0.0661905
-2906.0000000	0.0657143
-2905.0000000	0.0655000
-2904.0000000	0.0625000
-2903.0000000	0.0610000
-2902.0000000	0.0605000
-2901.0000000	0.0604762
-2900.0000000	0.0605000
-2899.0000000	0.0600000
-2898.0000000	0.0600000
-2897.0000000	0.0605263
-2896.0000000	0.0604762
-2895.0000000	0.0600000
-2894.0000000	0.0600000
-2893.0000000	0.0600000
-2892.0000000	0.0600000
-2891.0000000	0.0600000
-2890.0000000	0.0604762
-2889.0000000	0.0600000
-2888.0000000	0.0605000
-2887.0000000	0.0604762
-2886.0000000	0.0605000
-2885.0000000	0.0600000
-2884.0000000	0.0600000
-2883.0000000	0.0600000
-2882.0000000	0.0600000
-2881.0000000	0.0605000
-2880.0000000	0.0604762
-2879.0000000	0.0605000
-2878.0000000	0.0630000
-2877.0000000	0.0614286
-2876.0000000	0.0610000
-2875.0000000	0.0628571

-2874.0000000	0.0630000
-2873.0000000	0.0630000
-2872.0000000	0.0615000
-2871.0000000	0.0609524
-2870.0000000	0.0605000
-2869.0000000	0.0615000
-2868.0000000	0.0600000
-2867.0000000	0.0610000
-2866.0000000	0.0610000
-2865.0000000	0.0628571
-2864.0000000	0.0635000
-2863.0000000	0.0623809
-2862.0000000	0.0625000
-2861.0000000	0.0615000
-2860.0000000	0.0615000
-2859.0000000	0.0619047
-2858.0000000	0.0630000
-2857.0000000	0.0625000
-2856.0000000	0.0619048
-2855.0000000	0.0615000
-2854.0000000	0.0609524
-2853.0000000	0.0605000
-2852.0000000	0.0615000
-2851.0000000	0.0620000
-2850.0000000	0.0625000
-2849.0000000	0.0609524
-2848.0000000	0.0605000
-2847.0000000	0.0604762
-2846.0000000	0.0610000
-2845.0000000	0.0614286
-2844.0000000	0.0605000
-2843.0000000	0.0605000
-2842.0000000	0.0605000
-2841.0000000	0.0620000
-2840.0000000	0.0619048
-2839.0000000	0.0615000
-2838.0000000	0.0633333
-2837.0000000	0.0615000
-2836.0000000	0.0635000
-2835.0000000	0.0628571
-2834.0000000	0.0645000
-2833.0000000	0.0655000
-2832.0000000	0.0647619
-2831.0000000	0.0635000
-2830.0000000	0.0650000
-2829.0000000	0.0647619
-2828.0000000	0.0620000
-2827.0000000	0.0642857
-2826.0000000	0.0642105
-2825.0000000	0.0661905
-2824.0000000	0.0640000
-2823.0000000	0.0647619
-2822.0000000	0.0650000
-2821.0000000	0.0655000
-2820.0000000	0.0666667
-2819.0000000	0.0675000
-2818.0000000	0.0650000

-2817.0000000	0.0661905
-2816.0000000	0.0675000
-2815.0000000	0.0680953
-2814.0000000	0.0690000
-2813.0000000	0.0685000
-2812.0000000	0.0680000
-2811.0000000	0.0700000
-2810.0000000	0.0685000
-2809.0000000	0.0680000
-2808.0000000	0.0690476
-2807.0000000	0.0685000
-2806.0000000	0.0645000
-2805.0000000	0.0670000
-2804.0000000	0.0666667
-2803.0000000	0.0645000
-2802.0000000	0.0657143
-2801.0000000	0.0625000
-2800.0000000	0.0655000
-2799.0000000	0.0638095
-2798.0000000	0.0635000
-2797.0000000	0.0630000
-2796.0000000	0.0647619
-2795.0000000	0.0650000
-2794.0000000	0.0635000
-2793.0000000	0.0605000
-2792.0000000	0.0619047
-2791.0000000	0.0635000
-2790.0000000	0.0650000
-2789.0000000	0.0685714
-2788.0000000	0.0670000
-2787.0000000	0.0680952
-2786.0000000	0.0660000
-2785.0000000	0.0660000
-2784.0000000	0.0666667
-2783.0000000	0.0690000
-2782.0000000	0.0695000
-2781.0000000	0.0690000
-2780.0000000	0.0700000
-2779.0000000	0.0695238
-2778.0000000	0.0700000
-2777.0000000	0.0700000
-2776.0000000	0.0700000
-2775.0000000	0.0700000
-2774.0000000	0.0700000
-2773.0000000	0.0705000
-2772.0000000	0.0700000
-2771.0000000	0.0700000
-2770.0000000	0.0700000
-2769.0000000	0.0700000
-2768.0000000	0.0700000
-2767.0000000	0.0700000
-2766.0000000	0.0700000
-2765.0000000	0.0704762
-2764.0000000	0.0705000
-2763.0000000	0.0704762
-2762.0000000	0.0710000
-2761.0000000	0.0715000

-2760.0000000	0.0700000
-2759.0000000	0.0700000
-2758.0000000	0.0705000
-2757.0000000	0.0700000
-2756.0000000	0.0704762
-2755.0000000	0.0705000
-2754.0000000	0.0700000
-2753.0000000	0.0705000
-2752.0000000	0.0700000
-2751.0000000	0.0700000
-2750.0000000	0.0695238
-2749.0000000	0.0705000
-2748.0000000	0.0700000
-2747.0000000	0.0700000
-2746.0000000	0.0700000
-2745.0000000	0.0700000
-2744.0000000	0.0700000
-2743.0000000	0.0700000
-2742.0000000	0.0700000
-2741.0000000	0.0695238
-2740.0000000	0.0700000
-2739.0000000	0.0680952
-2738.0000000	0.0695000
-2737.0000000	0.0695000
-2736.0000000	0.0690476
-2735.0000000	0.0700000
-2734.0000000	0.0695000
-2733.0000000	0.0700000
-2732.0000000	0.0695238
-2731.0000000	0.0690000
-2730.0000000	0.0685000
-2729.0000000	0.0690476
-2728.0000000	0.0685000
-2727.0000000	0.0690476
-2726.0000000	0.0665000
-2725.0000000	0.0645000
-2724.0000000	0.0609524
-2723.0000000	0.0605000
-2722.0000000	0.0600000
-2721.0000000	0.0600000
-2720.0000000	0.0600000
-2719.0000000	0.0600000
-2718.0000000	0.0600000
-2717.0000000	0.0600000
-2716.0000000	0.0600000
-2715.0000000	0.0600000
-2714.0000000	0.0600000
-2713.0000000	0.0600000
-2712.0000000	0.0600000
-2711.0000000	0.0600000
-2710.0000000	0.0600000
-2709.0000000	0.0600000
-2708.0000000	0.0600000
-2707.0000000	0.0600000
-2706.0000000	0.0600000
-2705.0000000	0.0600000
-2704.0000000	0.0600000

-2703.0000000	0.0600000
-2702.0000000	0.0600000
-2701.0000000	0.0600000
-2700.0000000	0.0600000
-2699.0000000	0.0600000
-2698.0000000	0.0600000
-2697.0000000	0.0600000
-2696.0000000	0.0595238
-2695.0000000	0.0600000
-2694.0000000	0.0600000
-2693.0000000	0.0600000
-2692.0000000	0.0605000
-2691.0000000	0.0600000
-2690.0000000	0.0600000
-2689.0000000	0.0610000
-2688.0000000	0.0604762
-2687.0000000	0.0615000
-2686.0000000	0.0625000
-2685.0000000	0.0640000
-2684.0000000	0.0638095
-2683.0000000	0.0650000
-2682.0000000	0.0690000
-2681.0000000	0.0685714
-2680.0000000	0.0695000
-2679.0000000	0.0695000
-2678.0000000	0.0665000
-2677.0000000	0.0676191
-2676.0000000	0.0680000
-2675.0000000	0.0675000
-2674.0000000	0.0661905
-2673.0000000	0.0665000
-2672.0000000	0.0676190
-2671.0000000	0.0650000
-2670.0000000	0.0640000
-2669.0000000	0.0657143
-2668.0000000	0.0660000
-2667.0000000	0.0655000
-2666.0000000	0.0655000
-2665.0000000	0.0685714
-2664.0000000	0.0685000
-2663.0000000	0.0666667
-2662.0000000	0.0660000
-2661.0000000	0.0655000
-2660.0000000	0.0647619
-2659.0000000	0.0640000
-2658.0000000	0.0610000
-2657.0000000	0.0600000
-2656.0000000	0.0605000
-2655.0000000	0.0605000
-2654.0000000	0.0600000
-2653.0000000	0.0600000
-2652.0000000	0.0600000
-2651.0000000	0.0605000
-2650.0000000	0.0605000
-2649.0000000	0.0609524
-2648.0000000	0.0625000
-2647.0000000	0.0635000

-2646.0000000	0.0625000
-2645.0000000	0.0623810
-2644.0000000	0.0620000
-2643.0000000	0.0638095
-2642.0000000	0.0640000
-2641.0000000	0.0655000
-2640.0000000	0.0625000
-2639.0000000	0.0630000
-2638.0000000	0.0623809
-2637.0000000	0.0635000
-2636.0000000	0.0619048
-2635.0000000	0.0615000
-2634.0000000	0.0610000
-2633.0000000	0.0609524
-2632.0000000	0.0605000
-2631.0000000	0.0600000
-2630.0000000	0.0605000
-2629.0000000	0.0600000
-2628.0000000	0.0600000
-2627.0000000	0.0600000
-2626.0000000	0.0600000
-2625.0000000	0.0600000
-2624.0000000	0.0605000
-2623.0000000	0.0600000
-2622.0000000	0.0600000
-2621.0000000	0.0600000
-2620.0000000	0.0600000
-2619.0000000	0.0600000
-2618.0000000	0.0600000
-2617.0000000	0.0600000
-2616.0000000	0.0600000
-2615.0000000	0.0600000
-2614.0000000	0.0600000
-2613.0000000	0.0600000
-2612.0000000	0.0600000
-2611.0000000	0.0600000
-2610.0000000	0.0600000
-2609.0000000	0.0600000
-2608.0000000	0.0600000
-2607.0000000	0.0600000
-2606.0000000	0.0600000
-2605.0000000	0.0604762
-2604.0000000	0.0600000
-2603.0000000	0.0600000
-2602.0000000	0.0605000
-2601.0000000	0.0610000
-2600.0000000	0.0604762
-2599.0000000	0.0600000
-2598.0000000	0.0605000
-2597.0000000	0.0604762
-2596.0000000	0.0610000
-2595.0000000	0.0610000
-2594.0000000	0.0610000
-2593.0000000	0.0614286
-2592.0000000	0.0615000
-2591.0000000	0.0645000
-2590.0000000	0.0657143

-2589.0000000	0.0670000
-2588.0000000	0.0676190
-2587.0000000	0.0645000
-2586.0000000	0.0640000
-2585.0000000	0.0625000
-2584.0000000	0.0619047
-2583.0000000	0.0615000
-2582.0000000	0.0615000
-2581.0000000	0.0604762
-2580.0000000	0.0605000
-2579.0000000	0.0615000
-2578.0000000	0.0600000
-2577.0000000	0.0600000
-2576.0000000	0.0600000
-2575.0000000	0.0600000
-2574.0000000	0.0600000
-2573.0000000	0.0600000
-2572.0000000	0.0600000
-2571.0000000	0.0605000
-2570.0000000	0.0600000
-2569.0000000	0.0604762
-2568.0000000	0.0600000
-2567.0000000	0.0600000
-2566.0000000	0.0600000
-2565.0000000	0.0600000
-2564.0000000	0.0600000
-2563.0000000	0.0600000
-2562.0000000	0.0600000
-2561.0000000	0.0600000
-2560.0000000	0.0600000
-2559.0000000	0.0605000
-2558.0000000	0.0600000
-2557.0000000	0.0600000
-2556.0000000	0.0605000
-2555.0000000	0.0600000
-2554.0000000	0.0600000
-2553.0000000	0.0600000
-2552.0000000	0.0600000
-2551.0000000	0.0605000
-2550.0000000	0.0600000
-2549.0000000	0.0600000
-2548.0000000	0.0610000
-2547.0000000	0.0609524
-2546.0000000	0.0625000
-2545.0000000	0.0628571
-2544.0000000	0.0635000
-2543.0000000	0.0642857
-2542.0000000	0.0660000
-2541.0000000	0.0630000
-2540.0000000	0.0650000
-2539.0000000	0.0665000
-2538.0000000	0.0647619
-2537.0000000	0.0670000
-2536.0000000	0.0647619
-2535.0000000	0.0635000
-2534.0000000	0.0605000
-2533.0000000	0.0615000

-2532.0000000	0.0623809
-2531.0000000	0.0610000
-2530.0000000	0.0604762
-2529.0000000	0.0605000
-2528.0000000	0.0600000
-2527.0000000	0.0600000
-2526.0000000	0.0600000
-2525.0000000	0.0604762
-2524.0000000	0.0610000
-2523.0000000	0.0600000
-2522.0000000	0.0615000
-2521.0000000	0.0604762
-2520.0000000	0.0605000
-2519.0000000	0.0610000
-2518.0000000	0.0600000
-2517.0000000	0.0610000
-2516.0000000	0.0609524
-2515.0000000	0.0620000
-2514.0000000	0.0620000
-2513.0000000	0.0614286
-2512.0000000	0.0600000
-2511.0000000	0.0605000
-2510.0000000	0.0620000
-2509.0000000	0.0619047
-2508.0000000	0.0615000
-2507.0000000	0.0635000
-2506.0000000	0.0633333
-2505.0000000	0.0650000
-2504.0000000	0.0670000
-2503.0000000	0.0670000
-2502.0000000	0.0666667
-2501.0000000	0.0665000
-2500.0000000	0.0642857
-2499.0000000	0.0625000
-2498.0000000	0.0625000
-2497.0000000	0.0633333
-2496.0000000	0.0655000
-2495.0000000	0.0650000
-2494.0000000	0.0671429
-2493.0000000	0.0680000
-2492.0000000	0.0695000
-2491.0000000	0.0676190
-2490.0000000	0.0680000
-2489.0000000	0.0675000
-2488.0000000	0.0675000
-2487.0000000	0.0680952
-2486.0000000	0.0661905
-2485.0000000	0.0670000
-2484.0000000	0.0685000
-2483.0000000	0.0685000
-2482.0000000	0.0676190
-2481.0000000	0.0695000
-2480.0000000	0.0690000
-2479.0000000	0.0685000
-2478.0000000	0.0676190
-2477.0000000	0.0680953
-2476.0000000	0.0690000

-2475.0000000	0.0695000
-2474.0000000	0.0685000
-2473.0000000	0.0685000
-2472.0000000	0.0685714
-2471.0000000	0.0685000
-2470.0000000	0.0685714
-2469.0000000	0.0690000
-2468.0000000	0.0680000
-2467.0000000	0.0695238
-2466.0000000	0.0695000
-2465.0000000	0.0680000
-2464.0000000	0.0690000
-2463.0000000	0.0695238
-2462.0000000	0.0694737
-2461.0000000	0.0685714
-2460.0000000	0.0690476
-2459.0000000	0.0685000
-2458.0000000	0.0700000
-2457.0000000	0.0690476
-2456.0000000	0.0680000
-2455.0000000	0.0700000
-2454.0000000	0.0695238
-2453.0000000	0.0700000
-2452.0000000	0.0695238
-2451.0000000	0.0700000
-2450.0000000	0.0700000
-2449.0000000	0.0700000
-2448.0000000	0.0700000
-2447.0000000	0.0700000
-2446.0000000	0.0700000
-2445.0000000	0.0700000
-2444.0000000	0.0700000
-2443.0000000	0.0700000
-2442.0000000	0.0695238
-2441.0000000	0.0695000
-2440.0000000	0.0700000
-2439.0000000	0.0700000
-2438.0000000	0.0700000
-2437.0000000	0.0700000
-2436.0000000	0.0700000
-2435.0000000	0.0695000
-2434.0000000	0.0690476
-2433.0000000	0.0700000
-2432.0000000	0.0690000
-2431.0000000	0.0700000
-2430.0000000	0.0700000
-2429.0000000	0.0695000
-2428.0000000	0.0700000
-2427.0000000	0.0700000
-2426.0000000	0.0695000
-2425.0000000	0.0690476
-2424.0000000	0.0700000
-2423.0000000	0.0700000
-2422.0000000	0.0700000
-2421.0000000	0.0700000
-2420.0000000	0.0700000
-2419.0000000	0.0700000

-2418.0000000	0.0700000
-2417.0000000	0.0705000
-2416.0000000	0.0700000
-2415.0000000	0.0700000
-2414.0000000	0.0700000
-2413.0000000	0.0700000
-2412.0000000	0.0700000
-2411.0000000	0.0704762
-2410.0000000	0.0700000
-2409.0000000	0.0709524
-2408.0000000	0.0700000
-2407.0000000	0.0700000
-2406.0000000	0.0700000
-2405.0000000	0.0700000
-2404.0000000	0.0700000
-2403.0000000	0.0700000
-2402.0000000	0.0700000
-2401.0000000	0.0700000
-2400.0000000	0.0700000
-2399.0000000	0.0700000
-2398.0000000	0.0700000
-2397.0000000	0.0700000
-2396.0000000	0.0700000
-2395.0000000	0.0700000
-2394.0000000	0.0700000
-2393.0000000	0.0700000
-2392.0000000	0.0700000
-2391.0000000	0.0695238
-2390.0000000	0.0700000
-2389.0000000	0.0685000
-2388.0000000	0.0680000
-2387.0000000	0.0680952
-2386.0000000	0.0652381
-2385.0000000	0.0640000
-2384.0000000	0.0640000
-2383.0000000	0.0645000
-2382.0000000	0.0628571
-2381.0000000	0.0605000
-2380.0000000	0.0620000
-2379.0000000	0.0628571
-2378.0000000	0.0645000
-2377.0000000	0.0638095
-2376.0000000	0.0625000
-2375.0000000	0.0650000
-2374.0000000	0.0655000
-2373.0000000	0.0661905
-2372.0000000	0.0665000
-2371.0000000	0.0660000
-2370.0000000	0.0676190
-2369.0000000	0.0665000
-2368.0000000	0.0645000
-2367.0000000	0.0676191
-2366.0000000	0.0685000
-2365.0000000	0.0680000
-2364.0000000	0.0690000
-2363.0000000	0.0695238
-2362.0000000	0.0685000

-2361.0000000	0.0695238
-2360.0000000	0.0700000
-2359.0000000	0.0700000
-2358.0000000	0.0700000
-2357.0000000	0.0700000
-2356.0000000	0.0700000
-2355.0000000	0.0700000
-2354.0000000	0.0700000
-2353.0000000	0.0700000
-2352.0000000	0.0700000
-2351.0000000	0.0700000
-2350.0000000	0.0695238
-2349.0000000	0.0700000
-2348.0000000	0.0700000
-2347.0000000	0.0695238
-2346.0000000	0.0690000
-2345.0000000	0.0700000
-2344.0000000	0.0695000
-2343.0000000	0.0695238
-2342.0000000	0.0700000
-2341.0000000	0.0695000
-2340.0000000	0.0695238
-2339.0000000	0.0695000
-2338.0000000	0.0695000
-2337.0000000	0.0675000
-2336.0000000	0.0685714
-2335.0000000	0.0695000
-2334.0000000	0.0666667
-2333.0000000	0.0650000
-2332.0000000	0.0665000
-2331.0000000	0.0666667
-2330.0000000	0.0670000
-2329.0000000	0.0685000
-2328.0000000	0.0665000
-2327.0000000	0.0690476
-2326.0000000	0.0700000
-2325.0000000	0.0676191
-2324.0000000	0.0675000
-2323.0000000	0.0680000
-2322.0000000	0.0695000
-2321.0000000	0.0666667
-2320.0000000	0.0660000
-2319.0000000	0.0673684
-2318.0000000	0.0681818
-2317.0000000	0.0670000
-2316.0000000	0.0657143
-2315.0000000	0.0675000
-2314.0000000	0.0655000
-2313.0000000	0.0665000
-2312.0000000	0.0666667
-2311.0000000	0.0657143
-2310.0000000	0.0675000
-2309.0000000	0.0670000
-2308.0000000	0.0660000
-2307.0000000	0.0642857
-2306.0000000	0.0625000
-2305.0000000	0.0620000

-2304.0000000	0.0600000
-2303.0000000	0.0600000
-2302.0000000	0.0600000
-2301.0000000	0.0600000
-2300.0000000	0.0600000
-2299.0000000	0.0600000
-2298.0000000	0.0604762
-2297.0000000	0.0600000
-2296.0000000	0.0605000
-2295.0000000	0.0600000
-2294.0000000	0.0600000
-2293.0000000	0.0605000
-2292.0000000	0.0605000
-2291.0000000	0.0609524
-2290.0000000	0.0620000
-2289.0000000	0.0630000
-2288.0000000	0.0619048
-2287.0000000	0.0625000
-2286.0000000	0.0619048
-2285.0000000	0.0630000
-2284.0000000	0.0615000
-2283.0000000	0.0615000
-2282.0000000	0.0609524
-2281.0000000	0.0635000
-2280.0000000	0.0633333
-2279.0000000	0.0605000
-2278.0000000	0.0620000
-2277.0000000	0.0600000
-2276.0000000	0.0600000
-2275.0000000	0.0600000
-2274.0000000	0.0605000
-2273.0000000	0.0600000
-2272.0000000	0.0600000
-2271.0000000	0.0600000
-2270.0000000	0.0600000
-2269.0000000	0.0600000
-2268.0000000	0.0600000
-2267.0000000	0.0600000
-2266.0000000	0.0600000
-2265.0000000	0.0600000
-2264.0000000	0.0600000
-2263.0000000	0.0600000
-2262.0000000	0.0600000
-2261.0000000	0.0614286
-2260.0000000	0.0600000
-2259.0000000	0.0600000
-2258.0000000	0.0600000
-2257.0000000	0.0600000
-2256.0000000	0.0604762
-2255.0000000	0.0600000
-2254.0000000	0.0605000
-2253.0000000	0.0605000
-2252.0000000	0.0604762
-2251.0000000	0.0600000
-2250.0000000	0.0600000
-2249.0000000	0.0600000
-2248.0000000	0.0600000

-2247.0000000	0.0600000
-2246.0000000	0.0600000
-2245.0000000	0.0604762
-2244.0000000	0.0605000
-2243.0000000	0.0619048
-2242.0000000	0.0610000
-2241.0000000	0.0609524
-2240.0000000	0.0630000
-2239.0000000	0.0630000
-2238.0000000	0.0640000
-2237.0000000	0.0620000
-2236.0000000	0.0647619
-2235.0000000	0.0635000
-2234.0000000	0.0604762
-2233.0000000	0.0600000
-2232.0000000	0.0615000
-2231.0000000	0.0609524
-2230.0000000	0.0615000
-2229.0000000	0.0610000
-2228.0000000	0.0623809
-2227.0000000	0.0635000
-2226.0000000	0.0645000
-2225.0000000	0.0623810
-2224.0000000	0.0620000
-2223.0000000	0.0614286
-2222.0000000	0.0630000
-2221.0000000	0.0620000
-2220.0000000	0.0619048
-2219.0000000	0.0620000
-2218.0000000	0.0615000
-2217.0000000	0.0605000
-2216.0000000	0.0638095
-2215.0000000	0.0620000
-2214.0000000	0.0630000
-2213.0000000	0.0633333
-2212.0000000	0.0610000
-2211.0000000	0.0604762
-2210.0000000	0.0610000
-2209.0000000	0.0605000
-2208.0000000	0.0610000
-2207.0000000	0.0600000
-2206.0000000	0.0605000
-2205.0000000	0.0610000
-2204.0000000	0.0609524
-2203.0000000	0.0605000
-2202.0000000	0.0600000
-2201.0000000	0.0600000
-2200.0000000	0.0600000
-2199.0000000	0.0605000
-2198.0000000	0.0604762
-2197.0000000	0.0600000
-2196.0000000	0.0600000
-2195.0000000	0.0604762
-2194.0000000	0.0610000
-2193.0000000	0.0600000
-2192.0000000	0.0600000
-2191.0000000	0.0600000

-2190.0000000	0.0600000
-2189.0000000	0.0600000
-2188.0000000	0.0600000
-2187.0000000	0.0600000
-2186.0000000	0.0600000
-2185.0000000	0.0609524
-2184.0000000	0.0619048
-2183.0000000	0.0630000
-2182.0000000	0.0630000
-2181.0000000	0.0620000
-2180.0000000	0.0628571
-2179.0000000	0.0645000
-2178.0000000	0.0650000
-2177.0000000	0.0676190
-2176.0000000	0.0670000
-2175.0000000	0.0685000
-2174.0000000	0.0700000
-2173.0000000	0.0680000
-2172.0000000	0.0695000
-2171.0000000	0.0675000
-2170.0000000	0.0676191
-2169.0000000	0.0680000
-2168.0000000	0.0666667
-2167.0000000	0.0655000
-2166.0000000	0.0650000
-2165.0000000	0.0671429
-2164.0000000	0.0640000
-2163.0000000	0.0655000
-2162.0000000	0.0620000
-2161.0000000	0.0609524
-2160.0000000	0.0605000
-2159.0000000	0.0600000
-2158.0000000	0.0600000
-2157.0000000	0.0605000
-2156.0000000	0.0600000
-2155.0000000	0.0600000
-2154.0000000	0.0600000
-2153.0000000	0.0600000
-2152.0000000	0.0615000
-2151.0000000	0.0625000
-2150.0000000	0.0633333
-2149.0000000	0.0655000
-2148.0000000	0.0670000
-2147.0000000	0.0675000
-2146.0000000	0.0680952
-2145.0000000	0.0690000
-2144.0000000	0.0676190
-2143.0000000	0.0690000
-2142.0000000	0.0690000
-2141.0000000	0.0695238
-2140.0000000	0.0680000
-2139.0000000	0.0685000
-2138.0000000	0.0680000
-2137.0000000	0.0695238
-2136.0000000	0.0690000
-2135.0000000	0.0690476
-2134.0000000	0.0695000

-2133.0000000	0.0695000
-2132.0000000	0.0690476
-2131.0000000	0.0700000
-2130.0000000	0.0695000
-2129.0000000	0.0695238
-2128.0000000	0.0700000
-2127.0000000	0.0700000
-2126.0000000	0.0700000
-2125.0000000	0.0700000
-2124.0000000	0.0700000
-2123.0000000	0.0700000
-2122.0000000	0.0700000
-2121.0000000	0.0700000
-2120.0000000	0.0700000
-2119.0000000	0.0700000
-2118.0000000	0.0695000
-2117.0000000	0.0700000
-2116.0000000	0.0695238
-2115.0000000	0.0685000
-2114.0000000	0.0690476
-2113.0000000	0.0690000
-2112.0000000	0.0670000
-2111.0000000	0.0665000
-2110.0000000	0.0665000
-2109.0000000	0.0633333
-2108.0000000	0.0680000
-2107.0000000	0.0666667
-2106.0000000	0.0665000
-2105.0000000	0.0661905
-2104.0000000	0.0635000
-2103.0000000	0.0660000
-2102.0000000	0.0630000
-2101.0000000	0.0633333
-2100.0000000	0.0615000
-2099.0000000	0.0630000
-2098.0000000	0.0619047
-2097.0000000	0.0605000
-2096.0000000	0.0619048
-2095.0000000	0.0610000
-2094.0000000	0.0615000
-2093.0000000	0.0604762
-2092.0000000	0.0600000
-2091.0000000	0.0610000
-2090.0000000	0.0600000
-2089.0000000	0.0600000
-2088.0000000	0.0600000
-2087.0000000	0.0600000
-2086.0000000	0.0600000
-2085.0000000	0.0600000
-2084.0000000	0.0600000
-2083.0000000	0.0610000
-2082.0000000	0.0600000
-2081.0000000	0.0610000
-2080.0000000	0.0604762
-2079.0000000	0.0605000
-2078.0000000	0.0610000
-2077.0000000	0.0633333

-2076.0000000	0.0635000
-2075.0000000	0.0638095
-2074.0000000	0.0645000
-2073.0000000	0.0675000
-2072.0000000	0.0680000
-2071.0000000	0.0652381
-2070.0000000	0.0665000
-2069.0000000	0.0685000
-2068.0000000	0.0685714
-2067.0000000	0.0685000
-2066.0000000	0.0695000
-2065.0000000	0.0685714
-2064.0000000	0.0685000
-2063.0000000	0.0665000
-2062.0000000	0.0671429
-2061.0000000	0.0650000
-2060.0000000	0.0625000
-2059.0000000	0.0623809
-2058.0000000	0.0625000
-2057.0000000	0.0642857
-2056.0000000	0.0660000
-2055.0000000	0.0640000
-2054.0000000	0.0615000
-2053.0000000	0.0600000
-2052.0000000	0.0600000
-2051.0000000	0.0610000
-2050.0000000	0.0600000
-2049.0000000	0.0600000
-2048.0000000	0.0600000
-2047.0000000	0.0600000
-2046.0000000	0.0600000
-2045.0000000	0.0595238
-2044.0000000	0.0600000
-2043.0000000	0.0580000
-2042.0000000	0.0585000
-2041.0000000	0.0580952
-2040.0000000	0.0575000
-2039.0000000	0.0600000
-2038.0000000	0.0600000
-2037.0000000	0.0585000
-2036.0000000	0.0575000
-2035.0000000	0.0580000
-2034.0000000	0.0590476
-2033.0000000	0.0585000
-2032.0000000	0.0594737
-2031.0000000	0.0600000
-2030.0000000	0.0600000
-2029.0000000	0.0600000
-2028.0000000	0.0600000
-2027.0000000	0.0600000
-2026.0000000	0.0600000
-2025.0000000	0.0600000
-2024.0000000	0.0600000
-2023.0000000	0.0600000
-2022.0000000	0.0600000
-2021.0000000	0.0590000
-2020.0000000	0.0595000

-2019.0000000	0.0600000
-2018.0000000	0.0600000
-2017.0000000	0.0600000
-2016.0000000	0.0600000
-2015.0000000	0.0600000
-2014.0000000	0.0600000
-2013.0000000	0.0600000
-2012.0000000	0.0600000
-2011.0000000	0.0600000
-2010.0000000	0.0600000
-2009.0000000	0.0600000
-2008.0000000	0.0600000
-2007.0000000	0.0600000
-2006.0000000	0.0600000
-2005.0000000	0.0600000
-2004.0000000	0.0600000
-2003.0000000	0.0600000
-2002.0000000	0.0600000
-2001.0000000	0.0600000
-2000.0000000	0.0600000
-1999.0000000	0.0600000
-1998.0000000	0.0600000
-1997.0000000	0.0600000
-1996.0000000	0.0600000
-1995.0000000	0.0600000
-1994.0000000	0.0600000
-1993.0000000	0.0600000
-1992.0000000	0.0600000
-1991.0000000	0.0600000
-1990.0000000	0.0600000
-1989.0000000	0.0600000
-1988.0000000	0.0600000
-1987.0000000	0.0595238
-1986.0000000	0.0600000
-1985.0000000	0.0600000
-1984.0000000	0.0600000
-1983.0000000	0.0600000
-1982.0000000	0.0600000
-1981.0000000	0.0600000
-1980.0000000	0.0600000
-1979.0000000	0.0600000
-1978.0000000	0.0600000
-1977.0000000	0.0600000
-1976.0000000	0.0600000
-1975.0000000	0.0600000
-1974.0000000	0.0600000
-1973.0000000	0.0605000
-1972.0000000	0.0600000
-1971.0000000	0.0609524
-1970.0000000	0.0620000
-1969.0000000	0.0604762
-1968.0000000	0.0615000
-1967.0000000	0.0630000
-1966.0000000	0.0642857
-1965.0000000	0.0635000
-1964.0000000	0.0645000
-1963.0000000	0.0650000

-1962.0000000	0.0638095
-1961.0000000	0.0650000
-1960.0000000	0.0620000
-1959.0000000	0.0628571
-1958.0000000	0.0640000
-1957.0000000	0.0614286
-1956.0000000	0.0610000
-1955.0000000	0.0630000
-1954.0000000	0.0614286
-1953.0000000	0.0605000
-1952.0000000	0.0605000
-1951.0000000	0.0600000
-1950.0000000	0.0600000
-1949.0000000	0.0605000
-1948.0000000	0.0604762
-1947.0000000	0.0600000
-1946.0000000	0.0600000
-1945.0000000	0.0610000
-1944.0000000	0.0600000
-1943.0000000	0.0604762
-1942.0000000	0.0610000
-1941.0000000	0.0604762
-1940.0000000	0.0600000
-1939.0000000	0.0600000
-1938.0000000	0.0605000
-1937.0000000	0.0600000
-1936.0000000	0.0605000
-1935.0000000	0.0604762
-1934.0000000	0.0610000
-1933.0000000	0.0615000
-1932.0000000	0.0614286
-1931.0000000	0.0600000
-1930.0000000	0.0600000
-1929.0000000	0.0600000
-1928.0000000	0.0610000
-1927.0000000	0.0600000
-1926.0000000	0.0604762
-1925.0000000	0.0605000
-1924.0000000	0.0600000
-1923.0000000	0.0604762
-1922.0000000	0.0605000
-1921.0000000	0.0604762
-1920.0000000	0.0600000
-1919.0000000	0.0600000
-1918.0000000	0.0600000
-1917.0000000	0.0600000
-1916.0000000	0.0600000
-1915.0000000	0.0600000
-1914.0000000	0.0600000
-1913.0000000	0.0600000
-1912.0000000	0.0600000
-1911.0000000	0.0600000
-1910.0000000	0.0600000
-1909.0000000	0.0600000
-1908.0000000	0.0605000
-1907.0000000	0.0605000
-1906.0000000	0.0610000

-1905.0000000	0.0600000
-1904.0000000	0.0600000
-1903.0000000	0.0600000
-1902.0000000	0.0609524
-1901.0000000	0.0600000
-1900.0000000	0.0600000
-1899.0000000	0.0604762
-1898.0000000	0.0605000
-1897.0000000	0.0610000
-1896.0000000	0.0604762
-1895.0000000	0.0605000
-1894.0000000	0.0605000
-1893.0000000	0.0600000
-1892.0000000	0.0600000
-1891.0000000	0.0600000
-1890.0000000	0.0600000
-1889.0000000	0.0600000
-1888.0000000	0.0590000
-1887.0000000	0.0600000
-1886.0000000	0.0595000
-1885.0000000	0.0595000
-1884.0000000	0.0585000
-1883.0000000	0.0590000
-1882.0000000	0.0590476
-1881.0000000	0.0595000
-1880.0000000	0.0590476
-1879.0000000	0.0595000
-1878.0000000	0.0585714
-1877.0000000	0.0600000
-1876.0000000	0.0600000
-1875.0000000	0.0600000
-1874.0000000	0.0600000
-1873.0000000	0.0600000
-1872.0000000	0.0595238
-1871.0000000	0.0600000
-1870.0000000	0.0595000
-1869.0000000	0.0595238
-1868.0000000	0.0600000
-1867.0000000	0.0600000
-1866.0000000	0.0600000
-1865.0000000	0.0600000
-1864.0000000	0.0605000
-1863.0000000	0.0600000
-1862.0000000	0.0600000
-1861.0000000	0.0595000
-1860.0000000	0.0595000
-1859.0000000	0.0600000
-1858.0000000	0.0600000
-1857.0000000	0.0600000
-1856.0000000	0.0600000
-1855.0000000	0.0600000
-1854.0000000	0.0600000
-1853.0000000	0.0595238
-1852.0000000	0.0600000
-1851.0000000	0.0600000
-1850.0000000	0.0600000
-1849.0000000	0.0600000

-1848.0000000	0.0600000
-1847.0000000	0.0600000
-1846.0000000	0.0600000
-1845.0000000	0.0600000
-1844.0000000	0.0595238
-1843.0000000	0.0600000
-1842.0000000	0.0600000
-1841.0000000	0.0600000
-1840.0000000	0.0600000
-1839.0000000	0.0600000
-1838.0000000	0.0600000
-1837.0000000	0.0600000
-1836.0000000	0.0600000
-1835.0000000	0.0600000
-1834.0000000	0.0600000
-1833.0000000	0.0600000
-1832.0000000	0.0600000
-1831.0000000	0.0600000
-1830.0000000	0.0600000
-1829.0000000	0.0600000
-1828.0000000	0.0600000
-1827.0000000	0.0600000
-1826.0000000	0.0600000
-1825.0000000	0.0600000
-1824.0000000	0.0600000
-1823.0000000	0.0600000
-1822.0000000	0.0605000
-1821.0000000	0.0600000
-1820.0000000	0.0600000
-1819.0000000	0.0600000
-1818.0000000	0.0600000
-1817.0000000	0.0600000
-1816.0000000	0.0600000
-1815.0000000	0.0600000
-1814.0000000	0.0600000
-1813.0000000	0.0600000
-1812.0000000	0.0600000
-1811.0000000	0.0604762
-1810.0000000	0.0600000
-1809.0000000	0.0600000
-1808.0000000	0.0600000
-1807.0000000	0.0600000
-1806.0000000	0.0600000
-1805.0000000	0.0600000
-1804.0000000	0.0600000
-1803.0000000	0.0600000
-1802.0000000	0.0600000
-1801.0000000	0.0605000
-1800.0000000	0.0605000
-1799.0000000	0.0604762
-1798.0000000	0.0600000
-1797.0000000	0.0600000
-1796.0000000	0.0600000
-1795.0000000	0.0605000
-1794.0000000	0.0600000
-1793.0000000	0.0600000
-1792.0000000	0.0600000

-1791.0000000	0.0604762
-1790.0000000	0.0610000
-1789.0000000	0.0605000
-1788.0000000	0.0600000
-1787.0000000	0.0614286
-1786.0000000	0.0615000
-1785.0000000	0.0605000
-1784.0000000	0.0614286
-1783.0000000	0.0610000
-1782.0000000	0.0614286
-1781.0000000	0.0620000
-1780.0000000	0.0615000
-1779.0000000	0.0605000
-1778.0000000	0.0623809
-1777.0000000	0.0610000
-1776.0000000	0.0625000
-1775.0000000	0.0638095
-1774.0000000	0.0620000
-1773.0000000	0.0628571
-1772.0000000	0.0620000
-1771.0000000	0.0635000
-1770.0000000	0.0620000
-1769.0000000	0.0609524
-1768.0000000	0.0620000
-1767.0000000	0.0615000
-1766.0000000	0.0609524
-1765.0000000	0.0620000
-1764.0000000	0.0615000
-1763.0000000	0.0614286
-1762.0000000	0.0630000
-1761.0000000	0.0650000
-1760.0000000	0.0633333
-1759.0000000	0.0625000
-1758.0000000	0.0660000
-1757.0000000	0.0647619
-1756.0000000	0.0630000
-1755.0000000	0.0647619
-1754.0000000	0.0635000
-1753.0000000	0.0635000
-1752.0000000	0.0640000
-1751.0000000	0.0647619
-1750.0000000	0.0625000
-1749.0000000	0.0620000
-1748.0000000	0.0638095
-1747.0000000	0.0645000
-1746.0000000	0.0640000
-1745.0000000	0.0619047
-1744.0000000	0.0610000
-1743.0000000	0.0619047
-1742.0000000	0.0615000
-1741.0000000	0.0625000
-1740.0000000	0.0625000
-1739.0000000	0.0623809
-1738.0000000	0.0625000
-1737.0000000	0.0630000
-1736.0000000	0.0604762
-1735.0000000	0.0605000

-1734.0000000	0.0600000
-1733.0000000	0.0600000
-1732.0000000	0.0600000
-1731.0000000	0.0600000
-1730.0000000	0.0604762
-1729.0000000	0.0600000
-1728.0000000	0.0600000
-1727.0000000	0.0600000
-1726.0000000	0.0600000
-1725.0000000	0.0600000
-1724.0000000	0.0600000
-1723.0000000	0.0600000
-1722.0000000	0.0600000
-1721.0000000	0.0600000
-1720.0000000	0.0600000
-1719.0000000	0.0600000
-1718.0000000	0.0600000
-1717.0000000	0.0600000
-1716.0000000	0.0600000
-1715.0000000	0.0595238
-1714.0000000	0.0595000
-1713.0000000	0.0600000
-1712.0000000	0.0600000
-1711.0000000	0.0600000
-1710.0000000	0.0600000
-1709.0000000	0.0600000
-1708.0000000	0.0600000
-1707.0000000	0.0600000
-1706.0000000	0.0595238
-1705.0000000	0.0600000
-1704.0000000	0.0600000
-1703.0000000	0.0604762
-1702.0000000	0.0600000
-1701.0000000	0.0600000
-1700.0000000	0.0604762
-1699.0000000	0.0600000
-1698.0000000	0.0605000
-1697.0000000	0.0605000
-1696.0000000	0.0614286
-1695.0000000	0.0615000
-1694.0000000	0.0609524
-1693.0000000	0.0615000
-1692.0000000	0.0620000
-1691.0000000	0.0604762
-1690.0000000	0.0625000
-1689.0000000	0.0610000
-1688.0000000	0.0610000
-1687.0000000	0.0600000
-1686.0000000	0.0610000
-1685.0000000	0.0619048
-1684.0000000	0.0600000
-1683.0000000	0.0610000
-1682.0000000	0.0609524
-1681.0000000	0.0610000
-1680.0000000	0.0610000
-1679.0000000	0.0605000
-1678.0000000	0.0600000

-1677.0000000	0.0600000
-1676.0000000	0.0595238
-1675.0000000	0.0600000
-1674.0000000	0.0590000
-1673.0000000	0.0565000
-1672.0000000	0.0523810
-1671.0000000	0.0505000
-1670.0000000	0.0500000
-1669.0000000	0.0500000
-1668.0000000	0.0500000
-1667.0000000	0.0495238
-1666.0000000	0.0500000
-1665.0000000	0.0500000
-1664.0000000	0.0500000
-1663.0000000	0.0500000
-1662.0000000	0.0500000
-1661.0000000	0.0510000
-1660.0000000	0.0514286
-1659.0000000	0.0540000
-1658.0000000	0.0530000
-1657.0000000	0.0542857
-1656.0000000	0.0565000
-1655.0000000	0.0557143
-1654.0000000	0.0570000
-1653.0000000	0.0565000
-1652.0000000	0.0566667
-1651.0000000	0.0585000
-1650.0000000	0.0590000
-1649.0000000	0.0580000
-1648.0000000	0.0585714
-1647.0000000	0.0560000
-1646.0000000	0.0585714
-1645.0000000	0.0590000
-1644.0000000	0.0575000
-1643.0000000	0.0575000
-1642.0000000	0.0585714
-1641.0000000	0.0600000
-1640.0000000	0.0595000
-1639.0000000	0.0595238
-1638.0000000	0.0590000
-1637.0000000	0.0590000
-1636.0000000	0.0600000
-1635.0000000	0.0595000
-1634.0000000	0.0600000
-1633.0000000	0.0600000
-1632.0000000	0.0585000
-1631.0000000	0.0600000
-1630.0000000	0.0600000
-1629.0000000	0.0595000
-1628.0000000	0.0585714
-1627.0000000	0.0600000
-1626.0000000	0.0590000
-1625.0000000	0.0590000
-1624.0000000	0.0590476
-1623.0000000	0.0595000
-1622.0000000	0.0600000
-1621.0000000	0.0600000

-1620.0000000	0.0595000
-1619.0000000	0.0600000
-1618.0000000	0.0600000
-1617.0000000	0.0595000
-1616.0000000	0.0600000
-1615.0000000	0.0600000
-1614.0000000	0.0595000
-1613.0000000	0.0600000
-1612.0000000	0.0595238
-1611.0000000	0.0600000
-1610.0000000	0.0600000
-1609.0000000	0.0600000
-1608.0000000	0.0600000
-1607.0000000	0.0600000
-1606.0000000	0.0600000
-1605.0000000	0.0600000
-1604.0000000	0.0600000
-1603.0000000	0.0600000
-1602.0000000	0.0600000
-1601.0000000	0.0600000
-1600.0000000	0.0600000
-1599.0000000	0.0605000
-1598.0000000	0.0600000
-1597.0000000	0.0600000
-1596.0000000	0.0600000
-1595.0000000	0.0600000
-1594.0000000	0.0600000
-1593.0000000	0.0600000
-1592.0000000	0.0600000
-1591.0000000	0.0604762
-1590.0000000	0.0600000
-1589.0000000	0.0600000
-1588.0000000	0.0600000
-1587.0000000	0.0600000
-1586.0000000	0.0600000
-1585.0000000	0.0600000
-1584.0000000	0.0600000
-1583.0000000	0.0600000
-1582.0000000	0.0600000
-1581.0000000	0.0600000
-1580.0000000	0.0600000
-1579.0000000	0.0595238
-1578.0000000	0.0595000
-1577.0000000	0.0600000
-1576.0000000	0.0595238
-1575.0000000	0.0600000
-1574.0000000	0.0600000
-1573.0000000	0.0600000
-1572.0000000	0.0600000
-1571.0000000	0.0600000
-1570.0000000	0.0600000
-1569.0000000	0.0604762
-1568.0000000	0.0605000
-1567.0000000	0.0600000
-1566.0000000	0.0600000
-1565.0000000	0.0600000
-1564.0000000	0.0600000

-1563.0000000	0.0600000
-1562.0000000	0.0600000
-1561.0000000	0.0600000
-1560.0000000	0.0600000
-1559.0000000	0.0600000
-1558.0000000	0.0600000
-1557.0000000	0.0600000
-1556.0000000	0.0600000
-1555.0000000	0.0600000
-1554.0000000	0.0600000
-1553.0000000	0.0600000
-1552.0000000	0.0600000
-1551.0000000	0.0600000
-1550.0000000	0.0595238
-1549.0000000	0.0600000
-1548.0000000	0.0600000
-1547.0000000	0.0600000
-1546.0000000	0.0600000
-1545.0000000	0.0605000
-1544.0000000	0.0600000
-1543.0000000	0.0600000
-1542.0000000	0.0609524
-1541.0000000	0.0600000
-1540.0000000	0.0600000
-1539.0000000	0.0600000
-1538.0000000	0.0605000
-1537.0000000	0.0604762
-1536.0000000	0.0600000
-1535.0000000	0.0600000
-1534.0000000	0.0600000
-1533.0000000	0.0604762
-1532.0000000	0.0600000
-1531.0000000	0.0605000
-1530.0000000	0.0600000
-1529.0000000	0.0605000
-1528.0000000	0.0600000
-1527.0000000	0.0600000
-1526.0000000	0.0600000
-1525.0000000	0.0604762
-1524.0000000	0.0600000
-1523.0000000	0.0600000
-1522.0000000	0.0600000
-1521.0000000	0.0600000
-1520.0000000	0.0595238
-1519.0000000	0.0600000
-1518.0000000	0.0600000
-1517.0000000	0.0600000
-1516.0000000	0.0600000
-1515.0000000	0.0595000
-1514.0000000	0.0600000
-1513.0000000	0.0580000
-1512.0000000	0.0590476
-1511.0000000	0.0590000
-1510.0000000	0.0595238
-1509.0000000	0.0565000
-1508.0000000	0.0585000
-1507.0000000	0.0575000

-1506.0000000	0.0585714
-1505.0000000	0.0570000
-1504.0000000	0.0580000
-1503.0000000	0.0571428
-1502.0000000	0.0575000
-1501.0000000	0.0585714
-1500.0000000	0.0590000
-1499.0000000	0.0595000
-1498.0000000	0.0595000
-1497.0000000	0.0595238
-1496.0000000	0.0600000
-1495.0000000	0.0600000
-1494.0000000	0.0595238
-1493.0000000	0.0585000
-1492.0000000	0.0600000
-1491.0000000	0.0600000
-1490.0000000	0.0600000
-1489.0000000	0.0600000
-1488.0000000	0.0600000
-1487.0000000	0.0600000
-1486.0000000	0.0600000
-1485.0000000	0.0604762
-1484.0000000	0.0600000
-1483.0000000	0.0605000
-1482.0000000	0.0604762
-1481.0000000	0.0615000
-1480.0000000	0.0600000
-1479.0000000	0.0600000
-1478.0000000	0.0610000
-1477.0000000	0.0610000
-1476.0000000	0.0604762
-1475.0000000	0.0605000
-1474.0000000	0.0610000
-1473.0000000	0.0614286
-1472.0000000	0.0610000
-1471.0000000	0.0604762
-1470.0000000	0.0600000
-1469.0000000	0.0610000
-1468.0000000	0.0605000
-1467.0000000	0.0604762
-1466.0000000	0.0605000
-1465.0000000	0.0615000
-1464.0000000	0.0633333
-1463.0000000	0.0620000
-1462.0000000	0.0605000
-1461.0000000	0.0619048
-1460.0000000	0.0615000
-1459.0000000	0.0605000
-1458.0000000	0.0609524
-1457.0000000	0.0600000
-1456.0000000	0.0615000
-1455.0000000	0.0600000
-1454.0000000	0.0605000
-1453.0000000	0.0605000
-1452.0000000	0.0614286
-1451.0000000	0.0600000
-1450.0000000	0.0600000

-1449.0000000	0.0600000
-1448.0000000	0.0600000
-1447.0000000	0.0600000
-1446.0000000	0.0614286
-1445.0000000	0.0615000
-1444.0000000	0.0615000
-1443.0000000	0.0610000
-1442.0000000	0.0604762
-1441.0000000	0.0619048
-1440.0000000	0.0615000
-1439.0000000	0.0640000
-1438.0000000	0.0635000
-1437.0000000	0.0647619
-1436.0000000	0.0650000
-1435.0000000	0.0650000
-1434.0000000	0.0638095
-1433.0000000	0.0645000
-1432.0000000	0.0660000
-1431.0000000	0.0635000
-1430.0000000	0.0638095
-1429.0000000	0.0635000
-1428.0000000	0.0623810
-1427.0000000	0.0620000
-1426.0000000	0.0605000
-1425.0000000	0.0604762
-1424.0000000	0.0625000
-1423.0000000	0.0605000
-1422.0000000	0.0604762
-1421.0000000	0.0605000
-1420.0000000	0.0605000
-1419.0000000	0.0600000
-1418.0000000	0.0600000
-1417.0000000	0.0600000
-1416.0000000	0.0600000
-1415.0000000	0.0600000
-1414.0000000	0.0600000
-1413.0000000	0.0600000
-1412.0000000	0.0600000
-1411.0000000	0.0600000
-1410.0000000	0.0604762
-1409.0000000	0.0615000
-1408.0000000	0.0605000
-1407.0000000	0.0600000
-1406.0000000	0.0604762
-1405.0000000	0.0600000
-1404.0000000	0.0600000
-1403.0000000	0.0604762
-1402.0000000	0.0605000
-1401.0000000	0.0604762
-1400.0000000	0.0610000
-1399.0000000	0.0600000
-1398.0000000	0.0600000
-1397.0000000	0.0605000
-1396.0000000	0.0600000
-1395.0000000	0.0600000
-1394.0000000	0.0600000
-1393.0000000	0.0600000

-1392.0000000	0.0600000
-1391.0000000	0.0600000
-1390.0000000	0.0595000
-1389.0000000	0.0590476
-1388.0000000	0.0580000
-1387.0000000	0.0575000
-1386.0000000	0.0580000
-1385.0000000	0.0580952
-1384.0000000	0.0565000
-1383.0000000	0.0576190
-1382.0000000	0.0585000
-1381.0000000	0.0590000
-1380.0000000	0.0600000
-1379.0000000	0.0590000
-1378.0000000	0.0595000
-1377.0000000	0.0600000
-1376.0000000	0.0600000
-1375.0000000	0.0600000
-1374.0000000	0.0600000
-1373.0000000	0.0600000
-1372.0000000	0.0600000
-1371.0000000	0.0600000
-1370.0000000	0.0600000
-1369.0000000	0.0600000
-1368.0000000	0.0600000
-1367.0000000	0.0604762
-1366.0000000	0.0600000
-1365.0000000	0.0600000
-1364.0000000	0.0600000
-1363.0000000	0.0600000
-1362.0000000	0.0600000
-1361.0000000	0.0600000
-1360.0000000	0.0600000
-1359.0000000	0.0600000
-1358.0000000	0.0600000
-1357.0000000	0.0600000
-1356.0000000	0.0600000
-1355.0000000	0.0600000
-1354.0000000	0.0605000
-1353.0000000	0.0600000
-1352.0000000	0.0600000
-1351.0000000	0.0630000
-1350.0000000	0.0609524
-1349.0000000	0.0610000
-1348.0000000	0.0610000
-1347.0000000	0.0605000
-1346.0000000	0.0614286
-1345.0000000	0.0620000
-1344.0000000	0.0623810
-1343.0000000	0.0625000
-1342.0000000	0.0615000
-1341.0000000	0.0620000
-1340.0000000	0.0614286
-1339.0000000	0.0640000
-1338.0000000	0.0615000
-1337.0000000	0.0614286
-1336.0000000	0.0610000

-1335.0000000	0.0628571
-1334.0000000	0.0625000
-1333.0000000	0.0635000
-1332.0000000	0.0635000
-1331.0000000	0.0642857
-1330.0000000	0.0630000
-1329.0000000	0.0665000
-1328.0000000	0.0652381
-1327.0000000	0.0645000
-1326.0000000	0.0655000
-1325.0000000	0.0690476
-1324.0000000	0.0675000
-1323.0000000	0.0640000
-1322.0000000	0.0633333
-1321.0000000	0.0660000
-1320.0000000	0.0665000
-1319.0000000	0.0647619
-1318.0000000	0.0635000
-1317.0000000	0.0635000
-1316.0000000	0.0625000
-1315.0000000	0.0623809
-1314.0000000	0.0609524
-1313.0000000	0.0605000
-1312.0000000	0.0615000
-1311.0000000	0.0600000
-1310.0000000	0.0604762
-1309.0000000	0.0605000
-1308.0000000	0.0600000
-1307.0000000	0.0600000
-1306.0000000	0.0600000
-1305.0000000	0.0600000
-1304.0000000	0.0600000
-1303.0000000	0.0600000
-1302.0000000	0.0600000
-1301.0000000	0.0600000
-1300.0000000	0.0600000
-1299.0000000	0.0600000
-1298.0000000	0.0600000
-1297.0000000	0.0595000
-1296.0000000	0.0600000
-1295.0000000	0.0590476
-1294.0000000	0.0595000
-1293.0000000	0.0595000
-1292.0000000	0.0600000
-1291.0000000	0.0585000
-1290.0000000	0.0600000
-1289.0000000	0.0595000
-1288.0000000	0.0600000
-1287.0000000	0.0600000
-1286.0000000	0.0595238
-1285.0000000	0.0600000
-1284.0000000	0.0600000
-1283.0000000	0.0600000
-1282.0000000	0.0600000
-1281.0000000	0.0600000
-1280.0000000	0.0600000
-1279.0000000	0.0604762

-1278.0000000	0.0600000
-1277.0000000	0.0605000
-1276.0000000	0.0600000
-1275.0000000	0.0600000
-1274.0000000	0.0600000
-1273.0000000	0.0600000
-1272.0000000	0.0600000
-1271.0000000	0.0600000
-1270.0000000	0.0600000
-1269.0000000	0.0600000
-1268.0000000	0.0600000
-1267.0000000	0.0600000
-1266.0000000	0.0600000
-1265.0000000	0.0604762
-1264.0000000	0.0605000
-1263.0000000	0.0600000
-1262.0000000	0.0600000
-1261.0000000	0.0600000
-1260.0000000	0.0600000
-1259.0000000	0.0600000
-1258.0000000	0.0605000
-1257.0000000	0.0600000
-1256.0000000	0.0600000
-1255.0000000	0.0595000
-1254.0000000	0.0600000
-1253.0000000	0.0600000
-1252.0000000	0.0595000
-1251.0000000	0.0600000
-1250.0000000	0.0600000
-1249.0000000	0.0600000
-1248.0000000	0.0585000
-1247.0000000	0.0576190
-1246.0000000	0.0575000
-1245.0000000	0.0580000
-1244.0000000	0.0566667
-1243.0000000	0.0570000
-1242.0000000	0.0585000
-1241.0000000	0.0595000
-1240.0000000	0.0590476
-1239.0000000	0.0590000
-1238.0000000	0.0600000
-1237.0000000	0.0600000
-1236.0000000	0.0600000
-1235.0000000	0.0600000
-1234.0000000	0.0595000
-1233.0000000	0.0600000
-1232.0000000	0.0600000
-1231.0000000	0.0600000
-1230.0000000	0.0600000
-1229.0000000	0.0600000
-1228.0000000	0.0600000
-1227.0000000	0.0605000
-1226.0000000	0.0600000
-1225.0000000	0.0600000
-1224.0000000	0.0600000
-1223.0000000	0.0609524
-1222.0000000	0.0610000

-1221.0000000	0.0615000
-1220.0000000	0.0610000
-1219.0000000	0.0614286
-1218.0000000	0.0610000
-1217.0000000	0.0604762
-1216.0000000	0.0605000
-1215.0000000	0.0609524
-1214.0000000	0.0610000
-1213.0000000	0.0630000
-1212.0000000	0.0635000
-1211.0000000	0.0615000
-1210.0000000	0.0638095
-1209.0000000	0.0640000
-1208.0000000	0.0628571
-1207.0000000	0.0625000
-1206.0000000	0.0630000
-1205.0000000	0.0660000
-1204.0000000	0.0623810
-1203.0000000	0.0665000
-1202.0000000	0.0670000
-1201.0000000	0.0680952
-1200.0000000	0.0670000
-1199.0000000	0.0685714
-1198.0000000	0.0695000
-1197.0000000	0.0700000
-1196.0000000	0.0690000
-1195.0000000	0.0690476
-1194.0000000	0.0690000
-1193.0000000	0.0685000
-1192.0000000	0.0685714
-1191.0000000	0.0690000
-1190.0000000	0.0660000
-1189.0000000	0.0676190
-1188.0000000	0.0665000
-1187.0000000	0.0666667
-1186.0000000	0.0655000
-1185.0000000	0.0670000
-1184.0000000	0.0642857
-1183.0000000	0.0650000
-1182.0000000	0.0635000
-1181.0000000	0.0635000
-1180.0000000	0.0642857
-1179.0000000	0.0635000
-1178.0000000	0.0633333
-1177.0000000	0.0615000
-1176.0000000	0.0630000
-1175.0000000	0.0615000
-1174.0000000	0.0619048
-1173.0000000	0.0610000
-1172.0000000	0.0628571
-1171.0000000	0.0630000
-1170.0000000	0.0670000
-1169.0000000	0.0685714
-1168.0000000	0.0685000
-1167.0000000	0.0690000
-1166.0000000	0.0675000
-1165.0000000	0.0676190

-1164.0000000	0.0700000
-1163.0000000	0.0685714
-1162.0000000	0.0695000
-1161.0000000	0.0695000
-1160.0000000	0.0700000
-1159.0000000	0.0695000
-1158.0000000	0.0695238
-1157.0000000	0.0695000
-1156.0000000	0.0690476
-1155.0000000	0.0685000
-1154.0000000	0.0695000
-1153.0000000	0.0695238
-1152.0000000	0.0680000
-1151.0000000	0.0630000
-1150.0000000	0.0604762
-1149.0000000	0.0605000
-1148.0000000	0.0604762
-1147.0000000	0.0600000
-1146.0000000	0.0600000
-1145.0000000	0.0600000
-1144.0000000	0.0600000
-1143.0000000	0.0600000
-1142.0000000	0.0600000
-1141.0000000	0.0600000
-1140.0000000	0.0600000
-1139.0000000	0.0600000
-1138.0000000	0.0600000
-1137.0000000	0.0600000
-1136.0000000	0.0600000
-1135.0000000	0.0600000
-1134.0000000	0.0595000
-1133.0000000	0.0595000
-1132.0000000	0.0571428
-1131.0000000	0.0555000
-1130.0000000	0.0575000
-1129.0000000	0.0565000
-1128.0000000	0.0585714
-1127.0000000	0.0580000
-1126.0000000	0.0557143
-1125.0000000	0.0570000
-1124.0000000	0.0557143
-1123.0000000	0.0555000
-1122.0000000	0.0560000
-1121.0000000	0.0545000
-1120.0000000	0.0585714
-1119.0000000	0.0570000
-1118.0000000	0.0565000
-1117.0000000	0.0595238
-1116.0000000	0.0585000
-1115.0000000	0.0595000
-1114.0000000	0.0595000
-1113.0000000	0.0600000
-1112.0000000	0.0600000
-1111.0000000	0.0590000
-1110.0000000	0.0595238
-1109.0000000	0.0600000
-1108.0000000	0.0600000

-1107.0000000	0.0590000
-1106.0000000	0.0600000
-1105.0000000	0.0600000
-1104.0000000	0.0600000
-1103.0000000	0.0600000
-1102.0000000	0.0600000
-1101.0000000	0.0600000
-1100.0000000	0.0600000
-1099.0000000	0.0600000
-1098.0000000	0.0600000
-1097.0000000	0.0600000
-1096.0000000	0.0600000
-1095.0000000	0.0600000
-1094.0000000	0.0600000
-1093.0000000	0.0600000
-1092.0000000	0.0600000
-1091.0000000	0.0600000
-1090.0000000	0.0600000
-1089.0000000	0.0600000
-1088.0000000	0.0600000
-1087.0000000	0.0600000
-1086.0000000	0.0600000
-1085.0000000	0.0600000
-1084.0000000	0.0600000
-1083.0000000	0.0600000
-1082.0000000	0.0600000
-1081.0000000	0.0600000
-1080.0000000	0.0600000
-1079.0000000	0.0600000
-1078.0000000	0.0600000
-1077.0000000	0.0600000
-1076.0000000	0.0600000
-1075.0000000	0.0600000
-1074.0000000	0.0600000
-1073.0000000	0.0600000
-1072.0000000	0.0604762
-1071.0000000	0.0600000
-1070.0000000	0.0600000
-1069.0000000	0.0605000
-1068.0000000	0.0609524
-1067.0000000	0.0605000
-1066.0000000	0.0600000
-1065.0000000	0.0604762
-1064.0000000	0.0610000
-1063.0000000	0.0609524
-1062.0000000	0.0625000
-1061.0000000	0.0610000
-1060.0000000	0.0609524
-1059.0000000	0.0625000
-1058.0000000	0.0615000
-1057.0000000	0.0630000
-1056.0000000	0.0633333
-1055.0000000	0.0655000
-1054.0000000	0.0630000
-1053.0000000	0.0638095
-1052.0000000	0.0640000
-1051.0000000	0.0614286

-1050.0000000	0.0605000
-1049.0000000	0.0615000
-1048.0000000	0.0633333
-1047.0000000	0.0615000
-1046.0000000	0.0620000
-1045.0000000	0.0605000
-1044.0000000	0.0600000
-1043.0000000	0.0600000
-1042.0000000	0.0600000
-1041.0000000	0.0600000
-1040.0000000	0.0600000
-1039.0000000	0.0600000
-1038.0000000	0.0600000
-1037.0000000	0.0600000
-1036.0000000	0.0600000
-1035.0000000	0.0600000
-1034.0000000	0.0600000
-1033.0000000	0.0604762
-1032.0000000	0.0600000
-1031.0000000	0.0610000
-1030.0000000	0.0605000
-1029.0000000	0.0600000
-1028.0000000	0.0605000
-1027.0000000	0.0600000
-1026.0000000	0.0604762
-1025.0000000	0.0610000
-1024.0000000	0.0600000
-1023.0000000	0.0614286
-1022.0000000	0.0610000
-1021.0000000	0.0619048
-1020.0000000	0.0625000
-1019.0000000	0.0605000
-1018.0000000	0.0630000
-1017.0000000	0.0619048
-1016.0000000	0.0630000
-1015.0000000	0.0625000
-1014.0000000	0.0610000
-1013.0000000	0.0614286
-1012.0000000	0.0604762
-1011.0000000	0.0635000
-1010.0000000	0.0610000
-1009.0000000	0.0625000
-1008.0000000	0.0614286
-1007.0000000	0.0610000
-1006.0000000	0.0610000
-1005.0000000	0.0609524
-1004.0000000	0.0605000
-1003.0000000	0.0609524
-1002.0000000	0.0615000
-1001.0000000	0.0605000
-1000.0000000	0.0615000
-999.0000000	0.0628571
-998.0000000	0.0620000
-997.0000000	0.0638095
-996.0000000	0.0655000
-995.0000000	0.0625000
-994.0000000	0.0620000

-993.0000000	0.0633333
-992.0000000	0.0635000
-991.0000000	0.0615000
-990.0000000	0.0623809
-989.0000000	0.0600000
-988.0000000	0.0600000
-987.0000000	0.0600000
-986.0000000	0.0600000
-985.0000000	0.0600000
-984.0000000	0.0600000
-983.0000000	0.0600000
-982.0000000	0.0600000
-981.0000000	0.0600000
-980.0000000	0.0600000
-979.0000000	0.0600000
-978.0000000	0.0600000
-977.0000000	0.0600000
-976.0000000	0.0600000
-975.0000000	0.0600000
-974.0000000	0.0600000
-973.0000000	0.0600000
-972.0000000	0.0600000
-971.0000000	0.0600000
-970.0000000	0.0600000
-969.0000000	0.0600000
-968.0000000	0.0600000
-967.0000000	0.0600000
-966.0000000	0.0600000
-965.0000000	0.0600000
-964.0000000	0.0605000
-963.0000000	0.0604762
-962.0000000	0.0600000
-961.0000000	0.0604762
-960.0000000	0.0600000
-959.0000000	0.0600000
-958.0000000	0.0600000
-957.0000000	0.0600000
-956.0000000	0.0605000
-955.0000000	0.0600000
-954.0000000	0.0600000
-953.0000000	0.0600000
-952.0000000	0.0600000
-951.0000000	0.0600000
-950.0000000	0.0600000
-949.0000000	0.0600000
-948.0000000	0.0600000
-947.0000000	0.0600000
-946.0000000	0.0610000
-945.0000000	0.0604762
-944.0000000	0.0605000
-943.0000000	0.0605000
-942.0000000	0.0604762
-941.0000000	0.0600000
-940.0000000	0.0600000
-939.0000000	0.0600000
-938.0000000	0.0600000
-937.0000000	0.0600000

-936.0000000	0.0600000
-935.0000000	0.0600000
-934.0000000	0.0600000
-933.0000000	0.0600000
-932.0000000	0.0600000
-931.0000000	0.0600000
-930.0000000	0.0600000
-929.0000000	0.0600000
-928.0000000	0.0600000
-927.0000000	0.0600000
-926.0000000	0.0605000
-925.0000000	0.0605000
-924.0000000	0.0604762
-923.0000000	0.0610000
-922.0000000	0.0605000
-921.0000000	0.0600000
-920.0000000	0.0600000
-919.0000000	0.0600000
-918.0000000	0.0600000
-917.0000000	0.0604762
-916.0000000	0.0600000
-915.0000000	0.0604762
-914.0000000	0.0610000
-913.0000000	0.0609524
-912.0000000	0.0620000
-911.0000000	0.0600000
-910.0000000	0.0625000
-909.0000000	0.0605000
-908.0000000	0.0623809
-907.0000000	0.0615000
-906.0000000	0.0628571
-905.0000000	0.0630000
-904.0000000	0.0615000
-903.0000000	0.0620000
-902.0000000	0.0619047
-901.0000000	0.0605000
-900.0000000	0.0605000
-899.0000000	0.0600000
-898.0000000	0.0600000
-897.0000000	0.0604762
-896.0000000	0.0605000
-895.0000000	0.0605000
-894.0000000	0.0600000
-893.0000000	0.0604762
-892.0000000	0.0605000
-891.0000000	0.0610000
-890.0000000	0.0609524
-889.0000000	0.0615000
-888.0000000	0.0605000
-887.0000000	0.0610000
-886.0000000	0.0600000
-885.0000000	0.0600000
-884.0000000	0.0605000
-883.0000000	0.0600000
-882.0000000	0.0600000
-881.0000000	0.0600000
-880.0000000	0.0600000

-879.0000000	0.0600000
-878.0000000	0.0600000
-877.0000000	0.0600000
-876.0000000	0.0600000
-875.0000000	0.0600000
-874.0000000	0.0595000
-873.0000000	0.0600000
-872.0000000	0.0595238
-871.0000000	0.0585000
-870.0000000	0.0600000
-869.0000000	0.0600000
-868.0000000	0.0600000
-867.0000000	0.0600000
-866.0000000	0.0600000
-865.0000000	0.0595000
-864.0000000	0.0600000
-863.0000000	0.0600000
-862.0000000	0.0600000
-861.0000000	0.0600000
-860.0000000	0.0600000
-859.0000000	0.0600000
-858.0000000	0.0595000
-857.0000000	0.0595238
-856.0000000	0.0595000
-855.0000000	0.0590000
-854.0000000	0.0590476
-853.0000000	0.0580000
-852.0000000	0.0595238
-851.0000000	0.0590000
-850.0000000	0.0575000
-849.0000000	0.0590000
-848.0000000	0.0590000
-847.0000000	0.0590476
-846.0000000	0.0595238
-845.0000000	0.0595000
-844.0000000	0.0595000
-843.0000000	0.0595000
-842.0000000	0.0595238
-841.0000000	0.0600000
-840.0000000	0.0600000
-839.0000000	0.0600000
-838.0000000	0.0595238
-837.0000000	0.0595238
-836.0000000	0.0600000
-835.0000000	0.0600000
-834.0000000	0.0600000
-833.0000000	0.0600000
-832.0000000	0.0600000
-831.0000000	0.0600000
-830.0000000	0.0600000
-829.0000000	0.0605000
-828.0000000	0.0600000
-827.0000000	0.0600000
-826.0000000	0.0600000
-825.0000000	0.0600000
-824.0000000	0.0600000
-823.0000000	0.0600000

-822.0000000	0.0600000
-821.0000000	0.0600000
-820.0000000	0.0610000
-819.0000000	0.0610000
-818.0000000	0.0604762
-817.0000000	0.0605000
-816.0000000	0.0600000
-815.0000000	0.0609524
-814.0000000	0.0605000
-813.0000000	0.0600000
-812.0000000	0.0635000
-811.0000000	0.0628571
-810.0000000	0.0625000
-809.0000000	0.0645000
-808.0000000	0.0619048
-807.0000000	0.0620000
-806.0000000	0.0609524
-805.0000000	0.0620000
-804.0000000	0.0630000
-803.0000000	0.0610000
-802.0000000	0.0604762
-801.0000000	0.0605000
-800.0000000	0.0600000
-799.0000000	0.0600000
-798.0000000	0.0605000
-797.0000000	0.0604762
-796.0000000	0.0610000
-795.0000000	0.0610000
-794.0000000	0.0609524
-793.0000000	0.0610000
-792.0000000	0.0620000
-791.0000000	0.0628571
-790.0000000	0.0625000
-789.0000000	0.0610000
-788.0000000	0.0614286
-787.0000000	0.0640000
-786.0000000	0.0628571
-785.0000000	0.0645000
-784.0000000	0.0620000
-783.0000000	0.0625000
-782.0000000	0.0615000
-781.0000000	0.0619047
-780.0000000	0.0620000
-779.0000000	0.0609524
-778.0000000	0.0625000
-777.0000000	0.0604762
-776.0000000	0.0605000
-775.0000000	0.0610000
-774.0000000	0.0610000
-773.0000000	0.0600000
-772.0000000	0.0609524
-771.0000000	0.0610000
-770.0000000	0.0628571
-769.0000000	0.0645000
-768.0000000	0.0635000
-767.0000000	0.0670000
-766.0000000	0.0647619

-765.0000000	0.0655000
-764.0000000	0.0635000
-763.0000000	0.0652381
-762.0000000	0.0640000
-761.0000000	0.0657143
-760.0000000	0.0645000
-759.0000000	0.0655000
-758.0000000	0.0642857
-757.0000000	0.0645000
-756.0000000	0.0635000
-755.0000000	0.0642857
-754.0000000	0.0675000
-753.0000000	0.0655000
-752.0000000	0.0665000
-751.0000000	0.0676190
-750.0000000	0.0670000
-749.0000000	0.0680952
-748.0000000	0.0650000
-747.0000000	0.0640000
-746.0000000	0.0642857
-745.0000000	0.0655000
-744.0000000	0.0635000
-743.0000000	0.0642857
-742.0000000	0.0670000
-741.0000000	0.0675000
-740.0000000	0.0676190
-739.0000000	0.0680000
-738.0000000	0.0675000
-737.0000000	0.0675000
-736.0000000	0.0657143
-735.0000000	0.0660000
-734.0000000	0.0666667
-733.0000000	0.0650000
-732.0000000	0.0640000
-731.0000000	0.0628571
-730.0000000	0.0615000
-729.0000000	0.0605000
-728.0000000	0.0610000
-727.0000000	0.0614286
-726.0000000	0.0605000
-725.0000000	0.0600000
-724.0000000	0.0605000
-723.0000000	0.0600000
-722.0000000	0.0605000
-721.0000000	0.0610000
-720.0000000	0.0609524
-719.0000000	0.0628571
-718.0000000	0.0645000
-717.0000000	0.0660000
-716.0000000	0.0645000
-715.0000000	0.0657143
-714.0000000	0.0660000
-713.0000000	0.0665000
-712.0000000	0.0685000
-711.0000000	0.0671429
-710.0000000	0.0676190
-709.0000000	0.0670000

-708.0000000	0.0675000
-707.0000000	0.0670000
-706.0000000	0.0685714
-705.0000000	0.0670000
-704.0000000	0.0655000
-703.0000000	0.0633333
-702.0000000	0.0665000
-701.0000000	0.0666667
-700.0000000	0.0675000
-699.0000000	0.0695000
-698.0000000	0.0700000
-697.0000000	0.0700000
-696.0000000	0.0695000
-695.0000000	0.0700000
-694.0000000	0.0700000
-693.0000000	0.0695000
-692.0000000	0.0685000
-691.0000000	0.0695238
-690.0000000	0.0680000
-689.0000000	0.0670000
-688.0000000	0.0657143
-687.0000000	0.0645000
-686.0000000	0.0619048
-685.0000000	0.0620000
-684.0000000	0.0610000
-683.0000000	0.0620000
-682.0000000	0.0600000
-681.0000000	0.0600000
-680.0000000	0.0600000
-679.0000000	0.0600000
-678.0000000	0.0595000
-677.0000000	0.0600000
-676.0000000	0.0600000
-675.0000000	0.0600000
-674.0000000	0.0600000
-673.0000000	0.0600000
-672.0000000	0.0600000
-671.0000000	0.0600000
-670.0000000	0.0605000
-669.0000000	0.0600000
-668.0000000	0.0595000
-667.0000000	0.0600000
-666.0000000	0.0600000
-665.0000000	0.0600000
-664.0000000	0.0600000
-663.0000000	0.0600000
-662.0000000	0.0600000
-661.0000000	0.0600000
-660.0000000	0.0600000
-659.0000000	0.0600000
-658.0000000	0.0600000
-657.0000000	0.0600000
-656.0000000	0.0600000
-655.0000000	0.0600000
-654.0000000	0.0600000
-653.0000000	0.0600000
-652.0000000	0.0600000

-651.0000000	0.0605000
-650.0000000	0.0604762
-649.0000000	0.0620000
-648.0000000	0.0605000
-647.0000000	0.0610000
-646.0000000	0.0610000
-645.0000000	0.0600000
-644.0000000	0.0609524
-643.0000000	0.0609524
-642.0000000	0.0615000
-641.0000000	0.0635000
-640.0000000	0.0623810
-639.0000000	0.0615000
-638.0000000	0.0625000
-637.0000000	0.0625000
-636.0000000	0.0619048
-635.0000000	0.0630000
-634.0000000	0.0614286
-633.0000000	0.0610000
-632.0000000	0.0610000
-631.0000000	0.0630000
-630.0000000	0.0609524
-629.0000000	0.0630000
-628.0000000	0.0633333
-627.0000000	0.0620000
-626.0000000	0.0640000
-625.0000000	0.0633333
-624.0000000	0.0660000
-623.0000000	0.0660000
-622.0000000	0.0647619
-621.0000000	0.0645000
-620.0000000	0.0650000
-619.0000000	0.0676190
-618.0000000	0.0665000
-617.0000000	0.0655000
-616.0000000	0.0660000
-615.0000000	0.0652381
-614.0000000	0.0665000
-613.0000000	0.0661905
-612.0000000	0.0665000
-611.0000000	0.0652381
-610.0000000	0.0660000
-609.0000000	0.0660000
-608.0000000	0.0665000
-607.0000000	0.0675000
-606.0000000	0.0661905
-605.0000000	0.0660000
-604.0000000	0.0670000
-603.0000000	0.0665000
-602.0000000	0.0642857
-601.0000000	0.0655000
-600.0000000	0.0640000
-599.0000000	0.0650000
-598.0000000	0.0666667
-597.0000000	0.0633333
-596.0000000	0.0645000
-595.0000000	0.0670000

-594.0000000	0.0675000
-593.0000000	0.0671429
-592.0000000	0.0680000
-591.0000000	0.0655000
-590.0000000	0.0657143
-589.0000000	0.0665000
-588.0000000	0.0666667
-587.0000000	0.0645000
-586.0000000	0.0650000
-585.0000000	0.0640000
-584.0000000	0.0642857
-583.0000000	0.0630000
-582.0000000	0.0655000
-581.0000000	0.0661905
-580.0000000	0.0675000
-579.0000000	0.0655000
-578.0000000	0.0676191
-577.0000000	0.0680000
-576.0000000	0.0675000
-575.0000000	0.0647619
-574.0000000	0.0680000
-573.0000000	0.0671429
-572.0000000	0.0675000
-571.0000000	0.0665000
-570.0000000	0.0675000
-569.0000000	0.0671429
-568.0000000	0.0670000
-567.0000000	0.0675000
-566.0000000	0.0700000
-565.0000000	0.0660000
-564.0000000	0.0680952
-563.0000000	0.0670000
-562.0000000	0.0675000
-561.0000000	0.0680000
-560.0000000	0.0675000
-559.0000000	0.0652381
-558.0000000	0.0610000
-557.0000000	0.0619048
-556.0000000	0.0625000
-555.0000000	0.0610000
-554.0000000	0.0605000
-553.0000000	0.0609524
-552.0000000	0.0610000
-551.0000000	0.0605000
-550.0000000	0.0604762
-549.0000000	0.0633333
-548.0000000	0.0610000
-547.0000000	0.0610000
-546.0000000	0.0620000
-545.0000000	0.0623809
-544.0000000	0.0635000
-543.0000000	0.0660000
-542.0000000	0.0666667
-541.0000000	0.0675000
-540.0000000	0.0680000
-539.0000000	0.0685714
-538.0000000	0.0685000

-537.0000000	0.0685000
-536.0000000	0.0690476
-535.0000000	0.0670000
-534.0000000	0.0665000
-533.0000000	0.0657143
-532.0000000	0.0645000
-531.0000000	0.0610000
-530.0000000	0.0609524
-529.0000000	0.0620000
-528.0000000	0.0630000
-527.0000000	0.0614286
-526.0000000	0.0610000
-525.0000000	0.0610000
-524.0000000	0.0615000
-523.0000000	0.0609524
-522.0000000	0.0640000
-521.0000000	0.0645000
-520.0000000	0.0642857
-519.0000000	0.0650000
-518.0000000	0.0647619
-517.0000000	0.0640000
-516.0000000	0.0660000
-515.0000000	0.0645000
-514.0000000	0.0680952
-513.0000000	0.0680000
-512.0000000	0.0690000
-511.0000000	0.0685714
-510.0000000	0.0685000
-509.0000000	0.0690476
-508.0000000	0.0689474
-507.0000000	0.0685714
-506.0000000	0.0680952
-505.0000000	0.0685000
-504.0000000	0.0690000
-503.0000000	0.0700000
-502.0000000	0.0680952
-501.0000000	0.0695000
-500.0000000	0.0700000
-499.0000000	0.0685000
-498.0000000	0.0680000
-497.0000000	0.0680952
-496.0000000	0.0655000
-495.0000000	0.0675000
-494.0000000	0.0625000
-493.0000000	0.0619047
-492.0000000	0.0605000
-491.0000000	0.0614286
-490.0000000	0.0605000
-489.0000000	0.0600000
-488.0000000	0.0600000
-487.0000000	0.0600000
-486.0000000	0.0600000
-485.0000000	0.0605000
-484.0000000	0.0604762
-483.0000000	0.0605000
-482.0000000	0.0600000
-481.0000000	0.0605000

-480.0000000	0.0600000
-479.0000000	0.0600000
-478.0000000	0.0600000
-477.0000000	0.0605000
-476.0000000	0.0615000
-475.0000000	0.0604762
-474.0000000	0.0625000
-473.0000000	0.0633333
-472.0000000	0.0620000
-471.0000000	0.0640000
-470.0000000	0.0623810
-469.0000000	0.0640000
-468.0000000	0.0655000
-467.0000000	0.0655000
-466.0000000	0.0652381
-465.0000000	0.0625000
-464.0000000	0.0620000
-463.0000000	0.0610000
-462.0000000	0.0628571
-461.0000000	0.0609524
-460.0000000	0.0610000
-459.0000000	0.0600000
-458.0000000	0.0604762
-457.0000000	0.0610000
-456.0000000	0.0605000
-455.0000000	0.0605000
-454.0000000	0.0614286
-453.0000000	0.0615000
-452.0000000	0.0600000
-451.0000000	0.0609524
-450.0000000	0.0620000
-449.0000000	0.0605000
-448.0000000	0.0604762
-447.0000000	0.0615000
-446.0000000	0.0635000
-445.0000000	0.0647619
-444.0000000	0.0650000
-443.0000000	0.0630000
-442.0000000	0.0619048
-441.0000000	0.0625000
-440.0000000	0.0630000
-439.0000000	0.0619048
-438.0000000	0.0620000
-437.0000000	0.0625000
-436.0000000	0.0610000
-435.0000000	0.0604762
-434.0000000	0.0605000
-433.0000000	0.0615000
-432.0000000	0.0609524
-431.0000000	0.0620000
-430.0000000	0.0623809
-429.0000000	0.0625000
-428.0000000	0.0630000
-427.0000000	0.0609524
-426.0000000	0.0660000
-425.0000000	0.0615000
-424.0000000	0.0630000

-423.0000000	0.0619047
-422.0000000	0.0614286
-421.0000000	0.0610000
-420.0000000	0.0615000
-419.0000000	0.0620000
-418.0000000	0.0604762
-417.0000000	0.0625000
-416.0000000	0.0620000
-415.0000000	0.0623809
-414.0000000	0.0615000
-413.0000000	0.0620000
-412.0000000	0.0619048
-411.0000000	0.0635000
-410.0000000	0.0640000
-409.0000000	0.0657143
-408.0000000	0.0635000
-407.0000000	0.0650000
-406.0000000	0.0642857
-405.0000000	0.0660000
-404.0000000	0.0650000
-403.0000000	0.0625000
-402.0000000	0.0628571
-401.0000000	0.0610000
-400.0000000	0.0628571
-399.0000000	0.0630000
-398.0000000	0.0628571
-397.0000000	0.0600000
-396.0000000	0.0600000
-395.0000000	0.0605000
-394.0000000	0.0605000
-393.0000000	0.0604762
-392.0000000	0.0600000
-391.0000000	0.0604762
-390.0000000	0.0600000
-389.0000000	0.0605000
-388.0000000	0.0610000
-387.0000000	0.0609524
-386.0000000	0.0615000
-385.0000000	0.0620000
-384.0000000	0.0623809
-383.0000000	0.0605000
-382.0000000	0.0600000
-381.0000000	0.0600000
-380.0000000	0.0600000
-379.0000000	0.0600000
-378.0000000	0.0600000
-377.0000000	0.0600000
-376.0000000	0.0600000
-375.0000000	0.0600000
-374.0000000	0.0600000
-373.0000000	0.0600000
-372.0000000	0.0605000
-371.0000000	0.0605000
-370.0000000	0.0609524
-369.0000000	0.0600000
-368.0000000	0.0620000
-367.0000000	0.0633333

-366.0000000	0.0620000
-365.0000000	0.0620000
-364.0000000	0.0619048
-363.0000000	0.0610526
-362.0000000	0.0604762
-361.0000000	0.0604762
-360.0000000	0.0600000
-359.0000000	0.0595000
-358.0000000	0.0585000
-357.0000000	0.0590476
-356.0000000	0.0575000
-355.0000000	0.0590476
-354.0000000	0.0585000
-353.0000000	0.0580000
-352.0000000	0.0576190
-351.0000000	0.0575000
-350.0000000	0.0560000
-349.0000000	0.0550000
-348.0000000	0.0552381
-347.0000000	0.0575000
-346.0000000	0.0566667
-345.0000000	0.0565000
-344.0000000	0.0585000
-343.0000000	0.0595238
-342.0000000	0.0585000
-341.0000000	0.0600000
-340.0000000	0.0600000
-339.0000000	0.0600000
-338.0000000	0.0595000
-337.0000000	0.0600000
-336.0000000	0.0595000
-335.0000000	0.0600000
-334.0000000	0.0600000
-333.0000000	0.0600000
-332.0000000	0.0600000
-331.0000000	0.0600000
-330.0000000	0.0600000
-329.0000000	0.0600000
-328.0000000	0.0600000
-327.0000000	0.0600000
-326.0000000	0.0600000
-325.0000000	0.0600000
-324.0000000	0.0600000
-323.0000000	0.0590000
-322.0000000	0.0590476
-321.0000000	0.0600000
-320.0000000	0.0600000
-319.0000000	0.0600000
-318.0000000	0.0590476
-317.0000000	0.0595000
-316.0000000	0.0585000
-315.0000000	0.0585714
-314.0000000	0.0590000
-313.0000000	0.0595000
-312.0000000	0.0585714
-311.0000000	0.0600000
-310.0000000	0.0585000

-309.0000000	0.0600000
-308.0000000	0.0600000
-307.0000000	0.0600000
-306.0000000	0.0595000
-305.0000000	0.0595000
-304.0000000	0.0600000
-303.0000000	0.0600000
-302.0000000	0.0600000
-301.0000000	0.0600000
-300.0000000	0.0600000
-299.0000000	0.0595000
-298.0000000	0.0575000
-297.0000000	0.0580000
-296.0000000	0.0557143
-295.0000000	0.0571428
-294.0000000	0.0585000
-293.0000000	0.0590000
-292.0000000	0.0568421
-291.0000000	0.0580952
-290.0000000	0.0576190
-289.0000000	0.0595000
-288.0000000	0.0585714
-287.0000000	0.0580000
-286.0000000	0.0576190
-285.0000000	0.0590000
-284.0000000	0.0575000
-283.0000000	0.0580000
-282.0000000	0.0585714
-281.0000000	0.0590000
-280.0000000	0.0590000
-279.0000000	0.0576190
-278.0000000	0.0570000
-277.0000000	0.0590000
-276.0000000	0.0576190
-275.0000000	0.0590000
-274.0000000	0.0580000
-273.0000000	0.0571428
-272.0000000	0.0570000
-271.0000000	0.0566667
-270.0000000	0.0575000
-269.0000000	0.0555000
-268.0000000	0.0570000
-267.0000000	0.0575000
-266.0000000	0.0585714
-265.0000000	0.0590000
-264.0000000	0.0590476
-263.0000000	0.0595000
-262.0000000	0.0595238
-261.0000000	0.0595000
-260.0000000	0.0600000
-259.0000000	0.0595000
-258.0000000	0.0600000
-257.0000000	0.0595238
-256.0000000	0.0600000
-255.0000000	0.0600000
-254.0000000	0.0600000
-253.0000000	0.0600000

-252.0000000	0.0600000
-251.0000000	0.0600000
-250.0000000	0.0600000
-249.0000000	0.0600000
-248.0000000	0.0595238
-247.0000000	0.0600000
-246.0000000	0.0600000
-245.0000000	0.0600000
-244.0000000	0.0600000
-243.0000000	0.0600000
-242.0000000	0.0600000
-241.0000000	0.0600000
-240.0000000	0.0600000
-239.0000000	0.0600000
-238.0000000	0.0600000
-237.0000000	0.0600000
-236.0000000	0.0600000
-235.0000000	0.0600000
-234.0000000	0.0600000
-233.0000000	0.0600000
-232.0000000	0.0600000
-231.0000000	0.0604762
-230.0000000	0.0605000
-229.0000000	0.0600000
-228.0000000	0.0600000
-227.0000000	0.0595238
-226.0000000	0.0595238
-225.0000000	0.0590000
-224.0000000	0.0595000
-223.0000000	0.0600000
-222.0000000	0.0600000
-221.0000000	0.0595238
-220.0000000	0.0600000
-219.0000000	0.0600000
-218.0000000	0.0600000
-217.0000000	0.0600000
-216.0000000	0.0600000
-215.0000000	0.0600000
-214.0000000	0.0600000
-213.0000000	0.0600000
-212.0000000	0.0595238
-211.0000000	0.0600000
-210.0000000	0.0600000
-209.0000000	0.0595000
-208.0000000	0.0600000
-207.0000000	0.0600000
-206.0000000	0.0600000
-205.0000000	0.0595000
-204.0000000	0.0595000
-203.0000000	0.0600000
-202.0000000	0.0595000
-201.0000000	0.0585714
-200.0000000	0.0595000
-199.0000000	0.0600000
-198.0000000	0.0600000
-197.0000000	0.0600000
-196.0000000	0.0600000

-195.0000000	0.0604762
-194.0000000	0.0605000
-193.0000000	0.0600000
-192.0000000	0.0610000
-191.0000000	0.0600000
-190.0000000	0.0610000
-189.0000000	0.0610000
-188.0000000	0.0619047
-187.0000000	0.0605000
-186.0000000	0.0600000
-185.0000000	0.0600000
-184.0000000	0.0600000
-183.0000000	0.0614286
-182.0000000	0.0610000
-181.0000000	0.0620000
-180.0000000	0.0605000
-179.0000000	0.0600000
-178.0000000	0.0610000
-177.0000000	0.0605000
-176.0000000	0.0600000
-175.0000000	0.0615000
-174.0000000	0.0604762
-173.0000000	0.0610000
-172.0000000	0.0600000
-171.0000000	0.0604762
-170.0000000	0.0600000
-169.0000000	0.0605000
-168.0000000	0.0605000
-167.0000000	0.0604762
-166.0000000	0.0605000
-165.0000000	0.0604762
-164.0000000	0.0610000
-163.0000000	0.0600000
-162.0000000	0.0610000
-161.0000000	0.0610000
-160.0000000	0.0609524
-159.0000000	0.0623809
-158.0000000	0.0625000
-157.0000000	0.0615000
-156.0000000	0.0614286
-155.0000000	0.0600000
-154.0000000	0.0610000
-153.0000000	0.0610000
-152.0000000	0.0605000
-151.0000000	0.0604762
-150.0000000	0.0605000
-149.0000000	0.0600000
-148.0000000	0.0600000
-147.0000000	0.0600000
-146.0000000	0.0600000
-145.0000000	0.0600000
-144.0000000	0.0600000
-143.0000000	0.0600000
-142.0000000	0.0600000
-141.0000000	0.0595000
-140.0000000	0.0595238
-139.0000000	0.0595000

-138.0000000	0.0590476
-137.0000000	0.0590000
-136.0000000	0.0595000
-135.0000000	0.0590476
-134.0000000	0.0595000
-133.0000000	0.0595000
-132.0000000	0.0580000
-131.0000000	0.0538095
-130.0000000	0.0535000
-129.0000000	0.0519048
-128.0000000	0.0525000
-127.0000000	0.0535000
-126.0000000	0.0504762
-125.0000000	0.0505000
-124.0000000	0.0500000
-123.0000000	0.0500000
-122.0000000	0.0500000
-121.0000000	0.0500000
-120.0000000	0.0504762
-119.0000000	0.0505000
-118.0000000	0.0505000
-117.0000000	0.0515000
-116.0000000	0.0520000
-115.0000000	0.0542857
-114.0000000	0.0535000
-113.0000000	0.0547619
-112.0000000	0.0540000
-111.0000000	0.0560000
-110.0000000	0.0571428
-109.0000000	0.0565000
-108.0000000	0.0565000
-107.0000000	0.0566667
-106.0000000	0.0545000
-105.0000000	0.0540000
-104.0000000	0.0528571
-103.0000000	0.0540000
-102.0000000	0.0530000
-101.0000000	0.0530000
-100.0000000	0.0528571
-99.0000000	0.0525000
-98.0000000	0.0509524
-97.0000000	0.0500000
-96.0000000	0.0500000
-95.0000000	0.0504762
-94.0000000	0.0500000
-93.0000000	0.0500000
-92.0000000	0.0500000
-91.0000000	0.0500000
-90.0000000	0.0500000
-89.0000000	0.0484211
-88.0000000	0.0471429
-87.0000000	0.0480000
-86.0000000	0.0485714
-85.0000000	0.0495000
-84.0000000	0.0460000
-83.0000000	0.0457143
-82.0000000	0.0445000

-81.0000000	0.0452381
-80.0000000	0.0470000
-79.0000000	0.0470000
-78.0000000	0.0475000
-77.0000000	0.0461905
-76.0000000	0.0475000
-75.0000000	0.0470000
-74.0000000	0.0457143
-73.0000000	0.0460000
-72.0000000	0.0470000
-71.0000000	0.0466667
-70.0000000	0.0470000
-69.0000000	0.0475000
-68.0000000	0.0480952
-67.0000000	0.0485000
-66.0000000	0.0490000
-65.0000000	0.0476191
-64.0000000	0.0490000
-63.0000000	0.0485000
-62.0000000	0.0495000
-61.0000000	0.0490476
-60.0000000	0.0495000
-59.0000000	0.0490476
-58.0000000	0.0485000
-57.0000000	0.0480000
-56.0000000	0.0455000
-55.0000000	0.0433333
-54.0000000	0.0435000
-53.0000000	0.0410000
-52.0000000	0.0414286
-51.0000000	0.0410000
-50.0000000	0.0423810
-49.0000000	0.0430000
-48.0000000	0.0430000
-47.0000000	0.0450000
-46.0000000	0.0471429
-45.0000000	0.0450000
-44.0000000	0.0470000
-43.0000000	0.0461905
-42.0000000	0.0460000
-41.0000000	0.0466667
-40.0000000	0.0485000
-39.0000000	0.0490000
-38.0000000	0.0485714
-37.0000000	0.0485000
-36.0000000	0.0490000
-35.0000000	0.0480000
-34.0000000	0.0485714
-33.0000000	0.0490000
-32.0000000	0.0466667
-31.0000000	0.0460000
-30.0000000	0.0460000
-29.0000000	0.0461905
-28.0000000	0.0450000
-27.0000000	0.0445000
-26.0000000	0.0460000
-25.0000000	0.0457143

-24.0000000	0.0435000
-23.0000000	0.0430000
-22.0000000	0.0428571
-21.0000000	0.0435000
-20.0000000	0.0423810
-19.0000000	0.0415000
-18.0000000	0.0420000
-17.0000000	0.0415000
-16.0000000	0.0409524
-15.0000000	0.0405000
-14.0000000	0.0414286
-13.0000000	0.0415000
-12.0000000	0.0440000
-11.0000000	0.0435000
-10.0000000	0.0457143
-9.0000000	0.0485000
-8.0000000	0.0470000
-7.0000000	0.0476191
-6.0000000	0.0495000
-5.0000000	0.0485714
-4.0000000	0.0494737
-3.0000000	0.0485714
-2.0000000	0.0490476
-1.0000000	0.0500000
0.0000000	0.0495000
1.0000000	0.0485000
2.0000000	0.0495238
3.0000000	0.0485000
4.0000000	0.0470000
5.0000000	0.0466667
6.0000000	0.0460000
7.0000000	0.0466667
8.0000000	0.0425000
9.0000000	0.0445000
10.0000000	0.0447619
11.0000000	0.0455000
12.0000000	0.0455000
13.0000000	0.0455000
14.0000000	0.0447619
15.0000000	0.0485000
16.0000000	0.0465000
17.0000000	0.0447619
18.0000000	0.0455000
19.0000000	0.0435000
20.0000000	0.0461905
21.0000000	0.0430000
22.0000000	0.0455000
23.0000000	0.0423810
24.0000000	0.0445000
25.0000000	0.0430000
26.0000000	0.0428571
27.0000000	0.0440000
28.0000000	0.0430000
29.0000000	0.0433333
30.0000000	0.0430000
31.0000000	0.0420000
32.0000000	0.0409524

33.0000000	0.0405000
34.0000000	0.0430000
35.0000000	0.0440000
36.0000000	0.0438095
37.0000000	0.0450000
38.0000000	0.0447619
39.0000000	0.0455000
40.0000000	0.0445000
41.0000000	0.0442857
42.0000000	0.0465000
43.0000000	0.0455000
44.0000000	0.0485000
45.0000000	0.0485714
46.0000000	0.0485714
47.0000000	0.0485000
48.0000000	0.0485000
49.0000000	0.0485000
50.0000000	0.0490476
51.0000000	0.0490000
52.0000000	0.0495000
53.0000000	0.0495000
54.0000000	0.0495238
55.0000000	0.0495000
56.0000000	0.0485714
57.0000000	0.0495000
58.0000000	0.0490000
59.0000000	0.0500000
60.0000000	0.0500000
61.0000000	0.0495000
62.0000000	0.0500000
63.0000000	0.0500000
64.0000000	0.0500000
65.0000000	0.0500000
66.0000000	0.0495238
67.0000000	0.0490000
68.0000000	0.0485714
69.0000000	0.0460000
70.0000000	0.0466667
71.0000000	0.0435000
72.0000000	0.0415000
73.0000000	0.0445000
74.0000000	0.0404762
75.0000000	0.0415000
76.0000000	0.0410000
77.0000000	0.0423810
78.0000000	0.0430000
79.0000000	0.0425000
80.0000000	0.0440000
81.0000000	0.0433333
82.0000000	0.0450000
83.0000000	0.0452381
84.0000000	0.0450000
85.0000000	0.0425000
86.0000000	0.0423810
87.0000000	0.0425000
88.0000000	0.0415000
89.0000000	0.0445000

90.0000000	0.0423810
91.0000000	0.0415000
92.0000000	0.0419048
93.0000000	0.0415000
94.0000000	0.0400000
95.0000000	0.0419048
96.0000000	0.0410000
97.0000000	0.0430000
98.0000000	0.0433333
99.0000000	0.0430000
100.0000000	0.0425000
101.0000000	0.0414286
102.0000000	0.0420000
103.0000000	0.0410000
104.0000000	0.0410000
105.0000000	0.0409524
106.0000000	0.0420000
107.0000000	0.0419048
108.0000000	0.0420000
109.0000000	0.0440000
110.0000000	0.0414286
111.0000000	0.0430000
112.0000000	0.0410000
113.0000000	0.0415000
114.0000000	0.0438095
115.0000000	0.0433333
116.0000000	0.0425000
117.0000000	0.0420000
118.0000000	0.0425000
119.0000000	0.0425000
120.0000000	0.0423810
121.0000000	0.0435000
122.0000000	0.0428572
123.0000000	0.0435000
124.0000000	0.0450000
125.0000000	0.0455000
126.0000000	0.0461905
127.0000000	0.0455000
128.0000000	0.0460000
129.0000000	0.0471429
130.0000000	0.0475000
131.0000000	0.0480952
132.0000000	0.0495000
133.0000000	0.0500000
134.0000000	0.0490476
135.0000000	0.0495000
136.0000000	0.0490000
137.0000000	0.0500000
138.0000000	0.0490476
139.0000000	0.0500000
140.0000000	0.0495000
141.0000000	0.0490000
142.0000000	0.0500000
143.0000000	0.0500000
144.0000000	0.0500000
145.0000000	0.0500000
146.0000000	0.0500000

147.0000000	0.0500000
148.0000000	0.0500000
149.0000000	0.0500000
150.0000000	0.0500000
151.0000000	0.0500000
152.0000000	0.0500000
153.0000000	0.0500000
154.0000000	0.0500000
155.0000000	0.0500000
156.0000000	0.0500000
157.0000000	0.0500000
158.0000000	0.0500000
159.0000000	0.0500000
160.0000000	0.0500000
161.0000000	0.0505000
162.0000000	0.0500000
163.0000000	0.0500000
164.0000000	0.0500000
165.0000000	0.0500000
166.0000000	0.0500000
167.0000000	0.0500000
168.0000000	0.0500000
169.0000000	0.0500000
170.0000000	0.0500000
171.0000000	0.0500000
172.0000000	0.0500000
173.0000000	0.0495000
174.0000000	0.0500000
175.0000000	0.0500000
176.0000000	0.0500000
177.0000000	0.0500000
178.0000000	0.0500000
179.0000000	0.0500000
180.0000000	0.0500000
181.0000000	0.0500000
182.0000000	0.0500000
183.0000000	0.0504762
184.0000000	0.0500000
185.0000000	0.0500000
186.0000000	0.0500000
187.0000000	0.0500000
188.0000000	0.0505000
189.0000000	0.0509524
190.0000000	0.0515000
191.0000000	0.0515000
192.0000000	0.0533333
193.0000000	0.0520000
194.0000000	0.0520000
195.0000000	0.0547619
196.0000000	0.0535000
197.0000000	0.0525000
198.0000000	0.0519048
199.0000000	0.0520000
200.0000000	0.0500000
201.0000000	0.0515000
202.0000000	0.0519048
203.0000000	0.0509524

204.0000000	0.0500000
205.0000000	0.0500000
206.0000000	0.0505000
207.0000000	0.0500000
208.0000000	0.0500000
209.0000000	0.0500000
210.0000000	0.0500000
211.0000000	0.0500000
212.0000000	0.0500000
213.0000000	0.0500000
214.0000000	0.0500000
215.0000000	0.0500000
216.0000000	0.0500000
217.0000000	0.0500000
218.0000000	0.0500000
219.0000000	0.0495238
220.0000000	0.0500000
221.0000000	0.0500000
222.0000000	0.0500000
223.0000000	0.0500000
224.0000000	0.0500000
225.0000000	0.0510000
226.0000000	0.0514286
227.0000000	0.0500000
228.0000000	0.0500000
229.0000000	0.0505000
230.0000000	0.0500000
231.0000000	0.0505000
232.0000000	0.0519048
233.0000000	0.0510000
234.0000000	0.0523810
235.0000000	0.0515000
236.0000000	0.0500000
237.0000000	0.0504762
238.0000000	0.0500000
239.0000000	0.0500000
240.0000000	0.0500000
241.0000000	0.0509524
242.0000000	0.0500000
243.0000000	0.0504762
244.0000000	0.0505000
245.0000000	0.0505000
246.0000000	0.0500000
247.0000000	0.0500000
248.0000000	0.0500000
249.0000000	0.0505000
250.0000000	0.0500000
251.0000000	0.0505000
252.0000000	0.0514286
253.0000000	0.0500000
254.0000000	0.0530000
255.0000000	0.0557143
256.0000000	0.0565000
257.0000000	0.0585000
258.0000000	0.0590000
259.0000000	0.0595238
260.0000000	0.0600000

261.0000000	0.0595000
262.0000000	0.0600000
263.0000000	0.0600000
264.0000000	0.0600000
265.0000000	0.0600000
266.0000000	0.0600000
267.0000000	0.0600000
268.0000000	0.0595000
269.0000000	0.0595000
270.0000000	0.0600000
271.0000000	0.0595238
272.0000000	0.0595000
273.0000000	0.0600000
274.0000000	0.0600000
275.0000000	0.0600000
276.0000000	0.0600000
277.0000000	0.0600000
278.0000000	0.0595000
279.0000000	0.0600000
280.0000000	0.0595238
281.0000000	0.0600000
282.0000000	0.0600000
283.0000000	0.0600000
284.0000000	0.0600000
285.0000000	0.0600000
286.0000000	0.0590476
287.0000000	0.0600000
288.0000000	0.0595000
289.0000000	0.0600000
290.0000000	0.0600000
291.0000000	0.0590476
292.0000000	0.0600000
293.0000000	0.0600000
294.0000000	0.0600000
295.0000000	0.0600000
296.0000000	0.0600000
297.0000000	0.0600000
298.0000000	0.0590476
299.0000000	0.0590000
300.0000000	0.0600000
301.0000000	0.0600000
302.0000000	0.0595000
303.0000000	0.0600000
304.0000000	0.0600000
305.0000000	0.0600000
306.0000000	0.0600000
307.0000000	0.0595238
308.0000000	0.0600000
309.0000000	0.0600000
310.0000000	0.0595238
311.0000000	0.0580000
312.0000000	0.0595238
313.0000000	0.0600000
314.0000000	0.0600000
315.0000000	0.0600000
316.0000000	0.0600000
317.0000000	0.0600000

318.0000000	0.0600000
319.0000000	0.0600000
320.0000000	0.0600000
321.0000000	0.0605000
322.0000000	0.0604762
323.0000000	0.0600000
324.0000000	0.0600000
325.0000000	0.0619048
326.0000000	0.0600000
327.0000000	0.0605000
328.0000000	0.0623809
329.0000000	0.0630000
330.0000000	0.0620000
331.0000000	0.0633333
332.0000000	0.0615000
333.0000000	0.0620000
334.0000000	0.0633333
335.0000000	0.0635000
336.0000000	0.0635000
337.0000000	0.0615000
338.0000000	0.0609524
339.0000000	0.0605000
340.0000000	0.0614286
341.0000000	0.0605000
342.0000000	0.0605000
343.0000000	0.0619047
344.0000000	0.0605000
345.0000000	0.0605000
346.0000000	0.0605000
347.0000000	0.0619048
348.0000000	0.0605000
349.0000000	0.0633333
350.0000000	0.0615000
351.0000000	0.0635000
352.0000000	0.0657143
353.0000000	0.0640000
354.0000000	0.0670000
355.0000000	0.0680953
356.0000000	0.0684211
357.0000000	0.0685714
358.0000000	0.0700000
359.0000000	0.0700000
360.0000000	0.0695000
361.0000000	0.0690000
362.0000000	0.0690476
363.0000000	0.0675000
364.0000000	0.0676190
365.0000000	0.0655000
366.0000000	0.0655000
367.0000000	0.0660000
368.0000000	0.0680953
369.0000000	0.0660000
370.0000000	0.0671429
371.0000000	0.0665000
372.0000000	0.0652381
373.0000000	0.0665000
374.0000000	0.0650000

375.0000000	0.0615000
376.0000000	0.0660000
377.0000000	0.0633333
378.0000000	0.0625000
379.0000000	0.0638095
380.0000000	0.0650000
381.0000000	0.0640000
382.0000000	0.0650000
383.0000000	0.0638095
384.0000000	0.0645000
385.0000000	0.0660000
386.0000000	0.0661905
387.0000000	0.0650000
388.0000000	0.0671429
389.0000000	0.0675000
390.0000000	0.0670000
391.0000000	0.0685000
392.0000000	0.0647619
393.0000000	0.0685000
394.0000000	0.0676190
395.0000000	0.0685000
396.0000000	0.0675000
397.0000000	0.0670000
398.0000000	0.0661905
399.0000000	0.0680000
400.0000000	0.0676191
401.0000000	0.0680000
402.0000000	0.0660000
403.0000000	0.0676191
404.0000000	0.0665000
405.0000000	0.0670000
406.0000000	0.0655000
407.0000000	0.0661905
408.0000000	0.0670000
409.0000000	0.0671429
410.0000000	0.0655000
411.0000000	0.0650000
412.0000000	0.0666667
413.0000000	0.0645000
414.0000000	0.0650000
415.0000000	0.0635000
416.0000000	0.0642857
417.0000000	0.0633333
418.0000000	0.0605000
419.0000000	0.0620000
420.0000000	0.0610000
421.0000000	0.0605000
422.0000000	0.0600000
423.0000000	0.0605000
424.0000000	0.0600000
425.0000000	0.0600000
426.0000000	0.0600000
427.0000000	0.0595000
428.0000000	0.0571428
429.0000000	0.0590000
430.0000000	0.0580000
431.0000000	0.0590476

432.0000000	0.0580000
433.0000000	0.0580952
434.0000000	0.0555000
435.0000000	0.0545000
436.0000000	0.0535000
437.0000000	0.0557143
438.0000000	0.0540000
439.0000000	0.0550000
440.0000000	0.0566667
441.0000000	0.0545000
442.0000000	0.0545000
443.0000000	0.0552381
444.0000000	0.0520000
445.0000000	0.0510000
446.0000000	0.0523810
447.0000000	0.0505000
448.0000000	0.0509524
449.0000000	0.0500000
450.0000000	0.0500000
451.0000000	0.0500000
452.0000000	0.0504762
453.0000000	0.0505000
454.0000000	0.0500000
455.0000000	0.0500000
456.0000000	0.0500000
457.0000000	0.0500000
458.0000000	0.0495238
459.0000000	0.0500000
460.0000000	0.0500000
461.0000000	0.0500000
462.0000000	0.0495000
463.0000000	0.0500000
464.0000000	0.0500000
465.0000000	0.0500000
466.0000000	0.0500000
467.0000000	0.0500000
468.0000000	0.0500000
469.0000000	0.0500000
470.0000000	0.0505000
471.0000000	0.0510000
472.0000000	0.0510000
473.0000000	0.0509524
474.0000000	0.0530000
475.0000000	0.0540000
476.0000000	0.0519048
477.0000000	0.0515000
478.0000000	0.0519048
479.0000000	0.0525000
480.0000000	0.0535000
481.0000000	0.0545000
482.0000000	0.0547619
483.0000000	0.0550000
484.0000000	0.0535000
485.0000000	0.0552381
486.0000000	0.0535000
487.0000000	0.0530000
488.0000000	0.0504762

489.0000000	0.0515000
490.0000000	0.0510000
491.0000000	0.0504762
492.0000000	0.0505000
493.0000000	0.0510000
494.0000000	0.0504762
495.0000000	0.0505000
496.0000000	0.0500000
497.0000000	0.0500000
498.0000000	0.0500000
499.0000000	0.0500000
500.0000000	0.0500000
501.0000000	0.0500000
502.0000000	0.0500000
503.0000000	0.0505000
504.0000000	0.0500000
505.0000000	0.0504762
506.0000000	0.0500000
507.0000000	0.0509524
508.0000000	0.0500000
509.0000000	0.0523810
510.0000000	0.0535000
511.0000000	0.0540000
512.0000000	0.0542857
513.0000000	0.0550000
514.0000000	0.0535000
515.0000000	0.0523810
516.0000000	0.0520000
517.0000000	0.0540000
518.0000000	0.0525000
519.0000000	0.0514286
520.0000000	0.0515000
521.0000000	0.0500000
522.0000000	0.0500000
523.0000000	0.0500000
524.0000000	0.0495238
525.0000000	0.0495000
526.0000000	0.0490000
527.0000000	0.0490000
528.0000000	0.0480952
529.0000000	0.0495000
530.0000000	0.0495238
531.0000000	0.0500000
532.0000000	0.0495000
533.0000000	0.0500000
534.0000000	0.0500000
535.0000000	0.0495000
536.0000000	0.0500000
537.0000000	0.0505000
538.0000000	0.0520000
539.0000000	0.0514286
540.0000000	0.0510000
541.0000000	0.0505000
542.0000000	0.0505000
543.0000000	0.0523810
544.0000000	0.0530000
545.0000000	0.0523810

546.0000000	0.0555000
547.0000000	0.0530000
548.0000000	0.0528571
549.0000000	0.0510000
550.0000000	0.0505000
551.0000000	0.0505000
552.0000000	0.0514286
553.0000000	0.0520000
554.0000000	0.0509524
555.0000000	0.0500000
556.0000000	0.0505000
557.0000000	0.0514286
558.0000000	0.0530000
559.0000000	0.0505000
560.0000000	0.0509524
561.0000000	0.0505000
562.0000000	0.0500000
563.0000000	0.0500000
564.0000000	0.0500000
565.0000000	0.0500000
566.0000000	0.0500000
567.0000000	0.0500000
568.0000000	0.0500000
569.0000000	0.0500000
570.0000000	0.0500000
571.0000000	0.0500000
572.0000000	0.0500000
573.0000000	0.0500000
574.0000000	0.0500000
575.0000000	0.0500000
576.0000000	0.0500000
577.0000000	0.0500000
578.0000000	0.0500000
579.0000000	0.0500000
580.0000000	0.0500000
581.0000000	0.0500000
582.0000000	0.0495238
583.0000000	0.0490000
584.0000000	0.0480952
585.0000000	0.0490000
586.0000000	0.0490000
587.0000000	0.0495000
588.0000000	0.0495238
589.0000000	0.0495000
590.0000000	0.0495000
591.0000000	0.0490476
592.0000000	0.0500000
593.0000000	0.0500000
594.0000000	0.0490000
595.0000000	0.0500000
596.0000000	0.0500000
597.0000000	0.0500000
598.0000000	0.0500000
599.0000000	0.0500000
600.0000000	0.0500000
601.0000000	0.0500000
602.0000000	0.0495000

603.0000000	0.0500000
604.0000000	0.0495000
605.0000000	0.0490476
606.0000000	0.0500000
607.0000000	0.0495000
608.0000000	0.0480000
609.0000000	0.0500000
610.0000000	0.0485000
611.0000000	0.0475000
612.0000000	0.0480952
613.0000000	0.0460000
614.0000000	0.0452381
615.0000000	0.0465000
616.0000000	0.0465000
617.0000000	0.0485000
618.0000000	0.0471429
619.0000000	0.0460000
620.0000000	0.0445000
621.0000000	0.0447619
622.0000000	0.0445000
623.0000000	0.0455000
624.0000000	0.0442857
625.0000000	0.0450000
626.0000000	0.0455000
627.0000000	0.0452381
628.0000000	0.0455000
629.0000000	0.0466667
630.0000000	0.0420000
631.0000000	0.0460000
632.0000000	0.0440000
633.0000000	0.0476191
634.0000000	0.0460000
635.0000000	0.0460000
636.0000000	0.0447619
637.0000000	0.0475000
638.0000000	0.0485000
639.0000000	0.0465000
640.0000000	0.0490476
641.0000000	0.0490000
642.0000000	0.0490476
643.0000000	0.0495000
644.0000000	0.0495000
645.0000000	0.0500000
646.0000000	0.0500000
647.0000000	0.0490000
648.0000000	0.0500000
649.0000000	0.0500000
650.0000000	0.0500000
651.0000000	0.0500000
652.0000000	0.0500000
653.0000000	0.0500000
654.0000000	0.0495238
655.0000000	0.0500000
656.0000000	0.0500000
657.0000000	0.0500000
658.0000000	0.0500000
659.0000000	0.0490000

660.0000000	0.0500000
661.0000000	0.0500000
662.0000000	0.0500000
663.0000000	0.0490476
664.0000000	0.0485000
665.0000000	0.0480952
666.0000000	0.0485000
667.0000000	0.0480000
668.0000000	0.0475000
669.0000000	0.0495000
670.0000000	0.0471429
671.0000000	0.0485000
672.0000000	0.0480952
673.0000000	0.0480000
674.0000000	0.0485714
675.0000000	0.0470000
676.0000000	0.0475000
677.0000000	0.0460000
678.0000000	0.0425000
679.0000000	0.0423810
680.0000000	0.0435000
681.0000000	0.0419048
682.0000000	0.0425000
683.0000000	0.0430000
684.0000000	0.0430000
685.0000000	0.0433333
686.0000000	0.0430000
687.0000000	0.0445000
688.0000000	0.0452381
689.0000000	0.0465000
690.0000000	0.0476191
691.0000000	0.0490000
692.0000000	0.0475000
693.0000000	0.0485714
694.0000000	0.0480000
695.0000000	0.0485000
696.0000000	0.0475000
697.0000000	0.0485714
698.0000000	0.0495000
699.0000000	0.0490000
700.0000000	0.0480952
701.0000000	0.0490000
702.0000000	0.0485714
703.0000000	0.0485000
704.0000000	0.0490000
705.0000000	0.0500000
706.0000000	0.0495000
707.0000000	0.0500000
708.0000000	0.0495000
709.0000000	0.0495238
710.0000000	0.0495238
711.0000000	0.0495000
712.0000000	0.0500000
713.0000000	0.0495000
714.0000000	0.0495000
715.0000000	0.0500000
716.0000000	0.0495000

717.0000000	0.0500000
718.0000000	0.0500000
719.0000000	0.0490000
720.0000000	0.0476191
721.0000000	0.0480000
722.0000000	0.0470000
723.0000000	0.0480000
724.0000000	0.0471429
725.0000000	0.0460000
726.0000000	0.0452381
727.0000000	0.0460000
728.0000000	0.0450000
729.0000000	0.0450000
730.0000000	0.0466667
731.0000000	0.0475000
732.0000000	0.0475000
733.0000000	0.0442857
734.0000000	0.0470000
735.0000000	0.0452381
736.0000000	0.0470000
737.0000000	0.0470000
738.0000000	0.0455000
739.0000000	0.0457143
740.0000000	0.0460000
741.0000000	0.0480952
742.0000000	0.0495000
743.0000000	0.0495000
744.0000000	0.0495000
745.0000000	0.0490476
746.0000000	0.0495000
747.0000000	0.0495000
748.0000000	0.0480000
749.0000000	0.0480952
750.0000000	0.0490476
751.0000000	0.0485000
752.0000000	0.0485000
753.0000000	0.0490000
754.0000000	0.0476191
755.0000000	0.0475000
756.0000000	0.0490000
757.0000000	0.0490476
758.0000000	0.0485000
759.0000000	0.0480000
760.0000000	0.0476191
761.0000000	0.0455000
762.0000000	0.0457143
763.0000000	0.0460000
764.0000000	0.0465000
765.0000000	0.0466667
766.0000000	0.0475000
767.0000000	0.0480000
768.0000000	0.0485000
769.0000000	0.0476191
770.0000000	0.0485000
771.0000000	0.0485714
772.0000000	0.0485000
773.0000000	0.0480000

774.0000000	0.0490000
775.0000000	0.0480000
776.0000000	0.0485714
777.0000000	0.0475000
778.0000000	0.0490476
779.0000000	0.0500000
780.0000000	0.0500000
781.0000000	0.0500000
782.0000000	0.0495000
783.0000000	0.0500000
784.0000000	0.0500000
785.0000000	0.0500000
786.0000000	0.0500000
787.0000000	0.0495238
788.0000000	0.0500000
789.0000000	0.0500000
790.0000000	0.0500000
791.0000000	0.0500000
792.0000000	0.0500000
793.0000000	0.0500000
794.0000000	0.0490000
795.0000000	0.0500000
796.0000000	0.0495238
797.0000000	0.0485000
798.0000000	0.0485714
799.0000000	0.0490000
800.0000000	0.0495000
801.0000000	0.0490000
802.0000000	0.0500000
803.0000000	0.0495000
804.0000000	0.0500000
805.0000000	0.0500000
806.0000000	0.0500000
807.0000000	0.0495000
808.0000000	0.0500000
809.0000000	0.0500000
810.0000000	0.0495238
811.0000000	0.0500000
812.0000000	0.0500000
813.0000000	0.0500000
814.0000000	0.0500000
815.0000000	0.0500000
816.0000000	0.0500000
817.0000000	0.0500000
818.0000000	0.0500000
819.0000000	0.0500000
820.0000000	0.0500000
821.0000000	0.0500000
822.0000000	0.0500000
823.0000000	0.0500000
824.0000000	0.0500000
825.0000000	0.0500000
826.0000000	0.0500000
827.0000000	0.0505000
828.0000000	0.0500000
829.0000000	0.0500000
830.0000000	0.0500000

831.0000000	0.0500000
832.0000000	0.0504762
833.0000000	0.0500000
834.0000000	0.0500000
835.0000000	0.0505000
836.0000000	0.0500000
837.0000000	0.0500000
838.0000000	0.0500000
839.0000000	0.0495000
840.0000000	0.0485000
841.0000000	0.0480952
842.0000000	0.0480000
843.0000000	0.0465000
844.0000000	0.0475000
845.0000000	0.0461905
846.0000000	0.0470000
847.0000000	0.0466667
848.0000000	0.0475000
849.0000000	0.0470000
850.0000000	0.0452381
851.0000000	0.0465000
852.0000000	0.0445000
853.0000000	0.0460000
854.0000000	0.0452381
855.0000000	0.0440000
856.0000000	0.0457143
857.0000000	0.0480000
858.0000000	0.0445000
859.0000000	0.0490476
860.0000000	0.0475000
861.0000000	0.0470000
862.0000000	0.0476191
863.0000000	0.0465000
864.0000000	0.0490000
865.0000000	0.0455000
866.0000000	0.0480952
867.0000000	0.0495000
868.0000000	0.0461905
869.0000000	0.0475000
870.0000000	0.0480000
871.0000000	0.0457143
872.0000000	0.0455000
873.0000000	0.0450000
874.0000000	0.0450000
875.0000000	0.0438095
876.0000000	0.0455000
877.0000000	0.0438095
878.0000000	0.0440000
879.0000000	0.0455000
880.0000000	0.0465000
881.0000000	0.0471429
882.0000000	0.0480000
883.0000000	0.0470000
884.0000000	0.0490476
885.0000000	0.0480000
886.0000000	0.0500000
887.0000000	0.0495000

888.0000000	0.0495000
889.0000000	0.0485000
890.0000000	0.0490476
891.0000000	0.0490000
892.0000000	0.0475000
893.0000000	0.0461905
894.0000000	0.0460000
895.0000000	0.0470000
896.0000000	0.0476191
897.0000000	0.0485000
898.0000000	0.0495238
899.0000000	0.0480000
900.0000000	0.0475000
901.0000000	0.0480000
902.0000000	0.0471429
903.0000000	0.0485000
904.0000000	0.0475000
905.0000000	0.0485714
906.0000000	0.0475000
907.0000000	0.0490476
908.0000000	0.0480000
909.0000000	0.0480000
910.0000000	0.0485000
911.0000000	0.0495238
912.0000000	0.0480000
913.0000000	0.0480000
914.0000000	0.0500000
915.0000000	0.0495238
916.0000000	0.0500000
917.0000000	0.0495000
918.0000000	0.0485000
919.0000000	0.0485000
920.0000000	0.0480952
921.0000000	0.0460000
922.0000000	0.0480952
923.0000000	0.0485000
924.0000000	0.0480000
925.0000000	0.0480000
926.0000000	0.0480952
927.0000000	0.0485000
928.0000000	0.0460000
929.0000000	0.0466667
930.0000000	0.0490000
931.0000000	0.0485714
932.0000000	0.0490000
933.0000000	0.0485000
934.0000000	0.0470000
935.0000000	0.0485714
936.0000000	0.0480000
937.0000000	0.0485000
938.0000000	0.0490476
939.0000000	0.0470000
940.0000000	0.0495000
941.0000000	0.0485000
942.0000000	0.0476191
943.0000000	0.0480000
944.0000000	0.0490476

945.0000000	0.0500000
946.0000000	0.0500000
947.0000000	0.0475000
948.0000000	0.0500000
949.0000000	0.0500000
950.0000000	0.0500000
951.0000000	0.0495000
952.0000000	0.0500000
953.0000000	0.0500000
954.0000000	0.0495238
955.0000000	0.0500000
956.0000000	0.0500000
957.0000000	0.0500000
958.0000000	0.0495000
959.0000000	0.0476191
960.0000000	0.0460000
961.0000000	0.0470000
962.0000000	0.0457143
963.0000000	0.0455000
964.0000000	0.0430000
965.0000000	0.0452381
966.0000000	0.0455000
967.0000000	0.0476191
968.0000000	0.0460000
969.0000000	0.0475000
970.0000000	0.0480000
971.0000000	0.0495000
972.0000000	0.0480952
973.0000000	0.0480000
974.0000000	0.0476191
975.0000000	0.0490000
976.0000000	0.0495238
977.0000000	0.0480000
978.0000000	0.0495000
979.0000000	0.0490000
980.0000000	0.0480000
981.0000000	0.0490476
982.0000000	0.0490000
983.0000000	0.0480952
984.0000000	0.0485000
985.0000000	0.0475000
986.0000000	0.0480952
987.0000000	0.0495000
988.0000000	0.0490000
989.0000000	0.0480000
990.0000000	0.0485714
991.0000000	0.0465000
992.0000000	0.0457143
993.0000000	0.0435000
994.0000000	0.0425000
995.0000000	0.0438095
996.0000000	0.0420000
997.0000000	0.0425000
998.0000000	0.0414286
999.0000000	0.0420000
1000.0000000	0.0400000
1001.0000000	0.0400000

1002.0000000	0.0409524
1003.0000000	0.0414286
1004.0000000	0.0415000
1005.0000000	0.0405000
1006.0000000	0.0415000
1007.0000000	0.0423810
1008.0000000	0.0410000
1009.0000000	0.0430000
1010.0000000	0.0419048
1011.0000000	0.0410000
1012.0000000	0.0435000
1013.0000000	0.0425000
1014.0000000	0.0409524
1015.0000000	0.0405000
1016.0000000	0.0405000
1017.0000000	0.0400000
1018.0000000	0.0400000
1019.0000000	0.0400000
1020.0000000	0.0400000
1021.0000000	0.0405000
1022.0000000	0.0404762
1023.0000000	0.0405000
1024.0000000	0.0400000
1025.0000000	0.0400000
1026.0000000	0.0409524
1027.0000000	0.0400000
1028.0000000	0.0400000
1029.0000000	0.0400000
1030.0000000	0.0400000
1031.0000000	0.0415000
1032.0000000	0.0423810
1033.0000000	0.0415000
1034.0000000	0.0440000
1035.0000000	0.0438095
1036.0000000	0.0445000
1037.0000000	0.0419048
1038.0000000	0.0450000
1039.0000000	0.0435000
1040.0000000	0.0420000
1041.0000000	0.0430000
1042.0000000	0.0423810
1043.0000000	0.0428572
1044.0000000	0.0435000
1045.0000000	0.0435000
1046.0000000	0.0440000
1047.0000000	0.0419048
1048.0000000	0.0410000
1049.0000000	0.0420000
1050.0000000	0.0409524
1051.0000000	0.0405000
1052.0000000	0.0409524
1053.0000000	0.0405000
1054.0000000	0.0420000
1055.0000000	0.0414286
1056.0000000	0.0410000
1057.0000000	0.0415000
1058.0000000	0.0405000

1059.0000000	0.0409524
1060.0000000	0.0410000
1061.0000000	0.0415000
1062.0000000	0.0404762
1063.0000000	0.0405000
1064.0000000	0.0404762
1065.0000000	0.0400000
1066.0000000	0.0410000
1067.0000000	0.0414286
1068.0000000	0.0405000
1069.0000000	0.0405000
1070.0000000	0.0415000
1071.0000000	0.0419048
1072.0000000	0.0415000
1073.0000000	0.0423810
1074.0000000	0.0440000
1075.0000000	0.0415000
1076.0000000	0.0420000
1077.0000000	0.0405000
1078.0000000	0.0414286
1079.0000000	0.0410000
1080.0000000	0.0419048
1081.0000000	0.0405000
1082.0000000	0.0419048
1083.0000000	0.0410000
1084.0000000	0.0425000
1085.0000000	0.0410000
1086.0000000	0.0414286
1087.0000000	0.0425000
1088.0000000	0.0405000
1089.0000000	0.0423810
1090.0000000	0.0400000
1091.0000000	0.0404762
1092.0000000	0.0410000
1093.0000000	0.0400000
1094.0000000	0.0400000
1095.0000000	0.0400000
1096.0000000	0.0405000
1097.0000000	0.0400000
1098.0000000	0.0409524
1099.0000000	0.0405000
1100.0000000	0.0404762
1101.0000000	0.0405000
1102.0000000	0.0405000
1103.0000000	0.0400000
1104.0000000	0.0400000
1105.0000000	0.0405000
1106.0000000	0.0400000
1107.0000000	0.0404762
1108.0000000	0.0410000
1109.0000000	0.0405000
1110.0000000	0.0400000
1111.0000000	0.0400000
1112.0000000	0.0404762
1113.0000000	0.0400000
1114.0000000	0.0400000
1115.0000000	0.0400000

1116.0000000	0.0404762
1117.0000000	0.0405000
1118.0000000	0.0410000
1119.0000000	0.0400000
1120.0000000	0.0405000
1121.0000000	0.0405000
1122.0000000	0.0405000
1123.0000000	0.0404762
1124.0000000	0.0405000
1125.0000000	0.0395238
1126.0000000	0.0410000
1127.0000000	0.0400000
1128.0000000	0.0405000
1129.0000000	0.0400000
1130.0000000	0.0400000
1131.0000000	0.0400000
1132.0000000	0.0400000
1133.0000000	0.0400000
1134.0000000	0.0404762
1135.0000000	0.0400000
1136.0000000	0.0405000
1137.0000000	0.0400000
1138.0000000	0.0400000
1139.0000000	0.0400000
1140.0000000	0.0400000
1141.0000000	0.0405000
1142.0000000	0.0400000
1143.0000000	0.0404762
1144.0000000	0.0400000
1145.0000000	0.0400000
1146.0000000	0.0400000
1147.0000000	0.0400000
1148.0000000	0.0405000
1149.0000000	0.0400000
1150.0000000	0.0405000
1151.0000000	0.0400000
1152.0000000	0.0400000
1153.0000000	0.0400000
1154.0000000	0.0400000
1155.0000000	0.0400000
1156.0000000	0.0400000
1157.0000000	0.0400000
1158.0000000	0.0400000
1159.0000000	0.0400000
1160.0000000	0.0400000
1161.0000000	0.0400000
1162.0000000	0.0400000
1163.0000000	0.0410000
1164.0000000	0.0400000
1165.0000000	0.0400000
1166.0000000	0.0400000
1167.0000000	0.0400000
1168.0000000	0.0400000
1169.0000000	0.0400000
1170.0000000	0.0400000
1171.0000000	0.0400000
1172.0000000	0.0405000

1173.0000000	0.0400000
1174.0000000	0.0400000
1175.0000000	0.0400000
1176.0000000	0.0400000
1177.0000000	0.0400000
1178.0000000	0.0400000
1179.0000000	0.0404762
1180.0000000	0.0400000
1181.0000000	0.0400000
1182.0000000	0.0400000
1183.0000000	0.0400000
1184.0000000	0.0405000
1185.0000000	0.0400000
1186.0000000	0.0400000
1187.0000000	0.0400000
1188.0000000	0.0400000
1189.0000000	0.0400000
1190.0000000	0.0400000
1191.0000000	0.0400000
1192.0000000	0.0400000
1193.0000000	0.0400000
1194.0000000	0.0400000
1195.0000000	0.0400000
1196.0000000	0.0400000
1197.0000000	0.0405000
1198.0000000	0.0400000
1199.0000000	0.0400000
1200.0000000	0.0409524
1201.0000000	0.0410000
1202.0000000	0.0410000
1203.0000000	0.0409524
1204.0000000	0.0425000
1205.0000000	0.0430000
1206.0000000	0.0425000
1207.0000000	0.0423810
1208.0000000	0.0455000
1209.0000000	0.0442857
1210.0000000	0.0460000
1211.0000000	0.0425000
1212.0000000	0.0465000
1213.0000000	0.0452381
1214.0000000	0.0455000
1215.0000000	0.0471429
1216.0000000	0.0480000
1217.0000000	0.0475000
1218.0000000	0.0471429
1219.0000000	0.0465000
1220.0000000	0.0470000
1221.0000000	0.0460000
1222.0000000	0.0476191
1223.0000000	0.0460000
1224.0000000	0.0452381
1225.0000000	0.0445000
1226.0000000	0.0430000
1227.0000000	0.0420000
1228.0000000	0.0400000
1229.0000000	0.0405000

1230.0000000	0.0405000
1231.0000000	0.0409524
1232.0000000	0.0400000
1233.0000000	0.0405000
1234.0000000	0.0400000
1235.0000000	0.0400000
1236.0000000	0.0410000
1237.0000000	0.0404762
1238.0000000	0.0400000
1239.0000000	0.0400000
1240.0000000	0.0400000
1241.0000000	0.0400000
1242.0000000	0.0400000
1243.0000000	0.0400000
1244.0000000	0.0400000
1245.0000000	0.0400000
1246.0000000	0.0400000
1247.0000000	0.0400000
1248.0000000	0.0400000
1249.0000000	0.0400000
1250.0000000	0.0400000
1251.0000000	0.0400000
1252.0000000	0.0404762
1253.0000000	0.0405000
1254.0000000	0.0410000
1255.0000000	0.0400000
1256.0000000	0.0400000
1257.0000000	0.0415000
1258.0000000	0.0414286
1259.0000000	0.0400000
1260.0000000	0.0400000
1261.0000000	0.0400000
1262.0000000	0.0405000
1263.0000000	0.0405000
1264.0000000	0.0409524
1265.0000000	0.0425000
1266.0000000	0.0420000
1267.0000000	0.0447619
1268.0000000	0.0460000
1269.0000000	0.0460000
1270.0000000	0.0461905
1271.0000000	0.0470000
1272.0000000	0.0445000
1273.0000000	0.0460000
1274.0000000	0.0471429
1275.0000000	0.0470000
1276.0000000	0.0485714
1277.0000000	0.0475000
1278.0000000	0.0470000
1279.0000000	0.0480952
1280.0000000	0.0445000
1281.0000000	0.0460000
1282.0000000	0.0445000
1283.0000000	0.0438095
1284.0000000	0.0450000
1285.0000000	0.0461905
1286.0000000	0.0455000

1287.0000000	0.0420000
1288.0000000	0.0447619
1289.0000000	0.0445000
1290.0000000	0.0450000
1291.0000000	0.0442857
1292.0000000	0.0455000
1293.0000000	0.0450000
1294.0000000	0.0438095
1295.0000000	0.0435000
1296.0000000	0.0435000
1297.0000000	0.0423810
1298.0000000	0.0430000
1299.0000000	0.0405000
1300.0000000	0.0409524
1301.0000000	0.0410000
1302.0000000	0.0405000
1303.0000000	0.0405000
1304.0000000	0.0404762
1305.0000000	0.0410000
1306.0000000	0.0419048
1307.0000000	0.0405000
1308.0000000	0.0404762
1309.0000000	0.0405000
1310.0000000	0.0410000
1311.0000000	0.0410000
1312.0000000	0.0400000
1313.0000000	0.0400000
1314.0000000	0.0400000
1315.0000000	0.0400000
1316.0000000	0.0400000
1317.0000000	0.0400000
1318.0000000	0.0400000
1319.0000000	0.0400000
1320.0000000	0.0400000
1321.0000000	0.0400000
1322.0000000	0.0400000
1323.0000000	0.0400000
1324.0000000	0.0400000
1325.0000000	0.0405000
1326.0000000	0.0400000
1327.0000000	0.0405000
1328.0000000	0.0400000
1329.0000000	0.0400000
1330.0000000	0.0410000
1331.0000000	0.0404762
1332.0000000	0.0400000
1333.0000000	0.0400000
1334.0000000	0.0410000
1335.0000000	0.0404762
1336.0000000	0.0414286
1337.0000000	0.0410000
1338.0000000	0.0410000
1339.0000000	0.0419048
1340.0000000	0.0420000
1341.0000000	0.0405000
1342.0000000	0.0415000
1343.0000000	0.0419048

1344.0000000	0.0420000
1345.0000000	0.0404762
1346.0000000	0.0420000
1347.0000000	0.0405000
1348.0000000	0.0425000
1349.0000000	0.0433333
1350.0000000	0.0420000
1351.0000000	0.0420000
1352.0000000	0.0419048
1353.0000000	0.0415000
1354.0000000	0.0409524
1355.0000000	0.0405000
1356.0000000	0.0405000
1357.0000000	0.0405000
1358.0000000	0.0404762
1359.0000000	0.0400000
1360.0000000	0.0404762
1361.0000000	0.0425000
1362.0000000	0.0410000
1363.0000000	0.0410000
1364.0000000	0.0404762
1365.0000000	0.0415000
1366.0000000	0.0405000
1367.0000000	0.0423810
1368.0000000	0.0420000
1369.0000000	0.0414286
1370.0000000	0.0425000
1371.0000000	0.0415000
1372.0000000	0.0415000
1373.0000000	0.0428572
1374.0000000	0.0405000
1375.0000000	0.0415000
1376.0000000	0.0414286
1377.0000000	0.0415000
1378.0000000	0.0410000
1379.0000000	0.0433333
1380.0000000	0.0425000
1381.0000000	0.0435000
1382.0000000	0.0414286
1383.0000000	0.0445000
1384.0000000	0.0414286
1385.0000000	0.0420000
1386.0000000	0.0430000
1387.0000000	0.0455000
1388.0000000	0.0471429
1389.0000000	0.0475000
1390.0000000	0.0465000
1391.0000000	0.0452381
1392.0000000	0.0445000
1393.0000000	0.0470000
1394.0000000	0.0457143
1395.0000000	0.0480000
1396.0000000	0.0452381
1397.0000000	0.0478947
1398.0000000	0.0461905
1399.0000000	0.0455000
1400.0000000	0.0452381

1401.0000000	0.0475000
1402.0000000	0.0450000
1403.0000000	0.0442857
1404.0000000	0.0435000
1405.0000000	0.0447619
1406.0000000	0.0465000
1407.0000000	0.0460000
1408.0000000	0.0435000
1409.0000000	0.0457143
1410.0000000	0.0450000
1411.0000000	0.0445000
1412.0000000	0.0442857
1413.0000000	0.0435000
1414.0000000	0.0447619
1415.0000000	0.0420000
1416.0000000	0.0435000
1417.0000000	0.0460000
1418.0000000	0.0452381
1419.0000000	0.0495000
1420.0000000	0.0480000
1421.0000000	0.0480952
1422.0000000	0.0500000
1423.0000000	0.0500000
1424.0000000	0.0495238
1425.0000000	0.0490000
1426.0000000	0.0490000
1427.0000000	0.0500000
1428.0000000	0.0490000
1429.0000000	0.0495000
1430.0000000	0.0476191
1431.0000000	0.0475000
1432.0000000	0.0475000
1433.0000000	0.0480952
1434.0000000	0.0485000
1435.0000000	0.0460000
1436.0000000	0.0438095
1437.0000000	0.0440000
1438.0000000	0.0410000
1439.0000000	0.0420000
1440.0000000	0.0409524
1441.0000000	0.0400000
1442.0000000	0.0404762
1443.0000000	0.0400000
1444.0000000	0.0400000
1445.0000000	0.0423810
1446.0000000	0.0415000
1447.0000000	0.0425000
1448.0000000	0.0423810
1449.0000000	0.0455000
1450.0000000	0.0465000
1451.0000000	0.0480000
1452.0000000	0.0466667
1453.0000000	0.0455000
1454.0000000	0.0475000
1455.0000000	0.0452381
1456.0000000	0.0485000
1457.0000000	0.0471429

1458.0000000	0.0450000
1459.0000000	0.0450000
1460.0000000	0.0452381
1461.0000000	0.0480000
1462.0000000	0.0480000
1463.0000000	0.0470000
1464.0000000	0.0476191
1465.0000000	0.0460000
1466.0000000	0.0480952
1467.0000000	0.0465000
1468.0000000	0.0450000
1469.0000000	0.0430000
1470.0000000	0.0419048
1471.0000000	0.0445000
1472.0000000	0.0433333
1473.0000000	0.0410000
1474.0000000	0.0420000
1475.0000000	0.0423810
1476.0000000	0.0440000
1477.0000000	0.0450000
1478.0000000	0.0450000
1479.0000000	0.0438095
1480.0000000	0.0460000
1481.0000000	0.0457143
1482.0000000	0.0455000
1483.0000000	0.0485000
1484.0000000	0.0490000
1485.0000000	0.0490476
1486.0000000	0.0480000
1487.0000000	0.0475000
1488.0000000	0.0485714
1489.0000000	0.0480000
1490.0000000	0.0485000
1491.0000000	0.0500000
1492.0000000	0.0500000
1493.0000000	0.0500000
1494.0000000	0.0500000
1495.0000000	0.0500000
1496.0000000	0.0500000
1497.0000000	0.0500000
1498.0000000	0.0500000
1499.0000000	0.0500000
1500.0000000	0.0504762
1501.0000000	0.0500000
1502.0000000	0.0500000
1503.0000000	0.0500000
1504.0000000	0.0500000
1505.0000000	0.0500000
1506.0000000	0.0500000
1507.0000000	0.0500000
1508.0000000	0.0500000
1509.0000000	0.0500000
1510.0000000	0.0500000
1511.0000000	0.0500000
1512.0000000	0.0500000
1513.0000000	0.0500000
1514.0000000	0.0500000

1515.0000000	0.0500000
1516.0000000	0.0500000
1517.0000000	0.0500000
1518.0000000	0.0505000
1519.0000000	0.0500000
1520.0000000	0.0500000
1521.0000000	0.0500000
1522.0000000	0.0500000
1523.0000000	0.0500000
1524.0000000	0.0504762
1525.0000000	0.0500000
1526.0000000	0.0510000
1527.0000000	0.0514286
1528.0000000	0.0515000
1529.0000000	0.0520000
1530.0000000	0.0504762
1531.0000000	0.0500000
1532.0000000	0.0505000
1533.0000000	0.0528571
1534.0000000	0.0525000
1535.0000000	0.0510000
1536.0000000	0.0514286
1537.0000000	0.0515000
1538.0000000	0.0520000
1539.0000000	0.0520000
1540.0000000	0.0509524
1541.0000000	0.0514286
1542.0000000	0.0500000
1543.0000000	0.0500000
1544.0000000	0.0500000
1545.0000000	0.0504762
1546.0000000	0.0505000
1547.0000000	0.0500000
1548.0000000	0.0500000
1549.0000000	0.0500000
1550.0000000	0.0500000
1551.0000000	0.0500000
1552.0000000	0.0500000
1553.0000000	0.0500000
1554.0000000	0.0500000
1555.0000000	0.0495000
1556.0000000	0.0500000
1557.0000000	0.0490476
1558.0000000	0.0500000
1559.0000000	0.0500000
1560.0000000	0.0500000
1561.0000000	0.0500000
1562.0000000	0.0500000
1563.0000000	0.0504762
1564.0000000	0.0505000
1565.0000000	0.0509524
1566.0000000	0.0505000
1567.0000000	0.0505000
1568.0000000	0.0500000
1569.0000000	0.0504762
1570.0000000	0.0505000
1571.0000000	0.0510000

1572.0000000	0.0509524
1573.0000000	0.0510000
1574.0000000	0.0500000
1575.0000000	0.0500000
1576.0000000	0.0500000
1577.0000000	0.0500000
1578.0000000	0.0500000
1579.0000000	0.0500000
1580.0000000	0.0500000
1581.0000000	0.0500000
1582.0000000	0.0500000
1583.0000000	0.0495000
1584.0000000	0.0500000
1585.0000000	0.0500000
1586.0000000	0.0505263
1587.0000000	0.0504762
1588.0000000	0.0500000
1589.0000000	0.0505000
1590.0000000	0.0509524
1591.0000000	0.0500000
1592.0000000	0.0500000
1593.0000000	0.0504762
1594.0000000	0.0510000
1595.0000000	0.0505000
1596.0000000	0.0500000
1597.0000000	0.0510000
1598.0000000	0.0500000
1599.0000000	0.0500000
1600.0000000	0.0500000
1601.0000000	0.0500000
1602.0000000	0.0500000
1603.0000000	0.0500000
1604.0000000	0.0500000
1605.0000000	0.0495000
1606.0000000	0.0500000
1607.0000000	0.0500000
1608.0000000	0.0500000
1609.0000000	0.0500000
1610.0000000	0.0500000
1611.0000000	0.0500000
1612.0000000	0.0500000
1613.0000000	0.0500000
1614.0000000	0.0500000
1615.0000000	0.0500000
1616.0000000	0.0500000
1617.0000000	0.0500000
1618.0000000	0.0500000
1619.0000000	0.0500000
1620.0000000	0.0500000
1621.0000000	0.0500000
1622.0000000	0.0500000
1623.0000000	0.0500000
1624.0000000	0.0500000
1625.0000000	0.0505000
1626.0000000	0.0500000
1627.0000000	0.0500000
1628.0000000	0.0500000

1629.0000000	0.0500000
1630.0000000	0.0500000
1631.0000000	0.0500000
1632.0000000	0.0500000
1633.0000000	0.0495238
1634.0000000	0.0500000
1635.0000000	0.0500000
1636.0000000	0.0495238
1637.0000000	0.0495000
1638.0000000	0.0495238
1639.0000000	0.0500000
1640.0000000	0.0500000
1641.0000000	0.0500000
1642.0000000	0.0500000
1643.0000000	0.0500000
1644.0000000	0.0500000
1645.0000000	0.0500000
1646.0000000	0.0500000
1647.0000000	0.0500000
1648.0000000	0.0505000
1649.0000000	0.0500000
1650.0000000	0.0500000
1651.0000000	0.0500000
1652.0000000	0.0505000
1653.0000000	0.0500000
1654.0000000	0.0500000
1655.0000000	0.0500000
1656.0000000	0.0500000
1657.0000000	0.0500000
1658.0000000	0.0500000
1659.0000000	0.0505000
1660.0000000	0.0500000
1661.0000000	0.0505000
1662.0000000	0.0500000
1663.0000000	0.0500000
1664.0000000	0.0505000
1665.0000000	0.0510000
1666.0000000	0.0500000
1667.0000000	0.0505000
1668.0000000	0.0500000
1669.0000000	0.0509524
1670.0000000	0.0515000
1671.0000000	0.0504762
1672.0000000	0.0500000
1673.0000000	0.0505000
1674.0000000	0.0500000
1675.0000000	0.0500000
1676.0000000	0.0500000
1677.0000000	0.0500000
1678.0000000	0.0500000
1679.0000000	0.0500000
1680.0000000	0.0500000
1681.0000000	0.0500000
1682.0000000	0.0505000
1683.0000000	0.0500000
1684.0000000	0.0500000
1685.0000000	0.0500000

1686.0000000	0.0500000
1687.0000000	0.0500000
1688.0000000	0.0495000
1689.0000000	0.0500000
1690.0000000	0.0500000
1691.0000000	0.0495000
1692.0000000	0.0495000
1693.0000000	0.0500000
1694.0000000	0.0500000
1695.0000000	0.0505000
1696.0000000	0.0500000
1697.0000000	0.0500000
1698.0000000	0.0500000
1699.0000000	0.0500000
1700.0000000	0.0500000
1701.0000000	0.0500000
1702.0000000	0.0500000
1703.0000000	0.0500000
1704.0000000	0.0505000
1705.0000000	0.0500000
1706.0000000	0.0505000
1707.0000000	0.0500000
1708.0000000	0.0515000
1709.0000000	0.0505000
1710.0000000	0.0500000
1711.0000000	0.0505000
1712.0000000	0.0500000
1713.0000000	0.0510000
1714.0000000	0.0523810
1715.0000000	0.0525000
1716.0000000	0.0519048
1717.0000000	0.0530000
1718.0000000	0.0525000
1719.0000000	0.0505000
1720.0000000	0.0530000
1721.0000000	0.0533333
1722.0000000	0.0545000
1723.0000000	0.0561905
1724.0000000	0.0555000
1725.0000000	0.0530000
1726.0000000	0.0542857
1727.0000000	0.0580000
1728.0000000	0.0565000
1729.0000000	0.0595238
1730.0000000	0.0575000
1731.0000000	0.0585000
1732.0000000	0.0561905
1733.0000000	0.0575000
1734.0000000	0.0590000
1735.0000000	0.0570000
1736.0000000	0.0585714
1737.0000000	0.0575000
1738.0000000	0.0580952
1739.0000000	0.0585000
1740.0000000	0.0575000
1741.0000000	0.0580952
1742.0000000	0.0580000

1743.0000000	0.0555000
1744.0000000	0.0540000
1745.0000000	0.0566667
1746.0000000	0.0552381
1747.0000000	0.0530000
1748.0000000	0.0535000
1749.0000000	0.0545000
1750.0000000	0.0552381
1751.0000000	0.0530000
1752.0000000	0.0550000
1753.0000000	0.0557143
1754.0000000	0.0540000
1755.0000000	0.0530000
1756.0000000	0.0530000
1757.0000000	0.0538095
1758.0000000	0.0545000
1759.0000000	0.0547619
1760.0000000	0.0530000
1761.0000000	0.0525000
1762.0000000	0.0528571
1763.0000000	0.0535000
1764.0000000	0.0525000
1765.0000000	0.0505000
1766.0000000	0.0519048
1767.0000000	0.0525000
1768.0000000	0.0504762
1769.0000000	0.0510000
1770.0000000	0.0505000
1771.0000000	0.0515000
1772.0000000	0.0509524
1773.0000000	0.0515000
1774.0000000	0.0542857
1775.0000000	0.0540000
1776.0000000	0.0515000
1777.0000000	0.0538095
1778.0000000	0.0520000
1779.0000000	0.0520000
1780.0000000	0.0530000
1781.0000000	0.0523810
1782.0000000	0.0520000
1783.0000000	0.0510000
1784.0000000	0.0509524
1785.0000000	0.0515000
1786.0000000	0.0509524
1787.0000000	0.0515000
1788.0000000	0.0510000
1789.0000000	0.0505000
1790.0000000	0.0523810
1791.0000000	0.0525000
1792.0000000	0.0505000
1793.0000000	0.0514286
1794.0000000	0.0510000
1795.0000000	0.0528571
1796.0000000	0.0530000
1797.0000000	0.0555000
1798.0000000	0.0533333
1799.0000000	0.0550000

1800.0000000	0.0530000
1801.0000000	0.0550000
1802.0000000	0.0528571
1803.0000000	0.0535000
1804.0000000	0.0528571
1805.0000000	0.0525000
1806.0000000	0.0540000
1807.0000000	0.0545000
1808.0000000	0.0576190
1809.0000000	0.0560000
1810.0000000	0.0575000
1811.0000000	0.0542857
1812.0000000	0.0540000
1813.0000000	0.0528571
1814.0000000	0.0530000
1815.0000000	0.0510000
1816.0000000	0.0500000
1817.0000000	0.0500000
1818.0000000	0.0500000
1819.0000000	0.0500000
1820.0000000	0.0500000
1821.0000000	0.0500000
1822.0000000	0.0500000
1823.0000000	0.0500000
1824.0000000	0.0500000
1825.0000000	0.0510000
1826.0000000	0.0504762
1827.0000000	0.0500000
1828.0000000	0.0510000
1829.0000000	0.0514286
1830.0000000	0.0530000
1831.0000000	0.0530000
1832.0000000	0.0540000
1833.0000000	0.0533333
1834.0000000	0.0528571
1835.0000000	0.0525000
1836.0000000	0.0525000
1837.0000000	0.0550000
1838.0000000	0.0547619
1839.0000000	0.0540000
1840.0000000	0.0540000
1841.0000000	0.0542857
1842.0000000	0.0550000
1843.0000000	0.0552381
1844.0000000	0.0535000
1845.0000000	0.0545000
1846.0000000	0.0535000
1847.0000000	0.0523810
1848.0000000	0.0515000
1849.0000000	0.0505000
1850.0000000	0.0504762
1851.0000000	0.0510000
1852.0000000	0.0509524
1853.0000000	0.0510000
1854.0000000	0.0505000
1855.0000000	0.0515000
1856.0000000	0.0500000

1857.0000000	0.0500000
1858.0000000	0.0514286
1859.0000000	0.0515000
1860.0000000	0.0500000
1861.0000000	0.0505000
1862.0000000	0.0500000
1863.0000000	0.0515000
1864.0000000	0.0500000
1865.0000000	0.0509524
1866.0000000	0.0510000
1867.0000000	0.0523810
1868.0000000	0.0515000
1869.0000000	0.0525000
1870.0000000	0.0525000
1871.0000000	0.0505000
1872.0000000	0.0519048
1873.0000000	0.0530000
1874.0000000	0.0523810
1875.0000000	0.0535000
1876.0000000	0.0525000
1877.0000000	0.0510000
1878.0000000	0.0519048
1879.0000000	0.0510000
1880.0000000	0.0510000
1881.0000000	0.0509524
1882.0000000	0.0504762
1883.0000000	0.0505000
1884.0000000	0.0505000
1885.0000000	0.0510000
1886.0000000	0.0500000
1887.0000000	0.0505000
1888.0000000	0.0525000
1889.0000000	0.0510000
1890.0000000	0.0514286
1891.0000000	0.0510000
1892.0000000	0.0504762
1893.0000000	0.0530000
1894.0000000	0.0520000
1895.0000000	0.0533333
1896.0000000	0.0510000
1897.0000000	0.0515000
1898.0000000	0.0533333
1899.0000000	0.0535000
1900.0000000	0.0560000
1901.0000000	0.0533333
1902.0000000	0.0540000
1903.0000000	0.0523810
1904.0000000	0.0560000
1905.0000000	0.0535000
1906.0000000	0.0535000
1907.0000000	0.0535000
1908.0000000	0.0547619
1909.0000000	0.0555000
1910.0000000	0.0528571
1911.0000000	0.0560000
1912.0000000	0.0566667
1913.0000000	0.0545000

1914.0000000	0.0575000
1915.0000000	0.0585000
1916.0000000	0.0570000
1917.0000000	0.0542857
1918.0000000	0.0570000
1919.0000000	0.0528571
1920.0000000	0.0565000
1921.0000000	0.0555000
1922.0000000	0.0542857
1923.0000000	0.0550000
1924.0000000	0.0545000
1925.0000000	0.0555000
1926.0000000	0.0566667
1927.0000000	0.0565000
1928.0000000	0.0580952
1929.0000000	0.0575000
1930.0000000	0.0550000
1931.0000000	0.0566667
1932.0000000	0.0570000
1933.0000000	0.0580000
1934.0000000	0.0576190
1935.0000000	0.0590000
1936.0000000	0.0585000
1937.0000000	0.0570000
1938.0000000	0.0576190
1939.0000000	0.0590000
1940.0000000	0.0595238
1941.0000000	0.0575000
1942.0000000	0.0575000
1943.0000000	0.0585714
1944.0000000	0.0590000
1945.0000000	0.0555000
1946.0000000	0.0571428
1947.0000000	0.0540000
1948.0000000	0.0555000
1949.0000000	0.0565000
1950.0000000	0.0552381
1951.0000000	0.0565000
1952.0000000	0.0570000
1953.0000000	0.0585714
1954.0000000	0.0595000
1955.0000000	0.0595238
1956.0000000	0.0590000
1957.0000000	0.0585000
1958.0000000	0.0585714
1959.0000000	0.0590000
1960.0000000	0.0590000
1961.0000000	0.0570000
1962.0000000	0.0585714
1963.0000000	0.0570000
1964.0000000	0.0576190
1965.0000000	0.0580000
1966.0000000	0.0575000
1967.0000000	0.0580000
1968.0000000	0.0590000
1969.0000000	0.0576190
1970.0000000	0.0576190

1971.0000000	0.0585000
1972.0000000	0.0580000
1973.0000000	0.0580952
1974.0000000	0.0560000
1975.0000000	0.0555000
1976.0000000	0.0545000
1977.0000000	0.0565000
1978.0000000	0.0557143
1979.0000000	0.0576190
1980.0000000	0.0590000
1981.0000000	0.0575000
1982.0000000	0.0585000
1983.0000000	0.0571428
1984.0000000	0.0590000
1985.0000000	0.0590000
1986.0000000	0.0590476
1987.0000000	0.0595000
1988.0000000	0.0595000
1989.0000000	0.0600000
1990.0000000	0.0595000
1991.0000000	0.0600000
1992.0000000	0.0600000
1993.0000000	0.0600000
1994.0000000	0.0600000
1995.0000000	0.0600000
1996.0000000	0.0600000
1997.0000000	0.0595000
1998.0000000	0.0600000
1999.0000000	0.0600000
2000.0000000	0.0600000
2001.0000000	0.0595000
2002.0000000	0.0600000
2003.0000000	0.0600000
2004.0000000	0.0600000
2005.0000000	0.0600000
2006.0000000	0.0600000
2007.0000000	0.0600000
2008.0000000	0.0595000
2009.0000000	0.0600000
2010.0000000	0.0600000
2011.0000000	0.0600000
2012.0000000	0.0600000
2013.0000000	0.0600000
2014.0000000	0.0600000
2015.0000000	0.0600000
2016.0000000	0.0600000
2017.0000000	0.0600000
2018.0000000	0.0600000
2019.0000000	0.0600000
2020.0000000	0.0600000
2021.0000000	0.0600000
2022.0000000	0.0600000
2023.0000000	0.0600000
2024.0000000	0.0600000
2025.0000000	0.0595238
2026.0000000	0.0600000
2027.0000000	0.0595000

2028.0000000	0.0600000
2029.0000000	0.0595000
2030.0000000	0.0600000
2031.0000000	0.0595238
2032.0000000	0.0590000
2033.0000000	0.0595000
2034.0000000	0.0595238
2035.0000000	0.0595000
2036.0000000	0.0600000
2037.0000000	0.0595000
2038.0000000	0.0585714
2039.0000000	0.0590476
2040.0000000	0.0580000
2041.0000000	0.0595000
2042.0000000	0.0585000
2043.0000000	0.0590476
2044.0000000	0.0595000
2045.0000000	0.0595000
2046.0000000	0.0595000
2047.0000000	0.0595238
2048.0000000	0.0590476
2049.0000000	0.0600000
2050.0000000	0.0600000
2051.0000000	0.0600000
2052.0000000	0.0600000
2053.0000000	0.0600000
2054.0000000	0.0600000
2055.0000000	0.0600000
2056.0000000	0.0600000
2057.0000000	0.0600000
2058.0000000	0.0600000
2059.0000000	0.0604762
2060.0000000	0.0600000
2061.0000000	0.0604762
2062.0000000	0.0600000
2063.0000000	0.0600000
2064.0000000	0.0600000
2065.0000000	0.0605000
2066.0000000	0.0600000
2067.0000000	0.0600000
2068.0000000	0.0615000
2069.0000000	0.0605000
2070.0000000	0.0604762
2071.0000000	0.0610000
2072.0000000	0.0600000
2073.0000000	0.0615000
2074.0000000	0.0609524
2075.0000000	0.0620000
2076.0000000	0.0615000
2077.0000000	0.0614286
2078.0000000	0.0620000
2079.0000000	0.0623809
2080.0000000	0.0615000
2081.0000000	0.0620000
2082.0000000	0.0620000
2083.0000000	0.0619048
2084.0000000	0.0630000

2085.0000000	0.0630000
2086.0000000	0.0619048
2087.0000000	0.0655000
2088.0000000	0.0642857
2089.0000000	0.0675000
2090.0000000	0.0635000
2091.0000000	0.0638095
2092.0000000	0.0625000
2093.0000000	0.0625000
2094.0000000	0.0600000
2095.0000000	0.0619048
2096.0000000	0.0605000
2097.0000000	0.0600000
2098.0000000	0.0600000
2099.0000000	0.0600000
2100.0000000	0.0600000
2101.0000000	0.0600000
2102.0000000	0.0600000
2103.0000000	0.0600000
2104.0000000	0.0600000
2105.0000000	0.0600000
2106.0000000	0.0604762
2107.0000000	0.0600000
2108.0000000	0.0605000
2109.0000000	0.0604762
2110.0000000	0.0610000
2111.0000000	0.0610000
2112.0000000	0.0600000
2113.0000000	0.0609524
2114.0000000	0.0615000
2115.0000000	0.0614286
2116.0000000	0.0605000
2117.0000000	0.0615000
2118.0000000	0.0605000
2119.0000000	0.0600000
2120.0000000	0.0600000
2121.0000000	0.0600000
2122.0000000	0.0600000
2123.0000000	0.0600000
2124.0000000	0.0600000
2125.0000000	0.0600000
2126.0000000	0.0590000
2127.0000000	0.0595000
2128.0000000	0.0600000
2129.0000000	0.0600000
2130.0000000	0.0600000
2131.0000000	0.0600000
2132.0000000	0.0600000
2133.0000000	0.0600000
2134.0000000	0.0595000
2135.0000000	0.0600000
2136.0000000	0.0600000
2137.0000000	0.0595000
2138.0000000	0.0590000
2139.0000000	0.0595238
2140.0000000	0.0600000
2141.0000000	0.0590000

2142.0000000	0.0590000
2143.0000000	0.0600000
2144.0000000	0.0595000
2145.0000000	0.0590476
2146.0000000	0.0600000
2147.0000000	0.0600000
2148.0000000	0.0600000
2149.0000000	0.0600000
2150.0000000	0.0600000
2151.0000000	0.0600000
2152.0000000	0.0600000
2153.0000000	0.0600000
2154.0000000	0.0600000
2155.0000000	0.0600000
2156.0000000	0.0600000
2157.0000000	0.0600000
2158.0000000	0.0600000
2159.0000000	0.0600000
2160.0000000	0.0595238
2161.0000000	0.0600000
2162.0000000	0.0600000
2163.0000000	0.0600000
2164.0000000	0.0600000
2165.0000000	0.0600000
2166.0000000	0.0600000
2167.0000000	0.0600000
2168.0000000	0.0600000
2169.0000000	0.0600000
2170.0000000	0.0600000
2171.0000000	0.0600000
2172.0000000	0.0600000
2173.0000000	0.0600000
2174.0000000	0.0600000
2175.0000000	0.0600000
2176.0000000	0.0600000
2177.0000000	0.0600000
2178.0000000	0.0600000
2179.0000000	0.0600000
2180.0000000	0.0600000
2181.0000000	0.0600000
2182.0000000	0.0600000
2183.0000000	0.0600000
2184.0000000	0.0600000
2185.0000000	0.0600000
2186.0000000	0.0600000
2187.0000000	0.0600000
2188.0000000	0.0600000
2189.0000000	0.0600000
2190.0000000	0.0600000
2191.0000000	0.0600000
2192.0000000	0.0600000
2193.0000000	0.0600000
2194.0000000	0.0600000
2195.0000000	0.0600000
2196.0000000	0.0600000
2197.0000000	0.0600000
2198.0000000	0.0600000

2199.0000000	0.0600000
2200.0000000	0.0600000
2201.0000000	0.0600000
2202.0000000	0.0600000
2203.0000000	0.0600000
2204.0000000	0.0600000
2205.0000000	0.0600000
2206.0000000	0.0600000
2207.0000000	0.0600000
2208.0000000	0.0600000
2209.0000000	0.0600000
2210.0000000	0.0600000
2211.0000000	0.0600000
2212.0000000	0.0600000
2213.0000000	0.0600000
2214.0000000	0.0600000
2215.0000000	0.0600000
2216.0000000	0.0600000
2217.0000000	0.0600000
2218.0000000	0.0600000
2219.0000000	0.0600000
2220.0000000	0.0600000
2221.0000000	0.0600000
2222.0000000	0.0600000
2223.0000000	0.0600000
2224.0000000	0.0600000
2225.0000000	0.0600000
2226.0000000	0.0600000
2227.0000000	0.0605000
2228.0000000	0.0600000
2229.0000000	0.0605000
2230.0000000	0.0604762
2231.0000000	0.0600000
2232.0000000	0.0620000
2233.0000000	0.0604762
2234.0000000	0.0605000
2235.0000000	0.0610000
2236.0000000	0.0609524
2237.0000000	0.0640000
2238.0000000	0.0630000
2239.0000000	0.0625000
2240.0000000	0.0628571
2241.0000000	0.0625000
2242.0000000	0.0619047
2243.0000000	0.0640000
2244.0000000	0.0635000
2245.0000000	0.0609524
2246.0000000	0.0630000
2247.0000000	0.0620000
2248.0000000	0.0623810
2249.0000000	0.0620000
2250.0000000	0.0635000
2251.0000000	0.0620000
2252.0000000	0.0614286
2253.0000000	0.0615000
2254.0000000	0.0610000
2255.0000000	0.0619048

2256.0000000	0.0620000
2257.0000000	0.0614286
2258.0000000	0.0615000
2259.0000000	0.0610000
2260.0000000	0.0614286
2261.0000000	0.0615000
2262.0000000	0.0600000
2263.0000000	0.0600000
2264.0000000	0.0609524
2265.0000000	0.0605000
2266.0000000	0.0614286
2267.0000000	0.0600000
2268.0000000	0.0620000
2269.0000000	0.0625000
2270.0000000	0.0630000
2271.0000000	0.0614286
2272.0000000	0.0623809
2273.0000000	0.0630000
2274.0000000	0.0620000
2275.0000000	0.0623809
2276.0000000	0.0610000
2277.0000000	0.0610000
2278.0000000	0.0610000
2279.0000000	0.0619047
2280.0000000	0.0605000
2281.0000000	0.0610000
2282.0000000	0.0600000
2283.0000000	0.0600000
2284.0000000	0.0605000
2285.0000000	0.0609524
2286.0000000	0.0605000
2287.0000000	0.0600000
2288.0000000	0.0614286
2289.0000000	0.0610000
2290.0000000	0.0610000
2291.0000000	0.0604762
2292.0000000	0.0600000
2293.0000000	0.0600000
2294.0000000	0.0600000
2295.0000000	0.0605000
2296.0000000	0.0600000
2297.0000000	0.0600000
2298.0000000	0.0600000
2299.0000000	0.0600000
2300.0000000	0.0600000
2301.0000000	0.0610000
2302.0000000	0.0604762
2303.0000000	0.0600000
2304.0000000	0.0600000
2305.0000000	0.0604762
2306.0000000	0.0600000
2307.0000000	0.0600000
2308.0000000	0.0600000
2309.0000000	0.0600000
2310.0000000	0.0605000
2311.0000000	0.0600000
2312.0000000	0.0610000

2313.0000000	0.0600000
2314.0000000	0.0600000
2315.0000000	0.0605000
2316.0000000	0.0600000
2317.0000000	0.0600000
2318.0000000	0.0600000
2319.0000000	0.0600000
2320.0000000	0.0600000
2321.0000000	0.0600000
2322.0000000	0.0600000
2323.0000000	0.0600000
2324.0000000	0.0600000
2325.0000000	0.0600000
2326.0000000	0.0600000
2327.0000000	0.0600000
2328.0000000	0.0605000
2329.0000000	0.0600000
2330.0000000	0.0600000
2331.0000000	0.0605000
2332.0000000	0.0600000
2333.0000000	0.0604762
2334.0000000	0.0605000
2335.0000000	0.0610000
2336.0000000	0.0600000
2337.0000000	0.0600000
2338.0000000	0.0605000
2339.0000000	0.0600000
2340.0000000	0.0604762
2341.0000000	0.0609524
2342.0000000	0.0625000
2343.0000000	0.0635000
2344.0000000	0.0635000
2345.0000000	0.0652381
2346.0000000	0.0655000
2347.0000000	0.0625000
2348.0000000	0.0628571
2349.0000000	0.0630000
2350.0000000	0.0638095
2351.0000000	0.0635000
2352.0000000	0.0665000
2353.0000000	0.0680000
2354.0000000	0.0666667
2355.0000000	0.0680000
2356.0000000	0.0675000
2357.0000000	0.0666667
2358.0000000	0.0670000
2359.0000000	0.0670000
2360.0000000	0.0647619
2361.0000000	0.0635000
2362.0000000	0.0630000
2363.0000000	0.0657143
2364.0000000	0.0640000
2365.0000000	0.0614286
2366.0000000	0.0620000
2367.0000000	0.0640000
2368.0000000	0.0615000
2369.0000000	0.0600000

2370.0000000	0.0605000
2371.0000000	0.0605000
2372.0000000	0.0604762
2373.0000000	0.0610000
2374.0000000	0.0610000
2375.0000000	0.0600000
2376.0000000	0.0609524
2377.0000000	0.0610000
2378.0000000	0.0600000
2379.0000000	0.0600000
2380.0000000	0.0600000
2381.0000000	0.0600000
2382.0000000	0.0600000
2383.0000000	0.0600000
2384.0000000	0.0600000
2385.0000000	0.0600000
2386.0000000	0.0605000
2387.0000000	0.0610000
2388.0000000	0.0609524
2389.0000000	0.0610000
2390.0000000	0.0623809
2391.0000000	0.0605000
2392.0000000	0.0630000
2393.0000000	0.0619048
2394.0000000	0.0605000
2395.0000000	0.0600000
2396.0000000	0.0609524
2397.0000000	0.0610000
2398.0000000	0.0620000
2399.0000000	0.0609524
2400.0000000	0.0615000
2401.0000000	0.0625000
2402.0000000	0.0604762
2403.0000000	0.0605000
2404.0000000	0.0600000
2405.0000000	0.0610000
2406.0000000	0.0628571
2407.0000000	0.0635000
2408.0000000	0.0628571
2409.0000000	0.0660000
2410.0000000	0.0635000
2411.0000000	0.0628571
2412.0000000	0.0635000
2413.0000000	0.0625000
2414.0000000	0.0645000
2415.0000000	0.0642857
2416.0000000	0.0640000
2417.0000000	0.0647619
2418.0000000	0.0635000
2419.0000000	0.0660000
2420.0000000	0.0645000
2421.0000000	0.0642857
2422.0000000	0.0625000
2423.0000000	0.0625000
2424.0000000	0.0628571
2425.0000000	0.0645000
2426.0000000	0.0642857

2427.0000000	0.0680000
2428.0000000	0.0650000
2429.0000000	0.0671429
2430.0000000	0.0680000
2431.0000000	0.0675000
2432.0000000	0.0680000
2433.0000000	0.0676190
2434.0000000	0.0675000
2435.0000000	0.0690000
2436.0000000	0.0671429
2437.0000000	0.0680000
2438.0000000	0.0685714
2439.0000000	0.0685000
2440.0000000	0.0700000
2441.0000000	0.0700000
2442.0000000	0.0700000
2443.0000000	0.0695000
2444.0000000	0.0695000
2445.0000000	0.0695238
2446.0000000	0.0690000
2447.0000000	0.0700000
2448.0000000	0.0695000
2449.0000000	0.0690000
2450.0000000	0.0695000
2451.0000000	0.0695238
2452.0000000	0.0695000
2453.0000000	0.0666667
2454.0000000	0.0660000
2455.0000000	0.0685000
2456.0000000	0.0680000
2457.0000000	0.0685714
2458.0000000	0.0690000
2459.0000000	0.0695000
2460.0000000	0.0700000
2461.0000000	0.0690000
2462.0000000	0.0695238
2463.0000000	0.0695000
2464.0000000	0.0695000
2465.0000000	0.0700000
2466.0000000	0.0700000
2467.0000000	0.0695238
2468.0000000	0.0700000
2469.0000000	0.0700000
2470.0000000	0.0700000
2471.0000000	0.0700000
2472.0000000	0.0700000
2473.0000000	0.0700000
2474.0000000	0.0700000
2475.0000000	0.0700000
2476.0000000	0.0704762
2477.0000000	0.0700000
2478.0000000	0.0700000
2479.0000000	0.0700000
2480.0000000	0.0700000
2481.0000000	0.0700000
2482.0000000	0.0705000
2483.0000000	0.0700000

2484.0000000	0.0700000
2485.0000000	0.0705000
2486.0000000	0.0700000
2487.0000000	0.0700000
2488.0000000	0.0705000
2489.0000000	0.0705000
2490.0000000	0.0700000
2491.0000000	0.0700000
2492.0000000	0.0705000
2493.0000000	0.0704762
2494.0000000	0.0705000
2495.0000000	0.0700000
2496.0000000	0.0700000
2497.0000000	0.0700000
2498.0000000	0.0700000
2499.0000000	0.0700000
2500.0000000	0.0700000
2501.0000000	0.0700000
2502.0000000	0.0700000
2503.0000000	0.0705000
2504.0000000	0.0700000
2505.0000000	0.0700000
2506.0000000	0.0700000
2507.0000000	0.0705000
2508.0000000	0.0700000
2509.0000000	0.0705000
2510.0000000	0.0700000
2511.0000000	0.0700000
2512.0000000	0.0700000
2513.0000000	0.0700000
2514.0000000	0.0700000
2515.0000000	0.0700000
2516.0000000	0.0700000
2517.0000000	0.0700000
2518.0000000	0.0700000
2519.0000000	0.0700000
2520.0000000	0.0700000
2521.0000000	0.0700000
2522.0000000	0.0705000
2523.0000000	0.0700000
2524.0000000	0.0700000
2525.0000000	0.0700000
2526.0000000	0.0704762
2527.0000000	0.0700000
2528.0000000	0.0705000
2529.0000000	0.0704762
2530.0000000	0.0705000
2531.0000000	0.0710000
2532.0000000	0.0704762
2533.0000000	0.0705000
2534.0000000	0.0700000
2535.0000000	0.0709524
2536.0000000	0.0715000
2537.0000000	0.0710000
2538.0000000	0.0709524
2539.0000000	0.0710000
2540.0000000	0.0715000

2541.0000000	0.0709524
2542.0000000	0.0740000
2543.0000000	0.0720000
2544.0000000	0.0725000
2545.0000000	0.0733333
2546.0000000	0.0738095
2547.0000000	0.0735000
2548.0000000	0.0725000
2549.0000000	0.0740000
2550.0000000	0.0752381
2551.0000000	0.0745000
2552.0000000	0.0730000
2553.0000000	0.0742857
2554.0000000	0.0760000
2555.0000000	0.0780000
2556.0000000	0.0765000
2557.0000000	0.0761905
2558.0000000	0.0760000
2559.0000000	0.0752381
2560.0000000	0.0735000
2561.0000000	0.0710000
2562.0000000	0.0709524
2563.0000000	0.0710000
2564.0000000	0.0730000
2565.0000000	0.0709524
2566.0000000	0.0705000
2567.0000000	0.0700000
2568.0000000	0.0704762
2569.0000000	0.0710000
2570.0000000	0.0705000
2571.0000000	0.0710000
2572.0000000	0.0705000
2573.0000000	0.0709524
2574.0000000	0.0709524
2575.0000000	0.0705000
2576.0000000	0.0700000
2577.0000000	0.0700000
2578.0000000	0.0705000
2579.0000000	0.0700000
2580.0000000	0.0715000
2581.0000000	0.0714286
2582.0000000	0.0710000
2583.0000000	0.0715000
2584.0000000	0.0719048
2585.0000000	0.0720000
2586.0000000	0.0742857
2587.0000000	0.0715000
2588.0000000	0.0715000
2589.0000000	0.0705000
2590.0000000	0.0728572
2591.0000000	0.0735000
2592.0000000	0.0710000
2593.0000000	0.0733333
2594.0000000	0.0740000
2595.0000000	0.0725000
2596.0000000	0.0714286
2597.0000000	0.0705000

2598.0000000	0.0700000
2599.0000000	0.0700000
2600.0000000	0.0700000
2601.0000000	0.0700000
2602.0000000	0.0700000
2603.0000000	0.0700000
2604.0000000	0.0700000
2605.0000000	0.0700000
2606.0000000	0.0700000
2607.0000000	0.0700000
2608.0000000	0.0695000
2609.0000000	0.0695000
2610.0000000	0.0685000
2611.0000000	0.0685714
2612.0000000	0.0655000
2613.0000000	0.0665000
2614.0000000	0.0652381
2615.0000000	0.0650000
2616.0000000	0.0620000
2617.0000000	0.0619048
2618.0000000	0.0620000
2619.0000000	0.0630000
2620.0000000	0.0633333
2621.0000000	0.0640000
2622.0000000	0.0665000
2623.0000000	0.0671429
2624.0000000	0.0660000
2625.0000000	0.0685000
2626.0000000	0.0685714
2627.0000000	0.0680000
2628.0000000	0.0685000
2629.0000000	0.0700000
2630.0000000	0.0690000
2631.0000000	0.0695000
2632.0000000	0.0700000
2633.0000000	0.0700000
2634.0000000	0.0700000
2635.0000000	0.0705000
2636.0000000	0.0700000
2637.0000000	0.0700000
2638.0000000	0.0704762
2639.0000000	0.0700000
2640.0000000	0.0700000
2641.0000000	0.0700000
2642.0000000	0.0705000
2643.0000000	0.0700000
2644.0000000	0.0705000
2645.0000000	0.0710000
2646.0000000	0.0710000
2647.0000000	0.0700000
2648.0000000	0.0710000
2649.0000000	0.0700000
2650.0000000	0.0714286
2651.0000000	0.0700000
2652.0000000	0.0709524
2653.0000000	0.0700000
2654.0000000	0.0700000

2655.0000000	0.0700000
2656.0000000	0.0700000
2657.0000000	0.0695000
2658.0000000	0.0685000
2659.0000000	0.0680953
2660.0000000	0.0630000
2661.0000000	0.0615000
2662.0000000	0.0619048
2663.0000000	0.0625000
2664.0000000	0.0610000
2665.0000000	0.0623809
2666.0000000	0.0660000
2667.0000000	0.0655000
2668.0000000	0.0647619
2669.0000000	0.0640000
2670.0000000	0.0655000
2671.0000000	0.0680000
2672.0000000	0.0685714
2673.0000000	0.0685000
2674.0000000	0.0695238
2675.0000000	0.0700000
2676.0000000	0.0700000
2677.0000000	0.0700000
2678.0000000	0.0700000
2679.0000000	0.0700000
2680.0000000	0.0700000
2681.0000000	0.0700000
2682.0000000	0.0700000
2683.0000000	0.0700000
2684.0000000	0.0700000
2685.0000000	0.0700000
2686.0000000	0.0700000
2687.0000000	0.0705000
2688.0000000	0.0700000
2689.0000000	0.0700000
2690.0000000	0.0700000
2691.0000000	0.0700000
2692.0000000	0.0700000
2693.0000000	0.0705000
2694.0000000	0.0700000
2695.0000000	0.0709524
2696.0000000	0.0715000
2697.0000000	0.0705000
2698.0000000	0.0704762
2699.0000000	0.0705000
2700.0000000	0.0700000
2701.0000000	0.0704762
2702.0000000	0.0705000
2703.0000000	0.0710000
2704.0000000	0.0719048
2705.0000000	0.0720000
2706.0000000	0.0705000
2707.0000000	0.0725000
2708.0000000	0.0719048
2709.0000000	0.0725000
2710.0000000	0.0709524
2711.0000000	0.0710000

2712.0000000	0.0715000
2713.0000000	0.0700000
2714.0000000	0.0710000
2715.0000000	0.0715000
2716.0000000	0.0700000
2717.0000000	0.0704762
2718.0000000	0.0715000
2719.0000000	0.0714286
2720.0000000	0.0700000
2721.0000000	0.0705000
2722.0000000	0.0719048
2723.0000000	0.0710000
2724.0000000	0.0710000
2725.0000000	0.0705000
2726.0000000	0.0719048
2727.0000000	0.0700000
2728.0000000	0.0700000
2729.0000000	0.0700000
2730.0000000	0.0705000
2731.0000000	0.0700000
2732.0000000	0.0700000
2733.0000000	0.0700000
2734.0000000	0.0700000
2735.0000000	0.0695000
2736.0000000	0.0700000
2737.0000000	0.0700000
2738.0000000	0.0700000
2739.0000000	0.0695000
2740.0000000	0.0680952
2741.0000000	0.0690000
2742.0000000	0.0670000
2743.0000000	0.0695238
2744.0000000	0.0685000
2745.0000000	0.0680000
2746.0000000	0.0630000
2747.0000000	0.0623809
2748.0000000	0.0625000
2749.0000000	0.0635000
2750.0000000	0.0623809
2751.0000000	0.0610000
2752.0000000	0.0615000
2753.0000000	0.0604762
2754.0000000	0.0605000
2755.0000000	0.0600000
2756.0000000	0.0600000
2757.0000000	0.0600000
2758.0000000	0.0600000
2759.0000000	0.0600000
2760.0000000	0.0600000
2761.0000000	0.0600000
2762.0000000	0.0604762
2763.0000000	0.0635000
2764.0000000	0.0620000
2765.0000000	0.0633333
2766.0000000	0.0640000
2767.0000000	0.0645000
2768.0000000	0.0655000

2769.0000000	0.0657143
2770.0000000	0.0660000
2771.0000000	0.0661905
2772.0000000	0.0670000
2773.0000000	0.0670000
2774.0000000	0.0642857
2775.0000000	0.0645000
2776.0000000	0.0630000
2777.0000000	0.0625000
2778.0000000	0.0642857
2779.0000000	0.0661905
2780.0000000	0.0640000
2781.0000000	0.0635000
2782.0000000	0.0630000
2783.0000000	0.0614286
2784.0000000	0.0620000
2785.0000000	0.0610000
2786.0000000	0.0623809
2787.0000000	0.0625000
2788.0000000	0.0615000
2789.0000000	0.0619047
2790.0000000	0.0625000
2791.0000000	0.0635000
2792.0000000	0.0628571
2793.0000000	0.0640000
2794.0000000	0.0655000
2795.0000000	0.0657143
2796.0000000	0.0690000
2797.0000000	0.0670000
2798.0000000	0.0685714
2799.0000000	0.0670000
2800.0000000	0.0675000
2801.0000000	0.0666667
2802.0000000	0.0675000
2803.0000000	0.0680952
2804.0000000	0.0655000
2805.0000000	0.0640000
2806.0000000	0.0670000
2807.0000000	0.0638095
2808.0000000	0.0625000
2809.0000000	0.0625000
2810.0000000	0.0633333
2811.0000000	0.0605000
2812.0000000	0.0620000
2813.0000000	0.0620000
2814.0000000	0.0614286
2815.0000000	0.0630000
2816.0000000	0.0633333
2817.0000000	0.0625000
2818.0000000	0.0615000
2819.0000000	0.0623810
2820.0000000	0.0605000
2821.0000000	0.0630000
2822.0000000	0.0642857
2823.0000000	0.0660000
2824.0000000	0.0620000
2825.0000000	0.0633333

2826.0000000	0.0610000
2827.0000000	0.0605000
2828.0000000	0.0600000
2829.0000000	0.0600000
2830.0000000	0.0610000
2831.0000000	0.0604762
2832.0000000	0.0600000
2833.0000000	0.0610000
2834.0000000	0.0619048
2835.0000000	0.0630000
2836.0000000	0.0635000
2837.0000000	0.0640000
2838.0000000	0.0657143
2839.0000000	0.0638095
2840.0000000	0.0675000
2841.0000000	0.0665000
2842.0000000	0.0670000
2843.0000000	0.0675000
2844.0000000	0.0647619
2845.0000000	0.0665000
2846.0000000	0.0638095
2847.0000000	0.0655000
2848.0000000	0.0666667
2849.0000000	0.0675000
2850.0000000	0.0680000
2851.0000000	0.0680000
2852.0000000	0.0670000
2853.0000000	0.0652381
2854.0000000	0.0685000
2855.0000000	0.0676190
2856.0000000	0.0670000
2857.0000000	0.0645000
2858.0000000	0.0650000
2859.0000000	0.0647619
2860.0000000	0.0640000
2861.0000000	0.0650000
2862.0000000	0.0628571
2863.0000000	0.0665000
2864.0000000	0.0657143
2865.0000000	0.0670000
2866.0000000	0.0660000
2867.0000000	0.0690476
2868.0000000	0.0680000
2869.0000000	0.0690000
2870.0000000	0.0685000
2871.0000000	0.0680952
2872.0000000	0.0690000
2873.0000000	0.0695000
2874.0000000	0.0680952
2875.0000000	0.0695000
2876.0000000	0.0695000
2877.0000000	0.0700000
2878.0000000	0.0695000
2879.0000000	0.0690476
2880.0000000	0.0690000
2881.0000000	0.0675000
2882.0000000	0.0685000

2883.0000000	0.0695238
2884.0000000	0.0685000
2885.0000000	0.0700000
2886.0000000	0.0680952
2887.0000000	0.0675000
2888.0000000	0.0690000
2889.0000000	0.0690476
2890.0000000	0.0685000
2891.0000000	0.0695238
2892.0000000	0.0700000
2893.0000000	0.0685000
2894.0000000	0.0695000
2895.0000000	0.0680952
2896.0000000	0.0670000
2897.0000000	0.0695000
2898.0000000	0.0690476
2899.0000000	0.0670000
2900.0000000	0.0671429
2901.0000000	0.0665000
2902.0000000	0.0660000
2903.0000000	0.0665000
2904.0000000	0.0642857
2905.0000000	0.0645000
2906.0000000	0.0630000
2907.0000000	0.0661905
2908.0000000	0.0650000
2909.0000000	0.0628571
2910.0000000	0.0635000
2911.0000000	0.0620000
2912.0000000	0.0610000
2913.0000000	0.0619048
2914.0000000	0.0610000
2915.0000000	0.0605000
2916.0000000	0.0604762
2917.0000000	0.0610000
2918.0000000	0.0630000
2919.0000000	0.0623809
2920.0000000	0.0620000
2921.0000000	0.0630000
2922.0000000	0.0638095
2923.0000000	0.0665000
2924.0000000	0.0660000
2925.0000000	0.0671429
2926.0000000	0.0670000
2927.0000000	0.0670000
2928.0000000	0.0671429
2929.0000000	0.0685000
2930.0000000	0.0655000
2931.0000000	0.0657143
2932.0000000	0.0665000
2933.0000000	0.0650000
2934.0000000	0.0655000
2935.0000000	0.0657143
2936.0000000	0.0642857
2937.0000000	0.0630000
2938.0000000	0.0640000
2939.0000000	0.0615000

2940.0000000	0.0628571
2941.0000000	0.0640000
2942.0000000	0.0645000
2943.0000000	0.0657143
2944.0000000	0.0665000
2945.0000000	0.0676190
2946.0000000	0.0685000
2947.0000000	0.0675000
2948.0000000	0.0675000
2949.0000000	0.0695238
2950.0000000	0.0700000
2951.0000000	0.0700000
2952.0000000	0.0695238
2953.0000000	0.0695000
2954.0000000	0.0700000
2955.0000000	0.0700000
2956.0000000	0.0700000
2957.0000000	0.0700000
2958.0000000	0.0700000
2959.0000000	0.0700000
2960.0000000	0.0700000
2961.0000000	0.0700000
2962.0000000	0.0700000
2963.0000000	0.0700000
2964.0000000	0.0700000
2965.0000000	0.0700000
2966.0000000	0.0705000
2967.0000000	0.0700000
2968.0000000	0.0700000
2969.0000000	0.0700000
2970.0000000	0.0700000
2971.0000000	0.0700000
2972.0000000	0.0700000
2973.0000000	0.0700000
2974.0000000	0.0704762
2975.0000000	0.0705000
2976.0000000	0.0700000
2977.0000000	0.0705000
2978.0000000	0.0705000
2979.0000000	0.0714286
2980.0000000	0.0705000
2981.0000000	0.0700000
2982.0000000	0.0710000
2983.0000000	0.0709524
2984.0000000	0.0700000
2985.0000000	0.0705000
2986.0000000	0.0705000
2987.0000000	0.0710000
2988.0000000	0.0704762
2989.0000000	0.0705000
2990.0000000	0.0710000
2991.0000000	0.0704762
2992.0000000	0.0700000
2993.0000000	0.0705000
2994.0000000	0.0705000
2995.0000000	0.0700000
2996.0000000	0.0700000

2997.0000000	0.0700000
2998.0000000	0.0710000
2999.0000000	0.0700000
3000.0000000	0.0700000
3001.0000000	0.0700000
3002.0000000	0.0700000
3003.0000000	0.0700000
3004.0000000	0.0700000
3005.0000000	0.0705000
3006.0000000	0.0700000
3007.0000000	0.0700000
3008.0000000	0.0700000
3009.0000000	0.0700000
3010.0000000	0.0700000
3011.0000000	0.0700000
3012.0000000	0.0700000
3013.0000000	0.0700000
3014.0000000	0.0700000
3015.0000000	0.0700000
3016.0000000	0.0700000
3017.0000000	0.0700000
3018.0000000	0.0700000
3019.0000000	0.0700000
3020.0000000	0.0700000
3021.0000000	0.0695238
3022.0000000	0.0700000
3023.0000000	0.0700000
3024.0000000	0.0700000
3025.0000000	0.0700000
3026.0000000	0.0700000
3027.0000000	0.0700000
3028.0000000	0.0700000
3029.0000000	0.0700000
3030.0000000	0.0700000
3031.0000000	0.0700000
3032.0000000	0.0690000
3033.0000000	0.0690476
3034.0000000	0.0690000
3035.0000000	0.0700000
3036.0000000	0.0690000
3037.0000000	0.0690476
3038.0000000	0.0695000
3039.0000000	0.0690476
3040.0000000	0.0700000
3041.0000000	0.0700000
3042.0000000	0.0695000
3043.0000000	0.0700000
3044.0000000	0.0700000
3045.0000000	0.0700000
3046.0000000	0.0700000
3047.0000000	0.0700000
3048.0000000	0.0700000
3049.0000000	0.0700000
3050.0000000	0.0705000
3051.0000000	0.0705000
3052.0000000	0.0700000
3053.0000000	0.0705000

3054.0000000	0.0700000
3055.0000000	0.0700000
3056.0000000	0.0705000
3057.0000000	0.0700000
3058.0000000	0.0700000
3059.0000000	0.0715000
3060.0000000	0.0710000
3061.0000000	0.0704762
3062.0000000	0.0720000
3063.0000000	0.0710000
3064.0000000	0.0704762
3065.0000000	0.0710000
3066.0000000	0.0715000
3067.0000000	0.0709524
3068.0000000	0.0720000
3069.0000000	0.0742857
3070.0000000	0.0715000
3071.0000000	0.0735000
3072.0000000	0.0760000
3073.0000000	0.0752381
3074.0000000	0.0775000
3075.0000000	0.0785000
3076.0000000	0.0795000
3077.0000000	0.0785714
3078.0000000	0.0790476
3079.0000000	0.0785000
3080.0000000	0.0780000
3081.0000000	0.0785000
3082.0000000	0.0785714
3083.0000000	0.0795000
3084.0000000	0.0775000
3085.0000000	0.0790476
3086.0000000	0.0765000
3087.0000000	0.0775000
3088.0000000	0.0775000
3089.0000000	0.0780952
3090.0000000	0.0785000
3091.0000000	0.0752381
3092.0000000	0.0770000
3093.0000000	0.0790000
3094.0000000	0.0771429
3095.0000000	0.0785000
3096.0000000	0.0795000
3097.0000000	0.0780952
3098.0000000	0.0780000
3099.0000000	0.0770000
3100.0000000	0.0795238
3101.0000000	0.0780000
3102.0000000	0.0765000
3103.0000000	0.0770000
3104.0000000	0.0761905
3105.0000000	0.0780000
3106.0000000	0.0738095
3107.0000000	0.0725000
3108.0000000	0.0715000
3109.0000000	0.0714286
3110.0000000	0.0715000

3111.0000000	0.0715000
3112.0000000	0.0700000
3113.0000000	0.0700000
3114.0000000	0.0700000
3115.0000000	0.0700000
3116.0000000	0.0700000
3117.0000000	0.0700000
3118.0000000	0.0700000
3119.0000000	0.0700000
3120.0000000	0.0700000
3121.0000000	0.0700000
3122.0000000	0.0700000
3123.0000000	0.0700000
3124.0000000	0.0700000
3125.0000000	0.0700000
3126.0000000	0.0700000
3127.0000000	0.0700000
3128.0000000	0.0700000
3129.0000000	0.0700000
3130.0000000	0.0704762
3131.0000000	0.0710000
3132.0000000	0.0730000
3133.0000000	0.0730000
3134.0000000	0.0719048
3135.0000000	0.0720000
3136.0000000	0.0728572
3137.0000000	0.0720000
3138.0000000	0.0720000
3139.0000000	0.0709524
3140.0000000	0.0715000
3141.0000000	0.0705000
3142.0000000	0.0710000
3143.0000000	0.0704762
3144.0000000	0.0705000
3145.0000000	0.0719048
3146.0000000	0.0710000
3147.0000000	0.0720000
3148.0000000	0.0735000
3149.0000000	0.0719048
3150.0000000	0.0710000
3151.0000000	0.0725000
3152.0000000	0.0719048
3153.0000000	0.0700000
3154.0000000	0.0719048
3155.0000000	0.0705000
3156.0000000	0.0710000
3157.0000000	0.0700000
3158.0000000	0.0700000
3159.0000000	0.0700000
3160.0000000	0.0700000
3161.0000000	0.0700000
3162.0000000	0.0700000
3163.0000000	0.0705000
3164.0000000	0.0700000
3165.0000000	0.0700000
3166.0000000	0.0704762
3167.0000000	0.0710000

3168.0000000	0.0710000
3169.0000000	0.0704762
3170.0000000	0.0710000
3171.0000000	0.0705000
3172.0000000	0.0715000
3173.0000000	0.0714286
3174.0000000	0.0700000
3175.0000000	0.0700000
3176.0000000	0.0700000
3177.0000000	0.0700000
3178.0000000	0.0700000
3179.0000000	0.0700000
3180.0000000	0.0700000
3181.0000000	0.0700000
3182.0000000	0.0700000
3183.0000000	0.0700000
3184.0000000	0.0700000
3185.0000000	0.0700000
3186.0000000	0.0700000
3187.0000000	0.0700000
3188.0000000	0.0700000
3189.0000000	0.0705000
3190.0000000	0.0700000
3191.0000000	0.0700000
3192.0000000	0.0700000
3193.0000000	0.0700000
3194.0000000	0.0700000
3195.0000000	0.0700000
3196.0000000	0.0700000
3197.0000000	0.0700000
3198.0000000	0.0700000
3199.0000000	0.0700000
3200.0000000	0.0700000
3201.0000000	0.0700000
3202.0000000	0.0700000
3203.0000000	0.0700000
3204.0000000	0.0700000
3205.0000000	0.0700000
3206.0000000	0.0700000
3207.0000000	0.0700000
3208.0000000	0.0700000
3209.0000000	0.0704762
3210.0000000	0.0700000
3211.0000000	0.0700000
3212.0000000	0.0700000
3213.0000000	0.0700000
3214.0000000	0.0700000
3215.0000000	0.0700000
3216.0000000	0.0700000
3217.0000000	0.0700000
3218.0000000	0.0700000
3219.0000000	0.0700000
3220.0000000	0.0700000
3221.0000000	0.0700000
3222.0000000	0.0705000
3223.0000000	0.0700000
3224.0000000	0.0700000

3225.0000000	0.0705000
3226.0000000	0.0700000
3227.0000000	0.0700000
3228.0000000	0.0700000
3229.0000000	0.0700000
3230.0000000	0.0700000
3231.0000000	0.0700000
3232.0000000	0.0705000
3233.0000000	0.0709524
3234.0000000	0.0705000
3235.0000000	0.0715000
3236.0000000	0.0700000
3237.0000000	0.0710000
3238.0000000	0.0715000
3239.0000000	0.0705000
3240.0000000	0.0723810
3241.0000000	0.0720000
3242.0000000	0.0714286
3243.0000000	0.0710000
3244.0000000	0.0725000
3245.0000000	0.0723810
3246.0000000	0.0720000
3247.0000000	0.0735000
3248.0000000	0.0715000
3249.0000000	0.0728572
3250.0000000	0.0705000
3251.0000000	0.0709524
3252.0000000	0.0700000
3253.0000000	0.0709524
3254.0000000	0.0714286
3255.0000000	0.0715000
3256.0000000	0.0725000
3257.0000000	0.0710000
3258.0000000	0.0704762
3259.0000000	0.0705000
3260.0000000	0.0723810
3261.0000000	0.0710000
3262.0000000	0.0715000
3263.0000000	0.0719048
3264.0000000	0.0730000
3265.0000000	0.0710000
3266.0000000	0.0728572
3267.0000000	0.0735000
3268.0000000	0.0725000
3269.0000000	0.0735000
3270.0000000	0.0733333
3271.0000000	0.0770000
3272.0000000	0.0761905
3273.0000000	0.0765000
3274.0000000	0.0761905
3275.0000000	0.0730000
3276.0000000	0.0725000
3277.0000000	0.0715000
3278.0000000	0.0709524
3279.0000000	0.0700000
3280.0000000	0.0700000
3281.0000000	0.0700000

3282.0000000	0.0700000
3283.0000000	0.0700000
3284.0000000	0.0700000
3285.0000000	0.0700000
3286.0000000	0.0700000
3287.0000000	0.0700000
3288.0000000	0.0700000
3289.0000000	0.0700000
3290.0000000	0.0704762
3291.0000000	0.0700000
3292.0000000	0.0705000
3293.0000000	0.0700000
3294.0000000	0.0700000
3295.0000000	0.0700000
3296.0000000	0.0704762
3297.0000000	0.0705000
3298.0000000	0.0710000
3299.0000000	0.0705000
3300.0000000	0.0705000
3301.0000000	0.0723810
3302.0000000	0.0728572
3303.0000000	0.0710000
3304.0000000	0.0700000
3305.0000000	0.0709524
3306.0000000	0.0710000
3307.0000000	0.0705000
3308.0000000	0.0710000
3309.0000000	0.0704762
3310.0000000	0.0705000
3311.0000000	0.0700000
3312.0000000	0.0700000
3313.0000000	0.0700000
3314.0000000	0.0700000
3315.0000000	0.0704762
3316.0000000	0.0700000
3317.0000000	0.0700000
3318.0000000	0.0700000
3319.0000000	0.0700000
3320.0000000	0.0700000
3321.0000000	0.0700000
3322.0000000	0.0700000
3323.0000000	0.0700000
3324.0000000	0.0695238
3325.0000000	0.0700000
3326.0000000	0.0700000
3327.0000000	0.0700000
3328.0000000	0.0700000
3329.0000000	0.0700000
3330.0000000	0.0700000
3331.0000000	0.0700000
3332.0000000	0.0700000
3333.0000000	0.0700000
3334.0000000	0.0700000
3335.0000000	0.0700000
3336.0000000	0.0700000
3337.0000000	0.0700000
3338.0000000	0.0700000

3339.0000000	0.0700000
3340.0000000	0.0700000
3341.0000000	0.0700000
3342.0000000	0.0695238
3343.0000000	0.0700000
3344.0000000	0.0700000
3345.0000000	0.0700000
3346.0000000	0.0700000
3347.0000000	0.0700000
3348.0000000	0.0700000
3349.0000000	0.0700000
3350.0000000	0.0700000
3351.0000000	0.0700000
3352.0000000	0.0700000
3353.0000000	0.0710000
3354.0000000	0.0709524
3355.0000000	0.0715000
3356.0000000	0.0710000
3357.0000000	0.0704762
3358.0000000	0.0715000
3359.0000000	0.0705000
3360.0000000	0.0700000
3361.0000000	0.0700000
3362.0000000	0.0700000
3363.0000000	0.0700000
3364.0000000	0.0700000
3365.0000000	0.0700000
3366.0000000	0.0700000
3367.0000000	0.0700000
3368.0000000	0.0700000
3369.0000000	0.0700000
3370.0000000	0.0700000
3371.0000000	0.0700000
3372.0000000	0.0700000
3373.0000000	0.0700000
3374.0000000	0.0700000
3375.0000000	0.0700000
3376.0000000	0.0700000
3377.0000000	0.0700000
3378.0000000	0.0700000
3379.0000000	0.0700000
3380.0000000	0.0700000
3381.0000000	0.0700000
3382.0000000	0.0705000
3383.0000000	0.0700000
3384.0000000	0.0700000
3385.0000000	0.0700000
3386.0000000	0.0700000
3387.0000000	0.0700000
3388.0000000	0.0700000
3389.0000000	0.0710000
3390.0000000	0.0709524
3391.0000000	0.0705000
3392.0000000	0.0725000
3393.0000000	0.0709524
3394.0000000	0.0705000
3395.0000000	0.0710000

3396.0000000	0.0709524
3397.0000000	0.0730000
3398.0000000	0.0740000
3399.0000000	0.0757143
3400.0000000	0.0760000
3401.0000000	0.0740000
3402.0000000	0.0742857
3403.0000000	0.0740000
3404.0000000	0.0765000
3405.0000000	0.0775000
3406.0000000	0.0771429
3407.0000000	0.0775000
3408.0000000	0.0765000
3409.0000000	0.0766667
3410.0000000	0.0790000
3411.0000000	0.0785714
3412.0000000	0.0795000
3413.0000000	0.0755000
3414.0000000	0.0776191
3415.0000000	0.0775000
3416.0000000	0.0785000
3417.0000000	0.0800000
3418.0000000	0.0790476
3419.0000000	0.0800000
3420.0000000	0.0795000
3421.0000000	0.0800000
3422.0000000	0.0800000
3423.0000000	0.0800000
3424.0000000	0.0800000
3425.0000000	0.0795000
3426.0000000	0.0790476
3427.0000000	0.0790000
3428.0000000	0.0800000
3429.0000000	0.0800000
3430.0000000	0.0795238
3431.0000000	0.0790000
3432.0000000	0.0790476
3433.0000000	0.0790000
3434.0000000	0.0800000
3435.0000000	0.0795000
3436.0000000	0.0800000
3437.0000000	0.0795000
3438.0000000	0.0795000
3439.0000000	0.0800000
3440.0000000	0.0795000
3441.0000000	0.0795238
3442.0000000	0.0790000
3443.0000000	0.0790000
3444.0000000	0.0800000
3445.0000000	0.0800000
3446.0000000	0.0800000
3447.0000000	0.0800000
3448.0000000	0.0795238
3449.0000000	0.0795000
3450.0000000	0.0795000
3451.0000000	0.0800000
3452.0000000	0.0800000

3453.0000000	0.0795000
3454.0000000	0.0800000
3455.0000000	0.0800000
3456.0000000	0.0800000
3457.0000000	0.0800000
3458.0000000	0.0800000
3459.0000000	0.0800000
3460.0000000	0.0800000
3461.0000000	0.0800000
3462.0000000	0.0800000
3463.0000000	0.0800000
3464.0000000	0.0805000
3465.0000000	0.0805000
3466.0000000	0.0804762
3467.0000000	0.0805000
3468.0000000	0.0804762
3469.0000000	0.0820000
3470.0000000	0.0805000
3471.0000000	0.0800000
3472.0000000	0.0815000
3473.0000000	0.0805000
3474.0000000	0.0810000
3475.0000000	0.0800000
3476.0000000	0.0815000
3477.0000000	0.0809524
3478.0000000	0.0810000
3479.0000000	0.0820000
3480.0000000	0.0805000
3481.0000000	0.0804762
3482.0000000	0.0805000
3483.0000000	0.0805000
3484.0000000	0.0800000
3485.0000000	0.0800000
3486.0000000	0.0800000
3487.0000000	0.0800000
3488.0000000	0.0800000
3489.0000000	0.0800000
3490.0000000	0.0800000
3491.0000000	0.0795000
3492.0000000	0.0790000
3493.0000000	0.0795238
3494.0000000	0.0800000
3495.0000000	0.0790000
3496.0000000	0.0795000
3497.0000000	0.0800000
3498.0000000	0.0795000
3499.0000000	0.0790476
3500.0000000	0.0800000
3501.0000000	0.0800000
3502.0000000	0.0800000
3503.0000000	0.0800000
3504.0000000	0.0800000
3505.0000000	0.0800000
3506.0000000	0.0800000
3507.0000000	0.0800000
3508.0000000	0.0800000
3509.0000000	0.0800000

3510.0000000	0.0800000
3511.0000000	0.0800000
3512.0000000	0.0785000
3513.0000000	0.0785000
3514.0000000	0.0775000
3515.0000000	0.0738095
3516.0000000	0.0733333
3517.0000000	0.0720000
3518.0000000	0.0715000
3519.0000000	0.0715000
3520.0000000	0.0709524
3521.0000000	0.0730000
3522.0000000	0.0720000
3523.0000000	0.0733333
3524.0000000	0.0770000
3525.0000000	0.0775000
3526.0000000	0.0776191
3527.0000000	0.0780000
3528.0000000	0.0760000
3529.0000000	0.0738095
3530.0000000	0.0730000
3531.0000000	0.0742857
3532.0000000	0.0770000
3533.0000000	0.0780000
3534.0000000	0.0790000
3535.0000000	0.0800000
3536.0000000	0.0785000
3537.0000000	0.0795000
3538.0000000	0.0795238
3539.0000000	0.0795000
3540.0000000	0.0800000
3541.0000000	0.0800000
3542.0000000	0.0795238
3543.0000000	0.0780000
3544.0000000	0.0800000
3545.0000000	0.0800000
3546.0000000	0.0800000
3547.0000000	0.0800000
3548.0000000	0.0800000
3549.0000000	0.0800000
3550.0000000	0.0800000
3551.0000000	0.0800000
3552.0000000	0.0800000
3553.0000000	0.0800000
3554.0000000	0.0800000
3555.0000000	0.0800000
3556.0000000	0.0800000
3557.0000000	0.0800000
3558.0000000	0.0800000
3559.0000000	0.0800000
3560.0000000	0.0805000
3561.0000000	0.0805000
3562.0000000	0.0809524
3563.0000000	0.0810000
3564.0000000	0.0820000
3565.0000000	0.0819048
3566.0000000	0.0835000

3567.0000000	0.0840000
3568.0000000	0.0838095
3569.0000000	0.0840000
3570.0000000	0.0845000
3571.0000000	0.0845000
3572.0000000	0.0861905
3573.0000000	0.0860000
3574.0000000	0.0835000
3575.0000000	0.0847619
3576.0000000	0.0828572
3577.0000000	0.0850000
3578.0000000	0.0865000
3579.0000000	0.0865000
3580.0000000	0.0845000
3581.0000000	0.0852381
3582.0000000	0.0860000
3583.0000000	0.0861905
3584.0000000	0.0865000
3585.0000000	0.0865000
3586.0000000	0.0870000
3587.0000000	0.0847619
3588.0000000	0.0860000
3589.0000000	0.0855000
3590.0000000	0.0857143
3591.0000000	0.0885000
3592.0000000	0.0880953
3593.0000000	0.0865000
3594.0000000	0.0870000
3595.0000000	0.0865000
3596.0000000	0.0885714
3597.0000000	0.0870000
3598.0000000	0.0880000
3599.0000000	0.0885714
3600.0000000	0.0870000
3601.0000000	0.0845000
3602.0000000	0.0820000
3603.0000000	0.0819048
3604.0000000	0.0823810
3605.0000000	0.0830000
3606.0000000	0.0815000
3607.0000000	0.0819048
3608.0000000	0.0810000
3609.0000000	0.0805000
3610.0000000	0.0800000
3611.0000000	0.0814286
3612.0000000	0.0805000
3613.0000000	0.0825000
3614.0000000	0.0838095
3615.0000000	0.0855000
3616.0000000	0.0885000
3617.0000000	0.0871429
3618.0000000	0.0875000
3619.0000000	0.0865000
3620.0000000	0.0876191
3621.0000000	0.0860000
3622.0000000	0.0855000
3623.0000000	0.0842857

3624.0000000	0.0855000
3625.0000000	0.0875000
3626.0000000	0.0861905
3627.0000000	0.0860000
3628.0000000	0.0857143
3629.0000000	0.0860000
3630.0000000	0.0885000
3631.0000000	0.0880000
3632.0000000	0.0857143
3633.0000000	0.0875000
3634.0000000	0.0875000
3635.0000000	0.0876191
3636.0000000	0.0890000
3637.0000000	0.0890476
3638.0000000	0.0895000
3639.0000000	0.0895000
3640.0000000	0.0900000
3641.0000000	0.0895238
3642.0000000	0.0880000
3643.0000000	0.0895000
3644.0000000	0.0895238
3645.0000000	0.0900000
3646.0000000	0.0890000
3647.0000000	0.0876191
3648.0000000	0.0870000
3649.0000000	0.0885000
3650.0000000	0.0866667
3651.0000000	0.0820000
3652.0000000	0.0830000
3653.0000000	0.0819048
3654.0000000	0.0800000
3655.0000000	0.0800000
3656.0000000	0.0800000
3657.0000000	0.0800000
3658.0000000	0.0800000
3659.0000000	0.0800000
3660.0000000	0.0800000
3661.0000000	0.0800000
3662.0000000	0.0800000
3663.0000000	0.0800000
3664.0000000	0.0795238
3665.0000000	0.0795000
3666.0000000	0.0800000
3667.0000000	0.0800000
3668.0000000	0.0800000
3669.0000000	0.0800000
3670.0000000	0.0800000
3671.0000000	0.0800000
3672.0000000	0.0800000
3673.0000000	0.0795238
3674.0000000	0.0800000
3675.0000000	0.0800000
3676.0000000	0.0800000
3677.0000000	0.0800000
3678.0000000	0.0800000
3679.0000000	0.0800000
3680.0000000	0.0804762

3681.0000000	0.0805000
3682.0000000	0.0800000
3683.0000000	0.0800000
3684.0000000	0.0810000
3685.0000000	0.0800000
3686.0000000	0.0800000
3687.0000000	0.0810000
3688.0000000	0.0805000
3689.0000000	0.0800000
3690.0000000	0.0800000
3691.0000000	0.0800000
3692.0000000	0.0800000
3693.0000000	0.0800000
3694.0000000	0.0800000
3695.0000000	0.0800000
3696.0000000	0.0800000
3697.0000000	0.0800000
3698.0000000	0.0800000
3699.0000000	0.0800000
3700.0000000	0.0800000
3701.0000000	0.0800000
3702.0000000	0.0800000
3703.0000000	0.0800000
3704.0000000	0.0800000
3705.0000000	0.0800000
3706.0000000	0.0800000
3707.0000000	0.0800000
3708.0000000	0.0800000
3709.0000000	0.0800000
3710.0000000	0.0800000
3711.0000000	0.0800000
3712.0000000	0.0800000
3713.0000000	0.0800000
3714.0000000	0.0800000
3715.0000000	0.0800000
3716.0000000	0.0800000
3717.0000000	0.0800000
3718.0000000	0.0800000
3719.0000000	0.0800000
3720.0000000	0.0804762
3721.0000000	0.0800000
3722.0000000	0.0800000
3723.0000000	0.0800000
3724.0000000	0.0800000
3725.0000000	0.0800000
3726.0000000	0.0800000
3727.0000000	0.0800000
3728.0000000	0.0800000
3729.0000000	0.0800000
3730.0000000	0.0765000
3731.0000000	0.0745000
3732.0000000	0.0747619
3733.0000000	0.0725000
3734.0000000	0.0709524
3735.0000000	0.0710000
3736.0000000	0.0725000
3737.0000000	0.0725000

3738.0000000	0.0719048
3739.0000000	0.0710000
3740.0000000	0.0715000
3741.0000000	0.0728572
3742.0000000	0.0730000
3743.0000000	0.0742857
3744.0000000	0.0730000
3745.0000000	0.0750000
3746.0000000	0.0730000
3747.0000000	0.0733333
3748.0000000	0.0755000
3749.0000000	0.0755000
3750.0000000	0.0738095
3751.0000000	0.0760000
3752.0000000	0.0771429
3753.0000000	0.0775000
3754.0000000	0.0770000
3755.0000000	0.0775000
3756.0000000	0.0771429
3757.0000000	0.0785000
3758.0000000	0.0780000
3759.0000000	0.0776191
3760.0000000	0.0770000
3761.0000000	0.0760000
3762.0000000	0.0757143
3763.0000000	0.0790000
3764.0000000	0.0780952
3765.0000000	0.0770000
3766.0000000	0.0780000
3767.0000000	0.0785000
3768.0000000	0.0780952
3769.0000000	0.0775000
3770.0000000	0.0785714
3771.0000000	0.0780000
3772.0000000	0.0775000
3773.0000000	0.0780952
3774.0000000	0.0790000
3775.0000000	0.0800000
3776.0000000	0.0795000
3777.0000000	0.0800000
3778.0000000	0.0800000
3779.0000000	0.0800000
3780.0000000	0.0800000
3781.0000000	0.0800000
3782.0000000	0.0800000
3783.0000000	0.0800000
3784.0000000	0.0800000
3785.0000000	0.0800000
3786.0000000	0.0800000
3787.0000000	0.0800000
3788.0000000	0.0800000
3789.0000000	0.0800000
3790.0000000	0.0795000
3791.0000000	0.0800000
3792.0000000	0.0800000
3793.0000000	0.0800000
3794.0000000	0.0800000

3795.0000000	0.0790476
3796.0000000	0.0800000
3797.0000000	0.0800000
3798.0000000	0.0800000
3799.0000000	0.0800000
3800.0000000	0.0800000
3801.0000000	0.0800000
3802.0000000	0.0800000
3803.0000000	0.0800000
3804.0000000	0.0800000
3805.0000000	0.0800000
3806.0000000	0.0800000
3807.0000000	0.0800000
3808.0000000	0.0800000
3809.0000000	0.0800000
3810.0000000	0.0800000
3811.0000000	0.0800000
3812.0000000	0.0800000
3813.0000000	0.0800000
3814.0000000	0.0800000
3815.0000000	0.0800000
3816.0000000	0.0800000
3817.0000000	0.0805000
3818.0000000	0.0800000
3819.0000000	0.0800000
3820.0000000	0.0800000
3821.0000000	0.0800000
3822.0000000	0.0800000
3823.0000000	0.0800000
3824.0000000	0.0800000
3825.0000000	0.0790476
3826.0000000	0.0795000
3827.0000000	0.0760000
3828.0000000	0.0755000
3829.0000000	0.0766667
3830.0000000	0.0760000
3831.0000000	0.0776191
3832.0000000	0.0755000
3833.0000000	0.0757143
3834.0000000	0.0780000
3835.0000000	0.0775000
3836.0000000	0.0775000
3837.0000000	0.0785000
3838.0000000	0.0785714
3839.0000000	0.0785000
3840.0000000	0.0776191
3841.0000000	0.0800000
3842.0000000	0.0800000
3843.0000000	0.0795000
3844.0000000	0.0790476
3845.0000000	0.0800000
3846.0000000	0.0800000
3847.0000000	0.0800000
3848.0000000	0.0800000
3849.0000000	0.0790476
3850.0000000	0.0800000
3851.0000000	0.0785000

3852.0000000	0.0790476
3853.0000000	0.0790000
3854.0000000	0.0785000
3855.0000000	0.0785000
3856.0000000	0.0761905
3857.0000000	0.0760000
3858.0000000	0.0742857
3859.0000000	0.0735000
3860.0000000	0.0740000
3861.0000000	0.0738095
3862.0000000	0.0735000
3863.0000000	0.0750000
3864.0000000	0.0742857
3865.0000000	0.0750000
3866.0000000	0.0740000
3867.0000000	0.0725000
3868.0000000	0.0733333
3869.0000000	0.0738095
3870.0000000	0.0730000
3871.0000000	0.0740000
3872.0000000	0.0725000
3873.0000000	0.0750000
3874.0000000	0.0733333
3875.0000000	0.0720000
3876.0000000	0.0728572
3877.0000000	0.0730000
3878.0000000	0.0757143
3879.0000000	0.0755000
3880.0000000	0.0765000
3881.0000000	0.0755000
3882.0000000	0.0765000
3883.0000000	0.0761905
3884.0000000	0.0760000
3885.0000000	0.0766667
3886.0000000	0.0770000
3887.0000000	0.0765000
3888.0000000	0.0755000
3889.0000000	0.0747619
3890.0000000	0.0755000
3891.0000000	0.0745000
3892.0000000	0.0733333
3893.0000000	0.0780000
3894.0000000	0.0785714
3895.0000000	0.0780000
3896.0000000	0.0790000
3897.0000000	0.0790476
3898.0000000	0.0800000
3899.0000000	0.0785000
3900.0000000	0.0800000
3901.0000000	0.0800000
3902.0000000	0.0800000
3903.0000000	0.0800000
3904.0000000	0.0800000
3905.0000000	0.0800000
3906.0000000	0.0800000
3907.0000000	0.0800000
3908.0000000	0.0800000

3909.0000000	0.0800000
3910.0000000	0.0800000
3911.0000000	0.0800000
3912.0000000	0.0800000
3913.0000000	0.0800000
3914.0000000	0.0800000
3915.0000000	0.0800000
3916.0000000	0.0800000
3917.0000000	0.0805000
3918.0000000	0.0805000
3919.0000000	0.0800000
3920.0000000	0.0810000
3921.0000000	0.0800000
3922.0000000	0.0815000
3923.0000000	0.0815000
3924.0000000	0.0805000
3925.0000000	0.0833334
3926.0000000	0.0840000
3927.0000000	0.0830000
3928.0000000	0.0847619
3929.0000000	0.0850000
3930.0000000	0.0838095
3931.0000000	0.0805000
3932.0000000	0.0815000
3933.0000000	0.0805000
3934.0000000	0.0800000
3935.0000000	0.0800000
3936.0000000	0.0800000
3937.0000000	0.0800000
3938.0000000	0.0800000
3939.0000000	0.0804762
3940.0000000	0.0800000
3941.0000000	0.0800000
3942.0000000	0.0800000
3943.0000000	0.0800000
3944.0000000	0.0800000
3945.0000000	0.0800000
3946.0000000	0.0780952
3947.0000000	0.0765000
3948.0000000	0.0775000
3949.0000000	0.0771429
3950.0000000	0.0770000
3951.0000000	0.0750000
3952.0000000	0.0766667
3953.0000000	0.0755000
3954.0000000	0.0770000
3955.0000000	0.0747619
3956.0000000	0.0760000
3957.0000000	0.0742857
3958.0000000	0.0730000
3959.0000000	0.0720000
3960.0000000	0.0715000
3961.0000000	0.0719048
3962.0000000	0.0735000
3963.0000000	0.0705000
3964.0000000	0.0705000
3965.0000000	0.0704762

3966.0000000	0.0709524
3967.0000000	0.0710000
3968.0000000	0.0725000
3969.0000000	0.0719048
3970.0000000	0.0700000
3971.0000000	0.0705000
3972.0000000	0.0705000
3973.0000000	0.0728572
3974.0000000	0.0710000
3975.0000000	0.0704762
3976.0000000	0.0710000
3977.0000000	0.0700000
3978.0000000	0.0700000
3979.0000000	0.0705000
3980.0000000	0.0704762
3981.0000000	0.0700000
3982.0000000	0.0700000
3983.0000000	0.0705000
3984.0000000	0.0700000
3985.0000000	0.0700000
3986.0000000	0.0730000
3987.0000000	0.0710000
3988.0000000	0.0725000
3989.0000000	0.0728572
3990.0000000	0.0750000
3991.0000000	0.0747619
3992.0000000	0.0715000
3993.0000000	0.0705000
3994.0000000	0.0723810
3995.0000000	0.0725000
3996.0000000	0.0705000
3997.0000000	0.0719048
3998.0000000	0.0730000
3999.0000000	0.0715000
4000.0000000	0.0733333
4001.0000000	0.0730000
4002.0000000	0.0725000
4003.0000000	0.0740000
4004.0000000	0.0733333
4005.0000000	0.0770000
4006.0000000	0.0747619
4007.0000000	0.0745000
4008.0000000	0.0725000
4009.0000000	0.0709524
4010.0000000	0.0710000
4011.0000000	0.0710000
4012.0000000	0.0700000
4013.0000000	0.0714286
4014.0000000	0.0714286
4015.0000000	0.0710000
4016.0000000	0.0715000
4017.0000000	0.0710000
4018.0000000	0.0709524
4019.0000000	0.0700000
4020.0000000	0.0705000
4021.0000000	0.0700000
4022.0000000	0.0700000

4023.0000000	0.0705000
4024.0000000	0.0710000
4025.0000000	0.0700000
4026.0000000	0.0705000
4027.0000000	0.0709524
4028.0000000	0.0705000
4029.0000000	0.0700000
4030.0000000	0.0700000
4031.0000000	0.0700000
4032.0000000	0.0700000
4033.0000000	0.0700000
4034.0000000	0.0700000
4035.0000000	0.0700000
4036.0000000	0.0700000
4037.0000000	0.0700000
4038.0000000	0.0700000
4039.0000000	0.0700000
4040.0000000	0.0700000
4041.0000000	0.0700000
4042.0000000	0.0700000
4043.0000000	0.0700000
4044.0000000	0.0700000
4045.0000000	0.0700000
4046.0000000	0.0700000
4047.0000000	0.0700000
4048.0000000	0.0710000
4049.0000000	0.0700000
4050.0000000	0.0700000
4051.0000000	0.0720000
4052.0000000	0.0738095
4053.0000000	0.0755000
4054.0000000	0.0761905
4055.0000000	0.0775000
4056.0000000	0.0770000
4057.0000000	0.0765000
4058.0000000	0.0785714
4059.0000000	0.0755000
4060.0000000	0.0765000
4061.0000000	0.0766667
4062.0000000	0.0775000
4063.0000000	0.0776191
4064.0000000	0.0770000
4065.0000000	0.0775000
4066.0000000	0.0766667
4067.0000000	0.0780000
4068.0000000	0.0740000
4069.0000000	0.0740000
4070.0000000	0.0728572
4071.0000000	0.0715000
4072.0000000	0.0720000
4073.0000000	0.0714286
4074.0000000	0.0714286
4075.0000000	0.0705000
4076.0000000	0.0700000
4077.0000000	0.0700000
4078.0000000	0.0700000
4079.0000000	0.0709524

4080.0000000	0.0710000
4081.0000000	0.0725000
4082.0000000	0.0709524
4083.0000000	0.0715000
4084.0000000	0.0705000
4085.0000000	0.0700000
4086.0000000	0.0700000
4087.0000000	0.0700000
4088.0000000	0.0704762
4089.0000000	0.0705000
4090.0000000	0.0700000
4091.0000000	0.0700000
4092.0000000	0.0700000
4093.0000000	0.0700000
4094.0000000	0.0709524
4095.0000000	0.0710000
4096.0000000	0.0710000
4097.0000000	0.0704762
4098.0000000	0.0715000
4099.0000000	0.0700000
4100.0000000	0.0720000
4101.0000000	0.0719048
4102.0000000	0.0714286
4103.0000000	0.0710000
4104.0000000	0.0710000
4105.0000000	0.0700000
4106.0000000	0.0704762
4107.0000000	0.0705000
4108.0000000	0.0710000
4109.0000000	0.0704762
4110.0000000	0.0700000
4111.0000000	0.0700000
4112.0000000	0.0709524
4113.0000000	0.0705000
4114.0000000	0.0720000
4115.0000000	0.0719048
4116.0000000	0.0715000
4117.0000000	0.0715000
4118.0000000	0.0700000
4119.0000000	0.0700000
4120.0000000	0.0710000
4121.0000000	0.0700000
4122.0000000	0.0700000
4123.0000000	0.0700000
4124.0000000	0.0704762
4125.0000000	0.0700000
4126.0000000	0.0700000
4127.0000000	0.0700000
4128.0000000	0.0695000
4129.0000000	0.0690000
4130.0000000	0.0680000
4131.0000000	0.0661905
4132.0000000	0.0665000
4133.0000000	0.0647619
4134.0000000	0.0645000
4135.0000000	0.0633333
4136.0000000	0.0620000

4137.0000000	0.0600000
4138.0000000	0.0635000
4139.0000000	0.0610000
4140.0000000	0.0623809
4141.0000000	0.0630000
4142.0000000	0.0619048
4143.0000000	0.0630000
4144.0000000	0.0640000
4145.0000000	0.0625000
4146.0000000	0.0623809
4147.0000000	0.0635000
4148.0000000	0.0650000
4149.0000000	0.0628571
4150.0000000	0.0625000
4151.0000000	0.0628571
4152.0000000	0.0615000
4153.0000000	0.0630000
4154.0000000	0.0666667
4155.0000000	0.0650000
4156.0000000	0.0645000
4157.0000000	0.0625000
4158.0000000	0.0614286
4159.0000000	0.0620000
4160.0000000	0.0604762
4161.0000000	0.0605000
4162.0000000	0.0610000
4163.0000000	0.0600000
4164.0000000	0.0610000
4165.0000000	0.0605000
4166.0000000	0.0614286
4167.0000000	0.0615000
4168.0000000	0.0615000
4169.0000000	0.0610000
4170.0000000	0.0614286
4171.0000000	0.0604762
4172.0000000	0.0620000
4173.0000000	0.0615000
4174.0000000	0.0625000
4175.0000000	0.0620000
4176.0000000	0.0633333
4177.0000000	0.0650000
4178.0000000	0.0666667
4179.0000000	0.0680000
4180.0000000	0.0690476
4181.0000000	0.0685000
4182.0000000	0.0690000
4183.0000000	0.0690000
4184.0000000	0.0695000
4185.0000000	0.0690476
4186.0000000	0.0700000
4187.0000000	0.0695238
4188.0000000	0.0695000
4189.0000000	0.0700000
4190.0000000	0.0700000
4191.0000000	0.0700000
4192.0000000	0.0700000
4193.0000000	0.0700000

4194.0000000	0.0695238
4195.0000000	0.0700000
4196.0000000	0.0700000
4197.0000000	0.0700000
4198.0000000	0.0700000
4199.0000000	0.0695238
4200.0000000	0.0675000
4201.0000000	0.0690000
4202.0000000	0.0676190
4203.0000000	0.0660000
4204.0000000	0.0675000
4205.0000000	0.0695000
4206.0000000	0.0666667
4207.0000000	0.0675000
4208.0000000	0.0680000
4209.0000000	0.0676190
4210.0000000	0.0695000
4211.0000000	0.0690476
4212.0000000	0.0690000
4213.0000000	0.0700000
4214.0000000	0.0700000
4215.0000000	0.0700000
4216.0000000	0.0685000
4217.0000000	0.0700000
4218.0000000	0.0695238
4219.0000000	0.0675000
4220.0000000	0.0700000
4221.0000000	0.0700000
4222.0000000	0.0695000
4223.0000000	0.0680952
4224.0000000	0.0695000
4225.0000000	0.0695000
4226.0000000	0.0685714
4227.0000000	0.0675000
4228.0000000	0.0660000
4229.0000000	0.0690000
4230.0000000	0.0690476
4231.0000000	0.0675000
4232.0000000	0.0676191
4233.0000000	0.0685000
4234.0000000	0.0695000
4235.0000000	0.0680000
4236.0000000	0.0690000
4237.0000000	0.0690476
4238.0000000	0.0700000
4239.0000000	0.0700000
4240.0000000	0.0695000
4241.0000000	0.0695238
4242.0000000	0.0700000
4243.0000000	0.0695000
4244.0000000	0.0700000
4245.0000000	0.0700000
4246.0000000	0.0700000
4247.0000000	0.0700000
4248.0000000	0.0700000
4249.0000000	0.0700000
4250.0000000	0.0695000

4251.0000000	0.0700000
4252.0000000	0.0700000
4253.0000000	0.0700000
4254.0000000	0.0700000
4255.0000000	0.0700000
4256.0000000	0.0700000
4257.0000000	0.0700000
4258.0000000	0.0700000
4259.0000000	0.0700000
4260.0000000	0.0700000
4261.0000000	0.0700000
4262.0000000	0.0700000
4263.0000000	0.0700000
4264.0000000	0.0700000
4265.0000000	0.0700000
4266.0000000	0.0700000
4267.0000000	0.0695000
4268.0000000	0.0700000
4269.0000000	0.0700000
4270.0000000	0.0695000
4271.0000000	0.0690476
4272.0000000	0.0685000
4273.0000000	0.0680000
4274.0000000	0.0675000
4275.0000000	0.0657143
4276.0000000	0.0665000
4277.0000000	0.0635000
4278.0000000	0.0628571
4279.0000000	0.0620000
4280.0000000	0.0620000
4281.0000000	0.0645000
4282.0000000	0.0609524
4283.0000000	0.0625000
4284.0000000	0.0633333
4285.0000000	0.0625000
4286.0000000	0.0630000
4287.0000000	0.0633333
4288.0000000	0.0640000
4289.0000000	0.0615000
4290.0000000	0.0642857
4291.0000000	0.0630000
4292.0000000	0.0625000
4293.0000000	0.0633333
4294.0000000	0.0630000
4295.0000000	0.0630000
4296.0000000	0.0625000
4297.0000000	0.0628571
4298.0000000	0.0620000
4299.0000000	0.0609524
4300.0000000	0.0605000
4301.0000000	0.0620000
4302.0000000	0.0614286
4303.0000000	0.0605000
4304.0000000	0.0610000
4305.0000000	0.0620000
4306.0000000	0.0600000
4307.0000000	0.0614286

4308.0000000	0.0610000
4309.0000000	0.0600000
4310.0000000	0.0615000
4311.0000000	0.0604762
4312.0000000	0.0615000
4313.0000000	0.0600000
4314.0000000	0.0609524
4315.0000000	0.0610000
4316.0000000	0.0635000
4317.0000000	0.0628571
4318.0000000	0.0615000
4319.0000000	0.0640000
4320.0000000	0.0652381
4321.0000000	0.0635000
4322.0000000	0.0640000
4323.0000000	0.0628571
4324.0000000	0.0635000
4325.0000000	0.0620000
4326.0000000	0.0625000
4327.0000000	0.0642857
4328.0000000	0.0630000
4329.0000000	0.0623809
4330.0000000	0.0625000
4331.0000000	0.0628571
4332.0000000	0.0605000
4333.0000000	0.0610000
4334.0000000	0.0620000
4335.0000000	0.0615000
4336.0000000	0.0623809
4337.0000000	0.0650000
4338.0000000	0.0652381
4339.0000000	0.0640000
4340.0000000	0.0635000
4341.0000000	0.0630000
4342.0000000	0.0633333
4343.0000000	0.0630000
4344.0000000	0.0625000
4345.0000000	0.0623809
4346.0000000	0.0600000
4347.0000000	0.0604762
4348.0000000	0.0600000
4349.0000000	0.0600000
4350.0000000	0.0605000
4351.0000000	0.0600000
4352.0000000	0.0600000
4353.0000000	0.0605000
4354.0000000	0.0604762
4355.0000000	0.0605000
4356.0000000	0.0600000
4357.0000000	0.0600000
4358.0000000	0.0600000
4359.0000000	0.0600000
4360.0000000	0.0600000
4361.0000000	0.0600000
4362.0000000	0.0600000
4363.0000000	0.0600000
4364.0000000	0.0600000

4365.0000000	0.0600000
4366.0000000	0.0600000
4367.0000000	0.0600000
4368.0000000	0.0600000
4369.0000000	0.0600000
4370.0000000	0.0600000
4371.0000000	0.0600000
4372.0000000	0.0600000
4373.0000000	0.0600000
4374.0000000	0.0600000
4375.0000000	0.0600000
4376.0000000	0.0600000
4377.0000000	0.0604762
4378.0000000	0.0600000
4379.0000000	0.0600000
4380.0000000	0.0615000
4381.0000000	0.0623809
4382.0000000	0.0610000
4383.0000000	0.0614286
4384.0000000	0.0635000
4385.0000000	0.0620000
4386.0000000	0.0605000
4387.0000000	0.0633333
4388.0000000	0.0645000
4389.0000000	0.0665000
4390.0000000	0.0680952
4391.0000000	0.0695000
4392.0000000	0.0700000
4393.0000000	0.0685000
4394.0000000	0.0695000
4395.0000000	0.0700000
4396.0000000	0.0695000
4397.0000000	0.0695000
4398.0000000	0.0700000
4399.0000000	0.0700000
4400.0000000	0.0695000
4401.0000000	0.0700000
4402.0000000	0.0690000
4403.0000000	0.0690476
4404.0000000	0.0700000
4405.0000000	0.0695000
4406.0000000	0.0700000
4407.0000000	0.0695238
4408.0000000	0.0690000
4409.0000000	0.0700000
4410.0000000	0.0695000
4411.0000000	0.0695000
4412.0000000	0.0695238
4413.0000000	0.0685000
4414.0000000	0.0680952
4415.0000000	0.0685000
4416.0000000	0.0675000
4417.0000000	0.0657143
4418.0000000	0.0650000
4419.0000000	0.0640000
4420.0000000	0.0614286
4421.0000000	0.0605000

4422.0000000	0.0620000
4423.0000000	0.0604762
4424.0000000	0.0605000
4425.0000000	0.0610000
4426.0000000	0.0609524
4427.0000000	0.0625000
4428.0000000	0.0620000
4429.0000000	0.0623809
4430.0000000	0.0635000
4431.0000000	0.0610000
4432.0000000	0.0610000
4433.0000000	0.0609524
4434.0000000	0.0600000
4435.0000000	0.0600000
4436.0000000	0.0605000
4437.0000000	0.0600000
4438.0000000	0.0600000
4439.0000000	0.0600000
4440.0000000	0.0605000
4441.0000000	0.0605000
4442.0000000	0.0614286
4443.0000000	0.0615000
4444.0000000	0.0614286
4445.0000000	0.0620000
4446.0000000	0.0615000
4447.0000000	0.0609524
4448.0000000	0.0605000
4449.0000000	0.0605000
4450.0000000	0.0610000
4451.0000000	0.0600000
4452.0000000	0.0600000
4453.0000000	0.0600000
4454.0000000	0.0600000
4455.0000000	0.0600000
4456.0000000	0.0600000
4457.0000000	0.0600000
4458.0000000	0.0600000
4459.0000000	0.0600000
4460.0000000	0.0605000
4461.0000000	0.0605000
4462.0000000	0.0600000
4463.0000000	0.0600000
4464.0000000	0.0600000
4465.0000000	0.0600000
4466.0000000	0.0600000
4467.0000000	0.0600000
4468.0000000	0.0600000
4469.0000000	0.0600000
4470.0000000	0.0600000
4471.0000000	0.0600000
4472.0000000	0.0600000
4473.0000000	0.0600000
4474.0000000	0.0600000
4475.0000000	0.0600000
4476.0000000	0.0595000
4477.0000000	0.0600000
4478.0000000	0.0600000

4479.0000000	0.0600000
4480.0000000	0.0600000
4481.0000000	0.0600000
4482.0000000	0.0600000
4483.0000000	0.0600000
4484.0000000	0.0600000
4485.0000000	0.0600000
4486.0000000	0.0600000
4487.0000000	0.0600000
4488.0000000	0.0600000
4489.0000000	0.0600000
4490.0000000	0.0600000
4491.0000000	0.0600000
4492.0000000	0.0600000
4493.0000000	0.0600000
4494.0000000	0.0600000
4495.0000000	0.0600000
4496.0000000	0.0600000
4497.0000000	0.0600000
4498.0000000	0.0600000
4499.0000000	0.0600000
4500.0000000	0.0600000
4501.0000000	0.0600000
4502.0000000	0.0600000
4503.0000000	0.0600000
4504.0000000	0.0600000
4505.0000000	0.0600000
4506.0000000	0.0600000
4507.0000000	0.0600000
4508.0000000	0.0600000
4509.0000000	0.0600000
4510.0000000	0.0600000
4511.0000000	0.0600000
4512.0000000	0.0600000
4513.0000000	0.0605000
4514.0000000	0.0600000
4515.0000000	0.0605000
4516.0000000	0.0604762
4517.0000000	0.0600000
4518.0000000	0.0600000
4519.0000000	0.0604762
4520.0000000	0.0605000
4521.0000000	0.0605000
4522.0000000	0.0605000
4523.0000000	0.0609524
4524.0000000	0.0605000
4525.0000000	0.0615000
4526.0000000	0.0614286
4527.0000000	0.0615000
4528.0000000	0.0614286
4529.0000000	0.0635000
4530.0000000	0.0650000
4531.0000000	0.0655000
4532.0000000	0.0652381
4533.0000000	0.0640000
4534.0000000	0.0665000
4535.0000000	0.0642857

4536.0000000	0.0625000
4537.0000000	0.0650000
4538.0000000	0.0650000
4539.0000000	0.0647619
4540.0000000	0.0635000
4541.0000000	0.0614286
4542.0000000	0.0635000
4543.0000000	0.0645000
4544.0000000	0.0666667
4545.0000000	0.0645000
4546.0000000	0.0650000
4547.0000000	0.0647619
4548.0000000	0.0640000
4549.0000000	0.0660000
4550.0000000	0.0642857
4551.0000000	0.0650000
4552.0000000	0.0650000
4553.0000000	0.0633333
4554.0000000	0.0615000
4555.0000000	0.0615000
4556.0000000	0.0600000
4557.0000000	0.0600000
4558.0000000	0.0600000
4559.0000000	0.0600000
4560.0000000	0.0600000
4561.0000000	0.0600000
4562.0000000	0.0600000
4563.0000000	0.0600000
4564.0000000	0.0600000
4565.0000000	0.0600000
4566.0000000	0.0600000
4567.0000000	0.0600000
4568.0000000	0.0600000
4569.0000000	0.0600000
4570.0000000	0.0595000
4571.0000000	0.0595238
4572.0000000	0.0600000
4573.0000000	0.0595238
4574.0000000	0.0600000
4575.0000000	0.0605000
4576.0000000	0.0595000
4577.0000000	0.0600000
4578.0000000	0.0600000
4579.0000000	0.0600000
4580.0000000	0.0600000
4581.0000000	0.0600000
4582.0000000	0.0600000
4583.0000000	0.0590476
4584.0000000	0.0600000
4585.0000000	0.0600000
4586.0000000	0.0600000
4587.0000000	0.0600000
4588.0000000	0.0600000
4589.0000000	0.0600000
4590.0000000	0.0600000
4591.0000000	0.0600000
4592.0000000	0.0600000

4593.0000000	0.0600000
4594.0000000	0.0605000
4595.0000000	0.0600000
4596.0000000	0.0605000
4597.0000000	0.0600000
4598.0000000	0.0615000
4599.0000000	0.0609524
4600.0000000	0.0619048
4601.0000000	0.0625000
4602.0000000	0.0620000
4603.0000000	0.0615000
4604.0000000	0.0619048
4605.0000000	0.0605000
4606.0000000	0.0630000
4607.0000000	0.0610000
4608.0000000	0.0628571
4609.0000000	0.0623810
4610.0000000	0.0605000
4611.0000000	0.0635000
4612.0000000	0.0625000
4613.0000000	0.0623809
4614.0000000	0.0625000
4615.0000000	0.0610000
4616.0000000	0.0614286
4617.0000000	0.0610000
4618.0000000	0.0620000
4619.0000000	0.0604762
4620.0000000	0.0625000
4621.0000000	0.0615000
4622.0000000	0.0614286
4623.0000000	0.0630000
4624.0000000	0.0620000
4625.0000000	0.0604762
4626.0000000	0.0600000
4627.0000000	0.0610000
4628.0000000	0.0605000
4629.0000000	0.0600000
4630.0000000	0.0610000
4631.0000000	0.0623809
4632.0000000	0.0620000
4633.0000000	0.0605000
4634.0000000	0.0623810
4635.0000000	0.0625000
4636.0000000	0.0625000
4637.0000000	0.0625000
4638.0000000	0.0642857
4639.0000000	0.0635000
4640.0000000	0.0609524
4641.0000000	0.0630000
4642.0000000	0.0655000
4643.0000000	0.0650000
4644.0000000	0.0652381
4645.0000000	0.0635000
4646.0000000	0.0640000
4647.0000000	0.0623809
4648.0000000	0.0620000
4649.0000000	0.0619048

4650.0000000	0.0635000
4651.0000000	0.0655000
4652.0000000	0.0647619
4653.0000000	0.0640000
4654.0000000	0.0650000
4655.0000000	0.0625000
4656.0000000	0.0638095
4657.0000000	0.0630000
4658.0000000	0.0630000
4659.0000000	0.0633333
4660.0000000	0.0635000
4661.0000000	0.0623809
4662.0000000	0.0620000
4663.0000000	0.0620000
4664.0000000	0.0614286
4665.0000000	0.0610000
4666.0000000	0.0605000
4667.0000000	0.0620000
4668.0000000	0.0604762
4669.0000000	0.0605000
4670.0000000	0.0604762
4671.0000000	0.0605000
4672.0000000	0.0610000
4673.0000000	0.0600000
4674.0000000	0.0600000
4675.0000000	0.0600000
4676.0000000	0.0600000
4677.0000000	0.0590476
4678.0000000	0.0600000
4679.0000000	0.0595238
4680.0000000	0.0590000
4681.0000000	0.0590000
4682.0000000	0.0600000
4683.0000000	0.0595238
4684.0000000	0.0600000
4685.0000000	0.0600000
4686.0000000	0.0595238
4687.0000000	0.0600000
4688.0000000	0.0595238
4689.0000000	0.0600000
4690.0000000	0.0600000
4691.0000000	0.0595000
4692.0000000	0.0590476
4693.0000000	0.0595000
4694.0000000	0.0590000
4695.0000000	0.0585714
4696.0000000	0.0585000
4697.0000000	0.0595238
4698.0000000	0.0590000
4699.0000000	0.0595000
4700.0000000	0.0585714
4701.0000000	0.0590000
4702.0000000	0.0590000
4703.0000000	0.0595000
4704.0000000	0.0600000
4705.0000000	0.0595000
4706.0000000	0.0600000

4707.0000000	0.0595238
4708.0000000	0.0600000
4709.0000000	0.0595238
4710.0000000	0.0600000
4711.0000000	0.0595000
4712.0000000	0.0600000
4713.0000000	0.0595238
4714.0000000	0.0595000
4715.0000000	0.0595000
4716.0000000	0.0600000
4717.0000000	0.0595000
4718.0000000	0.0595000
4719.0000000	0.0595000
4720.0000000	0.0590476
4721.0000000	0.0590000
4722.0000000	0.0595238
4723.0000000	0.0600000
4724.0000000	0.0600000
4725.0000000	0.0600000
4726.0000000	0.0590000
4727.0000000	0.0600000
4728.0000000	0.0600000
4729.0000000	0.0600000
4730.0000000	0.0600000
4731.0000000	0.0595238
4732.0000000	0.0600000
4733.0000000	0.0590000
4734.0000000	0.0600000
4735.0000000	0.0595238
4736.0000000	0.0600000
4737.0000000	0.0600000
4738.0000000	0.0600000
4739.0000000	0.0600000
4740.0000000	0.0600000
4741.0000000	0.0600000
4742.0000000	0.0600000
4743.0000000	0.0600000
4744.0000000	0.0600000
4745.0000000	0.0600000
4746.0000000	0.0600000
4747.0000000	0.0600000
4748.0000000	0.0600000
4749.0000000	0.0600000
4750.0000000	0.0600000
4751.0000000	0.0600000
4752.0000000	0.0600000
4753.0000000	0.0600000
4754.0000000	0.0600000
4755.0000000	0.0600000
4756.0000000	0.0600000
4757.0000000	0.0605000
4758.0000000	0.0600000
4759.0000000	0.0600000
4760.0000000	0.0600000
4761.0000000	0.0600000
4762.0000000	0.0600000
4763.0000000	0.0600000

4764.0000000	0.0600000
4765.0000000	0.0600000
4766.0000000	0.0610000
4767.0000000	0.0600000
4768.0000000	0.0600000
4769.0000000	0.0600000
4770.0000000	0.0600000
4771.0000000	0.0600000
4772.0000000	0.0600000
4773.0000000	0.0600000
4774.0000000	0.0600000
4775.0000000	0.0600000
4776.0000000	0.0595238
4777.0000000	0.0600000
4778.0000000	0.0600000
4779.0000000	0.0600000
4780.0000000	0.0600000
4781.0000000	0.0600000
4782.0000000	0.0600000
4783.0000000	0.0600000
4784.0000000	0.0600000
4785.0000000	0.0600000
4786.0000000	0.0600000
4787.0000000	0.0600000
4788.0000000	0.0595238
4789.0000000	0.0590000
4790.0000000	0.0565000
4791.0000000	0.0565000
4792.0000000	0.0542857
4793.0000000	0.0550000
4794.0000000	0.0545000
4795.0000000	0.0557143
4796.0000000	0.0575000
4797.0000000	0.0547619
4798.0000000	0.0535000
4799.0000000	0.0540000
4800.0000000	0.0542857
4801.0000000	0.0540000
4802.0000000	0.0525000
4803.0000000	0.0515000
4804.0000000	0.0509524
4805.0000000	0.0509524
4806.0000000	0.0505000
4807.0000000	0.0505000
4808.0000000	0.0510000
4809.0000000	0.0500000
4810.0000000	0.0519048
4811.0000000	0.0525000
4812.0000000	0.0566667
4813.0000000	0.0530000
4814.0000000	0.0542857
4815.0000000	0.0525000
4816.0000000	0.0530000
4817.0000000	0.0525000
4818.0000000	0.0530000
4819.0000000	0.0528571
4820.0000000	0.0525000

4821.0000000	0.0523810
4822.0000000	0.0515000
4823.0000000	0.0515000
4824.0000000	0.0525000
4825.0000000	0.0509524
4826.0000000	0.0500000
4827.0000000	0.0520000
4828.0000000	0.0504762
4829.0000000	0.0510000
4830.0000000	0.0519048
4831.0000000	0.0505000
4832.0000000	0.0510000
4833.0000000	0.0510000
4834.0000000	0.0504762
4835.0000000	0.0500000
4836.0000000	0.0500000
4837.0000000	0.0504762
4838.0000000	0.0500000
4839.0000000	0.0500000
4840.0000000	0.0500000
4841.0000000	0.0500000
4842.0000000	0.0500000
4843.0000000	0.0500000
4844.0000000	0.0500000
4845.0000000	0.0500000
4846.0000000	0.0500000
4847.0000000	0.0500000
4848.0000000	0.0500000
4849.0000000	0.0495238
4850.0000000	0.0500000
4851.0000000	0.0500000
4852.0000000	0.0500000
4853.0000000	0.0500000
4854.0000000	0.0500000
4855.0000000	0.0495238
4856.0000000	0.0500000
4857.0000000	0.0500000
4858.0000000	0.0500000
4859.0000000	0.0500000
4860.0000000	0.0500000
4861.0000000	0.0495238
4862.0000000	0.0500000
4863.0000000	0.0500000
4864.0000000	0.0500000
4865.0000000	0.0505000
4866.0000000	0.0500000
4867.0000000	0.0500000
4868.0000000	0.0500000
4869.0000000	0.0500000
4870.0000000	0.0500000
4871.0000000	0.0500000
4872.0000000	0.0500000
4873.0000000	0.0500000
4874.0000000	0.0500000
4875.0000000	0.0500000
4876.0000000	0.0500000
4877.0000000	0.0500000

4878.0000000	0.0510000
4879.0000000	0.0500000
4880.0000000	0.0500000
4881.0000000	0.0500000
4882.0000000	0.0504762
4883.0000000	0.0505000
4884.0000000	0.0510000
4885.0000000	0.0509524
4886.0000000	0.0510000
4887.0000000	0.0500000
4888.0000000	0.0519048
4889.0000000	0.0505000
4890.0000000	0.0500000
4891.0000000	0.0500000
4892.0000000	0.0515000
4893.0000000	0.0504762
4894.0000000	0.0500000
4895.0000000	0.0500000
4896.0000000	0.0500000
4897.0000000	0.0519048
4898.0000000	0.0515000
4899.0000000	0.0500000
4900.0000000	0.0505000
4901.0000000	0.0500000
4902.0000000	0.0509524
4903.0000000	0.0515000
4904.0000000	0.0520000
4905.0000000	0.0523810
4906.0000000	0.0540000
4907.0000000	0.0530000
4908.0000000	0.0525000
4909.0000000	0.0519048
4910.0000000	0.0515000
4911.0000000	0.0520000
4912.0000000	0.0523810
4913.0000000	0.0520000
4914.0000000	0.0520000
4915.0000000	0.0515000
4916.0000000	0.0538095
4917.0000000	0.0515000
4918.0000000	0.0538095
4919.0000000	0.0530000
4920.0000000	0.0525000
4921.0000000	0.0519048
4922.0000000	0.0535000
4923.0000000	0.0555000
4924.0000000	0.0560000
4925.0000000	0.0557143
4926.0000000	0.0565000
4927.0000000	0.0557143
4928.0000000	0.0555000
4929.0000000	0.0535000
4930.0000000	0.0560000
4931.0000000	0.0533333
4932.0000000	0.0545000
4933.0000000	0.0533333
4934.0000000	0.0550000

4935.0000000	0.0535000
4936.0000000	0.0538095
4937.0000000	0.0555000
4938.0000000	0.0560000
4939.0000000	0.0545000
4940.0000000	0.0542857
4941.0000000	0.0550000
4942.0000000	0.0547619
4943.0000000	0.0560000
4944.0000000	0.0545000
4945.0000000	0.0557143
4946.0000000	0.0565000
4947.0000000	0.0570000
4948.0000000	0.0590000
4949.0000000	0.0566667
4950.0000000	0.0575000
4951.0000000	0.0580952
4952.0000000	0.0595000
4953.0000000	0.0595000
4954.0000000	0.0571428
4955.0000000	0.0580000
4956.0000000	0.0580000
4957.0000000	0.0580952
4958.0000000	0.0575000
4959.0000000	0.0580000
4960.0000000	0.0590000
4961.0000000	0.0580952
4962.0000000	0.0580000
4963.0000000	0.0585714
4964.0000000	0.0585000
4965.0000000	0.0585000
4966.0000000	0.0576190
4967.0000000	0.0590000
4968.0000000	0.0595000
4969.0000000	0.0600000
4970.0000000	0.0600000
4971.0000000	0.0600000
4972.0000000	0.0600000
4973.0000000	0.0600000
4974.0000000	0.0590000
4975.0000000	0.0600000
4976.0000000	0.0600000
4977.0000000	0.0600000
4978.0000000	0.0595000
4979.0000000	0.0595238
4980.0000000	0.0585000
4981.0000000	0.0600000
4982.0000000	0.0595000
4983.0000000	0.0595000
4984.0000000	0.0600000
4985.0000000	0.0585714
4986.0000000	0.0600000
4987.0000000	0.0600000
4988.0000000	0.0600000
4989.0000000	0.0600000
4990.0000000	0.0600000
4991.0000000	0.0600000

4992.0000000	0.0600000
4993.0000000	0.0600000
4994.0000000	0.0600000
4995.0000000	0.0585000
4996.0000000	0.0595000
4997.0000000	0.0595238
4998.0000000	0.0595000
4999.0000000	0.0590000
5000.0000000	0.0566667
5001.0000000	0.0570000
5002.0000000	0.0590476
5003.0000000	0.0570000
5004.0000000	0.0580000
5005.0000000	0.0580000
5006.0000000	0.0585714
5007.0000000	0.0555000
5008.0000000	0.0585000
5009.0000000	0.0566667
5010.0000000	0.0580000
5011.0000000	0.0566667
5012.0000000	0.0585000
5013.0000000	0.0570000
5014.0000000	0.0565000
5015.0000000	0.0561905
5016.0000000	0.0530000
5017.0000000	0.0505000
5018.0000000	0.0514286
5019.0000000	0.0500000
5020.0000000	0.0510000
5021.0000000	0.0504762
5022.0000000	0.0510000
5023.0000000	0.0510000
5024.0000000	0.0523810
5025.0000000	0.0510000
5026.0000000	0.0504762
5027.0000000	0.0515000
5028.0000000	0.0520000
5029.0000000	0.0525000
5030.0000000	0.0519048
5031.0000000	0.0520000
5032.0000000	0.0515000
5033.0000000	0.0514286
5034.0000000	0.0530000
5035.0000000	0.0500000
5036.0000000	0.0520000
5037.0000000	0.0509524
5038.0000000	0.0515000
5039.0000000	0.0519048
5040.0000000	0.0505000
5041.0000000	0.0500000
5042.0000000	0.0509524
5043.0000000	0.0510000
5044.0000000	0.0505000
5045.0000000	0.0504762
5046.0000000	0.0515000
5047.0000000	0.0540000
5048.0000000	0.0545000

5049.0000000	0.0557143
5050.0000000	0.0560000
5051.0000000	0.0576190
5052.0000000	0.0590000
5053.0000000	0.0595000
5054.0000000	0.0595238
5055.0000000	0.0595000
5056.0000000	0.0600000
5057.0000000	0.0590476
5058.0000000	0.0600000
5059.0000000	0.0600000
5060.0000000	0.0600000
5061.0000000	0.0600000
5062.0000000	0.0600000
5063.0000000	0.0600000
5064.0000000	0.0600000
5065.0000000	0.0600000
5066.0000000	0.0600000
5067.0000000	0.0600000
5068.0000000	0.0600000
5069.0000000	0.0600000
5070.0000000	0.0600000
5071.0000000	0.0595238
5072.0000000	0.0600000
5073.0000000	0.0595000
5074.0000000	0.0600000
5075.0000000	0.0600000
5076.0000000	0.0595238
5077.0000000	0.0590000
5078.0000000	0.0585714
5079.0000000	0.0585000
5080.0000000	0.0555000
5081.0000000	0.0565000
5082.0000000	0.0538095
5083.0000000	0.0530000
5084.0000000	0.0530000
5085.0000000	0.0576190
5086.0000000	0.0540000
5087.0000000	0.0580952
5088.0000000	0.0550000
5089.0000000	0.0575000
5090.0000000	0.0585714
5091.0000000	0.0565000
5092.0000000	0.0565000
5093.0000000	0.0590476
5094.0000000	0.0575000
5095.0000000	0.0575000
5096.0000000	0.0565000
5097.0000000	0.0566667
5098.0000000	0.0545000
5099.0000000	0.0542857
5100.0000000	0.0560000
5101.0000000	0.0520000
5102.0000000	0.0514286
5103.0000000	0.0510000
5104.0000000	0.0505000
5105.0000000	0.0505000

5106.0000000	0.0500000
5107.0000000	0.0500000
5108.0000000	0.0504762
5109.0000000	0.0500000
5110.0000000	0.0505000
5111.0000000	0.0500000
5112.0000000	0.0504762
5113.0000000	0.0500000
5114.0000000	0.0500000
5115.0000000	0.0500000
5116.0000000	0.0510000
5117.0000000	0.0504762
5118.0000000	0.0500000
5119.0000000	0.0500000
5120.0000000	0.0505000
5121.0000000	0.0519048
5122.0000000	0.0515000
5123.0000000	0.0509524
5124.0000000	0.0525000
5125.0000000	0.0515000
5126.0000000	0.0510000
5127.0000000	0.0514286
5128.0000000	0.0540000
5129.0000000	0.0535000
5130.0000000	0.0542857
5131.0000000	0.0535000
5132.0000000	0.0533333
5133.0000000	0.0545000
5134.0000000	0.0555000
5135.0000000	0.0555000
5136.0000000	0.0576190
5137.0000000	0.0580000
5138.0000000	0.0590476
5139.0000000	0.0580000
5140.0000000	0.0580000
5141.0000000	0.0595000
5142.0000000	0.0566667
5143.0000000	0.0585000
5144.0000000	0.0565000
5145.0000000	0.0566667
5146.0000000	0.0570000
5147.0000000	0.0600000
5148.0000000	0.0595000
5149.0000000	0.0585000
5150.0000000	0.0580952
5151.0000000	0.0585000
5152.0000000	0.0580000
5153.0000000	0.0580000
5154.0000000	0.0590476
5155.0000000	0.0600000
5156.0000000	0.0580000
5157.0000000	0.0571428
5158.0000000	0.0585000
5159.0000000	0.0576190
5160.0000000	0.0585000
5161.0000000	0.0580000
5162.0000000	0.0580952

5163.0000000	0.0585000
5164.0000000	0.0585000
5165.0000000	0.0580000
5166.0000000	0.0576190
5167.0000000	0.0585000
5168.0000000	0.0580952
5169.0000000	0.0575000
5170.0000000	0.0575000
5171.0000000	0.0560000
5172.0000000	0.0555000
5173.0000000	0.0557143
5174.0000000	0.0545000
5175.0000000	0.0514286
5176.0000000	0.0515000
5177.0000000	0.0514286
5178.0000000	0.0510000
5179.0000000	0.0500000
5180.0000000	0.0500000
5181.0000000	0.0500000
5182.0000000	0.0500000
5183.0000000	0.0500000
5184.0000000	0.0500000
5185.0000000	0.0500000
5186.0000000	0.0510000
5187.0000000	0.0509524
5188.0000000	0.0505000
5189.0000000	0.0505000
5190.0000000	0.0514286
5191.0000000	0.0500000
5192.0000000	0.0500000
5193.0000000	0.0500000
5194.0000000	0.0500000
5195.0000000	0.0510000
5196.0000000	0.0504762
5197.0000000	0.0510000
5198.0000000	0.0509524
5199.0000000	0.0510000
5200.0000000	0.0500000
5201.0000000	0.0505000
5202.0000000	0.0500000
5203.0000000	0.0500000
5204.0000000	0.0505000
5205.0000000	0.0510000
5206.0000000	0.0504762
5207.0000000	0.0510000
5208.0000000	0.0515000
5209.0000000	0.0528571
5210.0000000	0.0530000
5211.0000000	0.0514286
5212.0000000	0.0530000
5213.0000000	0.0535000
5214.0000000	0.0538095
5215.0000000	0.0535000
5216.0000000	0.0545000
5217.0000000	0.0545000
5218.0000000	0.0561905
5219.0000000	0.0560000

5220.0000000	0.0576190
5221.0000000	0.0580000
5222.0000000	0.0580000
5223.0000000	0.0595238
5224.0000000	0.0600000
5225.0000000	0.0600000
5226.0000000	0.0600000
5227.0000000	0.0590000
5228.0000000	0.0600000
5229.0000000	0.0600000
5230.0000000	0.0595000
5231.0000000	0.0595000
5232.0000000	0.0590000
5233.0000000	0.0576190
5234.0000000	0.0580000
5235.0000000	0.0585714
5236.0000000	0.0585000
5237.0000000	0.0585000
5238.0000000	0.0580952
5239.0000000	0.0580000
5240.0000000	0.0585000
5241.0000000	0.0575000
5242.0000000	0.0571428
5243.0000000	0.0565000
5244.0000000	0.0561905
5245.0000000	0.0545000
5246.0000000	0.0545000
5247.0000000	0.0561905
5248.0000000	0.0540000
5249.0000000	0.0545000
5250.0000000	0.0538095
5251.0000000	0.0520000
5252.0000000	0.0520000
5253.0000000	0.0515000
5254.0000000	0.0509524
5255.0000000	0.0530000
5256.0000000	0.0533333
5257.0000000	0.0515000
5258.0000000	0.0515000
5259.0000000	0.0538095
5260.0000000	0.0515000
5261.0000000	0.0510000
5262.0000000	0.0520000
5263.0000000	0.0528571
5264.0000000	0.0525000
5265.0000000	0.0533333
5266.0000000	0.0515000
5267.0000000	0.0515000
5268.0000000	0.0514286
5269.0000000	0.0535000
5270.0000000	0.0530000
5271.0000000	0.0520000
5272.0000000	0.0542857
5273.0000000	0.0520000
5274.0000000	0.0523810
5275.0000000	0.0515000
5276.0000000	0.0505000

5277.0000000	0.0500000
5278.0000000	0.0504762
5279.0000000	0.0520000
5280.0000000	0.0510000
5281.0000000	0.0509524
5282.0000000	0.0515000
5283.0000000	0.0514286
5284.0000000	0.0505000
5285.0000000	0.0510000
5286.0000000	0.0515000
5287.0000000	0.0504762
5288.0000000	0.0500000
5289.0000000	0.0500000
5290.0000000	0.0519048
5291.0000000	0.0505000
5292.0000000	0.0505000
5293.0000000	0.0509524
5294.0000000	0.0520000
5295.0000000	0.0519048
5296.0000000	0.0530000
5297.0000000	0.0515000
5298.0000000	0.0500000
5299.0000000	0.0514286
5300.0000000	0.0520000
5301.0000000	0.0515000
5302.0000000	0.0528571
5303.0000000	0.0550000
5304.0000000	0.0528571
5305.0000000	0.0530000
5306.0000000	0.0565000
5307.0000000	0.0555000
5308.0000000	0.0561905
5309.0000000	0.0570000
5310.0000000	0.0555000
5311.0000000	0.0561905
5312.0000000	0.0555000
5313.0000000	0.0561905
5314.0000000	0.0560000
5315.0000000	0.0575000
5316.0000000	0.0545000
5317.0000000	0.0566667
5318.0000000	0.0555000
5319.0000000	0.0560000
5320.0000000	0.0561905
5321.0000000	0.0570000
5322.0000000	0.0540000
5323.0000000	0.0561905
5324.0000000	0.0565000
5325.0000000	0.0570000
5326.0000000	0.0576190
5327.0000000	0.0565000
5328.0000000	0.0575000
5329.0000000	0.0566667
5330.0000000	0.0585000
5331.0000000	0.0575000
5332.0000000	0.0576190
5333.0000000	0.0580000

5334.0000000	0.0560000
5335.0000000	0.0566667
5336.0000000	0.0545000
5337.0000000	0.0555000
5338.0000000	0.0557143
5339.0000000	0.0540000
5340.0000000	0.0525000
5341.0000000	0.0523810
5342.0000000	0.0520000
5343.0000000	0.0514286
5344.0000000	0.0540000
5345.0000000	0.0520000
5346.0000000	0.0530000
5347.0000000	0.0523810
5348.0000000	0.0536842
5349.0000000	0.0542857
5350.0000000	0.0523810
5351.0000000	0.0550000
5352.0000000	0.0555000
5353.0000000	0.0542857
5354.0000000	0.0525000
5355.0000000	0.0545000
5356.0000000	0.0538095
5357.0000000	0.0555000
5358.0000000	0.0555000
5359.0000000	0.0542857
5360.0000000	0.0535000
5361.0000000	0.0525000
5362.0000000	0.0528571
5363.0000000	0.0545000
5364.0000000	0.0540000
5365.0000000	0.0547619
5366.0000000	0.0545000
5367.0000000	0.0555000
5368.0000000	0.0545000
5369.0000000	0.0552381
5370.0000000	0.0550000
5371.0000000	0.0538095
5372.0000000	0.0550000
5373.0000000	0.0560000
5374.0000000	0.0580952
5375.0000000	0.0590000
5376.0000000	0.0570000
5377.0000000	0.0585000
5378.0000000	0.0576190
5379.0000000	0.0580000
5380.0000000	0.0590476
5381.0000000	0.0585000
5382.0000000	0.0585000
5383.0000000	0.0585714
5384.0000000	0.0590000
5385.0000000	0.0580000
5386.0000000	0.0570000
5387.0000000	0.0571428
5388.0000000	0.0590000
5389.0000000	0.0580952
5390.0000000	0.0585000

5391.0000000	0.0585000
5392.0000000	0.0561905
5393.0000000	0.0570000
5394.0000000	0.0555000
5395.0000000	0.0561905
5396.0000000	0.0565000
5397.0000000	0.0560000
5398.0000000	0.0575000
5399.0000000	0.0566667
5400.0000000	0.0575000
5401.0000000	0.0590476
5402.0000000	0.0560000
5403.0000000	0.0585000
5404.0000000	0.0590476
5405.0000000	0.0595000
5406.0000000	0.0595000
5407.0000000	0.0595238
5408.0000000	0.0590000
5409.0000000	0.0585000
5410.0000000	0.0580952
5411.0000000	0.0590000
5412.0000000	0.0590000
5413.0000000	0.0595000
5414.0000000	0.0595238
5415.0000000	0.0595000
5416.0000000	0.0585714
5417.0000000	0.0595000
5418.0000000	0.0600000
5419.0000000	0.0590476
5420.0000000	0.0585000
5421.0000000	0.0585000
5422.0000000	0.0595000
5423.0000000	0.0600000
5424.0000000	0.0595000
5425.0000000	0.0590000
5426.0000000	0.0590476
5427.0000000	0.0595000
5428.0000000	0.0600000
5429.0000000	0.0600000
5430.0000000	0.0600000
5431.0000000	0.0595238
5432.0000000	0.0600000
5433.0000000	0.0600000
5434.0000000	0.0600000
5435.0000000	0.0595238
5436.0000000	0.0600000
5437.0000000	0.0595000
5438.0000000	0.0595238
5439.0000000	0.0590000
5440.0000000	0.0590476
5441.0000000	0.0580000
5442.0000000	0.0575000
5443.0000000	0.0565000
5444.0000000	0.0557143
5445.0000000	0.0555000
5446.0000000	0.0585000
5447.0000000	0.0590476

5448.0000000	0.0595000
5449.0000000	0.0585714
5450.0000000	0.0585000
5451.0000000	0.0600000
5452.0000000	0.0595000
5453.0000000	0.0600000
5454.0000000	0.0600000
5455.0000000	0.0600000
5456.0000000	0.0600000
5457.0000000	0.0600000
5458.0000000	0.0600000
5459.0000000	0.0600000
5460.0000000	0.0600000
5461.0000000	0.0600000
5462.0000000	0.0600000
5463.0000000	0.0600000
5464.0000000	0.0600000
5465.0000000	0.0600000
5466.0000000	0.0600000
5467.0000000	0.0600000
5468.0000000	0.0600000
5469.0000000	0.0600000
5470.0000000	0.0600000
5471.0000000	0.0600000
5472.0000000	0.0600000
5473.0000000	0.0600000
5474.0000000	0.0600000
5475.0000000	0.0604762
5476.0000000	0.0600000
5477.0000000	0.0600000
5478.0000000	0.0600000
5479.0000000	0.0600000
5480.0000000	0.0600000
5481.0000000	0.0600000
5482.0000000	0.0600000
5483.0000000	0.0600000
5484.0000000	0.0590000
5485.0000000	0.0600000
5486.0000000	0.0595238
5487.0000000	0.0600000
5488.0000000	0.0600000
5489.0000000	0.0590476
5490.0000000	0.0590000
5491.0000000	0.0595000
5492.0000000	0.0600000
5493.0000000	0.0595000
5494.0000000	0.0600000
5495.0000000	0.0600000
5496.0000000	0.0595000
5497.0000000	0.0600000
5498.0000000	0.0600000
5499.0000000	0.0600000
5500.0000000	0.0600000
5501.0000000	0.0605000
5502.0000000	0.0600000
5503.0000000	0.0600000
5504.0000000	0.0600000

5505.0000000	0.0600000
5506.0000000	0.0600000
5507.0000000	0.0600000
5508.0000000	0.0600000
5509.0000000	0.0600000
5510.0000000	0.0600000
5511.0000000	0.0605000
5512.0000000	0.0605000
5513.0000000	0.0600000
5514.0000000	0.0600000
5515.0000000	0.0600000
5516.0000000	0.0604762
5517.0000000	0.0605000
5518.0000000	0.0600000
5519.0000000	0.0600000
5520.0000000	0.0600000
5521.0000000	0.0600000
5522.0000000	0.0600000
5523.0000000	0.0600000
5524.0000000	0.0600000
5525.0000000	0.0600000
5526.0000000	0.0600000
5527.0000000	0.0595000
5528.0000000	0.0600000
5529.0000000	0.0600000
5530.0000000	0.0595000
5531.0000000	0.0600000
5532.0000000	0.0590000
5533.0000000	0.0590000
5534.0000000	0.0595000
5535.0000000	0.0595238
5536.0000000	0.0590000
5537.0000000	0.0585714
5538.0000000	0.0590000
5539.0000000	0.0580000
5540.0000000	0.0576190
5541.0000000	0.0580000
5542.0000000	0.0580000
5543.0000000	0.0590476
5544.0000000	0.0580000
5545.0000000	0.0585000
5546.0000000	0.0585714
5547.0000000	0.0580000
5548.0000000	0.0555000
5549.0000000	0.0555000
5550.0000000	0.0552381
5551.0000000	0.0565000
5552.0000000	0.0547619
5553.0000000	0.0540000
5554.0000000	0.0515000
5555.0000000	0.0514286
5556.0000000	0.0525000
5557.0000000	0.0525000
5558.0000000	0.0535000
5559.0000000	0.0509524
5560.0000000	0.0545000
5561.0000000	0.0542857

5562.0000000	0.0535000
5563.0000000	0.0540000
5564.0000000	0.0560000
5565.0000000	0.0571428
5566.0000000	0.0555000
5567.0000000	0.0550000
5568.0000000	0.0557143
5569.0000000	0.0570000
5570.0000000	0.0557143
5571.0000000	0.0550000
5572.0000000	0.0540000
5573.0000000	0.0520000
5574.0000000	0.0500000
5575.0000000	0.0505000
5576.0000000	0.0504762
5577.0000000	0.0505000
5578.0000000	0.0505000
5579.0000000	0.0500000
5580.0000000	0.0509524
5581.0000000	0.0505000
5582.0000000	0.0505000
5583.0000000	0.0500000
5584.0000000	0.0500000
5585.0000000	0.0504762
5586.0000000	0.0500000
5587.0000000	0.0500000
5588.0000000	0.0500000
5589.0000000	0.0500000
5590.0000000	0.0500000
5591.0000000	0.0500000
5592.0000000	0.0500000
5593.0000000	0.0500000
5594.0000000	0.0500000
5595.0000000	0.0500000
5596.0000000	0.0500000
5597.0000000	0.0500000
5598.0000000	0.0505000
5599.0000000	0.0500000
5600.0000000	0.0504762
5601.0000000	0.0505000
5602.0000000	0.0500000
5603.0000000	0.0500000
5604.0000000	0.0519048
5605.0000000	0.0505000
5606.0000000	0.0500000
5607.0000000	0.0500000
5608.0000000	0.0500000
5609.0000000	0.0505000
5610.0000000	0.0504762
5611.0000000	0.0505000
5612.0000000	0.0505000
5613.0000000	0.0500000
5614.0000000	0.0510000
5615.0000000	0.0500000
5616.0000000	0.0500000
5617.0000000	0.0500000
5618.0000000	0.0500000

5619.0000000	0.0500000
5620.0000000	0.0500000
5621.0000000	0.0500000
5622.0000000	0.0500000
5623.0000000	0.0500000
5624.0000000	0.0500000
5625.0000000	0.0500000
5626.0000000	0.0505000
5627.0000000	0.0500000
5628.0000000	0.0500000
5629.0000000	0.0505000
5630.0000000	0.0500000
5631.0000000	0.0500000
5632.0000000	0.0500000
5633.0000000	0.0500000
5634.0000000	0.0500000
5635.0000000	0.0500000
5636.0000000	0.0504762
5637.0000000	0.0500000
5638.0000000	0.0500000
5639.0000000	0.0500000
5640.0000000	0.0509524
5641.0000000	0.0500000
5642.0000000	0.0510000
5643.0000000	0.0500000
5644.0000000	0.0500000
5645.0000000	0.0500000
5646.0000000	0.0500000
5647.0000000	0.0510000
5648.0000000	0.0515000
5649.0000000	0.0519048
5650.0000000	0.0510000
5651.0000000	0.0520000
5652.0000000	0.0514286
5653.0000000	0.0510000
5654.0000000	0.0505000
5655.0000000	0.0505000
5656.0000000	0.0504762
5657.0000000	0.0505000
5658.0000000	0.0504762
5659.0000000	0.0505000
5660.0000000	0.0509524
5661.0000000	0.0515000
5662.0000000	0.0510000
5663.0000000	0.0510000
5664.0000000	0.0528571
5665.0000000	0.0520000
5666.0000000	0.0515000
5667.0000000	0.0533333
5668.0000000	0.0545000
5669.0000000	0.0535000
5670.0000000	0.0560000
5671.0000000	0.0547619
5672.0000000	0.0555000
5673.0000000	0.0538095
5674.0000000	0.0565000
5675.0000000	0.0545000

5676.0000000	0.0547619
5677.0000000	0.0570000
5678.0000000	0.0545000
5679.0000000	0.0550000
5680.0000000	0.0571428
5681.0000000	0.0570000
5682.0000000	0.0560000
5683.0000000	0.0533333
5684.0000000	0.0560000
5685.0000000	0.0566667
5686.0000000	0.0540000
5687.0000000	0.0575000
5688.0000000	0.0566667
5689.0000000	0.0570000
5690.0000000	0.0570000
5691.0000000	0.0570000
5692.0000000	0.0576190
5693.0000000	0.0570000
5694.0000000	0.0561905
5695.0000000	0.0560000
5696.0000000	0.0575000
5697.0000000	0.0580952
5698.0000000	0.0590000
5699.0000000	0.0560000
5700.0000000	0.0570000
5701.0000000	0.0571428
5702.0000000	0.0545000
5703.0000000	0.0576190
5704.0000000	0.0565000
5705.0000000	0.0547619
5706.0000000	0.0540000
5707.0000000	0.0545000
5708.0000000	0.0535000
5709.0000000	0.0535000
5710.0000000	0.0538095
5711.0000000	0.0560000
5712.0000000	0.0561905
5713.0000000	0.0565000
5714.0000000	0.0565000
5715.0000000	0.0565000
5716.0000000	0.0576190
5717.0000000	0.0560000
5718.0000000	0.0560000
5719.0000000	0.0595238
5720.0000000	0.0550000
5721.0000000	0.0576190
5722.0000000	0.0575000
5723.0000000	0.0585000
5724.0000000	0.0580952
5725.0000000	0.0595000
5726.0000000	0.0595000
5727.0000000	0.0585000
5728.0000000	0.0595238
5729.0000000	0.0595000
5730.0000000	0.0600000
5731.0000000	0.0600000
5732.0000000	0.0595238

5733.0000000	0.0600000
5734.0000000	0.0600000
5735.0000000	0.0590000
5736.0000000	0.0595238
5737.0000000	0.0600000
5738.0000000	0.0600000
5739.0000000	0.0600000
5740.0000000	0.0600000
5741.0000000	0.0600000
5742.0000000	0.0595238
5743.0000000	0.0600000
5744.0000000	0.0600000
5745.0000000	0.0600000
5746.0000000	0.0595238
5747.0000000	0.0600000
5748.0000000	0.0590476
5749.0000000	0.0600000
5750.0000000	0.0600000
5751.0000000	0.0600000
5752.0000000	0.0590000
5753.0000000	0.0595000
5754.0000000	0.0585000
5755.0000000	0.0561905
5756.0000000	0.0565000
5757.0000000	0.0585714
5758.0000000	0.0570000
5759.0000000	0.0580000
5760.0000000	0.0580000
5761.0000000	0.0590476
5762.0000000	0.0585000
5763.0000000	0.0590000
5764.0000000	0.0600000
5765.0000000	0.0590000
5766.0000000	0.0595238
5767.0000000	0.0595000
5768.0000000	0.0590000
5769.0000000	0.0595000
5770.0000000	0.0600000
5771.0000000	0.0590476
5772.0000000	0.0595000
5773.0000000	0.0600000
5774.0000000	0.0600000
5775.0000000	0.0600000
5776.0000000	0.0595238
5777.0000000	0.0595000
5778.0000000	0.0600000
5779.0000000	0.0600000
5780.0000000	0.0600000
5781.0000000	0.0595238
5782.0000000	0.0600000
5783.0000000	0.0595000
5784.0000000	0.0600000
5785.0000000	0.0600000
5786.0000000	0.0600000
5787.0000000	0.0600000
5788.0000000	0.0600000
5789.0000000	0.0600000

5790.0000000	0.0600000
5791.0000000	0.0595238
5792.0000000	0.0600000
5793.0000000	0.0600000
5794.0000000	0.0585000
5795.0000000	0.0600000
5796.0000000	0.0600000
5797.0000000	0.0595238
5798.0000000	0.0600000
5799.0000000	0.0600000
5800.0000000	0.0600000
5801.0000000	0.0595000
5802.0000000	0.0595238
5803.0000000	0.0595000
5804.0000000	0.0590000
5805.0000000	0.0600000
5806.0000000	0.0600000
5807.0000000	0.0595238
5808.0000000	0.0590000
5809.0000000	0.0600000
5810.0000000	0.0600000
5811.0000000	0.0600000
5812.0000000	0.0595000
5813.0000000	0.0595000
5814.0000000	0.0600000
5815.0000000	0.0600000
5816.0000000	0.0600000
5817.0000000	0.0600000
5818.0000000	0.0600000
5819.0000000	0.0600000
5820.0000000	0.0600000
5821.0000000	0.0590476
5822.0000000	0.0590000
5823.0000000	0.0595000
5824.0000000	0.0600000
5825.0000000	0.0600000
5826.0000000	0.0595000
5827.0000000	0.0580952
5828.0000000	0.0590000
5829.0000000	0.0561905
5830.0000000	0.0580000
5831.0000000	0.0585000
5832.0000000	0.0580000
5833.0000000	0.0590476
5834.0000000	0.0590000
5835.0000000	0.0590000
5836.0000000	0.0560000
5837.0000000	0.0576190
5838.0000000	0.0570000
5839.0000000	0.0547619
5840.0000000	0.0545000
5841.0000000	0.0552381
5842.0000000	0.0555000
5843.0000000	0.0565000
5844.0000000	0.0560000
5845.0000000	0.0552381
5846.0000000	0.0575000

5847.0000000	0.0580000
5848.0000000	0.0580000
5849.0000000	0.0595238
5850.0000000	0.0585000
5851.0000000	0.0590000
5852.0000000	0.0595238
5853.0000000	0.0570000
5854.0000000	0.0590476
5855.0000000	0.0590000
5856.0000000	0.0595000
5857.0000000	0.0566667
5858.0000000	0.0585000
5859.0000000	0.0590000
5860.0000000	0.0585000
5861.0000000	0.0580952
5862.0000000	0.0575000
5863.0000000	0.0580952
5864.0000000	0.0570000
5865.0000000	0.0575000
5866.0000000	0.0570000
5867.0000000	0.0575000
5868.0000000	0.0566667
5869.0000000	0.0542857
5870.0000000	0.0560000
5871.0000000	0.0530000
5872.0000000	0.0542857
5873.0000000	0.0535000
5874.0000000	0.0555000
5875.0000000	0.0545000
5876.0000000	0.0538095
5877.0000000	0.0555000
5878.0000000	0.0542857
5879.0000000	0.0540000
5880.0000000	0.0515000
5881.0000000	0.0509524
5882.0000000	0.0505000
5883.0000000	0.0500000
5884.0000000	0.0500000
5885.0000000	0.0504762
5886.0000000	0.0505000
5887.0000000	0.0500000
5888.0000000	0.0500000
5889.0000000	0.0505000
5890.0000000	0.0504762
5891.0000000	0.0500000
5892.0000000	0.0505000
5893.0000000	0.0523810
5894.0000000	0.0510000
5895.0000000	0.0505000
5896.0000000	0.0500000
5897.0000000	0.0500000
5898.0000000	0.0500000
5899.0000000	0.0504762
5900.0000000	0.0500000
5901.0000000	0.0505000
5902.0000000	0.0500000
5903.0000000	0.0500000

5904.0000000	0.0500000
5905.0000000	0.0500000
5906.0000000	0.0500000
5907.0000000	0.0500000
5908.0000000	0.0495238
5909.0000000	0.0495000
5910.0000000	0.0495000
5911.0000000	0.0495000
5912.0000000	0.0480000
5913.0000000	0.0490476
5914.0000000	0.0465000
5915.0000000	0.0480952
5916.0000000	0.0490000
5917.0000000	0.0476191
5918.0000000	0.0475000
5919.0000000	0.0485000
5920.0000000	0.0490000
5921.0000000	0.0476191
5922.0000000	0.0485000
5923.0000000	0.0490000
5924.0000000	0.0495238
5925.0000000	0.0500000
5926.0000000	0.0500000
5927.0000000	0.0500000
5928.0000000	0.0500000
5929.0000000	0.0500000
5930.0000000	0.0500000
5931.0000000	0.0500000
5932.0000000	0.0495000
5933.0000000	0.0500000
5934.0000000	0.0500000
5935.0000000	0.0500000
5936.0000000	0.0500000
5937.0000000	0.0504762
5938.0000000	0.0504762
5939.0000000	0.0510000
5940.0000000	0.0500000
5941.0000000	0.0505000
5942.0000000	0.0500000
5943.0000000	0.0510000
5944.0000000	0.0515000
5945.0000000	0.0519048
5946.0000000	0.0515000
5947.0000000	0.0514286
5948.0000000	0.0515000
5949.0000000	0.0505000
5950.0000000	0.0520000
5951.0000000	0.0528571
5952.0000000	0.0520000
5953.0000000	0.0530000
5954.0000000	0.0523810
5955.0000000	0.0500000
5956.0000000	0.0510000
5957.0000000	0.0504762
5958.0000000	0.0505000
5959.0000000	0.0510000
5960.0000000	0.0500000

5961.0000000	0.0500000
5962.0000000	0.0504762
5963.0000000	0.0500000
5964.0000000	0.0500000
5965.0000000	0.0510000
5966.0000000	0.0500000
5967.0000000	0.0525000
5968.0000000	0.0510000
5969.0000000	0.0523810
5970.0000000	0.0520000
5971.0000000	0.0535000
5972.0000000	0.0500000
5973.0000000	0.0509524
5974.0000000	0.0515000
5975.0000000	0.0510000
5976.0000000	0.0504762
5977.0000000	0.0515000
5978.0000000	0.0519048
5979.0000000	0.0515000
5980.0000000	0.0500000
5981.0000000	0.0504762
5982.0000000	0.0500000
5983.0000000	0.0505000
5984.0000000	0.0505000
5985.0000000	0.0500000
5986.0000000	0.0500000
5987.0000000	0.0504762
5988.0000000	0.0500000
5989.0000000	0.0500000
5990.0000000	0.0500000
5991.0000000	0.0500000
5992.0000000	0.0500000
5993.0000000	0.0500000
5994.0000000	0.0500000
5995.0000000	0.0500000
5996.0000000	0.0500000
5997.0000000	0.0500000
5998.0000000	0.0500000
5999.0000000	0.0500000
6000.0000000	0.0500000
6001.0000000	0.0500000
6002.0000000	0.0500000
6003.0000000	0.0500000
6004.0000000	0.0500000
6005.0000000	0.0500000
6006.0000000	0.0500000
6007.0000000	0.0504762
6008.0000000	0.0500000
6009.0000000	0.0500000
6010.0000000	0.0500000
6011.0000000	0.0500000
6012.0000000	0.0495238
6013.0000000	0.0495000
6014.0000000	0.0500000
6015.0000000	0.0500000
6016.0000000	0.0500000
6017.0000000	0.0500000

6018.0000000	0.0500000
6019.0000000	0.0500000
6020.0000000	0.0500000
6021.0000000	0.0500000
6022.0000000	0.0500000
6023.0000000	0.0504762
6024.0000000	0.0505000
6025.0000000	0.0505000
6026.0000000	0.0504762
6027.0000000	0.0500000
6028.0000000	0.0505000
6029.0000000	0.0515000
6030.0000000	0.0514286
6031.0000000	0.0515000
6032.0000000	0.0530000
6033.0000000	0.0528571
6034.0000000	0.0525000
6035.0000000	0.0538095
6036.0000000	0.0535000
6037.0000000	0.0535000
6038.0000000	0.0514286
6039.0000000	0.0525000
6040.0000000	0.0525000
6041.0000000	0.0530000
6042.0000000	0.0523810
6043.0000000	0.0510000
6044.0000000	0.0509524
6045.0000000	0.0520000
6046.0000000	0.0510000
6047.0000000	0.0505000
6048.0000000	0.0519048
6049.0000000	0.0545000
6050.0000000	0.0528571
6051.0000000	0.0530000
6052.0000000	0.0510000
6053.0000000	0.0540000
6054.0000000	0.0509524
6055.0000000	0.0510000
6056.0000000	0.0505000
6057.0000000	0.0514286
6058.0000000	0.0520000
6059.0000000	0.0514286
6060.0000000	0.0505000
6061.0000000	0.0530000
6062.0000000	0.0530000
6063.0000000	0.0545000
6064.0000000	0.0538095
6065.0000000	0.0545000
6066.0000000	0.0552381
6067.0000000	0.0560000
6068.0000000	0.0552381
6069.0000000	0.0545000
6070.0000000	0.0550000
6071.0000000	0.0540000
6072.0000000	0.0545000
6073.0000000	0.0552381
6074.0000000	0.0566667

6075.0000000	0.0540000
6076.0000000	0.0560000
6077.0000000	0.0560000
6078.0000000	0.0571428
6079.0000000	0.0570000
6080.0000000	0.0560000
6081.0000000	0.0576190
6082.0000000	0.0565000
6083.0000000	0.0547619
6084.0000000	0.0545000
6085.0000000	0.0545000
6086.0000000	0.0576190
6087.0000000	0.0580000
6088.0000000	0.0565000
6089.0000000	0.0570000
6090.0000000	0.0566667
6091.0000000	0.0565000
6092.0000000	0.0555000
6093.0000000	0.0547619
6094.0000000	0.0540000
6095.0000000	0.0542857
6096.0000000	0.0565000
6097.0000000	0.0535000
6098.0000000	0.0528571
6099.0000000	0.0545000
6100.0000000	0.0550000
6101.0000000	0.0560000
6102.0000000	0.0533333
6103.0000000	0.0550000
6104.0000000	0.0552381
6105.0000000	0.0535000
6106.0000000	0.0540000
6107.0000000	0.0525000
6108.0000000	0.0530000
6109.0000000	0.0514286
6110.0000000	0.0510000
6111.0000000	0.0504762
6112.0000000	0.0515000
6113.0000000	0.0530000
6114.0000000	0.0504762
6115.0000000	0.0515000
6116.0000000	0.0505000
6117.0000000	0.0509524
6118.0000000	0.0520000
6119.0000000	0.0515000
6120.0000000	0.0504762
6121.0000000	0.0535000
6122.0000000	0.0510000
6123.0000000	0.0519048
6124.0000000	0.0515000
6125.0000000	0.0525000
6126.0000000	0.0514286
6127.0000000	0.0535000
6128.0000000	0.0530000
6129.0000000	0.0552381
6130.0000000	0.0530000
6131.0000000	0.0520000

6132.0000000	0.0538095
6133.0000000	0.0530000
6134.0000000	0.0538095
6135.0000000	0.0520000
6136.0000000	0.0530000
6137.0000000	0.0540000
6138.0000000	0.0528571
6139.0000000	0.0560000
6140.0000000	0.0545000
6141.0000000	0.0555000
6142.0000000	0.0557143
6143.0000000	0.0552381
6144.0000000	0.0552632
6145.0000000	0.0566667
6146.0000000	0.0560000
6147.0000000	0.0580952
6148.0000000	0.0585000
6149.0000000	0.0595000
6150.0000000	0.0595238
6151.0000000	0.0590000
6152.0000000	0.0585000
6153.0000000	0.0595000
6154.0000000	0.0585714
6155.0000000	0.0585000
6156.0000000	0.0585714
6157.0000000	0.0590000
6158.0000000	0.0565000
6159.0000000	0.0585714
6160.0000000	0.0585000
6161.0000000	0.0570000
6162.0000000	0.0561905
6163.0000000	0.0570000
6164.0000000	0.0570000
6165.0000000	0.0566667
6166.0000000	0.0570000
6167.0000000	0.0545000
6168.0000000	0.0565000
6169.0000000	0.0571428
6170.0000000	0.0575000
6171.0000000	0.0571428
6172.0000000	0.0580000
6173.0000000	0.0555000
6174.0000000	0.0571428
6175.0000000	0.0585000
6176.0000000	0.0540000
6177.0000000	0.0545000
6178.0000000	0.0542857
6179.0000000	0.0540000
6180.0000000	0.0545000
6181.0000000	0.0533333
6182.0000000	0.0535000
6183.0000000	0.0542857
6184.0000000	0.0520000
6185.0000000	0.0515000
6186.0000000	0.0509524
6187.0000000	0.0510000
6188.0000000	0.0505000

6189.0000000	0.0505000
6190.0000000	0.0500000
6191.0000000	0.0510000
6192.0000000	0.0500000
6193.0000000	0.0500000
6194.0000000	0.0510000
6195.0000000	0.0500000
6196.0000000	0.0500000
6197.0000000	0.0510000
6198.0000000	0.0510000
6199.0000000	0.0509524
6200.0000000	0.0510000
6201.0000000	0.0505000
6202.0000000	0.0514286
6203.0000000	0.0505000
6204.0000000	0.0504762
6205.0000000	0.0520000
6206.0000000	0.0525000
6207.0000000	0.0520000
6208.0000000	0.0528571
6209.0000000	0.0515000
6210.0000000	0.0514286
6211.0000000	0.0530000
6212.0000000	0.0525000
6213.0000000	0.0515000
6214.0000000	0.0533333
6215.0000000	0.0530000
6216.0000000	0.0520000
6217.0000000	0.0528571
6218.0000000	0.0530000
6219.0000000	0.0538095
6220.0000000	0.0540000
6221.0000000	0.0520000
6222.0000000	0.0520000
6223.0000000	0.0547619
6224.0000000	0.0540000
6225.0000000	0.0550000
6226.0000000	0.0538095
6227.0000000	0.0550000
6228.0000000	0.0560000
6229.0000000	0.0535000
6230.0000000	0.0566667
6231.0000000	0.0585714
6232.0000000	0.0565000
6233.0000000	0.0560000
6234.0000000	0.0571428
6235.0000000	0.0555000
6236.0000000	0.0575000
6237.0000000	0.0580000
6238.0000000	0.0576190
6239.0000000	0.0560000
6240.0000000	0.0571428
6241.0000000	0.0595000
6242.0000000	0.0565000
6243.0000000	0.0550000
6244.0000000	0.0552381
6245.0000000	0.0575000

6246.0000000	0.0555000
6247.0000000	0.0538095
6248.0000000	0.0565000
6249.0000000	0.0542857
6250.0000000	0.0550000
6251.0000000	0.0550000
6252.0000000	0.0530000
6253.0000000	0.0533333
6254.0000000	0.0525000
6255.0000000	0.0535000
6256.0000000	0.0552381
6257.0000000	0.0560000
6258.0000000	0.0565000
6259.0000000	0.0557143
6260.0000000	0.0585000
6261.0000000	0.0550000
6262.0000000	0.0566667
6263.0000000	0.0570000
6264.0000000	0.0585000
6265.0000000	0.0585714
6266.0000000	0.0575000
6267.0000000	0.0585000
6268.0000000	0.0575000
6269.0000000	0.0576190
6270.0000000	0.0570000
6271.0000000	0.0585714
6272.0000000	0.0585000
6273.0000000	0.0580000
6274.0000000	0.0590476
6275.0000000	0.0590000
6276.0000000	0.0595000
6277.0000000	0.0595000
6278.0000000	0.0595238
6279.0000000	0.0590476
6280.0000000	0.0595000
6281.0000000	0.0600000
6282.0000000	0.0595000
6283.0000000	0.0590476
6284.0000000	0.0600000
6285.0000000	0.0595000
6286.0000000	0.0595238
6287.0000000	0.0590000
6288.0000000	0.0595000
6289.0000000	0.0600000
6290.0000000	0.0585000
6291.0000000	0.0590000
6292.0000000	0.0590000
6293.0000000	0.0585714
6294.0000000	0.0585000
6295.0000000	0.0585714
6296.0000000	0.0575000
6297.0000000	0.0575000
6298.0000000	0.0566667
6299.0000000	0.0585000
6300.0000000	0.0575000
6301.0000000	0.0557143
6302.0000000	0.0560000

6303.0000000	0.0565000
6304.0000000	0.0570000
6305.0000000	0.0571428
6306.0000000	0.0540000
6307.0000000	0.0552381
6308.0000000	0.0550000
6309.0000000	0.0550000
6310.0000000	0.0561905
6311.0000000	0.0540000
6312.0000000	0.0540000
6313.0000000	0.0560000
6314.0000000	0.0533333
6315.0000000	0.0510000
6316.0000000	0.0514286
6317.0000000	0.0530000
6318.0000000	0.0530000
6319.0000000	0.0523810
6320.0000000	0.0520000
6321.0000000	0.0520000
6322.0000000	0.0510000
6323.0000000	0.0509524
6324.0000000	0.0535000
6325.0000000	0.0514286
6326.0000000	0.0510000
6327.0000000	0.0525000
6328.0000000	0.0528571
6329.0000000	0.0535000
6330.0000000	0.0530000
6331.0000000	0.0538095
6332.0000000	0.0550000
6333.0000000	0.0545000
6334.0000000	0.0540000
6335.0000000	0.0547619
6336.0000000	0.0570000
6337.0000000	0.0561905
6338.0000000	0.0540000
6339.0000000	0.0580000
6340.0000000	0.0561905
6341.0000000	0.0555000
6342.0000000	0.0550000
6343.0000000	0.0566667
6344.0000000	0.0545000
6345.0000000	0.0550000
6346.0000000	0.0560000
6347.0000000	0.0547619
6348.0000000	0.0555000
6349.0000000	0.0550000
6350.0000000	0.0542857
6351.0000000	0.0515000
6352.0000000	0.0523810
6353.0000000	0.0525000
6354.0000000	0.0540000
6355.0000000	0.0538095
6356.0000000	0.0535000
6357.0000000	0.0540000
6358.0000000	0.0525000
6359.0000000	0.0509524

6360.0000000	0.0515000
6361.0000000	0.0525000
6362.0000000	0.0528571
6363.0000000	0.0540000
6364.0000000	0.0540000
6365.0000000	0.0545000
6366.0000000	0.0557143
6367.0000000	0.0547619
6368.0000000	0.0570000
6369.0000000	0.0555000
6370.0000000	0.0560000
6371.0000000	0.0533333
6372.0000000	0.0530000
6373.0000000	0.0535000
6374.0000000	0.0557143
6375.0000000	0.0540000
6376.0000000	0.0557143
6377.0000000	0.0550000
6378.0000000	0.0535000
6379.0000000	0.0550000
6380.0000000	0.0542857
6381.0000000	0.0545000
6382.0000000	0.0555000
6383.0000000	0.0557143
6384.0000000	0.0575000
6385.0000000	0.0555000
6386.0000000	0.0566667
6387.0000000	0.0575000
6388.0000000	0.0585000
6389.0000000	0.0595238
6390.0000000	0.0590000
6391.0000000	0.0595238
6392.0000000	0.0590000
6393.0000000	0.0595000
6394.0000000	0.0590000
6395.0000000	0.0590476
6396.0000000	0.0585000
6397.0000000	0.0580000
6398.0000000	0.0595238
6399.0000000	0.0590000
6400.0000000	0.0590476
6401.0000000	0.0595000
6402.0000000	0.0600000
6403.0000000	0.0595000
6404.0000000	0.0580952
6405.0000000	0.0595000
6406.0000000	0.0595000
6407.0000000	0.0595238
6408.0000000	0.0595000
6409.0000000	0.0595000
6410.0000000	0.0600000
6411.0000000	0.0600000
6412.0000000	0.0600000
6413.0000000	0.0600000
6414.0000000	0.0600000
6415.0000000	0.0595000
6416.0000000	0.0600000

6417.0000000	0.0595000
6418.0000000	0.0590000
6419.0000000	0.0580952
6420.0000000	0.0590000
6421.0000000	0.0595000
6422.0000000	0.0600000
6423.0000000	0.0595000
6424.0000000	0.0595000
6425.0000000	0.0600000
6426.0000000	0.0595000
6427.0000000	0.0600000
6428.0000000	0.0600000
6429.0000000	0.0585000
6430.0000000	0.0575000
6431.0000000	0.0576190
6432.0000000	0.0580000
6433.0000000	0.0575000
6434.0000000	0.0565000
6435.0000000	0.0552381
6436.0000000	0.0576190
6437.0000000	0.0575000
6438.0000000	0.0590000
6439.0000000	0.0570000
6440.0000000	0.0600000
6441.0000000	0.0595000
6442.0000000	0.0590000
6443.0000000	0.0595238
6444.0000000	0.0585000
6445.0000000	0.0590476
6446.0000000	0.0590000
6447.0000000	0.0585000
6448.0000000	0.0565000
6449.0000000	0.0557143
6450.0000000	0.0540000
6451.0000000	0.0545000
6452.0000000	0.0533333
6453.0000000	0.0520000
6454.0000000	0.0510000
6455.0000000	0.0533333
6456.0000000	0.0520000
6457.0000000	0.0515000
6458.0000000	0.0509524
6459.0000000	0.0515000
6460.0000000	0.0505000
6461.0000000	0.0509524
6462.0000000	0.0505000
6463.0000000	0.0510000
6464.0000000	0.0500000
6465.0000000	0.0515000
6466.0000000	0.0510000
6467.0000000	0.0509524
6468.0000000	0.0525000
6469.0000000	0.0505000
6470.0000000	0.0505000
6471.0000000	0.0514286
6472.0000000	0.0510000
6473.0000000	0.0510000

6474.0000000	0.0538095
6475.0000000	0.0535000
6476.0000000	0.0514286
6477.0000000	0.0500000
6478.0000000	0.0510000
6479.0000000	0.0520000
6480.0000000	0.0523810
6481.0000000	0.0530000
6482.0000000	0.0525000
6483.0000000	0.0504762
6484.0000000	0.0525000
6485.0000000	0.0535000
6486.0000000	0.0528571
6487.0000000	0.0515000
6488.0000000	0.0528571
6489.0000000	0.0525000
6490.0000000	0.0515000
6491.0000000	0.0500000
6492.0000000	0.0510000
6493.0000000	0.0510000
6494.0000000	0.0515000
6495.0000000	0.0519048
6496.0000000	0.0510000
6497.0000000	0.0509524
6498.0000000	0.0510000
6499.0000000	0.0520000
6500.0000000	0.0510000
6501.0000000	0.0533333
6502.0000000	0.0520000
6503.0000000	0.0520000
6504.0000000	0.0533333
6505.0000000	0.0535000
6506.0000000	0.0557143
6507.0000000	0.0545000
6508.0000000	0.0555000
6509.0000000	0.0555000
6510.0000000	0.0585714
6511.0000000	0.0565000
6512.0000000	0.0580000
6513.0000000	0.0576190
6514.0000000	0.0580000
6515.0000000	0.0580000
6516.0000000	0.0576190
6517.0000000	0.0590000
6518.0000000	0.0600000
6519.0000000	0.0585714
6520.0000000	0.0590000
6521.0000000	0.0600000
6522.0000000	0.0590476
6523.0000000	0.0600000
6524.0000000	0.0590476
6525.0000000	0.0595000
6526.0000000	0.0600000
6527.0000000	0.0595000
6528.0000000	0.0595238
6529.0000000	0.0595000
6530.0000000	0.0600000

6531.0000000	0.0585714
6532.0000000	0.0595000
6533.0000000	0.0600000
6534.0000000	0.0585000
6535.0000000	0.0585000
6536.0000000	0.0595238
6537.0000000	0.0600000
6538.0000000	0.0600000
6539.0000000	0.0600000
6540.0000000	0.0590476
6541.0000000	0.0600000
6542.0000000	0.0600000
6543.0000000	0.0600000
6544.0000000	0.0595000
6545.0000000	0.0600000
6546.0000000	0.0600000
6547.0000000	0.0595238
6548.0000000	0.0600000
6549.0000000	0.0600000
6550.0000000	0.0600000
6551.0000000	0.0600000
6552.0000000	0.0600000
6553.0000000	0.0600000
6554.0000000	0.0600000
6555.0000000	0.0600000
6556.0000000	0.0600000
6557.0000000	0.0600000
6558.0000000	0.0600000
6559.0000000	0.0600000
6560.0000000	0.0600000
6561.0000000	0.0600000
6562.0000000	0.0600000
6563.0000000	0.0600000
6564.0000000	0.0600000
6565.0000000	0.0600000
6566.0000000	0.0600000
6567.0000000	0.0600000
6568.0000000	0.0600000
6569.0000000	0.0600000
6570.0000000	0.0600000
6571.0000000	0.0600000
6572.0000000	0.0600000
6573.0000000	0.0600000
6574.0000000	0.0600000
6575.0000000	0.0600000
6576.0000000	0.0600000
6577.0000000	0.0600000
6578.0000000	0.0600000
6579.0000000	0.0595238
6580.0000000	0.0600000
6581.0000000	0.0595238
6582.0000000	0.0590000
6583.0000000	0.0600000
6584.0000000	0.0590000
6585.0000000	0.0576190
6586.0000000	0.0595000
6587.0000000	0.0590000

6588.0000000	0.0590476
6589.0000000	0.0600000
6590.0000000	0.0595000
6591.0000000	0.0595000
6592.0000000	0.0600000
6593.0000000	0.0600000
6594.0000000	0.0600000
6595.0000000	0.0600000
6596.0000000	0.0595000
6597.0000000	0.0595000
6598.0000000	0.0600000
6599.0000000	0.0585000
6600.0000000	0.0575000
6601.0000000	0.0590476
6602.0000000	0.0570000
6603.0000000	0.0565000
6604.0000000	0.0570000
6605.0000000	0.0566667
6606.0000000	0.0590000
6607.0000000	0.0585714
6608.0000000	0.0590000
6609.0000000	0.0575000
6610.0000000	0.0566667
6611.0000000	0.0565000
6612.0000000	0.0557143
6613.0000000	0.0550000
6614.0000000	0.0520000
6615.0000000	0.0500000
6616.0000000	0.0519048
6617.0000000	0.0505000
6618.0000000	0.0505000
6619.0000000	0.0500000
6620.0000000	0.0500000
6621.0000000	0.0500000
6622.0000000	0.0505000
6623.0000000	0.0500000
6624.0000000	0.0505000
6625.0000000	0.0514286
6626.0000000	0.0500000
6627.0000000	0.0515000
6628.0000000	0.0514286
6629.0000000	0.0510000
6630.0000000	0.0520000
6631.0000000	0.0519048
6632.0000000	0.0515000
6633.0000000	0.0515000
6634.0000000	0.0528571
6635.0000000	0.0525000
6636.0000000	0.0505000
6637.0000000	0.0509524
6638.0000000	0.0530000
6639.0000000	0.0525000
6640.0000000	0.0523810
6641.0000000	0.0510000
6642.0000000	0.0510000
6643.0000000	0.0523810
6644.0000000	0.0505000

6645.0000000	0.0505000
6646.0000000	0.0505000
6647.0000000	0.0500000
6648.0000000	0.0500000
6649.0000000	0.0500000
6650.0000000	0.0505000
6651.0000000	0.0515000
6652.0000000	0.0504762
6653.0000000	0.0505000
6654.0000000	0.0500000
6655.0000000	0.0523810
6656.0000000	0.0515000
6657.0000000	0.0514286
6658.0000000	0.0520000
6659.0000000	0.0515000
6660.0000000	0.0515000
6661.0000000	0.0500000
6662.0000000	0.0505000
6663.0000000	0.0510000
6664.0000000	0.0519048
6665.0000000	0.0515000
6666.0000000	0.0530000
6667.0000000	0.0545000
6668.0000000	0.0523810
6669.0000000	0.0520000
6670.0000000	0.0538095
6671.0000000	0.0540000
6672.0000000	0.0505000
6673.0000000	0.0519048
6674.0000000	0.0525000
6675.0000000	0.0510000
6676.0000000	0.0509524
6677.0000000	0.0525000
6678.0000000	0.0520000
6679.0000000	0.0533333
6680.0000000	0.0540000
6681.0000000	0.0535000
6682.0000000	0.0535000
6683.0000000	0.0542857
6684.0000000	0.0540000
6685.0000000	0.0535000
6686.0000000	0.0523810
6687.0000000	0.0515000
6688.0000000	0.0500000
6689.0000000	0.0505000
6690.0000000	0.0505000
6691.0000000	0.0500000
6692.0000000	0.0504762
6693.0000000	0.0500000
6694.0000000	0.0505000
6695.0000000	0.0500000
6696.0000000	0.0505000
6697.0000000	0.0510000
6698.0000000	0.0514286
6699.0000000	0.0505000
6700.0000000	0.0519048
6701.0000000	0.0520000

6702.0000000	0.0525000
6703.0000000	0.0520000
6704.0000000	0.0519048
6705.0000000	0.0515000
6706.0000000	0.0520000
6707.0000000	0.0528571
6708.0000000	0.0535000
6709.0000000	0.0523810
6710.0000000	0.0530000
6711.0000000	0.0535000
6712.0000000	0.0540000
6713.0000000	0.0538095
6714.0000000	0.0565000
6715.0000000	0.0570000
6716.0000000	0.0571428
6717.0000000	0.0575000
6718.0000000	0.0580952
6719.0000000	0.0575000
6720.0000000	0.0580000
6721.0000000	0.0580000
6722.0000000	0.0571428
6723.0000000	0.0575000
6724.0000000	0.0575000
6725.0000000	0.0580952
6726.0000000	0.0575000
6727.0000000	0.0585000
6728.0000000	0.0576190
6729.0000000	0.0565000
6730.0000000	0.0555000
6731.0000000	0.0557143
6732.0000000	0.0535000
6733.0000000	0.0530000
6734.0000000	0.0509524
6735.0000000	0.0525000
6736.0000000	0.0528571
6737.0000000	0.0515000
6738.0000000	0.0510000
6739.0000000	0.0510000
6740.0000000	0.0514286
6741.0000000	0.0510000
6742.0000000	0.0510000
6743.0000000	0.0509524
6744.0000000	0.0505000
6745.0000000	0.0514286
6746.0000000	0.0500000
6747.0000000	0.0505000
6748.0000000	0.0509524
6749.0000000	0.0525000
6750.0000000	0.0520000
6751.0000000	0.0500000
6752.0000000	0.0509524
6753.0000000	0.0505000
6754.0000000	0.0514286
6755.0000000	0.0510000
6756.0000000	0.0500000
6757.0000000	0.0505000
6758.0000000	0.0515000

6759.0000000	0.0500000
6760.0000000	0.0500000
6761.0000000	0.0500000
6762.0000000	0.0505000
6763.0000000	0.0500000
6764.0000000	0.0504762
6765.0000000	0.0500000
6766.0000000	0.0515000
6767.0000000	0.0505000
6768.0000000	0.0500000
6769.0000000	0.0500000
6770.0000000	0.0504762
6771.0000000	0.0515000
6772.0000000	0.0505000
6773.0000000	0.0514286
6774.0000000	0.0515000
6775.0000000	0.0505000
6776.0000000	0.0514286
6777.0000000	0.0515000
6778.0000000	0.0510000
6779.0000000	0.0504762
6780.0000000	0.0500000
6781.0000000	0.0505000
6782.0000000	0.0500000
6783.0000000	0.0509524
6784.0000000	0.0500000
6785.0000000	0.0500000
6786.0000000	0.0510000
6787.0000000	0.0505000
6788.0000000	0.0504762
6789.0000000	0.0510000
6790.0000000	0.0505000
6791.0000000	0.0510000
6792.0000000	0.0500000
6793.0000000	0.0500000
6794.0000000	0.0510000
6795.0000000	0.0510000
6796.0000000	0.0500000
6797.0000000	0.0509524
6798.0000000	0.0505000
6799.0000000	0.0505000
6800.0000000	0.0519048
6801.0000000	0.0525000
6802.0000000	0.0515000
6803.0000000	0.0510000
6804.0000000	0.0509524
6805.0000000	0.0515000
6806.0000000	0.0504762
6807.0000000	0.0515000
6808.0000000	0.0510000
6809.0000000	0.0509524
6810.0000000	0.0525000
6811.0000000	0.0505000
6812.0000000	0.0509524
6813.0000000	0.0505000
6814.0000000	0.0515000
6815.0000000	0.0504762

6816.0000000	0.0520000
6817.0000000	0.0510000
6818.0000000	0.0505000
6819.0000000	0.0509524
6820.0000000	0.0510000
6821.0000000	0.0505000
6822.0000000	0.0509524
6823.0000000	0.0510000
6824.0000000	0.0514286
6825.0000000	0.0520000
6826.0000000	0.0525000
6827.0000000	0.0520000
6828.0000000	0.0504762
6829.0000000	0.0505000
6830.0000000	0.0515000
6831.0000000	0.0519048
6832.0000000	0.0520000
6833.0000000	0.0523810
6834.0000000	0.0510000
6835.0000000	0.0545000
6836.0000000	0.0523810
6837.0000000	0.0520000
6838.0000000	0.0550000
6839.0000000	0.0535000
6840.0000000	0.0523810
6841.0000000	0.0520000
6842.0000000	0.0514286
6843.0000000	0.0535000
6844.0000000	0.0530000
6845.0000000	0.0509524
6846.0000000	0.0510000
6847.0000000	0.0510000
6848.0000000	0.0530000
6849.0000000	0.0514286
6850.0000000	0.0505000
6851.0000000	0.0510000
6852.0000000	0.0500000
6853.0000000	0.0500000
6854.0000000	0.0500000
6855.0000000	0.0505000
6856.0000000	0.0515000
6857.0000000	0.0510000
6858.0000000	0.0504762
6859.0000000	0.0500000
6860.0000000	0.0514286
6861.0000000	0.0500000
6862.0000000	0.0510000
6863.0000000	0.0510000
6864.0000000	0.0500000
6865.0000000	0.0500000
6866.0000000	0.0500000
6867.0000000	0.0500000
6868.0000000	0.0500000
6869.0000000	0.0504762
6870.0000000	0.0500000
6871.0000000	0.0500000
6872.0000000	0.0500000

6873.0000000	0.0500000
6874.0000000	0.0500000
6875.0000000	0.0500000
6876.0000000	0.0500000
6877.0000000	0.0500000
6878.0000000	0.0500000
6879.0000000	0.0500000
6880.0000000	0.0504762
6881.0000000	0.0500000
6882.0000000	0.0505000
6883.0000000	0.0500000
6884.0000000	0.0500000
6885.0000000	0.0504762
6886.0000000	0.0505000
6887.0000000	0.0505000
6888.0000000	0.0514286
6889.0000000	0.0500000
6890.0000000	0.0500000
6891.0000000	0.0504762
6892.0000000	0.0505000
6893.0000000	0.0500000
6894.0000000	0.0504762
6895.0000000	0.0500000
6896.0000000	0.0505000
6897.0000000	0.0500000
6898.0000000	0.0500000
6899.0000000	0.0505000
6900.0000000	0.0504762
6901.0000000	0.0500000
6902.0000000	0.0500000
6903.0000000	0.0500000
6904.0000000	0.0500000
6905.0000000	0.0500000
6906.0000000	0.0500000
6907.0000000	0.0500000
6908.0000000	0.0500000
6909.0000000	0.0500000
6910.0000000	0.0504762
6911.0000000	0.0500000
6912.0000000	0.0504762
6913.0000000	0.0510000
6914.0000000	0.0514286
6915.0000000	0.0510000
6916.0000000	0.0520000
6917.0000000	0.0505000
6918.0000000	0.0520000
6919.0000000	0.0523810
6920.0000000	0.0510000
6921.0000000	0.0557143
6922.0000000	0.0530000
6923.0000000	0.0540000
6924.0000000	0.0561905
6925.0000000	0.0525000
6926.0000000	0.0540000
6927.0000000	0.0570000
6928.0000000	0.0533333
6929.0000000	0.0570000

6930.0000000	0.0528571
6931.0000000	0.0525000
6932.0000000	0.0510000
6933.0000000	0.0519048
6934.0000000	0.0505000
6935.0000000	0.0520000
6936.0000000	0.0509524
6937.0000000	0.0505000
6938.0000000	0.0500000
6939.0000000	0.0500000
6940.0000000	0.0500000
6941.0000000	0.0500000
6942.0000000	0.0500000
6943.0000000	0.0500000
6944.0000000	0.0500000
6945.0000000	0.0504762
6946.0000000	0.0505000
6947.0000000	0.0505000
6948.0000000	0.0510000
6949.0000000	0.0519048
6950.0000000	0.0523810
6951.0000000	0.0520000
6952.0000000	0.0525000
6953.0000000	0.0525000
6954.0000000	0.0545000
6955.0000000	0.0528571
6956.0000000	0.0520000
6957.0000000	0.0538095
6958.0000000	0.0545000
6959.0000000	0.0533333
6960.0000000	0.0560000
6961.0000000	0.0545000
6962.0000000	0.0585000
6963.0000000	0.0580000
6964.0000000	0.0576190
6965.0000000	0.0595000
6966.0000000	0.0590476
6967.0000000	0.0580000
6968.0000000	0.0590000
6969.0000000	0.0590476
6970.0000000	0.0590000
6971.0000000	0.0595000
6972.0000000	0.0595000
6973.0000000	0.0600000
6974.0000000	0.0595000
6975.0000000	0.0600000
6976.0000000	0.0600000
6977.0000000	0.0600000
6978.0000000	0.0595238
6979.0000000	0.0595000
6980.0000000	0.0600000
6981.0000000	0.0600000
6982.0000000	0.0595000
6983.0000000	0.0595000
6984.0000000	0.0600000
6985.0000000	0.0600000
6986.0000000	0.0595000

6987.0000000	0.0600000
6988.0000000	0.0595238
6989.0000000	0.0600000
6990.0000000	0.0590476
6991.0000000	0.0600000
6992.0000000	0.0600000
6993.0000000	0.0600000
6994.0000000	0.0600000
6995.0000000	0.0600000
6996.0000000	0.0600000
6997.0000000	0.0600000
6998.0000000	0.0585000
6999.0000000	0.0590000
7000.0000000	0.0585714
7001.0000000	0.0595000
7002.0000000	0.0580952
7003.0000000	0.0590000
7004.0000000	0.0585000
7005.0000000	0.0585714
7006.0000000	0.0590000
7007.0000000	0.0590000
7008.0000000	0.0600000
7009.0000000	0.0585714
7010.0000000	0.0595000
7011.0000000	0.0595238
7012.0000000	0.0595000
7013.0000000	0.0600000
7014.0000000	0.0595000
7015.0000000	0.0600000
7016.0000000	0.0600000
7017.0000000	0.0595238
7018.0000000	0.0595000
7019.0000000	0.0600000
7020.0000000	0.0590476
7021.0000000	0.0595000
7022.0000000	0.0580000
7023.0000000	0.0565000
7024.0000000	0.0566667
7025.0000000	0.0555000
7026.0000000	0.0565000
7027.0000000	0.0547619
7028.0000000	0.0555000
7029.0000000	0.0542857
7030.0000000	0.0565000
7031.0000000	0.0570000
7032.0000000	0.0580000
7033.0000000	0.0566667
7034.0000000	0.0560000
7035.0000000	0.0560000
7036.0000000	0.0571428
7037.0000000	0.0575000
7038.0000000	0.0547619
7039.0000000	0.0550000
7040.0000000	0.0540000
7041.0000000	0.0528571
7042.0000000	0.0550000
7043.0000000	0.0540000

7044.0000000	0.0545000
7045.0000000	0.0533333
7046.0000000	0.0535000
7047.0000000	0.0557143
7048.0000000	0.0535000
7049.0000000	0.0540000
7050.0000000	0.0514286
7051.0000000	0.0560000
7052.0000000	0.0545000
7053.0000000	0.0555000
7054.0000000	0.0552381
7055.0000000	0.0565000
7056.0000000	0.0565000
7057.0000000	0.0557143
7058.0000000	0.0560000
7059.0000000	0.0550000
7060.0000000	0.0555000
7061.0000000	0.0538095
7062.0000000	0.0530000
7063.0000000	0.0509524
7064.0000000	0.0525000
7065.0000000	0.0523810
7066.0000000	0.0535000
7067.0000000	0.0515000
7068.0000000	0.0520000
7069.0000000	0.0528571
7070.0000000	0.0510000
7071.0000000	0.0530000
7072.0000000	0.0519048
7073.0000000	0.0535000
7074.0000000	0.0515000
7075.0000000	0.0520000
7076.0000000	0.0538095
7077.0000000	0.0545000
7078.0000000	0.0523810
7079.0000000	0.0515000
7080.0000000	0.0535000
7081.0000000	0.0514286
7082.0000000	0.0535000
7083.0000000	0.0550000
7084.0000000	0.0525000
7085.0000000	0.0542857
7086.0000000	0.0557143
7087.0000000	0.0540000
7088.0000000	0.0555000
7089.0000000	0.0535000
7090.0000000	0.0542857
7091.0000000	0.0535000
7092.0000000	0.0525000
7093.0000000	0.0547619
7094.0000000	0.0540000
7095.0000000	0.0515000
7096.0000000	0.0547619
7097.0000000	0.0530000
7098.0000000	0.0550000
7099.0000000	0.0533333
7100.0000000	0.0540000

7101.0000000	0.0550000
7102.0000000	0.0538095
7103.0000000	0.0560000
7104.0000000	0.0570000
7105.0000000	0.0580952
7106.0000000	0.0565000
7107.0000000	0.0565000
7108.0000000	0.0561905
7109.0000000	0.0565000
7110.0000000	0.0547619
7111.0000000	0.0570000
7112.0000000	0.0560000
7113.0000000	0.0570000
7114.0000000	0.0550000
7115.0000000	0.0542857
7116.0000000	0.0545000
7117.0000000	0.0557143
7118.0000000	0.0535000
7119.0000000	0.0530000
7120.0000000	0.0550000
7121.0000000	0.0552381
7122.0000000	0.0545000
7123.0000000	0.0565000
7124.0000000	0.0542857
7125.0000000	0.0540000
7126.0000000	0.0542857
7127.0000000	0.0560000
7128.0000000	0.0550000
7129.0000000	0.0557143
7130.0000000	0.0570000
7131.0000000	0.0570000
7132.0000000	0.0575000
7133.0000000	0.0580952
7134.0000000	0.0590000
7135.0000000	0.0590476
7136.0000000	0.0590000
7137.0000000	0.0595000
7138.0000000	0.0580952
7139.0000000	0.0585000
7140.0000000	0.0590000
7141.0000000	0.0595238
7142.0000000	0.0590000
7143.0000000	0.0585000
7144.0000000	0.0600000
7145.0000000	0.0595000
7146.0000000	0.0595238
7147.0000000	0.0600000
7148.0000000	0.0595000
7149.0000000	0.0600000
7150.0000000	0.0600000
7151.0000000	0.0600000
7152.0000000	0.0600000
7153.0000000	0.0600000
7154.0000000	0.0600000
7155.0000000	0.0595238
7156.0000000	0.0600000
7157.0000000	0.0600000

7158.0000000	0.0600000
7159.0000000	0.0600000
7160.0000000	0.0600000
7161.0000000	0.0600000
7162.0000000	0.0600000
7163.0000000	0.0595000
7164.0000000	0.0600000
7165.0000000	0.0600000
7166.0000000	0.0600000
7167.0000000	0.0600000
7168.0000000	0.0590000
7169.0000000	0.0595238
7170.0000000	0.0600000
7171.0000000	0.0576190
7172.0000000	0.0585000
7173.0000000	0.0590000
7174.0000000	0.0580952
7175.0000000	0.0570000
7176.0000000	0.0585000
7177.0000000	0.0585000
7178.0000000	0.0590476
7179.0000000	0.0590000
7180.0000000	0.0600000
7181.0000000	0.0590476
7182.0000000	0.0595000
7183.0000000	0.0600000
7184.0000000	0.0600000
7185.0000000	0.0600000
7186.0000000	0.0600000
7187.0000000	0.0600000
7188.0000000	0.0600000
7189.0000000	0.0600000
7190.0000000	0.0600000
7191.0000000	0.0600000
7192.0000000	0.0600000
7193.0000000	0.0600000
7194.0000000	0.0600000
7195.0000000	0.0595000
7196.0000000	0.0600000
7197.0000000	0.0600000
7198.0000000	0.0600000
7199.0000000	0.0600000
7200.0000000	0.0600000
7201.0000000	0.0600000
7202.0000000	0.0600000
7203.0000000	0.0600000
7204.0000000	0.0595000
7205.0000000	0.0590476
7206.0000000	0.0595000
7207.0000000	0.0600000
7208.0000000	0.0590000
7209.0000000	0.0595000
7210.0000000	0.0600000
7211.0000000	0.0600000
7212.0000000	0.0600000
7213.0000000	0.0585000
7214.0000000	0.0585714

7215.0000000	0.0580000
7216.0000000	0.0571428
7217.0000000	0.0590000
7218.0000000	0.0575000
7219.0000000	0.0550000
7220.0000000	0.0575000
7221.0000000	0.0571428
7222.0000000	0.0552381
7223.0000000	0.0575000
7224.0000000	0.0570000
7225.0000000	0.0560000
7226.0000000	0.0566667
7227.0000000	0.0580000
7228.0000000	0.0585000
7229.0000000	0.0580952
7230.0000000	0.0585000
7231.0000000	0.0595000
7232.0000000	0.0585714
7233.0000000	0.0600000
7234.0000000	0.0600000
7235.0000000	0.0600000
7236.0000000	0.0600000
7237.0000000	0.0595000
7238.0000000	0.0600000
7239.0000000	0.0600000
7240.0000000	0.0595000
7241.0000000	0.0600000
7242.0000000	0.0600000
7243.0000000	0.0600000
7244.0000000	0.0600000
7245.0000000	0.0600000
7246.0000000	0.0595238
7247.0000000	0.0590000
7248.0000000	0.0590000
7249.0000000	0.0590000
7250.0000000	0.0585714
7251.0000000	0.0560000
7252.0000000	0.0547619
7253.0000000	0.0545000
7254.0000000	0.0560000
7255.0000000	0.0530000
7256.0000000	0.0530000
7257.0000000	0.0500000
7258.0000000	0.0515000
7259.0000000	0.0504762
7260.0000000	0.0500000
7261.0000000	0.0509524
7262.0000000	0.0505000
7263.0000000	0.0515000
7264.0000000	0.0510000
7265.0000000	0.0505000
7266.0000000	0.0500000
7267.0000000	0.0505000
7268.0000000	0.0504762
7269.0000000	0.0500000
7270.0000000	0.0505000
7271.0000000	0.0509524

7272.0000000	0.0505000
7273.0000000	0.0500000
7274.0000000	0.0509524
7275.0000000	0.0515000
7276.0000000	0.0520000
7277.0000000	0.0509524
7278.0000000	0.0510000
7279.0000000	0.0515000
7280.0000000	0.0510000
7281.0000000	0.0504762
7282.0000000	0.0530000
7283.0000000	0.0509524
7284.0000000	0.0515000
7285.0000000	0.0520000
7286.0000000	0.0523810
7287.0000000	0.0510000
7288.0000000	0.0505000
7289.0000000	0.0500000
7290.0000000	0.0504762
7291.0000000	0.0504762
7292.0000000	0.0510000
7293.0000000	0.0515000
7294.0000000	0.0505000
7295.0000000	0.0510000
7296.0000000	0.0523810
7297.0000000	0.0530000
7298.0000000	0.0504762
7299.0000000	0.0530000
7300.0000000	0.0525000
7301.0000000	0.0515000
7302.0000000	0.0528571
7303.0000000	0.0540000
7304.0000000	0.0552381
7305.0000000	0.0535000
7306.0000000	0.0550000
7307.0000000	0.0528571
7308.0000000	0.0550000
7309.0000000	0.0530000
7310.0000000	0.0538095
7311.0000000	0.0530000
7312.0000000	0.0535000
7313.0000000	0.0552381
7314.0000000	0.0540000
7315.0000000	0.0560000
7316.0000000	0.0555000
7317.0000000	0.0565000
7318.0000000	0.0585714
7319.0000000	0.0565000
7320.0000000	0.0590476
7321.0000000	0.0585000
7322.0000000	0.0571428
7323.0000000	0.0595000
7324.0000000	0.0595000
7325.0000000	0.0595000
7326.0000000	0.0595238
7327.0000000	0.0595000
7328.0000000	0.0595000

7329.0000000	0.0595238
7330.0000000	0.0600000
7331.0000000	0.0600000
7332.0000000	0.0600000
7333.0000000	0.0600000
7334.0000000	0.0600000
7335.0000000	0.0600000
7336.0000000	0.0600000
7337.0000000	0.0600000
7338.0000000	0.0600000
7339.0000000	0.0600000
7340.0000000	0.0600000
7341.0000000	0.0600000
7342.0000000	0.0600000
7343.0000000	0.0600000
7344.0000000	0.0600000
7345.0000000	0.0600000
7346.0000000	0.0600000
7347.0000000	0.0600000
7348.0000000	0.0600000
7349.0000000	0.0600000
7350.0000000	0.0600000
7351.0000000	0.0600000
7352.0000000	0.0600000
7353.0000000	0.0600000
7354.0000000	0.0600000
7355.0000000	0.0600000
7356.0000000	0.0600000
7357.0000000	0.0600000
7358.0000000	0.0600000
7359.0000000	0.0600000
7360.0000000	0.0600000
7361.0000000	0.0600000
7362.0000000	0.0600000
7363.0000000	0.0600000
7364.0000000	0.0600000
7365.0000000	0.0600000
7366.0000000	0.0600000
7367.0000000	0.0600000
7368.0000000	0.0600000
7369.0000000	0.0600000
7370.0000000	0.0600000
7371.0000000	0.0600000
7372.0000000	0.0600000
7373.0000000	0.0600000
7374.0000000	0.0600000
7375.0000000	0.0600000
7376.0000000	0.0600000
7377.0000000	0.0600000
7378.0000000	0.0600000
7379.0000000	0.0600000
7380.0000000	0.0600000
7381.0000000	0.0600000
7382.0000000	0.0600000
7383.0000000	0.0600000
7384.0000000	0.0600000
7385.0000000	0.0595000

7386.0000000	0.0595238
7387.0000000	0.0595000
7388.0000000	0.0595238
7389.0000000	0.0600000
7390.0000000	0.0600000
7391.0000000	0.0600000
7392.0000000	0.0600000
7393.0000000	0.0600000
7394.0000000	0.0600000
7395.0000000	0.0600000
7396.0000000	0.0600000
7397.0000000	0.0600000
7398.0000000	0.0600000
7399.0000000	0.0595000
7400.0000000	0.0600000
7401.0000000	0.0585714
7402.0000000	0.0595000
7403.0000000	0.0585000
7404.0000000	0.0590476
7405.0000000	0.0565000
7406.0000000	0.0550000
7407.0000000	0.0550000
7408.0000000	0.0542857
7409.0000000	0.0560000
7410.0000000	0.0576190
7411.0000000	0.0560000
7412.0000000	0.0570000
7413.0000000	0.0571428
7414.0000000	0.0580000
7415.0000000	0.0585000
7416.0000000	0.0575000
7417.0000000	0.0590476
7418.0000000	0.0585000
7419.0000000	0.0595238
7420.0000000	0.0595000
7421.0000000	0.0595000
7422.0000000	0.0595000
7423.0000000	0.0600000
7424.0000000	0.0595000
7425.0000000	0.0600000
7426.0000000	0.0600000
7427.0000000	0.0600000
7428.0000000	0.0595000
7429.0000000	0.0595000
7430.0000000	0.0585000
7431.0000000	0.0585714
7432.0000000	0.0590000
7433.0000000	0.0585000
7434.0000000	0.0590000
7435.0000000	0.0590476
7436.0000000	0.0595000
7437.0000000	0.0600000
7438.0000000	0.0585000
7439.0000000	0.0585000
7440.0000000	0.0595238
7441.0000000	0.0590000
7442.0000000	0.0580000

7443.0000000	0.0571428
7444.0000000	0.0540000
7445.0000000	0.0560000
7446.0000000	0.0570000
7447.0000000	0.0557143
7448.0000000	0.0536842
7449.0000000	0.0550000
7450.0000000	0.0550000
7451.0000000	0.0550000
7452.0000000	0.0555000
7453.0000000	0.0538095
7454.0000000	0.0545000
7455.0000000	0.0547619
7456.0000000	0.0550000
7457.0000000	0.0565000
7458.0000000	0.0576190
7459.0000000	0.0535000
7460.0000000	0.0555000
7461.0000000	0.0565000
7462.0000000	0.0557143
7463.0000000	0.0565000
7464.0000000	0.0576190
7465.0000000	0.0545000
7466.0000000	0.0580000
7467.0000000	0.0576190
7468.0000000	0.0555000
7469.0000000	0.0555000
7470.0000000	0.0570000
7471.0000000	0.0571428
7472.0000000	0.0575000
7473.0000000	0.0561905
7474.0000000	0.0585000
7475.0000000	0.0580000
7476.0000000	0.0561905
7477.0000000	0.0550000
7478.0000000	0.0550000
7479.0000000	0.0547619
7480.0000000	0.0540000
7481.0000000	0.0540000
7482.0000000	0.0530000
7483.0000000	0.0542857
7484.0000000	0.0520000
7485.0000000	0.0535000
7486.0000000	0.0528571
7487.0000000	0.0530000
7488.0000000	0.0533333
7489.0000000	0.0540000
7490.0000000	0.0520000
7491.0000000	0.0523810
7492.0000000	0.0550000
7493.0000000	0.0550000
7494.0000000	0.0570000
7495.0000000	0.0552381
7496.0000000	0.0545000
7497.0000000	0.0580000
7498.0000000	0.0571428
7499.0000000	0.0585000

7500.0000000	0.0590476
7501.0000000	0.0580000
7502.0000000	0.0580000
7503.0000000	0.0571428
7504.0000000	0.0580000
7505.0000000	0.0585000
7506.0000000	0.0590000
7507.0000000	0.0590476
7508.0000000	0.0585000
7509.0000000	0.0585000
7510.0000000	0.0590476
7511.0000000	0.0595000
7512.0000000	0.0600000
7513.0000000	0.0600000
7514.0000000	0.0595238
7515.0000000	0.0600000
7516.0000000	0.0595000
7517.0000000	0.0600000
7518.0000000	0.0600000
7519.0000000	0.0600000
7520.0000000	0.0595000
7521.0000000	0.0600000
7522.0000000	0.0600000
7523.0000000	0.0600000
7524.0000000	0.0600000
7525.0000000	0.0595238
7526.0000000	0.0595000
7527.0000000	0.0600000
7528.0000000	0.0600000
7529.0000000	0.0600000
7530.0000000	0.0600000
7531.0000000	0.0600000
7532.0000000	0.0600000
7533.0000000	0.0600000
7534.0000000	0.0600000
7535.0000000	0.0600000
7536.0000000	0.0600000
7537.0000000	0.0600000
7538.0000000	0.0600000
7539.0000000	0.0595000
7540.0000000	0.0600000
7541.0000000	0.0600000
7542.0000000	0.0600000
7543.0000000	0.0595238
7544.0000000	0.0600000
7545.0000000	0.0600000
7546.0000000	0.0590476
7547.0000000	0.0595000
7548.0000000	0.0595238
7549.0000000	0.0600000
7550.0000000	0.0595000
7551.0000000	0.0590000
7552.0000000	0.0590476
7553.0000000	0.0595000
7554.0000000	0.0595000
7555.0000000	0.0590476
7556.0000000	0.0585000

7557.0000000	0.0595000
7558.0000000	0.0600000
7559.0000000	0.0595238
7560.0000000	0.0600000
7561.0000000	0.0600000
7562.0000000	0.0595000
7563.0000000	0.0590000
7564.0000000	0.0590476
7565.0000000	0.0580000
7566.0000000	0.0570000
7567.0000000	0.0571428
7568.0000000	0.0585000
7569.0000000	0.0585000
7570.0000000	0.0580952
7571.0000000	0.0585000
7572.0000000	0.0575000
7573.0000000	0.0580000
7574.0000000	0.0552381
7575.0000000	0.0555000
7576.0000000	0.0566667
7577.0000000	0.0540000
7578.0000000	0.0520000
7579.0000000	0.0533333
7580.0000000	0.0520000
7581.0000000	0.0510000
7582.0000000	0.0535000
7583.0000000	0.0533333
7584.0000000	0.0514286
7585.0000000	0.0535000
7586.0000000	0.0535000
7587.0000000	0.0525000
7588.0000000	0.0525000
7589.0000000	0.0538095
7590.0000000	0.0520000
7591.0000000	0.0542857
7592.0000000	0.0515000
7593.0000000	0.0528571
7594.0000000	0.0520000
7595.0000000	0.0535000
7596.0000000	0.0540000
7597.0000000	0.0520000
7598.0000000	0.0552381
7599.0000000	0.0520000
7600.0000000	0.0528571
7601.0000000	0.0520000
7602.0000000	0.0530000
7603.0000000	0.0560000
7604.0000000	0.0528571
7605.0000000	0.0520000
7606.0000000	0.0525000
7607.0000000	0.0547619
7608.0000000	0.0530000
7609.0000000	0.0557143
7610.0000000	0.0545000
7611.0000000	0.0555000
7612.0000000	0.0565000
7613.0000000	0.0538095

7614.0000000	0.0530000
7615.0000000	0.0552381
7616.0000000	0.0555000
7617.0000000	0.0550000
7618.0000000	0.0550000
7619.0000000	0.0557143
7620.0000000	0.0555000
7621.0000000	0.0550000
7622.0000000	0.0542857
7623.0000000	0.0565000
7624.0000000	0.0552381
7625.0000000	0.0555000
7626.0000000	0.0565000
7627.0000000	0.0570000
7628.0000000	0.0557143
7629.0000000	0.0565000
7630.0000000	0.0560000
7631.0000000	0.0566667
7632.0000000	0.0555000
7633.0000000	0.0560000
7634.0000000	0.0552381
7635.0000000	0.0540000
7636.0000000	0.0566667
7637.0000000	0.0530000
7638.0000000	0.0560000
7639.0000000	0.0535000
7640.0000000	0.0523810
7641.0000000	0.0515000
7642.0000000	0.0525000
7643.0000000	0.0528571
7644.0000000	0.0525000
7645.0000000	0.0523810
7646.0000000	0.0540000
7647.0000000	0.0540000
7648.0000000	0.0550000
7649.0000000	0.0557143
7650.0000000	0.0555000
7651.0000000	0.0570000
7652.0000000	0.0557143
7653.0000000	0.0555000
7654.0000000	0.0561905
7655.0000000	0.0545000
7656.0000000	0.0545000
7657.0000000	0.0545000
7658.0000000	0.0561905
7659.0000000	0.0580000
7660.0000000	0.0575000
7661.0000000	0.0557143
7662.0000000	0.0565000
7663.0000000	0.0570000
7664.0000000	0.0561905
7665.0000000	0.0565000
7666.0000000	0.0570000
7667.0000000	0.0580952
7668.0000000	0.0560000
7669.0000000	0.0570000
7670.0000000	0.0566667

7671.0000000	0.0555000
7672.0000000	0.0538095
7673.0000000	0.0550000
7674.0000000	0.0540000
7675.0000000	0.0540000
7676.0000000	0.0528571
7677.0000000	0.0530000
7678.0000000	0.0535000
7679.0000000	0.0519048
7680.0000000	0.0520000
7681.0000000	0.0504762
7682.0000000	0.0500000
7683.0000000	0.0505000
7684.0000000	0.0504762
7685.0000000	0.0505000
7686.0000000	0.0500000
7687.0000000	0.0515000
7688.0000000	0.0509524
7689.0000000	0.0500000
7690.0000000	0.0505000
7691.0000000	0.0519048
7692.0000000	0.0520000
7693.0000000	0.0520000
7694.0000000	0.0523810
7695.0000000	0.0525000
7696.0000000	0.0505000
7697.0000000	0.0523810
7698.0000000	0.0525000
7699.0000000	0.0520000
7700.0000000	0.0533333
7701.0000000	0.0525000
7702.0000000	0.0520000
7703.0000000	0.0520000
7704.0000000	0.0528571
7705.0000000	0.0520000
7706.0000000	0.0528571
7707.0000000	0.0520000
7708.0000000	0.0520000
7709.0000000	0.0525000
7710.0000000	0.0533333
7711.0000000	0.0520000
7712.0000000	0.0547619
7713.0000000	0.0505000
7714.0000000	0.0520000
7715.0000000	0.0509524
7716.0000000	0.0505000
7717.0000000	0.0505000
7718.0000000	0.0505000
7719.0000000	0.0504762
7720.0000000	0.0500000
7721.0000000	0.0505000
7722.0000000	0.0505000
7723.0000000	0.0500000
7724.0000000	0.0500000
7725.0000000	0.0500000
7726.0000000	0.0515000
7727.0000000	0.0500000

7728.0000000	0.0500000
7729.0000000	0.0525000
7730.0000000	0.0500000
7731.0000000	0.0515000
7732.0000000	0.0510000
7733.0000000	0.0500000
7734.0000000	0.0520000
7735.0000000	0.0510000
7736.0000000	0.0509524
7737.0000000	0.0510000
7738.0000000	0.0505000
7739.0000000	0.0515000
7740.0000000	0.0504762
7741.0000000	0.0515000
7742.0000000	0.0509524
7743.0000000	0.0505000
7744.0000000	0.0500000
7745.0000000	0.0504762
7746.0000000	0.0505000
7747.0000000	0.0510000
7748.0000000	0.0510000
7749.0000000	0.0519048
7750.0000000	0.0505000
7751.0000000	0.0509524
7752.0000000	0.0515000
7753.0000000	0.0510000
7754.0000000	0.0515000
7755.0000000	0.0500000
7756.0000000	0.0510000
7757.0000000	0.0510000
7758.0000000	0.0504762
7759.0000000	0.0510000
7760.0000000	0.0523810
7761.0000000	0.0505000
7762.0000000	0.0510000
7763.0000000	0.0520000
7764.0000000	0.0504762
7765.0000000	0.0505000
7766.0000000	0.0500000
7767.0000000	0.0500000
7768.0000000	0.0505000
7769.0000000	0.0500000
7770.0000000	0.0500000
7771.0000000	0.0500000
7772.0000000	0.0500000
7773.0000000	0.0500000
7774.0000000	0.0495000
7775.0000000	0.0500000
7776.0000000	0.0500000
7777.0000000	0.0500000
7778.0000000	0.0500000
7779.0000000	0.0500000
7780.0000000	0.0500000
7781.0000000	0.0500000
7782.0000000	0.0500000
7783.0000000	0.0500000
7784.0000000	0.0500000

7785.0000000	0.0500000
7786.0000000	0.0500000
7787.0000000	0.0495000
7788.0000000	0.0500000
7789.0000000	0.0500000
7790.0000000	0.0500000
7791.0000000	0.0495000
7792.0000000	0.0500000
7793.0000000	0.0495000
7794.0000000	0.0500000
7795.0000000	0.0495000
7796.0000000	0.0500000
7797.0000000	0.0500000
7798.0000000	0.0500000
7799.0000000	0.0505000
7800.0000000	0.0504762
7801.0000000	0.0500000
7802.0000000	0.0500000
7803.0000000	0.0500000
7804.0000000	0.0500000
7805.0000000	0.0500000
7806.0000000	0.0500000
7807.0000000	0.0495000
7808.0000000	0.0500000
7809.0000000	0.0500000
7810.0000000	0.0500000
7811.0000000	0.0500000
7812.0000000	0.0500000
7813.0000000	0.0500000
7814.0000000	0.0500000
7815.0000000	0.0500000
7816.0000000	0.0500000
7817.0000000	0.0500000
7818.0000000	0.0500000
7819.0000000	0.0500000
7820.0000000	0.0500000
7821.0000000	0.0500000
7822.0000000	0.0500000
7823.0000000	0.0500000
7824.0000000	0.0500000
7825.0000000	0.0500000
7826.0000000	0.0500000
7827.0000000	0.0500000
7828.0000000	0.0504762
7829.0000000	0.0500000
7830.0000000	0.0500000
7831.0000000	0.0500000
7832.0000000	0.0500000
7833.0000000	0.0500000
7834.0000000	0.0490000
7835.0000000	0.0490000
7836.0000000	0.0461905
7837.0000000	0.0445000
7838.0000000	0.0470000
7839.0000000	0.0461905
7840.0000000	0.0435000
7841.0000000	0.0447619

7842.0000000	0.0435000
7843.0000000	0.0455000
7844.0000000	0.0445000
7845.0000000	0.0455000
7846.0000000	0.0438095
7847.0000000	0.0440000
7848.0000000	0.0447619
7849.0000000	0.0420000
7850.0000000	0.0423810
7851.0000000	0.0445000
7852.0000000	0.0415000
7853.0000000	0.0445000
7854.0000000	0.0460000
7855.0000000	0.0457143
7856.0000000	0.0460000
7857.0000000	0.0447619
7858.0000000	0.0445000
7859.0000000	0.0445000
7860.0000000	0.0457143
7861.0000000	0.0440000
7862.0000000	0.0465000
7863.0000000	0.0460000
7864.0000000	0.0433333
7865.0000000	0.0435000
7866.0000000	0.0438095
7867.0000000	0.0440000
7868.0000000	0.0430000
7869.0000000	0.0409524
7870.0000000	0.0415000
7871.0000000	0.0435000
7872.0000000	0.0423810
7873.0000000	0.0420000
7874.0000000	0.0435000
7875.0000000	0.0415000
7876.0000000	0.0409524
7877.0000000	0.0423810
7878.0000000	0.0420000
7879.0000000	0.0410000
7880.0000000	0.0440000
7881.0000000	0.0447619
7882.0000000	0.0445000
7883.0000000	0.0435000
7884.0000000	0.0447619
7885.0000000	0.0440000
7886.0000000	0.0442857
7887.0000000	0.0450000
7888.0000000	0.0435000
7889.0000000	0.0425000
7890.0000000	0.0440000
7891.0000000	0.0438095
7892.0000000	0.0435000
7893.0000000	0.0433333
7894.0000000	0.0425000
7895.0000000	0.0420000
7896.0000000	0.0409524
7897.0000000	0.0425000
7898.0000000	0.0435000

7899.0000000	0.0405000
7900.0000000	0.0414286
7901.0000000	0.0415000
7902.0000000	0.0419048
7903.0000000	0.0425000
7904.0000000	0.0435000
7905.0000000	0.0415000
7906.0000000	0.0423810
7907.0000000	0.0420000
7908.0000000	0.0435000
7909.0000000	0.0452381
7910.0000000	0.0450000
7911.0000000	0.0442857
7912.0000000	0.0435000
7913.0000000	0.0450000
7914.0000000	0.0455000
7915.0000000	0.0476191
7916.0000000	0.0495000
7917.0000000	0.0490476
7918.0000000	0.0485000
7919.0000000	0.0495000
7920.0000000	0.0485000
7921.0000000	0.0500000
7922.0000000	0.0485000
7923.0000000	0.0495000
7924.0000000	0.0495238
7925.0000000	0.0500000
7926.0000000	0.0500000
7927.0000000	0.0500000
7928.0000000	0.0500000
7929.0000000	0.0500000
7930.0000000	0.0500000
7931.0000000	0.0500000
7932.0000000	0.0500000
7933.0000000	0.0500000
7934.0000000	0.0500000
7935.0000000	0.0505000
7936.0000000	0.0500000
7937.0000000	0.0505000
7938.0000000	0.0500000
7939.0000000	0.0500000
7940.0000000	0.0500000
7941.0000000	0.0500000
7942.0000000	0.0500000
7943.0000000	0.0510000
7944.0000000	0.0500000
7945.0000000	0.0500000
7946.0000000	0.0500000
7947.0000000	0.0500000
7948.0000000	0.0505000
7949.0000000	0.0500000
7950.0000000	0.0500000
7951.0000000	0.0500000
7952.0000000	0.0500000
7953.0000000	0.0500000
7954.0000000	0.0504762
7955.0000000	0.0505000

7956.0000000	0.0500000
7957.0000000	0.0500000
7958.0000000	0.0505000
7959.0000000	0.0500000
7960.0000000	0.0504762
7961.0000000	0.0500000
7962.0000000	0.0500000
7963.0000000	0.0500000
7964.0000000	0.0500000
7965.0000000	0.0504762
7966.0000000	0.0500000
7967.0000000	0.0495000
7968.0000000	0.0500000
7969.0000000	0.0500000
7970.0000000	0.0500000
7971.0000000	0.0500000
7972.0000000	0.0500000
7973.0000000	0.0500000
7974.0000000	0.0500000
7975.0000000	0.0500000
7976.0000000	0.0500000
7977.0000000	0.0500000
7978.0000000	0.0500000
7979.0000000	0.0500000
7980.0000000	0.0490000
7981.0000000	0.0500000
7982.0000000	0.0500000
7983.0000000	0.0500000
7984.0000000	0.0500000
7985.0000000	0.0495000
7986.0000000	0.0500000
7987.0000000	0.0500000
7988.0000000	0.0495000
7989.0000000	0.0490000
7990.0000000	0.0480952
7991.0000000	0.0465000
7992.0000000	0.0460000
7993.0000000	0.0476191
7994.0000000	0.0485000
7995.0000000	0.0470000
7996.0000000	0.0455000
7997.0000000	0.0457143
7998.0000000	0.0465000
7999.0000000	0.0447619
8000.0000000	0.0460000
8001.0000000	0.0471429
8002.0000000	0.0460000
8003.0000000	0.0480000
8004.0000000	0.0470000
8005.0000000	0.0466667
8006.0000000	0.0480000
8007.0000000	0.0480000
8008.0000000	0.0476191
8009.0000000	0.0485000
8010.0000000	0.0485000
8011.0000000	0.0470000
8012.0000000	0.0466667

8013.0000000	0.0485000
8014.0000000	0.0466667
8015.0000000	0.0470000
8016.0000000	0.0440000
8017.0000000	0.0438095
8018.0000000	0.0450000
8019.0000000	0.0455000
8020.0000000	0.0450000
8021.0000000	0.0466667
8022.0000000	0.0455000
8023.0000000	0.0457143
8024.0000000	0.0460000
8025.0000000	0.0465000
8026.0000000	0.0466667
8027.0000000	0.0470000
8028.0000000	0.0480000
8029.0000000	0.0476191
8030.0000000	0.0490000
8031.0000000	0.0475000
8032.0000000	0.0485000
8033.0000000	0.0476191
8034.0000000	0.0485000
8035.0000000	0.0490476
8036.0000000	0.0495000
8037.0000000	0.0490000
8038.0000000	0.0471429
8039.0000000	0.0475000
8040.0000000	0.0475000
8041.0000000	0.0475000
8042.0000000	0.0480952
8043.0000000	0.0485000
8044.0000000	0.0485714
8045.0000000	0.0490000
8046.0000000	0.0495238
8047.0000000	0.0500000
8048.0000000	0.0490000
8049.0000000	0.0500000
8050.0000000	0.0500000
8051.0000000	0.0495238
8052.0000000	0.0500000
8053.0000000	0.0495238
8054.0000000	0.0500000
8055.0000000	0.0500000
8056.0000000	0.0500000
8057.0000000	0.0495238
8058.0000000	0.0500000
8059.0000000	0.0500000
8060.0000000	0.0500000
8061.0000000	0.0495000
8062.0000000	0.0500000
8063.0000000	0.0500000
8064.0000000	0.0500000
8065.0000000	0.0495238
8066.0000000	0.0495000
8067.0000000	0.0495000
8068.0000000	0.0495000
8069.0000000	0.0490476

8070.0000000	0.0495000
8071.0000000	0.0495000
8072.0000000	0.0495238
8073.0000000	0.0500000
8074.0000000	0.0495238
8075.0000000	0.0500000
8076.0000000	0.0500000
8077.0000000	0.0500000
8078.0000000	0.0500000
8079.0000000	0.0500000
8080.0000000	0.0500000
8081.0000000	0.0500000
8082.0000000	0.0500000
8083.0000000	0.0500000
8084.0000000	0.0500000
8085.0000000	0.0500000
8086.0000000	0.0500000
8087.0000000	0.0500000
8088.0000000	0.0495000
8089.0000000	0.0495238
8090.0000000	0.0500000
8091.0000000	0.0490000
8092.0000000	0.0495238
8093.0000000	0.0500000
8094.0000000	0.0495000
8095.0000000	0.0495000
8096.0000000	0.0495238
8097.0000000	0.0500000
8098.0000000	0.0495238
8099.0000000	0.0495000
8100.0000000	0.0490000
8101.0000000	0.0495000
8102.0000000	0.0495238
8103.0000000	0.0490000
8104.0000000	0.0470000
8105.0000000	0.0471429
8106.0000000	0.0460000
8107.0000000	0.0452381
8108.0000000	0.0465000
8109.0000000	0.0470000
8110.0000000	0.0475000
8111.0000000	0.0447619
8112.0000000	0.0450000
8113.0000000	0.0440000
8114.0000000	0.0452381
8115.0000000	0.0440000
8116.0000000	0.0445000
8117.0000000	0.0447619
8118.0000000	0.0450000
8119.0000000	0.0440000
8120.0000000	0.0447619
8121.0000000	0.0460000
8122.0000000	0.0457143
8123.0000000	0.0465000
8124.0000000	0.0460000
8125.0000000	0.0460000
8126.0000000	0.0471429

8127.0000000	0.0475000
8128.0000000	0.0465000
8129.0000000	0.0480952
8130.0000000	0.0480000
8131.0000000	0.0475000
8132.0000000	0.0461905
8133.0000000	0.0470000
8134.0000000	0.0471429
8135.0000000	0.0480000
8136.0000000	0.0460000
8137.0000000	0.0460000
8138.0000000	0.0461905
8139.0000000	0.0470000
8140.0000000	0.0470000
8141.0000000	0.0461905
8142.0000000	0.0470000
8143.0000000	0.0476191
8144.0000000	0.0475000
8145.0000000	0.0490000
8146.0000000	0.0480000
8147.0000000	0.0475000
8148.0000000	0.0490476
8149.0000000	0.0490000
8150.0000000	0.0490476
8151.0000000	0.0490000
8152.0000000	0.0485714
8153.0000000	0.0490000
8154.0000000	0.0490000
8155.0000000	0.0470000
8156.0000000	0.0485000
8157.0000000	0.0495238
8158.0000000	0.0490000
8159.0000000	0.0490476
8160.0000000	0.0470000
8161.0000000	0.0475000
8162.0000000	0.0476191
8163.0000000	0.0495000
8164.0000000	0.0490000
8165.0000000	0.0495238
8166.0000000	0.0490000
8167.0000000	0.0485000
8168.0000000	0.0480952
8169.0000000	0.0490000
8170.0000000	0.0500000
8171.0000000	0.0495000
8172.0000000	0.0495000
8173.0000000	0.0495000
8174.0000000	0.0495238
8175.0000000	0.0490000
8176.0000000	0.0490000
8177.0000000	0.0495000
8178.0000000	0.0485714
8179.0000000	0.0495238
8180.0000000	0.0500000
8181.0000000	0.0500000
8182.0000000	0.0500000
8183.0000000	0.0500000

8184.0000000	0.0500000
8185.0000000	0.0500000
8186.0000000	0.0500000
8187.0000000	0.0500000
8188.0000000	0.0500000
8189.0000000	0.0495238
8190.0000000	0.0495000
8191.0000000	0.0500000
8192.0000000	0.0500000
8193.0000000	0.0500000
8194.0000000	0.0500000
8195.0000000	0.0500000
8196.0000000	0.0500000
8197.0000000	0.0500000
8198.0000000	0.0495238
8199.0000000	0.0500000
8200.0000000	0.0500000
8201.0000000	0.0500000
8202.0000000	0.0500000
8203.0000000	0.0500000
8204.0000000	0.0500000
8205.0000000	0.0500000
8206.0000000	0.0495000
8207.0000000	0.0500000
8208.0000000	0.0500000
8209.0000000	0.0500000
8210.0000000	0.0500000
8211.0000000	0.0500000
8212.0000000	0.0510000
8213.0000000	0.0500000
8214.0000000	0.0505000
8215.0000000	0.0505000
8216.0000000	0.0500000
8217.0000000	0.0500000
8218.0000000	0.0500000
8219.0000000	0.0500000
8220.0000000	0.0500000
8221.0000000	0.0500000
8222.0000000	0.0500000
8223.0000000	0.0500000
8224.0000000	0.0500000
8225.0000000	0.0500000
8226.0000000	0.0500000
8227.0000000	0.0480000
8228.0000000	0.0495238
8229.0000000	0.0470000
8230.0000000	0.0485000
8231.0000000	0.0500000
8232.0000000	0.0495000
8233.0000000	0.0500000
8234.0000000	0.0500000
8235.0000000	0.0495000
8236.0000000	0.0500000
8237.0000000	0.0495000
8238.0000000	0.0495238
8239.0000000	0.0500000
8240.0000000	0.0495238

8241.0000000	0.0495000
8242.0000000	0.0500000
8243.0000000	0.0500000
8244.0000000	0.0495000
8245.0000000	0.0495000
8246.0000000	0.0500000
8247.0000000	0.0500000
8248.0000000	0.0500000
8249.0000000	0.0500000
8250.0000000	0.0500000
8251.0000000	0.0500000
8252.0000000	0.0500000
8253.0000000	0.0500000
8254.0000000	0.0500000
8255.0000000	0.0500000
8256.0000000	0.0500000
8257.0000000	0.0500000
8258.0000000	0.0500000
8259.0000000	0.0500000
8260.0000000	0.0500000
8261.0000000	0.0500000
8262.0000000	0.0500000
8263.0000000	0.0500000
8264.0000000	0.0505000
8265.0000000	0.0500000
8266.0000000	0.0500000
8267.0000000	0.0500000
8268.0000000	0.0500000
8269.0000000	0.0500000
8270.0000000	0.0500000
8271.0000000	0.0500000
8272.0000000	0.0500000
8273.0000000	0.0500000
8274.0000000	0.0500000
8275.0000000	0.0500000
8276.0000000	0.0500000
8277.0000000	0.0495238
8278.0000000	0.0500000
8279.0000000	0.0495238
8280.0000000	0.0500000
8281.0000000	0.0495000
8282.0000000	0.0500000
8283.0000000	0.0500000
8284.0000000	0.0500000
8285.0000000	0.0495000
8286.0000000	0.0490476
8287.0000000	0.0500000
8288.0000000	0.0490476
8289.0000000	0.0490000
8290.0000000	0.0500000
8291.0000000	0.0490000
8292.0000000	0.0480952
8293.0000000	0.0490000
8294.0000000	0.0485000
8295.0000000	0.0471429
8296.0000000	0.0455000
8297.0000000	0.0480000

8298.0000000	0.0461905
8299.0000000	0.0470000
8300.0000000	0.0475000
8301.0000000	0.0476191
8302.0000000	0.0465000
8303.0000000	0.0475000
8304.0000000	0.0447619
8305.0000000	0.0455000
8306.0000000	0.0430000
8307.0000000	0.0433333
8308.0000000	0.0430000
8309.0000000	0.0425000
8310.0000000	0.0428571
8311.0000000	0.0440000
8312.0000000	0.0425000
8313.0000000	0.0430000
8314.0000000	0.0433333
8315.0000000	0.0450000
8316.0000000	0.0442857
8317.0000000	0.0440000
8318.0000000	0.0426316
8319.0000000	0.0454546
8320.0000000	0.0425000
8321.0000000	0.0435000
8322.0000000	0.0433333
8323.0000000	0.0435000
8324.0000000	0.0415000
8325.0000000	0.0419048
8326.0000000	0.0430000
8327.0000000	0.0415000
8328.0000000	0.0419048
8329.0000000	0.0410000
8330.0000000	0.0405000
8331.0000000	0.0414286
8332.0000000	0.0400000
8333.0000000	0.0410000
8334.0000000	0.0419048
8335.0000000	0.0415000
8336.0000000	0.0405000
8337.0000000	0.0428571
8338.0000000	0.0415000
8339.0000000	0.0415000
8340.0000000	0.0414286
8341.0000000	0.0425000
8342.0000000	0.0430000
8343.0000000	0.0425000
8344.0000000	0.0423810
8345.0000000	0.0420000
8346.0000000	0.0419048
8347.0000000	0.0420000
8348.0000000	0.0455000
8349.0000000	0.0433333
8350.0000000	0.0445000
8351.0000000	0.0450000
8352.0000000	0.0430000
8353.0000000	0.0423810
8354.0000000	0.0425000

8355.0000000	0.0433333
8356.0000000	0.0455000
8357.0000000	0.0445000
8358.0000000	0.0466667
8359.0000000	0.0445000
8360.0000000	0.0455000
8361.0000000	0.0465000
8362.0000000	0.0452381
8363.0000000	0.0465000
8364.0000000	0.0480952
8365.0000000	0.0480000
8366.0000000	0.0475000
8367.0000000	0.0471429
8368.0000000	0.0470000
8369.0000000	0.0490000
8370.0000000	0.0466667
8371.0000000	0.0475000
8372.0000000	0.0460000
8373.0000000	0.0480000
8374.0000000	0.0461905
8375.0000000	0.0445000
8376.0000000	0.0461905
8377.0000000	0.0450000
8378.0000000	0.0435000
8379.0000000	0.0452381
8380.0000000	0.0455000
8381.0000000	0.0480000
8382.0000000	0.0480000
8383.0000000	0.0461905
8384.0000000	0.0450000
8385.0000000	0.0461905
8386.0000000	0.0475000
8387.0000000	0.0475000
8388.0000000	0.0470000
8389.0000000	0.0452381
8390.0000000	0.0452632
8391.0000000	0.0466667
8392.0000000	0.0461905
8393.0000000	0.0440000
8394.0000000	0.0452381
8395.0000000	0.0470000
8396.0000000	0.0445000
8397.0000000	0.0465000
8398.0000000	0.0457143
8399.0000000	0.0460000
8400.0000000	0.0455000
8401.0000000	0.0447619
8402.0000000	0.0450000
8403.0000000	0.0452381
8404.0000000	0.0445000
8405.0000000	0.0435000
8406.0000000	0.0455000
8407.0000000	0.0461905
8408.0000000	0.0420000
8409.0000000	0.0445000
8410.0000000	0.0423810
8411.0000000	0.0445000

8412.0000000	0.0440000
8413.0000000	0.0447619
8414.0000000	0.0445000
8415.0000000	0.0461905
8416.0000000	0.0430000
8417.0000000	0.0430000
8418.0000000	0.0410000
8419.0000000	0.0428572
8420.0000000	0.0410000
8421.0000000	0.0425000
8422.0000000	0.0428572
8423.0000000	0.0415000
8424.0000000	0.0433333
8425.0000000	0.0425000
8426.0000000	0.0420000
8427.0000000	0.0420000
8428.0000000	0.0419048
8429.0000000	0.0415000
8430.0000000	0.0400000
8431.0000000	0.0400000
8432.0000000	0.0400000
8433.0000000	0.0410000
8434.0000000	0.0404762
8435.0000000	0.0415000
8436.0000000	0.0425000
8437.0000000	0.0414286
8438.0000000	0.0420000
8439.0000000	0.0433333
8440.0000000	0.0430000
8441.0000000	0.0440000
8442.0000000	0.0450000
8443.0000000	0.0452381
8444.0000000	0.0465000
8445.0000000	0.0475000
8446.0000000	0.0452381
8447.0000000	0.0430000
8448.0000000	0.0460000
8449.0000000	0.0460000
8450.0000000	0.0452381
8451.0000000	0.0435000
8452.0000000	0.0466667
8453.0000000	0.0445000
8454.0000000	0.0425000
8455.0000000	0.0428572
8456.0000000	0.0410000
8457.0000000	0.0415000
8458.0000000	0.0433333
8459.0000000	0.0435000
8460.0000000	0.0430000
8461.0000000	0.0420000
8462.0000000	0.0433333
8463.0000000	0.0425000
8464.0000000	0.0428572
8465.0000000	0.0425000
8466.0000000	0.0415000
8467.0000000	0.0442857
8468.0000000	0.0415000

8469.0000000	0.0405000
8470.0000000	0.0414286
8471.0000000	0.0415000
8472.0000000	0.0410000
8473.0000000	0.0409524
8474.0000000	0.0425000
8475.0000000	0.0414286
8476.0000000	0.0420000
8477.0000000	0.0415000
8478.0000000	0.0410000
8479.0000000	0.0410000
8480.0000000	0.0419048
8481.0000000	0.0410000
8482.0000000	0.0409524
8483.0000000	0.0405000
8484.0000000	0.0404762
8485.0000000	0.0410000
8486.0000000	0.0410000
8487.0000000	0.0410000
8488.0000000	0.0415000
8489.0000000	0.0404762
8490.0000000	0.0415000
8491.0000000	0.0419048
8492.0000000	0.0400000
8493.0000000	0.0410000
8494.0000000	0.0410000
8495.0000000	0.0409524
8496.0000000	0.0405000
8497.0000000	0.0410000
8498.0000000	0.0419048
8499.0000000	0.0435000
8500.0000000	0.0419048
8501.0000000	0.0400000
8502.0000000	0.0410000
8503.0000000	0.0409524
8504.0000000	0.0420000
8505.0000000	0.0405000
8506.0000000	0.0405000
8507.0000000	0.0414286
8508.0000000	0.0400000
8509.0000000	0.0410000
8510.0000000	0.0423810
8511.0000000	0.0420000
8512.0000000	0.0404762
8513.0000000	0.0420000
8514.0000000	0.0415000
8515.0000000	0.0409524
8516.0000000	0.0425000
8517.0000000	0.0425000
8518.0000000	0.0415000
8519.0000000	0.0409524
8520.0000000	0.0410000
8521.0000000	0.0414286
8522.0000000	0.0405000
8523.0000000	0.0405000
8524.0000000	0.0405000
8525.0000000	0.0404762

8526.0000000	0.0415000
8527.0000000	0.0409524
8528.0000000	0.0400000
8529.0000000	0.0405000
8530.0000000	0.0404762
8531.0000000	0.0410000
8532.0000000	0.0400000
8533.0000000	0.0410000
8534.0000000	0.0414286
8535.0000000	0.0420000
8536.0000000	0.0409524
8537.0000000	0.0415000
8538.0000000	0.0420000
8539.0000000	0.0420000
8540.0000000	0.0414286
8541.0000000	0.0420000
8542.0000000	0.0420000
8543.0000000	0.0423810
8544.0000000	0.0405000
8545.0000000	0.0419048
8546.0000000	0.0430000
8547.0000000	0.0415000
8548.0000000	0.0415000
8549.0000000	0.0428572
8550.0000000	0.0430000
8551.0000000	0.0419048
8552.0000000	0.0410000
8553.0000000	0.0405000
8554.0000000	0.0410000
8555.0000000	0.0433333
8556.0000000	0.0415000
8557.0000000	0.0410000
8558.0000000	0.0409524
8559.0000000	0.0415000
8560.0000000	0.0400000
8561.0000000	0.0405000
8562.0000000	0.0405000
8563.0000000	0.0400000
8564.0000000	0.0409524
8565.0000000	0.0410000
8566.0000000	0.0400000
8567.0000000	0.0400000
8568.0000000	0.0405000
8569.0000000	0.0400000
8570.0000000	0.0400000
8571.0000000	0.0415000
8572.0000000	0.0404762
8573.0000000	0.0400000
8574.0000000	0.0400000
8575.0000000	0.0400000
8576.0000000	0.0405000
8577.0000000	0.0405000
8578.0000000	0.0400000
8579.0000000	0.0404762
8580.0000000	0.0410000
8581.0000000	0.0409524
8582.0000000	0.0405000

8583.0000000	0.0415000
8584.0000000	0.0405000
8585.0000000	0.0405000
8586.0000000	0.0414286
8587.0000000	0.0425000
8588.0000000	0.0419048
8589.0000000	0.0420000
8590.0000000	0.0419048
8591.0000000	0.0410000
8592.0000000	0.0405000
8593.0000000	0.0410000
8594.0000000	0.0410000
8595.0000000	0.0414286
8596.0000000	0.0400000
8597.0000000	0.0404762
8598.0000000	0.0400000
8599.0000000	0.0400000
8600.0000000	0.0400000
8601.0000000	0.0400000
8602.0000000	0.0400000
8603.0000000	0.0400000
8604.0000000	0.0400000
8605.0000000	0.0400000
8606.0000000	0.0400000
8607.0000000	0.0400000
8608.0000000	0.0400000
8609.0000000	0.0400000
8610.0000000	0.0400000
8611.0000000	0.0400000
8612.0000000	0.0400000
8613.0000000	0.0400000
8614.0000000	0.0400000
8615.0000000	0.0400000
8616.0000000	0.0400000
8617.0000000	0.0400000
8618.0000000	0.0400000
8619.0000000	0.0400000
8620.0000000	0.0400000
8621.0000000	0.0400000
8622.0000000	0.0400000
8623.0000000	0.0400000
8624.0000000	0.0400000
8625.0000000	0.0400000
8626.0000000	0.0400000
8627.0000000	0.0400000
8628.0000000	0.0400000
8629.0000000	0.0395000
8630.0000000	0.0400000
8631.0000000	0.0400000
8632.0000000	0.0395000
8633.0000000	0.0400000
8634.0000000	0.0400000
8635.0000000	0.0400000
8636.0000000	0.0400000
8637.0000000	0.0395000
8638.0000000	0.0395000
8639.0000000	0.0400000

8640.0000000	0.0400000
8641.0000000	0.0400000
8642.0000000	0.0400000
8643.0000000	0.0400000
8644.0000000	0.0375000
8645.0000000	0.0400000
8646.0000000	0.0400000
8647.0000000	0.0400000
8648.0000000	0.0400000
8649.0000000	0.0400000
8650.0000000	0.0400000
8651.0000000	0.0395238
8652.0000000	0.0390000
8653.0000000	0.0390000
8654.0000000	0.0395000
8655.0000000	0.0376190
8656.0000000	0.0385000
8657.0000000	0.0395238
8658.0000000	0.0395000
8659.0000000	0.0390000
8660.0000000	0.0400000
8661.0000000	0.0395000
8662.0000000	0.0390000
8663.0000000	0.0390000
8664.0000000	0.0380952
8665.0000000	0.0380000
8666.0000000	0.0380000
8667.0000000	0.0390476
8668.0000000	0.0400000
8669.0000000	0.0390476
8670.0000000	0.0395000
8671.0000000	0.0390000
8672.0000000	0.0380952
8673.0000000	0.0380000
8674.0000000	0.0390000
8675.0000000	0.0385000
8676.0000000	0.0385714
8677.0000000	0.0375000
8678.0000000	0.0385714
8679.0000000	0.0375000
8680.0000000	0.0390476
8681.0000000	0.0389474
8682.0000000	0.0380952
8683.0000000	0.0380000
8684.0000000	0.0375000
8685.0000000	0.0361905
8686.0000000	0.0385000
8687.0000000	0.0371429
8688.0000000	0.0370000
8689.0000000	0.0390000
8690.0000000	0.0370000
8691.0000000	0.0371429
8692.0000000	0.0375000
8693.0000000	0.0365000
8694.0000000	0.0352381
8695.0000000	0.0330000
8696.0000000	0.0338095

8697.0000000	0.0345000
8698.0000000	0.0350000
8699.0000000	0.0355000
8700.0000000	0.0357143
8701.0000000	0.0345000
8702.0000000	0.0345000
8703.0000000	0.0338095
8704.0000000	0.0325000
8705.0000000	0.0305000
8706.0000000	0.0338095
8707.0000000	0.0335000
8708.0000000	0.0323809
8709.0000000	0.0340000
8710.0000000	0.0310000
8711.0000000	0.0355000
8712.0000000	0.0342857
8713.0000000	0.0335000
8714.0000000	0.0325000
8715.0000000	0.0328571
8716.0000000	0.0325000
8717.0000000	0.0319048
8718.0000000	0.0320000
8719.0000000	0.0320000
8720.0000000	0.0305000
8721.0000000	0.0319048
8722.0000000	0.0315000
8723.0000000	0.0315000
8724.0000000	0.0309524
8725.0000000	0.0300000
8726.0000000	0.0314286
8727.0000000	0.0320000
8728.0000000	0.0310000
8729.0000000	0.0330000
8730.0000000	0.0309524
8731.0000000	0.0315000
8732.0000000	0.0314286
8733.0000000	0.0320000
8734.0000000	0.0320000
8735.0000000	0.0310000
8736.0000000	0.0309524
8737.0000000	0.0315000
8738.0000000	0.0330000
8739.0000000	0.0309524
8740.0000000	0.0310000
8741.0000000	0.0323809
8742.0000000	0.0335000
8743.0000000	0.0325000
8744.0000000	0.0320000
8745.0000000	0.0328571
8746.0000000	0.0330000
8747.0000000	0.0335000
8748.0000000	0.0338095
8749.0000000	0.0335000
8750.0000000	0.0340000
8751.0000000	0.0325000
8752.0000000	0.0352381
8753.0000000	0.0330000

8754.0000000	0.0315000
8755.0000000	0.0333333
8756.0000000	0.0319048
8757.0000000	0.0325000
8758.0000000	0.0335000
8759.0000000	0.0310000
8760.0000000	0.0309524
8761.0000000	0.0315000
8762.0000000	0.0330000
8763.0000000	0.0340000
8764.0000000	0.0309524
8765.0000000	0.0325000
8766.0000000	0.0309524
8767.0000000	0.0335000
8768.0000000	0.0325000
8769.0000000	0.0319048
8770.0000000	0.0330000
8771.0000000	0.0335000
8772.0000000	0.0338095
8773.0000000	0.0310000
8774.0000000	0.0325000
8775.0000000	0.0328571
8776.0000000	0.0325000
8777.0000000	0.0333333
8778.0000000	0.0320000
8779.0000000	0.0325000
8780.0000000	0.0360000
8781.0000000	0.0330000
8782.0000000	0.0352381
8783.0000000	0.0340000
8784.0000000	0.0347619
8785.0000000	0.0365000
8786.0000000	0.0366667
8787.0000000	0.0360000
8788.0000000	0.0365000
8789.0000000	0.0350000
8790.0000000	0.0370000
8791.0000000	0.0385714
8792.0000000	0.0385000
8793.0000000	0.0395238
8794.0000000	0.0380000
8795.0000000	0.0385000
8796.0000000	0.0400000
8797.0000000	0.0395000
8798.0000000	0.0380000
8799.0000000	0.0390000
8800.0000000	0.0380952
8801.0000000	0.0385000
8802.0000000	0.0376191
8803.0000000	0.0385000
8804.0000000	0.0375000
8805.0000000	0.0376190
8806.0000000	0.0370000
8807.0000000	0.0365000
8808.0000000	0.0390476
8809.0000000	0.0395000
8810.0000000	0.0390000

8811.0000000	0.0395000
8812.0000000	0.0361905
8813.0000000	0.0357143
8814.0000000	0.0390000
8815.0000000	0.0370000
8816.0000000	0.0370000
8817.0000000	0.0371429
8818.0000000	0.0370000
8819.0000000	0.0365000
8820.0000000	0.0376190
8821.0000000	0.0380000
8822.0000000	0.0380000
8823.0000000	0.0380952
8824.0000000	0.0365000
8825.0000000	0.0365000
8826.0000000	0.0340000
8827.0000000	0.0347619
8828.0000000	0.0365000
8829.0000000	0.0357143
8830.0000000	0.0350000
8831.0000000	0.0365000
8832.0000000	0.0380952
8833.0000000	0.0365000
8834.0000000	0.0385000
8835.0000000	0.0395000
8836.0000000	0.0390476
8837.0000000	0.0370000
8838.0000000	0.0385714
8839.0000000	0.0385000
8840.0000000	0.0390000
8841.0000000	0.0400000
8842.0000000	0.0400000
8843.0000000	0.0380952
8844.0000000	0.0380952
8845.0000000	0.0375000
8846.0000000	0.0395000
8847.0000000	0.0390476
8848.0000000	0.0385000
8849.0000000	0.0380000
8850.0000000	0.0395000
8851.0000000	0.0395000
8852.0000000	0.0395238
8853.0000000	0.0390476
8854.0000000	0.0375000
8855.0000000	0.0385000
8856.0000000	0.0375000
8857.0000000	0.0390476
8858.0000000	0.0390000
8859.0000000	0.0395000
8860.0000000	0.0390476
8861.0000000	0.0395000
8862.0000000	0.0385714
8863.0000000	0.0400000
8864.0000000	0.0400000
8865.0000000	0.0385714
8866.0000000	0.0395000
8867.0000000	0.0380000

8868.0000000	0.0380952
8869.0000000	0.0385000
8870.0000000	0.0385000
8871.0000000	0.0370000
8872.0000000	0.0385714
8873.0000000	0.0385000
8874.0000000	0.0376191
8875.0000000	0.0370000
8876.0000000	0.0370000
8877.0000000	0.0371429
8878.0000000	0.0355000
8879.0000000	0.0375000
8880.0000000	0.0375000
8881.0000000	0.0385714
8882.0000000	0.0375000
8883.0000000	0.0371429
8884.0000000	0.0385000
8885.0000000	0.0375000
8886.0000000	0.0335000
8887.0000000	0.0365000
8888.0000000	0.0338095
8889.0000000	0.0340000
8890.0000000	0.0314286
8891.0000000	0.0325000
8892.0000000	0.0352381
8893.0000000	0.0355000
8894.0000000	0.0330000
8895.0000000	0.0330000
8896.0000000	0.0328571
8897.0000000	0.0340000
8898.0000000	0.0320000
8899.0000000	0.0360000
8900.0000000	0.0361905
8901.0000000	0.0361905
8902.0000000	0.0340000
8903.0000000	0.0345000
8904.0000000	0.0365000
8905.0000000	0.0347619
8906.0000000	0.0350000
8907.0000000	0.0360000
8908.0000000	0.0366667
8909.0000000	0.0355000
8910.0000000	0.0340000
8911.0000000	0.0338095
8912.0000000	0.0355000
8913.0000000	0.0357143
8914.0000000	0.0360000
8915.0000000	0.0365000
8916.0000000	0.0350000
8917.0000000	0.0347619
8918.0000000	0.0350000
8919.0000000	0.0360000
8920.0000000	0.0347619
8921.0000000	0.0375000
8922.0000000	0.0366667
8923.0000000	0.0385000
8924.0000000	0.0375000

8925.0000000	0.0365000
8926.0000000	0.0376190
8927.0000000	0.0380000
8928.0000000	0.0355000
8929.0000000	0.0376190
8930.0000000	0.0385000
8931.0000000	0.0390000
8932.0000000	0.0380952
8933.0000000	0.0390000
8934.0000000	0.0365000
8935.0000000	0.0366667
8936.0000000	0.0385000
8937.0000000	0.0385714
8938.0000000	0.0375000
8939.0000000	0.0375000
8940.0000000	0.0390000
8941.0000000	0.0371429
8942.0000000	0.0400000
8943.0000000	0.0400000
8944.0000000	0.0376191
8945.0000000	0.0385000
8946.0000000	0.0380000
8947.0000000	0.0370000
8948.0000000	0.0366667
8949.0000000	0.0360000
8950.0000000	0.0357143
8951.0000000	0.0345000
8952.0000000	0.0355000
8953.0000000	0.0347619
8954.0000000	0.0375000
8955.0000000	0.0335000
8956.0000000	0.0335000
8957.0000000	0.0342857
8958.0000000	0.0350000
8959.0000000	0.0335000
8960.0000000	0.0338095
8961.0000000	0.0325000
8962.0000000	0.0352381
8963.0000000	0.0330000
8964.0000000	0.0330000
8965.0000000	0.0338095
8966.0000000	0.0315000
8967.0000000	0.0335000
8968.0000000	0.0328571
8969.0000000	0.0335000
8970.0000000	0.0330000
8971.0000000	0.0345000
8972.0000000	0.0338095
8973.0000000	0.0345000
8974.0000000	0.0376190
8975.0000000	0.0345000
8976.0000000	0.0355000
8977.0000000	0.0360000
8978.0000000	0.0357143
8979.0000000	0.0335000
8980.0000000	0.0342857
8981.0000000	0.0350000

8982.0000000	0.0330000
8983.0000000	0.0333333
8984.0000000	0.0330000
8985.0000000	0.0335000
8986.0000000	0.0355000
8987.0000000	0.0342857
8988.0000000	0.0345000
8989.0000000	0.0333333
8990.0000000	0.0345000
8991.0000000	0.0350000
8992.0000000	0.0340000
8993.0000000	0.0333333
8994.0000000	0.0340000
8995.0000000	0.0345000
8996.0000000	0.0347619
8997.0000000	0.0340000
8998.0000000	0.0333333
8999.0000000	0.0335000
9000.0000000	0.0350000
9001.0000000	0.0342857
9002.0000000	0.0365000
9003.0000000	0.0360000
9004.0000000	0.0350000
9005.0000000	0.0342857
9006.0000000	0.0355000
9007.0000000	0.0315000
9008.0000000	0.0347619
9009.0000000	0.0345000
9010.0000000	0.0347619
9011.0000000	0.0360000
9012.0000000	0.0365000
9013.0000000	0.0357143
9014.0000000	0.0360000
9015.0000000	0.0335000
9016.0000000	0.0370000
9017.0000000	0.0347619
9018.0000000	0.0380000
9019.0000000	0.0361905
9020.0000000	0.0350000
9021.0000000	0.0380000
9022.0000000	0.0360000
9023.0000000	0.0357143
9024.0000000	0.0350000
9025.0000000	0.0361905
9026.0000000	0.0365000
9027.0000000	0.0365000
9028.0000000	0.0361905
9029.0000000	0.0400000
9030.0000000	0.0385000
9031.0000000	0.0365000
9032.0000000	0.0366667
9033.0000000	0.0380000
9034.0000000	0.0390476
9035.0000000	0.0385000
9036.0000000	0.0365000
9037.0000000	0.0385000
9038.0000000	0.0380952

9039.0000000	0.0370000
9040.0000000	0.0390000
9041.0000000	0.0385714
9042.0000000	0.0380000
9043.0000000	0.0361905
9044.0000000	0.0385000
9045.0000000	0.0365000
9046.0000000	0.0370000
9047.0000000	0.0380000
9048.0000000	0.0347619
9049.0000000	0.0371429
9050.0000000	0.0375000
9051.0000000	0.0375000
9052.0000000	0.0355000
9053.0000000	0.0371429
9054.0000000	0.0380000
9055.0000000	0.0365000
9056.0000000	0.0365000
9057.0000000	0.0361905
9058.0000000	0.0347619
9059.0000000	0.0360000
9060.0000000	0.0385000
9061.0000000	0.0375000
9062.0000000	0.0371429
9063.0000000	0.0370000
9064.0000000	0.0375000
9065.0000000	0.0380952
9066.0000000	0.0395000
9067.0000000	0.0375000
9068.0000000	0.0380952
9069.0000000	0.0380000
9070.0000000	0.0380000
9071.0000000	0.0390476
9072.0000000	0.0385000
9073.0000000	0.0380000
9074.0000000	0.0371429
9075.0000000	0.0385000
9076.0000000	0.0380000
9077.0000000	0.0395238
9078.0000000	0.0390000
9079.0000000	0.0365000
9080.0000000	0.0376190
9081.0000000	0.0375000
9082.0000000	0.0360000
9083.0000000	0.0385000
9084.0000000	0.0380952
9085.0000000	0.0370000
9086.0000000	0.0376190
9087.0000000	0.0375000
9088.0000000	0.0380000
9089.0000000	0.0385714
9090.0000000	0.0400000
9091.0000000	0.0395000
9092.0000000	0.0355000
9093.0000000	0.0380952
9094.0000000	0.0385000
9095.0000000	0.0352381

9096.0000000	0.0360000
9097.0000000	0.0380000
9098.0000000	0.0352381
9099.0000000	0.0355000
9100.0000000	0.0350000
9101.0000000	0.0352381
9102.0000000	0.0340000
9103.0000000	0.0345000
9104.0000000	0.0347619
9105.0000000	0.0355000
9106.0000000	0.0365000
9107.0000000	0.0361905
9108.0000000	0.0350000
9109.0000000	0.0370000
9110.0000000	0.0366667
9111.0000000	0.0360000
9112.0000000	0.0365000
9113.0000000	0.0361905
9114.0000000	0.0355000
9115.0000000	0.0373684
9116.0000000	0.0359091
9117.0000000	0.0345000
9118.0000000	0.0352381
9119.0000000	0.0380000
9120.0000000	0.0360000
9121.0000000	0.0365000
9122.0000000	0.0376190
9123.0000000	0.0390000
9124.0000000	0.0350000
9125.0000000	0.0390476
9126.0000000	0.0355000
9127.0000000	0.0385000
9128.0000000	0.0390000
9129.0000000	0.0371429
9130.0000000	0.0375000
9131.0000000	0.0385714
9132.0000000	0.0380000
9133.0000000	0.0380000
9134.0000000	0.0390476
9135.0000000	0.0395000
9136.0000000	0.0370000
9137.0000000	0.0380952
9138.0000000	0.0385000
9139.0000000	0.0390000
9140.0000000	0.0390476
9141.0000000	0.0385000
9142.0000000	0.0390000
9143.0000000	0.0395000
9144.0000000	0.0385000
9145.0000000	0.0390476
9146.0000000	0.0395238
9147.0000000	0.0385000
9148.0000000	0.0380000
9149.0000000	0.0395238
9150.0000000	0.0400000
9151.0000000	0.0400000
9152.0000000	0.0390000

9153.0000000	0.0395238
9154.0000000	0.0400000
9155.0000000	0.0400000
9156.0000000	0.0385000
9157.0000000	0.0375000
9158.0000000	0.0380952
9159.0000000	0.0380000
9160.0000000	0.0385000
9161.0000000	0.0375000
9162.0000000	0.0390476
9163.0000000	0.0360000
9164.0000000	0.0365000
9165.0000000	0.0357143
9166.0000000	0.0350000
9167.0000000	0.0370000
9168.0000000	0.0352381
9169.0000000	0.0350000
9170.0000000	0.0361905
9171.0000000	0.0360000
9172.0000000	0.0375000
9173.0000000	0.0370000
9174.0000000	0.0366667
9175.0000000	0.0345000
9176.0000000	0.0355000
9177.0000000	0.0361905
9178.0000000	0.0395000
9179.0000000	0.0376190
9180.0000000	0.0380000
9181.0000000	0.0390000
9182.0000000	0.0390000
9183.0000000	0.0380952
9184.0000000	0.0375000
9185.0000000	0.0375000
9186.0000000	0.0371429
9187.0000000	0.0375000
9188.0000000	0.0355000
9189.0000000	0.0357143
9190.0000000	0.0375000
9191.0000000	0.0360000
9192.0000000	0.0328571
9193.0000000	0.0340000
9194.0000000	0.0319048
9195.0000000	0.0335000
9196.0000000	0.0350000
9197.0000000	0.0330000
9198.0000000	0.0319048
9199.0000000	0.0315000
9200.0000000	0.0325000
9201.0000000	0.0333333
9202.0000000	0.0340000
9203.0000000	0.0335000
9204.0000000	0.0314286
9205.0000000	0.0340000
9206.0000000	0.0323809
9207.0000000	0.0340000
9208.0000000	0.0315000
9209.0000000	0.0310000

9210.0000000	0.0328571
9211.0000000	0.0300000
9212.0000000	0.0315000
9213.0000000	0.0304762
9214.0000000	0.0320000
9215.0000000	0.0323809
9216.0000000	0.0300000
9217.0000000	0.0320000
9218.0000000	0.0320000
9219.0000000	0.0319048
9220.0000000	0.0315000
9221.0000000	0.0310000
9222.0000000	0.0319048
9223.0000000	0.0320000
9224.0000000	0.0328571
9225.0000000	0.0325000
9226.0000000	0.0315000
9227.0000000	0.0305000
9228.0000000	0.0319048
9229.0000000	0.0320000
9230.0000000	0.0310000
9231.0000000	0.0314286
9232.0000000	0.0325000
9233.0000000	0.0325000
9234.0000000	0.0328571
9235.0000000	0.0330000
9236.0000000	0.0330000
9237.0000000	0.0309524
9238.0000000	0.0310000
9239.0000000	0.0335000
9240.0000000	0.0314286
9241.0000000	0.0310000
9242.0000000	0.0320000
9243.0000000	0.0300000
9244.0000000	0.0310000
9245.0000000	0.0310000
9246.0000000	0.0309524
9247.0000000	0.0315000
9248.0000000	0.0315000
9249.0000000	0.0325000
9250.0000000	0.0328571
9251.0000000	0.0320000
9252.0000000	0.0310000
9253.0000000	0.0319048
9254.0000000	0.0319048
9255.0000000	0.0315000
9256.0000000	0.0335000
9257.0000000	0.0320000
9258.0000000	0.0328571
9259.0000000	0.0325000
9260.0000000	0.0335000
9261.0000000	0.0340000
9262.0000000	0.0328571
9263.0000000	0.0340000
9264.0000000	0.0315000
9265.0000000	0.0333333
9266.0000000	0.0355000

9267.0000000	0.0323809
9268.0000000	0.0350000
9269.0000000	0.0350000
9270.0000000	0.0371429
9271.0000000	0.0355000
9272.0000000	0.0350000
9273.0000000	0.0360000
9274.0000000	0.0357143
9275.0000000	0.0350000
9276.0000000	0.0352381
9277.0000000	0.0375000
9278.0000000	0.0380000
9279.0000000	0.0385000
9280.0000000	0.0366667
9281.0000000	0.0370000
9282.0000000	0.0366667
9283.0000000	0.0360000
9284.0000000	0.0365000
9285.0000000	0.0361905
9286.0000000	0.0355000
9287.0000000	0.0360000
9288.0000000	0.0375000
9289.0000000	0.0366667
9290.0000000	0.0340000
9291.0000000	0.0355000
9292.0000000	0.0342857
9293.0000000	0.0325000
9294.0000000	0.0319048
9295.0000000	0.0325000
9296.0000000	0.0320000
9297.0000000	0.0325000
9298.0000000	0.0323809
9299.0000000	0.0315000
9300.0000000	0.0330000
9301.0000000	0.0319048
9302.0000000	0.0320000
9303.0000000	0.0314286
9304.0000000	0.0325000
9305.0000000	0.0325000
9306.0000000	0.0319048
9307.0000000	0.0315000
9308.0000000	0.0320000
9309.0000000	0.0315000
9310.0000000	0.0333333
9311.0000000	0.0320000
9312.0000000	0.0323809
9313.0000000	0.0330000
9314.0000000	0.0320000
9315.0000000	0.0319048
9316.0000000	0.0340000
9317.0000000	0.0345000
9318.0000000	0.0340000
9319.0000000	0.0342857
9320.0000000	0.0345000
9321.0000000	0.0352381
9322.0000000	0.0330000
9323.0000000	0.0345000

9324.0000000	0.0340000
9325.0000000	0.0342857
9326.0000000	0.0345000
9327.0000000	0.0335000
9328.0000000	0.0328571
9329.0000000	0.0340000
9330.0000000	0.0347619
9331.0000000	0.0350000
9332.0000000	0.0350000
9333.0000000	0.0355000
9334.0000000	0.0357143
9335.0000000	0.0340000
9336.0000000	0.0350000
9337.0000000	0.0347619
9338.0000000	0.0355000
9339.0000000	0.0340000
9340.0000000	0.0360000
9341.0000000	0.0338095
9342.0000000	0.0319048
9343.0000000	0.0340000
9344.0000000	0.0350000
9345.0000000	0.0350000
9346.0000000	0.0333333
9347.0000000	0.0350000
9348.0000000	0.0345000
9349.0000000	0.0335000
9350.0000000	0.0338095
9351.0000000	0.0342857
9352.0000000	0.0320000
9353.0000000	0.0340000
9354.0000000	0.0320000
9355.0000000	0.0304762
9356.0000000	0.0305000
9357.0000000	0.0320000
9358.0000000	0.0314286
9359.0000000	0.0315000
9360.0000000	0.0319048
9361.0000000	0.0320000
9362.0000000	0.0320000
9363.0000000	0.0315000
9364.0000000	0.0328571
9365.0000000	0.0330000
9366.0000000	0.0325000
9367.0000000	0.0323809
9368.0000000	0.0330000
9369.0000000	0.0330000
9370.0000000	0.0314286
9371.0000000	0.0300000
9372.0000000	0.0300000
9373.0000000	0.0304762
9374.0000000	0.0310000
9375.0000000	0.0304762
9376.0000000	0.0305000
9377.0000000	0.0305000
9378.0000000	0.0305000
9379.0000000	0.0309524
9380.0000000	0.0310000

9381.0000000	0.0300000
9382.0000000	0.0309524
9383.0000000	0.0310000
9384.0000000	0.0305000
9385.0000000	0.0305000
9386.0000000	0.0300000
9387.0000000	0.0300000
9388.0000000	0.0300000
9389.0000000	0.0309524
9390.0000000	0.0310000
9391.0000000	0.0300000
9392.0000000	0.0300000
9393.0000000	0.0300000
9394.0000000	0.0309524
9395.0000000	0.0315000
9396.0000000	0.0320000
9397.0000000	0.0315000
9398.0000000	0.0304762
9399.0000000	0.0315000
9400.0000000	0.0323810
9401.0000000	0.0330000
9402.0000000	0.0320000
9403.0000000	0.0328571
9404.0000000	0.0340000
9405.0000000	0.0340000
9406.0000000	0.0338095
9407.0000000	0.0325000
9408.0000000	0.0320000
9409.0000000	0.0323810
9410.0000000	0.0345000
9411.0000000	0.0314286
9412.0000000	0.0335000
9413.0000000	0.0325000
9414.0000000	0.0325000
9415.0000000	0.0320000
9416.0000000	0.0338095
9417.0000000	0.0335000
9418.0000000	0.0328571
9419.0000000	0.0305000
9420.0000000	0.0314286
9421.0000000	0.0320000
9422.0000000	0.0300000
9423.0000000	0.0305000
9424.0000000	0.0305000
9425.0000000	0.0304762
9426.0000000	0.0310000
9427.0000000	0.0304762
9428.0000000	0.0310000
9429.0000000	0.0305000
9430.0000000	0.0320000
9431.0000000	0.0319048
9432.0000000	0.0325000
9433.0000000	0.0320000
9434.0000000	0.0309524
9435.0000000	0.0310000
9436.0000000	0.0309524
9437.0000000	0.0310000

9438.0000000	0.0325000
9439.0000000	0.0319048
9440.0000000	0.0305000
9441.0000000	0.0315000
9442.0000000	0.0310000
9443.0000000	0.0309524
9444.0000000	0.0300000
9445.0000000	0.0300000
9446.0000000	0.0309524
9447.0000000	0.0305000
9448.0000000	0.0309524
9449.0000000	0.0300000
9450.0000000	0.0305000
9451.0000000	0.0304762
9452.0000000	0.0305000
9453.0000000	0.0310000
9454.0000000	0.0315000
9455.0000000	0.0314286
9456.0000000	0.0315000
9457.0000000	0.0320000
9458.0000000	0.0309524
9459.0000000	0.0315000
9460.0000000	0.0330000
9461.0000000	0.0338095
9462.0000000	0.0315000
9463.0000000	0.0333333
9464.0000000	0.0325000
9465.0000000	0.0325000
9466.0000000	0.0340000
9467.0000000	0.0338095
9468.0000000	0.0340000
9469.0000000	0.0325000
9470.0000000	0.0357143
9471.0000000	0.0340000
9472.0000000	0.0328571
9473.0000000	0.0345000
9474.0000000	0.0335000
9475.0000000	0.0345000
9476.0000000	0.0350000
9477.0000000	0.0333333
9478.0000000	0.0350000
9479.0000000	0.0352381
9480.0000000	0.0350000
9481.0000000	0.0338095
9482.0000000	0.0365000
9483.0000000	0.0360000
9484.0000000	0.0350000
9485.0000000	0.0360000
9486.0000000	0.0361905
9487.0000000	0.0352381
9488.0000000	0.0345000
9489.0000000	0.0355000
9490.0000000	0.0355000
9491.0000000	0.0338095
9492.0000000	0.0355000
9493.0000000	0.0340000
9494.0000000	0.0347619

9495.0000000	0.0330000
9496.0000000	0.0335000
9497.0000000	0.0338095
9498.0000000	0.0350000
9499.0000000	0.0357143
9500.0000000	0.0350000
9501.0000000	0.0375000
9502.0000000	0.0365000
9503.0000000	0.0347619
9504.0000000	0.0365000
9505.0000000	0.0360000
9506.0000000	0.0366667
9507.0000000	0.0350000
9508.0000000	0.0380952
9509.0000000	0.0380000
9510.0000000	0.0365000
9511.0000000	0.0380952
9512.0000000	0.0380000
9513.0000000	0.0365000
9514.0000000	0.0380000
9515.0000000	0.0357143
9516.0000000	0.0365000
9517.0000000	0.0333333
9518.0000000	0.0370000
9519.0000000	0.0370000
9520.0000000	0.0360000
9521.0000000	0.0365000
9522.0000000	0.0366667
9523.0000000	0.0365000
9524.0000000	0.0380952
9525.0000000	0.0325000
9526.0000000	0.0366667
9527.0000000	0.0345000
9528.0000000	0.0365000
9529.0000000	0.0350000
9530.0000000	0.0365000
9531.0000000	0.0371429
9532.0000000	0.0355000
9533.0000000	0.0347619
9534.0000000	0.0345000
9535.0000000	0.0345000
9536.0000000	0.0333333
9537.0000000	0.0350000
9538.0000000	0.0350000
9539.0000000	0.0366667
9540.0000000	0.0360000
9541.0000000	0.0345000
9542.0000000	0.0338095
9543.0000000	0.0340000
9544.0000000	0.0340000
9545.0000000	0.0338095
9546.0000000	0.0340000
9547.0000000	0.0340000
9548.0000000	0.0333333
9549.0000000	0.0326316
9550.0000000	0.0347619
9551.0000000	0.0355000

9552.0000000	0.0347619
9553.0000000	0.0335000
9554.0000000	0.0365000
9555.0000000	0.0357143
9556.0000000	0.0366667
9557.0000000	0.0335000
9558.0000000	0.0345000
9559.0000000	0.0375000
9560.0000000	0.0361905
9561.0000000	0.0355000
9562.0000000	0.0350000
9563.0000000	0.0357143
9564.0000000	0.0350000
9565.0000000	0.0350000
9566.0000000	0.0360000
9567.0000000	0.0361905
9568.0000000	0.0360000
9569.0000000	0.0361905
9570.0000000	0.0340000
9571.0000000	0.0345000
9572.0000000	0.0352381
9573.0000000	0.0370000
9574.0000000	0.0365000
9575.0000000	0.0371429
9576.0000000	0.0355000
9577.0000000	0.0355000
9578.0000000	0.0361905
9579.0000000	0.0360000
9580.0000000	0.0350000
9581.0000000	0.0350000
9582.0000000	0.0352381
9583.0000000	0.0365000
9584.0000000	0.0360000
9585.0000000	0.0361905
9586.0000000	0.0365000
9587.0000000	0.0342857
9588.0000000	0.0350000
9589.0000000	0.0365000
9590.0000000	0.0370000
9591.0000000	0.0357143
9592.0000000	0.0360000
9593.0000000	0.0345000
9594.0000000	0.0342857
9595.0000000	0.0355000
9596.0000000	0.0328571
9597.0000000	0.0345000
9598.0000000	0.0370000
9599.0000000	0.0352381
9600.0000000	0.0350000
9601.0000000	0.0345000
9602.0000000	0.0320000
9603.0000000	0.0338095
9604.0000000	0.0345000
9605.0000000	0.0323810
9606.0000000	0.0360000
9607.0000000	0.0350000
9608.0000000	0.0323809

9609.0000000	0.0350000
9610.0000000	0.0345000
9611.0000000	0.0325000
9612.0000000	0.0328571
9613.0000000	0.0345000
9614.0000000	0.0342857
9615.0000000	0.0335000
9616.0000000	0.0328571
9617.0000000	0.0325000
9618.0000000	0.0335000
9619.0000000	0.0315000
9620.0000000	0.0335000
9621.0000000	0.0328571
9622.0000000	0.0315000
9623.0000000	0.0347619
9624.0000000	0.0340000
9625.0000000	0.0335000
9626.0000000	0.0335000
9627.0000000	0.0333333
9628.0000000	0.0340000
9629.0000000	0.0315000
9630.0000000	0.0338095
9631.0000000	0.0340000
9632.0000000	0.0328571
9633.0000000	0.0335000
9634.0000000	0.0330000
9635.0000000	0.0320000
9636.0000000	0.0319048
9637.0000000	0.0315000
9638.0000000	0.0330000
9639.0000000	0.0328571
9640.0000000	0.0325000
9641.0000000	0.0330000
9642.0000000	0.0315000
9643.0000000	0.0323809
9644.0000000	0.0328571
9645.0000000	0.0320000
9646.0000000	0.0310000
9647.0000000	0.0309524
9648.0000000	0.0315000
9649.0000000	0.0300000
9650.0000000	0.0310000
9651.0000000	0.0300000
9652.0000000	0.0310000
9653.0000000	0.0304762
9654.0000000	0.0300000
9655.0000000	0.0315000
9656.0000000	0.0305000
9657.0000000	0.0304762
9658.0000000	0.0320000
9659.0000000	0.0320000
9660.0000000	0.0323809
9661.0000000	0.0320000
9662.0000000	0.0300000
9663.0000000	0.0309524
9664.0000000	0.0325000
9665.0000000	0.0335000

9666.0000000	0.0314286
9667.0000000	0.0315000
9668.0000000	0.0300000
9669.0000000	0.0315000
9670.0000000	0.0310000
9671.0000000	0.0300000
9672.0000000	0.0309524
9673.0000000	0.0305000
9674.0000000	0.0310000
9675.0000000	0.0309524
9676.0000000	0.0320000
9677.0000000	0.0328571
9678.0000000	0.0300000
9679.0000000	0.0300000
9680.0000000	0.0310000
9681.0000000	0.0315000
9682.0000000	0.0319048
9683.0000000	0.0320000
9684.0000000	0.0309524
9685.0000000	0.0315000
9686.0000000	0.0315000
9687.0000000	0.0347619
9688.0000000	0.0320000
9689.0000000	0.0330000
9690.0000000	0.0300000
9691.0000000	0.0314286
9692.0000000	0.0304762
9693.0000000	0.0305000
9694.0000000	0.0315000
9695.0000000	0.0305000
9696.0000000	0.0323809
9697.0000000	0.0315000
9698.0000000	0.0320000
9699.0000000	0.0304762
9700.0000000	0.0330000
9701.0000000	0.0325000
9702.0000000	0.0319048
9703.0000000	0.0330000
9704.0000000	0.0328571
9705.0000000	0.0310000
9706.0000000	0.0310000
9707.0000000	0.0330000
9708.0000000	0.0333333
9709.0000000	0.0325000
9710.0000000	0.0335000
9711.0000000	0.0333333
9712.0000000	0.0340000
9713.0000000	0.0328571
9714.0000000	0.0330000
9715.0000000	0.0335000
9716.0000000	0.0350000
9717.0000000	0.0335000
9718.0000000	0.0328571
9719.0000000	0.0330000
9720.0000000	0.0352381
9721.0000000	0.0330000
9722.0000000	0.0345000

9723.0000000	0.0314286
9724.0000000	0.0340000
9725.0000000	0.0350000
9726.0000000	0.0330000
9727.0000000	0.0338095
9728.0000000	0.0330000
9729.0000000	0.0338095
9730.0000000	0.0335000
9731.0000000	0.0325000
9732.0000000	0.0328571
9733.0000000	0.0340000
9734.0000000	0.0320000
9735.0000000	0.0340000
9736.0000000	0.0347619
9737.0000000	0.0350000
9738.0000000	0.0328571
9739.0000000	0.0330000
9740.0000000	0.0340000
9741.0000000	0.0314286
9742.0000000	0.0340000
9743.0000000	0.0330000
9744.0000000	0.0319048
9745.0000000	0.0335000
9746.0000000	0.0325000
9747.0000000	0.0340000
9748.0000000	0.0328571
9749.0000000	0.0335000
9750.0000000	0.0328571
9751.0000000	0.0335000
9752.0000000	0.0305000
9753.0000000	0.0347619
9754.0000000	0.0330000
9755.0000000	0.0335000
9756.0000000	0.0328571
9757.0000000	0.0340000
9758.0000000	0.0350000
9759.0000000	0.0350000
9760.0000000	0.0352381
9761.0000000	0.0330000
9762.0000000	0.0350000
9763.0000000	0.0366667
9764.0000000	0.0355000
9765.0000000	0.0342857
9766.0000000	0.0360000
9767.0000000	0.0350000
9768.0000000	0.0366667
9769.0000000	0.0370000
9770.0000000	0.0355000
9771.0000000	0.0360000
9772.0000000	0.0347619
9773.0000000	0.0355000
9774.0000000	0.0347619
9775.0000000	0.0330000
9776.0000000	0.0340000
9777.0000000	0.0325000
9778.0000000	0.0350000
9779.0000000	0.0338095

9780.0000000	0.0333333
9781.0000000	0.0330000
9782.0000000	0.0340000
9783.0000000	0.0352381
9784.0000000	0.0325000
9785.0000000	0.0360000
9786.0000000	0.0330000
9787.0000000	0.0338095
9788.0000000	0.0350000
9789.0000000	0.0340000
9790.0000000	0.0347619
9791.0000000	0.0345000
9792.0000000	0.0338095
9793.0000000	0.0335000
9794.0000000	0.0370000
9795.0000000	0.0360000
9796.0000000	0.0338095
9797.0000000	0.0345000
9798.0000000	0.0345000
9799.0000000	0.0371429
9800.0000000	0.0365000
9801.0000000	0.0366667
9802.0000000	0.0355000
9803.0000000	0.0350000
9804.0000000	0.0352381
9805.0000000	0.0370000
9806.0000000	0.0375000
9807.0000000	0.0375000
9808.0000000	0.0361905
9809.0000000	0.0385000
9810.0000000	0.0376191
9811.0000000	0.0380000
9812.0000000	0.0365000
9813.0000000	0.0371429
9814.0000000	0.0375000
9815.0000000	0.0385000
9816.0000000	0.0395000
9817.0000000	0.0371429
9818.0000000	0.0385000
9819.0000000	0.0385714
9820.0000000	0.0380000
9821.0000000	0.0370000
9822.0000000	0.0365000
9823.0000000	0.0385000
9824.0000000	0.0395238
9825.0000000	0.0390000
9826.0000000	0.0371429
9827.0000000	0.0380000
9828.0000000	0.0380952
9829.0000000	0.0375000
9830.0000000	0.0395000
9831.0000000	0.0390000
9832.0000000	0.0385714
9833.0000000	0.0380000
9834.0000000	0.0375000
9835.0000000	0.0385714
9836.0000000	0.0380000

9837.0000000	0.0385000
9838.0000000	0.0376190
9839.0000000	0.0380000
9840.0000000	0.0400000
9841.0000000	0.0400000
9842.0000000	0.0385000
9843.0000000	0.0400000
9844.0000000	0.0385714
9845.0000000	0.0390000
9846.0000000	0.0400000
9847.0000000	0.0385000
9848.0000000	0.0395238
9849.0000000	0.0390476
9850.0000000	0.0385000
9851.0000000	0.0400000
9852.0000000	0.0400000
9853.0000000	0.0395238
9854.0000000	0.0390000
9855.0000000	0.0400000
9856.0000000	0.0390476
9857.0000000	0.0395000
9858.0000000	0.0395238
9859.0000000	0.0400000
9860.0000000	0.0390000
9861.0000000	0.0395000
9862.0000000	0.0400000
9863.0000000	0.0385000
9864.0000000	0.0380000
9865.0000000	0.0385714
9866.0000000	0.0385000
9867.0000000	0.0390000
9868.0000000	0.0380952
9869.0000000	0.0365000
9870.0000000	0.0375000
9871.0000000	0.0380952
9872.0000000	0.0390000
9873.0000000	0.0380000
9874.0000000	0.0361905
9875.0000000	0.0375000
9876.0000000	0.0365000
9877.0000000	0.0390476
9878.0000000	0.0375000
9879.0000000	0.0370000
9880.0000000	0.0376190
9881.0000000	0.0360000
9882.0000000	0.0385000
9883.0000000	0.0395000
9884.0000000	0.0376190
9885.0000000	0.0390000
9886.0000000	0.0395000
9887.0000000	0.0395238
9888.0000000	0.0400000
9889.0000000	0.0395238
9890.0000000	0.0395000
9891.0000000	0.0400000
9892.0000000	0.0400000
9893.0000000	0.0390000

9894.0000000	0.0400000
9895.0000000	0.0390000
9896.0000000	0.0380952
9897.0000000	0.0395000
9898.0000000	0.0395238
9899.0000000	0.0400000
9900.0000000	0.0395000
9901.0000000	0.0395238
9902.0000000	0.0380000
9903.0000000	0.0390000
9904.0000000	0.0376190
9905.0000000	0.0375000
9906.0000000	0.0370000
9907.0000000	0.0357143
9908.0000000	0.0385000
9909.0000000	0.0375000
9910.0000000	0.0366667
9911.0000000	0.0370000
9912.0000000	0.0380000
9913.0000000	0.0370000
9914.0000000	0.0357143
9915.0000000	0.0355000
9916.0000000	0.0365000
9917.0000000	0.0390476
9918.0000000	0.0370000
9919.0000000	0.0366667
9920.0000000	0.0370000
9921.0000000	0.0385000
9922.0000000	0.0385000
9923.0000000	0.0376191
9924.0000000	0.0385000
9925.0000000	0.0376191
9926.0000000	0.0390000
9927.0000000	0.0370000
9928.0000000	0.0360000
9929.0000000	0.0366667
9930.0000000	0.0380000
9931.0000000	0.0365000
9932.0000000	0.0380952
9933.0000000	0.0370000
9934.0000000	0.0365000
9935.0000000	0.0376190
9936.0000000	0.0370000
9937.0000000	0.0371429
9938.0000000	0.0370000
9939.0000000	0.0370000
9940.0000000	0.0370000
9941.0000000	0.0380952
9942.0000000	0.0380000
9943.0000000	0.0355000
9944.0000000	0.0371429
9945.0000000	0.0385000
9946.0000000	0.0366667
9947.0000000	0.0370000
9948.0000000	0.0365000
9949.0000000	0.0376190
9950.0000000	0.0375000

9951.0000000	0.0375000
9952.0000000	0.0370000
9953.0000000	0.0357143
9954.0000000	0.0380000
9955.0000000	0.0385714
9956.0000000	0.0370000
9957.0000000	0.0380000
9958.0000000	0.0375000
9959.0000000	0.0385714
9960.0000000	0.0370000
9961.0000000	0.0361905
9962.0000000	0.0350000
9963.0000000	0.0360000
9964.0000000	0.0365000
9965.0000000	0.0380952
9966.0000000	0.0355000
9967.0000000	0.0350000
9968.0000000	0.0357143
9969.0000000	0.0325000
9970.0000000	0.0340000
9971.0000000	0.0376190
9972.0000000	0.0375000
9973.0000000	0.0350000
9974.0000000	0.0345000
9975.0000000	0.0376190
9976.0000000	0.0375000
9977.0000000	0.0361905
9978.0000000	0.0365000
9979.0000000	0.0345000
9980.0000000	0.0376190
9981.0000000	0.0380000
9982.0000000	0.0370000
9983.0000000	0.0380000
9984.0000000	0.0371429
9985.0000000	0.0361905
9986.0000000	0.0385000
9987.0000000	0.0375000
9988.0000000	0.0355000
9989.0000000	0.0352381
9990.0000000	0.0370000
9991.0000000	0.0380000
9992.0000000	0.0347619
9993.0000000	0.0380000
9994.0000000	0.0376190
9995.0000000	0.0370000
9996.0000000	0.0355000
9997.0000000	0.0335000
9998.0000000	0.0366667
9999.0000000	0.0350000
10000.0000000	0.0345000
10001.0000000	0.0338095
10002.0000000	0.0340000
10003.0000000	0.0330000
10004.0000000	0.0328571
10005.0000000	0.0325000
10006.0000000	0.0315000
10007.0000000	0.0314286

10008.0000000	0.0320000
10009.0000000	0.0314286
10010.0000000	0.0330000
10011.0000000	0.0315000
10012.0000000	0.0330000
10013.0000000	0.0333333
10014.0000000	0.0330000
10015.0000000	0.0330000
10016.0000000	0.0357143
10017.0000000	0.0340000
10018.0000000	0.0345000
10019.0000000	0.0355000
10020.0000000	0.0361905
10021.0000000	0.0340000
10022.0000000	0.0361905
10023.0000000	0.0340000
10024.0000000	0.0340000
10025.0000000	0.0338095
10026.0000000	0.0345000
10027.0000000	0.0350000
10028.0000000	0.0370000
10029.0000000	0.0347619
10030.0000000	0.0345000
10031.0000000	0.0357143
10032.0000000	0.0350000
10033.0000000	0.0345000
10034.0000000	0.0323809
10035.0000000	0.0350000
10036.0000000	0.0360000
10037.0000000	0.0352381
10038.0000000	0.0355000
10039.0000000	0.0345000
10040.0000000	0.0366667
10041.0000000	0.0365000
10042.0000000	0.0350000
10043.0000000	0.0376190
10044.0000000	0.0355000
10045.0000000	0.0345000
10046.0000000	0.0357143
10047.0000000	0.0375000
10048.0000000	0.0350000
10049.0000000	0.0385000
10050.0000000	0.0361905
10051.0000000	0.0365000
10052.0000000	0.0365000
10053.0000000	0.0366667
10054.0000000	0.0352381
10055.0000000	0.0355000
10056.0000000	0.0345000
10057.0000000	0.0350000
10058.0000000	0.0340000
10059.0000000	0.0342857
10060.0000000	0.0345000
10061.0000000	0.0323809
10062.0000000	0.0315000
10063.0000000	0.0330000
10064.0000000	0.0325000

10065.0000000	0.0347619
10066.0000000	0.0330000
10067.0000000	0.0330000
10068.0000000	0.0347619
10069.0000000	0.0335000
10070.0000000	0.0323809
10071.0000000	0.0330000
10072.0000000	0.0335000
10073.0000000	0.0333333
10074.0000000	0.0315000
10075.0000000	0.0335000
10076.0000000	0.0345000
10077.0000000	0.0347619
10078.0000000	0.0335000
10079.0000000	0.0350000
10080.0000000	0.0357143
10081.0000000	0.0345000
10082.0000000	0.0342857
10083.0000000	0.0330000
10084.0000000	0.0335000
10085.0000000	0.0333333
10086.0000000	0.0320000
10087.0000000	0.0340000
10088.0000000	0.0335000
10089.0000000	0.0333333
10090.0000000	0.0350000
10091.0000000	0.0330000
10092.0000000	0.0323809
10093.0000000	0.0315000
10094.0000000	0.0315000
10095.0000000	0.0323809
10096.0000000	0.0330000
10097.0000000	0.0323810
10098.0000000	0.0325000
10099.0000000	0.0325000
10100.0000000	0.0305000
10101.0000000	0.0304762
10102.0000000	0.0300000
10103.0000000	0.0325000
10104.0000000	0.0309524
10105.0000000	0.0320000
10106.0000000	0.0319048
10107.0000000	0.0315000
10108.0000000	0.0325000
10109.0000000	0.0345000
10110.0000000	0.0314286
10111.0000000	0.0320000
10112.0000000	0.0340000
10113.0000000	0.0338095
10114.0000000	0.0315000
10115.0000000	0.0333333
10116.0000000	0.0330000
10117.0000000	0.0335000
10118.0000000	0.0315000
10119.0000000	0.0328571
10120.0000000	0.0320000
10121.0000000	0.0340000

10122.0000000	0.0338095
10123.0000000	0.0340000
10124.0000000	0.0310000
10125.0000000	0.0342857
10126.0000000	0.0335000
10127.0000000	0.0335000
10128.0000000	0.0345000
10129.0000000	0.0323810
10130.0000000	0.0350000
10131.0000000	0.0342857
10132.0000000	0.0335000
10133.0000000	0.0345000
10134.0000000	0.0338095
10135.0000000	0.0345000
10136.0000000	0.0335000
10137.0000000	0.0342857
10138.0000000	0.0365000
10139.0000000	0.0335000
10140.0000000	0.0340000
10141.0000000	0.0352381
10142.0000000	0.0347619
10143.0000000	0.0335000
10144.0000000	0.0335000
10145.0000000	0.0330000
10146.0000000	0.0319048
10147.0000000	0.0320000
10148.0000000	0.0325000
10149.0000000	0.0319048
10150.0000000	0.0315000
10151.0000000	0.0309524
10152.0000000	0.0315000
10153.0000000	0.0305000
10154.0000000	0.0305000
10155.0000000	0.0305000
10156.0000000	0.0304762
10157.0000000	0.0305000
10158.0000000	0.0304762
10159.0000000	0.0305000
10160.0000000	0.0309524
10161.0000000	0.0325000
10162.0000000	0.0320000
10163.0000000	0.0305000
10164.0000000	0.0325000
10165.0000000	0.0304762
10166.0000000	0.0305000
10167.0000000	0.0342857
10168.0000000	0.0340000
10169.0000000	0.0320000
10170.0000000	0.0319048
10171.0000000	0.0315000
10172.0000000	0.0315000
10173.0000000	0.0319048
10174.0000000	0.0315000
10175.0000000	0.0320000
10176.0000000	0.0328571
10177.0000000	0.0310000
10178.0000000	0.0315000

10179.0000000	0.0305000
10180.0000000	0.0319048
10181.0000000	0.0310000
10182.0000000	0.0309524
10183.0000000	0.0305000
10184.0000000	0.0305000
10185.0000000	0.0305000
10186.0000000	0.0314286
10187.0000000	0.0310000
10188.0000000	0.0305000
10189.0000000	0.0304762
10190.0000000	0.0304762
10191.0000000	0.0305000
10192.0000000	0.0305000
10193.0000000	0.0300000
10194.0000000	0.0304762
10195.0000000	0.0300000
10196.0000000	0.0305000
10197.0000000	0.0305263
10198.0000000	0.0305000
10199.0000000	0.0315000
10200.0000000	0.0319048
10201.0000000	0.0300000
10202.0000000	0.0304762
10203.0000000	0.0315000
10204.0000000	0.0310000
10205.0000000	0.0314286
10206.0000000	0.0305000
10207.0000000	0.0310000
10208.0000000	0.0305000
10209.0000000	0.0309524
10210.0000000	0.0304762
10211.0000000	0.0325000
10212.0000000	0.0310000
10213.0000000	0.0315000
10214.0000000	0.0309524
10215.0000000	0.0320000
10216.0000000	0.0320000
10217.0000000	0.0319048
10218.0000000	0.0305000
10219.0000000	0.0325000
10220.0000000	0.0340000
10221.0000000	0.0342857
10222.0000000	0.0355000
10223.0000000	0.0338095
10224.0000000	0.0355000
10225.0000000	0.0325000
10226.0000000	0.0342857
10227.0000000	0.0335000
10228.0000000	0.0345000
10229.0000000	0.0338095
10230.0000000	0.0340000
10231.0000000	0.0350000
10232.0000000	0.0352381
10233.0000000	0.0350000
10234.0000000	0.0345000
10235.0000000	0.0360000

10236.0000000	0.0350000
10237.0000000	0.0352381
10238.0000000	0.0360000
10239.0000000	0.0371429
10240.0000000	0.0355000
10241.0000000	0.0352381
10242.0000000	0.0360000
10243.0000000	0.0370000
10244.0000000	0.0385000
10245.0000000	0.0376190
10246.0000000	0.0365000
10247.0000000	0.0365000
10248.0000000	0.0361905
10249.0000000	0.0365000
10250.0000000	0.0371429
10251.0000000	0.0370000
10252.0000000	0.0365000
10253.0000000	0.0375000
10254.0000000	0.0361905
10255.0000000	0.0370000
10256.0000000	0.0370000
10257.0000000	0.0347619
10258.0000000	0.0360000
10259.0000000	0.0342857
10260.0000000	0.0340000
10261.0000000	0.0360000
10262.0000000	0.0357143
10263.0000000	0.0335000
10264.0000000	0.0345000
10265.0000000	0.0335000
10266.0000000	0.0342857
10267.0000000	0.0340000
10268.0000000	0.0338095
10269.0000000	0.0336842
10270.0000000	0.0319048
10271.0000000	0.0338095
10272.0000000	0.0345000
10273.0000000	0.0335000
10274.0000000	0.0335000
10275.0000000	0.0323809
10276.0000000	0.0325000
10277.0000000	0.0325000
10278.0000000	0.0323809
10279.0000000	0.0330000
10280.0000000	0.0350000
10281.0000000	0.0347619
10282.0000000	0.0335000
10283.0000000	0.0325000
10284.0000000	0.0333333
10285.0000000	0.0340000
10286.0000000	0.0338095
10287.0000000	0.0330000
10288.0000000	0.0345000
10289.0000000	0.0330000
10290.0000000	0.0338095
10291.0000000	0.0335000
10292.0000000	0.0315000

10293.0000000	0.0328571
10294.0000000	0.0320000
10295.0000000	0.0305000
10296.0000000	0.0310000
10297.0000000	0.0319048
10298.0000000	0.0309524
10299.0000000	0.0300000
10300.0000000	0.0310000
10301.0000000	0.0310000
10302.0000000	0.0314286
10303.0000000	0.0315000
10304.0000000	0.0315000
10305.0000000	0.0304762
10306.0000000	0.0300000
10307.0000000	0.0304762
10308.0000000	0.0315000
10309.0000000	0.0305000
10310.0000000	0.0305000
10311.0000000	0.0309524
10312.0000000	0.0305000
10313.0000000	0.0305000
10314.0000000	0.0309524
10315.0000000	0.0320000
10316.0000000	0.0315000
10317.0000000	0.0304762
10318.0000000	0.0315000
10319.0000000	0.0315000
10320.0000000	0.0314286
10321.0000000	0.0305000
10322.0000000	0.0314286
10323.0000000	0.0305000
10324.0000000	0.0300000
10325.0000000	0.0315000
10326.0000000	0.0323809
10327.0000000	0.0315000
10328.0000000	0.0330000
10329.0000000	0.0314286
10330.0000000	0.0315000
10331.0000000	0.0314286
10332.0000000	0.0320000
10333.0000000	0.0325000
10334.0000000	0.0310000
10335.0000000	0.0325000
10336.0000000	0.0309524
10337.0000000	0.0310000
10338.0000000	0.0314286
10339.0000000	0.0320000
10340.0000000	0.0320000
10341.0000000	0.0340000
10342.0000000	0.0314286
10343.0000000	0.0310000
10344.0000000	0.0330000
10345.0000000	0.0319048
10346.0000000	0.0342857
10347.0000000	0.0330000
10348.0000000	0.0340000
10349.0000000	0.0335000

10350.0000000	0.0357143
10351.0000000	0.0345000
10352.0000000	0.0355000
10353.0000000	0.0361905
10354.0000000	0.0345000
10355.0000000	0.0350000
10356.0000000	0.0338095
10357.0000000	0.0350000
10358.0000000	0.0361905
10359.0000000	0.0350000
10360.0000000	0.0340000
10361.0000000	0.0365000
10362.0000000	0.0342857
10363.0000000	0.0340000
10364.0000000	0.0350000
10365.0000000	0.0357143
10366.0000000	0.0330000
10367.0000000	0.0366667
10368.0000000	0.0345000
10369.0000000	0.0360000
10370.0000000	0.0355000
10371.0000000	0.0365000
10372.0000000	0.0347619
10373.0000000	0.0345000
10374.0000000	0.0352381
10375.0000000	0.0345000
10376.0000000	0.0328571
10377.0000000	0.0350000
10378.0000000	0.0365000
10379.0000000	0.0350000
10380.0000000	0.0365000
10381.0000000	0.0347619
10382.0000000	0.0355000
10383.0000000	0.0357143
10384.0000000	0.0360000
10385.0000000	0.0350000
10386.0000000	0.0371429
10387.0000000	0.0350000
10388.0000000	0.0345000
10389.0000000	0.0330000
10390.0000000	0.0323809
10391.0000000	0.0320000
10392.0000000	0.0323809
10393.0000000	0.0335000
10394.0000000	0.0335000
10395.0000000	0.0338095
10396.0000000	0.0335000
10397.0000000	0.0335000
10398.0000000	0.0352381
10399.0000000	0.0320000
10400.0000000	0.0335000
10401.0000000	0.0330000
10402.0000000	0.0333333
10403.0000000	0.0320000
10404.0000000	0.0338095
10405.0000000	0.0350000
10406.0000000	0.0350000

10407.0000000	0.0338095
10408.0000000	0.0345000
10409.0000000	0.0345000
10410.0000000	0.0342857
10411.0000000	0.0360000
10412.0000000	0.0355000
10413.0000000	0.0370000
10414.0000000	0.0355000
10415.0000000	0.0333333
10416.0000000	0.0355000
10417.0000000	0.0357143
10418.0000000	0.0375000
10419.0000000	0.0342857
10420.0000000	0.0355000
10421.0000000	0.0345000
10422.0000000	0.0361905
10423.0000000	0.0345000
10424.0000000	0.0345000
10425.0000000	0.0345000
10426.0000000	0.0342857
10427.0000000	0.0340000
10428.0000000	0.0347619
10429.0000000	0.0335000
10430.0000000	0.0340000
10431.0000000	0.0325000
10432.0000000	0.0370000
10433.0000000	0.0333333
10434.0000000	0.0323809
10435.0000000	0.0330000
10436.0000000	0.0310000
10437.0000000	0.0342857
10438.0000000	0.0340000
10439.0000000	0.0315000
10440.0000000	0.0335000
10441.0000000	0.0320000
10442.0000000	0.0319048
10443.0000000	0.0345000
10444.0000000	0.0328571
10445.0000000	0.0310000
10446.0000000	0.0320000
10447.0000000	0.0328571
10448.0000000	0.0320000
10449.0000000	0.0335000
10450.0000000	0.0338095
10451.0000000	0.0320000
10452.0000000	0.0335000
10453.0000000	0.0338095
10454.0000000	0.0365000
10455.0000000	0.0328571
10456.0000000	0.0350000
10457.0000000	0.0350000
10458.0000000	0.0342857
10459.0000000	0.0360000
10460.0000000	0.0365000
10461.0000000	0.0355000
10462.0000000	0.0357143
10463.0000000	0.0370000

10464.0000000	0.0385714
10465.0000000	0.0365000
10466.0000000	0.0370000
10467.0000000	0.0371429
10468.0000000	0.0375000
10469.0000000	0.0370000
10470.0000000	0.0380000
10471.0000000	0.0376191
10472.0000000	0.0375000
10473.0000000	0.0380952
10474.0000000	0.0350000
10475.0000000	0.0380000
10476.0000000	0.0365000
10477.0000000	0.0370000
10478.0000000	0.0352381
10479.0000000	0.0350000
10480.0000000	0.0357143
10481.0000000	0.0355000
10482.0000000	0.0330000
10483.0000000	0.0338095
10484.0000000	0.0360000
10485.0000000	0.0375000
10486.0000000	0.0376191
10487.0000000	0.0365000
10488.0000000	0.0370000
10489.0000000	0.0380952
10490.0000000	0.0375000
10491.0000000	0.0370000
10492.0000000	0.0385714
10493.0000000	0.0395000
10494.0000000	0.0390000
10495.0000000	0.0395238
10496.0000000	0.0370000
10497.0000000	0.0370000
10498.0000000	0.0380952
10499.0000000	0.0375000
10500.0000000	0.0370000
10501.0000000	0.0355000
10502.0000000	0.0357143
10503.0000000	0.0361905
10504.0000000	0.0365000
10505.0000000	0.0370000
10506.0000000	0.0385000
10507.0000000	0.0361905
10508.0000000	0.0380000
10509.0000000	0.0370000
10510.0000000	0.0376190
10511.0000000	0.0395000
10512.0000000	0.0385714
10513.0000000	0.0385000
10514.0000000	0.0385000
10515.0000000	0.0395000
10516.0000000	0.0400000
10517.0000000	0.0395000
10518.0000000	0.0390000
10519.0000000	0.0395238
10520.0000000	0.0385000

10521.0000000	0.0400000
10522.0000000	0.0390476
10523.0000000	0.0395000
10524.0000000	0.0390000
10525.0000000	0.0380952
10526.0000000	0.0395000
10527.0000000	0.0390000
10528.0000000	0.0385714
10529.0000000	0.0370000
10530.0000000	0.0390000
10531.0000000	0.0366667
10532.0000000	0.0385000
10533.0000000	0.0365000
10534.0000000	0.0380952
10535.0000000	0.0380000
10536.0000000	0.0380000
10537.0000000	0.0400000
10538.0000000	0.0385714
10539.0000000	0.0400000
10540.0000000	0.0385000
10541.0000000	0.0395238
10542.0000000	0.0390000
10543.0000000	0.0390476
10544.0000000	0.0390000
10545.0000000	0.0395000
10546.0000000	0.0375000
10547.0000000	0.0390476
10548.0000000	0.0385000
10549.0000000	0.0400000
10550.0000000	0.0395238
10551.0000000	0.0390000
10552.0000000	0.0385714
10553.0000000	0.0385000
10554.0000000	0.0390000
10555.0000000	0.0385714
10556.0000000	0.0390000
10557.0000000	0.0395000
10558.0000000	0.0395000
10559.0000000	0.0395238
10560.0000000	0.0400000
10561.0000000	0.0395238
10562.0000000	0.0400000
10563.0000000	0.0395000
10564.0000000	0.0390476
10565.0000000	0.0400000
10566.0000000	0.0385000
10567.0000000	0.0375000
10568.0000000	0.0380952
10569.0000000	0.0380000
10570.0000000	0.0366667
10571.0000000	0.0375000
10572.0000000	0.0390000
10573.0000000	0.0371429
10574.0000000	0.0395000
10575.0000000	0.0390000
10576.0000000	0.0380000
10577.0000000	0.0380952

10578.0000000	0.0390000
10579.0000000	0.0400000
10580.0000000	0.0400000
10581.0000000	0.0400000
10582.0000000	0.0400000
10583.0000000	0.0395238
10584.0000000	0.0400000
10585.0000000	0.0395000
10586.0000000	0.0400000
10587.0000000	0.0400000
10588.0000000	0.0400000
10589.0000000	0.0395000
10590.0000000	0.0400000
10591.0000000	0.0400000
10592.0000000	0.0385000
10593.0000000	0.0400000
10594.0000000	0.0400000
10595.0000000	0.0395238
10596.0000000	0.0395000
10597.0000000	0.0400000
10598.0000000	0.0395238
10599.0000000	0.0400000
10600.0000000	0.0400000
10601.0000000	0.0405000
10602.0000000	0.0400000
10603.0000000	0.0400000
10604.0000000	0.0400000
10605.0000000	0.0395000
10606.0000000	0.0400000
10607.0000000	0.0400000
10608.0000000	0.0400000
10609.0000000	0.0400000
10610.0000000	0.0395000
10611.0000000	0.0395000
10612.0000000	0.0400000
10613.0000000	0.0400000
10614.0000000	0.0400000
10615.0000000	0.0395238
10616.0000000	0.0390000
10617.0000000	0.0395000
10618.0000000	0.0390000
10619.0000000	0.0395238
10620.0000000	0.0375000
10621.0000000	0.0385000
10622.0000000	0.0357143
10623.0000000	0.0375000
10624.0000000	0.0371429
10625.0000000	0.0355000
10626.0000000	0.0360000
10627.0000000	0.0350000
10628.0000000	0.0350000
10629.0000000	0.0352381
10630.0000000	0.0365000
10631.0000000	0.0347619
10632.0000000	0.0355000
10633.0000000	0.0333333
10634.0000000	0.0350000

10635.0000000	0.0355000
10636.0000000	0.0355000
10637.0000000	0.0355000
10638.0000000	0.0338095
10639.0000000	0.0360000
10640.0000000	0.0323809
10641.0000000	0.0325000
10642.0000000	0.0320000
10643.0000000	0.0323809
10644.0000000	0.0310000
10645.0000000	0.0335000
10646.0000000	0.0310000
10647.0000000	0.0323809
10648.0000000	0.0333333
10649.0000000	0.0325000
10650.0000000	0.0320000
10651.0000000	0.0335000
10652.0000000	0.0323809
10653.0000000	0.0330000
10654.0000000	0.0305000
10655.0000000	0.0309524
10656.0000000	0.0320000
10657.0000000	0.0340000
10658.0000000	0.0333333
10659.0000000	0.0340000
10660.0000000	0.0310000
10661.0000000	0.0319048
10662.0000000	0.0310000
10663.0000000	0.0335000
10664.0000000	0.0333333
10665.0000000	0.0320000
10666.0000000	0.0310000
10667.0000000	0.0309524
10668.0000000	0.0315000
10669.0000000	0.0310000
10670.0000000	0.0319048
10671.0000000	0.0315000
10672.0000000	0.0345000
10673.0000000	0.0330000
10674.0000000	0.0319048
10675.0000000	0.0310000
10676.0000000	0.0323809
10677.0000000	0.0305000
10678.0000000	0.0305000
10679.0000000	0.0314286
10680.0000000	0.0335000
10681.0000000	0.0330000
10682.0000000	0.0320000
10683.0000000	0.0319048
10684.0000000	0.0330000
10685.0000000	0.0328571
10686.0000000	0.0330000
10687.0000000	0.0330000
10688.0000000	0.0328571
10689.0000000	0.0330000
10690.0000000	0.0340000
10691.0000000	0.0323809

10692.0000000	0.0330000
10693.0000000	0.0315000
10694.0000000	0.0328571
10695.0000000	0.0330000
10696.0000000	0.0330000
10697.0000000	0.0338095
10698.0000000	0.0315000
10699.0000000	0.0320000
10700.0000000	0.0314286
10701.0000000	0.0345000
10702.0000000	0.0340000
10703.0000000	0.0328571
10704.0000000	0.0345000
10705.0000000	0.0335000
10706.0000000	0.0335000
10707.0000000	0.0352381
10708.0000000	0.0347619
10709.0000000	0.0355000
10710.0000000	0.0335000
10711.0000000	0.0335000
10712.0000000	0.0333333
10713.0000000	0.0335000
10714.0000000	0.0330000
10715.0000000	0.0328571
10716.0000000	0.0355000
10717.0000000	0.0320000
10718.0000000	0.0345000
10719.0000000	0.0333333
10720.0000000	0.0325000
10721.0000000	0.0335000
10722.0000000	0.0342857
10723.0000000	0.0315000
10724.0000000	0.0314286
10725.0000000	0.0325000
10726.0000000	0.0330000
10727.0000000	0.0338095
10728.0000000	0.0335000
10729.0000000	0.0325000
10730.0000000	0.0340000
10731.0000000	0.0347619
10732.0000000	0.0345000
10733.0000000	0.0355000
10734.0000000	0.0325000
10735.0000000	0.0352381
10736.0000000	0.0342857
10737.0000000	0.0335000
10738.0000000	0.0340000
10739.0000000	0.0342857
10740.0000000	0.0360000
10741.0000000	0.0365000
10742.0000000	0.0345000
10743.0000000	0.0328571
10744.0000000	0.0330000
10745.0000000	0.0320000
10746.0000000	0.0319048
10747.0000000	0.0320000
10748.0000000	0.0310000

10749.0000000	0.0314286
10750.0000000	0.0320000
10751.0000000	0.0320000
10752.0000000	0.0328571
10753.0000000	0.0335000
10754.0000000	0.0340000
10755.0000000	0.0309524
10756.0000000	0.0325000
10757.0000000	0.0330000
10758.0000000	0.0323809
10759.0000000	0.0325000
10760.0000000	0.0314286
10761.0000000	0.0325000
10762.0000000	0.0315000
10763.0000000	0.0325000
10764.0000000	0.0319048
10765.0000000	0.0340000
10766.0000000	0.0325000
10767.0000000	0.0314286
10768.0000000	0.0300000
10769.0000000	0.0309524
10770.0000000	0.0305000
10771.0000000	0.0315000
10772.0000000	0.0310000
10773.0000000	0.0314286
10774.0000000	0.0305000
10775.0000000	0.0315000
10776.0000000	0.0319048
10777.0000000	0.0320000
10778.0000000	0.0300000
10779.0000000	0.0319048
10780.0000000	0.0315000
10781.0000000	0.0315000
10782.0000000	0.0319048
10783.0000000	0.0320000
10784.0000000	0.0320000
10785.0000000	0.0323809
10786.0000000	0.0320000
10787.0000000	0.0320000
10788.0000000	0.0323809
10789.0000000	0.0315000
10790.0000000	0.0315000
10791.0000000	0.0328571
10792.0000000	0.0310000
10793.0000000	0.0315000
10794.0000000	0.0325000
10795.0000000	0.0319048
10796.0000000	0.0319048
10797.0000000	0.0325000
10798.0000000	0.0340000
10799.0000000	0.0345000
10800.0000000	0.0328571
10801.0000000	0.0325000
10802.0000000	0.0345000
10803.0000000	0.0323809
10804.0000000	0.0330000
10805.0000000	0.0323809

10806.0000000	0.0355000
10807.0000000	0.0315000
10808.0000000	0.0330000
10809.0000000	0.0335000
10810.0000000	0.0319048
10811.0000000	0.0330000
10812.0000000	0.0342857
10813.0000000	0.0350000
10814.0000000	0.0342857
10815.0000000	0.0355000
10816.0000000	0.0330000
10817.0000000	0.0345000
10818.0000000	0.0340000
10819.0000000	0.0352381
10820.0000000	0.0335000
10821.0000000	0.0357143
10822.0000000	0.0335000
10823.0000000	0.0360000
10824.0000000	0.0352381
10825.0000000	0.0355000
10826.0000000	0.0335000
10827.0000000	0.0342857
10828.0000000	0.0330000
10829.0000000	0.0345000
10830.0000000	0.0342857
10831.0000000	0.0355000
10832.0000000	0.0345000
10833.0000000	0.0360000
10834.0000000	0.0357143
10835.0000000	0.0340000
10836.0000000	0.0357143
10837.0000000	0.0360000
10838.0000000	0.0365000
10839.0000000	0.0350000
10840.0000000	0.0342857
10841.0000000	0.0345000
10842.0000000	0.0350000
10843.0000000	0.0342857
10844.0000000	0.0357143
10845.0000000	0.0360000
10846.0000000	0.0350000
10847.0000000	0.0360000
10848.0000000	0.0357143
10849.0000000	0.0375000
10850.0000000	0.0355000
10851.0000000	0.0365000
10852.0000000	0.0380952
10853.0000000	0.0375000
10854.0000000	0.0375000
10855.0000000	0.0361905
10856.0000000	0.0385000
10857.0000000	0.0376191
10858.0000000	0.0375000
10859.0000000	0.0375000
10860.0000000	0.0371429
10861.0000000	0.0385000
10862.0000000	0.0390000

10863.0000000	0.0390000
10864.0000000	0.0380952
10865.0000000	0.0365000
10866.0000000	0.0380952
10867.0000000	0.0390000
10868.0000000	0.0385000
10869.0000000	0.0375000
10870.0000000	0.0385714
10871.0000000	0.0390000
10872.0000000	0.0375000
10873.0000000	0.0385714
10874.0000000	0.0395000
10875.0000000	0.0395238
10876.0000000	0.0380000
10877.0000000	0.0385000
10878.0000000	0.0390000
10879.0000000	0.0385714
10880.0000000	0.0400000
10881.0000000	0.0395000
10882.0000000	0.0395238
10883.0000000	0.0395000
10884.0000000	0.0400000
10885.0000000	0.0400000
10886.0000000	0.0400000
10887.0000000	0.0400000
10888.0000000	0.0395238
10889.0000000	0.0400000
10890.0000000	0.0395000
10891.0000000	0.0395238
10892.0000000	0.0400000
10893.0000000	0.0395238
10894.0000000	0.0395000
10895.0000000	0.0400000
10896.0000000	0.0395238
10897.0000000	0.0400000
10898.0000000	0.0390000
10899.0000000	0.0395000
10900.0000000	0.0395238
10901.0000000	0.0395000
10902.0000000	0.0380952
10903.0000000	0.0390000
10904.0000000	0.0385000
10905.0000000	0.0395238
10906.0000000	0.0395000
10907.0000000	0.0385000
10908.0000000	0.0385000
10909.0000000	0.0395238
10910.0000000	0.0400000
10911.0000000	0.0395000
10912.0000000	0.0390476
10913.0000000	0.0390000
10914.0000000	0.0375000
10915.0000000	0.0380000
10916.0000000	0.0395238
10917.0000000	0.0380000
10918.0000000	0.0390476
10919.0000000	0.0389474

10920.0000000	0.0386364
10921.0000000	0.0385000
10922.0000000	0.0380000
10923.0000000	0.0375000
10924.0000000	0.0376190
10925.0000000	0.0385000
10926.0000000	0.0385000
10927.0000000	0.0376191
10928.0000000	0.0385000
10929.0000000	0.0390000
10930.0000000	0.0385000
10931.0000000	0.0395238
10932.0000000	0.0390476
10933.0000000	0.0395000
10934.0000000	0.0400000
10935.0000000	0.0390000
10936.0000000	0.0395238
10937.0000000	0.0400000
10938.0000000	0.0395000
10939.0000000	0.0400000
10940.0000000	0.0400000
10941.0000000	0.0400000
10942.0000000	0.0400000
10943.0000000	0.0395000
10944.0000000	0.0395000
10945.0000000	0.0390476
10946.0000000	0.0395000
10947.0000000	0.0400000
10948.0000000	0.0395238
10949.0000000	0.0395000
10950.0000000	0.0400000
10951.0000000	0.0395238
10952.0000000	0.0400000
10953.0000000	0.0395000
10954.0000000	0.0400000
10955.0000000	0.0390000
10956.0000000	0.0400000
10957.0000000	0.0385714
10958.0000000	0.0395000
10959.0000000	0.0390000
10960.0000000	0.0385000
10961.0000000	0.0380952
10962.0000000	0.0365000
10963.0000000	0.0361905
10964.0000000	0.0375000
10965.0000000	0.0371429
10966.0000000	0.0375000
10967.0000000	0.0375000
10968.0000000	0.0360000
10969.0000000	0.0380000
10970.0000000	0.0371429
10971.0000000	0.0375000
10972.0000000	0.0361905
10973.0000000	0.0365000
10974.0000000	0.0370000
10975.0000000	0.0375000
10976.0000000	0.0361905

10977.0000000	0.0385000
10978.0000000	0.0390000
10979.0000000	0.0371429
10980.0000000	0.0385000
10981.0000000	0.0361905
10982.0000000	0.0380000
10983.0000000	0.0375000
10984.0000000	0.0361905
10985.0000000	0.0375000
10986.0000000	0.0380000
10987.0000000	0.0380000
10988.0000000	0.0347619
10989.0000000	0.0350000
10990.0000000	0.0357143
10991.0000000	0.0350000
10992.0000000	0.0350000
10993.0000000	0.0375000
10994.0000000	0.0361905
10995.0000000	0.0360000
10996.0000000	0.0371429
10997.0000000	0.0360000
10998.0000000	0.0345000
10999.0000000	0.0345000
11000.0000000	0.0342857
11001.0000000	0.0357143
11002.0000000	0.0360000
11003.0000000	0.0345000
11004.0000000	0.0375000
11005.0000000	0.0360000
11006.0000000	0.0347619
11007.0000000	0.0330000
11008.0000000	0.0347619
11009.0000000	0.0335000
11010.0000000	0.0323809
11011.0000000	0.0330000
11012.0000000	0.0345000
11013.0000000	0.0350000
11014.0000000	0.0335000
11015.0000000	0.0328571
11016.0000000	0.0330000
11017.0000000	0.0342857
11018.0000000	0.0340000
11019.0000000	0.0345000
11020.0000000	0.0342857
11021.0000000	0.0345000
11022.0000000	0.0365000
11023.0000000	0.0360000
11024.0000000	0.0342857
11025.0000000	0.0350000
11026.0000000	0.0352381
11027.0000000	0.0315000
11028.0000000	0.0345000
11029.0000000	0.0342857
11030.0000000	0.0345000
11031.0000000	0.0330000
11032.0000000	0.0338095
11033.0000000	0.0315000

11034.0000000	0.0335000
11035.0000000	0.0345000
11036.0000000	0.0333333
11037.0000000	0.0320000
11038.0000000	0.0345000
11039.0000000	0.0352381
11040.0000000	0.0355000
11041.0000000	0.0352381
11042.0000000	0.0370000
11043.0000000	0.0345000
11044.0000000	0.0340000
11045.0000000	0.0347619
11046.0000000	0.0365000
11047.0000000	0.0360000
11048.0000000	0.0366667
11049.0000000	0.0360000
11050.0000000	0.0370000
11051.0000000	0.0371429
11052.0000000	0.0365000
11053.0000000	0.0347619
11054.0000000	0.0375000
11055.0000000	0.0335000
11056.0000000	0.0352381
11057.0000000	0.0375000
11058.0000000	0.0370000
11059.0000000	0.0345000
11060.0000000	0.0371429
11061.0000000	0.0380000
11062.0000000	0.0380952
11063.0000000	0.0375000
11064.0000000	0.0380000
11065.0000000	0.0375000
11066.0000000	0.0370000
11067.0000000	0.0380952
11068.0000000	0.0385000
11069.0000000	0.0376191
11070.0000000	0.0385000
11071.0000000	0.0380952
11072.0000000	0.0380000
11073.0000000	0.0380000
11074.0000000	0.0380000
11075.0000000	0.0360000
11076.0000000	0.0376190
11077.0000000	0.0395000
11078.0000000	0.0371429
11079.0000000	0.0370000
11080.0000000	0.0385000
11081.0000000	0.0366667
11082.0000000	0.0375000
11083.0000000	0.0380000
11084.0000000	0.0371429
11085.0000000	0.0395000
11086.0000000	0.0390000
11087.0000000	0.0390476
11088.0000000	0.0380000
11089.0000000	0.0376190
11090.0000000	0.0390000

11091.0000000	0.0385000
11092.0000000	0.0390000
11093.0000000	0.0400000
11094.0000000	0.0390000
11095.0000000	0.0375000
11096.0000000	0.0385714
11097.0000000	0.0390000
11098.0000000	0.0385714
11099.0000000	0.0385000
11100.0000000	0.0375000
11101.0000000	0.0376190
11102.0000000	0.0360000
11103.0000000	0.0375000
11104.0000000	0.0350000
11105.0000000	0.0366667
11106.0000000	0.0380000
11107.0000000	0.0361905
11108.0000000	0.0375000
11109.0000000	0.0365000
11110.0000000	0.0395000
11111.0000000	0.0375000
11112.0000000	0.0385714
11113.0000000	0.0375000
11114.0000000	0.0371429
11115.0000000	0.0390000
11116.0000000	0.0355000
11117.0000000	0.0376190
11118.0000000	0.0375000
11119.0000000	0.0365000
11120.0000000	0.0361905
11121.0000000	0.0355000
11122.0000000	0.0375000
11123.0000000	0.0376190
11124.0000000	0.0365000
11125.0000000	0.0380000
11126.0000000	0.0385000
11127.0000000	0.0371429
11128.0000000	0.0380000
11129.0000000	0.0400000
11130.0000000	0.0370000
11131.0000000	0.0385000
11132.0000000	0.0376190
11133.0000000	0.0365000
11134.0000000	0.0385000
11135.0000000	0.0380000
11136.0000000	0.0385714
11137.0000000	0.0385714
11138.0000000	0.0365000
11139.0000000	0.0385000
11140.0000000	0.0385000
11141.0000000	0.0385000
11142.0000000	0.0395238
11143.0000000	0.0395000
11144.0000000	0.0375000
11145.0000000	0.0361905
11146.0000000	0.0371429
11147.0000000	0.0385000

11148.0000000	0.0365000
11149.0000000	0.0370000
11150.0000000	0.0361905
11151.0000000	0.0370000
11152.0000000	0.0375000
11153.0000000	0.0376190
11154.0000000	0.0385000
11155.0000000	0.0375000
11156.0000000	0.0370000
11157.0000000	0.0376190
11158.0000000	0.0370000
11159.0000000	0.0376191
11160.0000000	0.0380000
11161.0000000	0.0380000
11162.0000000	0.0380952
11163.0000000	0.0390000
11164.0000000	0.0380000
11165.0000000	0.0390000
11166.0000000	0.0385714
11167.0000000	0.0385000
11168.0000000	0.0380952
11169.0000000	0.0360000
11170.0000000	0.0380000
11171.0000000	0.0375000
11172.0000000	0.0361905
11173.0000000	0.0355000
11174.0000000	0.0360000
11175.0000000	0.0357143
11176.0000000	0.0360000
11177.0000000	0.0371429
11178.0000000	0.0360000
11179.0000000	0.0350000
11180.0000000	0.0385000
11181.0000000	0.0352381
11182.0000000	0.0350000
11183.0000000	0.0335000
11184.0000000	0.0352381
11185.0000000	0.0355000
11186.0000000	0.0338095
11187.0000000	0.0330000
11188.0000000	0.0345000
11189.0000000	0.0338095
11190.0000000	0.0350000
11191.0000000	0.0330000
11192.0000000	0.0335000
11193.0000000	0.0352381
11194.0000000	0.0335000
11195.0000000	0.0328571
11196.0000000	0.0345000
11197.0000000	0.0335000
11198.0000000	0.0333333
11199.0000000	0.0320000
11200.0000000	0.0355000
11201.0000000	0.0330000
11202.0000000	0.0328571
11203.0000000	0.0335000
11204.0000000	0.0350000

11205.0000000	0.0352381
11206.0000000	0.0320000
11207.0000000	0.0352381
11208.0000000	0.0330000
11209.0000000	0.0335000
11210.0000000	0.0340000
11211.0000000	0.0328571
11212.0000000	0.0325000
11213.0000000	0.0304762
11214.0000000	0.0325000
11215.0000000	0.0310000
11216.0000000	0.0310000
11217.0000000	0.0309524
11218.0000000	0.0315000
11219.0000000	0.0310000
11220.0000000	0.0323809
11221.0000000	0.0330000
11222.0000000	0.0314286
11223.0000000	0.0315000
11224.0000000	0.0305000
11225.0000000	0.0300000
11226.0000000	0.0310000
11227.0000000	0.0315000
11228.0000000	0.0310000
11229.0000000	0.0304762
11230.0000000	0.0305000
11231.0000000	0.0320000
11232.0000000	0.0315000
11233.0000000	0.0319048
11234.0000000	0.0309524
11235.0000000	0.0305000
11236.0000000	0.0305000
11237.0000000	0.0314286
11238.0000000	0.0310000
11239.0000000	0.0315000
11240.0000000	0.0310000
11241.0000000	0.0323809
11242.0000000	0.0315000
11243.0000000	0.0315000
11244.0000000	0.0300000
11245.0000000	0.0315000
11246.0000000	0.0310000
11247.0000000	0.0314286
11248.0000000	0.0330000
11249.0000000	0.0325000
11250.0000000	0.0323809
11251.0000000	0.0300000
11252.0000000	0.0310000
11253.0000000	0.0309524
11254.0000000	0.0305000
11255.0000000	0.0315000
11256.0000000	0.0304762
11257.0000000	0.0305000
11258.0000000	0.0319048
11259.0000000	0.0320000
11260.0000000	0.0325000
11261.0000000	0.0330000

11262.0000000	0.0320000
11263.0000000	0.0319048
11264.0000000	0.0325000
11265.0000000	0.0323809
11266.0000000	0.0315000
11267.0000000	0.0325000
11268.0000000	0.0314286
11269.0000000	0.0315000
11270.0000000	0.0320000
11271.0000000	0.0325000
11272.0000000	0.0309524
11273.0000000	0.0315000
11274.0000000	0.0319048
11275.0000000	0.0320000
11276.0000000	0.0315000
11277.0000000	0.0319048
11278.0000000	0.0320000
11279.0000000	0.0310000
11280.0000000	0.0300000
11281.0000000	0.0319048
11282.0000000	0.0305000
11283.0000000	0.0314286
11284.0000000	0.0315000
11285.0000000	0.0315000
11286.0000000	0.0314286
11287.0000000	0.0310000
11288.0000000	0.0325000
11289.0000000	0.0319048
11290.0000000	0.0310000
11291.0000000	0.0330000
11292.0000000	0.0330000
11293.0000000	0.0323810
11294.0000000	0.0320000
11295.0000000	0.0338095
11296.0000000	0.0330000
11297.0000000	0.0320000
11298.0000000	0.0333333
11299.0000000	0.0350000
11300.0000000	0.0340000
11301.0000000	0.0357143
11302.0000000	0.0355000
11303.0000000	0.0345000
11304.0000000	0.0352381
11305.0000000	0.0360000
11306.0000000	0.0360000
11307.0000000	0.0365000
11308.0000000	0.0357143
11309.0000000	0.0375000
11310.0000000	0.0366667
11311.0000000	0.0380000
11312.0000000	0.0365000
11313.0000000	0.0385714
11314.0000000	0.0380000
11315.0000000	0.0385000
11316.0000000	0.0390000
11317.0000000	0.0385714
11318.0000000	0.0385000

11319.0000000	0.0376190
11320.0000000	0.0375000
11321.0000000	0.0385000
11322.0000000	0.0385714
11323.0000000	0.0395000
11324.0000000	0.0385000
11325.0000000	0.0385714
11326.0000000	0.0380000
11327.0000000	0.0385000
11328.0000000	0.0395238
11329.0000000	0.0370000
11330.0000000	0.0385000
11331.0000000	0.0380000
11332.0000000	0.0390476
11333.0000000	0.0375000
11334.0000000	0.0380952
11335.0000000	0.0385000
11336.0000000	0.0380000
11337.0000000	0.0390000
11338.0000000	0.0395238
11339.0000000	0.0390000
11340.0000000	0.0375000
11341.0000000	0.0400000
11342.0000000	0.0380952
11343.0000000	0.0400000
11344.0000000	0.0390000
11345.0000000	0.0390000
11346.0000000	0.0395238
11347.0000000	0.0400000
11348.0000000	0.0375000
11349.0000000	0.0395000
11350.0000000	0.0395238
11351.0000000	0.0400000
11352.0000000	0.0395000
11353.0000000	0.0385714
11354.0000000	0.0390000
11355.0000000	0.0390000
11356.0000000	0.0395238
11357.0000000	0.0385000
11358.0000000	0.0390476
11359.0000000	0.0390000
11360.0000000	0.0400000
11361.0000000	0.0390000
11362.0000000	0.0385714
11363.0000000	0.0390000
11364.0000000	0.0395238
11365.0000000	0.0380000
11366.0000000	0.0385000
11367.0000000	0.0390000
11368.0000000	0.0400000
11369.0000000	0.0390476
11370.0000000	0.0380000
11371.0000000	0.0380952
11372.0000000	0.0390000
11373.0000000	0.0385000
11374.0000000	0.0385714
11375.0000000	0.0390000

11376.0000000	0.0385000
11377.0000000	0.0380952
11378.0000000	0.0385000
11379.0000000	0.0385000
11380.0000000	0.0390476
11381.0000000	0.0385000
11382.0000000	0.0385000
11383.0000000	0.0380952
11384.0000000	0.0380000
11385.0000000	0.0385000
11386.0000000	0.0376190
11387.0000000	0.0375000
11388.0000000	0.0400000
11389.0000000	0.0385714
11390.0000000	0.0390000
11391.0000000	0.0385000
11392.0000000	0.0400000
11393.0000000	0.0395000
11394.0000000	0.0390476
11395.0000000	0.0395000
11396.0000000	0.0395000
11397.0000000	0.0395000
11398.0000000	0.0385714
11399.0000000	0.0380000
11400.0000000	0.0395000
11401.0000000	0.0395238
11402.0000000	0.0385000
11403.0000000	0.0395238
11404.0000000	0.0395000
11405.0000000	0.0395000
11406.0000000	0.0400000
11407.0000000	0.0400000
11408.0000000	0.0395000
11409.0000000	0.0400000
11410.0000000	0.0400000
11411.0000000	0.0400000
11412.0000000	0.0400000
11413.0000000	0.0390000
11414.0000000	0.0395238
11415.0000000	0.0395000
11416.0000000	0.0390476
11417.0000000	0.0400000
11418.0000000	0.0400000
11419.0000000	0.0390476
11420.0000000	0.0390000
11421.0000000	0.0400000
11422.0000000	0.0380952
11423.0000000	0.0380000
11424.0000000	0.0395000
11425.0000000	0.0385714
11426.0000000	0.0380000
11427.0000000	0.0390000
11428.0000000	0.0380000
11429.0000000	0.0390476
11430.0000000	0.0385000
11431.0000000	0.0395238
11432.0000000	0.0390000

11433.0000000	0.0390000
11434.0000000	0.0395238
11435.0000000	0.0390000
11436.0000000	0.0390000
11437.0000000	0.0395000
11438.0000000	0.0390476
11439.0000000	0.0390476
11440.0000000	0.0400000
11441.0000000	0.0395000
11442.0000000	0.0385000
11443.0000000	0.0385714
11444.0000000	0.0395000
11445.0000000	0.0390000
11446.0000000	0.0395238
11447.0000000	0.0395000
11448.0000000	0.0390000
11449.0000000	0.0390476
11450.0000000	0.0385000
11451.0000000	0.0395000
11452.0000000	0.0385000
11453.0000000	0.0395238
11454.0000000	0.0395000
11455.0000000	0.0385714
11456.0000000	0.0385000
11457.0000000	0.0385000
11458.0000000	0.0390000
11459.0000000	0.0390476
11460.0000000	0.0395000
11461.0000000	0.0390000
11462.0000000	0.0390476
11463.0000000	0.0400000
11464.0000000	0.0395238
11465.0000000	0.0395000
11466.0000000	0.0400000
11467.0000000	0.0395000
11468.0000000	0.0395000
11469.0000000	0.0395238
11470.0000000	0.0395238
11471.0000000	0.0400000
11472.0000000	0.0400000
11473.0000000	0.0380000
11474.0000000	0.0380952
11475.0000000	0.0395000
11476.0000000	0.0395000
11477.0000000	0.0390476
11478.0000000	0.0380000
11479.0000000	0.0390476
11480.0000000	0.0400000
11481.0000000	0.0400000
11482.0000000	0.0395238
11483.0000000	0.0395000
11484.0000000	0.0400000
11485.0000000	0.0390000
11486.0000000	0.0385714
11487.0000000	0.0395000
11488.0000000	0.0390000
11489.0000000	0.0395238

11490.0000000	0.0400000
11491.0000000	0.0390476
11492.0000000	0.0395000
11493.0000000	0.0390000
11494.0000000	0.0400000
11495.0000000	0.0395000
11496.0000000	0.0395000
11497.0000000	0.0395000
11498.0000000	0.0390476
11499.0000000	0.0400000
11500.0000000	0.0380952
11501.0000000	0.0400000
11502.0000000	0.0390000
11503.0000000	0.0395000
11504.0000000	0.0390476
11505.0000000	0.0395000
11506.0000000	0.0395000
11507.0000000	0.0390476
11508.0000000	0.0400000
11509.0000000	0.0385714
11510.0000000	0.0390000
11511.0000000	0.0390000
11512.0000000	0.0385000
11513.0000000	0.0380952
11514.0000000	0.0390000
11515.0000000	0.0390000
11516.0000000	0.0380952
11517.0000000	0.0380000
11518.0000000	0.0375000
11519.0000000	0.0371429
11520.0000000	0.0360000
11521.0000000	0.0350000
11522.0000000	0.0357143
11523.0000000	0.0370000
11524.0000000	0.0335000
11525.0000000	0.0338095
11526.0000000	0.0325000
11527.0000000	0.0338095
11528.0000000	0.0325000
11529.0000000	0.0315000
11530.0000000	0.0333333
11531.0000000	0.0305000
11532.0000000	0.0305000
11533.0000000	0.0305000
11534.0000000	0.0300000
11535.0000000	0.0300000
11536.0000000	0.0310000
11537.0000000	0.0304762
11538.0000000	0.0300000
11539.0000000	0.0300000
11540.0000000	0.0300000
11541.0000000	0.0315000
11542.0000000	0.0300000
11543.0000000	0.0304762
11544.0000000	0.0300000
11545.0000000	0.0300000
11546.0000000	0.0300000

11547.0000000	0.0300000
11548.0000000	0.0300000
11549.0000000	0.0300000
11550.0000000	0.0300000
11551.0000000	0.0300000
11552.0000000	0.0300000
11553.0000000	0.0300000
11554.0000000	0.0300000
11555.0000000	0.0304762
11556.0000000	0.0300000
11557.0000000	0.0300000
11558.0000000	0.0300000
11559.0000000	0.0300000
11560.0000000	0.0300000
11561.0000000	0.0309524
11562.0000000	0.0300000
11563.0000000	0.0305000
11564.0000000	0.0300000
11565.0000000	0.0300000
11566.0000000	0.0300000
11567.0000000	0.0300000
11568.0000000	0.0300000
11569.0000000	0.0305000
11570.0000000	0.0304762
11571.0000000	0.0300000
11572.0000000	0.0300000
11573.0000000	0.0300000
11574.0000000	0.0300000
11575.0000000	0.0300000
11576.0000000	0.0304762
11577.0000000	0.0300000
11578.0000000	0.0300000
11579.0000000	0.0300000
11580.0000000	0.0300000
11581.0000000	0.0305000
11582.0000000	0.0300000
11583.0000000	0.0300000
11584.0000000	0.0300000
11585.0000000	0.0300000
11586.0000000	0.0300000
11587.0000000	0.0300000
11588.0000000	0.0300000
11589.0000000	0.0300000
11590.0000000	0.0300000
11591.0000000	0.0300000
11592.0000000	0.0300000
11593.0000000	0.0300000
11594.0000000	0.0300000
11595.0000000	0.0300000
11596.0000000	0.0295000
11597.0000000	0.0300000
11598.0000000	0.0300000
11599.0000000	0.0300000
11600.0000000	0.0300000
11601.0000000	0.0300000
11602.0000000	0.0300000
11603.0000000	0.0300000

11604.0000000	0.0300000
11605.0000000	0.0300000
11606.0000000	0.0300000
11607.0000000	0.0300000
11608.0000000	0.0305000
11609.0000000	0.0295000
11610.0000000	0.0300000
11611.0000000	0.0295000
11612.0000000	0.0300000
11613.0000000	0.0300000
11614.0000000	0.0295000
11615.0000000	0.0300000
11616.0000000	0.0300000
11617.0000000	0.0300000
11618.0000000	0.0300000
11619.0000000	0.0290000
11620.0000000	0.0295000
11621.0000000	0.0295000
11622.0000000	0.0295238
11623.0000000	0.0290000
11624.0000000	0.0290000
11625.0000000	0.0285714
11626.0000000	0.0285000
11627.0000000	0.0280952
11628.0000000	0.0285000
11629.0000000	0.0275000
11630.0000000	0.0285714
11631.0000000	0.0290000
11632.0000000	0.0290000
11633.0000000	0.0290000
11634.0000000	0.0300000
11635.0000000	0.0290476
11636.0000000	0.0295000
11637.0000000	0.0285000
11638.0000000	0.0285000
11639.0000000	0.0280000
11640.0000000	0.0290476
11641.0000000	0.0285000
11642.0000000	0.0295000
11643.0000000	0.0285714
11644.0000000	0.0300000
11645.0000000	0.0284210
11646.0000000	0.0290476
11647.0000000	0.0285000
11648.0000000	0.0290000
11649.0000000	0.0276190
11650.0000000	0.0285000
11651.0000000	0.0285714
11652.0000000	0.0295000
11653.0000000	0.0285000
11654.0000000	0.0300000
11655.0000000	0.0290476
11656.0000000	0.0300000
11657.0000000	0.0275000
11658.0000000	0.0280952
11659.0000000	0.0285000
11660.0000000	0.0285714

11661.0000000	0.0290000
11662.0000000	0.0275000
11663.0000000	0.0300000
11664.0000000	0.0280952
11665.0000000	0.0285000
11666.0000000	0.0285000
11667.0000000	0.0285714
11668.0000000	0.0255000
11669.0000000	0.0275000
11670.0000000	0.0261905
11671.0000000	0.0260000
11672.0000000	0.0250000
11673.0000000	0.0242857
11674.0000000	0.0235000
11675.0000000	0.0242857
11676.0000000	0.0230000
11677.0000000	0.0245000
11678.0000000	0.0245000
11679.0000000	0.0242857
11680.0000000	0.0225000
11681.0000000	0.0235000
11682.0000000	0.0238095
11683.0000000	0.0245000
11684.0000000	0.0250000
11685.0000000	0.0271429
11686.0000000	0.0255000
11687.0000000	0.0261905
11688.0000000	0.0270000
11689.0000000	0.0270000
11690.0000000	0.0270000
11691.0000000	0.0271429
11692.0000000	0.0275000
11693.0000000	0.0265000
11694.0000000	0.0247619
11695.0000000	0.0255000
11696.0000000	0.0252381
11697.0000000	0.0270000
11698.0000000	0.0265000
11699.0000000	0.0255000
11700.0000000	0.0265000
11701.0000000	0.0266667
11702.0000000	0.0280000
11703.0000000	0.0280952
11704.0000000	0.0285000
11705.0000000	0.0271429
11706.0000000	0.0285000
11707.0000000	0.0285000
11708.0000000	0.0280000
11709.0000000	0.0280000
11710.0000000	0.0276190
11711.0000000	0.0290000
11712.0000000	0.0285714
11713.0000000	0.0280000
11714.0000000	0.0285000
11715.0000000	0.0290476
11716.0000000	0.0280000
11717.0000000	0.0285000

11718.0000000	0.0280952
11719.0000000	0.0280000
11720.0000000	0.0270000
11721.0000000	0.0271429
11722.0000000	0.0280000
11723.0000000	0.0290476
11724.0000000	0.0290000
11725.0000000	0.0295000
11726.0000000	0.0290000
11727.0000000	0.0290476
11728.0000000	0.0295000
11729.0000000	0.0295000
11730.0000000	0.0280000
11731.0000000	0.0285714
11732.0000000	0.0280952
11733.0000000	0.0260000
11734.0000000	0.0290000
11735.0000000	0.0276190
11736.0000000	0.0280000
11737.0000000	0.0275000
11738.0000000	0.0280000
11739.0000000	0.0276190
11740.0000000	0.0295000
11741.0000000	0.0285000
11742.0000000	0.0276190
11743.0000000	0.0285000
11744.0000000	0.0260000
11745.0000000	0.0270000
11746.0000000	0.0276190
11747.0000000	0.0260000
11748.0000000	0.0257143
11749.0000000	0.0250000
11750.0000000	0.0270000
11751.0000000	0.0271429
11752.0000000	0.0270000
11753.0000000	0.0270000
11754.0000000	0.0255000
11755.0000000	0.0247619
11756.0000000	0.0245000
11757.0000000	0.0271429
11758.0000000	0.0275000
11759.0000000	0.0260000
11760.0000000	0.0285000
11761.0000000	0.0261905
11762.0000000	0.0270000
11763.0000000	0.0276190
11764.0000000	0.0270000
11765.0000000	0.0275000
11766.0000000	0.0276190
11767.0000000	0.0270000
11768.0000000	0.0280000
11769.0000000	0.0270000
11770.0000000	0.0271429
11771.0000000	0.0285000
11772.0000000	0.0276190
11773.0000000	0.0270000
11774.0000000	0.0265000

11775.0000000	0.0271429
11776.0000000	0.0255000
11777.0000000	0.0275000
11778.0000000	0.0270000
11779.0000000	0.0280952
11780.0000000	0.0280000
11781.0000000	0.0276190
11782.0000000	0.0265000
11783.0000000	0.0265000
11784.0000000	0.0266667
11785.0000000	0.0270000
11786.0000000	0.0285000
11787.0000000	0.0271429
11788.0000000	0.0295000
11789.0000000	0.0285000
11790.0000000	0.0280000
11791.0000000	0.0295238
11792.0000000	0.0285000
11793.0000000	0.0300000
11794.0000000	0.0300000
11795.0000000	0.0290000
11796.0000000	0.0295238
11797.0000000	0.0295000
11798.0000000	0.0295000
11799.0000000	0.0295000
11800.0000000	0.0290476
11801.0000000	0.0275000
11802.0000000	0.0300000
11803.0000000	0.0275000
11804.0000000	0.0295000
11805.0000000	0.0295000
11806.0000000	0.0295000
11807.0000000	0.0280952
11808.0000000	0.0295000
11809.0000000	0.0300000
11810.0000000	0.0290000
11811.0000000	0.0290476
11812.0000000	0.0295000
11813.0000000	0.0285000
11814.0000000	0.0295000
11815.0000000	0.0300000
11816.0000000	0.0300000
11817.0000000	0.0290000
11818.0000000	0.0300000
11819.0000000	0.0300000
11820.0000000	0.0300000
11821.0000000	0.0295000
11822.0000000	0.0295000
11823.0000000	0.0300000
11824.0000000	0.0295000
11825.0000000	0.0295000
11826.0000000	0.0295000
11827.0000000	0.0290476
11828.0000000	0.0290000
11829.0000000	0.0295000
11830.0000000	0.0290476
11831.0000000	0.0280000

11832.0000000	0.0295238
11833.0000000	0.0290000
11834.0000000	0.0295000
11835.0000000	0.0295000
11836.0000000	0.0290476
11837.0000000	0.0300000
11838.0000000	0.0300000
11839.0000000	0.0290476
11840.0000000	0.0300000
11841.0000000	0.0290476
11842.0000000	0.0290000
11843.0000000	0.0300000
11844.0000000	0.0300000
11845.0000000	0.0300000
11846.0000000	0.0300000
11847.0000000	0.0300000
11848.0000000	0.0295238
11849.0000000	0.0300000
11850.0000000	0.0290000
11851.0000000	0.0290476
11852.0000000	0.0300000
11853.0000000	0.0290000
11854.0000000	0.0300000
11855.0000000	0.0285000
11856.0000000	0.0295238
11857.0000000	0.0295000
11858.0000000	0.0295000
11859.0000000	0.0285000
11860.0000000	0.0285714
11861.0000000	0.0300000
11862.0000000	0.0290000
11863.0000000	0.0285714
11864.0000000	0.0290000
11865.0000000	0.0280000
11866.0000000	0.0295000
11867.0000000	0.0290476
11868.0000000	0.0280000
11869.0000000	0.0271429
11870.0000000	0.0295000
11871.0000000	0.0280000
11872.0000000	0.0285714
11873.0000000	0.0290000
11874.0000000	0.0290000
11875.0000000	0.0290476
11876.0000000	0.0290000
11877.0000000	0.0295000
11878.0000000	0.0295238
11879.0000000	0.0285000
11880.0000000	0.0290000
11881.0000000	0.0290476
11882.0000000	0.0290000
11883.0000000	0.0300000
11884.0000000	0.0290476
11885.0000000	0.0275000
11886.0000000	0.0290000
11887.0000000	0.0276190
11888.0000000	0.0290000

11889.0000000	0.0295000
11890.0000000	0.0290476
11891.0000000	0.0285000
11892.0000000	0.0285714
11893.0000000	0.0295000
11894.0000000	0.0285000
11895.0000000	0.0275000
11896.0000000	0.0285000
11897.0000000	0.0290476
11898.0000000	0.0280000
11899.0000000	0.0280952
11900.0000000	0.0275000
11901.0000000	0.0285000
11902.0000000	0.0280952
11903.0000000	0.0265000
11904.0000000	0.0270000
11905.0000000	0.0260000
11906.0000000	0.0252381
11907.0000000	0.0250000
11908.0000000	0.0242857
11909.0000000	0.0275000
11910.0000000	0.0275000
11911.0000000	0.0261905
11912.0000000	0.0250000
11913.0000000	0.0245000
11914.0000000	0.0255000
11915.0000000	0.0266667
11916.0000000	0.0275000
11917.0000000	0.0261905
11918.0000000	0.0245000
11919.0000000	0.0265000
11920.0000000	0.0276190
11921.0000000	0.0250000
11922.0000000	0.0260000
11923.0000000	0.0228571
11924.0000000	0.0255000
11925.0000000	0.0260000
11926.0000000	0.0255000
11927.0000000	0.0252381
11928.0000000	0.0255000
11929.0000000	0.0266667
11930.0000000	0.0260000
11931.0000000	0.0260000
11932.0000000	0.0285714
11933.0000000	0.0270000
11934.0000000	0.0275000
11935.0000000	0.0275000
11936.0000000	0.0266667
11937.0000000	0.0280000
11938.0000000	0.0280952
11939.0000000	0.0275000
11940.0000000	0.0275000
11941.0000000	0.0275000
11942.0000000	0.0285714
11943.0000000	0.0280000
11944.0000000	0.0280952
11945.0000000	0.0265000

11946.0000000	0.0280000
11947.0000000	0.0285714
11948.0000000	0.0260000
11949.0000000	0.0275000
11950.0000000	0.0270000
11951.0000000	0.0257143
11952.0000000	0.0265000
11953.0000000	0.0276190
11954.0000000	0.0265000
11955.0000000	0.0260000
11956.0000000	0.0252381
11957.0000000	0.0260000
11958.0000000	0.0235000
11959.0000000	0.0255000
11960.0000000	0.0238095
11961.0000000	0.0240000
11962.0000000	0.0242857
11963.0000000	0.0240000
11964.0000000	0.0265000
11965.0000000	0.0265000
11966.0000000	0.0252381
11967.0000000	0.0255000
11968.0000000	0.0252381
11969.0000000	0.0265000
11970.0000000	0.0270000
11971.0000000	0.0265000
11972.0000000	0.0257143
11973.0000000	0.0255000
11974.0000000	0.0280000
11975.0000000	0.0280952
11976.0000000	0.0270000
11977.0000000	0.0295238
11978.0000000	0.0290000
11979.0000000	0.0280000
11980.0000000	0.0295000
11981.0000000	0.0300000
11982.0000000	0.0300000
11983.0000000	0.0290000
11984.0000000	0.0300000
11985.0000000	0.0300000
11986.0000000	0.0300000
11987.0000000	0.0290476
11988.0000000	0.0300000
11989.0000000	0.0300000
11990.0000000	0.0300000
11991.0000000	0.0300000
11992.0000000	0.0300000
11993.0000000	0.0300000
11994.0000000	0.0300000
11995.0000000	0.0300000
11996.0000000	0.0300000
11997.0000000	0.0300000
11998.0000000	0.0300000
11999.0000000	0.0300000
12000.0000000	0.0300000
12001.0000000	0.0300000
12002.0000000	0.0295000

12003.0000000	0.0300000
12004.0000000	0.0300000
12005.0000000	0.0300000
12006.0000000	0.0305263
12007.0000000	0.0300000
12008.0000000	0.0300000
12009.0000000	0.0300000
12010.0000000	0.0300000
12011.0000000	0.0300000
12012.0000000	0.0304762
12013.0000000	0.0300000
12014.0000000	0.0300000
12015.0000000	0.0300000
12016.0000000	0.0300000
12017.0000000	0.0300000
12018.0000000	0.0300000
12019.0000000	0.0300000
12020.0000000	0.0300000
12021.0000000	0.0300000
12022.0000000	0.0300000
12023.0000000	0.0300000
12024.0000000	0.0300000
12025.0000000	0.0300000
12026.0000000	0.0300000
12027.0000000	0.0300000
12028.0000000	0.0300000
12029.0000000	0.0300000
12030.0000000	0.0300000
12031.0000000	0.0300000
12032.0000000	0.0300000
12033.0000000	0.0300000
12034.0000000	0.0300000
12035.0000000	0.0300000
12036.0000000	0.0300000
12037.0000000	0.0300000
12038.0000000	0.0300000
12039.0000000	0.0300000
12040.0000000	0.0300000
12041.0000000	0.0300000
12042.0000000	0.0300000
12043.0000000	0.0300000
12044.0000000	0.0300000
12045.0000000	0.0300000
12046.0000000	0.0295000
12047.0000000	0.0300000
12048.0000000	0.0309524
12049.0000000	0.0300000
12050.0000000	0.0300000
12051.0000000	0.0305000
12052.0000000	0.0300000
12053.0000000	0.0300000
12054.0000000	0.0300000
12055.0000000	0.0300000
12056.0000000	0.0300000
12057.0000000	0.0300000
12058.0000000	0.0300000
12059.0000000	0.0300000

12060.0000000	0.0300000
12061.0000000	0.0310000
12062.0000000	0.0300000
12063.0000000	0.0300000
12064.0000000	0.0300000
12065.0000000	0.0300000
12066.0000000	0.0300000
12067.0000000	0.0300000
12068.0000000	0.0300000
12069.0000000	0.0305000
12070.0000000	0.0300000
12071.0000000	0.0305000
12072.0000000	0.0300000
12073.0000000	0.0300000
12074.0000000	0.0300000
12075.0000000	0.0300000
12076.0000000	0.0305000
12077.0000000	0.0309524
12078.0000000	0.0300000
12079.0000000	0.0300000
12080.0000000	0.0304762
12081.0000000	0.0305000
12082.0000000	0.0300000
12083.0000000	0.0304762
12084.0000000	0.0300000
12085.0000000	0.0300000
12086.0000000	0.0310000
12087.0000000	0.0300000
12088.0000000	0.0300000
12089.0000000	0.0304762
12090.0000000	0.0305000
12091.0000000	0.0305000
12092.0000000	0.0300000
12093.0000000	0.0300000
12094.0000000	0.0305000
12095.0000000	0.0300000
12096.0000000	0.0314286
12097.0000000	0.0305000
12098.0000000	0.0304762
12099.0000000	0.0300000
12100.0000000	0.0300000
12101.0000000	0.0300000
12102.0000000	0.0304762
12103.0000000	0.0310000
12104.0000000	0.0304762
12105.0000000	0.0315000
12106.0000000	0.0305000
12107.0000000	0.0300000
12108.0000000	0.0304762
12109.0000000	0.0300000
12110.0000000	0.0300000
12111.0000000	0.0304762
12112.0000000	0.0300000
12113.0000000	0.0304762
12114.0000000	0.0300000
12115.0000000	0.0305000
12116.0000000	0.0300000

12117.0000000	0.0304762
12118.0000000	0.0300000
12119.0000000	0.0300000
12120.0000000	0.0300000
12121.0000000	0.0300000
12122.0000000	0.0300000
12123.0000000	0.0300000
12124.0000000	0.0300000
12125.0000000	0.0300000
12126.0000000	0.0300000
12127.0000000	0.0300000
12128.0000000	0.0300000
12129.0000000	0.0300000
12130.0000000	0.0300000
12131.0000000	0.0300000
12132.0000000	0.0300000
12133.0000000	0.0305000
12134.0000000	0.0304762
12135.0000000	0.0300000
12136.0000000	0.0300000
12137.0000000	0.0300000
12138.0000000	0.0300000
12139.0000000	0.0300000
12140.0000000	0.0305000
12141.0000000	0.0300000
12142.0000000	0.0305000
12143.0000000	0.0300000
12144.0000000	0.0305000
12145.0000000	0.0305000
12146.0000000	0.0305000
12147.0000000	0.0300000
12148.0000000	0.0300000
12149.0000000	0.0300000
12150.0000000	0.0300000
12151.0000000	0.0300000
12152.0000000	0.0305000
12153.0000000	0.0314286
12154.0000000	0.0305000
12155.0000000	0.0305000
12156.0000000	0.0300000
12157.0000000	0.0310000
12158.0000000	0.0305000
12159.0000000	0.0319048
12160.0000000	0.0305000
12161.0000000	0.0309524
12162.0000000	0.0305000
12163.0000000	0.0320000
12164.0000000	0.0315000
12165.0000000	0.0314286
12166.0000000	0.0305000
12167.0000000	0.0310000
12168.0000000	0.0314286
12169.0000000	0.0300000
12170.0000000	0.0315000
12171.0000000	0.0328571
12172.0000000	0.0315000
12173.0000000	0.0314286

12174.0000000	0.0335000
12175.0000000	0.0330000
12176.0000000	0.0320000
12177.0000000	0.0314286
12178.0000000	0.0310000
12179.0000000	0.0310000
12180.0000000	0.0309524
12181.0000000	0.0305000
12182.0000000	0.0320000
12183.0000000	0.0320000
12184.0000000	0.0304762
12185.0000000	0.0305000
12186.0000000	0.0309524
12187.0000000	0.0300000
12188.0000000	0.0310000
12189.0000000	0.0309524
12190.0000000	0.0315000
12191.0000000	0.0305000
12192.0000000	0.0309524
12193.0000000	0.0325000
12194.0000000	0.0315000
12195.0000000	0.0309524
12196.0000000	0.0310000
12197.0000000	0.0310000
12198.0000000	0.0315000
12199.0000000	0.0309524
12200.0000000	0.0325000
12201.0000000	0.0323809
12202.0000000	0.0315000
12203.0000000	0.0320000
12204.0000000	0.0314286
12205.0000000	0.0320000
12206.0000000	0.0320000
12207.0000000	0.0315000
12208.0000000	0.0328571
12209.0000000	0.0320000
12210.0000000	0.0342857
12211.0000000	0.0320000
12212.0000000	0.0320000
12213.0000000	0.0342857
12214.0000000	0.0325000
12215.0000000	0.0315000
12216.0000000	0.0320000
12217.0000000	0.0333333
12218.0000000	0.0300000
12219.0000000	0.0320000
12220.0000000	0.0328571
12221.0000000	0.0310000
12222.0000000	0.0328571
12223.0000000	0.0325000
12224.0000000	0.0320000
12225.0000000	0.0328571
12226.0000000	0.0325000
12227.0000000	0.0305000
12228.0000000	0.0315000
12229.0000000	0.0323809
12230.0000000	0.0340000

12231.0000000	0.0333333
12232.0000000	0.0310000
12233.0000000	0.0330000
12234.0000000	0.0338095
12235.0000000	0.0330000
12236.0000000	0.0330000
12237.0000000	0.0340000
12238.0000000	0.0338095
12239.0000000	0.0340000
12240.0000000	0.0342857
12241.0000000	0.0360000
12242.0000000	0.0330000
12243.0000000	0.0360000
12244.0000000	0.0347619
12245.0000000	0.0350000
12246.0000000	0.0365000
12247.0000000	0.0328571
12248.0000000	0.0330000
12249.0000000	0.0333333
12250.0000000	0.0335000
12251.0000000	0.0350000
12252.0000000	0.0345000
12253.0000000	0.0357143
12254.0000000	0.0345000
12255.0000000	0.0350000
12256.0000000	0.0333333
12257.0000000	0.0350000
12258.0000000	0.0355000
12259.0000000	0.0333333
12260.0000000	0.0345000
12261.0000000	0.0361905
12262.0000000	0.0330000
12263.0000000	0.0370000
12264.0000000	0.0330000
12265.0000000	0.0333333
12266.0000000	0.0345000
12267.0000000	0.0340000
12268.0000000	0.0328571
12269.0000000	0.0325000
12270.0000000	0.0333333
12271.0000000	0.0320000
12272.0000000	0.0320000
12273.0000000	0.0335000
12274.0000000	0.0319048
12275.0000000	0.0315000
12276.0000000	0.0320000
12277.0000000	0.0328571
12278.0000000	0.0315000
12279.0000000	0.0323809
12280.0000000	0.0315000
12281.0000000	0.0300000
12282.0000000	0.0310000
12283.0000000	0.0304762
12284.0000000	0.0300000
12285.0000000	0.0300000
12286.0000000	0.0305000
12287.0000000	0.0305000

12288.0000000	0.0305000
12289.0000000	0.0300000
12290.0000000	0.0310000
12291.0000000	0.0300000
12292.0000000	0.0309524
12293.0000000	0.0305000
12294.0000000	0.0300000
12295.0000000	0.0310000
12296.0000000	0.0305000
12297.0000000	0.0300000
12298.0000000	0.0304762
12299.0000000	0.0305000
12300.0000000	0.0325000
12301.0000000	0.0323809
12302.0000000	0.0310000
12303.0000000	0.0310000
12304.0000000	0.0310000
12305.0000000	0.0304762
12306.0000000	0.0325000
12307.0000000	0.0310000
12308.0000000	0.0323809
12309.0000000	0.0333333
12310.0000000	0.0315000
12311.0000000	0.0310000
12312.0000000	0.0305000
12313.0000000	0.0319048
12314.0000000	0.0320000
12315.0000000	0.0320000
12316.0000000	0.0320000
12317.0000000	0.0309524
12318.0000000	0.0310000
12319.0000000	0.0323809
12320.0000000	0.0335000
12321.0000000	0.0320000
12322.0000000	0.0328571
12323.0000000	0.0330000
12324.0000000	0.0350000
12325.0000000	0.0328571
12326.0000000	0.0350000
12327.0000000	0.0345000
12328.0000000	0.0342857
12329.0000000	0.0345000
12330.0000000	0.0323810
12331.0000000	0.0335000
12332.0000000	0.0355000
12333.0000000	0.0320000
12334.0000000	0.0335000
12335.0000000	0.0342857
12336.0000000	0.0320000
12337.0000000	0.0347619
12338.0000000	0.0340000
12339.0000000	0.0342857
12340.0000000	0.0325000
12341.0000000	0.0340000
12342.0000000	0.0330000
12343.0000000	0.0350000
12344.0000000	0.0328571

12345.0000000	0.0340000
12346.0000000	0.0333333
12347.0000000	0.0320000
12348.0000000	0.0360000
12349.0000000	0.0328571
12350.0000000	0.0335000
12351.0000000	0.0340000
12352.0000000	0.0320000
12353.0000000	0.0357143
12354.0000000	0.0335000
12355.0000000	0.0342857
12356.0000000	0.0345000
12357.0000000	0.0335000
12358.0000000	0.0338095
12359.0000000	0.0330000
12360.0000000	0.0330000
12361.0000000	0.0300000
12362.0000000	0.0320000
12363.0000000	0.0320000
12364.0000000	0.0315000
12365.0000000	0.0323809
12366.0000000	0.0314286
12367.0000000	0.0310000
12368.0000000	0.0300000
12369.0000000	0.0305000
12370.0000000	0.0309524
12371.0000000	0.0300000
12372.0000000	0.0305000
12373.0000000	0.0309524
12374.0000000	0.0305000
12375.0000000	0.0310000
12376.0000000	0.0300000
12377.0000000	0.0300000
12378.0000000	0.0305000
12379.0000000	0.0305000
12380.0000000	0.0309524
12381.0000000	0.0305000
12382.0000000	0.0319048
12383.0000000	0.0300000
12384.0000000	0.0305000
12385.0000000	0.0304762
12386.0000000	0.0305000
12387.0000000	0.0300000
12388.0000000	0.0305000
12389.0000000	0.0300000
12390.0000000	0.0300000
12391.0000000	0.0300000
12392.0000000	0.0310000
12393.0000000	0.0300000
12394.0000000	0.0310000
12395.0000000	0.0300000
12396.0000000	0.0300000
12397.0000000	0.0300000
12398.0000000	0.0310000
12399.0000000	0.0315000
12400.0000000	0.0304762
12401.0000000	0.0305000

12402.0000000	0.0305000
12403.0000000	0.0310000
12404.0000000	0.0305000
12405.0000000	0.0309524
12406.0000000	0.0304762
12407.0000000	0.0310000
12408.0000000	0.0320000
12409.0000000	0.0305000
12410.0000000	0.0304762
12411.0000000	0.0315000
12412.0000000	0.0310000
12413.0000000	0.0304762
12414.0000000	0.0310000
12415.0000000	0.0309524
12416.0000000	0.0305000
12417.0000000	0.0315000
12418.0000000	0.0309524
12419.0000000	0.0320000
12420.0000000	0.0310000
12421.0000000	0.0314286
12422.0000000	0.0305000
12423.0000000	0.0305000
12424.0000000	0.0305000
12425.0000000	0.0319048
12426.0000000	0.0310000
12427.0000000	0.0314286
12428.0000000	0.0300000
12429.0000000	0.0305000
12430.0000000	0.0323809
12431.0000000	0.0325000
12432.0000000	0.0310000
12433.0000000	0.0315000
12434.0000000	0.0300000
12435.0000000	0.0310000
12436.0000000	0.0328571
12437.0000000	0.0325000
12438.0000000	0.0315000
12439.0000000	0.0310000
12440.0000000	0.0335000
12441.0000000	0.0309524
12442.0000000	0.0310000
12443.0000000	0.0319048
12444.0000000	0.0315000
12445.0000000	0.0314286
12446.0000000	0.0335000
12447.0000000	0.0330000
12448.0000000	0.0335000
12449.0000000	0.0319048
12450.0000000	0.0315000
12451.0000000	0.0320000
12452.0000000	0.0304762
12453.0000000	0.0330000
12454.0000000	0.0309524
12455.0000000	0.0305000
12456.0000000	0.0320000
12457.0000000	0.0310000
12458.0000000	0.0304762

12459.0000000	0.0325000
12460.0000000	0.0315000
12461.0000000	0.0314286
12462.0000000	0.0320000
12463.0000000	0.0320000
12464.0000000	0.0328571
12465.0000000	0.0325000
12466.0000000	0.0342857
12467.0000000	0.0340000
12468.0000000	0.0320000
12469.0000000	0.0325000
12470.0000000	0.0333333
12471.0000000	0.0315000
12472.0000000	0.0310000
12473.0000000	0.0323809
12474.0000000	0.0330000
12475.0000000	0.0328571
12476.0000000	0.0330000
12477.0000000	0.0320000
12478.0000000	0.0345000
12479.0000000	0.0323810
12480.0000000	0.0330000
12481.0000000	0.0325000
12482.0000000	0.0319048
12483.0000000	0.0330000
12484.0000000	0.0320000
12485.0000000	0.0320000
12486.0000000	0.0328571
12487.0000000	0.0310000
12488.0000000	0.0328571
12489.0000000	0.0305000
12490.0000000	0.0319048
12491.0000000	0.0320000
12492.0000000	0.0305000
12493.0000000	0.0300000
12494.0000000	0.0314286
12495.0000000	0.0305000
12496.0000000	0.0310000
12497.0000000	0.0323809
12498.0000000	0.0315000
12499.0000000	0.0325000
12500.0000000	0.0300000
12501.0000000	0.0300000
12502.0000000	0.0305000
12503.0000000	0.0304762
12504.0000000	0.0310000
12505.0000000	0.0305000
12506.0000000	0.0300000
12507.0000000	0.0300000
12508.0000000	0.0305000
12509.0000000	0.0300000
12510.0000000	0.0304762
12511.0000000	0.0320000
12512.0000000	0.0300000
12513.0000000	0.0304762
12514.0000000	0.0300000
12515.0000000	0.0309524

12516.0000000	0.0310000
12517.0000000	0.0310000
12518.0000000	0.0300000
12519.0000000	0.0305000
12520.0000000	0.0305000
12521.0000000	0.0300000
12522.0000000	0.0314286
12523.0000000	0.0300000
12524.0000000	0.0304762
12525.0000000	0.0305000
12526.0000000	0.0300000
12527.0000000	0.0304762
12528.0000000	0.0300000
12529.0000000	0.0305000
12530.0000000	0.0300000
12531.0000000	0.0309524
12532.0000000	0.0305000
12533.0000000	0.0319048
12534.0000000	0.0300000
12535.0000000	0.0300000
12536.0000000	0.0315000
12537.0000000	0.0315000
12538.0000000	0.0300000
12539.0000000	0.0310000
12540.0000000	0.0304762
12541.0000000	0.0305000
12542.0000000	0.0304762
12543.0000000	0.0310000
12544.0000000	0.0315000
12545.0000000	0.0305000
12546.0000000	0.0314286
12547.0000000	0.0315000
12548.0000000	0.0310000
12549.0000000	0.0309524
12550.0000000	0.0310000
12551.0000000	0.0304762
12552.0000000	0.0305000
12553.0000000	0.0315000
12554.0000000	0.0319048
12555.0000000	0.0315000
12556.0000000	0.0315000
12557.0000000	0.0325000
12558.0000000	0.0328571
12559.0000000	0.0310000
12560.0000000	0.0320000
12561.0000000	0.0342857
12562.0000000	0.0335000
12563.0000000	0.0347619
12564.0000000	0.0350000
12565.0000000	0.0340000
12566.0000000	0.0361905
12567.0000000	0.0355000
12568.0000000	0.0360000
12569.0000000	0.0370000
12570.0000000	0.0376191
12571.0000000	0.0357143
12572.0000000	0.0355000

12573.0000000	0.0330000
12574.0000000	0.0350000
12575.0000000	0.0315000
12576.0000000	0.0323809
12577.0000000	0.0325000
12578.0000000	0.0323809
12579.0000000	0.0340000
12580.0000000	0.0335000
12581.0000000	0.0314286
12582.0000000	0.0350000
12583.0000000	0.0335000
12584.0000000	0.0320000
12585.0000000	0.0328571
12586.0000000	0.0325000
12587.0000000	0.0328571
12588.0000000	0.0310000
12589.0000000	0.0315000
12590.0000000	0.0315000
12591.0000000	0.0309524
12592.0000000	0.0305000
12593.0000000	0.0310000
12594.0000000	0.0309524
12595.0000000	0.0310000
12596.0000000	0.0304762
12597.0000000	0.0310000
12598.0000000	0.0300000
12599.0000000	0.0305000
12600.0000000	0.0320000
12601.0000000	0.0309524
12602.0000000	0.0310000
12603.0000000	0.0304762
12604.0000000	0.0310000
12605.0000000	0.0305000
12606.0000000	0.0304762
12607.0000000	0.0305000
12608.0000000	0.0305000
12609.0000000	0.0300000
12610.0000000	0.0304762
12611.0000000	0.0300000
12612.0000000	0.0305000
12613.0000000	0.0305000
12614.0000000	0.0300000
12615.0000000	0.0300000
12616.0000000	0.0315000
12617.0000000	0.0315000
12618.0000000	0.0304762
12619.0000000	0.0300000
12620.0000000	0.0300000
12621.0000000	0.0309524
12622.0000000	0.0300000
12623.0000000	0.0304762
12624.0000000	0.0310000
12625.0000000	0.0300000
12626.0000000	0.0300000
12627.0000000	0.0300000
12628.0000000	0.0300000
12629.0000000	0.0300000

12630.0000000	0.0300000
12631.0000000	0.0310000
12632.0000000	0.0309524
12633.0000000	0.0315000
12634.0000000	0.0315000
12635.0000000	0.0300000
12636.0000000	0.0300000
12637.0000000	0.0304762
12638.0000000	0.0305000
12639.0000000	0.0300000
12640.0000000	0.0310000
12641.0000000	0.0305000
12642.0000000	0.0304762
12643.0000000	0.0305000
12644.0000000	0.0305000
12645.0000000	0.0305000
12646.0000000	0.0300000
12647.0000000	0.0305000
12648.0000000	0.0300000
12649.0000000	0.0310000
12650.0000000	0.0300000
12651.0000000	0.0300000
12652.0000000	0.0300000
12653.0000000	0.0300000
12654.0000000	0.0304762
12655.0000000	0.0300000
12656.0000000	0.0300000
12657.0000000	0.0304762
12658.0000000	0.0300000
12659.0000000	0.0300000
12660.0000000	0.0300000
12661.0000000	0.0300000
12662.0000000	0.0295000
12663.0000000	0.0295238
12664.0000000	0.0300000
12665.0000000	0.0300000
12666.0000000	0.0300000
12667.0000000	0.0300000
12668.0000000	0.0300000
12669.0000000	0.0300000
12670.0000000	0.0300000
12671.0000000	0.0300000
12672.0000000	0.0300000
12673.0000000	0.0300000
12674.0000000	0.0300000
12675.0000000	0.0304762
12676.0000000	0.0300000
12677.0000000	0.0300000
12678.0000000	0.0304762
12679.0000000	0.0300000
12680.0000000	0.0300000
12681.0000000	0.0300000
12682.0000000	0.0300000
12683.0000000	0.0300000
12684.0000000	0.0300000
12685.0000000	0.0300000
12686.0000000	0.0300000

12687.0000000	0.0300000
12688.0000000	0.0310000
12689.0000000	0.0300000
12690.0000000	0.0300000
12691.0000000	0.0300000
12692.0000000	0.0300000
12693.0000000	0.0304762
12694.0000000	0.0300000
12695.0000000	0.0300000
12696.0000000	0.0300000
12697.0000000	0.0300000
12698.0000000	0.0304762
12699.0000000	0.0300000
12700.0000000	0.0300000
12701.0000000	0.0300000
12702.0000000	0.0309524
12703.0000000	0.0305000
12704.0000000	0.0305000
12705.0000000	0.0300000
12706.0000000	0.0304762
12707.0000000	0.0300000
12708.0000000	0.0300000
12709.0000000	0.0300000
12710.0000000	0.0315000
12711.0000000	0.0300000
12712.0000000	0.0300000
12713.0000000	0.0305000
12714.0000000	0.0300000
12715.0000000	0.0305000
12716.0000000	0.0310000
12717.0000000	0.0305000
12718.0000000	0.0304762
12719.0000000	0.0300000
12720.0000000	0.0305000
12721.0000000	0.0304762
12722.0000000	0.0305000
12723.0000000	0.0323809
12724.0000000	0.0315000
12725.0000000	0.0335000
12726.0000000	0.0310000
12727.0000000	0.0304762
12728.0000000	0.0315000
12729.0000000	0.0315000
12730.0000000	0.0319048
12731.0000000	0.0325000
12732.0000000	0.0319048
12733.0000000	0.0310000
12734.0000000	0.0300000
12735.0000000	0.0315000
12736.0000000	0.0314286
12737.0000000	0.0320000
12738.0000000	0.0300000
12739.0000000	0.0304762
12740.0000000	0.0305000
12741.0000000	0.0310000
12742.0000000	0.0314286
12743.0000000	0.0310000

12744.0000000	0.0300000
12745.0000000	0.0304762
12746.0000000	0.0310000
12747.0000000	0.0309524
12748.0000000	0.0310000
12749.0000000	0.0315000
12750.0000000	0.0305000
12751.0000000	0.0328571
12752.0000000	0.0320000
12753.0000000	0.0305000
12754.0000000	0.0304762
12755.0000000	0.0310000
12756.0000000	0.0310000
12757.0000000	0.0309524
12758.0000000	0.0320000
12759.0000000	0.0320000
12760.0000000	0.0319048
12761.0000000	0.0330000
12762.0000000	0.0320000
12763.0000000	0.0323809
12764.0000000	0.0340000
12765.0000000	0.0340000
12766.0000000	0.0314286
12767.0000000	0.0315000
12768.0000000	0.0314286
12769.0000000	0.0310000
12770.0000000	0.0305000
12771.0000000	0.0315000
12772.0000000	0.0300000
12773.0000000	0.0310000
12774.0000000	0.0315000
12775.0000000	0.0314286
12776.0000000	0.0330000
12777.0000000	0.0319048
12778.0000000	0.0325000
12779.0000000	0.0310000
12780.0000000	0.0330000
12781.0000000	0.0333333
12782.0000000	0.0340000
12783.0000000	0.0330000
12784.0000000	0.0338095
12785.0000000	0.0345000
12786.0000000	0.0325000
12787.0000000	0.0319048
12788.0000000	0.0360000
12789.0000000	0.0340000
12790.0000000	0.0333333
12791.0000000	0.0355000
12792.0000000	0.0330000
12793.0000000	0.0352381
12794.0000000	0.0340000
12795.0000000	0.0340000
12796.0000000	0.0352381
12797.0000000	0.0340000
12798.0000000	0.0350000
12799.0000000	0.0347619
12800.0000000	0.0350000

12801.0000000	0.0355000
12802.0000000	0.0345000
12803.0000000	0.0342857
12804.0000000	0.0335000
12805.0000000	0.0330000
12806.0000000	0.0323809
12807.0000000	0.0328571
12808.0000000	0.0330000
12809.0000000	0.0300000
12810.0000000	0.0320000
12811.0000000	0.0323809
12812.0000000	0.0315000
12813.0000000	0.0310000
12814.0000000	0.0310000
12815.0000000	0.0328571
12816.0000000	0.0310000
12817.0000000	0.0314286
12818.0000000	0.0320000
12819.0000000	0.0315000
12820.0000000	0.0319048
12821.0000000	0.0330000
12822.0000000	0.0330000
12823.0000000	0.0323809
12824.0000000	0.0315000
12825.0000000	0.0335000
12826.0000000	0.0320000
12827.0000000	0.0304762
12828.0000000	0.0320000
12829.0000000	0.0314286
12830.0000000	0.0330000
12831.0000000	0.0320000
12832.0000000	0.0310000
12833.0000000	0.0333333
12834.0000000	0.0310000
12835.0000000	0.0309524
12836.0000000	0.0325000
12837.0000000	0.0305000
12838.0000000	0.0314286
12839.0000000	0.0315000
12840.0000000	0.0330000
12841.0000000	0.0320000
12842.0000000	0.0314286
12843.0000000	0.0300000
12844.0000000	0.0310000
12845.0000000	0.0309524
12846.0000000	0.0320000
12847.0000000	0.0309524
12848.0000000	0.0310000
12849.0000000	0.0330000
12850.0000000	0.0320000
12851.0000000	0.0323809
12852.0000000	0.0315000
12853.0000000	0.0325000
12854.0000000	0.0328571
12855.0000000	0.0335000
12856.0000000	0.0314286
12857.0000000	0.0315000

12858.0000000	0.0320000
12859.0000000	0.0319048
12860.0000000	0.0310000
12861.0000000	0.0335000
12862.0000000	0.0330000
12863.0000000	0.0333333
12864.0000000	0.0325000
12865.0000000	0.0323809
12866.0000000	0.0320000
12867.0000000	0.0325000
12868.0000000	0.0314286
12869.0000000	0.0315000
12870.0000000	0.0315000
12871.0000000	0.0310000
12872.0000000	0.0309524
12873.0000000	0.0315000
12874.0000000	0.0314286
12875.0000000	0.0320000
12876.0000000	0.0315000
12877.0000000	0.0320000
12878.0000000	0.0314286
12879.0000000	0.0320000
12880.0000000	0.0310000
12881.0000000	0.0319048
12882.0000000	0.0320000
12883.0000000	0.0309524
12884.0000000	0.0320000
12885.0000000	0.0335000
12886.0000000	0.0325000
12887.0000000	0.0314286
12888.0000000	0.0320000
12889.0000000	0.0325000
12890.0000000	0.0309524
12891.0000000	0.0310000
12892.0000000	0.0310000
12893.0000000	0.0309524
12894.0000000	0.0305000
12895.0000000	0.0314286
12896.0000000	0.0315000
12897.0000000	0.0310000
12898.0000000	0.0305000
12899.0000000	0.0309524
12900.0000000	0.0305000
12901.0000000	0.0320000
12902.0000000	0.0305000
12903.0000000	0.0319048
12904.0000000	0.0309524
12905.0000000	0.0310000
12906.0000000	0.0325000
12907.0000000	0.0310000
12908.0000000	0.0319048
12909.0000000	0.0325000
12910.0000000	0.0310000
12911.0000000	0.0314286
12912.0000000	0.0320000
12913.0000000	0.0323809
12914.0000000	0.0330000

12915.0000000	0.0325000
12916.0000000	0.0325000
12917.0000000	0.0314286
12918.0000000	0.0330000
12919.0000000	0.0319048
12920.0000000	0.0315000
12921.0000000	0.0320000
12922.0000000	0.0320000
12923.0000000	0.0314286
12924.0000000	0.0310000
12925.0000000	0.0315000
12926.0000000	0.0314286
12927.0000000	0.0315000
12928.0000000	0.0314286
12929.0000000	0.0310000
12930.0000000	0.0310000
12931.0000000	0.0305000
12932.0000000	0.0300000
12933.0000000	0.0314286
12934.0000000	0.0300000
12935.0000000	0.0304762
12936.0000000	0.0300000
12937.0000000	0.0300000
12938.0000000	0.0305000
12939.0000000	0.0304762
12940.0000000	0.0300000
12941.0000000	0.0300000
12942.0000000	0.0300000
12943.0000000	0.0300000
12944.0000000	0.0304762
12945.0000000	0.0305000
12946.0000000	0.0305000
12947.0000000	0.0300000
12948.0000000	0.0300000
12949.0000000	0.0300000
12950.0000000	0.0300000
12951.0000000	0.0300000
12952.0000000	0.0300000
12953.0000000	0.0300000
12954.0000000	0.0300000
12955.0000000	0.0300000
12956.0000000	0.0300000
12957.0000000	0.0305000
12958.0000000	0.0305000
12959.0000000	0.0304762
12960.0000000	0.0300000
12961.0000000	0.0300000
12962.0000000	0.0300000
12963.0000000	0.0305000
12964.0000000	0.0309524
12965.0000000	0.0310000
12966.0000000	0.0300000
12967.0000000	0.0305000
12968.0000000	0.0300000
12969.0000000	0.0300000
12970.0000000	0.0300000
12971.0000000	0.0300000

12972.0000000	0.0300000
12973.0000000	0.0300000
12974.0000000	0.0305000
12975.0000000	0.0300000
12976.0000000	0.0300000
12977.0000000	0.0300000
12978.0000000	0.0300000
12979.0000000	0.0300000
12980.0000000	0.0300000
12981.0000000	0.0300000
12982.0000000	0.0300000
12983.0000000	0.0300000
12984.0000000	0.0300000
12985.0000000	0.0300000
12986.0000000	0.0300000
12987.0000000	0.0300000
12988.0000000	0.0300000
12989.0000000	0.0300000
12990.0000000	0.0300000
12991.0000000	0.0300000
12992.0000000	0.0295238
12993.0000000	0.0300000
12994.0000000	0.0300000
12995.0000000	0.0300000
12996.0000000	0.0300000
12997.0000000	0.0295000
12998.0000000	0.0300000
12999.0000000	0.0300000
13000.0000000	0.0300000
13001.0000000	0.0300000
13002.0000000	0.0300000
13003.0000000	0.0300000
13004.0000000	0.0300000
13005.0000000	0.0300000
13006.0000000	0.0300000
13007.0000000	0.0300000
13008.0000000	0.0300000
13009.0000000	0.0295000
13010.0000000	0.0300000
13011.0000000	0.0295238
13012.0000000	0.0300000
13013.0000000	0.0300000
13014.0000000	0.0300000
13015.0000000	0.0300000
13016.0000000	0.0300000
13017.0000000	0.0300000
13018.0000000	0.0305000
13019.0000000	0.0300000
13020.0000000	0.0300000
13021.0000000	0.0300000
13022.0000000	0.0300000
13023.0000000	0.0309524
13024.0000000	0.0300000
13025.0000000	0.0300000
13026.0000000	0.0300000
13027.0000000	0.0305000
13028.0000000	0.0300000

13029.0000000	0.0300000
13030.0000000	0.0300000
13031.0000000	0.0300000
13032.0000000	0.0304762
13033.0000000	0.0300000
13034.0000000	0.0300000
13035.0000000	0.0300000
13036.0000000	0.0305000
13037.0000000	0.0300000
13038.0000000	0.0315000
13039.0000000	0.0300000
13040.0000000	0.0300000
13041.0000000	0.0300000
13042.0000000	0.0305000
13043.0000000	0.0300000
13044.0000000	0.0304762
13045.0000000	0.0315000
13046.0000000	0.0300000
13047.0000000	0.0300000
13048.0000000	0.0310000
13049.0000000	0.0305000
13050.0000000	0.0309524
13051.0000000	0.0305000
13052.0000000	0.0304762
13053.0000000	0.0320000
13054.0000000	0.0305000
13055.0000000	0.0305000
13056.0000000	0.0309524
13057.0000000	0.0300000
13058.0000000	0.0310000
13059.0000000	0.0323809
13060.0000000	0.0315000
13061.0000000	0.0309524
13062.0000000	0.0300000
13063.0000000	0.0305000
13064.0000000	0.0314286
13065.0000000	0.0300000
13066.0000000	0.0300000
13067.0000000	0.0300000
13068.0000000	0.0300000
13069.0000000	0.0325000
13070.0000000	0.0304762
13071.0000000	0.0320000
13072.0000000	0.0310000
13073.0000000	0.0320000
13074.0000000	0.0315000
13075.0000000	0.0300000
13076.0000000	0.0300000
13077.0000000	0.0300000
13078.0000000	0.0310000
13079.0000000	0.0304762
13080.0000000	0.0315000
13081.0000000	0.0305000
13082.0000000	0.0305000
13083.0000000	0.0314286
13084.0000000	0.0320000
13085.0000000	0.0325000

13086.0000000	0.0314286
13087.0000000	0.0305000
13088.0000000	0.0305000
13089.0000000	0.0309524
13090.0000000	0.0305000
13091.0000000	0.0310000
13092.0000000	0.0309524
13093.0000000	0.0325000
13094.0000000	0.0315000
13095.0000000	0.0333333
13096.0000000	0.0325000
13097.0000000	0.0310000
13098.0000000	0.0323809
13099.0000000	0.0330000
13100.0000000	0.0328571
13101.0000000	0.0320000
13102.0000000	0.0315000
13103.0000000	0.0345000
13104.0000000	0.0328571
13105.0000000	0.0330000
13106.0000000	0.0335000
13107.0000000	0.0350000
13108.0000000	0.0328571
13109.0000000	0.0347619
13110.0000000	0.0345000
13111.0000000	0.0345000
13112.0000000	0.0360000
13113.0000000	0.0366667
13114.0000000	0.0350000
13115.0000000	0.0370000
13116.0000000	0.0376190
13117.0000000	0.0375000
13118.0000000	0.0390000
13119.0000000	0.0370000
13120.0000000	0.0357143
13121.0000000	0.0385000
13122.0000000	0.0361905
13123.0000000	0.0385000
13124.0000000	0.0370000
13125.0000000	0.0376191
13126.0000000	0.0380000
13127.0000000	0.0380000
13128.0000000	0.0385714
13129.0000000	0.0375000
13130.0000000	0.0365000
13131.0000000	0.0366667
13132.0000000	0.0380000
13133.0000000	0.0360000
13134.0000000	0.0370000
13135.0000000	0.0366667
13136.0000000	0.0380000
13137.0000000	0.0376191
13138.0000000	0.0385000
13139.0000000	0.0380000
13140.0000000	0.0385714
13141.0000000	0.0390000
13142.0000000	0.0395000

13143.0000000	0.0380000
13144.0000000	0.0380952
13145.0000000	0.0375000
13146.0000000	0.0370000
13147.0000000	0.0357143
13148.0000000	0.0360000
13149.0000000	0.0366667
13150.0000000	0.0350000
13151.0000000	0.0350000
13152.0000000	0.0347619
13153.0000000	0.0360000
13154.0000000	0.0330000
13155.0000000	0.0345000
13156.0000000	0.0338095
13157.0000000	0.0340000
13158.0000000	0.0347619
13159.0000000	0.0340000
13160.0000000	0.0365000
13161.0000000	0.0338095
13162.0000000	0.0345000
13163.0000000	0.0325000
13164.0000000	0.0340000
13165.0000000	0.0342857
13166.0000000	0.0310000
13167.0000000	0.0328571
13168.0000000	0.0335000
13169.0000000	0.0360000
13170.0000000	0.0319048
13171.0000000	0.0325000
13172.0000000	0.0365000
13173.0000000	0.0350000
13174.0000000	0.0319048
13175.0000000	0.0375000
13176.0000000	0.0357143
13177.0000000	0.0350000
13178.0000000	0.0360000
13179.0000000	0.0350000
13180.0000000	0.0338095
13181.0000000	0.0350000
13182.0000000	0.0360000
13183.0000000	0.0342857
13184.0000000	0.0365000
13185.0000000	0.0357143
13186.0000000	0.0360000
13187.0000000	0.0375000
13188.0000000	0.0352381
13189.0000000	0.0380000
13190.0000000	0.0365000
13191.0000000	0.0375000
13192.0000000	0.0395238
13193.0000000	0.0380000
13194.0000000	0.0390000
13195.0000000	0.0395000
13196.0000000	0.0390476
13197.0000000	0.0395238
13198.0000000	0.0380000
13199.0000000	0.0385000

13200.0000000	0.0390476
13201.0000000	0.0390000
13202.0000000	0.0395000
13203.0000000	0.0400000
13204.0000000	0.0400000
13205.0000000	0.0385000
13206.0000000	0.0400000
13207.0000000	0.0400000
13208.0000000	0.0395000
13209.0000000	0.0395000
13210.0000000	0.0390476
13211.0000000	0.0400000
13212.0000000	0.0395000
13213.0000000	0.0400000
13214.0000000	0.0400000
13215.0000000	0.0395000
13216.0000000	0.0400000
13217.0000000	0.0400000
13218.0000000	0.0400000
13219.0000000	0.0385714
13220.0000000	0.0390000
13221.0000000	0.0400000
13222.0000000	0.0400000
13223.0000000	0.0395000
13224.0000000	0.0400000
13225.0000000	0.0395238
13226.0000000	0.0395000
13227.0000000	0.0390000
13228.0000000	0.0390476
13229.0000000	0.0400000
13230.0000000	0.0395238
13231.0000000	0.0390000
13232.0000000	0.0395000
13233.0000000	0.0400000
13234.0000000	0.0395000
13235.0000000	0.0395238
13236.0000000	0.0400000
13237.0000000	0.0390476
13238.0000000	0.0390000
13239.0000000	0.0395000
13240.0000000	0.0395238
13241.0000000	0.0400000
13242.0000000	0.0390000
13243.0000000	0.0390000
13244.0000000	0.0395238
13245.0000000	0.0395238
13246.0000000	0.0385000
13247.0000000	0.0385000
13248.0000000	0.0395000
13249.0000000	0.0390476
13250.0000000	0.0400000
13251.0000000	0.0390000
13252.0000000	0.0400000
13253.0000000	0.0400000
13254.0000000	0.0395000
13255.0000000	0.0390476
13256.0000000	0.0390000

13257.0000000	0.0390476
13258.0000000	0.0395000
13259.0000000	0.0390000
13260.0000000	0.0400000
13261.0000000	0.0400000
13262.0000000	0.0390000
13263.0000000	0.0400000
13264.0000000	0.0385714
13265.0000000	0.0395000
13266.0000000	0.0395238
13267.0000000	0.0390000
13268.0000000	0.0380000
13269.0000000	0.0400000
13270.0000000	0.0380000
13271.0000000	0.0395238
13272.0000000	0.0385000
13273.0000000	0.0380952
13274.0000000	0.0380000
13275.0000000	0.0395238
13276.0000000	0.0375000
13277.0000000	0.0375000
13278.0000000	0.0375000
13279.0000000	0.0380000
13280.0000000	0.0385714
13281.0000000	0.0370000
13282.0000000	0.0376191
13283.0000000	0.0375000
13284.0000000	0.0370000
13285.0000000	0.0371429
13286.0000000	0.0380000
13287.0000000	0.0375000
13288.0000000	0.0366667
13289.0000000	0.0365000
13290.0000000	0.0365000
13291.0000000	0.0371429
13292.0000000	0.0375000
13293.0000000	0.0360000
13294.0000000	0.0385714
13295.0000000	0.0375000
13296.0000000	0.0385000
13297.0000000	0.0390476
13298.0000000	0.0380000
13299.0000000	0.0390000
13300.0000000	0.0375000
13301.0000000	0.0390476
13302.0000000	0.0390000
13303.0000000	0.0385714
13304.0000000	0.0380000
13305.0000000	0.0385714
13306.0000000	0.0370000
13307.0000000	0.0400000
13308.0000000	0.0390000
13309.0000000	0.0395238
13310.0000000	0.0395000
13311.0000000	0.0400000
13312.0000000	0.0390000
13313.0000000	0.0390476

13314.0000000	0.0395000
13315.0000000	0.0385000
13316.0000000	0.0376190
13317.0000000	0.0395000
13318.0000000	0.0400000
13319.0000000	0.0390000
13320.0000000	0.0385000
13321.0000000	0.0390476
13322.0000000	0.0380000
13323.0000000	0.0395000
13324.0000000	0.0395000
13325.0000000	0.0395238
13326.0000000	0.0385000
13327.0000000	0.0395238
13328.0000000	0.0395000
13329.0000000	0.0370000
13330.0000000	0.0390000
13331.0000000	0.0375000
13332.0000000	0.0385714
13333.0000000	0.0380952
13334.0000000	0.0390000
13335.0000000	0.0395000
13336.0000000	0.0395238
13337.0000000	0.0390000
13338.0000000	0.0385000
13339.0000000	0.0375000
13340.0000000	0.0371429
13341.0000000	0.0380000
13342.0000000	0.0385714
13343.0000000	0.0365000
13344.0000000	0.0345000
13345.0000000	0.0333333
13346.0000000	0.0330000
13347.0000000	0.0340000
13348.0000000	0.0320000
13349.0000000	0.0328571
13350.0000000	0.0310000
13351.0000000	0.0340000
13352.0000000	0.0338095
13353.0000000	0.0320000
13354.0000000	0.0340000
13355.0000000	0.0342857
13356.0000000	0.0345000
13357.0000000	0.0328571
13358.0000000	0.0335000
13359.0000000	0.0355000
13360.0000000	0.0310000
13361.0000000	0.0314286
13362.0000000	0.0345000
13363.0000000	0.0320000
13364.0000000	0.0342857
13365.0000000	0.0325000
13366.0000000	0.0338095
13367.0000000	0.0345000
13368.0000000	0.0320000
13369.0000000	0.0320000
13370.0000000	0.0342857

13371.0000000	0.0335000
13372.0000000	0.0345000
13373.0000000	0.0347619
13374.0000000	0.0345000
13375.0000000	0.0320000
13376.0000000	0.0338095
13377.0000000	0.0350000
13378.0000000	0.0340000
13379.0000000	0.0347619
13380.0000000	0.0355000
13381.0000000	0.0352381
13382.0000000	0.0350000
13383.0000000	0.0365000
13384.0000000	0.0345000
13385.0000000	0.0352381
13386.0000000	0.0375000
13387.0000000	0.0350000
13388.0000000	0.0361905
13389.0000000	0.0355000
13390.0000000	0.0345000
13391.0000000	0.0357143
13392.0000000	0.0385000
13393.0000000	0.0366667
13394.0000000	0.0360000
13395.0000000	0.0360000
13396.0000000	0.0360000
13397.0000000	0.0357143
13398.0000000	0.0360000
13399.0000000	0.0355000
13400.0000000	0.0360000
13401.0000000	0.0361905
13402.0000000	0.0357143
13403.0000000	0.0355000
13404.0000000	0.0360000
13405.0000000	0.0365000
13406.0000000	0.0380952
13407.0000000	0.0375000
13408.0000000	0.0380000
13409.0000000	0.0390476
13410.0000000	0.0385000
13411.0000000	0.0385714
13412.0000000	0.0385000
13413.0000000	0.0395000
13414.0000000	0.0380000
13415.0000000	0.0395238
13416.0000000	0.0370000
13417.0000000	0.0385000
13418.0000000	0.0385714
13419.0000000	0.0380000
13420.0000000	0.0395000
13421.0000000	0.0380952
13422.0000000	0.0365000
13423.0000000	0.0380000
13424.0000000	0.0376190
13425.0000000	0.0385000
13426.0000000	0.0395000
13427.0000000	0.0385714

13428.0000000	0.0390000
13429.0000000	0.0400000
13430.0000000	0.0380952
13431.0000000	0.0400000
13432.0000000	0.0395000
13433.0000000	0.0395238
13434.0000000	0.0395000
13435.0000000	0.0400000
13436.0000000	0.0400000
13437.0000000	0.0390476
13438.0000000	0.0400000
13439.0000000	0.0395000
13440.0000000	0.0390476
13441.0000000	0.0400000
13442.0000000	0.0400000
13443.0000000	0.0395000
13444.0000000	0.0400000
13445.0000000	0.0395238
13446.0000000	0.0400000
13447.0000000	0.0400000
13448.0000000	0.0400000
13449.0000000	0.0400000
13450.0000000	0.0400000
13451.0000000	0.0400000
13452.0000000	0.0400000
13453.0000000	0.0400000
13454.0000000	0.0400000
13455.0000000	0.0400000
13456.0000000	0.0400000
13457.0000000	0.0400000
13458.0000000	0.0400000
13459.0000000	0.0400000
13460.0000000	0.0400000
13461.0000000	0.0400000
13462.0000000	0.0400000
13463.0000000	0.0400000
13464.0000000	0.0400000
13465.0000000	0.0400000
13466.0000000	0.0400000
13467.0000000	0.0400000
13468.0000000	0.0400000
13469.0000000	0.0400000
13470.0000000	0.0400000
13471.0000000	0.0400000
13472.0000000	0.0395238
13473.0000000	0.0400000
13474.0000000	0.0400000
13475.0000000	0.0400000
13476.0000000	0.0400000
13477.0000000	0.0400000
13478.0000000	0.0400000
13479.0000000	0.0400000
13480.0000000	0.0400000
13481.0000000	0.0400000
13482.0000000	0.0400000
13483.0000000	0.0400000
13484.0000000	0.0400000

13485.0000000	0.0400000
13486.0000000	0.0400000
13487.0000000	0.0400000
13488.0000000	0.0400000
13489.0000000	0.0400000
13490.0000000	0.0400000
13491.0000000	0.0400000
13492.0000000	0.0400000
13493.0000000	0.0400000
13494.0000000	0.0395000
13495.0000000	0.0395000
13496.0000000	0.0400000
13497.0000000	0.0400000
13498.0000000	0.0400000
13499.0000000	0.0400000
13500.0000000	0.0400000
13501.0000000	0.0400000
13502.0000000	0.0400000
13503.0000000	0.0395000
13504.0000000	0.0400000
13505.0000000	0.0400000
13506.0000000	0.0400000
13507.0000000	0.0400000
13508.0000000	0.0400000
13509.0000000	0.0400000
13510.0000000	0.0400000
13511.0000000	0.0400000
13512.0000000	0.0400000
13513.0000000	0.0395000
13514.0000000	0.0395000
13515.0000000	0.0400000
13516.0000000	0.0400000
13517.0000000	0.0400000
13518.0000000	0.0400000
13519.0000000	0.0400000
13520.0000000	0.0400000
13521.0000000	0.0400000
13522.0000000	0.0400000
13523.0000000	0.0395000
13524.0000000	0.0395238
13525.0000000	0.0395000
13526.0000000	0.0395000
13527.0000000	0.0390000
13528.0000000	0.0395238
13529.0000000	0.0390000
13530.0000000	0.0400000
13531.0000000	0.0390000
13532.0000000	0.0395000
13533.0000000	0.0395238
13534.0000000	0.0400000
13535.0000000	0.0400000
13536.0000000	0.0395000
13537.0000000	0.0390476
13538.0000000	0.0390476
13539.0000000	0.0400000
13540.0000000	0.0395000
13541.0000000	0.0395000

13542.0000000	0.0395238
13543.0000000	0.0400000
13544.0000000	0.0400000
13545.0000000	0.0385714
13546.0000000	0.0395000
13547.0000000	0.0400000
13548.0000000	0.0400000
13549.0000000	0.0400000
13550.0000000	0.0400000
13551.0000000	0.0395238
13552.0000000	0.0395000
13553.0000000	0.0400000
13554.0000000	0.0400000
13555.0000000	0.0400000
13556.0000000	0.0400000
13557.0000000	0.0400000
13558.0000000	0.0400000
13559.0000000	0.0400000
13560.0000000	0.0400000
13561.0000000	0.0395000
13562.0000000	0.0400000
13563.0000000	0.0400000
13564.0000000	0.0400000
13565.0000000	0.0400000
13566.0000000	0.0400000
13567.0000000	0.0400000
13568.0000000	0.0400000
13569.0000000	0.0400000
13570.0000000	0.0400000
13571.0000000	0.0400000
13572.0000000	0.0400000
13573.0000000	0.0400000
13574.0000000	0.0400000
13575.0000000	0.0400000
13576.0000000	0.0400000
13577.0000000	0.0400000
13578.0000000	0.0400000
13579.0000000	0.0400000
13580.0000000	0.0400000
13581.0000000	0.0400000
13582.0000000	0.0400000
13583.0000000	0.0400000
13584.0000000	0.0400000
13585.0000000	0.0400000
13586.0000000	0.0400000
13587.0000000	0.0400000
13588.0000000	0.0400000
13589.0000000	0.0400000
13590.0000000	0.0400000
13591.0000000	0.0400000
13592.0000000	0.0400000
13593.0000000	0.0400000
13594.0000000	0.0400000
13595.0000000	0.0405000
13596.0000000	0.0400000
13597.0000000	0.0400000
13598.0000000	0.0400000

13599.0000000	0.0400000
13600.0000000	0.0400000
13601.0000000	0.0410000
13602.0000000	0.0405000
13603.0000000	0.0400000
13604.0000000	0.0400000
13605.0000000	0.0400000
13606.0000000	0.0400000
13607.0000000	0.0404762
13608.0000000	0.0400000
13609.0000000	0.0400000
13610.0000000	0.0405000
13611.0000000	0.0400000
13612.0000000	0.0404762
13613.0000000	0.0400000
13614.0000000	0.0400000
13615.0000000	0.0400000
13616.0000000	0.0405000
13617.0000000	0.0400000
13618.0000000	0.0404762
13619.0000000	0.0400000
13620.0000000	0.0405000
13621.0000000	0.0404762
13622.0000000	0.0405000
13623.0000000	0.0400000
13624.0000000	0.0400000
13625.0000000	0.0400000
13626.0000000	0.0400000
13627.0000000	0.0400000
13628.0000000	0.0400000
13629.0000000	0.0400000
13630.0000000	0.0404762
13631.0000000	0.0405000
13632.0000000	0.0400000
13633.0000000	0.0400000
13634.0000000	0.0410000
13635.0000000	0.0400000
13636.0000000	0.0400000
13637.0000000	0.0410000
13638.0000000	0.0400000
13639.0000000	0.0400000
13640.0000000	0.0405000
13641.0000000	0.0410000
13642.0000000	0.0409524
13643.0000000	0.0410000
13644.0000000	0.0400000
13645.0000000	0.0409524
13646.0000000	0.0400000
13647.0000000	0.0405000
13648.0000000	0.0404762
13649.0000000	0.0400000
13650.0000000	0.0400000
13651.0000000	0.0400000
13652.0000000	0.0405000
13653.0000000	0.0400000
13654.0000000	0.0404762
13655.0000000	0.0400000

13656.0000000	0.0410000
13657.0000000	0.0400000
13658.0000000	0.0405000
13659.0000000	0.0404762
13660.0000000	0.0415000
13661.0000000	0.0400000
13662.0000000	0.0410000
13663.0000000	0.0405000
13664.0000000	0.0400000
13665.0000000	0.0410000
13666.0000000	0.0404762
13667.0000000	0.0410000
13668.0000000	0.0409524
13669.0000000	0.0410000
13670.0000000	0.0420000
13671.0000000	0.0410000
13672.0000000	0.0420000
13673.0000000	0.0428572
13674.0000000	0.0414286
13675.0000000	0.0425000
13676.0000000	0.0435000
13677.0000000	0.0435000
13678.0000000	0.0400000
13679.0000000	0.0415000
13680.0000000	0.0420000
13681.0000000	0.0409524
13682.0000000	0.0410000
13683.0000000	0.0415000
13684.0000000	0.0414286
13685.0000000	0.0420000
13686.0000000	0.0404762
13687.0000000	0.0405000
13688.0000000	0.0410000
13689.0000000	0.0400000
13690.0000000	0.0420000
13691.0000000	0.0404762
13692.0000000	0.0400000
13693.0000000	0.0405000
13694.0000000	0.0404762
13695.0000000	0.0404762
13696.0000000	0.0405000
13697.0000000	0.0400000
13698.0000000	0.0400000
13699.0000000	0.0410000
13700.0000000	0.0405000
13701.0000000	0.0405000
13702.0000000	0.0400000
13703.0000000	0.0400000
13704.0000000	0.0400000
13705.0000000	0.0400000
13706.0000000	0.0410000
13707.0000000	0.0405000
13708.0000000	0.0400000
13709.0000000	0.0400000
13710.0000000	0.0400000
13711.0000000	0.0400000
13712.0000000	0.0400000

13713.0000000	0.0400000
13714.0000000	0.0400000
13715.0000000	0.0400000
13716.0000000	0.0400000
13717.0000000	0.0400000
13718.0000000	0.0400000
13719.0000000	0.0400000
13720.0000000	0.0400000
13721.0000000	0.0400000
13722.0000000	0.0400000
13723.0000000	0.0400000
13724.0000000	0.0400000
13725.0000000	0.0400000
13726.0000000	0.0400000
13727.0000000	0.0400000
13728.0000000	0.0400000
13729.0000000	0.0400000
13730.0000000	0.0400000
13731.0000000	0.0400000
13732.0000000	0.0400000
13733.0000000	0.0400000
13734.0000000	0.0400000
13735.0000000	0.0400000
13736.0000000	0.0400000
13737.0000000	0.0400000
13738.0000000	0.0400000
13739.0000000	0.0400000
13740.0000000	0.0400000
13741.0000000	0.0400000
13742.0000000	0.0395238
13743.0000000	0.0400000
13744.0000000	0.0400000
13745.0000000	0.0400000
13746.0000000	0.0400000
13747.0000000	0.0400000
13748.0000000	0.0400000
13749.0000000	0.0400000
13750.0000000	0.0400000
13751.0000000	0.0395000
13752.0000000	0.0400000
13753.0000000	0.0400000
13754.0000000	0.0400000
13755.0000000	0.0400000
13756.0000000	0.0400000
13757.0000000	0.0400000
13758.0000000	0.0400000
13759.0000000	0.0400000
13760.0000000	0.0400000
13761.0000000	0.0405000
13762.0000000	0.0400000
13763.0000000	0.0400000
13764.0000000	0.0400000
13765.0000000	0.0400000
13766.0000000	0.0400000
13767.0000000	0.0400000
13768.0000000	0.0400000
13769.0000000	0.0404762

13770.0000000	0.0400000
13771.0000000	0.0400000
13772.0000000	0.0400000
13773.0000000	0.0400000
13774.0000000	0.0400000
13775.0000000	0.0405000
13776.0000000	0.0400000
13777.0000000	0.0400000
13778.0000000	0.0400000
13779.0000000	0.0400000
13780.0000000	0.0400000
13781.0000000	0.0400000
13782.0000000	0.0400000
13783.0000000	0.0400000
13784.0000000	0.0400000
13785.0000000	0.0400000
13786.0000000	0.0400000
13787.0000000	0.0400000
13788.0000000	0.0400000
13789.0000000	0.0405000
13790.0000000	0.0400000
13791.0000000	0.0400000
13792.0000000	0.0400000
13793.0000000	0.0400000
13794.0000000	0.0400000
13795.0000000	0.0400000
13796.0000000	0.0405000
13797.0000000	0.0400000
13798.0000000	0.0400000
13799.0000000	0.0400000
13800.0000000	0.0400000
13801.0000000	0.0400000
13802.0000000	0.0405000
13803.0000000	0.0400000
13804.0000000	0.0400000
13805.0000000	0.0400000
13806.0000000	0.0400000
13807.0000000	0.0400000
13808.0000000	0.0400000
13809.0000000	0.0400000
13810.0000000	0.0400000
13811.0000000	0.0400000
13812.0000000	0.0400000
13813.0000000	0.0400000
13814.0000000	0.0400000
13815.0000000	0.0400000
13816.0000000	0.0400000
13817.0000000	0.0395000
13818.0000000	0.0400000
13819.0000000	0.0400000
13820.0000000	0.0400000
13821.0000000	0.0400000
13822.0000000	0.0395238
13823.0000000	0.0395000
13824.0000000	0.0400000
13825.0000000	0.0400000
13826.0000000	0.0400000

13827.0000000	0.0400000
13828.0000000	0.0400000
13829.0000000	0.0400000
13830.0000000	0.0400000
13831.0000000	0.0400000
13832.0000000	0.0400000
13833.0000000	0.0400000
13834.0000000	0.0400000
13835.0000000	0.0400000
13836.0000000	0.0395000
13837.0000000	0.0395238
13838.0000000	0.0395000
13839.0000000	0.0400000
13840.0000000	0.0400000
13841.0000000	0.0395000
13842.0000000	0.0395000
13843.0000000	0.0400000
13844.0000000	0.0400000
13845.0000000	0.0400000
13846.0000000	0.0400000
13847.0000000	0.0395000
13848.0000000	0.0400000
13849.0000000	0.0395238
13850.0000000	0.0400000
13851.0000000	0.0400000
13852.0000000	0.0400000
13853.0000000	0.0400000
13854.0000000	0.0400000
13855.0000000	0.0400000
13856.0000000	0.0395000
13857.0000000	0.0400000
13858.0000000	0.0400000
13859.0000000	0.0400000
13860.0000000	0.0395000
13861.0000000	0.0400000
13862.0000000	0.0400000
13863.0000000	0.0400000
13864.0000000	0.0400000
13865.0000000	0.0400000
13866.0000000	0.0400000
13867.0000000	0.0400000
13868.0000000	0.0400000
13869.0000000	0.0400000
13870.0000000	0.0400000
13871.0000000	0.0400000
13872.0000000	0.0400000
13873.0000000	0.0400000
13874.0000000	0.0404762
13875.0000000	0.0400000
13876.0000000	0.0400000
13877.0000000	0.0400000
13878.0000000	0.0400000
13879.0000000	0.0400000
13880.0000000	0.0395000
13881.0000000	0.0400000
13882.0000000	0.0400000
13883.0000000	0.0400000

13884.0000000	0.0395000
13885.0000000	0.0400000
13886.0000000	0.0400000
13887.0000000	0.0400000
13888.0000000	0.0400000
13889.0000000	0.0400000
13890.0000000	0.0400000
13891.0000000	0.0395238
13892.0000000	0.0400000
13893.0000000	0.0395000
13894.0000000	0.0390476
13895.0000000	0.0395000
13896.0000000	0.0390000
13897.0000000	0.0390000
13898.0000000	0.0400000
13899.0000000	0.0400000
13900.0000000	0.0395238
13901.0000000	0.0400000
13902.0000000	0.0400000
13903.0000000	0.0400000
13904.0000000	0.0395238
13905.0000000	0.0395000
13906.0000000	0.0395000
13907.0000000	0.0390476
13908.0000000	0.0395000
13909.0000000	0.0400000
13910.0000000	0.0400000
13911.0000000	0.0400000
13912.0000000	0.0400000
13913.0000000	0.0400000
13914.0000000	0.0395000
13915.0000000	0.0400000
13916.0000000	0.0395238
13917.0000000	0.0390000
13918.0000000	0.0395000
13919.0000000	0.0400000
13920.0000000	0.0395000
13921.0000000	0.0400000
13922.0000000	0.0400000
13923.0000000	0.0395000
13924.0000000	0.0395238
13925.0000000	0.0400000
13926.0000000	0.0400000
13927.0000000	0.0395000
13928.0000000	0.0400000
13929.0000000	0.0395000
13930.0000000	0.0400000
13931.0000000	0.0395000
13932.0000000	0.0400000
13933.0000000	0.0395000
13934.0000000	0.0395238
13935.0000000	0.0395000
13936.0000000	0.0400000
13937.0000000	0.0400000
13938.0000000	0.0400000
13939.0000000	0.0395238
13940.0000000	0.0390000

13941.0000000	0.0395000
13942.0000000	0.0395000
13943.0000000	0.0395238
13944.0000000	0.0390000
13945.0000000	0.0395000
13946.0000000	0.0390476
13947.0000000	0.0385000
13948.0000000	0.0390000
13949.0000000	0.0395238
13950.0000000	0.0385000
13951.0000000	0.0390000
13952.0000000	0.0395238
13953.0000000	0.0400000
13954.0000000	0.0400000
13955.0000000	0.0400000
13956.0000000	0.0400000
13957.0000000	0.0400000
13958.0000000	0.0400000
13959.0000000	0.0400000
13960.0000000	0.0400000
13961.0000000	0.0400000
13962.0000000	0.0395000
13963.0000000	0.0400000
13964.0000000	0.0395238
13965.0000000	0.0400000
13966.0000000	0.0395000
13967.0000000	0.0400000
13968.0000000	0.0400000
13969.0000000	0.0400000
13970.0000000	0.0400000
13971.0000000	0.0395000
13972.0000000	0.0400000
13973.0000000	0.0400000
13974.0000000	0.0390000
13975.0000000	0.0400000
13976.0000000	0.0400000
13977.0000000	0.0390000
13978.0000000	0.0400000
13979.0000000	0.0395238
13980.0000000	0.0385000
13981.0000000	0.0400000
13982.0000000	0.0390476
13983.0000000	0.0400000
13984.0000000	0.0395238
13985.0000000	0.0400000
13986.0000000	0.0390000
13987.0000000	0.0380000
13988.0000000	0.0390476
13989.0000000	0.0400000
13990.0000000	0.0395000
13991.0000000	0.0395238
13992.0000000	0.0380000
13993.0000000	0.0395000
13994.0000000	0.0395000
13995.0000000	0.0400000
13996.0000000	0.0400000
13997.0000000	0.0400000

13998.0000000	0.0400000
13999.0000000	0.0385000
14000.0000000	0.0390476
14001.0000000	0.0380000
14002.0000000	0.0390000
14003.0000000	0.0390000
14004.0000000	0.0390476
14005.0000000	0.0400000
14006.0000000	0.0390000
14007.0000000	0.0390476
14008.0000000	0.0390000
14009.0000000	0.0385714
14010.0000000	0.0390000
14011.0000000	0.0400000
14012.0000000	0.0395238
14013.0000000	0.0390000
14014.0000000	0.0390000
14015.0000000	0.0366667
14016.0000000	0.0395000
14017.0000000	0.0365000
14018.0000000	0.0361905
14019.0000000	0.0365000
14020.0000000	0.0357143
14021.0000000	0.0375000
14022.0000000	0.0365000
14023.0000000	0.0345000
14024.0000000	0.0340000
14025.0000000	0.0352381
14026.0000000	0.0335000
14027.0000000	0.0338095
14028.0000000	0.0335000
14029.0000000	0.0323810
14030.0000000	0.0335000
14031.0000000	0.0360000
14032.0000000	0.0370000
14033.0000000	0.0330000
14034.0000000	0.0338095
14035.0000000	0.0350000
14036.0000000	0.0366667
14037.0000000	0.0335000
14038.0000000	0.0350000
14039.0000000	0.0350000
14040.0000000	0.0323809
14041.0000000	0.0350000
14042.0000000	0.0335000
14043.0000000	0.0333333
14044.0000000	0.0320000
14045.0000000	0.0323809
14046.0000000	0.0315000
14047.0000000	0.0305000
14048.0000000	0.0314286
14049.0000000	0.0325000
14050.0000000	0.0305000
14051.0000000	0.0325000
14052.0000000	0.0323809
14053.0000000	0.0335000
14054.0000000	0.0310000

14055.0000000	0.0309524
14056.0000000	0.0300000
14057.0000000	0.0309524
14058.0000000	0.0305000
14059.0000000	0.0300000
14060.0000000	0.0304762
14061.0000000	0.0305000
14062.0000000	0.0310000
14063.0000000	0.0305000
14064.0000000	0.0304762
14065.0000000	0.0300000
14066.0000000	0.0304762
14067.0000000	0.0300000
14068.0000000	0.0310000
14069.0000000	0.0305000
14070.0000000	0.0300000
14071.0000000	0.0300000
14072.0000000	0.0304762
14073.0000000	0.0305000
14074.0000000	0.0300000
14075.0000000	0.0300000
14076.0000000	0.0304762
14077.0000000	0.0320000
14078.0000000	0.0300000
14079.0000000	0.0314286
14080.0000000	0.0310000
14081.0000000	0.0304762
14082.0000000	0.0305000
14083.0000000	0.0310000
14084.0000000	0.0300000
14085.0000000	0.0320000
14086.0000000	0.0309524
14087.0000000	0.0315000
14088.0000000	0.0314286
14089.0000000	0.0310000
14090.0000000	0.0304762
14091.0000000	0.0300000
14092.0000000	0.0305000
14093.0000000	0.0305000
14094.0000000	0.0315000
14095.0000000	0.0304762
14096.0000000	0.0300000
14097.0000000	0.0305000
14098.0000000	0.0300000
14099.0000000	0.0300000
14100.0000000	0.0300000
14101.0000000	0.0300000
14102.0000000	0.0300000
14103.0000000	0.0300000
14104.0000000	0.0300000
14105.0000000	0.0300000
14106.0000000	0.0300000
14107.0000000	0.0300000
14108.0000000	0.0300000
14109.0000000	0.0305000
14110.0000000	0.0300000
14111.0000000	0.0300000

14112.0000000	0.0300000
14113.0000000	0.0300000
14114.0000000	0.0300000
14115.0000000	0.0300000
14116.0000000	0.0300000
14117.0000000	0.0300000
14118.0000000	0.0300000
14119.0000000	0.0300000
14120.0000000	0.0300000
14121.0000000	0.0300000
14122.0000000	0.0300000
14123.0000000	0.0295000
14124.0000000	0.0300000
14125.0000000	0.0305000
14126.0000000	0.0300000
14127.0000000	0.0300000
14128.0000000	0.0300000
14129.0000000	0.0300000
14130.0000000	0.0300000
14131.0000000	0.0300000
14132.0000000	0.0300000
14133.0000000	0.0300000
14134.0000000	0.0305000
14135.0000000	0.0300000
14136.0000000	0.0300000
14137.0000000	0.0300000
14138.0000000	0.0300000
14139.0000000	0.0300000
14140.0000000	0.0300000
14141.0000000	0.0300000
14142.0000000	0.0300000
14143.0000000	0.0305000
14144.0000000	0.0300000
14145.0000000	0.0300000
14146.0000000	0.0300000
14147.0000000	0.0300000
14148.0000000	0.0304762
14149.0000000	0.0300000
14150.0000000	0.0300000
14151.0000000	0.0300000
14152.0000000	0.0305000
14153.0000000	0.0300000
14154.0000000	0.0300000
14155.0000000	0.0300000
14156.0000000	0.0300000
14157.0000000	0.0300000
14158.0000000	0.0300000
14159.0000000	0.0300000
14160.0000000	0.0300000
14161.0000000	0.0300000
14162.0000000	0.0300000
14163.0000000	0.0300000
14164.0000000	0.0304762
14165.0000000	0.0300000
14166.0000000	0.0300000
14167.0000000	0.0300000
14168.0000000	0.0300000

14169.0000000	0.0304762
14170.0000000	0.0300000
14171.0000000	0.0300000
14172.0000000	0.0300000
14173.0000000	0.0305000
14174.0000000	0.0300000
14175.0000000	0.0300000
14176.0000000	0.0300000
14177.0000000	0.0300000
14178.0000000	0.0300000
14179.0000000	0.0300000
14180.0000000	0.0300000
14181.0000000	0.0304762
14182.0000000	0.0295000
14183.0000000	0.0300000
14184.0000000	0.0295238
14185.0000000	0.0300000
14186.0000000	0.0300000
14187.0000000	0.0300000
14188.0000000	0.0300000
14189.0000000	0.0300000
14190.0000000	0.0300000
14191.0000000	0.0300000
14192.0000000	0.0300000
14193.0000000	0.0295238
14194.0000000	0.0300000
14195.0000000	0.0300000
14196.0000000	0.0300000
14197.0000000	0.0300000
14198.0000000	0.0300000
14199.0000000	0.0300000
14200.0000000	0.0295238
14201.0000000	0.0295000
14202.0000000	0.0295000
14203.0000000	0.0295238
14204.0000000	0.0300000
14205.0000000	0.0295238
14206.0000000	0.0290000
14207.0000000	0.0290000
14208.0000000	0.0285000
14209.0000000	0.0285714
14210.0000000	0.0290000
14211.0000000	0.0290000
14212.0000000	0.0285714
14213.0000000	0.0265000
14214.0000000	0.0280952
14215.0000000	0.0285000
14216.0000000	0.0275000
14217.0000000	0.0295238
14218.0000000	0.0295000
14219.0000000	0.0290000
14220.0000000	0.0275000
14221.0000000	0.0271429
14222.0000000	0.0260000
14223.0000000	0.0257143
14224.0000000	0.0240000
14225.0000000	0.0257143

14226.0000000	0.0240000
14227.0000000	0.0255000
14228.0000000	0.0260000
14229.0000000	0.0265000
14230.0000000	0.0266667
14231.0000000	0.0240000
14232.0000000	0.0228571
14233.0000000	0.0235000
14234.0000000	0.0235000
14235.0000000	0.0235000
14236.0000000	0.0223810
14237.0000000	0.0240000
14238.0000000	0.0245000
14239.0000000	0.0233333
14240.0000000	0.0245000
14241.0000000	0.0223810
14242.0000000	0.0245000
14243.0000000	0.0225000
14244.0000000	0.0255000
14245.0000000	0.0252381
14246.0000000	0.0235000
14247.0000000	0.0245000
14248.0000000	0.0233333
14249.0000000	0.0240000
14250.0000000	0.0235000
14251.0000000	0.0230000
14252.0000000	0.0219048
14253.0000000	0.0214286
14254.0000000	0.0205000
14255.0000000	0.0225000
14256.0000000	0.0210000
14257.0000000	0.0200000
14258.0000000	0.0210000
14259.0000000	0.0215000
14260.0000000	0.0233333
14261.0000000	0.0205000
14262.0000000	0.0219048
14263.0000000	0.0215000
14264.0000000	0.0225000
14265.0000000	0.0230000
14266.0000000	0.0219048
14267.0000000	0.0225000
14268.0000000	0.0225000
14269.0000000	0.0233333
14270.0000000	0.0240000
14271.0000000	0.0247619
14272.0000000	0.0220000
14273.0000000	0.0235000
14274.0000000	0.0255000
14275.0000000	0.0238095
14276.0000000	0.0230000
14277.0000000	0.0219048
14278.0000000	0.0215000
14279.0000000	0.0215000
14280.0000000	0.0215000
14281.0000000	0.0219048
14282.0000000	0.0205000

14283.0000000	0.0210000
14284.0000000	0.0219048
14285.0000000	0.0220000
14286.0000000	0.0219048
14287.0000000	0.0210000
14288.0000000	0.0215000
14289.0000000	0.0210000
14290.0000000	0.0210000
14291.0000000	0.0223810
14292.0000000	0.0205000
14293.0000000	0.0204762
14294.0000000	0.0200000
14295.0000000	0.0205000
14296.0000000	0.0200000
14297.0000000	0.0209524
14298.0000000	0.0210000
14299.0000000	0.0215000
14300.0000000	0.0209524
14301.0000000	0.0209524
14302.0000000	0.0205000
14303.0000000	0.0215000
14304.0000000	0.0210000
14305.0000000	0.0200000
14306.0000000	0.0210000
14307.0000000	0.0215000
14308.0000000	0.0209524
14309.0000000	0.0210000
14310.0000000	0.0200000
14311.0000000	0.0204762
14312.0000000	0.0205000
14313.0000000	0.0215000
14314.0000000	0.0209524
14315.0000000	0.0200000
14316.0000000	0.0200000
14317.0000000	0.0204762
14318.0000000	0.0200000
14319.0000000	0.0205000
14320.0000000	0.0200000
14321.0000000	0.0200000
14322.0000000	0.0200000
14323.0000000	0.0205000
14324.0000000	0.0205000
14325.0000000	0.0200000
14326.0000000	0.0200000
14327.0000000	0.0200000
14328.0000000	0.0200000
14329.0000000	0.0209524
14330.0000000	0.0200000
14331.0000000	0.0205000
14332.0000000	0.0200000
14333.0000000	0.0205000
14334.0000000	0.0205000
14335.0000000	0.0205000
14336.0000000	0.0200000
14337.0000000	0.0205000
14338.0000000	0.0200000
14339.0000000	0.0200000

14340.0000000	0.0205000
14341.0000000	0.0200000
14342.0000000	0.0205000
14343.0000000	0.0200000
14344.0000000	0.0200000
14345.0000000	0.0200000
14346.0000000	0.0200000
14347.0000000	0.0204762
14348.0000000	0.0200000
14349.0000000	0.0200000
14350.0000000	0.0200000
14351.0000000	0.0200000
14352.0000000	0.0200000
14353.0000000	0.0200000
14354.0000000	0.0200000
14355.0000000	0.0195000
14356.0000000	0.0200000
14357.0000000	0.0200000
14358.0000000	0.0200000
14359.0000000	0.0200000
14360.0000000	0.0200000
14361.0000000	0.0200000
14362.0000000	0.0200000
14363.0000000	0.0200000
14364.0000000	0.0200000
14365.0000000	0.0200000
14366.0000000	0.0200000
14367.0000000	0.0200000
14368.0000000	0.0200000
14369.0000000	0.0200000
14370.0000000	0.0200000
14371.0000000	0.0200000
14372.0000000	0.0200000
14373.0000000	0.0200000
14374.0000000	0.0195238
14375.0000000	0.0200000
14376.0000000	0.0200000
14377.0000000	0.0200000
14378.0000000	0.0200000
14379.0000000	0.0200000
14380.0000000	0.0200000
14381.0000000	0.0200000
14382.0000000	0.0200000
14383.0000000	0.0200000
14384.0000000	0.0200000
14385.0000000	0.0195000
14386.0000000	0.0195000
14387.0000000	0.0200000
14388.0000000	0.0200000
14389.0000000	0.0195238
14390.0000000	0.0195000
14391.0000000	0.0200000
14392.0000000	0.0200000
14393.0000000	0.0200000
14394.0000000	0.0200000
14395.0000000	0.0200000
14396.0000000	0.0200000

14397.0000000	0.0200000
14398.0000000	0.0200000
14399.0000000	0.0200000
14400.0000000	0.0200000
14401.0000000	0.0200000
14402.0000000	0.0200000
14403.0000000	0.0195000
14404.0000000	0.0195000
14405.0000000	0.0200000
14406.0000000	0.0200000
14407.0000000	0.0200000
14408.0000000	0.0200000
14409.0000000	0.0200000
14410.0000000	0.0200000
14411.0000000	0.0200000
14412.0000000	0.0200000
14413.0000000	0.0200000
14414.0000000	0.0200000
14415.0000000	0.0195000
14416.0000000	0.0200000
14417.0000000	0.0200000
14418.0000000	0.0200000
14419.0000000	0.0200000
14420.0000000	0.0195000
14421.0000000	0.0200000
14422.0000000	0.0200000
14423.0000000	0.0200000
14424.0000000	0.0200000
14425.0000000	0.0200000
14426.0000000	0.0190476
14427.0000000	0.0195000
14428.0000000	0.0195238
14429.0000000	0.0195000
14430.0000000	0.0195000
14431.0000000	0.0195000
14432.0000000	0.0200000
14433.0000000	0.0195238
14434.0000000	0.0195000
14435.0000000	0.0195238
14436.0000000	0.0185000
14437.0000000	0.0171429
14438.0000000	0.0195000
14439.0000000	0.0185000
14440.0000000	0.0185000
14441.0000000	0.0180952
14442.0000000	0.0195000
14443.0000000	0.0190000
14444.0000000	0.0185714
14445.0000000	0.0190000
14446.0000000	0.0190000
14447.0000000	0.0200000
14448.0000000	0.0185000
14449.0000000	0.0180000
14450.0000000	0.0195238
14451.0000000	0.0190000
14452.0000000	0.0185000
14453.0000000	0.0190476

14454.0000000	0.0190000
14455.0000000	0.0200000
14456.0000000	0.0200000
14457.0000000	0.0176190
14458.0000000	0.0180952
14459.0000000	0.0195000
14460.0000000	0.0190000
14461.0000000	0.0195000
14462.0000000	0.0195238
14463.0000000	0.0195000
14464.0000000	0.0185000
14465.0000000	0.0200000
14466.0000000	0.0200000
14467.0000000	0.0195238
14468.0000000	0.0200000
14469.0000000	0.0195000
14470.0000000	0.0185000
14471.0000000	0.0195238
14472.0000000	0.0185000
14473.0000000	0.0200000
14474.0000000	0.0195238
14475.0000000	0.0195000
14476.0000000	0.0200000
14477.0000000	0.0195000
14478.0000000	0.0200000
14479.0000000	0.0190000
14480.0000000	0.0185714
14481.0000000	0.0200000
14482.0000000	0.0190000
14483.0000000	0.0185714
14484.0000000	0.0185000
14485.0000000	0.0190000
14486.0000000	0.0185714
14487.0000000	0.0160000
14488.0000000	0.0170000
14489.0000000	0.0190476
14490.0000000	0.0160000
14491.0000000	0.0170000
14492.0000000	0.0175000
14493.0000000	0.0176190
14494.0000000	0.0175000
14495.0000000	0.0185000
14496.0000000	0.0171429
14497.0000000	0.0170000
14498.0000000	0.0166667
14499.0000000	0.0170000
14500.0000000	0.0160000
14501.0000000	0.0190000
14502.0000000	0.0185714
14503.0000000	0.0180000
14504.0000000	0.0185000
14505.0000000	0.0185714
14506.0000000	0.0180000
14507.0000000	0.0176191
14508.0000000	0.0185000
14509.0000000	0.0180000
14510.0000000	0.0176190

14511.0000000	0.0170000
14512.0000000	0.0175000
14513.0000000	0.0175000
14514.0000000	0.0166667
14515.0000000	0.0185000
14516.0000000	0.0180952
14517.0000000	0.0180000
14518.0000000	0.0170000
14519.0000000	0.0176190
14520.0000000	0.0175000
14521.0000000	0.0165000
14522.0000000	0.0170000
14523.0000000	0.0166667
14524.0000000	0.0185000
14525.0000000	0.0157143
14526.0000000	0.0185000
14527.0000000	0.0190000
14528.0000000	0.0166667
14529.0000000	0.0180000
14530.0000000	0.0170000
14531.0000000	0.0180000
14532.0000000	0.0157143
14533.0000000	0.0185000
14534.0000000	0.0166667
14535.0000000	0.0175000
14536.0000000	0.0190000
14537.0000000	0.0180000
14538.0000000	0.0180952
14539.0000000	0.0180000
14540.0000000	0.0170000
14541.0000000	0.0171429
14542.0000000	0.0185000
14543.0000000	0.0180952
14544.0000000	0.0190000
14545.0000000	0.0175000
14546.0000000	0.0171429
14547.0000000	0.0170000
14548.0000000	0.0165000
14549.0000000	0.0175000
14550.0000000	0.0185714
14551.0000000	0.0170000
14552.0000000	0.0175000
14553.0000000	0.0161905
14554.0000000	0.0175000
14555.0000000	0.0176190
14556.0000000	0.0185000
14557.0000000	0.0180000
14558.0000000	0.0185714
14559.0000000	0.0195000
14560.0000000	0.0195000
14561.0000000	0.0185000
14562.0000000	0.0180952
14563.0000000	0.0180000
14564.0000000	0.0185714
14565.0000000	0.0180000
14566.0000000	0.0160000
14567.0000000	0.0185000

14568.0000000	0.0166667
14569.0000000	0.0180000
14570.0000000	0.0171429
14571.0000000	0.0155000
14572.0000000	0.0165000
14573.0000000	0.0155000
14574.0000000	0.0171429
14575.0000000	0.0180000
14576.0000000	0.0155000
14577.0000000	0.0166667
14578.0000000	0.0175000
14579.0000000	0.0166667
14580.0000000	0.0145000
14581.0000000	0.0155000
14582.0000000	0.0175000
14583.0000000	0.0155000
14584.0000000	0.0166667
14585.0000000	0.0155000
14586.0000000	0.0166667
14587.0000000	0.0180000
14588.0000000	0.0190000
14589.0000000	0.0180952
14590.0000000	0.0165000
14591.0000000	0.0175000
14592.0000000	0.0145000
14593.0000000	0.0166667
14594.0000000	0.0155000
14595.0000000	0.0147619
14596.0000000	0.0175000
14597.0000000	0.0160000
14598.0000000	0.0180952
14599.0000000	0.0160000
14600.0000000	0.0165000
14601.0000000	0.0170000
14602.0000000	0.0152381
14603.0000000	0.0166667
14604.0000000	0.0165000
14605.0000000	0.0155000
14606.0000000	0.0160000
14607.0000000	0.0142857
14608.0000000	0.0135000
14609.0000000	0.0155000
14610.0000000	0.0133333
14611.0000000	0.0145000
14612.0000000	0.0150000
14613.0000000	0.0152381
14614.0000000	0.0140000
14615.0000000	0.0155000
14616.0000000	0.0147619
14617.0000000	0.0150000
14618.0000000	0.0140000
14619.0000000	0.0147619
14620.0000000	0.0140000
14621.0000000	0.0145000
14622.0000000	0.0138095
14623.0000000	0.0140000
14624.0000000	0.0125000

14625.0000000	0.0152381
14626.0000000	0.0145000
14627.0000000	0.0155000
14628.0000000	0.0155000
14629.0000000	0.0133333
14630.0000000	0.0140000
14631.0000000	0.0157143
14632.0000000	0.0150000
14633.0000000	0.0160000
14634.0000000	0.0161905
14635.0000000	0.0155000
14636.0000000	0.0150000
14637.0000000	0.0145000
14638.0000000	0.0152381
14639.0000000	0.0140000
14640.0000000	0.0142857
14641.0000000	0.0150000
14642.0000000	0.0130000
14643.0000000	0.0152381
14644.0000000	0.0145000
14645.0000000	0.0120000
14646.0000000	0.0114286
14647.0000000	0.0145000
14648.0000000	0.0135000
14649.0000000	0.0119048
14650.0000000	0.0135000
14651.0000000	0.0135000
14652.0000000	0.0142857
14653.0000000	0.0135000
14654.0000000	0.0125000
14655.0000000	0.0147619
14656.0000000	0.0145000
14657.0000000	0.0125000
14658.0000000	0.0140000
14659.0000000	0.0128571
14660.0000000	0.0135000
14661.0000000	0.0165000
14662.0000000	0.0152381
14663.0000000	0.0138095
14664.0000000	0.0170000
14665.0000000	0.0145000
14666.0000000	0.0155000
14667.0000000	0.0150000
14668.0000000	0.0138095
14669.0000000	0.0160000
14670.0000000	0.0166667
14671.0000000	0.0155000
14672.0000000	0.0170000
14673.0000000	0.0155000
14674.0000000	0.0171429
14675.0000000	0.0155000
14676.0000000	0.0155000
14677.0000000	0.0133333
14678.0000000	0.0135000
14679.0000000	0.0138095
14680.0000000	0.0140000
14681.0000000	0.0160000

14682.0000000	0.0140000
14683.0000000	0.0119048
14684.0000000	0.0140000
14685.0000000	0.0140000
14686.0000000	0.0142857
14687.0000000	0.0160000
14688.0000000	0.0135000
14689.0000000	0.0140000
14690.0000000	0.0123810
14691.0000000	0.0142857
14692.0000000	0.0130000
14693.0000000	0.0130000
14694.0000000	0.0135000
14695.0000000	0.0133333
14696.0000000	0.0125000
14697.0000000	0.0135000
14698.0000000	0.0128571
14699.0000000	0.0135000
14700.0000000	0.0135000
14701.0000000	0.0138095
14702.0000000	0.0130000
14703.0000000	0.0135000
14704.0000000	0.0138095
14705.0000000	0.0140000
14706.0000000	0.0145000
14707.0000000	0.0133333
14708.0000000	0.0145000
14709.0000000	0.0115000
14710.0000000	0.0133333
14711.0000000	0.0125000
14712.0000000	0.0125000
14713.0000000	0.0142857
14714.0000000	0.0140000
14715.0000000	0.0138095
14716.0000000	0.0125000
14717.0000000	0.0110000
14718.0000000	0.0120000
14719.0000000	0.0138095
14720.0000000	0.0115000
14721.0000000	0.0110000
14722.0000000	0.0123810
14723.0000000	0.0120000
14724.0000000	0.0123810
14725.0000000	0.0145000
14726.0000000	0.0110000
14727.0000000	0.0125000
14728.0000000	0.0119048
14729.0000000	0.0130000
14730.0000000	0.0125000
14731.0000000	0.0114286
14732.0000000	0.0125000
14733.0000000	0.0120000
14734.0000000	0.0119048
14735.0000000	0.0120000
14736.0000000	0.0120000
14737.0000000	0.0123810
14738.0000000	0.0135000

14739.0000000	0.0130000
14740.0000000	0.0119048
14741.0000000	0.0125000
14742.0000000	0.0135000
14743.0000000	0.0123810
14744.0000000	0.0140000
14745.0000000	0.0145000
14746.0000000	0.0142857
14747.0000000	0.0120000
14748.0000000	0.0135000
14749.0000000	0.0120000
14750.0000000	0.0133333
14751.0000000	0.0123810
14752.0000000	0.0110000
14753.0000000	0.0115000
14754.0000000	0.0140000
14755.0000000	0.0133333
14756.0000000	0.0140000
14757.0000000	0.0145000
14758.0000000	0.0128571
14759.0000000	0.0135000
14760.0000000	0.0128571
14761.0000000	0.0135000
14762.0000000	0.0130000
14763.0000000	0.0120000
14764.0000000	0.0120000
14765.0000000	0.0142857
14766.0000000	0.0135000
14767.0000000	0.0147619
14768.0000000	0.0165000
14769.0000000	0.0123810
14770.0000000	0.0140000
14771.0000000	0.0125000
14772.0000000	0.0130000
14773.0000000	0.0125000
14774.0000000	0.0114286
14775.0000000	0.0130000
14776.0000000	0.0133333
14777.0000000	0.0130000
14778.0000000	0.0125000
14779.0000000	0.0128571
14780.0000000	0.0130000
14781.0000000	0.0135000
14782.0000000	0.0115000
14783.0000000	0.0128571
14784.0000000	0.0125000
14785.0000000	0.0128571
14786.0000000	0.0115000
14787.0000000	0.0120000
14788.0000000	0.0135000
14789.0000000	0.0114286
14790.0000000	0.0105000
14791.0000000	0.0128571
14792.0000000	0.0125000
14793.0000000	0.0130000
14794.0000000	0.0115000
14795.0000000	0.0109524

14796.0000000	0.0130000
14797.0000000	0.0120000
14798.0000000	0.0114286
14799.0000000	0.0105000
14800.0000000	0.0114286
14801.0000000	0.0110000
14802.0000000	0.0110000
14803.0000000	0.0109524
14804.0000000	0.0110000
14805.0000000	0.0105000
14806.0000000	0.0105000
14807.0000000	0.0100000
14808.0000000	0.0105000
14809.0000000	0.0120000
14810.0000000	0.0109524
14811.0000000	0.0105000
14812.0000000	0.0123810
14813.0000000	0.0105000
14814.0000000	0.0100000
14815.0000000	0.0109524
14816.0000000	0.0110000
14817.0000000	0.0100000
14818.0000000	0.0120000
14819.0000000	0.0119048
14820.0000000	0.0110000
14821.0000000	0.0100000
14822.0000000	0.0115000
14823.0000000	0.0105000
14824.0000000	0.0100000
14825.0000000	0.0104762
14826.0000000	0.0100000
14827.0000000	0.0105000
14828.0000000	0.0109524
14829.0000000	0.0100000
14830.0000000	0.0104545
14831.0000000	0.0100000
14832.0000000	0.0115000
14833.0000000	0.0100000
14834.0000000	0.0100000
14835.0000000	0.0100000
14836.0000000	0.0100000
14837.0000000	0.0100000
14838.0000000	0.0105000
14839.0000000	0.0104762
14840.0000000	0.0100000
14841.0000000	0.0100000
14842.0000000	0.0100000
14843.0000000	0.0100000
14844.0000000	0.0100000
14845.0000000	0.0105000
14846.0000000	0.0104762
14847.0000000	0.0100000
14848.0000000	0.0104762
14849.0000000	0.0100000
14850.0000000	0.0100000
14851.0000000	0.0100000
14852.0000000	0.0105000

14853.0000000	0.0100000
14854.0000000	0.0100000
14855.0000000	0.0100000
14856.0000000	0.0100000
14857.0000000	0.0100000
14858.0000000	0.0100000
14859.0000000	0.0100000
14860.0000000	0.0100000
14861.0000000	0.0100000
14862.0000000	0.0100000
14863.0000000	0.0100000
14864.0000000	0.0104762
14865.0000000	0.0100000
14866.0000000	0.0100000
14867.0000000	0.0100000
14868.0000000	0.0100000
14869.0000000	0.0100000
14870.0000000	0.0100000
14871.0000000	0.0104762
14872.0000000	0.0100000
14873.0000000	0.0100000
14874.0000000	0.0100000
14875.0000000	0.0100000
14876.0000000	0.0100000
14877.0000000	0.0100000
14878.0000000	0.0100000
14879.0000000	0.0100000
14880.0000000	0.0105000
14881.0000000	0.0100000
14882.0000000	0.0100000
14883.0000000	0.0105000
14884.0000000	0.0110000
14885.0000000	0.0105000
14886.0000000	0.0100000
14887.0000000	0.0100000
14888.0000000	0.0100000
14889.0000000	0.0100000
14890.0000000	0.0100000
14891.0000000	0.0100000
14892.0000000	0.0100000
14893.0000000	0.0105000
14894.0000000	0.0100000
14895.0000000	0.0100000
14896.0000000	0.0100000
14897.0000000	0.0100000
14898.0000000	0.0105000
14899.0000000	0.0105000
14900.0000000	0.0100000
14901.0000000	0.0100000
14902.0000000	0.0100000
14903.0000000	0.0104762
14904.0000000	0.0110000
14905.0000000	0.0100000
14906.0000000	0.0104762
14907.0000000	0.0100000
14908.0000000	0.0100000
14909.0000000	0.0100000

14910.0000000	0.0100000
14911.0000000	0.0105000
14912.0000000	0.0100000
14913.0000000	0.0100000
14914.0000000	0.0100000
14915.0000000	0.0100000
14916.0000000	0.0100000
14917.0000000	0.0100000
14918.0000000	0.0100000
14919.0000000	0.0100000
14920.0000000	0.0109524
14921.0000000	0.0100000
14922.0000000	0.0100000
14923.0000000	0.0100000
14924.0000000	0.0100000
14925.0000000	0.0104762
14926.0000000	0.0100000
14927.0000000	0.0100000
14928.0000000	0.0100000
14929.0000000	0.0100000
14930.0000000	0.0100000
14931.0000000	0.0100000
14932.0000000	0.0100000
14933.0000000	0.0100000
14934.0000000	0.0100000
14935.0000000	0.0100000
14936.0000000	0.0095238
14937.0000000	0.0100000
14938.0000000	0.0100000
14939.0000000	0.0100000
14940.0000000	0.0100000
14941.0000000	0.0100000
14942.0000000	0.0100000
14943.0000000	0.0100000
14944.0000000	0.0100000
14945.0000000	0.0100000
14946.0000000	0.0100000
14947.0000000	0.0100000
14948.0000000	0.0100000
14949.0000000	0.0100000
14950.0000000	0.0100000
14951.0000000	0.0100000
14952.0000000	0.0100000
14953.0000000	0.0105000
14954.0000000	0.0100000
14955.0000000	0.0100000
14956.0000000	0.0100000
14957.0000000	0.0100000
14958.0000000	0.0100000
14959.0000000	0.0105000
14960.0000000	0.0100000
14961.0000000	0.0100000
14962.0000000	0.0100000
14963.0000000	0.0104762
14964.0000000	0.0105000
14965.0000000	0.0104762
14966.0000000	0.0100000

14967.0000000	0.0100000
14968.0000000	0.0100000
14969.0000000	0.0100000
14970.0000000	0.0109524
14971.0000000	0.0100000
14972.0000000	0.0109524
14973.0000000	0.0105000
14974.0000000	0.0055000
14975.0000000	0.0100000
14976.0000000	0.0109524
14977.0000000	0.0110000
14978.0000000	0.0105000
14979.0000000	0.0104762
14980.0000000	0.0100000
14981.0000000	0.0100000
14982.0000000	0.0100000
14983.0000000	0.0105000
14984.0000000	0.0114286
14985.0000000	0.0100000
14986.0000000	0.0100000
14987.0000000	0.0100000
14988.0000000	0.0104762
14989.0000000	0.0105000
14990.0000000	0.0105000
14991.0000000	0.0100000
14992.0000000	0.0100000
14993.0000000	0.0100000
14994.0000000	0.0104762
14995.0000000	0.0100000
14996.0000000	0.0104762
14997.0000000	0.0100000
14998.0000000	0.0115000
14999.0000000	0.0105000
15000.0000000	0.0109524
15001.0000000	0.0105000
15002.0000000	0.0115000
15003.0000000	0.0109524
15004.0000000	0.0110000
15005.0000000	0.0110000
15006.0000000	0.0119048
15007.0000000	0.0105000
15008.0000000	0.0119048
15009.0000000	0.0115000
15010.0000000	0.0120000
15011.0000000	0.0125000
15012.0000000	0.0119048
15013.0000000	0.0125000
15014.0000000	0.0120000
15015.0000000	0.0119048
15016.0000000	0.0125000
15017.0000000	0.0114286
15018.0000000	0.0120000
15019.0000000	0.0120000
15020.0000000	0.0120000
15021.0000000	0.0130000
15022.0000000	0.0114286
15023.0000000	0.0125000

15024.0000000	0.0123810
15025.0000000	0.0120000
15026.0000000	0.0123810
15027.0000000	0.0130000
15028.0000000	0.0140000
15029.0000000	0.0135000
15030.0000000	0.0120000
15031.0000000	0.0133333
15032.0000000	0.0140000
15033.0000000	0.0133333
15034.0000000	0.0125000
15035.0000000	0.0145000
15036.0000000	0.0142857
15037.0000000	0.0140000
15038.0000000	0.0140000
15039.0000000	0.0152381
15040.0000000	0.0150000
15041.0000000	0.0150000
15042.0000000	0.0157143
15043.0000000	0.0155000
15044.0000000	0.0171429
15045.0000000	0.0165000
15046.0000000	0.0145000
15047.0000000	0.0160000
15048.0000000	0.0176190
15049.0000000	0.0165000
15050.0000000	0.0175000
15051.0000000	0.0180952
15052.0000000	0.0190000
15053.0000000	0.0171429
15054.0000000	0.0190000
15055.0000000	0.0175000
15056.0000000	0.0166667
15057.0000000	0.0165000
15058.0000000	0.0150000
15059.0000000	0.0155000
15060.0000000	0.0180952
15061.0000000	0.0190000
15062.0000000	0.0171429
15063.0000000	0.0175000
15064.0000000	0.0190000
15065.0000000	0.0175000
15066.0000000	0.0195000
15067.0000000	0.0171429
15068.0000000	0.0175000
15069.0000000	0.0166667
15070.0000000	0.0175000
15071.0000000	0.0175000
15072.0000000	0.0190476
15073.0000000	0.0180000
15074.0000000	0.0175000
15075.0000000	0.0175000
15076.0000000	0.0190476
15077.0000000	0.0190000
15078.0000000	0.0176190
15079.0000000	0.0190000
15080.0000000	0.0180000

15081.0000000	0.0176190
15082.0000000	0.0175000
15083.0000000	0.0190000
15084.0000000	0.0190476
15085.0000000	0.0190000
15086.0000000	0.0175000
15087.0000000	0.0180952
15088.0000000	0.0180000
15089.0000000	0.0175000
15090.0000000	0.0190000
15091.0000000	0.0180952
15092.0000000	0.0180000
15093.0000000	0.0166667
15094.0000000	0.0165000
15095.0000000	0.0160000
15096.0000000	0.0171429
15097.0000000	0.0180000
15098.0000000	0.0165000
15099.0000000	0.0145000
15100.0000000	0.0161905
15101.0000000	0.0152381
15102.0000000	0.0160000
15103.0000000	0.0190000
15104.0000000	0.0170000
15105.0000000	0.0171429
15106.0000000	0.0175000
15107.0000000	0.0175000
15108.0000000	0.0171429
15109.0000000	0.0175000
15110.0000000	0.0180000
15111.0000000	0.0165000
15112.0000000	0.0176190
15113.0000000	0.0175000
15114.0000000	0.0180952
15115.0000000	0.0145000
15116.0000000	0.0170000
15117.0000000	0.0166667
15118.0000000	0.0170000
15119.0000000	0.0165000
15120.0000000	0.0157143
15121.0000000	0.0180000
15122.0000000	0.0170000
15123.0000000	0.0157143
15124.0000000	0.0185000
15125.0000000	0.0165000
15126.0000000	0.0165000
15127.0000000	0.0152381
15128.0000000	0.0170000
15129.0000000	0.0155000
15130.0000000	0.0157143
15131.0000000	0.0170000
15132.0000000	0.0166667
15133.0000000	0.0145000
15134.0000000	0.0165000
15135.0000000	0.0145000
15136.0000000	0.0152381
15137.0000000	0.0140000

15138.0000000	0.0145000
15139.0000000	0.0147619
15140.0000000	0.0145000
15141.0000000	0.0138095
15142.0000000	0.0135000
15143.0000000	0.0140000
15144.0000000	0.0138095
15145.0000000	0.0125000
15146.0000000	0.0125000
15147.0000000	0.0145000
15148.0000000	0.0138095
15149.0000000	0.0135000
15150.0000000	0.0166667
15151.0000000	0.0145000
15152.0000000	0.0125000
15153.0000000	0.0157143
15154.0000000	0.0145000
15155.0000000	0.0140000
15156.0000000	0.0130000
15157.0000000	0.0161905
15158.0000000	0.0145000
15159.0000000	0.0142857
15160.0000000	0.0145000
15161.0000000	0.0150000
15162.0000000	0.0157143
15163.0000000	0.0155000
15164.0000000	0.0145000
15165.0000000	0.0160000
15166.0000000	0.0142857
15167.0000000	0.0155000
15168.0000000	0.0147619
15169.0000000	0.0150000
15170.0000000	0.0160000
15171.0000000	0.0160000
15172.0000000	0.0161905
15173.0000000	0.0160000
15174.0000000	0.0155000
15175.0000000	0.0147619
15176.0000000	0.0135000
15177.0000000	0.0161905
15178.0000000	0.0150000
15179.0000000	0.0160000
15180.0000000	0.0140000
15181.0000000	0.0147619
15182.0000000	0.0140000
15183.0000000	0.0140000
15184.0000000	0.0147619
15185.0000000	0.0130000
15186.0000000	0.0135000
15187.0000000	0.0145000
15188.0000000	0.0142857
15189.0000000	0.0161905
15190.0000000	0.0140000
15191.0000000	0.0115000
15192.0000000	0.0152381
15193.0000000	0.0130000
15194.0000000	0.0145000

15195.0000000	0.0140000
15196.0000000	0.0119048
15197.0000000	0.0140000
15198.0000000	0.0130000
15199.0000000	0.0138095
15200.0000000	0.0125000
15201.0000000	0.0115000
15202.0000000	0.0152381
15203.0000000	0.0145000
15204.0000000	0.0130000
15205.0000000	0.0128571
15206.0000000	0.0125000
15207.0000000	0.0120000
15208.0000000	0.0123810
15209.0000000	0.0115000
15210.0000000	0.0125000
15211.0000000	0.0119048
15212.0000000	0.0105000
15213.0000000	0.0110000
15214.0000000	0.0104762
15215.0000000	0.0110000
15216.0000000	0.0105000
15217.0000000	0.0105000
15218.0000000	0.0114286
15219.0000000	0.0110000
15220.0000000	0.0109524
15221.0000000	0.0110000
15222.0000000	0.0105000
15223.0000000	0.0119048
15224.0000000	0.0110000
15225.0000000	0.0110000
15226.0000000	0.0120000
15227.0000000	0.0109524
15228.0000000	0.0105000
15229.0000000	0.0104762
15230.0000000	0.0105000
15231.0000000	0.0100000
15232.0000000	0.0104762
15233.0000000	0.0100000
15234.0000000	0.0105000
15235.0000000	0.0110000
15236.0000000	0.0100000
15237.0000000	0.0100000
15238.0000000	0.0104762
15239.0000000	0.0105000
15240.0000000	0.0105000
15241.0000000	0.0104762
15242.0000000	0.0105000
15243.0000000	0.0105000
15244.0000000	0.0100000
15245.0000000	0.0110000
15246.0000000	0.0110000
15247.0000000	0.0104762
15248.0000000	0.0105000
15249.0000000	0.0120000
15250.0000000	0.0104762
15251.0000000	0.0125000

15252.0000000	0.0115000
15253.0000000	0.0109524
15254.0000000	0.0110000
15255.0000000	0.0110000
15256.0000000	0.0123810
15257.0000000	0.0125000
15258.0000000	0.0105000
15259.0000000	0.0109524
15260.0000000	0.0125000
15261.0000000	0.0120000
15262.0000000	0.0100000
15263.0000000	0.0142857
15264.0000000	0.0120000
15265.0000000	0.0109524
15266.0000000	0.0115000
15267.0000000	0.0115000
15268.0000000	0.0133333
15269.0000000	0.0135000
15270.0000000	0.0120000
15271.0000000	0.0120000
15272.0000000	0.0109524
15273.0000000	0.0120000
15274.0000000	0.0104762
15275.0000000	0.0110000
15276.0000000	0.0130000
15277.0000000	0.0119048
15278.0000000	0.0120000
15279.0000000	0.0110000
15280.0000000	0.0130000
15281.0000000	0.0123810
15282.0000000	0.0110000
15283.0000000	0.0135000
15284.0000000	0.0119048
15285.0000000	0.0120000
15286.0000000	0.0133333
15287.0000000	0.0110000
15288.0000000	0.0130000
15289.0000000	0.0109524
15290.0000000	0.0125000
15291.0000000	0.0115000
15292.0000000	0.0125000
15293.0000000	0.0128571
15294.0000000	0.0135000
15295.0000000	0.0140000
15296.0000000	0.0109524
15297.0000000	0.0119048
15298.0000000	0.0115000
15299.0000000	0.0125000
15300.0000000	0.0120000
15301.0000000	0.0125000
15302.0000000	0.0119048
15303.0000000	0.0130000
15304.0000000	0.0145000
15305.0000000	0.0119048
15306.0000000	0.0120000
15307.0000000	0.0140000
15308.0000000	0.0123810

15309.0000000	0.0120000
15310.0000000	0.0125000
15311.0000000	0.0138095
15312.0000000	0.0115000
15313.0000000	0.0123810
15314.0000000	0.0135000
15315.0000000	0.0125000
15316.0000000	0.0130000
15317.0000000	0.0123810
15318.0000000	0.0125000
15319.0000000	0.0140000
15320.0000000	0.0119048
15321.0000000	0.0135000
15322.0000000	0.0125000
15323.0000000	0.0130000
15324.0000000	0.0119048
15325.0000000	0.0119048
15326.0000000	0.0130000
15327.0000000	0.0140000
15328.0000000	0.0105000
15329.0000000	0.0119048
15330.0000000	0.0115000
15331.0000000	0.0115000
15332.0000000	0.0147619
15333.0000000	0.0140000
15334.0000000	0.0125000
15335.0000000	0.0133333
15336.0000000	0.0135000
15337.0000000	0.0130000
15338.0000000	0.0138095
15339.0000000	0.0125000
15340.0000000	0.0115000
15341.0000000	0.0109524
15342.0000000	0.0125000
15343.0000000	0.0110000
15344.0000000	0.0114286
15345.0000000	0.0110000
15346.0000000	0.0120000
15347.0000000	0.0119048
15348.0000000	0.0110000
15349.0000000	0.0119048
15350.0000000	0.0130000
15351.0000000	0.0115000
15352.0000000	0.0120000
15353.0000000	0.0128571
15354.0000000	0.0120000
15355.0000000	0.0115000
15356.0000000	0.0109524
15357.0000000	0.0135000
15358.0000000	0.0123810
15359.0000000	0.0125000
15360.0000000	0.0110000
15361.0000000	0.0105000
15362.0000000	0.0114286
15363.0000000	0.0120000
15364.0000000	0.0105000
15365.0000000	0.0114286

15366.0000000	0.0120000
15367.0000000	0.0120000
15368.0000000	0.0105000
15369.0000000	0.0119048
15370.0000000	0.0110000
15371.0000000	0.0110000
15372.0000000	0.0119048
15373.0000000	0.0110000
15374.0000000	0.0109524
15375.0000000	0.0115000
15376.0000000	0.0120000
15377.0000000	0.0109524
15378.0000000	0.0120000
15379.0000000	0.0105000
15380.0000000	0.0105000
15381.0000000	0.0109524
15382.0000000	0.0115000
15383.0000000	0.0125000
15384.0000000	0.0109524
15385.0000000	0.0110000
15386.0000000	0.0123810
15387.0000000	0.0115000
15388.0000000	0.0120000
15389.0000000	0.0109524
15390.0000000	0.0120000
15391.0000000	0.0120000
15392.0000000	0.0110000
15393.0000000	0.0104762
15394.0000000	0.0104762
15395.0000000	0.0110000
15396.0000000	0.0115000
15397.0000000	0.0110000
15398.0000000	0.0110000
15399.0000000	0.0109524
15400.0000000	0.0115000
15401.0000000	0.0100000
15402.0000000	0.0110000
15403.0000000	0.0105000
15404.0000000	0.0109524
15405.0000000	0.0100000
15406.0000000	0.0105000
15407.0000000	0.0115000
15408.0000000	0.0114286
15409.0000000	0.0110000
15410.0000000	0.0125000
15411.0000000	0.0109524
15412.0000000	0.0105000
15413.0000000	0.0114286
15414.0000000	0.0100000
15415.0000000	0.0110000
15416.0000000	0.0100000
15417.0000000	0.0104762
15418.0000000	0.0110000
15419.0000000	0.0109524
15420.0000000	0.0120000
15421.0000000	0.0115000
15422.0000000	0.0125000

15423.0000000	0.0119048
15424.0000000	0.0100000
15425.0000000	0.0104762
15426.0000000	0.0105000
15427.0000000	0.0115000
15428.0000000	0.0110000
15429.0000000	0.0119048
15430.0000000	0.0100000
15431.0000000	0.0105000
15432.0000000	0.0109524
15433.0000000	0.0100000
15434.0000000	0.0114286
15435.0000000	0.0105000
15436.0000000	0.0105000
15437.0000000	0.0119048
15438.0000000	0.0125000
15439.0000000	0.0105000
15440.0000000	0.0110000
15441.0000000	0.0109524
15442.0000000	0.0115000
15443.0000000	0.0105000
15444.0000000	0.0109524
15445.0000000	0.0115000
15446.0000000	0.0119048
15447.0000000	0.0115000
15448.0000000	0.0110000
15449.0000000	0.0109524
15450.0000000	0.0120000
15451.0000000	0.0100000
15452.0000000	0.0110000
15453.0000000	0.0114286
15454.0000000	0.0115000
15455.0000000	0.0123810
15456.0000000	0.0110000
15457.0000000	0.0115000
15458.0000000	0.0120000
15459.0000000	0.0109524
15460.0000000	0.0105000
15461.0000000	0.0105000
15462.0000000	0.0100000
15463.0000000	0.0110000
15464.0000000	0.0104762
15465.0000000	0.0115000
15466.0000000	0.0110000
15467.0000000	0.0105000
15468.0000000	0.0105000
15469.0000000	0.0104762
15470.0000000	0.0120000
15471.0000000	0.0109524
15472.0000000	0.0105000
15473.0000000	0.0105000
15474.0000000	0.0100000
15475.0000000	0.0110000
15476.0000000	0.0105000
15477.0000000	0.0114286
15478.0000000	0.0105000
15479.0000000	0.0120000

15480.0000000	0.0114286
15481.0000000	0.0115000
15482.0000000	0.0119048
15483.0000000	0.0115789
15484.0000000	0.0114286
15485.0000000	0.0104762
15486.0000000	0.0120000
15487.0000000	0.0105000
15488.0000000	0.0115000
15489.0000000	0.0100000
15490.0000000	0.0105000
15491.0000000	0.0109524
15492.0000000	0.0110000
15493.0000000	0.0105000
15494.0000000	0.0109524
15495.0000000	0.0105000
15496.0000000	0.0110000
15497.0000000	0.0115000
15498.0000000	0.0114286
15499.0000000	0.0105000
15500.0000000	0.0105000
15501.0000000	0.0119048
15502.0000000	0.0115000
15503.0000000	0.0120000
15504.0000000	0.0115000
15505.0000000	0.0123810
15506.0000000	0.0110000
15507.0000000	0.0119048
15508.0000000	0.0130000
15509.0000000	0.0110000
15510.0000000	0.0119048
15511.0000000	0.0110000
15512.0000000	0.0125000
15513.0000000	0.0114286
15514.0000000	0.0120000
15515.0000000	0.0100000
15516.0000000	0.0119048
15517.0000000	0.0105000
15518.0000000	0.0120000
15519.0000000	0.0115000
15520.0000000	0.0128571
15521.0000000	0.0105000
15522.0000000	0.0100000
15523.0000000	0.0100000
15524.0000000	0.0125000
15525.0000000	0.0114286
15526.0000000	0.0125000
15527.0000000	0.0115000
15528.0000000	0.0120000
15529.0000000	0.0123810
15530.0000000	0.0123810
15531.0000000	0.0105000
15532.0000000	0.0125000
15533.0000000	0.0115000
15534.0000000	0.0109524
15535.0000000	0.0110000
15536.0000000	0.0125000

15537.0000000	0.0119048
15538.0000000	0.0120000
15539.0000000	0.0110000
15540.0000000	0.0128571
15541.0000000	0.0120000
15542.0000000	0.0110000
15543.0000000	0.0128571
15544.0000000	0.0110000
15545.0000000	0.0105000
15546.0000000	0.0114286
15547.0000000	0.0115000
15548.0000000	0.0105000
15549.0000000	0.0115000
15550.0000000	0.0104762
15551.0000000	0.0100000
15552.0000000	0.0114286
15553.0000000	0.0110000
15554.0000000	0.0115000
15555.0000000	0.0104762
15556.0000000	0.0110000
15557.0000000	0.0105000
15558.0000000	0.0120000
15559.0000000	0.0104762
15560.0000000	0.0110000
15561.0000000	0.0109524
15562.0000000	0.0100000
15563.0000000	0.0115000
15564.0000000	0.0100000
15565.0000000	0.0123810
15566.0000000	0.0120000
15567.0000000	0.0110000
15568.0000000	0.0104762
15569.0000000	0.0110000
15570.0000000	0.0100000
15571.0000000	0.0110000
15572.0000000	0.0105000
15573.0000000	0.0105000
15574.0000000	0.0109524
15575.0000000	0.0105000
15576.0000000	0.0105000
15577.0000000	0.0119048
15578.0000000	0.0105000
15579.0000000	0.0104762
15580.0000000	0.0115000
15581.0000000	0.0115000
15582.0000000	0.0114286
15583.0000000	0.0120000
15584.0000000	0.0100000
15585.0000000	0.0115000
15586.0000000	0.0109524
15587.0000000	0.0115000
15588.0000000	0.0125000
15589.0000000	0.0123810
15590.0000000	0.0110000
15591.0000000	0.0109524
15592.0000000	0.0120000
15593.0000000	0.0110000

15594.0000000	0.0105000
15595.0000000	0.0100000
15596.0000000	0.0115000
15597.0000000	0.0115000
15598.0000000	0.0100000
15599.0000000	0.0109524
15600.0000000	0.0110000
15601.0000000	0.0110000
15602.0000000	0.0105000
15603.0000000	0.0105000
15604.0000000	0.0104762
15605.0000000	0.0100000
15606.0000000	0.0114286
15607.0000000	0.0100000
15608.0000000	0.0100000
15609.0000000	0.0115000
15610.0000000	0.0100000
15611.0000000	0.0110000
15612.0000000	0.0105000
15613.0000000	0.0100000
15614.0000000	0.0105000
15615.0000000	0.0104762
15616.0000000	0.0100000
15617.0000000	0.0105000
15618.0000000	0.0104762
15619.0000000	0.0100000
15620.0000000	0.0110000
15621.0000000	0.0100000
15622.0000000	0.0100000
15623.0000000	0.0105000
15624.0000000	0.0105000
15625.0000000	0.0100000
15626.0000000	0.0100000
15627.0000000	0.0110000
15628.0000000	0.0100000
15629.0000000	0.0105000
15630.0000000	0.0100000
15631.0000000	0.0100000
15632.0000000	0.0100000
15633.0000000	0.0100000
15634.0000000	0.0104762
15635.0000000	0.0105000
15636.0000000	0.0110000
15637.0000000	0.0109524
15638.0000000	0.0105000
15639.0000000	0.0100000
15640.0000000	0.0100000
15641.0000000	0.0100000
15642.0000000	0.0104762
15643.0000000	0.0100000
15644.0000000	0.0100000
15645.0000000	0.0100000
15646.0000000	0.0104762
15647.0000000	0.0105000
15648.0000000	0.0105000
15649.0000000	0.0104762
15650.0000000	0.0100000

15651.0000000	0.0109524
15652.0000000	0.0105000
15653.0000000	0.0100000
15654.0000000	0.0110000
15655.0000000	0.0105000
15656.0000000	0.0104762
15657.0000000	0.0110000
15658.0000000	0.0100000
15659.0000000	0.0100000
15660.0000000	0.0100000
15661.0000000	0.0110000
15662.0000000	0.0100000
15663.0000000	0.0100000
15664.0000000	0.0105000
15665.0000000	0.0100000
15666.0000000	0.0105000
15667.0000000	0.0109524
15668.0000000	0.0105000
15669.0000000	0.0100000
15670.0000000	0.0104762
15671.0000000	0.0100000
15672.0000000	0.0100000
15673.0000000	0.0104762
15674.0000000	0.0105000
15675.0000000	0.0115000
15676.0000000	0.0100000
15677.0000000	0.0110000
15678.0000000	0.0104762
15679.0000000	0.0105000
15680.0000000	0.0100000
15681.0000000	0.0100000
15682.0000000	0.0104762
15683.0000000	0.0100000
15684.0000000	0.0100000
15685.0000000	0.0100000
15686.0000000	0.0100000
15687.0000000	0.0104762
15688.0000000	0.0110000
15689.0000000	0.0100000
15690.0000000	0.0100000
15691.0000000	0.0105000
15692.0000000	0.0100000
15693.0000000	0.0100000
15694.0000000	0.0100000
15695.0000000	0.0100000
15696.0000000	0.0105000
15697.0000000	0.0104762
15698.0000000	0.0105000
15699.0000000	0.0110000
15700.0000000	0.0100000
15701.0000000	0.0100000
15702.0000000	0.0105000
15703.0000000	0.0100000
15704.0000000	0.0100000
15705.0000000	0.0105000
15706.0000000	0.0100000
15707.0000000	0.0105000

15708.0000000	0.0100000
15709.0000000	0.0100000
15710.0000000	0.0104762
15711.0000000	0.0100000
15712.0000000	0.0100000
15713.0000000	0.0100000
15714.0000000	0.0100000
15715.0000000	0.0100000
15716.0000000	0.0104762
15717.0000000	0.0105000
15718.0000000	0.0100000
15719.0000000	0.0100000
15720.0000000	0.0105000
15721.0000000	0.0100000
15722.0000000	0.0110000
15723.0000000	0.0105000
15724.0000000	0.0100000
15725.0000000	0.0114286
15726.0000000	0.0105000
15727.0000000	0.0104762
15728.0000000	0.0105000
15729.0000000	0.0100000
15730.0000000	0.0104762
15731.0000000	0.0100000
15732.0000000	0.0110000
15733.0000000	0.0110000
15734.0000000	0.0114286
15735.0000000	0.0109524
15736.0000000	0.0105000
15737.0000000	0.0115000
15738.0000000	0.0105000
15739.0000000	0.0100000
15740.0000000	0.0105000
15741.0000000	0.0100000
15742.0000000	0.0104762
15743.0000000	0.0100000
15744.0000000	0.0110000
15745.0000000	0.0110000
15746.0000000	0.0100000
15747.0000000	0.0105000
15748.0000000	0.0104762
15749.0000000	0.0105000
15750.0000000	0.0110000
15751.0000000	0.0109524
15752.0000000	0.0110000
15753.0000000	0.0115000
15754.0000000	0.0105000
15755.0000000	0.0104762
15756.0000000	0.0105000
15757.0000000	0.0100000
15758.0000000	0.0110000
15759.0000000	0.0100000
15760.0000000	0.0105000
15761.0000000	0.0100000
15762.0000000	0.0104762
15763.0000000	0.0100000
15764.0000000	0.0100000

15765.0000000	0.0105000
15766.0000000	0.0109524
15767.0000000	0.0110000
15768.0000000	0.0110000
15769.0000000	0.0125000
15770.0000000	0.0104762
15771.0000000	0.0105000
15772.0000000	0.0105000
15773.0000000	0.0109524
15774.0000000	0.0110000
15775.0000000	0.0109524
15776.0000000	0.0105000
15777.0000000	0.0105000
15778.0000000	0.0109524
15779.0000000	0.0115000
15780.0000000	0.0110000
15781.0000000	0.0115000
15782.0000000	0.0100000
15783.0000000	0.0110000
15784.0000000	0.0104762
15785.0000000	0.0100000
15786.0000000	0.0120000
15787.0000000	0.0109524
15788.0000000	0.0110000
15789.0000000	0.0105000
15790.0000000	0.0110000
15791.0000000	0.0104762
15792.0000000	0.0110000
15793.0000000	0.0130000
15794.0000000	0.0114286
15795.0000000	0.0110000
15796.0000000	0.0114286
15797.0000000	0.0110000
15798.0000000	0.0110000
15799.0000000	0.0105000
15800.0000000	0.0100000
15801.0000000	0.0110000
15802.0000000	0.0120000
15803.0000000	0.0109524
15804.0000000	0.0110000
15805.0000000	0.0120000
15806.0000000	0.0109524
15807.0000000	0.0105000
15808.0000000	0.0110000
15809.0000000	0.0109524
15810.0000000	0.0105000
15811.0000000	0.0109524
15812.0000000	0.0110000
15813.0000000	0.0110000
15814.0000000	0.0125000
15815.0000000	0.0104762
15816.0000000	0.0110000
15817.0000000	0.0105000
15818.0000000	0.0109524
15819.0000000	0.0115000
15820.0000000	0.0110000
15821.0000000	0.0115000

15822.0000000	0.0100000
15823.0000000	0.0109524
15824.0000000	0.0105000
15825.0000000	0.0115000
15826.0000000	0.0110000
15827.0000000	0.0119048
15828.0000000	0.0110000
15829.0000000	0.0110000
15830.0000000	0.0104762
15831.0000000	0.0100000
15832.0000000	0.0100000
15833.0000000	0.0100000
15834.0000000	0.0100000
15835.0000000	0.0115000
15836.0000000	0.0100000
15837.0000000	0.0110000
15838.0000000	0.0110000
15839.0000000	0.0104762
15840.0000000	0.0115000
15841.0000000	0.0100000
15842.0000000	0.0109524
15843.0000000	0.0110000
15844.0000000	0.0105000
15845.0000000	0.0109524
15846.0000000	0.0110000
15847.0000000	0.0105000
15848.0000000	0.0100000
15849.0000000	0.0100000
15850.0000000	0.0105000
15851.0000000	0.0100000
15852.0000000	0.0100000
15853.0000000	0.0100000
15854.0000000	0.0104762
15855.0000000	0.0110000
15856.0000000	0.0100000
15857.0000000	0.0104762
15858.0000000	0.0105000
15859.0000000	0.0100000
15860.0000000	0.0105000
15861.0000000	0.0104762
15862.0000000	0.0100000
15863.0000000	0.0104762
15864.0000000	0.0115000
15865.0000000	0.0100000
15866.0000000	0.0110000
15867.0000000	0.0100000
15868.0000000	0.0110000
15869.0000000	0.0105000
15870.0000000	0.0100000
15871.0000000	0.0105000
15872.0000000	0.0100000
15873.0000000	0.0105000
15874.0000000	0.0105000
15875.0000000	0.0104762
15876.0000000	0.0100000
15877.0000000	0.0115000
15878.0000000	0.0100000

15879.0000000	0.0110000
15880.0000000	0.0100000
15881.0000000	0.0105000
15882.0000000	0.0104762
15883.0000000	0.0100000
15884.0000000	0.0104762
15885.0000000	0.0110000
15886.0000000	0.0110000
15887.0000000	0.0114286
15888.0000000	0.0100000
15889.0000000	0.0100000
15890.0000000	0.0100000
15891.0000000	0.0104762
15892.0000000	0.0100000
15893.0000000	0.0100000
15894.0000000	0.0105000
15895.0000000	0.0100000
15896.0000000	0.0100000
15897.0000000	0.0100000
15898.0000000	0.0105000
15899.0000000	0.0100000
15900.0000000	0.0100000
15901.0000000	0.0100000
15902.0000000	0.0104762
15903.0000000	0.0115000
15904.0000000	0.0105000
15905.0000000	0.0100000
15906.0000000	0.0104762
15907.0000000	0.0105000
15908.0000000	0.0105000
15909.0000000	0.0104762
15910.0000000	0.0110000
15911.0000000	0.0100000
15912.0000000	0.0105000
15913.0000000	0.0100000
15914.0000000	0.0105000
15915.0000000	0.0104762
15916.0000000	0.0100000
15917.0000000	0.0100000
15918.0000000	0.0104762
15919.0000000	0.0100000
15920.0000000	0.0100000
15921.0000000	0.0104762
15922.0000000	0.0100000
15923.0000000	0.0104762
15924.0000000	0.0105000
15925.0000000	0.0105000
15926.0000000	0.0105000
15927.0000000	0.0109524
15928.0000000	0.0105000
15929.0000000	0.0100000
15930.0000000	0.0100000
15931.0000000	0.0105000
15932.0000000	0.0104762
15933.0000000	0.0100000
15934.0000000	0.0105000
15935.0000000	0.0105000

15936.0000000	0.0109524
15937.0000000	0.0105000
15938.0000000	0.0110000
15939.0000000	0.0109524
15940.0000000	0.0105000
15941.0000000	0.0100000
15942.0000000	0.0100000
15943.0000000	0.0100000
15944.0000000	0.0105000
15945.0000000	0.0104762
15946.0000000	0.0100000
15947.0000000	0.0114286
15948.0000000	0.0105000
15949.0000000	0.0110000
15950.0000000	0.0100000
15951.0000000	0.0100000
15952.0000000	0.0120000
15953.0000000	0.0110000
15954.0000000	0.0109524
15955.0000000	0.0110000
15956.0000000	0.0110000
15957.0000000	0.0115000
15958.0000000	0.0104762
15959.0000000	0.0100000
15960.0000000	0.0100000
15961.0000000	0.0100000
15962.0000000	0.0110000
15963.0000000	0.0100000
15964.0000000	0.0100000
15965.0000000	0.0105000
15966.0000000	0.0105000
15967.0000000	0.0100000
15968.0000000	0.0100000
15969.0000000	0.0100000
15970.0000000	0.0110000
15971.0000000	0.0100000
15972.0000000	0.0100000
15973.0000000	0.0100000
15974.0000000	0.0115000
15975.0000000	0.0104762
15976.0000000	0.0100000
15977.0000000	0.0100000
15978.0000000	0.0104762
15979.0000000	0.0110000
15980.0000000	0.0105000
15981.0000000	0.0104762
15982.0000000	0.0100000
15983.0000000	0.0104762
15984.0000000	0.0100000
15985.0000000	0.0110000
15986.0000000	0.0100000
15987.0000000	0.0110000
15988.0000000	0.0104762
15989.0000000	0.0100000
15990.0000000	0.0100000
15991.0000000	0.0104762
15992.0000000	0.0109524

15993.0000000	0.0100000
15994.0000000	0.0100000
15995.0000000	0.0110000
15996.0000000	0.0100000
15997.0000000	0.0104762
15998.0000000	0.0105000
15999.0000000	0.0109524
16000.0000000	0.0105000
16001.0000000	0.0110000
16002.0000000	0.0105000
16003.0000000	0.0109524
16004.0000000	0.0100000
16005.0000000	0.0100000
16006.0000000	0.0104762
16007.0000000	0.0105000
16008.0000000	0.0100000
16009.0000000	0.0110000
16010.0000000	0.0100000
16011.0000000	0.0114286
16012.0000000	0.0105000
16013.0000000	0.0100000
16014.0000000	0.0100000
16015.0000000	0.0104762
16016.0000000	0.0100000
16017.0000000	0.0105000
16018.0000000	0.0100000
16019.0000000	0.0100000
16020.0000000	0.0100000
16021.0000000	0.0100000
16022.0000000	0.0100000
16023.0000000	0.0100000
16024.0000000	0.0100000
16025.0000000	0.0100000
16026.0000000	0.0100000
16027.0000000	0.0100000
16028.0000000	0.0105000
16029.0000000	0.0105000
16030.0000000	0.0100000
16031.0000000	0.0100000
16032.0000000	0.0100000
16033.0000000	0.0100000
16034.0000000	0.0100000
16035.0000000	0.0100000
16036.0000000	0.0100000
16037.0000000	0.0100000
16038.0000000	0.0100000
16039.0000000	0.0100000
16040.0000000	0.0095000
16041.0000000	0.0090000
16042.0000000	0.0100000
16043.0000000	0.0095000
16044.0000000	0.0090476
16045.0000000	0.0095000
16046.0000000	0.0090000
16047.0000000	0.0100000
16048.0000000	0.0085714
16049.0000000	0.0095000

16050.0000000	0.0095000
16051.0000000	0.0080952
16052.0000000	0.0085000
16053.0000000	0.0095238
16054.0000000	0.0085000
16055.0000000	0.0090000
16056.0000000	0.0090476
16057.0000000	0.0085000
16058.0000000	0.0080000
16059.0000000	0.0080000
16060.0000000	0.0071429
16061.0000000	0.0070000
16062.0000000	0.0055000
16063.0000000	0.0076190
16064.0000000	0.0060000
16065.0000000	0.0052381
16066.0000000	0.0065000
16067.0000000	0.0080000
16068.0000000	0.0057143
16069.0000000	0.0065000
16070.0000000	0.0055000
16071.0000000	0.0055000
16072.0000000	0.0057143
16073.0000000	0.0060000
16074.0000000	0.0057143
16075.0000000	0.0060000
16076.0000000	0.0040000
16077.0000000	0.0060000
16078.0000000	0.0061905
16079.0000000	0.0065000
16080.0000000	0.0057143
16081.0000000	0.0055000
16082.0000000	0.0055000
16083.0000000	0.0060000
16084.0000000	0.0047619
16085.0000000	0.0035000
16086.0000000	0.0050000
16087.0000000	0.0057143
16088.0000000	0.0055000
16089.0000000	0.0042857
16090.0000000	0.0020000
16091.0000000	0.0040000
16092.0000000	0.0025000
16093.0000000	0.0050000
16094.0000000	0.0047619
16095.0000000	0.0030000
16096.0000000	0.0033333
16097.0000000	0.0020000
16098.0000000	0.0042857
16099.0000000	0.0025000
16100.0000000	0.0025000
16101.0000000	0.0035000
16102.0000000	0.0030000
16103.0000000	0.0023810
16104.0000000	0.0033333
16105.0000000	0.0015000
16106.0000000	0.0010000

16107.0000000	0.0015000
16108.0000000	0.0023810
16109.0000000	0.0010000
16110.0000000	0.0000000
16111.0000000	0.0023810
16112.0000000	0.0005000
16113.0000000	0.0019048
16114.0000000	0.0015000
16115.0000000	0.0020000
16116.0000000	0.0030000
16117.0000000	0.0023810
16118.0000000	0.0020000
16119.0000000	0.0025000
16120.0000000	0.0009524
16121.0000000	0.0020000
16122.0000000	0.0015000
16123.0000000	0.0019048
16124.0000000	0.0015000
16125.0000000	0.0015000
16126.0000000	0.0009524
16127.0000000	0.0010000
16128.0000000	0.0004762
16129.0000000	0.0015000
16130.0000000	0.0020000
16131.0000000	0.0005000
16132.0000000	0.0028571
16133.0000000	0.0000000
16134.0000000	0.0005000
16135.0000000	0.0004762
16136.0000000	0.0010000
16137.0000000	0.0005000
16138.0000000	0.0000000
16139.0000000	0.0000000
16140.0000000	0.0000000
16141.0000000	0.0004762
16142.0000000	0.0010000
16143.0000000	0.0000000
16144.0000000	0.0004762
16145.0000000	0.0000000
16146.0000000	0.0000000
16147.0000000	0.0000000
16148.0000000	0.0014286
16149.0000000	0.0005000
16150.0000000	0.0000000
16151.0000000	0.0005000
16152.0000000	0.0005000
16153.0000000	0.0000000
16154.0000000	0.0005000
16155.0000000	0.0000000
16156.0000000	0.0009524
16157.0000000	0.0000000
16158.0000000	0.0005000
16159.0000000	0.0000000
16160.0000000	0.0000000
16161.0000000	0.0000000
16162.0000000	0.0000000
16163.0000000	0.0005000

16164.0000000	0.0000000
16165.0000000	0.0000000
16166.0000000	0.0000000
16167.0000000	0.0000000
16168.0000000	0.0000000
16169.0000000	0.0000000
16170.0000000	0.0000000
16171.0000000	0.0000000
16172.0000000	0.0000000
16173.0000000	0.0000000
16174.0000000	0.0000000
16175.0000000	0.0000000
16176.0000000	0.0000000
16177.0000000	0.0000000
16178.0000000	0.0000000
16179.0000000	0.0000000
16180.0000000	0.0000000
16181.0000000	0.0000000
16182.0000000	0.0000000
16183.0000000	0.0000000
16184.0000000	0.0000000
16185.0000000	0.0000000
16186.0000000	0.0000000
16187.0000000	0.0000000
16188.0000000	0.0000000
16189.0000000	0.0000000
16190.0000000	0.0000000
16191.0000000	-0.0005000
16192.0000000	0.0000000
16193.0000000	0.0000000
16194.0000000	0.0000000
16195.0000000	0.0000000
16196.0000000	0.0000000
16197.0000000	0.0000000
16198.0000000	0.0000000
16199.0000000	0.0000000
16200.0000000	0.0000000
16201.0000000	0.0000000
16202.0000000	0.0000000
16203.0000000	0.0000000
16204.0000000	0.0000000
16205.0000000	-0.0005000
16206.0000000	0.0000000
16207.0000000	-0.0005000
16208.0000000	0.0000000
16209.0000000	0.0000000
16210.0000000	-0.0005000
16211.0000000	0.0000000
16212.0000000	-0.0005000
16213.0000000	0.0000000
16214.0000000	-0.0010000
16215.0000000	0.0000000
16216.0000000	0.0000000
16217.0000000	-0.0004762
16218.0000000	0.0000000
16219.0000000	-0.0010000
16220.0000000	0.0000000

16221.0000000	0.0000000
16222.0000000	-0.0004762
16223.0000000	-0.0010000
16224.0000000	-0.0010000
16225.0000000	-0.0004762
16226.0000000	0.0000000
16227.0000000	0.0000000
16228.0000000	0.0000000
16229.0000000	0.0000000
16230.0000000	-0.0005000
16231.0000000	0.0000000
16232.0000000	-0.0005000
16233.0000000	0.0000000
16234.0000000	-0.0004762
16235.0000000	-0.0005000
16236.0000000	-0.0005000
16237.0000000	-0.0010000
16238.0000000	-0.0004762
16239.0000000	0.0000000
16240.0000000	-0.0020000
16241.0000000	-0.0019048
16242.0000000	-0.0005000
16243.0000000	-0.0020000
16244.0000000	-0.0019048
16245.0000000	0.0000000
16246.0000000	-0.0010000
16247.0000000	-0.0004762
16248.0000000	-0.0015000
16249.0000000	-0.0014286
16250.0000000	-0.0015000
16251.0000000	-0.0005000
16252.0000000	-0.0010000
16253.0000000	-0.0014286
16254.0000000	0.0000000
16255.0000000	-0.0005000
16256.0000000	-0.0009524
16257.0000000	-0.0015000
16258.0000000	0.0000000
16259.0000000	-0.0025000
16260.0000000	-0.0004762
16261.0000000	-0.0014286
16262.0000000	-0.0005000
16263.0000000	-0.0005000
16264.0000000	0.0000000
16265.0000000	-0.0014286
16266.0000000	-0.0010000
16267.0000000	0.0000000
16268.0000000	-0.0004762
16269.0000000	0.0000000
16270.0000000	0.0000000
16271.0000000	0.0000000
16272.0000000	-0.0010000
16273.0000000	0.0000000
16274.0000000	-0.0004762
16275.0000000	-0.0010000
16276.0000000	-0.0005000
16277.0000000	0.0000000

16278.0000000	0.0000000
16279.0000000	-0.0004762
16280.0000000	-0.0005000
16281.0000000	-0.0010000
16282.0000000	-0.0005000
16283.0000000	-0.0014286
16284.0000000	-0.0025000
16285.0000000	-0.0005000
16286.0000000	-0.0014286
16287.0000000	0.0000000
16288.0000000	0.0000000
16289.0000000	-0.0014286
16290.0000000	-0.0005000
16291.0000000	-0.0010000
16292.0000000	-0.0009524
16293.0000000	-0.0010000
16294.0000000	-0.0005000
16295.0000000	-0.0004762
16296.0000000	-0.0015000
16297.0000000	-0.0020000
16298.0000000	-0.0020000
16299.0000000	-0.0014286
16300.0000000	-0.0005000
16301.0000000	0.0000000
16302.0000000	-0.0015000
16303.0000000	-0.0005000
16304.0000000	-0.0010000
16305.0000000	-0.0014286
16306.0000000	-0.0005000
16307.0000000	-0.0010000
16308.0000000	-0.0014286
16309.0000000	-0.0009524
16310.0000000	-0.0035000
16311.0000000	-0.0005000
16312.0000000	-0.0010000
16313.0000000	-0.0009524
16314.0000000	-0.0005000
16315.0000000	-0.0015000
16316.0000000	-0.0010000
16317.0000000	-0.0009524
16318.0000000	0.0000000
16319.0000000	0.0000000
16320.0000000	-0.0010000
16321.0000000	-0.0005000
16322.0000000	-0.0009524
16323.0000000	-0.0010000
16324.0000000	-0.0015000
16325.0000000	-0.0014286
16326.0000000	-0.0020000
16327.0000000	-0.0015000
16328.0000000	-0.0004762
16329.0000000	-0.0020000
16330.0000000	-0.0020000
16331.0000000	-0.0033333
16332.0000000	-0.0015000
16333.0000000	-0.0015000
16334.0000000	-0.0015000

16335.0000000	-0.0014286
16336.0000000	-0.0010000
16337.0000000	-0.0014286
16338.0000000	-0.0035000
16339.0000000	-0.0025000
16340.0000000	-0.0014286
16341.0000000	-0.0010000
16342.0000000	-0.0020000
16343.0000000	-0.0020000
16344.0000000	-0.0023810
16345.0000000	-0.0015000
16346.0000000	-0.0028571
16347.0000000	-0.0015000
16348.0000000	-0.0015000
16349.0000000	-0.0023810
16350.0000000	-0.0015000
16351.0000000	-0.0030000
16352.0000000	-0.0025000
16353.0000000	-0.0028571
16354.0000000	-0.0025000
16355.0000000	-0.0014286
16356.0000000	-0.0025000
16357.0000000	-0.0025000
16358.0000000	-0.0019048
16359.0000000	-0.0025000
16360.0000000	-0.0015000
16361.0000000	-0.0019048
16362.0000000	-0.0025000
16363.0000000	-0.0020000
16364.0000000	-0.0035000
16365.0000000	-0.0019048
16366.0000000	-0.0030000
16367.0000000	-0.0019048
16368.0000000	-0.0015000
16369.0000000	-0.0015000
16370.0000000	-0.0028571
16371.0000000	-0.0015000
16372.0000000	-0.0010000
16373.0000000	-0.0023810
16374.0000000	-0.0015000
16375.0000000	-0.0010000
16376.0000000	-0.0030000
16377.0000000	-0.0004762
16378.0000000	-0.0030000
16379.0000000	-0.0025000
16380.0000000	-0.0019048
16381.0000000	0.0000000
16382.0000000	-0.0015000
16383.0000000	-0.0023810
16384.0000000	-0.0030000
16385.0000000	-0.0004762
16386.0000000	-0.0010000
16387.0000000	-0.0015000
16388.0000000	-0.0020000
16389.0000000	-0.0014286
16390.0000000	-0.0005000
16391.0000000	-0.0015000

16392.0000000	-0.0019048
16393.0000000	-0.0015000
16394.0000000	-0.0010000
16395.0000000	-0.0010000
16396.0000000	-0.0009524
16397.0000000	0.0000000
16398.0000000	-0.0033333
16399.0000000	-0.0025000
16400.0000000	-0.0015000
16401.0000000	-0.0014286
16402.0000000	0.0000000
16403.0000000	-0.0015000
16404.0000000	-0.0010000
16405.0000000	-0.0019048
16406.0000000	-0.0004762
16407.0000000	-0.0010000
16408.0000000	-0.0005000
16409.0000000	-0.0025000
16410.0000000	-0.0014286
16411.0000000	-0.0005000
16412.0000000	-0.0010000
16413.0000000	-0.0019048
16414.0000000	-0.0010000
16415.0000000	-0.0015000
16416.0000000	-0.0023810
16417.0000000	-0.0005000
16418.0000000	-0.0005000
16419.0000000	-0.0023810
16420.0000000	-0.0025000
16421.0000000	-0.0010000
16422.0000000	-0.0009524
16423.0000000	-0.0005000
16424.0000000	-0.0005000
16425.0000000	-0.0023810
16426.0000000	-0.0010000
16427.0000000	-0.0010000
16428.0000000	-0.0014286
16429.0000000	-0.0010000
16430.0000000	0.0000000
16431.0000000	-0.0005000
16432.0000000	-0.0015000
16433.0000000	-0.0005000
16434.0000000	-0.0009524
16435.0000000	-0.0015000
16436.0000000	-0.0010000
16437.0000000	-0.0019048
16438.0000000	0.0000000
16439.0000000	0.0000000
16440.0000000	0.0000000
16441.0000000	0.0000000
16442.0000000	-0.0015000
16443.0000000	-0.0004762
16444.0000000	-0.0020000
16445.0000000	-0.0005000
16446.0000000	0.0000000
16447.0000000	0.0000000
16448.0000000	-0.0015000

16449.0000000	-0.0009524
16450.0000000	-0.0010000
16451.0000000	-0.0005000
16452.0000000	-0.0014286
16453.0000000	-0.0020000
16454.0000000	-0.0010000
16455.0000000	-0.0014286
16456.0000000	-0.0020000
16457.0000000	-0.0005000
16458.0000000	-0.0028571
16459.0000000	-0.0030000
16460.0000000	-0.0025000
16461.0000000	-0.0009524
16462.0000000	-0.0015000
16463.0000000	-0.0005000
16464.0000000	0.0000000
16465.0000000	-0.0009524
16466.0000000	-0.0014286
16467.0000000	-0.0005000
16468.0000000	-0.0020000
16469.0000000	-0.0030000
16470.0000000	-0.0020000
16471.0000000	-0.0009524
16472.0000000	-0.0020000
16473.0000000	-0.0019048
16474.0000000	-0.0030000
16475.0000000	-0.0014286
16476.0000000	-0.0015000
16477.0000000	-0.0025000
16478.0000000	-0.0020000
16479.0000000	-0.0025000
16480.0000000	-0.0033333
16481.0000000	-0.0015000
16482.0000000	-0.0019048
16483.0000000	-0.0020000
16484.0000000	-0.0025000
16485.0000000	-0.0035000
16486.0000000	-0.0023810
16487.0000000	-0.0030000
16488.0000000	-0.0025000
16489.0000000	-0.0042857
16490.0000000	-0.0005000
16491.0000000	-0.0038095
16492.0000000	-0.0030000
16493.0000000	-0.0040000
16494.0000000	-0.0042857
16495.0000000	-0.0025000
16496.0000000	-0.0035000
16497.0000000	-0.0033333
16498.0000000	-0.0020000
16499.0000000	-0.0025000
16500.0000000	-0.0030000
16501.0000000	-0.0038095
16502.0000000	-0.0035000
16503.0000000	-0.0025000
16504.0000000	-0.0052381
16505.0000000	-0.0025000

16506.0000000	-0.0038095
16507.0000000	-0.0055000
16508.0000000	-0.0045000
16509.0000000	-0.0040000
16510.0000000	-0.0052381
16511.0000000	-0.0040000
16512.0000000	-0.0055000
16513.0000000	-0.0047619
16514.0000000	-0.0045000
16515.0000000	-0.0055000
16516.0000000	-0.0047619
16517.0000000	-0.0060000
16518.0000000	-0.0061905
16519.0000000	-0.0075000
16520.0000000	-0.0070000
16521.0000000	-0.0060000
16522.0000000	-0.0066667
16523.0000000	-0.0075000
16524.0000000	-0.0080000
16525.0000000	-0.0057143
16526.0000000	-0.0075000
16527.0000000	-0.0066667
16528.0000000	-0.0050000
16529.0000000	-0.0060000
16530.0000000	-0.0080000
16531.0000000	-0.0066667
16532.0000000	-0.0050000
16533.0000000	-0.0080000
16534.0000000	-0.0076190
16535.0000000	-0.0068421
16536.0000000	-0.0077273
16537.0000000	-0.0075000
16538.0000000	-0.0090000
16539.0000000	-0.0070000
16540.0000000	-0.0071429
16541.0000000	-0.0075000
16542.0000000	-0.0075000
16543.0000000	-0.0061905
16544.0000000	-0.0085000
16545.0000000	-0.0070000
16546.0000000	-0.0076190
16547.0000000	-0.0075000
16548.0000000	-0.0055000
16549.0000000	-0.0090476
16550.0000000	-0.0075000
16551.0000000	-0.0080000
16552.0000000	-0.0085714
16553.0000000	-0.0070000
16554.0000000	-0.0076190
16555.0000000	-0.0070000
16556.0000000	-0.0075000
16557.0000000	-0.0085000
16558.0000000	-0.0080952
16559.0000000	-0.0080000
16560.0000000	-0.0085000
16561.0000000	-0.0095238
16562.0000000	-0.0085000

16563.0000000	-0.0085714
16564.0000000	-0.0090000
16565.0000000	-0.0095000
16566.0000000	-0.0095238
16567.0000000	-0.0090000
16568.0000000	-0.0085000
16569.0000000	-0.0100000
16570.0000000	-0.0085714
16571.0000000	-0.0080000
16572.0000000	-0.0076190
16573.0000000	-0.0095000
16574.0000000	-0.0090000
16575.0000000	-0.0100000
16576.0000000	-0.0095000
16577.0000000	-0.0095238
16578.0000000	-0.0100000
16579.0000000	-0.0095238
16580.0000000	-0.0085000
16581.0000000	-0.0095000
16582.0000000	-0.0090476
16583.0000000	-0.0090000
16584.0000000	-0.0090000
16585.0000000	-0.0100000
16586.0000000	-0.0100000
16587.0000000	-0.0095000
16588.0000000	-0.0095238
16589.0000000	-0.0100000
16590.0000000	-0.0100000
16591.0000000	-0.0100000
16592.0000000	-0.0100000
16593.0000000	-0.0100000
16594.0000000	-0.0100000
16595.0000000	-0.0100000
16596.0000000	-0.0100000
16597.0000000	-0.0100000
16598.0000000	-0.0100000
16599.0000000	-0.0100000
16600.0000000	-0.0100000
16601.0000000	-0.0100000
16602.0000000	-0.0100000
16603.0000000	-0.0095000
16604.0000000	-0.0100000
16605.0000000	-0.0100000
16606.0000000	-0.0100000
16607.0000000	-0.0100000
16608.0000000	-0.0100000
16609.0000000	-0.0100000
16610.0000000	-0.0100000
16611.0000000	-0.0100000
16612.0000000	-0.0100000
16613.0000000	-0.0100000
16614.0000000	-0.0100000
16615.0000000	-0.0100000
16616.0000000	-0.0100000
16617.0000000	-0.0100000
16618.0000000	-0.0095238
16619.0000000	-0.0100000

16620.0000000	-0.0100000
16621.0000000	-0.0100000
16622.0000000	-0.0100000
16623.0000000	-0.0100000
16624.0000000	-0.0100000
16625.0000000	-0.0100000
16626.0000000	-0.0100000
16627.0000000	-0.0100000
16628.0000000	-0.0100000
16629.0000000	-0.0100000
16630.0000000	-0.0100000
16631.0000000	-0.0100000
16632.0000000	-0.0100000
16633.0000000	-0.0100000
16634.0000000	-0.0100000
16635.0000000	-0.0100000
16636.0000000	-0.0100000
16637.0000000	-0.0100000
16638.0000000	-0.0100000
16639.0000000	-0.0100000
16640.0000000	-0.0104762
16641.0000000	-0.0105000
16642.0000000	-0.0100000
16643.0000000	-0.0100000
16644.0000000	-0.0100000
16645.0000000	-0.0100000
16646.0000000	-0.0100000
16647.0000000	-0.0100000
16648.0000000	-0.0110000
16649.0000000	-0.0109524
16650.0000000	-0.0100000
16651.0000000	-0.0114286
16652.0000000	-0.0100000
16653.0000000	-0.0105000
16654.0000000	-0.0104762
16655.0000000	-0.0120000
16656.0000000	-0.0120000
16657.0000000	-0.0115000
16658.0000000	-0.0109524
16659.0000000	-0.0105000
16660.0000000	-0.0114286
16661.0000000	-0.0105000
16662.0000000	-0.0105000
16663.0000000	-0.0109524
16664.0000000	-0.0120000
16665.0000000	-0.0120000
16666.0000000	-0.0115000
16667.0000000	-0.0123810
16668.0000000	-0.0120000
16669.0000000	-0.0120000
16670.0000000	-0.0128571
16671.0000000	-0.0114286
16672.0000000	-0.0115000
16673.0000000	-0.0120000
16674.0000000	-0.0120000
16675.0000000	-0.0130000
16676.0000000	-0.0128571

16677.0000000	-0.0125000
16678.0000000	-0.0119048
16679.0000000	-0.0125000
16680.0000000	-0.0135000
16681.0000000	-0.0110000
16682.0000000	-0.0133333
16683.0000000	-0.0130000
16684.0000000	-0.0130000
16685.0000000	-0.0142857
16686.0000000	-0.0130000
16687.0000000	-0.0142857
16688.0000000	-0.0140000
16689.0000000	-0.0175000
16690.0000000	-0.0157143
16691.0000000	-0.0130000
16692.0000000	-0.0165000
16693.0000000	-0.0155000
16694.0000000	-0.0142857
16695.0000000	-0.0145000
16696.0000000	-0.0150000
16697.0000000	-0.0150000
16698.0000000	-0.0142857
16699.0000000	-0.0171429
16700.0000000	-0.0165000
16701.0000000	-0.0165000
16702.0000000	-0.0171429
16703.0000000	-0.0175000
16704.0000000	-0.0160000
16705.0000000	-0.0175000
16706.0000000	-0.0166667
16707.0000000	-0.0175000
16708.0000000	-0.0170000
16709.0000000	-0.0161905
16710.0000000	-0.0175000
16711.0000000	-0.0190000
16712.0000000	-0.0176190
16713.0000000	-0.0190000
16714.0000000	-0.0185714
16715.0000000	-0.0190000
16716.0000000	-0.0185000
16717.0000000	-0.0190000
16718.0000000	-0.0180952
16719.0000000	-0.0185000
16720.0000000	-0.0175000
16721.0000000	-0.0200000
16722.0000000	-0.0190000
16723.0000000	-0.0190476
16724.0000000	-0.0190000
16725.0000000	-0.0185000
16726.0000000	-0.0200000
16727.0000000	-0.0180000
16728.0000000	-0.0200000
16729.0000000	-0.0190000
16730.0000000	-0.0190476
16731.0000000	-0.0190000
16732.0000000	-0.0200000
16733.0000000	-0.0200000

16734.0000000	-0.0195000
16735.0000000	-0.0195000
16736.0000000	-0.0200000
16737.0000000	-0.0190476
16738.0000000	-0.0195000
16739.0000000	-0.0200000
16740.0000000	-0.0200000
16741.0000000	-0.0195000
16742.0000000	-0.0200000
16743.0000000	-0.0195000
16744.0000000	-0.0190000
16745.0000000	-0.0200000
16746.0000000	-0.0200000
16747.0000000	-0.0195000
16748.0000000	-0.0190476
16749.0000000	-0.0195000
16750.0000000	-0.0200000
16751.0000000	-0.0195238
16752.0000000	-0.0195000
16753.0000000	-0.0200000
16754.0000000	-0.0200000
16755.0000000	-0.0200000
16756.0000000	-0.0200000
16757.0000000	-0.0200000
16758.0000000	-0.0200000
16759.0000000	-0.0200000
16760.0000000	-0.0200000
16761.0000000	-0.0200000
16762.0000000	-0.0200000
16763.0000000	-0.0195238
16764.0000000	-0.0200000
16765.0000000	-0.0200000
16766.0000000	-0.0200000
16767.0000000	-0.0200000
16768.0000000	-0.0200000
16769.0000000	-0.0200000
16770.0000000	-0.0200000
16771.0000000	-0.0200000
16772.0000000	-0.0200000
16773.0000000	-0.0200000
16774.0000000	-0.0200000
16775.0000000	-0.0200000
16776.0000000	-0.0200000
16777.0000000	-0.0200000
16778.0000000	-0.0205000
16779.0000000	-0.0200000
16780.0000000	-0.0200000
16781.0000000	-0.0200000
16782.0000000	-0.0200000
16783.0000000	-0.0200000
16784.0000000	-0.0200000
16785.0000000	-0.0200000
16786.0000000	-0.0200000
16787.0000000	-0.0200000
16788.0000000	-0.0200000
16789.0000000	-0.0200000
16790.0000000	-0.0204762

16791.0000000	-0.0200000
16792.0000000	-0.0200000
16793.0000000	-0.0200000
16794.0000000	-0.0200000
16795.0000000	-0.0200000
16796.0000000	-0.0200000
16797.0000000	-0.0204762
16798.0000000	-0.0200000
16799.0000000	-0.0204762
16800.0000000	-0.0210000
16801.0000000	-0.0210000
16802.0000000	-0.0205000
16803.0000000	-0.0214286
16804.0000000	-0.0200000
16805.0000000	-0.0210000
16806.0000000	-0.0214286
16807.0000000	-0.0204762
16808.0000000	-0.0205000
16809.0000000	-0.0205000
16810.0000000	-0.0215000
16811.0000000	-0.0204762
16812.0000000	-0.0215000
16813.0000000	-0.0240000
16814.0000000	-0.0219048
16815.0000000	-0.0215000
16816.0000000	-0.0205000
16817.0000000	-0.0205000
16818.0000000	-0.0219048
16819.0000000	-0.0225000
16820.0000000	-0.0219048
16821.0000000	-0.0210000
16822.0000000	-0.0230000
16823.0000000	-0.0238095
16824.0000000	-0.0225000
16825.0000000	-0.0220000
16826.0000000	-0.0240000
16827.0000000	-0.0223810
16828.0000000	-0.0240000
16829.0000000	-0.0247619
16830.0000000	-0.0230000
16831.0000000	-0.0255000
16832.0000000	-0.0230000
16833.0000000	-0.0225000
16834.0000000	-0.0238095
16835.0000000	-0.0235000
16836.0000000	-0.0242857
16837.0000000	-0.0250000
16838.0000000	-0.0252381
16839.0000000	-0.0250000
16840.0000000	-0.0250000
16841.0000000	-0.0270000
16842.0000000	-0.0257143
16843.0000000	-0.0245000
16844.0000000	-0.0260000
16845.0000000	-0.0252381
16846.0000000	-0.0275000
16847.0000000	-0.0280952

16848.0000000	-0.0270000
16849.0000000	-0.0270000
16850.0000000	-0.0250000
16851.0000000	-0.0252381
16852.0000000	-0.0275000
16853.0000000	-0.0260000
16854.0000000	-0.0266667
16855.0000000	-0.0285000
16856.0000000	-0.0238095
16857.0000000	-0.0250000
16858.0000000	-0.0280000
16859.0000000	-0.0266667
16860.0000000	-0.0265000
16861.0000000	-0.0270000
16862.0000000	-0.0275000
16863.0000000	-0.0276190
16864.0000000	-0.0280000
16865.0000000	-0.0271429
16866.0000000	-0.0265000
16867.0000000	-0.0275000
16868.0000000	-0.0276190
16869.0000000	-0.0280000
16870.0000000	-0.0280000
16871.0000000	-0.0295000
16872.0000000	-0.0280952
16873.0000000	-0.0295000
16874.0000000	-0.0300000
16875.0000000	-0.0285714
16876.0000000	-0.0290000
16877.0000000	-0.0295000
16878.0000000	-0.0290476
16879.0000000	-0.0300000
16880.0000000	-0.0290000
16881.0000000	-0.0290476
16882.0000000	-0.0295000
16883.0000000	-0.0290476
16884.0000000	-0.0300000
16885.0000000	-0.0290000
16886.0000000	-0.0295000
16887.0000000	-0.0290476
16888.0000000	-0.0295000
16889.0000000	-0.0295000
16890.0000000	-0.0300000
16891.0000000	-0.0300000
16892.0000000	-0.0300000
16893.0000000	-0.0300000
16894.0000000	-0.0300000
16895.0000000	-0.0300000
16896.0000000	-0.0300000
16897.0000000	-0.0300000
16898.0000000	-0.0295000
16899.0000000	-0.0295238
16900.0000000	-0.0300000
16901.0000000	-0.0300000
16902.0000000	-0.0300000
16903.0000000	-0.0300000
16904.0000000	-0.0300000

16905.0000000	-0.0300000
16906.0000000	-0.0300000
16907.0000000	-0.0300000
16908.0000000	-0.0300000
16909.0000000	-0.0295000
16910.0000000	-0.0300000
16911.0000000	-0.0300000
16912.0000000	-0.0300000
16913.0000000	-0.0300000
16914.0000000	-0.0300000
16915.0000000	-0.0300000
16916.0000000	-0.0300000
16917.0000000	-0.0304762
16918.0000000	-0.0300000
16919.0000000	-0.0300000
16920.0000000	-0.0300000
16921.0000000	-0.0300000
16922.0000000	-0.0300000
16923.0000000	-0.0300000
16924.0000000	-0.0305000
16925.0000000	-0.0300000
16926.0000000	-0.0300000
16927.0000000	-0.0300000
16928.0000000	-0.0300000
16929.0000000	-0.0300000
16930.0000000	-0.0305000
16931.0000000	-0.0300000
16932.0000000	-0.0300000
16933.0000000	-0.0300000
16934.0000000	-0.0305000
16935.0000000	-0.0300000
16936.0000000	-0.0300000
16937.0000000	-0.0300000
16938.0000000	-0.0300000
16939.0000000	-0.0304762
16940.0000000	-0.0310000
16941.0000000	-0.0305000
16942.0000000	-0.0300000
16943.0000000	-0.0304762
16944.0000000	-0.0310000
16945.0000000	-0.0305000
16946.0000000	-0.0305000
16947.0000000	-0.0304762
16948.0000000	-0.0315000
16949.0000000	-0.0320000
16950.0000000	-0.0305000
16951.0000000	-0.0314286
16952.0000000	-0.0325000
16953.0000000	-0.0315000
16954.0000000	-0.0314286
16955.0000000	-0.0320000
16956.0000000	-0.0319048
16957.0000000	-0.0315000
16958.0000000	-0.0310000
16959.0000000	-0.0314286
16960.0000000	-0.0310000
16961.0000000	-0.0305000

16962.0000000	-0.0315000
16963.0000000	-0.0304762
16964.0000000	-0.0310000
16965.0000000	-0.0300000
16966.0000000	-0.0310000
16967.0000000	-0.0310000
16968.0000000	-0.0305000
16969.0000000	-0.0304762
16970.0000000	-0.0300000
16971.0000000	-0.0310000
16972.0000000	-0.0309524
16973.0000000	-0.0305000
16974.0000000	-0.0304762
16975.0000000	-0.0300000
16976.0000000	-0.0310000
16977.0000000	-0.0305000
16978.0000000	-0.0304762
16979.0000000	-0.0305000
16980.0000000	-0.0305000
16981.0000000	-0.0309524
16982.0000000	-0.0305000
16983.0000000	-0.0300000
16984.0000000	-0.0305000
16985.0000000	-0.0315000
16986.0000000	-0.0310000
16987.0000000	-0.0323809
16988.0000000	-0.0315000
16989.0000000	-0.0310000
16990.0000000	-0.0309524
16991.0000000	-0.0300000
16992.0000000	-0.0304762
16993.0000000	-0.0325000
16994.0000000	-0.0305000
16995.0000000	-0.0314286
16996.0000000	-0.0315000
16997.0000000	-0.0310000
16998.0000000	-0.0315000
16999.0000000	-0.0314286
17000.0000000	-0.0300000
17001.0000000	-0.0320000
17002.0000000	-0.0300000
17003.0000000	-0.0315000
17004.0000000	-0.0304762
17005.0000000	-0.0320000
17006.0000000	-0.0310000
17007.0000000	-0.0315000
17008.0000000	-0.0300000
17009.0000000	-0.0305000
17010.0000000	-0.0305000
17011.0000000	-0.0309524
17012.0000000	-0.0310000
17013.0000000	-0.0310000
17014.0000000	-0.0314286
17015.0000000	-0.0325000
17016.0000000	-0.0320000
17017.0000000	-0.0323809
17018.0000000	-0.0325000

17019.0000000	-0.0304762
17020.0000000	-0.0320000
17021.0000000	-0.0320000
17022.0000000	-0.0310000
17023.0000000	-0.0314286
17024.0000000	-0.0325000
17025.0000000	-0.0315000
17026.0000000	-0.0328571
17027.0000000	-0.0325000
17028.0000000	-0.0315000
17029.0000000	-0.0320000
17030.0000000	-0.0319048
17031.0000000	-0.0325000
17032.0000000	-0.0338095
17033.0000000	-0.0315000
17034.0000000	-0.0330000
17035.0000000	-0.0319048
17036.0000000	-0.0330000
17037.0000000	-0.0315000
17038.0000000	-0.0340000
17039.0000000	-0.0328571
17040.0000000	-0.0310000
17041.0000000	-0.0314286
17042.0000000	-0.0320000
17043.0000000	-0.0325000
17044.0000000	-0.0314286
17045.0000000	-0.0335000
17046.0000000	-0.0345000
17047.0000000	-0.0342857
17048.0000000	-0.0335000
17049.0000000	-0.0340000
17050.0000000	-0.0352381
17051.0000000	-0.0365000
17052.0000000	-0.0360000
17053.0000000	-0.0333333
17054.0000000	-0.0355000
17055.0000000	-0.0350000
17056.0000000	-0.0357143
17057.0000000	-0.0370000
17058.0000000	-0.0355000
17059.0000000	-0.0335000
17060.0000000	-0.0342857
17061.0000000	-0.0350000
17062.0000000	-0.0357143
17063.0000000	-0.0340000
17064.0000000	-0.0347619
17065.0000000	-0.0350000
17066.0000000	-0.0330000
17067.0000000	-0.0335000
17068.0000000	-0.0350000
17069.0000000	-0.0366667
17070.0000000	-0.0350000
17071.0000000	-0.0366667
17072.0000000	-0.0345000
17073.0000000	-0.0340000
17074.0000000	-0.0350000
17075.0000000	-0.0342857

17076.0000000	-0.0315000
17077.0000000	-0.0345000
17078.0000000	-0.0333333
17079.0000000	-0.0340000
17080.0000000	-0.0338095
17081.0000000	-0.0345000
17082.0000000	-0.0340000
17083.0000000	-0.0323809
17084.0000000	-0.0330000
17085.0000000	-0.0355000
17086.0000000	-0.0350000
17087.0000000	-0.0357143
17088.0000000	-0.0360000
17089.0000000	-0.0342857
17090.0000000	-0.0360000
17091.0000000	-0.0340000
17092.0000000	-0.0366667
17093.0000000	-0.0360000
17094.0000000	-0.0375000
17095.0000000	-0.0371429
17096.0000000	-0.0375000
17097.0000000	-0.0365000
17098.0000000	-0.0370000
17099.0000000	-0.0361905
17100.0000000	-0.0380952
17101.0000000	-0.0380000
17102.0000000	-0.0380000
17103.0000000	-0.0375000
17104.0000000	-0.0375000
17105.0000000	-0.0376191
17106.0000000	-0.0375000
17107.0000000	-0.0376191
17108.0000000	-0.0385000
17109.0000000	-0.0366667
17110.0000000	-0.0380000
17111.0000000	-0.0355000
17112.0000000	-0.0360000
17113.0000000	-0.0355000
17114.0000000	-0.0361905
17115.0000000	-0.0360000
17116.0000000	-0.0355000
17117.0000000	-0.0347619
17118.0000000	-0.0340000
17119.0000000	-0.0355000
17120.0000000	-0.0352381
17121.0000000	-0.0360000
17122.0000000	-0.0345000
17123.0000000	-0.0333333
17124.0000000	-0.0355000
17125.0000000	-0.0342857
17126.0000000	-0.0315000
17127.0000000	-0.0345000
17128.0000000	-0.0328571
17129.0000000	-0.0345000
17130.0000000	-0.0350000
17131.0000000	-0.0320000
17132.0000000	-0.0323809

17133.0000000	-0.0330000
17134.0000000	-0.0350000
17135.0000000	-0.0333333
17136.0000000	-0.0345000
17137.0000000	-0.0325000
17138.0000000	-0.0328571
17139.0000000	-0.0320000
17140.0000000	-0.0333333
17141.0000000	-0.0345000
17142.0000000	-0.0355000
17143.0000000	-0.0345000
17144.0000000	-0.0357143
17145.0000000	-0.0335000
17146.0000000	-0.0330000
17147.0000000	-0.0347619
17148.0000000	-0.0320000
17149.0000000	-0.0345000
17150.0000000	-0.0323809
17151.0000000	-0.0325000
17152.0000000	-0.0347619
17153.0000000	-0.0330000
17154.0000000	-0.0345000
17155.0000000	-0.0325000
17156.0000000	-0.0328571
17157.0000000	-0.0340000
17158.0000000	-0.0335000
17159.0000000	-0.0319048
17160.0000000	-0.0325000
17161.0000000	-0.0328571
17162.0000000	-0.0315000
17163.0000000	-0.0355000
17164.0000000	-0.0330000
17165.0000000	-0.0315000
17166.0000000	-0.0347619
17167.0000000	-0.0335000
17168.0000000	-0.0333333
17169.0000000	-0.0330000
17170.0000000	-0.0352381
17171.0000000	-0.0340000
17172.0000000	-0.0330000
17173.0000000	-0.0335000
17174.0000000	-0.0360000
17175.0000000	-0.0328571
17176.0000000	-0.0360000
17177.0000000	-0.0352381
17178.0000000	-0.0340000
17179.0000000	-0.0340000
17180.0000000	-0.0361905
17181.0000000	-0.0360000
17182.0000000	-0.0345000
17183.0000000	-0.0357143
17184.0000000	-0.0365000
17185.0000000	-0.0360000
17186.0000000	-0.0347619
17187.0000000	-0.0365000
17188.0000000	-0.0345000
17189.0000000	-0.0347619

17190.0000000	-0.0335000
17191.0000000	-0.0350000
17192.0000000	-0.0361905
17193.0000000	-0.0365000
17194.0000000	-0.0350000
17195.0000000	-0.0365000
17196.0000000	-0.0342857
17197.0000000	-0.0352381
17198.0000000	-0.0345000
17199.0000000	-0.0360000
17200.0000000	-0.0361905
17201.0000000	-0.0360000
17202.0000000	-0.0360000
17203.0000000	-0.0375000
17204.0000000	-0.0366667
17205.0000000	-0.0380000
17206.0000000	-0.0380952
17207.0000000	-0.0375000
17208.0000000	-0.0380000
17209.0000000	-0.0385000
17210.0000000	-0.0380000
17211.0000000	-0.0366667
17212.0000000	-0.0365000
17213.0000000	-0.0380952
17214.0000000	-0.0380000
17215.0000000	-0.0370000
17216.0000000	-0.0366667
17217.0000000	-0.0370000
17218.0000000	-0.0365000
17219.0000000	-0.0385000
17220.0000000	-0.0376191
17221.0000000	-0.0340000
17222.0000000	-0.0371429
17223.0000000	-0.0370000
17224.0000000	-0.0370000
17225.0000000	-0.0375000
17226.0000000	-0.0376191
17227.0000000	-0.0365000
17228.0000000	-0.0376190
17229.0000000	-0.0355000
17230.0000000	-0.0375000
17231.0000000	-0.0371429
17232.0000000	-0.0350000
17233.0000000	-0.0350000
17234.0000000	-0.0345000
17235.0000000	-0.0361905
17236.0000000	-0.0352381
17237.0000000	-0.0345000
17238.0000000	-0.0350000
17239.0000000	-0.0325000
17240.0000000	-0.0342857
17241.0000000	-0.0360000
17242.0000000	-0.0355000
17243.0000000	-0.0335000
17244.0000000	-0.0347619
17245.0000000	-0.0345000
17246.0000000	-0.0357143

17247.0000000	-0.0360000
17248.0000000	-0.0370000
17249.0000000	-0.0376190
17250.0000000	-0.0350000
17251.0000000	-0.0360000
17252.0000000	-0.0380952
17253.0000000	-0.0365000
17254.0000000	-0.0345000
17255.0000000	-0.0325000
17256.0000000	-0.0352381
17257.0000000	-0.0350000
17258.0000000	-0.0352381
17259.0000000	-0.0350000
17260.0000000	-0.0355000
17261.0000000	-0.0380952
17262.0000000	-0.0365000
17263.0000000	-0.0370000
17264.0000000	-0.0370000
17265.0000000	-0.0371429
17266.0000000	-0.0355000
17267.0000000	-0.0366667
17268.0000000	-0.0355000
17269.0000000	-0.0365000
17270.0000000	-0.0355000
17271.0000000	-0.0371429
17272.0000000	-0.0370000
17273.0000000	-0.0380000
17274.0000000	-0.0376190
17275.0000000	-0.0375000
17276.0000000	-0.0366667
17277.0000000	-0.0365000
17278.0000000	-0.0375000
17279.0000000	-0.0375000
17280.0000000	-0.0366667
17281.0000000	-0.0380000
17282.0000000	-0.0370000
17283.0000000	-0.0366667
17284.0000000	-0.0375000
17285.0000000	-0.0385714
17286.0000000	-0.0365000
17287.0000000	-0.0370000
17288.0000000	-0.0338095
17289.0000000	-0.0375000
17290.0000000	-0.0355000
17291.0000000	-0.0360000
17292.0000000	-0.0361905
17293.0000000	-0.0360000
17294.0000000	-0.0366667
17295.0000000	-0.0360000
17296.0000000	-0.0370000
17297.0000000	-0.0371429
17298.0000000	-0.0355000
17299.0000000	-0.0370000
17300.0000000	-0.0365000
17301.0000000	-0.0371429
17302.0000000	-0.0370000
17303.0000000	-0.0370000

17304.0000000	-0.0361905
17305.0000000	-0.0365000
17306.0000000	-0.0366667
17307.0000000	-0.0355000
17308.0000000	-0.0370000
17309.0000000	-0.0365000
17310.0000000	-0.0380952
17311.0000000	-0.0385000
17312.0000000	-0.0376191
17313.0000000	-0.0385000
17314.0000000	-0.0395000
17315.0000000	-0.0385000
17316.0000000	-0.0371429
17317.0000000	-0.0375000
17318.0000000	-0.0380000
17319.0000000	-0.0385714
17320.0000000	-0.0370000
17321.0000000	-0.0376191
17322.0000000	-0.0385000
17323.0000000	-0.0380000
17324.0000000	-0.0380000
17325.0000000	-0.0385714
17326.0000000	-0.0385000
17327.0000000	-0.0385000
17328.0000000	-0.0385714
17329.0000000	-0.0375000
17330.0000000	-0.0375000
17331.0000000	-0.0375000
17332.0000000	-0.0390476
17333.0000000	-0.0380952
17334.0000000	-0.0375000
17335.0000000	-0.0385000
17336.0000000	-0.0366667
17337.0000000	-0.0390000
17338.0000000	-0.0390000
17339.0000000	-0.0380000
17340.0000000	-0.0366667
17341.0000000	-0.0360000
17342.0000000	-0.0380000
17343.0000000	-0.0380952
17344.0000000	-0.0380000
17345.0000000	-0.0390000
17346.0000000	-0.0385714
17347.0000000	-0.0395000
17348.0000000	-0.0385000
17349.0000000	-0.0395238
17350.0000000	-0.0390000
17351.0000000	-0.0390000
17352.0000000	-0.0390476
17353.0000000	-0.0390000
17354.0000000	-0.0395000
17355.0000000	-0.0395238
17356.0000000	-0.0390000
17357.0000000	-0.0390476
17358.0000000	-0.0385000
17359.0000000	-0.0390000
17360.0000000	-0.0390000

17361.0000000	-0.0395000
17362.0000000	-0.0400000
17363.0000000	-0.0385000
17364.0000000	-0.0395238
17365.0000000	-0.0400000
17366.0000000	-0.0400000
17367.0000000	-0.0395000
17368.0000000	-0.0400000
17369.0000000	-0.0395000
17370.0000000	-0.0395000
17371.0000000	-0.0395238
17372.0000000	-0.0395000
17373.0000000	-0.0400000
17374.0000000	-0.0400000
17375.0000000	-0.0400000
17376.0000000	-0.0390476
17377.0000000	-0.0390000
17378.0000000	-0.0400000
17379.0000000	-0.0395000
17380.0000000	-0.0395238
17381.0000000	-0.0400000
17382.0000000	-0.0395238
17383.0000000	-0.0390000
17384.0000000	-0.0395000
17385.0000000	-0.0390476
17386.0000000	-0.0400000
17387.0000000	-0.0400000
17388.0000000	-0.0395238
17389.0000000	-0.0400000
17390.0000000	-0.0395000
17391.0000000	-0.0400000
17392.0000000	-0.0400000
17393.0000000	-0.0400000
17394.0000000	-0.0400000
17395.0000000	-0.0400000
17396.0000000	-0.0400000
17397.0000000	-0.0400000
17398.0000000	-0.0395238
17399.0000000	-0.0400000
17400.0000000	-0.0400000
17401.0000000	-0.0400000
17402.0000000	-0.0400000
17403.0000000	-0.0400000
17404.0000000	-0.0400000
17405.0000000	-0.0400000
17406.0000000	-0.0404762
17407.0000000	-0.0400000
17408.0000000	-0.0400000
17409.0000000	-0.0400000
17410.0000000	-0.0404762
17411.0000000	-0.0400000
17412.0000000	-0.0400000
17413.0000000	-0.0400000
17414.0000000	-0.0400000
17415.0000000	-0.0400000
17416.0000000	-0.0400000
17417.0000000	-0.0400000

17418.0000000	-0.0395238
17419.0000000	-0.0400000
17420.0000000	-0.0400000
17421.0000000	-0.0400000
17422.0000000	-0.0405000
17423.0000000	-0.0400000
17424.0000000	-0.0400000
17425.0000000	-0.0395238
17426.0000000	-0.0400000
17427.0000000	-0.0400000
17428.0000000	-0.0400000
17429.0000000	-0.0400000
17430.0000000	-0.0400000
17431.0000000	-0.0400000
17432.0000000	-0.0400000
17433.0000000	-0.0400000
17434.0000000	-0.0400000
17435.0000000	-0.0400000
17436.0000000	-0.0400000
17437.0000000	-0.0400000
17438.0000000	-0.0400000
17439.0000000	-0.0400000
17440.0000000	-0.0400000
17441.0000000	-0.0400000
17442.0000000	-0.0400000
17443.0000000	-0.0400000
17444.0000000	-0.0400000
17445.0000000	-0.0400000
17446.0000000	-0.0400000
17447.0000000	-0.0400000
17448.0000000	-0.0400000
17449.0000000	-0.0400000
17450.0000000	-0.0400000
17451.0000000	-0.0400000
17452.0000000	-0.0400000
17453.0000000	-0.0400000
17454.0000000	-0.0404762
17455.0000000	-0.0405000
17456.0000000	-0.0400000
17457.0000000	-0.0405000
17458.0000000	-0.0400000
17459.0000000	-0.0400000
17460.0000000	-0.0400000
17461.0000000	-0.0400000
17462.0000000	-0.0400000
17463.0000000	-0.0400000
17464.0000000	-0.0405000
17465.0000000	-0.0400000
17466.0000000	-0.0400000
17467.0000000	-0.0405000
17468.0000000	-0.0400000
17469.0000000	-0.0405000
17470.0000000	-0.0409524
17471.0000000	-0.0405000
17472.0000000	-0.0404762
17473.0000000	-0.0410000
17474.0000000	-0.0405000

17475.0000000	-0.0410000
17476.0000000	-0.0409524
17477.0000000	-0.0405000
17478.0000000	-0.0420000
17479.0000000	-0.0419048
17480.0000000	-0.0405000
17481.0000000	-0.0414286
17482.0000000	-0.0405000
17483.0000000	-0.0405000
17484.0000000	-0.0410000
17485.0000000	-0.0410000
17486.0000000	-0.0404762
17487.0000000	-0.0420000
17488.0000000	-0.0404762
17489.0000000	-0.0425000
17490.0000000	-0.0409524
17491.0000000	-0.0415000
17492.0000000	-0.0420000
17493.0000000	-0.0410000
17494.0000000	-0.0409524
17495.0000000	-0.0430000
17496.0000000	-0.0430000
17497.0000000	-0.0419048
17498.0000000	-0.0415000
17499.0000000	-0.0409524
17500.0000000	-0.0420000
17501.0000000	-0.0430000
17502.0000000	-0.0410000
17503.0000000	-0.0423810
17504.0000000	-0.0405000
17505.0000000	-0.0420000
17506.0000000	-0.0423810
17507.0000000	-0.0425000
17508.0000000	-0.0414286
17509.0000000	-0.0420000
17510.0000000	-0.0425000
17511.0000000	-0.0400000
17512.0000000	-0.0415000
17513.0000000	-0.0405000
17514.0000000	-0.0430000
17515.0000000	-0.0428572
17516.0000000	-0.0410000
17517.0000000	-0.0435000
17518.0000000	-0.0419048
17519.0000000	-0.0410000
17520.0000000	-0.0414286
17521.0000000	-0.0420000
17522.0000000	-0.0430000
17523.0000000	-0.0415000
17524.0000000	-0.0428572
17525.0000000	-0.0410000
17526.0000000	-0.0405000
17527.0000000	-0.0428572
17528.0000000	-0.0410000
17529.0000000	-0.0410000
17530.0000000	-0.0423810
17531.0000000	-0.0415000

17532.0000000	-0.0420000
17533.0000000	-0.0423810
17534.0000000	-0.0415000
17535.0000000	-0.0404762
17536.0000000	-0.0410000
17537.0000000	-0.0405000
17538.0000000	-0.0415000
17539.0000000	-0.0404762
17540.0000000	-0.0405000
17541.0000000	-0.0410000
17542.0000000	-0.0400000
17543.0000000	-0.0405000
17544.0000000	-0.0405000
17545.0000000	-0.0400000
17546.0000000	-0.0414286
17547.0000000	-0.0400000
17548.0000000	-0.0400000
17549.0000000	-0.0400000
17550.0000000	-0.0410000
17551.0000000	-0.0400000
17552.0000000	-0.0400000
17553.0000000	-0.0400000
17554.0000000	-0.0404762
17555.0000000	-0.0400000
17556.0000000	-0.0400000
17557.0000000	-0.0400000
17558.0000000	-0.0400000
17559.0000000	-0.0400000
17560.0000000	-0.0400000
17561.0000000	-0.0400000
17562.0000000	-0.0400000
17563.0000000	-0.0400000
17564.0000000	-0.0400000
17565.0000000	-0.0400000
17566.0000000	-0.0400000
17567.0000000	-0.0400000
17568.0000000	-0.0400000
17569.0000000	-0.0400000
17570.0000000	-0.0400000
17571.0000000	-0.0400000
17572.0000000	-0.0400000
17573.0000000	-0.0400000
17574.0000000	-0.0400000
17575.0000000	-0.0400000
17576.0000000	-0.0400000
17577.0000000	-0.0400000
17578.0000000	-0.0400000
17579.0000000	-0.0400000
17580.0000000	-0.0400000
17581.0000000	-0.0404762
17582.0000000	-0.0410000
17583.0000000	-0.0400000
17584.0000000	-0.0400000
17585.0000000	-0.0400000
17586.0000000	-0.0405000
17587.0000000	-0.0400000
17588.0000000	-0.0400000

17589.0000000	-0.0400000
17590.0000000	-0.0400000
17591.0000000	-0.0400000
17592.0000000	-0.0400000
17593.0000000	-0.0400000
17594.0000000	-0.0400000
17595.0000000	-0.0405000
17596.0000000	-0.0400000
17597.0000000	-0.0400000
17598.0000000	-0.0405000
17599.0000000	-0.0404762
17600.0000000	-0.0400000
17601.0000000	-0.0400000
17602.0000000	-0.0400000
17603.0000000	-0.0400000
17604.0000000	-0.0400000
17605.0000000	-0.0405000
17606.0000000	-0.0400000
17607.0000000	-0.0400000
17608.0000000	-0.0404762
17609.0000000	-0.0400000
17610.0000000	-0.0400000
17611.0000000	-0.0404762
17612.0000000	-0.0400000
17613.0000000	-0.0405000
17614.0000000	-0.0415000
17615.0000000	-0.0400000
17616.0000000	-0.0410000
17617.0000000	-0.0409524
17618.0000000	-0.0400000
17619.0000000	-0.0400000
17620.0000000	-0.0400000
17621.0000000	-0.0404762
17622.0000000	-0.0400000
17623.0000000	-0.0414286
17624.0000000	-0.0400000
17625.0000000	-0.0410000
17626.0000000	-0.0409524
17627.0000000	-0.0405000
17628.0000000	-0.0400000
17629.0000000	-0.0400000
17630.0000000	-0.0400000
17631.0000000	-0.0410000
17632.0000000	-0.0409524
17633.0000000	-0.0400000
17634.0000000	-0.0400000
17635.0000000	-0.0400000
17636.0000000	-0.0400000
17637.0000000	-0.0400000
17638.0000000	-0.0400000
17639.0000000	-0.0404762
17640.0000000	-0.0400000
17641.0000000	-0.0404762
17642.0000000	-0.0400000
17643.0000000	-0.0405000
17644.0000000	-0.0410000
17645.0000000	-0.0404762

17646.0000000	-0.0415000
17647.0000000	-0.0404762
17648.0000000	-0.0405000
17649.0000000	-0.0405000
17650.0000000	-0.0400000
17651.0000000	-0.0409524
17652.0000000	-0.0405000
17653.0000000	-0.0405000
17654.0000000	-0.0400000
17655.0000000	-0.0400000
17656.0000000	-0.0400000
17657.0000000	-0.0395000
17658.0000000	-0.0400000
17659.0000000	-0.0400000
17660.0000000	-0.0400000
17661.0000000	-0.0400000
17662.0000000	-0.0400000
17663.0000000	-0.0400000
17664.0000000	-0.0400000
17665.0000000	-0.0400000
17666.0000000	-0.0400000
17667.0000000	-0.0400000
17668.0000000	-0.0400000
17669.0000000	-0.0400000
17670.0000000	-0.0400000
17671.0000000	-0.0400000
17672.0000000	-0.0400000
17673.0000000	-0.0400000
17674.0000000	-0.0395000
17675.0000000	-0.0400000
17676.0000000	-0.0405000
17677.0000000	-0.0400000
17678.0000000	-0.0400000
17679.0000000	-0.0400000
17680.0000000	-0.0400000
17681.0000000	-0.0400000
17682.0000000	-0.0400000
17683.0000000	-0.0400000
17684.0000000	-0.0400000
17685.0000000	-0.0400000
17686.0000000	-0.0400000
17687.0000000	-0.0400000
17688.0000000	-0.0400000
17689.0000000	-0.0400000
17690.0000000	-0.0400000
17691.0000000	-0.0400000
17692.0000000	-0.0400000
17693.0000000	-0.0400000
17694.0000000	-0.0400000
17695.0000000	-0.0400000
17696.0000000	-0.0400000
17697.0000000	-0.0400000
17698.0000000	-0.0400000
17699.0000000	-0.0400000
17700.0000000	-0.0400000
17701.0000000	-0.0400000
17702.0000000	-0.0404762

17703.0000000	-0.0400000
17704.0000000	-0.0400000
17705.0000000	-0.0400000
17706.0000000	-0.0400000
17707.0000000	-0.0400000
17708.0000000	-0.0400000
17709.0000000	-0.0400000
17710.0000000	-0.0400000
17711.0000000	-0.0400000
17712.0000000	-0.0400000
17713.0000000	-0.0400000
17714.0000000	-0.0400000
17715.0000000	-0.0400000
17716.0000000	-0.0400000
17717.0000000	-0.0400000
17718.0000000	-0.0400000
17719.0000000	-0.0395000
17720.0000000	-0.0400000
17721.0000000	-0.0400000
17722.0000000	-0.0400000
17723.0000000	-0.0395238
17724.0000000	-0.0400000
17725.0000000	-0.0400000
17726.0000000	-0.0400000
17727.0000000	-0.0400000
17728.0000000	-0.0400000
17729.0000000	-0.0400000
17730.0000000	-0.0400000
17731.0000000	-0.0385000
17732.0000000	-0.0400000
17733.0000000	-0.0400000
17734.0000000	-0.0395000
17735.0000000	-0.0390476
17736.0000000	-0.0400000
17737.0000000	-0.0400000
17738.0000000	-0.0400000
17739.0000000	-0.0400000
17740.0000000	-0.0400000
17741.0000000	-0.0400000
17742.0000000	-0.0400000
17743.0000000	-0.0395000
17744.0000000	-0.0395238
17745.0000000	-0.0400000
17746.0000000	-0.0400000
17747.0000000	-0.0390476
17748.0000000	-0.0400000
17749.0000000	-0.0400000
17750.0000000	-0.0400000
17751.0000000	-0.0400000
17752.0000000	-0.0400000
17753.0000000	-0.0395000
17754.0000000	-0.0395000
17755.0000000	-0.0400000
17756.0000000	-0.0395000
17757.0000000	-0.0400000
17758.0000000	-0.0400000
17759.0000000	-0.0400000

17760.0000000	-0.0395000
17761.0000000	-0.0400000
17762.0000000	-0.0400000
17763.0000000	-0.0400000
17764.0000000	-0.0400000
17765.0000000	-0.0400000
17766.0000000	-0.0400000
17767.0000000	-0.0395000
17768.0000000	-0.0400000
17769.0000000	-0.0400000
17770.0000000	-0.0400000
17771.0000000	-0.0400000
17772.0000000	-0.0400000
17773.0000000	-0.0400000
17774.0000000	-0.0400000
17775.0000000	-0.0400000
17776.0000000	-0.0400000
17777.0000000	-0.0400000
17778.0000000	-0.0400000
17779.0000000	-0.0400000
17780.0000000	-0.0400000
17781.0000000	-0.0400000
17782.0000000	-0.0400000
17783.0000000	-0.0400000
17784.0000000	-0.0395000
17785.0000000	-0.0400000
17786.0000000	-0.0400000
17787.0000000	-0.0400000
17788.0000000	-0.0400000
17789.0000000	-0.0400000
17790.0000000	-0.0400000
17791.0000000	-0.0400000
17792.0000000	-0.0400000
17793.0000000	-0.0400000
17794.0000000	-0.0400000
17795.0000000	-0.0400000
17796.0000000	-0.0400000
17797.0000000	-0.0395000
17798.0000000	-0.0400000
17799.0000000	-0.0400000
17800.0000000	-0.0400000
17801.0000000	-0.0400000
17802.0000000	-0.0400000
17803.0000000	-0.0385000
17804.0000000	-0.0390476
17805.0000000	-0.0400000
17806.0000000	-0.0395000
17807.0000000	-0.0400000
17808.0000000	-0.0395238
17809.0000000	-0.0390000
17810.0000000	-0.0390000
17811.0000000	-0.0385714
17812.0000000	-0.0380000
17813.0000000	-0.0385714
17814.0000000	-0.0380000
17815.0000000	-0.0390000
17816.0000000	-0.0375000

17817.0000000	-0.0390476
17818.0000000	-0.0395000
17819.0000000	-0.0380000
17820.0000000	-0.0385714
17821.0000000	-0.0400000
17822.0000000	-0.0376191
17823.0000000	-0.0390000
17824.0000000	-0.0395000
17825.0000000	-0.0370000
17826.0000000	-0.0385000
17827.0000000	-0.0380952
17828.0000000	-0.0370000
17829.0000000	-0.0366667
17830.0000000	-0.0365000
17831.0000000	-0.0385000
17832.0000000	-0.0352381
17833.0000000	-0.0365000
17834.0000000	-0.0375000
17835.0000000	-0.0380952
17836.0000000	-0.0385000
17837.0000000	-0.0385000
17838.0000000	-0.0385714
17839.0000000	-0.0385000
17840.0000000	-0.0380952
17841.0000000	-0.0375000
17842.0000000	-0.0400000
17843.0000000	-0.0385000
17844.0000000	-0.0400000
17845.0000000	-0.0395000
17846.0000000	-0.0395000
17847.0000000	-0.0400000
17848.0000000	-0.0400000
17849.0000000	-0.0390000
17850.0000000	-0.0395238
17851.0000000	-0.0400000
17852.0000000	-0.0400000
17853.0000000	-0.0395000
17854.0000000	-0.0385000
17855.0000000	-0.0385000
17856.0000000	-0.0390476
17857.0000000	-0.0385000
17858.0000000	-0.0395000
17859.0000000	-0.0390476
17860.0000000	-0.0375000
17861.0000000	-0.0390000
17862.0000000	-0.0380000
17863.0000000	-0.0352381
17864.0000000	-0.0365000
17865.0000000	-0.0371429
17866.0000000	-0.0375000
17867.0000000	-0.0375000
17868.0000000	-0.0361905
17869.0000000	-0.0385000
17870.0000000	-0.0385000
17871.0000000	-0.0380000
17872.0000000	-0.0366667
17873.0000000	-0.0350000

17874.0000000	-0.0357143
17875.0000000	-0.0380000
17876.0000000	-0.0390000
17877.0000000	-0.0380000
17878.0000000	-0.0347619
17879.0000000	-0.0360000
17880.0000000	-0.0371429
17881.0000000	-0.0360000
17882.0000000	-0.0355000
17883.0000000	-0.0357143
17884.0000000	-0.0345000
17885.0000000	-0.0365000
17886.0000000	-0.0385000
17887.0000000	-0.0376190
17888.0000000	-0.0375000
17889.0000000	-0.0380952
17890.0000000	-0.0375000
17891.0000000	-0.0385000
17892.0000000	-0.0385714
17893.0000000	-0.0385000
17894.0000000	-0.0380000
17895.0000000	-0.0375000
17896.0000000	-0.0376190
17897.0000000	-0.0385000
17898.0000000	-0.0370000
17899.0000000	-0.0380952
17900.0000000	-0.0360000
17901.0000000	-0.0371429
17902.0000000	-0.0370000
17903.0000000	-0.0375000
17904.0000000	-0.0376190
17905.0000000	-0.0355000
17906.0000000	-0.0360000
17907.0000000	-0.0350000
17908.0000000	-0.0370000
17909.0000000	-0.0371429
17910.0000000	-0.0371429
17911.0000000	-0.0375000
17912.0000000	-0.0350000
17913.0000000	-0.0361905
17914.0000000	-0.0365000
17915.0000000	-0.0375000
17916.0000000	-0.0345000
17917.0000000	-0.0352381
17918.0000000	-0.0370000
17919.0000000	-0.0361905
17920.0000000	-0.0355000
17921.0000000	-0.0350000
17922.0000000	-0.0360000
17923.0000000	-0.0371429
17924.0000000	-0.0375000
17925.0000000	-0.0360000
17926.0000000	-0.0361905
17927.0000000	-0.0360000
17928.0000000	-0.0361905
17929.0000000	-0.0350000
17930.0000000	-0.0340000

17931.0000000	-0.0345000
17932.0000000	-0.0333333
17933.0000000	-0.0355000
17934.0000000	-0.0360000
17935.0000000	-0.0357143
17936.0000000	-0.0350000
17937.0000000	-0.0365000
17938.0000000	-0.0366667
17939.0000000	-0.0355000
17940.0000000	-0.0357143
17941.0000000	-0.0345000
17942.0000000	-0.0330000
17943.0000000	-0.0325000
17944.0000000	-0.0328571
17945.0000000	-0.0335000
17946.0000000	-0.0330000
17947.0000000	-0.0314286
17948.0000000	-0.0305000
17949.0000000	-0.0319048
17950.0000000	-0.0320000
17951.0000000	-0.0320000
17952.0000000	-0.0310000
17953.0000000	-0.0309524
17954.0000000	-0.0335000
17955.0000000	-0.0310000
17956.0000000	-0.0309524
17957.0000000	-0.0328571
17958.0000000	-0.0305000
17959.0000000	-0.0320000
17960.0000000	-0.0325000
17961.0000000	-0.0335000
17962.0000000	-0.0323809
17963.0000000	-0.0330000
17964.0000000	-0.0314286
17965.0000000	-0.0325000
17966.0000000	-0.0330000
17967.0000000	-0.0325000
17968.0000000	-0.0314286
17969.0000000	-0.0320000
17970.0000000	-0.0320000
17971.0000000	-0.0319048
17972.0000000	-0.0330000
17973.0000000	-0.0304762
17974.0000000	-0.0315000
17975.0000000	-0.0330000
17976.0000000	-0.0315000
17977.0000000	-0.0319048
17978.0000000	-0.0305000
17979.0000000	-0.0325000
17980.0000000	-0.0314286
17981.0000000	-0.0335000
17982.0000000	-0.0330000
17983.0000000	-0.0335000
17984.0000000	-0.0333333
17985.0000000	-0.0330000
17986.0000000	-0.0323809
17987.0000000	-0.0335000

17988.0000000	-0.0352381
17989.0000000	-0.0345000
17990.0000000	-0.0350000
17991.0000000	-0.0360000
17992.0000000	-0.0347619
17993.0000000	-0.0335000
17994.0000000	-0.0355000
17995.0000000	-0.0350000
17996.0000000	-0.0357143
17997.0000000	-0.0360000
17998.0000000	-0.0347619
17999.0000000	-0.0330000
18000.0000000	-0.0350000
18001.0000000	-0.0347619
18002.0000000	-0.0365000
18003.0000000	-0.0350000
18004.0000000	-0.0342857
18005.0000000	-0.0345000
18006.0000000	-0.0340000
18007.0000000	-0.0357143
18008.0000000	-0.0355000
18009.0000000	-0.0342857
18010.0000000	-0.0345000
18011.0000000	-0.0335000
18012.0000000	-0.0335000
18013.0000000	-0.0340000
18014.0000000	-0.0314286
18015.0000000	-0.0325000
18016.0000000	-0.0304762
18017.0000000	-0.0315000
18018.0000000	-0.0304762
18019.0000000	-0.0315000
18020.0000000	-0.0300000
18021.0000000	-0.0300000
18022.0000000	-0.0305000
18023.0000000	-0.0300000
18024.0000000	-0.0300000
18025.0000000	-0.0300000
18026.0000000	-0.0300000
18027.0000000	-0.0295000
18028.0000000	-0.0300000
18029.0000000	-0.0305000
18030.0000000	-0.0300000
18031.0000000	-0.0300000
18032.0000000	-0.0304762
18033.0000000	-0.0300000
18034.0000000	-0.0309524
18035.0000000	-0.0300000
18036.0000000	-0.0300000
18037.0000000	-0.0300000
18038.0000000	-0.0300000
18039.0000000	-0.0310000
18040.0000000	-0.0300000
18041.0000000	-0.0305000
18042.0000000	-0.0300000
18043.0000000	-0.0305000
18044.0000000	-0.0300000

18045.0000000	-0.0304762
18046.0000000	-0.0320000
18047.0000000	-0.0305000
18048.0000000	-0.0300000
18049.0000000	-0.0304762
18050.0000000	-0.0300000
18051.0000000	-0.0310000
18052.0000000	-0.0304762
18053.0000000	-0.0315000
18054.0000000	-0.0305000
18055.0000000	-0.0304762
18056.0000000	-0.0305000
18057.0000000	-0.0300000
18058.0000000	-0.0305000
18059.0000000	-0.0300000
18060.0000000	-0.0300000
18061.0000000	-0.0300000
18062.0000000	-0.0300000
18063.0000000	-0.0300000
18064.0000000	-0.0300000
18065.0000000	-0.0300000
18066.0000000	-0.0300000
18067.0000000	-0.0305000
18068.0000000	-0.0300000
18069.0000000	-0.0300000
18070.0000000	-0.0300000
18071.0000000	-0.0300000
18072.0000000	-0.0305000
18073.0000000	-0.0300000
18074.0000000	-0.0300000
18075.0000000	-0.0300000
18076.0000000	-0.0300000
18077.0000000	-0.0300000
18078.0000000	-0.0300000
18079.0000000	-0.0300000
18080.0000000	-0.0300000
18081.0000000	-0.0300000
18082.0000000	-0.0305000
18083.0000000	-0.0300000
18084.0000000	-0.0300000
18085.0000000	-0.0300000
18086.0000000	-0.0300000
18087.0000000	-0.0305000
18088.0000000	-0.0300000
18089.0000000	-0.0300000
18090.0000000	-0.0300000
18091.0000000	-0.0300000
18092.0000000	-0.0300000
18093.0000000	-0.0300000
18094.0000000	-0.0300000
18095.0000000	-0.0300000
18096.0000000	-0.0300000
18097.0000000	-0.0300000
18098.0000000	-0.0305000
18099.0000000	-0.0300000
18100.0000000	-0.0300000
18101.0000000	-0.0300000

18102.0000000	-0.0300000
18103.0000000	-0.0305000
18104.0000000	-0.0300000
18105.0000000	-0.0300000
18106.0000000	-0.0300000
18107.0000000	-0.0300000
18108.0000000	-0.0300000
18109.0000000	-0.0300000
18110.0000000	-0.0305000
18111.0000000	-0.0300000
18112.0000000	-0.0300000
18113.0000000	-0.0300000
18114.0000000	-0.0300000
18115.0000000	-0.0304762
18116.0000000	-0.0300000
18117.0000000	-0.0300000
18118.0000000	-0.0300000
18119.0000000	-0.0305000
18120.0000000	-0.0300000
18121.0000000	-0.0300000
18122.0000000	-0.0300000
18123.0000000	-0.0300000
18124.0000000	-0.0304762
18125.0000000	-0.0300000
18126.0000000	-0.0300000
18127.0000000	-0.0300000
18128.0000000	-0.0300000
18129.0000000	-0.0300000
18130.0000000	-0.0300000
18131.0000000	-0.0300000
18132.0000000	-0.0295000
18133.0000000	-0.0300000
18134.0000000	-0.0300000
18135.0000000	-0.0300000
18136.0000000	-0.0300000
18137.0000000	-0.0300000
18138.0000000	-0.0300000
18139.0000000	-0.0305000
18140.0000000	-0.0295238
18141.0000000	-0.0300000
18142.0000000	-0.0300000
18143.0000000	-0.0300000
18144.0000000	-0.0300000
18145.0000000	-0.0300000
18146.0000000	-0.0300000
18147.0000000	-0.0300000
18148.0000000	-0.0300000
18149.0000000	-0.0300000
18150.0000000	-0.0295000
18151.0000000	-0.0295000
18152.0000000	-0.0300000
18153.0000000	-0.0300000
18154.0000000	-0.0300000
18155.0000000	-0.0300000
18156.0000000	-0.0300000
18157.0000000	-0.0300000
18158.0000000	-0.0300000

18159.0000000	-0.0300000
18160.0000000	-0.0300000
18161.0000000	-0.0295238
18162.0000000	-0.0300000
18163.0000000	-0.0300000
18164.0000000	-0.0300000
18165.0000000	-0.0295238
18166.0000000	-0.0300000
18167.0000000	-0.0300000
18168.0000000	-0.0300000
18169.0000000	-0.0300000
18170.0000000	-0.0295000
18171.0000000	-0.0290000
18172.0000000	-0.0295000
18173.0000000	-0.0295238
18174.0000000	-0.0300000
18175.0000000	-0.0300000
18176.0000000	-0.0300000
18177.0000000	-0.0285000
18178.0000000	-0.0300000
18179.0000000	-0.0300000
18180.0000000	-0.0300000
18181.0000000	-0.0295000
18182.0000000	-0.0300000
18183.0000000	-0.0295000
18184.0000000	-0.0300000
18185.0000000	-0.0300000
18186.0000000	-0.0290000
18187.0000000	-0.0300000
18188.0000000	-0.0300000
18189.0000000	-0.0300000
18190.0000000	-0.0295000
18191.0000000	-0.0300000
18192.0000000	-0.0300000
18193.0000000	-0.0300000
18194.0000000	-0.0300000
18195.0000000	-0.0300000
18196.0000000	-0.0300000
18197.0000000	-0.0295238
18198.0000000	-0.0300000
18199.0000000	-0.0300000
18200.0000000	-0.0300000
18201.0000000	-0.0300000
18202.0000000	-0.0300000
18203.0000000	-0.0300000
18204.0000000	-0.0300000
18205.0000000	-0.0300000
18206.0000000	-0.0300000
18207.0000000	-0.0300000
18208.0000000	-0.0300000
18209.0000000	-0.0300000
18210.0000000	-0.0300000
18211.0000000	-0.0300000
18212.0000000	-0.0300000
18213.0000000	-0.0300000
18214.0000000	-0.0300000
18215.0000000	-0.0300000

18216.0000000	-0.0300000
18217.0000000	-0.0300000
18218.0000000	-0.0300000
18219.0000000	-0.0300000
18220.0000000	-0.0300000
18221.0000000	-0.0300000
18222.0000000	-0.0300000
18223.0000000	-0.0300000
18224.0000000	-0.0295000
18225.0000000	-0.0300000
18226.0000000	-0.0295000
18227.0000000	-0.0300000
18228.0000000	-0.0300000
18229.0000000	-0.0300000
18230.0000000	-0.0300000
18231.0000000	-0.0300000
18232.0000000	-0.0300000
18233.0000000	-0.0300000
18234.0000000	-0.0295000
18235.0000000	-0.0290000
18236.0000000	-0.0295000
18237.0000000	-0.0300000
18238.0000000	-0.0300000
18239.0000000	-0.0295000
18240.0000000	-0.0295238
18241.0000000	-0.0300000
18242.0000000	-0.0300000
18243.0000000	-0.0295000
18244.0000000	-0.0300000
18245.0000000	-0.0300000
18246.0000000	-0.0295000
18247.0000000	-0.0300000
18248.0000000	-0.0290000
18249.0000000	-0.0300000
18250.0000000	-0.0300000
18251.0000000	-0.0300000
18252.0000000	-0.0300000
18253.0000000	-0.0300000
18254.0000000	-0.0300000
18255.0000000	-0.0290476
18256.0000000	-0.0300000
18257.0000000	-0.0300000
18258.0000000	-0.0300000
18259.0000000	-0.0300000
18260.0000000	-0.0300000
18261.0000000	-0.0295000
18262.0000000	-0.0300000
18263.0000000	-0.0300000
18264.0000000	-0.0300000
18265.0000000	-0.0300000
18266.0000000	-0.0300000
18267.0000000	-0.0300000
18268.0000000	-0.0300000
18269.0000000	-0.0300000
18270.0000000	-0.0300000
18271.0000000	-0.0300000
18272.0000000	-0.0300000

18273.0000000	-0.0295238
18274.0000000	-0.0300000
18275.0000000	-0.0300000
18276.0000000	-0.0300000
18277.0000000	-0.0300000
18278.0000000	-0.0300000
18279.0000000	-0.0295238
18280.0000000	-0.0295000
18281.0000000	-0.0300000
18282.0000000	-0.0300000
18283.0000000	-0.0300000
18284.0000000	-0.0300000
18285.0000000	-0.0300000
18286.0000000	-0.0300000
18287.0000000	-0.0300000
18288.0000000	-0.0300000
18289.0000000	-0.0295000
18290.0000000	-0.0300000
18291.0000000	-0.0300000
18292.0000000	-0.0290000
18293.0000000	-0.0295000
18294.0000000	-0.0300000
18295.0000000	-0.0295000
18296.0000000	-0.0295000
18297.0000000	-0.0300000
18298.0000000	-0.0300000
18299.0000000	-0.0295000
18300.0000000	-0.0295238
18301.0000000	-0.0290000
18302.0000000	-0.0285000
18303.0000000	-0.0285714
18304.0000000	-0.0295000
18305.0000000	-0.0290476
18306.0000000	-0.0290000
18307.0000000	-0.0300000
18308.0000000	-0.0295000
18309.0000000	-0.0295238
18310.0000000	-0.0290000
18311.0000000	-0.0290000
18312.0000000	-0.0276190
18313.0000000	-0.0280000
18314.0000000	-0.0275000
18315.0000000	-0.0285000
18316.0000000	-0.0290476
18317.0000000	-0.0275000
18318.0000000	-0.0290476
18319.0000000	-0.0285000
18320.0000000	-0.0270000
18321.0000000	-0.0271429
18322.0000000	-0.0275000
18323.0000000	-0.0270000
18324.0000000	-0.0275000
18325.0000000	-0.0266667
18326.0000000	-0.0280000
18327.0000000	-0.0265000
18328.0000000	-0.0261905
18329.0000000	-0.0235000

18330.0000000	-0.0261905
18331.0000000	-0.0265000
18332.0000000	-0.0245000
18333.0000000	-0.0271429
18334.0000000	-0.0260000
18335.0000000	-0.0240000
18336.0000000	-0.0245000
18337.0000000	-0.0257143
18338.0000000	-0.0240000
18339.0000000	-0.0247619
18340.0000000	-0.0230000
18341.0000000	-0.0260000
18342.0000000	-0.0223810
18343.0000000	-0.0255000
18344.0000000	-0.0235000
18345.0000000	-0.0250000
18346.0000000	-0.0271429
18347.0000000	-0.0260000
18348.0000000	-0.0247619
18349.0000000	-0.0260000
18350.0000000	-0.0238095
18351.0000000	-0.0265000
18352.0000000	-0.0250000
18353.0000000	-0.0245000
18354.0000000	-0.0250000
18355.0000000	-0.0242857
18356.0000000	-0.0255000
18357.0000000	-0.0242857
18358.0000000	-0.0235000
18359.0000000	-0.0255000
18360.0000000	-0.0220000
18361.0000000	-0.0252381
18362.0000000	-0.0255000
18363.0000000	-0.0240000
18364.0000000	-0.0238095
18365.0000000	-0.0235000
18366.0000000	-0.0233333
18367.0000000	-0.0260000
18368.0000000	-0.0260000
18369.0000000	-0.0252381
18370.0000000	-0.0240000
18371.0000000	-0.0260000
18372.0000000	-0.0250000
18373.0000000	-0.0238095
18374.0000000	-0.0250000
18375.0000000	-0.0255000
18376.0000000	-0.0240000
18377.0000000	-0.0242857
18378.0000000	-0.0228571
18379.0000000	-0.0250000
18380.0000000	-0.0235000
18381.0000000	-0.0233333
18382.0000000	-0.0240000
18383.0000000	-0.0250000
18384.0000000	-0.0225000
18385.0000000	-0.0242857
18386.0000000	-0.0240000

18387.0000000	-0.0252381
18388.0000000	-0.0225000
18389.0000000	-0.0230000
18390.0000000	-0.0245000
18391.0000000	-0.0238095
18392.0000000	-0.0225000
18393.0000000	-0.0228571
18394.0000000	-0.0225000
18395.0000000	-0.0230000
18396.0000000	-0.0238095
18397.0000000	-0.0235000
18398.0000000	-0.0245000
18399.0000000	-0.0225000
18400.0000000	-0.0204762
18401.0000000	-0.0235000
18402.0000000	-0.0219048
18403.0000000	-0.0230000
18404.0000000	-0.0250000
18405.0000000	-0.0240000
18406.0000000	-0.0219048
18407.0000000	-0.0225000
18408.0000000	-0.0215000
18409.0000000	-0.0233333
18410.0000000	-0.0215000
18411.0000000	-0.0228571
18412.0000000	-0.0215000
18413.0000000	-0.0220000
18414.0000000	-0.0225000
18415.0000000	-0.0235000
18416.0000000	-0.0219048
18417.0000000	-0.0230000
18418.0000000	-0.0233333
18419.0000000	-0.0240000
18420.0000000	-0.0225000
18421.0000000	-0.0228571
18422.0000000	-0.0220000
18423.0000000	-0.0235000
18424.0000000	-0.0225000
18425.0000000	-0.0228571
18426.0000000	-0.0238095
18427.0000000	-0.0225000
18428.0000000	-0.0255000
18429.0000000	-0.0255000
18430.0000000	-0.0242857
18431.0000000	-0.0255000
18432.0000000	-0.0250000
18433.0000000	-0.0261905
18434.0000000	-0.0255000
18435.0000000	-0.0230000
18436.0000000	-0.0257143
18437.0000000	-0.0265000
18438.0000000	-0.0252381
18439.0000000	-0.0270000
18440.0000000	-0.0260000
18441.0000000	-0.0265000
18442.0000000	-0.0280952
18443.0000000	-0.0275000

18444.0000000	-0.0270000
18445.0000000	-0.0280952
18446.0000000	-0.0260000
18447.0000000	-0.0261905
18448.0000000	-0.0300000
18449.0000000	-0.0275000
18450.0000000	-0.0260000
18451.0000000	-0.0280000
18452.0000000	-0.0233333
18453.0000000	-0.0250000
18454.0000000	-0.0261905
18455.0000000	-0.0240000
18456.0000000	-0.0252381
18457.0000000	-0.0260000
18458.0000000	-0.0275000
18459.0000000	-0.0265000
18460.0000000	-0.0255000
18461.0000000	-0.0242857
18462.0000000	-0.0255000
18463.0000000	-0.0223810
18464.0000000	-0.0265000
18465.0000000	-0.0270000
18466.0000000	-0.0266667
18467.0000000	-0.0250000
18468.0000000	-0.0270000
18469.0000000	-0.0271429
18470.0000000	-0.0270000
18471.0000000	-0.0285000
18472.0000000	-0.0247619
18473.0000000	-0.0260000
18474.0000000	-0.0261905
18475.0000000	-0.0255000
18476.0000000	-0.0270000
18477.0000000	-0.0265000
18478.0000000	-0.0266667
18479.0000000	-0.0260000
18480.0000000	-0.0255000
18481.0000000	-0.0261905
18482.0000000	-0.0270000
18483.0000000	-0.0290000
18484.0000000	-0.0280952
18485.0000000	-0.0270000
18486.0000000	-0.0285714
18487.0000000	-0.0284210
18488.0000000	-0.0261905
18489.0000000	-0.0260000
18490.0000000	-0.0271429
18491.0000000	-0.0240000
18492.0000000	-0.0250000
18493.0000000	-0.0266667
18494.0000000	-0.0265000
18495.0000000	-0.0260000
18496.0000000	-0.0250000
18497.0000000	-0.0257143
18498.0000000	-0.0245000
18499.0000000	-0.0252381
18500.0000000	-0.0250000

18501.0000000	-0.0220000
18502.0000000	-0.0228571
18503.0000000	-0.0255000
18504.0000000	-0.0255000
18505.0000000	-0.0250000
18506.0000000	-0.0247619
18507.0000000	-0.0260000
18508.0000000	-0.0266667
18509.0000000	-0.0240000
18510.0000000	-0.0230000
18511.0000000	-0.0230000
18512.0000000	-0.0245000
18513.0000000	-0.0261905
18514.0000000	-0.0252381
18515.0000000	-0.0225000
18516.0000000	-0.0230000
18517.0000000	-0.0228571
18518.0000000	-0.0235000
18519.0000000	-0.0260000
18520.0000000	-0.0250000
18521.0000000	-0.0228571
18522.0000000	-0.0230000
18523.0000000	-0.0242857
18524.0000000	-0.0260000
18525.0000000	-0.0230000
18526.0000000	-0.0242857
18527.0000000	-0.0270000
18528.0000000	-0.0275000
18529.0000000	-0.0245000
18530.0000000	-0.0266667
18531.0000000	-0.0265000
18532.0000000	-0.0260000
18533.0000000	-0.0271429
18534.0000000	-0.0250000
18535.0000000	-0.0276190
18536.0000000	-0.0290000
18537.0000000	-0.0280000
18538.0000000	-0.0280952
18539.0000000	-0.0280000
18540.0000000	-0.0295000
18541.0000000	-0.0275000
18542.0000000	-0.0295238
18543.0000000	-0.0290000
18544.0000000	-0.0280952
18545.0000000	-0.0290000
18546.0000000	-0.0280000
18547.0000000	-0.0285714
18548.0000000	-0.0295000
18549.0000000	-0.0275000
18550.0000000	-0.0295000
18551.0000000	-0.0285714
18552.0000000	-0.0290000
18553.0000000	-0.0276190
18554.0000000	-0.0295000
18555.0000000	-0.0300000
18556.0000000	-0.0295000
18557.0000000	-0.0295238

18558.0000000	-0.0290000
18559.0000000	-0.0300000
18560.0000000	-0.0290476
18561.0000000	-0.0295000
18562.0000000	-0.0295238
18563.0000000	-0.0285000
18564.0000000	-0.0280000
18565.0000000	-0.0290000
18566.0000000	-0.0285714
18567.0000000	-0.0295000
18568.0000000	-0.0285000
18569.0000000	-0.0300000
18570.0000000	-0.0290000
18571.0000000	-0.0285000
18572.0000000	-0.0300000
18573.0000000	-0.0290000
18574.0000000	-0.0295238
18575.0000000	-0.0295000
18576.0000000	-0.0280000
18577.0000000	-0.0300000
18578.0000000	-0.0300000
18579.0000000	-0.0295000
18580.0000000	-0.0300000
18581.0000000	-0.0280952
18582.0000000	-0.0300000
18583.0000000	-0.0295238
18584.0000000	-0.0290000
18585.0000000	-0.0300000
18586.0000000	-0.0295000
18587.0000000	-0.0300000
18588.0000000	-0.0295000
18589.0000000	-0.0300000
18590.0000000	-0.0290476
18591.0000000	-0.0295000
18592.0000000	-0.0290476
18593.0000000	-0.0300000
18594.0000000	-0.0295000
18595.0000000	-0.0290000
18596.0000000	-0.0295238
18597.0000000	-0.0295000
18598.0000000	-0.0300000
18599.0000000	-0.0295000
18600.0000000	-0.0300000
18601.0000000	-0.0285000
18602.0000000	-0.0290476
18603.0000000	-0.0295000
18604.0000000	-0.0285000
18605.0000000	-0.0295238
18606.0000000	-0.0290000
18607.0000000	-0.0300000
18608.0000000	-0.0295000
18609.0000000	-0.0295000
18610.0000000	-0.0300000
18611.0000000	-0.0300000
18612.0000000	-0.0290000
18613.0000000	-0.0300000
18614.0000000	-0.0300000

18615.0000000	-0.0300000
18616.0000000	-0.0300000
18617.0000000	-0.0295000
18618.0000000	-0.0300000
18619.0000000	-0.0300000
18620.0000000	-0.0300000
18621.0000000	-0.0300000
18622.0000000	-0.0300000
18623.0000000	-0.0300000
18624.0000000	-0.0300000
18625.0000000	-0.0300000
18626.0000000	-0.0300000
18627.0000000	-0.0300000
18628.0000000	-0.0300000
18629.0000000	-0.0300000
18630.0000000	-0.0300000
18631.0000000	-0.0300000
18632.0000000	-0.0300000
18633.0000000	-0.0305000
18634.0000000	-0.0300000
18635.0000000	-0.0300000
18636.0000000	-0.0300000
18637.0000000	-0.0300000
18638.0000000	-0.0300000
18639.0000000	-0.0305000
18640.0000000	-0.0300000
18641.0000000	-0.0300000
18642.0000000	-0.0300000
18643.0000000	-0.0300000
18644.0000000	-0.0300000
18645.0000000	-0.0300000
18646.0000000	-0.0300000
18647.0000000	-0.0300000
18648.0000000	-0.0304762
18649.0000000	-0.0300000
18650.0000000	-0.0300000
18651.0000000	-0.0300000
18652.0000000	-0.0300000
18653.0000000	-0.0300000
18654.0000000	-0.0300000
18655.0000000	-0.0300000
18656.0000000	-0.0300000
18657.0000000	-0.0300000
18658.0000000	-0.0300000
18659.0000000	-0.0300000
18660.0000000	-0.0300000
18661.0000000	-0.0300000
18662.0000000	-0.0300000
18663.0000000	-0.0300000
18664.0000000	-0.0300000
18665.0000000	-0.0300000
18666.0000000	-0.0300000
18667.0000000	-0.0300000
18668.0000000	-0.0304762
18669.0000000	-0.0300000
18670.0000000	-0.0300000
18671.0000000	-0.0300000

18672.0000000	-0.0305000
18673.0000000	-0.0300000
18674.0000000	-0.0295238
18675.0000000	-0.0300000
18676.0000000	-0.0300000
18677.0000000	-0.0300000
18678.0000000	-0.0300000
18679.0000000	-0.0300000
18680.0000000	-0.0300000
18681.0000000	-0.0300000
18682.0000000	-0.0300000
18683.0000000	-0.0300000
18684.0000000	-0.0295000
18685.0000000	-0.0295000
18686.0000000	-0.0300000
18687.0000000	-0.0300000
18688.0000000	-0.0300000
18689.0000000	-0.0300000
18690.0000000	-0.0300000
18691.0000000	-0.0300000
18692.0000000	-0.0300000
18693.0000000	-0.0300000
18694.0000000	-0.0300000
18695.0000000	-0.0300000
18696.0000000	-0.0295000
18697.0000000	-0.0300000
18698.0000000	-0.0304762
18699.0000000	-0.0300000
18700.0000000	-0.0300000
18701.0000000	-0.0295000
18702.0000000	-0.0300000
18703.0000000	-0.0300000
18704.0000000	-0.0300000
18705.0000000	-0.0295000
18706.0000000	-0.0295000
18707.0000000	-0.0300000
18708.0000000	-0.0300000
18709.0000000	-0.0295238
18710.0000000	-0.0300000
18711.0000000	-0.0300000
18712.0000000	-0.0300000
18713.0000000	-0.0300000
18714.0000000	-0.0300000
18715.0000000	-0.0300000
18716.0000000	-0.0300000
18717.0000000	-0.0300000
18718.0000000	-0.0300000
18719.0000000	-0.0300000
18720.0000000	-0.0300000
18721.0000000	-0.0300000
18722.0000000	-0.0300000
18723.0000000	-0.0300000
18724.0000000	-0.0300000
18725.0000000	-0.0300000
18726.0000000	-0.0300000
18727.0000000	-0.0300000
18728.0000000	-0.0300000

18729.0000000	-0.0300000
18730.0000000	-0.0300000
18731.0000000	-0.0300000
18732.0000000	-0.0295000
18733.0000000	-0.0300000
18734.0000000	-0.0295000
18735.0000000	-0.0300000
18736.0000000	-0.0300000
18737.0000000	-0.0300000
18738.0000000	-0.0300000
18739.0000000	-0.0300000
18740.0000000	-0.0300000
18741.0000000	-0.0300000
18742.0000000	-0.0300000
18743.0000000	-0.0300000
18744.0000000	-0.0300000
18745.0000000	-0.0300000
18746.0000000	-0.0300000
18747.0000000	-0.0300000
18748.0000000	-0.0300000
18749.0000000	-0.0300000
18750.0000000	-0.0300000
18751.0000000	-0.0300000
18752.0000000	-0.0295000
18753.0000000	-0.0300000
18754.0000000	-0.0300000
18755.0000000	-0.0300000
18756.0000000	-0.0300000
18757.0000000	-0.0300000
18758.0000000	-0.0300000
18759.0000000	-0.0300000
18760.0000000	-0.0300000
18761.0000000	-0.0300000
18762.0000000	-0.0300000
18763.0000000	-0.0300000
18764.0000000	-0.0300000
18765.0000000	-0.0300000
18766.0000000	-0.0300000
18767.0000000	-0.0300000
18768.0000000	-0.0300000
18769.0000000	-0.0295000
18770.0000000	-0.0300000
18771.0000000	-0.0295238
18772.0000000	-0.0290000
18773.0000000	-0.0300000
18774.0000000	-0.0300000
18775.0000000	-0.0290000
18776.0000000	-0.0300000
18777.0000000	-0.0300000
18778.0000000	-0.0295000
18779.0000000	-0.0295238
18780.0000000	-0.0290000
18781.0000000	-0.0295000
18782.0000000	-0.0295000
18783.0000000	-0.0285714
18784.0000000	-0.0285000
18785.0000000	-0.0280000

18786.0000000	-0.0290476
18787.0000000	-0.0275000
18788.0000000	-0.0290476
18789.0000000	-0.0280000
18790.0000000	-0.0285000
18791.0000000	-0.0290000
18792.0000000	-0.0290476
18793.0000000	-0.0290000
18794.0000000	-0.0290000
18795.0000000	-0.0295238
18796.0000000	-0.0295000
18797.0000000	-0.0295000
18798.0000000	-0.0295000
18799.0000000	-0.0295238
18800.0000000	-0.0295000
18801.0000000	-0.0280952
18802.0000000	-0.0280000
18803.0000000	-0.0280000
18804.0000000	-0.0285714
18805.0000000	-0.0280000
18806.0000000	-0.0275000
18807.0000000	-0.0280952
18808.0000000	-0.0290000
18809.0000000	-0.0300000
18810.0000000	-0.0285714
18811.0000000	-0.0290000
18812.0000000	-0.0290000
18813.0000000	-0.0280000
18814.0000000	-0.0257143
18815.0000000	-0.0265000
18816.0000000	-0.0290476
18817.0000000	-0.0270000
18818.0000000	-0.0255000
18819.0000000	-0.0271429
18820.0000000	-0.0265000
18821.0000000	-0.0260000
18822.0000000	-0.0260000
18823.0000000	-0.0261905
18824.0000000	-0.0265000
18825.0000000	-0.0260000
18826.0000000	-0.0252381
18827.0000000	-0.0260000
18828.0000000	-0.0280000
18829.0000000	-0.0257143
18830.0000000	-0.0265000
18831.0000000	-0.0261905
18832.0000000	-0.0245000
18833.0000000	-0.0275000
18834.0000000	-0.0261905
18835.0000000	-0.0270000
18836.0000000	-0.0265000
18837.0000000	-0.0245000
18838.0000000	-0.0252381
18839.0000000	-0.0230000
18840.0000000	-0.0223810
18841.0000000	-0.0230000
18842.0000000	-0.0245000

18843.0000000	-0.0245000
18844.0000000	-0.0233333
18845.0000000	-0.0230000
18846.0000000	-0.0245000
18847.0000000	-0.0242857
18848.0000000	-0.0215000
18849.0000000	-0.0219048
18850.0000000	-0.0220000
18851.0000000	-0.0220000
18852.0000000	-0.0210000
18853.0000000	-0.0219048
18854.0000000	-0.0215000
18855.0000000	-0.0214286
18856.0000000	-0.0200000
18857.0000000	-0.0210000
18858.0000000	-0.0225000
18859.0000000	-0.0228571
18860.0000000	-0.0210000
18861.0000000	-0.0220000
18862.0000000	-0.0214286
18863.0000000	-0.0220000
18864.0000000	-0.0225000
18865.0000000	-0.0219048
18866.0000000	-0.0235000
18867.0000000	-0.0219048
18868.0000000	-0.0225000
18869.0000000	-0.0235000
18870.0000000	-0.0220000
18871.0000000	-0.0233333
18872.0000000	-0.0230000
18873.0000000	-0.0235000
18874.0000000	-0.0238095
18875.0000000	-0.0230000
18876.0000000	-0.0223810
18877.0000000	-0.0270000
18878.0000000	-0.0220000
18879.0000000	-0.0233333
18880.0000000	-0.0235000
18881.0000000	-0.0230000
18882.0000000	-0.0225000
18883.0000000	-0.0214286
18884.0000000	-0.0230000
18885.0000000	-0.0223810
18886.0000000	-0.0245000
18887.0000000	-0.0250000
18888.0000000	-0.0220000
18889.0000000	-0.0225000
18890.0000000	-0.0223810
18891.0000000	-0.0215000
18892.0000000	-0.0223810
18893.0000000	-0.0215000
18894.0000000	-0.0214286
18895.0000000	-0.0225000
18896.0000000	-0.0220000
18897.0000000	-0.0215000
18898.0000000	-0.0228571
18899.0000000	-0.0210000

18900.0000000	-0.0215000
18901.0000000	-0.0219048
18902.0000000	-0.0210000
18903.0000000	-0.0205000
18904.0000000	-0.0214286
18905.0000000	-0.0210000
18906.0000000	-0.0210000
18907.0000000	-0.0200000
18908.0000000	-0.0215000
18909.0000000	-0.0220000
18910.0000000	-0.0209524
18911.0000000	-0.0210000
18912.0000000	-0.0210000
18913.0000000	-0.0235000
18914.0000000	-0.0209524
18915.0000000	-0.0219048
18916.0000000	-0.0215000
18917.0000000	-0.0200000
18918.0000000	-0.0220000
18919.0000000	-0.0204762
18920.0000000	-0.0225000
18921.0000000	-0.0215000
18922.0000000	-0.0209524
18923.0000000	-0.0215000
18924.0000000	-0.0219048
18925.0000000	-0.0230000
18926.0000000	-0.0225000
18927.0000000	-0.0220000
18928.0000000	-0.0214286
18929.0000000	-0.0210000
18930.0000000	-0.0230000
18931.0000000	-0.0219048
18932.0000000	-0.0225000
18933.0000000	-0.0225000
18934.0000000	-0.0215000
18935.0000000	-0.0219048
18936.0000000	-0.0205000
18937.0000000	-0.0204762
18938.0000000	-0.0220000
18939.0000000	-0.0204762
18940.0000000	-0.0210000
18941.0000000	-0.0225000
18942.0000000	-0.0215000
18943.0000000	-0.0223810
18944.0000000	-0.0225000
18945.0000000	-0.0215000
18946.0000000	-0.0209524
18947.0000000	-0.0205000
18948.0000000	-0.0210000
18949.0000000	-0.0210000
18950.0000000	-0.0204762
18951.0000000	-0.0220000
18952.0000000	-0.0215000
18953.0000000	-0.0209524
18954.0000000	-0.0205000
18955.0000000	-0.0200000
18956.0000000	-0.0200000

18957.0000000	-0.0200000
18958.0000000	-0.0200000
18959.0000000	-0.0209524
18960.0000000	-0.0210000
18961.0000000	-0.0205000
18962.0000000	-0.0219048
18963.0000000	-0.0215000
18964.0000000	-0.0209524
18965.0000000	-0.0205000
18966.0000000	-0.0210000
18967.0000000	-0.0219048
18968.0000000	-0.0205000
18969.0000000	-0.0200000
18970.0000000	-0.0215000
18971.0000000	-0.0219048
18972.0000000	-0.0215000
18973.0000000	-0.0219048
18974.0000000	-0.0210000
18975.0000000	-0.0220000
18976.0000000	-0.0204762
18977.0000000	-0.0215000
18978.0000000	-0.0210000
18979.0000000	-0.0205000
18980.0000000	-0.0219048
18981.0000000	-0.0210000
18982.0000000	-0.0204762
18983.0000000	-0.0230000
18984.0000000	-0.0214286
18985.0000000	-0.0210000
18986.0000000	-0.0215000
18987.0000000	-0.0205000
18988.0000000	-0.0215000
18989.0000000	-0.0204762
18990.0000000	-0.0210000
18991.0000000	-0.0200000
18992.0000000	-0.0205000
18993.0000000	-0.0220000
18994.0000000	-0.0210000
18995.0000000	-0.0204762
18996.0000000	-0.0205000
18997.0000000	-0.0200000
18998.0000000	-0.0209524
18999.0000000	-0.0200000
19000.0000000	-0.0200000
19001.0000000	-0.0210000
19002.0000000	-0.0210000
19003.0000000	-0.0204762
19004.0000000	-0.0200000
19005.0000000	-0.0200000
19006.0000000	-0.0215000
19007.0000000	-0.0200000
19008.0000000	-0.0205000
19009.0000000	-0.0205000
19010.0000000	-0.0200000
19011.0000000	-0.0210000
19012.0000000	-0.0204762
19013.0000000	-0.0210000

19014.0000000	-0.0200000
19015.0000000	-0.0200000
19016.0000000	-0.0200000
19017.0000000	-0.0200000
19018.0000000	-0.0205000
19019.0000000	-0.0204762
19020.0000000	-0.0205000
19021.0000000	-0.0200000
19022.0000000	-0.0200000
19023.0000000	-0.0205000
19024.0000000	-0.0205000
19025.0000000	-0.0200000
19026.0000000	-0.0200000
19027.0000000	-0.0204762
19028.0000000	-0.0200000
19029.0000000	-0.0205000
19030.0000000	-0.0200000
19031.0000000	-0.0200000
19032.0000000	-0.0205000
19033.0000000	-0.0205000
19034.0000000	-0.0200000
19035.0000000	-0.0200000
19036.0000000	-0.0200000
19037.0000000	-0.0210000
19038.0000000	-0.0200000
19039.0000000	-0.0200000
19040.0000000	-0.0200000
19041.0000000	-0.0200000
19042.0000000	-0.0200000
19043.0000000	-0.0200000
19044.0000000	-0.0200000
19045.0000000	-0.0200000
19046.0000000	-0.0200000
19047.0000000	-0.0205000
19048.0000000	-0.0200000
19049.0000000	-0.0200000
19050.0000000	-0.0200000
19051.0000000	-0.0200000
19052.0000000	-0.0200000
19053.0000000	-0.0200000
19054.0000000	-0.0200000
19055.0000000	-0.0195238
19056.0000000	-0.0200000
19057.0000000	-0.0200000
19058.0000000	-0.0200000
19059.0000000	-0.0200000
19060.0000000	-0.0200000
19061.0000000	-0.0200000
19062.0000000	-0.0200000
19063.0000000	-0.0200000
19064.0000000	-0.0200000
19065.0000000	-0.0200000
19066.0000000	-0.0200000
19067.0000000	-0.0200000
19068.0000000	-0.0195000
19069.0000000	-0.0200000
19070.0000000	-0.0200000

19071.0000000	-0.0200000
19072.0000000	-0.0200000
19073.0000000	-0.0200000
19074.0000000	-0.0200000
19075.0000000	-0.0200000
19076.0000000	-0.0200000
19077.0000000	-0.0200000
19078.0000000	-0.0200000
19079.0000000	-0.0200000
19080.0000000	-0.0195000
19081.0000000	-0.0200000
19082.0000000	-0.0200000
19083.0000000	-0.0200000
19084.0000000	-0.0200000
19085.0000000	-0.0200000
19086.0000000	-0.0195238
19087.0000000	-0.0200000
19088.0000000	-0.0200000
19089.0000000	-0.0200000
19090.0000000	-0.0200000
19091.0000000	-0.0200000
19092.0000000	-0.0200000
19093.0000000	-0.0200000
19094.0000000	-0.0200000
19095.0000000	-0.0200000
19096.0000000	-0.0200000
19097.0000000	-0.0200000
19098.0000000	-0.0200000
19099.0000000	-0.0200000
19100.0000000	-0.0200000
19101.0000000	-0.0200000
19102.0000000	-0.0200000
19103.0000000	-0.0200000
19104.0000000	-0.0200000
19105.0000000	-0.0195000
19106.0000000	-0.0200000
19107.0000000	-0.0200000
19108.0000000	-0.0200000
19109.0000000	-0.0195238
19110.0000000	-0.0195000
19111.0000000	-0.0200000
19112.0000000	-0.0200000
19113.0000000	-0.0195000
19114.0000000	-0.0200000
19115.0000000	-0.0190476
19116.0000000	-0.0195000
19117.0000000	-0.0195000
19118.0000000	-0.0190000
19119.0000000	-0.0195238
19120.0000000	-0.0195238
19121.0000000	-0.0195000
19122.0000000	-0.0190000
19123.0000000	-0.0200000
19124.0000000	-0.0200000
19125.0000000	-0.0195000
19126.0000000	-0.0190000
19127.0000000	-0.0190476

19128.0000000	-0.0185000
19129.0000000	-0.0190000
19130.0000000	-0.0185000
19131.0000000	-0.0171429
19132.0000000	-0.0185000
19133.0000000	-0.0180952
19134.0000000	-0.0190000
19135.0000000	-0.0185000
19136.0000000	-0.0176190
19137.0000000	-0.0165000
19138.0000000	-0.0185000
19139.0000000	-0.0180952
19140.0000000	-0.0190000
19141.0000000	-0.0165000
19142.0000000	-0.0171429
19143.0000000	-0.0175000
19144.0000000	-0.0160000
19145.0000000	-0.0170000
19146.0000000	-0.0180000
19147.0000000	-0.0176190
19148.0000000	-0.0171429
19149.0000000	-0.0155000
19150.0000000	-0.0180000
19151.0000000	-0.0166667
19152.0000000	-0.0150000
19153.0000000	-0.0165000
19154.0000000	-0.0165000
19155.0000000	-0.0185714
19156.0000000	-0.0180000
19157.0000000	-0.0170000
19158.0000000	-0.0176190
19159.0000000	-0.0165000
19160.0000000	-0.0152381
19161.0000000	-0.0150000
19162.0000000	-0.0180000
19163.0000000	-0.0175000
19164.0000000	-0.0171429
19165.0000000	-0.0175000
19166.0000000	-0.0165000
19167.0000000	-0.0176190
19168.0000000	-0.0155000
19169.0000000	-0.0166667
19170.0000000	-0.0160000
19171.0000000	-0.0130000
19172.0000000	-0.0152381
19173.0000000	-0.0135000
19174.0000000	-0.0160000
19175.0000000	-0.0150000
19176.0000000	-0.0119048
19177.0000000	-0.0155000
19178.0000000	-0.0152381
19179.0000000	-0.0155000
19180.0000000	-0.0140000
19181.0000000	-0.0147619
19182.0000000	-0.0130000
19183.0000000	-0.0135000
19184.0000000	-0.0130000

19185.0000000	-0.0142857
19186.0000000	-0.0130000
19187.0000000	-0.0138095
19188.0000000	-0.0145000
19189.0000000	-0.0125000
19190.0000000	-0.0135000
19191.0000000	-0.0142857
19192.0000000	-0.0135000
19193.0000000	-0.0130000
19194.0000000	-0.0104762
19195.0000000	-0.0140000
19196.0000000	-0.0123810
19197.0000000	-0.0125000
19198.0000000	-0.0125000
19199.0000000	-0.0140000
19200.0000000	-0.0119048
19201.0000000	-0.0120000
19202.0000000	-0.0105000
19203.0000000	-0.0133333
19204.0000000	-0.0130000
19205.0000000	-0.0130000
19206.0000000	-0.0123810
19207.0000000	-0.0125000
19208.0000000	-0.0119048
19209.0000000	-0.0115000
19210.0000000	-0.0100000
19211.0000000	-0.0110000
19212.0000000	-0.0128571
19213.0000000	-0.0105000
19214.0000000	-0.0110000
19215.0000000	-0.0104762
19216.0000000	-0.0105000
19217.0000000	-0.0104762
19218.0000000	-0.0105000
19219.0000000	-0.0100000
19220.0000000	-0.0105000
19221.0000000	-0.0104762
19222.0000000	-0.0105000
19223.0000000	-0.0100000
19224.0000000	-0.0109524
19225.0000000	-0.0105000
19226.0000000	-0.0100000
19227.0000000	-0.0100000
19228.0000000	-0.0110000
19229.0000000	-0.0105000
19230.0000000	-0.0100000
19231.0000000	-0.0100000
19232.0000000	-0.0105000
19233.0000000	-0.0100000
19234.0000000	-0.0100000
19235.0000000	-0.0110000
19236.0000000	-0.0100000
19237.0000000	-0.0100000
19238.0000000	-0.0100000
19239.0000000	-0.0100000
19240.0000000	-0.0100000
19241.0000000	-0.0100000

19242.0000000	-0.0100000
19243.0000000	-0.0100000
19244.0000000	-0.0100000
19245.0000000	-0.0100000
19246.0000000	-0.0100000
19247.0000000	-0.0100000
19248.0000000	-0.0100000
19249.0000000	-0.0100000
19250.0000000	-0.0100000
19251.0000000	-0.0100000
19252.0000000	-0.0100000
19253.0000000	-0.0100000
19254.0000000	-0.0100000
19255.0000000	-0.0100000
19256.0000000	-0.0100000
19257.0000000	-0.0100000
19258.0000000	-0.0100000
19259.0000000	-0.0100000
19260.0000000	-0.0100000
19261.0000000	-0.0100000
19262.0000000	-0.0100000
19263.0000000	-0.0100000
19264.0000000	-0.0100000
19265.0000000	-0.0100000
19266.0000000	-0.0100000
19267.0000000	-0.0100000
19268.0000000	-0.0100000
19269.0000000	-0.0100000
19270.0000000	-0.0100000
19271.0000000	-0.0100000
19272.0000000	-0.0095238
19273.0000000	-0.0100000
19274.0000000	-0.0100000
19275.0000000	-0.0100000
19276.0000000	-0.0100000
19277.0000000	-0.0105000
19278.0000000	-0.0100000
19279.0000000	-0.0100000
19280.0000000	-0.0100000
19281.0000000	-0.0100000
19282.0000000	-0.0100000
19283.0000000	-0.0100000
19284.0000000	-0.0100000
19285.0000000	-0.0100000
19286.0000000	-0.0100000
19287.0000000	-0.0100000
19288.0000000	-0.0100000
19289.0000000	-0.0100000
19290.0000000	-0.0100000
19291.0000000	-0.0100000
19292.0000000	-0.0100000
19293.0000000	-0.0100000
19294.0000000	-0.0100000
19295.0000000	-0.0100000
19296.0000000	-0.0100000
19297.0000000	-0.0100000
19298.0000000	-0.0100000

19299.0000000	-0.0100000
19300.0000000	-0.0100000
19301.0000000	-0.0100000
19302.0000000	-0.0100000
19303.0000000	-0.0100000
19304.0000000	-0.0100000
19305.0000000	-0.0100000
19306.0000000	-0.0100000
19307.0000000	-0.0100000
19308.0000000	-0.0100000
19309.0000000	-0.0100000
19310.0000000	-0.0100000
19311.0000000	-0.0100000
19312.0000000	-0.0100000
19313.0000000	-0.0100000
19314.0000000	-0.0100000
19315.0000000	-0.0100000
19316.0000000	-0.0100000
19317.0000000	-0.0100000
19318.0000000	-0.0100000
19319.0000000	-0.0100000
19320.0000000	-0.0100000
19321.0000000	-0.0100000
19322.0000000	-0.0100000
19323.0000000	-0.0100000
19324.0000000	-0.0104762
19325.0000000	-0.0100000
19326.0000000	-0.0100000
19327.0000000	-0.0100000
19328.0000000	-0.0100000
19329.0000000	-0.0100000
19330.0000000	-0.0100000
19331.0000000	-0.0100000
19332.0000000	-0.0095238
19333.0000000	-0.0100000
19334.0000000	-0.0100000
19335.0000000	-0.0100000
19336.0000000	-0.0095238
19337.0000000	-0.0100000
19338.0000000	-0.0100000
19339.0000000	-0.0095238
19340.0000000	-0.0090000
19341.0000000	-0.0100000
19342.0000000	-0.0100000
19343.0000000	-0.0100000
19344.0000000	-0.0100000
19345.0000000	-0.0100000
19346.0000000	-0.0100000
19347.0000000	-0.0100000
19348.0000000	-0.0100000
19349.0000000	-0.0090000
19350.0000000	-0.0100000
19351.0000000	-0.0090000
19352.0000000	-0.0095238
19353.0000000	-0.0095238
19354.0000000	-0.0095000
19355.0000000	-0.0100000

19356.0000000	-0.0100000
19357.0000000	-0.0100000
19358.0000000	-0.0100000
19359.0000000	-0.0090476
19360.0000000	-0.0100000
19361.0000000	-0.0080000
19362.0000000	-0.0095000
19363.0000000	-0.0095238
19364.0000000	-0.0095000
19365.0000000	-0.0095000
19366.0000000	-0.0090476
19367.0000000	-0.0085000
19368.0000000	-0.0095238
19369.0000000	-0.0100000
19370.0000000	-0.0100000
19371.0000000	-0.0095000
19372.0000000	-0.0095238
19373.0000000	-0.0090000
19374.0000000	-0.0100000
19375.0000000	-0.0100000
19376.0000000	-0.0085000
19377.0000000	-0.0095238
19378.0000000	-0.0095000
19379.0000000	-0.0080000
19380.0000000	-0.0100000
19381.0000000	-0.0075000
19382.0000000	-0.0085714
19383.0000000	-0.0085000
19384.0000000	-0.0085714
19385.0000000	-0.0085000
19386.0000000	-0.0080000
19387.0000000	-0.0075000
19388.0000000	-0.0085714
19389.0000000	-0.0100000
19390.0000000	-0.0095000
19391.0000000	-0.0085714
19392.0000000	-0.0090000
19393.0000000	-0.0080952
19394.0000000	-0.0085000
19395.0000000	-0.0085000
19396.0000000	-0.0066667
19397.0000000	-0.0075000
19398.0000000	-0.0060000
19399.0000000	-0.0070000
19400.0000000	-0.0052381
19401.0000000	-0.0080000
19402.0000000	-0.0061905
19403.0000000	-0.0090000
19404.0000000	-0.0060000
19405.0000000	-0.0061905
19406.0000000	-0.0065000
19407.0000000	-0.0070000
19408.0000000	-0.0066667
19409.0000000	-0.0050000
19410.0000000	-0.0055000
19411.0000000	-0.0066667
19412.0000000	-0.0045000

19413.0000000	-0.0052381
19414.0000000	-0.0050000
19415.0000000	-0.0035000
19416.0000000	-0.0050000
19417.0000000	-0.0040000
19418.0000000	-0.0057143
19419.0000000	-0.0030000
19420.0000000	-0.0028571
19421.0000000	-0.0050000
19422.0000000	-0.0033333
19423.0000000	-0.0030000
19424.0000000	-0.0035000
19425.0000000	-0.0035000
19426.0000000	-0.0030000
19427.0000000	-0.0038095
19428.0000000	-0.0015000
19429.0000000	-0.0014286
19430.0000000	-0.0015000
19431.0000000	-0.0025000
19432.0000000	-0.0023810
19433.0000000	-0.0010000
19434.0000000	-0.0015000
19435.0000000	-0.0030000
19436.0000000	-0.0019048
19437.0000000	-0.0010000
19438.0000000	-0.0023810
19439.0000000	-0.0015000
19440.0000000	-0.0020000
19441.0000000	-0.0009524
19442.0000000	-0.0005000
19443.0000000	-0.0005000
19444.0000000	-0.0004762
19445.0000000	-0.0010000
19446.0000000	0.0000000
19447.0000000	0.0000000
19448.0000000	-0.0004762
19449.0000000	-0.0005000
19450.0000000	-0.0010000
19451.0000000	-0.0004762
19452.0000000	-0.0005000
19453.0000000	-0.0004762
19454.0000000	0.0000000
19455.0000000	-0.0005000
19456.0000000	0.0000000
19457.0000000	0.0000000
19458.0000000	0.0000000
19459.0000000	0.0000000
19460.0000000	-0.0004762
19461.0000000	-0.0005000
19462.0000000	0.0000000
19463.0000000	0.0000000
19464.0000000	0.0000000
19465.0000000	-0.0004762
19466.0000000	0.0000000
19467.0000000	0.0000000
19468.0000000	-0.0004762
19469.0000000	0.0000000

19470.0000000	0.0000000
19471.0000000	0.0000000
19472.0000000	0.0000000
19473.0000000	-0.0005000
19474.0000000	0.0000000
19475.0000000	0.0000000
19476.0000000	0.0000000
19477.0000000	0.0000000
19478.0000000	0.0000000
19479.0000000	0.0000000
19480.0000000	0.0000000
19481.0000000	0.0000000
19482.0000000	0.0000000
19483.0000000	0.0000000
19484.0000000	0.0000000
19485.0000000	0.0000000
19486.0000000	0.0000000
19487.0000000	0.0000000
19488.0000000	0.0000000
19489.0000000	0.0000000
19490.0000000	0.0000000
19491.0000000	0.0000000
19492.0000000	0.0005000
19493.0000000	0.0000000
19494.0000000	0.0000000
19495.0000000	0.0000000
19496.0000000	0.0000000
19497.0000000	0.0000000
19498.0000000	0.0000000
19499.0000000	0.0000000
19500.0000000	0.0000000
19501.0000000	0.0000000
19502.0000000	0.0000000
19503.0000000	0.0004000
19504.0000000	0.0000000
19505.0000000	0.0009524
19506.0000000	0.0000000
19507.0000000	0.0000000
19508.0000000	0.0000000
19509.0000000	0.0005000
19510.0000000	0.0000000
19511.0000000	0.0005000
19512.0000000	0.0005000
19513.0000000	0.0000000
19514.0000000	0.0000000
19515.0000000	0.0010000
19516.0000000	0.0000000
19517.0000000	0.0004762
19518.0000000	0.0005000
19519.0000000	0.0009524
19520.0000000	0.0000000
19521.0000000	0.0000000
19522.0000000	0.0015000
19523.0000000	0.0015000
19524.0000000	0.0004762
19525.0000000	0.0005000
19526.0000000	0.0028571

19527.0000000	0.0010000
19528.0000000	0.0005000
19529.0000000	0.0004762
19530.0000000	0.0000000
19531.0000000	0.0000000
19532.0000000	0.0000000
19533.0000000	0.0005000
19534.0000000	0.0025000
19535.0000000	0.0004762
19536.0000000	0.0015000
19537.0000000	0.0015000
19538.0000000	0.0000000
19539.0000000	0.0015000
19540.0000000	0.0000000
19541.0000000	0.0014286
19542.0000000	0.0020000
19543.0000000	0.0025000
19544.0000000	0.0014286
19545.0000000	0.0010000
19546.0000000	0.0015000
19547.0000000	0.0020000
19548.0000000	0.0014286
19549.0000000	0.0023810
19550.0000000	0.0025000
19551.0000000	0.0000000
19552.0000000	0.0010000
19553.0000000	0.0009524
19554.0000000	0.0010000
19555.0000000	0.0010000
19556.0000000	0.0019048
19557.0000000	0.0010000
19558.0000000	0.0019048
19559.0000000	0.0030000
19560.0000000	0.0040000
19561.0000000	0.0030000
19562.0000000	0.0023810
19563.0000000	0.0020000
19564.0000000	0.0020000
19565.0000000	0.0014286
19566.0000000	0.0025000
19567.0000000	0.0025000
19568.0000000	0.0035000
19569.0000000	0.0042857
19570.0000000	0.0020000
19571.0000000	0.0038095
19572.0000000	0.0030000
19573.0000000	0.0033333
19574.0000000	0.0040000
19575.0000000	0.0030000
19576.0000000	0.0030000
19577.0000000	0.0028571
19578.0000000	0.0040000
19579.0000000	0.0040000
19580.0000000	0.0038095
19581.0000000	0.0045000
19582.0000000	0.0025000
19583.0000000	0.0035000

19584.0000000	0.0042857
19585.0000000	0.0040000
19586.0000000	0.0035000
19587.0000000	0.0042857
19588.0000000	0.0035000
19589.0000000	0.0061905
19590.0000000	0.0035000
19591.0000000	0.0040000
19592.0000000	0.0040000
19593.0000000	0.0023810
19594.0000000	0.0030000
19595.0000000	0.0030000
19596.0000000	0.0033333
19597.0000000	0.0035000
19598.0000000	0.0023810
19599.0000000	0.0030000
19600.0000000	0.0030000
19601.0000000	0.0028571
19602.0000000	0.0055000
19603.0000000	0.0045000
19604.0000000	0.0050000
19605.0000000	0.0047619
19606.0000000	0.0025000
19607.0000000	0.0042857
19608.0000000	0.0045000
19609.0000000	0.0050000
19610.0000000	0.0033333
19611.0000000	0.0045000
19612.0000000	0.0040000
19613.0000000	0.0030000
19614.0000000	0.0033333
19615.0000000	0.0060000
19616.0000000	0.0057143
19617.0000000	0.0055000
19618.0000000	0.0045000
19619.0000000	0.0042857
19620.0000000	0.0055000
19621.0000000	0.0050000
19622.0000000	0.0070000
19623.0000000	0.0076190
19624.0000000	0.0055000
19625.0000000	0.0071429
19626.0000000	0.0085000
19627.0000000	0.0070000
19628.0000000	0.0070000
19629.0000000	0.0066667
19630.0000000	0.0070000
19631.0000000	0.0090000
19632.0000000	0.0066667
19633.0000000	0.0075000
19634.0000000	0.0080952
19635.0000000	0.0075000
19636.0000000	0.0060000
19637.0000000	0.0071429
19638.0000000	0.0075000
19639.0000000	0.0095000
19640.0000000	0.0080000

19641.0000000	0.0090476
19642.0000000	0.0075000
19643.0000000	0.0085000
19644.0000000	0.0095238
19645.0000000	0.0080000
19646.0000000	0.0071429
19647.0000000	0.0094737
19648.0000000	0.0095238
19649.0000000	0.0095238
19650.0000000	0.0095000
19651.0000000	0.0090000
19652.0000000	0.0100000
19653.0000000	0.0095238
19654.0000000	0.0100000
19655.0000000	0.0090476
19656.0000000	0.0100000
19657.0000000	0.0100000
19658.0000000	0.0085000
19659.0000000	0.0100000
19660.0000000	0.0095000
19661.0000000	0.0090476
19662.0000000	0.0100000
19663.0000000	0.0100000
19664.0000000	0.0095000
19665.0000000	0.0100000
19666.0000000	0.0095000
19667.0000000	0.0100000
19668.0000000	0.0095238
19669.0000000	0.0100000
19670.0000000	0.0100000
19671.0000000	0.0095000
19672.0000000	0.0100000
19673.0000000	0.0100000
19674.0000000	0.0100000
19675.0000000	0.0100000
19676.0000000	0.0100000
19677.0000000	0.0100000
19678.0000000	0.0100000
19679.0000000	0.0100000
19680.0000000	0.0100000
19681.0000000	0.0100000
19682.0000000	0.0100000
19683.0000000	0.0100000
19684.0000000	0.0100000
19685.0000000	0.0100000
19686.0000000	0.0109524
19687.0000000	0.0100000
19688.0000000	0.0100000
19689.0000000	0.0100000
19690.0000000	0.0100000
19691.0000000	0.0100000
19692.0000000	0.0105000
19693.0000000	0.0100000
19694.0000000	0.0105000
19695.0000000	0.0100000
19696.0000000	0.0100000
19697.0000000	0.0100000

19698.0000000	0.0100000
19699.0000000	0.0100000
19700.0000000	0.0105000
19701.0000000	0.0100000
19702.0000000	0.0100000
19703.0000000	0.0105000
19704.0000000	0.0100000
19705.0000000	0.0110000
19706.0000000	0.0105000
19707.0000000	0.0119048
19708.0000000	0.0105000
19709.0000000	0.0105000
19710.0000000	0.0100000
19711.0000000	0.0100000
19712.0000000	0.0105000
19713.0000000	0.0119048
19714.0000000	0.0110000
19715.0000000	0.0105000
19716.0000000	0.0109524
19717.0000000	0.0110000
19718.0000000	0.0120000
19719.0000000	0.0100000
19720.0000000	0.0114286
19721.0000000	0.0125000
19722.0000000	0.0104762
19723.0000000	0.0130000
19724.0000000	0.0115000
19725.0000000	0.0104762
19726.0000000	0.0140000
19727.0000000	0.0115000
19728.0000000	0.0140000
19729.0000000	0.0133333
19730.0000000	0.0115000
19731.0000000	0.0123810
19732.0000000	0.0135000
19733.0000000	0.0130000
19734.0000000	0.0142857
19735.0000000	0.0135000
19736.0000000	0.0150000
19737.0000000	0.0142857
19738.0000000	0.0165000
19739.0000000	0.0145000
19740.0000000	0.0152381
19741.0000000	0.0170000
19742.0000000	0.0145000
19743.0000000	0.0152381
19744.0000000	0.0155000
19745.0000000	0.0165000
19746.0000000	0.0147619
19747.0000000	0.0165000
19748.0000000	0.0175000
19749.0000000	0.0171429
19750.0000000	0.0180000
19751.0000000	0.0160000
19752.0000000	0.0175000
19753.0000000	0.0171429
19754.0000000	0.0171429

19755.0000000	0.0165000
19756.0000000	0.0190000
19757.0000000	0.0175000
19758.0000000	0.0171429
19759.0000000	0.0155000
19760.0000000	0.0175000
19761.0000000	0.0176190
19762.0000000	0.0170000
19763.0000000	0.0185000
19764.0000000	0.0170000
19765.0000000	0.0190476
19766.0000000	0.0195000
19767.0000000	0.0180000
19768.0000000	0.0180952
19769.0000000	0.0180000
19770.0000000	0.0176190
19771.0000000	0.0185000
19772.0000000	0.0175000
19773.0000000	0.0171429
19774.0000000	0.0195000
19775.0000000	0.0195000
19776.0000000	0.0185000
19777.0000000	0.0190476
19778.0000000	0.0200000
19779.0000000	0.0195000
19780.0000000	0.0195000
19781.0000000	0.0190476
19782.0000000	0.0190476
19783.0000000	0.0190000
19784.0000000	0.0195000
19785.0000000	0.0195238
19786.0000000	0.0200000
19787.0000000	0.0195000
19788.0000000	0.0200000
19789.0000000	0.0195238
19790.0000000	0.0200000
19791.0000000	0.0195000
19792.0000000	0.0200000
19793.0000000	0.0200000
19794.0000000	0.0200000
19795.0000000	0.0200000
19796.0000000	0.0195000
19797.0000000	0.0200000
19798.0000000	0.0200000
19799.0000000	0.0200000
19800.0000000	0.0195000
19801.0000000	0.0190476
19802.0000000	0.0200000
19803.0000000	0.0200000
19804.0000000	0.0195238
19805.0000000	0.0200000
19806.0000000	0.0200000
19807.0000000	0.0200000
19808.0000000	0.0200000
19809.0000000	0.0200000
19810.0000000	0.0200000
19811.0000000	0.0200000

19812.0000000	0.0200000
19813.0000000	0.0200000
19814.0000000	0.0200000
19815.0000000	0.0200000
19816.0000000	0.0200000
19817.0000000	0.0200000
19818.0000000	0.0200000
19819.0000000	0.0200000
19820.0000000	0.0200000
19821.0000000	0.0200000
19822.0000000	0.0200000
19823.0000000	0.0200000
19824.0000000	0.0200000
19825.0000000	0.0200000
19826.0000000	0.0200000
19827.0000000	0.0200000
19828.0000000	0.0200000
19829.0000000	0.0200000
19830.0000000	0.0200000
19831.0000000	0.0204762
19832.0000000	0.0205000
19833.0000000	0.0200000
19834.0000000	0.0204762
19835.0000000	0.0205000
19836.0000000	0.0200000
19837.0000000	0.0200000
19838.0000000	0.0205000
19839.0000000	0.0200000
19840.0000000	0.0205000
19841.0000000	0.0204762
19842.0000000	0.0200000
19843.0000000	0.0210000
19844.0000000	0.0200000
19845.0000000	0.0205000
19846.0000000	0.0209524
19847.0000000	0.0210000
19848.0000000	0.0200000
19849.0000000	0.0219048
19850.0000000	0.0205000
19851.0000000	0.0214286
19852.0000000	0.0210000
19853.0000000	0.0210000
19854.0000000	0.0205000
19855.0000000	0.0205000
19856.0000000	0.0204762
19857.0000000	0.0210000
19858.0000000	0.0204762
19859.0000000	0.0210000
19860.0000000	0.0200000
19861.0000000	0.0210000
19862.0000000	0.0210000
19863.0000000	0.0215000
19864.0000000	0.0220000
19865.0000000	0.0214286
19866.0000000	0.0210000
19867.0000000	0.0209524
19868.0000000	0.0215000

19869.0000000	0.0215000
19870.0000000	0.0204762
19871.0000000	0.0210000
19872.0000000	0.0220000
19873.0000000	0.0223810
19874.0000000	0.0225000
19875.0000000	0.0225000
19876.0000000	0.0223810
19877.0000000	0.0225000
19878.0000000	0.0225000
19879.0000000	0.0230000
19880.0000000	0.0223810
19881.0000000	0.0225000
19882.0000000	0.0223810
19883.0000000	0.0240000
19884.0000000	0.0215000
19885.0000000	0.0240000
19886.0000000	0.0233333
19887.0000000	0.0235000
19888.0000000	0.0235000
19889.0000000	0.0242857
19890.0000000	0.0238095
19891.0000000	0.0250000
19892.0000000	0.0250000
19893.0000000	0.0255000
19894.0000000	0.0252381
19895.0000000	0.0255000
19896.0000000	0.0250000
19897.0000000	0.0242857
19898.0000000	0.0240000
19899.0000000	0.0255000
19900.0000000	0.0285000
19901.0000000	0.0257143
19902.0000000	0.0260000
19903.0000000	0.0252381
19904.0000000	0.0250000
19905.0000000	0.0255000
19906.0000000	0.0247619
19907.0000000	0.0240000
19908.0000000	0.0255000
19909.0000000	0.0240000
19910.0000000	0.0271429
19911.0000000	0.0255000
19912.0000000	0.0261905
19913.0000000	0.0250000
19914.0000000	0.0245000
19915.0000000	0.0240000
19916.0000000	0.0235000
19917.0000000	0.0252381
19918.0000000	0.0255000
19919.0000000	0.0238095
19920.0000000	0.0255000
19921.0000000	0.0242857
19922.0000000	0.0255000
19923.0000000	0.0260000
19924.0000000	0.0255000
19925.0000000	0.0247619

19926.0000000	0.0265000
19927.0000000	0.0260000
19928.0000000	0.0280952
19929.0000000	0.0265000
19930.0000000	0.0266667
19931.0000000	0.0260000
19932.0000000	0.0265000
19933.0000000	0.0285000
19934.0000000	0.0290476
19935.0000000	0.0270000
19936.0000000	0.0285000
19937.0000000	0.0280952
19938.0000000	0.0280000
19939.0000000	0.0285714
19940.0000000	0.0290000
19941.0000000	0.0280000
19942.0000000	0.0276190
19943.0000000	0.0280000
19944.0000000	0.0295000
19945.0000000	0.0290000
19946.0000000	0.0285714
19947.0000000	0.0295000
19948.0000000	0.0290476
19949.0000000	0.0300000
19950.0000000	0.0300000
19951.0000000	0.0295238
19952.0000000	0.0275000
19953.0000000	0.0280000
19954.0000000	0.0280000
19955.0000000	0.0285714
19956.0000000	0.0295000
19957.0000000	0.0295000
19958.0000000	0.0295238
19959.0000000	0.0285000
19960.0000000	0.0295000
19961.0000000	0.0300000
19962.0000000	0.0295000
19963.0000000	0.0295000
19964.0000000	0.0300000
19965.0000000	0.0295000
19966.0000000	0.0290476
19967.0000000	0.0295000
19968.0000000	0.0290000
19969.0000000	0.0300000
19970.0000000	0.0290476
19971.0000000	0.0295000
19972.0000000	0.0295000
19973.0000000	0.0290476
19974.0000000	0.0295000
19975.0000000	0.0295000
19976.0000000	0.0295000
19977.0000000	0.0300000
19978.0000000	0.0295238
19979.0000000	0.0285000
19980.0000000	0.0300000
19981.0000000	0.0290000
19982.0000000	0.0295238

19983.0000000	0.0300000
19984.0000000	0.0295000
19985.0000000	0.0285714
19986.0000000	0.0300000
19987.0000000	0.0280952
19988.0000000	0.0290000
19989.0000000	0.0290000
19990.0000000	0.0300000
19991.0000000	0.0290476
19992.0000000	0.0290000
19993.0000000	0.0295000
19994.0000000	0.0300000
19995.0000000	0.0300000
19996.0000000	0.0300000
19997.0000000	0.0300000
19998.0000000	0.0295000
19999.0000000	0.0300000
20000.0000000	0.0295238
20001.0000000	0.0285000
20002.0000000	0.0300000
20003.0000000	0.0300000
20004.0000000	0.0295000
20005.0000000	0.0295000
20006.0000000	0.0300000
20007.0000000	0.0300000
20008.0000000	0.0300000
20009.0000000	0.0295238
20010.0000000	0.0295000
20011.0000000	0.0295238
20012.0000000	0.0295000
20013.0000000	0.0300000
20014.0000000	0.0300000
20015.0000000	0.0300000
20016.0000000	0.0295238
20017.0000000	0.0300000
20018.0000000	0.0295238
20019.0000000	0.0300000
20020.0000000	0.0300000
20021.0000000	0.0300000
20022.0000000	0.0300000
20023.0000000	0.0300000
20024.0000000	0.0300000
20025.0000000	0.0300000
20026.0000000	0.0300000
20027.0000000	0.0295000
20028.0000000	0.0300000
20029.0000000	0.0300000
20030.0000000	0.0300000
20031.0000000	0.0295000
20032.0000000	0.0295000
20033.0000000	0.0300000
20034.0000000	0.0300000
20035.0000000	0.0290000
20036.0000000	0.0295238
20037.0000000	0.0300000
20038.0000000	0.0300000
20039.0000000	0.0300000

20040.0000000	0.0300000
20041.0000000	0.0300000
20042.0000000	0.0300000
20043.0000000	0.0300000
20044.0000000	0.0300000
20045.0000000	0.0300000
20046.0000000	0.0300000
20047.0000000	0.0300000
20048.0000000	0.0300000
20049.0000000	0.0300000
20050.0000000	0.0295000
20051.0000000	0.0300000
20052.0000000	0.0300000
20053.0000000	0.0300000
20054.0000000	0.0300000
20055.0000000	0.0300000
20056.0000000	0.0300000
20057.0000000	0.0300000
20058.0000000	0.0300000
20059.0000000	0.0300000
20060.0000000	0.0300000
20061.0000000	0.0300000
20062.0000000	0.0300000
20063.0000000	0.0300000
20064.0000000	0.0300000
20065.0000000	0.0300000
20066.0000000	0.0300000
20067.0000000	0.0300000
20068.0000000	0.0300000
20069.0000000	0.0295000
20070.0000000	0.0295238
20071.0000000	0.0300000
20072.0000000	0.0300000
20073.0000000	0.0290000
20074.0000000	0.0300000
20075.0000000	0.0300000
20076.0000000	0.0300000
20077.0000000	0.0295000
20078.0000000	0.0300000
20079.0000000	0.0300000
20080.0000000	0.0300000
20081.0000000	0.0300000
20082.0000000	0.0300000
20083.0000000	0.0300000
20084.0000000	0.0300000
20085.0000000	0.0300000
20086.0000000	0.0295000
20087.0000000	0.0300000
20088.0000000	0.0300000
20089.0000000	0.0300000
20090.0000000	0.0300000
20091.0000000	0.0295000
20092.0000000	0.0300000
20093.0000000	0.0300000
20094.0000000	0.0300000
20095.0000000	0.0300000
20096.0000000	0.0300000

20097.0000000	0.0300000
20098.0000000	0.0300000
20099.0000000	0.0300000
20100.0000000	0.0300000
20101.0000000	0.0300000
20102.0000000	0.0300000
20103.0000000	0.0300000
20104.0000000	0.0300000
20105.0000000	0.0300000
20106.0000000	0.0300000
20107.0000000	0.0300000
20108.0000000	0.0300000
20109.0000000	0.0300000
20110.0000000	0.0300000
20111.0000000	0.0300000
20112.0000000	0.0300000
20113.0000000	0.0300000
20114.0000000	0.0300000
20115.0000000	0.0300000
20116.0000000	0.0300000
20117.0000000	0.0300000
20118.0000000	0.0300000
20119.0000000	0.0300000
20120.0000000	0.0300000
20121.0000000	0.0300000
20122.0000000	0.0300000
20123.0000000	0.0300000
20124.0000000	0.0300000
20125.0000000	0.0300000
20126.0000000	0.0300000
20127.0000000	0.0300000
20128.0000000	0.0300000
20129.0000000	0.0300000
20130.0000000	0.0300000
20131.0000000	0.0300000
20132.0000000	0.0300000
20133.0000000	0.0300000
20134.0000000	0.0300000
20135.0000000	0.0305000
20136.0000000	0.0300000
20137.0000000	0.0300000
20138.0000000	0.0300000
20139.0000000	0.0300000
20140.0000000	0.0305000
20141.0000000	0.0300000
20142.0000000	0.0304762
20143.0000000	0.0310000
20144.0000000	0.0300000
20145.0000000	0.0304762
20146.0000000	0.0310000
20147.0000000	0.0300000
20148.0000000	0.0305000
20149.0000000	0.0315000
20150.0000000	0.0305000
20151.0000000	0.0309524
20152.0000000	0.0310000
20153.0000000	0.0310000

20154.0000000	0.0319048
20155.0000000	0.0315000
20156.0000000	0.0300000
20157.0000000	0.0325000
20158.0000000	0.0304762
20159.0000000	0.0305000
20160.0000000	0.0304762
20161.0000000	0.0315000
20162.0000000	0.0315000
20163.0000000	0.0309524
20164.0000000	0.0325000
20165.0000000	0.0305000
20166.0000000	0.0309524
20167.0000000	0.0300000
20168.0000000	0.0305000
20169.0000000	0.0323809
20170.0000000	0.0305000
20171.0000000	0.0330000
20172.0000000	0.0320000
20173.0000000	0.0323809
20174.0000000	0.0320000
20175.0000000	0.0323809
20176.0000000	0.0320000
20177.0000000	0.0330000
20178.0000000	0.0323809
20179.0000000	0.0330000
20180.0000000	0.0330000
20181.0000000	0.0330000
20182.0000000	0.0314286
20183.0000000	0.0319048
20184.0000000	0.0320000
20185.0000000	0.0315000
20186.0000000	0.0330000
20187.0000000	0.0323809
20188.0000000	0.0305000
20189.0000000	0.0325000
20190.0000000	0.0319048
20191.0000000	0.0315000
20192.0000000	0.0319048
20193.0000000	0.0330000
20194.0000000	0.0315000
20195.0000000	0.0330000
20196.0000000	0.0323809
20197.0000000	0.0325000
20198.0000000	0.0325000
20199.0000000	0.0323809
20200.0000000	0.0335000
20201.0000000	0.0345000
20202.0000000	0.0333333
20203.0000000	0.0320000
20204.0000000	0.0330000
20205.0000000	0.0330000
20206.0000000	0.0333333
20207.0000000	0.0355000
20208.0000000	0.0333333
20209.0000000	0.0340000
20210.0000000	0.0350000

20211.0000000	0.0345000
20212.0000000	0.0361905
20213.0000000	0.0325000
20214.0000000	0.0347619
20215.0000000	0.0340000
20216.0000000	0.0335000
20217.0000000	0.0320000
20218.0000000	0.0342857
20219.0000000	0.0340000
20220.0000000	0.0360000
20221.0000000	0.0328571
20222.0000000	0.0345000
20223.0000000	0.0352381
20224.0000000	0.0350000
20225.0000000	0.0345000
20226.0000000	0.0345000
20227.0000000	0.0357143
20228.0000000	0.0360000
20229.0000000	0.0360000
20230.0000000	0.0361905
20231.0000000	0.0355000
20232.0000000	0.0360000
20233.0000000	0.0380952
20234.0000000	0.0350000
20235.0000000	0.0376190
20236.0000000	0.0375000
20237.0000000	0.0380000
20238.0000000	0.0385714
20239.0000000	0.0370000
20240.0000000	0.0370000
20241.0000000	0.0360000
20242.0000000	0.0357143
20243.0000000	0.0375000
20244.0000000	0.0376190
20245.0000000	0.0370000
20246.0000000	0.0365000
20247.0000000	0.0370000
20248.0000000	0.0371429
20249.0000000	0.0375000
20250.0000000	0.0385000
20251.0000000	0.0385714
20252.0000000	0.0365000
20253.0000000	0.0380952
20254.0000000	0.0395000
20255.0000000	0.0390000
20256.0000000	0.0390000
20257.0000000	0.0366667
20258.0000000	0.0395000
20259.0000000	0.0390000
20260.0000000	0.0380952
20261.0000000	0.0390000
20262.0000000	0.0375000
20263.0000000	0.0390476
20264.0000000	0.0390000
20265.0000000	0.0385000
20266.0000000	0.0385714
20267.0000000	0.0390000

20268.0000000	0.0375000
20269.0000000	0.0390476
20270.0000000	0.0395000
20271.0000000	0.0380952
20272.0000000	0.0400000
20273.0000000	0.0395000
20274.0000000	0.0390000
20275.0000000	0.0395238
20276.0000000	0.0390000
20277.0000000	0.0395000
20278.0000000	0.0395238
20279.0000000	0.0400000
20280.0000000	0.0395238
20281.0000000	0.0400000
20282.0000000	0.0390000
20283.0000000	0.0395238
20284.0000000	0.0395000
20285.0000000	0.0390000
20286.0000000	0.0395000
20287.0000000	0.0385714
20288.0000000	0.0390000
20289.0000000	0.0395000
20290.0000000	0.0390476
20291.0000000	0.0390000
20292.0000000	0.0380000
20293.0000000	0.0380952
20294.0000000	0.0385000
20295.0000000	0.0395000
20296.0000000	0.0395238
20297.0000000	0.0370000
20298.0000000	0.0385000
20299.0000000	0.0395238
20300.0000000	0.0390000
20301.0000000	0.0390000
20302.0000000	0.0400000
20303.0000000	0.0390000
20304.0000000	0.0390000
20305.0000000	0.0400000
20306.0000000	0.0400000
20307.0000000	0.0395000
20308.0000000	0.0390000
20309.0000000	0.0395238
20310.0000000	0.0395000
20311.0000000	0.0395238
20312.0000000	0.0395000
20313.0000000	0.0385000
20314.0000000	0.0385714
20315.0000000	0.0400000
20316.0000000	0.0390000
20317.0000000	0.0395000
20318.0000000	0.0395238
20319.0000000	0.0390476
20320.0000000	0.0400000
20321.0000000	0.0400000
20322.0000000	0.0395000
20323.0000000	0.0395238
20324.0000000	0.0395000

20325.0000000	0.0395000
20326.0000000	0.0390476
20327.0000000	0.0385000
20328.0000000	0.0395000
20329.0000000	0.0395238
20330.0000000	0.0390000
20331.0000000	0.0400000
20332.0000000	0.0390476
20333.0000000	0.0395000
20334.0000000	0.0400000
20335.0000000	0.0400000
20336.0000000	0.0390000
20337.0000000	0.0395000
20338.0000000	0.0400000
20339.0000000	0.0400000
20340.0000000	0.0400000
20341.0000000	0.0400000
20342.0000000	0.0400000
20343.0000000	0.0400000
20344.0000000	0.0400000
20345.0000000	0.0400000
20346.0000000	0.0400000
20347.0000000	0.0395000
20348.0000000	0.0395238
20349.0000000	0.0400000
20350.0000000	0.0400000
20351.0000000	0.0395000
20352.0000000	0.0395000
20353.0000000	0.0400000
20354.0000000	0.0400000
20355.0000000	0.0400000
20356.0000000	0.0400000
20357.0000000	0.0400000
20358.0000000	0.0400000
20359.0000000	0.0395238
20360.0000000	0.0400000
20361.0000000	0.0395000
20362.0000000	0.0400000
20363.0000000	0.0400000
20364.0000000	0.0400000
20365.0000000	0.0400000
20366.0000000	0.0400000
20367.0000000	0.0400000
20368.0000000	0.0400000
20369.0000000	0.0400000
20370.0000000	0.0400000
20371.0000000	0.0400000
20372.0000000	0.0400000
20373.0000000	0.0400000
20374.0000000	0.0400000
20375.0000000	0.0400000
20376.0000000	0.0400000
20377.0000000	0.0400000
20378.0000000	0.0400000
20379.0000000	0.0400000
20380.0000000	0.0400000
20381.0000000	0.0400000

20382.0000000	0.0400000
20383.0000000	0.0400000
20384.0000000	0.0400000
20385.0000000	0.0395000
20386.0000000	0.0400000
20387.0000000	0.0400000
20388.0000000	0.0390476
20389.0000000	0.0395000
20390.0000000	0.0400000
20391.0000000	0.0400000
20392.0000000	0.0395000
20393.0000000	0.0385714
20394.0000000	0.0395000
20395.0000000	0.0395238
20396.0000000	0.0395000
20397.0000000	0.0390000
20398.0000000	0.0390000
20399.0000000	0.0400000
20400.0000000	0.0400000
20401.0000000	0.0395000
20402.0000000	0.0400000
20403.0000000	0.0395000
20404.0000000	0.0395238
20405.0000000	0.0400000
20406.0000000	0.0395000
20407.0000000	0.0400000
20408.0000000	0.0400000
20409.0000000	0.0385000
20410.0000000	0.0400000
20411.0000000	0.0390476
20412.0000000	0.0395000
20413.0000000	0.0395000
20414.0000000	0.0400000
20415.0000000	0.0395238
20416.0000000	0.0400000
20417.0000000	0.0400000
20418.0000000	0.0395000
20419.0000000	0.0400000
20420.0000000	0.0395000
20421.0000000	0.0395000
20422.0000000	0.0390000
20423.0000000	0.0390476
20424.0000000	0.0395000
20425.0000000	0.0395000
20426.0000000	0.0400000
20427.0000000	0.0395238
20428.0000000	0.0390000
20429.0000000	0.0395238
20430.0000000	0.0400000
20431.0000000	0.0400000
20432.0000000	0.0395238
20433.0000000	0.0400000
20434.0000000	0.0400000
20435.0000000	0.0400000
20436.0000000	0.0400000
20437.0000000	0.0395000
20438.0000000	0.0385714

20439.0000000	0.0395000
20440.0000000	0.0395238
20441.0000000	0.0395000
20442.0000000	0.0400000
20443.0000000	0.0400000
20444.0000000	0.0400000
20445.0000000	0.0400000
20446.0000000	0.0395000
20447.0000000	0.0400000
20448.0000000	0.0400000
20449.0000000	0.0400000
20450.0000000	0.0400000
20451.0000000	0.0400000
20452.0000000	0.0395000
20453.0000000	0.0400000
20454.0000000	0.0390476
20455.0000000	0.0400000
20456.0000000	0.0400000
20457.0000000	0.0400000
20458.0000000	0.0390000
20459.0000000	0.0395238
20460.0000000	0.0395000
20461.0000000	0.0400000
20462.0000000	0.0390000
20463.0000000	0.0390476
20464.0000000	0.0395000
20465.0000000	0.0400000
20466.0000000	0.0395000
20467.0000000	0.0395000
20468.0000000	0.0395238
20469.0000000	0.0395000
20470.0000000	0.0400000
20471.0000000	0.0395238
20472.0000000	0.0395000
20473.0000000	0.0400000
20474.0000000	0.0400000
20475.0000000	0.0400000
20476.0000000	0.0400000
20477.0000000	0.0390000
20478.0000000	0.0400000
20479.0000000	0.0395000
20480.0000000	0.0395238
20481.0000000	0.0395000
20482.0000000	0.0400000
20483.0000000	0.0400000
20484.0000000	0.0395238
20485.0000000	0.0400000
20486.0000000	0.0390000
20487.0000000	0.0400000
20488.0000000	0.0395000
20489.0000000	0.0400000
20490.0000000	0.0395238
20491.0000000	0.0400000
20492.0000000	0.0400000
20493.0000000	0.0400000
20494.0000000	0.0400000
20495.0000000	0.0400000

20496.0000000	0.0400000
20497.0000000	0.0400000
20498.0000000	0.0400000
20499.0000000	0.0400000
20500.0000000	0.0400000
20501.0000000	0.0390476
20502.0000000	0.0400000
20503.0000000	0.0400000
20504.0000000	0.0400000
20505.0000000	0.0400000
20506.0000000	0.0400000
20507.0000000	0.0400000
20508.0000000	0.0400000
20509.0000000	0.0400000
20510.0000000	0.0400000
20511.0000000	0.0400000
20512.0000000	0.0400000
20513.0000000	0.0400000
20514.0000000	0.0400000
20515.0000000	0.0400000
20516.0000000	0.0400000
20517.0000000	0.0400000
20518.0000000	0.0400000
20519.0000000	0.0400000
20520.0000000	0.0400000
20521.0000000	0.0400000
20522.0000000	0.0400000
20523.0000000	0.0400000
20524.0000000	0.0400000
20525.0000000	0.0400000
20526.0000000	0.0395000
20527.0000000	0.0400000
20528.0000000	0.0400000
20529.0000000	0.0395000
20530.0000000	0.0400000
20531.0000000	0.0400000
20532.0000000	0.0400000
20533.0000000	0.0400000
20534.0000000	0.0400000
20535.0000000	0.0400000
20536.0000000	0.0400000
20537.0000000	0.0400000
20538.0000000	0.0400000
20539.0000000	0.0400000
20540.0000000	0.0400000
20541.0000000	0.0400000
20542.0000000	0.0400000
20543.0000000	0.0400000
20544.0000000	0.0400000
20545.0000000	0.0400000
20546.0000000	0.0400000
20547.0000000	0.0405000
20548.0000000	0.0405000
20549.0000000	0.0400000
20550.0000000	0.0400000
20551.0000000	0.0400000
20552.0000000	0.0400000

20553.0000000	0.0404762
20554.0000000	0.0400000
20555.0000000	0.0404762
20556.0000000	0.0400000
20557.0000000	0.0400000
20558.0000000	0.0405000
20559.0000000	0.0400000
20560.0000000	0.0405000
20561.0000000	0.0400000
20562.0000000	0.0409524
20563.0000000	0.0400000
20564.0000000	0.0400000
20565.0000000	0.0400000
20566.0000000	0.0400000
20567.0000000	0.0400000
20568.0000000	0.0400000
20569.0000000	0.0400000
20570.0000000	0.0400000
20571.0000000	0.0400000
20572.0000000	0.0400000
20573.0000000	0.0400000
20574.0000000	0.0400000
20575.0000000	0.0400000
20576.0000000	0.0400000
20577.0000000	0.0400000
20578.0000000	0.0400000
20579.0000000	0.0400000
20580.0000000	0.0404762
20581.0000000	0.0405000
20582.0000000	0.0400000
20583.0000000	0.0400000
20584.0000000	0.0400000
20585.0000000	0.0400000
20586.0000000	0.0405000
20587.0000000	0.0400000
20588.0000000	0.0400000
20589.0000000	0.0400000
20590.0000000	0.0400000
20591.0000000	0.0405000
20592.0000000	0.0400000
20593.0000000	0.0400000
20594.0000000	0.0400000
20595.0000000	0.0400000
20596.0000000	0.0400000
20597.0000000	0.0415000
20598.0000000	0.0400000
20599.0000000	0.0400000
20600.0000000	0.0400000
20601.0000000	0.0405000
20602.0000000	0.0400000
20603.0000000	0.0400000
20604.0000000	0.0400000
20605.0000000	0.0400000
20606.0000000	0.0400000
20607.0000000	0.0400000
20608.0000000	0.0405000
20609.0000000	0.0405000

20610.0000000	0.0405000
20611.0000000	0.0400000
20612.0000000	0.0414286
20613.0000000	0.0405000
20614.0000000	0.0410000
20615.0000000	0.0400000
20616.0000000	0.0419048
20617.0000000	0.0405000
20618.0000000	0.0400000
20619.0000000	0.0414286
20620.0000000	0.0405000
20621.0000000	0.0410000
20622.0000000	0.0419048
20623.0000000	0.0405000
20624.0000000	0.0420000
20625.0000000	0.0419048
20626.0000000	0.0420000
20627.0000000	0.0425000
20628.0000000	0.0419048
20629.0000000	0.0420000
20630.0000000	0.0420000
20631.0000000	0.0404762
20632.0000000	0.0440000
20633.0000000	0.0430000
20634.0000000	0.0414286
20635.0000000	0.0435000
20636.0000000	0.0428572
20637.0000000	0.0425000
20638.0000000	0.0435000
20639.0000000	0.0425000
20640.0000000	0.0410000
20641.0000000	0.0423810
20642.0000000	0.0415000
20643.0000000	0.0414286
20644.0000000	0.0415000
20645.0000000	0.0410000
20646.0000000	0.0428572
20647.0000000	0.0420000
20648.0000000	0.0415000
20649.0000000	0.0420000
20650.0000000	0.0423810
20651.0000000	0.0435000
20652.0000000	0.0414286
20653.0000000	0.0430000
20654.0000000	0.0435000
20655.0000000	0.0430000
20656.0000000	0.0419048
20657.0000000	0.0410000
20658.0000000	0.0435000
20659.0000000	0.0433333
20660.0000000	0.0425000
20661.0000000	0.0438095
20662.0000000	0.0445000
20663.0000000	0.0440000
20664.0000000	0.0438095
20665.0000000	0.0430000
20666.0000000	0.0450000

20667.0000000	0.0425000
20668.0000000	0.0442857
20669.0000000	0.0475000
20670.0000000	0.0440000
20671.0000000	0.0428572
20672.0000000	0.0420000
20673.0000000	0.0423810
20674.0000000	0.0440000
20675.0000000	0.0440000
20676.0000000	0.0428572
20677.0000000	0.0420000
20678.0000000	0.0430000
20679.0000000	0.0430000
20680.0000000	0.0433333
20681.0000000	0.0425000
20682.0000000	0.0438095
20683.0000000	0.0445000
20684.0000000	0.0420000
20685.0000000	0.0425000
20686.0000000	0.0442857
20687.0000000	0.0445000
20688.0000000	0.0438095
20689.0000000	0.0430000
20690.0000000	0.0435000
20691.0000000	0.0452381
20692.0000000	0.0440000
20693.0000000	0.0440000
20694.0000000	0.0445000
20695.0000000	0.0433333
20696.0000000	0.0450000
20697.0000000	0.0452381
20698.0000000	0.0425000
20699.0000000	0.0445000
20700.0000000	0.0438095
20701.0000000	0.0425000
20702.0000000	0.0425000
20703.0000000	0.0420000
20704.0000000	0.0438095
20705.0000000	0.0450000
20706.0000000	0.0447619
20707.0000000	0.0435000
20708.0000000	0.0435000
20709.0000000	0.0430000
20710.0000000	0.0442857
20711.0000000	0.0430000
20712.0000000	0.0442857
20713.0000000	0.0445000
20714.0000000	0.0435000
20715.0000000	0.0450000
20716.0000000	0.0438095
20717.0000000	0.0440000
20718.0000000	0.0460000
20719.0000000	0.0442857
20720.0000000	0.0460000
20721.0000000	0.0428572
20722.0000000	0.0455000
20723.0000000	0.0450000

20724.0000000	0.0455000
20725.0000000	0.0447619
20726.0000000	0.0455000
20727.0000000	0.0480000
20728.0000000	0.0466667
20729.0000000	0.0440000
20730.0000000	0.0460000
20731.0000000	0.0461905
20732.0000000	0.0465000
20733.0000000	0.0435000
20734.0000000	0.0442857
20735.0000000	0.0430000
20736.0000000	0.0447619
20737.0000000	0.0445000
20738.0000000	0.0440000
20739.0000000	0.0450000
20740.0000000	0.0438095
20741.0000000	0.0460000
20742.0000000	0.0445000
20743.0000000	0.0438095
20744.0000000	0.0435000
20745.0000000	0.0425000
20746.0000000	0.0435000
20747.0000000	0.0442857
20748.0000000	0.0440000
20749.0000000	0.0442857
20750.0000000	0.0425000
20751.0000000	0.0440000
20752.0000000	0.0438095
20753.0000000	0.0450000
20754.0000000	0.0440000
20755.0000000	0.0445000
20756.0000000	0.0447619
20757.0000000	0.0435000
20758.0000000	0.0447619
20759.0000000	0.0460000
20760.0000000	0.0440000
20761.0000000	0.0447619
20762.0000000	0.0450000
20763.0000000	0.0435000
20764.0000000	0.0457143
20765.0000000	0.0445000
20766.0000000	0.0440000
20767.0000000	0.0438095
20768.0000000	0.0455000
20769.0000000	0.0470000
20770.0000000	0.0442857
20771.0000000	0.0460000
20772.0000000	0.0460000
20773.0000000	0.0433333
20774.0000000	0.0450000
20775.0000000	0.0470000
20776.0000000	0.0461905
20777.0000000	0.0468421
20778.0000000	0.0480952
20779.0000000	0.0438095
20780.0000000	0.0480000

20781.0000000	0.0476191
20782.0000000	0.0470000
20783.0000000	0.0470000
20784.0000000	0.0470000
20785.0000000	0.0461905
20786.0000000	0.0465000
20787.0000000	0.0475000
20788.0000000	0.0485714
20789.0000000	0.0470000
20790.0000000	0.0485000
20791.0000000	0.0480000
20792.0000000	0.0466667
20793.0000000	0.0485000
20794.0000000	0.0457143
20795.0000000	0.0475000
20796.0000000	0.0465000
20797.0000000	0.0471429
20798.0000000	0.0475000
20799.0000000	0.0465000
20800.0000000	0.0461905
20801.0000000	0.0470000
20802.0000000	0.0460000
20803.0000000	0.0485000
20804.0000000	0.0476191
20805.0000000	0.0485000
20806.0000000	0.0465000
20807.0000000	0.0480952
20808.0000000	0.0465000
20809.0000000	0.0480952
20810.0000000	0.0465000
20811.0000000	0.0470000
20812.0000000	0.0485714
20813.0000000	0.0480000
20814.0000000	0.0480000
20815.0000000	0.0465000
20816.0000000	0.0471429
20817.0000000	0.0471429
20818.0000000	0.0475000
20819.0000000	0.0490000
20820.0000000	0.0470000
20821.0000000	0.0480000
20822.0000000	0.0490476
20823.0000000	0.0475000
20824.0000000	0.0466667
20825.0000000	0.0475000
20826.0000000	0.0470000
20827.0000000	0.0471429
20828.0000000	0.0485000
20829.0000000	0.0480000
20830.0000000	0.0465000
20831.0000000	0.0466667
20832.0000000	0.0455000
20833.0000000	0.0452381
20834.0000000	0.0455000
20835.0000000	0.0455000
20836.0000000	0.0465000
20837.0000000	0.0466667

20838.0000000	0.0445000
20839.0000000	0.0445000
20840.0000000	0.0442857
20841.0000000	0.0465000
20842.0000000	0.0466667
20843.0000000	0.0465000
20844.0000000	0.0455000
20845.0000000	0.0450000
20846.0000000	0.0457143
20847.0000000	0.0455000
20848.0000000	0.0452381
20849.0000000	0.0460000
20850.0000000	0.0445000
20851.0000000	0.0450000
20852.0000000	0.0433333
20853.0000000	0.0440000
20854.0000000	0.0420000
20855.0000000	0.0438095
20856.0000000	0.0415000
20857.0000000	0.0428571
20858.0000000	0.0405000
20859.0000000	0.0425000
20860.0000000	0.0430000
20861.0000000	0.0419048
20862.0000000	0.0440000
20863.0000000	0.0435000
20864.0000000	0.0428572
20865.0000000	0.0430000
20866.0000000	0.0440000
20867.0000000	0.0433333
20868.0000000	0.0415000
20869.0000000	0.0438095
20870.0000000	0.0440000
20871.0000000	0.0425000
20872.0000000	0.0438095
20873.0000000	0.0440000
20874.0000000	0.0440000
20875.0000000	0.0435000
20876.0000000	0.0438095
20877.0000000	0.0440000
20878.0000000	0.0442857
20879.0000000	0.0435000
20880.0000000	0.0465000
20881.0000000	0.0465000
20882.0000000	0.0460000
20883.0000000	0.0466667
20884.0000000	0.0460000
20885.0000000	0.0457143
20886.0000000	0.0465000
20887.0000000	0.0490476
20888.0000000	0.0490000
20889.0000000	0.0485000
20890.0000000	0.0490000
20891.0000000	0.0475000
20892.0000000	0.0471429
20893.0000000	0.0485000
20894.0000000	0.0500000

20895.0000000	0.0495000
20896.0000000	0.0495000
20897.0000000	0.0490476
20898.0000000	0.0490000
20899.0000000	0.0500000
20900.0000000	0.0485714
20901.0000000	0.0490000
20902.0000000	0.0490000
20903.0000000	0.0500000
20904.0000000	0.0495000
20905.0000000	0.0495238
20906.0000000	0.0495000
20907.0000000	0.0495000
20908.0000000	0.0500000
20909.0000000	0.0500000
20910.0000000	0.0495000
20911.0000000	0.0500000
20912.0000000	0.0500000
20913.0000000	0.0500000
20914.0000000	0.0500000
20915.0000000	0.0495238
20916.0000000	0.0500000
20917.0000000	0.0500000
20918.0000000	0.0495000
20919.0000000	0.0500000
20920.0000000	0.0500000
20921.0000000	0.0500000
20922.0000000	0.0500000
20923.0000000	0.0500000
20924.0000000	0.0500000
20925.0000000	0.0500000
20926.0000000	0.0500000
20927.0000000	0.0495000
20928.0000000	0.0500000
20929.0000000	0.0500000
20930.0000000	0.0495238
20931.0000000	0.0495000
20932.0000000	0.0500000
20933.0000000	0.0500000
20934.0000000	0.0500000
20935.0000000	0.0495000
20936.0000000	0.0500000
20937.0000000	0.0500000
20938.0000000	0.0500000
20939.0000000	0.0500000
20940.0000000	0.0500000
20941.0000000	0.0500000
20942.0000000	0.0500000
20943.0000000	0.0500000
20944.0000000	0.0500000
20945.0000000	0.0500000
20946.0000000	0.0505000
20947.0000000	0.0505000
20948.0000000	0.0504762
20949.0000000	0.0500000
20950.0000000	0.0504762
20951.0000000	0.0505000

20952.0000000	0.0509524
20953.0000000	0.0510000
20954.0000000	0.0504762
20955.0000000	0.0500000
20956.0000000	0.0510000
20957.0000000	0.0514286
20958.0000000	0.0515000
20959.0000000	0.0505000
20960.0000000	0.0514286
20961.0000000	0.0515000
20962.0000000	0.0515000
20963.0000000	0.0504762
20964.0000000	0.0530000
20965.0000000	0.0505000
20966.0000000	0.0509524
20967.0000000	0.0515000
20968.0000000	0.0500000
20969.0000000	0.0514286
20970.0000000	0.0500000
20971.0000000	0.0505000
20972.0000000	0.0505000
20973.0000000	0.0500000
20974.0000000	0.0505000
20975.0000000	0.0500000
20976.0000000	0.0510000
20977.0000000	0.0500000
20978.0000000	0.0500000
20979.0000000	0.0500000
20980.0000000	0.0505000
20981.0000000	0.0500000
20982.0000000	0.0500000
20983.0000000	0.0500000
20984.0000000	0.0504762
20985.0000000	0.0505000
20986.0000000	0.0500000
20987.0000000	0.0505000
20988.0000000	0.0504762
20989.0000000	0.0500000
20990.0000000	0.0500000
20991.0000000	0.0504762
20992.0000000	0.0505000
20993.0000000	0.0504762
20994.0000000	0.0500000
20995.0000000	0.0500000
20996.0000000	0.0500000
20997.0000000	0.0505000
20998.0000000	0.0510000
20999.0000000	0.0509524
21000.0000000	0.0505000
21001.0000000	0.0504762
21002.0000000	0.0505000
21003.0000000	0.0520000
21004.0000000	0.0509524
21005.0000000	0.0520000
21006.0000000	0.0515000
21007.0000000	0.0509524
21008.0000000	0.0510000

21009.0000000	0.0535000
21010.0000000	0.0505000
21011.0000000	0.0523810
21012.0000000	0.0535000
21013.0000000	0.0523810
21014.0000000	0.0525000
21015.0000000	0.0560000
21016.0000000	0.0542857
21017.0000000	0.0525000
21018.0000000	0.0560000
21019.0000000	0.0545000
21020.0000000	0.0528571
21021.0000000	0.0545000
21022.0000000	0.0545000
21023.0000000	0.0566667
21024.0000000	0.0565000
21025.0000000	0.0561905
21026.0000000	0.0560000
21027.0000000	0.0580000
21028.0000000	0.0540000
21029.0000000	0.0538095
21030.0000000	0.0560000
21031.0000000	0.0552381
21032.0000000	0.0555000
21033.0000000	0.0545000
21034.0000000	0.0570000
21035.0000000	0.0552381
21036.0000000	0.0550000
21037.0000000	0.0540000
21038.0000000	0.0542857
21039.0000000	0.0540000
21040.0000000	0.0547619
21041.0000000	0.0545000
21042.0000000	0.0550000
21043.0000000	0.0538095
21044.0000000	0.0535000
21045.0000000	0.0535000
21046.0000000	0.0540000
21047.0000000	0.0519048
21048.0000000	0.0550000
21049.0000000	0.0550000
21050.0000000	0.0552381
21051.0000000	0.0560000
21052.0000000	0.0571428
21053.0000000	0.0550000
21054.0000000	0.0560000
21055.0000000	0.0561905
21056.0000000	0.0580000
21057.0000000	0.0555000
21058.0000000	0.0565000
21059.0000000	0.0557143
21060.0000000	0.0570000
21061.0000000	0.0571428
21062.0000000	0.0570000
21063.0000000	0.0545000
21064.0000000	0.0580000
21065.0000000	0.0557143

21066.0000000	0.0555000
21067.0000000	0.0565000
21068.0000000	0.0571428
21069.0000000	0.0560000
21070.0000000	0.0566667
21071.0000000	0.0555000
21072.0000000	0.0570000
21073.0000000	0.0565000
21074.0000000	0.0566667
21075.0000000	0.0550000
21076.0000000	0.0540000
21077.0000000	0.0557143
21078.0000000	0.0560000
21079.0000000	0.0560000
21080.0000000	0.0552381
21081.0000000	0.0540000
21082.0000000	0.0530000
21083.0000000	0.0533333
21084.0000000	0.0545000
21085.0000000	0.0555000
21086.0000000	0.0519048
21087.0000000	0.0550000
21088.0000000	0.0535000
21089.0000000	0.0538095
21090.0000000	0.0555000
21091.0000000	0.0550000
21092.0000000	0.0542857
21093.0000000	0.0550000
21094.0000000	0.0550000
21095.0000000	0.0538095
21096.0000000	0.0525000
21097.0000000	0.0525000
21098.0000000	0.0538095
21099.0000000	0.0540000
21100.0000000	0.0533333
21101.0000000	0.0530000
21102.0000000	0.0545000
21103.0000000	0.0520000
21104.0000000	0.0542857
21105.0000000	0.0525000
21106.0000000	0.0520000
21107.0000000	0.0519048
21108.0000000	0.0530000
21109.0000000	0.0530000
21110.0000000	0.0520000
21111.0000000	0.0509524
21112.0000000	0.0515000
21113.0000000	0.0514286
21114.0000000	0.0520000
21115.0000000	0.0515000
21116.0000000	0.0504762
21117.0000000	0.0525000
21118.0000000	0.0515000
21119.0000000	0.0523810
21120.0000000	0.0510000
21121.0000000	0.0515000
21122.0000000	0.0519048

21123.0000000	0.0525000
21124.0000000	0.0510000
21125.0000000	0.0510000
21126.0000000	0.0528571
21127.0000000	0.0500000
21128.0000000	0.0504762
21129.0000000	0.0500000
21130.0000000	0.0505000
21131.0000000	0.0514286
21132.0000000	0.0510000
21133.0000000	0.0510000
21134.0000000	0.0515000
21135.0000000	0.0514286
21136.0000000	0.0535000
21137.0000000	0.0515000
21138.0000000	0.0523810
21139.0000000	0.0520000
21140.0000000	0.0514286
21141.0000000	0.0530000
21142.0000000	0.0530000
21143.0000000	0.0523810
21144.0000000	0.0535000
21145.0000000	0.0540000
21146.0000000	0.0545000
21147.0000000	0.0519048
21148.0000000	0.0545000
21149.0000000	0.0528571
21150.0000000	0.0535000
21151.0000000	0.0535000
21152.0000000	0.0538095
21153.0000000	0.0540000
21154.0000000	0.0555000
21155.0000000	0.0545000
21156.0000000	0.0542857
21157.0000000	0.0550000
21158.0000000	0.0552381
21159.0000000	0.0525000
21160.0000000	0.0528571
21161.0000000	0.0530000
21162.0000000	0.0535000
21163.0000000	0.0520000
21164.0000000	0.0520000
21165.0000000	0.0504762
21166.0000000	0.0525000
21167.0000000	0.0528571
21168.0000000	0.0525000
21169.0000000	0.0545000
21170.0000000	0.0530000
21171.0000000	0.0519048
21172.0000000	0.0530000
21173.0000000	0.0540000
21174.0000000	0.0519048
21175.0000000	0.0525000
21176.0000000	0.0542857
21177.0000000	0.0530000
21178.0000000	0.0525000
21179.0000000	0.0515000

21180.0000000	0.0523810
21181.0000000	0.0525000
21182.0000000	0.0525000
21183.0000000	0.0538095
21184.0000000	0.0530000
21185.0000000	0.0540000
21186.0000000	0.0533333
21187.0000000	0.0530000
21188.0000000	0.0528571
21189.0000000	0.0530000
21190.0000000	0.0550000
21191.0000000	0.0533333
21192.0000000	0.0550000
21193.0000000	0.0565000
21194.0000000	0.0570000
21195.0000000	0.0566667
21196.0000000	0.0580000
21197.0000000	0.0566667
21198.0000000	0.0575000
21199.0000000	0.0580000
21200.0000000	0.0570000
21201.0000000	0.0580952
21202.0000000	0.0575000
21203.0000000	0.0595000
21204.0000000	0.0576190
21205.0000000	0.0600000
21206.0000000	0.0595238
21207.0000000	0.0595000
21208.0000000	0.0585000
21209.0000000	0.0595000
21210.0000000	0.0600000
21211.0000000	0.0585000
21212.0000000	0.0595238
21213.0000000	0.0595000
21214.0000000	0.0595000
21215.0000000	0.0600000
21216.0000000	0.0600000
21217.0000000	0.0600000
21218.0000000	0.0600000
21219.0000000	0.0600000
21220.0000000	0.0600000
21221.0000000	0.0600000
21222.0000000	0.0595000
21223.0000000	0.0600000
21224.0000000	0.0600000
21225.0000000	0.0595238
21226.0000000	0.0600000
21227.0000000	0.0595000
21228.0000000	0.0595238
21229.0000000	0.0600000
21230.0000000	0.0600000
21231.0000000	0.0600000
21232.0000000	0.0600000
21233.0000000	0.0600000
21234.0000000	0.0600000
21235.0000000	0.0600000
21236.0000000	0.0595238

21237.0000000	0.0600000
21238.0000000	0.0600000
21239.0000000	0.0600000
21240.0000000	0.0590476
21241.0000000	0.0595000
21242.0000000	0.0595000
21243.0000000	0.0600000
21244.0000000	0.0600000
21245.0000000	0.0600000
21246.0000000	0.0595238
21247.0000000	0.0600000
21248.0000000	0.0600000
21249.0000000	0.0600000
21250.0000000	0.0595000
21251.0000000	0.0600000
21252.0000000	0.0590476
21253.0000000	0.0595000
21254.0000000	0.0600000
21255.0000000	0.0600000
21256.0000000	0.0595000
21257.0000000	0.0600000
21258.0000000	0.0595000
21259.0000000	0.0600000
21260.0000000	0.0590000
21261.0000000	0.0600000
21262.0000000	0.0595238
21263.0000000	0.0600000
21264.0000000	0.0600000
21265.0000000	0.0600000
21266.0000000	0.0600000
21267.0000000	0.0600000
21268.0000000	0.0600000
21269.0000000	0.0600000
21270.0000000	0.0600000
21271.0000000	0.0600000
21272.0000000	0.0600000
21273.0000000	0.0600000
21274.0000000	0.0595000
21275.0000000	0.0600000
21276.0000000	0.0600000
21277.0000000	0.0600000
21278.0000000	0.0600000
21279.0000000	0.0600000
21280.0000000	0.0600000
21281.0000000	0.0600000
21282.0000000	0.0600000
21283.0000000	0.0600000
21284.0000000	0.0595000
21285.0000000	0.0600000
21286.0000000	0.0600000
21287.0000000	0.0600000
21288.0000000	0.0600000
21289.0000000	0.0600000
21290.0000000	0.0600000
21291.0000000	0.0600000
21292.0000000	0.0600000
21293.0000000	0.0600000

21294.0000000	0.0600000
21295.0000000	0.0595000
21296.0000000	0.0600000
21297.0000000	0.0600000
21298.0000000	0.0600000
21299.0000000	0.0600000
21300.0000000	0.0595238
21301.0000000	0.0600000
21302.0000000	0.0595000
21303.0000000	0.0600000
21304.0000000	0.0590000
21305.0000000	0.0585000
21306.0000000	0.0595000
21307.0000000	0.0590476
21308.0000000	0.0600000
21309.0000000	0.0571428
21310.0000000	0.0590000
21311.0000000	0.0570000
21312.0000000	0.0580952
21313.0000000	0.0575000
21314.0000000	0.0585000
21315.0000000	0.0585000
21316.0000000	0.0595238
21317.0000000	0.0570000
21318.0000000	0.0580952
21319.0000000	0.0580000
21320.0000000	0.0575000
21321.0000000	0.0590000
21322.0000000	0.0585000
21323.0000000	0.0595238
21324.0000000	0.0585714
21325.0000000	0.0585000
21326.0000000	0.0595000
21327.0000000	0.0595238
21328.0000000	0.0595000
21329.0000000	0.0595000
21330.0000000	0.0595000
21331.0000000	0.0595238
21332.0000000	0.0600000
21333.0000000	0.0600000
21334.0000000	0.0590000
21335.0000000	0.0600000
21336.0000000	0.0595238
21337.0000000	0.0590000
21338.0000000	0.0600000
21339.0000000	0.0585000
21340.0000000	0.0590476
21341.0000000	0.0595000
21342.0000000	0.0590000
21343.0000000	0.0595238
21344.0000000	0.0590000
21345.0000000	0.0571428
21346.0000000	0.0575000
21347.0000000	0.0565000
21348.0000000	0.0571428
21349.0000000	0.0565000
21350.0000000	0.0560000

21351.0000000	0.0555000
21352.0000000	0.0547619
21353.0000000	0.0525000
21354.0000000	0.0519048
21355.0000000	0.0535000
21356.0000000	0.0520000
21357.0000000	0.0542857
21358.0000000	0.0520000
21359.0000000	0.0530000
21360.0000000	0.0525000
21361.0000000	0.0528571
21362.0000000	0.0525000
21363.0000000	0.0538095
21364.0000000	0.0540000
21365.0000000	0.0525000
21366.0000000	0.0540000
21367.0000000	0.0535000
21368.0000000	0.0533333
21369.0000000	0.0510000
21370.0000000	0.0523810
21371.0000000	0.0530000
21372.0000000	0.0533333
21373.0000000	0.0535000
21374.0000000	0.0510000
21375.0000000	0.0530000
21376.0000000	0.0552381
21377.0000000	0.0550000
21378.0000000	0.0545000
21379.0000000	0.0538095
21380.0000000	0.0535000
21381.0000000	0.0535000
21382.0000000	0.0533333
21383.0000000	0.0530000
21384.0000000	0.0538095
21385.0000000	0.0515000
21386.0000000	0.0540000
21387.0000000	0.0505000
21388.0000000	0.0519048
21389.0000000	0.0540000
21390.0000000	0.0525000
21391.0000000	0.0538095
21392.0000000	0.0530000
21393.0000000	0.0547619
21394.0000000	0.0525000
21395.0000000	0.0530000
21396.0000000	0.0520000
21397.0000000	0.0533333
21398.0000000	0.0535000
21399.0000000	0.0540000
21400.0000000	0.0533333
21401.0000000	0.0535000
21402.0000000	0.0528571
21403.0000000	0.0540000
21404.0000000	0.0525000
21405.0000000	0.0540000
21406.0000000	0.0552381
21407.0000000	0.0545000

21408.0000000	0.0555000
21409.0000000	0.0533333
21410.0000000	0.0575000
21411.0000000	0.0585000
21412.0000000	0.0585714
21413.0000000	0.0570000
21414.0000000	0.0565000
21415.0000000	0.0576190
21416.0000000	0.0575000
21417.0000000	0.0565000
21418.0000000	0.0580952
21419.0000000	0.0560000
21420.0000000	0.0555000
21421.0000000	0.0566667
21422.0000000	0.0570000
21423.0000000	0.0570000
21424.0000000	0.0557143
21425.0000000	0.0550000
21426.0000000	0.0565000
21427.0000000	0.0560000
21428.0000000	0.0566667
21429.0000000	0.0570000
21430.0000000	0.0561905
21431.0000000	0.0585000
21432.0000000	0.0560000
21433.0000000	0.0576190
21434.0000000	0.0550000
21435.0000000	0.0570000
21436.0000000	0.0585714
21437.0000000	0.0570000
21438.0000000	0.0580000
21439.0000000	0.0565000
21440.0000000	0.0580952
21441.0000000	0.0560000
21442.0000000	0.0561905
21443.0000000	0.0570000
21444.0000000	0.0565000
21445.0000000	0.0566667
21446.0000000	0.0540000
21447.0000000	0.0555000
21448.0000000	0.0566667
21449.0000000	0.0550000
21450.0000000	0.0550000
21451.0000000	0.0542857
21452.0000000	0.0570000
21453.0000000	0.0555000
21454.0000000	0.0560000
21455.0000000	0.0557143
21456.0000000	0.0565000
21457.0000000	0.0555000
21458.0000000	0.0557143
21459.0000000	0.0545000
21460.0000000	0.0566667
21461.0000000	0.0540000
21462.0000000	0.0555000
21463.0000000	0.0566667
21464.0000000	0.0540000

21465.0000000	0.0560000
21466.0000000	0.0550000
21467.0000000	0.0552381
21468.0000000	0.0560000
21469.0000000	0.0538095
21470.0000000	0.0525000
21471.0000000	0.0525000
21472.0000000	0.0523810
21473.0000000	0.0520000
21474.0000000	0.0500000
21475.0000000	0.0520000
21476.0000000	0.0509524
21477.0000000	0.0510000
21478.0000000	0.0504762
21479.0000000	0.0500000
21480.0000000	0.0500000
21481.0000000	0.0509524
21482.0000000	0.0505000
21483.0000000	0.0510000
21484.0000000	0.0509524
21485.0000000	0.0500000
21486.0000000	0.0515000
21487.0000000	0.0510000
21488.0000000	0.0509524
21489.0000000	0.0505000
21490.0000000	0.0509524
21491.0000000	0.0505000
21492.0000000	0.0515000
21493.0000000	0.0509524
21494.0000000	0.0505000
21495.0000000	0.0510000
21496.0000000	0.0505000
21497.0000000	0.0519048
21498.0000000	0.0515000
21499.0000000	0.0514286
21500.0000000	0.0535000
21501.0000000	0.0525000
21502.0000000	0.0520000
21503.0000000	0.0514286
21504.0000000	0.0530000
21505.0000000	0.0528571
21506.0000000	0.0550000
21507.0000000	0.0535000
21508.0000000	0.0542857
21509.0000000	0.0530000
21510.0000000	0.0550000
21511.0000000	0.0540000
21512.0000000	0.0561905
21513.0000000	0.0565000
21514.0000000	0.0570000
21515.0000000	0.0542857
21516.0000000	0.0585000
21517.0000000	0.0560000
21518.0000000	0.0561905
21519.0000000	0.0575000
21520.0000000	0.0575000
21521.0000000	0.0571428

21522.0000000	0.0570000
21523.0000000	0.0570000
21524.0000000	0.0595238
21525.0000000	0.0575000
21526.0000000	0.0575000
21527.0000000	0.0580000
21528.0000000	0.0580952
21529.0000000	0.0580952
21530.0000000	0.0570000
21531.0000000	0.0575000
21532.0000000	0.0580000
21533.0000000	0.0590476
21534.0000000	0.0570000
21535.0000000	0.0565000
21536.0000000	0.0595238
21537.0000000	0.0580000
21538.0000000	0.0576190
21539.0000000	0.0575000
21540.0000000	0.0585000
21541.0000000	0.0595000
21542.0000000	0.0571428
21543.0000000	0.0590000
21544.0000000	0.0570000
21545.0000000	0.0590476
21546.0000000	0.0595000
21547.0000000	0.0595000
21548.0000000	0.0566667
21549.0000000	0.0590000
21550.0000000	0.0585000
21551.0000000	0.0595238
21552.0000000	0.0595000
21553.0000000	0.0580952
21554.0000000	0.0580000
21555.0000000	0.0575000
21556.0000000	0.0590000
21557.0000000	0.0580952
21558.0000000	0.0590000
21559.0000000	0.0595000
21560.0000000	0.0595238
21561.0000000	0.0585000
21562.0000000	0.0590000
21563.0000000	0.0595000
21564.0000000	0.0585714
21565.0000000	0.0590000
21566.0000000	0.0590000
21567.0000000	0.0585714
21568.0000000	0.0570000
21569.0000000	0.0590476
21570.0000000	0.0575000
21571.0000000	0.0570000
21572.0000000	0.0580000
21573.0000000	0.0571428
21574.0000000	0.0590000
21575.0000000	0.0580000
21576.0000000	0.0571428
21577.0000000	0.0575000
21578.0000000	0.0571428

21579.0000000	0.0555000
21580.0000000	0.0565000
21581.0000000	0.0552381
21582.0000000	0.0555000
21583.0000000	0.0545000
21584.0000000	0.0557143
21585.0000000	0.0535000
21586.0000000	0.0535000
21587.0000000	0.0557143
21588.0000000	0.0510000
21589.0000000	0.0542857
21590.0000000	0.0510000
21591.0000000	0.0525000
21592.0000000	0.0545000
21593.0000000	0.0530000
21594.0000000	0.0523810
21595.0000000	0.0525000
21596.0000000	0.0528571
21597.0000000	0.0535000
21598.0000000	0.0509524
21599.0000000	0.0515000
21600.0000000	0.0520000
21601.0000000	0.0525000
21602.0000000	0.0530000
21603.0000000	0.0519048
21604.0000000	0.0505000
21605.0000000	0.0523810
21606.0000000	0.0535000
21607.0000000	0.0525000
21608.0000000	0.0525000
21609.0000000	0.0514286
21610.0000000	0.0515000
21611.0000000	0.0525000
21612.0000000	0.0523810
21613.0000000	0.0520000
21614.0000000	0.0542857
21615.0000000	0.0540000
21616.0000000	0.0510000
21617.0000000	0.0519048
21618.0000000	0.0520000
21619.0000000	0.0525000
21620.0000000	0.0545000
21621.0000000	0.0528571
21622.0000000	0.0525000
21623.0000000	0.0540000
21624.0000000	0.0533333
21625.0000000	0.0550000
21626.0000000	0.0538095
21627.0000000	0.0535000
21628.0000000	0.0540000
21629.0000000	0.0547619
21630.0000000	0.0535000
21631.0000000	0.0540000
21632.0000000	0.0550000
21633.0000000	0.0538095
21634.0000000	0.0540000
21635.0000000	0.0547619

21636.0000000	0.0530000
21637.0000000	0.0540000
21638.0000000	0.0560000
21639.0000000	0.0528571
21640.0000000	0.0535000
21641.0000000	0.0538095
21642.0000000	0.0555000
21643.0000000	0.0530000
21644.0000000	0.0525000
21645.0000000	0.0547619
21646.0000000	0.0555000
21647.0000000	0.0545000
21648.0000000	0.0538095
21649.0000000	0.0550000
21650.0000000	0.0523810
21651.0000000	0.0565000
21652.0000000	0.0545000
21653.0000000	0.0535000
21654.0000000	0.0557143
21655.0000000	0.0540000
21656.0000000	0.0550000
21657.0000000	0.0557143
21658.0000000	0.0565000
21659.0000000	0.0566667
21660.0000000	0.0530000
21661.0000000	0.0575000
21662.0000000	0.0565000
21663.0000000	0.0533333
21664.0000000	0.0545000
21665.0000000	0.0538095
21666.0000000	0.0550000
21667.0000000	0.0550000
21668.0000000	0.0525000
21669.0000000	0.0533333
21670.0000000	0.0550000
21671.0000000	0.0555000
21672.0000000	0.0528571
21673.0000000	0.0540000
21674.0000000	0.0547619
21675.0000000	0.0550000
21676.0000000	0.0530000
21677.0000000	0.0547619
21678.0000000	0.0565000
21679.0000000	0.0550000
21680.0000000	0.0540000
21681.0000000	0.0538095
21682.0000000	0.0540000
21683.0000000	0.0555000
21684.0000000	0.0542857
21685.0000000	0.0545000
21686.0000000	0.0542857
21687.0000000	0.0550000
21688.0000000	0.0565000
21689.0000000	0.0547619
21690.0000000	0.0575000
21691.0000000	0.0565000
21692.0000000	0.0555000

21693.0000000	0.0571428
21694.0000000	0.0580000
21695.0000000	0.0561905
21696.0000000	0.0575000
21697.0000000	0.0560000
21698.0000000	0.0540000
21699.0000000	0.0540000
21700.0000000	0.0557143
21701.0000000	0.0555000
21702.0000000	0.0557143
21703.0000000	0.0545000
21704.0000000	0.0547619
21705.0000000	0.0545000
21706.0000000	0.0530000
21707.0000000	0.0535000
21708.0000000	0.0550000
21709.0000000	0.0547619
21710.0000000	0.0535000
21711.0000000	0.0528571
21712.0000000	0.0540000
21713.0000000	0.0520000
21714.0000000	0.0533333
21715.0000000	0.0520000
21716.0000000	0.0525000
21717.0000000	0.0533333
21718.0000000	0.0530000
21719.0000000	0.0530000
21720.0000000	0.0514286
21721.0000000	0.0525000
21722.0000000	0.0515000
21723.0000000	0.0528571
21724.0000000	0.0535000
21725.0000000	0.0505000
21726.0000000	0.0538095
21727.0000000	0.0535000
21728.0000000	0.0525000
21729.0000000	0.0528571
21730.0000000	0.0540000
21731.0000000	0.0525000
21732.0000000	0.0540000
21733.0000000	0.0547619
21734.0000000	0.0528571
21735.0000000	0.0525000
21736.0000000	0.0550000
21737.0000000	0.0535000
21738.0000000	0.0538095
21739.0000000	0.0535000
21740.0000000	0.0526316
21741.0000000	0.0540909
21742.0000000	0.0550000
21743.0000000	0.0535000
21744.0000000	0.0555000
21745.0000000	0.0547619
21746.0000000	0.0520000
21747.0000000	0.0542857
21748.0000000	0.0555000
21749.0000000	0.0545000

21750.0000000	0.0552381
21751.0000000	0.0540000
21752.0000000	0.0545000
21753.0000000	0.0538095
21754.0000000	0.0535000
21755.0000000	0.0535000
21756.0000000	0.0542857
21757.0000000	0.0545000
21758.0000000	0.0540000
21759.0000000	0.0545000
21760.0000000	0.0547619
21761.0000000	0.0550000
21762.0000000	0.0547619
21763.0000000	0.0550000
21764.0000000	0.0535000
21765.0000000	0.0547619
21766.0000000	0.0550000
21767.0000000	0.0550000
21768.0000000	0.0545000
21769.0000000	0.0547619
21770.0000000	0.0535000
21771.0000000	0.0530000
21772.0000000	0.0538095
21773.0000000	0.0540000
21774.0000000	0.0523810
21775.0000000	0.0525000
21776.0000000	0.0535000
21777.0000000	0.0528571
21778.0000000	0.0520000
21779.0000000	0.0520000
21780.0000000	0.0525000
21781.0000000	0.0523810
21782.0000000	0.0525000
21783.0000000	0.0542857
21784.0000000	0.0535000
21785.0000000	0.0525000
21786.0000000	0.0533333
21787.0000000	0.0540000
21788.0000000	0.0530000
21789.0000000	0.0535000
21790.0000000	0.0514286
21791.0000000	0.0515000
21792.0000000	0.0504762
21793.0000000	0.0505000
21794.0000000	0.0514286
21795.0000000	0.0505000
21796.0000000	0.0505000
21797.0000000	0.0510000
21798.0000000	0.0510000
21799.0000000	0.0528571
21800.0000000	0.0530000
21801.0000000	0.0519048
21802.0000000	0.0505000
21803.0000000	0.0515000
21804.0000000	0.0510000
21805.0000000	0.0519048
21806.0000000	0.0520000

21807.0000000	0.0520000
21808.0000000	0.0533333
21809.0000000	0.0525000
21810.0000000	0.0523810
21811.0000000	0.0535000
21812.0000000	0.0525000
21813.0000000	0.0525000
21814.0000000	0.0538095
21815.0000000	0.0545000
21816.0000000	0.0540000
21817.0000000	0.0538095
21818.0000000	0.0530000
21819.0000000	0.0570000
21820.0000000	0.0530000
21821.0000000	0.0519048
21822.0000000	0.0538095
21823.0000000	0.0545000
21824.0000000	0.0550000
21825.0000000	0.0535000
21826.0000000	0.0552381
21827.0000000	0.0510000
21828.0000000	0.0550000
21829.0000000	0.0547619
21830.0000000	0.0545000
21831.0000000	0.0542857
21832.0000000	0.0555000
21833.0000000	0.0550000
21834.0000000	0.0565000
21835.0000000	0.0557143
21836.0000000	0.0550000
21837.0000000	0.0565000
21838.0000000	0.0552381
21839.0000000	0.0555000
21840.0000000	0.0552381
21841.0000000	0.0580000
21842.0000000	0.0575000
21843.0000000	0.0570000
21844.0000000	0.0580952
21845.0000000	0.0580000
21846.0000000	0.0571428
21847.0000000	0.0560000
21848.0000000	0.0575000
21849.0000000	0.0570000
21850.0000000	0.0566667
21851.0000000	0.0555000
21852.0000000	0.0570000
21853.0000000	0.0571428
21854.0000000	0.0585000
21855.0000000	0.0576190
21856.0000000	0.0565000
21857.0000000	0.0555000
21858.0000000	0.0560000
21859.0000000	0.0545000
21860.0000000	0.0542857
21861.0000000	0.0540000
21862.0000000	0.0576190
21863.0000000	0.0560000

21864.0000000	0.0565000
21865.0000000	0.0547619
21866.0000000	0.0555000
21867.0000000	0.0560000
21868.0000000	0.0550000
21869.0000000	0.0547619
21870.0000000	0.0552381
21871.0000000	0.0570000
21872.0000000	0.0560000
21873.0000000	0.0545000
21874.0000000	0.0552381
21875.0000000	0.0575000
21876.0000000	0.0550000
21877.0000000	0.0576190
21878.0000000	0.0580000
21879.0000000	0.0555000
21880.0000000	0.0576190
21881.0000000	0.0585000
21882.0000000	0.0566667
21883.0000000	0.0565000
21884.0000000	0.0580000
21885.0000000	0.0550000
21886.0000000	0.0580952
21887.0000000	0.0560000
21888.0000000	0.0585000
21889.0000000	0.0585714
21890.0000000	0.0590000
21891.0000000	0.0585714
21892.0000000	0.0585000
21893.0000000	0.0575000
21894.0000000	0.0590000
21895.0000000	0.0570000
21896.0000000	0.0590476
21897.0000000	0.0590000
21898.0000000	0.0580952
21899.0000000	0.0575000
21900.0000000	0.0575000
21901.0000000	0.0595238
21902.0000000	0.0590000
21903.0000000	0.0575000
21904.0000000	0.0585000
21905.0000000	0.0580952
21906.0000000	0.0565000
21907.0000000	0.0595238
21908.0000000	0.0595000
21909.0000000	0.0590000
21910.0000000	0.0590476
21911.0000000	0.0595000
21912.0000000	0.0590000
21913.0000000	0.0595000
21914.0000000	0.0595238
21915.0000000	0.0600000
21916.0000000	0.0600000
21917.0000000	0.0595000
21918.0000000	0.0595000
21919.0000000	0.0585714
21920.0000000	0.0595000

21921.0000000	0.0585000
21922.0000000	0.0585714
21923.0000000	0.0595000
21924.0000000	0.0595000
21925.0000000	0.0590000
21926.0000000	0.0576190
21927.0000000	0.0580000
21928.0000000	0.0561905
21929.0000000	0.0555000
21930.0000000	0.0576190
21931.0000000	0.0560000
21932.0000000	0.0575000
21933.0000000	0.0555000
21934.0000000	0.0547619
21935.0000000	0.0545000
21936.0000000	0.0535000
21937.0000000	0.0540000
21938.0000000	0.0557143
21939.0000000	0.0555000
21940.0000000	0.0555000
21941.0000000	0.0552381
21942.0000000	0.0555000
21943.0000000	0.0538095
21944.0000000	0.0570000
21945.0000000	0.0555000
21946.0000000	0.0571428
21947.0000000	0.0560000
21948.0000000	0.0545000
21949.0000000	0.0550000
21950.0000000	0.0547619
21951.0000000	0.0555000
21952.0000000	0.0557143
21953.0000000	0.0540000
21954.0000000	0.0545000
21955.0000000	0.0515000
21956.0000000	0.0560000
21957.0000000	0.0547619
21958.0000000	0.0547619
21959.0000000	0.0550000
21960.0000000	0.0545000
21961.0000000	0.0557143
21962.0000000	0.0565000
21963.0000000	0.0560000
21964.0000000	0.0555000
21965.0000000	0.0566667
21966.0000000	0.0555000
21967.0000000	0.0561905
21968.0000000	0.0550000
21969.0000000	0.0550000
21970.0000000	0.0571428
21971.0000000	0.0550000
21972.0000000	0.0580000
21973.0000000	0.0550000
21974.0000000	0.0557143
21975.0000000	0.0570000
21976.0000000	0.0550000
21977.0000000	0.0547619

21978.0000000	0.0545000
21979.0000000	0.0540000
21980.0000000	0.0552381
21981.0000000	0.0570000
21982.0000000	0.0552381
21983.0000000	0.0540000
21984.0000000	0.0565000
21985.0000000	0.0560000
21986.0000000	0.0542857
21987.0000000	0.0560000
21988.0000000	0.0565000
21989.0000000	0.0552381
21990.0000000	0.0565000
21991.0000000	0.0552381
21992.0000000	0.0535000
21993.0000000	0.0550000
21994.0000000	0.0555000
21995.0000000	0.0552381
21996.0000000	0.0540000
21997.0000000	0.0530000
21998.0000000	0.0542857
21999.0000000	0.0540000
22000.0000000	0.0550000
22001.0000000	0.0566667
22002.0000000	0.0535000
22003.0000000	0.0565000
22004.0000000	0.0552381
22005.0000000	0.0560000
22006.0000000	0.0552381
22007.0000000	0.0545000
22008.0000000	0.0560000
22009.0000000	0.0550000
22010.0000000	0.0557143
22011.0000000	0.0535000
22012.0000000	0.0555000
22013.0000000	0.0561905
22014.0000000	0.0550000
22015.0000000	0.0565000
22016.0000000	0.0561905
22017.0000000	0.0535000
22018.0000000	0.0547619
22019.0000000	0.0570000
22020.0000000	0.0555000
22021.0000000	0.0565000
22022.0000000	0.0571428
22023.0000000	0.0555000
22024.0000000	0.0580000
22025.0000000	0.0575000
22026.0000000	0.0571428
22027.0000000	0.0590476
22028.0000000	0.0565000
22029.0000000	0.0590000
22030.0000000	0.0585000
22031.0000000	0.0566667
22032.0000000	0.0585000
22033.0000000	0.0573684
22034.0000000	0.0581818

22035.0000000	0.0585000
22036.0000000	0.0585714
22037.0000000	0.0585000
22038.0000000	0.0585000
22039.0000000	0.0595000
22040.0000000	0.0595238
22041.0000000	0.0580000
22042.0000000	0.0580000
22043.0000000	0.0600000
22044.0000000	0.0585000
22045.0000000	0.0600000
22046.0000000	0.0580952
22047.0000000	0.0580000
22048.0000000	0.0590000
22049.0000000	0.0595000
22050.0000000	0.0580952
22051.0000000	0.0590000
22052.0000000	0.0590476
22053.0000000	0.0585000
22054.0000000	0.0590000
22055.0000000	0.0585714
22056.0000000	0.0590000
22057.0000000	0.0585000
22058.0000000	0.0561905
22059.0000000	0.0560000
22060.0000000	0.0545000
22061.0000000	0.0545000
22062.0000000	0.0552381
22063.0000000	0.0555000
22064.0000000	0.0540000
22065.0000000	0.0542857
22066.0000000	0.0520000
22067.0000000	0.0519048
22068.0000000	0.0530000
22069.0000000	0.0545000
22070.0000000	0.0528571
22071.0000000	0.0520000
22072.0000000	0.0515000
22073.0000000	0.0525000
22074.0000000	0.0514286
22075.0000000	0.0550000
22076.0000000	0.0510000
22077.0000000	0.0519048
22078.0000000	0.0540000
22079.0000000	0.0523810
22080.0000000	0.0530000
22081.0000000	0.0560000
22082.0000000	0.0552381
22083.0000000	0.0530000
22084.0000000	0.0525000
22085.0000000	0.0530000
22086.0000000	0.0552381
22087.0000000	0.0545000
22088.0000000	0.0542857
22089.0000000	0.0545000
22090.0000000	0.0545000
22091.0000000	0.0540000

22092.0000000	0.0552381
22093.0000000	0.0570000
22094.0000000	0.0528571
22095.0000000	0.0530000
22096.0000000	0.0535000
22097.0000000	0.0542857
22098.0000000	0.0555000
22099.0000000	0.0560000
22100.0000000	0.0560000
22101.0000000	0.0547619
22102.0000000	0.0570000
22103.0000000	0.0560000
22104.0000000	0.0576190
22105.0000000	0.0570000
22106.0000000	0.0571428
22107.0000000	0.0580000
22108.0000000	0.0570000
22109.0000000	0.0560000
22110.0000000	0.0547619
22111.0000000	0.0570000
22112.0000000	0.0555000
22113.0000000	0.0590476
22114.0000000	0.0590000
22115.0000000	0.0561905
22116.0000000	0.0560000
22117.0000000	0.0580000
22118.0000000	0.0590476
22119.0000000	0.0575000
22120.0000000	0.0580000
22121.0000000	0.0565000
22122.0000000	0.0561905
22123.0000000	0.0560000
22124.0000000	0.0566667
22125.0000000	0.0555000
22126.0000000	0.0550000
22127.0000000	0.0557143
22128.0000000	0.0545000
22129.0000000	0.0565000
22130.0000000	0.0535000
22131.0000000	0.0538095
22132.0000000	0.0545000
22133.0000000	0.0538095
22134.0000000	0.0535000
22135.0000000	0.0555000
22136.0000000	0.0535000
22137.0000000	0.0550000
22138.0000000	0.0538095
22139.0000000	0.0535000
22140.0000000	0.0533333
22141.0000000	0.0530000
22142.0000000	0.0519048
22143.0000000	0.0515000
22144.0000000	0.0530000
22145.0000000	0.0510000
22146.0000000	0.0504762
22147.0000000	0.0510000
22148.0000000	0.0505000

22149.0000000	0.0504762
22150.0000000	0.0505000
22151.0000000	0.0500000
22152.0000000	0.0510000
22153.0000000	0.0500000
22154.0000000	0.0500000
22155.0000000	0.0500000
22156.0000000	0.0500000
22157.0000000	0.0500000
22158.0000000	0.0500000
22159.0000000	0.0500000
22160.0000000	0.0505000
22161.0000000	0.0500000
22162.0000000	0.0500000
22163.0000000	0.0500000
22164.0000000	0.0500000
22165.0000000	0.0500000
22166.0000000	0.0500000
22167.0000000	0.0500000
22168.0000000	0.0500000
22169.0000000	0.0500000
22170.0000000	0.0500000
22171.0000000	0.0500000
22172.0000000	0.0509524
22173.0000000	0.0500000
22174.0000000	0.0500000
22175.0000000	0.0500000
22176.0000000	0.0500000
22177.0000000	0.0500000
22178.0000000	0.0500000
22179.0000000	0.0500000
22180.0000000	0.0500000
22181.0000000	0.0500000
22182.0000000	0.0504762
22183.0000000	0.0505000
22184.0000000	0.0500000
22185.0000000	0.0500000
22186.0000000	0.0510000
22187.0000000	0.0509524
22188.0000000	0.0505000
22189.0000000	0.0515000
22190.0000000	0.0515000
22191.0000000	0.0510000
22192.0000000	0.0523810
22193.0000000	0.0510000
22194.0000000	0.0519048
22195.0000000	0.0515000
22196.0000000	0.0510000
22197.0000000	0.0520000
22198.0000000	0.0538095
22199.0000000	0.0540000
22200.0000000	0.0555000
22201.0000000	0.0538095
22202.0000000	0.0545000
22203.0000000	0.0533333
22204.0000000	0.0530000
22205.0000000	0.0515000

22206.0000000	0.0557143
22207.0000000	0.0560000
22208.0000000	0.0545000
22209.0000000	0.0560000
22210.0000000	0.0557143
22211.0000000	0.0540000
22212.0000000	0.0571428
22213.0000000	0.0590000
22214.0000000	0.0575000
22215.0000000	0.0580952
22216.0000000	0.0555000
22217.0000000	0.0580000
22218.0000000	0.0576190
22219.0000000	0.0590000
22220.0000000	0.0585000
22221.0000000	0.0576190
22222.0000000	0.0585000
22223.0000000	0.0576190
22224.0000000	0.0550000
22225.0000000	0.0570000
22226.0000000	0.0570000
22227.0000000	0.0575000
22228.0000000	0.0585714
22229.0000000	0.0580000
22230.0000000	0.0585714
22231.0000000	0.0580000
22232.0000000	0.0580952
22233.0000000	0.0570000
22234.0000000	0.0595000
22235.0000000	0.0590000
22236.0000000	0.0585000
22237.0000000	0.0590476
22238.0000000	0.0590000
22239.0000000	0.0580952
22240.0000000	0.0580000
22241.0000000	0.0585000
22242.0000000	0.0590000
22243.0000000	0.0585714
22244.0000000	0.0590000
22245.0000000	0.0590000
22246.0000000	0.0585714
22247.0000000	0.0595000
22248.0000000	0.0590476
22249.0000000	0.0595000
22250.0000000	0.0590000
22251.0000000	0.0590476
22252.0000000	0.0600000
22253.0000000	0.0590000
22254.0000000	0.0600000
22255.0000000	0.0600000
22256.0000000	0.0595000
22257.0000000	0.0600000
22258.0000000	0.0600000
22259.0000000	0.0600000
22260.0000000	0.0600000
22261.0000000	0.0600000
22262.0000000	0.0600000

22263.0000000	0.0600000
22264.0000000	0.0600000
22265.0000000	0.0600000
22266.0000000	0.0600000
22267.0000000	0.0600000
22268.0000000	0.0600000
22269.0000000	0.0600000
22270.0000000	0.0600000
22271.0000000	0.0600000
22272.0000000	0.0605000
22273.0000000	0.0604762
22274.0000000	0.0600000
22275.0000000	0.0600000
22276.0000000	0.0600000
22277.0000000	0.0600000
22278.0000000	0.0600000
22279.0000000	0.0600000
22280.0000000	0.0600000
22281.0000000	0.0605000
22282.0000000	0.0600000
22283.0000000	0.0600000
22284.0000000	0.0600000
22285.0000000	0.0600000
22286.0000000	0.0600000
22287.0000000	0.0600000
22288.0000000	0.0600000
22289.0000000	0.0604762
22290.0000000	0.0600000
22291.0000000	0.0600000
22292.0000000	0.0600000
22293.0000000	0.0600000
22294.0000000	0.0600000
22295.0000000	0.0600000
22296.0000000	0.0600000
22297.0000000	0.0600000
22298.0000000	0.0600000
22299.0000000	0.0600000
22300.0000000	0.0605000
22301.0000000	0.0600000
22302.0000000	0.0600000
22303.0000000	0.0600000
22304.0000000	0.0600000
22305.0000000	0.0600000
22306.0000000	0.0600000
22307.0000000	0.0600000
22308.0000000	0.0600000
22309.0000000	0.0600000
22310.0000000	0.0600000
22311.0000000	0.0600000
22312.0000000	0.0600000
22313.0000000	0.0600000
22314.0000000	0.0600000
22315.0000000	0.0600000
22316.0000000	0.0600000
22317.0000000	0.0600000
22318.0000000	0.0600000
22319.0000000	0.0600000

22320.0000000	0.0600000
22321.0000000	0.0600000
22322.0000000	0.0600000
22323.0000000	0.0600000
22324.0000000	0.0600000
22325.0000000	0.0600000
22326.0000000	0.0605000
22327.0000000	0.0600000
22328.0000000	0.0600000
22329.0000000	0.0600000
22330.0000000	0.0600000
22331.0000000	0.0600000
22332.0000000	0.0600000
22333.0000000	0.0600000
22334.0000000	0.0600000
22335.0000000	0.0600000
22336.0000000	0.0600000
22337.0000000	0.0600000
22338.0000000	0.0595000
22339.0000000	0.0600000
22340.0000000	0.0600000
22341.0000000	0.0600000
22342.0000000	0.0600000
22343.0000000	0.0595000
22344.0000000	0.0595000
22345.0000000	0.0600000
22346.0000000	0.0600000
22347.0000000	0.0600000
22348.0000000	0.0600000
22349.0000000	0.0600000
22350.0000000	0.0600000
22351.0000000	0.0600000
22352.0000000	0.0600000
22353.0000000	0.0600000
22354.0000000	0.0600000
22355.0000000	0.0600000
22356.0000000	0.0600000
22357.0000000	0.0600000
22358.0000000	0.0600000
22359.0000000	0.0600000
22360.0000000	0.0595000
22361.0000000	0.0600000
22362.0000000	0.0600000
22363.0000000	0.0595238
22364.0000000	0.0600000
22365.0000000	0.0595000
22366.0000000	0.0585000
22367.0000000	0.0595238
22368.0000000	0.0595238
22369.0000000	0.0580000
22370.0000000	0.0590000
22371.0000000	0.0595000
22372.0000000	0.0580952
22373.0000000	0.0595000
22374.0000000	0.0575000
22375.0000000	0.0590476
22376.0000000	0.0595000

22377.0000000	0.0590000
22378.0000000	0.0600000
22379.0000000	0.0590476
22380.0000000	0.0595000
22381.0000000	0.0585714
22382.0000000	0.0590000
22383.0000000	0.0600000
22384.0000000	0.0595238
22385.0000000	0.0585000
22386.0000000	0.0600000
22387.0000000	0.0590476
22388.0000000	0.0595000
22389.0000000	0.0600000
22390.0000000	0.0600000
22391.0000000	0.0600000
22392.0000000	0.0600000
22393.0000000	0.0600000
22394.0000000	0.0600000
22395.0000000	0.0600000
22396.0000000	0.0600000
22397.0000000	0.0600000
22398.0000000	0.0600000
22399.0000000	0.0595238
22400.0000000	0.0600000
22401.0000000	0.0600000
22402.0000000	0.0600000
22403.0000000	0.0600000
22404.0000000	0.0600000
22405.0000000	0.0595000
22406.0000000	0.0595238
22407.0000000	0.0600000
22408.0000000	0.0595000
22409.0000000	0.0600000
22410.0000000	0.0590000
22411.0000000	0.0590476
22412.0000000	0.0595000
22413.0000000	0.0600000
22414.0000000	0.0595000
22415.0000000	0.0600000
22416.0000000	0.0590000
22417.0000000	0.0600000
22418.0000000	0.0600000
22419.0000000	0.0600000
22420.0000000	0.0600000
22421.0000000	0.0600000
22422.0000000	0.0595000
22423.0000000	0.0595000
22424.0000000	0.0600000
22425.0000000	0.0600000
22426.0000000	0.0600000
22427.0000000	0.0595238
22428.0000000	0.0600000
22429.0000000	0.0590476
22430.0000000	0.0595000
22431.0000000	0.0590000
22432.0000000	0.0590000
22433.0000000	0.0585714

22434.0000000	0.0590000
22435.0000000	0.0580000
22436.0000000	0.0595238
22437.0000000	0.0600000
22438.0000000	0.0590000
22439.0000000	0.0585714
22440.0000000	0.0595000
22441.0000000	0.0575000
22442.0000000	0.0580952
22443.0000000	0.0600000
22444.0000000	0.0585000
22445.0000000	0.0590476
22446.0000000	0.0575000
22447.0000000	0.0585714
22448.0000000	0.0585000
22449.0000000	0.0570000
22450.0000000	0.0575000
22451.0000000	0.0580952
22452.0000000	0.0580000
22453.0000000	0.0555000
22454.0000000	0.0565000
22455.0000000	0.0576190
22456.0000000	0.0576190
22457.0000000	0.0565000
22458.0000000	0.0555000
22459.0000000	0.0561905
22460.0000000	0.0570000
22461.0000000	0.0575000
22462.0000000	0.0575000
22463.0000000	0.0585714
22464.0000000	0.0585000
22465.0000000	0.0585714
22466.0000000	0.0590000
22467.0000000	0.0590000
22468.0000000	0.0590000
22469.0000000	0.0595238
22470.0000000	0.0600000
22471.0000000	0.0600000
22472.0000000	0.0600000
22473.0000000	0.0595000
22474.0000000	0.0600000
22475.0000000	0.0600000
22476.0000000	0.0600000
22477.0000000	0.0600000
22478.0000000	0.0595238
22479.0000000	0.0600000
22480.0000000	0.0600000
22481.0000000	0.0600000
22482.0000000	0.0600000
22483.0000000	0.0600000
22484.0000000	0.0600000
22485.0000000	0.0600000
22486.0000000	0.0600000
22487.0000000	0.0600000
22488.0000000	0.0600000
22489.0000000	0.0600000
22490.0000000	0.0600000

22491.0000000	0.0600000
22492.0000000	0.0600000
22493.0000000	0.0600000
22494.0000000	0.0600000
22495.0000000	0.0600000
22496.0000000	0.0600000
22497.0000000	0.0600000
22498.0000000	0.0595000
22499.0000000	0.0600000
22500.0000000	0.0600000
22501.0000000	0.0600000
22502.0000000	0.0595000
22503.0000000	0.0600000
22504.0000000	0.0600000
22505.0000000	0.0600000
22506.0000000	0.0600000
22507.0000000	0.0600000
22508.0000000	0.0600000
22509.0000000	0.0600000
22510.0000000	0.0600000
22511.0000000	0.0600000
22512.0000000	0.0600000
22513.0000000	0.0600000
22514.0000000	0.0600000
22515.0000000	0.0600000
22516.0000000	0.0600000
22517.0000000	0.0600000
22518.0000000	0.0600000
22519.0000000	0.0600000
22520.0000000	0.0600000
22521.0000000	0.0600000
22522.0000000	0.0600000
22523.0000000	0.0600000
22524.0000000	0.0600000
22525.0000000	0.0600000
22526.0000000	0.0600000
22527.0000000	0.0600000
22528.0000000	0.0600000
22529.0000000	0.0600000
22530.0000000	0.0600000
22531.0000000	0.0600000
22532.0000000	0.0600000
22533.0000000	0.0600000
22534.0000000	0.0600000
22535.0000000	0.0600000
22536.0000000	0.0600000
22537.0000000	0.0600000
22538.0000000	0.0600000
22539.0000000	0.0600000
22540.0000000	0.0600000
22541.0000000	0.0600000
22542.0000000	0.0600000
22543.0000000	0.0600000
22544.0000000	0.0600000
22545.0000000	0.0600000
22546.0000000	0.0600000
22547.0000000	0.0600000

22548.0000000	0.0600000
22549.0000000	0.0600000
22550.0000000	0.0600000
22551.0000000	0.0600000
22552.0000000	0.0600000
22553.0000000	0.0600000
22554.0000000	0.0600000
22555.0000000	0.0605000
22556.0000000	0.0604762
22557.0000000	0.0600000
22558.0000000	0.0600000
22559.0000000	0.0600000
22560.0000000	0.0600000
22561.0000000	0.0600000
22562.0000000	0.0600000
22563.0000000	0.0600000
22564.0000000	0.0600000
22565.0000000	0.0605000
22566.0000000	0.0600000
22567.0000000	0.0600000
22568.0000000	0.0600000
22569.0000000	0.0600000
22570.0000000	0.0605000
22571.0000000	0.0600000
22572.0000000	0.0600000
22573.0000000	0.0600000
22574.0000000	0.0605000
22575.0000000	0.0604762
22576.0000000	0.0600000
22577.0000000	0.0600000
22578.0000000	0.0600000
22579.0000000	0.0600000
22580.0000000	0.0600000
22581.0000000	0.0605000
22582.0000000	0.0600000
22583.0000000	0.0600000
22584.0000000	0.0605000
22585.0000000	0.0600000
22586.0000000	0.0600000
22587.0000000	0.0600000
22588.0000000	0.0605000
22589.0000000	0.0600000
22590.0000000	0.0605000
22591.0000000	0.0600000
22592.0000000	0.0600000
22593.0000000	0.0600000
22594.0000000	0.0600000
22595.0000000	0.0600000
22596.0000000	0.0600000
22597.0000000	0.0605000
22598.0000000	0.0600000
22599.0000000	0.0600000
22600.0000000	0.0600000
22601.0000000	0.0600000
22602.0000000	0.0600000
22603.0000000	0.0600000
22604.0000000	0.0600000

22605.0000000	0.0600000
22606.0000000	0.0600000
22607.0000000	0.0600000
22608.0000000	0.0600000
22609.0000000	0.0600000
22610.0000000	0.0600000
22611.0000000	0.0590476
22612.0000000	0.0600000
22613.0000000	0.0595000
22614.0000000	0.0575000
22615.0000000	0.0585714
22616.0000000	0.0585714
22617.0000000	0.0595000
22618.0000000	0.0600000
22619.0000000	0.0585000
22620.0000000	0.0575000
22621.0000000	0.0580952
22622.0000000	0.0580000
22623.0000000	0.0580952
22624.0000000	0.0555000
22625.0000000	0.0571428
22626.0000000	0.0555000
22627.0000000	0.0565000
22628.0000000	0.0560000
22629.0000000	0.0570000
22630.0000000	0.0561905
22631.0000000	0.0560000
22632.0000000	0.0566667
22633.0000000	0.0560000
22634.0000000	0.0555000
22635.0000000	0.0576190
22636.0000000	0.0540000
22637.0000000	0.0555000
22638.0000000	0.0555000
22639.0000000	0.0557143
22640.0000000	0.0575000
22641.0000000	0.0557143
22642.0000000	0.0565000
22643.0000000	0.0565000
22644.0000000	0.0566667
22645.0000000	0.0565000
22646.0000000	0.0555000
22647.0000000	0.0542857
22648.0000000	0.0555000
22649.0000000	0.0550000
22650.0000000	0.0561905
22651.0000000	0.0540000
22652.0000000	0.0547619
22653.0000000	0.0550000
22654.0000000	0.0555000
22655.0000000	0.0555000
22656.0000000	0.0547619
22657.0000000	0.0550000
22658.0000000	0.0540000
22659.0000000	0.0552381
22660.0000000	0.0530000
22661.0000000	0.0552381

22662.0000000	0.0545000
22663.0000000	0.0540000
22664.0000000	0.0530000
22665.0000000	0.0520000
22666.0000000	0.0519048
22667.0000000	0.0525000
22668.0000000	0.0514286
22669.0000000	0.0505000
22670.0000000	0.0509524
22671.0000000	0.0530000
22672.0000000	0.0515000
22673.0000000	0.0535000
22674.0000000	0.0535000
22675.0000000	0.0528571
22676.0000000	0.0525000
22677.0000000	0.0533333
22678.0000000	0.0510000
22679.0000000	0.0535000
22680.0000000	0.0528571
22681.0000000	0.0515000
22682.0000000	0.0540000
22683.0000000	0.0533333
22684.0000000	0.0540000
22685.0000000	0.0550000
22686.0000000	0.0535000
22687.0000000	0.0557143
22688.0000000	0.0560000
22689.0000000	0.0542857
22690.0000000	0.0570000
22691.0000000	0.0575000
22692.0000000	0.0576190
22693.0000000	0.0560000
22694.0000000	0.0580000
22695.0000000	0.0575000
22696.0000000	0.0580952
22697.0000000	0.0595000
22698.0000000	0.0590000
22699.0000000	0.0585714
22700.0000000	0.0595238
22701.0000000	0.0600000
22702.0000000	0.0600000
22703.0000000	0.0600000
22704.0000000	0.0600000
22705.0000000	0.0590000
22706.0000000	0.0590000
22707.0000000	0.0576190
22708.0000000	0.0595000
22709.0000000	0.0595000
22710.0000000	0.0585000
22711.0000000	0.0590476
22712.0000000	0.0595000
22713.0000000	0.0600000
22714.0000000	0.0595000
22715.0000000	0.0590000
22716.0000000	0.0600000
22717.0000000	0.0590000
22718.0000000	0.0595000

22719.0000000	0.0585000
22720.0000000	0.0595238
22721.0000000	0.0600000
22722.0000000	0.0585714
22723.0000000	0.0595000
22724.0000000	0.0590000
22725.0000000	0.0595000
22726.0000000	0.0595000
22727.0000000	0.0600000
22728.0000000	0.0600000
22729.0000000	0.0595238
22730.0000000	0.0600000
22731.0000000	0.0600000
22732.0000000	0.0600000
22733.0000000	0.0600000
22734.0000000	0.0600000
22735.0000000	0.0600000
22736.0000000	0.0600000
22737.0000000	0.0600000
22738.0000000	0.0600000
22739.0000000	0.0600000
22740.0000000	0.0600000
22741.0000000	0.0600000
22742.0000000	0.0600000
22743.0000000	0.0600000
22744.0000000	0.0600000
22745.0000000	0.0600000
22746.0000000	0.0600000
22747.0000000	0.0600000
22748.0000000	0.0600000
22749.0000000	0.0600000
22750.0000000	0.0600000
22751.0000000	0.0600000
22752.0000000	0.0600000
22753.0000000	0.0600000
22754.0000000	0.0600000
22755.0000000	0.0600000
22756.0000000	0.0595238
22757.0000000	0.0600000
22758.0000000	0.0600000
22759.0000000	0.0595000
22760.0000000	0.0595000
22761.0000000	0.0590476
22762.0000000	0.0595000
22763.0000000	0.0595000
22764.0000000	0.0595000
22765.0000000	0.0590476
22766.0000000	0.0595000
22767.0000000	0.0600000
22768.0000000	0.0590476
22769.0000000	0.0590000
22770.0000000	0.0585000
22771.0000000	0.0585714
22772.0000000	0.0600000
22773.0000000	0.0595000
22774.0000000	0.0590476
22775.0000000	0.0595000

22776.0000000	0.0590000
22777.0000000	0.0595238
22778.0000000	0.0585000
22779.0000000	0.0595000
22780.0000000	0.0580952
22781.0000000	0.0595000
22782.0000000	0.0595000
22783.0000000	0.0600000
22784.0000000	0.0590000
22785.0000000	0.0575000
22786.0000000	0.0595000
22787.0000000	0.0600000
22788.0000000	0.0595238
22789.0000000	0.0600000
22790.0000000	0.0600000
22791.0000000	0.0585000
22792.0000000	0.0600000
22793.0000000	0.0600000
22794.0000000	0.0595000
22795.0000000	0.0595238
22796.0000000	0.0595000
22797.0000000	0.0595238
22798.0000000	0.0600000
22799.0000000	0.0600000
22800.0000000	0.0595000
22801.0000000	0.0600000
22802.0000000	0.0585000
22803.0000000	0.0585000
22804.0000000	0.0590476
22805.0000000	0.0590000
22806.0000000	0.0590000
22807.0000000	0.0580952
22808.0000000	0.0585000
22809.0000000	0.0595000
22810.0000000	0.0590476
22811.0000000	0.0600000
22812.0000000	0.0600000
22813.0000000	0.0600000
22814.0000000	0.0600000
22815.0000000	0.0595000
22816.0000000	0.0590476
22817.0000000	0.0590000
22818.0000000	0.0580000
22819.0000000	0.0585714
22820.0000000	0.0590000
22821.0000000	0.0590000
22822.0000000	0.0595238
22823.0000000	0.0590000
22824.0000000	0.0575000
22825.0000000	0.0570000
22826.0000000	0.0576190
22827.0000000	0.0590000
22828.0000000	0.0590476
22829.0000000	0.0595000
22830.0000000	0.0590000
22831.0000000	0.0590000
22832.0000000	0.0590476

22833.0000000	0.0595000
22834.0000000	0.0590000
22835.0000000	0.0600000
22836.0000000	0.0590476
22837.0000000	0.0590000
22838.0000000	0.0595000
22839.0000000	0.0595000
22840.0000000	0.0595238
22841.0000000	0.0590000
22842.0000000	0.0600000
22843.0000000	0.0595000
22844.0000000	0.0590476
22845.0000000	0.0580000
22846.0000000	0.0580000
22847.0000000	0.0595238
22848.0000000	0.0590000
22849.0000000	0.0580952
22850.0000000	0.0595000
22851.0000000	0.0600000
22852.0000000	0.0595238
22853.0000000	0.0590000
22854.0000000	0.0595000
22855.0000000	0.0585000
22856.0000000	0.0590476
22857.0000000	0.0595000
22858.0000000	0.0595238
22859.0000000	0.0600000
22860.0000000	0.0600000
22861.0000000	0.0585000
22862.0000000	0.0590476
22863.0000000	0.0600000
22864.0000000	0.0595000
22865.0000000	0.0600000
22866.0000000	0.0585000
22867.0000000	0.0595238
22868.0000000	0.0600000
22869.0000000	0.0600000
22870.0000000	0.0595000
22871.0000000	0.0595238
22872.0000000	0.0595000
22873.0000000	0.0600000
22874.0000000	0.0600000
22875.0000000	0.0600000
22876.0000000	0.0600000
22877.0000000	0.0600000
22878.0000000	0.0600000
22879.0000000	0.0595000
22880.0000000	0.0600000
22881.0000000	0.0595000
22882.0000000	0.0600000
22883.0000000	0.0595238
22884.0000000	0.0600000
22885.0000000	0.0600000
22886.0000000	0.0595000
22887.0000000	0.0590000
22888.0000000	0.0600000
22889.0000000	0.0590000

22890.0000000	0.0590000
22891.0000000	0.0590000
22892.0000000	0.0585714
22893.0000000	0.0585000
22894.0000000	0.0585714
22895.0000000	0.0570000
22896.0000000	0.0590000
22897.0000000	0.0590476
22898.0000000	0.0575000
22899.0000000	0.0575000
22900.0000000	0.0580000
22901.0000000	0.0580000
22902.0000000	0.0590476
22903.0000000	0.0580000
22904.0000000	0.0576190
22905.0000000	0.0560000
22906.0000000	0.0585000
22907.0000000	0.0585714
22908.0000000	0.0585000
22909.0000000	0.0570000
22910.0000000	0.0580952
22911.0000000	0.0580000
22912.0000000	0.0580952
22913.0000000	0.0585000
22914.0000000	0.0595000
22915.0000000	0.0585000
22916.0000000	0.0590476
22917.0000000	0.0580000
22918.0000000	0.0590000
22919.0000000	0.0580952
22920.0000000	0.0585000
22921.0000000	0.0600000
22922.0000000	0.0595000
22923.0000000	0.0595238
22924.0000000	0.0590000
22925.0000000	0.0580952
22926.0000000	0.0600000
22927.0000000	0.0595000
22928.0000000	0.0595238
22929.0000000	0.0600000
22930.0000000	0.0600000
22931.0000000	0.0600000
22932.0000000	0.0595238
22933.0000000	0.0590000
22934.0000000	0.0595238
22935.0000000	0.0595000
22936.0000000	0.0590000
22937.0000000	0.0595238
22938.0000000	0.0595000
22939.0000000	0.0585000
22940.0000000	0.0600000
22941.0000000	0.0600000
22942.0000000	0.0600000
22943.0000000	0.0600000
22944.0000000	0.0600000
22945.0000000	0.0600000
22946.0000000	0.0600000

22947.0000000	0.0600000
22948.0000000	0.0600000
22949.0000000	0.0595238
22950.0000000	0.0595000
22951.0000000	0.0600000
22952.0000000	0.0590000
22953.0000000	0.0600000
22954.0000000	0.0595000
22955.0000000	0.0600000
22956.0000000	0.0600000
22957.0000000	0.0600000
22958.0000000	0.0600000
22959.0000000	0.0600000
22960.0000000	0.0600000
22961.0000000	0.0600000
22962.0000000	0.0600000
22963.0000000	0.0600000
22964.0000000	0.0600000
22965.0000000	0.0600000
22966.0000000	0.0600000
22967.0000000	0.0600000
22968.0000000	0.0600000
22969.0000000	0.0600000
22970.0000000	0.0605000
22971.0000000	0.0600000
22972.0000000	0.0600000
22973.0000000	0.0600000
22974.0000000	0.0600000
22975.0000000	0.0600000
22976.0000000	0.0600000
22977.0000000	0.0600000
22978.0000000	0.0600000
22979.0000000	0.0600000
22980.0000000	0.0600000
22981.0000000	0.0600000
22982.0000000	0.0590476
22983.0000000	0.0600000
22984.0000000	0.0595000
22985.0000000	0.0600000
22986.0000000	0.0600000
22987.0000000	0.0600000
22988.0000000	0.0595238
22989.0000000	0.0590000
22990.0000000	0.0585000
22991.0000000	0.0600000
22992.0000000	0.0595238
22993.0000000	0.0595238
22994.0000000	0.0595000
22995.0000000	0.0580000
22996.0000000	0.0600000
22997.0000000	0.0585000
22998.0000000	0.0571428
22999.0000000	0.0585000
23000.0000000	0.0580952
23001.0000000	0.0595000
23002.0000000	0.0590476
23003.0000000	0.0585000

23004.0000000	0.0590000
23005.0000000	0.0590000
23006.0000000	0.0575000
23007.0000000	0.0571428
23008.0000000	0.0585000
23009.0000000	0.0590476
23010.0000000	0.0580000
23011.0000000	0.0570000
23012.0000000	0.0585714
23013.0000000	0.0580000
23014.0000000	0.0585000
23015.0000000	0.0600000
23016.0000000	0.0590476
23017.0000000	0.0580000
23018.0000000	0.0595238
23019.0000000	0.0585000
23020.0000000	0.0600000
23021.0000000	0.0580952
23022.0000000	0.0595000
23023.0000000	0.0580000
23024.0000000	0.0576190
23025.0000000	0.0580000
23026.0000000	0.0580000
23027.0000000	0.0570000
23028.0000000	0.0576190
23029.0000000	0.0580000
23030.0000000	0.0575000
23031.0000000	0.0571428
23032.0000000	0.0595000
23033.0000000	0.0585714
23034.0000000	0.0590000
23035.0000000	0.0580000
23036.0000000	0.0585000
23037.0000000	0.0585714
23038.0000000	0.0570000
23039.0000000	0.0560000
23040.0000000	0.0585714
23041.0000000	0.0580000
23042.0000000	0.0575000
23043.0000000	0.0566667
23044.0000000	0.0575000
23045.0000000	0.0557143
23046.0000000	0.0580000
23047.0000000	0.0570000
23048.0000000	0.0575000
23049.0000000	0.0566667
23050.0000000	0.0570000
23051.0000000	0.0570000
23052.0000000	0.0585714
23053.0000000	0.0565000
23054.0000000	0.0580952
23055.0000000	0.0560000
23056.0000000	0.0565000
23057.0000000	0.0570000
23058.0000000	0.0575000
23059.0000000	0.0576190
23060.0000000	0.0570000

23061.0000000	0.0561905
23062.0000000	0.0580000
23063.0000000	0.0585714
23064.0000000	0.0580000
23065.0000000	0.0585000
23066.0000000	0.0590000
23067.0000000	0.0570000
23068.0000000	0.0585714
23069.0000000	0.0590000
23070.0000000	0.0590476
23071.0000000	0.0575000
23072.0000000	0.0600000
23073.0000000	0.0580952
23074.0000000	0.0600000
23075.0000000	0.0595000
23076.0000000	0.0580952
23077.0000000	0.0595000
23078.0000000	0.0595000
23079.0000000	0.0600000
23080.0000000	0.0600000
23081.0000000	0.0590476
23082.0000000	0.0600000
23083.0000000	0.0595000
23084.0000000	0.0600000
23085.0000000	0.0595238
23086.0000000	0.0595000
23087.0000000	0.0600000
23088.0000000	0.0600000
23089.0000000	0.0600000
23090.0000000	0.0595238
23091.0000000	0.0600000
23092.0000000	0.0595000
23093.0000000	0.0595238
23094.0000000	0.0600000
23095.0000000	0.0595000
23096.0000000	0.0590000
23097.0000000	0.0600000
23098.0000000	0.0600000
23099.0000000	0.0600000
23100.0000000	0.0600000
23101.0000000	0.0600000
23102.0000000	0.0600000
23103.0000000	0.0600000
23104.0000000	0.0600000
23105.0000000	0.0595000
23106.0000000	0.0585714
23107.0000000	0.0595000
23108.0000000	0.0585000
23109.0000000	0.0595238
23110.0000000	0.0585000
23111.0000000	0.0595000
23112.0000000	0.0590476
23113.0000000	0.0600000
23114.0000000	0.0595000
23115.0000000	0.0600000
23116.0000000	0.0590000
23117.0000000	0.0590000

23118.0000000	0.0600000
23119.0000000	0.0595238
23120.0000000	0.0595000
23121.0000000	0.0600000
23122.0000000	0.0600000
23123.0000000	0.0600000
23124.0000000	0.0600000
23125.0000000	0.0595000
23126.0000000	0.0595000
23127.0000000	0.0600000
23128.0000000	0.0600000
23129.0000000	0.0600000
23130.0000000	0.0600000
23131.0000000	0.0600000
23132.0000000	0.0600000
23133.0000000	0.0600000
23134.0000000	0.0600000
23135.0000000	0.0600000
23136.0000000	0.0595238
23137.0000000	0.0600000
23138.0000000	0.0595238
23139.0000000	0.0600000
23140.0000000	0.0600000
23141.0000000	0.0600000
23142.0000000	0.0600000
23143.0000000	0.0600000
23144.0000000	0.0600000
23145.0000000	0.0600000
23146.0000000	0.0595000
23147.0000000	0.0600000
23148.0000000	0.0595000
23149.0000000	0.0600000
23150.0000000	0.0600000
23151.0000000	0.0600000
23152.0000000	0.0600000
23153.0000000	0.0600000
23154.0000000	0.0600000
23155.0000000	0.0600000
23156.0000000	0.0600000
23157.0000000	0.0600000
23158.0000000	0.0600000
23159.0000000	0.0600000
23160.0000000	0.0600000
23161.0000000	0.0605000
23162.0000000	0.0610000
23163.0000000	0.0600000
23164.0000000	0.0600000
23165.0000000	0.0605000
23166.0000000	0.0600000
23167.0000000	0.0600000
23168.0000000	0.0610000
23169.0000000	0.0600000
23170.0000000	0.0600000
23171.0000000	0.0600000
23172.0000000	0.0605000
23173.0000000	0.0600000
23174.0000000	0.0600000

23175.0000000	0.0600000
23176.0000000	0.0600000
23177.0000000	0.0610000
23178.0000000	0.0609524
23179.0000000	0.0605000
23180.0000000	0.0615000
23181.0000000	0.0604762
23182.0000000	0.0600000
23183.0000000	0.0610000
23184.0000000	0.0605000
23185.0000000	0.0623809
23186.0000000	0.0605000
23187.0000000	0.0604762
23188.0000000	0.0600000
23189.0000000	0.0600000
23190.0000000	0.0609524
23191.0000000	0.0610000
23192.0000000	0.0615000
23193.0000000	0.0610000
23194.0000000	0.0614286
23195.0000000	0.0630000
23196.0000000	0.0630000
23197.0000000	0.0609524
23198.0000000	0.0615000
23199.0000000	0.0604762
23200.0000000	0.0620000
23201.0000000	0.0625000
23202.0000000	0.0620000
23203.0000000	0.0633333
23204.0000000	0.0630000
23205.0000000	0.0628571
23206.0000000	0.0615000
23207.0000000	0.0615000
23208.0000000	0.0630000
23209.0000000	0.0623809
23210.0000000	0.0615000
23211.0000000	0.0610000
23212.0000000	0.0628571
23213.0000000	0.0620000
23214.0000000	0.0619048
23215.0000000	0.0620000
23216.0000000	0.0615000
23217.0000000	0.0609524
23218.0000000	0.0620000
23219.0000000	0.0615000
23220.0000000	0.0620000
23221.0000000	0.0619047
23222.0000000	0.0640000
23223.0000000	0.0645000
23224.0000000	0.0614286
23225.0000000	0.0640000
23226.0000000	0.0638095
23227.0000000	0.0620000
23228.0000000	0.0635000
23229.0000000	0.0657143
23230.0000000	0.0640000
23231.0000000	0.0645000

23232.0000000	0.0645000
23233.0000000	0.0647619
23234.0000000	0.0640000
23235.0000000	0.0630000
23236.0000000	0.0652381
23237.0000000	0.0650000
23238.0000000	0.0660000
23239.0000000	0.0652381
23240.0000000	0.0675000
23241.0000000	0.0650000
23242.0000000	0.0657143
23243.0000000	0.0665000
23244.0000000	0.0660000
23245.0000000	0.0652381
23246.0000000	0.0670000
23247.0000000	0.0640000
23248.0000000	0.0657143
23249.0000000	0.0640000
23250.0000000	0.0670000
23251.0000000	0.0633333
23252.0000000	0.0660000
23253.0000000	0.0665000
23254.0000000	0.0660000
23255.0000000	0.0671429
23256.0000000	0.0675000
23257.0000000	0.0647619
23258.0000000	0.0655000
23259.0000000	0.0640000
23260.0000000	0.0657143
23261.0000000	0.0660000
23262.0000000	0.0660000
23263.0000000	0.0650000
23264.0000000	0.0638095
23265.0000000	0.0671429
23266.0000000	0.0675000
23267.0000000	0.0665000
23268.0000000	0.0675000
23269.0000000	0.0666667
23270.0000000	0.0665000
23271.0000000	0.0660000
23272.0000000	0.0645000
23273.0000000	0.0652381
23274.0000000	0.0660000
23275.0000000	0.0652381
23276.0000000	0.0640000
23277.0000000	0.0650000
23278.0000000	0.0652381
23279.0000000	0.0670000
23280.0000000	0.0650000
23281.0000000	0.0642857
23282.0000000	0.0665000
23283.0000000	0.0640000
23284.0000000	0.0640000
23285.0000000	0.0647619
23286.0000000	0.0635000
23287.0000000	0.0633333
23288.0000000	0.0650000

23289.0000000	0.0625000
23290.0000000	0.0633333
23291.0000000	0.0620000
23292.0000000	0.0630000
23293.0000000	0.0642857
23294.0000000	0.0630000
23295.0000000	0.0620000
23296.0000000	0.0642857
23297.0000000	0.0635000
23298.0000000	0.0640000
23299.0000000	0.0620000
23300.0000000	0.0633333
23301.0000000	0.0625000
23302.0000000	0.0620000
23303.0000000	0.0628571
23304.0000000	0.0615000
23305.0000000	0.0619047
23306.0000000	0.0620000
23307.0000000	0.0605000
23308.0000000	0.0620000
23309.0000000	0.0609524
23310.0000000	0.0600000
23311.0000000	0.0610000
23312.0000000	0.0600000
23313.0000000	0.0625000
23314.0000000	0.0600000
23315.0000000	0.0600000
23316.0000000	0.0600000
23317.0000000	0.0604762
23318.0000000	0.0605000
23319.0000000	0.0600000
23320.0000000	0.0600000
23321.0000000	0.0604762
23322.0000000	0.0600000
23323.0000000	0.0600000
23324.0000000	0.0600000
23325.0000000	0.0600000
23326.0000000	0.0604762
23327.0000000	0.0600000
23328.0000000	0.0605000
23329.0000000	0.0600000
23330.0000000	0.0600000
23331.0000000	0.0600000
23332.0000000	0.0600000
23333.0000000	0.0600000
23334.0000000	0.0600000
23335.0000000	0.0600000
23336.0000000	0.0600000
23337.0000000	0.0600000
23338.0000000	0.0600000
23339.0000000	0.0600000
23340.0000000	0.0600000
23341.0000000	0.0600000
23342.0000000	0.0595000
23343.0000000	0.0600000
23344.0000000	0.0600000
23345.0000000	0.0600000

23346.0000000	0.0590000
23347.0000000	0.0595000
23348.0000000	0.0600000
23349.0000000	0.0600000
23350.0000000	0.0600000
23351.0000000	0.0600000
23352.0000000	0.0600000
23353.0000000	0.0595000
23354.0000000	0.0600000
23355.0000000	0.0600000
23356.0000000	0.0595000
23357.0000000	0.0600000
23358.0000000	0.0600000
23359.0000000	0.0600000
23360.0000000	0.0600000
23361.0000000	0.0600000
23362.0000000	0.0595000
23363.0000000	0.0600000
23364.0000000	0.0595000
23365.0000000	0.0600000
23366.0000000	0.0600000
23367.0000000	0.0600000
23368.0000000	0.0600000
23369.0000000	0.0600000
23370.0000000	0.0600000
23371.0000000	0.0600000
23372.0000000	0.0600000
23373.0000000	0.0600000
23374.0000000	0.0600000
23375.0000000	0.0600000
23376.0000000	0.0605000
23377.0000000	0.0600000
23378.0000000	0.0600000
23379.0000000	0.0600000
23380.0000000	0.0600000
23381.0000000	0.0604762
23382.0000000	0.0605000
23383.0000000	0.0600000
23384.0000000	0.0600000
23385.0000000	0.0610000
23386.0000000	0.0600000
23387.0000000	0.0600000
23388.0000000	0.0610000
23389.0000000	0.0605000
23390.0000000	0.0605000
23391.0000000	0.0600000
23392.0000000	0.0600000
23393.0000000	0.0609524
23394.0000000	0.0605000
23395.0000000	0.0604762
23396.0000000	0.0600000
23397.0000000	0.0600000
23398.0000000	0.0605000
23399.0000000	0.0605000
23400.0000000	0.0604762
23401.0000000	0.0610000
23402.0000000	0.0614286

23403.0000000	0.0605000
23404.0000000	0.0605000
23405.0000000	0.0614286
23406.0000000	0.0625000
23407.0000000	0.0615000
23408.0000000	0.0615000
23409.0000000	0.0604762
23410.0000000	0.0615000
23411.0000000	0.0614286
23412.0000000	0.0610000
23413.0000000	0.0620000
23414.0000000	0.0619047
23415.0000000	0.0605000
23416.0000000	0.0605000
23417.0000000	0.0604762
23418.0000000	0.0605000
23419.0000000	0.0610000
23420.0000000	0.0605000
23421.0000000	0.0604762
23422.0000000	0.0604762
23423.0000000	0.0600000
23424.0000000	0.0600000
23425.0000000	0.0605000
23426.0000000	0.0600000
23427.0000000	0.0605000
23428.0000000	0.0605000
23429.0000000	0.0600000
23430.0000000	0.0600000
23431.0000000	0.0600000
23432.0000000	0.0605000
23433.0000000	0.0600000
23434.0000000	0.0610000
23435.0000000	0.0605000
23436.0000000	0.0600000
23437.0000000	0.0605000
23438.0000000	0.0600000
23439.0000000	0.0605000
23440.0000000	0.0610000
23441.0000000	0.0600000
23442.0000000	0.0600000
23443.0000000	0.0600000
23444.0000000	0.0600000
23445.0000000	0.0604762
23446.0000000	0.0600000
23447.0000000	0.0600000
23448.0000000	0.0600000
23449.0000000	0.0600000
23450.0000000	0.0600000
23451.0000000	0.0600000
23452.0000000	0.0600000
23453.0000000	0.0600000
23454.0000000	0.0595000
23455.0000000	0.0600000
23456.0000000	0.0600000
23457.0000000	0.0600000
23458.0000000	0.0600000
23459.0000000	0.0600000

23460.0000000	0.0604762
23461.0000000	0.0600000
23462.0000000	0.0600000
23463.0000000	0.0600000
23464.0000000	0.0600000
23465.0000000	0.0600000
23466.0000000	0.0600000
23467.0000000	0.0600000
23468.0000000	0.0605000
23469.0000000	0.0600000
23470.0000000	0.0604762
23471.0000000	0.0600000
23472.0000000	0.0600000
23473.0000000	0.0605000
23474.0000000	0.0600000
23475.0000000	0.0600000
23476.0000000	0.0600000
23477.0000000	0.0604762
23478.0000000	0.0605000
23479.0000000	0.0600000
23480.0000000	0.0600000
23481.0000000	0.0619047
23482.0000000	0.0605000
23483.0000000	0.0604762
23484.0000000	0.0600000
23485.0000000	0.0605000
23486.0000000	0.0609524
23487.0000000	0.0610000
23488.0000000	0.0605000
23489.0000000	0.0625000
23490.0000000	0.0623810
23491.0000000	0.0620000
23492.0000000	0.0609524
23493.0000000	0.0610000
23494.0000000	0.0615000
23495.0000000	0.0615000
23496.0000000	0.0615000
23497.0000000	0.0604762
23498.0000000	0.0610000
23499.0000000	0.0604762
23500.0000000	0.0615000
23501.0000000	0.0609524
23502.0000000	0.0600000
23503.0000000	0.0610000
23504.0000000	0.0600000
23505.0000000	0.0609524
23506.0000000	0.0600000
23507.0000000	0.0605000
23508.0000000	0.0600000
23509.0000000	0.0600000
23510.0000000	0.0600000
23511.0000000	0.0600000
23512.0000000	0.0600000
23513.0000000	0.0600000
23514.0000000	0.0600000
23515.0000000	0.0600000
23516.0000000	0.0600000

23517.0000000	0.0600000
23518.0000000	0.0600000
23519.0000000	0.0600000
23520.0000000	0.0600000
23521.0000000	0.0600000
23522.0000000	0.0600000
23523.0000000	0.0600000
23524.0000000	0.0600000
23525.0000000	0.0600000
23526.0000000	0.0600000
23527.0000000	0.0600000
23528.0000000	0.0600000
23529.0000000	0.0600000
23530.0000000	0.0600000
23531.0000000	0.0604762
23532.0000000	0.0600000
23533.0000000	0.0600000
23534.0000000	0.0600000
23535.0000000	0.0604762
23536.0000000	0.0600000
23537.0000000	0.0600000
23538.0000000	0.0600000
23539.0000000	0.0600000
23540.0000000	0.0600000
23541.0000000	0.0600000
23542.0000000	0.0600000
23543.0000000	0.0600000
23544.0000000	0.0600000
23545.0000000	0.0600000
23546.0000000	0.0600000
23547.0000000	0.0600000
23548.0000000	0.0600000
23549.0000000	0.0600000
23550.0000000	0.0600000
23551.0000000	0.0600000
23552.0000000	0.0600000
23553.0000000	0.0604762
23554.0000000	0.0600000
23555.0000000	0.0600000
23556.0000000	0.0595000
23557.0000000	0.0600000
23558.0000000	0.0600000
23559.0000000	0.0600000
23560.0000000	0.0600000
23561.0000000	0.0595000
23562.0000000	0.0600000
23563.0000000	0.0600000
23564.0000000	0.0600000
23565.0000000	0.0595238
23566.0000000	0.0595000
23567.0000000	0.0600000
23568.0000000	0.0600000
23569.0000000	0.0600000
23570.0000000	0.0600000
23571.0000000	0.0600000
23572.0000000	0.0600000
23573.0000000	0.0600000

23574.0000000	0.0600000
23575.0000000	0.0600000
23576.0000000	0.0600000
23577.0000000	0.0600000
23578.0000000	0.0605000
23579.0000000	0.0600000
23580.0000000	0.0600000
23581.0000000	0.0600000
23582.0000000	0.0605000
23583.0000000	0.0600000
23584.0000000	0.0605000
23585.0000000	0.0605000
23586.0000000	0.0600000
23587.0000000	0.0600000
23588.0000000	0.0600000
23589.0000000	0.0600000
23590.0000000	0.0600000
23591.0000000	0.0610000
23592.0000000	0.0600000
23593.0000000	0.0605000
23594.0000000	0.0600000
23595.0000000	0.0600000
23596.0000000	0.0600000
23597.0000000	0.0600000
23598.0000000	0.0600000
23599.0000000	0.0610000
23600.0000000	0.0605000
23601.0000000	0.0600000
23602.0000000	0.0600000
23603.0000000	0.0600000
23604.0000000	0.0610000
23605.0000000	0.0609524
23606.0000000	0.0600000
23607.0000000	0.0600000
23608.0000000	0.0600000
23609.0000000	0.0605000
23610.0000000	0.0600000
23611.0000000	0.0600000
23612.0000000	0.0610000
23613.0000000	0.0605000
23614.0000000	0.0604762
23615.0000000	0.0600000
23616.0000000	0.0605000
23617.0000000	0.0604762
23618.0000000	0.0605000
23619.0000000	0.0609524
23620.0000000	0.0615000
23621.0000000	0.0600000
23622.0000000	0.0614286
23623.0000000	0.0610000
23624.0000000	0.0605000
23625.0000000	0.0615000
23626.0000000	0.0614286
23627.0000000	0.0610000
23628.0000000	0.0619047
23629.0000000	0.0600000
23630.0000000	0.0605000

23631.0000000	0.0605000
23632.0000000	0.0600000
23633.0000000	0.0610000
23634.0000000	0.0605000
23635.0000000	0.0609524
23636.0000000	0.0620000
23637.0000000	0.0609524
23638.0000000	0.0620000
23639.0000000	0.0620000
23640.0000000	0.0605000
23641.0000000	0.0609524
23642.0000000	0.0625000
23643.0000000	0.0614286
23644.0000000	0.0620000
23645.0000000	0.0615000
23646.0000000	0.0604762
23647.0000000	0.0610000
23648.0000000	0.0610000
23649.0000000	0.0625000
23650.0000000	0.0604762
23651.0000000	0.0610000
23652.0000000	0.0619047
23653.0000000	0.0610000
23654.0000000	0.0615000
23655.0000000	0.0610000
23656.0000000	0.0623810
23657.0000000	0.0625000
23658.0000000	0.0619048
23659.0000000	0.0615000
23660.0000000	0.0610000
23661.0000000	0.0615000
23662.0000000	0.0619047
23663.0000000	0.0600000
23664.0000000	0.0605000
23665.0000000	0.0619047
23666.0000000	0.0610000
23667.0000000	0.0619048
23668.0000000	0.0620000
23669.0000000	0.0610000
23670.0000000	0.0614286
23671.0000000	0.0625000
23672.0000000	0.0615000
23673.0000000	0.0625000
23674.0000000	0.0609524
23675.0000000	0.0610000
23676.0000000	0.0610000
23677.0000000	0.0609524
23678.0000000	0.0610000
23679.0000000	0.0605000
23680.0000000	0.0604762
23681.0000000	0.0600000
23682.0000000	0.0600000
23683.0000000	0.0600000
23684.0000000	0.0600000
23685.0000000	0.0600000
23686.0000000	0.0609524
23687.0000000	0.0600000

23688.0000000	0.0605000
23689.0000000	0.0604762
23690.0000000	0.0600000
23691.0000000	0.0600000
23692.0000000	0.0600000
23693.0000000	0.0600000
23694.0000000	0.0600000
23695.0000000	0.0600000
23696.0000000	0.0605000
23697.0000000	0.0600000
23698.0000000	0.0604762
23699.0000000	0.0600000
23700.0000000	0.0605000
23701.0000000	0.0605000
23702.0000000	0.0600000
23703.0000000	0.0600000
23704.0000000	0.0600000
23705.0000000	0.0605000
23706.0000000	0.0600000
23707.0000000	0.0600000
23708.0000000	0.0600000
23709.0000000	0.0600000
23710.0000000	0.0600000
23711.0000000	0.0610000
23712.0000000	0.0600000
23713.0000000	0.0604762
23714.0000000	0.0600000
23715.0000000	0.0605000
23716.0000000	0.0604762
23717.0000000	0.0605000
23718.0000000	0.0600000
23719.0000000	0.0600000
23720.0000000	0.0600000
23721.0000000	0.0600000
23722.0000000	0.0604762
23723.0000000	0.0605000
23724.0000000	0.0600000
23725.0000000	0.0609524
23726.0000000	0.0605000
23727.0000000	0.0604762
23728.0000000	0.0600000
23729.0000000	0.0610000
23730.0000000	0.0600000
23731.0000000	0.0609524
23732.0000000	0.0610000
23733.0000000	0.0610000
23734.0000000	0.0614286
23735.0000000	0.0615000
23736.0000000	0.0605000
23737.0000000	0.0610000
23738.0000000	0.0600000
23739.0000000	0.0615000
23740.0000000	0.0604762
23741.0000000	0.0600000
23742.0000000	0.0600000
23743.0000000	0.0604762
23744.0000000	0.0600000

23745.0000000	0.0610000
23746.0000000	0.0604762
23747.0000000	0.0620000
23748.0000000	0.0605000
23749.0000000	0.0623809
23750.0000000	0.0610000
23751.0000000	0.0615000
23752.0000000	0.0605000
23753.0000000	0.0609524
23754.0000000	0.0605000
23755.0000000	0.0604762
23756.0000000	0.0620000
23757.0000000	0.0610000
23758.0000000	0.0600000
23759.0000000	0.0605000
23760.0000000	0.0605000
23761.0000000	0.0610000
23762.0000000	0.0604762
23763.0000000	0.0604762
23764.0000000	0.0605000
23765.0000000	0.0605000
23766.0000000	0.0605000
23767.0000000	0.0605000
23768.0000000	0.0604762
23769.0000000	0.0600000
23770.0000000	0.0604762
23771.0000000	0.0610526
23772.0000000	0.0614286
23773.0000000	0.0609524
23774.0000000	0.0605000
23775.0000000	0.0600000
23776.0000000	0.0610000
23777.0000000	0.0604762
23778.0000000	0.0610000
23779.0000000	0.0600000
23780.0000000	0.0605000
23781.0000000	0.0625000
23782.0000000	0.0610000
23783.0000000	0.0604762
23784.0000000	0.0615000
23785.0000000	0.0600000
23786.0000000	0.0619048
23787.0000000	0.0615000
23788.0000000	0.0609524
23789.0000000	0.0620000
23790.0000000	0.0630000
23791.0000000	0.0620000
23792.0000000	0.0628571
23793.0000000	0.0625000
23794.0000000	0.0623809
23795.0000000	0.0625000
23796.0000000	0.0615000
23797.0000000	0.0630000
23798.0000000	0.0619048
23799.0000000	0.0625000
23800.0000000	0.0625000
23801.0000000	0.0638095

23802.0000000	0.0645000
23803.0000000	0.0623810
23804.0000000	0.0625000
23805.0000000	0.0640000
23806.0000000	0.0625000
23807.0000000	0.0652381
23808.0000000	0.0635000
23809.0000000	0.0635000
23810.0000000	0.0652381
23811.0000000	0.0640000
23812.0000000	0.0630000
23813.0000000	0.0633333
23814.0000000	0.0630000
23815.0000000	0.0628571
23816.0000000	0.0635000
23817.0000000	0.0640000
23818.0000000	0.0645000
23819.0000000	0.0623809
23820.0000000	0.0645000
23821.0000000	0.0630000
23822.0000000	0.0638095
23823.0000000	0.0635000
23824.0000000	0.0661905
23825.0000000	0.0655000
23826.0000000	0.0650000
23827.0000000	0.0655000
23828.0000000	0.0642857
23829.0000000	0.0655000
23830.0000000	0.0650000
23831.0000000	0.0628571
23832.0000000	0.0640000
23833.0000000	0.0638095
23834.0000000	0.0635000
23835.0000000	0.0640000
23836.0000000	0.0630000
23837.0000000	0.0633333
23838.0000000	0.0645000
23839.0000000	0.0635000
23840.0000000	0.0642857
23841.0000000	0.0645000
23842.0000000	0.0625000
23843.0000000	0.0623809
23844.0000000	0.0650000
23845.0000000	0.0650000
23846.0000000	0.0638095
23847.0000000	0.0620000
23848.0000000	0.0640000
23849.0000000	0.0647619
23850.0000000	0.0640000
23851.0000000	0.0619047
23852.0000000	0.0610000
23853.0000000	0.0620000
23854.0000000	0.0605000
23855.0000000	0.0609524
23856.0000000	0.0600000
23857.0000000	0.0605000
23858.0000000	0.0609524

23859.0000000	0.0600000
23860.0000000	0.0600000
23861.0000000	0.0600000
23862.0000000	0.0600000
23863.0000000	0.0600000
23864.0000000	0.0605000
23865.0000000	0.0600000
23866.0000000	0.0600000
23867.0000000	0.0600000
23868.0000000	0.0605000
23869.0000000	0.0600000
23870.0000000	0.0609524
23871.0000000	0.0600000
23872.0000000	0.0600000
23873.0000000	0.0625000
23874.0000000	0.0600000
23875.0000000	0.0605000
23876.0000000	0.0604762
23877.0000000	0.0605000
23878.0000000	0.0615000
23879.0000000	0.0609524
23880.0000000	0.0620000
23881.0000000	0.0620000
23882.0000000	0.0623809
23883.0000000	0.0610000
23884.0000000	0.0620000
23885.0000000	0.0623809
23886.0000000	0.0620000
23887.0000000	0.0615000
23888.0000000	0.0615000
23889.0000000	0.0614286
23890.0000000	0.0625000
23891.0000000	0.0619047
23892.0000000	0.0610000
23893.0000000	0.0625000
23894.0000000	0.0633333
23895.0000000	0.0630000
23896.0000000	0.0625000
23897.0000000	0.0630000
23898.0000000	0.0619048
23899.0000000	0.0625000
23900.0000000	0.0604762
23901.0000000	0.0605000
23902.0000000	0.0605000
23903.0000000	0.0604762
23904.0000000	0.0600000
23905.0000000	0.0605000
23906.0000000	0.0600000
23907.0000000	0.0615000
23908.0000000	0.0605000
23909.0000000	0.0600000
23910.0000000	0.0609524
23911.0000000	0.0605000
23912.0000000	0.0600000
23913.0000000	0.0600000
23914.0000000	0.0600000
23915.0000000	0.0604762

23916.0000000	0.0600000
23917.0000000	0.0600000
23918.0000000	0.0600000
23919.0000000	0.0600000
23920.0000000	0.0605000
23921.0000000	0.0600000
23922.0000000	0.0600000
23923.0000000	0.0609524
23924.0000000	0.0600000
23925.0000000	0.0610000
23926.0000000	0.0600000
23927.0000000	0.0605000
23928.0000000	0.0600000
23929.0000000	0.0610000
23930.0000000	0.0604762
23931.0000000	0.0600000
23932.0000000	0.0600000
23933.0000000	0.0605000
23934.0000000	0.0600000
23935.0000000	0.0600000
23936.0000000	0.0600000
23937.0000000	0.0604762
23938.0000000	0.0610000
23939.0000000	0.0604762
23940.0000000	0.0605000
23941.0000000	0.0610000
23942.0000000	0.0605000
23943.0000000	0.0609524
23944.0000000	0.0600000
23945.0000000	0.0600000
23946.0000000	0.0600000
23947.0000000	0.0600000
23948.0000000	0.0605000
23949.0000000	0.0600000
23950.0000000	0.0600000
23951.0000000	0.0600000
23952.0000000	0.0600000
23953.0000000	0.0605000
23954.0000000	0.0600000
23955.0000000	0.0600000
23956.0000000	0.0600000
23957.0000000	0.0600000
23958.0000000	0.0604762
23959.0000000	0.0605000
23960.0000000	0.0604762
23961.0000000	0.0600000
23962.0000000	0.0605000
23963.0000000	0.0610000
23964.0000000	0.0600000
23965.0000000	0.0600000
23966.0000000	0.0605000
23967.0000000	0.0600000
23968.0000000	0.0600000
23969.0000000	0.0600000
23970.0000000	0.0610000
23971.0000000	0.0610000
23972.0000000	0.0605000

23973.0000000	0.0600000
23974.0000000	0.0605000
23975.0000000	0.0619047
23976.0000000	0.0610000
23977.0000000	0.0605000
23978.0000000	0.0600000
23979.0000000	0.0604762
23980.0000000	0.0615000
23981.0000000	0.0610000
23982.0000000	0.0600000
23983.0000000	0.0605000
23984.0000000	0.0604762
23985.0000000	0.0605000
23986.0000000	0.0610000
23987.0000000	0.0610000
23988.0000000	0.0604762
23989.0000000	0.0605000
23990.0000000	0.0610000
23991.0000000	0.0614286
23992.0000000	0.0610000
23993.0000000	0.0610000
23994.0000000	0.0620000
23995.0000000	0.0604762
23996.0000000	0.0630000
23997.0000000	0.0609524
23998.0000000	0.0615000
23999.0000000	0.0614286
24000.0000000	0.0630000
24001.0000000	0.0625000
24002.0000000	0.0620000
24003.0000000	0.0623809
24004.0000000	0.0630000
24005.0000000	0.0625000
24006.0000000	0.0647619
24007.0000000	0.0625000
24008.0000000	0.0645000
24009.0000000	0.0623810
24010.0000000	0.0635000
24011.0000000	0.0633333
24012.0000000	0.0645000
24013.0000000	0.0630000
24014.0000000	0.0660000
24015.0000000	0.0642857
24016.0000000	0.0640000
24017.0000000	0.0650000
24018.0000000	0.0647619
24019.0000000	0.0660000
24020.0000000	0.0652381
24021.0000000	0.0655000
24022.0000000	0.0630000
24023.0000000	0.0635000
24024.0000000	0.0645000
24025.0000000	0.0633333
24026.0000000	0.0660000
24027.0000000	0.0666667
24028.0000000	0.0655000
24029.0000000	0.0633333

24030.0000000	0.0650000
24031.0000000	0.0635000
24032.0000000	0.0645000
24033.0000000	0.0630000
24034.0000000	0.0633333
24035.0000000	0.0645000
24036.0000000	0.0628571
24037.0000000	0.0625000
24038.0000000	0.0625000
24039.0000000	0.0638095
24040.0000000	0.0620000
24041.0000000	0.0630000
24042.0000000	0.0640000
24043.0000000	0.0628571
24044.0000000	0.0615000
24045.0000000	0.0609524
24046.0000000	0.0635000
24047.0000000	0.0610000
24048.0000000	0.0623810
24049.0000000	0.0615000
24050.0000000	0.0635000
24051.0000000	0.0619048
24052.0000000	0.0620000
24053.0000000	0.0615000
24054.0000000	0.0615000
24055.0000000	0.0609524
24056.0000000	0.0630000
24057.0000000	0.0614286
24058.0000000	0.0615000
24059.0000000	0.0640000
24060.0000000	0.0638095
24061.0000000	0.0645000
24062.0000000	0.0635000
24063.0000000	0.0619047
24064.0000000	0.0615000
24065.0000000	0.0625000
24066.0000000	0.0614286
24067.0000000	0.0615000
24068.0000000	0.0615000
24069.0000000	0.0625000
24070.0000000	0.0609524
24071.0000000	0.0620000
24072.0000000	0.0614286
24073.0000000	0.0605000
24074.0000000	0.0615000
24075.0000000	0.0600000
24076.0000000	0.0600000
24077.0000000	0.0605000
24078.0000000	0.0600000
24079.0000000	0.0600000
24080.0000000	0.0600000
24081.0000000	0.0600000
24082.0000000	0.0600000
24083.0000000	0.0600000
24084.0000000	0.0600000
24085.0000000	0.0600000
24086.0000000	0.0600000

24087.0000000	0.0600000
24088.0000000	0.0600000
24089.0000000	0.0600000
24090.0000000	0.0595238
24091.0000000	0.0600000
24092.0000000	0.0600000
24093.0000000	0.0595000
24094.0000000	0.0600000
24095.0000000	0.0600000
24096.0000000	0.0600000
24097.0000000	0.0595000
24098.0000000	0.0600000
24099.0000000	0.0600000
24100.0000000	0.0590000
24101.0000000	0.0600000
24102.0000000	0.0600000
24103.0000000	0.0600000
24104.0000000	0.0595000
24105.0000000	0.0600000
24106.0000000	0.0600000
24107.0000000	0.0595000
24108.0000000	0.0595238
24109.0000000	0.0600000
24110.0000000	0.0600000
24111.0000000	0.0590476
24112.0000000	0.0600000
24113.0000000	0.0595000
24114.0000000	0.0600000
24115.0000000	0.0595238
24116.0000000	0.0600000
24117.0000000	0.0595000
24118.0000000	0.0600000
24119.0000000	0.0600000
24120.0000000	0.0600000
24121.0000000	0.0600000
24122.0000000	0.0600000
24123.0000000	0.0600000
24124.0000000	0.0600000
24125.0000000	0.0600000
24126.0000000	0.0600000
24127.0000000	0.0595238
24128.0000000	0.0600000
24129.0000000	0.0600000
24130.0000000	0.0595000
24131.0000000	0.0600000
24132.0000000	0.0600000
24133.0000000	0.0595238
24134.0000000	0.0600000
24135.0000000	0.0600000
24136.0000000	0.0595000
24137.0000000	0.0595000
24138.0000000	0.0595000
24139.0000000	0.0595238
24140.0000000	0.0595000
24141.0000000	0.0595000
24142.0000000	0.0580952
24143.0000000	0.0590000

24144.0000000	0.0595000
24145.0000000	0.0585714
24146.0000000	0.0570000
24147.0000000	0.0585000
24148.0000000	0.0590476
24149.0000000	0.0590000
24150.0000000	0.0600000
24151.0000000	0.0580952
24152.0000000	0.0585000
24153.0000000	0.0580000
24154.0000000	0.0600000
24155.0000000	0.0585000
24156.0000000	0.0580952
24157.0000000	0.0580000
24158.0000000	0.0585000
24159.0000000	0.0590000
24160.0000000	0.0590476
24161.0000000	0.0590000
24162.0000000	0.0595000
24163.0000000	0.0580952
24164.0000000	0.0570000
24165.0000000	0.0585714
24166.0000000	0.0590000
24167.0000000	0.0585000
24168.0000000	0.0580000
24169.0000000	0.0580952
24170.0000000	0.0580000
24171.0000000	0.0585000
24172.0000000	0.0595238
24173.0000000	0.0575000
24174.0000000	0.0590000
24175.0000000	0.0595238
24176.0000000	0.0575000
24177.0000000	0.0585000
24178.0000000	0.0580000
24179.0000000	0.0600000
24180.0000000	0.0590000
24181.0000000	0.0590476
24182.0000000	0.0595000
24183.0000000	0.0585000
24184.0000000	0.0600000
24185.0000000	0.0595000
24186.0000000	0.0580000
24187.0000000	0.0590476
24188.0000000	0.0590000
24189.0000000	0.0585000
24190.0000000	0.0595000
24191.0000000	0.0576190
24192.0000000	0.0590000
24193.0000000	0.0580952
24194.0000000	0.0575000
24195.0000000	0.0585000
24196.0000000	0.0585714
24197.0000000	0.0595000
24198.0000000	0.0585000
24199.0000000	0.0576190
24200.0000000	0.0585000

24201.0000000	0.0600000
24202.0000000	0.0600000
24203.0000000	0.0585000
24204.0000000	0.0585000
24205.0000000	0.0590000
24206.0000000	0.0571428
24207.0000000	0.0590000
24208.0000000	0.0585714
24209.0000000	0.0590000
24210.0000000	0.0585000
24211.0000000	0.0571428
24212.0000000	0.0575000
24213.0000000	0.0570000
24214.0000000	0.0580000
24215.0000000	0.0585714
24216.0000000	0.0585000
24217.0000000	0.0566667
24218.0000000	0.0555000
24219.0000000	0.0555000
24220.0000000	0.0560000
24221.0000000	0.0566667
24222.0000000	0.0565000
24223.0000000	0.0580952
24224.0000000	0.0580000
24225.0000000	0.0575000
24226.0000000	0.0557143
24227.0000000	0.0555000
24228.0000000	0.0565000
24229.0000000	0.0570000
24230.0000000	0.0571428
24231.0000000	0.0600000
24232.0000000	0.0590476
24233.0000000	0.0590000
24234.0000000	0.0585000
24235.0000000	0.0576190
24236.0000000	0.0575000
24237.0000000	0.0575000
24238.0000000	0.0580000
24239.0000000	0.0571428
24240.0000000	0.0580000
24241.0000000	0.0571428
24242.0000000	0.0585000
24243.0000000	0.0570000
24244.0000000	0.0580952
24245.0000000	0.0590000
24246.0000000	0.0580000
24247.0000000	0.0576190
24248.0000000	0.0595000
24249.0000000	0.0570000
24250.0000000	0.0595000
24251.0000000	0.0595238
24252.0000000	0.0580000
24253.0000000	0.0576190
24254.0000000	0.0590000
24255.0000000	0.0575000
24256.0000000	0.0595238
24257.0000000	0.0595000

24258.0000000	0.0585000
24259.0000000	0.0595000
24260.0000000	0.0595238
24261.0000000	0.0590000
24262.0000000	0.0590476
24263.0000000	0.0585000
24264.0000000	0.0595000
24265.0000000	0.0595000
24266.0000000	0.0580000
24267.0000000	0.0585714
24268.0000000	0.0575000
24269.0000000	0.0585714
24270.0000000	0.0580000
24271.0000000	0.0571428
24272.0000000	0.0590000
24273.0000000	0.0590000
24274.0000000	0.0580000
24275.0000000	0.0580952
24276.0000000	0.0560000
24277.0000000	0.0580000
24278.0000000	0.0547619
24279.0000000	0.0570000
24280.0000000	0.0557143
24281.0000000	0.0555000
24282.0000000	0.0550000
24283.0000000	0.0560000
24284.0000000	0.0561905
24285.0000000	0.0555000
24286.0000000	0.0560000
24287.0000000	0.0571428
24288.0000000	0.0575000
24289.0000000	0.0565000
24290.0000000	0.0566667
24291.0000000	0.0570000
24292.0000000	0.0566667
24293.0000000	0.0555000
24294.0000000	0.0570000
24295.0000000	0.0580000
24296.0000000	0.0566667
24297.0000000	0.0560000
24298.0000000	0.0570000
24299.0000000	0.0571428
24300.0000000	0.0575000
24301.0000000	0.0557143
24302.0000000	0.0560000
24303.0000000	0.0565000
24304.0000000	0.0575000
24305.0000000	0.0571428
24306.0000000	0.0575000
24307.0000000	0.0580000
24308.0000000	0.0576190
24309.0000000	0.0575000
24310.0000000	0.0565000
24311.0000000	0.0576190
24312.0000000	0.0580000
24313.0000000	0.0585000
24314.0000000	0.0585714

24315.0000000	0.0590000
24316.0000000	0.0571428
24317.0000000	0.0580000
24318.0000000	0.0580000
24319.0000000	0.0580000
24320.0000000	0.0576190
24321.0000000	0.0590000
24322.0000000	0.0580000
24323.0000000	0.0590476
24324.0000000	0.0585000
24325.0000000	0.0580000
24326.0000000	0.0580000
24327.0000000	0.0595238
24328.0000000	0.0590000
24329.0000000	0.0595238
24330.0000000	0.0600000
24331.0000000	0.0600000
24332.0000000	0.0595238
24333.0000000	0.0595000
24334.0000000	0.0600000
24335.0000000	0.0590476
24336.0000000	0.0590000
24337.0000000	0.0595000
24338.0000000	0.0590000
24339.0000000	0.0595238
24340.0000000	0.0600000
24341.0000000	0.0600000
24342.0000000	0.0600000
24343.0000000	0.0600000
24344.0000000	0.0600000
24345.0000000	0.0600000
24346.0000000	0.0600000
24347.0000000	0.0595238
24348.0000000	0.0600000
24349.0000000	0.0600000
24350.0000000	0.0600000
24351.0000000	0.0600000
24352.0000000	0.0600000
24353.0000000	0.0595000
24354.0000000	0.0595000
24355.0000000	0.0595000
24356.0000000	0.0600000
24357.0000000	0.0585714
24358.0000000	0.0590000
24359.0000000	0.0585714
24360.0000000	0.0585000
24361.0000000	0.0585714
24362.0000000	0.0595000
24363.0000000	0.0585000
24364.0000000	0.0585000
24365.0000000	0.0580000
24366.0000000	0.0585714
24367.0000000	0.0590000
24368.0000000	0.0595238
24369.0000000	0.0590000
24370.0000000	0.0590000
24371.0000000	0.0590000

24372.0000000	0.0595238
24373.0000000	0.0585000
24374.0000000	0.0580000
24375.0000000	0.0585714
24376.0000000	0.0590000
24377.0000000	0.0585714
24378.0000000	0.0585000
24379.0000000	0.0580000
24380.0000000	0.0585714
24381.0000000	0.0590000
24382.0000000	0.0575000
24383.0000000	0.0580952
24384.0000000	0.0570000
24385.0000000	0.0590000
24386.0000000	0.0580000
24387.0000000	0.0571428
24388.0000000	0.0575000
24389.0000000	0.0571428
24390.0000000	0.0580000
24391.0000000	0.0585000
24392.0000000	0.0580952
24393.0000000	0.0590000
24394.0000000	0.0590000
24395.0000000	0.0580000
24396.0000000	0.0585714
24397.0000000	0.0575000
24398.0000000	0.0580952
24399.0000000	0.0590000
24400.0000000	0.0590000
24401.0000000	0.0580000
24402.0000000	0.0580952
24403.0000000	0.0580000
24404.0000000	0.0576190
24405.0000000	0.0595000
24406.0000000	0.0590000
24407.0000000	0.0590476
24408.0000000	0.0595000
24409.0000000	0.0585000
24410.0000000	0.0585000
24411.0000000	0.0585714
24412.0000000	0.0585000
24413.0000000	0.0600000
24414.0000000	0.0590000
24415.0000000	0.0590000
24416.0000000	0.0595000
24417.0000000	0.0595238
24418.0000000	0.0595000
24419.0000000	0.0585000
24420.0000000	0.0595238
24421.0000000	0.0590000
24422.0000000	0.0595238
24423.0000000	0.0585000
24424.0000000	0.0595000
24425.0000000	0.0580000
24426.0000000	0.0600000
24427.0000000	0.0590476
24428.0000000	0.0600000

24429.0000000	0.0595000
24430.0000000	0.0600000
24431.0000000	0.0600000
24432.0000000	0.0595238
24433.0000000	0.0595000
24434.0000000	0.0585000
24435.0000000	0.0590476
24436.0000000	0.0600000
24437.0000000	0.0595238
24438.0000000	0.0595000
24439.0000000	0.0585000
24440.0000000	0.0590476
24441.0000000	0.0590000
24442.0000000	0.0595000
24443.0000000	0.0595000
24444.0000000	0.0600000
24445.0000000	0.0580000
24446.0000000	0.0590000
24447.0000000	0.0595238
24448.0000000	0.0595000
24449.0000000	0.0595238
24450.0000000	0.0595000
24451.0000000	0.0590000
24452.0000000	0.0590476
24453.0000000	0.0590000
24454.0000000	0.0580000
24455.0000000	0.0600000
24456.0000000	0.0595238
24457.0000000	0.0600000
24458.0000000	0.0595000
24459.0000000	0.0576190
24460.0000000	0.0595000
24461.0000000	0.0595000
24462.0000000	0.0585000
24463.0000000	0.0600000
24464.0000000	0.0595000
24465.0000000	0.0590476
24466.0000000	0.0595000
24467.0000000	0.0590000
24468.0000000	0.0595238
24469.0000000	0.0585000
24470.0000000	0.0575000
24471.0000000	0.0590476
24472.0000000	0.0590000
24473.0000000	0.0595000
24474.0000000	0.0600000
24475.0000000	0.0595000
24476.0000000	0.0590000
24477.0000000	0.0595238
24478.0000000	0.0585000
24479.0000000	0.0595000
24480.0000000	0.0595238
24481.0000000	0.0595000
24482.0000000	0.0590000
24483.0000000	0.0595238
24484.0000000	0.0600000
24485.0000000	0.0595000

24486.0000000	0.0600000
24487.0000000	0.0600000
24488.0000000	0.0585714
24489.0000000	0.0595000
24490.0000000	0.0595000
24491.0000000	0.0600000
24492.0000000	0.0600000
24493.0000000	0.0594737
24494.0000000	0.0590476
24495.0000000	0.0585714
24496.0000000	0.0600000
24497.0000000	0.0595238
24498.0000000	0.0585000
24499.0000000	0.0595000
24500.0000000	0.0590000
24501.0000000	0.0585714
24502.0000000	0.0585000
24503.0000000	0.0595000
24504.0000000	0.0600000
24505.0000000	0.0590000
24506.0000000	0.0585000
24507.0000000	0.0600000
24508.0000000	0.0595238
24509.0000000	0.0595000
24510.0000000	0.0585714
24511.0000000	0.0575000
24512.0000000	0.0600000
24513.0000000	0.0595238
24514.0000000	0.0585000
24515.0000000	0.0595000
24516.0000000	0.0585714
24517.0000000	0.0595000
24518.0000000	0.0595000
24519.0000000	0.0595238
24520.0000000	0.0600000
24521.0000000	0.0590000
24522.0000000	0.0600000
24523.0000000	0.0595238
24524.0000000	0.0595000
24525.0000000	0.0600000
24526.0000000	0.0590000
24527.0000000	0.0595000
24528.0000000	0.0590476
24529.0000000	0.0595000
24530.0000000	0.0600000
24531.0000000	0.0595000
24532.0000000	0.0600000
24533.0000000	0.0600000
24534.0000000	0.0595238
24535.0000000	0.0600000
24536.0000000	0.0600000
24537.0000000	0.0595000
24538.0000000	0.0595238
24539.0000000	0.0600000
24540.0000000	0.0600000
24541.0000000	0.0600000
24542.0000000	0.0595000

24543.0000000	0.0600000
24544.0000000	0.0600000
24545.0000000	0.0595000
24546.0000000	0.0595000
24547.0000000	0.0600000
24548.0000000	0.0595000
24549.0000000	0.0595238
24550.0000000	0.0600000
24551.0000000	0.0600000
24552.0000000	0.0600000
24553.0000000	0.0595238
24554.0000000	0.0600000
24555.0000000	0.0595000
24556.0000000	0.0590476
24557.0000000	0.0595000
24558.0000000	0.0600000
24559.0000000	0.0600000
24560.0000000	0.0590000
24561.0000000	0.0600000
24562.0000000	0.0595238
24563.0000000	0.0595000
24564.0000000	0.0595238
24565.0000000	0.0600000
24566.0000000	0.0595000
24567.0000000	0.0600000
24568.0000000	0.0600000
24569.0000000	0.0600000
24570.0000000	0.0595000
24571.0000000	0.0600000
24572.0000000	0.0600000
24573.0000000	0.0600000
24574.0000000	0.0600000
24575.0000000	0.0600000
24576.0000000	0.0600000
24577.0000000	0.0595000
24578.0000000	0.0600000
24579.0000000	0.0600000
24580.0000000	0.0600000
24581.0000000	0.0600000
24582.0000000	0.0600000
24583.0000000	0.0600000
24584.0000000	0.0600000
24585.0000000	0.0600000
24586.0000000	0.0595000
24587.0000000	0.0600000
24588.0000000	0.0600000
24589.0000000	0.0600000
24590.0000000	0.0595000
24591.0000000	0.0590000
24592.0000000	0.0600000
24593.0000000	0.0600000
24594.0000000	0.0600000
24595.0000000	0.0600000
24596.0000000	0.0600000
24597.0000000	0.0595000
24598.0000000	0.0600000
24599.0000000	0.0595238

24600.0000000	0.0595000
24601.0000000	0.0600000
24602.0000000	0.0600000
24603.0000000	0.0600000
24604.0000000	0.0600000
24605.0000000	0.0595238
24606.0000000	0.0600000
24607.0000000	0.0600000
24608.0000000	0.0600000
24609.0000000	0.0595000
24610.0000000	0.0600000
24611.0000000	0.0600000
24612.0000000	0.0600000
24613.0000000	0.0600000
24614.0000000	0.0595000
24615.0000000	0.0600000
24616.0000000	0.0595000
24617.0000000	0.0590476
24618.0000000	0.0595000
24619.0000000	0.0590476
24620.0000000	0.0590000
24621.0000000	0.0585000
24622.0000000	0.0600000
24623.0000000	0.0576190
24624.0000000	0.0595000
24625.0000000	0.0595000
24626.0000000	0.0600000
24627.0000000	0.0595000
24628.0000000	0.0580000
24629.0000000	0.0585000
24630.0000000	0.0600000
24631.0000000	0.0595000
24632.0000000	0.0600000
24633.0000000	0.0600000
24634.0000000	0.0600000
24635.0000000	0.0595238
24636.0000000	0.0585000
24637.0000000	0.0585000
24638.0000000	0.0600000
24639.0000000	0.0595238
24640.0000000	0.0600000
24641.0000000	0.0595238
24642.0000000	0.0600000
24643.0000000	0.0595000
24644.0000000	0.0590476
24645.0000000	0.0595000
24646.0000000	0.0600000
24647.0000000	0.0600000
24648.0000000	0.0600000
24649.0000000	0.0600000
24650.0000000	0.0600000
24651.0000000	0.0600000
24652.0000000	0.0600000
24653.0000000	0.0600000
24654.0000000	0.0600000
24655.0000000	0.0600000
24656.0000000	0.0600000

24657.0000000	0.0595000
24658.0000000	0.0600000
24659.0000000	0.0600000
24660.0000000	0.0600000
24661.0000000	0.0600000
24662.0000000	0.0600000
24663.0000000	0.0600000
24664.0000000	0.0600000
24665.0000000	0.0600000
24666.0000000	0.0595000
24667.0000000	0.0600000
24668.0000000	0.0600000
24669.0000000	0.0600000
24670.0000000	0.0600000
24671.0000000	0.0600000
24672.0000000	0.0600000
24673.0000000	0.0600000
24674.0000000	0.0600000
24675.0000000	0.0600000
24676.0000000	0.0600000
24677.0000000	0.0600000
24678.0000000	0.0600000
24679.0000000	0.0600000
24680.0000000	0.0600000
24681.0000000	0.0600000
24682.0000000	0.0600000
24683.0000000	0.0600000
24684.0000000	0.0600000
24685.0000000	0.0600000
24686.0000000	0.0600000
24687.0000000	0.0600000
24688.0000000	0.0600000
24689.0000000	0.0600000
24690.0000000	0.0595238
24691.0000000	0.0595000
24692.0000000	0.0595000
24693.0000000	0.0595238
24694.0000000	0.0595000
24695.0000000	0.0600000
24696.0000000	0.0590000
24697.0000000	0.0595000
24698.0000000	0.0595000
24699.0000000	0.0585714
24700.0000000	0.0600000
24701.0000000	0.0600000
24702.0000000	0.0590476
24703.0000000	0.0600000
24704.0000000	0.0590000
24705.0000000	0.0590476
24706.0000000	0.0590000
24707.0000000	0.0595238
24708.0000000	0.0595000
24709.0000000	0.0595000
24710.0000000	0.0585000
24711.0000000	0.0600000
24712.0000000	0.0585000
24713.0000000	0.0590000

24714.0000000	0.0600000
24715.0000000	0.0590000
24716.0000000	0.0600000
24717.0000000	0.0600000
24718.0000000	0.0590000
24719.0000000	0.0580000
24720.0000000	0.0590476
24721.0000000	0.0575000
24722.0000000	0.0585000
24723.0000000	0.0585714
24724.0000000	0.0580000
24725.0000000	0.0580952
24726.0000000	0.0565000
24727.0000000	0.0570000
24728.0000000	0.0565000
24729.0000000	0.0566667
24730.0000000	0.0580000
24731.0000000	0.0590000
24732.0000000	0.0590476
24733.0000000	0.0585000
24734.0000000	0.0595000
24735.0000000	0.0566667
24736.0000000	0.0575000
24737.0000000	0.0580000
24738.0000000	0.0566667
24739.0000000	0.0575000
24740.0000000	0.0570000
24741.0000000	0.0571428
24742.0000000	0.0530000
24743.0000000	0.0550000
24744.0000000	0.0557143
24745.0000000	0.0570000
24746.0000000	0.0550000
24747.0000000	0.0552381
24748.0000000	0.0540000
24749.0000000	0.0585000
24750.0000000	0.0560000
24751.0000000	0.0552381
24752.0000000	0.0542857
24753.0000000	0.0550000
24754.0000000	0.0560000
24755.0000000	0.0545000
24756.0000000	0.0552381
24757.0000000	0.0545000
24758.0000000	0.0520000
24759.0000000	0.0550000
24760.0000000	0.0533333
24761.0000000	0.0533333
24762.0000000	0.0540000
24763.0000000	0.0510000
24764.0000000	0.0520000
24765.0000000	0.0535000
24766.0000000	0.0519048
24767.0000000	0.0530000
24768.0000000	0.0528571
24769.0000000	0.0515000
24770.0000000	0.0510000

24771.0000000	0.0523810
24772.0000000	0.0530000
24773.0000000	0.0505000
24774.0000000	0.0515000
24775.0000000	0.0514286
24776.0000000	0.0500000
24777.0000000	0.0504762
24778.0000000	0.0505000
24779.0000000	0.0500000
24780.0000000	0.0505000
24781.0000000	0.0500000
24782.0000000	0.0505000
24783.0000000	0.0504762
24784.0000000	0.0500000
24785.0000000	0.0500000
24786.0000000	0.0504762
24787.0000000	0.0500000
24788.0000000	0.0510000
24789.0000000	0.0500000
24790.0000000	0.0504762
24791.0000000	0.0510000
24792.0000000	0.0500000
24793.0000000	0.0505000
24794.0000000	0.0520000
24795.0000000	0.0500000
24796.0000000	0.0505000
24797.0000000	0.0500000
24798.0000000	0.0505000
24799.0000000	0.0509524
24800.0000000	0.0504762
24801.0000000	0.0510000
24802.0000000	0.0510000
24803.0000000	0.0500000
24804.0000000	0.0514286
24805.0000000	0.0505000
24806.0000000	0.0510000
24807.0000000	0.0514286
24808.0000000	0.0515000
24809.0000000	0.0510000
24810.0000000	0.0510000
24811.0000000	0.0509524
24812.0000000	0.0505000
24813.0000000	0.0509524
24814.0000000	0.0510000
24815.0000000	0.0505000
24816.0000000	0.0514286
24817.0000000	0.0510000
24818.0000000	0.0520000
24819.0000000	0.0505000
24820.0000000	0.0514286
24821.0000000	0.0510000
24822.0000000	0.0500000
24823.0000000	0.0500000
24824.0000000	0.0510000
24825.0000000	0.0500000
24826.0000000	0.0500000
24827.0000000	0.0504762

24828.0000000	0.0500000
24829.0000000	0.0500000
24830.0000000	0.0505000
24831.0000000	0.0500000
24832.0000000	0.0500000
24833.0000000	0.0510000
24834.0000000	0.0500000
24835.0000000	0.0500000
24836.0000000	0.0500000
24837.0000000	0.0515000
24838.0000000	0.0504762
24839.0000000	0.0500000
24840.0000000	0.0500000
24841.0000000	0.0500000
24842.0000000	0.0505000
24843.0000000	0.0500000
24844.0000000	0.0500000
24845.0000000	0.0500000
24846.0000000	0.0500000
24847.0000000	0.0500000
24848.0000000	0.0500000
24849.0000000	0.0500000
24850.0000000	0.0505000
24851.0000000	0.0500000
24852.0000000	0.0500000
24853.0000000	0.0500000
24854.0000000	0.0500000
24855.0000000	0.0505000
24856.0000000	0.0500000
24857.0000000	0.0500000
24858.0000000	0.0500000
24859.0000000	0.0500000
24860.0000000	0.0500000
24861.0000000	0.0500000
24862.0000000	0.0500000
24863.0000000	0.0510000
24864.0000000	0.0505000
24865.0000000	0.0504762
24866.0000000	0.0510000
24867.0000000	0.0500000
24868.0000000	0.0509524
24869.0000000	0.0500000
24870.0000000	0.0505000
24871.0000000	0.0504762
24872.0000000	0.0500000
24873.0000000	0.0510000
24874.0000000	0.0500000
24875.0000000	0.0500000
24876.0000000	0.0504762
24877.0000000	0.0510000
24878.0000000	0.0500000
24879.0000000	0.0500000
24880.0000000	0.0504762
24881.0000000	0.0520000
24882.0000000	0.0505000
24883.0000000	0.0509524
24884.0000000	0.0500000

24885.0000000	0.0500000
24886.0000000	0.0515000
24887.0000000	0.0519048
24888.0000000	0.0523810
24889.0000000	0.0520000
24890.0000000	0.0500000
24891.0000000	0.0510000
24892.0000000	0.0514286
24893.0000000	0.0505000
24894.0000000	0.0515000
24895.0000000	0.0514286
24896.0000000	0.0520000
24897.0000000	0.0505000
24898.0000000	0.0504762
24899.0000000	0.0500000
24900.0000000	0.0505000
24901.0000000	0.0504762
24902.0000000	0.0510000
24903.0000000	0.0510000
24904.0000000	0.0509524
24905.0000000	0.0510000
24906.0000000	0.0500000
24907.0000000	0.0504762
24908.0000000	0.0500000
24909.0000000	0.0510000
24910.0000000	0.0509524
24911.0000000	0.0520000
24912.0000000	0.0514286
24913.0000000	0.0520000
24914.0000000	0.0505000
24915.0000000	0.0500000
24916.0000000	0.0510000
24917.0000000	0.0514286
24918.0000000	0.0505000
24919.0000000	0.0509524
24920.0000000	0.0500000
24921.0000000	0.0504762
24922.0000000	0.0515000
24923.0000000	0.0510000
24924.0000000	0.0505000
24925.0000000	0.0505000
24926.0000000	0.0514286
24927.0000000	0.0505000
24928.0000000	0.0504762
24929.0000000	0.0500000
24930.0000000	0.0515000
24931.0000000	0.0500000
24932.0000000	0.0514286
24933.0000000	0.0500000
24934.0000000	0.0515000
24935.0000000	0.0509524
24936.0000000	0.0510000
24937.0000000	0.0500000
24938.0000000	0.0505000
24939.0000000	0.0505000
24940.0000000	0.0500000
24941.0000000	0.0505000

24942.0000000	0.0510000
24943.0000000	0.0505000
24944.0000000	0.0504762
24945.0000000	0.0500000
24946.0000000	0.0500000
24947.0000000	0.0500000
24948.0000000	0.0500000
24949.0000000	0.0504762
24950.0000000	0.0510000
24951.0000000	0.0500000
24952.0000000	0.0500000
24953.0000000	0.0500000
24954.0000000	0.0505000
24955.0000000	0.0505000
24956.0000000	0.0500000
24957.0000000	0.0504762
24958.0000000	0.0515000
24959.0000000	0.0500000
24960.0000000	0.0510000
24961.0000000	0.0500000
24962.0000000	0.0504762
24963.0000000	0.0505000
24964.0000000	0.0509524
24965.0000000	0.0505000
24966.0000000	0.0500000
24967.0000000	0.0500000
24968.0000000	0.0505000
24969.0000000	0.0500000
24970.0000000	0.0500000
24971.0000000	0.0500000
24972.0000000	0.0500000
24973.0000000	0.0500000
24974.0000000	0.0500000
24975.0000000	0.0505000
24976.0000000	0.0500000
24977.0000000	0.0510000
24978.0000000	0.0500000
24979.0000000	0.0500000
24980.0000000	0.0509524
24981.0000000	0.0505000
24982.0000000	0.0500000
24983.0000000	0.0505000
24984.0000000	0.0500000
24985.0000000	0.0500000
24986.0000000	0.0500000
24987.0000000	0.0505000
24988.0000000	0.0504762
24989.0000000	0.0510000
24990.0000000	0.0500000
24991.0000000	0.0495000
24992.0000000	0.0500000
24993.0000000	0.0510000
24994.0000000	0.0500000
24995.0000000	0.0500000
24996.0000000	0.0500000
24997.0000000	0.0500000
24998.0000000	0.0500000

24999.0000000	0.0500000
25000.0000000	0.0500000
25001.0000000	0.0500000
25002.0000000	0.0500000
25003.0000000	0.0505000
25004.0000000	0.0500000
25005.0000000	0.0500000
25006.0000000	0.0500000
25007.0000000	0.0500000
25008.0000000	0.0500000
25009.0000000	0.0500000
25010.0000000	0.0500000
25011.0000000	0.0500000
25012.0000000	0.0500000
25013.0000000	0.0500000
25014.0000000	0.0500000
25015.0000000	0.0500000
25016.0000000	0.0500000
25017.0000000	0.0500000
25018.0000000	0.0500000
25019.0000000	0.0500000
25020.0000000	0.0500000
25021.0000000	0.0500000
25022.0000000	0.0500000
25023.0000000	0.0500000
25024.0000000	0.0500000
25025.0000000	0.0504762
25026.0000000	0.0500000
25027.0000000	0.0500000
25028.0000000	0.0500000
25029.0000000	0.0500000
25030.0000000	0.0500000
25031.0000000	0.0500000
25032.0000000	0.0500000
25033.0000000	0.0500000
25034.0000000	0.0500000
25035.0000000	0.0500000
25036.0000000	0.0500000
25037.0000000	0.0500000
25038.0000000	0.0500000
25039.0000000	0.0500000
25040.0000000	0.0495238
25041.0000000	0.0500000
25042.0000000	0.0495000
25043.0000000	0.0500000
25044.0000000	0.0500000
25045.0000000	0.0495238
25046.0000000	0.0500000
25047.0000000	0.0490000
25048.0000000	0.0495238
25049.0000000	0.0500000
25050.0000000	0.0500000
25051.0000000	0.0500000
25052.0000000	0.0500000
25053.0000000	0.0500000
25054.0000000	0.0490000
25055.0000000	0.0500000

25056.0000000	0.0495000
25057.0000000	0.0490476
25058.0000000	0.0495000
25059.0000000	0.0500000
25060.0000000	0.0495000
25061.0000000	0.0471429
25062.0000000	0.0485000
25063.0000000	0.0470000
25064.0000000	0.0471429
25065.0000000	0.0450000
25066.0000000	0.0455000
25067.0000000	0.0460000
25068.0000000	0.0438095
25069.0000000	0.0450000
25070.0000000	0.0438095
25071.0000000	0.0430000
25072.0000000	0.0420000
25073.0000000	0.0419048
25074.0000000	0.0405000
25075.0000000	0.0415000
25076.0000000	0.0409524
25077.0000000	0.0410000
25078.0000000	0.0410000
25079.0000000	0.0433333
25080.0000000	0.0415000
25081.0000000	0.0410000
25082.0000000	0.0405000
25083.0000000	0.0414286
25084.0000000	0.0415000
25085.0000000	0.0409524
25086.0000000	0.0400000
25087.0000000	0.0420000
25088.0000000	0.0414286
25089.0000000	0.0415000
25090.0000000	0.0405000
25091.0000000	0.0400000
25092.0000000	0.0414286
25093.0000000	0.0409524
25094.0000000	0.0415000
25095.0000000	0.0415000
25096.0000000	0.0420000
25097.0000000	0.0409524
25098.0000000	0.0405000
25099.0000000	0.0420000
25100.0000000	0.0414286
25101.0000000	0.0425000
25102.0000000	0.0405000
25103.0000000	0.0428571
25104.0000000	0.0430000
25105.0000000	0.0430000
25106.0000000	0.0438095
25107.0000000	0.0425000
25108.0000000	0.0430000
25109.0000000	0.0428572
25110.0000000	0.0435000
25111.0000000	0.0450000
25112.0000000	0.0438095

25113.0000000	0.0420000
25114.0000000	0.0460000
25115.0000000	0.0423810
25116.0000000	0.0445000
25117.0000000	0.0420000
25118.0000000	0.0447619
25119.0000000	0.0445000
25120.0000000	0.0465000
25121.0000000	0.0455000
25122.0000000	0.0447619
25123.0000000	0.0450000
25124.0000000	0.0461905
25125.0000000	0.0465000
25126.0000000	0.0465000
25127.0000000	0.0460000
25128.0000000	0.0457143
25129.0000000	0.0480000
25130.0000000	0.0460000
25131.0000000	0.0476191
25132.0000000	0.0470000
25133.0000000	0.0452381
25134.0000000	0.0485000
25135.0000000	0.0490000
25136.0000000	0.0476191
25137.0000000	0.0475000
25138.0000000	0.0475000
25139.0000000	0.0475000
25140.0000000	0.0480952
25141.0000000	0.0470000
25142.0000000	0.0485000
25143.0000000	0.0490476
25144.0000000	0.0480000
25145.0000000	0.0475000
25146.0000000	0.0490476
25147.0000000	0.0500000
25148.0000000	0.0495238
25149.0000000	0.0485000
25150.0000000	0.0485000
25151.0000000	0.0495000
25152.0000000	0.0495238
25153.0000000	0.0495000
25154.0000000	0.0500000
25155.0000000	0.0495000
25156.0000000	0.0495000
25157.0000000	0.0500000
25158.0000000	0.0500000
25159.0000000	0.0495000
25160.0000000	0.0495000
25161.0000000	0.0495238
25162.0000000	0.0495000
25163.0000000	0.0495238
25164.0000000	0.0495000
25165.0000000	0.0495000
25166.0000000	0.0495000
25167.0000000	0.0495238
25168.0000000	0.0495000
25169.0000000	0.0495000

25170.0000000	0.0495238
25171.0000000	0.0495000
25172.0000000	0.0495000
25173.0000000	0.0500000
25174.0000000	0.0485000
25175.0000000	0.0495000
25176.0000000	0.0490476
25177.0000000	0.0490000
25178.0000000	0.0495000
25179.0000000	0.0500000
25180.0000000	0.0490000
25181.0000000	0.0490476
25182.0000000	0.0500000
25183.0000000	0.0490000
25184.0000000	0.0500000
25185.0000000	0.0485714
25186.0000000	0.0485000
25187.0000000	0.0490000
25188.0000000	0.0495238
25189.0000000	0.0490000
25190.0000000	0.0495000
25191.0000000	0.0485714
25192.0000000	0.0495000
25193.0000000	0.0495238
25194.0000000	0.0495000
25195.0000000	0.0495000
25196.0000000	0.0500000
25197.0000000	0.0495238
25198.0000000	0.0490000
25199.0000000	0.0475000
25200.0000000	0.0495238
25201.0000000	0.0495000
25202.0000000	0.0485000
25203.0000000	0.0475000
25204.0000000	0.0490476
25205.0000000	0.0495000
25206.0000000	0.0490476
25207.0000000	0.0490000
25208.0000000	0.0480000
25209.0000000	0.0485714
25210.0000000	0.0480000
25211.0000000	0.0475000
25212.0000000	0.0476191
25213.0000000	0.0490000
25214.0000000	0.0470000
25215.0000000	0.0476191
25216.0000000	0.0480000
25217.0000000	0.0480000
25218.0000000	0.0470000
25219.0000000	0.0480952
25220.0000000	0.0485000
25221.0000000	0.0480952
25222.0000000	0.0485000
25223.0000000	0.0480000
25224.0000000	0.0480952
25225.0000000	0.0485000
25226.0000000	0.0485000

25227.0000000	0.0480000
25228.0000000	0.0490476
25229.0000000	0.0480000
25230.0000000	0.0480952
25231.0000000	0.0500000
25232.0000000	0.0475000
25233.0000000	0.0476191
25234.0000000	0.0475000
25235.0000000	0.0480000
25236.0000000	0.0471429
25237.0000000	0.0475000
25238.0000000	0.0480000
25239.0000000	0.0476191
25240.0000000	0.0485000
25241.0000000	0.0480000
25242.0000000	0.0490476
25243.0000000	0.0485000
25244.0000000	0.0475000
25245.0000000	0.0480952
25246.0000000	0.0470000
25247.0000000	0.0485000
25248.0000000	0.0480000
25249.0000000	0.0480952
25250.0000000	0.0480000
25251.0000000	0.0485714
25252.0000000	0.0485000
25253.0000000	0.0476191
25254.0000000	0.0480000
25255.0000000	0.0470000
25256.0000000	0.0500000
25257.0000000	0.0485000
25258.0000000	0.0485714
25259.0000000	0.0490000
25260.0000000	0.0495238
25261.0000000	0.0490000
25262.0000000	0.0495000
25263.0000000	0.0485000
25264.0000000	0.0495238
25265.0000000	0.0495000
25266.0000000	0.0490000
25267.0000000	0.0490476
25268.0000000	0.0495000
25269.0000000	0.0480952
25270.0000000	0.0495000
25271.0000000	0.0495000
25272.0000000	0.0500000
25273.0000000	0.0490476
25274.0000000	0.0495000
25275.0000000	0.0495000
25276.0000000	0.0490476
25277.0000000	0.0490000
25278.0000000	0.0485714
25279.0000000	0.0500000
25280.0000000	0.0500000
25281.0000000	0.0500000
25282.0000000	0.0500000
25283.0000000	0.0490000

25284.0000000	0.0490476
25285.0000000	0.0495000
25286.0000000	0.0500000
25287.0000000	0.0495000
25288.0000000	0.0500000
25289.0000000	0.0495000
25290.0000000	0.0485714
25291.0000000	0.0490000
25292.0000000	0.0500000
25293.0000000	0.0500000
25294.0000000	0.0495238
25295.0000000	0.0485000
25296.0000000	0.0495000
25297.0000000	0.0490476
25298.0000000	0.0490476
25299.0000000	0.0490000
25300.0000000	0.0490000
25301.0000000	0.0490000
25302.0000000	0.0490000
25303.0000000	0.0490476
25304.0000000	0.0495000
25305.0000000	0.0490476
25306.0000000	0.0495000
25307.0000000	0.0495000
25308.0000000	0.0490000
25309.0000000	0.0495238
25310.0000000	0.0490000
25311.0000000	0.0485000
25312.0000000	0.0485714
25313.0000000	0.0485000
25314.0000000	0.0476191
25315.0000000	0.0495000
25316.0000000	0.0475000
25317.0000000	0.0471429
25318.0000000	0.0470000
25319.0000000	0.0480000
25320.0000000	0.0470000
25321.0000000	0.0466667
25322.0000000	0.0470000
25323.0000000	0.0475000
25324.0000000	0.0466667
25325.0000000	0.0455000
25326.0000000	0.0465000
25327.0000000	0.0452381
25328.0000000	0.0470000
25329.0000000	0.0476191
25330.0000000	0.0470000
25331.0000000	0.0465000
25332.0000000	0.0475000
25333.0000000	0.0466667
25334.0000000	0.0470000
25335.0000000	0.0485000
25336.0000000	0.0485714
25337.0000000	0.0475000
25338.0000000	0.0495000
25339.0000000	0.0476191
25340.0000000	0.0475000

25341.0000000	0.0476191
25342.0000000	0.0485000
25343.0000000	0.0475000
25344.0000000	0.0480000
25345.0000000	0.0485714
25346.0000000	0.0465000
25347.0000000	0.0475000
25348.0000000	0.0490476
25349.0000000	0.0470000
25350.0000000	0.0490476
25351.0000000	0.0475000
25352.0000000	0.0485000
25353.0000000	0.0485000
25354.0000000	0.0485000
25355.0000000	0.0471429
25356.0000000	0.0460000
25357.0000000	0.0466667
25358.0000000	0.0475000
25359.0000000	0.0471429
25360.0000000	0.0475000
25361.0000000	0.0480000
25362.0000000	0.0430000
25363.0000000	0.0480000
25364.0000000	0.0466667
25365.0000000	0.0485000
25366.0000000	0.0485714
25367.0000000	0.0485000
25368.0000000	0.0480000
25369.0000000	0.0471429
25370.0000000	0.0480000
25371.0000000	0.0470000
25372.0000000	0.0461905
25373.0000000	0.0460000
25374.0000000	0.0475000
25375.0000000	0.0461905
25376.0000000	0.0450000
25377.0000000	0.0457143
25378.0000000	0.0475000
25379.0000000	0.0480000
25380.0000000	0.0455000
25381.0000000	0.0461905
25382.0000000	0.0465000
25383.0000000	0.0475000
25384.0000000	0.0470000
25385.0000000	0.0457143
25386.0000000	0.0457143
25387.0000000	0.0465000
25388.0000000	0.0455000
25389.0000000	0.0471429
25390.0000000	0.0470000
25391.0000000	0.0480000
25392.0000000	0.0450000
25393.0000000	0.0466667
25394.0000000	0.0440000
25395.0000000	0.0455000
25396.0000000	0.0466667
25397.0000000	0.0465000

25398.0000000	0.0435000
25399.0000000	0.0465000
25400.0000000	0.0471429
25401.0000000	0.0455000
25402.0000000	0.0466667
25403.0000000	0.0450000
25404.0000000	0.0465000
25405.0000000	0.0452381
25406.0000000	0.0440000
25407.0000000	0.0455000
25408.0000000	0.0470000
25409.0000000	0.0442857
25410.0000000	0.0455000
25411.0000000	0.0466667
25412.0000000	0.0445000
25413.0000000	0.0455000
25414.0000000	0.0475000
25415.0000000	0.0461905
25416.0000000	0.0480000
25417.0000000	0.0452381
25418.0000000	0.0460000
25419.0000000	0.0450000
25420.0000000	0.0457143
25421.0000000	0.0465000
25422.0000000	0.0465000
25423.0000000	0.0450000
25424.0000000	0.0466667
25425.0000000	0.0465000
25426.0000000	0.0447619
25427.0000000	0.0470000
25428.0000000	0.0475000
25429.0000000	0.0457143
25430.0000000	0.0460000
25431.0000000	0.0445000
25432.0000000	0.0455000
25433.0000000	0.0471429
25434.0000000	0.0480000
25435.0000000	0.0457143
25436.0000000	0.0485000
25437.0000000	0.0465000
25438.0000000	0.0471429
25439.0000000	0.0475000
25440.0000000	0.0435000
25441.0000000	0.0461905
25442.0000000	0.0450000
25443.0000000	0.0465000
25444.0000000	0.0455000
25445.0000000	0.0442857
25446.0000000	0.0450000
25447.0000000	0.0461905
25448.0000000	0.0455000
25449.0000000	0.0465000
25450.0000000	0.0452381
25451.0000000	0.0450000
25452.0000000	0.0465000
25453.0000000	0.0430000
25454.0000000	0.0438095

25455.0000000	0.0445000
25456.0000000	0.0447619
25457.0000000	0.0435000
25458.0000000	0.0440000
25459.0000000	0.0455000
25460.0000000	0.0442857
25461.0000000	0.0460000
25462.0000000	0.0440000
25463.0000000	0.0419048
25464.0000000	0.0465000
25465.0000000	0.0433333
25466.0000000	0.0420000
25467.0000000	0.0450000
25468.0000000	0.0445000
25469.0000000	0.0457143
25470.0000000	0.0445000
25471.0000000	0.0435000
25472.0000000	0.0438095
25473.0000000	0.0450000
25474.0000000	0.0423810
25475.0000000	0.0445000
25476.0000000	0.0435000
25477.0000000	0.0428571
25478.0000000	0.0430000
25479.0000000	0.0430000
25480.0000000	0.0425000
25481.0000000	0.0419048
25482.0000000	0.0440000
25483.0000000	0.0425000
25484.0000000	0.0414286
25485.0000000	0.0420000
25486.0000000	0.0414286
25487.0000000	0.0400000
25488.0000000	0.0410000
25489.0000000	0.0420000
25490.0000000	0.0409524
25491.0000000	0.0425000
25492.0000000	0.0410000
25493.0000000	0.0414286
25494.0000000	0.0410000
25495.0000000	0.0404762
25496.0000000	0.0415000
25497.0000000	0.0400000
25498.0000000	0.0405000
25499.0000000	0.0414286
25500.0000000	0.0405000
25501.0000000	0.0405000
25502.0000000	0.0419048
25503.0000000	0.0425000
25504.0000000	0.0425000
25505.0000000	0.0433333
25506.0000000	0.0410000
25507.0000000	0.0415000
25508.0000000	0.0419048
25509.0000000	0.0425000
25510.0000000	0.0415000
25511.0000000	0.0423810

25512.0000000	0.0425000
25513.0000000	0.0430000
25514.0000000	0.0433333
25515.0000000	0.0460000
25516.0000000	0.0440000
25517.0000000	0.0442857
25518.0000000	0.0435000
25519.0000000	0.0440000
25520.0000000	0.0465000
25521.0000000	0.0466667
25522.0000000	0.0461905
25523.0000000	0.0465000
25524.0000000	0.0475000
25525.0000000	0.0455000
25526.0000000	0.0471429
25527.0000000	0.0475000
25528.0000000	0.0455000
25529.0000000	0.0480952
25530.0000000	0.0465000
25531.0000000	0.0485000
25532.0000000	0.0480952
25533.0000000	0.0465000
25534.0000000	0.0485000
25535.0000000	0.0480952
25536.0000000	0.0480000
25537.0000000	0.0490000
25538.0000000	0.0485714
25539.0000000	0.0485000
25540.0000000	0.0490000
25541.0000000	0.0485714
25542.0000000	0.0495000
25543.0000000	0.0485000
25544.0000000	0.0495238
25545.0000000	0.0480000
25546.0000000	0.0490000
25547.0000000	0.0476191
25548.0000000	0.0490000
25549.0000000	0.0480000
25550.0000000	0.0490000
25551.0000000	0.0461905
25552.0000000	0.0465000
25553.0000000	0.0476191
25554.0000000	0.0480000
25555.0000000	0.0475000
25556.0000000	0.0476191
25557.0000000	0.0485000
25558.0000000	0.0465000
25559.0000000	0.0470000
25560.0000000	0.0461905
25561.0000000	0.0470000
25562.0000000	0.0466667
25563.0000000	0.0470000
25564.0000000	0.0460000
25565.0000000	0.0447619
25566.0000000	0.0475000
25567.0000000	0.0450000
25568.0000000	0.0460000

25569.0000000	0.0457143
25570.0000000	0.0470000
25571.0000000	0.0480952
25572.0000000	0.0450000
25573.0000000	0.0470000
25574.0000000	0.0461905
25575.0000000	0.0470000
25576.0000000	0.0485000
25577.0000000	0.0452381
25578.0000000	0.0475000
25579.0000000	0.0450000
25580.0000000	0.0465000
25581.0000000	0.0461905
25582.0000000	0.0475000
25583.0000000	0.0471429
25584.0000000	0.0470000
25585.0000000	0.0480000
25586.0000000	0.0461905
25587.0000000	0.0475000
25588.0000000	0.0455000
25589.0000000	0.0480000
25590.0000000	0.0452381
25591.0000000	0.0480000
25592.0000000	0.0476191
25593.0000000	0.0455000
25594.0000000	0.0490000
25595.0000000	0.0460000
25596.0000000	0.0461905
25597.0000000	0.0475000
25598.0000000	0.0480000
25599.0000000	0.0461905
25600.0000000	0.0455000
25601.0000000	0.0457143
25602.0000000	0.0455000
25603.0000000	0.0450000
25604.0000000	0.0465000
25605.0000000	0.0452381
25606.0000000	0.0455000
25607.0000000	0.0455000
25608.0000000	0.0461905
25609.0000000	0.0455000
25610.0000000	0.0476191
25611.0000000	0.0460000
25612.0000000	0.0445000
25613.0000000	0.0457143
25614.0000000	0.0460000
25615.0000000	0.0455000
25616.0000000	0.0460000
25617.0000000	0.0457143
25618.0000000	0.0450000
25619.0000000	0.0450000
25620.0000000	0.0466667
25621.0000000	0.0450000
25622.0000000	0.0457143
25623.0000000	0.0450000
25624.0000000	0.0435000
25625.0000000	0.0450000

25626.0000000	0.0461905
25627.0000000	0.0450000
25628.0000000	0.0440000
25629.0000000	0.0438095
25630.0000000	0.0450000
25631.0000000	0.0442857
25632.0000000	0.0445000
25633.0000000	0.0455000
25634.0000000	0.0450000
25635.0000000	0.0442857
25636.0000000	0.0440000
25637.0000000	0.0447619
25638.0000000	0.0460000
25639.0000000	0.0440000
25640.0000000	0.0460000
25641.0000000	0.0419048
25642.0000000	0.0445000
25643.0000000	0.0435000
25644.0000000	0.0447619
25645.0000000	0.0460000
25646.0000000	0.0447619
25647.0000000	0.0430000
25648.0000000	0.0440000
25649.0000000	0.0430000
25650.0000000	0.0442857
25651.0000000	0.0440000
25652.0000000	0.0440000
25653.0000000	0.0438095
25654.0000000	0.0430000
25655.0000000	0.0450000
25656.0000000	0.0435000
25657.0000000	0.0447619
25658.0000000	0.0455000
25659.0000000	0.0442857
25660.0000000	0.0450000
25661.0000000	0.0450000
25662.0000000	0.0452381
25663.0000000	0.0445000
25664.0000000	0.0455000
25665.0000000	0.0442857
25666.0000000	0.0440000
25667.0000000	0.0435000
25668.0000000	0.0450000
25669.0000000	0.0457143
25670.0000000	0.0460000
25671.0000000	0.0485714
25672.0000000	0.0460000
25673.0000000	0.0475000
25674.0000000	0.0471429
25675.0000000	0.0470000
25676.0000000	0.0460000
25677.0000000	0.0476191
25678.0000000	0.0480000
25679.0000000	0.0480000
25680.0000000	0.0485714
25681.0000000	0.0485000
25682.0000000	0.0471429

25683.0000000	0.0485000
25684.0000000	0.0475000
25685.0000000	0.0485000
25686.0000000	0.0490000
25687.0000000	0.0485714
25688.0000000	0.0490000
25689.0000000	0.0490476
25690.0000000	0.0500000
25691.0000000	0.0485714
25692.0000000	0.0470000
25693.0000000	0.0490000
25694.0000000	0.0480000
25695.0000000	0.0490000
25696.0000000	0.0480952
25697.0000000	0.0485000
25698.0000000	0.0495238
25699.0000000	0.0485000
25700.0000000	0.0485000
25701.0000000	0.0500000
25702.0000000	0.0475000
25703.0000000	0.0475000
25704.0000000	0.0480000
25705.0000000	0.0490476
25706.0000000	0.0495000
25707.0000000	0.0495238
25708.0000000	0.0485000
25709.0000000	0.0495000
25710.0000000	0.0485714
25711.0000000	0.0490000
25712.0000000	0.0485000
25713.0000000	0.0480952
25714.0000000	0.0500000
25715.0000000	0.0485000
25716.0000000	0.0495000
25717.0000000	0.0490476
25718.0000000	0.0485714
25719.0000000	0.0490000
25720.0000000	0.0495000
25721.0000000	0.0490000
25722.0000000	0.0495238
25723.0000000	0.0485000
25724.0000000	0.0495000
25725.0000000	0.0490000
25726.0000000	0.0485714
25727.0000000	0.0500000
25728.0000000	0.0485000
25729.0000000	0.0490000
25730.0000000	0.0475000
25731.0000000	0.0490000
25732.0000000	0.0485714
25733.0000000	0.0475000
25734.0000000	0.0495238
25735.0000000	0.0465000
25736.0000000	0.0475000
25737.0000000	0.0452381
25738.0000000	0.0465000
25739.0000000	0.0425000

25740.0000000	0.0460000
25741.0000000	0.0452381
25742.0000000	0.0445000
25743.0000000	0.0461905
25744.0000000	0.0445000
25745.0000000	0.0485000
25746.0000000	0.0455000
25747.0000000	0.0452381
25748.0000000	0.0465000
25749.0000000	0.0465000
25750.0000000	0.0452381
25751.0000000	0.0475000
25752.0000000	0.0461905
25753.0000000	0.0480000
25754.0000000	0.0480000
25755.0000000	0.0475000
25756.0000000	0.0470000
25757.0000000	0.0471429
25758.0000000	0.0471429
25759.0000000	0.0470000
25760.0000000	0.0445000
25761.0000000	0.0460000
25762.0000000	0.0466667
25763.0000000	0.0455000
25764.0000000	0.0460000
25765.0000000	0.0452381
25766.0000000	0.0465000
25767.0000000	0.0442857
25768.0000000	0.0455000
25769.0000000	0.0480000
25770.0000000	0.0476191
25771.0000000	0.0455000
25772.0000000	0.0480000
25773.0000000	0.0455000
25774.0000000	0.0466667
25775.0000000	0.0465000
25776.0000000	0.0470000
25777.0000000	0.0457143
25778.0000000	0.0460000
25779.0000000	0.0466667
25780.0000000	0.0490000
25781.0000000	0.0440000
25782.0000000	0.0452381
25783.0000000	0.0460000
25784.0000000	0.0480000
25785.0000000	0.0470000
25786.0000000	0.0476191
25787.0000000	0.0485000
25788.0000000	0.0461905
25789.0000000	0.0465000
25790.0000000	0.0465000
25791.0000000	0.0485000
25792.0000000	0.0460000
25793.0000000	0.0471429
25794.0000000	0.0480000
25795.0000000	0.0452381
25796.0000000	0.0455000

25797.0000000	0.0471429
25798.0000000	0.0460000
25799.0000000	0.0465000
25800.0000000	0.0450000
25801.0000000	0.0452381
25802.0000000	0.0470000
25803.0000000	0.0485000
25804.0000000	0.0461905
25805.0000000	0.0460000
25806.0000000	0.0452381
25807.0000000	0.0455000
25808.0000000	0.0430000
25809.0000000	0.0430000
25810.0000000	0.0438095
25811.0000000	0.0425000
25812.0000000	0.0440000
25813.0000000	0.0414286
25814.0000000	0.0430000
25815.0000000	0.0423810
25816.0000000	0.0425000
25817.0000000	0.0420000
25818.0000000	0.0414286
25819.0000000	0.0420000
25820.0000000	0.0410000
25821.0000000	0.0415000
25822.0000000	0.0409524
25823.0000000	0.0405000
25824.0000000	0.0435000
25825.0000000	0.0419048
25826.0000000	0.0415000
25827.0000000	0.0428571
25828.0000000	0.0415000
25829.0000000	0.0430000
25830.0000000	0.0450000
25831.0000000	0.0433333
25832.0000000	0.0440000
25833.0000000	0.0445000
25834.0000000	0.0452381
25835.0000000	0.0445000
25836.0000000	0.0445000
25837.0000000	0.0450000
25838.0000000	0.0423810
25839.0000000	0.0440000
25840.0000000	0.0447619
25841.0000000	0.0455000
25842.0000000	0.0442857
25843.0000000	0.0440000
25844.0000000	0.0435000
25845.0000000	0.0415000
25846.0000000	0.0433333
25847.0000000	0.0420000
25848.0000000	0.0425000
25849.0000000	0.0409524
25850.0000000	0.0410000
25851.0000000	0.0410000
25852.0000000	0.0410000
25853.0000000	0.0409524

25854.0000000	0.0410000
25855.0000000	0.0414286
25856.0000000	0.0415000
25857.0000000	0.0430000
25858.0000000	0.0419048
25859.0000000	0.0410000
25860.0000000	0.0415000
25861.0000000	0.0445000
25862.0000000	0.0419048
25863.0000000	0.0420000
25864.0000000	0.0409524
25865.0000000	0.0410000
25866.0000000	0.0420000
25867.0000000	0.0414286
25868.0000000	0.0400000
25869.0000000	0.0425000
25870.0000000	0.0433333
25871.0000000	0.0420000
25872.0000000	0.0440000
25873.0000000	0.0450000
25874.0000000	0.0419048
25875.0000000	0.0445000
25876.0000000	0.0442857
25877.0000000	0.0460000
25878.0000000	0.0420000
25879.0000000	0.0438095
25880.0000000	0.0440000
25881.0000000	0.0435000
25882.0000000	0.0430000
25883.0000000	0.0433333
25884.0000000	0.0435000
25885.0000000	0.0442857
25886.0000000	0.0425000
25887.0000000	0.0435000
25888.0000000	0.0442857
25889.0000000	0.0440000
25890.0000000	0.0435000
25891.0000000	0.0425000
25892.0000000	0.0438095
25893.0000000	0.0440000
25894.0000000	0.0442857
25895.0000000	0.0430000
25896.0000000	0.0435000
25897.0000000	0.0440000
25898.0000000	0.0428572
25899.0000000	0.0435000
25900.0000000	0.0440000
25901.0000000	0.0423810
25902.0000000	0.0425000
25903.0000000	0.0438095
25904.0000000	0.0430000
25905.0000000	0.0430000
25906.0000000	0.0442857
25907.0000000	0.0430000
25908.0000000	0.0420000
25909.0000000	0.0445000
25910.0000000	0.0428572

25911.0000000	0.0425000
25912.0000000	0.0435000
25913.0000000	0.0428572
25914.0000000	0.0415000
25915.0000000	0.0428572
25916.0000000	0.0450000
25917.0000000	0.0445000
25918.0000000	0.0442857
25919.0000000	0.0425000
25920.0000000	0.0430000
25921.0000000	0.0440000
25922.0000000	0.0419048
25923.0000000	0.0435000
25924.0000000	0.0438095
25925.0000000	0.0440000
25926.0000000	0.0420000
25927.0000000	0.0440000
25928.0000000	0.0442857
25929.0000000	0.0415000
25930.0000000	0.0435000
25931.0000000	0.0419048
25932.0000000	0.0440000
25933.0000000	0.0442857
25934.0000000	0.0420000
25935.0000000	0.0430000
25936.0000000	0.0440000
25937.0000000	0.0447619
25938.0000000	0.0445000
25939.0000000	0.0440000
25940.0000000	0.0433333
25941.0000000	0.0430000
25942.0000000	0.0428572
25943.0000000	0.0450000
25944.0000000	0.0440000
25945.0000000	0.0450000
25946.0000000	0.0447619
25947.0000000	0.0445000
25948.0000000	0.0460000
25949.0000000	0.0447619
25950.0000000	0.0455000
25951.0000000	0.0430000
25952.0000000	0.0452381
25953.0000000	0.0475000
25954.0000000	0.0457143
25955.0000000	0.0435000
25956.0000000	0.0465000
25957.0000000	0.0440000
25958.0000000	0.0442857
25959.0000000	0.0465000
25960.0000000	0.0460000
25961.0000000	0.0475000
25962.0000000	0.0480952
25963.0000000	0.0438095
25964.0000000	0.0470000
25965.0000000	0.0490000
25966.0000000	0.0450000
25967.0000000	0.0466667

25968.0000000	0.0455000
25969.0000000	0.0450000
25970.0000000	0.0471429
25971.0000000	0.0465000
25972.0000000	0.0460000
25973.0000000	0.0452381
25974.0000000	0.0465000
25975.0000000	0.0455000
25976.0000000	0.0461905
25977.0000000	0.0460000
25978.0000000	0.0450000
25979.0000000	0.0466667
25980.0000000	0.0445000
25981.0000000	0.0430000
25982.0000000	0.0433333
25983.0000000	0.0440000
25984.0000000	0.0420000
25985.0000000	0.0423810
25986.0000000	0.0435000
25987.0000000	0.0445000
25988.0000000	0.0415000
25989.0000000	0.0428571
25990.0000000	0.0425000
25991.0000000	0.0438095
25992.0000000	0.0430000
25993.0000000	0.0410000
25994.0000000	0.0438095
25995.0000000	0.0440000
25996.0000000	0.0420000
25997.0000000	0.0420000
25998.0000000	0.0419048
25999.0000000	0.0430000
26000.0000000	0.0425000
26001.0000000	0.0433333
26002.0000000	0.0445000
26003.0000000	0.0457143
26004.0000000	0.0435000
26005.0000000	0.0430000
26006.0000000	0.0433333
26007.0000000	0.0465000
26008.0000000	0.0430000
26009.0000000	0.0455000
26010.0000000	0.0447619
26011.0000000	0.0420000
26012.0000000	0.0457143
26013.0000000	0.0440000
26014.0000000	0.0460000
26015.0000000	0.0433333
26016.0000000	0.0465000
26017.0000000	0.0435000
26018.0000000	0.0460000
26019.0000000	0.0433333
26020.0000000	0.0435000
26021.0000000	0.0457143
26022.0000000	0.0465000
26023.0000000	0.0457143
26024.0000000	0.0440000

26025.0000000	0.0450000
26026.0000000	0.0445000
26027.0000000	0.0450000
26028.0000000	0.0452381
26029.0000000	0.0445000
26030.0000000	0.0447619
26031.0000000	0.0445000
26032.0000000	0.0450000
26033.0000000	0.0455000
26034.0000000	0.0433333
26035.0000000	0.0445000
26036.0000000	0.0445000
26037.0000000	0.0447619
26038.0000000	0.0430000
26039.0000000	0.0447619
26040.0000000	0.0435000
26041.0000000	0.0425000
26042.0000000	0.0442857
26043.0000000	0.0440000
26044.0000000	0.0420000
26045.0000000	0.0415000
26046.0000000	0.0428572
26047.0000000	0.0415000
26048.0000000	0.0420000
26049.0000000	0.0414286
26050.0000000	0.0420000
26051.0000000	0.0409524
26052.0000000	0.0400000
26053.0000000	0.0405000
26054.0000000	0.0414286
26055.0000000	0.0400000
26056.0000000	0.0400000
26057.0000000	0.0400000
26058.0000000	0.0409524
26059.0000000	0.0400000
26060.0000000	0.0405000
26061.0000000	0.0405000
26062.0000000	0.0410000
26063.0000000	0.0405000
26064.0000000	0.0400000
26065.0000000	0.0410000
26066.0000000	0.0400000
26067.0000000	0.0404762
26068.0000000	0.0410000
26069.0000000	0.0404762
26070.0000000	0.0400000
26071.0000000	0.0405000
26072.0000000	0.0405000
26073.0000000	0.0414286
26074.0000000	0.0400000
26075.0000000	0.0400000
26076.0000000	0.0400000
26077.0000000	0.0405000
26078.0000000	0.0400000
26079.0000000	0.0404762
26080.0000000	0.0410000
26081.0000000	0.0410000

26082.0000000	0.0414286
26083.0000000	0.0420000
26084.0000000	0.0400000
26085.0000000	0.0400000
26086.0000000	0.0405000
26087.0000000	0.0410000
26088.0000000	0.0405000
26089.0000000	0.0414286
26090.0000000	0.0410000
26091.0000000	0.0414286
26092.0000000	0.0425000
26093.0000000	0.0420000
26094.0000000	0.0404762
26095.0000000	0.0410000
26096.0000000	0.0405000
26097.0000000	0.0415000
26098.0000000	0.0409524
26099.0000000	0.0419048
26100.0000000	0.0430000
26101.0000000	0.0425000
26102.0000000	0.0405000
26103.0000000	0.0419048
26104.0000000	0.0420000
26105.0000000	0.0415000
26106.0000000	0.0409524
26107.0000000	0.0415000
26108.0000000	0.0410000
26109.0000000	0.0423810
26110.0000000	0.0410000
26111.0000000	0.0419048
26112.0000000	0.0425000
26113.0000000	0.0420000
26114.0000000	0.0420000
26115.0000000	0.0419048
26116.0000000	0.0430000
26117.0000000	0.0420000
26118.0000000	0.0414286
26119.0000000	0.0415000
26120.0000000	0.0414286
26121.0000000	0.0410000
26122.0000000	0.0415000
26123.0000000	0.0415000
26124.0000000	0.0405000
26125.0000000	0.0409524
26126.0000000	0.0410000
26127.0000000	0.0404762
26128.0000000	0.0415000
26129.0000000	0.0409524
26130.0000000	0.0410000
26131.0000000	0.0400000
26132.0000000	0.0410000
26133.0000000	0.0405000
26134.0000000	0.0409524
26135.0000000	0.0415000
26136.0000000	0.0400000
26137.0000000	0.0405000
26138.0000000	0.0405000

26139.0000000	0.0400000
26140.0000000	0.0400000
26141.0000000	0.0415000
26142.0000000	0.0404762
26143.0000000	0.0405000
26144.0000000	0.0410000
26145.0000000	0.0404762
26146.0000000	0.0400000
26147.0000000	0.0404762
26148.0000000	0.0400000
26149.0000000	0.0420000
26150.0000000	0.0405000
26151.0000000	0.0409524
26152.0000000	0.0425000
26153.0000000	0.0415000
26154.0000000	0.0409524
26155.0000000	0.0410000
26156.0000000	0.0405000
26157.0000000	0.0404762
26158.0000000	0.0410000
26159.0000000	0.0404762
26160.0000000	0.0425000
26161.0000000	0.0405000
26162.0000000	0.0410000
26163.0000000	0.0414286
26164.0000000	0.0400000
26165.0000000	0.0410000
26166.0000000	0.0404762
26167.0000000	0.0410000
26168.0000000	0.0405000
26169.0000000	0.0405000
26170.0000000	0.0404762
26171.0000000	0.0405000
26172.0000000	0.0400000
26173.0000000	0.0400000
26174.0000000	0.0400000
26175.0000000	0.0404762
26176.0000000	0.0410000
26177.0000000	0.0400000
26178.0000000	0.0405000
26179.0000000	0.0409524
26180.0000000	0.0405000
26181.0000000	0.0400000
26182.0000000	0.0410000
26183.0000000	0.0410000
26184.0000000	0.0420000
26185.0000000	0.0405000
26186.0000000	0.0400000
26187.0000000	0.0404762
26188.0000000	0.0405000
26189.0000000	0.0400000
26190.0000000	0.0400000
26191.0000000	0.0410000
26192.0000000	0.0405000
26193.0000000	0.0405000
26194.0000000	0.0404762
26195.0000000	0.0400000

26196.0000000	0.0404762
26197.0000000	0.0405000
26198.0000000	0.0405000
26199.0000000	0.0409524
26200.0000000	0.0405000
26201.0000000	0.0410000
26202.0000000	0.0405000
26203.0000000	0.0409524
26204.0000000	0.0405000
26205.0000000	0.0410000
26206.0000000	0.0414286
26207.0000000	0.0420000
26208.0000000	0.0400000
26209.0000000	0.0416000
26210.0000000	0.0400000
26211.0000000	0.0409524
26212.0000000	0.0400000
26213.0000000	0.0400000
26214.0000000	0.0400000
26215.0000000	0.0409524
26216.0000000	0.0400000
26217.0000000	0.0409524
26218.0000000	0.0405000
26219.0000000	0.0400000
26220.0000000	0.0404762
26221.0000000	0.0400000
26222.0000000	0.0400000
26223.0000000	0.0400000
26224.0000000	0.0400000
26225.0000000	0.0400000
26226.0000000	0.0400000
26227.0000000	0.0400000
26228.0000000	0.0400000
26229.0000000	0.0400000
26230.0000000	0.0400000
26231.0000000	0.0400000
26232.0000000	0.0405000
26233.0000000	0.0400000
26234.0000000	0.0400000
26235.0000000	0.0400000
26236.0000000	0.0405000
26237.0000000	0.0400000
26238.0000000	0.0400000
26239.0000000	0.0400000
26240.0000000	0.0400000
26241.0000000	0.0405000
26242.0000000	0.0400000
26243.0000000	0.0400000
26244.0000000	0.0400000
26245.0000000	0.0400000
26246.0000000	0.0400000
26247.0000000	0.0400000
26248.0000000	0.0400000
26249.0000000	0.0400000
26250.0000000	0.0400000
26251.0000000	0.0400000
26252.0000000	0.0400000

26253.0000000	0.0400000
26254.0000000	0.0404762
26255.0000000	0.0400000
26256.0000000	0.0400000
26257.0000000	0.0400000
26258.0000000	0.0400000
26259.0000000	0.0400000
26260.0000000	0.0400000
26261.0000000	0.0400000
26262.0000000	0.0400000
26263.0000000	0.0400000
26264.0000000	0.0400000
26265.0000000	0.0400000
26266.0000000	0.0400000
26267.0000000	0.0400000
26268.0000000	0.0400000
26269.0000000	0.0400000
26270.0000000	0.0400000
26271.0000000	0.0400000
26272.0000000	0.0400000
26273.0000000	0.0400000
26274.0000000	0.0400000
26275.0000000	0.0400000
26276.0000000	0.0400000
26277.0000000	0.0400000
26278.0000000	0.0400000
26279.0000000	0.0400000
26280.0000000	0.0400000
26281.0000000	0.0400000
26282.0000000	0.0400000
26283.0000000	0.0400000
26284.0000000	0.0400000
26285.0000000	0.0400000
26286.0000000	0.0400000
26287.0000000	0.0400000
26288.0000000	0.0400000
26289.0000000	0.0395000
26290.0000000	0.0400000
26291.0000000	0.0400000
26292.0000000	0.0400000
26293.0000000	0.0400000
26294.0000000	0.0400000
26295.0000000	0.0395238
26296.0000000	0.0400000
26297.0000000	0.0395000
26298.0000000	0.0395000
26299.0000000	0.0400000
26300.0000000	0.0390000
26301.0000000	0.0390000
26302.0000000	0.0390000
26303.0000000	0.0385714
26304.0000000	0.0390000
26305.0000000	0.0390476
26306.0000000	0.0390000
26307.0000000	0.0395000
26308.0000000	0.0395238
26309.0000000	0.0395000

26310.0000000	0.0395000
26311.0000000	0.0390476
26312.0000000	0.0395000
26313.0000000	0.0385000
26314.0000000	0.0390476
26315.0000000	0.0390000
26316.0000000	0.0380000
26317.0000000	0.0380952
26318.0000000	0.0395000
26319.0000000	0.0400000
26320.0000000	0.0390000
26321.0000000	0.0395238
26322.0000000	0.0400000
26323.0000000	0.0385714
26324.0000000	0.0370000
26325.0000000	0.0390000
26326.0000000	0.0395238
26327.0000000	0.0395000
26328.0000000	0.0385000
26329.0000000	0.0390000
26330.0000000	0.0380952
26331.0000000	0.0390000
26332.0000000	0.0385714
26333.0000000	0.0380000
26334.0000000	0.0380000
26335.0000000	0.0390476
26336.0000000	0.0375000
26337.0000000	0.0375000
26338.0000000	0.0360000
26339.0000000	0.0380952
26340.0000000	0.0390000
26341.0000000	0.0385714
26342.0000000	0.0380000
26343.0000000	0.0350000
26344.0000000	0.0371429
26345.0000000	0.0385000
26346.0000000	0.0360000
26347.0000000	0.0357143
26348.0000000	0.0350000
26349.0000000	0.0365000
26350.0000000	0.0350000
26351.0000000	0.0366667
26352.0000000	0.0355000
26353.0000000	0.0352381
26354.0000000	0.0355000
26355.0000000	0.0340000
26356.0000000	0.0338095
26357.0000000	0.0335000
26358.0000000	0.0335000
26359.0000000	0.0314286
26360.0000000	0.0325000
26361.0000000	0.0330000
26362.0000000	0.0323809
26363.0000000	0.0325000
26364.0000000	0.0305000
26365.0000000	0.0325000
26366.0000000	0.0352381

26367.0000000	0.0325000
26368.0000000	0.0323809
26369.0000000	0.0335000
26370.0000000	0.0335000
26371.0000000	0.0323809
26372.0000000	0.0320000
26373.0000000	0.0345000
26374.0000000	0.0335000
26375.0000000	0.0314286
26376.0000000	0.0325000
26377.0000000	0.0325000
26378.0000000	0.0319048
26379.0000000	0.0305000
26380.0000000	0.0325000
26381.0000000	0.0314286
26382.0000000	0.0325000
26383.0000000	0.0338095
26384.0000000	0.0335000
26385.0000000	0.0325000
26386.0000000	0.0325000
26387.0000000	0.0338095
26388.0000000	0.0320000
26389.0000000	0.0320000
26390.0000000	0.0325000
26391.0000000	0.0323809
26392.0000000	0.0323809
26393.0000000	0.0325000
26394.0000000	0.0345000
26395.0000000	0.0335000
26396.0000000	0.0304762
26397.0000000	0.0315000
26398.0000000	0.0320000
26399.0000000	0.0309524
26400.0000000	0.0320000
26401.0000000	0.0314286
26402.0000000	0.0320000
26403.0000000	0.0345000
26404.0000000	0.0310000
26405.0000000	0.0333333
26406.0000000	0.0330000
26407.0000000	0.0335000
26408.0000000	0.0304762
26409.0000000	0.0325000
26410.0000000	0.0325000
26411.0000000	0.0314286
26412.0000000	0.0320000
26413.0000000	0.0325000
26414.0000000	0.0323810
26415.0000000	0.0320000
26416.0000000	0.0323809
26417.0000000	0.0325000
26418.0000000	0.0325000
26419.0000000	0.0320000
26420.0000000	0.0319048
26421.0000000	0.0330000
26422.0000000	0.0330000
26423.0000000	0.0328571

26424.0000000	0.0335000
26425.0000000	0.0310000
26426.0000000	0.0310000
26427.0000000	0.0323809
26428.0000000	0.0325000
26429.0000000	0.0333333
26430.0000000	0.0310000
26431.0000000	0.0320000
26432.0000000	0.0304762
26433.0000000	0.0325000
26434.0000000	0.0310000
26435.0000000	0.0314286
26436.0000000	0.0305000
26437.0000000	0.0320000
26438.0000000	0.0310000
26439.0000000	0.0300000
26440.0000000	0.0315000
26441.0000000	0.0309524
26442.0000000	0.0320000
26443.0000000	0.0310000
26444.0000000	0.0300000
26445.0000000	0.0310000
26446.0000000	0.0305000
26447.0000000	0.0314286
26448.0000000	0.0310000
26449.0000000	0.0305000
26450.0000000	0.0300000
26451.0000000	0.0314286
26452.0000000	0.0309524
26453.0000000	0.0315000
26454.0000000	0.0310000
26455.0000000	0.0305000
26456.0000000	0.0300000
26457.0000000	0.0304762
26458.0000000	0.0300000
26459.0000000	0.0304762
26460.0000000	0.0300000
26461.0000000	0.0304762
26462.0000000	0.0300000
26463.0000000	0.0300000
26464.0000000	0.0300000
26465.0000000	0.0300000
26466.0000000	0.0300000
26467.0000000	0.0300000
26468.0000000	0.0304762
26469.0000000	0.0300000
26470.0000000	0.0300000
26471.0000000	0.0300000
26472.0000000	0.0300000
26473.0000000	0.0300000
26474.0000000	0.0300000
26475.0000000	0.0300000
26476.0000000	0.0300000
26477.0000000	0.0300000
26478.0000000	0.0300000
26479.0000000	0.0300000
26480.0000000	0.0300000

26481.0000000	0.0300000
26482.0000000	0.0300000
26483.0000000	0.0300000
26484.0000000	0.0295000
26485.0000000	0.0300000
26486.0000000	0.0300000
26487.0000000	0.0300000
26488.0000000	0.0295000
26489.0000000	0.0300000
26490.0000000	0.0300000
26491.0000000	0.0300000
26492.0000000	0.0300000
26493.0000000	0.0300000
26494.0000000	0.0300000
26495.0000000	0.0300000
26496.0000000	0.0300000
26497.0000000	0.0300000
26498.0000000	0.0300000
26499.0000000	0.0300000
26500.0000000	0.0300000
26501.0000000	0.0300000
26502.0000000	0.0300000
26503.0000000	0.0300000
26504.0000000	0.0300000
26505.0000000	0.0300000
26506.0000000	0.0300000
26507.0000000	0.0300000
26508.0000000	0.0300000
26509.0000000	0.0300000
26510.0000000	0.0300000
26511.0000000	0.0300000
26512.0000000	0.0300000
26513.0000000	0.0300000
26514.0000000	0.0300000
26515.0000000	0.0300000
26516.0000000	0.0300000
26517.0000000	0.0305000
26518.0000000	0.0304762
26519.0000000	0.0300000
26520.0000000	0.0300000
26521.0000000	0.0300000
26522.0000000	0.0304545
26523.0000000	0.0300000
26524.0000000	0.0300000
26525.0000000	0.0300000
26526.0000000	0.0310000
26527.0000000	0.0300000
26528.0000000	0.0300000
26529.0000000	0.0300000
26530.0000000	0.0300000
26531.0000000	0.0300000
26532.0000000	0.0300000
26533.0000000	0.0300000
26534.0000000	0.0300000
26535.0000000	0.0300000
26536.0000000	0.0300000
26537.0000000	0.0300000

26538.0000000	0.0300000
26539.0000000	0.0300000
26540.0000000	0.0300000
26541.0000000	0.0300000
26542.0000000	0.0305000
26543.0000000	0.0300000
26544.0000000	0.0300000
26545.0000000	0.0300000
26546.0000000	0.0300000
26547.0000000	0.0304762
26548.0000000	0.0300000
26549.0000000	0.0300000
26550.0000000	0.0300000
26551.0000000	0.0300000
26552.0000000	0.0300000
26553.0000000	0.0300000
26554.0000000	0.0300000
26555.0000000	0.0300000
26556.0000000	0.0300000
26557.0000000	0.0300000
26558.0000000	0.0300000
26559.0000000	0.0300000
26560.0000000	0.0300000
26561.0000000	0.0300000
26562.0000000	0.0300000
26563.0000000	0.0300000
26564.0000000	0.0300000
26565.0000000	0.0300000
26566.0000000	0.0300000
26567.0000000	0.0300000
26568.0000000	0.0300000
26569.0000000	0.0300000
26570.0000000	0.0300000
26571.0000000	0.0295238
26572.0000000	0.0300000
26573.0000000	0.0300000
26574.0000000	0.0304762
26575.0000000	0.0300000
26576.0000000	0.0305000
26577.0000000	0.0300000
26578.0000000	0.0300000
26579.0000000	0.0300000
26580.0000000	0.0300000
26581.0000000	0.0300000
26582.0000000	0.0300000
26583.0000000	0.0300000
26584.0000000	0.0305000
26585.0000000	0.0300000
26586.0000000	0.0300000
26587.0000000	0.0300000
26588.0000000	0.0300000
26589.0000000	0.0300000
26590.0000000	0.0300000
26591.0000000	0.0300000
26592.0000000	0.0300000
26593.0000000	0.0300000
26594.0000000	0.0300000

26595.0000000	0.0300000
26596.0000000	0.0300000
26597.0000000	0.0300000
26598.0000000	0.0300000
26599.0000000	0.0305000
26600.0000000	0.0300000
26601.0000000	0.0300000
26602.0000000	0.0300000
26603.0000000	0.0300000
26604.0000000	0.0300000
26605.0000000	0.0300000
26606.0000000	0.0300000
26607.0000000	0.0300000
26608.0000000	0.0300000
26609.0000000	0.0300000
26610.0000000	0.0300000
26611.0000000	0.0300000
26612.0000000	0.0300000
26613.0000000	0.0300000
26614.0000000	0.0295000
26615.0000000	0.0295000
26616.0000000	0.0300000
26617.0000000	0.0300000
26618.0000000	0.0300000
26619.0000000	0.0300000
26620.0000000	0.0300000
26621.0000000	0.0300000
26622.0000000	0.0300000
26623.0000000	0.0300000
26624.0000000	0.0300000
26625.0000000	0.0300000
26626.0000000	0.0300000
26627.0000000	0.0300000
26628.0000000	0.0300000
26629.0000000	0.0300000
26630.0000000	0.0300000
26631.0000000	0.0300000
26632.0000000	0.0300000
26633.0000000	0.0300000
26634.0000000	0.0300000
26635.0000000	0.0300000
26636.0000000	0.0300000
26637.0000000	0.0300000
26638.0000000	0.0300000
26639.0000000	0.0300000
26640.0000000	0.0300000
26641.0000000	0.0300000
26642.0000000	0.0300000
26643.0000000	0.0300000
26644.0000000	0.0300000
26645.0000000	0.0305000
26646.0000000	0.0300000
26647.0000000	0.0300000
26648.0000000	0.0305000
26649.0000000	0.0300000
26650.0000000	0.0305000
26651.0000000	0.0300000

26652.0000000	0.0300000
26653.0000000	0.0300000
26654.0000000	0.0300000
26655.0000000	0.0300000
26656.0000000	0.0300000
26657.0000000	0.0300000
26658.0000000	0.0300000
26659.0000000	0.0300000
26660.0000000	0.0300000
26661.0000000	0.0300000
26662.0000000	0.0300000
26663.0000000	0.0300000
26664.0000000	0.0300000
26665.0000000	0.0300000
26666.0000000	0.0300000
26667.0000000	0.0300000
26668.0000000	0.0300000
26669.0000000	0.0300000
26670.0000000	0.0300000
26671.0000000	0.0304762
26672.0000000	0.0300000
26673.0000000	0.0300000
26674.0000000	0.0300000
26675.0000000	0.0300000
26676.0000000	0.0295238
26677.0000000	0.0300000
26678.0000000	0.0295000
26679.0000000	0.0300000
26680.0000000	0.0300000
26681.0000000	0.0295000
26682.0000000	0.0300000
26683.0000000	0.0300000
26684.0000000	0.0295000
26685.0000000	0.0300000
26686.0000000	0.0300000
26687.0000000	0.0300000
26688.0000000	0.0300000
26689.0000000	0.0300000
26690.0000000	0.0300000
26691.0000000	0.0295000
26692.0000000	0.0295238
26693.0000000	0.0300000
26694.0000000	0.0300000
26695.0000000	0.0300000
26696.0000000	0.0300000
26697.0000000	0.0300000
26698.0000000	0.0300000
26699.0000000	0.0300000
26700.0000000	0.0300000
26701.0000000	0.0300000
26702.0000000	0.0300000
26703.0000000	0.0300000
26704.0000000	0.0300000
26705.0000000	0.0300000
26706.0000000	0.0300000
26707.0000000	0.0300000
26708.0000000	0.0300000

26709.0000000	0.0300000
26710.0000000	0.0300000
26711.0000000	0.0300000
26712.0000000	0.0300000
26713.0000000	0.0300000
26714.0000000	0.0300000
26715.0000000	0.0300000
26716.0000000	0.0300000
26717.0000000	0.0300000
26718.0000000	0.0300000
26719.0000000	0.0300000
26720.0000000	0.0300000
26721.0000000	0.0300000
26722.0000000	0.0300000
26723.0000000	0.0300000
26724.0000000	0.0300000
26725.0000000	0.0300000
26726.0000000	0.0300000
26727.0000000	0.0300000
26728.0000000	0.0300000
26729.0000000	0.0295000
26730.0000000	0.0300000
26731.0000000	0.0300000
26732.0000000	0.0300000
26733.0000000	0.0300000
26734.0000000	0.0305000
26735.0000000	0.0300000
26736.0000000	0.0305000
26737.0000000	0.0300000
26738.0000000	0.0300000
26739.0000000	0.0300000
26740.0000000	0.0300000
26741.0000000	0.0300000
26742.0000000	0.0300000
26743.0000000	0.0300000
26744.0000000	0.0300000
26745.0000000	0.0300000
26746.0000000	0.0300000
26747.0000000	0.0300000
26748.0000000	0.0300000
26749.0000000	0.0300000
26750.0000000	0.0300000
26751.0000000	0.0300000
26752.0000000	0.0300000
26753.0000000	0.0300000
26754.0000000	0.0300000
26755.0000000	0.0300000
26756.0000000	0.0300000
26757.0000000	0.0300000
26758.0000000	0.0300000
26759.0000000	0.0300000
26760.0000000	0.0300000
26761.0000000	0.0300000
26762.0000000	0.0300000
26763.0000000	0.0300000
26764.0000000	0.0300000
26765.0000000	0.0300000

26766.0000000	0.0300000
26767.0000000	0.0300000
26768.0000000	0.0300000
26769.0000000	0.0300000
26770.0000000	0.0300000
26771.0000000	0.0300000
26772.0000000	0.0300000
26773.0000000	0.0300000
26774.0000000	0.0300000
26775.0000000	0.0305000
26776.0000000	0.0300000
26777.0000000	0.0295000
26778.0000000	0.0300000
26779.0000000	0.0300000
26780.0000000	0.0300000
26781.0000000	0.0300000
26782.0000000	0.0300000
26783.0000000	0.0300000
26784.0000000	0.0300000
26785.0000000	0.0300000
26786.0000000	0.0300000
26787.0000000	0.0300000
26788.0000000	0.0295238
26789.0000000	0.0300000
26790.0000000	0.0300000
26791.0000000	0.0300000
26792.0000000	0.0300000
26793.0000000	0.0300000
26794.0000000	0.0300000
26795.0000000	0.0300000
26796.0000000	0.0300000
26797.0000000	0.0295238
26798.0000000	0.0300000
26799.0000000	0.0300000
26800.0000000	0.0300000
26801.0000000	0.0295000
26802.0000000	0.0300000
26803.0000000	0.0300000
26804.0000000	0.0300000
26805.0000000	0.0300000
26806.0000000	0.0300000
26807.0000000	0.0300000
26808.0000000	0.0300000
26809.0000000	0.0300000
26810.0000000	0.0300000
26811.0000000	0.0300000
26812.0000000	0.0300000
26813.0000000	0.0295000
26814.0000000	0.0300000
26815.0000000	0.0300000
26816.0000000	0.0300000
26817.0000000	0.0300000
26818.0000000	0.0300000
26819.0000000	0.0295238
26820.0000000	0.0300000
26821.0000000	0.0295238
26822.0000000	0.0300000

26823.0000000	0.0300000
26824.0000000	0.0300000
26825.0000000	0.0300000
26826.0000000	0.0305000
26827.0000000	0.0300000
26828.0000000	0.0300000
26829.0000000	0.0300000
26830.0000000	0.0300000
26831.0000000	0.0300000
26832.0000000	0.0300000
26833.0000000	0.0305000
26834.0000000	0.0300000
26835.0000000	0.0305000
26836.0000000	0.0300000
26837.0000000	0.0300000
26838.0000000	0.0300000
26839.0000000	0.0300000
26840.0000000	0.0300000
26841.0000000	0.0300000
26842.0000000	0.0300000
26843.0000000	0.0300000
26844.0000000	0.0300000
26845.0000000	0.0300000
26846.0000000	0.0300000
26847.0000000	0.0300000
26848.0000000	0.0300000
26849.0000000	0.0300000
26850.0000000	0.0300000
26851.0000000	0.0300000
26852.0000000	0.0300000
26853.0000000	0.0300000
26854.0000000	0.0300000
26855.0000000	0.0300000
26856.0000000	0.0300000
26857.0000000	0.0305000
26858.0000000	0.0300000
26859.0000000	0.0300000
26860.0000000	0.0300000
26861.0000000	0.0300000
26862.0000000	0.0295000
26863.0000000	0.0300000
26864.0000000	0.0300000
26865.0000000	0.0300000
26866.0000000	0.0300000
26867.0000000	0.0300000
26868.0000000	0.0300000
26869.0000000	0.0300000
26870.0000000	0.0300000
26871.0000000	0.0300000
26872.0000000	0.0300000
26873.0000000	0.0300000
26874.0000000	0.0295000
26875.0000000	0.0300000
26876.0000000	0.0300000
26877.0000000	0.0300000
26878.0000000	0.0295000
26879.0000000	0.0300000

26880.0000000	0.0300000
26881.0000000	0.0300000
26882.0000000	0.0300000
26883.0000000	0.0295000
26884.0000000	0.0295238
26885.0000000	0.0295000
26886.0000000	0.0295000
26887.0000000	0.0300000
26888.0000000	0.0300000
26889.0000000	0.0290000
26890.0000000	0.0300000
26891.0000000	0.0300000
26892.0000000	0.0290000
26893.0000000	0.0295000
26894.0000000	0.0300000
26895.0000000	0.0290476
26896.0000000	0.0300000
26897.0000000	0.0300000
26898.0000000	0.0295000
26899.0000000	0.0300000
26900.0000000	0.0295000
26901.0000000	0.0300000
26902.0000000	0.0300000
26903.0000000	0.0300000
26904.0000000	0.0300000
26905.0000000	0.0300000
26906.0000000	0.0300000
26907.0000000	0.0295000
26908.0000000	0.0300000
26909.0000000	0.0300000
26910.0000000	0.0300000
26911.0000000	0.0300000
26912.0000000	0.0300000
26913.0000000	0.0300000
26914.0000000	0.0300000
26915.0000000	0.0300000
26916.0000000	0.0300000
26917.0000000	0.0300000
26918.0000000	0.0295238
26919.0000000	0.0300000
26920.0000000	0.0300000
26921.0000000	0.0300000
26922.0000000	0.0300000
26923.0000000	0.0300000
26924.0000000	0.0300000
26925.0000000	0.0300000
26926.0000000	0.0300000
26927.0000000	0.0300000
26928.0000000	0.0300000
26929.0000000	0.0300000
26930.0000000	0.0300000
26931.0000000	0.0300000
26932.0000000	0.0300000
26933.0000000	0.0300000
26934.0000000	0.0300000
26935.0000000	0.0300000
26936.0000000	0.0304762

26937.0000000	0.0300000
26938.0000000	0.0300000
26939.0000000	0.0300000
26940.0000000	0.0300000
26941.0000000	0.0300000
26942.0000000	0.0300000
26943.0000000	0.0300000
26944.0000000	0.0300000
26945.0000000	0.0300000
26946.0000000	0.0300000
26947.0000000	0.0300000
26948.0000000	0.0300000
26949.0000000	0.0295000
26950.0000000	0.0305000
26951.0000000	0.0300000
26952.0000000	0.0300000
26953.0000000	0.0300000
26954.0000000	0.0300000
26955.0000000	0.0295000
26956.0000000	0.0300000
26957.0000000	0.0300000
26958.0000000	0.0300000
26959.0000000	0.0300000
26960.0000000	0.0300000
26961.0000000	0.0295000
26962.0000000	0.0295000
26963.0000000	0.0300000
26964.0000000	0.0295238
26965.0000000	0.0295000
26966.0000000	0.0300000
26967.0000000	0.0300000
26968.0000000	0.0300000
26969.0000000	0.0295238
26970.0000000	0.0295000
26971.0000000	0.0290000
26972.0000000	0.0295238
26973.0000000	0.0300000
26974.0000000	0.0285000
26975.0000000	0.0300000
26976.0000000	0.0300000
26977.0000000	0.0290000
26978.0000000	0.0290476
26979.0000000	0.0295000
26980.0000000	0.0295000
26981.0000000	0.0290476
26982.0000000	0.0300000
26983.0000000	0.0300000
26984.0000000	0.0285000
26985.0000000	0.0295238
26986.0000000	0.0295000
26987.0000000	0.0300000
26988.0000000	0.0290000
26989.0000000	0.0295238
26990.0000000	0.0295000
26991.0000000	0.0300000
26992.0000000	0.0295000
26993.0000000	0.0300000

26994.0000000	0.0290476
26995.0000000	0.0295000
26996.0000000	0.0295238
26997.0000000	0.0295000
26998.0000000	0.0300000
26999.0000000	0.0295000
27000.0000000	0.0300000
27001.0000000	0.0300000
27002.0000000	0.0295000
27003.0000000	0.0300000
27004.0000000	0.0300000
27005.0000000	0.0290476
27006.0000000	0.0300000
27007.0000000	0.0300000
27008.0000000	0.0300000
27009.0000000	0.0295238
27010.0000000	0.0300000
27011.0000000	0.0300000
27012.0000000	0.0295238
27013.0000000	0.0295000
27014.0000000	0.0300000
27015.0000000	0.0300000
27016.0000000	0.0300000
27017.0000000	0.0300000
27018.0000000	0.0290000
27019.0000000	0.0300000
27020.0000000	0.0295000
27021.0000000	0.0300000
27022.0000000	0.0300000
27023.0000000	0.0300000
27024.0000000	0.0285714
27025.0000000	0.0300000
27026.0000000	0.0295454
27027.0000000	0.0300000
27028.0000000	0.0300000
27029.0000000	0.0300000
27030.0000000	0.0300000
27031.0000000	0.0300000
27032.0000000	0.0300000
27033.0000000	0.0295238
27034.0000000	0.0300000
27035.0000000	0.0300000
27036.0000000	0.0290000
27037.0000000	0.0290000
27038.0000000	0.0300000
27039.0000000	0.0300000
27040.0000000	0.0295000
27041.0000000	0.0300000
27042.0000000	0.0300000
27043.0000000	0.0300000
27044.0000000	0.0300000
27045.0000000	0.0295238
27046.0000000	0.0290000
27047.0000000	0.0300000
27048.0000000	0.0285714
27049.0000000	0.0285000
27050.0000000	0.0300000

27051.0000000	0.0280000
27052.0000000	0.0285000
27053.0000000	0.0280000
27054.0000000	0.0275000
27055.0000000	0.0295238
27056.0000000	0.0275000
27057.0000000	0.0271429
27058.0000000	0.0290000
27059.0000000	0.0295000
27060.0000000	0.0271429
27061.0000000	0.0290000
27062.0000000	0.0265000
27063.0000000	0.0285000
27064.0000000	0.0276190
27065.0000000	0.0280952
27066.0000000	0.0255000
27067.0000000	0.0265000
27068.0000000	0.0275000
27069.0000000	0.0266667
27070.0000000	0.0275000
27071.0000000	0.0270000
27072.0000000	0.0242857
27073.0000000	0.0265000
27074.0000000	0.0270000
27075.0000000	0.0266667
27076.0000000	0.0275000
27077.0000000	0.0280000
27078.0000000	0.0252381
27079.0000000	0.0275000
27080.0000000	0.0265000
27081.0000000	0.0276190
27082.0000000	0.0265000
27083.0000000	0.0275000
27084.0000000	0.0271429
27085.0000000	0.0250000
27086.0000000	0.0280000
27087.0000000	0.0276190
27088.0000000	0.0265000
27089.0000000	0.0280000
27090.0000000	0.0285000
27091.0000000	0.0276190
27092.0000000	0.0265000
27093.0000000	0.0295238
27094.0000000	0.0285000
27095.0000000	0.0280000
27096.0000000	0.0280952
27097.0000000	0.0270000
27098.0000000	0.0275000
27099.0000000	0.0270000
27100.0000000	0.0276190
27101.0000000	0.0270000
27102.0000000	0.0266667
27103.0000000	0.0260000
27104.0000000	0.0285000
27105.0000000	0.0271429
27106.0000000	0.0285000
27107.0000000	0.0275000

27108.0000000	0.0270000
27109.0000000	0.0285714
27110.0000000	0.0275000
27111.0000000	0.0295238
27112.0000000	0.0255000
27113.0000000	0.0290000
27114.0000000	0.0276190
27115.0000000	0.0275000
27116.0000000	0.0290000
27117.0000000	0.0266667
27118.0000000	0.0265000
27119.0000000	0.0300000
27120.0000000	0.0285000
27121.0000000	0.0280952
27122.0000000	0.0295000
27123.0000000	0.0290476
27124.0000000	0.0290000
27125.0000000	0.0290476
27126.0000000	0.0280000
27127.0000000	0.0280000
27128.0000000	0.0295000
27129.0000000	0.0290476
27130.0000000	0.0290000
27131.0000000	0.0300000
27132.0000000	0.0290000
27133.0000000	0.0295238
27134.0000000	0.0290000
27135.0000000	0.0285000
27136.0000000	0.0295238
27137.0000000	0.0290000
27138.0000000	0.0290476
27139.0000000	0.0300000
27140.0000000	0.0295000
27141.0000000	0.0295238
27142.0000000	0.0295000
27143.0000000	0.0295000
27144.0000000	0.0290000
27145.0000000	0.0290476
27146.0000000	0.0300000
27147.0000000	0.0295000
27148.0000000	0.0295238
27149.0000000	0.0285000
27150.0000000	0.0285000
27151.0000000	0.0295000
27152.0000000	0.0280952
27153.0000000	0.0271429
27154.0000000	0.0290000
27155.0000000	0.0285000
27156.0000000	0.0300000
27157.0000000	0.0280952
27158.0000000	0.0285000
27159.0000000	0.0285000
27160.0000000	0.0285714
27161.0000000	0.0285000
27162.0000000	0.0285714
27163.0000000	0.0285000
27164.0000000	0.0285000

27165.0000000	0.0290000
27166.0000000	0.0290476
27167.0000000	0.0300000
27168.0000000	0.0295000
27169.0000000	0.0280952
27170.0000000	0.0290000
27171.0000000	0.0285000
27172.0000000	0.0290476
27173.0000000	0.0300000
27174.0000000	0.0295000
27175.0000000	0.0285714
27176.0000000	0.0280000
27177.0000000	0.0295238
27178.0000000	0.0295000
27179.0000000	0.0295000
27180.0000000	0.0290000
27181.0000000	0.0290476
27182.0000000	0.0295000
27183.0000000	0.0285000
27184.0000000	0.0280952
27185.0000000	0.0280000
27186.0000000	0.0290476
27187.0000000	0.0285000
27188.0000000	0.0280000
27189.0000000	0.0275000
27190.0000000	0.0271429
27191.0000000	0.0285000
27192.0000000	0.0285000
27193.0000000	0.0285714
27194.0000000	0.0280000
27195.0000000	0.0290000
27196.0000000	0.0260000
27197.0000000	0.0290476
27198.0000000	0.0285000
27199.0000000	0.0290476
27200.0000000	0.0290000
27201.0000000	0.0290476
27202.0000000	0.0275000
27203.0000000	0.0280000
27204.0000000	0.0275000
27205.0000000	0.0290476
27206.0000000	0.0280000
27207.0000000	0.0295000
27208.0000000	0.0295238
27209.0000000	0.0285000
27210.0000000	0.0290000
27211.0000000	0.0280952
27212.0000000	0.0285000
27213.0000000	0.0271429
27214.0000000	0.0285000
27215.0000000	0.0285000
27216.0000000	0.0270000
27217.0000000	0.0276190
27218.0000000	0.0265000
27219.0000000	0.0275000
27220.0000000	0.0280000
27221.0000000	0.0276190

27222.0000000	0.0285714
27223.0000000	0.0275000
27224.0000000	0.0280000
27225.0000000	0.0245000
27226.0000000	0.0295000
27227.0000000	0.0271429
27228.0000000	0.0270000
27229.0000000	0.0280952
27230.0000000	0.0290000
27231.0000000	0.0276190
27232.0000000	0.0270000
27233.0000000	0.0280000
27234.0000000	0.0285000
27235.0000000	0.0275000
27236.0000000	0.0280952
27237.0000000	0.0280000
27238.0000000	0.0280952
27239.0000000	0.0285000
27240.0000000	0.0275000
27241.0000000	0.0285714
27242.0000000	0.0285000
27243.0000000	0.0290000
27244.0000000	0.0275000
27245.0000000	0.0290476
27246.0000000	0.0300000
27247.0000000	0.0290476
27248.0000000	0.0270000
27249.0000000	0.0295000
27250.0000000	0.0276190
27251.0000000	0.0280000
27252.0000000	0.0285000
27253.0000000	0.0276190
27254.0000000	0.0280000
27255.0000000	0.0285000
27256.0000000	0.0270000
27257.0000000	0.0280952
27258.0000000	0.0265000
27259.0000000	0.0290000
27260.0000000	0.0285714
27261.0000000	0.0275000
27262.0000000	0.0285714
27263.0000000	0.0280000
27264.0000000	0.0270000
27265.0000000	0.0276190
27266.0000000	0.0290000
27267.0000000	0.0265000
27268.0000000	0.0285000
27269.0000000	0.0280952
27270.0000000	0.0265000
27271.0000000	0.0285000
27272.0000000	0.0252381
27273.0000000	0.0270000
27274.0000000	0.0276190
27275.0000000	0.0280000
27276.0000000	0.0280000
27277.0000000	0.0276190
27278.0000000	0.0280000

27279.0000000	0.0290000
27280.0000000	0.0290000
27281.0000000	0.0257143
27282.0000000	0.0265000
27283.0000000	0.0266667
27284.0000000	0.0270000
27285.0000000	0.0275000
27286.0000000	0.0275000
27287.0000000	0.0266667
27288.0000000	0.0275000
27289.0000000	0.0247619
27290.0000000	0.0275000
27291.0000000	0.0265000
27292.0000000	0.0276190
27293.0000000	0.0260000
27294.0000000	0.0260000
27295.0000000	0.0235000
27296.0000000	0.0257143
27297.0000000	0.0240000
27298.0000000	0.0250000
27299.0000000	0.0247619
27300.0000000	0.0250000
27301.0000000	0.0260000
27302.0000000	0.0261905
27303.0000000	0.0235000
27304.0000000	0.0280000
27305.0000000	0.0247619
27306.0000000	0.0270000
27307.0000000	0.0270000
27308.0000000	0.0247619
27309.0000000	0.0230000
27310.0000000	0.0247619
27311.0000000	0.0260000
27312.0000000	0.0250000
27313.0000000	0.0257143
27314.0000000	0.0255000
27315.0000000	0.0255000
27316.0000000	0.0255000
27317.0000000	0.0260000
27318.0000000	0.0257143
27319.0000000	0.0252381
27320.0000000	0.0255000
27321.0000000	0.0260000
27322.0000000	0.0266667
27323.0000000	0.0255000
27324.0000000	0.0260000
27325.0000000	0.0245000
27326.0000000	0.0223810
27327.0000000	0.0265000
27328.0000000	0.0271429
27329.0000000	0.0270000
27330.0000000	0.0245000
27331.0000000	0.0265000
27332.0000000	0.0235000
27333.0000000	0.0233333
27334.0000000	0.0270000
27335.0000000	0.0257143

27336.0000000	0.0245000
27337.0000000	0.0261905
27338.0000000	0.0240000
27339.0000000	0.0235000
27340.0000000	0.0245000
27341.0000000	0.0242857
27342.0000000	0.0250000
27343.0000000	0.0240000
27344.0000000	0.0242857
27345.0000000	0.0210000
27346.0000000	0.0245000
27347.0000000	0.0220000
27348.0000000	0.0242857
27349.0000000	0.0240000
27350.0000000	0.0214286
27351.0000000	0.0225000
27352.0000000	0.0240000
27353.0000000	0.0214286
27354.0000000	0.0225000
27355.0000000	0.0215000
27356.0000000	0.0215000
27357.0000000	0.0209524
27358.0000000	0.0219048
27359.0000000	0.0210000
27360.0000000	0.0220000
27361.0000000	0.0220000
27362.0000000	0.0204762
27363.0000000	0.0215000
27364.0000000	0.0210000
27365.0000000	0.0200000
27366.0000000	0.0205000
27367.0000000	0.0219048
27368.0000000	0.0220000
27369.0000000	0.0225000
27370.0000000	0.0220000
27371.0000000	0.0214286
27372.0000000	0.0210000
27373.0000000	0.0215000
27374.0000000	0.0223810
27375.0000000	0.0225000
27376.0000000	0.0230000
27377.0000000	0.0219048
27378.0000000	0.0205000
27379.0000000	0.0230000
27380.0000000	0.0238095
27381.0000000	0.0210000
27382.0000000	0.0219048
27383.0000000	0.0220000
27384.0000000	0.0225000
27385.0000000	0.0215000
27386.0000000	0.0210000
27387.0000000	0.0223810
27388.0000000	0.0210000
27389.0000000	0.0210000
27390.0000000	0.0214286
27391.0000000	0.0210000
27392.0000000	0.0215000

27393.0000000	0.0204762
27394.0000000	0.0215000
27395.0000000	0.0205000
27396.0000000	0.0214286
27397.0000000	0.0215000
27398.0000000	0.0214286
27399.0000000	0.0200000
27400.0000000	0.0215000
27401.0000000	0.0204762
27402.0000000	0.0210000
27403.0000000	0.0210000
27404.0000000	0.0205000
27405.0000000	0.0200000
27406.0000000	0.0215000
27407.0000000	0.0204762
27408.0000000	0.0215000
27409.0000000	0.0210000
27410.0000000	0.0219048
27411.0000000	0.0215000
27412.0000000	0.0220000
27413.0000000	0.0214286
27414.0000000	0.0230000
27415.0000000	0.0205000
27416.0000000	0.0228571
27417.0000000	0.0225000
27418.0000000	0.0230000
27419.0000000	0.0204762
27420.0000000	0.0220000
27421.0000000	0.0220000
27422.0000000	0.0225000
27423.0000000	0.0209524
27424.0000000	0.0220000
27425.0000000	0.0214286
27426.0000000	0.0220000
27427.0000000	0.0210000
27428.0000000	0.0214286
27429.0000000	0.0210000
27430.0000000	0.0205000
27431.0000000	0.0230000
27432.0000000	0.0214286
27433.0000000	0.0210000
27434.0000000	0.0223810
27435.0000000	0.0225000
27436.0000000	0.0215000
27437.0000000	0.0200000
27438.0000000	0.0209524
27439.0000000	0.0210000
27440.0000000	0.0205000
27441.0000000	0.0204762
27442.0000000	0.0200000
27443.0000000	0.0209524
27444.0000000	0.0205000
27445.0000000	0.0205000
27446.0000000	0.0206250
27447.0000000	0.0212000
27448.0000000	0.0210000
27449.0000000	0.0204762

27450.0000000	0.0210000
27451.0000000	0.0210000
27452.0000000	0.0215000
27453.0000000	0.0209524
27454.0000000	0.0205000
27455.0000000	0.0204762
27456.0000000	0.0205000
27457.0000000	0.0205000
27458.0000000	0.0200000
27459.0000000	0.0205000
27460.0000000	0.0210000
27461.0000000	0.0215000
27462.0000000	0.0200000
27463.0000000	0.0200000
27464.0000000	0.0220000
27465.0000000	0.0200000
27466.0000000	0.0215000
27467.0000000	0.0210000
27468.0000000	0.0204762
27469.0000000	0.0210000
27470.0000000	0.0204762
27471.0000000	0.0220000
27472.0000000	0.0220000
27473.0000000	0.0210000
27474.0000000	0.0200000
27475.0000000	0.0205000
27476.0000000	0.0220000
27477.0000000	0.0214286
27478.0000000	0.0205000
27479.0000000	0.0214286
27480.0000000	0.0205000
27481.0000000	0.0215000
27482.0000000	0.0215000
27483.0000000	0.0215000
27484.0000000	0.0209524
27485.0000000	0.0210000
27486.0000000	0.0204762
27487.0000000	0.0205000
27488.0000000	0.0209524
27489.0000000	0.0215000
27490.0000000	0.0205000
27491.0000000	0.0215000
27492.0000000	0.0200000
27493.0000000	0.0209524
27494.0000000	0.0204762
27495.0000000	0.0205000
27496.0000000	0.0220000
27497.0000000	0.0210000
27498.0000000	0.0204762
27499.0000000	0.0205000
27500.0000000	0.0210000
27501.0000000	0.0219048
27502.0000000	0.0220000
27503.0000000	0.0215000
27504.0000000	0.0223810
27505.0000000	0.0210000
27506.0000000	0.0209524

27507.0000000	0.0210000
27508.0000000	0.0210000
27509.0000000	0.0215000
27510.0000000	0.0219048
27511.0000000	0.0215000
27512.0000000	0.0220000
27513.0000000	0.0205000
27514.0000000	0.0223810
27515.0000000	0.0210000
27516.0000000	0.0219048
27517.0000000	0.0235000
27518.0000000	0.0228571
27519.0000000	0.0225000
27520.0000000	0.0225000
27521.0000000	0.0220000
27522.0000000	0.0214286
27523.0000000	0.0215000
27524.0000000	0.0210000
27525.0000000	0.0219048
27526.0000000	0.0225000
27527.0000000	0.0215000
27528.0000000	0.0230000
27529.0000000	0.0209524
27530.0000000	0.0205000
27531.0000000	0.0204762
27532.0000000	0.0215000
27533.0000000	0.0220000
27534.0000000	0.0204762
27535.0000000	0.0200000
27536.0000000	0.0210000
27537.0000000	0.0209524
27538.0000000	0.0205000
27539.0000000	0.0205000
27540.0000000	0.0214286
27541.0000000	0.0200000
27542.0000000	0.0205000
27543.0000000	0.0219048
27544.0000000	0.0205000
27545.0000000	0.0215000
27546.0000000	0.0214286
27547.0000000	0.0205000
27548.0000000	0.0215000
27549.0000000	0.0200000
27550.0000000	0.0220000
27551.0000000	0.0215000
27552.0000000	0.0205000
27553.0000000	0.0209524
27554.0000000	0.0200000
27555.0000000	0.0205000
27556.0000000	0.0205000
27557.0000000	0.0220000
27558.0000000	0.0200000
27559.0000000	0.0210000
27560.0000000	0.0205000
27561.0000000	0.0200000
27562.0000000	0.0204762
27563.0000000	0.0200000

27564.0000000	0.0205000
27565.0000000	0.0205000
27566.0000000	0.0205000
27567.0000000	0.0210000
27568.0000000	0.0204762
27569.0000000	0.0210000
27570.0000000	0.0204762
27571.0000000	0.0220000
27572.0000000	0.0200000
27573.0000000	0.0215000
27574.0000000	0.0204762
27575.0000000	0.0200000
27576.0000000	0.0205000
27577.0000000	0.0204762
27578.0000000	0.0205000
27579.0000000	0.0214286
27580.0000000	0.0210000
27581.0000000	0.0205000
27582.0000000	0.0204762
27583.0000000	0.0215000
27584.0000000	0.0200000
27585.0000000	0.0215000
27586.0000000	0.0209524
27587.0000000	0.0205000
27588.0000000	0.0200000
27589.0000000	0.0209524
27590.0000000	0.0205000
27591.0000000	0.0210000
27592.0000000	0.0219048
27593.0000000	0.0210000
27594.0000000	0.0214286
27595.0000000	0.0225000
27596.0000000	0.0205000
27597.0000000	0.0225000
27598.0000000	0.0204762
27599.0000000	0.0210000
27600.0000000	0.0210000
27601.0000000	0.0200000
27602.0000000	0.0215000
27603.0000000	0.0205000
27604.0000000	0.0204762
27605.0000000	0.0210000
27606.0000000	0.0205000
27607.0000000	0.0214286
27608.0000000	0.0210000
27609.0000000	0.0205000
27610.0000000	0.0214286
27611.0000000	0.0220000
27612.0000000	0.0200000
27613.0000000	0.0209524
27614.0000000	0.0215000
27615.0000000	0.0214286
27616.0000000	0.0210000
27617.0000000	0.0210000
27618.0000000	0.0215000
27619.0000000	0.0219048
27620.0000000	0.0205000

27621.0000000	0.0210000
27622.0000000	0.0204762
27623.0000000	0.0210000
27624.0000000	0.0214286
27625.0000000	0.0210000
27626.0000000	0.0200000
27627.0000000	0.0200000
27628.0000000	0.0205000
27629.0000000	0.0204762
27630.0000000	0.0200000
27631.0000000	0.0209524
27632.0000000	0.0200000
27633.0000000	0.0200000
27634.0000000	0.0209524
27635.0000000	0.0200000
27636.0000000	0.0205000
27637.0000000	0.0200000
27638.0000000	0.0200000
27639.0000000	0.0205000
27640.0000000	0.0209524
27641.0000000	0.0205000
27642.0000000	0.0204762
27643.0000000	0.0200000
27644.0000000	0.0205000
27645.0000000	0.0200000
27646.0000000	0.0209524
27647.0000000	0.0205000
27648.0000000	0.0215000
27649.0000000	0.0205000
27650.0000000	0.0200000
27651.0000000	0.0200000
27652.0000000	0.0205000
27653.0000000	0.0200000
27654.0000000	0.0204762
27655.0000000	0.0200000
27656.0000000	0.0200000
27657.0000000	0.0200000
27658.0000000	0.0209524
27659.0000000	0.0200000
27660.0000000	0.0200000
27661.0000000	0.0200000
27662.0000000	0.0200000
27663.0000000	0.0200000
27664.0000000	0.0200000
27665.0000000	0.0204762
27666.0000000	0.0205000
27667.0000000	0.0200000
27668.0000000	0.0200000
27669.0000000	0.0205000
27670.0000000	0.0200000
27671.0000000	0.0200000
27672.0000000	0.0200000
27673.0000000	0.0200000
27674.0000000	0.0200000
27675.0000000	0.0200000
27676.0000000	0.0200000
27677.0000000	0.0200000

27678.0000000	0.0205000
27679.0000000	0.0200000
27680.0000000	0.0200000
27681.0000000	0.0200000
27682.0000000	0.0200000
27683.0000000	0.0200000
27684.0000000	0.0200000
27685.0000000	0.0200000
27686.0000000	0.0200000
27687.0000000	0.0200000
27688.0000000	0.0200000
27689.0000000	0.0200000
27690.0000000	0.0200000
27691.0000000	0.0200000
27692.0000000	0.0200000
27693.0000000	0.0200000
27694.0000000	0.0200000
27695.0000000	0.0200000
27696.0000000	0.0200000
27697.0000000	0.0200000
27698.0000000	0.0200000
27699.0000000	0.0200000
27700.0000000	0.0200000
27701.0000000	0.0200000
27702.0000000	0.0200000
27703.0000000	0.0200000
27704.0000000	0.0205000
27705.0000000	0.0200000
27706.0000000	0.0200000
27707.0000000	0.0200000
27708.0000000	0.0200000
27709.0000000	0.0200000
27710.0000000	0.0200000
27711.0000000	0.0200000
27712.0000000	0.0200000
27713.0000000	0.0205000
27714.0000000	0.0200000
27715.0000000	0.0200000
27716.0000000	0.0200000
27717.0000000	0.0200000
27718.0000000	0.0200000
27719.0000000	0.0204762
27720.0000000	0.0200000
27721.0000000	0.0204762
27722.0000000	0.0200000
27723.0000000	0.0205000
27724.0000000	0.0200000
27725.0000000	0.0204762
27726.0000000	0.0200000
27727.0000000	0.0200000
27728.0000000	0.0204762
27729.0000000	0.0200000
27730.0000000	0.0204762
27731.0000000	0.0200000
27732.0000000	0.0200000
27733.0000000	0.0205000
27734.0000000	0.0200000

27735.0000000	0.0200000
27736.0000000	0.0210000
27737.0000000	0.0204762
27738.0000000	0.0200000
27739.0000000	0.0200000
27740.0000000	0.0205000
27741.0000000	0.0200000
27742.0000000	0.0200000
27743.0000000	0.0200000
27744.0000000	0.0200000
27745.0000000	0.0200000
27746.0000000	0.0200000
27747.0000000	0.0200000
27748.0000000	0.0200000
27749.0000000	0.0200000
27750.0000000	0.0200000
27751.0000000	0.0200000
27752.0000000	0.0200000
27753.0000000	0.0200000
27754.0000000	0.0200000
27755.0000000	0.0204762
27756.0000000	0.0210000
27757.0000000	0.0200000
27758.0000000	0.0204762
27759.0000000	0.0200000
27760.0000000	0.0200000
27761.0000000	0.0205000
27762.0000000	0.0200000
27763.0000000	0.0200000
27764.0000000	0.0200000
27765.0000000	0.0200000
27766.0000000	0.0200000
27767.0000000	0.0200000
27768.0000000	0.0200000
27769.0000000	0.0200000
27770.0000000	0.0200000
27771.0000000	0.0200000
27772.0000000	0.0200000
27773.0000000	0.0200000
27774.0000000	0.0200000
27775.0000000	0.0200000
27776.0000000	0.0200000
27777.0000000	0.0200000
27778.0000000	0.0200000
27779.0000000	0.0195000
27780.0000000	0.0200000
27781.0000000	0.0200000
27782.0000000	0.0200000
27783.0000000	0.0200000
27784.0000000	0.0200000
27785.0000000	0.0200000
27786.0000000	0.0200000
27787.0000000	0.0200000
27788.0000000	0.0200000
27789.0000000	0.0200000
27790.0000000	0.0200000
27791.0000000	0.0200000

27792.0000000	0.0200000
27793.0000000	0.0200000
27794.0000000	0.0200000
27795.0000000	0.0200000
27796.0000000	0.0205000
27797.0000000	0.0204762
27798.0000000	0.0205000
27799.0000000	0.0200000
27800.0000000	0.0200000
27801.0000000	0.0200000
27802.0000000	0.0200000
27803.0000000	0.0200000
27804.0000000	0.0200000
27805.0000000	0.0200000
27806.0000000	0.0200000
27807.0000000	0.0200000
27808.0000000	0.0205000
27809.0000000	0.0204762
27810.0000000	0.0205000
27811.0000000	0.0204762
27812.0000000	0.0205000
27813.0000000	0.0200000
27814.0000000	0.0200000
27815.0000000	0.0200000
27816.0000000	0.0200000
27817.0000000	0.0200000
27818.0000000	0.0200000
27819.0000000	0.0200000
27820.0000000	0.0200000
27821.0000000	0.0200000
27822.0000000	0.0200000
27823.0000000	0.0205000
27824.0000000	0.0200000
27825.0000000	0.0200000
27826.0000000	0.0200000
27827.0000000	0.0200000
27828.0000000	0.0200000
27829.0000000	0.0215000
27830.0000000	0.0200000
27831.0000000	0.0200000
27832.0000000	0.0205000
27833.0000000	0.0200000
27834.0000000	0.0209524
27835.0000000	0.0200000
27836.0000000	0.0200000
27837.0000000	0.0200000
27838.0000000	0.0200000
27839.0000000	0.0204762
27840.0000000	0.0205000
27841.0000000	0.0200000
27842.0000000	0.0200000
27843.0000000	0.0200000
27844.0000000	0.0210000
27845.0000000	0.0200000
27846.0000000	0.0200000
27847.0000000	0.0210000
27848.0000000	0.0214286

27849.0000000	0.0200000
27850.0000000	0.0200000
27851.0000000	0.0214286
27852.0000000	0.0205000
27853.0000000	0.0210000
27854.0000000	0.0200000
27855.0000000	0.0210000
27856.0000000	0.0205000
27857.0000000	0.0214286
27858.0000000	0.0205000
27859.0000000	0.0210000
27860.0000000	0.0205000
27861.0000000	0.0209524
27862.0000000	0.0210000
27863.0000000	0.0209524
27864.0000000	0.0205000
27865.0000000	0.0205000
27866.0000000	0.0209524
27867.0000000	0.0215000
27868.0000000	0.0215000
27869.0000000	0.0210000
27870.0000000	0.0209524
27871.0000000	0.0215000
27872.0000000	0.0219048
27873.0000000	0.0215000
27874.0000000	0.0210000
27875.0000000	0.0209524
27876.0000000	0.0205000
27877.0000000	0.0205000
27878.0000000	0.0209524
27879.0000000	0.0215000
27880.0000000	0.0205000
27881.0000000	0.0200000
27882.0000000	0.0215000
27883.0000000	0.0205000
27884.0000000	0.0200000
27885.0000000	0.0200000
27886.0000000	0.0200000
27887.0000000	0.0200000
27888.0000000	0.0200000
27889.0000000	0.0205000
27890.0000000	0.0200000
27891.0000000	0.0204762
27892.0000000	0.0200000
27893.0000000	0.0205000
27894.0000000	0.0200000
27895.0000000	0.0200000
27896.0000000	0.0205000
27897.0000000	0.0205000
27898.0000000	0.0205000
27899.0000000	0.0200000
27900.0000000	0.0200000
27901.0000000	0.0200000
27902.0000000	0.0204762
27903.0000000	0.0200000
27904.0000000	0.0200000
27905.0000000	0.0200000

27906.0000000	0.0200000
27907.0000000	0.0200000
27908.0000000	0.0200000
27909.0000000	0.0200000
27910.0000000	0.0200000
27911.0000000	0.0200000
27912.0000000	0.0205000
27913.0000000	0.0200000
27914.0000000	0.0200000
27915.0000000	0.0200000
27916.0000000	0.0200000
27917.0000000	0.0200000
27918.0000000	0.0200000
27919.0000000	0.0200000
27920.0000000	0.0200000
27921.0000000	0.0200000
27922.0000000	0.0204762
27923.0000000	0.0200000
27924.0000000	0.0200000
27925.0000000	0.0205000
27926.0000000	0.0200000
27927.0000000	0.0200000
27928.0000000	0.0205000
27929.0000000	0.0205000
27930.0000000	0.0200000
27931.0000000	0.0205000
27932.0000000	0.0205000
27933.0000000	0.0200000
27934.0000000	0.0200000
27935.0000000	0.0200000
27936.0000000	0.0204762
27937.0000000	0.0205000
27938.0000000	0.0205000
27939.0000000	0.0200000
27940.0000000	0.0200000
27941.0000000	0.0200000
27942.0000000	0.0204762
27943.0000000	0.0200000
27944.0000000	0.0200000
27945.0000000	0.0200000
27946.0000000	0.0200000
27947.0000000	0.0204762
27948.0000000	0.0200000
27949.0000000	0.0200000
27950.0000000	0.0200000
27951.0000000	0.0200000
27952.0000000	0.0200000
27953.0000000	0.0215000
27954.0000000	0.0200000
27955.0000000	0.0205000
27956.0000000	0.0204762
27957.0000000	0.0210000
27958.0000000	0.0205000
27959.0000000	0.0210000
27960.0000000	0.0200000
27961.0000000	0.0200000
27962.0000000	0.0215000

27963.0000000	0.0204762
27964.0000000	0.0200000
27965.0000000	0.0215000
27966.0000000	0.0200000
27967.0000000	0.0205000
27968.0000000	0.0205000
27969.0000000	0.0205000
27970.0000000	0.0204762
27971.0000000	0.0220000
27972.0000000	0.0200000
27973.0000000	0.0215000
27974.0000000	0.0215000
27975.0000000	0.0200000
27976.0000000	0.0215000
27977.0000000	0.0205000
27978.0000000	0.0209524
27979.0000000	0.0220000
27980.0000000	0.0200000
27981.0000000	0.0210000
27982.0000000	0.0214286
27983.0000000	0.0214286
27984.0000000	0.0210000
27985.0000000	0.0200000
27986.0000000	0.0215000
27987.0000000	0.0204762
27988.0000000	0.0215000
27989.0000000	0.0205000
27990.0000000	0.0214286
27991.0000000	0.0215000
27992.0000000	0.0204762
27993.0000000	0.0220000
27994.0000000	0.0210000
27995.0000000	0.0215000
27996.0000000	0.0205000
27997.0000000	0.0223810
27998.0000000	0.0210000
27999.0000000	0.0214286
28000.0000000	0.0205000
28001.0000000	0.0205000
28002.0000000	0.0214286
28003.0000000	0.0210000
28004.0000000	0.0215000
28005.0000000	0.0205000
28006.0000000	0.0204762
28007.0000000	0.0215000
28008.0000000	0.0209524
28009.0000000	0.0205000
28010.0000000	0.0210000
28011.0000000	0.0214286
28012.0000000	0.0210000
28013.0000000	0.0220000
28014.0000000	0.0209524
28015.0000000	0.0200000
28016.0000000	0.0205000
28017.0000000	0.0204762
28018.0000000	0.0210000
28019.0000000	0.0200000

28020.0000000	0.0200000
28021.0000000	0.0209524
28022.0000000	0.0205000
28023.0000000	0.0204762
28024.0000000	0.0200000
28025.0000000	0.0205000
28026.0000000	0.0205000
28027.0000000	0.0204762
28028.0000000	0.0200000
28029.0000000	0.0200000
28030.0000000	0.0209524
28031.0000000	0.0209524
28032.0000000	0.0200000
28033.0000000	0.0200000
28034.0000000	0.0200000
28035.0000000	0.0204762
28036.0000000	0.0200000
28037.0000000	0.0205000
28038.0000000	0.0200000
28039.0000000	0.0205000
28040.0000000	0.0205263
28041.0000000	0.0209524
28042.0000000	0.0204762
28043.0000000	0.0220000
28044.0000000	0.0204762
28045.0000000	0.0205000
28046.0000000	0.0200000
28047.0000000	0.0204762
28048.0000000	0.0220000
28049.0000000	0.0205000
28050.0000000	0.0200000
28051.0000000	0.0200000
28052.0000000	0.0205000
28053.0000000	0.0204762
28054.0000000	0.0200000
28055.0000000	0.0205000
28056.0000000	0.0200000
28057.0000000	0.0200000
28058.0000000	0.0200000
28059.0000000	0.0200000
28060.0000000	0.0200000
28061.0000000	0.0205000
28062.0000000	0.0200000
28063.0000000	0.0200000
28064.0000000	0.0200000
28065.0000000	0.0200000
28066.0000000	0.0204762
28067.0000000	0.0205000
28068.0000000	0.0210000
28069.0000000	0.0204762
28070.0000000	0.0200000
28071.0000000	0.0204762
28072.0000000	0.0205000
28073.0000000	0.0210000
28074.0000000	0.0210000
28075.0000000	0.0200000
28076.0000000	0.0205000

28077.0000000	0.0210000
28078.0000000	0.0204762
28079.0000000	0.0200000
28080.0000000	0.0200000
28081.0000000	0.0210000
28082.0000000	0.0205000
28083.0000000	0.0209524
28084.0000000	0.0200000
28085.0000000	0.0205000
28086.0000000	0.0200000
28087.0000000	0.0204762
28088.0000000	0.0205000
28089.0000000	0.0209524
28090.0000000	0.0200000
28091.0000000	0.0215000
28092.0000000	0.0209524
28093.0000000	0.0210000
28094.0000000	0.0210000
28095.0000000	0.0200000
28096.0000000	0.0214286
28097.0000000	0.0200000
28098.0000000	0.0210000
28099.0000000	0.0200000
28100.0000000	0.0205000
28101.0000000	0.0205000
28102.0000000	0.0200000
28103.0000000	0.0215000
28104.0000000	0.0210000
28105.0000000	0.0209524
28106.0000000	0.0205000
28107.0000000	0.0200000
28108.0000000	0.0200000
28109.0000000	0.0214286
28110.0000000	0.0200000
28111.0000000	0.0214286
28112.0000000	0.0210000
28113.0000000	0.0200000
28114.0000000	0.0204762
28115.0000000	0.0200000
28116.0000000	0.0205000
28117.0000000	0.0205000
28118.0000000	0.0200000
28119.0000000	0.0204762
28120.0000000	0.0205000
28121.0000000	0.0220000
28122.0000000	0.0220000
28123.0000000	0.0200000
28124.0000000	0.0205000
28125.0000000	0.0220000
28126.0000000	0.0219048
28127.0000000	0.0205000
28128.0000000	0.0225000
28129.0000000	0.0228571
28130.0000000	0.0220000
28131.0000000	0.0225000
28132.0000000	0.0219048
28133.0000000	0.0205000

28134.0000000	0.0230000
28135.0000000	0.0233333
28136.0000000	0.0220000
28137.0000000	0.0215000
28138.0000000	0.0228571
28139.0000000	0.0230000
28140.0000000	0.0225000
28141.0000000	0.0247619
28142.0000000	0.0245000
28143.0000000	0.0220000
28144.0000000	0.0219048
28145.0000000	0.0220000
28146.0000000	0.0230000
28147.0000000	0.0240000
28148.0000000	0.0242857
28149.0000000	0.0245000
28150.0000000	0.0223810
28151.0000000	0.0235000
28152.0000000	0.0240000
28153.0000000	0.0242857
28154.0000000	0.0250000
28155.0000000	0.0240000
28156.0000000	0.0225000
28157.0000000	0.0252381
28158.0000000	0.0245000
28159.0000000	0.0261905
28160.0000000	0.0265000
28161.0000000	0.0245000
28162.0000000	0.0250000
28163.0000000	0.0238095
28164.0000000	0.0260000
28165.0000000	0.0255000
28166.0000000	0.0276190
28167.0000000	0.0240000
28168.0000000	0.0257143
28169.0000000	0.0265000
28170.0000000	0.0240000
28171.0000000	0.0271429
28172.0000000	0.0265000
28173.0000000	0.0260000
28174.0000000	0.0247619
28175.0000000	0.0260000
28176.0000000	0.0250000
28177.0000000	0.0257143
28178.0000000	0.0265000
28179.0000000	0.0260000
28180.0000000	0.0247619
28181.0000000	0.0275000
28182.0000000	0.0280000
28183.0000000	0.0257143
28184.0000000	0.0265000
28185.0000000	0.0275000
28186.0000000	0.0260000
28187.0000000	0.0261905
28188.0000000	0.0275000
28189.0000000	0.0261905
28190.0000000	0.0275000

28191.0000000	0.0270000
28192.0000000	0.0265000
28193.0000000	0.0271429
28194.0000000	0.0275000
28195.0000000	0.0280000
28196.0000000	0.0275000
28197.0000000	0.0295000
28198.0000000	0.0280952
28199.0000000	0.0275000
28200.0000000	0.0285000
28201.0000000	0.0295238
28202.0000000	0.0285000
28203.0000000	0.0260000
28204.0000000	0.0290476
28205.0000000	0.0280000
28206.0000000	0.0285000
28207.0000000	0.0295238
28208.0000000	0.0275000
28209.0000000	0.0280000
28210.0000000	0.0275000
28211.0000000	0.0300000
28212.0000000	0.0290000
28213.0000000	0.0290476
28214.0000000	0.0285000
28215.0000000	0.0275000
28216.0000000	0.0300000
28217.0000000	0.0290000
28218.0000000	0.0285000
28219.0000000	0.0290000
28220.0000000	0.0290476
28221.0000000	0.0290000
28222.0000000	0.0280952
28223.0000000	0.0285000
28224.0000000	0.0285000
28225.0000000	0.0280000
28226.0000000	0.0285714
28227.0000000	0.0290000
28228.0000000	0.0295238
28229.0000000	0.0270000
28230.0000000	0.0280000
28231.0000000	0.0261905
28232.0000000	0.0265000
28233.0000000	0.0290000
28234.0000000	0.0270000
28235.0000000	0.0271429
28236.0000000	0.0265000
28237.0000000	0.0271429
28238.0000000	0.0265000
28239.0000000	0.0265000
28240.0000000	0.0261905
28241.0000000	0.0280000
28242.0000000	0.0265000
28243.0000000	0.0265000
28244.0000000	0.0271429
28245.0000000	0.0280000
28246.0000000	0.0247619
28247.0000000	0.0275000

28248.0000000	0.0275000
28249.0000000	0.0270000
28250.0000000	0.0276190
28251.0000000	0.0275000
28252.0000000	0.0266667
28253.0000000	0.0275000
28254.0000000	0.0265000
28255.0000000	0.0285000
28256.0000000	0.0261905
28257.0000000	0.0240000
28258.0000000	0.0285000
28259.0000000	0.0280952
28260.0000000	0.0290000
28261.0000000	0.0285714
28262.0000000	0.0290000
28263.0000000	0.0280000
28264.0000000	0.0285000
28265.0000000	0.0280952
28266.0000000	0.0285000
28267.0000000	0.0295000
28268.0000000	0.0280952
28269.0000000	0.0290000
28270.0000000	0.0275000
28271.0000000	0.0290000
28272.0000000	0.0300000
28273.0000000	0.0285000
28274.0000000	0.0285714
28275.0000000	0.0285000
28276.0000000	0.0300000
28277.0000000	0.0290000
28278.0000000	0.0290000
28279.0000000	0.0290000
28280.0000000	0.0295238
28281.0000000	0.0295000
28282.0000000	0.0290000
28283.0000000	0.0285714
28284.0000000	0.0285000
28285.0000000	0.0290000
28286.0000000	0.0285714
28287.0000000	0.0280000
28288.0000000	0.0290000
28289.0000000	0.0295238
28290.0000000	0.0285000
28291.0000000	0.0275000
28292.0000000	0.0295238
28293.0000000	0.0285000
28294.0000000	0.0285000
28295.0000000	0.0280952
28296.0000000	0.0295000
28297.0000000	0.0300000
28298.0000000	0.0290000
28299.0000000	0.0295000
28300.0000000	0.0295000
28301.0000000	0.0290476
28302.0000000	0.0295000
28303.0000000	0.0285000
28304.0000000	0.0276190

28305.0000000	0.0290000
28306.0000000	0.0300000
28307.0000000	0.0285000
28308.0000000	0.0275000
28309.0000000	0.0295000
28310.0000000	0.0290476
28311.0000000	0.0285000
28312.0000000	0.0290000
28313.0000000	0.0295238
28314.0000000	0.0290000
28315.0000000	0.0290000
28316.0000000	0.0290476
28317.0000000	0.0290000
28318.0000000	0.0280000
28319.0000000	0.0300000
28320.0000000	0.0280000
28321.0000000	0.0275000
28322.0000000	0.0295238
28323.0000000	0.0295000
28324.0000000	0.0295000
28325.0000000	0.0295238
28326.0000000	0.0295000
28327.0000000	0.0290000
28328.0000000	0.0285714
28329.0000000	0.0295000
28330.0000000	0.0295000
28331.0000000	0.0290000
28332.0000000	0.0285714
28333.0000000	0.0300000
28334.0000000	0.0300000
28335.0000000	0.0295000
28336.0000000	0.0290000
28337.0000000	0.0300000
28338.0000000	0.0295000
28339.0000000	0.0295000
28340.0000000	0.0300000
28341.0000000	0.0295000
28342.0000000	0.0295000
28343.0000000	0.0290000
28344.0000000	0.0295238
28345.0000000	0.0295000
28346.0000000	0.0300000
28347.0000000	0.0300000
28348.0000000	0.0300000
28349.0000000	0.0300000
28350.0000000	0.0295000
28351.0000000	0.0300000
28352.0000000	0.0300000
28353.0000000	0.0290000
28354.0000000	0.0290000
28355.0000000	0.0300000
28356.0000000	0.0295238
28357.0000000	0.0295000
28358.0000000	0.0300000
28359.0000000	0.0290000
28360.0000000	0.0300000
28361.0000000	0.0295000

28362.0000000	0.0295238
28363.0000000	0.0300000
28364.0000000	0.0300000
28365.0000000	0.0295000
28366.0000000	0.0300000
28367.0000000	0.0300000
28368.0000000	0.0300000
28369.0000000	0.0290000
28370.0000000	0.0295000
28371.0000000	0.0300000
28372.0000000	0.0290000
28373.0000000	0.0300000
28374.0000000	0.0295000
28375.0000000	0.0300000
28376.0000000	0.0300000
28377.0000000	0.0295238
28378.0000000	0.0280000
28379.0000000	0.0300000
28380.0000000	0.0300000
28381.0000000	0.0285000
28382.0000000	0.0300000
28383.0000000	0.0295238
28384.0000000	0.0295000
28385.0000000	0.0285714
28386.0000000	0.0295000
28387.0000000	0.0290000
28388.0000000	0.0285714
28389.0000000	0.0300000
28390.0000000	0.0280000
28391.0000000	0.0300000
28392.0000000	0.0295238
28393.0000000	0.0290000
28394.0000000	0.0300000
28395.0000000	0.0295000
28396.0000000	0.0290000
28397.0000000	0.0300000
28398.0000000	0.0300000
28399.0000000	0.0300000
28400.0000000	0.0295000
28401.0000000	0.0295238
28402.0000000	0.0300000
28403.0000000	0.0300000
28404.0000000	0.0290000
28405.0000000	0.0300000
28406.0000000	0.0295000
28407.0000000	0.0300000
28408.0000000	0.0300000
28409.0000000	0.0300000
28410.0000000	0.0300000
28411.0000000	0.0300000
28412.0000000	0.0300000
28413.0000000	0.0300000
28414.0000000	0.0300000
28415.0000000	0.0300000
28416.0000000	0.0300000
28417.0000000	0.0300000
28418.0000000	0.0300000

28419.0000000	0.0300000
28420.0000000	0.0300000
28421.0000000	0.0295000
28422.0000000	0.0300000
28423.0000000	0.0295000
28424.0000000	0.0300000
28425.0000000	0.0300000
28426.0000000	0.0300000
28427.0000000	0.0300000
28428.0000000	0.0300000
28429.0000000	0.0300000
28430.0000000	0.0300000
28431.0000000	0.0300000
28432.0000000	0.0300000
28433.0000000	0.0300000
28434.0000000	0.0300000
28435.0000000	0.0300000
28436.0000000	0.0300000
28437.0000000	0.0309524
28438.0000000	0.0300000
28439.0000000	0.0305000
28440.0000000	0.0300000
28441.0000000	0.0300000
28442.0000000	0.0304762
28443.0000000	0.0305000
28444.0000000	0.0305000
28445.0000000	0.0300000
28446.0000000	0.0300000
28447.0000000	0.0300000
28448.0000000	0.0310000
28449.0000000	0.0304762
28450.0000000	0.0315000
28451.0000000	0.0310000
28452.0000000	0.0300000
28453.0000000	0.0305000
28454.0000000	0.0310000
28455.0000000	0.0304762
28456.0000000	0.0310000
28457.0000000	0.0309524
28458.0000000	0.0315000
28459.0000000	0.0305000
28460.0000000	0.0300000
28461.0000000	0.0300000
28462.0000000	0.0305000
28463.0000000	0.0300000
28464.0000000	0.0300000
28465.0000000	0.0305000
28466.0000000	0.0305000
28467.0000000	0.0305000
28468.0000000	0.0300000
28469.0000000	0.0304762
28470.0000000	0.0309524
28471.0000000	0.0300000
28472.0000000	0.0305000
28473.0000000	0.0304762
28474.0000000	0.0315000
28475.0000000	0.0305000

28476.0000000	0.0323809
28477.0000000	0.0305000
28478.0000000	0.0315000
28479.0000000	0.0300000
28480.0000000	0.0300000
28481.0000000	0.0305000
28482.0000000	0.0300000
28483.0000000	0.0300000
28484.0000000	0.0310000
28485.0000000	0.0300000
28486.0000000	0.0300000
28487.0000000	0.0310000
28488.0000000	0.0304762
28489.0000000	0.0300000
28490.0000000	0.0305000
28491.0000000	0.0300000
28492.0000000	0.0315000
28493.0000000	0.0309524
28494.0000000	0.0310000
28495.0000000	0.0310000
28496.0000000	0.0305000
28497.0000000	0.0310000
28498.0000000	0.0309524
28499.0000000	0.0330000
28500.0000000	0.0314286
28501.0000000	0.0310000
28502.0000000	0.0310000
28503.0000000	0.0300000
28504.0000000	0.0330000
28505.0000000	0.0315000
28506.0000000	0.0330000
28507.0000000	0.0323809
28508.0000000	0.0320000
28509.0000000	0.0328571
28510.0000000	0.0315000
28511.0000000	0.0330000
28512.0000000	0.0335000
28513.0000000	0.0328571
28514.0000000	0.0315000
28515.0000000	0.0305000
28516.0000000	0.0338095
28517.0000000	0.0350000
28518.0000000	0.0319048
28519.0000000	0.0335000
28520.0000000	0.0330000
28521.0000000	0.0361905
28522.0000000	0.0335000
28523.0000000	0.0360000
28524.0000000	0.0340000
28525.0000000	0.0347619
28526.0000000	0.0355000
28527.0000000	0.0340000
28528.0000000	0.0323809
28529.0000000	0.0340000
28530.0000000	0.0342857
28531.0000000	0.0345000
28532.0000000	0.0350000

28533.0000000	0.0338095
28534.0000000	0.0355000
28535.0000000	0.0320000
28536.0000000	0.0335000
28537.0000000	0.0342857
28538.0000000	0.0330000
28539.0000000	0.0325000
28540.0000000	0.0357143
28541.0000000	0.0360000
28542.0000000	0.0340000
28543.0000000	0.0338095
28544.0000000	0.0350000
28545.0000000	0.0361905
28546.0000000	0.0360000
28547.0000000	0.0350000
28548.0000000	0.0355000
28549.0000000	0.0371429
28550.0000000	0.0345000
28551.0000000	0.0360000
28552.0000000	0.0376190
28553.0000000	0.0360000
28554.0000000	0.0357143
28555.0000000	0.0355000
28556.0000000	0.0375000
28557.0000000	0.0375000
28558.0000000	0.0371429
28559.0000000	0.0360000
28560.0000000	0.0375000
28561.0000000	0.0376191
28562.0000000	0.0365000
28563.0000000	0.0376190
28564.0000000	0.0370000
28565.0000000	0.0365000
28566.0000000	0.0375000
28567.0000000	0.0370000
28568.0000000	0.0371429
28569.0000000	0.0371429
28570.0000000	0.0385000
28571.0000000	0.0380000
28572.0000000	0.0395000
28573.0000000	0.0385714
28574.0000000	0.0385000
28575.0000000	0.0375000
28576.0000000	0.0380952
28577.0000000	0.0400000
28578.0000000	0.0371429
28579.0000000	0.0390000
28580.0000000	0.0390000
28581.0000000	0.0385000
28582.0000000	0.0380952
28583.0000000	0.0390000
28584.0000000	0.0365000
28585.0000000	0.0385714
28586.0000000	0.0350000
28587.0000000	0.0360000
28588.0000000	0.0347619
28589.0000000	0.0365000

28590.0000000	0.0375000
28591.0000000	0.0380952
28592.0000000	0.0365000
28593.0000000	0.0361905
28594.0000000	0.0370000
28595.0000000	0.0360000
28596.0000000	0.0390000
28597.0000000	0.0366667
28598.0000000	0.0365000
28599.0000000	0.0375000
28600.0000000	0.0395238
28601.0000000	0.0380000
28602.0000000	0.0400000
28603.0000000	0.0375000
28604.0000000	0.0385714
28605.0000000	0.0380000
28606.0000000	0.0385714
28607.0000000	0.0395000
28608.0000000	0.0375000
28609.0000000	0.0395238
28610.0000000	0.0385000
28611.0000000	0.0390000
28612.0000000	0.0395238
28613.0000000	0.0390000
28614.0000000	0.0385000
28615.0000000	0.0390476
28616.0000000	0.0390000
28617.0000000	0.0390000
28618.0000000	0.0385000
28619.0000000	0.0390476
28620.0000000	0.0390000
28621.0000000	0.0400000
28622.0000000	0.0390000
28623.0000000	0.0395000
28624.0000000	0.0395238
28625.0000000	0.0400000
28626.0000000	0.0400000
28627.0000000	0.0395238
28628.0000000	0.0400000
28629.0000000	0.0395238
28630.0000000	0.0400000
28631.0000000	0.0400000
28632.0000000	0.0390000
28633.0000000	0.0400000
28634.0000000	0.0400000
28635.0000000	0.0395000
28636.0000000	0.0390476
28637.0000000	0.0395000
28638.0000000	0.0390476
28639.0000000	0.0400000
28640.0000000	0.0395000
28641.0000000	0.0400000
28642.0000000	0.0400000
28643.0000000	0.0400000
28644.0000000	0.0400000
28645.0000000	0.0400000
28646.0000000	0.0395000

28647.0000000	0.0400000
28648.0000000	0.0400000
28649.0000000	0.0400000
28650.0000000	0.0400000
28651.0000000	0.0400000
28652.0000000	0.0400000
28653.0000000	0.0400000
28654.0000000	0.0400000
28655.0000000	0.0400000
28656.0000000	0.0395000
28657.0000000	0.0395238
28658.0000000	0.0400000
28659.0000000	0.0400000
28660.0000000	0.0400000
28661.0000000	0.0400000
28662.0000000	0.0400000
28663.0000000	0.0400000
28664.0000000	0.0400000
28665.0000000	0.0400000
28666.0000000	0.0400000
28667.0000000	0.0400000
28668.0000000	0.0400000
28669.0000000	0.0400000
28670.0000000	0.0400000
28671.0000000	0.0400000
28672.0000000	0.0400000
28673.0000000	0.0400000
28674.0000000	0.0400000
28675.0000000	0.0395238
28676.0000000	0.0400000
28677.0000000	0.0400000
28678.0000000	0.0400000
28679.0000000	0.0400000
28680.0000000	0.0405000
28681.0000000	0.0400000
28682.0000000	0.0400000
28683.0000000	0.0400000
28684.0000000	0.0405000
28685.0000000	0.0400000
28686.0000000	0.0400000
28687.0000000	0.0405000
28688.0000000	0.0400000
28689.0000000	0.0410526
28690.0000000	0.0404546
28691.0000000	0.0405000
28692.0000000	0.0405000
28693.0000000	0.0410000
28694.0000000	0.0400000
28695.0000000	0.0405000
28696.0000000	0.0405000
28697.0000000	0.0400000
28698.0000000	0.0400000
28699.0000000	0.0400000
28700.0000000	0.0400000
28701.0000000	0.0400000
28702.0000000	0.0400000
28703.0000000	0.0410000

28704.0000000	0.0404762
28705.0000000	0.0400000
28706.0000000	0.0400000
28707.0000000	0.0405000
28708.0000000	0.0415000
28709.0000000	0.0400000
28710.0000000	0.0400000
28711.0000000	0.0400000
28712.0000000	0.0404762
28713.0000000	0.0405000
28714.0000000	0.0400000
28715.0000000	0.0400000
28716.0000000	0.0405000
28717.0000000	0.0414286
28718.0000000	0.0405000
28719.0000000	0.0400000
28720.0000000	0.0400000
28721.0000000	0.0404762
28722.0000000	0.0400000
28723.0000000	0.0400000
28724.0000000	0.0404762
28725.0000000	0.0405000
28726.0000000	0.0404762
28727.0000000	0.0405000
28728.0000000	0.0400000
28729.0000000	0.0409524
28730.0000000	0.0405000
28731.0000000	0.0405000
28732.0000000	0.0410000
28733.0000000	0.0404762
28734.0000000	0.0410000
28735.0000000	0.0409524
28736.0000000	0.0410000
28737.0000000	0.0400000
28738.0000000	0.0400000
28739.0000000	0.0405000
28740.0000000	0.0414286
28741.0000000	0.0415000
28742.0000000	0.0404762
28743.0000000	0.0410000
28744.0000000	0.0400000
28745.0000000	0.0410000
28746.0000000	0.0405000
28747.0000000	0.0405000
28748.0000000	0.0410000
28749.0000000	0.0404762
28750.0000000	0.0415000
28751.0000000	0.0400000
28752.0000000	0.0400000
28753.0000000	0.0405000
28754.0000000	0.0400000
28755.0000000	0.0400000
28756.0000000	0.0400000
28757.0000000	0.0400000
28758.0000000	0.0400000
28759.0000000	0.0400000
28760.0000000	0.0400000

28761.0000000	0.0400000
28762.0000000	0.0400000
28763.0000000	0.0400000
28764.0000000	0.0400000
28765.0000000	0.0400000
28766.0000000	0.0400000
28767.0000000	0.0400000
28768.0000000	0.0400000
28769.0000000	0.0400000
28770.0000000	0.0400000
28771.0000000	0.0400000
28772.0000000	0.0395000
28773.0000000	0.0400000
28774.0000000	0.0400000
28775.0000000	0.0400000
28776.0000000	0.0400000
28777.0000000	0.0400000
28778.0000000	0.0400000
28779.0000000	0.0400000
28780.0000000	0.0400000
28781.0000000	0.0400000
28782.0000000	0.0400000
28783.0000000	0.0400000
28784.0000000	0.0395000
28785.0000000	0.0395238
28786.0000000	0.0400000
28787.0000000	0.0395238
28788.0000000	0.0400000
28789.0000000	0.0400000
28790.0000000	0.0400000
28791.0000000	0.0400000
28792.0000000	0.0400000
28793.0000000	0.0400000
28794.0000000	0.0400000
28795.0000000	0.0400000
28796.0000000	0.0400000
28797.0000000	0.0395000
28798.0000000	0.0400000
28799.0000000	0.0400000
28800.0000000	0.0400000
28801.0000000	0.0400000
28802.0000000	0.0400000
28803.0000000	0.0400000
28804.0000000	0.0400000
28805.0000000	0.0400000
28806.0000000	0.0395000
28807.0000000	0.0400000
28808.0000000	0.0400000
28809.0000000	0.0395238
28810.0000000	0.0400000
28811.0000000	0.0400000
28812.0000000	0.0400000
28813.0000000	0.0400000
28814.0000000	0.0395238
28815.0000000	0.0400000
28816.0000000	0.0400000
28817.0000000	0.0400000

28818.0000000	0.0400000
28819.0000000	0.0395000
28820.0000000	0.0400000
28821.0000000	0.0400000
28822.0000000	0.0395000
28823.0000000	0.0395238
28824.0000000	0.0395000
28825.0000000	0.0395000
28826.0000000	0.0395238
28827.0000000	0.0400000
28828.0000000	0.0390000
28829.0000000	0.0400000
28830.0000000	0.0400000
28831.0000000	0.0400000
28832.0000000	0.0400000
28833.0000000	0.0400000
28834.0000000	0.0400000
28835.0000000	0.0400000
28836.0000000	0.0400000
28837.0000000	0.0390000
28838.0000000	0.0400000
28839.0000000	0.0400000
28840.0000000	0.0400000
28841.0000000	0.0400000
28842.0000000	0.0395000
28843.0000000	0.0395000
28844.0000000	0.0400000
28845.0000000	0.0400000
28846.0000000	0.0400000
28847.0000000	0.0400000
28848.0000000	0.0400000
28849.0000000	0.0400000
28850.0000000	0.0400000
28851.0000000	0.0400000
28852.0000000	0.0400000
28853.0000000	0.0400000
28854.0000000	0.0395000
28855.0000000	0.0400000
28856.0000000	0.0400000
28857.0000000	0.0400000
28858.0000000	0.0400000
28859.0000000	0.0400000
28860.0000000	0.0400000
28861.0000000	0.0400000
28862.0000000	0.0400000
28863.0000000	0.0400000
28864.0000000	0.0400000
28865.0000000	0.0400000
28866.0000000	0.0400000
28867.0000000	0.0400000
28868.0000000	0.0400000
28869.0000000	0.0400000
28870.0000000	0.0400000
28871.0000000	0.0400000
28872.0000000	0.0405000
28873.0000000	0.0400000
28874.0000000	0.0400000

28875.0000000	0.0400000
28876.0000000	0.0400000
28877.0000000	0.0400000
28878.0000000	0.0400000
28879.0000000	0.0400000
28880.0000000	0.0400000
28881.0000000	0.0400000
28882.0000000	0.0400000
28883.0000000	0.0405000
28884.0000000	0.0400000
28885.0000000	0.0405000
28886.0000000	0.0400000
28887.0000000	0.0400000
28888.0000000	0.0400000
28889.0000000	0.0400000
28890.0000000	0.0400000
28891.0000000	0.0400000
28892.0000000	0.0400000
28893.0000000	0.0400000
28894.0000000	0.0400000
28895.0000000	0.0400000
28896.0000000	0.0400000
28897.0000000	0.0400000
28898.0000000	0.0400000
28899.0000000	0.0400000
28900.0000000	0.0400000
28901.0000000	0.0395000
28902.0000000	0.0400000
28903.0000000	0.0400000
28904.0000000	0.0400000
28905.0000000	0.0400000
28906.0000000	0.0400000
28907.0000000	0.0400000
28908.0000000	0.0400000
28909.0000000	0.0400000
28910.0000000	0.0400000
28911.0000000	0.0400000
28912.0000000	0.0395000
28913.0000000	0.0395000
28914.0000000	0.0400000
28915.0000000	0.0400000
28916.0000000	0.0400000
28917.0000000	0.0400000
28918.0000000	0.0395000
28919.0000000	0.0400000
28920.0000000	0.0395238
28921.0000000	0.0400000
28922.0000000	0.0400000
28923.0000000	0.0400000
28924.0000000	0.0395000
28925.0000000	0.0400000
28926.0000000	0.0395238
28927.0000000	0.0400000
28928.0000000	0.0395000
28929.0000000	0.0400000
28930.0000000	0.0400000
28931.0000000	0.0400000

28932.0000000	0.0395238
28933.0000000	0.0400000
28934.0000000	0.0395000
28935.0000000	0.0400000
28936.0000000	0.0400000
28937.0000000	0.0400000
28938.0000000	0.0395238
28939.0000000	0.0400000
28940.0000000	0.0400000
28941.0000000	0.0400000
28942.0000000	0.0400000
28943.0000000	0.0400000
28944.0000000	0.0390000
28945.0000000	0.0400000
28946.0000000	0.0400000
28947.0000000	0.0400000
28948.0000000	0.0400000
28949.0000000	0.0400000
28950.0000000	0.0395238
28951.0000000	0.0400000
28952.0000000	0.0395000
28953.0000000	0.0395238
28954.0000000	0.0395000
28955.0000000	0.0400000
28956.0000000	0.0400000
28957.0000000	0.0400000
28958.0000000	0.0400000
28959.0000000	0.0395238
28960.0000000	0.0390000
28961.0000000	0.0395000
28962.0000000	0.0395238
28963.0000000	0.0395000
28964.0000000	0.0400000
28965.0000000	0.0385714
28966.0000000	0.0385000
28967.0000000	0.0395000
28968.0000000	0.0385714
28969.0000000	0.0380000
28970.0000000	0.0390476
28971.0000000	0.0380000
28972.0000000	0.0380000
28973.0000000	0.0375000
28974.0000000	0.0385714
28975.0000000	0.0395000
28976.0000000	0.0390000
28977.0000000	0.0390000
28978.0000000	0.0390476
28979.0000000	0.0375000
28980.0000000	0.0385000
28981.0000000	0.0385714
28982.0000000	0.0380000
28983.0000000	0.0395000
28984.0000000	0.0366667
28985.0000000	0.0370000
28986.0000000	0.0376190
28987.0000000	0.0380000
28988.0000000	0.0370000

28989.0000000	0.0347619
28990.0000000	0.0360000
28991.0000000	0.0375000
28992.0000000	0.0355000
28993.0000000	0.0352381
28994.0000000	0.0355000
28995.0000000	0.0355000
28996.0000000	0.0350000
28997.0000000	0.0342857
28998.0000000	0.0352381
28999.0000000	0.0335000
29000.0000000	0.0350000
29001.0000000	0.0347619
29002.0000000	0.0335000
29003.0000000	0.0350000
29004.0000000	0.0350000
29005.0000000	0.0357143
29006.0000000	0.0340000
29007.0000000	0.0323809
29008.0000000	0.0325000
29009.0000000	0.0345000
29010.0000000	0.0350000
29011.0000000	0.0333333
29012.0000000	0.0345000
29013.0000000	0.0347619
29014.0000000	0.0360000
29015.0000000	0.0350000
29016.0000000	0.0360000
29017.0000000	0.0352381
29018.0000000	0.0345000
29019.0000000	0.0340000
29020.0000000	0.0333333
29021.0000000	0.0335000
29022.0000000	0.0338095
29023.0000000	0.0320000
29024.0000000	0.0330000
29025.0000000	0.0320000
29026.0000000	0.0314286
29027.0000000	0.0325000
29028.0000000	0.0325000
29029.0000000	0.0328571
29030.0000000	0.0320000
29031.0000000	0.0338095
29032.0000000	0.0330000
29033.0000000	0.0345000
29034.0000000	0.0335000
29035.0000000	0.0333333
29036.0000000	0.0330000
29037.0000000	0.0325000
29038.0000000	0.0328571
29039.0000000	0.0330000
29040.0000000	0.0355000
29041.0000000	0.0342857
29042.0000000	0.0335000
29043.0000000	0.0360000
29044.0000000	0.0340000
29045.0000000	0.0328571

29046.0000000	0.0333333
29047.0000000	0.0335000
29048.0000000	0.0325000
29049.0000000	0.0345000
29050.0000000	0.0328571
29051.0000000	0.0336842
29052.0000000	0.0333333
29053.0000000	0.0338095
29054.0000000	0.0335000
29055.0000000	0.0330000
29056.0000000	0.0342857
29057.0000000	0.0335000
29058.0000000	0.0357143
29059.0000000	0.0345000
29060.0000000	0.0360000
29061.0000000	0.0360000
29062.0000000	0.0361905
29063.0000000	0.0360000
29064.0000000	0.0350000
29065.0000000	0.0333333
29066.0000000	0.0355000
29067.0000000	0.0333333
29068.0000000	0.0335000
29069.0000000	0.0340000
29070.0000000	0.0360000
29071.0000000	0.0345000
29072.0000000	0.0328571
29073.0000000	0.0345000
29074.0000000	0.0342857
29075.0000000	0.0350000
29076.0000000	0.0352381
29077.0000000	0.0350000
29078.0000000	0.0370000
29079.0000000	0.0345000
29080.0000000	0.0355000
29081.0000000	0.0347619
29082.0000000	0.0350000
29083.0000000	0.0342857
29084.0000000	0.0345000
29085.0000000	0.0360000
29086.0000000	0.0357143
29087.0000000	0.0350000
29088.0000000	0.0355000
29089.0000000	0.0371429
29090.0000000	0.0360000
29091.0000000	0.0365000
29092.0000000	0.0361905
29093.0000000	0.0365000
29094.0000000	0.0350000
29095.0000000	0.0357143
29096.0000000	0.0365000
29097.0000000	0.0350000
29098.0000000	0.0371429
29099.0000000	0.0355000
29100.0000000	0.0360000
29101.0000000	0.0375000
29102.0000000	0.0385714

29103.0000000	0.0370000
29104.0000000	0.0370000
29105.0000000	0.0366667
29106.0000000	0.0385714
29107.0000000	0.0375000
29108.0000000	0.0375000
29109.0000000	0.0360000
29110.0000000	0.0376190
29111.0000000	0.0380000
29112.0000000	0.0375000
29113.0000000	0.0371429
29114.0000000	0.0375000
29115.0000000	0.0390000
29116.0000000	0.0370000
29117.0000000	0.0385714
29118.0000000	0.0380000
29119.0000000	0.0390476
29120.0000000	0.0385000
29121.0000000	0.0385000
29122.0000000	0.0390476
29123.0000000	0.0370000
29124.0000000	0.0375000
29125.0000000	0.0380000
29126.0000000	0.0395238
29127.0000000	0.0385000
29128.0000000	0.0395238
29129.0000000	0.0395000
29130.0000000	0.0385000
29131.0000000	0.0390000
29132.0000000	0.0390000
29133.0000000	0.0395238
29134.0000000	0.0400000
29135.0000000	0.0400000
29136.0000000	0.0400000
29137.0000000	0.0395238
29138.0000000	0.0390000
29139.0000000	0.0400000
29140.0000000	0.0400000
29141.0000000	0.0400000
29142.0000000	0.0395000
29143.0000000	0.0395000
29144.0000000	0.0400000
29145.0000000	0.0400000
29146.0000000	0.0400000
29147.0000000	0.0400000
29148.0000000	0.0400000
29149.0000000	0.0400000
29150.0000000	0.0400000
29151.0000000	0.0400000
29152.0000000	0.0400000
29153.0000000	0.0395238
29154.0000000	0.0400000
29155.0000000	0.0400000
29156.0000000	0.0400000
29157.0000000	0.0400000
29158.0000000	0.0400000
29159.0000000	0.0400000

29160.0000000	0.0400000
29161.0000000	0.0400000
29162.0000000	0.0400000
29163.0000000	0.0400000
29164.0000000	0.0395238
29165.0000000	0.0400000
29166.0000000	0.0400000
29167.0000000	0.0400000
29168.0000000	0.0400000
29169.0000000	0.0400000
29170.0000000	0.0400000
29171.0000000	0.0400000
29172.0000000	0.0400000
29173.0000000	0.0400000
29174.0000000	0.0400000
29175.0000000	0.0400000
29176.0000000	0.0400000
29177.0000000	0.0400000
29178.0000000	0.0400000
29179.0000000	0.0400000
29180.0000000	0.0400000
29181.0000000	0.0400000
29182.0000000	0.0400000
29183.0000000	0.0400000
29184.0000000	0.0400000
29185.0000000	0.0400000
29186.0000000	0.0400000
29187.0000000	0.0400000
29188.0000000	0.0400000
29189.0000000	0.0400000
29190.0000000	0.0400000
29191.0000000	0.0400000
29192.0000000	0.0400000
29193.0000000	0.0400000
29194.0000000	0.0400000
29195.0000000	0.0400000
29196.0000000	0.0400000
29197.0000000	0.0400000
29198.0000000	0.0400000
29199.0000000	0.0400000
29200.0000000	0.0400000
29201.0000000	0.0400000
29202.0000000	0.0400000
29203.0000000	0.0400000
29204.0000000	0.0400000
29205.0000000	0.0400000
29206.0000000	0.0400000
29207.0000000	0.0404762
29208.0000000	0.0400000
29209.0000000	0.0405000
29210.0000000	0.0400000
29211.0000000	0.0400000
29212.0000000	0.0400000
29213.0000000	0.0400000
29214.0000000	0.0405000
29215.0000000	0.0410000
29216.0000000	0.0400000

29217.0000000	0.0400000
29218.0000000	0.0409524
29219.0000000	0.0410000
29220.0000000	0.0405000
29221.0000000	0.0405000
29222.0000000	0.0409524
29223.0000000	0.0410000
29224.0000000	0.0410000
29225.0000000	0.0419048
29226.0000000	0.0410000
29227.0000000	0.0414286
29228.0000000	0.0415000
29229.0000000	0.0425000
29230.0000000	0.0420000
29231.0000000	0.0420000
29232.0000000	0.0438095
29233.0000000	0.0430000
29234.0000000	0.0452381
29235.0000000	0.0430000
29236.0000000	0.0435000
29237.0000000	0.0435000
29238.0000000	0.0428572
29239.0000000	0.0430000
29240.0000000	0.0450000
29241.0000000	0.0442857
29242.0000000	0.0447619
29243.0000000	0.0455000
29244.0000000	0.0465000
29245.0000000	0.0460000
29246.0000000	0.0442857
29247.0000000	0.0465000
29248.0000000	0.0465000
29249.0000000	0.0475000
29250.0000000	0.0471429
29251.0000000	0.0480000
29252.0000000	0.0447619
29253.0000000	0.0455000
29254.0000000	0.0470000
29255.0000000	0.0457143
29256.0000000	0.0440000
29257.0000000	0.0455000
29258.0000000	0.0485714
29259.0000000	0.0465000
29260.0000000	0.0480000
29261.0000000	0.0480952
29262.0000000	0.0480000
29263.0000000	0.0485000
29264.0000000	0.0485714
29265.0000000	0.0485000
29266.0000000	0.0445000
29267.0000000	0.0475000
29268.0000000	0.0471429
29269.0000000	0.0470000
29270.0000000	0.0480952
29271.0000000	0.0465000
29272.0000000	0.0480000
29273.0000000	0.0480952

29274.0000000	0.0465000
29275.0000000	0.0475000
29276.0000000	0.0485000
29277.0000000	0.0490476
29278.0000000	0.0490000
29279.0000000	0.0490476
29280.0000000	0.0495000
29281.0000000	0.0485000
29282.0000000	0.0500000
29283.0000000	0.0495000
29284.0000000	0.0500000
29285.0000000	0.0495000
29286.0000000	0.0500000
29287.0000000	0.0500000
29288.0000000	0.0495238
29289.0000000	0.0495000
29290.0000000	0.0500000
29291.0000000	0.0500000
29292.0000000	0.0500000
29293.0000000	0.0500000
29294.0000000	0.0500000
29295.0000000	0.0505000
29296.0000000	0.0500000
29297.0000000	0.0500000
29298.0000000	0.0500000
29299.0000000	0.0500000
29300.0000000	0.0500000
29301.0000000	0.0500000
29302.0000000	0.0500000
29303.0000000	0.0500000
29304.0000000	0.0500000
29305.0000000	0.0500000
29306.0000000	0.0500000
29307.0000000	0.0504762
29308.0000000	0.0505000
29309.0000000	0.0500000
29310.0000000	0.0504762
29311.0000000	0.0500000
29312.0000000	0.0500000
29313.0000000	0.0504762
29314.0000000	0.0500000
29315.0000000	0.0500000
29316.0000000	0.0500000
29317.0000000	0.0505000
29318.0000000	0.0504762
29319.0000000	0.0500000
29320.0000000	0.0510000
29321.0000000	0.0500000
29322.0000000	0.0500000
29323.0000000	0.0500000
29324.0000000	0.0500000
29325.0000000	0.0509524
29326.0000000	0.0500000
29327.0000000	0.0505000
29328.0000000	0.0510000
29329.0000000	0.0500000
29330.0000000	0.0500000

29331.0000000	0.0505000
29332.0000000	0.0515000
29333.0000000	0.0500000
29334.0000000	0.0519048
29335.0000000	0.0515000
29336.0000000	0.0500000
29337.0000000	0.0510000
29338.0000000	0.0514286
29339.0000000	0.0519048
29340.0000000	0.0525000
29341.0000000	0.0500000
29342.0000000	0.0525000
29343.0000000	0.0533333
29344.0000000	0.0520000
29345.0000000	0.0505000
29346.0000000	0.0519048
29347.0000000	0.0510000
29348.0000000	0.0505000
29349.0000000	0.0523810
29350.0000000	0.0515000
29351.0000000	0.0525000
29352.0000000	0.0523810
29353.0000000	0.0540000
29354.0000000	0.0533333
29355.0000000	0.0555000
29356.0000000	0.0545000
29357.0000000	0.0525000
29358.0000000	0.0542857
29359.0000000	0.0560000
29360.0000000	0.0560000
29361.0000000	0.0542857
29362.0000000	0.0560000
29363.0000000	0.0552381
29364.0000000	0.0560000
29365.0000000	0.0550000
29366.0000000	0.0560000
29367.0000000	0.0566667
29368.0000000	0.0580000
29369.0000000	0.0555000
29370.0000000	0.0552381
29371.0000000	0.0550000
29372.0000000	0.0580000
29373.0000000	0.0570000
29374.0000000	0.0561905
29375.0000000	0.0585000
29376.0000000	0.0590000
29377.0000000	0.0580952
29378.0000000	0.0595238
29379.0000000	0.0580000
29380.0000000	0.0575000
29381.0000000	0.0590000
29382.0000000	0.0600000
29383.0000000	0.0580000
29384.0000000	0.0585000
29385.0000000	0.0585000
29386.0000000	0.0595238
29387.0000000	0.0590000

29388.0000000	0.0590476
29389.0000000	0.0600000
29390.0000000	0.0590000
29391.0000000	0.0595238
29392.0000000	0.0600000
29393.0000000	0.0595000
29394.0000000	0.0595238
29395.0000000	0.0590000
29396.0000000	0.0600000
29397.0000000	0.0595000
29398.0000000	0.0590476
29399.0000000	0.0590476
29400.0000000	0.0600000
29401.0000000	0.0595000
29402.0000000	0.0595000
29403.0000000	0.0595000
29404.0000000	0.0600000
29405.0000000	0.0590000
29406.0000000	0.0590476
29407.0000000	0.0590000
29408.0000000	0.0600000
29409.0000000	0.0590000
29410.0000000	0.0600000
29411.0000000	0.0600000
29412.0000000	0.0595000
29413.0000000	0.0595238
29414.0000000	0.0600000
29415.0000000	0.0595238
29416.0000000	0.0600000
29417.0000000	0.0600000
29418.0000000	0.0600000
29419.0000000	0.0595000
29420.0000000	0.0600000
29421.0000000	0.0600000
29422.0000000	0.0600000
29423.0000000	0.0600000
29424.0000000	0.0600000
29425.0000000	0.0600000
29426.0000000	0.0600000
29427.0000000	0.0600000
29428.0000000	0.0600000
29429.0000000	0.0600000
29430.0000000	0.0600000
29431.0000000	0.0600000
29432.0000000	0.0600000
29433.0000000	0.0600000
29434.0000000	0.0600000
29435.0000000	0.0600000
29436.0000000	0.0600000
29437.0000000	0.0600000
29438.0000000	0.0605000
29439.0000000	0.0604762
29440.0000000	0.0600000
29441.0000000	0.0600000
29442.0000000	0.0600000
29443.0000000	0.0600000
29444.0000000	0.0600000

29445.0000000	0.0600000
29446.0000000	0.0604762
29447.0000000	0.0610000
29448.0000000	0.0600000
29449.0000000	0.0600000
29450.0000000	0.0600000
29451.0000000	0.0609524
29452.0000000	0.0605000
29453.0000000	0.0600000
29454.0000000	0.0600000
29455.0000000	0.0605000
29456.0000000	0.0605000
29457.0000000	0.0600000
29458.0000000	0.0604762
29459.0000000	0.0600000
29460.0000000	0.0600000
29461.0000000	0.0600000
29462.0000000	0.0605000
29463.0000000	0.0600000
29464.0000000	0.0610000
29465.0000000	0.0609524
29466.0000000	0.0619048
29467.0000000	0.0630000
29468.0000000	0.0605000
29469.0000000	0.0614286
29470.0000000	0.0625000
29471.0000000	0.0625000
29472.0000000	0.0625000
29473.0000000	0.0625000
29474.0000000	0.0638095
29475.0000000	0.0645000
29476.0000000	0.0657143
29477.0000000	0.0655000
29478.0000000	0.0660000
29479.0000000	0.0642857
29480.0000000	0.0630000
29481.0000000	0.0635000
29482.0000000	0.0657143
29483.0000000	0.0630000
29484.0000000	0.0655000
29485.0000000	0.0633333
29486.0000000	0.0660000
29487.0000000	0.0638095
29488.0000000	0.0640000
29489.0000000	0.0660000
29490.0000000	0.0655000
29491.0000000	0.0666667
29492.0000000	0.0640000
29493.0000000	0.0665000
29494.0000000	0.0657143
29495.0000000	0.0655000
29496.0000000	0.0647619
29497.0000000	0.0660000
29498.0000000	0.0670000
29499.0000000	0.0638095
29500.0000000	0.0665000
29501.0000000	0.0650000

29502.0000000	0.0640000
29503.0000000	0.0680952
29504.0000000	0.0680000
29505.0000000	0.0661905
29506.0000000	0.0685000
29507.0000000	0.0690000
29508.0000000	0.0675000
29509.0000000	0.0685000
29510.0000000	0.0695238
29511.0000000	0.0695000
29512.0000000	0.0685714
29513.0000000	0.0700000
29514.0000000	0.0700000
29515.0000000	0.0700000
29516.0000000	0.0700000
29517.0000000	0.0695000
29518.0000000	0.0695238
29519.0000000	0.0700000
29520.0000000	0.0700000
29521.0000000	0.0690476
29522.0000000	0.0700000
29523.0000000	0.0700000
29524.0000000	0.0700000
29525.0000000	0.0700000
29526.0000000	0.0700000
29527.0000000	0.0700000
29528.0000000	0.0695000
29529.0000000	0.0700000
29530.0000000	0.0695238
29531.0000000	0.0700000
29532.0000000	0.0700000
29533.0000000	0.0700000
29534.0000000	0.0695238
29535.0000000	0.0700000
29536.0000000	0.0700000
29537.0000000	0.0700000
29538.0000000	0.0700000
29539.0000000	0.0700000
29540.0000000	0.0700000
29541.0000000	0.0700000
29542.0000000	0.0700000
29543.0000000	0.0700000
29544.0000000	0.0700000
29545.0000000	0.0700000
29546.0000000	0.0700000
29547.0000000	0.0700000
29548.0000000	0.0700000
29549.0000000	0.0700000
29550.0000000	0.0700000
29551.0000000	0.0700000
29552.0000000	0.0700000
29553.0000000	0.0700000
29554.0000000	0.0700000
29555.0000000	0.0700000
29556.0000000	0.0700000
29557.0000000	0.0700000
29558.0000000	0.0700000

29559.0000000	0.0700000
29560.0000000	0.0700000
29561.0000000	0.0705000
29562.0000000	0.0705000
29563.0000000	0.0700000
29564.0000000	0.0700000
29565.0000000	0.0700000
29566.0000000	0.0704762
29567.0000000	0.0705000
29568.0000000	0.0700000
29569.0000000	0.0705000
29570.0000000	0.0700000
29571.0000000	0.0700000
29572.0000000	0.0705000
29573.0000000	0.0709524
29574.0000000	0.0705000
29575.0000000	0.0700000
29576.0000000	0.0700000
29577.0000000	0.0715000
29578.0000000	0.0709524
29579.0000000	0.0720000
29580.0000000	0.0715000
29581.0000000	0.0715000
29582.0000000	0.0728572
29583.0000000	0.0730000
29584.0000000	0.0728572
29585.0000000	0.0720000
29586.0000000	0.0735000
29587.0000000	0.0742857
29588.0000000	0.0740000
29589.0000000	0.0720000
29590.0000000	0.0747619
29591.0000000	0.0745000
29592.0000000	0.0730000
29593.0000000	0.0742857
29594.0000000	0.0730000
29595.0000000	0.0760000
29596.0000000	0.0723810
29597.0000000	0.0735000
29598.0000000	0.0740000
29599.0000000	0.0750000
29600.0000000	0.0728572
29601.0000000	0.0715000
29602.0000000	0.0738095
29603.0000000	0.0740000
29604.0000000	0.0715000
29605.0000000	0.0733333
29606.0000000	0.0725000
29607.0000000	0.0730000
29608.0000000	0.0730000
29609.0000000	0.0747619
29610.0000000	0.0750000
29611.0000000	0.0733333
29612.0000000	0.0730000
29613.0000000	0.0745000
29614.0000000	0.0745000
29615.0000000	0.0728572

29616.0000000	0.0760000
29617.0000000	0.0720000
29618.0000000	0.0738095
29619.0000000	0.0740000
29620.0000000	0.0719048
29621.0000000	0.0750000
29622.0000000	0.0750000
29623.0000000	0.0742857
29624.0000000	0.0755000
29625.0000000	0.0730000
29626.0000000	0.0735000
29627.0000000	0.0733333
29628.0000000	0.0740000
29629.0000000	0.0750000
29630.0000000	0.0733333
29631.0000000	0.0720000
29632.0000000	0.0733333
29633.0000000	0.0725000
29634.0000000	0.0720000
29635.0000000	0.0733333
29636.0000000	0.0745000
29637.0000000	0.0745000
29638.0000000	0.0755000
29639.0000000	0.0738095
29640.0000000	0.0740000
29641.0000000	0.0755000
29642.0000000	0.0723810
29643.0000000	0.0740000
29644.0000000	0.0740000
29645.0000000	0.0747619
29646.0000000	0.0722222
29647.0000000	0.0750000
29648.0000000	0.0757143
29649.0000000	0.0740000
29650.0000000	0.0770588
29651.0000000	0.0766667
29652.0000000	0.0765000
29653.0000000	0.0745000
29654.0000000	0.0761905
29655.0000000	0.0760000
29656.0000000	0.0740000
29657.0000000	0.0714286
29658.0000000	0.0730000
29659.0000000	0.0755000
29660.0000000	0.0745000
29661.0000000	0.0728572
29662.0000000	0.0745000
29663.0000000	0.0757143
29664.0000000	0.0745000
29665.0000000	0.0750000
29666.0000000	0.0738095
29667.0000000	0.0745000
29668.0000000	0.0750000
29669.0000000	0.0760000
29670.0000000	0.0757143
29671.0000000	0.0761905
29672.0000000	0.0760000

29673.0000000	0.0730000
29674.0000000	0.0755000
29675.0000000	0.0752381
29676.0000000	0.0730000
29677.0000000	0.0750000
29678.0000000	0.0752381
29679.0000000	0.0740000
29680.0000000	0.0740000
29681.0000000	0.0738095
29682.0000000	0.0740000
29683.0000000	0.0745000
29684.0000000	0.0719048
29685.0000000	0.0730000
29686.0000000	0.0730000
29687.0000000	0.0723810
29688.0000000	0.0720000
29689.0000000	0.0730000
29690.0000000	0.0719048
29691.0000000	0.0710000
29692.0000000	0.0715000
29693.0000000	0.0733333
29694.0000000	0.0725000
29695.0000000	0.0728572
29696.0000000	0.0725000
29697.0000000	0.0715000
29698.0000000	0.0720000
29699.0000000	0.0725000
29700.0000000	0.0719048
29701.0000000	0.0715000
29702.0000000	0.0733333
29703.0000000	0.0720000
29704.0000000	0.0705000
29705.0000000	0.0740000
29706.0000000	0.0719048
29707.0000000	0.0725000
29708.0000000	0.0720000
29709.0000000	0.0723810
29710.0000000	0.0730000
29711.0000000	0.0719048
29712.0000000	0.0705000
29713.0000000	0.0715000
29714.0000000	0.0700000
29715.0000000	0.0719048
29716.0000000	0.0710000
29717.0000000	0.0710000
29718.0000000	0.0714286
29719.0000000	0.0715000
29720.0000000	0.0738095
29721.0000000	0.0715000
29722.0000000	0.0710000
29723.0000000	0.0723810
29724.0000000	0.0715000
29725.0000000	0.0720000
29726.0000000	0.0723810
29727.0000000	0.0755000
29728.0000000	0.0735000
29729.0000000	0.0735000

29730.0000000	0.0761905
29731.0000000	0.0752381
29732.0000000	0.0745000
29733.0000000	0.0745000
29734.0000000	0.0760000
29735.0000000	0.0750000
29736.0000000	0.0757143
29737.0000000	0.0760000
29738.0000000	0.0745000
29739.0000000	0.0733333
29740.0000000	0.0752381
29741.0000000	0.0720000
29742.0000000	0.0730000
29743.0000000	0.0715000
29744.0000000	0.0705000
29745.0000000	0.0714286
29746.0000000	0.0700000
29747.0000000	0.0715000
29748.0000000	0.0728572
29749.0000000	0.0710000
29750.0000000	0.0720000
29751.0000000	0.0723810
29752.0000000	0.0725000
29753.0000000	0.0730000
29754.0000000	0.0719048
29755.0000000	0.0715000
29756.0000000	0.0728572
29757.0000000	0.0740000
29758.0000000	0.0765000
29759.0000000	0.0747619
29760.0000000	0.0760000
29761.0000000	0.0770000
29762.0000000	0.0770000
29763.0000000	0.0790476
29764.0000000	0.0790000
29765.0000000	0.0790000
29766.0000000	0.0785714
29767.0000000	0.0780000
29768.0000000	0.0785000
29769.0000000	0.0785714
29770.0000000	0.0795000
29771.0000000	0.0780952
29772.0000000	0.0795000
29773.0000000	0.0795000
29774.0000000	0.0795000
29775.0000000	0.0795000
29776.0000000	0.0800000
29777.0000000	0.0800000
29778.0000000	0.0800000
29779.0000000	0.0790000
29780.0000000	0.0795000
29781.0000000	0.0800000
29782.0000000	0.0800000
29783.0000000	0.0790476
29784.0000000	0.0785000
29785.0000000	0.0785000
29786.0000000	0.0785000

29787.0000000	0.0780952
29788.0000000	0.0790000
29789.0000000	0.0795000
29790.0000000	0.0785714
29791.0000000	0.0790000
29792.0000000	0.0790476
29793.0000000	0.0790000
29794.0000000	0.0800000
29795.0000000	0.0795000
29796.0000000	0.0795000
29797.0000000	0.0795238
29798.0000000	0.0800000
29799.0000000	0.0795238
29800.0000000	0.0800000
29801.0000000	0.0795238
29802.0000000	0.0800000
29803.0000000	0.0795000
29804.0000000	0.0800000
29805.0000000	0.0800000
29806.0000000	0.0800000
29807.0000000	0.0800000
29808.0000000	0.0800000
29809.0000000	0.0800000
29810.0000000	0.0795000
29811.0000000	0.0795238
29812.0000000	0.0795000
29813.0000000	0.0800000
29814.0000000	0.0800000
29815.0000000	0.0800000
29816.0000000	0.0800000
29817.0000000	0.0800000
29818.0000000	0.0800000
29819.0000000	0.0795238
29820.0000000	0.0800000
29821.0000000	0.0800000
29822.0000000	0.0800000
29823.0000000	0.0800000
29824.0000000	0.0800000
29825.0000000	0.0800000
29826.0000000	0.0800000
29827.0000000	0.0800000
29828.0000000	0.0800000
29829.0000000	0.0800000
29830.0000000	0.0800000
29831.0000000	0.0800000
29832.0000000	0.0800000
29833.0000000	0.0800000
29834.0000000	0.0800000
29835.0000000	0.0800000
29836.0000000	0.0800000
29837.0000000	0.0800000
29838.0000000	0.0800000
29839.0000000	0.0800000
29840.0000000	0.0810000
29841.0000000	0.0800000
29842.0000000	0.0800000
29843.0000000	0.0800000

29844.0000000	0.0809524
29845.0000000	0.0800000
29846.0000000	0.0800000
29847.0000000	0.0800000
29848.0000000	0.0800000
29849.0000000	0.0805000
29850.0000000	0.0805000
29851.0000000	0.0804762
29852.0000000	0.0800000
29853.0000000	0.0800000
29854.0000000	0.0800000
29855.0000000	0.0805000
29856.0000000	0.0800000
29857.0000000	0.0800000
29858.0000000	0.0800000
29859.0000000	0.0800000
29860.0000000	0.0800000
29861.0000000	0.0805000
29862.0000000	0.0804762
29863.0000000	0.0800000
29864.0000000	0.0800000
29865.0000000	0.0800000
29866.0000000	0.0800000
29867.0000000	0.0804762
29868.0000000	0.0800000
29869.0000000	0.0800000
29870.0000000	0.0800000
29871.0000000	0.0800000
29872.0000000	0.0800000
29873.0000000	0.0800000
29874.0000000	0.0805000
29875.0000000	0.0800000
29876.0000000	0.0800000
29877.0000000	0.0805000
29878.0000000	0.0800000
29879.0000000	0.0800000
29880.0000000	0.0804762
29881.0000000	0.0800000
29882.0000000	0.0800000
29883.0000000	0.0800000
29884.0000000	0.0800000
29885.0000000	0.0825000
29886.0000000	0.0800000
29887.0000000	0.0800000
29888.0000000	0.0805000
29889.0000000	0.0809524
29890.0000000	0.0810000
29891.0000000	0.0805000
29892.0000000	0.0804762
29893.0000000	0.0800000
29894.0000000	0.0810000
29895.0000000	0.0804762
29896.0000000	0.0805000
29897.0000000	0.0810000
29898.0000000	0.0814286
29899.0000000	0.0810000
29900.0000000	0.0810000

29901.0000000	0.0815000
29902.0000000	0.0814286
29903.0000000	0.0810000
29904.0000000	0.0810000
29905.0000000	0.0804762
29906.0000000	0.0810000
29907.0000000	0.0804762
29908.0000000	0.0805000
29909.0000000	0.0810000
29910.0000000	0.0820000
29911.0000000	0.0804762
29912.0000000	0.0810000
29913.0000000	0.0810000
29914.0000000	0.0800000
29915.0000000	0.0810000
29916.0000000	0.0809524
29917.0000000	0.0810000
29918.0000000	0.0820000
29919.0000000	0.0828572
29920.0000000	0.0820000
29921.0000000	0.0825000
29922.0000000	0.0820000
29923.0000000	0.0823810
29924.0000000	0.0830000
29925.0000000	0.0833334
29926.0000000	0.0820000
29927.0000000	0.0835000
29928.0000000	0.0842857
29929.0000000	0.0855000
29930.0000000	0.0825000
29931.0000000	0.0865000
29932.0000000	0.0852381
29933.0000000	0.0840000
29934.0000000	0.0840000
29935.0000000	0.0866667
29936.0000000	0.0845000
29937.0000000	0.0866667
29938.0000000	0.0860000
29939.0000000	0.0885000
29940.0000000	0.0860000
29941.0000000	0.0857143
29942.0000000	0.0860000
29943.0000000	0.0852381
29944.0000000	0.0860000
29945.0000000	0.0860000
29946.0000000	0.0860000
29947.0000000	0.0880953
29948.0000000	0.0850000
29949.0000000	0.0870000
29950.0000000	0.0866667
29951.0000000	0.0865000
29952.0000000	0.0852381
29953.0000000	0.0875000
29954.0000000	0.0855000
29955.0000000	0.0842857
29956.0000000	0.0835000
29957.0000000	0.0840000

29958.0000000	0.0855000
29959.0000000	0.0838095
29960.0000000	0.0850000
29961.0000000	0.0840000
29962.0000000	0.0835000
29963.0000000	0.0857143
29964.0000000	0.0838095
29965.0000000	0.0870000
29966.0000000	0.0865000
29967.0000000	0.0871429
29968.0000000	0.0900000
29969.0000000	0.0890000
29970.0000000	0.0880000
29971.0000000	0.0890476
29972.0000000	0.0895000
29973.0000000	0.0890000
29974.0000000	0.0890476
29975.0000000	0.0890000
29976.0000000	0.0885000
29977.0000000	0.0900000
29978.0000000	0.0894445
29979.0000000	0.0900000
29980.0000000	0.0900000
29981.0000000	0.0885000
29982.0000000	0.0895000
29983.0000000	0.0890476
29984.0000000	0.0895000
29985.0000000	0.0890000
29986.0000000	0.0895238
29987.0000000	0.0890000
29988.0000000	0.0895000
29989.0000000	0.0900000
29990.0000000	0.0895000
29991.0000000	0.0895000
29992.0000000	0.0890000
29993.0000000	0.0890476
29994.0000000	0.0890000
29995.0000000	0.0890476
29996.0000000	0.0895000
29997.0000000	0.0895000
29998.0000000	0.0895238
29999.0000000	0.0900000
30000.0000000	0.0890000
30001.0000000	0.0900000
30002.0000000	0.0895238
30003.0000000	0.0900000
30004.0000000	0.0900000
30005.0000000	0.0900000
30006.0000000	0.0895000
30007.0000000	0.0895238
30008.0000000	0.0895000
30009.0000000	0.0900000
30010.0000000	0.0890000
30011.0000000	0.0890476
30012.0000000	0.0900000
30013.0000000	0.0890476
30014.0000000	0.0885000

30015.0000000	0.0885000
30016.0000000	0.0895238
30017.0000000	0.0880000
30018.0000000	0.0890000
30019.0000000	0.0895238
30020.0000000	0.0885000
30021.0000000	0.0885000
30022.0000000	0.0885714
30023.0000000	0.0885000
30024.0000000	0.0875000
30025.0000000	0.0871429
30026.0000000	0.0875000
30027.0000000	0.0880000
30028.0000000	0.0876191
30029.0000000	0.0885000
30030.0000000	0.0870000
30031.0000000	0.0880953
30032.0000000	0.0870000
30033.0000000	0.0830000
30034.0000000	0.0861905
30035.0000000	0.0835000
30036.0000000	0.0835000
30037.0000000	0.0845000
30038.0000000	0.0852381
30039.0000000	0.0855000
30040.0000000	0.0842857
30041.0000000	0.0840000
30042.0000000	0.0845000
30043.0000000	0.0847619
30044.0000000	0.0870000
30045.0000000	0.0850000
30046.0000000	0.0865000
30047.0000000	0.0876191
30048.0000000	0.0890000
30049.0000000	0.0890476
30050.0000000	0.0885000
30051.0000000	0.0875000
30052.0000000	0.0871429
30053.0000000	0.0880000
30054.0000000	0.0870000
30055.0000000	0.0871429
30056.0000000	0.0865000
30057.0000000	0.0870000
30058.0000000	0.0857143
30059.0000000	0.0855000
30060.0000000	0.0885000
30061.0000000	0.0876191
30062.0000000	0.0850000
30063.0000000	0.0880000
30064.0000000	0.0865000
30065.0000000	0.0871429
30066.0000000	0.0845000
30067.0000000	0.0875000
30068.0000000	0.0884211
30069.0000000	0.0881818
30070.0000000	0.0855000
30071.0000000	0.0876191

30072.0000000	0.0847619
30073.0000000	0.0850000
30074.0000000	0.0840000
30075.0000000	0.0855000
30076.0000000	0.0860000
30077.0000000	0.0857143
30078.0000000	0.0860000
30079.0000000	0.0855000
30080.0000000	0.0857143
30081.0000000	0.0845000
30082.0000000	0.0845000
30083.0000000	0.0857143
30084.0000000	0.0845000
30085.0000000	0.0875000
30086.0000000	0.0861905
30087.0000000	0.0840000
30088.0000000	0.0871429
30089.0000000	0.0855000
30090.0000000	0.0875000
30091.0000000	0.0875000
30092.0000000	0.0890476
30093.0000000	0.0900000
30094.0000000	0.0890000
30095.0000000	0.0895238
30096.0000000	0.0890000
30097.0000000	0.0890000
30098.0000000	0.0890000
30099.0000000	0.0900000
30100.0000000	0.0900000
30101.0000000	0.0900000
30102.0000000	0.0895000
30103.0000000	0.0900000
30104.0000000	0.0900000
30105.0000000	0.0900000
30106.0000000	0.0900000
30107.0000000	0.0900000
30108.0000000	0.0900000
30109.0000000	0.0900000
30110.0000000	0.0900000
30111.0000000	0.0905000
30112.0000000	0.0900000
30113.0000000	0.0909524
30114.0000000	0.0905000
30115.0000000	0.0900000
30116.0000000	0.0900000
30117.0000000	0.0900000
30118.0000000	0.0900000
30119.0000000	0.0900000
30120.0000000	0.0900000
30121.0000000	0.0900000
30122.0000000	0.0900000
30123.0000000	0.0900000
30124.0000000	0.0900000
30125.0000000	0.0900000
30126.0000000	0.0900000
30127.0000000	0.0900000
30128.0000000	0.0900000

30129.0000000	0.0900000
30130.0000000	0.0900000
30131.0000000	0.0904762
30132.0000000	0.0920000
30133.0000000	0.0914286
30134.0000000	0.0905000
30135.0000000	0.0910000
30136.0000000	0.0910000
30137.0000000	0.0925000
30138.0000000	0.0938095
30139.0000000	0.0935000
30140.0000000	0.0938095
30141.0000000	0.0960000
30142.0000000	0.0950000
30143.0000000	0.0976191
30144.0000000	0.0940000
30145.0000000	0.0940000
30146.0000000	0.0935000
30147.0000000	0.0923810
30148.0000000	0.0950000
30149.0000000	0.0919048
30150.0000000	0.0955000
30151.0000000	0.0970000
30152.0000000	0.0938095
30153.0000000	0.0935000
30154.0000000	0.0930000
30155.0000000	0.0942857
30156.0000000	0.0950000
30157.0000000	0.0960000
30158.0000000	0.0925000
30159.0000000	0.0947619
30160.0000000	0.0947619
30161.0000000	0.0930000
30162.0000000	0.0955000
30163.0000000	0.0965000
30164.0000000	0.0966667
30165.0000000	0.0950000
30166.0000000	0.0960000
30167.0000000	0.0961905
30168.0000000	0.0985000
30169.0000000	0.0966667
30170.0000000	0.0980000
30171.0000000	0.0975000
30172.0000000	0.0995000
30173.0000000	0.0975000
30174.0000000	0.0990476
30175.0000000	0.0995000
30176.0000000	0.0985714
30177.0000000	0.0985000
30178.0000000	0.0995000
30179.0000000	0.0990476
30180.0000000	0.1000000
30181.0000000	0.1000000
30182.0000000	0.1000000
30183.0000000	0.1000000
30184.0000000	0.0995000
30185.0000000	0.1000000

30186.0000000	0.1000000
30187.0000000	0.1000000
30188.0000000	0.1000000
30189.0000000	0.1000000
30190.0000000	0.1000000
30191.0000000	0.1000000
30192.0000000	0.1000000
30193.0000000	0.1000000
30194.0000000	0.1000000
30195.0000000	0.1005000
30196.0000000	0.1000000
30197.0000000	0.1000000
30198.0000000	0.1004762
30199.0000000	0.1000000
30200.0000000	0.1000000
30201.0000000	0.1005000
30202.0000000	0.1000000
30203.0000000	0.1015000
30204.0000000	0.1014286
30205.0000000	0.1010000
30206.0000000	0.1015000
30207.0000000	0.1005000
30208.0000000	0.1022727
30209.0000000	0.1025000
30210.0000000	0.1020000
30211.0000000	0.1035000
30212.0000000	0.1019048
30213.0000000	0.1015000
30214.0000000	0.1020000
30215.0000000	0.1010000
30216.0000000	0.1000000
30217.0000000	0.1010000
30218.0000000	0.1000000
30219.0000000	0.1009524
30220.0000000	0.1005000
30221.0000000	0.1019048
30222.0000000	0.1005000
30223.0000000	0.1000000
30224.0000000	0.1019048
30225.0000000	0.1025000
30226.0000000	0.1040000
30227.0000000	0.1035000
30228.0000000	0.1038095
30229.0000000	0.1055000
30230.0000000	0.1066667
30231.0000000	0.1065000
30232.0000000	0.1075000
30233.0000000	0.1090000
30234.0000000	0.1070000
30235.0000000	0.1090476
30236.0000000	0.1080000
30237.0000000	0.1095238
30238.0000000	0.1095000
30239.0000000	0.1090476
30240.0000000	0.1100000
30241.0000000	0.1085000
30242.0000000	0.1095000

30243.0000000	0.1085714
30244.0000000	0.1080000
30245.0000000	0.1085000
30246.0000000	0.1095238
30247.0000000	0.1090000
30248.0000000	0.1095238
30249.0000000	0.1100000
30250.0000000	0.1100000
30251.0000000	0.1095000
30252.0000000	0.1100000
30253.0000000	0.1100000
30254.0000000	0.1100000
30255.0000000	0.1100000
30256.0000000	0.1105000
30257.0000000	0.1090476
30258.0000000	0.1100000
30259.0000000	0.1100000
30260.0000000	0.1095238
30261.0000000	0.1100000
30262.0000000	0.1100000
30263.0000000	0.1100000
30264.0000000	0.1100000
30265.0000000	0.1100000
30266.0000000	0.1095238
30267.0000000	0.1100000
30268.0000000	0.1100000
30269.0000000	0.1100000
30270.0000000	0.1105000
30271.0000000	0.1110000
30272.0000000	0.1110000
30273.0000000	0.1114286
30274.0000000	0.1140000
30275.0000000	0.1130000
30276.0000000	0.1147619
30277.0000000	0.1175000
30278.0000000	0.1185000
30279.0000000	0.1190476
30280.0000000	0.1185000
30281.0000000	0.1200000
30282.0000000	0.1200000
30283.0000000	0.1200000
30284.0000000	0.1200000
30285.0000000	0.1200000
30286.0000000	0.1200000
30287.0000000	0.1200000
30288.0000000	0.1204762
30289.0000000	0.1210000
30290.0000000	0.1210000
30291.0000000	0.1228571
30292.0000000	0.1230000
30293.0000000	0.1220000
30294.0000000	0.1215000
30295.0000000	0.1238095
30296.0000000	0.1271429
30297.0000000	0.1255000
30298.0000000	0.1280000
30299.0000000	0.1285000

30300.0000000	0.1285714
30301.0000000	0.1295000
30302.0000000	0.1295000
30303.0000000	0.1300000
30304.0000000	0.1305000
30305.0000000	0.1304762
30306.0000000	0.1300000
30307.0000000	0.1305000
30308.0000000	0.1305000
30309.0000000	0.1323810
30310.0000000	0.1315000
30311.0000000	0.1305000
30312.0000000	0.1347619
30313.0000000	0.1360000
30314.0000000	0.1350000
30315.0000000	0.1385714
30316.0000000	0.1365000
30317.0000000	0.1400000
30318.0000000	0.1400000
30319.0000000	0.1400000
30320.0000000	0.1400000
30321.0000000	0.1400000
30322.0000000	0.1400000
30323.0000000	0.1400000
30324.0000000	0.1400000
30325.0000000	0.1400000
30326.0000000	0.1405000
30327.0000000	0.1404762
30328.0000000	0.1405000
30329.0000000	0.1405000
30330.0000000	0.1400000
30331.0000000	0.1405000
30332.0000000	0.1415000
30333.0000000	0.1435000
30334.0000000	0.1438096
30335.0000000	0.1430000
30336.0000000	0.1452381
30337.0000000	0.1420000
30338.0000000	0.1450000
30339.0000000	0.1405000
30340.0000000	0.1419048
30341.0000000	0.1440000
30342.0000000	0.1425000
30343.0000000	0.1428572
30344.0000000	0.1455000
30345.0000000	0.1480953
30346.0000000	0.1480000
30347.0000000	0.1475000
30348.0000000	0.1476191
30349.0000000	0.1485000
30350.0000000	0.1500000
30351.0000000	0.1495000
30352.0000000	0.1500000
30353.0000000	0.1500000
30354.0000000	0.1500000
30355.0000000	0.1500000
30356.0000000	0.1510000

30357.0000000	0.1500000
30358.0000000	0.1510000
30359.0000000	0.1510000
30360.0000000	0.1542857
30361.0000000	0.1530000
30362.0000000	0.1525000
30363.0000000	0.1540000
30364.0000000	0.1547619
30365.0000000	0.1575000
30366.0000000	0.1576191
30367.0000000	0.1575000
30368.0000000	0.1575000
30369.0000000	0.1575000
30370.0000000	0.1595238
30371.0000000	0.1595000
30372.0000000	0.1600000
30373.0000000	0.1595000
30374.0000000	0.1595000
30375.0000000	0.1600000
30376.0000000	0.1600000
30377.0000000	0.1600000
30378.0000000	0.1605000
30379.0000000	0.1600000
30380.0000000	0.1600000
30381.0000000	0.1600000
30382.0000000	0.1600000
30383.0000000	0.1600000
30384.0000000	0.1595238
30385.0000000	0.1600000
30386.0000000	0.1600000
30387.0000000	0.1600000
30388.0000000	0.1590476
30389.0000000	0.1600000
30390.0000000	0.1600000
30391.0000000	0.1600000
30392.0000000	0.1600000
30393.0000000	0.1595238
30394.0000000	0.1605000
30395.0000000	0.1600000
30396.0000000	0.1600000
30397.0000000	0.1600000
30398.0000000	0.1600000
30399.0000000	0.1600000
30400.0000000	0.1600000
30401.0000000	0.1600000
30402.0000000	0.1600000
30403.0000000	0.1600000
30404.0000000	0.1600000
30405.0000000	0.1600000
30406.0000000	0.1600000
30407.0000000	0.1600000
30408.0000000	0.1600000
30409.0000000	0.1600000
30410.0000000	0.1605000
30411.0000000	0.1610000
30412.0000000	0.1628572
30413.0000000	0.1635000

30414.0000000	0.1630000
30415.0000000	0.1623810
30416.0000000	0.1665000
30417.0000000	0.1670000
30418.0000000	0.1671429
30419.0000000	0.1680000
30420.0000000	0.1671429
30421.0000000	0.1680000
30422.0000000	0.1690000
30423.0000000	0.1695000
30424.0000000	0.1690476
30425.0000000	0.1700000
30426.0000000	0.1695000
30427.0000000	0.1690476
30428.0000000	0.1700000
30429.0000000	0.1700000
30430.0000000	0.1700000
30431.0000000	0.1695238
30432.0000000	0.1700000
30433.0000000	0.1700000
30434.0000000	0.1695000
30435.0000000	0.1680000
30436.0000000	0.1690476
30437.0000000	0.1700000
30438.0000000	0.1700000
30439.0000000	0.1700000
30440.0000000	0.1700000
30441.0000000	0.1700000
30442.0000000	0.1700000
30443.0000000	0.1700000
30444.0000000	0.1700000
30445.0000000	0.1700000
30446.0000000	0.1700000
30447.0000000	0.1700000
30448.0000000	0.1723810
30449.0000000	0.1725000
30450.0000000	0.1730000
30451.0000000	0.1750000
30452.0000000	0.1738096
30453.0000000	0.1750000
30454.0000000	0.1738096
30455.0000000	0.1740000
30456.0000000	0.1715000
30457.0000000	0.1714286
30458.0000000	0.1725000
30459.0000000	0.1750000
30460.0000000	0.1740000
30461.0000000	0.1761905
30462.0000000	0.1755000
30463.0000000	0.1747619
30464.0000000	0.1740000
30465.0000000	0.1728572
30466.0000000	0.1735000
30467.0000000	0.1735000
30468.0000000	0.1745000
30469.0000000	0.1755000
30470.0000000	0.1723810

30471.0000000	0.1730000
30472.0000000	0.1742858
30473.0000000	0.1725000
30474.0000000	0.1770000
30475.0000000	0.1770000
30476.0000000	0.1747619
30477.0000000	0.1785000
30478.0000000	0.1795000
30479.0000000	0.1771429
30480.0000000	0.1795000
30481.0000000	0.1795239
30482.0000000	0.1795000
30483.0000000	0.1800000
30484.0000000	0.1800000
30485.0000000	0.1800000
30486.0000000	0.1800000
30487.0000000	0.1800000
30488.0000000	0.1800000
30489.0000000	0.1810000
30490.0000000	0.1805000
30491.0000000	0.1814286
30492.0000000	0.1815000
30493.0000000	0.1814286
30494.0000000	0.1840000
30495.0000000	0.1825000
30496.0000000	0.1823810
30497.0000000	0.1850000
30498.0000000	0.1865000
30499.0000000	0.1870000
30500.0000000	0.1871429
30501.0000000	0.1876191
30502.0000000	0.1885000
30503.0000000	0.1880000
30504.0000000	0.1870000
30505.0000000	0.1875000
30506.0000000	0.1885715
30507.0000000	0.1875000
30508.0000000	0.1900000
30509.0000000	0.1895000
30510.0000000	0.1885715
30511.0000000	0.1885000
30512.0000000	0.1895000
30513.0000000	0.1890000
30514.0000000	0.1900000
30515.0000000	0.1895238
30516.0000000	0.1900000
30517.0000000	0.1900000
30518.0000000	0.1895000
30519.0000000	0.1900000
30520.0000000	0.1900000
30521.0000000	0.1895000
30522.0000000	0.1900000
30523.0000000	0.1895000
30524.0000000	0.1900000
30525.0000000	0.1900000
30526.0000000	0.1900000
30527.0000000	0.1900000

30528.0000000	0.1900000
30529.0000000	0.1900000
30530.0000000	0.1900000
30531.0000000	0.1900000
30532.0000000	0.1900000
30533.0000000	0.1900000
30534.0000000	0.1900000
30535.0000000	0.1900000
30536.0000000	0.1900000
30537.0000000	0.1900000
30538.0000000	0.1900000
30539.0000000	0.1900000
30540.0000000	0.1900000
30541.0000000	0.1900000
30542.0000000	0.1900000
30543.0000000	0.1900000
30544.0000000	0.1900000
30545.0000000	0.1900000
30546.0000000	0.1900000
30547.0000000	0.1900000
30548.0000000	0.1900000
30549.0000000	0.1900000
30550.0000000	0.1900000
30551.0000000	0.1904762
30552.0000000	0.1900000
30553.0000000	0.1900000
30554.0000000	0.1920000
30555.0000000	0.1905000
30556.0000000	0.1919048
30557.0000000	0.1910000
30558.0000000	0.1930000
30559.0000000	0.1920000
30560.0000000	0.1923810
30561.0000000	0.1940000
30562.0000000	0.1928572
30563.0000000	0.1940000
30564.0000000	0.1950000
30565.0000000	0.1940000
30566.0000000	0.1940000
30567.0000000	0.1971429
30568.0000000	0.1975000
30569.0000000	0.1947619
30570.0000000	0.1950000
30571.0000000	0.1942857
30572.0000000	0.1960000
30573.0000000	0.1920000
30574.0000000	0.1940000
30575.0000000	0.1920000
30576.0000000	0.1952381
30577.0000000	0.1940000
30578.0000000	0.1961905
30579.0000000	0.1945000
30580.0000000	0.1965000
30581.0000000	0.1938096
30582.0000000	0.1940000
30583.0000000	0.1950000
30584.0000000	0.1942857

30585.0000000	0.1920000
30586.0000000	0.1910000
30587.0000000	0.1904762
30588.0000000	0.1900000
30589.0000000	0.1900000
30590.0000000	0.1900000
30591.0000000	0.1900000
30592.0000000	0.1900000
30593.0000000	0.1900000
30594.0000000	0.1900000
30595.0000000	0.1900000
30596.0000000	0.1904762
30597.0000000	0.1900000
30598.0000000	0.1904762
30599.0000000	0.1900000
30600.0000000	0.1900000
30601.0000000	0.1909524
30602.0000000	0.1910000
30603.0000000	0.1905000
30604.0000000	0.1910000
30605.0000000	0.1914286
30606.0000000	0.1910000
30607.0000000	0.1940000
30608.0000000	0.1928572
30609.0000000	0.1950000
30610.0000000	0.1970000
30611.0000000	0.1980000
30612.0000000	0.1976191
30613.0000000	0.1985000
30614.0000000	0.1995238
30615.0000000	0.2000000
30616.0000000	0.2000000
30617.0000000	0.1995238
30618.0000000	0.2000000
30619.0000000	0.2000000
30620.0000000	0.2000000
30621.0000000	0.2000000
30622.0000000	0.2000000
30623.0000000	0.2000000
30624.0000000	0.2000000
30625.0000000	0.2000000
30626.0000000	0.2000000
30627.0000000	0.2000000
30628.0000000	0.2000000
30629.0000000	0.2009524
30630.0000000	0.2000000
30631.0000000	0.2000000
30632.0000000	0.2000000
30633.0000000	0.2010000
30634.0000000	0.2025000
30635.0000000	0.2005000
30636.0000000	0.2019048
30637.0000000	0.2028572
30638.0000000	0.2045000
30639.0000000	0.2060000
30640.0000000	0.2080000
30641.0000000	0.2080953

30642.0000000	0.2090000
30643.0000000	0.2080000
30644.0000000	0.2090476
30645.0000000	0.2095000
30646.0000000	0.2095000
30647.0000000	0.2090476
30648.0000000	0.2070000
30649.0000000	0.2055000
30650.0000000	0.2052381
30651.0000000	0.2050000
30652.0000000	0.2050000
30653.0000000	0.2042857
30654.0000000	0.2030000
30655.0000000	0.2050000
30656.0000000	0.2035000
30657.0000000	0.2023810
30658.0000000	0.2030000
30659.0000000	0.2023810
30660.0000000	0.2015000
30661.0000000	0.2010000
30662.0000000	0.2004762
30663.0000000	0.2000000
30664.0000000	0.2000000
30665.0000000	0.2000000
30666.0000000	0.2000000
30667.0000000	0.2000000
30668.0000000	0.2000000
30669.0000000	0.2000000
30670.0000000	0.2000000
30671.0000000	0.2000000
30672.0000000	0.2000000
30673.0000000	0.2000000
30674.0000000	0.2000000
30675.0000000	0.2000000
30676.0000000	0.2000000
30677.0000000	0.2000000
30678.0000000	0.2000000
30679.0000000	0.2000000
30680.0000000	0.2000000
30681.0000000	0.2000000
30682.0000000	0.2000000
30683.0000000	0.2000000
30684.0000000	0.2000000
30685.0000000	0.2000000
30686.0000000	0.2005000
30687.0000000	0.2004762
30688.0000000	0.2015000
30689.0000000	0.2038096
30690.0000000	0.2030000
30691.0000000	0.2040000
30692.0000000	0.2061905
30693.0000000	0.2050000
30694.0000000	0.2055000
30695.0000000	0.2070000
30696.0000000	0.2080953
30697.0000000	0.2085000
30698.0000000	0.2085715

30699.0000000	0.2090000
30700.0000000	0.2090000
30701.0000000	0.2090000
30702.0000000	0.2090476
30703.0000000	0.2085000
30704.0000000	0.2090000
30705.0000000	0.2100000
30706.0000000	0.2100000
30707.0000000	0.2090476
30708.0000000	0.2100000
30709.0000000	0.2100000
30710.0000000	0.2100000
30711.0000000	0.2100000
30712.0000000	0.2100000
30713.0000000	0.2100000
30714.0000000	0.2100000
30715.0000000	0.2100000
30716.0000000	0.2100000
30717.0000000	0.2095238
30718.0000000	0.2095000
30719.0000000	0.2095000
30720.0000000	0.2100000
30721.0000000	0.2095000
30722.0000000	0.2095000
30723.0000000	0.2095238
30724.0000000	0.2095000
30725.0000000	0.2076191
30726.0000000	0.2030000
30727.0000000	0.2055000
30728.0000000	0.2020000
30729.0000000	0.2004762
30730.0000000	0.2005000
30731.0000000	0.2000000
30732.0000000	0.2000000
30733.0000000	0.2000000
30734.0000000	0.1995000
30735.0000000	0.1995238
30736.0000000	0.2000000
30737.0000000	0.2000000
30738.0000000	0.2000000
30739.0000000	0.1995000
30740.0000000	0.2000000
30741.0000000	0.2000000
30742.0000000	0.2000000
30743.0000000	0.1995000
30744.0000000	0.1995238
30745.0000000	0.1995000
30746.0000000	0.2000000
30747.0000000	0.2000000
30748.0000000	0.2000000
30749.0000000	0.2005000
30750.0000000	0.2004762
30751.0000000	0.2000000
30752.0000000	0.2005000
30753.0000000	0.2014286
30754.0000000	0.2010000
30755.0000000	0.2015000

30756.0000000	0.2014286
30757.0000000	0.2010000
30758.0000000	0.2005000
30759.0000000	0.2023810
30760.0000000	0.2000000
30761.0000000	0.2030000
30762.0000000	0.2005000
30763.0000000	0.2009524
30764.0000000	0.2005000
30765.0000000	0.2014286
30766.0000000	0.2040000
30767.0000000	0.2045000
30768.0000000	0.2047619
30769.0000000	0.2075000
30770.0000000	0.2075000
30771.0000000	0.2085000
30772.0000000	0.2100000
30773.0000000	0.2095000
30774.0000000	0.2100000
30775.0000000	0.2100000
30776.0000000	0.2100000
30777.0000000	0.2100000
30778.0000000	0.2100000
30779.0000000	0.2105000
30780.0000000	0.2109524
30781.0000000	0.2100000
30782.0000000	0.2105000
30783.0000000	0.2100000
30784.0000000	0.2100000
30785.0000000	0.2100000
30786.0000000	0.2104762
30787.0000000	0.2100000
30788.0000000	0.2100000
30789.0000000	0.2100000
30790.0000000	0.2105000
30791.0000000	0.2100000
30792.0000000	0.2105000
30793.0000000	0.2109524
30794.0000000	0.2100000
30795.0000000	0.2109524
30796.0000000	0.2115000
30797.0000000	0.2138095
30798.0000000	0.2130000
30799.0000000	0.2130000
30800.0000000	0.2140000
30801.0000000	0.2140000
30802.0000000	0.2138095
30803.0000000	0.2145000
30804.0000000	0.2138095
30805.0000000	0.2145000
30806.0000000	0.2130000
30807.0000000	0.2125000
30808.0000000	0.2109524
30809.0000000	0.2110000
30810.0000000	0.2105000
30811.0000000	0.2104762
30812.0000000	0.2105000

30813.0000000	0.2100000
30814.0000000	0.2095000
30815.0000000	0.2070000
30816.0000000	0.2040000
30817.0000000	0.2014286
30818.0000000	0.2000000
30819.0000000	0.2000000
30820.0000000	0.2000000
30821.0000000	0.2000000
30822.0000000	0.2000000
30823.0000000	0.2000000
30824.0000000	0.2000000
30825.0000000	0.2000000
30826.0000000	0.2005000
30827.0000000	0.2000000
30828.0000000	0.2000000
30829.0000000	0.2000000
30830.0000000	0.2000000
30831.0000000	0.2000000
30832.0000000	0.2000000
30833.0000000	0.1995000
30834.0000000	0.1995238
30835.0000000	0.1985000
30836.0000000	0.1995000
30837.0000000	0.1995000
30838.0000000	0.2000000
30839.0000000	0.2005000
30840.0000000	0.2000000
30841.0000000	0.2000000
30842.0000000	0.1995238
30843.0000000	0.2000000
30844.0000000	0.1995000
30845.0000000	0.1995000
30846.0000000	0.2000000
30847.0000000	0.2000000
30848.0000000	0.2000000
30849.0000000	0.2000000
30850.0000000	0.2000000
30851.0000000	0.2000000
30852.0000000	0.2000000
30853.0000000	0.2000000
30854.0000000	0.2000000
30855.0000000	0.2000000
30856.0000000	0.2000000
30857.0000000	0.2000000
30858.0000000	0.2000000
30859.0000000	0.2000000
30860.0000000	0.2000000
30861.0000000	0.2000000
30862.0000000	0.1995238
30863.0000000	0.1995000
30864.0000000	0.1990000
30865.0000000	0.1995238
30866.0000000	0.1985000
30867.0000000	0.1985000
30868.0000000	0.1985715
30869.0000000	0.1950000

30870.0000000	0.1940000
30871.0000000	0.1935000
30872.0000000	0.1947619
30873.0000000	0.1957143
30874.0000000	0.1960000
30875.0000000	0.1960000
30876.0000000	0.1970000
30877.0000000	0.1942857
30878.0000000	0.1955000
30879.0000000	0.1940000
30880.0000000	0.1942857
30881.0000000	0.1945000
30882.0000000	0.1965000
30883.0000000	0.1961905
30884.0000000	0.1930000
30885.0000000	0.1919048
30886.0000000	0.1925000
30887.0000000	0.1930000
30888.0000000	0.1925000
30889.0000000	0.1938096
30890.0000000	0.1905000
30891.0000000	0.1905000
30892.0000000	0.1909524
30893.0000000	0.1915000
30894.0000000	0.1914286
30895.0000000	0.1915000
30896.0000000	0.1900000
30897.0000000	0.1905000
30898.0000000	0.1910000
30899.0000000	0.1904762
30900.0000000	0.1900000
30901.0000000	0.1900000
30902.0000000	0.1915000
30903.0000000	0.1900000
30904.0000000	0.1920000
30905.0000000	0.1925000
30906.0000000	0.1900000
30907.0000000	0.1920000
30908.0000000	0.1909524
30909.0000000	0.1900000
30910.0000000	0.1914286
30911.0000000	0.1930000
30912.0000000	0.1915000
30913.0000000	0.1952381
30914.0000000	0.1945000
30915.0000000	0.1925000
30916.0000000	0.1947619
30917.0000000	0.1965000
30918.0000000	0.1945000
30919.0000000	0.1957143
30920.0000000	0.1925000
30921.0000000	0.1952381
30922.0000000	0.1975000
30923.0000000	0.1930000
30924.0000000	0.1945000
30925.0000000	0.1928572
30926.0000000	0.1920000

30927.0000000	0.1920000
30928.0000000	0.1915000
30929.0000000	0.1919048
30930.0000000	0.1909524
30931.0000000	0.1900000
30932.0000000	0.1900000
30933.0000000	0.1900000
30934.0000000	0.1885000
30935.0000000	0.1870000
30936.0000000	0.1850000
30937.0000000	0.1819048
30938.0000000	0.1805000
30939.0000000	0.1800000
30940.0000000	0.1795239
30941.0000000	0.1795000
30942.0000000	0.1785000
30943.0000000	0.1790000
30944.0000000	0.1776191
30945.0000000	0.1750000
30946.0000000	0.1733334
30947.0000000	0.1705000
30948.0000000	0.1710000
30949.0000000	0.1704762
30950.0000000	0.1700000
30951.0000000	0.1700000
30952.0000000	0.1710000
30953.0000000	0.1700000
30954.0000000	0.1705000
30955.0000000	0.1719048
30956.0000000	0.1720000
30957.0000000	0.1720000
30958.0000000	0.1720000
30959.0000000	0.1714286
30960.0000000	0.1720000
30961.0000000	0.1719048
30962.0000000	0.1730000
30963.0000000	0.1710000
30964.0000000	0.1714286
30965.0000000	0.1710000
30966.0000000	0.1725000
30967.0000000	0.1715000
30968.0000000	0.1719048
30969.0000000	0.1760000
30970.0000000	0.1723810
30971.0000000	0.1740000
30972.0000000	0.1755001
30973.0000000	0.1752381
30974.0000000	0.1755000
30975.0000000	0.1730000
30976.0000000	0.1735000
30977.0000000	0.1752381
30978.0000000	0.1740000
30979.0000000	0.1747619
30980.0000000	0.1745000
30981.0000000	0.1740000
30982.0000000	0.1780953
30983.0000000	0.1755000

30984.0000000	0.1775000
30985.0000000	0.1761905
30986.0000000	0.1745000
30987.0000000	0.1755000
30988.0000000	0.1750000
30989.0000000	0.1709524
30990.0000000	0.1710000
30991.0000000	0.1700000
30992.0000000	0.1704762
30993.0000000	0.1705000
30994.0000000	0.1700000
30995.0000000	0.1700000
30996.0000000	0.1700000
30997.0000000	0.1700000
30998.0000000	0.1695238
30999.0000000	0.1700000
31000.0000000	0.1700000
31001.0000000	0.1700000
31002.0000000	0.1700000
31003.0000000	0.1700000
31004.0000000	0.1700000
31005.0000000	0.1700000
31006.0000000	0.1700000
31007.0000000	0.1700000
31008.0000000	0.1700000
31009.0000000	0.1700000
31010.0000000	0.1695000
31011.0000000	0.1700000
31012.0000000	0.1695000
31013.0000000	0.1690476
31014.0000000	0.1640000
31015.0000000	0.1610000
31016.0000000	0.1604762
31017.0000000	0.1620000
31018.0000000	0.1600000
31019.0000000	0.1600000
31020.0000000	0.1600000
31021.0000000	0.1590476
31022.0000000	0.1595000
31023.0000000	0.1545000
31024.0000000	0.1565000
31025.0000000	0.1561905
31026.0000000	0.1570000
31027.0000000	0.1570000
31028.0000000	0.1566667
31029.0000000	0.1580000
31030.0000000	0.1590476
31031.0000000	0.1580000
31032.0000000	0.1590000
31033.0000000	0.1590000
31034.0000000	0.1585715
31035.0000000	0.1600000
31036.0000000	0.1590000
31037.0000000	0.1585715
31038.0000000	0.1595000
31039.0000000	0.1595238
31040.0000000	0.1590000

31041.0000000	0.1570000
31042.0000000	0.1565000
31043.0000000	0.1576191
31044.0000000	0.1515000
31045.0000000	0.1510000
31046.0000000	0.1504762
31047.0000000	0.1500000
31048.0000000	0.1500000
31049.0000000	0.1500000
31050.0000000	0.1500000
31051.0000000	0.1500000
31052.0000000	0.1495238
31053.0000000	0.1500000
31054.0000000	0.1500000
31055.0000000	0.1500000
31056.0000000	0.1495000
31057.0000000	0.1485715
31058.0000000	0.1500000
31059.0000000	0.1495000
31060.0000000	0.1500000
31061.0000000	0.1485715
31062.0000000	0.1495000
31063.0000000	0.1480000
31064.0000000	0.1455000
31065.0000000	0.1442858
31066.0000000	0.1425000
31067.0000000	0.1404762
31068.0000000	0.1400000
31069.0000000	0.1404762
31070.0000000	0.1400000
31071.0000000	0.1395000
31072.0000000	0.1395000
31073.0000000	0.1390477
31074.0000000	0.1395000
31075.0000000	0.1395000
31076.0000000	0.1390000
31077.0000000	0.1390476
31078.0000000	0.1395000
31079.0000000	0.1395000
31080.0000000	0.1395238
31081.0000000	0.1395000
31082.0000000	0.1400000
31083.0000000	0.1400000
31084.0000000	0.1395000
31085.0000000	0.1400000
31086.0000000	0.1395000
31087.0000000	0.1400000
31088.0000000	0.1400000
31089.0000000	0.1400000
31090.0000000	0.1395000
31091.0000000	0.1400000
31092.0000000	0.1400000
31093.0000000	0.1400000
31094.0000000	0.1400000
31095.0000000	0.1400000
31096.0000000	0.1400000
31097.0000000	0.1400000

31098.0000000	0.1400000
31099.0000000	0.1400000
31100.0000000	0.1400000
31101.0000000	0.1395000
31102.0000000	0.1395000
31103.0000000	0.1375000
31104.0000000	0.1385715
31105.0000000	0.1365000
31106.0000000	0.1366667
31107.0000000	0.1390000
31108.0000000	0.1380000
31109.0000000	0.1390477
31110.0000000	0.1380000
31111.0000000	0.1345000
31112.0000000	0.1355000
31113.0000000	0.1352381
31114.0000000	0.1345000
31115.0000000	0.1380000
31116.0000000	0.1376191
31117.0000000	0.1385000
31118.0000000	0.1385715
31119.0000000	0.1395000
31120.0000000	0.1400000
31121.0000000	0.1400000
31122.0000000	0.1400000
31123.0000000	0.1400000
31124.0000000	0.1400000
31125.0000000	0.1400000
31126.0000000	0.1395000
31127.0000000	0.1400000
31128.0000000	0.1400000
31129.0000000	0.1400000
31130.0000000	0.1400000
31131.0000000	0.1400000
31132.0000000	0.1400000
31133.0000000	0.1400000
31134.0000000	0.1404762
31135.0000000	0.1400000
31136.0000000	0.1404762
31137.0000000	0.1400000
31138.0000000	0.1400000
31139.0000000	0.1405000
31140.0000000	0.1400000
31141.0000000	0.1400000
31142.0000000	0.1400000
31143.0000000	0.1400000
31144.0000000	0.1400000
31145.0000000	0.1400000
31146.0000000	0.1400000
31147.0000000	0.1400000
31148.0000000	0.1400000
31149.0000000	0.1376191
31150.0000000	0.1370000
31151.0000000	0.1375000
31152.0000000	0.1376191
31153.0000000	0.1380000
31154.0000000	0.1345000

31155.0000000	0.1347619
31156.0000000	0.1320000
31157.0000000	0.1338095
31158.0000000	0.1315000
31159.0000000	0.1320000
31160.0000000	0.1320000
31161.0000000	0.1333333
31162.0000000	0.1325000
31163.0000000	0.1345000
31164.0000000	0.1357143
31165.0000000	0.1340000
31166.0000000	0.1333333
31167.0000000	0.1330000
31168.0000000	0.1325000
31169.0000000	0.1350000
31170.0000000	0.1328572
31171.0000000	0.1330000
31172.0000000	0.1350000
31173.0000000	0.1366667
31174.0000000	0.1361905
31175.0000000	0.1355000
31176.0000000	0.1355000
31177.0000000	0.1355000
31178.0000000	0.1370000
31179.0000000	0.1376191
31180.0000000	0.1375000
31181.0000000	0.1395238
31182.0000000	0.1365000
31183.0000000	0.1370000
31184.0000000	0.1355000
31185.0000000	0.1338096
31186.0000000	0.1330000
31187.0000000	0.1330000
31188.0000000	0.1342857
31189.0000000	0.1345000
31190.0000000	0.1347619
31191.0000000	0.1315000
31192.0000000	0.1330000
31193.0000000	0.1320000
31194.0000000	0.1319048
31195.0000000	0.1310000
31196.0000000	0.1320000
31197.0000000	0.1314286
31198.0000000	0.1310000
31199.0000000	0.1315000
31200.0000000	0.1300000
31201.0000000	0.1300000
31202.0000000	0.1305000
31203.0000000	0.1300000
31204.0000000	0.1295000
31205.0000000	0.1300000
31206.0000000	0.1300000
31207.0000000	0.1300000
31208.0000000	0.1300000
31209.0000000	0.1300000
31210.0000000	0.1300000
31211.0000000	0.1300000

31212.0000000	0.1295000
31213.0000000	0.1295238
31214.0000000	0.1295000
31215.0000000	0.1290476
31216.0000000	0.1295000
31217.0000000	0.1290000
31218.0000000	0.1276191
31219.0000000	0.1290000
31220.0000000	0.1285000
31221.0000000	0.1261905
31222.0000000	0.1275000
31223.0000000	0.1285000
31224.0000000	0.1280000
31225.0000000	0.1276191
31226.0000000	0.1271428
31227.0000000	0.1280000
31228.0000000	0.1295000
31229.0000000	0.1280000
31230.0000000	0.1275000
31231.0000000	0.1247619
31232.0000000	0.1275000
31233.0000000	0.1242857
31234.0000000	0.1250000
31235.0000000	0.1257143
31236.0000000	0.1220000
31237.0000000	0.1240000
31238.0000000	0.1240000
31239.0000000	0.1250000
31240.0000000	0.1223809
31241.0000000	0.1235000
31242.0000000	0.1209524
31243.0000000	0.1215000
31244.0000000	0.1210000
31245.0000000	0.1204762
31246.0000000	0.1205000
31247.0000000	0.1210000
31248.0000000	0.1205000
31249.0000000	0.1200000
31250.0000000	0.1205000
31251.0000000	0.1200000
31252.0000000	0.1200000
31253.0000000	0.1200000
31254.0000000	0.1200000
31255.0000000	0.1200000
31256.0000000	0.1200000
31257.0000000	0.1190476
31258.0000000	0.1195000
31259.0000000	0.1190000
31260.0000000	0.1190000
31261.0000000	0.1200000
31262.0000000	0.1190000
31263.0000000	0.1171428
31264.0000000	0.1170000
31265.0000000	0.1165000
31266.0000000	0.1157143
31267.0000000	0.1155000
31268.0000000	0.1125000

31269.0000000	0.1109524
31270.0000000	0.1100000
31271.0000000	0.1100000
31272.0000000	0.1090476
31273.0000000	0.1090000
31274.0000000	0.1055000
31275.0000000	0.1020000
31276.0000000	0.1023810
31277.0000000	0.1040000
31278.0000000	0.1014286
31279.0000000	0.1015000
31280.0000000	0.1005000
31281.0000000	0.1014286
31282.0000000	0.1005000
31283.0000000	0.1000000
31284.0000000	0.1005000
31285.0000000	0.1004762
31286.0000000	0.1000000
31287.0000000	0.1000000
31288.0000000	0.1000000
31289.0000000	0.1000000
31290.0000000	0.1000000
31291.0000000	0.1000000
31292.0000000	0.1000000
31293.0000000	0.1000000
31294.0000000	0.0995000
31295.0000000	0.1000000
31296.0000000	0.0995238
31297.0000000	0.0995000
31298.0000000	0.0995000
31299.0000000	0.0995000
31300.0000000	0.0975000
31301.0000000	0.0990476
31302.0000000	0.0985714
31303.0000000	0.0980000
31304.0000000	0.0985000
31305.0000000	0.0970000
31306.0000000	0.0985714
31307.0000000	0.0975000
31308.0000000	0.0990000
31309.0000000	0.1000000
31310.0000000	0.1000000
31311.0000000	0.0975000
31312.0000000	0.0995000
31313.0000000	0.0995000
31314.0000000	0.0985714
31315.0000000	0.0975000
31316.0000000	0.0995000
31317.0000000	0.0985714
31318.0000000	0.0980000
31319.0000000	0.0985000
31320.0000000	0.0995000
31321.0000000	0.0971429
31322.0000000	0.0965000
31323.0000000	0.0945000
31324.0000000	0.0947619
31325.0000000	0.0925000

31326.0000000	0.0904762
31327.0000000	0.0905000
31328.0000000	0.0905000
31329.0000000	0.0905000
31330.0000000	0.0904762
31331.0000000	0.0900000
31332.0000000	0.0900000
31333.0000000	0.0900000
31334.0000000	0.0900000
31335.0000000	0.0900000
31336.0000000	0.0900000
31337.0000000	0.0890476
31338.0000000	0.0900000
31339.0000000	0.0861905
31340.0000000	0.0875000
31341.0000000	0.0876191
31342.0000000	0.0885000
31343.0000000	0.0860000
31344.0000000	0.0870000
31345.0000000	0.0871429
31346.0000000	0.0870000
31347.0000000	0.0865000
31348.0000000	0.0847619
31349.0000000	0.0845000
31350.0000000	0.0870000
31351.0000000	0.0866667
31352.0000000	0.0850000
31353.0000000	0.0880000
31354.0000000	0.0885715
31355.0000000	0.0875000
31356.0000000	0.0885000
31357.0000000	0.0871429
31358.0000000	0.0865000
31359.0000000	0.0875000
31360.0000000	0.0866667
31361.0000000	0.0870000
31362.0000000	0.0866667
31363.0000000	0.0860000
31364.0000000	0.0875000
31365.0000000	0.0885000
31366.0000000	0.0885714
31367.0000000	0.0895000
31368.0000000	0.0895000
31369.0000000	0.0880953
31370.0000000	0.0895000
31371.0000000	0.0885715
31372.0000000	0.0900000
31373.0000000	0.0890000
31374.0000000	0.0890000
31375.0000000	0.0895238
31376.0000000	0.0890000
31377.0000000	0.0880000
31378.0000000	0.0885714
31379.0000000	0.0875000
31380.0000000	0.0855000
31381.0000000	0.0842857
31382.0000000	0.0825000

31383.0000000	0.0835000
31384.0000000	0.0805000
31385.0000000	0.0819048
31386.0000000	0.0820000
31387.0000000	0.0809524
31388.0000000	0.0800000
31389.0000000	0.0805000
31390.0000000	0.0800000
31391.0000000	0.0800000
31392.0000000	0.0800000
31393.0000000	0.0800000
31394.0000000	0.0800000
31395.0000000	0.0800000
31396.0000000	0.0800000
31397.0000000	0.0800000
31398.0000000	0.0800000
31399.0000000	0.0795238
31400.0000000	0.0800000
31401.0000000	0.0800000
31402.0000000	0.0795238
31403.0000000	0.0795000
31404.0000000	0.0790000
31405.0000000	0.0800000
31406.0000000	0.0800000
31407.0000000	0.0800000
31408.0000000	0.0795238
31409.0000000	0.0800000
31410.0000000	0.0795000
31411.0000000	0.0800000
31412.0000000	0.0795238
31413.0000000	0.0800000
31414.0000000	0.0795238
31415.0000000	0.0800000
31416.0000000	0.0800000
31417.0000000	0.0800000
31418.0000000	0.0790000
31419.0000000	0.0795000
31420.0000000	0.0790000
31421.0000000	0.0790476
31422.0000000	0.0785000
31423.0000000	0.0776191
31424.0000000	0.0770000
31425.0000000	0.0770000
31426.0000000	0.0745000
31427.0000000	0.0738095
31428.0000000	0.0750000
31429.0000000	0.0747619
31430.0000000	0.0760000
31431.0000000	0.0755000
31432.0000000	0.0752381
31433.0000000	0.0740000
31434.0000000	0.0725000
31435.0000000	0.0740000
31436.0000000	0.0719048
31437.0000000	0.0710000
31438.0000000	0.0709524
31439.0000000	0.0720000

31440.0000000	0.0725000
31441.0000000	0.0710000
31442.0000000	0.0719048
31443.0000000	0.0715000
31444.0000000	0.0715000
31445.0000000	0.0709524
31446.0000000	0.0725000
31447.0000000	0.0709524
31448.0000000	0.0715000
31449.0000000	0.0705000
31450.0000000	0.0719048
31451.0000000	0.0715000
31452.0000000	0.0710000
31453.0000000	0.0719048
31454.0000000	0.0730000
31455.0000000	0.0715000
31456.0000000	0.0740000
31457.0000000	0.0719048
31458.0000000	0.0750000
31459.0000000	0.0742857
31460.0000000	0.0755000
31461.0000000	0.0750000
31462.0000000	0.0771429
31463.0000000	0.0780000
31464.0000000	0.0770000
31465.0000000	0.0770000
31466.0000000	0.0757143
31467.0000000	0.0760000
31468.0000000	0.0752381
31469.0000000	0.0765000
31470.0000000	0.0755000
31471.0000000	0.0755000
31472.0000000	0.0760000
31473.0000000	0.0766667
31474.0000000	0.0750000
31475.0000000	0.0757143
31476.0000000	0.0745000
31477.0000000	0.0719048
31478.0000000	0.0730000
31479.0000000	0.0715000
31480.0000000	0.0715000
31481.0000000	0.0738095
31482.0000000	0.0710000
31483.0000000	0.0720000
31484.0000000	0.0733333
31485.0000000	0.0710000
31486.0000000	0.0704762
31487.0000000	0.0710000
31488.0000000	0.0710000
31489.0000000	0.0710000
31490.0000000	0.0714286
31491.0000000	0.0705000
31492.0000000	0.0710000
31493.0000000	0.0704762
31494.0000000	0.0700000
31495.0000000	0.0700000
31496.0000000	0.0700000

31497.0000000	0.0700000
31498.0000000	0.0700000
31499.0000000	0.0700000
31500.0000000	0.0705000
31501.0000000	0.0700000
31502.0000000	0.0700000
31503.0000000	0.0705000
31504.0000000	0.0700000
31505.0000000	0.0704762
31506.0000000	0.0700000
31507.0000000	0.0700000
31508.0000000	0.0705000
31509.0000000	0.0700000
31510.0000000	0.0705000
31511.0000000	0.0705000
31512.0000000	0.0710000
31513.0000000	0.0700000
31514.0000000	0.0700000
31515.0000000	0.0700000
31516.0000000	0.0700000
31517.0000000	0.0704762
31518.0000000	0.0715000
31519.0000000	0.0710000
31520.0000000	0.0709524
31521.0000000	0.0700000
31522.0000000	0.0700000
31523.0000000	0.0705000
31524.0000000	0.0700000
31525.0000000	0.0705000
31526.0000000	0.0709524
31527.0000000	0.0705000
31528.0000000	0.0700000
31529.0000000	0.0700000
31530.0000000	0.0705000
31531.0000000	0.0705000
31532.0000000	0.0700000
31533.0000000	0.0704762
31534.0000000	0.0700000
31535.0000000	0.0709524
31536.0000000	0.0715000
31537.0000000	0.0710000
31538.0000000	0.0704762
31539.0000000	0.0710000
31540.0000000	0.0705000
31541.0000000	0.0704762
31542.0000000	0.0710000
31543.0000000	0.0715000
31544.0000000	0.0705000
31545.0000000	0.0700000
31546.0000000	0.0700000
31547.0000000	0.0700000
31548.0000000	0.0700000
31549.0000000	0.0700000
31550.0000000	0.0700000
31551.0000000	0.0700000
31552.0000000	0.0700000
31553.0000000	0.0700000

31554.0000000	0.0700000
31555.0000000	0.0700000
31556.0000000	0.0700000
31557.0000000	0.0700000
31558.0000000	0.0700000
31559.0000000	0.0700000
31560.0000000	0.0700000
31561.0000000	0.0700000
31562.0000000	0.0700000
31563.0000000	0.0700000
31564.0000000	0.0700000
31565.0000000	0.0700000
31566.0000000	0.0700000
31567.0000000	0.0700000
31568.0000000	0.0700000
31569.0000000	0.0700000
31570.0000000	0.0700000
31571.0000000	0.0685000
31572.0000000	0.0695238
31573.0000000	0.0685000
31574.0000000	0.0700000
31575.0000000	0.0700000
31576.0000000	0.0690000
31577.0000000	0.0695000
31578.0000000	0.0700000
31579.0000000	0.0690000
31580.0000000	0.0700000
31581.0000000	0.0690476
31582.0000000	0.0685000
31583.0000000	0.0695238
31584.0000000	0.0675000
31585.0000000	0.0665000
31586.0000000	0.0676191
31587.0000000	0.0660000
31588.0000000	0.0675000
31589.0000000	0.0655000
31590.0000000	0.0657143
31591.0000000	0.0635000
31592.0000000	0.0630000
31593.0000000	0.0623809
31594.0000000	0.0630000
31595.0000000	0.0638095
31596.0000000	0.0640000
31597.0000000	0.0635000
31598.0000000	0.0614286
31599.0000000	0.0635000
31600.0000000	0.0625000
31601.0000000	0.0640000
31602.0000000	0.0614286
31603.0000000	0.0605000
31604.0000000	0.0614286
31605.0000000	0.0605000
31606.0000000	0.0605000
31607.0000000	0.0605000
31608.0000000	0.0600000
31609.0000000	0.0610000
31610.0000000	0.0600000

31611.0000000	0.0600000
31612.0000000	0.0600000
31613.0000000	0.0600000
31614.0000000	0.0600000
31615.0000000	0.0600000
31616.0000000	0.0600000
31617.0000000	0.0600000
31618.0000000	0.0600000
31619.0000000	0.0600000
31620.0000000	0.0600000
31621.0000000	0.0600000
31622.0000000	0.0600000
31623.0000000	0.0590476
31624.0000000	0.0590000
31625.0000000	0.0575000
31626.0000000	0.0576190
31627.0000000	0.0560000
31628.0000000	0.0523810
31629.0000000	0.0535000
31630.0000000	0.0540000
31631.0000000	0.0540000
31632.0000000	0.0535000
31633.0000000	0.0519048
31634.0000000	0.0533333
31635.0000000	0.0515000
31636.0000000	0.0505000
31637.0000000	0.0510000
31638.0000000	0.0519048
31639.0000000	0.0505000
31640.0000000	0.0510000
31641.0000000	0.0509524
31642.0000000	0.0505000
31643.0000000	0.0528571
31644.0000000	0.0500000
31645.0000000	0.0510000
31646.0000000	0.0509524
31647.0000000	0.0510000
31648.0000000	0.0505000
31649.0000000	0.0510000
31650.0000000	0.0500000
31651.0000000	0.0500000
31652.0000000	0.0500000
31653.0000000	0.0500000
31654.0000000	0.0500000
31655.0000000	0.0500000
31656.0000000	0.0500000
31657.0000000	0.0500000
31658.0000000	0.0505000
31659.0000000	0.0504762
31660.0000000	0.0510000
31661.0000000	0.0500000
31662.0000000	0.0504762
31663.0000000	0.0500000
31664.0000000	0.0509524
31665.0000000	0.0500000
31666.0000000	0.0505000
31667.0000000	0.0505000

31668.0000000	0.0505000
31669.0000000	0.0504762
31670.0000000	0.0505000
31671.0000000	0.0500000
31672.0000000	0.0500000
31673.0000000	0.0500000
31674.0000000	0.0505000
31675.0000000	0.0505000
31676.0000000	0.0500000
31677.0000000	0.0505000
31678.0000000	0.0500000
31679.0000000	0.0500000
31680.0000000	0.0500000
31681.0000000	0.0500000
31682.0000000	0.0500000
31683.0000000	0.0500000
31684.0000000	0.0490000
31685.0000000	0.0490000
31686.0000000	0.0485714
31687.0000000	0.0470000
31688.0000000	0.0475000
31689.0000000	0.0471429
31690.0000000	0.0465000
31691.0000000	0.0465000
31692.0000000	0.0461905
31693.0000000	0.0465000
31694.0000000	0.0465000
31695.0000000	0.0452381
31696.0000000	0.0440000
31697.0000000	0.0445000
31698.0000000	0.0423810
31699.0000000	0.0425000
31700.0000000	0.0435000
31701.0000000	0.0428572
31702.0000000	0.0435000
31703.0000000	0.0438095
31704.0000000	0.0430000
31705.0000000	0.0425000
31706.0000000	0.0425000
31707.0000000	0.0423810
31708.0000000	0.0425000
31709.0000000	0.0425000
31710.0000000	0.0414286
31711.0000000	0.0430000
31712.0000000	0.0410000
31713.0000000	0.0415000
31714.0000000	0.0400000
31715.0000000	0.0410000
31716.0000000	0.0404762
31717.0000000	0.0410000
31718.0000000	0.0400000
31719.0000000	0.0400000
31720.0000000	0.0405000
31721.0000000	0.0405000
31722.0000000	0.0400000
31723.0000000	0.0400000
31724.0000000	0.0400000

31725.0000000	0.0400000
31726.0000000	0.0400000
31727.0000000	0.0400000
31728.0000000	0.0400000
31729.0000000	0.0400000
31730.0000000	0.0400000
31731.0000000	0.0400000
31732.0000000	0.0395000
31733.0000000	0.0385000
31734.0000000	0.0390476
31735.0000000	0.0370000
31736.0000000	0.0385000
31737.0000000	0.0395000
31738.0000000	0.0357143
31739.0000000	0.0355000
31740.0000000	0.0342857
31741.0000000	0.0345000
31742.0000000	0.0355000
31743.0000000	0.0333333
31744.0000000	0.0335000
31745.0000000	0.0320000
31746.0000000	0.0323809
31747.0000000	0.0340000
31748.0000000	0.0320000
31749.0000000	0.0320000
31750.0000000	0.0319048
31751.0000000	0.0310000
31752.0000000	0.0330000
31753.0000000	0.0323809
31754.0000000	0.0305000
31755.0000000	0.0304762
31756.0000000	0.0310000
31757.0000000	0.0300000
31758.0000000	0.0300000
31759.0000000	0.0300000
31760.0000000	0.0300000
31761.0000000	0.0300000
31762.0000000	0.0290476
31763.0000000	0.0300000
31764.0000000	0.0300000
31765.0000000	0.0280000
31766.0000000	0.0285000
31767.0000000	0.0285000
31768.0000000	0.0266667
31769.0000000	0.0270000
31770.0000000	0.0257143
31771.0000000	0.0255000
31772.0000000	0.0265000
31773.0000000	0.0240000
31774.0000000	0.0242857
31775.0000000	0.0220000
31776.0000000	0.0230000
31777.0000000	0.0219048
31778.0000000	0.0210000
31779.0000000	0.0228571
31780.0000000	0.0210000
31781.0000000	0.0210000

31782.0000000	0.0200000
31783.0000000	0.0204762
31784.0000000	0.0200000
31785.0000000	0.0200000
31786.0000000	0.0200000
31787.0000000	0.0200000
31788.0000000	0.0200000
31789.0000000	0.0200000
31790.0000000	0.0200000
31791.0000000	0.0200000
31792.0000000	0.0200000
31793.0000000	0.0200000
31794.0000000	0.0200000
31795.0000000	0.0200000
31796.0000000	0.0200000
31797.0000000	0.0200000
31798.0000000	0.0200000
31799.0000000	0.0195238
31800.0000000	0.0200000
31801.0000000	0.0190000
31802.0000000	0.0200000
31803.0000000	0.0195000
31804.0000000	0.0200000
31805.0000000	0.0190476
31806.0000000	0.0195000
31807.0000000	0.0200000
31808.0000000	0.0200000
31809.0000000	0.0180000
31810.0000000	0.0180000
31811.0000000	0.0185714
31812.0000000	0.0195000
31813.0000000	0.0195000
31814.0000000	0.0180000
31815.0000000	0.0200000
31816.0000000	0.0185000
31817.0000000	0.0195238
31818.0000000	0.0190000
31819.0000000	0.0190000
31820.0000000	0.0190476
31821.0000000	0.0170000
31822.0000000	0.0185000
31823.0000000	0.0180000
31824.0000000	0.0176190
31825.0000000	0.0165000
31826.0000000	0.0152381
31827.0000000	0.0170000
31828.0000000	0.0170000
31829.0000000	0.0166667
31830.0000000	0.0150000
31831.0000000	0.0170000
31832.0000000	0.0150000
31833.0000000	0.0157143
31834.0000000	0.0133333
31835.0000000	0.0145000
31836.0000000	0.0120000
31837.0000000	0.0160000
31838.0000000	0.0128571

31839.0000000	0.0135000
31840.0000000	0.0130000
31841.0000000	0.0128571
31842.0000000	0.0135000
31843.0000000	0.0115000
31844.0000000	0.0115000
31845.0000000	0.0133333
31846.0000000	0.0125000
31847.0000000	0.0114286
31848.0000000	0.0125000
31849.0000000	0.0140000
31850.0000000	0.0114286
31851.0000000	0.0120000
31852.0000000	0.0120000
31853.0000000	0.0110000
31854.0000000	0.0138095
31855.0000000	0.0115000
31856.0000000	0.0104762
31857.0000000	0.0125000
31858.0000000	0.0115000
31859.0000000	0.0100000
31860.0000000	0.0128571
31861.0000000	0.0110000
31862.0000000	0.0130000
31863.0000000	0.0109524
31864.0000000	0.0100000
31865.0000000	0.0104762
31866.0000000	0.0140000
31867.0000000	0.0115000
31868.0000000	0.0105000
31869.0000000	0.0114286
31870.0000000	0.0100000
31871.0000000	0.0110000
31872.0000000	0.0109524
31873.0000000	0.0105000
31874.0000000	0.0109524
31875.0000000	0.0110000
31876.0000000	0.0105000
31877.0000000	0.0104762
31878.0000000	0.0100000
31879.0000000	0.0105000
31880.0000000	0.0105000
31881.0000000	0.0109524
31882.0000000	0.0110000
31883.0000000	0.0100000
31884.0000000	0.0105000
31885.0000000	0.0100000
31886.0000000	0.0100000
31887.0000000	0.0100000
31888.0000000	0.0100000
31889.0000000	0.0100000
31890.0000000	0.0100000
31891.0000000	0.0100000
31892.0000000	0.0100000
31893.0000000	0.0100000
31894.0000000	0.0100000
31895.0000000	0.0100000

31896.0000000	0.0100000
31897.0000000	0.0100000
31898.0000000	0.0095000
31899.0000000	0.0100000
31900.0000000	0.0100000
31901.0000000	0.0100000
31902.0000000	0.0100000
31903.0000000	0.0100000
31904.0000000	0.0100000
31905.0000000	0.0095238
31906.0000000	0.0100000
31907.0000000	0.0095000
31908.0000000	0.0100000
31909.0000000	0.0100000
31910.0000000	0.0095238
31911.0000000	0.0100000
31912.0000000	0.0100000
31913.0000000	0.0095238
31914.0000000	0.0100000
31915.0000000	0.0095000
31916.0000000	0.0100000
31917.0000000	0.0100000
31918.0000000	0.0095000
31919.0000000	0.0100000
31920.0000000	0.0095000
31921.0000000	0.0100000
31922.0000000	0.0095238
31923.0000000	0.0100000
31924.0000000	0.0095000
31925.0000000	0.0100000
31926.0000000	0.0100000
31927.0000000	0.0090000
31928.0000000	0.0100000
31929.0000000	0.0100000
31930.0000000	0.0085000
31931.0000000	0.0095000
31932.0000000	0.0095238
31933.0000000	0.0085000
31934.0000000	0.0100000
31935.0000000	0.0080952
31936.0000000	0.0090000
31937.0000000	0.0085000
31938.0000000	0.0090476
31939.0000000	0.0095000
31940.0000000	0.0085000
31941.0000000	0.0100000
31942.0000000	0.0100000
31943.0000000	0.0090000
31944.0000000	0.0090476
31945.0000000	0.0090000
31946.0000000	0.0095238
31947.0000000	0.0100000
31948.0000000	0.0085000
31949.0000000	0.0090000
31950.0000000	0.0095000
31951.0000000	0.0090476
31952.0000000	0.0080000

31953.0000000	0.0090476
31954.0000000	0.0090000
31955.0000000	0.0090476
31956.0000000	0.0095000
31957.0000000	0.0090000
31958.0000000	0.0090000
31959.0000000	0.0095000
31960.0000000	0.0085714
31961.0000000	0.0095000
31962.0000000	0.0095238
31963.0000000	0.0090000
31964.0000000	0.0090000
31965.0000000	0.0090476
31966.0000000	0.0095000
31967.0000000	0.0090000
31968.0000000	0.0090000
31969.0000000	0.0080952
31970.0000000	0.0090000
31971.0000000	0.0090476
31972.0000000	0.0085000
31973.0000000	0.0085000
31974.0000000	0.0085714
31975.0000000	0.0085000
31976.0000000	0.0085000
31977.0000000	0.0090476
31978.0000000	0.0080000
31979.0000000	0.0075000
31980.0000000	0.0085000
31981.0000000	0.0100000
31982.0000000	0.0071429
31983.0000000	0.0090000
31984.0000000	0.0095000
31985.0000000	0.0090000
31986.0000000	0.0090476
31987.0000000	0.0080000
31988.0000000	0.0100000
31989.0000000	0.0095238
31990.0000000	0.0095000
31991.0000000	0.0100000
31992.0000000	0.0080000
31993.0000000	0.0090000
31994.0000000	0.0085000
31995.0000000	0.0090000
31996.0000000	0.0100000
31997.0000000	0.0095000
31998.0000000	0.0085714
31999.0000000	0.0080000
32000.0000000	0.0085714
32001.0000000	0.0085000
32002.0000000	0.0100000
32003.0000000	0.0095000
32004.0000000	0.0090000
32005.0000000	0.0095238
32006.0000000	0.0085000
32007.0000000	0.0085714
32008.0000000	0.0095000
32009.0000000	0.0095000

32010.0000000	0.0080952
32011.0000000	0.0080000
32012.0000000	0.0095000
32013.0000000	0.0090476
32014.0000000	0.0100000
32015.0000000	0.0085000
32016.0000000	0.0095238
32017.0000000	0.0095000
32018.0000000	0.0090000
32019.0000000	0.0095000
32020.0000000	0.0090476
32021.0000000	0.0085000
32022.0000000	0.0095238
32023.0000000	0.0100000
32024.0000000	0.0085000
32025.0000000	0.0085000
32026.0000000	0.0095238
32027.0000000	0.0100000
32028.0000000	0.0090000
32029.0000000	0.0095238
32030.0000000	0.0100000
32031.0000000	0.0095000
32032.0000000	0.0095000
32033.0000000	0.0095000
32034.0000000	0.0090476
32035.0000000	0.0095000
32036.0000000	0.0095000
32037.0000000	0.0090476
32038.0000000	0.0100000
32039.0000000	0.0095000
32040.0000000	0.0095000
32041.0000000	0.0080952
32042.0000000	0.0095000
32043.0000000	0.0090476
32044.0000000	0.0085000
32045.0000000	0.0090000
32046.0000000	0.0076190
32047.0000000	0.0095000
32048.0000000	0.0090000
32049.0000000	0.0090000
32050.0000000	0.0095238
32051.0000000	0.0085000
32052.0000000	0.0095238
32053.0000000	0.0090000
32054.0000000	0.0090000
32055.0000000	0.0090000
32056.0000000	0.0085000
32057.0000000	0.0085714
32058.0000000	0.0085000
32059.0000000	0.0090476
32060.0000000	0.0085000
32061.0000000	0.0076190
32062.0000000	0.0085000
32063.0000000	0.0085000
32064.0000000	0.0080000
32065.0000000	0.0080952
32066.0000000	0.0085000

32067.0000000	0.0085000
32068.0000000	0.0076190
32069.0000000	0.0085000
32070.0000000	0.0071429
32071.0000000	0.0080000
32072.0000000	0.0070000
32073.0000000	0.0070000
32074.0000000	0.0085714
32075.0000000	0.0085000
32076.0000000	0.0060000
32077.0000000	0.0071429
32078.0000000	0.0035000
32079.0000000	0.0066667
32080.0000000	0.0070000
32081.0000000	0.0060000
32082.0000000	0.0057143
32083.0000000	0.0060000
32084.0000000	0.0080000
32085.0000000	0.0050000
32086.0000000	0.0066667
32087.0000000	0.0035000
32088.0000000	0.0045000
32089.0000000	0.0042857
32090.0000000	0.0060000
32091.0000000	0.0047619
32092.0000000	0.0035000
32093.0000000	0.0040000
32094.0000000	0.0040000
32095.0000000	0.0033333
32096.0000000	0.0055000
32097.0000000	0.0020000
32098.0000000	0.0061905
32099.0000000	0.0050000
32100.0000000	0.0035000
32101.0000000	0.0028571
32102.0000000	0.0035000
32103.0000000	0.0045000
32104.0000000	0.0028571
32105.0000000	0.0035000
32106.0000000	0.0015000
32107.0000000	0.0028571
32108.0000000	0.0030000
32109.0000000	0.0035000
32110.0000000	0.0033333
32111.0000000	0.0030000
32112.0000000	0.0040000
32113.0000000	0.0019048
32114.0000000	0.0020000
32115.0000000	0.0035000
32116.0000000	0.0055000
32117.0000000	0.0038095
32118.0000000	0.0038095
32119.0000000	0.0040000
32120.0000000	0.0015000
32121.0000000	0.0050000
32122.0000000	0.0033333
32123.0000000	0.0025000

32124.0000000	0.0035000
32125.0000000	0.0033333
32126.0000000	0.0030000
32127.0000000	0.0033333
32128.0000000	0.0030000
32129.0000000	0.0035000
32130.0000000	0.0060000
32131.0000000	0.0023810
32132.0000000	0.0035000
32133.0000000	0.0035000
32134.0000000	0.0038095
32135.0000000	0.0045000
32136.0000000	0.0045000
32137.0000000	0.0057143
32138.0000000	0.0035000
32139.0000000	0.0035000
32140.0000000	0.0047619
32141.0000000	0.0030000
32142.0000000	0.0035000
32143.0000000	0.0033333
32144.0000000	0.0060000
32145.0000000	0.0040000
32146.0000000	0.0035000
32147.0000000	0.0047619
32148.0000000	0.0040000
32149.0000000	0.0033333
32150.0000000	0.0040000
32151.0000000	0.0060000
32152.0000000	0.0047619
32153.0000000	0.0025000
32154.0000000	0.0025000
32155.0000000	0.0050000
32156.0000000	0.0033333
32157.0000000	0.0045000
32158.0000000	0.0042857
32159.0000000	0.0045000
32160.0000000	0.0050000
32161.0000000	0.0065000
32162.0000000	0.0057143
32163.0000000	0.0060000
32164.0000000	0.0055000
32165.0000000	0.0014286
32166.0000000	0.0042857
32167.0000000	0.0065000
32168.0000000	0.0045000
32169.0000000	0.0050000
32170.0000000	0.0052381
32171.0000000	0.0055000
32172.0000000	0.0065000
32173.0000000	0.0045000
32174.0000000	0.0038095
32175.0000000	0.0050000
32176.0000000	0.0040000
32177.0000000	0.0038095
32178.0000000	0.0065000
32179.0000000	0.0038095
32180.0000000	0.0030000

32181.0000000	0.0055000
32182.0000000	0.0057143
32183.0000000	0.0050000
32184.0000000	0.0025000
32185.0000000	0.0025000
32186.0000000	0.0047619
32187.0000000	0.0045000
32188.0000000	0.0052381
32189.0000000	0.0060000
32190.0000000	0.0035000
32191.0000000	0.0045000
32192.0000000	0.0052381
32193.0000000	0.0075000
32194.0000000	0.0040000
32195.0000000	0.0038095
32196.0000000	0.0040000
32197.0000000	0.0033333
32198.0000000	0.0055000
32199.0000000	0.0045000
32200.0000000	0.0020000
32201.0000000	0.0033333
32202.0000000	0.0050000
32203.0000000	0.0035000
32204.0000000	0.0033333
32205.0000000	0.0050000
32206.0000000	0.0042857
32207.0000000	0.0020000
32208.0000000	0.0030000
32209.0000000	0.0030000
32210.0000000	0.0033333
32211.0000000	0.0035000
32212.0000000	0.0040000
32213.0000000	0.0019048
32214.0000000	0.0020000
32215.0000000	0.0023810
32216.0000000	0.0050000
32217.0000000	0.0030000
32218.0000000	0.0028571
32219.0000000	0.0020000
32220.0000000	0.0015000
32221.0000000	0.0045000
32222.0000000	0.0033333
32223.0000000	0.0040000
32224.0000000	0.0025000
32225.0000000	0.0047619
32226.0000000	0.0030000
32227.0000000	0.0023810
32228.0000000	0.0020000
32229.0000000	0.0030000
32230.0000000	0.0035000
32231.0000000	0.0009524
32232.0000000	0.0030000
32233.0000000	0.0025000
32234.0000000	0.0019048
32235.0000000	0.0020000
32236.0000000	0.0025000
32237.0000000	0.0014286

32238.0000000	0.0005000
32239.0000000	0.0045000
32240.0000000	0.0009524
32241.0000000	0.0005000
32242.0000000	0.0019048
32243.0000000	0.0010000
32244.0000000	0.0030000
32245.0000000	0.0015000
32246.0000000	0.0047619
32247.0000000	0.0020000
32248.0000000	0.0035000
32249.0000000	0.0009524
32250.0000000	0.0030000
32251.0000000	0.0025000
32252.0000000	0.0020000
32253.0000000	0.0000000
32254.0000000	0.0028571
32255.0000000	0.0010000
32256.0000000	0.0010000
32257.0000000	0.0015000
32258.0000000	0.0019048
32259.0000000	0.0020000
32260.0000000	0.0010000
32261.0000000	0.0010000
32262.0000000	0.0009524
32263.0000000	0.0010000
32264.0000000	0.0009524
32265.0000000	0.0005000
32266.0000000	0.0005000
32267.0000000	0.0014286
32268.0000000	0.0005000
32269.0000000	0.0005000
32270.0000000	0.0004762
32271.0000000	0.0005000
32272.0000000	0.0010000
32273.0000000	0.0000000
32274.0000000	0.0000000
32275.0000000	0.0005000
32276.0000000	0.0004762
32277.0000000	0.0000000
32278.0000000	0.0004762
32279.0000000	0.0005000
32280.0000000	0.0005000
32281.0000000	0.0000000
32282.0000000	0.0000000
32283.0000000	0.0000000
32284.0000000	0.0005000
32285.0000000	0.0004762
32286.0000000	0.0010000
32287.0000000	0.0004762
32288.0000000	0.0000000
32289.0000000	0.0005000
32290.0000000	0.0000000
32291.0000000	0.0000000
32292.0000000	0.0000000
32293.0000000	0.0000000
32294.0000000	0.0000000

32295.0000000	0.0000000
32296.0000000	0.0010000
32297.0000000	0.0000000
32298.0000000	0.0000000
32299.0000000	0.0000000
32300.0000000	0.0000000
32301.0000000	0.0000000
32302.0000000	0.0005000
32303.0000000	0.0000000
32304.0000000	0.0000000
32305.0000000	0.0000000
32306.0000000	0.0000000
32307.0000000	0.0000000
32308.0000000	0.0005000
32309.0000000	0.0000000
32310.0000000	0.0000000
32311.0000000	0.0000000
32312.0000000	0.0000000
32313.0000000	0.0000000
32314.0000000	0.0000000
32315.0000000	0.0000000
32316.0000000	0.0000000
32317.0000000	0.0000000
32318.0000000	0.0000000
32319.0000000	0.0000000
32320.0000000	0.0000000
32321.0000000	0.0000000
32322.0000000	0.0000000
32323.0000000	0.0000000
32324.0000000	0.0000000
32325.0000000	0.0000000
32326.0000000	0.0000000
32327.0000000	0.0000000
32328.0000000	0.0000000
32329.0000000	0.0000000
32330.0000000	0.0000000
32331.0000000	0.0000000
32332.0000000	0.0000000
32333.0000000	0.0000000
32334.0000000	0.0000000
32335.0000000	0.0000000
32336.0000000	0.0000000
32337.0000000	0.0000000
32338.0000000	0.0000000
32339.0000000	0.0000000
32340.0000000	-0.0005000
32341.0000000	0.0000000
32342.0000000	0.0000000
32343.0000000	-0.0005000
32344.0000000	0.0000000
32345.0000000	-0.0005000
32346.0000000	0.0000000
32347.0000000	0.0000000
32348.0000000	0.0000000
32349.0000000	-0.0010000
32350.0000000	0.0000000
32351.0000000	-0.0004762

32352.0000000	0.0000000
32353.0000000	-0.0005000
32354.0000000	0.0000000
32355.0000000	-0.0005000
32356.0000000	0.0000000
32357.0000000	-0.0005000
32358.0000000	0.0000000
32359.0000000	-0.0010000
32360.0000000	-0.0005000
32361.0000000	-0.0004762
32362.0000000	-0.0010000
32363.0000000	-0.0009524
32364.0000000	-0.0010000
32365.0000000	-0.0020000
32366.0000000	-0.0009524
32367.0000000	0.0000000
32368.0000000	-0.0015000
32369.0000000	-0.0010000
32370.0000000	-0.0009524
32371.0000000	-0.0010000
32372.0000000	-0.0015000
32373.0000000	-0.0009524
32374.0000000	-0.0020000
32375.0000000	-0.0004762
32376.0000000	-0.0010000
32377.0000000	-0.0015000
32378.0000000	-0.0028571
32379.0000000	-0.0020000
32380.0000000	-0.0025000
32381.0000000	-0.0015000
32382.0000000	-0.0009524
32383.0000000	-0.0020000
32384.0000000	-0.0033333
32385.0000000	-0.0040000
32386.0000000	-0.0030000
32387.0000000	-0.0015000
32388.0000000	-0.0010000
32389.0000000	-0.0019048
32390.0000000	-0.0035000
32391.0000000	-0.0033333
32392.0000000	-0.0045000
32393.0000000	-0.0033333
32394.0000000	-0.0020000
32395.0000000	-0.0050000
32396.0000000	-0.0045000
32397.0000000	-0.0040000
32398.0000000	-0.0038095
32399.0000000	-0.0045000
32400.0000000	-0.0038095
32401.0000000	-0.0040000
32402.0000000	-0.0055000
32403.0000000	-0.0071429
32404.0000000	-0.0050000
32405.0000000	-0.0065000
32406.0000000	-0.0052381
32407.0000000	-0.0050000
32408.0000000	-0.0060000

32409.0000000	-0.0057143
32410.0000000	-0.0045000
32411.0000000	-0.0033333
32412.0000000	-0.0035000
32413.0000000	-0.0055000
32414.0000000	-0.0076190
32415.0000000	-0.0055000
32416.0000000	-0.0040000
32417.0000000	-0.0055000
32418.0000000	-0.0057143
32419.0000000	-0.0040000
32420.0000000	-0.0055000
32421.0000000	-0.0055000
32422.0000000	-0.0061905
32423.0000000	-0.0055000
32424.0000000	-0.0071429
32425.0000000	-0.0065000
32426.0000000	-0.0060000
32427.0000000	-0.0038095
32428.0000000	-0.0050000
32429.0000000	-0.0070000
32430.0000000	-0.0057143
32431.0000000	-0.0070000
32432.0000000	-0.0070000
32433.0000000	-0.0080000
32434.0000000	-0.0080952
32435.0000000	-0.0080000
32436.0000000	-0.0042857
32437.0000000	-0.0055000
32438.0000000	-0.0065000
32439.0000000	-0.0057143
32440.0000000	-0.0070000
32441.0000000	-0.0080000
32442.0000000	-0.0070000
32443.0000000	-0.0057143
32444.0000000	-0.0075000
32445.0000000	-0.0076190
32446.0000000	-0.0050000
32447.0000000	-0.0055000
32448.0000000	-0.0070000
32449.0000000	-0.0052381
32450.0000000	-0.0070000
32451.0000000	-0.0050000
32452.0000000	-0.0066667
32453.0000000	-0.0060000
32454.0000000	-0.0061905
32455.0000000	-0.0065000
32456.0000000	-0.0040000
32457.0000000	-0.0060000
32458.0000000	-0.0052381
32459.0000000	-0.0065000
32460.0000000	-0.0055000
32461.0000000	-0.0052381
32462.0000000	-0.0060000
32463.0000000	-0.0055000
32464.0000000	-0.0047619
32465.0000000	-0.0045000

32466.0000000	-0.0065000
32467.0000000	-0.0047619
32468.0000000	-0.0055000
32469.0000000	-0.0038095
32470.0000000	-0.0030000
32471.0000000	-0.0065000
32472.0000000	-0.0040000
32473.0000000	-0.0066667
32474.0000000	-0.0040000
32475.0000000	-0.0035000
32476.0000000	-0.0042857
32477.0000000	-0.0065000
32478.0000000	-0.0055000
32479.0000000	-0.0038095
32480.0000000	-0.0035000
32481.0000000	-0.0040000
32482.0000000	-0.0019048
32483.0000000	-0.0015000
32484.0000000	-0.0047619
32485.0000000	-0.0040000
32486.0000000	-0.0025000
32487.0000000	-0.0030000
32488.0000000	-0.0052381
32489.0000000	-0.0035000
32490.0000000	-0.0030000
32491.0000000	-0.0014286
32492.0000000	-0.0020000
32493.0000000	-0.0020000
32494.0000000	-0.0014286
32495.0000000	-0.0015000
32496.0000000	-0.0045000
32497.0000000	-0.0019048
32498.0000000	-0.0025000
32499.0000000	-0.0019048
32500.0000000	-0.0035000
32501.0000000	-0.0025000
32502.0000000	-0.0030000
32503.0000000	-0.0033333
32504.0000000	-0.0010000
32505.0000000	-0.0030000
32506.0000000	-0.0009524
32507.0000000	-0.0025000
32508.0000000	-0.0005000
32509.0000000	-0.0014286
32510.0000000	-0.0015000
32511.0000000	-0.0020000
32512.0000000	-0.0019048
32513.0000000	-0.0020000
32514.0000000	-0.0010000
32515.0000000	-0.0023810
32516.0000000	-0.0020000
32517.0000000	-0.0005000
32518.0000000	-0.0014286
32519.0000000	-0.0010000
32520.0000000	-0.0010000
32521.0000000	-0.0023810
32522.0000000	-0.0005000

32523.0000000	-0.0010000
32524.0000000	-0.0009524
32525.0000000	-0.0025000
32526.0000000	-0.0010000
32527.0000000	-0.0009524
32528.0000000	-0.0015000
32529.0000000	-0.0019048
32530.0000000	-0.0015000
32531.0000000	-0.0005000
32532.0000000	0.0000000
32533.0000000	-0.0014286
32534.0000000	-0.0015000
32535.0000000	-0.0015000
32536.0000000	-0.0014286
32537.0000000	-0.0015000
32538.0000000	-0.0005000
32539.0000000	0.0000000
32540.0000000	-0.0009524
32541.0000000	0.0000000
32542.0000000	-0.0009524
32543.0000000	-0.0015000
32544.0000000	0.0000000
32545.0000000	-0.0004762
32546.0000000	-0.0005000
32547.0000000	-0.0005000
32548.0000000	0.0000000
32549.0000000	-0.0004762
32550.0000000	-0.0010000
32551.0000000	-0.0009524
32552.0000000	0.0000000
32553.0000000	0.0000000
32554.0000000	0.0000000
32555.0000000	-0.0005000
32556.0000000	-0.0005000
32557.0000000	0.0000000
32558.0000000	-0.0004762
32559.0000000	0.0000000
32560.0000000	0.0000000
32561.0000000	0.0000000
32562.0000000	0.0000000
32563.0000000	0.0000000
32564.0000000	0.0000000
32565.0000000	0.0000000
32566.0000000	0.0000000
32567.0000000	0.0000000
32568.0000000	0.0000000
32569.0000000	0.0000000
32570.0000000	0.0000000
32571.0000000	0.0000000
32572.0000000	0.0000000
32573.0000000	0.0000000
32574.0000000	0.0000000
32575.0000000	0.0000000
32576.0000000	0.0000000
32577.0000000	0.0000000
32578.0000000	0.0005000
32579.0000000	0.0000000

32580.0000000	0.0005000
32581.0000000	0.0000000
32582.0000000	0.0000000
32583.0000000	0.0000000
32584.0000000	0.0000000
32585.0000000	0.0000000
32586.0000000	0.0000000
32587.0000000	0.0000000
32588.0000000	0.0000000
32589.0000000	0.0000000
32590.0000000	0.0004762
32591.0000000	0.0005000
32592.0000000	0.0000000
32593.0000000	0.0000000
32594.0000000	0.0005000
32595.0000000	0.0010000
32596.0000000	0.0000000
32597.0000000	0.0004762
32598.0000000	0.0000000
32599.0000000	0.0005000
32600.0000000	0.0014286
32601.0000000	0.0005000
32602.0000000	0.0009524
32603.0000000	0.0020000
32604.0000000	0.0000000
32605.0000000	0.0015000
32606.0000000	0.0004762
32607.0000000	0.0000000
32608.0000000	0.0015000
32609.0000000	0.0019048
32610.0000000	0.0010000
32611.0000000	0.0014286
32612.0000000	0.0020000
32613.0000000	0.0020000
32614.0000000	0.0015000
32615.0000000	0.0019048
32616.0000000	0.0040000
32617.0000000	0.0045000
32618.0000000	0.0047619
32619.0000000	0.0060000
32620.0000000	0.0042857
32621.0000000	0.0050000
32622.0000000	0.0040000
32623.0000000	0.0050000
32624.0000000	0.0057143
32625.0000000	0.0070000
32626.0000000	0.0050000
32627.0000000	0.0071429
32628.0000000	0.0050000
32629.0000000	0.0070000
32630.0000000	0.0060000
32631.0000000	0.0076190
32632.0000000	0.0070000
32633.0000000	0.0057143
32634.0000000	0.0080000
32635.0000000	0.0060000
32636.0000000	0.0057143

32637.0000000	0.0080000
32638.0000000	0.0060000
32639.0000000	0.0090476
32640.0000000	0.0075000
32641.0000000	0.0080952
32642.0000000	0.0090000
32643.0000000	0.0080000
32644.0000000	0.0085000
32645.0000000	0.0085714
32646.0000000	0.0090000
32647.0000000	0.0095000
32648.0000000	0.0100000
32649.0000000	0.0100000
32650.0000000	0.0085714
32651.0000000	0.0080000
32652.0000000	0.0085000
32653.0000000	0.0095000
32654.0000000	0.0100000
32655.0000000	0.0095000
32656.0000000	0.0100000
32657.0000000	0.0100000
32658.0000000	0.0100000
32659.0000000	0.0100000
32660.0000000	0.0100000
32661.0000000	0.0100000
32662.0000000	0.0100000
32663.0000000	0.0100000
32664.0000000	0.0105000
32665.0000000	0.0095000
32666.0000000	0.0100000
32667.0000000	0.0095000
32668.0000000	0.0100000
32669.0000000	0.0100000
32670.0000000	0.0100000
32671.0000000	0.0100000
32672.0000000	0.0100000
32673.0000000	0.0105000
32674.0000000	0.0100000
32675.0000000	0.0100000
32676.0000000	0.0100000
32677.0000000	0.0100000
32678.0000000	0.0100000
32679.0000000	0.0100000
32680.0000000	0.0100000
32681.0000000	0.0104762
32682.0000000	0.0100000
32683.0000000	0.0100000
32684.0000000	0.0100000
32685.0000000	0.0105000
32686.0000000	0.0100000
32687.0000000	0.0104762
32688.0000000	0.0105000
32689.0000000	0.0100000
32690.0000000	0.0100000
32691.0000000	0.0104762
32692.0000000	0.0100000
32693.0000000	0.0100000

32694.0000000	0.0100000
32695.0000000	0.0100000
32696.0000000	0.0104762
32697.0000000	0.0110000
32698.0000000	0.0115000
32699.0000000	0.0119048
32700.0000000	0.0100000
32701.0000000	0.0105000
32702.0000000	0.0109524
32703.0000000	0.0110000
32704.0000000	0.0110000
32705.0000000	0.0105000
32706.0000000	0.0109524
32707.0000000	0.0120000
32708.0000000	0.0133333
32709.0000000	0.0120000
32710.0000000	0.0120000
32711.0000000	0.0133333
32712.0000000	0.0120000
32713.0000000	0.0125000
32714.0000000	0.0128571
32715.0000000	0.0130000
32716.0000000	0.0140000
32717.0000000	0.0138095
32718.0000000	0.0140000
32719.0000000	0.0165000
32720.0000000	0.0175000
32721.0000000	0.0157143
32722.0000000	0.0165000
32723.0000000	0.0157143
32724.0000000	0.0160000
32725.0000000	0.0170000
32726.0000000	0.0195238
32727.0000000	0.0175000
32728.0000000	0.0180000
32729.0000000	0.0175000
32730.0000000	0.0171429
32731.0000000	0.0190000
32732.0000000	0.0170000
32733.0000000	0.0176190
32734.0000000	0.0190000
32735.0000000	0.0190000
32736.0000000	0.0195238
32737.0000000	0.0195000
32738.0000000	0.0200000
32739.0000000	0.0195000
32740.0000000	0.0200000
32741.0000000	0.0190476
32742.0000000	0.0200000
32743.0000000	0.0195000
32744.0000000	0.0195000
32745.0000000	0.0200000
32746.0000000	0.0195000
32747.0000000	0.0200000
32748.0000000	0.0195000
32749.0000000	0.0200000
32750.0000000	0.0200000

32751.0000000	0.0200000
32752.0000000	0.0200000
32753.0000000	0.0200000
32754.0000000	0.0200000
32755.0000000	0.0200000
32756.0000000	0.0200000
32757.0000000	0.0200000
32758.0000000	0.0200000
32759.0000000	0.0200000
32760.0000000	0.0200000
32761.0000000	0.0200000
32762.0000000	0.0200000
32763.0000000	0.0200000
32764.0000000	0.0200000
32765.0000000	0.0200000
32766.0000000	0.0200000
32767.0000000	0.0200000
32768.0000000	0.0204762
32769.0000000	0.0205000
32770.0000000	0.0205000
32771.0000000	0.0215000
32772.0000000	0.0200000
32773.0000000	0.0215000
32774.0000000	0.0210000
32775.0000000	0.0200000
32776.0000000	0.0205000
32777.0000000	0.0214286
32778.0000000	0.0235000
32779.0000000	0.0235000
32780.0000000	0.0215000
32781.0000000	0.0223810
32782.0000000	0.0235000
32783.0000000	0.0230000
32784.0000000	0.0233333
32785.0000000	0.0245000
32786.0000000	0.0233333
32787.0000000	0.0240000
32788.0000000	0.0245000
32789.0000000	0.0245000
32790.0000000	0.0252381
32791.0000000	0.0265000
32792.0000000	0.0275000
32793.0000000	0.0266667
32794.0000000	0.0270000
32795.0000000	0.0290000
32796.0000000	0.0270000
32797.0000000	0.0280952
32798.0000000	0.0285714
32799.0000000	0.0290000
32800.0000000	0.0290000
32801.0000000	0.0290000
32802.0000000	0.0285714
32803.0000000	0.0290000
32804.0000000	0.0295000
32805.0000000	0.0300000
32806.0000000	0.0300000
32807.0000000	0.0295238

32808.0000000	0.0300000
32809.0000000	0.0300000
32810.0000000	0.0300000
32811.0000000	0.0300000
32812.0000000	0.0300000
32813.0000000	0.0295000
32814.0000000	0.0300000
32815.0000000	0.0295000
32816.0000000	0.0300000
32817.0000000	0.0300000
32818.0000000	0.0300000
32819.0000000	0.0300000
32820.0000000	0.0300000
32821.0000000	0.0300000
32822.0000000	0.0300000
32823.0000000	0.0300000
32824.0000000	0.0300000
32825.0000000	0.0300000
32826.0000000	0.0300000
32827.0000000	0.0304762
32828.0000000	0.0300000
32829.0000000	0.0300000
32830.0000000	0.0300000
32831.0000000	0.0300000
32832.0000000	0.0300000
32833.0000000	0.0300000
32834.0000000	0.0300000
32835.0000000	0.0300000
32836.0000000	0.0304762
32837.0000000	0.0305000
32838.0000000	0.0300000
32839.0000000	0.0310000
32840.0000000	0.0305000
32841.0000000	0.0304762
32842.0000000	0.0320000
32843.0000000	0.0305000
32844.0000000	0.0300000
32845.0000000	0.0309524
32846.0000000	0.0310000
32847.0000000	0.0309524
32848.0000000	0.0310000
32849.0000000	0.0320000
32850.0000000	0.0309524
32851.0000000	0.0325000
32852.0000000	0.0330000
32853.0000000	0.0338095
32854.0000000	0.0335000
32855.0000000	0.0335000
32856.0000000	0.0330000
32857.0000000	0.0357143
32858.0000000	0.0335000
32859.0000000	0.0347619
32860.0000000	0.0340000
32861.0000000	0.0355000
32862.0000000	0.0376190
32863.0000000	0.0355000
32864.0000000	0.0370000

32865.0000000	0.0365000
32866.0000000	0.0357143
32867.0000000	0.0350000
32868.0000000	0.0376190
32869.0000000	0.0385000
32870.0000000	0.0390000
32871.0000000	0.0366667
32872.0000000	0.0380000
32873.0000000	0.0375000
32874.0000000	0.0390000
32875.0000000	0.0395238
32876.0000000	0.0385000
32877.0000000	0.0400000
32878.0000000	0.0390000
32879.0000000	0.0395000
32880.0000000	0.0390000
32881.0000000	0.0395238
32882.0000000	0.0395000
32883.0000000	0.0400000
32884.0000000	0.0400000
32885.0000000	0.0400000
32886.0000000	0.0395000
32887.0000000	0.0400000
32888.0000000	0.0395000
32889.0000000	0.0400000
32890.0000000	0.0395000
32891.0000000	0.0400000
32892.0000000	0.0400000
32893.0000000	0.0400000
32894.0000000	0.0400000
32895.0000000	0.0400000
32896.0000000	0.0400000
32897.0000000	0.0400000
32898.0000000	0.0404762
32899.0000000	0.0405000
32900.0000000	0.0400000
32901.0000000	0.0400000
32902.0000000	0.0400000
32903.0000000	0.0400000
32904.0000000	0.0400000
32905.0000000	0.0404762
32906.0000000	0.0400000
32907.0000000	0.0400000
32908.0000000	0.0400000
32909.0000000	0.0400000
32910.0000000	0.0400000
32911.0000000	0.0404762
32912.0000000	0.0405000
32913.0000000	0.0404762
32914.0000000	0.0400000
32915.0000000	0.0405000
32916.0000000	0.0405000
32917.0000000	0.0404762
32918.0000000	0.0410000
32919.0000000	0.0410000
32920.0000000	0.0400000
32921.0000000	0.0415000

32922.0000000	0.0419048
32923.0000000	0.0410000
32924.0000000	0.0410000
32925.0000000	0.0410000
32926.0000000	0.0400000
32927.0000000	0.0420000
32928.0000000	0.0410000
32929.0000000	0.0423810
32930.0000000	0.0410000
32931.0000000	0.0400000
32932.0000000	0.0404762
32933.0000000	0.0410000
32934.0000000	0.0415000
32935.0000000	0.0419048
32936.0000000	0.0410000
32937.0000000	0.0409524
32938.0000000	0.0410000
32939.0000000	0.0430000
32940.0000000	0.0410000
32941.0000000	0.0428571
32942.0000000	0.0420000
32943.0000000	0.0428572
32944.0000000	0.0425000
32945.0000000	0.0425000
32946.0000000	0.0450000
32947.0000000	0.0438095
32948.0000000	0.0430000
32949.0000000	0.0440000
32950.0000000	0.0442857
32951.0000000	0.0450000
32952.0000000	0.0461905
32953.0000000	0.0455000
32954.0000000	0.0445000
32955.0000000	0.0445000
32956.0000000	0.0457143
32957.0000000	0.0450000
32958.0000000	0.0440000
32959.0000000	0.0438095
32960.0000000	0.0455000
32961.0000000	0.0450000
32962.0000000	0.0455000
32963.0000000	0.0447619
32964.0000000	0.0470000
32965.0000000	0.0461905
32966.0000000	0.0480000
32967.0000000	0.0475000
32968.0000000	0.0476191
32969.0000000	0.0485000
32970.0000000	0.0470000
32971.0000000	0.0471429
32972.0000000	0.0495000
32973.0000000	0.0485714
32974.0000000	0.0460000
32975.0000000	0.0495000
32976.0000000	0.0480000
32977.0000000	0.0466667
32978.0000000	0.0495000

32979.0000000	0.0490000
32980.0000000	0.0485000
32981.0000000	0.0495238
32982.0000000	0.0490476
32983.0000000	0.0495000
32984.0000000	0.0495000
32985.0000000	0.0500000
32986.0000000	0.0500000
32987.0000000	0.0495000
32988.0000000	0.0500000
32989.0000000	0.0495238
32990.0000000	0.0500000
32991.0000000	0.0490000
32992.0000000	0.0495000
32993.0000000	0.0500000
32994.0000000	0.0500000
32995.0000000	0.0500000
32996.0000000	0.0495000
32997.0000000	0.0500000
32998.0000000	0.0495238
32999.0000000	0.0500000
33000.0000000	0.0495000
33001.0000000	0.0495000
33002.0000000	0.0500000
33003.0000000	0.0500000
33004.0000000	0.0500000
33005.0000000	0.0500000
33006.0000000	0.0500000
33007.0000000	0.0500000
33008.0000000	0.0495000
33009.0000000	0.0500000
33010.0000000	0.0500000
33011.0000000	0.0500000
33012.0000000	0.0500000
33013.0000000	0.0500000
33014.0000000	0.0500000
33015.0000000	0.0500000
33016.0000000	0.0500000
33017.0000000	0.0500000
33018.0000000	0.0500000
33019.0000000	0.0500000
33020.0000000	0.0500000
33021.0000000	0.0500000
33022.0000000	0.0500000
33023.0000000	0.0500000
33024.0000000	0.0500000
33025.0000000	0.0500000
33026.0000000	0.0500000
33027.0000000	0.0500000
33028.0000000	0.0500000
33029.0000000	0.0500000
33030.0000000	0.0500000
33031.0000000	0.0500000
33032.0000000	0.0500000
33033.0000000	0.0500000
33034.0000000	0.0500000
33035.0000000	0.0505000

33036.0000000	0.0500000
33037.0000000	0.0500000
33038.0000000	0.0500000
33039.0000000	0.0500000
33040.0000000	0.0500000
33041.0000000	0.0500000
33042.0000000	0.0500000
33043.0000000	0.0500000
33044.0000000	0.0500000
33045.0000000	0.0500000
33046.0000000	0.0500000
33047.0000000	0.0500000
33048.0000000	0.0500000
33049.0000000	0.0500000
33050.0000000	0.0500000
33051.0000000	0.0500000
33052.0000000	0.0500000
33053.0000000	0.0504762
33054.0000000	0.0505000
33055.0000000	0.0500000
33056.0000000	0.0500000
33057.0000000	0.0500000
33058.0000000	0.0504762
33059.0000000	0.0500000
33060.0000000	0.0505000
33061.0000000	0.0500000
33062.0000000	0.0500000
33063.0000000	0.0505000
33064.0000000	0.0504762
33065.0000000	0.0505000
33066.0000000	0.0500000
33067.0000000	0.0500000
33068.0000000	0.0500000
33069.0000000	0.0505000
33070.0000000	0.0505000
33071.0000000	0.0514286
33072.0000000	0.0510000
33073.0000000	0.0500000
33074.0000000	0.0510000
33075.0000000	0.0510000
33076.0000000	0.0510000
33077.0000000	0.0514286
33078.0000000	0.0520000
33079.0000000	0.0520000
33080.0000000	0.0523810
33081.0000000	0.0525000
33082.0000000	0.0525000
33083.0000000	0.0514286
33084.0000000	0.0510000
33085.0000000	0.0540000
33086.0000000	0.0514286
33087.0000000	0.0550000
33088.0000000	0.0523810
33089.0000000	0.0520000
33090.0000000	0.0520000
33091.0000000	0.0550000
33092.0000000	0.0561905

33093.0000000	0.0535000
33094.0000000	0.0542857
33095.0000000	0.0550000
33096.0000000	0.0550000
33097.0000000	0.0555000
33098.0000000	0.0571428
33099.0000000	0.0530000
33100.0000000	0.0560000
33101.0000000	0.0547619
33102.0000000	0.0560000
33103.0000000	0.0557143
33104.0000000	0.0555000
33105.0000000	0.0570000
33106.0000000	0.0560000
33107.0000000	0.0590476
33108.0000000	0.0590000
33109.0000000	0.0565000
33110.0000000	0.0585714
33111.0000000	0.0585000
33112.0000000	0.0580000
33113.0000000	0.0570000
33114.0000000	0.0580952
33115.0000000	0.0565000
33116.0000000	0.0590476
33117.0000000	0.0585000
33118.0000000	0.0580952
33119.0000000	0.0590000
33120.0000000	0.0580000
33121.0000000	0.0580000
33122.0000000	0.0595238
33123.0000000	0.0595000
33124.0000000	0.0595000
33125.0000000	0.0595238
33126.0000000	0.0595000
33127.0000000	0.0590000
33128.0000000	0.0595238
33129.0000000	0.0600000
33130.0000000	0.0595000
33131.0000000	0.0595238
33132.0000000	0.0595000
33133.0000000	0.0595000
33134.0000000	0.0600000
33135.0000000	0.0595000
33136.0000000	0.0600000
33137.0000000	0.0600000
33138.0000000	0.0595000
33139.0000000	0.0600000
33140.0000000	0.0600000
33141.0000000	0.0600000
33142.0000000	0.0600000
33143.0000000	0.0595000
33144.0000000	0.0600000
33145.0000000	0.0595000
33146.0000000	0.0600000
33147.0000000	0.0600000
33148.0000000	0.0600000
33149.0000000	0.0600000

33150.0000000	0.0600000
33151.0000000	0.0600000
33152.0000000	0.0604762
33153.0000000	0.0600000
33154.0000000	0.0600000
33155.0000000	0.0600000
33156.0000000	0.0600000
33157.0000000	0.0600000
33158.0000000	0.0600000
33159.0000000	0.0600000
33160.0000000	0.0605000
[truncated: 398,040 more chars]
