# Supplementary material for: Periodic oscillations of atmospheric electric field during snowfall in the Tokyo metropolitan area
Source: Sci Rep. 2021 Jan 26;11:2280. doi: 10.1038/s41598-021-82091-w (PMC7838252; doi:10.1038/s41598-021-82091-w)
Supplement: Supplementary file 5 — Supplementary Information 5. [file 41598_2021_82091_MOESM5_ESM.pdf]

36020.0000000	0.0000000	-50.0000000
36020.0000000	0.2500000	-50.0000000
36020.0000000	0.5000000	-50.0000000
36020.0000000	0.7500000	-50.0000000
36020.0000000	1.0000000	-50.0000000
36020.0000000	1.2500000	-50.0000000
36020.0000000	1.5000000	-50.0000000
36020.0000000	1.7500000	-50.0000000
36020.0000000	2.0000000	-50.0000000
36020.0000000	2.2500000	-50.0000000
36020.0000000	2.5000000	-50.0000000
36020.0000000	2.7500000	-50.0000000
36020.0000000	3.0000000	-50.0000000
36020.0000000	3.2500000	-50.0000000
36020.0000000	3.5000000	-50.0000000
36020.0000000	3.7500000	-50.0000000
36020.0000000	4.0000000	-50.0000000
36020.0000000	4.2500000	-50.0000000
36020.0000000	4.5000000	-50.0000000
36020.0000000	4.7500000	-50.0000000
36020.0000000	5.0000000	-50.0000000
36020.0000000	5.2500000	-50.0000000
36020.0000000	5.5000000	-50.0000000
36020.0000000	5.7500000	-50.0000000
36020.0000000	6.0000000	-50.0000000
36020.0000000	6.2500000	-50.0000000
36020.0000000	6.5000000	-50.0000000
36020.0000000	6.7500000	-50.0000000
36020.0000000	7.0000000	-50.0000000
36020.0000000	7.2500000	-50.0000000
36020.0000000	7.5000000	-50.0000000
36020.0000000	7.7500000	-50.0000000
36020.0000000	8.0000000	-50.0000000
36020.0000000	8.2500000	-50.0000000
36020.0000000	8.5000000	-50.0000000
36020.0000000	8.7500000	-50.0000000
36020.0000000	9.0000000	-50.0000000
36020.0000000	9.2500000	-50.0000000
36020.0000000	9.5000000	-50.0000000
36020.0000000	9.7500000	-50.0000000
36020.0000000	10.0000000	-50.0000000
36020.0000000	10.2500000	-50.0000000
36020.0000000	10.5000000	-50.0000000
36020.0000000	10.7500000	-50.0000000
36020.0000000	11.0000000	-50.0000000
36020.0000000	11.2500000	-50.0000000
36020.0000000	11.5000000	-50.0000000
36020.0000000	11.7500000	-50.0000000
36020.0000000	12.0000000	-50.0000000
36020.0000000	12.2500000	-50.0000000
36020.0000000	12.5000000	-50.0000000
36020.0000000	12.7500000	-50.0000000
36020.0000000	13.0000000	-50.0000000
36020.0000000	13.2500000	-50.0000000
36020.0000000	13.5000000	-50.0000000
36020.0000000	13.7500000	-50.0000000
36020.0000000	14.0000000	-50.0000000
36020.0000000	14.2500000	-50.0000000

36020.0000000	14.5000000	-50.0000000
36020.0000000	14.7500000	-50.0000000
36020.0000000	15.0000000	-50.0000000
36050.0000000	0.0000000	-50.0000000
36050.0000000	0.2500000	-50.0000000
36050.0000000	0.5000000	-50.0000000
36050.0000000	0.7500000	-50.0000000
36050.0000000	1.0000000	-50.0000000
36050.0000000	1.2500000	-50.0000000
36050.0000000	1.5000000	-50.0000000
36050.0000000	1.7500000	-50.0000000
36050.0000000	2.0000000	-50.0000000
36050.0000000	2.2500000	-50.0000000
36050.0000000	2.5000000	-50.0000000
36050.0000000	2.7500000	-50.0000000
36050.0000000	3.0000000	-50.0000000
36050.0000000	3.2500000	-50.0000000
36050.0000000	3.5000000	-50.0000000
36050.0000000	3.7500000	-50.0000000
36050.0000000	4.0000000	-50.0000000
36050.0000000	4.2500000	-50.0000000
36050.0000000	4.5000000	-50.0000000
36050.0000000	4.7500000	-50.0000000
36050.0000000	5.0000000	-50.0000000
36050.0000000	5.2500000	-50.0000000
36050.0000000	5.5000000	-50.0000000
36050.0000000	5.7500000	-50.0000000
36050.0000000	6.0000000	-50.0000000
36050.0000000	6.2500000	-50.0000000
36050.0000000	6.5000000	-50.0000000
36050.0000000	6.7500000	-50.0000000
36050.0000000	7.0000000	-50.0000000
36050.0000000	7.2500000	-50.0000000
36050.0000000	7.5000000	-50.0000000
36050.0000000	7.7500000	-50.0000000
36050.0000000	8.0000000	-50.0000000
36050.0000000	8.2500000	-50.0000000
36050.0000000	8.5000000	-50.0000000
36050.0000000	8.7500000	-50.0000000
36050.0000000	9.0000000	-50.0000000
36050.0000000	9.2500000	-50.0000000
36050.0000000	9.5000000	-50.0000000
36050.0000000	9.7500000	-50.0000000
36050.0000000	10.0000000	-50.0000000
36050.0000000	10.2500000	-50.0000000
36050.0000000	10.5000000	-50.0000000
36050.0000000	10.7500000	-50.0000000
36050.0000000	11.0000000	-50.0000000
36050.0000000	11.2500000	-50.0000000
36050.0000000	11.5000000	-50.0000000
36050.0000000	11.7500000	-50.0000000
36050.0000000	12.0000000	-50.0000000
36050.0000000	12.2500000	-50.0000000
36050.0000000	12.5000000	-50.0000000
36050.0000000	12.7500000	-50.0000000
36050.0000000	13.0000000	-50.0000000
36050.0000000	13.2500000	-50.0000000
36050.0000000	13.5000000	-50.0000000

36050.0000000	13.7500000	-50.0000000
36050.0000000	14.0000000	-50.0000000
36050.0000000	14.2500000	-50.0000000
36050.0000000	14.5000000	-50.0000000
36050.0000000	14.7500000	-50.0000000
36050.0000000	15.0000000	-50.0000000
36080.0000000	0.0000000	-50.0000000
36080.0000000	0.2500000	-50.0000000
36080.0000000	0.5000000	-50.0000000
36080.0000000	0.7500000	-50.0000000
36080.0000000	1.0000000	-50.0000000
36080.0000000	1.2500000	-50.0000000
36080.0000000	1.5000000	-50.0000000
36080.0000000	1.7500000	-50.0000000
36080.0000000	2.0000000	-50.0000000
36080.0000000	2.2500000	-50.0000000
36080.0000000	2.5000000	-50.0000000
36080.0000000	2.7500000	-50.0000000
36080.0000000	3.0000000	-50.0000000
36080.0000000	3.2500000	-50.0000000
36080.0000000	3.5000000	-50.0000000
36080.0000000	3.7500000	-50.0000000
36080.0000000	4.0000000	-50.0000000
36080.0000000	4.2500000	-50.0000000
36080.0000000	4.5000000	-50.0000000
36080.0000000	4.7500000	-50.0000000
36080.0000000	5.0000000	-50.0000000
36080.0000000	5.2500000	-50.0000000
36080.0000000	5.5000000	-50.0000000
36080.0000000	5.7500000	-50.0000000
36080.0000000	6.0000000	-50.0000000
36080.0000000	6.2500000	-50.0000000
36080.0000000	6.5000000	-50.0000000
36080.0000000	6.7500000	-50.0000000
36080.0000000	7.0000000	-50.0000000
36080.0000000	7.2500000	-50.0000000
36080.0000000	7.5000000	-50.0000000
36080.0000000	7.7500000	-50.0000000
36080.0000000	8.0000000	-50.0000000
36080.0000000	8.2500000	-50.0000000
36080.0000000	8.5000000	-50.0000000
36080.0000000	8.7500000	-50.0000000
36080.0000000	9.0000000	-50.0000000
36080.0000000	9.2500000	-50.0000000
36080.0000000	9.5000000	-50.0000000
36080.0000000	9.7500000	-50.0000000
36080.0000000	10.0000000	-50.0000000
36080.0000000	10.2500000	-50.0000000
36080.0000000	10.5000000	-50.0000000
36080.0000000	10.7500000	-50.0000000
36080.0000000	11.0000000	-50.0000000
36080.0000000	11.2500000	-50.0000000
36080.0000000	11.5000000	-50.0000000
36080.0000000	11.7500000	-50.0000000
36080.0000000	12.0000000	-50.0000000
36080.0000000	12.2500000	-50.0000000
36080.0000000	12.5000000	-50.0000000
36080.0000000	12.7500000	-50.0000000

36080.0000000	13.0000000	-50.0000000
36080.0000000	13.2500000	-50.0000000
36080.0000000	13.5000000	-50.0000000
36080.0000000	13.7500000	-50.0000000
36080.0000000	14.0000000	-50.0000000
36080.0000000	14.2500000	-50.0000000
36080.0000000	14.5000000	-50.0000000
36080.0000000	14.7500000	-50.0000000
36080.0000000	15.0000000	-50.0000000
36110.0000000	0.0000000	-50.0000000
36110.0000000	0.2500000	-50.0000000
36110.0000000	0.5000000	-50.0000000
36110.0000000	0.7500000	-50.0000000
36110.0000000	1.0000000	-50.0000000
36110.0000000	1.2500000	-50.0000000
36110.0000000	1.5000000	-50.0000000
36110.0000000	1.7500000	-50.0000000
36110.0000000	2.0000000	-50.0000000
36110.0000000	2.2500000	-50.0000000
36110.0000000	2.5000000	-50.0000000
36110.0000000	2.7500000	-50.0000000
36110.0000000	3.0000000	-50.0000000
36110.0000000	3.2500000	-50.0000000
36110.0000000	3.5000000	-50.0000000
36110.0000000	3.7500000	-50.0000000
36110.0000000	4.0000000	-50.0000000
36110.0000000	4.2500000	-50.0000000
36110.0000000	4.5000000	-50.0000000
36110.0000000	4.7500000	-50.0000000
36110.0000000	5.0000000	-50.0000000
36110.0000000	5.2500000	-50.0000000
36110.0000000	5.5000000	-50.0000000
36110.0000000	5.7500000	-50.0000000
36110.0000000	6.0000000	-50.0000000
36110.0000000	6.2500000	-50.0000000
36110.0000000	6.5000000	-50.0000000
36110.0000000	6.7500000	-50.0000000
36110.0000000	7.0000000	-50.0000000
36110.0000000	7.2500000	-50.0000000
36110.0000000	7.5000000	-50.0000000
36110.0000000	7.7500000	-50.0000000
36110.0000000	8.0000000	-50.0000000
36110.0000000	8.2500000	-50.0000000
36110.0000000	8.5000000	-50.0000000
36110.0000000	8.7500000	-50.0000000
36110.0000000	9.0000000	-50.0000000
36110.0000000	9.2500000	-50.0000000
36110.0000000	9.5000000	-50.0000000
36110.0000000	9.7500000	-50.0000000
36110.0000000	10.0000000	-50.0000000
36110.0000000	10.2500000	-50.0000000
36110.0000000	10.5000000	-50.0000000
36110.0000000	10.7500000	-50.0000000
36110.0000000	11.0000000	-50.0000000
36110.0000000	11.2500000	-50.0000000
36110.0000000	11.5000000	-50.0000000
36110.0000000	11.7500000	-50.0000000
36110.0000000	12.0000000	-50.0000000

36110.0000000	12.2500000	-50.0000000
36110.0000000	12.5000000	-50.0000000
36110.0000000	12.7500000	-50.0000000
36110.0000000	13.0000000	-50.0000000
36110.0000000	13.2500000	-50.0000000
36110.0000000	13.5000000	-50.0000000
36110.0000000	13.7500000	-50.0000000
36110.0000000	14.0000000	-50.0000000
36110.0000000	14.2500000	-50.0000000
36110.0000000	14.5000000	-50.0000000
36110.0000000	14.7500000	-50.0000000
36110.0000000	15.0000000	-50.0000000
36140.0000000	0.0000000	-50.0000000
36140.0000000	0.2500000	-50.0000000
36140.0000000	0.5000000	-50.0000000
36140.0000000	0.7500000	-50.0000000
36140.0000000	1.0000000	-50.0000000
36140.0000000	1.2500000	-50.0000000
36140.0000000	1.5000000	-50.0000000
36140.0000000	1.7500000	-50.0000000
36140.0000000	2.0000000	-50.0000000
36140.0000000	2.2500000	-50.0000000
36140.0000000	2.5000000	-50.0000000
36140.0000000	2.7500000	-50.0000000
36140.0000000	3.0000000	-50.0000000
36140.0000000	3.2500000	-50.0000000
36140.0000000	3.5000000	-50.0000000
36140.0000000	3.7500000	-50.0000000
36140.0000000	4.0000000	-50.0000000
36140.0000000	4.2500000	-50.0000000
36140.0000000	4.5000000	-50.0000000
36140.0000000	4.7500000	-50.0000000
36140.0000000	5.0000000	-50.0000000
36140.0000000	5.2500000	-50.0000000
36140.0000000	5.5000000	-50.0000000
36140.0000000	5.7500000	-50.0000000
36140.0000000	6.0000000	-50.0000000
36140.0000000	6.2500000	-50.0000000
36140.0000000	6.5000000	-50.0000000
36140.0000000	6.7500000	-50.0000000
36140.0000000	7.0000000	-50.0000000
36140.0000000	7.2500000	-50.0000000
36140.0000000	7.5000000	-50.0000000
36140.0000000	7.7500000	-50.0000000
36140.0000000	8.0000000	-50.0000000
36140.0000000	8.2500000	-50.0000000
36140.0000000	8.5000000	-50.0000000
36140.0000000	8.7500000	-50.0000000
36140.0000000	9.0000000	-50.0000000
36140.0000000	9.2500000	-50.0000000
36140.0000000	9.5000000	-50.0000000
36140.0000000	9.7500000	-50.0000000
36140.0000000	10.0000000	-50.0000000
36140.0000000	10.2500000	-50.0000000
36140.0000000	10.5000000	-50.0000000
36140.0000000	10.7500000	-50.0000000
36140.0000000	11.0000000	-50.0000000
36140.0000000	11.2500000	-50.0000000

36140.0000000	11.5000000	-50.0000000
36140.0000000	11.7500000	-50.0000000
36140.0000000	12.0000000	-50.0000000
36140.0000000	12.2500000	-50.0000000
36140.0000000	12.5000000	-50.0000000
36140.0000000	12.7500000	-50.0000000
36140.0000000	13.0000000	-50.0000000
36140.0000000	13.2500000	-50.0000000
36140.0000000	13.5000000	-50.0000000
36140.0000000	13.7500000	-50.0000000
36140.0000000	14.0000000	-50.0000000
36140.0000000	14.2500000	-50.0000000
36140.0000000	14.5000000	-50.0000000
36140.0000000	14.7500000	-50.0000000
36140.0000000	15.0000000	-50.0000000
36170.0000000	0.0000000	-50.0000000
36170.0000000	0.2500000	-50.0000000
36170.0000000	0.5000000	-50.0000000
36170.0000000	0.7500000	-50.0000000
36170.0000000	1.0000000	-50.0000000
36170.0000000	1.2500000	-50.0000000
36170.0000000	1.5000000	-50.0000000
36170.0000000	1.7500000	-50.0000000
36170.0000000	2.0000000	-50.0000000
36170.0000000	2.2500000	-50.0000000
36170.0000000	2.5000000	-50.0000000
36170.0000000	2.7500000	-50.0000000
36170.0000000	3.0000000	-50.0000000
36170.0000000	3.2500000	-50.0000000
36170.0000000	3.5000000	-50.0000000
36170.0000000	3.7500000	-50.0000000
36170.0000000	4.0000000	-50.0000000
36170.0000000	4.2500000	-50.0000000
36170.0000000	4.5000000	-50.0000000
36170.0000000	4.7500000	-50.0000000
36170.0000000	5.0000000	-50.0000000
36170.0000000	5.2500000	-50.0000000
36170.0000000	5.5000000	-50.0000000
36170.0000000	5.7500000	-50.0000000
36170.0000000	6.0000000	-50.0000000
36170.0000000	6.2500000	-50.0000000
36170.0000000	6.5000000	-50.0000000
36170.0000000	6.7500000	-50.0000000
36170.0000000	7.0000000	-50.0000000
36170.0000000	7.2500000	-50.0000000
36170.0000000	7.5000000	-50.0000000
36170.0000000	7.7500000	-50.0000000
36170.0000000	8.0000000	-50.0000000
36170.0000000	8.2500000	-50.0000000
36170.0000000	8.5000000	-50.0000000
36170.0000000	8.7500000	-50.0000000
36170.0000000	9.0000000	-50.0000000
36170.0000000	9.2500000	-50.0000000
36170.0000000	9.5000000	-50.0000000
36170.0000000	9.7500000	-50.0000000
36170.0000000	10.0000000	-50.0000000
36170.0000000	10.2500000	-50.0000000
36170.0000000	10.5000000	-50.0000000

36170.0000000	10.7500000	-50.0000000
36170.0000000	11.0000000	-50.0000000
36170.0000000	11.2500000	-50.0000000
36170.0000000	11.5000000	-50.0000000
36170.0000000	11.7500000	-50.0000000
36170.0000000	12.0000000	-50.0000000
36170.0000000	12.2500000	-50.0000000
36170.0000000	12.5000000	-50.0000000
36170.0000000	12.7500000	-50.0000000
36170.0000000	13.0000000	-50.0000000
36170.0000000	13.2500000	-50.0000000
36170.0000000	13.5000000	-50.0000000
36170.0000000	13.7500000	-50.0000000
36170.0000000	14.0000000	-50.0000000
36170.0000000	14.2500000	-50.0000000
36170.0000000	14.5000000	-50.0000000
36170.0000000	14.7500000	-50.0000000
36170.0000000	15.0000000	-50.0000000
36200.0000000	0.0000000	-50.0000000
36200.0000000	0.2500000	-50.0000000
36200.0000000	0.5000000	-50.0000000
36200.0000000	0.7500000	-50.0000000
36200.0000000	1.0000000	-50.0000000
36200.0000000	1.2500000	-50.0000000
36200.0000000	1.5000000	-50.0000000
36200.0000000	1.7500000	-50.0000000
36200.0000000	2.0000000	-50.0000000
36200.0000000	2.2500000	-50.0000000
36200.0000000	2.5000000	-50.0000000
36200.0000000	2.7500000	-50.0000000
36200.0000000	3.0000000	-50.0000000
36200.0000000	3.2500000	-50.0000000
36200.0000000	3.5000000	-50.0000000
36200.0000000	3.7500000	-50.0000000
36200.0000000	4.0000000	-50.0000000
36200.0000000	4.2500000	-50.0000000
36200.0000000	4.5000000	-50.0000000
36200.0000000	4.7500000	-50.0000000
36200.0000000	5.0000000	-50.0000000
36200.0000000	5.2500000	-50.0000000
36200.0000000	5.5000000	-50.0000000
36200.0000000	5.7500000	-50.0000000
36200.0000000	6.0000000	-50.0000000
36200.0000000	6.2500000	-50.0000000
36200.0000000	6.5000000	-50.0000000
36200.0000000	6.7500000	-50.0000000
36200.0000000	7.0000000	-50.0000000
36200.0000000	7.2500000	-50.0000000
36200.0000000	7.5000000	-50.0000000
36200.0000000	7.7500000	-50.0000000
36200.0000000	8.0000000	-50.0000000
36200.0000000	8.2500000	-50.0000000
36200.0000000	8.5000000	-50.0000000
36200.0000000	8.7500000	-50.0000000
36200.0000000	9.0000000	-50.0000000
36200.0000000	9.2500000	-50.0000000
36200.0000000	9.5000000	-50.0000000
36200.0000000	9.7500000	-50.0000000

36200.0000000	10.0000000	-50.0000000
36200.0000000	10.2500000	-50.0000000
36200.0000000	10.5000000	-50.0000000
36200.0000000	10.7500000	-50.0000000
36200.0000000	11.0000000	-50.0000000
36200.0000000	11.2500000	-50.0000000
36200.0000000	11.5000000	-50.0000000
36200.0000000	11.7500000	-50.0000000
36200.0000000	12.0000000	-50.0000000
36200.0000000	12.2500000	-50.0000000
36200.0000000	12.5000000	-50.0000000
36200.0000000	12.7500000	-50.0000000
36200.0000000	13.0000000	-50.0000000
36200.0000000	13.2500000	-50.0000000
36200.0000000	13.5000000	-50.0000000
36200.0000000	13.7500000	-50.0000000
36200.0000000	14.0000000	-50.0000000
36200.0000000	14.2500000	-50.0000000
36200.0000000	14.5000000	-50.0000000
36200.0000000	14.7500000	-50.0000000
36200.0000000	15.0000000	-50.0000000
36230.0000000	0.0000000	-50.0000000
36230.0000000	0.2500000	-50.0000000
36230.0000000	0.5000000	-50.0000000
36230.0000000	0.7500000	-50.0000000
36230.0000000	1.0000000	-50.0000000
36230.0000000	1.2500000	-50.0000000
36230.0000000	1.5000000	-50.0000000
36230.0000000	1.7500000	-50.0000000
36230.0000000	2.0000000	-50.0000000
36230.0000000	2.2500000	-50.0000000
36230.0000000	2.5000000	-50.0000000
36230.0000000	2.7500000	-50.0000000
36230.0000000	3.0000000	-50.0000000
36230.0000000	3.2500000	-50.0000000
36230.0000000	3.5000000	-50.0000000
36230.0000000	3.7500000	-50.0000000
36230.0000000	4.0000000	-50.0000000
36230.0000000	4.2500000	-50.0000000
36230.0000000	4.5000000	-50.0000000
36230.0000000	4.7500000	-50.0000000
36230.0000000	5.0000000	-50.0000000
36230.0000000	5.2500000	-50.0000000
36230.0000000	5.5000000	-50.0000000
36230.0000000	5.7500000	-50.0000000
36230.0000000	6.0000000	-50.0000000
36230.0000000	6.2500000	-50.0000000
36230.0000000	6.5000000	-50.0000000
36230.0000000	6.7500000	-50.0000000
36230.0000000	7.0000000	-50.0000000
36230.0000000	7.2500000	-50.0000000
36230.0000000	7.5000000	-50.0000000
36230.0000000	7.7500000	-50.0000000
36230.0000000	8.0000000	-50.0000000
36230.0000000	8.2500000	-50.0000000
36230.0000000	8.5000000	-50.0000000
36230.0000000	8.7500000	-50.0000000
36230.0000000	9.0000000	-50.0000000

36230.0000000	9.2500000	-50.0000000
36230.0000000	9.5000000	-50.0000000
36230.0000000	9.7500000	-50.0000000
36230.0000000	10.0000000	-50.0000000
36230.0000000	10.2500000	-50.0000000
36230.0000000	10.5000000	-50.0000000
36230.0000000	10.7500000	-50.0000000
36230.0000000	11.0000000	-50.0000000
36230.0000000	11.2500000	-50.0000000
36230.0000000	11.5000000	-50.0000000
36230.0000000	11.7500000	-50.0000000
36230.0000000	12.0000000	-50.0000000
36230.0000000	12.2500000	-50.0000000
36230.0000000	12.5000000	-50.0000000
36230.0000000	12.7500000	-50.0000000
36230.0000000	13.0000000	-50.0000000
36230.0000000	13.2500000	-50.0000000
36230.0000000	13.5000000	-50.0000000
36230.0000000	13.7500000	-50.0000000
36230.0000000	14.0000000	-50.0000000
36230.0000000	14.2500000	-50.0000000
36230.0000000	14.5000000	-50.0000000
36230.0000000	14.7500000	-50.0000000
36230.0000000	15.0000000	-50.0000000
36260.0000000	0.0000000	-50.0000000
36260.0000000	0.2500000	-50.0000000
36260.0000000	0.5000000	-50.0000000
36260.0000000	0.7500000	-50.0000000
36260.0000000	1.0000000	-50.0000000
36260.0000000	1.2500000	-50.0000000
36260.0000000	1.5000000	-50.0000000
36260.0000000	1.7500000	-50.0000000
36260.0000000	2.0000000	-50.0000000
36260.0000000	2.2500000	-50.0000000
36260.0000000	2.5000000	-50.0000000
36260.0000000	2.7500000	-50.0000000
36260.0000000	3.0000000	-50.0000000
36260.0000000	3.2500000	-50.0000000
36260.0000000	3.5000000	-50.0000000
36260.0000000	3.7500000	-50.0000000
36260.0000000	4.0000000	-50.0000000
36260.0000000	4.2500000	-50.0000000
36260.0000000	4.5000000	-50.0000000
36260.0000000	4.7500000	-50.0000000
36260.0000000	5.0000000	-50.0000000
36260.0000000	5.2500000	-50.0000000
36260.0000000	5.5000000	-50.0000000
36260.0000000	5.7500000	-50.0000000
36260.0000000	6.0000000	-50.0000000
36260.0000000	6.2500000	-50.0000000
36260.0000000	6.5000000	-50.0000000
36260.0000000	6.7500000	-50.0000000
36260.0000000	7.0000000	-50.0000000
36260.0000000	7.2500000	-50.0000000
36260.0000000	7.5000000	-50.0000000
36260.0000000	7.7500000	-50.0000000
36260.0000000	8.0000000	-50.0000000
36260.0000000	8.2500000	-50.0000000

36260.0000000	8.5000000	-50.0000000
36260.0000000	8.7500000	-50.0000000
36260.0000000	9.0000000	-50.0000000
36260.0000000	9.2500000	-50.0000000
36260.0000000	9.5000000	-50.0000000
36260.0000000	9.7500000	-50.0000000
36260.0000000	10.0000000	-50.0000000
36260.0000000	10.2500000	-50.0000000
36260.0000000	10.5000000	-50.0000000
36260.0000000	10.7500000	-50.0000000
36260.0000000	11.0000000	-50.0000000
36260.0000000	11.2500000	-50.0000000
36260.0000000	11.5000000	-50.0000000
36260.0000000	11.7500000	-50.0000000
36260.0000000	12.0000000	-50.0000000
36260.0000000	12.2500000	-50.0000000
36260.0000000	12.5000000	-50.0000000
36260.0000000	12.7500000	-50.0000000
36260.0000000	13.0000000	-50.0000000
36260.0000000	13.2500000	-50.0000000
36260.0000000	13.5000000	-50.0000000
36260.0000000	13.7500000	-50.0000000
36260.0000000	14.0000000	-50.0000000
36260.0000000	14.2500000	-50.0000000
36260.0000000	14.5000000	-50.0000000
36260.0000000	14.7500000	-50.0000000
36260.0000000	15.0000000	-50.0000000
36290.0000000	0.0000000	-50.0000000
36290.0000000	0.2500000	-50.0000000
36290.0000000	0.5000000	-50.0000000
36290.0000000	0.7500000	-50.0000000
36290.0000000	1.0000000	-50.0000000
36290.0000000	1.2500000	-50.0000000
36290.0000000	1.5000000	-50.0000000
36290.0000000	1.7500000	-50.0000000
36290.0000000	2.0000000	-50.0000000
36290.0000000	2.2500000	-50.0000000
36290.0000000	2.5000000	-50.0000000
36290.0000000	2.7500000	-50.0000000
36290.0000000	3.0000000	-50.0000000
36290.0000000	3.2500000	-50.0000000
36290.0000000	3.5000000	-50.0000000
36290.0000000	3.7500000	-50.0000000
36290.0000000	4.0000000	-50.0000000
36290.0000000	4.2500000	-50.0000000
36290.0000000	4.5000000	-50.0000000
36290.0000000	4.7500000	-50.0000000
36290.0000000	5.0000000	-50.0000000
36290.0000000	5.2500000	-50.0000000
36290.0000000	5.5000000	-50.0000000
36290.0000000	5.7500000	-50.0000000
36290.0000000	6.0000000	-50.0000000
36290.0000000	6.2500000	-50.0000000
36290.0000000	6.5000000	-50.0000000
36290.0000000	6.7500000	-50.0000000
36290.0000000	7.0000000	-50.0000000
36290.0000000	7.2500000	-50.0000000
36290.0000000	7.5000000	-50.0000000

36290.0000000	7.7500000	-50.0000000
36290.0000000	8.0000000	-50.0000000
36290.0000000	8.2500000	-50.0000000
36290.0000000	8.5000000	-50.0000000
36290.0000000	8.7500000	-50.0000000
36290.0000000	9.0000000	-50.0000000
36290.0000000	9.2500000	-50.0000000
36290.0000000	9.5000000	-50.0000000
36290.0000000	9.7500000	-50.0000000
36290.0000000	10.0000000	-50.0000000
36290.0000000	10.2500000	-50.0000000
36290.0000000	10.5000000	-50.0000000
36290.0000000	10.7500000	-50.0000000
36290.0000000	11.0000000	-50.0000000
36290.0000000	11.2500000	-50.0000000
36290.0000000	11.5000000	-50.0000000
36290.0000000	11.7500000	-50.0000000
36290.0000000	12.0000000	-50.0000000
36290.0000000	12.2500000	-50.0000000
36290.0000000	12.5000000	-50.0000000
36290.0000000	12.7500000	-50.0000000
36290.0000000	13.0000000	-50.0000000
36290.0000000	13.2500000	-50.0000000
36290.0000000	13.5000000	-50.0000000
36290.0000000	13.7500000	-50.0000000
36290.0000000	14.0000000	-50.0000000
36290.0000000	14.2500000	-50.0000000
36290.0000000	14.5000000	-50.0000000
36290.0000000	14.7500000	-50.0000000
36290.0000000	15.0000000	-50.0000000
36320.0000000	0.0000000	-50.0000000
36320.0000000	0.2500000	-50.0000000
36320.0000000	0.5000000	-50.0000000
36320.0000000	0.7500000	-50.0000000
36320.0000000	1.0000000	-50.0000000
36320.0000000	1.2500000	-50.0000000
36320.0000000	1.5000000	-50.0000000
36320.0000000	1.7500000	-50.0000000
36320.0000000	2.0000000	-50.0000000
36320.0000000	2.2500000	-50.0000000
36320.0000000	2.5000000	-50.0000000
36320.0000000	2.7500000	-50.0000000
36320.0000000	3.0000000	-50.0000000
36320.0000000	3.2500000	-50.0000000
36320.0000000	3.5000000	-50.0000000
36320.0000000	3.7500000	-50.0000000
36320.0000000	4.0000000	-50.0000000
36320.0000000	4.2500000	-50.0000000
36320.0000000	4.5000000	-50.0000000
36320.0000000	4.7500000	-50.0000000
36320.0000000	5.0000000	-50.0000000
36320.0000000	5.2500000	-50.0000000
36320.0000000	5.5000000	-50.0000000
36320.0000000	5.7500000	-50.0000000
36320.0000000	6.0000000	-50.0000000
36320.0000000	6.2500000	-50.0000000
36320.0000000	6.5000000	-50.0000000
36320.0000000	6.7500000	-50.0000000

36320.0000000	7.0000000	-50.0000000
36320.0000000	7.2500000	-50.0000000
36320.0000000	7.5000000	-50.0000000
36320.0000000	7.7500000	-50.0000000
36320.0000000	8.0000000	-50.0000000
36320.0000000	8.2500000	-50.0000000
36320.0000000	8.5000000	-50.0000000
36320.0000000	8.7500000	-50.0000000
36320.0000000	9.0000000	-50.0000000
36320.0000000	9.2500000	-50.0000000
36320.0000000	9.5000000	-50.0000000
36320.0000000	9.7500000	-50.0000000
36320.0000000	10.0000000	-50.0000000
36320.0000000	10.2500000	-50.0000000
36320.0000000	10.5000000	-50.0000000
36320.0000000	10.7500000	-50.0000000
36320.0000000	11.0000000	-50.0000000
36320.0000000	11.2500000	-50.0000000
36320.0000000	11.5000000	-50.0000000
36320.0000000	11.7500000	-50.0000000
36320.0000000	12.0000000	-50.0000000
36320.0000000	12.2500000	-50.0000000
36320.0000000	12.5000000	-50.0000000
36320.0000000	12.7500000	-50.0000000
36320.0000000	13.0000000	-50.0000000
36320.0000000	13.2500000	-50.0000000
36320.0000000	13.5000000	-50.0000000
36320.0000000	13.7500000	-50.0000000
36320.0000000	14.0000000	-50.0000000
36320.0000000	14.2500000	-50.0000000
36320.0000000	14.5000000	-50.0000000
36320.0000000	14.7500000	-50.0000000
36320.0000000	15.0000000	-50.0000000
36350.0000000	0.0000000	-50.0000000
36350.0000000	0.2500000	-50.0000000
36350.0000000	0.5000000	-50.0000000
36350.0000000	0.7500000	-50.0000000
36350.0000000	1.0000000	-50.0000000
36350.0000000	1.2500000	-50.0000000
36350.0000000	1.5000000	-50.0000000
36350.0000000	1.7500000	-50.0000000
36350.0000000	2.0000000	-50.0000000
36350.0000000	2.2500000	-50.0000000
36350.0000000	2.5000000	-50.0000000
36350.0000000	2.7500000	-50.0000000
36350.0000000	3.0000000	-50.0000000
36350.0000000	3.2500000	-50.0000000
36350.0000000	3.5000000	-50.0000000
36350.0000000	3.7500000	-50.0000000
36350.0000000	4.0000000	-50.0000000
36350.0000000	4.2500000	-50.0000000
36350.0000000	4.5000000	-50.0000000
36350.0000000	4.7500000	-50.0000000
36350.0000000	5.0000000	-50.0000000
36350.0000000	5.2500000	-50.0000000
36350.0000000	5.5000000	-50.0000000
36350.0000000	5.7500000	-50.0000000
36350.0000000	6.0000000	-50.0000000

36350.0000000	6.2500000	-50.0000000
36350.0000000	6.5000000	-50.0000000
36350.0000000	6.7500000	-50.0000000
36350.0000000	7.0000000	-50.0000000
36350.0000000	7.2500000	-50.0000000
36350.0000000	7.5000000	-50.0000000
36350.0000000	7.7500000	-50.0000000
36350.0000000	8.0000000	-50.0000000
36350.0000000	8.2500000	-50.0000000
36350.0000000	8.5000000	-50.0000000
36350.0000000	8.7500000	-50.0000000
36350.0000000	9.0000000	-50.0000000
36350.0000000	9.2500000	-50.0000000
36350.0000000	9.5000000	-50.0000000
36350.0000000	9.7500000	-50.0000000
36350.0000000	10.0000000	-50.0000000
36350.0000000	10.2500000	-50.0000000
36350.0000000	10.5000000	-50.0000000
36350.0000000	10.7500000	-50.0000000
36350.0000000	11.0000000	-50.0000000
36350.0000000	11.2500000	-50.0000000
36350.0000000	11.5000000	-50.0000000
36350.0000000	11.7500000	-50.0000000
36350.0000000	12.0000000	-50.0000000
36350.0000000	12.2500000	-50.0000000
36350.0000000	12.5000000	-50.0000000
36350.0000000	12.7500000	-50.0000000
36350.0000000	13.0000000	-50.0000000
36350.0000000	13.2500000	-50.0000000
36350.0000000	13.5000000	-50.0000000
36350.0000000	13.7500000	-50.0000000
36350.0000000	14.0000000	-50.0000000
36350.0000000	14.2500000	-50.0000000
36350.0000000	14.5000000	-50.0000000
36350.0000000	14.7500000	-50.0000000
36350.0000000	15.0000000	-50.0000000
36380.0000000	0.0000000	-50.0000000
36380.0000000	0.2500000	-50.0000000
36380.0000000	0.5000000	-50.0000000
36380.0000000	0.7500000	-50.0000000
36380.0000000	1.0000000	-50.0000000
36380.0000000	1.2500000	-50.0000000
36380.0000000	1.5000000	-50.0000000
36380.0000000	1.7500000	-50.0000000
36380.0000000	2.0000000	-50.0000000
36380.0000000	2.2500000	-50.0000000
36380.0000000	2.5000000	-50.0000000
36380.0000000	2.7500000	-50.0000000
36380.0000000	3.0000000	-50.0000000
36380.0000000	3.2500000	-50.0000000
36380.0000000	3.5000000	-50.0000000
36380.0000000	3.7500000	-50.0000000
36380.0000000	4.0000000	-50.0000000
36380.0000000	4.2500000	-50.0000000
36380.0000000	4.5000000	-50.0000000
36380.0000000	4.7500000	-50.0000000
36380.0000000	5.0000000	-50.0000000
36380.0000000	5.2500000	-50.0000000

36380.0000000	5.5000000	-50.0000000
36380.0000000	5.7500000	-50.0000000
36380.0000000	6.0000000	-50.0000000
36380.0000000	6.2500000	-50.0000000
36380.0000000	6.5000000	-50.0000000
36380.0000000	6.7500000	-50.0000000
36380.0000000	7.0000000	-50.0000000
36380.0000000	7.2500000	-50.0000000
36380.0000000	7.5000000	-50.0000000
36380.0000000	7.7500000	-50.0000000
36380.0000000	8.0000000	-50.0000000
36380.0000000	8.2500000	-50.0000000
36380.0000000	8.5000000	-50.0000000
36380.0000000	8.7500000	-50.0000000
36380.0000000	9.0000000	-50.0000000
36380.0000000	9.2500000	-50.0000000
36380.0000000	9.5000000	-50.0000000
36380.0000000	9.7500000	-50.0000000
36380.0000000	10.0000000	-50.0000000
36380.0000000	10.2500000	-50.0000000
36380.0000000	10.5000000	-50.0000000
36380.0000000	10.7500000	-50.0000000
36380.0000000	11.0000000	-50.0000000
36380.0000000	11.2500000	-50.0000000
36380.0000000	11.5000000	-50.0000000
36380.0000000	11.7500000	-50.0000000
36380.0000000	12.0000000	-50.0000000
36380.0000000	12.2500000	-50.0000000
36380.0000000	12.5000000	-50.0000000
36380.0000000	12.7500000	-50.0000000
36380.0000000	13.0000000	-50.0000000
36380.0000000	13.2500000	-50.0000000
36380.0000000	13.5000000	-50.0000000
36380.0000000	13.7500000	-50.0000000
36380.0000000	14.0000000	-50.0000000
36380.0000000	14.2500000	-50.0000000
36380.0000000	14.5000000	-50.0000000
36380.0000000	14.7500000	-50.0000000
36380.0000000	15.0000000	-50.0000000
36410.0000000	0.0000000	-50.0000000
36410.0000000	0.2500000	-50.0000000
36410.0000000	0.5000000	-50.0000000
36410.0000000	0.7500000	-50.0000000
36410.0000000	1.0000000	-50.0000000
36410.0000000	1.2500000	-50.0000000
36410.0000000	1.5000000	-50.0000000
36410.0000000	1.7500000	-50.0000000
36410.0000000	2.0000000	-50.0000000
36410.0000000	2.2500000	-50.0000000
36410.0000000	2.5000000	-50.0000000
36410.0000000	2.7500000	-50.0000000
36410.0000000	3.0000000	-50.0000000
36410.0000000	3.2500000	-50.0000000
36410.0000000	3.5000000	-50.0000000
36410.0000000	3.7500000	-50.0000000
36410.0000000	4.0000000	-50.0000000
36410.0000000	4.2500000	-50.0000000
36410.0000000	4.5000000	-50.0000000

36410.0000000	4.7500000	-50.0000000
36410.0000000	5.0000000	-50.0000000
36410.0000000	5.2500000	-50.0000000
36410.0000000	5.5000000	-50.0000000
36410.0000000	5.7500000	-50.0000000
36410.0000000	6.0000000	-50.0000000
36410.0000000	6.2500000	-50.0000000
36410.0000000	6.5000000	-50.0000000
36410.0000000	6.7500000	-50.0000000
36410.0000000	7.0000000	-50.0000000
36410.0000000	7.2500000	-50.0000000
36410.0000000	7.5000000	-50.0000000
36410.0000000	7.7500000	-50.0000000
36410.0000000	8.0000000	-50.0000000
36410.0000000	8.2500000	-50.0000000
36410.0000000	8.5000000	-50.0000000
36410.0000000	8.7500000	-50.0000000
36410.0000000	9.0000000	-50.0000000
36410.0000000	9.2500000	-50.0000000
36410.0000000	9.5000000	-50.0000000
36410.0000000	9.7500000	-50.0000000
36410.0000000	10.0000000	-50.0000000
36410.0000000	10.2500000	-50.0000000
36410.0000000	10.5000000	-50.0000000
36410.0000000	10.7500000	-50.0000000
36410.0000000	11.0000000	-50.0000000
36410.0000000	11.2500000	-50.0000000
36410.0000000	11.5000000	-50.0000000
36410.0000000	11.7500000	-50.0000000
36410.0000000	12.0000000	-50.0000000
36410.0000000	12.2500000	-50.0000000
36410.0000000	12.5000000	-50.0000000
36410.0000000	12.7500000	-50.0000000
36410.0000000	13.0000000	-50.0000000
36410.0000000	13.2500000	-50.0000000
36410.0000000	13.5000000	-50.0000000
36410.0000000	13.7500000	-50.0000000
36410.0000000	14.0000000	-50.0000000
36410.0000000	14.2500000	-50.0000000
36410.0000000	14.5000000	-50.0000000
36410.0000000	14.7500000	-50.0000000
36410.0000000	15.0000000	-50.0000000
36440.0000000	0.0000000	-50.0000000
36440.0000000	0.2500000	-50.0000000
36440.0000000	0.5000000	-50.0000000
36440.0000000	0.7500000	-50.0000000
36440.0000000	1.0000000	-50.0000000
36440.0000000	1.2500000	-50.0000000
36440.0000000	1.5000000	-50.0000000
36440.0000000	1.7500000	-50.0000000
36440.0000000	2.0000000	-50.0000000
36440.0000000	2.2500000	-50.0000000
36440.0000000	2.5000000	-50.0000000
36440.0000000	2.7500000	-50.0000000
36440.0000000	3.0000000	-50.0000000
36440.0000000	3.2500000	-50.0000000
36440.0000000	3.5000000	-50.0000000
36440.0000000	3.7500000	-50.0000000

36440.0000000	4.0000000	-50.0000000
36440.0000000	4.2500000	-50.0000000
36440.0000000	4.5000000	-50.0000000
36440.0000000	4.7500000	-50.0000000
36440.0000000	5.0000000	-50.0000000
36440.0000000	5.2500000	-50.0000000
36440.0000000	5.5000000	-50.0000000
36440.0000000	5.7500000	-50.0000000
36440.0000000	6.0000000	-50.0000000
36440.0000000	6.2500000	-50.0000000
36440.0000000	6.5000000	-50.0000000
36440.0000000	6.7500000	-50.0000000
36440.0000000	7.0000000	-50.0000000
36440.0000000	7.2500000	-50.0000000
36440.0000000	7.5000000	-50.0000000
36440.0000000	7.7500000	-50.0000000
36440.0000000	8.0000000	-50.0000000
36440.0000000	8.2500000	-50.0000000
36440.0000000	8.5000000	-50.0000000
36440.0000000	8.7500000	-50.0000000
36440.0000000	9.0000000	-50.0000000
36440.0000000	9.2500000	-50.0000000
36440.0000000	9.5000000	-50.0000000
36440.0000000	9.7500000	-50.0000000
36440.0000000	10.0000000	-50.0000000
36440.0000000	10.2500000	-50.0000000
36440.0000000	10.5000000	-50.0000000
36440.0000000	10.7500000	-50.0000000
36440.0000000	11.0000000	-50.0000000
36440.0000000	11.2500000	-50.0000000
36440.0000000	11.5000000	-50.0000000
36440.0000000	11.7500000	-50.0000000
36440.0000000	12.0000000	-50.0000000
36440.0000000	12.2500000	-50.0000000
36440.0000000	12.5000000	-50.0000000
36440.0000000	12.7500000	-50.0000000
36440.0000000	13.0000000	-50.0000000
36440.0000000	13.2500000	-50.0000000
36440.0000000	13.5000000	-50.0000000
36440.0000000	13.7500000	-50.0000000
36440.0000000	14.0000000	-50.0000000
36440.0000000	14.2500000	-50.0000000
36440.0000000	14.5000000	-50.0000000
36440.0000000	14.7500000	-50.0000000
36440.0000000	15.0000000	-50.0000000
36470.0000000	0.0000000	-50.0000000
36470.0000000	0.2500000	-50.0000000
36470.0000000	0.5000000	-50.0000000
36470.0000000	0.7500000	-50.0000000
36470.0000000	1.0000000	-50.0000000
36470.0000000	1.2500000	-50.0000000
36470.0000000	1.5000000	-50.0000000
36470.0000000	1.7500000	-50.0000000
36470.0000000	2.0000000	-50.0000000
36470.0000000	2.2500000	-50.0000000
36470.0000000	2.5000000	-50.0000000
36470.0000000	2.7500000	-50.0000000
36470.0000000	3.0000000	-50.0000000

36470.0000000	3.2500000	-50.0000000
36470.0000000	3.5000000	-50.0000000
36470.0000000	3.7500000	-50.0000000
36470.0000000	4.0000000	-50.0000000
36470.0000000	4.2500000	-50.0000000
36470.0000000	4.5000000	-50.0000000
36470.0000000	4.7500000	-50.0000000
36470.0000000	5.0000000	-50.0000000
36470.0000000	5.2500000	-50.0000000
36470.0000000	5.5000000	-50.0000000
36470.0000000	5.7500000	-50.0000000
36470.0000000	6.0000000	-50.0000000
36470.0000000	6.2500000	-50.0000000
36470.0000000	6.5000000	-50.0000000
36470.0000000	6.7500000	-50.0000000
36470.0000000	7.0000000	-50.0000000
36470.0000000	7.2500000	-50.0000000
36470.0000000	7.5000000	-50.0000000
36470.0000000	7.7500000	-50.0000000
36470.0000000	8.0000000	-50.0000000
36470.0000000	8.2500000	-50.0000000
36470.0000000	8.5000000	-50.0000000
36470.0000000	8.7500000	-50.0000000
36470.0000000	9.0000000	-50.0000000
36470.0000000	9.2500000	-50.0000000
36470.0000000	9.5000000	-50.0000000
36470.0000000	9.7500000	-50.0000000
36470.0000000	10.0000000	-50.0000000
36470.0000000	10.2500000	-50.0000000
36470.0000000	10.5000000	-50.0000000
36470.0000000	10.7500000	-50.0000000
36470.0000000	11.0000000	-50.0000000
36470.0000000	11.2500000	-50.0000000
36470.0000000	11.5000000	-50.0000000
36470.0000000	11.7500000	-50.0000000
36470.0000000	12.0000000	-50.0000000
36470.0000000	12.2500000	-50.0000000
36470.0000000	12.5000000	-50.0000000
36470.0000000	12.7500000	-50.0000000
36470.0000000	13.0000000	-50.0000000
36470.0000000	13.2500000	-50.0000000
36470.0000000	13.5000000	-50.0000000
36470.0000000	13.7500000	-50.0000000
36470.0000000	14.0000000	-50.0000000
36470.0000000	14.2500000	-50.0000000
36470.0000000	14.5000000	-50.0000000
36470.0000000	14.7500000	-50.0000000
36470.0000000	15.0000000	-50.0000000
36500.0000000	0.0000000	-50.0000000
36500.0000000	0.2500000	-50.0000000
36500.0000000	0.5000000	-50.0000000
36500.0000000	0.7500000	-50.0000000
36500.0000000	1.0000000	-50.0000000
36500.0000000	1.2500000	-50.0000000
36500.0000000	1.5000000	-50.0000000
36500.0000000	1.7500000	-50.0000000
36500.0000000	2.0000000	-50.0000000
36500.0000000	2.2500000	-50.0000000

36500.0000000	2.5000000	-50.0000000
36500.0000000	2.7500000	-50.0000000
36500.0000000	3.0000000	-50.0000000
36500.0000000	3.2500000	-50.0000000
36500.0000000	3.5000000	-50.0000000
36500.0000000	3.7500000	-50.0000000
36500.0000000	4.0000000	-50.0000000
36500.0000000	4.2500000	-50.0000000
36500.0000000	4.5000000	-50.0000000
36500.0000000	4.7500000	-50.0000000
36500.0000000	5.0000000	-50.0000000
36500.0000000	5.2500000	-50.0000000
36500.0000000	5.5000000	-50.0000000
36500.0000000	5.7500000	-50.0000000
36500.0000000	6.0000000	-50.0000000
36500.0000000	6.2500000	-50.0000000
36500.0000000	6.5000000	-50.0000000
36500.0000000	6.7500000	-50.0000000
36500.0000000	7.0000000	-50.0000000
36500.0000000	7.2500000	-50.0000000
36500.0000000	7.5000000	-50.0000000
36500.0000000	7.7500000	-50.0000000
36500.0000000	8.0000000	-50.0000000
36500.0000000	8.2500000	-50.0000000
36500.0000000	8.5000000	-50.0000000
36500.0000000	8.7500000	-50.0000000
36500.0000000	9.0000000	-50.0000000
36500.0000000	9.2500000	-50.0000000
36500.0000000	9.5000000	-50.0000000
36500.0000000	9.7500000	-50.0000000
36500.0000000	10.0000000	-50.0000000
36500.0000000	10.2500000	-50.0000000
36500.0000000	10.5000000	-50.0000000
36500.0000000	10.7500000	-50.0000000
36500.0000000	11.0000000	-50.0000000
36500.0000000	11.2500000	-50.0000000
36500.0000000	11.5000000	-50.0000000
36500.0000000	11.7500000	-50.0000000
36500.0000000	12.0000000	-50.0000000
36500.0000000	12.2500000	-50.0000000
36500.0000000	12.5000000	-50.0000000
36500.0000000	12.7500000	-50.0000000
36500.0000000	13.0000000	-50.0000000
36500.0000000	13.2500000	-50.0000000
36500.0000000	13.5000000	-50.0000000
36500.0000000	13.7500000	-50.0000000
36500.0000000	14.0000000	-50.0000000
36500.0000000	14.2500000	-50.0000000
36500.0000000	14.5000000	-50.0000000
36500.0000000	14.7500000	-50.0000000
36500.0000000	15.0000000	-50.0000000
36530.0000000	0.0000000	-50.0000000
36530.0000000	0.2500000	-50.0000000
36530.0000000	0.5000000	-50.0000000
36530.0000000	0.7500000	-50.0000000
36530.0000000	1.0000000	-50.0000000
36530.0000000	1.2500000	-50.0000000
36530.0000000	1.5000000	-50.0000000

36530.0000000	1.7500000	-50.0000000
36530.0000000	2.0000000	-50.0000000
36530.0000000	2.2500000	-50.0000000
36530.0000000	2.5000000	-50.0000000
36530.0000000	2.7500000	-50.0000000
36530.0000000	3.0000000	-50.0000000
36530.0000000	3.2500000	-50.0000000
36530.0000000	3.5000000	-50.0000000
36530.0000000	3.7500000	-50.0000000
36530.0000000	4.0000000	-50.0000000
36530.0000000	4.2500000	-50.0000000
36530.0000000	4.5000000	-50.0000000
36530.0000000	4.7500000	-50.0000000
36530.0000000	5.0000000	-50.0000000
36530.0000000	5.2500000	-50.0000000
36530.0000000	5.5000000	-50.0000000
36530.0000000	5.7500000	-50.0000000
36530.0000000	6.0000000	-50.0000000
36530.0000000	6.2500000	-50.0000000
36530.0000000	6.5000000	-50.0000000
36530.0000000	6.7500000	-50.0000000
36530.0000000	7.0000000	-50.0000000
36530.0000000	7.2500000	-50.0000000
36530.0000000	7.5000000	-50.0000000
36530.0000000	7.7500000	-50.0000000
36530.0000000	8.0000000	-50.0000000
36530.0000000	8.2500000	-50.0000000
36530.0000000	8.5000000	-50.0000000
36530.0000000	8.7500000	-50.0000000
36530.0000000	9.0000000	-50.0000000
36530.0000000	9.2500000	-50.0000000
36530.0000000	9.5000000	-50.0000000
36530.0000000	9.7500000	-50.0000000
36530.0000000	10.0000000	-50.0000000
36530.0000000	10.2500000	-50.0000000
36530.0000000	10.5000000	-50.0000000
36530.0000000	10.7500000	-50.0000000
36530.0000000	11.0000000	-50.0000000
36530.0000000	11.2500000	-50.0000000
36530.0000000	11.5000000	-50.0000000
36530.0000000	11.7500000	-50.0000000
36530.0000000	12.0000000	-50.0000000
36530.0000000	12.2500000	-50.0000000
36530.0000000	12.5000000	-50.0000000
36530.0000000	12.7500000	-50.0000000
36530.0000000	13.0000000	-50.0000000
36530.0000000	13.2500000	-50.0000000
36530.0000000	13.5000000	-50.0000000
36530.0000000	13.7500000	-50.0000000
36530.0000000	14.0000000	-50.0000000
36530.0000000	14.2500000	-50.0000000
36530.0000000	14.5000000	-50.0000000
36530.0000000	14.7500000	-50.0000000
36530.0000000	15.0000000	-50.0000000
36560.0000000	0.0000000	-50.0000000
36560.0000000	0.2500000	-50.0000000
36560.0000000	0.5000000	-50.0000000
36560.0000000	0.7500000	-50.0000000

36560.0000000	1.0000000	-50.0000000
36560.0000000	1.2500000	-50.0000000
36560.0000000	1.5000000	-50.0000000
36560.0000000	1.7500000	-50.0000000
36560.0000000	2.0000000	-50.0000000
36560.0000000	2.2500000	-50.0000000
36560.0000000	2.5000000	-50.0000000
36560.0000000	2.7500000	-50.0000000
36560.0000000	3.0000000	-50.0000000
36560.0000000	3.2500000	-50.0000000
36560.0000000	3.5000000	-50.0000000
36560.0000000	3.7500000	-50.0000000
36560.0000000	4.0000000	-50.0000000
36560.0000000	4.2500000	-50.0000000
36560.0000000	4.5000000	-50.0000000
36560.0000000	4.7500000	-50.0000000
36560.0000000	5.0000000	-50.0000000
36560.0000000	5.2500000	-50.0000000
36560.0000000	5.5000000	-50.0000000
36560.0000000	5.7500000	-50.0000000
36560.0000000	6.0000000	-50.0000000
36560.0000000	6.2500000	-50.0000000
36560.0000000	6.5000000	-50.0000000
36560.0000000	6.7500000	-50.0000000
36560.0000000	7.0000000	-50.0000000
36560.0000000	7.2500000	-50.0000000
36560.0000000	7.5000000	-50.0000000
36560.0000000	7.7500000	-50.0000000
36560.0000000	8.0000000	-50.0000000
36560.0000000	8.2500000	-50.0000000
36560.0000000	8.5000000	-50.0000000
36560.0000000	8.7500000	-50.0000000
36560.0000000	9.0000000	-50.0000000
36560.0000000	9.2500000	-50.0000000
36560.0000000	9.5000000	-50.0000000
36560.0000000	9.7500000	-50.0000000
36560.0000000	10.0000000	-50.0000000
36560.0000000	10.2500000	-50.0000000
36560.0000000	10.5000000	-50.0000000
36560.0000000	10.7500000	-50.0000000
36560.0000000	11.0000000	-50.0000000
36560.0000000	11.2500000	-50.0000000
36560.0000000	11.5000000	-50.0000000
36560.0000000	11.7500000	-50.0000000
36560.0000000	12.0000000	-50.0000000
36560.0000000	12.2500000	-50.0000000
36560.0000000	12.5000000	-50.0000000
36560.0000000	12.7500000	-50.0000000
36560.0000000	13.0000000	-50.0000000
36560.0000000	13.2500000	-50.0000000
36560.0000000	13.5000000	-50.0000000
36560.0000000	13.7500000	-50.0000000
36560.0000000	14.0000000	-50.0000000
36560.0000000	14.2500000	-50.0000000
36560.0000000	14.5000000	-50.0000000
36560.0000000	14.7500000	-50.0000000
36560.0000000	15.0000000	-50.0000000
36590.0000000	0.0000000	-50.0000000

36590.0000000	0.2500000	-50.0000000
36590.0000000	0.5000000	-50.0000000
36590.0000000	0.7500000	-50.0000000
36590.0000000	1.0000000	-50.0000000
36590.0000000	1.2500000	-50.0000000
36590.0000000	1.5000000	-50.0000000
36590.0000000	1.7500000	-50.0000000
36590.0000000	2.0000000	-50.0000000
36590.0000000	2.2500000	-50.0000000
36590.0000000	2.5000000	-50.0000000
36590.0000000	2.7500000	-50.0000000
36590.0000000	3.0000000	-50.0000000
36590.0000000	3.2500000	-50.0000000
36590.0000000	3.5000000	-50.0000000
36590.0000000	3.7500000	-50.0000000
36590.0000000	4.0000000	-50.0000000
36590.0000000	4.2500000	-50.0000000
36590.0000000	4.5000000	-50.0000000
36590.0000000	4.7500000	-50.0000000
36590.0000000	5.0000000	-50.0000000
36590.0000000	5.2500000	-50.0000000
36590.0000000	5.5000000	-50.0000000
36590.0000000	5.7500000	-50.0000000
36590.0000000	6.0000000	-50.0000000
36590.0000000	6.2500000	-50.0000000
36590.0000000	6.5000000	-50.0000000
36590.0000000	6.7500000	-50.0000000
36590.0000000	7.0000000	-50.0000000
36590.0000000	7.2500000	-50.0000000
36590.0000000	7.5000000	-50.0000000
36590.0000000	7.7500000	-50.0000000
36590.0000000	8.0000000	-50.0000000
36590.0000000	8.2500000	-50.0000000
36590.0000000	8.5000000	-50.0000000
36590.0000000	8.7500000	-50.0000000
36590.0000000	9.0000000	-50.0000000
36590.0000000	9.2500000	-50.0000000
36590.0000000	9.5000000	-50.0000000
36590.0000000	9.7500000	-50.0000000
36590.0000000	10.0000000	-50.0000000
36590.0000000	10.2500000	-50.0000000
36590.0000000	10.5000000	-50.0000000
36590.0000000	10.7500000	-50.0000000
36590.0000000	11.0000000	-50.0000000
36590.0000000	11.2500000	-50.0000000
36590.0000000	11.5000000	-50.0000000
36590.0000000	11.7500000	-50.0000000
36590.0000000	12.0000000	-50.0000000
36590.0000000	12.2500000	-50.0000000
36590.0000000	12.5000000	-50.0000000
36590.0000000	12.7500000	-50.0000000
36590.0000000	13.0000000	-50.0000000
36590.0000000	13.2500000	-50.0000000
36590.0000000	13.5000000	-50.0000000
36590.0000000	13.7500000	-50.0000000
36590.0000000	14.0000000	-50.0000000
36590.0000000	14.2500000	-50.0000000
36590.0000000	14.5000000	-50.0000000

36590.0000000	14.7500000	-50.0000000
36590.0000000	15.0000000	-50.0000000
36620.0000000	0.0000000	-50.0000000
36620.0000000	0.2500000	-50.0000000
36620.0000000	0.5000000	-50.0000000
36620.0000000	0.7500000	-50.0000000
36620.0000000	1.0000000	-50.0000000
36620.0000000	1.2500000	-50.0000000
36620.0000000	1.5000000	-50.0000000
36620.0000000	1.7500000	-50.0000000
36620.0000000	2.0000000	-50.0000000
36620.0000000	2.2500000	-50.0000000
36620.0000000	2.5000000	-50.0000000
36620.0000000	2.7500000	-50.0000000
36620.0000000	3.0000000	-50.0000000
36620.0000000	3.2500000	-50.0000000
36620.0000000	3.5000000	-50.0000000
36620.0000000	3.7500000	-50.0000000
36620.0000000	4.0000000	-50.0000000
36620.0000000	4.2500000	-50.0000000
36620.0000000	4.5000000	-50.0000000
36620.0000000	4.7500000	-50.0000000
36620.0000000	5.0000000	-50.0000000
36620.0000000	5.2500000	-50.0000000
36620.0000000	5.5000000	-50.0000000
36620.0000000	5.7500000	-50.0000000
36620.0000000	6.0000000	-50.0000000
36620.0000000	6.2500000	-50.0000000
36620.0000000	6.5000000	-50.0000000
36620.0000000	6.7500000	-50.0000000
36620.0000000	7.0000000	-50.0000000
36620.0000000	7.2500000	-50.0000000
36620.0000000	7.5000000	-50.0000000
36620.0000000	7.7500000	-50.0000000
36620.0000000	8.0000000	-50.0000000
36620.0000000	8.2500000	-50.0000000
36620.0000000	8.5000000	-50.0000000
36620.0000000	8.7500000	-50.0000000
36620.0000000	9.0000000	-50.0000000
36620.0000000	9.2500000	-50.0000000
36620.0000000	9.5000000	-50.0000000
36620.0000000	9.7500000	-50.0000000
36620.0000000	10.0000000	-50.0000000
36620.0000000	10.2500000	-50.0000000
36620.0000000	10.5000000	-50.0000000
36620.0000000	10.7500000	-50.0000000
36620.0000000	11.0000000	-50.0000000
36620.0000000	11.2500000	-50.0000000
36620.0000000	11.5000000	-50.0000000
36620.0000000	11.7500000	-50.0000000
36620.0000000	12.0000000	-50.0000000
36620.0000000	12.2500000	-50.0000000
36620.0000000	12.5000000	-50.0000000
36620.0000000	12.7500000	-50.0000000
36620.0000000	13.0000000	-50.0000000
36620.0000000	13.2500000	-50.0000000
36620.0000000	13.5000000	-50.0000000
36620.0000000	13.7500000	-50.0000000

36620.0000000	14.0000000	-50.0000000
36620.0000000	14.2500000	-50.0000000
36620.0000000	14.5000000	-50.0000000
36620.0000000	14.7500000	-50.0000000
36620.0000000	15.0000000	-50.0000000
36650.0000000	0.0000000	-50.0000000
36650.0000000	0.2500000	-50.0000000
36650.0000000	0.5000000	-50.0000000
36650.0000000	0.7500000	-50.0000000
36650.0000000	1.0000000	-50.0000000
36650.0000000	1.2500000	-50.0000000
36650.0000000	1.5000000	-50.0000000
36650.0000000	1.7500000	-50.0000000
36650.0000000	2.0000000	-50.0000000
36650.0000000	2.2500000	-50.0000000
36650.0000000	2.5000000	-50.0000000
36650.0000000	2.7500000	-50.0000000
36650.0000000	3.0000000	-50.0000000
36650.0000000	3.2500000	-50.0000000
36650.0000000	3.5000000	-50.0000000
36650.0000000	3.7500000	-50.0000000
36650.0000000	4.0000000	-50.0000000
36650.0000000	4.2500000	-50.0000000
36650.0000000	4.5000000	-50.0000000
36650.0000000	4.7500000	-50.0000000
36650.0000000	5.0000000	-50.0000000
36650.0000000	5.2500000	-50.0000000
36650.0000000	5.5000000	-50.0000000
36650.0000000	5.7500000	-50.0000000
36650.0000000	6.0000000	-50.0000000
36650.0000000	6.2500000	-50.0000000
36650.0000000	6.5000000	-50.0000000
36650.0000000	6.7500000	-50.0000000
36650.0000000	7.0000000	-50.0000000
36650.0000000	7.2500000	-50.0000000
36650.0000000	7.5000000	-50.0000000
36650.0000000	7.7500000	-50.0000000
36650.0000000	8.0000000	-50.0000000
36650.0000000	8.2500000	-50.0000000
36650.0000000	8.5000000	-50.0000000
36650.0000000	8.7500000	-50.0000000
36650.0000000	9.0000000	-50.0000000
36650.0000000	9.2500000	-50.0000000
36650.0000000	9.5000000	-50.0000000
36650.0000000	9.7500000	-50.0000000
36650.0000000	10.0000000	-50.0000000
36650.0000000	10.2500000	-50.0000000
36650.0000000	10.5000000	-50.0000000
36650.0000000	10.7500000	-50.0000000
36650.0000000	11.0000000	-50.0000000
36650.0000000	11.2500000	-50.0000000
36650.0000000	11.5000000	-50.0000000
36650.0000000	11.7500000	-50.0000000
36650.0000000	12.0000000	-50.0000000
36650.0000000	12.2500000	-50.0000000
36650.0000000	12.5000000	-50.0000000
36650.0000000	12.7500000	-50.0000000
36650.0000000	13.0000000	-50.0000000

36650.0000000	13.2500000	-50.0000000
36650.0000000	13.5000000	-50.0000000
36650.0000000	13.7500000	-50.0000000
36650.0000000	14.0000000	-50.0000000
36650.0000000	14.2500000	-50.0000000
36650.0000000	14.5000000	-50.0000000
36650.0000000	14.7500000	-50.0000000
36650.0000000	15.0000000	-50.0000000
36680.0000000	0.0000000	-50.0000000
36680.0000000	0.2500000	-50.0000000
36680.0000000	0.5000000	-50.0000000
36680.0000000	0.7500000	-50.0000000
36680.0000000	1.0000000	-50.0000000
36680.0000000	1.2500000	-50.0000000
36680.0000000	1.5000000	-50.0000000
36680.0000000	1.7500000	-50.0000000
36680.0000000	2.0000000	-50.0000000
36680.0000000	2.2500000	-50.0000000
36680.0000000	2.5000000	-50.0000000
36680.0000000	2.7500000	-50.0000000
36680.0000000	3.0000000	-50.0000000
36680.0000000	3.2500000	-50.0000000
36680.0000000	3.5000000	-50.0000000
36680.0000000	3.7500000	-50.0000000
36680.0000000	4.0000000	-50.0000000
36680.0000000	4.2500000	-50.0000000
36680.0000000	4.5000000	-50.0000000
36680.0000000	4.7500000	-50.0000000
36680.0000000	5.0000000	-50.0000000
36680.0000000	5.2500000	-50.0000000
36680.0000000	5.5000000	-50.0000000
36680.0000000	5.7500000	-50.0000000
36680.0000000	6.0000000	-50.0000000
36680.0000000	6.2500000	-50.0000000
36680.0000000	6.5000000	-50.0000000
36680.0000000	6.7500000	-50.0000000
36680.0000000	7.0000000	-50.0000000
36680.0000000	7.2500000	-50.0000000
36680.0000000	7.5000000	-50.0000000
36680.0000000	7.7500000	-50.0000000
36680.0000000	8.0000000	-50.0000000
36680.0000000	8.2500000	-50.0000000
36680.0000000	8.5000000	-50.0000000
36680.0000000	8.7500000	-50.0000000
36680.0000000	9.0000000	-50.0000000
36680.0000000	9.2500000	-50.0000000
36680.0000000	9.5000000	-50.0000000
36680.0000000	9.7500000	-50.0000000
36680.0000000	10.0000000	-50.0000000
36680.0000000	10.2500000	-50.0000000
36680.0000000	10.5000000	-50.0000000
36680.0000000	10.7500000	-50.0000000
36680.0000000	11.0000000	-50.0000000
36680.0000000	11.2500000	-50.0000000
36680.0000000	11.5000000	-50.0000000
36680.0000000	11.7500000	-50.0000000
36680.0000000	12.0000000	-50.0000000
36680.0000000	12.2500000	-50.0000000

36680.0000000	12.5000000	-50.0000000
36680.0000000	12.7500000	-50.0000000
36680.0000000	13.0000000	-50.0000000
36680.0000000	13.2500000	-50.0000000
36680.0000000	13.5000000	-50.0000000
36680.0000000	13.7500000	-50.0000000
36680.0000000	14.0000000	-50.0000000
36680.0000000	14.2500000	-50.0000000
36680.0000000	14.5000000	-50.0000000
36680.0000000	14.7500000	-50.0000000
36680.0000000	15.0000000	-50.0000000
36710.0000000	0.0000000	-50.0000000
36710.0000000	0.2500000	-50.0000000
36710.0000000	0.5000000	-50.0000000
36710.0000000	0.7500000	-50.0000000
36710.0000000	1.0000000	-50.0000000
36710.0000000	1.2500000	-50.0000000
36710.0000000	1.5000000	-50.0000000
36710.0000000	1.7500000	-50.0000000
36710.0000000	2.0000000	-50.0000000
36710.0000000	2.2500000	-50.0000000
36710.0000000	2.5000000	-50.0000000
36710.0000000	2.7500000	-50.0000000
36710.0000000	3.0000000	-50.0000000
36710.0000000	3.2500000	-50.0000000
36710.0000000	3.5000000	-50.0000000
36710.0000000	3.7500000	-50.0000000
36710.0000000	4.0000000	-50.0000000
36710.0000000	4.2500000	-50.0000000
36710.0000000	4.5000000	-50.0000000
36710.0000000	4.7500000	-50.0000000
36710.0000000	5.0000000	-50.0000000
36710.0000000	5.2500000	-50.0000000
36710.0000000	5.5000000	-50.0000000
36710.0000000	5.7500000	-50.0000000
36710.0000000	6.0000000	-50.0000000
36710.0000000	6.2500000	-50.0000000
36710.0000000	6.5000000	-50.0000000
36710.0000000	6.7500000	-50.0000000
36710.0000000	7.0000000	-50.0000000
36710.0000000	7.2500000	-50.0000000
36710.0000000	7.5000000	-50.0000000
36710.0000000	7.7500000	-50.0000000
36710.0000000	8.0000000	-50.0000000
36710.0000000	8.2500000	-50.0000000
36710.0000000	8.5000000	-50.0000000
36710.0000000	8.7500000	-50.0000000
36710.0000000	9.0000000	-50.0000000
36710.0000000	9.2500000	-50.0000000
36710.0000000	9.5000000	-50.0000000
36710.0000000	9.7500000	-50.0000000
36710.0000000	10.0000000	-50.0000000
36710.0000000	10.2500000	-50.0000000
36710.0000000	10.5000000	-50.0000000
36710.0000000	10.7500000	-50.0000000
36710.0000000	11.0000000	-50.0000000
36710.0000000	11.2500000	-50.0000000
36710.0000000	11.5000000	-50.0000000

36710.0000000	11.7500000	-50.0000000
36710.0000000	12.0000000	-50.0000000
36710.0000000	12.2500000	-50.0000000
36710.0000000	12.5000000	-50.0000000
36710.0000000	12.7500000	-50.0000000
36710.0000000	13.0000000	-50.0000000
36710.0000000	13.2500000	-50.0000000
36710.0000000	13.5000000	-50.0000000
36710.0000000	13.7500000	-50.0000000
36710.0000000	14.0000000	-50.0000000
36710.0000000	14.2500000	-50.0000000
36710.0000000	14.5000000	-50.0000000
36710.0000000	14.7500000	-50.0000000
36710.0000000	15.0000000	-50.0000000
36740.0000000	0.0000000	-50.0000000
36740.0000000	0.2500000	-50.0000000
36740.0000000	0.5000000	-50.0000000
36740.0000000	0.7500000	-50.0000000
36740.0000000	1.0000000	-50.0000000
36740.0000000	1.2500000	-50.0000000
36740.0000000	1.5000000	-50.0000000
36740.0000000	1.7500000	-50.0000000
36740.0000000	2.0000000	-50.0000000
36740.0000000	2.2500000	-50.0000000
36740.0000000	2.5000000	-50.0000000
36740.0000000	2.7500000	-50.0000000
36740.0000000	3.0000000	-50.0000000
36740.0000000	3.2500000	-50.0000000
36740.0000000	3.5000000	-50.0000000
36740.0000000	3.7500000	-50.0000000
36740.0000000	4.0000000	-50.0000000
36740.0000000	4.2500000	-50.0000000
36740.0000000	4.5000000	-50.0000000
36740.0000000	4.7500000	-50.0000000
36740.0000000	5.0000000	-50.0000000
36740.0000000	5.2500000	-50.0000000
36740.0000000	5.5000000	-50.0000000
36740.0000000	5.7500000	-50.0000000
36740.0000000	6.0000000	-50.0000000
36740.0000000	6.2500000	-50.0000000
36740.0000000	6.5000000	-50.0000000
36740.0000000	6.7500000	-50.0000000
36740.0000000	7.0000000	-50.0000000
36740.0000000	7.2500000	-50.0000000
36740.0000000	7.5000000	-50.0000000
36740.0000000	7.7500000	-50.0000000
36740.0000000	8.0000000	-50.0000000
36740.0000000	8.2500000	-50.0000000
36740.0000000	8.5000000	-50.0000000
36740.0000000	8.7500000	-50.0000000
36740.0000000	9.0000000	-50.0000000
36740.0000000	9.2500000	-50.0000000
36740.0000000	9.5000000	-50.0000000
36740.0000000	9.7500000	-50.0000000
36740.0000000	10.0000000	-50.0000000
36740.0000000	10.2500000	-50.0000000
36740.0000000	10.5000000	-50.0000000
36740.0000000	10.7500000	-50.0000000

36740.0000000	11.0000000	-50.0000000
36740.0000000	11.2500000	-50.0000000
36740.0000000	11.5000000	-50.0000000
36740.0000000	11.7500000	-50.0000000
36740.0000000	12.0000000	-50.0000000
36740.0000000	12.2500000	-50.0000000
36740.0000000	12.5000000	-50.0000000
36740.0000000	12.7500000	-50.0000000
36740.0000000	13.0000000	-50.0000000
36740.0000000	13.2500000	-50.0000000
36740.0000000	13.5000000	-50.0000000
36740.0000000	13.7500000	-50.0000000
36740.0000000	14.0000000	-50.0000000
36740.0000000	14.2500000	-50.0000000
36740.0000000	14.5000000	-50.0000000
36740.0000000	14.7500000	-50.0000000
36740.0000000	15.0000000	-50.0000000
36770.0000000	0.0000000	-50.0000000
36770.0000000	0.2500000	-50.0000000
36770.0000000	0.5000000	-50.0000000
36770.0000000	0.7500000	-50.0000000
36770.0000000	1.0000000	-50.0000000
36770.0000000	1.2500000	-50.0000000
36770.0000000	1.5000000	-50.0000000
36770.0000000	1.7500000	-50.0000000
36770.0000000	2.0000000	-50.0000000
36770.0000000	2.2500000	-50.0000000
36770.0000000	2.5000000	-50.0000000
36770.0000000	2.7500000	-50.0000000
36770.0000000	3.0000000	-50.0000000
36770.0000000	3.2500000	-50.0000000
36770.0000000	3.5000000	-50.0000000
36770.0000000	3.7500000	-50.0000000
36770.0000000	4.0000000	-50.0000000
36770.0000000	4.2500000	-50.0000000
36770.0000000	4.5000000	-50.0000000
36770.0000000	4.7500000	-50.0000000
36770.0000000	5.0000000	-50.0000000
36770.0000000	5.2500000	-50.0000000
36770.0000000	5.5000000	-50.0000000
36770.0000000	5.7500000	-50.0000000
36770.0000000	6.0000000	-50.0000000
36770.0000000	6.2500000	-50.0000000
36770.0000000	6.5000000	-50.0000000
36770.0000000	6.7500000	-50.0000000
36770.0000000	7.0000000	-50.0000000
36770.0000000	7.2500000	-50.0000000
36770.0000000	7.5000000	-50.0000000
36770.0000000	7.7500000	-50.0000000
36770.0000000	8.0000000	-50.0000000
36770.0000000	8.2500000	-50.0000000
36770.0000000	8.5000000	-50.0000000
36770.0000000	8.7500000	-50.0000000
36770.0000000	9.0000000	-50.0000000
36770.0000000	9.2500000	-50.0000000
36770.0000000	9.5000000	-50.0000000
36770.0000000	9.7500000	-50.0000000
36770.0000000	10.0000000	-50.0000000

36770.0000000	10.2500000	-50.0000000
36770.0000000	10.5000000	-50.0000000
36770.0000000	10.7500000	-50.0000000
36770.0000000	11.0000000	-50.0000000
36770.0000000	11.2500000	-50.0000000
36770.0000000	11.5000000	-50.0000000
36770.0000000	11.7500000	-50.0000000
36770.0000000	12.0000000	-50.0000000
36770.0000000	12.2500000	-50.0000000
36770.0000000	12.5000000	-50.0000000
36770.0000000	12.7500000	-50.0000000
36770.0000000	13.0000000	-50.0000000
36770.0000000	13.2500000	-50.0000000
36770.0000000	13.5000000	-50.0000000
36770.0000000	13.7500000	-50.0000000
36770.0000000	14.0000000	-50.0000000
36770.0000000	14.2500000	-50.0000000
36770.0000000	14.5000000	-50.0000000
36770.0000000	14.7500000	-50.0000000
36770.0000000	15.0000000	-50.0000000
36800.0000000	0.0000000	-50.0000000
36800.0000000	0.2500000	-50.0000000
36800.0000000	0.5000000	-50.0000000
36800.0000000	0.7500000	-50.0000000
36800.0000000	1.0000000	-50.0000000
36800.0000000	1.2500000	-50.0000000
36800.0000000	1.5000000	-50.0000000
36800.0000000	1.7500000	-50.0000000
36800.0000000	2.0000000	-50.0000000
36800.0000000	2.2500000	-50.0000000
36800.0000000	2.5000000	-50.0000000
36800.0000000	2.7500000	-50.0000000
36800.0000000	3.0000000	-50.0000000
36800.0000000	3.2500000	-50.0000000
36800.0000000	3.5000000	-50.0000000
36800.0000000	3.7500000	-50.0000000
36800.0000000	4.0000000	-50.0000000
36800.0000000	4.2500000	-50.0000000
36800.0000000	4.5000000	-50.0000000
36800.0000000	4.7500000	-50.0000000
36800.0000000	5.0000000	-50.0000000
36800.0000000	5.2500000	-50.0000000
36800.0000000	5.5000000	-50.0000000
36800.0000000	5.7500000	-50.0000000
36800.0000000	6.0000000	-50.0000000
36800.0000000	6.2500000	-50.0000000
36800.0000000	6.5000000	-50.0000000
36800.0000000	6.7500000	-50.0000000
36800.0000000	7.0000000	-50.0000000
36800.0000000	7.2500000	-50.0000000
36800.0000000	7.5000000	-50.0000000
36800.0000000	7.7500000	-50.0000000
36800.0000000	8.0000000	-50.0000000
36800.0000000	8.2500000	-50.0000000
36800.0000000	8.5000000	-50.0000000
36800.0000000	8.7500000	-50.0000000
36800.0000000	9.0000000	-50.0000000
36800.0000000	9.2500000	-50.0000000

36800.0000000	9.5000000	-50.0000000
36800.0000000	9.7500000	-50.0000000
36800.0000000	10.0000000	-50.0000000
36800.0000000	10.2500000	-50.0000000
36800.0000000	10.5000000	-50.0000000
36800.0000000	10.7500000	-50.0000000
36800.0000000	11.0000000	-50.0000000
36800.0000000	11.2500000	-50.0000000
36800.0000000	11.5000000	-50.0000000
36800.0000000	11.7500000	-50.0000000
36800.0000000	12.0000000	-50.0000000
36800.0000000	12.2500000	-50.0000000
36800.0000000	12.5000000	-50.0000000
36800.0000000	12.7500000	-50.0000000
36800.0000000	13.0000000	-50.0000000
36800.0000000	13.2500000	-50.0000000
36800.0000000	13.5000000	-50.0000000
36800.0000000	13.7500000	-50.0000000
36800.0000000	14.0000000	-50.0000000
36800.0000000	14.2500000	-50.0000000
36800.0000000	14.5000000	-50.0000000
36800.0000000	14.7500000	-50.0000000
36800.0000000	15.0000000	-50.0000000
36830.0000000	0.0000000	-50.0000000
36830.0000000	0.2500000	-50.0000000
36830.0000000	0.5000000	-50.0000000
36830.0000000	0.7500000	-50.0000000
36830.0000000	1.0000000	-50.0000000
36830.0000000	1.2500000	-50.0000000
36830.0000000	1.5000000	-50.0000000
36830.0000000	1.7500000	-50.0000000
36830.0000000	2.0000000	-50.0000000
36830.0000000	2.2500000	-50.0000000
36830.0000000	2.5000000	-50.0000000
36830.0000000	2.7500000	-50.0000000
36830.0000000	3.0000000	-50.0000000
36830.0000000	3.2500000	-50.0000000
36830.0000000	3.5000000	-50.0000000
36830.0000000	3.7500000	-50.0000000
36830.0000000	4.0000000	-50.0000000
36830.0000000	4.2500000	-50.0000000
36830.0000000	4.5000000	-50.0000000
36830.0000000	4.7500000	-50.0000000
36830.0000000	5.0000000	-50.0000000
36830.0000000	5.2500000	-50.0000000
36830.0000000	5.5000000	-50.0000000
36830.0000000	5.7500000	-50.0000000
36830.0000000	6.0000000	-50.0000000
36830.0000000	6.2500000	-50.0000000
36830.0000000	6.5000000	-50.0000000
36830.0000000	6.7500000	-50.0000000
36830.0000000	7.0000000	-50.0000000
36830.0000000	7.2500000	-50.0000000
36830.0000000	7.5000000	-50.0000000
36830.0000000	7.7500000	-50.0000000
36830.0000000	8.0000000	-50.0000000
36830.0000000	8.2500000	-50.0000000
36830.0000000	8.5000000	-50.0000000

36830.0000000	8.7500000	-50.0000000
36830.0000000	9.0000000	-50.0000000
36830.0000000	9.2500000	-50.0000000
36830.0000000	9.5000000	-50.0000000
36830.0000000	9.7500000	-50.0000000
36830.0000000	10.0000000	-50.0000000
36830.0000000	10.2500000	-50.0000000
36830.0000000	10.5000000	-50.0000000
36830.0000000	10.7500000	-50.0000000
36830.0000000	11.0000000	-50.0000000
36830.0000000	11.2500000	-50.0000000
36830.0000000	11.5000000	-50.0000000
36830.0000000	11.7500000	-50.0000000
36830.0000000	12.0000000	-50.0000000
36830.0000000	12.2500000	-50.0000000
36830.0000000	12.5000000	-50.0000000
36830.0000000	12.7500000	-50.0000000
36830.0000000	13.0000000	-50.0000000
36830.0000000	13.2500000	-50.0000000
36830.0000000	13.5000000	-50.0000000
36830.0000000	13.7500000	-50.0000000
36830.0000000	14.0000000	-50.0000000
36830.0000000	14.2500000	-50.0000000
36830.0000000	14.5000000	-50.0000000
36830.0000000	14.7500000	-50.0000000
36830.0000000	15.0000000	-50.0000000
36860.0000000	0.0000000	-50.0000000
36860.0000000	0.2500000	-50.0000000
36860.0000000	0.5000000	-50.0000000
36860.0000000	0.7500000	-50.0000000
36860.0000000	1.0000000	-50.0000000
36860.0000000	1.2500000	-50.0000000
36860.0000000	1.5000000	-50.0000000
36860.0000000	1.7500000	-50.0000000
36860.0000000	2.0000000	-50.0000000
36860.0000000	2.2500000	-50.0000000
36860.0000000	2.5000000	-50.0000000
36860.0000000	2.7500000	-50.0000000
36860.0000000	3.0000000	-50.0000000
36860.0000000	3.2500000	-50.0000000
36860.0000000	3.5000000	-50.0000000
36860.0000000	3.7500000	-50.0000000
36860.0000000	4.0000000	-50.0000000
36860.0000000	4.2500000	-50.0000000
36860.0000000	4.5000000	-50.0000000
36860.0000000	4.7500000	-50.0000000
36860.0000000	5.0000000	-50.0000000
36860.0000000	5.2500000	-50.0000000
36860.0000000	5.5000000	-50.0000000
36860.0000000	5.7500000	-50.0000000
36860.0000000	6.0000000	-50.0000000
36860.0000000	6.2500000	-50.0000000
36860.0000000	6.5000000	-50.0000000
36860.0000000	6.7500000	-50.0000000
36860.0000000	7.0000000	-50.0000000
36860.0000000	7.2500000	-50.0000000
36860.0000000	7.5000000	-50.0000000
36860.0000000	7.7500000	-50.0000000

36860.0000000	8.0000000	-50.0000000
36860.0000000	8.2500000	-50.0000000
36860.0000000	8.5000000	-50.0000000
36860.0000000	8.7500000	-50.0000000
36860.0000000	9.0000000	-50.0000000
36860.0000000	9.2500000	-50.0000000
36860.0000000	9.5000000	-50.0000000
36860.0000000	9.7500000	-50.0000000
36860.0000000	10.0000000	-50.0000000
36860.0000000	10.2500000	-50.0000000
36860.0000000	10.5000000	-50.0000000
36860.0000000	10.7500000	-50.0000000
36860.0000000	11.0000000	-50.0000000
36860.0000000	11.2500000	-50.0000000
36860.0000000	11.5000000	-50.0000000
36860.0000000	11.7500000	-50.0000000
36860.0000000	12.0000000	-50.0000000
36860.0000000	12.2500000	-50.0000000
36860.0000000	12.5000000	-50.0000000
36860.0000000	12.7500000	-50.0000000
36860.0000000	13.0000000	-50.0000000
36860.0000000	13.2500000	-50.0000000
36860.0000000	13.5000000	-50.0000000
36860.0000000	13.7500000	-50.0000000
36860.0000000	14.0000000	-50.0000000
36860.0000000	14.2500000	-50.0000000
36860.0000000	14.5000000	-50.0000000
36860.0000000	14.7500000	-50.0000000
36860.0000000	15.0000000	-50.0000000
36890.0000000	0.0000000	-50.0000000
36890.0000000	0.2500000	-50.0000000
36890.0000000	0.5000000	-50.0000000
36890.0000000	0.7500000	-50.0000000
36890.0000000	1.0000000	-50.0000000
36890.0000000	1.2500000	-50.0000000
36890.0000000	1.5000000	-50.0000000
36890.0000000	1.7500000	-50.0000000
36890.0000000	2.0000000	-50.0000000
36890.0000000	2.2500000	-50.0000000
36890.0000000	2.5000000	-50.0000000
36890.0000000	2.7500000	-50.0000000
36890.0000000	3.0000000	-50.0000000
36890.0000000	3.2500000	-50.0000000
36890.0000000	3.5000000	-50.0000000
36890.0000000	3.7500000	-50.0000000
36890.0000000	4.0000000	-50.0000000
36890.0000000	4.2500000	-50.0000000
36890.0000000	4.5000000	-50.0000000
36890.0000000	4.7500000	-50.0000000
36890.0000000	5.0000000	-50.0000000
36890.0000000	5.2500000	-50.0000000
36890.0000000	5.5000000	-50.0000000
36890.0000000	5.7500000	-50.0000000
36890.0000000	6.0000000	-50.0000000
36890.0000000	6.2500000	-50.0000000
36890.0000000	6.5000000	-50.0000000
36890.0000000	6.7500000	-50.0000000
36890.0000000	7.0000000	-50.0000000

36890.0000000	7.2500000	-50.0000000
36890.0000000	7.5000000	-50.0000000
36890.0000000	7.7500000	-50.0000000
36890.0000000	8.0000000	-50.0000000
36890.0000000	8.2500000	-50.0000000
36890.0000000	8.5000000	-50.0000000
36890.0000000	8.7500000	-50.0000000
36890.0000000	9.0000000	-50.0000000
36890.0000000	9.2500000	-50.0000000
36890.0000000	9.5000000	-50.0000000
36890.0000000	9.7500000	-50.0000000
36890.0000000	10.0000000	-50.0000000
36890.0000000	10.2500000	-50.0000000
36890.0000000	10.5000000	-50.0000000
36890.0000000	10.7500000	-50.0000000
36890.0000000	11.0000000	-50.0000000
36890.0000000	11.2500000	-50.0000000
36890.0000000	11.5000000	-50.0000000
36890.0000000	11.7500000	-50.0000000
36890.0000000	12.0000000	-50.0000000
36890.0000000	12.2500000	-50.0000000
36890.0000000	12.5000000	-50.0000000
36890.0000000	12.7500000	-50.0000000
36890.0000000	13.0000000	-50.0000000
36890.0000000	13.2500000	-50.0000000
36890.0000000	13.5000000	-50.0000000
36890.0000000	13.7500000	-50.0000000
36890.0000000	14.0000000	-50.0000000
36890.0000000	14.2500000	-50.0000000
36890.0000000	14.5000000	-50.0000000
36890.0000000	14.7500000	-50.0000000
36890.0000000	15.0000000	-50.0000000
36920.0000000	0.0000000	-50.0000000
36920.0000000	0.2500000	-50.0000000
36920.0000000	0.5000000	-50.0000000
36920.0000000	0.7500000	-50.0000000
36920.0000000	1.0000000	-50.0000000
36920.0000000	1.2500000	-50.0000000
36920.0000000	1.5000000	-50.0000000
36920.0000000	1.7500000	-50.0000000
36920.0000000	2.0000000	-50.0000000
36920.0000000	2.2500000	-50.0000000
36920.0000000	2.5000000	-50.0000000
36920.0000000	2.7500000	-50.0000000
36920.0000000	3.0000000	-50.0000000
36920.0000000	3.2500000	-50.0000000
36920.0000000	3.5000000	-50.0000000
36920.0000000	3.7500000	-50.0000000
36920.0000000	4.0000000	-50.0000000
36920.0000000	4.2500000	-50.0000000
36920.0000000	4.5000000	-50.0000000
36920.0000000	4.7500000	-50.0000000
36920.0000000	5.0000000	-50.0000000
36920.0000000	5.2500000	-50.0000000
36920.0000000	5.5000000	-50.0000000
36920.0000000	5.7500000	-50.0000000
36920.0000000	6.0000000	-50.0000000
36920.0000000	6.2500000	-50.0000000

36920.0000000	6.5000000	-50.0000000
36920.0000000	6.7500000	-50.0000000
36920.0000000	7.0000000	-50.0000000
36920.0000000	7.2500000	-50.0000000
36920.0000000	7.5000000	-50.0000000
36920.0000000	7.7500000	-50.0000000
36920.0000000	8.0000000	-50.0000000
36920.0000000	8.2500000	-50.0000000
36920.0000000	8.5000000	-50.0000000
36920.0000000	8.7500000	-50.0000000
36920.0000000	9.0000000	-50.0000000
36920.0000000	9.2500000	-50.0000000
36920.0000000	9.5000000	-50.0000000
36920.0000000	9.7500000	-50.0000000
36920.0000000	10.0000000	-50.0000000
36920.0000000	10.2500000	-50.0000000
36920.0000000	10.5000000	-50.0000000
36920.0000000	10.7500000	-50.0000000
36920.0000000	11.0000000	-50.0000000
36920.0000000	11.2500000	-50.0000000
36920.0000000	11.5000000	-50.0000000
36920.0000000	11.7500000	-50.0000000
36920.0000000	12.0000000	-50.0000000
36920.0000000	12.2500000	-50.0000000
36920.0000000	12.5000000	-50.0000000
36920.0000000	12.7500000	-50.0000000
36920.0000000	13.0000000	-50.0000000
36920.0000000	13.2500000	-50.0000000
36920.0000000	13.5000000	-50.0000000
36920.0000000	13.7500000	-50.0000000
36920.0000000	14.0000000	-50.0000000
36920.0000000	14.2500000	-50.0000000
36920.0000000	14.5000000	-50.0000000
36920.0000000	14.7500000	-50.0000000
36920.0000000	15.0000000	-50.0000000
36950.0000000	0.0000000	-50.0000000
36950.0000000	0.2500000	-50.0000000
36950.0000000	0.5000000	-50.0000000
36950.0000000	0.7500000	-50.0000000
36950.0000000	1.0000000	-50.0000000
36950.0000000	1.2500000	-50.0000000
36950.0000000	1.5000000	-50.0000000
36950.0000000	1.7500000	-50.0000000
36950.0000000	2.0000000	-50.0000000
36950.0000000	2.2500000	-50.0000000
36950.0000000	2.5000000	-50.0000000
36950.0000000	2.7500000	-50.0000000
36950.0000000	3.0000000	-50.0000000
36950.0000000	3.2500000	-50.0000000
36950.0000000	3.5000000	-50.0000000
36950.0000000	3.7500000	-50.0000000
36950.0000000	4.0000000	-50.0000000
36950.0000000	4.2500000	-50.0000000
36950.0000000	4.5000000	-50.0000000
36950.0000000	4.7500000	-50.0000000
36950.0000000	5.0000000	-50.0000000
36950.0000000	5.2500000	-50.0000000
36950.0000000	5.5000000	-50.0000000

36950.0000000	5.7500000	-50.0000000
36950.0000000	6.0000000	-50.0000000
36950.0000000	6.2500000	-50.0000000
36950.0000000	6.5000000	-50.0000000
36950.0000000	6.7500000	-50.0000000
36950.0000000	7.0000000	-50.0000000
36950.0000000	7.2500000	-50.0000000
36950.0000000	7.5000000	-50.0000000
36950.0000000	7.7500000	-50.0000000
36950.0000000	8.0000000	-50.0000000
36950.0000000	8.2500000	-50.0000000
36950.0000000	8.5000000	-50.0000000
36950.0000000	8.7500000	-50.0000000
36950.0000000	9.0000000	-50.0000000
36950.0000000	9.2500000	-50.0000000
36950.0000000	9.5000000	-50.0000000
36950.0000000	9.7500000	-50.0000000
36950.0000000	10.0000000	-50.0000000
36950.0000000	10.2500000	-50.0000000
36950.0000000	10.5000000	-50.0000000
36950.0000000	10.7500000	-50.0000000
36950.0000000	11.0000000	-50.0000000
36950.0000000	11.2500000	-50.0000000
36950.0000000	11.5000000	-50.0000000
36950.0000000	11.7500000	-50.0000000
36950.0000000	12.0000000	-50.0000000
36950.0000000	12.2500000	-50.0000000
36950.0000000	12.5000000	-50.0000000
36950.0000000	12.7500000	-50.0000000
36950.0000000	13.0000000	-50.0000000
36950.0000000	13.2500000	-50.0000000
36950.0000000	13.5000000	-50.0000000
36950.0000000	13.7500000	-50.0000000
36950.0000000	14.0000000	-50.0000000
36950.0000000	14.2500000	-50.0000000
36950.0000000	14.5000000	-50.0000000
36950.0000000	14.7500000	-50.0000000
36950.0000000	15.0000000	-50.0000000
36980.0000000	0.0000000	-50.0000000
36980.0000000	0.2500000	-50.0000000
36980.0000000	0.5000000	-50.0000000
36980.0000000	0.7500000	-50.0000000
36980.0000000	1.0000000	-50.0000000
36980.0000000	1.2500000	-50.0000000
36980.0000000	1.5000000	-50.0000000
36980.0000000	1.7500000	-50.0000000
36980.0000000	2.0000000	-50.0000000
36980.0000000	2.2500000	-50.0000000
36980.0000000	2.5000000	-50.0000000
36980.0000000	2.7500000	-50.0000000
36980.0000000	3.0000000	-50.0000000
36980.0000000	3.2500000	-50.0000000
36980.0000000	3.5000000	-50.0000000
36980.0000000	3.7500000	-50.0000000
36980.0000000	4.0000000	-50.0000000
36980.0000000	4.2500000	-50.0000000
36980.0000000	4.5000000	-50.0000000
36980.0000000	4.7500000	-50.0000000

36980.0000000	5.0000000	-50.0000000
36980.0000000	5.2500000	-50.0000000
36980.0000000	5.5000000	-50.0000000
36980.0000000	5.7500000	-50.0000000
36980.0000000	6.0000000	-50.0000000
36980.0000000	6.2500000	-50.0000000
36980.0000000	6.5000000	-50.0000000
36980.0000000	6.7500000	-50.0000000
36980.0000000	7.0000000	-50.0000000
36980.0000000	7.2500000	-50.0000000
36980.0000000	7.5000000	-50.0000000
36980.0000000	7.7500000	-50.0000000
36980.0000000	8.0000000	-50.0000000
36980.0000000	8.2500000	-50.0000000
36980.0000000	8.5000000	-50.0000000
36980.0000000	8.7500000	-50.0000000
36980.0000000	9.0000000	-50.0000000
36980.0000000	9.2500000	-50.0000000
36980.0000000	9.5000000	-50.0000000
36980.0000000	9.7500000	-50.0000000
36980.0000000	10.0000000	-50.0000000
36980.0000000	10.2500000	-50.0000000
36980.0000000	10.5000000	-50.0000000
36980.0000000	10.7500000	-50.0000000
36980.0000000	11.0000000	-50.0000000
36980.0000000	11.2500000	-50.0000000
36980.0000000	11.5000000	-50.0000000
36980.0000000	11.7500000	-50.0000000
36980.0000000	12.0000000	-50.0000000
36980.0000000	12.2500000	-50.0000000
36980.0000000	12.5000000	-50.0000000
36980.0000000	12.7500000	-50.0000000
36980.0000000	13.0000000	-50.0000000
36980.0000000	13.2500000	-50.0000000
36980.0000000	13.5000000	-50.0000000
36980.0000000	13.7500000	-50.0000000
36980.0000000	14.0000000	-50.0000000
36980.0000000	14.2500000	-50.0000000
36980.0000000	14.5000000	-50.0000000
36980.0000000	14.7500000	-50.0000000
36980.0000000	15.0000000	-50.0000000
37010.0000000	0.0000000	-50.0000000
37010.0000000	0.2500000	-50.0000000
37010.0000000	0.5000000	-50.0000000
37010.0000000	0.7500000	-50.0000000
37010.0000000	1.0000000	-50.0000000
37010.0000000	1.2500000	-50.0000000
37010.0000000	1.5000000	-50.0000000
37010.0000000	1.7500000	-50.0000000
37010.0000000	2.0000000	-50.0000000
37010.0000000	2.2500000	-50.0000000
37010.0000000	2.5000000	-50.0000000
37010.0000000	2.7500000	-50.0000000
37010.0000000	3.0000000	-50.0000000
37010.0000000	3.2500000	-50.0000000
37010.0000000	3.5000000	-50.0000000
37010.0000000	3.7500000	-50.0000000
37010.0000000	4.0000000	-50.0000000

37010.0000000	4.2500000	-50.0000000
37010.0000000	4.5000000	-50.0000000
37010.0000000	4.7500000	-50.0000000
37010.0000000	5.0000000	-50.0000000
37010.0000000	5.2500000	-50.0000000
37010.0000000	5.5000000	-50.0000000
37010.0000000	5.7500000	-50.0000000
37010.0000000	6.0000000	-50.0000000
37010.0000000	6.2500000	-50.0000000
37010.0000000	6.5000000	-50.0000000
37010.0000000	6.7500000	-50.0000000
37010.0000000	7.0000000	-50.0000000
37010.0000000	7.2500000	-50.0000000
37010.0000000	7.5000000	-50.0000000
37010.0000000	7.7500000	-50.0000000
37010.0000000	8.0000000	-50.0000000
37010.0000000	8.2500000	-50.0000000
37010.0000000	8.5000000	-50.0000000
37010.0000000	8.7500000	-50.0000000
37010.0000000	9.0000000	-50.0000000
37010.0000000	9.2500000	-50.0000000
37010.0000000	9.5000000	-50.0000000
37010.0000000	9.7500000	-50.0000000
37010.0000000	10.0000000	-50.0000000
37010.0000000	10.2500000	-50.0000000
37010.0000000	10.5000000	-50.0000000
37010.0000000	10.7500000	-50.0000000
37010.0000000	11.0000000	-50.0000000
37010.0000000	11.2500000	-50.0000000
37010.0000000	11.5000000	-50.0000000
37010.0000000	11.7500000	-50.0000000
37010.0000000	12.0000000	-50.0000000
37010.0000000	12.2500000	-50.0000000
37010.0000000	12.5000000	-50.0000000
37010.0000000	12.7500000	-50.0000000
37010.0000000	13.0000000	-50.0000000
37010.0000000	13.2500000	-50.0000000
37010.0000000	13.5000000	-50.0000000
37010.0000000	13.7500000	-50.0000000
37010.0000000	14.0000000	-50.0000000
37010.0000000	14.2500000	-50.0000000
37010.0000000	14.5000000	-50.0000000
37010.0000000	14.7500000	-50.0000000
37010.0000000	15.0000000	-50.0000000
37040.0000000	0.0000000	-50.0000000
37040.0000000	0.2500000	-50.0000000
37040.0000000	0.5000000	-50.0000000
37040.0000000	0.7500000	-50.0000000
37040.0000000	1.0000000	-50.0000000
37040.0000000	1.2500000	-50.0000000
37040.0000000	1.5000000	-50.0000000
37040.0000000	1.7500000	-50.0000000
37040.0000000	2.0000000	-50.0000000
37040.0000000	2.2500000	-50.0000000
37040.0000000	2.5000000	-50.0000000
37040.0000000	2.7500000	-50.0000000
37040.0000000	3.0000000	-50.0000000
37040.0000000	3.2500000	-50.0000000

37040.0000000	3.5000000	-50.0000000
37040.0000000	3.7500000	-50.0000000
37040.0000000	4.0000000	-50.0000000
37040.0000000	4.2500000	-50.0000000
37040.0000000	4.5000000	-50.0000000
37040.0000000	4.7500000	-50.0000000
37040.0000000	5.0000000	-50.0000000
37040.0000000	5.2500000	-50.0000000
37040.0000000	5.5000000	-50.0000000
37040.0000000	5.7500000	-50.0000000
37040.0000000	6.0000000	-50.0000000
37040.0000000	6.2500000	-50.0000000
37040.0000000	6.5000000	-50.0000000
37040.0000000	6.7500000	-50.0000000
37040.0000000	7.0000000	-50.0000000
37040.0000000	7.2500000	-50.0000000
37040.0000000	7.5000000	-50.0000000
37040.0000000	7.7500000	-50.0000000
37040.0000000	8.0000000	-50.0000000
37040.0000000	8.2500000	-50.0000000
37040.0000000	8.5000000	-50.0000000
37040.0000000	8.7500000	-50.0000000
37040.0000000	9.0000000	-50.0000000
37040.0000000	9.2500000	-50.0000000
37040.0000000	9.5000000	-50.0000000
37040.0000000	9.7500000	-50.0000000
37040.0000000	10.0000000	-50.0000000
37040.0000000	10.2500000	-50.0000000
37040.0000000	10.5000000	-50.0000000
37040.0000000	10.7500000	-50.0000000
37040.0000000	11.0000000	-50.0000000
37040.0000000	11.2500000	-50.0000000
37040.0000000	11.5000000	-50.0000000
37040.0000000	11.7500000	-50.0000000
37040.0000000	12.0000000	-50.0000000
37040.0000000	12.2500000	-50.0000000
37040.0000000	12.5000000	-50.0000000
37040.0000000	12.7500000	-50.0000000
37040.0000000	13.0000000	-50.0000000
37040.0000000	13.2500000	-50.0000000
37040.0000000	13.5000000	-50.0000000
37040.0000000	13.7500000	-50.0000000
37040.0000000	14.0000000	-50.0000000
37040.0000000	14.2500000	-50.0000000
37040.0000000	14.5000000	-50.0000000
37040.0000000	14.7500000	-50.0000000
37040.0000000	15.0000000	-50.0000000
37070.0000000	0.0000000	-50.0000000
37070.0000000	0.2500000	-50.0000000
37070.0000000	0.5000000	-50.0000000
37070.0000000	0.7500000	-50.0000000
37070.0000000	1.0000000	-50.0000000
37070.0000000	1.2500000	-50.0000000
37070.0000000	1.5000000	-50.0000000
37070.0000000	1.7500000	-50.0000000
37070.0000000	2.0000000	-50.0000000
37070.0000000	2.2500000	-50.0000000
37070.0000000	2.5000000	-50.0000000

37070.0000000	2.7500000	-50.0000000
37070.0000000	3.0000000	-50.0000000
37070.0000000	3.2500000	-50.0000000
37070.0000000	3.5000000	-50.0000000
37070.0000000	3.7500000	-50.0000000
37070.0000000	4.0000000	-50.0000000
37070.0000000	4.2500000	-50.0000000
37070.0000000	4.5000000	-50.0000000
37070.0000000	4.7500000	-50.0000000
37070.0000000	5.0000000	-50.0000000
37070.0000000	5.2500000	-50.0000000
37070.0000000	5.5000000	-50.0000000
37070.0000000	5.7500000	-50.0000000
37070.0000000	6.0000000	-50.0000000
37070.0000000	6.2500000	-50.0000000
37070.0000000	6.5000000	-50.0000000
37070.0000000	6.7500000	-50.0000000
37070.0000000	7.0000000	-50.0000000
37070.0000000	7.2500000	-50.0000000
37070.0000000	7.5000000	-50.0000000
37070.0000000	7.7500000	-50.0000000
37070.0000000	8.0000000	-50.0000000
37070.0000000	8.2500000	-50.0000000
37070.0000000	8.5000000	-50.0000000
37070.0000000	8.7500000	-50.0000000
37070.0000000	9.0000000	-50.0000000
37070.0000000	9.2500000	-50.0000000
37070.0000000	9.5000000	-50.0000000
37070.0000000	9.7500000	-50.0000000
37070.0000000	10.0000000	-50.0000000
37070.0000000	10.2500000	-50.0000000
37070.0000000	10.5000000	-50.0000000
37070.0000000	10.7500000	-50.0000000
37070.0000000	11.0000000	-50.0000000
37070.0000000	11.2500000	-50.0000000
37070.0000000	11.5000000	-50.0000000
37070.0000000	11.7500000	-50.0000000
37070.0000000	12.0000000	-50.0000000
37070.0000000	12.2500000	-50.0000000
37070.0000000	12.5000000	-50.0000000
37070.0000000	12.7500000	-50.0000000
37070.0000000	13.0000000	-50.0000000
37070.0000000	13.2500000	-50.0000000
37070.0000000	13.5000000	-50.0000000
37070.0000000	13.7500000	-50.0000000
37070.0000000	14.0000000	-50.0000000
37070.0000000	14.2500000	-50.0000000
37070.0000000	14.5000000	-50.0000000
37070.0000000	14.7500000	-50.0000000
37070.0000000	15.0000000	-50.0000000
37100.0000000	0.0000000	-50.0000000
37100.0000000	0.2500000	-50.0000000
37100.0000000	0.5000000	-50.0000000
37100.0000000	0.7500000	-50.0000000
37100.0000000	1.0000000	-50.0000000
37100.0000000	1.2500000	-50.0000000
37100.0000000	1.5000000	-50.0000000
37100.0000000	1.7500000	-50.0000000

37100.0000000	2.0000000	-50.0000000
37100.0000000	2.2500000	-50.0000000
37100.0000000	2.5000000	-50.0000000
37100.0000000	2.7500000	-50.0000000
37100.0000000	3.0000000	-50.0000000
37100.0000000	3.2500000	-50.0000000
37100.0000000	3.5000000	-50.0000000
37100.0000000	3.7500000	-50.0000000
37100.0000000	4.0000000	-50.0000000
37100.0000000	4.2500000	-50.0000000
37100.0000000	4.5000000	-50.0000000
37100.0000000	4.7500000	-50.0000000
37100.0000000	5.0000000	-50.0000000
37100.0000000	5.2500000	-50.0000000
37100.0000000	5.5000000	-50.0000000
37100.0000000	5.7500000	-50.0000000
37100.0000000	6.0000000	-50.0000000
37100.0000000	6.2500000	-50.0000000
37100.0000000	6.5000000	-50.0000000
37100.0000000	6.7500000	-50.0000000
37100.0000000	7.0000000	-50.0000000
37100.0000000	7.2500000	-50.0000000
37100.0000000	7.5000000	-50.0000000
37100.0000000	7.7500000	-50.0000000
37100.0000000	8.0000000	-50.0000000
37100.0000000	8.2500000	-50.0000000
37100.0000000	8.5000000	-50.0000000
37100.0000000	8.7500000	-50.0000000
37100.0000000	9.0000000	-50.0000000
37100.0000000	9.2500000	-50.0000000
37100.0000000	9.5000000	-50.0000000
37100.0000000	9.7500000	-50.0000000
37100.0000000	10.0000000	-50.0000000
37100.0000000	10.2500000	-50.0000000
37100.0000000	10.5000000	-50.0000000
37100.0000000	10.7500000	-50.0000000
37100.0000000	11.0000000	-50.0000000
37100.0000000	11.2500000	-50.0000000
37100.0000000	11.5000000	-50.0000000
37100.0000000	11.7500000	-50.0000000
37100.0000000	12.0000000	-50.0000000
37100.0000000	12.2500000	-50.0000000
37100.0000000	12.5000000	-50.0000000
37100.0000000	12.7500000	-50.0000000
37100.0000000	13.0000000	-50.0000000
37100.0000000	13.2500000	-50.0000000
37100.0000000	13.5000000	-50.0000000
37100.0000000	13.7500000	-50.0000000
37100.0000000	14.0000000	-50.0000000
37100.0000000	14.2500000	-50.0000000
37100.0000000	14.5000000	-50.0000000
37100.0000000	14.7500000	-50.0000000
37100.0000000	15.0000000	-50.0000000
37130.0000000	0.0000000	-50.0000000
37130.0000000	0.2500000	-50.0000000
37130.0000000	0.5000000	-50.0000000
37130.0000000	0.7500000	-50.0000000
37130.0000000	1.0000000	-50.0000000

37130.0000000	1.2500000	-50.0000000
37130.0000000	1.5000000	-50.0000000
37130.0000000	1.7500000	-50.0000000
37130.0000000	2.0000000	-50.0000000
37130.0000000	2.2500000	-50.0000000
37130.0000000	2.5000000	-50.0000000
37130.0000000	2.7500000	-50.0000000
37130.0000000	3.0000000	-50.0000000
37130.0000000	3.2500000	-50.0000000
37130.0000000	3.5000000	-50.0000000
37130.0000000	3.7500000	-50.0000000
37130.0000000	4.0000000	-50.0000000
37130.0000000	4.2500000	-50.0000000
37130.0000000	4.5000000	-50.0000000
37130.0000000	4.7500000	-50.0000000
37130.0000000	5.0000000	-50.0000000
37130.0000000	5.2500000	-50.0000000
37130.0000000	5.5000000	-50.0000000
37130.0000000	5.7500000	-50.0000000
37130.0000000	6.0000000	-50.0000000
37130.0000000	6.2500000	-50.0000000
37130.0000000	6.5000000	-50.0000000
37130.0000000	6.7500000	-50.0000000
37130.0000000	7.0000000	-50.0000000
37130.0000000	7.2500000	-50.0000000
37130.0000000	7.5000000	-50.0000000
37130.0000000	7.7500000	-50.0000000
37130.0000000	8.0000000	-50.0000000
37130.0000000	8.2500000	-50.0000000
37130.0000000	8.5000000	-50.0000000
37130.0000000	8.7500000	-50.0000000
37130.0000000	9.0000000	-50.0000000
37130.0000000	9.2500000	-50.0000000
37130.0000000	9.5000000	-50.0000000
37130.0000000	9.7500000	-50.0000000
37130.0000000	10.0000000	-50.0000000
37130.0000000	10.2500000	-50.0000000
37130.0000000	10.5000000	-50.0000000
37130.0000000	10.7500000	-50.0000000
37130.0000000	11.0000000	-50.0000000
37130.0000000	11.2500000	-50.0000000
37130.0000000	11.5000000	-50.0000000
37130.0000000	11.7500000	-50.0000000
37130.0000000	12.0000000	-50.0000000
37130.0000000	12.2500000	-50.0000000
37130.0000000	12.5000000	-50.0000000
37130.0000000	12.7500000	-50.0000000
37130.0000000	13.0000000	-50.0000000
37130.0000000	13.2500000	-50.0000000
37130.0000000	13.5000000	-50.0000000
37130.0000000	13.7500000	-50.0000000
37130.0000000	14.0000000	-50.0000000
37130.0000000	14.2500000	-50.0000000
37130.0000000	14.5000000	-50.0000000
37130.0000000	14.7500000	-50.0000000
37130.0000000	15.0000000	-50.0000000
37160.0000000	0.0000000	-50.0000000
37160.0000000	0.2500000	-50.0000000

37160.0000000	0.5000000	-50.0000000
37160.0000000	0.7500000	-50.0000000
37160.0000000	1.0000000	-50.0000000
37160.0000000	1.2500000	-50.0000000
37160.0000000	1.5000000	-50.0000000
37160.0000000	1.7500000	-50.0000000
37160.0000000	2.0000000	-50.0000000
37160.0000000	2.2500000	-50.0000000
37160.0000000	2.5000000	-50.0000000
37160.0000000	2.7500000	-50.0000000
37160.0000000	3.0000000	-50.0000000
37160.0000000	3.2500000	-50.0000000
37160.0000000	3.5000000	-50.0000000
37160.0000000	3.7500000	-50.0000000
37160.0000000	4.0000000	-50.0000000
37160.0000000	4.2500000	-50.0000000
37160.0000000	4.5000000	-50.0000000
37160.0000000	4.7500000	-50.0000000
37160.0000000	5.0000000	-50.0000000
37160.0000000	5.2500000	-50.0000000
37160.0000000	5.5000000	-50.0000000
37160.0000000	5.7500000	-50.0000000
37160.0000000	6.0000000	-50.0000000
37160.0000000	6.2500000	-50.0000000
37160.0000000	6.5000000	-50.0000000
37160.0000000	6.7500000	-50.0000000
37160.0000000	7.0000000	-50.0000000
37160.0000000	7.2500000	-50.0000000
37160.0000000	7.5000000	-50.0000000
37160.0000000	7.7500000	-50.0000000
37160.0000000	8.0000000	-50.0000000
37160.0000000	8.2500000	-50.0000000
37160.0000000	8.5000000	-50.0000000
37160.0000000	8.7500000	-50.0000000
37160.0000000	9.0000000	-50.0000000
37160.0000000	9.2500000	-50.0000000
37160.0000000	9.5000000	-50.0000000
37160.0000000	9.7500000	-50.0000000
37160.0000000	10.0000000	-50.0000000
37160.0000000	10.2500000	-50.0000000
37160.0000000	10.5000000	-50.0000000
37160.0000000	10.7500000	-50.0000000
37160.0000000	11.0000000	-50.0000000
37160.0000000	11.2500000	-50.0000000
37160.0000000	11.5000000	-50.0000000
37160.0000000	11.7500000	-50.0000000
37160.0000000	12.0000000	-50.0000000
37160.0000000	12.2500000	-50.0000000
37160.0000000	12.5000000	-50.0000000
37160.0000000	12.7500000	-50.0000000
37160.0000000	13.0000000	-50.0000000
37160.0000000	13.2500000	-50.0000000
37160.0000000	13.5000000	-50.0000000
37160.0000000	13.7500000	-50.0000000
37160.0000000	14.0000000	-50.0000000
37160.0000000	14.2500000	-50.0000000
37160.0000000	14.5000000	-50.0000000
37160.0000000	14.7500000	-50.0000000

37160.0000000	15.0000000	-50.0000000
37190.0000000	0.0000000	-50.0000000
37190.0000000	0.2500000	-50.0000000
37190.0000000	0.5000000	-50.0000000
37190.0000000	0.7500000	-50.0000000
37190.0000000	1.0000000	-50.0000000
37190.0000000	1.2500000	-50.0000000
37190.0000000	1.5000000	-50.0000000
37190.0000000	1.7500000	-50.0000000
37190.0000000	2.0000000	-50.0000000
37190.0000000	2.2500000	-50.0000000
37190.0000000	2.5000000	-50.0000000
37190.0000000	2.7500000	-50.0000000
37190.0000000	3.0000000	-50.0000000
37190.0000000	3.2500000	-50.0000000
37190.0000000	3.5000000	-50.0000000
37190.0000000	3.7500000	-50.0000000
37190.0000000	4.0000000	-50.0000000
37190.0000000	4.2500000	-50.0000000
37190.0000000	4.5000000	-50.0000000
37190.0000000	4.7500000	-50.0000000
37190.0000000	5.0000000	-50.0000000
37190.0000000	5.2500000	-50.0000000
37190.0000000	5.5000000	-50.0000000
37190.0000000	5.7500000	-50.0000000
37190.0000000	6.0000000	-50.0000000
37190.0000000	6.2500000	-50.0000000
37190.0000000	6.5000000	-50.0000000
37190.0000000	6.7500000	-50.0000000
37190.0000000	7.0000000	-50.0000000
37190.0000000	7.2500000	-50.0000000
37190.0000000	7.5000000	-50.0000000
37190.0000000	7.7500000	-50.0000000
37190.0000000	8.0000000	-50.0000000
37190.0000000	8.2500000	-50.0000000
37190.0000000	8.5000000	-50.0000000
37190.0000000	8.7500000	-50.0000000
37190.0000000	9.0000000	-50.0000000
37190.0000000	9.2500000	-50.0000000
37190.0000000	9.5000000	-50.0000000
37190.0000000	9.7500000	-50.0000000
37190.0000000	10.0000000	-50.0000000
37190.0000000	10.2500000	-50.0000000
37190.0000000	10.5000000	-50.0000000
37190.0000000	10.7500000	-50.0000000
37190.0000000	11.0000000	-50.0000000
37190.0000000	11.2500000	-50.0000000
37190.0000000	11.5000000	-50.0000000
37190.0000000	11.7500000	-50.0000000
37190.0000000	12.0000000	-50.0000000
37190.0000000	12.2500000	-50.0000000
37190.0000000	12.5000000	-50.0000000
37190.0000000	12.7500000	-50.0000000
37190.0000000	13.0000000	-50.0000000
37190.0000000	13.2500000	-50.0000000
37190.0000000	13.5000000	-50.0000000
37190.0000000	13.7500000	-50.0000000
37190.0000000	14.0000000	-50.0000000

37190.0000000	14.2500000	-50.0000000
37190.0000000	14.5000000	-50.0000000
37190.0000000	14.7500000	-50.0000000
37190.0000000	15.0000000	-50.0000000
37220.0000000	0.0000000	-50.0000000
37220.0000000	0.2500000	-50.0000000
37220.0000000	0.5000000	-50.0000000
37220.0000000	0.7500000	-50.0000000
37220.0000000	1.0000000	-50.0000000
37220.0000000	1.2500000	-50.0000000
37220.0000000	1.5000000	-50.0000000
37220.0000000	1.7500000	-50.0000000
37220.0000000	2.0000000	-50.0000000
37220.0000000	2.2500000	-50.0000000
37220.0000000	2.5000000	-50.0000000
37220.0000000	2.7500000	-50.0000000
37220.0000000	3.0000000	-50.0000000
37220.0000000	3.2500000	-50.0000000
37220.0000000	3.5000000	-50.0000000
37220.0000000	3.7500000	-50.0000000
37220.0000000	4.0000000	-50.0000000
37220.0000000	4.2500000	-50.0000000
37220.0000000	4.5000000	-50.0000000
37220.0000000	4.7500000	-50.0000000
37220.0000000	5.0000000	-50.0000000
37220.0000000	5.2500000	-50.0000000
37220.0000000	5.5000000	-50.0000000
37220.0000000	5.7500000	-50.0000000
37220.0000000	6.0000000	-50.0000000
37220.0000000	6.2500000	-50.0000000
37220.0000000	6.5000000	-50.0000000
37220.0000000	6.7500000	-50.0000000
37220.0000000	7.0000000	-50.0000000
37220.0000000	7.2500000	-50.0000000
37220.0000000	7.5000000	-50.0000000
37220.0000000	7.7500000	-50.0000000
37220.0000000	8.0000000	-50.0000000
37220.0000000	8.2500000	-50.0000000
37220.0000000	8.5000000	-50.0000000
37220.0000000	8.7500000	-50.0000000
37220.0000000	9.0000000	-50.0000000
37220.0000000	9.2500000	-50.0000000
37220.0000000	9.5000000	-50.0000000
37220.0000000	9.7500000	-50.0000000
37220.0000000	10.0000000	-50.0000000
37220.0000000	10.2500000	-50.0000000
37220.0000000	10.5000000	-50.0000000
37220.0000000	10.7500000	-50.0000000
37220.0000000	11.0000000	-50.0000000
37220.0000000	11.2500000	-50.0000000
37220.0000000	11.5000000	-50.0000000
37220.0000000	11.7500000	-50.0000000
37220.0000000	12.0000000	-50.0000000
37220.0000000	12.2500000	-50.0000000
37220.0000000	12.5000000	-50.0000000
37220.0000000	12.7500000	-50.0000000
37220.0000000	13.0000000	-50.0000000
37220.0000000	13.2500000	-50.0000000

37220.0000000	13.5000000	-50.0000000
37220.0000000	13.7500000	-50.0000000
37220.0000000	14.0000000	-50.0000000
37220.0000000	14.2500000	-50.0000000
37220.0000000	14.5000000	-50.0000000
37220.0000000	14.7500000	-50.0000000
37220.0000000	15.0000000	-50.0000000
37250.0000000	0.0000000	-50.0000000
37250.0000000	0.2500000	-50.0000000
37250.0000000	0.5000000	-50.0000000
37250.0000000	0.7500000	-50.0000000
37250.0000000	1.0000000	-50.0000000
37250.0000000	1.2500000	-50.0000000
37250.0000000	1.5000000	-50.0000000
37250.0000000	1.7500000	-50.0000000
37250.0000000	2.0000000	-50.0000000
37250.0000000	2.2500000	-50.0000000
37250.0000000	2.5000000	-50.0000000
37250.0000000	2.7500000	-50.0000000
37250.0000000	3.0000000	-50.0000000
37250.0000000	3.2500000	-50.0000000
37250.0000000	3.5000000	-50.0000000
37250.0000000	3.7500000	-50.0000000
37250.0000000	4.0000000	-50.0000000
37250.0000000	4.2500000	-50.0000000
37250.0000000	4.5000000	-50.0000000
37250.0000000	4.7500000	-50.0000000
37250.0000000	5.0000000	-50.0000000
37250.0000000	5.2500000	-50.0000000
37250.0000000	5.5000000	-50.0000000
37250.0000000	5.7500000	-50.0000000
37250.0000000	6.0000000	-50.0000000
37250.0000000	6.2500000	-50.0000000
37250.0000000	6.5000000	-50.0000000
37250.0000000	6.7500000	-50.0000000
37250.0000000	7.0000000	-50.0000000
37250.0000000	7.2500000	-50.0000000
37250.0000000	7.5000000	-50.0000000
37250.0000000	7.7500000	-50.0000000
37250.0000000	8.0000000	-50.0000000
37250.0000000	8.2500000	-50.0000000
37250.0000000	8.5000000	-50.0000000
37250.0000000	8.7500000	-50.0000000
37250.0000000	9.0000000	-50.0000000
37250.0000000	9.2500000	-50.0000000
37250.0000000	9.5000000	-50.0000000
37250.0000000	9.7500000	-50.0000000
37250.0000000	10.0000000	-50.0000000
37250.0000000	10.2500000	-50.0000000
37250.0000000	10.5000000	-50.0000000
37250.0000000	10.7500000	-50.0000000
37250.0000000	11.0000000	-50.0000000
37250.0000000	11.2500000	-50.0000000
37250.0000000	11.5000000	-50.0000000
37250.0000000	11.7500000	-50.0000000
37250.0000000	12.0000000	-50.0000000
37250.0000000	12.2500000	-50.0000000
37250.0000000	12.5000000	-50.0000000

37250.0000000	12.7500000	-50.0000000
37250.0000000	13.0000000	-50.0000000
37250.0000000	13.2500000	-50.0000000
37250.0000000	13.5000000	-50.0000000
37250.0000000	13.7500000	-50.0000000
37250.0000000	14.0000000	-50.0000000
37250.0000000	14.2500000	-50.0000000
37250.0000000	14.5000000	-50.0000000
37250.0000000	14.7500000	-50.0000000
37250.0000000	15.0000000	-50.0000000
37280.0000000	0.0000000	-50.0000000
37280.0000000	0.2500000	-50.0000000
37280.0000000	0.5000000	-50.0000000
37280.0000000	0.7500000	-50.0000000
37280.0000000	1.0000000	-50.0000000
37280.0000000	1.2500000	-50.0000000
37280.0000000	1.5000000	-50.0000000
37280.0000000	1.7500000	-50.0000000
37280.0000000	2.0000000	-50.0000000
37280.0000000	2.2500000	-50.0000000
37280.0000000	2.5000000	-50.0000000
37280.0000000	2.7500000	-50.0000000
37280.0000000	3.0000000	-50.0000000
37280.0000000	3.2500000	-50.0000000
37280.0000000	3.5000000	-50.0000000
37280.0000000	3.7500000	-50.0000000
37280.0000000	4.0000000	-50.0000000
37280.0000000	4.2500000	-50.0000000
37280.0000000	4.5000000	-50.0000000
37280.0000000	4.7500000	-50.0000000
37280.0000000	5.0000000	-50.0000000
37280.0000000	5.2500000	-50.0000000
37280.0000000	5.5000000	-50.0000000
37280.0000000	5.7500000	-50.0000000
37280.0000000	6.0000000	-50.0000000
37280.0000000	6.2500000	-50.0000000
37280.0000000	6.5000000	-50.0000000
37280.0000000	6.7500000	-50.0000000
37280.0000000	7.0000000	-50.0000000
37280.0000000	7.2500000	-50.0000000
37280.0000000	7.5000000	-50.0000000
37280.0000000	7.7500000	-50.0000000
37280.0000000	8.0000000	-50.0000000
37280.0000000	8.2500000	-50.0000000
37280.0000000	8.5000000	-50.0000000
37280.0000000	8.7500000	-50.0000000
37280.0000000	9.0000000	-50.0000000
37280.0000000	9.2500000	-50.0000000
37280.0000000	9.5000000	-50.0000000
37280.0000000	9.7500000	-50.0000000
37280.0000000	10.0000000	-50.0000000
37280.0000000	10.2500000	-50.0000000
37280.0000000	10.5000000	-50.0000000
37280.0000000	10.7500000	-50.0000000
37280.0000000	11.0000000	-50.0000000
37280.0000000	11.2500000	-50.0000000
37280.0000000	11.5000000	-50.0000000
37280.0000000	11.7500000	-50.0000000

37280.0000000	12.0000000	-50.0000000
37280.0000000	12.2500000	-50.0000000
37280.0000000	12.5000000	-50.0000000
37280.0000000	12.7500000	-50.0000000
37280.0000000	13.0000000	-50.0000000
37280.0000000	13.2500000	-50.0000000
37280.0000000	13.5000000	-50.0000000
37280.0000000	13.7500000	-50.0000000
37280.0000000	14.0000000	-50.0000000
37280.0000000	14.2500000	-50.0000000
37280.0000000	14.5000000	-50.0000000
37280.0000000	14.7500000	-50.0000000
37280.0000000	15.0000000	-50.0000000
37310.0000000	0.0000000	-50.0000000
37310.0000000	0.2500000	-50.0000000
37310.0000000	0.5000000	-50.0000000
37310.0000000	0.7500000	-50.0000000
37310.0000000	1.0000000	-50.0000000
37310.0000000	1.2500000	-50.0000000
37310.0000000	1.5000000	-50.0000000
37310.0000000	1.7500000	-50.0000000
37310.0000000	2.0000000	-50.0000000
37310.0000000	2.2500000	-50.0000000
37310.0000000	2.5000000	-50.0000000
37310.0000000	2.7500000	-50.0000000
37310.0000000	3.0000000	-50.0000000
37310.0000000	3.2500000	-50.0000000
37310.0000000	3.5000000	-50.0000000
37310.0000000	3.7500000	-50.0000000
37310.0000000	4.0000000	-50.0000000
37310.0000000	4.2500000	-50.0000000
37310.0000000	4.5000000	-50.0000000
37310.0000000	4.7500000	-50.0000000
37310.0000000	5.0000000	-50.0000000
37310.0000000	5.2500000	-50.0000000
37310.0000000	5.5000000	-50.0000000
37310.0000000	5.7500000	-50.0000000
37310.0000000	6.0000000	-50.0000000
37310.0000000	6.2500000	-50.0000000
37310.0000000	6.5000000	-50.0000000
37310.0000000	6.7500000	-50.0000000
37310.0000000	7.0000000	-50.0000000
37310.0000000	7.2500000	-50.0000000
37310.0000000	7.5000000	-50.0000000
37310.0000000	7.7500000	-50.0000000
37310.0000000	8.0000000	-50.0000000
37310.0000000	8.2500000	-50.0000000
37310.0000000	8.5000000	-50.0000000
37310.0000000	8.7500000	-50.0000000
37310.0000000	9.0000000	-50.0000000
37310.0000000	9.2500000	-50.0000000
37310.0000000	9.5000000	-50.0000000
37310.0000000	9.7500000	-50.0000000
37310.0000000	10.0000000	-50.0000000
37310.0000000	10.2500000	-50.0000000
37310.0000000	10.5000000	-50.0000000
37310.0000000	10.7500000	-50.0000000
37310.0000000	11.0000000	-50.0000000

37310.0000000	11.2500000	-50.0000000
37310.0000000	11.5000000	-50.0000000
37310.0000000	11.7500000	-50.0000000
37310.0000000	12.0000000	-50.0000000
37310.0000000	12.2500000	-50.0000000
37310.0000000	12.5000000	-50.0000000
37310.0000000	12.7500000	-50.0000000
37310.0000000	13.0000000	-50.0000000
37310.0000000	13.2500000	-50.0000000
37310.0000000	13.5000000	-50.0000000
37310.0000000	13.7500000	-50.0000000
37310.0000000	14.0000000	-50.0000000
37310.0000000	14.2500000	-50.0000000
37310.0000000	14.5000000	-50.0000000
37310.0000000	14.7500000	-50.0000000
37310.0000000	15.0000000	-50.0000000
37340.0000000	0.0000000	-50.0000000
37340.0000000	0.2500000	-50.0000000
37340.0000000	0.5000000	-50.0000000
37340.0000000	0.7500000	-50.0000000
37340.0000000	1.0000000	-50.0000000
37340.0000000	1.2500000	-50.0000000
37340.0000000	1.5000000	-50.0000000
37340.0000000	1.7500000	-50.0000000
37340.0000000	2.0000000	-50.0000000
37340.0000000	2.2500000	-50.0000000
37340.0000000	2.5000000	-50.0000000
37340.0000000	2.7500000	-50.0000000
37340.0000000	3.0000000	-50.0000000
37340.0000000	3.2500000	-50.0000000
37340.0000000	3.5000000	-50.0000000
37340.0000000	3.7500000	-50.0000000
37340.0000000	4.0000000	-50.0000000
37340.0000000	4.2500000	-50.0000000
37340.0000000	4.5000000	-50.0000000
37340.0000000	4.7500000	-50.0000000
37340.0000000	5.0000000	-50.0000000
37340.0000000	5.2500000	-50.0000000
37340.0000000	5.5000000	-50.0000000
37340.0000000	5.7500000	-50.0000000
37340.0000000	6.0000000	-50.0000000
37340.0000000	6.2500000	-50.0000000
37340.0000000	6.5000000	-50.0000000
37340.0000000	6.7500000	-50.0000000
37340.0000000	7.0000000	-50.0000000
37340.0000000	7.2500000	-50.0000000
37340.0000000	7.5000000	-50.0000000
37340.0000000	7.7500000	-50.0000000
37340.0000000	8.0000000	-50.0000000
37340.0000000	8.2500000	-50.0000000
37340.0000000	8.5000000	-50.0000000
37340.0000000	8.7500000	-50.0000000
37340.0000000	9.0000000	-50.0000000
37340.0000000	9.2500000	-50.0000000
37340.0000000	9.5000000	-50.0000000
37340.0000000	9.7500000	-50.0000000
37340.0000000	10.0000000	-50.0000000
37340.0000000	10.2500000	-50.0000000

37340.0000000	10.5000000	-50.0000000
37340.0000000	10.7500000	-50.0000000
37340.0000000	11.0000000	-50.0000000
37340.0000000	11.2500000	-50.0000000
37340.0000000	11.5000000	-50.0000000
37340.0000000	11.7500000	-50.0000000
37340.0000000	12.0000000	-50.0000000
37340.0000000	12.2500000	-50.0000000
37340.0000000	12.5000000	-50.0000000
37340.0000000	12.7500000	-50.0000000
37340.0000000	13.0000000	-50.0000000
37340.0000000	13.2500000	-50.0000000
37340.0000000	13.5000000	-50.0000000
37340.0000000	13.7500000	-50.0000000
37340.0000000	14.0000000	-50.0000000
37340.0000000	14.2500000	-50.0000000
37340.0000000	14.5000000	-50.0000000
37340.0000000	14.7500000	-50.0000000
37340.0000000	15.0000000	-50.0000000
37370.0000000	0.0000000	-50.0000000
37370.0000000	0.2500000	-50.0000000
37370.0000000	0.5000000	-50.0000000
37370.0000000	0.7500000	-50.0000000
37370.0000000	1.0000000	-50.0000000
37370.0000000	1.2500000	-50.0000000
37370.0000000	1.5000000	-50.0000000
37370.0000000	1.7500000	-50.0000000
37370.0000000	2.0000000	-50.0000000
37370.0000000	2.2500000	-50.0000000
37370.0000000	2.5000000	-50.0000000
37370.0000000	2.7500000	-50.0000000
37370.0000000	3.0000000	-50.0000000
37370.0000000	3.2500000	-50.0000000
37370.0000000	3.5000000	-50.0000000
37370.0000000	3.7500000	-50.0000000
37370.0000000	4.0000000	-50.0000000
37370.0000000	4.2500000	-50.0000000
37370.0000000	4.5000000	-50.0000000
37370.0000000	4.7500000	-50.0000000
37370.0000000	5.0000000	-50.0000000
37370.0000000	5.2500000	-50.0000000
37370.0000000	5.5000000	-50.0000000
37370.0000000	5.7500000	-50.0000000
37370.0000000	6.0000000	-50.0000000
37370.0000000	6.2500000	-50.0000000
37370.0000000	6.5000000	-50.0000000
37370.0000000	6.7500000	-50.0000000
37370.0000000	7.0000000	-50.0000000
37370.0000000	7.2500000	-50.0000000
37370.0000000	7.5000000	-50.0000000
37370.0000000	7.7500000	-50.0000000
37370.0000000	8.0000000	-50.0000000
37370.0000000	8.2500000	-50.0000000
37370.0000000	8.5000000	-50.0000000
37370.0000000	8.7500000	-50.0000000
37370.0000000	9.0000000	-50.0000000
37370.0000000	9.2500000	-50.0000000
37370.0000000	9.5000000	-50.0000000

37370.0000000	9.7500000	-50.0000000
37370.0000000	10.0000000	-50.0000000
37370.0000000	10.2500000	-50.0000000
37370.0000000	10.5000000	-50.0000000
37370.0000000	10.7500000	-50.0000000
37370.0000000	11.0000000	-50.0000000
37370.0000000	11.2500000	-50.0000000
37370.0000000	11.5000000	-50.0000000
37370.0000000	11.7500000	-50.0000000
37370.0000000	12.0000000	-50.0000000
37370.0000000	12.2500000	-50.0000000
37370.0000000	12.5000000	-50.0000000
37370.0000000	12.7500000	-50.0000000
37370.0000000	13.0000000	-50.0000000
37370.0000000	13.2500000	-50.0000000
37370.0000000	13.5000000	-50.0000000
37370.0000000	13.7500000	-50.0000000
37370.0000000	14.0000000	-50.0000000
37370.0000000	14.2500000	-50.0000000
37370.0000000	14.5000000	-50.0000000
37370.0000000	14.7500000	-50.0000000
37370.0000000	15.0000000	-50.0000000
37400.0000000	0.0000000	-50.0000000
37400.0000000	0.2500000	-50.0000000
37400.0000000	0.5000000	-50.0000000
37400.0000000	0.7500000	-50.0000000
37400.0000000	1.0000000	-50.0000000
37400.0000000	1.2500000	-50.0000000
37400.0000000	1.5000000	-50.0000000
37400.0000000	1.7500000	-50.0000000
37400.0000000	2.0000000	-50.0000000
37400.0000000	2.2500000	-50.0000000
37400.0000000	2.5000000	-50.0000000
37400.0000000	2.7500000	-50.0000000
37400.0000000	3.0000000	-50.0000000
37400.0000000	3.2500000	-50.0000000
37400.0000000	3.5000000	-50.0000000
37400.0000000	3.7500000	-50.0000000
37400.0000000	4.0000000	-50.0000000
37400.0000000	4.2500000	-50.0000000
37400.0000000	4.5000000	-50.0000000
37400.0000000	4.7500000	-50.0000000
37400.0000000	5.0000000	-50.0000000
37400.0000000	5.2500000	-50.0000000
37400.0000000	5.5000000	-50.0000000
37400.0000000	5.7500000	-50.0000000
37400.0000000	6.0000000	-50.0000000
37400.0000000	6.2500000	-50.0000000
37400.0000000	6.5000000	-50.0000000
37400.0000000	6.7500000	-50.0000000
37400.0000000	7.0000000	-50.0000000
37400.0000000	7.2500000	-50.0000000
37400.0000000	7.5000000	-50.0000000
37400.0000000	7.7500000	-50.0000000
37400.0000000	8.0000000	-50.0000000
37400.0000000	8.2500000	-50.0000000
37400.0000000	8.5000000	-50.0000000
37400.0000000	8.7500000	-50.0000000

37400.0000000	9.0000000	-50.0000000
37400.0000000	9.2500000	-50.0000000
37400.0000000	9.5000000	-50.0000000
37400.0000000	9.7500000	-50.0000000
37400.0000000	10.0000000	-50.0000000
37400.0000000	10.2500000	-50.0000000
37400.0000000	10.5000000	-50.0000000
37400.0000000	10.7500000	-50.0000000
37400.0000000	11.0000000	-50.0000000
37400.0000000	11.2500000	-50.0000000
37400.0000000	11.5000000	-50.0000000
37400.0000000	11.7500000	-50.0000000
37400.0000000	12.0000000	-50.0000000
37400.0000000	12.2500000	-50.0000000
37400.0000000	12.5000000	-50.0000000
37400.0000000	12.7500000	-50.0000000
37400.0000000	13.0000000	-50.0000000
37400.0000000	13.2500000	-50.0000000
37400.0000000	13.5000000	-50.0000000
37400.0000000	13.7500000	-50.0000000
37400.0000000	14.0000000	-50.0000000
37400.0000000	14.2500000	-50.0000000
37400.0000000	14.5000000	-50.0000000
37400.0000000	14.7500000	-50.0000000
37400.0000000	15.0000000	-50.0000000
37430.0000000	0.0000000	-50.0000000
37430.0000000	0.2500000	-50.0000000
37430.0000000	0.5000000	-50.0000000
37430.0000000	0.7500000	-50.0000000
37430.0000000	1.0000000	-50.0000000
37430.0000000	1.2500000	-50.0000000
37430.0000000	1.5000000	-50.0000000
37430.0000000	1.7500000	-50.0000000
37430.0000000	2.0000000	-50.0000000
37430.0000000	2.2500000	-50.0000000
37430.0000000	2.5000000	-50.0000000
37430.0000000	2.7500000	-50.0000000
37430.0000000	3.0000000	-50.0000000
37430.0000000	3.2500000	-50.0000000
37430.0000000	3.5000000	-50.0000000
37430.0000000	3.7500000	-50.0000000
37430.0000000	4.0000000	-50.0000000
37430.0000000	4.2500000	-50.0000000
37430.0000000	4.5000000	-50.0000000
37430.0000000	4.7500000	-50.0000000
37430.0000000	5.0000000	-50.0000000
37430.0000000	5.2500000	-50.0000000
37430.0000000	5.5000000	-50.0000000
37430.0000000	5.7500000	-50.0000000
37430.0000000	6.0000000	-50.0000000
37430.0000000	6.2500000	-50.0000000
37430.0000000	6.5000000	-50.0000000
37430.0000000	6.7500000	-50.0000000
37430.0000000	7.0000000	-50.0000000
37430.0000000	7.2500000	-50.0000000
37430.0000000	7.5000000	-50.0000000
37430.0000000	7.7500000	-50.0000000
37430.0000000	8.0000000	-50.0000000

37430.0000000	8.2500000	-50.0000000
37430.0000000	8.5000000	-50.0000000
37430.0000000	8.7500000	-50.0000000
37430.0000000	9.0000000	-50.0000000
37430.0000000	9.2500000	-50.0000000
37430.0000000	9.5000000	-50.0000000
37430.0000000	9.7500000	-50.0000000
37430.0000000	10.0000000	-50.0000000
37430.0000000	10.2500000	-50.0000000
37430.0000000	10.5000000	-50.0000000
37430.0000000	10.7500000	-50.0000000
37430.0000000	11.0000000	-50.0000000
37430.0000000	11.2500000	-50.0000000
37430.0000000	11.5000000	-50.0000000
37430.0000000	11.7500000	-50.0000000
37430.0000000	12.0000000	-50.0000000
37430.0000000	12.2500000	-50.0000000
37430.0000000	12.5000000	-50.0000000
37430.0000000	12.7500000	-50.0000000
37430.0000000	13.0000000	-50.0000000
37430.0000000	13.2500000	-50.0000000
37430.0000000	13.5000000	-50.0000000
37430.0000000	13.7500000	-50.0000000
37430.0000000	14.0000000	-50.0000000
37430.0000000	14.2500000	-50.0000000
37430.0000000	14.5000000	-50.0000000
37430.0000000	14.7500000	-50.0000000
37430.0000000	15.0000000	-50.0000000
37460.0000000	0.0000000	-50.0000000
37460.0000000	0.2500000	-50.0000000
37460.0000000	0.5000000	-50.0000000
37460.0000000	0.7500000	-50.0000000
37460.0000000	1.0000000	-50.0000000
37460.0000000	1.2500000	-50.0000000
37460.0000000	1.5000000	-50.0000000
37460.0000000	1.7500000	-50.0000000
37460.0000000	2.0000000	-50.0000000
37460.0000000	2.2500000	-50.0000000
37460.0000000	2.5000000	-50.0000000
37460.0000000	2.7500000	-50.0000000
37460.0000000	3.0000000	-50.0000000
37460.0000000	3.2500000	-50.0000000
37460.0000000	3.5000000	-50.0000000
37460.0000000	3.7500000	-50.0000000
37460.0000000	4.0000000	-50.0000000
37460.0000000	4.2500000	-50.0000000
37460.0000000	4.5000000	-50.0000000
37460.0000000	4.7500000	-50.0000000
37460.0000000	5.0000000	-50.0000000
37460.0000000	5.2500000	-50.0000000
37460.0000000	5.5000000	-50.0000000
37460.0000000	5.7500000	-50.0000000
37460.0000000	6.0000000	-50.0000000
37460.0000000	6.2500000	-50.0000000
37460.0000000	6.5000000	-50.0000000
37460.0000000	6.7500000	-50.0000000
37460.0000000	7.0000000	-50.0000000
37460.0000000	7.2500000	-50.0000000

37460.0000000	7.5000000	-50.0000000
37460.0000000	7.7500000	-50.0000000
37460.0000000	8.0000000	-50.0000000
37460.0000000	8.2500000	-50.0000000
37460.0000000	8.5000000	-50.0000000
37460.0000000	8.7500000	-50.0000000
37460.0000000	9.0000000	-50.0000000
37460.0000000	9.2500000	-50.0000000
37460.0000000	9.5000000	-50.0000000
37460.0000000	9.7500000	-50.0000000
37460.0000000	10.0000000	-50.0000000
37460.0000000	10.2500000	-50.0000000
37460.0000000	10.5000000	-50.0000000
37460.0000000	10.7500000	-50.0000000
37460.0000000	11.0000000	-50.0000000
37460.0000000	11.2500000	-50.0000000
37460.0000000	11.5000000	-50.0000000
37460.0000000	11.7500000	-50.0000000
37460.0000000	12.0000000	-50.0000000
37460.0000000	12.2500000	-50.0000000
37460.0000000	12.5000000	-50.0000000
37460.0000000	12.7500000	-50.0000000
37460.0000000	13.0000000	-50.0000000
37460.0000000	13.2500000	-50.0000000
37460.0000000	13.5000000	-50.0000000
37460.0000000	13.7500000	-50.0000000
37460.0000000	14.0000000	-50.0000000
37460.0000000	14.2500000	-50.0000000
37460.0000000	14.5000000	-50.0000000
37460.0000000	14.7500000	-50.0000000
37460.0000000	15.0000000	-50.0000000
37490.0000000	0.0000000	-50.0000000
37490.0000000	0.2500000	-50.0000000
37490.0000000	0.5000000	-50.0000000
37490.0000000	0.7500000	-50.0000000
37490.0000000	1.0000000	-50.0000000
37490.0000000	1.2500000	-50.0000000
37490.0000000	1.5000000	-50.0000000
37490.0000000	1.7500000	-50.0000000
37490.0000000	2.0000000	-50.0000000
37490.0000000	2.2500000	-50.0000000
37490.0000000	2.5000000	-50.0000000
37490.0000000	2.7500000	-50.0000000
37490.0000000	3.0000000	-50.0000000
37490.0000000	3.2500000	-50.0000000
37490.0000000	3.5000000	-50.0000000
37490.0000000	3.7500000	-50.0000000
37490.0000000	4.0000000	-50.0000000
37490.0000000	4.2500000	-50.0000000
37490.0000000	4.5000000	-50.0000000
37490.0000000	4.7500000	-50.0000000
37490.0000000	5.0000000	-50.0000000
37490.0000000	5.2500000	-50.0000000
37490.0000000	5.5000000	-50.0000000
37490.0000000	5.7500000	-50.0000000
37490.0000000	6.0000000	-50.0000000
37490.0000000	6.2500000	-50.0000000
37490.0000000	6.5000000	-50.0000000

37490.0000000	6.7500000	-50.0000000
37490.0000000	7.0000000	-50.0000000
37490.0000000	7.2500000	-50.0000000
37490.0000000	7.5000000	-50.0000000
37490.0000000	7.7500000	-50.0000000
37490.0000000	8.0000000	-50.0000000
37490.0000000	8.2500000	-50.0000000
37490.0000000	8.5000000	-50.0000000
37490.0000000	8.7500000	-50.0000000
37490.0000000	9.0000000	-50.0000000
37490.0000000	9.2500000	-50.0000000
37490.0000000	9.5000000	-50.0000000
37490.0000000	9.7500000	-50.0000000
37490.0000000	10.0000000	-50.0000000
37490.0000000	10.2500000	-50.0000000
37490.0000000	10.5000000	-50.0000000
37490.0000000	10.7500000	-50.0000000
37490.0000000	11.0000000	-50.0000000
37490.0000000	11.2500000	-50.0000000
37490.0000000	11.5000000	-50.0000000
37490.0000000	11.7500000	-50.0000000
37490.0000000	12.0000000	-50.0000000
37490.0000000	12.2500000	-50.0000000
37490.0000000	12.5000000	-50.0000000
37490.0000000	12.7500000	-50.0000000
37490.0000000	13.0000000	-50.0000000
37490.0000000	13.2500000	-50.0000000
37490.0000000	13.5000000	-50.0000000
37490.0000000	13.7500000	-50.0000000
37490.0000000	14.0000000	-50.0000000
37490.0000000	14.2500000	-50.0000000
37490.0000000	14.5000000	-50.0000000
37490.0000000	14.7500000	-50.0000000
37490.0000000	15.0000000	-50.0000000
37520.0000000	0.0000000	-50.0000000
37520.0000000	0.2500000	-50.0000000
37520.0000000	0.5000000	-50.0000000
37520.0000000	0.7500000	-50.0000000
37520.0000000	1.0000000	-50.0000000
37520.0000000	1.2500000	-50.0000000
37520.0000000	1.5000000	-50.0000000
37520.0000000	1.7500000	-50.0000000
37520.0000000	2.0000000	-50.0000000
37520.0000000	2.2500000	-50.0000000
37520.0000000	2.5000000	-50.0000000
37520.0000000	2.7500000	-50.0000000
37520.0000000	3.0000000	-50.0000000
37520.0000000	3.2500000	-50.0000000
37520.0000000	3.5000000	-50.0000000
37520.0000000	3.7500000	-50.0000000
37520.0000000	4.0000000	-50.0000000
37520.0000000	4.2500000	-50.0000000
37520.0000000	4.5000000	-50.0000000
37520.0000000	4.7500000	-50.0000000
37520.0000000	5.0000000	-50.0000000
37520.0000000	5.2500000	-50.0000000
37520.0000000	5.5000000	-50.0000000
37520.0000000	5.7500000	-50.0000000

37520.0000000	6.0000000	-50.0000000
37520.0000000	6.2500000	-50.0000000
37520.0000000	6.5000000	-50.0000000
37520.0000000	6.7500000	-50.0000000
37520.0000000	7.0000000	-50.0000000
37520.0000000	7.2500000	-50.0000000
37520.0000000	7.5000000	-50.0000000
37520.0000000	7.7500000	-50.0000000
37520.0000000	8.0000000	-50.0000000
37520.0000000	8.2500000	-50.0000000
37520.0000000	8.5000000	-50.0000000
37520.0000000	8.7500000	-50.0000000
37520.0000000	9.0000000	-50.0000000
37520.0000000	9.2500000	-50.0000000
37520.0000000	9.5000000	-50.0000000
37520.0000000	9.7500000	-50.0000000
37520.0000000	10.0000000	-50.0000000
37520.0000000	10.2500000	-50.0000000
37520.0000000	10.5000000	-50.0000000
37520.0000000	10.7500000	-50.0000000
37520.0000000	11.0000000	-50.0000000
37520.0000000	11.2500000	-50.0000000
37520.0000000	11.5000000	-50.0000000
37520.0000000	11.7500000	-50.0000000
37520.0000000	12.0000000	-50.0000000
37520.0000000	12.2500000	-50.0000000
37520.0000000	12.5000000	-50.0000000
37520.0000000	12.7500000	-50.0000000
37520.0000000	13.0000000	-50.0000000
37520.0000000	13.2500000	-50.0000000
37520.0000000	13.5000000	-50.0000000
37520.0000000	13.7500000	-50.0000000
37520.0000000	14.0000000	-50.0000000
37520.0000000	14.2500000	-50.0000000
37520.0000000	14.5000000	-50.0000000
37520.0000000	14.7500000	-50.0000000
37520.0000000	15.0000000	-50.0000000
37550.0000000	0.0000000	-50.0000000
37550.0000000	0.2500000	-50.0000000
37550.0000000	0.5000000	-50.0000000
37550.0000000	0.7500000	-50.0000000
37550.0000000	1.0000000	-50.0000000
37550.0000000	1.2500000	-50.0000000
37550.0000000	1.5000000	-50.0000000
37550.0000000	1.7500000	-50.0000000
37550.0000000	2.0000000	-50.0000000
37550.0000000	2.2500000	-50.0000000
37550.0000000	2.5000000	-50.0000000
37550.0000000	2.7500000	-50.0000000
37550.0000000	3.0000000	-50.0000000
37550.0000000	3.2500000	-50.0000000
37550.0000000	3.5000000	-50.0000000
37550.0000000	3.7500000	-50.0000000
37550.0000000	4.0000000	-50.0000000
37550.0000000	4.2500000	-50.0000000
37550.0000000	4.5000000	-50.0000000
37550.0000000	4.7500000	-50.0000000
37550.0000000	5.0000000	-50.0000000

37550.0000000	5.2500000	-50.0000000
37550.0000000	5.5000000	-50.0000000
37550.0000000	5.7500000	-50.0000000
37550.0000000	6.0000000	-50.0000000
37550.0000000	6.2500000	-50.0000000
37550.0000000	6.5000000	-50.0000000
37550.0000000	6.7500000	-50.0000000
37550.0000000	7.0000000	-50.0000000
37550.0000000	7.2500000	-50.0000000
37550.0000000	7.5000000	-50.0000000
37550.0000000	7.7500000	-50.0000000
37550.0000000	8.0000000	-50.0000000
37550.0000000	8.2500000	-50.0000000
37550.0000000	8.5000000	-50.0000000
37550.0000000	8.7500000	-50.0000000
37550.0000000	9.0000000	-50.0000000
37550.0000000	9.2500000	-50.0000000
37550.0000000	9.5000000	-50.0000000
37550.0000000	9.7500000	-50.0000000
37550.0000000	10.0000000	-50.0000000
37550.0000000	10.2500000	-50.0000000
37550.0000000	10.5000000	-50.0000000
37550.0000000	10.7500000	-50.0000000
37550.0000000	11.0000000	-50.0000000
37550.0000000	11.2500000	-50.0000000
37550.0000000	11.5000000	-50.0000000
37550.0000000	11.7500000	-50.0000000
37550.0000000	12.0000000	-50.0000000
37550.0000000	12.2500000	-50.0000000
37550.0000000	12.5000000	-50.0000000
37550.0000000	12.7500000	-50.0000000
37550.0000000	13.0000000	-50.0000000
37550.0000000	13.2500000	-50.0000000
37550.0000000	13.5000000	-50.0000000
37550.0000000	13.7500000	-50.0000000
37550.0000000	14.0000000	-50.0000000
37550.0000000	14.2500000	-50.0000000
37550.0000000	14.5000000	-50.0000000
37550.0000000	14.7500000	-50.0000000
37550.0000000	15.0000000	-50.0000000
37580.0000000	0.0000000	-50.0000000
37580.0000000	0.2500000	-50.0000000
37580.0000000	0.5000000	-50.0000000
37580.0000000	0.7500000	-50.0000000
37580.0000000	1.0000000	-50.0000000
37580.0000000	1.2500000	-50.0000000
37580.0000000	1.5000000	-50.0000000
37580.0000000	1.7500000	-50.0000000
37580.0000000	2.0000000	-50.0000000
37580.0000000	2.2500000	-50.0000000
37580.0000000	2.5000000	-50.0000000
37580.0000000	2.7500000	-50.0000000
37580.0000000	3.0000000	-50.0000000
37580.0000000	3.2500000	-50.0000000
37580.0000000	3.5000000	-50.0000000
37580.0000000	3.7500000	-50.0000000
37580.0000000	4.0000000	-50.0000000
37580.0000000	4.2500000	-50.0000000

37580.0000000	4.5000000	-50.0000000
37580.0000000	4.7500000	-50.0000000
37580.0000000	5.0000000	-50.0000000
37580.0000000	5.2500000	-50.0000000
37580.0000000	5.5000000	-50.0000000
37580.0000000	5.7500000	-50.0000000
37580.0000000	6.0000000	-50.0000000
37580.0000000	6.2500000	-50.0000000
37580.0000000	6.5000000	-50.0000000
37580.0000000	6.7500000	-50.0000000
37580.0000000	7.0000000	-50.0000000
37580.0000000	7.2500000	-50.0000000
37580.0000000	7.5000000	-50.0000000
37580.0000000	7.7500000	-50.0000000
37580.0000000	8.0000000	-50.0000000
37580.0000000	8.2500000	-50.0000000
37580.0000000	8.5000000	-50.0000000
37580.0000000	8.7500000	-50.0000000
37580.0000000	9.0000000	-50.0000000
37580.0000000	9.2500000	-50.0000000
37580.0000000	9.5000000	-50.0000000
37580.0000000	9.7500000	-50.0000000
37580.0000000	10.0000000	-50.0000000
37580.0000000	10.2500000	-50.0000000
37580.0000000	10.5000000	-50.0000000
37580.0000000	10.7500000	-50.0000000
37580.0000000	11.0000000	-50.0000000
37580.0000000	11.2500000	-50.0000000
37580.0000000	11.5000000	-50.0000000
37580.0000000	11.7500000	-50.0000000
37580.0000000	12.0000000	-50.0000000
37580.0000000	12.2500000	-50.0000000
37580.0000000	12.5000000	-50.0000000
37580.0000000	12.7500000	-50.0000000
37580.0000000	13.0000000	-50.0000000
37580.0000000	13.2500000	-50.0000000
37580.0000000	13.5000000	-50.0000000
37580.0000000	13.7500000	-50.0000000
37580.0000000	14.0000000	-50.0000000
37580.0000000	14.2500000	-50.0000000
37580.0000000	14.5000000	-50.0000000
37580.0000000	14.7500000	-50.0000000
37580.0000000	15.0000000	-50.0000000
37610.0000000	0.0000000	-50.0000000
37610.0000000	0.2500000	-50.0000000
37610.0000000	0.5000000	-50.0000000
37610.0000000	0.7500000	-50.0000000
37610.0000000	1.0000000	-50.0000000
37610.0000000	1.2500000	-50.0000000
37610.0000000	1.5000000	-50.0000000
37610.0000000	1.7500000	-50.0000000
37610.0000000	2.0000000	-50.0000000
37610.0000000	2.2500000	-50.0000000
37610.0000000	2.5000000	-50.0000000
37610.0000000	2.7500000	-50.0000000
37610.0000000	3.0000000	-50.0000000
37610.0000000	3.2500000	-50.0000000
37610.0000000	3.5000000	-50.0000000

37610.0000000	3.7500000	-50.0000000
37610.0000000	4.0000000	-50.0000000
37610.0000000	4.2500000	-50.0000000
37610.0000000	4.5000000	-50.0000000
37610.0000000	4.7500000	-50.0000000
37610.0000000	5.0000000	-50.0000000
37610.0000000	5.2500000	-50.0000000
37610.0000000	5.5000000	-50.0000000
37610.0000000	5.7500000	-50.0000000
37610.0000000	6.0000000	-50.0000000
37610.0000000	6.2500000	-50.0000000
37610.0000000	6.5000000	-50.0000000
37610.0000000	6.7500000	-50.0000000
37610.0000000	7.0000000	-50.0000000
37610.0000000	7.2500000	-50.0000000
37610.0000000	7.5000000	-50.0000000
37610.0000000	7.7500000	-50.0000000
37610.0000000	8.0000000	-50.0000000
37610.0000000	8.2500000	-50.0000000
37610.0000000	8.5000000	-50.0000000
37610.0000000	8.7500000	-50.0000000
37610.0000000	9.0000000	-50.0000000
37610.0000000	9.2500000	-50.0000000
37610.0000000	9.5000000	-50.0000000
37610.0000000	9.7500000	-50.0000000
37610.0000000	10.0000000	-50.0000000
37610.0000000	10.2500000	-50.0000000
37610.0000000	10.5000000	-50.0000000
37610.0000000	10.7500000	-50.0000000
37610.0000000	11.0000000	-50.0000000
37610.0000000	11.2500000	-50.0000000
37610.0000000	11.5000000	-50.0000000
37610.0000000	11.7500000	-50.0000000
37610.0000000	12.0000000	-50.0000000
37610.0000000	12.2500000	-50.0000000
37610.0000000	12.5000000	-50.0000000
37610.0000000	12.7500000	-50.0000000
37610.0000000	13.0000000	-50.0000000
37610.0000000	13.2500000	-50.0000000
37610.0000000	13.5000000	-50.0000000
37610.0000000	13.7500000	-50.0000000
37610.0000000	14.0000000	-50.0000000
37610.0000000	14.2500000	-50.0000000
37610.0000000	14.5000000	-50.0000000
37610.0000000	14.7500000	-50.0000000
37610.0000000	15.0000000	-50.0000000
37640.0000000	0.0000000	-50.0000000
37640.0000000	0.2500000	-50.0000000
37640.0000000	0.5000000	-50.0000000
37640.0000000	0.7500000	-50.0000000
37640.0000000	1.0000000	-50.0000000
37640.0000000	1.2500000	-50.0000000
37640.0000000	1.5000000	-50.0000000
37640.0000000	1.7500000	-50.0000000
37640.0000000	2.0000000	-50.0000000
37640.0000000	2.2500000	-50.0000000
37640.0000000	2.5000000	-50.0000000
37640.0000000	2.7500000	-50.0000000

37640.0000000	3.0000000	-50.0000000
37640.0000000	3.2500000	-50.0000000
37640.0000000	3.5000000	-50.0000000
37640.0000000	3.7500000	-50.0000000
37640.0000000	4.0000000	-50.0000000
37640.0000000	4.2500000	-50.0000000
37640.0000000	4.5000000	-50.0000000
37640.0000000	4.7500000	-50.0000000
37640.0000000	5.0000000	-50.0000000
37640.0000000	5.2500000	-50.0000000
37640.0000000	5.5000000	-50.0000000
37640.0000000	5.7500000	-50.0000000
37640.0000000	6.0000000	-50.0000000
37640.0000000	6.2500000	-50.0000000
37640.0000000	6.5000000	-50.0000000
37640.0000000	6.7500000	-50.0000000
37640.0000000	7.0000000	-50.0000000
37640.0000000	7.2500000	-50.0000000
37640.0000000	7.5000000	-50.0000000
37640.0000000	7.7500000	-50.0000000
37640.0000000	8.0000000	-50.0000000
37640.0000000	8.2500000	-50.0000000
37640.0000000	8.5000000	-50.0000000
37640.0000000	8.7500000	-50.0000000
37640.0000000	9.0000000	-50.0000000
37640.0000000	9.2500000	-50.0000000
37640.0000000	9.5000000	-50.0000000
37640.0000000	9.7500000	-50.0000000
37640.0000000	10.0000000	-50.0000000
37640.0000000	10.2500000	-50.0000000
37640.0000000	10.5000000	-50.0000000
37640.0000000	10.7500000	-50.0000000
37640.0000000	11.0000000	-50.0000000
37640.0000000	11.2500000	-50.0000000
37640.0000000	11.5000000	-50.0000000
37640.0000000	11.7500000	-50.0000000
37640.0000000	12.0000000	-50.0000000
37640.0000000	12.2500000	-50.0000000
37640.0000000	12.5000000	-50.0000000
37640.0000000	12.7500000	-50.0000000
37640.0000000	13.0000000	-50.0000000
37640.0000000	13.2500000	-50.0000000
37640.0000000	13.5000000	-50.0000000
37640.0000000	13.7500000	-50.0000000
37640.0000000	14.0000000	-50.0000000
37640.0000000	14.2500000	-50.0000000
37640.0000000	14.5000000	-50.0000000
37640.0000000	14.7500000	-50.0000000
37640.0000000	15.0000000	-50.0000000
37670.0000000	0.0000000	-50.0000000
37670.0000000	0.2500000	-50.0000000
37670.0000000	0.5000000	-50.0000000
37670.0000000	0.7500000	-50.0000000
37670.0000000	1.0000000	-50.0000000
37670.0000000	1.2500000	-50.0000000
37670.0000000	1.5000000	-50.0000000
37670.0000000	1.7500000	-50.0000000
37670.0000000	2.0000000	-50.0000000

37670.0000000	2.2500000	-50.0000000
37670.0000000	2.5000000	-50.0000000
37670.0000000	2.7500000	-50.0000000
37670.0000000	3.0000000	-50.0000000
37670.0000000	3.2500000	-50.0000000
37670.0000000	3.5000000	-50.0000000
37670.0000000	3.7500000	-50.0000000
37670.0000000	4.0000000	-50.0000000
37670.0000000	4.2500000	-50.0000000
37670.0000000	4.5000000	-50.0000000
37670.0000000	4.7500000	-50.0000000
37670.0000000	5.0000000	-50.0000000
37670.0000000	5.2500000	-50.0000000
37670.0000000	5.5000000	-50.0000000
37670.0000000	5.7500000	-50.0000000
37670.0000000	6.0000000	-50.0000000
37670.0000000	6.2500000	-50.0000000
37670.0000000	6.5000000	-50.0000000
37670.0000000	6.7500000	-50.0000000
37670.0000000	7.0000000	-50.0000000
37670.0000000	7.2500000	-50.0000000
37670.0000000	7.5000000	-50.0000000
37670.0000000	7.7500000	-50.0000000
37670.0000000	8.0000000	-50.0000000
37670.0000000	8.2500000	-50.0000000
37670.0000000	8.5000000	-50.0000000
37670.0000000	8.7500000	-50.0000000
37670.0000000	9.0000000	-50.0000000
37670.0000000	9.2500000	-50.0000000
37670.0000000	9.5000000	-50.0000000
37670.0000000	9.7500000	-50.0000000
37670.0000000	10.0000000	-50.0000000
37670.0000000	10.2500000	-50.0000000
37670.0000000	10.5000000	-50.0000000
37670.0000000	10.7500000	-50.0000000
37670.0000000	11.0000000	-50.0000000
37670.0000000	11.2500000	-50.0000000
37670.0000000	11.5000000	-50.0000000
37670.0000000	11.7500000	-50.0000000
37670.0000000	12.0000000	-50.0000000
37670.0000000	12.2500000	-50.0000000
37670.0000000	12.5000000	-50.0000000
37670.0000000	12.7500000	-50.0000000
37670.0000000	13.0000000	-50.0000000
37670.0000000	13.2500000	-50.0000000
37670.0000000	13.5000000	-50.0000000
37670.0000000	13.7500000	-50.0000000
37670.0000000	14.0000000	-50.0000000
37670.0000000	14.2500000	-50.0000000
37670.0000000	14.5000000	-50.0000000
37670.0000000	14.7500000	-50.0000000
37670.0000000	15.0000000	-50.0000000
37700.0000000	0.0000000	-50.0000000
37700.0000000	0.2500000	-50.0000000
37700.0000000	0.5000000	-50.0000000
37700.0000000	0.7500000	-50.0000000
37700.0000000	1.0000000	-50.0000000
37700.0000000	1.2500000	-50.0000000

37700.0000000	1.5000000	-50.0000000
37700.0000000	1.7500000	-50.0000000
37700.0000000	2.0000000	-50.0000000
37700.0000000	2.2500000	-50.0000000
37700.0000000	2.5000000	-50.0000000
37700.0000000	2.7500000	-50.0000000
37700.0000000	3.0000000	-50.0000000
37700.0000000	3.2500000	-50.0000000
37700.0000000	3.5000000	-50.0000000
37700.0000000	3.7500000	-50.0000000
37700.0000000	4.0000000	-50.0000000
37700.0000000	4.2500000	-50.0000000
37700.0000000	4.5000000	-50.0000000
37700.0000000	4.7500000	-50.0000000
37700.0000000	5.0000000	-50.0000000
37700.0000000	5.2500000	-50.0000000
37700.0000000	5.5000000	-50.0000000
37700.0000000	5.7500000	-50.0000000
37700.0000000	6.0000000	-50.0000000
37700.0000000	6.2500000	-50.0000000
37700.0000000	6.5000000	-50.0000000
37700.0000000	6.7500000	-50.0000000
37700.0000000	7.0000000	-50.0000000
37700.0000000	7.2500000	-50.0000000
37700.0000000	7.5000000	-50.0000000
37700.0000000	7.7500000	-50.0000000
37700.0000000	8.0000000	-50.0000000
37700.0000000	8.2500000	-50.0000000
37700.0000000	8.5000000	-50.0000000
37700.0000000	8.7500000	-50.0000000
37700.0000000	9.0000000	-50.0000000
37700.0000000	9.2500000	-50.0000000
37700.0000000	9.5000000	-50.0000000
37700.0000000	9.7500000	-50.0000000
37700.0000000	10.0000000	-50.0000000
37700.0000000	10.2500000	-50.0000000
37700.0000000	10.5000000	-50.0000000
37700.0000000	10.7500000	-50.0000000
37700.0000000	11.0000000	-50.0000000
37700.0000000	11.2500000	-50.0000000
37700.0000000	11.5000000	-50.0000000
37700.0000000	11.7500000	-50.0000000
37700.0000000	12.0000000	-50.0000000
37700.0000000	12.2500000	-50.0000000
37700.0000000	12.5000000	-50.0000000
37700.0000000	12.7500000	-50.0000000
37700.0000000	13.0000000	-50.0000000
37700.0000000	13.2500000	-50.0000000
37700.0000000	13.5000000	-50.0000000
37700.0000000	13.7500000	-50.0000000
37700.0000000	14.0000000	-50.0000000
37700.0000000	14.2500000	-50.0000000
37700.0000000	14.5000000	-50.0000000
37700.0000000	14.7500000	-50.0000000
37700.0000000	15.0000000	-50.0000000
37730.0000000	0.0000000	-50.0000000
37730.0000000	0.2500000	-50.0000000
37730.0000000	0.5000000	-50.0000000

37730.0000000	0.7500000	-50.0000000
37730.0000000	1.0000000	-50.0000000
37730.0000000	1.2500000	-50.0000000
37730.0000000	1.5000000	-50.0000000
37730.0000000	1.7500000	-50.0000000
37730.0000000	2.0000000	-50.0000000
37730.0000000	2.2500000	-50.0000000
37730.0000000	2.5000000	-50.0000000
37730.0000000	2.7500000	-50.0000000
37730.0000000	3.0000000	-50.0000000
37730.0000000	3.2500000	-50.0000000
37730.0000000	3.5000000	-50.0000000
37730.0000000	3.7500000	-50.0000000
37730.0000000	4.0000000	-50.0000000
37730.0000000	4.2500000	-50.0000000
37730.0000000	4.5000000	-50.0000000
37730.0000000	4.7500000	-50.0000000
37730.0000000	5.0000000	-50.0000000
37730.0000000	5.2500000	-50.0000000
37730.0000000	5.5000000	-50.0000000
37730.0000000	5.7500000	-50.0000000
37730.0000000	6.0000000	-50.0000000
37730.0000000	6.2500000	-50.0000000
37730.0000000	6.5000000	-50.0000000
37730.0000000	6.7500000	-50.0000000
37730.0000000	7.0000000	-50.0000000
37730.0000000	7.2500000	-50.0000000
37730.0000000	7.5000000	-50.0000000
37730.0000000	7.7500000	-50.0000000
37730.0000000	8.0000000	-50.0000000
37730.0000000	8.2500000	-50.0000000
37730.0000000	8.5000000	-50.0000000
37730.0000000	8.7500000	-50.0000000
37730.0000000	9.0000000	-50.0000000
37730.0000000	9.2500000	-50.0000000
37730.0000000	9.5000000	-50.0000000
37730.0000000	9.7500000	-50.0000000
37730.0000000	10.0000000	-50.0000000
37730.0000000	10.2500000	-50.0000000
37730.0000000	10.5000000	-50.0000000
37730.0000000	10.7500000	-50.0000000
37730.0000000	11.0000000	-50.0000000
37730.0000000	11.2500000	-50.0000000
37730.0000000	11.5000000	-50.0000000
37730.0000000	11.7500000	-50.0000000
37730.0000000	12.0000000	-50.0000000
37730.0000000	12.2500000	-50.0000000
37730.0000000	12.5000000	-50.0000000
37730.0000000	12.7500000	-50.0000000
37730.0000000	13.0000000	-50.0000000
37730.0000000	13.2500000	-50.0000000
37730.0000000	13.5000000	-50.0000000
37730.0000000	13.7500000	-50.0000000
37730.0000000	14.0000000	-50.0000000
37730.0000000	14.2500000	-50.0000000
37730.0000000	14.5000000	-50.0000000
37730.0000000	14.7500000	-50.0000000
37730.0000000	15.0000000	-50.0000000

37760.0000000	0.0000000	-50.0000000
37760.0000000	0.2500000	-50.0000000
37760.0000000	0.5000000	-50.0000000
37760.0000000	0.7500000	-50.0000000
37760.0000000	1.0000000	-50.0000000
37760.0000000	1.2500000	-50.0000000
37760.0000000	1.5000000	-50.0000000
37760.0000000	1.7500000	-50.0000000
37760.0000000	2.0000000	-50.0000000
37760.0000000	2.2500000	-50.0000000
37760.0000000	2.5000000	-50.0000000
37760.0000000	2.7500000	-50.0000000
37760.0000000	3.0000000	-50.0000000
37760.0000000	3.2500000	-50.0000000
37760.0000000	3.5000000	-50.0000000
37760.0000000	3.7500000	-50.0000000
37760.0000000	4.0000000	-50.0000000
37760.0000000	4.2500000	-50.0000000
37760.0000000	4.5000000	-50.0000000
37760.0000000	4.7500000	-50.0000000
37760.0000000	5.0000000	-50.0000000
37760.0000000	5.2500000	-50.0000000
37760.0000000	5.5000000	-50.0000000
37760.0000000	5.7500000	-50.0000000
37760.0000000	6.0000000	-50.0000000
37760.0000000	6.2500000	-50.0000000
37760.0000000	6.5000000	-50.0000000
37760.0000000	6.7500000	-50.0000000
37760.0000000	7.0000000	-50.0000000
37760.0000000	7.2500000	-50.0000000
37760.0000000	7.5000000	-50.0000000
37760.0000000	7.7500000	-50.0000000
37760.0000000	8.0000000	-50.0000000
37760.0000000	8.2500000	-50.0000000
37760.0000000	8.5000000	-50.0000000
37760.0000000	8.7500000	-50.0000000
37760.0000000	9.0000000	-50.0000000
37760.0000000	9.2500000	-50.0000000
37760.0000000	9.5000000	-50.0000000
37760.0000000	9.7500000	-50.0000000
37760.0000000	10.0000000	-50.0000000
37760.0000000	10.2500000	-50.0000000
37760.0000000	10.5000000	-50.0000000
37760.0000000	10.7500000	-50.0000000
37760.0000000	11.0000000	-50.0000000
37760.0000000	11.2500000	-50.0000000
37760.0000000	11.5000000	-50.0000000
37760.0000000	11.7500000	-50.0000000
37760.0000000	12.0000000	-50.0000000
37760.0000000	12.2500000	-50.0000000
37760.0000000	12.5000000	-50.0000000
37760.0000000	12.7500000	-50.0000000
37760.0000000	13.0000000	-50.0000000
37760.0000000	13.2500000	-50.0000000
37760.0000000	13.5000000	-50.0000000
37760.0000000	13.7500000	-50.0000000
37760.0000000	14.0000000	-50.0000000
37760.0000000	14.2500000	-50.0000000

37760.0000000	14.5000000	-50.0000000
37760.0000000	14.7500000	-50.0000000
37760.0000000	15.0000000	-50.0000000
37790.0000000	0.0000000	-50.0000000
37790.0000000	0.2500000	-50.0000000
37790.0000000	0.5000000	-50.0000000
37790.0000000	0.7500000	-50.0000000
37790.0000000	1.0000000	-50.0000000
37790.0000000	1.2500000	-50.0000000
37790.0000000	1.5000000	-50.0000000
37790.0000000	1.7500000	-50.0000000
37790.0000000	2.0000000	-50.0000000
37790.0000000	2.2500000	-50.0000000
37790.0000000	2.5000000	-50.0000000
37790.0000000	2.7500000	-50.0000000
37790.0000000	3.0000000	-50.0000000
37790.0000000	3.2500000	-50.0000000
37790.0000000	3.5000000	-50.0000000
37790.0000000	3.7500000	-50.0000000
37790.0000000	4.0000000	-50.0000000
37790.0000000	4.2500000	-50.0000000
37790.0000000	4.5000000	-50.0000000
37790.0000000	4.7500000	-50.0000000
37790.0000000	5.0000000	-50.0000000
37790.0000000	5.2500000	-50.0000000
37790.0000000	5.5000000	-50.0000000
37790.0000000	5.7500000	-50.0000000
37790.0000000	6.0000000	-50.0000000
37790.0000000	6.2500000	-50.0000000
37790.0000000	6.5000000	-50.0000000
37790.0000000	6.7500000	-50.0000000
37790.0000000	7.0000000	-50.0000000
37790.0000000	7.2500000	-50.0000000
37790.0000000	7.5000000	-50.0000000
37790.0000000	7.7500000	-50.0000000
37790.0000000	8.0000000	-50.0000000
37790.0000000	8.2500000	-50.0000000
37790.0000000	8.5000000	-50.0000000
37790.0000000	8.7500000	-50.0000000
37790.0000000	9.0000000	-50.0000000
37790.0000000	9.2500000	-50.0000000
37790.0000000	9.5000000	-50.0000000
37790.0000000	9.7500000	-50.0000000
37790.0000000	10.0000000	-50.0000000
37790.0000000	10.2500000	-50.0000000
37790.0000000	10.5000000	-50.0000000
37790.0000000	10.7500000	-50.0000000
37790.0000000	11.0000000	-50.0000000
37790.0000000	11.2500000	-50.0000000
37790.0000000	11.5000000	-50.0000000
37790.0000000	11.7500000	-50.0000000
37790.0000000	12.0000000	-50.0000000
37790.0000000	12.2500000	-50.0000000
37790.0000000	12.5000000	-50.0000000
37790.0000000	12.7500000	-50.0000000
37790.0000000	13.0000000	-50.0000000
37790.0000000	13.2500000	-50.0000000
37790.0000000	13.5000000	-50.0000000

37790.0000000	13.7500000	-50.0000000
37790.0000000	14.0000000	-50.0000000
37790.0000000	14.2500000	-50.0000000
37790.0000000	14.5000000	-50.0000000
37790.0000000	14.7500000	-50.0000000
37790.0000000	15.0000000	-50.0000000
37820.0000000	0.0000000	-50.0000000
37820.0000000	0.2500000	-50.0000000
37820.0000000	0.5000000	-50.0000000
37820.0000000	0.7500000	-50.0000000
37820.0000000	1.0000000	-50.0000000
37820.0000000	1.2500000	-50.0000000
37820.0000000	1.5000000	-50.0000000
37820.0000000	1.7500000	-50.0000000
37820.0000000	2.0000000	-50.0000000
37820.0000000	2.2500000	-50.0000000
37820.0000000	2.5000000	-50.0000000
37820.0000000	2.7500000	-50.0000000
37820.0000000	3.0000000	-50.0000000
37820.0000000	3.2500000	-50.0000000
37820.0000000	3.5000000	-50.0000000
37820.0000000	3.7500000	-50.0000000
37820.0000000	4.0000000	-50.0000000
37820.0000000	4.2500000	-50.0000000
37820.0000000	4.5000000	-50.0000000
37820.0000000	4.7500000	-50.0000000
37820.0000000	5.0000000	-50.0000000
37820.0000000	5.2500000	-50.0000000
37820.0000000	5.5000000	-50.0000000
37820.0000000	5.7500000	-50.0000000
37820.0000000	6.0000000	-50.0000000
37820.0000000	6.2500000	-50.0000000
37820.0000000	6.5000000	-50.0000000
37820.0000000	6.7500000	-50.0000000
37820.0000000	7.0000000	-50.0000000
37820.0000000	7.2500000	-50.0000000
37820.0000000	7.5000000	-50.0000000
37820.0000000	7.7500000	-50.0000000
37820.0000000	8.0000000	-50.0000000
37820.0000000	8.2500000	-50.0000000
37820.0000000	8.5000000	-50.0000000
37820.0000000	8.7500000	-50.0000000
37820.0000000	9.0000000	-50.0000000
37820.0000000	9.2500000	-50.0000000
37820.0000000	9.5000000	-50.0000000
37820.0000000	9.7500000	-50.0000000
37820.0000000	10.0000000	-50.0000000
37820.0000000	10.2500000	-50.0000000
37820.0000000	10.5000000	-50.0000000
37820.0000000	10.7500000	-50.0000000
37820.0000000	11.0000000	-50.0000000
37820.0000000	11.2500000	-50.0000000
37820.0000000	11.5000000	-50.0000000
37820.0000000	11.7500000	-50.0000000
37820.0000000	12.0000000	-50.0000000
37820.0000000	12.2500000	-50.0000000
37820.0000000	12.5000000	-50.0000000
37820.0000000	12.7500000	-50.0000000

37820.0000000	13.0000000	-50.0000000
37820.0000000	13.2500000	-50.0000000
37820.0000000	13.5000000	-50.0000000
37820.0000000	13.7500000	-50.0000000
37820.0000000	14.0000000	-50.0000000
37820.0000000	14.2500000	-50.0000000
37820.0000000	14.5000000	-50.0000000
37820.0000000	14.7500000	-50.0000000
37820.0000000	15.0000000	-50.0000000
37850.0000000	0.0000000	-50.0000000
37850.0000000	0.2500000	-50.0000000
37850.0000000	0.5000000	-50.0000000
37850.0000000	0.7500000	-50.0000000
37850.0000000	1.0000000	-50.0000000
37850.0000000	1.2500000	-50.0000000
37850.0000000	1.5000000	-50.0000000
37850.0000000	1.7500000	-50.0000000
37850.0000000	2.0000000	-50.0000000
37850.0000000	2.2500000	-50.0000000
37850.0000000	2.5000000	-50.0000000
37850.0000000	2.7500000	-50.0000000
37850.0000000	3.0000000	-50.0000000
37850.0000000	3.2500000	-50.0000000
37850.0000000	3.5000000	-50.0000000
37850.0000000	3.7500000	-50.0000000
37850.0000000	4.0000000	-50.0000000
37850.0000000	4.2500000	-50.0000000
37850.0000000	4.5000000	-50.0000000
37850.0000000	4.7500000	-50.0000000
37850.0000000	5.0000000	-50.0000000
37850.0000000	5.2500000	-50.0000000
37850.0000000	5.5000000	-50.0000000
37850.0000000	5.7500000	-50.0000000
37850.0000000	6.0000000	-50.0000000
37850.0000000	6.2500000	-50.0000000
37850.0000000	6.5000000	-50.0000000
37850.0000000	6.7500000	-50.0000000
37850.0000000	7.0000000	-50.0000000
37850.0000000	7.2500000	-50.0000000
37850.0000000	7.5000000	-50.0000000
37850.0000000	7.7500000	-50.0000000
37850.0000000	8.0000000	-50.0000000
37850.0000000	8.2500000	-50.0000000
37850.0000000	8.5000000	-50.0000000
37850.0000000	8.7500000	-50.0000000
37850.0000000	9.0000000	-50.0000000
37850.0000000	9.2500000	-50.0000000
37850.0000000	9.5000000	-50.0000000
37850.0000000	9.7500000	-50.0000000
37850.0000000	10.0000000	-50.0000000
37850.0000000	10.2500000	-50.0000000
37850.0000000	10.5000000	-50.0000000
37850.0000000	10.7500000	-50.0000000
37850.0000000	11.0000000	-50.0000000
37850.0000000	11.2500000	-50.0000000
37850.0000000	11.5000000	-50.0000000
37850.0000000	11.7500000	-50.0000000
37850.0000000	12.0000000	-50.0000000

37850.0000000	12.2500000	-50.0000000
37850.0000000	12.5000000	-50.0000000
37850.0000000	12.7500000	-50.0000000
37850.0000000	13.0000000	-50.0000000
37850.0000000	13.2500000	-50.0000000
37850.0000000	13.5000000	-50.0000000
37850.0000000	13.7500000	-50.0000000
37850.0000000	14.0000000	-50.0000000
37850.0000000	14.2500000	-50.0000000
37850.0000000	14.5000000	-50.0000000
37850.0000000	14.7500000	-50.0000000
37850.0000000	15.0000000	-50.0000000
37880.0000000	0.0000000	-50.0000000
37880.0000000	0.2500000	-50.0000000
37880.0000000	0.5000000	-50.0000000
37880.0000000	0.7500000	-50.0000000
37880.0000000	1.0000000	-50.0000000
37880.0000000	1.2500000	-50.0000000
37880.0000000	1.5000000	-50.0000000
37880.0000000	1.7500000	-50.0000000
37880.0000000	2.0000000	-50.0000000
37880.0000000	2.2500000	-50.0000000
37880.0000000	2.5000000	-50.0000000
37880.0000000	2.7500000	-50.0000000
37880.0000000	3.0000000	-50.0000000
37880.0000000	3.2500000	-50.0000000
37880.0000000	3.5000000	-50.0000000
37880.0000000	3.7500000	-50.0000000
37880.0000000	4.0000000	-50.0000000
37880.0000000	4.2500000	-50.0000000
37880.0000000	4.5000000	-50.0000000
37880.0000000	4.7500000	-50.0000000
37880.0000000	5.0000000	-50.0000000
37880.0000000	5.2500000	-50.0000000
37880.0000000	5.5000000	-50.0000000
37880.0000000	5.7500000	-50.0000000
37880.0000000	6.0000000	-50.0000000
37880.0000000	6.2500000	-50.0000000
37880.0000000	6.5000000	-50.0000000
37880.0000000	6.7500000	-50.0000000
37880.0000000	7.0000000	-50.0000000
37880.0000000	7.2500000	-50.0000000
37880.0000000	7.5000000	-50.0000000
37880.0000000	7.7500000	-50.0000000
37880.0000000	8.0000000	-50.0000000
37880.0000000	8.2500000	-50.0000000
37880.0000000	8.5000000	-50.0000000
37880.0000000	8.7500000	-50.0000000
37880.0000000	9.0000000	-50.0000000
37880.0000000	9.2500000	-50.0000000
37880.0000000	9.5000000	-50.0000000
37880.0000000	9.7500000	-50.0000000
37880.0000000	10.0000000	-50.0000000
37880.0000000	10.2500000	-50.0000000
37880.0000000	10.5000000	-50.0000000
37880.0000000	10.7500000	-50.0000000
37880.0000000	11.0000000	-50.0000000
37880.0000000	11.2500000	-50.0000000

37880.0000000	11.5000000	-50.0000000
37880.0000000	11.7500000	-50.0000000
37880.0000000	12.0000000	-50.0000000
37880.0000000	12.2500000	-50.0000000
37880.0000000	12.5000000	-50.0000000
37880.0000000	12.7500000	-50.0000000
37880.0000000	13.0000000	-50.0000000
37880.0000000	13.2500000	-50.0000000
37880.0000000	13.5000000	-50.0000000
37880.0000000	13.7500000	-50.0000000
37880.0000000	14.0000000	-50.0000000
37880.0000000	14.2500000	-50.0000000
37880.0000000	14.5000000	-50.0000000
37880.0000000	14.7500000	-50.0000000
37880.0000000	15.0000000	-50.0000000
37910.0000000	0.0000000	-50.0000000
37910.0000000	0.2500000	-50.0000000
37910.0000000	0.5000000	-50.0000000
37910.0000000	0.7500000	-50.0000000
37910.0000000	1.0000000	-50.0000000
37910.0000000	1.2500000	-50.0000000
37910.0000000	1.5000000	-50.0000000
37910.0000000	1.7500000	-50.0000000
37910.0000000	2.0000000	-50.0000000
37910.0000000	2.2500000	-50.0000000
37910.0000000	2.5000000	-50.0000000
37910.0000000	2.7500000	-50.0000000
37910.0000000	3.0000000	-50.0000000
37910.0000000	3.2500000	-50.0000000
37910.0000000	3.5000000	-50.0000000
37910.0000000	3.7500000	-50.0000000
37910.0000000	4.0000000	-50.0000000
37910.0000000	4.2500000	-50.0000000
37910.0000000	4.5000000	-50.0000000
37910.0000000	4.7500000	-50.0000000
37910.0000000	5.0000000	-50.0000000
37910.0000000	5.2500000	-50.0000000
37910.0000000	5.5000000	-50.0000000
37910.0000000	5.7500000	-50.0000000
37910.0000000	6.0000000	-50.0000000
37910.0000000	6.2500000	-50.0000000
37910.0000000	6.5000000	-50.0000000
37910.0000000	6.7500000	-50.0000000
37910.0000000	7.0000000	-50.0000000
37910.0000000	7.2500000	-50.0000000
37910.0000000	7.5000000	-50.0000000
37910.0000000	7.7500000	-50.0000000
37910.0000000	8.0000000	-50.0000000
37910.0000000	8.2500000	-50.0000000
37910.0000000	8.5000000	-50.0000000
37910.0000000	8.7500000	-50.0000000
37910.0000000	9.0000000	-50.0000000
37910.0000000	9.2500000	-50.0000000
37910.0000000	9.5000000	-50.0000000
37910.0000000	9.7500000	-50.0000000
37910.0000000	10.0000000	-50.0000000
37910.0000000	10.2500000	-50.0000000
37910.0000000	10.5000000	-50.0000000

37910.0000000	10.7500000	-50.0000000
37910.0000000	11.0000000	-50.0000000
37910.0000000	11.2500000	-50.0000000
37910.0000000	11.5000000	-50.0000000
37910.0000000	11.7500000	-50.0000000
37910.0000000	12.0000000	-50.0000000
37910.0000000	12.2500000	-50.0000000
37910.0000000	12.5000000	-50.0000000
37910.0000000	12.7500000	-50.0000000
37910.0000000	13.0000000	-50.0000000
37910.0000000	13.2500000	-50.0000000
37910.0000000	13.5000000	-50.0000000
37910.0000000	13.7500000	-50.0000000
37910.0000000	14.0000000	-50.0000000
37910.0000000	14.2500000	-50.0000000
37910.0000000	14.5000000	-50.0000000
37910.0000000	14.7500000	-50.0000000
37910.0000000	15.0000000	-50.0000000
37940.0000000	0.0000000	-50.0000000
37940.0000000	0.2500000	-50.0000000
37940.0000000	0.5000000	-50.0000000
37940.0000000	0.7500000	-50.0000000
37940.0000000	1.0000000	-50.0000000
37940.0000000	1.2500000	-50.0000000
37940.0000000	1.5000000	-50.0000000
37940.0000000	1.7500000	-50.0000000
37940.0000000	2.0000000	-50.0000000
37940.0000000	2.2500000	-50.0000000
37940.0000000	2.5000000	-50.0000000
37940.0000000	2.7500000	-50.0000000
37940.0000000	3.0000000	-50.0000000
37940.0000000	3.2500000	-50.0000000
37940.0000000	3.5000000	-50.0000000
37940.0000000	3.7500000	-50.0000000
37940.0000000	4.0000000	-50.0000000
37940.0000000	4.2500000	-50.0000000
37940.0000000	4.5000000	-50.0000000
37940.0000000	4.7500000	-50.0000000
37940.0000000	5.0000000	-50.0000000
37940.0000000	5.2500000	-50.0000000
37940.0000000	5.5000000	-50.0000000
37940.0000000	5.7500000	-50.0000000
37940.0000000	6.0000000	-50.0000000
37940.0000000	6.2500000	-50.0000000
37940.0000000	6.5000000	-50.0000000
37940.0000000	6.7500000	-50.0000000
37940.0000000	7.0000000	-50.0000000
37940.0000000	7.2500000	-50.0000000
37940.0000000	7.5000000	-50.0000000
37940.0000000	7.7500000	-50.0000000
37940.0000000	8.0000000	-50.0000000
37940.0000000	8.2500000	-50.0000000
37940.0000000	8.5000000	-50.0000000
37940.0000000	8.7500000	-50.0000000
37940.0000000	9.0000000	-50.0000000
37940.0000000	9.2500000	-50.0000000
37940.0000000	9.5000000	-50.0000000
37940.0000000	9.7500000	-50.0000000

37940.0000000	10.0000000	-50.0000000
37940.0000000	10.2500000	-50.0000000
37940.0000000	10.5000000	-50.0000000
37940.0000000	10.7500000	-50.0000000
37940.0000000	11.0000000	-50.0000000
37940.0000000	11.2500000	-50.0000000
37940.0000000	11.5000000	-50.0000000
37940.0000000	11.7500000	-50.0000000
37940.0000000	12.0000000	-50.0000000
37940.0000000	12.2500000	-50.0000000
37940.0000000	12.5000000	-50.0000000
37940.0000000	12.7500000	-50.0000000
37940.0000000	13.0000000	-50.0000000
37940.0000000	13.2500000	-50.0000000
37940.0000000	13.5000000	-50.0000000
37940.0000000	13.7500000	-50.0000000
37940.0000000	14.0000000	-50.0000000
37940.0000000	14.2500000	-50.0000000
37940.0000000	14.5000000	-50.0000000
37940.0000000	14.7500000	-50.0000000
37940.0000000	15.0000000	-50.0000000
37970.0000000	0.0000000	-50.0000000
37970.0000000	0.2500000	-50.0000000
37970.0000000	0.5000000	-50.0000000
37970.0000000	0.7500000	-50.0000000
37970.0000000	1.0000000	-50.0000000
37970.0000000	1.2500000	-50.0000000
37970.0000000	1.5000000	-50.0000000
37970.0000000	1.7500000	-50.0000000
37970.0000000	2.0000000	-50.0000000
37970.0000000	2.2500000	-50.0000000
37970.0000000	2.5000000	-50.0000000
37970.0000000	2.7500000	-50.0000000
37970.0000000	3.0000000	-50.0000000
37970.0000000	3.2500000	-50.0000000
37970.0000000	3.5000000	-50.0000000
37970.0000000	3.7500000	-50.0000000
37970.0000000	4.0000000	-50.0000000
37970.0000000	4.2500000	-50.0000000
37970.0000000	4.5000000	-50.0000000
37970.0000000	4.7500000	-50.0000000
37970.0000000	5.0000000	-50.0000000
37970.0000000	5.2500000	-50.0000000
37970.0000000	5.5000000	-50.0000000
37970.0000000	5.7500000	-50.0000000
37970.0000000	6.0000000	-50.0000000
37970.0000000	6.2500000	-50.0000000
37970.0000000	6.5000000	-50.0000000
37970.0000000	6.7500000	-50.0000000
37970.0000000	7.0000000	-50.0000000
37970.0000000	7.2500000	-50.0000000
37970.0000000	7.5000000	-50.0000000
37970.0000000	7.7500000	-50.0000000
37970.0000000	8.0000000	-50.0000000
37970.0000000	8.2500000	-50.0000000
37970.0000000	8.5000000	-50.0000000
37970.0000000	8.7500000	-50.0000000
37970.0000000	9.0000000	-50.0000000

37970.0000000	9.2500000	-50.0000000
37970.0000000	9.5000000	-50.0000000
37970.0000000	9.7500000	-50.0000000
37970.0000000	10.0000000	-50.0000000
37970.0000000	10.2500000	-50.0000000
37970.0000000	10.5000000	-50.0000000
37970.0000000	10.7500000	-50.0000000
37970.0000000	11.0000000	-50.0000000
37970.0000000	11.2500000	-50.0000000
37970.0000000	11.5000000	-50.0000000
37970.0000000	11.7500000	-50.0000000
37970.0000000	12.0000000	-50.0000000
37970.0000000	12.2500000	-50.0000000
37970.0000000	12.5000000	-50.0000000
37970.0000000	12.7500000	-50.0000000
37970.0000000	13.0000000	-50.0000000
37970.0000000	13.2500000	-50.0000000
37970.0000000	13.5000000	-50.0000000
37970.0000000	13.7500000	-50.0000000
37970.0000000	14.0000000	-50.0000000
37970.0000000	14.2500000	-50.0000000
37970.0000000	14.5000000	-50.0000000
37970.0000000	14.7500000	-50.0000000
37970.0000000	15.0000000	-50.0000000
38000.0000000	0.0000000	-50.0000000
38000.0000000	0.2500000	-50.0000000
38000.0000000	0.5000000	-50.0000000
38000.0000000	0.7500000	-50.0000000
38000.0000000	1.0000000	-50.0000000
38000.0000000	1.2500000	-50.0000000
38000.0000000	1.5000000	-50.0000000
38000.0000000	1.7500000	-50.0000000
38000.0000000	2.0000000	-50.0000000
38000.0000000	2.2500000	-50.0000000
38000.0000000	2.5000000	-50.0000000
38000.0000000	2.7500000	-50.0000000
38000.0000000	3.0000000	-50.0000000
38000.0000000	3.2500000	-50.0000000
38000.0000000	3.5000000	-50.0000000
38000.0000000	3.7500000	-50.0000000
38000.0000000	4.0000000	-50.0000000
38000.0000000	4.2500000	-50.0000000
38000.0000000	4.5000000	-50.0000000
38000.0000000	4.7500000	-50.0000000
38000.0000000	5.0000000	-50.0000000
38000.0000000	5.2500000	-50.0000000
38000.0000000	5.5000000	-50.0000000
38000.0000000	5.7500000	-50.0000000
38000.0000000	6.0000000	-50.0000000
38000.0000000	6.2500000	-50.0000000
38000.0000000	6.5000000	-50.0000000
38000.0000000	6.7500000	-50.0000000
38000.0000000	7.0000000	-50.0000000
38000.0000000	7.2500000	-50.0000000
38000.0000000	7.5000000	-50.0000000
38000.0000000	7.7500000	-50.0000000
38000.0000000	8.0000000	-50.0000000
38000.0000000	8.2500000	-50.0000000

38000.0000000	8.5000000	-50.0000000
38000.0000000	8.7500000	-50.0000000
38000.0000000	9.0000000	-50.0000000
38000.0000000	9.2500000	-50.0000000
38000.0000000	9.5000000	-50.0000000
38000.0000000	9.7500000	-50.0000000
38000.0000000	10.0000000	-50.0000000
38000.0000000	10.2500000	-50.0000000
38000.0000000	10.5000000	-50.0000000
38000.0000000	10.7500000	-50.0000000
38000.0000000	11.0000000	-50.0000000
38000.0000000	11.2500000	-50.0000000
38000.0000000	11.5000000	-50.0000000
38000.0000000	11.7500000	-50.0000000
38000.0000000	12.0000000	-50.0000000
38000.0000000	12.2500000	-50.0000000
38000.0000000	12.5000000	-50.0000000
38000.0000000	12.7500000	-50.0000000
38000.0000000	13.0000000	-50.0000000
38000.0000000	13.2500000	-50.0000000
38000.0000000	13.5000000	-50.0000000
38000.0000000	13.7500000	-50.0000000
38000.0000000	14.0000000	-50.0000000
38000.0000000	14.2500000	-50.0000000
38000.0000000	14.5000000	-50.0000000
38000.0000000	14.7500000	-50.0000000
38000.0000000	15.0000000	-50.0000000
38030.0000000	0.0000000	-50.0000000
38030.0000000	0.2500000	-50.0000000
38030.0000000	0.5000000	-50.0000000
38030.0000000	0.7500000	-50.0000000
38030.0000000	1.0000000	-50.0000000
38030.0000000	1.2500000	-50.0000000
38030.0000000	1.5000000	-50.0000000
38030.0000000	1.7500000	-50.0000000
38030.0000000	2.0000000	-50.0000000
38030.0000000	2.2500000	-50.0000000
38030.0000000	2.5000000	-50.0000000
38030.0000000	2.7500000	-50.0000000
38030.0000000	3.0000000	-50.0000000
38030.0000000	3.2500000	-50.0000000
38030.0000000	3.5000000	-50.0000000
38030.0000000	3.7500000	-50.0000000
38030.0000000	4.0000000	-50.0000000
38030.0000000	4.2500000	-50.0000000
38030.0000000	4.5000000	-50.0000000
38030.0000000	4.7500000	-50.0000000
38030.0000000	5.0000000	-50.0000000
38030.0000000	5.2500000	-50.0000000
38030.0000000	5.5000000	-50.0000000
38030.0000000	5.7500000	-50.0000000
38030.0000000	6.0000000	-50.0000000
38030.0000000	6.2500000	-50.0000000
38030.0000000	6.5000000	-50.0000000
38030.0000000	6.7500000	-50.0000000
38030.0000000	7.0000000	-50.0000000
38030.0000000	7.2500000	-50.0000000
38030.0000000	7.5000000	-50.0000000

38030.0000000	7.7500000	-50.0000000
38030.0000000	8.0000000	-50.0000000
38030.0000000	8.2500000	-50.0000000
38030.0000000	8.5000000	-50.0000000
38030.0000000	8.7500000	-50.0000000
38030.0000000	9.0000000	-50.0000000
38030.0000000	9.2500000	-50.0000000
38030.0000000	9.5000000	-50.0000000
38030.0000000	9.7500000	-50.0000000
38030.0000000	10.0000000	-50.0000000
38030.0000000	10.2500000	-50.0000000
38030.0000000	10.5000000	-50.0000000
38030.0000000	10.7500000	-50.0000000
38030.0000000	11.0000000	-50.0000000
38030.0000000	11.2500000	-50.0000000
38030.0000000	11.5000000	-50.0000000
38030.0000000	11.7500000	-50.0000000
38030.0000000	12.0000000	-50.0000000
38030.0000000	12.2500000	-50.0000000
38030.0000000	12.5000000	-50.0000000
38030.0000000	12.7500000	-50.0000000
38030.0000000	13.0000000	-50.0000000
38030.0000000	13.2500000	-50.0000000
38030.0000000	13.5000000	-50.0000000
38030.0000000	13.7500000	-50.0000000
38030.0000000	14.0000000	-50.0000000
38030.0000000	14.2500000	-50.0000000
38030.0000000	14.5000000	-50.0000000
38030.0000000	14.7500000	-50.0000000
38030.0000000	15.0000000	-50.0000000
38060.0000000	0.0000000	-50.0000000
38060.0000000	0.2500000	-50.0000000
38060.0000000	0.5000000	-50.0000000
38060.0000000	0.7500000	-50.0000000
38060.0000000	1.0000000	-50.0000000
38060.0000000	1.2500000	-50.0000000
38060.0000000	1.5000000	-50.0000000
38060.0000000	1.7500000	-50.0000000
38060.0000000	2.0000000	-50.0000000
38060.0000000	2.2500000	-50.0000000
38060.0000000	2.5000000	-50.0000000
38060.0000000	2.7500000	-50.0000000
38060.0000000	3.0000000	-50.0000000
38060.0000000	3.2500000	-50.0000000
38060.0000000	3.5000000	-50.0000000
38060.0000000	3.7500000	-50.0000000
38060.0000000	4.0000000	-50.0000000
38060.0000000	4.2500000	-50.0000000
38060.0000000	4.5000000	-50.0000000
38060.0000000	4.7500000	-50.0000000
38060.0000000	5.0000000	-50.0000000
38060.0000000	5.2500000	-50.0000000
38060.0000000	5.5000000	-50.0000000
38060.0000000	5.7500000	-50.0000000
38060.0000000	6.0000000	-50.0000000
38060.0000000	6.2500000	-50.0000000
38060.0000000	6.5000000	-50.0000000
38060.0000000	6.7500000	-50.0000000

38060.0000000	7.0000000	-50.0000000
38060.0000000	7.2500000	-50.0000000
38060.0000000	7.5000000	-50.0000000
38060.0000000	7.7500000	-50.0000000
38060.0000000	8.0000000	-50.0000000
38060.0000000	8.2500000	-50.0000000
38060.0000000	8.5000000	-50.0000000
38060.0000000	8.7500000	-50.0000000
38060.0000000	9.0000000	-50.0000000
38060.0000000	9.2500000	-50.0000000
38060.0000000	9.5000000	-50.0000000
38060.0000000	9.7500000	-50.0000000
38060.0000000	10.0000000	-50.0000000
38060.0000000	10.2500000	-50.0000000
38060.0000000	10.5000000	-50.0000000
38060.0000000	10.7500000	-50.0000000
38060.0000000	11.0000000	-50.0000000
38060.0000000	11.2500000	-50.0000000
38060.0000000	11.5000000	-50.0000000
38060.0000000	11.7500000	-50.0000000
38060.0000000	12.0000000	-50.0000000
38060.0000000	12.2500000	-50.0000000
38060.0000000	12.5000000	-50.0000000
38060.0000000	12.7500000	-50.0000000
38060.0000000	13.0000000	-50.0000000
38060.0000000	13.2500000	-50.0000000
38060.0000000	13.5000000	-50.0000000
38060.0000000	13.7500000	-50.0000000
38060.0000000	14.0000000	-50.0000000
38060.0000000	14.2500000	-50.0000000
38060.0000000	14.5000000	-50.0000000
38060.0000000	14.7500000	-50.0000000
38060.0000000	15.0000000	-50.0000000
38090.0000000	0.0000000	-50.0000000
38090.0000000	0.2500000	-50.0000000
38090.0000000	0.5000000	-50.0000000
38090.0000000	0.7500000	-50.0000000
38090.0000000	1.0000000	-50.0000000
38090.0000000	1.2500000	-50.0000000
38090.0000000	1.5000000	-50.0000000
38090.0000000	1.7500000	-50.0000000
38090.0000000	2.0000000	-50.0000000
38090.0000000	2.2500000	-50.0000000
38090.0000000	2.5000000	-50.0000000
38090.0000000	2.7500000	-50.0000000
38090.0000000	3.0000000	-50.0000000
38090.0000000	3.2500000	-50.0000000
38090.0000000	3.5000000	-50.0000000
38090.0000000	3.7500000	-50.0000000
38090.0000000	4.0000000	-50.0000000
38090.0000000	4.2500000	-50.0000000
38090.0000000	4.5000000	-50.0000000
38090.0000000	4.7500000	-50.0000000
38090.0000000	5.0000000	-50.0000000
38090.0000000	5.2500000	-50.0000000
38090.0000000	5.5000000	-50.0000000
38090.0000000	5.7500000	-50.0000000
38090.0000000	6.0000000	-50.0000000

38090.0000000	6.2500000	-50.0000000
38090.0000000	6.5000000	-50.0000000
38090.0000000	6.7500000	-50.0000000
38090.0000000	7.0000000	-50.0000000
38090.0000000	7.2500000	-50.0000000
38090.0000000	7.5000000	-50.0000000
38090.0000000	7.7500000	-50.0000000
38090.0000000	8.0000000	-50.0000000
38090.0000000	8.2500000	-50.0000000
38090.0000000	8.5000000	-50.0000000
38090.0000000	8.7500000	-50.0000000
38090.0000000	9.0000000	-50.0000000
38090.0000000	9.2500000	-50.0000000
38090.0000000	9.5000000	-50.0000000
38090.0000000	9.7500000	-50.0000000
38090.0000000	10.0000000	-50.0000000
38090.0000000	10.2500000	-50.0000000
38090.0000000	10.5000000	-50.0000000
38090.0000000	10.7500000	-50.0000000
38090.0000000	11.0000000	-50.0000000
38090.0000000	11.2500000	-50.0000000
38090.0000000	11.5000000	-50.0000000
38090.0000000	11.7500000	-50.0000000
38090.0000000	12.0000000	-50.0000000
38090.0000000	12.2500000	-50.0000000
38090.0000000	12.5000000	-50.0000000
38090.0000000	12.7500000	-50.0000000
38090.0000000	13.0000000	-50.0000000
38090.0000000	13.2500000	-50.0000000
38090.0000000	13.5000000	-50.0000000
38090.0000000	13.7500000	-50.0000000
38090.0000000	14.0000000	-50.0000000
38090.0000000	14.2500000	-50.0000000
38090.0000000	14.5000000	-50.0000000
38090.0000000	14.7500000	-50.0000000
38090.0000000	15.0000000	-50.0000000
38120.0000000	0.0000000	-50.0000000
38120.0000000	0.2500000	-50.0000000
38120.0000000	0.5000000	-50.0000000
38120.0000000	0.7500000	-50.0000000
38120.0000000	1.0000000	-50.0000000
38120.0000000	1.2500000	-50.0000000
38120.0000000	1.5000000	-50.0000000
38120.0000000	1.7500000	-50.0000000
38120.0000000	2.0000000	-50.0000000
38120.0000000	2.2500000	-50.0000000
38120.0000000	2.5000000	-50.0000000
38120.0000000	2.7500000	-50.0000000
38120.0000000	3.0000000	-50.0000000
38120.0000000	3.2500000	-50.0000000
38120.0000000	3.5000000	-50.0000000
38120.0000000	3.7500000	-50.0000000
38120.0000000	4.0000000	-50.0000000
38120.0000000	4.2500000	-50.0000000
38120.0000000	4.5000000	-50.0000000
38120.0000000	4.7500000	-50.0000000
38120.0000000	5.0000000	-50.0000000
38120.0000000	5.2500000	-50.0000000

38120.0000000	5.5000000	-50.0000000
38120.0000000	5.7500000	-50.0000000
38120.0000000	6.0000000	-50.0000000
38120.0000000	6.2500000	-50.0000000
38120.0000000	6.5000000	-50.0000000
38120.0000000	6.7500000	-50.0000000
38120.0000000	7.0000000	-50.0000000
38120.0000000	7.2500000	-50.0000000
38120.0000000	7.5000000	-50.0000000
38120.0000000	7.7500000	-50.0000000
38120.0000000	8.0000000	-50.0000000
38120.0000000	8.2500000	-50.0000000
38120.0000000	8.5000000	-50.0000000
38120.0000000	8.7500000	-50.0000000
38120.0000000	9.0000000	-50.0000000
38120.0000000	9.2500000	-50.0000000
38120.0000000	9.5000000	-50.0000000
38120.0000000	9.7500000	-50.0000000
38120.0000000	10.0000000	-50.0000000
38120.0000000	10.2500000	-50.0000000
38120.0000000	10.5000000	-50.0000000
38120.0000000	10.7500000	-50.0000000
38120.0000000	11.0000000	-50.0000000
38120.0000000	11.2500000	-50.0000000
38120.0000000	11.5000000	-50.0000000
38120.0000000	11.7500000	-50.0000000
38120.0000000	12.0000000	-50.0000000
38120.0000000	12.2500000	-50.0000000
38120.0000000	12.5000000	-50.0000000
38120.0000000	12.7500000	-50.0000000
38120.0000000	13.0000000	-50.0000000
38120.0000000	13.2500000	-50.0000000
38120.0000000	13.5000000	-50.0000000
38120.0000000	13.7500000	-50.0000000
38120.0000000	14.0000000	-50.0000000
38120.0000000	14.2500000	-50.0000000
38120.0000000	14.5000000	-50.0000000
38120.0000000	14.7500000	-50.0000000
38120.0000000	15.0000000	-50.0000000
38150.0000000	0.0000000	-50.0000000
38150.0000000	0.2500000	-50.0000000
38150.0000000	0.5000000	-50.0000000
38150.0000000	0.7500000	-50.0000000
38150.0000000	1.0000000	-50.0000000
38150.0000000	1.2500000	-50.0000000
38150.0000000	1.5000000	-50.0000000
38150.0000000	1.7500000	-50.0000000
38150.0000000	2.0000000	-50.0000000
38150.0000000	2.2500000	-50.0000000
38150.0000000	2.5000000	-50.0000000
38150.0000000	2.7500000	-50.0000000
38150.0000000	3.0000000	-50.0000000
38150.0000000	3.2500000	-50.0000000
38150.0000000	3.5000000	-50.0000000
38150.0000000	3.7500000	-50.0000000
38150.0000000	4.0000000	-50.0000000
38150.0000000	4.2500000	-50.0000000
38150.0000000	4.5000000	-50.0000000

38150.0000000	4.7500000	-50.0000000
38150.0000000	5.0000000	-50.0000000
38150.0000000	5.2500000	-50.0000000
38150.0000000	5.5000000	-50.0000000
38150.0000000	5.7500000	-50.0000000
38150.0000000	6.0000000	-50.0000000
38150.0000000	6.2500000	-50.0000000
38150.0000000	6.5000000	-50.0000000
38150.0000000	6.7500000	-50.0000000
38150.0000000	7.0000000	-50.0000000
38150.0000000	7.2500000	-50.0000000
38150.0000000	7.5000000	-50.0000000
38150.0000000	7.7500000	-50.0000000
38150.0000000	8.0000000	-50.0000000
38150.0000000	8.2500000	-50.0000000
38150.0000000	8.5000000	-50.0000000
38150.0000000	8.7500000	-50.0000000
38150.0000000	9.0000000	-50.0000000
38150.0000000	9.2500000	-50.0000000
38150.0000000	9.5000000	-50.0000000
38150.0000000	9.7500000	-50.0000000
38150.0000000	10.0000000	-50.0000000
38150.0000000	10.2500000	-50.0000000
38150.0000000	10.5000000	-50.0000000
38150.0000000	10.7500000	-50.0000000
38150.0000000	11.0000000	-50.0000000
38150.0000000	11.2500000	-50.0000000
38150.0000000	11.5000000	-50.0000000
38150.0000000	11.7500000	-50.0000000
38150.0000000	12.0000000	-50.0000000
38150.0000000	12.2500000	-50.0000000
38150.0000000	12.5000000	-50.0000000
38150.0000000	12.7500000	-50.0000000
38150.0000000	13.0000000	-50.0000000
38150.0000000	13.2500000	-50.0000000
38150.0000000	13.5000000	-50.0000000
38150.0000000	13.7500000	-50.0000000
38150.0000000	14.0000000	-50.0000000
38150.0000000	14.2500000	-50.0000000
38150.0000000	14.5000000	-50.0000000
38150.0000000	14.7500000	-50.0000000
38150.0000000	15.0000000	-50.0000000
38180.0000000	0.0000000	-50.0000000
38180.0000000	0.2500000	-50.0000000
38180.0000000	0.5000000	-50.0000000
38180.0000000	0.7500000	-50.0000000
38180.0000000	1.0000000	-50.0000000
38180.0000000	1.2500000	-50.0000000
38180.0000000	1.5000000	-50.0000000
38180.0000000	1.7500000	-50.0000000
38180.0000000	2.0000000	-50.0000000
38180.0000000	2.2500000	-50.0000000
38180.0000000	2.5000000	-50.0000000
38180.0000000	2.7500000	-50.0000000
38180.0000000	3.0000000	-50.0000000
38180.0000000	3.2500000	-50.0000000
38180.0000000	3.5000000	-50.0000000
38180.0000000	3.7500000	-50.0000000

38180.0000000	4.0000000	-50.0000000
38180.0000000	4.2500000	-50.0000000
38180.0000000	4.5000000	-50.0000000
38180.0000000	4.7500000	-50.0000000
38180.0000000	5.0000000	-50.0000000
38180.0000000	5.2500000	-50.0000000
38180.0000000	5.5000000	-50.0000000
38180.0000000	5.7500000	-50.0000000
38180.0000000	6.0000000	-50.0000000
38180.0000000	6.2500000	-50.0000000
38180.0000000	6.5000000	-50.0000000
38180.0000000	6.7500000	-50.0000000
38180.0000000	7.0000000	-50.0000000
38180.0000000	7.2500000	-50.0000000
38180.0000000	7.5000000	-50.0000000
38180.0000000	7.7500000	-50.0000000
38180.0000000	8.0000000	-50.0000000
38180.0000000	8.2500000	-50.0000000
38180.0000000	8.5000000	-50.0000000
38180.0000000	8.7500000	-50.0000000
38180.0000000	9.0000000	-50.0000000
38180.0000000	9.2500000	-50.0000000
38180.0000000	9.5000000	-50.0000000
38180.0000000	9.7500000	-50.0000000
38180.0000000	10.0000000	-50.0000000
38180.0000000	10.2500000	-50.0000000
38180.0000000	10.5000000	-50.0000000
38180.0000000	10.7500000	-50.0000000
38180.0000000	11.0000000	-50.0000000
38180.0000000	11.2500000	-50.0000000
38180.0000000	11.5000000	-50.0000000
38180.0000000	11.7500000	-50.0000000
38180.0000000	12.0000000	-50.0000000
38180.0000000	12.2500000	-50.0000000
38180.0000000	12.5000000	-50.0000000
38180.0000000	12.7500000	-50.0000000
38180.0000000	13.0000000	-50.0000000
38180.0000000	13.2500000	-50.0000000
38180.0000000	13.5000000	-50.0000000
38180.0000000	13.7500000	-50.0000000
38180.0000000	14.0000000	-50.0000000
38180.0000000	14.2500000	-50.0000000
38180.0000000	14.5000000	-50.0000000
38180.0000000	14.7500000	-50.0000000
38180.0000000	15.0000000	-50.0000000
38210.0000000	0.0000000	-50.0000000
38210.0000000	0.2500000	-50.0000000
38210.0000000	0.5000000	-50.0000000
38210.0000000	0.7500000	-50.0000000
38210.0000000	1.0000000	-50.0000000
38210.0000000	1.2500000	-50.0000000
38210.0000000	1.5000000	-50.0000000
38210.0000000	1.7500000	-50.0000000
38210.0000000	2.0000000	-50.0000000
38210.0000000	2.2500000	-50.0000000
38210.0000000	2.5000000	-50.0000000
38210.0000000	2.7500000	-50.0000000
38210.0000000	3.0000000	-50.0000000

38210.0000000	3.2500000	-50.0000000
38210.0000000	3.5000000	-50.0000000
38210.0000000	3.7500000	-50.0000000
38210.0000000	4.0000000	-50.0000000
38210.0000000	4.2500000	-50.0000000
38210.0000000	4.5000000	-50.0000000
38210.0000000	4.7500000	-50.0000000
38210.0000000	5.0000000	-50.0000000
38210.0000000	5.2500000	-50.0000000
38210.0000000	5.5000000	-50.0000000
38210.0000000	5.7500000	-50.0000000
38210.0000000	6.0000000	-50.0000000
38210.0000000	6.2500000	-50.0000000
38210.0000000	6.5000000	-50.0000000
38210.0000000	6.7500000	-50.0000000
38210.0000000	7.0000000	-50.0000000
38210.0000000	7.2500000	-50.0000000
38210.0000000	7.5000000	-50.0000000
38210.0000000	7.7500000	-50.0000000
38210.0000000	8.0000000	-50.0000000
38210.0000000	8.2500000	-50.0000000
38210.0000000	8.5000000	-50.0000000
38210.0000000	8.7500000	-50.0000000
38210.0000000	9.0000000	-50.0000000
38210.0000000	9.2500000	-50.0000000
38210.0000000	9.5000000	-50.0000000
38210.0000000	9.7500000	-50.0000000
38210.0000000	10.0000000	-50.0000000
38210.0000000	10.2500000	-50.0000000
38210.0000000	10.5000000	-50.0000000
38210.0000000	10.7500000	-50.0000000
38210.0000000	11.0000000	-50.0000000
38210.0000000	11.2500000	-50.0000000
38210.0000000	11.5000000	-50.0000000
38210.0000000	11.7500000	-50.0000000
38210.0000000	12.0000000	-50.0000000
38210.0000000	12.2500000	-50.0000000
38210.0000000	12.5000000	-50.0000000
38210.0000000	12.7500000	-50.0000000
38210.0000000	13.0000000	-50.0000000
38210.0000000	13.2500000	-50.0000000
38210.0000000	13.5000000	-50.0000000
38210.0000000	13.7500000	-50.0000000
38210.0000000	14.0000000	-50.0000000
38210.0000000	14.2500000	-50.0000000
38210.0000000	14.5000000	-50.0000000
38210.0000000	14.7500000	-50.0000000
38210.0000000	15.0000000	-50.0000000
38240.0000000	0.0000000	-50.0000000
38240.0000000	0.2500000	-50.0000000
38240.0000000	0.5000000	-50.0000000
38240.0000000	0.7500000	-50.0000000
38240.0000000	1.0000000	-50.0000000
38240.0000000	1.2500000	-50.0000000
38240.0000000	1.5000000	-50.0000000
38240.0000000	1.7500000	-50.0000000
38240.0000000	2.0000000	-50.0000000
38240.0000000	2.2500000	-50.0000000

38240.0000000	2.5000000	-50.0000000
38240.0000000	2.7500000	-50.0000000
38240.0000000	3.0000000	-50.0000000
38240.0000000	3.2500000	-50.0000000
38240.0000000	3.5000000	-50.0000000
38240.0000000	3.7500000	-50.0000000
38240.0000000	4.0000000	-50.0000000
38240.0000000	4.2500000	-50.0000000
38240.0000000	4.5000000	-50.0000000
38240.0000000	4.7500000	-50.0000000
38240.0000000	5.0000000	-50.0000000
38240.0000000	5.2500000	-50.0000000
38240.0000000	5.5000000	-50.0000000
38240.0000000	5.7500000	-50.0000000
38240.0000000	6.0000000	-50.0000000
38240.0000000	6.2500000	-50.0000000
38240.0000000	6.5000000	-50.0000000
38240.0000000	6.7500000	-50.0000000
38240.0000000	7.0000000	-50.0000000
38240.0000000	7.2500000	-50.0000000
38240.0000000	7.5000000	-50.0000000
38240.0000000	7.7500000	-50.0000000
38240.0000000	8.0000000	-50.0000000
38240.0000000	8.2500000	-50.0000000
38240.0000000	8.5000000	-50.0000000
38240.0000000	8.7500000	-50.0000000
38240.0000000	9.0000000	-50.0000000
38240.0000000	9.2500000	-50.0000000
38240.0000000	9.5000000	-50.0000000
38240.0000000	9.7500000	-50.0000000
38240.0000000	10.0000000	-50.0000000
38240.0000000	10.2500000	-50.0000000
38240.0000000	10.5000000	-50.0000000
38240.0000000	10.7500000	-50.0000000
38240.0000000	11.0000000	-50.0000000
38240.0000000	11.2500000	-50.0000000
38240.0000000	11.5000000	-50.0000000
38240.0000000	11.7500000	-50.0000000
38240.0000000	12.0000000	-50.0000000
38240.0000000	12.2500000	-50.0000000
38240.0000000	12.5000000	-50.0000000
38240.0000000	12.7500000	-50.0000000
38240.0000000	13.0000000	-50.0000000
38240.0000000	13.2500000	-50.0000000
38240.0000000	13.5000000	-50.0000000
38240.0000000	13.7500000	-50.0000000
38240.0000000	14.0000000	-50.0000000
38240.0000000	14.2500000	-50.0000000
38240.0000000	14.5000000	-50.0000000
38240.0000000	14.7500000	-50.0000000
38240.0000000	15.0000000	-50.0000000
38270.0000000	0.0000000	-50.0000000
38270.0000000	0.2500000	-50.0000000
38270.0000000	0.5000000	-50.0000000
38270.0000000	0.7500000	-50.0000000
38270.0000000	1.0000000	-50.0000000
38270.0000000	1.2500000	-50.0000000
38270.0000000	1.5000000	-50.0000000

38270.0000000	1.7500000	-50.0000000
38270.0000000	2.0000000	-50.0000000
38270.0000000	2.2500000	-50.0000000
38270.0000000	2.5000000	-50.0000000
38270.0000000	2.7500000	-50.0000000
38270.0000000	3.0000000	-50.0000000
38270.0000000	3.2500000	-50.0000000
38270.0000000	3.5000000	-50.0000000
38270.0000000	3.7500000	-50.0000000
38270.0000000	4.0000000	-50.0000000
38270.0000000	4.2500000	-50.0000000
38270.0000000	4.5000000	-50.0000000
38270.0000000	4.7500000	-50.0000000
38270.0000000	5.0000000	-50.0000000
38270.0000000	5.2500000	-50.0000000
38270.0000000	5.5000000	-50.0000000
38270.0000000	5.7500000	-50.0000000
38270.0000000	6.0000000	-50.0000000
38270.0000000	6.2500000	-50.0000000
38270.0000000	6.5000000	-50.0000000
38270.0000000	6.7500000	-50.0000000
38270.0000000	7.0000000	-50.0000000
38270.0000000	7.2500000	-50.0000000
38270.0000000	7.5000000	-50.0000000
38270.0000000	7.7500000	-50.0000000
38270.0000000	8.0000000	-50.0000000
38270.0000000	8.2500000	-50.0000000
38270.0000000	8.5000000	-50.0000000
38270.0000000	8.7500000	-50.0000000
38270.0000000	9.0000000	-50.0000000
38270.0000000	9.2500000	-50.0000000
38270.0000000	9.5000000	-50.0000000
38270.0000000	9.7500000	-50.0000000
38270.0000000	10.0000000	-50.0000000
38270.0000000	10.2500000	-50.0000000
38270.0000000	10.5000000	-50.0000000
38270.0000000	10.7500000	-50.0000000
38270.0000000	11.0000000	-50.0000000
38270.0000000	11.2500000	-50.0000000
38270.0000000	11.5000000	-50.0000000
38270.0000000	11.7500000	-50.0000000
38270.0000000	12.0000000	-50.0000000
38270.0000000	12.2500000	-50.0000000
38270.0000000	12.5000000	-50.0000000
38270.0000000	12.7500000	-50.0000000
38270.0000000	13.0000000	-50.0000000
38270.0000000	13.2500000	-50.0000000
38270.0000000	13.5000000	-50.0000000
38270.0000000	13.7500000	-50.0000000
38270.0000000	14.0000000	-50.0000000
38270.0000000	14.2500000	-50.0000000
38270.0000000	14.5000000	-50.0000000
38270.0000000	14.7500000	-50.0000000
38270.0000000	15.0000000	-50.0000000
38300.0000000	0.0000000	-50.0000000
38300.0000000	0.2500000	-50.0000000
38300.0000000	0.5000000	-50.0000000
38300.0000000	0.7500000	-50.0000000

38300.0000000	1.0000000	-50.0000000
38300.0000000	1.2500000	-50.0000000
38300.0000000	1.5000000	-50.0000000
38300.0000000	1.7500000	-50.0000000
38300.0000000	2.0000000	-50.0000000
38300.0000000	2.2500000	-50.0000000
38300.0000000	2.5000000	-50.0000000
38300.0000000	2.7500000	-50.0000000
38300.0000000	3.0000000	-50.0000000
38300.0000000	3.2500000	-50.0000000
38300.0000000	3.5000000	-50.0000000
38300.0000000	3.7500000	-50.0000000
38300.0000000	4.0000000	-50.0000000
38300.0000000	4.2500000	-50.0000000
38300.0000000	4.5000000	-50.0000000
38300.0000000	4.7500000	-50.0000000
38300.0000000	5.0000000	-50.0000000
38300.0000000	5.2500000	-50.0000000
38300.0000000	5.5000000	-50.0000000
38300.0000000	5.7500000	-50.0000000
38300.0000000	6.0000000	-50.0000000
38300.0000000	6.2500000	-50.0000000
38300.0000000	6.5000000	-50.0000000
38300.0000000	6.7500000	-50.0000000
38300.0000000	7.0000000	-50.0000000
38300.0000000	7.2500000	-50.0000000
38300.0000000	7.5000000	-50.0000000
38300.0000000	7.7500000	-50.0000000
38300.0000000	8.0000000	-50.0000000
38300.0000000	8.2500000	-50.0000000
38300.0000000	8.5000000	-50.0000000
38300.0000000	8.7500000	-50.0000000
38300.0000000	9.0000000	-50.0000000
38300.0000000	9.2500000	-50.0000000
38300.0000000	9.5000000	-50.0000000
38300.0000000	9.7500000	-50.0000000
38300.0000000	10.0000000	-50.0000000
38300.0000000	10.2500000	-50.0000000
38300.0000000	10.5000000	-50.0000000
38300.0000000	10.7500000	-50.0000000
38300.0000000	11.0000000	-50.0000000
38300.0000000	11.2500000	-50.0000000
38300.0000000	11.5000000	-50.0000000
38300.0000000	11.7500000	-50.0000000
38300.0000000	12.0000000	-50.0000000
38300.0000000	12.2500000	-50.0000000
38300.0000000	12.5000000	-50.0000000
38300.0000000	12.7500000	-50.0000000
38300.0000000	13.0000000	-50.0000000
38300.0000000	13.2500000	-50.0000000
38300.0000000	13.5000000	-50.0000000
38300.0000000	13.7500000	-50.0000000
38300.0000000	14.0000000	-50.0000000
38300.0000000	14.2500000	-50.0000000
38300.0000000	14.5000000	-50.0000000
38300.0000000	14.7500000	-50.0000000
38300.0000000	15.0000000	-50.0000000
38330.0000000	0.0000000	-50.0000000

38330.0000000	0.2500000	-50.0000000
38330.0000000	0.5000000	-50.0000000
38330.0000000	0.7500000	-50.0000000
38330.0000000	1.0000000	-50.0000000
38330.0000000	1.2500000	-50.0000000
38330.0000000	1.5000000	-50.0000000
38330.0000000	1.7500000	-50.0000000
38330.0000000	2.0000000	-50.0000000
38330.0000000	2.2500000	-50.0000000
38330.0000000	2.5000000	-50.0000000
38330.0000000	2.7500000	-50.0000000
38330.0000000	3.0000000	-50.0000000
38330.0000000	3.2500000	-50.0000000
38330.0000000	3.5000000	-50.0000000
38330.0000000	3.7500000	-50.0000000
38330.0000000	4.0000000	-50.0000000
38330.0000000	4.2500000	-50.0000000
38330.0000000	4.5000000	-50.0000000
38330.0000000	4.7500000	-50.0000000
38330.0000000	5.0000000	-50.0000000
38330.0000000	5.2500000	-50.0000000
38330.0000000	5.5000000	-50.0000000
38330.0000000	5.7500000	-50.0000000
38330.0000000	6.0000000	-50.0000000
38330.0000000	6.2500000	-50.0000000
38330.0000000	6.5000000	-50.0000000
38330.0000000	6.7500000	-50.0000000
38330.0000000	7.0000000	-50.0000000
38330.0000000	7.2500000	-50.0000000
38330.0000000	7.5000000	-50.0000000
38330.0000000	7.7500000	-50.0000000
38330.0000000	8.0000000	-50.0000000
38330.0000000	8.2500000	-50.0000000
38330.0000000	8.5000000	-50.0000000
38330.0000000	8.7500000	-50.0000000
38330.0000000	9.0000000	-50.0000000
38330.0000000	9.2500000	-50.0000000
38330.0000000	9.5000000	-50.0000000
38330.0000000	9.7500000	-50.0000000
38330.0000000	10.0000000	-50.0000000
38330.0000000	10.2500000	-50.0000000
38330.0000000	10.5000000	-50.0000000
38330.0000000	10.7500000	-50.0000000
38330.0000000	11.0000000	-50.0000000
38330.0000000	11.2500000	-50.0000000
38330.0000000	11.5000000	-50.0000000
38330.0000000	11.7500000	-50.0000000
38330.0000000	12.0000000	-50.0000000
38330.0000000	12.2500000	-50.0000000
38330.0000000	12.5000000	-50.0000000
38330.0000000	12.7500000	-50.0000000
38330.0000000	13.0000000	-50.0000000
38330.0000000	13.2500000	-50.0000000
38330.0000000	13.5000000	-50.0000000
38330.0000000	13.7500000	-50.0000000
38330.0000000	14.0000000	-50.0000000
38330.0000000	14.2500000	-50.0000000
38330.0000000	14.5000000	-50.0000000

38330.0000000	14.7500000	-50.0000000
38330.0000000	15.0000000	-50.0000000
38360.0000000	0.0000000	-50.0000000
38360.0000000	0.2500000	-50.0000000
38360.0000000	0.5000000	-50.0000000
38360.0000000	0.7500000	-50.0000000
38360.0000000	1.0000000	-50.0000000
38360.0000000	1.2500000	-50.0000000
38360.0000000	1.5000000	-50.0000000
38360.0000000	1.7500000	-50.0000000
38360.0000000	2.0000000	-50.0000000
38360.0000000	2.2500000	-50.0000000
38360.0000000	2.5000000	-50.0000000
38360.0000000	2.7500000	-50.0000000
38360.0000000	3.0000000	-50.0000000
38360.0000000	3.2500000	-50.0000000
38360.0000000	3.5000000	-50.0000000
38360.0000000	3.7500000	-50.0000000
38360.0000000	4.0000000	-50.0000000
38360.0000000	4.2500000	-50.0000000
38360.0000000	4.5000000	-50.0000000
38360.0000000	4.7500000	-50.0000000
38360.0000000	5.0000000	-50.0000000
38360.0000000	5.2500000	-50.0000000
38360.0000000	5.5000000	-50.0000000
38360.0000000	5.7500000	-50.0000000
38360.0000000	6.0000000	-50.0000000
38360.0000000	6.2500000	-50.0000000
38360.0000000	6.5000000	-50.0000000
38360.0000000	6.7500000	-50.0000000
38360.0000000	7.0000000	-50.0000000
38360.0000000	7.2500000	-50.0000000
38360.0000000	7.5000000	-50.0000000
38360.0000000	7.7500000	-50.0000000
38360.0000000	8.0000000	-50.0000000
38360.0000000	8.2500000	-50.0000000
38360.0000000	8.5000000	-50.0000000
38360.0000000	8.7500000	-50.0000000
38360.0000000	9.0000000	-50.0000000
38360.0000000	9.2500000	-50.0000000
38360.0000000	9.5000000	-50.0000000
38360.0000000	9.7500000	-50.0000000
38360.0000000	10.0000000	-50.0000000
38360.0000000	10.2500000	-50.0000000
38360.0000000	10.5000000	-50.0000000
38360.0000000	10.7500000	-50.0000000
38360.0000000	11.0000000	-50.0000000
38360.0000000	11.2500000	-50.0000000
38360.0000000	11.5000000	-50.0000000
38360.0000000	11.7500000	-50.0000000
38360.0000000	12.0000000	-50.0000000
38360.0000000	12.2500000	-50.0000000
38360.0000000	12.5000000	-50.0000000
38360.0000000	12.7500000	-50.0000000
38360.0000000	13.0000000	-50.0000000
38360.0000000	13.2500000	-50.0000000
38360.0000000	13.5000000	-50.0000000
38360.0000000	13.7500000	-50.0000000

38360.0000000	14.0000000	-50.0000000
38360.0000000	14.2500000	-50.0000000
38360.0000000	14.5000000	-50.0000000
38360.0000000	14.7500000	-50.0000000
38360.0000000	15.0000000	-50.0000000
38390.0000000	0.0000000	-50.0000000
38390.0000000	0.2500000	-50.0000000
38390.0000000	0.5000000	-50.0000000
38390.0000000	0.7500000	-50.0000000
38390.0000000	1.0000000	-50.0000000
38390.0000000	1.2500000	-50.0000000
38390.0000000	1.5000000	-50.0000000
38390.0000000	1.7500000	-50.0000000
38390.0000000	2.0000000	-50.0000000
38390.0000000	2.2500000	-50.0000000
38390.0000000	2.5000000	-50.0000000
38390.0000000	2.7500000	-50.0000000
38390.0000000	3.0000000	-50.0000000
38390.0000000	3.2500000	-50.0000000
38390.0000000	3.5000000	-50.0000000
38390.0000000	3.7500000	-50.0000000
38390.0000000	4.0000000	-50.0000000
38390.0000000	4.2500000	-50.0000000
38390.0000000	4.5000000	-50.0000000
38390.0000000	4.7500000	-50.0000000
38390.0000000	5.0000000	-50.0000000
38390.0000000	5.2500000	-50.0000000
38390.0000000	5.5000000	-50.0000000
38390.0000000	5.7500000	-50.0000000
38390.0000000	6.0000000	-50.0000000
38390.0000000	6.2500000	-50.0000000
38390.0000000	6.5000000	-50.0000000
38390.0000000	6.7500000	-50.0000000
38390.0000000	7.0000000	-50.0000000
38390.0000000	7.2500000	-50.0000000
38390.0000000	7.5000000	-50.0000000
38390.0000000	7.7500000	-50.0000000
38390.0000000	8.0000000	-50.0000000
38390.0000000	8.2500000	-50.0000000
38390.0000000	8.5000000	-50.0000000
38390.0000000	8.7500000	-50.0000000
38390.0000000	9.0000000	-50.0000000
38390.0000000	9.2500000	-50.0000000
38390.0000000	9.5000000	-50.0000000
38390.0000000	9.7500000	-50.0000000
38390.0000000	10.0000000	-50.0000000
38390.0000000	10.2500000	-50.0000000
38390.0000000	10.5000000	-50.0000000
38390.0000000	10.7500000	-50.0000000
38390.0000000	11.0000000	-50.0000000
38390.0000000	11.2500000	-50.0000000
38390.0000000	11.5000000	-50.0000000
38390.0000000	11.7500000	-50.0000000
38390.0000000	12.0000000	-50.0000000
38390.0000000	12.2500000	-50.0000000
38390.0000000	12.5000000	-50.0000000
38390.0000000	12.7500000	-50.0000000
38390.0000000	13.0000000	-50.0000000

38390.0000000	13.2500000	-50.0000000
38390.0000000	13.5000000	-50.0000000
38390.0000000	13.7500000	-50.0000000
38390.0000000	14.0000000	-50.0000000
38390.0000000	14.2500000	-50.0000000
38390.0000000	14.5000000	-50.0000000
38390.0000000	14.7500000	-50.0000000
38390.0000000	15.0000000	-50.0000000
38420.0000000	0.0000000	-50.0000000
38420.0000000	0.2500000	-50.0000000
38420.0000000	0.5000000	-50.0000000
38420.0000000	0.7500000	-50.0000000
38420.0000000	1.0000000	-50.0000000
38420.0000000	1.2500000	-50.0000000
38420.0000000	1.5000000	-50.0000000
38420.0000000	1.7500000	-50.0000000
38420.0000000	2.0000000	-50.0000000
38420.0000000	2.2500000	-50.0000000
38420.0000000	2.5000000	-50.0000000
38420.0000000	2.7500000	-50.0000000
38420.0000000	3.0000000	-50.0000000
38420.0000000	3.2500000	-50.0000000
38420.0000000	3.5000000	-50.0000000
38420.0000000	3.7500000	-50.0000000
38420.0000000	4.0000000	-50.0000000
38420.0000000	4.2500000	-50.0000000
38420.0000000	4.5000000	-50.0000000
38420.0000000	4.7500000	-50.0000000
38420.0000000	5.0000000	-50.0000000
38420.0000000	5.2500000	-50.0000000
38420.0000000	5.5000000	-50.0000000
38420.0000000	5.7500000	-50.0000000
38420.0000000	6.0000000	-50.0000000
38420.0000000	6.2500000	-50.0000000
38420.0000000	6.5000000	-50.0000000
38420.0000000	6.7500000	-50.0000000
38420.0000000	7.0000000	-50.0000000
38420.0000000	7.2500000	-50.0000000
38420.0000000	7.5000000	-50.0000000
38420.0000000	7.7500000	-50.0000000
38420.0000000	8.0000000	-50.0000000
38420.0000000	8.2500000	-50.0000000
38420.0000000	8.5000000	-50.0000000
38420.0000000	8.7500000	-50.0000000
38420.0000000	9.0000000	-50.0000000
38420.0000000	9.2500000	-50.0000000
38420.0000000	9.5000000	-50.0000000
38420.0000000	9.7500000	-50.0000000
38420.0000000	10.0000000	-50.0000000
38420.0000000	10.2500000	-50.0000000
38420.0000000	10.5000000	-50.0000000
38420.0000000	10.7500000	-50.0000000
38420.0000000	11.0000000	-50.0000000
38420.0000000	11.2500000	-50.0000000
38420.0000000	11.5000000	-50.0000000
38420.0000000	11.7500000	-50.0000000
38420.0000000	12.0000000	-50.0000000
38420.0000000	12.2500000	-50.0000000

38420.0000000	12.5000000	-50.0000000
38420.0000000	12.7500000	-50.0000000
38420.0000000	13.0000000	-50.0000000
38420.0000000	13.2500000	-50.0000000
38420.0000000	13.5000000	-50.0000000
38420.0000000	13.7500000	-50.0000000
38420.0000000	14.0000000	-50.0000000
38420.0000000	14.2500000	-50.0000000
38420.0000000	14.5000000	-50.0000000
38420.0000000	14.7500000	-50.0000000
38420.0000000	15.0000000	-50.0000000
38450.0000000	0.0000000	-50.0000000
38450.0000000	0.2500000	-50.0000000
38450.0000000	0.5000000	-50.0000000
38450.0000000	0.7500000	-50.0000000
38450.0000000	1.0000000	-50.0000000
38450.0000000	1.2500000	-50.0000000
38450.0000000	1.5000000	-50.0000000
38450.0000000	1.7500000	-50.0000000
38450.0000000	2.0000000	-50.0000000
38450.0000000	2.2500000	-50.0000000
38450.0000000	2.5000000	-50.0000000
38450.0000000	2.7500000	-50.0000000
38450.0000000	3.0000000	-50.0000000
38450.0000000	3.2500000	-50.0000000
38450.0000000	3.5000000	-50.0000000
38450.0000000	3.7500000	-50.0000000
38450.0000000	4.0000000	-50.0000000
38450.0000000	4.2500000	-50.0000000
38450.0000000	4.5000000	-50.0000000
38450.0000000	4.7500000	-50.0000000
38450.0000000	5.0000000	-50.0000000
38450.0000000	5.2500000	-50.0000000
38450.0000000	5.5000000	-50.0000000
38450.0000000	5.7500000	-50.0000000
38450.0000000	6.0000000	-50.0000000
38450.0000000	6.2500000	-50.0000000
38450.0000000	6.5000000	-50.0000000
38450.0000000	6.7500000	-50.0000000
38450.0000000	7.0000000	-50.0000000
38450.0000000	7.2500000	-50.0000000
38450.0000000	7.5000000	-50.0000000
38450.0000000	7.7500000	-50.0000000
38450.0000000	8.0000000	-50.0000000
38450.0000000	8.2500000	-50.0000000
38450.0000000	8.5000000	-50.0000000
38450.0000000	8.7500000	-50.0000000
38450.0000000	9.0000000	-50.0000000
38450.0000000	9.2500000	-50.0000000
38450.0000000	9.5000000	-50.0000000
38450.0000000	9.7500000	-50.0000000
38450.0000000	10.0000000	-50.0000000
38450.0000000	10.2500000	-50.0000000
38450.0000000	10.5000000	-50.0000000
38450.0000000	10.7500000	-50.0000000
38450.0000000	11.0000000	-50.0000000
38450.0000000	11.2500000	-50.0000000
38450.0000000	11.5000000	-50.0000000

38450.0000000	11.7500000	-50.0000000
38450.0000000	12.0000000	-50.0000000
38450.0000000	12.2500000	-50.0000000
38450.0000000	12.5000000	-50.0000000
38450.0000000	12.7500000	-50.0000000
38450.0000000	13.0000000	-50.0000000
38450.0000000	13.2500000	-50.0000000
38450.0000000	13.5000000	-50.0000000
38450.0000000	13.7500000	-50.0000000
38450.0000000	14.0000000	-50.0000000
38450.0000000	14.2500000	-50.0000000
38450.0000000	14.5000000	-50.0000000
38450.0000000	14.7500000	-50.0000000
38450.0000000	15.0000000	-50.0000000
38480.0000000	0.0000000	-50.0000000
38480.0000000	0.2500000	-50.0000000
38480.0000000	0.5000000	-50.0000000
38480.0000000	0.7500000	-50.0000000
38480.0000000	1.0000000	-50.0000000
38480.0000000	1.2500000	-50.0000000
38480.0000000	1.5000000	-50.0000000
38480.0000000	1.7500000	-50.0000000
38480.0000000	2.0000000	-50.0000000
38480.0000000	2.2500000	-50.0000000
38480.0000000	2.5000000	-50.0000000
38480.0000000	2.7500000	-50.0000000
38480.0000000	3.0000000	-50.0000000
38480.0000000	3.2500000	-50.0000000
38480.0000000	3.5000000	-50.0000000
38480.0000000	3.7500000	-50.0000000
38480.0000000	4.0000000	-50.0000000
38480.0000000	4.2500000	-50.0000000
38480.0000000	4.5000000	-50.0000000
38480.0000000	4.7500000	-50.0000000
38480.0000000	5.0000000	-50.0000000
38480.0000000	5.2500000	-50.0000000
38480.0000000	5.5000000	-50.0000000
38480.0000000	5.7500000	-50.0000000
38480.0000000	6.0000000	-50.0000000
38480.0000000	6.2500000	-50.0000000
38480.0000000	6.5000000	-50.0000000
38480.0000000	6.7500000	-50.0000000
38480.0000000	7.0000000	-50.0000000
38480.0000000	7.2500000	-50.0000000
38480.0000000	7.5000000	-50.0000000
38480.0000000	7.7500000	-50.0000000
38480.0000000	8.0000000	-50.0000000
38480.0000000	8.2500000	-50.0000000
38480.0000000	8.5000000	-50.0000000
38480.0000000	8.7500000	-50.0000000
38480.0000000	9.0000000	-50.0000000
38480.0000000	9.2500000	-50.0000000
38480.0000000	9.5000000	-50.0000000
38480.0000000	9.7500000	-50.0000000
38480.0000000	10.0000000	-50.0000000
38480.0000000	10.2500000	-50.0000000
38480.0000000	10.5000000	-50.0000000
38480.0000000	10.7500000	-50.0000000

38480.0000000	11.0000000	-50.0000000
38480.0000000	11.2500000	-50.0000000
38480.0000000	11.5000000	-50.0000000
38480.0000000	11.7500000	-50.0000000
38480.0000000	12.0000000	-50.0000000
38480.0000000	12.2500000	-50.0000000
38480.0000000	12.5000000	-50.0000000
38480.0000000	12.7500000	-50.0000000
38480.0000000	13.0000000	-50.0000000
38480.0000000	13.2500000	-50.0000000
38480.0000000	13.5000000	-50.0000000
38480.0000000	13.7500000	-50.0000000
38480.0000000	14.0000000	-50.0000000
38480.0000000	14.2500000	-50.0000000
38480.0000000	14.5000000	-50.0000000
38480.0000000	14.7500000	-50.0000000
38480.0000000	15.0000000	-50.0000000
38510.0000000	0.0000000	-50.0000000
38510.0000000	0.2500000	-50.0000000
38510.0000000	0.5000000	-50.0000000
38510.0000000	0.7500000	-50.0000000
38510.0000000	1.0000000	-50.0000000
38510.0000000	1.2500000	-50.0000000
38510.0000000	1.5000000	-50.0000000
38510.0000000	1.7500000	-50.0000000
38510.0000000	2.0000000	-50.0000000
38510.0000000	2.2500000	-50.0000000
38510.0000000	2.5000000	-50.0000000
38510.0000000	2.7500000	-50.0000000
38510.0000000	3.0000000	-50.0000000
38510.0000000	3.2500000	-50.0000000
38510.0000000	3.5000000	-50.0000000
38510.0000000	3.7500000	-50.0000000
38510.0000000	4.0000000	-50.0000000
38510.0000000	4.2500000	-50.0000000
38510.0000000	4.5000000	-50.0000000
38510.0000000	4.7500000	-50.0000000
38510.0000000	5.0000000	-50.0000000
38510.0000000	5.2500000	-50.0000000
38510.0000000	5.5000000	-50.0000000
38510.0000000	5.7500000	-50.0000000
38510.0000000	6.0000000	-50.0000000
38510.0000000	6.2500000	-50.0000000
38510.0000000	6.5000000	-50.0000000
38510.0000000	6.7500000	-50.0000000
38510.0000000	7.0000000	-50.0000000
38510.0000000	7.2500000	-50.0000000
38510.0000000	7.5000000	-50.0000000
38510.0000000	7.7500000	-50.0000000
38510.0000000	8.0000000	-50.0000000
38510.0000000	8.2500000	-50.0000000
38510.0000000	8.5000000	-50.0000000
38510.0000000	8.7500000	-50.0000000
38510.0000000	9.0000000	-50.0000000
38510.0000000	9.2500000	-50.0000000
38510.0000000	9.5000000	-50.0000000
38510.0000000	9.7500000	-50.0000000
38510.0000000	10.0000000	-50.0000000

38510.0000000	10.2500000	-50.0000000
38510.0000000	10.5000000	-50.0000000
38510.0000000	10.7500000	-50.0000000
38510.0000000	11.0000000	-50.0000000
38510.0000000	11.2500000	-50.0000000
38510.0000000	11.5000000	-50.0000000
38510.0000000	11.7500000	-50.0000000
38510.0000000	12.0000000	-50.0000000
38510.0000000	12.2500000	-50.0000000
38510.0000000	12.5000000	-50.0000000
38510.0000000	12.7500000	-50.0000000
38510.0000000	13.0000000	-50.0000000
38510.0000000	13.2500000	-50.0000000
38510.0000000	13.5000000	-50.0000000
38510.0000000	13.7500000	-50.0000000
38510.0000000	14.0000000	-50.0000000
38510.0000000	14.2500000	-50.0000000
38510.0000000	14.5000000	-50.0000000
38510.0000000	14.7500000	-50.0000000
38510.0000000	15.0000000	-50.0000000
38540.0000000	0.0000000	-50.0000000
38540.0000000	0.2500000	-50.0000000
38540.0000000	0.5000000	-50.0000000
38540.0000000	0.7500000	-50.0000000
38540.0000000	1.0000000	-50.0000000
38540.0000000	1.2500000	-50.0000000
38540.0000000	1.5000000	-50.0000000
38540.0000000	1.7500000	-50.0000000
38540.0000000	2.0000000	-50.0000000
38540.0000000	2.2500000	-50.0000000
38540.0000000	2.5000000	-50.0000000
38540.0000000	2.7500000	-50.0000000
38540.0000000	3.0000000	-50.0000000
38540.0000000	3.2500000	-50.0000000
38540.0000000	3.5000000	-50.0000000
38540.0000000	3.7500000	-50.0000000
38540.0000000	4.0000000	-50.0000000
38540.0000000	4.2500000	-50.0000000
38540.0000000	4.5000000	-50.0000000
38540.0000000	4.7500000	-50.0000000
38540.0000000	5.0000000	-50.0000000
38540.0000000	5.2500000	-50.0000000
38540.0000000	5.5000000	-50.0000000
38540.0000000	5.7500000	-50.0000000
38540.0000000	6.0000000	-50.0000000
38540.0000000	6.2500000	-50.0000000
38540.0000000	6.5000000	-50.0000000
38540.0000000	6.7500000	-50.0000000
38540.0000000	7.0000000	-50.0000000
38540.0000000	7.2500000	-50.0000000
38540.0000000	7.5000000	-50.0000000
38540.0000000	7.7500000	-50.0000000
38540.0000000	8.0000000	-50.0000000
38540.0000000	8.2500000	-50.0000000
38540.0000000	8.5000000	-50.0000000
38540.0000000	8.7500000	-50.0000000
38540.0000000	9.0000000	-50.0000000
38540.0000000	9.2500000	-50.0000000

38540.0000000	9.5000000	-50.0000000
38540.0000000	9.7500000	-50.0000000
38540.0000000	10.0000000	-50.0000000
38540.0000000	10.2500000	-50.0000000
38540.0000000	10.5000000	-50.0000000
38540.0000000	10.7500000	-50.0000000
38540.0000000	11.0000000	-50.0000000
38540.0000000	11.2500000	-50.0000000
38540.0000000	11.5000000	-50.0000000
38540.0000000	11.7500000	-50.0000000
38540.0000000	12.0000000	-50.0000000
38540.0000000	12.2500000	-50.0000000
38540.0000000	12.5000000	-50.0000000
38540.0000000	12.7500000	-50.0000000
38540.0000000	13.0000000	-50.0000000
38540.0000000	13.2500000	-50.0000000
38540.0000000	13.5000000	-50.0000000
38540.0000000	13.7500000	-50.0000000
38540.0000000	14.0000000	-50.0000000
38540.0000000	14.2500000	-50.0000000
38540.0000000	14.5000000	-50.0000000
38540.0000000	14.7500000	-50.0000000
38540.0000000	15.0000000	-50.0000000
38570.0000000	0.0000000	-50.0000000
38570.0000000	0.2500000	-50.0000000
38570.0000000	0.5000000	-50.0000000
38570.0000000	0.7500000	-50.0000000
38570.0000000	1.0000000	-50.0000000
38570.0000000	1.2500000	-50.0000000
38570.0000000	1.5000000	-50.0000000
38570.0000000	1.7500000	-50.0000000
38570.0000000	2.0000000	-50.0000000
38570.0000000	2.2500000	-50.0000000
38570.0000000	2.5000000	-50.0000000
38570.0000000	2.7500000	-50.0000000
38570.0000000	3.0000000	-50.0000000
38570.0000000	3.2500000	-50.0000000
38570.0000000	3.5000000	-50.0000000
38570.0000000	3.7500000	-50.0000000
38570.0000000	4.0000000	-50.0000000
38570.0000000	4.2500000	-50.0000000
38570.0000000	4.5000000	-50.0000000
38570.0000000	4.7500000	-50.0000000
38570.0000000	5.0000000	-50.0000000
38570.0000000	5.2500000	-50.0000000
38570.0000000	5.5000000	-50.0000000
38570.0000000	5.7500000	-50.0000000
38570.0000000	6.0000000	-50.0000000
38570.0000000	6.2500000	-50.0000000
38570.0000000	6.5000000	-50.0000000
38570.0000000	6.7500000	-50.0000000
38570.0000000	7.0000000	-50.0000000
38570.0000000	7.2500000	-50.0000000
38570.0000000	7.5000000	-50.0000000
38570.0000000	7.7500000	-50.0000000
38570.0000000	8.0000000	-50.0000000
38570.0000000	8.2500000	-50.0000000
38570.0000000	8.5000000	-50.0000000

38570.0000000	8.7500000	-50.0000000
38570.0000000	9.0000000	-50.0000000
38570.0000000	9.2500000	-50.0000000
38570.0000000	9.5000000	-50.0000000
38570.0000000	9.7500000	-50.0000000
38570.0000000	10.0000000	-50.0000000
38570.0000000	10.2500000	-50.0000000
38570.0000000	10.5000000	-50.0000000
38570.0000000	10.7500000	-50.0000000
38570.0000000	11.0000000	-50.0000000
38570.0000000	11.2500000	-50.0000000
38570.0000000	11.5000000	-50.0000000
38570.0000000	11.7500000	-50.0000000
38570.0000000	12.0000000	-50.0000000
38570.0000000	12.2500000	-50.0000000
38570.0000000	12.5000000	-50.0000000
38570.0000000	12.7500000	-50.0000000
38570.0000000	13.0000000	-50.0000000
38570.0000000	13.2500000	-50.0000000
38570.0000000	13.5000000	-50.0000000
38570.0000000	13.7500000	-50.0000000
38570.0000000	14.0000000	-50.0000000
38570.0000000	14.2500000	-50.0000000
38570.0000000	14.5000000	-50.0000000
38570.0000000	14.7500000	-50.0000000
38570.0000000	15.0000000	-50.0000000
38600.0000000	0.0000000	-50.0000000
38600.0000000	0.2500000	-50.0000000
38600.0000000	0.5000000	-50.0000000
38600.0000000	0.7500000	-50.0000000
38600.0000000	1.0000000	-50.0000000
38600.0000000	1.2500000	-50.0000000
38600.0000000	1.5000000	-50.0000000
38600.0000000	1.7500000	-50.0000000
38600.0000000	2.0000000	-50.0000000
38600.0000000	2.2500000	-50.0000000
38600.0000000	2.5000000	-50.0000000
38600.0000000	2.7500000	-50.0000000
38600.0000000	3.0000000	-50.0000000
38600.0000000	3.2500000	-50.0000000
38600.0000000	3.5000000	-50.0000000
38600.0000000	3.7500000	-50.0000000
38600.0000000	4.0000000	-50.0000000
38600.0000000	4.2500000	-50.0000000
38600.0000000	4.5000000	-50.0000000
38600.0000000	4.7500000	-50.0000000
38600.0000000	5.0000000	-50.0000000
38600.0000000	5.2500000	-50.0000000
38600.0000000	5.5000000	-50.0000000
38600.0000000	5.7500000	-50.0000000
38600.0000000	6.0000000	-50.0000000
38600.0000000	6.2500000	-50.0000000
38600.0000000	6.5000000	-50.0000000
38600.0000000	6.7500000	-50.0000000
38600.0000000	7.0000000	-50.0000000
38600.0000000	7.2500000	-50.0000000
38600.0000000	7.5000000	-50.0000000
38600.0000000	7.7500000	-50.0000000

38600.0000000	8.0000000	-50.0000000
38600.0000000	8.2500000	-50.0000000
38600.0000000	8.5000000	-50.0000000
38600.0000000	8.7500000	-50.0000000
38600.0000000	9.0000000	-50.0000000
38600.0000000	9.2500000	-50.0000000
38600.0000000	9.5000000	-50.0000000
38600.0000000	9.7500000	-50.0000000
38600.0000000	10.0000000	-50.0000000
38600.0000000	10.2500000	-50.0000000
38600.0000000	10.5000000	-50.0000000
38600.0000000	10.7500000	-50.0000000
38600.0000000	11.0000000	-50.0000000
38600.0000000	11.2500000	-50.0000000
38600.0000000	11.5000000	-50.0000000
38600.0000000	11.7500000	-50.0000000
38600.0000000	12.0000000	-50.0000000
38600.0000000	12.2500000	-50.0000000
38600.0000000	12.5000000	-50.0000000
38600.0000000	12.7500000	-50.0000000
38600.0000000	13.0000000	-50.0000000
38600.0000000	13.2500000	-50.0000000
38600.0000000	13.5000000	-50.0000000
38600.0000000	13.7500000	-50.0000000
38600.0000000	14.0000000	-50.0000000
38600.0000000	14.2500000	-50.0000000
38600.0000000	14.5000000	-50.0000000
38600.0000000	14.7500000	-50.0000000
38600.0000000	15.0000000	-50.0000000
38630.0000000	0.0000000	-50.0000000
38630.0000000	0.2500000	-50.0000000
38630.0000000	0.5000000	-50.0000000
38630.0000000	0.7500000	-50.0000000
38630.0000000	1.0000000	-50.0000000
38630.0000000	1.2500000	-50.0000000
38630.0000000	1.5000000	-50.0000000
38630.0000000	1.7500000	-50.0000000
38630.0000000	2.0000000	-50.0000000
38630.0000000	2.2500000	-50.0000000
38630.0000000	2.5000000	-50.0000000
38630.0000000	2.7500000	-50.0000000
38630.0000000	3.0000000	-50.0000000
38630.0000000	3.2500000	-50.0000000
38630.0000000	3.5000000	-50.0000000
38630.0000000	3.7500000	-50.0000000
38630.0000000	4.0000000	-50.0000000
38630.0000000	4.2500000	-50.0000000
38630.0000000	4.5000000	-50.0000000
38630.0000000	4.7500000	-50.0000000
38630.0000000	5.0000000	-50.0000000
38630.0000000	5.2500000	-50.0000000
38630.0000000	5.5000000	-50.0000000
38630.0000000	5.7500000	-50.0000000
38630.0000000	6.0000000	-50.0000000
38630.0000000	6.2500000	-50.0000000
38630.0000000	6.5000000	-50.0000000
38630.0000000	6.7500000	-50.0000000
38630.0000000	7.0000000	-50.0000000

38630.0000000	7.2500000	-50.0000000
38630.0000000	7.5000000	-50.0000000
38630.0000000	7.7500000	-50.0000000
38630.0000000	8.0000000	-50.0000000
38630.0000000	8.2500000	-50.0000000
38630.0000000	8.5000000	-50.0000000
38630.0000000	8.7500000	-50.0000000
38630.0000000	9.0000000	-50.0000000
38630.0000000	9.2500000	-50.0000000
38630.0000000	9.5000000	-50.0000000
38630.0000000	9.7500000	-50.0000000
38630.0000000	10.0000000	-50.0000000
38630.0000000	10.2500000	-50.0000000
38630.0000000	10.5000000	-50.0000000
38630.0000000	10.7500000	-50.0000000
38630.0000000	11.0000000	-50.0000000
38630.0000000	11.2500000	-50.0000000
38630.0000000	11.5000000	-50.0000000
38630.0000000	11.7500000	-50.0000000
38630.0000000	12.0000000	-50.0000000
38630.0000000	12.2500000	-50.0000000
38630.0000000	12.5000000	-50.0000000
38630.0000000	12.7500000	-50.0000000
38630.0000000	13.0000000	-50.0000000
38630.0000000	13.2500000	-50.0000000
38630.0000000	13.5000000	-50.0000000
38630.0000000	13.7500000	-50.0000000
38630.0000000	14.0000000	-50.0000000
38630.0000000	14.2500000	-50.0000000
38630.0000000	14.5000000	-50.0000000
38630.0000000	14.7500000	-50.0000000
38630.0000000	15.0000000	-50.0000000
38660.0000000	0.0000000	-50.0000000
38660.0000000	0.2500000	-50.0000000
38660.0000000	0.5000000	-50.0000000
38660.0000000	0.7500000	-50.0000000
38660.0000000	1.0000000	-50.0000000
38660.0000000	1.2500000	-50.0000000
38660.0000000	1.5000000	-50.0000000
38660.0000000	1.7500000	-50.0000000
38660.0000000	2.0000000	-50.0000000
38660.0000000	2.2500000	-50.0000000
38660.0000000	2.5000000	-50.0000000
38660.0000000	2.7500000	-50.0000000
38660.0000000	3.0000000	-50.0000000
38660.0000000	3.2500000	-50.0000000
38660.0000000	3.5000000	-50.0000000
38660.0000000	3.7500000	-50.0000000
38660.0000000	4.0000000	-50.0000000
38660.0000000	4.2500000	-50.0000000
38660.0000000	4.5000000	-50.0000000
38660.0000000	4.7500000	-50.0000000
38660.0000000	5.0000000	-50.0000000
38660.0000000	5.2500000	-50.0000000
38660.0000000	5.5000000	-50.0000000
38660.0000000	5.7500000	-50.0000000
38660.0000000	6.0000000	-50.0000000
38660.0000000	6.2500000	-50.0000000

38660.0000000	6.5000000	-50.0000000
38660.0000000	6.7500000	-50.0000000
38660.0000000	7.0000000	-50.0000000
38660.0000000	7.2500000	-50.0000000
38660.0000000	7.5000000	-50.0000000
38660.0000000	7.7500000	-50.0000000
38660.0000000	8.0000000	-50.0000000
38660.0000000	8.2500000	-50.0000000
38660.0000000	8.5000000	-50.0000000
38660.0000000	8.7500000	-50.0000000
38660.0000000	9.0000000	-50.0000000
38660.0000000	9.2500000	-50.0000000
38660.0000000	9.5000000	-50.0000000
38660.0000000	9.7500000	-50.0000000
38660.0000000	10.0000000	-50.0000000
38660.0000000	10.2500000	-50.0000000
38660.0000000	10.5000000	-50.0000000
38660.0000000	10.7500000	-50.0000000
38660.0000000	11.0000000	-50.0000000
38660.0000000	11.2500000	-50.0000000
38660.0000000	11.5000000	-50.0000000
38660.0000000	11.7500000	-50.0000000
38660.0000000	12.0000000	-50.0000000
38660.0000000	12.2500000	-50.0000000
38660.0000000	12.5000000	-50.0000000
38660.0000000	12.7500000	-50.0000000
38660.0000000	13.0000000	-50.0000000
38660.0000000	13.2500000	-50.0000000
38660.0000000	13.5000000	-50.0000000
38660.0000000	13.7500000	-50.0000000
38660.0000000	14.0000000	-50.0000000
38660.0000000	14.2500000	-50.0000000
38660.0000000	14.5000000	-50.0000000
38660.0000000	14.7500000	-50.0000000
38660.0000000	15.0000000	-50.0000000
38690.0000000	0.0000000	-50.0000000
38690.0000000	0.2500000	-50.0000000
38690.0000000	0.5000000	-50.0000000
38690.0000000	0.7500000	-50.0000000
38690.0000000	1.0000000	-50.0000000
38690.0000000	1.2500000	-50.0000000
38690.0000000	1.5000000	-50.0000000
38690.0000000	1.7500000	-50.0000000
38690.0000000	2.0000000	-50.0000000
38690.0000000	2.2500000	-50.0000000
38690.0000000	2.5000000	-50.0000000
38690.0000000	2.7500000	-50.0000000
38690.0000000	3.0000000	-50.0000000
38690.0000000	3.2500000	-50.0000000
38690.0000000	3.5000000	-50.0000000
38690.0000000	3.7500000	-50.0000000
38690.0000000	4.0000000	-50.0000000
38690.0000000	4.2500000	-50.0000000
38690.0000000	4.5000000	-50.0000000
38690.0000000	4.7500000	-50.0000000
38690.0000000	5.0000000	-50.0000000
38690.0000000	5.2500000	-50.0000000
38690.0000000	5.5000000	-50.0000000

38690.0000000	5.7500000	-50.0000000
38690.0000000	6.0000000	-50.0000000
38690.0000000	6.2500000	-50.0000000
38690.0000000	6.5000000	-50.0000000
38690.0000000	6.7500000	-50.0000000
38690.0000000	7.0000000	-50.0000000
38690.0000000	7.2500000	-50.0000000
38690.0000000	7.5000000	-50.0000000
38690.0000000	7.7500000	-50.0000000
38690.0000000	8.0000000	-50.0000000
38690.0000000	8.2500000	-50.0000000
38690.0000000	8.5000000	-50.0000000
38690.0000000	8.7500000	-50.0000000
38690.0000000	9.0000000	-50.0000000
38690.0000000	9.2500000	-50.0000000
38690.0000000	9.5000000	-50.0000000
38690.0000000	9.7500000	-50.0000000
38690.0000000	10.0000000	-50.0000000
38690.0000000	10.2500000	-50.0000000
38690.0000000	10.5000000	-50.0000000
38690.0000000	10.7500000	-50.0000000
38690.0000000	11.0000000	-50.0000000
38690.0000000	11.2500000	-50.0000000
38690.0000000	11.5000000	-50.0000000
38690.0000000	11.7500000	-50.0000000
38690.0000000	12.0000000	-50.0000000
38690.0000000	12.2500000	-50.0000000
38690.0000000	12.5000000	-50.0000000
38690.0000000	12.7500000	-50.0000000
38690.0000000	13.0000000	-50.0000000
38690.0000000	13.2500000	-50.0000000
38690.0000000	13.5000000	-50.0000000
38690.0000000	13.7500000	-50.0000000
38690.0000000	14.0000000	-50.0000000
38690.0000000	14.2500000	-50.0000000
38690.0000000	14.5000000	-50.0000000
38690.0000000	14.7500000	-50.0000000
38690.0000000	15.0000000	-50.0000000
38720.0000000	0.0000000	-50.0000000
38720.0000000	0.2500000	-50.0000000
38720.0000000	0.5000000	-50.0000000
38720.0000000	0.7500000	-50.0000000
38720.0000000	1.0000000	-50.0000000
38720.0000000	1.2500000	-50.0000000
38720.0000000	1.5000000	-50.0000000
38720.0000000	1.7500000	-50.0000000
38720.0000000	2.0000000	-50.0000000
38720.0000000	2.2500000	-50.0000000
38720.0000000	2.5000000	-50.0000000
38720.0000000	2.7500000	-50.0000000
38720.0000000	3.0000000	-50.0000000
38720.0000000	3.2500000	-50.0000000
38720.0000000	3.5000000	-50.0000000
38720.0000000	3.7500000	-50.0000000
38720.0000000	4.0000000	-50.0000000
38720.0000000	4.2500000	-50.0000000
38720.0000000	4.5000000	-50.0000000
38720.0000000	4.7500000	-50.0000000

38720.0000000	5.0000000	-50.0000000
38720.0000000	5.2500000	-50.0000000
38720.0000000	5.5000000	-50.0000000
38720.0000000	5.7500000	-50.0000000
38720.0000000	6.0000000	-50.0000000
38720.0000000	6.2500000	-50.0000000
38720.0000000	6.5000000	-50.0000000
38720.0000000	6.7500000	-50.0000000
38720.0000000	7.0000000	-50.0000000
38720.0000000	7.2500000	-50.0000000
38720.0000000	7.5000000	-50.0000000
38720.0000000	7.7500000	-50.0000000
38720.0000000	8.0000000	-50.0000000
38720.0000000	8.2500000	-50.0000000
38720.0000000	8.5000000	-50.0000000
38720.0000000	8.7500000	-50.0000000
38720.0000000	9.0000000	-50.0000000
38720.0000000	9.2500000	-50.0000000
38720.0000000	9.5000000	-50.0000000
38720.0000000	9.7500000	-50.0000000
38720.0000000	10.0000000	-50.0000000
38720.0000000	10.2500000	-50.0000000
38720.0000000	10.5000000	-50.0000000
38720.0000000	10.7500000	-50.0000000
38720.0000000	11.0000000	-50.0000000
38720.0000000	11.2500000	-50.0000000
38720.0000000	11.5000000	-50.0000000
38720.0000000	11.7500000	-50.0000000
38720.0000000	12.0000000	-50.0000000
38720.0000000	12.2500000	-50.0000000
38720.0000000	12.5000000	-50.0000000
38720.0000000	12.7500000	-50.0000000
38720.0000000	13.0000000	-50.0000000
38720.0000000	13.2500000	-50.0000000
38720.0000000	13.5000000	-50.0000000
38720.0000000	13.7500000	-50.0000000
38720.0000000	14.0000000	-50.0000000
38720.0000000	14.2500000	-50.0000000
38720.0000000	14.5000000	-50.0000000
38720.0000000	14.7500000	-50.0000000
38720.0000000	15.0000000	-50.0000000
38750.0000000	0.0000000	-50.0000000
38750.0000000	0.2500000	-50.0000000
38750.0000000	0.5000000	-50.0000000
38750.0000000	0.7500000	-50.0000000
38750.0000000	1.0000000	-50.0000000
38750.0000000	1.2500000	-50.0000000
38750.0000000	1.5000000	-50.0000000
38750.0000000	1.7500000	-50.0000000
38750.0000000	2.0000000	-50.0000000
38750.0000000	2.2500000	-50.0000000
38750.0000000	2.5000000	-50.0000000
38750.0000000	2.7500000	-50.0000000
38750.0000000	3.0000000	-50.0000000
38750.0000000	3.2500000	-50.0000000
38750.0000000	3.5000000	-50.0000000
38750.0000000	3.7500000	-50.0000000
38750.0000000	4.0000000	-50.0000000

38750.0000000	4.2500000	-50.0000000
38750.0000000	4.5000000	-50.0000000
38750.0000000	4.7500000	-50.0000000
38750.0000000	5.0000000	-50.0000000
38750.0000000	5.2500000	-50.0000000
38750.0000000	5.5000000	-50.0000000
38750.0000000	5.7500000	-50.0000000
38750.0000000	6.0000000	-50.0000000
38750.0000000	6.2500000	-50.0000000
38750.0000000	6.5000000	-50.0000000
38750.0000000	6.7500000	-50.0000000
38750.0000000	7.0000000	-50.0000000
38750.0000000	7.2500000	-50.0000000
38750.0000000	7.5000000	-50.0000000
38750.0000000	7.7500000	-50.0000000
38750.0000000	8.0000000	-50.0000000
38750.0000000	8.2500000	-50.0000000
38750.0000000	8.5000000	-50.0000000
38750.0000000	8.7500000	-50.0000000
38750.0000000	9.0000000	-50.0000000
38750.0000000	9.2500000	-50.0000000
38750.0000000	9.5000000	-50.0000000
38750.0000000	9.7500000	-50.0000000
38750.0000000	10.0000000	-50.0000000
38750.0000000	10.2500000	-50.0000000
38750.0000000	10.5000000	-50.0000000
38750.0000000	10.7500000	-50.0000000
38750.0000000	11.0000000	-50.0000000
38750.0000000	11.2500000	-50.0000000
38750.0000000	11.5000000	-50.0000000
38750.0000000	11.7500000	-50.0000000
38750.0000000	12.0000000	-50.0000000
38750.0000000	12.2500000	-50.0000000
38750.0000000	12.5000000	-50.0000000
38750.0000000	12.7500000	-50.0000000
38750.0000000	13.0000000	-50.0000000
38750.0000000	13.2500000	-50.0000000
38750.0000000	13.5000000	-50.0000000
38750.0000000	13.7500000	-50.0000000
38750.0000000	14.0000000	-50.0000000
38750.0000000	14.2500000	-50.0000000
38750.0000000	14.5000000	-50.0000000
38750.0000000	14.7500000	-50.0000000
38750.0000000	15.0000000	-50.0000000
38780.0000000	0.0000000	-50.0000000
38780.0000000	0.2500000	-50.0000000
38780.0000000	0.5000000	-50.0000000
38780.0000000	0.7500000	-50.0000000
38780.0000000	1.0000000	-50.0000000
38780.0000000	1.2500000	-50.0000000
38780.0000000	1.5000000	-50.0000000
38780.0000000	1.7500000	-50.0000000
38780.0000000	2.0000000	-50.0000000
38780.0000000	2.2500000	-50.0000000
38780.0000000	2.5000000	-50.0000000
38780.0000000	2.7500000	-50.0000000
38780.0000000	3.0000000	-50.0000000
38780.0000000	3.2500000	-50.0000000

38780.0000000	3.5000000	-50.0000000
38780.0000000	3.7500000	-50.0000000
38780.0000000	4.0000000	-50.0000000
38780.0000000	4.2500000	-50.0000000
38780.0000000	4.5000000	-50.0000000
38780.0000000	4.7500000	-50.0000000
38780.0000000	5.0000000	-50.0000000
38780.0000000	5.2500000	-50.0000000
38780.0000000	5.5000000	-50.0000000
38780.0000000	5.7500000	-50.0000000
38780.0000000	6.0000000	-50.0000000
38780.0000000	6.2500000	-50.0000000
38780.0000000	6.5000000	-50.0000000
38780.0000000	6.7500000	-50.0000000
38780.0000000	7.0000000	-50.0000000
38780.0000000	7.2500000	-50.0000000
38780.0000000	7.5000000	-50.0000000
38780.0000000	7.7500000	-50.0000000
38780.0000000	8.0000000	-50.0000000
38780.0000000	8.2500000	-50.0000000
38780.0000000	8.5000000	-50.0000000
38780.0000000	8.7500000	-50.0000000
38780.0000000	9.0000000	-50.0000000
38780.0000000	9.2500000	-50.0000000
38780.0000000	9.5000000	-50.0000000
38780.0000000	9.7500000	-50.0000000
38780.0000000	10.0000000	-50.0000000
38780.0000000	10.2500000	-50.0000000
38780.0000000	10.5000000	-50.0000000
38780.0000000	10.7500000	-50.0000000
38780.0000000	11.0000000	-50.0000000
38780.0000000	11.2500000	-50.0000000
38780.0000000	11.5000000	-50.0000000
38780.0000000	11.7500000	-50.0000000
38780.0000000	12.0000000	-50.0000000
38780.0000000	12.2500000	-50.0000000
38780.0000000	12.5000000	-50.0000000
38780.0000000	12.7500000	-50.0000000
38780.0000000	13.0000000	-50.0000000
38780.0000000	13.2500000	-50.0000000
38780.0000000	13.5000000	-50.0000000
38780.0000000	13.7500000	-50.0000000
38780.0000000	14.0000000	-50.0000000
38780.0000000	14.2500000	-50.0000000
38780.0000000	14.5000000	-50.0000000
38780.0000000	14.7500000	-50.0000000
38780.0000000	15.0000000	-50.0000000
38810.0000000	0.0000000	-50.0000000
38810.0000000	0.2500000	-50.0000000
38810.0000000	0.5000000	-50.0000000
38810.0000000	0.7500000	-50.0000000
38810.0000000	1.0000000	-50.0000000
38810.0000000	1.2500000	-50.0000000
38810.0000000	1.5000000	-50.0000000
38810.0000000	1.7500000	-50.0000000
38810.0000000	2.0000000	-50.0000000
38810.0000000	2.2500000	-50.0000000
38810.0000000	2.5000000	-50.0000000

38810.0000000	2.7500000	-50.0000000
38810.0000000	3.0000000	-50.0000000
38810.0000000	3.2500000	-50.0000000
38810.0000000	3.5000000	-50.0000000
38810.0000000	3.7500000	-50.0000000
38810.0000000	4.0000000	-50.0000000
38810.0000000	4.2500000	-50.0000000
38810.0000000	4.5000000	-50.0000000
38810.0000000	4.7500000	-50.0000000
38810.0000000	5.0000000	-50.0000000
38810.0000000	5.2500000	-50.0000000
38810.0000000	5.5000000	-50.0000000
38810.0000000	5.7500000	-50.0000000
38810.0000000	6.0000000	-50.0000000
38810.0000000	6.2500000	-50.0000000
38810.0000000	6.5000000	-50.0000000
38810.0000000	6.7500000	-50.0000000
38810.0000000	7.0000000	-50.0000000
38810.0000000	7.2500000	-50.0000000
38810.0000000	7.5000000	-50.0000000
38810.0000000	7.7500000	-50.0000000
38810.0000000	8.0000000	-50.0000000
38810.0000000	8.2500000	-50.0000000
38810.0000000	8.5000000	-50.0000000
38810.0000000	8.7500000	-50.0000000
38810.0000000	9.0000000	-50.0000000
38810.0000000	9.2500000	-50.0000000
38810.0000000	9.5000000	-50.0000000
38810.0000000	9.7500000	-50.0000000
38810.0000000	10.0000000	-50.0000000
38810.0000000	10.2500000	-50.0000000
38810.0000000	10.5000000	-50.0000000
38810.0000000	10.7500000	-50.0000000
38810.0000000	11.0000000	-50.0000000
38810.0000000	11.2500000	-50.0000000
38810.0000000	11.5000000	-50.0000000
38810.0000000	11.7500000	-50.0000000
38810.0000000	12.0000000	-50.0000000
38810.0000000	12.2500000	-50.0000000
38810.0000000	12.5000000	-50.0000000
38810.0000000	12.7500000	-50.0000000
38810.0000000	13.0000000	-50.0000000
38810.0000000	13.2500000	-50.0000000
38810.0000000	13.5000000	-50.0000000
38810.0000000	13.7500000	-50.0000000
38810.0000000	14.0000000	-50.0000000
38810.0000000	14.2500000	-50.0000000
38810.0000000	14.5000000	-50.0000000
38810.0000000	14.7500000	-50.0000000
38810.0000000	15.0000000	-50.0000000
38840.0000000	0.0000000	-50.0000000
38840.0000000	0.2500000	-50.0000000
38840.0000000	0.5000000	-50.0000000
38840.0000000	0.7500000	-50.0000000
38840.0000000	1.0000000	-50.0000000
38840.0000000	1.2500000	-50.0000000
38840.0000000	1.5000000	-50.0000000
38840.0000000	1.7500000	-50.0000000

38840.0000000	2.0000000	-50.0000000
38840.0000000	2.2500000	-50.0000000
38840.0000000	2.5000000	-50.0000000
38840.0000000	2.7500000	-50.0000000
38840.0000000	3.0000000	-50.0000000
38840.0000000	3.2500000	-50.0000000
38840.0000000	3.5000000	-50.0000000
38840.0000000	3.7500000	-50.0000000
38840.0000000	4.0000000	-50.0000000
38840.0000000	4.2500000	-50.0000000
38840.0000000	4.5000000	-50.0000000
38840.0000000	4.7500000	-50.0000000
38840.0000000	5.0000000	-50.0000000
38840.0000000	5.2500000	-50.0000000
38840.0000000	5.5000000	-50.0000000
38840.0000000	5.7500000	-50.0000000
38840.0000000	6.0000000	-50.0000000
38840.0000000	6.2500000	-50.0000000
38840.0000000	6.5000000	-50.0000000
38840.0000000	6.7500000	-50.0000000
38840.0000000	7.0000000	-50.0000000
38840.0000000	7.2500000	-50.0000000
38840.0000000	7.5000000	-50.0000000
38840.0000000	7.7500000	-50.0000000
38840.0000000	8.0000000	-50.0000000
38840.0000000	8.2500000	-50.0000000
38840.0000000	8.5000000	-50.0000000
38840.0000000	8.7500000	-50.0000000
38840.0000000	9.0000000	-50.0000000
38840.0000000	9.2500000	-50.0000000
38840.0000000	9.5000000	-50.0000000
38840.0000000	9.7500000	-50.0000000
38840.0000000	10.0000000	-50.0000000
38840.0000000	10.2500000	-50.0000000
38840.0000000	10.5000000	-50.0000000
38840.0000000	10.7500000	-50.0000000
38840.0000000	11.0000000	-50.0000000
38840.0000000	11.2500000	-50.0000000
38840.0000000	11.5000000	-50.0000000
38840.0000000	11.7500000	-50.0000000
38840.0000000	12.0000000	-50.0000000
38840.0000000	12.2500000	-50.0000000
38840.0000000	12.5000000	-50.0000000
38840.0000000	12.7500000	-50.0000000
38840.0000000	13.0000000	-50.0000000
38840.0000000	13.2500000	-50.0000000
38840.0000000	13.5000000	-50.0000000
38840.0000000	13.7500000	-50.0000000
38840.0000000	14.0000000	-50.0000000
38840.0000000	14.2500000	-50.0000000
38840.0000000	14.5000000	-50.0000000
38840.0000000	14.7500000	-50.0000000
38840.0000000	15.0000000	-50.0000000
38870.0000000	0.0000000	-50.0000000
38870.0000000	0.2500000	-50.0000000
38870.0000000	0.5000000	-50.0000000
38870.0000000	0.7500000	-50.0000000
38870.0000000	1.0000000	-50.0000000

38870.0000000	1.2500000	-50.0000000
38870.0000000	1.5000000	-50.0000000
38870.0000000	1.7500000	-50.0000000
38870.0000000	2.0000000	-50.0000000
38870.0000000	2.2500000	-50.0000000
38870.0000000	2.5000000	-50.0000000
38870.0000000	2.7500000	-50.0000000
38870.0000000	3.0000000	-50.0000000
38870.0000000	3.2500000	-50.0000000
38870.0000000	3.5000000	-50.0000000
38870.0000000	3.7500000	-50.0000000
38870.0000000	4.0000000	-50.0000000
38870.0000000	4.2500000	-50.0000000
38870.0000000	4.5000000	-50.0000000
38870.0000000	4.7500000	-50.0000000
38870.0000000	5.0000000	-50.0000000
38870.0000000	5.2500000	-50.0000000
38870.0000000	5.5000000	-50.0000000
38870.0000000	5.7500000	-50.0000000
38870.0000000	6.0000000	-50.0000000
38870.0000000	6.2500000	-50.0000000
38870.0000000	6.5000000	-50.0000000
38870.0000000	6.7500000	-50.0000000
38870.0000000	7.0000000	-50.0000000
38870.0000000	7.2500000	-50.0000000
38870.0000000	7.5000000	-50.0000000
38870.0000000	7.7500000	-50.0000000
38870.0000000	8.0000000	-50.0000000
38870.0000000	8.2500000	-50.0000000
38870.0000000	8.5000000	-50.0000000
38870.0000000	8.7500000	-50.0000000
38870.0000000	9.0000000	-50.0000000
38870.0000000	9.2500000	-50.0000000
38870.0000000	9.5000000	-50.0000000
38870.0000000	9.7500000	-50.0000000
38870.0000000	10.0000000	-50.0000000
38870.0000000	10.2500000	-50.0000000
38870.0000000	10.5000000	-50.0000000
38870.0000000	10.7500000	-50.0000000
38870.0000000	11.0000000	-50.0000000
38870.0000000	11.2500000	-50.0000000
38870.0000000	11.5000000	-50.0000000
38870.0000000	11.7500000	-50.0000000
38870.0000000	12.0000000	-50.0000000
38870.0000000	12.2500000	-50.0000000
38870.0000000	12.5000000	-50.0000000
38870.0000000	12.7500000	-50.0000000
38870.0000000	13.0000000	-50.0000000
38870.0000000	13.2500000	-50.0000000
38870.0000000	13.5000000	-50.0000000
38870.0000000	13.7500000	-50.0000000
38870.0000000	14.0000000	-50.0000000
38870.0000000	14.2500000	-50.0000000
38870.0000000	14.5000000	-50.0000000
38870.0000000	14.7500000	-50.0000000
38870.0000000	15.0000000	-50.0000000
38900.0000000	0.0000000	-50.0000000
38900.0000000	0.2500000	-50.0000000

38900.0000000	0.5000000	-50.0000000
38900.0000000	0.7500000	-50.0000000
38900.0000000	1.0000000	-50.0000000
38900.0000000	1.2500000	-50.0000000
38900.0000000	1.5000000	-50.0000000
38900.0000000	1.7500000	-50.0000000
38900.0000000	2.0000000	-50.0000000
38900.0000000	2.2500000	-50.0000000
38900.0000000	2.5000000	-50.0000000
38900.0000000	2.7500000	-50.0000000
38900.0000000	3.0000000	-50.0000000
38900.0000000	3.2500000	-50.0000000
38900.0000000	3.5000000	-50.0000000
38900.0000000	3.7500000	-50.0000000
38900.0000000	4.0000000	-50.0000000
38900.0000000	4.2500000	-50.0000000
38900.0000000	4.5000000	-50.0000000
38900.0000000	4.7500000	-50.0000000
38900.0000000	5.0000000	-50.0000000
38900.0000000	5.2500000	-50.0000000
38900.0000000	5.5000000	-50.0000000
38900.0000000	5.7500000	-50.0000000
38900.0000000	6.0000000	-50.0000000
38900.0000000	6.2500000	-50.0000000
38900.0000000	6.5000000	-50.0000000
38900.0000000	6.7500000	-50.0000000
38900.0000000	7.0000000	-50.0000000
38900.0000000	7.2500000	-50.0000000
38900.0000000	7.5000000	-50.0000000
38900.0000000	7.7500000	-50.0000000
38900.0000000	8.0000000	-50.0000000
38900.0000000	8.2500000	-50.0000000
38900.0000000	8.5000000	-50.0000000
38900.0000000	8.7500000	-50.0000000
38900.0000000	9.0000000	-50.0000000
38900.0000000	9.2500000	-50.0000000
38900.0000000	9.5000000	-50.0000000
38900.0000000	9.7500000	-50.0000000
38900.0000000	10.0000000	-50.0000000
38900.0000000	10.2500000	-50.0000000
38900.0000000	10.5000000	-50.0000000
38900.0000000	10.7500000	-50.0000000
38900.0000000	11.0000000	-50.0000000
38900.0000000	11.2500000	-50.0000000
38900.0000000	11.5000000	-50.0000000
38900.0000000	11.7500000	-50.0000000
38900.0000000	12.0000000	-50.0000000
38900.0000000	12.2500000	-50.0000000
38900.0000000	12.5000000	-50.0000000
38900.0000000	12.7500000	-50.0000000
38900.0000000	13.0000000	-50.0000000
38900.0000000	13.2500000	-50.0000000
38900.0000000	13.5000000	-50.0000000
38900.0000000	13.7500000	-50.0000000
38900.0000000	14.0000000	-50.0000000
38900.0000000	14.2500000	-50.0000000
38900.0000000	14.5000000	-50.0000000
38900.0000000	14.7500000	-50.0000000

38900.0000000	15.0000000	-50.0000000
38930.0000000	0.0000000	-50.0000000
38930.0000000	0.2500000	-50.0000000
38930.0000000	0.5000000	-50.0000000
38930.0000000	0.7500000	-50.0000000
38930.0000000	1.0000000	-50.0000000
38930.0000000	1.2500000	-50.0000000
38930.0000000	1.5000000	-50.0000000
38930.0000000	1.7500000	-50.0000000
38930.0000000	2.0000000	-50.0000000
38930.0000000	2.2500000	-50.0000000
38930.0000000	2.5000000	-50.0000000
38930.0000000	2.7500000	-50.0000000
38930.0000000	3.0000000	-50.0000000
38930.0000000	3.2500000	-50.0000000
38930.0000000	3.5000000	-50.0000000
38930.0000000	3.7500000	-50.0000000
38930.0000000	4.0000000	-50.0000000
38930.0000000	4.2500000	-50.0000000
38930.0000000	4.5000000	-50.0000000
38930.0000000	4.7500000	-50.0000000
38930.0000000	5.0000000	-50.0000000
38930.0000000	5.2500000	-50.0000000
38930.0000000	5.5000000	-50.0000000
38930.0000000	5.7500000	-50.0000000
38930.0000000	6.0000000	-50.0000000
38930.0000000	6.2500000	-50.0000000
38930.0000000	6.5000000	-50.0000000
38930.0000000	6.7500000	-50.0000000
38930.0000000	7.0000000	-50.0000000
38930.0000000	7.2500000	-50.0000000
38930.0000000	7.5000000	-50.0000000
38930.0000000	7.7500000	-50.0000000
38930.0000000	8.0000000	-50.0000000
38930.0000000	8.2500000	-50.0000000
38930.0000000	8.5000000	-50.0000000
38930.0000000	8.7500000	-50.0000000
38930.0000000	9.0000000	-50.0000000
38930.0000000	9.2500000	-50.0000000
38930.0000000	9.5000000	-50.0000000
38930.0000000	9.7500000	-50.0000000
38930.0000000	10.0000000	-50.0000000
38930.0000000	10.2500000	-50.0000000
38930.0000000	10.5000000	-50.0000000
38930.0000000	10.7500000	-50.0000000
38930.0000000	11.0000000	-50.0000000
38930.0000000	11.2500000	-50.0000000
38930.0000000	11.5000000	-50.0000000
38930.0000000	11.7500000	-50.0000000
38930.0000000	12.0000000	-50.0000000
38930.0000000	12.2500000	-50.0000000
38930.0000000	12.5000000	-50.0000000
38930.0000000	12.7500000	-50.0000000
38930.0000000	13.0000000	-50.0000000
38930.0000000	13.2500000	-50.0000000
38930.0000000	13.5000000	-50.0000000
38930.0000000	13.7500000	-50.0000000
38930.0000000	14.0000000	-50.0000000

38930.0000000	14.2500000	-50.0000000
38930.0000000	14.5000000	-50.0000000
38930.0000000	14.7500000	-50.0000000
38930.0000000	15.0000000	-50.0000000
38960.0000000	0.0000000	-50.0000000
38960.0000000	0.2500000	-50.0000000
38960.0000000	0.5000000	-50.0000000
38960.0000000	0.7500000	-50.0000000
38960.0000000	1.0000000	-50.0000000
38960.0000000	1.2500000	-50.0000000
38960.0000000	1.5000000	-50.0000000
38960.0000000	1.7500000	-50.0000000
38960.0000000	2.0000000	-50.0000000
38960.0000000	2.2500000	-50.0000000
38960.0000000	2.5000000	-50.0000000
38960.0000000	2.7500000	-50.0000000
38960.0000000	3.0000000	-50.0000000
38960.0000000	3.2500000	-50.0000000
38960.0000000	3.5000000	-50.0000000
38960.0000000	3.7500000	-50.0000000
38960.0000000	4.0000000	-50.0000000
38960.0000000	4.2500000	-50.0000000
38960.0000000	4.5000000	-50.0000000
38960.0000000	4.7500000	-50.0000000
38960.0000000	5.0000000	-50.0000000
38960.0000000	5.2500000	-50.0000000
38960.0000000	5.5000000	-50.0000000
38960.0000000	5.7500000	-50.0000000
38960.0000000	6.0000000	-50.0000000
38960.0000000	6.2500000	-50.0000000
38960.0000000	6.5000000	-50.0000000
38960.0000000	6.7500000	-50.0000000
38960.0000000	7.0000000	-50.0000000
38960.0000000	7.2500000	-50.0000000
38960.0000000	7.5000000	-50.0000000
38960.0000000	7.7500000	-50.0000000
38960.0000000	8.0000000	-50.0000000
38960.0000000	8.2500000	-50.0000000
38960.0000000	8.5000000	-50.0000000
38960.0000000	8.7500000	-50.0000000
38960.0000000	9.0000000	-50.0000000
38960.0000000	9.2500000	-50.0000000
38960.0000000	9.5000000	-50.0000000
38960.0000000	9.7500000	-50.0000000
38960.0000000	10.0000000	-50.0000000
38960.0000000	10.2500000	-50.0000000
38960.0000000	10.5000000	-50.0000000
38960.0000000	10.7500000	-50.0000000
38960.0000000	11.0000000	-50.0000000
38960.0000000	11.2500000	-50.0000000
38960.0000000	11.5000000	-50.0000000
38960.0000000	11.7500000	-50.0000000
38960.0000000	12.0000000	-50.0000000
38960.0000000	12.2500000	-50.0000000
38960.0000000	12.5000000	-50.0000000
38960.0000000	12.7500000	-50.0000000
38960.0000000	13.0000000	-50.0000000
38960.0000000	13.2500000	-50.0000000

38960.0000000	13.5000000	-50.0000000
38960.0000000	13.7500000	-50.0000000
38960.0000000	14.0000000	-50.0000000
38960.0000000	14.2500000	-50.0000000
38960.0000000	14.5000000	-50.0000000
38960.0000000	14.7500000	-50.0000000
38960.0000000	15.0000000	-50.0000000
38990.0000000	0.0000000	-50.0000000
38990.0000000	0.2500000	-50.0000000
38990.0000000	0.5000000	-50.0000000
38990.0000000	0.7500000	-50.0000000
38990.0000000	1.0000000	-50.0000000
38990.0000000	1.2500000	-50.0000000
38990.0000000	1.5000000	-50.0000000
38990.0000000	1.7500000	-50.0000000
38990.0000000	2.0000000	-50.0000000
38990.0000000	2.2500000	-50.0000000
38990.0000000	2.5000000	-50.0000000
38990.0000000	2.7500000	-50.0000000
38990.0000000	3.0000000	-50.0000000
38990.0000000	3.2500000	-50.0000000
38990.0000000	3.5000000	-50.0000000
38990.0000000	3.7500000	-50.0000000
38990.0000000	4.0000000	-50.0000000
38990.0000000	4.2500000	-50.0000000
38990.0000000	4.5000000	-50.0000000
38990.0000000	4.7500000	-50.0000000
38990.0000000	5.0000000	-50.0000000
38990.0000000	5.2500000	-50.0000000
38990.0000000	5.5000000	-50.0000000
38990.0000000	5.7500000	-50.0000000
38990.0000000	6.0000000	-50.0000000
38990.0000000	6.2500000	-50.0000000
38990.0000000	6.5000000	-50.0000000
38990.0000000	6.7500000	-50.0000000
38990.0000000	7.0000000	-50.0000000
38990.0000000	7.2500000	-50.0000000
38990.0000000	7.5000000	-50.0000000
38990.0000000	7.7500000	-50.0000000
38990.0000000	8.0000000	-50.0000000
38990.0000000	8.2500000	-50.0000000
38990.0000000	8.5000000	-50.0000000
38990.0000000	8.7500000	-50.0000000
38990.0000000	9.0000000	-50.0000000
38990.0000000	9.2500000	-50.0000000
38990.0000000	9.5000000	-50.0000000
38990.0000000	9.7500000	-50.0000000
38990.0000000	10.0000000	-50.0000000
38990.0000000	10.2500000	-50.0000000
38990.0000000	10.5000000	-50.0000000
38990.0000000	10.7500000	-50.0000000
38990.0000000	11.0000000	-50.0000000
38990.0000000	11.2500000	-50.0000000
38990.0000000	11.5000000	-50.0000000
38990.0000000	11.7500000	-50.0000000
38990.0000000	12.0000000	-50.0000000
38990.0000000	12.2500000	-50.0000000
38990.0000000	12.5000000	-50.0000000

38990.0000000	12.7500000	-50.0000000
38990.0000000	13.0000000	-50.0000000
38990.0000000	13.2500000	-50.0000000
38990.0000000	13.5000000	-50.0000000
38990.0000000	13.7500000	-50.0000000
38990.0000000	14.0000000	-50.0000000
38990.0000000	14.2500000	-50.0000000
38990.0000000	14.5000000	-50.0000000
38990.0000000	14.7500000	-50.0000000
38990.0000000	15.0000000	-50.0000000
39020.0000000	0.0000000	-50.0000000
39020.0000000	0.2500000	-50.0000000
39020.0000000	0.5000000	-50.0000000
39020.0000000	0.7500000	-50.0000000
39020.0000000	1.0000000	-50.0000000
39020.0000000	1.2500000	-50.0000000
39020.0000000	1.5000000	-50.0000000
39020.0000000	1.7500000	-50.0000000
39020.0000000	2.0000000	-50.0000000
39020.0000000	2.2500000	-50.0000000
39020.0000000	2.5000000	-50.0000000
39020.0000000	2.7500000	-50.0000000
39020.0000000	3.0000000	-50.0000000
39020.0000000	3.2500000	-50.0000000
39020.0000000	3.5000000	-50.0000000
39020.0000000	3.7500000	-50.0000000
39020.0000000	4.0000000	-50.0000000
39020.0000000	4.2500000	-50.0000000
39020.0000000	4.5000000	-50.0000000
39020.0000000	4.7500000	-50.0000000
39020.0000000	5.0000000	-50.0000000
39020.0000000	5.2500000	-50.0000000
39020.0000000	5.5000000	-50.0000000
39020.0000000	5.7500000	-50.0000000
39020.0000000	6.0000000	-50.0000000
39020.0000000	6.2500000	-50.0000000
39020.0000000	6.5000000	-50.0000000
39020.0000000	6.7500000	-50.0000000
39020.0000000	7.0000000	-50.0000000
39020.0000000	7.2500000	-50.0000000
39020.0000000	7.5000000	-50.0000000
39020.0000000	7.7500000	-50.0000000
39020.0000000	8.0000000	-50.0000000
39020.0000000	8.2500000	-50.0000000
39020.0000000	8.5000000	-50.0000000
39020.0000000	8.7500000	-50.0000000
39020.0000000	9.0000000	-50.0000000
39020.0000000	9.2500000	-50.0000000
39020.0000000	9.5000000	-50.0000000
39020.0000000	9.7500000	-50.0000000
39020.0000000	10.0000000	-50.0000000
39020.0000000	10.2500000	-50.0000000
39020.0000000	10.5000000	-50.0000000
39020.0000000	10.7500000	-50.0000000
39020.0000000	11.0000000	-50.0000000
39020.0000000	11.2500000	-50.0000000
39020.0000000	11.5000000	-50.0000000
39020.0000000	11.7500000	-50.0000000

39020.0000000	12.0000000	-50.0000000
39020.0000000	12.2500000	-50.0000000
39020.0000000	12.5000000	-50.0000000
39020.0000000	12.7500000	-50.0000000
39020.0000000	13.0000000	-50.0000000
39020.0000000	13.2500000	-50.0000000
39020.0000000	13.5000000	-50.0000000
39020.0000000	13.7500000	-50.0000000
39020.0000000	14.0000000	-50.0000000
39020.0000000	14.2500000	-50.0000000
39020.0000000	14.5000000	-50.0000000
39020.0000000	14.7500000	-50.0000000
39020.0000000	15.0000000	-50.0000000
39050.0000000	0.0000000	-50.0000000
39050.0000000	0.2500000	-50.0000000
39050.0000000	0.5000000	-50.0000000
39050.0000000	0.7500000	-50.0000000
39050.0000000	1.0000000	-50.0000000
39050.0000000	1.2500000	-50.0000000
39050.0000000	1.5000000	-50.0000000
39050.0000000	1.7500000	-50.0000000
39050.0000000	2.0000000	-50.0000000
39050.0000000	2.2500000	-50.0000000
39050.0000000	2.5000000	-50.0000000
39050.0000000	2.7500000	-50.0000000
39050.0000000	3.0000000	-50.0000000
39050.0000000	3.2500000	-50.0000000
39050.0000000	3.5000000	-50.0000000
39050.0000000	3.7500000	-50.0000000
39050.0000000	4.0000000	-50.0000000
39050.0000000	4.2500000	-50.0000000
39050.0000000	4.5000000	-50.0000000
39050.0000000	4.7500000	-50.0000000
39050.0000000	5.0000000	-50.0000000
39050.0000000	5.2500000	-50.0000000
39050.0000000	5.5000000	-50.0000000
39050.0000000	5.7500000	-50.0000000
39050.0000000	6.0000000	-50.0000000
39050.0000000	6.2500000	-50.0000000
39050.0000000	6.5000000	-50.0000000
39050.0000000	6.7500000	-50.0000000
39050.0000000	7.0000000	-50.0000000
39050.0000000	7.2500000	-50.0000000
39050.0000000	7.5000000	-50.0000000
39050.0000000	7.7500000	-50.0000000
39050.0000000	8.0000000	-50.0000000
39050.0000000	8.2500000	-50.0000000
39050.0000000	8.5000000	-50.0000000
39050.0000000	8.7500000	-50.0000000
39050.0000000	9.0000000	-50.0000000
39050.0000000	9.2500000	-50.0000000
39050.0000000	9.5000000	-50.0000000
39050.0000000	9.7500000	-50.0000000
39050.0000000	10.0000000	-50.0000000
39050.0000000	10.2500000	-50.0000000
39050.0000000	10.5000000	-50.0000000
39050.0000000	10.7500000	-50.0000000
39050.0000000	11.0000000	-50.0000000

39050.0000000	11.2500000	-50.0000000
39050.0000000	11.5000000	-50.0000000
39050.0000000	11.7500000	-50.0000000
39050.0000000	12.0000000	-50.0000000
39050.0000000	12.2500000	-50.0000000
39050.0000000	12.5000000	-50.0000000
39050.0000000	12.7500000	-50.0000000
39050.0000000	13.0000000	-50.0000000
39050.0000000	13.2500000	-50.0000000
39050.0000000	13.5000000	-50.0000000
39050.0000000	13.7500000	-50.0000000
39050.0000000	14.0000000	-50.0000000
39050.0000000	14.2500000	-50.0000000
39050.0000000	14.5000000	-50.0000000
39050.0000000	14.7500000	-50.0000000
39050.0000000	15.0000000	-50.0000000
39080.0000000	0.0000000	-50.0000000
39080.0000000	0.2500000	-50.0000000
39080.0000000	0.5000000	-50.0000000
39080.0000000	0.7500000	-50.0000000
39080.0000000	1.0000000	-50.0000000
39080.0000000	1.2500000	-50.0000000
39080.0000000	1.5000000	-50.0000000
39080.0000000	1.7500000	-50.0000000
39080.0000000	2.0000000	-50.0000000
39080.0000000	2.2500000	-50.0000000
39080.0000000	2.5000000	-50.0000000
39080.0000000	2.7500000	-50.0000000
39080.0000000	3.0000000	-50.0000000
39080.0000000	3.2500000	-50.0000000
39080.0000000	3.5000000	-50.0000000
39080.0000000	3.7500000	-50.0000000
39080.0000000	4.0000000	-50.0000000
39080.0000000	4.2500000	-50.0000000
39080.0000000	4.5000000	-50.0000000
39080.0000000	4.7500000	-50.0000000
39080.0000000	5.0000000	-50.0000000
39080.0000000	5.2500000	-50.0000000
39080.0000000	5.5000000	-50.0000000
39080.0000000	5.7500000	-50.0000000
39080.0000000	6.0000000	-50.0000000
39080.0000000	6.2500000	-50.0000000
39080.0000000	6.5000000	-50.0000000
39080.0000000	6.7500000	-50.0000000
39080.0000000	7.0000000	-50.0000000
39080.0000000	7.2500000	-50.0000000
39080.0000000	7.5000000	-50.0000000
39080.0000000	7.7500000	-50.0000000
39080.0000000	8.0000000	-50.0000000
39080.0000000	8.2500000	-50.0000000
39080.0000000	8.5000000	-50.0000000
39080.0000000	8.7500000	-50.0000000
39080.0000000	9.0000000	-50.0000000
39080.0000000	9.2500000	-50.0000000
39080.0000000	9.5000000	-50.0000000
39080.0000000	9.7500000	-50.0000000
39080.0000000	10.0000000	-50.0000000
39080.0000000	10.2500000	-50.0000000

39080.0000000	10.5000000	-50.0000000
39080.0000000	10.7500000	-50.0000000
39080.0000000	11.0000000	-50.0000000
39080.0000000	11.2500000	-50.0000000
39080.0000000	11.5000000	-50.0000000
39080.0000000	11.7500000	-50.0000000
39080.0000000	12.0000000	-50.0000000
39080.0000000	12.2500000	-50.0000000
39080.0000000	12.5000000	-50.0000000
39080.0000000	12.7500000	-50.0000000
39080.0000000	13.0000000	-50.0000000
39080.0000000	13.2500000	-50.0000000
39080.0000000	13.5000000	-50.0000000
39080.0000000	13.7500000	-50.0000000
39080.0000000	14.0000000	-50.0000000
39080.0000000	14.2500000	-50.0000000
39080.0000000	14.5000000	-50.0000000
39080.0000000	14.7500000	-50.0000000
39080.0000000	15.0000000	-50.0000000
39110.0000000	0.0000000	-50.0000000
39110.0000000	0.2500000	-50.0000000
39110.0000000	0.5000000	-50.0000000
39110.0000000	0.7500000	-50.0000000
39110.0000000	1.0000000	-50.0000000
39110.0000000	1.2500000	-50.0000000
39110.0000000	1.5000000	-50.0000000
39110.0000000	1.7500000	-50.0000000
39110.0000000	2.0000000	-50.0000000
39110.0000000	2.2500000	-50.0000000
39110.0000000	2.5000000	-50.0000000
39110.0000000	2.7500000	-50.0000000
39110.0000000	3.0000000	-50.0000000
39110.0000000	3.2500000	-50.0000000
39110.0000000	3.5000000	-50.0000000
39110.0000000	3.7500000	-50.0000000
39110.0000000	4.0000000	-50.0000000
39110.0000000	4.2500000	-50.0000000
39110.0000000	4.5000000	-50.0000000
39110.0000000	4.7500000	-50.0000000
39110.0000000	5.0000000	-50.0000000
39110.0000000	5.2500000	-50.0000000
39110.0000000	5.5000000	-50.0000000
39110.0000000	5.7500000	-50.0000000
39110.0000000	6.0000000	-50.0000000
39110.0000000	6.2500000	-50.0000000
39110.0000000	6.5000000	-50.0000000
39110.0000000	6.7500000	-50.0000000
39110.0000000	7.0000000	-50.0000000
39110.0000000	7.2500000	-50.0000000
39110.0000000	7.5000000	-50.0000000
39110.0000000	7.7500000	-50.0000000
39110.0000000	8.0000000	-50.0000000
39110.0000000	8.2500000	-50.0000000
39110.0000000	8.5000000	-50.0000000
39110.0000000	8.7500000	-50.0000000
39110.0000000	9.0000000	-50.0000000
39110.0000000	9.2500000	-50.0000000
39110.0000000	9.5000000	-50.0000000

39110.0000000	9.7500000	-50.0000000
39110.0000000	10.0000000	-50.0000000
39110.0000000	10.2500000	-50.0000000
39110.0000000	10.5000000	-50.0000000
39110.0000000	10.7500000	-50.0000000
39110.0000000	11.0000000	-50.0000000
39110.0000000	11.2500000	-50.0000000
39110.0000000	11.5000000	-50.0000000
39110.0000000	11.7500000	-50.0000000
39110.0000000	12.0000000	-50.0000000
39110.0000000	12.2500000	-50.0000000
39110.0000000	12.5000000	-50.0000000
39110.0000000	12.7500000	-50.0000000
39110.0000000	13.0000000	-50.0000000
39110.0000000	13.2500000	-50.0000000
39110.0000000	13.5000000	-50.0000000
39110.0000000	13.7500000	-50.0000000
39110.0000000	14.0000000	-50.0000000
39110.0000000	14.2500000	-50.0000000
39110.0000000	14.5000000	-50.0000000
39110.0000000	14.7500000	-50.0000000
39110.0000000	15.0000000	-50.0000000
39140.0000000	0.0000000	-50.0000000
39140.0000000	0.2500000	-50.0000000
39140.0000000	0.5000000	-50.0000000
39140.0000000	0.7500000	-50.0000000
39140.0000000	1.0000000	-50.0000000
39140.0000000	1.2500000	-50.0000000
39140.0000000	1.5000000	-50.0000000
39140.0000000	1.7500000	-50.0000000
39140.0000000	2.0000000	-50.0000000
39140.0000000	2.2500000	-50.0000000
39140.0000000	2.5000000	-50.0000000
39140.0000000	2.7500000	-50.0000000
39140.0000000	3.0000000	-50.0000000
39140.0000000	3.2500000	-50.0000000
39140.0000000	3.5000000	-50.0000000
39140.0000000	3.7500000	-50.0000000
39140.0000000	4.0000000	-50.0000000
39140.0000000	4.2500000	-50.0000000
39140.0000000	4.5000000	-50.0000000
39140.0000000	4.7500000	-50.0000000
39140.0000000	5.0000000	-50.0000000
39140.0000000	5.2500000	-50.0000000
39140.0000000	5.5000000	-50.0000000
39140.0000000	5.7500000	-50.0000000
39140.0000000	6.0000000	-50.0000000
39140.0000000	6.2500000	-50.0000000
39140.0000000	6.5000000	-50.0000000
39140.0000000	6.7500000	-50.0000000
39140.0000000	7.0000000	-50.0000000
39140.0000000	7.2500000	-50.0000000
39140.0000000	7.5000000	-50.0000000
39140.0000000	7.7500000	-50.0000000
39140.0000000	8.0000000	-50.0000000
39140.0000000	8.2500000	-50.0000000
39140.0000000	8.5000000	-50.0000000
39140.0000000	8.7500000	-50.0000000

39140.0000000	9.0000000	-50.0000000
39140.0000000	9.2500000	-50.0000000
39140.0000000	9.5000000	-50.0000000
39140.0000000	9.7500000	-50.0000000
39140.0000000	10.0000000	-50.0000000
39140.0000000	10.2500000	-50.0000000
39140.0000000	10.5000000	-50.0000000
39140.0000000	10.7500000	-50.0000000
39140.0000000	11.0000000	-50.0000000
39140.0000000	11.2500000	-50.0000000
39140.0000000	11.5000000	-50.0000000
39140.0000000	11.7500000	-50.0000000
39140.0000000	12.0000000	-50.0000000
39140.0000000	12.2500000	-50.0000000
39140.0000000	12.5000000	-50.0000000
39140.0000000	12.7500000	-50.0000000
39140.0000000	13.0000000	-50.0000000
39140.0000000	13.2500000	-50.0000000
39140.0000000	13.5000000	-50.0000000
39140.0000000	13.7500000	-50.0000000
39140.0000000	14.0000000	-50.0000000
39140.0000000	14.2500000	-50.0000000
39140.0000000	14.5000000	-50.0000000
39140.0000000	14.7500000	-50.0000000
39140.0000000	15.0000000	-50.0000000
39170.0000000	0.0000000	-50.0000000
39170.0000000	0.2500000	-50.0000000
39170.0000000	0.5000000	-50.0000000
39170.0000000	0.7500000	-50.0000000
39170.0000000	1.0000000	-50.0000000
39170.0000000	1.2500000	-50.0000000
39170.0000000	1.5000000	-50.0000000
39170.0000000	1.7500000	-50.0000000
39170.0000000	2.0000000	-50.0000000
39170.0000000	2.2500000	-50.0000000
39170.0000000	2.5000000	-50.0000000
39170.0000000	2.7500000	-50.0000000
39170.0000000	3.0000000	-50.0000000
39170.0000000	3.2500000	-50.0000000
39170.0000000	3.5000000	-50.0000000
39170.0000000	3.7500000	-50.0000000
39170.0000000	4.0000000	-50.0000000
39170.0000000	4.2500000	-50.0000000
39170.0000000	4.5000000	-50.0000000
39170.0000000	4.7500000	-50.0000000
39170.0000000	5.0000000	-50.0000000
39170.0000000	5.2500000	-50.0000000
39170.0000000	5.5000000	-50.0000000
39170.0000000	5.7500000	-50.0000000
39170.0000000	6.0000000	-50.0000000
39170.0000000	6.2500000	-50.0000000
39170.0000000	6.5000000	-50.0000000
39170.0000000	6.7500000	-50.0000000
39170.0000000	7.0000000	-50.0000000
39170.0000000	7.2500000	-50.0000000
39170.0000000	7.5000000	-50.0000000
39170.0000000	7.7500000	-50.0000000
39170.0000000	8.0000000	-50.0000000

39170.0000000	8.2500000	-50.0000000
39170.0000000	8.5000000	-50.0000000
39170.0000000	8.7500000	-50.0000000
39170.0000000	9.0000000	-50.0000000
39170.0000000	9.2500000	-50.0000000
39170.0000000	9.5000000	-50.0000000
39170.0000000	9.7500000	-50.0000000
39170.0000000	10.0000000	-50.0000000
39170.0000000	10.2500000	-50.0000000
39170.0000000	10.5000000	-50.0000000
39170.0000000	10.7500000	-50.0000000
39170.0000000	11.0000000	-50.0000000
39170.0000000	11.2500000	-50.0000000
39170.0000000	11.5000000	-50.0000000
39170.0000000	11.7500000	-50.0000000
39170.0000000	12.0000000	-50.0000000
39170.0000000	12.2500000	-50.0000000
39170.0000000	12.5000000	-50.0000000
39170.0000000	12.7500000	-50.0000000
39170.0000000	13.0000000	-50.0000000
39170.0000000	13.2500000	-50.0000000
39170.0000000	13.5000000	-50.0000000
39170.0000000	13.7500000	-50.0000000
39170.0000000	14.0000000	-50.0000000
39170.0000000	14.2500000	-50.0000000
39170.0000000	14.5000000	-50.0000000
39170.0000000	14.7500000	-50.0000000
39170.0000000	15.0000000	-50.0000000
39200.0000000	0.0000000	-50.0000000
39200.0000000	0.2500000	-50.0000000
39200.0000000	0.5000000	-50.0000000
39200.0000000	0.7500000	-50.0000000
39200.0000000	1.0000000	-50.0000000
39200.0000000	1.2500000	-50.0000000
39200.0000000	1.5000000	-50.0000000
39200.0000000	1.7500000	-50.0000000
39200.0000000	2.0000000	-50.0000000
39200.0000000	2.2500000	-50.0000000
39200.0000000	2.5000000	-50.0000000
39200.0000000	2.7500000	-50.0000000
39200.0000000	3.0000000	-50.0000000
39200.0000000	3.2500000	-50.0000000
39200.0000000	3.5000000	-50.0000000
39200.0000000	3.7500000	-50.0000000
39200.0000000	4.0000000	-50.0000000
39200.0000000	4.2500000	-50.0000000
39200.0000000	4.5000000	-50.0000000
39200.0000000	4.7500000	-50.0000000
39200.0000000	5.0000000	-50.0000000
39200.0000000	5.2500000	-50.0000000
39200.0000000	5.5000000	-50.0000000
39200.0000000	5.7500000	-50.0000000
39200.0000000	6.0000000	-50.0000000
39200.0000000	6.2500000	-50.0000000
39200.0000000	6.5000000	-50.0000000
39200.0000000	6.7500000	-50.0000000
39200.0000000	7.0000000	-50.0000000
39200.0000000	7.2500000	-50.0000000

39200.0000000	7.5000000	-50.0000000
39200.0000000	7.7500000	-50.0000000
39200.0000000	8.0000000	-50.0000000
39200.0000000	8.2500000	-50.0000000
39200.0000000	8.5000000	-50.0000000
39200.0000000	8.7500000	-50.0000000
39200.0000000	9.0000000	-50.0000000
39200.0000000	9.2500000	-50.0000000
39200.0000000	9.5000000	-50.0000000
39200.0000000	9.7500000	-50.0000000
39200.0000000	10.0000000	-50.0000000
39200.0000000	10.2500000	-50.0000000
39200.0000000	10.5000000	-50.0000000
39200.0000000	10.7500000	-50.0000000
39200.0000000	11.0000000	-50.0000000
39200.0000000	11.2500000	-50.0000000
39200.0000000	11.5000000	-50.0000000
39200.0000000	11.7500000	-50.0000000
39200.0000000	12.0000000	-50.0000000
39200.0000000	12.2500000	-50.0000000
39200.0000000	12.5000000	-50.0000000
39200.0000000	12.7500000	-50.0000000
39200.0000000	13.0000000	-50.0000000
39200.0000000	13.2500000	-50.0000000
39200.0000000	13.5000000	-50.0000000
39200.0000000	13.7500000	-50.0000000
39200.0000000	14.0000000	-50.0000000
39200.0000000	14.2500000	-50.0000000
39200.0000000	14.5000000	-50.0000000
39200.0000000	14.7500000	-50.0000000
39200.0000000	15.0000000	-50.0000000
39230.0000000	0.0000000	-50.0000000
39230.0000000	0.2500000	-50.0000000
39230.0000000	0.5000000	-50.0000000
39230.0000000	0.7500000	-50.0000000
39230.0000000	1.0000000	-50.0000000
39230.0000000	1.2500000	-50.0000000
39230.0000000	1.5000000	-50.0000000
39230.0000000	1.7500000	-50.0000000
39230.0000000	2.0000000	-50.0000000
39230.0000000	2.2500000	-50.0000000
39230.0000000	2.5000000	-50.0000000
39230.0000000	2.7500000	-50.0000000
39230.0000000	3.0000000	-50.0000000
39230.0000000	3.2500000	-50.0000000
39230.0000000	3.5000000	-50.0000000
39230.0000000	3.7500000	-50.0000000
39230.0000000	4.0000000	-50.0000000
39230.0000000	4.2500000	-50.0000000
39230.0000000	4.5000000	-50.0000000
39230.0000000	4.7500000	-50.0000000
39230.0000000	5.0000000	-50.0000000
39230.0000000	5.2500000	-50.0000000
39230.0000000	5.5000000	-50.0000000
39230.0000000	5.7500000	-50.0000000
39230.0000000	6.0000000	-50.0000000
39230.0000000	6.2500000	-50.0000000
39230.0000000	6.5000000	-50.0000000

39230.0000000	6.7500000	-50.0000000
39230.0000000	7.0000000	-50.0000000
39230.0000000	7.2500000	-50.0000000
39230.0000000	7.5000000	-50.0000000
39230.0000000	7.7500000	-50.0000000
39230.0000000	8.0000000	-50.0000000
39230.0000000	8.2500000	-50.0000000
39230.0000000	8.5000000	-50.0000000
39230.0000000	8.7500000	-50.0000000
39230.0000000	9.0000000	-50.0000000
39230.0000000	9.2500000	-50.0000000
39230.0000000	9.5000000	-50.0000000
39230.0000000	9.7500000	-50.0000000
39230.0000000	10.0000000	-50.0000000
39230.0000000	10.2500000	-50.0000000
39230.0000000	10.5000000	-50.0000000
39230.0000000	10.7500000	-50.0000000
39230.0000000	11.0000000	-50.0000000
39230.0000000	11.2500000	-50.0000000
39230.0000000	11.5000000	-50.0000000
39230.0000000	11.7500000	-50.0000000
39230.0000000	12.0000000	-50.0000000
39230.0000000	12.2500000	-50.0000000
39230.0000000	12.5000000	-50.0000000
39230.0000000	12.7500000	-50.0000000
39230.0000000	13.0000000	-50.0000000
39230.0000000	13.2500000	-50.0000000
39230.0000000	13.5000000	-50.0000000
39230.0000000	13.7500000	-50.0000000
39230.0000000	14.0000000	-50.0000000
39230.0000000	14.2500000	-50.0000000
39230.0000000	14.5000000	-50.0000000
39230.0000000	14.7500000	-50.0000000
39230.0000000	15.0000000	-50.0000000
39260.0000000	0.0000000	-50.0000000
39260.0000000	0.2500000	-50.0000000
39260.0000000	0.5000000	-50.0000000
39260.0000000	0.7500000	-50.0000000
39260.0000000	1.0000000	-50.0000000
39260.0000000	1.2500000	-50.0000000
39260.0000000	1.5000000	-50.0000000
39260.0000000	1.7500000	-50.0000000
39260.0000000	2.0000000	-50.0000000
39260.0000000	2.2500000	-50.0000000
39260.0000000	2.5000000	-50.0000000
39260.0000000	2.7500000	-50.0000000
39260.0000000	3.0000000	-50.0000000
39260.0000000	3.2500000	-50.0000000
39260.0000000	3.5000000	-50.0000000
39260.0000000	3.7500000	-50.0000000
39260.0000000	4.0000000	-50.0000000
39260.0000000	4.2500000	-50.0000000
39260.0000000	4.5000000	-50.0000000
39260.0000000	4.7500000	-50.0000000
39260.0000000	5.0000000	-50.0000000
39260.0000000	5.2500000	-50.0000000
39260.0000000	5.5000000	-50.0000000
39260.0000000	5.7500000	-50.0000000

39260.0000000	6.0000000	-50.0000000
39260.0000000	6.2500000	-50.0000000
39260.0000000	6.5000000	-50.0000000
39260.0000000	6.7500000	-50.0000000
39260.0000000	7.0000000	-50.0000000
39260.0000000	7.2500000	-50.0000000
39260.0000000	7.5000000	-50.0000000
39260.0000000	7.7500000	-50.0000000
39260.0000000	8.0000000	-50.0000000
39260.0000000	8.2500000	-50.0000000
39260.0000000	8.5000000	-50.0000000
39260.0000000	8.7500000	-50.0000000
39260.0000000	9.0000000	-50.0000000
39260.0000000	9.2500000	-50.0000000
39260.0000000	9.5000000	-50.0000000
39260.0000000	9.7500000	-50.0000000
39260.0000000	10.0000000	-50.0000000
39260.0000000	10.2500000	-50.0000000
39260.0000000	10.5000000	-50.0000000
39260.0000000	10.7500000	-50.0000000
39260.0000000	11.0000000	-50.0000000
39260.0000000	11.2500000	-50.0000000
39260.0000000	11.5000000	-50.0000000
39260.0000000	11.7500000	-50.0000000
39260.0000000	12.0000000	-50.0000000
39260.0000000	12.2500000	-50.0000000
39260.0000000	12.5000000	-50.0000000
39260.0000000	12.7500000	-50.0000000
39260.0000000	13.0000000	-50.0000000
39260.0000000	13.2500000	-50.0000000
39260.0000000	13.5000000	-50.0000000
39260.0000000	13.7500000	-50.0000000
39260.0000000	14.0000000	-50.0000000
39260.0000000	14.2500000	-50.0000000
39260.0000000	14.5000000	-50.0000000
39260.0000000	14.7500000	-50.0000000
39260.0000000	15.0000000	-50.0000000
39290.0000000	0.0000000	-50.0000000
39290.0000000	0.2500000	-50.0000000
39290.0000000	0.5000000	-50.0000000
39290.0000000	0.7500000	-50.0000000
39290.0000000	1.0000000	-50.0000000
39290.0000000	1.2500000	-50.0000000
39290.0000000	1.5000000	-50.0000000
39290.0000000	1.7500000	-50.0000000
39290.0000000	2.0000000	-50.0000000
39290.0000000	2.2500000	-50.0000000
39290.0000000	2.5000000	-50.0000000
39290.0000000	2.7500000	-50.0000000
39290.0000000	3.0000000	-50.0000000
39290.0000000	3.2500000	-50.0000000
39290.0000000	3.5000000	-50.0000000
39290.0000000	3.7500000	-50.0000000
39290.0000000	4.0000000	-50.0000000
39290.0000000	4.2500000	-50.0000000
39290.0000000	4.5000000	-50.0000000
39290.0000000	4.7500000	-50.0000000
39290.0000000	5.0000000	-50.0000000

39290.0000000	5.2500000	-50.0000000
39290.0000000	5.5000000	-50.0000000
39290.0000000	5.7500000	-50.0000000
39290.0000000	6.0000000	-50.0000000
39290.0000000	6.2500000	-50.0000000
39290.0000000	6.5000000	-50.0000000
39290.0000000	6.7500000	-50.0000000
39290.0000000	7.0000000	-50.0000000
39290.0000000	7.2500000	-50.0000000
39290.0000000	7.5000000	-50.0000000
39290.0000000	7.7500000	-50.0000000
39290.0000000	8.0000000	-50.0000000
39290.0000000	8.2500000	-50.0000000
39290.0000000	8.5000000	-50.0000000
39290.0000000	8.7500000	-50.0000000
39290.0000000	9.0000000	-50.0000000
39290.0000000	9.2500000	-50.0000000
39290.0000000	9.5000000	-50.0000000
39290.0000000	9.7500000	-50.0000000
39290.0000000	10.0000000	-50.0000000
39290.0000000	10.2500000	-50.0000000
39290.0000000	10.5000000	-50.0000000
39290.0000000	10.7500000	-50.0000000
39290.0000000	11.0000000	-50.0000000
39290.0000000	11.2500000	-50.0000000
39290.0000000	11.5000000	-50.0000000
39290.0000000	11.7500000	-50.0000000
39290.0000000	12.0000000	-50.0000000
39290.0000000	12.2500000	-50.0000000
39290.0000000	12.5000000	-50.0000000
39290.0000000	12.7500000	-50.0000000
39290.0000000	13.0000000	-50.0000000
39290.0000000	13.2500000	-50.0000000
39290.0000000	13.5000000	-50.0000000
39290.0000000	13.7500000	-50.0000000
39290.0000000	14.0000000	-50.0000000
39290.0000000	14.2500000	-50.0000000
39290.0000000	14.5000000	-50.0000000
39290.0000000	14.7500000	-50.0000000
39290.0000000	15.0000000	-50.0000000
39320.0000000	0.0000000	-50.0000000
39320.0000000	0.2500000	-50.0000000
39320.0000000	0.5000000	-50.0000000
39320.0000000	0.7500000	-50.0000000
39320.0000000	1.0000000	-50.0000000
39320.0000000	1.2500000	-50.0000000
39320.0000000	1.5000000	-50.0000000
39320.0000000	1.7500000	-50.0000000
39320.0000000	2.0000000	-50.0000000
39320.0000000	2.2500000	-50.0000000
39320.0000000	2.5000000	-50.0000000
39320.0000000	2.7500000	-50.0000000
39320.0000000	3.0000000	-50.0000000
39320.0000000	3.2500000	-50.0000000
39320.0000000	3.5000000	-50.0000000
39320.0000000	3.7500000	-50.0000000
39320.0000000	4.0000000	-50.0000000
39320.0000000	4.2500000	-50.0000000

39320.0000000	4.5000000	-50.0000000
39320.0000000	4.7500000	-50.0000000
39320.0000000	5.0000000	-50.0000000
39320.0000000	5.2500000	-50.0000000
39320.0000000	5.5000000	-50.0000000
39320.0000000	5.7500000	-50.0000000
39320.0000000	6.0000000	-50.0000000
39320.0000000	6.2500000	-50.0000000
39320.0000000	6.5000000	-50.0000000
39320.0000000	6.7500000	-50.0000000
39320.0000000	7.0000000	-50.0000000
39320.0000000	7.2500000	-50.0000000
39320.0000000	7.5000000	-50.0000000
39320.0000000	7.7500000	-50.0000000
39320.0000000	8.0000000	-50.0000000
39320.0000000	8.2500000	-50.0000000
39320.0000000	8.5000000	-50.0000000
39320.0000000	8.7500000	-50.0000000
39320.0000000	9.0000000	-50.0000000
39320.0000000	9.2500000	-50.0000000
39320.0000000	9.5000000	-50.0000000
39320.0000000	9.7500000	-50.0000000
39320.0000000	10.0000000	-50.0000000
39320.0000000	10.2500000	-50.0000000
39320.0000000	10.5000000	-50.0000000
39320.0000000	10.7500000	-50.0000000
39320.0000000	11.0000000	-50.0000000
39320.0000000	11.2500000	-50.0000000
39320.0000000	11.5000000	-50.0000000
39320.0000000	11.7500000	-50.0000000
39320.0000000	12.0000000	-50.0000000
39320.0000000	12.2500000	-50.0000000
39320.0000000	12.5000000	-50.0000000
39320.0000000	12.7500000	-50.0000000
39320.0000000	13.0000000	-50.0000000
39320.0000000	13.2500000	-50.0000000
39320.0000000	13.5000000	-50.0000000
39320.0000000	13.7500000	-50.0000000
39320.0000000	14.0000000	-50.0000000
39320.0000000	14.2500000	-50.0000000
39320.0000000	14.5000000	-50.0000000
39320.0000000	14.7500000	-50.0000000
39320.0000000	15.0000000	-50.0000000
39350.0000000	0.0000000	-50.0000000
39350.0000000	0.2500000	-50.0000000
39350.0000000	0.5000000	-50.0000000
39350.0000000	0.7500000	-50.0000000
39350.0000000	1.0000000	-50.0000000
39350.0000000	1.2500000	-50.0000000
39350.0000000	1.5000000	-50.0000000
39350.0000000	1.7500000	-50.0000000
39350.0000000	2.0000000	-50.0000000
39350.0000000	2.2500000	-50.0000000
39350.0000000	2.5000000	-50.0000000
39350.0000000	2.7500000	-50.0000000
39350.0000000	3.0000000	-50.0000000
39350.0000000	3.2500000	-50.0000000
39350.0000000	3.5000000	-50.0000000

39350.0000000	3.7500000	-50.0000000
39350.0000000	4.0000000	-50.0000000
39350.0000000	4.2500000	-50.0000000
39350.0000000	4.5000000	-50.0000000
39350.0000000	4.7500000	-50.0000000
39350.0000000	5.0000000	-50.0000000
39350.0000000	5.2500000	-50.0000000
39350.0000000	5.5000000	-50.0000000
39350.0000000	5.7500000	-50.0000000
39350.0000000	6.0000000	-50.0000000
39350.0000000	6.2500000	-50.0000000
39350.0000000	6.5000000	-50.0000000
39350.0000000	6.7500000	-50.0000000
39350.0000000	7.0000000	-50.0000000
39350.0000000	7.2500000	-50.0000000
39350.0000000	7.5000000	-50.0000000
39350.0000000	7.7500000	-50.0000000
39350.0000000	8.0000000	-50.0000000
39350.0000000	8.2500000	-50.0000000
39350.0000000	8.5000000	-50.0000000
39350.0000000	8.7500000	-50.0000000
39350.0000000	9.0000000	-50.0000000
39350.0000000	9.2500000	-50.0000000
39350.0000000	9.5000000	-50.0000000
39350.0000000	9.7500000	-50.0000000
39350.0000000	10.0000000	-50.0000000
39350.0000000	10.2500000	-50.0000000
39350.0000000	10.5000000	-50.0000000
39350.0000000	10.7500000	-50.0000000
39350.0000000	11.0000000	-50.0000000
39350.0000000	11.2500000	-50.0000000
39350.0000000	11.5000000	-50.0000000
39350.0000000	11.7500000	-50.0000000
39350.0000000	12.0000000	-50.0000000
39350.0000000	12.2500000	-50.0000000
39350.0000000	12.5000000	-50.0000000
39350.0000000	12.7500000	-50.0000000
39350.0000000	13.0000000	-50.0000000
39350.0000000	13.2500000	-50.0000000
39350.0000000	13.5000000	-50.0000000
39350.0000000	13.7500000	-50.0000000
39350.0000000	14.0000000	-50.0000000
39350.0000000	14.2500000	-50.0000000
39350.0000000	14.5000000	-50.0000000
39350.0000000	14.7500000	-50.0000000
39350.0000000	15.0000000	-50.0000000
39380.0000000	0.0000000	-50.0000000
39380.0000000	0.2500000	-50.0000000
39380.0000000	0.5000000	-50.0000000
39380.0000000	0.7500000	-50.0000000
39380.0000000	1.0000000	-50.0000000
39380.0000000	1.2500000	-50.0000000
39380.0000000	1.5000000	-50.0000000
39380.0000000	1.7500000	-50.0000000
39380.0000000	2.0000000	-50.0000000
39380.0000000	2.2500000	-50.0000000
39380.0000000	2.5000000	-50.0000000
39380.0000000	2.7500000	-50.0000000

39380.0000000	3.0000000	-50.0000000
39380.0000000	3.2500000	-50.0000000
39380.0000000	3.5000000	-50.0000000
39380.0000000	3.7500000	-50.0000000
39380.0000000	4.0000000	-50.0000000
39380.0000000	4.2500000	-50.0000000
39380.0000000	4.5000000	-50.0000000
39380.0000000	4.7500000	-50.0000000
39380.0000000	5.0000000	-50.0000000
39380.0000000	5.2500000	-50.0000000
39380.0000000	5.5000000	-50.0000000
39380.0000000	5.7500000	-50.0000000
39380.0000000	6.0000000	-50.0000000
39380.0000000	6.2500000	-50.0000000
39380.0000000	6.5000000	-50.0000000
39380.0000000	6.7500000	-50.0000000
39380.0000000	7.0000000	-50.0000000
39380.0000000	7.2500000	-50.0000000
39380.0000000	7.5000000	-50.0000000
39380.0000000	7.7500000	-50.0000000
39380.0000000	8.0000000	-50.0000000
39380.0000000	8.2500000	-50.0000000
39380.0000000	8.5000000	-50.0000000
39380.0000000	8.7500000	-50.0000000
39380.0000000	9.0000000	-50.0000000
39380.0000000	9.2500000	-50.0000000
39380.0000000	9.5000000	-50.0000000
39380.0000000	9.7500000	-50.0000000
39380.0000000	10.0000000	-50.0000000
39380.0000000	10.2500000	-50.0000000
39380.0000000	10.5000000	-50.0000000
39380.0000000	10.7500000	-50.0000000
39380.0000000	11.0000000	-50.0000000
39380.0000000	11.2500000	-50.0000000
39380.0000000	11.5000000	-50.0000000
39380.0000000	11.7500000	-50.0000000
39380.0000000	12.0000000	-50.0000000
39380.0000000	12.2500000	-50.0000000
39380.0000000	12.5000000	-50.0000000
39380.0000000	12.7500000	-50.0000000
39380.0000000	13.0000000	-50.0000000
39380.0000000	13.2500000	-50.0000000
39380.0000000	13.5000000	-50.0000000
39380.0000000	13.7500000	-50.0000000
39380.0000000	14.0000000	-50.0000000
39380.0000000	14.2500000	-50.0000000
39380.0000000	14.5000000	-50.0000000
39380.0000000	14.7500000	-50.0000000
39380.0000000	15.0000000	-50.0000000
39410.0000000	0.0000000	-50.0000000
39410.0000000	0.2500000	-50.0000000
39410.0000000	0.5000000	-50.0000000
39410.0000000	0.7500000	-50.0000000
39410.0000000	1.0000000	-50.0000000
39410.0000000	1.2500000	-50.0000000
39410.0000000	1.5000000	-50.0000000
39410.0000000	1.7500000	-50.0000000
39410.0000000	2.0000000	-50.0000000

39410.0000000	2.2500000	-50.0000000
39410.0000000	2.5000000	-50.0000000
39410.0000000	2.7500000	-50.0000000
39410.0000000	3.0000000	-50.0000000
39410.0000000	3.2500000	-50.0000000
39410.0000000	3.5000000	-50.0000000
39410.0000000	3.7500000	-50.0000000
39410.0000000	4.0000000	-50.0000000
39410.0000000	4.2500000	-50.0000000
39410.0000000	4.5000000	-50.0000000
39410.0000000	4.7500000	-50.0000000
39410.0000000	5.0000000	-50.0000000
39410.0000000	5.2500000	-50.0000000
39410.0000000	5.5000000	-50.0000000
39410.0000000	5.7500000	-50.0000000
39410.0000000	6.0000000	-50.0000000
39410.0000000	6.2500000	-50.0000000
39410.0000000	6.5000000	-50.0000000
39410.0000000	6.7500000	-50.0000000
39410.0000000	7.0000000	-50.0000000
39410.0000000	7.2500000	-50.0000000
39410.0000000	7.5000000	-50.0000000
39410.0000000	7.7500000	-50.0000000
39410.0000000	8.0000000	-50.0000000
39410.0000000	8.2500000	-50.0000000
39410.0000000	8.5000000	-50.0000000
39410.0000000	8.7500000	-50.0000000
39410.0000000	9.0000000	-50.0000000
39410.0000000	9.2500000	-50.0000000
39410.0000000	9.5000000	-50.0000000
39410.0000000	9.7500000	-50.0000000
39410.0000000	10.0000000	-50.0000000
39410.0000000	10.2500000	-50.0000000
39410.0000000	10.5000000	-50.0000000
39410.0000000	10.7500000	-50.0000000
39410.0000000	11.0000000	-50.0000000
39410.0000000	11.2500000	-50.0000000
39410.0000000	11.5000000	-50.0000000
39410.0000000	11.7500000	-50.0000000
39410.0000000	12.0000000	-50.0000000
39410.0000000	12.2500000	-50.0000000
39410.0000000	12.5000000	-50.0000000
39410.0000000	12.7500000	-50.0000000
39410.0000000	13.0000000	-50.0000000
39410.0000000	13.2500000	-50.0000000
39410.0000000	13.5000000	-50.0000000
39410.0000000	13.7500000	-50.0000000
39410.0000000	14.0000000	-50.0000000
39410.0000000	14.2500000	-50.0000000
39410.0000000	14.5000000	-50.0000000
39410.0000000	14.7500000	-50.0000000
39410.0000000	15.0000000	-50.0000000
39440.0000000	0.0000000	-50.0000000
39440.0000000	0.2500000	-50.0000000
39440.0000000	0.5000000	-50.0000000
39440.0000000	0.7500000	-50.0000000
39440.0000000	1.0000000	-50.0000000
39440.0000000	1.2500000	-50.0000000

39440.0000000	1.5000000	-50.0000000
39440.0000000	1.7500000	-50.0000000
39440.0000000	2.0000000	-50.0000000
39440.0000000	2.2500000	-50.0000000
39440.0000000	2.5000000	-50.0000000
39440.0000000	2.7500000	-50.0000000
39440.0000000	3.0000000	-50.0000000
39440.0000000	3.2500000	-50.0000000
39440.0000000	3.5000000	-50.0000000
39440.0000000	3.7500000	-50.0000000
39440.0000000	4.0000000	-50.0000000
39440.0000000	4.2500000	-50.0000000
39440.0000000	4.5000000	-50.0000000
39440.0000000	4.7500000	-50.0000000
39440.0000000	5.0000000	-50.0000000
39440.0000000	5.2500000	-50.0000000
39440.0000000	5.5000000	-50.0000000
39440.0000000	5.7500000	-50.0000000
39440.0000000	6.0000000	-50.0000000
39440.0000000	6.2500000	-50.0000000
39440.0000000	6.5000000	-50.0000000
39440.0000000	6.7500000	-50.0000000
39440.0000000	7.0000000	-50.0000000
39440.0000000	7.2500000	-50.0000000
39440.0000000	7.5000000	-50.0000000
39440.0000000	7.7500000	-50.0000000
39440.0000000	8.0000000	-50.0000000
39440.0000000	8.2500000	-50.0000000
39440.0000000	8.5000000	-50.0000000
39440.0000000	8.7500000	-50.0000000
39440.0000000	9.0000000	-50.0000000
39440.0000000	9.2500000	-50.0000000
39440.0000000	9.5000000	-50.0000000
39440.0000000	9.7500000	-50.0000000
39440.0000000	10.0000000	-50.0000000
39440.0000000	10.2500000	-50.0000000
39440.0000000	10.5000000	-50.0000000
39440.0000000	10.7500000	-50.0000000
39440.0000000	11.0000000	-50.0000000
39440.0000000	11.2500000	-50.0000000
39440.0000000	11.5000000	-50.0000000
39440.0000000	11.7500000	-50.0000000
39440.0000000	12.0000000	-50.0000000
39440.0000000	12.2500000	-50.0000000
39440.0000000	12.5000000	-50.0000000
39440.0000000	12.7500000	-50.0000000
39440.0000000	13.0000000	-50.0000000
39440.0000000	13.2500000	-50.0000000
39440.0000000	13.5000000	-50.0000000
39440.0000000	13.7500000	-50.0000000
39440.0000000	14.0000000	-50.0000000
39440.0000000	14.2500000	-50.0000000
39440.0000000	14.5000000	-50.0000000
39440.0000000	14.7500000	-50.0000000
39440.0000000	15.0000000	-50.0000000
39470.0000000	0.0000000	-50.0000000
39470.0000000	0.2500000	-50.0000000
39470.0000000	0.5000000	-50.0000000

39470.0000000	0.7500000	-50.0000000
39470.0000000	1.0000000	-50.0000000
39470.0000000	1.2500000	-50.0000000
39470.0000000	1.5000000	-50.0000000
39470.0000000	1.7500000	-50.0000000
39470.0000000	2.0000000	-50.0000000
39470.0000000	2.2500000	-50.0000000
39470.0000000	2.5000000	-50.0000000
39470.0000000	2.7500000	-50.0000000
39470.0000000	3.0000000	-50.0000000
39470.0000000	3.2500000	-50.0000000
39470.0000000	3.5000000	-50.0000000
39470.0000000	3.7500000	-50.0000000
39470.0000000	4.0000000	-50.0000000
39470.0000000	4.2500000	-50.0000000
39470.0000000	4.5000000	-50.0000000
39470.0000000	4.7500000	-50.0000000
39470.0000000	5.0000000	-50.0000000
39470.0000000	5.2500000	-50.0000000
39470.0000000	5.5000000	-50.0000000
39470.0000000	5.7500000	-50.0000000
39470.0000000	6.0000000	-50.0000000
39470.0000000	6.2500000	-50.0000000
39470.0000000	6.5000000	-50.0000000
39470.0000000	6.7500000	-50.0000000
39470.0000000	7.0000000	-50.0000000
39470.0000000	7.2500000	-50.0000000
39470.0000000	7.5000000	-50.0000000
39470.0000000	7.7500000	-50.0000000
39470.0000000	8.0000000	-50.0000000
39470.0000000	8.2500000	-50.0000000
39470.0000000	8.5000000	-50.0000000
39470.0000000	8.7500000	-50.0000000
39470.0000000	9.0000000	-50.0000000
39470.0000000	9.2500000	-50.0000000
39470.0000000	9.5000000	-50.0000000
39470.0000000	9.7500000	-50.0000000
39470.0000000	10.0000000	-50.0000000
39470.0000000	10.2500000	-50.0000000
39470.0000000	10.5000000	-50.0000000
39470.0000000	10.7500000	-50.0000000
39470.0000000	11.0000000	-50.0000000
39470.0000000	11.2500000	-50.0000000
39470.0000000	11.5000000	-50.0000000
39470.0000000	11.7500000	-50.0000000
39470.0000000	12.0000000	-50.0000000
39470.0000000	12.2500000	-50.0000000
39470.0000000	12.5000000	-50.0000000
39470.0000000	12.7500000	-50.0000000
39470.0000000	13.0000000	-50.0000000
39470.0000000	13.2500000	-50.0000000
39470.0000000	13.5000000	-50.0000000
39470.0000000	13.7500000	-50.0000000
39470.0000000	14.0000000	-50.0000000
39470.0000000	14.2500000	-50.0000000
39470.0000000	14.5000000	-50.0000000
39470.0000000	14.7500000	-50.0000000
39470.0000000	15.0000000	-50.0000000

39500.0000000	0.0000000	-50.0000000
39500.0000000	0.2500000	-50.0000000
39500.0000000	0.5000000	-50.0000000
39500.0000000	0.7500000	-50.0000000
39500.0000000	1.0000000	-50.0000000
39500.0000000	1.2500000	-50.0000000
39500.0000000	1.5000000	-50.0000000
39500.0000000	1.7500000	-50.0000000
39500.0000000	2.0000000	-50.0000000
39500.0000000	2.2500000	-50.0000000
39500.0000000	2.5000000	-50.0000000
39500.0000000	2.7500000	-50.0000000
39500.0000000	3.0000000	-50.0000000
39500.0000000	3.2500000	-50.0000000
39500.0000000	3.5000000	-50.0000000
39500.0000000	3.7500000	-50.0000000
39500.0000000	4.0000000	-50.0000000
39500.0000000	4.2500000	-50.0000000
39500.0000000	4.5000000	-50.0000000
39500.0000000	4.7500000	-50.0000000
39500.0000000	5.0000000	-50.0000000
39500.0000000	5.2500000	-50.0000000
39500.0000000	5.5000000	-50.0000000
39500.0000000	5.7500000	-50.0000000
39500.0000000	6.0000000	-50.0000000
39500.0000000	6.2500000	-50.0000000
39500.0000000	6.5000000	-50.0000000
39500.0000000	6.7500000	-50.0000000
39500.0000000	7.0000000	-50.0000000
39500.0000000	7.2500000	-50.0000000
39500.0000000	7.5000000	-50.0000000
39500.0000000	7.7500000	-50.0000000
39500.0000000	8.0000000	-50.0000000
39500.0000000	8.2500000	-50.0000000
39500.0000000	8.5000000	-50.0000000
39500.0000000	8.7500000	-50.0000000
39500.0000000	9.0000000	-50.0000000
39500.0000000	9.2500000	-50.0000000
39500.0000000	9.5000000	-50.0000000
39500.0000000	9.7500000	-50.0000000
39500.0000000	10.0000000	-50.0000000
39500.0000000	10.2500000	-50.0000000
39500.0000000	10.5000000	-50.0000000
39500.0000000	10.7500000	-50.0000000
39500.0000000	11.0000000	-50.0000000
39500.0000000	11.2500000	-50.0000000
39500.0000000	11.5000000	-50.0000000
39500.0000000	11.7500000	-50.0000000
39500.0000000	12.0000000	-50.0000000
39500.0000000	12.2500000	-50.0000000
39500.0000000	12.5000000	-50.0000000
39500.0000000	12.7500000	-50.0000000
39500.0000000	13.0000000	-50.0000000
39500.0000000	13.2500000	-50.0000000
39500.0000000	13.5000000	-50.0000000
39500.0000000	13.7500000	-50.0000000
39500.0000000	14.0000000	-50.0000000
39500.0000000	14.2500000	-50.0000000

39500.0000000	14.5000000	-50.0000000
39500.0000000	14.7500000	-50.0000000
39500.0000000	15.0000000	-50.0000000
39530.0000000	0.0000000	-50.0000000
39530.0000000	0.2500000	-50.0000000
39530.0000000	0.5000000	-50.0000000
39530.0000000	0.7500000	-50.0000000
39530.0000000	1.0000000	-50.0000000
39530.0000000	1.2500000	-50.0000000
39530.0000000	1.5000000	-50.0000000
39530.0000000	1.7500000	-50.0000000
39530.0000000	2.0000000	-50.0000000
39530.0000000	2.2500000	-50.0000000
39530.0000000	2.5000000	-50.0000000
39530.0000000	2.7500000	-50.0000000
39530.0000000	3.0000000	-50.0000000
39530.0000000	3.2500000	-50.0000000
39530.0000000	3.5000000	-50.0000000
39530.0000000	3.7500000	-50.0000000
39530.0000000	4.0000000	-50.0000000
39530.0000000	4.2500000	-50.0000000
39530.0000000	4.5000000	-50.0000000
39530.0000000	4.7500000	-50.0000000
39530.0000000	5.0000000	-50.0000000
39530.0000000	5.2500000	-50.0000000
39530.0000000	5.5000000	-50.0000000
39530.0000000	5.7500000	-50.0000000
39530.0000000	6.0000000	-50.0000000
39530.0000000	6.2500000	-50.0000000
39530.0000000	6.5000000	-50.0000000
39530.0000000	6.7500000	-50.0000000
39530.0000000	7.0000000	-50.0000000
39530.0000000	7.2500000	-50.0000000
39530.0000000	7.5000000	-50.0000000
39530.0000000	7.7500000	-50.0000000
39530.0000000	8.0000000	-50.0000000
39530.0000000	8.2500000	-50.0000000
39530.0000000	8.5000000	-50.0000000
39530.0000000	8.7500000	-50.0000000
39530.0000000	9.0000000	-50.0000000
39530.0000000	9.2500000	-50.0000000
39530.0000000	9.5000000	-50.0000000
39530.0000000	9.7500000	-50.0000000
39530.0000000	10.0000000	-50.0000000
39530.0000000	10.2500000	-50.0000000
39530.0000000	10.5000000	-50.0000000
39530.0000000	10.7500000	-50.0000000
39530.0000000	11.0000000	-50.0000000
39530.0000000	11.2500000	-50.0000000
39530.0000000	11.5000000	-50.0000000
39530.0000000	11.7500000	-50.0000000
39530.0000000	12.0000000	-50.0000000
39530.0000000	12.2500000	-50.0000000
39530.0000000	12.5000000	-50.0000000
39530.0000000	12.7500000	-50.0000000
39530.0000000	13.0000000	-50.0000000
39530.0000000	13.2500000	-50.0000000
39530.0000000	13.5000000	-50.0000000

39530.0000000	13.7500000	-50.0000000
39530.0000000	14.0000000	-50.0000000
39530.0000000	14.2500000	-50.0000000
39530.0000000	14.5000000	-50.0000000
39530.0000000	14.7500000	-50.0000000
39530.0000000	15.0000000	-50.0000000
39560.0000000	0.0000000	-50.0000000
39560.0000000	0.2500000	-50.0000000
39560.0000000	0.5000000	-50.0000000
39560.0000000	0.7500000	-50.0000000
39560.0000000	1.0000000	-50.0000000
39560.0000000	1.2500000	-50.0000000
39560.0000000	1.5000000	-50.0000000
39560.0000000	1.7500000	-50.0000000
39560.0000000	2.0000000	-50.0000000
39560.0000000	2.2500000	-50.0000000
39560.0000000	2.5000000	-50.0000000
39560.0000000	2.7500000	-50.0000000
39560.0000000	3.0000000	-50.0000000
39560.0000000	3.2500000	-50.0000000
39560.0000000	3.5000000	-50.0000000
39560.0000000	3.7500000	-50.0000000
39560.0000000	4.0000000	-50.0000000
39560.0000000	4.2500000	-50.0000000
39560.0000000	4.5000000	-50.0000000
39560.0000000	4.7500000	-50.0000000
39560.0000000	5.0000000	-50.0000000
39560.0000000	5.2500000	-50.0000000
39560.0000000	5.5000000	-50.0000000
39560.0000000	5.7500000	-50.0000000
39560.0000000	6.0000000	-50.0000000
39560.0000000	6.2500000	-50.0000000
39560.0000000	6.5000000	-50.0000000
39560.0000000	6.7500000	-50.0000000
39560.0000000	7.0000000	-50.0000000
39560.0000000	7.2500000	-50.0000000
39560.0000000	7.5000000	-50.0000000
39560.0000000	7.7500000	-50.0000000
39560.0000000	8.0000000	-50.0000000
39560.0000000	8.2500000	-50.0000000
39560.0000000	8.5000000	-50.0000000
39560.0000000	8.7500000	-50.0000000
39560.0000000	9.0000000	-50.0000000
39560.0000000	9.2500000	-50.0000000
39560.0000000	9.5000000	-50.0000000
39560.0000000	9.7500000	-50.0000000
39560.0000000	10.0000000	-50.0000000
39560.0000000	10.2500000	-50.0000000
39560.0000000	10.5000000	-50.0000000
39560.0000000	10.7500000	-50.0000000
39560.0000000	11.0000000	-50.0000000
39560.0000000	11.2500000	-50.0000000
39560.0000000	11.5000000	-50.0000000
39560.0000000	11.7500000	-50.0000000
39560.0000000	12.0000000	-50.0000000
39560.0000000	12.2500000	-50.0000000
39560.0000000	12.5000000	-50.0000000
39560.0000000	12.7500000	-50.0000000

39560.0000000	13.0000000	-50.0000000
39560.0000000	13.2500000	-50.0000000
39560.0000000	13.5000000	-50.0000000
39560.0000000	13.7500000	-50.0000000
39560.0000000	14.0000000	-50.0000000
39560.0000000	14.2500000	-50.0000000
39560.0000000	14.5000000	-50.0000000
39560.0000000	14.7500000	-50.0000000
39560.0000000	15.0000000	-50.0000000
39590.0000000	0.0000000	-50.0000000
39590.0000000	0.2500000	-50.0000000
39590.0000000	0.5000000	-50.0000000
39590.0000000	0.7500000	-50.0000000
39590.0000000	1.0000000	-50.0000000
39590.0000000	1.2500000	-50.0000000
39590.0000000	1.5000000	-50.0000000
39590.0000000	1.7500000	-50.0000000
39590.0000000	2.0000000	-50.0000000
39590.0000000	2.2500000	-50.0000000
39590.0000000	2.5000000	-50.0000000
39590.0000000	2.7500000	-50.0000000
39590.0000000	3.0000000	-50.0000000
39590.0000000	3.2500000	-50.0000000
39590.0000000	3.5000000	-50.0000000
39590.0000000	3.7500000	-50.0000000
39590.0000000	4.0000000	-50.0000000
39590.0000000	4.2500000	-50.0000000
39590.0000000	4.5000000	-50.0000000
39590.0000000	4.7500000	-50.0000000
39590.0000000	5.0000000	-50.0000000
39590.0000000	5.2500000	-50.0000000
39590.0000000	5.5000000	-50.0000000
39590.0000000	5.7500000	-50.0000000
39590.0000000	6.0000000	-50.0000000
39590.0000000	6.2500000	-50.0000000
39590.0000000	6.5000000	-50.0000000
39590.0000000	6.7500000	-50.0000000
39590.0000000	7.0000000	-50.0000000
39590.0000000	7.2500000	-50.0000000
39590.0000000	7.5000000	-50.0000000
39590.0000000	7.7500000	-50.0000000
39590.0000000	8.0000000	-50.0000000
39590.0000000	8.2500000	-50.0000000
39590.0000000	8.5000000	-50.0000000
39590.0000000	8.7500000	-50.0000000
39590.0000000	9.0000000	-50.0000000
39590.0000000	9.2500000	-50.0000000
39590.0000000	9.5000000	-50.0000000
39590.0000000	9.7500000	-50.0000000
39590.0000000	10.0000000	-50.0000000
39590.0000000	10.2500000	-50.0000000
39590.0000000	10.5000000	-50.0000000
39590.0000000	10.7500000	-50.0000000
39590.0000000	11.0000000	-50.0000000
39590.0000000	11.2500000	-50.0000000
39590.0000000	11.5000000	-50.0000000
39590.0000000	11.7500000	-50.0000000
39590.0000000	12.0000000	-50.0000000

39590.0000000	12.2500000	-50.0000000
39590.0000000	12.5000000	-50.0000000
39590.0000000	12.7500000	-50.0000000
39590.0000000	13.0000000	-50.0000000
39590.0000000	13.2500000	-50.0000000
39590.0000000	13.5000000	-50.0000000
39590.0000000	13.7500000	-50.0000000
39590.0000000	14.0000000	-50.0000000
39590.0000000	14.2500000	-50.0000000
39590.0000000	14.5000000	-50.0000000
39590.0000000	14.7500000	-50.0000000
39590.0000000	15.0000000	-50.0000000
39620.0000000	0.0000000	-50.0000000
39620.0000000	0.2500000	-50.0000000
39620.0000000	0.5000000	-50.0000000
39620.0000000	0.7500000	-50.0000000
39620.0000000	1.0000000	-50.0000000
39620.0000000	1.2500000	-50.0000000
39620.0000000	1.5000000	-50.0000000
39620.0000000	1.7500000	-50.0000000
39620.0000000	2.0000000	-50.0000000
39620.0000000	2.2500000	-50.0000000
39620.0000000	2.5000000	-50.0000000
39620.0000000	2.7500000	-50.0000000
39620.0000000	3.0000000	-50.0000000
39620.0000000	3.2500000	-50.0000000
39620.0000000	3.5000000	-50.0000000
39620.0000000	3.7500000	-50.0000000
39620.0000000	4.0000000	-50.0000000
39620.0000000	4.2500000	-50.0000000
39620.0000000	4.5000000	-50.0000000
39620.0000000	4.7500000	-50.0000000
39620.0000000	5.0000000	-50.0000000
39620.0000000	5.2500000	-50.0000000
39620.0000000	5.5000000	-50.0000000
39620.0000000	5.7500000	-50.0000000
39620.0000000	6.0000000	-50.0000000
39620.0000000	6.2500000	-50.0000000
39620.0000000	6.5000000	-50.0000000
39620.0000000	6.7500000	-50.0000000
39620.0000000	7.0000000	-50.0000000
39620.0000000	7.2500000	-50.0000000
39620.0000000	7.5000000	-50.0000000
39620.0000000	7.7500000	-50.0000000
39620.0000000	8.0000000	-50.0000000
39620.0000000	8.2500000	-50.0000000
39620.0000000	8.5000000	-50.0000000
39620.0000000	8.7500000	-50.0000000
39620.0000000	9.0000000	-50.0000000
39620.0000000	9.2500000	-50.0000000
39620.0000000	9.5000000	-50.0000000
39620.0000000	9.7500000	-50.0000000
39620.0000000	10.0000000	-50.0000000
39620.0000000	10.2500000	-50.0000000
39620.0000000	10.5000000	-50.0000000
39620.0000000	10.7500000	-50.0000000
39620.0000000	11.0000000	-50.0000000
39620.0000000	11.2500000	-50.0000000

39620.0000000	11.5000000	-50.0000000
39620.0000000	11.7500000	-50.0000000
39620.0000000	12.0000000	-50.0000000
39620.0000000	12.2500000	-50.0000000
39620.0000000	12.5000000	-50.0000000
39620.0000000	12.7500000	-50.0000000
39620.0000000	13.0000000	-50.0000000
39620.0000000	13.2500000	-50.0000000
39620.0000000	13.5000000	-50.0000000
39620.0000000	13.7500000	-50.0000000
39620.0000000	14.0000000	-50.0000000
39620.0000000	14.2500000	-50.0000000
39620.0000000	14.5000000	-50.0000000
39620.0000000	14.7500000	-50.0000000
39620.0000000	15.0000000	-50.0000000
39650.0000000	0.0000000	-50.0000000
39650.0000000	0.2500000	-50.0000000
39650.0000000	0.5000000	-50.0000000
39650.0000000	0.7500000	-50.0000000
39650.0000000	1.0000000	-50.0000000
39650.0000000	1.2500000	-50.0000000
39650.0000000	1.5000000	-50.0000000
39650.0000000	1.7500000	-50.0000000
39650.0000000	2.0000000	-50.0000000
39650.0000000	2.2500000	-50.0000000
39650.0000000	2.5000000	-50.0000000
39650.0000000	2.7500000	-50.0000000
39650.0000000	3.0000000	-50.0000000
39650.0000000	3.2500000	-50.0000000
39650.0000000	3.5000000	-50.0000000
39650.0000000	3.7500000	-50.0000000
39650.0000000	4.0000000	-50.0000000
39650.0000000	4.2500000	-50.0000000
39650.0000000	4.5000000	-50.0000000
39650.0000000	4.7500000	-50.0000000
39650.0000000	5.0000000	-50.0000000
39650.0000000	5.2500000	-50.0000000
39650.0000000	5.5000000	-50.0000000
39650.0000000	5.7500000	-50.0000000
39650.0000000	6.0000000	-50.0000000
39650.0000000	6.2500000	-50.0000000
39650.0000000	6.5000000	-50.0000000
39650.0000000	6.7500000	-50.0000000
39650.0000000	7.0000000	-50.0000000
39650.0000000	7.2500000	-50.0000000
39650.0000000	7.5000000	-50.0000000
39650.0000000	7.7500000	-50.0000000
39650.0000000	8.0000000	-50.0000000
39650.0000000	8.2500000	-50.0000000
39650.0000000	8.5000000	-50.0000000
39650.0000000	8.7500000	-50.0000000
39650.0000000	9.0000000	-50.0000000
39650.0000000	9.2500000	-50.0000000
39650.0000000	9.5000000	-50.0000000
39650.0000000	9.7500000	-50.0000000
39650.0000000	10.0000000	-50.0000000
39650.0000000	10.2500000	-50.0000000
39650.0000000	10.5000000	-50.0000000

39650.0000000	10.7500000	-50.0000000
39650.0000000	11.0000000	-50.0000000
39650.0000000	11.2500000	-50.0000000
39650.0000000	11.5000000	-50.0000000
39650.0000000	11.7500000	-50.0000000
39650.0000000	12.0000000	-50.0000000
39650.0000000	12.2500000	-50.0000000
39650.0000000	12.5000000	-50.0000000
39650.0000000	12.7500000	-50.0000000
39650.0000000	13.0000000	-50.0000000
39650.0000000	13.2500000	-50.0000000
39650.0000000	13.5000000	-50.0000000
39650.0000000	13.7500000	-50.0000000
39650.0000000	14.0000000	-50.0000000
39650.0000000	14.2500000	-50.0000000
39650.0000000	14.5000000	-50.0000000
39650.0000000	14.7500000	-50.0000000
39650.0000000	15.0000000	-50.0000000
39680.0000000	0.0000000	-50.0000000
39680.0000000	0.2500000	-50.0000000
39680.0000000	0.5000000	-50.0000000
39680.0000000	0.7500000	-50.0000000
39680.0000000	1.0000000	-50.0000000
39680.0000000	1.2500000	-50.0000000
39680.0000000	1.5000000	-50.0000000
39680.0000000	1.7500000	-50.0000000
39680.0000000	2.0000000	-50.0000000
39680.0000000	2.2500000	-50.0000000
39680.0000000	2.5000000	-50.0000000
39680.0000000	2.7500000	-50.0000000
39680.0000000	3.0000000	-50.0000000
39680.0000000	3.2500000	-50.0000000
39680.0000000	3.5000000	-50.0000000
39680.0000000	3.7500000	-50.0000000
39680.0000000	4.0000000	-50.0000000
39680.0000000	4.2500000	-50.0000000
39680.0000000	4.5000000	-50.0000000
39680.0000000	4.7500000	-50.0000000
39680.0000000	5.0000000	-50.0000000
39680.0000000	5.2500000	-50.0000000
39680.0000000	5.5000000	-50.0000000
39680.0000000	5.7500000	-50.0000000
39680.0000000	6.0000000	-50.0000000
39680.0000000	6.2500000	-50.0000000
39680.0000000	6.5000000	-50.0000000
39680.0000000	6.7500000	-50.0000000
39680.0000000	7.0000000	-50.0000000
39680.0000000	7.2500000	-50.0000000
39680.0000000	7.5000000	-50.0000000
39680.0000000	7.7500000	-50.0000000
39680.0000000	8.0000000	-50.0000000
39680.0000000	8.2500000	-50.0000000
39680.0000000	8.5000000	-50.0000000
39680.0000000	8.7500000	-50.0000000
39680.0000000	9.0000000	-50.0000000
39680.0000000	9.2500000	-50.0000000
39680.0000000	9.5000000	-50.0000000
39680.0000000	9.7500000	-50.0000000

39680.0000000	10.0000000	-50.0000000
39680.0000000	10.2500000	-50.0000000
39680.0000000	10.5000000	-50.0000000
39680.0000000	10.7500000	-50.0000000
39680.0000000	11.0000000	-50.0000000
39680.0000000	11.2500000	-50.0000000
39680.0000000	11.5000000	-50.0000000
39680.0000000	11.7500000	-50.0000000
39680.0000000	12.0000000	-50.0000000
39680.0000000	12.2500000	-50.0000000
39680.0000000	12.5000000	-50.0000000
39680.0000000	12.7500000	-50.0000000
39680.0000000	13.0000000	-50.0000000
39680.0000000	13.2500000	-50.0000000
39680.0000000	13.5000000	-50.0000000
39680.0000000	13.7500000	-50.0000000
39680.0000000	14.0000000	-50.0000000
39680.0000000	14.2500000	-50.0000000
39680.0000000	14.5000000	-50.0000000
39680.0000000	14.7500000	-50.0000000
39680.0000000	15.0000000	-50.0000000
39710.0000000	0.0000000	-50.0000000
39710.0000000	0.2500000	-50.0000000
39710.0000000	0.5000000	-50.0000000
39710.0000000	0.7500000	-50.0000000
39710.0000000	1.0000000	-50.0000000
39710.0000000	1.2500000	-50.0000000
39710.0000000	1.5000000	-50.0000000
39710.0000000	1.7500000	-50.0000000
39710.0000000	2.0000000	-50.0000000
39710.0000000	2.2500000	-50.0000000
39710.0000000	2.5000000	-50.0000000
39710.0000000	2.7500000	-50.0000000
39710.0000000	3.0000000	-50.0000000
39710.0000000	3.2500000	-50.0000000
39710.0000000	3.5000000	-50.0000000
39710.0000000	3.7500000	-50.0000000
39710.0000000	4.0000000	-50.0000000
39710.0000000	4.2500000	-50.0000000
39710.0000000	4.5000000	-50.0000000
39710.0000000	4.7500000	-50.0000000
39710.0000000	5.0000000	-50.0000000
39710.0000000	5.2500000	-50.0000000
39710.0000000	5.5000000	-50.0000000
39710.0000000	5.7500000	-50.0000000
39710.0000000	6.0000000	-50.0000000
39710.0000000	6.2500000	-50.0000000
39710.0000000	6.5000000	-50.0000000
39710.0000000	6.7500000	-50.0000000
39710.0000000	7.0000000	-50.0000000
39710.0000000	7.2500000	-50.0000000
39710.0000000	7.5000000	-50.0000000
39710.0000000	7.7500000	-50.0000000
39710.0000000	8.0000000	-50.0000000
39710.0000000	8.2500000	-50.0000000
39710.0000000	8.5000000	-50.0000000
39710.0000000	8.7500000	-50.0000000
39710.0000000	9.0000000	-50.0000000

39710.0000000	9.2500000	-50.0000000
39710.0000000	9.5000000	-50.0000000
39710.0000000	9.7500000	-50.0000000
39710.0000000	10.0000000	-50.0000000
39710.0000000	10.2500000	-50.0000000
39710.0000000	10.5000000	-50.0000000
39710.0000000	10.7500000	-50.0000000
39710.0000000	11.0000000	-50.0000000
39710.0000000	11.2500000	-50.0000000
39710.0000000	11.5000000	-50.0000000
39710.0000000	11.7500000	-50.0000000
39710.0000000	12.0000000	-50.0000000
39710.0000000	12.2500000	-50.0000000
39710.0000000	12.5000000	-50.0000000
39710.0000000	12.7500000	-50.0000000
39710.0000000	13.0000000	-50.0000000
39710.0000000	13.2500000	-50.0000000
39710.0000000	13.5000000	-50.0000000
39710.0000000	13.7500000	-50.0000000
39710.0000000	14.0000000	-50.0000000
39710.0000000	14.2500000	-50.0000000
39710.0000000	14.5000000	-50.0000000
39710.0000000	14.7500000	-50.0000000
39710.0000000	15.0000000	-50.0000000
39740.0000000	0.0000000	-50.0000000
39740.0000000	0.2500000	-50.0000000
39740.0000000	0.5000000	-50.0000000
39740.0000000	0.7500000	-50.0000000
39740.0000000	1.0000000	-50.0000000
39740.0000000	1.2500000	-50.0000000
39740.0000000	1.5000000	-50.0000000
39740.0000000	1.7500000	-50.0000000
39740.0000000	2.0000000	-50.0000000
39740.0000000	2.2500000	-50.0000000
39740.0000000	2.5000000	-50.0000000
39740.0000000	2.7500000	-50.0000000
39740.0000000	3.0000000	-50.0000000
39740.0000000	3.2500000	-50.0000000
39740.0000000	3.5000000	-50.0000000
39740.0000000	3.7500000	-50.0000000
39740.0000000	4.0000000	-50.0000000
39740.0000000	4.2500000	-50.0000000
39740.0000000	4.5000000	-50.0000000
39740.0000000	4.7500000	-50.0000000
39740.0000000	5.0000000	-50.0000000
39740.0000000	5.2500000	-50.0000000
39740.0000000	5.5000000	-50.0000000
39740.0000000	5.7500000	-50.0000000
39740.0000000	6.0000000	-50.0000000
39740.0000000	6.2500000	-50.0000000
39740.0000000	6.5000000	-50.0000000
39740.0000000	6.7500000	-50.0000000
39740.0000000	7.0000000	-50.0000000
39740.0000000	7.2500000	-50.0000000
39740.0000000	7.5000000	-50.0000000
39740.0000000	7.7500000	-50.0000000
39740.0000000	8.0000000	-50.0000000
39740.0000000	8.2500000	-50.0000000

39740.0000000	8.5000000	-50.0000000
39740.0000000	8.7500000	-50.0000000
39740.0000000	9.0000000	-50.0000000
39740.0000000	9.2500000	-50.0000000
39740.0000000	9.5000000	-50.0000000
39740.0000000	9.7500000	-50.0000000
39740.0000000	10.0000000	-50.0000000
39740.0000000	10.2500000	-50.0000000
39740.0000000	10.5000000	-50.0000000
39740.0000000	10.7500000	-50.0000000
39740.0000000	11.0000000	-50.0000000
39740.0000000	11.2500000	-50.0000000
39740.0000000	11.5000000	-50.0000000
39740.0000000	11.7500000	-50.0000000
39740.0000000	12.0000000	-50.0000000
39740.0000000	12.2500000	-50.0000000
39740.0000000	12.5000000	-50.0000000
39740.0000000	12.7500000	-50.0000000
39740.0000000	13.0000000	-50.0000000
39740.0000000	13.2500000	-50.0000000
39740.0000000	13.5000000	-50.0000000
39740.0000000	13.7500000	-50.0000000
39740.0000000	14.0000000	-50.0000000
39740.0000000	14.2500000	-50.0000000
39740.0000000	14.5000000	-50.0000000
39740.0000000	14.7500000	-50.0000000
39740.0000000	15.0000000	-50.0000000
39770.0000000	0.0000000	-50.0000000
39770.0000000	0.2500000	-50.0000000
39770.0000000	0.5000000	-50.0000000
39770.0000000	0.7500000	-50.0000000
39770.0000000	1.0000000	-50.0000000
39770.0000000	1.2500000	-50.0000000
39770.0000000	1.5000000	-50.0000000
39770.0000000	1.7500000	-50.0000000
39770.0000000	2.0000000	-50.0000000
39770.0000000	2.2500000	-50.0000000
39770.0000000	2.5000000	-50.0000000
39770.0000000	2.7500000	-50.0000000
39770.0000000	3.0000000	-50.0000000
39770.0000000	3.2500000	-50.0000000
39770.0000000	3.5000000	-50.0000000
39770.0000000	3.7500000	-50.0000000
39770.0000000	4.0000000	-50.0000000
39770.0000000	4.2500000	-50.0000000
39770.0000000	4.5000000	-50.0000000
39770.0000000	4.7500000	-50.0000000
39770.0000000	5.0000000	-50.0000000
39770.0000000	5.2500000	-50.0000000
39770.0000000	5.5000000	-50.0000000
39770.0000000	5.7500000	-50.0000000
39770.0000000	6.0000000	-50.0000000
39770.0000000	6.2500000	-50.0000000
39770.0000000	6.5000000	-50.0000000
39770.0000000	6.7500000	-50.0000000
39770.0000000	7.0000000	-50.0000000
39770.0000000	7.2500000	-50.0000000
39770.0000000	7.5000000	-50.0000000

39770.0000000	7.7500000	-50.0000000
39770.0000000	8.0000000	-50.0000000
39770.0000000	8.2500000	-50.0000000
39770.0000000	8.5000000	-50.0000000
39770.0000000	8.7500000	-50.0000000
39770.0000000	9.0000000	-50.0000000
39770.0000000	9.2500000	-50.0000000
39770.0000000	9.5000000	-50.0000000
39770.0000000	9.7500000	-50.0000000
39770.0000000	10.0000000	-50.0000000
39770.0000000	10.2500000	-50.0000000
39770.0000000	10.5000000	-50.0000000
39770.0000000	10.7500000	-50.0000000
39770.0000000	11.0000000	-50.0000000
39770.0000000	11.2500000	-50.0000000
39770.0000000	11.5000000	-50.0000000
39770.0000000	11.7500000	-50.0000000
39770.0000000	12.0000000	-50.0000000
39770.0000000	12.2500000	-50.0000000
39770.0000000	12.5000000	-50.0000000
39770.0000000	12.7500000	-50.0000000
39770.0000000	13.0000000	-50.0000000
39770.0000000	13.2500000	-50.0000000
39770.0000000	13.5000000	-50.0000000
39770.0000000	13.7500000	-50.0000000
39770.0000000	14.0000000	-50.0000000
39770.0000000	14.2500000	-50.0000000
39770.0000000	14.5000000	-50.0000000
39770.0000000	14.7500000	-50.0000000
39770.0000000	15.0000000	-50.0000000
39800.0000000	0.0000000	-50.0000000
39800.0000000	0.2500000	-50.0000000
39800.0000000	0.5000000	-50.0000000
39800.0000000	0.7500000	-50.0000000
39800.0000000	1.0000000	-50.0000000
39800.0000000	1.2500000	-50.0000000
39800.0000000	1.5000000	-50.0000000
39800.0000000	1.7500000	-50.0000000
39800.0000000	2.0000000	-50.0000000
39800.0000000	2.2500000	-50.0000000
39800.0000000	2.5000000	-50.0000000
39800.0000000	2.7500000	-50.0000000
39800.0000000	3.0000000	-50.0000000
39800.0000000	3.2500000	-50.0000000
39800.0000000	3.5000000	-50.0000000
39800.0000000	3.7500000	-50.0000000
39800.0000000	4.0000000	-50.0000000
39800.0000000	4.2500000	-50.0000000
39800.0000000	4.5000000	-50.0000000
39800.0000000	4.7500000	-50.0000000
39800.0000000	5.0000000	-50.0000000
39800.0000000	5.2500000	-50.0000000
39800.0000000	5.5000000	-50.0000000
39800.0000000	5.7500000	-50.0000000
39800.0000000	6.0000000	-50.0000000
39800.0000000	6.2500000	-50.0000000
39800.0000000	6.5000000	-50.0000000
39800.0000000	6.7500000	-50.0000000

39800.0000000	7.0000000	-50.0000000
39800.0000000	7.2500000	-50.0000000
39800.0000000	7.5000000	-50.0000000
39800.0000000	7.7500000	-50.0000000
39800.0000000	8.0000000	-50.0000000
39800.0000000	8.2500000	-50.0000000
39800.0000000	8.5000000	-50.0000000
39800.0000000	8.7500000	-50.0000000
39800.0000000	9.0000000	-50.0000000
39800.0000000	9.2500000	-50.0000000
39800.0000000	9.5000000	-50.0000000
39800.0000000	9.7500000	-50.0000000
39800.0000000	10.0000000	-50.0000000
39800.0000000	10.2500000	-50.0000000
39800.0000000	10.5000000	-50.0000000
39800.0000000	10.7500000	-50.0000000
39800.0000000	11.0000000	-50.0000000
39800.0000000	11.2500000	-50.0000000
39800.0000000	11.5000000	-50.0000000
39800.0000000	11.7500000	-50.0000000
39800.0000000	12.0000000	-50.0000000
39800.0000000	12.2500000	-50.0000000
39800.0000000	12.5000000	-50.0000000
39800.0000000	12.7500000	-50.0000000
39800.0000000	13.0000000	-50.0000000
39800.0000000	13.2500000	-50.0000000
39800.0000000	13.5000000	-50.0000000
39800.0000000	13.7500000	-50.0000000
39800.0000000	14.0000000	-50.0000000
39800.0000000	14.2500000	-50.0000000
39800.0000000	14.5000000	-50.0000000
39800.0000000	14.7500000	-50.0000000
39800.0000000	15.0000000	-50.0000000
39830.0000000	0.0000000	-50.0000000
39830.0000000	0.2500000	-50.0000000
39830.0000000	0.5000000	-50.0000000
39830.0000000	0.7500000	-50.0000000
39830.0000000	1.0000000	-50.0000000
39830.0000000	1.2500000	-50.0000000
39830.0000000	1.5000000	-50.0000000
39830.0000000	1.7500000	-50.0000000
39830.0000000	2.0000000	-50.0000000
39830.0000000	2.2500000	-50.0000000
39830.0000000	2.5000000	-50.0000000
39830.0000000	2.7500000	-50.0000000
39830.0000000	3.0000000	-50.0000000
39830.0000000	3.2500000	-50.0000000
39830.0000000	3.5000000	-50.0000000
39830.0000000	3.7500000	-50.0000000
39830.0000000	4.0000000	-50.0000000
39830.0000000	4.2500000	-50.0000000
39830.0000000	4.5000000	-50.0000000
39830.0000000	4.7500000	-50.0000000
39830.0000000	5.0000000	-50.0000000
39830.0000000	5.2500000	-50.0000000
39830.0000000	5.5000000	-50.0000000
39830.0000000	5.7500000	-50.0000000
39830.0000000	6.0000000	-50.0000000

39830.0000000	6.2500000	-50.0000000
39830.0000000	6.5000000	-50.0000000
39830.0000000	6.7500000	-50.0000000
39830.0000000	7.0000000	-50.0000000
39830.0000000	7.2500000	-50.0000000
39830.0000000	7.5000000	-50.0000000
39830.0000000	7.7500000	-50.0000000
39830.0000000	8.0000000	-50.0000000
39830.0000000	8.2500000	-50.0000000
39830.0000000	8.5000000	-50.0000000
39830.0000000	8.7500000	-50.0000000
39830.0000000	9.0000000	-50.0000000
39830.0000000	9.2500000	-50.0000000
39830.0000000	9.5000000	-50.0000000
39830.0000000	9.7500000	-50.0000000
39830.0000000	10.0000000	-50.0000000
39830.0000000	10.2500000	-50.0000000
39830.0000000	10.5000000	-50.0000000
39830.0000000	10.7500000	-50.0000000
39830.0000000	11.0000000	-50.0000000
39830.0000000	11.2500000	-50.0000000
39830.0000000	11.5000000	-50.0000000
39830.0000000	11.7500000	-50.0000000
39830.0000000	12.0000000	-50.0000000
39830.0000000	12.2500000	-50.0000000
39830.0000000	12.5000000	-50.0000000
39830.0000000	12.7500000	-50.0000000
39830.0000000	13.0000000	-50.0000000
39830.0000000	13.2500000	-50.0000000
39830.0000000	13.5000000	-50.0000000
39830.0000000	13.7500000	-50.0000000
39830.0000000	14.0000000	-50.0000000
39830.0000000	14.2500000	-50.0000000
39830.0000000	14.5000000	-50.0000000
39830.0000000	14.7500000	-50.0000000
39830.0000000	15.0000000	-50.0000000
39860.0000000	0.0000000	-50.0000000
39860.0000000	0.2500000	-50.0000000
39860.0000000	0.5000000	-50.0000000
39860.0000000	0.7500000	-50.0000000
39860.0000000	1.0000000	-50.0000000
39860.0000000	1.2500000	-50.0000000
39860.0000000	1.5000000	-50.0000000
39860.0000000	1.7500000	-50.0000000
39860.0000000	2.0000000	-50.0000000
39860.0000000	2.2500000	-50.0000000
39860.0000000	2.5000000	-50.0000000
39860.0000000	2.7500000	-50.0000000
39860.0000000	3.0000000	-50.0000000
39860.0000000	3.2500000	-50.0000000
39860.0000000	3.5000000	-50.0000000
39860.0000000	3.7500000	-50.0000000
39860.0000000	4.0000000	-50.0000000
39860.0000000	4.2500000	-50.0000000
39860.0000000	4.5000000	-50.0000000
39860.0000000	4.7500000	-50.0000000
39860.0000000	5.0000000	-50.0000000
39860.0000000	5.2500000	-50.0000000

39860.0000000	5.5000000	-50.0000000
39860.0000000	5.7500000	-50.0000000
39860.0000000	6.0000000	-50.0000000
39860.0000000	6.2500000	-50.0000000
39860.0000000	6.5000000	-50.0000000
39860.0000000	6.7500000	-50.0000000
39860.0000000	7.0000000	-50.0000000
39860.0000000	7.2500000	-50.0000000
39860.0000000	7.5000000	-50.0000000
39860.0000000	7.7500000	-50.0000000
39860.0000000	8.0000000	-50.0000000
39860.0000000	8.2500000	-50.0000000
39860.0000000	8.5000000	-50.0000000
39860.0000000	8.7500000	-50.0000000
39860.0000000	9.0000000	-50.0000000
39860.0000000	9.2500000	-50.0000000
39860.0000000	9.5000000	-50.0000000
39860.0000000	9.7500000	-50.0000000
39860.0000000	10.0000000	-50.0000000
39860.0000000	10.2500000	-50.0000000
39860.0000000	10.5000000	-50.0000000
39860.0000000	10.7500000	-50.0000000
39860.0000000	11.0000000	-50.0000000
39860.0000000	11.2500000	-50.0000000
39860.0000000	11.5000000	-50.0000000
39860.0000000	11.7500000	-50.0000000
39860.0000000	12.0000000	-50.0000000
39860.0000000	12.2500000	-50.0000000
39860.0000000	12.5000000	-50.0000000
39860.0000000	12.7500000	-50.0000000
39860.0000000	13.0000000	-50.0000000
39860.0000000	13.2500000	-50.0000000
39860.0000000	13.5000000	-50.0000000
39860.0000000	13.7500000	-50.0000000
39860.0000000	14.0000000	-50.0000000
39860.0000000	14.2500000	-50.0000000
39860.0000000	14.5000000	-50.0000000
39860.0000000	14.7500000	-50.0000000
39860.0000000	15.0000000	-50.0000000
39890.0000000	0.0000000	-50.0000000
39890.0000000	0.2500000	-50.0000000
39890.0000000	0.5000000	-50.0000000
39890.0000000	0.7500000	-50.0000000
39890.0000000	1.0000000	-50.0000000
39890.0000000	1.2500000	-50.0000000
39890.0000000	1.5000000	-50.0000000
39890.0000000	1.7500000	-50.0000000
39890.0000000	2.0000000	-50.0000000
39890.0000000	2.2500000	-50.0000000
39890.0000000	2.5000000	-50.0000000
39890.0000000	2.7500000	-50.0000000
39890.0000000	3.0000000	-50.0000000
39890.0000000	3.2500000	-50.0000000
39890.0000000	3.5000000	-50.0000000
39890.0000000	3.7500000	-50.0000000
39890.0000000	4.0000000	-50.0000000
39890.0000000	4.2500000	-50.0000000
39890.0000000	4.5000000	-50.0000000

39890.0000000	4.7500000	-50.0000000
39890.0000000	5.0000000	-50.0000000
39890.0000000	5.2500000	-50.0000000
39890.0000000	5.5000000	-50.0000000
39890.0000000	5.7500000	-50.0000000
39890.0000000	6.0000000	-50.0000000
39890.0000000	6.2500000	-50.0000000
39890.0000000	6.5000000	-50.0000000
39890.0000000	6.7500000	-50.0000000
39890.0000000	7.0000000	-50.0000000
39890.0000000	7.2500000	-50.0000000
39890.0000000	7.5000000	-50.0000000
39890.0000000	7.7500000	-50.0000000
39890.0000000	8.0000000	-50.0000000
39890.0000000	8.2500000	-50.0000000
39890.0000000	8.5000000	-50.0000000
39890.0000000	8.7500000	-50.0000000
39890.0000000	9.0000000	-50.0000000
39890.0000000	9.2500000	-50.0000000
39890.0000000	9.5000000	-50.0000000
39890.0000000	9.7500000	-50.0000000
39890.0000000	10.0000000	-50.0000000
39890.0000000	10.2500000	-50.0000000
39890.0000000	10.5000000	-50.0000000
39890.0000000	10.7500000	-50.0000000
39890.0000000	11.0000000	-50.0000000
39890.0000000	11.2500000	-50.0000000
39890.0000000	11.5000000	-50.0000000
39890.0000000	11.7500000	-50.0000000
39890.0000000	12.0000000	-50.0000000
39890.0000000	12.2500000	-50.0000000
39890.0000000	12.5000000	-50.0000000
39890.0000000	12.7500000	-50.0000000
39890.0000000	13.0000000	-50.0000000
39890.0000000	13.2500000	-50.0000000
39890.0000000	13.5000000	-50.0000000
39890.0000000	13.7500000	-50.0000000
39890.0000000	14.0000000	-50.0000000
39890.0000000	14.2500000	-50.0000000
39890.0000000	14.5000000	-50.0000000
39890.0000000	14.7500000	-50.0000000
39890.0000000	15.0000000	-50.0000000
39920.0000000	0.0000000	-50.0000000
39920.0000000	0.2500000	-50.0000000
39920.0000000	0.5000000	-50.0000000
39920.0000000	0.7500000	-50.0000000
39920.0000000	1.0000000	-50.0000000
39920.0000000	1.2500000	-50.0000000
39920.0000000	1.5000000	-50.0000000
39920.0000000	1.7500000	-50.0000000
39920.0000000	2.0000000	-50.0000000
39920.0000000	2.2500000	-50.0000000
39920.0000000	2.5000000	-50.0000000
39920.0000000	2.7500000	-50.0000000
39920.0000000	3.0000000	-50.0000000
39920.0000000	3.2500000	-50.0000000
39920.0000000	3.5000000	-50.0000000
39920.0000000	3.7500000	-50.0000000

39920.0000000	4.0000000	-50.0000000
39920.0000000	4.2500000	-50.0000000
39920.0000000	4.5000000	-50.0000000
39920.0000000	4.7500000	-50.0000000
39920.0000000	5.0000000	-50.0000000
39920.0000000	5.2500000	-50.0000000
39920.0000000	5.5000000	-50.0000000
39920.0000000	5.7500000	-50.0000000
39920.0000000	6.0000000	-50.0000000
39920.0000000	6.2500000	-50.0000000
39920.0000000	6.5000000	-50.0000000
39920.0000000	6.7500000	-50.0000000
39920.0000000	7.0000000	-50.0000000
39920.0000000	7.2500000	-50.0000000
39920.0000000	7.5000000	-50.0000000
39920.0000000	7.7500000	-50.0000000
39920.0000000	8.0000000	-50.0000000
39920.0000000	8.2500000	-50.0000000
39920.0000000	8.5000000	-50.0000000
39920.0000000	8.7500000	-50.0000000
39920.0000000	9.0000000	-50.0000000
39920.0000000	9.2500000	-50.0000000
39920.0000000	9.5000000	-50.0000000
39920.0000000	9.7500000	-50.0000000
39920.0000000	10.0000000	-50.0000000
39920.0000000	10.2500000	-50.0000000
39920.0000000	10.5000000	-50.0000000
39920.0000000	10.7500000	-50.0000000
39920.0000000	11.0000000	-50.0000000
39920.0000000	11.2500000	-50.0000000
39920.0000000	11.5000000	-50.0000000
39920.0000000	11.7500000	-50.0000000
39920.0000000	12.0000000	-50.0000000
39920.0000000	12.2500000	-50.0000000
39920.0000000	12.5000000	-50.0000000
39920.0000000	12.7500000	-50.0000000
39920.0000000	13.0000000	-50.0000000
39920.0000000	13.2500000	-50.0000000
39920.0000000	13.5000000	-50.0000000
39920.0000000	13.7500000	-50.0000000
39920.0000000	14.0000000	-50.0000000
39920.0000000	14.2500000	-50.0000000
39920.0000000	14.5000000	-50.0000000
39920.0000000	14.7500000	-50.0000000
39920.0000000	15.0000000	-50.0000000
39950.0000000	0.0000000	-50.0000000
39950.0000000	0.2500000	-50.0000000
39950.0000000	0.5000000	-50.0000000
39950.0000000	0.7500000	-50.0000000
39950.0000000	1.0000000	-50.0000000
39950.0000000	1.2500000	-50.0000000
39950.0000000	1.5000000	-50.0000000
39950.0000000	1.7500000	-50.0000000
39950.0000000	2.0000000	-50.0000000
39950.0000000	2.2500000	-50.0000000
39950.0000000	2.5000000	-50.0000000
39950.0000000	2.7500000	-50.0000000
39950.0000000	3.0000000	-50.0000000

39950.0000000	3.2500000	-50.0000000
39950.0000000	3.5000000	-50.0000000
39950.0000000	3.7500000	-50.0000000
39950.0000000	4.0000000	-50.0000000
39950.0000000	4.2500000	-50.0000000
39950.0000000	4.5000000	-50.0000000
39950.0000000	4.7500000	-50.0000000
39950.0000000	5.0000000	-50.0000000
39950.0000000	5.2500000	-50.0000000
39950.0000000	5.5000000	-50.0000000
39950.0000000	5.7500000	-50.0000000
39950.0000000	6.0000000	-50.0000000
39950.0000000	6.2500000	-50.0000000
39950.0000000	6.5000000	-50.0000000
39950.0000000	6.7500000	-50.0000000
39950.0000000	7.0000000	-50.0000000
39950.0000000	7.2500000	-50.0000000
39950.0000000	7.5000000	-50.0000000
39950.0000000	7.7500000	-50.0000000
39950.0000000	8.0000000	-50.0000000
39950.0000000	8.2500000	-50.0000000
39950.0000000	8.5000000	-50.0000000
39950.0000000	8.7500000	-50.0000000
39950.0000000	9.0000000	-50.0000000
39950.0000000	9.2500000	-50.0000000
39950.0000000	9.5000000	-50.0000000
39950.0000000	9.7500000	-50.0000000
39950.0000000	10.0000000	-50.0000000
39950.0000000	10.2500000	-50.0000000
39950.0000000	10.5000000	-50.0000000
39950.0000000	10.7500000	-50.0000000
39950.0000000	11.0000000	-50.0000000
39950.0000000	11.2500000	-50.0000000
39950.0000000	11.5000000	-50.0000000
39950.0000000	11.7500000	-50.0000000
39950.0000000	12.0000000	-50.0000000
39950.0000000	12.2500000	-50.0000000
39950.0000000	12.5000000	-50.0000000
39950.0000000	12.7500000	-50.0000000
39950.0000000	13.0000000	-50.0000000
39950.0000000	13.2500000	-50.0000000
39950.0000000	13.5000000	-50.0000000
39950.0000000	13.7500000	-50.0000000
39950.0000000	14.0000000	-50.0000000
39950.0000000	14.2500000	-50.0000000
39950.0000000	14.5000000	-50.0000000
39950.0000000	14.7500000	-50.0000000
39950.0000000	15.0000000	-50.0000000
39980.0000000	0.0000000	-50.0000000
39980.0000000	0.2500000	-50.0000000
39980.0000000	0.5000000	-50.0000000
39980.0000000	0.7500000	-50.0000000
39980.0000000	1.0000000	-50.0000000
39980.0000000	1.2500000	-50.0000000
39980.0000000	1.5000000	-50.0000000
39980.0000000	1.7500000	-50.0000000
39980.0000000	2.0000000	-50.0000000
39980.0000000	2.2500000	-50.0000000

39980.0000000	2.5000000	-50.0000000
39980.0000000	2.7500000	-50.0000000
39980.0000000	3.0000000	-50.0000000
39980.0000000	3.2500000	-50.0000000
39980.0000000	3.5000000	-50.0000000
39980.0000000	3.7500000	-50.0000000
39980.0000000	4.0000000	-50.0000000
39980.0000000	4.2500000	-50.0000000
39980.0000000	4.5000000	-50.0000000
39980.0000000	4.7500000	-50.0000000
39980.0000000	5.0000000	-50.0000000
39980.0000000	5.2500000	-50.0000000
39980.0000000	5.5000000	-50.0000000
39980.0000000	5.7500000	-50.0000000
39980.0000000	6.0000000	-50.0000000
39980.0000000	6.2500000	-50.0000000
39980.0000000	6.5000000	-50.0000000
39980.0000000	6.7500000	-50.0000000
39980.0000000	7.0000000	-50.0000000
39980.0000000	7.2500000	-50.0000000
39980.0000000	7.5000000	-50.0000000
39980.0000000	7.7500000	-50.0000000
39980.0000000	8.0000000	-50.0000000
39980.0000000	8.2500000	-50.0000000
39980.0000000	8.5000000	-50.0000000
39980.0000000	8.7500000	-50.0000000
39980.0000000	9.0000000	-50.0000000
39980.0000000	9.2500000	-50.0000000
39980.0000000	9.5000000	-50.0000000
39980.0000000	9.7500000	-50.0000000
39980.0000000	10.0000000	-50.0000000
39980.0000000	10.2500000	-50.0000000
39980.0000000	10.5000000	-50.0000000
39980.0000000	10.7500000	-50.0000000
39980.0000000	11.0000000	-50.0000000
39980.0000000	11.2500000	-50.0000000
39980.0000000	11.5000000	-50.0000000
39980.0000000	11.7500000	-50.0000000
39980.0000000	12.0000000	-50.0000000
39980.0000000	12.2500000	-50.0000000
39980.0000000	12.5000000	-50.0000000
39980.0000000	12.7500000	-50.0000000
39980.0000000	13.0000000	-50.0000000
39980.0000000	13.2500000	-50.0000000
39980.0000000	13.5000000	-50.0000000
39980.0000000	13.7500000	-50.0000000
39980.0000000	14.0000000	-50.0000000
39980.0000000	14.2500000	-50.0000000
39980.0000000	14.5000000	-50.0000000
39980.0000000	14.7500000	-50.0000000
39980.0000000	15.0000000	-50.0000000
40010.0000000	0.0000000	-50.0000000
40010.0000000	0.2500000	-50.0000000
40010.0000000	0.5000000	-50.0000000
40010.0000000	0.7500000	-50.0000000
40010.0000000	1.0000000	-50.0000000
40010.0000000	1.2500000	-50.0000000
40010.0000000	1.5000000	-50.0000000

40010.0000000	1.7500000	-50.0000000
40010.0000000	2.0000000	-50.0000000
40010.0000000	2.2500000	-50.0000000
40010.0000000	2.5000000	-50.0000000
40010.0000000	2.7500000	-50.0000000
40010.0000000	3.0000000	-50.0000000
40010.0000000	3.2500000	-50.0000000
40010.0000000	3.5000000	-50.0000000
40010.0000000	3.7500000	-50.0000000
40010.0000000	4.0000000	-50.0000000
40010.0000000	4.2500000	-50.0000000
40010.0000000	4.5000000	-50.0000000
40010.0000000	4.7500000	-50.0000000
40010.0000000	5.0000000	-50.0000000
40010.0000000	5.2500000	-50.0000000
40010.0000000	5.5000000	-50.0000000
40010.0000000	5.7500000	-50.0000000
40010.0000000	6.0000000	-50.0000000
40010.0000000	6.2500000	-50.0000000
40010.0000000	6.5000000	-50.0000000
40010.0000000	6.7500000	-50.0000000
40010.0000000	7.0000000	-50.0000000
40010.0000000	7.2500000	-50.0000000
40010.0000000	7.5000000	-50.0000000
40010.0000000	7.7500000	-50.0000000
40010.0000000	8.0000000	-50.0000000
40010.0000000	8.2500000	-50.0000000
40010.0000000	8.5000000	-50.0000000
40010.0000000	8.7500000	-50.0000000
40010.0000000	9.0000000	-50.0000000
40010.0000000	9.2500000	-50.0000000
40010.0000000	9.5000000	-50.0000000
40010.0000000	9.7500000	-50.0000000
40010.0000000	10.0000000	-50.0000000
40010.0000000	10.2500000	-50.0000000
40010.0000000	10.5000000	-50.0000000
40010.0000000	10.7500000	-50.0000000
40010.0000000	11.0000000	-50.0000000
40010.0000000	11.2500000	-50.0000000
40010.0000000	11.5000000	-50.0000000
40010.0000000	11.7500000	-50.0000000
40010.0000000	12.0000000	-50.0000000
40010.0000000	12.2500000	-50.0000000
40010.0000000	12.5000000	-50.0000000
40010.0000000	12.7500000	-50.0000000
40010.0000000	13.0000000	-50.0000000
40010.0000000	13.2500000	-50.0000000
40010.0000000	13.5000000	-50.0000000
40010.0000000	13.7500000	-50.0000000
40010.0000000	14.0000000	-50.0000000
40010.0000000	14.2500000	-50.0000000
40010.0000000	14.5000000	-50.0000000
40010.0000000	14.7500000	-50.0000000
40010.0000000	15.0000000	-50.0000000
40040.0000000	0.0000000	-50.0000000
40040.0000000	0.2500000	-50.0000000
40040.0000000	0.5000000	-50.0000000
40040.0000000	0.7500000	-50.0000000

40040.0000000	1.0000000	-50.0000000
40040.0000000	1.2500000	-50.0000000
40040.0000000	1.5000000	-50.0000000
40040.0000000	1.7500000	-50.0000000
40040.0000000	2.0000000	-50.0000000
40040.0000000	2.2500000	-50.0000000
40040.0000000	2.5000000	-50.0000000
40040.0000000	2.7500000	-50.0000000
40040.0000000	3.0000000	-50.0000000
40040.0000000	3.2500000	-50.0000000
40040.0000000	3.5000000	-50.0000000
40040.0000000	3.7500000	-50.0000000
40040.0000000	4.0000000	-50.0000000
40040.0000000	4.2500000	-50.0000000
40040.0000000	4.5000000	-50.0000000
40040.0000000	4.7500000	-50.0000000
40040.0000000	5.0000000	-50.0000000
40040.0000000	5.2500000	-50.0000000
40040.0000000	5.5000000	-50.0000000
40040.0000000	5.7500000	-50.0000000
40040.0000000	6.0000000	-50.0000000
40040.0000000	6.2500000	-50.0000000
40040.0000000	6.5000000	-50.0000000
40040.0000000	6.7500000	-50.0000000
40040.0000000	7.0000000	-50.0000000
40040.0000000	7.2500000	-50.0000000
40040.0000000	7.5000000	-50.0000000
40040.0000000	7.7500000	-50.0000000
40040.0000000	8.0000000	-50.0000000
40040.0000000	8.2500000	-50.0000000
40040.0000000	8.5000000	-50.0000000
40040.0000000	8.7500000	-50.0000000
40040.0000000	9.0000000	-50.0000000
40040.0000000	9.2500000	-50.0000000
40040.0000000	9.5000000	-50.0000000
40040.0000000	9.7500000	-50.0000000
40040.0000000	10.0000000	-50.0000000
40040.0000000	10.2500000	-50.0000000
40040.0000000	10.5000000	-50.0000000
40040.0000000	10.7500000	-50.0000000
40040.0000000	11.0000000	-50.0000000
40040.0000000	11.2500000	-50.0000000
40040.0000000	11.5000000	-50.0000000
40040.0000000	11.7500000	-50.0000000
40040.0000000	12.0000000	-50.0000000
40040.0000000	12.2500000	-50.0000000
40040.0000000	12.5000000	-50.0000000
40040.0000000	12.7500000	-50.0000000
40040.0000000	13.0000000	-50.0000000
40040.0000000	13.2500000	-50.0000000
40040.0000000	13.5000000	-50.0000000
40040.0000000	13.7500000	-50.0000000
40040.0000000	14.0000000	-50.0000000
40040.0000000	14.2500000	-50.0000000
40040.0000000	14.5000000	-50.0000000
40040.0000000	14.7500000	-50.0000000
40040.0000000	15.0000000	-50.0000000
40070.0000000	0.0000000	-50.0000000

40070.0000000	0.2500000	-50.0000000
40070.0000000	0.5000000	-50.0000000
40070.0000000	0.7500000	-50.0000000
40070.0000000	1.0000000	-50.0000000
40070.0000000	1.2500000	-50.0000000
40070.0000000	1.5000000	-50.0000000
40070.0000000	1.7500000	-50.0000000
40070.0000000	2.0000000	-50.0000000
40070.0000000	2.2500000	-50.0000000
40070.0000000	2.5000000	-50.0000000
40070.0000000	2.7500000	-50.0000000
40070.0000000	3.0000000	-50.0000000
40070.0000000	3.2500000	-50.0000000
40070.0000000	3.5000000	-50.0000000
40070.0000000	3.7500000	-50.0000000
40070.0000000	4.0000000	-50.0000000
40070.0000000	4.2500000	-50.0000000
40070.0000000	4.5000000	-50.0000000
40070.0000000	4.7500000	-50.0000000
40070.0000000	5.0000000	-50.0000000
40070.0000000	5.2500000	-50.0000000
40070.0000000	5.5000000	-50.0000000
40070.0000000	5.7500000	-50.0000000
40070.0000000	6.0000000	-50.0000000
40070.0000000	6.2500000	-50.0000000
40070.0000000	6.5000000	-50.0000000
40070.0000000	6.7500000	-50.0000000
40070.0000000	7.0000000	-50.0000000
40070.0000000	7.2500000	-50.0000000
40070.0000000	7.5000000	-50.0000000
40070.0000000	7.7500000	-50.0000000
40070.0000000	8.0000000	-50.0000000
40070.0000000	8.2500000	-50.0000000
40070.0000000	8.5000000	-50.0000000
40070.0000000	8.7500000	-50.0000000
40070.0000000	9.0000000	-50.0000000
40070.0000000	9.2500000	-50.0000000
40070.0000000	9.5000000	-50.0000000
40070.0000000	9.7500000	-50.0000000
40070.0000000	10.0000000	-50.0000000
40070.0000000	10.2500000	-50.0000000
40070.0000000	10.5000000	-50.0000000
40070.0000000	10.7500000	-50.0000000
40070.0000000	11.0000000	-50.0000000
40070.0000000	11.2500000	-50.0000000
40070.0000000	11.5000000	-50.0000000
40070.0000000	11.7500000	-50.0000000
40070.0000000	12.0000000	-50.0000000
40070.0000000	12.2500000	-50.0000000
40070.0000000	12.5000000	-50.0000000
40070.0000000	12.7500000	-50.0000000
40070.0000000	13.0000000	-50.0000000
40070.0000000	13.2500000	-50.0000000
40070.0000000	13.5000000	-50.0000000
40070.0000000	13.7500000	-50.0000000
40070.0000000	14.0000000	-50.0000000
40070.0000000	14.2500000	-50.0000000
40070.0000000	14.5000000	-50.0000000

40070.0000000	14.7500000	-50.0000000
40070.0000000	15.0000000	-50.0000000
40100.0000000	0.0000000	-50.0000000
40100.0000000	0.2500000	-50.0000000
40100.0000000	0.5000000	-50.0000000
40100.0000000	0.7500000	-50.0000000
40100.0000000	1.0000000	-50.0000000
40100.0000000	1.2500000	-50.0000000
40100.0000000	1.5000000	-50.0000000
40100.0000000	1.7500000	-50.0000000
40100.0000000	2.0000000	-50.0000000
40100.0000000	2.2500000	-50.0000000
40100.0000000	2.5000000	-50.0000000
40100.0000000	2.7500000	-50.0000000
40100.0000000	3.0000000	-50.0000000
40100.0000000	3.2500000	-50.0000000
40100.0000000	3.5000000	-50.0000000
40100.0000000	3.7500000	-50.0000000
40100.0000000	4.0000000	-50.0000000
40100.0000000	4.2500000	-50.0000000
40100.0000000	4.5000000	-50.0000000
40100.0000000	4.7500000	-50.0000000
40100.0000000	5.0000000	-50.0000000
40100.0000000	5.2500000	-50.0000000
40100.0000000	5.5000000	-50.0000000
40100.0000000	5.7500000	-50.0000000
40100.0000000	6.0000000	-50.0000000
40100.0000000	6.2500000	-50.0000000
40100.0000000	6.5000000	-50.0000000
40100.0000000	6.7500000	-50.0000000
40100.0000000	7.0000000	-50.0000000
40100.0000000	7.2500000	-50.0000000
40100.0000000	7.5000000	-50.0000000
40100.0000000	7.7500000	-50.0000000
40100.0000000	8.0000000	-50.0000000
40100.0000000	8.2500000	-50.0000000
40100.0000000	8.5000000	-50.0000000
40100.0000000	8.7500000	-50.0000000
40100.0000000	9.0000000	-50.0000000
40100.0000000	9.2500000	-50.0000000
40100.0000000	9.5000000	-50.0000000
40100.0000000	9.7500000	-50.0000000
40100.0000000	10.0000000	-50.0000000
40100.0000000	10.2500000	-50.0000000
40100.0000000	10.5000000	-50.0000000
40100.0000000	10.7500000	-50.0000000
40100.0000000	11.0000000	-50.0000000
40100.0000000	11.2500000	-50.0000000
40100.0000000	11.5000000	-50.0000000
40100.0000000	11.7500000	-50.0000000
40100.0000000	12.0000000	-50.0000000
40100.0000000	12.2500000	-50.0000000
40100.0000000	12.5000000	-50.0000000
40100.0000000	12.7500000	-50.0000000
40100.0000000	13.0000000	-50.0000000
40100.0000000	13.2500000	-50.0000000
40100.0000000	13.5000000	-50.0000000
40100.0000000	13.7500000	-50.0000000

40100.0000000	14.0000000	-50.0000000
40100.0000000	14.2500000	-50.0000000
40100.0000000	14.5000000	-50.0000000
40100.0000000	14.7500000	-50.0000000
40100.0000000	15.0000000	-50.0000000
40130.0000000	0.0000000	-50.0000000
40130.0000000	0.2500000	-50.0000000
40130.0000000	0.5000000	-50.0000000
40130.0000000	0.7500000	-50.0000000
40130.0000000	1.0000000	-50.0000000
40130.0000000	1.2500000	-50.0000000
40130.0000000	1.5000000	-50.0000000
40130.0000000	1.7500000	-50.0000000
40130.0000000	2.0000000	-50.0000000
40130.0000000	2.2500000	-50.0000000
40130.0000000	2.5000000	-50.0000000
40130.0000000	2.7500000	-50.0000000
40130.0000000	3.0000000	-50.0000000
40130.0000000	3.2500000	-50.0000000
40130.0000000	3.5000000	-50.0000000
40130.0000000	3.7500000	-50.0000000
40130.0000000	4.0000000	-50.0000000
40130.0000000	4.2500000	-50.0000000
40130.0000000	4.5000000	-50.0000000
40130.0000000	4.7500000	-50.0000000
40130.0000000	5.0000000	-50.0000000
40130.0000000	5.2500000	-50.0000000
40130.0000000	5.5000000	-50.0000000
40130.0000000	5.7500000	-50.0000000
40130.0000000	6.0000000	-50.0000000
40130.0000000	6.2500000	-50.0000000
40130.0000000	6.5000000	-50.0000000
40130.0000000	6.7500000	-50.0000000
40130.0000000	7.0000000	-50.0000000
40130.0000000	7.2500000	-50.0000000
40130.0000000	7.5000000	-50.0000000
40130.0000000	7.7500000	-50.0000000
40130.0000000	8.0000000	-50.0000000
40130.0000000	8.2500000	-50.0000000
40130.0000000	8.5000000	-50.0000000
40130.0000000	8.7500000	-50.0000000
40130.0000000	9.0000000	-50.0000000
40130.0000000	9.2500000	-50.0000000
40130.0000000	9.5000000	-50.0000000
40130.0000000	9.7500000	-50.0000000
40130.0000000	10.0000000	-50.0000000
40130.0000000	10.2500000	-50.0000000
40130.0000000	10.5000000	-50.0000000
40130.0000000	10.7500000	-50.0000000
40130.0000000	11.0000000	-50.0000000
40130.0000000	11.2500000	-50.0000000
40130.0000000	11.5000000	-50.0000000
40130.0000000	11.7500000	-50.0000000
40130.0000000	12.0000000	-50.0000000
40130.0000000	12.2500000	-50.0000000
40130.0000000	12.5000000	-50.0000000
40130.0000000	12.7500000	-50.0000000
40130.0000000	13.0000000	-50.0000000

40130.0000000	13.2500000	-50.0000000
40130.0000000	13.5000000	-50.0000000
40130.0000000	13.7500000	-50.0000000
40130.0000000	14.0000000	-50.0000000
40130.0000000	14.2500000	-50.0000000
40130.0000000	14.5000000	-50.0000000
40130.0000000	14.7500000	-50.0000000
40130.0000000	15.0000000	-50.0000000
40160.0000000	0.0000000	-50.0000000
40160.0000000	0.2500000	-50.0000000
40160.0000000	0.5000000	-50.0000000
40160.0000000	0.7500000	-50.0000000
40160.0000000	1.0000000	-50.0000000
40160.0000000	1.2500000	-50.0000000
40160.0000000	1.5000000	-50.0000000
40160.0000000	1.7500000	-50.0000000
40160.0000000	2.0000000	-50.0000000
40160.0000000	2.2500000	-50.0000000
40160.0000000	2.5000000	-50.0000000
40160.0000000	2.7500000	-50.0000000
40160.0000000	3.0000000	-50.0000000
40160.0000000	3.2500000	-50.0000000
40160.0000000	3.5000000	-50.0000000
40160.0000000	3.7500000	-50.0000000
40160.0000000	4.0000000	-50.0000000
40160.0000000	4.2500000	-50.0000000
40160.0000000	4.5000000	-50.0000000
40160.0000000	4.7500000	-50.0000000
40160.0000000	5.0000000	-50.0000000
40160.0000000	5.2500000	-50.0000000
40160.0000000	5.5000000	-50.0000000
40160.0000000	5.7500000	-50.0000000
40160.0000000	6.0000000	-50.0000000
40160.0000000	6.2500000	-50.0000000
40160.0000000	6.5000000	-50.0000000
40160.0000000	6.7500000	-50.0000000
40160.0000000	7.0000000	-50.0000000
40160.0000000	7.2500000	-50.0000000
40160.0000000	7.5000000	-50.0000000
40160.0000000	7.7500000	-50.0000000
40160.0000000	8.0000000	-50.0000000
40160.0000000	8.2500000	-50.0000000
40160.0000000	8.5000000	-50.0000000
40160.0000000	8.7500000	-50.0000000
40160.0000000	9.0000000	-50.0000000
40160.0000000	9.2500000	-50.0000000
40160.0000000	9.5000000	-50.0000000
40160.0000000	9.7500000	-50.0000000
40160.0000000	10.0000000	-50.0000000
40160.0000000	10.2500000	-50.0000000
40160.0000000	10.5000000	-50.0000000
40160.0000000	10.7500000	-50.0000000
40160.0000000	11.0000000	-50.0000000
40160.0000000	11.2500000	-50.0000000
40160.0000000	11.5000000	-50.0000000
40160.0000000	11.7500000	-50.0000000
40160.0000000	12.0000000	-50.0000000
40160.0000000	12.2500000	-50.0000000

40160.0000000	12.5000000	-50.0000000
40160.0000000	12.7500000	-50.0000000
40160.0000000	13.0000000	-50.0000000
40160.0000000	13.2500000	-50.0000000
40160.0000000	13.5000000	-50.0000000
40160.0000000	13.7500000	-50.0000000
40160.0000000	14.0000000	-50.0000000
40160.0000000	14.2500000	-50.0000000
40160.0000000	14.5000000	-50.0000000
40160.0000000	14.7500000	-50.0000000
40160.0000000	15.0000000	-50.0000000
40190.0000000	0.0000000	-50.0000000
40190.0000000	0.2500000	-50.0000000
40190.0000000	0.5000000	-50.0000000
40190.0000000	0.7500000	-50.0000000
40190.0000000	1.0000000	-50.0000000
40190.0000000	1.2500000	-50.0000000
40190.0000000	1.5000000	-50.0000000
40190.0000000	1.7500000	-50.0000000
40190.0000000	2.0000000	-50.0000000
40190.0000000	2.2500000	-50.0000000
40190.0000000	2.5000000	-50.0000000
40190.0000000	2.7500000	-50.0000000
40190.0000000	3.0000000	-50.0000000
40190.0000000	3.2500000	-50.0000000
40190.0000000	3.5000000	-50.0000000
40190.0000000	3.7500000	-50.0000000
40190.0000000	4.0000000	-50.0000000
40190.0000000	4.2500000	-50.0000000
40190.0000000	4.5000000	-50.0000000
40190.0000000	4.7500000	-50.0000000
40190.0000000	5.0000000	-50.0000000
40190.0000000	5.2500000	-50.0000000
40190.0000000	5.5000000	-50.0000000
40190.0000000	5.7500000	-50.0000000
40190.0000000	6.0000000	-50.0000000
40190.0000000	6.2500000	-50.0000000
40190.0000000	6.5000000	-50.0000000
40190.0000000	6.7500000	-50.0000000
40190.0000000	7.0000000	-50.0000000
40190.0000000	7.2500000	-50.0000000
40190.0000000	7.5000000	-50.0000000
40190.0000000	7.7500000	-50.0000000
40190.0000000	8.0000000	-50.0000000
40190.0000000	8.2500000	-50.0000000
40190.0000000	8.5000000	-50.0000000
40190.0000000	8.7500000	-50.0000000
40190.0000000	9.0000000	-50.0000000
40190.0000000	9.2500000	-50.0000000
40190.0000000	9.5000000	-50.0000000
40190.0000000	9.7500000	-50.0000000
40190.0000000	10.0000000	-50.0000000
40190.0000000	10.2500000	-50.0000000
40190.0000000	10.5000000	-50.0000000
40190.0000000	10.7500000	-50.0000000
40190.0000000	11.0000000	-50.0000000
40190.0000000	11.2500000	-50.0000000
40190.0000000	11.5000000	-50.0000000

40190.0000000	11.7500000	-50.0000000
40190.0000000	12.0000000	-50.0000000
40190.0000000	12.2500000	-50.0000000
40190.0000000	12.5000000	-50.0000000
40190.0000000	12.7500000	-50.0000000
40190.0000000	13.0000000	-50.0000000
40190.0000000	13.2500000	-50.0000000
40190.0000000	13.5000000	-50.0000000
40190.0000000	13.7500000	-50.0000000
40190.0000000	14.0000000	-50.0000000
40190.0000000	14.2500000	-50.0000000
40190.0000000	14.5000000	-50.0000000
40190.0000000	14.7500000	-50.0000000
40190.0000000	15.0000000	-50.0000000
40220.0000000	0.0000000	-50.0000000
40220.0000000	0.2500000	-50.0000000
40220.0000000	0.5000000	-50.0000000
40220.0000000	0.7500000	-50.0000000
40220.0000000	1.0000000	-50.0000000
40220.0000000	1.2500000	-50.0000000
40220.0000000	1.5000000	-50.0000000
40220.0000000	1.7500000	-50.0000000
40220.0000000	2.0000000	-50.0000000
40220.0000000	2.2500000	-50.0000000
40220.0000000	2.5000000	-50.0000000
40220.0000000	2.7500000	-50.0000000
40220.0000000	3.0000000	-50.0000000
40220.0000000	3.2500000	-50.0000000
40220.0000000	3.5000000	-50.0000000
40220.0000000	3.7500000	-50.0000000
40220.0000000	4.0000000	-50.0000000
40220.0000000	4.2500000	-50.0000000
40220.0000000	4.5000000	-50.0000000
40220.0000000	4.7500000	-50.0000000
40220.0000000	5.0000000	-50.0000000
40220.0000000	5.2500000	-50.0000000
40220.0000000	5.5000000	-50.0000000
40220.0000000	5.7500000	-50.0000000
40220.0000000	6.0000000	-50.0000000
40220.0000000	6.2500000	-50.0000000
40220.0000000	6.5000000	-50.0000000
40220.0000000	6.7500000	-50.0000000
40220.0000000	7.0000000	-50.0000000
40220.0000000	7.2500000	-50.0000000
40220.0000000	7.5000000	-50.0000000
40220.0000000	7.7500000	-50.0000000
40220.0000000	8.0000000	-50.0000000
40220.0000000	8.2500000	-50.0000000
40220.0000000	8.5000000	-50.0000000
40220.0000000	8.7500000	-50.0000000
40220.0000000	9.0000000	-50.0000000
40220.0000000	9.2500000	-50.0000000
40220.0000000	9.5000000	-50.0000000
40220.0000000	9.7500000	-50.0000000
40220.0000000	10.0000000	-50.0000000
40220.0000000	10.2500000	-50.0000000
40220.0000000	10.5000000	-50.0000000
40220.0000000	10.7500000	-50.0000000

40220.0000000	11.0000000	-50.0000000
40220.0000000	11.2500000	-50.0000000
40220.0000000	11.5000000	-50.0000000
40220.0000000	11.7500000	-50.0000000
40220.0000000	12.0000000	-50.0000000
40220.0000000	12.2500000	-50.0000000
40220.0000000	12.5000000	-50.0000000
40220.0000000	12.7500000	-50.0000000
40220.0000000	13.0000000	-50.0000000
40220.0000000	13.2500000	-50.0000000
40220.0000000	13.5000000	-50.0000000
40220.0000000	13.7500000	-50.0000000
40220.0000000	14.0000000	-50.0000000
40220.0000000	14.2500000	-50.0000000
40220.0000000	14.5000000	-50.0000000
40220.0000000	14.7500000	-50.0000000
40220.0000000	15.0000000	-50.0000000
40250.0000000	0.0000000	-50.0000000
40250.0000000	0.2500000	-50.0000000
40250.0000000	0.5000000	-50.0000000
40250.0000000	0.7500000	-50.0000000
40250.0000000	1.0000000	-50.0000000
40250.0000000	1.2500000	-50.0000000
40250.0000000	1.5000000	-50.0000000
40250.0000000	1.7500000	-50.0000000
40250.0000000	2.0000000	-50.0000000
40250.0000000	2.2500000	-50.0000000
40250.0000000	2.5000000	-50.0000000
40250.0000000	2.7500000	-50.0000000
40250.0000000	3.0000000	-50.0000000
40250.0000000	3.2500000	-50.0000000
40250.0000000	3.5000000	-50.0000000
40250.0000000	3.7500000	-50.0000000
40250.0000000	4.0000000	-50.0000000
40250.0000000	4.2500000	-50.0000000
40250.0000000	4.5000000	-50.0000000
40250.0000000	4.7500000	-50.0000000
40250.0000000	5.0000000	-50.0000000
40250.0000000	5.2500000	-50.0000000
40250.0000000	5.5000000	-50.0000000
40250.0000000	5.7500000	-50.0000000
40250.0000000	6.0000000	-50.0000000
40250.0000000	6.2500000	-50.0000000
40250.0000000	6.5000000	-50.0000000
40250.0000000	6.7500000	-50.0000000
40250.0000000	7.0000000	-50.0000000
40250.0000000	7.2500000	-50.0000000
40250.0000000	7.5000000	-50.0000000
40250.0000000	7.7500000	-50.0000000
40250.0000000	8.0000000	-50.0000000
40250.0000000	8.2500000	-50.0000000
40250.0000000	8.5000000	-50.0000000
40250.0000000	8.7500000	-50.0000000
40250.0000000	9.0000000	-50.0000000
40250.0000000	9.2500000	-50.0000000
40250.0000000	9.5000000	-50.0000000
40250.0000000	9.7500000	-50.0000000
40250.0000000	10.0000000	-50.0000000

40250.0000000	10.2500000	-50.0000000
40250.0000000	10.5000000	-50.0000000
40250.0000000	10.7500000	-50.0000000
40250.0000000	11.0000000	-50.0000000
40250.0000000	11.2500000	-50.0000000
40250.0000000	11.5000000	-50.0000000
40250.0000000	11.7500000	-50.0000000
40250.0000000	12.0000000	-50.0000000
40250.0000000	12.2500000	-50.0000000
40250.0000000	12.5000000	-50.0000000
40250.0000000	12.7500000	-50.0000000
40250.0000000	13.0000000	-50.0000000
40250.0000000	13.2500000	-50.0000000
40250.0000000	13.5000000	-50.0000000
40250.0000000	13.7500000	-50.0000000
40250.0000000	14.0000000	-50.0000000
40250.0000000	14.2500000	-50.0000000
40250.0000000	14.5000000	-50.0000000
40250.0000000	14.7500000	-50.0000000
40250.0000000	15.0000000	-50.0000000
40280.0000000	0.0000000	-50.0000000
40280.0000000	0.2500000	-50.0000000
40280.0000000	0.5000000	-50.0000000
40280.0000000	0.7500000	-50.0000000
40280.0000000	1.0000000	-50.0000000
40280.0000000	1.2500000	-50.0000000
40280.0000000	1.5000000	-50.0000000
40280.0000000	1.7500000	-50.0000000
40280.0000000	2.0000000	-50.0000000
40280.0000000	2.2500000	-50.0000000
40280.0000000	2.5000000	-50.0000000
40280.0000000	2.7500000	-50.0000000
40280.0000000	3.0000000	-50.0000000
40280.0000000	3.2500000	-50.0000000
40280.0000000	3.5000000	-50.0000000
40280.0000000	3.7500000	-50.0000000
40280.0000000	4.0000000	-50.0000000
40280.0000000	4.2500000	-50.0000000
40280.0000000	4.5000000	-50.0000000
40280.0000000	4.7500000	-50.0000000
40280.0000000	5.0000000	-50.0000000
40280.0000000	5.2500000	-50.0000000
40280.0000000	5.5000000	-50.0000000
40280.0000000	5.7500000	-50.0000000
40280.0000000	6.0000000	-50.0000000
40280.0000000	6.2500000	-50.0000000
40280.0000000	6.5000000	-50.0000000
40280.0000000	6.7500000	-50.0000000
40280.0000000	7.0000000	-50.0000000
40280.0000000	7.2500000	-50.0000000
40280.0000000	7.5000000	-50.0000000
40280.0000000	7.7500000	-50.0000000
40280.0000000	8.0000000	-50.0000000
40280.0000000	8.2500000	-50.0000000
40280.0000000	8.5000000	-50.0000000
40280.0000000	8.7500000	-50.0000000
40280.0000000	9.0000000	-50.0000000
40280.0000000	9.2500000	-50.0000000

40280.0000000	9.5000000	-50.0000000
40280.0000000	9.7500000	-50.0000000
40280.0000000	10.0000000	-50.0000000
40280.0000000	10.2500000	-50.0000000
40280.0000000	10.5000000	-50.0000000
40280.0000000	10.7500000	-50.0000000
40280.0000000	11.0000000	-50.0000000
40280.0000000	11.2500000	-50.0000000
40280.0000000	11.5000000	-50.0000000
40280.0000000	11.7500000	-50.0000000
40280.0000000	12.0000000	-50.0000000
40280.0000000	12.2500000	-50.0000000
40280.0000000	12.5000000	-50.0000000
40280.0000000	12.7500000	-50.0000000
40280.0000000	13.0000000	-50.0000000
40280.0000000	13.2500000	-50.0000000
40280.0000000	13.5000000	-50.0000000
40280.0000000	13.7500000	-50.0000000
40280.0000000	14.0000000	-50.0000000
40280.0000000	14.2500000	-50.0000000
40280.0000000	14.5000000	-50.0000000
40280.0000000	14.7500000	-50.0000000
40280.0000000	15.0000000	-50.0000000
40310.0000000	0.0000000	-50.0000000
40310.0000000	0.2500000	-50.0000000
40310.0000000	0.5000000	-50.0000000
40310.0000000	0.7500000	-50.0000000
40310.0000000	1.0000000	-50.0000000
40310.0000000	1.2500000	-50.0000000
40310.0000000	1.5000000	-50.0000000
40310.0000000	1.7500000	-50.0000000
40310.0000000	2.0000000	-50.0000000
40310.0000000	2.2500000	-50.0000000
40310.0000000	2.5000000	-50.0000000
40310.0000000	2.7500000	-50.0000000
40310.0000000	3.0000000	-50.0000000
40310.0000000	3.2500000	-50.0000000
40310.0000000	3.5000000	-50.0000000
40310.0000000	3.7500000	-50.0000000
40310.0000000	4.0000000	-50.0000000
40310.0000000	4.2500000	-50.0000000
40310.0000000	4.5000000	-50.0000000
40310.0000000	4.7500000	-50.0000000
40310.0000000	5.0000000	-50.0000000
40310.0000000	5.2500000	-50.0000000
40310.0000000	5.5000000	-50.0000000
40310.0000000	5.7500000	-50.0000000
40310.0000000	6.0000000	-50.0000000
40310.0000000	6.2500000	-50.0000000
40310.0000000	6.5000000	-50.0000000
40310.0000000	6.7500000	-50.0000000
40310.0000000	7.0000000	-50.0000000
40310.0000000	7.2500000	-50.0000000
40310.0000000	7.5000000	-50.0000000
40310.0000000	7.7500000	-50.0000000
40310.0000000	8.0000000	-50.0000000
40310.0000000	8.2500000	-50.0000000
40310.0000000	8.5000000	-50.0000000

40310.0000000	8.7500000	-50.0000000
40310.0000000	9.0000000	-50.0000000
40310.0000000	9.2500000	-50.0000000
40310.0000000	9.5000000	-50.0000000
40310.0000000	9.7500000	-50.0000000
40310.0000000	10.0000000	-50.0000000
40310.0000000	10.2500000	-50.0000000
40310.0000000	10.5000000	-50.0000000
40310.0000000	10.7500000	-50.0000000
40310.0000000	11.0000000	-50.0000000
40310.0000000	11.2500000	-50.0000000
40310.0000000	11.5000000	-50.0000000
40310.0000000	11.7500000	-50.0000000
40310.0000000	12.0000000	-50.0000000
40310.0000000	12.2500000	-50.0000000
40310.0000000	12.5000000	-50.0000000
40310.0000000	12.7500000	-50.0000000
40310.0000000	13.0000000	-50.0000000
40310.0000000	13.2500000	-50.0000000
40310.0000000	13.5000000	-50.0000000
40310.0000000	13.7500000	-50.0000000
40310.0000000	14.0000000	-50.0000000
40310.0000000	14.2500000	-50.0000000
40310.0000000	14.5000000	-50.0000000
40310.0000000	14.7500000	-50.0000000
40310.0000000	15.0000000	-50.0000000
40340.0000000	0.0000000	-50.0000000
40340.0000000	0.2500000	-50.0000000
40340.0000000	0.5000000	-50.0000000
40340.0000000	0.7500000	-50.0000000
40340.0000000	1.0000000	-50.0000000
40340.0000000	1.2500000	-50.0000000
40340.0000000	1.5000000	-50.0000000
40340.0000000	1.7500000	-50.0000000
40340.0000000	2.0000000	-50.0000000
40340.0000000	2.2500000	-50.0000000
40340.0000000	2.5000000	-50.0000000
40340.0000000	2.7500000	-50.0000000
40340.0000000	3.0000000	-50.0000000
40340.0000000	3.2500000	-50.0000000
40340.0000000	3.5000000	-50.0000000
40340.0000000	3.7500000	-50.0000000
40340.0000000	4.0000000	-50.0000000
40340.0000000	4.2500000	-50.0000000
40340.0000000	4.5000000	-50.0000000
40340.0000000	4.7500000	-50.0000000
40340.0000000	5.0000000	-50.0000000
40340.0000000	5.2500000	-50.0000000
40340.0000000	5.5000000	-50.0000000
40340.0000000	5.7500000	-50.0000000
40340.0000000	6.0000000	-50.0000000
40340.0000000	6.2500000	-50.0000000
40340.0000000	6.5000000	-50.0000000
40340.0000000	6.7500000	-50.0000000
40340.0000000	7.0000000	-50.0000000
40340.0000000	7.2500000	-50.0000000
40340.0000000	7.5000000	-50.0000000
40340.0000000	7.7500000	-50.0000000

40340.0000000	8.0000000	-50.0000000
40340.0000000	8.2500000	-50.0000000
40340.0000000	8.5000000	-50.0000000
40340.0000000	8.7500000	-50.0000000
40340.0000000	9.0000000	-50.0000000
40340.0000000	9.2500000	-50.0000000
40340.0000000	9.5000000	-50.0000000
40340.0000000	9.7500000	-50.0000000
40340.0000000	10.0000000	-50.0000000
40340.0000000	10.2500000	-50.0000000
40340.0000000	10.5000000	-50.0000000
40340.0000000	10.7500000	-50.0000000
40340.0000000	11.0000000	-50.0000000
40340.0000000	11.2500000	-50.0000000
40340.0000000	11.5000000	-50.0000000
40340.0000000	11.7500000	-50.0000000
40340.0000000	12.0000000	-50.0000000
40340.0000000	12.2500000	-50.0000000
40340.0000000	12.5000000	-50.0000000
40340.0000000	12.7500000	-50.0000000
40340.0000000	13.0000000	-50.0000000
40340.0000000	13.2500000	-50.0000000
40340.0000000	13.5000000	-50.0000000
40340.0000000	13.7500000	-50.0000000
40340.0000000	14.0000000	-50.0000000
40340.0000000	14.2500000	-50.0000000
40340.0000000	14.5000000	-50.0000000
40340.0000000	14.7500000	-50.0000000
40340.0000000	15.0000000	-50.0000000
40370.0000000	0.0000000	-50.0000000
40370.0000000	0.2500000	-50.0000000
40370.0000000	0.5000000	-50.0000000
40370.0000000	0.7500000	-50.0000000
40370.0000000	1.0000000	-50.0000000
40370.0000000	1.2500000	-50.0000000
40370.0000000	1.5000000	-50.0000000
40370.0000000	1.7500000	-50.0000000
40370.0000000	2.0000000	-50.0000000
40370.0000000	2.2500000	-50.0000000
40370.0000000	2.5000000	-50.0000000
40370.0000000	2.7500000	-50.0000000
40370.0000000	3.0000000	-50.0000000
40370.0000000	3.2500000	-50.0000000
40370.0000000	3.5000000	-50.0000000
40370.0000000	3.7500000	-50.0000000
40370.0000000	4.0000000	-50.0000000
40370.0000000	4.2500000	-50.0000000
40370.0000000	4.5000000	-50.0000000
40370.0000000	4.7500000	-50.0000000
40370.0000000	5.0000000	-50.0000000
40370.0000000	5.2500000	-50.0000000
40370.0000000	5.5000000	-50.0000000
40370.0000000	5.7500000	-50.0000000
40370.0000000	6.0000000	-50.0000000
40370.0000000	6.2500000	-50.0000000
40370.0000000	6.5000000	-50.0000000
40370.0000000	6.7500000	-50.0000000
40370.0000000	7.0000000	-50.0000000

40370.0000000	7.2500000	-50.0000000
40370.0000000	7.5000000	-50.0000000
40370.0000000	7.7500000	-50.0000000
40370.0000000	8.0000000	-50.0000000
40370.0000000	8.2500000	-50.0000000
40370.0000000	8.5000000	-50.0000000
40370.0000000	8.7500000	-50.0000000
40370.0000000	9.0000000	-50.0000000
40370.0000000	9.2500000	-50.0000000
40370.0000000	9.5000000	-50.0000000
40370.0000000	9.7500000	-50.0000000
40370.0000000	10.0000000	-50.0000000
40370.0000000	10.2500000	-50.0000000
40370.0000000	10.5000000	-50.0000000
40370.0000000	10.7500000	-50.0000000
40370.0000000	11.0000000	-50.0000000
40370.0000000	11.2500000	-50.0000000
40370.0000000	11.5000000	-50.0000000
40370.0000000	11.7500000	-50.0000000
40370.0000000	12.0000000	-50.0000000
40370.0000000	12.2500000	-50.0000000
40370.0000000	12.5000000	-50.0000000
40370.0000000	12.7500000	-50.0000000
40370.0000000	13.0000000	-50.0000000
40370.0000000	13.2500000	-50.0000000
40370.0000000	13.5000000	-50.0000000
40370.0000000	13.7500000	-50.0000000
40370.0000000	14.0000000	-50.0000000
40370.0000000	14.2500000	-50.0000000
40370.0000000	14.5000000	-50.0000000
40370.0000000	14.7500000	-50.0000000
40370.0000000	15.0000000	-50.0000000
40400.0000000	0.0000000	-50.0000000
40400.0000000	0.2500000	-50.0000000
40400.0000000	0.5000000	-50.0000000
40400.0000000	0.7500000	-50.0000000
40400.0000000	1.0000000	-50.0000000
40400.0000000	1.2500000	-50.0000000
40400.0000000	1.5000000	-50.0000000
40400.0000000	1.7500000	-50.0000000
40400.0000000	2.0000000	-50.0000000
40400.0000000	2.2500000	-50.0000000
40400.0000000	2.5000000	-50.0000000
40400.0000000	2.7500000	-50.0000000
40400.0000000	3.0000000	-50.0000000
40400.0000000	3.2500000	-50.0000000
40400.0000000	3.5000000	-50.0000000
40400.0000000	3.7500000	-50.0000000
40400.0000000	4.0000000	-50.0000000
40400.0000000	4.2500000	-50.0000000
40400.0000000	4.5000000	-50.0000000
40400.0000000	4.7500000	-50.0000000
40400.0000000	5.0000000	-50.0000000
40400.0000000	5.2500000	-50.0000000
40400.0000000	5.5000000	-50.0000000
40400.0000000	5.7500000	-50.0000000
40400.0000000	6.0000000	-50.0000000
40400.0000000	6.2500000	-50.0000000

40400.0000000	6.5000000	-50.0000000
40400.0000000	6.7500000	-50.0000000
40400.0000000	7.0000000	-50.0000000
40400.0000000	7.2500000	-50.0000000
40400.0000000	7.5000000	-50.0000000
40400.0000000	7.7500000	-50.0000000
40400.0000000	8.0000000	-50.0000000
40400.0000000	8.2500000	-50.0000000
40400.0000000	8.5000000	-50.0000000
40400.0000000	8.7500000	-50.0000000
40400.0000000	9.0000000	-50.0000000
40400.0000000	9.2500000	-50.0000000
40400.0000000	9.5000000	-50.0000000
40400.0000000	9.7500000	-50.0000000
40400.0000000	10.0000000	-50.0000000
40400.0000000	10.2500000	-50.0000000
40400.0000000	10.5000000	-50.0000000
40400.0000000	10.7500000	-50.0000000
40400.0000000	11.0000000	-50.0000000
40400.0000000	11.2500000	-50.0000000
40400.0000000	11.5000000	-50.0000000
40400.0000000	11.7500000	-50.0000000
40400.0000000	12.0000000	-50.0000000
40400.0000000	12.2500000	-50.0000000
40400.0000000	12.5000000	-50.0000000
40400.0000000	12.7500000	-50.0000000
40400.0000000	13.0000000	-50.0000000
40400.0000000	13.2500000	-50.0000000
40400.0000000	13.5000000	-50.0000000
40400.0000000	13.7500000	-50.0000000
40400.0000000	14.0000000	-50.0000000
40400.0000000	14.2500000	-50.0000000
40400.0000000	14.5000000	-50.0000000
40400.0000000	14.7500000	-50.0000000
40400.0000000	15.0000000	-50.0000000
40430.0000000	0.0000000	-50.0000000
40430.0000000	0.2500000	-50.0000000
40430.0000000	0.5000000	-50.0000000
40430.0000000	0.7500000	-50.0000000
40430.0000000	1.0000000	-50.0000000
40430.0000000	1.2500000	-50.0000000
40430.0000000	1.5000000	-50.0000000
40430.0000000	1.7500000	-50.0000000
40430.0000000	2.0000000	-50.0000000
40430.0000000	2.2500000	-50.0000000
40430.0000000	2.5000000	-50.0000000
40430.0000000	2.7500000	-50.0000000
40430.0000000	3.0000000	-50.0000000
40430.0000000	3.2500000	-50.0000000
40430.0000000	3.5000000	-50.0000000
40430.0000000	3.7500000	-50.0000000
40430.0000000	4.0000000	-50.0000000
40430.0000000	4.2500000	-50.0000000
40430.0000000	4.5000000	-50.0000000
40430.0000000	4.7500000	-50.0000000
40430.0000000	5.0000000	-50.0000000
40430.0000000	5.2500000	-50.0000000
40430.0000000	5.5000000	-50.0000000

40430.0000000	5.7500000	-50.0000000
40430.0000000	6.0000000	-50.0000000
40430.0000000	6.2500000	-50.0000000
40430.0000000	6.5000000	-50.0000000
40430.0000000	6.7500000	-50.0000000
40430.0000000	7.0000000	-50.0000000
40430.0000000	7.2500000	-50.0000000
40430.0000000	7.5000000	-50.0000000
40430.0000000	7.7500000	-50.0000000
40430.0000000	8.0000000	-50.0000000
40430.0000000	8.2500000	-50.0000000
40430.0000000	8.5000000	-50.0000000
40430.0000000	8.7500000	-50.0000000
40430.0000000	9.0000000	-50.0000000
40430.0000000	9.2500000	-50.0000000
40430.0000000	9.5000000	-50.0000000
40430.0000000	9.7500000	-50.0000000
40430.0000000	10.0000000	-50.0000000
40430.0000000	10.2500000	-50.0000000
40430.0000000	10.5000000	-50.0000000
40430.0000000	10.7500000	-50.0000000
40430.0000000	11.0000000	-50.0000000
40430.0000000	11.2500000	-50.0000000
40430.0000000	11.5000000	-50.0000000
40430.0000000	11.7500000	-50.0000000
40430.0000000	12.0000000	-50.0000000
40430.0000000	12.2500000	-50.0000000
40430.0000000	12.5000000	-50.0000000
40430.0000000	12.7500000	-50.0000000
40430.0000000	13.0000000	-50.0000000
40430.0000000	13.2500000	-50.0000000
40430.0000000	13.5000000	-50.0000000
40430.0000000	13.7500000	-50.0000000
40430.0000000	14.0000000	-50.0000000
40430.0000000	14.2500000	-50.0000000
40430.0000000	14.5000000	-50.0000000
40430.0000000	14.7500000	-50.0000000
40430.0000000	15.0000000	-50.0000000
40460.0000000	0.0000000	-50.0000000
40460.0000000	0.2500000	-50.0000000
40460.0000000	0.5000000	-50.0000000
40460.0000000	0.7500000	-50.0000000
40460.0000000	1.0000000	-50.0000000
40460.0000000	1.2500000	-50.0000000
40460.0000000	1.5000000	-50.0000000
40460.0000000	1.7500000	-50.0000000
40460.0000000	2.0000000	-50.0000000
40460.0000000	2.2500000	-50.0000000
40460.0000000	2.5000000	-50.0000000
40460.0000000	2.7500000	-50.0000000
40460.0000000	3.0000000	-50.0000000
40460.0000000	3.2500000	-50.0000000
40460.0000000	3.5000000	-50.0000000
40460.0000000	3.7500000	-50.0000000
40460.0000000	4.0000000	-50.0000000
40460.0000000	4.2500000	-50.0000000
40460.0000000	4.5000000	-50.0000000
40460.0000000	4.7500000	-50.0000000

40460.0000000	5.0000000	-50.0000000
40460.0000000	5.2500000	-50.0000000
40460.0000000	5.5000000	-50.0000000
40460.0000000	5.7500000	-50.0000000
40460.0000000	6.0000000	-50.0000000
40460.0000000	6.2500000	-50.0000000
40460.0000000	6.5000000	-50.0000000
40460.0000000	6.7500000	-50.0000000
40460.0000000	7.0000000	-50.0000000
40460.0000000	7.2500000	-50.0000000
40460.0000000	7.5000000	-50.0000000
40460.0000000	7.7500000	-50.0000000
40460.0000000	8.0000000	-50.0000000
40460.0000000	8.2500000	-50.0000000
40460.0000000	8.5000000	-50.0000000
40460.0000000	8.7500000	-50.0000000
40460.0000000	9.0000000	-50.0000000
40460.0000000	9.2500000	-50.0000000
40460.0000000	9.5000000	-50.0000000
40460.0000000	9.7500000	-50.0000000
40460.0000000	10.0000000	-50.0000000
40460.0000000	10.2500000	-50.0000000
40460.0000000	10.5000000	-50.0000000
40460.0000000	10.7500000	-50.0000000
40460.0000000	11.0000000	-50.0000000
40460.0000000	11.2500000	-50.0000000
40460.0000000	11.5000000	-50.0000000
40460.0000000	11.7500000	-50.0000000
40460.0000000	12.0000000	-50.0000000
40460.0000000	12.2500000	-50.0000000
40460.0000000	12.5000000	-50.0000000
40460.0000000	12.7500000	-50.0000000
40460.0000000	13.0000000	-50.0000000
40460.0000000	13.2500000	-50.0000000
40460.0000000	13.5000000	-50.0000000
40460.0000000	13.7500000	-50.0000000
40460.0000000	14.0000000	-50.0000000
40460.0000000	14.2500000	-50.0000000
40460.0000000	14.5000000	-50.0000000
40460.0000000	14.7500000	-50.0000000
40460.0000000	15.0000000	-50.0000000
40490.0000000	0.0000000	-50.0000000
40490.0000000	0.2500000	-50.0000000
40490.0000000	0.5000000	-50.0000000
40490.0000000	0.7500000	-50.0000000
40490.0000000	1.0000000	-50.0000000
40490.0000000	1.2500000	-50.0000000
40490.0000000	1.5000000	-50.0000000
40490.0000000	1.7500000	-50.0000000
40490.0000000	2.0000000	-50.0000000
40490.0000000	2.2500000	-50.0000000
40490.0000000	2.5000000	-50.0000000
40490.0000000	2.7500000	-50.0000000
40490.0000000	3.0000000	-50.0000000
40490.0000000	3.2500000	-50.0000000
40490.0000000	3.5000000	-50.0000000
40490.0000000	3.7500000	-50.0000000
40490.0000000	4.0000000	-50.0000000

40490.0000000	4.2500000	-50.0000000
40490.0000000	4.5000000	-50.0000000
40490.0000000	4.7500000	-50.0000000
40490.0000000	5.0000000	-50.0000000
40490.0000000	5.2500000	-50.0000000
40490.0000000	5.5000000	-50.0000000
40490.0000000	5.7500000	-50.0000000
40490.0000000	6.0000000	-50.0000000
40490.0000000	6.2500000	-50.0000000
40490.0000000	6.5000000	-50.0000000
40490.0000000	6.7500000	-50.0000000
40490.0000000	7.0000000	-50.0000000
40490.0000000	7.2500000	-50.0000000
40490.0000000	7.5000000	-50.0000000
40490.0000000	7.7500000	-50.0000000
40490.0000000	8.0000000	-50.0000000
40490.0000000	8.2500000	-50.0000000
40490.0000000	8.5000000	-50.0000000
40490.0000000	8.7500000	-50.0000000
40490.0000000	9.0000000	-50.0000000
40490.0000000	9.2500000	-50.0000000
40490.0000000	9.5000000	-50.0000000
40490.0000000	9.7500000	-50.0000000
40490.0000000	10.0000000	-50.0000000
40490.0000000	10.2500000	-50.0000000
40490.0000000	10.5000000	-50.0000000
40490.0000000	10.7500000	-50.0000000
40490.0000000	11.0000000	-50.0000000
40490.0000000	11.2500000	-50.0000000
40490.0000000	11.5000000	-50.0000000
40490.0000000	11.7500000	-50.0000000
40490.0000000	12.0000000	-50.0000000
40490.0000000	12.2500000	-50.0000000
40490.0000000	12.5000000	-50.0000000
40490.0000000	12.7500000	-50.0000000
40490.0000000	13.0000000	-50.0000000
40490.0000000	13.2500000	-50.0000000
40490.0000000	13.5000000	-50.0000000
40490.0000000	13.7500000	-50.0000000
40490.0000000	14.0000000	-50.0000000
40490.0000000	14.2500000	-50.0000000
40490.0000000	14.5000000	-50.0000000
40490.0000000	14.7500000	-50.0000000
40490.0000000	15.0000000	-50.0000000
40520.0000000	0.0000000	-50.0000000
40520.0000000	0.2500000	-50.0000000
40520.0000000	0.5000000	-50.0000000
40520.0000000	0.7500000	-50.0000000
40520.0000000	1.0000000	-50.0000000
40520.0000000	1.2500000	-50.0000000
40520.0000000	1.5000000	-50.0000000
40520.0000000	1.7500000	-50.0000000
40520.0000000	2.0000000	-50.0000000
40520.0000000	2.2500000	-50.0000000
40520.0000000	2.5000000	-50.0000000
40520.0000000	2.7500000	-50.0000000
40520.0000000	3.0000000	-50.0000000
40520.0000000	3.2500000	-50.0000000

40520.0000000	3.5000000	-50.0000000
40520.0000000	3.7500000	-50.0000000
40520.0000000	4.0000000	-50.0000000
40520.0000000	4.2500000	-50.0000000
40520.0000000	4.5000000	-50.0000000
40520.0000000	4.7500000	-50.0000000
40520.0000000	5.0000000	-50.0000000
40520.0000000	5.2500000	-50.0000000
40520.0000000	5.5000000	-50.0000000
40520.0000000	5.7500000	-50.0000000
40520.0000000	6.0000000	-50.0000000
40520.0000000	6.2500000	-50.0000000
40520.0000000	6.5000000	-50.0000000
40520.0000000	6.7500000	-50.0000000
40520.0000000	7.0000000	-50.0000000
40520.0000000	7.2500000	-50.0000000
40520.0000000	7.5000000	-50.0000000
40520.0000000	7.7500000	-50.0000000
40520.0000000	8.0000000	-50.0000000
40520.0000000	8.2500000	-50.0000000
40520.0000000	8.5000000	-50.0000000
40520.0000000	8.7500000	-50.0000000
40520.0000000	9.0000000	-50.0000000
40520.0000000	9.2500000	-50.0000000
40520.0000000	9.5000000	-50.0000000
40520.0000000	9.7500000	-50.0000000
40520.0000000	10.0000000	-50.0000000
40520.0000000	10.2500000	-50.0000000
40520.0000000	10.5000000	-50.0000000
40520.0000000	10.7500000	-50.0000000
40520.0000000	11.0000000	-50.0000000
40520.0000000	11.2500000	-50.0000000
40520.0000000	11.5000000	-50.0000000
40520.0000000	11.7500000	-50.0000000
40520.0000000	12.0000000	-50.0000000
40520.0000000	12.2500000	-50.0000000
40520.0000000	12.5000000	-50.0000000
40520.0000000	12.7500000	-50.0000000
40520.0000000	13.0000000	-50.0000000
40520.0000000	13.2500000	-50.0000000
40520.0000000	13.5000000	-50.0000000
40520.0000000	13.7500000	-50.0000000
40520.0000000	14.0000000	-50.0000000
40520.0000000	14.2500000	-50.0000000
40520.0000000	14.5000000	-50.0000000
40520.0000000	14.7500000	-50.0000000
40520.0000000	15.0000000	-50.0000000
40550.0000000	0.0000000	-50.0000000
40550.0000000	0.2500000	-50.0000000
40550.0000000	0.5000000	-50.0000000
40550.0000000	0.7500000	-50.0000000
40550.0000000	1.0000000	-50.0000000
40550.0000000	1.2500000	-50.0000000
40550.0000000	1.5000000	-50.0000000
40550.0000000	1.7500000	-50.0000000
40550.0000000	2.0000000	-50.0000000
40550.0000000	2.2500000	-50.0000000
40550.0000000	2.5000000	-50.0000000

40550.0000000	2.7500000	-50.0000000
40550.0000000	3.0000000	-50.0000000
40550.0000000	3.2500000	-50.0000000
40550.0000000	3.5000000	-50.0000000
40550.0000000	3.7500000	-50.0000000
40550.0000000	4.0000000	-50.0000000
40550.0000000	4.2500000	-50.0000000
40550.0000000	4.5000000	-50.0000000
40550.0000000	4.7500000	-50.0000000
40550.0000000	5.0000000	-50.0000000
40550.0000000	5.2500000	-50.0000000
40550.0000000	5.5000000	-50.0000000
40550.0000000	5.7500000	-50.0000000
40550.0000000	6.0000000	-50.0000000
40550.0000000	6.2500000	-50.0000000
40550.0000000	6.5000000	-50.0000000
40550.0000000	6.7500000	-50.0000000
40550.0000000	7.0000000	-50.0000000
40550.0000000	7.2500000	-50.0000000
40550.0000000	7.5000000	-50.0000000
40550.0000000	7.7500000	-50.0000000
40550.0000000	8.0000000	-50.0000000
40550.0000000	8.2500000	-50.0000000
40550.0000000	8.5000000	-50.0000000
40550.0000000	8.7500000	-50.0000000
40550.0000000	9.0000000	-50.0000000
40550.0000000	9.2500000	-50.0000000
40550.0000000	9.5000000	-50.0000000
40550.0000000	9.7500000	-50.0000000
40550.0000000	10.0000000	-50.0000000
40550.0000000	10.2500000	-50.0000000
40550.0000000	10.5000000	-50.0000000
40550.0000000	10.7500000	-50.0000000
40550.0000000	11.0000000	-50.0000000
40550.0000000	11.2500000	-50.0000000
40550.0000000	11.5000000	-50.0000000
40550.0000000	11.7500000	-50.0000000
40550.0000000	12.0000000	-50.0000000
40550.0000000	12.2500000	-50.0000000
40550.0000000	12.5000000	-50.0000000
40550.0000000	12.7500000	-50.0000000
40550.0000000	13.0000000	-50.0000000
40550.0000000	13.2500000	-50.0000000
40550.0000000	13.5000000	-50.0000000
40550.0000000	13.7500000	-50.0000000
40550.0000000	14.0000000	-50.0000000
40550.0000000	14.2500000	-50.0000000
40550.0000000	14.5000000	-50.0000000
40550.0000000	14.7500000	-50.0000000
40550.0000000	15.0000000	-50.0000000
40580.0000000	0.0000000	-50.0000000
40580.0000000	0.2500000	-50.0000000
40580.0000000	0.5000000	-50.0000000
40580.0000000	0.7500000	-50.0000000
40580.0000000	1.0000000	-50.0000000
40580.0000000	1.2500000	-50.0000000
40580.0000000	1.5000000	-50.0000000
40580.0000000	1.7500000	-50.0000000

40580.0000000	2.0000000	-50.0000000
40580.0000000	2.2500000	-50.0000000
40580.0000000	2.5000000	-50.0000000
40580.0000000	2.7500000	-50.0000000
40580.0000000	3.0000000	-50.0000000
40580.0000000	3.2500000	-50.0000000
40580.0000000	3.5000000	-50.0000000
40580.0000000	3.7500000	-50.0000000
40580.0000000	4.0000000	-50.0000000
40580.0000000	4.2500000	-50.0000000
40580.0000000	4.5000000	-50.0000000
40580.0000000	4.7500000	-50.0000000
40580.0000000	5.0000000	-50.0000000
40580.0000000	5.2500000	-50.0000000
40580.0000000	5.5000000	-50.0000000
40580.0000000	5.7500000	-50.0000000
40580.0000000	6.0000000	-50.0000000
40580.0000000	6.2500000	-50.0000000
40580.0000000	6.5000000	-50.0000000
40580.0000000	6.7500000	-50.0000000
40580.0000000	7.0000000	-50.0000000
40580.0000000	7.2500000	-50.0000000
40580.0000000	7.5000000	-50.0000000
40580.0000000	7.7500000	-50.0000000
40580.0000000	8.0000000	-50.0000000
40580.0000000	8.2500000	-50.0000000
40580.0000000	8.5000000	-50.0000000
40580.0000000	8.7500000	-50.0000000
40580.0000000	9.0000000	-50.0000000
40580.0000000	9.2500000	-50.0000000
40580.0000000	9.5000000	-50.0000000
40580.0000000	9.7500000	-50.0000000
40580.0000000	10.0000000	-50.0000000
40580.0000000	10.2500000	-50.0000000
40580.0000000	10.5000000	-50.0000000
40580.0000000	10.7500000	-50.0000000
40580.0000000	11.0000000	-50.0000000
40580.0000000	11.2500000	-50.0000000
40580.0000000	11.5000000	-50.0000000
40580.0000000	11.7500000	-50.0000000
40580.0000000	12.0000000	-50.0000000
40580.0000000	12.2500000	-50.0000000
40580.0000000	12.5000000	-50.0000000
40580.0000000	12.7500000	-50.0000000
40580.0000000	13.0000000	-50.0000000
40580.0000000	13.2500000	-50.0000000
40580.0000000	13.5000000	-50.0000000
40580.0000000	13.7500000	-50.0000000
40580.0000000	14.0000000	-50.0000000
40580.0000000	14.2500000	-50.0000000
40580.0000000	14.5000000	-50.0000000
40580.0000000	14.7500000	-50.0000000
40580.0000000	15.0000000	-50.0000000
40610.0000000	0.0000000	-50.0000000
40610.0000000	0.2500000	-50.0000000
40610.0000000	0.5000000	-50.0000000
40610.0000000	0.7500000	-50.0000000
40610.0000000	1.0000000	-50.0000000

40610.0000000	1.2500000	-50.0000000
40610.0000000	1.5000000	-50.0000000
40610.0000000	1.7500000	-50.0000000
40610.0000000	2.0000000	-50.0000000
40610.0000000	2.2500000	-50.0000000
40610.0000000	2.5000000	-50.0000000
40610.0000000	2.7500000	-50.0000000
40610.0000000	3.0000000	-50.0000000
40610.0000000	3.2500000	-50.0000000
40610.0000000	3.5000000	-50.0000000
40610.0000000	3.7500000	-50.0000000
40610.0000000	4.0000000	-50.0000000
40610.0000000	4.2500000	-50.0000000
40610.0000000	4.5000000	-50.0000000
40610.0000000	4.7500000	-50.0000000
40610.0000000	5.0000000	-50.0000000
40610.0000000	5.2500000	-50.0000000
40610.0000000	5.5000000	-50.0000000
40610.0000000	5.7500000	-50.0000000
40610.0000000	6.0000000	-50.0000000
40610.0000000	6.2500000	-50.0000000
40610.0000000	6.5000000	-50.0000000
40610.0000000	6.7500000	-50.0000000
40610.0000000	7.0000000	-50.0000000
40610.0000000	7.2500000	-50.0000000
40610.0000000	7.5000000	-50.0000000
40610.0000000	7.7500000	-50.0000000
40610.0000000	8.0000000	-50.0000000
40610.0000000	8.2500000	-50.0000000
40610.0000000	8.5000000	-50.0000000
40610.0000000	8.7500000	-50.0000000
40610.0000000	9.0000000	-50.0000000
40610.0000000	9.2500000	-50.0000000
40610.0000000	9.5000000	-50.0000000
40610.0000000	9.7500000	-50.0000000
40610.0000000	10.0000000	-50.0000000
40610.0000000	10.2500000	-50.0000000
40610.0000000	10.5000000	-50.0000000
40610.0000000	10.7500000	-50.0000000
40610.0000000	11.0000000	-50.0000000
40610.0000000	11.2500000	-50.0000000
40610.0000000	11.5000000	-50.0000000
40610.0000000	11.7500000	-50.0000000
40610.0000000	12.0000000	-50.0000000
40610.0000000	12.2500000	-50.0000000
40610.0000000	12.5000000	-50.0000000
40610.0000000	12.7500000	-50.0000000
40610.0000000	13.0000000	-50.0000000
40610.0000000	13.2500000	-50.0000000
40610.0000000	13.5000000	-50.0000000
40610.0000000	13.7500000	-50.0000000
40610.0000000	14.0000000	-50.0000000
40610.0000000	14.2500000	-50.0000000
40610.0000000	14.5000000	-50.0000000
40610.0000000	14.7500000	-50.0000000
40610.0000000	15.0000000	-50.0000000
40640.0000000	0.0000000	-50.0000000
40640.0000000	0.2500000	-50.0000000

40640.0000000	0.5000000	-50.0000000
40640.0000000	0.7500000	-50.0000000
40640.0000000	1.0000000	-50.0000000
40640.0000000	1.2500000	-50.0000000
40640.0000000	1.5000000	-50.0000000
40640.0000000	1.7500000	-50.0000000
40640.0000000	2.0000000	-50.0000000
40640.0000000	2.2500000	-50.0000000
40640.0000000	2.5000000	-50.0000000
40640.0000000	2.7500000	-50.0000000
40640.0000000	3.0000000	-50.0000000
40640.0000000	3.2500000	-50.0000000
40640.0000000	3.5000000	-50.0000000
40640.0000000	3.7500000	-50.0000000
40640.0000000	4.0000000	-50.0000000
40640.0000000	4.2500000	-50.0000000
40640.0000000	4.5000000	-50.0000000
40640.0000000	4.7500000	-50.0000000
40640.0000000	5.0000000	-50.0000000
40640.0000000	5.2500000	-50.0000000
40640.0000000	5.5000000	-50.0000000
40640.0000000	5.7500000	-50.0000000
40640.0000000	6.0000000	-50.0000000
40640.0000000	6.2500000	-50.0000000
40640.0000000	6.5000000	-50.0000000
40640.0000000	6.7500000	-50.0000000
40640.0000000	7.0000000	-50.0000000
40640.0000000	7.2500000	-50.0000000
40640.0000000	7.5000000	-50.0000000
40640.0000000	7.7500000	-50.0000000
40640.0000000	8.0000000	-50.0000000
40640.0000000	8.2500000	-50.0000000
40640.0000000	8.5000000	-50.0000000
40640.0000000	8.7500000	-50.0000000
40640.0000000	9.0000000	-50.0000000
40640.0000000	9.2500000	-50.0000000
40640.0000000	9.5000000	-50.0000000
40640.0000000	9.7500000	-50.0000000
40640.0000000	10.0000000	-50.0000000
40640.0000000	10.2500000	-50.0000000
40640.0000000	10.5000000	-50.0000000
40640.0000000	10.7500000	-50.0000000
40640.0000000	11.0000000	-50.0000000
40640.0000000	11.2500000	-50.0000000
40640.0000000	11.5000000	-50.0000000
40640.0000000	11.7500000	-50.0000000
40640.0000000	12.0000000	-50.0000000
40640.0000000	12.2500000	-50.0000000
40640.0000000	12.5000000	-50.0000000
40640.0000000	12.7500000	-50.0000000
40640.0000000	13.0000000	-50.0000000
40640.0000000	13.2500000	-50.0000000
40640.0000000	13.5000000	-50.0000000
40640.0000000	13.7500000	-50.0000000
40640.0000000	14.0000000	-50.0000000
40640.0000000	14.2500000	-50.0000000
40640.0000000	14.5000000	-50.0000000
40640.0000000	14.7500000	-50.0000000

40640.0000000	15.0000000	-50.0000000
40670.0000000	0.0000000	-50.0000000
40670.0000000	0.2500000	-50.0000000
40670.0000000	0.5000000	-50.0000000
40670.0000000	0.7500000	-50.0000000
40670.0000000	1.0000000	-50.0000000
40670.0000000	1.2500000	-50.0000000
40670.0000000	1.5000000	-50.0000000
40670.0000000	1.7500000	-50.0000000
40670.0000000	2.0000000	-50.0000000
40670.0000000	2.2500000	-50.0000000
40670.0000000	2.5000000	-50.0000000
40670.0000000	2.7500000	-50.0000000
40670.0000000	3.0000000	-50.0000000
40670.0000000	3.2500000	-50.0000000
40670.0000000	3.5000000	-50.0000000
40670.0000000	3.7500000	-50.0000000
40670.0000000	4.0000000	-50.0000000
40670.0000000	4.2500000	-50.0000000
40670.0000000	4.5000000	-50.0000000
40670.0000000	4.7500000	-50.0000000
40670.0000000	5.0000000	-50.0000000
40670.0000000	5.2500000	-50.0000000
40670.0000000	5.5000000	-50.0000000
40670.0000000	5.7500000	-50.0000000
40670.0000000	6.0000000	-50.0000000
40670.0000000	6.2500000	-50.0000000
40670.0000000	6.5000000	-50.0000000
40670.0000000	6.7500000	-50.0000000
40670.0000000	7.0000000	-50.0000000
40670.0000000	7.2500000	-50.0000000
40670.0000000	7.5000000	-50.0000000
40670.0000000	7.7500000	-50.0000000
40670.0000000	8.0000000	-50.0000000
40670.0000000	8.2500000	-50.0000000
40670.0000000	8.5000000	-50.0000000
40670.0000000	8.7500000	-50.0000000
40670.0000000	9.0000000	-50.0000000
40670.0000000	9.2500000	-50.0000000
40670.0000000	9.5000000	-50.0000000
40670.0000000	9.7500000	-50.0000000
40670.0000000	10.0000000	-50.0000000
40670.0000000	10.2500000	-50.0000000
40670.0000000	10.5000000	-50.0000000
40670.0000000	10.7500000	-50.0000000
40670.0000000	11.0000000	-50.0000000
40670.0000000	11.2500000	-50.0000000
40670.0000000	11.5000000	-50.0000000
40670.0000000	11.7500000	-50.0000000
40670.0000000	12.0000000	-50.0000000
40670.0000000	12.2500000	-50.0000000
40670.0000000	12.5000000	-50.0000000
40670.0000000	12.7500000	-50.0000000
40670.0000000	13.0000000	-50.0000000
40670.0000000	13.2500000	-50.0000000
40670.0000000	13.5000000	-50.0000000
40670.0000000	13.7500000	-50.0000000
40670.0000000	14.0000000	-50.0000000

40670.0000000	14.2500000	-50.0000000
40670.0000000	14.5000000	-50.0000000
40670.0000000	14.7500000	-50.0000000
40670.0000000	15.0000000	-50.0000000
40700.0000000	0.0000000	-50.0000000
40700.0000000	0.2500000	-50.0000000
40700.0000000	0.5000000	-50.0000000
40700.0000000	0.7500000	-50.0000000
40700.0000000	1.0000000	-50.0000000
40700.0000000	1.2500000	-50.0000000
40700.0000000	1.5000000	-50.0000000
40700.0000000	1.7500000	-50.0000000
40700.0000000	2.0000000	-50.0000000
40700.0000000	2.2500000	-50.0000000
40700.0000000	2.5000000	-50.0000000
40700.0000000	2.7500000	-50.0000000
40700.0000000	3.0000000	-50.0000000
40700.0000000	3.2500000	-50.0000000
40700.0000000	3.5000000	-50.0000000
40700.0000000	3.7500000	-50.0000000
40700.0000000	4.0000000	-50.0000000
40700.0000000	4.2500000	-50.0000000
40700.0000000	4.5000000	-50.0000000
40700.0000000	4.7500000	-50.0000000
40700.0000000	5.0000000	-50.0000000
40700.0000000	5.2500000	-50.0000000
40700.0000000	5.5000000	-50.0000000
40700.0000000	5.7500000	-50.0000000
40700.0000000	6.0000000	-50.0000000
40700.0000000	6.2500000	-50.0000000
40700.0000000	6.5000000	-50.0000000
40700.0000000	6.7500000	-50.0000000
40700.0000000	7.0000000	-50.0000000
40700.0000000	7.2500000	-50.0000000
40700.0000000	7.5000000	-50.0000000
40700.0000000	7.7500000	-50.0000000
40700.0000000	8.0000000	-50.0000000
40700.0000000	8.2500000	-50.0000000
40700.0000000	8.5000000	-50.0000000
40700.0000000	8.7500000	-50.0000000
40700.0000000	9.0000000	-50.0000000
40700.0000000	9.2500000	-50.0000000
40700.0000000	9.5000000	-50.0000000
40700.0000000	9.7500000	-50.0000000
40700.0000000	10.0000000	-50.0000000
40700.0000000	10.2500000	-50.0000000
40700.0000000	10.5000000	-50.0000000
40700.0000000	10.7500000	-50.0000000
40700.0000000	11.0000000	-50.0000000
40700.0000000	11.2500000	-50.0000000
40700.0000000	11.5000000	-50.0000000
40700.0000000	11.7500000	-50.0000000
40700.0000000	12.0000000	-50.0000000
40700.0000000	12.2500000	-50.0000000
40700.0000000	12.5000000	-50.0000000
40700.0000000	12.7500000	-50.0000000
40700.0000000	13.0000000	-50.0000000
40700.0000000	13.2500000	-50.0000000

40700.0000000	13.5000000	-50.0000000
40700.0000000	13.7500000	-50.0000000
40700.0000000	14.0000000	-50.0000000
40700.0000000	14.2500000	-50.0000000
40700.0000000	14.5000000	-50.0000000
40700.0000000	14.7500000	-50.0000000
40700.0000000	15.0000000	-50.0000000
40730.0000000	0.0000000	-50.0000000
40730.0000000	0.2500000	-50.0000000
40730.0000000	0.5000000	-50.0000000
40730.0000000	0.7500000	-50.0000000
40730.0000000	1.0000000	-50.0000000
40730.0000000	1.2500000	-50.0000000
40730.0000000	1.5000000	-50.0000000
40730.0000000	1.7500000	-50.0000000
40730.0000000	2.0000000	-50.0000000
40730.0000000	2.2500000	-50.0000000
40730.0000000	2.5000000	-50.0000000
40730.0000000	2.7500000	-50.0000000
40730.0000000	3.0000000	-50.0000000
40730.0000000	3.2500000	-50.0000000
40730.0000000	3.5000000	-50.0000000
40730.0000000	3.7500000	-50.0000000
40730.0000000	4.0000000	-50.0000000
40730.0000000	4.2500000	-50.0000000
40730.0000000	4.5000000	-50.0000000
40730.0000000	4.7500000	-50.0000000
40730.0000000	5.0000000	-50.0000000
40730.0000000	5.2500000	-50.0000000
40730.0000000	5.5000000	-50.0000000
40730.0000000	5.7500000	-50.0000000
40730.0000000	6.0000000	-50.0000000
40730.0000000	6.2500000	-50.0000000
40730.0000000	6.5000000	-50.0000000
40730.0000000	6.7500000	-50.0000000
40730.0000000	7.0000000	-50.0000000
40730.0000000	7.2500000	-50.0000000
40730.0000000	7.5000000	-50.0000000
40730.0000000	7.7500000	-50.0000000
40730.0000000	8.0000000	-50.0000000
40730.0000000	8.2500000	-50.0000000
40730.0000000	8.5000000	-50.0000000
40730.0000000	8.7500000	-50.0000000
40730.0000000	9.0000000	-50.0000000
40730.0000000	9.2500000	-50.0000000
40730.0000000	9.5000000	-50.0000000
40730.0000000	9.7500000	-50.0000000
40730.0000000	10.0000000	-50.0000000
40730.0000000	10.2500000	-50.0000000
40730.0000000	10.5000000	-50.0000000
40730.0000000	10.7500000	-50.0000000
40730.0000000	11.0000000	-50.0000000
40730.0000000	11.2500000	-50.0000000
40730.0000000	11.5000000	-50.0000000
40730.0000000	11.7500000	-50.0000000
40730.0000000	12.0000000	-50.0000000
40730.0000000	12.2500000	-50.0000000
40730.0000000	12.5000000	-50.0000000

40730.0000000	12.7500000	-50.0000000
40730.0000000	13.0000000	-50.0000000
40730.0000000	13.2500000	-50.0000000
40730.0000000	13.5000000	-50.0000000
40730.0000000	13.7500000	-50.0000000
40730.0000000	14.0000000	-50.0000000
40730.0000000	14.2500000	-50.0000000
40730.0000000	14.5000000	-50.0000000
40730.0000000	14.7500000	-50.0000000
40730.0000000	15.0000000	-50.0000000
40760.0000000	0.0000000	-50.0000000
40760.0000000	0.2500000	-50.0000000
40760.0000000	0.5000000	-50.0000000
40760.0000000	0.7500000	-50.0000000
40760.0000000	1.0000000	-50.0000000
40760.0000000	1.2500000	-50.0000000
40760.0000000	1.5000000	-50.0000000
40760.0000000	1.7500000	-50.0000000
40760.0000000	2.0000000	-50.0000000
40760.0000000	2.2500000	-50.0000000
40760.0000000	2.5000000	-50.0000000
40760.0000000	2.7500000	-50.0000000
40760.0000000	3.0000000	-50.0000000
40760.0000000	3.2500000	-50.0000000
40760.0000000	3.5000000	-50.0000000
40760.0000000	3.7500000	-50.0000000
40760.0000000	4.0000000	-50.0000000
40760.0000000	4.2500000	-50.0000000
40760.0000000	4.5000000	-50.0000000
40760.0000000	4.7500000	-50.0000000
40760.0000000	5.0000000	-50.0000000
40760.0000000	5.2500000	-50.0000000
40760.0000000	5.5000000	-50.0000000
40760.0000000	5.7500000	-50.0000000
40760.0000000	6.0000000	-50.0000000
40760.0000000	6.2500000	-50.0000000
40760.0000000	6.5000000	-50.0000000
40760.0000000	6.7500000	-50.0000000
40760.0000000	7.0000000	-50.0000000
40760.0000000	7.2500000	-50.0000000
40760.0000000	7.5000000	-50.0000000
40760.0000000	7.7500000	-50.0000000
40760.0000000	8.0000000	-50.0000000
40760.0000000	8.2500000	-50.0000000
40760.0000000	8.5000000	-50.0000000
40760.0000000	8.7500000	-50.0000000
40760.0000000	9.0000000	-50.0000000
40760.0000000	9.2500000	-50.0000000
40760.0000000	9.5000000	-50.0000000
40760.0000000	9.7500000	-50.0000000
40760.0000000	10.0000000	-50.0000000
40760.0000000	10.2500000	-50.0000000
40760.0000000	10.5000000	-50.0000000
40760.0000000	10.7500000	-50.0000000
40760.0000000	11.0000000	-50.0000000
40760.0000000	11.2500000	-50.0000000
40760.0000000	11.5000000	-50.0000000
40760.0000000	11.7500000	-50.0000000

40760.0000000	12.0000000	-50.0000000
40760.0000000	12.2500000	-50.0000000
40760.0000000	12.5000000	-50.0000000
40760.0000000	12.7500000	-50.0000000
40760.0000000	13.0000000	-50.0000000
40760.0000000	13.2500000	-50.0000000
40760.0000000	13.5000000	-50.0000000
40760.0000000	13.7500000	-50.0000000
40760.0000000	14.0000000	-50.0000000
40760.0000000	14.2500000	-50.0000000
40760.0000000	14.5000000	-50.0000000
40760.0000000	14.7500000	-50.0000000
40760.0000000	15.0000000	-50.0000000
40790.0000000	0.0000000	-50.0000000
40790.0000000	0.2500000	-50.0000000
40790.0000000	0.5000000	-50.0000000
40790.0000000	0.7500000	-50.0000000
40790.0000000	1.0000000	-50.0000000
40790.0000000	1.2500000	-50.0000000
40790.0000000	1.5000000	-50.0000000
40790.0000000	1.7500000	-50.0000000
40790.0000000	2.0000000	-50.0000000
40790.0000000	2.2500000	-50.0000000
40790.0000000	2.5000000	-50.0000000
40790.0000000	2.7500000	-50.0000000
40790.0000000	3.0000000	-50.0000000
40790.0000000	3.2500000	-50.0000000
40790.0000000	3.5000000	-50.0000000
40790.0000000	3.7500000	-50.0000000
40790.0000000	4.0000000	-50.0000000
40790.0000000	4.2500000	-50.0000000
40790.0000000	4.5000000	-50.0000000
40790.0000000	4.7500000	-50.0000000
40790.0000000	5.0000000	-50.0000000
40790.0000000	5.2500000	-50.0000000
40790.0000000	5.5000000	-50.0000000
40790.0000000	5.7500000	-50.0000000
40790.0000000	6.0000000	-50.0000000
40790.0000000	6.2500000	-50.0000000
40790.0000000	6.5000000	-50.0000000
40790.0000000	6.7500000	-50.0000000
40790.0000000	7.0000000	-50.0000000
40790.0000000	7.2500000	-50.0000000
40790.0000000	7.5000000	-50.0000000
40790.0000000	7.7500000	-50.0000000
40790.0000000	8.0000000	-50.0000000
40790.0000000	8.2500000	-50.0000000
40790.0000000	8.5000000	-50.0000000
40790.0000000	8.7500000	-50.0000000
40790.0000000	9.0000000	-50.0000000
40790.0000000	9.2500000	-50.0000000
40790.0000000	9.5000000	-50.0000000
40790.0000000	9.7500000	-50.0000000
40790.0000000	10.0000000	-50.0000000
40790.0000000	10.2500000	-50.0000000
40790.0000000	10.5000000	-50.0000000
40790.0000000	10.7500000	-50.0000000
40790.0000000	11.0000000	-50.0000000

40790.0000000	11.2500000	-50.0000000
40790.0000000	11.5000000	-50.0000000
40790.0000000	11.7500000	-50.0000000
40790.0000000	12.0000000	-50.0000000
40790.0000000	12.2500000	-50.0000000
40790.0000000	12.5000000	-50.0000000
40790.0000000	12.7500000	-50.0000000
40790.0000000	13.0000000	-50.0000000
40790.0000000	13.2500000	-50.0000000
40790.0000000	13.5000000	-50.0000000
40790.0000000	13.7500000	-50.0000000
40790.0000000	14.0000000	-50.0000000
40790.0000000	14.2500000	-50.0000000
40790.0000000	14.5000000	-50.0000000
40790.0000000	14.7500000	-50.0000000
40790.0000000	15.0000000	-50.0000000
40820.0000000	0.0000000	-50.0000000
40820.0000000	0.2500000	-50.0000000
40820.0000000	0.5000000	-50.0000000
40820.0000000	0.7500000	-50.0000000
40820.0000000	1.0000000	-50.0000000
40820.0000000	1.2500000	-50.0000000
40820.0000000	1.5000000	-50.0000000
40820.0000000	1.7500000	-50.0000000
40820.0000000	2.0000000	-50.0000000
40820.0000000	2.2500000	-50.0000000
40820.0000000	2.5000000	-50.0000000
40820.0000000	2.7500000	-50.0000000
40820.0000000	3.0000000	-50.0000000
40820.0000000	3.2500000	-50.0000000
40820.0000000	3.5000000	-50.0000000
40820.0000000	3.7500000	-50.0000000
40820.0000000	4.0000000	-50.0000000
40820.0000000	4.2500000	-50.0000000
40820.0000000	4.5000000	-50.0000000
40820.0000000	4.7500000	-50.0000000
40820.0000000	5.0000000	-50.0000000
40820.0000000	5.2500000	-50.0000000
40820.0000000	5.5000000	-50.0000000
40820.0000000	5.7500000	-50.0000000
40820.0000000	6.0000000	-50.0000000
40820.0000000	6.2500000	-50.0000000
40820.0000000	6.5000000	-50.0000000
40820.0000000	6.7500000	-50.0000000
40820.0000000	7.0000000	-50.0000000
40820.0000000	7.2500000	-50.0000000
40820.0000000	7.5000000	-50.0000000
40820.0000000	7.7500000	-50.0000000
40820.0000000	8.0000000	-50.0000000
40820.0000000	8.2500000	-50.0000000
40820.0000000	8.5000000	-50.0000000
40820.0000000	8.7500000	-50.0000000
40820.0000000	9.0000000	-50.0000000
40820.0000000	9.2500000	-50.0000000
40820.0000000	9.5000000	-50.0000000
40820.0000000	9.7500000	-50.0000000
40820.0000000	10.0000000	-50.0000000
40820.0000000	10.2500000	-50.0000000

40820.0000000	10.5000000	-50.0000000
40820.0000000	10.7500000	-50.0000000
40820.0000000	11.0000000	-50.0000000
40820.0000000	11.2500000	-50.0000000
40820.0000000	11.5000000	-50.0000000
40820.0000000	11.7500000	-50.0000000
40820.0000000	12.0000000	-50.0000000
40820.0000000	12.2500000	-50.0000000
40820.0000000	12.5000000	-50.0000000
40820.0000000	12.7500000	-50.0000000
40820.0000000	13.0000000	-50.0000000
40820.0000000	13.2500000	-50.0000000
40820.0000000	13.5000000	-50.0000000
40820.0000000	13.7500000	-50.0000000
40820.0000000	14.0000000	-50.0000000
40820.0000000	14.2500000	-50.0000000
40820.0000000	14.5000000	-50.0000000
40820.0000000	14.7500000	-50.0000000
40820.0000000	15.0000000	-50.0000000
40850.0000000	0.0000000	-50.0000000
40850.0000000	0.2500000	-50.0000000
40850.0000000	0.5000000	-50.0000000
40850.0000000	0.7500000	-50.0000000
40850.0000000	1.0000000	-50.0000000
40850.0000000	1.2500000	-50.0000000
40850.0000000	1.5000000	-50.0000000
40850.0000000	1.7500000	-50.0000000
40850.0000000	2.0000000	-50.0000000
40850.0000000	2.2500000	-50.0000000
40850.0000000	2.5000000	-50.0000000
40850.0000000	2.7500000	-50.0000000
40850.0000000	3.0000000	-50.0000000
40850.0000000	3.2500000	-50.0000000
40850.0000000	3.5000000	-50.0000000
40850.0000000	3.7500000	-50.0000000
40850.0000000	4.0000000	-50.0000000
40850.0000000	4.2500000	-50.0000000
40850.0000000	4.5000000	-50.0000000
40850.0000000	4.7500000	-50.0000000
40850.0000000	5.0000000	-50.0000000
40850.0000000	5.2500000	-50.0000000
40850.0000000	5.5000000	-50.0000000
40850.0000000	5.7500000	-50.0000000
40850.0000000	6.0000000	-50.0000000
40850.0000000	6.2500000	-50.0000000
40850.0000000	6.5000000	-50.0000000
40850.0000000	6.7500000	-50.0000000
40850.0000000	7.0000000	-50.0000000
40850.0000000	7.2500000	-50.0000000
40850.0000000	7.5000000	-50.0000000
40850.0000000	7.7500000	-50.0000000
40850.0000000	8.0000000	-50.0000000
40850.0000000	8.2500000	-50.0000000
40850.0000000	8.5000000	-50.0000000
40850.0000000	8.7500000	-50.0000000
40850.0000000	9.0000000	-50.0000000
40850.0000000	9.2500000	-50.0000000
40850.0000000	9.5000000	-50.0000000

40850.0000000	9.7500000	-50.0000000
40850.0000000	10.0000000	-50.0000000
40850.0000000	10.2500000	-50.0000000
40850.0000000	10.5000000	-50.0000000
40850.0000000	10.7500000	-50.0000000
40850.0000000	11.0000000	-50.0000000
40850.0000000	11.2500000	-50.0000000
40850.0000000	11.5000000	-50.0000000
40850.0000000	11.7500000	-50.0000000
40850.0000000	12.0000000	-50.0000000
40850.0000000	12.2500000	-50.0000000
40850.0000000	12.5000000	-50.0000000
40850.0000000	12.7500000	-50.0000000
40850.0000000	13.0000000	-50.0000000
40850.0000000	13.2500000	-50.0000000
40850.0000000	13.5000000	-50.0000000
40850.0000000	13.7500000	-50.0000000
40850.0000000	14.0000000	-50.0000000
40850.0000000	14.2500000	-50.0000000
40850.0000000	14.5000000	-50.0000000
40850.0000000	14.7500000	-50.0000000
40850.0000000	15.0000000	-50.0000000
40880.0000000	0.0000000	-50.0000000
40880.0000000	0.2500000	-50.0000000
40880.0000000	0.5000000	-50.0000000
40880.0000000	0.7500000	-50.0000000
40880.0000000	1.0000000	-50.0000000
40880.0000000	1.2500000	-50.0000000
40880.0000000	1.5000000	-50.0000000
40880.0000000	1.7500000	-50.0000000
40880.0000000	2.0000000	-50.0000000
40880.0000000	2.2500000	-50.0000000
40880.0000000	2.5000000	-50.0000000
40880.0000000	2.7500000	-50.0000000
40880.0000000	3.0000000	-50.0000000
40880.0000000	3.2500000	-50.0000000
40880.0000000	3.5000000	-50.0000000
40880.0000000	3.7500000	-50.0000000
40880.0000000	4.0000000	-50.0000000
40880.0000000	4.2500000	-50.0000000
40880.0000000	4.5000000	-50.0000000
40880.0000000	4.7500000	-50.0000000
40880.0000000	5.0000000	-50.0000000
40880.0000000	5.2500000	-50.0000000
40880.0000000	5.5000000	-50.0000000
40880.0000000	5.7500000	-50.0000000
40880.0000000	6.0000000	-50.0000000
40880.0000000	6.2500000	-50.0000000
40880.0000000	6.5000000	-50.0000000
40880.0000000	6.7500000	-50.0000000
40880.0000000	7.0000000	-50.0000000
40880.0000000	7.2500000	-50.0000000
40880.0000000	7.5000000	-50.0000000
40880.0000000	7.7500000	-50.0000000
40880.0000000	8.0000000	-50.0000000
40880.0000000	8.2500000	-50.0000000
40880.0000000	8.5000000	-50.0000000
40880.0000000	8.7500000	-50.0000000

40880.0000000	9.0000000	-50.0000000
40880.0000000	9.2500000	-50.0000000
40880.0000000	9.5000000	-50.0000000
40880.0000000	9.7500000	-50.0000000
40880.0000000	10.0000000	-50.0000000
40880.0000000	10.2500000	-50.0000000
40880.0000000	10.5000000	-50.0000000
40880.0000000	10.7500000	-50.0000000
40880.0000000	11.0000000	-50.0000000
40880.0000000	11.2500000	-50.0000000
40880.0000000	11.5000000	-50.0000000
40880.0000000	11.7500000	-50.0000000
40880.0000000	12.0000000	-50.0000000
40880.0000000	12.2500000	-50.0000000
40880.0000000	12.5000000	-50.0000000
40880.0000000	12.7500000	-50.0000000
40880.0000000	13.0000000	-50.0000000
40880.0000000	13.2500000	-50.0000000
40880.0000000	13.5000000	-50.0000000
40880.0000000	13.7500000	-50.0000000
40880.0000000	14.0000000	-50.0000000
40880.0000000	14.2500000	-50.0000000
40880.0000000	14.5000000	-50.0000000
40880.0000000	14.7500000	-50.0000000
40880.0000000	15.0000000	-50.0000000
40910.0000000	0.0000000	-50.0000000
40910.0000000	0.2500000	-50.0000000
40910.0000000	0.5000000	-50.0000000
40910.0000000	0.7500000	-50.0000000
40910.0000000	1.0000000	-50.0000000
40910.0000000	1.2500000	-50.0000000
40910.0000000	1.5000000	-50.0000000
40910.0000000	1.7500000	-50.0000000
40910.0000000	2.0000000	-50.0000000
40910.0000000	2.2500000	-50.0000000
40910.0000000	2.5000000	-50.0000000
40910.0000000	2.7500000	-50.0000000
40910.0000000	3.0000000	-50.0000000
40910.0000000	3.2500000	-50.0000000
40910.0000000	3.5000000	-50.0000000
40910.0000000	3.7500000	-50.0000000
40910.0000000	4.0000000	-50.0000000
40910.0000000	4.2500000	-50.0000000
40910.0000000	4.5000000	-50.0000000
40910.0000000	4.7500000	-50.0000000
40910.0000000	5.0000000	-50.0000000
40910.0000000	5.2500000	-50.0000000
40910.0000000	5.5000000	-50.0000000
40910.0000000	5.7500000	-50.0000000
40910.0000000	6.0000000	-50.0000000
40910.0000000	6.2500000	-50.0000000
40910.0000000	6.5000000	-50.0000000
40910.0000000	6.7500000	-50.0000000
40910.0000000	7.0000000	-50.0000000
40910.0000000	7.2500000	-50.0000000
40910.0000000	7.5000000	-50.0000000
40910.0000000	7.7500000	-50.0000000
40910.0000000	8.0000000	-50.0000000

40910.0000000	8.2500000	-50.0000000
40910.0000000	8.5000000	-50.0000000
40910.0000000	8.7500000	-50.0000000
40910.0000000	9.0000000	-50.0000000
40910.0000000	9.2500000	-50.0000000
40910.0000000	9.5000000	-50.0000000
40910.0000000	9.7500000	-50.0000000
40910.0000000	10.0000000	-50.0000000
40910.0000000	10.2500000	-50.0000000
40910.0000000	10.5000000	-50.0000000
40910.0000000	10.7500000	-50.0000000
40910.0000000	11.0000000	-50.0000000
40910.0000000	11.2500000	-50.0000000
40910.0000000	11.5000000	-50.0000000
40910.0000000	11.7500000	-50.0000000
40910.0000000	12.0000000	-50.0000000
40910.0000000	12.2500000	-50.0000000
40910.0000000	12.5000000	-50.0000000
40910.0000000	12.7500000	-50.0000000
40910.0000000	13.0000000	-50.0000000
40910.0000000	13.2500000	-50.0000000
40910.0000000	13.5000000	-50.0000000
40910.0000000	13.7500000	-50.0000000
40910.0000000	14.0000000	-50.0000000
40910.0000000	14.2500000	-50.0000000
40910.0000000	14.5000000	-50.0000000
40910.0000000	14.7500000	-50.0000000
40910.0000000	15.0000000	-50.0000000
40940.0000000	0.0000000	-50.0000000
40940.0000000	0.2500000	-50.0000000
40940.0000000	0.5000000	-50.0000000
40940.0000000	0.7500000	-50.0000000
40940.0000000	1.0000000	-50.0000000
40940.0000000	1.2500000	-50.0000000
40940.0000000	1.5000000	-50.0000000
40940.0000000	1.7500000	-50.0000000
40940.0000000	2.0000000	-50.0000000
40940.0000000	2.2500000	-50.0000000
40940.0000000	2.5000000	-50.0000000
40940.0000000	2.7500000	-50.0000000
40940.0000000	3.0000000	-50.0000000
40940.0000000	3.2500000	-50.0000000
40940.0000000	3.5000000	-50.0000000
40940.0000000	3.7500000	-50.0000000
40940.0000000	4.0000000	-50.0000000
40940.0000000	4.2500000	-50.0000000
40940.0000000	4.5000000	-50.0000000
40940.0000000	4.7500000	-50.0000000
40940.0000000	5.0000000	-50.0000000
40940.0000000	5.2500000	-50.0000000
40940.0000000	5.5000000	-50.0000000
40940.0000000	5.7500000	-50.0000000
40940.0000000	6.0000000	-50.0000000
40940.0000000	6.2500000	-50.0000000
40940.0000000	6.5000000	-50.0000000
40940.0000000	6.7500000	-50.0000000
40940.0000000	7.0000000	-50.0000000
40940.0000000	7.2500000	-50.0000000

40940.0000000	7.5000000	-50.0000000
40940.0000000	7.7500000	-50.0000000
40940.0000000	8.0000000	-50.0000000
40940.0000000	8.2500000	-50.0000000
40940.0000000	8.5000000	-50.0000000
40940.0000000	8.7500000	-50.0000000
40940.0000000	9.0000000	-50.0000000
40940.0000000	9.2500000	-50.0000000
40940.0000000	9.5000000	-50.0000000
40940.0000000	9.7500000	-50.0000000
40940.0000000	10.0000000	-50.0000000
40940.0000000	10.2500000	-50.0000000
40940.0000000	10.5000000	-50.0000000
40940.0000000	10.7500000	-50.0000000
40940.0000000	11.0000000	-50.0000000
40940.0000000	11.2500000	-50.0000000
40940.0000000	11.5000000	-50.0000000
40940.0000000	11.7500000	-50.0000000
40940.0000000	12.0000000	-50.0000000
40940.0000000	12.2500000	-50.0000000
40940.0000000	12.5000000	-50.0000000
40940.0000000	12.7500000	-50.0000000
40940.0000000	13.0000000	-50.0000000
40940.0000000	13.2500000	-50.0000000
40940.0000000	13.5000000	-50.0000000
40940.0000000	13.7500000	-50.0000000
40940.0000000	14.0000000	-50.0000000
40940.0000000	14.2500000	-50.0000000
40940.0000000	14.5000000	-50.0000000
40940.0000000	14.7500000	-50.0000000
40940.0000000	15.0000000	-50.0000000
40970.0000000	0.0000000	-50.0000000
40970.0000000	0.2500000	-50.0000000
40970.0000000	0.5000000	-50.0000000
40970.0000000	0.7500000	-50.0000000
40970.0000000	1.0000000	-50.0000000
40970.0000000	1.2500000	-50.0000000
40970.0000000	1.5000000	-50.0000000
40970.0000000	1.7500000	-50.0000000
40970.0000000	2.0000000	-50.0000000
40970.0000000	2.2500000	-50.0000000
40970.0000000	2.5000000	-50.0000000
40970.0000000	2.7500000	-50.0000000
40970.0000000	3.0000000	-50.0000000
40970.0000000	3.2500000	-50.0000000
40970.0000000	3.5000000	-50.0000000
40970.0000000	3.7500000	-50.0000000
40970.0000000	4.0000000	-50.0000000
40970.0000000	4.2500000	-50.0000000
40970.0000000	4.5000000	-50.0000000
40970.0000000	4.7500000	-50.0000000
40970.0000000	5.0000000	-50.0000000
40970.0000000	5.2500000	-50.0000000
40970.0000000	5.5000000	-50.0000000
40970.0000000	5.7500000	-50.0000000
40970.0000000	6.0000000	-50.0000000
40970.0000000	6.2500000	-50.0000000
40970.0000000	6.5000000	-50.0000000

40970.0000000	6.7500000	-50.0000000
40970.0000000	7.0000000	-50.0000000
40970.0000000	7.2500000	-50.0000000
40970.0000000	7.5000000	-50.0000000
40970.0000000	7.7500000	-50.0000000
40970.0000000	8.0000000	-50.0000000
40970.0000000	8.2500000	-50.0000000
40970.0000000	8.5000000	-50.0000000
40970.0000000	8.7500000	-50.0000000
40970.0000000	9.0000000	-50.0000000
40970.0000000	9.2500000	-50.0000000
40970.0000000	9.5000000	-50.0000000
40970.0000000	9.7500000	-50.0000000
40970.0000000	10.0000000	-50.0000000
40970.0000000	10.2500000	-50.0000000
40970.0000000	10.5000000	-50.0000000
40970.0000000	10.7500000	-50.0000000
40970.0000000	11.0000000	-50.0000000
40970.0000000	11.2500000	-50.0000000
40970.0000000	11.5000000	-50.0000000
40970.0000000	11.7500000	-50.0000000
40970.0000000	12.0000000	-50.0000000
40970.0000000	12.2500000	-50.0000000
40970.0000000	12.5000000	-50.0000000
40970.0000000	12.7500000	-50.0000000
40970.0000000	13.0000000	-50.0000000
40970.0000000	13.2500000	-50.0000000
40970.0000000	13.5000000	-50.0000000
40970.0000000	13.7500000	-50.0000000
40970.0000000	14.0000000	-50.0000000
40970.0000000	14.2500000	-50.0000000
40970.0000000	14.5000000	-50.0000000
40970.0000000	14.7500000	-50.0000000
40970.0000000	15.0000000	-50.0000000
41000.0000000	0.0000000	-50.0000000
41000.0000000	0.2500000	-50.0000000
41000.0000000	0.5000000	-50.0000000
41000.0000000	0.7500000	-50.0000000
41000.0000000	1.0000000	-50.0000000
41000.0000000	1.2500000	-50.0000000
41000.0000000	1.5000000	-50.0000000
41000.0000000	1.7500000	-50.0000000
41000.0000000	2.0000000	-50.0000000
41000.0000000	2.2500000	-50.0000000
41000.0000000	2.5000000	-50.0000000
41000.0000000	2.7500000	-50.0000000
41000.0000000	3.0000000	-50.0000000
41000.0000000	3.2500000	-50.0000000
41000.0000000	3.5000000	-50.0000000
41000.0000000	3.7500000	-50.0000000
41000.0000000	4.0000000	-50.0000000
41000.0000000	4.2500000	-50.0000000
41000.0000000	4.5000000	-50.0000000
41000.0000000	4.7500000	-50.0000000
41000.0000000	5.0000000	-50.0000000
41000.0000000	5.2500000	-50.0000000
41000.0000000	5.5000000	-50.0000000
41000.0000000	5.7500000	-50.0000000

41000.0000000	6.0000000	-50.0000000
41000.0000000	6.2500000	-50.0000000
41000.0000000	6.5000000	-50.0000000
41000.0000000	6.7500000	-50.0000000
41000.0000000	7.0000000	-50.0000000
41000.0000000	7.2500000	-50.0000000
41000.0000000	7.5000000	-50.0000000
41000.0000000	7.7500000	-50.0000000
41000.0000000	8.0000000	-50.0000000
41000.0000000	8.2500000	-50.0000000
41000.0000000	8.5000000	-50.0000000
41000.0000000	8.7500000	-50.0000000
41000.0000000	9.0000000	-50.0000000
41000.0000000	9.2500000	-50.0000000
41000.0000000	9.5000000	-50.0000000
41000.0000000	9.7500000	-50.0000000
41000.0000000	10.0000000	-50.0000000
41000.0000000	10.2500000	-50.0000000
41000.0000000	10.5000000	-50.0000000
41000.0000000	10.7500000	-50.0000000
41000.0000000	11.0000000	-50.0000000
41000.0000000	11.2500000	-50.0000000
41000.0000000	11.5000000	-50.0000000
41000.0000000	11.7500000	-50.0000000
41000.0000000	12.0000000	-50.0000000
41000.0000000	12.2500000	-50.0000000
41000.0000000	12.5000000	-50.0000000
41000.0000000	12.7500000	-50.0000000
41000.0000000	13.0000000	-50.0000000
41000.0000000	13.2500000	-50.0000000
41000.0000000	13.5000000	-50.0000000
41000.0000000	13.7500000	-50.0000000
41000.0000000	14.0000000	-50.0000000
41000.0000000	14.2500000	-50.0000000
41000.0000000	14.5000000	-50.0000000
41000.0000000	14.7500000	-50.0000000
41000.0000000	15.0000000	-50.0000000
41030.0000000	0.0000000	-50.0000000
41030.0000000	0.2500000	-50.0000000
41030.0000000	0.5000000	-50.0000000
41030.0000000	0.7500000	-50.0000000
41030.0000000	1.0000000	-50.0000000
41030.0000000	1.2500000	-50.0000000
41030.0000000	1.5000000	-50.0000000
41030.0000000	1.7500000	-50.0000000
41030.0000000	2.0000000	-50.0000000
41030.0000000	2.2500000	-50.0000000
41030.0000000	2.5000000	-50.0000000
41030.0000000	2.7500000	-50.0000000
41030.0000000	3.0000000	-50.0000000
41030.0000000	3.2500000	-50.0000000
41030.0000000	3.5000000	-50.0000000
41030.0000000	3.7500000	-50.0000000
41030.0000000	4.0000000	-50.0000000
41030.0000000	4.2500000	-50.0000000
41030.0000000	4.5000000	-50.0000000
41030.0000000	4.7500000	-50.0000000
41030.0000000	5.0000000	-50.0000000

41030.0000000	5.2500000	-50.0000000
41030.0000000	5.5000000	-50.0000000
41030.0000000	5.7500000	-50.0000000
41030.0000000	6.0000000	-50.0000000
41030.0000000	6.2500000	-50.0000000
41030.0000000	6.5000000	-50.0000000
41030.0000000	6.7500000	-50.0000000
41030.0000000	7.0000000	-50.0000000
41030.0000000	7.2500000	-50.0000000
41030.0000000	7.5000000	-50.0000000
41030.0000000	7.7500000	-50.0000000
41030.0000000	8.0000000	-50.0000000
41030.0000000	8.2500000	-50.0000000
41030.0000000	8.5000000	-50.0000000
41030.0000000	8.7500000	-50.0000000
41030.0000000	9.0000000	-50.0000000
41030.0000000	9.2500000	-50.0000000
41030.0000000	9.5000000	-50.0000000
41030.0000000	9.7500000	-50.0000000
41030.0000000	10.0000000	-50.0000000
41030.0000000	10.2500000	-50.0000000
41030.0000000	10.5000000	-50.0000000
41030.0000000	10.7500000	-50.0000000
41030.0000000	11.0000000	-50.0000000
41030.0000000	11.2500000	-50.0000000
41030.0000000	11.5000000	-50.0000000
41030.0000000	11.7500000	-50.0000000
41030.0000000	12.0000000	-50.0000000
41030.0000000	12.2500000	-50.0000000
41030.0000000	12.5000000	-50.0000000
41030.0000000	12.7500000	-50.0000000
41030.0000000	13.0000000	-50.0000000
41030.0000000	13.2500000	-50.0000000
41030.0000000	13.5000000	-50.0000000
41030.0000000	13.7500000	-50.0000000
41030.0000000	14.0000000	-50.0000000
41030.0000000	14.2500000	-50.0000000
41030.0000000	14.5000000	-50.0000000
41030.0000000	14.7500000	-50.0000000
41030.0000000	15.0000000	-50.0000000
41060.0000000	0.0000000	-50.0000000
41060.0000000	0.2500000	-50.0000000
41060.0000000	0.5000000	-50.0000000
41060.0000000	0.7500000	-50.0000000
41060.0000000	1.0000000	-50.0000000
41060.0000000	1.2500000	-50.0000000
41060.0000000	1.5000000	-50.0000000
41060.0000000	1.7500000	-50.0000000
41060.0000000	2.0000000	-50.0000000
41060.0000000	2.2500000	-50.0000000
41060.0000000	2.5000000	-50.0000000
41060.0000000	2.7500000	-50.0000000
41060.0000000	3.0000000	-50.0000000
41060.0000000	3.2500000	-50.0000000
41060.0000000	3.5000000	-50.0000000
41060.0000000	3.7500000	-50.0000000
41060.0000000	4.0000000	-50.0000000
41060.0000000	4.2500000	-50.0000000

41060.0000000	4.5000000	-50.0000000
41060.0000000	4.7500000	-50.0000000
41060.0000000	5.0000000	-50.0000000
41060.0000000	5.2500000	-50.0000000
41060.0000000	5.5000000	-50.0000000
41060.0000000	5.7500000	-50.0000000
41060.0000000	6.0000000	-50.0000000
41060.0000000	6.2500000	-50.0000000
41060.0000000	6.5000000	-50.0000000
41060.0000000	6.7500000	-50.0000000
41060.0000000	7.0000000	-50.0000000
41060.0000000	7.2500000	-50.0000000
41060.0000000	7.5000000	-50.0000000
41060.0000000	7.7500000	-50.0000000
41060.0000000	8.0000000	-50.0000000
41060.0000000	8.2500000	-50.0000000
41060.0000000	8.5000000	-50.0000000
41060.0000000	8.7500000	-50.0000000
41060.0000000	9.0000000	-50.0000000
41060.0000000	9.2500000	-50.0000000
41060.0000000	9.5000000	-50.0000000
41060.0000000	9.7500000	-50.0000000
41060.0000000	10.0000000	-50.0000000
41060.0000000	10.2500000	-50.0000000
41060.0000000	10.5000000	-50.0000000
41060.0000000	10.7500000	-50.0000000
41060.0000000	11.0000000	-50.0000000
41060.0000000	11.2500000	-50.0000000
41060.0000000	11.5000000	-50.0000000
41060.0000000	11.7500000	-50.0000000
41060.0000000	12.0000000	-50.0000000
41060.0000000	12.2500000	-50.0000000
41060.0000000	12.5000000	-50.0000000
41060.0000000	12.7500000	-50.0000000
41060.0000000	13.0000000	-50.0000000
41060.0000000	13.2500000	-50.0000000
41060.0000000	13.5000000	-50.0000000
41060.0000000	13.7500000	-50.0000000
41060.0000000	14.0000000	-50.0000000
41060.0000000	14.2500000	-50.0000000
41060.0000000	14.5000000	-50.0000000
41060.0000000	14.7500000	-50.0000000
41060.0000000	15.0000000	-50.0000000
41090.0000000	0.0000000	-50.0000000
41090.0000000	0.2500000	-50.0000000
41090.0000000	0.5000000	-50.0000000
41090.0000000	0.7500000	-50.0000000
41090.0000000	1.0000000	-50.0000000
41090.0000000	1.2500000	-50.0000000
41090.0000000	1.5000000	-50.0000000
41090.0000000	1.7500000	-50.0000000
41090.0000000	2.0000000	-50.0000000
41090.0000000	2.2500000	-50.0000000
41090.0000000	2.5000000	-50.0000000
41090.0000000	2.7500000	-50.0000000
41090.0000000	3.0000000	-50.0000000
41090.0000000	3.2500000	-50.0000000
41090.0000000	3.5000000	-50.0000000

41090.0000000	3.7500000	-50.0000000
41090.0000000	4.0000000	-50.0000000
41090.0000000	4.2500000	-50.0000000
41090.0000000	4.5000000	-50.0000000
41090.0000000	4.7500000	-50.0000000
41090.0000000	5.0000000	-50.0000000
41090.0000000	5.2500000	-50.0000000
41090.0000000	5.5000000	-50.0000000
41090.0000000	5.7500000	-50.0000000
41090.0000000	6.0000000	-50.0000000
41090.0000000	6.2500000	-50.0000000
41090.0000000	6.5000000	-50.0000000
41090.0000000	6.7500000	-50.0000000
41090.0000000	7.0000000	-50.0000000
41090.0000000	7.2500000	-50.0000000
41090.0000000	7.5000000	-50.0000000
41090.0000000	7.7500000	-50.0000000
41090.0000000	8.0000000	-50.0000000
41090.0000000	8.2500000	-50.0000000
41090.0000000	8.5000000	-50.0000000
41090.0000000	8.7500000	-50.0000000
41090.0000000	9.0000000	-50.0000000
41090.0000000	9.2500000	-50.0000000
41090.0000000	9.5000000	-50.0000000
41090.0000000	9.7500000	-50.0000000
41090.0000000	10.0000000	-50.0000000
41090.0000000	10.2500000	-50.0000000
41090.0000000	10.5000000	-50.0000000
41090.0000000	10.7500000	-50.0000000
41090.0000000	11.0000000	-50.0000000
41090.0000000	11.2500000	-50.0000000
41090.0000000	11.5000000	-50.0000000
41090.0000000	11.7500000	-50.0000000
41090.0000000	12.0000000	-50.0000000
41090.0000000	12.2500000	-50.0000000
41090.0000000	12.5000000	-50.0000000
41090.0000000	12.7500000	-50.0000000
41090.0000000	13.0000000	-50.0000000
41090.0000000	13.2500000	-50.0000000
41090.0000000	13.5000000	-50.0000000
41090.0000000	13.7500000	-50.0000000
41090.0000000	14.0000000	-50.0000000
41090.0000000	14.2500000	-50.0000000
41090.0000000	14.5000000	-50.0000000
41090.0000000	14.7500000	-50.0000000
41090.0000000	15.0000000	-50.0000000
41120.0000000	0.0000000	-50.0000000
41120.0000000	0.2500000	-50.0000000
41120.0000000	0.5000000	-50.0000000
41120.0000000	0.7500000	-50.0000000
41120.0000000	1.0000000	-50.0000000
41120.0000000	1.2500000	-50.0000000
41120.0000000	1.5000000	-50.0000000
41120.0000000	1.7500000	-50.0000000
41120.0000000	2.0000000	-50.0000000
41120.0000000	2.2500000	-50.0000000
41120.0000000	2.5000000	-50.0000000
41120.0000000	2.7500000	-50.0000000

41120.0000000	3.0000000	-50.0000000
41120.0000000	3.2500000	-50.0000000
41120.0000000	3.5000000	-50.0000000
41120.0000000	3.7500000	-50.0000000
41120.0000000	4.0000000	-50.0000000
41120.0000000	4.2500000	-50.0000000
41120.0000000	4.5000000	-50.0000000
41120.0000000	4.7500000	-50.0000000
41120.0000000	5.0000000	-50.0000000
41120.0000000	5.2500000	-50.0000000
41120.0000000	5.5000000	-50.0000000
41120.0000000	5.7500000	-50.0000000
41120.0000000	6.0000000	-50.0000000
41120.0000000	6.2500000	-50.0000000
41120.0000000	6.5000000	-50.0000000
41120.0000000	6.7500000	-50.0000000
41120.0000000	7.0000000	-50.0000000
41120.0000000	7.2500000	-50.0000000
41120.0000000	7.5000000	-50.0000000
41120.0000000	7.7500000	-50.0000000
41120.0000000	8.0000000	-50.0000000
41120.0000000	8.2500000	-50.0000000
41120.0000000	8.5000000	-50.0000000
41120.0000000	8.7500000	-50.0000000
41120.0000000	9.0000000	-50.0000000
41120.0000000	9.2500000	-50.0000000
41120.0000000	9.5000000	-50.0000000
41120.0000000	9.7500000	-50.0000000
41120.0000000	10.0000000	-50.0000000
41120.0000000	10.2500000	-50.0000000
41120.0000000	10.5000000	-50.0000000
41120.0000000	10.7500000	-50.0000000
41120.0000000	11.0000000	-50.0000000
41120.0000000	11.2500000	-50.0000000
41120.0000000	11.5000000	-50.0000000
41120.0000000	11.7500000	-50.0000000
41120.0000000	12.0000000	-50.0000000
41120.0000000	12.2500000	-50.0000000
41120.0000000	12.5000000	-50.0000000
41120.0000000	12.7500000	-50.0000000
41120.0000000	13.0000000	-50.0000000
41120.0000000	13.2500000	-50.0000000
41120.0000000	13.5000000	-50.0000000
41120.0000000	13.7500000	-50.0000000
41120.0000000	14.0000000	-50.0000000
41120.0000000	14.2500000	-50.0000000
41120.0000000	14.5000000	-50.0000000
41120.0000000	14.7500000	-50.0000000
41120.0000000	15.0000000	-50.0000000
41150.0000000	0.0000000	-50.0000000
41150.0000000	0.2500000	-50.0000000
41150.0000000	0.5000000	-50.0000000
41150.0000000	0.7500000	-50.0000000
41150.0000000	1.0000000	-50.0000000
41150.0000000	1.2500000	-50.0000000
41150.0000000	1.5000000	-50.0000000
41150.0000000	1.7500000	-50.0000000
41150.0000000	2.0000000	-50.0000000

41150.0000000	2.2500000	-50.0000000
41150.0000000	2.5000000	-50.0000000
41150.0000000	2.7500000	-50.0000000
41150.0000000	3.0000000	-50.0000000
41150.0000000	3.2500000	-50.0000000
41150.0000000	3.5000000	-50.0000000
41150.0000000	3.7500000	-50.0000000
41150.0000000	4.0000000	-50.0000000
41150.0000000	4.2500000	-50.0000000
41150.0000000	4.5000000	-50.0000000
41150.0000000	4.7500000	-50.0000000
41150.0000000	5.0000000	-50.0000000
41150.0000000	5.2500000	-50.0000000
41150.0000000	5.5000000	-50.0000000
41150.0000000	5.7500000	-50.0000000
41150.0000000	6.0000000	-50.0000000
41150.0000000	6.2500000	-50.0000000
41150.0000000	6.5000000	-50.0000000
41150.0000000	6.7500000	-50.0000000
41150.0000000	7.0000000	-50.0000000
41150.0000000	7.2500000	-50.0000000
41150.0000000	7.5000000	-50.0000000
41150.0000000	7.7500000	-50.0000000
41150.0000000	8.0000000	-50.0000000
41150.0000000	8.2500000	-50.0000000
41150.0000000	8.5000000	-50.0000000
41150.0000000	8.7500000	-50.0000000
41150.0000000	9.0000000	-50.0000000
41150.0000000	9.2500000	-50.0000000
41150.0000000	9.5000000	-50.0000000
41150.0000000	9.7500000	-50.0000000
41150.0000000	10.0000000	-50.0000000
41150.0000000	10.2500000	-50.0000000
41150.0000000	10.5000000	-50.0000000
41150.0000000	10.7500000	-50.0000000
41150.0000000	11.0000000	-50.0000000
41150.0000000	11.2500000	-50.0000000
41150.0000000	11.5000000	-50.0000000
41150.0000000	11.7500000	-50.0000000
41150.0000000	12.0000000	-50.0000000
41150.0000000	12.2500000	-50.0000000
41150.0000000	12.5000000	-50.0000000
41150.0000000	12.7500000	-50.0000000
41150.0000000	13.0000000	-50.0000000
41150.0000000	13.2500000	-50.0000000
41150.0000000	13.5000000	-50.0000000
41150.0000000	13.7500000	-50.0000000
41150.0000000	14.0000000	-50.0000000
41150.0000000	14.2500000	-50.0000000
41150.0000000	14.5000000	-50.0000000
41150.0000000	14.7500000	-50.0000000
41150.0000000	15.0000000	-50.0000000
41180.0000000	0.0000000	-50.0000000
41180.0000000	0.2500000	-50.0000000
41180.0000000	0.5000000	-50.0000000
41180.0000000	0.7500000	-50.0000000
41180.0000000	1.0000000	-50.0000000
41180.0000000	1.2500000	-50.0000000

41180.0000000	1.5000000	-50.0000000
41180.0000000	1.7500000	-50.0000000
41180.0000000	2.0000000	-50.0000000
41180.0000000	2.2500000	-50.0000000
41180.0000000	2.5000000	-50.0000000
41180.0000000	2.7500000	-50.0000000
41180.0000000	3.0000000	-50.0000000
41180.0000000	3.2500000	-50.0000000
41180.0000000	3.5000000	-50.0000000
41180.0000000	3.7500000	-50.0000000
41180.0000000	4.0000000	-50.0000000
41180.0000000	4.2500000	-50.0000000
41180.0000000	4.5000000	-50.0000000
41180.0000000	4.7500000	-50.0000000
41180.0000000	5.0000000	-50.0000000
41180.0000000	5.2500000	-50.0000000
41180.0000000	5.5000000	-50.0000000
41180.0000000	5.7500000	-50.0000000
41180.0000000	6.0000000	-50.0000000
41180.0000000	6.2500000	-50.0000000
41180.0000000	6.5000000	-50.0000000
41180.0000000	6.7500000	-50.0000000
41180.0000000	7.0000000	-50.0000000
41180.0000000	7.2500000	-50.0000000
41180.0000000	7.5000000	-50.0000000
41180.0000000	7.7500000	-50.0000000
41180.0000000	8.0000000	-50.0000000
41180.0000000	8.2500000	-50.0000000
41180.0000000	8.5000000	-50.0000000
41180.0000000	8.7500000	-50.0000000
41180.0000000	9.0000000	-50.0000000
41180.0000000	9.2500000	-50.0000000
41180.0000000	9.5000000	-50.0000000
41180.0000000	9.7500000	-50.0000000
41180.0000000	10.0000000	-50.0000000
41180.0000000	10.2500000	-50.0000000
41180.0000000	10.5000000	-50.0000000
41180.0000000	10.7500000	-50.0000000
41180.0000000	11.0000000	-50.0000000
41180.0000000	11.2500000	-50.0000000
41180.0000000	11.5000000	-50.0000000
41180.0000000	11.7500000	-50.0000000
41180.0000000	12.0000000	-50.0000000
41180.0000000	12.2500000	-50.0000000
41180.0000000	12.5000000	-50.0000000
41180.0000000	12.7500000	-50.0000000
41180.0000000	13.0000000	-50.0000000
41180.0000000	13.2500000	-50.0000000
41180.0000000	13.5000000	-50.0000000
41180.0000000	13.7500000	-50.0000000
41180.0000000	14.0000000	-50.0000000
41180.0000000	14.2500000	-50.0000000
41180.0000000	14.5000000	-50.0000000
41180.0000000	14.7500000	-50.0000000
41180.0000000	15.0000000	-50.0000000
41210.0000000	0.0000000	-50.0000000
41210.0000000	0.2500000	-50.0000000
41210.0000000	0.5000000	-50.0000000

41210.0000000	0.7500000	-50.0000000
41210.0000000	1.0000000	-50.0000000
41210.0000000	1.2500000	-50.0000000
41210.0000000	1.5000000	-50.0000000
41210.0000000	1.7500000	-50.0000000
41210.0000000	2.0000000	-50.0000000
41210.0000000	2.2500000	-50.0000000
41210.0000000	2.5000000	-50.0000000
41210.0000000	2.7500000	-50.0000000
41210.0000000	3.0000000	-50.0000000
41210.0000000	3.2500000	-50.0000000
41210.0000000	3.5000000	-50.0000000
41210.0000000	3.7500000	-50.0000000
41210.0000000	4.0000000	-50.0000000
41210.0000000	4.2500000	-50.0000000
41210.0000000	4.5000000	-50.0000000
41210.0000000	4.7500000	-50.0000000
41210.0000000	5.0000000	-50.0000000
41210.0000000	5.2500000	-50.0000000
41210.0000000	5.5000000	-50.0000000
41210.0000000	5.7500000	-50.0000000
41210.0000000	6.0000000	-50.0000000
41210.0000000	6.2500000	-50.0000000
41210.0000000	6.5000000	-50.0000000
41210.0000000	6.7500000	-50.0000000
41210.0000000	7.0000000	-50.0000000
41210.0000000	7.2500000	-50.0000000
41210.0000000	7.5000000	-50.0000000
41210.0000000	7.7500000	-50.0000000
41210.0000000	8.0000000	-50.0000000
41210.0000000	8.2500000	-50.0000000
41210.0000000	8.5000000	-50.0000000
41210.0000000	8.7500000	-50.0000000
41210.0000000	9.0000000	-50.0000000
41210.0000000	9.2500000	-50.0000000
41210.0000000	9.5000000	-50.0000000
41210.0000000	9.7500000	-50.0000000
41210.0000000	10.0000000	-50.0000000
41210.0000000	10.2500000	-50.0000000
41210.0000000	10.5000000	-50.0000000
41210.0000000	10.7500000	-50.0000000
41210.0000000	11.0000000	-50.0000000
41210.0000000	11.2500000	-50.0000000
41210.0000000	11.5000000	-50.0000000
41210.0000000	11.7500000	-50.0000000
41210.0000000	12.0000000	-50.0000000
41210.0000000	12.2500000	-50.0000000
41210.0000000	12.5000000	-50.0000000
41210.0000000	12.7500000	-50.0000000
41210.0000000	13.0000000	-50.0000000
41210.0000000	13.2500000	-50.0000000
41210.0000000	13.5000000	-50.0000000
41210.0000000	13.7500000	-50.0000000
41210.0000000	14.0000000	-50.0000000
41210.0000000	14.2500000	-50.0000000
41210.0000000	14.5000000	-50.0000000
41210.0000000	14.7500000	-50.0000000
41210.0000000	15.0000000	-50.0000000

41240.0000000	0.0000000	-50.0000000
41240.0000000	0.2500000	-50.0000000
41240.0000000	0.5000000	-50.0000000
41240.0000000	0.7500000	-50.0000000
41240.0000000	1.0000000	-50.0000000
41240.0000000	1.2500000	-50.0000000
41240.0000000	1.5000000	-50.0000000
41240.0000000	1.7500000	-50.0000000
41240.0000000	2.0000000	-50.0000000
41240.0000000	2.2500000	-50.0000000
41240.0000000	2.5000000	-50.0000000
41240.0000000	2.7500000	-50.0000000
41240.0000000	3.0000000	-50.0000000
41240.0000000	3.2500000	-50.0000000
41240.0000000	3.5000000	-50.0000000
41240.0000000	3.7500000	-50.0000000
41240.0000000	4.0000000	-50.0000000
41240.0000000	4.2500000	-50.0000000
41240.0000000	4.5000000	-50.0000000
41240.0000000	4.7500000	-50.0000000
41240.0000000	5.0000000	-50.0000000
41240.0000000	5.2500000	-50.0000000
41240.0000000	5.5000000	-50.0000000
41240.0000000	5.7500000	-50.0000000
41240.0000000	6.0000000	-50.0000000
41240.0000000	6.2500000	-50.0000000
41240.0000000	6.5000000	-50.0000000
41240.0000000	6.7500000	-50.0000000
41240.0000000	7.0000000	-50.0000000
41240.0000000	7.2500000	-50.0000000
41240.0000000	7.5000000	-50.0000000
41240.0000000	7.7500000	-50.0000000
41240.0000000	8.0000000	-50.0000000
41240.0000000	8.2500000	-50.0000000
41240.0000000	8.5000000	-50.0000000
41240.0000000	8.7500000	-50.0000000
41240.0000000	9.0000000	-50.0000000
41240.0000000	9.2500000	-50.0000000
41240.0000000	9.5000000	-50.0000000
41240.0000000	9.7500000	-50.0000000
41240.0000000	10.0000000	-50.0000000
41240.0000000	10.2500000	-50.0000000
41240.0000000	10.5000000	-50.0000000
41240.0000000	10.7500000	-50.0000000
41240.0000000	11.0000000	-50.0000000
41240.0000000	11.2500000	-50.0000000
41240.0000000	11.5000000	-50.0000000
41240.0000000	11.7500000	-50.0000000
41240.0000000	12.0000000	-50.0000000
41240.0000000	12.2500000	-50.0000000
41240.0000000	12.5000000	-50.0000000
41240.0000000	12.7500000	-50.0000000
41240.0000000	13.0000000	-50.0000000
41240.0000000	13.2500000	-50.0000000
41240.0000000	13.5000000	-50.0000000
41240.0000000	13.7500000	-50.0000000
41240.0000000	14.0000000	-50.0000000
41240.0000000	14.2500000	-50.0000000

41240.0000000	14.5000000	-50.0000000
41240.0000000	14.7500000	-50.0000000
41240.0000000	15.0000000	-50.0000000
41270.0000000	0.0000000	-50.0000000
41270.0000000	0.2500000	-50.0000000
41270.0000000	0.5000000	-50.0000000
41270.0000000	0.7500000	-50.0000000
41270.0000000	1.0000000	-50.0000000
41270.0000000	1.2500000	-50.0000000
41270.0000000	1.5000000	-50.0000000
41270.0000000	1.7500000	-50.0000000
41270.0000000	2.0000000	-50.0000000
41270.0000000	2.2500000	-50.0000000
41270.0000000	2.5000000	-50.0000000
41270.0000000	2.7500000	-50.0000000
41270.0000000	3.0000000	-50.0000000
41270.0000000	3.2500000	-50.0000000
41270.0000000	3.5000000	-50.0000000
41270.0000000	3.7500000	-50.0000000
41270.0000000	4.0000000	-50.0000000
41270.0000000	4.2500000	-50.0000000
41270.0000000	4.5000000	-50.0000000
41270.0000000	4.7500000	-50.0000000
41270.0000000	5.0000000	-50.0000000
41270.0000000	5.2500000	-50.0000000
41270.0000000	5.5000000	-50.0000000
41270.0000000	5.7500000	-50.0000000
41270.0000000	6.0000000	-50.0000000
41270.0000000	6.2500000	-50.0000000
41270.0000000	6.5000000	-50.0000000
41270.0000000	6.7500000	-50.0000000
41270.0000000	7.0000000	-50.0000000
41270.0000000	7.2500000	-50.0000000
41270.0000000	7.5000000	-50.0000000
41270.0000000	7.7500000	-50.0000000
41270.0000000	8.0000000	-50.0000000
41270.0000000	8.2500000	-50.0000000
41270.0000000	8.5000000	-50.0000000
41270.0000000	8.7500000	-50.0000000
41270.0000000	9.0000000	-50.0000000
41270.0000000	9.2500000	-50.0000000
41270.0000000	9.5000000	-50.0000000
41270.0000000	9.7500000	-50.0000000
41270.0000000	10.0000000	-50.0000000
41270.0000000	10.2500000	-50.0000000
41270.0000000	10.5000000	-50.0000000
41270.0000000	10.7500000	-50.0000000
41270.0000000	11.0000000	-50.0000000
41270.0000000	11.2500000	-50.0000000
41270.0000000	11.5000000	-50.0000000
41270.0000000	11.7500000	-50.0000000
41270.0000000	12.0000000	-50.0000000
41270.0000000	12.2500000	-50.0000000
41270.0000000	12.5000000	-50.0000000
41270.0000000	12.7500000	-50.0000000
41270.0000000	13.0000000	-50.0000000
41270.0000000	13.2500000	-50.0000000
41270.0000000	13.5000000	-50.0000000

41270.0000000	13.7500000	-50.0000000
41270.0000000	14.0000000	-50.0000000
41270.0000000	14.2500000	-50.0000000
41270.0000000	14.5000000	-50.0000000
41270.0000000	14.7500000	-50.0000000
41270.0000000	15.0000000	-50.0000000
41300.0000000	0.0000000	-50.0000000
41300.0000000	0.2500000	-50.0000000
41300.0000000	0.5000000	-50.0000000
41300.0000000	0.7500000	-50.0000000
41300.0000000	1.0000000	-50.0000000
41300.0000000	1.2500000	-50.0000000
41300.0000000	1.5000000	-50.0000000
41300.0000000	1.7500000	-50.0000000
41300.0000000	2.0000000	-50.0000000
41300.0000000	2.2500000	-50.0000000
41300.0000000	2.5000000	-50.0000000
41300.0000000	2.7500000	-50.0000000
41300.0000000	3.0000000	-50.0000000
41300.0000000	3.2500000	-50.0000000
41300.0000000	3.5000000	-50.0000000
41300.0000000	3.7500000	-50.0000000
41300.0000000	4.0000000	-50.0000000
41300.0000000	4.2500000	-50.0000000
41300.0000000	4.5000000	-50.0000000
41300.0000000	4.7500000	-50.0000000
41300.0000000	5.0000000	-50.0000000
41300.0000000	5.2500000	-50.0000000
41300.0000000	5.5000000	-50.0000000
41300.0000000	5.7500000	-50.0000000
41300.0000000	6.0000000	-50.0000000
41300.0000000	6.2500000	-50.0000000
41300.0000000	6.5000000	-50.0000000
41300.0000000	6.7500000	-50.0000000
41300.0000000	7.0000000	-50.0000000
41300.0000000	7.2500000	-50.0000000
41300.0000000	7.5000000	-50.0000000
41300.0000000	7.7500000	-50.0000000
41300.0000000	8.0000000	-50.0000000
41300.0000000	8.2500000	-50.0000000
41300.0000000	8.5000000	-50.0000000
41300.0000000	8.7500000	-50.0000000
41300.0000000	9.0000000	-50.0000000
41300.0000000	9.2500000	-50.0000000
41300.0000000	9.5000000	-50.0000000
41300.0000000	9.7500000	-50.0000000
41300.0000000	10.0000000	-50.0000000
41300.0000000	10.2500000	-50.0000000
41300.0000000	10.5000000	-50.0000000
41300.0000000	10.7500000	-50.0000000
41300.0000000	11.0000000	-50.0000000
41300.0000000	11.2500000	-50.0000000
41300.0000000	11.5000000	-50.0000000
41300.0000000	11.7500000	-50.0000000
41300.0000000	12.0000000	-50.0000000
41300.0000000	12.2500000	-50.0000000
41300.0000000	12.5000000	-50.0000000
41300.0000000	12.7500000	-50.0000000

41300.0000000	13.0000000	-50.0000000
41300.0000000	13.2500000	-50.0000000
41300.0000000	13.5000000	-50.0000000
41300.0000000	13.7500000	-50.0000000
41300.0000000	14.0000000	-50.0000000
41300.0000000	14.2500000	-50.0000000
41300.0000000	14.5000000	-50.0000000
41300.0000000	14.7500000	-50.0000000
41300.0000000	15.0000000	-50.0000000
41330.0000000	0.0000000	-50.0000000
41330.0000000	0.2500000	-50.0000000
41330.0000000	0.5000000	-50.0000000
41330.0000000	0.7500000	-50.0000000
41330.0000000	1.0000000	-50.0000000
41330.0000000	1.2500000	-50.0000000
41330.0000000	1.5000000	-50.0000000
41330.0000000	1.7500000	-50.0000000
41330.0000000	2.0000000	-50.0000000
41330.0000000	2.2500000	-50.0000000
41330.0000000	2.5000000	-50.0000000
41330.0000000	2.7500000	-50.0000000
41330.0000000	3.0000000	-50.0000000
41330.0000000	3.2500000	-50.0000000
41330.0000000	3.5000000	-50.0000000
41330.0000000	3.7500000	-50.0000000
41330.0000000	4.0000000	-50.0000000
41330.0000000	4.2500000	-50.0000000
41330.0000000	4.5000000	-50.0000000
41330.0000000	4.7500000	-50.0000000
41330.0000000	5.0000000	-50.0000000
41330.0000000	5.2500000	-50.0000000
41330.0000000	5.5000000	-50.0000000
41330.0000000	5.7500000	-50.0000000
41330.0000000	6.0000000	-50.0000000
41330.0000000	6.2500000	-50.0000000
41330.0000000	6.5000000	-50.0000000
41330.0000000	6.7500000	-50.0000000
41330.0000000	7.0000000	-50.0000000
41330.0000000	7.2500000	-50.0000000
41330.0000000	7.5000000	-50.0000000
41330.0000000	7.7500000	-50.0000000
41330.0000000	8.0000000	-50.0000000
41330.0000000	8.2500000	-50.0000000
41330.0000000	8.5000000	-50.0000000
41330.0000000	8.7500000	-50.0000000
41330.0000000	9.0000000	-50.0000000
41330.0000000	9.2500000	-50.0000000
41330.0000000	9.5000000	-50.0000000
41330.0000000	9.7500000	-50.0000000
41330.0000000	10.0000000	-50.0000000
41330.0000000	10.2500000	-50.0000000
41330.0000000	10.5000000	-50.0000000
41330.0000000	10.7500000	-50.0000000
41330.0000000	11.0000000	-50.0000000
41330.0000000	11.2500000	-50.0000000
41330.0000000	11.5000000	-50.0000000
41330.0000000	11.7500000	-50.0000000
41330.0000000	12.0000000	-50.0000000

41330.0000000	12.2500000	-50.0000000
41330.0000000	12.5000000	-50.0000000
41330.0000000	12.7500000	-50.0000000
41330.0000000	13.0000000	-50.0000000
41330.0000000	13.2500000	-50.0000000
41330.0000000	13.5000000	-50.0000000
41330.0000000	13.7500000	-50.0000000
41330.0000000	14.0000000	-50.0000000
41330.0000000	14.2500000	-50.0000000
41330.0000000	14.5000000	-50.0000000
41330.0000000	14.7500000	-50.0000000
41330.0000000	15.0000000	-50.0000000
41360.0000000	0.0000000	-50.0000000
41360.0000000	0.2500000	-50.0000000
41360.0000000	0.5000000	-50.0000000
41360.0000000	0.7500000	-50.0000000
41360.0000000	1.0000000	-50.0000000
41360.0000000	1.2500000	-50.0000000
41360.0000000	1.5000000	-50.0000000
41360.0000000	1.7500000	-50.0000000
41360.0000000	2.0000000	-50.0000000
41360.0000000	2.2500000	-50.0000000
41360.0000000	2.5000000	-50.0000000
41360.0000000	2.7500000	-50.0000000
41360.0000000	3.0000000	-50.0000000
41360.0000000	3.2500000	-50.0000000
41360.0000000	3.5000000	-50.0000000
41360.0000000	3.7500000	-50.0000000
41360.0000000	4.0000000	-50.0000000
41360.0000000	4.2500000	-50.0000000
41360.0000000	4.5000000	-50.0000000
41360.0000000	4.7500000	-50.0000000
41360.0000000	5.0000000	-50.0000000
41360.0000000	5.2500000	-50.0000000
41360.0000000	5.5000000	-50.0000000
41360.0000000	5.7500000	-50.0000000
41360.0000000	6.0000000	-50.0000000
41360.0000000	6.2500000	-50.0000000
41360.0000000	6.5000000	-50.0000000
41360.0000000	6.7500000	-50.0000000
41360.0000000	7.0000000	-50.0000000
41360.0000000	7.2500000	-50.0000000
41360.0000000	7.5000000	-50.0000000
41360.0000000	7.7500000	-50.0000000
41360.0000000	8.0000000	-50.0000000
41360.0000000	8.2500000	-50.0000000
41360.0000000	8.5000000	-50.0000000
41360.0000000	8.7500000	-50.0000000
41360.0000000	9.0000000	-50.0000000
41360.0000000	9.2500000	-50.0000000
41360.0000000	9.5000000	-50.0000000
41360.0000000	9.7500000	-50.0000000
41360.0000000	10.0000000	-50.0000000
41360.0000000	10.2500000	-50.0000000
41360.0000000	10.5000000	-50.0000000
41360.0000000	10.7500000	-50.0000000
41360.0000000	11.0000000	-50.0000000
41360.0000000	11.2500000	-50.0000000

41360.0000000	11.5000000	-50.0000000
41360.0000000	11.7500000	-50.0000000
41360.0000000	12.0000000	-50.0000000
41360.0000000	12.2500000	-50.0000000
41360.0000000	12.5000000	-50.0000000
41360.0000000	12.7500000	-50.0000000
41360.0000000	13.0000000	-50.0000000
41360.0000000	13.2500000	-50.0000000
41360.0000000	13.5000000	-50.0000000
41360.0000000	13.7500000	-50.0000000
41360.0000000	14.0000000	-50.0000000
41360.0000000	14.2500000	-50.0000000
41360.0000000	14.5000000	-50.0000000
41360.0000000	14.7500000	-50.0000000
41360.0000000	15.0000000	-50.0000000
41390.0000000	0.0000000	-50.0000000
41390.0000000	0.2500000	-50.0000000
41390.0000000	0.5000000	-50.0000000
41390.0000000	0.7500000	-50.0000000
41390.0000000	1.0000000	-50.0000000
41390.0000000	1.2500000	-50.0000000
41390.0000000	1.5000000	-50.0000000
41390.0000000	1.7500000	-50.0000000
41390.0000000	2.0000000	-50.0000000
41390.0000000	2.2500000	-50.0000000
41390.0000000	2.5000000	-50.0000000
41390.0000000	2.7500000	-50.0000000
41390.0000000	3.0000000	-50.0000000
41390.0000000	3.2500000	-50.0000000
41390.0000000	3.5000000	-50.0000000
41390.0000000	3.7500000	-50.0000000
41390.0000000	4.0000000	-50.0000000
41390.0000000	4.2500000	-50.0000000
41390.0000000	4.5000000	-50.0000000
41390.0000000	4.7500000	-50.0000000
41390.0000000	5.0000000	-50.0000000
41390.0000000	5.2500000	-50.0000000
41390.0000000	5.5000000	-50.0000000
41390.0000000	5.7500000	-50.0000000
41390.0000000	6.0000000	-50.0000000
41390.0000000	6.2500000	-50.0000000
41390.0000000	6.5000000	-50.0000000
41390.0000000	6.7500000	-50.0000000
41390.0000000	7.0000000	-50.0000000
41390.0000000	7.2500000	-50.0000000
41390.0000000	7.5000000	-50.0000000
41390.0000000	7.7500000	-50.0000000
41390.0000000	8.0000000	-50.0000000
41390.0000000	8.2500000	-50.0000000
41390.0000000	8.5000000	-50.0000000
41390.0000000	8.7500000	-50.0000000
41390.0000000	9.0000000	-50.0000000
41390.0000000	9.2500000	-50.0000000
41390.0000000	9.5000000	-50.0000000
41390.0000000	9.7500000	-50.0000000
41390.0000000	10.0000000	-50.0000000
41390.0000000	10.2500000	-50.0000000
41390.0000000	10.5000000	-50.0000000

41390.0000000	10.7500000	-50.0000000
41390.0000000	11.0000000	-50.0000000
41390.0000000	11.2500000	-50.0000000
41390.0000000	11.5000000	-50.0000000
41390.0000000	11.7500000	-50.0000000
41390.0000000	12.0000000	-50.0000000
41390.0000000	12.2500000	-50.0000000
41390.0000000	12.5000000	-50.0000000
41390.0000000	12.7500000	-50.0000000
41390.0000000	13.0000000	-50.0000000
41390.0000000	13.2500000	-50.0000000
41390.0000000	13.5000000	-50.0000000
41390.0000000	13.7500000	-50.0000000
41390.0000000	14.0000000	-50.0000000
41390.0000000	14.2500000	-50.0000000
41390.0000000	14.5000000	-50.0000000
41390.0000000	14.7500000	-50.0000000
41390.0000000	15.0000000	-50.0000000
41420.0000000	0.0000000	-50.0000000
41420.0000000	0.2500000	-50.0000000
41420.0000000	0.5000000	-50.0000000
41420.0000000	0.7500000	-50.0000000
41420.0000000	1.0000000	-50.0000000
41420.0000000	1.2500000	-50.0000000
41420.0000000	1.5000000	-50.0000000
41420.0000000	1.7500000	-50.0000000
41420.0000000	2.0000000	-50.0000000
41420.0000000	2.2500000	-50.0000000
41420.0000000	2.5000000	-50.0000000
41420.0000000	2.7500000	-50.0000000
41420.0000000	3.0000000	-50.0000000
41420.0000000	3.2500000	-50.0000000
41420.0000000	3.5000000	-50.0000000
41420.0000000	3.7500000	-50.0000000
41420.0000000	4.0000000	-50.0000000
41420.0000000	4.2500000	-50.0000000
41420.0000000	4.5000000	-50.0000000
41420.0000000	4.7500000	-50.0000000
41420.0000000	5.0000000	-50.0000000
41420.0000000	5.2500000	-50.0000000
41420.0000000	5.5000000	-50.0000000
41420.0000000	5.7500000	-50.0000000
41420.0000000	6.0000000	-50.0000000
41420.0000000	6.2500000	-50.0000000
41420.0000000	6.5000000	-50.0000000
41420.0000000	6.7500000	-50.0000000
41420.0000000	7.0000000	-50.0000000
41420.0000000	7.2500000	-50.0000000
41420.0000000	7.5000000	-50.0000000
41420.0000000	7.7500000	-50.0000000
41420.0000000	8.0000000	-50.0000000
41420.0000000	8.2500000	-50.0000000
41420.0000000	8.5000000	-50.0000000
41420.0000000	8.7500000	-50.0000000
41420.0000000	9.0000000	-50.0000000
41420.0000000	9.2500000	-50.0000000
41420.0000000	9.5000000	-50.0000000
41420.0000000	9.7500000	-50.0000000

41420.0000000	10.0000000	-50.0000000
41420.0000000	10.2500000	-50.0000000
41420.0000000	10.5000000	-50.0000000
41420.0000000	10.7500000	-50.0000000
41420.0000000	11.0000000	-50.0000000
41420.0000000	11.2500000	-50.0000000
41420.0000000	11.5000000	-50.0000000
41420.0000000	11.7500000	-50.0000000
41420.0000000	12.0000000	-50.0000000
41420.0000000	12.2500000	-50.0000000
41420.0000000	12.5000000	-50.0000000
41420.0000000	12.7500000	-50.0000000
41420.0000000	13.0000000	-50.0000000
41420.0000000	13.2500000	-50.0000000
41420.0000000	13.5000000	-50.0000000
41420.0000000	13.7500000	-50.0000000
41420.0000000	14.0000000	-50.0000000
41420.0000000	14.2500000	-50.0000000
41420.0000000	14.5000000	-50.0000000
41420.0000000	14.7500000	-50.0000000
41420.0000000	15.0000000	-50.0000000
41450.0000000	0.0000000	-50.0000000
41450.0000000	0.2500000	-50.0000000
41450.0000000	0.5000000	-50.0000000
41450.0000000	0.7500000	-50.0000000
41450.0000000	1.0000000	-50.0000000
41450.0000000	1.2500000	-50.0000000
41450.0000000	1.5000000	-50.0000000
41450.0000000	1.7500000	-50.0000000
41450.0000000	2.0000000	-50.0000000
41450.0000000	2.2500000	-50.0000000
41450.0000000	2.5000000	-50.0000000
41450.0000000	2.7500000	-50.0000000
41450.0000000	3.0000000	-50.0000000
41450.0000000	3.2500000	-50.0000000
41450.0000000	3.5000000	-50.0000000
41450.0000000	3.7500000	-50.0000000
41450.0000000	4.0000000	-50.0000000
41450.0000000	4.2500000	-50.0000000
41450.0000000	4.5000000	-50.0000000
41450.0000000	4.7500000	-50.0000000
41450.0000000	5.0000000	-50.0000000
41450.0000000	5.2500000	-50.0000000
41450.0000000	5.5000000	-50.0000000
41450.0000000	5.7500000	-50.0000000
41450.0000000	6.0000000	-50.0000000
41450.0000000	6.2500000	-50.0000000
41450.0000000	6.5000000	-50.0000000
41450.0000000	6.7500000	-50.0000000
41450.0000000	7.0000000	-50.0000000
41450.0000000	7.2500000	-50.0000000
41450.0000000	7.5000000	-50.0000000
41450.0000000	7.7500000	-50.0000000
41450.0000000	8.0000000	-50.0000000
41450.0000000	8.2500000	-50.0000000
41450.0000000	8.5000000	-50.0000000
41450.0000000	8.7500000	-50.0000000
41450.0000000	9.0000000	-50.0000000

41450.0000000	9.2500000	-50.0000000
41450.0000000	9.5000000	-50.0000000
41450.0000000	9.7500000	-50.0000000
41450.0000000	10.0000000	-50.0000000
41450.0000000	10.2500000	-50.0000000
41450.0000000	10.5000000	-50.0000000
41450.0000000	10.7500000	-50.0000000
41450.0000000	11.0000000	-50.0000000
41450.0000000	11.2500000	-50.0000000
41450.0000000	11.5000000	-50.0000000
41450.0000000	11.7500000	-50.0000000
41450.0000000	12.0000000	-50.0000000
41450.0000000	12.2500000	-50.0000000
41450.0000000	12.5000000	-50.0000000
41450.0000000	12.7500000	-50.0000000
41450.0000000	13.0000000	-50.0000000
41450.0000000	13.2500000	-50.0000000
41450.0000000	13.5000000	-50.0000000
41450.0000000	13.7500000	-50.0000000
41450.0000000	14.0000000	-50.0000000
41450.0000000	14.2500000	-50.0000000
41450.0000000	14.5000000	-50.0000000
41450.0000000	14.7500000	-50.0000000
41450.0000000	15.0000000	-50.0000000
41480.0000000	0.0000000	-50.0000000
41480.0000000	0.2500000	-50.0000000
41480.0000000	0.5000000	-50.0000000
41480.0000000	0.7500000	-50.0000000
41480.0000000	1.0000000	-50.0000000
41480.0000000	1.2500000	-50.0000000
41480.0000000	1.5000000	-50.0000000
41480.0000000	1.7500000	-50.0000000
41480.0000000	2.0000000	-50.0000000
41480.0000000	2.2500000	-50.0000000
41480.0000000	2.5000000	-50.0000000
41480.0000000	2.7500000	-50.0000000
41480.0000000	3.0000000	-50.0000000
41480.0000000	3.2500000	-50.0000000
41480.0000000	3.5000000	-50.0000000
41480.0000000	3.7500000	-50.0000000
41480.0000000	4.0000000	-50.0000000
41480.0000000	4.2500000	-50.0000000
41480.0000000	4.5000000	-50.0000000
41480.0000000	4.7500000	-50.0000000
41480.0000000	5.0000000	-50.0000000
41480.0000000	5.2500000	-50.0000000
41480.0000000	5.5000000	-50.0000000
41480.0000000	5.7500000	-50.0000000
41480.0000000	6.0000000	-50.0000000
41480.0000000	6.2500000	-50.0000000
41480.0000000	6.5000000	-50.0000000
41480.0000000	6.7500000	-50.0000000
41480.0000000	7.0000000	-50.0000000
41480.0000000	7.2500000	-50.0000000
41480.0000000	7.5000000	-50.0000000
41480.0000000	7.7500000	-50.0000000
41480.0000000	8.0000000	-50.0000000
41480.0000000	8.2500000	-50.0000000

41480.0000000	8.5000000	-50.0000000
41480.0000000	8.7500000	-50.0000000
41480.0000000	9.0000000	-50.0000000
41480.0000000	9.2500000	-50.0000000
41480.0000000	9.5000000	-50.0000000
41480.0000000	9.7500000	-50.0000000
41480.0000000	10.0000000	-50.0000000
41480.0000000	10.2500000	-50.0000000
41480.0000000	10.5000000	-50.0000000
41480.0000000	10.7500000	-50.0000000
41480.0000000	11.0000000	-50.0000000
41480.0000000	11.2500000	-50.0000000
41480.0000000	11.5000000	-50.0000000
41480.0000000	11.7500000	-50.0000000
41480.0000000	12.0000000	-50.0000000
41480.0000000	12.2500000	-50.0000000
41480.0000000	12.5000000	-50.0000000
41480.0000000	12.7500000	-50.0000000
41480.0000000	13.0000000	-50.0000000
41480.0000000	13.2500000	-50.0000000
41480.0000000	13.5000000	-50.0000000
41480.0000000	13.7500000	-50.0000000
41480.0000000	14.0000000	-50.0000000
41480.0000000	14.2500000	-50.0000000
41480.0000000	14.5000000	-50.0000000
41480.0000000	14.7500000	-50.0000000
41480.0000000	15.0000000	-50.0000000
41510.0000000	0.0000000	-50.0000000
41510.0000000	0.2500000	-50.0000000
41510.0000000	0.5000000	-50.0000000
41510.0000000	0.7500000	-50.0000000
41510.0000000	1.0000000	-50.0000000
41510.0000000	1.2500000	-50.0000000
41510.0000000	1.5000000	-50.0000000
41510.0000000	1.7500000	-50.0000000
41510.0000000	2.0000000	-50.0000000
41510.0000000	2.2500000	-50.0000000
41510.0000000	2.5000000	-50.0000000
41510.0000000	2.7500000	-50.0000000
41510.0000000	3.0000000	-50.0000000
41510.0000000	3.2500000	-50.0000000
41510.0000000	3.5000000	-50.0000000
41510.0000000	3.7500000	-50.0000000
41510.0000000	4.0000000	-50.0000000
41510.0000000	4.2500000	-50.0000000
41510.0000000	4.5000000	-50.0000000
41510.0000000	4.7500000	-50.0000000
41510.0000000	5.0000000	-50.0000000
41510.0000000	5.2500000	-50.0000000
41510.0000000	5.5000000	-50.0000000
41510.0000000	5.7500000	-50.0000000
41510.0000000	6.0000000	-50.0000000
41510.0000000	6.2500000	-50.0000000
41510.0000000	6.5000000	-50.0000000
41510.0000000	6.7500000	-50.0000000
41510.0000000	7.0000000	-50.0000000
41510.0000000	7.2500000	-50.0000000
41510.0000000	7.5000000	-50.0000000

41510.0000000	7.7500000	-50.0000000
41510.0000000	8.0000000	-50.0000000
41510.0000000	8.2500000	-50.0000000
41510.0000000	8.5000000	-50.0000000
41510.0000000	8.7500000	-50.0000000
41510.0000000	9.0000000	-50.0000000
41510.0000000	9.2500000	-50.0000000
41510.0000000	9.5000000	-50.0000000
41510.0000000	9.7500000	-50.0000000
41510.0000000	10.0000000	-50.0000000
41510.0000000	10.2500000	-50.0000000
41510.0000000	10.5000000	-50.0000000
41510.0000000	10.7500000	-50.0000000
41510.0000000	11.0000000	-50.0000000
41510.0000000	11.2500000	-50.0000000
41510.0000000	11.5000000	-50.0000000
41510.0000000	11.7500000	-50.0000000
41510.0000000	12.0000000	-50.0000000
41510.0000000	12.2500000	-50.0000000
41510.0000000	12.5000000	-50.0000000
41510.0000000	12.7500000	-50.0000000
41510.0000000	13.0000000	-50.0000000
41510.0000000	13.2500000	-50.0000000
41510.0000000	13.5000000	-50.0000000
41510.0000000	13.7500000	-50.0000000
41510.0000000	14.0000000	-50.0000000
41510.0000000	14.2500000	-50.0000000
41510.0000000	14.5000000	-50.0000000
41510.0000000	14.7500000	-50.0000000
41510.0000000	15.0000000	-50.0000000
41540.0000000	0.0000000	-50.0000000
41540.0000000	0.2500000	-50.0000000
41540.0000000	0.5000000	-50.0000000
41540.0000000	0.7500000	-50.0000000
41540.0000000	1.0000000	-50.0000000
41540.0000000	1.2500000	-50.0000000
41540.0000000	1.5000000	-50.0000000
41540.0000000	1.7500000	-50.0000000
41540.0000000	2.0000000	-50.0000000
41540.0000000	2.2500000	-50.0000000
41540.0000000	2.5000000	-50.0000000
41540.0000000	2.7500000	-50.0000000
41540.0000000	3.0000000	-50.0000000
41540.0000000	3.2500000	-50.0000000
41540.0000000	3.5000000	-50.0000000
41540.0000000	3.7500000	-50.0000000
41540.0000000	4.0000000	-50.0000000
41540.0000000	4.2500000	-50.0000000
41540.0000000	4.5000000	-50.0000000
41540.0000000	4.7500000	-50.0000000
41540.0000000	5.0000000	-50.0000000
41540.0000000	5.2500000	-50.0000000
41540.0000000	5.5000000	-50.0000000
41540.0000000	5.7500000	-50.0000000
41540.0000000	6.0000000	-50.0000000
41540.0000000	6.2500000	-50.0000000
41540.0000000	6.5000000	-50.0000000
41540.0000000	6.7500000	-50.0000000

41540.0000000	7.0000000	-50.0000000
41540.0000000	7.2500000	-50.0000000
41540.0000000	7.5000000	-50.0000000
41540.0000000	7.7500000	-50.0000000
41540.0000000	8.0000000	-50.0000000
41540.0000000	8.2500000	-50.0000000
41540.0000000	8.5000000	-50.0000000
41540.0000000	8.7500000	-50.0000000
41540.0000000	9.0000000	-50.0000000
41540.0000000	9.2500000	-50.0000000
41540.0000000	9.5000000	-50.0000000
41540.0000000	9.7500000	-50.0000000
41540.0000000	10.0000000	-50.0000000
41540.0000000	10.2500000	-50.0000000
41540.0000000	10.5000000	-50.0000000
41540.0000000	10.7500000	-50.0000000
41540.0000000	11.0000000	-50.0000000
41540.0000000	11.2500000	-50.0000000
41540.0000000	11.5000000	-50.0000000
41540.0000000	11.7500000	-50.0000000
41540.0000000	12.0000000	-50.0000000
41540.0000000	12.2500000	-50.0000000
41540.0000000	12.5000000	-50.0000000
41540.0000000	12.7500000	-50.0000000
41540.0000000	13.0000000	-50.0000000
41540.0000000	13.2500000	-50.0000000
41540.0000000	13.5000000	-50.0000000
41540.0000000	13.7500000	-50.0000000
41540.0000000	14.0000000	-50.0000000
41540.0000000	14.2500000	-50.0000000
41540.0000000	14.5000000	-50.0000000
41540.0000000	14.7500000	-50.0000000
41540.0000000	15.0000000	-50.0000000
41570.0000000	0.0000000	-50.0000000
41570.0000000	0.2500000	-50.0000000
41570.0000000	0.5000000	-50.0000000
41570.0000000	0.7500000	-50.0000000
41570.0000000	1.0000000	-50.0000000
41570.0000000	1.2500000	-50.0000000
41570.0000000	1.5000000	-50.0000000
41570.0000000	1.7500000	-50.0000000
41570.0000000	2.0000000	-50.0000000
41570.0000000	2.2500000	-50.0000000
41570.0000000	2.5000000	-50.0000000
41570.0000000	2.7500000	-50.0000000
41570.0000000	3.0000000	-50.0000000
41570.0000000	3.2500000	-50.0000000
41570.0000000	3.5000000	-50.0000000
41570.0000000	3.7500000	-50.0000000
41570.0000000	4.0000000	-50.0000000
41570.0000000	4.2500000	-50.0000000
41570.0000000	4.5000000	-50.0000000
41570.0000000	4.7500000	-50.0000000
41570.0000000	5.0000000	-50.0000000
41570.0000000	5.2500000	-50.0000000
41570.0000000	5.5000000	-50.0000000
41570.0000000	5.7500000	-50.0000000
41570.0000000	6.0000000	-50.0000000

41570.0000000	6.2500000	-50.0000000
41570.0000000	6.5000000	-50.0000000
41570.0000000	6.7500000	-50.0000000
41570.0000000	7.0000000	-50.0000000
41570.0000000	7.2500000	-50.0000000
41570.0000000	7.5000000	-50.0000000
41570.0000000	7.7500000	-50.0000000
41570.0000000	8.0000000	-50.0000000
41570.0000000	8.2500000	-50.0000000
41570.0000000	8.5000000	-50.0000000
41570.0000000	8.7500000	-50.0000000
41570.0000000	9.0000000	-50.0000000
41570.0000000	9.2500000	-50.0000000
41570.0000000	9.5000000	-50.0000000
41570.0000000	9.7500000	-50.0000000
41570.0000000	10.0000000	-50.0000000
41570.0000000	10.2500000	-50.0000000
41570.0000000	10.5000000	-50.0000000
41570.0000000	10.7500000	-50.0000000
41570.0000000	11.0000000	-50.0000000
41570.0000000	11.2500000	-50.0000000
41570.0000000	11.5000000	-50.0000000
41570.0000000	11.7500000	-50.0000000
41570.0000000	12.0000000	-50.0000000
41570.0000000	12.2500000	-50.0000000
41570.0000000	12.5000000	-50.0000000
41570.0000000	12.7500000	-50.0000000
41570.0000000	13.0000000	-50.0000000
41570.0000000	13.2500000	-50.0000000
41570.0000000	13.5000000	-50.0000000
41570.0000000	13.7500000	-50.0000000
41570.0000000	14.0000000	-50.0000000
41570.0000000	14.2500000	-50.0000000
41570.0000000	14.5000000	-50.0000000
41570.0000000	14.7500000	-50.0000000
41570.0000000	15.0000000	-50.0000000
41600.0000000	0.0000000	-50.0000000
41600.0000000	0.2500000	-50.0000000
41600.0000000	0.5000000	-50.0000000
41600.0000000	0.7500000	-50.0000000
41600.0000000	1.0000000	-50.0000000
41600.0000000	1.2500000	-50.0000000
41600.0000000	1.5000000	-50.0000000
41600.0000000	1.7500000	-50.0000000
41600.0000000	2.0000000	-50.0000000
41600.0000000	2.2500000	-50.0000000
41600.0000000	2.5000000	-50.0000000
41600.0000000	2.7500000	-50.0000000
41600.0000000	3.0000000	-50.0000000
41600.0000000	3.2500000	-50.0000000
41600.0000000	3.5000000	-50.0000000
41600.0000000	3.7500000	-50.0000000
41600.0000000	4.0000000	-50.0000000
41600.0000000	4.2500000	-50.0000000
41600.0000000	4.5000000	-50.0000000
41600.0000000	4.7500000	-50.0000000
41600.0000000	5.0000000	-50.0000000
41600.0000000	5.2500000	-50.0000000

41600.0000000	5.5000000	-50.0000000
41600.0000000	5.7500000	-50.0000000
41600.0000000	6.0000000	-50.0000000
41600.0000000	6.2500000	-50.0000000
41600.0000000	6.5000000	-50.0000000
41600.0000000	6.7500000	-50.0000000
41600.0000000	7.0000000	-50.0000000
41600.0000000	7.2500000	-50.0000000
41600.0000000	7.5000000	-50.0000000
41600.0000000	7.7500000	-50.0000000
41600.0000000	8.0000000	-50.0000000
41600.0000000	8.2500000	-50.0000000
41600.0000000	8.5000000	-50.0000000
41600.0000000	8.7500000	-50.0000000
41600.0000000	9.0000000	-50.0000000
41600.0000000	9.2500000	-50.0000000
41600.0000000	9.5000000	-50.0000000
41600.0000000	9.7500000	-50.0000000
41600.0000000	10.0000000	-50.0000000
41600.0000000	10.2500000	-50.0000000
41600.0000000	10.5000000	-50.0000000
41600.0000000	10.7500000	-50.0000000
41600.0000000	11.0000000	-50.0000000
41600.0000000	11.2500000	-50.0000000
41600.0000000	11.5000000	-50.0000000
41600.0000000	11.7500000	-50.0000000
41600.0000000	12.0000000	-50.0000000
41600.0000000	12.2500000	-50.0000000
41600.0000000	12.5000000	-50.0000000
41600.0000000	12.7500000	-50.0000000
41600.0000000	13.0000000	-50.0000000
41600.0000000	13.2500000	-50.0000000
41600.0000000	13.5000000	-50.0000000
41600.0000000	13.7500000	-50.0000000
41600.0000000	14.0000000	-50.0000000
41600.0000000	14.2500000	-50.0000000
41600.0000000	14.5000000	-50.0000000
41600.0000000	14.7500000	-50.0000000
41600.0000000	15.0000000	-50.0000000
41630.0000000	0.0000000	-50.0000000
41630.0000000	0.2500000	-50.0000000
41630.0000000	0.5000000	-50.0000000
41630.0000000	0.7500000	-50.0000000
41630.0000000	1.0000000	-50.0000000
41630.0000000	1.2500000	-50.0000000
41630.0000000	1.5000000	-50.0000000
41630.0000000	1.7500000	-50.0000000
41630.0000000	2.0000000	-50.0000000
41630.0000000	2.2500000	-50.0000000
41630.0000000	2.5000000	-50.0000000
41630.0000000	2.7500000	-50.0000000
41630.0000000	3.0000000	-50.0000000
41630.0000000	3.2500000	-50.0000000
41630.0000000	3.5000000	-50.0000000
41630.0000000	3.7500000	-50.0000000
41630.0000000	4.0000000	-50.0000000
41630.0000000	4.2500000	-50.0000000
41630.0000000	4.5000000	-50.0000000

41630.0000000	4.7500000	-50.0000000
41630.0000000	5.0000000	-50.0000000
41630.0000000	5.2500000	-50.0000000
41630.0000000	5.5000000	-50.0000000
41630.0000000	5.7500000	-50.0000000
41630.0000000	6.0000000	-50.0000000
41630.0000000	6.2500000	-50.0000000
41630.0000000	6.5000000	-50.0000000
41630.0000000	6.7500000	-50.0000000
41630.0000000	7.0000000	-50.0000000
41630.0000000	7.2500000	-50.0000000
41630.0000000	7.5000000	-50.0000000
41630.0000000	7.7500000	-50.0000000
41630.0000000	8.0000000	-50.0000000
41630.0000000	8.2500000	-50.0000000
41630.0000000	8.5000000	-50.0000000
41630.0000000	8.7500000	-50.0000000
41630.0000000	9.0000000	-50.0000000
41630.0000000	9.2500000	-50.0000000
41630.0000000	9.5000000	-50.0000000
41630.0000000	9.7500000	-50.0000000
41630.0000000	10.0000000	-50.0000000
41630.0000000	10.2500000	-50.0000000
41630.0000000	10.5000000	-50.0000000
41630.0000000	10.7500000	-50.0000000
41630.0000000	11.0000000	-50.0000000
41630.0000000	11.2500000	-50.0000000
41630.0000000	11.5000000	-50.0000000
41630.0000000	11.7500000	-50.0000000
41630.0000000	12.0000000	-50.0000000
41630.0000000	12.2500000	-50.0000000
41630.0000000	12.5000000	-50.0000000
41630.0000000	12.7500000	-50.0000000
41630.0000000	13.0000000	-50.0000000
41630.0000000	13.2500000	-50.0000000
41630.0000000	13.5000000	-50.0000000
41630.0000000	13.7500000	-50.0000000
41630.0000000	14.0000000	-50.0000000
41630.0000000	14.2500000	-50.0000000
41630.0000000	14.5000000	-50.0000000
41630.0000000	14.7500000	-50.0000000
41630.0000000	15.0000000	-50.0000000
41660.0000000	0.0000000	-50.0000000
41660.0000000	0.2500000	-50.0000000
41660.0000000	0.5000000	-50.0000000
41660.0000000	0.7500000	-50.0000000
41660.0000000	1.0000000	-50.0000000
41660.0000000	1.2500000	-50.0000000
41660.0000000	1.5000000	-50.0000000
41660.0000000	1.7500000	-50.0000000
41660.0000000	2.0000000	-50.0000000
41660.0000000	2.2500000	-50.0000000
41660.0000000	2.5000000	-50.0000000
41660.0000000	2.7500000	-50.0000000
41660.0000000	3.0000000	-50.0000000
41660.0000000	3.2500000	-50.0000000
41660.0000000	3.5000000	-50.0000000
41660.0000000	3.7500000	-50.0000000

41660.0000000	4.0000000	-50.0000000
41660.0000000	4.2500000	-50.0000000
41660.0000000	4.5000000	-50.0000000
41660.0000000	4.7500000	-50.0000000
41660.0000000	5.0000000	-50.0000000
41660.0000000	5.2500000	-50.0000000
41660.0000000	5.5000000	-50.0000000
41660.0000000	5.7500000	-50.0000000
41660.0000000	6.0000000	-50.0000000
41660.0000000	6.2500000	-50.0000000
41660.0000000	6.5000000	-50.0000000
41660.0000000	6.7500000	-50.0000000
41660.0000000	7.0000000	-50.0000000
41660.0000000	7.2500000	-50.0000000
41660.0000000	7.5000000	-50.0000000
41660.0000000	7.7500000	-50.0000000
41660.0000000	8.0000000	-50.0000000
41660.0000000	8.2500000	-50.0000000
41660.0000000	8.5000000	-50.0000000
41660.0000000	8.7500000	-50.0000000
41660.0000000	9.0000000	-50.0000000
41660.0000000	9.2500000	-50.0000000
41660.0000000	9.5000000	-50.0000000
41660.0000000	9.7500000	-50.0000000
41660.0000000	10.0000000	-50.0000000
41660.0000000	10.2500000	-50.0000000
41660.0000000	10.5000000	-50.0000000
41660.0000000	10.7500000	-50.0000000
41660.0000000	11.0000000	-50.0000000
41660.0000000	11.2500000	-50.0000000
41660.0000000	11.5000000	-50.0000000
41660.0000000	11.7500000	-50.0000000
41660.0000000	12.0000000	-50.0000000
41660.0000000	12.2500000	-50.0000000
41660.0000000	12.5000000	-50.0000000
41660.0000000	12.7500000	-50.0000000
41660.0000000	13.0000000	-50.0000000
41660.0000000	13.2500000	-50.0000000
41660.0000000	13.5000000	-50.0000000
41660.0000000	13.7500000	-50.0000000
41660.0000000	14.0000000	-50.0000000
41660.0000000	14.2500000	-50.0000000
41660.0000000	14.5000000	-50.0000000
41660.0000000	14.7500000	-50.0000000
41660.0000000	15.0000000	-50.0000000
41690.0000000	0.0000000	-50.0000000
41690.0000000	0.2500000	-50.0000000
41690.0000000	0.5000000	-50.0000000
41690.0000000	0.7500000	-50.0000000
41690.0000000	1.0000000	-50.0000000
41690.0000000	1.2500000	-50.0000000
41690.0000000	1.5000000	-50.0000000
41690.0000000	1.7500000	-50.0000000
41690.0000000	2.0000000	-50.0000000
41690.0000000	2.2500000	-50.0000000
41690.0000000	2.5000000	-50.0000000
41690.0000000	2.7500000	-50.0000000
41690.0000000	3.0000000	-50.0000000

41690.0000000	3.2500000	-50.0000000
41690.0000000	3.5000000	-50.0000000
41690.0000000	3.7500000	-50.0000000
41690.0000000	4.0000000	-50.0000000
41690.0000000	4.2500000	-50.0000000
41690.0000000	4.5000000	-50.0000000
41690.0000000	4.7500000	-50.0000000
41690.0000000	5.0000000	-50.0000000
41690.0000000	5.2500000	-50.0000000
41690.0000000	5.5000000	-50.0000000
41690.0000000	5.7500000	-50.0000000
41690.0000000	6.0000000	-50.0000000
41690.0000000	6.2500000	-50.0000000
41690.0000000	6.5000000	-50.0000000
41690.0000000	6.7500000	-50.0000000
41690.0000000	7.0000000	-50.0000000
41690.0000000	7.2500000	-50.0000000
41690.0000000	7.5000000	-50.0000000
41690.0000000	7.7500000	-50.0000000
41690.0000000	8.0000000	-50.0000000
41690.0000000	8.2500000	-50.0000000
41690.0000000	8.5000000	-50.0000000
41690.0000000	8.7500000	-50.0000000
41690.0000000	9.0000000	-50.0000000
41690.0000000	9.2500000	-50.0000000
41690.0000000	9.5000000	-50.0000000
41690.0000000	9.7500000	-50.0000000
41690.0000000	10.0000000	-50.0000000
41690.0000000	10.2500000	-50.0000000
41690.0000000	10.5000000	-50.0000000
41690.0000000	10.7500000	-50.0000000
41690.0000000	11.0000000	-50.0000000
41690.0000000	11.2500000	-50.0000000
41690.0000000	11.5000000	-50.0000000
41690.0000000	11.7500000	-50.0000000
41690.0000000	12.0000000	-50.0000000
41690.0000000	12.2500000	-50.0000000
41690.0000000	12.5000000	-50.0000000
41690.0000000	12.7500000	-50.0000000
41690.0000000	13.0000000	-50.0000000
41690.0000000	13.2500000	-50.0000000
41690.0000000	13.5000000	-50.0000000
41690.0000000	13.7500000	-50.0000000
41690.0000000	14.0000000	-50.0000000
41690.0000000	14.2500000	-50.0000000
41690.0000000	14.5000000	-50.0000000
41690.0000000	14.7500000	-50.0000000
41690.0000000	15.0000000	-50.0000000
41720.0000000	0.0000000	-50.0000000
41720.0000000	0.2500000	-50.0000000
41720.0000000	0.5000000	-50.0000000
41720.0000000	0.7500000	-50.0000000
41720.0000000	1.0000000	-50.0000000
41720.0000000	1.2500000	-50.0000000
41720.0000000	1.5000000	-50.0000000
41720.0000000	1.7500000	-50.0000000
41720.0000000	2.0000000	-50.0000000
41720.0000000	2.2500000	-50.0000000

41720.0000000	2.5000000	-50.0000000
41720.0000000	2.7500000	-50.0000000
41720.0000000	3.0000000	-50.0000000
41720.0000000	3.2500000	-50.0000000
41720.0000000	3.5000000	-50.0000000
41720.0000000	3.7500000	-50.0000000
41720.0000000	4.0000000	-50.0000000
41720.0000000	4.2500000	-50.0000000
41720.0000000	4.5000000	-50.0000000
41720.0000000	4.7500000	-50.0000000
41720.0000000	5.0000000	-50.0000000
41720.0000000	5.2500000	-50.0000000
41720.0000000	5.5000000	-50.0000000
41720.0000000	5.7500000	-50.0000000
41720.0000000	6.0000000	-50.0000000
41720.0000000	6.2500000	-50.0000000
41720.0000000	6.5000000	-50.0000000
41720.0000000	6.7500000	-50.0000000
41720.0000000	7.0000000	-50.0000000
41720.0000000	7.2500000	-50.0000000
41720.0000000	7.5000000	-50.0000000
41720.0000000	7.7500000	-50.0000000
41720.0000000	8.0000000	-50.0000000
41720.0000000	8.2500000	-50.0000000
41720.0000000	8.5000000	-50.0000000
41720.0000000	8.7500000	-50.0000000
41720.0000000	9.0000000	-50.0000000
41720.0000000	9.2500000	-50.0000000
41720.0000000	9.5000000	-50.0000000
41720.0000000	9.7500000	-50.0000000
41720.0000000	10.0000000	-50.0000000
41720.0000000	10.2500000	-50.0000000
41720.0000000	10.5000000	-50.0000000
41720.0000000	10.7500000	-50.0000000
41720.0000000	11.0000000	-50.0000000
41720.0000000	11.2500000	-50.0000000
41720.0000000	11.5000000	-50.0000000
41720.0000000	11.7500000	-50.0000000
41720.0000000	12.0000000	-50.0000000
41720.0000000	12.2500000	-50.0000000
41720.0000000	12.5000000	-50.0000000
41720.0000000	12.7500000	-50.0000000
41720.0000000	13.0000000	-50.0000000
41720.0000000	13.2500000	-50.0000000
41720.0000000	13.5000000	-50.0000000
41720.0000000	13.7500000	-50.0000000
41720.0000000	14.0000000	-50.0000000
41720.0000000	14.2500000	-50.0000000
41720.0000000	14.5000000	-50.0000000
41720.0000000	14.7500000	-50.0000000
41720.0000000	15.0000000	-50.0000000
41750.0000000	0.0000000	-50.0000000
41750.0000000	0.2500000	-50.0000000
41750.0000000	0.5000000	-50.0000000
41750.0000000	0.7500000	-50.0000000
41750.0000000	1.0000000	-50.0000000
41750.0000000	1.2500000	-50.0000000
41750.0000000	1.5000000	-50.0000000

41750.0000000	1.7500000	-50.0000000
41750.0000000	2.0000000	-50.0000000
41750.0000000	2.2500000	-50.0000000
41750.0000000	2.5000000	-50.0000000
41750.0000000	2.7500000	-50.0000000
41750.0000000	3.0000000	-50.0000000
41750.0000000	3.2500000	-50.0000000
41750.0000000	3.5000000	-50.0000000
41750.0000000	3.7500000	-50.0000000
41750.0000000	4.0000000	-50.0000000
41750.0000000	4.2500000	-50.0000000
41750.0000000	4.5000000	-50.0000000
41750.0000000	4.7500000	-50.0000000
41750.0000000	5.0000000	-50.0000000
41750.0000000	5.2500000	-50.0000000
41750.0000000	5.5000000	-50.0000000
41750.0000000	5.7500000	-50.0000000
41750.0000000	6.0000000	-50.0000000
41750.0000000	6.2500000	-50.0000000
41750.0000000	6.5000000	-50.0000000
41750.0000000	6.7500000	-50.0000000
41750.0000000	7.0000000	-50.0000000
41750.0000000	7.2500000	-50.0000000
41750.0000000	7.5000000	-50.0000000
41750.0000000	7.7500000	-50.0000000
41750.0000000	8.0000000	-50.0000000
41750.0000000	8.2500000	-50.0000000
41750.0000000	8.5000000	-50.0000000
41750.0000000	8.7500000	-50.0000000
41750.0000000	9.0000000	-50.0000000
41750.0000000	9.2500000	-50.0000000
41750.0000000	9.5000000	-50.0000000
41750.0000000	9.7500000	-50.0000000
41750.0000000	10.0000000	-50.0000000
41750.0000000	10.2500000	-50.0000000
41750.0000000	10.5000000	-50.0000000
41750.0000000	10.7500000	-50.0000000
41750.0000000	11.0000000	-50.0000000
41750.0000000	11.2500000	-50.0000000
41750.0000000	11.5000000	-50.0000000
41750.0000000	11.7500000	-50.0000000
41750.0000000	12.0000000	-50.0000000
41750.0000000	12.2500000	-50.0000000
41750.0000000	12.5000000	-50.0000000
41750.0000000	12.7500000	-50.0000000
41750.0000000	13.0000000	-50.0000000
41750.0000000	13.2500000	-50.0000000
41750.0000000	13.5000000	-50.0000000
41750.0000000	13.7500000	-50.0000000
41750.0000000	14.0000000	-50.0000000
41750.0000000	14.2500000	-50.0000000
41750.0000000	14.5000000	-50.0000000
41750.0000000	14.7500000	-50.0000000
41750.0000000	15.0000000	-50.0000000
41780.0000000	0.0000000	-50.0000000
41780.0000000	0.2500000	-50.0000000
41780.0000000	0.5000000	-50.0000000
41780.0000000	0.7500000	-50.0000000

41780.0000000	1.0000000	-50.0000000
41780.0000000	1.2500000	-50.0000000
41780.0000000	1.5000000	-50.0000000
41780.0000000	1.7500000	-50.0000000
41780.0000000	2.0000000	-50.0000000
41780.0000000	2.2500000	-50.0000000
41780.0000000	2.5000000	-50.0000000
41780.0000000	2.7500000	-50.0000000
41780.0000000	3.0000000	-50.0000000
41780.0000000	3.2500000	-50.0000000
41780.0000000	3.5000000	-50.0000000
41780.0000000	3.7500000	-50.0000000
41780.0000000	4.0000000	-50.0000000
41780.0000000	4.2500000	-50.0000000
41780.0000000	4.5000000	-50.0000000
41780.0000000	4.7500000	-50.0000000
41780.0000000	5.0000000	-50.0000000
41780.0000000	5.2500000	-50.0000000
41780.0000000	5.5000000	-50.0000000
41780.0000000	5.7500000	-50.0000000
41780.0000000	6.0000000	-50.0000000
41780.0000000	6.2500000	-50.0000000
41780.0000000	6.5000000	-50.0000000
41780.0000000	6.7500000	-50.0000000
41780.0000000	7.0000000	-50.0000000
41780.0000000	7.2500000	-50.0000000
41780.0000000	7.5000000	-50.0000000
41780.0000000	7.7500000	-50.0000000
41780.0000000	8.0000000	-50.0000000
41780.0000000	8.2500000	-50.0000000
41780.0000000	8.5000000	-50.0000000
41780.0000000	8.7500000	-50.0000000
41780.0000000	9.0000000	-50.0000000
41780.0000000	9.2500000	-50.0000000
41780.0000000	9.5000000	-50.0000000
41780.0000000	9.7500000	-50.0000000
41780.0000000	10.0000000	-50.0000000
41780.0000000	10.2500000	-50.0000000
41780.0000000	10.5000000	-50.0000000
41780.0000000	10.7500000	-50.0000000
41780.0000000	11.0000000	-50.0000000
41780.0000000	11.2500000	-50.0000000
41780.0000000	11.5000000	-50.0000000
41780.0000000	11.7500000	-50.0000000
41780.0000000	12.0000000	-50.0000000
41780.0000000	12.2500000	-50.0000000
41780.0000000	12.5000000	-50.0000000
41780.0000000	12.7500000	-50.0000000
41780.0000000	13.0000000	-50.0000000
41780.0000000	13.2500000	-50.0000000
41780.0000000	13.5000000	-50.0000000
41780.0000000	13.7500000	-50.0000000
41780.0000000	14.0000000	-50.0000000
41780.0000000	14.2500000	-50.0000000
41780.0000000	14.5000000	-50.0000000
41780.0000000	14.7500000	-50.0000000
41780.0000000	15.0000000	-50.0000000
41810.0000000	0.0000000	-50.0000000

41810.0000000	0.2500000	-50.0000000
41810.0000000	0.5000000	-50.0000000
41810.0000000	0.7500000	-50.0000000
41810.0000000	1.0000000	-50.0000000
41810.0000000	1.2500000	-50.0000000
41810.0000000	1.5000000	-50.0000000
41810.0000000	1.7500000	-50.0000000
41810.0000000	2.0000000	-50.0000000
41810.0000000	2.2500000	-50.0000000
41810.0000000	2.5000000	-50.0000000
41810.0000000	2.7500000	-50.0000000
41810.0000000	3.0000000	-50.0000000
41810.0000000	3.2500000	-50.0000000
41810.0000000	3.5000000	-50.0000000
41810.0000000	3.7500000	-50.0000000
41810.0000000	4.0000000	-50.0000000
41810.0000000	4.2500000	-50.0000000
41810.0000000	4.5000000	-50.0000000
41810.0000000	4.7500000	-50.0000000
41810.0000000	5.0000000	-50.0000000
41810.0000000	5.2500000	-50.0000000
41810.0000000	5.5000000	-50.0000000
41810.0000000	5.7500000	-50.0000000
41810.0000000	6.0000000	-50.0000000
41810.0000000	6.2500000	-50.0000000
41810.0000000	6.5000000	-50.0000000
41810.0000000	6.7500000	-50.0000000
41810.0000000	7.0000000	-50.0000000
41810.0000000	7.2500000	-50.0000000
41810.0000000	7.5000000	-50.0000000
41810.0000000	7.7500000	-50.0000000
41810.0000000	8.0000000	-50.0000000
41810.0000000	8.2500000	-50.0000000
41810.0000000	8.5000000	-50.0000000
41810.0000000	8.7500000	-50.0000000
41810.0000000	9.0000000	-50.0000000
41810.0000000	9.2500000	-50.0000000
41810.0000000	9.5000000	-50.0000000
41810.0000000	9.7500000	-50.0000000
41810.0000000	10.0000000	-50.0000000
41810.0000000	10.2500000	-50.0000000
41810.0000000	10.5000000	-50.0000000
41810.0000000	10.7500000	-50.0000000
41810.0000000	11.0000000	-50.0000000
41810.0000000	11.2500000	-50.0000000
41810.0000000	11.5000000	-50.0000000
41810.0000000	11.7500000	-50.0000000
41810.0000000	12.0000000	-50.0000000
41810.0000000	12.2500000	-50.0000000
41810.0000000	12.5000000	-50.0000000
41810.0000000	12.7500000	-50.0000000
41810.0000000	13.0000000	-50.0000000
41810.0000000	13.2500000	-50.0000000
41810.0000000	13.5000000	-50.0000000
41810.0000000	13.7500000	-50.0000000
41810.0000000	14.0000000	-50.0000000
41810.0000000	14.2500000	-50.0000000
41810.0000000	14.5000000	-50.0000000

41810.0000000	14.7500000	-50.0000000
41810.0000000	15.0000000	-50.0000000
41840.0000000	0.0000000	-50.0000000
41840.0000000	0.2500000	-50.0000000
41840.0000000	0.5000000	-50.0000000
41840.0000000	0.7500000	-50.0000000
41840.0000000	1.0000000	-50.0000000
41840.0000000	1.2500000	-50.0000000
41840.0000000	1.5000000	-50.0000000
41840.0000000	1.7500000	-50.0000000
41840.0000000	2.0000000	-50.0000000
41840.0000000	2.2500000	-50.0000000
41840.0000000	2.5000000	-50.0000000
41840.0000000	2.7500000	-50.0000000
41840.0000000	3.0000000	-50.0000000
41840.0000000	3.2500000	-50.0000000
41840.0000000	3.5000000	-50.0000000
41840.0000000	3.7500000	-50.0000000
41840.0000000	4.0000000	-50.0000000
41840.0000000	4.2500000	-50.0000000
41840.0000000	4.5000000	-50.0000000
41840.0000000	4.7500000	-50.0000000
41840.0000000	5.0000000	-50.0000000
41840.0000000	5.2500000	-50.0000000
41840.0000000	5.5000000	-50.0000000
41840.0000000	5.7500000	-50.0000000
41840.0000000	6.0000000	-50.0000000
41840.0000000	6.2500000	-50.0000000
41840.0000000	6.5000000	-50.0000000
41840.0000000	6.7500000	-50.0000000
41840.0000000	7.0000000	-50.0000000
41840.0000000	7.2500000	-50.0000000
41840.0000000	7.5000000	-50.0000000
41840.0000000	7.7500000	-50.0000000
41840.0000000	8.0000000	-50.0000000
41840.0000000	8.2500000	-50.0000000
41840.0000000	8.5000000	-50.0000000
41840.0000000	8.7500000	-50.0000000
41840.0000000	9.0000000	-50.0000000
41840.0000000	9.2500000	-50.0000000
41840.0000000	9.5000000	-50.0000000
41840.0000000	9.7500000	-50.0000000
41840.0000000	10.0000000	-50.0000000
41840.0000000	10.2500000	-50.0000000
41840.0000000	10.5000000	-50.0000000
41840.0000000	10.7500000	-50.0000000
41840.0000000	11.0000000	-50.0000000
41840.0000000	11.2500000	-50.0000000
41840.0000000	11.5000000	-50.0000000
41840.0000000	11.7500000	-50.0000000
41840.0000000	12.0000000	-50.0000000
41840.0000000	12.2500000	-50.0000000
41840.0000000	12.5000000	-50.0000000
41840.0000000	12.7500000	-50.0000000
41840.0000000	13.0000000	-50.0000000
41840.0000000	13.2500000	-50.0000000
41840.0000000	13.5000000	-50.0000000
41840.0000000	13.7500000	-50.0000000

41840.0000000	14.0000000	-50.0000000
41840.0000000	14.2500000	-50.0000000
41840.0000000	14.5000000	-50.0000000
41840.0000000	14.7500000	-50.0000000
41840.0000000	15.0000000	-50.0000000
41870.0000000	0.0000000	-50.0000000
41870.0000000	0.2500000	-50.0000000
41870.0000000	0.5000000	-50.0000000
41870.0000000	0.7500000	-50.0000000
41870.0000000	1.0000000	-50.0000000
41870.0000000	1.2500000	-50.0000000
41870.0000000	1.5000000	-50.0000000
41870.0000000	1.7500000	-50.0000000
41870.0000000	2.0000000	-50.0000000
41870.0000000	2.2500000	-50.0000000
41870.0000000	2.5000000	-50.0000000
41870.0000000	2.7500000	-50.0000000
41870.0000000	3.0000000	-50.0000000
41870.0000000	3.2500000	-50.0000000
41870.0000000	3.5000000	-50.0000000
41870.0000000	3.7500000	-50.0000000
41870.0000000	4.0000000	-50.0000000
41870.0000000	4.2500000	-50.0000000
41870.0000000	4.5000000	-50.0000000
41870.0000000	4.7500000	-50.0000000
41870.0000000	5.0000000	-50.0000000
41870.0000000	5.2500000	-50.0000000
41870.0000000	5.5000000	-50.0000000
41870.0000000	5.7500000	-50.0000000
41870.0000000	6.0000000	-50.0000000
41870.0000000	6.2500000	-50.0000000
41870.0000000	6.5000000	-50.0000000
41870.0000000	6.7500000	-50.0000000
41870.0000000	7.0000000	-50.0000000
41870.0000000	7.2500000	-50.0000000
41870.0000000	7.5000000	-50.0000000
41870.0000000	7.7500000	-50.0000000
41870.0000000	8.0000000	-50.0000000
41870.0000000	8.2500000	-50.0000000
41870.0000000	8.5000000	-50.0000000
41870.0000000	8.7500000	-50.0000000
41870.0000000	9.0000000	-50.0000000
41870.0000000	9.2500000	-50.0000000
41870.0000000	9.5000000	-50.0000000
41870.0000000	9.7500000	-50.0000000
41870.0000000	10.0000000	-50.0000000
41870.0000000	10.2500000	-50.0000000
41870.0000000	10.5000000	-50.0000000
41870.0000000	10.7500000	-50.0000000
41870.0000000	11.0000000	-50.0000000
41870.0000000	11.2500000	-50.0000000
41870.0000000	11.5000000	-50.0000000
41870.0000000	11.7500000	-50.0000000
41870.0000000	12.0000000	-50.0000000
41870.0000000	12.2500000	-50.0000000
41870.0000000	12.5000000	-50.0000000
41870.0000000	12.7500000	-50.0000000
41870.0000000	13.0000000	-50.0000000

41870.0000000	13.2500000	-50.0000000
41870.0000000	13.5000000	-50.0000000
41870.0000000	13.7500000	-50.0000000
41870.0000000	14.0000000	-50.0000000
41870.0000000	14.2500000	-50.0000000
41870.0000000	14.5000000	-50.0000000
41870.0000000	14.7500000	-50.0000000
41870.0000000	15.0000000	-50.0000000
41900.0000000	0.0000000	-50.0000000
41900.0000000	0.2500000	-50.0000000
41900.0000000	0.5000000	-50.0000000
41900.0000000	0.7500000	-50.0000000
41900.0000000	1.0000000	-50.0000000
41900.0000000	1.2500000	-50.0000000
41900.0000000	1.5000000	-50.0000000
41900.0000000	1.7500000	-50.0000000
41900.0000000	2.0000000	-50.0000000
41900.0000000	2.2500000	-50.0000000
41900.0000000	2.5000000	-50.0000000
41900.0000000	2.7500000	-50.0000000
41900.0000000	3.0000000	-50.0000000
41900.0000000	3.2500000	-50.0000000
41900.0000000	3.5000000	-50.0000000
41900.0000000	3.7500000	-50.0000000
41900.0000000	4.0000000	-50.0000000
41900.0000000	4.2500000	-50.0000000
41900.0000000	4.5000000	-50.0000000
41900.0000000	4.7500000	-50.0000000
41900.0000000	5.0000000	-50.0000000
41900.0000000	5.2500000	-50.0000000
41900.0000000	5.5000000	-50.0000000
41900.0000000	5.7500000	-50.0000000
41900.0000000	6.0000000	-50.0000000
41900.0000000	6.2500000	-50.0000000
41900.0000000	6.5000000	-50.0000000
41900.0000000	6.7500000	-50.0000000
41900.0000000	7.0000000	-50.0000000
41900.0000000	7.2500000	-50.0000000
41900.0000000	7.5000000	-50.0000000
41900.0000000	7.7500000	-50.0000000
41900.0000000	8.0000000	-50.0000000
41900.0000000	8.2500000	-50.0000000
41900.0000000	8.5000000	-50.0000000
41900.0000000	8.7500000	-50.0000000
41900.0000000	9.0000000	-50.0000000
41900.0000000	9.2500000	-50.0000000
41900.0000000	9.5000000	-50.0000000
41900.0000000	9.7500000	-50.0000000
41900.0000000	10.0000000	-50.0000000
41900.0000000	10.2500000	-50.0000000
41900.0000000	10.5000000	-50.0000000
41900.0000000	10.7500000	-50.0000000
41900.0000000	11.0000000	-50.0000000
41900.0000000	11.2500000	-50.0000000
41900.0000000	11.5000000	-50.0000000
41900.0000000	11.7500000	-50.0000000
41900.0000000	12.0000000	-50.0000000
41900.0000000	12.2500000	-50.0000000

41900.0000000	12.5000000	-50.0000000
41900.0000000	12.7500000	-50.0000000
41900.0000000	13.0000000	-50.0000000
41900.0000000	13.2500000	-50.0000000
41900.0000000	13.5000000	-50.0000000
41900.0000000	13.7500000	-50.0000000
41900.0000000	14.0000000	-50.0000000
41900.0000000	14.2500000	-50.0000000
41900.0000000	14.5000000	-50.0000000
41900.0000000	14.7500000	-50.0000000
41900.0000000	15.0000000	-50.0000000
41930.0000000	0.0000000	-50.0000000
41930.0000000	0.2500000	-50.0000000
41930.0000000	0.5000000	-50.0000000
41930.0000000	0.7500000	-50.0000000
41930.0000000	1.0000000	-50.0000000
41930.0000000	1.2500000	-50.0000000
41930.0000000	1.5000000	-50.0000000
41930.0000000	1.7500000	-50.0000000
41930.0000000	2.0000000	-50.0000000
41930.0000000	2.2500000	-50.0000000
41930.0000000	2.5000000	-50.0000000
41930.0000000	2.7500000	-50.0000000
41930.0000000	3.0000000	-50.0000000
41930.0000000	3.2500000	-50.0000000
41930.0000000	3.5000000	-50.0000000
41930.0000000	3.7500000	-50.0000000
41930.0000000	4.0000000	-50.0000000
41930.0000000	4.2500000	-50.0000000
41930.0000000	4.5000000	-50.0000000
41930.0000000	4.7500000	-50.0000000
41930.0000000	5.0000000	-50.0000000
41930.0000000	5.2500000	-50.0000000
41930.0000000	5.5000000	-50.0000000
41930.0000000	5.7500000	-50.0000000
41930.0000000	6.0000000	-50.0000000
41930.0000000	6.2500000	-50.0000000
41930.0000000	6.5000000	-50.0000000
41930.0000000	6.7500000	-50.0000000
41930.0000000	7.0000000	-50.0000000
41930.0000000	7.2500000	-50.0000000
41930.0000000	7.5000000	-50.0000000
41930.0000000	7.7500000	-50.0000000
41930.0000000	8.0000000	-50.0000000
41930.0000000	8.2500000	-50.0000000
41930.0000000	8.5000000	-50.0000000
41930.0000000	8.7500000	-50.0000000
41930.0000000	9.0000000	-50.0000000
41930.0000000	9.2500000	-50.0000000
41930.0000000	9.5000000	-50.0000000
41930.0000000	9.7500000	-50.0000000
41930.0000000	10.0000000	-50.0000000
41930.0000000	10.2500000	-50.0000000
41930.0000000	10.5000000	-50.0000000
41930.0000000	10.7500000	-50.0000000
41930.0000000	11.0000000	-50.0000000
41930.0000000	11.2500000	-50.0000000
41930.0000000	11.5000000	-50.0000000

41930.0000000	11.7500000	-50.0000000
41930.0000000	12.0000000	-50.0000000
41930.0000000	12.2500000	-50.0000000
41930.0000000	12.5000000	-50.0000000
41930.0000000	12.7500000	-50.0000000
41930.0000000	13.0000000	-50.0000000
41930.0000000	13.2500000	-50.0000000
41930.0000000	13.5000000	-50.0000000
41930.0000000	13.7500000	-50.0000000
41930.0000000	14.0000000	-50.0000000
41930.0000000	14.2500000	-50.0000000
41930.0000000	14.5000000	-50.0000000
41930.0000000	14.7500000	-50.0000000
41930.0000000	15.0000000	-50.0000000
41960.0000000	0.0000000	-50.0000000
41960.0000000	0.2500000	-50.0000000
41960.0000000	0.5000000	-50.0000000
41960.0000000	0.7500000	-50.0000000
41960.0000000	1.0000000	-50.0000000
41960.0000000	1.2500000	-50.0000000
41960.0000000	1.5000000	-50.0000000
41960.0000000	1.7500000	-50.0000000
41960.0000000	2.0000000	-50.0000000
41960.0000000	2.2500000	-50.0000000
41960.0000000	2.5000000	-50.0000000
41960.0000000	2.7500000	-50.0000000
41960.0000000	3.0000000	-50.0000000
41960.0000000	3.2500000	-50.0000000
41960.0000000	3.5000000	-50.0000000
41960.0000000	3.7500000	-50.0000000
41960.0000000	4.0000000	-50.0000000
41960.0000000	4.2500000	-50.0000000
41960.0000000	4.5000000	-50.0000000
41960.0000000	4.7500000	-50.0000000
41960.0000000	5.0000000	-50.0000000
41960.0000000	5.2500000	-50.0000000
41960.0000000	5.5000000	-50.0000000
41960.0000000	5.7500000	-50.0000000
41960.0000000	6.0000000	-50.0000000
41960.0000000	6.2500000	-50.0000000
41960.0000000	6.5000000	-50.0000000
41960.0000000	6.7500000	-50.0000000
41960.0000000	7.0000000	-50.0000000
41960.0000000	7.2500000	-50.0000000
41960.0000000	7.5000000	-50.0000000
41960.0000000	7.7500000	-50.0000000
41960.0000000	8.0000000	-50.0000000
41960.0000000	8.2500000	-50.0000000
41960.0000000	8.5000000	-50.0000000
41960.0000000	8.7500000	-50.0000000
41960.0000000	9.0000000	-50.0000000
41960.0000000	9.2500000	-50.0000000
41960.0000000	9.5000000	-50.0000000
41960.0000000	9.7500000	-50.0000000
41960.0000000	10.0000000	-50.0000000
41960.0000000	10.2500000	-50.0000000
41960.0000000	10.5000000	-50.0000000
41960.0000000	10.7500000	-50.0000000

41960.0000000	11.0000000	-50.0000000
41960.0000000	11.2500000	-50.0000000
41960.0000000	11.5000000	-50.0000000
41960.0000000	11.7500000	-50.0000000
41960.0000000	12.0000000	-50.0000000
41960.0000000	12.2500000	-50.0000000
41960.0000000	12.5000000	-50.0000000
41960.0000000	12.7500000	-50.0000000
41960.0000000	13.0000000	-50.0000000
41960.0000000	13.2500000	-50.0000000
41960.0000000	13.5000000	-50.0000000
41960.0000000	13.7500000	-50.0000000
41960.0000000	14.0000000	-50.0000000
41960.0000000	14.2500000	-50.0000000
41960.0000000	14.5000000	-50.0000000
41960.0000000	14.7500000	-50.0000000
41960.0000000	15.0000000	-50.0000000
41990.0000000	0.0000000	-50.0000000
41990.0000000	0.2500000	-50.0000000
41990.0000000	0.5000000	-50.0000000
41990.0000000	0.7500000	-50.0000000
41990.0000000	1.0000000	-50.0000000
41990.0000000	1.2500000	-50.0000000
41990.0000000	1.5000000	-50.0000000
41990.0000000	1.7500000	-50.0000000
41990.0000000	2.0000000	-50.0000000
41990.0000000	2.2500000	-50.0000000
41990.0000000	2.5000000	-50.0000000
41990.0000000	2.7500000	-50.0000000
41990.0000000	3.0000000	-50.0000000
41990.0000000	3.2500000	-50.0000000
41990.0000000	3.5000000	-50.0000000
41990.0000000	3.7500000	-50.0000000
41990.0000000	4.0000000	-50.0000000
41990.0000000	4.2500000	-50.0000000
41990.0000000	4.5000000	-50.0000000
41990.0000000	4.7500000	-50.0000000
41990.0000000	5.0000000	-50.0000000
41990.0000000	5.2500000	-50.0000000
41990.0000000	5.5000000	-50.0000000
41990.0000000	5.7500000	-50.0000000
41990.0000000	6.0000000	-50.0000000
41990.0000000	6.2500000	-50.0000000
41990.0000000	6.5000000	-50.0000000
41990.0000000	6.7500000	-50.0000000
41990.0000000	7.0000000	-50.0000000
41990.0000000	7.2500000	-50.0000000
41990.0000000	7.5000000	-50.0000000
41990.0000000	7.7500000	-50.0000000
41990.0000000	8.0000000	-50.0000000
41990.0000000	8.2500000	-50.0000000
41990.0000000	8.5000000	-50.0000000
41990.0000000	8.7500000	-50.0000000
41990.0000000	9.0000000	-50.0000000
41990.0000000	9.2500000	-50.0000000
41990.0000000	9.5000000	-50.0000000
41990.0000000	9.7500000	-50.0000000
41990.0000000	10.0000000	-50.0000000

41990.0000000	10.2500000	-50.0000000
41990.0000000	10.5000000	-50.0000000
41990.0000000	10.7500000	-50.0000000
41990.0000000	11.0000000	-50.0000000
41990.0000000	11.2500000	-50.0000000
41990.0000000	11.5000000	-50.0000000
41990.0000000	11.7500000	-50.0000000
41990.0000000	12.0000000	-50.0000000
41990.0000000	12.2500000	-50.0000000
41990.0000000	12.5000000	-50.0000000
41990.0000000	12.7500000	-50.0000000
41990.0000000	13.0000000	-50.0000000
41990.0000000	13.2500000	-50.0000000
41990.0000000	13.5000000	-50.0000000
41990.0000000	13.7500000	-50.0000000
41990.0000000	14.0000000	-50.0000000
41990.0000000	14.2500000	-50.0000000
41990.0000000	14.5000000	-50.0000000
41990.0000000	14.7500000	-50.0000000
41990.0000000	15.0000000	-50.0000000
42020.0000000	0.0000000	-50.0000000
42020.0000000	0.2500000	-50.0000000
42020.0000000	0.5000000	-50.0000000
42020.0000000	0.7500000	-50.0000000
42020.0000000	1.0000000	-50.0000000
42020.0000000	1.2500000	-50.0000000
42020.0000000	1.5000000	-50.0000000
42020.0000000	1.7500000	-50.0000000
42020.0000000	2.0000000	-50.0000000
42020.0000000	2.2500000	-50.0000000
42020.0000000	2.5000000	-50.0000000
42020.0000000	2.7500000	-50.0000000
42020.0000000	3.0000000	-50.0000000
42020.0000000	3.2500000	-50.0000000
42020.0000000	3.5000000	-50.0000000
42020.0000000	3.7500000	-50.0000000
42020.0000000	4.0000000	-50.0000000
42020.0000000	4.2500000	-50.0000000
42020.0000000	4.5000000	-50.0000000
42020.0000000	4.7500000	-50.0000000
42020.0000000	5.0000000	-50.0000000
42020.0000000	5.2500000	-50.0000000
42020.0000000	5.5000000	-50.0000000
42020.0000000	5.7500000	-50.0000000
42020.0000000	6.0000000	-50.0000000
42020.0000000	6.2500000	-50.0000000
42020.0000000	6.5000000	-50.0000000
42020.0000000	6.7500000	-50.0000000
42020.0000000	7.0000000	-50.0000000
42020.0000000	7.2500000	-50.0000000
42020.0000000	7.5000000	-50.0000000
42020.0000000	7.7500000	-50.0000000
42020.0000000	8.0000000	-50.0000000
42020.0000000	8.2500000	-50.0000000
42020.0000000	8.5000000	-50.0000000
42020.0000000	8.7500000	-50.0000000
42020.0000000	9.0000000	-50.0000000
42020.0000000	9.2500000	-50.0000000

42020.0000000	9.5000000	-50.0000000
42020.0000000	9.7500000	-50.0000000
42020.0000000	10.0000000	-50.0000000
42020.0000000	10.2500000	-50.0000000
42020.0000000	10.5000000	-50.0000000
42020.0000000	10.7500000	-50.0000000
42020.0000000	11.0000000	-50.0000000
42020.0000000	11.2500000	-50.0000000
42020.0000000	11.5000000	-50.0000000
42020.0000000	11.7500000	-50.0000000
42020.0000000	12.0000000	-50.0000000
42020.0000000	12.2500000	-50.0000000
42020.0000000	12.5000000	-50.0000000
42020.0000000	12.7500000	-50.0000000
42020.0000000	13.0000000	-50.0000000
42020.0000000	13.2500000	-50.0000000
42020.0000000	13.5000000	-50.0000000
42020.0000000	13.7500000	-50.0000000
42020.0000000	14.0000000	-50.0000000
42020.0000000	14.2500000	-50.0000000
42020.0000000	14.5000000	-50.0000000
42020.0000000	14.7500000	-50.0000000
42020.0000000	15.0000000	-50.0000000
42050.0000000	0.0000000	-50.0000000
42050.0000000	0.2500000	-50.0000000
42050.0000000	0.5000000	-50.0000000
42050.0000000	0.7500000	-50.0000000
42050.0000000	1.0000000	-50.0000000
42050.0000000	1.2500000	-50.0000000
42050.0000000	1.5000000	-50.0000000
42050.0000000	1.7500000	-50.0000000
42050.0000000	2.0000000	-50.0000000
42050.0000000	2.2500000	-50.0000000
42050.0000000	2.5000000	-50.0000000
42050.0000000	2.7500000	-50.0000000
42050.0000000	3.0000000	-50.0000000
42050.0000000	3.2500000	-50.0000000
42050.0000000	3.5000000	-50.0000000
42050.0000000	3.7500000	-50.0000000
42050.0000000	4.0000000	-50.0000000
42050.0000000	4.2500000	-50.0000000
42050.0000000	4.5000000	-50.0000000
42050.0000000	4.7500000	-50.0000000
42050.0000000	5.0000000	-50.0000000
42050.0000000	5.2500000	-50.0000000
42050.0000000	5.5000000	-50.0000000
42050.0000000	5.7500000	-50.0000000
42050.0000000	6.0000000	-50.0000000
42050.0000000	6.2500000	-50.0000000
42050.0000000	6.5000000	-50.0000000
42050.0000000	6.7500000	-50.0000000
42050.0000000	7.0000000	-50.0000000
42050.0000000	7.2500000	-50.0000000
42050.0000000	7.5000000	-50.0000000
42050.0000000	7.7500000	-50.0000000
42050.0000000	8.0000000	-50.0000000
42050.0000000	8.2500000	-50.0000000
42050.0000000	8.5000000	-50.0000000

42050.0000000	8.7500000	-50.0000000
42050.0000000	9.0000000	-50.0000000
42050.0000000	9.2500000	-50.0000000
42050.0000000	9.5000000	-50.0000000
42050.0000000	9.7500000	-50.0000000
42050.0000000	10.0000000	-50.0000000
42050.0000000	10.2500000	-50.0000000
42050.0000000	10.5000000	-50.0000000
42050.0000000	10.7500000	-50.0000000
42050.0000000	11.0000000	-50.0000000
42050.0000000	11.2500000	-50.0000000
42050.0000000	11.5000000	-50.0000000
42050.0000000	11.7500000	-50.0000000
42050.0000000	12.0000000	-50.0000000
42050.0000000	12.2500000	-50.0000000
42050.0000000	12.5000000	-50.0000000
42050.0000000	12.7500000	-50.0000000
42050.0000000	13.0000000	-50.0000000
42050.0000000	13.2500000	-50.0000000
42050.0000000	13.5000000	-50.0000000
42050.0000000	13.7500000	-50.0000000
42050.0000000	14.0000000	-50.0000000
42050.0000000	14.2500000	-50.0000000
42050.0000000	14.5000000	-50.0000000
42050.0000000	14.7500000	-50.0000000
42050.0000000	15.0000000	-50.0000000
42080.0000000	0.0000000	-50.0000000
42080.0000000	0.2500000	-50.0000000
42080.0000000	0.5000000	-50.0000000
42080.0000000	0.7500000	-50.0000000
42080.0000000	1.0000000	-50.0000000
42080.0000000	1.2500000	-50.0000000
42080.0000000	1.5000000	-50.0000000
42080.0000000	1.7500000	-50.0000000
42080.0000000	2.0000000	-50.0000000
42080.0000000	2.2500000	-50.0000000
42080.0000000	2.5000000	-50.0000000
42080.0000000	2.7500000	-50.0000000
42080.0000000	3.0000000	-50.0000000
42080.0000000	3.2500000	-50.0000000
42080.0000000	3.5000000	-50.0000000
42080.0000000	3.7500000	-50.0000000
42080.0000000	4.0000000	-50.0000000
42080.0000000	4.2500000	-50.0000000
42080.0000000	4.5000000	-50.0000000
42080.0000000	4.7500000	-50.0000000
42080.0000000	5.0000000	-50.0000000
42080.0000000	5.2500000	-50.0000000
42080.0000000	5.5000000	-50.0000000
42080.0000000	5.7500000	-50.0000000
42080.0000000	6.0000000	-50.0000000
42080.0000000	6.2500000	-50.0000000
42080.0000000	6.5000000	-50.0000000
42080.0000000	6.7500000	-50.0000000
42080.0000000	7.0000000	-50.0000000
42080.0000000	7.2500000	-50.0000000
42080.0000000	7.5000000	-50.0000000
42080.0000000	7.7500000	-50.0000000

42080.0000000	8.0000000	-50.0000000
42080.0000000	8.2500000	-50.0000000
42080.0000000	8.5000000	-50.0000000
42080.0000000	8.7500000	-50.0000000
42080.0000000	9.0000000	-50.0000000
42080.0000000	9.2500000	-50.0000000
42080.0000000	9.5000000	-50.0000000
42080.0000000	9.7500000	-50.0000000
42080.0000000	10.0000000	-50.0000000
42080.0000000	10.2500000	-50.0000000
42080.0000000	10.5000000	-50.0000000
42080.0000000	10.7500000	-50.0000000
42080.0000000	11.0000000	-50.0000000
42080.0000000	11.2500000	-50.0000000
42080.0000000	11.5000000	-50.0000000
42080.0000000	11.7500000	-50.0000000
42080.0000000	12.0000000	-50.0000000
42080.0000000	12.2500000	-50.0000000
42080.0000000	12.5000000	-50.0000000
42080.0000000	12.7500000	-50.0000000
42080.0000000	13.0000000	-50.0000000
42080.0000000	13.2500000	-50.0000000
42080.0000000	13.5000000	-50.0000000
42080.0000000	13.7500000	-50.0000000
42080.0000000	14.0000000	-50.0000000
42080.0000000	14.2500000	-50.0000000
42080.0000000	14.5000000	-50.0000000
42080.0000000	14.7500000	-50.0000000
42080.0000000	15.0000000	-50.0000000
42110.0000000	0.0000000	-50.0000000
42110.0000000	0.2500000	-50.0000000
42110.0000000	0.5000000	-50.0000000
42110.0000000	0.7500000	-50.0000000
42110.0000000	1.0000000	-50.0000000
42110.0000000	1.2500000	-50.0000000
42110.0000000	1.5000000	-50.0000000
42110.0000000	1.7500000	-50.0000000
42110.0000000	2.0000000	-50.0000000
42110.0000000	2.2500000	-50.0000000
42110.0000000	2.5000000	-50.0000000
42110.0000000	2.7500000	-50.0000000
42110.0000000	3.0000000	-50.0000000
42110.0000000	3.2500000	-50.0000000
42110.0000000	3.5000000	-50.0000000
42110.0000000	3.7500000	-50.0000000
42110.0000000	4.0000000	-50.0000000
42110.0000000	4.2500000	-50.0000000
42110.0000000	4.5000000	-50.0000000
42110.0000000	4.7500000	-50.0000000
42110.0000000	5.0000000	-50.0000000
42110.0000000	5.2500000	-50.0000000
42110.0000000	5.5000000	-50.0000000
42110.0000000	5.7500000	-50.0000000
42110.0000000	6.0000000	-50.0000000
42110.0000000	6.2500000	-50.0000000
42110.0000000	6.5000000	-50.0000000
42110.0000000	6.7500000	-50.0000000
42110.0000000	7.0000000	-50.0000000

42110.0000000	7.2500000	-50.0000000
42110.0000000	7.5000000	-50.0000000
42110.0000000	7.7500000	-50.0000000
42110.0000000	8.0000000	-50.0000000
42110.0000000	8.2500000	-50.0000000
42110.0000000	8.5000000	-50.0000000
42110.0000000	8.7500000	-50.0000000
42110.0000000	9.0000000	-50.0000000
42110.0000000	9.2500000	-50.0000000
42110.0000000	9.5000000	-50.0000000
42110.0000000	9.7500000	-50.0000000
42110.0000000	10.0000000	-50.0000000
42110.0000000	10.2500000	-50.0000000
42110.0000000	10.5000000	-50.0000000
42110.0000000	10.7500000	-50.0000000
42110.0000000	11.0000000	-50.0000000
42110.0000000	11.2500000	-50.0000000
42110.0000000	11.5000000	-50.0000000
42110.0000000	11.7500000	-50.0000000
42110.0000000	12.0000000	-50.0000000
42110.0000000	12.2500000	-50.0000000
42110.0000000	12.5000000	-50.0000000
42110.0000000	12.7500000	-50.0000000
42110.0000000	13.0000000	-50.0000000
42110.0000000	13.2500000	-50.0000000
42110.0000000	13.5000000	-50.0000000
42110.0000000	13.7500000	-50.0000000
42110.0000000	14.0000000	-50.0000000
42110.0000000	14.2500000	-50.0000000
42110.0000000	14.5000000	-50.0000000
42110.0000000	14.7500000	-50.0000000
42110.0000000	15.0000000	-50.0000000
42140.0000000	0.0000000	-50.0000000
42140.0000000	0.2500000	-50.0000000
42140.0000000	0.5000000	-50.0000000
42140.0000000	0.7500000	-50.0000000
42140.0000000	1.0000000	-50.0000000
42140.0000000	1.2500000	-50.0000000
42140.0000000	1.5000000	-50.0000000
42140.0000000	1.7500000	-50.0000000
42140.0000000	2.0000000	-50.0000000
42140.0000000	2.2500000	-50.0000000
42140.0000000	2.5000000	-50.0000000
42140.0000000	2.7500000	-50.0000000
42140.0000000	3.0000000	-50.0000000
42140.0000000	3.2500000	-50.0000000
42140.0000000	3.5000000	-50.0000000
42140.0000000	3.7500000	-50.0000000
42140.0000000	4.0000000	-50.0000000
42140.0000000	4.2500000	-50.0000000
42140.0000000	4.5000000	-50.0000000
42140.0000000	4.7500000	-50.0000000
42140.0000000	5.0000000	-50.0000000
42140.0000000	5.2500000	-50.0000000
42140.0000000	5.5000000	-50.0000000
42140.0000000	5.7500000	-50.0000000
42140.0000000	6.0000000	-50.0000000
42140.0000000	6.2500000	-50.0000000

42140.0000000	6.5000000	-50.0000000
42140.0000000	6.7500000	-50.0000000
42140.0000000	7.0000000	-50.0000000
42140.0000000	7.2500000	-50.0000000
42140.0000000	7.5000000	-50.0000000
42140.0000000	7.7500000	-50.0000000
42140.0000000	8.0000000	-50.0000000
42140.0000000	8.2500000	-50.0000000
42140.0000000	8.5000000	-50.0000000
42140.0000000	8.7500000	-50.0000000
42140.0000000	9.0000000	-50.0000000
42140.0000000	9.2500000	-50.0000000
42140.0000000	9.5000000	-50.0000000
42140.0000000	9.7500000	-50.0000000
42140.0000000	10.0000000	-50.0000000
42140.0000000	10.2500000	-50.0000000
42140.0000000	10.5000000	-50.0000000
42140.0000000	10.7500000	-50.0000000
42140.0000000	11.0000000	-50.0000000
42140.0000000	11.2500000	-50.0000000
42140.0000000	11.5000000	-50.0000000
42140.0000000	11.7500000	-50.0000000
42140.0000000	12.0000000	-50.0000000
42140.0000000	12.2500000	-50.0000000
42140.0000000	12.5000000	-50.0000000
42140.0000000	12.7500000	-50.0000000
42140.0000000	13.0000000	-50.0000000
42140.0000000	13.2500000	-50.0000000
42140.0000000	13.5000000	-50.0000000
42140.0000000	13.7500000	-50.0000000
42140.0000000	14.0000000	-50.0000000
42140.0000000	14.2500000	-50.0000000
42140.0000000	14.5000000	-50.0000000
42140.0000000	14.7500000	-50.0000000
42140.0000000	15.0000000	-50.0000000
42170.0000000	0.0000000	-50.0000000
42170.0000000	0.2500000	-50.0000000
42170.0000000	0.5000000	-50.0000000
42170.0000000	0.7500000	-50.0000000
42170.0000000	1.0000000	-50.0000000
42170.0000000	1.2500000	-50.0000000
42170.0000000	1.5000000	-50.0000000
42170.0000000	1.7500000	-50.0000000
42170.0000000	2.0000000	-50.0000000
42170.0000000	2.2500000	-50.0000000
42170.0000000	2.5000000	-50.0000000
42170.0000000	2.7500000	-50.0000000
42170.0000000	3.0000000	-50.0000000
42170.0000000	3.2500000	-50.0000000
42170.0000000	3.5000000	-50.0000000
42170.0000000	3.7500000	-50.0000000
42170.0000000	4.0000000	-50.0000000
42170.0000000	4.2500000	-50.0000000
42170.0000000	4.5000000	-50.0000000
42170.0000000	4.7500000	-50.0000000
42170.0000000	5.0000000	-50.0000000
42170.0000000	5.2500000	-50.0000000
42170.0000000	5.5000000	-50.0000000

42170.0000000	5.7500000	-50.0000000
42170.0000000	6.0000000	-50.0000000
42170.0000000	6.2500000	-50.0000000
42170.0000000	6.5000000	-50.0000000
42170.0000000	6.7500000	-50.0000000
42170.0000000	7.0000000	-50.0000000
42170.0000000	7.2500000	-50.0000000
42170.0000000	7.5000000	-50.0000000
42170.0000000	7.7500000	-50.0000000
42170.0000000	8.0000000	-50.0000000
42170.0000000	8.2500000	-50.0000000
42170.0000000	8.5000000	-50.0000000
42170.0000000	8.7500000	-50.0000000
42170.0000000	9.0000000	-50.0000000
42170.0000000	9.2500000	-50.0000000
42170.0000000	9.5000000	-50.0000000
42170.0000000	9.7500000	-50.0000000
42170.0000000	10.0000000	-50.0000000
42170.0000000	10.2500000	-50.0000000
42170.0000000	10.5000000	-50.0000000
42170.0000000	10.7500000	-50.0000000
42170.0000000	11.0000000	-50.0000000
42170.0000000	11.2500000	-50.0000000
42170.0000000	11.5000000	-50.0000000
42170.0000000	11.7500000	-50.0000000
42170.0000000	12.0000000	-50.0000000
42170.0000000	12.2500000	-50.0000000
42170.0000000	12.5000000	-50.0000000
42170.0000000	12.7500000	-50.0000000
42170.0000000	13.0000000	-50.0000000
42170.0000000	13.2500000	-50.0000000
42170.0000000	13.5000000	-50.0000000
42170.0000000	13.7500000	-50.0000000
42170.0000000	14.0000000	-50.0000000
42170.0000000	14.2500000	-50.0000000
42170.0000000	14.5000000	-50.0000000
42170.0000000	14.7500000	-50.0000000
42170.0000000	15.0000000	-50.0000000
42200.0000000	0.0000000	-50.0000000
42200.0000000	0.2500000	-50.0000000
42200.0000000	0.5000000	-50.0000000
42200.0000000	0.7500000	-50.0000000
42200.0000000	1.0000000	-50.0000000
42200.0000000	1.2500000	-50.0000000
42200.0000000	1.5000000	-50.0000000
42200.0000000	1.7500000	-50.0000000
42200.0000000	2.0000000	-50.0000000
42200.0000000	2.2500000	-50.0000000
42200.0000000	2.5000000	-50.0000000
42200.0000000	2.7500000	-50.0000000
42200.0000000	3.0000000	-50.0000000
42200.0000000	3.2500000	-50.0000000
42200.0000000	3.5000000	-50.0000000
42200.0000000	3.7500000	-50.0000000
42200.0000000	4.0000000	-50.0000000
42200.0000000	4.2500000	-50.0000000
42200.0000000	4.5000000	-50.0000000
42200.0000000	4.7500000	-50.0000000

42200.0000000	5.0000000	-50.0000000
42200.0000000	5.2500000	-50.0000000
42200.0000000	5.5000000	-50.0000000
42200.0000000	5.7500000	-50.0000000
42200.0000000	6.0000000	-50.0000000
42200.0000000	6.2500000	-50.0000000
42200.0000000	6.5000000	-50.0000000
42200.0000000	6.7500000	-50.0000000
42200.0000000	7.0000000	-50.0000000
42200.0000000	7.2500000	-50.0000000
42200.0000000	7.5000000	-50.0000000
42200.0000000	7.7500000	-50.0000000
42200.0000000	8.0000000	-50.0000000
42200.0000000	8.2500000	-50.0000000
42200.0000000	8.5000000	-50.0000000
42200.0000000	8.7500000	-50.0000000
42200.0000000	9.0000000	-50.0000000
42200.0000000	9.2500000	-50.0000000
42200.0000000	9.5000000	-50.0000000
42200.0000000	9.7500000	-50.0000000
42200.0000000	10.0000000	-50.0000000
42200.0000000	10.2500000	-50.0000000
42200.0000000	10.5000000	-50.0000000
42200.0000000	10.7500000	-50.0000000
42200.0000000	11.0000000	-50.0000000
42200.0000000	11.2500000	-50.0000000
42200.0000000	11.5000000	-50.0000000
42200.0000000	11.7500000	-50.0000000
42200.0000000	12.0000000	-50.0000000
42200.0000000	12.2500000	-50.0000000
42200.0000000	12.5000000	-50.0000000
42200.0000000	12.7500000	-50.0000000
42200.0000000	13.0000000	-50.0000000
42200.0000000	13.2500000	-50.0000000
42200.0000000	13.5000000	-50.0000000
42200.0000000	13.7500000	-50.0000000
42200.0000000	14.0000000	-50.0000000
42200.0000000	14.2500000	-50.0000000
42200.0000000	14.5000000	-50.0000000
42200.0000000	14.7500000	-50.0000000
42200.0000000	15.0000000	-50.0000000
42230.0000000	0.0000000	-50.0000000
42230.0000000	0.2500000	-50.0000000
42230.0000000	0.5000000	-50.0000000
42230.0000000	0.7500000	-50.0000000
42230.0000000	1.0000000	-50.0000000
42230.0000000	1.2500000	-50.0000000
42230.0000000	1.5000000	-50.0000000
42230.0000000	1.7500000	-50.0000000
42230.0000000	2.0000000	-50.0000000
42230.0000000	2.2500000	-50.0000000
42230.0000000	2.5000000	-50.0000000
42230.0000000	2.7500000	-50.0000000
42230.0000000	3.0000000	-50.0000000
42230.0000000	3.2500000	-50.0000000
42230.0000000	3.5000000	-50.0000000
42230.0000000	3.7500000	-50.0000000
42230.0000000	4.0000000	-50.0000000

42230.0000000	4.2500000	-50.0000000
42230.0000000	4.5000000	-50.0000000
42230.0000000	4.7500000	-50.0000000
42230.0000000	5.0000000	-50.0000000
42230.0000000	5.2500000	-50.0000000
42230.0000000	5.5000000	-50.0000000
42230.0000000	5.7500000	-50.0000000
42230.0000000	6.0000000	-50.0000000
42230.0000000	6.2500000	-50.0000000
42230.0000000	6.5000000	-50.0000000
42230.0000000	6.7500000	-50.0000000
42230.0000000	7.0000000	-50.0000000
42230.0000000	7.2500000	-50.0000000
42230.0000000	7.5000000	-50.0000000
42230.0000000	7.7500000	-50.0000000
42230.0000000	8.0000000	-50.0000000
42230.0000000	8.2500000	-50.0000000
42230.0000000	8.5000000	-50.0000000
42230.0000000	8.7500000	-50.0000000
42230.0000000	9.0000000	-50.0000000
42230.0000000	9.2500000	-50.0000000
42230.0000000	9.5000000	-50.0000000
42230.0000000	9.7500000	-50.0000000
42230.0000000	10.0000000	-50.0000000
42230.0000000	10.2500000	-50.0000000
42230.0000000	10.5000000	-50.0000000
42230.0000000	10.7500000	-50.0000000
42230.0000000	11.0000000	-50.0000000
42230.0000000	11.2500000	-50.0000000
42230.0000000	11.5000000	-50.0000000
42230.0000000	11.7500000	-50.0000000
42230.0000000	12.0000000	-50.0000000
42230.0000000	12.2500000	-50.0000000
42230.0000000	12.5000000	-50.0000000
42230.0000000	12.7500000	-50.0000000
42230.0000000	13.0000000	-50.0000000
42230.0000000	13.2500000	-50.0000000
42230.0000000	13.5000000	-50.0000000
42230.0000000	13.7500000	-50.0000000
42230.0000000	14.0000000	-50.0000000
42230.0000000	14.2500000	-50.0000000
42230.0000000	14.5000000	-50.0000000
42230.0000000	14.7500000	-50.0000000
42230.0000000	15.0000000	-50.0000000
42260.0000000	0.0000000	-50.0000000
42260.0000000	0.2500000	-50.0000000
42260.0000000	0.5000000	-50.0000000
42260.0000000	0.7500000	-50.0000000
42260.0000000	1.0000000	-50.0000000
42260.0000000	1.2500000	-50.0000000
42260.0000000	1.5000000	-50.0000000
42260.0000000	1.7500000	-50.0000000
42260.0000000	2.0000000	-50.0000000
42260.0000000	2.2500000	-50.0000000
42260.0000000	2.5000000	-50.0000000
42260.0000000	2.7500000	-50.0000000
42260.0000000	3.0000000	-50.0000000
42260.0000000	3.2500000	-50.0000000

42260.0000000	3.5000000	-50.0000000
42260.0000000	3.7500000	-50.0000000
42260.0000000	4.0000000	-50.0000000
42260.0000000	4.2500000	-50.0000000
42260.0000000	4.5000000	-50.0000000
42260.0000000	4.7500000	-50.0000000
42260.0000000	5.0000000	-50.0000000
42260.0000000	5.2500000	-50.0000000
42260.0000000	5.5000000	-50.0000000
42260.0000000	5.7500000	-50.0000000
42260.0000000	6.0000000	-50.0000000
42260.0000000	6.2500000	-50.0000000
42260.0000000	6.5000000	-50.0000000
42260.0000000	6.7500000	-50.0000000
42260.0000000	7.0000000	-50.0000000
42260.0000000	7.2500000	-50.0000000
42260.0000000	7.5000000	-50.0000000
42260.0000000	7.7500000	-50.0000000
42260.0000000	8.0000000	-50.0000000
42260.0000000	8.2500000	-50.0000000
42260.0000000	8.5000000	-50.0000000
42260.0000000	8.7500000	-50.0000000
42260.0000000	9.0000000	-50.0000000
42260.0000000	9.2500000	-50.0000000
42260.0000000	9.5000000	-50.0000000
42260.0000000	9.7500000	-50.0000000
42260.0000000	10.0000000	-50.0000000
42260.0000000	10.2500000	-50.0000000
42260.0000000	10.5000000	-50.0000000
42260.0000000	10.7500000	-50.0000000
42260.0000000	11.0000000	-50.0000000
42260.0000000	11.2500000	-50.0000000
42260.0000000	11.5000000	-50.0000000
42260.0000000	11.7500000	-50.0000000
42260.0000000	12.0000000	-50.0000000
42260.0000000	12.2500000	-50.0000000
42260.0000000	12.5000000	-50.0000000
42260.0000000	12.7500000	-50.0000000
42260.0000000	13.0000000	-50.0000000
42260.0000000	13.2500000	-50.0000000
42260.0000000	13.5000000	-50.0000000
42260.0000000	13.7500000	-50.0000000
42260.0000000	14.0000000	-50.0000000
42260.0000000	14.2500000	-50.0000000
42260.0000000	14.5000000	-50.0000000
42260.0000000	14.7500000	-50.0000000
42260.0000000	15.0000000	-50.0000000
42290.0000000	0.0000000	-50.0000000
42290.0000000	0.2500000	-50.0000000
42290.0000000	0.5000000	-50.0000000
42290.0000000	0.7500000	-50.0000000
42290.0000000	1.0000000	-50.0000000
42290.0000000	1.2500000	-50.0000000
42290.0000000	1.5000000	-50.0000000
42290.0000000	1.7500000	-50.0000000
42290.0000000	2.0000000	-50.0000000
42290.0000000	2.2500000	-50.0000000
42290.0000000	2.5000000	-50.0000000

42290.0000000	2.7500000	-50.0000000
42290.0000000	3.0000000	-50.0000000
42290.0000000	3.2500000	-50.0000000
42290.0000000	3.5000000	-50.0000000
42290.0000000	3.7500000	-50.0000000
42290.0000000	4.0000000	-50.0000000
42290.0000000	4.2500000	-50.0000000
42290.0000000	4.5000000	-50.0000000
42290.0000000	4.7500000	-50.0000000
42290.0000000	5.0000000	-50.0000000
42290.0000000	5.2500000	-50.0000000
42290.0000000	5.5000000	-50.0000000
42290.0000000	5.7500000	-50.0000000
42290.0000000	6.0000000	-50.0000000
42290.0000000	6.2500000	-50.0000000
42290.0000000	6.5000000	-50.0000000
42290.0000000	6.7500000	-50.0000000
42290.0000000	7.0000000	-50.0000000
42290.0000000	7.2500000	-50.0000000
42290.0000000	7.5000000	-50.0000000
42290.0000000	7.7500000	-50.0000000
42290.0000000	8.0000000	-50.0000000
42290.0000000	8.2500000	-50.0000000
42290.0000000	8.5000000	-50.0000000
42290.0000000	8.7500000	-50.0000000
42290.0000000	9.0000000	-50.0000000
42290.0000000	9.2500000	-50.0000000
42290.0000000	9.5000000	-50.0000000
42290.0000000	9.7500000	-50.0000000
42290.0000000	10.0000000	-50.0000000
42290.0000000	10.2500000	-50.0000000
42290.0000000	10.5000000	-50.0000000
42290.0000000	10.7500000	-50.0000000
42290.0000000	11.0000000	-50.0000000
42290.0000000	11.2500000	-50.0000000
42290.0000000	11.5000000	-50.0000000
42290.0000000	11.7500000	-50.0000000
42290.0000000	12.0000000	-50.0000000
42290.0000000	12.2500000	-50.0000000
42290.0000000	12.5000000	-50.0000000
42290.0000000	12.7500000	-50.0000000
42290.0000000	13.0000000	-50.0000000
42290.0000000	13.2500000	-50.0000000
42290.0000000	13.5000000	-50.0000000
42290.0000000	13.7500000	-50.0000000
42290.0000000	14.0000000	-50.0000000
42290.0000000	14.2500000	-50.0000000
42290.0000000	14.5000000	-50.0000000
42290.0000000	14.7500000	-50.0000000
42290.0000000	15.0000000	-50.0000000
42320.0000000	0.0000000	-50.0000000
42320.0000000	0.2500000	-50.0000000
42320.0000000	0.5000000	-50.0000000
42320.0000000	0.7500000	-50.0000000
42320.0000000	1.0000000	-50.0000000
42320.0000000	1.2500000	-50.0000000
42320.0000000	1.5000000	-50.0000000
42320.0000000	1.7500000	-50.0000000

42320.0000000	2.0000000	-50.0000000
42320.0000000	2.2500000	-50.0000000
42320.0000000	2.5000000	-50.0000000
42320.0000000	2.7500000	-50.0000000
42320.0000000	3.0000000	-50.0000000
42320.0000000	3.2500000	-50.0000000
42320.0000000	3.5000000	-50.0000000
42320.0000000	3.7500000	-50.0000000
42320.0000000	4.0000000	-50.0000000
42320.0000000	4.2500000	-50.0000000
42320.0000000	4.5000000	-50.0000000
42320.0000000	4.7500000	-50.0000000
42320.0000000	5.0000000	-50.0000000
42320.0000000	5.2500000	-50.0000000
42320.0000000	5.5000000	-50.0000000
42320.0000000	5.7500000	-50.0000000
42320.0000000	6.0000000	-50.0000000
42320.0000000	6.2500000	-50.0000000
42320.0000000	6.5000000	-50.0000000
42320.0000000	6.7500000	-50.0000000
42320.0000000	7.0000000	-50.0000000
42320.0000000	7.2500000	-50.0000000
42320.0000000	7.5000000	-50.0000000
42320.0000000	7.7500000	-50.0000000
42320.0000000	8.0000000	-50.0000000
42320.0000000	8.2500000	-50.0000000
42320.0000000	8.5000000	-50.0000000
42320.0000000	8.7500000	-50.0000000
42320.0000000	9.0000000	-50.0000000
42320.0000000	9.2500000	-50.0000000
42320.0000000	9.5000000	-50.0000000
42320.0000000	9.7500000	-50.0000000
42320.0000000	10.0000000	-50.0000000
42320.0000000	10.2500000	-50.0000000
42320.0000000	10.5000000	-50.0000000
42320.0000000	10.7500000	-50.0000000
42320.0000000	11.0000000	-50.0000000
42320.0000000	11.2500000	-50.0000000
42320.0000000	11.5000000	-50.0000000
42320.0000000	11.7500000	-50.0000000
42320.0000000	12.0000000	-50.0000000
42320.0000000	12.2500000	-50.0000000
42320.0000000	12.5000000	-50.0000000
42320.0000000	12.7500000	-50.0000000
42320.0000000	13.0000000	-50.0000000
42320.0000000	13.2500000	-50.0000000
42320.0000000	13.5000000	-50.0000000
42320.0000000	13.7500000	-50.0000000
42320.0000000	14.0000000	-50.0000000
42320.0000000	14.2500000	-50.0000000
42320.0000000	14.5000000	-50.0000000
42320.0000000	14.7500000	-50.0000000
42320.0000000	15.0000000	-50.0000000
42350.0000000	0.0000000	-50.0000000
42350.0000000	0.2500000	-50.0000000
42350.0000000	0.5000000	-50.0000000
42350.0000000	0.7500000	-50.0000000
42350.0000000	1.0000000	-50.0000000

42350.0000000	1.2500000	-50.0000000
42350.0000000	1.5000000	-50.0000000
42350.0000000	1.7500000	-50.0000000
42350.0000000	2.0000000	-50.0000000
42350.0000000	2.2500000	-50.0000000
42350.0000000	2.5000000	-50.0000000
42350.0000000	2.7500000	-50.0000000
42350.0000000	3.0000000	-50.0000000
42350.0000000	3.2500000	-50.0000000
42350.0000000	3.5000000	-50.0000000
42350.0000000	3.7500000	-50.0000000
42350.0000000	4.0000000	-50.0000000
42350.0000000	4.2500000	-50.0000000
42350.0000000	4.5000000	-50.0000000
42350.0000000	4.7500000	-50.0000000
42350.0000000	5.0000000	-50.0000000
42350.0000000	5.2500000	-50.0000000
42350.0000000	5.5000000	-50.0000000
42350.0000000	5.7500000	-50.0000000
42350.0000000	6.0000000	-50.0000000
42350.0000000	6.2500000	-50.0000000
42350.0000000	6.5000000	-50.0000000
42350.0000000	6.7500000	-50.0000000
42350.0000000	7.0000000	-50.0000000
42350.0000000	7.2500000	-50.0000000
42350.0000000	7.5000000	-50.0000000
42350.0000000	7.7500000	-50.0000000
42350.0000000	8.0000000	-50.0000000
42350.0000000	8.2500000	-50.0000000
42350.0000000	8.5000000	-50.0000000
42350.0000000	8.7500000	-50.0000000
42350.0000000	9.0000000	-50.0000000
42350.0000000	9.2500000	-50.0000000
42350.0000000	9.5000000	-50.0000000
42350.0000000	9.7500000	-50.0000000
42350.0000000	10.0000000	-50.0000000
42350.0000000	10.2500000	-50.0000000
42350.0000000	10.5000000	-50.0000000
42350.0000000	10.7500000	-50.0000000
42350.0000000	11.0000000	-50.0000000
42350.0000000	11.2500000	-50.0000000
42350.0000000	11.5000000	-50.0000000
42350.0000000	11.7500000	-50.0000000
42350.0000000	12.0000000	-50.0000000
42350.0000000	12.2500000	-50.0000000
42350.0000000	12.5000000	-50.0000000
42350.0000000	12.7500000	-50.0000000
42350.0000000	13.0000000	-50.0000000
42350.0000000	13.2500000	-50.0000000
42350.0000000	13.5000000	-50.0000000
42350.0000000	13.7500000	-50.0000000
42350.0000000	14.0000000	-50.0000000
42350.0000000	14.2500000	-50.0000000
42350.0000000	14.5000000	-50.0000000
42350.0000000	14.7500000	-50.0000000
42350.0000000	15.0000000	-50.0000000
42380.0000000	0.0000000	-50.0000000
42380.0000000	0.2500000	-50.0000000

42380.0000000	0.5000000	-50.0000000
42380.0000000	0.7500000	-50.0000000
42380.0000000	1.0000000	-50.0000000
42380.0000000	1.2500000	-50.0000000
42380.0000000	1.5000000	-50.0000000
42380.0000000	1.7500000	-50.0000000
42380.0000000	2.0000000	-50.0000000
42380.0000000	2.2500000	-50.0000000
42380.0000000	2.5000000	-50.0000000
42380.0000000	2.7500000	-50.0000000
42380.0000000	3.0000000	-50.0000000
42380.0000000	3.2500000	-50.0000000
42380.0000000	3.5000000	-50.0000000
42380.0000000	3.7500000	-50.0000000
42380.0000000	4.0000000	-50.0000000
42380.0000000	4.2500000	-50.0000000
42380.0000000	4.5000000	-50.0000000
42380.0000000	4.7500000	-50.0000000
42380.0000000	5.0000000	-50.0000000
42380.0000000	5.2500000	-50.0000000
42380.0000000	5.5000000	-50.0000000
42380.0000000	5.7500000	-50.0000000
42380.0000000	6.0000000	-50.0000000
42380.0000000	6.2500000	-50.0000000
42380.0000000	6.5000000	-50.0000000
42380.0000000	6.7500000	-50.0000000
42380.0000000	7.0000000	-50.0000000
42380.0000000	7.2500000	-50.0000000
42380.0000000	7.5000000	-50.0000000
42380.0000000	7.7500000	-50.0000000
42380.0000000	8.0000000	-50.0000000
42380.0000000	8.2500000	-50.0000000
42380.0000000	8.5000000	-50.0000000
42380.0000000	8.7500000	-50.0000000
42380.0000000	9.0000000	-50.0000000
42380.0000000	9.2500000	-50.0000000
42380.0000000	9.5000000	-50.0000000
42380.0000000	9.7500000	-50.0000000
42380.0000000	10.0000000	-50.0000000
42380.0000000	10.2500000	-50.0000000
42380.0000000	10.5000000	-50.0000000
42380.0000000	10.7500000	-50.0000000
42380.0000000	11.0000000	-50.0000000
42380.0000000	11.2500000	-50.0000000
42380.0000000	11.5000000	-50.0000000
42380.0000000	11.7500000	-50.0000000
42380.0000000	12.0000000	-50.0000000
42380.0000000	12.2500000	-50.0000000
42380.0000000	12.5000000	-50.0000000
42380.0000000	12.7500000	-50.0000000
42380.0000000	13.0000000	-50.0000000
42380.0000000	13.2500000	-50.0000000
42380.0000000	13.5000000	-50.0000000
42380.0000000	13.7500000	-50.0000000
42380.0000000	14.0000000	-50.0000000
42380.0000000	14.2500000	-50.0000000
42380.0000000	14.5000000	-50.0000000
42380.0000000	14.7500000	-50.0000000

42380.0000000	15.0000000	-50.0000000
42410.0000000	0.0000000	-50.0000000
42410.0000000	0.2500000	-50.0000000
42410.0000000	0.5000000	-50.0000000
42410.0000000	0.7500000	-50.0000000
42410.0000000	1.0000000	-50.0000000
42410.0000000	1.2500000	-50.0000000
42410.0000000	1.5000000	-50.0000000
42410.0000000	1.7500000	-50.0000000
42410.0000000	2.0000000	-50.0000000
42410.0000000	2.2500000	-50.0000000
42410.0000000	2.5000000	-50.0000000
42410.0000000	2.7500000	-50.0000000
42410.0000000	3.0000000	-50.0000000
42410.0000000	3.2500000	-50.0000000
42410.0000000	3.5000000	-50.0000000
42410.0000000	3.7500000	-50.0000000
42410.0000000	4.0000000	-50.0000000
42410.0000000	4.2500000	-50.0000000
42410.0000000	4.5000000	-50.0000000
42410.0000000	4.7500000	-50.0000000
42410.0000000	5.0000000	-50.0000000
42410.0000000	5.2500000	-50.0000000
42410.0000000	5.5000000	-50.0000000
42410.0000000	5.7500000	-50.0000000
42410.0000000	6.0000000	-50.0000000
42410.0000000	6.2500000	-50.0000000
42410.0000000	6.5000000	-50.0000000
42410.0000000	6.7500000	-50.0000000
42410.0000000	7.0000000	-50.0000000
42410.0000000	7.2500000	-50.0000000
42410.0000000	7.5000000	-50.0000000
42410.0000000	7.7500000	-50.0000000
42410.0000000	8.0000000	-50.0000000
42410.0000000	8.2500000	-50.0000000
42410.0000000	8.5000000	-50.0000000
42410.0000000	8.7500000	-50.0000000
42410.0000000	9.0000000	-50.0000000
42410.0000000	9.2500000	-50.0000000
42410.0000000	9.5000000	-50.0000000
42410.0000000	9.7500000	-50.0000000
42410.0000000	10.0000000	-50.0000000
42410.0000000	10.2500000	-50.0000000
42410.0000000	10.5000000	-50.0000000
42410.0000000	10.7500000	-50.0000000
42410.0000000	11.0000000	-50.0000000
42410.0000000	11.2500000	-50.0000000
42410.0000000	11.5000000	-50.0000000
42410.0000000	11.7500000	-50.0000000
42410.0000000	12.0000000	-50.0000000
42410.0000000	12.2500000	-50.0000000
42410.0000000	12.5000000	-50.0000000
42410.0000000	12.7500000	-50.0000000
42410.0000000	13.0000000	-50.0000000
42410.0000000	13.2500000	-50.0000000
42410.0000000	13.5000000	-50.0000000
42410.0000000	13.7500000	-50.0000000
42410.0000000	14.0000000	-50.0000000

42410.0000000	14.2500000	-50.0000000
42410.0000000	14.5000000	-50.0000000
42410.0000000	14.7500000	-50.0000000
42410.0000000	15.0000000	-50.0000000
42440.0000000	0.0000000	-50.0000000
42440.0000000	0.2500000	-50.0000000
42440.0000000	0.5000000	-50.0000000
42440.0000000	0.7500000	-50.0000000
42440.0000000	1.0000000	-50.0000000
42440.0000000	1.2500000	-50.0000000
42440.0000000	1.5000000	-50.0000000
42440.0000000	1.7500000	-50.0000000
42440.0000000	2.0000000	-50.0000000
42440.0000000	2.2500000	-50.0000000
42440.0000000	2.5000000	-50.0000000
42440.0000000	2.7500000	-50.0000000
42440.0000000	3.0000000	-50.0000000
42440.0000000	3.2500000	-50.0000000
42440.0000000	3.5000000	-50.0000000
42440.0000000	3.7500000	-50.0000000
42440.0000000	4.0000000	-50.0000000
42440.0000000	4.2500000	-50.0000000
42440.0000000	4.5000000	-50.0000000
42440.0000000	4.7500000	-50.0000000
42440.0000000	5.0000000	-50.0000000
42440.0000000	5.2500000	-50.0000000
42440.0000000	5.5000000	-50.0000000
42440.0000000	5.7500000	-50.0000000
42440.0000000	6.0000000	-50.0000000
42440.0000000	6.2500000	-50.0000000
42440.0000000	6.5000000	-50.0000000
42440.0000000	6.7500000	-50.0000000
42440.0000000	7.0000000	-50.0000000
42440.0000000	7.2500000	-50.0000000
42440.0000000	7.5000000	-50.0000000
42440.0000000	7.7500000	-50.0000000
42440.0000000	8.0000000	-50.0000000
42440.0000000	8.2500000	-50.0000000
42440.0000000	8.5000000	-50.0000000
42440.0000000	8.7500000	-50.0000000
42440.0000000	9.0000000	-50.0000000
42440.0000000	9.2500000	-50.0000000
42440.0000000	9.5000000	-50.0000000
42440.0000000	9.7500000	-50.0000000
42440.0000000	10.0000000	-50.0000000
42440.0000000	10.2500000	-50.0000000
42440.0000000	10.5000000	-50.0000000
42440.0000000	10.7500000	-50.0000000
42440.0000000	11.0000000	-50.0000000
42440.0000000	11.2500000	-50.0000000
42440.0000000	11.5000000	-50.0000000
42440.0000000	11.7500000	-50.0000000
42440.0000000	12.0000000	-50.0000000
42440.0000000	12.2500000	-50.0000000
42440.0000000	12.5000000	-50.0000000
42440.0000000	12.7500000	-50.0000000
42440.0000000	13.0000000	-50.0000000
42440.0000000	13.2500000	-50.0000000

42440.0000000	13.5000000	-50.0000000
42440.0000000	13.7500000	-50.0000000
42440.0000000	14.0000000	-50.0000000
42440.0000000	14.2500000	-50.0000000
42440.0000000	14.5000000	-50.0000000
42440.0000000	14.7500000	-50.0000000
42440.0000000	15.0000000	-50.0000000
42470.0000000	0.0000000	-50.0000000
42470.0000000	0.2500000	-50.0000000
42470.0000000	0.5000000	-50.0000000
42470.0000000	0.7500000	-50.0000000
42470.0000000	1.0000000	-50.0000000
42470.0000000	1.2500000	-50.0000000
42470.0000000	1.5000000	-50.0000000
42470.0000000	1.7500000	-50.0000000
42470.0000000	2.0000000	-50.0000000
42470.0000000	2.2500000	-50.0000000
42470.0000000	2.5000000	-50.0000000
42470.0000000	2.7500000	-50.0000000
42470.0000000	3.0000000	-50.0000000
42470.0000000	3.2500000	-50.0000000
42470.0000000	3.5000000	-50.0000000
42470.0000000	3.7500000	-50.0000000
42470.0000000	4.0000000	-50.0000000
42470.0000000	4.2500000	-50.0000000
42470.0000000	4.5000000	-50.0000000
42470.0000000	4.7500000	-50.0000000
42470.0000000	5.0000000	-50.0000000
42470.0000000	5.2500000	-50.0000000
42470.0000000	5.5000000	-50.0000000
42470.0000000	5.7500000	-50.0000000
42470.0000000	6.0000000	-50.0000000
42470.0000000	6.2500000	-50.0000000
42470.0000000	6.5000000	-50.0000000
42470.0000000	6.7500000	-50.0000000
42470.0000000	7.0000000	-50.0000000
42470.0000000	7.2500000	-50.0000000
42470.0000000	7.5000000	-50.0000000
42470.0000000	7.7500000	-50.0000000
42470.0000000	8.0000000	-50.0000000
42470.0000000	8.2500000	-50.0000000
42470.0000000	8.5000000	-50.0000000
42470.0000000	8.7500000	-50.0000000
42470.0000000	9.0000000	-50.0000000
42470.0000000	9.2500000	-50.0000000
42470.0000000	9.5000000	-50.0000000
42470.0000000	9.7500000	-50.0000000
42470.0000000	10.0000000	-50.0000000
42470.0000000	10.2500000	-50.0000000
42470.0000000	10.5000000	-50.0000000
42470.0000000	10.7500000	-50.0000000
42470.0000000	11.0000000	-50.0000000
42470.0000000	11.2500000	-50.0000000
42470.0000000	11.5000000	-50.0000000
42470.0000000	11.7500000	-50.0000000
42470.0000000	12.0000000	-50.0000000
42470.0000000	12.2500000	-50.0000000
42470.0000000	12.5000000	-50.0000000

42470.0000000	12.7500000	-50.0000000
42470.0000000	13.0000000	-50.0000000
42470.0000000	13.2500000	-50.0000000
42470.0000000	13.5000000	-50.0000000
42470.0000000	13.7500000	-50.0000000
42470.0000000	14.0000000	-50.0000000
42470.0000000	14.2500000	-50.0000000
42470.0000000	14.5000000	-50.0000000
42470.0000000	14.7500000	-50.0000000
42470.0000000	15.0000000	-50.0000000
42500.0000000	0.0000000	-50.0000000
42500.0000000	0.2500000	-50.0000000
42500.0000000	0.5000000	-50.0000000
42500.0000000	0.7500000	-50.0000000
42500.0000000	1.0000000	-50.0000000
42500.0000000	1.2500000	-50.0000000
42500.0000000	1.5000000	-50.0000000
42500.0000000	1.7500000	-50.0000000
42500.0000000	2.0000000	-50.0000000
42500.0000000	2.2500000	-50.0000000
42500.0000000	2.5000000	-50.0000000
42500.0000000	2.7500000	-50.0000000
42500.0000000	3.0000000	-50.0000000
42500.0000000	3.2500000	-50.0000000
42500.0000000	3.5000000	-50.0000000
42500.0000000	3.7500000	-50.0000000
42500.0000000	4.0000000	-50.0000000
42500.0000000	4.2500000	-50.0000000
42500.0000000	4.5000000	-50.0000000
42500.0000000	4.7500000	-50.0000000
42500.0000000	5.0000000	-50.0000000
42500.0000000	5.2500000	-50.0000000
42500.0000000	5.5000000	-50.0000000
42500.0000000	5.7500000	-50.0000000
42500.0000000	6.0000000	-50.0000000
42500.0000000	6.2500000	-50.0000000
42500.0000000	6.5000000	-50.0000000
42500.0000000	6.7500000	-50.0000000
42500.0000000	7.0000000	-50.0000000
42500.0000000	7.2500000	-50.0000000
42500.0000000	7.5000000	-50.0000000
42500.0000000	7.7500000	-50.0000000
42500.0000000	8.0000000	-50.0000000
42500.0000000	8.2500000	-50.0000000
42500.0000000	8.5000000	-50.0000000
42500.0000000	8.7500000	-50.0000000
42500.0000000	9.0000000	-50.0000000
42500.0000000	9.2500000	-50.0000000
42500.0000000	9.5000000	-50.0000000
42500.0000000	9.7500000	-50.0000000
42500.0000000	10.0000000	-50.0000000
42500.0000000	10.2500000	-50.0000000
42500.0000000	10.5000000	-50.0000000
42500.0000000	10.7500000	-50.0000000
42500.0000000	11.0000000	-50.0000000
42500.0000000	11.2500000	-50.0000000
42500.0000000	11.5000000	-50.0000000
42500.0000000	11.7500000	-50.0000000

42500.0000000	12.0000000	-50.0000000
42500.0000000	12.2500000	-50.0000000
42500.0000000	12.5000000	-50.0000000
42500.0000000	12.7500000	-50.0000000
42500.0000000	13.0000000	-50.0000000
42500.0000000	13.2500000	-50.0000000
42500.0000000	13.5000000	-50.0000000
42500.0000000	13.7500000	-50.0000000
42500.0000000	14.0000000	-50.0000000
42500.0000000	14.2500000	-50.0000000
42500.0000000	14.5000000	-50.0000000
42500.0000000	14.7500000	-50.0000000
42500.0000000	15.0000000	-50.0000000
42530.0000000	0.0000000	-50.0000000
42530.0000000	0.2500000	-50.0000000
42530.0000000	0.5000000	-50.0000000
42530.0000000	0.7500000	-50.0000000
42530.0000000	1.0000000	-50.0000000
42530.0000000	1.2500000	-50.0000000
42530.0000000	1.5000000	-50.0000000
42530.0000000	1.7500000	-50.0000000
42530.0000000	2.0000000	-50.0000000
42530.0000000	2.2500000	-50.0000000
42530.0000000	2.5000000	-50.0000000
42530.0000000	2.7500000	-50.0000000
42530.0000000	3.0000000	-50.0000000
42530.0000000	3.2500000	-50.0000000
42530.0000000	3.5000000	-50.0000000
42530.0000000	3.7500000	-50.0000000
42530.0000000	4.0000000	-50.0000000
42530.0000000	4.2500000	-50.0000000
42530.0000000	4.5000000	-50.0000000
42530.0000000	4.7500000	-50.0000000
42530.0000000	5.0000000	-50.0000000
42530.0000000	5.2500000	-50.0000000
42530.0000000	5.5000000	-50.0000000
42530.0000000	5.7500000	-50.0000000
42530.0000000	6.0000000	-50.0000000
42530.0000000	6.2500000	-50.0000000
42530.0000000	6.5000000	-50.0000000
42530.0000000	6.7500000	-50.0000000
42530.0000000	7.0000000	-50.0000000
42530.0000000	7.2500000	-50.0000000
42530.0000000	7.5000000	-50.0000000
42530.0000000	7.7500000	-50.0000000
42530.0000000	8.0000000	-50.0000000
42530.0000000	8.2500000	-50.0000000
42530.0000000	8.5000000	-50.0000000
42530.0000000	8.7500000	-50.0000000
42530.0000000	9.0000000	-50.0000000
42530.0000000	9.2500000	-50.0000000
42530.0000000	9.5000000	-50.0000000
42530.0000000	9.7500000	-50.0000000
42530.0000000	10.0000000	-50.0000000
42530.0000000	10.2500000	-50.0000000
42530.0000000	10.5000000	-50.0000000
42530.0000000	10.7500000	-50.0000000
42530.0000000	11.0000000	-50.0000000

42530.0000000	11.2500000	-50.0000000
42530.0000000	11.5000000	-50.0000000
42530.0000000	11.7500000	-50.0000000
42530.0000000	12.0000000	-50.0000000
42530.0000000	12.2500000	-50.0000000
42530.0000000	12.5000000	-50.0000000
42530.0000000	12.7500000	-50.0000000
42530.0000000	13.0000000	-50.0000000
42530.0000000	13.2500000	-50.0000000
42530.0000000	13.5000000	-50.0000000
42530.0000000	13.7500000	-50.0000000
42530.0000000	14.0000000	-50.0000000
42530.0000000	14.2500000	-50.0000000
42530.0000000	14.5000000	-50.0000000
42530.0000000	14.7500000	-50.0000000
42530.0000000	15.0000000	-50.0000000
42560.0000000	0.0000000	-50.0000000
42560.0000000	0.2500000	-50.0000000
42560.0000000	0.5000000	-50.0000000
42560.0000000	0.7500000	-50.0000000
42560.0000000	1.0000000	-50.0000000
42560.0000000	1.2500000	-50.0000000
42560.0000000	1.5000000	-50.0000000
42560.0000000	1.7500000	-50.0000000
42560.0000000	2.0000000	-50.0000000
42560.0000000	2.2500000	-50.0000000
42560.0000000	2.5000000	-50.0000000
42560.0000000	2.7500000	-50.0000000
42560.0000000	3.0000000	-50.0000000
42560.0000000	3.2500000	-50.0000000
42560.0000000	3.5000000	-50.0000000
42560.0000000	3.7500000	-50.0000000
42560.0000000	4.0000000	-50.0000000
42560.0000000	4.2500000	-50.0000000
42560.0000000	4.5000000	-50.0000000
42560.0000000	4.7500000	-50.0000000
42560.0000000	5.0000000	-50.0000000
42560.0000000	5.2500000	-50.0000000
42560.0000000	5.5000000	-50.0000000
42560.0000000	5.7500000	-50.0000000
42560.0000000	6.0000000	-50.0000000
42560.0000000	6.2500000	-50.0000000
42560.0000000	6.5000000	-50.0000000
42560.0000000	6.7500000	-50.0000000
42560.0000000	7.0000000	-50.0000000
42560.0000000	7.2500000	-50.0000000
42560.0000000	7.5000000	-50.0000000
42560.0000000	7.7500000	-50.0000000
42560.0000000	8.0000000	-50.0000000
42560.0000000	8.2500000	-50.0000000
42560.0000000	8.5000000	-50.0000000
42560.0000000	8.7500000	-50.0000000
42560.0000000	9.0000000	-50.0000000
42560.0000000	9.2500000	-50.0000000
42560.0000000	9.5000000	-50.0000000
42560.0000000	9.7500000	-50.0000000
42560.0000000	10.0000000	-50.0000000
42560.0000000	10.2500000	-50.0000000

42560.0000000	10.5000000	-50.0000000
42560.0000000	10.7500000	-50.0000000
42560.0000000	11.0000000	-50.0000000
42560.0000000	11.2500000	-50.0000000
42560.0000000	11.5000000	-50.0000000
42560.0000000	11.7500000	-50.0000000
42560.0000000	12.0000000	-50.0000000
42560.0000000	12.2500000	-50.0000000
42560.0000000	12.5000000	-50.0000000
42560.0000000	12.7500000	-50.0000000
42560.0000000	13.0000000	-50.0000000
42560.0000000	13.2500000	-50.0000000
42560.0000000	13.5000000	-50.0000000
42560.0000000	13.7500000	-50.0000000
42560.0000000	14.0000000	-50.0000000
42560.0000000	14.2500000	-50.0000000
42560.0000000	14.5000000	-50.0000000
42560.0000000	14.7500000	-50.0000000
42560.0000000	15.0000000	-50.0000000
42590.0000000	0.0000000	-50.0000000
42590.0000000	0.2500000	-50.0000000
42590.0000000	0.5000000	-50.0000000
42590.0000000	0.7500000	-50.0000000
42590.0000000	1.0000000	-50.0000000
42590.0000000	1.2500000	-50.0000000
42590.0000000	1.5000000	-50.0000000
42590.0000000	1.7500000	-50.0000000
42590.0000000	2.0000000	-50.0000000
42590.0000000	2.2500000	-50.0000000
42590.0000000	2.5000000	-50.0000000
42590.0000000	2.7500000	-50.0000000
42590.0000000	3.0000000	-50.0000000
42590.0000000	3.2500000	-50.0000000
42590.0000000	3.5000000	-50.0000000
42590.0000000	3.7500000	-50.0000000
42590.0000000	4.0000000	-50.0000000
42590.0000000	4.2500000	-50.0000000
42590.0000000	4.5000000	-50.0000000
42590.0000000	4.7500000	-50.0000000
42590.0000000	5.0000000	-50.0000000
42590.0000000	5.2500000	-50.0000000
42590.0000000	5.5000000	-50.0000000
42590.0000000	5.7500000	-50.0000000
42590.0000000	6.0000000	-50.0000000
42590.0000000	6.2500000	-50.0000000
42590.0000000	6.5000000	-50.0000000
42590.0000000	6.7500000	-50.0000000
42590.0000000	7.0000000	-50.0000000
42590.0000000	7.2500000	-50.0000000
42590.0000000	7.5000000	-50.0000000
42590.0000000	7.7500000	-50.0000000
42590.0000000	8.0000000	-50.0000000
42590.0000000	8.2500000	-50.0000000
42590.0000000	8.5000000	-50.0000000
42590.0000000	8.7500000	-50.0000000
42590.0000000	9.0000000	-50.0000000
42590.0000000	9.2500000	-50.0000000
42590.0000000	9.5000000	-50.0000000

42590.0000000	9.7500000	-50.0000000
42590.0000000	10.0000000	-50.0000000
42590.0000000	10.2500000	-50.0000000
42590.0000000	10.5000000	-50.0000000
42590.0000000	10.7500000	-50.0000000
42590.0000000	11.0000000	-50.0000000
42590.0000000	11.2500000	-50.0000000
42590.0000000	11.5000000	-50.0000000
42590.0000000	11.7500000	-50.0000000
42590.0000000	12.0000000	-50.0000000
42590.0000000	12.2500000	-50.0000000
42590.0000000	12.5000000	-50.0000000
42590.0000000	12.7500000	-50.0000000
42590.0000000	13.0000000	-50.0000000
42590.0000000	13.2500000	-50.0000000
42590.0000000	13.5000000	-50.0000000
42590.0000000	13.7500000	-50.0000000
42590.0000000	14.0000000	-50.0000000
42590.0000000	14.2500000	-50.0000000
42590.0000000	14.5000000	-50.0000000
42590.0000000	14.7500000	-50.0000000
42590.0000000	15.0000000	-50.0000000
42620.0000000	0.0000000	-50.0000000
42620.0000000	0.2500000	-50.0000000
42620.0000000	0.5000000	-50.0000000
42620.0000000	0.7500000	-50.0000000
42620.0000000	1.0000000	-50.0000000
42620.0000000	1.2500000	-50.0000000
42620.0000000	1.5000000	-50.0000000
42620.0000000	1.7500000	-50.0000000
42620.0000000	2.0000000	-50.0000000
42620.0000000	2.2500000	-50.0000000
42620.0000000	2.5000000	-50.0000000
42620.0000000	2.7500000	-50.0000000
42620.0000000	3.0000000	-50.0000000
42620.0000000	3.2500000	-50.0000000
42620.0000000	3.5000000	-50.0000000
42620.0000000	3.7500000	-50.0000000
42620.0000000	4.0000000	-50.0000000
42620.0000000	4.2500000	-50.0000000
42620.0000000	4.5000000	-50.0000000
42620.0000000	4.7500000	-50.0000000
42620.0000000	5.0000000	-50.0000000
42620.0000000	5.2500000	-50.0000000
42620.0000000	5.5000000	-50.0000000
42620.0000000	5.7500000	-50.0000000
42620.0000000	6.0000000	-50.0000000
42620.0000000	6.2500000	-50.0000000
42620.0000000	6.5000000	-50.0000000
42620.0000000	6.7500000	-50.0000000
42620.0000000	7.0000000	-50.0000000
42620.0000000	7.2500000	-50.0000000
42620.0000000	7.5000000	-50.0000000
42620.0000000	7.7500000	-50.0000000
42620.0000000	8.0000000	-50.0000000
42620.0000000	8.2500000	-50.0000000
42620.0000000	8.5000000	-50.0000000
42620.0000000	8.7500000	-50.0000000

42620.0000000	9.0000000	-50.0000000
42620.0000000	9.2500000	-50.0000000
42620.0000000	9.5000000	-50.0000000
42620.0000000	9.7500000	-50.0000000
42620.0000000	10.0000000	-50.0000000
42620.0000000	10.2500000	-50.0000000
42620.0000000	10.5000000	-50.0000000
42620.0000000	10.7500000	-50.0000000
42620.0000000	11.0000000	-50.0000000
42620.0000000	11.2500000	-50.0000000
42620.0000000	11.5000000	-50.0000000
42620.0000000	11.7500000	-50.0000000
42620.0000000	12.0000000	-50.0000000
42620.0000000	12.2500000	-50.0000000
42620.0000000	12.5000000	-50.0000000
42620.0000000	12.7500000	-50.0000000
42620.0000000	13.0000000	-50.0000000
42620.0000000	13.2500000	-50.0000000
42620.0000000	13.5000000	-50.0000000
42620.0000000	13.7500000	-50.0000000
42620.0000000	14.0000000	-50.0000000
42620.0000000	14.2500000	-50.0000000
42620.0000000	14.5000000	-50.0000000
42620.0000000	14.7500000	-50.0000000
42620.0000000	15.0000000	-50.0000000
42650.0000000	0.0000000	-50.0000000
42650.0000000	0.2500000	-50.0000000
42650.0000000	0.5000000	-50.0000000
42650.0000000	0.7500000	-50.0000000
42650.0000000	1.0000000	-50.0000000
42650.0000000	1.2500000	-50.0000000
42650.0000000	1.5000000	-50.0000000
42650.0000000	1.7500000	-50.0000000
42650.0000000	2.0000000	-50.0000000
42650.0000000	2.2500000	-50.0000000
42650.0000000	2.5000000	-50.0000000
42650.0000000	2.7500000	-50.0000000
42650.0000000	3.0000000	-50.0000000
42650.0000000	3.2500000	-50.0000000
42650.0000000	3.5000000	-50.0000000
42650.0000000	3.7500000	-50.0000000
42650.0000000	4.0000000	-50.0000000
42650.0000000	4.2500000	-50.0000000
42650.0000000	4.5000000	-50.0000000
42650.0000000	4.7500000	-50.0000000
42650.0000000	5.0000000	-50.0000000
42650.0000000	5.2500000	-50.0000000
42650.0000000	5.5000000	-50.0000000
42650.0000000	5.7500000	-50.0000000
42650.0000000	6.0000000	-50.0000000
42650.0000000	6.2500000	-50.0000000
42650.0000000	6.5000000	-50.0000000
42650.0000000	6.7500000	-50.0000000
42650.0000000	7.0000000	-50.0000000
42650.0000000	7.2500000	-50.0000000
42650.0000000	7.5000000	-50.0000000
42650.0000000	7.7500000	-50.0000000
42650.0000000	8.0000000	-50.0000000

42650.0000000	8.2500000	-50.0000000
42650.0000000	8.5000000	-50.0000000
42650.0000000	8.7500000	-50.0000000
42650.0000000	9.0000000	-50.0000000
42650.0000000	9.2500000	-50.0000000
42650.0000000	9.5000000	-50.0000000
42650.0000000	9.7500000	-50.0000000
42650.0000000	10.0000000	-50.0000000
42650.0000000	10.2500000	-50.0000000
42650.0000000	10.5000000	-50.0000000
42650.0000000	10.7500000	-50.0000000
42650.0000000	11.0000000	-50.0000000
42650.0000000	11.2500000	-50.0000000
42650.0000000	11.5000000	-50.0000000
42650.0000000	11.7500000	-50.0000000
42650.0000000	12.0000000	-50.0000000
42650.0000000	12.2500000	-50.0000000
42650.0000000	12.5000000	-50.0000000
42650.0000000	12.7500000	-50.0000000
42650.0000000	13.0000000	-50.0000000
42650.0000000	13.2500000	-50.0000000
42650.0000000	13.5000000	-50.0000000
42650.0000000	13.7500000	-50.0000000
42650.0000000	14.0000000	-50.0000000
42650.0000000	14.2500000	-50.0000000
42650.0000000	14.5000000	-50.0000000
42650.0000000	14.7500000	-50.0000000
42650.0000000	15.0000000	-50.0000000
42680.0000000	0.0000000	-50.0000000
42680.0000000	0.2500000	-50.0000000
42680.0000000	0.5000000	-50.0000000
42680.0000000	0.7500000	-50.0000000
42680.0000000	1.0000000	-50.0000000
42680.0000000	1.2500000	-50.0000000
42680.0000000	1.5000000	-50.0000000
42680.0000000	1.7500000	-50.0000000
42680.0000000	2.0000000	-50.0000000
42680.0000000	2.2500000	-50.0000000
42680.0000000	2.5000000	-50.0000000
42680.0000000	2.7500000	-50.0000000
42680.0000000	3.0000000	-50.0000000
42680.0000000	3.2500000	-50.0000000
42680.0000000	3.5000000	-50.0000000
42680.0000000	3.7500000	-50.0000000
42680.0000000	4.0000000	-50.0000000
42680.0000000	4.2500000	-50.0000000
42680.0000000	4.5000000	-50.0000000
42680.0000000	4.7500000	-50.0000000
42680.0000000	5.0000000	-50.0000000
42680.0000000	5.2500000	-50.0000000
42680.0000000	5.5000000	-50.0000000
42680.0000000	5.7500000	-50.0000000
42680.0000000	6.0000000	-50.0000000
42680.0000000	6.2500000	-50.0000000
42680.0000000	6.5000000	-50.0000000
42680.0000000	6.7500000	-50.0000000
42680.0000000	7.0000000	-50.0000000
42680.0000000	7.2500000	-50.0000000

42680.0000000	7.5000000	-50.0000000
42680.0000000	7.7500000	-50.0000000
42680.0000000	8.0000000	-50.0000000
42680.0000000	8.2500000	-50.0000000
42680.0000000	8.5000000	-50.0000000
42680.0000000	8.7500000	-50.0000000
42680.0000000	9.0000000	-50.0000000
42680.0000000	9.2500000	-50.0000000
42680.0000000	9.5000000	-50.0000000
42680.0000000	9.7500000	-50.0000000
42680.0000000	10.0000000	-50.0000000
42680.0000000	10.2500000	-50.0000000
42680.0000000	10.5000000	-50.0000000
42680.0000000	10.7500000	-50.0000000
42680.0000000	11.0000000	-50.0000000
42680.0000000	11.2500000	-50.0000000
42680.0000000	11.5000000	-50.0000000
42680.0000000	11.7500000	-50.0000000
42680.0000000	12.0000000	-50.0000000
42680.0000000	12.2500000	-50.0000000
42680.0000000	12.5000000	-50.0000000
42680.0000000	12.7500000	-50.0000000
42680.0000000	13.0000000	-50.0000000
42680.0000000	13.2500000	-50.0000000
42680.0000000	13.5000000	-50.0000000
42680.0000000	13.7500000	-50.0000000
42680.0000000	14.0000000	-50.0000000
42680.0000000	14.2500000	-50.0000000
42680.0000000	14.5000000	-50.0000000
42680.0000000	14.7500000	-50.0000000
42680.0000000	15.0000000	-50.0000000
42710.0000000	0.0000000	-50.0000000
42710.0000000	0.2500000	-50.0000000
42710.0000000	0.5000000	-50.0000000
42710.0000000	0.7500000	-50.0000000
42710.0000000	1.0000000	-50.0000000
42710.0000000	1.2500000	-50.0000000
42710.0000000	1.5000000	-50.0000000
42710.0000000	1.7500000	-50.0000000
42710.0000000	2.0000000	-50.0000000
42710.0000000	2.2500000	-50.0000000
42710.0000000	2.5000000	-50.0000000
42710.0000000	2.7500000	-50.0000000
42710.0000000	3.0000000	-50.0000000
42710.0000000	3.2500000	-50.0000000
42710.0000000	3.5000000	-50.0000000
42710.0000000	3.7500000	-50.0000000
42710.0000000	4.0000000	-50.0000000
42710.0000000	4.2500000	-50.0000000
42710.0000000	4.5000000	-50.0000000
42710.0000000	4.7500000	-50.0000000
42710.0000000	5.0000000	-50.0000000
42710.0000000	5.2500000	-50.0000000
42710.0000000	5.5000000	-50.0000000
42710.0000000	5.7500000	-50.0000000
42710.0000000	6.0000000	-50.0000000
42710.0000000	6.2500000	-50.0000000
42710.0000000	6.5000000	-50.0000000

42710.0000000	6.7500000	-50.0000000
42710.0000000	7.0000000	-50.0000000
42710.0000000	7.2500000	-50.0000000
42710.0000000	7.5000000	-50.0000000
42710.0000000	7.7500000	-50.0000000
42710.0000000	8.0000000	-50.0000000
42710.0000000	8.2500000	-50.0000000
42710.0000000	8.5000000	-50.0000000
42710.0000000	8.7500000	-50.0000000
42710.0000000	9.0000000	-50.0000000
42710.0000000	9.2500000	-50.0000000
42710.0000000	9.5000000	-50.0000000
42710.0000000	9.7500000	-50.0000000
42710.0000000	10.0000000	-50.0000000
42710.0000000	10.2500000	-50.0000000
42710.0000000	10.5000000	-50.0000000
42710.0000000	10.7500000	-50.0000000
42710.0000000	11.0000000	-50.0000000
42710.0000000	11.2500000	-50.0000000
42710.0000000	11.5000000	-50.0000000
42710.0000000	11.7500000	-50.0000000
42710.0000000	12.0000000	-50.0000000
42710.0000000	12.2500000	-50.0000000
42710.0000000	12.5000000	-50.0000000
42710.0000000	12.7500000	-50.0000000
42710.0000000	13.0000000	-50.0000000
42710.0000000	13.2500000	-50.0000000
42710.0000000	13.5000000	-50.0000000
42710.0000000	13.7500000	-50.0000000
42710.0000000	14.0000000	-50.0000000
42710.0000000	14.2500000	-50.0000000
42710.0000000	14.5000000	-50.0000000
42710.0000000	14.7500000	-50.0000000
42710.0000000	15.0000000	-50.0000000
42740.0000000	0.0000000	-50.0000000
42740.0000000	0.2500000	-50.0000000
42740.0000000	0.5000000	-50.0000000
42740.0000000	0.7500000	-50.0000000
42740.0000000	1.0000000	-50.0000000
42740.0000000	1.2500000	-50.0000000
42740.0000000	1.5000000	-50.0000000
42740.0000000	1.7500000	-50.0000000
42740.0000000	2.0000000	-50.0000000
42740.0000000	2.2500000	-50.0000000
42740.0000000	2.5000000	-50.0000000
42740.0000000	2.7500000	-50.0000000
42740.0000000	3.0000000	-50.0000000
42740.0000000	3.2500000	-50.0000000
42740.0000000	3.5000000	-50.0000000
42740.0000000	3.7500000	-50.0000000
42740.0000000	4.0000000	-50.0000000
42740.0000000	4.2500000	-50.0000000
42740.0000000	4.5000000	-50.0000000
42740.0000000	4.7500000	-50.0000000
42740.0000000	5.0000000	-50.0000000
42740.0000000	5.2500000	-50.0000000
42740.0000000	5.5000000	-50.0000000
42740.0000000	5.7500000	-50.0000000

42740.0000000	6.0000000	-50.0000000
42740.0000000	6.2500000	-50.0000000
42740.0000000	6.5000000	-50.0000000
42740.0000000	6.7500000	-50.0000000
42740.0000000	7.0000000	-50.0000000
42740.0000000	7.2500000	-50.0000000
42740.0000000	7.5000000	-50.0000000
42740.0000000	7.7500000	-50.0000000
42740.0000000	8.0000000	-50.0000000
42740.0000000	8.2500000	-50.0000000
42740.0000000	8.5000000	-50.0000000
42740.0000000	8.7500000	-50.0000000
42740.0000000	9.0000000	-50.0000000
42740.0000000	9.2500000	-50.0000000
42740.0000000	9.5000000	-50.0000000
42740.0000000	9.7500000	-50.0000000
42740.0000000	10.0000000	-50.0000000
42740.0000000	10.2500000	-50.0000000
42740.0000000	10.5000000	-50.0000000
42740.0000000	10.7500000	-50.0000000
42740.0000000	11.0000000	-50.0000000
42740.0000000	11.2500000	-50.0000000
42740.0000000	11.5000000	-50.0000000
42740.0000000	11.7500000	-50.0000000
42740.0000000	12.0000000	-50.0000000
42740.0000000	12.2500000	-50.0000000
42740.0000000	12.5000000	-50.0000000
42740.0000000	12.7500000	-50.0000000
42740.0000000	13.0000000	-50.0000000
42740.0000000	13.2500000	-50.0000000
42740.0000000	13.5000000	-50.0000000
42740.0000000	13.7500000	-50.0000000
42740.0000000	14.0000000	-50.0000000
42740.0000000	14.2500000	-50.0000000
42740.0000000	14.5000000	-50.0000000
42740.0000000	14.7500000	-50.0000000
42740.0000000	15.0000000	-50.0000000
42770.0000000	0.0000000	-50.0000000
42770.0000000	0.2500000	-50.0000000
42770.0000000	0.5000000	-50.0000000
42770.0000000	0.7500000	-50.0000000
42770.0000000	1.0000000	-50.0000000
42770.0000000	1.2500000	-50.0000000
42770.0000000	1.5000000	-50.0000000
42770.0000000	1.7500000	-50.0000000
42770.0000000	2.0000000	-50.0000000
42770.0000000	2.2500000	-50.0000000
42770.0000000	2.5000000	-50.0000000
42770.0000000	2.7500000	-50.0000000
42770.0000000	3.0000000	-50.0000000
42770.0000000	3.2500000	-50.0000000
42770.0000000	3.5000000	-50.0000000
42770.0000000	3.7500000	-50.0000000
42770.0000000	4.0000000	-50.0000000
42770.0000000	4.2500000	-50.0000000
42770.0000000	4.5000000	-50.0000000
42770.0000000	4.7500000	-50.0000000
42770.0000000	5.0000000	-50.0000000

42770.0000000	5.2500000	-50.0000000
42770.0000000	5.5000000	-50.0000000
42770.0000000	5.7500000	-50.0000000
42770.0000000	6.0000000	-50.0000000
42770.0000000	6.2500000	-50.0000000
42770.0000000	6.5000000	-50.0000000
42770.0000000	6.7500000	-50.0000000
42770.0000000	7.0000000	-50.0000000
42770.0000000	7.2500000	-50.0000000
42770.0000000	7.5000000	-50.0000000
42770.0000000	7.7500000	-50.0000000
42770.0000000	8.0000000	-50.0000000
42770.0000000	8.2500000	-50.0000000
42770.0000000	8.5000000	-50.0000000
42770.0000000	8.7500000	-50.0000000
42770.0000000	9.0000000	-50.0000000
42770.0000000	9.2500000	-50.0000000
42770.0000000	9.5000000	-50.0000000
42770.0000000	9.7500000	-50.0000000
42770.0000000	10.0000000	-50.0000000
42770.0000000	10.2500000	-50.0000000
42770.0000000	10.5000000	-50.0000000
42770.0000000	10.7500000	-50.0000000
42770.0000000	11.0000000	-50.0000000
42770.0000000	11.2500000	-50.0000000
42770.0000000	11.5000000	-50.0000000
42770.0000000	11.7500000	-50.0000000
42770.0000000	12.0000000	-50.0000000
42770.0000000	12.2500000	-50.0000000
42770.0000000	12.5000000	-50.0000000
42770.0000000	12.7500000	-50.0000000
42770.0000000	13.0000000	-50.0000000
42770.0000000	13.2500000	-50.0000000
42770.0000000	13.5000000	-50.0000000
42770.0000000	13.7500000	-50.0000000
42770.0000000	14.0000000	-50.0000000
42770.0000000	14.2500000	-50.0000000
42770.0000000	14.5000000	-50.0000000
42770.0000000	14.7500000	-50.0000000
42770.0000000	15.0000000	-50.0000000
42800.0000000	0.0000000	-50.0000000
42800.0000000	0.2500000	-50.0000000
42800.0000000	0.5000000	-50.0000000
42800.0000000	0.7500000	-50.0000000
42800.0000000	1.0000000	-50.0000000
42800.0000000	1.2500000	-50.0000000
42800.0000000	1.5000000	-50.0000000
42800.0000000	1.7500000	-50.0000000
42800.0000000	2.0000000	-50.0000000
42800.0000000	2.2500000	-50.0000000
42800.0000000	2.5000000	-50.0000000
42800.0000000	2.7500000	-50.0000000
42800.0000000	3.0000000	-50.0000000
42800.0000000	3.2500000	-50.0000000
42800.0000000	3.5000000	-50.0000000
42800.0000000	3.7500000	-50.0000000
42800.0000000	4.0000000	-50.0000000
42800.0000000	4.2500000	-50.0000000

42800.0000000	4.5000000	-50.0000000
42800.0000000	4.7500000	-50.0000000
42800.0000000	5.0000000	-50.0000000
42800.0000000	5.2500000	-50.0000000
42800.0000000	5.5000000	-50.0000000
42800.0000000	5.7500000	-50.0000000
42800.0000000	6.0000000	-50.0000000
42800.0000000	6.2500000	-50.0000000
42800.0000000	6.5000000	-50.0000000
42800.0000000	6.7500000	-50.0000000
42800.0000000	7.0000000	-50.0000000
42800.0000000	7.2500000	-50.0000000
42800.0000000	7.5000000	-50.0000000
42800.0000000	7.7500000	-50.0000000
42800.0000000	8.0000000	-50.0000000
42800.0000000	8.2500000	-50.0000000
42800.0000000	8.5000000	-50.0000000
42800.0000000	8.7500000	-50.0000000
42800.0000000	9.0000000	-50.0000000
42800.0000000	9.2500000	-50.0000000
42800.0000000	9.5000000	-50.0000000
42800.0000000	9.7500000	-50.0000000
42800.0000000	10.0000000	-50.0000000
42800.0000000	10.2500000	-50.0000000
42800.0000000	10.5000000	-50.0000000
42800.0000000	10.7500000	-50.0000000
42800.0000000	11.0000000	-50.0000000
42800.0000000	11.2500000	-50.0000000
42800.0000000	11.5000000	-50.0000000
42800.0000000	11.7500000	-50.0000000
42800.0000000	12.0000000	-50.0000000
42800.0000000	12.2500000	-50.0000000
42800.0000000	12.5000000	-50.0000000
42800.0000000	12.7500000	-50.0000000
42800.0000000	13.0000000	-50.0000000
42800.0000000	13.2500000	-50.0000000
42800.0000000	13.5000000	-50.0000000
42800.0000000	13.7500000	-50.0000000
42800.0000000	14.0000000	-50.0000000
42800.0000000	14.2500000	-50.0000000
42800.0000000	14.5000000	-50.0000000
42800.0000000	14.7500000	-50.0000000
42800.0000000	15.0000000	-50.0000000
42830.0000000	0.0000000	-50.0000000
42830.0000000	0.2500000	-50.0000000
42830.0000000	0.5000000	-50.0000000
42830.0000000	0.7500000	-50.0000000
42830.0000000	1.0000000	-50.0000000
42830.0000000	1.2500000	-50.0000000
42830.0000000	1.5000000	-50.0000000
42830.0000000	1.7500000	-50.0000000
42830.0000000	2.0000000	-50.0000000
42830.0000000	2.2500000	-50.0000000
42830.0000000	2.5000000	-50.0000000
42830.0000000	2.7500000	-50.0000000
42830.0000000	3.0000000	-50.0000000
42830.0000000	3.2500000	-50.0000000
42830.0000000	3.5000000	-50.0000000

42830.0000000	3.7500000	-50.0000000
42830.0000000	4.0000000	-50.0000000
42830.0000000	4.2500000	-50.0000000
42830.0000000	4.5000000	-50.0000000
42830.0000000	4.7500000	-50.0000000
42830.0000000	5.0000000	-50.0000000
42830.0000000	5.2500000	-50.0000000
42830.0000000	5.5000000	-50.0000000
42830.0000000	5.7500000	-50.0000000
42830.0000000	6.0000000	-50.0000000
42830.0000000	6.2500000	-50.0000000
42830.0000000	6.5000000	-50.0000000
42830.0000000	6.7500000	-50.0000000
42830.0000000	7.0000000	-50.0000000
42830.0000000	7.2500000	-50.0000000
42830.0000000	7.5000000	-50.0000000
42830.0000000	7.7500000	-50.0000000
42830.0000000	8.0000000	-50.0000000
42830.0000000	8.2500000	-50.0000000
42830.0000000	8.5000000	-50.0000000
42830.0000000	8.7500000	-50.0000000
42830.0000000	9.0000000	-50.0000000
42830.0000000	9.2500000	-50.0000000
42830.0000000	9.5000000	-50.0000000
42830.0000000	9.7500000	-50.0000000
42830.0000000	10.0000000	-50.0000000
42830.0000000	10.2500000	-50.0000000
42830.0000000	10.5000000	-50.0000000
42830.0000000	10.7500000	-50.0000000
42830.0000000	11.0000000	-50.0000000
42830.0000000	11.2500000	-50.0000000
42830.0000000	11.5000000	-50.0000000
42830.0000000	11.7500000	-50.0000000
42830.0000000	12.0000000	-50.0000000
42830.0000000	12.2500000	-50.0000000
42830.0000000	12.5000000	-50.0000000
42830.0000000	12.7500000	-50.0000000
42830.0000000	13.0000000	-50.0000000
42830.0000000	13.2500000	-50.0000000
42830.0000000	13.5000000	-50.0000000
42830.0000000	13.7500000	-50.0000000
42830.0000000	14.0000000	-50.0000000
42830.0000000	14.2500000	-50.0000000
42830.0000000	14.5000000	-50.0000000
42830.0000000	14.7500000	-50.0000000
42830.0000000	15.0000000	-50.0000000
42860.0000000	0.0000000	-50.0000000
42860.0000000	0.2500000	-50.0000000
42860.0000000	0.5000000	-50.0000000
42860.0000000	0.7500000	-50.0000000
42860.0000000	1.0000000	-50.0000000
42860.0000000	1.2500000	-50.0000000
42860.0000000	1.5000000	-50.0000000
42860.0000000	1.7500000	-50.0000000
42860.0000000	2.0000000	-50.0000000
42860.0000000	2.2500000	-50.0000000
42860.0000000	2.5000000	-50.0000000
42860.0000000	2.7500000	-50.0000000

42860.0000000	3.0000000	-50.0000000
42860.0000000	3.2500000	-50.0000000
42860.0000000	3.5000000	-50.0000000
42860.0000000	3.7500000	-50.0000000
42860.0000000	4.0000000	-50.0000000
42860.0000000	4.2500000	-50.0000000
42860.0000000	4.5000000	-50.0000000
42860.0000000	4.7500000	-50.0000000
42860.0000000	5.0000000	-50.0000000
42860.0000000	5.2500000	-50.0000000
42860.0000000	5.5000000	-50.0000000
42860.0000000	5.7500000	-50.0000000
42860.0000000	6.0000000	-50.0000000
42860.0000000	6.2500000	-50.0000000
42860.0000000	6.5000000	-50.0000000
42860.0000000	6.7500000	-50.0000000
42860.0000000	7.0000000	-50.0000000
42860.0000000	7.2500000	-50.0000000
42860.0000000	7.5000000	-50.0000000
42860.0000000	7.7500000	-50.0000000
42860.0000000	8.0000000	-50.0000000
42860.0000000	8.2500000	-50.0000000
42860.0000000	8.5000000	-50.0000000
42860.0000000	8.7500000	-50.0000000
42860.0000000	9.0000000	-50.0000000
42860.0000000	9.2500000	-50.0000000
42860.0000000	9.5000000	-50.0000000
42860.0000000	9.7500000	-50.0000000
42860.0000000	10.0000000	-50.0000000
42860.0000000	10.2500000	-50.0000000
42860.0000000	10.5000000	-50.0000000
42860.0000000	10.7500000	-50.0000000
42860.0000000	11.0000000	-50.0000000
42860.0000000	11.2500000	-50.0000000
42860.0000000	11.5000000	-50.0000000
42860.0000000	11.7500000	-50.0000000
42860.0000000	12.0000000	-50.0000000
42860.0000000	12.2500000	-50.0000000
42860.0000000	12.5000000	-50.0000000
42860.0000000	12.7500000	-50.0000000
42860.0000000	13.0000000	-50.0000000
42860.0000000	13.2500000	-50.0000000
42860.0000000	13.5000000	-50.0000000
42860.0000000	13.7500000	-50.0000000
42860.0000000	14.0000000	-50.0000000
42860.0000000	14.2500000	-50.0000000
42860.0000000	14.5000000	-50.0000000
42860.0000000	14.7500000	-50.0000000
42860.0000000	15.0000000	-50.0000000
42890.0000000	0.0000000	-50.0000000
42890.0000000	0.2500000	-50.0000000
42890.0000000	0.5000000	-50.0000000
42890.0000000	0.7500000	-50.0000000
42890.0000000	1.0000000	-50.0000000
42890.0000000	1.2500000	-50.0000000
42890.0000000	1.5000000	-50.0000000
42890.0000000	1.7500000	-50.0000000
42890.0000000	2.0000000	-50.0000000

42890.0000000	2.2500000	-50.0000000
42890.0000000	2.5000000	-50.0000000
42890.0000000	2.7500000	-50.0000000
42890.0000000	3.0000000	-50.0000000
42890.0000000	3.2500000	-50.0000000
42890.0000000	3.5000000	-50.0000000
42890.0000000	3.7500000	-50.0000000
42890.0000000	4.0000000	-50.0000000
42890.0000000	4.2500000	-50.0000000
42890.0000000	4.5000000	-50.0000000
42890.0000000	4.7500000	-50.0000000
42890.0000000	5.0000000	-50.0000000
42890.0000000	5.2500000	-50.0000000
42890.0000000	5.5000000	-50.0000000
42890.0000000	5.7500000	-50.0000000
42890.0000000	6.0000000	-50.0000000
42890.0000000	6.2500000	-50.0000000
42890.0000000	6.5000000	-50.0000000
42890.0000000	6.7500000	-50.0000000
42890.0000000	7.0000000	-50.0000000
42890.0000000	7.2500000	-50.0000000
42890.0000000	7.5000000	-50.0000000
42890.0000000	7.7500000	-50.0000000
42890.0000000	8.0000000	-50.0000000
42890.0000000	8.2500000	-50.0000000
42890.0000000	8.5000000	-50.0000000
42890.0000000	8.7500000	-50.0000000
42890.0000000	9.0000000	-50.0000000
42890.0000000	9.2500000	-50.0000000
42890.0000000	9.5000000	-50.0000000
42890.0000000	9.7500000	-50.0000000
42890.0000000	10.0000000	-50.0000000
42890.0000000	10.2500000	-50.0000000
42890.0000000	10.5000000	-50.0000000
42890.0000000	10.7500000	-50.0000000
42890.0000000	11.0000000	-50.0000000
42890.0000000	11.2500000	-50.0000000
42890.0000000	11.5000000	-50.0000000
42890.0000000	11.7500000	-50.0000000
42890.0000000	12.0000000	-50.0000000
42890.0000000	12.2500000	-50.0000000
42890.0000000	12.5000000	-50.0000000
42890.0000000	12.7500000	-50.0000000
42890.0000000	13.0000000	-50.0000000
42890.0000000	13.2500000	-50.0000000
42890.0000000	13.5000000	-50.0000000
42890.0000000	13.7500000	-50.0000000
42890.0000000	14.0000000	-50.0000000
42890.0000000	14.2500000	-50.0000000
42890.0000000	14.5000000	-50.0000000
42890.0000000	14.7500000	-50.0000000
42890.0000000	15.0000000	-50.0000000
42920.0000000	0.0000000	-50.0000000
42920.0000000	0.2500000	-50.0000000
42920.0000000	0.5000000	-50.0000000
42920.0000000	0.7500000	-50.0000000
42920.0000000	1.0000000	-50.0000000
42920.0000000	1.2500000	-50.0000000

42920.0000000	1.5000000	-50.0000000
42920.0000000	1.7500000	-50.0000000
42920.0000000	2.0000000	-50.0000000
42920.0000000	2.2500000	-50.0000000
42920.0000000	2.5000000	-50.0000000
42920.0000000	2.7500000	-50.0000000
42920.0000000	3.0000000	-50.0000000
42920.0000000	3.2500000	-50.0000000
42920.0000000	3.5000000	-50.0000000
42920.0000000	3.7500000	-50.0000000
42920.0000000	4.0000000	-50.0000000
42920.0000000	4.2500000	-50.0000000
42920.0000000	4.5000000	-50.0000000
42920.0000000	4.7500000	-50.0000000
42920.0000000	5.0000000	-50.0000000
42920.0000000	5.2500000	-50.0000000
42920.0000000	5.5000000	-50.0000000
42920.0000000	5.7500000	-50.0000000
42920.0000000	6.0000000	-50.0000000
42920.0000000	6.2500000	-50.0000000
42920.0000000	6.5000000	-50.0000000
42920.0000000	6.7500000	-50.0000000
42920.0000000	7.0000000	-50.0000000
42920.0000000	7.2500000	-50.0000000
42920.0000000	7.5000000	-50.0000000
42920.0000000	7.7500000	-50.0000000
42920.0000000	8.0000000	-50.0000000
42920.0000000	8.2500000	-50.0000000
42920.0000000	8.5000000	-50.0000000
42920.0000000	8.7500000	-50.0000000
42920.0000000	9.0000000	-50.0000000
42920.0000000	9.2500000	-50.0000000
42920.0000000	9.5000000	-50.0000000
42920.0000000	9.7500000	-50.0000000
42920.0000000	10.0000000	-50.0000000
42920.0000000	10.2500000	-50.0000000
42920.0000000	10.5000000	-50.0000000
42920.0000000	10.7500000	-50.0000000
42920.0000000	11.0000000	-50.0000000
42920.0000000	11.2500000	-50.0000000
42920.0000000	11.5000000	-50.0000000
42920.0000000	11.7500000	-50.0000000
42920.0000000	12.0000000	-50.0000000
42920.0000000	12.2500000	-50.0000000
42920.0000000	12.5000000	-50.0000000
42920.0000000	12.7500000	-50.0000000
42920.0000000	13.0000000	-50.0000000
42920.0000000	13.2500000	-50.0000000
42920.0000000	13.5000000	-50.0000000
42920.0000000	13.7500000	-50.0000000
42920.0000000	14.0000000	-50.0000000
42920.0000000	14.2500000	-50.0000000
42920.0000000	14.5000000	-50.0000000
42920.0000000	14.7500000	-50.0000000
42920.0000000	15.0000000	-50.0000000
42950.0000000	0.0000000	-50.0000000
42950.0000000	0.2500000	-50.0000000
42950.0000000	0.5000000	-50.0000000

42950.0000000	0.7500000	-50.0000000
42950.0000000	1.0000000	-50.0000000
42950.0000000	1.2500000	-50.0000000
42950.0000000	1.5000000	-50.0000000
42950.0000000	1.7500000	-50.0000000
42950.0000000	2.0000000	-50.0000000
42950.0000000	2.2500000	-50.0000000
42950.0000000	2.5000000	-50.0000000
42950.0000000	2.7500000	-50.0000000
42950.0000000	3.0000000	-50.0000000
42950.0000000	3.2500000	-50.0000000
42950.0000000	3.5000000	-50.0000000
42950.0000000	3.7500000	-50.0000000
42950.0000000	4.0000000	-50.0000000
42950.0000000	4.2500000	-50.0000000
42950.0000000	4.5000000	-50.0000000
42950.0000000	4.7500000	-50.0000000
42950.0000000	5.0000000	-50.0000000
42950.0000000	5.2500000	-50.0000000
42950.0000000	5.5000000	-50.0000000
42950.0000000	5.7500000	-50.0000000
42950.0000000	6.0000000	-50.0000000
42950.0000000	6.2500000	-50.0000000
42950.0000000	6.5000000	-50.0000000
42950.0000000	6.7500000	-50.0000000
42950.0000000	7.0000000	-50.0000000
42950.0000000	7.2500000	-50.0000000
42950.0000000	7.5000000	-50.0000000
42950.0000000	7.7500000	-50.0000000
42950.0000000	8.0000000	-50.0000000
42950.0000000	8.2500000	-50.0000000
42950.0000000	8.5000000	-50.0000000
42950.0000000	8.7500000	-50.0000000
42950.0000000	9.0000000	-50.0000000
42950.0000000	9.2500000	-50.0000000
42950.0000000	9.5000000	-50.0000000
42950.0000000	9.7500000	-50.0000000
42950.0000000	10.0000000	-50.0000000
42950.0000000	10.2500000	-50.0000000
42950.0000000	10.5000000	-50.0000000
42950.0000000	10.7500000	-50.0000000
42950.0000000	11.0000000	-50.0000000
42950.0000000	11.2500000	-50.0000000
42950.0000000	11.5000000	-50.0000000
42950.0000000	11.7500000	-50.0000000
42950.0000000	12.0000000	-50.0000000
42950.0000000	12.2500000	-50.0000000
42950.0000000	12.5000000	-50.0000000
42950.0000000	12.7500000	-50.0000000
42950.0000000	13.0000000	-50.0000000
42950.0000000	13.2500000	-50.0000000
42950.0000000	13.5000000	-50.0000000
42950.0000000	13.7500000	-50.0000000
42950.0000000	14.0000000	-50.0000000
42950.0000000	14.2500000	-50.0000000
42950.0000000	14.5000000	-50.0000000
42950.0000000	14.7500000	-50.0000000
42950.0000000	15.0000000	-50.0000000

42980.0000000	0.0000000	-50.0000000
42980.0000000	0.2500000	-50.0000000
42980.0000000	0.5000000	-50.0000000
42980.0000000	0.7500000	-50.0000000
42980.0000000	1.0000000	-50.0000000
42980.0000000	1.2500000	-50.0000000
42980.0000000	1.5000000	-50.0000000
42980.0000000	1.7500000	-50.0000000
42980.0000000	2.0000000	-50.0000000
42980.0000000	2.2500000	-50.0000000
42980.0000000	2.5000000	-50.0000000
42980.0000000	2.7500000	-50.0000000
42980.0000000	3.0000000	-50.0000000
42980.0000000	3.2500000	-50.0000000
42980.0000000	3.5000000	-50.0000000
42980.0000000	3.7500000	-50.0000000
42980.0000000	4.0000000	-50.0000000
42980.0000000	4.2500000	-50.0000000
42980.0000000	4.5000000	-50.0000000
42980.0000000	4.7500000	-50.0000000
42980.0000000	5.0000000	-50.0000000
42980.0000000	5.2500000	-50.0000000
42980.0000000	5.5000000	-50.0000000
42980.0000000	5.7500000	-50.0000000
42980.0000000	6.0000000	-50.0000000
42980.0000000	6.2500000	-50.0000000
42980.0000000	6.5000000	-50.0000000
42980.0000000	6.7500000	-50.0000000
42980.0000000	7.0000000	-50.0000000
42980.0000000	7.2500000	-50.0000000
42980.0000000	7.5000000	-50.0000000
42980.0000000	7.7500000	-50.0000000
42980.0000000	8.0000000	-50.0000000
42980.0000000	8.2500000	-50.0000000
42980.0000000	8.5000000	-50.0000000
42980.0000000	8.7500000	-50.0000000
42980.0000000	9.0000000	-50.0000000
42980.0000000	9.2500000	-50.0000000
42980.0000000	9.5000000	-50.0000000
42980.0000000	9.7500000	-50.0000000
42980.0000000	10.0000000	-50.0000000
42980.0000000	10.2500000	-50.0000000
42980.0000000	10.5000000	-50.0000000
42980.0000000	10.7500000	-50.0000000
42980.0000000	11.0000000	-50.0000000
42980.0000000	11.2500000	-50.0000000
42980.0000000	11.5000000	-50.0000000
42980.0000000	11.7500000	-50.0000000
42980.0000000	12.0000000	-50.0000000
42980.0000000	12.2500000	-50.0000000
42980.0000000	12.5000000	-50.0000000
42980.0000000	12.7500000	-50.0000000
42980.0000000	13.0000000	-50.0000000
42980.0000000	13.2500000	-50.0000000
42980.0000000	13.5000000	-50.0000000
42980.0000000	13.7500000	-50.0000000
42980.0000000	14.0000000	-50.0000000
42980.0000000	14.2500000	-50.0000000

42980.0000000	14.5000000	-50.0000000
42980.0000000	14.7500000	-50.0000000
42980.0000000	15.0000000	-50.0000000
43010.0000000	0.0000000	-50.0000000
43010.0000000	0.2500000	-50.0000000
43010.0000000	0.5000000	-50.0000000
43010.0000000	0.7500000	-50.0000000
43010.0000000	1.0000000	-50.0000000
43010.0000000	1.2500000	-50.0000000
43010.0000000	1.5000000	-50.0000000
43010.0000000	1.7500000	-50.0000000
43010.0000000	2.0000000	-50.0000000
43010.0000000	2.2500000	-50.0000000
43010.0000000	2.5000000	-50.0000000
43010.0000000	2.7500000	-50.0000000
43010.0000000	3.0000000	-50.0000000
43010.0000000	3.2500000	-50.0000000
43010.0000000	3.5000000	-50.0000000
43010.0000000	3.7500000	-50.0000000
43010.0000000	4.0000000	-50.0000000
43010.0000000	4.2500000	-50.0000000
43010.0000000	4.5000000	-50.0000000
43010.0000000	4.7500000	-50.0000000
43010.0000000	5.0000000	-50.0000000
43010.0000000	5.2500000	-50.0000000
43010.0000000	5.5000000	-50.0000000
43010.0000000	5.7500000	-50.0000000
43010.0000000	6.0000000	-50.0000000
43010.0000000	6.2500000	-50.0000000
43010.0000000	6.5000000	-50.0000000
43010.0000000	6.7500000	-50.0000000
43010.0000000	7.0000000	-50.0000000
43010.0000000	7.2500000	-50.0000000
43010.0000000	7.5000000	-50.0000000
43010.0000000	7.7500000	-50.0000000
43010.0000000	8.0000000	-50.0000000
43010.0000000	8.2500000	-50.0000000
43010.0000000	8.5000000	-50.0000000
43010.0000000	8.7500000	-50.0000000
43010.0000000	9.0000000	-50.0000000
43010.0000000	9.2500000	-50.0000000
43010.0000000	9.5000000	-50.0000000
43010.0000000	9.7500000	-50.0000000
43010.0000000	10.0000000	-50.0000000
43010.0000000	10.2500000	-50.0000000
43010.0000000	10.5000000	-50.0000000
43010.0000000	10.7500000	-50.0000000
43010.0000000	11.0000000	-50.0000000
43010.0000000	11.2500000	-50.0000000
43010.0000000	11.5000000	-50.0000000
43010.0000000	11.7500000	-50.0000000
43010.0000000	12.0000000	-50.0000000
43010.0000000	12.2500000	-50.0000000
43010.0000000	12.5000000	-50.0000000
43010.0000000	12.7500000	-50.0000000
43010.0000000	13.0000000	-50.0000000
43010.0000000	13.2500000	-50.0000000
43010.0000000	13.5000000	-50.0000000

43010.0000000	13.7500000	-50.0000000
43010.0000000	14.0000000	-50.0000000
43010.0000000	14.2500000	-50.0000000
43010.0000000	14.5000000	-50.0000000
43010.0000000	14.7500000	-50.0000000
43010.0000000	15.0000000	-50.0000000
43040.0000000	0.0000000	-50.0000000
43040.0000000	0.2500000	-50.0000000
43040.0000000	0.5000000	-50.0000000
43040.0000000	0.7500000	-50.0000000
43040.0000000	1.0000000	-50.0000000
43040.0000000	1.2500000	-50.0000000
43040.0000000	1.5000000	-50.0000000
43040.0000000	1.7500000	-50.0000000
43040.0000000	2.0000000	-50.0000000
43040.0000000	2.2500000	-50.0000000
43040.0000000	2.5000000	-50.0000000
43040.0000000	2.7500000	-50.0000000
43040.0000000	3.0000000	-50.0000000
43040.0000000	3.2500000	-50.0000000
43040.0000000	3.5000000	-50.0000000
43040.0000000	3.7500000	-50.0000000
43040.0000000	4.0000000	-50.0000000
43040.0000000	4.2500000	-50.0000000
43040.0000000	4.5000000	-50.0000000
43040.0000000	4.7500000	-50.0000000
43040.0000000	5.0000000	-50.0000000
43040.0000000	5.2500000	-50.0000000
43040.0000000	5.5000000	-50.0000000
43040.0000000	5.7500000	-50.0000000
43040.0000000	6.0000000	-50.0000000
43040.0000000	6.2500000	-50.0000000
43040.0000000	6.5000000	-50.0000000
43040.0000000	6.7500000	-50.0000000
43040.0000000	7.0000000	-50.0000000
43040.0000000	7.2500000	-50.0000000
43040.0000000	7.5000000	-50.0000000
43040.0000000	7.7500000	-50.0000000
43040.0000000	8.0000000	-50.0000000
43040.0000000	8.2500000	-50.0000000
43040.0000000	8.5000000	-50.0000000
43040.0000000	8.7500000	-50.0000000
43040.0000000	9.0000000	-50.0000000
43040.0000000	9.2500000	-50.0000000
43040.0000000	9.5000000	-50.0000000
43040.0000000	9.7500000	-50.0000000
43040.0000000	10.0000000	-50.0000000
43040.0000000	10.2500000	-50.0000000
43040.0000000	10.5000000	-50.0000000
43040.0000000	10.7500000	-50.0000000
43040.0000000	11.0000000	-50.0000000
43040.0000000	11.2500000	-50.0000000
43040.0000000	11.5000000	-50.0000000
43040.0000000	11.7500000	-50.0000000
43040.0000000	12.0000000	-50.0000000
43040.0000000	12.2500000	-50.0000000
43040.0000000	12.5000000	-50.0000000
43040.0000000	12.7500000	-50.0000000

43040.0000000	13.0000000	-50.0000000
43040.0000000	13.2500000	-50.0000000
43040.0000000	13.5000000	-50.0000000
43040.0000000	13.7500000	-50.0000000
43040.0000000	14.0000000	-50.0000000
43040.0000000	14.2500000	-50.0000000
43040.0000000	14.5000000	-50.0000000
43040.0000000	14.7500000	-50.0000000
43040.0000000	15.0000000	-50.0000000
43070.0000000	0.0000000	-50.0000000
43070.0000000	0.2500000	-50.0000000
43070.0000000	0.5000000	-50.0000000
43070.0000000	0.7500000	-50.0000000
43070.0000000	1.0000000	-50.0000000
43070.0000000	1.2500000	-50.0000000
43070.0000000	1.5000000	-50.0000000
43070.0000000	1.7500000	-50.0000000
43070.0000000	2.0000000	-50.0000000
43070.0000000	2.2500000	-50.0000000
43070.0000000	2.5000000	-50.0000000
43070.0000000	2.7500000	-50.0000000
43070.0000000	3.0000000	-50.0000000
43070.0000000	3.2500000	-50.0000000
43070.0000000	3.5000000	-50.0000000
43070.0000000	3.7500000	-50.0000000
43070.0000000	4.0000000	-50.0000000
43070.0000000	4.2500000	-50.0000000
43070.0000000	4.5000000	-50.0000000
43070.0000000	4.7500000	-50.0000000
43070.0000000	5.0000000	-50.0000000
43070.0000000	5.2500000	-50.0000000
43070.0000000	5.5000000	-50.0000000
43070.0000000	5.7500000	-50.0000000
43070.0000000	6.0000000	-50.0000000
43070.0000000	6.2500000	-50.0000000
43070.0000000	6.5000000	-50.0000000
43070.0000000	6.7500000	-50.0000000
43070.0000000	7.0000000	-50.0000000
43070.0000000	7.2500000	-50.0000000
43070.0000000	7.5000000	-50.0000000
43070.0000000	7.7500000	-50.0000000
43070.0000000	8.0000000	-50.0000000
43070.0000000	8.2500000	-50.0000000
43070.0000000	8.5000000	-50.0000000
43070.0000000	8.7500000	-50.0000000
43070.0000000	9.0000000	-50.0000000
43070.0000000	9.2500000	-50.0000000
43070.0000000	9.5000000	-50.0000000
43070.0000000	9.7500000	-50.0000000
43070.0000000	10.0000000	-50.0000000
43070.0000000	10.2500000	-50.0000000
43070.0000000	10.5000000	-50.0000000
43070.0000000	10.7500000	-50.0000000
43070.0000000	11.0000000	-50.0000000
43070.0000000	11.2500000	-50.0000000
43070.0000000	11.5000000	-50.0000000
43070.0000000	11.7500000	-50.0000000
43070.0000000	12.0000000	-50.0000000

43070.0000000	12.2500000	-50.0000000
43070.0000000	12.5000000	-50.0000000
43070.0000000	12.7500000	-50.0000000
43070.0000000	13.0000000	-50.0000000
43070.0000000	13.2500000	-50.0000000
43070.0000000	13.5000000	-50.0000000
43070.0000000	13.7500000	-50.0000000
43070.0000000	14.0000000	-50.0000000
43070.0000000	14.2500000	-50.0000000
43070.0000000	14.5000000	-50.0000000
43070.0000000	14.7500000	-50.0000000
43070.0000000	15.0000000	-50.0000000
43100.0000000	0.0000000	-50.0000000
43100.0000000	0.2500000	-50.0000000
43100.0000000	0.5000000	-50.0000000
43100.0000000	0.7500000	-50.0000000
43100.0000000	1.0000000	-50.0000000
43100.0000000	1.2500000	-50.0000000
43100.0000000	1.5000000	-50.0000000
43100.0000000	1.7500000	-50.0000000
43100.0000000	2.0000000	-50.0000000
43100.0000000	2.2500000	-50.0000000
43100.0000000	2.5000000	-50.0000000
43100.0000000	2.7500000	-50.0000000
43100.0000000	3.0000000	-50.0000000
43100.0000000	3.2500000	-50.0000000
43100.0000000	3.5000000	-50.0000000
43100.0000000	3.7500000	-50.0000000
43100.0000000	4.0000000	-50.0000000
43100.0000000	4.2500000	-50.0000000
43100.0000000	4.5000000	-50.0000000
43100.0000000	4.7500000	-50.0000000
43100.0000000	5.0000000	-50.0000000
43100.0000000	5.2500000	-50.0000000
43100.0000000	5.5000000	-50.0000000
43100.0000000	5.7500000	-50.0000000
43100.0000000	6.0000000	-50.0000000
43100.0000000	6.2500000	-50.0000000
43100.0000000	6.5000000	-50.0000000
43100.0000000	6.7500000	-50.0000000
43100.0000000	7.0000000	-50.0000000
43100.0000000	7.2500000	-50.0000000
43100.0000000	7.5000000	-50.0000000
43100.0000000	7.7500000	-50.0000000
43100.0000000	8.0000000	-50.0000000
43100.0000000	8.2500000	-50.0000000
43100.0000000	8.5000000	-50.0000000
43100.0000000	8.7500000	-50.0000000
43100.0000000	9.0000000	-50.0000000
43100.0000000	9.2500000	-50.0000000
43100.0000000	9.5000000	-50.0000000
43100.0000000	9.7500000	-50.0000000
43100.0000000	10.0000000	-50.0000000
43100.0000000	10.2500000	-50.0000000
43100.0000000	10.5000000	-50.0000000
43100.0000000	10.7500000	-50.0000000
43100.0000000	11.0000000	-50.0000000
43100.0000000	11.2500000	-50.0000000

43100.0000000	11.5000000	-50.0000000
43100.0000000	11.7500000	-50.0000000
43100.0000000	12.0000000	-50.0000000
43100.0000000	12.2500000	-50.0000000
43100.0000000	12.5000000	-50.0000000
43100.0000000	12.7500000	-50.0000000
43100.0000000	13.0000000	-50.0000000
43100.0000000	13.2500000	-50.0000000
43100.0000000	13.5000000	-50.0000000
43100.0000000	13.7500000	-50.0000000
43100.0000000	14.0000000	-50.0000000
43100.0000000	14.2500000	-50.0000000
43100.0000000	14.5000000	-50.0000000
43100.0000000	14.7500000	-50.0000000
43100.0000000	15.0000000	-50.0000000
43130.0000000	0.0000000	-50.0000000
43130.0000000	0.2500000	-50.0000000
43130.0000000	0.5000000	-50.0000000
43130.0000000	0.7500000	-50.0000000
43130.0000000	1.0000000	-50.0000000
43130.0000000	1.2500000	-50.0000000
43130.0000000	1.5000000	-50.0000000
43130.0000000	1.7500000	-50.0000000
43130.0000000	2.0000000	-50.0000000
43130.0000000	2.2500000	-50.0000000
43130.0000000	2.5000000	-50.0000000
43130.0000000	2.7500000	-50.0000000
43130.0000000	3.0000000	-50.0000000
43130.0000000	3.2500000	-50.0000000
43130.0000000	3.5000000	-50.0000000
43130.0000000	3.7500000	-50.0000000
43130.0000000	4.0000000	-50.0000000
43130.0000000	4.2500000	-50.0000000
43130.0000000	4.5000000	-50.0000000
43130.0000000	4.7500000	-50.0000000
43130.0000000	5.0000000	-50.0000000
43130.0000000	5.2500000	-50.0000000
43130.0000000	5.5000000	-50.0000000
43130.0000000	5.7500000	-50.0000000
43130.0000000	6.0000000	-50.0000000
43130.0000000	6.2500000	-50.0000000
43130.0000000	6.5000000	-50.0000000
43130.0000000	6.7500000	-50.0000000
43130.0000000	7.0000000	-50.0000000
43130.0000000	7.2500000	-50.0000000
43130.0000000	7.5000000	-50.0000000
43130.0000000	7.7500000	-50.0000000
43130.0000000	8.0000000	-50.0000000
43130.0000000	8.2500000	-50.0000000
43130.0000000	8.5000000	-50.0000000
43130.0000000	8.7500000	-50.0000000
43130.0000000	9.0000000	-50.0000000
43130.0000000	9.2500000	-50.0000000
43130.0000000	9.5000000	-50.0000000
43130.0000000	9.7500000	-50.0000000
43130.0000000	10.0000000	-50.0000000
43130.0000000	10.2500000	-50.0000000
43130.0000000	10.5000000	-50.0000000

43130.0000000	10.7500000	-50.0000000
43130.0000000	11.0000000	-50.0000000
43130.0000000	11.2500000	-50.0000000
43130.0000000	11.5000000	-50.0000000
43130.0000000	11.7500000	-50.0000000
43130.0000000	12.0000000	-50.0000000
43130.0000000	12.2500000	-50.0000000
43130.0000000	12.5000000	-50.0000000
43130.0000000	12.7500000	-50.0000000
43130.0000000	13.0000000	-50.0000000
43130.0000000	13.2500000	-50.0000000
43130.0000000	13.5000000	-50.0000000
43130.0000000	13.7500000	-50.0000000
43130.0000000	14.0000000	-50.0000000
43130.0000000	14.2500000	-50.0000000
43130.0000000	14.5000000	-50.0000000
43130.0000000	14.7500000	-50.0000000
43130.0000000	15.0000000	-50.0000000
43160.0000000	0.0000000	-50.0000000
43160.0000000	0.2500000	-50.0000000
43160.0000000	0.5000000	-50.0000000
43160.0000000	0.7500000	-50.0000000
43160.0000000	1.0000000	-50.0000000
43160.0000000	1.2500000	-50.0000000
43160.0000000	1.5000000	-50.0000000
43160.0000000	1.7500000	-50.0000000
43160.0000000	2.0000000	-50.0000000
43160.0000000	2.2500000	-50.0000000
43160.0000000	2.5000000	-50.0000000
43160.0000000	2.7500000	-50.0000000
43160.0000000	3.0000000	-50.0000000
43160.0000000	3.2500000	-50.0000000
43160.0000000	3.5000000	-50.0000000
43160.0000000	3.7500000	-50.0000000
43160.0000000	4.0000000	-50.0000000
43160.0000000	4.2500000	-50.0000000
43160.0000000	4.5000000	-50.0000000
43160.0000000	4.7500000	-50.0000000
43160.0000000	5.0000000	-50.0000000
43160.0000000	5.2500000	-50.0000000
43160.0000000	5.5000000	-50.0000000
43160.0000000	5.7500000	-50.0000000
43160.0000000	6.0000000	-50.0000000
43160.0000000	6.2500000	-50.0000000
43160.0000000	6.5000000	-50.0000000
43160.0000000	6.7500000	-50.0000000
43160.0000000	7.0000000	-50.0000000
43160.0000000	7.2500000	-50.0000000
43160.0000000	7.5000000	-50.0000000
43160.0000000	7.7500000	-50.0000000
43160.0000000	8.0000000	-50.0000000
43160.0000000	8.2500000	-50.0000000
43160.0000000	8.5000000	-50.0000000
43160.0000000	8.7500000	-50.0000000
43160.0000000	9.0000000	-50.0000000
43160.0000000	9.2500000	-50.0000000
43160.0000000	9.5000000	-50.0000000
43160.0000000	9.7500000	-50.0000000

43160.0000000	10.0000000	-50.0000000
43160.0000000	10.2500000	-50.0000000
43160.0000000	10.5000000	-50.0000000
43160.0000000	10.7500000	-50.0000000
43160.0000000	11.0000000	-50.0000000
43160.0000000	11.2500000	-50.0000000
43160.0000000	11.5000000	-50.0000000
43160.0000000	11.7500000	-50.0000000
43160.0000000	12.0000000	-50.0000000
43160.0000000	12.2500000	-50.0000000
43160.0000000	12.5000000	-50.0000000
43160.0000000	12.7500000	-50.0000000
43160.0000000	13.0000000	-50.0000000
43160.0000000	13.2500000	-50.0000000
43160.0000000	13.5000000	-50.0000000
43160.0000000	13.7500000	-50.0000000
43160.0000000	14.0000000	-50.0000000
43160.0000000	14.2500000	-50.0000000
43160.0000000	14.5000000	-50.0000000
43160.0000000	14.7500000	-50.0000000
43160.0000000	15.0000000	-50.0000000
43190.0000000	0.0000000	-50.0000000
43190.0000000	0.2500000	-50.0000000
43190.0000000	0.5000000	-50.0000000
43190.0000000	0.7500000	-50.0000000
43190.0000000	1.0000000	-50.0000000
43190.0000000	1.2500000	-50.0000000
43190.0000000	1.5000000	-50.0000000
43190.0000000	1.7500000	-50.0000000
43190.0000000	2.0000000	-50.0000000
43190.0000000	2.2500000	-50.0000000
43190.0000000	2.5000000	-50.0000000
43190.0000000	2.7500000	-50.0000000
43190.0000000	3.0000000	-50.0000000
43190.0000000	3.2500000	-50.0000000
43190.0000000	3.5000000	-50.0000000
43190.0000000	3.7500000	-50.0000000
43190.0000000	4.0000000	-50.0000000
43190.0000000	4.2500000	-50.0000000
43190.0000000	4.5000000	-50.0000000
43190.0000000	4.7500000	-50.0000000
43190.0000000	5.0000000	-50.0000000
43190.0000000	5.2500000	-50.0000000
43190.0000000	5.5000000	-50.0000000
43190.0000000	5.7500000	-50.0000000
43190.0000000	6.0000000	-50.0000000
43190.0000000	6.2500000	-50.0000000
43190.0000000	6.5000000	-50.0000000
43190.0000000	6.7500000	-50.0000000
43190.0000000	7.0000000	-50.0000000
43190.0000000	7.2500000	-50.0000000
43190.0000000	7.5000000	-50.0000000
43190.0000000	7.7500000	-50.0000000
43190.0000000	8.0000000	-50.0000000
43190.0000000	8.2500000	-50.0000000
43190.0000000	8.5000000	-50.0000000
43190.0000000	8.7500000	-50.0000000
43190.0000000	9.0000000	-50.0000000

43190.0000000	9.2500000	-50.0000000
43190.0000000	9.5000000	-50.0000000
43190.0000000	9.7500000	-50.0000000
43190.0000000	10.0000000	-50.0000000
43190.0000000	10.2500000	-50.0000000
43190.0000000	10.5000000	-50.0000000
43190.0000000	10.7500000	-50.0000000
43190.0000000	11.0000000	-50.0000000
43190.0000000	11.2500000	-50.0000000
43190.0000000	11.5000000	-50.0000000
43190.0000000	11.7500000	-50.0000000
43190.0000000	12.0000000	-50.0000000
43190.0000000	12.2500000	-50.0000000
43190.0000000	12.5000000	-50.0000000
43190.0000000	12.7500000	-50.0000000
43190.0000000	13.0000000	-50.0000000
43190.0000000	13.2500000	-50.0000000
43190.0000000	13.5000000	-50.0000000
43190.0000000	13.7500000	-50.0000000
43190.0000000	14.0000000	-50.0000000
43190.0000000	14.2500000	-50.0000000
43190.0000000	14.5000000	-50.0000000
43190.0000000	14.7500000	-50.0000000
43190.0000000	15.0000000	-50.0000000
43220.0000000	0.0000000	-50.0000000
43220.0000000	0.2500000	-50.0000000
43220.0000000	0.5000000	-50.0000000
43220.0000000	0.7500000	-50.0000000
43220.0000000	1.0000000	-50.0000000
43220.0000000	1.2500000	-50.0000000
43220.0000000	1.5000000	-50.0000000
43220.0000000	1.7500000	-50.0000000
43220.0000000	2.0000000	-50.0000000
43220.0000000	2.2500000	-50.0000000
43220.0000000	2.5000000	-50.0000000
43220.0000000	2.7500000	-50.0000000
43220.0000000	3.0000000	-50.0000000
43220.0000000	3.2500000	-50.0000000
43220.0000000	3.5000000	-50.0000000
43220.0000000	3.7500000	-50.0000000
43220.0000000	4.0000000	-50.0000000
43220.0000000	4.2500000	-50.0000000
43220.0000000	4.5000000	-50.0000000
43220.0000000	4.7500000	-50.0000000
43220.0000000	5.0000000	-50.0000000
43220.0000000	5.2500000	-50.0000000
43220.0000000	5.5000000	-50.0000000
43220.0000000	5.7500000	-50.0000000
43220.0000000	6.0000000	-50.0000000
43220.0000000	6.2500000	-50.0000000
43220.0000000	6.5000000	-50.0000000
43220.0000000	6.7500000	-50.0000000
43220.0000000	7.0000000	-50.0000000
43220.0000000	7.2500000	-50.0000000
43220.0000000	7.5000000	-50.0000000
43220.0000000	7.7500000	-50.0000000
43220.0000000	8.0000000	-50.0000000
43220.0000000	8.2500000	-50.0000000

43220.0000000	8.5000000	-50.0000000
43220.0000000	8.7500000	-50.0000000
43220.0000000	9.0000000	-50.0000000
43220.0000000	9.2500000	-50.0000000
43220.0000000	9.5000000	-50.0000000
43220.0000000	9.7500000	-50.0000000
43220.0000000	10.0000000	-50.0000000
43220.0000000	10.2500000	-50.0000000
43220.0000000	10.5000000	-50.0000000
43220.0000000	10.7500000	-50.0000000
43220.0000000	11.0000000	-50.0000000
43220.0000000	11.2500000	-50.0000000
43220.0000000	11.5000000	-50.0000000
43220.0000000	11.7500000	-50.0000000
43220.0000000	12.0000000	-50.0000000
43220.0000000	12.2500000	-50.0000000
43220.0000000	12.5000000	-50.0000000
43220.0000000	12.7500000	-50.0000000
43220.0000000	13.0000000	-50.0000000
43220.0000000	13.2500000	-50.0000000
43220.0000000	13.5000000	-50.0000000
43220.0000000	13.7500000	-50.0000000
43220.0000000	14.0000000	-50.0000000
43220.0000000	14.2500000	-50.0000000
43220.0000000	14.5000000	-50.0000000
43220.0000000	14.7500000	-50.0000000
43220.0000000	15.0000000	-50.0000000
43250.0000000	0.0000000	-50.0000000
43250.0000000	0.2500000	-50.0000000
43250.0000000	0.5000000	-50.0000000
43250.0000000	0.7500000	-50.0000000
43250.0000000	1.0000000	-50.0000000
43250.0000000	1.2500000	-50.0000000
43250.0000000	1.5000000	-50.0000000
43250.0000000	1.7500000	-50.0000000
43250.0000000	2.0000000	-50.0000000
43250.0000000	2.2500000	-50.0000000
43250.0000000	2.5000000	-50.0000000
43250.0000000	2.7500000	-50.0000000
43250.0000000	3.0000000	-50.0000000
43250.0000000	3.2500000	-50.0000000
43250.0000000	3.5000000	-50.0000000
43250.0000000	3.7500000	-50.0000000
43250.0000000	4.0000000	-50.0000000
43250.0000000	4.2500000	-50.0000000
43250.0000000	4.5000000	-50.0000000
43250.0000000	4.7500000	-50.0000000
43250.0000000	5.0000000	-50.0000000
43250.0000000	5.2500000	-50.0000000
43250.0000000	5.5000000	-50.0000000
43250.0000000	5.7500000	-50.0000000
43250.0000000	6.0000000	-50.0000000
43250.0000000	6.2500000	-50.0000000
43250.0000000	6.5000000	-50.0000000
43250.0000000	6.7500000	-50.0000000
43250.0000000	7.0000000	-50.0000000
43250.0000000	7.2500000	-50.0000000
43250.0000000	7.5000000	-50.0000000

43250.0000000	7.7500000	-50.0000000
43250.0000000	8.0000000	-50.0000000
43250.0000000	8.2500000	-50.0000000
43250.0000000	8.5000000	-50.0000000
43250.0000000	8.7500000	-50.0000000
43250.0000000	9.0000000	-50.0000000
43250.0000000	9.2500000	-50.0000000
43250.0000000	9.5000000	-50.0000000
43250.0000000	9.7500000	-50.0000000
43250.0000000	10.0000000	-50.0000000
43250.0000000	10.2500000	-50.0000000
43250.0000000	10.5000000	-50.0000000
43250.0000000	10.7500000	-50.0000000
43250.0000000	11.0000000	-50.0000000
43250.0000000	11.2500000	-50.0000000
43250.0000000	11.5000000	-50.0000000
43250.0000000	11.7500000	-50.0000000
43250.0000000	12.0000000	-50.0000000
43250.0000000	12.2500000	-50.0000000
43250.0000000	12.5000000	-50.0000000
43250.0000000	12.7500000	-50.0000000
43250.0000000	13.0000000	-50.0000000
43250.0000000	13.2500000	-50.0000000
43250.0000000	13.5000000	-50.0000000
43250.0000000	13.7500000	-50.0000000
43250.0000000	14.0000000	-50.0000000
43250.0000000	14.2500000	-50.0000000
43250.0000000	14.5000000	-50.0000000
43250.0000000	14.7500000	-50.0000000
43250.0000000	15.0000000	-50.0000000
43280.0000000	0.0000000	-50.0000000
43280.0000000	0.2500000	-50.0000000
43280.0000000	0.5000000	-50.0000000
43280.0000000	0.7500000	-50.0000000
43280.0000000	1.0000000	-50.0000000
43280.0000000	1.2500000	-50.0000000
43280.0000000	1.5000000	-50.0000000
43280.0000000	1.7500000	-50.0000000
43280.0000000	2.0000000	-50.0000000
43280.0000000	2.2500000	-50.0000000
43280.0000000	2.5000000	-50.0000000
43280.0000000	2.7500000	-50.0000000
43280.0000000	3.0000000	-50.0000000
43280.0000000	3.2500000	-50.0000000
43280.0000000	3.5000000	-50.0000000
43280.0000000	3.7500000	-50.0000000
43280.0000000	4.0000000	-50.0000000
43280.0000000	4.2500000	-50.0000000
43280.0000000	4.5000000	-50.0000000
43280.0000000	4.7500000	-50.0000000
43280.0000000	5.0000000	-50.0000000
43280.0000000	5.2500000	-50.0000000
43280.0000000	5.5000000	-50.0000000
43280.0000000	5.7500000	-50.0000000
43280.0000000	6.0000000	-50.0000000
43280.0000000	6.2500000	-50.0000000
43280.0000000	6.5000000	-50.0000000
43280.0000000	6.7500000	-50.0000000

43280.0000000	7.0000000	-50.0000000
43280.0000000	7.2500000	-50.0000000
43280.0000000	7.5000000	-50.0000000
43280.0000000	7.7500000	-50.0000000
43280.0000000	8.0000000	-50.0000000
43280.0000000	8.2500000	-50.0000000
43280.0000000	8.5000000	-50.0000000
43280.0000000	8.7500000	-50.0000000
43280.0000000	9.0000000	-50.0000000
43280.0000000	9.2500000	-50.0000000
43280.0000000	9.5000000	-50.0000000
43280.0000000	9.7500000	-50.0000000
43280.0000000	10.0000000	-50.0000000
43280.0000000	10.2500000	-50.0000000
43280.0000000	10.5000000	-50.0000000
43280.0000000	10.7500000	-50.0000000
43280.0000000	11.0000000	-50.0000000
43280.0000000	11.2500000	-50.0000000
43280.0000000	11.5000000	-50.0000000
43280.0000000	11.7500000	-50.0000000
43280.0000000	12.0000000	-50.0000000
43280.0000000	12.2500000	-50.0000000
43280.0000000	12.5000000	-50.0000000
43280.0000000	12.7500000	-50.0000000
43280.0000000	13.0000000	-50.0000000
43280.0000000	13.2500000	-50.0000000
43280.0000000	13.5000000	-50.0000000
43280.0000000	13.7500000	-50.0000000
43280.0000000	14.0000000	-50.0000000
43280.0000000	14.2500000	-50.0000000
43280.0000000	14.5000000	-50.0000000
43280.0000000	14.7500000	-50.0000000
43280.0000000	15.0000000	-50.0000000
43310.0000000	0.0000000	-50.0000000
43310.0000000	0.2500000	-50.0000000
43310.0000000	0.5000000	-50.0000000
43310.0000000	0.7500000	-50.0000000
43310.0000000	1.0000000	-50.0000000
43310.0000000	1.2500000	-50.0000000
43310.0000000	1.5000000	-50.0000000
43310.0000000	1.7500000	-50.0000000
43310.0000000	2.0000000	-50.0000000
43310.0000000	2.2500000	-50.0000000
43310.0000000	2.5000000	-50.0000000
43310.0000000	2.7500000	-50.0000000
43310.0000000	3.0000000	-50.0000000
43310.0000000	3.2500000	-50.0000000
43310.0000000	3.5000000	-50.0000000
43310.0000000	3.7500000	-50.0000000
43310.0000000	4.0000000	-50.0000000
43310.0000000	4.2500000	-50.0000000
43310.0000000	4.5000000	-50.0000000
43310.0000000	4.7500000	-50.0000000
43310.0000000	5.0000000	-50.0000000
43310.0000000	5.2500000	-50.0000000
43310.0000000	5.5000000	-50.0000000
43310.0000000	5.7500000	-50.0000000
43310.0000000	6.0000000	-50.0000000

43310.0000000	6.2500000	-50.0000000
43310.0000000	6.5000000	-50.0000000
43310.0000000	6.7500000	-50.0000000
43310.0000000	7.0000000	-50.0000000
43310.0000000	7.2500000	-50.0000000
43310.0000000	7.5000000	-50.0000000
43310.0000000	7.7500000	-50.0000000
43310.0000000	8.0000000	-50.0000000
43310.0000000	8.2500000	-50.0000000
43310.0000000	8.5000000	-50.0000000
43310.0000000	8.7500000	-50.0000000
43310.0000000	9.0000000	-50.0000000
43310.0000000	9.2500000	-50.0000000
43310.0000000	9.5000000	-50.0000000
43310.0000000	9.7500000	-50.0000000
43310.0000000	10.0000000	-50.0000000
43310.0000000	10.2500000	-50.0000000
43310.0000000	10.5000000	-50.0000000
43310.0000000	10.7500000	-50.0000000
43310.0000000	11.0000000	-50.0000000
43310.0000000	11.2500000	-50.0000000
43310.0000000	11.5000000	-50.0000000
43310.0000000	11.7500000	-50.0000000
43310.0000000	12.0000000	-50.0000000
43310.0000000	12.2500000	-50.0000000
43310.0000000	12.5000000	-50.0000000
43310.0000000	12.7500000	-50.0000000
43310.0000000	13.0000000	-50.0000000
43310.0000000	13.2500000	-50.0000000
43310.0000000	13.5000000	-50.0000000
43310.0000000	13.7500000	-50.0000000
43310.0000000	14.0000000	-50.0000000
43310.0000000	14.2500000	-50.0000000
43310.0000000	14.5000000	-50.0000000
43310.0000000	14.7500000	-50.0000000
43310.0000000	15.0000000	-50.0000000
43340.0000000	0.0000000	-50.0000000
43340.0000000	0.2500000	-50.0000000
43340.0000000	0.5000000	-50.0000000
43340.0000000	0.7500000	-50.0000000
43340.0000000	1.0000000	-50.0000000
43340.0000000	1.2500000	-50.0000000
43340.0000000	1.5000000	-50.0000000
43340.0000000	1.7500000	-50.0000000
43340.0000000	2.0000000	-50.0000000
43340.0000000	2.2500000	-50.0000000
43340.0000000	2.5000000	-50.0000000
43340.0000000	2.7500000	-50.0000000
43340.0000000	3.0000000	-50.0000000
43340.0000000	3.2500000	-50.0000000
43340.0000000	3.5000000	-50.0000000
43340.0000000	3.7500000	-50.0000000
43340.0000000	4.0000000	-50.0000000
43340.0000000	4.2500000	-50.0000000
43340.0000000	4.5000000	-50.0000000
43340.0000000	4.7500000	-50.0000000
43340.0000000	5.0000000	-50.0000000
43340.0000000	5.2500000	-50.0000000

43340.0000000	5.5000000	-50.0000000
43340.0000000	5.7500000	-50.0000000
43340.0000000	6.0000000	-50.0000000
43340.0000000	6.2500000	-50.0000000
43340.0000000	6.5000000	-50.0000000
43340.0000000	6.7500000	-50.0000000
43340.0000000	7.0000000	-50.0000000
43340.0000000	7.2500000	-50.0000000
43340.0000000	7.5000000	-50.0000000
43340.0000000	7.7500000	-50.0000000
43340.0000000	8.0000000	-50.0000000
43340.0000000	8.2500000	-50.0000000
43340.0000000	8.5000000	-50.0000000
43340.0000000	8.7500000	-50.0000000
43340.0000000	9.0000000	-50.0000000
43340.0000000	9.2500000	-50.0000000
43340.0000000	9.5000000	-50.0000000
43340.0000000	9.7500000	-50.0000000
43340.0000000	10.0000000	-50.0000000
43340.0000000	10.2500000	-50.0000000
43340.0000000	10.5000000	-50.0000000
43340.0000000	10.7500000	-50.0000000
43340.0000000	11.0000000	-50.0000000
43340.0000000	11.2500000	-50.0000000
43340.0000000	11.5000000	-50.0000000
43340.0000000	11.7500000	-50.0000000
43340.0000000	12.0000000	-50.0000000
43340.0000000	12.2500000	-50.0000000
43340.0000000	12.5000000	-50.0000000
43340.0000000	12.7500000	-50.0000000
43340.0000000	13.0000000	-50.0000000
43340.0000000	13.2500000	-50.0000000
43340.0000000	13.5000000	-50.0000000
43340.0000000	13.7500000	-50.0000000
43340.0000000	14.0000000	-50.0000000
43340.0000000	14.2500000	-50.0000000
43340.0000000	14.5000000	-50.0000000
43340.0000000	14.7500000	-50.0000000
43340.0000000	15.0000000	-50.0000000
43370.0000000	0.0000000	-50.0000000
43370.0000000	0.2500000	-50.0000000
43370.0000000	0.5000000	-50.0000000
43370.0000000	0.7500000	-50.0000000
43370.0000000	1.0000000	-50.0000000
43370.0000000	1.2500000	-50.0000000
43370.0000000	1.5000000	-50.0000000
43370.0000000	1.7500000	-50.0000000
43370.0000000	2.0000000	-50.0000000
43370.0000000	2.2500000	-50.0000000
43370.0000000	2.5000000	-50.0000000
43370.0000000	2.7500000	-50.0000000
43370.0000000	3.0000000	-50.0000000
43370.0000000	3.2500000	-50.0000000
43370.0000000	3.5000000	-50.0000000
43370.0000000	3.7500000	-50.0000000
43370.0000000	4.0000000	-50.0000000
43370.0000000	4.2500000	-50.0000000
43370.0000000	4.5000000	-50.0000000

43370.0000000	4.7500000	-50.0000000
43370.0000000	5.0000000	-50.0000000
43370.0000000	5.2500000	-50.0000000
43370.0000000	5.5000000	-50.0000000
43370.0000000	5.7500000	-50.0000000
43370.0000000	6.0000000	-50.0000000
43370.0000000	6.2500000	-50.0000000
43370.0000000	6.5000000	-50.0000000
43370.0000000	6.7500000	-50.0000000
43370.0000000	7.0000000	-50.0000000
43370.0000000	7.2500000	-50.0000000
43370.0000000	7.5000000	-50.0000000
43370.0000000	7.7500000	-50.0000000
43370.0000000	8.0000000	-50.0000000
43370.0000000	8.2500000	-50.0000000
43370.0000000	8.5000000	-50.0000000
43370.0000000	8.7500000	-50.0000000
43370.0000000	9.0000000	-50.0000000
43370.0000000	9.2500000	-50.0000000
43370.0000000	9.5000000	-50.0000000
43370.0000000	9.7500000	-50.0000000
43370.0000000	10.0000000	-50.0000000
43370.0000000	10.2500000	-50.0000000
43370.0000000	10.5000000	-50.0000000
43370.0000000	10.7500000	-50.0000000
43370.0000000	11.0000000	-50.0000000
43370.0000000	11.2500000	-50.0000000
43370.0000000	11.5000000	-50.0000000
43370.0000000	11.7500000	-50.0000000
43370.0000000	12.0000000	-50.0000000
43370.0000000	12.2500000	-50.0000000
43370.0000000	12.5000000	-50.0000000
43370.0000000	12.7500000	-50.0000000
43370.0000000	13.0000000	-50.0000000
43370.0000000	13.2500000	-50.0000000
43370.0000000	13.5000000	-50.0000000
43370.0000000	13.7500000	-50.0000000
43370.0000000	14.0000000	-50.0000000
43370.0000000	14.2500000	-50.0000000
43370.0000000	14.5000000	-50.0000000
43370.0000000	14.7500000	-50.0000000
43370.0000000	15.0000000	-50.0000000
43400.0000000	0.0000000	-50.0000000
43400.0000000	0.2500000	-50.0000000
43400.0000000	0.5000000	-50.0000000
43400.0000000	0.7500000	-50.0000000
43400.0000000	1.0000000	-50.0000000
43400.0000000	1.2500000	-50.0000000
43400.0000000	1.5000000	-50.0000000
43400.0000000	1.7500000	-50.0000000
43400.0000000	2.0000000	-50.0000000
43400.0000000	2.2500000	-50.0000000
43400.0000000	2.5000000	-50.0000000
43400.0000000	2.7500000	-50.0000000
43400.0000000	3.0000000	-50.0000000
43400.0000000	3.2500000	-50.0000000
43400.0000000	3.5000000	-50.0000000
43400.0000000	3.7500000	-50.0000000

43400.0000000	4.0000000	-50.0000000
43400.0000000	4.2500000	-50.0000000
43400.0000000	4.5000000	-50.0000000
43400.0000000	4.7500000	-50.0000000
43400.0000000	5.0000000	-50.0000000
43400.0000000	5.2500000	-50.0000000
43400.0000000	5.5000000	-50.0000000
43400.0000000	5.7500000	-50.0000000
43400.0000000	6.0000000	-50.0000000
43400.0000000	6.2500000	-50.0000000
43400.0000000	6.5000000	-50.0000000
43400.0000000	6.7500000	-50.0000000
43400.0000000	7.0000000	-50.0000000
43400.0000000	7.2500000	-50.0000000
43400.0000000	7.5000000	-50.0000000
43400.0000000	7.7500000	-50.0000000
43400.0000000	8.0000000	-50.0000000
43400.0000000	8.2500000	-50.0000000
43400.0000000	8.5000000	-50.0000000
43400.0000000	8.7500000	-50.0000000
43400.0000000	9.0000000	-50.0000000
43400.0000000	9.2500000	-50.0000000
43400.0000000	9.5000000	-50.0000000
43400.0000000	9.7500000	-50.0000000
43400.0000000	10.0000000	-50.0000000
43400.0000000	10.2500000	-50.0000000
43400.0000000	10.5000000	-50.0000000
43400.0000000	10.7500000	-50.0000000
43400.0000000	11.0000000	-50.0000000
43400.0000000	11.2500000	-50.0000000
43400.0000000	11.5000000	-50.0000000
43400.0000000	11.7500000	-50.0000000
43400.0000000	12.0000000	-50.0000000
43400.0000000	12.2500000	-50.0000000
43400.0000000	12.5000000	-50.0000000
43400.0000000	12.7500000	-50.0000000
43400.0000000	13.0000000	-50.0000000
43400.0000000	13.2500000	-50.0000000
43400.0000000	13.5000000	-50.0000000
43400.0000000	13.7500000	-50.0000000
43400.0000000	14.0000000	-50.0000000
43400.0000000	14.2500000	-50.0000000
43400.0000000	14.5000000	-50.0000000
43400.0000000	14.7500000	-50.0000000
43400.0000000	15.0000000	-50.0000000
43430.0000000	0.0000000	-50.0000000
43430.0000000	0.2500000	-50.0000000
43430.0000000	0.5000000	-50.0000000
43430.0000000	0.7500000	-50.0000000
43430.0000000	1.0000000	-50.0000000
43430.0000000	1.2500000	-50.0000000
43430.0000000	1.5000000	-50.0000000
43430.0000000	1.7500000	-50.0000000
43430.0000000	2.0000000	-50.0000000
43430.0000000	2.2500000	-50.0000000
43430.0000000	2.5000000	-50.0000000
43430.0000000	2.7500000	-50.0000000
43430.0000000	3.0000000	-50.0000000

43430.0000000	3.2500000	-50.0000000
43430.0000000	3.5000000	-50.0000000
43430.0000000	3.7500000	-50.0000000
43430.0000000	4.0000000	-50.0000000
43430.0000000	4.2500000	-50.0000000
43430.0000000	4.5000000	-50.0000000
43430.0000000	4.7500000	-50.0000000
43430.0000000	5.0000000	-50.0000000
43430.0000000	5.2500000	-50.0000000
43430.0000000	5.5000000	-50.0000000
43430.0000000	5.7500000	-50.0000000
43430.0000000	6.0000000	-50.0000000
43430.0000000	6.2500000	-50.0000000
43430.0000000	6.5000000	-50.0000000
43430.0000000	6.7500000	-50.0000000
43430.0000000	7.0000000	-50.0000000
43430.0000000	7.2500000	-50.0000000
43430.0000000	7.5000000	-50.0000000
43430.0000000	7.7500000	-50.0000000
43430.0000000	8.0000000	-50.0000000
43430.0000000	8.2500000	-50.0000000
43430.0000000	8.5000000	-50.0000000
43430.0000000	8.7500000	-50.0000000
43430.0000000	9.0000000	-50.0000000
43430.0000000	9.2500000	-50.0000000
43430.0000000	9.5000000	-50.0000000
43430.0000000	9.7500000	-50.0000000
43430.0000000	10.0000000	-50.0000000
43430.0000000	10.2500000	-50.0000000
43430.0000000	10.5000000	-50.0000000
43430.0000000	10.7500000	-50.0000000
43430.0000000	11.0000000	-50.0000000
43430.0000000	11.2500000	-50.0000000
43430.0000000	11.5000000	-50.0000000
43430.0000000	11.7500000	-50.0000000
43430.0000000	12.0000000	-50.0000000
43430.0000000	12.2500000	-50.0000000
43430.0000000	12.5000000	-50.0000000
43430.0000000	12.7500000	-50.0000000
43430.0000000	13.0000000	-50.0000000
43430.0000000	13.2500000	-50.0000000
43430.0000000	13.5000000	-50.0000000
43430.0000000	13.7500000	-50.0000000
43430.0000000	14.0000000	-50.0000000
43430.0000000	14.2500000	-50.0000000
43430.0000000	14.5000000	-50.0000000
43430.0000000	14.7500000	-50.0000000
43430.0000000	15.0000000	-50.0000000
43460.0000000	0.0000000	-50.0000000
43460.0000000	0.2500000	-50.0000000
43460.0000000	0.5000000	-50.0000000
43460.0000000	0.7500000	-50.0000000
43460.0000000	1.0000000	-50.0000000
43460.0000000	1.2500000	-50.0000000
43460.0000000	1.5000000	-50.0000000
43460.0000000	1.7500000	-50.0000000
43460.0000000	2.0000000	-50.0000000
43460.0000000	2.2500000	-50.0000000

43460.0000000	2.5000000	-50.0000000
43460.0000000	2.7500000	-50.0000000
43460.0000000	3.0000000	-50.0000000
43460.0000000	3.2500000	-50.0000000
43460.0000000	3.5000000	-50.0000000
43460.0000000	3.7500000	-50.0000000
43460.0000000	4.0000000	-50.0000000
43460.0000000	4.2500000	-50.0000000
43460.0000000	4.5000000	-50.0000000
43460.0000000	4.7500000	-50.0000000
43460.0000000	5.0000000	-50.0000000
43460.0000000	5.2500000	-50.0000000
43460.0000000	5.5000000	-50.0000000
43460.0000000	5.7500000	-50.0000000
43460.0000000	6.0000000	-50.0000000
43460.0000000	6.2500000	-50.0000000
43460.0000000	6.5000000	-50.0000000
43460.0000000	6.7500000	-50.0000000
43460.0000000	7.0000000	-50.0000000
43460.0000000	7.2500000	-50.0000000
43460.0000000	7.5000000	-50.0000000
43460.0000000	7.7500000	-50.0000000
43460.0000000	8.0000000	-50.0000000
43460.0000000	8.2500000	-50.0000000
43460.0000000	8.5000000	-50.0000000
43460.0000000	8.7500000	-50.0000000
43460.0000000	9.0000000	-50.0000000
43460.0000000	9.2500000	-50.0000000
43460.0000000	9.5000000	-50.0000000
43460.0000000	9.7500000	-50.0000000
43460.0000000	10.0000000	-50.0000000
43460.0000000	10.2500000	-50.0000000
43460.0000000	10.5000000	-50.0000000
43460.0000000	10.7500000	-50.0000000
43460.0000000	11.0000000	-50.0000000
43460.0000000	11.2500000	-50.0000000
43460.0000000	11.5000000	-50.0000000
43460.0000000	11.7500000	-50.0000000
43460.0000000	12.0000000	-50.0000000
43460.0000000	12.2500000	-50.0000000
43460.0000000	12.5000000	-50.0000000
43460.0000000	12.7500000	-50.0000000
43460.0000000	13.0000000	-50.0000000
43460.0000000	13.2500000	-50.0000000
43460.0000000	13.5000000	-50.0000000
43460.0000000	13.7500000	-50.0000000
43460.0000000	14.0000000	-50.0000000
43460.0000000	14.2500000	-50.0000000
43460.0000000	14.5000000	-50.0000000
43460.0000000	14.7500000	-50.0000000
43460.0000000	15.0000000	-50.0000000
43490.0000000	0.0000000	-50.0000000
43490.0000000	0.2500000	-50.0000000
43490.0000000	0.5000000	-50.0000000
43490.0000000	0.7500000	-50.0000000
43490.0000000	1.0000000	-50.0000000
43490.0000000	1.2500000	-50.0000000
43490.0000000	1.5000000	-50.0000000

43490.0000000	1.7500000	-50.0000000
43490.0000000	2.0000000	-50.0000000
43490.0000000	2.2500000	-50.0000000
43490.0000000	2.5000000	-50.0000000
43490.0000000	2.7500000	-50.0000000
43490.0000000	3.0000000	-50.0000000
43490.0000000	3.2500000	-50.0000000
43490.0000000	3.5000000	-50.0000000
43490.0000000	3.7500000	-50.0000000
43490.0000000	4.0000000	-50.0000000
43490.0000000	4.2500000	-50.0000000
43490.0000000	4.5000000	-50.0000000
43490.0000000	4.7500000	-50.0000000
43490.0000000	5.0000000	-50.0000000
43490.0000000	5.2500000	-50.0000000
43490.0000000	5.5000000	-50.0000000
43490.0000000	5.7500000	-50.0000000
43490.0000000	6.0000000	-50.0000000
43490.0000000	6.2500000	-50.0000000
43490.0000000	6.5000000	-50.0000000
43490.0000000	6.7500000	-50.0000000
43490.0000000	7.0000000	-50.0000000
43490.0000000	7.2500000	-50.0000000
43490.0000000	7.5000000	-50.0000000
43490.0000000	7.7500000	-50.0000000
43490.0000000	8.0000000	-50.0000000
43490.0000000	8.2500000	-50.0000000
43490.0000000	8.5000000	-50.0000000
43490.0000000	8.7500000	-50.0000000
43490.0000000	9.0000000	-50.0000000
43490.0000000	9.2500000	-50.0000000
43490.0000000	9.5000000	-50.0000000
43490.0000000	9.7500000	-50.0000000
43490.0000000	10.0000000	-50.0000000
43490.0000000	10.2500000	-50.0000000
43490.0000000	10.5000000	-50.0000000
43490.0000000	10.7500000	-50.0000000
43490.0000000	11.0000000	-50.0000000
43490.0000000	11.2500000	-50.0000000
43490.0000000	11.5000000	-50.0000000
43490.0000000	11.7500000	-50.0000000
43490.0000000	12.0000000	-50.0000000
43490.0000000	12.2500000	-50.0000000
43490.0000000	12.5000000	-50.0000000
43490.0000000	12.7500000	-50.0000000
43490.0000000	13.0000000	-50.0000000
43490.0000000	13.2500000	-50.0000000
43490.0000000	13.5000000	-50.0000000
43490.0000000	13.7500000	-50.0000000
43490.0000000	14.0000000	-50.0000000
43490.0000000	14.2500000	-50.0000000
43490.0000000	14.5000000	-50.0000000
43490.0000000	14.7500000	-50.0000000
43490.0000000	15.0000000	-50.0000000
43520.0000000	0.0000000	-50.0000000
43520.0000000	0.2500000	-50.0000000
43520.0000000	0.5000000	-50.0000000
43520.0000000	0.7500000	-50.0000000

43520.0000000	1.0000000	-50.0000000
43520.0000000	1.2500000	-50.0000000
43520.0000000	1.5000000	-50.0000000
43520.0000000	1.7500000	-50.0000000
43520.0000000	2.0000000	-50.0000000
43520.0000000	2.2500000	-50.0000000
43520.0000000	2.5000000	-50.0000000
43520.0000000	2.7500000	-50.0000000
43520.0000000	3.0000000	-50.0000000
43520.0000000	3.2500000	-50.0000000
43520.0000000	3.5000000	-50.0000000
43520.0000000	3.7500000	-50.0000000
43520.0000000	4.0000000	-50.0000000
43520.0000000	4.2500000	-50.0000000
43520.0000000	4.5000000	-50.0000000
43520.0000000	4.7500000	-50.0000000
43520.0000000	5.0000000	-50.0000000
43520.0000000	5.2500000	-50.0000000
43520.0000000	5.5000000	-50.0000000
43520.0000000	5.7500000	-50.0000000
43520.0000000	6.0000000	-50.0000000
43520.0000000	6.2500000	-50.0000000
43520.0000000	6.5000000	-50.0000000
43520.0000000	6.7500000	-50.0000000
43520.0000000	7.0000000	-50.0000000
43520.0000000	7.2500000	-50.0000000
43520.0000000	7.5000000	-50.0000000
43520.0000000	7.7500000	-50.0000000
43520.0000000	8.0000000	-50.0000000
43520.0000000	8.2500000	-50.0000000
43520.0000000	8.5000000	-50.0000000
43520.0000000	8.7500000	-50.0000000
43520.0000000	9.0000000	-50.0000000
43520.0000000	9.2500000	-50.0000000
43520.0000000	9.5000000	-50.0000000
43520.0000000	9.7500000	-50.0000000
43520.0000000	10.0000000	-50.0000000
43520.0000000	10.2500000	-50.0000000
43520.0000000	10.5000000	-50.0000000
43520.0000000	10.7500000	-50.0000000
43520.0000000	11.0000000	-50.0000000
43520.0000000	11.2500000	-50.0000000
43520.0000000	11.5000000	-50.0000000
43520.0000000	11.7500000	-50.0000000
43520.0000000	12.0000000	-50.0000000
43520.0000000	12.2500000	-50.0000000
43520.0000000	12.5000000	-50.0000000
43520.0000000	12.7500000	-50.0000000
43520.0000000	13.0000000	-50.0000000
43520.0000000	13.2500000	-50.0000000
43520.0000000	13.5000000	-50.0000000
43520.0000000	13.7500000	-50.0000000
43520.0000000	14.0000000	-50.0000000
43520.0000000	14.2500000	-50.0000000
43520.0000000	14.5000000	-50.0000000
43520.0000000	14.7500000	-50.0000000
43520.0000000	15.0000000	-50.0000000
43550.0000000	0.0000000	-50.0000000

43550.0000000	0.2500000	-50.0000000
43550.0000000	0.5000000	-50.0000000
43550.0000000	0.7500000	-50.0000000
43550.0000000	1.0000000	-50.0000000
43550.0000000	1.2500000	-50.0000000
43550.0000000	1.5000000	-50.0000000
43550.0000000	1.7500000	-50.0000000
43550.0000000	2.0000000	-50.0000000
43550.0000000	2.2500000	-50.0000000
43550.0000000	2.5000000	-50.0000000
43550.0000000	2.7500000	-50.0000000
43550.0000000	3.0000000	-50.0000000
43550.0000000	3.2500000	-50.0000000
43550.0000000	3.5000000	-50.0000000
43550.0000000	3.7500000	-50.0000000
43550.0000000	4.0000000	-50.0000000
43550.0000000	4.2500000	-50.0000000
43550.0000000	4.5000000	-50.0000000
43550.0000000	4.7500000	-50.0000000
43550.0000000	5.0000000	-50.0000000
43550.0000000	5.2500000	-50.0000000
43550.0000000	5.5000000	-50.0000000
43550.0000000	5.7500000	-50.0000000
43550.0000000	6.0000000	-50.0000000
43550.0000000	6.2500000	-50.0000000
43550.0000000	6.5000000	-50.0000000
43550.0000000	6.7500000	-50.0000000
43550.0000000	7.0000000	-50.0000000
43550.0000000	7.2500000	-50.0000000
43550.0000000	7.5000000	-50.0000000
43550.0000000	7.7500000	-50.0000000
43550.0000000	8.0000000	-50.0000000
43550.0000000	8.2500000	-50.0000000
43550.0000000	8.5000000	-50.0000000
43550.0000000	8.7500000	-50.0000000
43550.0000000	9.0000000	-50.0000000
43550.0000000	9.2500000	-50.0000000
43550.0000000	9.5000000	-50.0000000
43550.0000000	9.7500000	-50.0000000
43550.0000000	10.0000000	-50.0000000
43550.0000000	10.2500000	-50.0000000
43550.0000000	10.5000000	-50.0000000
43550.0000000	10.7500000	-50.0000000
43550.0000000	11.0000000	-50.0000000
43550.0000000	11.2500000	-50.0000000
43550.0000000	11.5000000	-50.0000000
43550.0000000	11.7500000	-50.0000000
43550.0000000	12.0000000	-50.0000000
43550.0000000	12.2500000	-50.0000000
43550.0000000	12.5000000	-50.0000000
43550.0000000	12.7500000	-50.0000000
43550.0000000	13.0000000	-50.0000000
43550.0000000	13.2500000	-50.0000000
43550.0000000	13.5000000	-50.0000000
43550.0000000	13.7500000	-50.0000000
43550.0000000	14.0000000	-50.0000000
43550.0000000	14.2500000	-50.0000000
43550.0000000	14.5000000	-50.0000000

43550.0000000	14.7500000	-50.0000000
43550.0000000	15.0000000	-50.0000000
43580.0000000	0.0000000	-50.0000000
43580.0000000	0.2500000	-50.0000000
43580.0000000	0.5000000	-50.0000000
43580.0000000	0.7500000	-50.0000000
43580.0000000	1.0000000	-50.0000000
43580.0000000	1.2500000	-50.0000000
43580.0000000	1.5000000	-50.0000000
43580.0000000	1.7500000	-50.0000000
43580.0000000	2.0000000	-50.0000000
43580.0000000	2.2500000	-50.0000000
43580.0000000	2.5000000	-50.0000000
43580.0000000	2.7500000	-50.0000000
43580.0000000	3.0000000	-50.0000000
43580.0000000	3.2500000	-50.0000000
43580.0000000	3.5000000	-50.0000000
43580.0000000	3.7500000	-50.0000000
43580.0000000	4.0000000	-50.0000000
43580.0000000	4.2500000	-50.0000000
43580.0000000	4.5000000	-50.0000000
43580.0000000	4.7500000	-50.0000000
43580.0000000	5.0000000	-50.0000000
43580.0000000	5.2500000	-50.0000000
43580.0000000	5.5000000	-50.0000000
43580.0000000	5.7500000	-50.0000000
43580.0000000	6.0000000	-50.0000000
43580.0000000	6.2500000	-50.0000000
43580.0000000	6.5000000	-50.0000000
43580.0000000	6.7500000	-50.0000000
43580.0000000	7.0000000	-50.0000000
43580.0000000	7.2500000	-50.0000000
43580.0000000	7.5000000	-50.0000000
43580.0000000	7.7500000	-50.0000000
43580.0000000	8.0000000	-50.0000000
43580.0000000	8.2500000	-50.0000000
43580.0000000	8.5000000	-50.0000000
43580.0000000	8.7500000	-50.0000000
43580.0000000	9.0000000	-50.0000000
43580.0000000	9.2500000	-50.0000000
43580.0000000	9.5000000	-50.0000000
43580.0000000	9.7500000	-50.0000000
43580.0000000	10.0000000	-50.0000000
43580.0000000	10.2500000	-50.0000000
43580.0000000	10.5000000	-50.0000000
43580.0000000	10.7500000	-50.0000000
43580.0000000	11.0000000	-50.0000000
43580.0000000	11.2500000	-50.0000000
43580.0000000	11.5000000	-50.0000000
43580.0000000	11.7500000	-50.0000000
43580.0000000	12.0000000	-50.0000000
43580.0000000	12.2500000	-50.0000000
43580.0000000	12.5000000	-50.0000000
43580.0000000	12.7500000	-50.0000000
43580.0000000	13.0000000	-50.0000000
43580.0000000	13.2500000	-50.0000000
43580.0000000	13.5000000	-50.0000000
43580.0000000	13.7500000	-50.0000000

43580.0000000	14.0000000	-50.0000000
43580.0000000	14.2500000	-50.0000000
43580.0000000	14.5000000	-50.0000000
43580.0000000	14.7500000	-50.0000000
43580.0000000	15.0000000	-50.0000000
43610.0000000	0.0000000	-50.0000000
43610.0000000	0.2500000	-50.0000000
43610.0000000	0.5000000	-50.0000000
43610.0000000	0.7500000	-50.0000000
43610.0000000	1.0000000	-50.0000000
43610.0000000	1.2500000	-50.0000000
43610.0000000	1.5000000	-50.0000000
43610.0000000	1.7500000	-50.0000000
43610.0000000	2.0000000	-50.0000000
43610.0000000	2.2500000	-50.0000000
43610.0000000	2.5000000	-50.0000000
43610.0000000	2.7500000	-50.0000000
43610.0000000	3.0000000	-50.0000000
43610.0000000	3.2500000	-50.0000000
43610.0000000	3.5000000	-50.0000000
43610.0000000	3.7500000	-50.0000000
43610.0000000	4.0000000	-50.0000000
43610.0000000	4.2500000	-50.0000000
43610.0000000	4.5000000	-50.0000000
43610.0000000	4.7500000	-50.0000000
43610.0000000	5.0000000	-50.0000000
43610.0000000	5.2500000	-50.0000000
43610.0000000	5.5000000	-50.0000000
43610.0000000	5.7500000	-50.0000000
43610.0000000	6.0000000	-50.0000000
43610.0000000	6.2500000	-50.0000000
43610.0000000	6.5000000	-50.0000000
43610.0000000	6.7500000	-50.0000000
43610.0000000	7.0000000	-50.0000000
43610.0000000	7.2500000	-50.0000000
43610.0000000	7.5000000	-50.0000000
43610.0000000	7.7500000	-50.0000000
43610.0000000	8.0000000	-50.0000000
43610.0000000	8.2500000	-50.0000000
43610.0000000	8.5000000	-50.0000000
43610.0000000	8.7500000	-50.0000000
43610.0000000	9.0000000	-50.0000000
43610.0000000	9.2500000	-50.0000000
43610.0000000	9.5000000	-50.0000000
43610.0000000	9.7500000	-50.0000000
43610.0000000	10.0000000	-50.0000000
43610.0000000	10.2500000	-50.0000000
43610.0000000	10.5000000	-50.0000000
43610.0000000	10.7500000	-50.0000000
43610.0000000	11.0000000	-50.0000000
43610.0000000	11.2500000	-50.0000000
43610.0000000	11.5000000	-50.0000000
43610.0000000	11.7500000	-50.0000000
43610.0000000	12.0000000	-50.0000000
43610.0000000	12.2500000	-50.0000000
43610.0000000	12.5000000	-50.0000000
43610.0000000	12.7500000	-50.0000000
43610.0000000	13.0000000	-50.0000000

43610.0000000	13.2500000	-50.0000000
43610.0000000	13.5000000	-50.0000000
43610.0000000	13.7500000	-50.0000000
43610.0000000	14.0000000	-50.0000000
43610.0000000	14.2500000	-50.0000000
43610.0000000	14.5000000	-50.0000000
43610.0000000	14.7500000	-50.0000000
43610.0000000	15.0000000	-50.0000000
43640.0000000	0.0000000	-50.0000000
43640.0000000	0.2500000	-50.0000000
43640.0000000	0.5000000	-50.0000000
43640.0000000	0.7500000	-50.0000000
43640.0000000	1.0000000	-50.0000000
43640.0000000	1.2500000	-50.0000000
43640.0000000	1.5000000	-50.0000000
43640.0000000	1.7500000	-50.0000000
43640.0000000	2.0000000	-50.0000000
43640.0000000	2.2500000	-50.0000000
43640.0000000	2.5000000	-50.0000000
43640.0000000	2.7500000	-50.0000000
43640.0000000	3.0000000	-50.0000000
43640.0000000	3.2500000	-50.0000000
43640.0000000	3.5000000	-50.0000000
43640.0000000	3.7500000	-50.0000000
43640.0000000	4.0000000	-50.0000000
43640.0000000	4.2500000	-50.0000000
43640.0000000	4.5000000	-50.0000000
43640.0000000	4.7500000	-50.0000000
43640.0000000	5.0000000	-50.0000000
43640.0000000	5.2500000	-50.0000000
43640.0000000	5.5000000	-50.0000000
43640.0000000	5.7500000	-50.0000000
43640.0000000	6.0000000	-50.0000000
43640.0000000	6.2500000	-50.0000000
43640.0000000	6.5000000	-50.0000000
43640.0000000	6.7500000	-50.0000000
43640.0000000	7.0000000	-50.0000000
43640.0000000	7.2500000	-50.0000000
43640.0000000	7.5000000	-50.0000000
43640.0000000	7.7500000	-50.0000000
43640.0000000	8.0000000	-50.0000000
43640.0000000	8.2500000	-50.0000000
43640.0000000	8.5000000	-50.0000000
43640.0000000	8.7500000	-50.0000000
43640.0000000	9.0000000	-50.0000000
43640.0000000	9.2500000	-50.0000000
43640.0000000	9.5000000	-50.0000000
43640.0000000	9.7500000	-50.0000000
43640.0000000	10.0000000	-50.0000000
43640.0000000	10.2500000	-50.0000000
43640.0000000	10.5000000	-50.0000000
43640.0000000	10.7500000	-50.0000000
43640.0000000	11.0000000	-50.0000000
43640.0000000	11.2500000	-50.0000000
43640.0000000	11.5000000	-50.0000000
43640.0000000	11.7500000	-50.0000000
43640.0000000	12.0000000	-50.0000000
43640.0000000	12.2500000	-50.0000000

43640.0000000	12.5000000	-50.0000000
43640.0000000	12.7500000	-50.0000000
43640.0000000	13.0000000	-50.0000000
43640.0000000	13.2500000	-50.0000000
43640.0000000	13.5000000	-50.0000000
43640.0000000	13.7500000	-50.0000000
43640.0000000	14.0000000	-50.0000000
43640.0000000	14.2500000	-50.0000000
43640.0000000	14.5000000	-50.0000000
43640.0000000	14.7500000	-50.0000000
43640.0000000	15.0000000	-50.0000000
43670.0000000	0.0000000	-50.0000000
43670.0000000	0.2500000	-50.0000000
43670.0000000	0.5000000	-50.0000000
43670.0000000	0.7500000	-50.0000000
43670.0000000	1.0000000	-50.0000000
43670.0000000	1.2500000	-50.0000000
43670.0000000	1.5000000	-50.0000000
43670.0000000	1.7500000	-50.0000000
43670.0000000	2.0000000	-50.0000000
43670.0000000	2.2500000	-50.0000000
43670.0000000	2.5000000	-50.0000000
43670.0000000	2.7500000	-50.0000000
43670.0000000	3.0000000	-50.0000000
43670.0000000	3.2500000	-50.0000000
43670.0000000	3.5000000	-50.0000000
43670.0000000	3.7500000	-50.0000000
43670.0000000	4.0000000	-50.0000000
43670.0000000	4.2500000	-50.0000000
43670.0000000	4.5000000	-50.0000000
43670.0000000	4.7500000	-50.0000000
43670.0000000	5.0000000	-50.0000000
43670.0000000	5.2500000	-50.0000000
43670.0000000	5.5000000	-50.0000000
43670.0000000	5.7500000	-50.0000000
43670.0000000	6.0000000	-50.0000000
43670.0000000	6.2500000	-50.0000000
43670.0000000	6.5000000	-50.0000000
43670.0000000	6.7500000	-50.0000000
43670.0000000	7.0000000	-50.0000000
43670.0000000	7.2500000	-50.0000000
43670.0000000	7.5000000	-50.0000000
43670.0000000	7.7500000	-50.0000000
43670.0000000	8.0000000	-50.0000000
43670.0000000	8.2500000	-50.0000000
43670.0000000	8.5000000	-50.0000000
43670.0000000	8.7500000	-50.0000000
43670.0000000	9.0000000	-50.0000000
43670.0000000	9.2500000	-50.0000000
43670.0000000	9.5000000	-50.0000000
43670.0000000	9.7500000	-50.0000000
43670.0000000	10.0000000	-50.0000000
43670.0000000	10.2500000	-50.0000000
43670.0000000	10.5000000	-50.0000000
43670.0000000	10.7500000	-50.0000000
43670.0000000	11.0000000	-50.0000000
43670.0000000	11.2500000	-50.0000000
43670.0000000	11.5000000	-50.0000000

43670.0000000	11.7500000	-50.0000000
43670.0000000	12.0000000	-50.0000000
43670.0000000	12.2500000	-50.0000000
43670.0000000	12.5000000	-50.0000000
43670.0000000	12.7500000	-50.0000000
43670.0000000	13.0000000	-50.0000000
43670.0000000	13.2500000	-50.0000000
43670.0000000	13.5000000	-50.0000000
43670.0000000	13.7500000	-50.0000000
43670.0000000	14.0000000	-50.0000000
43670.0000000	14.2500000	-50.0000000
43670.0000000	14.5000000	-50.0000000
43670.0000000	14.7500000	-50.0000000
43670.0000000	15.0000000	-50.0000000
43700.0000000	0.0000000	-50.0000000
43700.0000000	0.2500000	-50.0000000
43700.0000000	0.5000000	-50.0000000
43700.0000000	0.7500000	-50.0000000
43700.0000000	1.0000000	-50.0000000
43700.0000000	1.2500000	-50.0000000
43700.0000000	1.5000000	-50.0000000
43700.0000000	1.7500000	-50.0000000
43700.0000000	2.0000000	-50.0000000
43700.0000000	2.2500000	-50.0000000
43700.0000000	2.5000000	-50.0000000
43700.0000000	2.7500000	-50.0000000
43700.0000000	3.0000000	-50.0000000
43700.0000000	3.2500000	-50.0000000
43700.0000000	3.5000000	-50.0000000
43700.0000000	3.7500000	-50.0000000
43700.0000000	4.0000000	-50.0000000
43700.0000000	4.2500000	-50.0000000
43700.0000000	4.5000000	-50.0000000
43700.0000000	4.7500000	-50.0000000
43700.0000000	5.0000000	-50.0000000
43700.0000000	5.2500000	-50.0000000
43700.0000000	5.5000000	-50.0000000
43700.0000000	5.7500000	-50.0000000
43700.0000000	6.0000000	-50.0000000
43700.0000000	6.2500000	-50.0000000
43700.0000000	6.5000000	-50.0000000
43700.0000000	6.7500000	-50.0000000
43700.0000000	7.0000000	-50.0000000
43700.0000000	7.2500000	-50.0000000
43700.0000000	7.5000000	-50.0000000
43700.0000000	7.7500000	-50.0000000
43700.0000000	8.0000000	-50.0000000
43700.0000000	8.2500000	-50.0000000
43700.0000000	8.5000000	-50.0000000
43700.0000000	8.7500000	-50.0000000
43700.0000000	9.0000000	-50.0000000
43700.0000000	9.2500000	-50.0000000
43700.0000000	9.5000000	-50.0000000
43700.0000000	9.7500000	-50.0000000
43700.0000000	10.0000000	-50.0000000
43700.0000000	10.2500000	-50.0000000
43700.0000000	10.5000000	-50.0000000
43700.0000000	10.7500000	-50.0000000

43700.0000000	11.0000000	-50.0000000
43700.0000000	11.2500000	-50.0000000
43700.0000000	11.5000000	-50.0000000
43700.0000000	11.7500000	-50.0000000
43700.0000000	12.0000000	-50.0000000
43700.0000000	12.2500000	-50.0000000
43700.0000000	12.5000000	-50.0000000
43700.0000000	12.7500000	-50.0000000
43700.0000000	13.0000000	-50.0000000
43700.0000000	13.2500000	-50.0000000
43700.0000000	13.5000000	-50.0000000
43700.0000000	13.7500000	-50.0000000
43700.0000000	14.0000000	-50.0000000
43700.0000000	14.2500000	-50.0000000
43700.0000000	14.5000000	-50.0000000
43700.0000000	14.7500000	-50.0000000
43700.0000000	15.0000000	-50.0000000
43730.0000000	0.0000000	-50.0000000
43730.0000000	0.2500000	-50.0000000
43730.0000000	0.5000000	-50.0000000
43730.0000000	0.7500000	-50.0000000
43730.0000000	1.0000000	-50.0000000
43730.0000000	1.2500000	-50.0000000
43730.0000000	1.5000000	-50.0000000
43730.0000000	1.7500000	-50.0000000
43730.0000000	2.0000000	-50.0000000
43730.0000000	2.2500000	-50.0000000
43730.0000000	2.5000000	-50.0000000
43730.0000000	2.7500000	-50.0000000
43730.0000000	3.0000000	-50.0000000
43730.0000000	3.2500000	-50.0000000
43730.0000000	3.5000000	-50.0000000
43730.0000000	3.7500000	-50.0000000
43730.0000000	4.0000000	-50.0000000
43730.0000000	4.2500000	-50.0000000
43730.0000000	4.5000000	-50.0000000
43730.0000000	4.7500000	-50.0000000
43730.0000000	5.0000000	-50.0000000
43730.0000000	5.2500000	-50.0000000
43730.0000000	5.5000000	-50.0000000
43730.0000000	5.7500000	-50.0000000
43730.0000000	6.0000000	-50.0000000
43730.0000000	6.2500000	-50.0000000
43730.0000000	6.5000000	-50.0000000
43730.0000000	6.7500000	-50.0000000
43730.0000000	7.0000000	-50.0000000
43730.0000000	7.2500000	-50.0000000
43730.0000000	7.5000000	-50.0000000
43730.0000000	7.7500000	-50.0000000
43730.0000000	8.0000000	-50.0000000
43730.0000000	8.2500000	-50.0000000
43730.0000000	8.5000000	-50.0000000
43730.0000000	8.7500000	-50.0000000
43730.0000000	9.0000000	-50.0000000
43730.0000000	9.2500000	-50.0000000
43730.0000000	9.5000000	-50.0000000
43730.0000000	9.7500000	-50.0000000
43730.0000000	10.0000000	-50.0000000

43730.0000000	10.2500000	-50.0000000
43730.0000000	10.5000000	-50.0000000
43730.0000000	10.7500000	-50.0000000
43730.0000000	11.0000000	-50.0000000
43730.0000000	11.2500000	-50.0000000
43730.0000000	11.5000000	-50.0000000
43730.0000000	11.7500000	-50.0000000
43730.0000000	12.0000000	-50.0000000
43730.0000000	12.2500000	-50.0000000
43730.0000000	12.5000000	-50.0000000
43730.0000000	12.7500000	-50.0000000
43730.0000000	13.0000000	-50.0000000
43730.0000000	13.2500000	-50.0000000
43730.0000000	13.5000000	-50.0000000
43730.0000000	13.7500000	-50.0000000
43730.0000000	14.0000000	-50.0000000
43730.0000000	14.2500000	-50.0000000
43730.0000000	14.5000000	-50.0000000
43730.0000000	14.7500000	-50.0000000
43730.0000000	15.0000000	-50.0000000
43760.0000000	0.0000000	-50.0000000
43760.0000000	0.2500000	-50.0000000
43760.0000000	0.5000000	-50.0000000
43760.0000000	0.7500000	-50.0000000
43760.0000000	1.0000000	-50.0000000
43760.0000000	1.2500000	-50.0000000
43760.0000000	1.5000000	-50.0000000
43760.0000000	1.7500000	-50.0000000
43760.0000000	2.0000000	-50.0000000
43760.0000000	2.2500000	-50.0000000
43760.0000000	2.5000000	-50.0000000
43760.0000000	2.7500000	-50.0000000
43760.0000000	3.0000000	-50.0000000
43760.0000000	3.2500000	-50.0000000
43760.0000000	3.5000000	-50.0000000
43760.0000000	3.7500000	-50.0000000
43760.0000000	4.0000000	-50.0000000
43760.0000000	4.2500000	-50.0000000
43760.0000000	4.5000000	-50.0000000
43760.0000000	4.7500000	-50.0000000
43760.0000000	5.0000000	-50.0000000
43760.0000000	5.2500000	-50.0000000
43760.0000000	5.5000000	-50.0000000
43760.0000000	5.7500000	-50.0000000
43760.0000000	6.0000000	-50.0000000
43760.0000000	6.2500000	-50.0000000
43760.0000000	6.5000000	-50.0000000
43760.0000000	6.7500000	-50.0000000
43760.0000000	7.0000000	-50.0000000
43760.0000000	7.2500000	-50.0000000
43760.0000000	7.5000000	-50.0000000
43760.0000000	7.7500000	-50.0000000
43760.0000000	8.0000000	-50.0000000
43760.0000000	8.2500000	-50.0000000
43760.0000000	8.5000000	-50.0000000
43760.0000000	8.7500000	-50.0000000
43760.0000000	9.0000000	-50.0000000
43760.0000000	9.2500000	-50.0000000

43760.0000000	9.5000000	-50.0000000
43760.0000000	9.7500000	-50.0000000
43760.0000000	10.0000000	-50.0000000
43760.0000000	10.2500000	-50.0000000
43760.0000000	10.5000000	-50.0000000
43760.0000000	10.7500000	-50.0000000
43760.0000000	11.0000000	-50.0000000
43760.0000000	11.2500000	-50.0000000
43760.0000000	11.5000000	-50.0000000
43760.0000000	11.7500000	-50.0000000
43760.0000000	12.0000000	-50.0000000
43760.0000000	12.2500000	-50.0000000
43760.0000000	12.5000000	-50.0000000
43760.0000000	12.7500000	-50.0000000
43760.0000000	13.0000000	-50.0000000
43760.0000000	13.2500000	-50.0000000
43760.0000000	13.5000000	-50.0000000
43760.0000000	13.7500000	-50.0000000
43760.0000000	14.0000000	-50.0000000
43760.0000000	14.2500000	-50.0000000
43760.0000000	14.5000000	-50.0000000
43760.0000000	14.7500000	-50.0000000
43760.0000000	15.0000000	-50.0000000
43790.0000000	0.0000000	-50.0000000
43790.0000000	0.2500000	-50.0000000
43790.0000000	0.5000000	-50.0000000
43790.0000000	0.7500000	-50.0000000
43790.0000000	1.0000000	-50.0000000
43790.0000000	1.2500000	-50.0000000
43790.0000000	1.5000000	-50.0000000
43790.0000000	1.7500000	-50.0000000
43790.0000000	2.0000000	-50.0000000
43790.0000000	2.2500000	-50.0000000
43790.0000000	2.5000000	-50.0000000
43790.0000000	2.7500000	-50.0000000
43790.0000000	3.0000000	-50.0000000
43790.0000000	3.2500000	-50.0000000
43790.0000000	3.5000000	-50.0000000
43790.0000000	3.7500000	-50.0000000
43790.0000000	4.0000000	-50.0000000
43790.0000000	4.2500000	-50.0000000
43790.0000000	4.5000000	-50.0000000
43790.0000000	4.7500000	-50.0000000
43790.0000000	5.0000000	-50.0000000
43790.0000000	5.2500000	-50.0000000
43790.0000000	5.5000000	-50.0000000
43790.0000000	5.7500000	-50.0000000
43790.0000000	6.0000000	-50.0000000
43790.0000000	6.2500000	-50.0000000
43790.0000000	6.5000000	-50.0000000
43790.0000000	6.7500000	-50.0000000
43790.0000000	7.0000000	-50.0000000
43790.0000000	7.2500000	-50.0000000
43790.0000000	7.5000000	-50.0000000
43790.0000000	7.7500000	-50.0000000
43790.0000000	8.0000000	-50.0000000
43790.0000000	8.2500000	-50.0000000
43790.0000000	8.5000000	-50.0000000

43790.0000000	8.7500000	-50.0000000
43790.0000000	9.0000000	-50.0000000
43790.0000000	9.2500000	-50.0000000
43790.0000000	9.5000000	-50.0000000
43790.0000000	9.7500000	-50.0000000
43790.0000000	10.0000000	-50.0000000
43790.0000000	10.2500000	-50.0000000
43790.0000000	10.5000000	-50.0000000
43790.0000000	10.7500000	-50.0000000
43790.0000000	11.0000000	-50.0000000
43790.0000000	11.2500000	-50.0000000
43790.0000000	11.5000000	-50.0000000
43790.0000000	11.7500000	-50.0000000
43790.0000000	12.0000000	-50.0000000
43790.0000000	12.2500000	-50.0000000
43790.0000000	12.5000000	-50.0000000
43790.0000000	12.7500000	-50.0000000
43790.0000000	13.0000000	-50.0000000
43790.0000000	13.2500000	-50.0000000
43790.0000000	13.5000000	-50.0000000
43790.0000000	13.7500000	-50.0000000
43790.0000000	14.0000000	-50.0000000
43790.0000000	14.2500000	-50.0000000
43790.0000000	14.5000000	-50.0000000
43790.0000000	14.7500000	-50.0000000
43790.0000000	15.0000000	-50.0000000
43820.0000000	0.0000000	-50.0000000
43820.0000000	0.2500000	-50.0000000
43820.0000000	0.5000000	-50.0000000
43820.0000000	0.7500000	-50.0000000
43820.0000000	1.0000000	-50.0000000
43820.0000000	1.2500000	-50.0000000
43820.0000000	1.5000000	-50.0000000
43820.0000000	1.7500000	-50.0000000
43820.0000000	2.0000000	-50.0000000
43820.0000000	2.2500000	-50.0000000
43820.0000000	2.5000000	-50.0000000
43820.0000000	2.7500000	-50.0000000
43820.0000000	3.0000000	-50.0000000
43820.0000000	3.2500000	-50.0000000
43820.0000000	3.5000000	-50.0000000
43820.0000000	3.7500000	-50.0000000
43820.0000000	4.0000000	-50.0000000
43820.0000000	4.2500000	-50.0000000
43820.0000000	4.5000000	-50.0000000
43820.0000000	4.7500000	-50.0000000
43820.0000000	5.0000000	-50.0000000
43820.0000000	5.2500000	-50.0000000
43820.0000000	5.5000000	-50.0000000
43820.0000000	5.7500000	-50.0000000
43820.0000000	6.0000000	-50.0000000
43820.0000000	6.2500000	-50.0000000
43820.0000000	6.5000000	-50.0000000
43820.0000000	6.7500000	-50.0000000
43820.0000000	7.0000000	-50.0000000
43820.0000000	7.2500000	-50.0000000
43820.0000000	7.5000000	-50.0000000
43820.0000000	7.7500000	-50.0000000

43820.0000000	8.0000000	-50.0000000
43820.0000000	8.2500000	-50.0000000
43820.0000000	8.5000000	-50.0000000
43820.0000000	8.7500000	-50.0000000
43820.0000000	9.0000000	-50.0000000
43820.0000000	9.2500000	-50.0000000
43820.0000000	9.5000000	-50.0000000
43820.0000000	9.7500000	-50.0000000
43820.0000000	10.0000000	-50.0000000
43820.0000000	10.2500000	-50.0000000
43820.0000000	10.5000000	-50.0000000
43820.0000000	10.7500000	-50.0000000
43820.0000000	11.0000000	-50.0000000
43820.0000000	11.2500000	-50.0000000
43820.0000000	11.5000000	-50.0000000
43820.0000000	11.7500000	-50.0000000
43820.0000000	12.0000000	-50.0000000
43820.0000000	12.2500000	-50.0000000
43820.0000000	12.5000000	-50.0000000
43820.0000000	12.7500000	-50.0000000
43820.0000000	13.0000000	-50.0000000
43820.0000000	13.2500000	-50.0000000
43820.0000000	13.5000000	-50.0000000
43820.0000000	13.7500000	-50.0000000
43820.0000000	14.0000000	-50.0000000
43820.0000000	14.2500000	-50.0000000
43820.0000000	14.5000000	-50.0000000
43820.0000000	14.7500000	-50.0000000
43820.0000000	15.0000000	-50.0000000
43850.0000000	0.0000000	-50.0000000
43850.0000000	0.2500000	-50.0000000
43850.0000000	0.5000000	-50.0000000
43850.0000000	0.7500000	-50.0000000
43850.0000000	1.0000000	-50.0000000
43850.0000000	1.2500000	-50.0000000
43850.0000000	1.5000000	-50.0000000
43850.0000000	1.7500000	-50.0000000
43850.0000000	2.0000000	-50.0000000
43850.0000000	2.2500000	-50.0000000
43850.0000000	2.5000000	-50.0000000
43850.0000000	2.7500000	-50.0000000
43850.0000000	3.0000000	-50.0000000
43850.0000000	3.2500000	-50.0000000
43850.0000000	3.5000000	-50.0000000
43850.0000000	3.7500000	-50.0000000
43850.0000000	4.0000000	-50.0000000
43850.0000000	4.2500000	-50.0000000
43850.0000000	4.5000000	-50.0000000
43850.0000000	4.7500000	-50.0000000
43850.0000000	5.0000000	-50.0000000
43850.0000000	5.2500000	-50.0000000
43850.0000000	5.5000000	-50.0000000
43850.0000000	5.7500000	-50.0000000
43850.0000000	6.0000000	-50.0000000
43850.0000000	6.2500000	-50.0000000
43850.0000000	6.5000000	-50.0000000
43850.0000000	6.7500000	-50.0000000
43850.0000000	7.0000000	-50.0000000

43850.0000000	7.2500000	-50.0000000
43850.0000000	7.5000000	-50.0000000
43850.0000000	7.7500000	-50.0000000
43850.0000000	8.0000000	-50.0000000
43850.0000000	8.2500000	-50.0000000
43850.0000000	8.5000000	-50.0000000
43850.0000000	8.7500000	-50.0000000
43850.0000000	9.0000000	-50.0000000
43850.0000000	9.2500000	-50.0000000
43850.0000000	9.5000000	-50.0000000
43850.0000000	9.7500000	-50.0000000
43850.0000000	10.0000000	-50.0000000
43850.0000000	10.2500000	-50.0000000
43850.0000000	10.5000000	-50.0000000
43850.0000000	10.7500000	-50.0000000
43850.0000000	11.0000000	-50.0000000
43850.0000000	11.2500000	-50.0000000
43850.0000000	11.5000000	-50.0000000
43850.0000000	11.7500000	-50.0000000
43850.0000000	12.0000000	-50.0000000
43850.0000000	12.2500000	-50.0000000
43850.0000000	12.5000000	-50.0000000
43850.0000000	12.7500000	-50.0000000
43850.0000000	13.0000000	-50.0000000
43850.0000000	13.2500000	-50.0000000
43850.0000000	13.5000000	-50.0000000
43850.0000000	13.7500000	-50.0000000
43850.0000000	14.0000000	-50.0000000
43850.0000000	14.2500000	-50.0000000
43850.0000000	14.5000000	-50.0000000
43850.0000000	14.7500000	-50.0000000
43850.0000000	15.0000000	-50.0000000
43880.0000000	0.0000000	-50.0000000
43880.0000000	0.2500000	-50.0000000
43880.0000000	0.5000000	-50.0000000
43880.0000000	0.7500000	-50.0000000
43880.0000000	1.0000000	-50.0000000
43880.0000000	1.2500000	-50.0000000
43880.0000000	1.5000000	-50.0000000
43880.0000000	1.7500000	-50.0000000
43880.0000000	2.0000000	-50.0000000
43880.0000000	2.2500000	-50.0000000
43880.0000000	2.5000000	-50.0000000
43880.0000000	2.7500000	-50.0000000
43880.0000000	3.0000000	-50.0000000
43880.0000000	3.2500000	-50.0000000
43880.0000000	3.5000000	-50.0000000
43880.0000000	3.7500000	-50.0000000
43880.0000000	4.0000000	-50.0000000
43880.0000000	4.2500000	-50.0000000
43880.0000000	4.5000000	-50.0000000
43880.0000000	4.7500000	-50.0000000
43880.0000000	5.0000000	-50.0000000
43880.0000000	5.2500000	-50.0000000
43880.0000000	5.5000000	-50.0000000
43880.0000000	5.7500000	-50.0000000
43880.0000000	6.0000000	-50.0000000
43880.0000000	6.2500000	-50.0000000

43880.0000000	6.5000000	-50.0000000
43880.0000000	6.7500000	-50.0000000
43880.0000000	7.0000000	-50.0000000
43880.0000000	7.2500000	-50.0000000
43880.0000000	7.5000000	-50.0000000
43880.0000000	7.7500000	-50.0000000
43880.0000000	8.0000000	-50.0000000
43880.0000000	8.2500000	-50.0000000
43880.0000000	8.5000000	-50.0000000
43880.0000000	8.7500000	-50.0000000
43880.0000000	9.0000000	-50.0000000
43880.0000000	9.2500000	-50.0000000
43880.0000000	9.5000000	-50.0000000
43880.0000000	9.7500000	-50.0000000
43880.0000000	10.0000000	-50.0000000
43880.0000000	10.2500000	-50.0000000
43880.0000000	10.5000000	-50.0000000
43880.0000000	10.7500000	-50.0000000
43880.0000000	11.0000000	-50.0000000
43880.0000000	11.2500000	-50.0000000
43880.0000000	11.5000000	-50.0000000
43880.0000000	11.7500000	-50.0000000
43880.0000000	12.0000000	-50.0000000
43880.0000000	12.2500000	-50.0000000
43880.0000000	12.5000000	-50.0000000
43880.0000000	12.7500000	-50.0000000
43880.0000000	13.0000000	-50.0000000
43880.0000000	13.2500000	-50.0000000
43880.0000000	13.5000000	-50.0000000
43880.0000000	13.7500000	-50.0000000
43880.0000000	14.0000000	-50.0000000
43880.0000000	14.2500000	-50.0000000
43880.0000000	14.5000000	-50.0000000
43880.0000000	14.7500000	-50.0000000
43880.0000000	15.0000000	-50.0000000
43910.0000000	0.0000000	-50.0000000
43910.0000000	0.2500000	-50.0000000
43910.0000000	0.5000000	-50.0000000
43910.0000000	0.7500000	-50.0000000
43910.0000000	1.0000000	-50.0000000
43910.0000000	1.2500000	-50.0000000
43910.0000000	1.5000000	-50.0000000
43910.0000000	1.7500000	-50.0000000
43910.0000000	2.0000000	-50.0000000
43910.0000000	2.2500000	-50.0000000
43910.0000000	2.5000000	-50.0000000
43910.0000000	2.7500000	-50.0000000
43910.0000000	3.0000000	-50.0000000
43910.0000000	3.2500000	-50.0000000
43910.0000000	3.5000000	-50.0000000
43910.0000000	3.7500000	-50.0000000
43910.0000000	4.0000000	-50.0000000
43910.0000000	4.2500000	-50.0000000
43910.0000000	4.5000000	-50.0000000
43910.0000000	4.7500000	-50.0000000
43910.0000000	5.0000000	-50.0000000
43910.0000000	5.2500000	-50.0000000
43910.0000000	5.5000000	-50.0000000

43910.0000000	5.7500000	-50.0000000
43910.0000000	6.0000000	-50.0000000
43910.0000000	6.2500000	-50.0000000
43910.0000000	6.5000000	-50.0000000
43910.0000000	6.7500000	-50.0000000
43910.0000000	7.0000000	-50.0000000
43910.0000000	7.2500000	-50.0000000
43910.0000000	7.5000000	-50.0000000
43910.0000000	7.7500000	-50.0000000
43910.0000000	8.0000000	-50.0000000
43910.0000000	8.2500000	-50.0000000
43910.0000000	8.5000000	-50.0000000
43910.0000000	8.7500000	-50.0000000
43910.0000000	9.0000000	-50.0000000
43910.0000000	9.2500000	-50.0000000
43910.0000000	9.5000000	-50.0000000
43910.0000000	9.7500000	-50.0000000
43910.0000000	10.0000000	-50.0000000
43910.0000000	10.2500000	-50.0000000
43910.0000000	10.5000000	-50.0000000
43910.0000000	10.7500000	-50.0000000
43910.0000000	11.0000000	-50.0000000
43910.0000000	11.2500000	-50.0000000
43910.0000000	11.5000000	-50.0000000
43910.0000000	11.7500000	-50.0000000
43910.0000000	12.0000000	-50.0000000
43910.0000000	12.2500000	-50.0000000
43910.0000000	12.5000000	-50.0000000
43910.0000000	12.7500000	-50.0000000
43910.0000000	13.0000000	-50.0000000
43910.0000000	13.2500000	-50.0000000
43910.0000000	13.5000000	-50.0000000
43910.0000000	13.7500000	-50.0000000
43910.0000000	14.0000000	-50.0000000
43910.0000000	14.2500000	-50.0000000
43910.0000000	14.5000000	-50.0000000
43910.0000000	14.7500000	-50.0000000
43910.0000000	15.0000000	-50.0000000
43940.0000000	0.0000000	-50.0000000
43940.0000000	0.2500000	-50.0000000
43940.0000000	0.5000000	-50.0000000
43940.0000000	0.7500000	-50.0000000
43940.0000000	1.0000000	-50.0000000
43940.0000000	1.2500000	-50.0000000
43940.0000000	1.5000000	-50.0000000
43940.0000000	1.7500000	-50.0000000
43940.0000000	2.0000000	-50.0000000
43940.0000000	2.2500000	-50.0000000
43940.0000000	2.5000000	-50.0000000
43940.0000000	2.7500000	-50.0000000
43940.0000000	3.0000000	-50.0000000
43940.0000000	3.2500000	-50.0000000
43940.0000000	3.5000000	-50.0000000
43940.0000000	3.7500000	-50.0000000
43940.0000000	4.0000000	-50.0000000
43940.0000000	4.2500000	-50.0000000
43940.0000000	4.5000000	-50.0000000
43940.0000000	4.7500000	-50.0000000

43940.0000000	5.0000000	-50.0000000
43940.0000000	5.2500000	-50.0000000
43940.0000000	5.5000000	-50.0000000
43940.0000000	5.7500000	-50.0000000
43940.0000000	6.0000000	-50.0000000
43940.0000000	6.2500000	-50.0000000
43940.0000000	6.5000000	-50.0000000
43940.0000000	6.7500000	-50.0000000
43940.0000000	7.0000000	-50.0000000
43940.0000000	7.2500000	-50.0000000
43940.0000000	7.5000000	-50.0000000
43940.0000000	7.7500000	-50.0000000
43940.0000000	8.0000000	-50.0000000
43940.0000000	8.2500000	-50.0000000
43940.0000000	8.5000000	-50.0000000
43940.0000000	8.7500000	-50.0000000
43940.0000000	9.0000000	-50.0000000
43940.0000000	9.2500000	-50.0000000
43940.0000000	9.5000000	-50.0000000
43940.0000000	9.7500000	-50.0000000
43940.0000000	10.0000000	-50.0000000
43940.0000000	10.2500000	-50.0000000
43940.0000000	10.5000000	-50.0000000
43940.0000000	10.7500000	-50.0000000
43940.0000000	11.0000000	-50.0000000
43940.0000000	11.2500000	-50.0000000
43940.0000000	11.5000000	-50.0000000
43940.0000000	11.7500000	-50.0000000
43940.0000000	12.0000000	-50.0000000
43940.0000000	12.2500000	-50.0000000
43940.0000000	12.5000000	-50.0000000
43940.0000000	12.7500000	-50.0000000
43940.0000000	13.0000000	-50.0000000
43940.0000000	13.2500000	-50.0000000
43940.0000000	13.5000000	-50.0000000
43940.0000000	13.7500000	-50.0000000
43940.0000000	14.0000000	-50.0000000
43940.0000000	14.2500000	-50.0000000
43940.0000000	14.5000000	-50.0000000
43940.0000000	14.7500000	-50.0000000
43940.0000000	15.0000000	-50.0000000
43970.0000000	0.0000000	-50.0000000
43970.0000000	0.2500000	-50.0000000
43970.0000000	0.5000000	-50.0000000
43970.0000000	0.7500000	-50.0000000
43970.0000000	1.0000000	-50.0000000
43970.0000000	1.2500000	-50.0000000
43970.0000000	1.5000000	-50.0000000
43970.0000000	1.7500000	-50.0000000
43970.0000000	2.0000000	-50.0000000
43970.0000000	2.2500000	-50.0000000
43970.0000000	2.5000000	-50.0000000
43970.0000000	2.7500000	-50.0000000
43970.0000000	3.0000000	-50.0000000
43970.0000000	3.2500000	-50.0000000
43970.0000000	3.5000000	-50.0000000
43970.0000000	3.7500000	-50.0000000
43970.0000000	4.0000000	-50.0000000

43970.0000000	4.2500000	-50.0000000
43970.0000000	4.5000000	-50.0000000
43970.0000000	4.7500000	-50.0000000
43970.0000000	5.0000000	-50.0000000
43970.0000000	5.2500000	-50.0000000
43970.0000000	5.5000000	-50.0000000
43970.0000000	5.7500000	-50.0000000
43970.0000000	6.0000000	-50.0000000
43970.0000000	6.2500000	-50.0000000
43970.0000000	6.5000000	-50.0000000
43970.0000000	6.7500000	-50.0000000
43970.0000000	7.0000000	-50.0000000
43970.0000000	7.2500000	-50.0000000
43970.0000000	7.5000000	-50.0000000
43970.0000000	7.7500000	-50.0000000
43970.0000000	8.0000000	-50.0000000
43970.0000000	8.2500000	-50.0000000
43970.0000000	8.5000000	-50.0000000
43970.0000000	8.7500000	-50.0000000
43970.0000000	9.0000000	-50.0000000
43970.0000000	9.2500000	-50.0000000
43970.0000000	9.5000000	-50.0000000
43970.0000000	9.7500000	-50.0000000
43970.0000000	10.0000000	-50.0000000
43970.0000000	10.2500000	-50.0000000
43970.0000000	10.5000000	-50.0000000
43970.0000000	10.7500000	-50.0000000
43970.0000000	11.0000000	-50.0000000
43970.0000000	11.2500000	-50.0000000
43970.0000000	11.5000000	-50.0000000
43970.0000000	11.7500000	-50.0000000
43970.0000000	12.0000000	-50.0000000
43970.0000000	12.2500000	-50.0000000
43970.0000000	12.5000000	-50.0000000
43970.0000000	12.7500000	-50.0000000
43970.0000000	13.0000000	-50.0000000
43970.0000000	13.2500000	-50.0000000
43970.0000000	13.5000000	-50.0000000
43970.0000000	13.7500000	-50.0000000
43970.0000000	14.0000000	-50.0000000
43970.0000000	14.2500000	-50.0000000
43970.0000000	14.5000000	-50.0000000
43970.0000000	14.7500000	-50.0000000
43970.0000000	15.0000000	-50.0000000
44000.0000000	0.0000000	-50.0000000
44000.0000000	0.2500000	-50.0000000
44000.0000000	0.5000000	-50.0000000
44000.0000000	0.7500000	-50.0000000
44000.0000000	1.0000000	-50.0000000
44000.0000000	1.2500000	-50.0000000
44000.0000000	1.5000000	-50.0000000
44000.0000000	1.7500000	-50.0000000
44000.0000000	2.0000000	-50.0000000
44000.0000000	2.2500000	-50.0000000
44000.0000000	2.5000000	-50.0000000
44000.0000000	2.7500000	-50.0000000
44000.0000000	3.0000000	-50.0000000
44000.0000000	3.2500000	-50.0000000

44000.0000000	3.5000000	-50.0000000
44000.0000000	3.7500000	-50.0000000
44000.0000000	4.0000000	-50.0000000
44000.0000000	4.2500000	-50.0000000
44000.0000000	4.5000000	-50.0000000
44000.0000000	4.7500000	-50.0000000
44000.0000000	5.0000000	-50.0000000
44000.0000000	5.2500000	-50.0000000
44000.0000000	5.5000000	-50.0000000
44000.0000000	5.7500000	-50.0000000
44000.0000000	6.0000000	-50.0000000
44000.0000000	6.2500000	-50.0000000
44000.0000000	6.5000000	-50.0000000
44000.0000000	6.7500000	-50.0000000
44000.0000000	7.0000000	-50.0000000
44000.0000000	7.2500000	-50.0000000
44000.0000000	7.5000000	-50.0000000
44000.0000000	7.7500000	-50.0000000
44000.0000000	8.0000000	-50.0000000
44000.0000000	8.2500000	-50.0000000
44000.0000000	8.5000000	-50.0000000
44000.0000000	8.7500000	-50.0000000
44000.0000000	9.0000000	-50.0000000
44000.0000000	9.2500000	-50.0000000
44000.0000000	9.5000000	-50.0000000
44000.0000000	9.7500000	-50.0000000
44000.0000000	10.0000000	-50.0000000
44000.0000000	10.2500000	-50.0000000
44000.0000000	10.5000000	-50.0000000
44000.0000000	10.7500000	-50.0000000
44000.0000000	11.0000000	-50.0000000
44000.0000000	11.2500000	-50.0000000
44000.0000000	11.5000000	-50.0000000
44000.0000000	11.7500000	-50.0000000
44000.0000000	12.0000000	-50.0000000
44000.0000000	12.2500000	-50.0000000
44000.0000000	12.5000000	-50.0000000
44000.0000000	12.7500000	-50.0000000
44000.0000000	13.0000000	-50.0000000
44000.0000000	13.2500000	-50.0000000
44000.0000000	13.5000000	-50.0000000
44000.0000000	13.7500000	-50.0000000
44000.0000000	14.0000000	-50.0000000
44000.0000000	14.2500000	-50.0000000
44000.0000000	14.5000000	-50.0000000
44000.0000000	14.7500000	-50.0000000
44000.0000000	15.0000000	-50.0000000
44030.0000000	0.0000000	-50.0000000
44030.0000000	0.2500000	-50.0000000
44030.0000000	0.5000000	-50.0000000
44030.0000000	0.7500000	-50.0000000
44030.0000000	1.0000000	-50.0000000
44030.0000000	1.2500000	-50.0000000
44030.0000000	1.5000000	-50.0000000
44030.0000000	1.7500000	-50.0000000
44030.0000000	2.0000000	-50.0000000
44030.0000000	2.2500000	-50.0000000
44030.0000000	2.5000000	-50.0000000

44030.0000000	2.7500000	-50.0000000
44030.0000000	3.0000000	-50.0000000
44030.0000000	3.2500000	-50.0000000
44030.0000000	3.5000000	-50.0000000
44030.0000000	3.7500000	-50.0000000
44030.0000000	4.0000000	-50.0000000
44030.0000000	4.2500000	-50.0000000
44030.0000000	4.5000000	-50.0000000
44030.0000000	4.7500000	-50.0000000
44030.0000000	5.0000000	-50.0000000
44030.0000000	5.2500000	-50.0000000
44030.0000000	5.5000000	-50.0000000
44030.0000000	5.7500000	-50.0000000
44030.0000000	6.0000000	-50.0000000
44030.0000000	6.2500000	-50.0000000
44030.0000000	6.5000000	-50.0000000
44030.0000000	6.7500000	-50.0000000
44030.0000000	7.0000000	-50.0000000
44030.0000000	7.2500000	-50.0000000
44030.0000000	7.5000000	-50.0000000
44030.0000000	7.7500000	-50.0000000
44030.0000000	8.0000000	-50.0000000
44030.0000000	8.2500000	-50.0000000
44030.0000000	8.5000000	-50.0000000
44030.0000000	8.7500000	-50.0000000
44030.0000000	9.0000000	-50.0000000
44030.0000000	9.2500000	-50.0000000
44030.0000000	9.5000000	-50.0000000
44030.0000000	9.7500000	-50.0000000
44030.0000000	10.0000000	-50.0000000
44030.0000000	10.2500000	-50.0000000
44030.0000000	10.5000000	-50.0000000
44030.0000000	10.7500000	-50.0000000
44030.0000000	11.0000000	-50.0000000
44030.0000000	11.2500000	-50.0000000
44030.0000000	11.5000000	-50.0000000
44030.0000000	11.7500000	-50.0000000
44030.0000000	12.0000000	-50.0000000
44030.0000000	12.2500000	-50.0000000
44030.0000000	12.5000000	-50.0000000
44030.0000000	12.7500000	-50.0000000
44030.0000000	13.0000000	-50.0000000
44030.0000000	13.2500000	-50.0000000
44030.0000000	13.5000000	-50.0000000
44030.0000000	13.7500000	-50.0000000
44030.0000000	14.0000000	-50.0000000
44030.0000000	14.2500000	-50.0000000
44030.0000000	14.5000000	-50.0000000
44030.0000000	14.7500000	-50.0000000
44030.0000000	15.0000000	-50.0000000
44060.0000000	0.0000000	-50.0000000
44060.0000000	0.2500000	-50.0000000
44060.0000000	0.5000000	-50.0000000
44060.0000000	0.7500000	-50.0000000
44060.0000000	1.0000000	-50.0000000
44060.0000000	1.2500000	-50.0000000
44060.0000000	1.5000000	-50.0000000
44060.0000000	1.7500000	-50.0000000

44060.0000000	2.0000000	-50.0000000
44060.0000000	2.2500000	-50.0000000
44060.0000000	2.5000000	-50.0000000
44060.0000000	2.7500000	-50.0000000
44060.0000000	3.0000000	-50.0000000
44060.0000000	3.2500000	-50.0000000
44060.0000000	3.5000000	-50.0000000
44060.0000000	3.7500000	-50.0000000
44060.0000000	4.0000000	-50.0000000
44060.0000000	4.2500000	-50.0000000
44060.0000000	4.5000000	-50.0000000
44060.0000000	4.7500000	-50.0000000
44060.0000000	5.0000000	-50.0000000
44060.0000000	5.2500000	-50.0000000
44060.0000000	5.5000000	-50.0000000
44060.0000000	5.7500000	-50.0000000
44060.0000000	6.0000000	-50.0000000
44060.0000000	6.2500000	-50.0000000
44060.0000000	6.5000000	-50.0000000
44060.0000000	6.7500000	-50.0000000
44060.0000000	7.0000000	-50.0000000
44060.0000000	7.2500000	-50.0000000
44060.0000000	7.5000000	-50.0000000
44060.0000000	7.7500000	-50.0000000
44060.0000000	8.0000000	-50.0000000
44060.0000000	8.2500000	-50.0000000
44060.0000000	8.5000000	-50.0000000
44060.0000000	8.7500000	-50.0000000
44060.0000000	9.0000000	-50.0000000
44060.0000000	9.2500000	-50.0000000
44060.0000000	9.5000000	-50.0000000
44060.0000000	9.7500000	-50.0000000
44060.0000000	10.0000000	-50.0000000
44060.0000000	10.2500000	-50.0000000
44060.0000000	10.5000000	-50.0000000
44060.0000000	10.7500000	-50.0000000
44060.0000000	11.0000000	-50.0000000
44060.0000000	11.2500000	-50.0000000
44060.0000000	11.5000000	-50.0000000
44060.0000000	11.7500000	-50.0000000
44060.0000000	12.0000000	-50.0000000
44060.0000000	12.2500000	-50.0000000
44060.0000000	12.5000000	-50.0000000
44060.0000000	12.7500000	-50.0000000
44060.0000000	13.0000000	-50.0000000
44060.0000000	13.2500000	-50.0000000
44060.0000000	13.5000000	-50.0000000
44060.0000000	13.7500000	-50.0000000
44060.0000000	14.0000000	-50.0000000
44060.0000000	14.2500000	-50.0000000
44060.0000000	14.5000000	-50.0000000
44060.0000000	14.7500000	-50.0000000
44060.0000000	15.0000000	-50.0000000
44090.0000000	0.0000000	-50.0000000
44090.0000000	0.2500000	-50.0000000
44090.0000000	0.5000000	-50.0000000
44090.0000000	0.7500000	-50.0000000
44090.0000000	1.0000000	-50.0000000

44090.0000000	1.2500000	-50.0000000
44090.0000000	1.5000000	-50.0000000
44090.0000000	1.7500000	-50.0000000
44090.0000000	2.0000000	-50.0000000
44090.0000000	2.2500000	-50.0000000
44090.0000000	2.5000000	-50.0000000
44090.0000000	2.7500000	-50.0000000
44090.0000000	3.0000000	-50.0000000
44090.0000000	3.2500000	-50.0000000
44090.0000000	3.5000000	-50.0000000
44090.0000000	3.7500000	-50.0000000
44090.0000000	4.0000000	-50.0000000
44090.0000000	4.2500000	-50.0000000
44090.0000000	4.5000000	-50.0000000
44090.0000000	4.7500000	-50.0000000
44090.0000000	5.0000000	-50.0000000
44090.0000000	5.2500000	-50.0000000
44090.0000000	5.5000000	-50.0000000
44090.0000000	5.7500000	-50.0000000
44090.0000000	6.0000000	-50.0000000
44090.0000000	6.2500000	-50.0000000
44090.0000000	6.5000000	-50.0000000
44090.0000000	6.7500000	-50.0000000
44090.0000000	7.0000000	-50.0000000
44090.0000000	7.2500000	-50.0000000
44090.0000000	7.5000000	-50.0000000
44090.0000000	7.7500000	-50.0000000
44090.0000000	8.0000000	-50.0000000
44090.0000000	8.2500000	-50.0000000
44090.0000000	8.5000000	-50.0000000
44090.0000000	8.7500000	-50.0000000
44090.0000000	9.0000000	-50.0000000
44090.0000000	9.2500000	-50.0000000
44090.0000000	9.5000000	-50.0000000
44090.0000000	9.7500000	-50.0000000
44090.0000000	10.0000000	-50.0000000
44090.0000000	10.2500000	-50.0000000
44090.0000000	10.5000000	-50.0000000
44090.0000000	10.7500000	-50.0000000
44090.0000000	11.0000000	-50.0000000
44090.0000000	11.2500000	-50.0000000
44090.0000000	11.5000000	-50.0000000
44090.0000000	11.7500000	-50.0000000
44090.0000000	12.0000000	-50.0000000
44090.0000000	12.2500000	-50.0000000
44090.0000000	12.5000000	-50.0000000
44090.0000000	12.7500000	-50.0000000
44090.0000000	13.0000000	-50.0000000
44090.0000000	13.2500000	-50.0000000
44090.0000000	13.5000000	-50.0000000
44090.0000000	13.7500000	-50.0000000
44090.0000000	14.0000000	-50.0000000
44090.0000000	14.2500000	-50.0000000
44090.0000000	14.5000000	-50.0000000
44090.0000000	14.7500000	-50.0000000
44090.0000000	15.0000000	-50.0000000
44120.0000000	0.0000000	-50.0000000
44120.0000000	0.2500000	-50.0000000

44120.0000000	0.5000000	-50.0000000
44120.0000000	0.7500000	-50.0000000
44120.0000000	1.0000000	-50.0000000
44120.0000000	1.2500000	-50.0000000
44120.0000000	1.5000000	-50.0000000
44120.0000000	1.7500000	-50.0000000
44120.0000000	2.0000000	-50.0000000
44120.0000000	2.2500000	-50.0000000
44120.0000000	2.5000000	-50.0000000
44120.0000000	2.7500000	-50.0000000
44120.0000000	3.0000000	-50.0000000
44120.0000000	3.2500000	-50.0000000
44120.0000000	3.5000000	-50.0000000
44120.0000000	3.7500000	-50.0000000
44120.0000000	4.0000000	-50.0000000
44120.0000000	4.2500000	-50.0000000
44120.0000000	4.5000000	-50.0000000
44120.0000000	4.7500000	-50.0000000
44120.0000000	5.0000000	-50.0000000
44120.0000000	5.2500000	-50.0000000
44120.0000000	5.5000000	-50.0000000
44120.0000000	5.7500000	-50.0000000
44120.0000000	6.0000000	-50.0000000
44120.0000000	6.2500000	-50.0000000
44120.0000000	6.5000000	-50.0000000
44120.0000000	6.7500000	-50.0000000
44120.0000000	7.0000000	-50.0000000
44120.0000000	7.2500000	-50.0000000
44120.0000000	7.5000000	-50.0000000
44120.0000000	7.7500000	-50.0000000
44120.0000000	8.0000000	-50.0000000
44120.0000000	8.2500000	-50.0000000
44120.0000000	8.5000000	-50.0000000
44120.0000000	8.7500000	-50.0000000
44120.0000000	9.0000000	-50.0000000
44120.0000000	9.2500000	-50.0000000
44120.0000000	9.5000000	-50.0000000
44120.0000000	9.7500000	-50.0000000
44120.0000000	10.0000000	-50.0000000
44120.0000000	10.2500000	-50.0000000
44120.0000000	10.5000000	-50.0000000
44120.0000000	10.7500000	-50.0000000
44120.0000000	11.0000000	-50.0000000
44120.0000000	11.2500000	-50.0000000
44120.0000000	11.5000000	-50.0000000
44120.0000000	11.7500000	-50.0000000
44120.0000000	12.0000000	-50.0000000
44120.0000000	12.2500000	-50.0000000
44120.0000000	12.5000000	-50.0000000
44120.0000000	12.7500000	-50.0000000
44120.0000000	13.0000000	-50.0000000
44120.0000000	13.2500000	-50.0000000
44120.0000000	13.5000000	-50.0000000
44120.0000000	13.7500000	-50.0000000
44120.0000000	14.0000000	-50.0000000
44120.0000000	14.2500000	-50.0000000
44120.0000000	14.5000000	-50.0000000
44120.0000000	14.7500000	-50.0000000

44120.0000000	15.0000000	-50.0000000
44150.0000000	0.0000000	-50.0000000
44150.0000000	0.2500000	-50.0000000
44150.0000000	0.5000000	-50.0000000
44150.0000000	0.7500000	-50.0000000
44150.0000000	1.0000000	-50.0000000
44150.0000000	1.2500000	-50.0000000
44150.0000000	1.5000000	-50.0000000
44150.0000000	1.7500000	-50.0000000
44150.0000000	2.0000000	-50.0000000
44150.0000000	2.2500000	-50.0000000
44150.0000000	2.5000000	-50.0000000
44150.0000000	2.7500000	-50.0000000
44150.0000000	3.0000000	-50.0000000
44150.0000000	3.2500000	-50.0000000
44150.0000000	3.5000000	-50.0000000
44150.0000000	3.7500000	-50.0000000
44150.0000000	4.0000000	-50.0000000
44150.0000000	4.2500000	-50.0000000
44150.0000000	4.5000000	-50.0000000
44150.0000000	4.7500000	-50.0000000
44150.0000000	5.0000000	-50.0000000
44150.0000000	5.2500000	-50.0000000
44150.0000000	5.5000000	-50.0000000
44150.0000000	5.7500000	-50.0000000
44150.0000000	6.0000000	-50.0000000
44150.0000000	6.2500000	-50.0000000
44150.0000000	6.5000000	-50.0000000
44150.0000000	6.7500000	-50.0000000
44150.0000000	7.0000000	-50.0000000
44150.0000000	7.2500000	-50.0000000
44150.0000000	7.5000000	-50.0000000
44150.0000000	7.7500000	-50.0000000
44150.0000000	8.0000000	-50.0000000
44150.0000000	8.2500000	-50.0000000
44150.0000000	8.5000000	-50.0000000
44150.0000000	8.7500000	-50.0000000
44150.0000000	9.0000000	-50.0000000
44150.0000000	9.2500000	-50.0000000
44150.0000000	9.5000000	-50.0000000
44150.0000000	9.7500000	-50.0000000
44150.0000000	10.0000000	-50.0000000
44150.0000000	10.2500000	-50.0000000
44150.0000000	10.5000000	-50.0000000
44150.0000000	10.7500000	-50.0000000
44150.0000000	11.0000000	-50.0000000
44150.0000000	11.2500000	-50.0000000
44150.0000000	11.5000000	-50.0000000
44150.0000000	11.7500000	-50.0000000
44150.0000000	12.0000000	-50.0000000
44150.0000000	12.2500000	-50.0000000
44150.0000000	12.5000000	-50.0000000
44150.0000000	12.7500000	-50.0000000
44150.0000000	13.0000000	-50.0000000
44150.0000000	13.2500000	-50.0000000
44150.0000000	13.5000000	-50.0000000
44150.0000000	13.7500000	-50.0000000
44150.0000000	14.0000000	-50.0000000

44150.0000000	14.2500000	-50.0000000
44150.0000000	14.5000000	-50.0000000
44150.0000000	14.7500000	-50.0000000
44150.0000000	15.0000000	-50.0000000
44180.0000000	0.0000000	-50.0000000
44180.0000000	0.2500000	-50.0000000
44180.0000000	0.5000000	-50.0000000
44180.0000000	0.7500000	-50.0000000
44180.0000000	1.0000000	-50.0000000
44180.0000000	1.2500000	-50.0000000
44180.0000000	1.5000000	-50.0000000
44180.0000000	1.7500000	-50.0000000
44180.0000000	2.0000000	-50.0000000
44180.0000000	2.2500000	-50.0000000
44180.0000000	2.5000000	-50.0000000
44180.0000000	2.7500000	-50.0000000
44180.0000000	3.0000000	-50.0000000
44180.0000000	3.2500000	-50.0000000
44180.0000000	3.5000000	-50.0000000
44180.0000000	3.7500000	-50.0000000
44180.0000000	4.0000000	-50.0000000
44180.0000000	4.2500000	-50.0000000
44180.0000000	4.5000000	-50.0000000
44180.0000000	4.7500000	-50.0000000
44180.0000000	5.0000000	-50.0000000
44180.0000000	5.2500000	-50.0000000
44180.0000000	5.5000000	-50.0000000
44180.0000000	5.7500000	-50.0000000
44180.0000000	6.0000000	-50.0000000
44180.0000000	6.2500000	-50.0000000
44180.0000000	6.5000000	-50.0000000
44180.0000000	6.7500000	-50.0000000
44180.0000000	7.0000000	-50.0000000
44180.0000000	7.2500000	-50.0000000
44180.0000000	7.5000000	-50.0000000
44180.0000000	7.7500000	-50.0000000
44180.0000000	8.0000000	-50.0000000
44180.0000000	8.2500000	-50.0000000
44180.0000000	8.5000000	-50.0000000
44180.0000000	8.7500000	-50.0000000
44180.0000000	9.0000000	-50.0000000
44180.0000000	9.2500000	-50.0000000
44180.0000000	9.5000000	-50.0000000
44180.0000000	9.7500000	-50.0000000
44180.0000000	10.0000000	-50.0000000
44180.0000000	10.2500000	-50.0000000
44180.0000000	10.5000000	-50.0000000
44180.0000000	10.7500000	-50.0000000
44180.0000000	11.0000000	-50.0000000
44180.0000000	11.2500000	-50.0000000
44180.0000000	11.5000000	-50.0000000
44180.0000000	11.7500000	-50.0000000
44180.0000000	12.0000000	-50.0000000
44180.0000000	12.2500000	-50.0000000
44180.0000000	12.5000000	-50.0000000
44180.0000000	12.7500000	-50.0000000
44180.0000000	13.0000000	-50.0000000
44180.0000000	13.2500000	-50.0000000

44180.0000000	13.5000000	-50.0000000
44180.0000000	13.7500000	-50.0000000
44180.0000000	14.0000000	-50.0000000
44180.0000000	14.2500000	-50.0000000
44180.0000000	14.5000000	-50.0000000
44180.0000000	14.7500000	-50.0000000
44180.0000000	15.0000000	-50.0000000
44210.0000000	0.0000000	-50.0000000
44210.0000000	0.2500000	-50.0000000
44210.0000000	0.5000000	-50.0000000
44210.0000000	0.7500000	-50.0000000
44210.0000000	1.0000000	-50.0000000
44210.0000000	1.2500000	-50.0000000
44210.0000000	1.5000000	-50.0000000
44210.0000000	1.7500000	-50.0000000
44210.0000000	2.0000000	-50.0000000
44210.0000000	2.2500000	-50.0000000
44210.0000000	2.5000000	-50.0000000
44210.0000000	2.7500000	-50.0000000
44210.0000000	3.0000000	-50.0000000
44210.0000000	3.2500000	-50.0000000
44210.0000000	3.5000000	-50.0000000
44210.0000000	3.7500000	-50.0000000
44210.0000000	4.0000000	-50.0000000
44210.0000000	4.2500000	-50.0000000
44210.0000000	4.5000000	-50.0000000
44210.0000000	4.7500000	-50.0000000
44210.0000000	5.0000000	-50.0000000
44210.0000000	5.2500000	-50.0000000
44210.0000000	5.5000000	-50.0000000
44210.0000000	5.7500000	-50.0000000
44210.0000000	6.0000000	-50.0000000
44210.0000000	6.2500000	-50.0000000
44210.0000000	6.5000000	-50.0000000
44210.0000000	6.7500000	-50.0000000
44210.0000000	7.0000000	-50.0000000
44210.0000000	7.2500000	-50.0000000
44210.0000000	7.5000000	-50.0000000
44210.0000000	7.7500000	-50.0000000
44210.0000000	8.0000000	-50.0000000
44210.0000000	8.2500000	-50.0000000
44210.0000000	8.5000000	-50.0000000
44210.0000000	8.7500000	-50.0000000
44210.0000000	9.0000000	-50.0000000
44210.0000000	9.2500000	-50.0000000
44210.0000000	9.5000000	-50.0000000
44210.0000000	9.7500000	-50.0000000
44210.0000000	10.0000000	-50.0000000
44210.0000000	10.2500000	-50.0000000
44210.0000000	10.5000000	-50.0000000
44210.0000000	10.7500000	-50.0000000
44210.0000000	11.0000000	-50.0000000
44210.0000000	11.2500000	-50.0000000
44210.0000000	11.5000000	-50.0000000
44210.0000000	11.7500000	-50.0000000
44210.0000000	12.0000000	-50.0000000
44210.0000000	12.2500000	-50.0000000
44210.0000000	12.5000000	-50.0000000

44210.0000000	12.7500000	-50.0000000
44210.0000000	13.0000000	-50.0000000
44210.0000000	13.2500000	-50.0000000
44210.0000000	13.5000000	-50.0000000
44210.0000000	13.7500000	-50.0000000
44210.0000000	14.0000000	-50.0000000
44210.0000000	14.2500000	-50.0000000
44210.0000000	14.5000000	-50.0000000
44210.0000000	14.7500000	-50.0000000
44210.0000000	15.0000000	-50.0000000
44240.0000000	0.0000000	-50.0000000
44240.0000000	0.2500000	-50.0000000
44240.0000000	0.5000000	-50.0000000
44240.0000000	0.7500000	-50.0000000
44240.0000000	1.0000000	-50.0000000
44240.0000000	1.2500000	-50.0000000
44240.0000000	1.5000000	-50.0000000
44240.0000000	1.7500000	-50.0000000
44240.0000000	2.0000000	-50.0000000
44240.0000000	2.2500000	-50.0000000
44240.0000000	2.5000000	-50.0000000
44240.0000000	2.7500000	-50.0000000
44240.0000000	3.0000000	-50.0000000
44240.0000000	3.2500000	-50.0000000
44240.0000000	3.5000000	-50.0000000
44240.0000000	3.7500000	-50.0000000
44240.0000000	4.0000000	-50.0000000
44240.0000000	4.2500000	-50.0000000
44240.0000000	4.5000000	-50.0000000
44240.0000000	4.7500000	-50.0000000
44240.0000000	5.0000000	-50.0000000
44240.0000000	5.2500000	-50.0000000
44240.0000000	5.5000000	-50.0000000
44240.0000000	5.7500000	-50.0000000
44240.0000000	6.0000000	-50.0000000
44240.0000000	6.2500000	-50.0000000
44240.0000000	6.5000000	-50.0000000
44240.0000000	6.7500000	-50.0000000
44240.0000000	7.0000000	-50.0000000
44240.0000000	7.2500000	-50.0000000
44240.0000000	7.5000000	-50.0000000
44240.0000000	7.7500000	-50.0000000
44240.0000000	8.0000000	-50.0000000
44240.0000000	8.2500000	-50.0000000
44240.0000000	8.5000000	-50.0000000
44240.0000000	8.7500000	-50.0000000
44240.0000000	9.0000000	-50.0000000
44240.0000000	9.2500000	-50.0000000
44240.0000000	9.5000000	-50.0000000
44240.0000000	9.7500000	-50.0000000
44240.0000000	10.0000000	-50.0000000
44240.0000000	10.2500000	-50.0000000
44240.0000000	10.5000000	-50.0000000
44240.0000000	10.7500000	-50.0000000
44240.0000000	11.0000000	-50.0000000
44240.0000000	11.2500000	-50.0000000
44240.0000000	11.5000000	-50.0000000
44240.0000000	11.7500000	-50.0000000

44240.0000000	12.0000000	-50.0000000
44240.0000000	12.2500000	-50.0000000
44240.0000000	12.5000000	-50.0000000
44240.0000000	12.7500000	-50.0000000
44240.0000000	13.0000000	-50.0000000
44240.0000000	13.2500000	-50.0000000
44240.0000000	13.5000000	-50.0000000
44240.0000000	13.7500000	-50.0000000
44240.0000000	14.0000000	-50.0000000
44240.0000000	14.2500000	-50.0000000
44240.0000000	14.5000000	-50.0000000
44240.0000000	14.7500000	-50.0000000
44240.0000000	15.0000000	-50.0000000
44270.0000000	0.0000000	-50.0000000
44270.0000000	0.2500000	-50.0000000
44270.0000000	0.5000000	-50.0000000
44270.0000000	0.7500000	-50.0000000
44270.0000000	1.0000000	-50.0000000
44270.0000000	1.2500000	-50.0000000
44270.0000000	1.5000000	-50.0000000
44270.0000000	1.7500000	-50.0000000
44270.0000000	2.0000000	-50.0000000
44270.0000000	2.2500000	-50.0000000
44270.0000000	2.5000000	-50.0000000
44270.0000000	2.7500000	-50.0000000
44270.0000000	3.0000000	-50.0000000
44270.0000000	3.2500000	-50.0000000
44270.0000000	3.5000000	-50.0000000
44270.0000000	3.7500000	-50.0000000
44270.0000000	4.0000000	-50.0000000
44270.0000000	4.2500000	-50.0000000
44270.0000000	4.5000000	-50.0000000
44270.0000000	4.7500000	-50.0000000
44270.0000000	5.0000000	-50.0000000
44270.0000000	5.2500000	-50.0000000
44270.0000000	5.5000000	-50.0000000
44270.0000000	5.7500000	-50.0000000
44270.0000000	6.0000000	-50.0000000
44270.0000000	6.2500000	-50.0000000
44270.0000000	6.5000000	-50.0000000
44270.0000000	6.7500000	-50.0000000
44270.0000000	7.0000000	-50.0000000
44270.0000000	7.2500000	-50.0000000
44270.0000000	7.5000000	-50.0000000
44270.0000000	7.7500000	-50.0000000
44270.0000000	8.0000000	-50.0000000
44270.0000000	8.2500000	-50.0000000
44270.0000000	8.5000000	-50.0000000
44270.0000000	8.7500000	-50.0000000
44270.0000000	9.0000000	-50.0000000
44270.0000000	9.2500000	-50.0000000
44270.0000000	9.5000000	-50.0000000
44270.0000000	9.7500000	-50.0000000
44270.0000000	10.0000000	-50.0000000
44270.0000000	10.2500000	-50.0000000
44270.0000000	10.5000000	-50.0000000
44270.0000000	10.7500000	-50.0000000
44270.0000000	11.0000000	-50.0000000

44270.0000000	11.2500000	-50.0000000
44270.0000000	11.5000000	-50.0000000
44270.0000000	11.7500000	-50.0000000
44270.0000000	12.0000000	-50.0000000
44270.0000000	12.2500000	-50.0000000
44270.0000000	12.5000000	-50.0000000
44270.0000000	12.7500000	-50.0000000
44270.0000000	13.0000000	-50.0000000
44270.0000000	13.2500000	-50.0000000
44270.0000000	13.5000000	-50.0000000
44270.0000000	13.7500000	-50.0000000
44270.0000000	14.0000000	-50.0000000
44270.0000000	14.2500000	-50.0000000
44270.0000000	14.5000000	-50.0000000
44270.0000000	14.7500000	-50.0000000
44270.0000000	15.0000000	-50.0000000
44300.0000000	0.0000000	-50.0000000
44300.0000000	0.2500000	-50.0000000
44300.0000000	0.5000000	-50.0000000
44300.0000000	0.7500000	-50.0000000
44300.0000000	1.0000000	-50.0000000
44300.0000000	1.2500000	-50.0000000
44300.0000000	1.5000000	-50.0000000
44300.0000000	1.7500000	-50.0000000
44300.0000000	2.0000000	-50.0000000
44300.0000000	2.2500000	-50.0000000
44300.0000000	2.5000000	-50.0000000
44300.0000000	2.7500000	-50.0000000
44300.0000000	3.0000000	-50.0000000
44300.0000000	3.2500000	-50.0000000
44300.0000000	3.5000000	-50.0000000
44300.0000000	3.7500000	-50.0000000
44300.0000000	4.0000000	-50.0000000
44300.0000000	4.2500000	-50.0000000
44300.0000000	4.5000000	-50.0000000
44300.0000000	4.7500000	-50.0000000
44300.0000000	5.0000000	-50.0000000
44300.0000000	5.2500000	-50.0000000
44300.0000000	5.5000000	-50.0000000
44300.0000000	5.7500000	-50.0000000
44300.0000000	6.0000000	-50.0000000
44300.0000000	6.2500000	-50.0000000
44300.0000000	6.5000000	-50.0000000
44300.0000000	6.7500000	-50.0000000
44300.0000000	7.0000000	-50.0000000
44300.0000000	7.2500000	-50.0000000
44300.0000000	7.5000000	-50.0000000
44300.0000000	7.7500000	-50.0000000
44300.0000000	8.0000000	-50.0000000
44300.0000000	8.2500000	-50.0000000
44300.0000000	8.5000000	-50.0000000
44300.0000000	8.7500000	-50.0000000
44300.0000000	9.0000000	-50.0000000
44300.0000000	9.2500000	-50.0000000
44300.0000000	9.5000000	-50.0000000
44300.0000000	9.7500000	-50.0000000
44300.0000000	10.0000000	-50.0000000
44300.0000000	10.2500000	-50.0000000

44300.0000000	10.5000000	-50.0000000
44300.0000000	10.7500000	-50.0000000
44300.0000000	11.0000000	-50.0000000
44300.0000000	11.2500000	-50.0000000
44300.0000000	11.5000000	-50.0000000
44300.0000000	11.7500000	-50.0000000
44300.0000000	12.0000000	-50.0000000
44300.0000000	12.2500000	-50.0000000
44300.0000000	12.5000000	-50.0000000
44300.0000000	12.7500000	-50.0000000
44300.0000000	13.0000000	-50.0000000
44300.0000000	13.2500000	-50.0000000
44300.0000000	13.5000000	-50.0000000
44300.0000000	13.7500000	-50.0000000
44300.0000000	14.0000000	-50.0000000
44300.0000000	14.2500000	-50.0000000
44300.0000000	14.5000000	-50.0000000
44300.0000000	14.7500000	-50.0000000
44300.0000000	15.0000000	-50.0000000
44330.0000000	0.0000000	-50.0000000
44330.0000000	0.2500000	-50.0000000
44330.0000000	0.5000000	-50.0000000
44330.0000000	0.7500000	-50.0000000
44330.0000000	1.0000000	-50.0000000
44330.0000000	1.2500000	-50.0000000
44330.0000000	1.5000000	-50.0000000
44330.0000000	1.7500000	-50.0000000
44330.0000000	2.0000000	-50.0000000
44330.0000000	2.2500000	-50.0000000
44330.0000000	2.5000000	-50.0000000
44330.0000000	2.7500000	-50.0000000
44330.0000000	3.0000000	-50.0000000
44330.0000000	3.2500000	-50.0000000
44330.0000000	3.5000000	-50.0000000
44330.0000000	3.7500000	-50.0000000
44330.0000000	4.0000000	-50.0000000
44330.0000000	4.2500000	-50.0000000
44330.0000000	4.5000000	-50.0000000
44330.0000000	4.7500000	-50.0000000
44330.0000000	5.0000000	-50.0000000
44330.0000000	5.2500000	-50.0000000
44330.0000000	5.5000000	-50.0000000
44330.0000000	5.7500000	-50.0000000
44330.0000000	6.0000000	-50.0000000
44330.0000000	6.2500000	-50.0000000
44330.0000000	6.5000000	-50.0000000
44330.0000000	6.7500000	-50.0000000
44330.0000000	7.0000000	-50.0000000
44330.0000000	7.2500000	-50.0000000
44330.0000000	7.5000000	-50.0000000
44330.0000000	7.7500000	-50.0000000
44330.0000000	8.0000000	-50.0000000
44330.0000000	8.2500000	-50.0000000
44330.0000000	8.5000000	-50.0000000
44330.0000000	8.7500000	-50.0000000
44330.0000000	9.0000000	-50.0000000
44330.0000000	9.2500000	-50.0000000
44330.0000000	9.5000000	-50.0000000

44330.0000000	9.7500000	-50.0000000
44330.0000000	10.0000000	-50.0000000
44330.0000000	10.2500000	-50.0000000
44330.0000000	10.5000000	-50.0000000
44330.0000000	10.7500000	-50.0000000
44330.0000000	11.0000000	-50.0000000
44330.0000000	11.2500000	-50.0000000
44330.0000000	11.5000000	-50.0000000
44330.0000000	11.7500000	-50.0000000
44330.0000000	12.0000000	-50.0000000
44330.0000000	12.2500000	-50.0000000
44330.0000000	12.5000000	-50.0000000
44330.0000000	12.7500000	-50.0000000
44330.0000000	13.0000000	-50.0000000
44330.0000000	13.2500000	-50.0000000
44330.0000000	13.5000000	-50.0000000
44330.0000000	13.7500000	-50.0000000
44330.0000000	14.0000000	-50.0000000
44330.0000000	14.2500000	-50.0000000
44330.0000000	14.5000000	-50.0000000
44330.0000000	14.7500000	-50.0000000
44330.0000000	15.0000000	-50.0000000
44360.0000000	0.0000000	-50.0000000
44360.0000000	0.2500000	-50.0000000
44360.0000000	0.5000000	-50.0000000
44360.0000000	0.7500000	-50.0000000
44360.0000000	1.0000000	-50.0000000
44360.0000000	1.2500000	-50.0000000
44360.0000000	1.5000000	-50.0000000
44360.0000000	1.7500000	-50.0000000
44360.0000000	2.0000000	-50.0000000
44360.0000000	2.2500000	-50.0000000
44360.0000000	2.5000000	-50.0000000
44360.0000000	2.7500000	-50.0000000
44360.0000000	3.0000000	-50.0000000
44360.0000000	3.2500000	-50.0000000
44360.0000000	3.5000000	-50.0000000
44360.0000000	3.7500000	-50.0000000
44360.0000000	4.0000000	-50.0000000
44360.0000000	4.2500000	-50.0000000
44360.0000000	4.5000000	-50.0000000
44360.0000000	4.7500000	-50.0000000
44360.0000000	5.0000000	-50.0000000
44360.0000000	5.2500000	-50.0000000
44360.0000000	5.5000000	-50.0000000
44360.0000000	5.7500000	-50.0000000
44360.0000000	6.0000000	-50.0000000
44360.0000000	6.2500000	-50.0000000
44360.0000000	6.5000000	-50.0000000
44360.0000000	6.7500000	-50.0000000
44360.0000000	7.0000000	-50.0000000
44360.0000000	7.2500000	-50.0000000
44360.0000000	7.5000000	-50.0000000
44360.0000000	7.7500000	-50.0000000
44360.0000000	8.0000000	-50.0000000
44360.0000000	8.2500000	-50.0000000
44360.0000000	8.5000000	-50.0000000
44360.0000000	8.7500000	-50.0000000

44360.0000000	9.0000000	-50.0000000
44360.0000000	9.2500000	-50.0000000
44360.0000000	9.5000000	-50.0000000
44360.0000000	9.7500000	-50.0000000
44360.0000000	10.0000000	-50.0000000
44360.0000000	10.2500000	-50.0000000
44360.0000000	10.5000000	-50.0000000
44360.0000000	10.7500000	-50.0000000
44360.0000000	11.0000000	-50.0000000
44360.0000000	11.2500000	-50.0000000
44360.0000000	11.5000000	-50.0000000
44360.0000000	11.7500000	-50.0000000
44360.0000000	12.0000000	-50.0000000
44360.0000000	12.2500000	-50.0000000
44360.0000000	12.5000000	-50.0000000
44360.0000000	12.7500000	-50.0000000
44360.0000000	13.0000000	-50.0000000
44360.0000000	13.2500000	-50.0000000
44360.0000000	13.5000000	-50.0000000
44360.0000000	13.7500000	-50.0000000
44360.0000000	14.0000000	-50.0000000
44360.0000000	14.2500000	-50.0000000
44360.0000000	14.5000000	-50.0000000
44360.0000000	14.7500000	-50.0000000
44360.0000000	15.0000000	-50.0000000
44390.0000000	0.0000000	-50.0000000
44390.0000000	0.2500000	-50.0000000
44390.0000000	0.5000000	-50.0000000
44390.0000000	0.7500000	-50.0000000
44390.0000000	1.0000000	-50.0000000
44390.0000000	1.2500000	-50.0000000
44390.0000000	1.5000000	-50.0000000
44390.0000000	1.7500000	-50.0000000
44390.0000000	2.0000000	-50.0000000
44390.0000000	2.2500000	-50.0000000
44390.0000000	2.5000000	-50.0000000
44390.0000000	2.7500000	-50.0000000
44390.0000000	3.0000000	-50.0000000
44390.0000000	3.2500000	-50.0000000
44390.0000000	3.5000000	-50.0000000
44390.0000000	3.7500000	-50.0000000
44390.0000000	4.0000000	-50.0000000
44390.0000000	4.2500000	-50.0000000
44390.0000000	4.5000000	-50.0000000
44390.0000000	4.7500000	-50.0000000
44390.0000000	5.0000000	-50.0000000
44390.0000000	5.2500000	-50.0000000
44390.0000000	5.5000000	-50.0000000
44390.0000000	5.7500000	-50.0000000
44390.0000000	6.0000000	-50.0000000
44390.0000000	6.2500000	-50.0000000
44390.0000000	6.5000000	-50.0000000
44390.0000000	6.7500000	-50.0000000
44390.0000000	7.0000000	-50.0000000
44390.0000000	7.2500000	-50.0000000
44390.0000000	7.5000000	-50.0000000
44390.0000000	7.7500000	-50.0000000
44390.0000000	8.0000000	-50.0000000

44390.0000000	8.2500000	-50.0000000
44390.0000000	8.5000000	-50.0000000
44390.0000000	8.7500000	-50.0000000
44390.0000000	9.0000000	-50.0000000
44390.0000000	9.2500000	-50.0000000
44390.0000000	9.5000000	-50.0000000
44390.0000000	9.7500000	-50.0000000
44390.0000000	10.0000000	-50.0000000
44390.0000000	10.2500000	-50.0000000
44390.0000000	10.5000000	-50.0000000
44390.0000000	10.7500000	-50.0000000
44390.0000000	11.0000000	-50.0000000
44390.0000000	11.2500000	-50.0000000
44390.0000000	11.5000000	-50.0000000
44390.0000000	11.7500000	-50.0000000
44390.0000000	12.0000000	-50.0000000
44390.0000000	12.2500000	-50.0000000
44390.0000000	12.5000000	-50.0000000
44390.0000000	12.7500000	-50.0000000
44390.0000000	13.0000000	-50.0000000
44390.0000000	13.2500000	-50.0000000
44390.0000000	13.5000000	-50.0000000
44390.0000000	13.7500000	-50.0000000
44390.0000000	14.0000000	-50.0000000
44390.0000000	14.2500000	-50.0000000
44390.0000000	14.5000000	-50.0000000
44390.0000000	14.7500000	-50.0000000
44390.0000000	15.0000000	-50.0000000
44420.0000000	0.0000000	-50.0000000
44420.0000000	0.2500000	-50.0000000
44420.0000000	0.5000000	-50.0000000
44420.0000000	0.7500000	-50.0000000
44420.0000000	1.0000000	-50.0000000
44420.0000000	1.2500000	-50.0000000
44420.0000000	1.5000000	-50.0000000
44420.0000000	1.7500000	-50.0000000
44420.0000000	2.0000000	-50.0000000
44420.0000000	2.2500000	-50.0000000
44420.0000000	2.5000000	-50.0000000
44420.0000000	2.7500000	-50.0000000
44420.0000000	3.0000000	-50.0000000
44420.0000000	3.2500000	-50.0000000
44420.0000000	3.5000000	-50.0000000
44420.0000000	3.7500000	-50.0000000
44420.0000000	4.0000000	-50.0000000
44420.0000000	4.2500000	-50.0000000
44420.0000000	4.5000000	-50.0000000
44420.0000000	4.7500000	-50.0000000
44420.0000000	5.0000000	-50.0000000
44420.0000000	5.2500000	-50.0000000
44420.0000000	5.5000000	-50.0000000
44420.0000000	5.7500000	-50.0000000
44420.0000000	6.0000000	-50.0000000
44420.0000000	6.2500000	-50.0000000
44420.0000000	6.5000000	-50.0000000
44420.0000000	6.7500000	-50.0000000
44420.0000000	7.0000000	-50.0000000
44420.0000000	7.2500000	-50.0000000

44420.0000000	7.5000000	-50.0000000
44420.0000000	7.7500000	-50.0000000
44420.0000000	8.0000000	-50.0000000
44420.0000000	8.2500000	-50.0000000
44420.0000000	8.5000000	-50.0000000
44420.0000000	8.7500000	-50.0000000
44420.0000000	9.0000000	-50.0000000
44420.0000000	9.2500000	-50.0000000
44420.0000000	9.5000000	-50.0000000
44420.0000000	9.7500000	-50.0000000
44420.0000000	10.0000000	-50.0000000
44420.0000000	10.2500000	-50.0000000
44420.0000000	10.5000000	-50.0000000
44420.0000000	10.7500000	-50.0000000
44420.0000000	11.0000000	-50.0000000
44420.0000000	11.2500000	-50.0000000
44420.0000000	11.5000000	-50.0000000
44420.0000000	11.7500000	-50.0000000
44420.0000000	12.0000000	-50.0000000
44420.0000000	12.2500000	-50.0000000
44420.0000000	12.5000000	-50.0000000
44420.0000000	12.7500000	-50.0000000
44420.0000000	13.0000000	-50.0000000
44420.0000000	13.2500000	-50.0000000
44420.0000000	13.5000000	-50.0000000
44420.0000000	13.7500000	-50.0000000
44420.0000000	14.0000000	-50.0000000
44420.0000000	14.2500000	-50.0000000
44420.0000000	14.5000000	-50.0000000
44420.0000000	14.7500000	-50.0000000
44420.0000000	15.0000000	-50.0000000
44450.0000000	0.0000000	-50.0000000
44450.0000000	0.2500000	-50.0000000
44450.0000000	0.5000000	-50.0000000
44450.0000000	0.7500000	-50.0000000
44450.0000000	1.0000000	-50.0000000
44450.0000000	1.2500000	-50.0000000
44450.0000000	1.5000000	-50.0000000
44450.0000000	1.7500000	-50.0000000
44450.0000000	2.0000000	-50.0000000
44450.0000000	2.2500000	-50.0000000
44450.0000000	2.5000000	-50.0000000
44450.0000000	2.7500000	-50.0000000
44450.0000000	3.0000000	-50.0000000
44450.0000000	3.2500000	-50.0000000
44450.0000000	3.5000000	-50.0000000
44450.0000000	3.7500000	-50.0000000
44450.0000000	4.0000000	-50.0000000
44450.0000000	4.2500000	-50.0000000
44450.0000000	4.5000000	-50.0000000
44450.0000000	4.7500000	-50.0000000
44450.0000000	5.0000000	-50.0000000
44450.0000000	5.2500000	-50.0000000
44450.0000000	5.5000000	-50.0000000
44450.0000000	5.7500000	-50.0000000
44450.0000000	6.0000000	-50.0000000
44450.0000000	6.2500000	-50.0000000
44450.0000000	6.5000000	-50.0000000

44450.0000000	6.7500000	-50.0000000
44450.0000000	7.0000000	-50.0000000
44450.0000000	7.2500000	-50.0000000
44450.0000000	7.5000000	-50.0000000
44450.0000000	7.7500000	-50.0000000
44450.0000000	8.0000000	-50.0000000
44450.0000000	8.2500000	-50.0000000
44450.0000000	8.5000000	-50.0000000
44450.0000000	8.7500000	-50.0000000
44450.0000000	9.0000000	-50.0000000
44450.0000000	9.2500000	-50.0000000
44450.0000000	9.5000000	-50.0000000
44450.0000000	9.7500000	-50.0000000
44450.0000000	10.0000000	-50.0000000
44450.0000000	10.2500000	-50.0000000
44450.0000000	10.5000000	-50.0000000
44450.0000000	10.7500000	-50.0000000
44450.0000000	11.0000000	-50.0000000
44450.0000000	11.2500000	-50.0000000
44450.0000000	11.5000000	-50.0000000
44450.0000000	11.7500000	-50.0000000
44450.0000000	12.0000000	-50.0000000
44450.0000000	12.2500000	-50.0000000
44450.0000000	12.5000000	-50.0000000
44450.0000000	12.7500000	-50.0000000
44450.0000000	13.0000000	-50.0000000
44450.0000000	13.2500000	-50.0000000
44450.0000000	13.5000000	-50.0000000
44450.0000000	13.7500000	-50.0000000
44450.0000000	14.0000000	-50.0000000
44450.0000000	14.2500000	-50.0000000
44450.0000000	14.5000000	-50.0000000
44450.0000000	14.7500000	-50.0000000
44450.0000000	15.0000000	-50.0000000
44480.0000000	0.0000000	-50.0000000
44480.0000000	0.2500000	-50.0000000
44480.0000000	0.5000000	-50.0000000
44480.0000000	0.7500000	-50.0000000
44480.0000000	1.0000000	-50.0000000
44480.0000000	1.2500000	-50.0000000
44480.0000000	1.5000000	-50.0000000
44480.0000000	1.7500000	-50.0000000
44480.0000000	2.0000000	-50.0000000
44480.0000000	2.2500000	-50.0000000
44480.0000000	2.5000000	-50.0000000
44480.0000000	2.7500000	-50.0000000
44480.0000000	3.0000000	-50.0000000
44480.0000000	3.2500000	-50.0000000
44480.0000000	3.5000000	-50.0000000
44480.0000000	3.7500000	-50.0000000
44480.0000000	4.0000000	-50.0000000
44480.0000000	4.2500000	-50.0000000
44480.0000000	4.5000000	-50.0000000
44480.0000000	4.7500000	-50.0000000
44480.0000000	5.0000000	-50.0000000
44480.0000000	5.2500000	-50.0000000
44480.0000000	5.5000000	-50.0000000
44480.0000000	5.7500000	-50.0000000

44480.0000000	6.0000000	-50.0000000
44480.0000000	6.2500000	-50.0000000
44480.0000000	6.5000000	-50.0000000
44480.0000000	6.7500000	-50.0000000
44480.0000000	7.0000000	-50.0000000
44480.0000000	7.2500000	-50.0000000
44480.0000000	7.5000000	-50.0000000
44480.0000000	7.7500000	-50.0000000
44480.0000000	8.0000000	-50.0000000
44480.0000000	8.2500000	-50.0000000
44480.0000000	8.5000000	-50.0000000
44480.0000000	8.7500000	-50.0000000
44480.0000000	9.0000000	-50.0000000
44480.0000000	9.2500000	-50.0000000
44480.0000000	9.5000000	-50.0000000
44480.0000000	9.7500000	-50.0000000
44480.0000000	10.0000000	-50.0000000
44480.0000000	10.2500000	-50.0000000
44480.0000000	10.5000000	-50.0000000
44480.0000000	10.7500000	-50.0000000
44480.0000000	11.0000000	-50.0000000
44480.0000000	11.2500000	-50.0000000
44480.0000000	11.5000000	-50.0000000
44480.0000000	11.7500000	-50.0000000
44480.0000000	12.0000000	-50.0000000
44480.0000000	12.2500000	-50.0000000
44480.0000000	12.5000000	-50.0000000
44480.0000000	12.7500000	-50.0000000
44480.0000000	13.0000000	-50.0000000
44480.0000000	13.2500000	-50.0000000
44480.0000000	13.5000000	-50.0000000
44480.0000000	13.7500000	-50.0000000
44480.0000000	14.0000000	-50.0000000
44480.0000000	14.2500000	-50.0000000
44480.0000000	14.5000000	-50.0000000
44480.0000000	14.7500000	-50.0000000
44480.0000000	15.0000000	-50.0000000
44510.0000000	0.0000000	-50.0000000
44510.0000000	0.2500000	-50.0000000
44510.0000000	0.5000000	-50.0000000
44510.0000000	0.7500000	-50.0000000
44510.0000000	1.0000000	-50.0000000
44510.0000000	1.2500000	-50.0000000
44510.0000000	1.5000000	-50.0000000
44510.0000000	1.7500000	-50.0000000
44510.0000000	2.0000000	-50.0000000
44510.0000000	2.2500000	-50.0000000
44510.0000000	2.5000000	-50.0000000
44510.0000000	2.7500000	-50.0000000
44510.0000000	3.0000000	-50.0000000
44510.0000000	3.2500000	-50.0000000
44510.0000000	3.5000000	-50.0000000
44510.0000000	3.7500000	-50.0000000
44510.0000000	4.0000000	-50.0000000
44510.0000000	4.2500000	-50.0000000
44510.0000000	4.5000000	-50.0000000
44510.0000000	4.7500000	-50.0000000
44510.0000000	5.0000000	-50.0000000

44510.0000000	5.2500000	-50.0000000
44510.0000000	5.5000000	-50.0000000
44510.0000000	5.7500000	-50.0000000
44510.0000000	6.0000000	-50.0000000
44510.0000000	6.2500000	-50.0000000
44510.0000000	6.5000000	-50.0000000
44510.0000000	6.7500000	-50.0000000
44510.0000000	7.0000000	-50.0000000
44510.0000000	7.2500000	-50.0000000
44510.0000000	7.5000000	-50.0000000
44510.0000000	7.7500000	-50.0000000
44510.0000000	8.0000000	-50.0000000
44510.0000000	8.2500000	-50.0000000
44510.0000000	8.5000000	-50.0000000
44510.0000000	8.7500000	-50.0000000
44510.0000000	9.0000000	-50.0000000
44510.0000000	9.2500000	-50.0000000
44510.0000000	9.5000000	-50.0000000
44510.0000000	9.7500000	-50.0000000
44510.0000000	10.0000000	-50.0000000
44510.0000000	10.2500000	-50.0000000
44510.0000000	10.5000000	-50.0000000
44510.0000000	10.7500000	-50.0000000
44510.0000000	11.0000000	-50.0000000
44510.0000000	11.2500000	-50.0000000
44510.0000000	11.5000000	-50.0000000
44510.0000000	11.7500000	-50.0000000
44510.0000000	12.0000000	-50.0000000
44510.0000000	12.2500000	-50.0000000
44510.0000000	12.5000000	-50.0000000
44510.0000000	12.7500000	-50.0000000
44510.0000000	13.0000000	-50.0000000
44510.0000000	13.2500000	-50.0000000
44510.0000000	13.5000000	-50.0000000
44510.0000000	13.7500000	-50.0000000
44510.0000000	14.0000000	-50.0000000
44510.0000000	14.2500000	-50.0000000
44510.0000000	14.5000000	-50.0000000
44510.0000000	14.7500000	-50.0000000
44510.0000000	15.0000000	-50.0000000
44540.0000000	0.0000000	-50.0000000
44540.0000000	0.2500000	-50.0000000
44540.0000000	0.5000000	-50.0000000
44540.0000000	0.7500000	-50.0000000
44540.0000000	1.0000000	-50.0000000
44540.0000000	1.2500000	-50.0000000
44540.0000000	1.5000000	-50.0000000
44540.0000000	1.7500000	-50.0000000
44540.0000000	2.0000000	-50.0000000
44540.0000000	2.2500000	-50.0000000
44540.0000000	2.5000000	-50.0000000
44540.0000000	2.7500000	-50.0000000
44540.0000000	3.0000000	-50.0000000
44540.0000000	3.2500000	-50.0000000
44540.0000000	3.5000000	-50.0000000
44540.0000000	3.7500000	-50.0000000
44540.0000000	4.0000000	-50.0000000
44540.0000000	4.2500000	-50.0000000

44540.0000000	4.5000000	-50.0000000
44540.0000000	4.7500000	-50.0000000
44540.0000000	5.0000000	-50.0000000
44540.0000000	5.2500000	-50.0000000
44540.0000000	5.5000000	-50.0000000
44540.0000000	5.7500000	-50.0000000
44540.0000000	6.0000000	-50.0000000
44540.0000000	6.2500000	-50.0000000
44540.0000000	6.5000000	-50.0000000
44540.0000000	6.7500000	-50.0000000
44540.0000000	7.0000000	-50.0000000
44540.0000000	7.2500000	-50.0000000
44540.0000000	7.5000000	-50.0000000
44540.0000000	7.7500000	-50.0000000
44540.0000000	8.0000000	-50.0000000
44540.0000000	8.2500000	-50.0000000
44540.0000000	8.5000000	-50.0000000
44540.0000000	8.7500000	-50.0000000
44540.0000000	9.0000000	-50.0000000
44540.0000000	9.2500000	-50.0000000
44540.0000000	9.5000000	-50.0000000
44540.0000000	9.7500000	-50.0000000
44540.0000000	10.0000000	-50.0000000
44540.0000000	10.2500000	-50.0000000
44540.0000000	10.5000000	-50.0000000
44540.0000000	10.7500000	-50.0000000
44540.0000000	11.0000000	-50.0000000
44540.0000000	11.2500000	-50.0000000
44540.0000000	11.5000000	-50.0000000
44540.0000000	11.7500000	-50.0000000
44540.0000000	12.0000000	-50.0000000
44540.0000000	12.2500000	-50.0000000
44540.0000000	12.5000000	-50.0000000
44540.0000000	12.7500000	-50.0000000
44540.0000000	13.0000000	-50.0000000
44540.0000000	13.2500000	-50.0000000
44540.0000000	13.5000000	-50.0000000
44540.0000000	13.7500000	-50.0000000
44540.0000000	14.0000000	-50.0000000
44540.0000000	14.2500000	-50.0000000
44540.0000000	14.5000000	-50.0000000
44540.0000000	14.7500000	-50.0000000
44540.0000000	15.0000000	-50.0000000
44570.0000000	0.0000000	-50.0000000
44570.0000000	0.2500000	-50.0000000
44570.0000000	0.5000000	-50.0000000
44570.0000000	0.7500000	-50.0000000
44570.0000000	1.0000000	-50.0000000
44570.0000000	1.2500000	-50.0000000
44570.0000000	1.5000000	-50.0000000
44570.0000000	1.7500000	-50.0000000
44570.0000000	2.0000000	-50.0000000
44570.0000000	2.2500000	-50.0000000
44570.0000000	2.5000000	-50.0000000
44570.0000000	2.7500000	-50.0000000
44570.0000000	3.0000000	-50.0000000
44570.0000000	3.2500000	-50.0000000
44570.0000000	3.5000000	-50.0000000

44570.0000000	3.7500000	-50.0000000
44570.0000000	4.0000000	-50.0000000
44570.0000000	4.2500000	-50.0000000
44570.0000000	4.5000000	-50.0000000
44570.0000000	4.7500000	-50.0000000
44570.0000000	5.0000000	-50.0000000
44570.0000000	5.2500000	-50.0000000
44570.0000000	5.5000000	-50.0000000
44570.0000000	5.7500000	-50.0000000
44570.0000000	6.0000000	-50.0000000
44570.0000000	6.2500000	-50.0000000
44570.0000000	6.5000000	-50.0000000
44570.0000000	6.7500000	-50.0000000
44570.0000000	7.0000000	-50.0000000
44570.0000000	7.2500000	-50.0000000
44570.0000000	7.5000000	-50.0000000
44570.0000000	7.7500000	-50.0000000
44570.0000000	8.0000000	-50.0000000
44570.0000000	8.2500000	-50.0000000
44570.0000000	8.5000000	-50.0000000
44570.0000000	8.7500000	-50.0000000
44570.0000000	9.0000000	-50.0000000
44570.0000000	9.2500000	-50.0000000
44570.0000000	9.5000000	-50.0000000
44570.0000000	9.7500000	-50.0000000
44570.0000000	10.0000000	-50.0000000
44570.0000000	10.2500000	-50.0000000
44570.0000000	10.5000000	-50.0000000
44570.0000000	10.7500000	-50.0000000
44570.0000000	11.0000000	-50.0000000
44570.0000000	11.2500000	-50.0000000
44570.0000000	11.5000000	-50.0000000
44570.0000000	11.7500000	-50.0000000
44570.0000000	12.0000000	-50.0000000
44570.0000000	12.2500000	-50.0000000
44570.0000000	12.5000000	-50.0000000
44570.0000000	12.7500000	-50.0000000
44570.0000000	13.0000000	-50.0000000
44570.0000000	13.2500000	-50.0000000
44570.0000000	13.5000000	-50.0000000
44570.0000000	13.7500000	-50.0000000
44570.0000000	14.0000000	-50.0000000
44570.0000000	14.2500000	-50.0000000
44570.0000000	14.5000000	-50.0000000
44570.0000000	14.7500000	-50.0000000
44570.0000000	15.0000000	-50.0000000
44600.0000000	0.0000000	-50.0000000
44600.0000000	0.2500000	-50.0000000
44600.0000000	0.5000000	-50.0000000
44600.0000000	0.7500000	-50.0000000
44600.0000000	1.0000000	-50.0000000
44600.0000000	1.2500000	-50.0000000
44600.0000000	1.5000000	-50.0000000
44600.0000000	1.7500000	-50.0000000
44600.0000000	2.0000000	-50.0000000
44600.0000000	2.2500000	-50.0000000
44600.0000000	2.5000000	-50.0000000
44600.0000000	2.7500000	-50.0000000

44600.0000000	3.0000000	-50.0000000
44600.0000000	3.2500000	-50.0000000
44600.0000000	3.5000000	-50.0000000
44600.0000000	3.7500000	-50.0000000
44600.0000000	4.0000000	-50.0000000
44600.0000000	4.2500000	-50.0000000
44600.0000000	4.5000000	-50.0000000
44600.0000000	4.7500000	-50.0000000
44600.0000000	5.0000000	-50.0000000
44600.0000000	5.2500000	-50.0000000
44600.0000000	5.5000000	-50.0000000
44600.0000000	5.7500000	-50.0000000
44600.0000000	6.0000000	-50.0000000
44600.0000000	6.2500000	-50.0000000
44600.0000000	6.5000000	-50.0000000
44600.0000000	6.7500000	-50.0000000
44600.0000000	7.0000000	-50.0000000
44600.0000000	7.2500000	-50.0000000
44600.0000000	7.5000000	-50.0000000
44600.0000000	7.7500000	-50.0000000
44600.0000000	8.0000000	-50.0000000
44600.0000000	8.2500000	-50.0000000
44600.0000000	8.5000000	-50.0000000
44600.0000000	8.7500000	-50.0000000
44600.0000000	9.0000000	-50.0000000
44600.0000000	9.2500000	-50.0000000
44600.0000000	9.5000000	-50.0000000
44600.0000000	9.7500000	-50.0000000
44600.0000000	10.0000000	-50.0000000
44600.0000000	10.2500000	-50.0000000
44600.0000000	10.5000000	-50.0000000
44600.0000000	10.7500000	-50.0000000
44600.0000000	11.0000000	-50.0000000
44600.0000000	11.2500000	-50.0000000
44600.0000000	11.5000000	-50.0000000
44600.0000000	11.7500000	-50.0000000
44600.0000000	12.0000000	-50.0000000
44600.0000000	12.2500000	-50.0000000
44600.0000000	12.5000000	-50.0000000
44600.0000000	12.7500000	-50.0000000
44600.0000000	13.0000000	-50.0000000
44600.0000000	13.2500000	-50.0000000
44600.0000000	13.5000000	-50.0000000
44600.0000000	13.7500000	-50.0000000
44600.0000000	14.0000000	-50.0000000
44600.0000000	14.2500000	-50.0000000
44600.0000000	14.5000000	-50.0000000
44600.0000000	14.7500000	-50.0000000
44600.0000000	15.0000000	-50.0000000
44630.0000000	0.0000000	-50.0000000
44630.0000000	0.2500000	-50.0000000
44630.0000000	0.5000000	-50.0000000
44630.0000000	0.7500000	-50.0000000
44630.0000000	1.0000000	-50.0000000
44630.0000000	1.2500000	-50.0000000
44630.0000000	1.5000000	-50.0000000
44630.0000000	1.7500000	-50.0000000
44630.0000000	2.0000000	-50.0000000

44630.0000000	2.2500000	-50.0000000
44630.0000000	2.5000000	-50.0000000
44630.0000000	2.7500000	-50.0000000
44630.0000000	3.0000000	-50.0000000
44630.0000000	3.2500000	-50.0000000
44630.0000000	3.5000000	-50.0000000
44630.0000000	3.7500000	-50.0000000
44630.0000000	4.0000000	-50.0000000
44630.0000000	4.2500000	-50.0000000
44630.0000000	4.5000000	-50.0000000
44630.0000000	4.7500000	-50.0000000
44630.0000000	5.0000000	-50.0000000
44630.0000000	5.2500000	-50.0000000
44630.0000000	5.5000000	-50.0000000
44630.0000000	5.7500000	-50.0000000
44630.0000000	6.0000000	-50.0000000
44630.0000000	6.2500000	-50.0000000
44630.0000000	6.5000000	-50.0000000
44630.0000000	6.7500000	-50.0000000
44630.0000000	7.0000000	-50.0000000
44630.0000000	7.2500000	-50.0000000
44630.0000000	7.5000000	-50.0000000
44630.0000000	7.7500000	-50.0000000
44630.0000000	8.0000000	-50.0000000
44630.0000000	8.2500000	-50.0000000
44630.0000000	8.5000000	-50.0000000
44630.0000000	8.7500000	-50.0000000
44630.0000000	9.0000000	-50.0000000
44630.0000000	9.2500000	-50.0000000
44630.0000000	9.5000000	-50.0000000
44630.0000000	9.7500000	-50.0000000
44630.0000000	10.0000000	-50.0000000
44630.0000000	10.2500000	-50.0000000
44630.0000000	10.5000000	-50.0000000
44630.0000000	10.7500000	-50.0000000
44630.0000000	11.0000000	-50.0000000
44630.0000000	11.2500000	-50.0000000
44630.0000000	11.5000000	-50.0000000
44630.0000000	11.7500000	-50.0000000
44630.0000000	12.0000000	-50.0000000
44630.0000000	12.2500000	-50.0000000
44630.0000000	12.5000000	-50.0000000
44630.0000000	12.7500000	-50.0000000
44630.0000000	13.0000000	-50.0000000
44630.0000000	13.2500000	-50.0000000
44630.0000000	13.5000000	-50.0000000
44630.0000000	13.7500000	-50.0000000
44630.0000000	14.0000000	-50.0000000
44630.0000000	14.2500000	-50.0000000
44630.0000000	14.5000000	-50.0000000
44630.0000000	14.7500000	-50.0000000
44630.0000000	15.0000000	-50.0000000
44660.0000000	0.0000000	-50.0000000
44660.0000000	0.2500000	-50.0000000
44660.0000000	0.5000000	-50.0000000
44660.0000000	0.7500000	-50.0000000
44660.0000000	1.0000000	-50.0000000
44660.0000000	1.2500000	-50.0000000

44660.0000000	1.5000000	-50.0000000
44660.0000000	1.7500000	-50.0000000
44660.0000000	2.0000000	-50.0000000
44660.0000000	2.2500000	-50.0000000
44660.0000000	2.5000000	-50.0000000
44660.0000000	2.7500000	-50.0000000
44660.0000000	3.0000000	-50.0000000
44660.0000000	3.2500000	-50.0000000
44660.0000000	3.5000000	-50.0000000
44660.0000000	3.7500000	-50.0000000
44660.0000000	4.0000000	-50.0000000
44660.0000000	4.2500000	-50.0000000
44660.0000000	4.5000000	-50.0000000
44660.0000000	4.7500000	-50.0000000
44660.0000000	5.0000000	-50.0000000
44660.0000000	5.2500000	-50.0000000
44660.0000000	5.5000000	-50.0000000
44660.0000000	5.7500000	-50.0000000
44660.0000000	6.0000000	-50.0000000
44660.0000000	6.2500000	-50.0000000
44660.0000000	6.5000000	-50.0000000
44660.0000000	6.7500000	-50.0000000
44660.0000000	7.0000000	-50.0000000
44660.0000000	7.2500000	-50.0000000
44660.0000000	7.5000000	-50.0000000
44660.0000000	7.7500000	-50.0000000
44660.0000000	8.0000000	-50.0000000
44660.0000000	8.2500000	-50.0000000
44660.0000000	8.5000000	-50.0000000
44660.0000000	8.7500000	-50.0000000
44660.0000000	9.0000000	-50.0000000
44660.0000000	9.2500000	-50.0000000
44660.0000000	9.5000000	-50.0000000
44660.0000000	9.7500000	-50.0000000
44660.0000000	10.0000000	-50.0000000
44660.0000000	10.2500000	-50.0000000
44660.0000000	10.5000000	-50.0000000
44660.0000000	10.7500000	-50.0000000
44660.0000000	11.0000000	-50.0000000
44660.0000000	11.2500000	-50.0000000
44660.0000000	11.5000000	-50.0000000
44660.0000000	11.7500000	-50.0000000
44660.0000000	12.0000000	-50.0000000
44660.0000000	12.2500000	-50.0000000
44660.0000000	12.5000000	-50.0000000
44660.0000000	12.7500000	-50.0000000
44660.0000000	13.0000000	-50.0000000
44660.0000000	13.2500000	-50.0000000
44660.0000000	13.5000000	-50.0000000
44660.0000000	13.7500000	-50.0000000
44660.0000000	14.0000000	-50.0000000
44660.0000000	14.2500000	-50.0000000
44660.0000000	14.5000000	-50.0000000
44660.0000000	14.7500000	-50.0000000
44660.0000000	15.0000000	-50.0000000
44690.0000000	0.0000000	-50.0000000
44690.0000000	0.2500000	-50.0000000
44690.0000000	0.5000000	-50.0000000

44690.0000000	0.7500000	-50.0000000
44690.0000000	1.0000000	-50.0000000
44690.0000000	1.2500000	-50.0000000
44690.0000000	1.5000000	-50.0000000
44690.0000000	1.7500000	-50.0000000
44690.0000000	2.0000000	-50.0000000
44690.0000000	2.2500000	-50.0000000
44690.0000000	2.5000000	-50.0000000
44690.0000000	2.7500000	-50.0000000
44690.0000000	3.0000000	-50.0000000
44690.0000000	3.2500000	-50.0000000
44690.0000000	3.5000000	-50.0000000
44690.0000000	3.7500000	-50.0000000
44690.0000000	4.0000000	-50.0000000
44690.0000000	4.2500000	-50.0000000
44690.0000000	4.5000000	-50.0000000
44690.0000000	4.7500000	-50.0000000
44690.0000000	5.0000000	-50.0000000
44690.0000000	5.2500000	-50.0000000
44690.0000000	5.5000000	-50.0000000
44690.0000000	5.7500000	-50.0000000
44690.0000000	6.0000000	-50.0000000
44690.0000000	6.2500000	-50.0000000
44690.0000000	6.5000000	-50.0000000
44690.0000000	6.7500000	-50.0000000
44690.0000000	7.0000000	-50.0000000
44690.0000000	7.2500000	-50.0000000
44690.0000000	7.5000000	-50.0000000
44690.0000000	7.7500000	-50.0000000
44690.0000000	8.0000000	-50.0000000
44690.0000000	8.2500000	-50.0000000
44690.0000000	8.5000000	-50.0000000
44690.0000000	8.7500000	-50.0000000
44690.0000000	9.0000000	-50.0000000
44690.0000000	9.2500000	-50.0000000
44690.0000000	9.5000000	-50.0000000
44690.0000000	9.7500000	-50.0000000
44690.0000000	10.0000000	-50.0000000
44690.0000000	10.2500000	-50.0000000
44690.0000000	10.5000000	-50.0000000
44690.0000000	10.7500000	-50.0000000
44690.0000000	11.0000000	-50.0000000
44690.0000000	11.2500000	-50.0000000
44690.0000000	11.5000000	-50.0000000
44690.0000000	11.7500000	-50.0000000
44690.0000000	12.0000000	-50.0000000
44690.0000000	12.2500000	-50.0000000
44690.0000000	12.5000000	-50.0000000
44690.0000000	12.7500000	-50.0000000
44690.0000000	13.0000000	-50.0000000
44690.0000000	13.2500000	-50.0000000
44690.0000000	13.5000000	-50.0000000
44690.0000000	13.7500000	-50.0000000
44690.0000000	14.0000000	-50.0000000
44690.0000000	14.2500000	-50.0000000
44690.0000000	14.5000000	-50.0000000
44690.0000000	14.7500000	-50.0000000
44690.0000000	15.0000000	-50.0000000

44720.0000000	0.0000000	-50.0000000
44720.0000000	0.2500000	-50.0000000
44720.0000000	0.5000000	-50.0000000
44720.0000000	0.7500000	-50.0000000
44720.0000000	1.0000000	-50.0000000
44720.0000000	1.2500000	-50.0000000
44720.0000000	1.5000000	-50.0000000
44720.0000000	1.7500000	-50.0000000
44720.0000000	2.0000000	-50.0000000
44720.0000000	2.2500000	-50.0000000
44720.0000000	2.5000000	-50.0000000
44720.0000000	2.7500000	-50.0000000
44720.0000000	3.0000000	-50.0000000
44720.0000000	3.2500000	-50.0000000
44720.0000000	3.5000000	-50.0000000
44720.0000000	3.7500000	-50.0000000
44720.0000000	4.0000000	-50.0000000
44720.0000000	4.2500000	-50.0000000
44720.0000000	4.5000000	-50.0000000
44720.0000000	4.7500000	-50.0000000
44720.0000000	5.0000000	-50.0000000
44720.0000000	5.2500000	-50.0000000
44720.0000000	5.5000000	-50.0000000
44720.0000000	5.7500000	-50.0000000
44720.0000000	6.0000000	-50.0000000
44720.0000000	6.2500000	-50.0000000
44720.0000000	6.5000000	-50.0000000
44720.0000000	6.7500000	-50.0000000
44720.0000000	7.0000000	-50.0000000
44720.0000000	7.2500000	-50.0000000
44720.0000000	7.5000000	-50.0000000
44720.0000000	7.7500000	-50.0000000
44720.0000000	8.0000000	-50.0000000
44720.0000000	8.2500000	-50.0000000
44720.0000000	8.5000000	-50.0000000
44720.0000000	8.7500000	-50.0000000
44720.0000000	9.0000000	-50.0000000
44720.0000000	9.2500000	-50.0000000
44720.0000000	9.5000000	-50.0000000
44720.0000000	9.7500000	-50.0000000
44720.0000000	10.0000000	-50.0000000
44720.0000000	10.2500000	-50.0000000
44720.0000000	10.5000000	-50.0000000
44720.0000000	10.7500000	-50.0000000
44720.0000000	11.0000000	-50.0000000
44720.0000000	11.2500000	-50.0000000
44720.0000000	11.5000000	-50.0000000
44720.0000000	11.7500000	-50.0000000
44720.0000000	12.0000000	-50.0000000
44720.0000000	12.2500000	-50.0000000
44720.0000000	12.5000000	-50.0000000
44720.0000000	12.7500000	-50.0000000
44720.0000000	13.0000000	-50.0000000
44720.0000000	13.2500000	-50.0000000
44720.0000000	13.5000000	-50.0000000
44720.0000000	13.7500000	-50.0000000
44720.0000000	14.0000000	-50.0000000
44720.0000000	14.2500000	-50.0000000

44720.0000000	14.5000000	-50.0000000
44720.0000000	14.7500000	-50.0000000
44720.0000000	15.0000000	-50.0000000
44750.0000000	0.0000000	-50.0000000
44750.0000000	0.2500000	-50.0000000
44750.0000000	0.5000000	-50.0000000
44750.0000000	0.7500000	-50.0000000
44750.0000000	1.0000000	-50.0000000
44750.0000000	1.2500000	-50.0000000
44750.0000000	1.5000000	-50.0000000
44750.0000000	1.7500000	-50.0000000
44750.0000000	2.0000000	-50.0000000
44750.0000000	2.2500000	-50.0000000
44750.0000000	2.5000000	-50.0000000
44750.0000000	2.7500000	-50.0000000
44750.0000000	3.0000000	-50.0000000
44750.0000000	3.2500000	-50.0000000
44750.0000000	3.5000000	-50.0000000
44750.0000000	3.7500000	-50.0000000
44750.0000000	4.0000000	-50.0000000
44750.0000000	4.2500000	-50.0000000
44750.0000000	4.5000000	-50.0000000
44750.0000000	4.7500000	-50.0000000
44750.0000000	5.0000000	-50.0000000
44750.0000000	5.2500000	-50.0000000
44750.0000000	5.5000000	-50.0000000
44750.0000000	5.7500000	-50.0000000
44750.0000000	6.0000000	-50.0000000
44750.0000000	6.2500000	-50.0000000
44750.0000000	6.5000000	-50.0000000
44750.0000000	6.7500000	-50.0000000
44750.0000000	7.0000000	-50.0000000
44750.0000000	7.2500000	-50.0000000
44750.0000000	7.5000000	-50.0000000
44750.0000000	7.7500000	-50.0000000
44750.0000000	8.0000000	-50.0000000
44750.0000000	8.2500000	-50.0000000
44750.0000000	8.5000000	-50.0000000
44750.0000000	8.7500000	-50.0000000
44750.0000000	9.0000000	-50.0000000
44750.0000000	9.2500000	-50.0000000
44750.0000000	9.5000000	-50.0000000
44750.0000000	9.7500000	-50.0000000
44750.0000000	10.0000000	-50.0000000
44750.0000000	10.2500000	-50.0000000
44750.0000000	10.5000000	-50.0000000
44750.0000000	10.7500000	-50.0000000
44750.0000000	11.0000000	-50.0000000
44750.0000000	11.2500000	-50.0000000
44750.0000000	11.5000000	-50.0000000
44750.0000000	11.7500000	-50.0000000
44750.0000000	12.0000000	-50.0000000
44750.0000000	12.2500000	-50.0000000
44750.0000000	12.5000000	-50.0000000
44750.0000000	12.7500000	-50.0000000
44750.0000000	13.0000000	-50.0000000
44750.0000000	13.2500000	-50.0000000
44750.0000000	13.5000000	-50.0000000

44750.0000000	13.7500000	-50.0000000
44750.0000000	14.0000000	-50.0000000
44750.0000000	14.2500000	-50.0000000
44750.0000000	14.5000000	-50.0000000
44750.0000000	14.7500000	-50.0000000
44750.0000000	15.0000000	-50.0000000
44780.0000000	0.0000000	-50.0000000
44780.0000000	0.2500000	-50.0000000
44780.0000000	0.5000000	-50.0000000
44780.0000000	0.7500000	-50.0000000
44780.0000000	1.0000000	-50.0000000
44780.0000000	1.2500000	-50.0000000
44780.0000000	1.5000000	-50.0000000
44780.0000000	1.7500000	-50.0000000
44780.0000000	2.0000000	-50.0000000
44780.0000000	2.2500000	-50.0000000
44780.0000000	2.5000000	-50.0000000
44780.0000000	2.7500000	-50.0000000
44780.0000000	3.0000000	-50.0000000
44780.0000000	3.2500000	-50.0000000
44780.0000000	3.5000000	-50.0000000
44780.0000000	3.7500000	-50.0000000
44780.0000000	4.0000000	-50.0000000
44780.0000000	4.2500000	-50.0000000
44780.0000000	4.5000000	-50.0000000
44780.0000000	4.7500000	-50.0000000
44780.0000000	5.0000000	-50.0000000
44780.0000000	5.2500000	-50.0000000
44780.0000000	5.5000000	-50.0000000
44780.0000000	5.7500000	-50.0000000
44780.0000000	6.0000000	-50.0000000
44780.0000000	6.2500000	-50.0000000
44780.0000000	6.5000000	-50.0000000
44780.0000000	6.7500000	-50.0000000
44780.0000000	7.0000000	-50.0000000
44780.0000000	7.2500000	-50.0000000
44780.0000000	7.5000000	-50.0000000
44780.0000000	7.7500000	-50.0000000
44780.0000000	8.0000000	-50.0000000
44780.0000000	8.2500000	-50.0000000
44780.0000000	8.5000000	-50.0000000
44780.0000000	8.7500000	-50.0000000
44780.0000000	9.0000000	-50.0000000
44780.0000000	9.2500000	-50.0000000
44780.0000000	9.5000000	-50.0000000
44780.0000000	9.7500000	-50.0000000
44780.0000000	10.0000000	-50.0000000
44780.0000000	10.2500000	-50.0000000
44780.0000000	10.5000000	-50.0000000
44780.0000000	10.7500000	-50.0000000
44780.0000000	11.0000000	-50.0000000
44780.0000000	11.2500000	-50.0000000
44780.0000000	11.5000000	-50.0000000
44780.0000000	11.7500000	-50.0000000
44780.0000000	12.0000000	-50.0000000
44780.0000000	12.2500000	-50.0000000
44780.0000000	12.5000000	-50.0000000
44780.0000000	12.7500000	-50.0000000

44780.0000000	13.0000000	-50.0000000
44780.0000000	13.2500000	-50.0000000
44780.0000000	13.5000000	-50.0000000
44780.0000000	13.7500000	-50.0000000
44780.0000000	14.0000000	-50.0000000
44780.0000000	14.2500000	-50.0000000
44780.0000000	14.5000000	-50.0000000
44780.0000000	14.7500000	-50.0000000
44780.0000000	15.0000000	-50.0000000
44810.0000000	0.0000000	-50.0000000
44810.0000000	0.2500000	-50.0000000
44810.0000000	0.5000000	-50.0000000
44810.0000000	0.7500000	-50.0000000
44810.0000000	1.0000000	-50.0000000
44810.0000000	1.2500000	-50.0000000
44810.0000000	1.5000000	-50.0000000
44810.0000000	1.7500000	-50.0000000
44810.0000000	2.0000000	-50.0000000
44810.0000000	2.2500000	-50.0000000
44810.0000000	2.5000000	-50.0000000
44810.0000000	2.7500000	-50.0000000
44810.0000000	3.0000000	-50.0000000
44810.0000000	3.2500000	-50.0000000
44810.0000000	3.5000000	-50.0000000
44810.0000000	3.7500000	-50.0000000
44810.0000000	4.0000000	-50.0000000
44810.0000000	4.2500000	-50.0000000
44810.0000000	4.5000000	-50.0000000
44810.0000000	4.7500000	-50.0000000
44810.0000000	5.0000000	-50.0000000
44810.0000000	5.2500000	-50.0000000
44810.0000000	5.5000000	-50.0000000
44810.0000000	5.7500000	-50.0000000
44810.0000000	6.0000000	-50.0000000
44810.0000000	6.2500000	-50.0000000
44810.0000000	6.5000000	-50.0000000
44810.0000000	6.7500000	-50.0000000
44810.0000000	7.0000000	-50.0000000
44810.0000000	7.2500000	-50.0000000
44810.0000000	7.5000000	-50.0000000
44810.0000000	7.7500000	-50.0000000
44810.0000000	8.0000000	-50.0000000
44810.0000000	8.2500000	-50.0000000
44810.0000000	8.5000000	-50.0000000
44810.0000000	8.7500000	-50.0000000
44810.0000000	9.0000000	-50.0000000
44810.0000000	9.2500000	-50.0000000
44810.0000000	9.5000000	-50.0000000
44810.0000000	9.7500000	-50.0000000
44810.0000000	10.0000000	-50.0000000
44810.0000000	10.2500000	-50.0000000
44810.0000000	10.5000000	-50.0000000
44810.0000000	10.7500000	-50.0000000
44810.0000000	11.0000000	-50.0000000
44810.0000000	11.2500000	-50.0000000
44810.0000000	11.5000000	-50.0000000
44810.0000000	11.7500000	-50.0000000
44810.0000000	12.0000000	-50.0000000

44810.0000000	12.2500000	-50.0000000
44810.0000000	12.5000000	-50.0000000
44810.0000000	12.7500000	-50.0000000
44810.0000000	13.0000000	-50.0000000
44810.0000000	13.2500000	-50.0000000
44810.0000000	13.5000000	-50.0000000
44810.0000000	13.7500000	-50.0000000
44810.0000000	14.0000000	-50.0000000
44810.0000000	14.2500000	-50.0000000
44810.0000000	14.5000000	-50.0000000
44810.0000000	14.7500000	-50.0000000
44810.0000000	15.0000000	-50.0000000
44840.0000000	0.0000000	-50.0000000
44840.0000000	0.2500000	-50.0000000
44840.0000000	0.5000000	-50.0000000
44840.0000000	0.7500000	-50.0000000
44840.0000000	1.0000000	-50.0000000
44840.0000000	1.2500000	-50.0000000
44840.0000000	1.5000000	-50.0000000
44840.0000000	1.7500000	-50.0000000
44840.0000000	2.0000000	-50.0000000
44840.0000000	2.2500000	-50.0000000
44840.0000000	2.5000000	-50.0000000
44840.0000000	2.7500000	-50.0000000
44840.0000000	3.0000000	-50.0000000
44840.0000000	3.2500000	-50.0000000
44840.0000000	3.5000000	-50.0000000
44840.0000000	3.7500000	-50.0000000
44840.0000000	4.0000000	-50.0000000
44840.0000000	4.2500000	-50.0000000
44840.0000000	4.5000000	-50.0000000
44840.0000000	4.7500000	-50.0000000
44840.0000000	5.0000000	-50.0000000
44840.0000000	5.2500000	-50.0000000
44840.0000000	5.5000000	-50.0000000
44840.0000000	5.7500000	-50.0000000
44840.0000000	6.0000000	-50.0000000
44840.0000000	6.2500000	-50.0000000
44840.0000000	6.5000000	-50.0000000
44840.0000000	6.7500000	-50.0000000
44840.0000000	7.0000000	-50.0000000
44840.0000000	7.2500000	-50.0000000
44840.0000000	7.5000000	-50.0000000
44840.0000000	7.7500000	-50.0000000
44840.0000000	8.0000000	-50.0000000
44840.0000000	8.2500000	-50.0000000
44840.0000000	8.5000000	-50.0000000
44840.0000000	8.7500000	-50.0000000
44840.0000000	9.0000000	-50.0000000
44840.0000000	9.2500000	-50.0000000
44840.0000000	9.5000000	-50.0000000
44840.0000000	9.7500000	-50.0000000
44840.0000000	10.0000000	-50.0000000
44840.0000000	10.2500000	-50.0000000
44840.0000000	10.5000000	-50.0000000
44840.0000000	10.7500000	-50.0000000
44840.0000000	11.0000000	-50.0000000
44840.0000000	11.2500000	-50.0000000

44840.0000000	11.5000000	-50.0000000
44840.0000000	11.7500000	-50.0000000
44840.0000000	12.0000000	-50.0000000
44840.0000000	12.2500000	-50.0000000
44840.0000000	12.5000000	-50.0000000
44840.0000000	12.7500000	-50.0000000
44840.0000000	13.0000000	-50.0000000
44840.0000000	13.2500000	-50.0000000
44840.0000000	13.5000000	-50.0000000
44840.0000000	13.7500000	-50.0000000
44840.0000000	14.0000000	-50.0000000
44840.0000000	14.2500000	-50.0000000
44840.0000000	14.5000000	-50.0000000
44840.0000000	14.7500000	-50.0000000
44840.0000000	15.0000000	-50.0000000
44870.0000000	0.0000000	-50.0000000
44870.0000000	0.2500000	-50.0000000
44870.0000000	0.5000000	-50.0000000
44870.0000000	0.7500000	-50.0000000
44870.0000000	1.0000000	-50.0000000
44870.0000000	1.2500000	-50.0000000
44870.0000000	1.5000000	-50.0000000
44870.0000000	1.7500000	-50.0000000
44870.0000000	2.0000000	-50.0000000
44870.0000000	2.2500000	-50.0000000
44870.0000000	2.5000000	-50.0000000
44870.0000000	2.7500000	-50.0000000
44870.0000000	3.0000000	-50.0000000
44870.0000000	3.2500000	-50.0000000
44870.0000000	3.5000000	-50.0000000
44870.0000000	3.7500000	-50.0000000
44870.0000000	4.0000000	-50.0000000
44870.0000000	4.2500000	-50.0000000
44870.0000000	4.5000000	-50.0000000
44870.0000000	4.7500000	-50.0000000
44870.0000000	5.0000000	-50.0000000
44870.0000000	5.2500000	-50.0000000
44870.0000000	5.5000000	-50.0000000
44870.0000000	5.7500000	-50.0000000
44870.0000000	6.0000000	-50.0000000
44870.0000000	6.2500000	-50.0000000
44870.0000000	6.5000000	-50.0000000
44870.0000000	6.7500000	-50.0000000
44870.0000000	7.0000000	-50.0000000
44870.0000000	7.2500000	-50.0000000
44870.0000000	7.5000000	-50.0000000
44870.0000000	7.7500000	-50.0000000
44870.0000000	8.0000000	-50.0000000
44870.0000000	8.2500000	-50.0000000
44870.0000000	8.5000000	-50.0000000
44870.0000000	8.7500000	-50.0000000
44870.0000000	9.0000000	-50.0000000
44870.0000000	9.2500000	-50.0000000
44870.0000000	9.5000000	-50.0000000
44870.0000000	9.7500000	-50.0000000
44870.0000000	10.0000000	-50.0000000
44870.0000000	10.2500000	-50.0000000
44870.0000000	10.5000000	-50.0000000

44870.0000000	10.7500000	-50.0000000
44870.0000000	11.0000000	-50.0000000
44870.0000000	11.2500000	-50.0000000
44870.0000000	11.5000000	-50.0000000
44870.0000000	11.7500000	-50.0000000
44870.0000000	12.0000000	-50.0000000
44870.0000000	12.2500000	-50.0000000
44870.0000000	12.5000000	-50.0000000
44870.0000000	12.7500000	-50.0000000
44870.0000000	13.0000000	-50.0000000
44870.0000000	13.2500000	-50.0000000
44870.0000000	13.5000000	-50.0000000
44870.0000000	13.7500000	-50.0000000
44870.0000000	14.0000000	-50.0000000
44870.0000000	14.2500000	-50.0000000
44870.0000000	14.5000000	-50.0000000
44870.0000000	14.7500000	-50.0000000
44870.0000000	15.0000000	-50.0000000
44900.0000000	0.0000000	-50.0000000
44900.0000000	0.2500000	-50.0000000
44900.0000000	0.5000000	-50.0000000
44900.0000000	0.7500000	-50.0000000
44900.0000000	1.0000000	-50.0000000
44900.0000000	1.2500000	-50.0000000
44900.0000000	1.5000000	-50.0000000
44900.0000000	1.7500000	-50.0000000
44900.0000000	2.0000000	-50.0000000
44900.0000000	2.2500000	-50.0000000
44900.0000000	2.5000000	-50.0000000
44900.0000000	2.7500000	-50.0000000
44900.0000000	3.0000000	-50.0000000
44900.0000000	3.2500000	-50.0000000
44900.0000000	3.5000000	-50.0000000
44900.0000000	3.7500000	-50.0000000
44900.0000000	4.0000000	-50.0000000
44900.0000000	4.2500000	-50.0000000
44900.0000000	4.5000000	-50.0000000
44900.0000000	4.7500000	-50.0000000
44900.0000000	5.0000000	-50.0000000
44900.0000000	5.2500000	-50.0000000
44900.0000000	5.5000000	-50.0000000
44900.0000000	5.7500000	-50.0000000
44900.0000000	6.0000000	-50.0000000
44900.0000000	6.2500000	-50.0000000
44900.0000000	6.5000000	-50.0000000
44900.0000000	6.7500000	-50.0000000
44900.0000000	7.0000000	-50.0000000
44900.0000000	7.2500000	-50.0000000
44900.0000000	7.5000000	-50.0000000
44900.0000000	7.7500000	-50.0000000
44900.0000000	8.0000000	-50.0000000
44900.0000000	8.2500000	-50.0000000
44900.0000000	8.5000000	-50.0000000
44900.0000000	8.7500000	-50.0000000
44900.0000000	9.0000000	-50.0000000
44900.0000000	9.2500000	-50.0000000
44900.0000000	9.5000000	-50.0000000
44900.0000000	9.7500000	-50.0000000

44900.0000000	10.0000000	-50.0000000
44900.0000000	10.2500000	-50.0000000
44900.0000000	10.5000000	-50.0000000
44900.0000000	10.7500000	-50.0000000
44900.0000000	11.0000000	-50.0000000
44900.0000000	11.2500000	-50.0000000
44900.0000000	11.5000000	-50.0000000
44900.0000000	11.7500000	-50.0000000
44900.0000000	12.0000000	-50.0000000
44900.0000000	12.2500000	-50.0000000
44900.0000000	12.5000000	-50.0000000
44900.0000000	12.7500000	-50.0000000
44900.0000000	13.0000000	-50.0000000
44900.0000000	13.2500000	-50.0000000
44900.0000000	13.5000000	-50.0000000
44900.0000000	13.7500000	-50.0000000
44900.0000000	14.0000000	-50.0000000
44900.0000000	14.2500000	-50.0000000
44900.0000000	14.5000000	-50.0000000
44900.0000000	14.7500000	-50.0000000
44900.0000000	15.0000000	-50.0000000
44930.0000000	0.0000000	-50.0000000
44930.0000000	0.2500000	-50.0000000
44930.0000000	0.5000000	-50.0000000
44930.0000000	0.7500000	-50.0000000
44930.0000000	1.0000000	-50.0000000
44930.0000000	1.2500000	-50.0000000
44930.0000000	1.5000000	-50.0000000
44930.0000000	1.7500000	-50.0000000
44930.0000000	2.0000000	-50.0000000
44930.0000000	2.2500000	-50.0000000
44930.0000000	2.5000000	-50.0000000
44930.0000000	2.7500000	-50.0000000
44930.0000000	3.0000000	-50.0000000
44930.0000000	3.2500000	-50.0000000
44930.0000000	3.5000000	-50.0000000
44930.0000000	3.7500000	-50.0000000
44930.0000000	4.0000000	-50.0000000
44930.0000000	4.2500000	-50.0000000
44930.0000000	4.5000000	-50.0000000
44930.0000000	4.7500000	-50.0000000
44930.0000000	5.0000000	-50.0000000
44930.0000000	5.2500000	-50.0000000
44930.0000000	5.5000000	-50.0000000
44930.0000000	5.7500000	-50.0000000
44930.0000000	6.0000000	-50.0000000
44930.0000000	6.2500000	-50.0000000
44930.0000000	6.5000000	-50.0000000
44930.0000000	6.7500000	-50.0000000
44930.0000000	7.0000000	-50.0000000
44930.0000000	7.2500000	-50.0000000
44930.0000000	7.5000000	-50.0000000
44930.0000000	7.7500000	-50.0000000
44930.0000000	8.0000000	-50.0000000
44930.0000000	8.2500000	-50.0000000
44930.0000000	8.5000000	-50.0000000
44930.0000000	8.7500000	-50.0000000
44930.0000000	9.0000000	-50.0000000

44930.0000000	9.2500000	-50.0000000
44930.0000000	9.5000000	-50.0000000
44930.0000000	9.7500000	-50.0000000
44930.0000000	10.0000000	-50.0000000
44930.0000000	10.2500000	-50.0000000
44930.0000000	10.5000000	-50.0000000
44930.0000000	10.7500000	-50.0000000
44930.0000000	11.0000000	-50.0000000
44930.0000000	11.2500000	-50.0000000
44930.0000000	11.5000000	-50.0000000
44930.0000000	11.7500000	-50.0000000
44930.0000000	12.0000000	-50.0000000
44930.0000000	12.2500000	-50.0000000
44930.0000000	12.5000000	-50.0000000
44930.0000000	12.7500000	-50.0000000
44930.0000000	13.0000000	-50.0000000
44930.0000000	13.2500000	-50.0000000
44930.0000000	13.5000000	-50.0000000
44930.0000000	13.7500000	-50.0000000
44930.0000000	14.0000000	-50.0000000
44930.0000000	14.2500000	-50.0000000
44930.0000000	14.5000000	-50.0000000
44930.0000000	14.7500000	-50.0000000
44930.0000000	15.0000000	-50.0000000
44960.0000000	0.0000000	-50.0000000
44960.0000000	0.2500000	-50.0000000
44960.0000000	0.5000000	-50.0000000
44960.0000000	0.7500000	-50.0000000
44960.0000000	1.0000000	-50.0000000
44960.0000000	1.2500000	-50.0000000
44960.0000000	1.5000000	-50.0000000
44960.0000000	1.7500000	-50.0000000
44960.0000000	2.0000000	15.7500000
44960.0000000	2.2500000	-50.0000000
44960.0000000	2.5000000	-50.0000000
44960.0000000	2.7500000	-50.0000000
44960.0000000	3.0000000	-50.0000000
44960.0000000	3.2500000	-50.0000000
44960.0000000	3.5000000	-50.0000000
44960.0000000	3.7500000	-50.0000000
44960.0000000	4.0000000	-50.0000000
44960.0000000	4.2500000	-50.0000000
44960.0000000	4.5000000	-50.0000000
44960.0000000	4.7500000	-50.0000000
44960.0000000	5.0000000	-50.0000000
44960.0000000	5.2500000	-50.0000000
44960.0000000	5.5000000	-50.0000000
44960.0000000	5.7500000	-50.0000000
44960.0000000	6.0000000	-50.0000000
44960.0000000	6.2500000	-50.0000000
44960.0000000	6.5000000	-50.0000000
44960.0000000	6.7500000	-50.0000000
44960.0000000	7.0000000	-50.0000000
44960.0000000	7.2500000	-50.0000000
44960.0000000	7.5000000	-50.0000000
44960.0000000	7.7500000	-50.0000000
44960.0000000	8.0000000	-50.0000000
44960.0000000	8.2500000	-50.0000000

44960.0000000	8.5000000	-50.0000000
44960.0000000	8.7500000	-50.0000000
44960.0000000	9.0000000	-50.0000000
44960.0000000	9.2500000	-50.0000000
44960.0000000	9.5000000	-50.0000000
44960.0000000	9.7500000	-50.0000000
44960.0000000	10.0000000	-50.0000000
44960.0000000	10.2500000	-50.0000000
44960.0000000	10.5000000	-50.0000000
44960.0000000	10.7500000	-50.0000000
44960.0000000	11.0000000	-50.0000000
44960.0000000	11.2500000	-50.0000000
44960.0000000	11.5000000	-50.0000000
44960.0000000	11.7500000	-50.0000000
44960.0000000	12.0000000	-50.0000000
44960.0000000	12.2500000	-50.0000000
44960.0000000	12.5000000	-50.0000000
44960.0000000	12.7500000	-50.0000000
44960.0000000	13.0000000	-50.0000000
44960.0000000	13.2500000	-50.0000000
44960.0000000	13.5000000	-50.0000000
44960.0000000	13.7500000	-50.0000000
44960.0000000	14.0000000	-50.0000000
44960.0000000	14.2500000	-50.0000000
44960.0000000	14.5000000	-50.0000000
44960.0000000	14.7500000	-50.0000000
44960.0000000	15.0000000	-50.0000000
44990.0000000	0.0000000	-50.0000000
44990.0000000	0.2500000	-50.0000000
44990.0000000	0.5000000	-50.0000000
44990.0000000	0.7500000	-50.0000000
44990.0000000	1.0000000	-50.0000000
44990.0000000	1.2500000	-50.0000000
44990.0000000	1.5000000	-50.0000000
44990.0000000	1.7500000	-50.0000000
44990.0000000	2.0000000	-50.0000000
44990.0000000	2.2500000	-50.0000000
44990.0000000	2.5000000	-50.0000000
44990.0000000	2.7500000	-50.0000000
44990.0000000	3.0000000	-50.0000000
44990.0000000	3.2500000	-50.0000000
44990.0000000	3.5000000	-50.0000000
44990.0000000	3.7500000	-50.0000000
44990.0000000	4.0000000	-50.0000000
44990.0000000	4.2500000	-50.0000000
44990.0000000	4.5000000	-50.0000000
44990.0000000	4.7500000	-50.0000000
44990.0000000	5.0000000	-50.0000000
44990.0000000	5.2500000	-50.0000000
44990.0000000	5.5000000	-50.0000000
44990.0000000	5.7500000	-50.0000000
44990.0000000	6.0000000	-50.0000000
44990.0000000	6.2500000	-50.0000000
44990.0000000	6.5000000	-50.0000000
44990.0000000	6.7500000	-50.0000000
44990.0000000	7.0000000	-50.0000000
44990.0000000	7.2500000	-50.0000000
44990.0000000	7.5000000	-50.0000000

44990.0000000	7.7500000	-50.0000000
44990.0000000	8.0000000	-50.0000000
44990.0000000	8.2500000	-50.0000000
44990.0000000	8.5000000	-50.0000000
44990.0000000	8.7500000	-50.0000000
44990.0000000	9.0000000	-50.0000000
44990.0000000	9.2500000	-50.0000000
44990.0000000	9.5000000	-50.0000000
44990.0000000	9.7500000	-50.0000000
44990.0000000	10.0000000	-50.0000000
44990.0000000	10.2500000	-50.0000000
44990.0000000	10.5000000	-50.0000000
44990.0000000	10.7500000	-50.0000000
44990.0000000	11.0000000	-50.0000000
44990.0000000	11.2500000	-50.0000000
44990.0000000	11.5000000	-50.0000000
44990.0000000	11.7500000	-50.0000000
44990.0000000	12.0000000	-50.0000000
44990.0000000	12.2500000	-50.0000000
44990.0000000	12.5000000	-50.0000000
44990.0000000	12.7500000	-50.0000000
44990.0000000	13.0000000	-50.0000000
44990.0000000	13.2500000	-50.0000000
44990.0000000	13.5000000	-50.0000000
44990.0000000	13.7500000	-50.0000000
44990.0000000	14.0000000	-50.0000000
44990.0000000	14.2500000	-50.0000000
44990.0000000	14.5000000	-50.0000000
44990.0000000	14.7500000	-50.0000000
44990.0000000	15.0000000	-50.0000000
45020.0000000	0.0000000	-50.0000000
45020.0000000	0.2500000	-50.0000000
45020.0000000	0.5000000	-50.0000000
45020.0000000	0.7500000	-50.0000000
45020.0000000	1.0000000	-50.0000000
45020.0000000	1.2500000	-50.0000000
45020.0000000	1.5000000	-50.0000000
45020.0000000	1.7500000	-50.0000000
45020.0000000	2.0000000	-50.0000000
45020.0000000	2.2500000	-50.0000000
45020.0000000	2.5000000	-50.0000000
45020.0000000	2.7500000	-50.0000000
45020.0000000	3.0000000	-50.0000000
45020.0000000	3.2500000	-50.0000000
45020.0000000	3.5000000	-50.0000000
45020.0000000	3.7500000	-50.0000000
45020.0000000	4.0000000	-50.0000000
45020.0000000	4.2500000	-50.0000000
45020.0000000	4.5000000	-50.0000000
45020.0000000	4.7500000	-50.0000000
45020.0000000	5.0000000	-50.0000000
45020.0000000	5.2500000	-50.0000000
45020.0000000	5.5000000	-50.0000000
45020.0000000	5.7500000	-50.0000000
45020.0000000	6.0000000	-50.0000000
45020.0000000	6.2500000	-50.0000000
45020.0000000	6.5000000	-50.0000000
45020.0000000	6.7500000	-50.0000000

45020.0000000	7.0000000	-50.0000000
45020.0000000	7.2500000	-50.0000000
45020.0000000	7.5000000	-50.0000000
45020.0000000	7.7500000	-50.0000000
45020.0000000	8.0000000	-50.0000000
45020.0000000	8.2500000	-50.0000000
45020.0000000	8.5000000	-50.0000000
45020.0000000	8.7500000	-50.0000000
45020.0000000	9.0000000	-50.0000000
45020.0000000	9.2500000	-50.0000000
45020.0000000	9.5000000	-50.0000000
45020.0000000	9.7500000	-50.0000000
45020.0000000	10.0000000	-50.0000000
45020.0000000	10.2500000	-50.0000000
45020.0000000	10.5000000	-50.0000000
45020.0000000	10.7500000	-50.0000000
45020.0000000	11.0000000	-50.0000000
45020.0000000	11.2500000	-50.0000000
45020.0000000	11.5000000	-50.0000000
45020.0000000	11.7500000	-50.0000000
45020.0000000	12.0000000	-50.0000000
45020.0000000	12.2500000	-50.0000000
45020.0000000	12.5000000	-50.0000000
45020.0000000	12.7500000	-50.0000000
45020.0000000	13.0000000	-50.0000000
45020.0000000	13.2500000	-50.0000000
45020.0000000	13.5000000	-50.0000000
45020.0000000	13.7500000	-50.0000000
45020.0000000	14.0000000	-50.0000000
45020.0000000	14.2500000	-50.0000000
45020.0000000	14.5000000	-50.0000000
45020.0000000	14.7500000	-50.0000000
45020.0000000	15.0000000	-50.0000000
45050.0000000	0.0000000	-50.0000000
45050.0000000	0.2500000	-50.0000000
45050.0000000	0.5000000	-50.0000000
45050.0000000	0.7500000	-50.0000000
45050.0000000	1.0000000	-50.0000000
45050.0000000	1.2500000	-50.0000000
45050.0000000	1.5000000	-50.0000000
45050.0000000	1.7500000	-50.0000000
45050.0000000	2.0000000	-50.0000000
45050.0000000	2.2500000	-50.0000000
45050.0000000	2.5000000	-50.0000000
45050.0000000	2.7500000	-50.0000000
45050.0000000	3.0000000	-50.0000000
45050.0000000	3.2500000	-50.0000000
45050.0000000	3.5000000	-50.0000000
45050.0000000	3.7500000	-50.0000000
45050.0000000	4.0000000	-50.0000000
45050.0000000	4.2500000	-50.0000000
45050.0000000	4.5000000	-50.0000000
45050.0000000	4.7500000	-50.0000000
45050.0000000	5.0000000	-50.0000000
45050.0000000	5.2500000	-50.0000000
45050.0000000	5.5000000	-50.0000000
45050.0000000	5.7500000	-50.0000000
45050.0000000	6.0000000	-50.0000000

45050.0000000	6.2500000	-50.0000000
45050.0000000	6.5000000	-50.0000000
45050.0000000	6.7500000	-50.0000000
45050.0000000	7.0000000	-50.0000000
45050.0000000	7.2500000	-50.0000000
45050.0000000	7.5000000	-50.0000000
45050.0000000	7.7500000	-50.0000000
45050.0000000	8.0000000	-50.0000000
45050.0000000	8.2500000	-50.0000000
45050.0000000	8.5000000	-50.0000000
45050.0000000	8.7500000	-50.0000000
45050.0000000	9.0000000	-50.0000000
45050.0000000	9.2500000	-50.0000000
45050.0000000	9.5000000	-50.0000000
45050.0000000	9.7500000	-50.0000000
45050.0000000	10.0000000	-50.0000000
45050.0000000	10.2500000	-50.0000000
45050.0000000	10.5000000	-50.0000000
45050.0000000	10.7500000	-50.0000000
45050.0000000	11.0000000	-50.0000000
45050.0000000	11.2500000	-50.0000000
45050.0000000	11.5000000	-50.0000000
45050.0000000	11.7500000	-50.0000000
45050.0000000	12.0000000	-50.0000000
45050.0000000	12.2500000	-50.0000000
45050.0000000	12.5000000	-50.0000000
45050.0000000	12.7500000	-50.0000000
45050.0000000	13.0000000	-50.0000000
45050.0000000	13.2500000	-50.0000000
45050.0000000	13.5000000	-50.0000000
45050.0000000	13.7500000	-50.0000000
45050.0000000	14.0000000	-50.0000000
45050.0000000	14.2500000	-50.0000000
45050.0000000	14.5000000	-50.0000000
45050.0000000	14.7500000	-50.0000000
45050.0000000	15.0000000	-50.0000000
45080.0000000	0.0000000	-50.0000000
45080.0000000	0.2500000	-50.0000000
45080.0000000	0.5000000	-50.0000000
45080.0000000	0.7500000	-50.0000000
45080.0000000	1.0000000	-50.0000000
45080.0000000	1.2500000	-50.0000000
45080.0000000	1.5000000	-50.0000000
45080.0000000	1.7500000	-50.0000000
45080.0000000	2.0000000	-50.0000000
45080.0000000	2.2500000	-50.0000000
45080.0000000	2.5000000	-50.0000000
45080.0000000	2.7500000	-50.0000000
45080.0000000	3.0000000	-50.0000000
45080.0000000	3.2500000	-50.0000000
45080.0000000	3.5000000	-50.0000000
45080.0000000	3.7500000	-50.0000000
45080.0000000	4.0000000	-50.0000000
45080.0000000	4.2500000	-50.0000000
45080.0000000	4.5000000	-50.0000000
45080.0000000	4.7500000	-50.0000000
45080.0000000	5.0000000	-50.0000000
45080.0000000	5.2500000	-50.0000000

45080.0000000	5.5000000	-50.0000000
45080.0000000	5.7500000	-50.0000000
45080.0000000	6.0000000	-50.0000000
45080.0000000	6.2500000	-50.0000000
45080.0000000	6.5000000	-50.0000000
45080.0000000	6.7500000	-50.0000000
45080.0000000	7.0000000	-50.0000000
45080.0000000	7.2500000	-50.0000000
45080.0000000	7.5000000	-50.0000000
45080.0000000	7.7500000	-50.0000000
45080.0000000	8.0000000	-50.0000000
45080.0000000	8.2500000	-50.0000000
45080.0000000	8.5000000	-50.0000000
45080.0000000	8.7500000	-50.0000000
45080.0000000	9.0000000	-50.0000000
45080.0000000	9.2500000	-50.0000000
45080.0000000	9.5000000	-50.0000000
45080.0000000	9.7500000	-50.0000000
45080.0000000	10.0000000	-50.0000000
45080.0000000	10.2500000	-50.0000000
45080.0000000	10.5000000	-50.0000000
45080.0000000	10.7500000	-50.0000000
45080.0000000	11.0000000	-50.0000000
45080.0000000	11.2500000	-50.0000000
45080.0000000	11.5000000	-50.0000000
45080.0000000	11.7500000	-50.0000000
45080.0000000	12.0000000	-50.0000000
45080.0000000	12.2500000	-50.0000000
45080.0000000	12.5000000	-50.0000000
45080.0000000	12.7500000	-50.0000000
45080.0000000	13.0000000	-50.0000000
45080.0000000	13.2500000	-50.0000000
45080.0000000	13.5000000	-50.0000000
45080.0000000	13.7500000	-50.0000000
45080.0000000	14.0000000	-50.0000000
45080.0000000	14.2500000	-50.0000000
45080.0000000	14.5000000	-50.0000000
45080.0000000	14.7500000	-50.0000000
45080.0000000	15.0000000	-50.0000000
45110.0000000	0.0000000	-50.0000000
45110.0000000	0.2500000	-50.0000000
45110.0000000	0.5000000	-50.0000000
45110.0000000	0.7500000	-50.0000000
45110.0000000	1.0000000	-50.0000000
45110.0000000	1.2500000	16.5699997
45110.0000000	1.5000000	15.7100000
45110.0000000	1.7500000	-50.0000000
45110.0000000	2.0000000	-50.0000000
45110.0000000	2.2500000	-50.0000000
45110.0000000	2.5000000	-50.0000000
45110.0000000	2.7500000	-50.0000000
45110.0000000	3.0000000	-50.0000000
45110.0000000	3.2500000	-50.0000000
45110.0000000	3.5000000	-50.0000000
45110.0000000	3.7500000	-50.0000000
45110.0000000	4.0000000	-50.0000000
45110.0000000	4.2500000	-50.0000000
45110.0000000	4.5000000	-50.0000000

45110.0000000	4.7500000	-50.0000000
45110.0000000	5.0000000	-50.0000000
45110.0000000	5.2500000	-50.0000000
45110.0000000	5.5000000	-50.0000000
45110.0000000	5.7500000	-50.0000000
45110.0000000	6.0000000	-50.0000000
45110.0000000	6.2500000	-50.0000000
45110.0000000	6.5000000	-50.0000000
45110.0000000	6.7500000	-50.0000000
45110.0000000	7.0000000	-50.0000000
45110.0000000	7.2500000	-50.0000000
45110.0000000	7.5000000	-50.0000000
45110.0000000	7.7500000	-50.0000000
45110.0000000	8.0000000	-50.0000000
45110.0000000	8.2500000	-50.0000000
45110.0000000	8.5000000	-50.0000000
45110.0000000	8.7500000	-50.0000000
45110.0000000	9.0000000	-50.0000000
45110.0000000	9.2500000	-50.0000000
45110.0000000	9.5000000	-50.0000000
45110.0000000	9.7500000	-50.0000000
45110.0000000	10.0000000	-50.0000000
45110.0000000	10.2500000	-50.0000000
45110.0000000	10.5000000	-50.0000000
45110.0000000	10.7500000	-50.0000000
45110.0000000	11.0000000	-50.0000000
45110.0000000	11.2500000	-50.0000000
45110.0000000	11.5000000	-50.0000000
45110.0000000	11.7500000	-50.0000000
45110.0000000	12.0000000	-50.0000000
45110.0000000	12.2500000	-50.0000000
45110.0000000	12.5000000	-50.0000000
45110.0000000	12.7500000	-50.0000000
45110.0000000	13.0000000	-50.0000000
45110.0000000	13.2500000	-50.0000000
45110.0000000	13.5000000	-50.0000000
45110.0000000	13.7500000	-50.0000000
45110.0000000	14.0000000	-50.0000000
45110.0000000	14.2500000	-50.0000000
45110.0000000	14.5000000	-50.0000000
45110.0000000	14.7500000	-50.0000000
45110.0000000	15.0000000	-50.0000000
45140.0000000	0.0000000	-50.0000000
45140.0000000	0.2500000	-50.0000000
45140.0000000	0.5000000	-50.0000000
45140.0000000	0.7500000	-50.0000000
45140.0000000	1.0000000	-50.0000000
45140.0000000	1.2500000	-50.0000000
45140.0000000	1.5000000	-50.0000000
45140.0000000	1.7500000	-50.0000000
45140.0000000	2.0000000	-50.0000000
45140.0000000	2.2500000	-50.0000000
45140.0000000	2.5000000	-50.0000000
45140.0000000	2.7500000	-50.0000000
45140.0000000	3.0000000	-50.0000000
45140.0000000	3.2500000	-50.0000000
45140.0000000	3.5000000	-50.0000000
45140.0000000	3.7500000	-50.0000000

45140.0000000	4.0000000	-50.0000000
45140.0000000	4.2500000	-50.0000000
45140.0000000	4.5000000	-50.0000000
45140.0000000	4.7500000	-50.0000000
45140.0000000	5.0000000	-50.0000000
45140.0000000	5.2500000	-50.0000000
45140.0000000	5.5000000	-50.0000000
45140.0000000	5.7500000	-50.0000000
45140.0000000	6.0000000	-50.0000000
45140.0000000	6.2500000	-50.0000000
45140.0000000	6.5000000	-50.0000000
45140.0000000	6.7500000	-50.0000000
45140.0000000	7.0000000	-50.0000000
45140.0000000	7.2500000	-50.0000000
45140.0000000	7.5000000	-50.0000000
45140.0000000	7.7500000	-50.0000000
45140.0000000	8.0000000	-50.0000000
45140.0000000	8.2500000	-50.0000000
45140.0000000	8.5000000	-50.0000000
45140.0000000	8.7500000	-50.0000000
45140.0000000	9.0000000	-50.0000000
45140.0000000	9.2500000	-50.0000000
45140.0000000	9.5000000	-50.0000000
45140.0000000	9.7500000	-50.0000000
45140.0000000	10.0000000	-50.0000000
45140.0000000	10.2500000	-50.0000000
45140.0000000	10.5000000	-50.0000000
45140.0000000	10.7500000	-50.0000000
45140.0000000	11.0000000	-50.0000000
45140.0000000	11.2500000	-50.0000000
45140.0000000	11.5000000	-50.0000000
45140.0000000	11.7500000	-50.0000000
45140.0000000	12.0000000	-50.0000000
45140.0000000	12.2500000	-50.0000000
45140.0000000	12.5000000	-50.0000000
45140.0000000	12.7500000	-50.0000000
45140.0000000	13.0000000	-50.0000000
45140.0000000	13.2500000	-50.0000000
45140.0000000	13.5000000	-50.0000000
45140.0000000	13.7500000	-50.0000000
45140.0000000	14.0000000	-50.0000000
45140.0000000	14.2500000	-50.0000000
45140.0000000	14.5000000	-50.0000000
45140.0000000	14.7500000	-50.0000000
45140.0000000	15.0000000	-50.0000000
45170.0000000	0.0000000	-50.0000000
45170.0000000	0.2500000	-50.0000000
45170.0000000	0.5000000	-50.0000000
45170.0000000	0.7500000	-50.0000000
45170.0000000	1.0000000	-50.0000000
45170.0000000	1.2500000	-50.0000000
45170.0000000	1.5000000	-50.0000000
45170.0000000	1.7500000	-50.0000000
45170.0000000	2.0000000	-50.0000000
45170.0000000	2.2500000	-50.0000000
45170.0000000	2.5000000	-50.0000000
45170.0000000	2.7500000	-50.0000000
45170.0000000	3.0000000	-50.0000000

45170.0000000	3.2500000	-50.0000000
45170.0000000	3.5000000	-50.0000000
45170.0000000	3.7500000	-50.0000000
45170.0000000	4.0000000	-50.0000000
45170.0000000	4.2500000	-50.0000000
45170.0000000	4.5000000	-50.0000000
45170.0000000	4.7500000	-50.0000000
45170.0000000	5.0000000	-50.0000000
45170.0000000	5.2500000	-50.0000000
45170.0000000	5.5000000	-50.0000000
45170.0000000	5.7500000	-50.0000000
45170.0000000	6.0000000	-50.0000000
45170.0000000	6.2500000	-50.0000000
45170.0000000	6.5000000	-50.0000000
45170.0000000	6.7500000	-50.0000000
45170.0000000	7.0000000	-50.0000000
45170.0000000	7.2500000	-50.0000000
45170.0000000	7.5000000	-50.0000000
45170.0000000	7.7500000	-50.0000000
45170.0000000	8.0000000	-50.0000000
45170.0000000	8.2500000	-50.0000000
45170.0000000	8.5000000	-50.0000000
45170.0000000	8.7500000	-50.0000000
45170.0000000	9.0000000	-50.0000000
45170.0000000	9.2500000	-50.0000000
45170.0000000	9.5000000	-50.0000000
45170.0000000	9.7500000	-50.0000000
45170.0000000	10.0000000	-50.0000000
45170.0000000	10.2500000	-50.0000000
45170.0000000	10.5000000	-50.0000000
45170.0000000	10.7500000	-50.0000000
45170.0000000	11.0000000	-50.0000000
45170.0000000	11.2500000	-50.0000000
45170.0000000	11.5000000	-50.0000000
45170.0000000	11.7500000	-50.0000000
45170.0000000	12.0000000	-50.0000000
45170.0000000	12.2500000	-50.0000000
45170.0000000	12.5000000	-50.0000000
45170.0000000	12.7500000	-50.0000000
45170.0000000	13.0000000	-50.0000000
45170.0000000	13.2500000	-50.0000000
45170.0000000	13.5000000	-50.0000000
45170.0000000	13.7500000	-50.0000000
45170.0000000	14.0000000	-50.0000000
45170.0000000	14.2500000	-50.0000000
45170.0000000	14.5000000	-50.0000000
45170.0000000	14.7500000	-50.0000000
45170.0000000	15.0000000	-50.0000000
45200.0000000	0.0000000	-50.0000000
45200.0000000	0.2500000	-50.0000000
45200.0000000	0.5000000	-50.0000000
45200.0000000	0.7500000	-50.0000000
45200.0000000	1.0000000	-50.0000000
45200.0000000	1.2500000	-50.0000000
45200.0000000	1.5000000	-50.0000000
45200.0000000	1.7500000	-50.0000000
45200.0000000	2.0000000	-50.0000000
45200.0000000	2.2500000	-50.0000000

45200.0000000	2.5000000	-50.0000000
45200.0000000	2.7500000	-50.0000000
45200.0000000	3.0000000	-50.0000000
45200.0000000	3.2500000	-50.0000000
45200.0000000	3.5000000	-50.0000000
45200.0000000	3.7500000	-50.0000000
45200.0000000	4.0000000	-50.0000000
45200.0000000	4.2500000	-50.0000000
45200.0000000	4.5000000	-50.0000000
45200.0000000	4.7500000	-50.0000000
45200.0000000	5.0000000	-50.0000000
45200.0000000	5.2500000	-50.0000000
45200.0000000	5.5000000	-50.0000000
45200.0000000	5.7500000	-50.0000000
45200.0000000	6.0000000	-50.0000000
45200.0000000	6.2500000	-50.0000000
45200.0000000	6.5000000	-50.0000000
45200.0000000	6.7500000	-50.0000000
45200.0000000	7.0000000	-50.0000000
45200.0000000	7.2500000	-50.0000000
45200.0000000	7.5000000	-50.0000000
45200.0000000	7.7500000	-50.0000000
45200.0000000	8.0000000	-50.0000000
45200.0000000	8.2500000	-50.0000000
45200.0000000	8.5000000	-50.0000000
45200.0000000	8.7500000	-50.0000000
45200.0000000	9.0000000	-50.0000000
45200.0000000	9.2500000	-50.0000000
45200.0000000	9.5000000	-50.0000000
45200.0000000	9.7500000	-50.0000000
45200.0000000	10.0000000	-50.0000000
45200.0000000	10.2500000	-50.0000000
45200.0000000	10.5000000	-50.0000000
45200.0000000	10.7500000	-50.0000000
45200.0000000	11.0000000	-50.0000000
45200.0000000	11.2500000	-50.0000000
45200.0000000	11.5000000	-50.0000000
45200.0000000	11.7500000	-50.0000000
45200.0000000	12.0000000	-50.0000000
45200.0000000	12.2500000	-50.0000000
45200.0000000	12.5000000	-50.0000000
45200.0000000	12.7500000	-50.0000000
45200.0000000	13.0000000	-50.0000000
45200.0000000	13.2500000	-50.0000000
45200.0000000	13.5000000	-50.0000000
45200.0000000	13.7500000	-50.0000000
45200.0000000	14.0000000	-50.0000000
45200.0000000	14.2500000	-50.0000000
45200.0000000	14.5000000	-50.0000000
45200.0000000	14.7500000	-50.0000000
45200.0000000	15.0000000	-50.0000000
45230.0000000	0.0000000	-50.0000000
45230.0000000	0.2500000	-50.0000000
45230.0000000	0.5000000	-50.0000000
45230.0000000	0.7500000	-50.0000000
45230.0000000	1.0000000	-50.0000000
45230.0000000	1.2500000	-50.0000000
45230.0000000	1.5000000	-50.0000000

45230.0000000	1.7500000	-50.0000000
45230.0000000	2.0000000	-50.0000000
45230.0000000	2.2500000	-50.0000000
45230.0000000	2.5000000	-50.0000000
45230.0000000	2.7500000	-50.0000000
45230.0000000	3.0000000	-50.0000000
45230.0000000	3.2500000	-50.0000000
45230.0000000	3.5000000	-50.0000000
45230.0000000	3.7500000	-50.0000000
45230.0000000	4.0000000	-50.0000000
45230.0000000	4.2500000	-50.0000000
45230.0000000	4.5000000	-50.0000000
45230.0000000	4.7500000	-50.0000000
45230.0000000	5.0000000	-50.0000000
45230.0000000	5.2500000	-50.0000000
45230.0000000	5.5000000	-50.0000000
45230.0000000	5.7500000	-50.0000000
45230.0000000	6.0000000	-50.0000000
45230.0000000	6.2500000	-50.0000000
45230.0000000	6.5000000	-50.0000000
45230.0000000	6.7500000	-50.0000000
45230.0000000	7.0000000	-50.0000000
45230.0000000	7.2500000	-50.0000000
45230.0000000	7.5000000	-50.0000000
45230.0000000	7.7500000	-50.0000000
45230.0000000	8.0000000	-50.0000000
45230.0000000	8.2500000	-50.0000000
45230.0000000	8.5000000	-50.0000000
45230.0000000	8.7500000	-50.0000000
45230.0000000	9.0000000	-50.0000000
45230.0000000	9.2500000	-50.0000000
45230.0000000	9.5000000	-50.0000000
45230.0000000	9.7500000	-50.0000000
45230.0000000	10.0000000	-50.0000000
45230.0000000	10.2500000	-50.0000000
45230.0000000	10.5000000	-50.0000000
45230.0000000	10.7500000	-50.0000000
45230.0000000	11.0000000	-50.0000000
45230.0000000	11.2500000	-50.0000000
45230.0000000	11.5000000	-50.0000000
45230.0000000	11.7500000	-50.0000000
45230.0000000	12.0000000	-50.0000000
45230.0000000	12.2500000	-50.0000000
45230.0000000	12.5000000	-50.0000000
45230.0000000	12.7500000	-50.0000000
45230.0000000	13.0000000	-50.0000000
45230.0000000	13.2500000	-50.0000000
45230.0000000	13.5000000	-50.0000000
45230.0000000	13.7500000	-50.0000000
45230.0000000	14.0000000	-50.0000000
45230.0000000	14.2500000	-50.0000000
45230.0000000	14.5000000	-50.0000000
45230.0000000	14.7500000	-50.0000000
45230.0000000	15.0000000	-50.0000000
45260.0000000	0.0000000	-50.0000000
45260.0000000	0.2500000	-50.0000000
45260.0000000	0.5000000	-50.0000000
45260.0000000	0.7500000	-50.0000000

45260.0000000	1.0000000	-50.0000000
45260.0000000	1.2500000	-50.0000000
45260.0000000	1.5000000	-50.0000000
45260.0000000	1.7500000	-50.0000000
45260.0000000	2.0000000	-50.0000000
45260.0000000	2.2500000	-50.0000000
45260.0000000	2.5000000	-50.0000000
45260.0000000	2.7500000	-50.0000000
45260.0000000	3.0000000	-50.0000000
45260.0000000	3.2500000	-50.0000000
45260.0000000	3.5000000	-50.0000000
45260.0000000	3.7500000	-50.0000000
45260.0000000	4.0000000	-50.0000000
45260.0000000	4.2500000	-50.0000000
45260.0000000	4.5000000	-50.0000000
45260.0000000	4.7500000	-50.0000000
45260.0000000	5.0000000	-50.0000000
45260.0000000	5.2500000	-50.0000000
45260.0000000	5.5000000	-50.0000000
45260.0000000	5.7500000	-50.0000000
45260.0000000	6.0000000	-50.0000000
45260.0000000	6.2500000	-50.0000000
45260.0000000	6.5000000	-50.0000000
45260.0000000	6.7500000	-50.0000000
45260.0000000	7.0000000	-50.0000000
45260.0000000	7.2500000	-50.0000000
45260.0000000	7.5000000	-50.0000000
45260.0000000	7.7500000	-50.0000000
45260.0000000	8.0000000	-50.0000000
45260.0000000	8.2500000	-50.0000000
45260.0000000	8.5000000	-50.0000000
45260.0000000	8.7500000	-50.0000000
45260.0000000	9.0000000	-50.0000000
45260.0000000	9.2500000	-50.0000000
45260.0000000	9.5000000	-50.0000000
45260.0000000	9.7500000	-50.0000000
45260.0000000	10.0000000	-50.0000000
45260.0000000	10.2500000	-50.0000000
45260.0000000	10.5000000	-50.0000000
45260.0000000	10.7500000	-50.0000000
45260.0000000	11.0000000	-50.0000000
45260.0000000	11.2500000	-50.0000000
45260.0000000	11.5000000	-50.0000000
45260.0000000	11.7500000	-50.0000000
45260.0000000	12.0000000	-50.0000000
45260.0000000	12.2500000	-50.0000000
45260.0000000	12.5000000	-50.0000000
45260.0000000	12.7500000	-50.0000000
45260.0000000	13.0000000	-50.0000000
45260.0000000	13.2500000	-50.0000000
45260.0000000	13.5000000	-50.0000000
45260.0000000	13.7500000	-50.0000000
45260.0000000	14.0000000	-50.0000000
45260.0000000	14.2500000	-50.0000000
45260.0000000	14.5000000	-50.0000000
45260.0000000	14.7500000	-50.0000000
45260.0000000	15.0000000	-50.0000000
45290.0000000	0.0000000	-50.0000000

45290.0000000	0.2500000	-50.0000000
45290.0000000	0.5000000	-50.0000000
45290.0000000	0.7500000	15.5799999
45290.0000000	1.0000000	-50.0000000
45290.0000000	1.2500000	-50.0000000
45290.0000000	1.5000000	-50.0000000
45290.0000000	1.7500000	-50.0000000
45290.0000000	2.0000000	-50.0000000
45290.0000000	2.2500000	-50.0000000
45290.0000000	2.5000000	-50.0000000
45290.0000000	2.7500000	-50.0000000
45290.0000000	3.0000000	-50.0000000
45290.0000000	3.2500000	-50.0000000
45290.0000000	3.5000000	-50.0000000
45290.0000000	3.7500000	-50.0000000
45290.0000000	4.0000000	-50.0000000
45290.0000000	4.2500000	-50.0000000
45290.0000000	4.5000000	-50.0000000
45290.0000000	4.7500000	-50.0000000
45290.0000000	5.0000000	-50.0000000
45290.0000000	5.2500000	-50.0000000
45290.0000000	5.5000000	-50.0000000
45290.0000000	5.7500000	-50.0000000
45290.0000000	6.0000000	-50.0000000
45290.0000000	6.2500000	-50.0000000
45290.0000000	6.5000000	-50.0000000
45290.0000000	6.7500000	-50.0000000
45290.0000000	7.0000000	-50.0000000
45290.0000000	7.2500000	-50.0000000
45290.0000000	7.5000000	-50.0000000
45290.0000000	7.7500000	-50.0000000
45290.0000000	8.0000000	-50.0000000
45290.0000000	8.2500000	-50.0000000
45290.0000000	8.5000000	-50.0000000
45290.0000000	8.7500000	-50.0000000
45290.0000000	9.0000000	-50.0000000
45290.0000000	9.2500000	-50.0000000
45290.0000000	9.5000000	-50.0000000
45290.0000000	9.7500000	-50.0000000
45290.0000000	10.0000000	-50.0000000
45290.0000000	10.2500000	-50.0000000
45290.0000000	10.5000000	-50.0000000
45290.0000000	10.7500000	-50.0000000
45290.0000000	11.0000000	-50.0000000
45290.0000000	11.2500000	-50.0000000
45290.0000000	11.5000000	-50.0000000
45290.0000000	11.7500000	-50.0000000
45290.0000000	12.0000000	-50.0000000
45290.0000000	12.2500000	-50.0000000
45290.0000000	12.5000000	-50.0000000
45290.0000000	12.7500000	-50.0000000
45290.0000000	13.0000000	-50.0000000
45290.0000000	13.2500000	-50.0000000
45290.0000000	13.5000000	-50.0000000
45290.0000000	13.7500000	-50.0000000
45290.0000000	14.0000000	-50.0000000
45290.0000000	14.2500000	-50.0000000
45290.0000000	14.5000000	-50.0000000

45290.0000000	14.7500000	-50.0000000
45290.0000000	15.0000000	-50.0000000
45320.0000000	0.0000000	-50.0000000
45320.0000000	0.2500000	-50.0000000
45320.0000000	0.5000000	-50.0000000
45320.0000000	0.7500000	-50.0000000
45320.0000000	1.0000000	-50.0000000
45320.0000000	1.2500000	-50.0000000
45320.0000000	1.5000000	-50.0000000
45320.0000000	1.7500000	-50.0000000
45320.0000000	2.0000000	-50.0000000
45320.0000000	2.2500000	-50.0000000
45320.0000000	2.5000000	-50.0000000
45320.0000000	2.7500000	-50.0000000
45320.0000000	3.0000000	-50.0000000
45320.0000000	3.2500000	-50.0000000
45320.0000000	3.5000000	-50.0000000
45320.0000000	3.7500000	-50.0000000
45320.0000000	4.0000000	-50.0000000
45320.0000000	4.2500000	-50.0000000
45320.0000000	4.5000000	-50.0000000
45320.0000000	4.7500000	-50.0000000
45320.0000000	5.0000000	-50.0000000
45320.0000000	5.2500000	-50.0000000
45320.0000000	5.5000000	-50.0000000
45320.0000000	5.7500000	-50.0000000
45320.0000000	6.0000000	-50.0000000
45320.0000000	6.2500000	-50.0000000
45320.0000000	6.5000000	-50.0000000
45320.0000000	6.7500000	-50.0000000
45320.0000000	7.0000000	-50.0000000
45320.0000000	7.2500000	-50.0000000
45320.0000000	7.5000000	-50.0000000
45320.0000000	7.7500000	-50.0000000
45320.0000000	8.0000000	-50.0000000
45320.0000000	8.2500000	-50.0000000
45320.0000000	8.5000000	-50.0000000
45320.0000000	8.7500000	-50.0000000
45320.0000000	9.0000000	-50.0000000
45320.0000000	9.2500000	-50.0000000
45320.0000000	9.5000000	-50.0000000
45320.0000000	9.7500000	-50.0000000
45320.0000000	10.0000000	-50.0000000
45320.0000000	10.2500000	-50.0000000
45320.0000000	10.5000000	-50.0000000
45320.0000000	10.7500000	-50.0000000
45320.0000000	11.0000000	-50.0000000
45320.0000000	11.2500000	-50.0000000
45320.0000000	11.5000000	-50.0000000
45320.0000000	11.7500000	-50.0000000
45320.0000000	12.0000000	-50.0000000
45320.0000000	12.2500000	-50.0000000
45320.0000000	12.5000000	-50.0000000
45320.0000000	12.7500000	-50.0000000
45320.0000000	13.0000000	-50.0000000
45320.0000000	13.2500000	-50.0000000
45320.0000000	13.5000000	-50.0000000
45320.0000000	13.7500000	-50.0000000

45320.0000000	14.0000000	-50.0000000
45320.0000000	14.2500000	-50.0000000
45320.0000000	14.5000000	-50.0000000
45320.0000000	14.7500000	-50.0000000
45320.0000000	15.0000000	-50.0000000
45350.0000000	0.0000000	-50.0000000
45350.0000000	0.2500000	-50.0000000
45350.0000000	0.5000000	-50.0000000
45350.0000000	0.7500000	16.5300007
45350.0000000	1.0000000	-50.0000000
45350.0000000	1.2500000	-50.0000000
45350.0000000	1.5000000	-50.0000000
45350.0000000	1.7500000	-50.0000000
45350.0000000	2.0000000	-50.0000000
45350.0000000	2.2500000	-50.0000000
45350.0000000	2.5000000	-50.0000000
45350.0000000	2.7500000	-50.0000000
45350.0000000	3.0000000	-50.0000000
45350.0000000	3.2500000	-50.0000000
45350.0000000	3.5000000	-50.0000000
45350.0000000	3.7500000	-50.0000000
45350.0000000	4.0000000	-50.0000000
45350.0000000	4.2500000	-50.0000000
45350.0000000	4.5000000	-50.0000000
45350.0000000	4.7500000	-50.0000000
45350.0000000	5.0000000	-50.0000000
45350.0000000	5.2500000	-50.0000000
45350.0000000	5.5000000	-50.0000000
45350.0000000	5.7500000	-50.0000000
45350.0000000	6.0000000	-50.0000000
45350.0000000	6.2500000	-50.0000000
45350.0000000	6.5000000	-50.0000000
45350.0000000	6.7500000	-50.0000000
45350.0000000	7.0000000	-50.0000000
45350.0000000	7.2500000	-50.0000000
45350.0000000	7.5000000	-50.0000000
45350.0000000	7.7500000	-50.0000000
45350.0000000	8.0000000	-50.0000000
45350.0000000	8.2500000	-50.0000000
45350.0000000	8.5000000	-50.0000000
45350.0000000	8.7500000	-50.0000000
45350.0000000	9.0000000	-50.0000000
45350.0000000	9.2500000	-50.0000000
45350.0000000	9.5000000	-50.0000000
45350.0000000	9.7500000	-50.0000000
45350.0000000	10.0000000	-50.0000000
45350.0000000	10.2500000	-50.0000000
45350.0000000	10.5000000	-50.0000000
45350.0000000	10.7500000	-50.0000000
45350.0000000	11.0000000	-50.0000000
45350.0000000	11.2500000	-50.0000000
45350.0000000	11.5000000	-50.0000000
45350.0000000	11.7500000	-50.0000000
45350.0000000	12.0000000	-50.0000000
45350.0000000	12.2500000	-50.0000000
45350.0000000	12.5000000	-50.0000000
45350.0000000	12.7500000	-50.0000000
45350.0000000	13.0000000	-50.0000000

45350.0000000	13.2500000	-50.0000000
45350.0000000	13.5000000	-50.0000000
45350.0000000	13.7500000	-50.0000000
45350.0000000	14.0000000	-50.0000000
45350.0000000	14.2500000	-50.0000000
45350.0000000	14.5000000	-50.0000000
45350.0000000	14.7500000	-50.0000000
45350.0000000	15.0000000	-50.0000000
45380.0000000	0.0000000	-50.0000000
45380.0000000	0.2500000	-50.0000000
45380.0000000	0.5000000	-50.0000000
45380.0000000	0.7500000	-50.0000000
45380.0000000	1.0000000	-50.0000000
45380.0000000	1.2500000	-50.0000000
45380.0000000	1.5000000	-50.0000000
45380.0000000	1.7500000	-50.0000000
45380.0000000	2.0000000	-50.0000000
45380.0000000	2.2500000	-50.0000000
45380.0000000	2.5000000	-50.0000000
45380.0000000	2.7500000	-50.0000000
45380.0000000	3.0000000	-50.0000000
45380.0000000	3.2500000	-50.0000000
45380.0000000	3.5000000	-50.0000000
45380.0000000	3.7500000	-50.0000000
45380.0000000	4.0000000	-50.0000000
45380.0000000	4.2500000	-50.0000000
45380.0000000	4.5000000	-50.0000000
45380.0000000	4.7500000	-50.0000000
45380.0000000	5.0000000	-50.0000000
45380.0000000	5.2500000	-50.0000000
45380.0000000	5.5000000	-50.0000000
45380.0000000	5.7500000	-50.0000000
45380.0000000	6.0000000	-50.0000000
45380.0000000	6.2500000	-50.0000000
45380.0000000	6.5000000	-50.0000000
45380.0000000	6.7500000	-50.0000000
45380.0000000	7.0000000	-50.0000000
45380.0000000	7.2500000	-50.0000000
45380.0000000	7.5000000	-50.0000000
45380.0000000	7.7500000	-50.0000000
45380.0000000	8.0000000	-50.0000000
45380.0000000	8.2500000	-50.0000000
45380.0000000	8.5000000	-50.0000000
45380.0000000	8.7500000	-50.0000000
45380.0000000	9.0000000	-50.0000000
45380.0000000	9.2500000	-50.0000000
45380.0000000	9.5000000	-50.0000000
45380.0000000	9.7500000	-50.0000000
45380.0000000	10.0000000	-50.0000000
45380.0000000	10.2500000	-50.0000000
45380.0000000	10.5000000	-50.0000000
45380.0000000	10.7500000	-50.0000000
45380.0000000	11.0000000	-50.0000000
45380.0000000	11.2500000	-50.0000000
45380.0000000	11.5000000	-50.0000000
45380.0000000	11.7500000	-50.0000000
45380.0000000	12.0000000	-50.0000000
45380.0000000	12.2500000	-50.0000000

45380.0000000	12.5000000	-50.0000000
45380.0000000	12.7500000	-50.0000000
45380.0000000	13.0000000	-50.0000000
45380.0000000	13.2500000	-50.0000000
45380.0000000	13.5000000	-50.0000000
45380.0000000	13.7500000	-50.0000000
45380.0000000	14.0000000	-50.0000000
45380.0000000	14.2500000	-50.0000000
45380.0000000	14.5000000	-50.0000000
45380.0000000	14.7500000	-50.0000000
45380.0000000	15.0000000	-50.0000000
45410.0000000	0.0000000	-50.0000000
45410.0000000	0.2500000	-50.0000000
45410.0000000	0.5000000	-50.0000000
45410.0000000	0.7500000	-50.0000000
45410.0000000	1.0000000	-50.0000000
45410.0000000	1.2500000	-50.0000000
45410.0000000	1.5000000	-50.0000000
45410.0000000	1.7500000	-50.0000000
45410.0000000	2.0000000	-50.0000000
45410.0000000	2.2500000	-50.0000000
45410.0000000	2.5000000	-50.0000000
45410.0000000	2.7500000	-50.0000000
45410.0000000	3.0000000	-50.0000000
45410.0000000	3.2500000	-50.0000000
45410.0000000	3.5000000	-50.0000000
45410.0000000	3.7500000	-50.0000000
45410.0000000	4.0000000	-50.0000000
45410.0000000	4.2500000	-50.0000000
45410.0000000	4.5000000	-50.0000000
45410.0000000	4.7500000	-50.0000000
45410.0000000	5.0000000	-50.0000000
45410.0000000	5.2500000	-50.0000000
45410.0000000	5.5000000	-50.0000000
45410.0000000	5.7500000	-50.0000000
45410.0000000	6.0000000	-50.0000000
45410.0000000	6.2500000	-50.0000000
45410.0000000	6.5000000	-50.0000000
45410.0000000	6.7500000	-50.0000000
45410.0000000	7.0000000	-50.0000000
45410.0000000	7.2500000	-50.0000000
45410.0000000	7.5000000	-50.0000000
45410.0000000	7.7500000	-50.0000000
45410.0000000	8.0000000	-50.0000000
45410.0000000	8.2500000	-50.0000000
45410.0000000	8.5000000	-50.0000000
45410.0000000	8.7500000	-50.0000000
45410.0000000	9.0000000	-50.0000000
45410.0000000	9.2500000	-50.0000000
45410.0000000	9.5000000	-50.0000000
45410.0000000	9.7500000	-50.0000000
45410.0000000	10.0000000	-50.0000000
45410.0000000	10.2500000	-50.0000000
45410.0000000	10.5000000	-50.0000000
45410.0000000	10.7500000	-50.0000000
45410.0000000	11.0000000	-50.0000000
45410.0000000	11.2500000	-50.0000000
45410.0000000	11.5000000	-50.0000000

45410.0000000	11.7500000	-50.0000000
45410.0000000	12.0000000	-50.0000000
45410.0000000	12.2500000	-50.0000000
45410.0000000	12.5000000	-50.0000000
45410.0000000	12.7500000	-50.0000000
45410.0000000	13.0000000	-50.0000000
45410.0000000	13.2500000	-50.0000000
45410.0000000	13.5000000	-50.0000000
45410.0000000	13.7500000	-50.0000000
45410.0000000	14.0000000	-50.0000000
45410.0000000	14.2500000	-50.0000000
45410.0000000	14.5000000	-50.0000000
45410.0000000	14.7500000	-50.0000000
45410.0000000	15.0000000	-50.0000000
45440.0000000	0.0000000	-50.0000000
45440.0000000	0.2500000	-50.0000000
45440.0000000	0.5000000	-50.0000000
45440.0000000	0.7500000	-50.0000000
45440.0000000	1.0000000	-50.0000000
45440.0000000	1.2500000	-50.0000000
45440.0000000	1.5000000	-50.0000000
45440.0000000	1.7500000	-50.0000000
45440.0000000	2.0000000	-50.0000000
45440.0000000	2.2500000	-50.0000000
45440.0000000	2.5000000	-50.0000000
45440.0000000	2.7500000	-50.0000000
45440.0000000	3.0000000	-50.0000000
45440.0000000	3.2500000	-50.0000000
45440.0000000	3.5000000	-50.0000000
45440.0000000	3.7500000	-50.0000000
45440.0000000	4.0000000	-50.0000000
45440.0000000	4.2500000	-50.0000000
45440.0000000	4.5000000	-50.0000000
45440.0000000	4.7500000	-50.0000000
45440.0000000	5.0000000	-50.0000000
45440.0000000	5.2500000	-50.0000000
45440.0000000	5.5000000	-50.0000000
45440.0000000	5.7500000	-50.0000000
45440.0000000	6.0000000	-50.0000000
45440.0000000	6.2500000	-50.0000000
45440.0000000	6.5000000	-50.0000000
45440.0000000	6.7500000	-50.0000000
45440.0000000	7.0000000	-50.0000000
45440.0000000	7.2500000	-50.0000000
45440.0000000	7.5000000	-50.0000000
45440.0000000	7.7500000	-50.0000000
45440.0000000	8.0000000	-50.0000000
45440.0000000	8.2500000	-50.0000000
45440.0000000	8.5000000	-50.0000000
45440.0000000	8.7500000	-50.0000000
45440.0000000	9.0000000	-50.0000000
45440.0000000	9.2500000	-50.0000000
45440.0000000	9.5000000	-50.0000000
45440.0000000	9.7500000	-50.0000000
45440.0000000	10.0000000	-50.0000000
45440.0000000	10.2500000	-50.0000000
45440.0000000	10.5000000	-50.0000000
45440.0000000	10.7500000	-50.0000000

45440.0000000	11.0000000	-50.0000000
45440.0000000	11.2500000	-50.0000000
45440.0000000	11.5000000	-50.0000000
45440.0000000	11.7500000	-50.0000000
45440.0000000	12.0000000	-50.0000000
45440.0000000	12.2500000	-50.0000000
45440.0000000	12.5000000	-50.0000000
45440.0000000	12.7500000	-50.0000000
45440.0000000	13.0000000	-50.0000000
45440.0000000	13.2500000	-50.0000000
45440.0000000	13.5000000	-50.0000000
45440.0000000	13.7500000	-50.0000000
45440.0000000	14.0000000	-50.0000000
45440.0000000	14.2500000	-50.0000000
45440.0000000	14.5000000	-50.0000000
45440.0000000	14.7500000	-50.0000000
45440.0000000	15.0000000	-50.0000000
45470.0000000	0.0000000	-50.0000000
45470.0000000	0.2500000	-50.0000000
45470.0000000	0.5000000	-50.0000000
45470.0000000	0.7500000	-50.0000000
45470.0000000	1.0000000	-50.0000000
45470.0000000	1.2500000	-50.0000000
45470.0000000	1.5000000	-50.0000000
45470.0000000	1.7500000	-50.0000000
45470.0000000	2.0000000	-50.0000000
45470.0000000	2.2500000	-50.0000000
45470.0000000	2.5000000	-50.0000000
45470.0000000	2.7500000	-50.0000000
45470.0000000	3.0000000	-50.0000000
45470.0000000	3.2500000	-50.0000000
45470.0000000	3.5000000	-50.0000000
45470.0000000	3.7500000	-50.0000000
45470.0000000	4.0000000	-50.0000000
45470.0000000	4.2500000	-50.0000000
45470.0000000	4.5000000	-50.0000000
45470.0000000	4.7500000	-50.0000000
45470.0000000	5.0000000	-50.0000000
45470.0000000	5.2500000	-50.0000000
45470.0000000	5.5000000	-50.0000000
45470.0000000	5.7500000	-50.0000000
45470.0000000	6.0000000	-50.0000000
45470.0000000	6.2500000	-50.0000000
45470.0000000	6.5000000	-50.0000000
45470.0000000	6.7500000	-50.0000000
45470.0000000	7.0000000	-50.0000000
45470.0000000	7.2500000	-50.0000000
45470.0000000	7.5000000	-50.0000000
45470.0000000	7.7500000	-50.0000000
45470.0000000	8.0000000	-50.0000000
45470.0000000	8.2500000	-50.0000000
45470.0000000	8.5000000	-50.0000000
45470.0000000	8.7500000	-50.0000000
45470.0000000	9.0000000	-50.0000000
45470.0000000	9.2500000	-50.0000000
45470.0000000	9.5000000	-50.0000000
45470.0000000	9.7500000	-50.0000000
45470.0000000	10.0000000	-50.0000000

45470.0000000	10.2500000	-50.0000000
45470.0000000	10.5000000	-50.0000000
45470.0000000	10.7500000	-50.0000000
45470.0000000	11.0000000	-50.0000000
45470.0000000	11.2500000	-50.0000000
45470.0000000	11.5000000	-50.0000000
45470.0000000	11.7500000	-50.0000000
45470.0000000	12.0000000	-50.0000000
45470.0000000	12.2500000	-50.0000000
45470.0000000	12.5000000	-50.0000000
45470.0000000	12.7500000	-50.0000000
45470.0000000	13.0000000	-50.0000000
45470.0000000	13.2500000	-50.0000000
45470.0000000	13.5000000	-50.0000000
45470.0000000	13.7500000	-50.0000000
45470.0000000	14.0000000	-50.0000000
45470.0000000	14.2500000	-50.0000000
45470.0000000	14.5000000	-50.0000000
45470.0000000	14.7500000	-50.0000000
45470.0000000	15.0000000	-50.0000000
45500.0000000	0.0000000	-50.0000000
45500.0000000	0.2500000	-50.0000000
45500.0000000	0.5000000	-50.0000000
45500.0000000	0.7500000	-50.0000000
45500.0000000	1.0000000	-50.0000000
45500.0000000	1.2500000	-50.0000000
45500.0000000	1.5000000	-50.0000000
45500.0000000	1.7500000	-50.0000000
45500.0000000	2.0000000	-50.0000000
45500.0000000	2.2500000	-50.0000000
45500.0000000	2.5000000	-50.0000000
45500.0000000	2.7500000	-50.0000000
45500.0000000	3.0000000	-50.0000000
45500.0000000	3.2500000	-50.0000000
45500.0000000	3.5000000	-50.0000000
45500.0000000	3.7500000	-50.0000000
45500.0000000	4.0000000	-50.0000000
45500.0000000	4.2500000	-50.0000000
45500.0000000	4.5000000	-50.0000000
45500.0000000	4.7500000	-50.0000000
45500.0000000	5.0000000	-50.0000000
45500.0000000	5.2500000	-50.0000000
45500.0000000	5.5000000	-50.0000000
45500.0000000	5.7500000	-50.0000000
45500.0000000	6.0000000	-50.0000000
45500.0000000	6.2500000	-50.0000000
45500.0000000	6.5000000	-50.0000000
45500.0000000	6.7500000	-50.0000000
45500.0000000	7.0000000	-50.0000000
45500.0000000	7.2500000	-50.0000000
45500.0000000	7.5000000	-50.0000000
45500.0000000	7.7500000	-50.0000000
45500.0000000	8.0000000	-50.0000000
45500.0000000	8.2500000	-50.0000000
45500.0000000	8.5000000	-50.0000000
45500.0000000	8.7500000	-50.0000000
45500.0000000	9.0000000	-50.0000000
45500.0000000	9.2500000	-50.0000000

45500.0000000	9.5000000	-50.0000000
45500.0000000	9.7500000	-50.0000000
45500.0000000	10.0000000	-50.0000000
45500.0000000	10.2500000	-50.0000000
45500.0000000	10.5000000	-50.0000000
45500.0000000	10.7500000	-50.0000000
45500.0000000	11.0000000	-50.0000000
45500.0000000	11.2500000	-50.0000000
45500.0000000	11.5000000	-50.0000000
45500.0000000	11.7500000	-50.0000000
45500.0000000	12.0000000	-50.0000000
45500.0000000	12.2500000	-50.0000000
45500.0000000	12.5000000	-50.0000000
45500.0000000	12.7500000	-50.0000000
45500.0000000	13.0000000	-50.0000000
45500.0000000	13.2500000	-50.0000000
45500.0000000	13.5000000	-50.0000000
45500.0000000	13.7500000	-50.0000000
45500.0000000	14.0000000	-50.0000000
45500.0000000	14.2500000	-50.0000000
45500.0000000	14.5000000	-50.0000000
45500.0000000	14.7500000	-50.0000000
45500.0000000	15.0000000	-50.0000000
45530.0000000	0.0000000	-50.0000000
45530.0000000	0.2500000	-50.0000000
45530.0000000	0.5000000	-50.0000000
45530.0000000	0.7500000	-50.0000000
45530.0000000	1.0000000	-50.0000000
45530.0000000	1.2500000	-50.0000000
45530.0000000	1.5000000	-50.0000000
45530.0000000	1.7500000	-50.0000000
45530.0000000	2.0000000	-50.0000000
45530.0000000	2.2500000	-50.0000000
45530.0000000	2.5000000	-50.0000000
45530.0000000	2.7500000	-50.0000000
45530.0000000	3.0000000	-50.0000000
45530.0000000	3.2500000	-50.0000000
45530.0000000	3.5000000	-50.0000000
45530.0000000	3.7500000	-50.0000000
45530.0000000	4.0000000	-50.0000000
45530.0000000	4.2500000	-50.0000000
45530.0000000	4.5000000	-50.0000000
45530.0000000	4.7500000	-50.0000000
45530.0000000	5.0000000	-50.0000000
45530.0000000	5.2500000	-50.0000000
45530.0000000	5.5000000	-50.0000000
45530.0000000	5.7500000	-50.0000000
45530.0000000	6.0000000	-50.0000000
45530.0000000	6.2500000	-50.0000000
45530.0000000	6.5000000	-50.0000000
45530.0000000	6.7500000	-50.0000000
45530.0000000	7.0000000	-50.0000000
45530.0000000	7.2500000	-50.0000000
45530.0000000	7.5000000	-50.0000000
45530.0000000	7.7500000	-50.0000000
45530.0000000	8.0000000	-50.0000000
45530.0000000	8.2500000	-50.0000000
45530.0000000	8.5000000	-50.0000000

45530.0000000	8.7500000	-50.0000000
45530.0000000	9.0000000	-50.0000000
45530.0000000	9.2500000	-50.0000000
45530.0000000	9.5000000	-50.0000000
45530.0000000	9.7500000	-50.0000000
45530.0000000	10.0000000	-50.0000000
45530.0000000	10.2500000	-50.0000000
45530.0000000	10.5000000	-50.0000000
45530.0000000	10.7500000	-50.0000000
45530.0000000	11.0000000	-50.0000000
45530.0000000	11.2500000	-50.0000000
45530.0000000	11.5000000	-50.0000000
45530.0000000	11.7500000	-50.0000000
45530.0000000	12.0000000	-50.0000000
45530.0000000	12.2500000	-50.0000000
45530.0000000	12.5000000	-50.0000000
45530.0000000	12.7500000	-50.0000000
45530.0000000	13.0000000	-50.0000000
45530.0000000	13.2500000	-50.0000000
45530.0000000	13.5000000	-50.0000000
45530.0000000	13.7500000	-50.0000000
45530.0000000	14.0000000	-50.0000000
45530.0000000	14.2500000	-50.0000000
45530.0000000	14.5000000	-50.0000000
45530.0000000	14.7500000	-50.0000000
45530.0000000	15.0000000	-50.0000000
45560.0000000	0.0000000	-50.0000000
45560.0000000	0.2500000	-50.0000000
45560.0000000	0.5000000	-50.0000000
45560.0000000	0.7500000	-50.0000000
45560.0000000	1.0000000	-50.0000000
45560.0000000	1.2500000	-50.0000000
45560.0000000	1.5000000	-50.0000000
45560.0000000	1.7500000	-50.0000000
45560.0000000	2.0000000	-50.0000000
45560.0000000	2.2500000	-50.0000000
45560.0000000	2.5000000	-50.0000000
45560.0000000	2.7500000	-50.0000000
45560.0000000	3.0000000	-50.0000000
45560.0000000	3.2500000	-50.0000000
45560.0000000	3.5000000	-50.0000000
45560.0000000	3.7500000	-50.0000000
45560.0000000	4.0000000	-50.0000000
45560.0000000	4.2500000	-50.0000000
45560.0000000	4.5000000	-50.0000000
45560.0000000	4.7500000	-50.0000000
45560.0000000	5.0000000	-50.0000000
45560.0000000	5.2500000	-50.0000000
45560.0000000	5.5000000	-50.0000000
45560.0000000	5.7500000	-50.0000000
45560.0000000	6.0000000	-50.0000000
45560.0000000	6.2500000	-50.0000000
45560.0000000	6.5000000	-50.0000000
45560.0000000	6.7500000	-50.0000000
45560.0000000	7.0000000	-50.0000000
45560.0000000	7.2500000	-50.0000000
45560.0000000	7.5000000	-50.0000000
45560.0000000	7.7500000	-50.0000000

45560.0000000	8.0000000	-50.0000000
45560.0000000	8.2500000	-50.0000000
45560.0000000	8.5000000	-50.0000000
45560.0000000	8.7500000	-50.0000000
45560.0000000	9.0000000	-50.0000000
45560.0000000	9.2500000	-50.0000000
45560.0000000	9.5000000	-50.0000000
45560.0000000	9.7500000	-50.0000000
45560.0000000	10.0000000	-50.0000000
45560.0000000	10.2500000	-50.0000000
45560.0000000	10.5000000	-50.0000000
45560.0000000	10.7500000	-50.0000000
45560.0000000	11.0000000	-50.0000000
45560.0000000	11.2500000	-50.0000000
45560.0000000	11.5000000	-50.0000000
45560.0000000	11.7500000	-50.0000000
45560.0000000	12.0000000	-50.0000000
45560.0000000	12.2500000	-50.0000000
45560.0000000	12.5000000	-50.0000000
45560.0000000	12.7500000	-50.0000000
45560.0000000	13.0000000	-50.0000000
45560.0000000	13.2500000	-50.0000000
45560.0000000	13.5000000	-50.0000000
45560.0000000	13.7500000	-50.0000000
45560.0000000	14.0000000	-50.0000000
45560.0000000	14.2500000	-50.0000000
45560.0000000	14.5000000	-50.0000000
45560.0000000	14.7500000	-50.0000000
45560.0000000	15.0000000	-50.0000000
45590.0000000	0.0000000	-50.0000000
45590.0000000	0.2500000	-50.0000000
45590.0000000	0.5000000	-50.0000000
45590.0000000	0.7500000	-50.0000000
45590.0000000	1.0000000	-50.0000000
45590.0000000	1.2500000	-50.0000000
45590.0000000	1.5000000	-50.0000000
45590.0000000	1.7500000	-50.0000000
45590.0000000	2.0000000	-50.0000000
45590.0000000	2.2500000	-50.0000000
45590.0000000	2.5000000	-50.0000000
45590.0000000	2.7500000	-50.0000000
45590.0000000	3.0000000	-50.0000000
45590.0000000	3.2500000	-50.0000000
45590.0000000	3.5000000	-50.0000000
45590.0000000	3.7500000	-50.0000000
45590.0000000	4.0000000	-50.0000000
45590.0000000	4.2500000	-50.0000000
45590.0000000	4.5000000	-50.0000000
45590.0000000	4.7500000	-50.0000000
45590.0000000	5.0000000	-50.0000000
45590.0000000	5.2500000	-50.0000000
45590.0000000	5.5000000	-50.0000000
45590.0000000	5.7500000	-50.0000000
45590.0000000	6.0000000	-50.0000000
45590.0000000	6.2500000	-50.0000000
45590.0000000	6.5000000	-50.0000000
45590.0000000	6.7500000	-50.0000000
45590.0000000	7.0000000	-50.0000000

45590.0000000	7.2500000	-50.0000000
45590.0000000	7.5000000	-50.0000000
45590.0000000	7.7500000	-50.0000000
45590.0000000	8.0000000	-50.0000000
45590.0000000	8.2500000	-50.0000000
45590.0000000	8.5000000	-50.0000000
45590.0000000	8.7500000	-50.0000000
45590.0000000	9.0000000	-50.0000000
45590.0000000	9.2500000	-50.0000000
45590.0000000	9.5000000	-50.0000000
45590.0000000	9.7500000	-50.0000000
45590.0000000	10.0000000	-50.0000000
45590.0000000	10.2500000	-50.0000000
45590.0000000	10.5000000	-50.0000000
45590.0000000	10.7500000	-50.0000000
45590.0000000	11.0000000	-50.0000000
45590.0000000	11.2500000	-50.0000000
45590.0000000	11.5000000	-50.0000000
45590.0000000	11.7500000	-50.0000000
45590.0000000	12.0000000	-50.0000000
45590.0000000	12.2500000	-50.0000000
45590.0000000	12.5000000	-50.0000000
45590.0000000	12.7500000	-50.0000000
45590.0000000	13.0000000	-50.0000000
45590.0000000	13.2500000	-50.0000000
45590.0000000	13.5000000	-50.0000000
45590.0000000	13.7500000	-50.0000000
45590.0000000	14.0000000	-50.0000000
45590.0000000	14.2500000	-50.0000000
45590.0000000	14.5000000	-50.0000000
45590.0000000	14.7500000	-50.0000000
45590.0000000	15.0000000	-50.0000000
45620.0000000	0.0000000	-50.0000000
45620.0000000	0.2500000	-50.0000000
45620.0000000	0.5000000	-50.0000000
45620.0000000	0.7500000	-50.0000000
45620.0000000	1.0000000	-50.0000000
45620.0000000	1.2500000	-50.0000000
45620.0000000	1.5000000	-50.0000000
45620.0000000	1.7500000	-50.0000000
45620.0000000	2.0000000	-50.0000000
45620.0000000	2.2500000	-50.0000000
45620.0000000	2.5000000	-50.0000000
45620.0000000	2.7500000	-50.0000000
45620.0000000	3.0000000	-50.0000000
45620.0000000	3.2500000	-50.0000000
45620.0000000	3.5000000	-50.0000000
45620.0000000	3.7500000	-50.0000000
45620.0000000	4.0000000	-50.0000000
45620.0000000	4.2500000	-50.0000000
45620.0000000	4.5000000	-50.0000000
45620.0000000	4.7500000	-50.0000000
45620.0000000	5.0000000	-50.0000000
45620.0000000	5.2500000	-50.0000000
45620.0000000	5.5000000	-50.0000000
45620.0000000	5.7500000	-50.0000000
45620.0000000	6.0000000	-50.0000000
45620.0000000	6.2500000	-50.0000000

45620.0000000	6.5000000	-50.0000000
45620.0000000	6.7500000	-50.0000000
45620.0000000	7.0000000	-50.0000000
45620.0000000	7.2500000	-50.0000000
45620.0000000	7.5000000	-50.0000000
45620.0000000	7.7500000	-50.0000000
45620.0000000	8.0000000	-50.0000000
45620.0000000	8.2500000	-50.0000000
45620.0000000	8.5000000	-50.0000000
45620.0000000	8.7500000	-50.0000000
45620.0000000	9.0000000	-50.0000000
45620.0000000	9.2500000	-50.0000000
45620.0000000	9.5000000	-50.0000000
45620.0000000	9.7500000	-50.0000000
45620.0000000	10.0000000	-50.0000000
45620.0000000	10.2500000	-50.0000000
45620.0000000	10.5000000	-50.0000000
45620.0000000	10.7500000	-50.0000000
45620.0000000	11.0000000	-50.0000000
45620.0000000	11.2500000	-50.0000000
45620.0000000	11.5000000	-50.0000000
45620.0000000	11.7500000	-50.0000000
45620.0000000	12.0000000	-50.0000000
45620.0000000	12.2500000	-50.0000000
45620.0000000	12.5000000	-50.0000000
45620.0000000	12.7500000	-50.0000000
45620.0000000	13.0000000	-50.0000000
45620.0000000	13.2500000	-50.0000000
45620.0000000	13.5000000	-50.0000000
45620.0000000	13.7500000	-50.0000000
45620.0000000	14.0000000	-50.0000000
45620.0000000	14.2500000	-50.0000000
45620.0000000	14.5000000	-50.0000000
45620.0000000	14.7500000	-50.0000000
45620.0000000	15.0000000	-50.0000000
45650.0000000	0.0000000	-50.0000000
45650.0000000	0.2500000	-50.0000000
45650.0000000	0.5000000	-50.0000000
45650.0000000	0.7500000	-50.0000000
45650.0000000	1.0000000	-50.0000000
45650.0000000	1.2500000	-50.0000000
45650.0000000	1.5000000	-50.0000000
45650.0000000	1.7500000	-50.0000000
45650.0000000	2.0000000	-50.0000000
45650.0000000	2.2500000	-50.0000000
45650.0000000	2.5000000	-50.0000000
45650.0000000	2.7500000	-50.0000000
45650.0000000	3.0000000	-50.0000000
45650.0000000	3.2500000	-50.0000000
45650.0000000	3.5000000	-50.0000000
45650.0000000	3.7500000	-50.0000000
45650.0000000	4.0000000	-50.0000000
45650.0000000	4.2500000	-50.0000000
45650.0000000	4.5000000	-50.0000000
45650.0000000	4.7500000	-50.0000000
45650.0000000	5.0000000	-50.0000000
45650.0000000	5.2500000	-50.0000000
45650.0000000	5.5000000	-50.0000000

45650.0000000	5.7500000	-50.0000000
45650.0000000	6.0000000	-50.0000000
45650.0000000	6.2500000	-50.0000000
45650.0000000	6.5000000	-50.0000000
45650.0000000	6.7500000	-50.0000000
45650.0000000	7.0000000	-50.0000000
45650.0000000	7.2500000	-50.0000000
45650.0000000	7.5000000	-50.0000000
45650.0000000	7.7500000	-50.0000000
45650.0000000	8.0000000	-50.0000000
45650.0000000	8.2500000	-50.0000000
45650.0000000	8.5000000	-50.0000000
45650.0000000	8.7500000	-50.0000000
45650.0000000	9.0000000	-50.0000000
45650.0000000	9.2500000	-50.0000000
45650.0000000	9.5000000	-50.0000000
45650.0000000	9.7500000	-50.0000000
45650.0000000	10.0000000	-50.0000000
45650.0000000	10.2500000	-50.0000000
45650.0000000	10.5000000	-50.0000000
45650.0000000	10.7500000	-50.0000000
45650.0000000	11.0000000	-50.0000000
45650.0000000	11.2500000	-50.0000000
45650.0000000	11.5000000	-50.0000000
45650.0000000	11.7500000	-50.0000000
45650.0000000	12.0000000	-50.0000000
45650.0000000	12.2500000	-50.0000000
45650.0000000	12.5000000	-50.0000000
45650.0000000	12.7500000	-50.0000000
45650.0000000	13.0000000	-50.0000000
45650.0000000	13.2500000	-50.0000000
45650.0000000	13.5000000	-50.0000000
45650.0000000	13.7500000	-50.0000000
45650.0000000	14.0000000	-50.0000000
45650.0000000	14.2500000	-50.0000000
45650.0000000	14.5000000	-50.0000000
45650.0000000	14.7500000	-50.0000000
45650.0000000	15.0000000	-50.0000000
45680.0000000	0.0000000	-50.0000000
45680.0000000	0.2500000	-50.0000000
45680.0000000	0.5000000	-50.0000000
45680.0000000	0.7500000	-50.0000000
45680.0000000	1.0000000	-50.0000000
45680.0000000	1.2500000	-50.0000000
45680.0000000	1.5000000	-50.0000000
45680.0000000	1.7500000	-50.0000000
45680.0000000	2.0000000	-50.0000000
45680.0000000	2.2500000	-50.0000000
45680.0000000	2.5000000	-50.0000000
45680.0000000	2.7500000	-50.0000000
45680.0000000	3.0000000	-50.0000000
45680.0000000	3.2500000	-50.0000000
45680.0000000	3.5000000	-50.0000000
45680.0000000	3.7500000	-50.0000000
45680.0000000	4.0000000	-50.0000000
45680.0000000	4.2500000	-50.0000000
45680.0000000	4.5000000	-50.0000000
45680.0000000	4.7500000	-50.0000000

45680.0000000	5.0000000	-50.0000000
45680.0000000	5.2500000	-50.0000000
45680.0000000	5.5000000	-50.0000000
45680.0000000	5.7500000	-50.0000000
45680.0000000	6.0000000	-50.0000000
45680.0000000	6.2500000	-50.0000000
45680.0000000	6.5000000	-50.0000000
45680.0000000	6.7500000	-50.0000000
45680.0000000	7.0000000	-50.0000000
45680.0000000	7.2500000	-50.0000000
45680.0000000	7.5000000	-50.0000000
45680.0000000	7.7500000	-50.0000000
45680.0000000	8.0000000	-50.0000000
45680.0000000	8.2500000	-50.0000000
45680.0000000	8.5000000	-50.0000000
45680.0000000	8.7500000	-50.0000000
45680.0000000	9.0000000	-50.0000000
45680.0000000	9.2500000	-50.0000000
45680.0000000	9.5000000	-50.0000000
45680.0000000	9.7500000	-50.0000000
45680.0000000	10.0000000	-50.0000000
45680.0000000	10.2500000	-50.0000000
45680.0000000	10.5000000	-50.0000000
45680.0000000	10.7500000	-50.0000000
45680.0000000	11.0000000	-50.0000000
45680.0000000	11.2500000	-50.0000000
45680.0000000	11.5000000	-50.0000000
45680.0000000	11.7500000	-50.0000000
45680.0000000	12.0000000	-50.0000000
45680.0000000	12.2500000	-50.0000000
45680.0000000	12.5000000	-50.0000000
45680.0000000	12.7500000	-50.0000000
45680.0000000	13.0000000	-50.0000000
45680.0000000	13.2500000	-50.0000000
45680.0000000	13.5000000	-50.0000000
45680.0000000	13.7500000	-50.0000000
45680.0000000	14.0000000	-50.0000000
45680.0000000	14.2500000	-50.0000000
45680.0000000	14.5000000	-50.0000000
45680.0000000	14.7500000	-50.0000000
45680.0000000	15.0000000	-50.0000000
45710.0000000	0.0000000	-50.0000000
45710.0000000	0.2500000	-50.0000000
45710.0000000	0.5000000	-50.0000000
45710.0000000	0.7500000	-50.0000000
45710.0000000	1.0000000	-50.0000000
45710.0000000	1.2500000	-50.0000000
45710.0000000	1.5000000	-50.0000000
45710.0000000	1.7500000	-50.0000000
45710.0000000	2.0000000	-50.0000000
45710.0000000	2.2500000	-50.0000000
45710.0000000	2.5000000	-50.0000000
45710.0000000	2.7500000	-50.0000000
45710.0000000	3.0000000	-50.0000000
45710.0000000	3.2500000	-50.0000000
45710.0000000	3.5000000	-50.0000000
45710.0000000	3.7500000	-50.0000000
45710.0000000	4.0000000	-50.0000000

45710.0000000	4.2500000	-50.0000000
45710.0000000	4.5000000	-50.0000000
45710.0000000	4.7500000	-50.0000000
45710.0000000	5.0000000	-50.0000000
45710.0000000	5.2500000	-50.0000000
45710.0000000	5.5000000	-50.0000000
45710.0000000	5.7500000	-50.0000000
45710.0000000	6.0000000	-50.0000000
45710.0000000	6.2500000	-50.0000000
45710.0000000	6.5000000	-50.0000000
45710.0000000	6.7500000	-50.0000000
45710.0000000	7.0000000	-50.0000000
45710.0000000	7.2500000	-50.0000000
45710.0000000	7.5000000	-50.0000000
45710.0000000	7.7500000	-50.0000000
45710.0000000	8.0000000	-50.0000000
45710.0000000	8.2500000	-50.0000000
45710.0000000	8.5000000	-50.0000000
45710.0000000	8.7500000	-50.0000000
45710.0000000	9.0000000	-50.0000000
45710.0000000	9.2500000	-50.0000000
45710.0000000	9.5000000	-50.0000000
45710.0000000	9.7500000	-50.0000000
45710.0000000	10.0000000	-50.0000000
45710.0000000	10.2500000	-50.0000000
45710.0000000	10.5000000	-50.0000000
45710.0000000	10.7500000	-50.0000000
45710.0000000	11.0000000	-50.0000000
45710.0000000	11.2500000	-50.0000000
45710.0000000	11.5000000	-50.0000000
45710.0000000	11.7500000	-50.0000000
45710.0000000	12.0000000	-50.0000000
45710.0000000	12.2500000	-50.0000000
45710.0000000	12.5000000	-50.0000000
45710.0000000	12.7500000	-50.0000000
45710.0000000	13.0000000	-50.0000000
45710.0000000	13.2500000	-50.0000000
45710.0000000	13.5000000	-50.0000000
45710.0000000	13.7500000	-50.0000000
45710.0000000	14.0000000	-50.0000000
45710.0000000	14.2500000	-50.0000000
45710.0000000	14.5000000	-50.0000000
45710.0000000	14.7500000	-50.0000000
45710.0000000	15.0000000	-50.0000000
45740.0000000	0.0000000	-50.0000000
45740.0000000	0.2500000	-50.0000000
45740.0000000	0.5000000	-50.0000000
45740.0000000	0.7500000	-50.0000000
45740.0000000	1.0000000	-50.0000000
45740.0000000	1.2500000	-50.0000000
45740.0000000	1.5000000	-50.0000000
45740.0000000	1.7500000	-50.0000000
45740.0000000	2.0000000	-50.0000000
45740.0000000	2.2500000	-50.0000000
45740.0000000	2.5000000	-50.0000000
45740.0000000	2.7500000	-50.0000000
45740.0000000	3.0000000	-50.0000000
45740.0000000	3.2500000	-50.0000000

45740.0000000	3.5000000	-50.0000000
45740.0000000	3.7500000	-50.0000000
45740.0000000	4.0000000	-50.0000000
45740.0000000	4.2500000	-50.0000000
45740.0000000	4.5000000	-50.0000000
45740.0000000	4.7500000	-50.0000000
45740.0000000	5.0000000	-50.0000000
45740.0000000	5.2500000	-50.0000000
45740.0000000	5.5000000	-50.0000000
45740.0000000	5.7500000	-50.0000000
45740.0000000	6.0000000	-50.0000000
45740.0000000	6.2500000	-50.0000000
45740.0000000	6.5000000	-50.0000000
45740.0000000	6.7500000	-50.0000000
45740.0000000	7.0000000	-50.0000000
45740.0000000	7.2500000	-50.0000000
45740.0000000	7.5000000	-50.0000000
45740.0000000	7.7500000	-50.0000000
45740.0000000	8.0000000	-50.0000000
45740.0000000	8.2500000	-50.0000000
45740.0000000	8.5000000	-50.0000000
45740.0000000	8.7500000	-50.0000000
45740.0000000	9.0000000	-50.0000000
45740.0000000	9.2500000	-50.0000000
45740.0000000	9.5000000	-50.0000000
45740.0000000	9.7500000	-50.0000000
45740.0000000	10.0000000	-50.0000000
45740.0000000	10.2500000	-50.0000000
45740.0000000	10.5000000	-50.0000000
45740.0000000	10.7500000	-50.0000000
45740.0000000	11.0000000	-50.0000000
45740.0000000	11.2500000	-50.0000000
45740.0000000	11.5000000	-50.0000000
45740.0000000	11.7500000	-50.0000000
45740.0000000	12.0000000	-50.0000000
45740.0000000	12.2500000	-50.0000000
45740.0000000	12.5000000	-50.0000000
45740.0000000	12.7500000	-50.0000000
45740.0000000	13.0000000	-50.0000000
45740.0000000	13.2500000	-50.0000000
45740.0000000	13.5000000	-50.0000000
45740.0000000	13.7500000	-50.0000000
45740.0000000	14.0000000	-50.0000000
45740.0000000	14.2500000	-50.0000000
45740.0000000	14.5000000	-50.0000000
45740.0000000	14.7500000	-50.0000000
45740.0000000	15.0000000	-50.0000000
45770.0000000	0.0000000	-50.0000000
45770.0000000	0.2500000	-50.0000000
45770.0000000	0.5000000	-50.0000000
45770.0000000	0.7500000	-50.0000000
45770.0000000	1.0000000	-50.0000000
45770.0000000	1.2500000	-50.0000000
45770.0000000	1.5000000	-50.0000000
45770.0000000	1.7500000	-50.0000000
45770.0000000	2.0000000	-50.0000000
45770.0000000	2.2500000	-50.0000000
45770.0000000	2.5000000	-50.0000000

45770.0000000	2.7500000	-50.0000000
45770.0000000	3.0000000	-50.0000000
45770.0000000	3.2500000	-50.0000000
45770.0000000	3.5000000	-50.0000000
45770.0000000	3.7500000	-50.0000000
45770.0000000	4.0000000	-50.0000000
45770.0000000	4.2500000	-50.0000000
45770.0000000	4.5000000	-50.0000000
45770.0000000	4.7500000	-50.0000000
45770.0000000	5.0000000	-50.0000000
45770.0000000	5.2500000	-50.0000000
45770.0000000	5.5000000	-50.0000000
45770.0000000	5.7500000	-50.0000000
45770.0000000	6.0000000	-50.0000000
45770.0000000	6.2500000	-50.0000000
45770.0000000	6.5000000	-50.0000000
45770.0000000	6.7500000	-50.0000000
45770.0000000	7.0000000	-50.0000000
45770.0000000	7.2500000	-50.0000000
45770.0000000	7.5000000	-50.0000000
45770.0000000	7.7500000	-50.0000000
45770.0000000	8.0000000	-50.0000000
45770.0000000	8.2500000	-50.0000000
45770.0000000	8.5000000	-50.0000000
45770.0000000	8.7500000	-50.0000000
45770.0000000	9.0000000	-50.0000000
45770.0000000	9.2500000	-50.0000000
45770.0000000	9.5000000	-50.0000000
45770.0000000	9.7500000	-50.0000000
45770.0000000	10.0000000	-50.0000000
45770.0000000	10.2500000	-50.0000000
45770.0000000	10.5000000	-50.0000000
45770.0000000	10.7500000	-50.0000000
45770.0000000	11.0000000	-50.0000000
45770.0000000	11.2500000	-50.0000000
45770.0000000	11.5000000	-50.0000000
45770.0000000	11.7500000	-50.0000000
45770.0000000	12.0000000	-50.0000000
45770.0000000	12.2500000	-50.0000000
45770.0000000	12.5000000	-50.0000000
45770.0000000	12.7500000	-50.0000000
45770.0000000	13.0000000	-50.0000000
45770.0000000	13.2500000	-50.0000000
45770.0000000	13.5000000	-50.0000000
45770.0000000	13.7500000	-50.0000000
45770.0000000	14.0000000	-50.0000000
45770.0000000	14.2500000	-50.0000000
45770.0000000	14.5000000	-50.0000000
45770.0000000	14.7500000	-50.0000000
45770.0000000	15.0000000	-50.0000000
45800.0000000	0.0000000	-50.0000000
45800.0000000	0.2500000	-50.0000000
45800.0000000	0.5000000	-50.0000000
45800.0000000	0.7500000	-50.0000000
45800.0000000	1.0000000	-50.0000000
45800.0000000	1.2500000	-50.0000000
45800.0000000	1.5000000	-50.0000000
45800.0000000	1.7500000	-50.0000000

45800.0000000	2.0000000	-50.0000000
45800.0000000	2.2500000	-50.0000000
45800.0000000	2.5000000	-50.0000000
45800.0000000	2.7500000	-50.0000000
45800.0000000	3.0000000	-50.0000000
45800.0000000	3.2500000	-50.0000000
45800.0000000	3.5000000	-50.0000000
45800.0000000	3.7500000	-50.0000000
45800.0000000	4.0000000	-50.0000000
45800.0000000	4.2500000	-50.0000000
45800.0000000	4.5000000	-50.0000000
45800.0000000	4.7500000	-50.0000000
45800.0000000	5.0000000	-50.0000000
45800.0000000	5.2500000	-50.0000000
45800.0000000	5.5000000	-50.0000000
45800.0000000	5.7500000	-50.0000000
45800.0000000	6.0000000	-50.0000000
45800.0000000	6.2500000	-50.0000000
45800.0000000	6.5000000	-50.0000000
45800.0000000	6.7500000	-50.0000000
45800.0000000	7.0000000	-50.0000000
45800.0000000	7.2500000	-50.0000000
45800.0000000	7.5000000	-50.0000000
45800.0000000	7.7500000	-50.0000000
45800.0000000	8.0000000	-50.0000000
45800.0000000	8.2500000	-50.0000000
45800.0000000	8.5000000	-50.0000000
45800.0000000	8.7500000	-50.0000000
45800.0000000	9.0000000	-50.0000000
45800.0000000	9.2500000	-50.0000000
45800.0000000	9.5000000	-50.0000000
45800.0000000	9.7500000	-50.0000000
45800.0000000	10.0000000	-50.0000000
45800.0000000	10.2500000	-50.0000000
45800.0000000	10.5000000	-50.0000000
45800.0000000	10.7500000	-50.0000000
45800.0000000	11.0000000	-50.0000000
45800.0000000	11.2500000	-50.0000000
45800.0000000	11.5000000	-50.0000000
45800.0000000	11.7500000	-50.0000000
45800.0000000	12.0000000	-50.0000000
45800.0000000	12.2500000	-50.0000000
45800.0000000	12.5000000	-50.0000000
45800.0000000	12.7500000	-50.0000000
45800.0000000	13.0000000	-50.0000000
45800.0000000	13.2500000	-50.0000000
45800.0000000	13.5000000	-50.0000000
45800.0000000	13.7500000	-50.0000000
45800.0000000	14.0000000	-50.0000000
45800.0000000	14.2500000	-50.0000000
45800.0000000	14.5000000	-50.0000000
45800.0000000	14.7500000	-50.0000000
45800.0000000	15.0000000	-50.0000000
45830.0000000	0.0000000	-50.0000000
45830.0000000	0.2500000	-50.0000000
45830.0000000	0.5000000	-50.0000000
45830.0000000	0.7500000	-50.0000000
45830.0000000	1.0000000	-50.0000000

45830.0000000	1.2500000	-50.0000000
45830.0000000	1.5000000	-50.0000000
45830.0000000	1.7500000	-50.0000000
45830.0000000	2.0000000	-50.0000000
45830.0000000	2.2500000	-50.0000000
45830.0000000	2.5000000	-50.0000000
45830.0000000	2.7500000	-50.0000000
45830.0000000	3.0000000	-50.0000000
45830.0000000	3.2500000	-50.0000000
45830.0000000	3.5000000	-50.0000000
45830.0000000	3.7500000	-50.0000000
45830.0000000	4.0000000	-50.0000000
45830.0000000	4.2500000	-50.0000000
45830.0000000	4.5000000	-50.0000000
45830.0000000	4.7500000	-50.0000000
45830.0000000	5.0000000	-50.0000000
45830.0000000	5.2500000	-50.0000000
45830.0000000	5.5000000	-50.0000000
45830.0000000	5.7500000	-50.0000000
45830.0000000	6.0000000	-50.0000000
45830.0000000	6.2500000	-50.0000000
45830.0000000	6.5000000	-50.0000000
45830.0000000	6.7500000	-50.0000000
45830.0000000	7.0000000	-50.0000000
45830.0000000	7.2500000	-50.0000000
45830.0000000	7.5000000	-50.0000000
45830.0000000	7.7500000	-50.0000000
45830.0000000	8.0000000	-50.0000000
45830.0000000	8.2500000	-50.0000000
45830.0000000	8.5000000	-50.0000000
45830.0000000	8.7500000	-50.0000000
45830.0000000	9.0000000	-50.0000000
45830.0000000	9.2500000	-50.0000000
45830.0000000	9.5000000	-50.0000000
45830.0000000	9.7500000	-50.0000000
45830.0000000	10.0000000	-50.0000000
45830.0000000	10.2500000	-50.0000000
45830.0000000	10.5000000	-50.0000000
45830.0000000	10.7500000	-50.0000000
45830.0000000	11.0000000	-50.0000000
45830.0000000	11.2500000	-50.0000000
45830.0000000	11.5000000	-50.0000000
45830.0000000	11.7500000	-50.0000000
45830.0000000	12.0000000	-50.0000000
45830.0000000	12.2500000	-50.0000000
45830.0000000	12.5000000	-50.0000000
45830.0000000	12.7500000	-50.0000000
45830.0000000	13.0000000	-50.0000000
45830.0000000	13.2500000	-50.0000000
45830.0000000	13.5000000	-50.0000000
45830.0000000	13.7500000	-50.0000000
45830.0000000	14.0000000	-50.0000000
45830.0000000	14.2500000	-50.0000000
45830.0000000	14.5000000	-50.0000000
45830.0000000	14.7500000	-50.0000000
45830.0000000	15.0000000	-50.0000000
45860.0000000	0.0000000	-50.0000000
45860.0000000	0.2500000	-50.0000000

45860.0000000	0.5000000	-50.0000000
45860.0000000	0.7500000	-50.0000000
45860.0000000	1.0000000	-50.0000000
45860.0000000	1.2500000	-50.0000000
45860.0000000	1.5000000	-50.0000000
45860.0000000	1.7500000	-50.0000000
45860.0000000	2.0000000	-50.0000000
45860.0000000	2.2500000	-50.0000000
45860.0000000	2.5000000	-50.0000000
45860.0000000	2.7500000	-50.0000000
45860.0000000	3.0000000	-50.0000000
45860.0000000	3.2500000	-50.0000000
45860.0000000	3.5000000	-50.0000000
45860.0000000	3.7500000	-50.0000000
45860.0000000	4.0000000	-50.0000000
45860.0000000	4.2500000	-50.0000000
45860.0000000	4.5000000	-50.0000000
45860.0000000	4.7500000	-50.0000000
45860.0000000	5.0000000	-50.0000000
45860.0000000	5.2500000	-50.0000000
45860.0000000	5.5000000	-50.0000000
45860.0000000	5.7500000	-50.0000000
45860.0000000	6.0000000	-50.0000000
45860.0000000	6.2500000	-50.0000000
45860.0000000	6.5000000	-50.0000000
45860.0000000	6.7500000	-50.0000000
45860.0000000	7.0000000	-50.0000000
45860.0000000	7.2500000	-50.0000000
45860.0000000	7.5000000	-50.0000000
45860.0000000	7.7500000	-50.0000000
45860.0000000	8.0000000	-50.0000000
45860.0000000	8.2500000	-50.0000000
45860.0000000	8.5000000	-50.0000000
45860.0000000	8.7500000	-50.0000000
45860.0000000	9.0000000	-50.0000000
45860.0000000	9.2500000	-50.0000000
45860.0000000	9.5000000	-50.0000000
45860.0000000	9.7500000	-50.0000000
45860.0000000	10.0000000	-50.0000000
45860.0000000	10.2500000	-50.0000000
45860.0000000	10.5000000	-50.0000000
45860.0000000	10.7500000	-50.0000000
45860.0000000	11.0000000	-50.0000000
45860.0000000	11.2500000	-50.0000000
45860.0000000	11.5000000	-50.0000000
45860.0000000	11.7500000	-50.0000000
45860.0000000	12.0000000	-50.0000000
45860.0000000	12.2500000	-50.0000000
45860.0000000	12.5000000	-50.0000000
45860.0000000	12.7500000	-50.0000000
45860.0000000	13.0000000	-50.0000000
45860.0000000	13.2500000	-50.0000000
45860.0000000	13.5000000	-50.0000000
45860.0000000	13.7500000	-50.0000000
45860.0000000	14.0000000	-50.0000000
45860.0000000	14.2500000	-50.0000000
45860.0000000	14.5000000	-50.0000000
45860.0000000	14.7500000	-50.0000000

45860.0000000	15.0000000	-50.0000000
45890.0000000	0.0000000	-50.0000000
45890.0000000	0.2500000	-50.0000000
45890.0000000	0.5000000	-50.0000000
45890.0000000	0.7500000	-50.0000000
45890.0000000	1.0000000	-50.0000000
45890.0000000	1.2500000	-50.0000000
45890.0000000	1.5000000	-50.0000000
45890.0000000	1.7500000	-50.0000000
45890.0000000	2.0000000	-50.0000000
45890.0000000	2.2500000	-50.0000000
45890.0000000	2.5000000	-50.0000000
45890.0000000	2.7500000	-50.0000000
45890.0000000	3.0000000	-50.0000000
45890.0000000	3.2500000	-50.0000000
45890.0000000	3.5000000	-50.0000000
45890.0000000	3.7500000	-50.0000000
45890.0000000	4.0000000	-50.0000000
45890.0000000	4.2500000	-50.0000000
45890.0000000	4.5000000	-50.0000000
45890.0000000	4.7500000	-50.0000000
45890.0000000	5.0000000	-50.0000000
45890.0000000	5.2500000	-50.0000000
45890.0000000	5.5000000	-50.0000000
45890.0000000	5.7500000	-50.0000000
45890.0000000	6.0000000	-50.0000000
45890.0000000	6.2500000	-50.0000000
45890.0000000	6.5000000	-50.0000000
45890.0000000	6.7500000	-50.0000000
45890.0000000	7.0000000	-50.0000000
45890.0000000	7.2500000	-50.0000000
45890.0000000	7.5000000	-50.0000000
45890.0000000	7.7500000	-50.0000000
45890.0000000	8.0000000	-50.0000000
45890.0000000	8.2500000	-50.0000000
45890.0000000	8.5000000	-50.0000000
45890.0000000	8.7500000	-50.0000000
45890.0000000	9.0000000	-50.0000000
45890.0000000	9.2500000	-50.0000000
45890.0000000	9.5000000	-50.0000000
45890.0000000	9.7500000	-50.0000000
45890.0000000	10.0000000	-50.0000000
45890.0000000	10.2500000	-50.0000000
45890.0000000	10.5000000	-50.0000000
45890.0000000	10.7500000	-50.0000000
45890.0000000	11.0000000	-50.0000000
45890.0000000	11.2500000	-50.0000000
45890.0000000	11.5000000	-50.0000000
45890.0000000	11.7500000	-50.0000000
45890.0000000	12.0000000	-50.0000000
45890.0000000	12.2500000	-50.0000000
45890.0000000	12.5000000	-50.0000000
45890.0000000	12.7500000	-50.0000000
45890.0000000	13.0000000	-50.0000000
45890.0000000	13.2500000	-50.0000000
45890.0000000	13.5000000	-50.0000000
45890.0000000	13.7500000	-50.0000000
45890.0000000	14.0000000	-50.0000000

45890.0000000	14.2500000	-50.0000000
45890.0000000	14.5000000	-50.0000000
45890.0000000	14.7500000	-50.0000000
45890.0000000	15.0000000	-50.0000000
45920.0000000	0.0000000	-50.0000000
45920.0000000	0.2500000	-50.0000000
45920.0000000	0.5000000	-50.0000000
45920.0000000	0.7500000	-50.0000000
45920.0000000	1.0000000	-50.0000000
45920.0000000	1.2500000	-50.0000000
45920.0000000	1.5000000	-50.0000000
45920.0000000	1.7500000	-50.0000000
45920.0000000	2.0000000	-50.0000000
45920.0000000	2.2500000	-50.0000000
45920.0000000	2.5000000	-50.0000000
45920.0000000	2.7500000	-50.0000000
45920.0000000	3.0000000	-50.0000000
45920.0000000	3.2500000	-50.0000000
45920.0000000	3.5000000	-50.0000000
45920.0000000	3.7500000	-50.0000000
45920.0000000	4.0000000	-50.0000000
45920.0000000	4.2500000	-50.0000000
45920.0000000	4.5000000	-50.0000000
45920.0000000	4.7500000	-50.0000000
45920.0000000	5.0000000	-50.0000000
45920.0000000	5.2500000	-50.0000000
45920.0000000	5.5000000	-50.0000000
45920.0000000	5.7500000	-50.0000000
45920.0000000	6.0000000	-50.0000000
45920.0000000	6.2500000	-50.0000000
45920.0000000	6.5000000	-50.0000000
45920.0000000	6.7500000	-50.0000000
45920.0000000	7.0000000	-50.0000000
45920.0000000	7.2500000	-50.0000000
45920.0000000	7.5000000	-50.0000000
45920.0000000	7.7500000	-50.0000000
45920.0000000	8.0000000	-50.0000000
45920.0000000	8.2500000	-50.0000000
45920.0000000	8.5000000	-50.0000000
45920.0000000	8.7500000	-50.0000000
45920.0000000	9.0000000	-50.0000000
45920.0000000	9.2500000	-50.0000000
45920.0000000	9.5000000	-50.0000000
45920.0000000	9.7500000	-50.0000000
45920.0000000	10.0000000	-50.0000000
45920.0000000	10.2500000	-50.0000000
45920.0000000	10.5000000	-50.0000000
45920.0000000	10.7500000	-50.0000000
45920.0000000	11.0000000	-50.0000000
45920.0000000	11.2500000	-50.0000000
45920.0000000	11.5000000	-50.0000000
45920.0000000	11.7500000	-50.0000000
45920.0000000	12.0000000	-50.0000000
45920.0000000	12.2500000	-50.0000000
45920.0000000	12.5000000	-50.0000000
45920.0000000	12.7500000	-50.0000000
45920.0000000	13.0000000	-50.0000000
45920.0000000	13.2500000	-50.0000000

45920.0000000	13.5000000	-50.0000000
45920.0000000	13.7500000	-50.0000000
45920.0000000	14.0000000	-50.0000000
45920.0000000	14.2500000	-50.0000000
45920.0000000	14.5000000	-50.0000000
45920.0000000	14.7500000	-50.0000000
45920.0000000	15.0000000	-50.0000000
45950.0000000	0.0000000	-50.0000000
45950.0000000	0.2500000	-50.0000000
45950.0000000	0.5000000	-50.0000000
45950.0000000	0.7500000	-50.0000000
45950.0000000	1.0000000	-50.0000000
45950.0000000	1.2500000	-50.0000000
45950.0000000	1.5000000	-50.0000000
45950.0000000	1.7500000	-50.0000000
45950.0000000	2.0000000	-50.0000000
45950.0000000	2.2500000	-50.0000000
45950.0000000	2.5000000	-50.0000000
45950.0000000	2.7500000	-50.0000000
45950.0000000	3.0000000	-50.0000000
45950.0000000	3.2500000	-50.0000000
45950.0000000	3.5000000	-50.0000000
45950.0000000	3.7500000	-50.0000000
45950.0000000	4.0000000	-50.0000000
45950.0000000	4.2500000	-50.0000000
45950.0000000	4.5000000	-50.0000000
45950.0000000	4.7500000	-50.0000000
45950.0000000	5.0000000	-50.0000000
45950.0000000	5.2500000	-50.0000000
45950.0000000	5.5000000	-50.0000000
45950.0000000	5.7500000	-50.0000000
45950.0000000	6.0000000	-50.0000000
45950.0000000	6.2500000	-50.0000000
45950.0000000	6.5000000	-50.0000000
45950.0000000	6.7500000	-50.0000000
45950.0000000	7.0000000	-50.0000000
45950.0000000	7.2500000	-50.0000000
45950.0000000	7.5000000	-50.0000000
45950.0000000	7.7500000	-50.0000000
45950.0000000	8.0000000	-50.0000000
45950.0000000	8.2500000	-50.0000000
45950.0000000	8.5000000	-50.0000000
45950.0000000	8.7500000	-50.0000000
45950.0000000	9.0000000	-50.0000000
45950.0000000	9.2500000	-50.0000000
45950.0000000	9.5000000	-50.0000000
45950.0000000	9.7500000	-50.0000000
45950.0000000	10.0000000	-50.0000000
45950.0000000	10.2500000	-50.0000000
45950.0000000	10.5000000	-50.0000000
45950.0000000	10.7500000	-50.0000000
45950.0000000	11.0000000	-50.0000000
45950.0000000	11.2500000	-50.0000000
45950.0000000	11.5000000	-50.0000000
45950.0000000	11.7500000	-50.0000000
45950.0000000	12.0000000	-50.0000000
45950.0000000	12.2500000	-50.0000000
45950.0000000	12.5000000	-50.0000000

45950.0000000	12.7500000	-50.0000000
45950.0000000	13.0000000	-50.0000000
45950.0000000	13.2500000	-50.0000000
45950.0000000	13.5000000	-50.0000000
45950.0000000	13.7500000	-50.0000000
45950.0000000	14.0000000	-50.0000000
45950.0000000	14.2500000	-50.0000000
45950.0000000	14.5000000	-50.0000000
45950.0000000	14.7500000	-50.0000000
45950.0000000	15.0000000	-50.0000000
45980.0000000	0.0000000	-50.0000000
45980.0000000	0.2500000	-50.0000000
45980.0000000	0.5000000	-50.0000000
45980.0000000	0.7500000	-50.0000000
45980.0000000	1.0000000	-50.0000000
45980.0000000	1.2500000	-50.0000000
45980.0000000	1.5000000	-50.0000000
45980.0000000	1.7500000	-50.0000000
45980.0000000	2.0000000	-50.0000000
45980.0000000	2.2500000	-50.0000000
45980.0000000	2.5000000	-50.0000000
45980.0000000	2.7500000	-50.0000000
45980.0000000	3.0000000	-50.0000000
45980.0000000	3.2500000	-50.0000000
45980.0000000	3.5000000	-50.0000000
45980.0000000	3.7500000	-50.0000000
45980.0000000	4.0000000	-50.0000000
45980.0000000	4.2500000	-50.0000000
45980.0000000	4.5000000	-50.0000000
45980.0000000	4.7500000	-50.0000000
45980.0000000	5.0000000	-50.0000000
45980.0000000	5.2500000	-50.0000000
45980.0000000	5.5000000	-50.0000000
45980.0000000	5.7500000	-50.0000000
45980.0000000	6.0000000	-50.0000000
45980.0000000	6.2500000	-50.0000000
45980.0000000	6.5000000	-50.0000000
45980.0000000	6.7500000	-50.0000000
45980.0000000	7.0000000	-50.0000000
45980.0000000	7.2500000	-50.0000000
45980.0000000	7.5000000	-50.0000000
45980.0000000	7.7500000	-50.0000000
45980.0000000	8.0000000	-50.0000000
45980.0000000	8.2500000	-50.0000000
45980.0000000	8.5000000	-50.0000000
45980.0000000	8.7500000	-50.0000000
45980.0000000	9.0000000	-50.0000000
45980.0000000	9.2500000	-50.0000000
45980.0000000	9.5000000	-50.0000000
45980.0000000	9.7500000	-50.0000000
45980.0000000	10.0000000	-50.0000000
45980.0000000	10.2500000	-50.0000000
45980.0000000	10.5000000	-50.0000000
45980.0000000	10.7500000	-50.0000000
45980.0000000	11.0000000	-50.0000000
45980.0000000	11.2500000	-50.0000000
45980.0000000	11.5000000	-50.0000000
45980.0000000	11.7500000	-50.0000000

45980.0000000	12.0000000	-50.0000000
45980.0000000	12.2500000	-50.0000000
45980.0000000	12.5000000	-50.0000000
45980.0000000	12.7500000	-50.0000000
45980.0000000	13.0000000	-50.0000000
45980.0000000	13.2500000	-50.0000000
45980.0000000	13.5000000	-50.0000000
45980.0000000	13.7500000	-50.0000000
45980.0000000	14.0000000	-50.0000000
45980.0000000	14.2500000	-50.0000000
45980.0000000	14.5000000	-50.0000000
45980.0000000	14.7500000	-50.0000000
45980.0000000	15.0000000	-50.0000000
46010.0000000	0.0000000	-50.0000000
46010.0000000	0.2500000	-50.0000000
46010.0000000	0.5000000	-50.0000000
46010.0000000	0.7500000	-50.0000000
46010.0000000	1.0000000	-50.0000000
46010.0000000	1.2500000	-50.0000000
46010.0000000	1.5000000	-50.0000000
46010.0000000	1.7500000	-50.0000000
46010.0000000	2.0000000	-50.0000000
46010.0000000	2.2500000	-50.0000000
46010.0000000	2.5000000	-50.0000000
46010.0000000	2.7500000	-50.0000000
46010.0000000	3.0000000	-50.0000000
46010.0000000	3.2500000	-50.0000000
46010.0000000	3.5000000	-50.0000000
46010.0000000	3.7500000	-50.0000000
46010.0000000	4.0000000	-50.0000000
46010.0000000	4.2500000	-50.0000000
46010.0000000	4.5000000	-50.0000000
46010.0000000	4.7500000	-50.0000000
46010.0000000	5.0000000	-50.0000000
46010.0000000	5.2500000	-50.0000000
46010.0000000	5.5000000	-50.0000000
46010.0000000	5.7500000	-50.0000000
46010.0000000	6.0000000	-50.0000000
46010.0000000	6.2500000	-50.0000000
46010.0000000	6.5000000	-50.0000000
46010.0000000	6.7500000	-50.0000000
46010.0000000	7.0000000	-50.0000000
46010.0000000	7.2500000	-50.0000000
46010.0000000	7.5000000	-50.0000000
46010.0000000	7.7500000	-50.0000000
46010.0000000	8.0000000	-50.0000000
46010.0000000	8.2500000	-50.0000000
46010.0000000	8.5000000	-50.0000000
46010.0000000	8.7500000	-50.0000000
46010.0000000	9.0000000	-50.0000000
46010.0000000	9.2500000	-50.0000000
46010.0000000	9.5000000	-50.0000000
46010.0000000	9.7500000	-50.0000000
46010.0000000	10.0000000	-50.0000000
46010.0000000	10.2500000	-50.0000000
46010.0000000	10.5000000	-50.0000000
46010.0000000	10.7500000	-50.0000000
46010.0000000	11.0000000	-50.0000000

46010.0000000	11.2500000	-50.0000000
46010.0000000	11.5000000	-50.0000000
46010.0000000	11.7500000	-50.0000000
46010.0000000	12.0000000	-50.0000000
46010.0000000	12.2500000	-50.0000000
46010.0000000	12.5000000	-50.0000000
46010.0000000	12.7500000	-50.0000000
46010.0000000	13.0000000	-50.0000000
46010.0000000	13.2500000	-50.0000000
46010.0000000	13.5000000	-50.0000000
46010.0000000	13.7500000	-50.0000000
46010.0000000	14.0000000	-50.0000000
46010.0000000	14.2500000	-50.0000000
46010.0000000	14.5000000	-50.0000000
46010.0000000	14.7500000	-50.0000000
46010.0000000	15.0000000	-50.0000000
46040.0000000	0.0000000	-50.0000000
46040.0000000	0.2500000	-50.0000000
46040.0000000	0.5000000	-50.0000000
46040.0000000	0.7500000	-50.0000000
46040.0000000	1.0000000	-50.0000000
46040.0000000	1.2500000	-50.0000000
46040.0000000	1.5000000	-50.0000000
46040.0000000	1.7500000	-50.0000000
46040.0000000	2.0000000	-50.0000000
46040.0000000	2.2500000	-50.0000000
46040.0000000	2.5000000	-50.0000000
46040.0000000	2.7500000	-50.0000000
46040.0000000	3.0000000	-50.0000000
46040.0000000	3.2500000	-50.0000000
46040.0000000	3.5000000	-50.0000000
46040.0000000	3.7500000	-50.0000000
46040.0000000	4.0000000	-50.0000000
46040.0000000	4.2500000	-50.0000000
46040.0000000	4.5000000	-50.0000000
46040.0000000	4.7500000	-50.0000000
46040.0000000	5.0000000	-50.0000000
46040.0000000	5.2500000	-50.0000000
46040.0000000	5.5000000	-50.0000000
46040.0000000	5.7500000	-50.0000000
46040.0000000	6.0000000	-50.0000000
46040.0000000	6.2500000	-50.0000000
46040.0000000	6.5000000	-50.0000000
46040.0000000	6.7500000	-50.0000000
46040.0000000	7.0000000	-50.0000000
46040.0000000	7.2500000	-50.0000000
46040.0000000	7.5000000	-50.0000000
46040.0000000	7.7500000	-50.0000000
46040.0000000	8.0000000	-50.0000000
46040.0000000	8.2500000	-50.0000000
46040.0000000	8.5000000	-50.0000000
46040.0000000	8.7500000	-50.0000000
46040.0000000	9.0000000	-50.0000000
46040.0000000	9.2500000	-50.0000000
46040.0000000	9.5000000	-50.0000000
46040.0000000	9.7500000	-50.0000000
46040.0000000	10.0000000	-50.0000000
46040.0000000	10.2500000	-50.0000000

46040.0000000	10.5000000	-50.0000000
46040.0000000	10.7500000	-50.0000000
46040.0000000	11.0000000	-50.0000000
46040.0000000	11.2500000	-50.0000000
46040.0000000	11.5000000	-50.0000000
46040.0000000	11.7500000	-50.0000000
46040.0000000	12.0000000	-50.0000000
46040.0000000	12.2500000	-50.0000000
46040.0000000	12.5000000	-50.0000000
46040.0000000	12.7500000	-50.0000000
46040.0000000	13.0000000	-50.0000000
46040.0000000	13.2500000	-50.0000000
46040.0000000	13.5000000	-50.0000000
46040.0000000	13.7500000	-50.0000000
46040.0000000	14.0000000	-50.0000000
46040.0000000	14.2500000	-50.0000000
46040.0000000	14.5000000	-50.0000000
46040.0000000	14.7500000	-50.0000000
46040.0000000	15.0000000	-50.0000000
46070.0000000	0.0000000	-50.0000000
46070.0000000	0.2500000	-50.0000000
46070.0000000	0.5000000	-50.0000000
46070.0000000	0.7500000	-50.0000000
46070.0000000	1.0000000	-50.0000000
46070.0000000	1.2500000	-50.0000000
46070.0000000	1.5000000	-50.0000000
46070.0000000	1.7500000	-50.0000000
46070.0000000	2.0000000	-50.0000000
46070.0000000	2.2500000	-50.0000000
46070.0000000	2.5000000	-50.0000000
46070.0000000	2.7500000	-50.0000000
46070.0000000	3.0000000	-50.0000000
46070.0000000	3.2500000	-50.0000000
46070.0000000	3.5000000	-50.0000000
46070.0000000	3.7500000	-50.0000000
46070.0000000	4.0000000	-50.0000000
46070.0000000	4.2500000	-50.0000000
46070.0000000	4.5000000	-50.0000000
46070.0000000	4.7500000	-50.0000000
46070.0000000	5.0000000	-50.0000000
46070.0000000	5.2500000	-50.0000000
46070.0000000	5.5000000	-50.0000000
46070.0000000	5.7500000	-50.0000000
46070.0000000	6.0000000	-50.0000000
46070.0000000	6.2500000	-50.0000000
46070.0000000	6.5000000	-50.0000000
46070.0000000	6.7500000	-50.0000000
46070.0000000	7.0000000	-50.0000000
46070.0000000	7.2500000	-50.0000000
46070.0000000	7.5000000	-50.0000000
46070.0000000	7.7500000	-50.0000000
46070.0000000	8.0000000	-50.0000000
46070.0000000	8.2500000	-50.0000000
46070.0000000	8.5000000	-50.0000000
46070.0000000	8.7500000	-50.0000000
46070.0000000	9.0000000	-50.0000000
46070.0000000	9.2500000	-50.0000000
46070.0000000	9.5000000	-50.0000000

46070.0000000	9.7500000	-50.0000000
46070.0000000	10.0000000	-50.0000000
46070.0000000	10.2500000	-50.0000000
46070.0000000	10.5000000	-50.0000000
46070.0000000	10.7500000	-50.0000000
46070.0000000	11.0000000	-50.0000000
46070.0000000	11.2500000	-50.0000000
46070.0000000	11.5000000	-50.0000000
46070.0000000	11.7500000	-50.0000000
46070.0000000	12.0000000	-50.0000000
46070.0000000	12.2500000	-50.0000000
46070.0000000	12.5000000	-50.0000000
46070.0000000	12.7500000	-50.0000000
46070.0000000	13.0000000	-50.0000000
46070.0000000	13.2500000	-50.0000000
46070.0000000	13.5000000	-50.0000000
46070.0000000	13.7500000	-50.0000000
46070.0000000	14.0000000	-50.0000000
46070.0000000	14.2500000	-50.0000000
46070.0000000	14.5000000	-50.0000000
46070.0000000	14.7500000	-50.0000000
46070.0000000	15.0000000	-50.0000000
46100.0000000	0.0000000	-50.0000000
46100.0000000	0.2500000	-50.0000000
46100.0000000	0.5000000	-50.0000000
46100.0000000	0.7500000	15.7200003
46100.0000000	1.0000000	-50.0000000
46100.0000000	1.2500000	-50.0000000
46100.0000000	1.5000000	-50.0000000
46100.0000000	1.7500000	-50.0000000
46100.0000000	2.0000000	-50.0000000
46100.0000000	2.2500000	-50.0000000
46100.0000000	2.5000000	-50.0000000
46100.0000000	2.7500000	-50.0000000
46100.0000000	3.0000000	-50.0000000
46100.0000000	3.2500000	-50.0000000
46100.0000000	3.5000000	-50.0000000
46100.0000000	3.7500000	-50.0000000
46100.0000000	4.0000000	-50.0000000
46100.0000000	4.2500000	-50.0000000
46100.0000000	4.5000000	-50.0000000
46100.0000000	4.7500000	-50.0000000
46100.0000000	5.0000000	-50.0000000
46100.0000000	5.2500000	-50.0000000
46100.0000000	5.5000000	-50.0000000
46100.0000000	5.7500000	-50.0000000
46100.0000000	6.0000000	-50.0000000
46100.0000000	6.2500000	-50.0000000
46100.0000000	6.5000000	-50.0000000
46100.0000000	6.7500000	-50.0000000
46100.0000000	7.0000000	-50.0000000
46100.0000000	7.2500000	-50.0000000
46100.0000000	7.5000000	-50.0000000
46100.0000000	7.7500000	-50.0000000
46100.0000000	8.0000000	-50.0000000
46100.0000000	8.2500000	-50.0000000
46100.0000000	8.5000000	-50.0000000
46100.0000000	8.7500000	-50.0000000

46100.0000000	9.0000000	-50.0000000
46100.0000000	9.2500000	-50.0000000
46100.0000000	9.5000000	-50.0000000
46100.0000000	9.7500000	-50.0000000
46100.0000000	10.0000000	-50.0000000
46100.0000000	10.2500000	-50.0000000
46100.0000000	10.5000000	-50.0000000
46100.0000000	10.7500000	-50.0000000
46100.0000000	11.0000000	-50.0000000
46100.0000000	11.2500000	-50.0000000
46100.0000000	11.5000000	-50.0000000
46100.0000000	11.7500000	-50.0000000
46100.0000000	12.0000000	-50.0000000
46100.0000000	12.2500000	-50.0000000
46100.0000000	12.5000000	-50.0000000
46100.0000000	12.7500000	-50.0000000
46100.0000000	13.0000000	-50.0000000
46100.0000000	13.2500000	-50.0000000
46100.0000000	13.5000000	-50.0000000
46100.0000000	13.7500000	-50.0000000
46100.0000000	14.0000000	-50.0000000
46100.0000000	14.2500000	-50.0000000
46100.0000000	14.5000000	-50.0000000
46100.0000000	14.7500000	-50.0000000
46100.0000000	15.0000000	-50.0000000
46130.0000000	0.0000000	-50.0000000
46130.0000000	0.2500000	-50.0000000
46130.0000000	0.5000000	-50.0000000
46130.0000000	0.7500000	19.5400009
46130.0000000	1.0000000	16.6299992
46130.0000000	1.2500000	-50.0000000
46130.0000000	1.5000000	-50.0000000
46130.0000000	1.7500000	-50.0000000
46130.0000000	2.0000000	-50.0000000
46130.0000000	2.2500000	-50.0000000
46130.0000000	2.5000000	-50.0000000
46130.0000000	2.7500000	-50.0000000
46130.0000000	3.0000000	-50.0000000
46130.0000000	3.2500000	-50.0000000
46130.0000000	3.5000000	-50.0000000
46130.0000000	3.7500000	-50.0000000
46130.0000000	4.0000000	-50.0000000
46130.0000000	4.2500000	-50.0000000
46130.0000000	4.5000000	-50.0000000
46130.0000000	4.7500000	-50.0000000
46130.0000000	5.0000000	-50.0000000
46130.0000000	5.2500000	-50.0000000
46130.0000000	5.5000000	-50.0000000
46130.0000000	5.7500000	-50.0000000
46130.0000000	6.0000000	-50.0000000
46130.0000000	6.2500000	-50.0000000
46130.0000000	6.5000000	-50.0000000
46130.0000000	6.7500000	-50.0000000
46130.0000000	7.0000000	-50.0000000
46130.0000000	7.2500000	-50.0000000
46130.0000000	7.5000000	-50.0000000
46130.0000000	7.7500000	-50.0000000
46130.0000000	8.0000000	-50.0000000

46130.0000000	8.2500000	-50.0000000
46130.0000000	8.5000000	-50.0000000
46130.0000000	8.7500000	-50.0000000
46130.0000000	9.0000000	-50.0000000
46130.0000000	9.2500000	-50.0000000
46130.0000000	9.5000000	-50.0000000
46130.0000000	9.7500000	-50.0000000
46130.0000000	10.0000000	-50.0000000
46130.0000000	10.2500000	-50.0000000
46130.0000000	10.5000000	-50.0000000
46130.0000000	10.7500000	-50.0000000
46130.0000000	11.0000000	-50.0000000
46130.0000000	11.2500000	-50.0000000
46130.0000000	11.5000000	-50.0000000
46130.0000000	11.7500000	-50.0000000
46130.0000000	12.0000000	-50.0000000
46130.0000000	12.2500000	-50.0000000
46130.0000000	12.5000000	-50.0000000
46130.0000000	12.7500000	-50.0000000
46130.0000000	13.0000000	-50.0000000
46130.0000000	13.2500000	-50.0000000
46130.0000000	13.5000000	-50.0000000
46130.0000000	13.7500000	-50.0000000
46130.0000000	14.0000000	-50.0000000
46130.0000000	14.2500000	-50.0000000
46130.0000000	14.5000000	-50.0000000
46130.0000000	14.7500000	-50.0000000
46130.0000000	15.0000000	-50.0000000
46160.0000000	0.0000000	-50.0000000
46160.0000000	0.2500000	-50.0000000
46160.0000000	0.5000000	-50.0000000
46160.0000000	0.7500000	16.2800007
46160.0000000	1.0000000	-50.0000000
46160.0000000	1.2500000	-50.0000000
46160.0000000	1.5000000	-50.0000000
46160.0000000	1.7500000	-50.0000000
46160.0000000	2.0000000	-50.0000000
46160.0000000	2.2500000	-50.0000000
46160.0000000	2.5000000	-50.0000000
46160.0000000	2.7500000	-50.0000000
46160.0000000	3.0000000	-50.0000000
46160.0000000	3.2500000	-50.0000000
46160.0000000	3.5000000	-50.0000000
46160.0000000	3.7500000	-50.0000000
46160.0000000	4.0000000	-50.0000000
46160.0000000	4.2500000	-50.0000000
46160.0000000	4.5000000	-50.0000000
46160.0000000	4.7500000	-50.0000000
46160.0000000	5.0000000	-50.0000000
46160.0000000	5.2500000	-50.0000000
46160.0000000	5.5000000	-50.0000000
46160.0000000	5.7500000	-50.0000000
46160.0000000	6.0000000	-50.0000000
46160.0000000	6.2500000	-50.0000000
46160.0000000	6.5000000	-50.0000000
46160.0000000	6.7500000	-50.0000000
46160.0000000	7.0000000	-50.0000000
46160.0000000	7.2500000	-50.0000000

46160.0000000	7.5000000	-50.0000000
46160.0000000	7.7500000	-50.0000000
46160.0000000	8.0000000	-50.0000000
46160.0000000	8.2500000	-50.0000000
46160.0000000	8.5000000	-50.0000000
46160.0000000	8.7500000	-50.0000000
46160.0000000	9.0000000	-50.0000000
46160.0000000	9.2500000	-50.0000000
46160.0000000	9.5000000	-50.0000000
46160.0000000	9.7500000	-50.0000000
46160.0000000	10.0000000	-50.0000000
46160.0000000	10.2500000	-50.0000000
46160.0000000	10.5000000	-50.0000000
46160.0000000	10.7500000	-50.0000000
46160.0000000	11.0000000	-50.0000000
46160.0000000	11.2500000	-50.0000000
46160.0000000	11.5000000	-50.0000000
46160.0000000	11.7500000	-50.0000000
46160.0000000	12.0000000	-50.0000000
46160.0000000	12.2500000	-50.0000000
46160.0000000	12.5000000	-50.0000000
46160.0000000	12.7500000	-50.0000000
46160.0000000	13.0000000	-50.0000000
46160.0000000	13.2500000	-50.0000000
46160.0000000	13.5000000	-50.0000000
46160.0000000	13.7500000	-50.0000000
46160.0000000	14.0000000	-50.0000000
46160.0000000	14.2500000	-50.0000000
46160.0000000	14.5000000	-50.0000000
46160.0000000	14.7500000	-50.0000000
46160.0000000	15.0000000	-50.0000000
46190.0000000	0.0000000	-50.0000000
46190.0000000	0.2500000	-50.0000000
46190.0000000	0.5000000	-50.0000000
46190.0000000	0.7500000	-50.0000000
46190.0000000	1.0000000	-50.0000000
46190.0000000	1.2500000	-50.0000000
46190.0000000	1.5000000	-50.0000000
46190.0000000	1.7500000	-50.0000000
46190.0000000	2.0000000	-50.0000000
46190.0000000	2.2500000	-50.0000000
46190.0000000	2.5000000	-50.0000000
46190.0000000	2.7500000	-50.0000000
46190.0000000	3.0000000	-50.0000000
46190.0000000	3.2500000	-50.0000000
46190.0000000	3.5000000	-50.0000000
46190.0000000	3.7500000	-50.0000000
46190.0000000	4.0000000	-50.0000000
46190.0000000	4.2500000	-50.0000000
46190.0000000	4.5000000	-50.0000000
46190.0000000	4.7500000	-50.0000000
46190.0000000	5.0000000	-50.0000000
46190.0000000	5.2500000	-50.0000000
46190.0000000	5.5000000	-50.0000000
46190.0000000	5.7500000	-50.0000000
46190.0000000	6.0000000	-50.0000000
46190.0000000	6.2500000	-50.0000000
46190.0000000	6.5000000	-50.0000000

46190.0000000	6.7500000	-50.0000000
46190.0000000	7.0000000	-50.0000000
46190.0000000	7.2500000	-50.0000000
46190.0000000	7.5000000	-50.0000000
46190.0000000	7.7500000	-50.0000000
46190.0000000	8.0000000	-50.0000000
46190.0000000	8.2500000	-50.0000000
46190.0000000	8.5000000	-50.0000000
46190.0000000	8.7500000	-50.0000000
46190.0000000	9.0000000	-50.0000000
46190.0000000	9.2500000	-50.0000000
46190.0000000	9.5000000	-50.0000000
46190.0000000	9.7500000	-50.0000000
46190.0000000	10.0000000	-50.0000000
46190.0000000	10.2500000	-50.0000000
46190.0000000	10.5000000	-50.0000000
46190.0000000	10.7500000	-50.0000000
46190.0000000	11.0000000	-50.0000000
46190.0000000	11.2500000	-50.0000000
46190.0000000	11.5000000	-50.0000000
46190.0000000	11.7500000	-50.0000000
46190.0000000	12.0000000	-50.0000000
46190.0000000	12.2500000	-50.0000000
46190.0000000	12.5000000	-50.0000000
46190.0000000	12.7500000	-50.0000000
46190.0000000	13.0000000	-50.0000000
46190.0000000	13.2500000	-50.0000000
46190.0000000	13.5000000	-50.0000000
46190.0000000	13.7500000	-50.0000000
46190.0000000	14.0000000	-50.0000000
46190.0000000	14.2500000	-50.0000000
46190.0000000	14.5000000	-50.0000000
46190.0000000	14.7500000	-50.0000000
46190.0000000	15.0000000	-50.0000000
46220.0000000	0.0000000	-50.0000000
46220.0000000	0.2500000	-50.0000000
46220.0000000	0.5000000	-50.0000000
46220.0000000	0.7500000	15.0000000
46220.0000000	1.0000000	-50.0000000
46220.0000000	1.2500000	-50.0000000
46220.0000000	1.5000000	-50.0000000
46220.0000000	1.7500000	-50.0000000
46220.0000000	2.0000000	-50.0000000
46220.0000000	2.2500000	-50.0000000
46220.0000000	2.5000000	-50.0000000
46220.0000000	2.7500000	-50.0000000
46220.0000000	3.0000000	-50.0000000
46220.0000000	3.2500000	-50.0000000
46220.0000000	3.5000000	-50.0000000
46220.0000000	3.7500000	-50.0000000
46220.0000000	4.0000000	-50.0000000
46220.0000000	4.2500000	-50.0000000
46220.0000000	4.5000000	-50.0000000
46220.0000000	4.7500000	-50.0000000
46220.0000000	5.0000000	-50.0000000
46220.0000000	5.2500000	-50.0000000
46220.0000000	5.5000000	-50.0000000
46220.0000000	5.7500000	-50.0000000

46220.0000000	6.0000000	-50.0000000
46220.0000000	6.2500000	-50.0000000
46220.0000000	6.5000000	-50.0000000
46220.0000000	6.7500000	-50.0000000
46220.0000000	7.0000000	-50.0000000
46220.0000000	7.2500000	-50.0000000
46220.0000000	7.5000000	-50.0000000
46220.0000000	7.7500000	-50.0000000
46220.0000000	8.0000000	-50.0000000
46220.0000000	8.2500000	-50.0000000
46220.0000000	8.5000000	-50.0000000
46220.0000000	8.7500000	-50.0000000
46220.0000000	9.0000000	-50.0000000
46220.0000000	9.2500000	-50.0000000
46220.0000000	9.5000000	-50.0000000
46220.0000000	9.7500000	-50.0000000
46220.0000000	10.0000000	-50.0000000
46220.0000000	10.2500000	-50.0000000
46220.0000000	10.5000000	-50.0000000
46220.0000000	10.7500000	-50.0000000
46220.0000000	11.0000000	-50.0000000
46220.0000000	11.2500000	-50.0000000
46220.0000000	11.5000000	-50.0000000
46220.0000000	11.7500000	-50.0000000
46220.0000000	12.0000000	-50.0000000
46220.0000000	12.2500000	-50.0000000
46220.0000000	12.5000000	-50.0000000
46220.0000000	12.7500000	-50.0000000
46220.0000000	13.0000000	-50.0000000
46220.0000000	13.2500000	-50.0000000
46220.0000000	13.5000000	-50.0000000
46220.0000000	13.7500000	-50.0000000
46220.0000000	14.0000000	-50.0000000
46220.0000000	14.2500000	-50.0000000
46220.0000000	14.5000000	-50.0000000
46220.0000000	14.7500000	-50.0000000
46220.0000000	15.0000000	-50.0000000
46250.0000000	0.0000000	-50.0000000
46250.0000000	0.2500000	-50.0000000
46250.0000000	0.5000000	-50.0000000
46250.0000000	0.7500000	-50.0000000
46250.0000000	1.0000000	-50.0000000
46250.0000000	1.2500000	-50.0000000
46250.0000000	1.5000000	-50.0000000
46250.0000000	1.7500000	-50.0000000
46250.0000000	2.0000000	-50.0000000
46250.0000000	2.2500000	-50.0000000
46250.0000000	2.5000000	-50.0000000
46250.0000000	2.7500000	-50.0000000
46250.0000000	3.0000000	-50.0000000
46250.0000000	3.2500000	-50.0000000
46250.0000000	3.5000000	-50.0000000
46250.0000000	3.7500000	-50.0000000
46250.0000000	4.0000000	-50.0000000
46250.0000000	4.2500000	-50.0000000
46250.0000000	4.5000000	-50.0000000
46250.0000000	4.7500000	-50.0000000
46250.0000000	5.0000000	-50.0000000

46250.0000000	5.2500000	-50.0000000
46250.0000000	5.5000000	-50.0000000
46250.0000000	5.7500000	-50.0000000
46250.0000000	6.0000000	-50.0000000
46250.0000000	6.2500000	-50.0000000
46250.0000000	6.5000000	-50.0000000
46250.0000000	6.7500000	-50.0000000
46250.0000000	7.0000000	-50.0000000
46250.0000000	7.2500000	-50.0000000
46250.0000000	7.5000000	-50.0000000
46250.0000000	7.7500000	-50.0000000
46250.0000000	8.0000000	-50.0000000
46250.0000000	8.2500000	-50.0000000
46250.0000000	8.5000000	-50.0000000
46250.0000000	8.7500000	-50.0000000
46250.0000000	9.0000000	-50.0000000
46250.0000000	9.2500000	-50.0000000
46250.0000000	9.5000000	-50.0000000
46250.0000000	9.7500000	-50.0000000
46250.0000000	10.0000000	-50.0000000
46250.0000000	10.2500000	-50.0000000
46250.0000000	10.5000000	-50.0000000
46250.0000000	10.7500000	-50.0000000
46250.0000000	11.0000000	-50.0000000
46250.0000000	11.2500000	-50.0000000
46250.0000000	11.5000000	-50.0000000
46250.0000000	11.7500000	-50.0000000
46250.0000000	12.0000000	-50.0000000
46250.0000000	12.2500000	-50.0000000
46250.0000000	12.5000000	-50.0000000
46250.0000000	12.7500000	-50.0000000
46250.0000000	13.0000000	-50.0000000
46250.0000000	13.2500000	-50.0000000
46250.0000000	13.5000000	-50.0000000
46250.0000000	13.7500000	-50.0000000
46250.0000000	14.0000000	-50.0000000
46250.0000000	14.2500000	-50.0000000
46250.0000000	14.5000000	-50.0000000
46250.0000000	14.7500000	-50.0000000
46250.0000000	15.0000000	-50.0000000
46280.0000000	0.0000000	-50.0000000
46280.0000000	0.2500000	-50.0000000
46280.0000000	0.5000000	-50.0000000
46280.0000000	0.7500000	-50.0000000
46280.0000000	1.0000000	-50.0000000
46280.0000000	1.2500000	-50.0000000
46280.0000000	1.5000000	-50.0000000
46280.0000000	1.7500000	-50.0000000
46280.0000000	2.0000000	-50.0000000
46280.0000000	2.2500000	-50.0000000
46280.0000000	2.5000000	-50.0000000
46280.0000000	2.7500000	-50.0000000
46280.0000000	3.0000000	-50.0000000
46280.0000000	3.2500000	-50.0000000
46280.0000000	3.5000000	-50.0000000
46280.0000000	3.7500000	-50.0000000
46280.0000000	4.0000000	-50.0000000
46280.0000000	4.2500000	-50.0000000

46280.0000000	4.5000000	-50.0000000
46280.0000000	4.7500000	-50.0000000
46280.0000000	5.0000000	-50.0000000
46280.0000000	5.2500000	-50.0000000
46280.0000000	5.5000000	-50.0000000
46280.0000000	5.7500000	-50.0000000
46280.0000000	6.0000000	-50.0000000
46280.0000000	6.2500000	-50.0000000
46280.0000000	6.5000000	-50.0000000
46280.0000000	6.7500000	-50.0000000
46280.0000000	7.0000000	-50.0000000
46280.0000000	7.2500000	-50.0000000
46280.0000000	7.5000000	-50.0000000
46280.0000000	7.7500000	-50.0000000
46280.0000000	8.0000000	-50.0000000
46280.0000000	8.2500000	-50.0000000
46280.0000000	8.5000000	-50.0000000
46280.0000000	8.7500000	-50.0000000
46280.0000000	9.0000000	-50.0000000
46280.0000000	9.2500000	-50.0000000
46280.0000000	9.5000000	-50.0000000
46280.0000000	9.7500000	-50.0000000
46280.0000000	10.0000000	-50.0000000
46280.0000000	10.2500000	-50.0000000
46280.0000000	10.5000000	-50.0000000
46280.0000000	10.7500000	-50.0000000
46280.0000000	11.0000000	-50.0000000
46280.0000000	11.2500000	-50.0000000
46280.0000000	11.5000000	-50.0000000
46280.0000000	11.7500000	-50.0000000
46280.0000000	12.0000000	-50.0000000
46280.0000000	12.2500000	-50.0000000
46280.0000000	12.5000000	-50.0000000
46280.0000000	12.7500000	-50.0000000
46280.0000000	13.0000000	-50.0000000
46280.0000000	13.2500000	-50.0000000
46280.0000000	13.5000000	-50.0000000
46280.0000000	13.7500000	-50.0000000
46280.0000000	14.0000000	-50.0000000
46280.0000000	14.2500000	-50.0000000
46280.0000000	14.5000000	-50.0000000
46280.0000000	14.7500000	-50.0000000
46280.0000000	15.0000000	-50.0000000
46310.0000000	0.0000000	-50.0000000
46310.0000000	0.2500000	-50.0000000
46310.0000000	0.5000000	-50.0000000
46310.0000000	0.7500000	-50.0000000
46310.0000000	1.0000000	-50.0000000
46310.0000000	1.2500000	-50.0000000
46310.0000000	1.5000000	-50.0000000
46310.0000000	1.7500000	-50.0000000
46310.0000000	2.0000000	-50.0000000
46310.0000000	2.2500000	-50.0000000
46310.0000000	2.5000000	-50.0000000
46310.0000000	2.7500000	-50.0000000
46310.0000000	3.0000000	-50.0000000
46310.0000000	3.2500000	-50.0000000
46310.0000000	3.5000000	-50.0000000

46310.0000000	3.7500000	-50.0000000
46310.0000000	4.0000000	-50.0000000
46310.0000000	4.2500000	-50.0000000
46310.0000000	4.5000000	-50.0000000
46310.0000000	4.7500000	-50.0000000
46310.0000000	5.0000000	-50.0000000
46310.0000000	5.2500000	-50.0000000
46310.0000000	5.5000000	-50.0000000
46310.0000000	5.7500000	-50.0000000
46310.0000000	6.0000000	-50.0000000
46310.0000000	6.2500000	-50.0000000
46310.0000000	6.5000000	-50.0000000
46310.0000000	6.7500000	-50.0000000
46310.0000000	7.0000000	-50.0000000
46310.0000000	7.2500000	-50.0000000
46310.0000000	7.5000000	-50.0000000
46310.0000000	7.7500000	-50.0000000
46310.0000000	8.0000000	-50.0000000
46310.0000000	8.2500000	-50.0000000
46310.0000000	8.5000000	-50.0000000
46310.0000000	8.7500000	-50.0000000
46310.0000000	9.0000000	-50.0000000
46310.0000000	9.2500000	-50.0000000
46310.0000000	9.5000000	-50.0000000
46310.0000000	9.7500000	-50.0000000
46310.0000000	10.0000000	-50.0000000
46310.0000000	10.2500000	-50.0000000
46310.0000000	10.5000000	-50.0000000
46310.0000000	10.7500000	-50.0000000
46310.0000000	11.0000000	-50.0000000
46310.0000000	11.2500000	-50.0000000
46310.0000000	11.5000000	-50.0000000
46310.0000000	11.7500000	-50.0000000
46310.0000000	12.0000000	-50.0000000
46310.0000000	12.2500000	-50.0000000
46310.0000000	12.5000000	-50.0000000
46310.0000000	12.7500000	-50.0000000
46310.0000000	13.0000000	-50.0000000
46310.0000000	13.2500000	-50.0000000
46310.0000000	13.5000000	-50.0000000
46310.0000000	13.7500000	-50.0000000
46310.0000000	14.0000000	-50.0000000
46310.0000000	14.2500000	-50.0000000
46310.0000000	14.5000000	-50.0000000
46310.0000000	14.7500000	-50.0000000
46310.0000000	15.0000000	-50.0000000
46340.0000000	0.0000000	-50.0000000
46340.0000000	0.2500000	-50.0000000
46340.0000000	0.5000000	-50.0000000
46340.0000000	0.7500000	-50.0000000
46340.0000000	1.0000000	-50.0000000
46340.0000000	1.2500000	-50.0000000
46340.0000000	1.5000000	-50.0000000
46340.0000000	1.7500000	-50.0000000
46340.0000000	2.0000000	-50.0000000
46340.0000000	2.2500000	-50.0000000
46340.0000000	2.5000000	-50.0000000
46340.0000000	2.7500000	-50.0000000

46340.0000000	3.0000000	-50.0000000
46340.0000000	3.2500000	-50.0000000
46340.0000000	3.5000000	-50.0000000
46340.0000000	3.7500000	-50.0000000
46340.0000000	4.0000000	-50.0000000
46340.0000000	4.2500000	-50.0000000
46340.0000000	4.5000000	-50.0000000
46340.0000000	4.7500000	-50.0000000
46340.0000000	5.0000000	-50.0000000
46340.0000000	5.2500000	-50.0000000
46340.0000000	5.5000000	-50.0000000
46340.0000000	5.7500000	-50.0000000
46340.0000000	6.0000000	-50.0000000
46340.0000000	6.2500000	-50.0000000
46340.0000000	6.5000000	-50.0000000
46340.0000000	6.7500000	-50.0000000
46340.0000000	7.0000000	-50.0000000
46340.0000000	7.2500000	-50.0000000
46340.0000000	7.5000000	-50.0000000
46340.0000000	7.7500000	-50.0000000
46340.0000000	8.0000000	-50.0000000
46340.0000000	8.2500000	-50.0000000
46340.0000000	8.5000000	-50.0000000
46340.0000000	8.7500000	-50.0000000
46340.0000000	9.0000000	-50.0000000
46340.0000000	9.2500000	-50.0000000
46340.0000000	9.5000000	-50.0000000
46340.0000000	9.7500000	-50.0000000
46340.0000000	10.0000000	-50.0000000
46340.0000000	10.2500000	-50.0000000
46340.0000000	10.5000000	-50.0000000
46340.0000000	10.7500000	-50.0000000
46340.0000000	11.0000000	-50.0000000
46340.0000000	11.2500000	-50.0000000
46340.0000000	11.5000000	-50.0000000
46340.0000000	11.7500000	-50.0000000
46340.0000000	12.0000000	-50.0000000
46340.0000000	12.2500000	-50.0000000
46340.0000000	12.5000000	-50.0000000
46340.0000000	12.7500000	-50.0000000
46340.0000000	13.0000000	-50.0000000
46340.0000000	13.2500000	-50.0000000
46340.0000000	13.5000000	-50.0000000
46340.0000000	13.7500000	-50.0000000
46340.0000000	14.0000000	-50.0000000
46340.0000000	14.2500000	-50.0000000
46340.0000000	14.5000000	-50.0000000
46340.0000000	14.7500000	-50.0000000
46340.0000000	15.0000000	-50.0000000
46370.0000000	0.0000000	-50.0000000
46370.0000000	0.2500000	-50.0000000
46370.0000000	0.5000000	-50.0000000
46370.0000000	0.7500000	-50.0000000
46370.0000000	1.0000000	-50.0000000
46370.0000000	1.2500000	-50.0000000
46370.0000000	1.5000000	-50.0000000
46370.0000000	1.7500000	-50.0000000
46370.0000000	2.0000000	-50.0000000

46370.0000000	2.2500000	-50.0000000
46370.0000000	2.5000000	-50.0000000
46370.0000000	2.7500000	-50.0000000
46370.0000000	3.0000000	-50.0000000
46370.0000000	3.2500000	-50.0000000
46370.0000000	3.5000000	-50.0000000
46370.0000000	3.7500000	-50.0000000
46370.0000000	4.0000000	-50.0000000
46370.0000000	4.2500000	-50.0000000
46370.0000000	4.5000000	-50.0000000
46370.0000000	4.7500000	-50.0000000
46370.0000000	5.0000000	-50.0000000
46370.0000000	5.2500000	-50.0000000
46370.0000000	5.5000000	-50.0000000
46370.0000000	5.7500000	-50.0000000
46370.0000000	6.0000000	-50.0000000
46370.0000000	6.2500000	-50.0000000
46370.0000000	6.5000000	-50.0000000
46370.0000000	6.7500000	-50.0000000
46370.0000000	7.0000000	-50.0000000
46370.0000000	7.2500000	-50.0000000
46370.0000000	7.5000000	-50.0000000
46370.0000000	7.7500000	-50.0000000
46370.0000000	8.0000000	-50.0000000
46370.0000000	8.2500000	-50.0000000
46370.0000000	8.5000000	-50.0000000
46370.0000000	8.7500000	-50.0000000
46370.0000000	9.0000000	-50.0000000
46370.0000000	9.2500000	-50.0000000
46370.0000000	9.5000000	-50.0000000
46370.0000000	9.7500000	-50.0000000
46370.0000000	10.0000000	-50.0000000
46370.0000000	10.2500000	-50.0000000
46370.0000000	10.5000000	-50.0000000
46370.0000000	10.7500000	-50.0000000
46370.0000000	11.0000000	-50.0000000
46370.0000000	11.2500000	-50.0000000
46370.0000000	11.5000000	-50.0000000
46370.0000000	11.7500000	-50.0000000
46370.0000000	12.0000000	-50.0000000
46370.0000000	12.2500000	-50.0000000
46370.0000000	12.5000000	-50.0000000
46370.0000000	12.7500000	-50.0000000
46370.0000000	13.0000000	-50.0000000
46370.0000000	13.2500000	-50.0000000
46370.0000000	13.5000000	-50.0000000
46370.0000000	13.7500000	-50.0000000
46370.0000000	14.0000000	-50.0000000
46370.0000000	14.2500000	-50.0000000
46370.0000000	14.5000000	-50.0000000
46370.0000000	14.7500000	-50.0000000
46370.0000000	15.0000000	-50.0000000
46400.0000000	0.0000000	-50.0000000
46400.0000000	0.2500000	-50.0000000
46400.0000000	0.5000000	-50.0000000
46400.0000000	0.7500000	-50.0000000
46400.0000000	1.0000000	-50.0000000
46400.0000000	1.2500000	-50.0000000

46400.0000000	1.5000000	-50.0000000
46400.0000000	1.7500000	-50.0000000
46400.0000000	2.0000000	-50.0000000
46400.0000000	2.2500000	-50.0000000
46400.0000000	2.5000000	-50.0000000
46400.0000000	2.7500000	-50.0000000
46400.0000000	3.0000000	-50.0000000
46400.0000000	3.2500000	-50.0000000
46400.0000000	3.5000000	-50.0000000
46400.0000000	3.7500000	-50.0000000
46400.0000000	4.0000000	-50.0000000
46400.0000000	4.2500000	-50.0000000
46400.0000000	4.5000000	-50.0000000
46400.0000000	4.7500000	-50.0000000
46400.0000000	5.0000000	-50.0000000
46400.0000000	5.2500000	-50.0000000
46400.0000000	5.5000000	-50.0000000
46400.0000000	5.7500000	-50.0000000
46400.0000000	6.0000000	-50.0000000
46400.0000000	6.2500000	-50.0000000
46400.0000000	6.5000000	-50.0000000
46400.0000000	6.7500000	-50.0000000
46400.0000000	7.0000000	-50.0000000
46400.0000000	7.2500000	-50.0000000
46400.0000000	7.5000000	-50.0000000
46400.0000000	7.7500000	-50.0000000
46400.0000000	8.0000000	-50.0000000
46400.0000000	8.2500000	-50.0000000
46400.0000000	8.5000000	-50.0000000
46400.0000000	8.7500000	-50.0000000
46400.0000000	9.0000000	-50.0000000
46400.0000000	9.2500000	-50.0000000
46400.0000000	9.5000000	-50.0000000
46400.0000000	9.7500000	-50.0000000
46400.0000000	10.0000000	-50.0000000
46400.0000000	10.2500000	-50.0000000
46400.0000000	10.5000000	-50.0000000
46400.0000000	10.7500000	-50.0000000
46400.0000000	11.0000000	-50.0000000
46400.0000000	11.2500000	-50.0000000
46400.0000000	11.5000000	-50.0000000
46400.0000000	11.7500000	-50.0000000
46400.0000000	12.0000000	-50.0000000
46400.0000000	12.2500000	-50.0000000
46400.0000000	12.5000000	-50.0000000
46400.0000000	12.7500000	-50.0000000
46400.0000000	13.0000000	-50.0000000
46400.0000000	13.2500000	-50.0000000
46400.0000000	13.5000000	-50.0000000
46400.0000000	13.7500000	-50.0000000
46400.0000000	14.0000000	-50.0000000
46400.0000000	14.2500000	-50.0000000
46400.0000000	14.5000000	-50.0000000
46400.0000000	14.7500000	-50.0000000
46400.0000000	15.0000000	-50.0000000
46430.0000000	0.0000000	-50.0000000
46430.0000000	0.2500000	-50.0000000
46430.0000000	0.5000000	-50.0000000

46430.0000000	0.7500000	-50.0000000
46430.0000000	1.0000000	-50.0000000
46430.0000000	1.2500000	-50.0000000
46430.0000000	1.5000000	-50.0000000
46430.0000000	1.7500000	-50.0000000
46430.0000000	2.0000000	-50.0000000
46430.0000000	2.2500000	-50.0000000
46430.0000000	2.5000000	-50.0000000
46430.0000000	2.7500000	-50.0000000
46430.0000000	3.0000000	-50.0000000
46430.0000000	3.2500000	-50.0000000
46430.0000000	3.5000000	-50.0000000
46430.0000000	3.7500000	-50.0000000
46430.0000000	4.0000000	-50.0000000
46430.0000000	4.2500000	-50.0000000
46430.0000000	4.5000000	-50.0000000
46430.0000000	4.7500000	-50.0000000
46430.0000000	5.0000000	-50.0000000
46430.0000000	5.2500000	-50.0000000
46430.0000000	5.5000000	-50.0000000
46430.0000000	5.7500000	-50.0000000
46430.0000000	6.0000000	-50.0000000
46430.0000000	6.2500000	-50.0000000
46430.0000000	6.5000000	-50.0000000
46430.0000000	6.7500000	-50.0000000
46430.0000000	7.0000000	-50.0000000
46430.0000000	7.2500000	-50.0000000
46430.0000000	7.5000000	-50.0000000
46430.0000000	7.7500000	-50.0000000
46430.0000000	8.0000000	-50.0000000
46430.0000000	8.2500000	-50.0000000
46430.0000000	8.5000000	-50.0000000
46430.0000000	8.7500000	-50.0000000
46430.0000000	9.0000000	-50.0000000
46430.0000000	9.2500000	-50.0000000
46430.0000000	9.5000000	-50.0000000
46430.0000000	9.7500000	-50.0000000
46430.0000000	10.0000000	-50.0000000
46430.0000000	10.2500000	-50.0000000
46430.0000000	10.5000000	-50.0000000
46430.0000000	10.7500000	-50.0000000
46430.0000000	11.0000000	-50.0000000
46430.0000000	11.2500000	-50.0000000
46430.0000000	11.5000000	-50.0000000
46430.0000000	11.7500000	-50.0000000
46430.0000000	12.0000000	-50.0000000
46430.0000000	12.2500000	-50.0000000
46430.0000000	12.5000000	-50.0000000
46430.0000000	12.7500000	-50.0000000
46430.0000000	13.0000000	-50.0000000
46430.0000000	13.2500000	-50.0000000
46430.0000000	13.5000000	-50.0000000
46430.0000000	13.7500000	-50.0000000
46430.0000000	14.0000000	-50.0000000
46430.0000000	14.2500000	-50.0000000
46430.0000000	14.5000000	-50.0000000
46430.0000000	14.7500000	-50.0000000
46430.0000000	15.0000000	-50.0000000

46460.0000000	0.0000000	-50.0000000
46460.0000000	0.2500000	-50.0000000
46460.0000000	0.5000000	-50.0000000
46460.0000000	0.7500000	-50.0000000
46460.0000000	1.0000000	-50.0000000
46460.0000000	1.2500000	-50.0000000
46460.0000000	1.5000000	-50.0000000
46460.0000000	1.7500000	-50.0000000
46460.0000000	2.0000000	-50.0000000
46460.0000000	2.2500000	-50.0000000
46460.0000000	2.5000000	-50.0000000
46460.0000000	2.7500000	-50.0000000
46460.0000000	3.0000000	-50.0000000
46460.0000000	3.2500000	-50.0000000
46460.0000000	3.5000000	-50.0000000
46460.0000000	3.7500000	-50.0000000
46460.0000000	4.0000000	-50.0000000
46460.0000000	4.2500000	-50.0000000
46460.0000000	4.5000000	-50.0000000
46460.0000000	4.7500000	-50.0000000
46460.0000000	5.0000000	-50.0000000
46460.0000000	5.2500000	-50.0000000
46460.0000000	5.5000000	-50.0000000
46460.0000000	5.7500000	-50.0000000
46460.0000000	6.0000000	-50.0000000
46460.0000000	6.2500000	-50.0000000
46460.0000000	6.5000000	-50.0000000
46460.0000000	6.7500000	-50.0000000
46460.0000000	7.0000000	-50.0000000
46460.0000000	7.2500000	-50.0000000
46460.0000000	7.5000000	-50.0000000
46460.0000000	7.7500000	-50.0000000
46460.0000000	8.0000000	-50.0000000
46460.0000000	8.2500000	-50.0000000
46460.0000000	8.5000000	-50.0000000
46460.0000000	8.7500000	-50.0000000
46460.0000000	9.0000000	-50.0000000
46460.0000000	9.2500000	-50.0000000
46460.0000000	9.5000000	-50.0000000
46460.0000000	9.7500000	-50.0000000
46460.0000000	10.0000000	-50.0000000
46460.0000000	10.2500000	-50.0000000
46460.0000000	10.5000000	-50.0000000
46460.0000000	10.7500000	-50.0000000
46460.0000000	11.0000000	-50.0000000
46460.0000000	11.2500000	-50.0000000
46460.0000000	11.5000000	-50.0000000
46460.0000000	11.7500000	-50.0000000
46460.0000000	12.0000000	-50.0000000
46460.0000000	12.2500000	-50.0000000
46460.0000000	12.5000000	-50.0000000
46460.0000000	12.7500000	-50.0000000
46460.0000000	13.0000000	-50.0000000
46460.0000000	13.2500000	-50.0000000
46460.0000000	13.5000000	-50.0000000
46460.0000000	13.7500000	-50.0000000
46460.0000000	14.0000000	-50.0000000
46460.0000000	14.2500000	-50.0000000

46460.0000000	14.5000000	-50.0000000
46460.0000000	14.7500000	-50.0000000
46460.0000000	15.0000000	-50.0000000
46490.0000000	0.0000000	-50.0000000
46490.0000000	0.2500000	-50.0000000
46490.0000000	0.5000000	-50.0000000
46490.0000000	0.7500000	-50.0000000
46490.0000000	1.0000000	-50.0000000
46490.0000000	1.2500000	-50.0000000
46490.0000000	1.5000000	-50.0000000
46490.0000000	1.7500000	-50.0000000
46490.0000000	2.0000000	-50.0000000
46490.0000000	2.2500000	-50.0000000
46490.0000000	2.5000000	-50.0000000
46490.0000000	2.7500000	-50.0000000
46490.0000000	3.0000000	-50.0000000
46490.0000000	3.2500000	-50.0000000
46490.0000000	3.5000000	-50.0000000
46490.0000000	3.7500000	-50.0000000
46490.0000000	4.0000000	-50.0000000
46490.0000000	4.2500000	-50.0000000
46490.0000000	4.5000000	-50.0000000
46490.0000000	4.7500000	-50.0000000
46490.0000000	5.0000000	-50.0000000
46490.0000000	5.2500000	-50.0000000
46490.0000000	5.5000000	-50.0000000
46490.0000000	5.7500000	-50.0000000
46490.0000000	6.0000000	-50.0000000
46490.0000000	6.2500000	-50.0000000
46490.0000000	6.5000000	-50.0000000
46490.0000000	6.7500000	-50.0000000
46490.0000000	7.0000000	-50.0000000
46490.0000000	7.2500000	-50.0000000
46490.0000000	7.5000000	-50.0000000
46490.0000000	7.7500000	-50.0000000
46490.0000000	8.0000000	-50.0000000
46490.0000000	8.2500000	-50.0000000
46490.0000000	8.5000000	-50.0000000
46490.0000000	8.7500000	-50.0000000
46490.0000000	9.0000000	-50.0000000
46490.0000000	9.2500000	-50.0000000
46490.0000000	9.5000000	-50.0000000
46490.0000000	9.7500000	-50.0000000
46490.0000000	10.0000000	-50.0000000
46490.0000000	10.2500000	-50.0000000
46490.0000000	10.5000000	-50.0000000
46490.0000000	10.7500000	-50.0000000
46490.0000000	11.0000000	-50.0000000
46490.0000000	11.2500000	-50.0000000
46490.0000000	11.5000000	-50.0000000
46490.0000000	11.7500000	-50.0000000
46490.0000000	12.0000000	-50.0000000
46490.0000000	12.2500000	-50.0000000
46490.0000000	12.5000000	-50.0000000
46490.0000000	12.7500000	-50.0000000
46490.0000000	13.0000000	-50.0000000
46490.0000000	13.2500000	-50.0000000
46490.0000000	13.5000000	-50.0000000

46490.0000000	13.7500000	-50.0000000
46490.0000000	14.0000000	-50.0000000
46490.0000000	14.2500000	-50.0000000
46490.0000000	14.5000000	-50.0000000
46490.0000000	14.7500000	-50.0000000
46490.0000000	15.0000000	-50.0000000
46520.0000000	0.0000000	-50.0000000
46520.0000000	0.2500000	-50.0000000
46520.0000000	0.5000000	-50.0000000
46520.0000000	0.7500000	-50.0000000
46520.0000000	1.0000000	-50.0000000
46520.0000000	1.2500000	-50.0000000
46520.0000000	1.5000000	-50.0000000
46520.0000000	1.7500000	-50.0000000
46520.0000000	2.0000000	-50.0000000
46520.0000000	2.2500000	-50.0000000
46520.0000000	2.5000000	-50.0000000
46520.0000000	2.7500000	-50.0000000
46520.0000000	3.0000000	-50.0000000
46520.0000000	3.2500000	-50.0000000
46520.0000000	3.5000000	-50.0000000
46520.0000000	3.7500000	-50.0000000
46520.0000000	4.0000000	-50.0000000
46520.0000000	4.2500000	-50.0000000
46520.0000000	4.5000000	-50.0000000
46520.0000000	4.7500000	-50.0000000
46520.0000000	5.0000000	-50.0000000
46520.0000000	5.2500000	-50.0000000
46520.0000000	5.5000000	-50.0000000
46520.0000000	5.7500000	-50.0000000
46520.0000000	6.0000000	-50.0000000
46520.0000000	6.2500000	-50.0000000
46520.0000000	6.5000000	-50.0000000
46520.0000000	6.7500000	-50.0000000
46520.0000000	7.0000000	-50.0000000
46520.0000000	7.2500000	-50.0000000
46520.0000000	7.5000000	-50.0000000
46520.0000000	7.7500000	-50.0000000
46520.0000000	8.0000000	-50.0000000
46520.0000000	8.2500000	-50.0000000
46520.0000000	8.5000000	-50.0000000
46520.0000000	8.7500000	-50.0000000
46520.0000000	9.0000000	-50.0000000
46520.0000000	9.2500000	-50.0000000
46520.0000000	9.5000000	-50.0000000
46520.0000000	9.7500000	-50.0000000
46520.0000000	10.0000000	-50.0000000
46520.0000000	10.2500000	-50.0000000
46520.0000000	10.5000000	-50.0000000
46520.0000000	10.7500000	-50.0000000
46520.0000000	11.0000000	-50.0000000
46520.0000000	11.2500000	-50.0000000
46520.0000000	11.5000000	-50.0000000
46520.0000000	11.7500000	-50.0000000
46520.0000000	12.0000000	-50.0000000
46520.0000000	12.2500000	-50.0000000
46520.0000000	12.5000000	-50.0000000
46520.0000000	12.7500000	-50.0000000

46520.0000000	13.0000000	-50.0000000
46520.0000000	13.2500000	-50.0000000
46520.0000000	13.5000000	-50.0000000
46520.0000000	13.7500000	-50.0000000
46520.0000000	14.0000000	-50.0000000
46520.0000000	14.2500000	-50.0000000
46520.0000000	14.5000000	-50.0000000
46520.0000000	14.7500000	-50.0000000
46520.0000000	15.0000000	-50.0000000
46550.0000000	0.0000000	-50.0000000
46550.0000000	0.2500000	-50.0000000
46550.0000000	0.5000000	-50.0000000
46550.0000000	0.7500000	-50.0000000
46550.0000000	1.0000000	-50.0000000
46550.0000000	1.2500000	-50.0000000
46550.0000000	1.5000000	-50.0000000
46550.0000000	1.7500000	-50.0000000
46550.0000000	2.0000000	-50.0000000
46550.0000000	2.2500000	-50.0000000
46550.0000000	2.5000000	-50.0000000
46550.0000000	2.7500000	-50.0000000
46550.0000000	3.0000000	-50.0000000
46550.0000000	3.2500000	-50.0000000
46550.0000000	3.5000000	-50.0000000
46550.0000000	3.7500000	-50.0000000
46550.0000000	4.0000000	-50.0000000
46550.0000000	4.2500000	-50.0000000
46550.0000000	4.5000000	-50.0000000
46550.0000000	4.7500000	-50.0000000
46550.0000000	5.0000000	-50.0000000
46550.0000000	5.2500000	-50.0000000
46550.0000000	5.5000000	-50.0000000
46550.0000000	5.7500000	-50.0000000
46550.0000000	6.0000000	-50.0000000
46550.0000000	6.2500000	-50.0000000
46550.0000000	6.5000000	-50.0000000
46550.0000000	6.7500000	-50.0000000
46550.0000000	7.0000000	-50.0000000
46550.0000000	7.2500000	-50.0000000
46550.0000000	7.5000000	-50.0000000
46550.0000000	7.7500000	-50.0000000
46550.0000000	8.0000000	-50.0000000
46550.0000000	8.2500000	-50.0000000
46550.0000000	8.5000000	-50.0000000
46550.0000000	8.7500000	-50.0000000
46550.0000000	9.0000000	-50.0000000
46550.0000000	9.2500000	-50.0000000
46550.0000000	9.5000000	-50.0000000
46550.0000000	9.7500000	-50.0000000
46550.0000000	10.0000000	-50.0000000
46550.0000000	10.2500000	-50.0000000
46550.0000000	10.5000000	-50.0000000
46550.0000000	10.7500000	-50.0000000
46550.0000000	11.0000000	-50.0000000
46550.0000000	11.2500000	-50.0000000
46550.0000000	11.5000000	-50.0000000
46550.0000000	11.7500000	-50.0000000
46550.0000000	12.0000000	-50.0000000

46550.0000000	12.2500000	-50.0000000
46550.0000000	12.5000000	-50.0000000
46550.0000000	12.7500000	-50.0000000
46550.0000000	13.0000000	-50.0000000
46550.0000000	13.2500000	-50.0000000
46550.0000000	13.5000000	-50.0000000
46550.0000000	13.7500000	-50.0000000
46550.0000000	14.0000000	-50.0000000
46550.0000000	14.2500000	-50.0000000
46550.0000000	14.5000000	-50.0000000
46550.0000000	14.7500000	-50.0000000
46550.0000000	15.0000000	-50.0000000
46580.0000000	0.0000000	-50.0000000
46580.0000000	0.2500000	-50.0000000
46580.0000000	0.5000000	-50.0000000
46580.0000000	0.7500000	-50.0000000
46580.0000000	1.0000000	-50.0000000
46580.0000000	1.2500000	-50.0000000
46580.0000000	1.5000000	-50.0000000
46580.0000000	1.7500000	-50.0000000
46580.0000000	2.0000000	-50.0000000
46580.0000000	2.2500000	-50.0000000
46580.0000000	2.5000000	-50.0000000
46580.0000000	2.7500000	-50.0000000
46580.0000000	3.0000000	-50.0000000
46580.0000000	3.2500000	-50.0000000
46580.0000000	3.5000000	-50.0000000
46580.0000000	3.7500000	-50.0000000
46580.0000000	4.0000000	-50.0000000
46580.0000000	4.2500000	-50.0000000
46580.0000000	4.5000000	-50.0000000
46580.0000000	4.7500000	-50.0000000
46580.0000000	5.0000000	-50.0000000
46580.0000000	5.2500000	-50.0000000
46580.0000000	5.5000000	-50.0000000
46580.0000000	5.7500000	-50.0000000
46580.0000000	6.0000000	-50.0000000
46580.0000000	6.2500000	-50.0000000
46580.0000000	6.5000000	-50.0000000
46580.0000000	6.7500000	-50.0000000
46580.0000000	7.0000000	-50.0000000
46580.0000000	7.2500000	-50.0000000
46580.0000000	7.5000000	-50.0000000
46580.0000000	7.7500000	-50.0000000
46580.0000000	8.0000000	-50.0000000
46580.0000000	8.2500000	-50.0000000
46580.0000000	8.5000000	-50.0000000
46580.0000000	8.7500000	-50.0000000
46580.0000000	9.0000000	-50.0000000
46580.0000000	9.2500000	-50.0000000
46580.0000000	9.5000000	-50.0000000
46580.0000000	9.7500000	-50.0000000
46580.0000000	10.0000000	-50.0000000
46580.0000000	10.2500000	-50.0000000
46580.0000000	10.5000000	-50.0000000
46580.0000000	10.7500000	-50.0000000
46580.0000000	11.0000000	-50.0000000
46580.0000000	11.2500000	-50.0000000

46580.0000000	11.5000000	-50.0000000
46580.0000000	11.7500000	-50.0000000
46580.0000000	12.0000000	-50.0000000
46580.0000000	12.2500000	-50.0000000
46580.0000000	12.5000000	-50.0000000
46580.0000000	12.7500000	-50.0000000
46580.0000000	13.0000000	-50.0000000
46580.0000000	13.2500000	-50.0000000
46580.0000000	13.5000000	-50.0000000
46580.0000000	13.7500000	-50.0000000
46580.0000000	14.0000000	-50.0000000
46580.0000000	14.2500000	-50.0000000
46580.0000000	14.5000000	-50.0000000
46580.0000000	14.7500000	-50.0000000
46580.0000000	15.0000000	-50.0000000
46610.0000000	0.0000000	-50.0000000
46610.0000000	0.2500000	-50.0000000
46610.0000000	0.5000000	-50.0000000
46610.0000000	0.7500000	-50.0000000
46610.0000000	1.0000000	-50.0000000
46610.0000000	1.2500000	-50.0000000
46610.0000000	1.5000000	-50.0000000
46610.0000000	1.7500000	-50.0000000
46610.0000000	2.0000000	-50.0000000
46610.0000000	2.2500000	-50.0000000
46610.0000000	2.5000000	-50.0000000
46610.0000000	2.7500000	-50.0000000
46610.0000000	3.0000000	-50.0000000
46610.0000000	3.2500000	-50.0000000
46610.0000000	3.5000000	-50.0000000
46610.0000000	3.7500000	-50.0000000
46610.0000000	4.0000000	-50.0000000
46610.0000000	4.2500000	-50.0000000
46610.0000000	4.5000000	-50.0000000
46610.0000000	4.7500000	-50.0000000
46610.0000000	5.0000000	-50.0000000
46610.0000000	5.2500000	-50.0000000
46610.0000000	5.5000000	-50.0000000
46610.0000000	5.7500000	-50.0000000
46610.0000000	6.0000000	-50.0000000
46610.0000000	6.2500000	-50.0000000
46610.0000000	6.5000000	-50.0000000
46610.0000000	6.7500000	-50.0000000
46610.0000000	7.0000000	-50.0000000
46610.0000000	7.2500000	-50.0000000
46610.0000000	7.5000000	-50.0000000
46610.0000000	7.7500000	-50.0000000
46610.0000000	8.0000000	-50.0000000
46610.0000000	8.2500000	-50.0000000
46610.0000000	8.5000000	-50.0000000
46610.0000000	8.7500000	-50.0000000
46610.0000000	9.0000000	-50.0000000
46610.0000000	9.2500000	-50.0000000
46610.0000000	9.5000000	-50.0000000
46610.0000000	9.7500000	-50.0000000
46610.0000000	10.0000000	-50.0000000
46610.0000000	10.2500000	-50.0000000
46610.0000000	10.5000000	-50.0000000

46610.0000000	10.7500000	-50.0000000
46610.0000000	11.0000000	-50.0000000
46610.0000000	11.2500000	-50.0000000
46610.0000000	11.5000000	-50.0000000
46610.0000000	11.7500000	-50.0000000
46610.0000000	12.0000000	-50.0000000
46610.0000000	12.2500000	-50.0000000
46610.0000000	12.5000000	-50.0000000
46610.0000000	12.7500000	-50.0000000
46610.0000000	13.0000000	-50.0000000
46610.0000000	13.2500000	-50.0000000
46610.0000000	13.5000000	-50.0000000
46610.0000000	13.7500000	-50.0000000
46610.0000000	14.0000000	-50.0000000
46610.0000000	14.2500000	-50.0000000
46610.0000000	14.5000000	-50.0000000
46610.0000000	14.7500000	-50.0000000
46610.0000000	15.0000000	-50.0000000
46640.0000000	0.0000000	-50.0000000
46640.0000000	0.2500000	-50.0000000
46640.0000000	0.5000000	-50.0000000
46640.0000000	0.7500000	-50.0000000
46640.0000000	1.0000000	-50.0000000
46640.0000000	1.2500000	-50.0000000
46640.0000000	1.5000000	-50.0000000
46640.0000000	1.7500000	-50.0000000
46640.0000000	2.0000000	-50.0000000
46640.0000000	2.2500000	-50.0000000
46640.0000000	2.5000000	-50.0000000
46640.0000000	2.7500000	-50.0000000
46640.0000000	3.0000000	-50.0000000
46640.0000000	3.2500000	-50.0000000
46640.0000000	3.5000000	-50.0000000
46640.0000000	3.7500000	-50.0000000
46640.0000000	4.0000000	-50.0000000
46640.0000000	4.2500000	-50.0000000
46640.0000000	4.5000000	-50.0000000
46640.0000000	4.7500000	-50.0000000
46640.0000000	5.0000000	-50.0000000
46640.0000000	5.2500000	-50.0000000
46640.0000000	5.5000000	-50.0000000
46640.0000000	5.7500000	-50.0000000
46640.0000000	6.0000000	-50.0000000
46640.0000000	6.2500000	-50.0000000
46640.0000000	6.5000000	-50.0000000
46640.0000000	6.7500000	-50.0000000
46640.0000000	7.0000000	-50.0000000
46640.0000000	7.2500000	-50.0000000
46640.0000000	7.5000000	-50.0000000
46640.0000000	7.7500000	-50.0000000
46640.0000000	8.0000000	-50.0000000
46640.0000000	8.2500000	-50.0000000
46640.0000000	8.5000000	-50.0000000
46640.0000000	8.7500000	-50.0000000
46640.0000000	9.0000000	-50.0000000
46640.0000000	9.2500000	-50.0000000
46640.0000000	9.5000000	-50.0000000
46640.0000000	9.7500000	-50.0000000

46640.0000000	10.0000000	-50.0000000
46640.0000000	10.2500000	-50.0000000
46640.0000000	10.5000000	-50.0000000
46640.0000000	10.7500000	-50.0000000
46640.0000000	11.0000000	-50.0000000
46640.0000000	11.2500000	-50.0000000
46640.0000000	11.5000000	-50.0000000
46640.0000000	11.7500000	-50.0000000
46640.0000000	12.0000000	-50.0000000
46640.0000000	12.2500000	-50.0000000
46640.0000000	12.5000000	-50.0000000
46640.0000000	12.7500000	-50.0000000
46640.0000000	13.0000000	-50.0000000
46640.0000000	13.2500000	-50.0000000
46640.0000000	13.5000000	-50.0000000
46640.0000000	13.7500000	-50.0000000
46640.0000000	14.0000000	-50.0000000
46640.0000000	14.2500000	-50.0000000
46640.0000000	14.5000000	-50.0000000
46640.0000000	14.7500000	-50.0000000
46640.0000000	15.0000000	-50.0000000
46670.0000000	0.0000000	-50.0000000
46670.0000000	0.2500000	-50.0000000
46670.0000000	0.5000000	-50.0000000
46670.0000000	0.7500000	-50.0000000
46670.0000000	1.0000000	-50.0000000
46670.0000000	1.2500000	-50.0000000
46670.0000000	1.5000000	-50.0000000
46670.0000000	1.7500000	-50.0000000
46670.0000000	2.0000000	-50.0000000
46670.0000000	2.2500000	-50.0000000
46670.0000000	2.5000000	-50.0000000
46670.0000000	2.7500000	-50.0000000
46670.0000000	3.0000000	-50.0000000
46670.0000000	3.2500000	-50.0000000
46670.0000000	3.5000000	-50.0000000
46670.0000000	3.7500000	-50.0000000
46670.0000000	4.0000000	-50.0000000
46670.0000000	4.2500000	-50.0000000
46670.0000000	4.5000000	-50.0000000
46670.0000000	4.7500000	-50.0000000
46670.0000000	5.0000000	-50.0000000
46670.0000000	5.2500000	-50.0000000
46670.0000000	5.5000000	-50.0000000
46670.0000000	5.7500000	-50.0000000
46670.0000000	6.0000000	-50.0000000
46670.0000000	6.2500000	-50.0000000
46670.0000000	6.5000000	-50.0000000
46670.0000000	6.7500000	-50.0000000
46670.0000000	7.0000000	-50.0000000
46670.0000000	7.2500000	-50.0000000
46670.0000000	7.5000000	-50.0000000
46670.0000000	7.7500000	-50.0000000
46670.0000000	8.0000000	-50.0000000
46670.0000000	8.2500000	-50.0000000
46670.0000000	8.5000000	-50.0000000
46670.0000000	8.7500000	-50.0000000
46670.0000000	9.0000000	-50.0000000

46670.0000000	9.2500000	-50.0000000
46670.0000000	9.5000000	-50.0000000
46670.0000000	9.7500000	-50.0000000
46670.0000000	10.0000000	-50.0000000
46670.0000000	10.2500000	-50.0000000
46670.0000000	10.5000000	-50.0000000
46670.0000000	10.7500000	-50.0000000
46670.0000000	11.0000000	-50.0000000
46670.0000000	11.2500000	-50.0000000
46670.0000000	11.5000000	-50.0000000
46670.0000000	11.7500000	-50.0000000
46670.0000000	12.0000000	-50.0000000
46670.0000000	12.2500000	-50.0000000
46670.0000000	12.5000000	-50.0000000
46670.0000000	12.7500000	-50.0000000
46670.0000000	13.0000000	-50.0000000
46670.0000000	13.2500000	-50.0000000
46670.0000000	13.5000000	-50.0000000
46670.0000000	13.7500000	-50.0000000
46670.0000000	14.0000000	-50.0000000
46670.0000000	14.2500000	-50.0000000
46670.0000000	14.5000000	-50.0000000
46670.0000000	14.7500000	-50.0000000
46670.0000000	15.0000000	-50.0000000
46700.0000000	0.0000000	-50.0000000
46700.0000000	0.2500000	-50.0000000
46700.0000000	0.5000000	-50.0000000
46700.0000000	0.7500000	-50.0000000
46700.0000000	1.0000000	-50.0000000
46700.0000000	1.2500000	-50.0000000
46700.0000000	1.5000000	-50.0000000
46700.0000000	1.7500000	-50.0000000
46700.0000000	2.0000000	-50.0000000
46700.0000000	2.2500000	-50.0000000
46700.0000000	2.5000000	-50.0000000
46700.0000000	2.7500000	-50.0000000
46700.0000000	3.0000000	-50.0000000
46700.0000000	3.2500000	-50.0000000
46700.0000000	3.5000000	-50.0000000
46700.0000000	3.7500000	-50.0000000
46700.0000000	4.0000000	-50.0000000
46700.0000000	4.2500000	-50.0000000
46700.0000000	4.5000000	-50.0000000
46700.0000000	4.7500000	-50.0000000
46700.0000000	5.0000000	-50.0000000
46700.0000000	5.2500000	-50.0000000
46700.0000000	5.5000000	-50.0000000
46700.0000000	5.7500000	-50.0000000
46700.0000000	6.0000000	-50.0000000
46700.0000000	6.2500000	-50.0000000
46700.0000000	6.5000000	-50.0000000
46700.0000000	6.7500000	-50.0000000
46700.0000000	7.0000000	-50.0000000
46700.0000000	7.2500000	-50.0000000
46700.0000000	7.5000000	-50.0000000
46700.0000000	7.7500000	-50.0000000
46700.0000000	8.0000000	-50.0000000
46700.0000000	8.2500000	-50.0000000

46700.0000000	8.5000000	-50.0000000
46700.0000000	8.7500000	-50.0000000
46700.0000000	9.0000000	-50.0000000
46700.0000000	9.2500000	-50.0000000
46700.0000000	9.5000000	-50.0000000
46700.0000000	9.7500000	-50.0000000
46700.0000000	10.0000000	-50.0000000
46700.0000000	10.2500000	-50.0000000
46700.0000000	10.5000000	-50.0000000
46700.0000000	10.7500000	-50.0000000
46700.0000000	11.0000000	-50.0000000
46700.0000000	11.2500000	-50.0000000
46700.0000000	11.5000000	-50.0000000
46700.0000000	11.7500000	-50.0000000
46700.0000000	12.0000000	-50.0000000
46700.0000000	12.2500000	-50.0000000
46700.0000000	12.5000000	-50.0000000
46700.0000000	12.7500000	-50.0000000
46700.0000000	13.0000000	-50.0000000
46700.0000000	13.2500000	-50.0000000
46700.0000000	13.5000000	-50.0000000
46700.0000000	13.7500000	-50.0000000
46700.0000000	14.0000000	-50.0000000
46700.0000000	14.2500000	-50.0000000
46700.0000000	14.5000000	-50.0000000
46700.0000000	14.7500000	-50.0000000
46700.0000000	15.0000000	-50.0000000
46730.0000000	0.0000000	-50.0000000
46730.0000000	0.2500000	-50.0000000
46730.0000000	0.5000000	-50.0000000
46730.0000000	0.7500000	-50.0000000
46730.0000000	1.0000000	-50.0000000
46730.0000000	1.2500000	-50.0000000
46730.0000000	1.5000000	-50.0000000
46730.0000000	1.7500000	-50.0000000
46730.0000000	2.0000000	-50.0000000
46730.0000000	2.2500000	-50.0000000
46730.0000000	2.5000000	-50.0000000
46730.0000000	2.7500000	-50.0000000
46730.0000000	3.0000000	-50.0000000
46730.0000000	3.2500000	-50.0000000
46730.0000000	3.5000000	-50.0000000
46730.0000000	3.7500000	-50.0000000
46730.0000000	4.0000000	-50.0000000
46730.0000000	4.2500000	-50.0000000
46730.0000000	4.5000000	-50.0000000
46730.0000000	4.7500000	-50.0000000
46730.0000000	5.0000000	-50.0000000
46730.0000000	5.2500000	-50.0000000
46730.0000000	5.5000000	-50.0000000
46730.0000000	5.7500000	-50.0000000
46730.0000000	6.0000000	-50.0000000
46730.0000000	6.2500000	-50.0000000
46730.0000000	6.5000000	-50.0000000
46730.0000000	6.7500000	-50.0000000
46730.0000000	7.0000000	-50.0000000
46730.0000000	7.2500000	-50.0000000
46730.0000000	7.5000000	-50.0000000

46730.0000000	7.7500000	-50.0000000
46730.0000000	8.0000000	-50.0000000
46730.0000000	8.2500000	-50.0000000
46730.0000000	8.5000000	-50.0000000
46730.0000000	8.7500000	-50.0000000
46730.0000000	9.0000000	-50.0000000
46730.0000000	9.2500000	-50.0000000
46730.0000000	9.5000000	-50.0000000
46730.0000000	9.7500000	-50.0000000
46730.0000000	10.0000000	-50.0000000
46730.0000000	10.2500000	-50.0000000
46730.0000000	10.5000000	-50.0000000
46730.0000000	10.7500000	-50.0000000
46730.0000000	11.0000000	-50.0000000
46730.0000000	11.2500000	-50.0000000
46730.0000000	11.5000000	-50.0000000
46730.0000000	11.7500000	-50.0000000
46730.0000000	12.0000000	-50.0000000
46730.0000000	12.2500000	-50.0000000
46730.0000000	12.5000000	-50.0000000
46730.0000000	12.7500000	-50.0000000
46730.0000000	13.0000000	-50.0000000
46730.0000000	13.2500000	-50.0000000
46730.0000000	13.5000000	-50.0000000
46730.0000000	13.7500000	-50.0000000
46730.0000000	14.0000000	-50.0000000
46730.0000000	14.2500000	-50.0000000
46730.0000000	14.5000000	-50.0000000
46730.0000000	14.7500000	-50.0000000
46730.0000000	15.0000000	-50.0000000
46760.0000000	0.0000000	-50.0000000
46760.0000000	0.2500000	-50.0000000
46760.0000000	0.5000000	-50.0000000
46760.0000000	0.7500000	-50.0000000
46760.0000000	1.0000000	-50.0000000
46760.0000000	1.2500000	-50.0000000
46760.0000000	1.5000000	-50.0000000
46760.0000000	1.7500000	-50.0000000
46760.0000000	2.0000000	-50.0000000
46760.0000000	2.2500000	-50.0000000
46760.0000000	2.5000000	-50.0000000
46760.0000000	2.7500000	-50.0000000
46760.0000000	3.0000000	-50.0000000
46760.0000000	3.2500000	-50.0000000
46760.0000000	3.5000000	-50.0000000
46760.0000000	3.7500000	-50.0000000
46760.0000000	4.0000000	-50.0000000
46760.0000000	4.2500000	-50.0000000
46760.0000000	4.5000000	-50.0000000
46760.0000000	4.7500000	-50.0000000
46760.0000000	5.0000000	-50.0000000
46760.0000000	5.2500000	-50.0000000
46760.0000000	5.5000000	-50.0000000
46760.0000000	5.7500000	-50.0000000
46760.0000000	6.0000000	-50.0000000
46760.0000000	6.2500000	-50.0000000
46760.0000000	6.5000000	-50.0000000
46760.0000000	6.7500000	-50.0000000

46760.0000000	7.0000000	-50.0000000
46760.0000000	7.2500000	-50.0000000
46760.0000000	7.5000000	-50.0000000
46760.0000000	7.7500000	-50.0000000
46760.0000000	8.0000000	-50.0000000
46760.0000000	8.2500000	-50.0000000
46760.0000000	8.5000000	-50.0000000
46760.0000000	8.7500000	-50.0000000
46760.0000000	9.0000000	-50.0000000
46760.0000000	9.2500000	-50.0000000
46760.0000000	9.5000000	-50.0000000
46760.0000000	9.7500000	-50.0000000
46760.0000000	10.0000000	-50.0000000
46760.0000000	10.2500000	-50.0000000
46760.0000000	10.5000000	-50.0000000
46760.0000000	10.7500000	-50.0000000
46760.0000000	11.0000000	-50.0000000
46760.0000000	11.2500000	-50.0000000
46760.0000000	11.5000000	-50.0000000
46760.0000000	11.7500000	-50.0000000
46760.0000000	12.0000000	-50.0000000
46760.0000000	12.2500000	-50.0000000
46760.0000000	12.5000000	-50.0000000
46760.0000000	12.7500000	-50.0000000
46760.0000000	13.0000000	-50.0000000
46760.0000000	13.2500000	-50.0000000
46760.0000000	13.5000000	-50.0000000
46760.0000000	13.7500000	-50.0000000
46760.0000000	14.0000000	-50.0000000
46760.0000000	14.2500000	-50.0000000
46760.0000000	14.5000000	-50.0000000
46760.0000000	14.7500000	-50.0000000
46760.0000000	15.0000000	-50.0000000
46790.0000000	0.0000000	-50.0000000
46790.0000000	0.2500000	-50.0000000
46790.0000000	0.5000000	-50.0000000
46790.0000000	0.7500000	-50.0000000
46790.0000000	1.0000000	-50.0000000
46790.0000000	1.2500000	-50.0000000
46790.0000000	1.5000000	-50.0000000
46790.0000000	1.7500000	-50.0000000
46790.0000000	2.0000000	-50.0000000
46790.0000000	2.2500000	-50.0000000
46790.0000000	2.5000000	-50.0000000
46790.0000000	2.7500000	-50.0000000
46790.0000000	3.0000000	-50.0000000
46790.0000000	3.2500000	-50.0000000
46790.0000000	3.5000000	-50.0000000
46790.0000000	3.7500000	-50.0000000
46790.0000000	4.0000000	-50.0000000
46790.0000000	4.2500000	-50.0000000
46790.0000000	4.5000000	-50.0000000
46790.0000000	4.7500000	-50.0000000
46790.0000000	5.0000000	-50.0000000
46790.0000000	5.2500000	-50.0000000
46790.0000000	5.5000000	-50.0000000
46790.0000000	5.7500000	-50.0000000
46790.0000000	6.0000000	-50.0000000

46790.0000000	6.2500000	-50.0000000
46790.0000000	6.5000000	-50.0000000
46790.0000000	6.7500000	-50.0000000
46790.0000000	7.0000000	-50.0000000
46790.0000000	7.2500000	-50.0000000
46790.0000000	7.5000000	-50.0000000
46790.0000000	7.7500000	-50.0000000
46790.0000000	8.0000000	-50.0000000
46790.0000000	8.2500000	-50.0000000
46790.0000000	8.5000000	-50.0000000
46790.0000000	8.7500000	-50.0000000
46790.0000000	9.0000000	-50.0000000
46790.0000000	9.2500000	-50.0000000
46790.0000000	9.5000000	-50.0000000
46790.0000000	9.7500000	-50.0000000
46790.0000000	10.0000000	-50.0000000
46790.0000000	10.2500000	-50.0000000
46790.0000000	10.5000000	-50.0000000
46790.0000000	10.7500000	-50.0000000
46790.0000000	11.0000000	-50.0000000
46790.0000000	11.2500000	-50.0000000
46790.0000000	11.5000000	-50.0000000
46790.0000000	11.7500000	-50.0000000
46790.0000000	12.0000000	-50.0000000
46790.0000000	12.2500000	-50.0000000
46790.0000000	12.5000000	-50.0000000
46790.0000000	12.7500000	-50.0000000
46790.0000000	13.0000000	-50.0000000
46790.0000000	13.2500000	-50.0000000
46790.0000000	13.5000000	-50.0000000
46790.0000000	13.7500000	-50.0000000
46790.0000000	14.0000000	-50.0000000
46790.0000000	14.2500000	-50.0000000
46790.0000000	14.5000000	-50.0000000
46790.0000000	14.7500000	-50.0000000
46790.0000000	15.0000000	-50.0000000
46820.0000000	0.0000000	-50.0000000
46820.0000000	0.2500000	-50.0000000
46820.0000000	0.5000000	-50.0000000
46820.0000000	0.7500000	-50.0000000
46820.0000000	1.0000000	-50.0000000
46820.0000000	1.2500000	-50.0000000
46820.0000000	1.5000000	-50.0000000
46820.0000000	1.7500000	-50.0000000
46820.0000000	2.0000000	-50.0000000
46820.0000000	2.2500000	-50.0000000
46820.0000000	2.5000000	-50.0000000
46820.0000000	2.7500000	-50.0000000
46820.0000000	3.0000000	-50.0000000
46820.0000000	3.2500000	-50.0000000
46820.0000000	3.5000000	-50.0000000
46820.0000000	3.7500000	-50.0000000
46820.0000000	4.0000000	-50.0000000
46820.0000000	4.2500000	-50.0000000
46820.0000000	4.5000000	-50.0000000
46820.0000000	4.7500000	-50.0000000
46820.0000000	5.0000000	-50.0000000
46820.0000000	5.2500000	-50.0000000

46820.0000000	5.5000000	-50.0000000
46820.0000000	5.7500000	-50.0000000
46820.0000000	6.0000000	-50.0000000
46820.0000000	6.2500000	-50.0000000
46820.0000000	6.5000000	-50.0000000
46820.0000000	6.7500000	-50.0000000
46820.0000000	7.0000000	-50.0000000
46820.0000000	7.2500000	-50.0000000
46820.0000000	7.5000000	-50.0000000
46820.0000000	7.7500000	-50.0000000
46820.0000000	8.0000000	-50.0000000
46820.0000000	8.2500000	-50.0000000
46820.0000000	8.5000000	-50.0000000
46820.0000000	8.7500000	-50.0000000
46820.0000000	9.0000000	-50.0000000
46820.0000000	9.2500000	-50.0000000
46820.0000000	9.5000000	-50.0000000
46820.0000000	9.7500000	-50.0000000
46820.0000000	10.0000000	-50.0000000
46820.0000000	10.2500000	-50.0000000
46820.0000000	10.5000000	-50.0000000
46820.0000000	10.7500000	-50.0000000
46820.0000000	11.0000000	-50.0000000
46820.0000000	11.2500000	-50.0000000
46820.0000000	11.5000000	-50.0000000
46820.0000000	11.7500000	-50.0000000
46820.0000000	12.0000000	-50.0000000
46820.0000000	12.2500000	-50.0000000
46820.0000000	12.5000000	-50.0000000
46820.0000000	12.7500000	-50.0000000
46820.0000000	13.0000000	-50.0000000
46820.0000000	13.2500000	-50.0000000
46820.0000000	13.5000000	-50.0000000
46820.0000000	13.7500000	-50.0000000
46820.0000000	14.0000000	-50.0000000
46820.0000000	14.2500000	-50.0000000
46820.0000000	14.5000000	-50.0000000
46820.0000000	14.7500000	-50.0000000
46820.0000000	15.0000000	-50.0000000
46850.0000000	0.0000000	-50.0000000
46850.0000000	0.2500000	-50.0000000
46850.0000000	0.5000000	-50.0000000
46850.0000000	0.7500000	-50.0000000
46850.0000000	1.0000000	-50.0000000
46850.0000000	1.2500000	-50.0000000
46850.0000000	1.5000000	-50.0000000
46850.0000000	1.7500000	-50.0000000
46850.0000000	2.0000000	-50.0000000
46850.0000000	2.2500000	-50.0000000
46850.0000000	2.5000000	-50.0000000
46850.0000000	2.7500000	-50.0000000
46850.0000000	3.0000000	-50.0000000
46850.0000000	3.2500000	-50.0000000
46850.0000000	3.5000000	-50.0000000
46850.0000000	3.7500000	-50.0000000
46850.0000000	4.0000000	-50.0000000
46850.0000000	4.2500000	-50.0000000
46850.0000000	4.5000000	-50.0000000

46850.0000000	4.7500000	-50.0000000
46850.0000000	5.0000000	-50.0000000
46850.0000000	5.2500000	-50.0000000
46850.0000000	5.5000000	-50.0000000
46850.0000000	5.7500000	-50.0000000
46850.0000000	6.0000000	-50.0000000
46850.0000000	6.2500000	-50.0000000
46850.0000000	6.5000000	-50.0000000
46850.0000000	6.7500000	-50.0000000
46850.0000000	7.0000000	-50.0000000
46850.0000000	7.2500000	-50.0000000
46850.0000000	7.5000000	-50.0000000
46850.0000000	7.7500000	-50.0000000
46850.0000000	8.0000000	-50.0000000
46850.0000000	8.2500000	-50.0000000
46850.0000000	8.5000000	-50.0000000
46850.0000000	8.7500000	-50.0000000
46850.0000000	9.0000000	-50.0000000
46850.0000000	9.2500000	-50.0000000
46850.0000000	9.5000000	-50.0000000
46850.0000000	9.7500000	-50.0000000
46850.0000000	10.0000000	-50.0000000
46850.0000000	10.2500000	-50.0000000
46850.0000000	10.5000000	-50.0000000
46850.0000000	10.7500000	-50.0000000
46850.0000000	11.0000000	-50.0000000
46850.0000000	11.2500000	-50.0000000
46850.0000000	11.5000000	-50.0000000
46850.0000000	11.7500000	-50.0000000
46850.0000000	12.0000000	-50.0000000
46850.0000000	12.2500000	-50.0000000
46850.0000000	12.5000000	-50.0000000
46850.0000000	12.7500000	-50.0000000
46850.0000000	13.0000000	-50.0000000
46850.0000000	13.2500000	-50.0000000
46850.0000000	13.5000000	-50.0000000
46850.0000000	13.7500000	-50.0000000
46850.0000000	14.0000000	-50.0000000
46850.0000000	14.2500000	-50.0000000
46850.0000000	14.5000000	-50.0000000
46850.0000000	14.7500000	-50.0000000
46850.0000000	15.0000000	-50.0000000
46880.0000000	0.0000000	-50.0000000
46880.0000000	0.2500000	-50.0000000
46880.0000000	0.5000000	-50.0000000
46880.0000000	0.7500000	-50.0000000
46880.0000000	1.0000000	-50.0000000
46880.0000000	1.2500000	-50.0000000
46880.0000000	1.5000000	-50.0000000
46880.0000000	1.7500000	-50.0000000
46880.0000000	2.0000000	-50.0000000
46880.0000000	2.2500000	-50.0000000
46880.0000000	2.5000000	-50.0000000
46880.0000000	2.7500000	-50.0000000
46880.0000000	3.0000000	-50.0000000
46880.0000000	3.2500000	-50.0000000
46880.0000000	3.5000000	-50.0000000
46880.0000000	3.7500000	-50.0000000

46880.0000000	4.0000000	-50.0000000
46880.0000000	4.2500000	-50.0000000
46880.0000000	4.5000000	-50.0000000
46880.0000000	4.7500000	-50.0000000
46880.0000000	5.0000000	-50.0000000
46880.0000000	5.2500000	-50.0000000
46880.0000000	5.5000000	-50.0000000
46880.0000000	5.7500000	-50.0000000
46880.0000000	6.0000000	-50.0000000
46880.0000000	6.2500000	-50.0000000
46880.0000000	6.5000000	-50.0000000
46880.0000000	6.7500000	-50.0000000
46880.0000000	7.0000000	-50.0000000
46880.0000000	7.2500000	-50.0000000
46880.0000000	7.5000000	-50.0000000
46880.0000000	7.7500000	-50.0000000
46880.0000000	8.0000000	-50.0000000
46880.0000000	8.2500000	-50.0000000
46880.0000000	8.5000000	-50.0000000
46880.0000000	8.7500000	-50.0000000
46880.0000000	9.0000000	-50.0000000
46880.0000000	9.2500000	-50.0000000
46880.0000000	9.5000000	-50.0000000
46880.0000000	9.7500000	-50.0000000
46880.0000000	10.0000000	-50.0000000
46880.0000000	10.2500000	-50.0000000
46880.0000000	10.5000000	-50.0000000
46880.0000000	10.7500000	-50.0000000
46880.0000000	11.0000000	-50.0000000
46880.0000000	11.2500000	-50.0000000
46880.0000000	11.5000000	-50.0000000
46880.0000000	11.7500000	-50.0000000
46880.0000000	12.0000000	-50.0000000
46880.0000000	12.2500000	-50.0000000
46880.0000000	12.5000000	-50.0000000
46880.0000000	12.7500000	-50.0000000
46880.0000000	13.0000000	-50.0000000
46880.0000000	13.2500000	-50.0000000
46880.0000000	13.5000000	-50.0000000
46880.0000000	13.7500000	-50.0000000
46880.0000000	14.0000000	-50.0000000
46880.0000000	14.2500000	-50.0000000
46880.0000000	14.5000000	-50.0000000
46880.0000000	14.7500000	-50.0000000
46880.0000000	15.0000000	-50.0000000
46910.0000000	0.0000000	-50.0000000
46910.0000000	0.2500000	-50.0000000
46910.0000000	0.5000000	-50.0000000
46910.0000000	0.7500000	15.4899998
46910.0000000	1.0000000	-50.0000000
46910.0000000	1.2500000	-50.0000000
46910.0000000	1.5000000	-50.0000000
46910.0000000	1.7500000	-50.0000000
46910.0000000	2.0000000	-50.0000000
46910.0000000	2.2500000	-50.0000000
46910.0000000	2.5000000	-50.0000000
46910.0000000	2.7500000	-50.0000000
46910.0000000	3.0000000	-50.0000000

46910.0000000	3.2500000	-50.0000000
46910.0000000	3.5000000	-50.0000000
46910.0000000	3.7500000	-50.0000000
46910.0000000	4.0000000	-50.0000000
46910.0000000	4.2500000	-50.0000000
46910.0000000	4.5000000	-50.0000000
46910.0000000	4.7500000	-50.0000000
46910.0000000	5.0000000	-50.0000000
46910.0000000	5.2500000	-50.0000000
46910.0000000	5.5000000	-50.0000000
46910.0000000	5.7500000	-50.0000000
46910.0000000	6.0000000	-50.0000000
46910.0000000	6.2500000	-50.0000000
46910.0000000	6.5000000	-50.0000000
46910.0000000	6.7500000	-50.0000000
46910.0000000	7.0000000	-50.0000000
46910.0000000	7.2500000	-50.0000000
46910.0000000	7.5000000	-50.0000000
46910.0000000	7.7500000	-50.0000000
46910.0000000	8.0000000	-50.0000000
46910.0000000	8.2500000	-50.0000000
46910.0000000	8.5000000	-50.0000000
46910.0000000	8.7500000	-50.0000000
46910.0000000	9.0000000	-50.0000000
46910.0000000	9.2500000	-50.0000000
46910.0000000	9.5000000	-50.0000000
46910.0000000	9.7500000	-50.0000000
46910.0000000	10.0000000	-50.0000000
46910.0000000	10.2500000	-50.0000000
46910.0000000	10.5000000	-50.0000000
46910.0000000	10.7500000	-50.0000000
46910.0000000	11.0000000	-50.0000000
46910.0000000	11.2500000	-50.0000000
46910.0000000	11.5000000	-50.0000000
46910.0000000	11.7500000	-50.0000000
46910.0000000	12.0000000	-50.0000000
46910.0000000	12.2500000	-50.0000000
46910.0000000	12.5000000	-50.0000000
46910.0000000	12.7500000	-50.0000000
46910.0000000	13.0000000	-50.0000000
46910.0000000	13.2500000	-50.0000000
46910.0000000	13.5000000	-50.0000000
46910.0000000	13.7500000	-50.0000000
46910.0000000	14.0000000	-50.0000000
46910.0000000	14.2500000	-50.0000000
46910.0000000	14.5000000	-50.0000000
46910.0000000	14.7500000	-50.0000000
46910.0000000	15.0000000	-50.0000000
46940.0000000	0.0000000	-50.0000000
46940.0000000	0.2500000	-50.0000000
46940.0000000	0.5000000	-50.0000000
46940.0000000	0.7500000	-50.0000000
46940.0000000	1.0000000	-50.0000000
46940.0000000	1.2500000	-50.0000000
46940.0000000	1.5000000	-50.0000000
46940.0000000	1.7500000	-50.0000000
46940.0000000	2.0000000	-50.0000000
46940.0000000	2.2500000	-50.0000000

46940.0000000	2.5000000	-50.0000000
46940.0000000	2.7500000	-50.0000000
46940.0000000	3.0000000	-50.0000000
46940.0000000	3.2500000	-50.0000000
46940.0000000	3.5000000	-50.0000000
46940.0000000	3.7500000	-50.0000000
46940.0000000	4.0000000	-50.0000000
46940.0000000	4.2500000	-50.0000000
46940.0000000	4.5000000	-50.0000000
46940.0000000	4.7500000	-50.0000000
46940.0000000	5.0000000	-50.0000000
46940.0000000	5.2500000	-50.0000000
46940.0000000	5.5000000	-50.0000000
46940.0000000	5.7500000	-50.0000000
46940.0000000	6.0000000	-50.0000000
46940.0000000	6.2500000	-50.0000000
46940.0000000	6.5000000	-50.0000000
46940.0000000	6.7500000	-50.0000000
46940.0000000	7.0000000	-50.0000000
46940.0000000	7.2500000	-50.0000000
46940.0000000	7.5000000	-50.0000000
46940.0000000	7.7500000	-50.0000000
46940.0000000	8.0000000	-50.0000000
46940.0000000	8.2500000	-50.0000000
46940.0000000	8.5000000	-50.0000000
46940.0000000	8.7500000	-50.0000000
46940.0000000	9.0000000	-50.0000000
46940.0000000	9.2500000	-50.0000000
46940.0000000	9.5000000	-50.0000000
46940.0000000	9.7500000	-50.0000000
46940.0000000	10.0000000	-50.0000000
46940.0000000	10.2500000	-50.0000000
46940.0000000	10.5000000	-50.0000000
46940.0000000	10.7500000	-50.0000000
46940.0000000	11.0000000	-50.0000000
46940.0000000	11.2500000	-50.0000000
46940.0000000	11.5000000	-50.0000000
46940.0000000	11.7500000	-50.0000000
46940.0000000	12.0000000	-50.0000000
46940.0000000	12.2500000	-50.0000000
46940.0000000	12.5000000	-50.0000000
46940.0000000	12.7500000	-50.0000000
46940.0000000	13.0000000	-50.0000000
46940.0000000	13.2500000	-50.0000000
46940.0000000	13.5000000	-50.0000000
46940.0000000	13.7500000	-50.0000000
46940.0000000	14.0000000	-50.0000000
46940.0000000	14.2500000	-50.0000000
46940.0000000	14.5000000	-50.0000000
46940.0000000	14.7500000	-50.0000000
46940.0000000	15.0000000	-50.0000000
46970.0000000	0.0000000	-50.0000000
46970.0000000	0.2500000	-50.0000000
46970.0000000	0.5000000	-50.0000000
46970.0000000	0.7500000	-50.0000000
46970.0000000	1.0000000	-50.0000000
46970.0000000	1.2500000	-50.0000000
46970.0000000	1.5000000	-50.0000000

46970.0000000	1.7500000	-50.0000000
46970.0000000	2.0000000	-50.0000000
46970.0000000	2.2500000	-50.0000000
46970.0000000	2.5000000	-50.0000000
46970.0000000	2.7500000	-50.0000000
46970.0000000	3.0000000	-50.0000000
46970.0000000	3.2500000	-50.0000000
46970.0000000	3.5000000	-50.0000000
46970.0000000	3.7500000	-50.0000000
46970.0000000	4.0000000	-50.0000000
46970.0000000	4.2500000	-50.0000000
46970.0000000	4.5000000	-50.0000000
46970.0000000	4.7500000	-50.0000000
46970.0000000	5.0000000	-50.0000000
46970.0000000	5.2500000	-50.0000000
46970.0000000	5.5000000	-50.0000000
46970.0000000	5.7500000	-50.0000000
46970.0000000	6.0000000	-50.0000000
46970.0000000	6.2500000	-50.0000000
46970.0000000	6.5000000	-50.0000000
46970.0000000	6.7500000	-50.0000000
46970.0000000	7.0000000	-50.0000000
46970.0000000	7.2500000	-50.0000000
46970.0000000	7.5000000	-50.0000000
46970.0000000	7.7500000	-50.0000000
46970.0000000	8.0000000	-50.0000000
46970.0000000	8.2500000	-50.0000000
46970.0000000	8.5000000	-50.0000000
46970.0000000	8.7500000	-50.0000000
46970.0000000	9.0000000	-50.0000000
46970.0000000	9.2500000	-50.0000000
46970.0000000	9.5000000	-50.0000000
46970.0000000	9.7500000	-50.0000000
46970.0000000	10.0000000	-50.0000000
46970.0000000	10.2500000	-50.0000000
46970.0000000	10.5000000	-50.0000000
46970.0000000	10.7500000	-50.0000000
46970.0000000	11.0000000	-50.0000000
46970.0000000	11.2500000	-50.0000000
46970.0000000	11.5000000	-50.0000000
46970.0000000	11.7500000	-50.0000000
46970.0000000	12.0000000	-50.0000000
46970.0000000	12.2500000	-50.0000000
46970.0000000	12.5000000	-50.0000000
46970.0000000	12.7500000	-50.0000000
46970.0000000	13.0000000	-50.0000000
46970.0000000	13.2500000	-50.0000000
46970.0000000	13.5000000	-50.0000000
46970.0000000	13.7500000	-50.0000000
46970.0000000	14.0000000	-50.0000000
46970.0000000	14.2500000	-50.0000000
46970.0000000	14.5000000	-50.0000000
46970.0000000	14.7500000	-50.0000000
46970.0000000	15.0000000	-50.0000000
47000.0000000	0.0000000	-50.0000000
47000.0000000	0.2500000	-50.0000000
47000.0000000	0.5000000	-50.0000000
47000.0000000	0.7500000	-50.0000000

47000.0000000	1.0000000	-50.0000000
47000.0000000	1.2500000	-50.0000000
47000.0000000	1.5000000	-50.0000000
47000.0000000	1.7500000	-50.0000000
47000.0000000	2.0000000	-50.0000000
47000.0000000	2.2500000	-50.0000000
47000.0000000	2.5000000	-50.0000000
47000.0000000	2.7500000	-50.0000000
47000.0000000	3.0000000	-50.0000000
47000.0000000	3.2500000	-50.0000000
47000.0000000	3.5000000	-50.0000000
47000.0000000	3.7500000	-50.0000000
47000.0000000	4.0000000	-50.0000000
47000.0000000	4.2500000	-50.0000000
47000.0000000	4.5000000	-50.0000000
47000.0000000	4.7500000	-50.0000000
47000.0000000	5.0000000	-50.0000000
47000.0000000	5.2500000	-50.0000000
47000.0000000	5.5000000	-50.0000000
47000.0000000	5.7500000	-50.0000000
47000.0000000	6.0000000	-50.0000000
47000.0000000	6.2500000	-50.0000000
47000.0000000	6.5000000	-50.0000000
47000.0000000	6.7500000	-50.0000000
47000.0000000	7.0000000	-50.0000000
47000.0000000	7.2500000	-50.0000000
47000.0000000	7.5000000	-50.0000000
47000.0000000	7.7500000	-50.0000000
47000.0000000	8.0000000	-50.0000000
47000.0000000	8.2500000	-50.0000000
47000.0000000	8.5000000	-50.0000000
47000.0000000	8.7500000	-50.0000000
47000.0000000	9.0000000	-50.0000000
47000.0000000	9.2500000	-50.0000000
47000.0000000	9.5000000	-50.0000000
47000.0000000	9.7500000	-50.0000000
47000.0000000	10.0000000	-50.0000000
47000.0000000	10.2500000	-50.0000000
47000.0000000	10.5000000	-50.0000000
47000.0000000	10.7500000	-50.0000000
47000.0000000	11.0000000	-50.0000000
47000.0000000	11.2500000	-50.0000000
47000.0000000	11.5000000	-50.0000000
47000.0000000	11.7500000	-50.0000000
47000.0000000	12.0000000	-50.0000000
47000.0000000	12.2500000	-50.0000000
47000.0000000	12.5000000	-50.0000000
47000.0000000	12.7500000	-50.0000000
47000.0000000	13.0000000	-50.0000000
47000.0000000	13.2500000	-50.0000000
47000.0000000	13.5000000	-50.0000000
47000.0000000	13.7500000	-50.0000000
47000.0000000	14.0000000	-50.0000000
47000.0000000	14.2500000	-50.0000000
47000.0000000	14.5000000	-50.0000000
47000.0000000	14.7500000	-50.0000000
47000.0000000	15.0000000	-50.0000000
47030.0000000	0.0000000	-50.0000000

47030.0000000	0.2500000	-50.0000000
47030.0000000	0.5000000	-50.0000000
47030.0000000	0.7500000	-50.0000000
47030.0000000	1.0000000	-50.0000000
47030.0000000	1.2500000	-50.0000000
47030.0000000	1.5000000	-50.0000000
47030.0000000	1.7500000	-50.0000000
47030.0000000	2.0000000	-50.0000000
47030.0000000	2.2500000	-50.0000000
47030.0000000	2.5000000	-50.0000000
47030.0000000	2.7500000	-50.0000000
47030.0000000	3.0000000	-50.0000000
47030.0000000	3.2500000	-50.0000000
47030.0000000	3.5000000	-50.0000000
47030.0000000	3.7500000	-50.0000000
47030.0000000	4.0000000	-50.0000000
47030.0000000	4.2500000	-50.0000000
47030.0000000	4.5000000	-50.0000000
47030.0000000	4.7500000	-50.0000000
47030.0000000	5.0000000	-50.0000000
47030.0000000	5.2500000	-50.0000000
47030.0000000	5.5000000	-50.0000000
47030.0000000	5.7500000	-50.0000000
47030.0000000	6.0000000	-50.0000000
47030.0000000	6.2500000	-50.0000000
47030.0000000	6.5000000	-50.0000000
47030.0000000	6.7500000	-50.0000000
47030.0000000	7.0000000	-50.0000000
47030.0000000	7.2500000	-50.0000000
47030.0000000	7.5000000	-50.0000000
47030.0000000	7.7500000	-50.0000000
47030.0000000	8.0000000	-50.0000000
47030.0000000	8.2500000	-50.0000000
47030.0000000	8.5000000	-50.0000000
47030.0000000	8.7500000	-50.0000000
47030.0000000	9.0000000	-50.0000000
47030.0000000	9.2500000	-50.0000000
47030.0000000	9.5000000	-50.0000000
47030.0000000	9.7500000	-50.0000000
47030.0000000	10.0000000	-50.0000000
47030.0000000	10.2500000	-50.0000000
47030.0000000	10.5000000	-50.0000000
47030.0000000	10.7500000	-50.0000000
47030.0000000	11.0000000	-50.0000000
47030.0000000	11.2500000	-50.0000000
47030.0000000	11.5000000	-50.0000000
47030.0000000	11.7500000	-50.0000000
47030.0000000	12.0000000	-50.0000000
47030.0000000	12.2500000	-50.0000000
47030.0000000	12.5000000	-50.0000000
47030.0000000	12.7500000	-50.0000000
47030.0000000	13.0000000	-50.0000000
47030.0000000	13.2500000	-50.0000000
47030.0000000	13.5000000	-50.0000000
47030.0000000	13.7500000	-50.0000000
47030.0000000	14.0000000	-50.0000000
47030.0000000	14.2500000	-50.0000000
47030.0000000	14.5000000	-50.0000000

47030.0000000	14.7500000	-50.0000000
47030.0000000	15.0000000	-50.0000000
47060.0000000	0.0000000	-50.0000000
47060.0000000	0.2500000	-50.0000000
47060.0000000	0.5000000	-50.0000000
47060.0000000	0.7500000	-50.0000000
47060.0000000	1.0000000	-50.0000000
47060.0000000	1.2500000	-50.0000000
47060.0000000	1.5000000	-50.0000000
47060.0000000	1.7500000	-50.0000000
47060.0000000	2.0000000	-50.0000000
47060.0000000	2.2500000	-50.0000000
47060.0000000	2.5000000	-50.0000000
47060.0000000	2.7500000	-50.0000000
47060.0000000	3.0000000	-50.0000000
47060.0000000	3.2500000	-50.0000000
47060.0000000	3.5000000	-50.0000000
47060.0000000	3.7500000	-50.0000000
47060.0000000	4.0000000	-50.0000000
47060.0000000	4.2500000	-50.0000000
47060.0000000	4.5000000	-50.0000000
47060.0000000	4.7500000	-50.0000000
47060.0000000	5.0000000	-50.0000000
47060.0000000	5.2500000	-50.0000000
47060.0000000	5.5000000	-50.0000000
47060.0000000	5.7500000	-50.0000000
47060.0000000	6.0000000	-50.0000000
47060.0000000	6.2500000	-50.0000000
47060.0000000	6.5000000	-50.0000000
47060.0000000	6.7500000	-50.0000000
47060.0000000	7.0000000	-50.0000000
47060.0000000	7.2500000	-50.0000000
47060.0000000	7.5000000	-50.0000000
47060.0000000	7.7500000	-50.0000000
47060.0000000	8.0000000	-50.0000000
47060.0000000	8.2500000	-50.0000000
47060.0000000	8.5000000	-50.0000000
47060.0000000	8.7500000	-50.0000000
47060.0000000	9.0000000	-50.0000000
47060.0000000	9.2500000	-50.0000000
47060.0000000	9.5000000	-50.0000000
47060.0000000	9.7500000	-50.0000000
47060.0000000	10.0000000	-50.0000000
47060.0000000	10.2500000	-50.0000000
47060.0000000	10.5000000	-50.0000000
47060.0000000	10.7500000	-50.0000000
47060.0000000	11.0000000	-50.0000000
47060.0000000	11.2500000	-50.0000000
47060.0000000	11.5000000	-50.0000000
47060.0000000	11.7500000	-50.0000000
47060.0000000	12.0000000	-50.0000000
47060.0000000	12.2500000	-50.0000000
47060.0000000	12.5000000	-50.0000000
47060.0000000	12.7500000	-50.0000000
47060.0000000	13.0000000	-50.0000000
47060.0000000	13.2500000	-50.0000000
47060.0000000	13.5000000	-50.0000000
47060.0000000	13.7500000	-50.0000000

47060.0000000	14.0000000	-50.0000000
47060.0000000	14.2500000	-50.0000000
47060.0000000	14.5000000	-50.0000000
47060.0000000	14.7500000	-50.0000000
47060.0000000	15.0000000	-50.0000000
47090.0000000	0.0000000	-50.0000000
47090.0000000	0.2500000	-50.0000000
47090.0000000	0.5000000	-50.0000000
47090.0000000	0.7500000	-50.0000000
47090.0000000	1.0000000	-50.0000000
47090.0000000	1.2500000	-50.0000000
47090.0000000	1.5000000	-50.0000000
47090.0000000	1.7500000	-50.0000000
47090.0000000	2.0000000	-50.0000000
47090.0000000	2.2500000	-50.0000000
47090.0000000	2.5000000	-50.0000000
47090.0000000	2.7500000	-50.0000000
47090.0000000	3.0000000	-50.0000000
47090.0000000	3.2500000	-50.0000000
47090.0000000	3.5000000	-50.0000000
47090.0000000	3.7500000	-50.0000000
47090.0000000	4.0000000	-50.0000000
47090.0000000	4.2500000	-50.0000000
47090.0000000	4.5000000	-50.0000000
47090.0000000	4.7500000	-50.0000000
47090.0000000	5.0000000	-50.0000000
47090.0000000	5.2500000	-50.0000000
47090.0000000	5.5000000	-50.0000000
47090.0000000	5.7500000	-50.0000000
47090.0000000	6.0000000	-50.0000000
47090.0000000	6.2500000	-50.0000000
47090.0000000	6.5000000	-50.0000000
47090.0000000	6.7500000	-50.0000000
47090.0000000	7.0000000	-50.0000000
47090.0000000	7.2500000	-50.0000000
47090.0000000	7.5000000	-50.0000000
47090.0000000	7.7500000	-50.0000000
47090.0000000	8.0000000	-50.0000000
47090.0000000	8.2500000	-50.0000000
47090.0000000	8.5000000	-50.0000000
47090.0000000	8.7500000	-50.0000000
47090.0000000	9.0000000	-50.0000000
47090.0000000	9.2500000	-50.0000000
47090.0000000	9.5000000	-50.0000000
47090.0000000	9.7500000	-50.0000000
47090.0000000	10.0000000	-50.0000000
47090.0000000	10.2500000	-50.0000000
47090.0000000	10.5000000	-50.0000000
47090.0000000	10.7500000	-50.0000000
47090.0000000	11.0000000	-50.0000000
47090.0000000	11.2500000	-50.0000000
47090.0000000	11.5000000	-50.0000000
47090.0000000	11.7500000	-50.0000000
47090.0000000	12.0000000	-50.0000000
47090.0000000	12.2500000	-50.0000000
47090.0000000	12.5000000	-50.0000000
47090.0000000	12.7500000	-50.0000000
47090.0000000	13.0000000	-50.0000000

47090.0000000	13.2500000	-50.0000000
47090.0000000	13.5000000	-50.0000000
47090.0000000	13.7500000	-50.0000000
47090.0000000	14.0000000	-50.0000000
47090.0000000	14.2500000	-50.0000000
47090.0000000	14.5000000	-50.0000000
47090.0000000	14.7500000	-50.0000000
47090.0000000	15.0000000	-50.0000000
47120.0000000	0.0000000	-50.0000000
47120.0000000	0.2500000	-50.0000000
47120.0000000	0.5000000	-50.0000000
47120.0000000	0.7500000	-50.0000000
47120.0000000	1.0000000	-50.0000000
47120.0000000	1.2500000	-50.0000000
47120.0000000	1.5000000	-50.0000000
47120.0000000	1.7500000	-50.0000000
47120.0000000	2.0000000	-50.0000000
47120.0000000	2.2500000	-50.0000000
47120.0000000	2.5000000	-50.0000000
47120.0000000	2.7500000	-50.0000000
47120.0000000	3.0000000	-50.0000000
47120.0000000	3.2500000	-50.0000000
47120.0000000	3.5000000	-50.0000000
47120.0000000	3.7500000	-50.0000000
47120.0000000	4.0000000	-50.0000000
47120.0000000	4.2500000	-50.0000000
47120.0000000	4.5000000	-50.0000000
47120.0000000	4.7500000	-50.0000000
47120.0000000	5.0000000	-50.0000000
47120.0000000	5.2500000	-50.0000000
47120.0000000	5.5000000	-50.0000000
47120.0000000	5.7500000	-50.0000000
47120.0000000	6.0000000	-50.0000000
47120.0000000	6.2500000	-50.0000000
47120.0000000	6.5000000	-50.0000000
47120.0000000	6.7500000	-50.0000000
47120.0000000	7.0000000	-50.0000000
47120.0000000	7.2500000	-50.0000000
47120.0000000	7.5000000	-50.0000000
47120.0000000	7.7500000	-50.0000000
47120.0000000	8.0000000	-50.0000000
47120.0000000	8.2500000	-50.0000000
47120.0000000	8.5000000	-50.0000000
47120.0000000	8.7500000	-50.0000000
47120.0000000	9.0000000	-50.0000000
47120.0000000	9.2500000	-50.0000000
47120.0000000	9.5000000	-50.0000000
47120.0000000	9.7500000	-50.0000000
47120.0000000	10.0000000	-50.0000000
47120.0000000	10.2500000	-50.0000000
47120.0000000	10.5000000	-50.0000000
47120.0000000	10.7500000	-50.0000000
47120.0000000	11.0000000	-50.0000000
47120.0000000	11.2500000	-50.0000000
47120.0000000	11.5000000	-50.0000000
47120.0000000	11.7500000	-50.0000000
47120.0000000	12.0000000	-50.0000000
47120.0000000	12.2500000	-50.0000000

47120.0000000	12.5000000	-50.0000000
47120.0000000	12.7500000	-50.0000000
47120.0000000	13.0000000	-50.0000000
47120.0000000	13.2500000	-50.0000000
47120.0000000	13.5000000	-50.0000000
47120.0000000	13.7500000	-50.0000000
47120.0000000	14.0000000	-50.0000000
47120.0000000	14.2500000	-50.0000000
47120.0000000	14.5000000	-50.0000000
47120.0000000	14.7500000	-50.0000000
47120.0000000	15.0000000	-50.0000000
47150.0000000	0.0000000	-50.0000000
47150.0000000	0.2500000	-50.0000000
47150.0000000	0.5000000	-50.0000000
47150.0000000	0.7500000	15.5200005
47150.0000000	1.0000000	16.8600006
47150.0000000	1.2500000	-50.0000000
47150.0000000	1.5000000	-50.0000000
47150.0000000	1.7500000	-50.0000000
47150.0000000	2.0000000	-50.0000000
47150.0000000	2.2500000	-50.0000000
47150.0000000	2.5000000	-50.0000000
47150.0000000	2.7500000	-50.0000000
47150.0000000	3.0000000	-50.0000000
47150.0000000	3.2500000	-50.0000000
47150.0000000	3.5000000	-50.0000000
47150.0000000	3.7500000	-50.0000000
47150.0000000	4.0000000	-50.0000000
47150.0000000	4.2500000	-50.0000000
47150.0000000	4.5000000	-50.0000000
47150.0000000	4.7500000	-50.0000000
47150.0000000	5.0000000	-50.0000000
47150.0000000	5.2500000	-50.0000000
47150.0000000	5.5000000	-50.0000000
47150.0000000	5.7500000	-50.0000000
47150.0000000	6.0000000	-50.0000000
47150.0000000	6.2500000	-50.0000000
47150.0000000	6.5000000	-50.0000000
47150.0000000	6.7500000	-50.0000000
47150.0000000	7.0000000	-50.0000000
47150.0000000	7.2500000	-50.0000000
47150.0000000	7.5000000	-50.0000000
47150.0000000	7.7500000	-50.0000000
47150.0000000	8.0000000	-50.0000000
47150.0000000	8.2500000	-50.0000000
47150.0000000	8.5000000	-50.0000000
47150.0000000	8.7500000	-50.0000000
47150.0000000	9.0000000	-50.0000000
47150.0000000	9.2500000	-50.0000000
47150.0000000	9.5000000	-50.0000000
47150.0000000	9.7500000	-50.0000000
47150.0000000	10.0000000	-50.0000000
47150.0000000	10.2500000	-50.0000000
47150.0000000	10.5000000	-50.0000000
47150.0000000	10.7500000	-50.0000000
47150.0000000	11.0000000	-50.0000000
47150.0000000	11.2500000	-50.0000000
47150.0000000	11.5000000	-50.0000000

47150.0000000	11.7500000	-50.0000000
47150.0000000	12.0000000	-50.0000000
47150.0000000	12.2500000	-50.0000000
47150.0000000	12.5000000	-50.0000000
47150.0000000	12.7500000	-50.0000000
47150.0000000	13.0000000	-50.0000000
47150.0000000	13.2500000	-50.0000000
47150.0000000	13.5000000	-50.0000000
47150.0000000	13.7500000	-50.0000000
47150.0000000	14.0000000	-50.0000000
47150.0000000	14.2500000	-50.0000000
47150.0000000	14.5000000	-50.0000000
47150.0000000	14.7500000	-50.0000000
47150.0000000	15.0000000	-50.0000000
47180.0000000	0.0000000	-50.0000000
47180.0000000	0.2500000	-50.0000000
47180.0000000	0.5000000	-50.0000000
47180.0000000	0.7500000	-50.0000000
47180.0000000	1.0000000	15.1300001
47180.0000000	1.2500000	-50.0000000
47180.0000000	1.5000000	-50.0000000
47180.0000000	1.7500000	-50.0000000
47180.0000000	2.0000000	-50.0000000
47180.0000000	2.2500000	-50.0000000
47180.0000000	2.5000000	-50.0000000
47180.0000000	2.7500000	-50.0000000
47180.0000000	3.0000000	-50.0000000
47180.0000000	3.2500000	-50.0000000
47180.0000000	3.5000000	-50.0000000
47180.0000000	3.7500000	-50.0000000
47180.0000000	4.0000000	-50.0000000
47180.0000000	4.2500000	-50.0000000
47180.0000000	4.5000000	-50.0000000
47180.0000000	4.7500000	-50.0000000
47180.0000000	5.0000000	-50.0000000
47180.0000000	5.2500000	-50.0000000
47180.0000000	5.5000000	-50.0000000
47180.0000000	5.7500000	-50.0000000
47180.0000000	6.0000000	-50.0000000
47180.0000000	6.2500000	-50.0000000
47180.0000000	6.5000000	-50.0000000
47180.0000000	6.7500000	-50.0000000
47180.0000000	7.0000000	-50.0000000
47180.0000000	7.2500000	-50.0000000
47180.0000000	7.5000000	-50.0000000
47180.0000000	7.7500000	-50.0000000
47180.0000000	8.0000000	-50.0000000
47180.0000000	8.2500000	-50.0000000
47180.0000000	8.5000000	-50.0000000
47180.0000000	8.7500000	-50.0000000
47180.0000000	9.0000000	-50.0000000
47180.0000000	9.2500000	-50.0000000
47180.0000000	9.5000000	-50.0000000
47180.0000000	9.7500000	-50.0000000
47180.0000000	10.0000000	-50.0000000
47180.0000000	10.2500000	-50.0000000
47180.0000000	10.5000000	-50.0000000
47180.0000000	10.7500000	-50.0000000

47180.0000000	11.0000000	-50.0000000
47180.0000000	11.2500000	-50.0000000
47180.0000000	11.5000000	-50.0000000
47180.0000000	11.7500000	-50.0000000
47180.0000000	12.0000000	-50.0000000
47180.0000000	12.2500000	-50.0000000
47180.0000000	12.5000000	-50.0000000
47180.0000000	12.7500000	-50.0000000
47180.0000000	13.0000000	-50.0000000
47180.0000000	13.2500000	-50.0000000
47180.0000000	13.5000000	-50.0000000
47180.0000000	13.7500000	-50.0000000
47180.0000000	14.0000000	-50.0000000
47180.0000000	14.2500000	-50.0000000
47180.0000000	14.5000000	-50.0000000
47180.0000000	14.7500000	-50.0000000
47180.0000000	15.0000000	-50.0000000
47210.0000000	0.0000000	-50.0000000
47210.0000000	0.2500000	-50.0000000
47210.0000000	0.5000000	-50.0000000
47210.0000000	0.7500000	-50.0000000
47210.0000000	1.0000000	-50.0000000
47210.0000000	1.2500000	-50.0000000
47210.0000000	1.5000000	-50.0000000
47210.0000000	1.7500000	-50.0000000
47210.0000000	2.0000000	-50.0000000
47210.0000000	2.2500000	-50.0000000
47210.0000000	2.5000000	-50.0000000
47210.0000000	2.7500000	-50.0000000
47210.0000000	3.0000000	-50.0000000
47210.0000000	3.2500000	-50.0000000
47210.0000000	3.5000000	-50.0000000
47210.0000000	3.7500000	-50.0000000
47210.0000000	4.0000000	-50.0000000
47210.0000000	4.2500000	-50.0000000
47210.0000000	4.5000000	-50.0000000
47210.0000000	4.7500000	-50.0000000
47210.0000000	5.0000000	-50.0000000
47210.0000000	5.2500000	-50.0000000
47210.0000000	5.5000000	-50.0000000
47210.0000000	5.7500000	-50.0000000
47210.0000000	6.0000000	-50.0000000
47210.0000000	6.2500000	-50.0000000
47210.0000000	6.5000000	-50.0000000
47210.0000000	6.7500000	-50.0000000
47210.0000000	7.0000000	-50.0000000
47210.0000000	7.2500000	-50.0000000
47210.0000000	7.5000000	-50.0000000
47210.0000000	7.7500000	-50.0000000
47210.0000000	8.0000000	-50.0000000
47210.0000000	8.2500000	-50.0000000
47210.0000000	8.5000000	-50.0000000
47210.0000000	8.7500000	-50.0000000
47210.0000000	9.0000000	-50.0000000
47210.0000000	9.2500000	-50.0000000
47210.0000000	9.5000000	-50.0000000
47210.0000000	9.7500000	-50.0000000
47210.0000000	10.0000000	-50.0000000

47210.0000000	10.2500000	-50.0000000
47210.0000000	10.5000000	-50.0000000
47210.0000000	10.7500000	-50.0000000
47210.0000000	11.0000000	-50.0000000
47210.0000000	11.2500000	-50.0000000
47210.0000000	11.5000000	-50.0000000
47210.0000000	11.7500000	-50.0000000
47210.0000000	12.0000000	-50.0000000
47210.0000000	12.2500000	-50.0000000
47210.0000000	12.5000000	-50.0000000
47210.0000000	12.7500000	-50.0000000
47210.0000000	13.0000000	-50.0000000
47210.0000000	13.2500000	-50.0000000
47210.0000000	13.5000000	-50.0000000
47210.0000000	13.7500000	-50.0000000
47210.0000000	14.0000000	-50.0000000
47210.0000000	14.2500000	-50.0000000
47210.0000000	14.5000000	-50.0000000
47210.0000000	14.7500000	-50.0000000
47210.0000000	15.0000000	-50.0000000
47240.0000000	0.0000000	-50.0000000
47240.0000000	0.2500000	-50.0000000
47240.0000000	0.5000000	-50.0000000
47240.0000000	0.7500000	17.2399998
47240.0000000	1.0000000	16.4699993
47240.0000000	1.2500000	-50.0000000
47240.0000000	1.5000000	-50.0000000
47240.0000000	1.7500000	-50.0000000
47240.0000000	2.0000000	-50.0000000
47240.0000000	2.2500000	-50.0000000
47240.0000000	2.5000000	-50.0000000
47240.0000000	2.7500000	-50.0000000
47240.0000000	3.0000000	-50.0000000
47240.0000000	3.2500000	-50.0000000
47240.0000000	3.5000000	-50.0000000
47240.0000000	3.7500000	-50.0000000
47240.0000000	4.0000000	-50.0000000
47240.0000000	4.2500000	-50.0000000
47240.0000000	4.5000000	-50.0000000
47240.0000000	4.7500000	-50.0000000
47240.0000000	5.0000000	-50.0000000
47240.0000000	5.2500000	-50.0000000
47240.0000000	5.5000000	-50.0000000
47240.0000000	5.7500000	-50.0000000
47240.0000000	6.0000000	-50.0000000
47240.0000000	6.2500000	-50.0000000
47240.0000000	6.5000000	-50.0000000
47240.0000000	6.7500000	-50.0000000
47240.0000000	7.0000000	-50.0000000
47240.0000000	7.2500000	-50.0000000
47240.0000000	7.5000000	-50.0000000
47240.0000000	7.7500000	-50.0000000
47240.0000000	8.0000000	-50.0000000
47240.0000000	8.2500000	-50.0000000
47240.0000000	8.5000000	-50.0000000
47240.0000000	8.7500000	-50.0000000
47240.0000000	9.0000000	-50.0000000
47240.0000000	9.2500000	-50.0000000

47240.0000000	9.5000000	-50.0000000
47240.0000000	9.7500000	-50.0000000
47240.0000000	10.0000000	-50.0000000
47240.0000000	10.2500000	-50.0000000
47240.0000000	10.5000000	-50.0000000
47240.0000000	10.7500000	-50.0000000
47240.0000000	11.0000000	-50.0000000
47240.0000000	11.2500000	-50.0000000
47240.0000000	11.5000000	-50.0000000
47240.0000000	11.7500000	-50.0000000
47240.0000000	12.0000000	-50.0000000
47240.0000000	12.2500000	-50.0000000
47240.0000000	12.5000000	-50.0000000
47240.0000000	12.7500000	-50.0000000
47240.0000000	13.0000000	-50.0000000
47240.0000000	13.2500000	-50.0000000
47240.0000000	13.5000000	-50.0000000
47240.0000000	13.7500000	-50.0000000
47240.0000000	14.0000000	-50.0000000
47240.0000000	14.2500000	-50.0000000
47240.0000000	14.5000000	-50.0000000
47240.0000000	14.7500000	-50.0000000
47240.0000000	15.0000000	-50.0000000
47270.0000000	0.0000000	-50.0000000
47270.0000000	0.2500000	-50.0000000
47270.0000000	0.5000000	-50.0000000
47270.0000000	0.7500000	18.1299992
47270.0000000	1.0000000	17.8299999
47270.0000000	1.2500000	-50.0000000
47270.0000000	1.5000000	-50.0000000
47270.0000000	1.7500000	-50.0000000
47270.0000000	2.0000000	-50.0000000
47270.0000000	2.2500000	-50.0000000
47270.0000000	2.5000000	-50.0000000
47270.0000000	2.7500000	-50.0000000
47270.0000000	3.0000000	-50.0000000
47270.0000000	3.2500000	-50.0000000
47270.0000000	3.5000000	-50.0000000
47270.0000000	3.7500000	-50.0000000
47270.0000000	4.0000000	-50.0000000
47270.0000000	4.2500000	-50.0000000
47270.0000000	4.5000000	-50.0000000
47270.0000000	4.7500000	-50.0000000
47270.0000000	5.0000000	-50.0000000
47270.0000000	5.2500000	-50.0000000
47270.0000000	5.5000000	-50.0000000
47270.0000000	5.7500000	-50.0000000
47270.0000000	6.0000000	-50.0000000
47270.0000000	6.2500000	-50.0000000
47270.0000000	6.5000000	-50.0000000
47270.0000000	6.7500000	-50.0000000
47270.0000000	7.0000000	-50.0000000
47270.0000000	7.2500000	-50.0000000
47270.0000000	7.5000000	-50.0000000
47270.0000000	7.7500000	-50.0000000
47270.0000000	8.0000000	-50.0000000
47270.0000000	8.2500000	-50.0000000
47270.0000000	8.5000000	-50.0000000

47270.0000000	8.7500000	-50.0000000
47270.0000000	9.0000000	-50.0000000
47270.0000000	9.2500000	-50.0000000
47270.0000000	9.5000000	-50.0000000
47270.0000000	9.7500000	-50.0000000
47270.0000000	10.0000000	-50.0000000
47270.0000000	10.2500000	-50.0000000
47270.0000000	10.5000000	-50.0000000
47270.0000000	10.7500000	-50.0000000
47270.0000000	11.0000000	-50.0000000
47270.0000000	11.2500000	-50.0000000
47270.0000000	11.5000000	-50.0000000
47270.0000000	11.7500000	-50.0000000
47270.0000000	12.0000000	-50.0000000
47270.0000000	12.2500000	-50.0000000
47270.0000000	12.5000000	-50.0000000
47270.0000000	12.7500000	-50.0000000
47270.0000000	13.0000000	-50.0000000
47270.0000000	13.2500000	-50.0000000
47270.0000000	13.5000000	-50.0000000
47270.0000000	13.7500000	-50.0000000
47270.0000000	14.0000000	-50.0000000
47270.0000000	14.2500000	-50.0000000
47270.0000000	14.5000000	-50.0000000
47270.0000000	14.7500000	-50.0000000
47270.0000000	15.0000000	-50.0000000
47300.0000000	0.0000000	-50.0000000
47300.0000000	0.2500000	-50.0000000
47300.0000000	0.5000000	-50.0000000
47300.0000000	0.7500000	16.0799999
47300.0000000	1.0000000	15.5200005
47300.0000000	1.2500000	-50.0000000
47300.0000000	1.5000000	-50.0000000
47300.0000000	1.7500000	-50.0000000
47300.0000000	2.0000000	-50.0000000
47300.0000000	2.2500000	-50.0000000
47300.0000000	2.5000000	-50.0000000
47300.0000000	2.7500000	-50.0000000
47300.0000000	3.0000000	-50.0000000
47300.0000000	3.2500000	-50.0000000
47300.0000000	3.5000000	-50.0000000
47300.0000000	3.7500000	-50.0000000
47300.0000000	4.0000000	-50.0000000
47300.0000000	4.2500000	-50.0000000
47300.0000000	4.5000000	-50.0000000
47300.0000000	4.7500000	-50.0000000
47300.0000000	5.0000000	-50.0000000
47300.0000000	5.2500000	-50.0000000
47300.0000000	5.5000000	-50.0000000
47300.0000000	5.7500000	-50.0000000
47300.0000000	6.0000000	-50.0000000
47300.0000000	6.2500000	-50.0000000
47300.0000000	6.5000000	-50.0000000
47300.0000000	6.7500000	-50.0000000
47300.0000000	7.0000000	-50.0000000
47300.0000000	7.2500000	-50.0000000
47300.0000000	7.5000000	-50.0000000
47300.0000000	7.7500000	-50.0000000

47300.0000000	8.0000000	-50.0000000
47300.0000000	8.2500000	-50.0000000
47300.0000000	8.5000000	-50.0000000
47300.0000000	8.7500000	-50.0000000
47300.0000000	9.0000000	-50.0000000
47300.0000000	9.2500000	-50.0000000
47300.0000000	9.5000000	-50.0000000
47300.0000000	9.7500000	-50.0000000
47300.0000000	10.0000000	-50.0000000
47300.0000000	10.2500000	-50.0000000
47300.0000000	10.5000000	-50.0000000
47300.0000000	10.7500000	-50.0000000
47300.0000000	11.0000000	-50.0000000
47300.0000000	11.2500000	-50.0000000
47300.0000000	11.5000000	-50.0000000
47300.0000000	11.7500000	-50.0000000
47300.0000000	12.0000000	-50.0000000
47300.0000000	12.2500000	-50.0000000
47300.0000000	12.5000000	-50.0000000
47300.0000000	12.7500000	-50.0000000
47300.0000000	13.0000000	-50.0000000
47300.0000000	13.2500000	-50.0000000
47300.0000000	13.5000000	-50.0000000
47300.0000000	13.7500000	-50.0000000
47300.0000000	14.0000000	-50.0000000
47300.0000000	14.2500000	-50.0000000
47300.0000000	14.5000000	-50.0000000
47300.0000000	14.7500000	-50.0000000
47300.0000000	15.0000000	-50.0000000
47330.0000000	0.0000000	-50.0000000
47330.0000000	0.2500000	-50.0000000
47330.0000000	0.5000000	-50.0000000
47330.0000000	0.7500000	17.0799999
47330.0000000	1.0000000	15.2399998
47330.0000000	1.2500000	-50.0000000
47330.0000000	1.5000000	-50.0000000
47330.0000000	1.7500000	-50.0000000
47330.0000000	2.0000000	-50.0000000
47330.0000000	2.2500000	-50.0000000
47330.0000000	2.5000000	-50.0000000
47330.0000000	2.7500000	-50.0000000
47330.0000000	3.0000000	-50.0000000
47330.0000000	3.2500000	-50.0000000
47330.0000000	3.5000000	-50.0000000
47330.0000000	3.7500000	-50.0000000
47330.0000000	4.0000000	-50.0000000
47330.0000000	4.2500000	-50.0000000
47330.0000000	4.5000000	-50.0000000
47330.0000000	4.7500000	-50.0000000
47330.0000000	5.0000000	-50.0000000
47330.0000000	5.2500000	-50.0000000
47330.0000000	5.5000000	-50.0000000
47330.0000000	5.7500000	-50.0000000
47330.0000000	6.0000000	-50.0000000
47330.0000000	6.2500000	-50.0000000
47330.0000000	6.5000000	-50.0000000
47330.0000000	6.7500000	-50.0000000
47330.0000000	7.0000000	-50.0000000

47330.0000000	7.2500000	-50.0000000
47330.0000000	7.5000000	-50.0000000
47330.0000000	7.7500000	-50.0000000
47330.0000000	8.0000000	-50.0000000
47330.0000000	8.2500000	-50.0000000
47330.0000000	8.5000000	-50.0000000
47330.0000000	8.7500000	-50.0000000
47330.0000000	9.0000000	-50.0000000
47330.0000000	9.2500000	-50.0000000
47330.0000000	9.5000000	-50.0000000
47330.0000000	9.7500000	-50.0000000
47330.0000000	10.0000000	-50.0000000
47330.0000000	10.2500000	-50.0000000
47330.0000000	10.5000000	-50.0000000
47330.0000000	10.7500000	-50.0000000
47330.0000000	11.0000000	-50.0000000
47330.0000000	11.2500000	-50.0000000
47330.0000000	11.5000000	-50.0000000
47330.0000000	11.7500000	-50.0000000
47330.0000000	12.0000000	-50.0000000
47330.0000000	12.2500000	-50.0000000
47330.0000000	12.5000000	-50.0000000
47330.0000000	12.7500000	-50.0000000
47330.0000000	13.0000000	-50.0000000
47330.0000000	13.2500000	-50.0000000
47330.0000000	13.5000000	-50.0000000
47330.0000000	13.7500000	-50.0000000
47330.0000000	14.0000000	-50.0000000
47330.0000000	14.2500000	-50.0000000
47330.0000000	14.5000000	-50.0000000
47330.0000000	14.7500000	-50.0000000
47330.0000000	15.0000000	-50.0000000
47360.0000000	0.0000000	-50.0000000
47360.0000000	0.2500000	-50.0000000
47360.0000000	0.5000000	15.6599998
47360.0000000	0.7500000	19.7900009
47360.0000000	1.0000000	16.0499992
47360.0000000	1.2500000	-50.0000000
47360.0000000	1.5000000	-50.0000000
47360.0000000	1.7500000	-50.0000000
47360.0000000	2.0000000	-50.0000000
47360.0000000	2.2500000	-50.0000000
47360.0000000	2.5000000	-50.0000000
47360.0000000	2.7500000	-50.0000000
47360.0000000	3.0000000	-50.0000000
47360.0000000	3.2500000	-50.0000000
47360.0000000	3.5000000	-50.0000000
47360.0000000	3.7500000	-50.0000000
47360.0000000	4.0000000	-50.0000000
47360.0000000	4.2500000	-50.0000000
47360.0000000	4.5000000	-50.0000000
47360.0000000	4.7500000	-50.0000000
47360.0000000	5.0000000	-50.0000000
47360.0000000	5.2500000	-50.0000000
47360.0000000	5.5000000	-50.0000000
47360.0000000	5.7500000	-50.0000000
47360.0000000	6.0000000	-50.0000000
47360.0000000	6.2500000	-50.0000000

47360.0000000	6.5000000	-50.0000000
47360.0000000	6.7500000	-50.0000000
47360.0000000	7.0000000	-50.0000000
47360.0000000	7.2500000	-50.0000000
47360.0000000	7.5000000	-50.0000000
47360.0000000	7.7500000	-50.0000000
47360.0000000	8.0000000	-50.0000000
47360.0000000	8.2500000	-50.0000000
47360.0000000	8.5000000	-50.0000000
47360.0000000	8.7500000	-50.0000000
47360.0000000	9.0000000	-50.0000000
47360.0000000	9.2500000	-50.0000000
47360.0000000	9.5000000	-50.0000000
47360.0000000	9.7500000	-50.0000000
47360.0000000	10.0000000	-50.0000000
47360.0000000	10.2500000	-50.0000000
47360.0000000	10.5000000	-50.0000000
47360.0000000	10.7500000	-50.0000000
47360.0000000	11.0000000	-50.0000000
47360.0000000	11.2500000	-50.0000000
47360.0000000	11.5000000	-50.0000000
47360.0000000	11.7500000	-50.0000000
47360.0000000	12.0000000	-50.0000000
47360.0000000	12.2500000	-50.0000000
47360.0000000	12.5000000	-50.0000000
47360.0000000	12.7500000	-50.0000000
47360.0000000	13.0000000	-50.0000000
47360.0000000	13.2500000	-50.0000000
47360.0000000	13.5000000	-50.0000000
47360.0000000	13.7500000	-50.0000000
47360.0000000	14.0000000	-50.0000000
47360.0000000	14.2500000	-50.0000000
47360.0000000	14.5000000	-50.0000000
47360.0000000	14.7500000	-50.0000000
47360.0000000	15.0000000	-50.0000000
47390.0000000	0.0000000	-50.0000000
47390.0000000	0.2500000	-50.0000000
47390.0000000	0.5000000	-50.0000000
47390.0000000	0.7500000	18.9699993
47390.0000000	1.0000000	16.5599995
47390.0000000	1.2500000	-50.0000000
47390.0000000	1.5000000	-50.0000000
47390.0000000	1.7500000	-50.0000000
47390.0000000	2.0000000	-50.0000000
47390.0000000	2.2500000	-50.0000000
47390.0000000	2.5000000	-50.0000000
47390.0000000	2.7500000	-50.0000000
47390.0000000	3.0000000	-50.0000000
47390.0000000	3.2500000	-50.0000000
47390.0000000	3.5000000	-50.0000000
47390.0000000	3.7500000	-50.0000000
47390.0000000	4.0000000	-50.0000000
47390.0000000	4.2500000	-50.0000000
47390.0000000	4.5000000	-50.0000000
47390.0000000	4.7500000	-50.0000000
47390.0000000	5.0000000	-50.0000000
47390.0000000	5.2500000	-50.0000000
47390.0000000	5.5000000	-50.0000000

47390.0000000	5.7500000	-50.0000000
47390.0000000	6.0000000	-50.0000000
47390.0000000	6.2500000	-50.0000000
47390.0000000	6.5000000	-50.0000000
47390.0000000	6.7500000	-50.0000000
47390.0000000	7.0000000	-50.0000000
47390.0000000	7.2500000	-50.0000000
47390.0000000	7.5000000	-50.0000000
47390.0000000	7.7500000	-50.0000000
47390.0000000	8.0000000	-50.0000000
47390.0000000	8.2500000	-50.0000000
47390.0000000	8.5000000	-50.0000000
47390.0000000	8.7500000	-50.0000000
47390.0000000	9.0000000	-50.0000000
47390.0000000	9.2500000	-50.0000000
47390.0000000	9.5000000	-50.0000000
47390.0000000	9.7500000	-50.0000000
47390.0000000	10.0000000	-50.0000000
47390.0000000	10.2500000	-50.0000000
47390.0000000	10.5000000	-50.0000000
47390.0000000	10.7500000	-50.0000000
47390.0000000	11.0000000	-50.0000000
47390.0000000	11.2500000	-50.0000000
47390.0000000	11.5000000	-50.0000000
47390.0000000	11.7500000	-50.0000000
47390.0000000	12.0000000	-50.0000000
47390.0000000	12.2500000	-50.0000000
47390.0000000	12.5000000	-50.0000000
47390.0000000	12.7500000	-50.0000000
47390.0000000	13.0000000	-50.0000000
47390.0000000	13.2500000	-50.0000000
47390.0000000	13.5000000	-50.0000000
47390.0000000	13.7500000	-50.0000000
47390.0000000	14.0000000	-50.0000000
47390.0000000	14.2500000	-50.0000000
47390.0000000	14.5000000	-50.0000000
47390.0000000	14.7500000	-50.0000000
47390.0000000	15.0000000	-50.0000000
47420.0000000	0.0000000	-50.0000000
47420.0000000	0.2500000	-50.0000000
47420.0000000	0.5000000	-50.0000000
47420.0000000	0.7500000	16.7099991
47420.0000000	1.0000000	-50.0000000
47420.0000000	1.2500000	-50.0000000
47420.0000000	1.5000000	-50.0000000
47420.0000000	1.7500000	-50.0000000
47420.0000000	2.0000000	-50.0000000
47420.0000000	2.2500000	-50.0000000
47420.0000000	2.5000000	-50.0000000
47420.0000000	2.7500000	-50.0000000
47420.0000000	3.0000000	-50.0000000
47420.0000000	3.2500000	-50.0000000
47420.0000000	3.5000000	-50.0000000
47420.0000000	3.7500000	-50.0000000
47420.0000000	4.0000000	-50.0000000
47420.0000000	4.2500000	-50.0000000
47420.0000000	4.5000000	-50.0000000
47420.0000000	4.7500000	-50.0000000

47420.0000000	5.0000000	-50.0000000
47420.0000000	5.2500000	-50.0000000
47420.0000000	5.5000000	-50.0000000
47420.0000000	5.7500000	-50.0000000
47420.0000000	6.0000000	-50.0000000
47420.0000000	6.2500000	-50.0000000
47420.0000000	6.5000000	-50.0000000
47420.0000000	6.7500000	-50.0000000
47420.0000000	7.0000000	-50.0000000
47420.0000000	7.2500000	-50.0000000
47420.0000000	7.5000000	-50.0000000
47420.0000000	7.7500000	-50.0000000
47420.0000000	8.0000000	-50.0000000
47420.0000000	8.2500000	-50.0000000
47420.0000000	8.5000000	-50.0000000
47420.0000000	8.7500000	-50.0000000
47420.0000000	9.0000000	-50.0000000
47420.0000000	9.2500000	-50.0000000
47420.0000000	9.5000000	-50.0000000
47420.0000000	9.7500000	-50.0000000
47420.0000000	10.0000000	-50.0000000
47420.0000000	10.2500000	-50.0000000
47420.0000000	10.5000000	-50.0000000
47420.0000000	10.7500000	-50.0000000
47420.0000000	11.0000000	-50.0000000
47420.0000000	11.2500000	-50.0000000
47420.0000000	11.5000000	-50.0000000
47420.0000000	11.7500000	-50.0000000
47420.0000000	12.0000000	-50.0000000
47420.0000000	12.2500000	-50.0000000
47420.0000000	12.5000000	-50.0000000
47420.0000000	12.7500000	-50.0000000
47420.0000000	13.0000000	-50.0000000
47420.0000000	13.2500000	-50.0000000
47420.0000000	13.5000000	-50.0000000
47420.0000000	13.7500000	-50.0000000
47420.0000000	14.0000000	-50.0000000
47420.0000000	14.2500000	-50.0000000
47420.0000000	14.5000000	-50.0000000
47420.0000000	14.7500000	-50.0000000
47420.0000000	15.0000000	-50.0000000
47450.0000000	0.0000000	-50.0000000
47450.0000000	0.2500000	-50.0000000
47450.0000000	0.5000000	-50.0000000
47450.0000000	0.7500000	15.6700001
47450.0000000	1.0000000	-50.0000000
47450.0000000	1.2500000	-50.0000000
47450.0000000	1.5000000	-50.0000000
47450.0000000	1.7500000	-50.0000000
47450.0000000	2.0000000	-50.0000000
47450.0000000	2.2500000	-50.0000000
47450.0000000	2.5000000	-50.0000000
47450.0000000	2.7500000	-50.0000000
47450.0000000	3.0000000	-50.0000000
47450.0000000	3.2500000	-50.0000000
47450.0000000	3.5000000	-50.0000000
47450.0000000	3.7500000	-50.0000000
47450.0000000	4.0000000	-50.0000000

47450.0000000	4.2500000	-50.0000000
47450.0000000	4.5000000	-50.0000000
47450.0000000	4.7500000	-50.0000000
47450.0000000	5.0000000	-50.0000000
47450.0000000	5.2500000	-50.0000000
47450.0000000	5.5000000	-50.0000000
47450.0000000	5.7500000	-50.0000000
47450.0000000	6.0000000	-50.0000000
47450.0000000	6.2500000	-50.0000000
47450.0000000	6.5000000	-50.0000000
47450.0000000	6.7500000	-50.0000000
47450.0000000	7.0000000	-50.0000000
47450.0000000	7.2500000	-50.0000000
47450.0000000	7.5000000	-50.0000000
47450.0000000	7.7500000	-50.0000000
47450.0000000	8.0000000	-50.0000000
47450.0000000	8.2500000	-50.0000000
47450.0000000	8.5000000	-50.0000000
47450.0000000	8.7500000	-50.0000000
47450.0000000	9.0000000	-50.0000000
47450.0000000	9.2500000	-50.0000000
47450.0000000	9.5000000	-50.0000000
47450.0000000	9.7500000	-50.0000000
47450.0000000	10.0000000	-50.0000000
47450.0000000	10.2500000	-50.0000000
47450.0000000	10.5000000	-50.0000000
47450.0000000	10.7500000	-50.0000000
47450.0000000	11.0000000	-50.0000000
47450.0000000	11.2500000	-50.0000000
47450.0000000	11.5000000	-50.0000000
47450.0000000	11.7500000	-50.0000000
47450.0000000	12.0000000	-50.0000000
47450.0000000	12.2500000	-50.0000000
47450.0000000	12.5000000	-50.0000000
47450.0000000	12.7500000	-50.0000000
47450.0000000	13.0000000	-50.0000000
47450.0000000	13.2500000	-50.0000000
47450.0000000	13.5000000	-50.0000000
47450.0000000	13.7500000	-50.0000000
47450.0000000	14.0000000	-50.0000000
47450.0000000	14.2500000	-50.0000000
47450.0000000	14.5000000	-50.0000000
47450.0000000	14.7500000	-50.0000000
47450.0000000	15.0000000	-50.0000000
47480.0000000	0.0000000	-50.0000000
47480.0000000	0.2500000	-50.0000000
47480.0000000	0.5000000	-50.0000000
47480.0000000	0.7500000	17.2399998
47480.0000000	1.0000000	-50.0000000
47480.0000000	1.2500000	-50.0000000
47480.0000000	1.5000000	-50.0000000
47480.0000000	1.7500000	-50.0000000
47480.0000000	2.0000000	-50.0000000
47480.0000000	2.2500000	-50.0000000
47480.0000000	2.5000000	-50.0000000
47480.0000000	2.7500000	-50.0000000
47480.0000000	3.0000000	-50.0000000
47480.0000000	3.2500000	-50.0000000

47480.0000000	3.5000000	-50.0000000
47480.0000000	3.7500000	-50.0000000
47480.0000000	4.0000000	-50.0000000
47480.0000000	4.2500000	-50.0000000
47480.0000000	4.5000000	-50.0000000
47480.0000000	4.7500000	-50.0000000
47480.0000000	5.0000000	-50.0000000
47480.0000000	5.2500000	-50.0000000
47480.0000000	5.5000000	-50.0000000
47480.0000000	5.7500000	-50.0000000
47480.0000000	6.0000000	-50.0000000
47480.0000000	6.2500000	-50.0000000
47480.0000000	6.5000000	-50.0000000
47480.0000000	6.7500000	-50.0000000
47480.0000000	7.0000000	-50.0000000
47480.0000000	7.2500000	-50.0000000
47480.0000000	7.5000000	-50.0000000
47480.0000000	7.7500000	-50.0000000
47480.0000000	8.0000000	-50.0000000
47480.0000000	8.2500000	-50.0000000
47480.0000000	8.5000000	-50.0000000
47480.0000000	8.7500000	-50.0000000
47480.0000000	9.0000000	-50.0000000
47480.0000000	9.2500000	-50.0000000
47480.0000000	9.5000000	-50.0000000
47480.0000000	9.7500000	-50.0000000
47480.0000000	10.0000000	-50.0000000
47480.0000000	10.2500000	-50.0000000
47480.0000000	10.5000000	-50.0000000
47480.0000000	10.7500000	-50.0000000
47480.0000000	11.0000000	-50.0000000
47480.0000000	11.2500000	-50.0000000
47480.0000000	11.5000000	-50.0000000
47480.0000000	11.7500000	-50.0000000
47480.0000000	12.0000000	-50.0000000
47480.0000000	12.2500000	-50.0000000
47480.0000000	12.5000000	-50.0000000
47480.0000000	12.7500000	-50.0000000
47480.0000000	13.0000000	-50.0000000
47480.0000000	13.2500000	-50.0000000
47480.0000000	13.5000000	-50.0000000
47480.0000000	13.7500000	-50.0000000
47480.0000000	14.0000000	-50.0000000
47480.0000000	14.2500000	-50.0000000
47480.0000000	14.5000000	-50.0000000
47480.0000000	14.7500000	-50.0000000
47480.0000000	15.0000000	-50.0000000
47510.0000000	0.0000000	-50.0000000
47510.0000000	0.2500000	-50.0000000
47510.0000000	0.5000000	-50.0000000
47510.0000000	0.7500000	-50.0000000
47510.0000000	1.0000000	-50.0000000
47510.0000000	1.2500000	-50.0000000
47510.0000000	1.5000000	-50.0000000
47510.0000000	1.7500000	-50.0000000
47510.0000000	2.0000000	-50.0000000
47510.0000000	2.2500000	-50.0000000
47510.0000000	2.5000000	-50.0000000

47510.0000000	2.7500000	-50.0000000
47510.0000000	3.0000000	-50.0000000
47510.0000000	3.2500000	-50.0000000
47510.0000000	3.5000000	-50.0000000
47510.0000000	3.7500000	-50.0000000
47510.0000000	4.0000000	-50.0000000
47510.0000000	4.2500000	-50.0000000
47510.0000000	4.5000000	-50.0000000
47510.0000000	4.7500000	-50.0000000
47510.0000000	5.0000000	-50.0000000
47510.0000000	5.2500000	-50.0000000
47510.0000000	5.5000000	-50.0000000
47510.0000000	5.7500000	-50.0000000
47510.0000000	6.0000000	-50.0000000
47510.0000000	6.2500000	-50.0000000
47510.0000000	6.5000000	-50.0000000
47510.0000000	6.7500000	-50.0000000
47510.0000000	7.0000000	-50.0000000
47510.0000000	7.2500000	-50.0000000
47510.0000000	7.5000000	-50.0000000
47510.0000000	7.7500000	-50.0000000
47510.0000000	8.0000000	-50.0000000
47510.0000000	8.2500000	-50.0000000
47510.0000000	8.5000000	-50.0000000
47510.0000000	8.7500000	-50.0000000
47510.0000000	9.0000000	-50.0000000
47510.0000000	9.2500000	-50.0000000
47510.0000000	9.5000000	-50.0000000
47510.0000000	9.7500000	-50.0000000
47510.0000000	10.0000000	-50.0000000
47510.0000000	10.2500000	-50.0000000
47510.0000000	10.5000000	-50.0000000
47510.0000000	10.7500000	-50.0000000
47510.0000000	11.0000000	-50.0000000
47510.0000000	11.2500000	-50.0000000
47510.0000000	11.5000000	-50.0000000
47510.0000000	11.7500000	-50.0000000
47510.0000000	12.0000000	-50.0000000
47510.0000000	12.2500000	-50.0000000
47510.0000000	12.5000000	-50.0000000
47510.0000000	12.7500000	-50.0000000
47510.0000000	13.0000000	-50.0000000
47510.0000000	13.2500000	-50.0000000
47510.0000000	13.5000000	-50.0000000
47510.0000000	13.7500000	-50.0000000
47510.0000000	14.0000000	-50.0000000
47510.0000000	14.2500000	-50.0000000
47510.0000000	14.5000000	-50.0000000
47510.0000000	14.7500000	-50.0000000
47510.0000000	15.0000000	-50.0000000
47540.0000000	0.0000000	-50.0000000
47540.0000000	0.2500000	-50.0000000
47540.0000000	0.5000000	15.7100000
47540.0000000	0.7500000	17.6599998
47540.0000000	1.0000000	15.4399996
47540.0000000	1.2500000	-50.0000000
47540.0000000	1.5000000	-50.0000000
47540.0000000	1.7500000	-50.0000000

47540.0000000	2.0000000	-50.0000000
47540.0000000	2.2500000	-50.0000000
47540.0000000	2.5000000	-50.0000000
47540.0000000	2.7500000	-50.0000000
47540.0000000	3.0000000	-50.0000000
47540.0000000	3.2500000	-50.0000000
47540.0000000	3.5000000	-50.0000000
47540.0000000	3.7500000	-50.0000000
47540.0000000	4.0000000	-50.0000000
47540.0000000	4.2500000	-50.0000000
47540.0000000	4.5000000	-50.0000000
47540.0000000	4.7500000	-50.0000000
47540.0000000	5.0000000	-50.0000000
47540.0000000	5.2500000	-50.0000000
47540.0000000	5.5000000	-50.0000000
47540.0000000	5.7500000	-50.0000000
47540.0000000	6.0000000	-50.0000000
47540.0000000	6.2500000	-50.0000000
47540.0000000	6.5000000	-50.0000000
47540.0000000	6.7500000	-50.0000000
47540.0000000	7.0000000	-50.0000000
47540.0000000	7.2500000	-50.0000000
47540.0000000	7.5000000	-50.0000000
47540.0000000	7.7500000	-50.0000000
47540.0000000	8.0000000	-50.0000000
47540.0000000	8.2500000	-50.0000000
47540.0000000	8.5000000	-50.0000000
47540.0000000	8.7500000	-50.0000000
47540.0000000	9.0000000	-50.0000000
47540.0000000	9.2500000	-50.0000000
47540.0000000	9.5000000	-50.0000000
47540.0000000	9.7500000	-50.0000000
47540.0000000	10.0000000	-50.0000000
47540.0000000	10.2500000	-50.0000000
47540.0000000	10.5000000	-50.0000000
47540.0000000	10.7500000	-50.0000000
47540.0000000	11.0000000	-50.0000000
47540.0000000	11.2500000	-50.0000000
47540.0000000	11.5000000	-50.0000000
47540.0000000	11.7500000	-50.0000000
47540.0000000	12.0000000	-50.0000000
47540.0000000	12.2500000	-50.0000000
47540.0000000	12.5000000	-50.0000000
47540.0000000	12.7500000	-50.0000000
47540.0000000	13.0000000	-50.0000000
47540.0000000	13.2500000	-50.0000000
47540.0000000	13.5000000	-50.0000000
47540.0000000	13.7500000	-50.0000000
47540.0000000	14.0000000	-50.0000000
47540.0000000	14.2500000	-50.0000000
47540.0000000	14.5000000	-50.0000000
47540.0000000	14.7500000	-50.0000000
47540.0000000	15.0000000	-50.0000000
47570.0000000	0.0000000	-50.0000000
47570.0000000	0.2500000	-50.0000000
47570.0000000	0.5000000	-50.0000000
47570.0000000	0.7500000	17.7500000
47570.0000000	1.0000000	16.1399994

47570.0000000	1.2500000	-50.0000000
47570.0000000	1.5000000	-50.0000000
47570.0000000	1.7500000	-50.0000000
47570.0000000	2.0000000	-50.0000000
47570.0000000	2.2500000	-50.0000000
47570.0000000	2.5000000	-50.0000000
47570.0000000	2.7500000	-50.0000000
47570.0000000	3.0000000	-50.0000000
47570.0000000	3.2500000	-50.0000000
47570.0000000	3.5000000	-50.0000000
47570.0000000	3.7500000	-50.0000000
47570.0000000	4.0000000	-50.0000000
47570.0000000	4.2500000	-50.0000000
47570.0000000	4.5000000	-50.0000000
47570.0000000	4.7500000	-50.0000000
47570.0000000	5.0000000	-50.0000000
47570.0000000	5.2500000	-50.0000000
47570.0000000	5.5000000	-50.0000000
47570.0000000	5.7500000	-50.0000000
47570.0000000	6.0000000	-50.0000000
47570.0000000	6.2500000	-50.0000000
47570.0000000	6.5000000	-50.0000000
47570.0000000	6.7500000	-50.0000000
47570.0000000	7.0000000	-50.0000000
47570.0000000	7.2500000	-50.0000000
47570.0000000	7.5000000	-50.0000000
47570.0000000	7.7500000	-50.0000000
47570.0000000	8.0000000	-50.0000000
47570.0000000	8.2500000	-50.0000000
47570.0000000	8.5000000	-50.0000000
47570.0000000	8.7500000	-50.0000000
47570.0000000	9.0000000	-50.0000000
47570.0000000	9.2500000	-50.0000000
47570.0000000	9.5000000	-50.0000000
47570.0000000	9.7500000	-50.0000000
47570.0000000	10.0000000	-50.0000000
47570.0000000	10.2500000	-50.0000000
47570.0000000	10.5000000	-50.0000000
47570.0000000	10.7500000	-50.0000000
47570.0000000	11.0000000	-50.0000000
47570.0000000	11.2500000	-50.0000000
47570.0000000	11.5000000	-50.0000000
47570.0000000	11.7500000	-50.0000000
47570.0000000	12.0000000	-50.0000000
47570.0000000	12.2500000	-50.0000000
47570.0000000	12.5000000	-50.0000000
47570.0000000	12.7500000	-50.0000000
47570.0000000	13.0000000	-50.0000000
47570.0000000	13.2500000	-50.0000000
47570.0000000	13.5000000	-50.0000000
47570.0000000	13.7500000	-50.0000000
47570.0000000	14.0000000	-50.0000000
47570.0000000	14.2500000	-50.0000000
47570.0000000	14.5000000	-50.0000000
47570.0000000	14.7500000	-50.0000000
47570.0000000	15.0000000	-50.0000000
47600.0000000	0.0000000	-50.0000000
47600.0000000	0.2500000	-50.0000000

47600.0000000	0.5000000	16.2099991
47600.0000000	0.7500000	19.6800003
47600.0000000	1.0000000	16.6800003
47600.0000000	1.2500000	-50.0000000
47600.0000000	1.5000000	-50.0000000
47600.0000000	1.7500000	-50.0000000
47600.0000000	2.0000000	-50.0000000
47600.0000000	2.2500000	-50.0000000
47600.0000000	2.5000000	-50.0000000
47600.0000000	2.7500000	-50.0000000
47600.0000000	3.0000000	-50.0000000
47600.0000000	3.2500000	-50.0000000
47600.0000000	3.5000000	-50.0000000
47600.0000000	3.7500000	-50.0000000
47600.0000000	4.0000000	-50.0000000
47600.0000000	4.2500000	-50.0000000
47600.0000000	4.5000000	-50.0000000
47600.0000000	4.7500000	-50.0000000
47600.0000000	5.0000000	-50.0000000
47600.0000000	5.2500000	-50.0000000
47600.0000000	5.5000000	-50.0000000
47600.0000000	5.7500000	-50.0000000
47600.0000000	6.0000000	-50.0000000
47600.0000000	6.2500000	-50.0000000
47600.0000000	6.5000000	-50.0000000
47600.0000000	6.7500000	-50.0000000
47600.0000000	7.0000000	-50.0000000
47600.0000000	7.2500000	-50.0000000
47600.0000000	7.5000000	-50.0000000
47600.0000000	7.7500000	-50.0000000
47600.0000000	8.0000000	-50.0000000
47600.0000000	8.2500000	-50.0000000
47600.0000000	8.5000000	-50.0000000
47600.0000000	8.7500000	-50.0000000
47600.0000000	9.0000000	-50.0000000
47600.0000000	9.2500000	-50.0000000
47600.0000000	9.5000000	-50.0000000
47600.0000000	9.7500000	-50.0000000
47600.0000000	10.0000000	-50.0000000
47600.0000000	10.2500000	-50.0000000
47600.0000000	10.5000000	-50.0000000
47600.0000000	10.7500000	-50.0000000
47600.0000000	11.0000000	-50.0000000
47600.0000000	11.2500000	-50.0000000
47600.0000000	11.5000000	-50.0000000
47600.0000000	11.7500000	-50.0000000
47600.0000000	12.0000000	-50.0000000
47600.0000000	12.2500000	-50.0000000
47600.0000000	12.5000000	-50.0000000
47600.0000000	12.7500000	-50.0000000
47600.0000000	13.0000000	-50.0000000
47600.0000000	13.2500000	-50.0000000
47600.0000000	13.5000000	-50.0000000
47600.0000000	13.7500000	-50.0000000
47600.0000000	14.0000000	-50.0000000
47600.0000000	14.2500000	-50.0000000
47600.0000000	14.5000000	-50.0000000
47600.0000000	14.7500000	-50.0000000

47600.0000000	15.0000000	-50.0000000
47630.0000000	0.0000000	-50.0000000
47630.0000000	0.2500000	-50.0000000
47630.0000000	0.5000000	-50.0000000
47630.0000000	0.7500000	19.3299999
47630.0000000	1.0000000	17.3400002
47630.0000000	1.2500000	-50.0000000
47630.0000000	1.5000000	-50.0000000
47630.0000000	1.7500000	-50.0000000
47630.0000000	2.0000000	-50.0000000
47630.0000000	2.2500000	-50.0000000
47630.0000000	2.5000000	-50.0000000
47630.0000000	2.7500000	-50.0000000
47630.0000000	3.0000000	-50.0000000
47630.0000000	3.2500000	-50.0000000
47630.0000000	3.5000000	-50.0000000
47630.0000000	3.7500000	-50.0000000
47630.0000000	4.0000000	-50.0000000
47630.0000000	4.2500000	-50.0000000
47630.0000000	4.5000000	-50.0000000
47630.0000000	4.7500000	-50.0000000
47630.0000000	5.0000000	-50.0000000
47630.0000000	5.2500000	-50.0000000
47630.0000000	5.5000000	-50.0000000
47630.0000000	5.7500000	-50.0000000
47630.0000000	6.0000000	-50.0000000
47630.0000000	6.2500000	-50.0000000
47630.0000000	6.5000000	-50.0000000
47630.0000000	6.7500000	-50.0000000
47630.0000000	7.0000000	-50.0000000
47630.0000000	7.2500000	-50.0000000
47630.0000000	7.5000000	-50.0000000
47630.0000000	7.7500000	-50.0000000
47630.0000000	8.0000000	-50.0000000
47630.0000000	8.2500000	-50.0000000
47630.0000000	8.5000000	-50.0000000
47630.0000000	8.7500000	-50.0000000
47630.0000000	9.0000000	-50.0000000
47630.0000000	9.2500000	-50.0000000
47630.0000000	9.5000000	-50.0000000
47630.0000000	9.7500000	-50.0000000
47630.0000000	10.0000000	-50.0000000
47630.0000000	10.2500000	-50.0000000
47630.0000000	10.5000000	-50.0000000
47630.0000000	10.7500000	-50.0000000
47630.0000000	11.0000000	-50.0000000
47630.0000000	11.2500000	-50.0000000
47630.0000000	11.5000000	-50.0000000
47630.0000000	11.7500000	-50.0000000
47630.0000000	12.0000000	-50.0000000
47630.0000000	12.2500000	-50.0000000
47630.0000000	12.5000000	-50.0000000
47630.0000000	12.7500000	-50.0000000
47630.0000000	13.0000000	-50.0000000
47630.0000000	13.2500000	-50.0000000
47630.0000000	13.5000000	-50.0000000
47630.0000000	13.7500000	-50.0000000
47630.0000000	14.0000000	-50.0000000

47630.0000000	14.2500000	-50.0000000
47630.0000000	14.5000000	-50.0000000
47630.0000000	14.7500000	-50.0000000
47630.0000000	15.0000000	-50.0000000
47660.0000000	0.0000000	-50.0000000
47660.0000000	0.2500000	-50.0000000
47660.0000000	0.5000000	-50.0000000
47660.0000000	0.7500000	-50.0000000
47660.0000000	1.0000000	-50.0000000
47660.0000000	1.2500000	-50.0000000
47660.0000000	1.5000000	-50.0000000
47660.0000000	1.7500000	-50.0000000
47660.0000000	2.0000000	-50.0000000
47660.0000000	2.2500000	-50.0000000
47660.0000000	2.5000000	-50.0000000
47660.0000000	2.7500000	-50.0000000
47660.0000000	3.0000000	-50.0000000
47660.0000000	3.2500000	-50.0000000
47660.0000000	3.5000000	-50.0000000
47660.0000000	3.7500000	-50.0000000
47660.0000000	4.0000000	-50.0000000
47660.0000000	4.2500000	-50.0000000
47660.0000000	4.5000000	-50.0000000
47660.0000000	4.7500000	-50.0000000
47660.0000000	5.0000000	-50.0000000
47660.0000000	5.2500000	-50.0000000
47660.0000000	5.5000000	-50.0000000
47660.0000000	5.7500000	-50.0000000
47660.0000000	6.0000000	-50.0000000
47660.0000000	6.2500000	-50.0000000
47660.0000000	6.5000000	-50.0000000
47660.0000000	6.7500000	-50.0000000
47660.0000000	7.0000000	-50.0000000
47660.0000000	7.2500000	-50.0000000
47660.0000000	7.5000000	-50.0000000
47660.0000000	7.7500000	-50.0000000
47660.0000000	8.0000000	-50.0000000
47660.0000000	8.2500000	-50.0000000
47660.0000000	8.5000000	-50.0000000
47660.0000000	8.7500000	-50.0000000
47660.0000000	9.0000000	-50.0000000
47660.0000000	9.2500000	-50.0000000
47660.0000000	9.5000000	-50.0000000
47660.0000000	9.7500000	-50.0000000
47660.0000000	10.0000000	-50.0000000
47660.0000000	10.2500000	-50.0000000
47660.0000000	10.5000000	-50.0000000
47660.0000000	10.7500000	-50.0000000
47660.0000000	11.0000000	-50.0000000
47660.0000000	11.2500000	-50.0000000
47660.0000000	11.5000000	-50.0000000
47660.0000000	11.7500000	-50.0000000
47660.0000000	12.0000000	-50.0000000
47660.0000000	12.2500000	-50.0000000
47660.0000000	12.5000000	-50.0000000
47660.0000000	12.7500000	-50.0000000
47660.0000000	13.0000000	-50.0000000
47660.0000000	13.2500000	-50.0000000

47660.0000000	13.5000000	-50.0000000
47660.0000000	13.7500000	-50.0000000
47660.0000000	14.0000000	-50.0000000
47660.0000000	14.2500000	-50.0000000
47660.0000000	14.5000000	-50.0000000
47660.0000000	14.7500000	-50.0000000
47660.0000000	15.0000000	-50.0000000
47690.0000000	0.0000000	-50.0000000
47690.0000000	0.2500000	-50.0000000
47690.0000000	0.5000000	15.5000000
47690.0000000	0.7500000	17.8999996
47690.0000000	1.0000000	-50.0000000
47690.0000000	1.2500000	-50.0000000
47690.0000000	1.5000000	-50.0000000
47690.0000000	1.7500000	-50.0000000
47690.0000000	2.0000000	-50.0000000
47690.0000000	2.2500000	-50.0000000
47690.0000000	2.5000000	-50.0000000
47690.0000000	2.7500000	-50.0000000
47690.0000000	3.0000000	-50.0000000
47690.0000000	3.2500000	-50.0000000
47690.0000000	3.5000000	-50.0000000
47690.0000000	3.7500000	-50.0000000
47690.0000000	4.0000000	-50.0000000
47690.0000000	4.2500000	-50.0000000
47690.0000000	4.5000000	-50.0000000
47690.0000000	4.7500000	-50.0000000
47690.0000000	5.0000000	-50.0000000
47690.0000000	5.2500000	-50.0000000
47690.0000000	5.5000000	-50.0000000
47690.0000000	5.7500000	-50.0000000
47690.0000000	6.0000000	-50.0000000
47690.0000000	6.2500000	-50.0000000
47690.0000000	6.5000000	-50.0000000
47690.0000000	6.7500000	-50.0000000
47690.0000000	7.0000000	-50.0000000
47690.0000000	7.2500000	-50.0000000
47690.0000000	7.5000000	-50.0000000
47690.0000000	7.7500000	-50.0000000
47690.0000000	8.0000000	-50.0000000
47690.0000000	8.2500000	-50.0000000
47690.0000000	8.5000000	-50.0000000
47690.0000000	8.7500000	-50.0000000
47690.0000000	9.0000000	-50.0000000
47690.0000000	9.2500000	-50.0000000
47690.0000000	9.5000000	-50.0000000
47690.0000000	9.7500000	-50.0000000
47690.0000000	10.0000000	-50.0000000
47690.0000000	10.2500000	-50.0000000
47690.0000000	10.5000000	-50.0000000
47690.0000000	10.7500000	-50.0000000
47690.0000000	11.0000000	-50.0000000
47690.0000000	11.2500000	-50.0000000
47690.0000000	11.5000000	-50.0000000
47690.0000000	11.7500000	-50.0000000
47690.0000000	12.0000000	-50.0000000
47690.0000000	12.2500000	-50.0000000
47690.0000000	12.5000000	-50.0000000

47690.0000000	12.7500000	-50.0000000
47690.0000000	13.0000000	-50.0000000
47690.0000000	13.2500000	-50.0000000
47690.0000000	13.5000000	-50.0000000
47690.0000000	13.7500000	-50.0000000
47690.0000000	14.0000000	-50.0000000
47690.0000000	14.2500000	-50.0000000
47690.0000000	14.5000000	-50.0000000
47690.0000000	14.7500000	-50.0000000
47690.0000000	15.0000000	-50.0000000
47720.0000000	0.0000000	-50.0000000
47720.0000000	0.2500000	-50.0000000
47720.0000000	0.5000000	-50.0000000
47720.0000000	0.7500000	17.7000008
47720.0000000	1.0000000	-50.0000000
47720.0000000	1.2500000	-50.0000000
47720.0000000	1.5000000	-50.0000000
47720.0000000	1.7500000	-50.0000000
47720.0000000	2.0000000	-50.0000000
47720.0000000	2.2500000	-50.0000000
47720.0000000	2.5000000	-50.0000000
47720.0000000	2.7500000	-50.0000000
47720.0000000	3.0000000	-50.0000000
47720.0000000	3.2500000	-50.0000000
47720.0000000	3.5000000	-50.0000000
47720.0000000	3.7500000	-50.0000000
47720.0000000	4.0000000	-50.0000000
47720.0000000	4.2500000	-50.0000000
47720.0000000	4.5000000	-50.0000000
47720.0000000	4.7500000	-50.0000000
47720.0000000	5.0000000	-50.0000000
47720.0000000	5.2500000	-50.0000000
47720.0000000	5.5000000	-50.0000000
47720.0000000	5.7500000	-50.0000000
47720.0000000	6.0000000	-50.0000000
47720.0000000	6.2500000	-50.0000000
47720.0000000	6.5000000	-50.0000000
47720.0000000	6.7500000	-50.0000000
47720.0000000	7.0000000	-50.0000000
47720.0000000	7.2500000	-50.0000000
47720.0000000	7.5000000	-50.0000000
47720.0000000	7.7500000	-50.0000000
47720.0000000	8.0000000	-50.0000000
47720.0000000	8.2500000	-50.0000000
47720.0000000	8.5000000	-50.0000000
47720.0000000	8.7500000	-50.0000000
47720.0000000	9.0000000	-50.0000000
47720.0000000	9.2500000	-50.0000000
47720.0000000	9.5000000	-50.0000000
47720.0000000	9.7500000	-50.0000000
47720.0000000	10.0000000	-50.0000000
47720.0000000	10.2500000	-50.0000000
47720.0000000	10.5000000	-50.0000000
47720.0000000	10.7500000	-50.0000000
47720.0000000	11.0000000	-50.0000000
47720.0000000	11.2500000	-50.0000000
47720.0000000	11.5000000	-50.0000000
47720.0000000	11.7500000	-50.0000000

47720.0000000	12.0000000	-50.0000000
47720.0000000	12.2500000	-50.0000000
47720.0000000	12.5000000	-50.0000000
47720.0000000	12.7500000	-50.0000000
47720.0000000	13.0000000	-50.0000000
47720.0000000	13.2500000	-50.0000000
47720.0000000	13.5000000	-50.0000000
47720.0000000	13.7500000	-50.0000000
47720.0000000	14.0000000	-50.0000000
47720.0000000	14.2500000	-50.0000000
47720.0000000	14.5000000	-50.0000000
47720.0000000	14.7500000	-50.0000000
47720.0000000	15.0000000	-50.0000000
47750.0000000	0.0000000	-50.0000000
47750.0000000	0.2500000	-50.0000000
47750.0000000	0.5000000	-50.0000000
47750.0000000	0.7500000	17.1900005
47750.0000000	1.0000000	-50.0000000
47750.0000000	1.2500000	-50.0000000
47750.0000000	1.5000000	-50.0000000
47750.0000000	1.7500000	-50.0000000
47750.0000000	2.0000000	-50.0000000
47750.0000000	2.2500000	-50.0000000
47750.0000000	2.5000000	-50.0000000
47750.0000000	2.7500000	-50.0000000
47750.0000000	3.0000000	-50.0000000
47750.0000000	3.2500000	-50.0000000
47750.0000000	3.5000000	-50.0000000
47750.0000000	3.7500000	-50.0000000
47750.0000000	4.0000000	-50.0000000
47750.0000000	4.2500000	-50.0000000
47750.0000000	4.5000000	-50.0000000
47750.0000000	4.7500000	-50.0000000
47750.0000000	5.0000000	-50.0000000
47750.0000000	5.2500000	-50.0000000
47750.0000000	5.5000000	-50.0000000
47750.0000000	5.7500000	-50.0000000
47750.0000000	6.0000000	-50.0000000
47750.0000000	6.2500000	-50.0000000
47750.0000000	6.5000000	-50.0000000
47750.0000000	6.7500000	-50.0000000
47750.0000000	7.0000000	-50.0000000
47750.0000000	7.2500000	-50.0000000
47750.0000000	7.5000000	-50.0000000
47750.0000000	7.7500000	-50.0000000
47750.0000000	8.0000000	-50.0000000
47750.0000000	8.2500000	-50.0000000
47750.0000000	8.5000000	-50.0000000
47750.0000000	8.7500000	-50.0000000
47750.0000000	9.0000000	-50.0000000
47750.0000000	9.2500000	-50.0000000
47750.0000000	9.5000000	-50.0000000
47750.0000000	9.7500000	-50.0000000
47750.0000000	10.0000000	-50.0000000
47750.0000000	10.2500000	-50.0000000
47750.0000000	10.5000000	-50.0000000
47750.0000000	10.7500000	-50.0000000
47750.0000000	11.0000000	-50.0000000

47750.0000000	11.2500000	-50.0000000
47750.0000000	11.5000000	-50.0000000
47750.0000000	11.7500000	-50.0000000
47750.0000000	12.0000000	-50.0000000
47750.0000000	12.2500000	-50.0000000
47750.0000000	12.5000000	-50.0000000
47750.0000000	12.7500000	-50.0000000
47750.0000000	13.0000000	-50.0000000
47750.0000000	13.2500000	-50.0000000
47750.0000000	13.5000000	-50.0000000
47750.0000000	13.7500000	-50.0000000
47750.0000000	14.0000000	-50.0000000
47750.0000000	14.2500000	-50.0000000
47750.0000000	14.5000000	-50.0000000
47750.0000000	14.7500000	-50.0000000
47750.0000000	15.0000000	-50.0000000
47780.0000000	0.0000000	-50.0000000
47780.0000000	0.2500000	-50.0000000
47780.0000000	0.5000000	-50.0000000
47780.0000000	0.7500000	15.7700005
47780.0000000	1.0000000	-50.0000000
47780.0000000	1.2500000	-50.0000000
47780.0000000	1.5000000	-50.0000000
47780.0000000	1.7500000	-50.0000000
47780.0000000	2.0000000	-50.0000000
47780.0000000	2.2500000	-50.0000000
47780.0000000	2.5000000	-50.0000000
47780.0000000	2.7500000	-50.0000000
47780.0000000	3.0000000	-50.0000000
47780.0000000	3.2500000	-50.0000000
47780.0000000	3.5000000	-50.0000000
47780.0000000	3.7500000	-50.0000000
47780.0000000	4.0000000	-50.0000000
47780.0000000	4.2500000	-50.0000000
47780.0000000	4.5000000	-50.0000000
47780.0000000	4.7500000	-50.0000000
47780.0000000	5.0000000	-50.0000000
47780.0000000	5.2500000	-50.0000000
47780.0000000	5.5000000	-50.0000000
47780.0000000	5.7500000	-50.0000000
47780.0000000	6.0000000	-50.0000000
47780.0000000	6.2500000	-50.0000000
47780.0000000	6.5000000	-50.0000000
47780.0000000	6.7500000	-50.0000000
47780.0000000	7.0000000	-50.0000000
47780.0000000	7.2500000	-50.0000000
47780.0000000	7.5000000	-50.0000000
47780.0000000	7.7500000	-50.0000000
47780.0000000	8.0000000	-50.0000000
47780.0000000	8.2500000	-50.0000000
47780.0000000	8.5000000	-50.0000000
47780.0000000	8.7500000	-50.0000000
47780.0000000	9.0000000	-50.0000000
47780.0000000	9.2500000	-50.0000000
47780.0000000	9.5000000	-50.0000000
47780.0000000	9.7500000	-50.0000000
47780.0000000	10.0000000	-50.0000000
47780.0000000	10.2500000	-50.0000000

47780.0000000	10.5000000	-50.0000000
47780.0000000	10.7500000	-50.0000000
47780.0000000	11.0000000	-50.0000000
47780.0000000	11.2500000	-50.0000000
47780.0000000	11.5000000	-50.0000000
47780.0000000	11.7500000	-50.0000000
47780.0000000	12.0000000	-50.0000000
47780.0000000	12.2500000	-50.0000000
47780.0000000	12.5000000	-50.0000000
47780.0000000	12.7500000	-50.0000000
47780.0000000	13.0000000	-50.0000000
47780.0000000	13.2500000	-50.0000000
47780.0000000	13.5000000	-50.0000000
47780.0000000	13.7500000	-50.0000000
47780.0000000	14.0000000	-50.0000000
47780.0000000	14.2500000	-50.0000000
47780.0000000	14.5000000	-50.0000000
47780.0000000	14.7500000	-50.0000000
47780.0000000	15.0000000	-50.0000000
47810.0000000	0.0000000	-50.0000000
47810.0000000	0.2500000	-50.0000000
47810.0000000	0.5000000	-50.0000000
47810.0000000	0.7500000	-50.0000000
47810.0000000	1.0000000	-50.0000000
47810.0000000	1.2500000	-50.0000000
47810.0000000	1.5000000	-50.0000000
47810.0000000	1.7500000	-50.0000000
47810.0000000	2.0000000	-50.0000000
47810.0000000	2.2500000	-50.0000000
47810.0000000	2.5000000	-50.0000000
47810.0000000	2.7500000	-50.0000000
47810.0000000	3.0000000	-50.0000000
47810.0000000	3.2500000	-50.0000000
47810.0000000	3.5000000	-50.0000000
47810.0000000	3.7500000	-50.0000000
47810.0000000	4.0000000	-50.0000000
47810.0000000	4.2500000	-50.0000000
47810.0000000	4.5000000	-50.0000000
47810.0000000	4.7500000	-50.0000000
47810.0000000	5.0000000	-50.0000000
47810.0000000	5.2500000	-50.0000000
47810.0000000	5.5000000	-50.0000000
47810.0000000	5.7500000	-50.0000000
47810.0000000	6.0000000	-50.0000000
47810.0000000	6.2500000	-50.0000000
47810.0000000	6.5000000	-50.0000000
47810.0000000	6.7500000	-50.0000000
47810.0000000	7.0000000	-50.0000000
47810.0000000	7.2500000	-50.0000000
47810.0000000	7.5000000	-50.0000000
47810.0000000	7.7500000	-50.0000000
47810.0000000	8.0000000	-50.0000000
47810.0000000	8.2500000	-50.0000000
47810.0000000	8.5000000	-50.0000000
47810.0000000	8.7500000	-50.0000000
47810.0000000	9.0000000	-50.0000000
47810.0000000	9.2500000	-50.0000000
47810.0000000	9.5000000	-50.0000000

47810.0000000	9.7500000	-50.0000000
47810.0000000	10.0000000	-50.0000000
47810.0000000	10.2500000	-50.0000000
47810.0000000	10.5000000	-50.0000000
47810.0000000	10.7500000	-50.0000000
47810.0000000	11.0000000	-50.0000000
47810.0000000	11.2500000	-50.0000000
47810.0000000	11.5000000	-50.0000000
47810.0000000	11.7500000	-50.0000000
47810.0000000	12.0000000	-50.0000000
47810.0000000	12.2500000	-50.0000000
47810.0000000	12.5000000	-50.0000000
47810.0000000	12.7500000	-50.0000000
47810.0000000	13.0000000	-50.0000000
47810.0000000	13.2500000	-50.0000000
47810.0000000	13.5000000	-50.0000000
47810.0000000	13.7500000	-50.0000000
47810.0000000	14.0000000	-50.0000000
47810.0000000	14.2500000	-50.0000000
47810.0000000	14.5000000	-50.0000000
47810.0000000	14.7500000	-50.0000000
47810.0000000	15.0000000	-50.0000000
47840.0000000	0.0000000	-50.0000000
47840.0000000	0.2500000	-50.0000000
47840.0000000	0.5000000	-50.0000000
47840.0000000	0.7500000	-50.0000000
47840.0000000	1.0000000	-50.0000000
47840.0000000	1.2500000	-50.0000000
47840.0000000	1.5000000	-50.0000000
47840.0000000	1.7500000	-50.0000000
47840.0000000	2.0000000	-50.0000000
47840.0000000	2.2500000	-50.0000000
47840.0000000	2.5000000	-50.0000000
47840.0000000	2.7500000	-50.0000000
47840.0000000	3.0000000	-50.0000000
47840.0000000	3.2500000	-50.0000000
47840.0000000	3.5000000	-50.0000000
47840.0000000	3.7500000	-50.0000000
47840.0000000	4.0000000	-50.0000000
47840.0000000	4.2500000	-50.0000000
47840.0000000	4.5000000	-50.0000000
47840.0000000	4.7500000	-50.0000000
47840.0000000	5.0000000	-50.0000000
47840.0000000	5.2500000	-50.0000000
47840.0000000	5.5000000	-50.0000000
47840.0000000	5.7500000	-50.0000000
47840.0000000	6.0000000	-50.0000000
47840.0000000	6.2500000	-50.0000000
47840.0000000	6.5000000	-50.0000000
47840.0000000	6.7500000	-50.0000000
47840.0000000	7.0000000	-50.0000000
47840.0000000	7.2500000	-50.0000000
47840.0000000	7.5000000	-50.0000000
47840.0000000	7.7500000	-50.0000000
47840.0000000	8.0000000	-50.0000000
47840.0000000	8.2500000	-50.0000000
47840.0000000	8.5000000	-50.0000000
47840.0000000	8.7500000	-50.0000000

47840.0000000	9.0000000	-50.0000000
47840.0000000	9.2500000	-50.0000000
47840.0000000	9.5000000	-50.0000000
47840.0000000	9.7500000	-50.0000000
47840.0000000	10.0000000	-50.0000000
47840.0000000	10.2500000	-50.0000000
47840.0000000	10.5000000	-50.0000000
47840.0000000	10.7500000	-50.0000000
47840.0000000	11.0000000	-50.0000000
47840.0000000	11.2500000	-50.0000000
47840.0000000	11.5000000	-50.0000000
47840.0000000	11.7500000	-50.0000000
47840.0000000	12.0000000	-50.0000000
47840.0000000	12.2500000	-50.0000000
47840.0000000	12.5000000	-50.0000000
47840.0000000	12.7500000	-50.0000000
47840.0000000	13.0000000	-50.0000000
47840.0000000	13.2500000	-50.0000000
47840.0000000	13.5000000	-50.0000000
47840.0000000	13.7500000	-50.0000000
47840.0000000	14.0000000	-50.0000000
47840.0000000	14.2500000	-50.0000000
47840.0000000	14.5000000	-50.0000000
47840.0000000	14.7500000	-50.0000000
47840.0000000	15.0000000	-50.0000000
47870.0000000	0.0000000	-50.0000000
47870.0000000	0.2500000	-50.0000000
47870.0000000	0.5000000	-50.0000000
47870.0000000	0.7500000	-50.0000000
47870.0000000	1.0000000	-50.0000000
47870.0000000	1.2500000	-50.0000000
47870.0000000	1.5000000	-50.0000000
47870.0000000	1.7500000	-50.0000000
47870.0000000	2.0000000	-50.0000000
47870.0000000	2.2500000	-50.0000000
47870.0000000	2.5000000	-50.0000000
47870.0000000	2.7500000	-50.0000000
47870.0000000	3.0000000	-50.0000000
47870.0000000	3.2500000	-50.0000000
47870.0000000	3.5000000	-50.0000000
47870.0000000	3.7500000	-50.0000000
47870.0000000	4.0000000	-50.0000000
47870.0000000	4.2500000	-50.0000000
47870.0000000	4.5000000	-50.0000000
47870.0000000	4.7500000	-50.0000000
47870.0000000	5.0000000	-50.0000000
47870.0000000	5.2500000	-50.0000000
47870.0000000	5.5000000	-50.0000000
47870.0000000	5.7500000	-50.0000000
47870.0000000	6.0000000	-50.0000000
47870.0000000	6.2500000	-50.0000000
47870.0000000	6.5000000	-50.0000000
47870.0000000	6.7500000	-50.0000000
47870.0000000	7.0000000	-50.0000000
47870.0000000	7.2500000	-50.0000000
47870.0000000	7.5000000	-50.0000000
47870.0000000	7.7500000	-50.0000000
47870.0000000	8.0000000	-50.0000000

47870.0000000	8.2500000	-50.0000000
47870.0000000	8.5000000	-50.0000000
47870.0000000	8.7500000	-50.0000000
47870.0000000	9.0000000	-50.0000000
47870.0000000	9.2500000	-50.0000000
47870.0000000	9.5000000	-50.0000000
47870.0000000	9.7500000	-50.0000000
47870.0000000	10.0000000	-50.0000000
47870.0000000	10.2500000	-50.0000000
47870.0000000	10.5000000	-50.0000000
47870.0000000	10.7500000	-50.0000000
47870.0000000	11.0000000	-50.0000000
47870.0000000	11.2500000	-50.0000000
47870.0000000	11.5000000	-50.0000000
47870.0000000	11.7500000	-50.0000000
47870.0000000	12.0000000	-50.0000000
47870.0000000	12.2500000	-50.0000000
47870.0000000	12.5000000	-50.0000000
47870.0000000	12.7500000	-50.0000000
47870.0000000	13.0000000	-50.0000000
47870.0000000	13.2500000	-50.0000000
47870.0000000	13.5000000	-50.0000000
47870.0000000	13.7500000	-50.0000000
47870.0000000	14.0000000	-50.0000000
47870.0000000	14.2500000	-50.0000000
47870.0000000	14.5000000	-50.0000000
47870.0000000	14.7500000	-50.0000000
47870.0000000	15.0000000	-50.0000000
47900.0000000	0.0000000	-50.0000000
47900.0000000	0.2500000	-50.0000000
47900.0000000	0.5000000	-50.0000000
47900.0000000	0.7500000	-50.0000000
47900.0000000	1.0000000	-50.0000000
47900.0000000	1.2500000	-50.0000000
47900.0000000	1.5000000	-50.0000000
47900.0000000	1.7500000	-50.0000000
47900.0000000	2.0000000	-50.0000000
47900.0000000	2.2500000	-50.0000000
47900.0000000	2.5000000	-50.0000000
47900.0000000	2.7500000	-50.0000000
47900.0000000	3.0000000	-50.0000000
47900.0000000	3.2500000	-50.0000000
47900.0000000	3.5000000	-50.0000000
47900.0000000	3.7500000	-50.0000000
47900.0000000	4.0000000	-50.0000000
47900.0000000	4.2500000	-50.0000000
47900.0000000	4.5000000	-50.0000000
47900.0000000	4.7500000	-50.0000000
47900.0000000	5.0000000	-50.0000000
47900.0000000	5.2500000	-50.0000000
47900.0000000	5.5000000	-50.0000000
47900.0000000	5.7500000	-50.0000000
47900.0000000	6.0000000	-50.0000000
47900.0000000	6.2500000	-50.0000000
47900.0000000	6.5000000	-50.0000000
47900.0000000	6.7500000	-50.0000000
47900.0000000	7.0000000	-50.0000000
47900.0000000	7.2500000	-50.0000000

47900.0000000	7.5000000	-50.0000000
47900.0000000	7.7500000	-50.0000000
47900.0000000	8.0000000	-50.0000000
47900.0000000	8.2500000	-50.0000000
47900.0000000	8.5000000	-50.0000000
47900.0000000	8.7500000	-50.0000000
47900.0000000	9.0000000	-50.0000000
47900.0000000	9.2500000	-50.0000000
47900.0000000	9.5000000	-50.0000000
47900.0000000	9.7500000	-50.0000000
47900.0000000	10.0000000	-50.0000000
47900.0000000	10.2500000	-50.0000000
47900.0000000	10.5000000	-50.0000000
47900.0000000	10.7500000	-50.0000000
47900.0000000	11.0000000	-50.0000000
47900.0000000	11.2500000	-50.0000000
47900.0000000	11.5000000	-50.0000000
47900.0000000	11.7500000	-50.0000000
47900.0000000	12.0000000	-50.0000000
47900.0000000	12.2500000	-50.0000000
47900.0000000	12.5000000	-50.0000000
47900.0000000	12.7500000	-50.0000000
47900.0000000	13.0000000	-50.0000000
47900.0000000	13.2500000	-50.0000000
47900.0000000	13.5000000	-50.0000000
47900.0000000	13.7500000	-50.0000000
47900.0000000	14.0000000	-50.0000000
47900.0000000	14.2500000	-50.0000000
47900.0000000	14.5000000	-50.0000000
47900.0000000	14.7500000	-50.0000000
47900.0000000	15.0000000	-50.0000000
47930.0000000	0.0000000	-50.0000000
47930.0000000	0.2500000	-50.0000000
47930.0000000	0.5000000	-50.0000000
47930.0000000	0.7500000	-50.0000000
47930.0000000	1.0000000	-50.0000000
47930.0000000	1.2500000	-50.0000000
47930.0000000	1.5000000	-50.0000000
47930.0000000	1.7500000	-50.0000000
47930.0000000	2.0000000	-50.0000000
47930.0000000	2.2500000	-50.0000000
47930.0000000	2.5000000	-50.0000000
47930.0000000	2.7500000	-50.0000000
47930.0000000	3.0000000	-50.0000000
47930.0000000	3.2500000	-50.0000000
47930.0000000	3.5000000	-50.0000000
47930.0000000	3.7500000	-50.0000000
47930.0000000	4.0000000	-50.0000000
47930.0000000	4.2500000	-50.0000000
47930.0000000	4.5000000	-50.0000000
47930.0000000	4.7500000	-50.0000000
47930.0000000	5.0000000	-50.0000000
47930.0000000	5.2500000	-50.0000000
47930.0000000	5.5000000	-50.0000000
47930.0000000	5.7500000	-50.0000000
47930.0000000	6.0000000	-50.0000000
47930.0000000	6.2500000	-50.0000000
47930.0000000	6.5000000	-50.0000000

47930.0000000	6.7500000	-50.0000000
47930.0000000	7.0000000	-50.0000000
47930.0000000	7.2500000	-50.0000000
47930.0000000	7.5000000	-50.0000000
47930.0000000	7.7500000	-50.0000000
47930.0000000	8.0000000	-50.0000000
47930.0000000	8.2500000	-50.0000000
47930.0000000	8.5000000	-50.0000000
47930.0000000	8.7500000	-50.0000000
47930.0000000	9.0000000	-50.0000000
47930.0000000	9.2500000	-50.0000000
47930.0000000	9.5000000	-50.0000000
47930.0000000	9.7500000	-50.0000000
47930.0000000	10.0000000	-50.0000000
47930.0000000	10.2500000	-50.0000000
47930.0000000	10.5000000	-50.0000000
47930.0000000	10.7500000	-50.0000000
47930.0000000	11.0000000	-50.0000000
47930.0000000	11.2500000	-50.0000000
47930.0000000	11.5000000	-50.0000000
47930.0000000	11.7500000	-50.0000000
47930.0000000	12.0000000	-50.0000000
47930.0000000	12.2500000	-50.0000000
47930.0000000	12.5000000	-50.0000000
47930.0000000	12.7500000	-50.0000000
47930.0000000	13.0000000	-50.0000000
47930.0000000	13.2500000	-50.0000000
47930.0000000	13.5000000	-50.0000000
47930.0000000	13.7500000	-50.0000000
47930.0000000	14.0000000	-50.0000000
47930.0000000	14.2500000	-50.0000000
47930.0000000	14.5000000	-50.0000000
47930.0000000	14.7500000	-50.0000000
47930.0000000	15.0000000	-50.0000000
47960.0000000	0.0000000	-50.0000000
47960.0000000	0.2500000	-50.0000000
47960.0000000	0.5000000	-50.0000000
47960.0000000	0.7500000	-50.0000000
47960.0000000	1.0000000	-50.0000000
47960.0000000	1.2500000	-50.0000000
47960.0000000	1.5000000	-50.0000000
47960.0000000	1.7500000	-50.0000000
47960.0000000	2.0000000	-50.0000000
47960.0000000	2.2500000	-50.0000000
47960.0000000	2.5000000	-50.0000000
47960.0000000	2.7500000	-50.0000000
47960.0000000	3.0000000	-50.0000000
47960.0000000	3.2500000	-50.0000000
47960.0000000	3.5000000	-50.0000000
47960.0000000	3.7500000	-50.0000000
47960.0000000	4.0000000	-50.0000000
47960.0000000	4.2500000	-50.0000000
47960.0000000	4.5000000	-50.0000000
47960.0000000	4.7500000	-50.0000000
47960.0000000	5.0000000	-50.0000000
47960.0000000	5.2500000	-50.0000000
47960.0000000	5.5000000	-50.0000000
47960.0000000	5.7500000	-50.0000000

47960.0000000	6.0000000	-50.0000000
47960.0000000	6.2500000	-50.0000000
47960.0000000	6.5000000	-50.0000000
47960.0000000	6.7500000	-50.0000000
47960.0000000	7.0000000	-50.0000000
47960.0000000	7.2500000	-50.0000000
47960.0000000	7.5000000	-50.0000000
47960.0000000	7.7500000	-50.0000000
47960.0000000	8.0000000	-50.0000000
47960.0000000	8.2500000	-50.0000000
47960.0000000	8.5000000	-50.0000000
47960.0000000	8.7500000	-50.0000000
47960.0000000	9.0000000	-50.0000000
47960.0000000	9.2500000	-50.0000000
47960.0000000	9.5000000	-50.0000000
47960.0000000	9.7500000	-50.0000000
47960.0000000	10.0000000	-50.0000000
47960.0000000	10.2500000	-50.0000000
47960.0000000	10.5000000	-50.0000000
47960.0000000	10.7500000	-50.0000000
47960.0000000	11.0000000	-50.0000000
47960.0000000	11.2500000	-50.0000000
47960.0000000	11.5000000	-50.0000000
47960.0000000	11.7500000	-50.0000000
47960.0000000	12.0000000	-50.0000000
47960.0000000	12.2500000	-50.0000000
47960.0000000	12.5000000	-50.0000000
47960.0000000	12.7500000	-50.0000000
47960.0000000	13.0000000	-50.0000000
47960.0000000	13.2500000	-50.0000000
47960.0000000	13.5000000	-50.0000000
47960.0000000	13.7500000	-50.0000000
47960.0000000	14.0000000	-50.0000000
47960.0000000	14.2500000	-50.0000000
47960.0000000	14.5000000	-50.0000000
47960.0000000	14.7500000	-50.0000000
47960.0000000	15.0000000	-50.0000000
47990.0000000	0.0000000	-50.0000000
47990.0000000	0.2500000	-50.0000000
47990.0000000	0.5000000	-50.0000000
47990.0000000	0.7500000	-50.0000000
47990.0000000	1.0000000	-50.0000000
47990.0000000	1.2500000	-50.0000000
47990.0000000	1.5000000	-50.0000000
47990.0000000	1.7500000	-50.0000000
47990.0000000	2.0000000	-50.0000000
47990.0000000	2.2500000	-50.0000000
47990.0000000	2.5000000	-50.0000000
47990.0000000	2.7500000	-50.0000000
47990.0000000	3.0000000	-50.0000000
47990.0000000	3.2500000	-50.0000000
47990.0000000	3.5000000	-50.0000000
47990.0000000	3.7500000	-50.0000000
47990.0000000	4.0000000	-50.0000000
47990.0000000	4.2500000	-50.0000000
47990.0000000	4.5000000	-50.0000000
47990.0000000	4.7500000	-50.0000000
47990.0000000	5.0000000	-50.0000000

47990.0000000	5.2500000	-50.0000000
47990.0000000	5.5000000	-50.0000000
47990.0000000	5.7500000	-50.0000000
47990.0000000	6.0000000	-50.0000000
47990.0000000	6.2500000	-50.0000000
47990.0000000	6.5000000	-50.0000000
47990.0000000	6.7500000	-50.0000000
47990.0000000	7.0000000	-50.0000000
47990.0000000	7.2500000	-50.0000000
47990.0000000	7.5000000	-50.0000000
47990.0000000	7.7500000	-50.0000000
47990.0000000	8.0000000	-50.0000000
47990.0000000	8.2500000	-50.0000000
47990.0000000	8.5000000	-50.0000000
47990.0000000	8.7500000	-50.0000000
47990.0000000	9.0000000	-50.0000000
47990.0000000	9.2500000	-50.0000000
47990.0000000	9.5000000	-50.0000000
47990.0000000	9.7500000	-50.0000000
47990.0000000	10.0000000	-50.0000000
47990.0000000	10.2500000	-50.0000000
47990.0000000	10.5000000	-50.0000000
47990.0000000	10.7500000	-50.0000000
47990.0000000	11.0000000	-50.0000000
47990.0000000	11.2500000	-50.0000000
47990.0000000	11.5000000	-50.0000000
47990.0000000	11.7500000	-50.0000000
47990.0000000	12.0000000	-50.0000000
47990.0000000	12.2500000	-50.0000000
47990.0000000	12.5000000	-50.0000000
47990.0000000	12.7500000	-50.0000000
47990.0000000	13.0000000	-50.0000000
47990.0000000	13.2500000	-50.0000000
47990.0000000	13.5000000	-50.0000000
47990.0000000	13.7500000	-50.0000000
47990.0000000	14.0000000	-50.0000000
47990.0000000	14.2500000	-50.0000000
47990.0000000	14.5000000	-50.0000000
47990.0000000	14.7500000	-50.0000000
47990.0000000	15.0000000	-50.0000000
48020.0000000	0.0000000	-50.0000000
48020.0000000	0.2500000	-50.0000000
48020.0000000	0.5000000	-50.0000000
48020.0000000	0.7500000	-50.0000000
48020.0000000	1.0000000	-50.0000000
48020.0000000	1.2500000	-50.0000000
48020.0000000	1.5000000	-50.0000000
48020.0000000	1.7500000	-50.0000000
48020.0000000	2.0000000	-50.0000000
48020.0000000	2.2500000	-50.0000000
48020.0000000	2.5000000	-50.0000000
48020.0000000	2.7500000	-50.0000000
48020.0000000	3.0000000	-50.0000000
48020.0000000	3.2500000	-50.0000000
48020.0000000	3.5000000	-50.0000000
48020.0000000	3.7500000	-50.0000000
48020.0000000	4.0000000	-50.0000000
48020.0000000	4.2500000	-50.0000000

48020.0000000	4.5000000	-50.0000000
48020.0000000	4.7500000	-50.0000000
48020.0000000	5.0000000	-50.0000000
48020.0000000	5.2500000	-50.0000000
48020.0000000	5.5000000	-50.0000000
48020.0000000	5.7500000	-50.0000000
48020.0000000	6.0000000	-50.0000000
48020.0000000	6.2500000	-50.0000000
48020.0000000	6.5000000	-50.0000000
48020.0000000	6.7500000	-50.0000000
48020.0000000	7.0000000	-50.0000000
48020.0000000	7.2500000	-50.0000000
48020.0000000	7.5000000	-50.0000000
48020.0000000	7.7500000	-50.0000000
48020.0000000	8.0000000	-50.0000000
48020.0000000	8.2500000	-50.0000000
48020.0000000	8.5000000	-50.0000000
48020.0000000	8.7500000	-50.0000000
48020.0000000	9.0000000	-50.0000000
48020.0000000	9.2500000	-50.0000000
48020.0000000	9.5000000	-50.0000000
48020.0000000	9.7500000	-50.0000000
48020.0000000	10.0000000	-50.0000000
48020.0000000	10.2500000	-50.0000000
48020.0000000	10.5000000	-50.0000000
48020.0000000	10.7500000	-50.0000000
48020.0000000	11.0000000	-50.0000000
48020.0000000	11.2500000	-50.0000000
48020.0000000	11.5000000	-50.0000000
48020.0000000	11.7500000	-50.0000000
48020.0000000	12.0000000	-50.0000000
48020.0000000	12.2500000	-50.0000000
48020.0000000	12.5000000	-50.0000000
48020.0000000	12.7500000	-50.0000000
48020.0000000	13.0000000	-50.0000000
48020.0000000	13.2500000	-50.0000000
48020.0000000	13.5000000	-50.0000000
48020.0000000	13.7500000	-50.0000000
48020.0000000	14.0000000	-50.0000000
48020.0000000	14.2500000	-50.0000000
48020.0000000	14.5000000	-50.0000000
48020.0000000	14.7500000	-50.0000000
48020.0000000	15.0000000	-50.0000000
48050.0000000	0.0000000	-50.0000000
48050.0000000	0.2500000	-50.0000000
48050.0000000	0.5000000	-50.0000000
48050.0000000	0.7500000	-50.0000000
48050.0000000	1.0000000	-50.0000000
48050.0000000	1.2500000	-50.0000000
48050.0000000	1.5000000	-50.0000000
48050.0000000	1.7500000	-50.0000000
48050.0000000	2.0000000	-50.0000000
48050.0000000	2.2500000	-50.0000000
48050.0000000	2.5000000	-50.0000000
48050.0000000	2.7500000	-50.0000000
48050.0000000	3.0000000	-50.0000000
48050.0000000	3.2500000	-50.0000000
48050.0000000	3.5000000	-50.0000000

48050.0000000	3.7500000	-50.0000000
48050.0000000	4.0000000	-50.0000000
48050.0000000	4.2500000	-50.0000000
48050.0000000	4.5000000	-50.0000000
48050.0000000	4.7500000	-50.0000000
48050.0000000	5.0000000	-50.0000000
48050.0000000	5.2500000	-50.0000000
48050.0000000	5.5000000	-50.0000000
48050.0000000	5.7500000	-50.0000000
48050.0000000	6.0000000	-50.0000000
48050.0000000	6.2500000	-50.0000000
48050.0000000	6.5000000	-50.0000000
48050.0000000	6.7500000	-50.0000000
48050.0000000	7.0000000	-50.0000000
48050.0000000	7.2500000	-50.0000000
48050.0000000	7.5000000	-50.0000000
48050.0000000	7.7500000	-50.0000000
48050.0000000	8.0000000	-50.0000000
48050.0000000	8.2500000	-50.0000000
48050.0000000	8.5000000	-50.0000000
48050.0000000	8.7500000	-50.0000000
48050.0000000	9.0000000	-50.0000000
48050.0000000	9.2500000	-50.0000000
48050.0000000	9.5000000	-50.0000000
48050.0000000	9.7500000	-50.0000000
48050.0000000	10.0000000	-50.0000000
48050.0000000	10.2500000	-50.0000000
48050.0000000	10.5000000	-50.0000000
48050.0000000	10.7500000	-50.0000000
48050.0000000	11.0000000	-50.0000000
48050.0000000	11.2500000	-50.0000000
48050.0000000	11.5000000	-50.0000000
48050.0000000	11.7500000	-50.0000000
48050.0000000	12.0000000	-50.0000000
48050.0000000	12.2500000	-50.0000000
48050.0000000	12.5000000	-50.0000000
48050.0000000	12.7500000	-50.0000000
48050.0000000	13.0000000	-50.0000000
48050.0000000	13.2500000	-50.0000000
48050.0000000	13.5000000	-50.0000000
48050.0000000	13.7500000	-50.0000000
48050.0000000	14.0000000	-50.0000000
48050.0000000	14.2500000	-50.0000000
48050.0000000	14.5000000	-50.0000000
48050.0000000	14.7500000	-50.0000000
48050.0000000	15.0000000	-50.0000000
48080.0000000	0.0000000	-50.0000000
48080.0000000	0.2500000	-50.0000000
48080.0000000	0.5000000	-50.0000000
48080.0000000	0.7500000	-50.0000000
48080.0000000	1.0000000	-50.0000000
48080.0000000	1.2500000	-50.0000000
48080.0000000	1.5000000	-50.0000000
48080.0000000	1.7500000	-50.0000000
48080.0000000	2.0000000	-50.0000000
48080.0000000	2.2500000	-50.0000000
48080.0000000	2.5000000	-50.0000000
48080.0000000	2.7500000	-50.0000000

48080.0000000	3.0000000	-50.0000000
48080.0000000	3.2500000	-50.0000000
48080.0000000	3.5000000	-50.0000000
48080.0000000	3.7500000	-50.0000000
48080.0000000	4.0000000	-50.0000000
48080.0000000	4.2500000	-50.0000000
48080.0000000	4.5000000	-50.0000000
48080.0000000	4.7500000	-50.0000000
48080.0000000	5.0000000	-50.0000000
48080.0000000	5.2500000	-50.0000000
48080.0000000	5.5000000	-50.0000000
48080.0000000	5.7500000	-50.0000000
48080.0000000	6.0000000	-50.0000000
48080.0000000	6.2500000	-50.0000000
48080.0000000	6.5000000	-50.0000000
48080.0000000	6.7500000	-50.0000000
48080.0000000	7.0000000	-50.0000000
48080.0000000	7.2500000	-50.0000000
48080.0000000	7.5000000	-50.0000000
48080.0000000	7.7500000	-50.0000000
48080.0000000	8.0000000	-50.0000000
48080.0000000	8.2500000	-50.0000000
48080.0000000	8.5000000	-50.0000000
48080.0000000	8.7500000	-50.0000000
48080.0000000	9.0000000	-50.0000000
48080.0000000	9.2500000	-50.0000000
48080.0000000	9.5000000	-50.0000000
48080.0000000	9.7500000	-50.0000000
48080.0000000	10.0000000	-50.0000000
48080.0000000	10.2500000	-50.0000000
48080.0000000	10.5000000	-50.0000000
48080.0000000	10.7500000	-50.0000000
48080.0000000	11.0000000	-50.0000000
48080.0000000	11.2500000	-50.0000000
48080.0000000	11.5000000	-50.0000000
48080.0000000	11.7500000	-50.0000000
48080.0000000	12.0000000	-50.0000000
48080.0000000	12.2500000	-50.0000000
48080.0000000	12.5000000	-50.0000000
48080.0000000	12.7500000	-50.0000000
48080.0000000	13.0000000	-50.0000000
48080.0000000	13.2500000	-50.0000000
48080.0000000	13.5000000	-50.0000000
48080.0000000	13.7500000	-50.0000000
48080.0000000	14.0000000	-50.0000000
48080.0000000	14.2500000	-50.0000000
48080.0000000	14.5000000	-50.0000000
48080.0000000	14.7500000	-50.0000000
48080.0000000	15.0000000	-50.0000000
48110.0000000	0.0000000	-50.0000000
48110.0000000	0.2500000	-50.0000000
48110.0000000	0.5000000	-50.0000000
48110.0000000	0.7500000	-50.0000000
48110.0000000	1.0000000	-50.0000000
48110.0000000	1.2500000	-50.0000000
48110.0000000	1.5000000	-50.0000000
48110.0000000	1.7500000	-50.0000000
48110.0000000	2.0000000	-50.0000000

48110.0000000	2.2500000	-50.0000000
48110.0000000	2.5000000	-50.0000000
48110.0000000	2.7500000	-50.0000000
48110.0000000	3.0000000	-50.0000000
48110.0000000	3.2500000	-50.0000000
48110.0000000	3.5000000	-50.0000000
48110.0000000	3.7500000	-50.0000000
48110.0000000	4.0000000	-50.0000000
48110.0000000	4.2500000	-50.0000000
48110.0000000	4.5000000	-50.0000000
48110.0000000	4.7500000	-50.0000000
48110.0000000	5.0000000	-50.0000000
48110.0000000	5.2500000	-50.0000000
48110.0000000	5.5000000	-50.0000000
48110.0000000	5.7500000	-50.0000000
48110.0000000	6.0000000	-50.0000000
48110.0000000	6.2500000	-50.0000000
48110.0000000	6.5000000	-50.0000000
48110.0000000	6.7500000	-50.0000000
48110.0000000	7.0000000	-50.0000000
48110.0000000	7.2500000	-50.0000000
48110.0000000	7.5000000	-50.0000000
48110.0000000	7.7500000	-50.0000000
48110.0000000	8.0000000	-50.0000000
48110.0000000	8.2500000	-50.0000000
48110.0000000	8.5000000	-50.0000000
48110.0000000	8.7500000	-50.0000000
48110.0000000	9.0000000	-50.0000000
48110.0000000	9.2500000	-50.0000000
48110.0000000	9.5000000	-50.0000000
48110.0000000	9.7500000	-50.0000000
48110.0000000	10.0000000	-50.0000000
48110.0000000	10.2500000	-50.0000000
48110.0000000	10.5000000	-50.0000000
48110.0000000	10.7500000	-50.0000000
48110.0000000	11.0000000	-50.0000000
48110.0000000	11.2500000	-50.0000000
48110.0000000	11.5000000	-50.0000000
48110.0000000	11.7500000	-50.0000000
48110.0000000	12.0000000	-50.0000000
48110.0000000	12.2500000	-50.0000000
48110.0000000	12.5000000	-50.0000000
48110.0000000	12.7500000	-50.0000000
48110.0000000	13.0000000	-50.0000000
48110.0000000	13.2500000	-50.0000000
48110.0000000	13.5000000	-50.0000000
48110.0000000	13.7500000	-50.0000000
48110.0000000	14.0000000	-50.0000000
48110.0000000	14.2500000	-50.0000000
48110.0000000	14.5000000	-50.0000000
48110.0000000	14.7500000	-50.0000000
48110.0000000	15.0000000	-50.0000000
48140.0000000	0.0000000	-50.0000000
48140.0000000	0.2500000	-50.0000000
48140.0000000	0.5000000	-50.0000000
48140.0000000	0.7500000	-50.0000000
48140.0000000	1.0000000	-50.0000000
48140.0000000	1.2500000	-50.0000000

48140.0000000	1.5000000	-50.0000000
48140.0000000	1.7500000	-50.0000000
48140.0000000	2.0000000	-50.0000000
48140.0000000	2.2500000	-50.0000000
48140.0000000	2.5000000	-50.0000000
48140.0000000	2.7500000	-50.0000000
48140.0000000	3.0000000	-50.0000000
48140.0000000	3.2500000	-50.0000000
48140.0000000	3.5000000	-50.0000000
48140.0000000	3.7500000	-50.0000000
48140.0000000	4.0000000	-50.0000000
48140.0000000	4.2500000	-50.0000000
48140.0000000	4.5000000	-50.0000000
48140.0000000	4.7500000	-50.0000000
48140.0000000	5.0000000	-50.0000000
48140.0000000	5.2500000	-50.0000000
48140.0000000	5.5000000	-50.0000000
48140.0000000	5.7500000	-50.0000000
48140.0000000	6.0000000	-50.0000000
48140.0000000	6.2500000	-50.0000000
48140.0000000	6.5000000	-50.0000000
48140.0000000	6.7500000	-50.0000000
48140.0000000	7.0000000	-50.0000000
48140.0000000	7.2500000	-50.0000000
48140.0000000	7.5000000	-50.0000000
48140.0000000	7.7500000	-50.0000000
48140.0000000	8.0000000	-50.0000000
48140.0000000	8.2500000	-50.0000000
48140.0000000	8.5000000	-50.0000000
48140.0000000	8.7500000	-50.0000000
48140.0000000	9.0000000	-50.0000000
48140.0000000	9.2500000	-50.0000000
48140.0000000	9.5000000	-50.0000000
48140.0000000	9.7500000	-50.0000000
48140.0000000	10.0000000	-50.0000000
48140.0000000	10.2500000	-50.0000000
48140.0000000	10.5000000	-50.0000000
48140.0000000	10.7500000	-50.0000000
48140.0000000	11.0000000	-50.0000000
48140.0000000	11.2500000	-50.0000000
48140.0000000	11.5000000	-50.0000000
48140.0000000	11.7500000	-50.0000000
48140.0000000	12.0000000	-50.0000000
48140.0000000	12.2500000	-50.0000000
48140.0000000	12.5000000	-50.0000000
48140.0000000	12.7500000	-50.0000000
48140.0000000	13.0000000	-50.0000000
48140.0000000	13.2500000	-50.0000000
48140.0000000	13.5000000	-50.0000000
48140.0000000	13.7500000	-50.0000000
48140.0000000	14.0000000	-50.0000000
48140.0000000	14.2500000	-50.0000000
48140.0000000	14.5000000	-50.0000000
48140.0000000	14.7500000	-50.0000000
48140.0000000	15.0000000	-50.0000000
48170.0000000	0.0000000	-50.0000000
48170.0000000	0.2500000	-50.0000000
48170.0000000	0.5000000	-50.0000000

48170.0000000	0.7500000	-50.0000000
48170.0000000	1.0000000	-50.0000000
48170.0000000	1.2500000	-50.0000000
48170.0000000	1.5000000	-50.0000000
48170.0000000	1.7500000	-50.0000000
48170.0000000	2.0000000	-50.0000000
48170.0000000	2.2500000	-50.0000000
48170.0000000	2.5000000	-50.0000000
48170.0000000	2.7500000	-50.0000000
48170.0000000	3.0000000	-50.0000000
48170.0000000	3.2500000	-50.0000000
48170.0000000	3.5000000	-50.0000000
48170.0000000	3.7500000	-50.0000000
48170.0000000	4.0000000	-50.0000000
48170.0000000	4.2500000	-50.0000000
48170.0000000	4.5000000	-50.0000000
48170.0000000	4.7500000	-50.0000000
48170.0000000	5.0000000	-50.0000000
48170.0000000	5.2500000	-50.0000000
48170.0000000	5.5000000	-50.0000000
48170.0000000	5.7500000	-50.0000000
48170.0000000	6.0000000	-50.0000000
48170.0000000	6.2500000	-50.0000000
48170.0000000	6.5000000	-50.0000000
48170.0000000	6.7500000	-50.0000000
48170.0000000	7.0000000	-50.0000000
48170.0000000	7.2500000	-50.0000000
48170.0000000	7.5000000	-50.0000000
48170.0000000	7.7500000	-50.0000000
48170.0000000	8.0000000	-50.0000000
48170.0000000	8.2500000	-50.0000000
48170.0000000	8.5000000	-50.0000000
48170.0000000	8.7500000	-50.0000000
48170.0000000	9.0000000	-50.0000000
48170.0000000	9.2500000	-50.0000000
48170.0000000	9.5000000	-50.0000000
48170.0000000	9.7500000	-50.0000000
48170.0000000	10.0000000	-50.0000000
48170.0000000	10.2500000	-50.0000000
48170.0000000	10.5000000	-50.0000000
48170.0000000	10.7500000	-50.0000000
48170.0000000	11.0000000	-50.0000000
48170.0000000	11.2500000	-50.0000000
48170.0000000	11.5000000	-50.0000000
48170.0000000	11.7500000	-50.0000000
48170.0000000	12.0000000	-50.0000000
48170.0000000	12.2500000	-50.0000000
48170.0000000	12.5000000	-50.0000000
48170.0000000	12.7500000	-50.0000000
48170.0000000	13.0000000	-50.0000000
48170.0000000	13.2500000	-50.0000000
48170.0000000	13.5000000	-50.0000000
48170.0000000	13.7500000	-50.0000000
48170.0000000	14.0000000	-50.0000000
48170.0000000	14.2500000	-50.0000000
48170.0000000	14.5000000	-50.0000000
48170.0000000	14.7500000	-50.0000000
48170.0000000	15.0000000	-50.0000000

48200.0000000	0.0000000	-50.0000000
48200.0000000	0.2500000	-50.0000000
48200.0000000	0.5000000	-50.0000000
48200.0000000	0.7500000	-50.0000000
48200.0000000	1.0000000	-50.0000000
48200.0000000	1.2500000	-50.0000000
48200.0000000	1.5000000	-50.0000000
48200.0000000	1.7500000	-50.0000000
48200.0000000	2.0000000	-50.0000000
48200.0000000	2.2500000	-50.0000000
48200.0000000	2.5000000	-50.0000000
48200.0000000	2.7500000	-50.0000000
48200.0000000	3.0000000	-50.0000000
48200.0000000	3.2500000	-50.0000000
48200.0000000	3.5000000	-50.0000000
48200.0000000	3.7500000	-50.0000000
48200.0000000	4.0000000	-50.0000000
48200.0000000	4.2500000	-50.0000000
48200.0000000	4.5000000	-50.0000000
48200.0000000	4.7500000	-50.0000000
48200.0000000	5.0000000	-50.0000000
48200.0000000	5.2500000	-50.0000000
48200.0000000	5.5000000	-50.0000000
48200.0000000	5.7500000	-50.0000000
48200.0000000	6.0000000	-50.0000000
48200.0000000	6.2500000	-50.0000000
48200.0000000	6.5000000	-50.0000000
48200.0000000	6.7500000	-50.0000000
48200.0000000	7.0000000	-50.0000000
48200.0000000	7.2500000	-50.0000000
48200.0000000	7.5000000	-50.0000000
48200.0000000	7.7500000	-50.0000000
48200.0000000	8.0000000	-50.0000000
48200.0000000	8.2500000	-50.0000000
48200.0000000	8.5000000	-50.0000000
48200.0000000	8.7500000	-50.0000000
48200.0000000	9.0000000	-50.0000000
48200.0000000	9.2500000	-50.0000000
48200.0000000	9.5000000	-50.0000000
48200.0000000	9.7500000	-50.0000000
48200.0000000	10.0000000	-50.0000000
48200.0000000	10.2500000	-50.0000000
48200.0000000	10.5000000	-50.0000000
48200.0000000	10.7500000	-50.0000000
48200.0000000	11.0000000	-50.0000000
48200.0000000	11.2500000	-50.0000000
48200.0000000	11.5000000	-50.0000000
48200.0000000	11.7500000	-50.0000000
48200.0000000	12.0000000	-50.0000000
48200.0000000	12.2500000	-50.0000000
48200.0000000	12.5000000	-50.0000000
48200.0000000	12.7500000	-50.0000000
48200.0000000	13.0000000	-50.0000000
48200.0000000	13.2500000	-50.0000000
48200.0000000	13.5000000	-50.0000000
48200.0000000	13.7500000	-50.0000000
48200.0000000	14.0000000	-50.0000000
48200.0000000	14.2500000	-50.0000000

48200.0000000	14.5000000	-50.0000000
48200.0000000	14.7500000	-50.0000000
48200.0000000	15.0000000	-50.0000000
48230.0000000	0.0000000	-50.0000000
48230.0000000	0.2500000	-50.0000000
48230.0000000	0.5000000	-50.0000000
48230.0000000	0.7500000	-50.0000000
48230.0000000	1.0000000	-50.0000000
48230.0000000	1.2500000	-50.0000000
48230.0000000	1.5000000	-50.0000000
48230.0000000	1.7500000	-50.0000000
48230.0000000	2.0000000	-50.0000000
48230.0000000	2.2500000	-50.0000000
48230.0000000	2.5000000	-50.0000000
48230.0000000	2.7500000	-50.0000000
48230.0000000	3.0000000	-50.0000000
48230.0000000	3.2500000	-50.0000000
48230.0000000	3.5000000	-50.0000000
48230.0000000	3.7500000	-50.0000000
48230.0000000	4.0000000	-50.0000000
48230.0000000	4.2500000	-50.0000000
48230.0000000	4.5000000	-50.0000000
48230.0000000	4.7500000	-50.0000000
48230.0000000	5.0000000	-50.0000000
48230.0000000	5.2500000	-50.0000000
48230.0000000	5.5000000	-50.0000000
48230.0000000	5.7500000	-50.0000000
48230.0000000	6.0000000	-50.0000000
48230.0000000	6.2500000	-50.0000000
48230.0000000	6.5000000	-50.0000000
48230.0000000	6.7500000	-50.0000000
48230.0000000	7.0000000	-50.0000000
48230.0000000	7.2500000	-50.0000000
48230.0000000	7.5000000	-50.0000000
48230.0000000	7.7500000	-50.0000000
48230.0000000	8.0000000	-50.0000000
48230.0000000	8.2500000	-50.0000000
48230.0000000	8.5000000	-50.0000000
48230.0000000	8.7500000	-50.0000000
48230.0000000	9.0000000	-50.0000000
48230.0000000	9.2500000	-50.0000000
48230.0000000	9.5000000	-50.0000000
48230.0000000	9.7500000	-50.0000000
48230.0000000	10.0000000	-50.0000000
48230.0000000	10.2500000	-50.0000000
48230.0000000	10.5000000	-50.0000000
48230.0000000	10.7500000	-50.0000000
48230.0000000	11.0000000	-50.0000000
48230.0000000	11.2500000	-50.0000000
48230.0000000	11.5000000	-50.0000000
48230.0000000	11.7500000	-50.0000000
48230.0000000	12.0000000	-50.0000000
48230.0000000	12.2500000	-50.0000000
48230.0000000	12.5000000	-50.0000000
48230.0000000	12.7500000	-50.0000000
48230.0000000	13.0000000	-50.0000000
48230.0000000	13.2500000	-50.0000000
48230.0000000	13.5000000	-50.0000000

48230.0000000	13.7500000	-50.0000000
48230.0000000	14.0000000	-50.0000000
48230.0000000	14.2500000	-50.0000000
48230.0000000	14.5000000	-50.0000000
48230.0000000	14.7500000	-50.0000000
48230.0000000	15.0000000	-50.0000000
48260.0000000	0.0000000	-50.0000000
48260.0000000	0.2500000	-50.0000000
48260.0000000	0.5000000	-50.0000000
48260.0000000	0.7500000	-50.0000000
48260.0000000	1.0000000	-50.0000000
48260.0000000	1.2500000	-50.0000000
48260.0000000	1.5000000	-50.0000000
48260.0000000	1.7500000	-50.0000000
48260.0000000	2.0000000	-50.0000000
48260.0000000	2.2500000	-50.0000000
48260.0000000	2.5000000	-50.0000000
48260.0000000	2.7500000	-50.0000000
48260.0000000	3.0000000	-50.0000000
48260.0000000	3.2500000	-50.0000000
48260.0000000	3.5000000	-50.0000000
48260.0000000	3.7500000	-50.0000000
48260.0000000	4.0000000	-50.0000000
48260.0000000	4.2500000	-50.0000000
48260.0000000	4.5000000	-50.0000000
48260.0000000	4.7500000	-50.0000000
48260.0000000	5.0000000	-50.0000000
48260.0000000	5.2500000	-50.0000000
48260.0000000	5.5000000	-50.0000000
48260.0000000	5.7500000	-50.0000000
48260.0000000	6.0000000	-50.0000000
48260.0000000	6.2500000	-50.0000000
48260.0000000	6.5000000	-50.0000000
48260.0000000	6.7500000	-50.0000000
48260.0000000	7.0000000	-50.0000000
48260.0000000	7.2500000	-50.0000000
48260.0000000	7.5000000	-50.0000000
48260.0000000	7.7500000	-50.0000000
48260.0000000	8.0000000	-50.0000000
48260.0000000	8.2500000	-50.0000000
48260.0000000	8.5000000	-50.0000000
48260.0000000	8.7500000	-50.0000000
48260.0000000	9.0000000	-50.0000000
48260.0000000	9.2500000	-50.0000000
48260.0000000	9.5000000	-50.0000000
48260.0000000	9.7500000	-50.0000000
48260.0000000	10.0000000	-50.0000000
48260.0000000	10.2500000	-50.0000000
48260.0000000	10.5000000	-50.0000000
48260.0000000	10.7500000	-50.0000000
48260.0000000	11.0000000	-50.0000000
48260.0000000	11.2500000	-50.0000000
48260.0000000	11.5000000	-50.0000000
48260.0000000	11.7500000	-50.0000000
48260.0000000	12.0000000	-50.0000000
48260.0000000	12.2500000	-50.0000000
48260.0000000	12.5000000	-50.0000000
48260.0000000	12.7500000	-50.0000000

48260.0000000	13.0000000	-50.0000000
48260.0000000	13.2500000	-50.0000000
48260.0000000	13.5000000	-50.0000000
48260.0000000	13.7500000	-50.0000000
48260.0000000	14.0000000	-50.0000000
48260.0000000	14.2500000	-50.0000000
48260.0000000	14.5000000	-50.0000000
48260.0000000	14.7500000	-50.0000000
48260.0000000	15.0000000	-50.0000000
48290.0000000	0.0000000	-50.0000000
48290.0000000	0.2500000	-50.0000000
48290.0000000	0.5000000	-50.0000000
48290.0000000	0.7500000	-50.0000000
48290.0000000	1.0000000	-50.0000000
48290.0000000	1.2500000	-50.0000000
48290.0000000	1.5000000	-50.0000000
48290.0000000	1.7500000	-50.0000000
48290.0000000	2.0000000	-50.0000000
48290.0000000	2.2500000	-50.0000000
48290.0000000	2.5000000	-50.0000000
48290.0000000	2.7500000	-50.0000000
48290.0000000	3.0000000	-50.0000000
48290.0000000	3.2500000	-50.0000000
48290.0000000	3.5000000	-50.0000000
48290.0000000	3.7500000	-50.0000000
48290.0000000	4.0000000	-50.0000000
48290.0000000	4.2500000	-50.0000000
48290.0000000	4.5000000	-50.0000000
48290.0000000	4.7500000	-50.0000000
48290.0000000	5.0000000	-50.0000000
48290.0000000	5.2500000	-50.0000000
48290.0000000	5.5000000	-50.0000000
48290.0000000	5.7500000	-50.0000000
48290.0000000	6.0000000	-50.0000000
48290.0000000	6.2500000	-50.0000000
48290.0000000	6.5000000	-50.0000000
48290.0000000	6.7500000	-50.0000000
48290.0000000	7.0000000	-50.0000000
48290.0000000	7.2500000	-50.0000000
48290.0000000	7.5000000	-50.0000000
48290.0000000	7.7500000	-50.0000000
48290.0000000	8.0000000	-50.0000000
48290.0000000	8.2500000	-50.0000000
48290.0000000	8.5000000	-50.0000000
48290.0000000	8.7500000	-50.0000000
48290.0000000	9.0000000	-50.0000000
48290.0000000	9.2500000	-50.0000000
48290.0000000	9.5000000	-50.0000000
48290.0000000	9.7500000	-50.0000000
48290.0000000	10.0000000	-50.0000000
48290.0000000	10.2500000	-50.0000000
48290.0000000	10.5000000	-50.0000000
48290.0000000	10.7500000	-50.0000000
48290.0000000	11.0000000	-50.0000000
48290.0000000	11.2500000	-50.0000000
48290.0000000	11.5000000	-50.0000000
48290.0000000	11.7500000	-50.0000000
48290.0000000	12.0000000	-50.0000000

48290.0000000	12.2500000	-50.0000000
48290.0000000	12.5000000	-50.0000000
48290.0000000	12.7500000	-50.0000000
48290.0000000	13.0000000	-50.0000000
48290.0000000	13.2500000	-50.0000000
48290.0000000	13.5000000	-50.0000000
48290.0000000	13.7500000	-50.0000000
48290.0000000	14.0000000	-50.0000000
48290.0000000	14.2500000	-50.0000000
48290.0000000	14.5000000	-50.0000000
48290.0000000	14.7500000	-50.0000000
48290.0000000	15.0000000	-50.0000000
48320.0000000	0.0000000	-50.0000000
48320.0000000	0.2500000	-50.0000000
48320.0000000	0.5000000	-50.0000000
48320.0000000	0.7500000	-50.0000000
48320.0000000	1.0000000	-50.0000000
48320.0000000	1.2500000	-50.0000000
48320.0000000	1.5000000	-50.0000000
48320.0000000	1.7500000	-50.0000000
48320.0000000	2.0000000	-50.0000000
48320.0000000	2.2500000	-50.0000000
48320.0000000	2.5000000	-50.0000000
48320.0000000	2.7500000	-50.0000000
48320.0000000	3.0000000	-50.0000000
48320.0000000	3.2500000	-50.0000000
48320.0000000	3.5000000	-50.0000000
48320.0000000	3.7500000	-50.0000000
48320.0000000	4.0000000	-50.0000000
48320.0000000	4.2500000	-50.0000000
48320.0000000	4.5000000	-50.0000000
48320.0000000	4.7500000	-50.0000000
48320.0000000	5.0000000	-50.0000000
48320.0000000	5.2500000	-50.0000000
48320.0000000	5.5000000	-50.0000000
48320.0000000	5.7500000	-50.0000000
48320.0000000	6.0000000	-50.0000000
48320.0000000	6.2500000	-50.0000000
48320.0000000	6.5000000	-50.0000000
48320.0000000	6.7500000	-50.0000000
48320.0000000	7.0000000	-50.0000000
48320.0000000	7.2500000	-50.0000000
48320.0000000	7.5000000	-50.0000000
48320.0000000	7.7500000	-50.0000000
48320.0000000	8.0000000	-50.0000000
48320.0000000	8.2500000	-50.0000000
48320.0000000	8.5000000	-50.0000000
48320.0000000	8.7500000	-50.0000000
48320.0000000	9.0000000	-50.0000000
48320.0000000	9.2500000	-50.0000000
48320.0000000	9.5000000	-50.0000000
48320.0000000	9.7500000	-50.0000000
48320.0000000	10.0000000	-50.0000000
48320.0000000	10.2500000	-50.0000000
48320.0000000	10.5000000	-50.0000000
48320.0000000	10.7500000	-50.0000000
48320.0000000	11.0000000	-50.0000000
48320.0000000	11.2500000	-50.0000000

48320.0000000	11.5000000	-50.0000000
48320.0000000	11.7500000	-50.0000000
48320.0000000	12.0000000	-50.0000000
48320.0000000	12.2500000	-50.0000000
48320.0000000	12.5000000	-50.0000000
48320.0000000	12.7500000	-50.0000000
48320.0000000	13.0000000	-50.0000000
48320.0000000	13.2500000	-50.0000000
48320.0000000	13.5000000	-50.0000000
48320.0000000	13.7500000	-50.0000000
48320.0000000	14.0000000	-50.0000000
48320.0000000	14.2500000	-50.0000000
48320.0000000	14.5000000	-50.0000000
48320.0000000	14.7500000	-50.0000000
48320.0000000	15.0000000	-50.0000000
48350.0000000	0.0000000	-50.0000000
48350.0000000	0.2500000	-50.0000000
48350.0000000	0.5000000	-50.0000000
48350.0000000	0.7500000	-50.0000000
48350.0000000	1.0000000	-50.0000000
48350.0000000	1.2500000	-50.0000000
48350.0000000	1.5000000	-50.0000000
48350.0000000	1.7500000	-50.0000000
48350.0000000	2.0000000	-50.0000000
48350.0000000	2.2500000	-50.0000000
48350.0000000	2.5000000	-50.0000000
48350.0000000	2.7500000	-50.0000000
48350.0000000	3.0000000	-50.0000000
48350.0000000	3.2500000	-50.0000000
48350.0000000	3.5000000	-50.0000000
48350.0000000	3.7500000	-50.0000000
48350.0000000	4.0000000	-50.0000000
48350.0000000	4.2500000	-50.0000000
48350.0000000	4.5000000	-50.0000000
48350.0000000	4.7500000	-50.0000000
48350.0000000	5.0000000	-50.0000000
48350.0000000	5.2500000	-50.0000000
48350.0000000	5.5000000	-50.0000000
48350.0000000	5.7500000	-50.0000000
48350.0000000	6.0000000	-50.0000000
48350.0000000	6.2500000	-50.0000000
48350.0000000	6.5000000	-50.0000000
48350.0000000	6.7500000	-50.0000000
48350.0000000	7.0000000	-50.0000000
48350.0000000	7.2500000	-50.0000000
48350.0000000	7.5000000	-50.0000000
48350.0000000	7.7500000	-50.0000000
48350.0000000	8.0000000	-50.0000000
48350.0000000	8.2500000	-50.0000000
48350.0000000	8.5000000	-50.0000000
48350.0000000	8.7500000	-50.0000000
48350.0000000	9.0000000	-50.0000000
48350.0000000	9.2500000	-50.0000000
48350.0000000	9.5000000	-50.0000000
48350.0000000	9.7500000	-50.0000000
48350.0000000	10.0000000	-50.0000000
48350.0000000	10.2500000	-50.0000000
48350.0000000	10.5000000	-50.0000000

48350.0000000	10.7500000	-50.0000000
48350.0000000	11.0000000	-50.0000000
48350.0000000	11.2500000	-50.0000000
48350.0000000	11.5000000	-50.0000000
48350.0000000	11.7500000	-50.0000000
48350.0000000	12.0000000	-50.0000000
48350.0000000	12.2500000	-50.0000000
48350.0000000	12.5000000	-50.0000000
48350.0000000	12.7500000	-50.0000000
48350.0000000	13.0000000	-50.0000000
48350.0000000	13.2500000	-50.0000000
48350.0000000	13.5000000	-50.0000000
48350.0000000	13.7500000	-50.0000000
48350.0000000	14.0000000	-50.0000000
48350.0000000	14.2500000	-50.0000000
48350.0000000	14.5000000	-50.0000000
48350.0000000	14.7500000	-50.0000000
48350.0000000	15.0000000	-50.0000000
48380.0000000	0.0000000	-50.0000000
48380.0000000	0.2500000	-50.0000000
48380.0000000	0.5000000	-50.0000000
48380.0000000	0.7500000	-50.0000000
48380.0000000	1.0000000	-50.0000000
48380.0000000	1.2500000	-50.0000000
48380.0000000	1.5000000	-50.0000000
48380.0000000	1.7500000	-50.0000000
48380.0000000	2.0000000	-50.0000000
48380.0000000	2.2500000	-50.0000000
48380.0000000	2.5000000	-50.0000000
48380.0000000	2.7500000	-50.0000000
48380.0000000	3.0000000	-50.0000000
48380.0000000	3.2500000	-50.0000000
48380.0000000	3.5000000	-50.0000000
48380.0000000	3.7500000	-50.0000000
48380.0000000	4.0000000	-50.0000000
48380.0000000	4.2500000	-50.0000000
48380.0000000	4.5000000	-50.0000000
48380.0000000	4.7500000	-50.0000000
48380.0000000	5.0000000	-50.0000000
48380.0000000	5.2500000	-50.0000000
48380.0000000	5.5000000	-50.0000000
48380.0000000	5.7500000	-50.0000000
48380.0000000	6.0000000	-50.0000000
48380.0000000	6.2500000	-50.0000000
48380.0000000	6.5000000	-50.0000000
48380.0000000	6.7500000	-50.0000000
48380.0000000	7.0000000	-50.0000000
48380.0000000	7.2500000	-50.0000000
48380.0000000	7.5000000	-50.0000000
48380.0000000	7.7500000	-50.0000000
48380.0000000	8.0000000	-50.0000000
48380.0000000	8.2500000	-50.0000000
48380.0000000	8.5000000	-50.0000000
48380.0000000	8.7500000	-50.0000000
48380.0000000	9.0000000	-50.0000000
48380.0000000	9.2500000	-50.0000000
48380.0000000	9.5000000	-50.0000000
48380.0000000	9.7500000	-50.0000000

48380.0000000	10.0000000	-50.0000000
48380.0000000	10.2500000	-50.0000000
48380.0000000	10.5000000	-50.0000000
48380.0000000	10.7500000	-50.0000000
48380.0000000	11.0000000	-50.0000000
48380.0000000	11.2500000	-50.0000000
48380.0000000	11.5000000	-50.0000000
48380.0000000	11.7500000	-50.0000000
48380.0000000	12.0000000	-50.0000000
48380.0000000	12.2500000	-50.0000000
48380.0000000	12.5000000	-50.0000000
48380.0000000	12.7500000	-50.0000000
48380.0000000	13.0000000	-50.0000000
48380.0000000	13.2500000	-50.0000000
48380.0000000	13.5000000	-50.0000000
48380.0000000	13.7500000	-50.0000000
48380.0000000	14.0000000	-50.0000000
48380.0000000	14.2500000	-50.0000000
48380.0000000	14.5000000	-50.0000000
48380.0000000	14.7500000	-50.0000000
48380.0000000	15.0000000	-50.0000000
48410.0000000	0.0000000	-50.0000000
48410.0000000	0.2500000	-50.0000000
48410.0000000	0.5000000	-50.0000000
48410.0000000	0.7500000	-50.0000000
48410.0000000	1.0000000	-50.0000000
48410.0000000	1.2500000	-50.0000000
48410.0000000	1.5000000	-50.0000000
48410.0000000	1.7500000	-50.0000000
48410.0000000	2.0000000	-50.0000000
48410.0000000	2.2500000	-50.0000000
48410.0000000	2.5000000	-50.0000000
48410.0000000	2.7500000	-50.0000000
48410.0000000	3.0000000	-50.0000000
48410.0000000	3.2500000	-50.0000000
48410.0000000	3.5000000	-50.0000000
48410.0000000	3.7500000	-50.0000000
48410.0000000	4.0000000	-50.0000000
48410.0000000	4.2500000	-50.0000000
48410.0000000	4.5000000	-50.0000000
48410.0000000	4.7500000	-50.0000000
48410.0000000	5.0000000	-50.0000000
48410.0000000	5.2500000	-50.0000000
48410.0000000	5.5000000	-50.0000000
48410.0000000	5.7500000	-50.0000000
48410.0000000	6.0000000	-50.0000000
48410.0000000	6.2500000	-50.0000000
48410.0000000	6.5000000	-50.0000000
48410.0000000	6.7500000	-50.0000000
48410.0000000	7.0000000	-50.0000000
48410.0000000	7.2500000	-50.0000000
48410.0000000	7.5000000	-50.0000000
48410.0000000	7.7500000	-50.0000000
48410.0000000	8.0000000	-50.0000000
48410.0000000	8.2500000	-50.0000000
48410.0000000	8.5000000	-50.0000000
48410.0000000	8.7500000	-50.0000000
48410.0000000	9.0000000	-50.0000000

48410.0000000	9.2500000	-50.0000000
48410.0000000	9.5000000	-50.0000000
48410.0000000	9.7500000	-50.0000000
48410.0000000	10.0000000	-50.0000000
48410.0000000	10.2500000	-50.0000000
48410.0000000	10.5000000	-50.0000000
48410.0000000	10.7500000	-50.0000000
48410.0000000	11.0000000	-50.0000000
48410.0000000	11.2500000	-50.0000000
48410.0000000	11.5000000	-50.0000000
48410.0000000	11.7500000	-50.0000000
48410.0000000	12.0000000	-50.0000000
48410.0000000	12.2500000	-50.0000000
48410.0000000	12.5000000	-50.0000000
48410.0000000	12.7500000	-50.0000000
48410.0000000	13.0000000	-50.0000000
48410.0000000	13.2500000	-50.0000000
48410.0000000	13.5000000	-50.0000000
48410.0000000	13.7500000	-50.0000000
48410.0000000	14.0000000	-50.0000000
48410.0000000	14.2500000	-50.0000000
48410.0000000	14.5000000	-50.0000000
48410.0000000	14.7500000	-50.0000000
48410.0000000	15.0000000	-50.0000000
48440.0000000	0.0000000	-50.0000000
48440.0000000	0.2500000	-50.0000000
48440.0000000	0.5000000	-50.0000000
48440.0000000	0.7500000	-50.0000000
48440.0000000	1.0000000	-50.0000000
48440.0000000	1.2500000	-50.0000000
48440.0000000	1.5000000	-50.0000000
48440.0000000	1.7500000	-50.0000000
48440.0000000	2.0000000	-50.0000000
48440.0000000	2.2500000	-50.0000000
48440.0000000	2.5000000	-50.0000000
48440.0000000	2.7500000	-50.0000000
48440.0000000	3.0000000	-50.0000000
48440.0000000	3.2500000	-50.0000000
48440.0000000	3.5000000	-50.0000000
48440.0000000	3.7500000	-50.0000000
48440.0000000	4.0000000	-50.0000000
48440.0000000	4.2500000	-50.0000000
48440.0000000	4.5000000	-50.0000000
48440.0000000	4.7500000	-50.0000000
48440.0000000	5.0000000	-50.0000000
48440.0000000	5.2500000	-50.0000000
48440.0000000	5.5000000	-50.0000000
48440.0000000	5.7500000	-50.0000000
48440.0000000	6.0000000	-50.0000000
48440.0000000	6.2500000	-50.0000000
48440.0000000	6.5000000	-50.0000000
48440.0000000	6.7500000	-50.0000000
48440.0000000	7.0000000	-50.0000000
48440.0000000	7.2500000	-50.0000000
48440.0000000	7.5000000	-50.0000000
48440.0000000	7.7500000	-50.0000000
48440.0000000	8.0000000	-50.0000000
48440.0000000	8.2500000	-50.0000000

48440.0000000	8.5000000	-50.0000000
48440.0000000	8.7500000	-50.0000000
48440.0000000	9.0000000	-50.0000000
48440.0000000	9.2500000	-50.0000000
48440.0000000	9.5000000	-50.0000000
48440.0000000	9.7500000	-50.0000000
48440.0000000	10.0000000	-50.0000000
48440.0000000	10.2500000	-50.0000000
48440.0000000	10.5000000	-50.0000000
48440.0000000	10.7500000	-50.0000000
48440.0000000	11.0000000	-50.0000000
48440.0000000	11.2500000	-50.0000000
48440.0000000	11.5000000	-50.0000000
48440.0000000	11.7500000	-50.0000000
48440.0000000	12.0000000	-50.0000000
48440.0000000	12.2500000	-50.0000000
48440.0000000	12.5000000	-50.0000000
48440.0000000	12.7500000	-50.0000000
48440.0000000	13.0000000	-50.0000000
48440.0000000	13.2500000	-50.0000000
48440.0000000	13.5000000	-50.0000000
48440.0000000	13.7500000	-50.0000000
48440.0000000	14.0000000	-50.0000000
48440.0000000	14.2500000	-50.0000000
48440.0000000	14.5000000	-50.0000000
48440.0000000	14.7500000	-50.0000000
48440.0000000	15.0000000	-50.0000000
48470.0000000	0.0000000	-50.0000000
48470.0000000	0.2500000	-50.0000000
48470.0000000	0.5000000	-50.0000000
48470.0000000	0.7500000	-50.0000000
48470.0000000	1.0000000	-50.0000000
48470.0000000	1.2500000	-50.0000000
48470.0000000	1.5000000	-50.0000000
48470.0000000	1.7500000	-50.0000000
48470.0000000	2.0000000	-50.0000000
48470.0000000	2.2500000	-50.0000000
48470.0000000	2.5000000	-50.0000000
48470.0000000	2.7500000	-50.0000000
48470.0000000	3.0000000	-50.0000000
48470.0000000	3.2500000	-50.0000000
48470.0000000	3.5000000	-50.0000000
48470.0000000	3.7500000	-50.0000000
48470.0000000	4.0000000	-50.0000000
48470.0000000	4.2500000	-50.0000000
48470.0000000	4.5000000	-50.0000000
48470.0000000	4.7500000	-50.0000000
48470.0000000	5.0000000	-50.0000000
48470.0000000	5.2500000	-50.0000000
48470.0000000	5.5000000	-50.0000000
48470.0000000	5.7500000	-50.0000000
48470.0000000	6.0000000	-50.0000000
48470.0000000	6.2500000	-50.0000000
48470.0000000	6.5000000	-50.0000000
48470.0000000	6.7500000	-50.0000000
48470.0000000	7.0000000	-50.0000000
48470.0000000	7.2500000	-50.0000000
48470.0000000	7.5000000	-50.0000000

48470.0000000	7.7500000	-50.0000000
48470.0000000	8.0000000	-50.0000000
48470.0000000	8.2500000	-50.0000000
48470.0000000	8.5000000	-50.0000000
48470.0000000	8.7500000	-50.0000000
48470.0000000	9.0000000	-50.0000000
48470.0000000	9.2500000	-50.0000000
48470.0000000	9.5000000	-50.0000000
48470.0000000	9.7500000	-50.0000000
48470.0000000	10.0000000	-50.0000000
48470.0000000	10.2500000	-50.0000000
48470.0000000	10.5000000	-50.0000000
48470.0000000	10.7500000	-50.0000000
48470.0000000	11.0000000	-50.0000000
48470.0000000	11.2500000	-50.0000000
48470.0000000	11.5000000	-50.0000000
48470.0000000	11.7500000	-50.0000000
48470.0000000	12.0000000	-50.0000000
48470.0000000	12.2500000	-50.0000000
48470.0000000	12.5000000	-50.0000000
48470.0000000	12.7500000	-50.0000000
48470.0000000	13.0000000	-50.0000000
48470.0000000	13.2500000	-50.0000000
48470.0000000	13.5000000	-50.0000000
48470.0000000	13.7500000	-50.0000000
48470.0000000	14.0000000	-50.0000000
48470.0000000	14.2500000	-50.0000000
48470.0000000	14.5000000	-50.0000000
48470.0000000	14.7500000	-50.0000000
48470.0000000	15.0000000	-50.0000000
48500.0000000	0.0000000	-50.0000000
48500.0000000	0.2500000	-50.0000000
48500.0000000	0.5000000	-50.0000000
48500.0000000	0.7500000	-50.0000000
48500.0000000	1.0000000	-50.0000000
48500.0000000	1.2500000	-50.0000000
48500.0000000	1.5000000	-50.0000000
48500.0000000	1.7500000	-50.0000000
48500.0000000	2.0000000	15.0200005
48500.0000000	2.2500000	-50.0000000
48500.0000000	2.5000000	-50.0000000
48500.0000000	2.7500000	-50.0000000
48500.0000000	3.0000000	-50.0000000
48500.0000000	3.2500000	-50.0000000
48500.0000000	3.5000000	-50.0000000
48500.0000000	3.7500000	-50.0000000
48500.0000000	4.0000000	-50.0000000
48500.0000000	4.2500000	-50.0000000
48500.0000000	4.5000000	-50.0000000
48500.0000000	4.7500000	-50.0000000
48500.0000000	5.0000000	-50.0000000
48500.0000000	5.2500000	-50.0000000
48500.0000000	5.5000000	-50.0000000
48500.0000000	5.7500000	-50.0000000
48500.0000000	6.0000000	-50.0000000
48500.0000000	6.2500000	-50.0000000
48500.0000000	6.5000000	-50.0000000
48500.0000000	6.7500000	-50.0000000

48500.0000000	7.0000000	-50.0000000
48500.0000000	7.2500000	-50.0000000
48500.0000000	7.5000000	-50.0000000
48500.0000000	7.7500000	-50.0000000
48500.0000000	8.0000000	-50.0000000
48500.0000000	8.2500000	-50.0000000
48500.0000000	8.5000000	-50.0000000
48500.0000000	8.7500000	-50.0000000
48500.0000000	9.0000000	-50.0000000
48500.0000000	9.2500000	-50.0000000
48500.0000000	9.5000000	-50.0000000
48500.0000000	9.7500000	-50.0000000
48500.0000000	10.0000000	-50.0000000
48500.0000000	10.2500000	-50.0000000
48500.0000000	10.5000000	-50.0000000
48500.0000000	10.7500000	-50.0000000
48500.0000000	11.0000000	-50.0000000
48500.0000000	11.2500000	-50.0000000
48500.0000000	11.5000000	-50.0000000
48500.0000000	11.7500000	-50.0000000
48500.0000000	12.0000000	-50.0000000
48500.0000000	12.2500000	-50.0000000
48500.0000000	12.5000000	-50.0000000
48500.0000000	12.7500000	-50.0000000
48500.0000000	13.0000000	-50.0000000
48500.0000000	13.2500000	-50.0000000
48500.0000000	13.5000000	-50.0000000
48500.0000000	13.7500000	-50.0000000
48500.0000000	14.0000000	-50.0000000
48500.0000000	14.2500000	-50.0000000
48500.0000000	14.5000000	-50.0000000
48500.0000000	14.7500000	-50.0000000
48500.0000000	15.0000000	-50.0000000
48530.0000000	0.0000000	-50.0000000
48530.0000000	0.2500000	-50.0000000
48530.0000000	0.5000000	-50.0000000
48530.0000000	0.7500000	-50.0000000
48530.0000000	1.0000000	-50.0000000
48530.0000000	1.2500000	-50.0000000
48530.0000000	1.5000000	-50.0000000
48530.0000000	1.7500000	-50.0000000
48530.0000000	2.0000000	15.3299999
48530.0000000	2.2500000	-50.0000000
48530.0000000	2.5000000	-50.0000000
48530.0000000	2.7500000	-50.0000000
48530.0000000	3.0000000	-50.0000000
48530.0000000	3.2500000	-50.0000000
48530.0000000	3.5000000	-50.0000000
48530.0000000	3.7500000	-50.0000000
48530.0000000	4.0000000	-50.0000000
48530.0000000	4.2500000	-50.0000000
48530.0000000	4.5000000	-50.0000000
48530.0000000	4.7500000	-50.0000000
48530.0000000	5.0000000	-50.0000000
48530.0000000	5.2500000	-50.0000000
48530.0000000	5.5000000	-50.0000000
48530.0000000	5.7500000	-50.0000000
48530.0000000	6.0000000	-50.0000000

48530.0000000	6.2500000	-50.0000000
48530.0000000	6.5000000	-50.0000000
48530.0000000	6.7500000	-50.0000000
48530.0000000	7.0000000	-50.0000000
48530.0000000	7.2500000	-50.0000000
48530.0000000	7.5000000	-50.0000000
48530.0000000	7.7500000	-50.0000000
48530.0000000	8.0000000	-50.0000000
48530.0000000	8.2500000	-50.0000000
48530.0000000	8.5000000	-50.0000000
48530.0000000	8.7500000	-50.0000000
48530.0000000	9.0000000	-50.0000000
48530.0000000	9.2500000	-50.0000000
48530.0000000	9.5000000	-50.0000000
48530.0000000	9.7500000	-50.0000000
48530.0000000	10.0000000	-50.0000000
48530.0000000	10.2500000	-50.0000000
48530.0000000	10.5000000	-50.0000000
48530.0000000	10.7500000	-50.0000000
48530.0000000	11.0000000	-50.0000000
48530.0000000	11.2500000	-50.0000000
48530.0000000	11.5000000	-50.0000000
48530.0000000	11.7500000	-50.0000000
48530.0000000	12.0000000	-50.0000000
48530.0000000	12.2500000	-50.0000000
48530.0000000	12.5000000	-50.0000000
48530.0000000	12.7500000	-50.0000000
48530.0000000	13.0000000	-50.0000000
48530.0000000	13.2500000	-50.0000000
48530.0000000	13.5000000	-50.0000000
48530.0000000	13.7500000	-50.0000000
48530.0000000	14.0000000	-50.0000000
48530.0000000	14.2500000	-50.0000000
48530.0000000	14.5000000	-50.0000000
48530.0000000	14.7500000	-50.0000000
48530.0000000	15.0000000	-50.0000000
48560.0000000	0.0000000	-50.0000000
48560.0000000	0.2500000	-50.0000000
48560.0000000	0.5000000	-50.0000000
48560.0000000	0.7500000	-50.0000000
48560.0000000	1.0000000	-50.0000000
48560.0000000	1.2500000	-50.0000000
48560.0000000	1.5000000	-50.0000000
48560.0000000	1.7500000	15.8400002
48560.0000000	2.0000000	-50.0000000
48560.0000000	2.2500000	-50.0000000
48560.0000000	2.5000000	-50.0000000
48560.0000000	2.7500000	-50.0000000
48560.0000000	3.0000000	-50.0000000
48560.0000000	3.2500000	-50.0000000
48560.0000000	3.5000000	-50.0000000
48560.0000000	3.7500000	-50.0000000
48560.0000000	4.0000000	-50.0000000
48560.0000000	4.2500000	-50.0000000
48560.0000000	4.5000000	-50.0000000
48560.0000000	4.7500000	-50.0000000
48560.0000000	5.0000000	-50.0000000
48560.0000000	5.2500000	-50.0000000

48560.0000000	5.5000000	-50.0000000
48560.0000000	5.7500000	-50.0000000
48560.0000000	6.0000000	-50.0000000
48560.0000000	6.2500000	-50.0000000
48560.0000000	6.5000000	-50.0000000
48560.0000000	6.7500000	-50.0000000
48560.0000000	7.0000000	-50.0000000
48560.0000000	7.2500000	-50.0000000
48560.0000000	7.5000000	-50.0000000
48560.0000000	7.7500000	-50.0000000
48560.0000000	8.0000000	-50.0000000
48560.0000000	8.2500000	-50.0000000
48560.0000000	8.5000000	-50.0000000
48560.0000000	8.7500000	-50.0000000
48560.0000000	9.0000000	-50.0000000
48560.0000000	9.2500000	-50.0000000
48560.0000000	9.5000000	-50.0000000
48560.0000000	9.7500000	-50.0000000
48560.0000000	10.0000000	-50.0000000
48560.0000000	10.2500000	-50.0000000
48560.0000000	10.5000000	-50.0000000
48560.0000000	10.7500000	-50.0000000
48560.0000000	11.0000000	-50.0000000
48560.0000000	11.2500000	-50.0000000
48560.0000000	11.5000000	-50.0000000
48560.0000000	11.7500000	-50.0000000
48560.0000000	12.0000000	-50.0000000
48560.0000000	12.2500000	-50.0000000
48560.0000000	12.5000000	-50.0000000
48560.0000000	12.7500000	-50.0000000
48560.0000000	13.0000000	-50.0000000
48560.0000000	13.2500000	-50.0000000
48560.0000000	13.5000000	-50.0000000
48560.0000000	13.7500000	-50.0000000
48560.0000000	14.0000000	-50.0000000
48560.0000000	14.2500000	-50.0000000
48560.0000000	14.5000000	-50.0000000
48560.0000000	14.7500000	-50.0000000
48560.0000000	15.0000000	-50.0000000
48590.0000000	0.0000000	-50.0000000
48590.0000000	0.2500000	-50.0000000
48590.0000000	0.5000000	-50.0000000
48590.0000000	0.7500000	-50.0000000
48590.0000000	1.0000000	-50.0000000
48590.0000000	1.2500000	-50.0000000
48590.0000000	1.5000000	-50.0000000
48590.0000000	1.7500000	-50.0000000
48590.0000000	2.0000000	-50.0000000
48590.0000000	2.2500000	-50.0000000
48590.0000000	2.5000000	-50.0000000
48590.0000000	2.7500000	-50.0000000
48590.0000000	3.0000000	-50.0000000
48590.0000000	3.2500000	-50.0000000
48590.0000000	3.5000000	-50.0000000
48590.0000000	3.7500000	-50.0000000
48590.0000000	4.0000000	-50.0000000
48590.0000000	4.2500000	-50.0000000
48590.0000000	4.5000000	-50.0000000

48590.0000000	4.7500000	-50.0000000
48590.0000000	5.0000000	-50.0000000
48590.0000000	5.2500000	-50.0000000
48590.0000000	5.5000000	-50.0000000
48590.0000000	5.7500000	-50.0000000
48590.0000000	6.0000000	-50.0000000
48590.0000000	6.2500000	-50.0000000
48590.0000000	6.5000000	-50.0000000
48590.0000000	6.7500000	-50.0000000
48590.0000000	7.0000000	-50.0000000
48590.0000000	7.2500000	-50.0000000
48590.0000000	7.5000000	-50.0000000
48590.0000000	7.7500000	-50.0000000
48590.0000000	8.0000000	-50.0000000
48590.0000000	8.2500000	-50.0000000
48590.0000000	8.5000000	-50.0000000
48590.0000000	8.7500000	-50.0000000
48590.0000000	9.0000000	-50.0000000
48590.0000000	9.2500000	-50.0000000
48590.0000000	9.5000000	-50.0000000
48590.0000000	9.7500000	-50.0000000
48590.0000000	10.0000000	-50.0000000
48590.0000000	10.2500000	-50.0000000
48590.0000000	10.5000000	-50.0000000
48590.0000000	10.7500000	-50.0000000
48590.0000000	11.0000000	-50.0000000
48590.0000000	11.2500000	-50.0000000
48590.0000000	11.5000000	-50.0000000
48590.0000000	11.7500000	-50.0000000
48590.0000000	12.0000000	-50.0000000
48590.0000000	12.2500000	-50.0000000
48590.0000000	12.5000000	-50.0000000
48590.0000000	12.7500000	-50.0000000
48590.0000000	13.0000000	-50.0000000
48590.0000000	13.2500000	-50.0000000
48590.0000000	13.5000000	-50.0000000
48590.0000000	13.7500000	-50.0000000
48590.0000000	14.0000000	-50.0000000
48590.0000000	14.2500000	-50.0000000
48590.0000000	14.5000000	-50.0000000
48590.0000000	14.7500000	-50.0000000
48590.0000000	15.0000000	-50.0000000
48620.0000000	0.0000000	-50.0000000
48620.0000000	0.2500000	-50.0000000
48620.0000000	0.5000000	-50.0000000
48620.0000000	0.7500000	-50.0000000
48620.0000000	1.0000000	-50.0000000
48620.0000000	1.2500000	-50.0000000
48620.0000000	1.5000000	-50.0000000
48620.0000000	1.7500000	-50.0000000
48620.0000000	2.0000000	-50.0000000
48620.0000000	2.2500000	-50.0000000
48620.0000000	2.5000000	-50.0000000
48620.0000000	2.7500000	-50.0000000
48620.0000000	3.0000000	-50.0000000
48620.0000000	3.2500000	-50.0000000
48620.0000000	3.5000000	-50.0000000
48620.0000000	3.7500000	-50.0000000

48620.0000000	4.0000000	-50.0000000
48620.0000000	4.2500000	-50.0000000
48620.0000000	4.5000000	-50.0000000
48620.0000000	4.7500000	-50.0000000
48620.0000000	5.0000000	-50.0000000
48620.0000000	5.2500000	-50.0000000
48620.0000000	5.5000000	-50.0000000
48620.0000000	5.7500000	-50.0000000
48620.0000000	6.0000000	-50.0000000
48620.0000000	6.2500000	-50.0000000
48620.0000000	6.5000000	-50.0000000
48620.0000000	6.7500000	-50.0000000
48620.0000000	7.0000000	-50.0000000
48620.0000000	7.2500000	-50.0000000
48620.0000000	7.5000000	-50.0000000
48620.0000000	7.7500000	-50.0000000
48620.0000000	8.0000000	-50.0000000
48620.0000000	8.2500000	-50.0000000
48620.0000000	8.5000000	-50.0000000
48620.0000000	8.7500000	-50.0000000
48620.0000000	9.0000000	-50.0000000
48620.0000000	9.2500000	-50.0000000
48620.0000000	9.5000000	-50.0000000
48620.0000000	9.7500000	-50.0000000
48620.0000000	10.0000000	-50.0000000
48620.0000000	10.2500000	-50.0000000
48620.0000000	10.5000000	-50.0000000
48620.0000000	10.7500000	-50.0000000
48620.0000000	11.0000000	-50.0000000
48620.0000000	11.2500000	-50.0000000
48620.0000000	11.5000000	-50.0000000
48620.0000000	11.7500000	-50.0000000
48620.0000000	12.0000000	-50.0000000
48620.0000000	12.2500000	-50.0000000
48620.0000000	12.5000000	-50.0000000
48620.0000000	12.7500000	-50.0000000
48620.0000000	13.0000000	-50.0000000
48620.0000000	13.2500000	-50.0000000
48620.0000000	13.5000000	-50.0000000
48620.0000000	13.7500000	-50.0000000
48620.0000000	14.0000000	-50.0000000
48620.0000000	14.2500000	-50.0000000
48620.0000000	14.5000000	-50.0000000
48620.0000000	14.7500000	-50.0000000
48620.0000000	15.0000000	-50.0000000
48650.0000000	0.0000000	-50.0000000
48650.0000000	0.2500000	-50.0000000
48650.0000000	0.5000000	-50.0000000
48650.0000000	0.7500000	-50.0000000
48650.0000000	1.0000000	-50.0000000
48650.0000000	1.2500000	-50.0000000
48650.0000000	1.5000000	-50.0000000
48650.0000000	1.7500000	-50.0000000
48650.0000000	2.0000000	-50.0000000
48650.0000000	2.2500000	-50.0000000
48650.0000000	2.5000000	-50.0000000
48650.0000000	2.7500000	-50.0000000
48650.0000000	3.0000000	-50.0000000

48650.0000000	3.2500000	-50.0000000
48650.0000000	3.5000000	-50.0000000
48650.0000000	3.7500000	-50.0000000
48650.0000000	4.0000000	-50.0000000
48650.0000000	4.2500000	-50.0000000
48650.0000000	4.5000000	-50.0000000
48650.0000000	4.7500000	-50.0000000
48650.0000000	5.0000000	-50.0000000
48650.0000000	5.2500000	-50.0000000
48650.0000000	5.5000000	-50.0000000
48650.0000000	5.7500000	-50.0000000
48650.0000000	6.0000000	-50.0000000
48650.0000000	6.2500000	-50.0000000
48650.0000000	6.5000000	-50.0000000
48650.0000000	6.7500000	-50.0000000
48650.0000000	7.0000000	-50.0000000
48650.0000000	7.2500000	-50.0000000
48650.0000000	7.5000000	-50.0000000
48650.0000000	7.7500000	-50.0000000
48650.0000000	8.0000000	-50.0000000
48650.0000000	8.2500000	-50.0000000
48650.0000000	8.5000000	-50.0000000
48650.0000000	8.7500000	-50.0000000
48650.0000000	9.0000000	-50.0000000
48650.0000000	9.2500000	-50.0000000
48650.0000000	9.5000000	-50.0000000
48650.0000000	9.7500000	-50.0000000
48650.0000000	10.0000000	-50.0000000
48650.0000000	10.2500000	-50.0000000
48650.0000000	10.5000000	-50.0000000
48650.0000000	10.7500000	-50.0000000
48650.0000000	11.0000000	-50.0000000
48650.0000000	11.2500000	-50.0000000
48650.0000000	11.5000000	-50.0000000
48650.0000000	11.7500000	-50.0000000
48650.0000000	12.0000000	-50.0000000
48650.0000000	12.2500000	-50.0000000
48650.0000000	12.5000000	-50.0000000
48650.0000000	12.7500000	-50.0000000
48650.0000000	13.0000000	-50.0000000
48650.0000000	13.2500000	-50.0000000
48650.0000000	13.5000000	-50.0000000
48650.0000000	13.7500000	-50.0000000
48650.0000000	14.0000000	-50.0000000
48650.0000000	14.2500000	-50.0000000
48650.0000000	14.5000000	-50.0000000
48650.0000000	14.7500000	-50.0000000
48650.0000000	15.0000000	-50.0000000
48680.0000000	0.0000000	-50.0000000
48680.0000000	0.2500000	-50.0000000
48680.0000000	0.5000000	-50.0000000
48680.0000000	0.7500000	-50.0000000
48680.0000000	1.0000000	-50.0000000
48680.0000000	1.2500000	-50.0000000
48680.0000000	1.5000000	-50.0000000
48680.0000000	1.7500000	-50.0000000
48680.0000000	2.0000000	-50.0000000
48680.0000000	2.2500000	-50.0000000

48680.0000000	2.5000000	-50.0000000
48680.0000000	2.7500000	-50.0000000
48680.0000000	3.0000000	-50.0000000
48680.0000000	3.2500000	-50.0000000
48680.0000000	3.5000000	-50.0000000
48680.0000000	3.7500000	-50.0000000
48680.0000000	4.0000000	-50.0000000
48680.0000000	4.2500000	-50.0000000
48680.0000000	4.5000000	-50.0000000
48680.0000000	4.7500000	-50.0000000
48680.0000000	5.0000000	-50.0000000
48680.0000000	5.2500000	-50.0000000
48680.0000000	5.5000000	-50.0000000
48680.0000000	5.7500000	-50.0000000
48680.0000000	6.0000000	-50.0000000
48680.0000000	6.2500000	-50.0000000
48680.0000000	6.5000000	-50.0000000
48680.0000000	6.7500000	-50.0000000
48680.0000000	7.0000000	-50.0000000
48680.0000000	7.2500000	-50.0000000
48680.0000000	7.5000000	-50.0000000
48680.0000000	7.7500000	-50.0000000
48680.0000000	8.0000000	-50.0000000
48680.0000000	8.2500000	-50.0000000
48680.0000000	8.5000000	-50.0000000
48680.0000000	8.7500000	-50.0000000
48680.0000000	9.0000000	-50.0000000
48680.0000000	9.2500000	-50.0000000
48680.0000000	9.5000000	-50.0000000
48680.0000000	9.7500000	-50.0000000
48680.0000000	10.0000000	-50.0000000
48680.0000000	10.2500000	-50.0000000
48680.0000000	10.5000000	-50.0000000
48680.0000000	10.7500000	-50.0000000
48680.0000000	11.0000000	-50.0000000
48680.0000000	11.2500000	-50.0000000
48680.0000000	11.5000000	-50.0000000
48680.0000000	11.7500000	-50.0000000
48680.0000000	12.0000000	-50.0000000
48680.0000000	12.2500000	-50.0000000
48680.0000000	12.5000000	-50.0000000
48680.0000000	12.7500000	-50.0000000
48680.0000000	13.0000000	-50.0000000
48680.0000000	13.2500000	-50.0000000
48680.0000000	13.5000000	-50.0000000
48680.0000000	13.7500000	-50.0000000
48680.0000000	14.0000000	-50.0000000
48680.0000000	14.2500000	-50.0000000
48680.0000000	14.5000000	-50.0000000
48680.0000000	14.7500000	-50.0000000
48680.0000000	15.0000000	-50.0000000
48710.0000000	0.0000000	-50.0000000
48710.0000000	0.2500000	-50.0000000
48710.0000000	0.5000000	-50.0000000
48710.0000000	0.7500000	-50.0000000
48710.0000000	1.0000000	-50.0000000
48710.0000000	1.2500000	15.4200001
48710.0000000	1.5000000	-50.0000000

48710.0000000	1.7500000	-50.0000000
48710.0000000	2.0000000	-50.0000000
48710.0000000	2.2500000	-50.0000000
48710.0000000	2.5000000	-50.0000000
48710.0000000	2.7500000	-50.0000000
48710.0000000	3.0000000	-50.0000000
48710.0000000	3.2500000	-50.0000000
48710.0000000	3.5000000	-50.0000000
48710.0000000	3.7500000	-50.0000000
48710.0000000	4.0000000	-50.0000000
48710.0000000	4.2500000	-50.0000000
48710.0000000	4.5000000	-50.0000000
48710.0000000	4.7500000	-50.0000000
48710.0000000	5.0000000	-50.0000000
48710.0000000	5.2500000	-50.0000000
48710.0000000	5.5000000	-50.0000000
48710.0000000	5.7500000	-50.0000000
48710.0000000	6.0000000	-50.0000000
48710.0000000	6.2500000	-50.0000000
48710.0000000	6.5000000	-50.0000000
48710.0000000	6.7500000	-50.0000000
48710.0000000	7.0000000	-50.0000000
48710.0000000	7.2500000	-50.0000000
48710.0000000	7.5000000	-50.0000000
48710.0000000	7.7500000	-50.0000000
48710.0000000	8.0000000	-50.0000000
48710.0000000	8.2500000	-50.0000000
48710.0000000	8.5000000	-50.0000000
48710.0000000	8.7500000	-50.0000000
48710.0000000	9.0000000	-50.0000000
48710.0000000	9.2500000	-50.0000000
48710.0000000	9.5000000	-50.0000000
48710.0000000	9.7500000	-50.0000000
48710.0000000	10.0000000	-50.0000000
48710.0000000	10.2500000	-50.0000000
48710.0000000	10.5000000	-50.0000000
48710.0000000	10.7500000	-50.0000000
48710.0000000	11.0000000	-50.0000000
48710.0000000	11.2500000	-50.0000000
48710.0000000	11.5000000	-50.0000000
48710.0000000	11.7500000	-50.0000000
48710.0000000	12.0000000	-50.0000000
48710.0000000	12.2500000	-50.0000000
48710.0000000	12.5000000	-50.0000000
48710.0000000	12.7500000	-50.0000000
48710.0000000	13.0000000	-50.0000000
48710.0000000	13.2500000	-50.0000000
48710.0000000	13.5000000	-50.0000000
48710.0000000	13.7500000	-50.0000000
48710.0000000	14.0000000	-50.0000000
48710.0000000	14.2500000	-50.0000000
48710.0000000	14.5000000	-50.0000000
48710.0000000	14.7500000	-50.0000000
48710.0000000	15.0000000	-50.0000000
48740.0000000	0.0000000	-50.0000000
48740.0000000	0.2500000	-50.0000000
48740.0000000	0.5000000	-50.0000000
48740.0000000	0.7500000	-50.0000000

48740.0000000	1.0000000	-50.0000000
48740.0000000	1.2500000	17.4899998
48740.0000000	1.5000000	-50.0000000
48740.0000000	1.7500000	-50.0000000
48740.0000000	2.0000000	-50.0000000
48740.0000000	2.2500000	-50.0000000
48740.0000000	2.5000000	-50.0000000
48740.0000000	2.7500000	-50.0000000
48740.0000000	3.0000000	-50.0000000
48740.0000000	3.2500000	-50.0000000
48740.0000000	3.5000000	-50.0000000
48740.0000000	3.7500000	-50.0000000
48740.0000000	4.0000000	-50.0000000
48740.0000000	4.2500000	-50.0000000
48740.0000000	4.5000000	-50.0000000
48740.0000000	4.7500000	-50.0000000
48740.0000000	5.0000000	-50.0000000
48740.0000000	5.2500000	-50.0000000
48740.0000000	5.5000000	-50.0000000
48740.0000000	5.7500000	-50.0000000
48740.0000000	6.0000000	-50.0000000
48740.0000000	6.2500000	-50.0000000
48740.0000000	6.5000000	-50.0000000
48740.0000000	6.7500000	-50.0000000
48740.0000000	7.0000000	-50.0000000
48740.0000000	7.2500000	-50.0000000
48740.0000000	7.5000000	-50.0000000
48740.0000000	7.7500000	-50.0000000
48740.0000000	8.0000000	-50.0000000
48740.0000000	8.2500000	-50.0000000
48740.0000000	8.5000000	-50.0000000
48740.0000000	8.7500000	-50.0000000
48740.0000000	9.0000000	-50.0000000
48740.0000000	9.2500000	-50.0000000
48740.0000000	9.5000000	-50.0000000
48740.0000000	9.7500000	-50.0000000
48740.0000000	10.0000000	-50.0000000
48740.0000000	10.2500000	-50.0000000
48740.0000000	10.5000000	-50.0000000
48740.0000000	10.7500000	-50.0000000
48740.0000000	11.0000000	-50.0000000
48740.0000000	11.2500000	-50.0000000
48740.0000000	11.5000000	-50.0000000
48740.0000000	11.7500000	-50.0000000
48740.0000000	12.0000000	-50.0000000
48740.0000000	12.2500000	-50.0000000
48740.0000000	12.5000000	-50.0000000
48740.0000000	12.7500000	-50.0000000
48740.0000000	13.0000000	-50.0000000
48740.0000000	13.2500000	-50.0000000
48740.0000000	13.5000000	-50.0000000
48740.0000000	13.7500000	-50.0000000
48740.0000000	14.0000000	-50.0000000
48740.0000000	14.2500000	-50.0000000
48740.0000000	14.5000000	-50.0000000
48740.0000000	14.7500000	-50.0000000
48740.0000000	15.0000000	-50.0000000
48770.0000000	0.0000000	-50.0000000

48770.0000000	0.2500000	-50.0000000
48770.0000000	0.5000000	-50.0000000
48770.0000000	0.7500000	-50.0000000
48770.0000000	1.0000000	-50.0000000
48770.0000000	1.2500000	17.4300003
48770.0000000	1.5000000	-50.0000000
48770.0000000	1.7500000	-50.0000000
48770.0000000	2.0000000	-50.0000000
48770.0000000	2.2500000	-50.0000000
48770.0000000	2.5000000	-50.0000000
48770.0000000	2.7500000	-50.0000000
48770.0000000	3.0000000	-50.0000000
48770.0000000	3.2500000	-50.0000000
48770.0000000	3.5000000	-50.0000000
48770.0000000	3.7500000	-50.0000000
48770.0000000	4.0000000	-50.0000000
48770.0000000	4.2500000	-50.0000000
48770.0000000	4.5000000	-50.0000000
48770.0000000	4.7500000	-50.0000000
48770.0000000	5.0000000	-50.0000000
48770.0000000	5.2500000	-50.0000000
48770.0000000	5.5000000	-50.0000000
48770.0000000	5.7500000	-50.0000000
48770.0000000	6.0000000	-50.0000000
48770.0000000	6.2500000	-50.0000000
48770.0000000	6.5000000	-50.0000000
48770.0000000	6.7500000	-50.0000000
48770.0000000	7.0000000	-50.0000000
48770.0000000	7.2500000	-50.0000000
48770.0000000	7.5000000	-50.0000000
48770.0000000	7.7500000	-50.0000000
48770.0000000	8.0000000	-50.0000000
48770.0000000	8.2500000	-50.0000000
48770.0000000	8.5000000	-50.0000000
48770.0000000	8.7500000	-50.0000000
48770.0000000	9.0000000	-50.0000000
48770.0000000	9.2500000	-50.0000000
48770.0000000	9.5000000	-50.0000000
48770.0000000	9.7500000	-50.0000000
48770.0000000	10.0000000	-50.0000000
48770.0000000	10.2500000	-50.0000000
48770.0000000	10.5000000	-50.0000000
48770.0000000	10.7500000	-50.0000000
48770.0000000	11.0000000	-50.0000000
48770.0000000	11.2500000	-50.0000000
48770.0000000	11.5000000	-50.0000000
48770.0000000	11.7500000	-50.0000000
48770.0000000	12.0000000	-50.0000000
48770.0000000	12.2500000	-50.0000000
48770.0000000	12.5000000	-50.0000000
48770.0000000	12.7500000	-50.0000000
48770.0000000	13.0000000	-50.0000000
48770.0000000	13.2500000	-50.0000000
48770.0000000	13.5000000	-50.0000000
48770.0000000	13.7500000	-50.0000000
48770.0000000	14.0000000	-50.0000000
48770.0000000	14.2500000	-50.0000000
48770.0000000	14.5000000	-50.0000000

48770.0000000	14.7500000	-50.0000000
48770.0000000	15.0000000	-50.0000000
48800.0000000	0.0000000	-50.0000000
48800.0000000	0.2500000	-50.0000000
48800.0000000	0.5000000	-50.0000000
48800.0000000	0.7500000	-50.0000000
48800.0000000	1.0000000	-50.0000000
48800.0000000	1.2500000	-50.0000000
48800.0000000	1.5000000	-50.0000000
48800.0000000	1.7500000	-50.0000000
48800.0000000	2.0000000	-50.0000000
48800.0000000	2.2500000	-50.0000000
48800.0000000	2.5000000	-50.0000000
48800.0000000	2.7500000	-50.0000000
48800.0000000	3.0000000	-50.0000000
48800.0000000	3.2500000	-50.0000000
48800.0000000	3.5000000	-50.0000000
48800.0000000	3.7500000	-50.0000000
48800.0000000	4.0000000	-50.0000000
48800.0000000	4.2500000	-50.0000000
48800.0000000	4.5000000	-50.0000000
48800.0000000	4.7500000	-50.0000000
48800.0000000	5.0000000	-50.0000000
48800.0000000	5.2500000	-50.0000000
48800.0000000	5.5000000	-50.0000000
48800.0000000	5.7500000	-50.0000000
48800.0000000	6.0000000	-50.0000000
48800.0000000	6.2500000	-50.0000000
48800.0000000	6.5000000	-50.0000000
48800.0000000	6.7500000	-50.0000000
48800.0000000	7.0000000	-50.0000000
48800.0000000	7.2500000	-50.0000000
48800.0000000	7.5000000	-50.0000000
48800.0000000	7.7500000	-50.0000000
48800.0000000	8.0000000	-50.0000000
48800.0000000	8.2500000	-50.0000000
48800.0000000	8.5000000	-50.0000000
48800.0000000	8.7500000	-50.0000000
48800.0000000	9.0000000	-50.0000000
48800.0000000	9.2500000	-50.0000000
48800.0000000	9.5000000	-50.0000000
48800.0000000	9.7500000	-50.0000000
48800.0000000	10.0000000	-50.0000000
48800.0000000	10.2500000	-50.0000000
48800.0000000	10.5000000	-50.0000000
48800.0000000	10.7500000	-50.0000000
48800.0000000	11.0000000	-50.0000000
48800.0000000	11.2500000	-50.0000000
48800.0000000	11.5000000	-50.0000000
48800.0000000	11.7500000	-50.0000000
48800.0000000	12.0000000	-50.0000000
48800.0000000	12.2500000	-50.0000000
48800.0000000	12.5000000	-50.0000000
48800.0000000	12.7500000	-50.0000000
48800.0000000	13.0000000	-50.0000000
48800.0000000	13.2500000	-50.0000000
48800.0000000	13.5000000	-50.0000000
48800.0000000	13.7500000	-50.0000000

48800.0000000	14.0000000	-50.0000000
48800.0000000	14.2500000	-50.0000000
48800.0000000	14.5000000	-50.0000000
48800.0000000	14.7500000	-50.0000000
48800.0000000	15.0000000	-50.0000000
48830.0000000	0.0000000	-50.0000000
48830.0000000	0.2500000	-50.0000000
48830.0000000	0.5000000	-50.0000000
48830.0000000	0.7500000	16.7399998
48830.0000000	1.0000000	16.9400005
48830.0000000	1.2500000	-50.0000000
48830.0000000	1.5000000	-50.0000000
48830.0000000	1.7500000	-50.0000000
48830.0000000	2.0000000	-50.0000000
48830.0000000	2.2500000	-50.0000000
48830.0000000	2.5000000	-50.0000000
48830.0000000	2.7500000	-50.0000000
48830.0000000	3.0000000	-50.0000000
48830.0000000	3.2500000	-50.0000000
48830.0000000	3.5000000	-50.0000000
48830.0000000	3.7500000	-50.0000000
48830.0000000	4.0000000	-50.0000000
48830.0000000	4.2500000	-50.0000000
48830.0000000	4.5000000	-50.0000000
48830.0000000	4.7500000	-50.0000000
48830.0000000	5.0000000	-50.0000000
48830.0000000	5.2500000	-50.0000000
48830.0000000	5.5000000	-50.0000000
48830.0000000	5.7500000	-50.0000000
48830.0000000	6.0000000	-50.0000000
48830.0000000	6.2500000	-50.0000000
48830.0000000	6.5000000	-50.0000000
48830.0000000	6.7500000	-50.0000000
48830.0000000	7.0000000	-50.0000000
48830.0000000	7.2500000	-50.0000000
48830.0000000	7.5000000	-50.0000000
48830.0000000	7.7500000	-50.0000000
48830.0000000	8.0000000	-50.0000000
48830.0000000	8.2500000	-50.0000000
48830.0000000	8.5000000	-50.0000000
48830.0000000	8.7500000	-50.0000000
48830.0000000	9.0000000	-50.0000000
48830.0000000	9.2500000	-50.0000000
48830.0000000	9.5000000	-50.0000000
48830.0000000	9.7500000	-50.0000000
48830.0000000	10.0000000	-50.0000000
48830.0000000	10.2500000	-50.0000000
48830.0000000	10.5000000	-50.0000000
48830.0000000	10.7500000	-50.0000000
48830.0000000	11.0000000	-50.0000000
48830.0000000	11.2500000	-50.0000000
48830.0000000	11.5000000	-50.0000000
48830.0000000	11.7500000	-50.0000000
48830.0000000	12.0000000	-50.0000000
48830.0000000	12.2500000	-50.0000000
48830.0000000	12.5000000	-50.0000000
48830.0000000	12.7500000	-50.0000000
48830.0000000	13.0000000	-50.0000000

48830.0000000	13.2500000	-50.0000000
48830.0000000	13.5000000	-50.0000000
48830.0000000	13.7500000	-50.0000000
48830.0000000	14.0000000	-50.0000000
48830.0000000	14.2500000	-50.0000000
48830.0000000	14.5000000	-50.0000000
48830.0000000	14.7500000	-50.0000000
48830.0000000	15.0000000	-50.0000000
48860.0000000	0.0000000	-50.0000000
48860.0000000	0.2500000	-50.0000000
48860.0000000	0.5000000	-50.0000000
48860.0000000	0.7500000	-50.0000000
48860.0000000	1.0000000	16.0499992
48860.0000000	1.2500000	-50.0000000
48860.0000000	1.5000000	-50.0000000
48860.0000000	1.7500000	-50.0000000
48860.0000000	2.0000000	-50.0000000
48860.0000000	2.2500000	-50.0000000
48860.0000000	2.5000000	-50.0000000
48860.0000000	2.7500000	-50.0000000
48860.0000000	3.0000000	-50.0000000
48860.0000000	3.2500000	-50.0000000
48860.0000000	3.5000000	-50.0000000
48860.0000000	3.7500000	-50.0000000
48860.0000000	4.0000000	-50.0000000
48860.0000000	4.2500000	-50.0000000
48860.0000000	4.5000000	-50.0000000
48860.0000000	4.7500000	-50.0000000
48860.0000000	5.0000000	-50.0000000
48860.0000000	5.2500000	-50.0000000
48860.0000000	5.5000000	-50.0000000
48860.0000000	5.7500000	-50.0000000
48860.0000000	6.0000000	-50.0000000
48860.0000000	6.2500000	-50.0000000
48860.0000000	6.5000000	-50.0000000
48860.0000000	6.7500000	-50.0000000
48860.0000000	7.0000000	-50.0000000
48860.0000000	7.2500000	-50.0000000
48860.0000000	7.5000000	-50.0000000
48860.0000000	7.7500000	-50.0000000
48860.0000000	8.0000000	-50.0000000
48860.0000000	8.2500000	-50.0000000
48860.0000000	8.5000000	-50.0000000
48860.0000000	8.7500000	-50.0000000
48860.0000000	9.0000000	-50.0000000
48860.0000000	9.2500000	-50.0000000
48860.0000000	9.5000000	-50.0000000
48860.0000000	9.7500000	-50.0000000
48860.0000000	10.0000000	-50.0000000
48860.0000000	10.2500000	-50.0000000
48860.0000000	10.5000000	-50.0000000
48860.0000000	10.7500000	-50.0000000
48860.0000000	11.0000000	-50.0000000
48860.0000000	11.2500000	-50.0000000
48860.0000000	11.5000000	-50.0000000
48860.0000000	11.7500000	-50.0000000
48860.0000000	12.0000000	-50.0000000
48860.0000000	12.2500000	-50.0000000

48860.0000000	12.5000000	-50.0000000
48860.0000000	12.7500000	-50.0000000
48860.0000000	13.0000000	-50.0000000
48860.0000000	13.2500000	-50.0000000
48860.0000000	13.5000000	-50.0000000
48860.0000000	13.7500000	-50.0000000
48860.0000000	14.0000000	-50.0000000
48860.0000000	14.2500000	-50.0000000
48860.0000000	14.5000000	-50.0000000
48860.0000000	14.7500000	-50.0000000
48860.0000000	15.0000000	-50.0000000
48890.0000000	0.0000000	-50.0000000
48890.0000000	0.2500000	-50.0000000
48890.0000000	0.5000000	-50.0000000
48890.0000000	0.7500000	-50.0000000
48890.0000000	1.0000000	16.5300007
48890.0000000	1.2500000	-50.0000000
48890.0000000	1.5000000	-50.0000000
48890.0000000	1.7500000	-50.0000000
48890.0000000	2.0000000	-50.0000000
48890.0000000	2.2500000	-50.0000000
48890.0000000	2.5000000	-50.0000000
48890.0000000	2.7500000	-50.0000000
48890.0000000	3.0000000	-50.0000000
48890.0000000	3.2500000	-50.0000000
48890.0000000	3.5000000	-50.0000000
48890.0000000	3.7500000	-50.0000000
48890.0000000	4.0000000	-50.0000000
48890.0000000	4.2500000	-50.0000000
48890.0000000	4.5000000	-50.0000000
48890.0000000	4.7500000	-50.0000000
48890.0000000	5.0000000	-50.0000000
48890.0000000	5.2500000	-50.0000000
48890.0000000	5.5000000	-50.0000000
48890.0000000	5.7500000	-50.0000000
48890.0000000	6.0000000	-50.0000000
48890.0000000	6.2500000	-50.0000000
48890.0000000	6.5000000	-50.0000000
48890.0000000	6.7500000	-50.0000000
48890.0000000	7.0000000	-50.0000000
48890.0000000	7.2500000	-50.0000000
48890.0000000	7.5000000	-50.0000000
48890.0000000	7.7500000	-50.0000000
48890.0000000	8.0000000	-50.0000000
48890.0000000	8.2500000	-50.0000000
48890.0000000	8.5000000	-50.0000000
48890.0000000	8.7500000	-50.0000000
48890.0000000	9.0000000	-50.0000000
48890.0000000	9.2500000	-50.0000000
48890.0000000	9.5000000	-50.0000000
48890.0000000	9.7500000	-50.0000000
48890.0000000	10.0000000	-50.0000000
48890.0000000	10.2500000	-50.0000000
48890.0000000	10.5000000	-50.0000000
48890.0000000	10.7500000	-50.0000000
48890.0000000	11.0000000	-50.0000000
48890.0000000	11.2500000	-50.0000000
48890.0000000	11.5000000	-50.0000000

48890.0000000	11.7500000	-50.0000000
48890.0000000	12.0000000	-50.0000000
48890.0000000	12.2500000	-50.0000000
48890.0000000	12.5000000	-50.0000000
48890.0000000	12.7500000	-50.0000000
48890.0000000	13.0000000	-50.0000000
48890.0000000	13.2500000	-50.0000000
48890.0000000	13.5000000	-50.0000000
48890.0000000	13.7500000	-50.0000000
48890.0000000	14.0000000	-50.0000000
48890.0000000	14.2500000	-50.0000000
48890.0000000	14.5000000	-50.0000000
48890.0000000	14.7500000	-50.0000000
48890.0000000	15.0000000	-50.0000000
48920.0000000	0.0000000	-50.0000000
48920.0000000	0.2500000	-50.0000000
48920.0000000	0.5000000	-50.0000000
48920.0000000	0.7500000	18.5000000
48920.0000000	1.0000000	18.9899998
48920.0000000	1.2500000	-50.0000000
48920.0000000	1.5000000	-50.0000000
48920.0000000	1.7500000	-50.0000000
48920.0000000	2.0000000	-50.0000000
48920.0000000	2.2500000	-50.0000000
48920.0000000	2.5000000	-50.0000000
48920.0000000	2.7500000	-50.0000000
48920.0000000	3.0000000	-50.0000000
48920.0000000	3.2500000	-50.0000000
48920.0000000	3.5000000	-50.0000000
48920.0000000	3.7500000	-50.0000000
48920.0000000	4.0000000	-50.0000000
48920.0000000	4.2500000	-50.0000000
48920.0000000	4.5000000	-50.0000000
48920.0000000	4.7500000	-50.0000000
48920.0000000	5.0000000	-50.0000000
48920.0000000	5.2500000	-50.0000000
48920.0000000	5.5000000	-50.0000000
48920.0000000	5.7500000	-50.0000000
48920.0000000	6.0000000	-50.0000000
48920.0000000	6.2500000	-50.0000000
48920.0000000	6.5000000	-50.0000000
48920.0000000	6.7500000	-50.0000000
48920.0000000	7.0000000	-50.0000000
48920.0000000	7.2500000	-50.0000000
48920.0000000	7.5000000	-50.0000000
48920.0000000	7.7500000	-50.0000000
48920.0000000	8.0000000	-50.0000000
48920.0000000	8.2500000	-50.0000000
48920.0000000	8.5000000	-50.0000000
48920.0000000	8.7500000	-50.0000000
48920.0000000	9.0000000	-50.0000000
48920.0000000	9.2500000	-50.0000000
48920.0000000	9.5000000	-50.0000000
48920.0000000	9.7500000	-50.0000000
48920.0000000	10.0000000	-50.0000000
48920.0000000	10.2500000	-50.0000000
48920.0000000	10.5000000	-50.0000000
48920.0000000	10.7500000	-50.0000000

48920.0000000	11.0000000	-50.0000000
48920.0000000	11.2500000	-50.0000000
48920.0000000	11.5000000	-50.0000000
48920.0000000	11.7500000	-50.0000000
48920.0000000	12.0000000	-50.0000000
48920.0000000	12.2500000	-50.0000000
48920.0000000	12.5000000	-50.0000000
48920.0000000	12.7500000	-50.0000000
48920.0000000	13.0000000	-50.0000000
48920.0000000	13.2500000	-50.0000000
48920.0000000	13.5000000	-50.0000000
48920.0000000	13.7500000	-50.0000000
48920.0000000	14.0000000	-50.0000000
48920.0000000	14.2500000	-50.0000000
48920.0000000	14.5000000	-50.0000000
48920.0000000	14.7500000	-50.0000000
48920.0000000	15.0000000	-50.0000000
48950.0000000	0.0000000	-50.0000000
48950.0000000	0.2500000	-50.0000000
48950.0000000	0.5000000	15.9700003
48950.0000000	0.7500000	23.7399998
48950.0000000	1.0000000	21.5599995
48950.0000000	1.2500000	-50.0000000
48950.0000000	1.5000000	-50.0000000
48950.0000000	1.7500000	-50.0000000
48950.0000000	2.0000000	-50.0000000
48950.0000000	2.2500000	-50.0000000
48950.0000000	2.5000000	-50.0000000
48950.0000000	2.7500000	-50.0000000
48950.0000000	3.0000000	-50.0000000
48950.0000000	3.2500000	-50.0000000
48950.0000000	3.5000000	-50.0000000
48950.0000000	3.7500000	-50.0000000
48950.0000000	4.0000000	-50.0000000
48950.0000000	4.2500000	-50.0000000
48950.0000000	4.5000000	-50.0000000
48950.0000000	4.7500000	-50.0000000
48950.0000000	5.0000000	-50.0000000
48950.0000000	5.2500000	-50.0000000
48950.0000000	5.5000000	-50.0000000
48950.0000000	5.7500000	-50.0000000
48950.0000000	6.0000000	-50.0000000
48950.0000000	6.2500000	-50.0000000
48950.0000000	6.5000000	-50.0000000
48950.0000000	6.7500000	-50.0000000
48950.0000000	7.0000000	-50.0000000
48950.0000000	7.2500000	-50.0000000
48950.0000000	7.5000000	-50.0000000
48950.0000000	7.7500000	-50.0000000
48950.0000000	8.0000000	-50.0000000
48950.0000000	8.2500000	-50.0000000
48950.0000000	8.5000000	-50.0000000
48950.0000000	8.7500000	-50.0000000
48950.0000000	9.0000000	-50.0000000
48950.0000000	9.2500000	-50.0000000
48950.0000000	9.5000000	-50.0000000
48950.0000000	9.7500000	-50.0000000
48950.0000000	10.0000000	-50.0000000

48950.0000000	10.2500000	-50.0000000
48950.0000000	10.5000000	-50.0000000
48950.0000000	10.7500000	-50.0000000
48950.0000000	11.0000000	-50.0000000
48950.0000000	11.2500000	-50.0000000
48950.0000000	11.5000000	-50.0000000
48950.0000000	11.7500000	-50.0000000
48950.0000000	12.0000000	-50.0000000
48950.0000000	12.2500000	-50.0000000
48950.0000000	12.5000000	-50.0000000
48950.0000000	12.7500000	-50.0000000
48950.0000000	13.0000000	-50.0000000
48950.0000000	13.2500000	-50.0000000
48950.0000000	13.5000000	-50.0000000
48950.0000000	13.7500000	-50.0000000
48950.0000000	14.0000000	-50.0000000
48950.0000000	14.2500000	-50.0000000
48950.0000000	14.5000000	-50.0000000
48950.0000000	14.7500000	-50.0000000
48950.0000000	15.0000000	-50.0000000
48980.0000000	0.0000000	-50.0000000
48980.0000000	0.2500000	-50.0000000
48980.0000000	0.5000000	19.8299999
48980.0000000	0.7500000	24.2000008
48980.0000000	1.0000000	18.0499992
48980.0000000	1.2500000	-50.0000000
48980.0000000	1.5000000	-50.0000000
48980.0000000	1.7500000	-50.0000000
48980.0000000	2.0000000	-50.0000000
48980.0000000	2.2500000	-50.0000000
48980.0000000	2.5000000	-50.0000000
48980.0000000	2.7500000	-50.0000000
48980.0000000	3.0000000	-50.0000000
48980.0000000	3.2500000	-50.0000000
48980.0000000	3.5000000	-50.0000000
48980.0000000	3.7500000	-50.0000000
48980.0000000	4.0000000	-50.0000000
48980.0000000	4.2500000	-50.0000000
48980.0000000	4.5000000	-50.0000000
48980.0000000	4.7500000	-50.0000000
48980.0000000	5.0000000	-50.0000000
48980.0000000	5.2500000	-50.0000000
48980.0000000	5.5000000	-50.0000000
48980.0000000	5.7500000	-50.0000000
48980.0000000	6.0000000	-50.0000000
48980.0000000	6.2500000	-50.0000000
48980.0000000	6.5000000	-50.0000000
48980.0000000	6.7500000	-50.0000000
48980.0000000	7.0000000	-50.0000000
48980.0000000	7.2500000	-50.0000000
48980.0000000	7.5000000	-50.0000000
48980.0000000	7.7500000	-50.0000000
48980.0000000	8.0000000	-50.0000000
48980.0000000	8.2500000	-50.0000000
48980.0000000	8.5000000	-50.0000000
48980.0000000	8.7500000	-50.0000000
48980.0000000	9.0000000	-50.0000000
48980.0000000	9.2500000	-50.0000000

48980.0000000	9.5000000	-50.0000000
48980.0000000	9.7500000	-50.0000000
48980.0000000	10.0000000	-50.0000000
48980.0000000	10.2500000	-50.0000000
48980.0000000	10.5000000	-50.0000000
48980.0000000	10.7500000	-50.0000000
48980.0000000	11.0000000	-50.0000000
48980.0000000	11.2500000	-50.0000000
48980.0000000	11.5000000	-50.0000000
48980.0000000	11.7500000	-50.0000000
48980.0000000	12.0000000	-50.0000000
48980.0000000	12.2500000	-50.0000000
48980.0000000	12.5000000	-50.0000000
48980.0000000	12.7500000	-50.0000000
48980.0000000	13.0000000	-50.0000000
48980.0000000	13.2500000	-50.0000000
48980.0000000	13.5000000	-50.0000000
48980.0000000	13.7500000	-50.0000000
48980.0000000	14.0000000	-50.0000000
48980.0000000	14.2500000	-50.0000000
48980.0000000	14.5000000	-50.0000000
48980.0000000	14.7500000	-50.0000000
48980.0000000	15.0000000	-50.0000000
49010.0000000	0.0000000	-50.0000000
49010.0000000	0.2500000	-50.0000000
49010.0000000	0.5000000	22.3999996
49010.0000000	0.7500000	25.1100006
49010.0000000	1.0000000	18.9300003
49010.0000000	1.2500000	-50.0000000
49010.0000000	1.5000000	-50.0000000
49010.0000000	1.7500000	-50.0000000
49010.0000000	2.0000000	-50.0000000
49010.0000000	2.2500000	-50.0000000
49010.0000000	2.5000000	-50.0000000
49010.0000000	2.7500000	-50.0000000
49010.0000000	3.0000000	-50.0000000
49010.0000000	3.2500000	-50.0000000
49010.0000000	3.5000000	-50.0000000
49010.0000000	3.7500000	-50.0000000
49010.0000000	4.0000000	-50.0000000
49010.0000000	4.2500000	-50.0000000
49010.0000000	4.5000000	-50.0000000
49010.0000000	4.7500000	-50.0000000
49010.0000000	5.0000000	-50.0000000
49010.0000000	5.2500000	-50.0000000
49010.0000000	5.5000000	-50.0000000
49010.0000000	5.7500000	-50.0000000
49010.0000000	6.0000000	-50.0000000
49010.0000000	6.2500000	-50.0000000
49010.0000000	6.5000000	-50.0000000
49010.0000000	6.7500000	-50.0000000
49010.0000000	7.0000000	-50.0000000
49010.0000000	7.2500000	-50.0000000
49010.0000000	7.5000000	-50.0000000
49010.0000000	7.7500000	-50.0000000
49010.0000000	8.0000000	-50.0000000
49010.0000000	8.2500000	-50.0000000
49010.0000000	8.5000000	-50.0000000

49010.0000000	8.7500000	-50.0000000
49010.0000000	9.0000000	-50.0000000
49010.0000000	9.2500000	-50.0000000
49010.0000000	9.5000000	-50.0000000
49010.0000000	9.7500000	-50.0000000
49010.0000000	10.0000000	-50.0000000
49010.0000000	10.2500000	-50.0000000
49010.0000000	10.5000000	-50.0000000
49010.0000000	10.7500000	-50.0000000
49010.0000000	11.0000000	-50.0000000
49010.0000000	11.2500000	-50.0000000
49010.0000000	11.5000000	-50.0000000
49010.0000000	11.7500000	-50.0000000
49010.0000000	12.0000000	-50.0000000
49010.0000000	12.2500000	-50.0000000
49010.0000000	12.5000000	-50.0000000
49010.0000000	12.7500000	-50.0000000
49010.0000000	13.0000000	-50.0000000
49010.0000000	13.2500000	-50.0000000
49010.0000000	13.5000000	-50.0000000
49010.0000000	13.7500000	-50.0000000
49010.0000000	14.0000000	-50.0000000
49010.0000000	14.2500000	-50.0000000
49010.0000000	14.5000000	-50.0000000
49010.0000000	14.7500000	-50.0000000
49010.0000000	15.0000000	-50.0000000
49040.0000000	0.0000000	-50.0000000
49040.0000000	0.2500000	-50.0000000
49040.0000000	0.5000000	23.3500004
49040.0000000	0.7500000	26.2500000
49040.0000000	1.0000000	18.9599991
49040.0000000	1.2500000	-50.0000000
49040.0000000	1.5000000	-50.0000000
49040.0000000	1.7500000	-50.0000000
49040.0000000	2.0000000	-50.0000000
49040.0000000	2.2500000	-50.0000000
49040.0000000	2.5000000	-50.0000000
49040.0000000	2.7500000	-50.0000000
49040.0000000	3.0000000	-50.0000000
49040.0000000	3.2500000	-50.0000000
49040.0000000	3.5000000	-50.0000000
49040.0000000	3.7500000	-50.0000000
49040.0000000	4.0000000	-50.0000000
49040.0000000	4.2500000	-50.0000000
49040.0000000	4.5000000	-50.0000000
49040.0000000	4.7500000	-50.0000000
49040.0000000	5.0000000	-50.0000000
49040.0000000	5.2500000	-50.0000000
49040.0000000	5.5000000	-50.0000000
49040.0000000	5.7500000	-50.0000000
49040.0000000	6.0000000	-50.0000000
49040.0000000	6.2500000	-50.0000000
49040.0000000	6.5000000	-50.0000000
49040.0000000	6.7500000	-50.0000000
49040.0000000	7.0000000	-50.0000000
49040.0000000	7.2500000	-50.0000000
49040.0000000	7.5000000	-50.0000000
49040.0000000	7.7500000	-50.0000000

49040.0000000	8.0000000	-50.0000000
49040.0000000	8.2500000	-50.0000000
49040.0000000	8.5000000	-50.0000000
49040.0000000	8.7500000	-50.0000000
49040.0000000	9.0000000	-50.0000000
49040.0000000	9.2500000	-50.0000000
49040.0000000	9.5000000	-50.0000000
49040.0000000	9.7500000	-50.0000000
49040.0000000	10.0000000	-50.0000000
49040.0000000	10.2500000	-50.0000000
49040.0000000	10.5000000	-50.0000000
49040.0000000	10.7500000	-50.0000000
49040.0000000	11.0000000	-50.0000000
49040.0000000	11.2500000	-50.0000000
49040.0000000	11.5000000	-50.0000000
49040.0000000	11.7500000	-50.0000000
49040.0000000	12.0000000	-50.0000000
49040.0000000	12.2500000	-50.0000000
49040.0000000	12.5000000	-50.0000000
49040.0000000	12.7500000	-50.0000000
49040.0000000	13.0000000	-50.0000000
49040.0000000	13.2500000	-50.0000000
49040.0000000	13.5000000	-50.0000000
49040.0000000	13.7500000	-50.0000000
49040.0000000	14.0000000	-50.0000000
49040.0000000	14.2500000	-50.0000000
49040.0000000	14.5000000	-50.0000000
49040.0000000	14.7500000	-50.0000000
49040.0000000	15.0000000	-50.0000000
49070.0000000	0.0000000	-50.0000000
49070.0000000	0.2500000	-50.0000000
49070.0000000	0.5000000	16.3999996
49070.0000000	0.7500000	19.5900002
49070.0000000	1.0000000	15.1999998
49070.0000000	1.2500000	-50.0000000
49070.0000000	1.5000000	-50.0000000
49070.0000000	1.7500000	-50.0000000
49070.0000000	2.0000000	-50.0000000
49070.0000000	2.2500000	-50.0000000
49070.0000000	2.5000000	-50.0000000
49070.0000000	2.7500000	-50.0000000
49070.0000000	3.0000000	-50.0000000
49070.0000000	3.2500000	-50.0000000
49070.0000000	3.5000000	-50.0000000
49070.0000000	3.7500000	-50.0000000
49070.0000000	4.0000000	-50.0000000
49070.0000000	4.2500000	-50.0000000
49070.0000000	4.5000000	-50.0000000
49070.0000000	4.7500000	-50.0000000
49070.0000000	5.0000000	-50.0000000
49070.0000000	5.2500000	-50.0000000
49070.0000000	5.5000000	-50.0000000
49070.0000000	5.7500000	-50.0000000
49070.0000000	6.0000000	-50.0000000
49070.0000000	6.2500000	-50.0000000
49070.0000000	6.5000000	-50.0000000
49070.0000000	6.7500000	-50.0000000
49070.0000000	7.0000000	-50.0000000

49070.0000000	7.2500000	-50.0000000
49070.0000000	7.5000000	-50.0000000
49070.0000000	7.7500000	-50.0000000
49070.0000000	8.0000000	-50.0000000
49070.0000000	8.2500000	-50.0000000
49070.0000000	8.5000000	-50.0000000
49070.0000000	8.7500000	-50.0000000
49070.0000000	9.0000000	-50.0000000
49070.0000000	9.2500000	-50.0000000
49070.0000000	9.5000000	-50.0000000
49070.0000000	9.7500000	-50.0000000
49070.0000000	10.0000000	-50.0000000
49070.0000000	10.2500000	-50.0000000
49070.0000000	10.5000000	-50.0000000
49070.0000000	10.7500000	-50.0000000
49070.0000000	11.0000000	-50.0000000
49070.0000000	11.2500000	-50.0000000
49070.0000000	11.5000000	-50.0000000
49070.0000000	11.7500000	-50.0000000
49070.0000000	12.0000000	-50.0000000
49070.0000000	12.2500000	-50.0000000
49070.0000000	12.5000000	-50.0000000
49070.0000000	12.7500000	-50.0000000
49070.0000000	13.0000000	-50.0000000
49070.0000000	13.2500000	-50.0000000
49070.0000000	13.5000000	-50.0000000
49070.0000000	13.7500000	-50.0000000
49070.0000000	14.0000000	-50.0000000
49070.0000000	14.2500000	-50.0000000
49070.0000000	14.5000000	-50.0000000
49070.0000000	14.7500000	-50.0000000
49070.0000000	15.0000000	-50.0000000
49100.0000000	0.0000000	-50.0000000
49100.0000000	0.2500000	-50.0000000
49100.0000000	0.5000000	20.7999992
49100.0000000	0.7500000	20.3500004
49100.0000000	1.0000000	-50.0000000
49100.0000000	1.2500000	-50.0000000
49100.0000000	1.5000000	-50.0000000
49100.0000000	1.7500000	-50.0000000
49100.0000000	2.0000000	-50.0000000
49100.0000000	2.2500000	-50.0000000
49100.0000000	2.5000000	-50.0000000
49100.0000000	2.7500000	-50.0000000
49100.0000000	3.0000000	-50.0000000
49100.0000000	3.2500000	-50.0000000
49100.0000000	3.5000000	-50.0000000
49100.0000000	3.7500000	-50.0000000
49100.0000000	4.0000000	-50.0000000
49100.0000000	4.2500000	-50.0000000
49100.0000000	4.5000000	-50.0000000
49100.0000000	4.7500000	-50.0000000
49100.0000000	5.0000000	-50.0000000
49100.0000000	5.2500000	-50.0000000
49100.0000000	5.5000000	-50.0000000
49100.0000000	5.7500000	-50.0000000
49100.0000000	6.0000000	-50.0000000
49100.0000000	6.2500000	-50.0000000

49100.0000000	6.5000000	-50.0000000
49100.0000000	6.7500000	-50.0000000
49100.0000000	7.0000000	-50.0000000
49100.0000000	7.2500000	-50.0000000
49100.0000000	7.5000000	-50.0000000
49100.0000000	7.7500000	-50.0000000
49100.0000000	8.0000000	-50.0000000
49100.0000000	8.2500000	-50.0000000
49100.0000000	8.5000000	-50.0000000
49100.0000000	8.7500000	-50.0000000
49100.0000000	9.0000000	-50.0000000
49100.0000000	9.2500000	-50.0000000
49100.0000000	9.5000000	-50.0000000
49100.0000000	9.7500000	-50.0000000
49100.0000000	10.0000000	-50.0000000
49100.0000000	10.2500000	-50.0000000
49100.0000000	10.5000000	-50.0000000
49100.0000000	10.7500000	-50.0000000
49100.0000000	11.0000000	-50.0000000
49100.0000000	11.2500000	-50.0000000
49100.0000000	11.5000000	-50.0000000
49100.0000000	11.7500000	-50.0000000
49100.0000000	12.0000000	-50.0000000
49100.0000000	12.2500000	-50.0000000
49100.0000000	12.5000000	-50.0000000
49100.0000000	12.7500000	-50.0000000
49100.0000000	13.0000000	-50.0000000
49100.0000000	13.2500000	-50.0000000
49100.0000000	13.5000000	-50.0000000
49100.0000000	13.7500000	-50.0000000
49100.0000000	14.0000000	-50.0000000
49100.0000000	14.2500000	-50.0000000
49100.0000000	14.5000000	-50.0000000
49100.0000000	14.7500000	-50.0000000
49100.0000000	15.0000000	-50.0000000
49130.0000000	0.0000000	-50.0000000
49130.0000000	0.2500000	-50.0000000
49130.0000000	0.5000000	16.0499992
49130.0000000	0.7500000	18.2800007
49130.0000000	1.0000000	-50.0000000
49130.0000000	1.2500000	-50.0000000
49130.0000000	1.5000000	-50.0000000
49130.0000000	1.7500000	-50.0000000
49130.0000000	2.0000000	-50.0000000
49130.0000000	2.2500000	-50.0000000
49130.0000000	2.5000000	-50.0000000
49130.0000000	2.7500000	-50.0000000
49130.0000000	3.0000000	-50.0000000
49130.0000000	3.2500000	-50.0000000
49130.0000000	3.5000000	-50.0000000
49130.0000000	3.7500000	-50.0000000
49130.0000000	4.0000000	-50.0000000
49130.0000000	4.2500000	-50.0000000
49130.0000000	4.5000000	-50.0000000
49130.0000000	4.7500000	-50.0000000
49130.0000000	5.0000000	-50.0000000
49130.0000000	5.2500000	-50.0000000
49130.0000000	5.5000000	-50.0000000

49130.0000000	5.7500000	-50.0000000
49130.0000000	6.0000000	-50.0000000
49130.0000000	6.2500000	-50.0000000
49130.0000000	6.5000000	-50.0000000
49130.0000000	6.7500000	-50.0000000
49130.0000000	7.0000000	-50.0000000
49130.0000000	7.2500000	-50.0000000
49130.0000000	7.5000000	-50.0000000
49130.0000000	7.7500000	-50.0000000
49130.0000000	8.0000000	-50.0000000
49130.0000000	8.2500000	-50.0000000
49130.0000000	8.5000000	-50.0000000
49130.0000000	8.7500000	-50.0000000
49130.0000000	9.0000000	-50.0000000
49130.0000000	9.2500000	-50.0000000
49130.0000000	9.5000000	-50.0000000
49130.0000000	9.7500000	-50.0000000
49130.0000000	10.0000000	-50.0000000
49130.0000000	10.2500000	-50.0000000
49130.0000000	10.5000000	-50.0000000
49130.0000000	10.7500000	-50.0000000
49130.0000000	11.0000000	-50.0000000
49130.0000000	11.2500000	-50.0000000
49130.0000000	11.5000000	-50.0000000
49130.0000000	11.7500000	-50.0000000
49130.0000000	12.0000000	-50.0000000
49130.0000000	12.2500000	-50.0000000
49130.0000000	12.5000000	-50.0000000
49130.0000000	12.7500000	-50.0000000
49130.0000000	13.0000000	-50.0000000
49130.0000000	13.2500000	-50.0000000
49130.0000000	13.5000000	-50.0000000
49130.0000000	13.7500000	-50.0000000
49130.0000000	14.0000000	-50.0000000
49130.0000000	14.2500000	-50.0000000
49130.0000000	14.5000000	-50.0000000
49130.0000000	14.7500000	-50.0000000
49130.0000000	15.0000000	-50.0000000
49160.0000000	0.0000000	-50.0000000
49160.0000000	0.2500000	-50.0000000
49160.0000000	0.5000000	-50.0000000
49160.0000000	0.7500000	-50.0000000
49160.0000000	1.0000000	-50.0000000
49160.0000000	1.2500000	-50.0000000
49160.0000000	1.5000000	-50.0000000
49160.0000000	1.7500000	-50.0000000
49160.0000000	2.0000000	-50.0000000
49160.0000000	2.2500000	-50.0000000
49160.0000000	2.5000000	-50.0000000
49160.0000000	2.7500000	-50.0000000
49160.0000000	3.0000000	-50.0000000
49160.0000000	3.2500000	-50.0000000
49160.0000000	3.5000000	-50.0000000
49160.0000000	3.7500000	-50.0000000
49160.0000000	4.0000000	-50.0000000
49160.0000000	4.2500000	-50.0000000
49160.0000000	4.5000000	-50.0000000
49160.0000000	4.7500000	-50.0000000

49160.0000000	5.0000000	-50.0000000
49160.0000000	5.2500000	-50.0000000
49160.0000000	5.5000000	-50.0000000
49160.0000000	5.7500000	-50.0000000
49160.0000000	6.0000000	-50.0000000
49160.0000000	6.2500000	-50.0000000
49160.0000000	6.5000000	-50.0000000
49160.0000000	6.7500000	-50.0000000
49160.0000000	7.0000000	-50.0000000
49160.0000000	7.2500000	-50.0000000
49160.0000000	7.5000000	-50.0000000
49160.0000000	7.7500000	-50.0000000
49160.0000000	8.0000000	-50.0000000
49160.0000000	8.2500000	-50.0000000
49160.0000000	8.5000000	-50.0000000
49160.0000000	8.7500000	-50.0000000
49160.0000000	9.0000000	-50.0000000
49160.0000000	9.2500000	-50.0000000
49160.0000000	9.5000000	-50.0000000
49160.0000000	9.7500000	-50.0000000
49160.0000000	10.0000000	-50.0000000
49160.0000000	10.2500000	-50.0000000
49160.0000000	10.5000000	-50.0000000
49160.0000000	10.7500000	-50.0000000
49160.0000000	11.0000000	-50.0000000
49160.0000000	11.2500000	-50.0000000
49160.0000000	11.5000000	-50.0000000
49160.0000000	11.7500000	-50.0000000
49160.0000000	12.0000000	-50.0000000
49160.0000000	12.2500000	-50.0000000
49160.0000000	12.5000000	-50.0000000
49160.0000000	12.7500000	-50.0000000
49160.0000000	13.0000000	-50.0000000
49160.0000000	13.2500000	-50.0000000
49160.0000000	13.5000000	-50.0000000
49160.0000000	13.7500000	-50.0000000
49160.0000000	14.0000000	-50.0000000
49160.0000000	14.2500000	-50.0000000
49160.0000000	14.5000000	-50.0000000
49160.0000000	14.7500000	-50.0000000
49160.0000000	15.0000000	-50.0000000
49190.0000000	0.0000000	-50.0000000
49190.0000000	0.2500000	-50.0000000
49190.0000000	0.5000000	-50.0000000
49190.0000000	0.7500000	-50.0000000
49190.0000000	1.0000000	-50.0000000
49190.0000000	1.2500000	-50.0000000
49190.0000000	1.5000000	-50.0000000
49190.0000000	1.7500000	-50.0000000
49190.0000000	2.0000000	-50.0000000
49190.0000000	2.2500000	-50.0000000
49190.0000000	2.5000000	-50.0000000
49190.0000000	2.7500000	-50.0000000
49190.0000000	3.0000000	-50.0000000
49190.0000000	3.2500000	-50.0000000
49190.0000000	3.5000000	-50.0000000
49190.0000000	3.7500000	-50.0000000
49190.0000000	4.0000000	-50.0000000

49190.0000000	4.2500000	-50.0000000
49190.0000000	4.5000000	-50.0000000
49190.0000000	4.7500000	-50.0000000
49190.0000000	5.0000000	-50.0000000
49190.0000000	5.2500000	-50.0000000
49190.0000000	5.5000000	-50.0000000
49190.0000000	5.7500000	-50.0000000
49190.0000000	6.0000000	-50.0000000
49190.0000000	6.2500000	-50.0000000
49190.0000000	6.5000000	-50.0000000
49190.0000000	6.7500000	-50.0000000
49190.0000000	7.0000000	-50.0000000
49190.0000000	7.2500000	-50.0000000
49190.0000000	7.5000000	-50.0000000
49190.0000000	7.7500000	-50.0000000
49190.0000000	8.0000000	-50.0000000
49190.0000000	8.2500000	-50.0000000
49190.0000000	8.5000000	-50.0000000
49190.0000000	8.7500000	-50.0000000
49190.0000000	9.0000000	-50.0000000
49190.0000000	9.2500000	-50.0000000
49190.0000000	9.5000000	-50.0000000
49190.0000000	9.7500000	-50.0000000
49190.0000000	10.0000000	-50.0000000
49190.0000000	10.2500000	-50.0000000
49190.0000000	10.5000000	-50.0000000
49190.0000000	10.7500000	-50.0000000
49190.0000000	11.0000000	-50.0000000
49190.0000000	11.2500000	-50.0000000
49190.0000000	11.5000000	-50.0000000
49190.0000000	11.7500000	-50.0000000
49190.0000000	12.0000000	-50.0000000
49190.0000000	12.2500000	-50.0000000
49190.0000000	12.5000000	-50.0000000
49190.0000000	12.7500000	-50.0000000
49190.0000000	13.0000000	-50.0000000
49190.0000000	13.2500000	-50.0000000
49190.0000000	13.5000000	-50.0000000
49190.0000000	13.7500000	-50.0000000
49190.0000000	14.0000000	-50.0000000
49190.0000000	14.2500000	-50.0000000
49190.0000000	14.5000000	-50.0000000
49190.0000000	14.7500000	-50.0000000
49190.0000000	15.0000000	-50.0000000
49220.0000000	0.0000000	-50.0000000
49220.0000000	0.2500000	-50.0000000
49220.0000000	0.5000000	-50.0000000
49220.0000000	0.7500000	-50.0000000
49220.0000000	1.0000000	-50.0000000
49220.0000000	1.2500000	-50.0000000
49220.0000000	1.5000000	-50.0000000
49220.0000000	1.7500000	-50.0000000
49220.0000000	2.0000000	-50.0000000
49220.0000000	2.2500000	-50.0000000
49220.0000000	2.5000000	-50.0000000
49220.0000000	2.7500000	-50.0000000
49220.0000000	3.0000000	-50.0000000
49220.0000000	3.2500000	-50.0000000

49220.0000000	3.5000000	-50.0000000
49220.0000000	3.7500000	-50.0000000
49220.0000000	4.0000000	-50.0000000
49220.0000000	4.2500000	-50.0000000
49220.0000000	4.5000000	-50.0000000
49220.0000000	4.7500000	-50.0000000
49220.0000000	5.0000000	-50.0000000
49220.0000000	5.2500000	-50.0000000
49220.0000000	5.5000000	-50.0000000
49220.0000000	5.7500000	-50.0000000
49220.0000000	6.0000000	-50.0000000
49220.0000000	6.2500000	-50.0000000
49220.0000000	6.5000000	-50.0000000
49220.0000000	6.7500000	-50.0000000
49220.0000000	7.0000000	-50.0000000
49220.0000000	7.2500000	-50.0000000
49220.0000000	7.5000000	-50.0000000
49220.0000000	7.7500000	-50.0000000
49220.0000000	8.0000000	-50.0000000
49220.0000000	8.2500000	-50.0000000
49220.0000000	8.5000000	-50.0000000
49220.0000000	8.7500000	-50.0000000
49220.0000000	9.0000000	-50.0000000
49220.0000000	9.2500000	-50.0000000
49220.0000000	9.5000000	-50.0000000
49220.0000000	9.7500000	-50.0000000
49220.0000000	10.0000000	-50.0000000
49220.0000000	10.2500000	-50.0000000
49220.0000000	10.5000000	-50.0000000
49220.0000000	10.7500000	-50.0000000
49220.0000000	11.0000000	-50.0000000
49220.0000000	11.2500000	-50.0000000
49220.0000000	11.5000000	-50.0000000
49220.0000000	11.7500000	-50.0000000
49220.0000000	12.0000000	-50.0000000
49220.0000000	12.2500000	-50.0000000
49220.0000000	12.5000000	-50.0000000
49220.0000000	12.7500000	-50.0000000
49220.0000000	13.0000000	-50.0000000
49220.0000000	13.2500000	-50.0000000
49220.0000000	13.5000000	-50.0000000
49220.0000000	13.7500000	-50.0000000
49220.0000000	14.0000000	-50.0000000
49220.0000000	14.2500000	-50.0000000
49220.0000000	14.5000000	-50.0000000
49220.0000000	14.7500000	-50.0000000
49220.0000000	15.0000000	-50.0000000
49250.0000000	0.0000000	-50.0000000
49250.0000000	0.2500000	-50.0000000
49250.0000000	0.5000000	-50.0000000
49250.0000000	0.7500000	-50.0000000
49250.0000000	1.0000000	-50.0000000
49250.0000000	1.2500000	-50.0000000
49250.0000000	1.5000000	-50.0000000
49250.0000000	1.7500000	-50.0000000
49250.0000000	2.0000000	-50.0000000
49250.0000000	2.2500000	-50.0000000
49250.0000000	2.5000000	-50.0000000

49250.0000000	2.7500000	-50.0000000
49250.0000000	3.0000000	-50.0000000
49250.0000000	3.2500000	-50.0000000
49250.0000000	3.5000000	-50.0000000
49250.0000000	3.7500000	-50.0000000
49250.0000000	4.0000000	-50.0000000
49250.0000000	4.2500000	-50.0000000
49250.0000000	4.5000000	-50.0000000
49250.0000000	4.7500000	-50.0000000
49250.0000000	5.0000000	-50.0000000
49250.0000000	5.2500000	-50.0000000
49250.0000000	5.5000000	-50.0000000
49250.0000000	5.7500000	-50.0000000
49250.0000000	6.0000000	-50.0000000
49250.0000000	6.2500000	-50.0000000
49250.0000000	6.5000000	-50.0000000
49250.0000000	6.7500000	-50.0000000
49250.0000000	7.0000000	-50.0000000
49250.0000000	7.2500000	-50.0000000
49250.0000000	7.5000000	-50.0000000
49250.0000000	7.7500000	-50.0000000
49250.0000000	8.0000000	-50.0000000
49250.0000000	8.2500000	-50.0000000
49250.0000000	8.5000000	-50.0000000
49250.0000000	8.7500000	-50.0000000
49250.0000000	9.0000000	-50.0000000
49250.0000000	9.2500000	-50.0000000
49250.0000000	9.5000000	-50.0000000
49250.0000000	9.7500000	-50.0000000
49250.0000000	10.0000000	-50.0000000
49250.0000000	10.2500000	-50.0000000
49250.0000000	10.5000000	-50.0000000
49250.0000000	10.7500000	-50.0000000
49250.0000000	11.0000000	-50.0000000
49250.0000000	11.2500000	-50.0000000
49250.0000000	11.5000000	-50.0000000
49250.0000000	11.7500000	-50.0000000
49250.0000000	12.0000000	-50.0000000
49250.0000000	12.2500000	-50.0000000
49250.0000000	12.5000000	-50.0000000
49250.0000000	12.7500000	-50.0000000
49250.0000000	13.0000000	-50.0000000
49250.0000000	13.2500000	-50.0000000
49250.0000000	13.5000000	-50.0000000
49250.0000000	13.7500000	-50.0000000
49250.0000000	14.0000000	-50.0000000
49250.0000000	14.2500000	-50.0000000
49250.0000000	14.5000000	-50.0000000
49250.0000000	14.7500000	-50.0000000
49250.0000000	15.0000000	-50.0000000
49280.0000000	0.0000000	-50.0000000
49280.0000000	0.2500000	-50.0000000
49280.0000000	0.5000000	-50.0000000
49280.0000000	0.7500000	16.4200001
49280.0000000	1.0000000	15.5500002
49280.0000000	1.2500000	-50.0000000
49280.0000000	1.5000000	-50.0000000
49280.0000000	1.7500000	-50.0000000

49280.0000000	2.0000000	-50.0000000
49280.0000000	2.2500000	-50.0000000
49280.0000000	2.5000000	-50.0000000
49280.0000000	2.7500000	-50.0000000
49280.0000000	3.0000000	-50.0000000
49280.0000000	3.2500000	-50.0000000
49280.0000000	3.5000000	-50.0000000
49280.0000000	3.7500000	-50.0000000
49280.0000000	4.0000000	-50.0000000
49280.0000000	4.2500000	-50.0000000
49280.0000000	4.5000000	-50.0000000
49280.0000000	4.7500000	-50.0000000
49280.0000000	5.0000000	-50.0000000
49280.0000000	5.2500000	-50.0000000
49280.0000000	5.5000000	-50.0000000
49280.0000000	5.7500000	-50.0000000
49280.0000000	6.0000000	-50.0000000
49280.0000000	6.2500000	-50.0000000
49280.0000000	6.5000000	-50.0000000
49280.0000000	6.7500000	-50.0000000
49280.0000000	7.0000000	-50.0000000
49280.0000000	7.2500000	-50.0000000
49280.0000000	7.5000000	-50.0000000
49280.0000000	7.7500000	-50.0000000
49280.0000000	8.0000000	-50.0000000
49280.0000000	8.2500000	-50.0000000
49280.0000000	8.5000000	-50.0000000
49280.0000000	8.7500000	-50.0000000
49280.0000000	9.0000000	-50.0000000
49280.0000000	9.2500000	-50.0000000
49280.0000000	9.5000000	-50.0000000
49280.0000000	9.7500000	-50.0000000
49280.0000000	10.0000000	-50.0000000
49280.0000000	10.2500000	-50.0000000
49280.0000000	10.5000000	-50.0000000
49280.0000000	10.7500000	-50.0000000
49280.0000000	11.0000000	-50.0000000
49280.0000000	11.2500000	-50.0000000
49280.0000000	11.5000000	-50.0000000
49280.0000000	11.7500000	-50.0000000
49280.0000000	12.0000000	-50.0000000
49280.0000000	12.2500000	-50.0000000
49280.0000000	12.5000000	-50.0000000
49280.0000000	12.7500000	-50.0000000
49280.0000000	13.0000000	-50.0000000
49280.0000000	13.2500000	-50.0000000
49280.0000000	13.5000000	-50.0000000
49280.0000000	13.7500000	-50.0000000
49280.0000000	14.0000000	-50.0000000
49280.0000000	14.2500000	-50.0000000
49280.0000000	14.5000000	-50.0000000
49280.0000000	14.7500000	-50.0000000
49280.0000000	15.0000000	-50.0000000
49310.0000000	0.0000000	-50.0000000
49310.0000000	0.2500000	-50.0000000
49310.0000000	0.5000000	-50.0000000
49310.0000000	0.7500000	-50.0000000
49310.0000000	1.0000000	-50.0000000

49310.0000000	1.2500000	-50.0000000
49310.0000000	1.5000000	-50.0000000
49310.0000000	1.7500000	-50.0000000
49310.0000000	2.0000000	-50.0000000
49310.0000000	2.2500000	-50.0000000
49310.0000000	2.5000000	-50.0000000
49310.0000000	2.7500000	-50.0000000
49310.0000000	3.0000000	-50.0000000
49310.0000000	3.2500000	-50.0000000
49310.0000000	3.5000000	-50.0000000
49310.0000000	3.7500000	-50.0000000
49310.0000000	4.0000000	-50.0000000
49310.0000000	4.2500000	-50.0000000
49310.0000000	4.5000000	-50.0000000
49310.0000000	4.7500000	-50.0000000
49310.0000000	5.0000000	-50.0000000
49310.0000000	5.2500000	-50.0000000
49310.0000000	5.5000000	-50.0000000
49310.0000000	5.7500000	-50.0000000
49310.0000000	6.0000000	-50.0000000
49310.0000000	6.2500000	-50.0000000
49310.0000000	6.5000000	-50.0000000
49310.0000000	6.7500000	-50.0000000
49310.0000000	7.0000000	-50.0000000
49310.0000000	7.2500000	-50.0000000
49310.0000000	7.5000000	-50.0000000
49310.0000000	7.7500000	-50.0000000
49310.0000000	8.0000000	-50.0000000
49310.0000000	8.2500000	-50.0000000
49310.0000000	8.5000000	-50.0000000
49310.0000000	8.7500000	-50.0000000
49310.0000000	9.0000000	-50.0000000
49310.0000000	9.2500000	-50.0000000
49310.0000000	9.5000000	-50.0000000
49310.0000000	9.7500000	-50.0000000
49310.0000000	10.0000000	-50.0000000
49310.0000000	10.2500000	-50.0000000
49310.0000000	10.5000000	-50.0000000
49310.0000000	10.7500000	-50.0000000
49310.0000000	11.0000000	-50.0000000
49310.0000000	11.2500000	-50.0000000
49310.0000000	11.5000000	-50.0000000
49310.0000000	11.7500000	-50.0000000
49310.0000000	12.0000000	-50.0000000
49310.0000000	12.2500000	-50.0000000
49310.0000000	12.5000000	-50.0000000
49310.0000000	12.7500000	-50.0000000
49310.0000000	13.0000000	-50.0000000
49310.0000000	13.2500000	-50.0000000
49310.0000000	13.5000000	-50.0000000
49310.0000000	13.7500000	-50.0000000
49310.0000000	14.0000000	-50.0000000
49310.0000000	14.2500000	-50.0000000
49310.0000000	14.5000000	-50.0000000
49310.0000000	14.7500000	-50.0000000
49310.0000000	15.0000000	-50.0000000
49340.0000000	0.0000000	-50.0000000
49340.0000000	0.2500000	-50.0000000

49340.0000000	0.5000000	-50.0000000
49340.0000000	0.7500000	16.4799995
49340.0000000	1.0000000	-50.0000000
49340.0000000	1.2500000	-50.0000000
49340.0000000	1.5000000	-50.0000000
49340.0000000	1.7500000	-50.0000000
49340.0000000	2.0000000	-50.0000000
49340.0000000	2.2500000	-50.0000000
49340.0000000	2.5000000	-50.0000000
49340.0000000	2.7500000	-50.0000000
49340.0000000	3.0000000	-50.0000000
49340.0000000	3.2500000	-50.0000000
49340.0000000	3.5000000	-50.0000000
49340.0000000	3.7500000	-50.0000000
49340.0000000	4.0000000	-50.0000000
49340.0000000	4.2500000	-50.0000000
49340.0000000	4.5000000	-50.0000000
49340.0000000	4.7500000	-50.0000000
49340.0000000	5.0000000	-50.0000000
49340.0000000	5.2500000	-50.0000000
49340.0000000	5.5000000	-50.0000000
49340.0000000	5.7500000	-50.0000000
49340.0000000	6.0000000	-50.0000000
49340.0000000	6.2500000	-50.0000000
49340.0000000	6.5000000	-50.0000000
49340.0000000	6.7500000	-50.0000000
49340.0000000	7.0000000	-50.0000000
49340.0000000	7.2500000	-50.0000000
49340.0000000	7.5000000	-50.0000000
49340.0000000	7.7500000	-50.0000000
49340.0000000	8.0000000	-50.0000000
49340.0000000	8.2500000	-50.0000000
49340.0000000	8.5000000	-50.0000000
49340.0000000	8.7500000	-50.0000000
49340.0000000	9.0000000	-50.0000000
49340.0000000	9.2500000	-50.0000000
49340.0000000	9.5000000	-50.0000000
49340.0000000	9.7500000	-50.0000000
49340.0000000	10.0000000	-50.0000000
49340.0000000	10.2500000	-50.0000000
49340.0000000	10.5000000	-50.0000000
49340.0000000	10.7500000	-50.0000000
49340.0000000	11.0000000	-50.0000000
49340.0000000	11.2500000	-50.0000000
49340.0000000	11.5000000	-50.0000000
49340.0000000	11.7500000	-50.0000000
49340.0000000	12.0000000	-50.0000000
49340.0000000	12.2500000	-50.0000000
49340.0000000	12.5000000	-50.0000000
49340.0000000	12.7500000	-50.0000000
49340.0000000	13.0000000	-50.0000000
49340.0000000	13.2500000	-50.0000000
49340.0000000	13.5000000	-50.0000000
49340.0000000	13.7500000	-50.0000000
49340.0000000	14.0000000	-50.0000000
49340.0000000	14.2500000	-50.0000000
49340.0000000	14.5000000	-50.0000000
49340.0000000	14.7500000	-50.0000000

49340.0000000	15.0000000	-50.0000000
49370.0000000	0.0000000	-50.0000000
49370.0000000	0.2500000	-50.0000000
49370.0000000	0.5000000	-50.0000000
49370.0000000	0.7500000	-50.0000000
49370.0000000	1.0000000	-50.0000000
49370.0000000	1.2500000	-50.0000000
49370.0000000	1.5000000	-50.0000000
49370.0000000	1.7500000	-50.0000000
49370.0000000	2.0000000	-50.0000000
49370.0000000	2.2500000	-50.0000000
49370.0000000	2.5000000	-50.0000000
49370.0000000	2.7500000	-50.0000000
49370.0000000	3.0000000	-50.0000000
49370.0000000	3.2500000	-50.0000000
49370.0000000	3.5000000	-50.0000000
49370.0000000	3.7500000	-50.0000000
49370.0000000	4.0000000	-50.0000000
49370.0000000	4.2500000	-50.0000000
49370.0000000	4.5000000	-50.0000000
49370.0000000	4.7500000	-50.0000000
49370.0000000	5.0000000	-50.0000000
49370.0000000	5.2500000	-50.0000000
49370.0000000	5.5000000	-50.0000000
49370.0000000	5.7500000	-50.0000000
49370.0000000	6.0000000	-50.0000000
49370.0000000	6.2500000	-50.0000000
49370.0000000	6.5000000	-50.0000000
49370.0000000	6.7500000	-50.0000000
49370.0000000	7.0000000	-50.0000000
49370.0000000	7.2500000	-50.0000000
49370.0000000	7.5000000	-50.0000000
49370.0000000	7.7500000	-50.0000000
49370.0000000	8.0000000	-50.0000000
49370.0000000	8.2500000	-50.0000000
49370.0000000	8.5000000	-50.0000000
49370.0000000	8.7500000	-50.0000000
49370.0000000	9.0000000	-50.0000000
49370.0000000	9.2500000	-50.0000000
49370.0000000	9.5000000	-50.0000000
49370.0000000	9.7500000	-50.0000000
49370.0000000	10.0000000	-50.0000000
49370.0000000	10.2500000	-50.0000000
49370.0000000	10.5000000	-50.0000000
49370.0000000	10.7500000	-50.0000000
49370.0000000	11.0000000	-50.0000000
49370.0000000	11.2500000	-50.0000000
49370.0000000	11.5000000	-50.0000000
49370.0000000	11.7500000	-50.0000000
49370.0000000	12.0000000	-50.0000000
49370.0000000	12.2500000	-50.0000000
49370.0000000	12.5000000	-50.0000000
49370.0000000	12.7500000	-50.0000000
49370.0000000	13.0000000	-50.0000000
49370.0000000	13.2500000	-50.0000000
49370.0000000	13.5000000	-50.0000000
49370.0000000	13.7500000	-50.0000000
49370.0000000	14.0000000	-50.0000000

49370.0000000	14.2500000	-50.0000000
49370.0000000	14.5000000	-50.0000000
49370.0000000	14.7500000	-50.0000000
49370.0000000	15.0000000	-50.0000000
49400.0000000	0.0000000	-50.0000000
49400.0000000	0.2500000	-50.0000000
49400.0000000	0.5000000	-50.0000000
49400.0000000	0.7500000	17.5900002
49400.0000000	1.0000000	-50.0000000
49400.0000000	1.2500000	-50.0000000
49400.0000000	1.5000000	-50.0000000
49400.0000000	1.7500000	-50.0000000
49400.0000000	2.0000000	-50.0000000
49400.0000000	2.2500000	-50.0000000
49400.0000000	2.5000000	-50.0000000
49400.0000000	2.7500000	-50.0000000
49400.0000000	3.0000000	-50.0000000
49400.0000000	3.2500000	-50.0000000
49400.0000000	3.5000000	-50.0000000
49400.0000000	3.7500000	-50.0000000
49400.0000000	4.0000000	-50.0000000
49400.0000000	4.2500000	-50.0000000
49400.0000000	4.5000000	-50.0000000
49400.0000000	4.7500000	-50.0000000
49400.0000000	5.0000000	-50.0000000
49400.0000000	5.2500000	-50.0000000
49400.0000000	5.5000000	-50.0000000
49400.0000000	5.7500000	-50.0000000
49400.0000000	6.0000000	-50.0000000
49400.0000000	6.2500000	-50.0000000
49400.0000000	6.5000000	-50.0000000
49400.0000000	6.7500000	-50.0000000
49400.0000000	7.0000000	-50.0000000
49400.0000000	7.2500000	-50.0000000
49400.0000000	7.5000000	-50.0000000
49400.0000000	7.7500000	-50.0000000
49400.0000000	8.0000000	-50.0000000
49400.0000000	8.2500000	-50.0000000
49400.0000000	8.5000000	-50.0000000
49400.0000000	8.7500000	-50.0000000
49400.0000000	9.0000000	-50.0000000
49400.0000000	9.2500000	-50.0000000
49400.0000000	9.5000000	-50.0000000
49400.0000000	9.7500000	-50.0000000
49400.0000000	10.0000000	-50.0000000
49400.0000000	10.2500000	-50.0000000
49400.0000000	10.5000000	-50.0000000
49400.0000000	10.7500000	-50.0000000
49400.0000000	11.0000000	-50.0000000
49400.0000000	11.2500000	-50.0000000
49400.0000000	11.5000000	-50.0000000
49400.0000000	11.7500000	-50.0000000
49400.0000000	12.0000000	-50.0000000
49400.0000000	12.2500000	-50.0000000
49400.0000000	12.5000000	-50.0000000
49400.0000000	12.7500000	-50.0000000
49400.0000000	13.0000000	-50.0000000
49400.0000000	13.2500000	-50.0000000

49400.0000000	13.5000000	-50.0000000
49400.0000000	13.7500000	-50.0000000
49400.0000000	14.0000000	-50.0000000
49400.0000000	14.2500000	-50.0000000
49400.0000000	14.5000000	-50.0000000
49400.0000000	14.7500000	-50.0000000
49400.0000000	15.0000000	-50.0000000
49430.0000000	0.0000000	-50.0000000
49430.0000000	0.2500000	-50.0000000
49430.0000000	0.5000000	-50.0000000
49430.0000000	0.7500000	16.2399998
49430.0000000	1.0000000	-50.0000000
49430.0000000	1.2500000	-50.0000000
49430.0000000	1.5000000	-50.0000000
49430.0000000	1.7500000	-50.0000000
49430.0000000	2.0000000	-50.0000000
49430.0000000	2.2500000	-50.0000000
49430.0000000	2.5000000	-50.0000000
49430.0000000	2.7500000	-50.0000000
49430.0000000	3.0000000	-50.0000000
49430.0000000	3.2500000	-50.0000000
49430.0000000	3.5000000	-50.0000000
49430.0000000	3.7500000	-50.0000000
49430.0000000	4.0000000	-50.0000000
49430.0000000	4.2500000	-50.0000000
49430.0000000	4.5000000	-50.0000000
49430.0000000	4.7500000	-50.0000000
49430.0000000	5.0000000	-50.0000000
49430.0000000	5.2500000	-50.0000000
49430.0000000	5.5000000	-50.0000000
49430.0000000	5.7500000	-50.0000000
49430.0000000	6.0000000	-50.0000000
49430.0000000	6.2500000	-50.0000000
49430.0000000	6.5000000	-50.0000000
49430.0000000	6.7500000	-50.0000000
49430.0000000	7.0000000	-50.0000000
49430.0000000	7.2500000	-50.0000000
49430.0000000	7.5000000	-50.0000000
49430.0000000	7.7500000	-50.0000000
49430.0000000	8.0000000	-50.0000000
49430.0000000	8.2500000	-50.0000000
49430.0000000	8.5000000	-50.0000000
49430.0000000	8.7500000	-50.0000000
49430.0000000	9.0000000	-50.0000000
49430.0000000	9.2500000	-50.0000000
49430.0000000	9.5000000	-50.0000000
49430.0000000	9.7500000	-50.0000000
49430.0000000	10.0000000	-50.0000000
49430.0000000	10.2500000	-50.0000000
49430.0000000	10.5000000	-50.0000000
49430.0000000	10.7500000	-50.0000000
49430.0000000	11.0000000	-50.0000000
49430.0000000	11.2500000	-50.0000000
49430.0000000	11.5000000	-50.0000000
49430.0000000	11.7500000	-50.0000000
49430.0000000	12.0000000	-50.0000000
49430.0000000	12.2500000	-50.0000000
49430.0000000	12.5000000	-50.0000000

49430.0000000	12.7500000	-50.0000000
49430.0000000	13.0000000	-50.0000000
49430.0000000	13.2500000	-50.0000000
49430.0000000	13.5000000	-50.0000000
49430.0000000	13.7500000	-50.0000000
49430.0000000	14.0000000	-50.0000000
49430.0000000	14.2500000	-50.0000000
49430.0000000	14.5000000	-50.0000000
49430.0000000	14.7500000	-50.0000000
49430.0000000	15.0000000	-50.0000000
49460.0000000	0.0000000	-50.0000000
49460.0000000	0.2500000	-50.0000000
49460.0000000	0.5000000	-50.0000000
49460.0000000	0.7500000	-50.0000000
49460.0000000	1.0000000	-50.0000000
49460.0000000	1.2500000	-50.0000000
49460.0000000	1.5000000	-50.0000000
49460.0000000	1.7500000	-50.0000000
49460.0000000	2.0000000	-50.0000000
49460.0000000	2.2500000	-50.0000000
49460.0000000	2.5000000	-50.0000000
49460.0000000	2.7500000	-50.0000000
49460.0000000	3.0000000	-50.0000000
49460.0000000	3.2500000	-50.0000000
49460.0000000	3.5000000	-50.0000000
49460.0000000	3.7500000	-50.0000000
49460.0000000	4.0000000	-50.0000000
49460.0000000	4.2500000	-50.0000000
49460.0000000	4.5000000	-50.0000000
49460.0000000	4.7500000	-50.0000000
49460.0000000	5.0000000	-50.0000000
49460.0000000	5.2500000	-50.0000000
49460.0000000	5.5000000	-50.0000000
49460.0000000	5.7500000	-50.0000000
49460.0000000	6.0000000	-50.0000000
49460.0000000	6.2500000	-50.0000000
49460.0000000	6.5000000	-50.0000000
49460.0000000	6.7500000	-50.0000000
49460.0000000	7.0000000	-50.0000000
49460.0000000	7.2500000	-50.0000000
49460.0000000	7.5000000	-50.0000000
49460.0000000	7.7500000	-50.0000000
49460.0000000	8.0000000	-50.0000000
49460.0000000	8.2500000	-50.0000000
49460.0000000	8.5000000	-50.0000000
49460.0000000	8.7500000	-50.0000000
49460.0000000	9.0000000	-50.0000000
49460.0000000	9.2500000	-50.0000000
49460.0000000	9.5000000	-50.0000000
49460.0000000	9.7500000	-50.0000000
49460.0000000	10.0000000	-50.0000000
49460.0000000	10.2500000	-50.0000000
49460.0000000	10.5000000	-50.0000000
49460.0000000	10.7500000	-50.0000000
49460.0000000	11.0000000	-50.0000000
49460.0000000	11.2500000	-50.0000000
49460.0000000	11.5000000	-50.0000000
49460.0000000	11.7500000	-50.0000000

49460.0000000	12.0000000	-50.0000000
49460.0000000	12.2500000	-50.0000000
49460.0000000	12.5000000	-50.0000000
49460.0000000	12.7500000	-50.0000000
49460.0000000	13.0000000	-50.0000000
49460.0000000	13.2500000	-50.0000000
49460.0000000	13.5000000	-50.0000000
49460.0000000	13.7500000	-50.0000000
49460.0000000	14.0000000	-50.0000000
49460.0000000	14.2500000	-50.0000000
49460.0000000	14.5000000	-50.0000000
49460.0000000	14.7500000	-50.0000000
49460.0000000	15.0000000	-50.0000000
49490.0000000	0.0000000	-50.0000000
49490.0000000	0.2500000	-50.0000000
49490.0000000	0.5000000	-50.0000000
49490.0000000	0.7500000	15.9399996
49490.0000000	1.0000000	-50.0000000
49490.0000000	1.2500000	-50.0000000
49490.0000000	1.5000000	-50.0000000
49490.0000000	1.7500000	-50.0000000
49490.0000000	2.0000000	-50.0000000
49490.0000000	2.2500000	-50.0000000
49490.0000000	2.5000000	-50.0000000
49490.0000000	2.7500000	-50.0000000
49490.0000000	3.0000000	-50.0000000
49490.0000000	3.2500000	-50.0000000
49490.0000000	3.5000000	-50.0000000
49490.0000000	3.7500000	-50.0000000
49490.0000000	4.0000000	-50.0000000
49490.0000000	4.2500000	-50.0000000
49490.0000000	4.5000000	-50.0000000
49490.0000000	4.7500000	-50.0000000
49490.0000000	5.0000000	-50.0000000
49490.0000000	5.2500000	-50.0000000
49490.0000000	5.5000000	-50.0000000
49490.0000000	5.7500000	-50.0000000
49490.0000000	6.0000000	-50.0000000
49490.0000000	6.2500000	-50.0000000
49490.0000000	6.5000000	-50.0000000
49490.0000000	6.7500000	-50.0000000
49490.0000000	7.0000000	-50.0000000
49490.0000000	7.2500000	-50.0000000
49490.0000000	7.5000000	-50.0000000
49490.0000000	7.7500000	-50.0000000
49490.0000000	8.0000000	-50.0000000
49490.0000000	8.2500000	-50.0000000
49490.0000000	8.5000000	-50.0000000
49490.0000000	8.7500000	-50.0000000
49490.0000000	9.0000000	-50.0000000
49490.0000000	9.2500000	-50.0000000
49490.0000000	9.5000000	-50.0000000
49490.0000000	9.7500000	-50.0000000
49490.0000000	10.0000000	-50.0000000
49490.0000000	10.2500000	-50.0000000
49490.0000000	10.5000000	-50.0000000
49490.0000000	10.7500000	-50.0000000
49490.0000000	11.0000000	-50.0000000

49490.0000000	11.2500000	-50.0000000
49490.0000000	11.5000000	-50.0000000
49490.0000000	11.7500000	-50.0000000
49490.0000000	12.0000000	-50.0000000
49490.0000000	12.2500000	-50.0000000
49490.0000000	12.5000000	-50.0000000
49490.0000000	12.7500000	-50.0000000
49490.0000000	13.0000000	-50.0000000
49490.0000000	13.2500000	-50.0000000
49490.0000000	13.5000000	-50.0000000
49490.0000000	13.7500000	-50.0000000
49490.0000000	14.0000000	-50.0000000
49490.0000000	14.2500000	-50.0000000
49490.0000000	14.5000000	-50.0000000
49490.0000000	14.7500000	-50.0000000
49490.0000000	15.0000000	-50.0000000
49520.0000000	0.0000000	-50.0000000
49520.0000000	0.2500000	-50.0000000
49520.0000000	0.5000000	-50.0000000
49520.0000000	0.7500000	17.4699993
49520.0000000	1.0000000	-50.0000000
49520.0000000	1.2500000	-50.0000000
49520.0000000	1.5000000	-50.0000000
49520.0000000	1.7500000	-50.0000000
49520.0000000	2.0000000	-50.0000000
49520.0000000	2.2500000	-50.0000000
49520.0000000	2.5000000	-50.0000000
49520.0000000	2.7500000	-50.0000000
49520.0000000	3.0000000	-50.0000000
49520.0000000	3.2500000	-50.0000000
49520.0000000	3.5000000	-50.0000000
49520.0000000	3.7500000	-50.0000000
49520.0000000	4.0000000	-50.0000000
49520.0000000	4.2500000	-50.0000000
49520.0000000	4.5000000	-50.0000000
49520.0000000	4.7500000	-50.0000000
49520.0000000	5.0000000	-50.0000000
49520.0000000	5.2500000	-50.0000000
49520.0000000	5.5000000	-50.0000000
49520.0000000	5.7500000	-50.0000000
49520.0000000	6.0000000	-50.0000000
49520.0000000	6.2500000	-50.0000000
49520.0000000	6.5000000	-50.0000000
49520.0000000	6.7500000	-50.0000000
49520.0000000	7.0000000	-50.0000000
49520.0000000	7.2500000	-50.0000000
49520.0000000	7.5000000	-50.0000000
49520.0000000	7.7500000	-50.0000000
49520.0000000	8.0000000	-50.0000000
49520.0000000	8.2500000	-50.0000000
49520.0000000	8.5000000	-50.0000000
49520.0000000	8.7500000	-50.0000000
49520.0000000	9.0000000	-50.0000000
49520.0000000	9.2500000	-50.0000000
49520.0000000	9.5000000	-50.0000000
49520.0000000	9.7500000	-50.0000000
49520.0000000	10.0000000	-50.0000000
49520.0000000	10.2500000	-50.0000000

49520.0000000	10.5000000	-50.0000000
49520.0000000	10.7500000	-50.0000000
49520.0000000	11.0000000	-50.0000000
49520.0000000	11.2500000	-50.0000000
49520.0000000	11.5000000	-50.0000000
49520.0000000	11.7500000	-50.0000000
49520.0000000	12.0000000	-50.0000000
49520.0000000	12.2500000	-50.0000000
49520.0000000	12.5000000	-50.0000000
49520.0000000	12.7500000	-50.0000000
49520.0000000	13.0000000	-50.0000000
49520.0000000	13.2500000	-50.0000000
49520.0000000	13.5000000	-50.0000000
49520.0000000	13.7500000	-50.0000000
49520.0000000	14.0000000	-50.0000000
49520.0000000	14.2500000	-50.0000000
49520.0000000	14.5000000	-50.0000000
49520.0000000	14.7500000	-50.0000000
49520.0000000	15.0000000	-50.0000000
49550.0000000	0.0000000	-50.0000000
49550.0000000	0.2500000	-50.0000000
49550.0000000	0.5000000	-50.0000000
49550.0000000	0.7500000	-50.0000000
49550.0000000	1.0000000	-50.0000000
49550.0000000	1.2500000	-50.0000000
49550.0000000	1.5000000	-50.0000000
49550.0000000	1.7500000	-50.0000000
49550.0000000	2.0000000	-50.0000000
49550.0000000	2.2500000	-50.0000000
49550.0000000	2.5000000	-50.0000000
49550.0000000	2.7500000	-50.0000000
49550.0000000	3.0000000	-50.0000000
49550.0000000	3.2500000	-50.0000000
49550.0000000	3.5000000	-50.0000000
49550.0000000	3.7500000	-50.0000000
49550.0000000	4.0000000	-50.0000000
49550.0000000	4.2500000	-50.0000000
49550.0000000	4.5000000	-50.0000000
49550.0000000	4.7500000	-50.0000000
49550.0000000	5.0000000	-50.0000000
49550.0000000	5.2500000	-50.0000000
49550.0000000	5.5000000	-50.0000000
49550.0000000	5.7500000	-50.0000000
49550.0000000	6.0000000	-50.0000000
49550.0000000	6.2500000	-50.0000000
49550.0000000	6.5000000	-50.0000000
49550.0000000	6.7500000	-50.0000000
49550.0000000	7.0000000	-50.0000000
49550.0000000	7.2500000	-50.0000000
49550.0000000	7.5000000	-50.0000000
49550.0000000	7.7500000	-50.0000000
49550.0000000	8.0000000	-50.0000000
49550.0000000	8.2500000	-50.0000000
49550.0000000	8.5000000	-50.0000000
49550.0000000	8.7500000	-50.0000000
49550.0000000	9.0000000	-50.0000000
49550.0000000	9.2500000	-50.0000000
49550.0000000	9.5000000	-50.0000000

49550.0000000	9.7500000	-50.0000000
49550.0000000	10.0000000	-50.0000000
49550.0000000	10.2500000	-50.0000000
49550.0000000	10.5000000	-50.0000000
49550.0000000	10.7500000	-50.0000000
49550.0000000	11.0000000	-50.0000000
49550.0000000	11.2500000	-50.0000000
49550.0000000	11.5000000	-50.0000000
49550.0000000	11.7500000	-50.0000000
49550.0000000	12.0000000	-50.0000000
49550.0000000	12.2500000	-50.0000000
49550.0000000	12.5000000	-50.0000000
49550.0000000	12.7500000	-50.0000000
49550.0000000	13.0000000	-50.0000000
49550.0000000	13.2500000	-50.0000000
49550.0000000	13.5000000	-50.0000000
49550.0000000	13.7500000	-50.0000000
49550.0000000	14.0000000	-50.0000000
49550.0000000	14.2500000	-50.0000000
49550.0000000	14.5000000	-50.0000000
49550.0000000	14.7500000	-50.0000000
49550.0000000	15.0000000	-50.0000000
49580.0000000	0.0000000	-50.0000000
49580.0000000	0.2500000	-50.0000000
49580.0000000	0.5000000	-50.0000000
49580.0000000	0.7500000	17.0200005
49580.0000000	1.0000000	-50.0000000
49580.0000000	1.2500000	-50.0000000
49580.0000000	1.5000000	-50.0000000
49580.0000000	1.7500000	-50.0000000
49580.0000000	2.0000000	-50.0000000
49580.0000000	2.2500000	-50.0000000
49580.0000000	2.5000000	-50.0000000
49580.0000000	2.7500000	-50.0000000
49580.0000000	3.0000000	-50.0000000
49580.0000000	3.2500000	-50.0000000
49580.0000000	3.5000000	-50.0000000
49580.0000000	3.7500000	-50.0000000
49580.0000000	4.0000000	-50.0000000
49580.0000000	4.2500000	-50.0000000
49580.0000000	4.5000000	-50.0000000
49580.0000000	4.7500000	-50.0000000
49580.0000000	5.0000000	-50.0000000
49580.0000000	5.2500000	-50.0000000
49580.0000000	5.5000000	-50.0000000
49580.0000000	5.7500000	-50.0000000
49580.0000000	6.0000000	-50.0000000
49580.0000000	6.2500000	-50.0000000
49580.0000000	6.5000000	-50.0000000
49580.0000000	6.7500000	-50.0000000
49580.0000000	7.0000000	-50.0000000
49580.0000000	7.2500000	-50.0000000
49580.0000000	7.5000000	-50.0000000
49580.0000000	7.7500000	-50.0000000
49580.0000000	8.0000000	-50.0000000
49580.0000000	8.2500000	-50.0000000
49580.0000000	8.5000000	-50.0000000
49580.0000000	8.7500000	-50.0000000

49580.0000000	9.0000000	-50.0000000
49580.0000000	9.2500000	-50.0000000
49580.0000000	9.5000000	-50.0000000
49580.0000000	9.7500000	-50.0000000
49580.0000000	10.0000000	-50.0000000
49580.0000000	10.2500000	-50.0000000
49580.0000000	10.5000000	-50.0000000
49580.0000000	10.7500000	-50.0000000
49580.0000000	11.0000000	-50.0000000
49580.0000000	11.2500000	-50.0000000
49580.0000000	11.5000000	-50.0000000
49580.0000000	11.7500000	-50.0000000
49580.0000000	12.0000000	-50.0000000
49580.0000000	12.2500000	-50.0000000
49580.0000000	12.5000000	-50.0000000
49580.0000000	12.7500000	-50.0000000
49580.0000000	13.0000000	-50.0000000
49580.0000000	13.2500000	-50.0000000
49580.0000000	13.5000000	-50.0000000
49580.0000000	13.7500000	-50.0000000
49580.0000000	14.0000000	-50.0000000
49580.0000000	14.2500000	-50.0000000
49580.0000000	14.5000000	-50.0000000
49580.0000000	14.7500000	-50.0000000
49580.0000000	15.0000000	-50.0000000
49610.0000000	0.0000000	-50.0000000
49610.0000000	0.2500000	-50.0000000
49610.0000000	0.5000000	-50.0000000
49610.0000000	0.7500000	-50.0000000
49610.0000000	1.0000000	-50.0000000
49610.0000000	1.2500000	-50.0000000
49610.0000000	1.5000000	-50.0000000
49610.0000000	1.7500000	-50.0000000
49610.0000000	2.0000000	-50.0000000
49610.0000000	2.2500000	-50.0000000
49610.0000000	2.5000000	-50.0000000
49610.0000000	2.7500000	-50.0000000
49610.0000000	3.0000000	-50.0000000
49610.0000000	3.2500000	-50.0000000
49610.0000000	3.5000000	-50.0000000
49610.0000000	3.7500000	-50.0000000
49610.0000000	4.0000000	-50.0000000
49610.0000000	4.2500000	-50.0000000
49610.0000000	4.5000000	-50.0000000
49610.0000000	4.7500000	-50.0000000
49610.0000000	5.0000000	-50.0000000
49610.0000000	5.2500000	-50.0000000
49610.0000000	5.5000000	-50.0000000
49610.0000000	5.7500000	-50.0000000
49610.0000000	6.0000000	-50.0000000
49610.0000000	6.2500000	-50.0000000
49610.0000000	6.5000000	-50.0000000
49610.0000000	6.7500000	-50.0000000
49610.0000000	7.0000000	-50.0000000
49610.0000000	7.2500000	-50.0000000
49610.0000000	7.5000000	-50.0000000
49610.0000000	7.7500000	-50.0000000
49610.0000000	8.0000000	-50.0000000

49610.0000000	8.2500000	-50.0000000
49610.0000000	8.5000000	-50.0000000
49610.0000000	8.7500000	-50.0000000
49610.0000000	9.0000000	-50.0000000
49610.0000000	9.2500000	-50.0000000
49610.0000000	9.5000000	-50.0000000
49610.0000000	9.7500000	-50.0000000
49610.0000000	10.0000000	-50.0000000
49610.0000000	10.2500000	-50.0000000
49610.0000000	10.5000000	-50.0000000
49610.0000000	10.7500000	-50.0000000
49610.0000000	11.0000000	-50.0000000
49610.0000000	11.2500000	-50.0000000
49610.0000000	11.5000000	-50.0000000
49610.0000000	11.7500000	-50.0000000
49610.0000000	12.0000000	-50.0000000
49610.0000000	12.2500000	-50.0000000
49610.0000000	12.5000000	-50.0000000
49610.0000000	12.7500000	-50.0000000
49610.0000000	13.0000000	-50.0000000
49610.0000000	13.2500000	-50.0000000
49610.0000000	13.5000000	-50.0000000
49610.0000000	13.7500000	-50.0000000
49610.0000000	14.0000000	-50.0000000
49610.0000000	14.2500000	-50.0000000
49610.0000000	14.5000000	-50.0000000
49610.0000000	14.7500000	-50.0000000
49610.0000000	15.0000000	-50.0000000
49640.0000000	0.0000000	-50.0000000
49640.0000000	0.2500000	-50.0000000
49640.0000000	0.5000000	15.4099998
49640.0000000	0.7500000	19.7099991
49640.0000000	1.0000000	-50.0000000
49640.0000000	1.2500000	-50.0000000
49640.0000000	1.5000000	-50.0000000
49640.0000000	1.7500000	-50.0000000
49640.0000000	2.0000000	-50.0000000
49640.0000000	2.2500000	-50.0000000
49640.0000000	2.5000000	-50.0000000
49640.0000000	2.7500000	-50.0000000
49640.0000000	3.0000000	-50.0000000
49640.0000000	3.2500000	-50.0000000
49640.0000000	3.5000000	-50.0000000
49640.0000000	3.7500000	-50.0000000
49640.0000000	4.0000000	-50.0000000
49640.0000000	4.2500000	-50.0000000
49640.0000000	4.5000000	-50.0000000
49640.0000000	4.7500000	-50.0000000
49640.0000000	5.0000000	-50.0000000
49640.0000000	5.2500000	-50.0000000
49640.0000000	5.5000000	-50.0000000
49640.0000000	5.7500000	-50.0000000
49640.0000000	6.0000000	-50.0000000
49640.0000000	6.2500000	-50.0000000
49640.0000000	6.5000000	-50.0000000
49640.0000000	6.7500000	-50.0000000
49640.0000000	7.0000000	-50.0000000
49640.0000000	7.2500000	-50.0000000

49640.0000000	7.5000000	-50.0000000
49640.0000000	7.7500000	-50.0000000
49640.0000000	8.0000000	-50.0000000
49640.0000000	8.2500000	-50.0000000
49640.0000000	8.5000000	-50.0000000
49640.0000000	8.7500000	-50.0000000
49640.0000000	9.0000000	-50.0000000
49640.0000000	9.2500000	-50.0000000
49640.0000000	9.5000000	-50.0000000
49640.0000000	9.7500000	-50.0000000
49640.0000000	10.0000000	-50.0000000
49640.0000000	10.2500000	-50.0000000
49640.0000000	10.5000000	-50.0000000
49640.0000000	10.7500000	-50.0000000
49640.0000000	11.0000000	-50.0000000
49640.0000000	11.2500000	-50.0000000
49640.0000000	11.5000000	-50.0000000
49640.0000000	11.7500000	-50.0000000
49640.0000000	12.0000000	-50.0000000
49640.0000000	12.2500000	-50.0000000
49640.0000000	12.5000000	-50.0000000
49640.0000000	12.7500000	-50.0000000
49640.0000000	13.0000000	-50.0000000
49640.0000000	13.2500000	-50.0000000
49640.0000000	13.5000000	-50.0000000
49640.0000000	13.7500000	-50.0000000
49640.0000000	14.0000000	-50.0000000
49640.0000000	14.2500000	-50.0000000
49640.0000000	14.5000000	-50.0000000
49640.0000000	14.7500000	-50.0000000
49640.0000000	15.0000000	-50.0000000
49670.0000000	0.0000000	-50.0000000
49670.0000000	0.2500000	-50.0000000
49670.0000000	0.5000000	-50.0000000
49670.0000000	0.7500000	16.2999992
49670.0000000	1.0000000	-50.0000000
49670.0000000	1.2500000	-50.0000000
49670.0000000	1.5000000	-50.0000000
49670.0000000	1.7500000	-50.0000000
49670.0000000	2.0000000	-50.0000000
49670.0000000	2.2500000	-50.0000000
49670.0000000	2.5000000	-50.0000000
49670.0000000	2.7500000	-50.0000000
49670.0000000	3.0000000	-50.0000000
49670.0000000	3.2500000	-50.0000000
49670.0000000	3.5000000	-50.0000000
49670.0000000	3.7500000	-50.0000000
49670.0000000	4.0000000	-50.0000000
49670.0000000	4.2500000	-50.0000000
49670.0000000	4.5000000	-50.0000000
49670.0000000	4.7500000	-50.0000000
49670.0000000	5.0000000	-50.0000000
49670.0000000	5.2500000	-50.0000000
49670.0000000	5.5000000	-50.0000000
49670.0000000	5.7500000	-50.0000000
49670.0000000	6.0000000	-50.0000000
49670.0000000	6.2500000	-50.0000000
49670.0000000	6.5000000	-50.0000000

49670.0000000	6.7500000	-50.0000000
49670.0000000	7.0000000	-50.0000000
49670.0000000	7.2500000	-50.0000000
49670.0000000	7.5000000	-50.0000000
49670.0000000	7.7500000	-50.0000000
49670.0000000	8.0000000	-50.0000000
49670.0000000	8.2500000	-50.0000000
49670.0000000	8.5000000	-50.0000000
49670.0000000	8.7500000	-50.0000000
49670.0000000	9.0000000	-50.0000000
49670.0000000	9.2500000	-50.0000000
49670.0000000	9.5000000	-50.0000000
49670.0000000	9.7500000	-50.0000000
49670.0000000	10.0000000	-50.0000000
49670.0000000	10.2500000	-50.0000000
49670.0000000	10.5000000	-50.0000000
49670.0000000	10.7500000	-50.0000000
49670.0000000	11.0000000	-50.0000000
49670.0000000	11.2500000	-50.0000000
49670.0000000	11.5000000	-50.0000000
49670.0000000	11.7500000	-50.0000000
49670.0000000	12.0000000	-50.0000000
49670.0000000	12.2500000	-50.0000000
49670.0000000	12.5000000	-50.0000000
49670.0000000	12.7500000	-50.0000000
49670.0000000	13.0000000	-50.0000000
49670.0000000	13.2500000	-50.0000000
49670.0000000	13.5000000	-50.0000000
49670.0000000	13.7500000	-50.0000000
49670.0000000	14.0000000	-50.0000000
49670.0000000	14.2500000	-50.0000000
49670.0000000	14.5000000	-50.0000000
49670.0000000	14.7500000	-50.0000000
49670.0000000	15.0000000	-50.0000000
49700.0000000	0.0000000	-50.0000000
49700.0000000	0.2500000	-50.0000000
49700.0000000	0.5000000	-50.0000000
49700.0000000	0.7500000	16.2399998
49700.0000000	1.0000000	-50.0000000
49700.0000000	1.2500000	-50.0000000
49700.0000000	1.5000000	-50.0000000
49700.0000000	1.7500000	-50.0000000
49700.0000000	2.0000000	-50.0000000
49700.0000000	2.2500000	-50.0000000
49700.0000000	2.5000000	-50.0000000
49700.0000000	2.7500000	-50.0000000
49700.0000000	3.0000000	-50.0000000
49700.0000000	3.2500000	-50.0000000
49700.0000000	3.5000000	-50.0000000
49700.0000000	3.7500000	-50.0000000
49700.0000000	4.0000000	-50.0000000
49700.0000000	4.2500000	-50.0000000
49700.0000000	4.5000000	-50.0000000
49700.0000000	4.7500000	-50.0000000
49700.0000000	5.0000000	-50.0000000
49700.0000000	5.2500000	-50.0000000
49700.0000000	5.5000000	-50.0000000
49700.0000000	5.7500000	-50.0000000

49700.0000000	6.0000000	-50.0000000
49700.0000000	6.2500000	-50.0000000
49700.0000000	6.5000000	-50.0000000
49700.0000000	6.7500000	-50.0000000
49700.0000000	7.0000000	-50.0000000
49700.0000000	7.2500000	-50.0000000
49700.0000000	7.5000000	-50.0000000
49700.0000000	7.7500000	-50.0000000
49700.0000000	8.0000000	-50.0000000
49700.0000000	8.2500000	-50.0000000
49700.0000000	8.5000000	-50.0000000
49700.0000000	8.7500000	-50.0000000
49700.0000000	9.0000000	-50.0000000
49700.0000000	9.2500000	-50.0000000
49700.0000000	9.5000000	-50.0000000
49700.0000000	9.7500000	-50.0000000
49700.0000000	10.0000000	-50.0000000
49700.0000000	10.2500000	-50.0000000
49700.0000000	10.5000000	-50.0000000
49700.0000000	10.7500000	-50.0000000
49700.0000000	11.0000000	-50.0000000
49700.0000000	11.2500000	-50.0000000
49700.0000000	11.5000000	-50.0000000
49700.0000000	11.7500000	-50.0000000
49700.0000000	12.0000000	-50.0000000
49700.0000000	12.2500000	-50.0000000
49700.0000000	12.5000000	-50.0000000
49700.0000000	12.7500000	-50.0000000
49700.0000000	13.0000000	-50.0000000
49700.0000000	13.2500000	-50.0000000
49700.0000000	13.5000000	-50.0000000
49700.0000000	13.7500000	-50.0000000
49700.0000000	14.0000000	-50.0000000
49700.0000000	14.2500000	-50.0000000
49700.0000000	14.5000000	-50.0000000
49700.0000000	14.7500000	-50.0000000
49700.0000000	15.0000000	-50.0000000
49730.0000000	0.0000000	-50.0000000
49730.0000000	0.2500000	-50.0000000
49730.0000000	0.5000000	-50.0000000
49730.0000000	0.7500000	17.2299995
49730.0000000	1.0000000	-50.0000000
49730.0000000	1.2500000	-50.0000000
49730.0000000	1.5000000	-50.0000000
49730.0000000	1.7500000	-50.0000000
49730.0000000	2.0000000	-50.0000000
49730.0000000	2.2500000	-50.0000000
49730.0000000	2.5000000	-50.0000000
49730.0000000	2.7500000	-50.0000000
49730.0000000	3.0000000	-50.0000000
49730.0000000	3.2500000	-50.0000000
49730.0000000	3.5000000	-50.0000000
49730.0000000	3.7500000	-50.0000000
49730.0000000	4.0000000	-50.0000000
49730.0000000	4.2500000	-50.0000000
49730.0000000	4.5000000	-50.0000000
49730.0000000	4.7500000	-50.0000000
49730.0000000	5.0000000	-50.0000000

49730.0000000	5.2500000	-50.0000000
49730.0000000	5.5000000	-50.0000000
49730.0000000	5.7500000	-50.0000000
49730.0000000	6.0000000	-50.0000000
49730.0000000	6.2500000	-50.0000000
49730.0000000	6.5000000	-50.0000000
49730.0000000	6.7500000	-50.0000000
49730.0000000	7.0000000	-50.0000000
49730.0000000	7.2500000	-50.0000000
49730.0000000	7.5000000	-50.0000000
49730.0000000	7.7500000	-50.0000000
49730.0000000	8.0000000	-50.0000000
49730.0000000	8.2500000	-50.0000000
49730.0000000	8.5000000	-50.0000000
49730.0000000	8.7500000	-50.0000000
49730.0000000	9.0000000	-50.0000000
49730.0000000	9.2500000	-50.0000000
49730.0000000	9.5000000	-50.0000000
49730.0000000	9.7500000	-50.0000000
49730.0000000	10.0000000	-50.0000000
49730.0000000	10.2500000	-50.0000000
49730.0000000	10.5000000	-50.0000000
49730.0000000	10.7500000	-50.0000000
49730.0000000	11.0000000	-50.0000000
49730.0000000	11.2500000	-50.0000000
49730.0000000	11.5000000	-50.0000000
49730.0000000	11.7500000	-50.0000000
49730.0000000	12.0000000	-50.0000000
49730.0000000	12.2500000	-50.0000000
49730.0000000	12.5000000	-50.0000000
49730.0000000	12.7500000	-50.0000000
49730.0000000	13.0000000	-50.0000000
49730.0000000	13.2500000	-50.0000000
49730.0000000	13.5000000	-50.0000000
49730.0000000	13.7500000	-50.0000000
49730.0000000	14.0000000	-50.0000000
49730.0000000	14.2500000	-50.0000000
49730.0000000	14.5000000	-50.0000000
49730.0000000	14.7500000	-50.0000000
49730.0000000	15.0000000	-50.0000000
49760.0000000	0.0000000	-50.0000000
49760.0000000	0.2500000	-50.0000000
49760.0000000	0.5000000	-50.0000000
49760.0000000	0.7500000	17.2399998
49760.0000000	1.0000000	-50.0000000
49760.0000000	1.2500000	-50.0000000
49760.0000000	1.5000000	-50.0000000
49760.0000000	1.7500000	-50.0000000
49760.0000000	2.0000000	-50.0000000
49760.0000000	2.2500000	-50.0000000
49760.0000000	2.5000000	-50.0000000
49760.0000000	2.7500000	-50.0000000
49760.0000000	3.0000000	-50.0000000
49760.0000000	3.2500000	-50.0000000
49760.0000000	3.5000000	-50.0000000
49760.0000000	3.7500000	-50.0000000
49760.0000000	4.0000000	-50.0000000
49760.0000000	4.2500000	-50.0000000

49760.0000000	4.5000000	-50.0000000
49760.0000000	4.7500000	-50.0000000
49760.0000000	5.0000000	-50.0000000
49760.0000000	5.2500000	-50.0000000
49760.0000000	5.5000000	-50.0000000
49760.0000000	5.7500000	-50.0000000
49760.0000000	6.0000000	-50.0000000
49760.0000000	6.2500000	-50.0000000
49760.0000000	6.5000000	-50.0000000
49760.0000000	6.7500000	-50.0000000
49760.0000000	7.0000000	-50.0000000
49760.0000000	7.2500000	-50.0000000
49760.0000000	7.5000000	-50.0000000
49760.0000000	7.7500000	-50.0000000
49760.0000000	8.0000000	-50.0000000
49760.0000000	8.2500000	-50.0000000
49760.0000000	8.5000000	-50.0000000
49760.0000000	8.7500000	-50.0000000
49760.0000000	9.0000000	-50.0000000
49760.0000000	9.2500000	-50.0000000
49760.0000000	9.5000000	-50.0000000
49760.0000000	9.7500000	-50.0000000
49760.0000000	10.0000000	-50.0000000
49760.0000000	10.2500000	-50.0000000
49760.0000000	10.5000000	-50.0000000
49760.0000000	10.7500000	-50.0000000
49760.0000000	11.0000000	-50.0000000
49760.0000000	11.2500000	-50.0000000
49760.0000000	11.5000000	-50.0000000
49760.0000000	11.7500000	-50.0000000
49760.0000000	12.0000000	-50.0000000
49760.0000000	12.2500000	-50.0000000
49760.0000000	12.5000000	-50.0000000
49760.0000000	12.7500000	-50.0000000
49760.0000000	13.0000000	-50.0000000
49760.0000000	13.2500000	-50.0000000
49760.0000000	13.5000000	-50.0000000
49760.0000000	13.7500000	-50.0000000
49760.0000000	14.0000000	-50.0000000
49760.0000000	14.2500000	-50.0000000
49760.0000000	14.5000000	-50.0000000
49760.0000000	14.7500000	-50.0000000
49760.0000000	15.0000000	-50.0000000
49790.0000000	0.0000000	-50.0000000
49790.0000000	0.2500000	-50.0000000
49790.0000000	0.5000000	15.0500002
49790.0000000	0.7500000	16.7299995
49790.0000000	1.0000000	-50.0000000
49790.0000000	1.2500000	-50.0000000
49790.0000000	1.5000000	-50.0000000
49790.0000000	1.7500000	-50.0000000
49790.0000000	2.0000000	-50.0000000
49790.0000000	2.2500000	-50.0000000
49790.0000000	2.5000000	-50.0000000
49790.0000000	2.7500000	-50.0000000
49790.0000000	3.0000000	-50.0000000
49790.0000000	3.2500000	-50.0000000
49790.0000000	3.5000000	-50.0000000

49790.0000000	3.7500000	-50.0000000
49790.0000000	4.0000000	-50.0000000
49790.0000000	4.2500000	-50.0000000
49790.0000000	4.5000000	-50.0000000
49790.0000000	4.7500000	-50.0000000
49790.0000000	5.0000000	-50.0000000
49790.0000000	5.2500000	-50.0000000
49790.0000000	5.5000000	-50.0000000
49790.0000000	5.7500000	-50.0000000
49790.0000000	6.0000000	-50.0000000
49790.0000000	6.2500000	-50.0000000
49790.0000000	6.5000000	-50.0000000
49790.0000000	6.7500000	-50.0000000
49790.0000000	7.0000000	-50.0000000
49790.0000000	7.2500000	-50.0000000
49790.0000000	7.5000000	-50.0000000
49790.0000000	7.7500000	-50.0000000
49790.0000000	8.0000000	-50.0000000
49790.0000000	8.2500000	-50.0000000
49790.0000000	8.5000000	-50.0000000
49790.0000000	8.7500000	-50.0000000
49790.0000000	9.0000000	-50.0000000
49790.0000000	9.2500000	-50.0000000
49790.0000000	9.5000000	-50.0000000
49790.0000000	9.7500000	-50.0000000
49790.0000000	10.0000000	-50.0000000
49790.0000000	10.2500000	-50.0000000
49790.0000000	10.5000000	-50.0000000
49790.0000000	10.7500000	-50.0000000
49790.0000000	11.0000000	-50.0000000
49790.0000000	11.2500000	-50.0000000
49790.0000000	11.5000000	-50.0000000
49790.0000000	11.7500000	-50.0000000
49790.0000000	12.0000000	-50.0000000
49790.0000000	12.2500000	-50.0000000
49790.0000000	12.5000000	-50.0000000
49790.0000000	12.7500000	-50.0000000
49790.0000000	13.0000000	-50.0000000
49790.0000000	13.2500000	-50.0000000
49790.0000000	13.5000000	-50.0000000
49790.0000000	13.7500000	-50.0000000
49790.0000000	14.0000000	-50.0000000
49790.0000000	14.2500000	-50.0000000
49790.0000000	14.5000000	-50.0000000
49790.0000000	14.7500000	-50.0000000
49790.0000000	15.0000000	-50.0000000
49820.0000000	0.0000000	-50.0000000
49820.0000000	0.2500000	-50.0000000
49820.0000000	0.5000000	15.9799995
49820.0000000	0.7500000	19.1900005
49820.0000000	1.0000000	15.4499998
49820.0000000	1.2500000	-50.0000000
49820.0000000	1.5000000	-50.0000000
49820.0000000	1.7500000	-50.0000000
49820.0000000	2.0000000	-50.0000000
49820.0000000	2.2500000	-50.0000000
49820.0000000	2.5000000	-50.0000000
49820.0000000	2.7500000	-50.0000000

49820.0000000	3.0000000	-50.0000000
49820.0000000	3.2500000	-50.0000000
49820.0000000	3.5000000	-50.0000000
49820.0000000	3.7500000	-50.0000000
49820.0000000	4.0000000	-50.0000000
49820.0000000	4.2500000	-50.0000000
49820.0000000	4.5000000	-50.0000000
49820.0000000	4.7500000	-50.0000000
49820.0000000	5.0000000	-50.0000000
49820.0000000	5.2500000	-50.0000000
49820.0000000	5.5000000	-50.0000000
49820.0000000	5.7500000	-50.0000000
49820.0000000	6.0000000	-50.0000000
49820.0000000	6.2500000	-50.0000000
49820.0000000	6.5000000	-50.0000000
49820.0000000	6.7500000	-50.0000000
49820.0000000	7.0000000	-50.0000000
49820.0000000	7.2500000	-50.0000000
49820.0000000	7.5000000	-50.0000000
49820.0000000	7.7500000	-50.0000000
49820.0000000	8.0000000	-50.0000000
49820.0000000	8.2500000	-50.0000000
49820.0000000	8.5000000	-50.0000000
49820.0000000	8.7500000	-50.0000000
49820.0000000	9.0000000	-50.0000000
49820.0000000	9.2500000	-50.0000000
49820.0000000	9.5000000	-50.0000000
49820.0000000	9.7500000	-50.0000000
49820.0000000	10.0000000	-50.0000000
49820.0000000	10.2500000	-50.0000000
49820.0000000	10.5000000	-50.0000000
49820.0000000	10.7500000	-50.0000000
49820.0000000	11.0000000	-50.0000000
49820.0000000	11.2500000	-50.0000000
49820.0000000	11.5000000	-50.0000000
49820.0000000	11.7500000	-50.0000000
49820.0000000	12.0000000	-50.0000000
49820.0000000	12.2500000	-50.0000000
49820.0000000	12.5000000	-50.0000000
49820.0000000	12.7500000	-50.0000000
49820.0000000	13.0000000	-50.0000000
49820.0000000	13.2500000	-50.0000000
49820.0000000	13.5000000	-50.0000000
49820.0000000	13.7500000	-50.0000000
49820.0000000	14.0000000	-50.0000000
49820.0000000	14.2500000	-50.0000000
49820.0000000	14.5000000	-50.0000000
49820.0000000	14.7500000	-50.0000000
49820.0000000	15.0000000	-50.0000000
49850.0000000	0.0000000	-50.0000000
49850.0000000	0.2500000	-50.0000000
49850.0000000	0.5000000	16.1900005
49850.0000000	0.7500000	19.9099998
49850.0000000	1.0000000	-50.0000000
49850.0000000	1.2500000	-50.0000000
49850.0000000	1.5000000	-50.0000000
49850.0000000	1.7500000	-50.0000000
49850.0000000	2.0000000	-50.0000000

49850.0000000	2.2500000	-50.0000000
49850.0000000	2.5000000	-50.0000000
49850.0000000	2.7500000	-50.0000000
49850.0000000	3.0000000	-50.0000000
49850.0000000	3.2500000	-50.0000000
49850.0000000	3.5000000	-50.0000000
49850.0000000	3.7500000	-50.0000000
49850.0000000	4.0000000	-50.0000000
49850.0000000	4.2500000	-50.0000000
49850.0000000	4.5000000	-50.0000000
49850.0000000	4.7500000	-50.0000000
49850.0000000	5.0000000	-50.0000000
49850.0000000	5.2500000	-50.0000000
49850.0000000	5.5000000	-50.0000000
49850.0000000	5.7500000	-50.0000000
49850.0000000	6.0000000	-50.0000000
49850.0000000	6.2500000	-50.0000000
49850.0000000	6.5000000	-50.0000000
49850.0000000	6.7500000	-50.0000000
49850.0000000	7.0000000	-50.0000000
49850.0000000	7.2500000	-50.0000000
49850.0000000	7.5000000	-50.0000000
49850.0000000	7.7500000	-50.0000000
49850.0000000	8.0000000	-50.0000000
49850.0000000	8.2500000	-50.0000000
49850.0000000	8.5000000	-50.0000000
49850.0000000	8.7500000	-50.0000000
49850.0000000	9.0000000	-50.0000000
49850.0000000	9.2500000	-50.0000000
49850.0000000	9.5000000	-50.0000000
49850.0000000	9.7500000	-50.0000000
49850.0000000	10.0000000	-50.0000000
49850.0000000	10.2500000	-50.0000000
49850.0000000	10.5000000	-50.0000000
49850.0000000	10.7500000	-50.0000000
49850.0000000	11.0000000	-50.0000000
49850.0000000	11.2500000	-50.0000000
49850.0000000	11.5000000	-50.0000000
49850.0000000	11.7500000	-50.0000000
49850.0000000	12.0000000	-50.0000000
49850.0000000	12.2500000	-50.0000000
49850.0000000	12.5000000	-50.0000000
49850.0000000	12.7500000	-50.0000000
49850.0000000	13.0000000	-50.0000000
49850.0000000	13.2500000	-50.0000000
49850.0000000	13.5000000	-50.0000000
49850.0000000	13.7500000	-50.0000000
49850.0000000	14.0000000	-50.0000000
49850.0000000	14.2500000	-50.0000000
49850.0000000	14.5000000	-50.0000000
49850.0000000	14.7500000	-50.0000000
49850.0000000	15.0000000	-50.0000000
49880.0000000	0.0000000	-50.0000000
49880.0000000	0.2500000	-50.0000000
49880.0000000	0.5000000	-50.0000000
49880.0000000	0.7500000	16.1399994
49880.0000000	1.0000000	-50.0000000
49880.0000000	1.2500000	-50.0000000

49880.0000000	1.5000000	-50.0000000
49880.0000000	1.7500000	-50.0000000
49880.0000000	2.0000000	-50.0000000
49880.0000000	2.2500000	-50.0000000
49880.0000000	2.5000000	-50.0000000
49880.0000000	2.7500000	-50.0000000
49880.0000000	3.0000000	-50.0000000
49880.0000000	3.2500000	-50.0000000
49880.0000000	3.5000000	-50.0000000
49880.0000000	3.7500000	-50.0000000
49880.0000000	4.0000000	-50.0000000
49880.0000000	4.2500000	-50.0000000
49880.0000000	4.5000000	-50.0000000
49880.0000000	4.7500000	-50.0000000
49880.0000000	5.0000000	-50.0000000
49880.0000000	5.2500000	-50.0000000
49880.0000000	5.5000000	-50.0000000
49880.0000000	5.7500000	-50.0000000
49880.0000000	6.0000000	-50.0000000
49880.0000000	6.2500000	-50.0000000
49880.0000000	6.5000000	-50.0000000
49880.0000000	6.7500000	-50.0000000
49880.0000000	7.0000000	-50.0000000
49880.0000000	7.2500000	-50.0000000
49880.0000000	7.5000000	-50.0000000
49880.0000000	7.7500000	-50.0000000
49880.0000000	8.0000000	-50.0000000
49880.0000000	8.2500000	-50.0000000
49880.0000000	8.5000000	-50.0000000
49880.0000000	8.7500000	-50.0000000
49880.0000000	9.0000000	-50.0000000
49880.0000000	9.2500000	-50.0000000
49880.0000000	9.5000000	-50.0000000
49880.0000000	9.7500000	-50.0000000
49880.0000000	10.0000000	-50.0000000
49880.0000000	10.2500000	-50.0000000
49880.0000000	10.5000000	-50.0000000
49880.0000000	10.7500000	-50.0000000
49880.0000000	11.0000000	-50.0000000
49880.0000000	11.2500000	-50.0000000
49880.0000000	11.5000000	-50.0000000
49880.0000000	11.7500000	-50.0000000
49880.0000000	12.0000000	-50.0000000
49880.0000000	12.2500000	-50.0000000
49880.0000000	12.5000000	-50.0000000
49880.0000000	12.7500000	-50.0000000
49880.0000000	13.0000000	-50.0000000
49880.0000000	13.2500000	-50.0000000
49880.0000000	13.5000000	-50.0000000
49880.0000000	13.7500000	-50.0000000
49880.0000000	14.0000000	-50.0000000
49880.0000000	14.2500000	-50.0000000
49880.0000000	14.5000000	-50.0000000
49880.0000000	14.7500000	-50.0000000
49880.0000000	15.0000000	-50.0000000
49910.0000000	0.0000000	-50.0000000
49910.0000000	0.2500000	-50.0000000
49910.0000000	0.5000000	-50.0000000

49910.0000000	0.7500000	16.9899998
49910.0000000	1.0000000	-50.0000000
49910.0000000	1.2500000	-50.0000000
49910.0000000	1.5000000	-50.0000000
49910.0000000	1.7500000	-50.0000000
49910.0000000	2.0000000	-50.0000000
49910.0000000	2.2500000	-50.0000000
49910.0000000	2.5000000	-50.0000000
49910.0000000	2.7500000	-50.0000000
49910.0000000	3.0000000	-50.0000000
49910.0000000	3.2500000	-50.0000000
49910.0000000	3.5000000	-50.0000000
49910.0000000	3.7500000	-50.0000000
49910.0000000	4.0000000	-50.0000000
49910.0000000	4.2500000	-50.0000000
49910.0000000	4.5000000	-50.0000000
49910.0000000	4.7500000	-50.0000000
49910.0000000	5.0000000	-50.0000000
49910.0000000	5.2500000	-50.0000000
49910.0000000	5.5000000	-50.0000000
49910.0000000	5.7500000	-50.0000000
49910.0000000	6.0000000	-50.0000000
49910.0000000	6.2500000	-50.0000000
49910.0000000	6.5000000	-50.0000000
49910.0000000	6.7500000	-50.0000000
49910.0000000	7.0000000	-50.0000000
49910.0000000	7.2500000	-50.0000000
49910.0000000	7.5000000	-50.0000000
49910.0000000	7.7500000	-50.0000000
49910.0000000	8.0000000	-50.0000000
49910.0000000	8.2500000	-50.0000000
49910.0000000	8.5000000	-50.0000000
49910.0000000	8.7500000	-50.0000000
49910.0000000	9.0000000	-50.0000000
49910.0000000	9.2500000	-50.0000000
49910.0000000	9.5000000	-50.0000000
49910.0000000	9.7500000	-50.0000000
49910.0000000	10.0000000	-50.0000000
49910.0000000	10.2500000	-50.0000000
49910.0000000	10.5000000	-50.0000000
49910.0000000	10.7500000	-50.0000000
49910.0000000	11.0000000	-50.0000000
49910.0000000	11.2500000	-50.0000000
49910.0000000	11.5000000	-50.0000000
49910.0000000	11.7500000	-50.0000000
49910.0000000	12.0000000	-50.0000000
49910.0000000	12.2500000	-50.0000000
49910.0000000	12.5000000	-50.0000000
49910.0000000	12.7500000	-50.0000000
49910.0000000	13.0000000	-50.0000000
49910.0000000	13.2500000	-50.0000000
49910.0000000	13.5000000	-50.0000000
49910.0000000	13.7500000	-50.0000000
49910.0000000	14.0000000	-50.0000000
49910.0000000	14.2500000	-50.0000000
49910.0000000	14.5000000	-50.0000000
49910.0000000	14.7500000	-50.0000000
49910.0000000	15.0000000	-50.0000000

49940.0000000	0.0000000	-50.0000000
49940.0000000	0.2500000	-50.0000000
49940.0000000	0.5000000	-50.0000000
49940.0000000	0.7500000	17.6200008
49940.0000000	1.0000000	-50.0000000
49940.0000000	1.2500000	-50.0000000
49940.0000000	1.5000000	-50.0000000
49940.0000000	1.7500000	-50.0000000
49940.0000000	2.0000000	-50.0000000
49940.0000000	2.2500000	-50.0000000
49940.0000000	2.5000000	-50.0000000
49940.0000000	2.7500000	-50.0000000
49940.0000000	3.0000000	-50.0000000
49940.0000000	3.2500000	-50.0000000
49940.0000000	3.5000000	-50.0000000
49940.0000000	3.7500000	15.8699999
49940.0000000	4.0000000	-50.0000000
49940.0000000	4.2500000	-50.0000000
49940.0000000	4.5000000	-50.0000000
49940.0000000	4.7500000	-50.0000000
49940.0000000	5.0000000	-50.0000000
49940.0000000	5.2500000	-50.0000000
49940.0000000	5.5000000	-50.0000000
49940.0000000	5.7500000	-50.0000000
49940.0000000	6.0000000	-50.0000000
49940.0000000	6.2500000	-50.0000000
49940.0000000	6.5000000	-50.0000000
49940.0000000	6.7500000	-50.0000000
49940.0000000	7.0000000	-50.0000000
49940.0000000	7.2500000	-50.0000000
49940.0000000	7.5000000	-50.0000000
49940.0000000	7.7500000	-50.0000000
49940.0000000	8.0000000	-50.0000000
49940.0000000	8.2500000	-50.0000000
49940.0000000	8.5000000	-50.0000000
49940.0000000	8.7500000	-50.0000000
49940.0000000	9.0000000	-50.0000000
49940.0000000	9.2500000	-50.0000000
49940.0000000	9.5000000	-50.0000000
49940.0000000	9.7500000	-50.0000000
49940.0000000	10.0000000	-50.0000000
49940.0000000	10.2500000	-50.0000000
49940.0000000	10.5000000	-50.0000000
49940.0000000	10.7500000	-50.0000000
49940.0000000	11.0000000	-50.0000000
49940.0000000	11.2500000	-50.0000000
49940.0000000	11.5000000	-50.0000000
49940.0000000	11.7500000	-50.0000000
49940.0000000	12.0000000	-50.0000000
49940.0000000	12.2500000	-50.0000000
49940.0000000	12.5000000	-50.0000000
49940.0000000	12.7500000	-50.0000000
49940.0000000	13.0000000	-50.0000000
49940.0000000	13.2500000	-50.0000000
49940.0000000	13.5000000	-50.0000000
49940.0000000	13.7500000	-50.0000000
49940.0000000	14.0000000	-50.0000000
49940.0000000	14.2500000	-50.0000000

49940.0000000	14.5000000	-50.0000000
49940.0000000	14.7500000	-50.0000000
49940.0000000	15.0000000	-50.0000000
49970.0000000	0.0000000	-50.0000000
49970.0000000	0.2500000	-50.0000000
49970.0000000	0.5000000	-50.0000000
49970.0000000	0.7500000	19.7900009
49970.0000000	1.0000000	15.8999996
49970.0000000	1.2500000	-50.0000000
49970.0000000	1.5000000	-50.0000000
49970.0000000	1.7500000	-50.0000000
49970.0000000	2.0000000	-50.0000000
49970.0000000	2.2500000	-50.0000000
49970.0000000	2.5000000	-50.0000000
49970.0000000	2.7500000	-50.0000000
49970.0000000	3.0000000	-50.0000000
49970.0000000	3.2500000	16.3799992
49970.0000000	3.5000000	-50.0000000
49970.0000000	3.7500000	-50.0000000
49970.0000000	4.0000000	-50.0000000
49970.0000000	4.2500000	-50.0000000
49970.0000000	4.5000000	-50.0000000
49970.0000000	4.7500000	-50.0000000
49970.0000000	5.0000000	-50.0000000
49970.0000000	5.2500000	-50.0000000
49970.0000000	5.5000000	-50.0000000
49970.0000000	5.7500000	-50.0000000
49970.0000000	6.0000000	-50.0000000
49970.0000000	6.2500000	-50.0000000
49970.0000000	6.5000000	-50.0000000
49970.0000000	6.7500000	-50.0000000
49970.0000000	7.0000000	-50.0000000
49970.0000000	7.2500000	-50.0000000
49970.0000000	7.5000000	-50.0000000
49970.0000000	7.7500000	-50.0000000
49970.0000000	8.0000000	-50.0000000
49970.0000000	8.2500000	-50.0000000
49970.0000000	8.5000000	-50.0000000
49970.0000000	8.7500000	-50.0000000
49970.0000000	9.0000000	-50.0000000
49970.0000000	9.2500000	-50.0000000
49970.0000000	9.5000000	-50.0000000
49970.0000000	9.7500000	-50.0000000
49970.0000000	10.0000000	-50.0000000
49970.0000000	10.2500000	-50.0000000
49970.0000000	10.5000000	-50.0000000
49970.0000000	10.7500000	-50.0000000
49970.0000000	11.0000000	-50.0000000
49970.0000000	11.2500000	-50.0000000
49970.0000000	11.5000000	-50.0000000
49970.0000000	11.7500000	-50.0000000
49970.0000000	12.0000000	-50.0000000
49970.0000000	12.2500000	-50.0000000
49970.0000000	12.5000000	-50.0000000
49970.0000000	12.7500000	-50.0000000
49970.0000000	13.0000000	-50.0000000
49970.0000000	13.2500000	-50.0000000
49970.0000000	13.5000000	-50.0000000

49970.0000000	13.7500000	-50.0000000
49970.0000000	14.0000000	-50.0000000
49970.0000000	14.2500000	-50.0000000
49970.0000000	14.5000000	-50.0000000
49970.0000000	14.7500000	-50.0000000
49970.0000000	15.0000000	-50.0000000
50000.0000000	0.0000000	-50.0000000
50000.0000000	0.2500000	-50.0000000
50000.0000000	0.5000000	-50.0000000
50000.0000000	0.7500000	18.5799999
50000.0000000	1.0000000	17.2299995
50000.0000000	1.2500000	-50.0000000
50000.0000000	1.5000000	-50.0000000
50000.0000000	1.7500000	-50.0000000
50000.0000000	2.0000000	-50.0000000
50000.0000000	2.2500000	-50.0000000
50000.0000000	2.5000000	-50.0000000
50000.0000000	2.7500000	-50.0000000
50000.0000000	3.0000000	-50.0000000
50000.0000000	3.2500000	-50.0000000
50000.0000000	3.5000000	-50.0000000
50000.0000000	3.7500000	-50.0000000
50000.0000000	4.0000000	-50.0000000
50000.0000000	4.2500000	-50.0000000
50000.0000000	4.5000000	-50.0000000
50000.0000000	4.7500000	-50.0000000
50000.0000000	5.0000000	-50.0000000
50000.0000000	5.2500000	-50.0000000
50000.0000000	5.5000000	-50.0000000
50000.0000000	5.7500000	-50.0000000
50000.0000000	6.0000000	-50.0000000
50000.0000000	6.2500000	-50.0000000
50000.0000000	6.5000000	-50.0000000
50000.0000000	6.7500000	-50.0000000
50000.0000000	7.0000000	-50.0000000
50000.0000000	7.2500000	-50.0000000
50000.0000000	7.5000000	-50.0000000
50000.0000000	7.7500000	-50.0000000
50000.0000000	8.0000000	-50.0000000
50000.0000000	8.2500000	-50.0000000
50000.0000000	8.5000000	-50.0000000
50000.0000000	8.7500000	-50.0000000
50000.0000000	9.0000000	-50.0000000
50000.0000000	9.2500000	-50.0000000
50000.0000000	9.5000000	-50.0000000
50000.0000000	9.7500000	-50.0000000
50000.0000000	10.0000000	-50.0000000
50000.0000000	10.2500000	-50.0000000
50000.0000000	10.5000000	-50.0000000
50000.0000000	10.7500000	-50.0000000
50000.0000000	11.0000000	-50.0000000
50000.0000000	11.2500000	-50.0000000
50000.0000000	11.5000000	-50.0000000
50000.0000000	11.7500000	-50.0000000
50000.0000000	12.0000000	-50.0000000
50000.0000000	12.2500000	-50.0000000
50000.0000000	12.5000000	-50.0000000
50000.0000000	12.7500000	-50.0000000

50000.0000000	13.0000000	-50.0000000
50000.0000000	13.2500000	-50.0000000
50000.0000000	13.5000000	-50.0000000
50000.0000000	13.7500000	-50.0000000
50000.0000000	14.0000000	-50.0000000
50000.0000000	14.2500000	-50.0000000
50000.0000000	14.5000000	-50.0000000
50000.0000000	14.7500000	-50.0000000
50000.0000000	15.0000000	-50.0000000
50030.0000000	0.0000000	-50.0000000
50030.0000000	0.2500000	-50.0000000
50030.0000000	0.5000000	17.8400002
50030.0000000	0.7500000	22.1599998
50030.0000000	1.0000000	17.0499992
50030.0000000	1.2500000	-50.0000000
50030.0000000	1.5000000	-50.0000000
50030.0000000	1.7500000	-50.0000000
50030.0000000	2.0000000	-50.0000000
50030.0000000	2.2500000	-50.0000000
50030.0000000	2.5000000	-50.0000000
50030.0000000	2.7500000	-50.0000000
50030.0000000	3.0000000	-50.0000000
50030.0000000	3.2500000	-50.0000000
50030.0000000	3.5000000	-50.0000000
50030.0000000	3.7500000	-50.0000000
50030.0000000	4.0000000	-50.0000000
50030.0000000	4.2500000	-50.0000000
50030.0000000	4.5000000	-50.0000000
50030.0000000	4.7500000	-50.0000000
50030.0000000	5.0000000	-50.0000000
50030.0000000	5.2500000	-50.0000000
50030.0000000	5.5000000	-50.0000000
50030.0000000	5.7500000	-50.0000000
50030.0000000	6.0000000	-50.0000000
50030.0000000	6.2500000	-50.0000000
50030.0000000	6.5000000	-50.0000000
50030.0000000	6.7500000	-50.0000000
50030.0000000	7.0000000	-50.0000000
50030.0000000	7.2500000	-50.0000000
50030.0000000	7.5000000	-50.0000000
50030.0000000	7.7500000	-50.0000000
50030.0000000	8.0000000	-50.0000000
50030.0000000	8.2500000	-50.0000000
50030.0000000	8.5000000	-50.0000000
50030.0000000	8.7500000	-50.0000000
50030.0000000	9.0000000	-50.0000000
50030.0000000	9.2500000	-50.0000000
50030.0000000	9.5000000	-50.0000000
50030.0000000	9.7500000	-50.0000000
50030.0000000	10.0000000	-50.0000000
50030.0000000	10.2500000	-50.0000000
50030.0000000	10.5000000	-50.0000000
50030.0000000	10.7500000	-50.0000000
50030.0000000	11.0000000	-50.0000000
50030.0000000	11.2500000	-50.0000000
50030.0000000	11.5000000	-50.0000000
50030.0000000	11.7500000	-50.0000000
50030.0000000	12.0000000	-50.0000000

50030.0000000	12.2500000	-50.0000000
50030.0000000	12.5000000	-50.0000000
50030.0000000	12.7500000	-50.0000000
50030.0000000	13.0000000	-50.0000000
50030.0000000	13.2500000	-50.0000000
50030.0000000	13.5000000	-50.0000000
50030.0000000	13.7500000	-50.0000000
50030.0000000	14.0000000	-50.0000000
50030.0000000	14.2500000	-50.0000000
50030.0000000	14.5000000	-50.0000000
50030.0000000	14.7500000	-50.0000000
50030.0000000	15.0000000	-50.0000000
50060.0000000	0.0000000	-50.0000000
50060.0000000	0.2500000	-50.0000000
50060.0000000	0.5000000	16.2700005
50060.0000000	0.7500000	21.1399994
50060.0000000	1.0000000	17.2500000
50060.0000000	1.2500000	-50.0000000
50060.0000000	1.5000000	-50.0000000
50060.0000000	1.7500000	-50.0000000
50060.0000000	2.0000000	-50.0000000
50060.0000000	2.2500000	-50.0000000
50060.0000000	2.5000000	-50.0000000
50060.0000000	2.7500000	-50.0000000
50060.0000000	3.0000000	-50.0000000
50060.0000000	3.2500000	16.5000000
50060.0000000	3.5000000	-50.0000000
50060.0000000	3.7500000	-50.0000000
50060.0000000	4.0000000	-50.0000000
50060.0000000	4.2500000	-50.0000000
50060.0000000	4.5000000	-50.0000000
50060.0000000	4.7500000	-50.0000000
50060.0000000	5.0000000	-50.0000000
50060.0000000	5.2500000	-50.0000000
50060.0000000	5.5000000	-50.0000000
50060.0000000	5.7500000	-50.0000000
50060.0000000	6.0000000	-50.0000000
50060.0000000	6.2500000	-50.0000000
50060.0000000	6.5000000	-50.0000000
50060.0000000	6.7500000	-50.0000000
50060.0000000	7.0000000	-50.0000000
50060.0000000	7.2500000	-50.0000000
50060.0000000	7.5000000	-50.0000000
50060.0000000	7.7500000	-50.0000000
50060.0000000	8.0000000	-50.0000000
50060.0000000	8.2500000	-50.0000000
50060.0000000	8.5000000	-50.0000000
50060.0000000	8.7500000	-50.0000000
50060.0000000	9.0000000	-50.0000000
50060.0000000	9.2500000	-50.0000000
50060.0000000	9.5000000	-50.0000000
50060.0000000	9.7500000	-50.0000000
50060.0000000	10.0000000	-50.0000000
50060.0000000	10.2500000	-50.0000000
50060.0000000	10.5000000	-50.0000000
50060.0000000	10.7500000	-50.0000000
50060.0000000	11.0000000	-50.0000000
50060.0000000	11.2500000	-50.0000000

50060.0000000	11.5000000	-50.0000000
50060.0000000	11.7500000	-50.0000000
50060.0000000	12.0000000	-50.0000000
50060.0000000	12.2500000	-50.0000000
50060.0000000	12.5000000	-50.0000000
50060.0000000	12.7500000	-50.0000000
50060.0000000	13.0000000	-50.0000000
50060.0000000	13.2500000	-50.0000000
50060.0000000	13.5000000	-50.0000000
50060.0000000	13.7500000	-50.0000000
50060.0000000	14.0000000	-50.0000000
50060.0000000	14.2500000	-50.0000000
50060.0000000	14.5000000	-50.0000000
50060.0000000	14.7500000	-50.0000000
50060.0000000	15.0000000	-50.0000000
50090.0000000	0.0000000	-50.0000000
50090.0000000	0.2500000	-50.0000000
50090.0000000	0.5000000	16.5599995
50090.0000000	0.7500000	21.0599995
50090.0000000	1.0000000	16.6700001
50090.0000000	1.2500000	-50.0000000
50090.0000000	1.5000000	-50.0000000
50090.0000000	1.7500000	-50.0000000
50090.0000000	2.0000000	-50.0000000
50090.0000000	2.2500000	-50.0000000
50090.0000000	2.5000000	-50.0000000
50090.0000000	2.7500000	-50.0000000
50090.0000000	3.0000000	-50.0000000
50090.0000000	3.2500000	-50.0000000
50090.0000000	3.5000000	-50.0000000
50090.0000000	3.7500000	-50.0000000
50090.0000000	4.0000000	-50.0000000
50090.0000000	4.2500000	-50.0000000
50090.0000000	4.5000000	-50.0000000
50090.0000000	4.7500000	-50.0000000
50090.0000000	5.0000000	-50.0000000
50090.0000000	5.2500000	-50.0000000
50090.0000000	5.5000000	-50.0000000
50090.0000000	5.7500000	-50.0000000
50090.0000000	6.0000000	-50.0000000
50090.0000000	6.2500000	-50.0000000
50090.0000000	6.5000000	-50.0000000
50090.0000000	6.7500000	-50.0000000
50090.0000000	7.0000000	-50.0000000
50090.0000000	7.2500000	-50.0000000
50090.0000000	7.5000000	-50.0000000
50090.0000000	7.7500000	-50.0000000
50090.0000000	8.0000000	-50.0000000
50090.0000000	8.2500000	-50.0000000
50090.0000000	8.5000000	-50.0000000
50090.0000000	8.7500000	-50.0000000
50090.0000000	9.0000000	-50.0000000
50090.0000000	9.2500000	-50.0000000
50090.0000000	9.5000000	-50.0000000
50090.0000000	9.7500000	-50.0000000
50090.0000000	10.0000000	-50.0000000
50090.0000000	10.2500000	-50.0000000
50090.0000000	10.5000000	-50.0000000

50090.0000000	10.7500000	-50.0000000
50090.0000000	11.0000000	-50.0000000
50090.0000000	11.2500000	-50.0000000
50090.0000000	11.5000000	-50.0000000
50090.0000000	11.7500000	-50.0000000
50090.0000000	12.0000000	-50.0000000
50090.0000000	12.2500000	-50.0000000
50090.0000000	12.5000000	-50.0000000
50090.0000000	12.7500000	-50.0000000
50090.0000000	13.0000000	-50.0000000
50090.0000000	13.2500000	-50.0000000
50090.0000000	13.5000000	-50.0000000
50090.0000000	13.7500000	-50.0000000
50090.0000000	14.0000000	-50.0000000
50090.0000000	14.2500000	-50.0000000
50090.0000000	14.5000000	-50.0000000
50090.0000000	14.7500000	-50.0000000
50090.0000000	15.0000000	-50.0000000
50120.0000000	0.0000000	-50.0000000
50120.0000000	0.2500000	-50.0000000
50120.0000000	0.5000000	-50.0000000
50120.0000000	0.7500000	19.4699993
50120.0000000	1.0000000	15.6599998
50120.0000000	1.2500000	-50.0000000
50120.0000000	1.5000000	-50.0000000
50120.0000000	1.7500000	-50.0000000
50120.0000000	2.0000000	-50.0000000
50120.0000000	2.2500000	15.6599998
50120.0000000	2.5000000	15.0400000
50120.0000000	2.7500000	-50.0000000
50120.0000000	3.0000000	-50.0000000
50120.0000000	3.2500000	-50.0000000
50120.0000000	3.5000000	-50.0000000
50120.0000000	3.7500000	-50.0000000
50120.0000000	4.0000000	-50.0000000
50120.0000000	4.2500000	-50.0000000
50120.0000000	4.5000000	-50.0000000
50120.0000000	4.7500000	-50.0000000
50120.0000000	5.0000000	-50.0000000
50120.0000000	5.2500000	-50.0000000
50120.0000000	5.5000000	-50.0000000
50120.0000000	5.7500000	-50.0000000
50120.0000000	6.0000000	-50.0000000
50120.0000000	6.2500000	-50.0000000
50120.0000000	6.5000000	-50.0000000
50120.0000000	6.7500000	-50.0000000
50120.0000000	7.0000000	-50.0000000
50120.0000000	7.2500000	-50.0000000
50120.0000000	7.5000000	-50.0000000
50120.0000000	7.7500000	-50.0000000
50120.0000000	8.0000000	-50.0000000
50120.0000000	8.2500000	-50.0000000
50120.0000000	8.5000000	-50.0000000
50120.0000000	8.7500000	-50.0000000
50120.0000000	9.0000000	-50.0000000
50120.0000000	9.2500000	-50.0000000
50120.0000000	9.5000000	-50.0000000
50120.0000000	9.7500000	-50.0000000

50120.0000000	10.0000000	-50.0000000
50120.0000000	10.2500000	-50.0000000
50120.0000000	10.5000000	-50.0000000
50120.0000000	10.7500000	-50.0000000
50120.0000000	11.0000000	-50.0000000
50120.0000000	11.2500000	-50.0000000
50120.0000000	11.5000000	-50.0000000
50120.0000000	11.7500000	-50.0000000
50120.0000000	12.0000000	-50.0000000
50120.0000000	12.2500000	-50.0000000
50120.0000000	12.5000000	-50.0000000
50120.0000000	12.7500000	-50.0000000
50120.0000000	13.0000000	-50.0000000
50120.0000000	13.2500000	-50.0000000
50120.0000000	13.5000000	-50.0000000
50120.0000000	13.7500000	-50.0000000
50120.0000000	14.0000000	-50.0000000
50120.0000000	14.2500000	-50.0000000
50120.0000000	14.5000000	-50.0000000
50120.0000000	14.7500000	-50.0000000
50120.0000000	15.0000000	-50.0000000
50150.0000000	0.0000000	-50.0000000
50150.0000000	0.2500000	-50.0000000
50150.0000000	0.5000000	16.1800003
50150.0000000	0.7500000	19.9599991
50150.0000000	1.0000000	16.9200001
50150.0000000	1.2500000	-50.0000000
50150.0000000	1.5000000	-50.0000000
50150.0000000	1.7500000	-50.0000000
50150.0000000	2.0000000	-50.0000000
50150.0000000	2.2500000	-50.0000000
50150.0000000	2.5000000	-50.0000000
50150.0000000	2.7500000	-50.0000000
50150.0000000	3.0000000	-50.0000000
50150.0000000	3.2500000	-50.0000000
50150.0000000	3.5000000	-50.0000000
50150.0000000	3.7500000	-50.0000000
50150.0000000	4.0000000	-50.0000000
50150.0000000	4.2500000	-50.0000000
50150.0000000	4.5000000	-50.0000000
50150.0000000	4.7500000	-50.0000000
50150.0000000	5.0000000	-50.0000000
50150.0000000	5.2500000	-50.0000000
50150.0000000	5.5000000	-50.0000000
50150.0000000	5.7500000	-50.0000000
50150.0000000	6.0000000	-50.0000000
50150.0000000	6.2500000	-50.0000000
50150.0000000	6.5000000	-50.0000000
50150.0000000	6.7500000	-50.0000000
50150.0000000	7.0000000	-50.0000000
50150.0000000	7.2500000	-50.0000000
50150.0000000	7.5000000	-50.0000000
50150.0000000	7.7500000	-50.0000000
50150.0000000	8.0000000	-50.0000000
50150.0000000	8.2500000	-50.0000000
50150.0000000	8.5000000	-50.0000000
50150.0000000	8.7500000	-50.0000000
50150.0000000	9.0000000	-50.0000000

50150.0000000	9.2500000	-50.0000000
50150.0000000	9.5000000	-50.0000000
50150.0000000	9.7500000	-50.0000000
50150.0000000	10.0000000	-50.0000000
50150.0000000	10.2500000	-50.0000000
50150.0000000	10.5000000	-50.0000000
50150.0000000	10.7500000	-50.0000000
50150.0000000	11.0000000	-50.0000000
50150.0000000	11.2500000	-50.0000000
50150.0000000	11.5000000	-50.0000000
50150.0000000	11.7500000	-50.0000000
50150.0000000	12.0000000	-50.0000000
50150.0000000	12.2500000	-50.0000000
50150.0000000	12.5000000	-50.0000000
50150.0000000	12.7500000	-50.0000000
50150.0000000	13.0000000	-50.0000000
50150.0000000	13.2500000	-50.0000000
50150.0000000	13.5000000	-50.0000000
50150.0000000	13.7500000	-50.0000000
50150.0000000	14.0000000	-50.0000000
50150.0000000	14.2500000	-50.0000000
50150.0000000	14.5000000	-50.0000000
50150.0000000	14.7500000	-50.0000000
50150.0000000	15.0000000	-50.0000000
50180.0000000	0.0000000	-50.0000000
50180.0000000	0.2500000	-50.0000000
50180.0000000	0.5000000	-50.0000000
50180.0000000	0.7500000	17.0200005
50180.0000000	1.0000000	15.8000002
50180.0000000	1.2500000	-50.0000000
50180.0000000	1.5000000	-50.0000000
50180.0000000	1.7500000	-50.0000000
50180.0000000	2.0000000	-50.0000000
50180.0000000	2.2500000	-50.0000000
50180.0000000	2.5000000	-50.0000000
50180.0000000	2.7500000	-50.0000000
50180.0000000	3.0000000	-50.0000000
50180.0000000	3.2500000	-50.0000000
50180.0000000	3.5000000	-50.0000000
50180.0000000	3.7500000	-50.0000000
50180.0000000	4.0000000	-50.0000000
50180.0000000	4.2500000	-50.0000000
50180.0000000	4.5000000	-50.0000000
50180.0000000	4.7500000	-50.0000000
50180.0000000	5.0000000	-50.0000000
50180.0000000	5.2500000	-50.0000000
50180.0000000	5.5000000	-50.0000000
50180.0000000	5.7500000	-50.0000000
50180.0000000	6.0000000	-50.0000000
50180.0000000	6.2500000	-50.0000000
50180.0000000	6.5000000	-50.0000000
50180.0000000	6.7500000	-50.0000000
50180.0000000	7.0000000	-50.0000000
50180.0000000	7.2500000	-50.0000000
50180.0000000	7.5000000	-50.0000000
50180.0000000	7.7500000	-50.0000000
50180.0000000	8.0000000	-50.0000000
50180.0000000	8.2500000	-50.0000000

50180.0000000	8.5000000	-50.0000000
50180.0000000	8.7500000	-50.0000000
50180.0000000	9.0000000	-50.0000000
50180.0000000	9.2500000	-50.0000000
50180.0000000	9.5000000	-50.0000000
50180.0000000	9.7500000	-50.0000000
50180.0000000	10.0000000	-50.0000000
50180.0000000	10.2500000	-50.0000000
50180.0000000	10.5000000	-50.0000000
50180.0000000	10.7500000	-50.0000000
50180.0000000	11.0000000	-50.0000000
50180.0000000	11.2500000	-50.0000000
50180.0000000	11.5000000	-50.0000000
50180.0000000	11.7500000	-50.0000000
50180.0000000	12.0000000	-50.0000000
50180.0000000	12.2500000	-50.0000000
50180.0000000	12.5000000	-50.0000000
50180.0000000	12.7500000	-50.0000000
50180.0000000	13.0000000	-50.0000000
50180.0000000	13.2500000	-50.0000000
50180.0000000	13.5000000	-50.0000000
50180.0000000	13.7500000	-50.0000000
50180.0000000	14.0000000	-50.0000000
50180.0000000	14.2500000	-50.0000000
50180.0000000	14.5000000	-50.0000000
50180.0000000	14.7500000	-50.0000000
50180.0000000	15.0000000	-50.0000000
50210.0000000	0.0000000	-50.0000000
50210.0000000	0.2500000	-50.0000000
50210.0000000	0.5000000	19.0900002
50210.0000000	0.7500000	22.5499992
50210.0000000	1.0000000	17.0799999
50210.0000000	1.2500000	-50.0000000
50210.0000000	1.5000000	-50.0000000
50210.0000000	1.7500000	-50.0000000
50210.0000000	2.0000000	-50.0000000
50210.0000000	2.2500000	-50.0000000
50210.0000000	2.5000000	-50.0000000
50210.0000000	2.7500000	-50.0000000
50210.0000000	3.0000000	-50.0000000
50210.0000000	3.2500000	-50.0000000
50210.0000000	3.5000000	-50.0000000
50210.0000000	3.7500000	-50.0000000
50210.0000000	4.0000000	-50.0000000
50210.0000000	4.2500000	-50.0000000
50210.0000000	4.5000000	-50.0000000
50210.0000000	4.7500000	-50.0000000
50210.0000000	5.0000000	-50.0000000
50210.0000000	5.2500000	-50.0000000
50210.0000000	5.5000000	-50.0000000
50210.0000000	5.7500000	-50.0000000
50210.0000000	6.0000000	-50.0000000
50210.0000000	6.2500000	-50.0000000
50210.0000000	6.5000000	-50.0000000
50210.0000000	6.7500000	-50.0000000
50210.0000000	7.0000000	-50.0000000
50210.0000000	7.2500000	-50.0000000
50210.0000000	7.5000000	-50.0000000

50210.0000000	7.7500000	-50.0000000
50210.0000000	8.0000000	-50.0000000
50210.0000000	8.2500000	-50.0000000
50210.0000000	8.5000000	-50.0000000
50210.0000000	8.7500000	-50.0000000
50210.0000000	9.0000000	-50.0000000
50210.0000000	9.2500000	-50.0000000
50210.0000000	9.5000000	-50.0000000
50210.0000000	9.7500000	-50.0000000
50210.0000000	10.0000000	-50.0000000
50210.0000000	10.2500000	-50.0000000
50210.0000000	10.5000000	-50.0000000
50210.0000000	10.7500000	-50.0000000
50210.0000000	11.0000000	-50.0000000
50210.0000000	11.2500000	-50.0000000
50210.0000000	11.5000000	-50.0000000
50210.0000000	11.7500000	-50.0000000
50210.0000000	12.0000000	-50.0000000
50210.0000000	12.2500000	-50.0000000
50210.0000000	12.5000000	-50.0000000
50210.0000000	12.7500000	-50.0000000
50210.0000000	13.0000000	-50.0000000
50210.0000000	13.2500000	-50.0000000
50210.0000000	13.5000000	-50.0000000
50210.0000000	13.7500000	-50.0000000
50210.0000000	14.0000000	-50.0000000
50210.0000000	14.2500000	-50.0000000
50210.0000000	14.5000000	-50.0000000
50210.0000000	14.7500000	-50.0000000
50210.0000000	15.0000000	-50.0000000
50240.0000000	0.0000000	-50.0000000
50240.0000000	0.2500000	-50.0000000
50240.0000000	0.5000000	15.4099998
50240.0000000	0.7500000	19.4300003
50240.0000000	1.0000000	16.0000000
50240.0000000	1.2500000	-50.0000000
50240.0000000	1.5000000	-50.0000000
50240.0000000	1.7500000	-50.0000000
50240.0000000	2.0000000	-50.0000000
50240.0000000	2.2500000	-50.0000000
50240.0000000	2.5000000	-50.0000000
50240.0000000	2.7500000	-50.0000000
50240.0000000	3.0000000	-50.0000000
50240.0000000	3.2500000	-50.0000000
50240.0000000	3.5000000	15.4200001
50240.0000000	3.7500000	-50.0000000
50240.0000000	4.0000000	-50.0000000
50240.0000000	4.2500000	-50.0000000
50240.0000000	4.5000000	-50.0000000
50240.0000000	4.7500000	-50.0000000
50240.0000000	5.0000000	-50.0000000
50240.0000000	5.2500000	-50.0000000
50240.0000000	5.5000000	-50.0000000
50240.0000000	5.7500000	-50.0000000
50240.0000000	6.0000000	-50.0000000
50240.0000000	6.2500000	-50.0000000
50240.0000000	6.5000000	-50.0000000
50240.0000000	6.7500000	-50.0000000

50240.0000000	7.0000000	-50.0000000
50240.0000000	7.2500000	-50.0000000
50240.0000000	7.5000000	-50.0000000
50240.0000000	7.7500000	-50.0000000
50240.0000000	8.0000000	-50.0000000
50240.0000000	8.2500000	-50.0000000
50240.0000000	8.5000000	-50.0000000
50240.0000000	8.7500000	-50.0000000
50240.0000000	9.0000000	-50.0000000
50240.0000000	9.2500000	-50.0000000
50240.0000000	9.5000000	-50.0000000
50240.0000000	9.7500000	-50.0000000
50240.0000000	10.0000000	-50.0000000
50240.0000000	10.2500000	-50.0000000
50240.0000000	10.5000000	-50.0000000
50240.0000000	10.7500000	-50.0000000
50240.0000000	11.0000000	-50.0000000
50240.0000000	11.2500000	-50.0000000
50240.0000000	11.5000000	-50.0000000
50240.0000000	11.7500000	-50.0000000
50240.0000000	12.0000000	-50.0000000
50240.0000000	12.2500000	-50.0000000
50240.0000000	12.5000000	-50.0000000
50240.0000000	12.7500000	-50.0000000
50240.0000000	13.0000000	-50.0000000
50240.0000000	13.2500000	-50.0000000
50240.0000000	13.5000000	-50.0000000
50240.0000000	13.7500000	-50.0000000
50240.0000000	14.0000000	-50.0000000
50240.0000000	14.2500000	-50.0000000
50240.0000000	14.5000000	-50.0000000
50240.0000000	14.7500000	-50.0000000
50240.0000000	15.0000000	-50.0000000
50270.0000000	0.0000000	-50.0000000
50270.0000000	0.2500000	-50.0000000
50270.0000000	0.5000000	17.1000004
50270.0000000	0.7500000	20.4899998
50270.0000000	1.0000000	17.0100002
50270.0000000	1.2500000	-50.0000000
50270.0000000	1.5000000	-50.0000000
50270.0000000	1.7500000	16.4699993
50270.0000000	2.0000000	16.5699997
50270.0000000	2.2500000	15.1300001
50270.0000000	2.5000000	-50.0000000
50270.0000000	2.7500000	-50.0000000
50270.0000000	3.0000000	-50.0000000
50270.0000000	3.2500000	-50.0000000
50270.0000000	3.5000000	-50.0000000
50270.0000000	3.7500000	-50.0000000
50270.0000000	4.0000000	-50.0000000
50270.0000000	4.2500000	-50.0000000
50270.0000000	4.5000000	-50.0000000
50270.0000000	4.7500000	-50.0000000
50270.0000000	5.0000000	-50.0000000
50270.0000000	5.2500000	-50.0000000
50270.0000000	5.5000000	-50.0000000
50270.0000000	5.7500000	-50.0000000
50270.0000000	6.0000000	-50.0000000

50270.0000000	6.2500000	-50.0000000
50270.0000000	6.5000000	-50.0000000
50270.0000000	6.7500000	-50.0000000
50270.0000000	7.0000000	-50.0000000
50270.0000000	7.2500000	-50.0000000
50270.0000000	7.5000000	-50.0000000
50270.0000000	7.7500000	-50.0000000
50270.0000000	8.0000000	-50.0000000
50270.0000000	8.2500000	-50.0000000
50270.0000000	8.5000000	-50.0000000
50270.0000000	8.7500000	-50.0000000
50270.0000000	9.0000000	-50.0000000
50270.0000000	9.2500000	-50.0000000
50270.0000000	9.5000000	-50.0000000
50270.0000000	9.7500000	-50.0000000
50270.0000000	10.0000000	-50.0000000
50270.0000000	10.2500000	-50.0000000
50270.0000000	10.5000000	-50.0000000
50270.0000000	10.7500000	-50.0000000
50270.0000000	11.0000000	-50.0000000
50270.0000000	11.2500000	-50.0000000
50270.0000000	11.5000000	-50.0000000
50270.0000000	11.7500000	-50.0000000
50270.0000000	12.0000000	-50.0000000
50270.0000000	12.2500000	-50.0000000
50270.0000000	12.5000000	-50.0000000
50270.0000000	12.7500000	-50.0000000
50270.0000000	13.0000000	-50.0000000
50270.0000000	13.2500000	-50.0000000
50270.0000000	13.5000000	-50.0000000
50270.0000000	13.7500000	-50.0000000
50270.0000000	14.0000000	-50.0000000
50270.0000000	14.2500000	-50.0000000
50270.0000000	14.5000000	-50.0000000
50270.0000000	14.7500000	-50.0000000
50270.0000000	15.0000000	-50.0000000
50300.0000000	0.0000000	-50.0000000
50300.0000000	0.2500000	-50.0000000
50300.0000000	0.5000000	19.0300007
50300.0000000	0.7500000	22.8099995
50300.0000000	1.0000000	18.6599998
50300.0000000	1.2500000	15.8000002
50300.0000000	1.5000000	16.7900009
50300.0000000	1.7500000	16.4200001
50300.0000000	2.0000000	-50.0000000
50300.0000000	2.2500000	-50.0000000
50300.0000000	2.5000000	-50.0000000
50300.0000000	2.7500000	-50.0000000
50300.0000000	3.0000000	-50.0000000
50300.0000000	3.2500000	-50.0000000
50300.0000000	3.5000000	-50.0000000
50300.0000000	3.7500000	-50.0000000
50300.0000000	4.0000000	-50.0000000
50300.0000000	4.2500000	-50.0000000
50300.0000000	4.5000000	-50.0000000
50300.0000000	4.7500000	-50.0000000
50300.0000000	5.0000000	-50.0000000
50300.0000000	5.2500000	-50.0000000

50300.0000000	5.5000000	-50.0000000
50300.0000000	5.7500000	-50.0000000
50300.0000000	6.0000000	-50.0000000
50300.0000000	6.2500000	-50.0000000
50300.0000000	6.5000000	-50.0000000
50300.0000000	6.7500000	-50.0000000
50300.0000000	7.0000000	-50.0000000
50300.0000000	7.2500000	-50.0000000
50300.0000000	7.5000000	-50.0000000
50300.0000000	7.7500000	-50.0000000
50300.0000000	8.0000000	-50.0000000
50300.0000000	8.2500000	-50.0000000
50300.0000000	8.5000000	-50.0000000
50300.0000000	8.7500000	-50.0000000
50300.0000000	9.0000000	-50.0000000
50300.0000000	9.2500000	-50.0000000
50300.0000000	9.5000000	-50.0000000
50300.0000000	9.7500000	-50.0000000
50300.0000000	10.0000000	-50.0000000
50300.0000000	10.2500000	-50.0000000
50300.0000000	10.5000000	-50.0000000
50300.0000000	10.7500000	-50.0000000
50300.0000000	11.0000000	-50.0000000
50300.0000000	11.2500000	-50.0000000
50300.0000000	11.5000000	-50.0000000
50300.0000000	11.7500000	-50.0000000
50300.0000000	12.0000000	-50.0000000
50300.0000000	12.2500000	-50.0000000
50300.0000000	12.5000000	-50.0000000
50300.0000000	12.7500000	-50.0000000
50300.0000000	13.0000000	-50.0000000
50300.0000000	13.2500000	-50.0000000
50300.0000000	13.5000000	-50.0000000
50300.0000000	13.7500000	-50.0000000
50300.0000000	14.0000000	-50.0000000
50300.0000000	14.2500000	-50.0000000
50300.0000000	14.5000000	-50.0000000
50300.0000000	14.7500000	-50.0000000
50300.0000000	15.0000000	-50.0000000
50330.0000000	0.0000000	-50.0000000
50330.0000000	0.2500000	-50.0000000
50330.0000000	0.5000000	16.1000004
50330.0000000	0.7500000	19.2399998
50330.0000000	1.0000000	15.4200001
50330.0000000	1.2500000	15.5200005
50330.0000000	1.5000000	-50.0000000
50330.0000000	1.7500000	-50.0000000
50330.0000000	2.0000000	-50.0000000
50330.0000000	2.2500000	-50.0000000
50330.0000000	2.5000000	-50.0000000
50330.0000000	2.7500000	-50.0000000
50330.0000000	3.0000000	-50.0000000
50330.0000000	3.2500000	-50.0000000
50330.0000000	3.5000000	-50.0000000
50330.0000000	3.7500000	-50.0000000
50330.0000000	4.0000000	15.6499996
50330.0000000	4.2500000	-50.0000000
50330.0000000	4.5000000	-50.0000000

50330.0000000	4.7500000	-50.0000000
50330.0000000	5.0000000	-50.0000000
50330.0000000	5.2500000	-50.0000000
50330.0000000	5.5000000	-50.0000000
50330.0000000	5.7500000	-50.0000000
50330.0000000	6.0000000	-50.0000000
50330.0000000	6.2500000	-50.0000000
50330.0000000	6.5000000	-50.0000000
50330.0000000	6.7500000	-50.0000000
50330.0000000	7.0000000	-50.0000000
50330.0000000	7.2500000	-50.0000000
50330.0000000	7.5000000	-50.0000000
50330.0000000	7.7500000	-50.0000000
50330.0000000	8.0000000	-50.0000000
50330.0000000	8.2500000	-50.0000000
50330.0000000	8.5000000	-50.0000000
50330.0000000	8.7500000	-50.0000000
50330.0000000	9.0000000	-50.0000000
50330.0000000	9.2500000	-50.0000000
50330.0000000	9.5000000	-50.0000000
50330.0000000	9.7500000	-50.0000000
50330.0000000	10.0000000	-50.0000000
50330.0000000	10.2500000	-50.0000000
50330.0000000	10.5000000	-50.0000000
50330.0000000	10.7500000	-50.0000000
50330.0000000	11.0000000	-50.0000000
50330.0000000	11.2500000	-50.0000000
50330.0000000	11.5000000	-50.0000000
50330.0000000	11.7500000	-50.0000000
50330.0000000	12.0000000	-50.0000000
50330.0000000	12.2500000	-50.0000000
50330.0000000	12.5000000	-50.0000000
50330.0000000	12.7500000	-50.0000000
50330.0000000	13.0000000	-50.0000000
50330.0000000	13.2500000	-50.0000000
50330.0000000	13.5000000	-50.0000000
50330.0000000	13.7500000	-50.0000000
50330.0000000	14.0000000	-50.0000000
50330.0000000	14.2500000	-50.0000000
50330.0000000	14.5000000	-50.0000000
50330.0000000	14.7500000	-50.0000000
50330.0000000	15.0000000	-50.0000000
50360.0000000	0.0000000	-50.0000000
50360.0000000	0.2500000	-50.0000000
50360.0000000	0.5000000	15.2900000
50360.0000000	0.7500000	19.0400009
50360.0000000	1.0000000	-50.0000000
50360.0000000	1.2500000	-50.0000000
50360.0000000	1.5000000	15.4700003
50360.0000000	1.7500000	17.1499996
50360.0000000	2.0000000	15.1999998
50360.0000000	2.2500000	-50.0000000
50360.0000000	2.5000000	15.1000004
50360.0000000	2.7500000	-50.0000000
50360.0000000	3.0000000	-50.0000000
50360.0000000	3.2500000	-50.0000000
50360.0000000	3.5000000	-50.0000000
50360.0000000	3.7500000	15.6599998

50360.0000000	4.0000000	-50.0000000
50360.0000000	4.2500000	-50.0000000
50360.0000000	4.5000000	-50.0000000
50360.0000000	4.7500000	-50.0000000
50360.0000000	5.0000000	-50.0000000
50360.0000000	5.2500000	-50.0000000
50360.0000000	5.5000000	-50.0000000
50360.0000000	5.7500000	-50.0000000
50360.0000000	6.0000000	-50.0000000
50360.0000000	6.2500000	-50.0000000
50360.0000000	6.5000000	-50.0000000
50360.0000000	6.7500000	-50.0000000
50360.0000000	7.0000000	-50.0000000
50360.0000000	7.2500000	-50.0000000
50360.0000000	7.5000000	-50.0000000
50360.0000000	7.7500000	-50.0000000
50360.0000000	8.0000000	-50.0000000
50360.0000000	8.2500000	-50.0000000
50360.0000000	8.5000000	-50.0000000
50360.0000000	8.7500000	-50.0000000
50360.0000000	9.0000000	-50.0000000
50360.0000000	9.2500000	-50.0000000
50360.0000000	9.5000000	-50.0000000
50360.0000000	9.7500000	-50.0000000
50360.0000000	10.0000000	-50.0000000
50360.0000000	10.2500000	-50.0000000
50360.0000000	10.5000000	-50.0000000
50360.0000000	10.7500000	-50.0000000
50360.0000000	11.0000000	-50.0000000
50360.0000000	11.2500000	-50.0000000
50360.0000000	11.5000000	-50.0000000
50360.0000000	11.7500000	-50.0000000
50360.0000000	12.0000000	-50.0000000
50360.0000000	12.2500000	-50.0000000
50360.0000000	12.5000000	-50.0000000
50360.0000000	12.7500000	-50.0000000
50360.0000000	13.0000000	-50.0000000
50360.0000000	13.2500000	-50.0000000
50360.0000000	13.5000000	-50.0000000
50360.0000000	13.7500000	-50.0000000
50360.0000000	14.0000000	-50.0000000
50360.0000000	14.2500000	-50.0000000
50360.0000000	14.5000000	-50.0000000
50360.0000000	14.7500000	-50.0000000
50360.0000000	15.0000000	-50.0000000
50390.0000000	0.0000000	-50.0000000
50390.0000000	0.2500000	-50.0000000
50390.0000000	0.5000000	15.7200003
50390.0000000	0.7500000	16.7000008
50390.0000000	1.0000000	-50.0000000
50390.0000000	1.2500000	15.2100000
50390.0000000	1.5000000	17.5599995
50390.0000000	1.7500000	16.1599998
50390.0000000	2.0000000	-50.0000000
50390.0000000	2.2500000	-50.0000000
50390.0000000	2.5000000	-50.0000000
50390.0000000	2.7500000	-50.0000000
50390.0000000	3.0000000	-50.0000000

50390.0000000	3.2500000	-50.0000000
50390.0000000	3.5000000	-50.0000000
50390.0000000	3.7500000	-50.0000000
50390.0000000	4.0000000	-50.0000000
50390.0000000	4.2500000	-50.0000000
50390.0000000	4.5000000	-50.0000000
50390.0000000	4.7500000	-50.0000000
50390.0000000	5.0000000	-50.0000000
50390.0000000	5.2500000	-50.0000000
50390.0000000	5.5000000	-50.0000000
50390.0000000	5.7500000	-50.0000000
50390.0000000	6.0000000	-50.0000000
50390.0000000	6.2500000	-50.0000000
50390.0000000	6.5000000	-50.0000000
50390.0000000	6.7500000	-50.0000000
50390.0000000	7.0000000	-50.0000000
50390.0000000	7.2500000	-50.0000000
50390.0000000	7.5000000	-50.0000000
50390.0000000	7.7500000	-50.0000000
50390.0000000	8.0000000	-50.0000000
50390.0000000	8.2500000	-50.0000000
50390.0000000	8.5000000	-50.0000000
50390.0000000	8.7500000	-50.0000000
50390.0000000	9.0000000	-50.0000000
50390.0000000	9.2500000	-50.0000000
50390.0000000	9.5000000	-50.0000000
50390.0000000	9.7500000	-50.0000000
50390.0000000	10.0000000	-50.0000000
50390.0000000	10.2500000	-50.0000000
50390.0000000	10.5000000	-50.0000000
50390.0000000	10.7500000	-50.0000000
50390.0000000	11.0000000	-50.0000000
50390.0000000	11.2500000	-50.0000000
50390.0000000	11.5000000	-50.0000000
50390.0000000	11.7500000	-50.0000000
50390.0000000	12.0000000	-50.0000000
50390.0000000	12.2500000	-50.0000000
50390.0000000	12.5000000	-50.0000000
50390.0000000	12.7500000	-50.0000000
50390.0000000	13.0000000	-50.0000000
50390.0000000	13.2500000	-50.0000000
50390.0000000	13.5000000	-50.0000000
50390.0000000	13.7500000	-50.0000000
50390.0000000	14.0000000	-50.0000000
50390.0000000	14.2500000	-50.0000000
50390.0000000	14.5000000	-50.0000000
50390.0000000	14.7500000	-50.0000000
50390.0000000	15.0000000	-50.0000000
50420.0000000	0.0000000	-50.0000000
50420.0000000	0.2500000	-50.0000000
50420.0000000	0.5000000	15.4300003
50420.0000000	0.7500000	18.9200001
50420.0000000	1.0000000	15.3599997
50420.0000000	1.2500000	16.5400009
50420.0000000	1.5000000	15.0200005
50420.0000000	1.7500000	-50.0000000
50420.0000000	2.0000000	-50.0000000
50420.0000000	2.2500000	-50.0000000

50420.0000000	2.5000000	-50.0000000
50420.0000000	2.7500000	-50.0000000
50420.0000000	3.0000000	-50.0000000
50420.0000000	3.2500000	-50.0000000
50420.0000000	3.5000000	-50.0000000
50420.0000000	3.7500000	-50.0000000
50420.0000000	4.0000000	-50.0000000
50420.0000000	4.2500000	-50.0000000
50420.0000000	4.5000000	-50.0000000
50420.0000000	4.7500000	-50.0000000
50420.0000000	5.0000000	-50.0000000
50420.0000000	5.2500000	-50.0000000
50420.0000000	5.5000000	-50.0000000
50420.0000000	5.7500000	-50.0000000
50420.0000000	6.0000000	-50.0000000
50420.0000000	6.2500000	-50.0000000
50420.0000000	6.5000000	-50.0000000
50420.0000000	6.7500000	-50.0000000
50420.0000000	7.0000000	-50.0000000
50420.0000000	7.2500000	-50.0000000
50420.0000000	7.5000000	-50.0000000
50420.0000000	7.7500000	-50.0000000
50420.0000000	8.0000000	-50.0000000
50420.0000000	8.2500000	-50.0000000
50420.0000000	8.5000000	-50.0000000
50420.0000000	8.7500000	-50.0000000
50420.0000000	9.0000000	-50.0000000
50420.0000000	9.2500000	-50.0000000
50420.0000000	9.5000000	-50.0000000
50420.0000000	9.7500000	-50.0000000
50420.0000000	10.0000000	-50.0000000
50420.0000000	10.2500000	-50.0000000
50420.0000000	10.5000000	-50.0000000
50420.0000000	10.7500000	-50.0000000
50420.0000000	11.0000000	-50.0000000
50420.0000000	11.2500000	-50.0000000
50420.0000000	11.5000000	-50.0000000
50420.0000000	11.7500000	-50.0000000
50420.0000000	12.0000000	-50.0000000
50420.0000000	12.2500000	-50.0000000
50420.0000000	12.5000000	-50.0000000
50420.0000000	12.7500000	-50.0000000
50420.0000000	13.0000000	-50.0000000
50420.0000000	13.2500000	-50.0000000
50420.0000000	13.5000000	-50.0000000
50420.0000000	13.7500000	-50.0000000
50420.0000000	14.0000000	-50.0000000
50420.0000000	14.2500000	-50.0000000
50420.0000000	14.5000000	-50.0000000
50420.0000000	14.7500000	-50.0000000
50420.0000000	15.0000000	-50.0000000
50450.0000000	0.0000000	-50.0000000
50450.0000000	0.2500000	-50.0000000
50450.0000000	0.5000000	-50.0000000
50450.0000000	0.7500000	18.1700001
50450.0000000	1.0000000	16.0000000
50450.0000000	1.2500000	15.8800001
50450.0000000	1.5000000	16.6000004

50450.0000000	1.7500000	16.0900002
50450.0000000	2.0000000	-50.0000000
50450.0000000	2.2500000	-50.0000000
50450.0000000	2.5000000	-50.0000000
50450.0000000	2.7500000	-50.0000000
50450.0000000	3.0000000	-50.0000000
50450.0000000	3.2500000	15.9399996
50450.0000000	3.5000000	-50.0000000
50450.0000000	3.7500000	-50.0000000
50450.0000000	4.0000000	-50.0000000
50450.0000000	4.2500000	-50.0000000
50450.0000000	4.5000000	-50.0000000
50450.0000000	4.7500000	-50.0000000
50450.0000000	5.0000000	-50.0000000
50450.0000000	5.2500000	-50.0000000
50450.0000000	5.5000000	-50.0000000
50450.0000000	5.7500000	-50.0000000
50450.0000000	6.0000000	-50.0000000
50450.0000000	6.2500000	-50.0000000
50450.0000000	6.5000000	-50.0000000
50450.0000000	6.7500000	-50.0000000
50450.0000000	7.0000000	-50.0000000
50450.0000000	7.2500000	-50.0000000
50450.0000000	7.5000000	-50.0000000
50450.0000000	7.7500000	-50.0000000
50450.0000000	8.0000000	-50.0000000
50450.0000000	8.2500000	-50.0000000
50450.0000000	8.5000000	-50.0000000
50450.0000000	8.7500000	-50.0000000
50450.0000000	9.0000000	-50.0000000
50450.0000000	9.2500000	-50.0000000
50450.0000000	9.5000000	-50.0000000
50450.0000000	9.7500000	-50.0000000
50450.0000000	10.0000000	-50.0000000
50450.0000000	10.2500000	-50.0000000
50450.0000000	10.5000000	-50.0000000
50450.0000000	10.7500000	-50.0000000
50450.0000000	11.0000000	-50.0000000
50450.0000000	11.2500000	-50.0000000
50450.0000000	11.5000000	-50.0000000
50450.0000000	11.7500000	-50.0000000
50450.0000000	12.0000000	-50.0000000
50450.0000000	12.2500000	-50.0000000
50450.0000000	12.5000000	-50.0000000
50450.0000000	12.7500000	-50.0000000
50450.0000000	13.0000000	-50.0000000
50450.0000000	13.2500000	-50.0000000
50450.0000000	13.5000000	-50.0000000
50450.0000000	13.7500000	-50.0000000
50450.0000000	14.0000000	-50.0000000
50450.0000000	14.2500000	-50.0000000
50450.0000000	14.5000000	-50.0000000
50450.0000000	14.7500000	-50.0000000
50450.0000000	15.0000000	-50.0000000
50480.0000000	0.0000000	-50.0000000
50480.0000000	0.2500000	-50.0000000
50480.0000000	0.5000000	16.8899994
50480.0000000	0.7500000	19.9899998

50480.0000000	1.0000000	16.3799992
50480.0000000	1.2500000	15.3599997
50480.0000000	1.5000000	15.8100004
50480.0000000	1.7500000	-50.0000000
50480.0000000	2.0000000	-50.0000000
50480.0000000	2.2500000	-50.0000000
50480.0000000	2.5000000	-50.0000000
50480.0000000	2.7500000	-50.0000000
50480.0000000	3.0000000	-50.0000000
50480.0000000	3.2500000	-50.0000000
50480.0000000	3.5000000	-50.0000000
50480.0000000	3.7500000	-50.0000000
50480.0000000	4.0000000	-50.0000000
50480.0000000	4.2500000	-50.0000000
50480.0000000	4.5000000	-50.0000000
50480.0000000	4.7500000	-50.0000000
50480.0000000	5.0000000	-50.0000000
50480.0000000	5.2500000	-50.0000000
50480.0000000	5.5000000	-50.0000000
50480.0000000	5.7500000	-50.0000000
50480.0000000	6.0000000	-50.0000000
50480.0000000	6.2500000	-50.0000000
50480.0000000	6.5000000	-50.0000000
50480.0000000	6.7500000	-50.0000000
50480.0000000	7.0000000	-50.0000000
50480.0000000	7.2500000	-50.0000000
50480.0000000	7.5000000	-50.0000000
50480.0000000	7.7500000	-50.0000000
50480.0000000	8.0000000	-50.0000000
50480.0000000	8.2500000	-50.0000000
50480.0000000	8.5000000	-50.0000000
50480.0000000	8.7500000	-50.0000000
50480.0000000	9.0000000	-50.0000000
50480.0000000	9.2500000	-50.0000000
50480.0000000	9.5000000	-50.0000000
50480.0000000	9.7500000	-50.0000000
50480.0000000	10.0000000	-50.0000000
50480.0000000	10.2500000	-50.0000000
50480.0000000	10.5000000	-50.0000000
50480.0000000	10.7500000	-50.0000000
50480.0000000	11.0000000	-50.0000000
50480.0000000	11.2500000	-50.0000000
50480.0000000	11.5000000	-50.0000000
50480.0000000	11.7500000	-50.0000000
50480.0000000	12.0000000	-50.0000000
50480.0000000	12.2500000	-50.0000000
50480.0000000	12.5000000	-50.0000000
50480.0000000	12.7500000	-50.0000000
50480.0000000	13.0000000	-50.0000000
50480.0000000	13.2500000	-50.0000000
50480.0000000	13.5000000	-50.0000000
50480.0000000	13.7500000	-50.0000000
50480.0000000	14.0000000	-50.0000000
50480.0000000	14.2500000	-50.0000000
50480.0000000	14.5000000	-50.0000000
50480.0000000	14.7500000	-50.0000000
50480.0000000	15.0000000	-50.0000000
50510.0000000	0.0000000	-50.0000000

50510.0000000	0.2500000	-50.0000000
50510.0000000	0.5000000	-50.0000000
50510.0000000	0.7500000	18.3099995
50510.0000000	1.0000000	15.7200003
50510.0000000	1.2500000	17.1399994
50510.0000000	1.5000000	17.8899994
50510.0000000	1.7500000	16.8199997
50510.0000000	2.0000000	17.9799995
50510.0000000	2.2500000	-50.0000000
50510.0000000	2.5000000	-50.0000000
50510.0000000	2.7500000	-50.0000000
50510.0000000	3.0000000	-50.0000000
50510.0000000	3.2500000	-50.0000000
50510.0000000	3.5000000	-50.0000000
50510.0000000	3.7500000	-50.0000000
50510.0000000	4.0000000	-50.0000000
50510.0000000	4.2500000	-50.0000000
50510.0000000	4.5000000	-50.0000000
50510.0000000	4.7500000	-50.0000000
50510.0000000	5.0000000	-50.0000000
50510.0000000	5.2500000	-50.0000000
50510.0000000	5.5000000	-50.0000000
50510.0000000	5.7500000	-50.0000000
50510.0000000	6.0000000	-50.0000000
50510.0000000	6.2500000	-50.0000000
50510.0000000	6.5000000	-50.0000000
50510.0000000	6.7500000	-50.0000000
50510.0000000	7.0000000	-50.0000000
50510.0000000	7.2500000	-50.0000000
50510.0000000	7.5000000	-50.0000000
50510.0000000	7.7500000	-50.0000000
50510.0000000	8.0000000	-50.0000000
50510.0000000	8.2500000	-50.0000000
50510.0000000	8.5000000	-50.0000000
50510.0000000	8.7500000	-50.0000000
50510.0000000	9.0000000	-50.0000000
50510.0000000	9.2500000	-50.0000000
50510.0000000	9.5000000	-50.0000000
50510.0000000	9.7500000	-50.0000000
50510.0000000	10.0000000	-50.0000000
50510.0000000	10.2500000	-50.0000000
50510.0000000	10.5000000	-50.0000000
50510.0000000	10.7500000	-50.0000000
50510.0000000	11.0000000	-50.0000000
50510.0000000	11.2500000	-50.0000000
50510.0000000	11.5000000	-50.0000000
50510.0000000	11.7500000	-50.0000000
50510.0000000	12.0000000	-50.0000000
50510.0000000	12.2500000	-50.0000000
50510.0000000	12.5000000	-50.0000000
50510.0000000	12.7500000	-50.0000000
50510.0000000	13.0000000	-50.0000000
50510.0000000	13.2500000	-50.0000000
50510.0000000	13.5000000	-50.0000000
50510.0000000	13.7500000	-50.0000000
50510.0000000	14.0000000	-50.0000000
50510.0000000	14.2500000	-50.0000000
50510.0000000	14.5000000	-50.0000000

50510.0000000	14.7500000	-50.0000000
50510.0000000	15.0000000	-50.0000000
50540.0000000	0.0000000	-50.0000000
50540.0000000	0.2500000	-50.0000000
50540.0000000	0.5000000	19.2199993
50540.0000000	0.7500000	24.3899994
50540.0000000	1.0000000	20.2999992
50540.0000000	1.2500000	17.9200001
50540.0000000	1.5000000	16.6000004
50540.0000000	1.7500000	17.5300007
50540.0000000	2.0000000	17.2399998
50540.0000000	2.2500000	-50.0000000
50540.0000000	2.5000000	-50.0000000
50540.0000000	2.7500000	-50.0000000
50540.0000000	3.0000000	-50.0000000
50540.0000000	3.2500000	-50.0000000
50540.0000000	3.5000000	-50.0000000
50540.0000000	3.7500000	-50.0000000
50540.0000000	4.0000000	-50.0000000
50540.0000000	4.2500000	-50.0000000
50540.0000000	4.5000000	-50.0000000
50540.0000000	4.7500000	-50.0000000
50540.0000000	5.0000000	-50.0000000
50540.0000000	5.2500000	-50.0000000
50540.0000000	5.5000000	-50.0000000
50540.0000000	5.7500000	-50.0000000
50540.0000000	6.0000000	-50.0000000
50540.0000000	6.2500000	-50.0000000
50540.0000000	6.5000000	-50.0000000
50540.0000000	6.7500000	-50.0000000
50540.0000000	7.0000000	-50.0000000
50540.0000000	7.2500000	-50.0000000
50540.0000000	7.5000000	-50.0000000
50540.0000000	7.7500000	-50.0000000
50540.0000000	8.0000000	-50.0000000
50540.0000000	8.2500000	-50.0000000
50540.0000000	8.5000000	-50.0000000
50540.0000000	8.7500000	-50.0000000
50540.0000000	9.0000000	-50.0000000
50540.0000000	9.2500000	-50.0000000
50540.0000000	9.5000000	-50.0000000
50540.0000000	9.7500000	-50.0000000
50540.0000000	10.0000000	-50.0000000
50540.0000000	10.2500000	-50.0000000
50540.0000000	10.5000000	-50.0000000
50540.0000000	10.7500000	-50.0000000
50540.0000000	11.0000000	-50.0000000
50540.0000000	11.2500000	-50.0000000
50540.0000000	11.5000000	-50.0000000
50540.0000000	11.7500000	-50.0000000
50540.0000000	12.0000000	-50.0000000
50540.0000000	12.2500000	-50.0000000
50540.0000000	12.5000000	-50.0000000
50540.0000000	12.7500000	-50.0000000
50540.0000000	13.0000000	-50.0000000
50540.0000000	13.2500000	-50.0000000
50540.0000000	13.5000000	-50.0000000
50540.0000000	13.7500000	-50.0000000

50540.0000000	14.0000000	-50.0000000
50540.0000000	14.2500000	-50.0000000
50540.0000000	14.5000000	-50.0000000
50540.0000000	14.7500000	-50.0000000
50540.0000000	15.0000000	-50.0000000
50570.0000000	0.0000000	-50.0000000
50570.0000000	0.2500000	-50.0000000
50570.0000000	0.5000000	16.2000008
50570.0000000	0.7500000	21.2600002
50570.0000000	1.0000000	17.9699993
50570.0000000	1.2500000	15.2399998
50570.0000000	1.5000000	16.9200001
50570.0000000	1.7500000	17.3999996
50570.0000000	2.0000000	15.1700001
50570.0000000	2.2500000	-50.0000000
50570.0000000	2.5000000	-50.0000000
50570.0000000	2.7500000	-50.0000000
50570.0000000	3.0000000	-50.0000000
50570.0000000	3.2500000	-50.0000000
50570.0000000	3.5000000	-50.0000000
50570.0000000	3.7500000	-50.0000000
50570.0000000	4.0000000	-50.0000000
50570.0000000	4.2500000	-50.0000000
50570.0000000	4.5000000	-50.0000000
50570.0000000	4.7500000	-50.0000000
50570.0000000	5.0000000	-50.0000000
50570.0000000	5.2500000	-50.0000000
50570.0000000	5.5000000	-50.0000000
50570.0000000	5.7500000	-50.0000000
50570.0000000	6.0000000	-50.0000000
50570.0000000	6.2500000	-50.0000000
50570.0000000	6.5000000	-50.0000000
50570.0000000	6.7500000	-50.0000000
50570.0000000	7.0000000	-50.0000000
50570.0000000	7.2500000	-50.0000000
50570.0000000	7.5000000	-50.0000000
50570.0000000	7.7500000	-50.0000000
50570.0000000	8.0000000	-50.0000000
50570.0000000	8.2500000	-50.0000000
50570.0000000	8.5000000	-50.0000000
50570.0000000	8.7500000	-50.0000000
50570.0000000	9.0000000	-50.0000000
50570.0000000	9.2500000	-50.0000000
50570.0000000	9.5000000	-50.0000000
50570.0000000	9.7500000	-50.0000000
50570.0000000	10.0000000	-50.0000000
50570.0000000	10.2500000	-50.0000000
50570.0000000	10.5000000	-50.0000000
50570.0000000	10.7500000	-50.0000000
50570.0000000	11.0000000	-50.0000000
50570.0000000	11.2500000	-50.0000000
50570.0000000	11.5000000	-50.0000000
50570.0000000	11.7500000	-50.0000000
50570.0000000	12.0000000	-50.0000000
50570.0000000	12.2500000	-50.0000000
50570.0000000	12.5000000	-50.0000000
50570.0000000	12.7500000	-50.0000000
50570.0000000	13.0000000	-50.0000000

50570.0000000	13.2500000	-50.0000000
50570.0000000	13.5000000	-50.0000000
50570.0000000	13.7500000	-50.0000000
50570.0000000	14.0000000	-50.0000000
50570.0000000	14.2500000	-50.0000000
50570.0000000	14.5000000	-50.0000000
50570.0000000	14.7500000	-50.0000000
50570.0000000	15.0000000	-50.0000000
50600.0000000	0.0000000	-50.0000000
50600.0000000	0.2500000	-50.0000000
50600.0000000	0.5000000	15.3199997
50600.0000000	0.7500000	20.4400005
50600.0000000	1.0000000	19.8099995
50600.0000000	1.2500000	18.7500000
50600.0000000	1.5000000	16.6200008
50600.0000000	1.7500000	16.3999996
50600.0000000	2.0000000	-50.0000000
50600.0000000	2.2500000	-50.0000000
50600.0000000	2.5000000	-50.0000000
50600.0000000	2.7500000	-50.0000000
50600.0000000	3.0000000	-50.0000000
50600.0000000	3.2500000	-50.0000000
50600.0000000	3.5000000	-50.0000000
50600.0000000	3.7500000	-50.0000000
50600.0000000	4.0000000	-50.0000000
50600.0000000	4.2500000	-50.0000000
50600.0000000	4.5000000	-50.0000000
50600.0000000	4.7500000	-50.0000000
50600.0000000	5.0000000	-50.0000000
50600.0000000	5.2500000	-50.0000000
50600.0000000	5.5000000	-50.0000000
50600.0000000	5.7500000	-50.0000000
50600.0000000	6.0000000	-50.0000000
50600.0000000	6.2500000	-50.0000000
50600.0000000	6.5000000	-50.0000000
50600.0000000	6.7500000	-50.0000000
50600.0000000	7.0000000	-50.0000000
50600.0000000	7.2500000	-50.0000000
50600.0000000	7.5000000	-50.0000000
50600.0000000	7.7500000	-50.0000000
50600.0000000	8.0000000	-50.0000000
50600.0000000	8.2500000	-50.0000000
50600.0000000	8.5000000	-50.0000000
50600.0000000	8.7500000	-50.0000000
50600.0000000	9.0000000	-50.0000000
50600.0000000	9.2500000	-50.0000000
50600.0000000	9.5000000	-50.0000000
50600.0000000	9.7500000	-50.0000000
50600.0000000	10.0000000	-50.0000000
50600.0000000	10.2500000	-50.0000000
50600.0000000	10.5000000	-50.0000000
50600.0000000	10.7500000	-50.0000000
50600.0000000	11.0000000	-50.0000000
50600.0000000	11.2500000	-50.0000000
50600.0000000	11.5000000	-50.0000000
50600.0000000	11.7500000	-50.0000000
50600.0000000	12.0000000	-50.0000000
50600.0000000	12.2500000	-50.0000000

50600.0000000	12.5000000	-50.0000000
50600.0000000	12.7500000	-50.0000000
50600.0000000	13.0000000	-50.0000000
50600.0000000	13.2500000	-50.0000000
50600.0000000	13.5000000	-50.0000000
50600.0000000	13.7500000	-50.0000000
50600.0000000	14.0000000	-50.0000000
50600.0000000	14.2500000	-50.0000000
50600.0000000	14.5000000	-50.0000000
50600.0000000	14.7500000	-50.0000000
50600.0000000	15.0000000	-50.0000000
50630.0000000	0.0000000	-50.0000000
50630.0000000	0.2500000	-50.0000000
50630.0000000	0.5000000	15.4899998
50630.0000000	0.7500000	20.1200008
50630.0000000	1.0000000	18.3899994
50630.0000000	1.2500000	19.5900002
50630.0000000	1.5000000	18.4799995
50630.0000000	1.7500000	16.0100002
50630.0000000	2.0000000	-50.0000000
50630.0000000	2.2500000	-50.0000000
50630.0000000	2.5000000	-50.0000000
50630.0000000	2.7500000	-50.0000000
50630.0000000	3.0000000	-50.0000000
50630.0000000	3.2500000	-50.0000000
50630.0000000	3.5000000	-50.0000000
50630.0000000	3.7500000	-50.0000000
50630.0000000	4.0000000	-50.0000000
50630.0000000	4.2500000	-50.0000000
50630.0000000	4.5000000	-50.0000000
50630.0000000	4.7500000	-50.0000000
50630.0000000	5.0000000	-50.0000000
50630.0000000	5.2500000	-50.0000000
50630.0000000	5.5000000	-50.0000000
50630.0000000	5.7500000	-50.0000000
50630.0000000	6.0000000	-50.0000000
50630.0000000	6.2500000	-50.0000000
50630.0000000	6.5000000	-50.0000000
50630.0000000	6.7500000	-50.0000000
50630.0000000	7.0000000	-50.0000000
50630.0000000	7.2500000	-50.0000000
50630.0000000	7.5000000	-50.0000000
50630.0000000	7.7500000	-50.0000000
50630.0000000	8.0000000	-50.0000000
50630.0000000	8.2500000	-50.0000000
50630.0000000	8.5000000	-50.0000000
50630.0000000	8.7500000	-50.0000000
50630.0000000	9.0000000	-50.0000000
50630.0000000	9.2500000	-50.0000000
50630.0000000	9.5000000	-50.0000000
50630.0000000	9.7500000	-50.0000000
50630.0000000	10.0000000	-50.0000000
50630.0000000	10.2500000	-50.0000000
50630.0000000	10.5000000	-50.0000000
50630.0000000	10.7500000	-50.0000000
50630.0000000	11.0000000	-50.0000000
50630.0000000	11.2500000	-50.0000000
50630.0000000	11.5000000	-50.0000000

50630.0000000	11.7500000	-50.0000000
50630.0000000	12.0000000	-50.0000000
50630.0000000	12.2500000	-50.0000000
50630.0000000	12.5000000	-50.0000000
50630.0000000	12.7500000	-50.0000000
50630.0000000	13.0000000	-50.0000000
50630.0000000	13.2500000	-50.0000000
50630.0000000	13.5000000	-50.0000000
50630.0000000	13.7500000	-50.0000000
50630.0000000	14.0000000	-50.0000000
50630.0000000	14.2500000	-50.0000000
50630.0000000	14.5000000	-50.0000000
50630.0000000	14.7500000	-50.0000000
50630.0000000	15.0000000	-50.0000000
50660.0000000	0.0000000	-50.0000000
50660.0000000	0.2500000	-50.0000000
50660.0000000	0.5000000	20.6100006
50660.0000000	0.7500000	23.9300003
50660.0000000	1.0000000	18.7000008
50660.0000000	1.2500000	15.2799997
50660.0000000	1.5000000	16.0200005
50660.0000000	1.7500000	15.9700003
50660.0000000	2.0000000	-50.0000000
50660.0000000	2.2500000	-50.0000000
50660.0000000	2.5000000	-50.0000000
50660.0000000	2.7500000	-50.0000000
50660.0000000	3.0000000	-50.0000000
50660.0000000	3.2500000	-50.0000000
50660.0000000	3.5000000	-50.0000000
50660.0000000	3.7500000	-50.0000000
50660.0000000	4.0000000	-50.0000000
50660.0000000	4.2500000	-50.0000000
50660.0000000	4.5000000	-50.0000000
50660.0000000	4.7500000	-50.0000000
50660.0000000	5.0000000	-50.0000000
50660.0000000	5.2500000	-50.0000000
50660.0000000	5.5000000	-50.0000000
50660.0000000	5.7500000	-50.0000000
50660.0000000	6.0000000	-50.0000000
50660.0000000	6.2500000	-50.0000000
50660.0000000	6.5000000	-50.0000000
50660.0000000	6.7500000	-50.0000000
50660.0000000	7.0000000	-50.0000000
50660.0000000	7.2500000	-50.0000000
50660.0000000	7.5000000	-50.0000000
50660.0000000	7.7500000	-50.0000000
50660.0000000	8.0000000	-50.0000000
50660.0000000	8.2500000	-50.0000000
50660.0000000	8.5000000	-50.0000000
50660.0000000	8.7500000	-50.0000000
50660.0000000	9.0000000	-50.0000000
50660.0000000	9.2500000	-50.0000000
50660.0000000	9.5000000	-50.0000000
50660.0000000	9.7500000	-50.0000000
50660.0000000	10.0000000	-50.0000000
50660.0000000	10.2500000	-50.0000000
50660.0000000	10.5000000	-50.0000000
50660.0000000	10.7500000	-50.0000000

50660.0000000	11.0000000	-50.0000000
50660.0000000	11.2500000	-50.0000000
50660.0000000	11.5000000	-50.0000000
50660.0000000	11.7500000	-50.0000000
50660.0000000	12.0000000	-50.0000000
50660.0000000	12.2500000	-50.0000000
50660.0000000	12.5000000	-50.0000000
50660.0000000	12.7500000	-50.0000000
50660.0000000	13.0000000	-50.0000000
50660.0000000	13.2500000	-50.0000000
50660.0000000	13.5000000	-50.0000000
50660.0000000	13.7500000	-50.0000000
50660.0000000	14.0000000	-50.0000000
50660.0000000	14.2500000	-50.0000000
50660.0000000	14.5000000	-50.0000000
50660.0000000	14.7500000	-50.0000000
50660.0000000	15.0000000	-50.0000000
50690.0000000	0.0000000	-50.0000000
50690.0000000	0.2500000	-50.0000000
50690.0000000	0.5000000	18.3099995
50690.0000000	0.7500000	21.1700001
50690.0000000	1.0000000	18.3500004
50690.0000000	1.2500000	18.8600006
50690.0000000	1.5000000	18.1900005
50690.0000000	1.7500000	16.6299992
50690.0000000	2.0000000	-50.0000000
50690.0000000	2.2500000	-50.0000000
50690.0000000	2.5000000	-50.0000000
50690.0000000	2.7500000	-50.0000000
50690.0000000	3.0000000	-50.0000000
50690.0000000	3.2500000	-50.0000000
50690.0000000	3.5000000	-50.0000000
50690.0000000	3.7500000	-50.0000000
50690.0000000	4.0000000	15.6800003
50690.0000000	4.2500000	-50.0000000
50690.0000000	4.5000000	-50.0000000
50690.0000000	4.7500000	-50.0000000
50690.0000000	5.0000000	-50.0000000
50690.0000000	5.2500000	-50.0000000
50690.0000000	5.5000000	-50.0000000
50690.0000000	5.7500000	-50.0000000
50690.0000000	6.0000000	-50.0000000
50690.0000000	6.2500000	-50.0000000
50690.0000000	6.5000000	-50.0000000
50690.0000000	6.7500000	-50.0000000
50690.0000000	7.0000000	-50.0000000
50690.0000000	7.2500000	-50.0000000
50690.0000000	7.5000000	-50.0000000
50690.0000000	7.7500000	-50.0000000
50690.0000000	8.0000000	-50.0000000
50690.0000000	8.2500000	-50.0000000
50690.0000000	8.5000000	-50.0000000
50690.0000000	8.7500000	-50.0000000
50690.0000000	9.0000000	-50.0000000
50690.0000000	9.2500000	-50.0000000
50690.0000000	9.5000000	-50.0000000
50690.0000000	9.7500000	-50.0000000
50690.0000000	10.0000000	-50.0000000

50690.0000000	10.2500000	-50.0000000
50690.0000000	10.5000000	-50.0000000
50690.0000000	10.7500000	-50.0000000
50690.0000000	11.0000000	-50.0000000
50690.0000000	11.2500000	-50.0000000
50690.0000000	11.5000000	-50.0000000
50690.0000000	11.7500000	-50.0000000
50690.0000000	12.0000000	-50.0000000
50690.0000000	12.2500000	-50.0000000
50690.0000000	12.5000000	-50.0000000
50690.0000000	12.7500000	-50.0000000
50690.0000000	13.0000000	-50.0000000
50690.0000000	13.2500000	-50.0000000
50690.0000000	13.5000000	-50.0000000
50690.0000000	13.7500000	-50.0000000
50690.0000000	14.0000000	-50.0000000
50690.0000000	14.2500000	-50.0000000
50690.0000000	14.5000000	-50.0000000
50690.0000000	14.7500000	-50.0000000
50690.0000000	15.0000000	-50.0000000
50720.0000000	0.0000000	-50.0000000
50720.0000000	0.2500000	-50.0000000
50720.0000000	0.5000000	21.2000008
50720.0000000	0.7500000	25.7600002
50720.0000000	1.0000000	20.5100002
50720.0000000	1.2500000	15.2500000
50720.0000000	1.5000000	15.7799997
50720.0000000	1.7500000	16.9899998
50720.0000000	2.0000000	-50.0000000
50720.0000000	2.2500000	-50.0000000
50720.0000000	2.5000000	-50.0000000
50720.0000000	2.7500000	-50.0000000
50720.0000000	3.0000000	-50.0000000
50720.0000000	3.2500000	-50.0000000
50720.0000000	3.5000000	-50.0000000
50720.0000000	3.7500000	-50.0000000
50720.0000000	4.0000000	-50.0000000
50720.0000000	4.2500000	-50.0000000
50720.0000000	4.5000000	-50.0000000
50720.0000000	4.7500000	-50.0000000
50720.0000000	5.0000000	-50.0000000
50720.0000000	5.2500000	-50.0000000
50720.0000000	5.5000000	-50.0000000
50720.0000000	5.7500000	-50.0000000
50720.0000000	6.0000000	-50.0000000
50720.0000000	6.2500000	-50.0000000
50720.0000000	6.5000000	-50.0000000
50720.0000000	6.7500000	-50.0000000
50720.0000000	7.0000000	-50.0000000
50720.0000000	7.2500000	-50.0000000
50720.0000000	7.5000000	-50.0000000
50720.0000000	7.7500000	-50.0000000
50720.0000000	8.0000000	-50.0000000
50720.0000000	8.2500000	-50.0000000
50720.0000000	8.5000000	-50.0000000
50720.0000000	8.7500000	-50.0000000
50720.0000000	9.0000000	-50.0000000
50720.0000000	9.2500000	-50.0000000

50720.0000000	9.5000000	-50.0000000
50720.0000000	9.7500000	-50.0000000
50720.0000000	10.0000000	-50.0000000
50720.0000000	10.2500000	-50.0000000
50720.0000000	10.5000000	-50.0000000
50720.0000000	10.7500000	-50.0000000
50720.0000000	11.0000000	-50.0000000
50720.0000000	11.2500000	-50.0000000
50720.0000000	11.5000000	-50.0000000
50720.0000000	11.7500000	-50.0000000
50720.0000000	12.0000000	-50.0000000
50720.0000000	12.2500000	-50.0000000
50720.0000000	12.5000000	-50.0000000
50720.0000000	12.7500000	-50.0000000
50720.0000000	13.0000000	-50.0000000
50720.0000000	13.2500000	-50.0000000
50720.0000000	13.5000000	-50.0000000
50720.0000000	13.7500000	-50.0000000
50720.0000000	14.0000000	-50.0000000
50720.0000000	14.2500000	-50.0000000
50720.0000000	14.5000000	-50.0000000
50720.0000000	14.7500000	-50.0000000
50720.0000000	15.0000000	-50.0000000
50750.0000000	0.0000000	-50.0000000
50750.0000000	0.2500000	-50.0000000
50750.0000000	0.5000000	22.2800007
50750.0000000	0.7500000	25.3299999
50750.0000000	1.0000000	19.1599998
50750.0000000	1.2500000	17.7900009
50750.0000000	1.5000000	19.7700005
50750.0000000	1.7500000	20.1499996
50750.0000000	2.0000000	16.5499992
50750.0000000	2.2500000	-50.0000000
50750.0000000	2.5000000	-50.0000000
50750.0000000	2.7500000	-50.0000000
50750.0000000	3.0000000	-50.0000000
50750.0000000	3.2500000	-50.0000000
50750.0000000	3.5000000	-50.0000000
50750.0000000	3.7500000	-50.0000000
50750.0000000	4.0000000	-50.0000000
50750.0000000	4.2500000	-50.0000000
50750.0000000	4.5000000	-50.0000000
50750.0000000	4.7500000	-50.0000000
50750.0000000	5.0000000	-50.0000000
50750.0000000	5.2500000	-50.0000000
50750.0000000	5.5000000	-50.0000000
50750.0000000	5.7500000	-50.0000000
50750.0000000	6.0000000	-50.0000000
50750.0000000	6.2500000	-50.0000000
50750.0000000	6.5000000	-50.0000000
50750.0000000	6.7500000	-50.0000000
50750.0000000	7.0000000	-50.0000000
50750.0000000	7.2500000	-50.0000000
50750.0000000	7.5000000	-50.0000000
50750.0000000	7.7500000	-50.0000000
50750.0000000	8.0000000	-50.0000000
50750.0000000	8.2500000	-50.0000000
50750.0000000	8.5000000	-50.0000000

50750.0000000	8.7500000	-50.0000000
50750.0000000	9.0000000	-50.0000000
50750.0000000	9.2500000	-50.0000000
50750.0000000	9.5000000	-50.0000000
50750.0000000	9.7500000	-50.0000000
50750.0000000	10.0000000	-50.0000000
50750.0000000	10.2500000	-50.0000000
50750.0000000	10.5000000	-50.0000000
50750.0000000	10.7500000	-50.0000000
50750.0000000	11.0000000	-50.0000000
50750.0000000	11.2500000	-50.0000000
50750.0000000	11.5000000	-50.0000000
50750.0000000	11.7500000	-50.0000000
50750.0000000	12.0000000	-50.0000000
50750.0000000	12.2500000	-50.0000000
50750.0000000	12.5000000	-50.0000000
50750.0000000	12.7500000	-50.0000000
50750.0000000	13.0000000	-50.0000000
50750.0000000	13.2500000	-50.0000000
50750.0000000	13.5000000	-50.0000000
50750.0000000	13.7500000	-50.0000000
50750.0000000	14.0000000	-50.0000000
50750.0000000	14.2500000	-50.0000000
50750.0000000	14.5000000	-50.0000000
50750.0000000	14.7500000	-50.0000000
50750.0000000	15.0000000	-50.0000000
50780.0000000	0.0000000	-50.0000000
50780.0000000	0.2500000	-50.0000000
50780.0000000	0.5000000	20.2000008
50780.0000000	0.7500000	22.2999992
50780.0000000	1.0000000	18.8799992
50780.0000000	1.2500000	16.5400009
50780.0000000	1.5000000	15.3100004
50780.0000000	1.7500000	15.0600004
50780.0000000	2.0000000	-50.0000000
50780.0000000	2.2500000	-50.0000000
50780.0000000	2.5000000	-50.0000000
50780.0000000	2.7500000	-50.0000000
50780.0000000	3.0000000	-50.0000000
50780.0000000	3.2500000	-50.0000000
50780.0000000	3.5000000	-50.0000000
50780.0000000	3.7500000	-50.0000000
50780.0000000	4.0000000	-50.0000000
50780.0000000	4.2500000	-50.0000000
50780.0000000	4.5000000	-50.0000000
50780.0000000	4.7500000	-50.0000000
50780.0000000	5.0000000	-50.0000000
50780.0000000	5.2500000	-50.0000000
50780.0000000	5.5000000	-50.0000000
50780.0000000	5.7500000	-50.0000000
50780.0000000	6.0000000	-50.0000000
50780.0000000	6.2500000	-50.0000000
50780.0000000	6.5000000	-50.0000000
50780.0000000	6.7500000	-50.0000000
50780.0000000	7.0000000	-50.0000000
50780.0000000	7.2500000	-50.0000000
50780.0000000	7.5000000	-50.0000000
50780.0000000	7.7500000	-50.0000000

50780.0000000	8.0000000	-50.0000000
50780.0000000	8.2500000	-50.0000000
50780.0000000	8.5000000	-50.0000000
50780.0000000	8.7500000	-50.0000000
50780.0000000	9.0000000	-50.0000000
50780.0000000	9.2500000	-50.0000000
50780.0000000	9.5000000	-50.0000000
50780.0000000	9.7500000	-50.0000000
50780.0000000	10.0000000	-50.0000000
50780.0000000	10.2500000	-50.0000000
50780.0000000	10.5000000	-50.0000000
50780.0000000	10.7500000	-50.0000000
50780.0000000	11.0000000	-50.0000000
50780.0000000	11.2500000	-50.0000000
50780.0000000	11.5000000	-50.0000000
50780.0000000	11.7500000	-50.0000000
50780.0000000	12.0000000	-50.0000000
50780.0000000	12.2500000	-50.0000000
50780.0000000	12.5000000	-50.0000000
50780.0000000	12.7500000	-50.0000000
50780.0000000	13.0000000	-50.0000000
50780.0000000	13.2500000	-50.0000000
50780.0000000	13.5000000	-50.0000000
50780.0000000	13.7500000	-50.0000000
50780.0000000	14.0000000	-50.0000000
50780.0000000	14.2500000	-50.0000000
50780.0000000	14.5000000	-50.0000000
50780.0000000	14.7500000	-50.0000000
50780.0000000	15.0000000	-50.0000000
50810.0000000	0.0000000	-50.0000000
50810.0000000	0.2500000	-50.0000000
50810.0000000	0.5000000	21.5100002
50810.0000000	0.7500000	24.6200008
50810.0000000	1.0000000	19.7700005
50810.0000000	1.2500000	18.3799992
50810.0000000	1.5000000	20.5000000
50810.0000000	1.7500000	19.1200008
50810.0000000	2.0000000	15.4399996
50810.0000000	2.2500000	-50.0000000
50810.0000000	2.5000000	-50.0000000
50810.0000000	2.7500000	-50.0000000
50810.0000000	3.0000000	15.0600004
50810.0000000	3.2500000	-50.0000000
50810.0000000	3.5000000	-50.0000000
50810.0000000	3.7500000	-50.0000000
50810.0000000	4.0000000	18.7900009
50810.0000000	4.2500000	-50.0000000
50810.0000000	4.5000000	-50.0000000
50810.0000000	4.7500000	-50.0000000
50810.0000000	5.0000000	-50.0000000
50810.0000000	5.2500000	-50.0000000
50810.0000000	5.5000000	-50.0000000
50810.0000000	5.7500000	-50.0000000
50810.0000000	6.0000000	-50.0000000
50810.0000000	6.2500000	-50.0000000
50810.0000000	6.5000000	-50.0000000
50810.0000000	6.7500000	-50.0000000
50810.0000000	7.0000000	-50.0000000

50810.0000000	7.2500000	-50.0000000
50810.0000000	7.5000000	-50.0000000
50810.0000000	7.7500000	-50.0000000
50810.0000000	8.0000000	-50.0000000
50810.0000000	8.2500000	-50.0000000
50810.0000000	8.5000000	-50.0000000
50810.0000000	8.7500000	-50.0000000
50810.0000000	9.0000000	-50.0000000
50810.0000000	9.2500000	-50.0000000
50810.0000000	9.5000000	-50.0000000
50810.0000000	9.7500000	-50.0000000
50810.0000000	10.0000000	-50.0000000
50810.0000000	10.2500000	-50.0000000
50810.0000000	10.5000000	-50.0000000
50810.0000000	10.7500000	-50.0000000
50810.0000000	11.0000000	-50.0000000
50810.0000000	11.2500000	-50.0000000
50810.0000000	11.5000000	-50.0000000
50810.0000000	11.7500000	-50.0000000
50810.0000000	12.0000000	-50.0000000
50810.0000000	12.2500000	-50.0000000
50810.0000000	12.5000000	-50.0000000
50810.0000000	12.7500000	-50.0000000
50810.0000000	13.0000000	-50.0000000
50810.0000000	13.2500000	-50.0000000
50810.0000000	13.5000000	-50.0000000
50810.0000000	13.7500000	-50.0000000
50810.0000000	14.0000000	-50.0000000
50810.0000000	14.2500000	-50.0000000
50810.0000000	14.5000000	-50.0000000
50810.0000000	14.7500000	-50.0000000
50810.0000000	15.0000000	-50.0000000
50840.0000000	0.0000000	-50.0000000
50840.0000000	0.2500000	-50.0000000
50840.0000000	0.5000000	22.2999992
50840.0000000	0.7500000	23.2600002
50840.0000000	1.0000000	17.9400005
50840.0000000	1.2500000	15.9399996
50840.0000000	1.5000000	16.7900009
50840.0000000	1.7500000	15.3000002
50840.0000000	2.0000000	-50.0000000
50840.0000000	2.2500000	-50.0000000
50840.0000000	2.5000000	-50.0000000
50840.0000000	2.7500000	-50.0000000
50840.0000000	3.0000000	-50.0000000
50840.0000000	3.2500000	-50.0000000
50840.0000000	3.5000000	-50.0000000
50840.0000000	3.7500000	-50.0000000
50840.0000000	4.0000000	-50.0000000
50840.0000000	4.2500000	-50.0000000
50840.0000000	4.5000000	-50.0000000
50840.0000000	4.7500000	-50.0000000
50840.0000000	5.0000000	-50.0000000
50840.0000000	5.2500000	-50.0000000
50840.0000000	5.5000000	-50.0000000
50840.0000000	5.7500000	-50.0000000
50840.0000000	6.0000000	-50.0000000
50840.0000000	6.2500000	-50.0000000

50840.0000000	6.5000000	-50.0000000
50840.0000000	6.7500000	-50.0000000
50840.0000000	7.0000000	-50.0000000
50840.0000000	7.2500000	-50.0000000
50840.0000000	7.5000000	-50.0000000
50840.0000000	7.7500000	-50.0000000
50840.0000000	8.0000000	-50.0000000
50840.0000000	8.2500000	-50.0000000
50840.0000000	8.5000000	-50.0000000
50840.0000000	8.7500000	-50.0000000
50840.0000000	9.0000000	-50.0000000
50840.0000000	9.2500000	-50.0000000
50840.0000000	9.5000000	-50.0000000
50840.0000000	9.7500000	-50.0000000
50840.0000000	10.0000000	-50.0000000
50840.0000000	10.2500000	-50.0000000
50840.0000000	10.5000000	-50.0000000
50840.0000000	10.7500000	-50.0000000
50840.0000000	11.0000000	-50.0000000
50840.0000000	11.2500000	-50.0000000
50840.0000000	11.5000000	-50.0000000
50840.0000000	11.7500000	-50.0000000
50840.0000000	12.0000000	-50.0000000
50840.0000000	12.2500000	-50.0000000
50840.0000000	12.5000000	-50.0000000
50840.0000000	12.7500000	-50.0000000
50840.0000000	13.0000000	-50.0000000
50840.0000000	13.2500000	-50.0000000
50840.0000000	13.5000000	-50.0000000
50840.0000000	13.7500000	-50.0000000
50840.0000000	14.0000000	-50.0000000
50840.0000000	14.2500000	-50.0000000
50840.0000000	14.5000000	-50.0000000
50840.0000000	14.7500000	-50.0000000
50840.0000000	15.0000000	-50.0000000
50870.0000000	0.0000000	-50.0000000
50870.0000000	0.2500000	-50.0000000
50870.0000000	0.5000000	21.7199993
50870.0000000	0.7500000	21.9899998
50870.0000000	1.0000000	18.6000004
50870.0000000	1.2500000	17.1100006
50870.0000000	1.5000000	15.1800003
50870.0000000	1.7500000	16.0499992
50870.0000000	2.0000000	-50.0000000
50870.0000000	2.2500000	-50.0000000
50870.0000000	2.5000000	-50.0000000
50870.0000000	2.7500000	-50.0000000
50870.0000000	3.0000000	-50.0000000
50870.0000000	3.2500000	-50.0000000
50870.0000000	3.5000000	-50.0000000
50870.0000000	3.7500000	-50.0000000
50870.0000000	4.0000000	-50.0000000
50870.0000000	4.2500000	-50.0000000
50870.0000000	4.5000000	-50.0000000
50870.0000000	4.7500000	-50.0000000
50870.0000000	5.0000000	-50.0000000
50870.0000000	5.2500000	-50.0000000
50870.0000000	5.5000000	-50.0000000

50870.0000000	5.7500000	-50.0000000
50870.0000000	6.0000000	-50.0000000
50870.0000000	6.2500000	-50.0000000
50870.0000000	6.5000000	-50.0000000
50870.0000000	6.7500000	-50.0000000
50870.0000000	7.0000000	-50.0000000
50870.0000000	7.2500000	-50.0000000
50870.0000000	7.5000000	-50.0000000
50870.0000000	7.7500000	-50.0000000
50870.0000000	8.0000000	-50.0000000
50870.0000000	8.2500000	-50.0000000
50870.0000000	8.5000000	-50.0000000
50870.0000000	8.7500000	-50.0000000
50870.0000000	9.0000000	-50.0000000
50870.0000000	9.2500000	-50.0000000
50870.0000000	9.5000000	-50.0000000
50870.0000000	9.7500000	-50.0000000
50870.0000000	10.0000000	-50.0000000
50870.0000000	10.2500000	-50.0000000
50870.0000000	10.5000000	-50.0000000
50870.0000000	10.7500000	-50.0000000
50870.0000000	11.0000000	-50.0000000
50870.0000000	11.2500000	-50.0000000
50870.0000000	11.5000000	-50.0000000
50870.0000000	11.7500000	-50.0000000
50870.0000000	12.0000000	-50.0000000
50870.0000000	12.2500000	-50.0000000
50870.0000000	12.5000000	-50.0000000
50870.0000000	12.7500000	-50.0000000
50870.0000000	13.0000000	-50.0000000
50870.0000000	13.2500000	-50.0000000
50870.0000000	13.5000000	-50.0000000
50870.0000000	13.7500000	-50.0000000
50870.0000000	14.0000000	-50.0000000
50870.0000000	14.2500000	-50.0000000
50870.0000000	14.5000000	-50.0000000
50870.0000000	14.7500000	-50.0000000
50870.0000000	15.0000000	-50.0000000
50900.0000000	0.0000000	-50.0000000
50900.0000000	0.2500000	-50.0000000
50900.0000000	0.5000000	22.8299999
50900.0000000	0.7500000	24.0000000
50900.0000000	1.0000000	19.3299999
50900.0000000	1.2500000	17.9599991
50900.0000000	1.5000000	17.1800003
50900.0000000	1.7500000	-50.0000000
50900.0000000	2.0000000	-50.0000000
50900.0000000	2.2500000	-50.0000000
50900.0000000	2.5000000	-50.0000000
50900.0000000	2.7500000	-50.0000000
50900.0000000	3.0000000	-50.0000000
50900.0000000	3.2500000	-50.0000000
50900.0000000	3.5000000	-50.0000000
50900.0000000	3.7500000	-50.0000000
50900.0000000	4.0000000	-50.0000000
50900.0000000	4.2500000	-50.0000000
50900.0000000	4.5000000	-50.0000000
50900.0000000	4.7500000	-50.0000000

50900.0000000	5.0000000	-50.0000000
50900.0000000	5.2500000	-50.0000000
50900.0000000	5.5000000	-50.0000000
50900.0000000	5.7500000	-50.0000000
50900.0000000	6.0000000	-50.0000000
50900.0000000	6.2500000	-50.0000000
50900.0000000	6.5000000	-50.0000000
50900.0000000	6.7500000	-50.0000000
50900.0000000	7.0000000	-50.0000000
50900.0000000	7.2500000	-50.0000000
50900.0000000	7.5000000	-50.0000000
50900.0000000	7.7500000	-50.0000000
50900.0000000	8.0000000	-50.0000000
50900.0000000	8.2500000	-50.0000000
50900.0000000	8.5000000	-50.0000000
50900.0000000	8.7500000	-50.0000000
50900.0000000	9.0000000	-50.0000000
50900.0000000	9.2500000	-50.0000000
50900.0000000	9.5000000	-50.0000000
50900.0000000	9.7500000	-50.0000000
50900.0000000	10.0000000	-50.0000000
50900.0000000	10.2500000	-50.0000000
50900.0000000	10.5000000	-50.0000000
50900.0000000	10.7500000	-50.0000000
50900.0000000	11.0000000	-50.0000000
50900.0000000	11.2500000	-50.0000000
50900.0000000	11.5000000	-50.0000000
50900.0000000	11.7500000	-50.0000000
50900.0000000	12.0000000	-50.0000000
50900.0000000	12.2500000	-50.0000000
50900.0000000	12.5000000	-50.0000000
50900.0000000	12.7500000	-50.0000000
50900.0000000	13.0000000	-50.0000000
50900.0000000	13.2500000	-50.0000000
50900.0000000	13.5000000	-50.0000000
50900.0000000	13.7500000	-50.0000000
50900.0000000	14.0000000	-50.0000000
50900.0000000	14.2500000	-50.0000000
50900.0000000	14.5000000	-50.0000000
50900.0000000	14.7500000	-50.0000000
50900.0000000	15.0000000	-50.0000000
50930.0000000	0.0000000	-50.0000000
50930.0000000	0.2500000	-50.0000000
50930.0000000	0.5000000	20.2399998
50930.0000000	0.7500000	22.0200005
50930.0000000	1.0000000	16.2199993
50930.0000000	1.2500000	15.7500000
50930.0000000	1.5000000	15.4700003
50930.0000000	1.7500000	-50.0000000
50930.0000000	2.0000000	-50.0000000
50930.0000000	2.2500000	-50.0000000
50930.0000000	2.5000000	-50.0000000
50930.0000000	2.7500000	-50.0000000
50930.0000000	3.0000000	-50.0000000
50930.0000000	3.2500000	-50.0000000
50930.0000000	3.5000000	-50.0000000
50930.0000000	3.7500000	-50.0000000
50930.0000000	4.0000000	-50.0000000

50930.0000000	4.2500000	-50.0000000
50930.0000000	4.5000000	-50.0000000
50930.0000000	4.7500000	-50.0000000
50930.0000000	5.0000000	-50.0000000
50930.0000000	5.2500000	-50.0000000
50930.0000000	5.5000000	-50.0000000
50930.0000000	5.7500000	-50.0000000
50930.0000000	6.0000000	-50.0000000
50930.0000000	6.2500000	-50.0000000
50930.0000000	6.5000000	-50.0000000
50930.0000000	6.7500000	-50.0000000
50930.0000000	7.0000000	-50.0000000
50930.0000000	7.2500000	-50.0000000
50930.0000000	7.5000000	-50.0000000
50930.0000000	7.7500000	-50.0000000
50930.0000000	8.0000000	-50.0000000
50930.0000000	8.2500000	-50.0000000
50930.0000000	8.5000000	-50.0000000
50930.0000000	8.7500000	-50.0000000
50930.0000000	9.0000000	-50.0000000
50930.0000000	9.2500000	-50.0000000
50930.0000000	9.5000000	-50.0000000
50930.0000000	9.7500000	-50.0000000
50930.0000000	10.0000000	-50.0000000
50930.0000000	10.2500000	-50.0000000
50930.0000000	10.5000000	-50.0000000
50930.0000000	10.7500000	-50.0000000
50930.0000000	11.0000000	-50.0000000
50930.0000000	11.2500000	-50.0000000
50930.0000000	11.5000000	-50.0000000
50930.0000000	11.7500000	-50.0000000
50930.0000000	12.0000000	-50.0000000
50930.0000000	12.2500000	-50.0000000
50930.0000000	12.5000000	-50.0000000
50930.0000000	12.7500000	-50.0000000
50930.0000000	13.0000000	-50.0000000
50930.0000000	13.2500000	-50.0000000
50930.0000000	13.5000000	-50.0000000
50930.0000000	13.7500000	-50.0000000
50930.0000000	14.0000000	-50.0000000
50930.0000000	14.2500000	-50.0000000
50930.0000000	14.5000000	-50.0000000
50930.0000000	14.7500000	-50.0000000
50930.0000000	15.0000000	-50.0000000
50960.0000000	0.0000000	-50.0000000
50960.0000000	0.2500000	-50.0000000
50960.0000000	0.5000000	18.6800003
50960.0000000	0.7500000	21.4200001
50960.0000000	1.0000000	19.1000004
50960.0000000	1.2500000	-50.0000000
50960.0000000	1.5000000	-50.0000000
50960.0000000	1.7500000	-50.0000000
50960.0000000	2.0000000	-50.0000000
50960.0000000	2.2500000	-50.0000000
50960.0000000	2.5000000	-50.0000000
50960.0000000	2.7500000	-50.0000000
50960.0000000	3.0000000	-50.0000000
50960.0000000	3.2500000	16.2500000

50960.0000000	3.5000000	-50.0000000
50960.0000000	3.7500000	-50.0000000
50960.0000000	4.0000000	-50.0000000
50960.0000000	4.2500000	-50.0000000
50960.0000000	4.5000000	-50.0000000
50960.0000000	4.7500000	-50.0000000
50960.0000000	5.0000000	-50.0000000
50960.0000000	5.2500000	-50.0000000
50960.0000000	5.5000000	-50.0000000
50960.0000000	5.7500000	-50.0000000
50960.0000000	6.0000000	-50.0000000
50960.0000000	6.2500000	-50.0000000
50960.0000000	6.5000000	-50.0000000
50960.0000000	6.7500000	-50.0000000
50960.0000000	7.0000000	-50.0000000
50960.0000000	7.2500000	-50.0000000
50960.0000000	7.5000000	-50.0000000
50960.0000000	7.7500000	-50.0000000
50960.0000000	8.0000000	-50.0000000
50960.0000000	8.2500000	-50.0000000
50960.0000000	8.5000000	-50.0000000
50960.0000000	8.7500000	-50.0000000
50960.0000000	9.0000000	-50.0000000
50960.0000000	9.2500000	-50.0000000
50960.0000000	9.5000000	-50.0000000
50960.0000000	9.7500000	-50.0000000
50960.0000000	10.0000000	-50.0000000
50960.0000000	10.2500000	-50.0000000
50960.0000000	10.5000000	-50.0000000
50960.0000000	10.7500000	-50.0000000
50960.0000000	11.0000000	-50.0000000
50960.0000000	11.2500000	-50.0000000
50960.0000000	11.5000000	-50.0000000
50960.0000000	11.7500000	-50.0000000
50960.0000000	12.0000000	-50.0000000
50960.0000000	12.2500000	-50.0000000
50960.0000000	12.5000000	-50.0000000
50960.0000000	12.7500000	-50.0000000
50960.0000000	13.0000000	-50.0000000
50960.0000000	13.2500000	-50.0000000
50960.0000000	13.5000000	-50.0000000
50960.0000000	13.7500000	-50.0000000
50960.0000000	14.0000000	-50.0000000
50960.0000000	14.2500000	-50.0000000
50960.0000000	14.5000000	-50.0000000
50960.0000000	14.7500000	-50.0000000
50960.0000000	15.0000000	-50.0000000
50990.0000000	0.0000000	-50.0000000
50990.0000000	0.2500000	-50.0000000
50990.0000000	0.5000000	22.6000004
50990.0000000	0.7500000	24.8099995
50990.0000000	1.0000000	19.7999992
50990.0000000	1.2500000	-50.0000000
50990.0000000	1.5000000	-50.0000000
50990.0000000	1.7500000	15.1499996
50990.0000000	2.0000000	-50.0000000
50990.0000000	2.2500000	-50.0000000
50990.0000000	2.5000000	-50.0000000

50990.0000000	2.7500000	-50.0000000
50990.0000000	3.0000000	-50.0000000
50990.0000000	3.2500000	-50.0000000
50990.0000000	3.5000000	-50.0000000
50990.0000000	3.7500000	-50.0000000
50990.0000000	4.0000000	-50.0000000
50990.0000000	4.2500000	-50.0000000
50990.0000000	4.5000000	-50.0000000
50990.0000000	4.7500000	-50.0000000
50990.0000000	5.0000000	-50.0000000
50990.0000000	5.2500000	-50.0000000
50990.0000000	5.5000000	-50.0000000
50990.0000000	5.7500000	-50.0000000
50990.0000000	6.0000000	-50.0000000
50990.0000000	6.2500000	-50.0000000
50990.0000000	6.5000000	-50.0000000
50990.0000000	6.7500000	-50.0000000
50990.0000000	7.0000000	-50.0000000
50990.0000000	7.2500000	-50.0000000
50990.0000000	7.5000000	-50.0000000
50990.0000000	7.7500000	-50.0000000
50990.0000000	8.0000000	-50.0000000
50990.0000000	8.2500000	-50.0000000
50990.0000000	8.5000000	-50.0000000
50990.0000000	8.7500000	-50.0000000
50990.0000000	9.0000000	-50.0000000
50990.0000000	9.2500000	-50.0000000
50990.0000000	9.5000000	-50.0000000
50990.0000000	9.7500000	-50.0000000
50990.0000000	10.0000000	-50.0000000
50990.0000000	10.2500000	-50.0000000
50990.0000000	10.5000000	-50.0000000
50990.0000000	10.7500000	-50.0000000
50990.0000000	11.0000000	-50.0000000
50990.0000000	11.2500000	-50.0000000
50990.0000000	11.5000000	-50.0000000
50990.0000000	11.7500000	-50.0000000
50990.0000000	12.0000000	-50.0000000
50990.0000000	12.2500000	-50.0000000
50990.0000000	12.5000000	-50.0000000
50990.0000000	12.7500000	-50.0000000
50990.0000000	13.0000000	-50.0000000
50990.0000000	13.2500000	-50.0000000
50990.0000000	13.5000000	-50.0000000
50990.0000000	13.7500000	-50.0000000
50990.0000000	14.0000000	-50.0000000
50990.0000000	14.2500000	-50.0000000
50990.0000000	14.5000000	-50.0000000
50990.0000000	14.7500000	-50.0000000
50990.0000000	15.0000000	-50.0000000
51020.0000000	0.0000000	-50.0000000
51020.0000000	0.2500000	-50.0000000
51020.0000000	0.5000000	21.0400009
51020.0000000	0.7500000	23.3600006
51020.0000000	1.0000000	18.3500004
51020.0000000	1.2500000	-50.0000000
51020.0000000	1.5000000	-50.0000000
51020.0000000	1.7500000	-50.0000000

51020.0000000	2.0000000	-50.0000000
51020.0000000	2.2500000	-50.0000000
51020.0000000	2.5000000	-50.0000000
51020.0000000	2.7500000	-50.0000000
51020.0000000	3.0000000	-50.0000000
51020.0000000	3.2500000	16.2500000
51020.0000000	3.5000000	16.0499992
51020.0000000	3.7500000	15.7399998
51020.0000000	4.0000000	-50.0000000
51020.0000000	4.2500000	-50.0000000
51020.0000000	4.5000000	-50.0000000
51020.0000000	4.7500000	-50.0000000
51020.0000000	5.0000000	-50.0000000
51020.0000000	5.2500000	-50.0000000
51020.0000000	5.5000000	-50.0000000
51020.0000000	5.7500000	-50.0000000
51020.0000000	6.0000000	-50.0000000
51020.0000000	6.2500000	-50.0000000
51020.0000000	6.5000000	-50.0000000
51020.0000000	6.7500000	-50.0000000
51020.0000000	7.0000000	-50.0000000
51020.0000000	7.2500000	-50.0000000
51020.0000000	7.5000000	-50.0000000
51020.0000000	7.7500000	-50.0000000
51020.0000000	8.0000000	-50.0000000
51020.0000000	8.2500000	-50.0000000
51020.0000000	8.5000000	-50.0000000
51020.0000000	8.7500000	-50.0000000
51020.0000000	9.0000000	-50.0000000
51020.0000000	9.2500000	-50.0000000
51020.0000000	9.5000000	-50.0000000
51020.0000000	9.7500000	-50.0000000
51020.0000000	10.0000000	-50.0000000
51020.0000000	10.2500000	-50.0000000
51020.0000000	10.5000000	-50.0000000
51020.0000000	10.7500000	-50.0000000
51020.0000000	11.0000000	-50.0000000
51020.0000000	11.2500000	-50.0000000
51020.0000000	11.5000000	-50.0000000
51020.0000000	11.7500000	-50.0000000
51020.0000000	12.0000000	-50.0000000
51020.0000000	12.2500000	-50.0000000
51020.0000000	12.5000000	-50.0000000
51020.0000000	12.7500000	-50.0000000
51020.0000000	13.0000000	-50.0000000
51020.0000000	13.2500000	-50.0000000
51020.0000000	13.5000000	-50.0000000
51020.0000000	13.7500000	-50.0000000
51020.0000000	14.0000000	-50.0000000
51020.0000000	14.2500000	-50.0000000
51020.0000000	14.5000000	-50.0000000
51020.0000000	14.7500000	-50.0000000
51020.0000000	15.0000000	-50.0000000
51050.0000000	0.0000000	-50.0000000
51050.0000000	0.2500000	-50.0000000
51050.0000000	0.5000000	22.6200008
51050.0000000	0.7500000	24.8199997
51050.0000000	1.0000000	19.1100006

51050.0000000	1.2500000	15.8800001
51050.0000000	1.5000000	-50.0000000
51050.0000000	1.7500000	-50.0000000
51050.0000000	2.0000000	-50.0000000
51050.0000000	2.2500000	-50.0000000
51050.0000000	2.5000000	-50.0000000
51050.0000000	2.7500000	-50.0000000
51050.0000000	3.0000000	-50.0000000
51050.0000000	3.2500000	-50.0000000
51050.0000000	3.5000000	-50.0000000
51050.0000000	3.7500000	-50.0000000
51050.0000000	4.0000000	-50.0000000
51050.0000000	4.2500000	-50.0000000
51050.0000000	4.5000000	-50.0000000
51050.0000000	4.7500000	-50.0000000
51050.0000000	5.0000000	-50.0000000
51050.0000000	5.2500000	-50.0000000
51050.0000000	5.5000000	-50.0000000
51050.0000000	5.7500000	-50.0000000
51050.0000000	6.0000000	-50.0000000
51050.0000000	6.2500000	-50.0000000
51050.0000000	6.5000000	-50.0000000
51050.0000000	6.7500000	-50.0000000
51050.0000000	7.0000000	-50.0000000
51050.0000000	7.2500000	-50.0000000
51050.0000000	7.5000000	-50.0000000
51050.0000000	7.7500000	-50.0000000
51050.0000000	8.0000000	-50.0000000
51050.0000000	8.2500000	-50.0000000
51050.0000000	8.5000000	-50.0000000
51050.0000000	8.7500000	-50.0000000
51050.0000000	9.0000000	-50.0000000
51050.0000000	9.2500000	-50.0000000
51050.0000000	9.5000000	-50.0000000
51050.0000000	9.7500000	-50.0000000
51050.0000000	10.0000000	-50.0000000
51050.0000000	10.2500000	-50.0000000
51050.0000000	10.5000000	-50.0000000
51050.0000000	10.7500000	-50.0000000
51050.0000000	11.0000000	-50.0000000
51050.0000000	11.2500000	-50.0000000
51050.0000000	11.5000000	-50.0000000
51050.0000000	11.7500000	-50.0000000
51050.0000000	12.0000000	-50.0000000
51050.0000000	12.2500000	-50.0000000
51050.0000000	12.5000000	-50.0000000
51050.0000000	12.7500000	-50.0000000
51050.0000000	13.0000000	-50.0000000
51050.0000000	13.2500000	-50.0000000
51050.0000000	13.5000000	-50.0000000
51050.0000000	13.7500000	-50.0000000
51050.0000000	14.0000000	-50.0000000
51050.0000000	14.2500000	-50.0000000
51050.0000000	14.5000000	-50.0000000
51050.0000000	14.7500000	-50.0000000
51050.0000000	15.0000000	-50.0000000
51080.0000000	0.0000000	-50.0000000
51080.0000000	0.2500000	-50.0000000

51080.0000000	0.5000000	24.0400009
51080.0000000	0.7500000	25.8099995
51080.0000000	1.0000000	20.0100002
51080.0000000	1.2500000	16.4599991
51080.0000000	1.5000000	15.3699999
51080.0000000	1.7500000	-50.0000000
51080.0000000	2.0000000	-50.0000000
51080.0000000	2.2500000	-50.0000000
51080.0000000	2.5000000	-50.0000000
51080.0000000	2.7500000	-50.0000000
51080.0000000	3.0000000	-50.0000000
51080.0000000	3.2500000	-50.0000000
51080.0000000	3.5000000	-50.0000000
51080.0000000	3.7500000	-50.0000000
51080.0000000	4.0000000	-50.0000000
51080.0000000	4.2500000	-50.0000000
51080.0000000	4.5000000	-50.0000000
51080.0000000	4.7500000	-50.0000000
51080.0000000	5.0000000	-50.0000000
51080.0000000	5.2500000	-50.0000000
51080.0000000	5.5000000	-50.0000000
51080.0000000	5.7500000	-50.0000000
51080.0000000	6.0000000	-50.0000000
51080.0000000	6.2500000	-50.0000000
51080.0000000	6.5000000	-50.0000000
51080.0000000	6.7500000	-50.0000000
51080.0000000	7.0000000	-50.0000000
51080.0000000	7.2500000	-50.0000000
51080.0000000	7.5000000	-50.0000000
51080.0000000	7.7500000	-50.0000000
51080.0000000	8.0000000	-50.0000000
51080.0000000	8.2500000	-50.0000000
51080.0000000	8.5000000	-50.0000000
51080.0000000	8.7500000	-50.0000000
51080.0000000	9.0000000	-50.0000000
51080.0000000	9.2500000	-50.0000000
51080.0000000	9.5000000	-50.0000000
51080.0000000	9.7500000	-50.0000000
51080.0000000	10.0000000	-50.0000000
51080.0000000	10.2500000	-50.0000000
51080.0000000	10.5000000	-50.0000000
51080.0000000	10.7500000	-50.0000000
51080.0000000	11.0000000	-50.0000000
51080.0000000	11.2500000	-50.0000000
51080.0000000	11.5000000	-50.0000000
51080.0000000	11.7500000	-50.0000000
51080.0000000	12.0000000	-50.0000000
51080.0000000	12.2500000	-50.0000000
51080.0000000	12.5000000	-50.0000000
51080.0000000	12.7500000	-50.0000000
51080.0000000	13.0000000	-50.0000000
51080.0000000	13.2500000	-50.0000000
51080.0000000	13.5000000	-50.0000000
51080.0000000	13.7500000	-50.0000000
51080.0000000	14.0000000	-50.0000000
51080.0000000	14.2500000	-50.0000000
51080.0000000	14.5000000	-50.0000000
51080.0000000	14.7500000	-50.0000000

51080.0000000	15.0000000	-50.0000000
51110.0000000	0.0000000	-50.0000000
51110.0000000	0.2500000	-50.0000000
51110.0000000	0.5000000	23.5300007
51110.0000000	0.7500000	24.7999992
51110.0000000	1.0000000	19.8700008
51110.0000000	1.2500000	15.0699997
51110.0000000	1.5000000	-50.0000000
51110.0000000	1.7500000	15.6400003
51110.0000000	2.0000000	-50.0000000
51110.0000000	2.2500000	-50.0000000
51110.0000000	2.5000000	-50.0000000
51110.0000000	2.7500000	-50.0000000
51110.0000000	3.0000000	-50.0000000
51110.0000000	3.2500000	-50.0000000
51110.0000000	3.5000000	-50.0000000
51110.0000000	3.7500000	-50.0000000
51110.0000000	4.0000000	-50.0000000
51110.0000000	4.2500000	-50.0000000
51110.0000000	4.5000000	-50.0000000
51110.0000000	4.7500000	-50.0000000
51110.0000000	5.0000000	-50.0000000
51110.0000000	5.2500000	-50.0000000
51110.0000000	5.5000000	-50.0000000
51110.0000000	5.7500000	-50.0000000
51110.0000000	6.0000000	-50.0000000
51110.0000000	6.2500000	-50.0000000
51110.0000000	6.5000000	-50.0000000
51110.0000000	6.7500000	-50.0000000
51110.0000000	7.0000000	-50.0000000
51110.0000000	7.2500000	-50.0000000
51110.0000000	7.5000000	-50.0000000
51110.0000000	7.7500000	-50.0000000
51110.0000000	8.0000000	-50.0000000
51110.0000000	8.2500000	-50.0000000
51110.0000000	8.5000000	-50.0000000
51110.0000000	8.7500000	-50.0000000
51110.0000000	9.0000000	-50.0000000
51110.0000000	9.2500000	-50.0000000
51110.0000000	9.5000000	-50.0000000
51110.0000000	9.7500000	-50.0000000
51110.0000000	10.0000000	-50.0000000
51110.0000000	10.2500000	-50.0000000
51110.0000000	10.5000000	-50.0000000
51110.0000000	10.7500000	-50.0000000
51110.0000000	11.0000000	-50.0000000
51110.0000000	11.2500000	-50.0000000
51110.0000000	11.5000000	-50.0000000
51110.0000000	11.7500000	-50.0000000
51110.0000000	12.0000000	-50.0000000
51110.0000000	12.2500000	-50.0000000
51110.0000000	12.5000000	-50.0000000
51110.0000000	12.7500000	-50.0000000
51110.0000000	13.0000000	-50.0000000
51110.0000000	13.2500000	-50.0000000
51110.0000000	13.5000000	-50.0000000
51110.0000000	13.7500000	-50.0000000
51110.0000000	14.0000000	-50.0000000

51110.0000000	14.2500000	-50.0000000
51110.0000000	14.5000000	-50.0000000
51110.0000000	14.7500000	-50.0000000
51110.0000000	15.0000000	-50.0000000
51140.0000000	0.0000000	-50.0000000
51140.0000000	0.2500000	-50.0000000
51140.0000000	0.5000000	24.6200008
51140.0000000	0.7500000	26.8999996
51140.0000000	1.0000000	19.7299995
51140.0000000	1.2500000	-50.0000000
51140.0000000	1.5000000	15.3500004
51140.0000000	1.7500000	15.3699999
51140.0000000	2.0000000	15.7299995
51140.0000000	2.2500000	17.6399994
51140.0000000	2.5000000	15.6400003
51140.0000000	2.7500000	-50.0000000
51140.0000000	3.0000000	-50.0000000
51140.0000000	3.2500000	-50.0000000
51140.0000000	3.5000000	-50.0000000
51140.0000000	3.7500000	-50.0000000
51140.0000000	4.0000000	-50.0000000
51140.0000000	4.2500000	-50.0000000
51140.0000000	4.5000000	-50.0000000
51140.0000000	4.7500000	-50.0000000
51140.0000000	5.0000000	-50.0000000
51140.0000000	5.2500000	-50.0000000
51140.0000000	5.5000000	-50.0000000
51140.0000000	5.7500000	-50.0000000
51140.0000000	6.0000000	-50.0000000
51140.0000000	6.2500000	-50.0000000
51140.0000000	6.5000000	-50.0000000
51140.0000000	6.7500000	-50.0000000
51140.0000000	7.0000000	-50.0000000
51140.0000000	7.2500000	-50.0000000
51140.0000000	7.5000000	-50.0000000
51140.0000000	7.7500000	-50.0000000
51140.0000000	8.0000000	-50.0000000
51140.0000000	8.2500000	-50.0000000
51140.0000000	8.5000000	-50.0000000
51140.0000000	8.7500000	-50.0000000
51140.0000000	9.0000000	-50.0000000
51140.0000000	9.2500000	-50.0000000
51140.0000000	9.5000000	-50.0000000
51140.0000000	9.7500000	-50.0000000
51140.0000000	10.0000000	-50.0000000
51140.0000000	10.2500000	-50.0000000
51140.0000000	10.5000000	-50.0000000
51140.0000000	10.7500000	-50.0000000
51140.0000000	11.0000000	-50.0000000
51140.0000000	11.2500000	-50.0000000
51140.0000000	11.5000000	-50.0000000
51140.0000000	11.7500000	-50.0000000
51140.0000000	12.0000000	-50.0000000
51140.0000000	12.2500000	-50.0000000
51140.0000000	12.5000000	-50.0000000
51140.0000000	12.7500000	-50.0000000
51140.0000000	13.0000000	-50.0000000
51140.0000000	13.2500000	-50.0000000

51140.0000000	13.5000000	-50.0000000
51140.0000000	13.7500000	-50.0000000
51140.0000000	14.0000000	-50.0000000
51140.0000000	14.2500000	-50.0000000
51140.0000000	14.5000000	-50.0000000
51140.0000000	14.7500000	-50.0000000
51140.0000000	15.0000000	-50.0000000
51170.0000000	0.0000000	-50.0000000
51170.0000000	0.2500000	-50.0000000
51170.0000000	0.5000000	23.6700001
51170.0000000	0.7500000	25.1299992
51170.0000000	1.0000000	19.4500008
51170.0000000	1.2500000	17.3799992
51170.0000000	1.5000000	15.4200001
51170.0000000	1.7500000	15.5100002
51170.0000000	2.0000000	16.0699997
51170.0000000	2.2500000	15.7900000
51170.0000000	2.5000000	-50.0000000
51170.0000000	2.7500000	-50.0000000
51170.0000000	3.0000000	16.2000008
51170.0000000	3.2500000	16.0499992
51170.0000000	3.5000000	-50.0000000
51170.0000000	3.7500000	-50.0000000
51170.0000000	4.0000000	-50.0000000
51170.0000000	4.2500000	-50.0000000
51170.0000000	4.5000000	-50.0000000
51170.0000000	4.7500000	-50.0000000
51170.0000000	5.0000000	-50.0000000
51170.0000000	5.2500000	-50.0000000
51170.0000000	5.5000000	-50.0000000
51170.0000000	5.7500000	-50.0000000
51170.0000000	6.0000000	-50.0000000
51170.0000000	6.2500000	-50.0000000
51170.0000000	6.5000000	-50.0000000
51170.0000000	6.7500000	-50.0000000
51170.0000000	7.0000000	-50.0000000
51170.0000000	7.2500000	-50.0000000
51170.0000000	7.5000000	-50.0000000
51170.0000000	7.7500000	-50.0000000
51170.0000000	8.0000000	-50.0000000
51170.0000000	8.2500000	-50.0000000
51170.0000000	8.5000000	-50.0000000
51170.0000000	8.7500000	-50.0000000
51170.0000000	9.0000000	-50.0000000
51170.0000000	9.2500000	-50.0000000
51170.0000000	9.5000000	-50.0000000
51170.0000000	9.7500000	-50.0000000
51170.0000000	10.0000000	-50.0000000
51170.0000000	10.2500000	-50.0000000
51170.0000000	10.5000000	-50.0000000
51170.0000000	10.7500000	-50.0000000
51170.0000000	11.0000000	-50.0000000
51170.0000000	11.2500000	-50.0000000
51170.0000000	11.5000000	-50.0000000
51170.0000000	11.7500000	-50.0000000
51170.0000000	12.0000000	-50.0000000
51170.0000000	12.2500000	-50.0000000
51170.0000000	12.5000000	-50.0000000

51170.0000000	12.7500000	-50.0000000
51170.0000000	13.0000000	-50.0000000
51170.0000000	13.2500000	-50.0000000
51170.0000000	13.5000000	-50.0000000
51170.0000000	13.7500000	-50.0000000
51170.0000000	14.0000000	-50.0000000
51170.0000000	14.2500000	-50.0000000
51170.0000000	14.5000000	-50.0000000
51170.0000000	14.7500000	-50.0000000
51170.0000000	15.0000000	-50.0000000
51200.0000000	0.0000000	-50.0000000
51200.0000000	0.2500000	-50.0000000
51200.0000000	0.5000000	21.9200001
51200.0000000	0.7500000	24.0499992
51200.0000000	1.0000000	17.4500008
51200.0000000	1.2500000	15.6199999
51200.0000000	1.5000000	15.1899996
51200.0000000	1.7500000	15.2100000
51200.0000000	2.0000000	15.1800003
51200.0000000	2.2500000	-50.0000000
51200.0000000	2.5000000	-50.0000000
51200.0000000	2.7500000	-50.0000000
51200.0000000	3.0000000	-50.0000000
51200.0000000	3.2500000	-50.0000000
51200.0000000	3.5000000	16.0000000
51200.0000000	3.7500000	15.2799997
51200.0000000	4.0000000	-50.0000000
51200.0000000	4.2500000	-50.0000000
51200.0000000	4.5000000	-50.0000000
51200.0000000	4.7500000	-50.0000000
51200.0000000	5.0000000	-50.0000000
51200.0000000	5.2500000	-50.0000000
51200.0000000	5.5000000	-50.0000000
51200.0000000	5.7500000	-50.0000000
51200.0000000	6.0000000	-50.0000000
51200.0000000	6.2500000	-50.0000000
51200.0000000	6.5000000	-50.0000000
51200.0000000	6.7500000	-50.0000000
51200.0000000	7.0000000	-50.0000000
51200.0000000	7.2500000	-50.0000000
51200.0000000	7.5000000	-50.0000000
51200.0000000	7.7500000	-50.0000000
51200.0000000	8.0000000	-50.0000000
51200.0000000	8.2500000	-50.0000000
51200.0000000	8.5000000	-50.0000000
51200.0000000	8.7500000	-50.0000000
51200.0000000	9.0000000	-50.0000000
51200.0000000	9.2500000	-50.0000000
51200.0000000	9.5000000	-50.0000000
51200.0000000	9.7500000	-50.0000000
51200.0000000	10.0000000	-50.0000000
51200.0000000	10.2500000	-50.0000000
51200.0000000	10.5000000	-50.0000000
51200.0000000	10.7500000	-50.0000000
51200.0000000	11.0000000	-50.0000000
51200.0000000	11.2500000	-50.0000000
51200.0000000	11.5000000	-50.0000000
51200.0000000	11.7500000	-50.0000000

51200.0000000	12.0000000	-50.0000000
51200.0000000	12.2500000	-50.0000000
51200.0000000	12.5000000	-50.0000000
51200.0000000	12.7500000	-50.0000000
51200.0000000	13.0000000	-50.0000000
51200.0000000	13.2500000	-50.0000000
51200.0000000	13.5000000	-50.0000000
51200.0000000	13.7500000	-50.0000000
51200.0000000	14.0000000	-50.0000000
51200.0000000	14.2500000	-50.0000000
51200.0000000	14.5000000	-50.0000000
51200.0000000	14.7500000	-50.0000000
51200.0000000	15.0000000	-50.0000000
51230.0000000	0.0000000	-50.0000000
51230.0000000	0.2500000	-50.0000000
51230.0000000	0.5000000	24.8299999
51230.0000000	0.7500000	26.4899998
51230.0000000	1.0000000	17.7500000
51230.0000000	1.2500000	-50.0000000
51230.0000000	1.5000000	15.9799995
51230.0000000	1.7500000	15.4899998
51230.0000000	2.0000000	15.1400003
51230.0000000	2.2500000	15.0900002
51230.0000000	2.5000000	-50.0000000
51230.0000000	2.7500000	-50.0000000
51230.0000000	3.0000000	-50.0000000
51230.0000000	3.2500000	-50.0000000
51230.0000000	3.5000000	15.1199999
51230.0000000	3.7500000	-50.0000000
51230.0000000	4.0000000	-50.0000000
51230.0000000	4.2500000	-50.0000000
51230.0000000	4.5000000	-50.0000000
51230.0000000	4.7500000	-50.0000000
51230.0000000	5.0000000	-50.0000000
51230.0000000	5.2500000	-50.0000000
51230.0000000	5.5000000	-50.0000000
51230.0000000	5.7500000	-50.0000000
51230.0000000	6.0000000	-50.0000000
51230.0000000	6.2500000	-50.0000000
51230.0000000	6.5000000	-50.0000000
51230.0000000	6.7500000	-50.0000000
51230.0000000	7.0000000	-50.0000000
51230.0000000	7.2500000	-50.0000000
51230.0000000	7.5000000	-50.0000000
51230.0000000	7.7500000	-50.0000000
51230.0000000	8.0000000	-50.0000000
51230.0000000	8.2500000	-50.0000000
51230.0000000	8.5000000	-50.0000000
51230.0000000	8.7500000	-50.0000000
51230.0000000	9.0000000	-50.0000000
51230.0000000	9.2500000	-50.0000000
51230.0000000	9.5000000	-50.0000000
51230.0000000	9.7500000	-50.0000000
51230.0000000	10.0000000	-50.0000000
51230.0000000	10.2500000	-50.0000000
51230.0000000	10.5000000	-50.0000000
51230.0000000	10.7500000	-50.0000000
51230.0000000	11.0000000	-50.0000000

51230.0000000	11.2500000	-50.0000000
51230.0000000	11.5000000	-50.0000000
51230.0000000	11.7500000	-50.0000000
51230.0000000	12.0000000	-50.0000000
51230.0000000	12.2500000	-50.0000000
51230.0000000	12.5000000	-50.0000000
51230.0000000	12.7500000	-50.0000000
51230.0000000	13.0000000	-50.0000000
51230.0000000	13.2500000	-50.0000000
51230.0000000	13.5000000	-50.0000000
51230.0000000	13.7500000	-50.0000000
51230.0000000	14.0000000	-50.0000000
51230.0000000	14.2500000	-50.0000000
51230.0000000	14.5000000	-50.0000000
51230.0000000	14.7500000	-50.0000000
51230.0000000	15.0000000	-50.0000000
51260.0000000	0.0000000	-50.0000000
51260.0000000	0.2500000	-50.0000000
51260.0000000	0.5000000	21.7500000
51260.0000000	0.7500000	24.2900009
51260.0000000	1.0000000	19.5799999
51260.0000000	1.2500000	16.0000000
51260.0000000	1.5000000	18.0499992
51260.0000000	1.7500000	16.2099991
51260.0000000	2.0000000	15.0500002
51260.0000000	2.2500000	-50.0000000
51260.0000000	2.5000000	-50.0000000
51260.0000000	2.7500000	-50.0000000
51260.0000000	3.0000000	-50.0000000
51260.0000000	3.2500000	-50.0000000
51260.0000000	3.5000000	-50.0000000
51260.0000000	3.7500000	-50.0000000
51260.0000000	4.0000000	-50.0000000
51260.0000000	4.2500000	-50.0000000
51260.0000000	4.5000000	-50.0000000
51260.0000000	4.7500000	-50.0000000
51260.0000000	5.0000000	-50.0000000
51260.0000000	5.2500000	-50.0000000
51260.0000000	5.5000000	-50.0000000
51260.0000000	5.7500000	-50.0000000
51260.0000000	6.0000000	-50.0000000
51260.0000000	6.2500000	-50.0000000
51260.0000000	6.5000000	-50.0000000
51260.0000000	6.7500000	-50.0000000
51260.0000000	7.0000000	-50.0000000
51260.0000000	7.2500000	-50.0000000
51260.0000000	7.5000000	-50.0000000
51260.0000000	7.7500000	-50.0000000
51260.0000000	8.0000000	-50.0000000
51260.0000000	8.2500000	-50.0000000
51260.0000000	8.5000000	-50.0000000
51260.0000000	8.7500000	-50.0000000
51260.0000000	9.0000000	-50.0000000
51260.0000000	9.2500000	-50.0000000
51260.0000000	9.5000000	-50.0000000
51260.0000000	9.7500000	-50.0000000
51260.0000000	10.0000000	-50.0000000
51260.0000000	10.2500000	-50.0000000

51260.0000000	10.5000000	-50.0000000
51260.0000000	10.7500000	-50.0000000
51260.0000000	11.0000000	-50.0000000
51260.0000000	11.2500000	-50.0000000
51260.0000000	11.5000000	-50.0000000
51260.0000000	11.7500000	-50.0000000
51260.0000000	12.0000000	-50.0000000
51260.0000000	12.2500000	-50.0000000
51260.0000000	12.5000000	-50.0000000
51260.0000000	12.7500000	-50.0000000
51260.0000000	13.0000000	-50.0000000
51260.0000000	13.2500000	-50.0000000
51260.0000000	13.5000000	-50.0000000
51260.0000000	13.7500000	-50.0000000
51260.0000000	14.0000000	-50.0000000
51260.0000000	14.2500000	-50.0000000
51260.0000000	14.5000000	-50.0000000
51260.0000000	14.7500000	-50.0000000
51260.0000000	15.0000000	-50.0000000
51290.0000000	0.0000000	-50.0000000
51290.0000000	0.2500000	-50.0000000
51290.0000000	0.5000000	19.1499996
51290.0000000	0.7500000	21.0699997
51290.0000000	1.0000000	15.4600000
51290.0000000	1.2500000	15.1599998
51290.0000000	1.5000000	16.3899994
51290.0000000	1.7500000	16.2800007
51290.0000000	2.0000000	15.4899998
51290.0000000	2.2500000	-50.0000000
51290.0000000	2.5000000	-50.0000000
51290.0000000	2.7500000	-50.0000000
51290.0000000	3.0000000	-50.0000000
51290.0000000	3.2500000	-50.0000000
51290.0000000	3.5000000	-50.0000000
51290.0000000	3.7500000	-50.0000000
51290.0000000	4.0000000	-50.0000000
51290.0000000	4.2500000	-50.0000000
51290.0000000	4.5000000	-50.0000000
51290.0000000	4.7500000	-50.0000000
51290.0000000	5.0000000	-50.0000000
51290.0000000	5.2500000	-50.0000000
51290.0000000	5.5000000	-50.0000000
51290.0000000	5.7500000	-50.0000000
51290.0000000	6.0000000	-50.0000000
51290.0000000	6.2500000	-50.0000000
51290.0000000	6.5000000	-50.0000000
51290.0000000	6.7500000	-50.0000000
51290.0000000	7.0000000	-50.0000000
51290.0000000	7.2500000	-50.0000000
51290.0000000	7.5000000	-50.0000000
51290.0000000	7.7500000	-50.0000000
51290.0000000	8.0000000	-50.0000000
51290.0000000	8.2500000	-50.0000000
51290.0000000	8.5000000	-50.0000000
51290.0000000	8.7500000	-50.0000000
51290.0000000	9.0000000	-50.0000000
51290.0000000	9.2500000	-50.0000000
51290.0000000	9.5000000	-50.0000000

51290.0000000	9.7500000	-50.0000000
51290.0000000	10.0000000	-50.0000000
51290.0000000	10.2500000	-50.0000000
51290.0000000	10.5000000	-50.0000000
51290.0000000	10.7500000	-50.0000000
51290.0000000	11.0000000	-50.0000000
51290.0000000	11.2500000	-50.0000000
51290.0000000	11.5000000	-50.0000000
51290.0000000	11.7500000	-50.0000000
51290.0000000	12.0000000	-50.0000000
51290.0000000	12.2500000	-50.0000000
51290.0000000	12.5000000	-50.0000000
51290.0000000	12.7500000	-50.0000000
51290.0000000	13.0000000	-50.0000000
51290.0000000	13.2500000	-50.0000000
51290.0000000	13.5000000	-50.0000000
51290.0000000	13.7500000	-50.0000000
51290.0000000	14.0000000	-50.0000000
51290.0000000	14.2500000	-50.0000000
51290.0000000	14.5000000	-50.0000000
51290.0000000	14.7500000	-50.0000000
51290.0000000	15.0000000	-50.0000000
51320.0000000	0.0000000	-50.0000000
51320.0000000	0.2500000	-50.0000000
51320.0000000	0.5000000	21.5200005
51320.0000000	0.7500000	23.6100006
51320.0000000	1.0000000	15.9700003
51320.0000000	1.2500000	-50.0000000
51320.0000000	1.5000000	15.0500002
51320.0000000	1.7500000	15.8999996
51320.0000000	2.0000000	15.8999996
51320.0000000	2.2500000	16.8999996
51320.0000000	2.5000000	-50.0000000
51320.0000000	2.7500000	-50.0000000
51320.0000000	3.0000000	-50.0000000
51320.0000000	3.2500000	-50.0000000
51320.0000000	3.5000000	-50.0000000
51320.0000000	3.7500000	-50.0000000
51320.0000000	4.0000000	-50.0000000
51320.0000000	4.2500000	-50.0000000
51320.0000000	4.5000000	-50.0000000
51320.0000000	4.7500000	-50.0000000
51320.0000000	5.0000000	-50.0000000
51320.0000000	5.2500000	-50.0000000
51320.0000000	5.5000000	-50.0000000
51320.0000000	5.7500000	-50.0000000
51320.0000000	6.0000000	-50.0000000
51320.0000000	6.2500000	-50.0000000
51320.0000000	6.5000000	-50.0000000
51320.0000000	6.7500000	-50.0000000
51320.0000000	7.0000000	-50.0000000
51320.0000000	7.2500000	-50.0000000
51320.0000000	7.5000000	-50.0000000
51320.0000000	7.7500000	-50.0000000
51320.0000000	8.0000000	-50.0000000
51320.0000000	8.2500000	-50.0000000
51320.0000000	8.5000000	-50.0000000
51320.0000000	8.7500000	-50.0000000

51320.0000000	9.0000000	-50.0000000
51320.0000000	9.2500000	-50.0000000
51320.0000000	9.5000000	-50.0000000
51320.0000000	9.7500000	-50.0000000
51320.0000000	10.0000000	-50.0000000
51320.0000000	10.2500000	-50.0000000
51320.0000000	10.5000000	-50.0000000
51320.0000000	10.7500000	-50.0000000
51320.0000000	11.0000000	-50.0000000
51320.0000000	11.2500000	-50.0000000
51320.0000000	11.5000000	-50.0000000
51320.0000000	11.7500000	-50.0000000
51320.0000000	12.0000000	-50.0000000
51320.0000000	12.2500000	-50.0000000
51320.0000000	12.5000000	-50.0000000
51320.0000000	12.7500000	-50.0000000
51320.0000000	13.0000000	-50.0000000
51320.0000000	13.2500000	-50.0000000
51320.0000000	13.5000000	-50.0000000
51320.0000000	13.7500000	-50.0000000
51320.0000000	14.0000000	-50.0000000
51320.0000000	14.2500000	-50.0000000
51320.0000000	14.5000000	-50.0000000
51320.0000000	14.7500000	-50.0000000
51320.0000000	15.0000000	-50.0000000
51350.0000000	0.0000000	-50.0000000
51350.0000000	0.2500000	15.7799997
51350.0000000	0.5000000	25.0599995
51350.0000000	0.7500000	25.4799995
51350.0000000	1.0000000	18.7099991
51350.0000000	1.2500000	16.9300003
51350.0000000	1.5000000	15.7900000
51350.0000000	1.7500000	-50.0000000
51350.0000000	2.0000000	-50.0000000
51350.0000000	2.2500000	-50.0000000
51350.0000000	2.5000000	-50.0000000
51350.0000000	2.7500000	-50.0000000
51350.0000000	3.0000000	-50.0000000
51350.0000000	3.2500000	-50.0000000
51350.0000000	3.5000000	-50.0000000
51350.0000000	3.7500000	-50.0000000
51350.0000000	4.0000000	17.5900002
51350.0000000	4.2500000	-50.0000000
51350.0000000	4.5000000	-50.0000000
51350.0000000	4.7500000	-50.0000000
51350.0000000	5.0000000	-50.0000000
51350.0000000	5.2500000	-50.0000000
51350.0000000	5.5000000	-50.0000000
51350.0000000	5.7500000	-50.0000000
51350.0000000	6.0000000	-50.0000000
51350.0000000	6.2500000	-50.0000000
51350.0000000	6.5000000	-50.0000000
51350.0000000	6.7500000	-50.0000000
51350.0000000	7.0000000	-50.0000000
51350.0000000	7.2500000	-50.0000000
51350.0000000	7.5000000	-50.0000000
51350.0000000	7.7500000	-50.0000000
51350.0000000	8.0000000	-50.0000000

51350.0000000	8.2500000	-50.0000000
51350.0000000	8.5000000	-50.0000000
51350.0000000	8.7500000	-50.0000000
51350.0000000	9.0000000	-50.0000000
51350.0000000	9.2500000	-50.0000000
51350.0000000	9.5000000	-50.0000000
51350.0000000	9.7500000	-50.0000000
51350.0000000	10.0000000	-50.0000000
51350.0000000	10.2500000	-50.0000000
51350.0000000	10.5000000	-50.0000000
51350.0000000	10.7500000	-50.0000000
51350.0000000	11.0000000	-50.0000000
51350.0000000	11.2500000	-50.0000000
51350.0000000	11.5000000	-50.0000000
51350.0000000	11.7500000	-50.0000000
51350.0000000	12.0000000	-50.0000000
51350.0000000	12.2500000	-50.0000000
51350.0000000	12.5000000	-50.0000000
51350.0000000	12.7500000	-50.0000000
51350.0000000	13.0000000	-50.0000000
51350.0000000	13.2500000	-50.0000000
51350.0000000	13.5000000	-50.0000000
51350.0000000	13.7500000	-50.0000000
51350.0000000	14.0000000	-50.0000000
51350.0000000	14.2500000	-50.0000000
51350.0000000	14.5000000	-50.0000000
51350.0000000	14.7500000	-50.0000000
51350.0000000	15.0000000	-50.0000000
51380.0000000	0.0000000	-50.0000000
51380.0000000	0.2500000	-50.0000000
51380.0000000	0.5000000	23.7500000
51380.0000000	0.7500000	24.7900009
51380.0000000	1.0000000	18.5400009
51380.0000000	1.2500000	17.0300007
51380.0000000	1.5000000	18.4099998
51380.0000000	1.7500000	17.4699993
51380.0000000	2.0000000	16.2999992
51380.0000000	2.2500000	-50.0000000
51380.0000000	2.5000000	-50.0000000
51380.0000000	2.7500000	-50.0000000
51380.0000000	3.0000000	-50.0000000
51380.0000000	3.2500000	16.1700001
51380.0000000	3.5000000	-50.0000000
51380.0000000	3.7500000	-50.0000000
51380.0000000	4.0000000	15.2399998
51380.0000000	4.2500000	-50.0000000
51380.0000000	4.5000000	-50.0000000
51380.0000000	4.7500000	-50.0000000
51380.0000000	5.0000000	-50.0000000
51380.0000000	5.2500000	-50.0000000
51380.0000000	5.5000000	-50.0000000
51380.0000000	5.7500000	-50.0000000
51380.0000000	6.0000000	-50.0000000
51380.0000000	6.2500000	-50.0000000
51380.0000000	6.5000000	-50.0000000
51380.0000000	6.7500000	-50.0000000
51380.0000000	7.0000000	-50.0000000
51380.0000000	7.2500000	-50.0000000

51380.0000000	7.5000000	-50.0000000
51380.0000000	7.7500000	-50.0000000
51380.0000000	8.0000000	-50.0000000
51380.0000000	8.2500000	-50.0000000
51380.0000000	8.5000000	-50.0000000
51380.0000000	8.7500000	-50.0000000
51380.0000000	9.0000000	-50.0000000
51380.0000000	9.2500000	-50.0000000
51380.0000000	9.5000000	-50.0000000
51380.0000000	9.7500000	-50.0000000
51380.0000000	10.0000000	-50.0000000
51380.0000000	10.2500000	-50.0000000
51380.0000000	10.5000000	-50.0000000
51380.0000000	10.7500000	-50.0000000
51380.0000000	11.0000000	-50.0000000
51380.0000000	11.2500000	-50.0000000
51380.0000000	11.5000000	-50.0000000
51380.0000000	11.7500000	-50.0000000
51380.0000000	12.0000000	-50.0000000
51380.0000000	12.2500000	-50.0000000
51380.0000000	12.5000000	-50.0000000
51380.0000000	12.7500000	-50.0000000
51380.0000000	13.0000000	-50.0000000
51380.0000000	13.2500000	-50.0000000
51380.0000000	13.5000000	-50.0000000
51380.0000000	13.7500000	-50.0000000
51380.0000000	14.0000000	-50.0000000
51380.0000000	14.2500000	-50.0000000
51380.0000000	14.5000000	-50.0000000
51380.0000000	14.7500000	-50.0000000
51380.0000000	15.0000000	-50.0000000
51410.0000000	0.0000000	-50.0000000
51410.0000000	0.2500000	-50.0000000
51410.0000000	0.5000000	23.7500000
51410.0000000	0.7500000	23.3600006
51410.0000000	1.0000000	17.6200008
51410.0000000	1.2500000	15.9300003
51410.0000000	1.5000000	15.4600000
51410.0000000	1.7500000	16.2199993
51410.0000000	2.0000000	16.1499996
51410.0000000	2.2500000	15.0400000
51410.0000000	2.5000000	-50.0000000
51410.0000000	2.7500000	-50.0000000
51410.0000000	3.0000000	-50.0000000
51410.0000000	3.2500000	-50.0000000
51410.0000000	3.5000000	-50.0000000
51410.0000000	3.7500000	-50.0000000
51410.0000000	4.0000000	-50.0000000
51410.0000000	4.2500000	-50.0000000
51410.0000000	4.5000000	-50.0000000
51410.0000000	4.7500000	-50.0000000
51410.0000000	5.0000000	-50.0000000
51410.0000000	5.2500000	-50.0000000
51410.0000000	5.5000000	-50.0000000
51410.0000000	5.7500000	-50.0000000
51410.0000000	6.0000000	-50.0000000
51410.0000000	6.2500000	-50.0000000
51410.0000000	6.5000000	-50.0000000

51410.0000000	6.7500000	-50.0000000
51410.0000000	7.0000000	-50.0000000
51410.0000000	7.2500000	-50.0000000
51410.0000000	7.5000000	-50.0000000
51410.0000000	7.7500000	-50.0000000
51410.0000000	8.0000000	-50.0000000
51410.0000000	8.2500000	-50.0000000
51410.0000000	8.5000000	-50.0000000
51410.0000000	8.7500000	-50.0000000
51410.0000000	9.0000000	-50.0000000
51410.0000000	9.2500000	-50.0000000
51410.0000000	9.5000000	-50.0000000
51410.0000000	9.7500000	-50.0000000
51410.0000000	10.0000000	-50.0000000
51410.0000000	10.2500000	-50.0000000
51410.0000000	10.5000000	-50.0000000
51410.0000000	10.7500000	-50.0000000
51410.0000000	11.0000000	-50.0000000
51410.0000000	11.2500000	-50.0000000
51410.0000000	11.5000000	-50.0000000
51410.0000000	11.7500000	-50.0000000
51410.0000000	12.0000000	-50.0000000
51410.0000000	12.2500000	-50.0000000
51410.0000000	12.5000000	-50.0000000
51410.0000000	12.7500000	-50.0000000
51410.0000000	13.0000000	-50.0000000
51410.0000000	13.2500000	-50.0000000
51410.0000000	13.5000000	-50.0000000
51410.0000000	13.7500000	-50.0000000
51410.0000000	14.0000000	-50.0000000
51410.0000000	14.2500000	-50.0000000
51410.0000000	14.5000000	-50.0000000
51410.0000000	14.7500000	-50.0000000
51410.0000000	15.0000000	-50.0000000
51440.0000000	0.0000000	-50.0000000
51440.0000000	0.2500000	-50.0000000
51440.0000000	0.5000000	20.9599991
51440.0000000	0.7500000	22.3500004
51440.0000000	1.0000000	16.5799999
51440.0000000	1.2500000	-50.0000000
51440.0000000	1.5000000	-50.0000000
51440.0000000	1.7500000	15.0699997
51440.0000000	2.0000000	-50.0000000
51440.0000000	2.2500000	-50.0000000
51440.0000000	2.5000000	-50.0000000
51440.0000000	2.7500000	-50.0000000
51440.0000000	3.0000000	15.4799995
51440.0000000	3.2500000	16.2399998
51440.0000000	3.5000000	-50.0000000
51440.0000000	3.7500000	-50.0000000
51440.0000000	4.0000000	-50.0000000
51440.0000000	4.2500000	15.2799997
51440.0000000	4.5000000	-50.0000000
51440.0000000	4.7500000	-50.0000000
51440.0000000	5.0000000	-50.0000000
51440.0000000	5.2500000	-50.0000000
51440.0000000	5.5000000	-50.0000000
51440.0000000	5.7500000	-50.0000000

51440.0000000	6.0000000	-50.0000000
51440.0000000	6.2500000	-50.0000000
51440.0000000	6.5000000	-50.0000000
51440.0000000	6.7500000	-50.0000000
51440.0000000	7.0000000	-50.0000000
51440.0000000	7.2500000	-50.0000000
51440.0000000	7.5000000	-50.0000000
51440.0000000	7.7500000	-50.0000000
51440.0000000	8.0000000	-50.0000000
51440.0000000	8.2500000	-50.0000000
51440.0000000	8.5000000	-50.0000000
51440.0000000	8.7500000	-50.0000000
51440.0000000	9.0000000	-50.0000000
51440.0000000	9.2500000	-50.0000000
51440.0000000	9.5000000	-50.0000000
51440.0000000	9.7500000	-50.0000000
51440.0000000	10.0000000	-50.0000000
51440.0000000	10.2500000	-50.0000000
51440.0000000	10.5000000	-50.0000000
51440.0000000	10.7500000	-50.0000000
51440.0000000	11.0000000	-50.0000000
51440.0000000	11.2500000	-50.0000000
51440.0000000	11.5000000	-50.0000000
51440.0000000	11.7500000	-50.0000000
51440.0000000	12.0000000	-50.0000000
51440.0000000	12.2500000	-50.0000000
51440.0000000	12.5000000	-50.0000000
51440.0000000	12.7500000	-50.0000000
51440.0000000	13.0000000	-50.0000000
51440.0000000	13.2500000	-50.0000000
51440.0000000	13.5000000	-50.0000000
51440.0000000	13.7500000	-50.0000000
51440.0000000	14.0000000	-50.0000000
51440.0000000	14.2500000	-50.0000000
51440.0000000	14.5000000	-50.0000000
51440.0000000	14.7500000	-50.0000000
51440.0000000	15.0000000	-50.0000000
51470.0000000	0.0000000	-50.0000000
51470.0000000	0.2500000	-50.0000000
51470.0000000	0.5000000	22.9899998
51470.0000000	0.7500000	23.8700008
51470.0000000	1.0000000	18.5000000
51470.0000000	1.2500000	16.2600002
51470.0000000	1.5000000	-50.0000000
51470.0000000	1.7500000	-50.0000000
51470.0000000	2.0000000	-50.0000000
51470.0000000	2.2500000	-50.0000000
51470.0000000	2.5000000	-50.0000000
51470.0000000	2.7500000	-50.0000000
51470.0000000	3.0000000	-50.0000000
51470.0000000	3.2500000	-50.0000000
51470.0000000	3.5000000	-50.0000000
51470.0000000	3.7500000	-50.0000000
51470.0000000	4.0000000	-50.0000000
51470.0000000	4.2500000	-50.0000000
51470.0000000	4.5000000	-50.0000000
51470.0000000	4.7500000	-50.0000000
51470.0000000	5.0000000	-50.0000000

51470.0000000	5.2500000	-50.0000000
51470.0000000	5.5000000	-50.0000000
51470.0000000	5.7500000	-50.0000000
51470.0000000	6.0000000	-50.0000000
51470.0000000	6.2500000	-50.0000000
51470.0000000	6.5000000	-50.0000000
51470.0000000	6.7500000	-50.0000000
51470.0000000	7.0000000	-50.0000000
51470.0000000	7.2500000	-50.0000000
51470.0000000	7.5000000	-50.0000000
51470.0000000	7.7500000	-50.0000000
51470.0000000	8.0000000	-50.0000000
51470.0000000	8.2500000	-50.0000000
51470.0000000	8.5000000	-50.0000000
51470.0000000	8.7500000	-50.0000000
51470.0000000	9.0000000	-50.0000000
51470.0000000	9.2500000	-50.0000000
51470.0000000	9.5000000	-50.0000000
51470.0000000	9.7500000	-50.0000000
51470.0000000	10.0000000	-50.0000000
51470.0000000	10.2500000	-50.0000000
51470.0000000	10.5000000	-50.0000000
51470.0000000	10.7500000	-50.0000000
51470.0000000	11.0000000	-50.0000000
51470.0000000	11.2500000	-50.0000000
51470.0000000	11.5000000	-50.0000000
51470.0000000	11.7500000	-50.0000000
51470.0000000	12.0000000	-50.0000000
51470.0000000	12.2500000	-50.0000000
51470.0000000	12.5000000	-50.0000000
51470.0000000	12.7500000	-50.0000000
51470.0000000	13.0000000	-50.0000000
51470.0000000	13.2500000	-50.0000000
51470.0000000	13.5000000	-50.0000000
51470.0000000	13.7500000	-50.0000000
51470.0000000	14.0000000	-50.0000000
51470.0000000	14.2500000	-50.0000000
51470.0000000	14.5000000	-50.0000000
51470.0000000	14.7500000	-50.0000000
51470.0000000	15.0000000	-50.0000000
51500.0000000	0.0000000	-50.0000000
51500.0000000	0.2500000	-50.0000000
51500.0000000	0.5000000	17.9699993
51500.0000000	0.7500000	20.4899998
51500.0000000	1.0000000	17.2199993
51500.0000000	1.2500000	16.8099995
51500.0000000	1.5000000	15.6899996
51500.0000000	1.7500000	-50.0000000
51500.0000000	2.0000000	-50.0000000
51500.0000000	2.2500000	-50.0000000
51500.0000000	2.5000000	-50.0000000
51500.0000000	2.7500000	-50.0000000
51500.0000000	3.0000000	-50.0000000
51500.0000000	3.2500000	-50.0000000
51500.0000000	3.5000000	15.2600002
51500.0000000	3.7500000	16.8899994
51500.0000000	4.0000000	17.7199993
51500.0000000	4.2500000	-50.0000000

51500.0000000	4.5000000	-50.0000000
51500.0000000	4.7500000	-50.0000000
51500.0000000	5.0000000	-50.0000000
51500.0000000	5.2500000	-50.0000000
51500.0000000	5.5000000	-50.0000000
51500.0000000	5.7500000	-50.0000000
51500.0000000	6.0000000	-50.0000000
51500.0000000	6.2500000	-50.0000000
51500.0000000	6.5000000	-50.0000000
51500.0000000	6.7500000	-50.0000000
51500.0000000	7.0000000	-50.0000000
51500.0000000	7.2500000	-50.0000000
51500.0000000	7.5000000	-50.0000000
51500.0000000	7.7500000	-50.0000000
51500.0000000	8.0000000	-50.0000000
51500.0000000	8.2500000	-50.0000000
51500.0000000	8.5000000	-50.0000000
51500.0000000	8.7500000	-50.0000000
51500.0000000	9.0000000	-50.0000000
51500.0000000	9.2500000	-50.0000000
51500.0000000	9.5000000	-50.0000000
51500.0000000	9.7500000	-50.0000000
51500.0000000	10.0000000	-50.0000000
51500.0000000	10.2500000	-50.0000000
51500.0000000	10.5000000	-50.0000000
51500.0000000	10.7500000	-50.0000000
51500.0000000	11.0000000	-50.0000000
51500.0000000	11.2500000	-50.0000000
51500.0000000	11.5000000	-50.0000000
51500.0000000	11.7500000	-50.0000000
51500.0000000	12.0000000	-50.0000000
51500.0000000	12.2500000	-50.0000000
51500.0000000	12.5000000	-50.0000000
51500.0000000	12.7500000	-50.0000000
51500.0000000	13.0000000	-50.0000000
51500.0000000	13.2500000	-50.0000000
51500.0000000	13.5000000	-50.0000000
51500.0000000	13.7500000	-50.0000000
51500.0000000	14.0000000	-50.0000000
51500.0000000	14.2500000	-50.0000000
51500.0000000	14.5000000	-50.0000000
51500.0000000	14.7500000	-50.0000000
51500.0000000	15.0000000	-50.0000000
51530.0000000	0.0000000	-50.0000000
51530.0000000	0.2500000	-50.0000000
51530.0000000	0.5000000	19.3600006
51530.0000000	0.7500000	20.9200001
51530.0000000	1.0000000	16.4599991
51530.0000000	1.2500000	15.2399998
51530.0000000	1.5000000	-50.0000000
51530.0000000	1.7500000	-50.0000000
51530.0000000	2.0000000	-50.0000000
51530.0000000	2.2500000	-50.0000000
51530.0000000	2.5000000	-50.0000000
51530.0000000	2.7500000	-50.0000000
51530.0000000	3.0000000	-50.0000000
51530.0000000	3.2500000	15.2200003
51530.0000000	3.5000000	-50.0000000

51530.0000000	3.7500000	-50.0000000
51530.0000000	4.0000000	-50.0000000
51530.0000000	4.2500000	-50.0000000
51530.0000000	4.5000000	-50.0000000
51530.0000000	4.7500000	-50.0000000
51530.0000000	5.0000000	-50.0000000
51530.0000000	5.2500000	-50.0000000
51530.0000000	5.5000000	-50.0000000
51530.0000000	5.7500000	-50.0000000
51530.0000000	6.0000000	-50.0000000
51530.0000000	6.2500000	-50.0000000
51530.0000000	6.5000000	-50.0000000
51530.0000000	6.7500000	-50.0000000
51530.0000000	7.0000000	-50.0000000
51530.0000000	7.2500000	-50.0000000
51530.0000000	7.5000000	-50.0000000
51530.0000000	7.7500000	-50.0000000
51530.0000000	8.0000000	-50.0000000
51530.0000000	8.2500000	-50.0000000
51530.0000000	8.5000000	-50.0000000
51530.0000000	8.7500000	-50.0000000
51530.0000000	9.0000000	-50.0000000
51530.0000000	9.2500000	-50.0000000
51530.0000000	9.5000000	-50.0000000
51530.0000000	9.7500000	-50.0000000
51530.0000000	10.0000000	-50.0000000
51530.0000000	10.2500000	-50.0000000
51530.0000000	10.5000000	-50.0000000
51530.0000000	10.7500000	-50.0000000
51530.0000000	11.0000000	-50.0000000
51530.0000000	11.2500000	-50.0000000
51530.0000000	11.5000000	-50.0000000
51530.0000000	11.7500000	-50.0000000
51530.0000000	12.0000000	-50.0000000
51530.0000000	12.2500000	-50.0000000
51530.0000000	12.5000000	-50.0000000
51530.0000000	12.7500000	-50.0000000
51530.0000000	13.0000000	-50.0000000
51530.0000000	13.2500000	-50.0000000
51530.0000000	13.5000000	-50.0000000
51530.0000000	13.7500000	-50.0000000
51530.0000000	14.0000000	-50.0000000
51530.0000000	14.2500000	-50.0000000
51530.0000000	14.5000000	-50.0000000
51530.0000000	14.7500000	-50.0000000
51530.0000000	15.0000000	-50.0000000
51560.0000000	0.0000000	-50.0000000
51560.0000000	0.2500000	-50.0000000
51560.0000000	0.5000000	17.6200008
51560.0000000	0.7500000	21.3099995
51560.0000000	1.0000000	18.2600002
51560.0000000	1.2500000	16.5900002
51560.0000000	1.5000000	-50.0000000
51560.0000000	1.7500000	-50.0000000
51560.0000000	2.0000000	-50.0000000
51560.0000000	2.2500000	-50.0000000
51560.0000000	2.5000000	-50.0000000
51560.0000000	2.7500000	-50.0000000

51560.0000000	3.0000000	-50.0000000
51560.0000000	3.2500000	-50.0000000
51560.0000000	3.5000000	-50.0000000
51560.0000000	3.7500000	-50.0000000
51560.0000000	4.0000000	-50.0000000
51560.0000000	4.2500000	-50.0000000
51560.0000000	4.5000000	-50.0000000
51560.0000000	4.7500000	-50.0000000
51560.0000000	5.0000000	-50.0000000
51560.0000000	5.2500000	-50.0000000
51560.0000000	5.5000000	-50.0000000
51560.0000000	5.7500000	-50.0000000
51560.0000000	6.0000000	-50.0000000
51560.0000000	6.2500000	-50.0000000
51560.0000000	6.5000000	-50.0000000
51560.0000000	6.7500000	-50.0000000
51560.0000000	7.0000000	-50.0000000
51560.0000000	7.2500000	-50.0000000
51560.0000000	7.5000000	-50.0000000
51560.0000000	7.7500000	-50.0000000
51560.0000000	8.0000000	-50.0000000
51560.0000000	8.2500000	-50.0000000
51560.0000000	8.5000000	-50.0000000
51560.0000000	8.7500000	-50.0000000
51560.0000000	9.0000000	-50.0000000
51560.0000000	9.2500000	-50.0000000
51560.0000000	9.5000000	-50.0000000
51560.0000000	9.7500000	-50.0000000
51560.0000000	10.0000000	-50.0000000
51560.0000000	10.2500000	-50.0000000
51560.0000000	10.5000000	-50.0000000
51560.0000000	10.7500000	-50.0000000
51560.0000000	11.0000000	-50.0000000
51560.0000000	11.2500000	-50.0000000
51560.0000000	11.5000000	-50.0000000
51560.0000000	11.7500000	-50.0000000
51560.0000000	12.0000000	-50.0000000
51560.0000000	12.2500000	-50.0000000
51560.0000000	12.5000000	-50.0000000
51560.0000000	12.7500000	-50.0000000
51560.0000000	13.0000000	-50.0000000
51560.0000000	13.2500000	-50.0000000
51560.0000000	13.5000000	-50.0000000
51560.0000000	13.7500000	-50.0000000
51560.0000000	14.0000000	-50.0000000
51560.0000000	14.2500000	-50.0000000
51560.0000000	14.5000000	-50.0000000
51560.0000000	14.7500000	-50.0000000
51560.0000000	15.0000000	-50.0000000
51590.0000000	0.0000000	-50.0000000
51590.0000000	0.2500000	-50.0000000
51590.0000000	0.5000000	22.3099995
51590.0000000	0.7500000	25.0000000
51590.0000000	1.0000000	19.6700001
51590.0000000	1.2500000	-50.0000000
51590.0000000	1.5000000	-50.0000000
51590.0000000	1.7500000	-50.0000000
51590.0000000	2.0000000	-50.0000000

51590.0000000	2.2500000	-50.0000000
51590.0000000	2.5000000	-50.0000000
51590.0000000	2.7500000	15.6199999
51590.0000000	3.0000000	15.9899998
51590.0000000	3.2500000	16.2199993
51590.0000000	3.5000000	-50.0000000
51590.0000000	3.7500000	-50.0000000
51590.0000000	4.0000000	15.9899998
51590.0000000	4.2500000	15.0299997
51590.0000000	4.5000000	-50.0000000
51590.0000000	4.7500000	-50.0000000
51590.0000000	5.0000000	-50.0000000
51590.0000000	5.2500000	-50.0000000
51590.0000000	5.5000000	-50.0000000
51590.0000000	5.7500000	-50.0000000
51590.0000000	6.0000000	-50.0000000
51590.0000000	6.2500000	-50.0000000
51590.0000000	6.5000000	-50.0000000
51590.0000000	6.7500000	-50.0000000
51590.0000000	7.0000000	-50.0000000
51590.0000000	7.2500000	-50.0000000
51590.0000000	7.5000000	-50.0000000
51590.0000000	7.7500000	-50.0000000
51590.0000000	8.0000000	-50.0000000
51590.0000000	8.2500000	-50.0000000
51590.0000000	8.5000000	-50.0000000
51590.0000000	8.7500000	-50.0000000
51590.0000000	9.0000000	-50.0000000
51590.0000000	9.2500000	-50.0000000
51590.0000000	9.5000000	-50.0000000
51590.0000000	9.7500000	-50.0000000
51590.0000000	10.0000000	-50.0000000
51590.0000000	10.2500000	-50.0000000
51590.0000000	10.5000000	-50.0000000
51590.0000000	10.7500000	-50.0000000
51590.0000000	11.0000000	-50.0000000
51590.0000000	11.2500000	-50.0000000
51590.0000000	11.5000000	-50.0000000
51590.0000000	11.7500000	-50.0000000
51590.0000000	12.0000000	-50.0000000
51590.0000000	12.2500000	-50.0000000
51590.0000000	12.5000000	-50.0000000
51590.0000000	12.7500000	-50.0000000
51590.0000000	13.0000000	-50.0000000
51590.0000000	13.2500000	-50.0000000
51590.0000000	13.5000000	-50.0000000
51590.0000000	13.7500000	-50.0000000
51590.0000000	14.0000000	-50.0000000
51590.0000000	14.2500000	-50.0000000
51590.0000000	14.5000000	-50.0000000
51590.0000000	14.7500000	-50.0000000
51590.0000000	15.0000000	-50.0000000
51620.0000000	0.0000000	-50.0000000
51620.0000000	0.2500000	-50.0000000
51620.0000000	0.5000000	21.7600002
51620.0000000	0.7500000	22.8199997
51620.0000000	1.0000000	16.2800007
51620.0000000	1.2500000	17.5799999

51620.0000000	1.5000000	-50.0000000
51620.0000000	1.7500000	-50.0000000
51620.0000000	2.0000000	-50.0000000
51620.0000000	2.2500000	-50.0000000
51620.0000000	2.5000000	-50.0000000
51620.0000000	2.7500000	-50.0000000
51620.0000000	3.0000000	-50.0000000
51620.0000000	3.2500000	15.2900000
51620.0000000	3.5000000	-50.0000000
51620.0000000	3.7500000	-50.0000000
51620.0000000	4.0000000	-50.0000000
51620.0000000	4.2500000	-50.0000000
51620.0000000	4.5000000	-50.0000000
51620.0000000	4.7500000	-50.0000000
51620.0000000	5.0000000	-50.0000000
51620.0000000	5.2500000	-50.0000000
51620.0000000	5.5000000	-50.0000000
51620.0000000	5.7500000	-50.0000000
51620.0000000	6.0000000	-50.0000000
51620.0000000	6.2500000	-50.0000000
51620.0000000	6.5000000	-50.0000000
51620.0000000	6.7500000	-50.0000000
51620.0000000	7.0000000	-50.0000000
51620.0000000	7.2500000	-50.0000000
51620.0000000	7.5000000	-50.0000000
51620.0000000	7.7500000	-50.0000000
51620.0000000	8.0000000	-50.0000000
51620.0000000	8.2500000	-50.0000000
51620.0000000	8.5000000	-50.0000000
51620.0000000	8.7500000	-50.0000000
51620.0000000	9.0000000	-50.0000000
51620.0000000	9.2500000	-50.0000000
51620.0000000	9.5000000	-50.0000000
51620.0000000	9.7500000	-50.0000000
51620.0000000	10.0000000	-50.0000000
51620.0000000	10.2500000	-50.0000000
51620.0000000	10.5000000	-50.0000000
51620.0000000	10.7500000	-50.0000000
51620.0000000	11.0000000	-50.0000000
51620.0000000	11.2500000	-50.0000000
51620.0000000	11.5000000	-50.0000000
51620.0000000	11.7500000	-50.0000000
51620.0000000	12.0000000	-50.0000000
51620.0000000	12.2500000	-50.0000000
51620.0000000	12.5000000	-50.0000000
51620.0000000	12.7500000	-50.0000000
51620.0000000	13.0000000	-50.0000000
51620.0000000	13.2500000	-50.0000000
51620.0000000	13.5000000	-50.0000000
51620.0000000	13.7500000	-50.0000000
51620.0000000	14.0000000	-50.0000000
51620.0000000	14.2500000	-50.0000000
51620.0000000	14.5000000	-50.0000000
51620.0000000	14.7500000	-50.0000000
51620.0000000	15.0000000	-50.0000000
51650.0000000	0.0000000	-50.0000000
51650.0000000	0.2500000	-50.0000000
51650.0000000	0.5000000	24.1000004

51650.0000000	0.7500000	25.6200008
51650.0000000	1.0000000	17.3400002
51650.0000000	1.2500000	-50.0000000
51650.0000000	1.5000000	-50.0000000
51650.0000000	1.7500000	-50.0000000
51650.0000000	2.0000000	-50.0000000
51650.0000000	2.2500000	-50.0000000
51650.0000000	2.5000000	-50.0000000
51650.0000000	2.7500000	-50.0000000
51650.0000000	3.0000000	-50.0000000
51650.0000000	3.2500000	-50.0000000
51650.0000000	3.5000000	-50.0000000
51650.0000000	3.7500000	-50.0000000
51650.0000000	4.0000000	15.2100000
51650.0000000	4.2500000	-50.0000000
51650.0000000	4.5000000	-50.0000000
51650.0000000	4.7500000	-50.0000000
51650.0000000	5.0000000	-50.0000000
51650.0000000	5.2500000	-50.0000000
51650.0000000	5.5000000	-50.0000000
51650.0000000	5.7500000	-50.0000000
51650.0000000	6.0000000	-50.0000000
51650.0000000	6.2500000	-50.0000000
51650.0000000	6.5000000	-50.0000000
51650.0000000	6.7500000	-50.0000000
51650.0000000	7.0000000	-50.0000000
51650.0000000	7.2500000	-50.0000000
51650.0000000	7.5000000	-50.0000000
51650.0000000	7.7500000	-50.0000000
51650.0000000	8.0000000	-50.0000000
51650.0000000	8.2500000	-50.0000000
51650.0000000	8.5000000	-50.0000000
51650.0000000	8.7500000	-50.0000000
51650.0000000	9.0000000	-50.0000000
51650.0000000	9.2500000	-50.0000000
51650.0000000	9.5000000	-50.0000000
51650.0000000	9.7500000	-50.0000000
51650.0000000	10.0000000	-50.0000000
51650.0000000	10.2500000	-50.0000000
51650.0000000	10.5000000	-50.0000000
51650.0000000	10.7500000	-50.0000000
51650.0000000	11.0000000	-50.0000000
51650.0000000	11.2500000	-50.0000000
51650.0000000	11.5000000	-50.0000000
51650.0000000	11.7500000	-50.0000000
51650.0000000	12.0000000	-50.0000000
51650.0000000	12.2500000	-50.0000000
51650.0000000	12.5000000	-50.0000000
51650.0000000	12.7500000	-50.0000000
51650.0000000	13.0000000	-50.0000000
51650.0000000	13.2500000	-50.0000000
51650.0000000	13.5000000	-50.0000000
51650.0000000	13.7500000	-50.0000000
51650.0000000	14.0000000	-50.0000000
51650.0000000	14.2500000	-50.0000000
51650.0000000	14.5000000	-50.0000000
51650.0000000	14.7500000	-50.0000000
51650.0000000	15.0000000	-50.0000000

51680.0000000	0.0000000	-50.0000000
51680.0000000	0.2500000	-50.0000000
51680.0000000	0.5000000	21.3899994
51680.0000000	0.7500000	23.4599991
51680.0000000	1.0000000	17.7500000
51680.0000000	1.2500000	-50.0000000
51680.0000000	1.5000000	15.4600000
51680.0000000	1.7500000	-50.0000000
51680.0000000	2.0000000	-50.0000000
51680.0000000	2.2500000	-50.0000000
51680.0000000	2.5000000	-50.0000000
51680.0000000	2.7500000	-50.0000000
51680.0000000	3.0000000	-50.0000000
51680.0000000	3.2500000	-50.0000000
51680.0000000	3.5000000	-50.0000000
51680.0000000	3.7500000	-50.0000000
51680.0000000	4.0000000	-50.0000000
51680.0000000	4.2500000	-50.0000000
51680.0000000	4.5000000	-50.0000000
51680.0000000	4.7500000	-50.0000000
51680.0000000	5.0000000	-50.0000000
51680.0000000	5.2500000	-50.0000000
51680.0000000	5.5000000	-50.0000000
51680.0000000	5.7500000	-50.0000000
51680.0000000	6.0000000	-50.0000000
51680.0000000	6.2500000	-50.0000000
51680.0000000	6.5000000	-50.0000000
51680.0000000	6.7500000	-50.0000000
51680.0000000	7.0000000	-50.0000000
51680.0000000	7.2500000	-50.0000000
51680.0000000	7.5000000	-50.0000000
51680.0000000	7.7500000	-50.0000000
51680.0000000	8.0000000	-50.0000000
51680.0000000	8.2500000	-50.0000000
51680.0000000	8.5000000	-50.0000000
51680.0000000	8.7500000	-50.0000000
51680.0000000	9.0000000	-50.0000000
51680.0000000	9.2500000	-50.0000000
51680.0000000	9.5000000	-50.0000000
51680.0000000	9.7500000	-50.0000000
51680.0000000	10.0000000	-50.0000000
51680.0000000	10.2500000	-50.0000000
51680.0000000	10.5000000	-50.0000000
51680.0000000	10.7500000	-50.0000000
51680.0000000	11.0000000	-50.0000000
51680.0000000	11.2500000	-50.0000000
51680.0000000	11.5000000	-50.0000000
51680.0000000	11.7500000	-50.0000000
51680.0000000	12.0000000	-50.0000000
51680.0000000	12.2500000	-50.0000000
51680.0000000	12.5000000	-50.0000000
51680.0000000	12.7500000	-50.0000000
51680.0000000	13.0000000	-50.0000000
51680.0000000	13.2500000	-50.0000000
51680.0000000	13.5000000	-50.0000000
51680.0000000	13.7500000	-50.0000000
51680.0000000	14.0000000	-50.0000000
51680.0000000	14.2500000	-50.0000000

51680.0000000	14.5000000	-50.0000000
51680.0000000	14.7500000	-50.0000000
51680.0000000	15.0000000	-50.0000000
51710.0000000	0.0000000	-50.0000000
51710.0000000	0.2500000	-50.0000000
51710.0000000	0.5000000	22.5000000
51710.0000000	0.7500000	24.3899994
51710.0000000	1.0000000	17.0000000
51710.0000000	1.2500000	-50.0000000
51710.0000000	1.5000000	-50.0000000
51710.0000000	1.7500000	-50.0000000
51710.0000000	2.0000000	-50.0000000
51710.0000000	2.2500000	-50.0000000
51710.0000000	2.5000000	-50.0000000
51710.0000000	2.7500000	16.3600006
51710.0000000	3.0000000	-50.0000000
51710.0000000	3.2500000	-50.0000000
51710.0000000	3.5000000	-50.0000000
51710.0000000	3.7500000	-50.0000000
51710.0000000	4.0000000	-50.0000000
51710.0000000	4.2500000	-50.0000000
51710.0000000	4.5000000	-50.0000000
51710.0000000	4.7500000	-50.0000000
51710.0000000	5.0000000	-50.0000000
51710.0000000	5.2500000	-50.0000000
51710.0000000	5.5000000	-50.0000000
51710.0000000	5.7500000	-50.0000000
51710.0000000	6.0000000	-50.0000000
51710.0000000	6.2500000	-50.0000000
51710.0000000	6.5000000	-50.0000000
51710.0000000	6.7500000	-50.0000000
51710.0000000	7.0000000	-50.0000000
51710.0000000	7.2500000	-50.0000000
51710.0000000	7.5000000	-50.0000000
51710.0000000	7.7500000	-50.0000000
51710.0000000	8.0000000	-50.0000000
51710.0000000	8.2500000	-50.0000000
51710.0000000	8.5000000	-50.0000000
51710.0000000	8.7500000	-50.0000000
51710.0000000	9.0000000	-50.0000000
51710.0000000	9.2500000	-50.0000000
51710.0000000	9.5000000	-50.0000000
51710.0000000	9.7500000	-50.0000000
51710.0000000	10.0000000	-50.0000000
51710.0000000	10.2500000	-50.0000000
51710.0000000	10.5000000	-50.0000000
51710.0000000	10.7500000	-50.0000000
51710.0000000	11.0000000	-50.0000000
51710.0000000	11.2500000	-50.0000000
51710.0000000	11.5000000	-50.0000000
51710.0000000	11.7500000	-50.0000000
51710.0000000	12.0000000	-50.0000000
51710.0000000	12.2500000	-50.0000000
51710.0000000	12.5000000	-50.0000000
51710.0000000	12.7500000	-50.0000000
51710.0000000	13.0000000	-50.0000000
51710.0000000	13.2500000	-50.0000000
51710.0000000	13.5000000	-50.0000000

51710.0000000	13.7500000	-50.0000000
51710.0000000	14.0000000	-50.0000000
51710.0000000	14.2500000	-50.0000000
51710.0000000	14.5000000	-50.0000000
51710.0000000	14.7500000	-50.0000000
51710.0000000	15.0000000	-50.0000000
51740.0000000	0.0000000	-50.0000000
51740.0000000	0.2500000	-50.0000000
51740.0000000	0.5000000	22.7399998
51740.0000000	0.7500000	24.0200005
51740.0000000	1.0000000	18.2700005
51740.0000000	1.2500000	17.2099991
51740.0000000	1.5000000	-50.0000000
51740.0000000	1.7500000	-50.0000000
51740.0000000	2.0000000	-50.0000000
51740.0000000	2.2500000	-50.0000000
51740.0000000	2.5000000	-50.0000000
51740.0000000	2.7500000	-50.0000000
51740.0000000	3.0000000	-50.0000000
51740.0000000	3.2500000	-50.0000000
51740.0000000	3.5000000	-50.0000000
51740.0000000	3.7500000	-50.0000000
51740.0000000	4.0000000	16.5300007
51740.0000000	4.2500000	-50.0000000
51740.0000000	4.5000000	-50.0000000
51740.0000000	4.7500000	-50.0000000
51740.0000000	5.0000000	-50.0000000
51740.0000000	5.2500000	-50.0000000
51740.0000000	5.5000000	-50.0000000
51740.0000000	5.7500000	-50.0000000
51740.0000000	6.0000000	-50.0000000
51740.0000000	6.2500000	-50.0000000
51740.0000000	6.5000000	-50.0000000
51740.0000000	6.7500000	-50.0000000
51740.0000000	7.0000000	-50.0000000
51740.0000000	7.2500000	-50.0000000
51740.0000000	7.5000000	-50.0000000
51740.0000000	7.7500000	-50.0000000
51740.0000000	8.0000000	-50.0000000
51740.0000000	8.2500000	-50.0000000
51740.0000000	8.5000000	-50.0000000
51740.0000000	8.7500000	-50.0000000
51740.0000000	9.0000000	-50.0000000
51740.0000000	9.2500000	-50.0000000
51740.0000000	9.5000000	-50.0000000
51740.0000000	9.7500000	-50.0000000
51740.0000000	10.0000000	-50.0000000
51740.0000000	10.2500000	-50.0000000
51740.0000000	10.5000000	-50.0000000
51740.0000000	10.7500000	-50.0000000
51740.0000000	11.0000000	-50.0000000
51740.0000000	11.2500000	-50.0000000
51740.0000000	11.5000000	-50.0000000
51740.0000000	11.7500000	-50.0000000
51740.0000000	12.0000000	-50.0000000
51740.0000000	12.2500000	-50.0000000
51740.0000000	12.5000000	-50.0000000
51740.0000000	12.7500000	-50.0000000

51740.0000000	13.0000000	-50.0000000
51740.0000000	13.2500000	-50.0000000
51740.0000000	13.5000000	-50.0000000
51740.0000000	13.7500000	-50.0000000
51740.0000000	14.0000000	-50.0000000
51740.0000000	14.2500000	-50.0000000
51740.0000000	14.5000000	-50.0000000
51740.0000000	14.7500000	-50.0000000
51740.0000000	15.0000000	-50.0000000
51770.0000000	0.0000000	-50.0000000
51770.0000000	0.2500000	-50.0000000
51770.0000000	0.5000000	22.1299992
51770.0000000	0.7500000	24.2700005
51770.0000000	1.0000000	18.7999992
51770.0000000	1.2500000	-50.0000000
51770.0000000	1.5000000	-50.0000000
51770.0000000	1.7500000	-50.0000000
51770.0000000	2.0000000	-50.0000000
51770.0000000	2.2500000	-50.0000000
51770.0000000	2.5000000	-50.0000000
51770.0000000	2.7500000	-50.0000000
51770.0000000	3.0000000	-50.0000000
51770.0000000	3.2500000	-50.0000000
51770.0000000	3.5000000	16.5300007
51770.0000000	3.7500000	16.4899998
51770.0000000	4.0000000	16.3799992
51770.0000000	4.2500000	-50.0000000
51770.0000000	4.5000000	-50.0000000
51770.0000000	4.7500000	-50.0000000
51770.0000000	5.0000000	-50.0000000
51770.0000000	5.2500000	-50.0000000
51770.0000000	5.5000000	-50.0000000
51770.0000000	5.7500000	-50.0000000
51770.0000000	6.0000000	-50.0000000
51770.0000000	6.2500000	-50.0000000
51770.0000000	6.5000000	-50.0000000
51770.0000000	6.7500000	-50.0000000
51770.0000000	7.0000000	-50.0000000
51770.0000000	7.2500000	-50.0000000
51770.0000000	7.5000000	-50.0000000
51770.0000000	7.7500000	-50.0000000
51770.0000000	8.0000000	-50.0000000
51770.0000000	8.2500000	-50.0000000
51770.0000000	8.5000000	-50.0000000
51770.0000000	8.7500000	-50.0000000
51770.0000000	9.0000000	-50.0000000
51770.0000000	9.2500000	-50.0000000
51770.0000000	9.5000000	-50.0000000
51770.0000000	9.7500000	-50.0000000
51770.0000000	10.0000000	-50.0000000
51770.0000000	10.2500000	-50.0000000
51770.0000000	10.5000000	-50.0000000
51770.0000000	10.7500000	-50.0000000
51770.0000000	11.0000000	-50.0000000
51770.0000000	11.2500000	-50.0000000
51770.0000000	11.5000000	-50.0000000
51770.0000000	11.7500000	-50.0000000
51770.0000000	12.0000000	-50.0000000

51770.0000000	12.2500000	-50.0000000
51770.0000000	12.5000000	-50.0000000
51770.0000000	12.7500000	-50.0000000
51770.0000000	13.0000000	-50.0000000
51770.0000000	13.2500000	-50.0000000
51770.0000000	13.5000000	-50.0000000
51770.0000000	13.7500000	-50.0000000
51770.0000000	14.0000000	-50.0000000
51770.0000000	14.2500000	-50.0000000
51770.0000000	14.5000000	-50.0000000
51770.0000000	14.7500000	-50.0000000
51770.0000000	15.0000000	-50.0000000
51800.0000000	0.0000000	-50.0000000
51800.0000000	0.2500000	-50.0000000
51800.0000000	0.5000000	21.8500004
51800.0000000	0.7500000	24.0900002
51800.0000000	1.0000000	19.1599998
51800.0000000	1.2500000	16.9899998
51800.0000000	1.5000000	15.3299999
51800.0000000	1.7500000	-50.0000000
51800.0000000	2.0000000	-50.0000000
51800.0000000	2.2500000	-50.0000000
51800.0000000	2.5000000	-50.0000000
51800.0000000	2.7500000	-50.0000000
51800.0000000	3.0000000	-50.0000000
51800.0000000	3.2500000	-50.0000000
51800.0000000	3.5000000	15.1899996
51800.0000000	3.7500000	15.0100002
51800.0000000	4.0000000	-50.0000000
51800.0000000	4.2500000	-50.0000000
51800.0000000	4.5000000	-50.0000000
51800.0000000	4.7500000	-50.0000000
51800.0000000	5.0000000	-50.0000000
51800.0000000	5.2500000	-50.0000000
51800.0000000	5.5000000	-50.0000000
51800.0000000	5.7500000	-50.0000000
51800.0000000	6.0000000	-50.0000000
51800.0000000	6.2500000	-50.0000000
51800.0000000	6.5000000	-50.0000000
51800.0000000	6.7500000	-50.0000000
51800.0000000	7.0000000	-50.0000000
51800.0000000	7.2500000	-50.0000000
51800.0000000	7.5000000	-50.0000000
51800.0000000	7.7500000	-50.0000000
51800.0000000	8.0000000	-50.0000000
51800.0000000	8.2500000	-50.0000000
51800.0000000	8.5000000	-50.0000000
51800.0000000	8.7500000	-50.0000000
51800.0000000	9.0000000	-50.0000000
51800.0000000	9.2500000	-50.0000000
51800.0000000	9.5000000	-50.0000000
51800.0000000	9.7500000	-50.0000000
51800.0000000	10.0000000	-50.0000000
51800.0000000	10.2500000	-50.0000000
51800.0000000	10.5000000	-50.0000000
51800.0000000	10.7500000	-50.0000000
51800.0000000	11.0000000	-50.0000000
51800.0000000	11.2500000	-50.0000000

51800.0000000	11.5000000	-50.0000000
51800.0000000	11.7500000	-50.0000000
51800.0000000	12.0000000	-50.0000000
51800.0000000	12.2500000	-50.0000000
51800.0000000	12.5000000	-50.0000000
51800.0000000	12.7500000	-50.0000000
51800.0000000	13.0000000	-50.0000000
51800.0000000	13.2500000	-50.0000000
51800.0000000	13.5000000	-50.0000000
51800.0000000	13.7500000	-50.0000000
51800.0000000	14.0000000	-50.0000000
51800.0000000	14.2500000	-50.0000000
51800.0000000	14.5000000	-50.0000000
51800.0000000	14.7500000	-50.0000000
51800.0000000	15.0000000	-50.0000000
51830.0000000	0.0000000	-50.0000000
51830.0000000	0.2500000	-50.0000000
51830.0000000	0.5000000	20.7700005
51830.0000000	0.7500000	24.2099991
51830.0000000	1.0000000	19.5799999
51830.0000000	1.2500000	15.4600000
51830.0000000	1.5000000	-50.0000000
51830.0000000	1.7500000	-50.0000000
51830.0000000	2.0000000	-50.0000000
51830.0000000	2.2500000	-50.0000000
51830.0000000	2.5000000	-50.0000000
51830.0000000	2.7500000	-50.0000000
51830.0000000	3.0000000	-50.0000000
51830.0000000	3.2500000	-50.0000000
51830.0000000	3.5000000	-50.0000000
51830.0000000	3.7500000	-50.0000000
51830.0000000	4.0000000	-50.0000000
51830.0000000	4.2500000	-50.0000000
51830.0000000	4.5000000	-50.0000000
51830.0000000	4.7500000	-50.0000000
51830.0000000	5.0000000	-50.0000000
51830.0000000	5.2500000	-50.0000000
51830.0000000	5.5000000	-50.0000000
51830.0000000	5.7500000	-50.0000000
51830.0000000	6.0000000	-50.0000000
51830.0000000	6.2500000	-50.0000000
51830.0000000	6.5000000	-50.0000000
51830.0000000	6.7500000	-50.0000000
51830.0000000	7.0000000	-50.0000000
51830.0000000	7.2500000	-50.0000000
51830.0000000	7.5000000	-50.0000000
51830.0000000	7.7500000	-50.0000000
51830.0000000	8.0000000	-50.0000000
51830.0000000	8.2500000	-50.0000000
51830.0000000	8.5000000	-50.0000000
51830.0000000	8.7500000	-50.0000000
51830.0000000	9.0000000	-50.0000000
51830.0000000	9.2500000	-50.0000000
51830.0000000	9.5000000	-50.0000000
51830.0000000	9.7500000	-50.0000000
51830.0000000	10.0000000	-50.0000000
51830.0000000	10.2500000	-50.0000000
51830.0000000	10.5000000	-50.0000000

51830.0000000	10.7500000	-50.0000000
51830.0000000	11.0000000	-50.0000000
51830.0000000	11.2500000	-50.0000000
51830.0000000	11.5000000	-50.0000000
51830.0000000	11.7500000	-50.0000000
51830.0000000	12.0000000	-50.0000000
51830.0000000	12.2500000	-50.0000000
51830.0000000	12.5000000	-50.0000000
51830.0000000	12.7500000	-50.0000000
51830.0000000	13.0000000	-50.0000000
51830.0000000	13.2500000	-50.0000000
51830.0000000	13.5000000	-50.0000000
51830.0000000	13.7500000	-50.0000000
51830.0000000	14.0000000	-50.0000000
51830.0000000	14.2500000	-50.0000000
51830.0000000	14.5000000	-50.0000000
51830.0000000	14.7500000	-50.0000000
51830.0000000	15.0000000	-50.0000000
51860.0000000	0.0000000	-50.0000000
51860.0000000	0.2500000	-50.0000000
51860.0000000	0.5000000	22.7299995
51860.0000000	0.7500000	24.6000004
51860.0000000	1.0000000	17.7800007
51860.0000000	1.2500000	-50.0000000
51860.0000000	1.5000000	-50.0000000
51860.0000000	1.7500000	-50.0000000
51860.0000000	2.0000000	-50.0000000
51860.0000000	2.2500000	-50.0000000
51860.0000000	2.5000000	-50.0000000
51860.0000000	2.7500000	-50.0000000
51860.0000000	3.0000000	-50.0000000
51860.0000000	3.2500000	-50.0000000
51860.0000000	3.5000000	-50.0000000
51860.0000000	3.7500000	-50.0000000
51860.0000000	4.0000000	-50.0000000
51860.0000000	4.2500000	-50.0000000
51860.0000000	4.5000000	-50.0000000
51860.0000000	4.7500000	-50.0000000
51860.0000000	5.0000000	-50.0000000
51860.0000000	5.2500000	-50.0000000
51860.0000000	5.5000000	-50.0000000
51860.0000000	5.7500000	-50.0000000
51860.0000000	6.0000000	-50.0000000
51860.0000000	6.2500000	-50.0000000
51860.0000000	6.5000000	-50.0000000
51860.0000000	6.7500000	-50.0000000
51860.0000000	7.0000000	-50.0000000
51860.0000000	7.2500000	-50.0000000
51860.0000000	7.5000000	-50.0000000
51860.0000000	7.7500000	-50.0000000
51860.0000000	8.0000000	-50.0000000
51860.0000000	8.2500000	-50.0000000
51860.0000000	8.5000000	-50.0000000
51860.0000000	8.7500000	-50.0000000
51860.0000000	9.0000000	-50.0000000
51860.0000000	9.2500000	-50.0000000
51860.0000000	9.5000000	-50.0000000
51860.0000000	9.7500000	-50.0000000

51860.0000000	10.0000000	-50.0000000
51860.0000000	10.2500000	-50.0000000
51860.0000000	10.5000000	-50.0000000
51860.0000000	10.7500000	-50.0000000
51860.0000000	11.0000000	-50.0000000
51860.0000000	11.2500000	-50.0000000
51860.0000000	11.5000000	-50.0000000
51860.0000000	11.7500000	-50.0000000
51860.0000000	12.0000000	-50.0000000
51860.0000000	12.2500000	-50.0000000
51860.0000000	12.5000000	-50.0000000
51860.0000000	12.7500000	-50.0000000
51860.0000000	13.0000000	-50.0000000
51860.0000000	13.2500000	-50.0000000
51860.0000000	13.5000000	-50.0000000
51860.0000000	13.7500000	-50.0000000
51860.0000000	14.0000000	-50.0000000
51860.0000000	14.2500000	-50.0000000
51860.0000000	14.5000000	-50.0000000
51860.0000000	14.7500000	-50.0000000
51860.0000000	15.0000000	-50.0000000
51890.0000000	0.0000000	-50.0000000
51890.0000000	0.2500000	-50.0000000
51890.0000000	0.5000000	20.5499992
51890.0000000	0.7500000	23.3500004
51890.0000000	1.0000000	18.3799992
51890.0000000	1.2500000	16.8400002
51890.0000000	1.5000000	-50.0000000
51890.0000000	1.7500000	-50.0000000
51890.0000000	2.0000000	-50.0000000
51890.0000000	2.2500000	-50.0000000
51890.0000000	2.5000000	-50.0000000
51890.0000000	2.7500000	-50.0000000
51890.0000000	3.0000000	-50.0000000
51890.0000000	3.2500000	-50.0000000
51890.0000000	3.5000000	-50.0000000
51890.0000000	3.7500000	-50.0000000
51890.0000000	4.0000000	15.5299997
51890.0000000	4.2500000	15.2799997
51890.0000000	4.5000000	-50.0000000
51890.0000000	4.7500000	-50.0000000
51890.0000000	5.0000000	-50.0000000
51890.0000000	5.2500000	-50.0000000
51890.0000000	5.5000000	-50.0000000
51890.0000000	5.7500000	-50.0000000
51890.0000000	6.0000000	-50.0000000
51890.0000000	6.2500000	-50.0000000
51890.0000000	6.5000000	-50.0000000
51890.0000000	6.7500000	-50.0000000
51890.0000000	7.0000000	-50.0000000
51890.0000000	7.2500000	-50.0000000
51890.0000000	7.5000000	-50.0000000
51890.0000000	7.7500000	-50.0000000
51890.0000000	8.0000000	-50.0000000
51890.0000000	8.2500000	-50.0000000
51890.0000000	8.5000000	-50.0000000
51890.0000000	8.7500000	-50.0000000
51890.0000000	9.0000000	-50.0000000

51890.0000000	9.2500000	-50.0000000
51890.0000000	9.5000000	-50.0000000
51890.0000000	9.7500000	-50.0000000
51890.0000000	10.0000000	-50.0000000
51890.0000000	10.2500000	-50.0000000
51890.0000000	10.5000000	-50.0000000
51890.0000000	10.7500000	-50.0000000
51890.0000000	11.0000000	-50.0000000
51890.0000000	11.2500000	-50.0000000
51890.0000000	11.5000000	-50.0000000
51890.0000000	11.7500000	-50.0000000
51890.0000000	12.0000000	-50.0000000
51890.0000000	12.2500000	-50.0000000
51890.0000000	12.5000000	-50.0000000
51890.0000000	12.7500000	-50.0000000
51890.0000000	13.0000000	-50.0000000
51890.0000000	13.2500000	-50.0000000
51890.0000000	13.5000000	-50.0000000
51890.0000000	13.7500000	-50.0000000
51890.0000000	14.0000000	-50.0000000
51890.0000000	14.2500000	-50.0000000
51890.0000000	14.5000000	-50.0000000
51890.0000000	14.7500000	-50.0000000
51890.0000000	15.0000000	-50.0000000
51920.0000000	0.0000000	-50.0000000
51920.0000000	0.2500000	-50.0000000
51920.0000000	0.5000000	21.4699993
51920.0000000	0.7500000	24.4500008
51920.0000000	1.0000000	18.6000004
51920.0000000	1.2500000	-50.0000000
51920.0000000	1.5000000	-50.0000000
51920.0000000	1.7500000	-50.0000000
51920.0000000	2.0000000	-50.0000000
51920.0000000	2.2500000	-50.0000000
51920.0000000	2.5000000	-50.0000000
51920.0000000	2.7500000	-50.0000000
51920.0000000	3.0000000	-50.0000000
51920.0000000	3.2500000	-50.0000000
51920.0000000	3.5000000	15.0000000
51920.0000000	3.7500000	-50.0000000
51920.0000000	4.0000000	-50.0000000
51920.0000000	4.2500000	-50.0000000
51920.0000000	4.5000000	-50.0000000
51920.0000000	4.7500000	-50.0000000
51920.0000000	5.0000000	-50.0000000
51920.0000000	5.2500000	-50.0000000
51920.0000000	5.5000000	-50.0000000
51920.0000000	5.7500000	-50.0000000
51920.0000000	6.0000000	-50.0000000
51920.0000000	6.2500000	-50.0000000
51920.0000000	6.5000000	-50.0000000
51920.0000000	6.7500000	-50.0000000
51920.0000000	7.0000000	-50.0000000
51920.0000000	7.2500000	-50.0000000
51920.0000000	7.5000000	-50.0000000
51920.0000000	7.7500000	-50.0000000
51920.0000000	8.0000000	-50.0000000
51920.0000000	8.2500000	-50.0000000

51920.0000000	8.5000000	-50.0000000
51920.0000000	8.7500000	-50.0000000
51920.0000000	9.0000000	-50.0000000
51920.0000000	9.2500000	-50.0000000
51920.0000000	9.5000000	-50.0000000
51920.0000000	9.7500000	-50.0000000
51920.0000000	10.0000000	-50.0000000
51920.0000000	10.2500000	-50.0000000
51920.0000000	10.5000000	-50.0000000
51920.0000000	10.7500000	-50.0000000
51920.0000000	11.0000000	-50.0000000
51920.0000000	11.2500000	-50.0000000
51920.0000000	11.5000000	-50.0000000
51920.0000000	11.7500000	-50.0000000
51920.0000000	12.0000000	-50.0000000
51920.0000000	12.2500000	-50.0000000
51920.0000000	12.5000000	-50.0000000
51920.0000000	12.7500000	-50.0000000
51920.0000000	13.0000000	-50.0000000
51920.0000000	13.2500000	-50.0000000
51920.0000000	13.5000000	-50.0000000
51920.0000000	13.7500000	-50.0000000
51920.0000000	14.0000000	-50.0000000
51920.0000000	14.2500000	-50.0000000
51920.0000000	14.5000000	-50.0000000
51920.0000000	14.7500000	-50.0000000
51920.0000000	15.0000000	-50.0000000
51950.0000000	0.0000000	-50.0000000
51950.0000000	0.2500000	-50.0000000
51950.0000000	0.5000000	21.6599998
51950.0000000	0.7500000	23.8999996
51950.0000000	1.0000000	15.4200001
51950.0000000	1.2500000	-50.0000000
51950.0000000	1.5000000	-50.0000000
51950.0000000	1.7500000	-50.0000000
51950.0000000	2.0000000	15.0000000
51950.0000000	2.2500000	-50.0000000
51950.0000000	2.5000000	-50.0000000
51950.0000000	2.7500000	-50.0000000
51950.0000000	3.0000000	-50.0000000
51950.0000000	3.2500000	17.4200001
51950.0000000	3.5000000	18.1499996
51950.0000000	3.7500000	-50.0000000
51950.0000000	4.0000000	-50.0000000
51950.0000000	4.2500000	-50.0000000
51950.0000000	4.5000000	-50.0000000
51950.0000000	4.7500000	-50.0000000
51950.0000000	5.0000000	-50.0000000
51950.0000000	5.2500000	-50.0000000
51950.0000000	5.5000000	-50.0000000
51950.0000000	5.7500000	-50.0000000
51950.0000000	6.0000000	-50.0000000
51950.0000000	6.2500000	-50.0000000
51950.0000000	6.5000000	-50.0000000
51950.0000000	6.7500000	-50.0000000
51950.0000000	7.0000000	-50.0000000
51950.0000000	7.2500000	-50.0000000
51950.0000000	7.5000000	-50.0000000

51950.0000000	7.7500000	-50.0000000
51950.0000000	8.0000000	-50.0000000
51950.0000000	8.2500000	-50.0000000
51950.0000000	8.5000000	-50.0000000
51950.0000000	8.7500000	-50.0000000
51950.0000000	9.0000000	-50.0000000
51950.0000000	9.2500000	-50.0000000
51950.0000000	9.5000000	-50.0000000
51950.0000000	9.7500000	-50.0000000
51950.0000000	10.0000000	-50.0000000
51950.0000000	10.2500000	-50.0000000
51950.0000000	10.5000000	-50.0000000
51950.0000000	10.7500000	-50.0000000
51950.0000000	11.0000000	-50.0000000
51950.0000000	11.2500000	-50.0000000
51950.0000000	11.5000000	-50.0000000
51950.0000000	11.7500000	-50.0000000
51950.0000000	12.0000000	-50.0000000
51950.0000000	12.2500000	-50.0000000
51950.0000000	12.5000000	-50.0000000
51950.0000000	12.7500000	-50.0000000
51950.0000000	13.0000000	-50.0000000
51950.0000000	13.2500000	-50.0000000
51950.0000000	13.5000000	-50.0000000
51950.0000000	13.7500000	-50.0000000
51950.0000000	14.0000000	-50.0000000
51950.0000000	14.2500000	-50.0000000
51950.0000000	14.5000000	-50.0000000
51950.0000000	14.7500000	-50.0000000
51950.0000000	15.0000000	-50.0000000
51980.0000000	0.0000000	-50.0000000
51980.0000000	0.2500000	-50.0000000
51980.0000000	0.5000000	20.9899998
51980.0000000	0.7500000	22.7299995
51980.0000000	1.0000000	16.1299992
51980.0000000	1.2500000	15.0200005
51980.0000000	1.5000000	15.3500004
51980.0000000	1.7500000	-50.0000000
51980.0000000	2.0000000	-50.0000000
51980.0000000	2.2500000	-50.0000000
51980.0000000	2.5000000	-50.0000000
51980.0000000	2.7500000	-50.0000000
51980.0000000	3.0000000	15.8599997
51980.0000000	3.2500000	15.0400000
51980.0000000	3.5000000	17.2099991
51980.0000000	3.7500000	15.7100000
51980.0000000	4.0000000	18.0499992
51980.0000000	4.2500000	-50.0000000
51980.0000000	4.5000000	-50.0000000
51980.0000000	4.7500000	-50.0000000
51980.0000000	5.0000000	-50.0000000
51980.0000000	5.2500000	-50.0000000
51980.0000000	5.5000000	-50.0000000
51980.0000000	5.7500000	-50.0000000
51980.0000000	6.0000000	-50.0000000
51980.0000000	6.2500000	-50.0000000
51980.0000000	6.5000000	-50.0000000
51980.0000000	6.7500000	-50.0000000

51980.0000000	7.0000000	-50.0000000
51980.0000000	7.2500000	-50.0000000
51980.0000000	7.5000000	-50.0000000
51980.0000000	7.7500000	-50.0000000
51980.0000000	8.0000000	-50.0000000
51980.0000000	8.2500000	-50.0000000
51980.0000000	8.5000000	-50.0000000
51980.0000000	8.7500000	-50.0000000
51980.0000000	9.0000000	-50.0000000
51980.0000000	9.2500000	-50.0000000
51980.0000000	9.5000000	-50.0000000
51980.0000000	9.7500000	-50.0000000
51980.0000000	10.0000000	-50.0000000
51980.0000000	10.2500000	-50.0000000
51980.0000000	10.5000000	-50.0000000
51980.0000000	10.7500000	-50.0000000
51980.0000000	11.0000000	-50.0000000
51980.0000000	11.2500000	-50.0000000
51980.0000000	11.5000000	-50.0000000
51980.0000000	11.7500000	-50.0000000
51980.0000000	12.0000000	-50.0000000
51980.0000000	12.2500000	-50.0000000
51980.0000000	12.5000000	-50.0000000
51980.0000000	12.7500000	-50.0000000
51980.0000000	13.0000000	-50.0000000
51980.0000000	13.2500000	-50.0000000
51980.0000000	13.5000000	-50.0000000
51980.0000000	13.7500000	-50.0000000
51980.0000000	14.0000000	-50.0000000
51980.0000000	14.2500000	-50.0000000
51980.0000000	14.5000000	-50.0000000
51980.0000000	14.7500000	-50.0000000
51980.0000000	15.0000000	-50.0000000
52010.0000000	0.0000000	-50.0000000
52010.0000000	0.2500000	-50.0000000
52010.0000000	0.5000000	20.2999992
52010.0000000	0.7500000	22.0300007
52010.0000000	1.0000000	-50.0000000
52010.0000000	1.2500000	-50.0000000
52010.0000000	1.5000000	-50.0000000
52010.0000000	1.7500000	-50.0000000
52010.0000000	2.0000000	-50.0000000
52010.0000000	2.2500000	-50.0000000
52010.0000000	2.5000000	16.7099991
52010.0000000	2.7500000	-50.0000000
52010.0000000	3.0000000	15.1999998
52010.0000000	3.2500000	15.1300001
52010.0000000	3.5000000	16.8700008
52010.0000000	3.7500000	-50.0000000
52010.0000000	4.0000000	-50.0000000
52010.0000000	4.2500000	-50.0000000
52010.0000000	4.5000000	-50.0000000
52010.0000000	4.7500000	-50.0000000
52010.0000000	5.0000000	-50.0000000
52010.0000000	5.2500000	-50.0000000
52010.0000000	5.5000000	-50.0000000
52010.0000000	5.7500000	-50.0000000
52010.0000000	6.0000000	-50.0000000

52010.0000000	6.2500000	-50.0000000
52010.0000000	6.5000000	-50.0000000
52010.0000000	6.7500000	-50.0000000
52010.0000000	7.0000000	-50.0000000
52010.0000000	7.2500000	-50.0000000
52010.0000000	7.5000000	-50.0000000
52010.0000000	7.7500000	-50.0000000
52010.0000000	8.0000000	-50.0000000
52010.0000000	8.2500000	-50.0000000
52010.0000000	8.5000000	-50.0000000
52010.0000000	8.7500000	-50.0000000
52010.0000000	9.0000000	-50.0000000
52010.0000000	9.2500000	-50.0000000
52010.0000000	9.5000000	-50.0000000
52010.0000000	9.7500000	-50.0000000
52010.0000000	10.0000000	-50.0000000
52010.0000000	10.2500000	-50.0000000
52010.0000000	10.5000000	-50.0000000
52010.0000000	10.7500000	-50.0000000
52010.0000000	11.0000000	-50.0000000
52010.0000000	11.2500000	-50.0000000
52010.0000000	11.5000000	-50.0000000
52010.0000000	11.7500000	-50.0000000
52010.0000000	12.0000000	-50.0000000
52010.0000000	12.2500000	-50.0000000
52010.0000000	12.5000000	-50.0000000
52010.0000000	12.7500000	-50.0000000
52010.0000000	13.0000000	-50.0000000
52010.0000000	13.2500000	-50.0000000
52010.0000000	13.5000000	-50.0000000
52010.0000000	13.7500000	-50.0000000
52010.0000000	14.0000000	-50.0000000
52010.0000000	14.2500000	-50.0000000
52010.0000000	14.5000000	-50.0000000
52010.0000000	14.7500000	-50.0000000
52010.0000000	15.0000000	-50.0000000
52040.0000000	0.0000000	-50.0000000
52040.0000000	0.2500000	-50.0000000
52040.0000000	0.5000000	21.6299992
52040.0000000	0.7500000	22.5699997
52040.0000000	1.0000000	15.0299997
52040.0000000	1.2500000	-50.0000000
52040.0000000	1.5000000	-50.0000000
52040.0000000	1.7500000	-50.0000000
52040.0000000	2.0000000	-50.0000000
52040.0000000	2.2500000	-50.0000000
52040.0000000	2.5000000	-50.0000000
52040.0000000	2.7500000	15.0799999
52040.0000000	3.0000000	-50.0000000
52040.0000000	3.2500000	-50.0000000
52040.0000000	3.5000000	-50.0000000
52040.0000000	3.7500000	15.3599997
52040.0000000	4.0000000	17.0100002
52040.0000000	4.2500000	15.3100004
52040.0000000	4.5000000	-50.0000000
52040.0000000	4.7500000	-50.0000000
52040.0000000	5.0000000	-50.0000000
52040.0000000	5.2500000	-50.0000000

52040.0000000	5.5000000	-50.0000000
52040.0000000	5.7500000	-50.0000000
52040.0000000	6.0000000	-50.0000000
52040.0000000	6.2500000	-50.0000000
52040.0000000	6.5000000	-50.0000000
52040.0000000	6.7500000	-50.0000000
52040.0000000	7.0000000	-50.0000000
52040.0000000	7.2500000	-50.0000000
52040.0000000	7.5000000	-50.0000000
52040.0000000	7.7500000	-50.0000000
52040.0000000	8.0000000	-50.0000000
52040.0000000	8.2500000	-50.0000000
52040.0000000	8.5000000	-50.0000000
52040.0000000	8.7500000	-50.0000000
52040.0000000	9.0000000	-50.0000000
52040.0000000	9.2500000	-50.0000000
52040.0000000	9.5000000	-50.0000000
52040.0000000	9.7500000	-50.0000000
52040.0000000	10.0000000	-50.0000000
52040.0000000	10.2500000	-50.0000000
52040.0000000	10.5000000	-50.0000000
52040.0000000	10.7500000	-50.0000000
52040.0000000	11.0000000	-50.0000000
52040.0000000	11.2500000	-50.0000000
52040.0000000	11.5000000	-50.0000000
52040.0000000	11.7500000	-50.0000000
52040.0000000	12.0000000	-50.0000000
52040.0000000	12.2500000	-50.0000000
52040.0000000	12.5000000	-50.0000000
52040.0000000	12.7500000	-50.0000000
52040.0000000	13.0000000	-50.0000000
52040.0000000	13.2500000	-50.0000000
52040.0000000	13.5000000	-50.0000000
52040.0000000	13.7500000	-50.0000000
52040.0000000	14.0000000	-50.0000000
52040.0000000	14.2500000	-50.0000000
52040.0000000	14.5000000	-50.0000000
52040.0000000	14.7500000	-50.0000000
52040.0000000	15.0000000	-50.0000000
52070.0000000	0.0000000	-50.0000000
52070.0000000	0.2500000	-50.0000000
52070.0000000	0.5000000	21.1200008
52070.0000000	0.7500000	22.3099995
52070.0000000	1.0000000	-50.0000000
52070.0000000	1.2500000	-50.0000000
52070.0000000	1.5000000	-50.0000000
52070.0000000	1.7500000	-50.0000000
52070.0000000	2.0000000	-50.0000000
52070.0000000	2.2500000	-50.0000000
52070.0000000	2.5000000	-50.0000000
52070.0000000	2.7500000	-50.0000000
52070.0000000	3.0000000	-50.0000000
52070.0000000	3.2500000	16.0699997
52070.0000000	3.5000000	19.6399994
52070.0000000	3.7500000	17.4500008
52070.0000000	4.0000000	16.0100002
52070.0000000	4.2500000	-50.0000000
52070.0000000	4.5000000	-50.0000000

52070.0000000	4.7500000	-50.0000000
52070.0000000	5.0000000	-50.0000000
52070.0000000	5.2500000	-50.0000000
52070.0000000	5.5000000	-50.0000000
52070.0000000	5.7500000	-50.0000000
52070.0000000	6.0000000	-50.0000000
52070.0000000	6.2500000	-50.0000000
52070.0000000	6.5000000	-50.0000000
52070.0000000	6.7500000	-50.0000000
52070.0000000	7.0000000	-50.0000000
52070.0000000	7.2500000	-50.0000000
52070.0000000	7.5000000	-50.0000000
52070.0000000	7.7500000	-50.0000000
52070.0000000	8.0000000	-50.0000000
52070.0000000	8.2500000	-50.0000000
52070.0000000	8.5000000	-50.0000000
52070.0000000	8.7500000	-50.0000000
52070.0000000	9.0000000	-50.0000000
52070.0000000	9.2500000	-50.0000000
52070.0000000	9.5000000	-50.0000000
52070.0000000	9.7500000	-50.0000000
52070.0000000	10.0000000	-50.0000000
52070.0000000	10.2500000	-50.0000000
52070.0000000	10.5000000	-50.0000000
52070.0000000	10.7500000	-50.0000000
52070.0000000	11.0000000	-50.0000000
52070.0000000	11.2500000	-50.0000000
52070.0000000	11.5000000	-50.0000000
52070.0000000	11.7500000	-50.0000000
52070.0000000	12.0000000	-50.0000000
52070.0000000	12.2500000	-50.0000000
52070.0000000	12.5000000	-50.0000000
52070.0000000	12.7500000	-50.0000000
52070.0000000	13.0000000	-50.0000000
52070.0000000	13.2500000	-50.0000000
52070.0000000	13.5000000	-50.0000000
52070.0000000	13.7500000	-50.0000000
52070.0000000	14.0000000	-50.0000000
52070.0000000	14.2500000	-50.0000000
52070.0000000	14.5000000	-50.0000000
52070.0000000	14.7500000	-50.0000000
52070.0000000	15.0000000	-50.0000000
52100.0000000	0.0000000	-50.0000000
52100.0000000	0.2500000	-50.0000000
52100.0000000	0.5000000	21.3700008
52100.0000000	0.7500000	21.3700008
52100.0000000	1.0000000	15.3900003
52100.0000000	1.2500000	-50.0000000
52100.0000000	1.5000000	-50.0000000
52100.0000000	1.7500000	-50.0000000
52100.0000000	2.0000000	15.6599998
52100.0000000	2.2500000	-50.0000000
52100.0000000	2.5000000	15.3199997
52100.0000000	2.7500000	-50.0000000
52100.0000000	3.0000000	-50.0000000
52100.0000000	3.2500000	15.7200003
52100.0000000	3.5000000	15.1400003
52100.0000000	3.7500000	16.5599995

52100.0000000	4.0000000	17.3199997
52100.0000000	4.2500000	-50.0000000
52100.0000000	4.5000000	-50.0000000
52100.0000000	4.7500000	-50.0000000
52100.0000000	5.0000000	-50.0000000
52100.0000000	5.2500000	-50.0000000
52100.0000000	5.5000000	-50.0000000
52100.0000000	5.7500000	-50.0000000
52100.0000000	6.0000000	-50.0000000
52100.0000000	6.2500000	-50.0000000
52100.0000000	6.5000000	-50.0000000
52100.0000000	6.7500000	-50.0000000
52100.0000000	7.0000000	-50.0000000
52100.0000000	7.2500000	-50.0000000
52100.0000000	7.5000000	-50.0000000
52100.0000000	7.7500000	-50.0000000
52100.0000000	8.0000000	-50.0000000
52100.0000000	8.2500000	-50.0000000
52100.0000000	8.5000000	-50.0000000
52100.0000000	8.7500000	-50.0000000
52100.0000000	9.0000000	-50.0000000
52100.0000000	9.2500000	-50.0000000
52100.0000000	9.5000000	-50.0000000
52100.0000000	9.7500000	-50.0000000
52100.0000000	10.0000000	-50.0000000
52100.0000000	10.2500000	-50.0000000
52100.0000000	10.5000000	-50.0000000
52100.0000000	10.7500000	-50.0000000
52100.0000000	11.0000000	-50.0000000
52100.0000000	11.2500000	-50.0000000
52100.0000000	11.5000000	-50.0000000
52100.0000000	11.7500000	-50.0000000
52100.0000000	12.0000000	-50.0000000
52100.0000000	12.2500000	-50.0000000
52100.0000000	12.5000000	-50.0000000
52100.0000000	12.7500000	-50.0000000
52100.0000000	13.0000000	-50.0000000
52100.0000000	13.2500000	-50.0000000
52100.0000000	13.5000000	-50.0000000
52100.0000000	13.7500000	-50.0000000
52100.0000000	14.0000000	-50.0000000
52100.0000000	14.2500000	-50.0000000
52100.0000000	14.5000000	-50.0000000
52100.0000000	14.7500000	-50.0000000
52100.0000000	15.0000000	-50.0000000
52130.0000000	0.0000000	-50.0000000
52130.0000000	0.2500000	-50.0000000
52130.0000000	0.5000000	18.5400009
52130.0000000	0.7500000	19.1200008
52130.0000000	1.0000000	-50.0000000
52130.0000000	1.2500000	-50.0000000
52130.0000000	1.5000000	-50.0000000
52130.0000000	1.7500000	16.1100006
52130.0000000	2.0000000	16.5599995
52130.0000000	2.2500000	16.9699993
52130.0000000	2.5000000	-50.0000000
52130.0000000	2.7500000	15.6300001
52130.0000000	3.0000000	15.5100002

52130.0000000	3.2500000	15.4300003
52130.0000000	3.5000000	15.1099997
52130.0000000	3.7500000	-50.0000000
52130.0000000	4.0000000	15.2600002
52130.0000000	4.2500000	-50.0000000
52130.0000000	4.5000000	-50.0000000
52130.0000000	4.7500000	-50.0000000
52130.0000000	5.0000000	-50.0000000
52130.0000000	5.2500000	-50.0000000
52130.0000000	5.5000000	-50.0000000
52130.0000000	5.7500000	-50.0000000
52130.0000000	6.0000000	-50.0000000
52130.0000000	6.2500000	-50.0000000
52130.0000000	6.5000000	-50.0000000
52130.0000000	6.7500000	-50.0000000
52130.0000000	7.0000000	-50.0000000
52130.0000000	7.2500000	-50.0000000
52130.0000000	7.5000000	-50.0000000
52130.0000000	7.7500000	-50.0000000
52130.0000000	8.0000000	-50.0000000
52130.0000000	8.2500000	-50.0000000
52130.0000000	8.5000000	-50.0000000
52130.0000000	8.7500000	-50.0000000
52130.0000000	9.0000000	-50.0000000
52130.0000000	9.2500000	-50.0000000
52130.0000000	9.5000000	-50.0000000
52130.0000000	9.7500000	-50.0000000
52130.0000000	10.0000000	-50.0000000
52130.0000000	10.2500000	-50.0000000
52130.0000000	10.5000000	-50.0000000
52130.0000000	10.7500000	-50.0000000
52130.0000000	11.0000000	-50.0000000
52130.0000000	11.2500000	-50.0000000
52130.0000000	11.5000000	-50.0000000
52130.0000000	11.7500000	-50.0000000
52130.0000000	12.0000000	-50.0000000
52130.0000000	12.2500000	-50.0000000
52130.0000000	12.5000000	-50.0000000
52130.0000000	12.7500000	-50.0000000
52130.0000000	13.0000000	-50.0000000
52130.0000000	13.2500000	-50.0000000
52130.0000000	13.5000000	-50.0000000
52130.0000000	13.7500000	-50.0000000
52130.0000000	14.0000000	-50.0000000
52130.0000000	14.2500000	-50.0000000
52130.0000000	14.5000000	-50.0000000
52130.0000000	14.7500000	-50.0000000
52130.0000000	15.0000000	-50.0000000
52160.0000000	0.0000000	-50.0000000
52160.0000000	0.2500000	-50.0000000
52160.0000000	0.5000000	17.8799992
52160.0000000	0.7500000	20.2399998
52160.0000000	1.0000000	-50.0000000
52160.0000000	1.2500000	-50.0000000
52160.0000000	1.5000000	-50.0000000
52160.0000000	1.7500000	17.6100006
52160.0000000	2.0000000	15.8900003
52160.0000000	2.2500000	-50.0000000

52160.0000000	2.5000000	-50.0000000
52160.0000000	2.7500000	-50.0000000
52160.0000000	3.0000000	15.3900003
52160.0000000	3.2500000	17.0699997
52160.0000000	3.5000000	15.5400000
52160.0000000	3.7500000	15.5699997
52160.0000000	4.0000000	16.1700001
52160.0000000	4.2500000	-50.0000000
52160.0000000	4.5000000	-50.0000000
52160.0000000	4.7500000	-50.0000000
52160.0000000	5.0000000	-50.0000000
52160.0000000	5.2500000	-50.0000000
52160.0000000	5.5000000	-50.0000000
52160.0000000	5.7500000	-50.0000000
52160.0000000	6.0000000	-50.0000000
52160.0000000	6.2500000	-50.0000000
52160.0000000	6.5000000	-50.0000000
52160.0000000	6.7500000	-50.0000000
52160.0000000	7.0000000	-50.0000000
52160.0000000	7.2500000	-50.0000000
52160.0000000	7.5000000	-50.0000000
52160.0000000	7.7500000	-50.0000000
52160.0000000	8.0000000	-50.0000000
52160.0000000	8.2500000	-50.0000000
52160.0000000	8.5000000	-50.0000000
52160.0000000	8.7500000	-50.0000000
52160.0000000	9.0000000	-50.0000000
52160.0000000	9.2500000	-50.0000000
52160.0000000	9.5000000	-50.0000000
52160.0000000	9.7500000	-50.0000000
52160.0000000	10.0000000	-50.0000000
52160.0000000	10.2500000	-50.0000000
52160.0000000	10.5000000	-50.0000000
52160.0000000	10.7500000	-50.0000000
52160.0000000	11.0000000	-50.0000000
52160.0000000	11.2500000	-50.0000000
52160.0000000	11.5000000	-50.0000000
52160.0000000	11.7500000	-50.0000000
52160.0000000	12.0000000	-50.0000000
52160.0000000	12.2500000	-50.0000000
52160.0000000	12.5000000	-50.0000000
52160.0000000	12.7500000	-50.0000000
52160.0000000	13.0000000	-50.0000000
52160.0000000	13.2500000	-50.0000000
52160.0000000	13.5000000	-50.0000000
52160.0000000	13.7500000	-50.0000000
52160.0000000	14.0000000	-50.0000000
52160.0000000	14.2500000	-50.0000000
52160.0000000	14.5000000	-50.0000000
52160.0000000	14.7500000	-50.0000000
52160.0000000	15.0000000	-50.0000000
52190.0000000	0.0000000	-50.0000000
52190.0000000	0.2500000	-50.0000000
52190.0000000	0.5000000	20.0400009
52190.0000000	0.7500000	21.7999992
52190.0000000	1.0000000	15.1199999
52190.0000000	1.2500000	-50.0000000
52190.0000000	1.5000000	-50.0000000

52190.0000000	1.7500000	-50.0000000
52190.0000000	2.0000000	-50.0000000
52190.0000000	2.2500000	-50.0000000
52190.0000000	2.5000000	15.2399998
52190.0000000	2.7500000	16.2500000
52190.0000000	3.0000000	15.8599997
52190.0000000	3.2500000	18.2299995
52190.0000000	3.5000000	19.6299992
52190.0000000	3.7500000	18.4799995
52190.0000000	4.0000000	15.6499996
52190.0000000	4.2500000	-50.0000000
52190.0000000	4.5000000	-50.0000000
52190.0000000	4.7500000	-50.0000000
52190.0000000	5.0000000	-50.0000000
52190.0000000	5.2500000	-50.0000000
52190.0000000	5.5000000	-50.0000000
52190.0000000	5.7500000	-50.0000000
52190.0000000	6.0000000	-50.0000000
52190.0000000	6.2500000	-50.0000000
52190.0000000	6.5000000	-50.0000000
52190.0000000	6.7500000	-50.0000000
52190.0000000	7.0000000	-50.0000000
52190.0000000	7.2500000	-50.0000000
52190.0000000	7.5000000	-50.0000000
52190.0000000	7.7500000	-50.0000000
52190.0000000	8.0000000	-50.0000000
52190.0000000	8.2500000	-50.0000000
52190.0000000	8.5000000	-50.0000000
52190.0000000	8.7500000	-50.0000000
52190.0000000	9.0000000	-50.0000000
52190.0000000	9.2500000	-50.0000000
52190.0000000	9.5000000	-50.0000000
52190.0000000	9.7500000	-50.0000000
52190.0000000	10.0000000	-50.0000000
52190.0000000	10.2500000	-50.0000000
52190.0000000	10.5000000	-50.0000000
52190.0000000	10.7500000	-50.0000000
52190.0000000	11.0000000	-50.0000000
52190.0000000	11.2500000	-50.0000000
52190.0000000	11.5000000	-50.0000000
52190.0000000	11.7500000	-50.0000000
52190.0000000	12.0000000	-50.0000000
52190.0000000	12.2500000	-50.0000000
52190.0000000	12.5000000	-50.0000000
52190.0000000	12.7500000	-50.0000000
52190.0000000	13.0000000	-50.0000000
52190.0000000	13.2500000	-50.0000000
52190.0000000	13.5000000	-50.0000000
52190.0000000	13.7500000	-50.0000000
52190.0000000	14.0000000	-50.0000000
52190.0000000	14.2500000	-50.0000000
52190.0000000	14.5000000	-50.0000000
52190.0000000	14.7500000	-50.0000000
52190.0000000	15.0000000	-50.0000000
52220.0000000	0.0000000	-50.0000000
52220.0000000	0.2500000	-50.0000000
52220.0000000	0.5000000	-50.0000000
52220.0000000	0.7500000	17.4500008

52220.0000000	1.0000000	-50.0000000
52220.0000000	1.2500000	-50.0000000
52220.0000000	1.5000000	15.0699997
52220.0000000	1.7500000	-50.0000000
52220.0000000	2.0000000	-50.0000000
52220.0000000	2.2500000	-50.0000000
52220.0000000	2.5000000	-50.0000000
52220.0000000	2.7500000	-50.0000000
52220.0000000	3.0000000	-50.0000000
52220.0000000	3.2500000	-50.0000000
52220.0000000	3.5000000	-50.0000000
52220.0000000	3.7500000	-50.0000000
52220.0000000	4.0000000	17.5699997
52220.0000000	4.2500000	-50.0000000
52220.0000000	4.5000000	-50.0000000
52220.0000000	4.7500000	-50.0000000
52220.0000000	5.0000000	-50.0000000
52220.0000000	5.2500000	-50.0000000
52220.0000000	5.5000000	-50.0000000
52220.0000000	5.7500000	-50.0000000
52220.0000000	6.0000000	-50.0000000
52220.0000000	6.2500000	-50.0000000
52220.0000000	6.5000000	-50.0000000
52220.0000000	6.7500000	-50.0000000
52220.0000000	7.0000000	-50.0000000
52220.0000000	7.2500000	-50.0000000
52220.0000000	7.5000000	-50.0000000
52220.0000000	7.7500000	-50.0000000
52220.0000000	8.0000000	-50.0000000
52220.0000000	8.2500000	-50.0000000
52220.0000000	8.5000000	-50.0000000
52220.0000000	8.7500000	-50.0000000
52220.0000000	9.0000000	-50.0000000
52220.0000000	9.2500000	-50.0000000
52220.0000000	9.5000000	-50.0000000
52220.0000000	9.7500000	-50.0000000
52220.0000000	10.0000000	-50.0000000
52220.0000000	10.2500000	-50.0000000
52220.0000000	10.5000000	-50.0000000
52220.0000000	10.7500000	-50.0000000
52220.0000000	11.0000000	-50.0000000
52220.0000000	11.2500000	-50.0000000
52220.0000000	11.5000000	-50.0000000
52220.0000000	11.7500000	-50.0000000
52220.0000000	12.0000000	-50.0000000
52220.0000000	12.2500000	-50.0000000
52220.0000000	12.5000000	-50.0000000
52220.0000000	12.7500000	-50.0000000
52220.0000000	13.0000000	-50.0000000
52220.0000000	13.2500000	-50.0000000
52220.0000000	13.5000000	-50.0000000
52220.0000000	13.7500000	-50.0000000
52220.0000000	14.0000000	-50.0000000
52220.0000000	14.2500000	-50.0000000
52220.0000000	14.5000000	-50.0000000
52220.0000000	14.7500000	-50.0000000
52220.0000000	15.0000000	-50.0000000
52250.0000000	0.0000000	-50.0000000

52250.0000000	0.2500000	-50.0000000
52250.0000000	0.5000000	18.9300003
52250.0000000	0.7500000	18.4099998
52250.0000000	1.0000000	-50.0000000
52250.0000000	1.2500000	-50.0000000
52250.0000000	1.5000000	-50.0000000
52250.0000000	1.7500000	-50.0000000
52250.0000000	2.0000000	-50.0000000
52250.0000000	2.2500000	-50.0000000
52250.0000000	2.5000000	-50.0000000
52250.0000000	2.7500000	-50.0000000
52250.0000000	3.0000000	-50.0000000
52250.0000000	3.2500000	-50.0000000
52250.0000000	3.5000000	17.1499996
52250.0000000	3.7500000	18.7000008
52250.0000000	4.0000000	-50.0000000
52250.0000000	4.2500000	-50.0000000
52250.0000000	4.5000000	-50.0000000
52250.0000000	4.7500000	-50.0000000
52250.0000000	5.0000000	-50.0000000
52250.0000000	5.2500000	-50.0000000
52250.0000000	5.5000000	-50.0000000
52250.0000000	5.7500000	-50.0000000
52250.0000000	6.0000000	-50.0000000
52250.0000000	6.2500000	-50.0000000
52250.0000000	6.5000000	-50.0000000
52250.0000000	6.7500000	-50.0000000
52250.0000000	7.0000000	-50.0000000
52250.0000000	7.2500000	-50.0000000
52250.0000000	7.5000000	-50.0000000
52250.0000000	7.7500000	-50.0000000
52250.0000000	8.0000000	-50.0000000
52250.0000000	8.2500000	-50.0000000
52250.0000000	8.5000000	-50.0000000
52250.0000000	8.7500000	-50.0000000
52250.0000000	9.0000000	-50.0000000
52250.0000000	9.2500000	-50.0000000
52250.0000000	9.5000000	-50.0000000
52250.0000000	9.7500000	-50.0000000
52250.0000000	10.0000000	-50.0000000
52250.0000000	10.2500000	-50.0000000
52250.0000000	10.5000000	-50.0000000
52250.0000000	10.7500000	-50.0000000
52250.0000000	11.0000000	-50.0000000
52250.0000000	11.2500000	-50.0000000
52250.0000000	11.5000000	-50.0000000
52250.0000000	11.7500000	-50.0000000
52250.0000000	12.0000000	-50.0000000
52250.0000000	12.2500000	-50.0000000
52250.0000000	12.5000000	-50.0000000
52250.0000000	12.7500000	-50.0000000
52250.0000000	13.0000000	-50.0000000
52250.0000000	13.2500000	-50.0000000
52250.0000000	13.5000000	-50.0000000
52250.0000000	13.7500000	-50.0000000
52250.0000000	14.0000000	-50.0000000
52250.0000000	14.2500000	-50.0000000
52250.0000000	14.5000000	-50.0000000

52250.0000000	14.7500000	-50.0000000
52250.0000000	15.0000000	-50.0000000
52280.0000000	0.0000000	-50.0000000
52280.0000000	0.2500000	-50.0000000
52280.0000000	0.5000000	20.1800003
52280.0000000	0.7500000	21.6399994
52280.0000000	1.0000000	17.4200001
52280.0000000	1.2500000	15.0000000
52280.0000000	1.5000000	-50.0000000
52280.0000000	1.7500000	-50.0000000
52280.0000000	2.0000000	-50.0000000
52280.0000000	2.2500000	-50.0000000
52280.0000000	2.5000000	-50.0000000
52280.0000000	2.7500000	-50.0000000
52280.0000000	3.0000000	16.4500008
52280.0000000	3.2500000	17.4500008
52280.0000000	3.5000000	15.3100004
52280.0000000	3.7500000	18.0900002
52280.0000000	4.0000000	-50.0000000
52280.0000000	4.2500000	-50.0000000
52280.0000000	4.5000000	-50.0000000
52280.0000000	4.7500000	-50.0000000
52280.0000000	5.0000000	-50.0000000
52280.0000000	5.2500000	-50.0000000
52280.0000000	5.5000000	-50.0000000
52280.0000000	5.7500000	-50.0000000
52280.0000000	6.0000000	-50.0000000
52280.0000000	6.2500000	-50.0000000
52280.0000000	6.5000000	-50.0000000
52280.0000000	6.7500000	-50.0000000
52280.0000000	7.0000000	-50.0000000
52280.0000000	7.2500000	-50.0000000
52280.0000000	7.5000000	-50.0000000
52280.0000000	7.7500000	-50.0000000
52280.0000000	8.0000000	-50.0000000
52280.0000000	8.2500000	-50.0000000
52280.0000000	8.5000000	-50.0000000
52280.0000000	8.7500000	-50.0000000
52280.0000000	9.0000000	-50.0000000
52280.0000000	9.2500000	-50.0000000
52280.0000000	9.5000000	-50.0000000
52280.0000000	9.7500000	-50.0000000
52280.0000000	10.0000000	-50.0000000
52280.0000000	10.2500000	-50.0000000
52280.0000000	10.5000000	-50.0000000
52280.0000000	10.7500000	-50.0000000
52280.0000000	11.0000000	-50.0000000
52280.0000000	11.2500000	-50.0000000
52280.0000000	11.5000000	-50.0000000
52280.0000000	11.7500000	-50.0000000
52280.0000000	12.0000000	-50.0000000
52280.0000000	12.2500000	-50.0000000
52280.0000000	12.5000000	-50.0000000
52280.0000000	12.7500000	-50.0000000
52280.0000000	13.0000000	-50.0000000
52280.0000000	13.2500000	-50.0000000
52280.0000000	13.5000000	-50.0000000
52280.0000000	13.7500000	-50.0000000

52280.0000000	14.0000000	-50.0000000
52280.0000000	14.2500000	-50.0000000
52280.0000000	14.5000000	-50.0000000
52280.0000000	14.7500000	-50.0000000
52280.0000000	15.0000000	-50.0000000
52310.0000000	0.0000000	-50.0000000
52310.0000000	0.2500000	-50.0000000
52310.0000000	0.5000000	18.2900009
52310.0000000	0.7500000	19.3799992
52310.0000000	1.0000000	-50.0000000
52310.0000000	1.2500000	15.6700001
52310.0000000	1.5000000	16.7700005
52310.0000000	1.7500000	17.7500000
52310.0000000	2.0000000	15.6899996
52310.0000000	2.2500000	-50.0000000
52310.0000000	2.5000000	-50.0000000
52310.0000000	2.7500000	-50.0000000
52310.0000000	3.0000000	-50.0000000
52310.0000000	3.2500000	16.4200001
52310.0000000	3.5000000	17.0100002
52310.0000000	3.7500000	16.7999992
52310.0000000	4.0000000	-50.0000000
52310.0000000	4.2500000	-50.0000000
52310.0000000	4.5000000	15.2600002
52310.0000000	4.7500000	-50.0000000
52310.0000000	5.0000000	-50.0000000
52310.0000000	5.2500000	-50.0000000
52310.0000000	5.5000000	-50.0000000
52310.0000000	5.7500000	-50.0000000
52310.0000000	6.0000000	-50.0000000
52310.0000000	6.2500000	-50.0000000
52310.0000000	6.5000000	-50.0000000
52310.0000000	6.7500000	-50.0000000
52310.0000000	7.0000000	-50.0000000
52310.0000000	7.2500000	-50.0000000
52310.0000000	7.5000000	-50.0000000
52310.0000000	7.7500000	-50.0000000
52310.0000000	8.0000000	-50.0000000
52310.0000000	8.2500000	-50.0000000
52310.0000000	8.5000000	-50.0000000
52310.0000000	8.7500000	-50.0000000
52310.0000000	9.0000000	-50.0000000
52310.0000000	9.2500000	-50.0000000
52310.0000000	9.5000000	-50.0000000
52310.0000000	9.7500000	-50.0000000
52310.0000000	10.0000000	-50.0000000
52310.0000000	10.2500000	-50.0000000
52310.0000000	10.5000000	-50.0000000
52310.0000000	10.7500000	-50.0000000
52310.0000000	11.0000000	-50.0000000
52310.0000000	11.2500000	-50.0000000
52310.0000000	11.5000000	-50.0000000
52310.0000000	11.7500000	-50.0000000
52310.0000000	12.0000000	-50.0000000
52310.0000000	12.2500000	-50.0000000
52310.0000000	12.5000000	-50.0000000
52310.0000000	12.7500000	-50.0000000
52310.0000000	13.0000000	-50.0000000

52310.0000000	13.2500000	-50.0000000
52310.0000000	13.5000000	-50.0000000
52310.0000000	13.7500000	-50.0000000
52310.0000000	14.0000000	-50.0000000
52310.0000000	14.2500000	-50.0000000
52310.0000000	14.5000000	-50.0000000
52310.0000000	14.7500000	-50.0000000
52310.0000000	15.0000000	-50.0000000
52340.0000000	0.0000000	-50.0000000
52340.0000000	0.2500000	-50.0000000
52340.0000000	0.5000000	21.9400005
52340.0000000	0.7500000	23.7999992
52340.0000000	1.0000000	18.5599995
52340.0000000	1.2500000	17.8600006
52340.0000000	1.5000000	16.9599991
52340.0000000	1.7500000	16.0900002
52340.0000000	2.0000000	-50.0000000
52340.0000000	2.2500000	-50.0000000
52340.0000000	2.5000000	-50.0000000
52340.0000000	2.7500000	-50.0000000
52340.0000000	3.0000000	15.4899998
52340.0000000	3.2500000	16.9699993
52340.0000000	3.5000000	19.0100002
52340.0000000	3.7500000	16.5400009
52340.0000000	4.0000000	15.3000002
52340.0000000	4.2500000	15.9200001
52340.0000000	4.5000000	-50.0000000
52340.0000000	4.7500000	-50.0000000
52340.0000000	5.0000000	-50.0000000
52340.0000000	5.2500000	-50.0000000
52340.0000000	5.5000000	-50.0000000
52340.0000000	5.7500000	-50.0000000
52340.0000000	6.0000000	-50.0000000
52340.0000000	6.2500000	-50.0000000
52340.0000000	6.5000000	-50.0000000
52340.0000000	6.7500000	-50.0000000
52340.0000000	7.0000000	-50.0000000
52340.0000000	7.2500000	-50.0000000
52340.0000000	7.5000000	-50.0000000
52340.0000000	7.7500000	-50.0000000
52340.0000000	8.0000000	-50.0000000
52340.0000000	8.2500000	-50.0000000
52340.0000000	8.5000000	-50.0000000
52340.0000000	8.7500000	-50.0000000
52340.0000000	9.0000000	-50.0000000
52340.0000000	9.2500000	-50.0000000
52340.0000000	9.5000000	-50.0000000
52340.0000000	9.7500000	-50.0000000
52340.0000000	10.0000000	-50.0000000
52340.0000000	10.2500000	-50.0000000
52340.0000000	10.5000000	-50.0000000
52340.0000000	10.7500000	-50.0000000
52340.0000000	11.0000000	-50.0000000
52340.0000000	11.2500000	-50.0000000
52340.0000000	11.5000000	-50.0000000
52340.0000000	11.7500000	-50.0000000
52340.0000000	12.0000000	-50.0000000
52340.0000000	12.2500000	-50.0000000

52340.0000000	12.5000000	-50.0000000
52340.0000000	12.7500000	-50.0000000
52340.0000000	13.0000000	-50.0000000
52340.0000000	13.2500000	-50.0000000
52340.0000000	13.5000000	-50.0000000
52340.0000000	13.7500000	-50.0000000
52340.0000000	14.0000000	-50.0000000
52340.0000000	14.2500000	-50.0000000
52340.0000000	14.5000000	-50.0000000
52340.0000000	14.7500000	-50.0000000
52340.0000000	15.0000000	-50.0000000
52370.0000000	0.0000000	-50.0000000
52370.0000000	0.2500000	-50.0000000
52370.0000000	0.5000000	19.9899998
52370.0000000	0.7500000	21.2999992
52370.0000000	1.0000000	16.3299999
52370.0000000	1.2500000	17.4799995
52370.0000000	1.5000000	16.2399998
52370.0000000	1.7500000	15.5600004
52370.0000000	2.0000000	-50.0000000
52370.0000000	2.2500000	-50.0000000
52370.0000000	2.5000000	-50.0000000
52370.0000000	2.7500000	15.8500004
52370.0000000	3.0000000	17.0200005
52370.0000000	3.2500000	18.1399994
52370.0000000	3.5000000	18.9699993
52370.0000000	3.7500000	17.3299999
52370.0000000	4.0000000	16.8799992
52370.0000000	4.2500000	-50.0000000
52370.0000000	4.5000000	-50.0000000
52370.0000000	4.7500000	-50.0000000
52370.0000000	5.0000000	-50.0000000
52370.0000000	5.2500000	-50.0000000
52370.0000000	5.5000000	-50.0000000
52370.0000000	5.7500000	-50.0000000
52370.0000000	6.0000000	-50.0000000
52370.0000000	6.2500000	-50.0000000
52370.0000000	6.5000000	-50.0000000
52370.0000000	6.7500000	-50.0000000
52370.0000000	7.0000000	-50.0000000
52370.0000000	7.2500000	-50.0000000
52370.0000000	7.5000000	-50.0000000
52370.0000000	7.7500000	-50.0000000
52370.0000000	8.0000000	-50.0000000
52370.0000000	8.2500000	-50.0000000
52370.0000000	8.5000000	-50.0000000
52370.0000000	8.7500000	-50.0000000
52370.0000000	9.0000000	-50.0000000
52370.0000000	9.2500000	-50.0000000
52370.0000000	9.5000000	-50.0000000
52370.0000000	9.7500000	-50.0000000
52370.0000000	10.0000000	-50.0000000
52370.0000000	10.2500000	-50.0000000
52370.0000000	10.5000000	-50.0000000
52370.0000000	10.7500000	-50.0000000
52370.0000000	11.0000000	-50.0000000
52370.0000000	11.2500000	-50.0000000
52370.0000000	11.5000000	-50.0000000

52370.0000000	11.7500000	-50.0000000
52370.0000000	12.0000000	-50.0000000
52370.0000000	12.2500000	-50.0000000
52370.0000000	12.5000000	-50.0000000
52370.0000000	12.7500000	-50.0000000
52370.0000000	13.0000000	-50.0000000
52370.0000000	13.2500000	-50.0000000
52370.0000000	13.5000000	-50.0000000
52370.0000000	13.7500000	-50.0000000
52370.0000000	14.0000000	-50.0000000
52370.0000000	14.2500000	-50.0000000
52370.0000000	14.5000000	-50.0000000
52370.0000000	14.7500000	-50.0000000
52370.0000000	15.0000000	-50.0000000
52400.0000000	0.0000000	-50.0000000
52400.0000000	0.2500000	-50.0000000
52400.0000000	0.5000000	17.9200001
52400.0000000	0.7500000	19.1499996
52400.0000000	1.0000000	15.9700003
52400.0000000	1.2500000	15.7700005
52400.0000000	1.5000000	15.0500002
52400.0000000	1.7500000	-50.0000000
52400.0000000	2.0000000	15.4899998
52400.0000000	2.2500000	15.3900003
52400.0000000	2.5000000	-50.0000000
52400.0000000	2.7500000	15.2200003
52400.0000000	3.0000000	17.1000004
52400.0000000	3.2500000	15.8400002
52400.0000000	3.5000000	20.6100006
52400.0000000	3.7500000	18.4899998
52400.0000000	4.0000000	16.7199993
52400.0000000	4.2500000	15.6000004
52400.0000000	4.5000000	-50.0000000
52400.0000000	4.7500000	15.7700005
52400.0000000	5.0000000	-50.0000000
52400.0000000	5.2500000	-50.0000000
52400.0000000	5.5000000	-50.0000000
52400.0000000	5.7500000	-50.0000000
52400.0000000	6.0000000	-50.0000000
52400.0000000	6.2500000	-50.0000000
52400.0000000	6.5000000	-50.0000000
52400.0000000	6.7500000	-50.0000000
52400.0000000	7.0000000	-50.0000000
52400.0000000	7.2500000	-50.0000000
52400.0000000	7.5000000	-50.0000000
52400.0000000	7.7500000	-50.0000000
52400.0000000	8.0000000	-50.0000000
52400.0000000	8.2500000	-50.0000000
52400.0000000	8.5000000	-50.0000000
52400.0000000	8.7500000	-50.0000000
52400.0000000	9.0000000	-50.0000000
52400.0000000	9.2500000	-50.0000000
52400.0000000	9.5000000	-50.0000000
52400.0000000	9.7500000	-50.0000000
52400.0000000	10.0000000	-50.0000000
52400.0000000	10.2500000	-50.0000000
52400.0000000	10.5000000	-50.0000000
52400.0000000	10.7500000	-50.0000000

52400.0000000	11.0000000	-50.0000000
52400.0000000	11.2500000	-50.0000000
52400.0000000	11.5000000	-50.0000000
52400.0000000	11.7500000	-50.0000000
52400.0000000	12.0000000	-50.0000000
52400.0000000	12.2500000	-50.0000000
52400.0000000	12.5000000	-50.0000000
52400.0000000	12.7500000	-50.0000000
52400.0000000	13.0000000	-50.0000000
52400.0000000	13.2500000	-50.0000000
52400.0000000	13.5000000	-50.0000000
52400.0000000	13.7500000	-50.0000000
52400.0000000	14.0000000	-50.0000000
52400.0000000	14.2500000	-50.0000000
52400.0000000	14.5000000	-50.0000000
52400.0000000	14.7500000	-50.0000000
52400.0000000	15.0000000	-50.0000000
52430.0000000	0.0000000	-50.0000000
52430.0000000	0.2500000	-50.0000000
52430.0000000	0.5000000	18.7900009
52430.0000000	0.7500000	20.7600002
52430.0000000	1.0000000	-50.0000000
52430.0000000	1.2500000	-50.0000000
52430.0000000	1.5000000	-50.0000000
52430.0000000	1.7500000	-50.0000000
52430.0000000	2.0000000	-50.0000000
52430.0000000	2.2500000	-50.0000000
52430.0000000	2.5000000	16.7199993
52430.0000000	2.7500000	16.5900002
52430.0000000	3.0000000	16.0900002
52430.0000000	3.2500000	-50.0000000
52430.0000000	3.5000000	15.0500002
52430.0000000	3.7500000	16.3700008
52430.0000000	4.0000000	-50.0000000
52430.0000000	4.2500000	15.5699997
52430.0000000	4.5000000	15.1800003
52430.0000000	4.7500000	-50.0000000
52430.0000000	5.0000000	-50.0000000
52430.0000000	5.2500000	-50.0000000
52430.0000000	5.5000000	-50.0000000
52430.0000000	5.7500000	-50.0000000
52430.0000000	6.0000000	-50.0000000
52430.0000000	6.2500000	-50.0000000
52430.0000000	6.5000000	-50.0000000
52430.0000000	6.7500000	-50.0000000
52430.0000000	7.0000000	-50.0000000
52430.0000000	7.2500000	-50.0000000
52430.0000000	7.5000000	-50.0000000
52430.0000000	7.7500000	-50.0000000
52430.0000000	8.0000000	-50.0000000
52430.0000000	8.2500000	-50.0000000
52430.0000000	8.5000000	-50.0000000
52430.0000000	8.7500000	-50.0000000
52430.0000000	9.0000000	-50.0000000
52430.0000000	9.2500000	-50.0000000
52430.0000000	9.5000000	-50.0000000
52430.0000000	9.7500000	-50.0000000
52430.0000000	10.0000000	-50.0000000

52430.0000000	10.2500000	-50.0000000
52430.0000000	10.5000000	-50.0000000
52430.0000000	10.7500000	-50.0000000
52430.0000000	11.0000000	-50.0000000
52430.0000000	11.2500000	-50.0000000
52430.0000000	11.5000000	-50.0000000
52430.0000000	11.7500000	-50.0000000
52430.0000000	12.0000000	-50.0000000
52430.0000000	12.2500000	-50.0000000
52430.0000000	12.5000000	-50.0000000
52430.0000000	12.7500000	-50.0000000
52430.0000000	13.0000000	-50.0000000
52430.0000000	13.2500000	-50.0000000
52430.0000000	13.5000000	-50.0000000
52430.0000000	13.7500000	-50.0000000
52430.0000000	14.0000000	-50.0000000
52430.0000000	14.2500000	-50.0000000
52430.0000000	14.5000000	-50.0000000
52430.0000000	14.7500000	-50.0000000
52430.0000000	15.0000000	-50.0000000
52460.0000000	0.0000000	-50.0000000
52460.0000000	0.2500000	-50.0000000
52460.0000000	0.5000000	18.2800007
52460.0000000	0.7500000	19.1800003
52460.0000000	1.0000000	16.9899998
52460.0000000	1.2500000	15.9899998
52460.0000000	1.5000000	-50.0000000
52460.0000000	1.7500000	-50.0000000
52460.0000000	2.0000000	-50.0000000
52460.0000000	2.2500000	-50.0000000
52460.0000000	2.5000000	-50.0000000
52460.0000000	2.7500000	-50.0000000
52460.0000000	3.0000000	17.1700001
52460.0000000	3.2500000	15.2799997
52460.0000000	3.5000000	-50.0000000
52460.0000000	3.7500000	17.5799999
52460.0000000	4.0000000	19.5599995
52460.0000000	4.2500000	16.4300003
52460.0000000	4.5000000	-50.0000000
52460.0000000	4.7500000	15.1400003
52460.0000000	5.0000000	-50.0000000
52460.0000000	5.2500000	-50.0000000
52460.0000000	5.5000000	-50.0000000
52460.0000000	5.7500000	-50.0000000
52460.0000000	6.0000000	-50.0000000
52460.0000000	6.2500000	-50.0000000
52460.0000000	6.5000000	-50.0000000
52460.0000000	6.7500000	-50.0000000
52460.0000000	7.0000000	-50.0000000
52460.0000000	7.2500000	-50.0000000
52460.0000000	7.5000000	-50.0000000
52460.0000000	7.7500000	-50.0000000
52460.0000000	8.0000000	-50.0000000
52460.0000000	8.2500000	-50.0000000
52460.0000000	8.5000000	-50.0000000
52460.0000000	8.7500000	-50.0000000
52460.0000000	9.0000000	-50.0000000
52460.0000000	9.2500000	-50.0000000

52460.0000000	9.5000000	-50.0000000
52460.0000000	9.7500000	-50.0000000
52460.0000000	10.0000000	-50.0000000
52460.0000000	10.2500000	-50.0000000
52460.0000000	10.5000000	-50.0000000
52460.0000000	10.7500000	-50.0000000
52460.0000000	11.0000000	-50.0000000
52460.0000000	11.2500000	-50.0000000
52460.0000000	11.5000000	-50.0000000
52460.0000000	11.7500000	-50.0000000
52460.0000000	12.0000000	-50.0000000
52460.0000000	12.2500000	-50.0000000
52460.0000000	12.5000000	-50.0000000
52460.0000000	12.7500000	-50.0000000
52460.0000000	13.0000000	-50.0000000
52460.0000000	13.2500000	-50.0000000
52460.0000000	13.5000000	-50.0000000
52460.0000000	13.7500000	-50.0000000
52460.0000000	14.0000000	-50.0000000
52460.0000000	14.2500000	-50.0000000
52460.0000000	14.5000000	-50.0000000
52460.0000000	14.7500000	-50.0000000
52460.0000000	15.0000000	-50.0000000
52490.0000000	0.0000000	-50.0000000
52490.0000000	0.2500000	-50.0000000
52490.0000000	0.5000000	20.3700008
52490.0000000	0.7500000	21.5100002
52490.0000000	1.0000000	15.3100004
52490.0000000	1.2500000	-50.0000000
52490.0000000	1.5000000	-50.0000000
52490.0000000	1.7500000	-50.0000000
52490.0000000	2.0000000	-50.0000000
52490.0000000	2.2500000	15.6199999
52490.0000000	2.5000000	-50.0000000
52490.0000000	2.7500000	-50.0000000
52490.0000000	3.0000000	16.5699997
52490.0000000	3.2500000	-50.0000000
52490.0000000	3.5000000	15.0500002
52490.0000000	3.7500000	18.1599998
52490.0000000	4.0000000	18.3500004
52490.0000000	4.2500000	-50.0000000
52490.0000000	4.5000000	-50.0000000
52490.0000000	4.7500000	-50.0000000
52490.0000000	5.0000000	-50.0000000
52490.0000000	5.2500000	-50.0000000
52490.0000000	5.5000000	-50.0000000
52490.0000000	5.7500000	-50.0000000
52490.0000000	6.0000000	-50.0000000
52490.0000000	6.2500000	-50.0000000
52490.0000000	6.5000000	-50.0000000
52490.0000000	6.7500000	-50.0000000
52490.0000000	7.0000000	-50.0000000
52490.0000000	7.2500000	-50.0000000
52490.0000000	7.5000000	-50.0000000
52490.0000000	7.7500000	-50.0000000
52490.0000000	8.0000000	-50.0000000
52490.0000000	8.2500000	-50.0000000
52490.0000000	8.5000000	-50.0000000

52490.0000000	8.7500000	-50.0000000
52490.0000000	9.0000000	-50.0000000
52490.0000000	9.2500000	-50.0000000
52490.0000000	9.5000000	-50.0000000
52490.0000000	9.7500000	-50.0000000
52490.0000000	10.0000000	-50.0000000
52490.0000000	10.2500000	-50.0000000
52490.0000000	10.5000000	-50.0000000
52490.0000000	10.7500000	-50.0000000
52490.0000000	11.0000000	-50.0000000
52490.0000000	11.2500000	-50.0000000
52490.0000000	11.5000000	-50.0000000
52490.0000000	11.7500000	-50.0000000
52490.0000000	12.0000000	-50.0000000
52490.0000000	12.2500000	-50.0000000
52490.0000000	12.5000000	-50.0000000
52490.0000000	12.7500000	-50.0000000
52490.0000000	13.0000000	-50.0000000
52490.0000000	13.2500000	-50.0000000
52490.0000000	13.5000000	-50.0000000
52490.0000000	13.7500000	-50.0000000
52490.0000000	14.0000000	-50.0000000
52490.0000000	14.2500000	-50.0000000
52490.0000000	14.5000000	-50.0000000
52490.0000000	14.7500000	-50.0000000
52490.0000000	15.0000000	-50.0000000
52520.0000000	0.0000000	-50.0000000
52520.0000000	0.2500000	-50.0000000
52520.0000000	0.5000000	17.1399994
52520.0000000	0.7500000	19.6800003
52520.0000000	1.0000000	17.5900002
52520.0000000	1.2500000	16.2600002
52520.0000000	1.5000000	15.8400002
52520.0000000	1.7500000	-50.0000000
52520.0000000	2.0000000	-50.0000000
52520.0000000	2.2500000	-50.0000000
52520.0000000	2.5000000	-50.0000000
52520.0000000	2.7500000	-50.0000000
52520.0000000	3.0000000	-50.0000000
52520.0000000	3.2500000	-50.0000000
52520.0000000	3.5000000	-50.0000000
52520.0000000	3.7500000	16.1200008
52520.0000000	4.0000000	-50.0000000
52520.0000000	4.2500000	16.2900009
52520.0000000	4.5000000	15.0699997
52520.0000000	4.7500000	-50.0000000
52520.0000000	5.0000000	-50.0000000
52520.0000000	5.2500000	-50.0000000
52520.0000000	5.5000000	-50.0000000
52520.0000000	5.7500000	-50.0000000
52520.0000000	6.0000000	-50.0000000
52520.0000000	6.2500000	-50.0000000
52520.0000000	6.5000000	-50.0000000
52520.0000000	6.7500000	-50.0000000
52520.0000000	7.0000000	-50.0000000
52520.0000000	7.2500000	-50.0000000
52520.0000000	7.5000000	-50.0000000
52520.0000000	7.7500000	-50.0000000

52520.0000000	8.0000000	-50.0000000
52520.0000000	8.2500000	-50.0000000
52520.0000000	8.5000000	-50.0000000
52520.0000000	8.7500000	-50.0000000
52520.0000000	9.0000000	-50.0000000
52520.0000000	9.2500000	-50.0000000
52520.0000000	9.5000000	-50.0000000
52520.0000000	9.7500000	-50.0000000
52520.0000000	10.0000000	-50.0000000
52520.0000000	10.2500000	-50.0000000
52520.0000000	10.5000000	-50.0000000
52520.0000000	10.7500000	-50.0000000
52520.0000000	11.0000000	-50.0000000
52520.0000000	11.2500000	-50.0000000
52520.0000000	11.5000000	-50.0000000
52520.0000000	11.7500000	-50.0000000
52520.0000000	12.0000000	-50.0000000
52520.0000000	12.2500000	-50.0000000
52520.0000000	12.5000000	-50.0000000
52520.0000000	12.7500000	-50.0000000
52520.0000000	13.0000000	-50.0000000
52520.0000000	13.2500000	-50.0000000
52520.0000000	13.5000000	-50.0000000
52520.0000000	13.7500000	-50.0000000
52520.0000000	14.0000000	-50.0000000
52520.0000000	14.2500000	-50.0000000
52520.0000000	14.5000000	-50.0000000
52520.0000000	14.7500000	-50.0000000
52520.0000000	15.0000000	-50.0000000
52550.0000000	0.0000000	-50.0000000
52550.0000000	0.2500000	-50.0000000
52550.0000000	0.5000000	17.2299995
52550.0000000	0.7500000	19.9300003
52550.0000000	1.0000000	15.5799999
52550.0000000	1.2500000	-50.0000000
52550.0000000	1.5000000	-50.0000000
52550.0000000	1.7500000	-50.0000000
52550.0000000	2.0000000	-50.0000000
52550.0000000	2.2500000	-50.0000000
52550.0000000	2.5000000	15.6999998
52550.0000000	2.7500000	-50.0000000
52550.0000000	3.0000000	-50.0000000
52550.0000000	3.2500000	15.9099998
52550.0000000	3.5000000	16.7199993
52550.0000000	3.7500000	15.5699997
52550.0000000	4.0000000	15.7700005
52550.0000000	4.2500000	-50.0000000
52550.0000000	4.5000000	16.2299995
52550.0000000	4.7500000	15.2399998
52550.0000000	5.0000000	-50.0000000
52550.0000000	5.2500000	-50.0000000
52550.0000000	5.5000000	-50.0000000
52550.0000000	5.7500000	-50.0000000
52550.0000000	6.0000000	-50.0000000
52550.0000000	6.2500000	-50.0000000
52550.0000000	6.5000000	-50.0000000
52550.0000000	6.7500000	-50.0000000
52550.0000000	7.0000000	-50.0000000

52550.0000000	7.2500000	-50.0000000
52550.0000000	7.5000000	-50.0000000
52550.0000000	7.7500000	-50.0000000
52550.0000000	8.0000000	-50.0000000
52550.0000000	8.2500000	-50.0000000
52550.0000000	8.5000000	-50.0000000
52550.0000000	8.7500000	-50.0000000
52550.0000000	9.0000000	-50.0000000
52550.0000000	9.2500000	-50.0000000
52550.0000000	9.5000000	-50.0000000
52550.0000000	9.7500000	-50.0000000
52550.0000000	10.0000000	-50.0000000
52550.0000000	10.2500000	-50.0000000
52550.0000000	10.5000000	-50.0000000
52550.0000000	10.7500000	-50.0000000
52550.0000000	11.0000000	-50.0000000
52550.0000000	11.2500000	-50.0000000
52550.0000000	11.5000000	-50.0000000
52550.0000000	11.7500000	-50.0000000
52550.0000000	12.0000000	-50.0000000
52550.0000000	12.2500000	-50.0000000
52550.0000000	12.5000000	-50.0000000
52550.0000000	12.7500000	-50.0000000
52550.0000000	13.0000000	-50.0000000
52550.0000000	13.2500000	-50.0000000
52550.0000000	13.5000000	-50.0000000
52550.0000000	13.7500000	-50.0000000
52550.0000000	14.0000000	-50.0000000
52550.0000000	14.2500000	-50.0000000
52550.0000000	14.5000000	-50.0000000
52550.0000000	14.7500000	-50.0000000
52550.0000000	15.0000000	-50.0000000
52580.0000000	0.0000000	-50.0000000
52580.0000000	0.2500000	-50.0000000
52580.0000000	0.5000000	18.5900002
52580.0000000	0.7500000	20.4500008
52580.0000000	1.0000000	15.8500004
52580.0000000	1.2500000	15.6999998
52580.0000000	1.5000000	-50.0000000
52580.0000000	1.7500000	-50.0000000
52580.0000000	2.0000000	-50.0000000
52580.0000000	2.2500000	-50.0000000
52580.0000000	2.5000000	-50.0000000
52580.0000000	2.7500000	15.2399998
52580.0000000	3.0000000	-50.0000000
52580.0000000	3.2500000	16.1900005
52580.0000000	3.5000000	18.5300007
52580.0000000	3.7500000	18.0599995
52580.0000000	4.0000000	16.7399998
52580.0000000	4.2500000	17.1499996
52580.0000000	4.5000000	15.7799997
52580.0000000	4.7500000	-50.0000000
52580.0000000	5.0000000	-50.0000000
52580.0000000	5.2500000	-50.0000000
52580.0000000	5.5000000	-50.0000000
52580.0000000	5.7500000	-50.0000000
52580.0000000	6.0000000	-50.0000000
52580.0000000	6.2500000	-50.0000000

52580.0000000	6.5000000	-50.0000000
52580.0000000	6.7500000	-50.0000000
52580.0000000	7.0000000	-50.0000000
52580.0000000	7.2500000	-50.0000000
52580.0000000	7.5000000	-50.0000000
52580.0000000	7.7500000	-50.0000000
52580.0000000	8.0000000	-50.0000000
52580.0000000	8.2500000	-50.0000000
52580.0000000	8.5000000	-50.0000000
52580.0000000	8.7500000	-50.0000000
52580.0000000	9.0000000	-50.0000000
52580.0000000	9.2500000	-50.0000000
52580.0000000	9.5000000	-50.0000000
52580.0000000	9.7500000	-50.0000000
52580.0000000	10.0000000	-50.0000000
52580.0000000	10.2500000	-50.0000000
52580.0000000	10.5000000	-50.0000000
52580.0000000	10.7500000	-50.0000000
52580.0000000	11.0000000	-50.0000000
52580.0000000	11.2500000	-50.0000000
52580.0000000	11.5000000	-50.0000000
52580.0000000	11.7500000	-50.0000000
52580.0000000	12.0000000	-50.0000000
52580.0000000	12.2500000	-50.0000000
52580.0000000	12.5000000	-50.0000000
52580.0000000	12.7500000	-50.0000000
52580.0000000	13.0000000	-50.0000000
52580.0000000	13.2500000	-50.0000000
52580.0000000	13.5000000	-50.0000000
52580.0000000	13.7500000	-50.0000000
52580.0000000	14.0000000	-50.0000000
52580.0000000	14.2500000	-50.0000000
52580.0000000	14.5000000	-50.0000000
52580.0000000	14.7500000	-50.0000000
52580.0000000	15.0000000	-50.0000000
52610.0000000	0.0000000	-50.0000000
52610.0000000	0.2500000	-50.0000000
52610.0000000	0.5000000	19.7900009
52610.0000000	0.7500000	21.2399998
52610.0000000	1.0000000	17.2999992
52610.0000000	1.2500000	15.2200003
52610.0000000	1.5000000	15.5699997
52610.0000000	1.7500000	-50.0000000
52610.0000000	2.0000000	-50.0000000
52610.0000000	2.2500000	-50.0000000
52610.0000000	2.5000000	16.9099998
52610.0000000	2.7500000	-50.0000000
52610.0000000	3.0000000	-50.0000000
52610.0000000	3.2500000	-50.0000000
52610.0000000	3.5000000	18.7500000
52610.0000000	3.7500000	18.7700005
52610.0000000	4.0000000	17.2800007
52610.0000000	4.2500000	16.4699993
52610.0000000	4.5000000	15.0100002
52610.0000000	4.7500000	15.9499998
52610.0000000	5.0000000	-50.0000000
52610.0000000	5.2500000	-50.0000000
52610.0000000	5.5000000	-50.0000000

52610.0000000	5.7500000	-50.0000000
52610.0000000	6.0000000	-50.0000000
52610.0000000	6.2500000	-50.0000000
52610.0000000	6.5000000	-50.0000000
52610.0000000	6.7500000	-50.0000000
52610.0000000	7.0000000	-50.0000000
52610.0000000	7.2500000	-50.0000000
52610.0000000	7.5000000	-50.0000000
52610.0000000	7.7500000	-50.0000000
52610.0000000	8.0000000	-50.0000000
52610.0000000	8.2500000	-50.0000000
52610.0000000	8.5000000	-50.0000000
52610.0000000	8.7500000	-50.0000000
52610.0000000	9.0000000	-50.0000000
52610.0000000	9.2500000	-50.0000000
52610.0000000	9.5000000	-50.0000000
52610.0000000	9.7500000	-50.0000000
52610.0000000	10.0000000	-50.0000000
52610.0000000	10.2500000	-50.0000000
52610.0000000	10.5000000	-50.0000000
52610.0000000	10.7500000	-50.0000000
52610.0000000	11.0000000	-50.0000000
52610.0000000	11.2500000	-50.0000000
52610.0000000	11.5000000	-50.0000000
52610.0000000	11.7500000	-50.0000000
52610.0000000	12.0000000	-50.0000000
52610.0000000	12.2500000	-50.0000000
52610.0000000	12.5000000	-50.0000000
52610.0000000	12.7500000	-50.0000000
52610.0000000	13.0000000	-50.0000000
52610.0000000	13.2500000	-50.0000000
52610.0000000	13.5000000	-50.0000000
52610.0000000	13.7500000	-50.0000000
52610.0000000	14.0000000	-50.0000000
52610.0000000	14.2500000	-50.0000000
52610.0000000	14.5000000	-50.0000000
52610.0000000	14.7500000	-50.0000000
52610.0000000	15.0000000	-50.0000000
52640.0000000	0.0000000	-50.0000000
52640.0000000	0.2500000	-50.0000000
52640.0000000	0.5000000	19.5100002
52640.0000000	0.7500000	20.6000004
52640.0000000	1.0000000	15.4300003
52640.0000000	1.2500000	18.4899998
52640.0000000	1.5000000	16.2399998
52640.0000000	1.7500000	-50.0000000
52640.0000000	2.0000000	-50.0000000
52640.0000000	2.2500000	-50.0000000
52640.0000000	2.5000000	16.3099995
52640.0000000	2.7500000	15.5299997
52640.0000000	3.0000000	-50.0000000
52640.0000000	3.2500000	15.3299999
52640.0000000	3.5000000	20.0699997
52640.0000000	3.7500000	16.8299999
52640.0000000	4.0000000	18.6499996
52640.0000000	4.2500000	16.3799992
52640.0000000	4.5000000	-50.0000000
52640.0000000	4.7500000	-50.0000000

52640.0000000	5.0000000	-50.0000000
52640.0000000	5.2500000	-50.0000000
52640.0000000	5.5000000	-50.0000000
52640.0000000	5.7500000	-50.0000000
52640.0000000	6.0000000	-50.0000000
52640.0000000	6.2500000	-50.0000000
52640.0000000	6.5000000	-50.0000000
52640.0000000	6.7500000	-50.0000000
52640.0000000	7.0000000	-50.0000000
52640.0000000	7.2500000	-50.0000000
52640.0000000	7.5000000	-50.0000000
52640.0000000	7.7500000	-50.0000000
52640.0000000	8.0000000	-50.0000000
52640.0000000	8.2500000	-50.0000000
52640.0000000	8.5000000	-50.0000000
52640.0000000	8.7500000	-50.0000000
52640.0000000	9.0000000	-50.0000000
52640.0000000	9.2500000	-50.0000000
52640.0000000	9.5000000	-50.0000000
52640.0000000	9.7500000	-50.0000000
52640.0000000	10.0000000	-50.0000000
52640.0000000	10.2500000	-50.0000000
52640.0000000	10.5000000	-50.0000000
52640.0000000	10.7500000	-50.0000000
52640.0000000	11.0000000	-50.0000000
52640.0000000	11.2500000	-50.0000000
52640.0000000	11.5000000	-50.0000000
52640.0000000	11.7500000	-50.0000000
52640.0000000	12.0000000	-50.0000000
52640.0000000	12.2500000	-50.0000000
52640.0000000	12.5000000	-50.0000000
52640.0000000	12.7500000	-50.0000000
52640.0000000	13.0000000	-50.0000000
52640.0000000	13.2500000	-50.0000000
52640.0000000	13.5000000	-50.0000000
52640.0000000	13.7500000	-50.0000000
52640.0000000	14.0000000	-50.0000000
52640.0000000	14.2500000	-50.0000000
52640.0000000	14.5000000	-50.0000000
52640.0000000	14.7500000	-50.0000000
52640.0000000	15.0000000	-50.0000000
52670.0000000	0.0000000	-50.0000000
52670.0000000	0.2500000	-50.0000000
52670.0000000	0.5000000	19.5799999
52670.0000000	0.7500000	20.5900002
52670.0000000	1.0000000	-50.0000000
52670.0000000	1.2500000	-50.0000000
52670.0000000	1.5000000	-50.0000000
52670.0000000	1.7500000	-50.0000000
52670.0000000	2.0000000	-50.0000000
52670.0000000	2.2500000	-50.0000000
52670.0000000	2.5000000	-50.0000000
52670.0000000	2.7500000	-50.0000000
52670.0000000	3.0000000	-50.0000000
52670.0000000	3.2500000	15.4700003
52670.0000000	3.5000000	18.1800003
52670.0000000	3.7500000	18.7199993
52670.0000000	4.0000000	17.2700005

52670.0000000	4.2500000	16.0900002
52670.0000000	4.5000000	16.5799999
52670.0000000	4.7500000	15.6300001
52670.0000000	5.0000000	-50.0000000
52670.0000000	5.2500000	-50.0000000
52670.0000000	5.5000000	-50.0000000
52670.0000000	5.7500000	-50.0000000
52670.0000000	6.0000000	-50.0000000
52670.0000000	6.2500000	-50.0000000
52670.0000000	6.5000000	-50.0000000
52670.0000000	6.7500000	-50.0000000
52670.0000000	7.0000000	-50.0000000
52670.0000000	7.2500000	-50.0000000
52670.0000000	7.5000000	-50.0000000
52670.0000000	7.7500000	-50.0000000
52670.0000000	8.0000000	-50.0000000
52670.0000000	8.2500000	-50.0000000
52670.0000000	8.5000000	-50.0000000
52670.0000000	8.7500000	-50.0000000
52670.0000000	9.0000000	-50.0000000
52670.0000000	9.2500000	-50.0000000
52670.0000000	9.5000000	-50.0000000
52670.0000000	9.7500000	-50.0000000
52670.0000000	10.0000000	-50.0000000
52670.0000000	10.2500000	-50.0000000
52670.0000000	10.5000000	-50.0000000
52670.0000000	10.7500000	-50.0000000
52670.0000000	11.0000000	-50.0000000
52670.0000000	11.2500000	-50.0000000
52670.0000000	11.5000000	-50.0000000
52670.0000000	11.7500000	-50.0000000
52670.0000000	12.0000000	-50.0000000
52670.0000000	12.2500000	-50.0000000
52670.0000000	12.5000000	-50.0000000
52670.0000000	12.7500000	-50.0000000
52670.0000000	13.0000000	-50.0000000
52670.0000000	13.2500000	-50.0000000
52670.0000000	13.5000000	-50.0000000
52670.0000000	13.7500000	-50.0000000
52670.0000000	14.0000000	-50.0000000
52670.0000000	14.2500000	-50.0000000
52670.0000000	14.5000000	-50.0000000
52670.0000000	14.7500000	-50.0000000
52670.0000000	15.0000000	-50.0000000
52700.0000000	0.0000000	-50.0000000
52700.0000000	0.2500000	-50.0000000
52700.0000000	0.5000000	18.9899998
52700.0000000	0.7500000	19.6100006
52700.0000000	1.0000000	-50.0000000
52700.0000000	1.2500000	16.4099998
52700.0000000	1.5000000	-50.0000000
52700.0000000	1.7500000	-50.0000000
52700.0000000	2.0000000	-50.0000000
52700.0000000	2.2500000	-50.0000000
52700.0000000	2.5000000	-50.0000000
52700.0000000	2.7500000	-50.0000000
52700.0000000	3.0000000	-50.0000000
52700.0000000	3.2500000	-50.0000000

52700.0000000	3.5000000	16.0400009
52700.0000000	3.7500000	17.3999996
52700.0000000	4.0000000	17.4500008
52700.0000000	4.2500000	16.1100006
52700.0000000	4.5000000	-50.0000000
52700.0000000	4.7500000	-50.0000000
52700.0000000	5.0000000	-50.0000000
52700.0000000	5.2500000	-50.0000000
52700.0000000	5.5000000	-50.0000000
52700.0000000	5.7500000	-50.0000000
52700.0000000	6.0000000	-50.0000000
52700.0000000	6.2500000	-50.0000000
52700.0000000	6.5000000	-50.0000000
52700.0000000	6.7500000	-50.0000000
52700.0000000	7.0000000	-50.0000000
52700.0000000	7.2500000	-50.0000000
52700.0000000	7.5000000	-50.0000000
52700.0000000	7.7500000	-50.0000000
52700.0000000	8.0000000	-50.0000000
52700.0000000	8.2500000	-50.0000000
52700.0000000	8.5000000	-50.0000000
52700.0000000	8.7500000	-50.0000000
52700.0000000	9.0000000	-50.0000000
52700.0000000	9.2500000	-50.0000000
52700.0000000	9.5000000	-50.0000000
52700.0000000	9.7500000	-50.0000000
52700.0000000	10.0000000	-50.0000000
52700.0000000	10.2500000	-50.0000000
52700.0000000	10.5000000	-50.0000000
52700.0000000	10.7500000	-50.0000000
52700.0000000	11.0000000	-50.0000000
52700.0000000	11.2500000	-50.0000000
52700.0000000	11.5000000	-50.0000000
52700.0000000	11.7500000	-50.0000000
52700.0000000	12.0000000	-50.0000000
52700.0000000	12.2500000	-50.0000000
52700.0000000	12.5000000	-50.0000000
52700.0000000	12.7500000	-50.0000000
52700.0000000	13.0000000	-50.0000000
52700.0000000	13.2500000	-50.0000000
52700.0000000	13.5000000	-50.0000000
52700.0000000	13.7500000	-50.0000000
52700.0000000	14.0000000	-50.0000000
52700.0000000	14.2500000	-50.0000000
52700.0000000	14.5000000	-50.0000000
52700.0000000	14.7500000	-50.0000000
52700.0000000	15.0000000	-50.0000000
52730.0000000	0.0000000	-50.0000000
52730.0000000	0.2500000	-50.0000000
52730.0000000	0.5000000	19.3600006
52730.0000000	0.7500000	21.3600006
52730.0000000	1.0000000	15.5500002
52730.0000000	1.2500000	-50.0000000
52730.0000000	1.5000000	-50.0000000
52730.0000000	1.7500000	-50.0000000
52730.0000000	2.0000000	-50.0000000
52730.0000000	2.2500000	-50.0000000
52730.0000000	2.5000000	-50.0000000

52730.0000000	2.7500000	-50.0000000
52730.0000000	3.0000000	-50.0000000
52730.0000000	3.2500000	-50.0000000
52730.0000000	3.5000000	-50.0000000
52730.0000000	3.7500000	16.1900005
52730.0000000	4.0000000	19.8099995
52730.0000000	4.2500000	15.9799995
52730.0000000	4.5000000	16.6700001
52730.0000000	4.7500000	-50.0000000
52730.0000000	5.0000000	-50.0000000
52730.0000000	5.2500000	-50.0000000
52730.0000000	5.5000000	-50.0000000
52730.0000000	5.7500000	-50.0000000
52730.0000000	6.0000000	-50.0000000
52730.0000000	6.2500000	-50.0000000
52730.0000000	6.5000000	-50.0000000
52730.0000000	6.7500000	-50.0000000
52730.0000000	7.0000000	-50.0000000
52730.0000000	7.2500000	-50.0000000
52730.0000000	7.5000000	-50.0000000
52730.0000000	7.7500000	-50.0000000
52730.0000000	8.0000000	-50.0000000
52730.0000000	8.2500000	-50.0000000
52730.0000000	8.5000000	-50.0000000
52730.0000000	8.7500000	-50.0000000
52730.0000000	9.0000000	-50.0000000
52730.0000000	9.2500000	-50.0000000
52730.0000000	9.5000000	-50.0000000
52730.0000000	9.7500000	-50.0000000
52730.0000000	10.0000000	-50.0000000
52730.0000000	10.2500000	-50.0000000
52730.0000000	10.5000000	-50.0000000
52730.0000000	10.7500000	-50.0000000
52730.0000000	11.0000000	-50.0000000
52730.0000000	11.2500000	-50.0000000
52730.0000000	11.5000000	-50.0000000
52730.0000000	11.7500000	-50.0000000
52730.0000000	12.0000000	-50.0000000
52730.0000000	12.2500000	-50.0000000
52730.0000000	12.5000000	-50.0000000
52730.0000000	12.7500000	-50.0000000
52730.0000000	13.0000000	-50.0000000
52730.0000000	13.2500000	-50.0000000
52730.0000000	13.5000000	-50.0000000
52730.0000000	13.7500000	-50.0000000
52730.0000000	14.0000000	-50.0000000
52730.0000000	14.2500000	-50.0000000
52730.0000000	14.5000000	-50.0000000
52730.0000000	14.7500000	-50.0000000
52730.0000000	15.0000000	-50.0000000
52760.0000000	0.0000000	-50.0000000
52760.0000000	0.2500000	-50.0000000
52760.0000000	0.5000000	18.6800003
52760.0000000	0.7500000	19.2299995
52760.0000000	1.0000000	-50.0000000
52760.0000000	1.2500000	-50.0000000
52760.0000000	1.5000000	-50.0000000
52760.0000000	1.7500000	-50.0000000

52760.0000000	2.0000000	-50.0000000
52760.0000000	2.2500000	-50.0000000
52760.0000000	2.5000000	-50.0000000
52760.0000000	2.7500000	-50.0000000
52760.0000000	3.0000000	-50.0000000
52760.0000000	3.2500000	16.2700005
52760.0000000	3.5000000	18.4500008
52760.0000000	3.7500000	19.6100006
52760.0000000	4.0000000	19.6100006
52760.0000000	4.2500000	20.4899998
52760.0000000	4.5000000	17.7600002
52760.0000000	4.7500000	-50.0000000
52760.0000000	5.0000000	-50.0000000
52760.0000000	5.2500000	-50.0000000
52760.0000000	5.5000000	-50.0000000
52760.0000000	5.7500000	-50.0000000
52760.0000000	6.0000000	-50.0000000
52760.0000000	6.2500000	-50.0000000
52760.0000000	6.5000000	-50.0000000
52760.0000000	6.7500000	-50.0000000
52760.0000000	7.0000000	-50.0000000
52760.0000000	7.2500000	-50.0000000
52760.0000000	7.5000000	-50.0000000
52760.0000000	7.7500000	-50.0000000
52760.0000000	8.0000000	-50.0000000
52760.0000000	8.2500000	-50.0000000
52760.0000000	8.5000000	-50.0000000
52760.0000000	8.7500000	-50.0000000
52760.0000000	9.0000000	-50.0000000
52760.0000000	9.2500000	-50.0000000
52760.0000000	9.5000000	-50.0000000
52760.0000000	9.7500000	-50.0000000
52760.0000000	10.0000000	-50.0000000
52760.0000000	10.2500000	-50.0000000
52760.0000000	10.5000000	-50.0000000
52760.0000000	10.7500000	-50.0000000
52760.0000000	11.0000000	-50.0000000
52760.0000000	11.2500000	-50.0000000
52760.0000000	11.5000000	-50.0000000
52760.0000000	11.7500000	-50.0000000
52760.0000000	12.0000000	-50.0000000
52760.0000000	12.2500000	-50.0000000
52760.0000000	12.5000000	-50.0000000
52760.0000000	12.7500000	-50.0000000
52760.0000000	13.0000000	-50.0000000
52760.0000000	13.2500000	-50.0000000
52760.0000000	13.5000000	-50.0000000
52760.0000000	13.7500000	-50.0000000
52760.0000000	14.0000000	-50.0000000
52760.0000000	14.2500000	-50.0000000
52760.0000000	14.5000000	-50.0000000
52760.0000000	14.7500000	-50.0000000
52760.0000000	15.0000000	-50.0000000
52790.0000000	0.0000000	-50.0000000
52790.0000000	0.2500000	-50.0000000
52790.0000000	0.5000000	23.4899998
52790.0000000	0.7500000	24.4200001
52790.0000000	1.0000000	16.6399994

52790.0000000	1.2500000	-50.0000000
52790.0000000	1.5000000	-50.0000000
52790.0000000	1.7500000	-50.0000000
52790.0000000	2.0000000	-50.0000000
52790.0000000	2.2500000	-50.0000000
52790.0000000	2.5000000	-50.0000000
52790.0000000	2.7500000	-50.0000000
52790.0000000	3.0000000	-50.0000000
52790.0000000	3.2500000	16.4400005
52790.0000000	3.5000000	18.2700005
52790.0000000	3.7500000	17.7299995
52790.0000000	4.0000000	17.0200005
52790.0000000	4.2500000	16.6900005
52790.0000000	4.5000000	-50.0000000
52790.0000000	4.7500000	-50.0000000
52790.0000000	5.0000000	-50.0000000
52790.0000000	5.2500000	-50.0000000
52790.0000000	5.5000000	-50.0000000
52790.0000000	5.7500000	-50.0000000
52790.0000000	6.0000000	-50.0000000
52790.0000000	6.2500000	-50.0000000
52790.0000000	6.5000000	-50.0000000
52790.0000000	6.7500000	-50.0000000
52790.0000000	7.0000000	-50.0000000
52790.0000000	7.2500000	-50.0000000
52790.0000000	7.5000000	-50.0000000
52790.0000000	7.7500000	-50.0000000
52790.0000000	8.0000000	-50.0000000
52790.0000000	8.2500000	-50.0000000
52790.0000000	8.5000000	-50.0000000
52790.0000000	8.7500000	-50.0000000
52790.0000000	9.0000000	-50.0000000
52790.0000000	9.2500000	-50.0000000
52790.0000000	9.5000000	-50.0000000
52790.0000000	9.7500000	-50.0000000
52790.0000000	10.0000000	-50.0000000
52790.0000000	10.2500000	-50.0000000
52790.0000000	10.5000000	-50.0000000
52790.0000000	10.7500000	-50.0000000
52790.0000000	11.0000000	-50.0000000
52790.0000000	11.2500000	-50.0000000
52790.0000000	11.5000000	-50.0000000
52790.0000000	11.7500000	-50.0000000
52790.0000000	12.0000000	-50.0000000
52790.0000000	12.2500000	-50.0000000
52790.0000000	12.5000000	-50.0000000
52790.0000000	12.7500000	-50.0000000
52790.0000000	13.0000000	-50.0000000
52790.0000000	13.2500000	-50.0000000
52790.0000000	13.5000000	-50.0000000
52790.0000000	13.7500000	-50.0000000
52790.0000000	14.0000000	-50.0000000
52790.0000000	14.2500000	-50.0000000
52790.0000000	14.5000000	-50.0000000
52790.0000000	14.7500000	-50.0000000
52790.0000000	15.0000000	-50.0000000
52820.0000000	0.0000000	-50.0000000
52820.0000000	0.2500000	-50.0000000

52820.0000000	0.5000000	21.5499992
52820.0000000	0.7500000	22.8400002
52820.0000000	1.0000000	15.8999996
52820.0000000	1.2500000	-50.0000000
52820.0000000	1.5000000	-50.0000000
52820.0000000	1.7500000	-50.0000000
52820.0000000	2.0000000	-50.0000000
52820.0000000	2.2500000	-50.0000000
52820.0000000	2.5000000	-50.0000000
52820.0000000	2.7500000	-50.0000000
52820.0000000	3.0000000	-50.0000000
52820.0000000	3.2500000	-50.0000000
52820.0000000	3.5000000	15.4300003
52820.0000000	3.7500000	16.0400009
52820.0000000	4.0000000	17.9699993
52820.0000000	4.2500000	-50.0000000
52820.0000000	4.5000000	16.7600002
52820.0000000	4.7500000	-50.0000000
52820.0000000	5.0000000	-50.0000000
52820.0000000	5.2500000	-50.0000000
52820.0000000	5.5000000	-50.0000000
52820.0000000	5.7500000	-50.0000000
52820.0000000	6.0000000	-50.0000000
52820.0000000	6.2500000	-50.0000000
52820.0000000	6.5000000	-50.0000000
52820.0000000	6.7500000	-50.0000000
52820.0000000	7.0000000	-50.0000000
52820.0000000	7.2500000	-50.0000000
52820.0000000	7.5000000	-50.0000000
52820.0000000	7.7500000	-50.0000000
52820.0000000	8.0000000	-50.0000000
52820.0000000	8.2500000	-50.0000000
52820.0000000	8.5000000	-50.0000000
52820.0000000	8.7500000	-50.0000000
52820.0000000	9.0000000	-50.0000000
52820.0000000	9.2500000	-50.0000000
52820.0000000	9.5000000	-50.0000000
52820.0000000	9.7500000	-50.0000000
52820.0000000	10.0000000	-50.0000000
52820.0000000	10.2500000	-50.0000000
52820.0000000	10.5000000	-50.0000000
52820.0000000	10.7500000	-50.0000000
52820.0000000	11.0000000	-50.0000000
52820.0000000	11.2500000	-50.0000000
52820.0000000	11.5000000	-50.0000000
52820.0000000	11.7500000	-50.0000000
52820.0000000	12.0000000	-50.0000000
52820.0000000	12.2500000	-50.0000000
52820.0000000	12.5000000	-50.0000000
52820.0000000	12.7500000	-50.0000000
52820.0000000	13.0000000	-50.0000000
52820.0000000	13.2500000	-50.0000000
52820.0000000	13.5000000	-50.0000000
52820.0000000	13.7500000	-50.0000000
52820.0000000	14.0000000	-50.0000000
52820.0000000	14.2500000	-50.0000000
52820.0000000	14.5000000	-50.0000000
52820.0000000	14.7500000	-50.0000000

52820.0000000	15.0000000	-50.0000000
52850.0000000	0.0000000	-50.0000000
52850.0000000	0.2500000	-50.0000000
52850.0000000	0.5000000	19.7099991
52850.0000000	0.7500000	20.9899998
52850.0000000	1.0000000	15.7399998
52850.0000000	1.2500000	-50.0000000
52850.0000000	1.5000000	-50.0000000
52850.0000000	1.7500000	-50.0000000
52850.0000000	2.0000000	-50.0000000
52850.0000000	2.2500000	-50.0000000
52850.0000000	2.5000000	-50.0000000
52850.0000000	2.7500000	17.1700001
52850.0000000	3.0000000	16.8700008
52850.0000000	3.2500000	17.9699993
52850.0000000	3.5000000	17.5599995
52850.0000000	3.7500000	17.8799992
52850.0000000	4.0000000	19.8899994
52850.0000000	4.2500000	15.3100004
52850.0000000	4.5000000	15.1199999
52850.0000000	4.7500000	-50.0000000
52850.0000000	5.0000000	-50.0000000
52850.0000000	5.2500000	-50.0000000
52850.0000000	5.5000000	-50.0000000
52850.0000000	5.7500000	-50.0000000
52850.0000000	6.0000000	-50.0000000
52850.0000000	6.2500000	-50.0000000
52850.0000000	6.5000000	-50.0000000
52850.0000000	6.7500000	-50.0000000
52850.0000000	7.0000000	-50.0000000
52850.0000000	7.2500000	-50.0000000
52850.0000000	7.5000000	-50.0000000
52850.0000000	7.7500000	-50.0000000
52850.0000000	8.0000000	-50.0000000
52850.0000000	8.2500000	-50.0000000
52850.0000000	8.5000000	-50.0000000
52850.0000000	8.7500000	-50.0000000
52850.0000000	9.0000000	-50.0000000
52850.0000000	9.2500000	-50.0000000
52850.0000000	9.5000000	-50.0000000
52850.0000000	9.7500000	-50.0000000
52850.0000000	10.0000000	-50.0000000
52850.0000000	10.2500000	-50.0000000
52850.0000000	10.5000000	-50.0000000
52850.0000000	10.7500000	-50.0000000
52850.0000000	11.0000000	-50.0000000
52850.0000000	11.2500000	-50.0000000
52850.0000000	11.5000000	-50.0000000
52850.0000000	11.7500000	-50.0000000
52850.0000000	12.0000000	-50.0000000
52850.0000000	12.2500000	-50.0000000
52850.0000000	12.5000000	-50.0000000
52850.0000000	12.7500000	-50.0000000
52850.0000000	13.0000000	-50.0000000
52850.0000000	13.2500000	-50.0000000
52850.0000000	13.5000000	-50.0000000
52850.0000000	13.7500000	-50.0000000
52850.0000000	14.0000000	-50.0000000

52850.0000000	14.2500000	-50.0000000
52850.0000000	14.5000000	-50.0000000
52850.0000000	14.7500000	-50.0000000
52850.0000000	15.0000000	-50.0000000
52880.0000000	0.0000000	-50.0000000
52880.0000000	0.2500000	-50.0000000
52880.0000000	0.5000000	19.2199993
52880.0000000	0.7500000	20.8299999
52880.0000000	1.0000000	18.1399994
52880.0000000	1.2500000	17.9300003
52880.0000000	1.5000000	-50.0000000
52880.0000000	1.7500000	-50.0000000
52880.0000000	2.0000000	-50.0000000
52880.0000000	2.2500000	-50.0000000
52880.0000000	2.5000000	-50.0000000
52880.0000000	2.7500000	-50.0000000
52880.0000000	3.0000000	-50.0000000
52880.0000000	3.2500000	16.2399998
52880.0000000	3.5000000	17.3500004
52880.0000000	3.7500000	16.1100006
52880.0000000	4.0000000	16.7999992
52880.0000000	4.2500000	-50.0000000
52880.0000000	4.5000000	15.2700005
52880.0000000	4.7500000	-50.0000000
52880.0000000	5.0000000	-50.0000000
52880.0000000	5.2500000	-50.0000000
52880.0000000	5.5000000	-50.0000000
52880.0000000	5.7500000	-50.0000000
52880.0000000	6.0000000	-50.0000000
52880.0000000	6.2500000	-50.0000000
52880.0000000	6.5000000	-50.0000000
52880.0000000	6.7500000	-50.0000000
52880.0000000	7.0000000	-50.0000000
52880.0000000	7.2500000	-50.0000000
52880.0000000	7.5000000	-50.0000000
52880.0000000	7.7500000	-50.0000000
52880.0000000	8.0000000	-50.0000000
52880.0000000	8.2500000	-50.0000000
52880.0000000	8.5000000	-50.0000000
52880.0000000	8.7500000	-50.0000000
52880.0000000	9.0000000	-50.0000000
52880.0000000	9.2500000	-50.0000000
52880.0000000	9.5000000	-50.0000000
52880.0000000	9.7500000	-50.0000000
52880.0000000	10.0000000	-50.0000000
52880.0000000	10.2500000	-50.0000000
52880.0000000	10.5000000	-50.0000000
52880.0000000	10.7500000	-50.0000000
52880.0000000	11.0000000	-50.0000000
52880.0000000	11.2500000	-50.0000000
52880.0000000	11.5000000	-50.0000000
52880.0000000	11.7500000	-50.0000000
52880.0000000	12.0000000	-50.0000000
52880.0000000	12.2500000	-50.0000000
52880.0000000	12.5000000	-50.0000000
52880.0000000	12.7500000	-50.0000000
52880.0000000	13.0000000	-50.0000000
52880.0000000	13.2500000	-50.0000000

52880.0000000	13.5000000	-50.0000000
52880.0000000	13.7500000	-50.0000000
52880.0000000	14.0000000	-50.0000000
52880.0000000	14.2500000	-50.0000000
52880.0000000	14.5000000	-50.0000000
52880.0000000	14.7500000	-50.0000000
52880.0000000	15.0000000	-50.0000000
52910.0000000	0.0000000	-50.0000000
52910.0000000	0.2500000	-50.0000000
52910.0000000	0.5000000	19.3799992
52910.0000000	0.7500000	21.2399998
52910.0000000	1.0000000	16.4500008
52910.0000000	1.2500000	-50.0000000
52910.0000000	1.5000000	-50.0000000
52910.0000000	1.7500000	-50.0000000
52910.0000000	2.0000000	-50.0000000
52910.0000000	2.2500000	-50.0000000
52910.0000000	2.5000000	-50.0000000
52910.0000000	2.7500000	-50.0000000
52910.0000000	3.0000000	-50.0000000
52910.0000000	3.2500000	17.3799992
52910.0000000	3.5000000	19.5699997
52910.0000000	3.7500000	16.3500004
52910.0000000	4.0000000	-50.0000000
52910.0000000	4.2500000	17.1200008
52910.0000000	4.5000000	16.4500008
52910.0000000	4.7500000	17.8199997
52910.0000000	5.0000000	-50.0000000
52910.0000000	5.2500000	-50.0000000
52910.0000000	5.5000000	-50.0000000
52910.0000000	5.7500000	-50.0000000
52910.0000000	6.0000000	-50.0000000
52910.0000000	6.2500000	-50.0000000
52910.0000000	6.5000000	-50.0000000
52910.0000000	6.7500000	-50.0000000
52910.0000000	7.0000000	-50.0000000
52910.0000000	7.2500000	-50.0000000
52910.0000000	7.5000000	-50.0000000
52910.0000000	7.7500000	-50.0000000
52910.0000000	8.0000000	-50.0000000
52910.0000000	8.2500000	-50.0000000
52910.0000000	8.5000000	-50.0000000
52910.0000000	8.7500000	-50.0000000
52910.0000000	9.0000000	-50.0000000
52910.0000000	9.2500000	-50.0000000
52910.0000000	9.5000000	-50.0000000
52910.0000000	9.7500000	-50.0000000
52910.0000000	10.0000000	-50.0000000
52910.0000000	10.2500000	-50.0000000
52910.0000000	10.5000000	-50.0000000
52910.0000000	10.7500000	-50.0000000
52910.0000000	11.0000000	-50.0000000
52910.0000000	11.2500000	-50.0000000
52910.0000000	11.5000000	-50.0000000
52910.0000000	11.7500000	-50.0000000
52910.0000000	12.0000000	-50.0000000
52910.0000000	12.2500000	-50.0000000
52910.0000000	12.5000000	-50.0000000

52910.0000000	12.7500000	-50.0000000
52910.0000000	13.0000000	-50.0000000
52910.0000000	13.2500000	-50.0000000
52910.0000000	13.5000000	-50.0000000
52910.0000000	13.7500000	-50.0000000
52910.0000000	14.0000000	-50.0000000
52910.0000000	14.2500000	-50.0000000
52910.0000000	14.5000000	-50.0000000
52910.0000000	14.7500000	-50.0000000
52910.0000000	15.0000000	-50.0000000
52940.0000000	0.0000000	-50.0000000
52940.0000000	0.2500000	-50.0000000
52940.0000000	0.5000000	19.7299995
52940.0000000	0.7500000	20.6800003
52940.0000000	1.0000000	16.5200005
52940.0000000	1.2500000	-50.0000000
52940.0000000	1.5000000	-50.0000000
52940.0000000	1.7500000	-50.0000000
52940.0000000	2.0000000	-50.0000000
52940.0000000	2.2500000	-50.0000000
52940.0000000	2.5000000	-50.0000000
52940.0000000	2.7500000	-50.0000000
52940.0000000	3.0000000	15.5699997
52940.0000000	3.2500000	16.3999996
52940.0000000	3.5000000	-50.0000000
52940.0000000	3.7500000	-50.0000000
52940.0000000	4.0000000	-50.0000000
52940.0000000	4.2500000	18.4099998
52940.0000000	4.5000000	-50.0000000
52940.0000000	4.7500000	-50.0000000
52940.0000000	5.0000000	15.6499996
52940.0000000	5.2500000	-50.0000000
52940.0000000	5.5000000	-50.0000000
52940.0000000	5.7500000	-50.0000000
52940.0000000	6.0000000	-50.0000000
52940.0000000	6.2500000	-50.0000000
52940.0000000	6.5000000	-50.0000000
52940.0000000	6.7500000	-50.0000000
52940.0000000	7.0000000	-50.0000000
52940.0000000	7.2500000	-50.0000000
52940.0000000	7.5000000	-50.0000000
52940.0000000	7.7500000	-50.0000000
52940.0000000	8.0000000	-50.0000000
52940.0000000	8.2500000	-50.0000000
52940.0000000	8.5000000	-50.0000000
52940.0000000	8.7500000	-50.0000000
52940.0000000	9.0000000	-50.0000000
52940.0000000	9.2500000	-50.0000000
52940.0000000	9.5000000	-50.0000000
52940.0000000	9.7500000	-50.0000000
52940.0000000	10.0000000	-50.0000000
52940.0000000	10.2500000	-50.0000000
52940.0000000	10.5000000	-50.0000000
52940.0000000	10.7500000	-50.0000000
52940.0000000	11.0000000	-50.0000000
52940.0000000	11.2500000	-50.0000000
52940.0000000	11.5000000	-50.0000000
52940.0000000	11.7500000	-50.0000000

52940.0000000	12.0000000	-50.0000000
52940.0000000	12.2500000	-50.0000000
52940.0000000	12.5000000	-50.0000000
52940.0000000	12.7500000	-50.0000000
52940.0000000	13.0000000	-50.0000000
52940.0000000	13.2500000	-50.0000000
52940.0000000	13.5000000	-50.0000000
52940.0000000	13.7500000	-50.0000000
52940.0000000	14.0000000	-50.0000000
52940.0000000	14.2500000	-50.0000000
52940.0000000	14.5000000	-50.0000000
52940.0000000	14.7500000	-50.0000000
52940.0000000	15.0000000	-50.0000000
52970.0000000	0.0000000	-50.0000000
52970.0000000	0.2500000	-50.0000000
52970.0000000	0.5000000	19.1700001
52970.0000000	0.7500000	21.2399998
52970.0000000	1.0000000	15.4399996
52970.0000000	1.2500000	-50.0000000
52970.0000000	1.5000000	-50.0000000
52970.0000000	1.7500000	-50.0000000
52970.0000000	2.0000000	-50.0000000
52970.0000000	2.2500000	-50.0000000
52970.0000000	2.5000000	16.0900002
52970.0000000	2.7500000	-50.0000000
52970.0000000	3.0000000	16.6499996
52970.0000000	3.2500000	15.7500000
52970.0000000	3.5000000	18.8700008
52970.0000000	3.7500000	17.1399994
52970.0000000	4.0000000	-50.0000000
52970.0000000	4.2500000	-50.0000000
52970.0000000	4.5000000	16.4699993
52970.0000000	4.7500000	16.6900005
52970.0000000	5.0000000	-50.0000000
52970.0000000	5.2500000	-50.0000000
52970.0000000	5.5000000	-50.0000000
52970.0000000	5.7500000	-50.0000000
52970.0000000	6.0000000	-50.0000000
52970.0000000	6.2500000	-50.0000000
52970.0000000	6.5000000	-50.0000000
52970.0000000	6.7500000	-50.0000000
52970.0000000	7.0000000	-50.0000000
52970.0000000	7.2500000	-50.0000000
52970.0000000	7.5000000	-50.0000000
52970.0000000	7.7500000	-50.0000000
52970.0000000	8.0000000	-50.0000000
52970.0000000	8.2500000	-50.0000000
52970.0000000	8.5000000	-50.0000000
52970.0000000	8.7500000	-50.0000000
52970.0000000	9.0000000	-50.0000000
52970.0000000	9.2500000	-50.0000000
52970.0000000	9.5000000	-50.0000000
52970.0000000	9.7500000	-50.0000000
52970.0000000	10.0000000	-50.0000000
52970.0000000	10.2500000	-50.0000000
52970.0000000	10.5000000	-50.0000000
52970.0000000	10.7500000	-50.0000000
52970.0000000	11.0000000	-50.0000000

52970.0000000	11.2500000	-50.0000000
52970.0000000	11.5000000	-50.0000000
52970.0000000	11.7500000	-50.0000000
52970.0000000	12.0000000	-50.0000000
52970.0000000	12.2500000	-50.0000000
52970.0000000	12.5000000	-50.0000000
52970.0000000	12.7500000	-50.0000000
52970.0000000	13.0000000	-50.0000000
52970.0000000	13.2500000	-50.0000000
52970.0000000	13.5000000	-50.0000000
52970.0000000	13.7500000	-50.0000000
52970.0000000	14.0000000	-50.0000000
52970.0000000	14.2500000	-50.0000000
52970.0000000	14.5000000	-50.0000000
52970.0000000	14.7500000	-50.0000000
52970.0000000	15.0000000	-50.0000000
53000.0000000	0.0000000	-50.0000000
53000.0000000	0.2500000	-50.0000000
53000.0000000	0.5000000	20.1299992
53000.0000000	0.7500000	20.9200001
53000.0000000	1.0000000	15.5900002
53000.0000000	1.2500000	-50.0000000
53000.0000000	1.5000000	-50.0000000
53000.0000000	1.7500000	-50.0000000
53000.0000000	2.0000000	-50.0000000
53000.0000000	2.2500000	15.8100004
53000.0000000	2.5000000	-50.0000000
53000.0000000	2.7500000	-50.0000000
53000.0000000	3.0000000	15.7900000
53000.0000000	3.2500000	15.4799995
53000.0000000	3.5000000	17.4799995
53000.0000000	3.7500000	15.8500004
53000.0000000	4.0000000	16.0200005
53000.0000000	4.2500000	16.2000008
53000.0000000	4.5000000	18.4500008
53000.0000000	4.7500000	16.1399994
53000.0000000	5.0000000	-50.0000000
53000.0000000	5.2500000	-50.0000000
53000.0000000	5.5000000	-50.0000000
53000.0000000	5.7500000	-50.0000000
53000.0000000	6.0000000	-50.0000000
53000.0000000	6.2500000	-50.0000000
53000.0000000	6.5000000	-50.0000000
53000.0000000	6.7500000	-50.0000000
53000.0000000	7.0000000	-50.0000000
53000.0000000	7.2500000	-50.0000000
53000.0000000	7.5000000	-50.0000000
53000.0000000	7.7500000	-50.0000000
53000.0000000	8.0000000	-50.0000000
53000.0000000	8.2500000	-50.0000000
53000.0000000	8.5000000	-50.0000000
53000.0000000	8.7500000	-50.0000000
53000.0000000	9.0000000	-50.0000000
53000.0000000	9.2500000	-50.0000000
53000.0000000	9.5000000	-50.0000000
53000.0000000	9.7500000	-50.0000000
53000.0000000	10.0000000	-50.0000000
53000.0000000	10.2500000	-50.0000000

53000.0000000	10.5000000	-50.0000000
53000.0000000	10.7500000	-50.0000000
53000.0000000	11.0000000	-50.0000000
53000.0000000	11.2500000	-50.0000000
53000.0000000	11.5000000	-50.0000000
53000.0000000	11.7500000	-50.0000000
53000.0000000	12.0000000	-50.0000000
53000.0000000	12.2500000	-50.0000000
53000.0000000	12.5000000	-50.0000000
53000.0000000	12.7500000	-50.0000000
53000.0000000	13.0000000	-50.0000000
53000.0000000	13.2500000	-50.0000000
53000.0000000	13.5000000	-50.0000000
53000.0000000	13.7500000	-50.0000000
53000.0000000	14.0000000	-50.0000000
53000.0000000	14.2500000	-50.0000000
53000.0000000	14.5000000	-50.0000000
53000.0000000	14.7500000	-50.0000000
53000.0000000	15.0000000	-50.0000000
53030.0000000	0.0000000	-50.0000000
53030.0000000	0.2500000	-50.0000000
53030.0000000	0.5000000	21.4400005
53030.0000000	0.7500000	23.5000000
53030.0000000	1.0000000	17.1200008
53030.0000000	1.2500000	-50.0000000
53030.0000000	1.5000000	-50.0000000
53030.0000000	1.7500000	15.3100004
53030.0000000	2.0000000	15.3000002
53030.0000000	2.2500000	-50.0000000
53030.0000000	2.5000000	-50.0000000
53030.0000000	2.7500000	15.5500002
53030.0000000	3.0000000	17.2900009
53030.0000000	3.2500000	-50.0000000
53030.0000000	3.5000000	-50.0000000
53030.0000000	3.7500000	-50.0000000
53030.0000000	4.0000000	17.6299992
53030.0000000	4.2500000	17.9099998
53030.0000000	4.5000000	15.9499998
53030.0000000	4.7500000	-50.0000000
53030.0000000	5.0000000	-50.0000000
53030.0000000	5.2500000	-50.0000000
53030.0000000	5.5000000	-50.0000000
53030.0000000	5.7500000	-50.0000000
53030.0000000	6.0000000	-50.0000000
53030.0000000	6.2500000	-50.0000000
53030.0000000	6.5000000	-50.0000000
53030.0000000	6.7500000	-50.0000000
53030.0000000	7.0000000	-50.0000000
53030.0000000	7.2500000	-50.0000000
53030.0000000	7.5000000	-50.0000000
53030.0000000	7.7500000	-50.0000000
53030.0000000	8.0000000	-50.0000000
53030.0000000	8.2500000	-50.0000000
53030.0000000	8.5000000	-50.0000000
53030.0000000	8.7500000	-50.0000000
53030.0000000	9.0000000	-50.0000000
53030.0000000	9.2500000	-50.0000000
53030.0000000	9.5000000	-50.0000000

53030.0000000	9.7500000	-50.0000000
53030.0000000	10.0000000	-50.0000000
53030.0000000	10.2500000	-50.0000000
53030.0000000	10.5000000	-50.0000000
53030.0000000	10.7500000	-50.0000000
53030.0000000	11.0000000	-50.0000000
53030.0000000	11.2500000	-50.0000000
53030.0000000	11.5000000	-50.0000000
53030.0000000	11.7500000	-50.0000000
53030.0000000	12.0000000	-50.0000000
53030.0000000	12.2500000	-50.0000000
53030.0000000	12.5000000	-50.0000000
53030.0000000	12.7500000	-50.0000000
53030.0000000	13.0000000	-50.0000000
53030.0000000	13.2500000	-50.0000000
53030.0000000	13.5000000	-50.0000000
53030.0000000	13.7500000	-50.0000000
53030.0000000	14.0000000	-50.0000000
53030.0000000	14.2500000	-50.0000000
53030.0000000	14.5000000	-50.0000000
53030.0000000	14.7500000	-50.0000000
53030.0000000	15.0000000	-50.0000000
53060.0000000	0.0000000	-50.0000000
53060.0000000	0.2500000	-50.0000000
53060.0000000	0.5000000	17.0900002
53060.0000000	0.7500000	18.8299999
53060.0000000	1.0000000	-50.0000000
53060.0000000	1.2500000	-50.0000000
53060.0000000	1.5000000	-50.0000000
53060.0000000	1.7500000	-50.0000000
53060.0000000	2.0000000	-50.0000000
53060.0000000	2.2500000	-50.0000000
53060.0000000	2.5000000	-50.0000000
53060.0000000	2.7500000	-50.0000000
53060.0000000	3.0000000	16.5799999
53060.0000000	3.2500000	-50.0000000
53060.0000000	3.5000000	-50.0000000
53060.0000000	3.7500000	17.0799999
53060.0000000	4.0000000	17.7600002
53060.0000000	4.2500000	19.9200001
53060.0000000	4.5000000	17.6499996
53060.0000000	4.7500000	-50.0000000
53060.0000000	5.0000000	-50.0000000
53060.0000000	5.2500000	-50.0000000
53060.0000000	5.5000000	-50.0000000
53060.0000000	5.7500000	-50.0000000
53060.0000000	6.0000000	-50.0000000
53060.0000000	6.2500000	-50.0000000
53060.0000000	6.5000000	-50.0000000
53060.0000000	6.7500000	-50.0000000
53060.0000000	7.0000000	-50.0000000
53060.0000000	7.2500000	-50.0000000
53060.0000000	7.5000000	-50.0000000
53060.0000000	7.7500000	-50.0000000
53060.0000000	8.0000000	-50.0000000
53060.0000000	8.2500000	-50.0000000
53060.0000000	8.5000000	-50.0000000
53060.0000000	8.7500000	-50.0000000

53060.0000000	9.0000000	-50.0000000
53060.0000000	9.2500000	-50.0000000
53060.0000000	9.5000000	-50.0000000
53060.0000000	9.7500000	-50.0000000
53060.0000000	10.0000000	-50.0000000
53060.0000000	10.2500000	-50.0000000
53060.0000000	10.5000000	-50.0000000
53060.0000000	10.7500000	-50.0000000
53060.0000000	11.0000000	-50.0000000
53060.0000000	11.2500000	-50.0000000
53060.0000000	11.5000000	-50.0000000
53060.0000000	11.7500000	-50.0000000
53060.0000000	12.0000000	-50.0000000
53060.0000000	12.2500000	-50.0000000
53060.0000000	12.5000000	-50.0000000
53060.0000000	12.7500000	-50.0000000
53060.0000000	13.0000000	-50.0000000
53060.0000000	13.2500000	-50.0000000
53060.0000000	13.5000000	-50.0000000
53060.0000000	13.7500000	-50.0000000
53060.0000000	14.0000000	-50.0000000
53060.0000000	14.2500000	-50.0000000
53060.0000000	14.5000000	-50.0000000
53060.0000000	14.7500000	-50.0000000
53060.0000000	15.0000000	-50.0000000
53090.0000000	0.0000000	-50.0000000
53090.0000000	0.2500000	-50.0000000
53090.0000000	0.5000000	-50.0000000
53090.0000000	0.7500000	15.9799995
53090.0000000	1.0000000	-50.0000000
53090.0000000	1.2500000	-50.0000000
53090.0000000	1.5000000	-50.0000000
53090.0000000	1.7500000	-50.0000000
53090.0000000	2.0000000	-50.0000000
53090.0000000	2.2500000	-50.0000000
53090.0000000	2.5000000	19.7099991
53090.0000000	2.7500000	21.1200008
53090.0000000	3.0000000	16.2000008
53090.0000000	3.2500000	16.6599998
53090.0000000	3.5000000	16.9899998
53090.0000000	3.7500000	-50.0000000
53090.0000000	4.0000000	16.2999992
53090.0000000	4.2500000	17.4599991
53090.0000000	4.5000000	18.5400009
53090.0000000	4.7500000	15.0900002
53090.0000000	5.0000000	-50.0000000
53090.0000000	5.2500000	-50.0000000
53090.0000000	5.5000000	-50.0000000
53090.0000000	5.7500000	-50.0000000
53090.0000000	6.0000000	-50.0000000
53090.0000000	6.2500000	-50.0000000
53090.0000000	6.5000000	-50.0000000
53090.0000000	6.7500000	-50.0000000
53090.0000000	7.0000000	-50.0000000
53090.0000000	7.2500000	-50.0000000
53090.0000000	7.5000000	-50.0000000
53090.0000000	7.7500000	-50.0000000
53090.0000000	8.0000000	-50.0000000

53090.0000000	8.2500000	-50.0000000
53090.0000000	8.5000000	-50.0000000
53090.0000000	8.7500000	-50.0000000
53090.0000000	9.0000000	-50.0000000
53090.0000000	9.2500000	-50.0000000
53090.0000000	9.5000000	-50.0000000
53090.0000000	9.7500000	-50.0000000
53090.0000000	10.0000000	-50.0000000
53090.0000000	10.2500000	-50.0000000
53090.0000000	10.5000000	-50.0000000
53090.0000000	10.7500000	-50.0000000
53090.0000000	11.0000000	-50.0000000
53090.0000000	11.2500000	-50.0000000
53090.0000000	11.5000000	-50.0000000
53090.0000000	11.7500000	-50.0000000
53090.0000000	12.0000000	-50.0000000
53090.0000000	12.2500000	-50.0000000
53090.0000000	12.5000000	-50.0000000
53090.0000000	12.7500000	-50.0000000
53090.0000000	13.0000000	-50.0000000
53090.0000000	13.2500000	-50.0000000
53090.0000000	13.5000000	-50.0000000
53090.0000000	13.7500000	-50.0000000
53090.0000000	14.0000000	-50.0000000
53090.0000000	14.2500000	-50.0000000
53090.0000000	14.5000000	-50.0000000
53090.0000000	14.7500000	-50.0000000
53090.0000000	15.0000000	-50.0000000
53120.0000000	0.0000000	-50.0000000
53120.0000000	0.2500000	-50.0000000
53120.0000000	0.5000000	21.1000004
53120.0000000	0.7500000	22.1299992
53120.0000000	1.0000000	-50.0000000
53120.0000000	1.2500000	-50.0000000
53120.0000000	1.5000000	15.9399996
53120.0000000	1.7500000	-50.0000000
53120.0000000	2.0000000	-50.0000000
53120.0000000	2.2500000	15.1400003
53120.0000000	2.5000000	16.3899994
53120.0000000	2.7500000	16.7700005
53120.0000000	3.0000000	16.6599998
53120.0000000	3.2500000	-50.0000000
53120.0000000	3.5000000	15.9099998
53120.0000000	3.7500000	16.3700008
53120.0000000	4.0000000	17.7500000
53120.0000000	4.2500000	18.6299992
53120.0000000	4.5000000	19.4699993
53120.0000000	4.7500000	-50.0000000
53120.0000000	5.0000000	-50.0000000
53120.0000000	5.2500000	-50.0000000
53120.0000000	5.5000000	-50.0000000
53120.0000000	5.7500000	-50.0000000
53120.0000000	6.0000000	-50.0000000
53120.0000000	6.2500000	-50.0000000
53120.0000000	6.5000000	-50.0000000
53120.0000000	6.7500000	-50.0000000
53120.0000000	7.0000000	-50.0000000
53120.0000000	7.2500000	-50.0000000

53120.0000000	7.5000000	-50.0000000
53120.0000000	7.7500000	-50.0000000
53120.0000000	8.0000000	-50.0000000
53120.0000000	8.2500000	-50.0000000
53120.0000000	8.5000000	-50.0000000
53120.0000000	8.7500000	-50.0000000
53120.0000000	9.0000000	-50.0000000
53120.0000000	9.2500000	-50.0000000
53120.0000000	9.5000000	-50.0000000
53120.0000000	9.7500000	-50.0000000
53120.0000000	10.0000000	-50.0000000
53120.0000000	10.2500000	-50.0000000
53120.0000000	10.5000000	-50.0000000
53120.0000000	10.7500000	-50.0000000
53120.0000000	11.0000000	-50.0000000
53120.0000000	11.2500000	-50.0000000
53120.0000000	11.5000000	-50.0000000
53120.0000000	11.7500000	-50.0000000
53120.0000000	12.0000000	-50.0000000
53120.0000000	12.2500000	-50.0000000
53120.0000000	12.5000000	-50.0000000
53120.0000000	12.7500000	-50.0000000
53120.0000000	13.0000000	-50.0000000
53120.0000000	13.2500000	-50.0000000
53120.0000000	13.5000000	-50.0000000
53120.0000000	13.7500000	-50.0000000
53120.0000000	14.0000000	-50.0000000
53120.0000000	14.2500000	-50.0000000
53120.0000000	14.5000000	-50.0000000
53120.0000000	14.7500000	-50.0000000
53120.0000000	15.0000000	-50.0000000
53150.0000000	0.0000000	-50.0000000
53150.0000000	0.2500000	-50.0000000
53150.0000000	0.5000000	18.1900005
53150.0000000	0.7500000	19.8400002
53150.0000000	1.0000000	-50.0000000
53150.0000000	1.2500000	-50.0000000
53150.0000000	1.5000000	15.5299997
53150.0000000	1.7500000	16.0599995
53150.0000000	2.0000000	15.5100002
53150.0000000	2.2500000	-50.0000000
53150.0000000	2.5000000	17.0100002
53150.0000000	2.7500000	17.1299992
53150.0000000	3.0000000	15.6300001
53150.0000000	3.2500000	15.8999996
53150.0000000	3.5000000	-50.0000000
53150.0000000	3.7500000	16.8600006
53150.0000000	4.0000000	16.5599995
53150.0000000	4.2500000	17.3500004
53150.0000000	4.5000000	20.1900005
53150.0000000	4.7500000	17.4599991
53150.0000000	5.0000000	-50.0000000
53150.0000000	5.2500000	-50.0000000
53150.0000000	5.5000000	-50.0000000
53150.0000000	5.7500000	-50.0000000
53150.0000000	6.0000000	-50.0000000
53150.0000000	6.2500000	-50.0000000
53150.0000000	6.5000000	-50.0000000

53150.0000000	6.7500000	-50.0000000
53150.0000000	7.0000000	-50.0000000
53150.0000000	7.2500000	-50.0000000
53150.0000000	7.5000000	-50.0000000
53150.0000000	7.7500000	-50.0000000
53150.0000000	8.0000000	-50.0000000
53150.0000000	8.2500000	-50.0000000
53150.0000000	8.5000000	-50.0000000
53150.0000000	8.7500000	-50.0000000
53150.0000000	9.0000000	-50.0000000
53150.0000000	9.2500000	-50.0000000
53150.0000000	9.5000000	-50.0000000
53150.0000000	9.7500000	-50.0000000
53150.0000000	10.0000000	-50.0000000
53150.0000000	10.2500000	-50.0000000
53150.0000000	10.5000000	-50.0000000
53150.0000000	10.7500000	-50.0000000
53150.0000000	11.0000000	-50.0000000
53150.0000000	11.2500000	-50.0000000
53150.0000000	11.5000000	-50.0000000
53150.0000000	11.7500000	-50.0000000
53150.0000000	12.0000000	-50.0000000
53150.0000000	12.2500000	-50.0000000
53150.0000000	12.5000000	-50.0000000
53150.0000000	12.7500000	-50.0000000
53150.0000000	13.0000000	-50.0000000
53150.0000000	13.2500000	-50.0000000
53150.0000000	13.5000000	-50.0000000
53150.0000000	13.7500000	-50.0000000
53150.0000000	14.0000000	-50.0000000
53150.0000000	14.2500000	-50.0000000
53150.0000000	14.5000000	-50.0000000
53150.0000000	14.7500000	-50.0000000
53150.0000000	15.0000000	-50.0000000
53180.0000000	0.0000000	-50.0000000
53180.0000000	0.2500000	-50.0000000
53180.0000000	0.5000000	21.4400005
53180.0000000	0.7500000	22.0000000
53180.0000000	1.0000000	-50.0000000
53180.0000000	1.2500000	-50.0000000
53180.0000000	1.5000000	-50.0000000
53180.0000000	1.7500000	16.1200008
53180.0000000	2.0000000	16.4599991
53180.0000000	2.2500000	17.1000004
53180.0000000	2.5000000	16.3400002
53180.0000000	2.7500000	15.3900003
53180.0000000	3.0000000	17.7399998
53180.0000000	3.2500000	15.5500002
53180.0000000	3.5000000	-50.0000000
53180.0000000	3.7500000	16.4400005
53180.0000000	4.0000000	18.7399998
53180.0000000	4.2500000	18.1299992
53180.0000000	4.5000000	20.7099991
53180.0000000	4.7500000	16.4300003
53180.0000000	5.0000000	-50.0000000
53180.0000000	5.2500000	-50.0000000
53180.0000000	5.5000000	-50.0000000
53180.0000000	5.7500000	-50.0000000

53180.0000000	6.0000000	-50.0000000
53180.0000000	6.2500000	-50.0000000
53180.0000000	6.5000000	-50.0000000
53180.0000000	6.7500000	-50.0000000
53180.0000000	7.0000000	-50.0000000
53180.0000000	7.2500000	-50.0000000
53180.0000000	7.5000000	-50.0000000
53180.0000000	7.7500000	-50.0000000
53180.0000000	8.0000000	-50.0000000
53180.0000000	8.2500000	-50.0000000
53180.0000000	8.5000000	-50.0000000
53180.0000000	8.7500000	-50.0000000
53180.0000000	9.0000000	-50.0000000
53180.0000000	9.2500000	-50.0000000
53180.0000000	9.5000000	-50.0000000
53180.0000000	9.7500000	-50.0000000
53180.0000000	10.0000000	-50.0000000
53180.0000000	10.2500000	-50.0000000
53180.0000000	10.5000000	-50.0000000
53180.0000000	10.7500000	-50.0000000
53180.0000000	11.0000000	-50.0000000
53180.0000000	11.2500000	-50.0000000
53180.0000000	11.5000000	-50.0000000
53180.0000000	11.7500000	-50.0000000
53180.0000000	12.0000000	-50.0000000
53180.0000000	12.2500000	-50.0000000
53180.0000000	12.5000000	-50.0000000
53180.0000000	12.7500000	-50.0000000
53180.0000000	13.0000000	-50.0000000
53180.0000000	13.2500000	-50.0000000
53180.0000000	13.5000000	-50.0000000
53180.0000000	13.7500000	-50.0000000
53180.0000000	14.0000000	-50.0000000
53180.0000000	14.2500000	-50.0000000
53180.0000000	14.5000000	-50.0000000
53180.0000000	14.7500000	-50.0000000
53180.0000000	15.0000000	-50.0000000
53210.0000000	0.0000000	-50.0000000
53210.0000000	0.2500000	-50.0000000
53210.0000000	0.5000000	19.2299995
53210.0000000	0.7500000	19.5400009
53210.0000000	1.0000000	-50.0000000
53210.0000000	1.2500000	-50.0000000
53210.0000000	1.5000000	-50.0000000
53210.0000000	1.7500000	15.8800001
53210.0000000	2.0000000	16.2299995
53210.0000000	2.2500000	17.2399998
53210.0000000	2.5000000	17.1399994
53210.0000000	2.7500000	17.2199993
53210.0000000	3.0000000	15.9200001
53210.0000000	3.2500000	-50.0000000
53210.0000000	3.5000000	-50.0000000
53210.0000000	3.7500000	17.5599995
53210.0000000	4.0000000	19.9099998
53210.0000000	4.2500000	21.8199997
53210.0000000	4.5000000	19.8899994
53210.0000000	4.7500000	15.0200005
53210.0000000	5.0000000	-50.0000000

53210.0000000	5.2500000	-50.0000000
53210.0000000	5.5000000	-50.0000000
53210.0000000	5.7500000	-50.0000000
53210.0000000	6.0000000	-50.0000000
53210.0000000	6.2500000	-50.0000000
53210.0000000	6.5000000	-50.0000000
53210.0000000	6.7500000	-50.0000000
53210.0000000	7.0000000	-50.0000000
53210.0000000	7.2500000	-50.0000000
53210.0000000	7.5000000	-50.0000000
53210.0000000	7.7500000	-50.0000000
53210.0000000	8.0000000	-50.0000000
53210.0000000	8.2500000	-50.0000000
53210.0000000	8.5000000	-50.0000000
53210.0000000	8.7500000	-50.0000000
53210.0000000	9.0000000	-50.0000000
53210.0000000	9.2500000	-50.0000000
53210.0000000	9.5000000	-50.0000000
53210.0000000	9.7500000	-50.0000000
53210.0000000	10.0000000	-50.0000000
53210.0000000	10.2500000	-50.0000000
53210.0000000	10.5000000	-50.0000000
53210.0000000	10.7500000	-50.0000000
53210.0000000	11.0000000	-50.0000000
53210.0000000	11.2500000	-50.0000000
53210.0000000	11.5000000	-50.0000000
53210.0000000	11.7500000	-50.0000000
53210.0000000	12.0000000	-50.0000000
53210.0000000	12.2500000	-50.0000000
53210.0000000	12.5000000	-50.0000000
53210.0000000	12.7500000	-50.0000000
53210.0000000	13.0000000	-50.0000000
53210.0000000	13.2500000	-50.0000000
53210.0000000	13.5000000	-50.0000000
53210.0000000	13.7500000	-50.0000000
53210.0000000	14.0000000	-50.0000000
53210.0000000	14.2500000	-50.0000000
53210.0000000	14.5000000	-50.0000000
53210.0000000	14.7500000	-50.0000000
53210.0000000	15.0000000	-50.0000000
53240.0000000	0.0000000	-50.0000000
53240.0000000	0.2500000	-50.0000000
53240.0000000	0.5000000	19.2700005
53240.0000000	0.7500000	20.0900002
53240.0000000	1.0000000	-50.0000000
53240.0000000	1.2500000	-50.0000000
53240.0000000	1.5000000	15.1400003
53240.0000000	1.7500000	15.7500000
53240.0000000	2.0000000	18.3799992
53240.0000000	2.2500000	17.9400005
53240.0000000	2.5000000	-50.0000000
53240.0000000	2.7500000	15.8699999
53240.0000000	3.0000000	16.3299999
53240.0000000	3.2500000	16.3099995
53240.0000000	3.5000000	15.5000000
53240.0000000	3.7500000	15.9099998
53240.0000000	4.0000000	18.3600006
53240.0000000	4.2500000	19.8299999

53240.0000000	4.5000000	20.0100002
53240.0000000	4.7500000	17.7600002
53240.0000000	5.0000000	-50.0000000
53240.0000000	5.2500000	-50.0000000
53240.0000000	5.5000000	-50.0000000
53240.0000000	5.7500000	-50.0000000
53240.0000000	6.0000000	-50.0000000
53240.0000000	6.2500000	-50.0000000
53240.0000000	6.5000000	-50.0000000
53240.0000000	6.7500000	-50.0000000
53240.0000000	7.0000000	-50.0000000
53240.0000000	7.2500000	-50.0000000
53240.0000000	7.5000000	-50.0000000
53240.0000000	7.7500000	-50.0000000
53240.0000000	8.0000000	-50.0000000
53240.0000000	8.2500000	-50.0000000
53240.0000000	8.5000000	-50.0000000
53240.0000000	8.7500000	-50.0000000
53240.0000000	9.0000000	-50.0000000
53240.0000000	9.2500000	-50.0000000
53240.0000000	9.5000000	-50.0000000
53240.0000000	9.7500000	-50.0000000
53240.0000000	10.0000000	-50.0000000
53240.0000000	10.2500000	-50.0000000
53240.0000000	10.5000000	-50.0000000
53240.0000000	10.7500000	-50.0000000
53240.0000000	11.0000000	-50.0000000
53240.0000000	11.2500000	-50.0000000
53240.0000000	11.5000000	-50.0000000
53240.0000000	11.7500000	-50.0000000
53240.0000000	12.0000000	-50.0000000
53240.0000000	12.2500000	-50.0000000
53240.0000000	12.5000000	-50.0000000
53240.0000000	12.7500000	-50.0000000
53240.0000000	13.0000000	-50.0000000
53240.0000000	13.2500000	-50.0000000
53240.0000000	13.5000000	-50.0000000
53240.0000000	13.7500000	-50.0000000
53240.0000000	14.0000000	-50.0000000
53240.0000000	14.2500000	-50.0000000
53240.0000000	14.5000000	-50.0000000
53240.0000000	14.7500000	-50.0000000
53240.0000000	15.0000000	-50.0000000
53270.0000000	0.0000000	-50.0000000
53270.0000000	0.2500000	-50.0000000
53270.0000000	0.5000000	16.9099998
53270.0000000	0.7500000	18.8500004
53270.0000000	1.0000000	15.0200005
53270.0000000	1.2500000	16.3099995
53270.0000000	1.5000000	15.3800001
53270.0000000	1.7500000	-50.0000000
53270.0000000	2.0000000	16.7700005
53270.0000000	2.2500000	-50.0000000
53270.0000000	2.5000000	-50.0000000
53270.0000000	2.7500000	16.8400002
53270.0000000	3.0000000	15.4600000
53270.0000000	3.2500000	-50.0000000
53270.0000000	3.5000000	-50.0000000

53270.0000000	3.7500000	18.0699997
53270.0000000	4.0000000	19.8700008
53270.0000000	4.2500000	20.7900009
53270.0000000	4.5000000	18.4500008
53270.0000000	4.7500000	19.1100006
53270.0000000	5.0000000	-50.0000000
53270.0000000	5.2500000	-50.0000000
53270.0000000	5.5000000	-50.0000000
53270.0000000	5.7500000	-50.0000000
53270.0000000	6.0000000	-50.0000000
53270.0000000	6.2500000	-50.0000000
53270.0000000	6.5000000	-50.0000000
53270.0000000	6.7500000	-50.0000000
53270.0000000	7.0000000	-50.0000000
53270.0000000	7.2500000	-50.0000000
53270.0000000	7.5000000	-50.0000000
53270.0000000	7.7500000	-50.0000000
53270.0000000	8.0000000	-50.0000000
53270.0000000	8.2500000	-50.0000000
53270.0000000	8.5000000	-50.0000000
53270.0000000	8.7500000	-50.0000000
53270.0000000	9.0000000	-50.0000000
53270.0000000	9.2500000	-50.0000000
53270.0000000	9.5000000	-50.0000000
53270.0000000	9.7500000	-50.0000000
53270.0000000	10.0000000	-50.0000000
53270.0000000	10.2500000	-50.0000000
53270.0000000	10.5000000	-50.0000000
53270.0000000	10.7500000	-50.0000000
53270.0000000	11.0000000	-50.0000000
53270.0000000	11.2500000	-50.0000000
53270.0000000	11.5000000	-50.0000000
53270.0000000	11.7500000	-50.0000000
53270.0000000	12.0000000	-50.0000000
53270.0000000	12.2500000	-50.0000000
53270.0000000	12.5000000	-50.0000000
53270.0000000	12.7500000	-50.0000000
53270.0000000	13.0000000	-50.0000000
53270.0000000	13.2500000	-50.0000000
53270.0000000	13.5000000	-50.0000000
53270.0000000	13.7500000	-50.0000000
53270.0000000	14.0000000	-50.0000000
53270.0000000	14.2500000	-50.0000000
53270.0000000	14.5000000	-50.0000000
53270.0000000	14.7500000	-50.0000000
53270.0000000	15.0000000	-50.0000000
53300.0000000	0.0000000	-50.0000000
53300.0000000	0.2500000	-50.0000000
53300.0000000	0.5000000	22.1900005
53300.0000000	0.7500000	22.7500000
53300.0000000	1.0000000	-50.0000000
53300.0000000	1.2500000	-50.0000000
53300.0000000	1.5000000	-50.0000000
53300.0000000	1.7500000	17.5000000
53300.0000000	2.0000000	18.0599995
53300.0000000	2.2500000	18.5300007
53300.0000000	2.5000000	-50.0000000
53300.0000000	2.7500000	-50.0000000

53300.0000000	3.0000000	16.0499992
53300.0000000	3.2500000	-50.0000000
53300.0000000	3.5000000	15.1999998
53300.0000000	3.7500000	17.8099995
53300.0000000	4.0000000	17.6499996
53300.0000000	4.2500000	16.2199993
53300.0000000	4.5000000	17.9599991
53300.0000000	4.7500000	18.8400002
53300.0000000	5.0000000	-50.0000000
53300.0000000	5.2500000	-50.0000000
53300.0000000	5.5000000	-50.0000000
53300.0000000	5.7500000	-50.0000000
53300.0000000	6.0000000	-50.0000000
53300.0000000	6.2500000	-50.0000000
53300.0000000	6.5000000	-50.0000000
53300.0000000	6.7500000	-50.0000000
53300.0000000	7.0000000	-50.0000000
53300.0000000	7.2500000	-50.0000000
53300.0000000	7.5000000	-50.0000000
53300.0000000	7.7500000	-50.0000000
53300.0000000	8.0000000	-50.0000000
53300.0000000	8.2500000	-50.0000000
53300.0000000	8.5000000	-50.0000000
53300.0000000	8.7500000	-50.0000000
53300.0000000	9.0000000	-50.0000000
53300.0000000	9.2500000	-50.0000000
53300.0000000	9.5000000	-50.0000000
53300.0000000	9.7500000	-50.0000000
53300.0000000	10.0000000	-50.0000000
53300.0000000	10.2500000	-50.0000000
53300.0000000	10.5000000	-50.0000000
53300.0000000	10.7500000	-50.0000000
53300.0000000	11.0000000	-50.0000000
53300.0000000	11.2500000	-50.0000000
53300.0000000	11.5000000	-50.0000000
53300.0000000	11.7500000	-50.0000000
53300.0000000	12.0000000	-50.0000000
53300.0000000	12.2500000	-50.0000000
53300.0000000	12.5000000	-50.0000000
53300.0000000	12.7500000	-50.0000000
53300.0000000	13.0000000	-50.0000000
53300.0000000	13.2500000	-50.0000000
53300.0000000	13.5000000	-50.0000000
53300.0000000	13.7500000	-50.0000000
53300.0000000	14.0000000	-50.0000000
53300.0000000	14.2500000	-50.0000000
53300.0000000	14.5000000	-50.0000000
53300.0000000	14.7500000	-50.0000000
53300.0000000	15.0000000	-50.0000000
53330.0000000	0.0000000	-50.0000000
53330.0000000	0.2500000	-50.0000000
53330.0000000	0.5000000	21.2099991
53330.0000000	0.7500000	21.7600002
53330.0000000	1.0000000	16.5200005
53330.0000000	1.2500000	17.0100002
53330.0000000	1.5000000	15.3500004
53330.0000000	1.7500000	-50.0000000
53330.0000000	2.0000000	16.2099991

53330.0000000	2.2500000	15.5699997
53330.0000000	2.5000000	18.2000008
53330.0000000	2.7500000	18.0100002
53330.0000000	3.0000000	17.2199993
53330.0000000	3.2500000	17.9799995
53330.0000000	3.5000000	-50.0000000
53330.0000000	3.7500000	18.3600006
53330.0000000	4.0000000	20.5200005
53330.0000000	4.2500000	20.6000004
53330.0000000	4.5000000	18.1000004
53330.0000000	4.7500000	16.5499992
53330.0000000	5.0000000	15.3199997
53330.0000000	5.2500000	-50.0000000
53330.0000000	5.5000000	-50.0000000
53330.0000000	5.7500000	-50.0000000
53330.0000000	6.0000000	-50.0000000
53330.0000000	6.2500000	-50.0000000
53330.0000000	6.5000000	-50.0000000
53330.0000000	6.7500000	-50.0000000
53330.0000000	7.0000000	-50.0000000
53330.0000000	7.2500000	-50.0000000
53330.0000000	7.5000000	-50.0000000
53330.0000000	7.7500000	-50.0000000
53330.0000000	8.0000000	-50.0000000
53330.0000000	8.2500000	-50.0000000
53330.0000000	8.5000000	-50.0000000
53330.0000000	8.7500000	-50.0000000
53330.0000000	9.0000000	-50.0000000
53330.0000000	9.2500000	-50.0000000
53330.0000000	9.5000000	-50.0000000
53330.0000000	9.7500000	-50.0000000
53330.0000000	10.0000000	-50.0000000
53330.0000000	10.2500000	-50.0000000
53330.0000000	10.5000000	-50.0000000
53330.0000000	10.7500000	-50.0000000
53330.0000000	11.0000000	-50.0000000
53330.0000000	11.2500000	-50.0000000
53330.0000000	11.5000000	-50.0000000
53330.0000000	11.7500000	-50.0000000
53330.0000000	12.0000000	-50.0000000
53330.0000000	12.2500000	-50.0000000
53330.0000000	12.5000000	-50.0000000
53330.0000000	12.7500000	-50.0000000
53330.0000000	13.0000000	-50.0000000
53330.0000000	13.2500000	-50.0000000
53330.0000000	13.5000000	-50.0000000
53330.0000000	13.7500000	-50.0000000
53330.0000000	14.0000000	-50.0000000
53330.0000000	14.2500000	-50.0000000
53330.0000000	14.5000000	-50.0000000
53330.0000000	14.7500000	-50.0000000
53330.0000000	15.0000000	-50.0000000
53360.0000000	0.0000000	-50.0000000
53360.0000000	0.2500000	-50.0000000
53360.0000000	0.5000000	18.2999992
53360.0000000	0.7500000	19.9300003
53360.0000000	1.0000000	16.2099991
53360.0000000	1.2500000	16.3700008

53360.0000000	1.5000000	15.4300003
53360.0000000	1.7500000	16.5699997
53360.0000000	2.0000000	17.5000000
53360.0000000	2.2500000	17.4500008
53360.0000000	2.5000000	15.4499998
53360.0000000	2.7500000	16.8099995
53360.0000000	3.0000000	17.3899994
53360.0000000	3.2500000	16.2500000
53360.0000000	3.5000000	15.0500002
53360.0000000	3.7500000	18.1499996
53360.0000000	4.0000000	18.8799992
53360.0000000	4.2500000	20.0599995
53360.0000000	4.5000000	19.5300007
53360.0000000	4.7500000	15.8699999
53360.0000000	5.0000000	-50.0000000
53360.0000000	5.2500000	-50.0000000
53360.0000000	5.5000000	-50.0000000
53360.0000000	5.7500000	-50.0000000
53360.0000000	6.0000000	-50.0000000
53360.0000000	6.2500000	-50.0000000
53360.0000000	6.5000000	-50.0000000
53360.0000000	6.7500000	-50.0000000
53360.0000000	7.0000000	-50.0000000
53360.0000000	7.2500000	-50.0000000
53360.0000000	7.5000000	-50.0000000
53360.0000000	7.7500000	-50.0000000
53360.0000000	8.0000000	-50.0000000
53360.0000000	8.2500000	-50.0000000
53360.0000000	8.5000000	-50.0000000
53360.0000000	8.7500000	-50.0000000
53360.0000000	9.0000000	-50.0000000
53360.0000000	9.2500000	-50.0000000
53360.0000000	9.5000000	-50.0000000
53360.0000000	9.7500000	-50.0000000
53360.0000000	10.0000000	-50.0000000
53360.0000000	10.2500000	-50.0000000
53360.0000000	10.5000000	-50.0000000
53360.0000000	10.7500000	-50.0000000
53360.0000000	11.0000000	-50.0000000
53360.0000000	11.2500000	-50.0000000
53360.0000000	11.5000000	-50.0000000
53360.0000000	11.7500000	-50.0000000
53360.0000000	12.0000000	-50.0000000
53360.0000000	12.2500000	-50.0000000
53360.0000000	12.5000000	-50.0000000
53360.0000000	12.7500000	-50.0000000
53360.0000000	13.0000000	-50.0000000
53360.0000000	13.2500000	-50.0000000
53360.0000000	13.5000000	-50.0000000
53360.0000000	13.7500000	-50.0000000
53360.0000000	14.0000000	-50.0000000
53360.0000000	14.2500000	-50.0000000
53360.0000000	14.5000000	-50.0000000
53360.0000000	14.7500000	-50.0000000
53360.0000000	15.0000000	-50.0000000
53390.0000000	0.0000000	-50.0000000
53390.0000000	0.2500000	-50.0000000
53390.0000000	0.5000000	20.3799992

53390.0000000	0.7500000	21.5499992
53390.0000000	1.0000000	17.5100002
53390.0000000	1.2500000	18.5400009
53390.0000000	1.5000000	17.5499992
53390.0000000	1.7500000	16.5699997
53390.0000000	2.0000000	17.3500004
53390.0000000	2.2500000	17.4200001
53390.0000000	2.5000000	16.4799995
53390.0000000	2.7500000	18.7700005
53390.0000000	3.0000000	18.7600002
53390.0000000	3.2500000	16.4899998
53390.0000000	3.5000000	-50.0000000
53390.0000000	3.7500000	17.2099991
53390.0000000	4.0000000	20.2800007
53390.0000000	4.2500000	20.6499996
53390.0000000	4.5000000	20.3199997
53390.0000000	4.7500000	19.1100006
53390.0000000	5.0000000	15.1700001
53390.0000000	5.2500000	-50.0000000
53390.0000000	5.5000000	-50.0000000
53390.0000000	5.7500000	-50.0000000
53390.0000000	6.0000000	-50.0000000
53390.0000000	6.2500000	-50.0000000
53390.0000000	6.5000000	-50.0000000
53390.0000000	6.7500000	-50.0000000
53390.0000000	7.0000000	-50.0000000
53390.0000000	7.2500000	-50.0000000
53390.0000000	7.5000000	-50.0000000
53390.0000000	7.7500000	-50.0000000
53390.0000000	8.0000000	-50.0000000
53390.0000000	8.2500000	-50.0000000
53390.0000000	8.5000000	-50.0000000
53390.0000000	8.7500000	-50.0000000
53390.0000000	9.0000000	-50.0000000
53390.0000000	9.2500000	-50.0000000
53390.0000000	9.5000000	-50.0000000
53390.0000000	9.7500000	-50.0000000
53390.0000000	10.0000000	-50.0000000
53390.0000000	10.2500000	-50.0000000
53390.0000000	10.5000000	-50.0000000
53390.0000000	10.7500000	-50.0000000
53390.0000000	11.0000000	-50.0000000
53390.0000000	11.2500000	-50.0000000
53390.0000000	11.5000000	-50.0000000
53390.0000000	11.7500000	-50.0000000
53390.0000000	12.0000000	-50.0000000
53390.0000000	12.2500000	-50.0000000
53390.0000000	12.5000000	-50.0000000
53390.0000000	12.7500000	-50.0000000
53390.0000000	13.0000000	-50.0000000
53390.0000000	13.2500000	-50.0000000
53390.0000000	13.5000000	-50.0000000
53390.0000000	13.7500000	-50.0000000
53390.0000000	14.0000000	-50.0000000
53390.0000000	14.2500000	-50.0000000
53390.0000000	14.5000000	-50.0000000
53390.0000000	14.7500000	-50.0000000
53390.0000000	15.0000000	-50.0000000

53420.0000000	0.0000000	-50.0000000
53420.0000000	0.2500000	-50.0000000
53420.0000000	0.5000000	18.7000008
53420.0000000	0.7500000	19.9599991
53420.0000000	1.0000000	15.3400002
53420.0000000	1.2500000	15.0600004
53420.0000000	1.5000000	16.2000008
53420.0000000	1.7500000	18.0900002
53420.0000000	2.0000000	18.3899994
53420.0000000	2.2500000	18.3600006
53420.0000000	2.5000000	16.7299995
53420.0000000	2.7500000	15.6099997
53420.0000000	3.0000000	17.5300007
53420.0000000	3.2500000	15.1899996
53420.0000000	3.5000000	16.4500008
53420.0000000	3.7500000	17.1499996
53420.0000000	4.0000000	17.7199993
53420.0000000	4.2500000	16.6100006
53420.0000000	4.5000000	18.1100006
53420.0000000	4.7500000	16.8999996
53420.0000000	5.0000000	-50.0000000
53420.0000000	5.2500000	-50.0000000
53420.0000000	5.5000000	-50.0000000
53420.0000000	5.7500000	-50.0000000
53420.0000000	6.0000000	-50.0000000
53420.0000000	6.2500000	-50.0000000
53420.0000000	6.5000000	-50.0000000
53420.0000000	6.7500000	-50.0000000
53420.0000000	7.0000000	-50.0000000
53420.0000000	7.2500000	-50.0000000
53420.0000000	7.5000000	-50.0000000
53420.0000000	7.7500000	-50.0000000
53420.0000000	8.0000000	-50.0000000
53420.0000000	8.2500000	-50.0000000
53420.0000000	8.5000000	-50.0000000
53420.0000000	8.7500000	-50.0000000
53420.0000000	9.0000000	-50.0000000
53420.0000000	9.2500000	-50.0000000
53420.0000000	9.5000000	-50.0000000
53420.0000000	9.7500000	-50.0000000
53420.0000000	10.0000000	-50.0000000
53420.0000000	10.2500000	-50.0000000
53420.0000000	10.5000000	-50.0000000
53420.0000000	10.7500000	-50.0000000
53420.0000000	11.0000000	-50.0000000
53420.0000000	11.2500000	-50.0000000
53420.0000000	11.5000000	-50.0000000
53420.0000000	11.7500000	-50.0000000
53420.0000000	12.0000000	-50.0000000
53420.0000000	12.2500000	-50.0000000
53420.0000000	12.5000000	-50.0000000
53420.0000000	12.7500000	-50.0000000
53420.0000000	13.0000000	-50.0000000
53420.0000000	13.2500000	-50.0000000
53420.0000000	13.5000000	-50.0000000
53420.0000000	13.7500000	-50.0000000
53420.0000000	14.0000000	-50.0000000
53420.0000000	14.2500000	-50.0000000

53420.0000000	14.5000000	-50.0000000
53420.0000000	14.7500000	-50.0000000
53420.0000000	15.0000000	-50.0000000
53450.0000000	0.0000000	-50.0000000
53450.0000000	0.2500000	-50.0000000
53450.0000000	0.5000000	17.3600006
53450.0000000	0.7500000	19.6499996
53450.0000000	1.0000000	16.6700001
53450.0000000	1.2500000	17.2099991
53450.0000000	1.5000000	17.1399994
53450.0000000	1.7500000	17.9099998
53450.0000000	2.0000000	18.3500004
53450.0000000	2.2500000	-50.0000000
53450.0000000	2.5000000	15.3699999
53450.0000000	2.7500000	16.6800003
53450.0000000	3.0000000	-50.0000000
53450.0000000	3.2500000	15.7799997
53450.0000000	3.5000000	15.8800001
53450.0000000	3.7500000	17.9500008
53450.0000000	4.0000000	19.8799992
53450.0000000	4.2500000	20.2299995
53450.0000000	4.5000000	16.0499992
53450.0000000	4.7500000	15.7299995
53450.0000000	5.0000000	-50.0000000
53450.0000000	5.2500000	-50.0000000
53450.0000000	5.5000000	-50.0000000
53450.0000000	5.7500000	-50.0000000
53450.0000000	6.0000000	-50.0000000
53450.0000000	6.2500000	-50.0000000
53450.0000000	6.5000000	-50.0000000
53450.0000000	6.7500000	-50.0000000
53450.0000000	7.0000000	-50.0000000
53450.0000000	7.2500000	-50.0000000
53450.0000000	7.5000000	-50.0000000
53450.0000000	7.7500000	-50.0000000
53450.0000000	8.0000000	-50.0000000
53450.0000000	8.2500000	-50.0000000
53450.0000000	8.5000000	-50.0000000
53450.0000000	8.7500000	-50.0000000
53450.0000000	9.0000000	-50.0000000
53450.0000000	9.2500000	-50.0000000
53450.0000000	9.5000000	-50.0000000
53450.0000000	9.7500000	-50.0000000
53450.0000000	10.0000000	-50.0000000
53450.0000000	10.2500000	-50.0000000
53450.0000000	10.5000000	-50.0000000
53450.0000000	10.7500000	-50.0000000
53450.0000000	11.0000000	-50.0000000
53450.0000000	11.2500000	-50.0000000
53450.0000000	11.5000000	-50.0000000
53450.0000000	11.7500000	-50.0000000
53450.0000000	12.0000000	-50.0000000
53450.0000000	12.2500000	-50.0000000
53450.0000000	12.5000000	-50.0000000
53450.0000000	12.7500000	-50.0000000
53450.0000000	13.0000000	-50.0000000
53450.0000000	13.2500000	-50.0000000
53450.0000000	13.5000000	-50.0000000

53450.0000000	13.7500000	-50.0000000
53450.0000000	14.0000000	-50.0000000
53450.0000000	14.2500000	-50.0000000
53450.0000000	14.5000000	-50.0000000
53450.0000000	14.7500000	-50.0000000
53450.0000000	15.0000000	-50.0000000
53480.0000000	0.0000000	-50.0000000
53480.0000000	0.2500000	-50.0000000
53480.0000000	0.5000000	16.0900002
53480.0000000	0.7500000	18.1800003
53480.0000000	1.0000000	15.5200005
53480.0000000	1.2500000	-50.0000000
53480.0000000	1.5000000	17.3299999
53480.0000000	1.7500000	18.3099995
53480.0000000	2.0000000	17.9699993
53480.0000000	2.2500000	18.2500000
53480.0000000	2.5000000	15.7200003
53480.0000000	2.7500000	15.4600000
53480.0000000	3.0000000	17.8799992
53480.0000000	3.2500000	17.4400005
53480.0000000	3.5000000	-50.0000000
53480.0000000	3.7500000	17.4799995
53480.0000000	4.0000000	19.8400002
53480.0000000	4.2500000	18.7700005
53480.0000000	4.5000000	17.8299999
53480.0000000	4.7500000	16.0699997
53480.0000000	5.0000000	-50.0000000
53480.0000000	5.2500000	-50.0000000
53480.0000000	5.5000000	-50.0000000
53480.0000000	5.7500000	-50.0000000
53480.0000000	6.0000000	-50.0000000
53480.0000000	6.2500000	-50.0000000
53480.0000000	6.5000000	-50.0000000
53480.0000000	6.7500000	-50.0000000
53480.0000000	7.0000000	-50.0000000
53480.0000000	7.2500000	-50.0000000
53480.0000000	7.5000000	-50.0000000
53480.0000000	7.7500000	-50.0000000
53480.0000000	8.0000000	-50.0000000
53480.0000000	8.2500000	-50.0000000
53480.0000000	8.5000000	-50.0000000
53480.0000000	8.7500000	-50.0000000
53480.0000000	9.0000000	-50.0000000
53480.0000000	9.2500000	-50.0000000
53480.0000000	9.5000000	-50.0000000
53480.0000000	9.7500000	-50.0000000
53480.0000000	10.0000000	-50.0000000
53480.0000000	10.2500000	-50.0000000
53480.0000000	10.5000000	-50.0000000
53480.0000000	10.7500000	-50.0000000
53480.0000000	11.0000000	-50.0000000
53480.0000000	11.2500000	-50.0000000
53480.0000000	11.5000000	-50.0000000
53480.0000000	11.7500000	-50.0000000
53480.0000000	12.0000000	-50.0000000
53480.0000000	12.2500000	-50.0000000
53480.0000000	12.5000000	-50.0000000
53480.0000000	12.7500000	-50.0000000

53480.0000000	13.0000000	-50.0000000
53480.0000000	13.2500000	-50.0000000
53480.0000000	13.5000000	-50.0000000
53480.0000000	13.7500000	-50.0000000
53480.0000000	14.0000000	-50.0000000
53480.0000000	14.2500000	-50.0000000
53480.0000000	14.5000000	-50.0000000
53480.0000000	14.7500000	-50.0000000
53480.0000000	15.0000000	-50.0000000
53510.0000000	0.0000000	-50.0000000
53510.0000000	0.2500000	-50.0000000
53510.0000000	0.5000000	15.3900003
53510.0000000	0.7500000	17.1200008
53510.0000000	1.0000000	15.5400000
53510.0000000	1.2500000	16.6700001
53510.0000000	1.5000000	17.8799992
53510.0000000	1.7500000	17.1599998
53510.0000000	2.0000000	16.9500008
53510.0000000	2.2500000	17.1000004
53510.0000000	2.5000000	16.9500008
53510.0000000	2.7500000	17.9099998
53510.0000000	3.0000000	17.7900009
53510.0000000	3.2500000	15.5299997
53510.0000000	3.5000000	-50.0000000
53510.0000000	3.7500000	16.8999996
53510.0000000	4.0000000	18.3600006
53510.0000000	4.2500000	16.1599998
53510.0000000	4.5000000	17.8999996
53510.0000000	4.7500000	-50.0000000
53510.0000000	5.0000000	-50.0000000
53510.0000000	5.2500000	-50.0000000
53510.0000000	5.5000000	-50.0000000
53510.0000000	5.7500000	-50.0000000
53510.0000000	6.0000000	-50.0000000
53510.0000000	6.2500000	-50.0000000
53510.0000000	6.5000000	-50.0000000
53510.0000000	6.7500000	-50.0000000
53510.0000000	7.0000000	-50.0000000
53510.0000000	7.2500000	-50.0000000
53510.0000000	7.5000000	-50.0000000
53510.0000000	7.7500000	-50.0000000
53510.0000000	8.0000000	-50.0000000
53510.0000000	8.2500000	-50.0000000
53510.0000000	8.5000000	-50.0000000
53510.0000000	8.7500000	-50.0000000
53510.0000000	9.0000000	-50.0000000
53510.0000000	9.2500000	-50.0000000
53510.0000000	9.5000000	-50.0000000
53510.0000000	9.7500000	-50.0000000
53510.0000000	10.0000000	-50.0000000
53510.0000000	10.2500000	-50.0000000
53510.0000000	10.5000000	-50.0000000
53510.0000000	10.7500000	-50.0000000
53510.0000000	11.0000000	-50.0000000
53510.0000000	11.2500000	-50.0000000
53510.0000000	11.5000000	-50.0000000
53510.0000000	11.7500000	-50.0000000
53510.0000000	12.0000000	-50.0000000

53510.0000000	12.2500000	-50.0000000
53510.0000000	12.5000000	-50.0000000
53510.0000000	12.7500000	-50.0000000
53510.0000000	13.0000000	-50.0000000
53510.0000000	13.2500000	-50.0000000
53510.0000000	13.5000000	-50.0000000
53510.0000000	13.7500000	-50.0000000
53510.0000000	14.0000000	-50.0000000
53510.0000000	14.2500000	-50.0000000
53510.0000000	14.5000000	-50.0000000
53510.0000000	14.7500000	-50.0000000
53510.0000000	15.0000000	-50.0000000
53540.0000000	0.0000000	-50.0000000
53540.0000000	0.2500000	-50.0000000
53540.0000000	0.5000000	19.7399998
53540.0000000	0.7500000	20.5100002
53540.0000000	1.0000000	16.3099995
53540.0000000	1.2500000	16.8799992
53540.0000000	1.5000000	18.1000004
53540.0000000	1.7500000	17.5900002
53540.0000000	2.0000000	18.6200008
53540.0000000	2.2500000	16.8899994
53540.0000000	2.5000000	15.3999996
53540.0000000	2.7500000	17.2700005
53540.0000000	3.0000000	17.5000000
53540.0000000	3.2500000	18.8199997
53540.0000000	3.5000000	17.3400002
53540.0000000	3.7500000	16.2600002
53540.0000000	4.0000000	17.4699993
53540.0000000	4.2500000	18.8199997
53540.0000000	4.5000000	19.4799995
53540.0000000	4.7500000	18.1900005
53540.0000000	5.0000000	-50.0000000
53540.0000000	5.2500000	-50.0000000
53540.0000000	5.5000000	-50.0000000
53540.0000000	5.7500000	-50.0000000
53540.0000000	6.0000000	-50.0000000
53540.0000000	6.2500000	-50.0000000
53540.0000000	6.5000000	-50.0000000
53540.0000000	6.7500000	-50.0000000
53540.0000000	7.0000000	-50.0000000
53540.0000000	7.2500000	-50.0000000
53540.0000000	7.5000000	-50.0000000
53540.0000000	7.7500000	-50.0000000
53540.0000000	8.0000000	-50.0000000
53540.0000000	8.2500000	-50.0000000
53540.0000000	8.5000000	-50.0000000
53540.0000000	8.7500000	-50.0000000
53540.0000000	9.0000000	-50.0000000
53540.0000000	9.2500000	-50.0000000
53540.0000000	9.5000000	-50.0000000
53540.0000000	9.7500000	-50.0000000
53540.0000000	10.0000000	-50.0000000
53540.0000000	10.2500000	-50.0000000
53540.0000000	10.5000000	-50.0000000
53540.0000000	10.7500000	-50.0000000
53540.0000000	11.0000000	-50.0000000
53540.0000000	11.2500000	-50.0000000

53540.0000000	11.5000000	-50.0000000
53540.0000000	11.7500000	-50.0000000
53540.0000000	12.0000000	-50.0000000
53540.0000000	12.2500000	-50.0000000
53540.0000000	12.5000000	-50.0000000
53540.0000000	12.7500000	-50.0000000
53540.0000000	13.0000000	-50.0000000
53540.0000000	13.2500000	-50.0000000
53540.0000000	13.5000000	-50.0000000
53540.0000000	13.7500000	-50.0000000
53540.0000000	14.0000000	-50.0000000
53540.0000000	14.2500000	-50.0000000
53540.0000000	14.5000000	-50.0000000
53540.0000000	14.7500000	-50.0000000
53540.0000000	15.0000000	-50.0000000
53570.0000000	0.0000000	-50.0000000
53570.0000000	0.2500000	-50.0000000
53570.0000000	0.5000000	18.7800007
53570.0000000	0.7500000	21.0499992
53570.0000000	1.0000000	17.9599991
53570.0000000	1.2500000	18.7199993
53570.0000000	1.5000000	20.5599995
53570.0000000	1.7500000	19.1599998
53570.0000000	2.0000000	17.3999996
53570.0000000	2.2500000	18.4599991
53570.0000000	2.5000000	18.5000000
53570.0000000	2.7500000	16.3799992
53570.0000000	3.0000000	-50.0000000
53570.0000000	3.2500000	-50.0000000
53570.0000000	3.5000000	19.0100002
53570.0000000	3.7500000	17.9699993
53570.0000000	4.0000000	15.8999996
53570.0000000	4.2500000	18.0599995
53570.0000000	4.5000000	17.6200008
53570.0000000	4.7500000	15.0200005
53570.0000000	5.0000000	-50.0000000
53570.0000000	5.2500000	-50.0000000
53570.0000000	5.5000000	-50.0000000
53570.0000000	5.7500000	-50.0000000
53570.0000000	6.0000000	-50.0000000
53570.0000000	6.2500000	-50.0000000
53570.0000000	6.5000000	-50.0000000
53570.0000000	6.7500000	-50.0000000
53570.0000000	7.0000000	-50.0000000
53570.0000000	7.2500000	-50.0000000
53570.0000000	7.5000000	-50.0000000
53570.0000000	7.7500000	-50.0000000
53570.0000000	8.0000000	-50.0000000
53570.0000000	8.2500000	-50.0000000
53570.0000000	8.5000000	-50.0000000
53570.0000000	8.7500000	-50.0000000
53570.0000000	9.0000000	-50.0000000
53570.0000000	9.2500000	-50.0000000
53570.0000000	9.5000000	-50.0000000
53570.0000000	9.7500000	-50.0000000
53570.0000000	10.0000000	-50.0000000
53570.0000000	10.2500000	-50.0000000
53570.0000000	10.5000000	-50.0000000

53570.0000000	10.7500000	-50.0000000
53570.0000000	11.0000000	-50.0000000
53570.0000000	11.2500000	-50.0000000
53570.0000000	11.5000000	-50.0000000
53570.0000000	11.7500000	-50.0000000
53570.0000000	12.0000000	-50.0000000
53570.0000000	12.2500000	-50.0000000
53570.0000000	12.5000000	-50.0000000
53570.0000000	12.7500000	-50.0000000
53570.0000000	13.0000000	-50.0000000
53570.0000000	13.2500000	-50.0000000
53570.0000000	13.5000000	-50.0000000
53570.0000000	13.7500000	-50.0000000
53570.0000000	14.0000000	-50.0000000
53570.0000000	14.2500000	-50.0000000
53570.0000000	14.5000000	-50.0000000
53570.0000000	14.7500000	-50.0000000
53570.0000000	15.0000000	-50.0000000
53600.0000000	0.0000000	-50.0000000
53600.0000000	0.2500000	-50.0000000
53600.0000000	0.5000000	18.9500008
53600.0000000	0.7500000	19.7000008
53600.0000000	1.0000000	18.5799999
53600.0000000	1.2500000	18.1700001
53600.0000000	1.5000000	18.8999996
53600.0000000	1.7500000	18.8299999
53600.0000000	2.0000000	17.4599991
53600.0000000	2.2500000	15.6199999
53600.0000000	2.5000000	-50.0000000
53600.0000000	2.7500000	-50.0000000
53600.0000000	3.0000000	-50.0000000
53600.0000000	3.2500000	16.8700008
53600.0000000	3.5000000	15.1000004
53600.0000000	3.7500000	17.0900002
53600.0000000	4.0000000	17.2700005
53600.0000000	4.2500000	18.4400005
53600.0000000	4.5000000	18.3700008
53600.0000000	4.7500000	18.2399998
53600.0000000	5.0000000	-50.0000000
53600.0000000	5.2500000	-50.0000000
53600.0000000	5.5000000	-50.0000000
53600.0000000	5.7500000	-50.0000000
53600.0000000	6.0000000	-50.0000000
53600.0000000	6.2500000	-50.0000000
53600.0000000	6.5000000	-50.0000000
53600.0000000	6.7500000	-50.0000000
53600.0000000	7.0000000	-50.0000000
53600.0000000	7.2500000	-50.0000000
53600.0000000	7.5000000	-50.0000000
53600.0000000	7.7500000	-50.0000000
53600.0000000	8.0000000	-50.0000000
53600.0000000	8.2500000	-50.0000000
53600.0000000	8.5000000	-50.0000000
53600.0000000	8.7500000	-50.0000000
53600.0000000	9.0000000	-50.0000000
53600.0000000	9.2500000	-50.0000000
53600.0000000	9.5000000	-50.0000000
53600.0000000	9.7500000	-50.0000000

53600.0000000	10.0000000	-50.0000000
53600.0000000	10.2500000	-50.0000000
53600.0000000	10.5000000	-50.0000000
53600.0000000	10.7500000	-50.0000000
53600.0000000	11.0000000	-50.0000000
53600.0000000	11.2500000	-50.0000000
53600.0000000	11.5000000	-50.0000000
53600.0000000	11.7500000	-50.0000000
53600.0000000	12.0000000	-50.0000000
53600.0000000	12.2500000	-50.0000000
53600.0000000	12.5000000	-50.0000000
53600.0000000	12.7500000	-50.0000000
53600.0000000	13.0000000	-50.0000000
53600.0000000	13.2500000	-50.0000000
53600.0000000	13.5000000	-50.0000000
53600.0000000	13.7500000	-50.0000000
53600.0000000	14.0000000	-50.0000000
53600.0000000	14.2500000	-50.0000000
53600.0000000	14.5000000	-50.0000000
53600.0000000	14.7500000	-50.0000000
53600.0000000	15.0000000	-50.0000000
53630.0000000	0.0000000	-50.0000000
53630.0000000	0.2500000	-50.0000000
53630.0000000	0.5000000	19.1200008
53630.0000000	0.7500000	21.0799999
53630.0000000	1.0000000	17.4200001
53630.0000000	1.2500000	19.7000008
53630.0000000	1.5000000	18.0400009
53630.0000000	1.7500000	16.1000004
53630.0000000	2.0000000	17.0200005
53630.0000000	2.2500000	17.8999996
53630.0000000	2.5000000	16.2099991
53630.0000000	2.7500000	-50.0000000
53630.0000000	3.0000000	-50.0000000
53630.0000000	3.2500000	17.2800007
53630.0000000	3.5000000	18.5000000
53630.0000000	3.7500000	19.6499996
53630.0000000	4.0000000	18.7600002
53630.0000000	4.2500000	18.7199993
53630.0000000	4.5000000	20.0799999
53630.0000000	4.7500000	16.3299999
53630.0000000	5.0000000	-50.0000000
53630.0000000	5.2500000	-50.0000000
53630.0000000	5.5000000	-50.0000000
53630.0000000	5.7500000	-50.0000000
53630.0000000	6.0000000	-50.0000000
53630.0000000	6.2500000	-50.0000000
53630.0000000	6.5000000	-50.0000000
53630.0000000	6.7500000	-50.0000000
53630.0000000	7.0000000	-50.0000000
53630.0000000	7.2500000	-50.0000000
53630.0000000	7.5000000	-50.0000000
53630.0000000	7.7500000	-50.0000000
53630.0000000	8.0000000	-50.0000000
53630.0000000	8.2500000	-50.0000000
53630.0000000	8.5000000	-50.0000000
53630.0000000	8.7500000	-50.0000000
53630.0000000	9.0000000	-50.0000000

53630.0000000	9.2500000	-50.0000000
53630.0000000	9.5000000	-50.0000000
53630.0000000	9.7500000	-50.0000000
53630.0000000	10.0000000	-50.0000000
53630.0000000	10.2500000	-50.0000000
53630.0000000	10.5000000	-50.0000000
53630.0000000	10.7500000	-50.0000000
53630.0000000	11.0000000	-50.0000000
53630.0000000	11.2500000	-50.0000000
53630.0000000	11.5000000	-50.0000000
53630.0000000	11.7500000	-50.0000000
53630.0000000	12.0000000	-50.0000000
53630.0000000	12.2500000	-50.0000000
53630.0000000	12.5000000	-50.0000000
53630.0000000	12.7500000	-50.0000000
53630.0000000	13.0000000	-50.0000000
53630.0000000	13.2500000	-50.0000000
53630.0000000	13.5000000	-50.0000000
53630.0000000	13.7500000	-50.0000000
53630.0000000	14.0000000	-50.0000000
53630.0000000	14.2500000	-50.0000000
53630.0000000	14.5000000	-50.0000000
53630.0000000	14.7500000	-50.0000000
53630.0000000	15.0000000	-50.0000000
53660.0000000	0.0000000	-50.0000000
53660.0000000	0.2500000	-50.0000000
53660.0000000	0.5000000	18.4500008
53660.0000000	0.7500000	20.4500008
53660.0000000	1.0000000	18.6399994
53660.0000000	1.2500000	16.0400009
53660.0000000	1.5000000	16.9400005
53660.0000000	1.7500000	18.0000000
53660.0000000	2.0000000	17.7299995
53660.0000000	2.2500000	16.9400005
53660.0000000	2.5000000	16.9099998
53660.0000000	2.7500000	17.0100002
53660.0000000	3.0000000	16.9200001
53660.0000000	3.2500000	17.0900002
53660.0000000	3.5000000	17.5599995
53660.0000000	3.7500000	20.0799999
53660.0000000	4.0000000	17.5300007
53660.0000000	4.2500000	18.5900002
53660.0000000	4.5000000	18.0599995
53660.0000000	4.7500000	-50.0000000
53660.0000000	5.0000000	-50.0000000
53660.0000000	5.2500000	-50.0000000
53660.0000000	5.5000000	-50.0000000
53660.0000000	5.7500000	-50.0000000
53660.0000000	6.0000000	-50.0000000
53660.0000000	6.2500000	-50.0000000
53660.0000000	6.5000000	-50.0000000
53660.0000000	6.7500000	-50.0000000
53660.0000000	7.0000000	-50.0000000
53660.0000000	7.2500000	-50.0000000
53660.0000000	7.5000000	-50.0000000
53660.0000000	7.7500000	-50.0000000
53660.0000000	8.0000000	-50.0000000
53660.0000000	8.2500000	-50.0000000

53660.0000000	8.5000000	-50.0000000
53660.0000000	8.7500000	-50.0000000
53660.0000000	9.0000000	-50.0000000
53660.0000000	9.2500000	-50.0000000
53660.0000000	9.5000000	-50.0000000
53660.0000000	9.7500000	-50.0000000
53660.0000000	10.0000000	-50.0000000
53660.0000000	10.2500000	-50.0000000
53660.0000000	10.5000000	-50.0000000
53660.0000000	10.7500000	-50.0000000
53660.0000000	11.0000000	-50.0000000
53660.0000000	11.2500000	-50.0000000
53660.0000000	11.5000000	-50.0000000
53660.0000000	11.7500000	-50.0000000
53660.0000000	12.0000000	-50.0000000
53660.0000000	12.2500000	-50.0000000
53660.0000000	12.5000000	-50.0000000
53660.0000000	12.7500000	-50.0000000
53660.0000000	13.0000000	-50.0000000
53660.0000000	13.2500000	-50.0000000
53660.0000000	13.5000000	-50.0000000
53660.0000000	13.7500000	-50.0000000
53660.0000000	14.0000000	-50.0000000
53660.0000000	14.2500000	-50.0000000
53660.0000000	14.5000000	-50.0000000
53660.0000000	14.7500000	-50.0000000
53660.0000000	15.0000000	-50.0000000
53690.0000000	0.0000000	-50.0000000
53690.0000000	0.2500000	-50.0000000
53690.0000000	0.5000000	21.0699997
53690.0000000	0.7500000	22.1700001
53690.0000000	1.0000000	18.3299999
53690.0000000	1.2500000	19.6900005
53690.0000000	1.5000000	19.3199997
53690.0000000	1.7500000	18.2800007
53690.0000000	2.0000000	18.3099995
53690.0000000	2.2500000	17.6100006
53690.0000000	2.5000000	16.6000004
53690.0000000	2.7500000	18.7000008
53690.0000000	3.0000000	15.1999998
53690.0000000	3.2500000	15.9700003
53690.0000000	3.5000000	-50.0000000
53690.0000000	3.7500000	20.3099995
53690.0000000	4.0000000	19.5100002
53690.0000000	4.2500000	16.9500008
53690.0000000	4.5000000	19.6299992
53690.0000000	4.7500000	15.2700005
53690.0000000	5.0000000	-50.0000000
53690.0000000	5.2500000	-50.0000000
53690.0000000	5.5000000	-50.0000000
53690.0000000	5.7500000	-50.0000000
53690.0000000	6.0000000	-50.0000000
53690.0000000	6.2500000	-50.0000000
53690.0000000	6.5000000	-50.0000000
53690.0000000	6.7500000	-50.0000000
53690.0000000	7.0000000	-50.0000000
53690.0000000	7.2500000	-50.0000000
53690.0000000	7.5000000	-50.0000000

53690.0000000	7.7500000	-50.0000000
53690.0000000	8.0000000	-50.0000000
53690.0000000	8.2500000	-50.0000000
53690.0000000	8.5000000	-50.0000000
53690.0000000	8.7500000	-50.0000000
53690.0000000	9.0000000	-50.0000000
53690.0000000	9.2500000	-50.0000000
53690.0000000	9.5000000	-50.0000000
53690.0000000	9.7500000	-50.0000000
53690.0000000	10.0000000	-50.0000000
53690.0000000	10.2500000	-50.0000000
53690.0000000	10.5000000	-50.0000000
53690.0000000	10.7500000	-50.0000000
53690.0000000	11.0000000	-50.0000000
53690.0000000	11.2500000	-50.0000000
53690.0000000	11.5000000	-50.0000000
53690.0000000	11.7500000	-50.0000000
53690.0000000	12.0000000	-50.0000000
53690.0000000	12.2500000	-50.0000000
53690.0000000	12.5000000	-50.0000000
53690.0000000	12.7500000	-50.0000000
53690.0000000	13.0000000	-50.0000000
53690.0000000	13.2500000	-50.0000000
53690.0000000	13.5000000	-50.0000000
53690.0000000	13.7500000	-50.0000000
53690.0000000	14.0000000	-50.0000000
53690.0000000	14.2500000	-50.0000000
53690.0000000	14.5000000	-50.0000000
53690.0000000	14.7500000	-50.0000000
53690.0000000	15.0000000	-50.0000000
53720.0000000	0.0000000	-50.0000000
53720.0000000	0.2500000	-50.0000000
53720.0000000	0.5000000	19.2099991
53720.0000000	0.7500000	21.0900002
53720.0000000	1.0000000	15.4499998
53720.0000000	1.2500000	19.8799992
53720.0000000	1.5000000	20.4300003
53720.0000000	1.7500000	18.3799992
53720.0000000	2.0000000	17.6399994
53720.0000000	2.2500000	19.0699997
53720.0000000	2.5000000	16.7500000
53720.0000000	2.7500000	15.7600002
53720.0000000	3.0000000	15.6300001
53720.0000000	3.2500000	17.3299999
53720.0000000	3.5000000	17.8099995
53720.0000000	3.7500000	15.8400002
53720.0000000	4.0000000	16.7399998
53720.0000000	4.2500000	15.8500004
53720.0000000	4.5000000	16.8400002
53720.0000000	4.7500000	15.2799997
53720.0000000	5.0000000	-50.0000000
53720.0000000	5.2500000	-50.0000000
53720.0000000	5.5000000	-50.0000000
53720.0000000	5.7500000	-50.0000000
53720.0000000	6.0000000	-50.0000000
53720.0000000	6.2500000	-50.0000000
53720.0000000	6.5000000	-50.0000000
53720.0000000	6.7500000	-50.0000000

53720.0000000	7.0000000	-50.0000000
53720.0000000	7.2500000	-50.0000000
53720.0000000	7.5000000	-50.0000000
53720.0000000	7.7500000	-50.0000000
53720.0000000	8.0000000	-50.0000000
53720.0000000	8.2500000	-50.0000000
53720.0000000	8.5000000	-50.0000000
53720.0000000	8.7500000	-50.0000000
53720.0000000	9.0000000	-50.0000000
53720.0000000	9.2500000	-50.0000000
53720.0000000	9.5000000	-50.0000000
53720.0000000	9.7500000	-50.0000000
53720.0000000	10.0000000	-50.0000000
53720.0000000	10.2500000	-50.0000000
53720.0000000	10.5000000	-50.0000000
53720.0000000	10.7500000	-50.0000000
53720.0000000	11.0000000	-50.0000000
53720.0000000	11.2500000	-50.0000000
53720.0000000	11.5000000	-50.0000000
53720.0000000	11.7500000	-50.0000000
53720.0000000	12.0000000	-50.0000000
53720.0000000	12.2500000	-50.0000000
53720.0000000	12.5000000	-50.0000000
53720.0000000	12.7500000	-50.0000000
53720.0000000	13.0000000	-50.0000000
53720.0000000	13.2500000	-50.0000000
53720.0000000	13.5000000	-50.0000000
53720.0000000	13.7500000	-50.0000000
53720.0000000	14.0000000	-50.0000000
53720.0000000	14.2500000	-50.0000000
53720.0000000	14.5000000	-50.0000000
53720.0000000	14.7500000	-50.0000000
53720.0000000	15.0000000	-50.0000000
53750.0000000	0.0000000	-50.0000000
53750.0000000	0.2500000	-50.0000000
53750.0000000	0.5000000	21.0599995
53750.0000000	0.7500000	23.0699997
53750.0000000	1.0000000	16.6499996
53750.0000000	1.2500000	19.3899994
53750.0000000	1.5000000	20.4200001
53750.0000000	1.7500000	17.2900009
53750.0000000	2.0000000	18.3199997
53750.0000000	2.2500000	18.4599991
53750.0000000	2.5000000	16.0699997
53750.0000000	2.7500000	-50.0000000
53750.0000000	3.0000000	-50.0000000
53750.0000000	3.2500000	17.5699997
53750.0000000	3.5000000	16.5200005
53750.0000000	3.7500000	17.0100002
53750.0000000	4.0000000	19.1299992
53750.0000000	4.2500000	18.5499992
53750.0000000	4.5000000	15.4600000
53750.0000000	4.7500000	-50.0000000
53750.0000000	5.0000000	-50.0000000
53750.0000000	5.2500000	-50.0000000
53750.0000000	5.5000000	-50.0000000
53750.0000000	5.7500000	-50.0000000
53750.0000000	6.0000000	-50.0000000

53750.0000000	6.2500000	-50.0000000
53750.0000000	6.5000000	-50.0000000
53750.0000000	6.7500000	-50.0000000
53750.0000000	7.0000000	-50.0000000
53750.0000000	7.2500000	-50.0000000
53750.0000000	7.5000000	-50.0000000
53750.0000000	7.7500000	-50.0000000
53750.0000000	8.0000000	-50.0000000
53750.0000000	8.2500000	-50.0000000
53750.0000000	8.5000000	-50.0000000
53750.0000000	8.7500000	-50.0000000
53750.0000000	9.0000000	-50.0000000
53750.0000000	9.2500000	-50.0000000
53750.0000000	9.5000000	-50.0000000
53750.0000000	9.7500000	-50.0000000
53750.0000000	10.0000000	-50.0000000
53750.0000000	10.2500000	-50.0000000
53750.0000000	10.5000000	-50.0000000
53750.0000000	10.7500000	-50.0000000
53750.0000000	11.0000000	-50.0000000
53750.0000000	11.2500000	-50.0000000
53750.0000000	11.5000000	-50.0000000
53750.0000000	11.7500000	-50.0000000
53750.0000000	12.0000000	-50.0000000
53750.0000000	12.2500000	-50.0000000
53750.0000000	12.5000000	-50.0000000
53750.0000000	12.7500000	-50.0000000
53750.0000000	13.0000000	-50.0000000
53750.0000000	13.2500000	-50.0000000
53750.0000000	13.5000000	-50.0000000
53750.0000000	13.7500000	-50.0000000
53750.0000000	14.0000000	-50.0000000
53750.0000000	14.2500000	-50.0000000
53750.0000000	14.5000000	-50.0000000
53750.0000000	14.7500000	-50.0000000
53750.0000000	15.0000000	-50.0000000
53780.0000000	0.0000000	-50.0000000
53780.0000000	0.2500000	-50.0000000
53780.0000000	0.5000000	25.3700008
53780.0000000	0.7500000	26.5300007
53780.0000000	1.0000000	18.0599995
53780.0000000	1.2500000	18.6499996
53780.0000000	1.5000000	17.6299992
53780.0000000	1.7500000	17.1700001
53780.0000000	2.0000000	18.1800003
53780.0000000	2.2500000	18.0499992
53780.0000000	2.5000000	15.5699997
53780.0000000	2.7500000	-50.0000000
53780.0000000	3.0000000	15.8100004
53780.0000000	3.2500000	17.8700008
53780.0000000	3.5000000	18.1100006
53780.0000000	3.7500000	18.5200005
53780.0000000	4.0000000	19.6700001
53780.0000000	4.2500000	18.4400005
53780.0000000	4.5000000	17.1499996
53780.0000000	4.7500000	15.7100000
53780.0000000	5.0000000	-50.0000000
53780.0000000	5.2500000	-50.0000000

53780.0000000	5.5000000	-50.0000000
53780.0000000	5.7500000	-50.0000000
53780.0000000	6.0000000	-50.0000000
53780.0000000	6.2500000	-50.0000000
53780.0000000	6.5000000	-50.0000000
53780.0000000	6.7500000	-50.0000000
53780.0000000	7.0000000	-50.0000000
53780.0000000	7.2500000	-50.0000000
53780.0000000	7.5000000	-50.0000000
53780.0000000	7.7500000	-50.0000000
53780.0000000	8.0000000	-50.0000000
53780.0000000	8.2500000	-50.0000000
53780.0000000	8.5000000	-50.0000000
53780.0000000	8.7500000	-50.0000000
53780.0000000	9.0000000	-50.0000000
53780.0000000	9.2500000	-50.0000000
53780.0000000	9.5000000	-50.0000000
53780.0000000	9.7500000	-50.0000000
53780.0000000	10.0000000	-50.0000000
53780.0000000	10.2500000	-50.0000000
53780.0000000	10.5000000	-50.0000000
53780.0000000	10.7500000	-50.0000000
53780.0000000	11.0000000	-50.0000000
53780.0000000	11.2500000	-50.0000000
53780.0000000	11.5000000	-50.0000000
53780.0000000	11.7500000	-50.0000000
53780.0000000	12.0000000	-50.0000000
53780.0000000	12.2500000	-50.0000000
53780.0000000	12.5000000	-50.0000000
53780.0000000	12.7500000	-50.0000000
53780.0000000	13.0000000	-50.0000000
53780.0000000	13.2500000	-50.0000000
53780.0000000	13.5000000	-50.0000000
53780.0000000	13.7500000	-50.0000000
53780.0000000	14.0000000	-50.0000000
53780.0000000	14.2500000	-50.0000000
53780.0000000	14.5000000	-50.0000000
53780.0000000	14.7500000	-50.0000000
53780.0000000	15.0000000	-50.0000000
53810.0000000	0.0000000	-50.0000000
53810.0000000	0.2500000	-50.0000000
53810.0000000	0.5000000	20.5100002
53810.0000000	0.7500000	22.3299999
53810.0000000	1.0000000	19.6000004
53810.0000000	1.2500000	19.3099995
53810.0000000	1.5000000	19.0200005
53810.0000000	1.7500000	18.6700001
53810.0000000	2.0000000	19.8999996
53810.0000000	2.2500000	20.3099995
53810.0000000	2.5000000	17.9799995
53810.0000000	2.7500000	17.1700001
53810.0000000	3.0000000	18.5300007
53810.0000000	3.2500000	15.1800003
53810.0000000	3.5000000	17.0200005
53810.0000000	3.7500000	18.4799995
53810.0000000	4.0000000	17.3899994
53810.0000000	4.2500000	17.5000000
53810.0000000	4.5000000	16.8999996

53810.0000000	4.7500000	-50.0000000
53810.0000000	5.0000000	-50.0000000
53810.0000000	5.2500000	-50.0000000
53810.0000000	5.5000000	-50.0000000
53810.0000000	5.7500000	-50.0000000
53810.0000000	6.0000000	-50.0000000
53810.0000000	6.2500000	-50.0000000
53810.0000000	6.5000000	-50.0000000
53810.0000000	6.7500000	-50.0000000
53810.0000000	7.0000000	-50.0000000
53810.0000000	7.2500000	-50.0000000
53810.0000000	7.5000000	-50.0000000
53810.0000000	7.7500000	-50.0000000
53810.0000000	8.0000000	-50.0000000
53810.0000000	8.2500000	-50.0000000
53810.0000000	8.5000000	-50.0000000
53810.0000000	8.7500000	-50.0000000
53810.0000000	9.0000000	-50.0000000
53810.0000000	9.2500000	-50.0000000
53810.0000000	9.5000000	-50.0000000
53810.0000000	9.7500000	-50.0000000
53810.0000000	10.0000000	-50.0000000
53810.0000000	10.2500000	-50.0000000
53810.0000000	10.5000000	-50.0000000
53810.0000000	10.7500000	-50.0000000
53810.0000000	11.0000000	-50.0000000
53810.0000000	11.2500000	-50.0000000
53810.0000000	11.5000000	-50.0000000
53810.0000000	11.7500000	-50.0000000
53810.0000000	12.0000000	-50.0000000
53810.0000000	12.2500000	-50.0000000
53810.0000000	12.5000000	-50.0000000
53810.0000000	12.7500000	-50.0000000
53810.0000000	13.0000000	-50.0000000
53810.0000000	13.2500000	-50.0000000
53810.0000000	13.5000000	-50.0000000
53810.0000000	13.7500000	-50.0000000
53810.0000000	14.0000000	-50.0000000
53810.0000000	14.2500000	-50.0000000
53810.0000000	14.5000000	-50.0000000
53810.0000000	14.7500000	-50.0000000
53810.0000000	15.0000000	-50.0000000
53840.0000000	0.0000000	-50.0000000
53840.0000000	0.2500000	-50.0000000
53840.0000000	0.5000000	23.7600002
53840.0000000	0.7500000	24.4799995
53840.0000000	1.0000000	19.7099991
53840.0000000	1.2500000	19.0799999
53840.0000000	1.5000000	17.5599995
53840.0000000	1.7500000	18.3199997
53840.0000000	2.0000000	19.8799992
53840.0000000	2.2500000	21.1100006
53840.0000000	2.5000000	17.8500004
53840.0000000	2.7500000	18.2299995
53840.0000000	3.0000000	20.2399998
53840.0000000	3.2500000	17.7000008
53840.0000000	3.5000000	17.1800003
53840.0000000	3.7500000	16.2299995

53840.0000000	4.0000000	17.6200008
53840.0000000	4.2500000	17.7299995
53840.0000000	4.5000000	19.4099998
53840.0000000	4.7500000	-50.0000000
53840.0000000	5.0000000	-50.0000000
53840.0000000	5.2500000	-50.0000000
53840.0000000	5.5000000	-50.0000000
53840.0000000	5.7500000	-50.0000000
53840.0000000	6.0000000	-50.0000000
53840.0000000	6.2500000	-50.0000000
53840.0000000	6.5000000	-50.0000000
53840.0000000	6.7500000	-50.0000000
53840.0000000	7.0000000	-50.0000000
53840.0000000	7.2500000	-50.0000000
53840.0000000	7.5000000	-50.0000000
53840.0000000	7.7500000	-50.0000000
53840.0000000	8.0000000	-50.0000000
53840.0000000	8.2500000	-50.0000000
53840.0000000	8.5000000	-50.0000000
53840.0000000	8.7500000	-50.0000000
53840.0000000	9.0000000	-50.0000000
53840.0000000	9.2500000	-50.0000000
53840.0000000	9.5000000	-50.0000000
53840.0000000	9.7500000	-50.0000000
53840.0000000	10.0000000	-50.0000000
53840.0000000	10.2500000	-50.0000000
53840.0000000	10.5000000	-50.0000000
53840.0000000	10.7500000	-50.0000000
53840.0000000	11.0000000	-50.0000000
53840.0000000	11.2500000	-50.0000000
53840.0000000	11.5000000	-50.0000000
53840.0000000	11.7500000	-50.0000000
53840.0000000	12.0000000	-50.0000000
53840.0000000	12.2500000	-50.0000000
53840.0000000	12.5000000	-50.0000000
53840.0000000	12.7500000	-50.0000000
53840.0000000	13.0000000	-50.0000000
53840.0000000	13.2500000	-50.0000000
53840.0000000	13.5000000	-50.0000000
53840.0000000	13.7500000	-50.0000000
53840.0000000	14.0000000	-50.0000000
53840.0000000	14.2500000	-50.0000000
53840.0000000	14.5000000	-50.0000000
53840.0000000	14.7500000	-50.0000000
53840.0000000	15.0000000	-50.0000000
53870.0000000	0.0000000	-50.0000000
53870.0000000	0.2500000	-50.0000000
53870.0000000	0.5000000	24.6499996
53870.0000000	0.7500000	25.5699997
53870.0000000	1.0000000	17.3999996
53870.0000000	1.2500000	16.4699993
53870.0000000	1.5000000	17.9200001
53870.0000000	1.7500000	20.3199997
53870.0000000	2.0000000	20.2600002
53870.0000000	2.2500000	19.6499996
53870.0000000	2.5000000	20.0799999
53870.0000000	2.7500000	19.3899994
53870.0000000	3.0000000	19.1299992

53870.0000000	3.2500000	17.0300007
53870.0000000	3.5000000	15.0799999
53870.0000000	3.7500000	17.7299995
53870.0000000	4.0000000	20.5400009
53870.0000000	4.2500000	18.0200005
53870.0000000	4.5000000	18.2700005
53870.0000000	4.7500000	-50.0000000
53870.0000000	5.0000000	-50.0000000
53870.0000000	5.2500000	-50.0000000
53870.0000000	5.5000000	-50.0000000
53870.0000000	5.7500000	-50.0000000
53870.0000000	6.0000000	-50.0000000
53870.0000000	6.2500000	-50.0000000
53870.0000000	6.5000000	-50.0000000
53870.0000000	6.7500000	-50.0000000
53870.0000000	7.0000000	-50.0000000
53870.0000000	7.2500000	-50.0000000
53870.0000000	7.5000000	-50.0000000
53870.0000000	7.7500000	-50.0000000
53870.0000000	8.0000000	-50.0000000
53870.0000000	8.2500000	-50.0000000
53870.0000000	8.5000000	-50.0000000
53870.0000000	8.7500000	-50.0000000
53870.0000000	9.0000000	-50.0000000
53870.0000000	9.2500000	-50.0000000
53870.0000000	9.5000000	-50.0000000
53870.0000000	9.7500000	-50.0000000
53870.0000000	10.0000000	-50.0000000
53870.0000000	10.2500000	-50.0000000
53870.0000000	10.5000000	-50.0000000
53870.0000000	10.7500000	-50.0000000
53870.0000000	11.0000000	-50.0000000
53870.0000000	11.2500000	-50.0000000
53870.0000000	11.5000000	-50.0000000
53870.0000000	11.7500000	-50.0000000
53870.0000000	12.0000000	-50.0000000
53870.0000000	12.2500000	-50.0000000
53870.0000000	12.5000000	-50.0000000
53870.0000000	12.7500000	-50.0000000
53870.0000000	13.0000000	-50.0000000
53870.0000000	13.2500000	-50.0000000
53870.0000000	13.5000000	-50.0000000
53870.0000000	13.7500000	-50.0000000
53870.0000000	14.0000000	-50.0000000
53870.0000000	14.2500000	-50.0000000
53870.0000000	14.5000000	-50.0000000
53870.0000000	14.7500000	-50.0000000
53870.0000000	15.0000000	-50.0000000
53900.0000000	0.0000000	-50.0000000
53900.0000000	0.2500000	-50.0000000
53900.0000000	0.5000000	22.4200001
53900.0000000	0.7500000	23.8899994
53900.0000000	1.0000000	18.3899994
53900.0000000	1.2500000	16.1299992
53900.0000000	1.5000000	18.4599991
53900.0000000	1.7500000	18.8500004
53900.0000000	2.0000000	18.9500008
53900.0000000	2.2500000	19.4699993

53900.0000000	2.5000000	21.1700001
53900.0000000	2.7500000	18.1000004
53900.0000000	3.0000000	17.8899994
53900.0000000	3.2500000	15.6800003
53900.0000000	3.5000000	-50.0000000
53900.0000000	3.7500000	-50.0000000
53900.0000000	4.0000000	16.2099991
53900.0000000	4.2500000	15.9200001
53900.0000000	4.5000000	-50.0000000
53900.0000000	4.7500000	-50.0000000
53900.0000000	5.0000000	-50.0000000
53900.0000000	5.2500000	-50.0000000
53900.0000000	5.5000000	-50.0000000
53900.0000000	5.7500000	-50.0000000
53900.0000000	6.0000000	-50.0000000
53900.0000000	6.2500000	-50.0000000
53900.0000000	6.5000000	-50.0000000
53900.0000000	6.7500000	-50.0000000
53900.0000000	7.0000000	-50.0000000
53900.0000000	7.2500000	-50.0000000
53900.0000000	7.5000000	-50.0000000
53900.0000000	7.7500000	-50.0000000
53900.0000000	8.0000000	-50.0000000
53900.0000000	8.2500000	-50.0000000
53900.0000000	8.5000000	-50.0000000
53900.0000000	8.7500000	-50.0000000
53900.0000000	9.0000000	-50.0000000
53900.0000000	9.2500000	-50.0000000
53900.0000000	9.5000000	-50.0000000
53900.0000000	9.7500000	-50.0000000
53900.0000000	10.0000000	-50.0000000
53900.0000000	10.2500000	-50.0000000
53900.0000000	10.5000000	-50.0000000
53900.0000000	10.7500000	-50.0000000
53900.0000000	11.0000000	-50.0000000
53900.0000000	11.2500000	-50.0000000
53900.0000000	11.5000000	-50.0000000
53900.0000000	11.7500000	-50.0000000
53900.0000000	12.0000000	-50.0000000
53900.0000000	12.2500000	-50.0000000
53900.0000000	12.5000000	-50.0000000
53900.0000000	12.7500000	-50.0000000
53900.0000000	13.0000000	-50.0000000
53900.0000000	13.2500000	-50.0000000
53900.0000000	13.5000000	-50.0000000
53900.0000000	13.7500000	-50.0000000
53900.0000000	14.0000000	-50.0000000
53900.0000000	14.2500000	-50.0000000
53900.0000000	14.5000000	-50.0000000
53900.0000000	14.7500000	-50.0000000
53900.0000000	15.0000000	-50.0000000
53930.0000000	0.0000000	-50.0000000
53930.0000000	0.2500000	-50.0000000
53930.0000000	0.5000000	24.2500000
53930.0000000	0.7500000	25.0699997
53930.0000000	1.0000000	21.5699997
53930.0000000	1.2500000	20.2000008
53930.0000000	1.5000000	18.8199997

53930.0000000	1.7500000	20.0699997
53930.0000000	2.0000000	20.2099991
53930.0000000	2.2500000	19.4400005
53930.0000000	2.5000000	19.4200001
53930.0000000	2.7500000	18.1900005
53930.0000000	3.0000000	-50.0000000
53930.0000000	3.2500000	17.4300003
53930.0000000	3.5000000	18.1000004
53930.0000000	3.7500000	17.6499996
53930.0000000	4.0000000	17.9500008
53930.0000000	4.2500000	17.2399998
53930.0000000	4.5000000	15.8000002
53930.0000000	4.7500000	-50.0000000
53930.0000000	5.0000000	-50.0000000
53930.0000000	5.2500000	-50.0000000
53930.0000000	5.5000000	-50.0000000
53930.0000000	5.7500000	-50.0000000
53930.0000000	6.0000000	-50.0000000
53930.0000000	6.2500000	-50.0000000
53930.0000000	6.5000000	-50.0000000
53930.0000000	6.7500000	-50.0000000
53930.0000000	7.0000000	-50.0000000
53930.0000000	7.2500000	-50.0000000
53930.0000000	7.5000000	-50.0000000
53930.0000000	7.7500000	-50.0000000
53930.0000000	8.0000000	-50.0000000
53930.0000000	8.2500000	-50.0000000
53930.0000000	8.5000000	-50.0000000
53930.0000000	8.7500000	-50.0000000
53930.0000000	9.0000000	-50.0000000
53930.0000000	9.2500000	-50.0000000
53930.0000000	9.5000000	-50.0000000
53930.0000000	9.7500000	-50.0000000
53930.0000000	10.0000000	-50.0000000
53930.0000000	10.2500000	-50.0000000
53930.0000000	10.5000000	-50.0000000
53930.0000000	10.7500000	-50.0000000
53930.0000000	11.0000000	-50.0000000
53930.0000000	11.2500000	-50.0000000
53930.0000000	11.5000000	-50.0000000
53930.0000000	11.7500000	-50.0000000
53930.0000000	12.0000000	-50.0000000
53930.0000000	12.2500000	-50.0000000
53930.0000000	12.5000000	-50.0000000
53930.0000000	12.7500000	-50.0000000
53930.0000000	13.0000000	-50.0000000
53930.0000000	13.2500000	-50.0000000
53930.0000000	13.5000000	-50.0000000
53930.0000000	13.7500000	-50.0000000
53930.0000000	14.0000000	-50.0000000
53930.0000000	14.2500000	-50.0000000
53930.0000000	14.5000000	-50.0000000
53930.0000000	14.7500000	-50.0000000
53930.0000000	15.0000000	-50.0000000
53960.0000000	0.0000000	-50.0000000
53960.0000000	0.2500000	-50.0000000
53960.0000000	0.5000000	22.5000000
53960.0000000	0.7500000	24.1599998

53960.0000000	1.0000000	21.7999992
53960.0000000	1.2500000	19.2099991
53960.0000000	1.5000000	18.3700008
53960.0000000	1.7500000	19.2299995
53960.0000000	2.0000000	19.2299995
53960.0000000	2.2500000	19.5400009
53960.0000000	2.5000000	16.7399998
53960.0000000	2.7500000	19.9099998
53960.0000000	3.0000000	18.9699993
53960.0000000	3.2500000	16.1499996
53960.0000000	3.5000000	18.8099995
53960.0000000	3.7500000	18.7299995
53960.0000000	4.0000000	17.9899998
53960.0000000	4.2500000	16.0699997
53960.0000000	4.5000000	17.8400002
53960.0000000	4.7500000	16.7399998
53960.0000000	5.0000000	-50.0000000
53960.0000000	5.2500000	-50.0000000
53960.0000000	5.5000000	-50.0000000
53960.0000000	5.7500000	-50.0000000
53960.0000000	6.0000000	-50.0000000
53960.0000000	6.2500000	-50.0000000
53960.0000000	6.5000000	-50.0000000
53960.0000000	6.7500000	-50.0000000
53960.0000000	7.0000000	-50.0000000
53960.0000000	7.2500000	-50.0000000
53960.0000000	7.5000000	-50.0000000
53960.0000000	7.7500000	-50.0000000
53960.0000000	8.0000000	-50.0000000
53960.0000000	8.2500000	-50.0000000
53960.0000000	8.5000000	-50.0000000
53960.0000000	8.7500000	-50.0000000
53960.0000000	9.0000000	-50.0000000
53960.0000000	9.2500000	-50.0000000
53960.0000000	9.5000000	-50.0000000
53960.0000000	9.7500000	-50.0000000
53960.0000000	10.0000000	-50.0000000
53960.0000000	10.2500000	-50.0000000
53960.0000000	10.5000000	-50.0000000
53960.0000000	10.7500000	-50.0000000
53960.0000000	11.0000000	-50.0000000
53960.0000000	11.2500000	-50.0000000
53960.0000000	11.5000000	-50.0000000
53960.0000000	11.7500000	-50.0000000
53960.0000000	12.0000000	-50.0000000
53960.0000000	12.2500000	-50.0000000
53960.0000000	12.5000000	-50.0000000
53960.0000000	12.7500000	-50.0000000
53960.0000000	13.0000000	-50.0000000
53960.0000000	13.2500000	-50.0000000
53960.0000000	13.5000000	-50.0000000
53960.0000000	13.7500000	-50.0000000
53960.0000000	14.0000000	-50.0000000
53960.0000000	14.2500000	-50.0000000
53960.0000000	14.5000000	-50.0000000
53960.0000000	14.7500000	-50.0000000
53960.0000000	15.0000000	-50.0000000
53990.0000000	0.0000000	-50.0000000

53990.0000000	0.2500000	-50.0000000
53990.0000000	0.5000000	23.7299995
53990.0000000	0.7500000	24.0599995
53990.0000000	1.0000000	19.0699997
53990.0000000	1.2500000	19.2999992
53990.0000000	1.5000000	19.8299999
53990.0000000	1.7500000	19.3299999
53990.0000000	2.0000000	19.1399994
53990.0000000	2.2500000	19.3999996
53990.0000000	2.5000000	18.9799995
53990.0000000	2.7500000	16.2199993
53990.0000000	3.0000000	15.2900000
53990.0000000	3.2500000	16.7299995
53990.0000000	3.5000000	17.6700001
53990.0000000	3.7500000	17.3799992
53990.0000000	4.0000000	19.4400005
53990.0000000	4.2500000	21.7600002
53990.0000000	4.5000000	18.6499996
53990.0000000	4.7500000	-50.0000000
53990.0000000	5.0000000	-50.0000000
53990.0000000	5.2500000	-50.0000000
53990.0000000	5.5000000	-50.0000000
53990.0000000	5.7500000	-50.0000000
53990.0000000	6.0000000	-50.0000000
53990.0000000	6.2500000	-50.0000000
53990.0000000	6.5000000	-50.0000000
53990.0000000	6.7500000	-50.0000000
53990.0000000	7.0000000	-50.0000000
53990.0000000	7.2500000	-50.0000000
53990.0000000	7.5000000	-50.0000000
53990.0000000	7.7500000	-50.0000000
53990.0000000	8.0000000	-50.0000000
53990.0000000	8.2500000	-50.0000000
53990.0000000	8.5000000	-50.0000000
53990.0000000	8.7500000	-50.0000000
53990.0000000	9.0000000	-50.0000000
53990.0000000	9.2500000	-50.0000000
53990.0000000	9.5000000	-50.0000000
53990.0000000	9.7500000	-50.0000000
53990.0000000	10.0000000	-50.0000000
53990.0000000	10.2500000	-50.0000000
53990.0000000	10.5000000	-50.0000000
53990.0000000	10.7500000	-50.0000000
53990.0000000	11.0000000	-50.0000000
53990.0000000	11.2500000	-50.0000000
53990.0000000	11.5000000	-50.0000000
53990.0000000	11.7500000	-50.0000000
53990.0000000	12.0000000	-50.0000000
53990.0000000	12.2500000	-50.0000000
53990.0000000	12.5000000	-50.0000000
53990.0000000	12.7500000	-50.0000000
53990.0000000	13.0000000	-50.0000000
53990.0000000	13.2500000	-50.0000000
53990.0000000	13.5000000	-50.0000000
53990.0000000	13.7500000	-50.0000000
53990.0000000	14.0000000	-50.0000000
53990.0000000	14.2500000	-50.0000000
53990.0000000	14.5000000	-50.0000000

53990.0000000	14.7500000	-50.0000000
53990.0000000	15.0000000	-50.0000000
54020.0000000	0.0000000	-50.0000000
54020.0000000	0.2500000	-50.0000000
54020.0000000	0.5000000	24.7299995
54020.0000000	0.7500000	26.1200008
54020.0000000	1.0000000	20.5599995
54020.0000000	1.2500000	19.5000000
54020.0000000	1.5000000	20.6200008
54020.0000000	1.7500000	18.7399998
54020.0000000	2.0000000	18.1800003
54020.0000000	2.2500000	20.0000000
54020.0000000	2.5000000	18.4400005
54020.0000000	2.7500000	18.0699997
54020.0000000	3.0000000	15.4099998
54020.0000000	3.2500000	15.8999996
54020.0000000	3.5000000	16.0100002
54020.0000000	3.7500000	17.2299995
54020.0000000	4.0000000	17.8199997
54020.0000000	4.2500000	17.8600006
54020.0000000	4.5000000	15.6199999
54020.0000000	4.7500000	15.8100004
54020.0000000	5.0000000	-50.0000000
54020.0000000	5.2500000	-50.0000000
54020.0000000	5.5000000	-50.0000000
54020.0000000	5.7500000	-50.0000000
54020.0000000	6.0000000	-50.0000000
54020.0000000	6.2500000	-50.0000000
54020.0000000	6.5000000	-50.0000000
54020.0000000	6.7500000	-50.0000000
54020.0000000	7.0000000	-50.0000000
54020.0000000	7.2500000	-50.0000000
54020.0000000	7.5000000	-50.0000000
54020.0000000	7.7500000	-50.0000000
54020.0000000	8.0000000	-50.0000000
54020.0000000	8.2500000	-50.0000000
54020.0000000	8.5000000	-50.0000000
54020.0000000	8.7500000	-50.0000000
54020.0000000	9.0000000	-50.0000000
54020.0000000	9.2500000	-50.0000000
54020.0000000	9.5000000	-50.0000000
54020.0000000	9.7500000	-50.0000000
54020.0000000	10.0000000	-50.0000000
54020.0000000	10.2500000	-50.0000000
54020.0000000	10.5000000	-50.0000000
54020.0000000	10.7500000	-50.0000000
54020.0000000	11.0000000	-50.0000000
54020.0000000	11.2500000	-50.0000000
54020.0000000	11.5000000	-50.0000000
54020.0000000	11.7500000	-50.0000000
54020.0000000	12.0000000	-50.0000000
54020.0000000	12.2500000	-50.0000000
54020.0000000	12.5000000	-50.0000000
54020.0000000	12.7500000	-50.0000000
54020.0000000	13.0000000	-50.0000000
54020.0000000	13.2500000	-50.0000000
54020.0000000	13.5000000	-50.0000000
54020.0000000	13.7500000	-50.0000000

54020.0000000	14.0000000	-50.0000000
54020.0000000	14.2500000	-50.0000000
54020.0000000	14.5000000	-50.0000000
54020.0000000	14.7500000	-50.0000000
54020.0000000	15.0000000	-50.0000000
54050.0000000	0.0000000	-50.0000000
54050.0000000	0.2500000	-50.0000000
54050.0000000	0.5000000	22.9099998
54050.0000000	0.7500000	24.5200005
54050.0000000	1.0000000	18.9599991
54050.0000000	1.2500000	18.2099991
54050.0000000	1.5000000	17.3899994
54050.0000000	1.7500000	18.5400009
54050.0000000	2.0000000	19.8400002
54050.0000000	2.2500000	20.5100002
54050.0000000	2.5000000	17.8899994
54050.0000000	2.7500000	18.0699997
54050.0000000	3.0000000	17.4799995
54050.0000000	3.2500000	15.8199997
54050.0000000	3.5000000	16.5400009
54050.0000000	3.7500000	16.4599991
54050.0000000	4.0000000	15.9600000
54050.0000000	4.2500000	15.9300003
54050.0000000	4.5000000	19.2199993
54050.0000000	4.7500000	15.5100002
54050.0000000	5.0000000	-50.0000000
54050.0000000	5.2500000	-50.0000000
54050.0000000	5.5000000	-50.0000000
54050.0000000	5.7500000	-50.0000000
54050.0000000	6.0000000	-50.0000000
54050.0000000	6.2500000	-50.0000000
54050.0000000	6.5000000	-50.0000000
54050.0000000	6.7500000	-50.0000000
54050.0000000	7.0000000	-50.0000000
54050.0000000	7.2500000	-50.0000000
54050.0000000	7.5000000	-50.0000000
54050.0000000	7.7500000	-50.0000000
54050.0000000	8.0000000	-50.0000000
54050.0000000	8.2500000	-50.0000000
54050.0000000	8.5000000	-50.0000000
54050.0000000	8.7500000	-50.0000000
54050.0000000	9.0000000	-50.0000000
54050.0000000	9.2500000	-50.0000000
54050.0000000	9.5000000	-50.0000000
54050.0000000	9.7500000	-50.0000000
54050.0000000	10.0000000	-50.0000000
54050.0000000	10.2500000	-50.0000000
54050.0000000	10.5000000	-50.0000000
54050.0000000	10.7500000	-50.0000000
54050.0000000	11.0000000	-50.0000000
54050.0000000	11.2500000	-50.0000000
54050.0000000	11.5000000	-50.0000000
54050.0000000	11.7500000	-50.0000000
54050.0000000	12.0000000	-50.0000000
54050.0000000	12.2500000	-50.0000000
54050.0000000	12.5000000	-50.0000000
54050.0000000	12.7500000	-50.0000000
54050.0000000	13.0000000	-50.0000000

54050.0000000	13.2500000	-50.0000000
54050.0000000	13.5000000	-50.0000000
54050.0000000	13.7500000	-50.0000000
54050.0000000	14.0000000	-50.0000000
54050.0000000	14.2500000	-50.0000000
54050.0000000	14.5000000	-50.0000000
54050.0000000	14.7500000	-50.0000000
54050.0000000	15.0000000	-50.0000000
54080.0000000	0.0000000	-50.0000000
54080.0000000	0.2500000	17.1499996
54080.0000000	0.5000000	25.5499992
54080.0000000	0.7500000	25.5000000
54080.0000000	1.0000000	18.7900009
54080.0000000	1.2500000	17.0000000
54080.0000000	1.5000000	19.0699997
54080.0000000	1.7500000	19.9500008
54080.0000000	2.0000000	19.4400005
54080.0000000	2.2500000	18.9300003
54080.0000000	2.5000000	18.9400005
54080.0000000	2.7500000	15.8999996
54080.0000000	3.0000000	16.8999996
54080.0000000	3.2500000	17.3199997
54080.0000000	3.5000000	15.3800001
54080.0000000	3.7500000	-50.0000000
54080.0000000	4.0000000	16.0100002
54080.0000000	4.2500000	19.0400009
54080.0000000	4.5000000	17.1299992
54080.0000000	4.7500000	-50.0000000
54080.0000000	5.0000000	-50.0000000
54080.0000000	5.2500000	-50.0000000
54080.0000000	5.5000000	-50.0000000
54080.0000000	5.7500000	-50.0000000
54080.0000000	6.0000000	-50.0000000
54080.0000000	6.2500000	-50.0000000
54080.0000000	6.5000000	-50.0000000
54080.0000000	6.7500000	-50.0000000
54080.0000000	7.0000000	-50.0000000
54080.0000000	7.2500000	-50.0000000
54080.0000000	7.5000000	-50.0000000
54080.0000000	7.7500000	-50.0000000
54080.0000000	8.0000000	-50.0000000
54080.0000000	8.2500000	-50.0000000
54080.0000000	8.5000000	-50.0000000
54080.0000000	8.7500000	-50.0000000
54080.0000000	9.0000000	-50.0000000
54080.0000000	9.2500000	-50.0000000
54080.0000000	9.5000000	-50.0000000
54080.0000000	9.7500000	-50.0000000
54080.0000000	10.0000000	-50.0000000
54080.0000000	10.2500000	-50.0000000
54080.0000000	10.5000000	-50.0000000
54080.0000000	10.7500000	-50.0000000
54080.0000000	11.0000000	-50.0000000
54080.0000000	11.2500000	-50.0000000
54080.0000000	11.5000000	-50.0000000
54080.0000000	11.7500000	-50.0000000
54080.0000000	12.0000000	-50.0000000
54080.0000000	12.2500000	-50.0000000

54080.0000000	12.5000000	-50.0000000
54080.0000000	12.7500000	-50.0000000
54080.0000000	13.0000000	-50.0000000
54080.0000000	13.2500000	-50.0000000
54080.0000000	13.5000000	-50.0000000
54080.0000000	13.7500000	-50.0000000
54080.0000000	14.0000000	-50.0000000
54080.0000000	14.2500000	-50.0000000
54080.0000000	14.5000000	-50.0000000
54080.0000000	14.7500000	-50.0000000
54080.0000000	15.0000000	-50.0000000
54110.0000000	0.0000000	-50.0000000
54110.0000000	0.2500000	17.2900009
54110.0000000	0.5000000	26.2600002
54110.0000000	0.7500000	26.0400009
54110.0000000	1.0000000	20.4500008
54110.0000000	1.2500000	22.6599998
54110.0000000	1.5000000	22.3999996
54110.0000000	1.7500000	18.6499996
54110.0000000	2.0000000	18.2600002
54110.0000000	2.2500000	19.5000000
54110.0000000	2.5000000	17.9300003
54110.0000000	2.7500000	-50.0000000
54110.0000000	3.0000000	-50.0000000
54110.0000000	3.2500000	15.7500000
54110.0000000	3.5000000	15.6300001
54110.0000000	3.7500000	15.5000000
54110.0000000	4.0000000	17.0900002
54110.0000000	4.2500000	18.4500008
54110.0000000	4.5000000	18.1000004
54110.0000000	4.7500000	15.6400003
54110.0000000	5.0000000	-50.0000000
54110.0000000	5.2500000	-50.0000000
54110.0000000	5.5000000	-50.0000000
54110.0000000	5.7500000	-50.0000000
54110.0000000	6.0000000	-50.0000000
54110.0000000	6.2500000	-50.0000000
54110.0000000	6.5000000	-50.0000000
54110.0000000	6.7500000	-50.0000000
54110.0000000	7.0000000	-50.0000000
54110.0000000	7.2500000	-50.0000000
54110.0000000	7.5000000	-50.0000000
54110.0000000	7.7500000	-50.0000000
54110.0000000	8.0000000	-50.0000000
54110.0000000	8.2500000	-50.0000000
54110.0000000	8.5000000	-50.0000000
54110.0000000	8.7500000	-50.0000000
54110.0000000	9.0000000	-50.0000000
54110.0000000	9.2500000	-50.0000000
54110.0000000	9.5000000	-50.0000000
54110.0000000	9.7500000	-50.0000000
54110.0000000	10.0000000	-50.0000000
54110.0000000	10.2500000	-50.0000000
54110.0000000	10.5000000	-50.0000000
54110.0000000	10.7500000	-50.0000000
54110.0000000	11.0000000	-50.0000000
54110.0000000	11.2500000	-50.0000000
54110.0000000	11.5000000	-50.0000000

54110.0000000	11.7500000	-50.0000000
54110.0000000	12.0000000	-50.0000000
54110.0000000	12.2500000	-50.0000000
54110.0000000	12.5000000	-50.0000000
54110.0000000	12.7500000	-50.0000000
54110.0000000	13.0000000	-50.0000000
54110.0000000	13.2500000	-50.0000000
54110.0000000	13.5000000	-50.0000000
54110.0000000	13.7500000	-50.0000000
54110.0000000	14.0000000	-50.0000000
54110.0000000	14.2500000	-50.0000000
54110.0000000	14.5000000	-50.0000000
54110.0000000	14.7500000	-50.0000000
54110.0000000	15.0000000	-50.0000000
54140.0000000	0.0000000	-50.0000000
54140.0000000	0.2500000	16.6700001
54140.0000000	0.5000000	26.0499992
54140.0000000	0.7500000	25.8899994
54140.0000000	1.0000000	20.9300003
54140.0000000	1.2500000	20.4300003
54140.0000000	1.5000000	19.2500000
54140.0000000	1.7500000	20.2800007
54140.0000000	2.0000000	21.0599995
54140.0000000	2.2500000	19.1399994
54140.0000000	2.5000000	-50.0000000
54140.0000000	2.7500000	18.1100006
54140.0000000	3.0000000	18.4400005
54140.0000000	3.2500000	16.6700001
54140.0000000	3.5000000	15.3100004
54140.0000000	3.7500000	-50.0000000
54140.0000000	4.0000000	18.2999992
54140.0000000	4.2500000	18.6000004
54140.0000000	4.5000000	18.0200005
54140.0000000	4.7500000	-50.0000000
54140.0000000	5.0000000	-50.0000000
54140.0000000	5.2500000	-50.0000000
54140.0000000	5.5000000	-50.0000000
54140.0000000	5.7500000	-50.0000000
54140.0000000	6.0000000	-50.0000000
54140.0000000	6.2500000	-50.0000000
54140.0000000	6.5000000	-50.0000000
54140.0000000	6.7500000	-50.0000000
54140.0000000	7.0000000	-50.0000000
54140.0000000	7.2500000	-50.0000000
54140.0000000	7.5000000	-50.0000000
54140.0000000	7.7500000	-50.0000000
54140.0000000	8.0000000	-50.0000000
54140.0000000	8.2500000	-50.0000000
54140.0000000	8.5000000	-50.0000000
54140.0000000	8.7500000	-50.0000000
54140.0000000	9.0000000	-50.0000000
54140.0000000	9.2500000	-50.0000000
54140.0000000	9.5000000	-50.0000000
54140.0000000	9.7500000	-50.0000000
54140.0000000	10.0000000	-50.0000000
54140.0000000	10.2500000	-50.0000000
54140.0000000	10.5000000	-50.0000000
54140.0000000	10.7500000	-50.0000000

54140.0000000	11.0000000	-50.0000000
54140.0000000	11.2500000	-50.0000000
54140.0000000	11.5000000	-50.0000000
54140.0000000	11.7500000	-50.0000000
54140.0000000	12.0000000	-50.0000000
54140.0000000	12.2500000	-50.0000000
54140.0000000	12.5000000	-50.0000000
54140.0000000	12.7500000	-50.0000000
54140.0000000	13.0000000	-50.0000000
54140.0000000	13.2500000	-50.0000000
54140.0000000	13.5000000	-50.0000000
54140.0000000	13.7500000	-50.0000000
54140.0000000	14.0000000	-50.0000000
54140.0000000	14.2500000	-50.0000000
54140.0000000	14.5000000	-50.0000000
54140.0000000	14.7500000	-50.0000000
54140.0000000	15.0000000	-50.0000000
54170.0000000	0.0000000	-50.0000000
54170.0000000	0.2500000	18.9099998
54170.0000000	0.5000000	27.6000004
54170.0000000	0.7500000	27.2900009
54170.0000000	1.0000000	19.9799995
54170.0000000	1.2500000	18.3899994
54170.0000000	1.5000000	21.1900005
54170.0000000	1.7500000	20.4500008
54170.0000000	2.0000000	19.5499992
54170.0000000	2.2500000	19.0400009
54170.0000000	2.5000000	18.7500000
54170.0000000	2.7500000	18.1900005
54170.0000000	3.0000000	16.9400005
54170.0000000	3.2500000	16.0499992
54170.0000000	3.5000000	16.9200001
54170.0000000	3.7500000	-50.0000000
54170.0000000	4.0000000	17.3999996
54170.0000000	4.2500000	16.5699997
54170.0000000	4.5000000	17.2099991
54170.0000000	4.7500000	-50.0000000
54170.0000000	5.0000000	-50.0000000
54170.0000000	5.2500000	-50.0000000
54170.0000000	5.5000000	-50.0000000
54170.0000000	5.7500000	-50.0000000
54170.0000000	6.0000000	-50.0000000
54170.0000000	6.2500000	-50.0000000
54170.0000000	6.5000000	-50.0000000
54170.0000000	6.7500000	-50.0000000
54170.0000000	7.0000000	-50.0000000
54170.0000000	7.2500000	-50.0000000
54170.0000000	7.5000000	-50.0000000
54170.0000000	7.7500000	-50.0000000
54170.0000000	8.0000000	-50.0000000
54170.0000000	8.2500000	-50.0000000
54170.0000000	8.5000000	-50.0000000
54170.0000000	8.7500000	-50.0000000
54170.0000000	9.0000000	-50.0000000
54170.0000000	9.2500000	-50.0000000
54170.0000000	9.5000000	-50.0000000
54170.0000000	9.7500000	-50.0000000
54170.0000000	10.0000000	-50.0000000

54170.0000000	10.2500000	-50.0000000
54170.0000000	10.5000000	-50.0000000
54170.0000000	10.7500000	-50.0000000
54170.0000000	11.0000000	-50.0000000
54170.0000000	11.2500000	-50.0000000
54170.0000000	11.5000000	-50.0000000
54170.0000000	11.7500000	-50.0000000
54170.0000000	12.0000000	-50.0000000
54170.0000000	12.2500000	-50.0000000
54170.0000000	12.5000000	-50.0000000
54170.0000000	12.7500000	-50.0000000
54170.0000000	13.0000000	-50.0000000
54170.0000000	13.2500000	-50.0000000
54170.0000000	13.5000000	-50.0000000
54170.0000000	13.7500000	-50.0000000
54170.0000000	14.0000000	-50.0000000
54170.0000000	14.2500000	-50.0000000
54170.0000000	14.5000000	-50.0000000
54170.0000000	14.7500000	-50.0000000
54170.0000000	15.0000000	-50.0000000
54200.0000000	0.0000000	-50.0000000
54200.0000000	0.2500000	18.6299992
54200.0000000	0.5000000	28.9300003
54200.0000000	0.7500000	28.8799992
54200.0000000	1.0000000	20.6299992
54200.0000000	1.2500000	19.6200008
54200.0000000	1.5000000	19.7900009
54200.0000000	1.7500000	20.0400009
54200.0000000	2.0000000	19.9799995
54200.0000000	2.2500000	18.1900005
54200.0000000	2.5000000	16.8899994
54200.0000000	2.7500000	-50.0000000
54200.0000000	3.0000000	-50.0000000
54200.0000000	3.2500000	16.7099991
54200.0000000	3.5000000	17.5799999
54200.0000000	3.7500000	17.0200005
54200.0000000	4.0000000	15.3199997
54200.0000000	4.2500000	17.8400002
54200.0000000	4.5000000	16.3400002
54200.0000000	4.7500000	16.0300007
54200.0000000	5.0000000	-50.0000000
54200.0000000	5.2500000	-50.0000000
54200.0000000	5.5000000	-50.0000000
54200.0000000	5.7500000	-50.0000000
54200.0000000	6.0000000	-50.0000000
54200.0000000	6.2500000	-50.0000000
54200.0000000	6.5000000	-50.0000000
54200.0000000	6.7500000	-50.0000000
54200.0000000	7.0000000	-50.0000000
54200.0000000	7.2500000	-50.0000000
54200.0000000	7.5000000	-50.0000000
54200.0000000	7.7500000	-50.0000000
54200.0000000	8.0000000	-50.0000000
54200.0000000	8.2500000	-50.0000000
54200.0000000	8.5000000	-50.0000000
54200.0000000	8.7500000	-50.0000000
54200.0000000	9.0000000	-50.0000000
54200.0000000	9.2500000	-50.0000000

54200.0000000	9.5000000	-50.0000000
54200.0000000	9.7500000	-50.0000000
54200.0000000	10.0000000	-50.0000000
54200.0000000	10.2500000	-50.0000000
54200.0000000	10.5000000	-50.0000000
54200.0000000	10.7500000	-50.0000000
54200.0000000	11.0000000	-50.0000000
54200.0000000	11.2500000	-50.0000000
54200.0000000	11.5000000	-50.0000000
54200.0000000	11.7500000	-50.0000000
54200.0000000	12.0000000	-50.0000000
54200.0000000	12.2500000	-50.0000000
54200.0000000	12.5000000	-50.0000000
54200.0000000	12.7500000	-50.0000000
54200.0000000	13.0000000	-50.0000000
54200.0000000	13.2500000	-50.0000000
54200.0000000	13.5000000	-50.0000000
54200.0000000	13.7500000	-50.0000000
54200.0000000	14.0000000	-50.0000000
54200.0000000	14.2500000	-50.0000000
54200.0000000	14.5000000	-50.0000000
54200.0000000	14.7500000	-50.0000000
54200.0000000	15.0000000	-50.0000000
54230.0000000	0.0000000	-50.0000000
54230.0000000	0.2500000	15.7600002
54230.0000000	0.5000000	24.6900005
54230.0000000	0.7500000	24.9599991
54230.0000000	1.0000000	21.1000004
54230.0000000	1.2500000	19.8799992
54230.0000000	1.5000000	19.5499992
54230.0000000	1.7500000	20.6000004
54230.0000000	2.0000000	19.8799992
54230.0000000	2.2500000	17.0599995
54230.0000000	2.5000000	15.6000004
54230.0000000	2.7500000	18.1000004
54230.0000000	3.0000000	18.8500004
54230.0000000	3.2500000	16.1700001
54230.0000000	3.5000000	-50.0000000
54230.0000000	3.7500000	-50.0000000
54230.0000000	4.0000000	18.3999996
54230.0000000	4.2500000	18.7900009
54230.0000000	4.5000000	15.1700001
54230.0000000	4.7500000	-50.0000000
54230.0000000	5.0000000	-50.0000000
54230.0000000	5.2500000	-50.0000000
54230.0000000	5.5000000	-50.0000000
54230.0000000	5.7500000	-50.0000000
54230.0000000	6.0000000	-50.0000000
54230.0000000	6.2500000	-50.0000000
54230.0000000	6.5000000	-50.0000000
54230.0000000	6.7500000	-50.0000000
54230.0000000	7.0000000	-50.0000000
54230.0000000	7.2500000	-50.0000000
54230.0000000	7.5000000	-50.0000000
54230.0000000	7.7500000	-50.0000000
54230.0000000	8.0000000	-50.0000000
54230.0000000	8.2500000	-50.0000000
54230.0000000	8.5000000	-50.0000000

54230.0000000	8.7500000	-50.0000000
54230.0000000	9.0000000	-50.0000000
54230.0000000	9.2500000	-50.0000000
54230.0000000	9.5000000	-50.0000000
54230.0000000	9.7500000	-50.0000000
54230.0000000	10.0000000	-50.0000000
54230.0000000	10.2500000	-50.0000000
54230.0000000	10.5000000	-50.0000000
54230.0000000	10.7500000	-50.0000000
54230.0000000	11.0000000	-50.0000000
54230.0000000	11.2500000	-50.0000000
54230.0000000	11.5000000	-50.0000000
54230.0000000	11.7500000	-50.0000000
54230.0000000	12.0000000	-50.0000000
54230.0000000	12.2500000	-50.0000000
54230.0000000	12.5000000	-50.0000000
54230.0000000	12.7500000	-50.0000000
54230.0000000	13.0000000	-50.0000000
54230.0000000	13.2500000	-50.0000000
54230.0000000	13.5000000	-50.0000000
54230.0000000	13.7500000	-50.0000000
54230.0000000	14.0000000	-50.0000000
54230.0000000	14.2500000	-50.0000000
54230.0000000	14.5000000	-50.0000000
54230.0000000	14.7500000	-50.0000000
54230.0000000	15.0000000	-50.0000000
54260.0000000	0.0000000	-50.0000000
54260.0000000	0.2500000	15.4899998
54260.0000000	0.5000000	26.1700001
54260.0000000	0.7500000	26.4099998
54260.0000000	1.0000000	20.2099991
54260.0000000	1.2500000	21.1900005
54260.0000000	1.5000000	19.5699997
54260.0000000	1.7500000	19.7700005
54260.0000000	2.0000000	19.4300003
54260.0000000	2.2500000	17.7500000
54260.0000000	2.5000000	18.1599998
54260.0000000	2.7500000	18.2000008
54260.0000000	3.0000000	15.9600000
54260.0000000	3.2500000	17.3899994
54260.0000000	3.5000000	16.8600006
54260.0000000	3.7500000	-50.0000000
54260.0000000	4.0000000	16.1100006
54260.0000000	4.2500000	16.6900005
54260.0000000	4.5000000	19.2700005
54260.0000000	4.7500000	-50.0000000
54260.0000000	5.0000000	-50.0000000
54260.0000000	5.2500000	-50.0000000
54260.0000000	5.5000000	-50.0000000
54260.0000000	5.7500000	-50.0000000
54260.0000000	6.0000000	-50.0000000
54260.0000000	6.2500000	-50.0000000
54260.0000000	6.5000000	-50.0000000
54260.0000000	6.7500000	-50.0000000
54260.0000000	7.0000000	-50.0000000
54260.0000000	7.2500000	-50.0000000
54260.0000000	7.5000000	-50.0000000
54260.0000000	7.7500000	-50.0000000

54260.0000000	8.0000000	-50.0000000
54260.0000000	8.2500000	-50.0000000
54260.0000000	8.5000000	-50.0000000
54260.0000000	8.7500000	-50.0000000
54260.0000000	9.0000000	-50.0000000
54260.0000000	9.2500000	-50.0000000
54260.0000000	9.5000000	-50.0000000
54260.0000000	9.7500000	-50.0000000
54260.0000000	10.0000000	-50.0000000
54260.0000000	10.2500000	-50.0000000
54260.0000000	10.5000000	-50.0000000
54260.0000000	10.7500000	-50.0000000
54260.0000000	11.0000000	-50.0000000
54260.0000000	11.2500000	-50.0000000
54260.0000000	11.5000000	-50.0000000
54260.0000000	11.7500000	-50.0000000
54260.0000000	12.0000000	-50.0000000
54260.0000000	12.2500000	-50.0000000
54260.0000000	12.5000000	-50.0000000
54260.0000000	12.7500000	-50.0000000
54260.0000000	13.0000000	-50.0000000
54260.0000000	13.2500000	-50.0000000
54260.0000000	13.5000000	-50.0000000
54260.0000000	13.7500000	-50.0000000
54260.0000000	14.0000000	-50.0000000
54260.0000000	14.2500000	-50.0000000
54260.0000000	14.5000000	-50.0000000
54260.0000000	14.7500000	-50.0000000
54260.0000000	15.0000000	-50.0000000
54290.0000000	0.0000000	-50.0000000
54290.0000000	0.2500000	-50.0000000
54290.0000000	0.5000000	23.1000004
54290.0000000	0.7500000	23.4099998
54290.0000000	1.0000000	20.8299999
54290.0000000	1.2500000	20.9400005
54290.0000000	1.5000000	18.3799992
54290.0000000	1.7500000	18.5699997
54290.0000000	2.0000000	18.0900002
54290.0000000	2.2500000	18.8799992
54290.0000000	2.5000000	16.3500004
54290.0000000	2.7500000	15.8699999
54290.0000000	3.0000000	16.1399994
54290.0000000	3.2500000	16.8600006
54290.0000000	3.5000000	17.2199993
54290.0000000	3.7500000	16.5400009
54290.0000000	4.0000000	16.4500008
54290.0000000	4.2500000	17.6399994
54290.0000000	4.5000000	15.9700003
54290.0000000	4.7500000	-50.0000000
54290.0000000	5.0000000	-50.0000000
54290.0000000	5.2500000	-50.0000000
54290.0000000	5.5000000	-50.0000000
54290.0000000	5.7500000	-50.0000000
54290.0000000	6.0000000	-50.0000000
54290.0000000	6.2500000	-50.0000000
54290.0000000	6.5000000	-50.0000000
54290.0000000	6.7500000	-50.0000000
54290.0000000	7.0000000	-50.0000000

54290.0000000	7.2500000	-50.0000000
54290.0000000	7.5000000	-50.0000000
54290.0000000	7.7500000	-50.0000000
54290.0000000	8.0000000	-50.0000000
54290.0000000	8.2500000	-50.0000000
54290.0000000	8.5000000	-50.0000000
54290.0000000	8.7500000	-50.0000000
54290.0000000	9.0000000	-50.0000000
54290.0000000	9.2500000	-50.0000000
54290.0000000	9.5000000	-50.0000000
54290.0000000	9.7500000	-50.0000000
54290.0000000	10.0000000	-50.0000000
54290.0000000	10.2500000	-50.0000000
54290.0000000	10.5000000	-50.0000000
54290.0000000	10.7500000	-50.0000000
54290.0000000	11.0000000	-50.0000000
54290.0000000	11.2500000	-50.0000000
54290.0000000	11.5000000	-50.0000000
54290.0000000	11.7500000	-50.0000000
54290.0000000	12.0000000	-50.0000000
54290.0000000	12.2500000	-50.0000000
54290.0000000	12.5000000	-50.0000000
54290.0000000	12.7500000	-50.0000000
54290.0000000	13.0000000	-50.0000000
54290.0000000	13.2500000	-50.0000000
54290.0000000	13.5000000	-50.0000000
54290.0000000	13.7500000	-50.0000000
54290.0000000	14.0000000	-50.0000000
54290.0000000	14.2500000	-50.0000000
54290.0000000	14.5000000	-50.0000000
54290.0000000	14.7500000	-50.0000000
54290.0000000	15.0000000	-50.0000000
54320.0000000	0.0000000	-50.0000000
54320.0000000	0.2500000	-50.0000000
54320.0000000	0.5000000	24.0699997
54320.0000000	0.7500000	24.7500000
54320.0000000	1.0000000	22.6800003
54320.0000000	1.2500000	21.0499992
54320.0000000	1.5000000	17.8799992
54320.0000000	1.7500000	18.0699997
54320.0000000	2.0000000	19.0100002
54320.0000000	2.2500000	17.9899998
54320.0000000	2.5000000	15.8999996
54320.0000000	2.7500000	15.3699999
54320.0000000	3.0000000	17.9699993
54320.0000000	3.2500000	16.6100006
54320.0000000	3.5000000	17.5699997
54320.0000000	3.7500000	18.8899994
54320.0000000	4.0000000	17.6700001
54320.0000000	4.2500000	15.8900003
54320.0000000	4.5000000	17.8899994
54320.0000000	4.7500000	-50.0000000
54320.0000000	5.0000000	-50.0000000
54320.0000000	5.2500000	-50.0000000
54320.0000000	5.5000000	-50.0000000
54320.0000000	5.7500000	-50.0000000
54320.0000000	6.0000000	-50.0000000
54320.0000000	6.2500000	-50.0000000

54320.0000000	6.5000000	-50.0000000
54320.0000000	6.7500000	-50.0000000
54320.0000000	7.0000000	-50.0000000
54320.0000000	7.2500000	-50.0000000
54320.0000000	7.5000000	-50.0000000
54320.0000000	7.7500000	-50.0000000
54320.0000000	8.0000000	-50.0000000
54320.0000000	8.2500000	-50.0000000
54320.0000000	8.5000000	-50.0000000
54320.0000000	8.7500000	-50.0000000
54320.0000000	9.0000000	-50.0000000
54320.0000000	9.2500000	-50.0000000
54320.0000000	9.5000000	-50.0000000
54320.0000000	9.7500000	-50.0000000
54320.0000000	10.0000000	-50.0000000
54320.0000000	10.2500000	-50.0000000
54320.0000000	10.5000000	-50.0000000
54320.0000000	10.7500000	-50.0000000
54320.0000000	11.0000000	-50.0000000
54320.0000000	11.2500000	-50.0000000
54320.0000000	11.5000000	-50.0000000
54320.0000000	11.7500000	-50.0000000
54320.0000000	12.0000000	-50.0000000
54320.0000000	12.2500000	-50.0000000
54320.0000000	12.5000000	-50.0000000
54320.0000000	12.7500000	-50.0000000
54320.0000000	13.0000000	-50.0000000
54320.0000000	13.2500000	-50.0000000
54320.0000000	13.5000000	-50.0000000
54320.0000000	13.7500000	-50.0000000
54320.0000000	14.0000000	-50.0000000
54320.0000000	14.2500000	-50.0000000
54320.0000000	14.5000000	-50.0000000
54320.0000000	14.7500000	-50.0000000
54320.0000000	15.0000000	-50.0000000
54350.0000000	0.0000000	-50.0000000
54350.0000000	0.2500000	-50.0000000
54350.0000000	0.5000000	22.7700005
54350.0000000	0.7500000	23.3999996
54350.0000000	1.0000000	19.4500008
54350.0000000	1.2500000	20.7600002
54350.0000000	1.5000000	19.6499996
54350.0000000	1.7500000	18.6800003
54350.0000000	2.0000000	18.7500000
54350.0000000	2.2500000	21.3799992
54350.0000000	2.5000000	21.2800007
54350.0000000	2.7500000	17.8700008
54350.0000000	3.0000000	16.5200005
54350.0000000	3.2500000	17.1000004
54350.0000000	3.5000000	17.1700001
54350.0000000	3.7500000	16.4899998
54350.0000000	4.0000000	16.7099991
54350.0000000	4.2500000	16.2000008
54350.0000000	4.5000000	17.1700001
54350.0000000	4.7500000	-50.0000000
54350.0000000	5.0000000	-50.0000000
54350.0000000	5.2500000	-50.0000000
54350.0000000	5.5000000	-50.0000000

54350.0000000	5.7500000	-50.0000000
54350.0000000	6.0000000	-50.0000000
54350.0000000	6.2500000	-50.0000000
54350.0000000	6.5000000	-50.0000000
54350.0000000	6.7500000	-50.0000000
54350.0000000	7.0000000	-50.0000000
54350.0000000	7.2500000	-50.0000000
54350.0000000	7.5000000	-50.0000000
54350.0000000	7.7500000	-50.0000000
54350.0000000	8.0000000	-50.0000000
54350.0000000	8.2500000	-50.0000000
54350.0000000	8.5000000	-50.0000000
54350.0000000	8.7500000	-50.0000000
54350.0000000	9.0000000	-50.0000000
54350.0000000	9.2500000	-50.0000000
54350.0000000	9.5000000	-50.0000000
54350.0000000	9.7500000	-50.0000000
54350.0000000	10.0000000	-50.0000000
54350.0000000	10.2500000	-50.0000000
54350.0000000	10.5000000	-50.0000000
54350.0000000	10.7500000	-50.0000000
54350.0000000	11.0000000	-50.0000000
54350.0000000	11.2500000	-50.0000000
54350.0000000	11.5000000	-50.0000000
54350.0000000	11.7500000	-50.0000000
54350.0000000	12.0000000	-50.0000000
54350.0000000	12.2500000	-50.0000000
54350.0000000	12.5000000	-50.0000000
54350.0000000	12.7500000	-50.0000000
54350.0000000	13.0000000	-50.0000000
54350.0000000	13.2500000	-50.0000000
54350.0000000	13.5000000	-50.0000000
54350.0000000	13.7500000	-50.0000000
54350.0000000	14.0000000	-50.0000000
54350.0000000	14.2500000	-50.0000000
54350.0000000	14.5000000	-50.0000000
54350.0000000	14.7500000	-50.0000000
54350.0000000	15.0000000	-50.0000000
54380.0000000	0.0000000	-50.0000000
54380.0000000	0.2500000	-50.0000000
54380.0000000	0.5000000	25.7099991
54380.0000000	0.7500000	26.6399994
54380.0000000	1.0000000	20.4899998
54380.0000000	1.2500000	22.1599998
54380.0000000	1.5000000	20.5200005
54380.0000000	1.7500000	17.9500008
54380.0000000	2.0000000	18.3500004
54380.0000000	2.2500000	17.8999996
54380.0000000	2.5000000	-50.0000000
54380.0000000	2.7500000	18.6599998
54380.0000000	3.0000000	18.0300007
54380.0000000	3.2500000	18.2399998
54380.0000000	3.5000000	16.6200008
54380.0000000	3.7500000	16.3400002
54380.0000000	4.0000000	18.3099995
54380.0000000	4.2500000	17.6599998
54380.0000000	4.5000000	15.1000004
54380.0000000	4.7500000	-50.0000000

54380.0000000	5.0000000	-50.0000000
54380.0000000	5.2500000	-50.0000000
54380.0000000	5.5000000	-50.0000000
54380.0000000	5.7500000	-50.0000000
54380.0000000	6.0000000	-50.0000000
54380.0000000	6.2500000	-50.0000000
54380.0000000	6.5000000	-50.0000000
54380.0000000	6.7500000	-50.0000000
54380.0000000	7.0000000	-50.0000000
54380.0000000	7.2500000	-50.0000000
54380.0000000	7.5000000	-50.0000000
54380.0000000	7.7500000	-50.0000000
54380.0000000	8.0000000	-50.0000000
54380.0000000	8.2500000	-50.0000000
54380.0000000	8.5000000	-50.0000000
54380.0000000	8.7500000	-50.0000000
54380.0000000	9.0000000	-50.0000000
54380.0000000	9.2500000	-50.0000000
54380.0000000	9.5000000	-50.0000000
54380.0000000	9.7500000	-50.0000000
54380.0000000	10.0000000	-50.0000000
54380.0000000	10.2500000	-50.0000000
54380.0000000	10.5000000	-50.0000000
54380.0000000	10.7500000	-50.0000000
54380.0000000	11.0000000	-50.0000000
54380.0000000	11.2500000	-50.0000000
54380.0000000	11.5000000	-50.0000000
54380.0000000	11.7500000	-50.0000000
54380.0000000	12.0000000	-50.0000000
54380.0000000	12.2500000	-50.0000000
54380.0000000	12.5000000	-50.0000000
54380.0000000	12.7500000	-50.0000000
54380.0000000	13.0000000	-50.0000000
54380.0000000	13.2500000	-50.0000000
54380.0000000	13.5000000	-50.0000000
54380.0000000	13.7500000	-50.0000000
54380.0000000	14.0000000	-50.0000000
54380.0000000	14.2500000	-50.0000000
54380.0000000	14.5000000	-50.0000000
54380.0000000	14.7500000	-50.0000000
54380.0000000	15.0000000	-50.0000000
54410.0000000	0.0000000	-50.0000000
54410.0000000	0.2500000	16.3500004
54410.0000000	0.5000000	24.7000008
54410.0000000	0.7500000	24.3600006
54410.0000000	1.0000000	21.5300007
54410.0000000	1.2500000	18.1599998
54410.0000000	1.5000000	17.5699997
54410.0000000	1.7500000	18.2099991
54410.0000000	2.0000000	18.4799995
54410.0000000	2.2500000	19.1499996
54410.0000000	2.5000000	20.4400005
54410.0000000	2.7500000	20.7399998
54410.0000000	3.0000000	21.1800003
54410.0000000	3.2500000	19.7399998
54410.0000000	3.5000000	17.9899998
54410.0000000	3.7500000	16.4799995
54410.0000000	4.0000000	16.4400005

54410.0000000	4.2500000	18.3899994
54410.0000000	4.5000000	17.7399998
54410.0000000	4.7500000	-50.0000000
54410.0000000	5.0000000	-50.0000000
54410.0000000	5.2500000	-50.0000000
54410.0000000	5.5000000	-50.0000000
54410.0000000	5.7500000	-50.0000000
54410.0000000	6.0000000	-50.0000000
54410.0000000	6.2500000	-50.0000000
54410.0000000	6.5000000	-50.0000000
54410.0000000	6.7500000	-50.0000000
54410.0000000	7.0000000	-50.0000000
54410.0000000	7.2500000	-50.0000000
54410.0000000	7.5000000	-50.0000000
54410.0000000	7.7500000	-50.0000000
54410.0000000	8.0000000	-50.0000000
54410.0000000	8.2500000	-50.0000000
54410.0000000	8.5000000	-50.0000000
54410.0000000	8.7500000	-50.0000000
54410.0000000	9.0000000	-50.0000000
54410.0000000	9.2500000	-50.0000000
54410.0000000	9.5000000	-50.0000000
54410.0000000	9.7500000	-50.0000000
54410.0000000	10.0000000	-50.0000000
54410.0000000	10.2500000	-50.0000000
54410.0000000	10.5000000	-50.0000000
54410.0000000	10.7500000	-50.0000000
54410.0000000	11.0000000	-50.0000000
54410.0000000	11.2500000	-50.0000000
54410.0000000	11.5000000	-50.0000000
54410.0000000	11.7500000	-50.0000000
54410.0000000	12.0000000	-50.0000000
54410.0000000	12.2500000	-50.0000000
54410.0000000	12.5000000	-50.0000000
54410.0000000	12.7500000	-50.0000000
54410.0000000	13.0000000	-50.0000000
54410.0000000	13.2500000	-50.0000000
54410.0000000	13.5000000	-50.0000000
54410.0000000	13.7500000	-50.0000000
54410.0000000	14.0000000	-50.0000000
54410.0000000	14.2500000	-50.0000000
54410.0000000	14.5000000	-50.0000000
54410.0000000	14.7500000	-50.0000000
54410.0000000	15.0000000	-50.0000000
54440.0000000	0.0000000	-50.0000000
54440.0000000	0.2500000	19.0499992
54440.0000000	0.5000000	27.2800007
54440.0000000	0.7500000	26.6000004
54440.0000000	1.0000000	21.5000000
54440.0000000	1.2500000	21.4200001
54440.0000000	1.5000000	19.0599995
54440.0000000	1.7500000	18.4799995
54440.0000000	2.0000000	17.6100006
54440.0000000	2.2500000	17.7900009
54440.0000000	2.5000000	17.4899998
54440.0000000	2.7500000	20.2500000
54440.0000000	3.0000000	20.2999992
54440.0000000	3.2500000	17.0400009

54440.0000000	3.5000000	17.2600002
54440.0000000	3.7500000	16.7500000
54440.0000000	4.0000000	16.1499996
54440.0000000	4.2500000	16.6800003
54440.0000000	4.5000000	20.4799995
54440.0000000	4.7500000	15.1199999
54440.0000000	5.0000000	-50.0000000
54440.0000000	5.2500000	-50.0000000
54440.0000000	5.5000000	-50.0000000
54440.0000000	5.7500000	-50.0000000
54440.0000000	6.0000000	-50.0000000
54440.0000000	6.2500000	-50.0000000
54440.0000000	6.5000000	-50.0000000
54440.0000000	6.7500000	-50.0000000
54440.0000000	7.0000000	-50.0000000
54440.0000000	7.2500000	-50.0000000
54440.0000000	7.5000000	-50.0000000
54440.0000000	7.7500000	-50.0000000
54440.0000000	8.0000000	-50.0000000
54440.0000000	8.2500000	-50.0000000
54440.0000000	8.5000000	-50.0000000
54440.0000000	8.7500000	-50.0000000
54440.0000000	9.0000000	-50.0000000
54440.0000000	9.2500000	-50.0000000
54440.0000000	9.5000000	-50.0000000
54440.0000000	9.7500000	-50.0000000
54440.0000000	10.0000000	-50.0000000
54440.0000000	10.2500000	-50.0000000
54440.0000000	10.5000000	-50.0000000
54440.0000000	10.7500000	-50.0000000
54440.0000000	11.0000000	-50.0000000
54440.0000000	11.2500000	-50.0000000
54440.0000000	11.5000000	-50.0000000
54440.0000000	11.7500000	-50.0000000
54440.0000000	12.0000000	-50.0000000
54440.0000000	12.2500000	-50.0000000
54440.0000000	12.5000000	-50.0000000
54440.0000000	12.7500000	-50.0000000
54440.0000000	13.0000000	-50.0000000
54440.0000000	13.2500000	-50.0000000
54440.0000000	13.5000000	-50.0000000
54440.0000000	13.7500000	-50.0000000
54440.0000000	14.0000000	-50.0000000
54440.0000000	14.2500000	-50.0000000
54440.0000000	14.5000000	-50.0000000
54440.0000000	14.7500000	-50.0000000
54440.0000000	15.0000000	-50.0000000
54470.0000000	0.0000000	-50.0000000
54470.0000000	0.2500000	23.4400005
54470.0000000	0.5000000	31.8299999
54470.0000000	0.7500000	30.3199997
54470.0000000	1.0000000	23.6200008
54470.0000000	1.2500000	19.9899998
54470.0000000	1.5000000	18.3500004
54470.0000000	1.7500000	18.8099995
54470.0000000	2.0000000	20.0400009
54470.0000000	2.2500000	18.5599995
54470.0000000	2.5000000	19.5000000

54470.0000000	2.7500000	20.0000000
54470.0000000	3.0000000	19.2800007
54470.0000000	3.2500000	18.9599991
54470.0000000	3.5000000	18.7399998
54470.0000000	3.7500000	16.2199993
54470.0000000	4.0000000	17.6299992
54470.0000000	4.2500000	18.7800007
54470.0000000	4.5000000	15.1000004
54470.0000000	4.7500000	-50.0000000
54470.0000000	5.0000000	-50.0000000
54470.0000000	5.2500000	-50.0000000
54470.0000000	5.5000000	-50.0000000
54470.0000000	5.7500000	-50.0000000
54470.0000000	6.0000000	-50.0000000
54470.0000000	6.2500000	-50.0000000
54470.0000000	6.5000000	-50.0000000
54470.0000000	6.7500000	-50.0000000
54470.0000000	7.0000000	-50.0000000
54470.0000000	7.2500000	-50.0000000
54470.0000000	7.5000000	-50.0000000
54470.0000000	7.7500000	-50.0000000
54470.0000000	8.0000000	-50.0000000
54470.0000000	8.2500000	-50.0000000
54470.0000000	8.5000000	-50.0000000
54470.0000000	8.7500000	-50.0000000
54470.0000000	9.0000000	-50.0000000
54470.0000000	9.2500000	-50.0000000
54470.0000000	9.5000000	-50.0000000
54470.0000000	9.7500000	-50.0000000
54470.0000000	10.0000000	-50.0000000
54470.0000000	10.2500000	-50.0000000
54470.0000000	10.5000000	-50.0000000
54470.0000000	10.7500000	-50.0000000
54470.0000000	11.0000000	-50.0000000
54470.0000000	11.2500000	-50.0000000
54470.0000000	11.5000000	-50.0000000
54470.0000000	11.7500000	-50.0000000
54470.0000000	12.0000000	-50.0000000
54470.0000000	12.2500000	-50.0000000
54470.0000000	12.5000000	-50.0000000
54470.0000000	12.7500000	-50.0000000
54470.0000000	13.0000000	-50.0000000
54470.0000000	13.2500000	-50.0000000
54470.0000000	13.5000000	-50.0000000
54470.0000000	13.7500000	-50.0000000
54470.0000000	14.0000000	-50.0000000
54470.0000000	14.2500000	-50.0000000
54470.0000000	14.5000000	-50.0000000
54470.0000000	14.7500000	-50.0000000
54470.0000000	15.0000000	-50.0000000
54500.0000000	0.0000000	-50.0000000
54500.0000000	0.2500000	19.5100002
54500.0000000	0.5000000	28.3500004
54500.0000000	0.7500000	27.4099998
54500.0000000	1.0000000	21.2399998
54500.0000000	1.2500000	20.1700001
54500.0000000	1.5000000	20.2199993
54500.0000000	1.7500000	19.2399998

54500.0000000	2.0000000	18.0400009
54500.0000000	2.2500000	17.7600002
54500.0000000	2.5000000	17.7299995
54500.0000000	2.7500000	18.1599998
54500.0000000	3.0000000	17.0400009
54500.0000000	3.2500000	16.0900002
54500.0000000	3.5000000	18.8799992
54500.0000000	3.7500000	17.7299995
54500.0000000	4.0000000	17.7700005
54500.0000000	4.2500000	18.6200008
54500.0000000	4.5000000	17.9300003
54500.0000000	4.7500000	-50.0000000
54500.0000000	5.0000000	-50.0000000
54500.0000000	5.2500000	-50.0000000
54500.0000000	5.5000000	-50.0000000
54500.0000000	5.7500000	-50.0000000
54500.0000000	6.0000000	-50.0000000
54500.0000000	6.2500000	-50.0000000
54500.0000000	6.5000000	-50.0000000
54500.0000000	6.7500000	-50.0000000
54500.0000000	7.0000000	-50.0000000
54500.0000000	7.2500000	-50.0000000
54500.0000000	7.5000000	-50.0000000
54500.0000000	7.7500000	-50.0000000
54500.0000000	8.0000000	-50.0000000
54500.0000000	8.2500000	-50.0000000
54500.0000000	8.5000000	-50.0000000
54500.0000000	8.7500000	-50.0000000
54500.0000000	9.0000000	-50.0000000
54500.0000000	9.2500000	-50.0000000
54500.0000000	9.5000000	-50.0000000
54500.0000000	9.7500000	-50.0000000
54500.0000000	10.0000000	-50.0000000
54500.0000000	10.2500000	-50.0000000
54500.0000000	10.5000000	-50.0000000
54500.0000000	10.7500000	-50.0000000
54500.0000000	11.0000000	-50.0000000
54500.0000000	11.2500000	-50.0000000
54500.0000000	11.5000000	-50.0000000
54500.0000000	11.7500000	-50.0000000
54500.0000000	12.0000000	-50.0000000
54500.0000000	12.2500000	-50.0000000
54500.0000000	12.5000000	-50.0000000
54500.0000000	12.7500000	-50.0000000
54500.0000000	13.0000000	-50.0000000
54500.0000000	13.2500000	-50.0000000
54500.0000000	13.5000000	-50.0000000
54500.0000000	13.7500000	-50.0000000
54500.0000000	14.0000000	-50.0000000
54500.0000000	14.2500000	-50.0000000
54500.0000000	14.5000000	-50.0000000
54500.0000000	14.7500000	-50.0000000
54500.0000000	15.0000000	-50.0000000
54530.0000000	0.0000000	-50.0000000
54530.0000000	0.2500000	18.0200005
54530.0000000	0.5000000	25.3199997
54530.0000000	0.7500000	24.2000008
54530.0000000	1.0000000	19.5300007

54530.0000000	1.2500000	18.6800003
54530.0000000	1.5000000	19.3899994
54530.0000000	1.7500000	17.7500000
54530.0000000	2.0000000	18.4799995
54530.0000000	2.2500000	19.7299995
54530.0000000	2.5000000	19.9200001
54530.0000000	2.7500000	19.6800003
54530.0000000	3.0000000	19.9099998
54530.0000000	3.2500000	17.8899994
54530.0000000	3.5000000	15.9499998
54530.0000000	3.7500000	16.8899994
54530.0000000	4.0000000	16.2299995
54530.0000000	4.2500000	17.1299992
54530.0000000	4.5000000	16.2800007
54530.0000000	4.7500000	-50.0000000
54530.0000000	5.0000000	-50.0000000
54530.0000000	5.2500000	-50.0000000
54530.0000000	5.5000000	-50.0000000
54530.0000000	5.7500000	-50.0000000
54530.0000000	6.0000000	-50.0000000
54530.0000000	6.2500000	-50.0000000
54530.0000000	6.5000000	-50.0000000
54530.0000000	6.7500000	-50.0000000
54530.0000000	7.0000000	-50.0000000
54530.0000000	7.2500000	-50.0000000
54530.0000000	7.5000000	-50.0000000
54530.0000000	7.7500000	-50.0000000
54530.0000000	8.0000000	-50.0000000
54530.0000000	8.2500000	-50.0000000
54530.0000000	8.5000000	-50.0000000
54530.0000000	8.7500000	-50.0000000
54530.0000000	9.0000000	-50.0000000
54530.0000000	9.2500000	-50.0000000
54530.0000000	9.5000000	-50.0000000
54530.0000000	9.7500000	-50.0000000
54530.0000000	10.0000000	-50.0000000
54530.0000000	10.2500000	-50.0000000
54530.0000000	10.5000000	-50.0000000
54530.0000000	10.7500000	-50.0000000
54530.0000000	11.0000000	-50.0000000
54530.0000000	11.2500000	-50.0000000
54530.0000000	11.5000000	-50.0000000
54530.0000000	11.7500000	-50.0000000
54530.0000000	12.0000000	-50.0000000
54530.0000000	12.2500000	-50.0000000
54530.0000000	12.5000000	-50.0000000
54530.0000000	12.7500000	-50.0000000
54530.0000000	13.0000000	-50.0000000
54530.0000000	13.2500000	-50.0000000
54530.0000000	13.5000000	-50.0000000
54530.0000000	13.7500000	-50.0000000
54530.0000000	14.0000000	-50.0000000
54530.0000000	14.2500000	-50.0000000
54530.0000000	14.5000000	-50.0000000
54530.0000000	14.7500000	-50.0000000
54530.0000000	15.0000000	-50.0000000
54560.0000000	0.0000000	-50.0000000
54560.0000000	0.2500000	20.4300003

54560.0000000	0.5000000	29.1399994
54560.0000000	0.7500000	27.7299995
54560.0000000	1.0000000	19.9099998
54560.0000000	1.2500000	20.0200005
54560.0000000	1.5000000	18.7600002
54560.0000000	1.7500000	20.2700005
54560.0000000	2.0000000	19.9500008
54560.0000000	2.2500000	19.9099998
54560.0000000	2.5000000	20.2099991
54560.0000000	2.7500000	19.5000000
54560.0000000	3.0000000	19.7999992
54560.0000000	3.2500000	20.3099995
54560.0000000	3.5000000	17.5799999
54560.0000000	3.7500000	17.2099991
54560.0000000	4.0000000	19.4899998
54560.0000000	4.2500000	17.9200001
54560.0000000	4.5000000	17.5300007
54560.0000000	4.7500000	-50.0000000
54560.0000000	5.0000000	-50.0000000
54560.0000000	5.2500000	-50.0000000
54560.0000000	5.5000000	-50.0000000
54560.0000000	5.7500000	-50.0000000
54560.0000000	6.0000000	-50.0000000
54560.0000000	6.2500000	-50.0000000
54560.0000000	6.5000000	-50.0000000
54560.0000000	6.7500000	-50.0000000
54560.0000000	7.0000000	-50.0000000
54560.0000000	7.2500000	-50.0000000
54560.0000000	7.5000000	-50.0000000
54560.0000000	7.7500000	-50.0000000
54560.0000000	8.0000000	-50.0000000
54560.0000000	8.2500000	-50.0000000
54560.0000000	8.5000000	-50.0000000
54560.0000000	8.7500000	-50.0000000
54560.0000000	9.0000000	-50.0000000
54560.0000000	9.2500000	-50.0000000
54560.0000000	9.5000000	-50.0000000
54560.0000000	9.7500000	-50.0000000
54560.0000000	10.0000000	-50.0000000
54560.0000000	10.2500000	-50.0000000
54560.0000000	10.5000000	-50.0000000
54560.0000000	10.7500000	-50.0000000
54560.0000000	11.0000000	-50.0000000
54560.0000000	11.2500000	-50.0000000
54560.0000000	11.5000000	-50.0000000
54560.0000000	11.7500000	-50.0000000
54560.0000000	12.0000000	-50.0000000
54560.0000000	12.2500000	-50.0000000
54560.0000000	12.5000000	-50.0000000
54560.0000000	12.7500000	-50.0000000
54560.0000000	13.0000000	-50.0000000
54560.0000000	13.2500000	-50.0000000
54560.0000000	13.5000000	-50.0000000
54560.0000000	13.7500000	-50.0000000
54560.0000000	14.0000000	-50.0000000
54560.0000000	14.2500000	-50.0000000
54560.0000000	14.5000000	-50.0000000
54560.0000000	14.7500000	-50.0000000

54560.0000000	15.0000000	-50.0000000
54590.0000000	0.0000000	-50.0000000
54590.0000000	0.2500000	19.8400002
54590.0000000	0.5000000	27.0100002
54590.0000000	0.7500000	25.4599991
54590.0000000	1.0000000	19.1000004
54590.0000000	1.2500000	18.2399998
54590.0000000	1.5000000	17.9699993
54590.0000000	1.7500000	19.1299992
54590.0000000	2.0000000	18.6499996
54590.0000000	2.2500000	19.5699997
54590.0000000	2.5000000	18.7199993
54590.0000000	2.7500000	19.6900005
54590.0000000	3.0000000	20.8700008
54590.0000000	3.2500000	22.0400009
54590.0000000	3.5000000	17.7099991
54590.0000000	3.7500000	15.1199999
54590.0000000	4.0000000	16.1299992
54590.0000000	4.2500000	19.5200005
54590.0000000	4.5000000	15.3400002
54590.0000000	4.7500000	-50.0000000
54590.0000000	5.0000000	-50.0000000
54590.0000000	5.2500000	-50.0000000
54590.0000000	5.5000000	-50.0000000
54590.0000000	5.7500000	-50.0000000
54590.0000000	6.0000000	-50.0000000
54590.0000000	6.2500000	-50.0000000
54590.0000000	6.5000000	-50.0000000
54590.0000000	6.7500000	-50.0000000
54590.0000000	7.0000000	-50.0000000
54590.0000000	7.2500000	-50.0000000
54590.0000000	7.5000000	-50.0000000
54590.0000000	7.7500000	-50.0000000
54590.0000000	8.0000000	-50.0000000
54590.0000000	8.2500000	-50.0000000
54590.0000000	8.5000000	-50.0000000
54590.0000000	8.7500000	-50.0000000
54590.0000000	9.0000000	-50.0000000
54590.0000000	9.2500000	-50.0000000
54590.0000000	9.5000000	-50.0000000
54590.0000000	9.7500000	-50.0000000
54590.0000000	10.0000000	-50.0000000
54590.0000000	10.2500000	-50.0000000
54590.0000000	10.5000000	-50.0000000
54590.0000000	10.7500000	-50.0000000
54590.0000000	11.0000000	-50.0000000
54590.0000000	11.2500000	-50.0000000
54590.0000000	11.5000000	-50.0000000
54590.0000000	11.7500000	-50.0000000
54590.0000000	12.0000000	-50.0000000
54590.0000000	12.2500000	-50.0000000
54590.0000000	12.5000000	-50.0000000
54590.0000000	12.7500000	-50.0000000
54590.0000000	13.0000000	-50.0000000
54590.0000000	13.2500000	-50.0000000
54590.0000000	13.5000000	-50.0000000
54590.0000000	13.7500000	-50.0000000
54590.0000000	14.0000000	-50.0000000

54590.0000000	14.2500000	-50.0000000
54590.0000000	14.5000000	-50.0000000
54590.0000000	14.7500000	-50.0000000
54590.0000000	15.0000000	-50.0000000
54620.0000000	0.0000000	-50.0000000
54620.0000000	0.2500000	23.3199997
54620.0000000	0.5000000	31.3899994
54620.0000000	0.7500000	29.9400005
54620.0000000	1.0000000	19.5599995
54620.0000000	1.2500000	18.4300003
54620.0000000	1.5000000	19.4200001
54620.0000000	1.7500000	19.9799995
54620.0000000	2.0000000	19.7000008
54620.0000000	2.2500000	20.1000004
54620.0000000	2.5000000	19.0400009
54620.0000000	2.7500000	18.1399994
54620.0000000	3.0000000	16.8700008
54620.0000000	3.2500000	18.8299999
54620.0000000	3.5000000	17.7399998
54620.0000000	3.7500000	16.6299992
54620.0000000	4.0000000	16.7500000
54620.0000000	4.2500000	16.9799995
54620.0000000	4.5000000	-50.0000000
54620.0000000	4.7500000	-50.0000000
54620.0000000	5.0000000	-50.0000000
54620.0000000	5.2500000	-50.0000000
54620.0000000	5.5000000	-50.0000000
54620.0000000	5.7500000	-50.0000000
54620.0000000	6.0000000	-50.0000000
54620.0000000	6.2500000	-50.0000000
54620.0000000	6.5000000	-50.0000000
54620.0000000	6.7500000	-50.0000000
54620.0000000	7.0000000	-50.0000000
54620.0000000	7.2500000	-50.0000000
54620.0000000	7.5000000	-50.0000000
54620.0000000	7.7500000	-50.0000000
54620.0000000	8.0000000	-50.0000000
54620.0000000	8.2500000	-50.0000000
54620.0000000	8.5000000	-50.0000000
54620.0000000	8.7500000	-50.0000000
54620.0000000	9.0000000	-50.0000000
54620.0000000	9.2500000	-50.0000000
54620.0000000	9.5000000	-50.0000000
54620.0000000	9.7500000	-50.0000000
54620.0000000	10.0000000	-50.0000000
54620.0000000	10.2500000	-50.0000000
54620.0000000	10.5000000	-50.0000000
54620.0000000	10.7500000	-50.0000000
54620.0000000	11.0000000	-50.0000000
54620.0000000	11.2500000	-50.0000000
54620.0000000	11.5000000	-50.0000000
54620.0000000	11.7500000	-50.0000000
54620.0000000	12.0000000	-50.0000000
54620.0000000	12.2500000	-50.0000000
54620.0000000	12.5000000	-50.0000000
54620.0000000	12.7500000	-50.0000000
54620.0000000	13.0000000	-50.0000000
54620.0000000	13.2500000	-50.0000000

54620.0000000	13.5000000	-50.0000000
54620.0000000	13.7500000	-50.0000000
54620.0000000	14.0000000	-50.0000000
54620.0000000	14.2500000	-50.0000000
54620.0000000	14.5000000	-50.0000000
54620.0000000	14.7500000	-50.0000000
54620.0000000	15.0000000	-50.0000000
54650.0000000	0.0000000	-50.0000000
54650.0000000	0.2500000	15.7200003
54650.0000000	0.5000000	25.4400005
54650.0000000	0.7500000	26.6700001
54650.0000000	1.0000000	22.9099998
54650.0000000	1.2500000	20.0599995
54650.0000000	1.5000000	18.4599991
54650.0000000	1.7500000	18.2800007
54650.0000000	2.0000000	20.5499992
54650.0000000	2.2500000	21.7700005
54650.0000000	2.5000000	19.1900005
54650.0000000	2.7500000	18.5100002
54650.0000000	3.0000000	19.2199993
54650.0000000	3.2500000	18.0100002
54650.0000000	3.5000000	17.2900009
54650.0000000	3.7500000	17.8099995
54650.0000000	4.0000000	17.6000004
54650.0000000	4.2500000	20.0499992
54650.0000000	4.5000000	15.2700005
54650.0000000	4.7500000	-50.0000000
54650.0000000	5.0000000	-50.0000000
54650.0000000	5.2500000	-50.0000000
54650.0000000	5.5000000	-50.0000000
54650.0000000	5.7500000	-50.0000000
54650.0000000	6.0000000	-50.0000000
54650.0000000	6.2500000	-50.0000000
54650.0000000	6.5000000	-50.0000000
54650.0000000	6.7500000	-50.0000000
54650.0000000	7.0000000	-50.0000000
54650.0000000	7.2500000	-50.0000000
54650.0000000	7.5000000	-50.0000000
54650.0000000	7.7500000	-50.0000000
54650.0000000	8.0000000	-50.0000000
54650.0000000	8.2500000	-50.0000000
54650.0000000	8.5000000	-50.0000000
54650.0000000	8.7500000	-50.0000000
54650.0000000	9.0000000	-50.0000000
54650.0000000	9.2500000	-50.0000000
54650.0000000	9.5000000	-50.0000000
54650.0000000	9.7500000	-50.0000000
54650.0000000	10.0000000	-50.0000000
54650.0000000	10.2500000	-50.0000000
54650.0000000	10.5000000	-50.0000000
54650.0000000	10.7500000	-50.0000000
54650.0000000	11.0000000	-50.0000000
54650.0000000	11.2500000	-50.0000000
54650.0000000	11.5000000	-50.0000000
54650.0000000	11.7500000	-50.0000000
54650.0000000	12.0000000	-50.0000000
54650.0000000	12.2500000	-50.0000000
54650.0000000	12.5000000	-50.0000000

54650.0000000	12.7500000	-50.0000000
54650.0000000	13.0000000	-50.0000000
54650.0000000	13.2500000	-50.0000000
54650.0000000	13.5000000	-50.0000000
54650.0000000	13.7500000	-50.0000000
54650.0000000	14.0000000	-50.0000000
54650.0000000	14.2500000	-50.0000000
54650.0000000	14.5000000	-50.0000000
54650.0000000	14.7500000	-50.0000000
54650.0000000	15.0000000	-50.0000000
54680.0000000	0.0000000	-50.0000000
54680.0000000	0.2500000	18.0000000
54680.0000000	0.5000000	26.2900009
54680.0000000	0.7500000	24.8299999
54680.0000000	1.0000000	19.6700001
54680.0000000	1.2500000	19.4300003
54680.0000000	1.5000000	18.2900009
54680.0000000	1.7500000	20.1800003
54680.0000000	2.0000000	21.0000000
54680.0000000	2.2500000	22.4400005
54680.0000000	2.5000000	21.2999992
54680.0000000	2.7500000	21.9599991
54680.0000000	3.0000000	19.3999996
54680.0000000	3.2500000	16.9400005
54680.0000000	3.5000000	15.8299999
54680.0000000	3.7500000	-50.0000000
54680.0000000	4.0000000	16.6200008
54680.0000000	4.2500000	17.3999996
54680.0000000	4.5000000	16.3799992
54680.0000000	4.7500000	-50.0000000
54680.0000000	5.0000000	-50.0000000
54680.0000000	5.2500000	-50.0000000
54680.0000000	5.5000000	-50.0000000
54680.0000000	5.7500000	-50.0000000
54680.0000000	6.0000000	-50.0000000
54680.0000000	6.2500000	-50.0000000
54680.0000000	6.5000000	-50.0000000
54680.0000000	6.7500000	-50.0000000
54680.0000000	7.0000000	-50.0000000
54680.0000000	7.2500000	-50.0000000
54680.0000000	7.5000000	-50.0000000
54680.0000000	7.7500000	-50.0000000
54680.0000000	8.0000000	-50.0000000
54680.0000000	8.2500000	-50.0000000
54680.0000000	8.5000000	-50.0000000
54680.0000000	8.7500000	-50.0000000
54680.0000000	9.0000000	-50.0000000
54680.0000000	9.2500000	-50.0000000
54680.0000000	9.5000000	-50.0000000
54680.0000000	9.7500000	-50.0000000
54680.0000000	10.0000000	-50.0000000
54680.0000000	10.2500000	-50.0000000
54680.0000000	10.5000000	-50.0000000
54680.0000000	10.7500000	-50.0000000
54680.0000000	11.0000000	-50.0000000
54680.0000000	11.2500000	-50.0000000
54680.0000000	11.5000000	-50.0000000
54680.0000000	11.7500000	-50.0000000

54680.0000000	12.0000000	-50.0000000
54680.0000000	12.2500000	-50.0000000
54680.0000000	12.5000000	-50.0000000
54680.0000000	12.7500000	-50.0000000
54680.0000000	13.0000000	-50.0000000
54680.0000000	13.2500000	-50.0000000
54680.0000000	13.5000000	-50.0000000
54680.0000000	13.7500000	-50.0000000
54680.0000000	14.0000000	-50.0000000
54680.0000000	14.2500000	-50.0000000
54680.0000000	14.5000000	-50.0000000
54680.0000000	14.7500000	-50.0000000
54680.0000000	15.0000000	-50.0000000
54710.0000000	0.0000000	-50.0000000
54710.0000000	0.2500000	18.7000008
54710.0000000	0.5000000	27.8700008
54710.0000000	0.7500000	27.1299992
54710.0000000	1.0000000	21.4300003
54710.0000000	1.2500000	17.4899998
54710.0000000	1.5000000	19.3400002
54710.0000000	1.7500000	18.6800003
54710.0000000	2.0000000	19.3799992
54710.0000000	2.2500000	18.4899998
54710.0000000	2.5000000	19.6100006
54710.0000000	2.7500000	18.4200001
54710.0000000	3.0000000	16.8500004
54710.0000000	3.2500000	18.0900002
54710.0000000	3.5000000	17.1800003
54710.0000000	3.7500000	16.4899998
54710.0000000	4.0000000	19.9099998
54710.0000000	4.2500000	19.3600006
54710.0000000	4.5000000	16.7500000
54710.0000000	4.7500000	-50.0000000
54710.0000000	5.0000000	-50.0000000
54710.0000000	5.2500000	-50.0000000
54710.0000000	5.5000000	-50.0000000
54710.0000000	5.7500000	-50.0000000
54710.0000000	6.0000000	-50.0000000
54710.0000000	6.2500000	-50.0000000
54710.0000000	6.5000000	-50.0000000
54710.0000000	6.7500000	-50.0000000
54710.0000000	7.0000000	-50.0000000
54710.0000000	7.2500000	-50.0000000
54710.0000000	7.5000000	-50.0000000
54710.0000000	7.7500000	-50.0000000
54710.0000000	8.0000000	-50.0000000
54710.0000000	8.2500000	-50.0000000
54710.0000000	8.5000000	-50.0000000
54710.0000000	8.7500000	-50.0000000
54710.0000000	9.0000000	-50.0000000
54710.0000000	9.2500000	-50.0000000
54710.0000000	9.5000000	-50.0000000
54710.0000000	9.7500000	-50.0000000
54710.0000000	10.0000000	-50.0000000
54710.0000000	10.2500000	-50.0000000
54710.0000000	10.5000000	-50.0000000
54710.0000000	10.7500000	-50.0000000
54710.0000000	11.0000000	-50.0000000

54710.0000000	11.2500000	-50.0000000
54710.0000000	11.5000000	-50.0000000
54710.0000000	11.7500000	-50.0000000
54710.0000000	12.0000000	-50.0000000
54710.0000000	12.2500000	-50.0000000
54710.0000000	12.5000000	-50.0000000
54710.0000000	12.7500000	-50.0000000
54710.0000000	13.0000000	-50.0000000
54710.0000000	13.2500000	-50.0000000
54710.0000000	13.5000000	-50.0000000
54710.0000000	13.7500000	-50.0000000
54710.0000000	14.0000000	-50.0000000
54710.0000000	14.2500000	-50.0000000
54710.0000000	14.5000000	-50.0000000
54710.0000000	14.7500000	-50.0000000
54710.0000000	15.0000000	-50.0000000
54740.0000000	0.0000000	-50.0000000
54740.0000000	0.2500000	22.7800007
54740.0000000	0.5000000	31.3600006
54740.0000000	0.7500000	30.5699997
54740.0000000	1.0000000	21.5499992
54740.0000000	1.2500000	18.4300003
54740.0000000	1.5000000	19.0100002
54740.0000000	1.7500000	19.6499996
54740.0000000	2.0000000	19.7800007
54740.0000000	2.2500000	18.7700005
54740.0000000	2.5000000	21.2700005
54740.0000000	2.7500000	18.6599998
54740.0000000	3.0000000	16.7399998
54740.0000000	3.2500000	17.9400005
54740.0000000	3.5000000	19.1800003
54740.0000000	3.7500000	17.5300007
54740.0000000	4.0000000	17.1299992
54740.0000000	4.2500000	17.7399998
54740.0000000	4.5000000	-50.0000000
54740.0000000	4.7500000	-50.0000000
54740.0000000	5.0000000	-50.0000000
54740.0000000	5.2500000	-50.0000000
54740.0000000	5.5000000	-50.0000000
54740.0000000	5.7500000	-50.0000000
54740.0000000	6.0000000	-50.0000000
54740.0000000	6.2500000	-50.0000000
54740.0000000	6.5000000	-50.0000000
54740.0000000	6.7500000	-50.0000000
54740.0000000	7.0000000	-50.0000000
54740.0000000	7.2500000	-50.0000000
54740.0000000	7.5000000	-50.0000000
54740.0000000	7.7500000	-50.0000000
54740.0000000	8.0000000	-50.0000000
54740.0000000	8.2500000	-50.0000000
54740.0000000	8.5000000	-50.0000000
54740.0000000	8.7500000	-50.0000000
54740.0000000	9.0000000	-50.0000000
54740.0000000	9.2500000	-50.0000000
54740.0000000	9.5000000	-50.0000000
54740.0000000	9.7500000	-50.0000000
54740.0000000	10.0000000	-50.0000000
54740.0000000	10.2500000	-50.0000000

54740.0000000	10.5000000	-50.0000000
54740.0000000	10.7500000	-50.0000000
54740.0000000	11.0000000	-50.0000000
54740.0000000	11.2500000	-50.0000000
54740.0000000	11.5000000	-50.0000000
54740.0000000	11.7500000	-50.0000000
54740.0000000	12.0000000	-50.0000000
54740.0000000	12.2500000	-50.0000000
54740.0000000	12.5000000	-50.0000000
54740.0000000	12.7500000	-50.0000000
54740.0000000	13.0000000	-50.0000000
54740.0000000	13.2500000	-50.0000000
54740.0000000	13.5000000	-50.0000000
54740.0000000	13.7500000	-50.0000000
54740.0000000	14.0000000	-50.0000000
54740.0000000	14.2500000	-50.0000000
54740.0000000	14.5000000	-50.0000000
54740.0000000	14.7500000	-50.0000000
54740.0000000	15.0000000	-50.0000000
54770.0000000	0.0000000	-50.0000000
54770.0000000	0.2500000	21.0400009
54770.0000000	0.5000000	27.2099991
54770.0000000	0.7500000	25.1499996
54770.0000000	1.0000000	19.4300003
54770.0000000	1.2500000	18.4799995
54770.0000000	1.5000000	20.5499992
54770.0000000	1.7500000	22.0699997
54770.0000000	2.0000000	20.3600006
54770.0000000	2.2500000	18.5200005
54770.0000000	2.5000000	16.8400002
54770.0000000	2.7500000	16.9200001
54770.0000000	3.0000000	15.9899998
54770.0000000	3.2500000	17.7399998
54770.0000000	3.5000000	18.4099998
54770.0000000	3.7500000	18.5499992
54770.0000000	4.0000000	17.0000000
54770.0000000	4.2500000	18.2099991
54770.0000000	4.5000000	15.6300001
54770.0000000	4.7500000	-50.0000000
54770.0000000	5.0000000	-50.0000000
54770.0000000	5.2500000	-50.0000000
54770.0000000	5.5000000	-50.0000000
54770.0000000	5.7500000	-50.0000000
54770.0000000	6.0000000	-50.0000000
54770.0000000	6.2500000	-50.0000000
54770.0000000	6.5000000	-50.0000000
54770.0000000	6.7500000	-50.0000000
54770.0000000	7.0000000	-50.0000000
54770.0000000	7.2500000	-50.0000000
54770.0000000	7.5000000	-50.0000000
54770.0000000	7.7500000	-50.0000000
54770.0000000	8.0000000	-50.0000000
54770.0000000	8.2500000	-50.0000000
54770.0000000	8.5000000	-50.0000000
54770.0000000	8.7500000	-50.0000000
54770.0000000	9.0000000	-50.0000000
54770.0000000	9.2500000	-50.0000000
54770.0000000	9.5000000	-50.0000000

54770.0000000	9.7500000	-50.0000000
54770.0000000	10.0000000	-50.0000000
54770.0000000	10.2500000	-50.0000000
54770.0000000	10.5000000	-50.0000000
54770.0000000	10.7500000	-50.0000000
54770.0000000	11.0000000	-50.0000000
54770.0000000	11.2500000	-50.0000000
54770.0000000	11.5000000	-50.0000000
54770.0000000	11.7500000	-50.0000000
54770.0000000	12.0000000	-50.0000000
54770.0000000	12.2500000	-50.0000000
54770.0000000	12.5000000	-50.0000000
54770.0000000	12.7500000	-50.0000000
54770.0000000	13.0000000	-50.0000000
54770.0000000	13.2500000	-50.0000000
54770.0000000	13.5000000	-50.0000000
54770.0000000	13.7500000	-50.0000000
54770.0000000	14.0000000	-50.0000000
54770.0000000	14.2500000	-50.0000000
54770.0000000	14.5000000	-50.0000000
54770.0000000	14.7500000	-50.0000000
54770.0000000	15.0000000	-50.0000000
54800.0000000	0.0000000	-50.0000000
54800.0000000	0.2500000	-50.0000000
54800.0000000	0.5000000	24.0300007
54800.0000000	0.7500000	25.7999992
54800.0000000	1.0000000	21.0200005
54800.0000000	1.2500000	19.1499996
54800.0000000	1.5000000	18.5900002
54800.0000000	1.7500000	18.0400009
54800.0000000	2.0000000	17.0200005
54800.0000000	2.2500000	19.6599998
54800.0000000	2.5000000	17.7099991
54800.0000000	2.7500000	17.7399998
54800.0000000	3.0000000	18.2900009
54800.0000000	3.2500000	16.7199993
54800.0000000	3.5000000	18.0799999
54800.0000000	3.7500000	18.6100006
54800.0000000	4.0000000	16.4500008
54800.0000000	4.2500000	20.3199997
54800.0000000	4.5000000	19.0699997
54800.0000000	4.7500000	17.8799992
54800.0000000	5.0000000	-50.0000000
54800.0000000	5.2500000	-50.0000000
54800.0000000	5.5000000	-50.0000000
54800.0000000	5.7500000	-50.0000000
54800.0000000	6.0000000	-50.0000000
54800.0000000	6.2500000	-50.0000000
54800.0000000	6.5000000	-50.0000000
54800.0000000	6.7500000	-50.0000000
54800.0000000	7.0000000	-50.0000000
54800.0000000	7.2500000	-50.0000000
54800.0000000	7.5000000	-50.0000000
54800.0000000	7.7500000	-50.0000000
54800.0000000	8.0000000	-50.0000000
54800.0000000	8.2500000	-50.0000000
54800.0000000	8.5000000	-50.0000000
54800.0000000	8.7500000	-50.0000000

54800.0000000	9.0000000	-50.0000000
54800.0000000	9.2500000	-50.0000000
54800.0000000	9.5000000	-50.0000000
54800.0000000	9.7500000	-50.0000000
54800.0000000	10.0000000	-50.0000000
54800.0000000	10.2500000	-50.0000000
54800.0000000	10.5000000	-50.0000000
54800.0000000	10.7500000	-50.0000000
54800.0000000	11.0000000	-50.0000000
54800.0000000	11.2500000	-50.0000000
54800.0000000	11.5000000	-50.0000000
54800.0000000	11.7500000	-50.0000000
54800.0000000	12.0000000	-50.0000000
54800.0000000	12.2500000	-50.0000000
54800.0000000	12.5000000	-50.0000000
54800.0000000	12.7500000	-50.0000000
54800.0000000	13.0000000	-50.0000000
54800.0000000	13.2500000	-50.0000000
54800.0000000	13.5000000	-50.0000000
54800.0000000	13.7500000	-50.0000000
54800.0000000	14.0000000	-50.0000000
54800.0000000	14.2500000	-50.0000000
54800.0000000	14.5000000	-50.0000000
54800.0000000	14.7500000	-50.0000000
54800.0000000	15.0000000	-50.0000000
54830.0000000	0.0000000	-50.0000000
54830.0000000	0.2500000	15.3299999
54830.0000000	0.5000000	24.3899994
54830.0000000	0.7500000	25.4799995
54830.0000000	1.0000000	21.2000008
54830.0000000	1.2500000	20.1200008
54830.0000000	1.5000000	20.0200005
54830.0000000	1.7500000	20.6000004
54830.0000000	2.0000000	19.9400005
54830.0000000	2.2500000	17.0699997
54830.0000000	2.5000000	17.4899998
54830.0000000	2.7500000	18.6499996
54830.0000000	3.0000000	18.8999996
54830.0000000	3.2500000	21.2600002
54830.0000000	3.5000000	18.7399998
54830.0000000	3.7500000	-50.0000000
54830.0000000	4.0000000	15.3400002
54830.0000000	4.2500000	15.6599998
54830.0000000	4.5000000	15.1400003
54830.0000000	4.7500000	16.8600006
54830.0000000	5.0000000	-50.0000000
54830.0000000	5.2500000	-50.0000000
54830.0000000	5.5000000	-50.0000000
54830.0000000	5.7500000	-50.0000000
54830.0000000	6.0000000	-50.0000000
54830.0000000	6.2500000	-50.0000000
54830.0000000	6.5000000	-50.0000000
54830.0000000	6.7500000	-50.0000000
54830.0000000	7.0000000	-50.0000000
54830.0000000	7.2500000	-50.0000000
54830.0000000	7.5000000	-50.0000000
54830.0000000	7.7500000	-50.0000000
54830.0000000	8.0000000	-50.0000000

54830.0000000	8.2500000	-50.0000000
54830.0000000	8.5000000	-50.0000000
54830.0000000	8.7500000	-50.0000000
54830.0000000	9.0000000	-50.0000000
54830.0000000	9.2500000	-50.0000000
54830.0000000	9.5000000	-50.0000000
54830.0000000	9.7500000	-50.0000000
54830.0000000	10.0000000	-50.0000000
54830.0000000	10.2500000	-50.0000000
54830.0000000	10.5000000	-50.0000000
54830.0000000	10.7500000	-50.0000000
54830.0000000	11.0000000	-50.0000000
54830.0000000	11.2500000	-50.0000000
54830.0000000	11.5000000	-50.0000000
54830.0000000	11.7500000	-50.0000000
54830.0000000	12.0000000	-50.0000000
54830.0000000	12.2500000	-50.0000000
54830.0000000	12.5000000	-50.0000000
54830.0000000	12.7500000	-50.0000000
54830.0000000	13.0000000	-50.0000000
54830.0000000	13.2500000	-50.0000000
54830.0000000	13.5000000	-50.0000000
54830.0000000	13.7500000	-50.0000000
54830.0000000	14.0000000	-50.0000000
54830.0000000	14.2500000	-50.0000000
54830.0000000	14.5000000	-50.0000000
54830.0000000	14.7500000	-50.0000000
54830.0000000	15.0000000	-50.0000000
54860.0000000	0.0000000	-50.0000000
54860.0000000	0.2500000	17.6800003
54860.0000000	0.5000000	26.5599995
54860.0000000	0.7500000	27.3899994
54860.0000000	1.0000000	22.1700001
54860.0000000	1.2500000	21.4400005
54860.0000000	1.5000000	22.3899994
54860.0000000	1.7500000	21.8799992
54860.0000000	2.0000000	20.0200005
54860.0000000	2.2500000	19.2800007
54860.0000000	2.5000000	17.9899998
54860.0000000	2.7500000	18.9200001
54860.0000000	3.0000000	16.1599998
54860.0000000	3.2500000	17.0100002
54860.0000000	3.5000000	15.0699997
54860.0000000	3.7500000	16.1700001
54860.0000000	4.0000000	16.9200001
54860.0000000	4.2500000	17.3700008
54860.0000000	4.5000000	18.5499992
54860.0000000	4.7500000	17.0599995
54860.0000000	5.0000000	-50.0000000
54860.0000000	5.2500000	-50.0000000
54860.0000000	5.5000000	-50.0000000
54860.0000000	5.7500000	-50.0000000
54860.0000000	6.0000000	-50.0000000
54860.0000000	6.2500000	-50.0000000
54860.0000000	6.5000000	-50.0000000
54860.0000000	6.7500000	-50.0000000
54860.0000000	7.0000000	-50.0000000
54860.0000000	7.2500000	-50.0000000

54860.0000000	7.5000000	-50.0000000
54860.0000000	7.7500000	-50.0000000
54860.0000000	8.0000000	-50.0000000
54860.0000000	8.2500000	-50.0000000
54860.0000000	8.5000000	-50.0000000
54860.0000000	8.7500000	-50.0000000
54860.0000000	9.0000000	-50.0000000
54860.0000000	9.2500000	-50.0000000
54860.0000000	9.5000000	-50.0000000
54860.0000000	9.7500000	-50.0000000
54860.0000000	10.0000000	-50.0000000
54860.0000000	10.2500000	-50.0000000
54860.0000000	10.5000000	-50.0000000
54860.0000000	10.7500000	-50.0000000
54860.0000000	11.0000000	-50.0000000
54860.0000000	11.2500000	-50.0000000
54860.0000000	11.5000000	-50.0000000
54860.0000000	11.7500000	-50.0000000
54860.0000000	12.0000000	-50.0000000
54860.0000000	12.2500000	-50.0000000
54860.0000000	12.5000000	-50.0000000
54860.0000000	12.7500000	-50.0000000
54860.0000000	13.0000000	-50.0000000
54860.0000000	13.2500000	-50.0000000
54860.0000000	13.5000000	-50.0000000
54860.0000000	13.7500000	-50.0000000
54860.0000000	14.0000000	-50.0000000
54860.0000000	14.2500000	-50.0000000
54860.0000000	14.5000000	-50.0000000
54860.0000000	14.7500000	-50.0000000
54860.0000000	15.0000000	-50.0000000
54890.0000000	0.0000000	-50.0000000
54890.0000000	0.2500000	16.4300003
54890.0000000	0.5000000	25.8899994
54890.0000000	0.7500000	24.7000008
54890.0000000	1.0000000	17.5799999
54890.0000000	1.2500000	18.4699993
54890.0000000	1.5000000	20.4200001
54890.0000000	1.7500000	19.6499996
54890.0000000	2.0000000	18.0000000
54890.0000000	2.2500000	19.1000004
54890.0000000	2.5000000	16.1599998
54890.0000000	2.7500000	18.3199997
54890.0000000	3.0000000	19.4899998
54890.0000000	3.2500000	17.8400002
54890.0000000	3.5000000	18.9400005
54890.0000000	3.7500000	17.9899998
54890.0000000	4.0000000	15.8199997
54890.0000000	4.2500000	-50.0000000
54890.0000000	4.5000000	-50.0000000
54890.0000000	4.7500000	-50.0000000
54890.0000000	5.0000000	-50.0000000
54890.0000000	5.2500000	-50.0000000
54890.0000000	5.5000000	-50.0000000
54890.0000000	5.7500000	-50.0000000
54890.0000000	6.0000000	-50.0000000
54890.0000000	6.2500000	-50.0000000
54890.0000000	6.5000000	-50.0000000

54890.0000000	6.7500000	-50.0000000
54890.0000000	7.0000000	-50.0000000
54890.0000000	7.2500000	-50.0000000
54890.0000000	7.5000000	-50.0000000
54890.0000000	7.7500000	-50.0000000
54890.0000000	8.0000000	-50.0000000
54890.0000000	8.2500000	-50.0000000
54890.0000000	8.5000000	-50.0000000
54890.0000000	8.7500000	-50.0000000
54890.0000000	9.0000000	-50.0000000
54890.0000000	9.2500000	-50.0000000
54890.0000000	9.5000000	-50.0000000
54890.0000000	9.7500000	-50.0000000
54890.0000000	10.0000000	-50.0000000
54890.0000000	10.2500000	-50.0000000
54890.0000000	10.5000000	-50.0000000
54890.0000000	10.7500000	-50.0000000
54890.0000000	11.0000000	-50.0000000
54890.0000000	11.2500000	-50.0000000
54890.0000000	11.5000000	-50.0000000
54890.0000000	11.7500000	-50.0000000
54890.0000000	12.0000000	-50.0000000
54890.0000000	12.2500000	-50.0000000
54890.0000000	12.5000000	-50.0000000
54890.0000000	12.7500000	-50.0000000
54890.0000000	13.0000000	-50.0000000
54890.0000000	13.2500000	-50.0000000
54890.0000000	13.5000000	-50.0000000
54890.0000000	13.7500000	-50.0000000
54890.0000000	14.0000000	-50.0000000
54890.0000000	14.2500000	-50.0000000
54890.0000000	14.5000000	-50.0000000
54890.0000000	14.7500000	-50.0000000
54890.0000000	15.0000000	-50.0000000
54920.0000000	0.0000000	-50.0000000
54920.0000000	0.2500000	-50.0000000
54920.0000000	0.5000000	25.0000000
54920.0000000	0.7500000	25.0200005
54920.0000000	1.0000000	19.1700001
54920.0000000	1.2500000	17.0100002
54920.0000000	1.5000000	18.3700008
54920.0000000	1.7500000	18.8700008
54920.0000000	2.0000000	18.6200008
54920.0000000	2.2500000	19.0599995
54920.0000000	2.5000000	18.5100002
54920.0000000	2.7500000	16.7700005
54920.0000000	3.0000000	17.0799999
54920.0000000	3.2500000	18.8500004
54920.0000000	3.5000000	18.7000008
54920.0000000	3.7500000	18.0799999
54920.0000000	4.0000000	17.3600006
54920.0000000	4.2500000	16.9899998
54920.0000000	4.5000000	16.7000008
54920.0000000	4.7500000	17.1000004
54920.0000000	5.0000000	-50.0000000
54920.0000000	5.2500000	-50.0000000
54920.0000000	5.5000000	-50.0000000
54920.0000000	5.7500000	-50.0000000

54920.0000000	6.0000000	-50.0000000
54920.0000000	6.2500000	-50.0000000
54920.0000000	6.5000000	-50.0000000
54920.0000000	6.7500000	-50.0000000
54920.0000000	7.0000000	-50.0000000
54920.0000000	7.2500000	-50.0000000
54920.0000000	7.5000000	-50.0000000
54920.0000000	7.7500000	-50.0000000
54920.0000000	8.0000000	-50.0000000
54920.0000000	8.2500000	-50.0000000
54920.0000000	8.5000000	-50.0000000
54920.0000000	8.7500000	-50.0000000
54920.0000000	9.0000000	-50.0000000
54920.0000000	9.2500000	-50.0000000
54920.0000000	9.5000000	-50.0000000
54920.0000000	9.7500000	-50.0000000
54920.0000000	10.0000000	-50.0000000
54920.0000000	10.2500000	-50.0000000
54920.0000000	10.5000000	-50.0000000
54920.0000000	10.7500000	-50.0000000
54920.0000000	11.0000000	-50.0000000
54920.0000000	11.2500000	-50.0000000
54920.0000000	11.5000000	-50.0000000
54920.0000000	11.7500000	-50.0000000
54920.0000000	12.0000000	-50.0000000
54920.0000000	12.2500000	-50.0000000
54920.0000000	12.5000000	-50.0000000
54920.0000000	12.7500000	-50.0000000
54920.0000000	13.0000000	-50.0000000
54920.0000000	13.2500000	-50.0000000
54920.0000000	13.5000000	-50.0000000
54920.0000000	13.7500000	-50.0000000
54920.0000000	14.0000000	-50.0000000
54920.0000000	14.2500000	-50.0000000
54920.0000000	14.5000000	-50.0000000
54920.0000000	14.7500000	-50.0000000
54920.0000000	15.0000000	-50.0000000
54950.0000000	0.0000000	-50.0000000
54950.0000000	0.2500000	-50.0000000
54950.0000000	0.5000000	23.8199997
54950.0000000	0.7500000	24.3799992
54950.0000000	1.0000000	17.9599991
54950.0000000	1.2500000	18.9799995
54950.0000000	1.5000000	20.9699993
54950.0000000	1.7500000	19.4799995
54950.0000000	2.0000000	18.6700001
54950.0000000	2.2500000	19.3600006
54950.0000000	2.5000000	18.3400002
54950.0000000	2.7500000	20.3899994
54950.0000000	3.0000000	22.9500008
54950.0000000	3.2500000	20.6900005
54950.0000000	3.5000000	17.8299999
54950.0000000	3.7500000	17.0000000
54950.0000000	4.0000000	15.4300003
54950.0000000	4.2500000	16.9500008
54950.0000000	4.5000000	17.0599995
54950.0000000	4.7500000	-50.0000000
54950.0000000	5.0000000	-50.0000000

54950.0000000	5.2500000	-50.0000000
54950.0000000	5.5000000	-50.0000000
54950.0000000	5.7500000	-50.0000000
54950.0000000	6.0000000	-50.0000000
54950.0000000	6.2500000	-50.0000000
54950.0000000	6.5000000	-50.0000000
54950.0000000	6.7500000	-50.0000000
54950.0000000	7.0000000	-50.0000000
54950.0000000	7.2500000	-50.0000000
54950.0000000	7.5000000	-50.0000000
54950.0000000	7.7500000	-50.0000000
54950.0000000	8.0000000	-50.0000000
54950.0000000	8.2500000	-50.0000000
54950.0000000	8.5000000	-50.0000000
54950.0000000	8.7500000	-50.0000000
54950.0000000	9.0000000	-50.0000000
54950.0000000	9.2500000	-50.0000000
54950.0000000	9.5000000	-50.0000000
54950.0000000	9.7500000	-50.0000000
54950.0000000	10.0000000	-50.0000000
54950.0000000	10.2500000	-50.0000000
54950.0000000	10.5000000	-50.0000000
54950.0000000	10.7500000	-50.0000000
54950.0000000	11.0000000	-50.0000000
54950.0000000	11.2500000	-50.0000000
54950.0000000	11.5000000	-50.0000000
54950.0000000	11.7500000	-50.0000000
54950.0000000	12.0000000	-50.0000000
54950.0000000	12.2500000	-50.0000000
54950.0000000	12.5000000	-50.0000000
54950.0000000	12.7500000	-50.0000000
54950.0000000	13.0000000	-50.0000000
54950.0000000	13.2500000	-50.0000000
54950.0000000	13.5000000	-50.0000000
54950.0000000	13.7500000	-50.0000000
54950.0000000	14.0000000	-50.0000000
54950.0000000	14.2500000	-50.0000000
54950.0000000	14.5000000	-50.0000000
54950.0000000	14.7500000	-50.0000000
54950.0000000	15.0000000	-50.0000000
54980.0000000	0.0000000	-50.0000000
54980.0000000	0.2500000	15.3500004
54980.0000000	0.5000000	24.2199993
54980.0000000	0.7500000	24.1299992
54980.0000000	1.0000000	19.3400002
54980.0000000	1.2500000	19.3299999
54980.0000000	1.5000000	17.9200001
54980.0000000	1.7500000	20.6399994
54980.0000000	2.0000000	20.0699997
54980.0000000	2.2500000	17.8600006
54980.0000000	2.5000000	18.5400009
54980.0000000	2.7500000	19.3199997
54980.0000000	3.0000000	20.5699997
54980.0000000	3.2500000	19.4699993
54980.0000000	3.5000000	18.6599998
54980.0000000	3.7500000	19.2099991
54980.0000000	4.0000000	18.1100006
54980.0000000	4.2500000	16.8500004

54980.0000000	4.5000000	15.3800001
54980.0000000	4.7500000	-50.0000000
54980.0000000	5.0000000	-50.0000000
54980.0000000	5.2500000	-50.0000000
54980.0000000	5.5000000	-50.0000000
54980.0000000	5.7500000	-50.0000000
54980.0000000	6.0000000	-50.0000000
54980.0000000	6.2500000	-50.0000000
54980.0000000	6.5000000	-50.0000000
54980.0000000	6.7500000	-50.0000000
54980.0000000	7.0000000	-50.0000000
54980.0000000	7.2500000	-50.0000000
54980.0000000	7.5000000	-50.0000000
54980.0000000	7.7500000	-50.0000000
54980.0000000	8.0000000	-50.0000000
54980.0000000	8.2500000	-50.0000000
54980.0000000	8.5000000	-50.0000000
54980.0000000	8.7500000	-50.0000000
54980.0000000	9.0000000	-50.0000000
54980.0000000	9.2500000	-50.0000000
54980.0000000	9.5000000	-50.0000000
54980.0000000	9.7500000	-50.0000000
54980.0000000	10.0000000	-50.0000000
54980.0000000	10.2500000	-50.0000000
54980.0000000	10.5000000	-50.0000000
54980.0000000	10.7500000	-50.0000000
54980.0000000	11.0000000	-50.0000000
54980.0000000	11.2500000	-50.0000000
54980.0000000	11.5000000	-50.0000000
54980.0000000	11.7500000	-50.0000000
54980.0000000	12.0000000	-50.0000000
54980.0000000	12.2500000	-50.0000000
54980.0000000	12.5000000	-50.0000000
54980.0000000	12.7500000	-50.0000000
54980.0000000	13.0000000	-50.0000000
54980.0000000	13.2500000	-50.0000000
54980.0000000	13.5000000	-50.0000000
54980.0000000	13.7500000	-50.0000000
54980.0000000	14.0000000	-50.0000000
54980.0000000	14.2500000	-50.0000000
54980.0000000	14.5000000	-50.0000000
54980.0000000	14.7500000	-50.0000000
54980.0000000	15.0000000	-50.0000000
55010.0000000	0.0000000	-50.0000000
55010.0000000	0.2500000	19.4099998
55010.0000000	0.5000000	27.4699993
55010.0000000	0.7500000	26.1800003
55010.0000000	1.0000000	20.1000004
55010.0000000	1.2500000	20.5900002
55010.0000000	1.5000000	19.7099991
55010.0000000	1.7500000	18.1800003
55010.0000000	2.0000000	18.6100006
55010.0000000	2.2500000	17.8400002
55010.0000000	2.5000000	17.6599998
55010.0000000	2.7500000	20.5000000
55010.0000000	3.0000000	19.4500008
55010.0000000	3.2500000	16.8500004
55010.0000000	3.5000000	18.8700008

55010.0000000	3.7500000	19.6100006
55010.0000000	4.0000000	16.7299995
55010.0000000	4.2500000	15.3699999
55010.0000000	4.5000000	15.8800001
55010.0000000	4.7500000	-50.0000000
55010.0000000	5.0000000	-50.0000000
55010.0000000	5.2500000	-50.0000000
55010.0000000	5.5000000	-50.0000000
55010.0000000	5.7500000	-50.0000000
55010.0000000	6.0000000	-50.0000000
55010.0000000	6.2500000	-50.0000000
55010.0000000	6.5000000	-50.0000000
55010.0000000	6.7500000	-50.0000000
55010.0000000	7.0000000	-50.0000000
55010.0000000	7.2500000	-50.0000000
55010.0000000	7.5000000	-50.0000000
55010.0000000	7.7500000	-50.0000000
55010.0000000	8.0000000	-50.0000000
55010.0000000	8.2500000	-50.0000000
55010.0000000	8.5000000	-50.0000000
55010.0000000	8.7500000	-50.0000000
55010.0000000	9.0000000	-50.0000000
55010.0000000	9.2500000	-50.0000000
55010.0000000	9.5000000	-50.0000000
55010.0000000	9.7500000	-50.0000000
55010.0000000	10.0000000	-50.0000000
55010.0000000	10.2500000	-50.0000000
55010.0000000	10.5000000	-50.0000000
55010.0000000	10.7500000	-50.0000000
55010.0000000	11.0000000	-50.0000000
55010.0000000	11.2500000	-50.0000000
55010.0000000	11.5000000	-50.0000000
55010.0000000	11.7500000	-50.0000000
55010.0000000	12.0000000	-50.0000000
55010.0000000	12.2500000	-50.0000000
55010.0000000	12.5000000	-50.0000000
55010.0000000	12.7500000	-50.0000000
55010.0000000	13.0000000	-50.0000000
55010.0000000	13.2500000	-50.0000000
55010.0000000	13.5000000	-50.0000000
55010.0000000	13.7500000	-50.0000000
55010.0000000	14.0000000	-50.0000000
55010.0000000	14.2500000	-50.0000000
55010.0000000	14.5000000	-50.0000000
55010.0000000	14.7500000	-50.0000000
55010.0000000	15.0000000	-50.0000000
55040.0000000	0.0000000	-50.0000000
55040.0000000	0.2500000	17.1900005
55040.0000000	0.5000000	25.5799999
55040.0000000	0.7500000	24.9500008
55040.0000000	1.0000000	20.8500004
55040.0000000	1.2500000	18.9500008
55040.0000000	1.5000000	19.8600006
55040.0000000	1.7500000	18.7000008
55040.0000000	2.0000000	17.9799995
55040.0000000	2.2500000	18.9699993
55040.0000000	2.5000000	19.0400009
55040.0000000	2.7500000	19.1900005

55040.0000000	3.0000000	19.9899998
55040.0000000	3.2500000	17.8799992
55040.0000000	3.5000000	19.7900009
55040.0000000	3.7500000	21.2900009
55040.0000000	4.0000000	16.6000004
55040.0000000	4.2500000	16.3999996
55040.0000000	4.5000000	15.8699999
55040.0000000	4.7500000	-50.0000000
55040.0000000	5.0000000	-50.0000000
55040.0000000	5.2500000	-50.0000000
55040.0000000	5.5000000	-50.0000000
55040.0000000	5.7500000	-50.0000000
55040.0000000	6.0000000	-50.0000000
55040.0000000	6.2500000	-50.0000000
55040.0000000	6.5000000	-50.0000000
55040.0000000	6.7500000	-50.0000000
55040.0000000	7.0000000	-50.0000000
55040.0000000	7.2500000	-50.0000000
55040.0000000	7.5000000	-50.0000000
55040.0000000	7.7500000	-50.0000000
55040.0000000	8.0000000	-50.0000000
55040.0000000	8.2500000	-50.0000000
55040.0000000	8.5000000	-50.0000000
55040.0000000	8.7500000	-50.0000000
55040.0000000	9.0000000	-50.0000000
55040.0000000	9.2500000	-50.0000000
55040.0000000	9.5000000	-50.0000000
55040.0000000	9.7500000	-50.0000000
55040.0000000	10.0000000	-50.0000000
55040.0000000	10.2500000	-50.0000000
55040.0000000	10.5000000	-50.0000000
55040.0000000	10.7500000	-50.0000000
55040.0000000	11.0000000	-50.0000000
55040.0000000	11.2500000	-50.0000000
55040.0000000	11.5000000	-50.0000000
55040.0000000	11.7500000	-50.0000000
55040.0000000	12.0000000	-50.0000000
55040.0000000	12.2500000	-50.0000000
55040.0000000	12.5000000	-50.0000000
55040.0000000	12.7500000	-50.0000000
55040.0000000	13.0000000	-50.0000000
55040.0000000	13.2500000	-50.0000000
55040.0000000	13.5000000	-50.0000000
55040.0000000	13.7500000	-50.0000000
55040.0000000	14.0000000	-50.0000000
55040.0000000	14.2500000	-50.0000000
55040.0000000	14.5000000	-50.0000000
55040.0000000	14.7500000	-50.0000000
55040.0000000	15.0000000	-50.0000000
55070.0000000	0.0000000	-50.0000000
55070.0000000	0.2500000	16.0799999
55070.0000000	0.5000000	25.1399994
55070.0000000	0.7500000	25.4899998
55070.0000000	1.0000000	18.6900005
55070.0000000	1.2500000	16.5400009
55070.0000000	1.5000000	17.9899998
55070.0000000	1.7500000	17.9300003
55070.0000000	2.0000000	19.1599998

55070.0000000	2.2500000	20.3299999
55070.0000000	2.5000000	19.8299999
55070.0000000	2.7500000	20.3500004
55070.0000000	3.0000000	18.6100006
55070.0000000	3.2500000	17.1299992
55070.0000000	3.5000000	18.9099998
55070.0000000	3.7500000	18.0000000
55070.0000000	4.0000000	17.1000004
55070.0000000	4.2500000	18.0100002
55070.0000000	4.5000000	16.6700001
55070.0000000	4.7500000	-50.0000000
55070.0000000	5.0000000	-50.0000000
55070.0000000	5.2500000	-50.0000000
55070.0000000	5.5000000	-50.0000000
55070.0000000	5.7500000	-50.0000000
55070.0000000	6.0000000	-50.0000000
55070.0000000	6.2500000	-50.0000000
55070.0000000	6.5000000	-50.0000000
55070.0000000	6.7500000	-50.0000000
55070.0000000	7.0000000	-50.0000000
55070.0000000	7.2500000	-50.0000000
55070.0000000	7.5000000	-50.0000000
55070.0000000	7.7500000	-50.0000000
55070.0000000	8.0000000	-50.0000000
55070.0000000	8.2500000	-50.0000000
55070.0000000	8.5000000	-50.0000000
55070.0000000	8.7500000	-50.0000000
55070.0000000	9.0000000	-50.0000000
55070.0000000	9.2500000	-50.0000000
55070.0000000	9.5000000	-50.0000000
55070.0000000	9.7500000	-50.0000000
55070.0000000	10.0000000	-50.0000000
55070.0000000	10.2500000	-50.0000000
55070.0000000	10.5000000	-50.0000000
55070.0000000	10.7500000	-50.0000000
55070.0000000	11.0000000	-50.0000000
55070.0000000	11.2500000	-50.0000000
55070.0000000	11.5000000	-50.0000000
55070.0000000	11.7500000	-50.0000000
55070.0000000	12.0000000	-50.0000000
55070.0000000	12.2500000	-50.0000000
55070.0000000	12.5000000	-50.0000000
55070.0000000	12.7500000	-50.0000000
55070.0000000	13.0000000	-50.0000000
55070.0000000	13.2500000	-50.0000000
55070.0000000	13.5000000	-50.0000000
55070.0000000	13.7500000	-50.0000000
55070.0000000	14.0000000	-50.0000000
55070.0000000	14.2500000	-50.0000000
55070.0000000	14.5000000	-50.0000000
55070.0000000	14.7500000	-50.0000000
55070.0000000	15.0000000	-50.0000000
55100.0000000	0.0000000	-50.0000000
55100.0000000	0.2500000	16.9799995
55100.0000000	0.5000000	25.4300003
55100.0000000	0.7500000	24.7900009
55100.0000000	1.0000000	20.3400002
55100.0000000	1.2500000	17.6900005

55100.0000000	1.5000000	18.0300007
55100.0000000	1.7500000	18.7299995
55100.0000000	2.0000000	18.2000008
55100.0000000	2.2500000	21.1700001
55100.0000000	2.5000000	19.7700005
55100.0000000	2.7500000	18.2500000
55100.0000000	3.0000000	17.7500000
55100.0000000	3.2500000	17.3700008
55100.0000000	3.5000000	19.0499992
55100.0000000	3.7500000	17.7500000
55100.0000000	4.0000000	20.5400009
55100.0000000	4.2500000	17.3299999
55100.0000000	4.5000000	15.2900000
55100.0000000	4.7500000	-50.0000000
55100.0000000	5.0000000	-50.0000000
55100.0000000	5.2500000	-50.0000000
55100.0000000	5.5000000	-50.0000000
55100.0000000	5.7500000	-50.0000000
55100.0000000	6.0000000	-50.0000000
55100.0000000	6.2500000	-50.0000000
55100.0000000	6.5000000	-50.0000000
55100.0000000	6.7500000	-50.0000000
55100.0000000	7.0000000	-50.0000000
55100.0000000	7.2500000	-50.0000000
55100.0000000	7.5000000	-50.0000000
55100.0000000	7.7500000	-50.0000000
55100.0000000	8.0000000	-50.0000000
55100.0000000	8.2500000	-50.0000000
55100.0000000	8.5000000	-50.0000000
55100.0000000	8.7500000	-50.0000000
55100.0000000	9.0000000	-50.0000000
55100.0000000	9.2500000	-50.0000000
55100.0000000	9.5000000	-50.0000000
55100.0000000	9.7500000	-50.0000000
55100.0000000	10.0000000	-50.0000000
55100.0000000	10.2500000	-50.0000000
55100.0000000	10.5000000	-50.0000000
55100.0000000	10.7500000	-50.0000000
55100.0000000	11.0000000	-50.0000000
55100.0000000	11.2500000	-50.0000000
55100.0000000	11.5000000	-50.0000000
55100.0000000	11.7500000	-50.0000000
55100.0000000	12.0000000	-50.0000000
55100.0000000	12.2500000	-50.0000000
55100.0000000	12.5000000	-50.0000000
55100.0000000	12.7500000	-50.0000000
55100.0000000	13.0000000	-50.0000000
55100.0000000	13.2500000	-50.0000000
55100.0000000	13.5000000	-50.0000000
55100.0000000	13.7500000	-50.0000000
55100.0000000	14.0000000	-50.0000000
55100.0000000	14.2500000	-50.0000000
55100.0000000	14.5000000	-50.0000000
55100.0000000	14.7500000	-50.0000000
55100.0000000	15.0000000	-50.0000000
55130.0000000	0.0000000	-50.0000000
55130.0000000	0.2500000	-50.0000000
55130.0000000	0.5000000	24.2500000

55130.0000000	0.7500000	26.3899994
55130.0000000	1.0000000	22.0200005
55130.0000000	1.2500000	20.2800007
55130.0000000	1.5000000	18.2600002
55130.0000000	1.7500000	18.1599998
55130.0000000	2.0000000	18.4300003
55130.0000000	2.2500000	18.9899998
55130.0000000	2.5000000	20.2800007
55130.0000000	2.7500000	18.7399998
55130.0000000	3.0000000	18.2900009
55130.0000000	3.2500000	18.0300007
55130.0000000	3.5000000	17.9500008
55130.0000000	3.7500000	-50.0000000
55130.0000000	4.0000000	18.3500004
55130.0000000	4.2500000	18.4200001
55130.0000000	4.5000000	18.0499992
55130.0000000	4.7500000	-50.0000000
55130.0000000	5.0000000	-50.0000000
55130.0000000	5.2500000	-50.0000000
55130.0000000	5.5000000	-50.0000000
55130.0000000	5.7500000	-50.0000000
55130.0000000	6.0000000	-50.0000000
55130.0000000	6.2500000	-50.0000000
55130.0000000	6.5000000	-50.0000000
55130.0000000	6.7500000	-50.0000000
55130.0000000	7.0000000	-50.0000000
55130.0000000	7.2500000	-50.0000000
55130.0000000	7.5000000	-50.0000000
55130.0000000	7.7500000	-50.0000000
55130.0000000	8.0000000	-50.0000000
55130.0000000	8.2500000	-50.0000000
55130.0000000	8.5000000	-50.0000000
55130.0000000	8.7500000	-50.0000000
55130.0000000	9.0000000	-50.0000000
55130.0000000	9.2500000	-50.0000000
55130.0000000	9.5000000	-50.0000000
55130.0000000	9.7500000	-50.0000000
55130.0000000	10.0000000	-50.0000000
55130.0000000	10.2500000	-50.0000000
55130.0000000	10.5000000	-50.0000000
55130.0000000	10.7500000	-50.0000000
55130.0000000	11.0000000	-50.0000000
55130.0000000	11.2500000	-50.0000000
55130.0000000	11.5000000	-50.0000000
55130.0000000	11.7500000	-50.0000000
55130.0000000	12.0000000	-50.0000000
55130.0000000	12.2500000	-50.0000000
55130.0000000	12.5000000	-50.0000000
55130.0000000	12.7500000	-50.0000000
55130.0000000	13.0000000	-50.0000000
55130.0000000	13.2500000	-50.0000000
55130.0000000	13.5000000	-50.0000000
55130.0000000	13.7500000	-50.0000000
55130.0000000	14.0000000	-50.0000000
55130.0000000	14.2500000	-50.0000000
55130.0000000	14.5000000	-50.0000000
55130.0000000	14.7500000	-50.0000000
55130.0000000	15.0000000	-50.0000000

55160.0000000	0.0000000	-50.0000000
55160.0000000	0.2500000	15.9399996
55160.0000000	0.5000000	25.5200005
55160.0000000	0.7500000	25.8700008
55160.0000000	1.0000000	21.6499996
55160.0000000	1.2500000	21.8799992
55160.0000000	1.5000000	19.9599991
55160.0000000	1.7500000	19.3999996
55160.0000000	2.0000000	19.7199993
55160.0000000	2.2500000	21.2500000
55160.0000000	2.5000000	19.8899994
55160.0000000	2.7500000	16.8999996
55160.0000000	3.0000000	16.3999996
55160.0000000	3.2500000	18.4300003
55160.0000000	3.5000000	16.8500004
55160.0000000	3.7500000	18.5499992
55160.0000000	4.0000000	18.3500004
55160.0000000	4.2500000	16.0200005
55160.0000000	4.5000000	-50.0000000
55160.0000000	4.7500000	-50.0000000
55160.0000000	5.0000000	-50.0000000
55160.0000000	5.2500000	-50.0000000
55160.0000000	5.5000000	-50.0000000
55160.0000000	5.7500000	-50.0000000
55160.0000000	6.0000000	-50.0000000
55160.0000000	6.2500000	-50.0000000
55160.0000000	6.5000000	-50.0000000
55160.0000000	6.7500000	-50.0000000
55160.0000000	7.0000000	-50.0000000
55160.0000000	7.2500000	-50.0000000
55160.0000000	7.5000000	-50.0000000
55160.0000000	7.7500000	-50.0000000
55160.0000000	8.0000000	-50.0000000
55160.0000000	8.2500000	-50.0000000
55160.0000000	8.5000000	-50.0000000
55160.0000000	8.7500000	-50.0000000
55160.0000000	9.0000000	-50.0000000
55160.0000000	9.2500000	-50.0000000
55160.0000000	9.5000000	-50.0000000
55160.0000000	9.7500000	-50.0000000
55160.0000000	10.0000000	-50.0000000
55160.0000000	10.2500000	-50.0000000
55160.0000000	10.5000000	-50.0000000
55160.0000000	10.7500000	-50.0000000
55160.0000000	11.0000000	-50.0000000
55160.0000000	11.2500000	-50.0000000
55160.0000000	11.5000000	-50.0000000
55160.0000000	11.7500000	-50.0000000
55160.0000000	12.0000000	-50.0000000
55160.0000000	12.2500000	-50.0000000
55160.0000000	12.5000000	-50.0000000
55160.0000000	12.7500000	-50.0000000
55160.0000000	13.0000000	-50.0000000
55160.0000000	13.2500000	-50.0000000
55160.0000000	13.5000000	-50.0000000
55160.0000000	13.7500000	-50.0000000
55160.0000000	14.0000000	-50.0000000
55160.0000000	14.2500000	-50.0000000

55160.0000000	14.5000000	-50.0000000
55160.0000000	14.7500000	-50.0000000
55160.0000000	15.0000000	-50.0000000
55190.0000000	0.0000000	-50.0000000
55190.0000000	0.2500000	15.4099998
55190.0000000	0.5000000	23.9300003
55190.0000000	0.7500000	24.1299992
55190.0000000	1.0000000	20.0699997
55190.0000000	1.2500000	17.3299999
55190.0000000	1.5000000	16.0200005
55190.0000000	1.7500000	16.9699993
55190.0000000	2.0000000	18.7900009
55190.0000000	2.2500000	21.0599995
55190.0000000	2.5000000	21.3899994
55190.0000000	2.7500000	21.8400002
55190.0000000	3.0000000	20.2600002
55190.0000000	3.2500000	18.1000004
55190.0000000	3.5000000	18.9099998
55190.0000000	3.7500000	17.9500008
55190.0000000	4.0000000	16.3400002
55190.0000000	4.2500000	16.5300007
55190.0000000	4.5000000	17.3400002
55190.0000000	4.7500000	15.5400000
55190.0000000	5.0000000	-50.0000000
55190.0000000	5.2500000	-50.0000000
55190.0000000	5.5000000	-50.0000000
55190.0000000	5.7500000	-50.0000000
55190.0000000	6.0000000	-50.0000000
55190.0000000	6.2500000	-50.0000000
55190.0000000	6.5000000	-50.0000000
55190.0000000	6.7500000	-50.0000000
55190.0000000	7.0000000	-50.0000000
55190.0000000	7.2500000	-50.0000000
55190.0000000	7.5000000	-50.0000000
55190.0000000	7.7500000	-50.0000000
55190.0000000	8.0000000	-50.0000000
55190.0000000	8.2500000	-50.0000000
55190.0000000	8.5000000	-50.0000000
55190.0000000	8.7500000	-50.0000000
55190.0000000	9.0000000	-50.0000000
55190.0000000	9.2500000	-50.0000000
55190.0000000	9.5000000	-50.0000000
55190.0000000	9.7500000	-50.0000000
55190.0000000	10.0000000	-50.0000000
55190.0000000	10.2500000	-50.0000000
55190.0000000	10.5000000	-50.0000000
55190.0000000	10.7500000	-50.0000000
55190.0000000	11.0000000	-50.0000000
55190.0000000	11.2500000	-50.0000000
55190.0000000	11.5000000	-50.0000000
55190.0000000	11.7500000	-50.0000000
55190.0000000	12.0000000	-50.0000000
55190.0000000	12.2500000	-50.0000000
55190.0000000	12.5000000	-50.0000000
55190.0000000	12.7500000	-50.0000000
55190.0000000	13.0000000	-50.0000000
55190.0000000	13.2500000	-50.0000000
55190.0000000	13.5000000	-50.0000000

55190.0000000	13.7500000	-50.0000000
55190.0000000	14.0000000	-50.0000000
55190.0000000	14.2500000	-50.0000000
55190.0000000	14.5000000	-50.0000000
55190.0000000	14.7500000	-50.0000000
55190.0000000	15.0000000	-50.0000000
55220.0000000	0.0000000	-50.0000000
55220.0000000	0.2500000	16.1399994
55220.0000000	0.5000000	25.3299999
55220.0000000	0.7500000	25.2999992
55220.0000000	1.0000000	20.6800003
55220.0000000	1.2500000	21.3199997
55220.0000000	1.5000000	21.6299992
55220.0000000	1.7500000	18.8700008
55220.0000000	2.0000000	20.4400005
55220.0000000	2.2500000	18.9500008
55220.0000000	2.5000000	19.5400009
55220.0000000	2.7500000	20.7399998
55220.0000000	3.0000000	19.0900002
55220.0000000	3.2500000	17.3299999
55220.0000000	3.5000000	19.1399994
55220.0000000	3.7500000	19.2399998
55220.0000000	4.0000000	17.5799999
55220.0000000	4.2500000	17.5599995
55220.0000000	4.5000000	17.6200008
55220.0000000	4.7500000	15.8500004
55220.0000000	5.0000000	-50.0000000
55220.0000000	5.2500000	-50.0000000
55220.0000000	5.5000000	-50.0000000
55220.0000000	5.7500000	-50.0000000
55220.0000000	6.0000000	-50.0000000
55220.0000000	6.2500000	-50.0000000
55220.0000000	6.5000000	-50.0000000
55220.0000000	6.7500000	-50.0000000
55220.0000000	7.0000000	-50.0000000
55220.0000000	7.2500000	-50.0000000
55220.0000000	7.5000000	-50.0000000
55220.0000000	7.7500000	-50.0000000
55220.0000000	8.0000000	-50.0000000
55220.0000000	8.2500000	-50.0000000
55220.0000000	8.5000000	-50.0000000
55220.0000000	8.7500000	-50.0000000
55220.0000000	9.0000000	-50.0000000
55220.0000000	9.2500000	-50.0000000
55220.0000000	9.5000000	-50.0000000
55220.0000000	9.7500000	-50.0000000
55220.0000000	10.0000000	-50.0000000
55220.0000000	10.2500000	-50.0000000
55220.0000000	10.5000000	-50.0000000
55220.0000000	10.7500000	-50.0000000
55220.0000000	11.0000000	-50.0000000
55220.0000000	11.2500000	-50.0000000
55220.0000000	11.5000000	-50.0000000
55220.0000000	11.7500000	-50.0000000
55220.0000000	12.0000000	-50.0000000
55220.0000000	12.2500000	-50.0000000
55220.0000000	12.5000000	-50.0000000
55220.0000000	12.7500000	-50.0000000

55220.0000000	13.0000000	-50.0000000
55220.0000000	13.2500000	-50.0000000
55220.0000000	13.5000000	-50.0000000
55220.0000000	13.7500000	-50.0000000
55220.0000000	14.0000000	-50.0000000
55220.0000000	14.2500000	-50.0000000
55220.0000000	14.5000000	-50.0000000
55220.0000000	14.7500000	-50.0000000
55220.0000000	15.0000000	-50.0000000
55250.0000000	0.0000000	-50.0000000
55250.0000000	0.2500000	-50.0000000
55250.0000000	0.5000000	24.9099998
55250.0000000	0.7500000	26.1800003
55250.0000000	1.0000000	22.1800003
55250.0000000	1.2500000	20.7299995
55250.0000000	1.5000000	18.8099995
55250.0000000	1.7500000	18.2600002
55250.0000000	2.0000000	18.1200008
55250.0000000	2.2500000	17.0599995
55250.0000000	2.5000000	18.7299995
55250.0000000	2.7500000	16.9899998
55250.0000000	3.0000000	20.3400002
55250.0000000	3.2500000	18.9300003
55250.0000000	3.5000000	19.0599995
55250.0000000	3.7500000	17.6599998
55250.0000000	4.0000000	18.1100006
55250.0000000	4.2500000	17.6100006
55250.0000000	4.5000000	17.8500004
55250.0000000	4.7500000	15.4099998
55250.0000000	5.0000000	-50.0000000
55250.0000000	5.2500000	-50.0000000
55250.0000000	5.5000000	-50.0000000
55250.0000000	5.7500000	-50.0000000
55250.0000000	6.0000000	-50.0000000
55250.0000000	6.2500000	-50.0000000
55250.0000000	6.5000000	-50.0000000
55250.0000000	6.7500000	-50.0000000
55250.0000000	7.0000000	-50.0000000
55250.0000000	7.2500000	-50.0000000
55250.0000000	7.5000000	-50.0000000
55250.0000000	7.7500000	-50.0000000
55250.0000000	8.0000000	-50.0000000
55250.0000000	8.2500000	-50.0000000
55250.0000000	8.5000000	-50.0000000
55250.0000000	8.7500000	-50.0000000
55250.0000000	9.0000000	-50.0000000
55250.0000000	9.2500000	-50.0000000
55250.0000000	9.5000000	-50.0000000
55250.0000000	9.7500000	-50.0000000
55250.0000000	10.0000000	-50.0000000
55250.0000000	10.2500000	-50.0000000
55250.0000000	10.5000000	-50.0000000
55250.0000000	10.7500000	-50.0000000
55250.0000000	11.0000000	-50.0000000
55250.0000000	11.2500000	-50.0000000
55250.0000000	11.5000000	-50.0000000
55250.0000000	11.7500000	-50.0000000
55250.0000000	12.0000000	-50.0000000

55250.0000000	12.2500000	-50.0000000
55250.0000000	12.5000000	-50.0000000
55250.0000000	12.7500000	-50.0000000
55250.0000000	13.0000000	-50.0000000
55250.0000000	13.2500000	-50.0000000
55250.0000000	13.5000000	-50.0000000
55250.0000000	13.7500000	-50.0000000
55250.0000000	14.0000000	-50.0000000
55250.0000000	14.2500000	-50.0000000
55250.0000000	14.5000000	-50.0000000
55250.0000000	14.7500000	-50.0000000
55250.0000000	15.0000000	-50.0000000
55280.0000000	0.0000000	-50.0000000
55280.0000000	0.2500000	-50.0000000
55280.0000000	0.5000000	23.1700001
55280.0000000	0.7500000	23.9599991
55280.0000000	1.0000000	21.1499996
55280.0000000	1.2500000	20.6000004
55280.0000000	1.5000000	19.3500004
55280.0000000	1.7500000	19.4699993
55280.0000000	2.0000000	17.3999996
55280.0000000	2.2500000	16.5200005
55280.0000000	2.5000000	17.6000004
55280.0000000	2.7500000	21.1200008
55280.0000000	3.0000000	21.4300003
55280.0000000	3.2500000	19.7399998
55280.0000000	3.5000000	18.5799999
55280.0000000	3.7500000	18.0699997
55280.0000000	4.0000000	19.6399994
55280.0000000	4.2500000	18.6800003
55280.0000000	4.5000000	19.2199993
55280.0000000	4.7500000	-50.0000000
55280.0000000	5.0000000	-50.0000000
55280.0000000	5.2500000	-50.0000000
55280.0000000	5.5000000	-50.0000000
55280.0000000	5.7500000	-50.0000000
55280.0000000	6.0000000	-50.0000000
55280.0000000	6.2500000	-50.0000000
55280.0000000	6.5000000	-50.0000000
55280.0000000	6.7500000	-50.0000000
55280.0000000	7.0000000	-50.0000000
55280.0000000	7.2500000	-50.0000000
55280.0000000	7.5000000	-50.0000000
55280.0000000	7.7500000	-50.0000000
55280.0000000	8.0000000	-50.0000000
55280.0000000	8.2500000	-50.0000000
55280.0000000	8.5000000	-50.0000000
55280.0000000	8.7500000	-50.0000000
55280.0000000	9.0000000	-50.0000000
55280.0000000	9.2500000	-50.0000000
55280.0000000	9.5000000	-50.0000000
55280.0000000	9.7500000	-50.0000000
55280.0000000	10.0000000	-50.0000000
55280.0000000	10.2500000	-50.0000000
55280.0000000	10.5000000	-50.0000000
55280.0000000	10.7500000	-50.0000000
55280.0000000	11.0000000	-50.0000000
55280.0000000	11.2500000	-50.0000000

55280.0000000	11.5000000	-50.0000000
55280.0000000	11.7500000	-50.0000000
55280.0000000	12.0000000	-50.0000000
55280.0000000	12.2500000	-50.0000000
55280.0000000	12.5000000	-50.0000000
55280.0000000	12.7500000	-50.0000000
55280.0000000	13.0000000	-50.0000000
55280.0000000	13.2500000	-50.0000000
55280.0000000	13.5000000	-50.0000000
55280.0000000	13.7500000	-50.0000000
55280.0000000	14.0000000	-50.0000000
55280.0000000	14.2500000	-50.0000000
55280.0000000	14.5000000	-50.0000000
55280.0000000	14.7500000	-50.0000000
55280.0000000	15.0000000	-50.0000000
55310.0000000	0.0000000	-50.0000000
55310.0000000	0.2500000	18.1299992
55310.0000000	0.5000000	27.5799999
55310.0000000	0.7500000	26.8700008
55310.0000000	1.0000000	19.2199993
55310.0000000	1.2500000	17.4599991
55310.0000000	1.5000000	18.1900005
55310.0000000	1.7500000	19.6800003
55310.0000000	2.0000000	20.0699997
55310.0000000	2.2500000	18.5300007
55310.0000000	2.5000000	18.8199997
55310.0000000	2.7500000	18.5599995
55310.0000000	3.0000000	18.3700008
55310.0000000	3.2500000	16.6800003
55310.0000000	3.5000000	21.9599991
55310.0000000	3.7500000	21.2399998
55310.0000000	4.0000000	18.7700005
55310.0000000	4.2500000	18.6499996
55310.0000000	4.5000000	19.2199993
55310.0000000	4.7500000	-50.0000000
55310.0000000	5.0000000	-50.0000000
55310.0000000	5.2500000	-50.0000000
55310.0000000	5.5000000	-50.0000000
55310.0000000	5.7500000	-50.0000000
55310.0000000	6.0000000	-50.0000000
55310.0000000	6.2500000	-50.0000000
55310.0000000	6.5000000	-50.0000000
55310.0000000	6.7500000	-50.0000000
55310.0000000	7.0000000	-50.0000000
55310.0000000	7.2500000	-50.0000000
55310.0000000	7.5000000	-50.0000000
55310.0000000	7.7500000	-50.0000000
55310.0000000	8.0000000	-50.0000000
55310.0000000	8.2500000	-50.0000000
55310.0000000	8.5000000	-50.0000000
55310.0000000	8.7500000	-50.0000000
55310.0000000	9.0000000	-50.0000000
55310.0000000	9.2500000	-50.0000000
55310.0000000	9.5000000	-50.0000000
55310.0000000	9.7500000	-50.0000000
55310.0000000	10.0000000	-50.0000000
55310.0000000	10.2500000	-50.0000000
55310.0000000	10.5000000	-50.0000000

55310.0000000	10.7500000	-50.0000000
55310.0000000	11.0000000	-50.0000000
55310.0000000	11.2500000	-50.0000000
55310.0000000	11.5000000	-50.0000000
55310.0000000	11.7500000	-50.0000000
55310.0000000	12.0000000	-50.0000000
55310.0000000	12.2500000	-50.0000000
55310.0000000	12.5000000	-50.0000000
55310.0000000	12.7500000	-50.0000000
55310.0000000	13.0000000	-50.0000000
55310.0000000	13.2500000	-50.0000000
55310.0000000	13.5000000	-50.0000000
55310.0000000	13.7500000	-50.0000000
55310.0000000	14.0000000	-50.0000000
55310.0000000	14.2500000	-50.0000000
55310.0000000	14.5000000	-50.0000000
55310.0000000	14.7500000	-50.0000000
55310.0000000	15.0000000	-50.0000000
55340.0000000	0.0000000	-50.0000000
55340.0000000	0.2500000	22.3899994
55340.0000000	0.5000000	28.8700008
55340.0000000	0.7500000	26.9200001
55340.0000000	1.0000000	20.0100002
55340.0000000	1.2500000	21.1000004
55340.0000000	1.5000000	21.2099991
55340.0000000	1.7500000	17.1700001
55340.0000000	2.0000000	18.1100006
55340.0000000	2.2500000	18.7399998
55340.0000000	2.5000000	20.4200001
55340.0000000	2.7500000	20.8400002
55340.0000000	3.0000000	21.4099998
55340.0000000	3.2500000	17.6800003
55340.0000000	3.5000000	19.0799999
55340.0000000	3.7500000	19.4400005
55340.0000000	4.0000000	17.4699993
55340.0000000	4.2500000	15.4499998
55340.0000000	4.5000000	15.5200005
55340.0000000	4.7500000	15.9799995
55340.0000000	5.0000000	-50.0000000
55340.0000000	5.2500000	-50.0000000
55340.0000000	5.5000000	-50.0000000
55340.0000000	5.7500000	-50.0000000
55340.0000000	6.0000000	-50.0000000
55340.0000000	6.2500000	-50.0000000
55340.0000000	6.5000000	-50.0000000
55340.0000000	6.7500000	-50.0000000
55340.0000000	7.0000000	-50.0000000
55340.0000000	7.2500000	-50.0000000
55340.0000000	7.5000000	-50.0000000
55340.0000000	7.7500000	-50.0000000
55340.0000000	8.0000000	-50.0000000
55340.0000000	8.2500000	-50.0000000
55340.0000000	8.5000000	-50.0000000
55340.0000000	8.7500000	-50.0000000
55340.0000000	9.0000000	-50.0000000
55340.0000000	9.2500000	-50.0000000
55340.0000000	9.5000000	-50.0000000
55340.0000000	9.7500000	-50.0000000

55340.0000000	10.0000000	-50.0000000
55340.0000000	10.2500000	-50.0000000
55340.0000000	10.5000000	-50.0000000
55340.0000000	10.7500000	-50.0000000
55340.0000000	11.0000000	-50.0000000
55340.0000000	11.2500000	-50.0000000
55340.0000000	11.5000000	-50.0000000
55340.0000000	11.7500000	-50.0000000
55340.0000000	12.0000000	-50.0000000
55340.0000000	12.2500000	-50.0000000
55340.0000000	12.5000000	-50.0000000
55340.0000000	12.7500000	-50.0000000
55340.0000000	13.0000000	-50.0000000
55340.0000000	13.2500000	-50.0000000
55340.0000000	13.5000000	-50.0000000
55340.0000000	13.7500000	-50.0000000
55340.0000000	14.0000000	-50.0000000
55340.0000000	14.2500000	-50.0000000
55340.0000000	14.5000000	-50.0000000
55340.0000000	14.7500000	-50.0000000
55340.0000000	15.0000000	-50.0000000
55370.0000000	0.0000000	-50.0000000
55370.0000000	0.2500000	19.5000000
55370.0000000	0.5000000	28.7600002
55370.0000000	0.7500000	28.1200008
55370.0000000	1.0000000	18.4300003
55370.0000000	1.2500000	18.2000008
55370.0000000	1.5000000	20.0599995
55370.0000000	1.7500000	20.5200005
55370.0000000	2.0000000	19.1499996
55370.0000000	2.2500000	18.7299995
55370.0000000	2.5000000	21.6100006
55370.0000000	2.7500000	22.7600002
55370.0000000	3.0000000	21.3899994
55370.0000000	3.2500000	19.2800007
55370.0000000	3.5000000	18.3299999
55370.0000000	3.7500000	17.5499992
55370.0000000	4.0000000	15.8000002
55370.0000000	4.2500000	16.4899998
55370.0000000	4.5000000	17.6299992
55370.0000000	4.7500000	-50.0000000
55370.0000000	5.0000000	-50.0000000
55370.0000000	5.2500000	-50.0000000
55370.0000000	5.5000000	-50.0000000
55370.0000000	5.7500000	-50.0000000
55370.0000000	6.0000000	-50.0000000
55370.0000000	6.2500000	-50.0000000
55370.0000000	6.5000000	-50.0000000
55370.0000000	6.7500000	-50.0000000
55370.0000000	7.0000000	-50.0000000
55370.0000000	7.2500000	-50.0000000
55370.0000000	7.5000000	-50.0000000
55370.0000000	7.7500000	-50.0000000
55370.0000000	8.0000000	-50.0000000
55370.0000000	8.2500000	-50.0000000
55370.0000000	8.5000000	-50.0000000
55370.0000000	8.7500000	-50.0000000
55370.0000000	9.0000000	-50.0000000

55370.0000000	9.2500000	-50.0000000
55370.0000000	9.5000000	-50.0000000
55370.0000000	9.7500000	-50.0000000
55370.0000000	10.0000000	-50.0000000
55370.0000000	10.2500000	-50.0000000
55370.0000000	10.5000000	-50.0000000
55370.0000000	10.7500000	-50.0000000
55370.0000000	11.0000000	-50.0000000
55370.0000000	11.2500000	-50.0000000
55370.0000000	11.5000000	-50.0000000
55370.0000000	11.7500000	-50.0000000
55370.0000000	12.0000000	-50.0000000
55370.0000000	12.2500000	-50.0000000
55370.0000000	12.5000000	-50.0000000
55370.0000000	12.7500000	-50.0000000
55370.0000000	13.0000000	-50.0000000
55370.0000000	13.2500000	-50.0000000
55370.0000000	13.5000000	-50.0000000
55370.0000000	13.7500000	-50.0000000
55370.0000000	14.0000000	-50.0000000
55370.0000000	14.2500000	-50.0000000
55370.0000000	14.5000000	-50.0000000
55370.0000000	14.7500000	-50.0000000
55370.0000000	15.0000000	-50.0000000
55400.0000000	0.0000000	-50.0000000
55400.0000000	0.2500000	18.5000000
55400.0000000	0.5000000	27.2500000
55400.0000000	0.7500000	27.0100002
55400.0000000	1.0000000	22.3799992
55400.0000000	1.2500000	21.0499992
55400.0000000	1.5000000	19.1100006
55400.0000000	1.7500000	19.6299992
55400.0000000	2.0000000	19.8299999
55400.0000000	2.2500000	19.3700008
55400.0000000	2.5000000	19.8700008
55400.0000000	2.7500000	20.6900005
55400.0000000	3.0000000	19.3299999
55400.0000000	3.2500000	17.1800003
55400.0000000	3.5000000	17.8099995
55400.0000000	3.7500000	20.8199997
55400.0000000	4.0000000	18.0000000
55400.0000000	4.2500000	17.0200005
55400.0000000	4.5000000	17.6399994
55400.0000000	4.7500000	16.8199997
55400.0000000	5.0000000	-50.0000000
55400.0000000	5.2500000	-50.0000000
55400.0000000	5.5000000	-50.0000000
55400.0000000	5.7500000	-50.0000000
55400.0000000	6.0000000	-50.0000000
55400.0000000	6.2500000	-50.0000000
55400.0000000	6.5000000	-50.0000000
55400.0000000	6.7500000	-50.0000000
55400.0000000	7.0000000	-50.0000000
55400.0000000	7.2500000	-50.0000000
55400.0000000	7.5000000	-50.0000000
55400.0000000	7.7500000	-50.0000000
55400.0000000	8.0000000	-50.0000000
55400.0000000	8.2500000	-50.0000000

55400.0000000	8.5000000	-50.0000000
55400.0000000	8.7500000	-50.0000000
55400.0000000	9.0000000	-50.0000000
55400.0000000	9.2500000	-50.0000000
55400.0000000	9.5000000	-50.0000000
55400.0000000	9.7500000	-50.0000000
55400.0000000	10.0000000	-50.0000000
55400.0000000	10.2500000	-50.0000000
55400.0000000	10.5000000	-50.0000000
55400.0000000	10.7500000	-50.0000000
55400.0000000	11.0000000	-50.0000000
55400.0000000	11.2500000	-50.0000000
55400.0000000	11.5000000	-50.0000000
55400.0000000	11.7500000	-50.0000000
55400.0000000	12.0000000	-50.0000000
55400.0000000	12.2500000	-50.0000000
55400.0000000	12.5000000	-50.0000000
55400.0000000	12.7500000	-50.0000000
55400.0000000	13.0000000	-50.0000000
55400.0000000	13.2500000	-50.0000000
55400.0000000	13.5000000	-50.0000000
55400.0000000	13.7500000	-50.0000000
55400.0000000	14.0000000	-50.0000000
55400.0000000	14.2500000	-50.0000000
55400.0000000	14.5000000	-50.0000000
55400.0000000	14.7500000	-50.0000000
55400.0000000	15.0000000	-50.0000000
55430.0000000	0.0000000	-50.0000000
55430.0000000	0.2500000	18.8400002
55430.0000000	0.5000000	28.8199997
55430.0000000	0.7500000	28.6800003
55430.0000000	1.0000000	18.7700005
55430.0000000	1.2500000	15.8599997
55430.0000000	1.5000000	18.6599998
55430.0000000	1.7500000	20.1499996
55430.0000000	2.0000000	19.5200005
55430.0000000	2.2500000	21.3799992
55430.0000000	2.5000000	21.1399994
55430.0000000	2.7500000	17.9300003
55430.0000000	3.0000000	19.2299995
55430.0000000	3.2500000	20.8799992
55430.0000000	3.5000000	19.9300003
55430.0000000	3.7500000	19.9300003
55430.0000000	4.0000000	21.1000004
55430.0000000	4.2500000	18.6000004
55430.0000000	4.5000000	18.3600006
55430.0000000	4.7500000	15.7100000
55430.0000000	5.0000000	-50.0000000
55430.0000000	5.2500000	-50.0000000
55430.0000000	5.5000000	-50.0000000
55430.0000000	5.7500000	-50.0000000
55430.0000000	6.0000000	-50.0000000
55430.0000000	6.2500000	-50.0000000
55430.0000000	6.5000000	-50.0000000
55430.0000000	6.7500000	-50.0000000
55430.0000000	7.0000000	-50.0000000
55430.0000000	7.2500000	-50.0000000
55430.0000000	7.5000000	-50.0000000

55430.0000000	7.7500000	-50.0000000
55430.0000000	8.0000000	-50.0000000
55430.0000000	8.2500000	-50.0000000
55430.0000000	8.5000000	-50.0000000
55430.0000000	8.7500000	-50.0000000
55430.0000000	9.0000000	-50.0000000
55430.0000000	9.2500000	-50.0000000
55430.0000000	9.5000000	-50.0000000
55430.0000000	9.7500000	-50.0000000
55430.0000000	10.0000000	-50.0000000
55430.0000000	10.2500000	-50.0000000
55430.0000000	10.5000000	-50.0000000
55430.0000000	10.7500000	-50.0000000
55430.0000000	11.0000000	-50.0000000
55430.0000000	11.2500000	-50.0000000
55430.0000000	11.5000000	-50.0000000
55430.0000000	11.7500000	-50.0000000
55430.0000000	12.0000000	-50.0000000
55430.0000000	12.2500000	-50.0000000
55430.0000000	12.5000000	-50.0000000
55430.0000000	12.7500000	-50.0000000
55430.0000000	13.0000000	-50.0000000
55430.0000000	13.2500000	-50.0000000
55430.0000000	13.5000000	-50.0000000
55430.0000000	13.7500000	-50.0000000
55430.0000000	14.0000000	-50.0000000
55430.0000000	14.2500000	-50.0000000
55430.0000000	14.5000000	-50.0000000
55430.0000000	14.7500000	-50.0000000
55430.0000000	15.0000000	-50.0000000
55460.0000000	0.0000000	-50.0000000
55460.0000000	0.2500000	16.0200005
55460.0000000	0.5000000	25.5300007
55460.0000000	0.7500000	26.3700008
55460.0000000	1.0000000	20.4300003
55460.0000000	1.2500000	18.3199997
55460.0000000	1.5000000	20.4200001
55460.0000000	1.7500000	18.8400002
55460.0000000	2.0000000	19.8199997
55460.0000000	2.2500000	19.1900005
55460.0000000	2.5000000	19.4699993
55460.0000000	2.7500000	20.8600006
55460.0000000	3.0000000	21.9300003
55460.0000000	3.2500000	20.2900009
55460.0000000	3.5000000	19.2900009
55460.0000000	3.7500000	19.0100002
55460.0000000	4.0000000	18.2600002
55460.0000000	4.2500000	16.3500004
55460.0000000	4.5000000	18.5799999
55460.0000000	4.7500000	-50.0000000
55460.0000000	5.0000000	-50.0000000
55460.0000000	5.2500000	-50.0000000
55460.0000000	5.5000000	-50.0000000
55460.0000000	5.7500000	-50.0000000
55460.0000000	6.0000000	-50.0000000
55460.0000000	6.2500000	-50.0000000
55460.0000000	6.5000000	-50.0000000
55460.0000000	6.7500000	-50.0000000

55460.0000000	7.0000000	-50.0000000
55460.0000000	7.2500000	-50.0000000
55460.0000000	7.5000000	-50.0000000
55460.0000000	7.7500000	-50.0000000
55460.0000000	8.0000000	-50.0000000
55460.0000000	8.2500000	-50.0000000
55460.0000000	8.5000000	-50.0000000
55460.0000000	8.7500000	-50.0000000
55460.0000000	9.0000000	-50.0000000
55460.0000000	9.2500000	-50.0000000
55460.0000000	9.5000000	-50.0000000
55460.0000000	9.7500000	-50.0000000
55460.0000000	10.0000000	-50.0000000
55460.0000000	10.2500000	-50.0000000
55460.0000000	10.5000000	-50.0000000
55460.0000000	10.7500000	-50.0000000
55460.0000000	11.0000000	-50.0000000
55460.0000000	11.2500000	-50.0000000
55460.0000000	11.5000000	-50.0000000
55460.0000000	11.7500000	-50.0000000
55460.0000000	12.0000000	-50.0000000
55460.0000000	12.2500000	-50.0000000
55460.0000000	12.5000000	-50.0000000
55460.0000000	12.7500000	-50.0000000
55460.0000000	13.0000000	-50.0000000
55460.0000000	13.2500000	-50.0000000
55460.0000000	13.5000000	-50.0000000
55460.0000000	13.7500000	-50.0000000
55460.0000000	14.0000000	-50.0000000
55460.0000000	14.2500000	-50.0000000
55460.0000000	14.5000000	-50.0000000
55460.0000000	14.7500000	-50.0000000
55460.0000000	15.0000000	-50.0000000
55490.0000000	0.0000000	-50.0000000
55490.0000000	0.2500000	18.9799995
55490.0000000	0.5000000	27.7000008
55490.0000000	0.7500000	27.0499992
55490.0000000	1.0000000	19.9699993
55490.0000000	1.2500000	17.8999996
55490.0000000	1.5000000	18.5300007
55490.0000000	1.7500000	18.1599998
55490.0000000	2.0000000	19.1800003
55490.0000000	2.2500000	19.1200008
55490.0000000	2.5000000	18.6700001
55490.0000000	2.7500000	16.3500004
55490.0000000	3.0000000	17.0200005
55490.0000000	3.2500000	20.4099998
55490.0000000	3.5000000	19.2700005
55490.0000000	3.7500000	16.9599991
55490.0000000	4.0000000	18.5100002
55490.0000000	4.2500000	18.0799999
55490.0000000	4.5000000	18.5100002
55490.0000000	4.7500000	15.2100000
55490.0000000	5.0000000	-50.0000000
55490.0000000	5.2500000	-50.0000000
55490.0000000	5.5000000	-50.0000000
55490.0000000	5.7500000	-50.0000000
55490.0000000	6.0000000	-50.0000000

55490.0000000	6.2500000	-50.0000000
55490.0000000	6.5000000	-50.0000000
55490.0000000	6.7500000	-50.0000000
55490.0000000	7.0000000	-50.0000000
55490.0000000	7.2500000	-50.0000000
55490.0000000	7.5000000	-50.0000000
55490.0000000	7.7500000	-50.0000000
55490.0000000	8.0000000	-50.0000000
55490.0000000	8.2500000	-50.0000000
55490.0000000	8.5000000	-50.0000000
55490.0000000	8.7500000	-50.0000000
55490.0000000	9.0000000	-50.0000000
55490.0000000	9.2500000	-50.0000000
55490.0000000	9.5000000	-50.0000000
55490.0000000	9.7500000	-50.0000000
55490.0000000	10.0000000	-50.0000000
55490.0000000	10.2500000	-50.0000000
55490.0000000	10.5000000	-50.0000000
55490.0000000	10.7500000	-50.0000000
55490.0000000	11.0000000	-50.0000000
55490.0000000	11.2500000	-50.0000000
55490.0000000	11.5000000	-50.0000000
55490.0000000	11.7500000	-50.0000000
55490.0000000	12.0000000	-50.0000000
55490.0000000	12.2500000	-50.0000000
55490.0000000	12.5000000	-50.0000000
55490.0000000	12.7500000	-50.0000000
55490.0000000	13.0000000	-50.0000000
55490.0000000	13.2500000	-50.0000000
55490.0000000	13.5000000	-50.0000000
55490.0000000	13.7500000	-50.0000000
55490.0000000	14.0000000	-50.0000000
55490.0000000	14.2500000	-50.0000000
55490.0000000	14.5000000	-50.0000000
55490.0000000	14.7500000	-50.0000000
55490.0000000	15.0000000	-50.0000000
55520.0000000	0.0000000	-50.0000000
55520.0000000	0.2500000	16.8700008
55520.0000000	0.5000000	25.6000004
55520.0000000	0.7500000	25.0400009
55520.0000000	1.0000000	18.9899998
55520.0000000	1.2500000	18.2299995
55520.0000000	1.5000000	18.2000008
55520.0000000	1.7500000	19.4799995
55520.0000000	2.0000000	20.0000000
55520.0000000	2.2500000	19.6299992
55520.0000000	2.5000000	21.5799999
55520.0000000	2.7500000	21.2299995
55520.0000000	3.0000000	18.7199993
55520.0000000	3.2500000	17.7600002
55520.0000000	3.5000000	19.1499996
55520.0000000	3.7500000	18.8899994
55520.0000000	4.0000000	18.2399998
55520.0000000	4.2500000	15.9399996
55520.0000000	4.5000000	17.0699997
55520.0000000	4.7500000	16.7800007
55520.0000000	5.0000000	-50.0000000
55520.0000000	5.2500000	-50.0000000

55520.0000000	5.5000000	-50.0000000
55520.0000000	5.7500000	-50.0000000
55520.0000000	6.0000000	-50.0000000
55520.0000000	6.2500000	-50.0000000
55520.0000000	6.5000000	-50.0000000
55520.0000000	6.7500000	-50.0000000
55520.0000000	7.0000000	-50.0000000
55520.0000000	7.2500000	-50.0000000
55520.0000000	7.5000000	-50.0000000
55520.0000000	7.7500000	-50.0000000
55520.0000000	8.0000000	-50.0000000
55520.0000000	8.2500000	-50.0000000
55520.0000000	8.5000000	-50.0000000
55520.0000000	8.7500000	-50.0000000
55520.0000000	9.0000000	-50.0000000
55520.0000000	9.2500000	-50.0000000
55520.0000000	9.5000000	-50.0000000
55520.0000000	9.7500000	-50.0000000
55520.0000000	10.0000000	-50.0000000
55520.0000000	10.2500000	-50.0000000
55520.0000000	10.5000000	-50.0000000
55520.0000000	10.7500000	-50.0000000
55520.0000000	11.0000000	-50.0000000
55520.0000000	11.2500000	-50.0000000
55520.0000000	11.5000000	-50.0000000
55520.0000000	11.7500000	-50.0000000
55520.0000000	12.0000000	-50.0000000
55520.0000000	12.2500000	-50.0000000
55520.0000000	12.5000000	-50.0000000
55520.0000000	12.7500000	-50.0000000
55520.0000000	13.0000000	-50.0000000
55520.0000000	13.2500000	-50.0000000
55520.0000000	13.5000000	-50.0000000
55520.0000000	13.7500000	-50.0000000
55520.0000000	14.0000000	-50.0000000
55520.0000000	14.2500000	-50.0000000
55520.0000000	14.5000000	-50.0000000
55520.0000000	14.7500000	-50.0000000
55520.0000000	15.0000000	-50.0000000
55550.0000000	0.0000000	-50.0000000
55550.0000000	0.2500000	17.7299995
55550.0000000	0.5000000	24.7399998
55550.0000000	0.7500000	24.1900005
55550.0000000	1.0000000	18.4500008
55550.0000000	1.2500000	19.2500000
55550.0000000	1.5000000	19.0499992
55550.0000000	1.7500000	20.6599998
55550.0000000	2.0000000	20.7999992
55550.0000000	2.2500000	17.7900009
55550.0000000	2.5000000	17.2600002
55550.0000000	2.7500000	20.1499996
55550.0000000	3.0000000	21.1499996
55550.0000000	3.2500000	19.1399994
55550.0000000	3.5000000	19.8299999
55550.0000000	3.7500000	19.2800007
55550.0000000	4.0000000	18.9400005
55550.0000000	4.2500000	18.2500000
55550.0000000	4.5000000	-50.0000000

55550.0000000	4.7500000	-50.0000000
55550.0000000	5.0000000	-50.0000000
55550.0000000	5.2500000	-50.0000000
55550.0000000	5.5000000	-50.0000000
55550.0000000	5.7500000	-50.0000000
55550.0000000	6.0000000	-50.0000000
55550.0000000	6.2500000	-50.0000000
55550.0000000	6.5000000	-50.0000000
55550.0000000	6.7500000	-50.0000000
55550.0000000	7.0000000	-50.0000000
55550.0000000	7.2500000	-50.0000000
55550.0000000	7.5000000	-50.0000000
55550.0000000	7.7500000	-50.0000000
55550.0000000	8.0000000	-50.0000000
55550.0000000	8.2500000	-50.0000000
55550.0000000	8.5000000	-50.0000000
55550.0000000	8.7500000	-50.0000000
55550.0000000	9.0000000	-50.0000000
55550.0000000	9.2500000	-50.0000000
55550.0000000	9.5000000	-50.0000000
55550.0000000	9.7500000	-50.0000000
55550.0000000	10.0000000	-50.0000000
55550.0000000	10.2500000	-50.0000000
55550.0000000	10.5000000	-50.0000000
55550.0000000	10.7500000	-50.0000000
55550.0000000	11.0000000	-50.0000000
55550.0000000	11.2500000	-50.0000000
55550.0000000	11.5000000	-50.0000000
55550.0000000	11.7500000	-50.0000000
55550.0000000	12.0000000	-50.0000000
55550.0000000	12.2500000	-50.0000000
55550.0000000	12.5000000	-50.0000000
55550.0000000	12.7500000	-50.0000000
55550.0000000	13.0000000	-50.0000000
55550.0000000	13.2500000	-50.0000000
55550.0000000	13.5000000	-50.0000000
55550.0000000	13.7500000	-50.0000000
55550.0000000	14.0000000	-50.0000000
55550.0000000	14.2500000	-50.0000000
55550.0000000	14.5000000	-50.0000000
55550.0000000	14.7500000	-50.0000000
55550.0000000	15.0000000	-50.0000000
55580.0000000	0.0000000	-50.0000000
55580.0000000	0.2500000	20.7900009
55580.0000000	0.5000000	28.1800003
55580.0000000	0.7500000	25.5499992
55580.0000000	1.0000000	15.9099998
55580.0000000	1.2500000	20.5400009
55580.0000000	1.5000000	20.3500004
55580.0000000	1.7500000	18.5100002
55580.0000000	2.0000000	19.7000008
55580.0000000	2.2500000	19.7999992
55580.0000000	2.5000000	19.2700005
55580.0000000	2.7500000	17.7999992
55580.0000000	3.0000000	20.8700008
55580.0000000	3.2500000	22.1900005
55580.0000000	3.5000000	20.9400005
55580.0000000	3.7500000	19.0699997

55580.0000000	4.0000000	19.0599995
55580.0000000	4.2500000	18.3899994
55580.0000000	4.5000000	15.2500000
55580.0000000	4.7500000	-50.0000000
55580.0000000	5.0000000	-50.0000000
55580.0000000	5.2500000	-50.0000000
55580.0000000	5.5000000	-50.0000000
55580.0000000	5.7500000	-50.0000000
55580.0000000	6.0000000	-50.0000000
55580.0000000	6.2500000	-50.0000000
55580.0000000	6.5000000	-50.0000000
55580.0000000	6.7500000	-50.0000000
55580.0000000	7.0000000	-50.0000000
55580.0000000	7.2500000	-50.0000000
55580.0000000	7.5000000	-50.0000000
55580.0000000	7.7500000	-50.0000000
55580.0000000	8.0000000	-50.0000000
55580.0000000	8.2500000	-50.0000000
55580.0000000	8.5000000	-50.0000000
55580.0000000	8.7500000	-50.0000000
55580.0000000	9.0000000	-50.0000000
55580.0000000	9.2500000	-50.0000000
55580.0000000	9.5000000	-50.0000000
55580.0000000	9.7500000	-50.0000000
55580.0000000	10.0000000	-50.0000000
55580.0000000	10.2500000	-50.0000000
55580.0000000	10.5000000	-50.0000000
55580.0000000	10.7500000	-50.0000000
55580.0000000	11.0000000	-50.0000000
55580.0000000	11.2500000	-50.0000000
55580.0000000	11.5000000	-50.0000000
55580.0000000	11.7500000	-50.0000000
55580.0000000	12.0000000	-50.0000000
55580.0000000	12.2500000	-50.0000000
55580.0000000	12.5000000	-50.0000000
55580.0000000	12.7500000	-50.0000000
55580.0000000	13.0000000	-50.0000000
55580.0000000	13.2500000	-50.0000000
55580.0000000	13.5000000	-50.0000000
55580.0000000	13.7500000	-50.0000000
55580.0000000	14.0000000	-50.0000000
55580.0000000	14.2500000	-50.0000000
55580.0000000	14.5000000	-50.0000000
55580.0000000	14.7500000	-50.0000000
55580.0000000	15.0000000	-50.0000000
55610.0000000	0.0000000	-50.0000000
55610.0000000	0.2500000	23.7399998
55610.0000000	0.5000000	30.9400005
55610.0000000	0.7500000	28.6100006
55610.0000000	1.0000000	19.7199993
55610.0000000	1.2500000	16.6299992
55610.0000000	1.5000000	19.2999992
55610.0000000	1.7500000	19.7299995
55610.0000000	2.0000000	20.6200008
55610.0000000	2.2500000	20.5100002
55610.0000000	2.5000000	19.5100002
55610.0000000	2.7500000	20.3299999
55610.0000000	3.0000000	21.0699997

55610.0000000	3.2500000	18.1700001
55610.0000000	3.5000000	16.6800003
55610.0000000	3.7500000	15.4600000
55610.0000000	4.0000000	17.8799992
55610.0000000	4.2500000	16.3799992
55610.0000000	4.5000000	15.3800001
55610.0000000	4.7500000	-50.0000000
55610.0000000	5.0000000	-50.0000000
55610.0000000	5.2500000	-50.0000000
55610.0000000	5.5000000	-50.0000000
55610.0000000	5.7500000	-50.0000000
55610.0000000	6.0000000	-50.0000000
55610.0000000	6.2500000	-50.0000000
55610.0000000	6.5000000	-50.0000000
55610.0000000	6.7500000	-50.0000000
55610.0000000	7.0000000	-50.0000000
55610.0000000	7.2500000	-50.0000000
55610.0000000	7.5000000	-50.0000000
55610.0000000	7.7500000	-50.0000000
55610.0000000	8.0000000	-50.0000000
55610.0000000	8.2500000	-50.0000000
55610.0000000	8.5000000	-50.0000000
55610.0000000	8.7500000	-50.0000000
55610.0000000	9.0000000	-50.0000000
55610.0000000	9.2500000	-50.0000000
55610.0000000	9.5000000	-50.0000000
55610.0000000	9.7500000	-50.0000000
55610.0000000	10.0000000	-50.0000000
55610.0000000	10.2500000	-50.0000000
55610.0000000	10.5000000	-50.0000000
55610.0000000	10.7500000	-50.0000000
55610.0000000	11.0000000	-50.0000000
55610.0000000	11.2500000	-50.0000000
55610.0000000	11.5000000	-50.0000000
55610.0000000	11.7500000	-50.0000000
55610.0000000	12.0000000	-50.0000000
55610.0000000	12.2500000	-50.0000000
55610.0000000	12.5000000	-50.0000000
55610.0000000	12.7500000	-50.0000000
55610.0000000	13.0000000	-50.0000000
55610.0000000	13.2500000	-50.0000000
55610.0000000	13.5000000	-50.0000000
55610.0000000	13.7500000	-50.0000000
55610.0000000	14.0000000	-50.0000000
55610.0000000	14.2500000	-50.0000000
55610.0000000	14.5000000	-50.0000000
55610.0000000	14.7500000	-50.0000000
55610.0000000	15.0000000	-50.0000000
55640.0000000	0.0000000	-50.0000000
55640.0000000	0.2500000	20.6800003
55640.0000000	0.5000000	28.7999992
55640.0000000	0.7500000	27.2299995
55640.0000000	1.0000000	18.8700008
55640.0000000	1.2500000	18.3700008
55640.0000000	1.5000000	19.0400009
55640.0000000	1.7500000	20.6900005
55640.0000000	2.0000000	20.2800007
55640.0000000	2.2500000	20.0300007

55640.0000000	2.5000000	20.0400009
55640.0000000	2.7500000	17.1200008
55640.0000000	3.0000000	17.9200001
55640.0000000	3.2500000	18.6000004
55640.0000000	3.5000000	17.1200008
55640.0000000	3.7500000	18.3400002
55640.0000000	4.0000000	19.6700001
55640.0000000	4.2500000	16.7299995
55640.0000000	4.5000000	19.5699997
55640.0000000	4.7500000	-50.0000000
55640.0000000	5.0000000	-50.0000000
55640.0000000	5.2500000	-50.0000000
55640.0000000	5.5000000	-50.0000000
55640.0000000	5.7500000	-50.0000000
55640.0000000	6.0000000	-50.0000000
55640.0000000	6.2500000	-50.0000000
55640.0000000	6.5000000	-50.0000000
55640.0000000	6.7500000	-50.0000000
55640.0000000	7.0000000	-50.0000000
55640.0000000	7.2500000	-50.0000000
55640.0000000	7.5000000	-50.0000000
55640.0000000	7.7500000	-50.0000000
55640.0000000	8.0000000	-50.0000000
55640.0000000	8.2500000	-50.0000000
55640.0000000	8.5000000	-50.0000000
55640.0000000	8.7500000	-50.0000000
55640.0000000	9.0000000	-50.0000000
55640.0000000	9.2500000	-50.0000000
55640.0000000	9.5000000	-50.0000000
55640.0000000	9.7500000	-50.0000000
55640.0000000	10.0000000	-50.0000000
55640.0000000	10.2500000	-50.0000000
55640.0000000	10.5000000	-50.0000000
55640.0000000	10.7500000	-50.0000000
55640.0000000	11.0000000	-50.0000000
55640.0000000	11.2500000	-50.0000000
55640.0000000	11.5000000	-50.0000000
55640.0000000	11.7500000	-50.0000000
55640.0000000	12.0000000	-50.0000000
55640.0000000	12.2500000	-50.0000000
55640.0000000	12.5000000	-50.0000000
55640.0000000	12.7500000	-50.0000000
55640.0000000	13.0000000	-50.0000000
55640.0000000	13.2500000	-50.0000000
55640.0000000	13.5000000	-50.0000000
55640.0000000	13.7500000	-50.0000000
55640.0000000	14.0000000	-50.0000000
55640.0000000	14.2500000	-50.0000000
55640.0000000	14.5000000	-50.0000000
55640.0000000	14.7500000	-50.0000000
55640.0000000	15.0000000	-50.0000000
55670.0000000	0.0000000	-50.0000000
55670.0000000	0.2500000	21.1800003
55670.0000000	0.5000000	27.0900002
55670.0000000	0.7500000	24.1299992
55670.0000000	1.0000000	18.2900009
55670.0000000	1.2500000	16.5200005
55670.0000000	1.5000000	18.9500008

55670.0000000	1.7500000	20.2999992
55670.0000000	2.0000000	20.9200001
55670.0000000	2.2500000	19.4300003
55670.0000000	2.5000000	21.0000000
55670.0000000	2.7500000	23.8600006
55670.0000000	3.0000000	20.1499996
55670.0000000	3.2500000	19.5400009
55670.0000000	3.5000000	18.3199997
55670.0000000	3.7500000	18.2399998
55670.0000000	4.0000000	18.1100006
55670.0000000	4.2500000	17.5100002
55670.0000000	4.5000000	18.0000000
55670.0000000	4.7500000	-50.0000000
55670.0000000	5.0000000	-50.0000000
55670.0000000	5.2500000	-50.0000000
55670.0000000	5.5000000	-50.0000000
55670.0000000	5.7500000	-50.0000000
55670.0000000	6.0000000	-50.0000000
55670.0000000	6.2500000	-50.0000000
55670.0000000	6.5000000	-50.0000000
55670.0000000	6.7500000	-50.0000000
55670.0000000	7.0000000	-50.0000000
55670.0000000	7.2500000	-50.0000000
55670.0000000	7.5000000	-50.0000000
55670.0000000	7.7500000	-50.0000000
55670.0000000	8.0000000	-50.0000000
55670.0000000	8.2500000	-50.0000000
55670.0000000	8.5000000	-50.0000000
55670.0000000	8.7500000	-50.0000000
55670.0000000	9.0000000	-50.0000000
55670.0000000	9.2500000	-50.0000000
55670.0000000	9.5000000	-50.0000000
55670.0000000	9.7500000	-50.0000000
55670.0000000	10.0000000	-50.0000000
55670.0000000	10.2500000	-50.0000000
55670.0000000	10.5000000	-50.0000000
55670.0000000	10.7500000	-50.0000000
55670.0000000	11.0000000	-50.0000000
55670.0000000	11.2500000	-50.0000000
55670.0000000	11.5000000	-50.0000000
55670.0000000	11.7500000	-50.0000000
55670.0000000	12.0000000	-50.0000000
55670.0000000	12.2500000	-50.0000000
55670.0000000	12.5000000	-50.0000000
55670.0000000	12.7500000	-50.0000000
55670.0000000	13.0000000	-50.0000000
55670.0000000	13.2500000	-50.0000000
55670.0000000	13.5000000	-50.0000000
55670.0000000	13.7500000	-50.0000000
55670.0000000	14.0000000	-50.0000000
55670.0000000	14.2500000	-50.0000000
55670.0000000	14.5000000	-50.0000000
55670.0000000	14.7500000	-50.0000000
55670.0000000	15.0000000	-50.0000000
55700.0000000	0.0000000	-50.0000000
55700.0000000	0.2500000	17.7500000
55700.0000000	0.5000000	26.1599998
55700.0000000	0.7500000	25.3700008

55700.0000000	1.0000000	22.7600002
55700.0000000	1.2500000	22.5799999
55700.0000000	1.5000000	19.3199997
55700.0000000	1.7500000	18.2999992
55700.0000000	2.0000000	19.2900009
55700.0000000	2.2500000	20.3500004
55700.0000000	2.5000000	20.2000008
55700.0000000	2.7500000	22.3099995
55700.0000000	3.0000000	19.7700005
55700.0000000	3.2500000	17.9400005
55700.0000000	3.5000000	16.5400009
55700.0000000	3.7500000	17.5799999
55700.0000000	4.0000000	17.7800007
55700.0000000	4.2500000	20.3099995
55700.0000000	4.5000000	-50.0000000
55700.0000000	4.7500000	-50.0000000
55700.0000000	5.0000000	-50.0000000
55700.0000000	5.2500000	-50.0000000
55700.0000000	5.5000000	-50.0000000
55700.0000000	5.7500000	-50.0000000
55700.0000000	6.0000000	-50.0000000
55700.0000000	6.2500000	-50.0000000
55700.0000000	6.5000000	-50.0000000
55700.0000000	6.7500000	-50.0000000
55700.0000000	7.0000000	-50.0000000
55700.0000000	7.2500000	-50.0000000
55700.0000000	7.5000000	-50.0000000
55700.0000000	7.7500000	-50.0000000
55700.0000000	8.0000000	-50.0000000
55700.0000000	8.2500000	-50.0000000
55700.0000000	8.5000000	-50.0000000
55700.0000000	8.7500000	-50.0000000
55700.0000000	9.0000000	-50.0000000
55700.0000000	9.2500000	-50.0000000
55700.0000000	9.5000000	-50.0000000
55700.0000000	9.7500000	-50.0000000
55700.0000000	10.0000000	-50.0000000
55700.0000000	10.2500000	-50.0000000
55700.0000000	10.5000000	-50.0000000
55700.0000000	10.7500000	-50.0000000
55700.0000000	11.0000000	-50.0000000
55700.0000000	11.2500000	-50.0000000
55700.0000000	11.5000000	-50.0000000
55700.0000000	11.7500000	-50.0000000
55700.0000000	12.0000000	-50.0000000
55700.0000000	12.2500000	-50.0000000
55700.0000000	12.5000000	-50.0000000
55700.0000000	12.7500000	-50.0000000
55700.0000000	13.0000000	-50.0000000
55700.0000000	13.2500000	-50.0000000
55700.0000000	13.5000000	-50.0000000
55700.0000000	13.7500000	-50.0000000
55700.0000000	14.0000000	-50.0000000
55700.0000000	14.2500000	-50.0000000
55700.0000000	14.5000000	-50.0000000
55700.0000000	14.7500000	-50.0000000
55700.0000000	15.0000000	-50.0000000
55730.0000000	0.0000000	-50.0000000

55730.0000000	0.2500000	-50.0000000
55730.0000000	0.5000000	22.1100006
55730.0000000	0.7500000	23.1499996
55730.0000000	1.0000000	18.8299999
55730.0000000	1.2500000	19.7299995
55730.0000000	1.5000000	20.6000004
55730.0000000	1.7500000	20.7000008
55730.0000000	2.0000000	20.6200008
55730.0000000	2.2500000	21.3899994
55730.0000000	2.5000000	20.5200005
55730.0000000	2.7500000	21.6299992
55730.0000000	3.0000000	16.7199993
55730.0000000	3.2500000	16.8199997
55730.0000000	3.5000000	20.3400002
55730.0000000	3.7500000	19.2700005
55730.0000000	4.0000000	19.8099995
55730.0000000	4.2500000	19.3400002
55730.0000000	4.5000000	17.8999996
55730.0000000	4.7500000	-50.0000000
55730.0000000	5.0000000	-50.0000000
55730.0000000	5.2500000	-50.0000000
55730.0000000	5.5000000	-50.0000000
55730.0000000	5.7500000	-50.0000000
55730.0000000	6.0000000	-50.0000000
55730.0000000	6.2500000	-50.0000000
55730.0000000	6.5000000	-50.0000000
55730.0000000	6.7500000	-50.0000000
55730.0000000	7.0000000	-50.0000000
55730.0000000	7.2500000	-50.0000000
55730.0000000	7.5000000	-50.0000000
55730.0000000	7.7500000	-50.0000000
55730.0000000	8.0000000	-50.0000000
55730.0000000	8.2500000	-50.0000000
55730.0000000	8.5000000	-50.0000000
55730.0000000	8.7500000	-50.0000000
55730.0000000	9.0000000	-50.0000000
55730.0000000	9.2500000	-50.0000000
55730.0000000	9.5000000	-50.0000000
55730.0000000	9.7500000	-50.0000000
55730.0000000	10.0000000	-50.0000000
55730.0000000	10.2500000	-50.0000000
55730.0000000	10.5000000	-50.0000000
55730.0000000	10.7500000	-50.0000000
55730.0000000	11.0000000	-50.0000000
55730.0000000	11.2500000	-50.0000000
55730.0000000	11.5000000	-50.0000000
55730.0000000	11.7500000	-50.0000000
55730.0000000	12.0000000	-50.0000000
55730.0000000	12.2500000	-50.0000000
55730.0000000	12.5000000	-50.0000000
55730.0000000	12.7500000	-50.0000000
55730.0000000	13.0000000	-50.0000000
55730.0000000	13.2500000	-50.0000000
55730.0000000	13.5000000	-50.0000000
55730.0000000	13.7500000	-50.0000000
55730.0000000	14.0000000	-50.0000000
55730.0000000	14.2500000	-50.0000000
55730.0000000	14.5000000	-50.0000000

55730.0000000	14.7500000	-50.0000000
55730.0000000	15.0000000	-50.0000000
55760.0000000	0.0000000	-50.0000000
55760.0000000	0.2500000	15.7200003
55760.0000000	0.5000000	23.7199993
55760.0000000	0.7500000	24.2399998
55760.0000000	1.0000000	17.7199993
55760.0000000	1.2500000	19.7099991
55760.0000000	1.5000000	20.6800003
55760.0000000	1.7500000	20.9500008
55760.0000000	2.0000000	21.0900002
55760.0000000	2.2500000	20.6599998
55760.0000000	2.5000000	19.7999992
55760.0000000	2.7500000	20.3600006
55760.0000000	3.0000000	19.8500004
55760.0000000	3.2500000	19.3999996
55760.0000000	3.5000000	18.0699997
55760.0000000	3.7500000	18.1700001
55760.0000000	4.0000000	17.6200008
55760.0000000	4.2500000	18.9599991
55760.0000000	4.5000000	16.8500004
55760.0000000	4.7500000	16.0699997
55760.0000000	5.0000000	-50.0000000
55760.0000000	5.2500000	-50.0000000
55760.0000000	5.5000000	-50.0000000
55760.0000000	5.7500000	-50.0000000
55760.0000000	6.0000000	-50.0000000
55760.0000000	6.2500000	-50.0000000
55760.0000000	6.5000000	-50.0000000
55760.0000000	6.7500000	-50.0000000
55760.0000000	7.0000000	-50.0000000
55760.0000000	7.2500000	-50.0000000
55760.0000000	7.5000000	-50.0000000
55760.0000000	7.7500000	-50.0000000
55760.0000000	8.0000000	-50.0000000
55760.0000000	8.2500000	-50.0000000
55760.0000000	8.5000000	-50.0000000
55760.0000000	8.7500000	-50.0000000
55760.0000000	9.0000000	-50.0000000
55760.0000000	9.2500000	-50.0000000
55760.0000000	9.5000000	-50.0000000
55760.0000000	9.7500000	-50.0000000
55760.0000000	10.0000000	-50.0000000
55760.0000000	10.2500000	-50.0000000
55760.0000000	10.5000000	-50.0000000
55760.0000000	10.7500000	-50.0000000
55760.0000000	11.0000000	-50.0000000
55760.0000000	11.2500000	-50.0000000
55760.0000000	11.5000000	-50.0000000
55760.0000000	11.7500000	-50.0000000
55760.0000000	12.0000000	-50.0000000
55760.0000000	12.2500000	-50.0000000
55760.0000000	12.5000000	-50.0000000
55760.0000000	12.7500000	-50.0000000
55760.0000000	13.0000000	-50.0000000
55760.0000000	13.2500000	-50.0000000
55760.0000000	13.5000000	-50.0000000
55760.0000000	13.7500000	-50.0000000

55760.0000000	14.0000000	-50.0000000
55760.0000000	14.2500000	-50.0000000
55760.0000000	14.5000000	-50.0000000
55760.0000000	14.7500000	-50.0000000
55760.0000000	15.0000000	-50.0000000
55790.0000000	0.0000000	-50.0000000
55790.0000000	0.2500000	17.3799992
55790.0000000	0.5000000	26.3199997
55790.0000000	0.7500000	25.8099995
55790.0000000	1.0000000	18.0799999
55790.0000000	1.2500000	17.5699997
55790.0000000	1.5000000	20.6200008
55790.0000000	1.7500000	20.2500000
55790.0000000	2.0000000	20.6200008
55790.0000000	2.2500000	21.9899998
55790.0000000	2.5000000	21.1000004
55790.0000000	2.7500000	18.7500000
55790.0000000	3.0000000	18.3299999
55790.0000000	3.2500000	19.8400002
55790.0000000	3.5000000	19.2299995
55790.0000000	3.7500000	18.9200001
55790.0000000	4.0000000	17.9899998
55790.0000000	4.2500000	19.2900009
55790.0000000	4.5000000	-50.0000000
55790.0000000	4.7500000	-50.0000000
55790.0000000	5.0000000	-50.0000000
55790.0000000	5.2500000	-50.0000000
55790.0000000	5.5000000	-50.0000000
55790.0000000	5.7500000	-50.0000000
55790.0000000	6.0000000	-50.0000000
55790.0000000	6.2500000	-50.0000000
55790.0000000	6.5000000	-50.0000000
55790.0000000	6.7500000	-50.0000000
55790.0000000	7.0000000	-50.0000000
55790.0000000	7.2500000	-50.0000000
55790.0000000	7.5000000	-50.0000000
55790.0000000	7.7500000	-50.0000000
55790.0000000	8.0000000	-50.0000000
55790.0000000	8.2500000	-50.0000000
55790.0000000	8.5000000	-50.0000000
55790.0000000	8.7500000	-50.0000000
55790.0000000	9.0000000	-50.0000000
55790.0000000	9.2500000	-50.0000000
55790.0000000	9.5000000	-50.0000000
55790.0000000	9.7500000	-50.0000000
55790.0000000	10.0000000	-50.0000000
55790.0000000	10.2500000	-50.0000000
55790.0000000	10.5000000	-50.0000000
55790.0000000	10.7500000	-50.0000000
55790.0000000	11.0000000	-50.0000000
55790.0000000	11.2500000	-50.0000000
55790.0000000	11.5000000	-50.0000000
55790.0000000	11.7500000	-50.0000000
55790.0000000	12.0000000	-50.0000000
55790.0000000	12.2500000	-50.0000000
55790.0000000	12.5000000	-50.0000000
55790.0000000	12.7500000	-50.0000000
55790.0000000	13.0000000	-50.0000000

55790.0000000	13.2500000	-50.0000000
55790.0000000	13.5000000	-50.0000000
55790.0000000	13.7500000	-50.0000000
55790.0000000	14.0000000	-50.0000000
55790.0000000	14.2500000	-50.0000000
55790.0000000	14.5000000	-50.0000000
55790.0000000	14.7500000	-50.0000000
55790.0000000	15.0000000	-50.0000000
55820.0000000	0.0000000	-50.0000000
55820.0000000	0.2500000	-50.0000000
55820.0000000	0.5000000	24.3600006
55820.0000000	0.7500000	24.8999996
55820.0000000	1.0000000	19.8600006
55820.0000000	1.2500000	19.8899994
55820.0000000	1.5000000	21.0100002
55820.0000000	1.7500000	20.5499992
55820.0000000	2.0000000	21.6299992
55820.0000000	2.2500000	19.5100002
55820.0000000	2.5000000	20.1800003
55820.0000000	2.7500000	17.9699993
55820.0000000	3.0000000	18.7999992
55820.0000000	3.2500000	20.3500004
55820.0000000	3.5000000	19.5799999
55820.0000000	3.7500000	18.8899994
55820.0000000	4.0000000	15.8299999
55820.0000000	4.2500000	17.8600006
55820.0000000	4.5000000	19.4599991
55820.0000000	4.7500000	-50.0000000
55820.0000000	5.0000000	-50.0000000
55820.0000000	5.2500000	-50.0000000
55820.0000000	5.5000000	-50.0000000
55820.0000000	5.7500000	-50.0000000
55820.0000000	6.0000000	-50.0000000
55820.0000000	6.2500000	-50.0000000
55820.0000000	6.5000000	-50.0000000
55820.0000000	6.7500000	-50.0000000
55820.0000000	7.0000000	-50.0000000
55820.0000000	7.2500000	-50.0000000
55820.0000000	7.5000000	-50.0000000
55820.0000000	7.7500000	-50.0000000
55820.0000000	8.0000000	-50.0000000
55820.0000000	8.2500000	-50.0000000
55820.0000000	8.5000000	-50.0000000
55820.0000000	8.7500000	-50.0000000
55820.0000000	9.0000000	-50.0000000
55820.0000000	9.2500000	-50.0000000
55820.0000000	9.5000000	-50.0000000
55820.0000000	9.7500000	-50.0000000
55820.0000000	10.0000000	-50.0000000
55820.0000000	10.2500000	-50.0000000
55820.0000000	10.5000000	-50.0000000
55820.0000000	10.7500000	-50.0000000
55820.0000000	11.0000000	-50.0000000
55820.0000000	11.2500000	-50.0000000
55820.0000000	11.5000000	-50.0000000
55820.0000000	11.7500000	-50.0000000
55820.0000000	12.0000000	-50.0000000
55820.0000000	12.2500000	-50.0000000

55820.0000000	12.5000000	-50.0000000
55820.0000000	12.7500000	-50.0000000
55820.0000000	13.0000000	-50.0000000
55820.0000000	13.2500000	-50.0000000
55820.0000000	13.5000000	-50.0000000
55820.0000000	13.7500000	-50.0000000
55820.0000000	14.0000000	-50.0000000
55820.0000000	14.2500000	-50.0000000
55820.0000000	14.5000000	-50.0000000
55820.0000000	14.7500000	-50.0000000
55820.0000000	15.0000000	-50.0000000
55850.0000000	0.0000000	-50.0000000
55850.0000000	0.2500000	15.1199999
55850.0000000	0.5000000	25.7600002
55850.0000000	0.7500000	26.2700005
55850.0000000	1.0000000	18.5499992
55850.0000000	1.2500000	17.8299999
55850.0000000	1.5000000	19.9400005
55850.0000000	1.7500000	19.1000004
55850.0000000	2.0000000	19.9200001
55850.0000000	2.2500000	18.0699997
55850.0000000	2.5000000	19.3700008
55850.0000000	2.7500000	17.7299995
55850.0000000	3.0000000	18.5900002
55850.0000000	3.2500000	19.0900002
55850.0000000	3.5000000	21.3600006
55850.0000000	3.7500000	20.2199993
55850.0000000	4.0000000	17.0100002
55850.0000000	4.2500000	15.4499998
55850.0000000	4.5000000	15.5900002
55850.0000000	4.7500000	-50.0000000
55850.0000000	5.0000000	-50.0000000
55850.0000000	5.2500000	-50.0000000
55850.0000000	5.5000000	-50.0000000
55850.0000000	5.7500000	-50.0000000
55850.0000000	6.0000000	-50.0000000
55850.0000000	6.2500000	-50.0000000
55850.0000000	6.5000000	-50.0000000
55850.0000000	6.7500000	-50.0000000
55850.0000000	7.0000000	-50.0000000
55850.0000000	7.2500000	-50.0000000
55850.0000000	7.5000000	-50.0000000
55850.0000000	7.7500000	-50.0000000
55850.0000000	8.0000000	-50.0000000
55850.0000000	8.2500000	-50.0000000
55850.0000000	8.5000000	-50.0000000
55850.0000000	8.7500000	-50.0000000
55850.0000000	9.0000000	-50.0000000
55850.0000000	9.2500000	-50.0000000
55850.0000000	9.5000000	-50.0000000
55850.0000000	9.7500000	-50.0000000
55850.0000000	10.0000000	-50.0000000
55850.0000000	10.2500000	-50.0000000
55850.0000000	10.5000000	-50.0000000
55850.0000000	10.7500000	-50.0000000
55850.0000000	11.0000000	-50.0000000
55850.0000000	11.2500000	-50.0000000
55850.0000000	11.5000000	-50.0000000

55850.0000000	11.7500000	-50.0000000
55850.0000000	12.0000000	-50.0000000
55850.0000000	12.2500000	-50.0000000
55850.0000000	12.5000000	-50.0000000
55850.0000000	12.7500000	-50.0000000
55850.0000000	13.0000000	-50.0000000
55850.0000000	13.2500000	-50.0000000
55850.0000000	13.5000000	-50.0000000
55850.0000000	13.7500000	-50.0000000
55850.0000000	14.0000000	-50.0000000
55850.0000000	14.2500000	-50.0000000
55850.0000000	14.5000000	-50.0000000
55850.0000000	14.7500000	-50.0000000
55850.0000000	15.0000000	-50.0000000
55880.0000000	0.0000000	-50.0000000
55880.0000000	0.2500000	17.9400005
55880.0000000	0.5000000	26.4500008
55880.0000000	0.7500000	25.2399998
55880.0000000	1.0000000	19.6599998
55880.0000000	1.2500000	20.2199993
55880.0000000	1.5000000	19.8400002
55880.0000000	1.7500000	21.5699997
55880.0000000	2.0000000	20.0499992
55880.0000000	2.2500000	18.6000004
55880.0000000	2.5000000	18.9599991
55880.0000000	2.7500000	17.5499992
55880.0000000	3.0000000	17.5499992
55880.0000000	3.2500000	20.3700008
55880.0000000	3.5000000	20.0900002
55880.0000000	3.7500000	16.8400002
55880.0000000	4.0000000	17.4799995
55880.0000000	4.2500000	15.9099998
55880.0000000	4.5000000	-50.0000000
55880.0000000	4.7500000	-50.0000000
55880.0000000	5.0000000	-50.0000000
55880.0000000	5.2500000	-50.0000000
55880.0000000	5.5000000	-50.0000000
55880.0000000	5.7500000	-50.0000000
55880.0000000	6.0000000	-50.0000000
55880.0000000	6.2500000	-50.0000000
55880.0000000	6.5000000	-50.0000000
55880.0000000	6.7500000	-50.0000000
55880.0000000	7.0000000	-50.0000000
55880.0000000	7.2500000	-50.0000000
55880.0000000	7.5000000	-50.0000000
55880.0000000	7.7500000	-50.0000000
55880.0000000	8.0000000	-50.0000000
55880.0000000	8.2500000	-50.0000000
55880.0000000	8.5000000	-50.0000000
55880.0000000	8.7500000	-50.0000000
55880.0000000	9.0000000	-50.0000000
55880.0000000	9.2500000	-50.0000000
55880.0000000	9.5000000	-50.0000000
55880.0000000	9.7500000	-50.0000000
55880.0000000	10.0000000	-50.0000000
55880.0000000	10.2500000	-50.0000000
55880.0000000	10.5000000	-50.0000000
55880.0000000	10.7500000	-50.0000000

55880.0000000	11.0000000	-50.0000000
55880.0000000	11.2500000	-50.0000000
55880.0000000	11.5000000	-50.0000000
55880.0000000	11.7500000	-50.0000000
55880.0000000	12.0000000	-50.0000000
55880.0000000	12.2500000	-50.0000000
55880.0000000	12.5000000	-50.0000000
55880.0000000	12.7500000	-50.0000000
55880.0000000	13.0000000	-50.0000000
55880.0000000	13.2500000	-50.0000000
55880.0000000	13.5000000	-50.0000000
55880.0000000	13.7500000	-50.0000000
55880.0000000	14.0000000	-50.0000000
55880.0000000	14.2500000	-50.0000000
55880.0000000	14.5000000	-50.0000000
55880.0000000	14.7500000	-50.0000000
55880.0000000	15.0000000	-50.0000000
55910.0000000	0.0000000	-50.0000000
55910.0000000	0.2500000	19.6399994
55910.0000000	0.5000000	29.2099991
55910.0000000	0.7500000	29.4200001
55910.0000000	1.0000000	22.3700008
55910.0000000	1.2500000	22.3600006
55910.0000000	1.5000000	21.7999992
55910.0000000	1.7500000	21.4699993
55910.0000000	2.0000000	20.9300003
55910.0000000	2.2500000	19.6299992
55910.0000000	2.5000000	20.6100006
55910.0000000	2.7500000	20.2700005
55910.0000000	3.0000000	20.7600002
55910.0000000	3.2500000	20.1200008
55910.0000000	3.5000000	17.5100002
55910.0000000	3.7500000	18.0200005
55910.0000000	4.0000000	15.7100000
55910.0000000	4.2500000	16.4699993
55910.0000000	4.5000000	16.6100006
55910.0000000	4.7500000	-50.0000000
55910.0000000	5.0000000	-50.0000000
55910.0000000	5.2500000	-50.0000000
55910.0000000	5.5000000	-50.0000000
55910.0000000	5.7500000	-50.0000000
55910.0000000	6.0000000	-50.0000000
55910.0000000	6.2500000	-50.0000000
55910.0000000	6.5000000	-50.0000000
55910.0000000	6.7500000	-50.0000000
55910.0000000	7.0000000	-50.0000000
55910.0000000	7.2500000	-50.0000000
55910.0000000	7.5000000	-50.0000000
55910.0000000	7.7500000	-50.0000000
55910.0000000	8.0000000	-50.0000000
55910.0000000	8.2500000	-50.0000000
55910.0000000	8.5000000	-50.0000000
55910.0000000	8.7500000	-50.0000000
55910.0000000	9.0000000	-50.0000000
55910.0000000	9.2500000	-50.0000000
55910.0000000	9.5000000	-50.0000000
55910.0000000	9.7500000	-50.0000000
55910.0000000	10.0000000	-50.0000000

55910.0000000	10.2500000	-50.0000000
55910.0000000	10.5000000	-50.0000000
55910.0000000	10.7500000	-50.0000000
55910.0000000	11.0000000	-50.0000000
55910.0000000	11.2500000	-50.0000000
55910.0000000	11.5000000	-50.0000000
55910.0000000	11.7500000	-50.0000000
55910.0000000	12.0000000	-50.0000000
55910.0000000	12.2500000	-50.0000000
55910.0000000	12.5000000	-50.0000000
55910.0000000	12.7500000	-50.0000000
55910.0000000	13.0000000	-50.0000000
55910.0000000	13.2500000	-50.0000000
55910.0000000	13.5000000	-50.0000000
55910.0000000	13.7500000	-50.0000000
55910.0000000	14.0000000	-50.0000000
55910.0000000	14.2500000	-50.0000000
55910.0000000	14.5000000	-50.0000000
55910.0000000	14.7500000	-50.0000000
55910.0000000	15.0000000	-50.0000000
55940.0000000	0.0000000	-50.0000000
55940.0000000	0.2500000	19.9500008
55940.0000000	0.5000000	27.5000000
55940.0000000	0.7500000	25.3500004
55940.0000000	1.0000000	18.6599998
55940.0000000	1.2500000	17.2199993
55940.0000000	1.5000000	20.5400009
55940.0000000	1.7500000	20.6100006
55940.0000000	2.0000000	20.2999992
55940.0000000	2.2500000	20.8099995
55940.0000000	2.5000000	20.9400005
55940.0000000	2.7500000	18.5900002
55940.0000000	3.0000000	17.6700001
55940.0000000	3.2500000	18.9899998
55940.0000000	3.5000000	20.2199993
55940.0000000	3.7500000	19.1000004
55940.0000000	4.0000000	16.2399998
55940.0000000	4.2500000	15.5200005
55940.0000000	4.5000000	15.4700003
55940.0000000	4.7500000	-50.0000000
55940.0000000	5.0000000	-50.0000000
55940.0000000	5.2500000	-50.0000000
55940.0000000	5.5000000	-50.0000000
55940.0000000	5.7500000	-50.0000000
55940.0000000	6.0000000	-50.0000000
55940.0000000	6.2500000	-50.0000000
55940.0000000	6.5000000	-50.0000000
55940.0000000	6.7500000	-50.0000000
55940.0000000	7.0000000	-50.0000000
55940.0000000	7.2500000	-50.0000000
55940.0000000	7.5000000	-50.0000000
55940.0000000	7.7500000	-50.0000000
55940.0000000	8.0000000	-50.0000000
55940.0000000	8.2500000	-50.0000000
55940.0000000	8.5000000	-50.0000000
55940.0000000	8.7500000	-50.0000000
55940.0000000	9.0000000	-50.0000000
55940.0000000	9.2500000	-50.0000000

55940.0000000	9.5000000	-50.0000000
55940.0000000	9.7500000	-50.0000000
55940.0000000	10.0000000	-50.0000000
55940.0000000	10.2500000	-50.0000000
55940.0000000	10.5000000	-50.0000000
55940.0000000	10.7500000	-50.0000000
55940.0000000	11.0000000	-50.0000000
55940.0000000	11.2500000	-50.0000000
55940.0000000	11.5000000	-50.0000000
55940.0000000	11.7500000	-50.0000000
55940.0000000	12.0000000	-50.0000000
55940.0000000	12.2500000	-50.0000000
55940.0000000	12.5000000	-50.0000000
55940.0000000	12.7500000	-50.0000000
55940.0000000	13.0000000	-50.0000000
55940.0000000	13.2500000	-50.0000000
55940.0000000	13.5000000	-50.0000000
55940.0000000	13.7500000	-50.0000000
55940.0000000	14.0000000	-50.0000000
55940.0000000	14.2500000	-50.0000000
55940.0000000	14.5000000	-50.0000000
55940.0000000	14.7500000	-50.0000000
55940.0000000	15.0000000	-50.0000000
55970.0000000	0.0000000	-50.0000000
55970.0000000	0.2500000	18.6900005
55970.0000000	0.5000000	27.7000008
55970.0000000	0.7500000	26.9699993
55970.0000000	1.0000000	20.7500000
55970.0000000	1.2500000	21.3999996
55970.0000000	1.5000000	21.6000004
55970.0000000	1.7500000	18.8799992
55970.0000000	2.0000000	19.5400009
55970.0000000	2.2500000	21.5300007
55970.0000000	2.5000000	20.1200008
55970.0000000	2.7500000	19.1499996
55970.0000000	3.0000000	19.4599991
55970.0000000	3.2500000	18.8799992
55970.0000000	3.5000000	17.1000004
55970.0000000	3.7500000	15.5100002
55970.0000000	4.0000000	16.7900009
55970.0000000	4.2500000	15.8699999
55970.0000000	4.5000000	-50.0000000
55970.0000000	4.7500000	-50.0000000
55970.0000000	5.0000000	-50.0000000
55970.0000000	5.2500000	-50.0000000
55970.0000000	5.5000000	-50.0000000
55970.0000000	5.7500000	-50.0000000
55970.0000000	6.0000000	-50.0000000
55970.0000000	6.2500000	-50.0000000
55970.0000000	6.5000000	-50.0000000
55970.0000000	6.7500000	-50.0000000
55970.0000000	7.0000000	-50.0000000
55970.0000000	7.2500000	-50.0000000
55970.0000000	7.5000000	-50.0000000
55970.0000000	7.7500000	-50.0000000
55970.0000000	8.0000000	-50.0000000
55970.0000000	8.2500000	-50.0000000
55970.0000000	8.5000000	-50.0000000

55970.0000000	8.7500000	-50.0000000
55970.0000000	9.0000000	-50.0000000
55970.0000000	9.2500000	-50.0000000
55970.0000000	9.5000000	-50.0000000
55970.0000000	9.7500000	-50.0000000
55970.0000000	10.0000000	-50.0000000
55970.0000000	10.2500000	-50.0000000
55970.0000000	10.5000000	-50.0000000
55970.0000000	10.7500000	-50.0000000
55970.0000000	11.0000000	-50.0000000
55970.0000000	11.2500000	-50.0000000
55970.0000000	11.5000000	-50.0000000
55970.0000000	11.7500000	-50.0000000
55970.0000000	12.0000000	-50.0000000
55970.0000000	12.2500000	-50.0000000
55970.0000000	12.5000000	-50.0000000
55970.0000000	12.7500000	-50.0000000
55970.0000000	13.0000000	-50.0000000
55970.0000000	13.2500000	-50.0000000
55970.0000000	13.5000000	-50.0000000
55970.0000000	13.7500000	-50.0000000
55970.0000000	14.0000000	-50.0000000
55970.0000000	14.2500000	-50.0000000
55970.0000000	14.5000000	-50.0000000
55970.0000000	14.7500000	-50.0000000
55970.0000000	15.0000000	-50.0000000
56000.0000000	0.0000000	-50.0000000
56000.0000000	0.2500000	15.3699999
56000.0000000	0.5000000	24.2999992
56000.0000000	0.7500000	23.8299999
56000.0000000	1.0000000	20.7999992
56000.0000000	1.2500000	21.8500004
56000.0000000	1.5000000	20.9599991
56000.0000000	1.7500000	20.1000004
56000.0000000	2.0000000	20.1800003
56000.0000000	2.2500000	20.6200008
56000.0000000	2.5000000	19.5699997
56000.0000000	2.7500000	19.7600002
56000.0000000	3.0000000	18.7900009
56000.0000000	3.2500000	17.8899994
56000.0000000	3.5000000	19.4500008
56000.0000000	3.7500000	18.4300003
56000.0000000	4.0000000	15.5799999
56000.0000000	4.2500000	-50.0000000
56000.0000000	4.5000000	-50.0000000
56000.0000000	4.7500000	-50.0000000
56000.0000000	5.0000000	-50.0000000
56000.0000000	5.2500000	-50.0000000
56000.0000000	5.5000000	-50.0000000
56000.0000000	5.7500000	-50.0000000
56000.0000000	6.0000000	-50.0000000
56000.0000000	6.2500000	-50.0000000
56000.0000000	6.5000000	-50.0000000
56000.0000000	6.7500000	-50.0000000
56000.0000000	7.0000000	-50.0000000
56000.0000000	7.2500000	-50.0000000
56000.0000000	7.5000000	-50.0000000
56000.0000000	7.7500000	-50.0000000

56000.0000000	8.0000000	-50.0000000
56000.0000000	8.2500000	-50.0000000
56000.0000000	8.5000000	-50.0000000
56000.0000000	8.7500000	-50.0000000
56000.0000000	9.0000000	-50.0000000
56000.0000000	9.2500000	-50.0000000
56000.0000000	9.5000000	-50.0000000
56000.0000000	9.7500000	-50.0000000
56000.0000000	10.0000000	-50.0000000
56000.0000000	10.2500000	-50.0000000
56000.0000000	10.5000000	-50.0000000
56000.0000000	10.7500000	-50.0000000
56000.0000000	11.0000000	-50.0000000
56000.0000000	11.2500000	-50.0000000
56000.0000000	11.5000000	-50.0000000
56000.0000000	11.7500000	-50.0000000
56000.0000000	12.0000000	-50.0000000
56000.0000000	12.2500000	-50.0000000
56000.0000000	12.5000000	-50.0000000
56000.0000000	12.7500000	-50.0000000
56000.0000000	13.0000000	-50.0000000
56000.0000000	13.2500000	-50.0000000
56000.0000000	13.5000000	-50.0000000
56000.0000000	13.7500000	-50.0000000
56000.0000000	14.0000000	-50.0000000
56000.0000000	14.2500000	-50.0000000
56000.0000000	14.5000000	-50.0000000
56000.0000000	14.7500000	-50.0000000
56000.0000000	15.0000000	-50.0000000
56030.0000000	0.0000000	-50.0000000
56030.0000000	0.2500000	20.5499992
56030.0000000	0.5000000	28.0400009
56030.0000000	0.7500000	26.0000000
56030.0000000	1.0000000	19.9300003
56030.0000000	1.2500000	20.0699997
56030.0000000	1.5000000	21.3899994
56030.0000000	1.7500000	21.3799992
56030.0000000	2.0000000	21.9500008
56030.0000000	2.2500000	21.2700005
56030.0000000	2.5000000	20.8799992
56030.0000000	2.7500000	21.3400002
56030.0000000	3.0000000	18.6800003
56030.0000000	3.2500000	21.0400009
56030.0000000	3.5000000	19.7600002
56030.0000000	3.7500000	19.6599998
56030.0000000	4.0000000	15.7900000
56030.0000000	4.2500000	-50.0000000
56030.0000000	4.5000000	-50.0000000
56030.0000000	4.7500000	-50.0000000
56030.0000000	5.0000000	-50.0000000
56030.0000000	5.2500000	-50.0000000
56030.0000000	5.5000000	-50.0000000
56030.0000000	5.7500000	-50.0000000
56030.0000000	6.0000000	-50.0000000
56030.0000000	6.2500000	-50.0000000
56030.0000000	6.5000000	-50.0000000
56030.0000000	6.7500000	-50.0000000
56030.0000000	7.0000000	-50.0000000

56030.0000000	7.2500000	-50.0000000
56030.0000000	7.5000000	-50.0000000
56030.0000000	7.7500000	-50.0000000
56030.0000000	8.0000000	-50.0000000
56030.0000000	8.2500000	-50.0000000
56030.0000000	8.5000000	-50.0000000
56030.0000000	8.7500000	-50.0000000
56030.0000000	9.0000000	-50.0000000
56030.0000000	9.2500000	-50.0000000
56030.0000000	9.5000000	-50.0000000
56030.0000000	9.7500000	-50.0000000
56030.0000000	10.0000000	-50.0000000
56030.0000000	10.2500000	-50.0000000
56030.0000000	10.5000000	-50.0000000
56030.0000000	10.7500000	-50.0000000
56030.0000000	11.0000000	-50.0000000
56030.0000000	11.2500000	-50.0000000
56030.0000000	11.5000000	-50.0000000
56030.0000000	11.7500000	-50.0000000
56030.0000000	12.0000000	-50.0000000
56030.0000000	12.2500000	-50.0000000
56030.0000000	12.5000000	-50.0000000
56030.0000000	12.7500000	-50.0000000
56030.0000000	13.0000000	-50.0000000
56030.0000000	13.2500000	-50.0000000
56030.0000000	13.5000000	-50.0000000
56030.0000000	13.7500000	-50.0000000
56030.0000000	14.0000000	-50.0000000
56030.0000000	14.2500000	-50.0000000
56030.0000000	14.5000000	-50.0000000
56030.0000000	14.7500000	-50.0000000
56030.0000000	15.0000000	-50.0000000
56060.0000000	0.0000000	-50.0000000
56060.0000000	0.2500000	-50.0000000
56060.0000000	0.5000000	24.6499996
56060.0000000	0.7500000	24.6700001
56060.0000000	1.0000000	19.7700005
56060.0000000	1.2500000	20.4699993
56060.0000000	1.5000000	20.8799992
56060.0000000	1.7500000	19.6800003
56060.0000000	2.0000000	19.4899998
56060.0000000	2.2500000	20.3799992
56060.0000000	2.5000000	20.4099998
56060.0000000	2.7500000	18.3899994
56060.0000000	3.0000000	20.6299992
56060.0000000	3.2500000	19.0000000
56060.0000000	3.5000000	18.5900002
56060.0000000	3.7500000	20.9599991
56060.0000000	4.0000000	20.2099991
56060.0000000	4.2500000	16.8400002
56060.0000000	4.5000000	-50.0000000
56060.0000000	4.7500000	-50.0000000
56060.0000000	5.0000000	-50.0000000
56060.0000000	5.2500000	-50.0000000
56060.0000000	5.5000000	-50.0000000
56060.0000000	5.7500000	-50.0000000
56060.0000000	6.0000000	-50.0000000
56060.0000000	6.2500000	-50.0000000

56060.0000000	6.5000000	-50.0000000
56060.0000000	6.7500000	-50.0000000
56060.0000000	7.0000000	-50.0000000
56060.0000000	7.2500000	-50.0000000
56060.0000000	7.5000000	-50.0000000
56060.0000000	7.7500000	-50.0000000
56060.0000000	8.0000000	-50.0000000
56060.0000000	8.2500000	-50.0000000
56060.0000000	8.5000000	-50.0000000
56060.0000000	8.7500000	-50.0000000
56060.0000000	9.0000000	-50.0000000
56060.0000000	9.2500000	-50.0000000
56060.0000000	9.5000000	-50.0000000
56060.0000000	9.7500000	-50.0000000
56060.0000000	10.0000000	-50.0000000
56060.0000000	10.2500000	-50.0000000
56060.0000000	10.5000000	-50.0000000
56060.0000000	10.7500000	-50.0000000
56060.0000000	11.0000000	-50.0000000
56060.0000000	11.2500000	-50.0000000
56060.0000000	11.5000000	-50.0000000
56060.0000000	11.7500000	-50.0000000
56060.0000000	12.0000000	-50.0000000
56060.0000000	12.2500000	-50.0000000
56060.0000000	12.5000000	-50.0000000
56060.0000000	12.7500000	-50.0000000
56060.0000000	13.0000000	-50.0000000
56060.0000000	13.2500000	-50.0000000
56060.0000000	13.5000000	-50.0000000
56060.0000000	13.7500000	-50.0000000
56060.0000000	14.0000000	-50.0000000
56060.0000000	14.2500000	-50.0000000
56060.0000000	14.5000000	-50.0000000
56060.0000000	14.7500000	-50.0000000
56060.0000000	15.0000000	-50.0000000
56090.0000000	0.0000000	-50.0000000
56090.0000000	0.2500000	19.3400002
56090.0000000	0.5000000	26.4599991
56090.0000000	0.7500000	24.5699997
56090.0000000	1.0000000	19.7199993
56090.0000000	1.2500000	19.8600006
56090.0000000	1.5000000	20.6200008
56090.0000000	1.7500000	19.6800003
56090.0000000	2.0000000	20.2900009
56090.0000000	2.2500000	22.9500008
56090.0000000	2.5000000	20.7000008
56090.0000000	2.7500000	22.5900002
56090.0000000	3.0000000	20.8799992
56090.0000000	3.2500000	20.1000004
56090.0000000	3.5000000	17.6299992
56090.0000000	3.7500000	17.5499992
56090.0000000	4.0000000	17.5200005
56090.0000000	4.2500000	15.9200001
56090.0000000	4.5000000	16.0499992
56090.0000000	4.7500000	-50.0000000
56090.0000000	5.0000000	-50.0000000
56090.0000000	5.2500000	-50.0000000
56090.0000000	5.5000000	-50.0000000

56090.0000000	5.7500000	-50.0000000
56090.0000000	6.0000000	-50.0000000
56090.0000000	6.2500000	-50.0000000
56090.0000000	6.5000000	-50.0000000
56090.0000000	6.7500000	-50.0000000
56090.0000000	7.0000000	-50.0000000
56090.0000000	7.2500000	-50.0000000
56090.0000000	7.5000000	-50.0000000
56090.0000000	7.7500000	-50.0000000
56090.0000000	8.0000000	-50.0000000
56090.0000000	8.2500000	-50.0000000
56090.0000000	8.5000000	-50.0000000
56090.0000000	8.7500000	-50.0000000
56090.0000000	9.0000000	-50.0000000
56090.0000000	9.2500000	-50.0000000
56090.0000000	9.5000000	-50.0000000
56090.0000000	9.7500000	-50.0000000
56090.0000000	10.0000000	-50.0000000
56090.0000000	10.2500000	-50.0000000
56090.0000000	10.5000000	-50.0000000
56090.0000000	10.7500000	-50.0000000
56090.0000000	11.0000000	-50.0000000
56090.0000000	11.2500000	-50.0000000
56090.0000000	11.5000000	-50.0000000
56090.0000000	11.7500000	-50.0000000
56090.0000000	12.0000000	-50.0000000
56090.0000000	12.2500000	-50.0000000
56090.0000000	12.5000000	-50.0000000
56090.0000000	12.7500000	-50.0000000
56090.0000000	13.0000000	-50.0000000
56090.0000000	13.2500000	-50.0000000
56090.0000000	13.5000000	-50.0000000
56090.0000000	13.7500000	-50.0000000
56090.0000000	14.0000000	-50.0000000
56090.0000000	14.2500000	-50.0000000
56090.0000000	14.5000000	-50.0000000
56090.0000000	14.7500000	-50.0000000
56090.0000000	15.0000000	-50.0000000
56120.0000000	0.0000000	-50.0000000
56120.0000000	0.2500000	18.7000008
56120.0000000	0.5000000	27.1399994
56120.0000000	0.7500000	26.5499992
56120.0000000	1.0000000	20.5599995
56120.0000000	1.2500000	22.2000008
56120.0000000	1.5000000	20.6800003
56120.0000000	1.7500000	19.0100002
56120.0000000	2.0000000	20.2700005
56120.0000000	2.2500000	19.2000008
56120.0000000	2.5000000	17.7999992
56120.0000000	2.7500000	18.8700008
56120.0000000	3.0000000	20.2900009
56120.0000000	3.2500000	18.8999996
56120.0000000	3.5000000	21.3600006
56120.0000000	3.7500000	19.3700008
56120.0000000	4.0000000	17.4400005
56120.0000000	4.2500000	18.1499996
56120.0000000	4.5000000	15.1700001
56120.0000000	4.7500000	-50.0000000

56120.0000000	5.0000000	-50.0000000
56120.0000000	5.2500000	-50.0000000
56120.0000000	5.5000000	-50.0000000
56120.0000000	5.7500000	-50.0000000
56120.0000000	6.0000000	-50.0000000
56120.0000000	6.2500000	-50.0000000
56120.0000000	6.5000000	-50.0000000
56120.0000000	6.7500000	-50.0000000
56120.0000000	7.0000000	-50.0000000
56120.0000000	7.2500000	-50.0000000
56120.0000000	7.5000000	-50.0000000
56120.0000000	7.7500000	-50.0000000
56120.0000000	8.0000000	-50.0000000
56120.0000000	8.2500000	-50.0000000
56120.0000000	8.5000000	-50.0000000
56120.0000000	8.7500000	-50.0000000
56120.0000000	9.0000000	-50.0000000
56120.0000000	9.2500000	-50.0000000
56120.0000000	9.5000000	-50.0000000
56120.0000000	9.7500000	-50.0000000
56120.0000000	10.0000000	-50.0000000
56120.0000000	10.2500000	-50.0000000
56120.0000000	10.5000000	-50.0000000
56120.0000000	10.7500000	-50.0000000
56120.0000000	11.0000000	-50.0000000
56120.0000000	11.2500000	-50.0000000
56120.0000000	11.5000000	-50.0000000
56120.0000000	11.7500000	-50.0000000
56120.0000000	12.0000000	-50.0000000
56120.0000000	12.2500000	-50.0000000
56120.0000000	12.5000000	-50.0000000
56120.0000000	12.7500000	-50.0000000
56120.0000000	13.0000000	-50.0000000
56120.0000000	13.2500000	-50.0000000
56120.0000000	13.5000000	-50.0000000
56120.0000000	13.7500000	-50.0000000
56120.0000000	14.0000000	-50.0000000
56120.0000000	14.2500000	-50.0000000
56120.0000000	14.5000000	-50.0000000
56120.0000000	14.7500000	-50.0000000
56120.0000000	15.0000000	-50.0000000
56150.0000000	0.0000000	-50.0000000
56150.0000000	0.2500000	17.4500008
56150.0000000	0.5000000	27.7000008
56150.0000000	0.7500000	27.1499996
56150.0000000	1.0000000	22.1900005
56150.0000000	1.2500000	22.1100006
56150.0000000	1.5000000	21.6499996
56150.0000000	1.7500000	20.0000000
56150.0000000	2.0000000	20.6200008
56150.0000000	2.2500000	20.7500000
56150.0000000	2.5000000	20.9799995
56150.0000000	2.7500000	19.5499992
56150.0000000	3.0000000	19.2900009
56150.0000000	3.2500000	19.7399998
56150.0000000	3.5000000	19.0300007
56150.0000000	3.7500000	15.3599997
56150.0000000	4.0000000	17.7800007

56150.0000000	4.2500000	16.4200001
56150.0000000	4.5000000	17.6299992
56150.0000000	4.7500000	15.4600000
56150.0000000	5.0000000	-50.0000000
56150.0000000	5.2500000	-50.0000000
56150.0000000	5.5000000	-50.0000000
56150.0000000	5.7500000	-50.0000000
56150.0000000	6.0000000	-50.0000000
56150.0000000	6.2500000	-50.0000000
56150.0000000	6.5000000	-50.0000000
56150.0000000	6.7500000	-50.0000000
56150.0000000	7.0000000	-50.0000000
56150.0000000	7.2500000	-50.0000000
56150.0000000	7.5000000	-50.0000000
56150.0000000	7.7500000	-50.0000000
56150.0000000	8.0000000	-50.0000000
56150.0000000	8.2500000	-50.0000000
56150.0000000	8.5000000	-50.0000000
56150.0000000	8.7500000	-50.0000000
56150.0000000	9.0000000	-50.0000000
56150.0000000	9.2500000	-50.0000000
56150.0000000	9.5000000	-50.0000000
56150.0000000	9.7500000	-50.0000000
56150.0000000	10.0000000	-50.0000000
56150.0000000	10.2500000	-50.0000000
56150.0000000	10.5000000	-50.0000000
56150.0000000	10.7500000	-50.0000000
56150.0000000	11.0000000	-50.0000000
56150.0000000	11.2500000	-50.0000000
56150.0000000	11.5000000	-50.0000000
56150.0000000	11.7500000	-50.0000000
56150.0000000	12.0000000	-50.0000000
56150.0000000	12.2500000	-50.0000000
56150.0000000	12.5000000	-50.0000000
56150.0000000	12.7500000	-50.0000000
56150.0000000	13.0000000	-50.0000000
56150.0000000	13.2500000	-50.0000000
56150.0000000	13.5000000	-50.0000000
56150.0000000	13.7500000	-50.0000000
56150.0000000	14.0000000	-50.0000000
56150.0000000	14.2500000	-50.0000000
56150.0000000	14.5000000	-50.0000000
56150.0000000	14.7500000	-50.0000000
56150.0000000	15.0000000	-50.0000000
56180.0000000	0.0000000	-50.0000000
56180.0000000	0.2500000	18.2000008
56180.0000000	0.5000000	25.4200001
56180.0000000	0.7500000	25.1499996
56180.0000000	1.0000000	20.5499992
56180.0000000	1.2500000	20.5499992
56180.0000000	1.5000000	21.4400005
56180.0000000	1.7500000	20.6499996
56180.0000000	2.0000000	19.8299999
56180.0000000	2.2500000	19.1499996
56180.0000000	2.5000000	19.2900009
56180.0000000	2.7500000	21.4099998
56180.0000000	3.0000000	22.1399994
56180.0000000	3.2500000	20.1700001

56180.0000000	3.5000000	18.5000000
56180.0000000	3.7500000	16.0300007
56180.0000000	4.0000000	15.4399996
56180.0000000	4.2500000	15.1700001
56180.0000000	4.5000000	18.5699997
56180.0000000	4.7500000	15.6700001
56180.0000000	5.0000000	-50.0000000
56180.0000000	5.2500000	-50.0000000
56180.0000000	5.5000000	-50.0000000
56180.0000000	5.7500000	-50.0000000
56180.0000000	6.0000000	-50.0000000
56180.0000000	6.2500000	-50.0000000
56180.0000000	6.5000000	-50.0000000
56180.0000000	6.7500000	-50.0000000
56180.0000000	7.0000000	-50.0000000
56180.0000000	7.2500000	-50.0000000
56180.0000000	7.5000000	-50.0000000
56180.0000000	7.7500000	-50.0000000
56180.0000000	8.0000000	-50.0000000
56180.0000000	8.2500000	-50.0000000
56180.0000000	8.5000000	-50.0000000
56180.0000000	8.7500000	-50.0000000
56180.0000000	9.0000000	-50.0000000
56180.0000000	9.2500000	-50.0000000
56180.0000000	9.5000000	-50.0000000
56180.0000000	9.7500000	-50.0000000
56180.0000000	10.0000000	-50.0000000
56180.0000000	10.2500000	-50.0000000
56180.0000000	10.5000000	-50.0000000
56180.0000000	10.7500000	-50.0000000
56180.0000000	11.0000000	-50.0000000
56180.0000000	11.2500000	-50.0000000
56180.0000000	11.5000000	-50.0000000
56180.0000000	11.7500000	-50.0000000
56180.0000000	12.0000000	-50.0000000
56180.0000000	12.2500000	-50.0000000
56180.0000000	12.5000000	-50.0000000
56180.0000000	12.7500000	-50.0000000
56180.0000000	13.0000000	-50.0000000
56180.0000000	13.2500000	-50.0000000
56180.0000000	13.5000000	-50.0000000
56180.0000000	13.7500000	-50.0000000
56180.0000000	14.0000000	-50.0000000
56180.0000000	14.2500000	-50.0000000
56180.0000000	14.5000000	-50.0000000
56180.0000000	14.7500000	-50.0000000
56180.0000000	15.0000000	-50.0000000
56210.0000000	0.0000000	-50.0000000
56210.0000000	0.2500000	17.8600006
56210.0000000	0.5000000	27.3999996
56210.0000000	0.7500000	27.3600006
56210.0000000	1.0000000	21.2399998
56210.0000000	1.2500000	20.5699997
56210.0000000	1.5000000	21.4400005
56210.0000000	1.7500000	20.9599991
56210.0000000	2.0000000	19.5699997
56210.0000000	2.2500000	16.6700001
56210.0000000	2.5000000	19.5599995

56210.0000000	2.7500000	20.3999996
56210.0000000	3.0000000	18.7299995
56210.0000000	3.2500000	20.2399998
56210.0000000	3.5000000	18.5300007
56210.0000000	3.7500000	15.3900003
56210.0000000	4.0000000	15.1700001
56210.0000000	4.2500000	-50.0000000
56210.0000000	4.5000000	15.3000002
56210.0000000	4.7500000	-50.0000000
56210.0000000	5.0000000	-50.0000000
56210.0000000	5.2500000	-50.0000000
56210.0000000	5.5000000	-50.0000000
56210.0000000	5.7500000	-50.0000000
56210.0000000	6.0000000	-50.0000000
56210.0000000	6.2500000	-50.0000000
56210.0000000	6.5000000	-50.0000000
56210.0000000	6.7500000	-50.0000000
56210.0000000	7.0000000	-50.0000000
56210.0000000	7.2500000	-50.0000000
56210.0000000	7.5000000	-50.0000000
56210.0000000	7.7500000	-50.0000000
56210.0000000	8.0000000	-50.0000000
56210.0000000	8.2500000	-50.0000000
56210.0000000	8.5000000	-50.0000000
56210.0000000	8.7500000	-50.0000000
56210.0000000	9.0000000	-50.0000000
56210.0000000	9.2500000	-50.0000000
56210.0000000	9.5000000	-50.0000000
56210.0000000	9.7500000	-50.0000000
56210.0000000	10.0000000	-50.0000000
56210.0000000	10.2500000	-50.0000000
56210.0000000	10.5000000	-50.0000000
56210.0000000	10.7500000	-50.0000000
56210.0000000	11.0000000	-50.0000000
56210.0000000	11.2500000	-50.0000000
56210.0000000	11.5000000	-50.0000000
56210.0000000	11.7500000	-50.0000000
56210.0000000	12.0000000	-50.0000000
56210.0000000	12.2500000	-50.0000000
56210.0000000	12.5000000	-50.0000000
56210.0000000	12.7500000	-50.0000000
56210.0000000	13.0000000	-50.0000000
56210.0000000	13.2500000	-50.0000000
56210.0000000	13.5000000	-50.0000000
56210.0000000	13.7500000	-50.0000000
56210.0000000	14.0000000	-50.0000000
56210.0000000	14.2500000	-50.0000000
56210.0000000	14.5000000	-50.0000000
56210.0000000	14.7500000	-50.0000000
56210.0000000	15.0000000	-50.0000000
56240.0000000	0.0000000	-50.0000000
56240.0000000	0.2500000	22.9899998
56240.0000000	0.5000000	30.8600006
56240.0000000	0.7500000	28.7600002
56240.0000000	1.0000000	23.1800003
56240.0000000	1.2500000	20.2999992
56240.0000000	1.5000000	22.1499996
56240.0000000	1.7500000	21.8199997

56240.0000000	2.0000000	20.8899994
56240.0000000	2.2500000	22.0799999
56240.0000000	2.5000000	21.3899994
56240.0000000	2.7500000	19.7900009
56240.0000000	3.0000000	19.9099998
56240.0000000	3.2500000	18.8199997
56240.0000000	3.5000000	18.6100006
56240.0000000	3.7500000	-50.0000000
56240.0000000	4.0000000	-50.0000000
56240.0000000	4.2500000	16.1599998
56240.0000000	4.5000000	18.7199993
56240.0000000	4.7500000	15.2399998
56240.0000000	5.0000000	-50.0000000
56240.0000000	5.2500000	-50.0000000
56240.0000000	5.5000000	-50.0000000
56240.0000000	5.7500000	-50.0000000
56240.0000000	6.0000000	-50.0000000
56240.0000000	6.2500000	-50.0000000
56240.0000000	6.5000000	-50.0000000
56240.0000000	6.7500000	-50.0000000
56240.0000000	7.0000000	-50.0000000
56240.0000000	7.2500000	-50.0000000
56240.0000000	7.5000000	-50.0000000
56240.0000000	7.7500000	-50.0000000
56240.0000000	8.0000000	-50.0000000
56240.0000000	8.2500000	-50.0000000
56240.0000000	8.5000000	-50.0000000
56240.0000000	8.7500000	-50.0000000
56240.0000000	9.0000000	-50.0000000
56240.0000000	9.2500000	-50.0000000
56240.0000000	9.5000000	-50.0000000
56240.0000000	9.7500000	-50.0000000
56240.0000000	10.0000000	-50.0000000
56240.0000000	10.2500000	-50.0000000
56240.0000000	10.5000000	-50.0000000
56240.0000000	10.7500000	-50.0000000
56240.0000000	11.0000000	-50.0000000
56240.0000000	11.2500000	-50.0000000
56240.0000000	11.5000000	-50.0000000
56240.0000000	11.7500000	-50.0000000
56240.0000000	12.0000000	-50.0000000
56240.0000000	12.2500000	-50.0000000
56240.0000000	12.5000000	-50.0000000
56240.0000000	12.7500000	-50.0000000
56240.0000000	13.0000000	-50.0000000
56240.0000000	13.2500000	-50.0000000
56240.0000000	13.5000000	-50.0000000
56240.0000000	13.7500000	-50.0000000
56240.0000000	14.0000000	-50.0000000
56240.0000000	14.2500000	-50.0000000
56240.0000000	14.5000000	-50.0000000
56240.0000000	14.7500000	-50.0000000
56240.0000000	15.0000000	-50.0000000
56270.0000000	0.0000000	-50.0000000
56270.0000000	0.2500000	19.1100006
56270.0000000	0.5000000	26.8199997
56270.0000000	0.7500000	25.1599998
56270.0000000	1.0000000	19.5499992

56270.0000000	1.2500000	18.2399998
56270.0000000	1.5000000	19.6299992
56270.0000000	1.7500000	21.0699997
56270.0000000	2.0000000	20.5900002
56270.0000000	2.2500000	18.7500000
56270.0000000	2.5000000	19.9500008
56270.0000000	2.7500000	20.5499992
56270.0000000	3.0000000	18.9899998
56270.0000000	3.2500000	16.3700008
56270.0000000	3.5000000	17.1700001
56270.0000000	3.7500000	18.0900002
56270.0000000	4.0000000	16.2500000
56270.0000000	4.2500000	16.8799992
56270.0000000	4.5000000	16.6499996
56270.0000000	4.7500000	-50.0000000
56270.0000000	5.0000000	-50.0000000
56270.0000000	5.2500000	-50.0000000
56270.0000000	5.5000000	-50.0000000
56270.0000000	5.7500000	-50.0000000
56270.0000000	6.0000000	-50.0000000
56270.0000000	6.2500000	-50.0000000
56270.0000000	6.5000000	-50.0000000
56270.0000000	6.7500000	-50.0000000
56270.0000000	7.0000000	-50.0000000
56270.0000000	7.2500000	-50.0000000
56270.0000000	7.5000000	-50.0000000
56270.0000000	7.7500000	-50.0000000
56270.0000000	8.0000000	-50.0000000
56270.0000000	8.2500000	-50.0000000
56270.0000000	8.5000000	-50.0000000
56270.0000000	8.7500000	-50.0000000
56270.0000000	9.0000000	-50.0000000
56270.0000000	9.2500000	-50.0000000
56270.0000000	9.5000000	-50.0000000
56270.0000000	9.7500000	-50.0000000
56270.0000000	10.0000000	-50.0000000
56270.0000000	10.2500000	-50.0000000
56270.0000000	10.5000000	-50.0000000
56270.0000000	10.7500000	-50.0000000
56270.0000000	11.0000000	-50.0000000
56270.0000000	11.2500000	-50.0000000
56270.0000000	11.5000000	-50.0000000
56270.0000000	11.7500000	-50.0000000
56270.0000000	12.0000000	-50.0000000
56270.0000000	12.2500000	-50.0000000
56270.0000000	12.5000000	-50.0000000
56270.0000000	12.7500000	-50.0000000
56270.0000000	13.0000000	-50.0000000
56270.0000000	13.2500000	-50.0000000
56270.0000000	13.5000000	-50.0000000
56270.0000000	13.7500000	-50.0000000
56270.0000000	14.0000000	-50.0000000
56270.0000000	14.2500000	-50.0000000
56270.0000000	14.5000000	-50.0000000
56270.0000000	14.7500000	-50.0000000
56270.0000000	15.0000000	-50.0000000
56300.0000000	0.0000000	-50.0000000
56300.0000000	0.2500000	-50.0000000

56300.0000000	0.5000000	21.2900009
56300.0000000	0.7500000	25.1599998
56300.0000000	1.0000000	24.5599995
56300.0000000	1.2500000	20.3299999
56300.0000000	1.5000000	21.1700001
56300.0000000	1.7500000	20.6399994
56300.0000000	2.0000000	19.7299995
56300.0000000	2.2500000	18.8700008
56300.0000000	2.5000000	18.4500008
56300.0000000	2.7500000	18.2399998
56300.0000000	3.0000000	20.0400009
56300.0000000	3.2500000	19.6200008
56300.0000000	3.5000000	18.5699997
56300.0000000	3.7500000	-50.0000000
56300.0000000	4.0000000	16.5000000
56300.0000000	4.2500000	18.2299995
56300.0000000	4.5000000	16.1200008
56300.0000000	4.7500000	-50.0000000
56300.0000000	5.0000000	-50.0000000
56300.0000000	5.2500000	-50.0000000
56300.0000000	5.5000000	-50.0000000
56300.0000000	5.7500000	-50.0000000
56300.0000000	6.0000000	-50.0000000
56300.0000000	6.2500000	-50.0000000
56300.0000000	6.5000000	-50.0000000
56300.0000000	6.7500000	-50.0000000
56300.0000000	7.0000000	-50.0000000
56300.0000000	7.2500000	-50.0000000
56300.0000000	7.5000000	-50.0000000
56300.0000000	7.7500000	-50.0000000
56300.0000000	8.0000000	-50.0000000
56300.0000000	8.2500000	-50.0000000
56300.0000000	8.5000000	-50.0000000
56300.0000000	8.7500000	-50.0000000
56300.0000000	9.0000000	-50.0000000
56300.0000000	9.2500000	-50.0000000
56300.0000000	9.5000000	-50.0000000
56300.0000000	9.7500000	-50.0000000
56300.0000000	10.0000000	-50.0000000
56300.0000000	10.2500000	-50.0000000
56300.0000000	10.5000000	-50.0000000
56300.0000000	10.7500000	-50.0000000
56300.0000000	11.0000000	-50.0000000
56300.0000000	11.2500000	-50.0000000
56300.0000000	11.5000000	-50.0000000
56300.0000000	11.7500000	-50.0000000
56300.0000000	12.0000000	-50.0000000
56300.0000000	12.2500000	-50.0000000
56300.0000000	12.5000000	-50.0000000
56300.0000000	12.7500000	-50.0000000
56300.0000000	13.0000000	-50.0000000
56300.0000000	13.2500000	-50.0000000
56300.0000000	13.5000000	-50.0000000
56300.0000000	13.7500000	-50.0000000
56300.0000000	14.0000000	-50.0000000
56300.0000000	14.2500000	-50.0000000
56300.0000000	14.5000000	-50.0000000
56300.0000000	14.7500000	-50.0000000

56300.0000000	15.0000000	-50.0000000
56330.0000000	0.0000000	-50.0000000
56330.0000000	0.2500000	21.1900005
56330.0000000	0.5000000	28.7900009
56330.0000000	0.7500000	27.4099998
56330.0000000	1.0000000	22.8400002
56330.0000000	1.2500000	18.5599995
56330.0000000	1.5000000	21.8299999
56330.0000000	1.7500000	21.0100002
56330.0000000	2.0000000	19.4500008
56330.0000000	2.2500000	20.0200005
56330.0000000	2.5000000	19.4500008
56330.0000000	2.7500000	19.1200008
56330.0000000	3.0000000	18.3799992
56330.0000000	3.2500000	18.4599991
56330.0000000	3.5000000	15.8599997
56330.0000000	3.7500000	16.2000008
56330.0000000	4.0000000	16.2099991
56330.0000000	4.2500000	19.0200005
56330.0000000	4.5000000	19.1100006
56330.0000000	4.7500000	17.6800003
56330.0000000	5.0000000	-50.0000000
56330.0000000	5.2500000	-50.0000000
56330.0000000	5.5000000	-50.0000000
56330.0000000	5.7500000	-50.0000000
56330.0000000	6.0000000	-50.0000000
56330.0000000	6.2500000	-50.0000000
56330.0000000	6.5000000	-50.0000000
56330.0000000	6.7500000	-50.0000000
56330.0000000	7.0000000	-50.0000000
56330.0000000	7.2500000	-50.0000000
56330.0000000	7.5000000	-50.0000000
56330.0000000	7.7500000	-50.0000000
56330.0000000	8.0000000	-50.0000000
56330.0000000	8.2500000	-50.0000000
56330.0000000	8.5000000	-50.0000000
56330.0000000	8.7500000	-50.0000000
56330.0000000	9.0000000	-50.0000000
56330.0000000	9.2500000	-50.0000000
56330.0000000	9.5000000	-50.0000000
56330.0000000	9.7500000	-50.0000000
56330.0000000	10.0000000	-50.0000000
56330.0000000	10.2500000	-50.0000000
56330.0000000	10.5000000	-50.0000000
56330.0000000	10.7500000	-50.0000000
56330.0000000	11.0000000	-50.0000000
56330.0000000	11.2500000	-50.0000000
56330.0000000	11.5000000	-50.0000000
56330.0000000	11.7500000	-50.0000000
56330.0000000	12.0000000	-50.0000000
56330.0000000	12.2500000	-50.0000000
56330.0000000	12.5000000	-50.0000000
56330.0000000	12.7500000	-50.0000000
56330.0000000	13.0000000	-50.0000000
56330.0000000	13.2500000	-50.0000000
56330.0000000	13.5000000	-50.0000000
56330.0000000	13.7500000	-50.0000000
56330.0000000	14.0000000	-50.0000000

56330.0000000	14.2500000	-50.0000000
56330.0000000	14.5000000	-50.0000000
56330.0000000	14.7500000	-50.0000000
56330.0000000	15.0000000	-50.0000000
56360.0000000	0.0000000	-50.0000000
56360.0000000	0.2500000	19.8600006
56360.0000000	0.5000000	27.2299995
56360.0000000	0.7500000	26.5799999
56360.0000000	1.0000000	21.7299995
56360.0000000	1.2500000	19.9899998
56360.0000000	1.5000000	21.3999996
56360.0000000	1.7500000	20.3799992
56360.0000000	2.0000000	18.4599991
56360.0000000	2.2500000	19.3799992
56360.0000000	2.5000000	21.3700008
56360.0000000	2.7500000	21.8199997
56360.0000000	3.0000000	21.4400005
56360.0000000	3.2500000	17.7099991
56360.0000000	3.5000000	17.7399998
56360.0000000	3.7500000	19.0699997
56360.0000000	4.0000000	16.5900002
56360.0000000	4.2500000	16.1599998
56360.0000000	4.5000000	16.5400009
56360.0000000	4.7500000	-50.0000000
56360.0000000	5.0000000	-50.0000000
56360.0000000	5.2500000	-50.0000000
56360.0000000	5.5000000	-50.0000000
56360.0000000	5.7500000	-50.0000000
56360.0000000	6.0000000	-50.0000000
56360.0000000	6.2500000	-50.0000000
56360.0000000	6.5000000	-50.0000000
56360.0000000	6.7500000	-50.0000000
56360.0000000	7.0000000	-50.0000000
56360.0000000	7.2500000	-50.0000000
56360.0000000	7.5000000	-50.0000000
56360.0000000	7.7500000	-50.0000000
56360.0000000	8.0000000	-50.0000000
56360.0000000	8.2500000	-50.0000000
56360.0000000	8.5000000	-50.0000000
56360.0000000	8.7500000	-50.0000000
56360.0000000	9.0000000	-50.0000000
56360.0000000	9.2500000	-50.0000000
56360.0000000	9.5000000	-50.0000000
56360.0000000	9.7500000	-50.0000000
56360.0000000	10.0000000	-50.0000000
56360.0000000	10.2500000	-50.0000000
56360.0000000	10.5000000	-50.0000000
56360.0000000	10.7500000	-50.0000000
56360.0000000	11.0000000	-50.0000000
56360.0000000	11.2500000	-50.0000000
56360.0000000	11.5000000	-50.0000000
56360.0000000	11.7500000	-50.0000000
56360.0000000	12.0000000	-50.0000000
56360.0000000	12.2500000	-50.0000000
56360.0000000	12.5000000	-50.0000000
56360.0000000	12.7500000	-50.0000000
56360.0000000	13.0000000	-50.0000000
56360.0000000	13.2500000	-50.0000000

56360.0000000	13.5000000	-50.0000000
56360.0000000	13.7500000	-50.0000000
56360.0000000	14.0000000	-50.0000000
56360.0000000	14.2500000	-50.0000000
56360.0000000	14.5000000	-50.0000000
56360.0000000	14.7500000	-50.0000000
56360.0000000	15.0000000	-50.0000000
56390.0000000	0.0000000	-50.0000000
56390.0000000	0.2500000	18.7999992
56390.0000000	0.5000000	27.9300003
56390.0000000	0.7500000	27.2099991
56390.0000000	1.0000000	20.2199993
56390.0000000	1.2500000	19.8700008
56390.0000000	1.5000000	20.3400002
56390.0000000	1.7500000	21.0499992
56390.0000000	2.0000000	19.8099995
56390.0000000	2.2500000	19.5499992
56390.0000000	2.5000000	18.5499992
56390.0000000	2.7500000	20.5100002
56390.0000000	3.0000000	21.1000004
56390.0000000	3.2500000	19.1100006
56390.0000000	3.5000000	19.0599995
56390.0000000	3.7500000	18.8500004
56390.0000000	4.0000000	17.4300003
56390.0000000	4.2500000	17.4300003
56390.0000000	4.5000000	17.5799999
56390.0000000	4.7500000	-50.0000000
56390.0000000	5.0000000	-50.0000000
56390.0000000	5.2500000	-50.0000000
56390.0000000	5.5000000	-50.0000000
56390.0000000	5.7500000	-50.0000000
56390.0000000	6.0000000	-50.0000000
56390.0000000	6.2500000	-50.0000000
56390.0000000	6.5000000	-50.0000000
56390.0000000	6.7500000	-50.0000000
56390.0000000	7.0000000	-50.0000000
56390.0000000	7.2500000	-50.0000000
56390.0000000	7.5000000	-50.0000000
56390.0000000	7.7500000	-50.0000000
56390.0000000	8.0000000	-50.0000000
56390.0000000	8.2500000	-50.0000000
56390.0000000	8.5000000	-50.0000000
56390.0000000	8.7500000	-50.0000000
56390.0000000	9.0000000	-50.0000000
56390.0000000	9.2500000	-50.0000000
56390.0000000	9.5000000	-50.0000000
56390.0000000	9.7500000	-50.0000000
56390.0000000	10.0000000	-50.0000000
56390.0000000	10.2500000	-50.0000000
56390.0000000	10.5000000	-50.0000000
56390.0000000	10.7500000	-50.0000000
56390.0000000	11.0000000	-50.0000000
56390.0000000	11.2500000	-50.0000000
56390.0000000	11.5000000	-50.0000000
56390.0000000	11.7500000	-50.0000000
56390.0000000	12.0000000	-50.0000000
56390.0000000	12.2500000	-50.0000000
56390.0000000	12.5000000	-50.0000000

56390.0000000	12.7500000	-50.0000000
56390.0000000	13.0000000	-50.0000000
56390.0000000	13.2500000	-50.0000000
56390.0000000	13.5000000	-50.0000000
56390.0000000	13.7500000	-50.0000000
56390.0000000	14.0000000	-50.0000000
56390.0000000	14.2500000	-50.0000000
56390.0000000	14.5000000	-50.0000000
56390.0000000	14.7500000	-50.0000000
56390.0000000	15.0000000	-50.0000000
56420.0000000	0.0000000	-50.0000000
56420.0000000	0.2500000	18.4200001
56420.0000000	0.5000000	26.5799999
56420.0000000	0.7500000	26.0400009
56420.0000000	1.0000000	22.1299992
56420.0000000	1.2500000	22.7999992
56420.0000000	1.5000000	21.9200001
56420.0000000	1.7500000	20.1900005
56420.0000000	2.0000000	19.6499996
56420.0000000	2.2500000	19.1800003
56420.0000000	2.5000000	21.4599991
56420.0000000	2.7500000	20.7900009
56420.0000000	3.0000000	20.6499996
56420.0000000	3.2500000	19.1200008
56420.0000000	3.5000000	-50.0000000
56420.0000000	3.7500000	15.2799997
56420.0000000	4.0000000	16.6000004
56420.0000000	4.2500000	16.8999996
56420.0000000	4.5000000	15.9499998
56420.0000000	4.7500000	15.9600000
56420.0000000	5.0000000	-50.0000000
56420.0000000	5.2500000	-50.0000000
56420.0000000	5.5000000	-50.0000000
56420.0000000	5.7500000	-50.0000000
56420.0000000	6.0000000	-50.0000000
56420.0000000	6.2500000	-50.0000000
56420.0000000	6.5000000	-50.0000000
56420.0000000	6.7500000	-50.0000000
56420.0000000	7.0000000	-50.0000000
56420.0000000	7.2500000	-50.0000000
56420.0000000	7.5000000	-50.0000000
56420.0000000	7.7500000	-50.0000000
56420.0000000	8.0000000	-50.0000000
56420.0000000	8.2500000	-50.0000000
56420.0000000	8.5000000	-50.0000000
56420.0000000	8.7500000	-50.0000000
56420.0000000	9.0000000	-50.0000000
56420.0000000	9.2500000	-50.0000000
56420.0000000	9.5000000	-50.0000000
56420.0000000	9.7500000	-50.0000000
56420.0000000	10.0000000	-50.0000000
56420.0000000	10.2500000	-50.0000000
56420.0000000	10.5000000	-50.0000000
56420.0000000	10.7500000	-50.0000000
56420.0000000	11.0000000	-50.0000000
56420.0000000	11.2500000	-50.0000000
56420.0000000	11.5000000	-50.0000000
56420.0000000	11.7500000	-50.0000000

56420.0000000	12.0000000	-50.0000000
56420.0000000	12.2500000	-50.0000000
56420.0000000	12.5000000	-50.0000000
56420.0000000	12.7500000	-50.0000000
56420.0000000	13.0000000	-50.0000000
56420.0000000	13.2500000	-50.0000000
56420.0000000	13.5000000	-50.0000000
56420.0000000	13.7500000	-50.0000000
56420.0000000	14.0000000	-50.0000000
56420.0000000	14.2500000	-50.0000000
56420.0000000	14.5000000	-50.0000000
56420.0000000	14.7500000	-50.0000000
56420.0000000	15.0000000	-50.0000000
56450.0000000	0.0000000	-50.0000000
56450.0000000	0.2500000	20.7199993
56450.0000000	0.5000000	27.8799992
56450.0000000	0.7500000	26.6000004
56450.0000000	1.0000000	20.2600002
56450.0000000	1.2500000	19.2999992
56450.0000000	1.5000000	20.6499996
56450.0000000	1.7500000	21.6299992
56450.0000000	2.0000000	20.7299995
56450.0000000	2.2500000	21.6599998
56450.0000000	2.5000000	20.0400009
56450.0000000	2.7500000	18.5799999
56450.0000000	3.0000000	17.5599995
56450.0000000	3.2500000	18.2099991
56450.0000000	3.5000000	16.3400002
56450.0000000	3.7500000	17.6100006
56450.0000000	4.0000000	19.2999992
56450.0000000	4.2500000	19.7600002
56450.0000000	4.5000000	21.8999996
56450.0000000	4.7500000	17.1200008
56450.0000000	5.0000000	-50.0000000
56450.0000000	5.2500000	-50.0000000
56450.0000000	5.5000000	-50.0000000
56450.0000000	5.7500000	-50.0000000
56450.0000000	6.0000000	-50.0000000
56450.0000000	6.2500000	-50.0000000
56450.0000000	6.5000000	-50.0000000
56450.0000000	6.7500000	-50.0000000
56450.0000000	7.0000000	-50.0000000
56450.0000000	7.2500000	-50.0000000
56450.0000000	7.5000000	-50.0000000
56450.0000000	7.7500000	-50.0000000
56450.0000000	8.0000000	-50.0000000
56450.0000000	8.2500000	-50.0000000
56450.0000000	8.5000000	-50.0000000
56450.0000000	8.7500000	-50.0000000
56450.0000000	9.0000000	-50.0000000
56450.0000000	9.2500000	-50.0000000
56450.0000000	9.5000000	-50.0000000
56450.0000000	9.7500000	-50.0000000
56450.0000000	10.0000000	-50.0000000
56450.0000000	10.2500000	-50.0000000
56450.0000000	10.5000000	-50.0000000
56450.0000000	10.7500000	-50.0000000
56450.0000000	11.0000000	-50.0000000

56450.0000000	11.2500000	-50.0000000
56450.0000000	11.5000000	-50.0000000
56450.0000000	11.7500000	-50.0000000
56450.0000000	12.0000000	-50.0000000
56450.0000000	12.2500000	-50.0000000
56450.0000000	12.5000000	-50.0000000
56450.0000000	12.7500000	-50.0000000
56450.0000000	13.0000000	-50.0000000
56450.0000000	13.2500000	-50.0000000
56450.0000000	13.5000000	-50.0000000
56450.0000000	13.7500000	-50.0000000
56450.0000000	14.0000000	-50.0000000
56450.0000000	14.2500000	-50.0000000
56450.0000000	14.5000000	-50.0000000
56450.0000000	14.7500000	-50.0000000
56450.0000000	15.0000000	-50.0000000
56480.0000000	0.0000000	-50.0000000
56480.0000000	0.2500000	17.5900002
56480.0000000	0.5000000	25.2099991
56480.0000000	0.7500000	24.5300007
56480.0000000	1.0000000	20.6299992
56480.0000000	1.2500000	21.7399998
56480.0000000	1.5000000	20.9400005
56480.0000000	1.7500000	21.3899994
56480.0000000	2.0000000	19.4200001
56480.0000000	2.2500000	20.4200001
56480.0000000	2.5000000	18.8099995
56480.0000000	2.7500000	18.8099995
56480.0000000	3.0000000	17.8700008
56480.0000000	3.2500000	18.7000008
56480.0000000	3.5000000	18.2800007
56480.0000000	3.7500000	-50.0000000
56480.0000000	4.0000000	16.3199997
56480.0000000	4.2500000	19.4300003
56480.0000000	4.5000000	16.6100006
56480.0000000	4.7500000	-50.0000000
56480.0000000	5.0000000	-50.0000000
56480.0000000	5.2500000	-50.0000000
56480.0000000	5.5000000	-50.0000000
56480.0000000	5.7500000	-50.0000000
56480.0000000	6.0000000	-50.0000000
56480.0000000	6.2500000	-50.0000000
56480.0000000	6.5000000	-50.0000000
56480.0000000	6.7500000	-50.0000000
56480.0000000	7.0000000	-50.0000000
56480.0000000	7.2500000	-50.0000000
56480.0000000	7.5000000	-50.0000000
56480.0000000	7.7500000	-50.0000000
56480.0000000	8.0000000	-50.0000000
56480.0000000	8.2500000	-50.0000000
56480.0000000	8.5000000	-50.0000000
56480.0000000	8.7500000	-50.0000000
56480.0000000	9.0000000	-50.0000000
56480.0000000	9.2500000	-50.0000000
56480.0000000	9.5000000	-50.0000000
56480.0000000	9.7500000	-50.0000000
56480.0000000	10.0000000	-50.0000000
56480.0000000	10.2500000	-50.0000000

56480.0000000	10.5000000	-50.0000000
56480.0000000	10.7500000	-50.0000000
56480.0000000	11.0000000	-50.0000000
56480.0000000	11.2500000	-50.0000000
56480.0000000	11.5000000	-50.0000000
56480.0000000	11.7500000	-50.0000000
56480.0000000	12.0000000	-50.0000000
56480.0000000	12.2500000	-50.0000000
56480.0000000	12.5000000	-50.0000000
56480.0000000	12.7500000	-50.0000000
56480.0000000	13.0000000	-50.0000000
56480.0000000	13.2500000	-50.0000000
56480.0000000	13.5000000	-50.0000000
56480.0000000	13.7500000	-50.0000000
56480.0000000	14.0000000	-50.0000000
56480.0000000	14.2500000	-50.0000000
56480.0000000	14.5000000	-50.0000000
56480.0000000	14.7500000	-50.0000000
56480.0000000	15.0000000	-50.0000000
56510.0000000	0.0000000	-50.0000000
56510.0000000	0.2500000	22.7299995
56510.0000000	0.5000000	30.0200005
56510.0000000	0.7500000	27.6000004
56510.0000000	1.0000000	21.0300007
56510.0000000	1.2500000	22.6000004
56510.0000000	1.5000000	20.2000008
56510.0000000	1.7500000	18.4300003
56510.0000000	2.0000000	20.3299999
56510.0000000	2.2500000	21.7999992
56510.0000000	2.5000000	20.4899998
56510.0000000	2.7500000	18.3600006
56510.0000000	3.0000000	18.1499996
56510.0000000	3.2500000	17.3700008
56510.0000000	3.5000000	16.7199993
56510.0000000	3.7500000	18.5799999
56510.0000000	4.0000000	17.9699993
56510.0000000	4.2500000	18.1100006
56510.0000000	4.5000000	-50.0000000
56510.0000000	4.7500000	-50.0000000
56510.0000000	5.0000000	-50.0000000
56510.0000000	5.2500000	-50.0000000
56510.0000000	5.5000000	-50.0000000
56510.0000000	5.7500000	-50.0000000
56510.0000000	6.0000000	-50.0000000
56510.0000000	6.2500000	-50.0000000
56510.0000000	6.5000000	-50.0000000
56510.0000000	6.7500000	-50.0000000
56510.0000000	7.0000000	-50.0000000
56510.0000000	7.2500000	-50.0000000
56510.0000000	7.5000000	-50.0000000
56510.0000000	7.7500000	-50.0000000
56510.0000000	8.0000000	-50.0000000
56510.0000000	8.2500000	-50.0000000
56510.0000000	8.5000000	-50.0000000
56510.0000000	8.7500000	-50.0000000
56510.0000000	9.0000000	-50.0000000
56510.0000000	9.2500000	-50.0000000
56510.0000000	9.5000000	-50.0000000

56510.0000000	9.7500000	-50.0000000
56510.0000000	10.0000000	-50.0000000
56510.0000000	10.2500000	-50.0000000
56510.0000000	10.5000000	-50.0000000
56510.0000000	10.7500000	-50.0000000
56510.0000000	11.0000000	-50.0000000
56510.0000000	11.2500000	-50.0000000
56510.0000000	11.5000000	-50.0000000
56510.0000000	11.7500000	-50.0000000
56510.0000000	12.0000000	-50.0000000
56510.0000000	12.2500000	-50.0000000
56510.0000000	12.5000000	-50.0000000
56510.0000000	12.7500000	-50.0000000
56510.0000000	13.0000000	-50.0000000
56510.0000000	13.2500000	-50.0000000
56510.0000000	13.5000000	-50.0000000
56510.0000000	13.7500000	-50.0000000
56510.0000000	14.0000000	-50.0000000
56510.0000000	14.2500000	-50.0000000
56510.0000000	14.5000000	-50.0000000
56510.0000000	14.7500000	-50.0000000
56510.0000000	15.0000000	-50.0000000
56540.0000000	0.0000000	-50.0000000
56540.0000000	0.2500000	19.8099995
56540.0000000	0.5000000	27.4899998
56540.0000000	0.7500000	26.3899994
56540.0000000	1.0000000	20.7999992
56540.0000000	1.2500000	19.8899994
56540.0000000	1.5000000	19.7500000
56540.0000000	1.7500000	21.2500000
56540.0000000	2.0000000	20.6900005
56540.0000000	2.2500000	19.7600002
56540.0000000	2.5000000	19.1000004
56540.0000000	2.7500000	15.1999998
56540.0000000	3.0000000	17.0799999
56540.0000000	3.2500000	18.1200008
56540.0000000	3.5000000	17.5799999
56540.0000000	3.7500000	17.4200001
56540.0000000	4.0000000	16.6499996
56540.0000000	4.2500000	17.5300007
56540.0000000	4.5000000	17.7399998
56540.0000000	4.7500000	15.3199997
56540.0000000	5.0000000	-50.0000000
56540.0000000	5.2500000	-50.0000000
56540.0000000	5.5000000	-50.0000000
56540.0000000	5.7500000	-50.0000000
56540.0000000	6.0000000	-50.0000000
56540.0000000	6.2500000	-50.0000000
56540.0000000	6.5000000	-50.0000000
56540.0000000	6.7500000	-50.0000000
56540.0000000	7.0000000	-50.0000000
56540.0000000	7.2500000	-50.0000000
56540.0000000	7.5000000	-50.0000000
56540.0000000	7.7500000	-50.0000000
56540.0000000	8.0000000	-50.0000000
56540.0000000	8.2500000	-50.0000000
56540.0000000	8.5000000	-50.0000000
56540.0000000	8.7500000	-50.0000000

56540.0000000	9.0000000	-50.0000000
56540.0000000	9.2500000	-50.0000000
56540.0000000	9.5000000	-50.0000000
56540.0000000	9.7500000	-50.0000000
56540.0000000	10.0000000	-50.0000000
56540.0000000	10.2500000	-50.0000000
56540.0000000	10.5000000	-50.0000000
56540.0000000	10.7500000	-50.0000000
56540.0000000	11.0000000	-50.0000000
56540.0000000	11.2500000	-50.0000000
56540.0000000	11.5000000	-50.0000000
56540.0000000	11.7500000	-50.0000000
56540.0000000	12.0000000	-50.0000000
56540.0000000	12.2500000	-50.0000000
56540.0000000	12.5000000	-50.0000000
56540.0000000	12.7500000	-50.0000000
56540.0000000	13.0000000	-50.0000000
56540.0000000	13.2500000	-50.0000000
56540.0000000	13.5000000	-50.0000000
56540.0000000	13.7500000	-50.0000000
56540.0000000	14.0000000	-50.0000000
56540.0000000	14.2500000	-50.0000000
56540.0000000	14.5000000	-50.0000000
56540.0000000	14.7500000	-50.0000000
56540.0000000	15.0000000	-50.0000000
56570.0000000	0.0000000	-50.0000000
56570.0000000	0.2500000	21.4599991
56570.0000000	0.5000000	29.0699997
56570.0000000	0.7500000	27.5599995
56570.0000000	1.0000000	21.0599995
56570.0000000	1.2500000	21.1700001
56570.0000000	1.5000000	21.2399998
56570.0000000	1.7500000	21.3899994
56570.0000000	2.0000000	19.9300003
56570.0000000	2.2500000	19.7399998
56570.0000000	2.5000000	19.1299992
56570.0000000	2.7500000	19.8999996
56570.0000000	3.0000000	20.6700001
56570.0000000	3.2500000	17.9699993
56570.0000000	3.5000000	-50.0000000
56570.0000000	3.7500000	16.7199993
56570.0000000	4.0000000	20.5499992
56570.0000000	4.2500000	17.0200005
56570.0000000	4.5000000	16.7099991
56570.0000000	4.7500000	-50.0000000
56570.0000000	5.0000000	-50.0000000
56570.0000000	5.2500000	-50.0000000
56570.0000000	5.5000000	-50.0000000
56570.0000000	5.7500000	-50.0000000
56570.0000000	6.0000000	-50.0000000
56570.0000000	6.2500000	-50.0000000
56570.0000000	6.5000000	-50.0000000
56570.0000000	6.7500000	-50.0000000
56570.0000000	7.0000000	-50.0000000
56570.0000000	7.2500000	-50.0000000
56570.0000000	7.5000000	-50.0000000
56570.0000000	7.7500000	-50.0000000
56570.0000000	8.0000000	-50.0000000

56570.0000000	8.2500000	-50.0000000
56570.0000000	8.5000000	-50.0000000
56570.0000000	8.7500000	-50.0000000
56570.0000000	9.0000000	-50.0000000
56570.0000000	9.2500000	-50.0000000
56570.0000000	9.5000000	-50.0000000
56570.0000000	9.7500000	-50.0000000
56570.0000000	10.0000000	-50.0000000
56570.0000000	10.2500000	-50.0000000
56570.0000000	10.5000000	-50.0000000
56570.0000000	10.7500000	-50.0000000
56570.0000000	11.0000000	-50.0000000
56570.0000000	11.2500000	-50.0000000
56570.0000000	11.5000000	-50.0000000
56570.0000000	11.7500000	-50.0000000
56570.0000000	12.0000000	-50.0000000
56570.0000000	12.2500000	-50.0000000
56570.0000000	12.5000000	-50.0000000
56570.0000000	12.7500000	-50.0000000
56570.0000000	13.0000000	-50.0000000
56570.0000000	13.2500000	-50.0000000
56570.0000000	13.5000000	-50.0000000
56570.0000000	13.7500000	-50.0000000
56570.0000000	14.0000000	-50.0000000
56570.0000000	14.2500000	-50.0000000
56570.0000000	14.5000000	-50.0000000
56570.0000000	14.7500000	-50.0000000
56570.0000000	15.0000000	-50.0000000
56600.0000000	0.0000000	-50.0000000
56600.0000000	0.2500000	20.3999996
56600.0000000	0.5000000	26.9899998
56600.0000000	0.7500000	25.1800003
56600.0000000	1.0000000	22.0699997
56600.0000000	1.2500000	22.6800003
56600.0000000	1.5000000	21.4899998
56600.0000000	1.7500000	19.1000004
56600.0000000	2.0000000	18.0799999
56600.0000000	2.2500000	19.7800007
56600.0000000	2.5000000	18.3500004
56600.0000000	2.7500000	19.8500004
56600.0000000	3.0000000	15.4200001
56600.0000000	3.2500000	19.3700008
56600.0000000	3.5000000	19.1000004
56600.0000000	3.7500000	17.5599995
56600.0000000	4.0000000	18.8099995
56600.0000000	4.2500000	17.2500000
56600.0000000	4.5000000	19.1299992
56600.0000000	4.7500000	16.4300003
56600.0000000	5.0000000	-50.0000000
56600.0000000	5.2500000	-50.0000000
56600.0000000	5.5000000	-50.0000000
56600.0000000	5.7500000	-50.0000000
56600.0000000	6.0000000	-50.0000000
56600.0000000	6.2500000	-50.0000000
56600.0000000	6.5000000	-50.0000000
56600.0000000	6.7500000	-50.0000000
56600.0000000	7.0000000	-50.0000000
56600.0000000	7.2500000	-50.0000000

56600.0000000	7.5000000	-50.0000000
56600.0000000	7.7500000	-50.0000000
56600.0000000	8.0000000	-50.0000000
56600.0000000	8.2500000	-50.0000000
56600.0000000	8.5000000	-50.0000000
56600.0000000	8.7500000	-50.0000000
56600.0000000	9.0000000	-50.0000000
56600.0000000	9.2500000	-50.0000000
56600.0000000	9.5000000	-50.0000000
56600.0000000	9.7500000	-50.0000000
56600.0000000	10.0000000	-50.0000000
56600.0000000	10.2500000	-50.0000000
56600.0000000	10.5000000	-50.0000000
56600.0000000	10.7500000	-50.0000000
56600.0000000	11.0000000	-50.0000000
56600.0000000	11.2500000	-50.0000000
56600.0000000	11.5000000	-50.0000000
56600.0000000	11.7500000	-50.0000000
56600.0000000	12.0000000	-50.0000000
56600.0000000	12.2500000	-50.0000000
56600.0000000	12.5000000	-50.0000000
56600.0000000	12.7500000	-50.0000000
56600.0000000	13.0000000	-50.0000000
56600.0000000	13.2500000	-50.0000000
56600.0000000	13.5000000	-50.0000000
56600.0000000	13.7500000	-50.0000000
56600.0000000	14.0000000	-50.0000000
56600.0000000	14.2500000	-50.0000000
56600.0000000	14.5000000	-50.0000000
56600.0000000	14.7500000	-50.0000000
56600.0000000	15.0000000	-50.0000000
56630.0000000	0.0000000	-50.0000000
56630.0000000	0.2500000	17.0200005
56630.0000000	0.5000000	25.8299999
56630.0000000	0.7500000	25.5599995
56630.0000000	1.0000000	22.1100006
56630.0000000	1.2500000	20.5300007
56630.0000000	1.5000000	21.2600002
56630.0000000	1.7500000	20.1200008
56630.0000000	2.0000000	19.9099998
56630.0000000	2.2500000	18.9699993
56630.0000000	2.5000000	20.0599995
56630.0000000	2.7500000	16.6700001
56630.0000000	3.0000000	17.7800007
56630.0000000	3.2500000	16.9300003
56630.0000000	3.5000000	19.2099991
56630.0000000	3.7500000	17.8600006
56630.0000000	4.0000000	18.0799999
56630.0000000	4.2500000	17.5000000
56630.0000000	4.5000000	18.4699993
56630.0000000	4.7500000	-50.0000000
56630.0000000	5.0000000	-50.0000000
56630.0000000	5.2500000	-50.0000000
56630.0000000	5.5000000	-50.0000000
56630.0000000	5.7500000	-50.0000000
56630.0000000	6.0000000	-50.0000000
56630.0000000	6.2500000	-50.0000000
56630.0000000	6.5000000	-50.0000000

56630.0000000	6.7500000	-50.0000000
56630.0000000	7.0000000	-50.0000000
56630.0000000	7.2500000	-50.0000000
56630.0000000	7.5000000	-50.0000000
56630.0000000	7.7500000	-50.0000000
56630.0000000	8.0000000	-50.0000000
56630.0000000	8.2500000	-50.0000000
56630.0000000	8.5000000	-50.0000000
56630.0000000	8.7500000	-50.0000000
56630.0000000	9.0000000	-50.0000000
56630.0000000	9.2500000	-50.0000000
56630.0000000	9.5000000	-50.0000000
56630.0000000	9.7500000	-50.0000000
56630.0000000	10.0000000	-50.0000000
56630.0000000	10.2500000	-50.0000000
56630.0000000	10.5000000	-50.0000000
56630.0000000	10.7500000	-50.0000000
56630.0000000	11.0000000	-50.0000000
56630.0000000	11.2500000	-50.0000000
56630.0000000	11.5000000	-50.0000000
56630.0000000	11.7500000	-50.0000000
56630.0000000	12.0000000	-50.0000000
56630.0000000	12.2500000	-50.0000000
56630.0000000	12.5000000	-50.0000000
56630.0000000	12.7500000	-50.0000000
56630.0000000	13.0000000	-50.0000000
56630.0000000	13.2500000	-50.0000000
56630.0000000	13.5000000	-50.0000000
56630.0000000	13.7500000	-50.0000000
56630.0000000	14.0000000	-50.0000000
56630.0000000	14.2500000	-50.0000000
56630.0000000	14.5000000	-50.0000000
56630.0000000	14.7500000	-50.0000000
56630.0000000	15.0000000	-50.0000000
56660.0000000	0.0000000	-50.0000000
56660.0000000	0.2500000	22.3600006
56660.0000000	0.5000000	28.4099998
56660.0000000	0.7500000	25.7000008
56660.0000000	1.0000000	20.6700001
56660.0000000	1.2500000	17.7900009
56660.0000000	1.5000000	19.1399994
56660.0000000	1.7500000	21.3799992
56660.0000000	2.0000000	19.6499996
56660.0000000	2.2500000	19.6700001
56660.0000000	2.5000000	19.6100006
56660.0000000	2.7500000	17.9599991
56660.0000000	3.0000000	16.4699993
56660.0000000	3.2500000	19.9400005
56660.0000000	3.5000000	20.9699993
56660.0000000	3.7500000	21.3600006
56660.0000000	4.0000000	20.2000008
56660.0000000	4.2500000	18.9899998
56660.0000000	4.5000000	17.6499996
56660.0000000	4.7500000	15.3500004
56660.0000000	5.0000000	-50.0000000
56660.0000000	5.2500000	-50.0000000
56660.0000000	5.5000000	-50.0000000
56660.0000000	5.7500000	-50.0000000

56660.0000000	6.0000000	-50.0000000
56660.0000000	6.2500000	-50.0000000
56660.0000000	6.5000000	-50.0000000
56660.0000000	6.7500000	-50.0000000
56660.0000000	7.0000000	-50.0000000
56660.0000000	7.2500000	-50.0000000
56660.0000000	7.5000000	-50.0000000
56660.0000000	7.7500000	-50.0000000
56660.0000000	8.0000000	-50.0000000
56660.0000000	8.2500000	-50.0000000
56660.0000000	8.5000000	-50.0000000
56660.0000000	8.7500000	-50.0000000
56660.0000000	9.0000000	-50.0000000
56660.0000000	9.2500000	-50.0000000
56660.0000000	9.5000000	-50.0000000
56660.0000000	9.7500000	-50.0000000
56660.0000000	10.0000000	-50.0000000
56660.0000000	10.2500000	-50.0000000
56660.0000000	10.5000000	-50.0000000
56660.0000000	10.7500000	-50.0000000
56660.0000000	11.0000000	-50.0000000
56660.0000000	11.2500000	-50.0000000
56660.0000000	11.5000000	-50.0000000
56660.0000000	11.7500000	-50.0000000
56660.0000000	12.0000000	-50.0000000
56660.0000000	12.2500000	-50.0000000
56660.0000000	12.5000000	-50.0000000
56660.0000000	12.7500000	-50.0000000
56660.0000000	13.0000000	-50.0000000
56660.0000000	13.2500000	-50.0000000
56660.0000000	13.5000000	-50.0000000
56660.0000000	13.7500000	-50.0000000
56660.0000000	14.0000000	-50.0000000
56660.0000000	14.2500000	-50.0000000
56660.0000000	14.5000000	-50.0000000
56660.0000000	14.7500000	-50.0000000
56660.0000000	15.0000000	-50.0000000
56690.0000000	0.0000000	-50.0000000
56690.0000000	0.2500000	15.5600004
56690.0000000	0.5000000	25.3299999
56690.0000000	0.7500000	25.2500000
56690.0000000	1.0000000	21.0799999
56690.0000000	1.2500000	21.7700005
56690.0000000	1.5000000	20.3700008
56690.0000000	1.7500000	20.9699993
56690.0000000	2.0000000	20.4500008
56690.0000000	2.2500000	21.4599991
56690.0000000	2.5000000	20.4500008
56690.0000000	2.7500000	18.4899998
56690.0000000	3.0000000	16.6499996
56690.0000000	3.2500000	19.0599995
56690.0000000	3.5000000	18.8899994
56690.0000000	3.7500000	21.1700001
56690.0000000	4.0000000	19.2399998
56690.0000000	4.2500000	17.1100006
56690.0000000	4.5000000	16.2800007
56690.0000000	4.7500000	16.2800007
56690.0000000	5.0000000	-50.0000000

56690.0000000	5.2500000	-50.0000000
56690.0000000	5.5000000	-50.0000000
56690.0000000	5.7500000	-50.0000000
56690.0000000	6.0000000	-50.0000000
56690.0000000	6.2500000	-50.0000000
56690.0000000	6.5000000	-50.0000000
56690.0000000	6.7500000	-50.0000000
56690.0000000	7.0000000	-50.0000000
56690.0000000	7.2500000	-50.0000000
56690.0000000	7.5000000	-50.0000000
56690.0000000	7.7500000	-50.0000000
56690.0000000	8.0000000	-50.0000000
56690.0000000	8.2500000	-50.0000000
56690.0000000	8.5000000	-50.0000000
56690.0000000	8.7500000	-50.0000000
56690.0000000	9.0000000	-50.0000000
56690.0000000	9.2500000	-50.0000000
56690.0000000	9.5000000	-50.0000000
56690.0000000	9.7500000	-50.0000000
56690.0000000	10.0000000	-50.0000000
56690.0000000	10.2500000	-50.0000000
56690.0000000	10.5000000	-50.0000000
56690.0000000	10.7500000	-50.0000000
56690.0000000	11.0000000	-50.0000000
56690.0000000	11.2500000	-50.0000000
56690.0000000	11.5000000	-50.0000000
56690.0000000	11.7500000	-50.0000000
56690.0000000	12.0000000	-50.0000000
56690.0000000	12.2500000	-50.0000000
56690.0000000	12.5000000	-50.0000000
56690.0000000	12.7500000	-50.0000000
56690.0000000	13.0000000	-50.0000000
56690.0000000	13.2500000	-50.0000000
56690.0000000	13.5000000	-50.0000000
56690.0000000	13.7500000	-50.0000000
56690.0000000	14.0000000	-50.0000000
56690.0000000	14.2500000	-50.0000000
56690.0000000	14.5000000	-50.0000000
56690.0000000	14.7500000	-50.0000000
56690.0000000	15.0000000	-50.0000000
56720.0000000	0.0000000	-50.0000000
56720.0000000	0.2500000	19.3999996
56720.0000000	0.5000000	28.2500000
56720.0000000	0.7500000	27.1299992
56720.0000000	1.0000000	19.8899994
56720.0000000	1.2500000	20.2800007
56720.0000000	1.5000000	20.7000008
56720.0000000	1.7500000	21.3999996
56720.0000000	2.0000000	20.8400002
56720.0000000	2.2500000	19.1100006
56720.0000000	2.5000000	18.1000004
56720.0000000	2.7500000	17.7900009
56720.0000000	3.0000000	17.0599995
56720.0000000	3.2500000	18.8999996
56720.0000000	3.5000000	17.9200001
56720.0000000	3.7500000	19.5599995
56720.0000000	4.0000000	19.0599995
56720.0000000	4.2500000	17.6100006

56720.0000000	4.5000000	19.1200008
56720.0000000	4.7500000	16.5900002
56720.0000000	5.0000000	-50.0000000
56720.0000000	5.2500000	-50.0000000
56720.0000000	5.5000000	-50.0000000
56720.0000000	5.7500000	-50.0000000
56720.0000000	6.0000000	-50.0000000
56720.0000000	6.2500000	-50.0000000
56720.0000000	6.5000000	-50.0000000
56720.0000000	6.7500000	-50.0000000
56720.0000000	7.0000000	-50.0000000
56720.0000000	7.2500000	-50.0000000
56720.0000000	7.5000000	-50.0000000
56720.0000000	7.7500000	-50.0000000
56720.0000000	8.0000000	-50.0000000
56720.0000000	8.2500000	-50.0000000
56720.0000000	8.5000000	-50.0000000
56720.0000000	8.7500000	-50.0000000
56720.0000000	9.0000000	-50.0000000
56720.0000000	9.2500000	-50.0000000
56720.0000000	9.5000000	-50.0000000
56720.0000000	9.7500000	-50.0000000
56720.0000000	10.0000000	-50.0000000
56720.0000000	10.2500000	-50.0000000
56720.0000000	10.5000000	-50.0000000
56720.0000000	10.7500000	-50.0000000
56720.0000000	11.0000000	-50.0000000
56720.0000000	11.2500000	-50.0000000
56720.0000000	11.5000000	-50.0000000
56720.0000000	11.7500000	-50.0000000
56720.0000000	12.0000000	-50.0000000
56720.0000000	12.2500000	-50.0000000
56720.0000000	12.5000000	-50.0000000
56720.0000000	12.7500000	-50.0000000
56720.0000000	13.0000000	-50.0000000
56720.0000000	13.2500000	-50.0000000
56720.0000000	13.5000000	-50.0000000
56720.0000000	13.7500000	-50.0000000
56720.0000000	14.0000000	-50.0000000
56720.0000000	14.2500000	-50.0000000
56720.0000000	14.5000000	-50.0000000
56720.0000000	14.7500000	-50.0000000
56720.0000000	15.0000000	-50.0000000
56750.0000000	0.0000000	-50.0000000
56750.0000000	0.2500000	25.3899994
56750.0000000	0.5000000	31.8199997
56750.0000000	0.7500000	28.9699993
56750.0000000	1.0000000	20.8799992
56750.0000000	1.2500000	19.7099991
56750.0000000	1.5000000	19.0200005
56750.0000000	1.7500000	18.3700008
56750.0000000	2.0000000	19.2600002
56750.0000000	2.2500000	19.6800003
56750.0000000	2.5000000	19.7000008
56750.0000000	2.7500000	15.6599998
56750.0000000	3.0000000	19.6200008
56750.0000000	3.2500000	20.4599991
56750.0000000	3.5000000	21.3700008

56750.0000000	3.7500000	18.9699993
56750.0000000	4.0000000	17.9699993
56750.0000000	4.2500000	-50.0000000
56750.0000000	4.5000000	18.9500008
56750.0000000	4.7500000	16.9899998
56750.0000000	5.0000000	-50.0000000
56750.0000000	5.2500000	-50.0000000
56750.0000000	5.5000000	-50.0000000
56750.0000000	5.7500000	-50.0000000
56750.0000000	6.0000000	-50.0000000
56750.0000000	6.2500000	-50.0000000
56750.0000000	6.5000000	-50.0000000
56750.0000000	6.7500000	-50.0000000
56750.0000000	7.0000000	-50.0000000
56750.0000000	7.2500000	-50.0000000
56750.0000000	7.5000000	-50.0000000
56750.0000000	7.7500000	-50.0000000
56750.0000000	8.0000000	-50.0000000
56750.0000000	8.2500000	-50.0000000
56750.0000000	8.5000000	-50.0000000
56750.0000000	8.7500000	-50.0000000
56750.0000000	9.0000000	-50.0000000
56750.0000000	9.2500000	-50.0000000
56750.0000000	9.5000000	-50.0000000
56750.0000000	9.7500000	-50.0000000
56750.0000000	10.0000000	-50.0000000
56750.0000000	10.2500000	-50.0000000
56750.0000000	10.5000000	-50.0000000
56750.0000000	10.7500000	-50.0000000
56750.0000000	11.0000000	-50.0000000
56750.0000000	11.2500000	-50.0000000
56750.0000000	11.5000000	-50.0000000
56750.0000000	11.7500000	-50.0000000
56750.0000000	12.0000000	-50.0000000
56750.0000000	12.2500000	-50.0000000
56750.0000000	12.5000000	-50.0000000
56750.0000000	12.7500000	-50.0000000
56750.0000000	13.0000000	-50.0000000
56750.0000000	13.2500000	-50.0000000
56750.0000000	13.5000000	-50.0000000
56750.0000000	13.7500000	-50.0000000
56750.0000000	14.0000000	-50.0000000
56750.0000000	14.2500000	-50.0000000
56750.0000000	14.5000000	-50.0000000
56750.0000000	14.7500000	-50.0000000
56750.0000000	15.0000000	-50.0000000
56780.0000000	0.0000000	-50.0000000
56780.0000000	0.2500000	20.5400009
56780.0000000	0.5000000	27.3799992
56780.0000000	0.7500000	25.2700005
56780.0000000	1.0000000	19.9099998
56780.0000000	1.2500000	23.6599998
56780.0000000	1.5000000	22.7099991
56780.0000000	1.7500000	19.4699993
56780.0000000	2.0000000	19.2700005
56780.0000000	2.2500000	21.1299992
56780.0000000	2.5000000	20.4200001
56780.0000000	2.7500000	19.7099991

56780.0000000	3.0000000	17.2099991
56780.0000000	3.2500000	18.0900002
56780.0000000	3.5000000	19.7600002
56780.0000000	3.7500000	21.2999992
56780.0000000	4.0000000	19.0599995
56780.0000000	4.2500000	18.2399998
56780.0000000	4.5000000	15.2200003
56780.0000000	4.7500000	-50.0000000
56780.0000000	5.0000000	-50.0000000
56780.0000000	5.2500000	-50.0000000
56780.0000000	5.5000000	-50.0000000
56780.0000000	5.7500000	-50.0000000
56780.0000000	6.0000000	-50.0000000
56780.0000000	6.2500000	-50.0000000
56780.0000000	6.5000000	-50.0000000
56780.0000000	6.7500000	-50.0000000
56780.0000000	7.0000000	-50.0000000
56780.0000000	7.2500000	-50.0000000
56780.0000000	7.5000000	-50.0000000
56780.0000000	7.7500000	-50.0000000
56780.0000000	8.0000000	-50.0000000
56780.0000000	8.2500000	-50.0000000
56780.0000000	8.5000000	-50.0000000
56780.0000000	8.7500000	-50.0000000
56780.0000000	9.0000000	-50.0000000
56780.0000000	9.2500000	-50.0000000
56780.0000000	9.5000000	-50.0000000
56780.0000000	9.7500000	-50.0000000
56780.0000000	10.0000000	-50.0000000
56780.0000000	10.2500000	-50.0000000
56780.0000000	10.5000000	-50.0000000
56780.0000000	10.7500000	-50.0000000
56780.0000000	11.0000000	-50.0000000
56780.0000000	11.2500000	-50.0000000
56780.0000000	11.5000000	-50.0000000
56780.0000000	11.7500000	-50.0000000
56780.0000000	12.0000000	-50.0000000
56780.0000000	12.2500000	-50.0000000
56780.0000000	12.5000000	-50.0000000
56780.0000000	12.7500000	-50.0000000
56780.0000000	13.0000000	-50.0000000
56780.0000000	13.2500000	-50.0000000
56780.0000000	13.5000000	-50.0000000
56780.0000000	13.7500000	-50.0000000
56780.0000000	14.0000000	-50.0000000
56780.0000000	14.2500000	-50.0000000
56780.0000000	14.5000000	-50.0000000
56780.0000000	14.7500000	-50.0000000
56780.0000000	15.0000000	-50.0000000
56810.0000000	0.0000000	-50.0000000
56810.0000000	0.2500000	19.0000000
56810.0000000	0.5000000	26.4300003
56810.0000000	0.7500000	25.8799992
56810.0000000	1.0000000	22.5400009
56810.0000000	1.2500000	21.2800007
56810.0000000	1.5000000	20.2000008
56810.0000000	1.7500000	19.7900009
56810.0000000	2.0000000	19.5499992

56810.0000000	2.2500000	19.7099991
56810.0000000	2.5000000	21.8099995
56810.0000000	2.7500000	18.8400002
56810.0000000	3.0000000	18.4699993
56810.0000000	3.2500000	21.7199993
56810.0000000	3.5000000	20.5499992
56810.0000000	3.7500000	20.3099995
56810.0000000	4.0000000	19.7099991
56810.0000000	4.2500000	16.3099995
56810.0000000	4.5000000	17.3799992
56810.0000000	4.7500000	15.6899996
56810.0000000	5.0000000	-50.0000000
56810.0000000	5.2500000	-50.0000000
56810.0000000	5.5000000	-50.0000000
56810.0000000	5.7500000	-50.0000000
56810.0000000	6.0000000	-50.0000000
56810.0000000	6.2500000	-50.0000000
56810.0000000	6.5000000	-50.0000000
56810.0000000	6.7500000	-50.0000000
56810.0000000	7.0000000	-50.0000000
56810.0000000	7.2500000	-50.0000000
56810.0000000	7.5000000	-50.0000000
56810.0000000	7.7500000	-50.0000000
56810.0000000	8.0000000	-50.0000000
56810.0000000	8.2500000	-50.0000000
56810.0000000	8.5000000	-50.0000000
56810.0000000	8.7500000	-50.0000000
56810.0000000	9.0000000	-50.0000000
56810.0000000	9.2500000	-50.0000000
56810.0000000	9.5000000	-50.0000000
56810.0000000	9.7500000	-50.0000000
56810.0000000	10.0000000	-50.0000000
56810.0000000	10.2500000	-50.0000000
56810.0000000	10.5000000	-50.0000000
56810.0000000	10.7500000	-50.0000000
56810.0000000	11.0000000	-50.0000000
56810.0000000	11.2500000	-50.0000000
56810.0000000	11.5000000	-50.0000000
56810.0000000	11.7500000	-50.0000000
56810.0000000	12.0000000	-50.0000000
56810.0000000	12.2500000	-50.0000000
56810.0000000	12.5000000	-50.0000000
56810.0000000	12.7500000	-50.0000000
56810.0000000	13.0000000	-50.0000000
56810.0000000	13.2500000	-50.0000000
56810.0000000	13.5000000	-50.0000000
56810.0000000	13.7500000	-50.0000000
56810.0000000	14.0000000	-50.0000000
56810.0000000	14.2500000	-50.0000000
56810.0000000	14.5000000	-50.0000000
56810.0000000	14.7500000	-50.0000000
56810.0000000	15.0000000	-50.0000000
56840.0000000	0.0000000	-50.0000000
56840.0000000	0.2500000	19.4200001
56840.0000000	0.5000000	27.4899998
56840.0000000	0.7500000	25.9099998
56840.0000000	1.0000000	19.8199997
56840.0000000	1.2500000	18.7800007

56840.0000000	1.5000000	19.2900009
56840.0000000	1.7500000	20.8799992
56840.0000000	2.0000000	20.8299999
56840.0000000	2.2500000	19.4300003
56840.0000000	2.5000000	19.3199997
56840.0000000	2.7500000	22.0200005
56840.0000000	3.0000000	21.1299992
56840.0000000	3.2500000	19.7000008
56840.0000000	3.5000000	19.5300007
56840.0000000	3.7500000	20.8600006
56840.0000000	4.0000000	16.7900009
56840.0000000	4.2500000	16.3600006
56840.0000000	4.5000000	17.3999996
56840.0000000	4.7500000	15.0299997
56840.0000000	5.0000000	-50.0000000
56840.0000000	5.2500000	-50.0000000
56840.0000000	5.5000000	-50.0000000
56840.0000000	5.7500000	-50.0000000
56840.0000000	6.0000000	-50.0000000
56840.0000000	6.2500000	-50.0000000
56840.0000000	6.5000000	-50.0000000
56840.0000000	6.7500000	-50.0000000
56840.0000000	7.0000000	-50.0000000
56840.0000000	7.2500000	-50.0000000
56840.0000000	7.5000000	-50.0000000
56840.0000000	7.7500000	-50.0000000
56840.0000000	8.0000000	-50.0000000
56840.0000000	8.2500000	-50.0000000
56840.0000000	8.5000000	-50.0000000
56840.0000000	8.7500000	-50.0000000
56840.0000000	9.0000000	-50.0000000
56840.0000000	9.2500000	-50.0000000
56840.0000000	9.5000000	-50.0000000
56840.0000000	9.7500000	-50.0000000
56840.0000000	10.0000000	-50.0000000
56840.0000000	10.2500000	-50.0000000
56840.0000000	10.5000000	-50.0000000
56840.0000000	10.7500000	-50.0000000
56840.0000000	11.0000000	-50.0000000
56840.0000000	11.2500000	-50.0000000
56840.0000000	11.5000000	-50.0000000
56840.0000000	11.7500000	-50.0000000
56840.0000000	12.0000000	-50.0000000
56840.0000000	12.2500000	-50.0000000
56840.0000000	12.5000000	-50.0000000
56840.0000000	12.7500000	-50.0000000
56840.0000000	13.0000000	-50.0000000
56840.0000000	13.2500000	-50.0000000
56840.0000000	13.5000000	-50.0000000
56840.0000000	13.7500000	-50.0000000
56840.0000000	14.0000000	-50.0000000
56840.0000000	14.2500000	-50.0000000
56840.0000000	14.5000000	-50.0000000
56840.0000000	14.7500000	-50.0000000
56840.0000000	15.0000000	-50.0000000
56870.0000000	0.0000000	-50.0000000
56870.0000000	0.2500000	21.5799999
56870.0000000	0.5000000	29.0699997

56870.0000000	0.7500000	27.1000004
56870.0000000	1.0000000	21.6000004
56870.0000000	1.2500000	21.6299992
56870.0000000	1.5000000	19.0200005
56870.0000000	1.7500000	18.0699997
56870.0000000	2.0000000	20.3700008
56870.0000000	2.2500000	20.9599991
56870.0000000	2.5000000	19.9400005
56870.0000000	2.7500000	19.8799992
56870.0000000	3.0000000	16.6399994
56870.0000000	3.2500000	17.4200001
56870.0000000	3.5000000	19.5200005
56870.0000000	3.7500000	19.6200008
56870.0000000	4.0000000	19.1700001
56870.0000000	4.2500000	15.8999996
56870.0000000	4.5000000	15.9300003
56870.0000000	4.7500000	-50.0000000
56870.0000000	5.0000000	-50.0000000
56870.0000000	5.2500000	-50.0000000
56870.0000000	5.5000000	-50.0000000
56870.0000000	5.7500000	-50.0000000
56870.0000000	6.0000000	-50.0000000
56870.0000000	6.2500000	-50.0000000
56870.0000000	6.5000000	-50.0000000
56870.0000000	6.7500000	-50.0000000
56870.0000000	7.0000000	-50.0000000
56870.0000000	7.2500000	-50.0000000
56870.0000000	7.5000000	-50.0000000
56870.0000000	7.7500000	-50.0000000
56870.0000000	8.0000000	-50.0000000
56870.0000000	8.2500000	-50.0000000
56870.0000000	8.5000000	-50.0000000
56870.0000000	8.7500000	-50.0000000
56870.0000000	9.0000000	-50.0000000
56870.0000000	9.2500000	-50.0000000
56870.0000000	9.5000000	-50.0000000
56870.0000000	9.7500000	-50.0000000
56870.0000000	10.0000000	-50.0000000
56870.0000000	10.2500000	-50.0000000
56870.0000000	10.5000000	-50.0000000
56870.0000000	10.7500000	-50.0000000
56870.0000000	11.0000000	-50.0000000
56870.0000000	11.2500000	-50.0000000
56870.0000000	11.5000000	-50.0000000
56870.0000000	11.7500000	-50.0000000
56870.0000000	12.0000000	-50.0000000
56870.0000000	12.2500000	-50.0000000
56870.0000000	12.5000000	-50.0000000
56870.0000000	12.7500000	-50.0000000
56870.0000000	13.0000000	-50.0000000
56870.0000000	13.2500000	-50.0000000
56870.0000000	13.5000000	-50.0000000
56870.0000000	13.7500000	-50.0000000
56870.0000000	14.0000000	-50.0000000
56870.0000000	14.2500000	-50.0000000
56870.0000000	14.5000000	-50.0000000
56870.0000000	14.7500000	-50.0000000
56870.0000000	15.0000000	-50.0000000

56900.0000000	0.0000000	-50.0000000
56900.0000000	0.2500000	23.9400005
56900.0000000	0.5000000	31.8400002
56900.0000000	0.7500000	29.8199997
56900.0000000	1.0000000	20.9500008
56900.0000000	1.2500000	20.0799999
56900.0000000	1.5000000	19.5499992
56900.0000000	1.7500000	20.1499996
56900.0000000	2.0000000	21.2299995
56900.0000000	2.2500000	18.5300007
56900.0000000	2.5000000	20.3700008
56900.0000000	2.7500000	18.2000008
56900.0000000	3.0000000	18.4699993
56900.0000000	3.2500000	18.2999992
56900.0000000	3.5000000	18.8199997
56900.0000000	3.7500000	18.5699997
56900.0000000	4.0000000	18.5699997
56900.0000000	4.2500000	16.6800003
56900.0000000	4.5000000	15.2799997
56900.0000000	4.7500000	-50.0000000
56900.0000000	5.0000000	-50.0000000
56900.0000000	5.2500000	-50.0000000
56900.0000000	5.5000000	-50.0000000
56900.0000000	5.7500000	-50.0000000
56900.0000000	6.0000000	-50.0000000
56900.0000000	6.2500000	-50.0000000
56900.0000000	6.5000000	-50.0000000
56900.0000000	6.7500000	-50.0000000
56900.0000000	7.0000000	-50.0000000
56900.0000000	7.2500000	-50.0000000
56900.0000000	7.5000000	-50.0000000
56900.0000000	7.7500000	-50.0000000
56900.0000000	8.0000000	-50.0000000
56900.0000000	8.2500000	-50.0000000
56900.0000000	8.5000000	-50.0000000
56900.0000000	8.7500000	-50.0000000
56900.0000000	9.0000000	-50.0000000
56900.0000000	9.2500000	-50.0000000
56900.0000000	9.5000000	-50.0000000
56900.0000000	9.7500000	-50.0000000
56900.0000000	10.0000000	-50.0000000
56900.0000000	10.2500000	-50.0000000
56900.0000000	10.5000000	-50.0000000
56900.0000000	10.7500000	-50.0000000
56900.0000000	11.0000000	-50.0000000
56900.0000000	11.2500000	-50.0000000
56900.0000000	11.5000000	-50.0000000
56900.0000000	11.7500000	-50.0000000
56900.0000000	12.0000000	-50.0000000
56900.0000000	12.2500000	-50.0000000
56900.0000000	12.5000000	-50.0000000
56900.0000000	12.7500000	-50.0000000
56900.0000000	13.0000000	-50.0000000
56900.0000000	13.2500000	-50.0000000
56900.0000000	13.5000000	-50.0000000
56900.0000000	13.7500000	-50.0000000
56900.0000000	14.0000000	-50.0000000
56900.0000000	14.2500000	-50.0000000

56900.0000000	14.5000000	-50.0000000
56900.0000000	14.7500000	-50.0000000
56900.0000000	15.0000000	-50.0000000
56930.0000000	0.0000000	-50.0000000
56930.0000000	0.2500000	16.8799992
56930.0000000	0.5000000	25.1200008
56930.0000000	0.7500000	24.5300007
56930.0000000	1.0000000	21.0200005
56930.0000000	1.2500000	19.5300007
56930.0000000	1.5000000	20.0000000
56930.0000000	1.7500000	18.2399998
56930.0000000	2.0000000	19.9699993
56930.0000000	2.2500000	21.4300003
56930.0000000	2.5000000	19.3500004
56930.0000000	2.7500000	17.8600006
56930.0000000	3.0000000	18.8199997
56930.0000000	3.2500000	17.7099991
56930.0000000	3.5000000	18.7600002
56930.0000000	3.7500000	19.7199993
56930.0000000	4.0000000	17.1499996
56930.0000000	4.2500000	17.4899998
56930.0000000	4.5000000	15.8199997
56930.0000000	4.7500000	-50.0000000
56930.0000000	5.0000000	-50.0000000
56930.0000000	5.2500000	-50.0000000
56930.0000000	5.5000000	-50.0000000
56930.0000000	5.7500000	-50.0000000
56930.0000000	6.0000000	-50.0000000
56930.0000000	6.2500000	-50.0000000
56930.0000000	6.5000000	-50.0000000
56930.0000000	6.7500000	-50.0000000
56930.0000000	7.0000000	-50.0000000
56930.0000000	7.2500000	-50.0000000
56930.0000000	7.5000000	-50.0000000
56930.0000000	7.7500000	-50.0000000
56930.0000000	8.0000000	-50.0000000
56930.0000000	8.2500000	-50.0000000
56930.0000000	8.5000000	-50.0000000
56930.0000000	8.7500000	-50.0000000
56930.0000000	9.0000000	-50.0000000
56930.0000000	9.2500000	-50.0000000
56930.0000000	9.5000000	-50.0000000
56930.0000000	9.7500000	-50.0000000
56930.0000000	10.0000000	-50.0000000
56930.0000000	10.2500000	-50.0000000
56930.0000000	10.5000000	-50.0000000
56930.0000000	10.7500000	-50.0000000
56930.0000000	11.0000000	-50.0000000
56930.0000000	11.2500000	-50.0000000
56930.0000000	11.5000000	-50.0000000
56930.0000000	11.7500000	-50.0000000
56930.0000000	12.0000000	-50.0000000
56930.0000000	12.2500000	-50.0000000
56930.0000000	12.5000000	-50.0000000
56930.0000000	12.7500000	-50.0000000
56930.0000000	13.0000000	-50.0000000
56930.0000000	13.2500000	-50.0000000
56930.0000000	13.5000000	-50.0000000

56930.0000000	13.7500000	-50.0000000
56930.0000000	14.0000000	-50.0000000
56930.0000000	14.2500000	-50.0000000
56930.0000000	14.5000000	-50.0000000
56930.0000000	14.7500000	-50.0000000
56930.0000000	15.0000000	-50.0000000
56960.0000000	0.0000000	-50.0000000
56960.0000000	0.2500000	20.3299999
56960.0000000	0.5000000	26.6900005
56960.0000000	0.7500000	24.5300007
56960.0000000	1.0000000	21.3400002
56960.0000000	1.2500000	20.2399998
56960.0000000	1.5000000	20.3899994
56960.0000000	1.7500000	20.0100002
56960.0000000	2.0000000	20.5699997
56960.0000000	2.2500000	19.8700008
56960.0000000	2.5000000	17.9400005
56960.0000000	2.7500000	16.7399998
56960.0000000	3.0000000	20.0100002
56960.0000000	3.2500000	21.0200005
56960.0000000	3.5000000	19.6700001
56960.0000000	3.7500000	19.9400005
56960.0000000	4.0000000	17.1599998
56960.0000000	4.2500000	-50.0000000
56960.0000000	4.5000000	16.4599991
56960.0000000	4.7500000	15.0100002
56960.0000000	5.0000000	-50.0000000
56960.0000000	5.2500000	-50.0000000
56960.0000000	5.5000000	-50.0000000
56960.0000000	5.7500000	-50.0000000
56960.0000000	6.0000000	-50.0000000
56960.0000000	6.2500000	-50.0000000
56960.0000000	6.5000000	-50.0000000
56960.0000000	6.7500000	-50.0000000
56960.0000000	7.0000000	-50.0000000
56960.0000000	7.2500000	-50.0000000
56960.0000000	7.5000000	-50.0000000
56960.0000000	7.7500000	-50.0000000
56960.0000000	8.0000000	-50.0000000
56960.0000000	8.2500000	-50.0000000
56960.0000000	8.5000000	-50.0000000
56960.0000000	8.7500000	-50.0000000
56960.0000000	9.0000000	-50.0000000
56960.0000000	9.2500000	-50.0000000
56960.0000000	9.5000000	-50.0000000
56960.0000000	9.7500000	-50.0000000
56960.0000000	10.0000000	-50.0000000
56960.0000000	10.2500000	-50.0000000
56960.0000000	10.5000000	-50.0000000
56960.0000000	10.7500000	-50.0000000
56960.0000000	11.0000000	-50.0000000
56960.0000000	11.2500000	-50.0000000
56960.0000000	11.5000000	-50.0000000
56960.0000000	11.7500000	-50.0000000
56960.0000000	12.0000000	-50.0000000
56960.0000000	12.2500000	-50.0000000
56960.0000000	12.5000000	-50.0000000
56960.0000000	12.7500000	-50.0000000

56960.0000000	13.0000000	-50.0000000
56960.0000000	13.2500000	-50.0000000
56960.0000000	13.5000000	-50.0000000
56960.0000000	13.7500000	-50.0000000
56960.0000000	14.0000000	-50.0000000
56960.0000000	14.2500000	-50.0000000
56960.0000000	14.5000000	-50.0000000
56960.0000000	14.7500000	-50.0000000
56960.0000000	15.0000000	-50.0000000
56990.0000000	0.0000000	-50.0000000
56990.0000000	0.2500000	19.5599995
56990.0000000	0.5000000	27.0200005
56990.0000000	0.7500000	25.9400005
56990.0000000	1.0000000	19.4500008
56990.0000000	1.2500000	22.1399994
56990.0000000	1.5000000	20.2800007
56990.0000000	1.7500000	19.0200005
56990.0000000	2.0000000	20.3799992
56990.0000000	2.2500000	21.3400002
56990.0000000	2.5000000	20.6800003
56990.0000000	2.7500000	17.9899998
56990.0000000	3.0000000	18.7500000
56990.0000000	3.2500000	20.7299995
56990.0000000	3.5000000	19.5799999
56990.0000000	3.7500000	18.4500008
56990.0000000	4.0000000	18.1399994
56990.0000000	4.2500000	17.4400005
56990.0000000	4.5000000	17.9699993
56990.0000000	4.7500000	-50.0000000
56990.0000000	5.0000000	-50.0000000
56990.0000000	5.2500000	-50.0000000
56990.0000000	5.5000000	-50.0000000
56990.0000000	5.7500000	-50.0000000
56990.0000000	6.0000000	-50.0000000
56990.0000000	6.2500000	-50.0000000
56990.0000000	6.5000000	-50.0000000
56990.0000000	6.7500000	-50.0000000
56990.0000000	7.0000000	-50.0000000
56990.0000000	7.2500000	-50.0000000
56990.0000000	7.5000000	-50.0000000
56990.0000000	7.7500000	-50.0000000
56990.0000000	8.0000000	-50.0000000
56990.0000000	8.2500000	-50.0000000
56990.0000000	8.5000000	-50.0000000
56990.0000000	8.7500000	-50.0000000
56990.0000000	9.0000000	-50.0000000
56990.0000000	9.2500000	-50.0000000
56990.0000000	9.5000000	-50.0000000
56990.0000000	9.7500000	-50.0000000
56990.0000000	10.0000000	-50.0000000
56990.0000000	10.2500000	-50.0000000
56990.0000000	10.5000000	-50.0000000
56990.0000000	10.7500000	-50.0000000
56990.0000000	11.0000000	-50.0000000
56990.0000000	11.2500000	-50.0000000
56990.0000000	11.5000000	-50.0000000
56990.0000000	11.7500000	-50.0000000
56990.0000000	12.0000000	-50.0000000

56990.0000000	12.2500000	-50.0000000
56990.0000000	12.5000000	-50.0000000
56990.0000000	12.7500000	-50.0000000
56990.0000000	13.0000000	-50.0000000
56990.0000000	13.2500000	-50.0000000
56990.0000000	13.5000000	-50.0000000
56990.0000000	13.7500000	-50.0000000
56990.0000000	14.0000000	-50.0000000
56990.0000000	14.2500000	-50.0000000
56990.0000000	14.5000000	-50.0000000
56990.0000000	14.7500000	-50.0000000
56990.0000000	15.0000000	-50.0000000
57020.0000000	0.0000000	-50.0000000
57020.0000000	0.2500000	24.8899994
57020.0000000	0.5000000	31.9799995
57020.0000000	0.7500000	29.4099998
57020.0000000	1.0000000	21.3199997
57020.0000000	1.2500000	20.6100006
57020.0000000	1.5000000	20.7900009
57020.0000000	1.7500000	21.1399994
57020.0000000	2.0000000	20.5799999
57020.0000000	2.2500000	21.7800007
57020.0000000	2.5000000	22.4099998
57020.0000000	2.7500000	23.4300003
57020.0000000	3.0000000	23.3999996
57020.0000000	3.2500000	20.8400002
57020.0000000	3.5000000	16.2800007
57020.0000000	3.7500000	17.4099998
57020.0000000	4.0000000	18.2299995
57020.0000000	4.2500000	18.7999992
57020.0000000	4.5000000	15.2399998
57020.0000000	4.7500000	16.9899998
57020.0000000	5.0000000	-50.0000000
57020.0000000	5.2500000	-50.0000000
57020.0000000	5.5000000	-50.0000000
57020.0000000	5.7500000	-50.0000000
57020.0000000	6.0000000	-50.0000000
57020.0000000	6.2500000	-50.0000000
57020.0000000	6.5000000	-50.0000000
57020.0000000	6.7500000	-50.0000000
57020.0000000	7.0000000	-50.0000000
57020.0000000	7.2500000	-50.0000000
57020.0000000	7.5000000	-50.0000000
57020.0000000	7.7500000	-50.0000000
57020.0000000	8.0000000	-50.0000000
57020.0000000	8.2500000	-50.0000000
57020.0000000	8.5000000	-50.0000000
57020.0000000	8.7500000	-50.0000000
57020.0000000	9.0000000	-50.0000000
57020.0000000	9.2500000	-50.0000000
57020.0000000	9.5000000	-50.0000000
57020.0000000	9.7500000	-50.0000000
57020.0000000	10.0000000	-50.0000000
57020.0000000	10.2500000	-50.0000000
57020.0000000	10.5000000	-50.0000000
57020.0000000	10.7500000	-50.0000000
57020.0000000	11.0000000	-50.0000000
57020.0000000	11.2500000	-50.0000000

57020.0000000	11.5000000	-50.0000000
57020.0000000	11.7500000	-50.0000000
57020.0000000	12.0000000	-50.0000000
57020.0000000	12.2500000	-50.0000000
57020.0000000	12.5000000	-50.0000000
57020.0000000	12.7500000	-50.0000000
57020.0000000	13.0000000	-50.0000000
57020.0000000	13.2500000	-50.0000000
57020.0000000	13.5000000	-50.0000000
57020.0000000	13.7500000	-50.0000000
57020.0000000	14.0000000	-50.0000000
57020.0000000	14.2500000	-50.0000000
57020.0000000	14.5000000	-50.0000000
57020.0000000	14.7500000	-50.0000000
57020.0000000	15.0000000	-50.0000000
57050.0000000	0.0000000	-50.0000000
57050.0000000	0.2500000	18.8600006
57050.0000000	0.5000000	26.1100006
57050.0000000	0.7500000	24.5699997
57050.0000000	1.0000000	19.7299995
57050.0000000	1.2500000	20.5799999
57050.0000000	1.5000000	21.2099991
57050.0000000	1.7500000	19.5499992
57050.0000000	2.0000000	20.9799995
57050.0000000	2.2500000	21.3899994
57050.0000000	2.5000000	18.1200008
57050.0000000	2.7500000	21.3899994
57050.0000000	3.0000000	21.8600006
57050.0000000	3.2500000	19.3099995
57050.0000000	3.5000000	18.1800003
57050.0000000	3.7500000	18.3600006
57050.0000000	4.0000000	19.0599995
57050.0000000	4.2500000	15.9300003
57050.0000000	4.5000000	18.0000000
57050.0000000	4.7500000	16.0799999
57050.0000000	5.0000000	-50.0000000
57050.0000000	5.2500000	-50.0000000
57050.0000000	5.5000000	-50.0000000
57050.0000000	5.7500000	-50.0000000
57050.0000000	6.0000000	-50.0000000
57050.0000000	6.2500000	-50.0000000
57050.0000000	6.5000000	-50.0000000
57050.0000000	6.7500000	-50.0000000
57050.0000000	7.0000000	-50.0000000
57050.0000000	7.2500000	-50.0000000
57050.0000000	7.5000000	-50.0000000
57050.0000000	7.7500000	-50.0000000
57050.0000000	8.0000000	-50.0000000
57050.0000000	8.2500000	-50.0000000
57050.0000000	8.5000000	-50.0000000
57050.0000000	8.7500000	-50.0000000
57050.0000000	9.0000000	-50.0000000
57050.0000000	9.2500000	-50.0000000
57050.0000000	9.5000000	-50.0000000
57050.0000000	9.7500000	-50.0000000
57050.0000000	10.0000000	-50.0000000
57050.0000000	10.2500000	-50.0000000
57050.0000000	10.5000000	-50.0000000

57050.0000000	10.7500000	-50.0000000
57050.0000000	11.0000000	-50.0000000
57050.0000000	11.2500000	-50.0000000
57050.0000000	11.5000000	-50.0000000
57050.0000000	11.7500000	-50.0000000
57050.0000000	12.0000000	-50.0000000
57050.0000000	12.2500000	-50.0000000
57050.0000000	12.5000000	-50.0000000
57050.0000000	12.7500000	-50.0000000
57050.0000000	13.0000000	-50.0000000
57050.0000000	13.2500000	-50.0000000
57050.0000000	13.5000000	-50.0000000
57050.0000000	13.7500000	-50.0000000
57050.0000000	14.0000000	-50.0000000
57050.0000000	14.2500000	-50.0000000
57050.0000000	14.5000000	-50.0000000
57050.0000000	14.7500000	-50.0000000
57050.0000000	15.0000000	-50.0000000
57080.0000000	0.0000000	-50.0000000
57080.0000000	0.2500000	-50.0000000
57080.0000000	0.5000000	22.1000004
57080.0000000	0.7500000	23.2000008
57080.0000000	1.0000000	21.7399998
57080.0000000	1.2500000	21.0400009
57080.0000000	1.5000000	20.8899994
57080.0000000	1.7500000	20.5499992
57080.0000000	2.0000000	20.3500004
57080.0000000	2.2500000	20.5400009
57080.0000000	2.5000000	21.1800003
57080.0000000	2.7500000	20.7299995
57080.0000000	3.0000000	19.3899994
57080.0000000	3.2500000	19.7399998
57080.0000000	3.5000000	20.7800007
57080.0000000	3.7500000	20.7800007
57080.0000000	4.0000000	18.9099998
57080.0000000	4.2500000	17.6200008
57080.0000000	4.5000000	17.1299992
57080.0000000	4.7500000	-50.0000000
57080.0000000	5.0000000	-50.0000000
57080.0000000	5.2500000	-50.0000000
57080.0000000	5.5000000	-50.0000000
57080.0000000	5.7500000	-50.0000000
57080.0000000	6.0000000	-50.0000000
57080.0000000	6.2500000	-50.0000000
57080.0000000	6.5000000	-50.0000000
57080.0000000	6.7500000	-50.0000000
57080.0000000	7.0000000	-50.0000000
57080.0000000	7.2500000	-50.0000000
57080.0000000	7.5000000	-50.0000000
57080.0000000	7.7500000	-50.0000000
57080.0000000	8.0000000	-50.0000000
57080.0000000	8.2500000	-50.0000000
57080.0000000	8.5000000	-50.0000000
57080.0000000	8.7500000	-50.0000000
57080.0000000	9.0000000	-50.0000000
57080.0000000	9.2500000	-50.0000000
57080.0000000	9.5000000	-50.0000000
57080.0000000	9.7500000	-50.0000000

57080.0000000	10.0000000	-50.0000000
57080.0000000	10.2500000	-50.0000000
57080.0000000	10.5000000	-50.0000000
57080.0000000	10.7500000	-50.0000000
57080.0000000	11.0000000	-50.0000000
57080.0000000	11.2500000	-50.0000000
57080.0000000	11.5000000	-50.0000000
57080.0000000	11.7500000	-50.0000000
57080.0000000	12.0000000	-50.0000000
57080.0000000	12.2500000	-50.0000000
57080.0000000	12.5000000	-50.0000000
57080.0000000	12.7500000	-50.0000000
57080.0000000	13.0000000	-50.0000000
57080.0000000	13.2500000	-50.0000000
57080.0000000	13.5000000	-50.0000000
57080.0000000	13.7500000	-50.0000000
57080.0000000	14.0000000	-50.0000000
57080.0000000	14.2500000	-50.0000000
57080.0000000	14.5000000	-50.0000000
57080.0000000	14.7500000	-50.0000000
57080.0000000	15.0000000	-50.0000000
57110.0000000	0.0000000	-50.0000000
57110.0000000	0.2500000	-50.0000000
57110.0000000	0.5000000	22.8700008
57110.0000000	0.7500000	23.9400005
57110.0000000	1.0000000	23.5400009
57110.0000000	1.2500000	23.6000004
57110.0000000	1.5000000	22.0799999
57110.0000000	1.7500000	20.2999992
57110.0000000	2.0000000	20.7900009
57110.0000000	2.2500000	19.6700001
57110.0000000	2.5000000	18.2700005
57110.0000000	2.7500000	19.9500008
57110.0000000	3.0000000	19.1100006
57110.0000000	3.2500000	19.3299999
57110.0000000	3.5000000	20.3400002
57110.0000000	3.7500000	20.7000008
57110.0000000	4.0000000	18.6599998
57110.0000000	4.2500000	15.5200005
57110.0000000	4.5000000	17.6100006
57110.0000000	4.7500000	17.7399998
57110.0000000	5.0000000	-50.0000000
57110.0000000	5.2500000	-50.0000000
57110.0000000	5.5000000	-50.0000000
57110.0000000	5.7500000	-50.0000000
57110.0000000	6.0000000	-50.0000000
57110.0000000	6.2500000	-50.0000000
57110.0000000	6.5000000	-50.0000000
57110.0000000	6.7500000	-50.0000000
57110.0000000	7.0000000	-50.0000000
57110.0000000	7.2500000	-50.0000000
57110.0000000	7.5000000	-50.0000000
57110.0000000	7.7500000	-50.0000000
57110.0000000	8.0000000	-50.0000000
57110.0000000	8.2500000	-50.0000000
57110.0000000	8.5000000	-50.0000000
57110.0000000	8.7500000	-50.0000000
57110.0000000	9.0000000	-50.0000000

57110.0000000	9.2500000	-50.0000000
57110.0000000	9.5000000	-50.0000000
57110.0000000	9.7500000	-50.0000000
57110.0000000	10.0000000	-50.0000000
57110.0000000	10.2500000	-50.0000000
57110.0000000	10.5000000	-50.0000000
57110.0000000	10.7500000	-50.0000000
57110.0000000	11.0000000	-50.0000000
57110.0000000	11.2500000	-50.0000000
57110.0000000	11.5000000	-50.0000000
57110.0000000	11.7500000	-50.0000000
57110.0000000	12.0000000	-50.0000000
57110.0000000	12.2500000	-50.0000000
57110.0000000	12.5000000	-50.0000000
57110.0000000	12.7500000	-50.0000000
57110.0000000	13.0000000	-50.0000000
57110.0000000	13.2500000	-50.0000000
57110.0000000	13.5000000	-50.0000000
57110.0000000	13.7500000	-50.0000000
57110.0000000	14.0000000	-50.0000000
57110.0000000	14.2500000	-50.0000000
57110.0000000	14.5000000	-50.0000000
57110.0000000	14.7500000	-50.0000000
57110.0000000	15.0000000	-50.0000000
57140.0000000	0.0000000	-50.0000000
57140.0000000	0.2500000	18.6800003
57140.0000000	0.5000000	26.7800007
57140.0000000	0.7500000	25.3899994
57140.0000000	1.0000000	20.5000000
57140.0000000	1.2500000	20.3500004
57140.0000000	1.5000000	20.9200001
57140.0000000	1.7500000	22.4500008
57140.0000000	2.0000000	21.0699997
57140.0000000	2.2500000	21.3199997
57140.0000000	2.5000000	20.5599995
57140.0000000	2.7500000	21.6100006
57140.0000000	3.0000000	19.3299999
57140.0000000	3.2500000	19.5300007
57140.0000000	3.5000000	19.0599995
57140.0000000	3.7500000	16.7999992
57140.0000000	4.0000000	17.9899998
57140.0000000	4.2500000	15.7799997
57140.0000000	4.5000000	18.1599998
57140.0000000	4.7500000	-50.0000000
57140.0000000	5.0000000	-50.0000000
57140.0000000	5.2500000	-50.0000000
57140.0000000	5.5000000	-50.0000000
57140.0000000	5.7500000	-50.0000000
57140.0000000	6.0000000	-50.0000000
57140.0000000	6.2500000	-50.0000000
57140.0000000	6.5000000	-50.0000000
57140.0000000	6.7500000	-50.0000000
57140.0000000	7.0000000	-50.0000000
57140.0000000	7.2500000	-50.0000000
57140.0000000	7.5000000	-50.0000000
57140.0000000	7.7500000	-50.0000000
57140.0000000	8.0000000	-50.0000000
57140.0000000	8.2500000	-50.0000000

57140.0000000	8.5000000	-50.0000000
57140.0000000	8.7500000	-50.0000000
57140.0000000	9.0000000	-50.0000000
57140.0000000	9.2500000	-50.0000000
57140.0000000	9.5000000	-50.0000000
57140.0000000	9.7500000	-50.0000000
57140.0000000	10.0000000	-50.0000000
57140.0000000	10.2500000	-50.0000000
57140.0000000	10.5000000	-50.0000000
57140.0000000	10.7500000	-50.0000000
57140.0000000	11.0000000	-50.0000000
57140.0000000	11.2500000	-50.0000000
57140.0000000	11.5000000	-50.0000000
57140.0000000	11.7500000	-50.0000000
57140.0000000	12.0000000	-50.0000000
57140.0000000	12.2500000	-50.0000000
57140.0000000	12.5000000	-50.0000000
57140.0000000	12.7500000	-50.0000000
57140.0000000	13.0000000	-50.0000000
57140.0000000	13.2500000	-50.0000000
57140.0000000	13.5000000	-50.0000000
57140.0000000	13.7500000	-50.0000000
57140.0000000	14.0000000	-50.0000000
57140.0000000	14.2500000	-50.0000000
57140.0000000	14.5000000	-50.0000000
57140.0000000	14.7500000	-50.0000000
57140.0000000	15.0000000	-50.0000000
57170.0000000	0.0000000	-50.0000000
57170.0000000	0.2500000	21.2199993
57170.0000000	0.5000000	27.2500000
57170.0000000	0.7500000	25.2299995
57170.0000000	1.0000000	22.7999992
57170.0000000	1.2500000	24.6299992
57170.0000000	1.5000000	23.7500000
57170.0000000	1.7500000	21.6800003
57170.0000000	2.0000000	19.8999996
57170.0000000	2.2500000	18.1299992
57170.0000000	2.5000000	19.8199997
57170.0000000	2.7500000	23.6200008
57170.0000000	3.0000000	23.4699993
57170.0000000	3.2500000	20.1200008
57170.0000000	3.5000000	22.2000008
57170.0000000	3.7500000	17.6900005
57170.0000000	4.0000000	18.0799999
57170.0000000	4.2500000	16.4500008
57170.0000000	4.5000000	19.8700008
57170.0000000	4.7500000	-50.0000000
57170.0000000	5.0000000	-50.0000000
57170.0000000	5.2500000	-50.0000000
57170.0000000	5.5000000	-50.0000000
57170.0000000	5.7500000	-50.0000000
57170.0000000	6.0000000	-50.0000000
57170.0000000	6.2500000	-50.0000000
57170.0000000	6.5000000	-50.0000000
57170.0000000	6.7500000	-50.0000000
57170.0000000	7.0000000	-50.0000000
57170.0000000	7.2500000	-50.0000000
57170.0000000	7.5000000	-50.0000000

57170.0000000	7.7500000	-50.0000000
57170.0000000	8.0000000	-50.0000000
57170.0000000	8.2500000	-50.0000000
57170.0000000	8.5000000	-50.0000000
57170.0000000	8.7500000	-50.0000000
57170.0000000	9.0000000	-50.0000000
57170.0000000	9.2500000	-50.0000000
57170.0000000	9.5000000	-50.0000000
57170.0000000	9.7500000	-50.0000000
57170.0000000	10.0000000	-50.0000000
57170.0000000	10.2500000	-50.0000000
57170.0000000	10.5000000	-50.0000000
57170.0000000	10.7500000	-50.0000000
57170.0000000	11.0000000	-50.0000000
57170.0000000	11.2500000	-50.0000000
57170.0000000	11.5000000	-50.0000000
57170.0000000	11.7500000	-50.0000000
57170.0000000	12.0000000	-50.0000000
57170.0000000	12.2500000	-50.0000000
57170.0000000	12.5000000	-50.0000000
57170.0000000	12.7500000	-50.0000000
57170.0000000	13.0000000	-50.0000000
57170.0000000	13.2500000	-50.0000000
57170.0000000	13.5000000	-50.0000000
57170.0000000	13.7500000	-50.0000000
57170.0000000	14.0000000	-50.0000000
57170.0000000	14.2500000	-50.0000000
57170.0000000	14.5000000	-50.0000000
57170.0000000	14.7500000	-50.0000000
57170.0000000	15.0000000	-50.0000000
57200.0000000	0.0000000	-50.0000000
57200.0000000	0.2500000	21.1599998
57200.0000000	0.5000000	27.5200005
57200.0000000	0.7500000	25.1499996
57200.0000000	1.0000000	20.9699993
57200.0000000	1.2500000	19.0100002
57200.0000000	1.5000000	20.9300003
57200.0000000	1.7500000	20.7900009
57200.0000000	2.0000000	20.5400009
57200.0000000	2.2500000	19.1700001
57200.0000000	2.5000000	20.8400002
57200.0000000	2.7500000	18.5699997
57200.0000000	3.0000000	19.3400002
57200.0000000	3.2500000	18.1499996
57200.0000000	3.5000000	17.5699997
57200.0000000	3.7500000	16.4300003
57200.0000000	4.0000000	16.6399994
57200.0000000	4.2500000	17.0799999
57200.0000000	4.5000000	17.5200005
57200.0000000	4.7500000	-50.0000000
57200.0000000	5.0000000	-50.0000000
57200.0000000	5.2500000	-50.0000000
57200.0000000	5.5000000	-50.0000000
57200.0000000	5.7500000	-50.0000000
57200.0000000	6.0000000	-50.0000000
57200.0000000	6.2500000	-50.0000000
57200.0000000	6.5000000	-50.0000000
57200.0000000	6.7500000	-50.0000000

57200.0000000	7.0000000	-50.0000000
57200.0000000	7.2500000	-50.0000000
57200.0000000	7.5000000	-50.0000000
57200.0000000	7.7500000	-50.0000000
57200.0000000	8.0000000	-50.0000000
57200.0000000	8.2500000	-50.0000000
57200.0000000	8.5000000	-50.0000000
57200.0000000	8.7500000	-50.0000000
57200.0000000	9.0000000	-50.0000000
57200.0000000	9.2500000	-50.0000000
57200.0000000	9.5000000	-50.0000000
57200.0000000	9.7500000	-50.0000000
57200.0000000	10.0000000	-50.0000000
57200.0000000	10.2500000	-50.0000000
57200.0000000	10.5000000	-50.0000000
57200.0000000	10.7500000	-50.0000000
57200.0000000	11.0000000	-50.0000000
57200.0000000	11.2500000	-50.0000000
57200.0000000	11.5000000	-50.0000000
57200.0000000	11.7500000	-50.0000000
57200.0000000	12.0000000	-50.0000000
57200.0000000	12.2500000	-50.0000000
57200.0000000	12.5000000	-50.0000000
57200.0000000	12.7500000	-50.0000000
57200.0000000	13.0000000	-50.0000000
57200.0000000	13.2500000	-50.0000000
57200.0000000	13.5000000	-50.0000000
57200.0000000	13.7500000	-50.0000000
57200.0000000	14.0000000	-50.0000000
57200.0000000	14.2500000	-50.0000000
57200.0000000	14.5000000	-50.0000000
57200.0000000	14.7500000	-50.0000000
57200.0000000	15.0000000	-50.0000000
57230.0000000	0.0000000	-50.0000000
57230.0000000	0.2500000	19.5799999
57230.0000000	0.5000000	26.5699997
57230.0000000	0.7500000	25.4400005
57230.0000000	1.0000000	21.8700008
57230.0000000	1.2500000	20.1599998
57230.0000000	1.5000000	22.0499992
57230.0000000	1.7500000	21.4699993
57230.0000000	2.0000000	19.6499996
57230.0000000	2.2500000	19.1399994
57230.0000000	2.5000000	17.2000008
57230.0000000	2.7500000	19.1599998
57230.0000000	3.0000000	18.7900009
57230.0000000	3.2500000	19.5200005
57230.0000000	3.5000000	19.5100002
57230.0000000	3.7500000	19.7399998
57230.0000000	4.0000000	18.5599995
57230.0000000	4.2500000	16.8600006
57230.0000000	4.5000000	17.2600002
57230.0000000	4.7500000	16.6700001
57230.0000000	5.0000000	-50.0000000
57230.0000000	5.2500000	-50.0000000
57230.0000000	5.5000000	-50.0000000
57230.0000000	5.7500000	-50.0000000
57230.0000000	6.0000000	-50.0000000

57230.0000000	6.2500000	-50.0000000
57230.0000000	6.5000000	-50.0000000
57230.0000000	6.7500000	-50.0000000
57230.0000000	7.0000000	-50.0000000
57230.0000000	7.2500000	-50.0000000
57230.0000000	7.5000000	-50.0000000
57230.0000000	7.7500000	-50.0000000
57230.0000000	8.0000000	-50.0000000
57230.0000000	8.2500000	-50.0000000
57230.0000000	8.5000000	-50.0000000
57230.0000000	8.7500000	-50.0000000
57230.0000000	9.0000000	-50.0000000
57230.0000000	9.2500000	-50.0000000
57230.0000000	9.5000000	-50.0000000
57230.0000000	9.7500000	-50.0000000
57230.0000000	10.0000000	-50.0000000
57230.0000000	10.2500000	-50.0000000
57230.0000000	10.5000000	-50.0000000
57230.0000000	10.7500000	-50.0000000
57230.0000000	11.0000000	-50.0000000
57230.0000000	11.2500000	-50.0000000
57230.0000000	11.5000000	-50.0000000
57230.0000000	11.7500000	-50.0000000
57230.0000000	12.0000000	-50.0000000
57230.0000000	12.2500000	-50.0000000
57230.0000000	12.5000000	-50.0000000
57230.0000000	12.7500000	-50.0000000
57230.0000000	13.0000000	-50.0000000
57230.0000000	13.2500000	-50.0000000
57230.0000000	13.5000000	-50.0000000
57230.0000000	13.7500000	-50.0000000
57230.0000000	14.0000000	-50.0000000
57230.0000000	14.2500000	-50.0000000
57230.0000000	14.5000000	-50.0000000
57230.0000000	14.7500000	-50.0000000
57230.0000000	15.0000000	-50.0000000
57260.0000000	0.0000000	-50.0000000
57260.0000000	0.2500000	19.5400009
57260.0000000	0.5000000	26.5499992
57260.0000000	0.7500000	24.4200001
57260.0000000	1.0000000	20.6299992
57260.0000000	1.2500000	21.8600006
57260.0000000	1.5000000	22.1700001
57260.0000000	1.7500000	20.7099991
57260.0000000	2.0000000	21.0699997
57260.0000000	2.2500000	20.2700005
57260.0000000	2.5000000	24.1299992
57260.0000000	2.7500000	23.5699997
57260.0000000	3.0000000	19.2099991
57260.0000000	3.2500000	20.3400002
57260.0000000	3.5000000	16.1900005
57260.0000000	3.7500000	19.0599995
57260.0000000	4.0000000	18.4899998
57260.0000000	4.2500000	16.2500000
57260.0000000	4.5000000	17.3700008
57260.0000000	4.7500000	-50.0000000
57260.0000000	5.0000000	-50.0000000
57260.0000000	5.2500000	-50.0000000

57260.0000000	5.5000000	-50.0000000
57260.0000000	5.7500000	-50.0000000
57260.0000000	6.0000000	-50.0000000
57260.0000000	6.2500000	-50.0000000
57260.0000000	6.5000000	-50.0000000
57260.0000000	6.7500000	-50.0000000
57260.0000000	7.0000000	-50.0000000
57260.0000000	7.2500000	-50.0000000
57260.0000000	7.5000000	-50.0000000
57260.0000000	7.7500000	-50.0000000
57260.0000000	8.0000000	-50.0000000
57260.0000000	8.2500000	-50.0000000
57260.0000000	8.5000000	-50.0000000
57260.0000000	8.7500000	-50.0000000
57260.0000000	9.0000000	-50.0000000
57260.0000000	9.2500000	-50.0000000
57260.0000000	9.5000000	-50.0000000
57260.0000000	9.7500000	-50.0000000
57260.0000000	10.0000000	-50.0000000
57260.0000000	10.2500000	-50.0000000
57260.0000000	10.5000000	-50.0000000
57260.0000000	10.7500000	-50.0000000
57260.0000000	11.0000000	-50.0000000
57260.0000000	11.2500000	-50.0000000
57260.0000000	11.5000000	-50.0000000
57260.0000000	11.7500000	-50.0000000
57260.0000000	12.0000000	-50.0000000
57260.0000000	12.2500000	-50.0000000
57260.0000000	12.5000000	-50.0000000
57260.0000000	12.7500000	-50.0000000
57260.0000000	13.0000000	-50.0000000
57260.0000000	13.2500000	-50.0000000
57260.0000000	13.5000000	-50.0000000
57260.0000000	13.7500000	-50.0000000
57260.0000000	14.0000000	-50.0000000
57260.0000000	14.2500000	-50.0000000
57260.0000000	14.5000000	-50.0000000
57260.0000000	14.7500000	-50.0000000
57260.0000000	15.0000000	-50.0000000
57290.0000000	0.0000000	-50.0000000
57290.0000000	0.2500000	21.9200001
57290.0000000	0.5000000	29.1700001
57290.0000000	0.7500000	27.0100002
57290.0000000	1.0000000	21.9500008
57290.0000000	1.2500000	22.2000008
57290.0000000	1.5000000	21.8099995
57290.0000000	1.7500000	21.5300007
57290.0000000	2.0000000	18.8500004
57290.0000000	2.2500000	19.8099995
57290.0000000	2.5000000	22.8299999
57290.0000000	2.7500000	22.4899998
57290.0000000	3.0000000	20.8700008
57290.0000000	3.2500000	22.2199993
57290.0000000	3.5000000	20.6800003
57290.0000000	3.7500000	21.4099998
57290.0000000	4.0000000	18.3500004
57290.0000000	4.2500000	18.1700001
57290.0000000	4.5000000	15.5000000

57290.0000000	4.7500000	-50.0000000
57290.0000000	5.0000000	-50.0000000
57290.0000000	5.2500000	-50.0000000
57290.0000000	5.5000000	-50.0000000
57290.0000000	5.7500000	-50.0000000
57290.0000000	6.0000000	-50.0000000
57290.0000000	6.2500000	-50.0000000
57290.0000000	6.5000000	-50.0000000
57290.0000000	6.7500000	-50.0000000
57290.0000000	7.0000000	-50.0000000
57290.0000000	7.2500000	-50.0000000
57290.0000000	7.5000000	-50.0000000
57290.0000000	7.7500000	-50.0000000
57290.0000000	8.0000000	-50.0000000
57290.0000000	8.2500000	-50.0000000
57290.0000000	8.5000000	-50.0000000
57290.0000000	8.7500000	-50.0000000
57290.0000000	9.0000000	-50.0000000
57290.0000000	9.2500000	-50.0000000
57290.0000000	9.5000000	-50.0000000
57290.0000000	9.7500000	-50.0000000
57290.0000000	10.0000000	-50.0000000
57290.0000000	10.2500000	-50.0000000
57290.0000000	10.5000000	-50.0000000
57290.0000000	10.7500000	-50.0000000
57290.0000000	11.0000000	-50.0000000
57290.0000000	11.2500000	-50.0000000
57290.0000000	11.5000000	-50.0000000
57290.0000000	11.7500000	-50.0000000
57290.0000000	12.0000000	-50.0000000
57290.0000000	12.2500000	-50.0000000
57290.0000000	12.5000000	-50.0000000
57290.0000000	12.7500000	-50.0000000
57290.0000000	13.0000000	-50.0000000
57290.0000000	13.2500000	-50.0000000
57290.0000000	13.5000000	-50.0000000
57290.0000000	13.7500000	-50.0000000
57290.0000000	14.0000000	-50.0000000
57290.0000000	14.2500000	-50.0000000
57290.0000000	14.5000000	-50.0000000
57290.0000000	14.7500000	-50.0000000
57290.0000000	15.0000000	-50.0000000
57320.0000000	0.0000000	-50.0000000
57320.0000000	0.2500000	20.9099998
57320.0000000	0.5000000	27.7500000
57320.0000000	0.7500000	25.5400009
57320.0000000	1.0000000	19.8799992
57320.0000000	1.2500000	22.4300003
57320.0000000	1.5000000	22.4300003
57320.0000000	1.7500000	21.0799999
57320.0000000	2.0000000	20.4400005
57320.0000000	2.2500000	20.1100006
57320.0000000	2.5000000	20.7900009
57320.0000000	2.7500000	22.9500008
57320.0000000	3.0000000	21.0100002
57320.0000000	3.2500000	20.5000000
57320.0000000	3.5000000	16.8700008
57320.0000000	3.7500000	17.6499996

57320.0000000	4.0000000	16.3099995
57320.0000000	4.2500000	16.1900005
57320.0000000	4.5000000	16.3099995
57320.0000000	4.7500000	-50.0000000
57320.0000000	5.0000000	-50.0000000
57320.0000000	5.2500000	-50.0000000
57320.0000000	5.5000000	-50.0000000
57320.0000000	5.7500000	-50.0000000
57320.0000000	6.0000000	-50.0000000
57320.0000000	6.2500000	-50.0000000
57320.0000000	6.5000000	-50.0000000
57320.0000000	6.7500000	-50.0000000
57320.0000000	7.0000000	-50.0000000
57320.0000000	7.2500000	-50.0000000
57320.0000000	7.5000000	-50.0000000
57320.0000000	7.7500000	-50.0000000
57320.0000000	8.0000000	-50.0000000
57320.0000000	8.2500000	-50.0000000
57320.0000000	8.5000000	-50.0000000
57320.0000000	8.7500000	-50.0000000
57320.0000000	9.0000000	-50.0000000
57320.0000000	9.2500000	-50.0000000
57320.0000000	9.5000000	-50.0000000
57320.0000000	9.7500000	-50.0000000
57320.0000000	10.0000000	-50.0000000
57320.0000000	10.2500000	-50.0000000
57320.0000000	10.5000000	-50.0000000
57320.0000000	10.7500000	-50.0000000
57320.0000000	11.0000000	-50.0000000
57320.0000000	11.2500000	-50.0000000
57320.0000000	11.5000000	-50.0000000
57320.0000000	11.7500000	-50.0000000
57320.0000000	12.0000000	-50.0000000
57320.0000000	12.2500000	-50.0000000
57320.0000000	12.5000000	-50.0000000
57320.0000000	12.7500000	-50.0000000
57320.0000000	13.0000000	-50.0000000
57320.0000000	13.2500000	-50.0000000
57320.0000000	13.5000000	-50.0000000
57320.0000000	13.7500000	-50.0000000
57320.0000000	14.0000000	-50.0000000
57320.0000000	14.2500000	-50.0000000
57320.0000000	14.5000000	-50.0000000
57320.0000000	14.7500000	-50.0000000
57320.0000000	15.0000000	-50.0000000
57350.0000000	0.0000000	-50.0000000
57350.0000000	0.2500000	18.1399994
57350.0000000	0.5000000	25.1299992
57350.0000000	0.7500000	24.5499992
57350.0000000	1.0000000	21.9200001
57350.0000000	1.2500000	21.7999992
57350.0000000	1.5000000	21.3400002
57350.0000000	1.7500000	20.2199993
57350.0000000	2.0000000	17.8899994
57350.0000000	2.2500000	20.5300007
57350.0000000	2.5000000	20.5499992
57350.0000000	2.7500000	19.8899994
57350.0000000	3.0000000	20.7399998

57350.0000000	3.2500000	20.4699993
57350.0000000	3.5000000	18.5499992
57350.0000000	3.7500000	16.5200005
57350.0000000	4.0000000	17.9500008
57350.0000000	4.2500000	17.1900005
57350.0000000	4.5000000	17.8199997
57350.0000000	4.7500000	-50.0000000
57350.0000000	5.0000000	-50.0000000
57350.0000000	5.2500000	-50.0000000
57350.0000000	5.5000000	-50.0000000
57350.0000000	5.7500000	-50.0000000
57350.0000000	6.0000000	-50.0000000
57350.0000000	6.2500000	-50.0000000
57350.0000000	6.5000000	-50.0000000
57350.0000000	6.7500000	-50.0000000
57350.0000000	7.0000000	-50.0000000
57350.0000000	7.2500000	-50.0000000
57350.0000000	7.5000000	-50.0000000
57350.0000000	7.7500000	-50.0000000
57350.0000000	8.0000000	-50.0000000
57350.0000000	8.2500000	-50.0000000
57350.0000000	8.5000000	-50.0000000
57350.0000000	8.7500000	-50.0000000
57350.0000000	9.0000000	-50.0000000
57350.0000000	9.2500000	-50.0000000
57350.0000000	9.5000000	-50.0000000
57350.0000000	9.7500000	-50.0000000
57350.0000000	10.0000000	-50.0000000
57350.0000000	10.2500000	-50.0000000
57350.0000000	10.5000000	-50.0000000
57350.0000000	10.7500000	-50.0000000
57350.0000000	11.0000000	-50.0000000
57350.0000000	11.2500000	-50.0000000
57350.0000000	11.5000000	-50.0000000
57350.0000000	11.7500000	-50.0000000
57350.0000000	12.0000000	-50.0000000
57350.0000000	12.2500000	-50.0000000
57350.0000000	12.5000000	-50.0000000
57350.0000000	12.7500000	-50.0000000
57350.0000000	13.0000000	-50.0000000
57350.0000000	13.2500000	-50.0000000
57350.0000000	13.5000000	-50.0000000
57350.0000000	13.7500000	-50.0000000
57350.0000000	14.0000000	-50.0000000
57350.0000000	14.2500000	-50.0000000
57350.0000000	14.5000000	-50.0000000
57350.0000000	14.7500000	-50.0000000
57350.0000000	15.0000000	-50.0000000
57380.0000000	0.0000000	-50.0000000
57380.0000000	0.2500000	17.0699997
57380.0000000	0.5000000	24.6100006
57380.0000000	0.7500000	24.1299992
57380.0000000	1.0000000	21.1299992
57380.0000000	1.2500000	21.8600006
57380.0000000	1.5000000	21.6499996
57380.0000000	1.7500000	20.3999996
57380.0000000	2.0000000	21.6499996
57380.0000000	2.2500000	19.4599991

57380.0000000	2.5000000	19.1599998
57380.0000000	2.7500000	18.5300007
57380.0000000	3.0000000	19.8400002
57380.0000000	3.2500000	21.1399994
57380.0000000	3.5000000	19.5799999
57380.0000000	3.7500000	19.7500000
57380.0000000	4.0000000	17.1700001
57380.0000000	4.2500000	15.1999998
57380.0000000	4.5000000	17.8600006
57380.0000000	4.7500000	-50.0000000
57380.0000000	5.0000000	-50.0000000
57380.0000000	5.2500000	-50.0000000
57380.0000000	5.5000000	-50.0000000
57380.0000000	5.7500000	-50.0000000
57380.0000000	6.0000000	-50.0000000
57380.0000000	6.2500000	-50.0000000
57380.0000000	6.5000000	-50.0000000
57380.0000000	6.7500000	-50.0000000
57380.0000000	7.0000000	-50.0000000
57380.0000000	7.2500000	-50.0000000
57380.0000000	7.5000000	-50.0000000
57380.0000000	7.7500000	-50.0000000
57380.0000000	8.0000000	-50.0000000
57380.0000000	8.2500000	-50.0000000
57380.0000000	8.5000000	-50.0000000
57380.0000000	8.7500000	-50.0000000
57380.0000000	9.0000000	-50.0000000
57380.0000000	9.2500000	-50.0000000
57380.0000000	9.5000000	-50.0000000
57380.0000000	9.7500000	-50.0000000
57380.0000000	10.0000000	-50.0000000
57380.0000000	10.2500000	-50.0000000
57380.0000000	10.5000000	-50.0000000
57380.0000000	10.7500000	-50.0000000
57380.0000000	11.0000000	-50.0000000
57380.0000000	11.2500000	-50.0000000
57380.0000000	11.5000000	-50.0000000
57380.0000000	11.7500000	-50.0000000
57380.0000000	12.0000000	-50.0000000
57380.0000000	12.2500000	-50.0000000
57380.0000000	12.5000000	-50.0000000
57380.0000000	12.7500000	-50.0000000
57380.0000000	13.0000000	-50.0000000
57380.0000000	13.2500000	-50.0000000
57380.0000000	13.5000000	-50.0000000
57380.0000000	13.7500000	-50.0000000
57380.0000000	14.0000000	-50.0000000
57380.0000000	14.2500000	-50.0000000
57380.0000000	14.5000000	-50.0000000
57380.0000000	14.7500000	-50.0000000
57380.0000000	15.0000000	-50.0000000
57410.0000000	0.0000000	-50.0000000
57410.0000000	0.2500000	19.8899994
57410.0000000	0.5000000	26.8299999
57410.0000000	0.7500000	25.0599995
57410.0000000	1.0000000	22.9200001
57410.0000000	1.2500000	23.6700001
57410.0000000	1.5000000	22.3899994

57410.0000000	1.7500000	21.8700008
57410.0000000	2.0000000	20.3899994
57410.0000000	2.2500000	18.6000004
57410.0000000	2.5000000	19.1000004
57410.0000000	2.7500000	20.3299999
57410.0000000	3.0000000	19.7199993
57410.0000000	3.2500000	19.6399994
57410.0000000	3.5000000	18.5000000
57410.0000000	3.7500000	19.0799999
57410.0000000	4.0000000	18.2000008
57410.0000000	4.2500000	-50.0000000
57410.0000000	4.5000000	16.5000000
57410.0000000	4.7500000	-50.0000000
57410.0000000	5.0000000	-50.0000000
57410.0000000	5.2500000	-50.0000000
57410.0000000	5.5000000	-50.0000000
57410.0000000	5.7500000	-50.0000000
57410.0000000	6.0000000	-50.0000000
57410.0000000	6.2500000	-50.0000000
57410.0000000	6.5000000	-50.0000000
57410.0000000	6.7500000	-50.0000000
57410.0000000	7.0000000	-50.0000000
57410.0000000	7.2500000	-50.0000000
57410.0000000	7.5000000	-50.0000000
57410.0000000	7.7500000	-50.0000000
57410.0000000	8.0000000	-50.0000000
57410.0000000	8.2500000	-50.0000000
57410.0000000	8.5000000	-50.0000000
57410.0000000	8.7500000	-50.0000000
57410.0000000	9.0000000	-50.0000000
57410.0000000	9.2500000	-50.0000000
57410.0000000	9.5000000	-50.0000000
57410.0000000	9.7500000	-50.0000000
57410.0000000	10.0000000	-50.0000000
57410.0000000	10.2500000	-50.0000000
57410.0000000	10.5000000	-50.0000000
57410.0000000	10.7500000	-50.0000000
57410.0000000	11.0000000	-50.0000000
57410.0000000	11.2500000	-50.0000000
57410.0000000	11.5000000	-50.0000000
57410.0000000	11.7500000	-50.0000000
57410.0000000	12.0000000	-50.0000000
57410.0000000	12.2500000	-50.0000000
57410.0000000	12.5000000	-50.0000000
57410.0000000	12.7500000	-50.0000000
57410.0000000	13.0000000	-50.0000000
57410.0000000	13.2500000	-50.0000000
57410.0000000	13.5000000	-50.0000000
57410.0000000	13.7500000	-50.0000000
57410.0000000	14.0000000	-50.0000000
57410.0000000	14.2500000	-50.0000000
57410.0000000	14.5000000	-50.0000000
57410.0000000	14.7500000	-50.0000000
57410.0000000	15.0000000	-50.0000000
57440.0000000	0.0000000	-50.0000000
57440.0000000	0.2500000	17.4099998
57440.0000000	0.5000000	24.8899994
57440.0000000	0.7500000	23.9300003

57440.0000000	1.0000000	21.7700005
57440.0000000	1.2500000	21.9099998
57440.0000000	1.5000000	21.2600002
57440.0000000	1.7500000	20.2800007
57440.0000000	2.0000000	20.5200005
57440.0000000	2.2500000	20.4500008
57440.0000000	2.5000000	18.7299995
57440.0000000	2.7500000	18.0699997
57440.0000000	3.0000000	20.2600002
57440.0000000	3.2500000	20.1399994
57440.0000000	3.5000000	17.1700001
57440.0000000	3.7500000	-50.0000000
57440.0000000	4.0000000	15.6599998
57440.0000000	4.2500000	16.0100002
57440.0000000	4.5000000	16.5699997
57440.0000000	4.7500000	-50.0000000
57440.0000000	5.0000000	-50.0000000
57440.0000000	5.2500000	-50.0000000
57440.0000000	5.5000000	-50.0000000
57440.0000000	5.7500000	-50.0000000
57440.0000000	6.0000000	-50.0000000
57440.0000000	6.2500000	-50.0000000
57440.0000000	6.5000000	-50.0000000
57440.0000000	6.7500000	-50.0000000
57440.0000000	7.0000000	-50.0000000
57440.0000000	7.2500000	-50.0000000
57440.0000000	7.5000000	-50.0000000
57440.0000000	7.7500000	-50.0000000
57440.0000000	8.0000000	-50.0000000
57440.0000000	8.2500000	-50.0000000
57440.0000000	8.5000000	-50.0000000
57440.0000000	8.7500000	-50.0000000
57440.0000000	9.0000000	-50.0000000
57440.0000000	9.2500000	-50.0000000
57440.0000000	9.5000000	-50.0000000
57440.0000000	9.7500000	-50.0000000
57440.0000000	10.0000000	-50.0000000
57440.0000000	10.2500000	-50.0000000
57440.0000000	10.5000000	-50.0000000
57440.0000000	10.7500000	-50.0000000
57440.0000000	11.0000000	-50.0000000
57440.0000000	11.2500000	-50.0000000
57440.0000000	11.5000000	-50.0000000
57440.0000000	11.7500000	-50.0000000
57440.0000000	12.0000000	-50.0000000
57440.0000000	12.2500000	-50.0000000
57440.0000000	12.5000000	-50.0000000
57440.0000000	12.7500000	-50.0000000
57440.0000000	13.0000000	-50.0000000
57440.0000000	13.2500000	-50.0000000
57440.0000000	13.5000000	-50.0000000
57440.0000000	13.7500000	-50.0000000
57440.0000000	14.0000000	-50.0000000
57440.0000000	14.2500000	-50.0000000
57440.0000000	14.5000000	-50.0000000
57440.0000000	14.7500000	-50.0000000
57440.0000000	15.0000000	-50.0000000
57470.0000000	0.0000000	-50.0000000

57470.0000000	0.2500000	21.4099998
57470.0000000	0.5000000	27.9799995
57470.0000000	0.7500000	25.5300007
57470.0000000	1.0000000	20.6900005
57470.0000000	1.2500000	21.2000008
57470.0000000	1.5000000	22.3899994
57470.0000000	1.7500000	21.0799999
57470.0000000	2.0000000	20.3799992
57470.0000000	2.2500000	21.7099991
57470.0000000	2.5000000	19.3500004
57470.0000000	2.7500000	19.5200005
57470.0000000	3.0000000	18.9599991
57470.0000000	3.2500000	18.7099991
57470.0000000	3.5000000	17.8799992
57470.0000000	3.7500000	15.8100004
57470.0000000	4.0000000	15.2600002
57470.0000000	4.2500000	18.1800003
57470.0000000	4.5000000	16.2199993
57470.0000000	4.7500000	-50.0000000
57470.0000000	5.0000000	-50.0000000
57470.0000000	5.2500000	-50.0000000
57470.0000000	5.5000000	-50.0000000
57470.0000000	5.7500000	-50.0000000
57470.0000000	6.0000000	-50.0000000
57470.0000000	6.2500000	-50.0000000
57470.0000000	6.5000000	-50.0000000
57470.0000000	6.7500000	-50.0000000
57470.0000000	7.0000000	-50.0000000
57470.0000000	7.2500000	-50.0000000
57470.0000000	7.5000000	-50.0000000
57470.0000000	7.7500000	-50.0000000
57470.0000000	8.0000000	-50.0000000
57470.0000000	8.2500000	-50.0000000
57470.0000000	8.5000000	-50.0000000
57470.0000000	8.7500000	-50.0000000
57470.0000000	9.0000000	-50.0000000
57470.0000000	9.2500000	-50.0000000
57470.0000000	9.5000000	-50.0000000
57470.0000000	9.7500000	-50.0000000
57470.0000000	10.0000000	-50.0000000
57470.0000000	10.2500000	-50.0000000
57470.0000000	10.5000000	-50.0000000
57470.0000000	10.7500000	-50.0000000
57470.0000000	11.0000000	-50.0000000
57470.0000000	11.2500000	-50.0000000
57470.0000000	11.5000000	-50.0000000
57470.0000000	11.7500000	-50.0000000
57470.0000000	12.0000000	-50.0000000
57470.0000000	12.2500000	-50.0000000
57470.0000000	12.5000000	-50.0000000
57470.0000000	12.7500000	-50.0000000
57470.0000000	13.0000000	-50.0000000
57470.0000000	13.2500000	-50.0000000
57470.0000000	13.5000000	-50.0000000
57470.0000000	13.7500000	-50.0000000
57470.0000000	14.0000000	-50.0000000
57470.0000000	14.2500000	-50.0000000
57470.0000000	14.5000000	-50.0000000

57470.0000000	14.7500000	-50.0000000
57470.0000000	15.0000000	-50.0000000
57500.0000000	0.0000000	-50.0000000
57500.0000000	0.2500000	17.6100006
57500.0000000	0.5000000	25.2500000
57500.0000000	0.7500000	25.0900002
57500.0000000	1.0000000	22.9300003
57500.0000000	1.2500000	20.5900002
57500.0000000	1.5000000	20.4699993
57500.0000000	1.7500000	20.3999996
57500.0000000	2.0000000	19.5100002
57500.0000000	2.2500000	21.5799999
57500.0000000	2.5000000	23.5000000
57500.0000000	2.7500000	21.8799992
57500.0000000	3.0000000	19.1499996
57500.0000000	3.2500000	17.1000004
57500.0000000	3.5000000	16.5900002
57500.0000000	3.7500000	16.3799992
57500.0000000	4.0000000	17.4400005
57500.0000000	4.2500000	16.1900005
57500.0000000	4.5000000	15.2500000
57500.0000000	4.7500000	-50.0000000
57500.0000000	5.0000000	-50.0000000
57500.0000000	5.2500000	-50.0000000
57500.0000000	5.5000000	-50.0000000
57500.0000000	5.7500000	-50.0000000
57500.0000000	6.0000000	-50.0000000
57500.0000000	6.2500000	-50.0000000
57500.0000000	6.5000000	-50.0000000
57500.0000000	6.7500000	-50.0000000
57500.0000000	7.0000000	-50.0000000
57500.0000000	7.2500000	-50.0000000
57500.0000000	7.5000000	-50.0000000
57500.0000000	7.7500000	-50.0000000
57500.0000000	8.0000000	-50.0000000
57500.0000000	8.2500000	-50.0000000
57500.0000000	8.5000000	-50.0000000
57500.0000000	8.7500000	-50.0000000
57500.0000000	9.0000000	-50.0000000
57500.0000000	9.2500000	-50.0000000
57500.0000000	9.5000000	-50.0000000
57500.0000000	9.7500000	-50.0000000
57500.0000000	10.0000000	-50.0000000
57500.0000000	10.2500000	-50.0000000
57500.0000000	10.5000000	-50.0000000
57500.0000000	10.7500000	-50.0000000
57500.0000000	11.0000000	-50.0000000
57500.0000000	11.2500000	-50.0000000
57500.0000000	11.5000000	-50.0000000
57500.0000000	11.7500000	-50.0000000
57500.0000000	12.0000000	-50.0000000
57500.0000000	12.2500000	-50.0000000
57500.0000000	12.5000000	-50.0000000
57500.0000000	12.7500000	-50.0000000
57500.0000000	13.0000000	-50.0000000
57500.0000000	13.2500000	-50.0000000
57500.0000000	13.5000000	-50.0000000
57500.0000000	13.7500000	-50.0000000

57500.0000000	14.0000000	-50.0000000
57500.0000000	14.2500000	-50.0000000
57500.0000000	14.5000000	-50.0000000
57500.0000000	14.7500000	-50.0000000
57500.0000000	15.0000000	-50.0000000
57530.0000000	0.0000000	-50.0000000
57530.0000000	0.2500000	19.1299992
57530.0000000	0.5000000	24.7900009
57530.0000000	0.7500000	23.3799992
57530.0000000	1.0000000	20.8099995
57530.0000000	1.2500000	22.3600006
57530.0000000	1.5000000	21.8700008
57530.0000000	1.7500000	21.9300003
57530.0000000	2.0000000	21.5499992
57530.0000000	2.2500000	21.2299995
57530.0000000	2.5000000	21.0499992
57530.0000000	2.7500000	18.9599991
57530.0000000	3.0000000	18.7800007
57530.0000000	3.2500000	21.4699993
57530.0000000	3.5000000	19.7900009
57530.0000000	3.7500000	17.6900005
57530.0000000	4.0000000	15.1400003
57530.0000000	4.2500000	15.8500004
57530.0000000	4.5000000	-50.0000000
57530.0000000	4.7500000	-50.0000000
57530.0000000	5.0000000	-50.0000000
57530.0000000	5.2500000	-50.0000000
57530.0000000	5.5000000	-50.0000000
57530.0000000	5.7500000	-50.0000000
57530.0000000	6.0000000	-50.0000000
57530.0000000	6.2500000	-50.0000000
57530.0000000	6.5000000	-50.0000000
57530.0000000	6.7500000	-50.0000000
57530.0000000	7.0000000	-50.0000000
57530.0000000	7.2500000	-50.0000000
57530.0000000	7.5000000	-50.0000000
57530.0000000	7.7500000	-50.0000000
57530.0000000	8.0000000	-50.0000000
57530.0000000	8.2500000	-50.0000000
57530.0000000	8.5000000	-50.0000000
57530.0000000	8.7500000	-50.0000000
57530.0000000	9.0000000	-50.0000000
57530.0000000	9.2500000	-50.0000000
57530.0000000	9.5000000	-50.0000000
57530.0000000	9.7500000	-50.0000000
57530.0000000	10.0000000	-50.0000000
57530.0000000	10.2500000	-50.0000000
57530.0000000	10.5000000	-50.0000000
57530.0000000	10.7500000	-50.0000000
57530.0000000	11.0000000	-50.0000000
57530.0000000	11.2500000	-50.0000000
57530.0000000	11.5000000	-50.0000000
57530.0000000	11.7500000	-50.0000000
57530.0000000	12.0000000	-50.0000000
57530.0000000	12.2500000	-50.0000000
57530.0000000	12.5000000	-50.0000000
57530.0000000	12.7500000	-50.0000000
57530.0000000	13.0000000	-50.0000000

57530.0000000	13.2500000	-50.0000000
57530.0000000	13.5000000	-50.0000000
57530.0000000	13.7500000	-50.0000000
57530.0000000	14.0000000	-50.0000000
57530.0000000	14.2500000	-50.0000000
57530.0000000	14.5000000	-50.0000000
57530.0000000	14.7500000	-50.0000000
57530.0000000	15.0000000	-50.0000000
57560.0000000	0.0000000	-50.0000000
57560.0000000	0.2500000	15.5600004
57560.0000000	0.5000000	24.0300007
57560.0000000	0.7500000	24.1299992
57560.0000000	1.0000000	22.6299992
57560.0000000	1.2500000	22.2999992
57560.0000000	1.5000000	21.8299999
57560.0000000	1.7500000	21.4899998
57560.0000000	2.0000000	21.3400002
57560.0000000	2.2500000	20.8099995
57560.0000000	2.5000000	21.8299999
57560.0000000	2.7500000	21.9599991
57560.0000000	3.0000000	16.9599991
57560.0000000	3.2500000	20.6700001
57560.0000000	3.5000000	20.5300007
57560.0000000	3.7500000	17.8600006
57560.0000000	4.0000000	-50.0000000
57560.0000000	4.2500000	15.7200003
57560.0000000	4.5000000	-50.0000000
57560.0000000	4.7500000	-50.0000000
57560.0000000	5.0000000	-50.0000000
57560.0000000	5.2500000	-50.0000000
57560.0000000	5.5000000	-50.0000000
57560.0000000	5.7500000	-50.0000000
57560.0000000	6.0000000	-50.0000000
57560.0000000	6.2500000	-50.0000000
57560.0000000	6.5000000	-50.0000000
57560.0000000	6.7500000	-50.0000000
57560.0000000	7.0000000	-50.0000000
57560.0000000	7.2500000	-50.0000000
57560.0000000	7.5000000	-50.0000000
57560.0000000	7.7500000	-50.0000000
57560.0000000	8.0000000	-50.0000000
57560.0000000	8.2500000	-50.0000000
57560.0000000	8.5000000	-50.0000000
57560.0000000	8.7500000	-50.0000000
57560.0000000	9.0000000	-50.0000000
57560.0000000	9.2500000	-50.0000000
57560.0000000	9.5000000	-50.0000000
57560.0000000	9.7500000	-50.0000000
57560.0000000	10.0000000	-50.0000000
57560.0000000	10.2500000	-50.0000000
57560.0000000	10.5000000	-50.0000000
57560.0000000	10.7500000	-50.0000000
57560.0000000	11.0000000	-50.0000000
57560.0000000	11.2500000	-50.0000000
57560.0000000	11.5000000	-50.0000000
57560.0000000	11.7500000	-50.0000000
57560.0000000	12.0000000	-50.0000000
57560.0000000	12.2500000	-50.0000000

57560.0000000	12.5000000	-50.0000000
57560.0000000	12.7500000	-50.0000000
57560.0000000	13.0000000	-50.0000000
57560.0000000	13.2500000	-50.0000000
57560.0000000	13.5000000	-50.0000000
57560.0000000	13.7500000	-50.0000000
57560.0000000	14.0000000	-50.0000000
57560.0000000	14.2500000	-50.0000000
57560.0000000	14.5000000	-50.0000000
57560.0000000	14.7500000	-50.0000000
57560.0000000	15.0000000	-50.0000000
57590.0000000	0.0000000	-50.0000000
57590.0000000	0.2500000	25.0599995
57590.0000000	0.5000000	30.9699993
57590.0000000	0.7500000	27.6399994
57590.0000000	1.0000000	20.9300003
57590.0000000	1.2500000	21.0100002
57590.0000000	1.5000000	21.8400002
57590.0000000	1.7500000	20.9500008
57590.0000000	2.0000000	21.3400002
57590.0000000	2.2500000	19.6499996
57590.0000000	2.5000000	19.9300003
57590.0000000	2.7500000	21.1399994
57590.0000000	3.0000000	19.1800003
57590.0000000	3.2500000	18.7900009
57590.0000000	3.5000000	19.6399994
57590.0000000	3.7500000	17.2800007
57590.0000000	4.0000000	-50.0000000
57590.0000000	4.2500000	-50.0000000
57590.0000000	4.5000000	-50.0000000
57590.0000000	4.7500000	-50.0000000
57590.0000000	5.0000000	-50.0000000
57590.0000000	5.2500000	-50.0000000
57590.0000000	5.5000000	-50.0000000
57590.0000000	5.7500000	-50.0000000
57590.0000000	6.0000000	-50.0000000
57590.0000000	6.2500000	-50.0000000
57590.0000000	6.5000000	-50.0000000
57590.0000000	6.7500000	-50.0000000
57590.0000000	7.0000000	-50.0000000
57590.0000000	7.2500000	-50.0000000
57590.0000000	7.5000000	-50.0000000
57590.0000000	7.7500000	-50.0000000
57590.0000000	8.0000000	-50.0000000
57590.0000000	8.2500000	-50.0000000
57590.0000000	8.5000000	-50.0000000
57590.0000000	8.7500000	-50.0000000
57590.0000000	9.0000000	-50.0000000
57590.0000000	9.2500000	-50.0000000
57590.0000000	9.5000000	-50.0000000
57590.0000000	9.7500000	-50.0000000
57590.0000000	10.0000000	-50.0000000
57590.0000000	10.2500000	-50.0000000
57590.0000000	10.5000000	-50.0000000
57590.0000000	10.7500000	-50.0000000
57590.0000000	11.0000000	-50.0000000
57590.0000000	11.2500000	-50.0000000
57590.0000000	11.5000000	-50.0000000

57590.0000000	11.7500000	-50.0000000
57590.0000000	12.0000000	-50.0000000
57590.0000000	12.2500000	-50.0000000
57590.0000000	12.5000000	-50.0000000
57590.0000000	12.7500000	-50.0000000
57590.0000000	13.0000000	-50.0000000
57590.0000000	13.2500000	-50.0000000
57590.0000000	13.5000000	-50.0000000
57590.0000000	13.7500000	-50.0000000
57590.0000000	14.0000000	-50.0000000
57590.0000000	14.2500000	-50.0000000
57590.0000000	14.5000000	-50.0000000
57590.0000000	14.7500000	-50.0000000
57590.0000000	15.0000000	-50.0000000
57620.0000000	0.0000000	-50.0000000
57620.0000000	0.2500000	18.3600006
57620.0000000	0.5000000	24.6599998
57620.0000000	0.7500000	24.0100002
57620.0000000	1.0000000	21.1399994
57620.0000000	1.2500000	19.5799999
57620.0000000	1.5000000	20.6399994
57620.0000000	1.7500000	21.0100002
57620.0000000	2.0000000	20.1900005
57620.0000000	2.2500000	23.2600002
57620.0000000	2.5000000	22.2199993
57620.0000000	2.7500000	21.4699993
57620.0000000	3.0000000	19.1000004
57620.0000000	3.2500000	19.5699997
57620.0000000	3.5000000	17.5499992
57620.0000000	3.7500000	15.8999996
57620.0000000	4.0000000	15.0500002
57620.0000000	4.2500000	-50.0000000
57620.0000000	4.5000000	-50.0000000
57620.0000000	4.7500000	-50.0000000
57620.0000000	5.0000000	-50.0000000
57620.0000000	5.2500000	-50.0000000
57620.0000000	5.5000000	-50.0000000
57620.0000000	5.7500000	-50.0000000
57620.0000000	6.0000000	-50.0000000
57620.0000000	6.2500000	-50.0000000
57620.0000000	6.5000000	-50.0000000
57620.0000000	6.7500000	-50.0000000
57620.0000000	7.0000000	-50.0000000
57620.0000000	7.2500000	-50.0000000
57620.0000000	7.5000000	-50.0000000
57620.0000000	7.7500000	-50.0000000
57620.0000000	8.0000000	-50.0000000
57620.0000000	8.2500000	-50.0000000
57620.0000000	8.5000000	-50.0000000
57620.0000000	8.7500000	-50.0000000
57620.0000000	9.0000000	-50.0000000
57620.0000000	9.2500000	-50.0000000
57620.0000000	9.5000000	-50.0000000
57620.0000000	9.7500000	-50.0000000
57620.0000000	10.0000000	-50.0000000
57620.0000000	10.2500000	-50.0000000
57620.0000000	10.5000000	-50.0000000
57620.0000000	10.7500000	-50.0000000

57620.0000000	11.0000000	-50.0000000
57620.0000000	11.2500000	-50.0000000
57620.0000000	11.5000000	-50.0000000
57620.0000000	11.7500000	-50.0000000
57620.0000000	12.0000000	-50.0000000
57620.0000000	12.2500000	-50.0000000
57620.0000000	12.5000000	-50.0000000
57620.0000000	12.7500000	-50.0000000
57620.0000000	13.0000000	-50.0000000
57620.0000000	13.2500000	-50.0000000
57620.0000000	13.5000000	-50.0000000
57620.0000000	13.7500000	-50.0000000
57620.0000000	14.0000000	-50.0000000
57620.0000000	14.2500000	-50.0000000
57620.0000000	14.5000000	-50.0000000
57620.0000000	14.7500000	-50.0000000
57620.0000000	15.0000000	-50.0000000
57650.0000000	0.0000000	-50.0000000
57650.0000000	0.2500000	17.1900005
57650.0000000	0.5000000	23.8899994
57650.0000000	0.7500000	24.9799995
57650.0000000	1.0000000	23.6200008
57650.0000000	1.2500000	22.2800007
57650.0000000	1.5000000	21.5499992
57650.0000000	1.7500000	20.5400009
57650.0000000	2.0000000	20.9799995
57650.0000000	2.2500000	20.4500008
57650.0000000	2.5000000	20.6599998
57650.0000000	2.7500000	16.6599998
57650.0000000	3.0000000	15.0699997
57650.0000000	3.2500000	17.0100002
57650.0000000	3.5000000	19.6299992
57650.0000000	3.7500000	17.8999996
57650.0000000	4.0000000	15.1400003
57650.0000000	4.2500000	15.0799999
57650.0000000	4.5000000	-50.0000000
57650.0000000	4.7500000	-50.0000000
57650.0000000	5.0000000	-50.0000000
57650.0000000	5.2500000	-50.0000000
57650.0000000	5.5000000	-50.0000000
57650.0000000	5.7500000	-50.0000000
57650.0000000	6.0000000	-50.0000000
57650.0000000	6.2500000	-50.0000000
57650.0000000	6.5000000	-50.0000000
57650.0000000	6.7500000	-50.0000000
57650.0000000	7.0000000	-50.0000000
57650.0000000	7.2500000	-50.0000000
57650.0000000	7.5000000	-50.0000000
57650.0000000	7.7500000	-50.0000000
57650.0000000	8.0000000	-50.0000000
57650.0000000	8.2500000	-50.0000000
57650.0000000	8.5000000	-50.0000000
57650.0000000	8.7500000	-50.0000000
57650.0000000	9.0000000	-50.0000000
57650.0000000	9.2500000	-50.0000000
57650.0000000	9.5000000	-50.0000000
57650.0000000	9.7500000	-50.0000000
57650.0000000	10.0000000	-50.0000000

57650.0000000	10.2500000	-50.0000000
57650.0000000	10.5000000	-50.0000000
57650.0000000	10.7500000	-50.0000000
57650.0000000	11.0000000	-50.0000000
57650.0000000	11.2500000	-50.0000000
57650.0000000	11.5000000	-50.0000000
57650.0000000	11.7500000	-50.0000000
57650.0000000	12.0000000	-50.0000000
57650.0000000	12.2500000	-50.0000000
57650.0000000	12.5000000	-50.0000000
57650.0000000	12.7500000	-50.0000000
57650.0000000	13.0000000	-50.0000000
57650.0000000	13.2500000	-50.0000000
57650.0000000	13.5000000	-50.0000000
57650.0000000	13.7500000	-50.0000000
57650.0000000	14.0000000	-50.0000000
57650.0000000	14.2500000	-50.0000000
57650.0000000	14.5000000	-50.0000000
57650.0000000	14.7500000	-50.0000000
57650.0000000	15.0000000	-50.0000000
57680.0000000	0.0000000	-50.0000000
57680.0000000	0.2500000	18.2099991
57680.0000000	0.5000000	25.0100002
57680.0000000	0.7500000	22.8400002
57680.0000000	1.0000000	18.0699997
57680.0000000	1.2500000	20.0300007
57680.0000000	1.5000000	22.2199993
57680.0000000	1.7500000	23.1700001
57680.0000000	2.0000000	20.2099991
57680.0000000	2.2500000	20.0000000
57680.0000000	2.5000000	22.1800003
57680.0000000	2.7500000	23.1499996
57680.0000000	3.0000000	20.5200005
57680.0000000	3.2500000	17.4799995
57680.0000000	3.5000000	16.8899994
57680.0000000	3.7500000	20.3099995
57680.0000000	4.0000000	17.7900009
57680.0000000	4.2500000	16.5900002
57680.0000000	4.5000000	-50.0000000
57680.0000000	4.7500000	-50.0000000
57680.0000000	5.0000000	-50.0000000
57680.0000000	5.2500000	-50.0000000
57680.0000000	5.5000000	-50.0000000
57680.0000000	5.7500000	-50.0000000
57680.0000000	6.0000000	-50.0000000
57680.0000000	6.2500000	-50.0000000
57680.0000000	6.5000000	-50.0000000
57680.0000000	6.7500000	-50.0000000
57680.0000000	7.0000000	-50.0000000
57680.0000000	7.2500000	-50.0000000
57680.0000000	7.5000000	-50.0000000
57680.0000000	7.7500000	-50.0000000
57680.0000000	8.0000000	-50.0000000
57680.0000000	8.2500000	-50.0000000
57680.0000000	8.5000000	-50.0000000
57680.0000000	8.7500000	-50.0000000
57680.0000000	9.0000000	-50.0000000
57680.0000000	9.2500000	-50.0000000

57680.0000000	9.5000000	-50.0000000
57680.0000000	9.7500000	-50.0000000
57680.0000000	10.0000000	-50.0000000
57680.0000000	10.2500000	-50.0000000
57680.0000000	10.5000000	-50.0000000
57680.0000000	10.7500000	-50.0000000
57680.0000000	11.0000000	-50.0000000
57680.0000000	11.2500000	-50.0000000
57680.0000000	11.5000000	-50.0000000
57680.0000000	11.7500000	-50.0000000
57680.0000000	12.0000000	-50.0000000
57680.0000000	12.2500000	-50.0000000
57680.0000000	12.5000000	-50.0000000
57680.0000000	12.7500000	-50.0000000
57680.0000000	13.0000000	-50.0000000
57680.0000000	13.2500000	-50.0000000
57680.0000000	13.5000000	-50.0000000
57680.0000000	13.7500000	-50.0000000
57680.0000000	14.0000000	-50.0000000
57680.0000000	14.2500000	-50.0000000
57680.0000000	14.5000000	-50.0000000
57680.0000000	14.7500000	-50.0000000
57680.0000000	15.0000000	-50.0000000
57710.0000000	0.0000000	-50.0000000
57710.0000000	0.2500000	16.9099998
57710.0000000	0.5000000	24.6599998
57710.0000000	0.7500000	23.8999996
57710.0000000	1.0000000	19.9099998
57710.0000000	1.2500000	21.2800007
57710.0000000	1.5000000	20.6900005
57710.0000000	1.7500000	20.4099998
57710.0000000	2.0000000	20.1200008
57710.0000000	2.2500000	17.9300003
57710.0000000	2.5000000	17.6399994
57710.0000000	2.7500000	20.5000000
57710.0000000	3.0000000	18.5799999
57710.0000000	3.2500000	20.1000004
57710.0000000	3.5000000	17.4899998
57710.0000000	3.7500000	-50.0000000
57710.0000000	4.0000000	-50.0000000
57710.0000000	4.2500000	-50.0000000
57710.0000000	4.5000000	-50.0000000
57710.0000000	4.7500000	-50.0000000
57710.0000000	5.0000000	-50.0000000
57710.0000000	5.2500000	-50.0000000
57710.0000000	5.5000000	-50.0000000
57710.0000000	5.7500000	-50.0000000
57710.0000000	6.0000000	-50.0000000
57710.0000000	6.2500000	-50.0000000
57710.0000000	6.5000000	-50.0000000
57710.0000000	6.7500000	-50.0000000
57710.0000000	7.0000000	-50.0000000
57710.0000000	7.2500000	-50.0000000
57710.0000000	7.5000000	-50.0000000
57710.0000000	7.7500000	-50.0000000
57710.0000000	8.0000000	-50.0000000
57710.0000000	8.2500000	-50.0000000
57710.0000000	8.5000000	-50.0000000

57710.0000000	8.7500000	-50.0000000
57710.0000000	9.0000000	-50.0000000
57710.0000000	9.2500000	-50.0000000
57710.0000000	9.5000000	-50.0000000
57710.0000000	9.7500000	-50.0000000
57710.0000000	10.0000000	-50.0000000
57710.0000000	10.2500000	-50.0000000
57710.0000000	10.5000000	-50.0000000
57710.0000000	10.7500000	-50.0000000
57710.0000000	11.0000000	-50.0000000
57710.0000000	11.2500000	-50.0000000
57710.0000000	11.5000000	-50.0000000
57710.0000000	11.7500000	-50.0000000
57710.0000000	12.0000000	-50.0000000
57710.0000000	12.2500000	-50.0000000
57710.0000000	12.5000000	-50.0000000
57710.0000000	12.7500000	-50.0000000
57710.0000000	13.0000000	-50.0000000
57710.0000000	13.2500000	-50.0000000
57710.0000000	13.5000000	-50.0000000
57710.0000000	13.7500000	-50.0000000
57710.0000000	14.0000000	-50.0000000
57710.0000000	14.2500000	-50.0000000
57710.0000000	14.5000000	-50.0000000
57710.0000000	14.7500000	-50.0000000
57710.0000000	15.0000000	-50.0000000
57740.0000000	0.0000000	-50.0000000
57740.0000000	0.2500000	-50.0000000
57740.0000000	0.5000000	22.6399994
57740.0000000	0.7500000	24.2700005
57740.0000000	1.0000000	24.1000004
57740.0000000	1.2500000	21.3500004
57740.0000000	1.5000000	21.5499992
57740.0000000	1.7500000	21.8799992
57740.0000000	2.0000000	20.5599995
57740.0000000	2.2500000	18.8999996
57740.0000000	2.5000000	20.0200005
57740.0000000	2.7500000	21.9899998
57740.0000000	3.0000000	21.3500004
57740.0000000	3.2500000	20.3799992
57740.0000000	3.5000000	16.1200008
57740.0000000	3.7500000	-50.0000000
57740.0000000	4.0000000	16.2900009
57740.0000000	4.2500000	-50.0000000
57740.0000000	4.5000000	15.1000004
57740.0000000	4.7500000	-50.0000000
57740.0000000	5.0000000	-50.0000000
57740.0000000	5.2500000	-50.0000000
57740.0000000	5.5000000	-50.0000000
57740.0000000	5.7500000	-50.0000000
57740.0000000	6.0000000	-50.0000000
57740.0000000	6.2500000	-50.0000000
57740.0000000	6.5000000	-50.0000000
57740.0000000	6.7500000	-50.0000000
57740.0000000	7.0000000	-50.0000000
57740.0000000	7.2500000	-50.0000000
57740.0000000	7.5000000	-50.0000000
57740.0000000	7.7500000	-50.0000000

57740.0000000	8.0000000	-50.0000000
57740.0000000	8.2500000	-50.0000000
57740.0000000	8.5000000	-50.0000000
57740.0000000	8.7500000	-50.0000000
57740.0000000	9.0000000	-50.0000000
57740.0000000	9.2500000	-50.0000000
57740.0000000	9.5000000	-50.0000000
57740.0000000	9.7500000	-50.0000000
57740.0000000	10.0000000	-50.0000000
57740.0000000	10.2500000	-50.0000000
57740.0000000	10.5000000	-50.0000000
57740.0000000	10.7500000	-50.0000000
57740.0000000	11.0000000	-50.0000000
57740.0000000	11.2500000	-50.0000000
57740.0000000	11.5000000	-50.0000000
57740.0000000	11.7500000	-50.0000000
57740.0000000	12.0000000	-50.0000000
57740.0000000	12.2500000	-50.0000000
57740.0000000	12.5000000	-50.0000000
57740.0000000	12.7500000	-50.0000000
57740.0000000	13.0000000	-50.0000000
57740.0000000	13.2500000	-50.0000000
57740.0000000	13.5000000	-50.0000000
57740.0000000	13.7500000	-50.0000000
57740.0000000	14.0000000	-50.0000000
57740.0000000	14.2500000	-50.0000000
57740.0000000	14.5000000	-50.0000000
57740.0000000	14.7500000	-50.0000000
57740.0000000	15.0000000	-50.0000000
57770.0000000	0.0000000	-50.0000000
57770.0000000	0.2500000	-50.0000000
57770.0000000	0.5000000	22.0900002
57770.0000000	0.7500000	22.2600002
57770.0000000	1.0000000	21.0000000
57770.0000000	1.2500000	22.1299992
57770.0000000	1.5000000	20.7199993
57770.0000000	1.7500000	20.2900009
57770.0000000	2.0000000	20.2800007
57770.0000000	2.2500000	20.2999992
57770.0000000	2.5000000	20.8099995
57770.0000000	2.7500000	18.1299992
57770.0000000	3.0000000	15.8199997
57770.0000000	3.2500000	18.0599995
57770.0000000	3.5000000	18.1200008
57770.0000000	3.7500000	-50.0000000
57770.0000000	4.0000000	-50.0000000
57770.0000000	4.2500000	-50.0000000
57770.0000000	4.5000000	-50.0000000
57770.0000000	4.7500000	-50.0000000
57770.0000000	5.0000000	-50.0000000
57770.0000000	5.2500000	-50.0000000
57770.0000000	5.5000000	-50.0000000
57770.0000000	5.7500000	-50.0000000
57770.0000000	6.0000000	-50.0000000
57770.0000000	6.2500000	-50.0000000
57770.0000000	6.5000000	-50.0000000
57770.0000000	6.7500000	-50.0000000
57770.0000000	7.0000000	-50.0000000

57770.0000000	7.2500000	-50.0000000
57770.0000000	7.5000000	-50.0000000
57770.0000000	7.7500000	-50.0000000
57770.0000000	8.0000000	-50.0000000
57770.0000000	8.2500000	-50.0000000
57770.0000000	8.5000000	-50.0000000
57770.0000000	8.7500000	-50.0000000
57770.0000000	9.0000000	-50.0000000
57770.0000000	9.2500000	-50.0000000
57770.0000000	9.5000000	-50.0000000
57770.0000000	9.7500000	-50.0000000
57770.0000000	10.0000000	-50.0000000
57770.0000000	10.2500000	-50.0000000
57770.0000000	10.5000000	-50.0000000
57770.0000000	10.7500000	-50.0000000
57770.0000000	11.0000000	-50.0000000
57770.0000000	11.2500000	-50.0000000
57770.0000000	11.5000000	-50.0000000
57770.0000000	11.7500000	-50.0000000
57770.0000000	12.0000000	-50.0000000
57770.0000000	12.2500000	-50.0000000
57770.0000000	12.5000000	-50.0000000
57770.0000000	12.7500000	-50.0000000
57770.0000000	13.0000000	-50.0000000
57770.0000000	13.2500000	-50.0000000
57770.0000000	13.5000000	-50.0000000
57770.0000000	13.7500000	-50.0000000
57770.0000000	14.0000000	-50.0000000
57770.0000000	14.2500000	-50.0000000
57770.0000000	14.5000000	-50.0000000
57770.0000000	14.7500000	-50.0000000
57770.0000000	15.0000000	-50.0000000
57800.0000000	0.0000000	-50.0000000
57800.0000000	0.2500000	-50.0000000
57800.0000000	0.5000000	21.5100002
57800.0000000	0.7500000	22.9699993
57800.0000000	1.0000000	21.1599998
57800.0000000	1.2500000	20.8700008
57800.0000000	1.5000000	22.5200005
57800.0000000	1.7500000	23.5300007
57800.0000000	2.0000000	20.9699993
57800.0000000	2.2500000	21.1200008
57800.0000000	2.5000000	19.7199993
57800.0000000	2.7500000	17.6000004
57800.0000000	3.0000000	17.6800003
57800.0000000	3.2500000	17.4699993
57800.0000000	3.5000000	17.8600006
57800.0000000	3.7500000	15.8900003
57800.0000000	4.0000000	-50.0000000
57800.0000000	4.2500000	-50.0000000
57800.0000000	4.5000000	-50.0000000
57800.0000000	4.7500000	-50.0000000
57800.0000000	5.0000000	-50.0000000
57800.0000000	5.2500000	-50.0000000
57800.0000000	5.5000000	-50.0000000
57800.0000000	5.7500000	-50.0000000
57800.0000000	6.0000000	-50.0000000
57800.0000000	6.2500000	-50.0000000

57800.0000000	6.5000000	-50.0000000
57800.0000000	6.7500000	-50.0000000
57800.0000000	7.0000000	-50.0000000
57800.0000000	7.2500000	-50.0000000
57800.0000000	7.5000000	-50.0000000
57800.0000000	7.7500000	-50.0000000
57800.0000000	8.0000000	-50.0000000
57800.0000000	8.2500000	-50.0000000
57800.0000000	8.5000000	-50.0000000
57800.0000000	8.7500000	-50.0000000
57800.0000000	9.0000000	-50.0000000
57800.0000000	9.2500000	-50.0000000
57800.0000000	9.5000000	-50.0000000
57800.0000000	9.7500000	-50.0000000
57800.0000000	10.0000000	-50.0000000
57800.0000000	10.2500000	-50.0000000
57800.0000000	10.5000000	-50.0000000
57800.0000000	10.7500000	-50.0000000
57800.0000000	11.0000000	-50.0000000
57800.0000000	11.2500000	-50.0000000
57800.0000000	11.5000000	-50.0000000
57800.0000000	11.7500000	-50.0000000
57800.0000000	12.0000000	-50.0000000
57800.0000000	12.2500000	-50.0000000
57800.0000000	12.5000000	-50.0000000
57800.0000000	12.7500000	-50.0000000
57800.0000000	13.0000000	-50.0000000
57800.0000000	13.2500000	-50.0000000
57800.0000000	13.5000000	-50.0000000
57800.0000000	13.7500000	-50.0000000
57800.0000000	14.0000000	-50.0000000
57800.0000000	14.2500000	-50.0000000
57800.0000000	14.5000000	-50.0000000
57800.0000000	14.7500000	-50.0000000
57800.0000000	15.0000000	-50.0000000
57830.0000000	0.0000000	-50.0000000
57830.0000000	0.2500000	-50.0000000
57830.0000000	0.5000000	21.7600002
57830.0000000	0.7500000	22.5799999
57830.0000000	1.0000000	20.4699993
57830.0000000	1.2500000	20.6599998
57830.0000000	1.5000000	21.2000008
57830.0000000	1.7500000	22.6200008
57830.0000000	2.0000000	20.5400009
57830.0000000	2.2500000	19.6299992
57830.0000000	2.5000000	19.1000004
57830.0000000	2.7500000	19.6499996
57830.0000000	3.0000000	19.3700008
57830.0000000	3.2500000	18.9500008
57830.0000000	3.5000000	16.7000008
57830.0000000	3.7500000	16.3500004
57830.0000000	4.0000000	-50.0000000
57830.0000000	4.2500000	-50.0000000
57830.0000000	4.5000000	17.8600006
57830.0000000	4.7500000	-50.0000000
57830.0000000	5.0000000	-50.0000000
57830.0000000	5.2500000	-50.0000000
57830.0000000	5.5000000	-50.0000000

57830.0000000	5.7500000	-50.0000000
57830.0000000	6.0000000	-50.0000000
57830.0000000	6.2500000	-50.0000000
57830.0000000	6.5000000	-50.0000000
57830.0000000	6.7500000	-50.0000000
57830.0000000	7.0000000	-50.0000000
57830.0000000	7.2500000	-50.0000000
57830.0000000	7.5000000	-50.0000000
57830.0000000	7.7500000	-50.0000000
57830.0000000	8.0000000	-50.0000000
57830.0000000	8.2500000	-50.0000000
57830.0000000	8.5000000	-50.0000000
57830.0000000	8.7500000	-50.0000000
57830.0000000	9.0000000	-50.0000000
57830.0000000	9.2500000	-50.0000000
57830.0000000	9.5000000	-50.0000000
57830.0000000	9.7500000	-50.0000000
57830.0000000	10.0000000	-50.0000000
57830.0000000	10.2500000	-50.0000000
57830.0000000	10.5000000	-50.0000000
57830.0000000	10.7500000	-50.0000000
57830.0000000	11.0000000	-50.0000000
57830.0000000	11.2500000	-50.0000000
57830.0000000	11.5000000	-50.0000000
57830.0000000	11.7500000	-50.0000000
57830.0000000	12.0000000	-50.0000000
57830.0000000	12.2500000	-50.0000000
57830.0000000	12.5000000	-50.0000000
57830.0000000	12.7500000	-50.0000000
57830.0000000	13.0000000	-50.0000000
57830.0000000	13.2500000	-50.0000000
57830.0000000	13.5000000	-50.0000000
57830.0000000	13.7500000	-50.0000000
57830.0000000	14.0000000	-50.0000000
57830.0000000	14.2500000	-50.0000000
57830.0000000	14.5000000	-50.0000000
57830.0000000	14.7500000	-50.0000000
57830.0000000	15.0000000	-50.0000000
57860.0000000	0.0000000	-50.0000000
57860.0000000	0.2500000	19.5499992
57860.0000000	0.5000000	26.1200008
57860.0000000	0.7500000	25.5699997
57860.0000000	1.0000000	24.0799999
57860.0000000	1.2500000	21.7000008
57860.0000000	1.5000000	20.9599991
57860.0000000	1.7500000	21.1499996
57860.0000000	2.0000000	19.6299992
57860.0000000	2.2500000	20.6100006
57860.0000000	2.5000000	19.2700005
57860.0000000	2.7500000	18.1599998
57860.0000000	3.0000000	19.2000008
57860.0000000	3.2500000	18.2900009
57860.0000000	3.5000000	17.3700008
57860.0000000	3.7500000	-50.0000000
57860.0000000	4.0000000	15.2900000
57860.0000000	4.2500000	16.8500004
57860.0000000	4.5000000	-50.0000000
57860.0000000	4.7500000	-50.0000000

57860.0000000	5.0000000	-50.0000000
57860.0000000	5.2500000	-50.0000000
57860.0000000	5.5000000	-50.0000000
57860.0000000	5.7500000	-50.0000000
57860.0000000	6.0000000	-50.0000000
57860.0000000	6.2500000	-50.0000000
57860.0000000	6.5000000	-50.0000000
57860.0000000	6.7500000	-50.0000000
57860.0000000	7.0000000	-50.0000000
57860.0000000	7.2500000	-50.0000000
57860.0000000	7.5000000	-50.0000000
57860.0000000	7.7500000	-50.0000000
57860.0000000	8.0000000	-50.0000000
57860.0000000	8.2500000	-50.0000000
57860.0000000	8.5000000	-50.0000000
57860.0000000	8.7500000	-50.0000000
57860.0000000	9.0000000	-50.0000000
57860.0000000	9.2500000	-50.0000000
57860.0000000	9.5000000	-50.0000000
57860.0000000	9.7500000	-50.0000000
57860.0000000	10.0000000	-50.0000000
57860.0000000	10.2500000	-50.0000000
57860.0000000	10.5000000	-50.0000000
57860.0000000	10.7500000	-50.0000000
57860.0000000	11.0000000	-50.0000000
57860.0000000	11.2500000	-50.0000000
57860.0000000	11.5000000	-50.0000000
57860.0000000	11.7500000	-50.0000000
57860.0000000	12.0000000	-50.0000000
57860.0000000	12.2500000	-50.0000000
57860.0000000	12.5000000	-50.0000000
57860.0000000	12.7500000	-50.0000000
57860.0000000	13.0000000	-50.0000000
57860.0000000	13.2500000	-50.0000000
57860.0000000	13.5000000	-50.0000000
57860.0000000	13.7500000	-50.0000000
57860.0000000	14.0000000	-50.0000000
57860.0000000	14.2500000	-50.0000000
57860.0000000	14.5000000	-50.0000000
57860.0000000	14.7500000	-50.0000000
57860.0000000	15.0000000	-50.0000000
57890.0000000	0.0000000	-50.0000000
57890.0000000	0.2500000	17.4300003
57890.0000000	0.5000000	23.7700005
57890.0000000	0.7500000	22.0599995
57890.0000000	1.0000000	21.8199997
57890.0000000	1.2500000	20.3199997
57890.0000000	1.5000000	21.4500008
57890.0000000	1.7500000	19.6599998
57890.0000000	2.0000000	19.8799992
57890.0000000	2.2500000	19.7099991
57890.0000000	2.5000000	20.5400009
57890.0000000	2.7500000	18.4699993
57890.0000000	3.0000000	18.2000008
57890.0000000	3.2500000	18.6299992
57890.0000000	3.5000000	18.4699993
57890.0000000	3.7500000	16.6800003
57890.0000000	4.0000000	18.0599995

57890.0000000	4.2500000	-50.0000000
57890.0000000	4.5000000	-50.0000000
57890.0000000	4.7500000	-50.0000000
57890.0000000	5.0000000	-50.0000000
57890.0000000	5.2500000	-50.0000000
57890.0000000	5.5000000	-50.0000000
57890.0000000	5.7500000	-50.0000000
57890.0000000	6.0000000	-50.0000000
57890.0000000	6.2500000	-50.0000000
57890.0000000	6.5000000	-50.0000000
57890.0000000	6.7500000	-50.0000000
57890.0000000	7.0000000	-50.0000000
57890.0000000	7.2500000	-50.0000000
57890.0000000	7.5000000	-50.0000000
57890.0000000	7.7500000	-50.0000000
57890.0000000	8.0000000	-50.0000000
57890.0000000	8.2500000	-50.0000000
57890.0000000	8.5000000	-50.0000000
57890.0000000	8.7500000	-50.0000000
57890.0000000	9.0000000	-50.0000000
57890.0000000	9.2500000	-50.0000000
57890.0000000	9.5000000	-50.0000000
57890.0000000	9.7500000	-50.0000000
57890.0000000	10.0000000	-50.0000000
57890.0000000	10.2500000	-50.0000000
57890.0000000	10.5000000	-50.0000000
57890.0000000	10.7500000	-50.0000000
57890.0000000	11.0000000	-50.0000000
57890.0000000	11.2500000	-50.0000000
57890.0000000	11.5000000	-50.0000000
57890.0000000	11.7500000	-50.0000000
57890.0000000	12.0000000	-50.0000000
57890.0000000	12.2500000	-50.0000000
57890.0000000	12.5000000	-50.0000000
57890.0000000	12.7500000	-50.0000000
57890.0000000	13.0000000	-50.0000000
57890.0000000	13.2500000	-50.0000000
57890.0000000	13.5000000	-50.0000000
57890.0000000	13.7500000	-50.0000000
57890.0000000	14.0000000	-50.0000000
57890.0000000	14.2500000	-50.0000000
57890.0000000	14.5000000	-50.0000000
57890.0000000	14.7500000	-50.0000000
57890.0000000	15.0000000	-50.0000000
57920.0000000	0.0000000	-50.0000000
57920.0000000	0.2500000	23.1900005
57920.0000000	0.5000000	29.7700005
57920.0000000	0.7500000	27.9699993
57920.0000000	1.0000000	22.4899998
57920.0000000	1.2500000	19.5599995
57920.0000000	1.5000000	20.8700008
57920.0000000	1.7500000	19.9099998
57920.0000000	2.0000000	20.5100002
57920.0000000	2.2500000	19.0599995
57920.0000000	2.5000000	21.2299995
57920.0000000	2.7500000	20.9500008
57920.0000000	3.0000000	18.3799992
57920.0000000	3.2500000	17.2099991

57920.0000000	3.5000000	-50.0000000
57920.0000000	3.7500000	17.2399998
57920.0000000	4.0000000	16.7399998
57920.0000000	4.2500000	17.0799999
57920.0000000	4.5000000	15.2299995
57920.0000000	4.7500000	-50.0000000
57920.0000000	5.0000000	-50.0000000
57920.0000000	5.2500000	-50.0000000
57920.0000000	5.5000000	-50.0000000
57920.0000000	5.7500000	-50.0000000
57920.0000000	6.0000000	-50.0000000
57920.0000000	6.2500000	-50.0000000
57920.0000000	6.5000000	-50.0000000
57920.0000000	6.7500000	-50.0000000
57920.0000000	7.0000000	-50.0000000
57920.0000000	7.2500000	-50.0000000
57920.0000000	7.5000000	-50.0000000
57920.0000000	7.7500000	-50.0000000
57920.0000000	8.0000000	-50.0000000
57920.0000000	8.2500000	-50.0000000
57920.0000000	8.5000000	-50.0000000
57920.0000000	8.7500000	-50.0000000
57920.0000000	9.0000000	-50.0000000
57920.0000000	9.2500000	-50.0000000
57920.0000000	9.5000000	-50.0000000
57920.0000000	9.7500000	-50.0000000
57920.0000000	10.0000000	-50.0000000
57920.0000000	10.2500000	-50.0000000
57920.0000000	10.5000000	-50.0000000
57920.0000000	10.7500000	-50.0000000
57920.0000000	11.0000000	-50.0000000
57920.0000000	11.2500000	-50.0000000
57920.0000000	11.5000000	-50.0000000
57920.0000000	11.7500000	-50.0000000
57920.0000000	12.0000000	-50.0000000
57920.0000000	12.2500000	-50.0000000
57920.0000000	12.5000000	-50.0000000
57920.0000000	12.7500000	-50.0000000
57920.0000000	13.0000000	-50.0000000
57920.0000000	13.2500000	-50.0000000
57920.0000000	13.5000000	-50.0000000
57920.0000000	13.7500000	-50.0000000
57920.0000000	14.0000000	-50.0000000
57920.0000000	14.2500000	-50.0000000
57920.0000000	14.5000000	-50.0000000
57920.0000000	14.7500000	-50.0000000
57920.0000000	15.0000000	-50.0000000
57950.0000000	0.0000000	-50.0000000
57950.0000000	0.2500000	17.0000000
57950.0000000	0.5000000	24.2700005
57950.0000000	0.7500000	25.1900005
57950.0000000	1.0000000	22.2900009
57950.0000000	1.2500000	20.6499996
57950.0000000	1.5000000	21.9599991
57950.0000000	1.7500000	20.1599998
57950.0000000	2.0000000	18.3199997
57950.0000000	2.2500000	18.4699993
57950.0000000	2.5000000	18.8299999

57950.0000000	2.7500000	17.7800007
57950.0000000	3.0000000	17.0300007
57950.0000000	3.2500000	18.0200005
57950.0000000	3.5000000	15.9499998
57950.0000000	3.7500000	-50.0000000
57950.0000000	4.0000000	16.7700005
57950.0000000	4.2500000	16.7700005
57950.0000000	4.5000000	15.1999998
57950.0000000	4.7500000	-50.0000000
57950.0000000	5.0000000	-50.0000000
57950.0000000	5.2500000	-50.0000000
57950.0000000	5.5000000	-50.0000000
57950.0000000	5.7500000	-50.0000000
57950.0000000	6.0000000	-50.0000000
57950.0000000	6.2500000	-50.0000000
57950.0000000	6.5000000	-50.0000000
57950.0000000	6.7500000	-50.0000000
57950.0000000	7.0000000	-50.0000000
57950.0000000	7.2500000	-50.0000000
57950.0000000	7.5000000	-50.0000000
57950.0000000	7.7500000	-50.0000000
57950.0000000	8.0000000	-50.0000000
57950.0000000	8.2500000	-50.0000000
57950.0000000	8.5000000	-50.0000000
57950.0000000	8.7500000	-50.0000000
57950.0000000	9.0000000	-50.0000000
57950.0000000	9.2500000	-50.0000000
57950.0000000	9.5000000	-50.0000000
57950.0000000	9.7500000	-50.0000000
57950.0000000	10.0000000	-50.0000000
57950.0000000	10.2500000	-50.0000000
57950.0000000	10.5000000	-50.0000000
57950.0000000	10.7500000	-50.0000000
57950.0000000	11.0000000	-50.0000000
57950.0000000	11.2500000	-50.0000000
57950.0000000	11.5000000	-50.0000000
57950.0000000	11.7500000	-50.0000000
57950.0000000	12.0000000	-50.0000000
57950.0000000	12.2500000	-50.0000000
57950.0000000	12.5000000	-50.0000000
57950.0000000	12.7500000	-50.0000000
57950.0000000	13.0000000	-50.0000000
57950.0000000	13.2500000	-50.0000000
57950.0000000	13.5000000	-50.0000000
57950.0000000	13.7500000	-50.0000000
57950.0000000	14.0000000	-50.0000000
57950.0000000	14.2500000	-50.0000000
57950.0000000	14.5000000	-50.0000000
57950.0000000	14.7500000	-50.0000000
57950.0000000	15.0000000	-50.0000000
57980.0000000	0.0000000	-50.0000000
57980.0000000	0.2500000	19.4300003
57980.0000000	0.5000000	26.6200008
57980.0000000	0.7500000	25.7299995
57980.0000000	1.0000000	23.0799999
57980.0000000	1.2500000	21.9200001
57980.0000000	1.5000000	20.2500000
57980.0000000	1.7500000	20.3400002

57980.0000000	2.0000000	21.2500000
57980.0000000	2.2500000	19.6299992
57980.0000000	2.5000000	19.3099995
57980.0000000	2.7500000	20.3500004
57980.0000000	3.0000000	19.1800003
57980.0000000	3.2500000	16.8500004
57980.0000000	3.5000000	17.4400005
57980.0000000	3.7500000	15.8299999
57980.0000000	4.0000000	18.0599995
57980.0000000	4.2500000	18.5499992
57980.0000000	4.5000000	15.1899996
57980.0000000	4.7500000	-50.0000000
57980.0000000	5.0000000	-50.0000000
57980.0000000	5.2500000	-50.0000000
57980.0000000	5.5000000	-50.0000000
57980.0000000	5.7500000	-50.0000000
57980.0000000	6.0000000	-50.0000000
57980.0000000	6.2500000	-50.0000000
57980.0000000	6.5000000	-50.0000000
57980.0000000	6.7500000	-50.0000000
57980.0000000	7.0000000	-50.0000000
57980.0000000	7.2500000	-50.0000000
57980.0000000	7.5000000	-50.0000000
57980.0000000	7.7500000	-50.0000000
57980.0000000	8.0000000	-50.0000000
57980.0000000	8.2500000	-50.0000000
57980.0000000	8.5000000	-50.0000000
57980.0000000	8.7500000	-50.0000000
57980.0000000	9.0000000	-50.0000000
57980.0000000	9.2500000	-50.0000000
57980.0000000	9.5000000	-50.0000000
57980.0000000	9.7500000	-50.0000000
57980.0000000	10.0000000	-50.0000000
57980.0000000	10.2500000	-50.0000000
57980.0000000	10.5000000	-50.0000000
57980.0000000	10.7500000	-50.0000000
57980.0000000	11.0000000	-50.0000000
57980.0000000	11.2500000	-50.0000000
57980.0000000	11.5000000	-50.0000000
57980.0000000	11.7500000	-50.0000000
57980.0000000	12.0000000	-50.0000000
57980.0000000	12.2500000	-50.0000000
57980.0000000	12.5000000	-50.0000000
57980.0000000	12.7500000	-50.0000000
57980.0000000	13.0000000	-50.0000000
57980.0000000	13.2500000	-50.0000000
57980.0000000	13.5000000	-50.0000000
57980.0000000	13.7500000	-50.0000000
57980.0000000	14.0000000	-50.0000000
57980.0000000	14.2500000	-50.0000000
57980.0000000	14.5000000	-50.0000000
57980.0000000	14.7500000	-50.0000000
57980.0000000	15.0000000	-50.0000000
58010.0000000	0.0000000	-50.0000000
58010.0000000	0.2500000	19.7399998
58010.0000000	0.5000000	26.2199993
58010.0000000	0.7500000	24.6599998
58010.0000000	1.0000000	20.0699997

58010.0000000	1.2500000	19.6399994
58010.0000000	1.5000000	19.3899994
58010.0000000	1.7500000	20.8700008
58010.0000000	2.0000000	21.7999992
58010.0000000	2.2500000	23.7199993
58010.0000000	2.5000000	21.7800007
58010.0000000	2.7500000	19.4799995
58010.0000000	3.0000000	19.0100002
58010.0000000	3.2500000	18.4799995
58010.0000000	3.5000000	16.8899994
58010.0000000	3.7500000	17.0799999
58010.0000000	4.0000000	18.7299995
58010.0000000	4.2500000	15.0100002
58010.0000000	4.5000000	-50.0000000
58010.0000000	4.7500000	-50.0000000
58010.0000000	5.0000000	-50.0000000
58010.0000000	5.2500000	-50.0000000
58010.0000000	5.5000000	-50.0000000
58010.0000000	5.7500000	-50.0000000
58010.0000000	6.0000000	-50.0000000
58010.0000000	6.2500000	-50.0000000
58010.0000000	6.5000000	-50.0000000
58010.0000000	6.7500000	-50.0000000
58010.0000000	7.0000000	-50.0000000
58010.0000000	7.2500000	-50.0000000
58010.0000000	7.5000000	-50.0000000
58010.0000000	7.7500000	-50.0000000
58010.0000000	8.0000000	-50.0000000
58010.0000000	8.2500000	-50.0000000
58010.0000000	8.5000000	-50.0000000
58010.0000000	8.7500000	-50.0000000
58010.0000000	9.0000000	-50.0000000
58010.0000000	9.2500000	-50.0000000
58010.0000000	9.5000000	-50.0000000
58010.0000000	9.7500000	-50.0000000
58010.0000000	10.0000000	-50.0000000
58010.0000000	10.2500000	-50.0000000
58010.0000000	10.5000000	-50.0000000
58010.0000000	10.7500000	-50.0000000
58010.0000000	11.0000000	-50.0000000
58010.0000000	11.2500000	-50.0000000
58010.0000000	11.5000000	-50.0000000
58010.0000000	11.7500000	-50.0000000
58010.0000000	12.0000000	-50.0000000
58010.0000000	12.2500000	-50.0000000
58010.0000000	12.5000000	-50.0000000
58010.0000000	12.7500000	-50.0000000
58010.0000000	13.0000000	-50.0000000
58010.0000000	13.2500000	-50.0000000
58010.0000000	13.5000000	-50.0000000
58010.0000000	13.7500000	-50.0000000
58010.0000000	14.0000000	-50.0000000
58010.0000000	14.2500000	-50.0000000
58010.0000000	14.5000000	-50.0000000
58010.0000000	14.7500000	-50.0000000
58010.0000000	15.0000000	-50.0000000
58040.0000000	0.0000000	-50.0000000
58040.0000000	0.2500000	19.3700008

58040.0000000	0.5000000	26.2299995
58040.0000000	0.7500000	24.7600002
58040.0000000	1.0000000	20.4699993
58040.0000000	1.2500000	21.3299999
58040.0000000	1.5000000	20.4300003
58040.0000000	1.7500000	20.3999996
58040.0000000	2.0000000	20.4400005
[truncated: 381,640 more chars]
