# Supplementary material for: Periodic oscillations of atmospheric electric field during snowfall in the Tokyo metropolitan area
Source: Sci Rep. 2021 Jan 26;11:2280. doi: 10.1038/s41598-021-82091-w (PMC7838252; doi:10.1038/s41598-021-82091-w)
Supplement: Supplementary file 7 — Supplementary Information 7. [file 41598_2021_82091_MOESM7_ESM.pdf]

15.0000000	0.2928000	-3.5220201
15.0000000	0.3416000	-3.6263306
15.0000000	0.3904000	-3.8717675
15.0000000	0.4392000	-3.9699423
15.0000000	0.4880000	-4.4240007
15.0000000	0.5368000	-3.8963113
15.0000000	0.5856000	-4.7614765
15.0000000	0.6344000	-3.6999617
15.0000000	0.6832000	-3.5711074
15.0000000	0.7320000	-3.0925055
15.0000000	0.7808000	-1.7548742
15.0000000	0.8296000	-1.7487383
15.0000000	0.8784000	-1.6382914
15.0000000	0.9272000	-1.6444273
15.0000000	0.9760000	-1.5339808
15.0000000	1.0247999	-1.4419422
15.0000000	1.0735999	-1.2885437
15.0000000	1.1223999	-1.2885437
15.0000000	1.1711999	-1.2824078
15.0000000	1.2200000	-1.3928547
15.0000000	1.2688000	-1.3314953
15.0000000	1.3176000	-1.3437672
15.0000000	1.3664000	-1.5401168
15.0000000	1.4152000	-1.5585246
15.0000000	1.4640000	-1.5646605
15.0000000	1.5128000	-1.5523887
15.0000000	1.5616000	-1.6444273
15.0000000	1.6104000	-1.5953403
15.0000000	1.6592000	-1.1044660
15.0000000	1.7079999	-1.7732821
15.0000000	1.7567999	-1.7671461
15.0000000	1.8055999	-1.2149129
15.0000000	1.8543999	-1.2333207
15.0000000	1.9031999	-1.2394567
15.0000000	1.9519999	-1.1412816
15.0000000	2.0007999	-1.1412816
15.0000000	2.0495999	-1.2394567
15.0000000	2.0983999	-1.2271848
15.0000000	2.1471999	-1.2271848
15.0000000	2.1959999	-1.2333207
15.0000000	2.2447999	-1.3192234
15.0000000	2.2935998	-1.3437672
15.0000000	2.3423998	-1.3499031
15.0000000	2.3912001	-1.4296703
15.0000000	2.4400001	-1.1903691
15.0000000	2.4888000	-1.2210488
15.0000000	2.5376000	-1.2087770
15.0000000	2.5864000	-1.0553789
15.0000000	2.6352000	-1.0737867
15.0000000	2.6840000	-1.0369711
15.0000000	2.7328000	-1.0369711
15.0000000	2.7816000	-1.0431070
15.0000000	2.8304000	-1.0983305
15.0000000	2.8792000	-1.0921946
15.0000000	2.9280000	-1.0737867
15.0000000	2.9768000	-1.0921946
15.0000000	3.0256000	-1.0737867

15.0000000	3.0743999	-1.0492430
15.0000000	3.1231999	-1.0983305
15.0000000	3.1719999	-1.1167378
15.0000000	3.2207999	-0.9142528
15.0000000	3.2695999	-1.0369711
15.0000000	3.3183999	-1.0369711
15.0000000	3.3671999	-1.0246992
15.0000000	3.4159999	-1.0246992
15.0000000	3.4647999	-0.9572039
15.0000000	3.5135999	-1.0553789
15.0000000	3.5623999	-1.0676508
15.0000000	3.6111999	-0.9878836
15.0000000	3.6599998	-0.8467574
15.0000000	3.7087998	-0.8651652
15.0000000	3.7575998	-0.8467574
15.0000000	3.8063998	-0.7792621
15.0000000	3.8551998	-0.7792621
15.0000000	3.9039998	-0.7853980
15.0000000	3.9527998	-0.7853980
15.0000000	4.0016003	-0.8774371
15.0000000	4.0503998	-0.8774371
15.0000000	4.0991998	-0.8099418
15.0000000	4.1479998	-0.8038058
15.0000000	4.1967998	-0.8835731
15.0000000	4.2455997	-0.7608547
15.0000000	4.2943997	-0.7485828
15.0000000	4.3431997	-0.6810875
15.0000000	4.3919997	-0.6810875
15.0000000	4.4407997	-0.6749516
15.0000000	4.4895997	-0.6688156
15.0000000	4.5383997	-0.7608547
15.0000000	4.5871997	-0.7608547
15.0000000	4.6359997	-0.7608547
15.0000000	4.6847997	-0.8528934
15.0000000	4.7336001	-0.8344855
15.0000000	4.7824001	-0.3865633
15.0000000	4.8312001	-0.4417863
15.0000000	4.8800001	-0.9081168
15.0000000	4.9288001	-0.9265246
15.0000000	4.9776001	-0.7608547
15.0000000	5.0264001	-0.4417863
15.0000000	5.0752001	-0.4356503
15.0000000	5.1240001	-0.5767770
15.0000000	5.1728001	-0.5829129
15.0000000	5.2216001	-0.7117672
15.0000000	5.2704000	613586.0000000
15.0000000	5.3192000	613586.0000000
15.0000000	5.3680000	613586.0000000
15.0000000	5.4168000	613586.0000000
15.0000000	5.4656000	613586.0000000
15.0000000	5.5144000	613586.0000000
15.0000000	5.5632000	613586.0000000
15.0000000	5.6120000	613586.0000000
15.0000000	5.6608000	613586.0000000
15.0000000	5.7096000	613586.0000000
15.0000000	5.7584000	-0.6442719
15.0000000	5.8072000	-0.6626797

15.0000000	5.8559999	-0.6872234
15.0000000	5.9047999	613586.0000000
15.0000000	5.9535999	613586.0000000
15.0000000	6.0023999	613586.0000000
15.0166664	0.2928000	-4.6817093
15.0166664	0.3416000	-4.6019425
15.0166664	0.3904000	-4.6080785
15.0166664	0.4392000	-3.9453988
15.0166664	0.4880000	-3.5588355
15.0166664	0.5368000	-4.2890105
15.0166664	0.5856000	-4.3749132
15.0166664	0.6344000	-4.4485445
15.0166664	0.6832000	-2.7243500
15.0166664	0.7320000	-2.2212043
15.0166664	0.7808000	-1.7180586
15.0166664	0.8296000	-1.7057867
15.0166664	0.8784000	-1.7978253
15.0166664	0.9272000	-1.5401168
15.0166664	0.9760000	-1.5401168
15.0166664	1.0247999	-1.2333207
15.0166664	1.0735999	-1.2149129
15.0166664	1.1223999	-1.3621750
15.0166664	1.1711999	-1.3560390
15.0166664	1.2200000	-1.3560390
15.0166664	1.2688000	-1.9634953
15.0166664	1.3176000	-2.1291652
15.0166664	1.3664000	-2.1291652
15.0166664	1.4152000	-1.9328160
15.0166664	1.4640000	-1.8837285
15.0166664	1.5128000	-1.8837285
15.0166664	1.5616000	-2.0616703
15.0166664	1.6104000	-1.7303305
15.0166664	1.6592000	-1.7426023
15.0166664	1.7079999	-1.7794180
15.0166664	1.7567999	-1.8960004
15.0166664	1.8055999	-1.8960004
15.0166664	1.8543999	-1.6812429
15.0166664	1.9031999	-1.6751070
15.0166664	1.9519999	-1.3805828
15.0166664	2.0007999	-1.3499031
15.0166664	2.0495999	-1.3376312
15.0166664	2.0983999	-1.2271848
15.0166664	2.1471999	-1.3192234
15.0166664	2.1959999	-1.3253593
15.0166664	2.2447999	-1.3376312
15.0166664	2.2935998	-1.3744469
15.0166664	2.3423998	-1.2394567
15.0166664	2.3912001	-1.3621750
15.0166664	2.4400001	-1.2824078
15.0166664	2.4888000	-1.2824078
15.0166664	2.5376000	-1.2640004
15.0166664	2.5864000	-1.2517285
15.0166664	2.6352000	-1.2578645
15.0166664	2.6840000	-1.2701364
15.0166664	2.7328000	-1.2762718
15.0166664	2.7816000	-1.0737867
15.0166664	2.8304000	-1.1228738

15.0166664	2.8792000	-1.1290097
15.0166664	2.9280000	-1.1228738
15.0166664	2.9768000	-1.1167378
15.0166664	3.0256000	-1.1290097
15.0166664	3.0743999	-1.1167378
15.0166664	3.1231999	-1.0983305
15.0166664	3.1719999	-1.0921946
15.0166664	3.2207999	-1.1780972
15.0166664	3.2695999	-1.0553789
15.0166664	3.3183999	-1.0737867
15.0166664	3.3671999	-1.0737867
15.0166664	3.4159999	-1.1474175
15.0166664	3.4647999	-1.0308352
15.0166664	3.5135999	-1.0185633
15.0166664	3.5623999	-0.9449320
15.0166664	3.6111999	-0.9387960
15.0166664	3.6599998	-0.9326606
15.0166664	3.7087998	-0.9265246
15.0166664	3.7575998	-0.9203887
15.0166664	3.8063998	-0.8099418
15.0166664	3.8551998	-0.8222136
15.0166664	3.9039998	-0.8222136
15.0166664	3.9527998	-0.8467574
15.0166664	4.0016003	-0.8467574
15.0166664	4.0503998	-0.8467574
15.0166664	4.0991998	-0.8467574
15.0166664	4.1479998	-0.7792621
15.0166664	4.1967998	-0.7853980
15.0166664	4.2455997	-0.7731261
15.0166664	4.2943997	-0.8344855
15.0166664	4.3431997	-0.8344855
15.0166664	4.3919997	-0.6442719
15.0166664	4.4407997	-0.6135921
15.0166664	4.4895997	-0.7669902
15.0166664	4.5383997	-0.6442719
15.0166664	4.5871997	-0.6197281
15.0166664	4.6359997	-0.6810875
15.0166664	4.6847997	-0.6810875
15.0166664	4.7336001	-0.6442719
15.0166664	4.7824001	-0.4847379
15.0166664	4.8312001	-0.5951843
15.0166664	4.8800001	-0.5951843
15.0166664	4.9288001	-0.5890489
15.0166664	4.9776001	-0.3436117
15.0166664	5.0264001	-0.3865633
15.0166664	5.0752001	-0.4786019
15.0166664	5.1240001	-0.4786019
15.0166664	5.1728001	-0.7485828
15.0166664	5.2216001	-0.6504078
15.0166664	5.2704000	613586.0000000
15.0166664	5.3192000	613586.0000000
15.0166664	5.3680000	613586.0000000
15.0166664	5.4168000	613586.0000000
15.0166664	5.4656000	613586.0000000
15.0166664	5.5144000	613586.0000000
15.0166664	5.5632000	613586.0000000
15.0166664	5.6120000	613586.0000000

15.0166664	5.6608000	613586.0000000
15.0166664	5.7096000	613586.0000000
15.0166664	5.7584000	-1.7241945
15.0166664	5.8072000	-1.6014762
15.0166664	5.8559999	-1.1842332
15.0166664	5.9047999	-1.0799227
15.0166664	5.9535999	-0.9081168
15.0166664	6.0023999	-0.9019809
15.0333338	0.2928000	-4.4424086
15.0333338	0.3416000	-4.4424086
15.0333338	0.3904000	-3.9515345
15.0333338	0.4392000	-4.7307968
15.0333338	0.4880000	-4.7307968
15.0333338	0.5368000	-4.7369328
15.0333338	0.5856000	-4.1049328
15.0333338	0.6344000	-4.1049328
15.0333338	0.6832000	-4.1049328
15.0333338	0.7320000	-2.5832238
15.0333338	0.7808000	-2.4420974
15.0333338	0.8296000	-2.2641559
15.0333338	0.8784000	-2.2518840
15.0333338	0.9272000	-2.2396121
15.0333338	0.9760000	-2.3439226
15.0333338	1.0247999	-2.5770879
15.0333338	1.0735999	-2.2886994
15.0333338	1.1223999	-2.5034568
15.0333338	1.1711999	-2.6998062
15.0333338	1.2200000	-2.6936703
15.0333338	1.2688000	-2.6077673
15.0333338	1.3176000	-2.4114180
15.0333338	1.3664000	-2.4175539
15.0333338	1.4152000	-2.2212043
15.0333338	1.4640000	-2.0739422
15.0333338	1.5128000	-1.7303305
15.0333338	1.5616000	-1.7241945
15.0333338	1.6104000	-1.7241945
15.0333338	1.6592000	-1.3683109
15.0333338	1.7079999	-1.7487383
15.0333338	1.7567999	-1.7548742
15.0333338	1.8055999	-1.6689711
15.0333338	1.8543999	-1.6628351
15.0333338	1.9031999	-1.7364664
15.0333338	1.9519999	-1.5462527
15.0333338	2.0007999	-1.5830684
15.0333338	2.0495999	-1.5953403
15.0333338	2.0983999	-1.3008156
15.0333338	2.1471999	-1.2824078
15.0333338	2.1959999	-1.4726214
15.0333338	2.2447999	-1.7180586
15.0333338	2.2935998	-1.1903691
15.0333338	2.3423998	-1.1903691
15.0333338	2.3912001	-1.1780972
15.0333338	2.4400001	-1.4051266
15.0333338	2.4888000	-1.5462527
15.0333338	2.5376000	-1.4664855
15.0333338	2.5864000	-1.4848933
15.0333338	2.6352000	-1.0431070

15.0333338	2.6840000	-1.1351457
15.0333338	2.7328000	-1.1351457
15.0333338	2.7816000	-1.1290097
15.0333338	2.8304000	-1.1658254
15.0333338	2.8792000	-1.1044660
15.0333338	2.9280000	-1.0860586
15.0333338	2.9768000	-1.0860586
15.0333338	3.0256000	-1.0860586
15.0333338	3.0743999	-1.0799227
15.0333338	3.1231999	-1.0799227
15.0333338	3.1719999	-1.0860586
15.0333338	3.2207999	-1.0001554
15.0333338	3.2695999	-1.0431070
15.0333338	3.3183999	-1.0369711
15.0333338	3.3671999	-1.0431070
15.0333338	3.4159999	-0.9940195
15.0333338	3.4647999	-0.9633398
15.0333338	3.5135999	-0.9572039
15.0333338	3.5623999	-0.9572039
15.0333338	3.6111999	-0.9694757
15.0333338	3.6599998	-0.9694757
15.0333338	3.7087998	-0.9694757
15.0333338	3.7575998	-0.9756117
15.0333338	3.8063998	-0.9756117
15.0333338	3.8551998	-0.9756117
15.0333338	3.9039998	-0.9019809
15.0333338	3.9527998	-0.9019809
15.0333338	4.0016003	-0.8774371
15.0333338	4.0503998	-0.8467574
15.0333338	4.0991998	-0.8467574
15.0333338	4.1479998	-0.7853980
15.0333338	4.1967998	-0.7915339
15.0333338	4.2455997	-0.6933594
15.0333338	4.2943997	-0.7792621
15.0333338	4.3431997	-0.7853980
15.0333338	4.3919997	-0.6320000
15.0333338	4.4407997	-0.6320000
15.0333338	4.4895997	-0.7179031
15.0333338	4.5383997	-0.7056313
15.0333338	4.5871997	-0.5767770
15.0333338	4.6359997	-0.6013203
15.0333338	4.6847997	-0.6013203
15.0333338	4.7336001	-0.4417863
15.0333338	4.7824001	-0.4908738
15.0333338	4.8312001	-0.3681555
15.0333338	4.8800001	-0.3742914
15.0333338	4.9288001	-0.3742914
15.0333338	4.9776001	-0.3988352
15.0333338	5.0264001	-0.3988352
15.0333338	5.0752001	-0.4049711
15.0333338	5.1240001	-0.4172430
15.0333338	5.1728001	-0.7424469
15.0333338	5.2216001	-0.7240391
15.0333338	5.2704000	613586.0000000
15.0333338	5.3192000	613586.0000000
15.0333338	5.3680000	613586.0000000
15.0333338	5.4168000	613586.0000000

15.0333338	5.4656000	613586.0000000
15.0333338	5.5144000	613586.0000000
15.0333338	5.5632000	613586.0000000
15.0333338	5.6120000	613586.0000000
15.0333338	5.6608000	613586.0000000
15.0333338	5.7096000	613586.0000000
15.0333338	5.7584000	-0.9694757
15.0333338	5.8072000	-0.9142528
15.0333338	5.8559999	-0.8528934
15.0333338	5.9047999	-0.9203887
15.0333338	5.9535999	-0.8958449
15.0333338	6.0023999	-0.9081168
15.0500002	0.2928000	-3.7060976
15.0500002	0.3416000	-4.3319616
15.0500002	0.3904000	-4.4424086
15.0500002	0.4392000	-4.8412437
15.0500002	0.4880000	-5.3566608
15.0500002	0.5368000	-5.1419039
15.0500002	0.5856000	-5.1480398
15.0500002	0.6344000	-4.0251656
15.0500002	0.6832000	-4.0251656
15.0500002	0.7320000	-2.6323111
15.0500002	0.7808000	-2.6445830
15.0500002	0.8296000	-2.6445830
15.0500002	0.8784000	-2.5893598
15.0500002	0.9272000	-2.6016314
15.0500002	0.9760000	-2.7366219
15.0500002	1.0247999	-2.8838840
15.0500002	1.0735999	3.0925050
15.0500002	1.1223999	-2.0862141
15.0500002	1.1711999	-2.0923500
15.0500002	1.2200000	-2.1046219
15.0500002	1.2688000	-2.1046219
15.0500002	1.3176000	-2.0862141
15.0500002	1.3664000	-2.0739422
15.0500002	1.4152000	-1.9266801
15.0500002	1.4640000	-1.7119226
15.0500002	1.5128000	-1.6996508
15.0500002	1.5616000	-1.6996508
15.0500002	1.6104000	-1.4971652
15.0500002	1.6592000	-1.4971652
15.0500002	1.7079999	-1.8837285
15.0500002	1.7567999	-1.7978253
15.0500002	1.8055999	-1.7916894
15.0500002	1.8543999	-1.6076121
15.0500002	1.9031999	-1.4542136
15.0500002	1.9519999	-1.3928547
15.0500002	2.0007999	-1.3867188
15.0500002	2.0495999	-1.3683109
15.0500002	2.0983999	-1.2271848
15.0500002	2.1471999	-1.5278449
15.0500002	2.1959999	-1.5278449
15.0500002	2.2447999	-1.3560390
15.0500002	2.2935998	-1.3560390
15.0500002	2.3423998	-1.2271848
15.0500002	2.3912001	-1.1780972
15.0500002	2.4400001	-1.1965051

15.0500002	2.4888000	-1.3621750
15.0500002	2.5376000	-1.3621750
15.0500002	2.5864000	-1.0983305
15.0500002	2.6352000	-1.1965051
15.0500002	2.6840000	-1.1965051
15.0500002	2.7328000	-1.1965051
15.0500002	2.7816000	-1.1535535
15.0500002	2.8304000	-1.2640004
15.0500002	2.8792000	-1.2517285
15.0500002	2.9280000	-1.1965051
15.0500002	2.9768000	-1.1412816
15.0500002	3.0256000	-1.0492430
15.0500002	3.0743999	-1.0737867
15.0500002	3.1231999	-1.0799227
15.0500002	3.1719999	-0.9817476
15.0500002	3.2207999	-0.9756117
15.0500002	3.2695999	-0.9633398
15.0500002	3.3183999	-0.9817476
15.0500002	3.3671999	-1.0062914
15.0500002	3.4159999	-1.1351457
15.0500002	3.4647999	-1.1044660
15.0500002	3.5135999	-1.1290097
15.0500002	3.5623999	-0.9694757
15.0500002	3.6111999	-0.9694757
15.0500002	3.6599998	-0.9694757
15.0500002	3.7087998	-0.9817476
15.0500002	3.7575998	-0.8406215
15.0500002	3.8063998	-0.8160777
15.0500002	3.8551998	-0.8222136
15.0500002	3.9039998	-0.8222136
15.0500002	3.9527998	-0.8222136
15.0500002	4.0016003	-0.8222136
15.0500002	4.0503998	-0.8222136
15.0500002	4.0991998	-0.8406215
15.0500002	4.1479998	-0.9387960
15.0500002	4.1967998	-0.8897090
15.0500002	4.2455997	-0.7547188
15.0500002	4.2943997	-0.7363110
15.0500002	4.3431997	-0.7485828
15.0500002	4.3919997	-0.7424469
15.0500002	4.4407997	-0.6504078
15.0500002	4.4895997	-0.6442719
15.0500002	4.5383997	-0.7547188
15.0500002	4.5871997	-0.6320000
15.0500002	4.6359997	-0.5276895
15.0500002	4.6847997	-0.6135921
15.0500002	4.7336001	-0.4295144
15.0500002	4.7824001	-0.3558836
15.0500002	4.8312001	-0.3681555
15.0500002	4.8800001	-0.5583692
15.0500002	4.9288001	-0.6994953
15.0500002	4.9776001	-0.6074562
15.0500002	5.0264001	-0.6135921
15.0500002	5.0752001	-0.3804274
15.0500002	5.1240001	-0.2699804
15.0500002	5.1728001	-0.1411262
15.0500002	5.2216001	613586.0000000

15.0500002	5.2704000	613586.0000000
15.0500002	5.3192000	613586.0000000
15.0500002	5.3680000	613586.0000000
15.0500002	5.4168000	613586.0000000
15.0500002	5.4656000	613586.0000000
15.0500002	5.5144000	613586.0000000
15.0500002	5.5632000	613586.0000000
15.0500002	5.6120000	613586.0000000
15.0500002	5.6608000	613586.0000000
15.0500002	5.7096000	613586.0000000
15.0500002	5.7584000	613586.0000000
15.0500002	5.8072000	613586.0000000
15.0500002	5.8559999	613586.0000000
15.0500002	5.9047999	-0.8344855
15.0500002	5.9535999	613586.0000000
15.0500002	6.0023999	613586.0000000
15.0666666	0.2928000	-4.1601563
15.0666666	0.3416000	-4.4117289
15.0666666	0.3904000	-4.4055929
15.0666666	0.4392000	-4.8412437
15.0666666	0.4880000	-4.8412437
15.0666666	0.5368000	-4.4424086
15.0666666	0.5856000	-4.3626413
15.0666666	0.6344000	-4.1356125
15.0666666	0.6832000	-3.6570103
15.0666666	0.7320000	-2.8102529
15.0666666	0.7808000	-2.8102529
15.0666666	0.8296000	-2.5095925
15.0666666	0.8784000	-2.3500586
15.0666666	0.9272000	-2.5709519
15.0666666	0.9760000	-2.7734373
15.0666666	1.0247999	-2.8163888
15.0666666	1.0735999	-2.6323111
15.0666666	1.1223999	-2.8347967
15.0666666	1.1711999	-2.8163888
15.0666666	1.2200000	-2.8102529
15.0666666	1.2688000	-1.7057867
15.0666666	1.3176000	-1.4112625
15.0666666	1.3664000	-1.4726214
15.0666666	1.4152000	-1.4726214
15.0666666	1.4640000	-1.5278449
15.0666666	1.5128000	-1.5217090
15.0666666	1.5616000	-1.5094371
15.0666666	1.6104000	-1.3928547
15.0666666	1.6592000	-1.7732821
15.0666666	1.7079999	-1.8775926
15.0666666	1.7567999	-1.3314953
15.0666666	1.8055999	-1.3192234
15.0666666	1.8543999	-1.1658254
15.0666666	1.9031999	-1.1412816
15.0666666	1.9519999	-1.2946796
15.0666666	2.0007999	-1.1658254
15.0666666	2.0495999	-1.1228738
15.0666666	2.0983999	-1.2271848
15.0666666	2.1471999	-1.4051266
15.0666666	2.1959999	-1.1965051
15.0666666	2.2447999	-1.1167378

15.0666666	2.2935998	-1.5462527
15.0666666	2.3423998	-1.5462527
15.0666666	2.3912001	-1.5401168
15.0666666	2.4400001	-1.3192234
15.0666666	2.4888000	-1.3192234
15.0666666	2.5376000	-1.4051266
15.0666666	2.5864000	-1.4051266
15.0666666	2.6352000	-1.2517285
15.0666666	2.6840000	-1.2394567
15.0666666	2.7328000	-1.3069515
15.0666666	2.7816000	-1.3008156
15.0666666	2.8304000	-1.0001554
15.0666666	2.8792000	-1.1780972
15.0666666	2.9280000	-1.1596894
15.0666666	2.9768000	-1.1535535
15.0666666	3.0256000	-1.1535535
15.0666666	3.0743999	-1.0001554
15.0666666	3.1231999	-1.1351457
15.0666666	3.1719999	-1.1290097
15.0666666	3.2207999	-1.1106019
15.0666666	3.2695999	-1.0921946
15.0666666	3.3183999	-1.1412816
15.0666666	3.3671999	-1.1474175
15.0666666	3.4159999	-0.9756117
15.0666666	3.4647999	-0.9756117
15.0666666	3.5135999	-1.0553789
15.0666666	3.5623999	-0.9817476
15.0666666	3.6111999	-1.0001554
15.0666666	3.6599998	-0.9449320
15.0666666	3.7087998	-1.0308352
15.0666666	3.7575998	-0.9326606
15.0666666	3.8063998	-0.9387960
15.0666666	3.8551998	-0.9387960
15.0666666	3.9039998	-0.9572039
15.0666666	3.9527998	-0.8222136
15.0666666	4.0016003	-0.7547188
15.0666666	4.0503998	-0.8406215
15.0666666	4.0991998	-0.8467574
15.0666666	4.1479998	-0.8467574
15.0666666	4.1967998	-0.8651652
15.0666666	4.2455997	-0.7547188
15.0666666	4.2943997	-0.6994953
15.0666666	4.3431997	-0.6994953
15.0666666	4.3919997	-0.8467574
15.0666666	4.4407997	-0.7485828
15.0666666	4.4895997	-0.6320000
15.0666666	4.5383997	-0.6442719
15.0666666	4.5871997	-0.5951843
15.0666666	4.6359997	-0.6994953
15.0666666	4.6847997	-0.6872234
15.0666666	4.7336001	-0.6810875
15.0666666	4.7824001	-0.6565437
15.0666666	4.8312001	-0.6688156
15.0666666	4.8800001	-0.5460973
15.0666666	4.9288001	-0.5522332
15.0666666	4.9776001	-0.6074562
15.0666666	5.0264001	-0.8344855

15.0666666	5.0752001	613586.0000000
15.0666666	5.1240001	-1.1903691
15.0666666	5.1728001	613586.0000000
15.0666666	5.2216001	613586.0000000
15.0666666	5.2704000	613586.0000000
15.0666666	5.3192000	613586.0000000
15.0666666	5.3680000	613586.0000000
15.0666666	5.4168000	613586.0000000
15.0666666	5.4656000	613586.0000000
15.0666666	5.5144000	613586.0000000
15.0666666	5.5632000	613586.0000000
15.0666666	5.6120000	613586.0000000
15.0666666	5.6608000	613586.0000000
15.0666666	5.7096000	613586.0000000
15.0666666	5.7584000	613586.0000000
15.0666666	5.8072000	-0.3681555
15.0666666	5.8559999	613586.0000000
15.0666666	5.9047999	613586.0000000
15.0666666	5.9535999	613586.0000000
15.0666666	6.0023999	613586.0000000
15.0833330	0.2928000	-4.4853601
15.0833330	0.3416000	-4.3258257
15.0833330	0.3904000	-4.9210105
15.0833330	0.4392000	-4.9271464
15.0833330	0.4880000	-4.3380976
15.0833330	0.5368000	-4.7246609
15.0833330	0.5856000	-4.6633015
15.0833330	0.6344000	-4.4485445
15.0833330	0.6832000	-3.1845441
15.0833330	0.7320000	-2.2334762
15.0833330	0.7808000	-2.2334762
15.0833330	0.8296000	-2.0309906
15.0833330	0.8784000	-1.9450879
15.0833330	0.9272000	-1.8898644
15.0833330	0.9760000	-1.8960004
15.0833330	1.0247999	-1.8898644
15.0833330	1.0735999	-1.8898644
15.0833330	1.1223999	-1.5830684
15.0833330	1.1711999	-1.5769324
15.0833330	1.2200000	-1.1596894
15.0833330	1.2688000	-1.3805828
15.0833330	1.3176000	-1.6996508
15.0833330	1.3664000	-2.0371265
15.0833330	1.4152000	-1.7794180
15.0833330	1.4640000	-1.6076121
15.0833330	1.5128000	-1.4173985
15.0833330	1.5616000	-1.3069515
15.0833330	1.6104000	-1.3867188
15.0833330	1.6592000	-1.4726214
15.0833330	1.7079999	-1.3683109
15.0833330	1.7567999	-1.3683109
15.0833330	1.8055999	-1.3683109
15.0833330	1.8543999	-1.6751070
15.0833330	1.9031999	-1.6935148
15.0833330	1.9519999	-1.5585246
15.0833330	2.0007999	-1.3499031
15.0833330	2.0495999	-1.3376312

15.0833330	2.0983999	-1.3314953
15.0833330	2.1471999	-1.3314953
15.0833330	2.1959999	-1.2762718
15.0833330	2.2447999	-1.3560390
15.0833330	2.2935998	-1.3560390
15.0833330	2.3423998	-1.3499031
15.0833330	2.3912001	-1.3867188
15.0833330	2.4400001	-1.3069515
15.0833330	2.4888000	-1.3069515
15.0833330	2.5376000	-1.3560390
15.0833330	2.5864000	-1.1351457
15.0833330	2.6352000	-1.2824078
15.0833330	2.6840000	-1.1474175
15.0833330	2.7328000	-1.1719613
15.0833330	2.7816000	-1.2517285
15.0833330	2.8304000	-1.1719613
15.0833330	2.8792000	-1.1658254
15.0833330	2.9280000	-1.1780972
15.0833330	2.9768000	-1.1842332
15.0833330	3.0256000	-1.1842332
15.0833330	3.0743999	-1.2640004
15.0833330	3.1231999	-1.2701364
15.0833330	3.1719999	-1.2701364
15.0833330	3.2207999	-1.0921946
15.0833330	3.2695999	-1.1903691
15.0833330	3.3183999	-1.1842332
15.0833330	3.3671999	-1.1842332
15.0833330	3.4159999	-1.2149129
15.0833330	3.4647999	-1.2210488
15.0833330	3.5135999	-0.8958449
15.0833330	3.5623999	-0.9387960
15.0833330	3.6111999	-0.9449320
15.0833330	3.6599998	-1.0185633
15.0833330	3.7087998	-0.9387960
15.0833330	3.7575998	-0.9449320
15.0833330	3.8063998	-0.9326606
15.0833330	3.8551998	-0.9694757
15.0833330	3.9039998	-0.8651652
15.0833330	3.9527998	-0.7731261
15.0833330	4.0016003	-0.7731261
15.0833330	4.0503998	-0.7915339
15.0833330	4.0991998	-0.7915339
15.0833330	4.1479998	-0.7915339
15.0833330	4.1967998	-0.7853980
15.0833330	4.2455997	-0.7485828
15.0833330	4.2943997	-0.7424469
15.0833330	4.3431997	-0.6320000
15.0833330	4.3919997	-0.5522332
15.0833330	4.4407997	-0.6872234
15.0833330	4.4895997	-0.6933594
15.0833330	4.5383997	-0.6381359
15.0833330	4.5871997	-0.7485828
15.0833330	4.6359997	-0.4970098
15.0833330	4.6847997	-0.5706410
15.0833330	4.7336001	-0.5829129
15.0833330	4.7824001	-0.4601941
15.0833330	4.8312001	-0.4540582

15.0833330	4.8800001	-0.3374758
15.0833330	4.9288001	-0.2208934
15.0833330	4.9776001	-0.2331653
15.0833330	5.0264001	-0.4970098
15.0833330	5.0752001	-0.5092816
15.0833330	5.1240001	-0.6749516
15.0833330	5.1728001	-0.5276895
15.0833330	5.2216001	613586.0000000
15.0833330	5.2704000	613586.0000000
15.0833330	5.3192000	613586.0000000
15.0833330	5.3680000	613586.0000000
15.0833330	5.4168000	613586.0000000
15.0833330	5.4656000	613586.0000000
15.0833330	5.5144000	613586.0000000
15.0833330	5.5632000	613586.0000000
15.0833330	5.6120000	613586.0000000
15.0833330	5.6608000	613586.0000000
15.0833330	5.7096000	613586.0000000
15.0833330	5.7584000	613586.0000000
15.0833330	5.8072000	613586.0000000
15.0833330	5.8559999	613586.0000000
15.0833330	5.9047999	613586.0000000
15.0833330	5.9535999	613586.0000000
15.0833330	6.0023999	613586.0000000
15.1000004	0.2928000	-3.9085832
15.1000004	0.3416000	-3.9024472
15.1000004	0.3904000	-3.7183695
15.1000004	0.4392000	-3.9331269
15.1000004	0.4880000	-3.9331269
15.1000004	0.5368000	-4.2460589
15.1000004	0.5856000	-3.7367773
15.1000004	0.6344000	-3.7429132
15.1000004	0.6832000	-3.7429132
15.1000004	0.7320000	-1.9144082
15.1000004	0.7808000	-1.9144082
15.1000004	0.8296000	-1.2271848
15.1000004	0.8784000	-1.2210488
15.1000004	0.9272000	-1.2762718
15.1000004	0.9760000	-1.1412816
15.1000004	1.0247999	-1.2824078
15.1000004	1.0735999	-1.2885437
15.1000004	1.1223999	-1.3253593
15.1000004	1.1711999	-1.7180586
15.1000004	1.2200000	-1.5155730
15.1000004	1.2688000	-1.2271848
15.1000004	1.3176000	-1.2701364
15.1000004	1.3664000	-1.3744469
15.1000004	1.4152000	-1.3253593
15.1000004	1.4640000	-1.3621750
15.1000004	1.5128000	-1.1842332
15.1000004	1.5616000	-1.3437672
15.1000004	1.6104000	-1.3192234
15.1000004	1.6592000	-1.2578645
15.1000004	1.7079999	-0.9817476
15.1000004	1.7567999	-1.1780972
15.1000004	1.8055999	-0.8222136
15.1000004	1.8543999	-0.8222136

15.1000004	1.9031999	-1.0492430
15.1000004	1.9519999	-1.3314953
15.1000004	2.0007999	-1.3437672
15.1000004	2.0495999	-1.3805828
15.1000004	2.0983999	-1.3928547
15.1000004	2.1471999	-1.3376312
15.1000004	2.1959999	-1.3008156
15.1000004	2.2447999	-1.3253593
15.1000004	2.2935998	-1.3621750
15.1000004	2.3423998	-1.3069515
15.1000004	2.3912001	-1.3130875
15.1000004	2.4400001	-1.3867188
15.1000004	2.4888000	-1.3314953
15.1000004	2.5376000	-1.1290097
15.1000004	2.5864000	-1.1228738
15.1000004	2.6352000	-1.1228738
15.1000004	2.6840000	-1.2885437
15.1000004	2.7328000	-1.1596894
15.1000004	2.7816000	-1.1106019
15.1000004	2.8304000	-1.2885437
15.1000004	2.8792000	-1.3069515
15.1000004	2.9280000	-1.3130875
15.1000004	2.9768000	-1.2824078
15.1000004	3.0256000	-1.2824078
15.1000004	3.0743999	-1.2885437
15.1000004	3.1231999	-1.2333207
15.1000004	3.1719999	-1.1658254
15.1000004	3.2207999	-1.0246992
15.1000004	3.2695999	-1.0124273
15.1000004	3.3183999	-1.0553789
15.1000004	3.3671999	-0.9694757
15.1000004	3.4159999	-0.9694757
15.1000004	3.4647999	-0.8651652
15.1000004	3.5135999	-0.8958449
15.1000004	3.5623999	-1.0492430
15.1000004	3.6111999	-0.8099418
15.1000004	3.6599998	-0.7363110
15.1000004	3.7087998	-0.7117672
15.1000004	3.7575998	-0.7117672
15.1000004	3.8063998	-0.8467574
15.1000004	3.8551998	-0.8467574
15.1000004	3.9039998	-0.8528934
15.1000004	3.9527998	-0.8038058
15.1000004	4.0016003	-0.8835731
15.1000004	4.0503998	-0.8406215
15.1000004	4.0991998	-0.8406215
15.1000004	4.1479998	-0.7056313
15.1000004	4.1967998	-0.7240391
15.1000004	4.2455997	-0.7363110
15.1000004	4.2943997	-0.7179031
15.1000004	4.3431997	-0.7179031
15.1000004	4.3919997	-0.7179031
15.1000004	4.4407997	-0.4847379
15.1000004	4.4895997	-0.3374758
15.1000004	4.5383997	-0.4172430
15.1000004	4.5871997	-0.5706410
15.1000004	4.6359997	-0.4479222

15.1000004	4.6847997	-0.6626797
15.1000004	4.7336001	-0.6626797
15.1000004	4.7824001	-0.4111071
15.1000004	4.8312001	-0.4049711
15.1000004	4.8800001	-0.4049711
15.1000004	4.9288001	-0.5890489
15.1000004	4.9776001	-0.4111071
15.1000004	5.0264001	-0.3558836
15.1000004	5.0752001	-0.6074562
15.1000004	5.1240001	-0.6258640
15.1000004	5.1728001	613586.0000000
15.1000004	5.2216001	613586.0000000
15.1000004	5.2704000	613586.0000000
15.1000004	5.3192000	613586.0000000
15.1000004	5.3680000	613586.0000000
15.1000004	5.4168000	613586.0000000
15.1000004	5.4656000	613586.0000000
15.1000004	5.5144000	613586.0000000
15.1000004	5.5632000	613586.0000000
15.1000004	5.6120000	613586.0000000
15.1000004	5.6608000	-1.0553789
15.1000004	5.7096000	613586.0000000
15.1000004	5.7584000	613586.0000000
15.1000004	5.8072000	613586.0000000
15.1000004	5.8559999	613586.0000000
15.1000004	5.9047999	613586.0000000
15.1000004	5.9535999	613586.0000000
15.1000004	6.0023999	613586.0000000
15.1166668	0.2928000	-4.7185249
15.1166668	0.3416000	-4.3994570
15.1166668	0.3904000	-4.3503695
15.1166668	0.4392000	-3.4545248
15.1166668	0.4880000	-4.7614765
15.1166668	0.5368000	-4.5405831
15.1166668	0.5856000	-4.5283113
15.1166668	0.6344000	-3.5833793
15.1166668	0.6832000	-2.7795732
15.1166668	0.7320000	-1.9205441
15.1166668	0.7808000	-1.8100972
15.1166668	0.8296000	-1.1474175
15.1166668	0.8784000	-1.1412816
15.1166668	0.9272000	-1.2640004
15.1166668	0.9760000	-1.2640004
15.1166668	1.0247999	-1.2578645
15.1166668	1.0735999	-1.1412816
15.1166668	1.1223999	-1.1903691
15.1166668	1.1711999	-1.8100972
15.1166668	1.2200000	-1.7916894
15.1166668	1.2688000	-0.9756117
15.1166668	1.3176000	-0.9817476
15.1166668	1.3664000	-1.4296703
15.1166668	1.4152000	-1.6505632
15.1166668	1.4640000	-1.7732821
15.1166668	1.5128000	-1.7732821
15.1166668	1.5616000	-1.8653207
15.1166668	1.6104000	-1.8653207
15.1166668	1.6592000	-1.9757671

15.1166668	1.7079999	-1.7487383
15.1166668	1.7567999	-1.6137476
15.1166668	1.8055999	-1.6260195
15.1166668	1.8543999	-1.6321554
15.1166668	1.9031999	-1.6321554
15.1166668	1.9519999	-1.3253593
15.1166668	2.0007999	-1.3253593
15.1166668	2.0495999	-1.3253593
15.1166668	2.0983999	-1.3683109
15.1166668	2.1471999	-1.3437672
15.1166668	2.1959999	-1.3437672
15.1166668	2.2447999	-1.3376312
15.1166668	2.2935998	-1.3253593
15.1166668	2.3423998	-1.3130875
15.1166668	2.3912001	-1.1842332
15.1166668	2.4400001	-1.2946796
15.1166668	2.4888000	-1.2946796
15.1166668	2.5376000	-1.2824078
15.1166668	2.5864000	-1.2824078
15.1166668	2.6352000	-0.9510679
15.1166668	2.6840000	-0.9449320
15.1166668	2.7328000	-1.2333207
15.1166668	2.7816000	-1.3683109
15.1166668	2.8304000	-1.3560390
15.1166668	2.8792000	-1.0860586
15.1166668	2.9280000	-1.0921946
15.1166668	2.9768000	-1.0001554
15.1166668	3.0256000	-0.9510679
15.1166668	3.0743999	-1.1228738
15.1166668	3.1231999	-0.9510679
15.1166668	3.1719999	-1.1412816
15.1166668	3.2207999	-1.0676508
15.1166668	3.2695999	-0.9694757
15.1166668	3.3183999	-0.9633398
15.1166668	3.3671999	-0.8222136
15.1166668	3.4159999	-0.8713012
15.1166668	3.4647999	-0.7117672
15.1166668	3.5135999	-0.9510679
15.1166668	3.5623999	-0.9572039
15.1166668	3.6111999	-0.7547188
15.1166668	3.6599998	-0.8283496
15.1166668	3.7087998	-0.8467574
15.1166668	3.7575998	-0.8344855
15.1166668	3.8063998	-0.7424469
15.1166668	3.8551998	-0.7240391
15.1166668	3.9039998	-0.7363110
15.1166668	3.9527998	-0.6688156
15.1166668	4.0016003	-0.6688156
15.1166668	4.0503998	-0.6994953
15.1166668	4.0991998	-0.8038058
15.1166668	4.1479998	-0.7915339
15.1166668	4.1967998	-0.6994953
15.1166668	4.2455997	-0.6872234
15.1166668	4.2943997	-0.6626797
15.1166668	4.3431997	-0.6504078
15.1166668	4.3919997	-0.5890489
15.1166668	4.4407997	-0.5829129

15.1166668	4.4895997	-0.4847379
15.1166668	4.5383997	-0.4847379
15.1166668	4.5871997	-0.5706410
15.1166668	4.6359997	-0.4295144
15.1166668	4.6847997	-0.2331653
15.1166668	4.7336001	-0.5092816
15.1166668	4.7824001	-0.5154176
15.1166668	4.8312001	-0.5767770
15.1166668	4.8800001	-0.5767770
15.1166668	4.9288001	-0.5215535
15.1166668	4.9776001	-0.5583692
15.1166668	5.0264001	-0.5767770
15.1166668	5.0752001	-0.6749516
15.1166668	5.1240001	-0.4847379
15.1166668	5.1728001	613586.0000000
15.1166668	5.2216001	613586.0000000
15.1166668	5.2704000	613586.0000000
15.1166668	5.3192000	613586.0000000
15.1166668	5.3680000	613586.0000000
15.1166668	5.4168000	613586.0000000
15.1166668	5.4656000	613586.0000000
15.1166668	5.5144000	613586.0000000
15.1166668	5.5632000	613586.0000000
15.1166668	5.6120000	613586.0000000
15.1166668	5.6608000	613586.0000000
15.1166668	5.7096000	613586.0000000
15.1166668	5.7584000	613586.0000000
15.1166668	5.8072000	613586.0000000
15.1166668	5.8559999	613586.0000000
15.1166668	5.9047999	613586.0000000
15.1166668	5.9535999	613586.0000000
15.1166668	6.0023999	613586.0000000
15.1333332	0.2928000	-4.7430687
15.1333332	0.3416000	-3.7245054
15.1333332	0.3904000	-4.3442335
15.1333332	0.4392000	-4.1908360
15.1333332	0.4880000	-4.1969719
15.1333332	0.5368000	-3.6201947
15.1333332	0.5856000	-3.3993015
15.1333332	0.6344000	-3.3993015
15.1333332	0.6832000	-2.6261752
15.1333332	0.7320000	-2.0739422
15.1333332	0.7808000	-2.0555344
15.1333332	0.8296000	-1.9144082
15.1333332	0.8784000	-1.2333207
15.1333332	0.9272000	-1.5523887
15.1333332	0.9760000	-1.6566992
15.1333332	1.0247999	-1.5155730
15.1333332	1.0735999	-1.5585246
15.1333332	1.1223999	-1.7794180
15.1333332	1.1711999	-1.7671461
15.1333332	1.2200000	-2.1966605
15.1333332	1.2688000	-2.1843886
15.1333332	1.3176000	2.9513793
15.1333332	1.3664000	-2.8900199
15.1333332	1.4152000	-2.4727771
15.1333332	1.4640000	-2.6077673

15.1333332	1.5128000	-2.6077673
15.1333332	1.5616000	-2.3684664
15.1333332	1.6104000	-2.3009713
15.1333332	1.6592000	-2.1230292
15.1333332	1.7079999	-2.1905246
15.1333332	1.7567999	-1.8653207
15.1333332	1.8055999	-1.8285050
15.1333332	1.8543999	-1.6566992
15.1333332	1.9031999	-1.4971652
15.1333332	1.9519999	-1.6260195
15.1333332	2.0007999	-1.3989906
15.1333332	2.0495999	-1.2517285
15.1333332	2.0983999	-1.1474175
15.1333332	2.1471999	-1.1596894
15.1333332	2.1959999	-1.1719613
15.1333332	2.2447999	-1.5953403
15.1333332	2.2935998	-1.4726214
15.1333332	2.3423998	-1.6321554
15.1333332	2.3912001	-1.3192234
15.1333332	2.4400001	-1.3130875
15.1333332	2.4888000	-1.3805828
15.1333332	2.5376000	-1.2149129
15.1333332	2.5864000	-1.2149129
15.1333332	2.6352000	-1.2762718
15.1333332	2.6840000	-1.1106019
15.1333332	2.7328000	-1.0799227
15.1333332	2.7816000	-1.0860586
15.1333332	2.8304000	-1.0492430
15.1333332	2.8792000	-1.0308352
15.1333332	2.9280000	-1.1719613
15.1333332	2.9768000	-1.1228738
15.1333332	3.0256000	-1.1290097
15.1333332	3.0743999	-1.1290097
15.1333332	3.1231999	-1.1290097
15.1333332	3.1719999	-1.1228738
15.1333332	3.2207999	-1.2149129
15.1333332	3.2695999	-1.0860586
15.1333332	3.3183999	-1.0799227
15.1333332	3.3671999	-1.0369711
15.1333332	3.4159999	-1.0431070
15.1333332	3.4647999	-0.8651652
15.1333332	3.5135999	-1.2394567
15.1333332	3.5623999	-0.9449320
15.1333332	3.6111999	-0.9449320
15.1333332	3.6599998	-1.0246992
15.1333332	3.7087998	-1.0246992
15.1333332	3.7575998	-0.8160777
15.1333332	3.8063998	-0.8222136
15.1333332	3.8551998	-0.8283496
15.1333332	3.9039998	-0.8283496
15.1333332	3.9527998	-0.8038058
15.1333332	4.0016003	-0.8897090
15.1333332	4.0503998	-0.8958449
15.1333332	4.0991998	-0.8344855
15.1333332	4.1479998	-0.9510679
15.1333332	4.1967998	-0.9694757
15.1333332	4.2455997	-0.8038058

15.1333332	4.2943997	-0.8038058
15.1333332	4.3431997	-0.9203887
15.1333332	4.3919997	-0.7976699
15.1333332	4.4407997	-0.7424469
15.1333332	4.4895997	-0.8099418
15.1333332	4.5383997	-0.8038058
15.1333332	4.5871997	-0.7976699
15.1333332	4.6359997	-0.6135921
15.1333332	4.6847997	-0.6135921
15.1333332	4.7336001	-0.6197281
15.1333332	4.7824001	-0.6135921
15.1333332	4.8312001	-0.6320000
15.1333332	4.8800001	-0.7547188
15.1333332	4.9288001	-0.8590293
15.1333332	4.9776001	-0.2515731
15.1333332	5.0264001	-0.2454371
15.1333332	5.0752001	-0.3620195
15.1333332	5.1240001	-0.3742914
15.1333332	5.1728001	-0.3620195
15.1333332	5.2216001	613586.0000000
15.1333332	5.2704000	613586.0000000
15.1333332	5.3192000	613586.0000000
15.1333332	5.3680000	613586.0000000
15.1333332	5.4168000	613586.0000000
15.1333332	5.4656000	613586.0000000
15.1333332	5.5144000	613586.0000000
15.1333332	5.5632000	613586.0000000
15.1333332	5.6120000	613586.0000000
15.1333332	5.6608000	613586.0000000
15.1333332	5.7096000	613586.0000000
15.1333332	5.7584000	613586.0000000
15.1333332	5.8072000	613586.0000000
15.1333332	5.8559999	613586.0000000
15.1333332	5.9047999	613586.0000000
15.1333332	5.9535999	613586.0000000
15.1333332	6.0023999	613586.0000000
15.1499996	0.2928000	-4.7430687
15.1499996	0.3416000	-3.8410881
15.1499996	0.3904000	-4.6571655
15.1499996	0.4392000	-3.8901753
15.1499996	0.4880000	-4.7307968
15.1499996	0.5368000	-4.7307968
15.1499996	0.5856000	-4.0006218
15.1499996	0.6344000	-2.7366219
15.1499996	0.6832000	-2.7427578
15.1499996	0.7320000	-2.7611654
15.1499996	0.7808000	-2.4236896
15.1499996	0.8296000	-2.1598449
15.1499996	0.8784000	-2.1598449
15.1499996	0.9272000	-2.3255150
15.1499996	0.9760000	-2.4666412
15.1499996	1.0247999	-2.4605052
15.1499996	1.0735999	-2.4482334
15.1499996	1.1223999	-1.4419422
15.1499996	1.1711999	-1.6935148
15.1499996	1.2200000	-1.7671461
15.1499996	1.2688000	-2.6016314

15.1499996	1.3176000	-2.7059422
15.1499996	1.3664000	-2.8961558
15.1499996	1.4152000	-2.8961558
15.1499996	1.4640000	-2.6077673
15.1499996	1.5128000	-2.6016314
15.1499996	1.5616000	-2.1537089
15.1499996	1.6104000	-2.1475730
15.1499996	1.6592000	-1.6566992
15.1499996	1.7079999	-2.0555344
15.1499996	1.7567999	-2.0064468
15.1499996	1.8055999	-1.8162332
15.1499996	1.8543999	-1.8162332
15.1499996	1.9031999	-1.6566992
15.1499996	1.9519999	-1.4664855
15.1499996	2.0007999	-1.4051266
15.1499996	2.0495999	-1.4296703
15.1499996	2.0983999	-1.4419422
15.1499996	2.1471999	-1.4664855
15.1499996	2.1959999	-1.3867188
15.1499996	2.2447999	-1.3499031
15.1499996	2.2935998	-1.3437672
15.1499996	2.3423998	-1.3683109
15.1499996	2.3912001	-1.3683109
15.1499996	2.4400001	-1.2762718
15.1499996	2.4888000	-1.3989906
15.1499996	2.5376000	-1.3069515
15.1499996	2.5864000	-1.0921946
15.1499996	2.6352000	-1.1719613
15.1499996	2.6840000	-1.1719613
15.1499996	2.7328000	-1.1842332
15.1499996	2.7816000	-1.0185633
15.1499996	2.8304000	-1.0369711
15.1499996	2.8792000	-1.3621750
15.1499996	2.9280000	-1.2333207
15.1499996	2.9768000	-1.2824078
15.1499996	3.0256000	-1.3008156
15.1499996	3.0743999	-1.1903691
15.1499996	3.1231999	-1.1903691
15.1499996	3.1719999	-1.1842332
15.1499996	3.2207999	-1.1658254
15.1499996	3.2695999	-1.1474175
15.1499996	3.3183999	-1.1106019
15.1499996	3.3671999	-1.0737867
15.1499996	3.4159999	-1.0308352
15.1499996	3.4647999	-1.0124273
15.1499996	3.5135999	-0.8099418
15.1499996	3.5623999	-1.0001554
15.1499996	3.6111999	-0.7731261
15.1499996	3.6599998	-0.9081168
15.1499996	3.7087998	-0.9081168
15.1499996	3.7575998	-0.8590293
15.1499996	3.8063998	-0.8651652
15.1499996	3.8551998	-0.8528934
15.1499996	3.9039998	-0.7669902
15.1499996	3.9527998	-0.9878836
15.1499996	4.0016003	-0.9756117
15.1499996	4.0503998	-0.9756117

15.1499996	4.0991998	-0.8406215
15.1499996	4.1479998	-0.6872234
15.1499996	4.1967998	-0.5706410
15.1499996	4.2455997	-0.8099418
15.1499996	4.2943997	-0.6074562
15.1499996	4.3431997	-0.4663301
15.1499996	4.3919997	-0.6565437
15.1499996	4.4407997	-0.6074562
15.1499996	4.4895997	-0.3190680
15.1499996	4.5383997	-0.3190680
15.1499996	4.5871997	-0.3252039
15.1499996	4.6359997	-0.2454371
15.1499996	4.6847997	-0.5276895
15.1499996	4.7336001	-0.5829129
15.1499996	4.7824001	-0.5460973
15.1499996	4.8312001	-0.7056313
15.1499996	4.8800001	-0.5829129
15.1499996	4.9288001	-0.6074562
15.1499996	4.9776001	-0.6074562
15.1499996	5.0264001	-0.4601941
15.1499996	5.0752001	-0.4172430
15.1499996	5.1240001	-0.4172430
15.1499996	5.1728001	-0.4233789
15.1499996	5.2216001	-0.2147574
15.1499996	5.2704000	-0.2147574
15.1499996	5.3192000	613586.0000000
15.1499996	5.3680000	613586.0000000
15.1499996	5.4168000	613586.0000000
15.1499996	5.4656000	613586.0000000
15.1499996	5.5144000	613586.0000000
15.1499996	5.5632000	613586.0000000
15.1499996	5.6120000	613586.0000000
15.1499996	5.6608000	613586.0000000
15.1499996	5.7096000	613586.0000000
15.1499996	5.7584000	613586.0000000
15.1499996	5.8072000	613586.0000000
15.1499996	5.8559999	613586.0000000
15.1499996	5.9047999	613586.0000000
15.1499996	5.9535999	613586.0000000
15.1499996	6.0023999	613586.0000000
15.1666670	0.2928000	-5.1848555
15.1666670	0.3416000	-5.1787195
15.1666670	0.3904000	-5.1787195
15.1666670	0.4392000	-4.5221753
15.1666670	0.4880000	-4.7492046
15.1666670	0.5368000	-4.5712628
15.1666670	0.5856000	-3.9085832
15.1666670	0.6344000	-3.5404277
15.1666670	0.6832000	-2.5893598
15.1666670	0.7320000	-2.9022918
15.1666670	0.7808000	-2.9022918
15.1666670	0.8296000	-2.8041170
15.1666670	0.8784000	-2.4052820
15.1666670	0.9272000	-2.5464082
15.1666670	0.9760000	-2.6261752
15.1666670	1.0247999	-2.3807383
15.1666670	1.0735999	-2.4052820

15.1666670	1.1223999	-2.4175539
15.1666670	1.1711999	-2.4727771
15.1666670	1.2200000	-2.2273402
15.1666670	1.2688000	-0.9449320
15.1666670	1.3176000	-0.9449320
15.1666670	1.3664000	-2.0432625
15.1666670	1.4152000	-1.9450879
15.1666670	1.4640000	-1.9573593
15.1666670	1.5128000	-1.9512239
15.1666670	1.5616000	-1.8960004
15.1666670	1.6104000	-1.9144082
15.1666670	1.6592000	-1.9205441
15.1666670	1.7079999	-1.5523887
15.1666670	1.7567999	-1.5523887
15.1666670	1.8055999	-1.5401168
15.1666670	1.8543999	-1.3621750
15.1666670	1.9031999	-1.5523887
15.1666670	1.9519999	-1.3805828
15.1666670	2.0007999	-1.3744469
15.1666670	2.0495999	-1.3744469
15.1666670	2.0983999	-1.4480777
15.1666670	2.1471999	-1.4480777
15.1666670	2.1959999	-1.3928547
15.1666670	2.2447999	-1.3130875
15.1666670	2.2935998	-1.3008156
15.1666670	2.3423998	-1.2762718
15.1666670	2.3912001	-1.3989906
15.1666670	2.4400001	-1.3928547
15.1666670	2.4888000	-1.3008156
15.1666670	2.5376000	-1.3069515
15.1666670	2.5864000	-1.2701364
15.1666670	2.6352000	-1.2517285
15.1666670	2.6840000	-1.1412816
15.1666670	2.7328000	-1.1412816
15.1666670	2.7816000	-1.0799227
15.1666670	2.8304000	-1.4419422
15.1666670	2.8792000	-1.4358063
15.1666670	2.9280000	-1.4296703
15.1666670	2.9768000	-1.3989906
15.1666670	3.0256000	-1.4112625
15.1666670	3.0743999	-1.3928547
15.1666670	3.1231999	-1.3744469
15.1666670	3.1719999	-1.3683109
15.1666670	3.2207999	-1.3069515
15.1666670	3.2695999	-1.3069515
15.1666670	3.3183999	-1.3376312
15.1666670	3.3671999	-1.3376312
15.1666670	3.4159999	-1.2455926
15.1666670	3.4647999	-1.1903691
15.1666670	3.5135999	-1.1903691
15.1666670	3.5623999	-1.3008156
15.1666670	3.6111999	-1.1412816
15.1666670	3.6599998	-1.0921946
15.1666670	3.7087998	-1.1290097
15.1666670	3.7575998	-1.2026410
15.1666670	3.8063998	-1.0246992
15.1666670	3.8551998	-1.0308352

15.1666670	3.9039998	-1.1412816
15.1666670	3.9527998	-0.9326606
15.1666670	4.0016003	-1.0860586
15.1666670	4.0503998	-0.8528934
15.1666670	4.0991998	-1.0246992
15.1666670	4.1479998	-0.8958449
15.1666670	4.1967998	-0.8958449
15.1666670	4.2455997	-1.3130875
15.1666670	4.2943997	-1.2087770
15.1666670	4.3431997	-1.2210488
15.1666670	4.3919997	-1.3253593
15.1666670	4.4407997	-1.2640004
15.1666670	4.4895997	-0.7424469
15.1666670	4.5383997	-0.9940195
15.1666670	4.5871997	-0.7363110
15.1666670	4.6359997	-0.5031457
15.1666670	4.6847997	-0.5092816
15.1666670	4.7336001	-0.5154176
15.1666670	4.7824001	-0.4540582
15.1666670	4.8312001	-0.5522332
15.1666670	4.8800001	-0.4356503
15.1666670	4.9288001	-0.4356503
15.1666670	4.9776001	-0.3067961
15.1666670	5.0264001	-0.3558836
15.1666670	5.0752001	-0.3497477
15.1666670	5.1240001	-0.3006601
15.1666670	5.1728001	613586.0000000
15.1666670	5.2216001	-0.4786019
15.1666670	5.2704000	-0.4724660
15.1666670	5.3192000	613586.0000000
15.1666670	5.3680000	613586.0000000
15.1666670	5.4168000	613586.0000000
15.1666670	5.4656000	613586.0000000
15.1666670	5.5144000	613586.0000000
15.1666670	5.5632000	613586.0000000
15.1666670	5.6120000	613586.0000000
15.1666670	5.6608000	613586.0000000
15.1666670	5.7096000	613586.0000000
15.1666670	5.7584000	613586.0000000
15.1666670	5.8072000	613586.0000000
15.1666670	5.8559999	613586.0000000
15.1666670	5.9047999	613586.0000000
15.1666670	5.9535999	613586.0000000
15.1666670	6.0023999	613586.0000000
15.1833334	0.2928000	-4.1540203
15.1833334	0.3416000	-4.1662922
15.1833334	0.3904000	-4.6387582
15.1833334	0.4392000	-4.7001171
15.1833334	0.4880000	-4.7001171
15.1833334	0.5368000	-5.2707582
15.1833334	0.5856000	-4.0435734
15.1833334	0.6344000	-4.0435734
15.1833334	0.6832000	-3.3563499
15.1833334	0.7320000	-2.7734373
15.1833334	0.7808000	-2.2396121
15.1833334	0.8296000	-2.2518840
15.1833334	0.8784000	-2.4666412

15.1833334	0.9272000	-2.4359615
15.1833334	0.9760000	-2.3623304
15.1833334	1.0247999	-2.3746023
15.1833334	1.0735999	-2.4420974
15.1833334	1.1223999	-2.4359615
15.1833334	1.1711999	-2.5034568
15.1833334	1.2200000	-2.4973209
15.1833334	1.2688000	-1.8469129
15.1833334	1.3176000	-1.8775926
15.1833334	1.3664000	-1.6566992
15.1833334	1.4152000	-1.6628351
15.1833334	1.4640000	-1.5892043
15.1833334	1.5128000	-1.5892043
15.1833334	1.5616000	-1.6689711
15.1833334	1.6104000	-1.4910293
15.1833334	1.6592000	-1.4787574
15.1833334	1.7079999	-1.5830684
15.1833334	1.7567999	-1.5892043
15.1833334	1.8055999	-1.3376312
15.1833334	1.8543999	-1.3867188
15.1833334	1.9031999	-1.4542136
15.1833334	1.9519999	-1.4358063
15.1833334	2.0007999	-1.5278449
15.1833334	2.0495999	-1.4603496
15.1833334	2.0983999	-1.4480777
15.1833334	2.1471999	-1.4419422
15.1833334	2.1959999	-1.2087770
15.1833334	2.2447999	-1.2087770
15.1833334	2.2935998	-1.2149129
15.1833334	2.3423998	-1.4358063
15.1833334	2.3912001	-1.4296703
15.1833334	2.4400001	-1.3560390
15.1833334	2.4888000	-1.3560390
15.1833334	2.5376000	-1.3499031
15.1833334	2.5864000	-1.3560390
15.1833334	2.6352000	-1.3560390
15.1833334	2.6840000	-1.3621750
15.1833334	2.7328000	-1.2517285
15.1833334	2.7816000	-1.3192234
15.1833334	2.8304000	-1.3192234
15.1833334	2.8792000	-1.3499031
15.1833334	2.9280000	-1.3499031
15.1833334	2.9768000	-1.2333207
15.1833334	3.0256000	-1.2333207
15.1833334	3.0743999	-1.2333207
15.1833334	3.1231999	-1.1228738
15.1833334	3.1719999	-1.1351457
15.1833334	3.2207999	-1.1290097
15.1833334	3.2695999	-1.1044660
15.1833334	3.3183999	-1.1106019
15.1833334	3.3671999	-1.0983305
15.1833334	3.4159999	-1.0921946
15.1833334	3.4647999	-0.9387960
15.1833334	3.5135999	-1.0431070
15.1833334	3.5623999	-0.9449320
15.1833334	3.6111999	-0.8528934
15.1833334	3.6599998	-0.8467574

15.1833334	3.7087998	-0.8467574
15.1833334	3.7575998	-0.7301750
15.1833334	3.8063998	-0.7669902
15.1833334	3.8551998	-0.7669902
15.1833334	3.9039998	-0.6320000
15.1833334	3.9527998	-0.6504078
15.1833334	4.0016003	-0.6688156
15.1833334	4.0503998	-0.7547188
15.1833334	4.0991998	-0.7608547
15.1833334	4.1479998	-0.6810875
15.1833334	4.1967998	-0.7179031
15.1833334	4.2455997	-0.5399613
15.1833334	4.2943997	-0.3067961
15.1833334	4.3431997	-0.2331653
15.1833334	4.3919997	-0.2331653
15.1833334	4.4407997	-0.5829129
15.1833334	4.4895997	-0.5645051
15.1833334	4.5383997	-0.9940195
15.1833334	4.5871997	-0.8038058
15.1833334	4.6359997	-0.8038058
15.1833334	4.6847997	-0.9572039
15.1833334	4.7336001	-0.5031457
15.1833334	4.7824001	-0.5092816
15.1833334	4.8312001	-0.4295144
15.1833334	4.8800001	-0.6320000
15.1833334	4.9288001	-0.4049711
15.1833334	4.9776001	-0.2577085
15.1833334	5.0264001	-0.5031457
15.1833334	5.0752001	-0.1595340
15.1833334	5.1240001	-0.0245438
15.1833334	5.1728001	-0.0306797
15.1833334	5.2216001	0.1779418
15.1833334	5.2704000	0.1779418
15.1833334	5.3192000	613586.0000000
15.1833334	5.3680000	613586.0000000
15.1833334	5.4168000	613586.0000000
15.1833334	5.4656000	613586.0000000
15.1833334	5.5144000	613586.0000000
15.1833334	5.5632000	613586.0000000
15.1833334	5.6120000	613586.0000000
15.1833334	5.6608000	613586.0000000
15.1833334	5.7096000	613586.0000000
15.1833334	5.7584000	613586.0000000
15.1833334	5.8072000	613586.0000000
15.1833334	5.8559999	613586.0000000
15.1833334	5.9047999	613586.0000000
15.1833334	5.9535999	613586.0000000
15.1833334	6.0023999	613586.0000000
15.1999998	0.2928000	-4.3074183
15.1999998	0.3416000	-5.0989523
15.1999998	0.3904000	-4.4117289
15.1999998	0.4392000	-4.4055929
15.1999998	0.4880000	-4.8289719
15.1999998	0.5368000	-5.1541758
15.1999998	0.5856000	-4.9087386
15.1999998	0.6344000	-3.4483888
15.1999998	0.6832000	-2.4789131

15.1999998	0.7320000	-2.4789131
15.1999998	0.7808000	-2.3132432
15.1999998	0.8296000	-2.2334762
15.1999998	0.8784000	-1.9941750
15.1999998	0.9272000	-2.0064468
15.1999998	0.9760000	-1.9512239
15.1999998	1.0247999	-1.8469129
15.1999998	1.0735999	-1.9205441
15.1999998	1.1223999	-1.9205441
15.1999998	1.1711999	-1.9205441
15.1999998	1.2200000	-2.3009713
15.1999998	1.2688000	-2.1659808
15.1999998	1.3176000	-1.4173985
15.1999998	1.3664000	-1.3989906
15.1999998	1.4152000	-1.5217090
15.1999998	1.4640000	-1.3989906
15.1999998	1.5128000	-1.4112625
15.1999998	1.5616000	-1.4235344
15.1999998	1.6104000	-1.2885437
15.1999998	1.6592000	-1.4296703
15.1999998	1.7079999	-1.2149129
15.1999998	1.7567999	-1.5339808
15.1999998	1.8055999	-1.3560390
15.1999998	1.8543999	-1.3437672
15.1999998	1.9031999	-1.3376312
15.1999998	1.9519999	-1.3376312
15.1999998	2.0007999	-1.3499031
15.1999998	2.0495999	-1.2946796
15.1999998	2.0983999	-1.2762718
15.1999998	2.1471999	-1.5094371
15.1999998	2.1959999	-1.5155730
15.1999998	2.2447999	-1.5892043
15.1999998	2.2935998	-1.4419422
15.1999998	2.3423998	-1.3928547
15.1999998	2.3912001	-1.3928547
15.1999998	2.4400001	-1.3928547
15.1999998	2.4888000	-1.2946796
15.1999998	2.5376000	-1.3008156
15.1999998	2.5864000	-1.4358063
15.1999998	2.6352000	-1.3621750
15.1999998	2.6840000	-1.3560390
15.1999998	2.7328000	-1.4235344
15.1999998	2.7816000	-1.4051266
15.1999998	2.8304000	-1.3989906
15.1999998	2.8792000	-1.4664855
15.1999998	2.9280000	-1.5094371
15.1999998	2.9768000	-1.4542136
15.1999998	3.0256000	-1.5523887
15.1999998	3.0743999	-1.5707965
15.1999998	3.1231999	-1.4235344
15.1999998	3.1719999	-1.4112625
15.1999998	3.2207999	-1.3437672
15.1999998	3.2695999	-1.3376312
15.1999998	3.3183999	-1.2762718
15.1999998	3.3671999	-1.2701364
15.1999998	3.4159999	-1.2149129
15.1999998	3.4647999	-1.0737867

15.1999998	3.5135999	-1.0553789
15.1999998	3.5623999	-1.0615149
15.1999998	3.6111999	-1.0553789
15.1999998	3.6599998	-1.0553789
15.1999998	3.7087998	-0.8897090
15.1999998	3.7575998	-0.8774371
15.1999998	3.8063998	-0.8897090
15.1999998	3.8551998	-0.9142528
15.1999998	3.9039998	-0.9081168
15.1999998	3.9527998	-0.8344855
15.1999998	4.0016003	-0.7669902
15.1999998	4.0503998	-0.7669902
15.1999998	4.0991998	-0.7669902
15.1999998	4.1479998	-0.7179031
15.1999998	4.1967998	-0.7179031
15.1999998	4.2455997	-0.6135921
15.1999998	4.2943997	-0.6197281
15.1999998	4.3431997	-0.4908738
15.1999998	4.3919997	-0.7363110
15.1999998	4.4407997	-0.7363110
15.1999998	4.4895997	-0.7179031
15.1999998	4.5383997	-0.5890489
15.1999998	4.5871997	-0.5829129
15.1999998	4.6359997	-0.5215535
15.1999998	4.6847997	-0.0490875
15.1999998	4.7336001	-0.2945242
15.1999998	4.7824001	-0.4847379
15.1999998	4.8312001	-0.5031457
15.1999998	4.8800001	-0.6565437
15.1999998	4.9288001	-0.4847379
15.1999998	4.9776001	-0.2270293
15.1999998	5.0264001	-0.4295144
15.1999998	5.0752001	-0.4663301
15.1999998	5.1240001	-0.4049711
15.1999998	5.1728001	-0.3804274
15.1999998	5.2216001	-0.0368156
15.1999998	5.2704000	-0.2208934
15.1999998	5.3192000	-0.2147574
15.1999998	5.3680000	-0.3313398
15.1999998	5.4168000	613586.0000000
15.1999998	5.4656000	613586.0000000
15.1999998	5.5144000	613586.0000000
15.1999998	5.5632000	613586.0000000
15.1999998	5.6120000	613586.0000000
15.1999998	5.6608000	613586.0000000
15.1999998	5.7096000	613586.0000000
15.1999998	5.7584000	613586.0000000
15.1999998	5.8072000	613586.0000000
15.1999998	5.8559999	613586.0000000
15.1999998	5.9047999	613586.0000000
15.1999998	5.9535999	613586.0000000
15.1999998	6.0023999	613586.0000000
15.2166662	0.2928000	-4.8351078
15.2166662	0.3416000	-4.5589910
15.2166662	0.3904000	-4.0435734
15.2166662	0.4392000	-5.0314569
15.2166662	0.4880000	-4.1969719

15.2166662	0.5368000	-4.7614765
15.2166662	0.5856000	-4.3994570
15.2166662	0.6344000	-2.3377867
15.2166662	0.6832000	-2.3377867
15.2166662	0.7320000	-1.6935148
15.2166662	0.7808000	-1.6935148
15.2166662	0.8296000	-1.6935148
15.2166662	0.8784000	-1.3989906
15.2166662	0.9272000	-1.3989906
15.2166662	0.9760000	-1.4910293
15.2166662	1.0247999	-1.5646605
15.2166662	1.0735999	-1.6689711
15.2166662	1.1223999	-1.6321554
15.2166662	1.1711999	-1.6198835
15.2166662	1.2200000	-2.1046219
15.2166662	1.2688000	-2.1046219
15.2166662	1.3176000	-1.8837285
15.2166662	1.3664000	-1.8714566
15.2166662	1.4152000	-1.6751070
15.2166662	1.4640000	-1.5462527
15.2166662	1.5128000	-1.1842332
15.2166662	1.5616000	-1.2762718
15.2166662	1.6104000	-1.1719613
15.2166662	1.6592000	-1.1167378
15.2166662	1.7079999	-1.2455926
15.2166662	1.7567999	-1.2946796
15.2166662	1.8055999	-1.2578645
15.2166662	1.8543999	-1.2640004
15.2166662	1.9031999	-1.3621750
15.2166662	1.9519999	-1.5585246
15.2166662	2.0007999	-1.5462527
15.2166662	2.0495999	-1.5462527
15.2166662	2.0983999	-1.5646605
15.2166662	2.1471999	-1.5646605
15.2166662	2.1959999	-1.4112625
15.2166662	2.2447999	-1.5401168
15.2166662	2.2935998	-1.7732821
15.2166662	2.3423998	-1.5278449
15.2166662	2.3912001	-1.5278449
15.2166662	2.4400001	-1.4235344
15.2166662	2.4888000	-1.4173985
15.2166662	2.5376000	-1.4603496
15.2166662	2.5864000	-1.2885437
15.2166662	2.6352000	-1.3683109
15.2166662	2.6840000	-1.5585246
15.2166662	2.7328000	-1.5585246
15.2166662	2.7816000	-1.6444273
15.2166662	2.8304000	-1.4051266
15.2166662	2.8792000	-1.4112625
15.2166662	2.9280000	-1.4173985
15.2166662	2.9768000	-1.4235344
15.2166662	3.0256000	-1.4296703
15.2166662	3.0743999	-1.4603496
15.2166662	3.1231999	-1.4542136
15.2166662	3.1719999	-1.3560390
15.2166662	3.2207999	-1.3499031
15.2166662	3.2695999	-1.3560390

15.2166662	3.3183999	-1.2394567
15.2166662	3.3671999	-1.2333207
15.2166662	3.4159999	-1.2333207
15.2166662	3.4647999	-1.2578645
15.2166662	3.5135999	-1.2640004
15.2166662	3.5623999	-1.0737867
15.2166662	3.6111999	-1.0737867
15.2166662	3.6599998	-0.9694757
15.2166662	3.7087998	-0.9817476
15.2166662	3.7575998	-0.9449320
15.2166662	3.8063998	-0.9387960
15.2166662	3.8551998	-0.9756117
15.2166662	3.9039998	-0.9756117
15.2166662	3.9527998	-1.0062914
15.2166662	4.0016003	-1.0062914
15.2166662	4.0503998	-0.9940195
15.2166662	4.0991998	-0.9878836
15.2166662	4.1479998	-0.9203887
15.2166662	4.1967998	-0.7792621
15.2166662	4.2455997	-0.9142528
15.2166662	4.2943997	-0.9203887
15.2166662	4.3431997	-0.9203887
15.2166662	4.3919997	-0.7117672
15.2166662	4.4407997	-0.8099418
15.2166662	4.4895997	-0.7363110
15.2166662	4.5383997	-0.7363110
15.2166662	4.5871997	-0.7240391
15.2166662	4.6359997	-0.8099418
15.2166662	4.6847997	-0.6442719
15.2166662	4.7336001	-0.6320000
15.2166662	4.7824001	-0.3865633
15.2166662	4.8312001	-0.4479222
15.2166662	4.8800001	-0.4479222
15.2166662	4.9288001	-0.3067961
15.2166662	4.9776001	-0.3804274
15.2166662	5.0264001	-0.3681555
15.2166662	5.0752001	-0.9142528
15.2166662	5.1240001	-0.9142528
15.2166662	5.1728001	-0.6565437
15.2166662	5.2216001	-0.6565437
15.2166662	5.2704000	-0.4786019
15.2166662	5.3192000	-0.4786019
15.2166662	5.3680000	613586.0000000
15.2166662	5.4168000	613586.0000000
15.2166662	5.4656000	613586.0000000
15.2166662	5.5144000	613586.0000000
15.2166662	5.5632000	613586.0000000
15.2166662	5.6120000	613586.0000000
15.2166662	5.6608000	613586.0000000
15.2166662	5.7096000	613586.0000000
15.2166662	5.7584000	613586.0000000
15.2166662	5.8072000	613586.0000000
15.2166662	5.8559999	613586.0000000
15.2166662	5.9047999	613586.0000000
15.2166662	5.9535999	613586.0000000
15.2166662	6.0023999	613586.0000000
15.2333336	0.2928000	-4.4362726

15.2333336	0.3416000	-4.4240007
15.2333336	0.3904000	-4.5589910
15.2333336	0.4392000	-4.5099039
15.2333336	0.4880000	-4.2399230
15.2333336	0.5368000	-3.3563499
15.2333336	0.5856000	-3.9515345
15.2333336	0.6344000	-3.5956511
15.2333336	0.6832000	-1.9880390
15.2333336	0.7320000	-1.9389520
15.2333336	0.7808000	-1.7057867
15.2333336	0.8296000	-1.2824078
15.2333336	0.8784000	-1.2762718
15.2333336	0.9272000	-1.2762718
15.2333336	0.9760000	-0.8467574
15.2333336	1.0247999	-0.3436117
15.2333336	1.0735999	0.0490875
15.2333336	1.1223999	-1.7548742
15.2333336	1.1711999	-1.8960004
15.2333336	1.2200000	-2.2027965
15.2333336	1.2688000	-1.6566992
15.2333336	1.3176000	-1.3314953
15.2333336	1.3664000	-1.3192234
15.2333336	1.4152000	-1.3437672
15.2333336	1.4640000	-1.4910293
15.2333336	1.5128000	-1.4910293
15.2333336	1.5616000	-1.5646605
15.2333336	1.6104000	-1.5646605
15.2333336	1.6592000	-1.4480777
15.2333336	1.7079999	-1.4419422
15.2333336	1.7567999	-1.3683109
15.2333336	1.8055999	-1.3744469
15.2333336	1.8543999	-1.2762718
15.2333336	1.9031999	-1.5278449
15.2333336	1.9519999	-1.5585246
15.2333336	2.0007999	-1.5462527
15.2333336	2.0495999	-1.5462527
15.2333336	2.0983999	-1.5462527
15.2333336	2.1471999	-1.5646605
15.2333336	2.1959999	-1.5646605
15.2333336	2.2447999	-1.6321554
15.2333336	2.2935998	-1.4910293
15.2333336	2.3423998	-1.5523887
15.2333336	2.3912001	-1.5462527
15.2333336	2.4400001	-1.5401168
15.2333336	2.4888000	-1.5155730
15.2333336	2.5376000	-1.4726214
15.2333336	2.5864000	-1.4726214
15.2333336	2.6352000	-1.4726214
15.2333336	2.6840000	-1.5278449
15.2333336	2.7328000	-1.5707965
15.2333336	2.7816000	-1.4664855
15.2333336	2.8304000	-1.4051266
15.2333336	2.8792000	-1.5033011
15.2333336	2.9280000	-1.4542136
15.2333336	2.9768000	-1.4542136
15.2333336	3.0256000	-1.4603496
15.2333336	3.0743999	-1.3192234

15.2333336	3.1231999	-1.3253593
15.2333336	3.1719999	-1.2885437
15.2333336	3.2207999	-1.2885437
15.2333336	3.2695999	-1.2885437
15.2333336	3.3183999	-1.1780972
15.2333336	3.3671999	-1.2701364
15.2333336	3.4159999	-1.1228738
15.2333336	3.4647999	-1.0983305
15.2333336	3.5135999	-1.0983305
15.2333336	3.5623999	-0.9265246
15.2333336	3.6111999	-0.9142528
15.2333336	3.6599998	-0.9142528
15.2333336	3.7087998	-0.9081168
15.2333336	3.7575998	-0.8099418
15.2333336	3.8063998	-0.8651652
15.2333336	3.8551998	-0.7792621
15.2333336	3.9039998	-0.7792621
15.2333336	3.9527998	-0.6994953
15.2333336	4.0016003	-0.6994953
15.2333336	4.0503998	-0.7179031
15.2333336	4.0991998	-0.6135921
15.2333336	4.1479998	-0.5951843
15.2333336	4.1967998	-0.6074562
15.2333336	4.2455997	-0.5215535
15.2333336	4.2943997	-0.5215535
15.2333336	4.3431997	-0.5338254
15.2333336	4.3919997	-0.4786019
15.2333336	4.4407997	-0.5092816
15.2333336	4.4895997	-0.4724660
15.2333336	4.5383997	-0.5215535
15.2333336	4.5871997	-0.7485828
15.2333336	4.6359997	-0.7485828
15.2333336	4.6847997	-0.7547188
15.2333336	4.7336001	-0.6626797
15.2333336	4.7824001	-0.5829129
15.2333336	4.8312001	-0.5890489
15.2333336	4.8800001	-0.6135921
15.2333336	4.9288001	-0.6258640
15.2333336	4.9776001	-0.8528934
15.2333336	5.0264001	-0.6688156
15.2333336	5.0752001	-0.4786019
15.2333336	5.1240001	-0.4601941
15.2333336	5.1728001	-0.4970098
15.2333336	5.2216001	-0.3558836
15.2333336	5.2704000	-0.2454371
15.2333336	5.3192000	-0.2393012
15.2333336	5.3680000	613586.0000000
15.2333336	5.4168000	613586.0000000
15.2333336	5.4656000	613586.0000000
15.2333336	5.5144000	613586.0000000
15.2333336	5.5632000	613586.0000000
15.2333336	5.6120000	613586.0000000
15.2333336	5.6608000	613586.0000000
15.2333336	5.7096000	613586.0000000
15.2333336	5.7584000	613586.0000000
15.2333336	5.8072000	613586.0000000
15.2333336	5.8559999	613586.0000000

15.2333336	5.9047999	613586.0000000
15.2333336	5.9535999	613586.0000000
15.2333336	6.0023999	613586.0000000
15.2500000	0.2928000	-4.5037680
15.2500000	0.3416000	-3.3870296
15.2500000	0.3904000	-3.3808937
15.2500000	0.4392000	-3.3808937
15.2500000	0.4880000	-4.8657870
15.2500000	0.5368000	-4.0681171
15.2500000	0.5856000	-3.5895152
15.2500000	0.6344000	-3.2459035
15.2500000	0.6832000	-3.0495539
15.2500000	0.7320000	-1.5646605
15.2500000	0.7808000	-1.3683109
15.2500000	0.8296000	-1.3744469
15.2500000	0.8784000	-1.0860586
15.2500000	0.9272000	-1.1842332
15.2500000	0.9760000	-1.1780972
15.2500000	1.0247999	-0.9142528
15.2500000	1.0735999	-0.9203887
15.2500000	1.1223999	-1.1903691
15.2500000	1.1711999	-1.6444273
15.2500000	1.2200000	-1.5830684
15.2500000	1.2688000	-1.2824078
15.2500000	1.3176000	-1.1780972
15.2500000	1.3664000	-2.6936703
15.2500000	1.4152000	-1.8898644
15.2500000	1.4640000	-1.7364664
15.2500000	1.5128000	-1.7241945
15.2500000	1.5616000	-1.7241945
15.2500000	1.6104000	-1.7364664
15.2500000	1.6592000	-1.6873789
15.2500000	1.7079999	-1.6996508
15.2500000	1.7567999	-1.6444273
15.2500000	1.8055999	-1.6628351
15.2500000	1.8543999	-1.5278449
15.2500000	1.9031999	-1.5769324
15.2500000	1.9519999	-1.5892043
15.2500000	2.0007999	-1.5892043
15.2500000	2.0495999	-1.5523887
15.2500000	2.0983999	-1.5523887
15.2500000	2.1471999	-1.4787574
15.2500000	2.1959999	-1.4664855
15.2500000	2.2447999	-1.5892043
15.2500000	2.2935998	-1.6382914
15.2500000	2.3423998	-1.4664855
15.2500000	2.3912001	-1.5094371
15.2500000	2.4400001	-1.4358063
15.2500000	2.4888000	-1.4235344
15.2500000	2.5376000	-1.4542136
15.2500000	2.5864000	-1.4542136
15.2500000	2.6352000	-1.5278449
15.2500000	2.6840000	-1.4051266
15.2500000	2.7328000	-1.3928547
15.2500000	2.7816000	-1.3928547
15.2500000	2.8304000	-1.4848933
15.2500000	2.8792000	-1.4971652

15.2500000	2.9280000	-1.4603496
15.2500000	2.9768000	-1.3560390
15.2500000	3.0256000	-1.4542136
15.2500000	3.0743999	-1.3621750
15.2500000	3.1231999	-1.2394567
15.2500000	3.1719999	-1.3069515
15.2500000	3.2207999	-1.2087770
15.2500000	3.2695999	-1.3069515
15.2500000	3.3183999	-1.1044660
15.2500000	3.3671999	-1.1044660
15.2500000	3.4159999	-1.0921946
15.2500000	3.4647999	-1.1412816
15.2500000	3.5135999	-1.0737867
15.2500000	3.5623999	-1.0062914
15.2500000	3.6111999	-0.9265246
15.2500000	3.6599998	-0.9142528
15.2500000	3.7087998	-0.8958449
15.2500000	3.7575998	-0.8160777
15.2500000	3.8063998	-0.8222136
15.2500000	3.8551998	-0.8160777
15.2500000	3.9039998	-0.8099418
15.2500000	3.9527998	-0.7424469
15.2500000	4.0016003	-0.8283496
15.2500000	4.0503998	-0.6872234
15.2500000	4.0991998	-0.6320000
15.2500000	4.1479998	-0.6320000
15.2500000	4.1967998	-0.5645051
15.2500000	4.2455997	-0.5583692
15.2500000	4.2943997	-0.5645051
15.2500000	4.3431997	-0.4847379
15.2500000	4.3919997	-0.5460973
15.2500000	4.4407997	-0.5522332
15.2500000	4.4895997	-0.5829129
15.2500000	4.5383997	-0.5890489
15.2500000	4.5871997	-0.7547188
15.2500000	4.6359997	-0.5031457
15.2500000	4.6847997	-0.5399613
15.2500000	4.7336001	-0.7240391
15.2500000	4.7824001	-0.7240391
15.2500000	4.8312001	-0.5338254
15.2500000	4.8800001	-0.5460973
15.2500000	4.9288001	-0.5215535
15.2500000	4.9776001	-0.5031457
15.2500000	5.0264001	-0.5706410
15.2500000	5.0752001	-0.5645051
15.2500000	5.1240001	-0.5276895
15.2500000	5.1728001	-0.3190680
15.2500000	5.2216001	-0.4233789
15.2500000	5.2704000	-0.4233789
15.2500000	5.3192000	-0.4786019
15.2500000	5.3680000	613586.0000000
15.2500000	5.4168000	613586.0000000
15.2500000	5.4656000	613586.0000000
15.2500000	5.5144000	613586.0000000
15.2500000	5.5632000	613586.0000000
15.2500000	5.6120000	613586.0000000
15.2500000	5.6608000	613586.0000000

15.2500000	5.7096000	613586.0000000
15.2500000	5.7584000	613586.0000000
15.2500000	5.8072000	613586.0000000
15.2500000	5.8559999	613586.0000000
15.2500000	5.9047999	613586.0000000
15.2500000	5.9535999	613586.0000000
15.2500000	6.0023999	613586.0000000
15.2666664	0.2928000	-5.0191851
15.2666664	0.3416000	-3.0556898
15.2666664	0.3904000	-3.8779035
15.2666664	0.4392000	-3.8840394
15.2666664	0.4880000	-4.0865250
15.2666664	0.5368000	-4.0987968
15.2666664	0.5856000	-3.4913404
15.2666664	0.6344000	-2.7795732
15.2666664	0.6832000	-2.7918451
15.2666664	0.7320000	-2.7918451
15.2666664	0.7808000	-1.7487383
15.2666664	0.8296000	-1.7241945
15.2666664	0.8784000	-1.6076121
15.2666664	0.9272000	-2.1475730
15.2666664	0.9760000	-2.3009713
15.2666664	1.0247999	-2.3071072
15.2666664	1.0735999	-1.6751070
15.2666664	1.1223999	-2.2580199
15.2666664	1.1711999	-1.9757671
15.2666664	1.2200000	-2.0862141
15.2666664	1.2688000	-2.5832238
15.2666664	1.3176000	-2.9268355
15.2666664	1.3664000	-2.5525441
15.2666664	1.4152000	-2.3561945
15.2666664	1.4640000	-2.1598449
15.2666664	1.5128000	-1.8530488
15.2666664	1.5616000	-1.7303305
15.2666664	1.6104000	-2.0003109
15.2666664	1.6592000	-1.9205441
15.2666664	1.7079999	-1.9021363
15.2666664	1.7567999	-1.7548742
15.2666664	1.8055999	-2.0555344
15.2666664	1.8543999	-2.0493984
15.2666664	1.9031999	-1.8039613
15.2666664	1.9519999	-1.6014762
15.2666664	2.0007999	-1.6137476
15.2666664	2.0495999	-1.5707965
15.2666664	2.0983999	-1.5769324
15.2666664	2.1471999	-1.6935148
15.2666664	2.1959999	-1.4173985
15.2666664	2.2447999	-1.5094371
15.2666664	2.2935998	-1.4971652
15.2666664	2.3423998	-1.5646605
15.2666664	2.3912001	-1.5892043
15.2666664	2.4400001	-1.4726214
15.2666664	2.4888000	-1.4726214
15.2666664	2.5376000	-1.4787574
15.2666664	2.5864000	-1.4112625
15.2666664	2.6352000	-1.4051266
15.2666664	2.6840000	-1.4051266

15.2666664	2.7328000	-1.5462527
15.2666664	2.7816000	-1.5462527
15.2666664	2.8304000	-1.5830684
15.2666664	2.8792000	-1.3560390
15.2666664	2.9280000	-1.2946796
15.2666664	2.9768000	-1.2762718
15.2666664	3.0256000	-1.2824078
15.2666664	3.0743999	-1.2394567
15.2666664	3.1231999	-1.3253593
15.2666664	3.1719999	-1.2762718
15.2666664	3.2207999	-1.2087770
15.2666664	3.2695999	-1.2087770
15.2666664	3.3183999	-1.1596894
15.2666664	3.3671999	-1.1780972
15.2666664	3.4159999	-1.1780972
15.2666664	3.4647999	-1.1719613
15.2666664	3.5135999	-1.1719613
15.2666664	3.5623999	-1.0983305
15.2666664	3.6111999	-0.8344855
15.2666664	3.6599998	-0.8344855
15.2666664	3.7087998	-0.8713012
15.2666664	3.7575998	-0.9510679
15.2666664	3.8063998	-0.9510679
15.2666664	3.8551998	-0.9387960
15.2666664	3.9039998	-0.9265246
15.2666664	3.9527998	-0.7485828
15.2666664	4.0016003	-0.8160777
15.2666664	4.0503998	-0.7669902
15.2666664	4.0991998	-0.6749516
15.2666664	4.1479998	-0.7853980
15.2666664	4.1967998	-0.5829129
15.2666664	4.2455997	-0.6872234
15.2666664	4.2943997	-0.6933594
15.2666664	4.3431997	-0.7179031
15.2666664	4.3919997	-0.5645051
15.2666664	4.4407997	-0.5829129
15.2666664	4.4895997	-0.5890489
15.2666664	4.5383997	-0.6013203
15.2666664	4.5871997	-0.6381359
15.2666664	4.6359997	-0.6320000
15.2666664	4.6847997	-0.6381359
15.2666664	4.7336001	-0.7608547
15.2666664	4.7824001	-0.5706410
15.2666664	4.8312001	-0.5583692
15.2666664	4.8800001	-0.4663301
15.2666664	4.9288001	-0.3374758
15.2666664	4.9776001	-0.3436117
15.2666664	5.0264001	-0.3436117
15.2666664	5.0752001	-0.1656699
15.2666664	5.1240001	-0.2270293
15.2666664	5.1728001	-0.1288543
15.2666664	5.2216001	-0.1963496
15.2666664	5.2704000	-0.1963496
15.2666664	5.3192000	-0.1595340
15.2666664	5.3680000	613586.0000000
15.2666664	5.4168000	613586.0000000
15.2666664	5.4656000	613586.0000000

15.2666664	5.5144000	613586.0000000
15.2666664	5.5632000	613586.0000000
15.2666664	5.6120000	613586.0000000
15.2666664	5.6608000	613586.0000000
15.2666664	5.7096000	613586.0000000
15.2666664	5.7584000	613586.0000000
15.2666664	5.8072000	613586.0000000
15.2666664	5.8559999	613586.0000000
15.2666664	5.9047999	613586.0000000
15.2666664	5.9535999	613586.0000000
15.2666664	6.0023999	613586.0000000
15.2833338	0.2928000	-3.4606607
15.2833338	0.3416000	-4.4301367
15.2833338	0.3904000	-4.3012824
15.2833338	0.4392000	-4.8596516
15.2833338	0.4880000	-4.8535156
15.2833338	0.5368000	-5.7186804
15.2833338	0.5856000	-4.8719230
15.2833338	0.6344000	-4.2767386
15.2833338	0.6832000	-2.3500586
15.2833338	0.7320000	-2.3500586
15.2833338	0.7808000	-2.3439226
15.2833338	0.8296000	-2.0616703
15.2833338	0.8784000	-2.0616703
15.2833338	0.9272000	-2.4052820
15.2833338	0.9760000	-2.4052820
15.2833338	1.0247999	-2.7243500
15.2833338	1.0735999	-1.6076121
15.2833338	1.1223999	-1.6137476
15.2833338	1.1711999	-2.7059422
15.2833338	1.2200000	-2.4114180
15.2833338	1.2688000	-2.5954955
15.2833338	1.3176000	-2.3991461
15.2833338	1.3664000	-2.3316510
15.2833338	1.4152000	-2.1537089
15.2833338	1.4640000	-1.8407769
15.2833338	1.5128000	-1.6076121
15.2833338	1.5616000	-1.6076121
15.2833338	1.6104000	-1.9941750
15.2833338	1.6592000	-1.9450879
15.2833338	1.7079999	-1.9450879
15.2833338	1.7567999	-1.8407769
15.2833338	1.8055999	-1.4664855
15.2833338	1.8543999	-1.4664855
15.2833338	1.9031999	-1.5523887
15.2833338	1.9519999	-1.4910293
15.2833338	2.0007999	-1.4910293
15.2833338	2.0495999	-1.4910293
15.2833338	2.0983999	-1.6812429
15.2833338	2.1471999	-1.5401168
15.2833338	2.1959999	-1.5278449
15.2833338	2.2447999	-1.5707965
15.2833338	2.2935998	-1.5707965
15.2833338	2.3423998	-1.5033011
15.2833338	2.3912001	-1.4173985
15.2833338	2.4400001	-1.5830684
15.2833338	2.4888000	-1.5769324

15.2833338	2.5376000	-1.5707965
15.2833338	2.5864000	-1.5585246
15.2833338	2.6352000	-1.4173985
15.2833338	2.6840000	-1.4603496
15.2833338	2.7328000	-1.4603496
15.2833338	2.7816000	-1.3621750
15.2833338	2.8304000	-1.4112625
15.2833338	2.8792000	-1.3560390
15.2833338	2.9280000	-1.2394567
15.2833338	2.9768000	-1.2394567
15.2833338	3.0256000	-1.2394567
15.2833338	3.0743999	-1.2824078
15.2833338	3.1231999	-1.2824078
15.2833338	3.1719999	-1.2087770
15.2833338	3.2207999	-1.2087770
15.2833338	3.2695999	-1.2210488
15.2833338	3.3183999	-1.2210488
15.2833338	3.3671999	-1.1106019
15.2833338	3.4159999	-1.0921946
15.2833338	3.4647999	-0.9940195
15.2833338	3.5135999	-1.1106019
15.2833338	3.5623999	-1.1106019
15.2833338	3.6111999	-1.1228738
15.2833338	3.6599998	-1.2149129
15.2833338	3.7087998	-1.0431070
15.2833338	3.7575998	-0.8835731
15.2833338	3.8063998	-0.8897090
15.2833338	3.8551998	-0.8897090
15.2833338	3.9039998	-0.8467574
15.2833338	3.9527998	-0.8344855
15.2833338	4.0016003	-0.6688156
15.2833338	4.0503998	-0.7301750
15.2833338	4.0991998	-0.8038058
15.2833338	4.1479998	-0.8222136
15.2833338	4.1967998	-0.8222136
15.2833338	4.2455997	-0.7608547
15.2833338	4.2943997	-0.7547188
15.2833338	4.3431997	-0.6197281
15.2833338	4.3919997	-0.7915339
15.2833338	4.4407997	-0.6810875
15.2833338	4.4895997	-0.6810875
15.2833338	4.5383997	-0.7240391
15.2833338	4.5871997	-0.5829129
15.2833338	4.6359997	-0.7363110
15.2833338	4.6847997	-0.7485828
15.2833338	4.7336001	-0.6626797
15.2833338	4.7824001	-0.6688156
15.2833338	4.8312001	-0.6810875
15.2833338	4.8800001	-1.0001554
15.2833338	4.9288001	-0.8835731
15.2833338	4.9776001	-0.8835731
15.2833338	5.0264001	-0.9019809
15.2833338	5.0752001	-0.9019809
15.2833338	5.1240001	-1.0062914
15.2833338	5.1728001	-1.0062914
15.2833338	5.2216001	613586.0000000
15.2833338	5.2704000	613586.0000000

15.2833338	5.3192000	613586.0000000
15.2833338	5.3680000	613586.0000000
15.2833338	5.4168000	613586.0000000
15.2833338	5.4656000	613586.0000000
15.2833338	5.5144000	613586.0000000
15.2833338	5.5632000	613586.0000000
15.2833338	5.6120000	613586.0000000
15.2833338	5.6608000	613586.0000000
15.2833338	5.7096000	613586.0000000
15.2833338	5.7584000	613586.0000000
15.2833338	5.8072000	613586.0000000
15.2833338	5.8559999	613586.0000000
15.2833338	5.9047999	613586.0000000
15.2833338	5.9535999	613586.0000000
15.2833338	6.0023999	613586.0000000
15.3000002	0.2928000	-4.8964667
15.3000002	0.3416000	-4.8964667
15.3000002	0.3904000	-5.2339425
15.3000002	0.4392000	-5.6327777
15.3000002	0.4880000	-5.0928164
15.3000002	0.5368000	-5.5284672
15.3000002	0.5856000	-4.6571655
15.3000002	0.6344000	-4.1662922
15.3000002	0.6832000	-1.9144082
15.3000002	0.7320000	-2.1721168
15.3000002	0.7808000	-2.3500586
15.3000002	0.8296000	-2.3500586
15.3000002	0.8784000	-2.5034568
15.3000002	0.9272000	-2.5095925
15.3000002	0.9760000	-2.5832238
15.3000002	1.0247999	-2.7734373
15.3000002	1.0735999	-2.6875343
15.3000002	1.1223999	-2.8409326
15.3000002	1.1711999	-2.8409326
15.3000002	1.2200000	-1.7303305
15.3000002	1.2688000	-1.7241945
15.3000002	1.3176000	-1.4051266
15.3000002	1.3664000	-1.4235344
15.3000002	1.4152000	-1.6444273
15.3000002	1.4640000	-1.6382914
15.3000002	1.5128000	-1.6566992
15.3000002	1.5616000	-1.6198835
15.3000002	1.6104000	-1.6505632
15.3000002	1.6592000	-1.6076121
15.3000002	1.7079999	-1.6014762
15.3000002	1.7567999	-1.6260195
15.3000002	1.8055999	-1.6260195
15.3000002	1.8543999	-1.6260195
15.3000002	1.9031999	-1.5217090
15.3000002	1.9519999	-1.5094371
15.3000002	2.0007999	-1.4971652
15.3000002	2.0495999	-1.4726214
15.3000002	2.0983999	-1.2333207
15.3000002	2.1471999	-1.5523887
15.3000002	2.1959999	-1.4296703
15.3000002	2.2447999	-1.4910293
15.3000002	2.2935998	-1.5462527

15.3000002	2.3423998	-1.5462527
15.3000002	2.3912001	-1.4848933
15.3000002	2.4400001	-1.4848933
15.3000002	2.4888000	-1.4848933
15.3000002	2.5376000	-1.5339808
15.3000002	2.5864000	-1.5278449
15.3000002	2.6352000	-1.4480777
15.3000002	2.6840000	-1.4419422
15.3000002	2.7328000	-1.3192234
15.3000002	2.7816000	-1.3130875
15.3000002	2.8304000	-1.3008156
15.3000002	2.8792000	-1.2026410
15.3000002	2.9280000	-1.3130875
15.3000002	2.9768000	-1.3130875
15.3000002	3.0256000	-1.3130875
15.3000002	3.0743999	-1.3130875
15.3000002	3.1231999	-1.2701364
15.3000002	3.1719999	-1.2394567
15.3000002	3.2207999	-1.2762718
15.3000002	3.2695999	-1.1903691
15.3000002	3.3183999	-1.0983305
15.3000002	3.3671999	-1.1167378
15.3000002	3.4159999	-1.0185633
15.3000002	3.4647999	-0.9265246
15.3000002	3.5135999	-0.9265246
15.3000002	3.5623999	-0.9878836
15.3000002	3.6111999	-0.8406215
15.3000002	3.6599998	-0.8835731
15.3000002	3.7087998	-0.7547188
15.3000002	3.7575998	-0.7240391
15.3000002	3.8063998	-0.7301750
15.3000002	3.8551998	-0.7853980
15.3000002	3.9039998	-0.6810875
15.3000002	3.9527998	-0.6381359
15.3000002	4.0016003	-0.7056313
15.3000002	4.0503998	-0.7853980
15.3000002	4.0991998	-0.7853980
15.3000002	4.1479998	-0.6197281
15.3000002	4.1967998	-0.6749516
15.3000002	4.2455997	-0.6688156
15.3000002	4.2943997	-0.6626797
15.3000002	4.3431997	-0.7056313
15.3000002	4.3919997	-0.7547188
15.3000002	4.4407997	-0.4724660
15.3000002	4.4895997	-0.3558836
15.3000002	4.5383997	-0.5460973
15.3000002	4.5871997	-0.5522332
15.3000002	4.6359997	-0.4786019
15.3000002	4.6847997	-0.4970098
15.3000002	4.7336001	-0.5767770
15.3000002	4.7824001	-0.6565437
15.3000002	4.8312001	-0.7608547
15.3000002	4.8800001	-0.7608547
15.3000002	4.9288001	-0.8467574
15.3000002	4.9776001	-0.9203887
15.3000002	5.0264001	-0.6872234
15.3000002	5.0752001	-0.5767770

15.3000002	5.1240001	-0.5767770
15.3000002	5.1728001	-0.2024856
15.3000002	5.2216001	613586.0000000
15.3000002	5.2704000	613586.0000000
15.3000002	5.3192000	613586.0000000
15.3000002	5.3680000	613586.0000000
15.3000002	5.4168000	613586.0000000
15.3000002	5.4656000	613586.0000000
15.3000002	5.5144000	613586.0000000
15.3000002	5.5632000	613586.0000000
15.3000002	5.6120000	613586.0000000
15.3000002	5.6608000	613586.0000000
15.3000002	5.7096000	613586.0000000
15.3000002	5.7584000	613586.0000000
15.3000002	5.8072000	613586.0000000
15.3000002	5.8559999	613586.0000000
15.3000002	5.9047999	613586.0000000
15.3000002	5.9535999	613586.0000000
15.3000002	6.0023999	613586.0000000
15.3166666	0.2928000	-4.6142144
15.3166666	0.3416000	-4.2644668
15.3166666	0.3904000	-4.2644668
15.3166666	0.4392000	-4.1540203
15.3166666	0.4880000	-4.1110687
15.3166666	0.5368000	-4.4055929
15.3166666	0.5856000	-4.3933210
15.3166666	0.6344000	-4.3933210
15.3166666	0.6832000	-3.1784084
15.3166666	0.7320000	-1.7732821
15.3166666	0.7808000	-1.7732821
15.3166666	0.8296000	-1.3928547
15.3166666	0.8784000	-1.4910293
15.3166666	0.9272000	-1.5278449
15.3166666	0.9760000	-1.4296703
15.3166666	1.0247999	-1.4296703
15.3166666	1.0735999	-1.3192234
15.3166666	1.1223999	-1.3253593
15.3166666	1.1711999	-1.0860586
15.3166666	1.2200000	-1.6137476
15.3166666	1.2688000	-1.4296703
15.3166666	1.3176000	-1.4358063
15.3166666	1.3664000	-0.7424469
15.3166666	1.4152000	-0.7424469
15.3166666	1.4640000	-1.1044660
15.3166666	1.5128000	-0.9817476
15.3166666	1.5616000	-1.2210488
15.3166666	1.6104000	-1.5094371
15.3166666	1.6592000	-1.5094371
15.3166666	1.7079999	-1.5033011
15.3166666	1.7567999	-1.5217090
15.3166666	1.8055999	-1.4787574
15.3166666	1.8543999	-1.4726214
15.3166666	1.9031999	-1.4664855
15.3166666	1.9519999	-1.4726214
15.3166666	2.0007999	-1.4664855
15.3166666	2.0495999	-1.5462527
15.3166666	2.0983999	-1.5401168

15.3166666	2.1471999	-1.5401168
15.3166666	2.1959999	-1.5401168
15.3166666	2.2447999	-1.6628351
15.3166666	2.2935998	-1.6260195
15.3166666	2.3423998	-1.6198835
15.3166666	2.3912001	-1.5646605
15.3166666	2.4400001	-1.4358063
15.3166666	2.4888000	-1.4419422
15.3166666	2.5376000	-1.4664855
15.3166666	2.5864000	-1.4726214
15.3166666	2.6352000	-1.3437672
15.3166666	2.6840000	-1.3376312
15.3166666	2.7328000	-1.4235344
15.3166666	2.7816000	-1.4296703
15.3166666	2.8304000	-1.2578645
15.3166666	2.8792000	-1.3130875
15.3166666	2.9280000	-1.4051266
15.3166666	2.9768000	-1.3253593
15.3166666	3.0256000	-1.3437672
15.3166666	3.0743999	-1.1535535
15.3166666	3.1231999	-1.2640004
15.3166666	3.1719999	-1.2640004
15.3166666	3.2207999	-1.2271848
15.3166666	3.2695999	-1.1228738
15.3166666	3.3183999	-1.1719613
15.3166666	3.3671999	-1.0001554
15.3166666	3.4159999	-0.9326606
15.3166666	3.4647999	-0.9387960
15.3166666	3.5135999	-0.8958449
15.3166666	3.5623999	-0.9142528
15.3166666	3.6111999	-0.7240391
15.3166666	3.6599998	-0.7179031
15.3166666	3.7087998	-0.5583692
15.3166666	3.7575998	-0.5583692
15.3166666	3.8063998	-0.6994953
15.3166666	3.8551998	-0.6933594
15.3166666	3.9039998	-0.5767770
15.3166666	3.9527998	-0.7485828
15.3166666	4.0016003	-0.5215535
15.3166666	4.0503998	-0.6197281
15.3166666	4.0991998	-0.5829129
15.3166666	4.1479998	-0.4908738
15.3166666	4.1967998	-0.5154176
15.3166666	4.2455997	-0.5215535
15.3166666	4.2943997	-0.5215535
15.3166666	4.3431997	-0.6320000
15.3166666	4.3919997	-0.5829129
15.3166666	4.4407997	-0.7731261
15.3166666	4.4895997	-0.8467574
15.3166666	4.5383997	-0.8406215
15.3166666	4.5871997	-0.6197281
15.3166666	4.6359997	-0.3436117
15.3166666	4.6847997	-0.8038058
15.3166666	4.7336001	-0.4295144
15.3166666	4.7824001	-0.4970098
15.3166666	4.8312001	-0.2699804
15.3166666	4.8800001	-0.4601941

15.3166666	4.9288001	-0.4601941
15.3166666	4.9776001	-0.4724660
15.3166666	5.0264001	-0.2024856
15.3166666	5.0752001	-0.1963496
15.3166666	5.1240001	-0.2393012
15.3166666	5.1728001	-0.2208934
15.3166666	5.2216001	-0.3681555
15.3166666	5.2704000	-0.2945242
15.3166666	5.3192000	0.0736313
15.3166666	5.3680000	0.0797672
15.3166666	5.4168000	613586.0000000
15.3166666	5.4656000	613586.0000000
15.3166666	5.5144000	613586.0000000
15.3166666	5.5632000	613586.0000000
15.3166666	5.6120000	613586.0000000
15.3166666	5.6608000	613586.0000000
15.3166666	5.7096000	613586.0000000
15.3166666	5.7584000	613586.0000000
15.3166666	5.8072000	613586.0000000
15.3166666	5.8559999	613586.0000000
15.3166666	5.9047999	613586.0000000
15.3166666	5.9535999	613586.0000000
15.3166666	6.0023999	613586.0000000
15.3333330	0.2928000	-4.2521949
15.3333330	0.3416000	-4.4362726
15.3333330	0.3904000	-4.4362726
15.3333330	0.4392000	-4.5528550
15.3333330	0.4880000	-3.3808937
15.3333330	0.5368000	-4.2092433
15.3333330	0.5856000	-4.3687773
15.3333330	0.6344000	-3.8717675
15.3333330	0.6832000	-2.6936703
15.3333330	0.7320000	-1.5339808
15.3333330	0.7808000	-1.3683109
15.3333330	0.8296000	-1.3621750
15.3333330	0.8784000	-1.1596894
15.3333330	0.9272000	-1.3621750
15.3333330	0.9760000	-1.3499031
15.3333330	1.0247999	-1.3314953
15.3333330	1.0735999	-1.7057867
15.3333330	1.1223999	-1.4971652
15.3333330	1.1711999	-1.4603496
15.3333330	1.2200000	-1.2394567
15.3333330	1.2688000	-1.2455926
15.3333330	1.3176000	-1.1719613
15.3333330	1.3664000	-1.6198835
15.3333330	1.4152000	-1.3989906
15.3333330	1.4640000	-1.2517285
15.3333330	1.5128000	-1.2333207
15.3333330	1.5616000	-1.3069515
15.3333330	1.6104000	-1.3130875
15.3333330	1.6592000	-1.5892043
15.3333330	1.7079999	-1.5155730
15.3333330	1.7567999	-1.5033011
15.3333330	1.8055999	-1.4664855
15.3333330	1.8543999	-1.4726214
15.3333330	1.9031999	-1.5094371

15.3333330	1.9519999	-1.6812429
15.3333330	2.0007999	-1.6444273
15.3333330	2.0495999	-1.6444273
15.3333330	2.0983999	-1.5646605
15.3333330	2.1471999	-1.4910293
15.3333330	2.1959999	-1.5339808
15.3333330	2.2447999	-1.3928547
15.3333330	2.2935998	-1.3376312
15.3333330	2.3423998	-1.4112625
15.3333330	2.3912001	-1.2640004
15.3333330	2.4400001	-1.2517285
15.3333330	2.4888000	-1.2517285
15.3333330	2.5376000	-1.4112625
15.3333330	2.5864000	-1.4173985
15.3333330	2.6352000	-1.4173985
15.3333330	2.6840000	-1.4296703
15.3333330	2.7328000	-1.4542136
15.3333330	2.7816000	-1.3805828
15.3333330	2.8304000	-1.4358063
15.3333330	2.8792000	-1.3376312
15.3333330	2.9280000	-1.3437672
15.3333330	2.9768000	-1.3499031
15.3333330	3.0256000	-1.3192234
15.3333330	3.0743999	-1.1842332
15.3333330	3.1231999	-1.1719613
15.3333330	3.1719999	-1.1842332
15.3333330	3.2207999	-1.1719613
15.3333330	3.2695999	-1.1780972
15.3333330	3.3183999	-1.1719613
15.3333330	3.3671999	-1.1842332
15.3333330	3.4159999	-1.1780972
15.3333330	3.4647999	-1.0308352
15.3333330	3.5135999	-1.0860586
15.3333330	3.5623999	-0.8651652
15.3333330	3.6111999	-0.9142528
15.3333330	3.6599998	-1.0185633
15.3333330	3.7087998	-0.8160777
15.3333330	3.7575998	-0.8713012
15.3333330	3.8063998	-0.8528934
15.3333330	3.8551998	-0.7976699
15.3333330	3.9039998	-0.7240391
15.3333330	3.9527998	-0.6994953
15.3333330	4.0016003	-0.8590293
15.3333330	4.0503998	-0.7915339
15.3333330	4.0991998	-0.6504078
15.3333330	4.1479998	-0.6810875
15.3333330	4.1967998	-0.6504078
15.3333330	4.2455997	-0.4786019
15.3333330	4.2943997	-0.5338254
15.3333330	4.3431997	-0.4356503
15.3333330	4.3919997	-0.4356503
15.3333330	4.4407997	-0.5767770
15.3333330	4.4895997	-0.5460973
15.3333330	4.5383997	-0.4049711
15.3333330	4.5871997	-0.4111071
15.3333330	4.6359997	-0.6626797
15.3333330	4.6847997	-0.6688156

15.3333330	4.7336001	-0.7792621
15.3333330	4.7824001	-0.7792621
15.3333330	4.8312001	-0.6135921
15.3333330	4.8800001	-0.6074562
15.3333330	4.9288001	-0.8467574
15.3333330	4.9776001	-0.7731261
15.3333330	5.0264001	-0.7915339
15.3333330	5.0752001	-0.5583692
15.3333330	5.1240001	-0.5645051
15.3333330	5.1728001	-0.6135921
15.3333330	5.2216001	-0.5767770
15.3333330	5.2704000	-0.5522332
15.3333330	5.3192000	-0.3742914
15.3333330	5.3680000	613586.0000000
15.3333330	5.4168000	613586.0000000
15.3333330	5.4656000	613586.0000000
15.3333330	5.5144000	613586.0000000
15.3333330	5.5632000	613586.0000000
15.3333330	5.6120000	613586.0000000
15.3333330	5.6608000	613586.0000000
15.3333330	5.7096000	613586.0000000
15.3333330	5.7584000	613586.0000000
15.3333330	5.8072000	613586.0000000
15.3333330	5.8559999	613586.0000000
15.3333330	5.9047999	613586.0000000
15.3333330	5.9535999	613586.0000000
15.3333330	6.0023999	613586.0000000
15.3500004	0.2928000	-4.9271464
15.3500004	0.3416000	-4.9332824
15.3500004	0.3904000	-3.9638064
15.3500004	0.4392000	-4.5651269
15.3500004	0.4880000	-4.2644668
15.3500004	0.5368000	-4.2460589
15.3500004	0.5856000	-4.9332824
15.3500004	0.6344000	-3.2213597
15.3500004	0.6832000	-2.4850490
15.3500004	0.7320000	-2.1659808
15.3500004	0.7808000	-1.7916894
15.3500004	0.8296000	-1.3437672
15.3500004	0.8784000	-1.0308352
15.3500004	0.9272000	-0.8222136
15.3500004	0.9760000	-0.7301750
15.3500004	1.0247999	-0.7240391
15.3500004	1.0735999	-0.8099418
15.3500004	1.1223999	-1.3437672
15.3500004	1.1711999	-1.8100972
15.3500004	1.2200000	-1.1903691
15.3500004	1.2688000	-0.8099418
15.3500004	1.3176000	-0.2699804
15.3500004	1.3664000	0.1902137
15.3500004	1.4152000	0.1104465
15.3500004	1.4640000	-1.5892043
15.3500004	1.5128000	-1.6628351
15.3500004	1.5616000	-1.5278449
15.3500004	1.6104000	-1.5462527
15.3500004	1.6592000	-1.5094371
15.3500004	1.7079999	-1.3499031

15.3500004	1.7567999	-1.4480777
15.3500004	1.8055999	-1.4419422
15.3500004	1.8543999	-1.4480777
15.3500004	1.9031999	-1.5401168
15.3500004	1.9519999	-1.5339808
15.3500004	2.0007999	-1.5339808
15.3500004	2.0495999	-1.5278449
15.3500004	2.0983999	-1.4296703
15.3500004	2.1471999	-1.5953403
15.3500004	2.1959999	-1.4848933
15.3500004	2.2447999	-1.4848933
15.3500004	2.2935998	-1.2394567
15.3500004	2.3423998	-1.2578645
15.3500004	2.3912001	-1.4480777
15.3500004	2.4400001	-1.3437672
15.3500004	2.4888000	-1.4726214
15.3500004	2.5376000	-1.4664855
15.3500004	2.5864000	-1.4542136
15.3500004	2.6352000	-1.3560390
15.3500004	2.6840000	-1.3499031
15.3500004	2.7328000	-1.3499031
15.3500004	2.7816000	-1.2394567
15.3500004	2.8304000	-1.2394567
15.3500004	2.8792000	-1.0921946
15.3500004	2.9280000	-1.2210488
15.3500004	2.9768000	-1.1474175
15.3500004	3.0256000	-1.0615149
15.3500004	3.0743999	-1.1658254
15.3500004	3.1231999	-1.1167378
15.3500004	3.1719999	-1.1290097
15.3500004	3.2207999	-1.1351457
15.3500004	3.2695999	-1.1228738
15.3500004	3.3183999	-1.1167378
15.3500004	3.3671999	-1.0676508
15.3500004	3.4159999	-0.9449320
15.3500004	3.4647999	-0.9449320
15.3500004	3.5135999	-0.9081168
15.3500004	3.5623999	-0.9203887
15.3500004	3.6111999	-0.9326606
15.3500004	3.6599998	-0.8774371
15.3500004	3.7087998	-0.8958449
15.3500004	3.7575998	-0.7792621
15.3500004	3.8063998	-0.7853980
15.3500004	3.8551998	-0.7731261
15.3500004	3.9039998	-0.7792621
15.3500004	3.9527998	-0.7853980
15.3500004	4.0016003	-0.7853980
15.3500004	4.0503998	-0.6994953
15.3500004	4.0991998	-0.7915339
15.3500004	4.1479998	-0.6442719
15.3500004	4.1967998	-0.6381359
15.3500004	4.2455997	-0.7731261
15.3500004	4.2943997	-0.7301750
15.3500004	4.3431997	-0.7240391
15.3500004	4.3919997	-0.5829129
15.3500004	4.4407997	-0.5829129
15.3500004	4.4895997	-0.6810875

15.3500004	4.5383997	-0.7485828
15.3500004	4.5871997	-0.8897090
15.3500004	4.6359997	-0.8897090
15.3500004	4.6847997	-1.0737867
15.3500004	4.7336001	-0.8651652
15.3500004	4.7824001	-1.0001554
15.3500004	4.8312001	-0.7301750
15.3500004	4.8800001	-0.7301750
15.3500004	4.9288001	-0.7301750
15.3500004	4.9776001	-0.5583692
15.3500004	5.0264001	-0.5706410
15.3500004	5.0752001	-0.5583692
15.3500004	5.1240001	-0.5460973
15.3500004	5.1728001	-0.6135921
15.3500004	5.2216001	-0.5706410
15.3500004	5.2704000	613586.0000000
15.3500004	5.3192000	613586.0000000
15.3500004	5.3680000	613586.0000000
15.3500004	5.4168000	613586.0000000
15.3500004	5.4656000	613586.0000000
15.3500004	5.5144000	613586.0000000
15.3500004	5.5632000	613586.0000000
15.3500004	5.6120000	613586.0000000
15.3500004	5.6608000	613586.0000000
15.3500004	5.7096000	613586.0000000
15.3500004	5.7584000	613586.0000000
15.3500004	5.8072000	613586.0000000
15.3500004	5.8559999	613586.0000000
15.3500004	5.9047999	613586.0000000
15.3500004	5.9535999	613586.0000000
15.3500004	6.0023999	613586.0000000
15.3666668	0.2928000	-4.2767386
15.3666668	0.3416000	-4.3380976
15.3666668	0.3904000	-4.3319616
15.3666668	0.4392000	-4.3626413
15.3666668	0.4880000	-4.3626413
15.3666668	0.5368000	-4.3626413
15.3666668	0.5856000	-4.4669523
15.3666668	0.6344000	-3.9147191
15.3666668	0.6832000	-2.9391072
15.3666668	0.7320000	-2.1168938
15.3666668	0.7808000	-1.3928547
15.3666668	0.8296000	-1.3928547
15.3666668	0.8784000	-1.0737867
15.3666668	0.9272000	-1.0799227
15.3666668	0.9760000	-1.0062914
15.3666668	1.0247999	-0.8038058
15.3666668	1.0735999	-0.6197281
15.3666668	1.1223999	-0.5092816
15.3666668	1.1711999	-0.2147574
15.3666668	1.2200000	-1.3805828
15.3666668	1.2688000	-2.6261752
15.3666668	1.3176000	-2.6261752
15.3666668	1.3664000	-2.9943306
15.3666668	1.4152000	-2.3255150
15.3666668	1.4640000	-2.3746023
15.3666668	1.5128000	-2.1843886

15.3666668	1.5616000	-1.9205441
15.3666668	1.6104000	-1.8591847
15.3666668	1.6592000	-1.7978253
15.3666668	1.7079999	-1.5278449
15.3666668	1.7567999	-1.5217090
15.3666668	1.8055999	-1.4664855
15.3666668	1.8543999	-1.3744469
15.3666668	1.9031999	-1.3683109
15.3666668	1.9519999	-1.4542136
15.3666668	2.0007999	-1.4051266
15.3666668	2.0495999	-1.4051266
15.3666668	2.0983999	-1.5217090
15.3666668	2.1471999	-1.4910293
15.3666668	2.1959999	-1.3805828
15.3666668	2.2447999	-1.3867188
15.3666668	2.2935998	-1.4173985
15.3666668	2.3423998	-1.4235344
15.3666668	2.3912001	-1.3683109
15.3666668	2.4400001	-1.3437672
15.3666668	2.4888000	-1.3437672
15.3666668	2.5376000	-1.3069515
15.3666668	2.5864000	-1.2578645
15.3666668	2.6352000	-1.3499031
15.3666668	2.6840000	-1.1658254
15.3666668	2.7328000	-1.3008156
15.3666668	2.7816000	-1.2333207
15.3666668	2.8304000	-1.2394567
15.3666668	2.8792000	-1.2578645
15.3666668	2.9280000	-1.1412816
15.3666668	2.9768000	-1.0124273
15.3666668	3.0256000	-1.1228738
15.3666668	3.0743999	-1.1412816
15.3666668	3.1231999	-1.1412816
15.3666668	3.1719999	-1.1412816
15.3666668	3.2207999	-1.1412816
15.3666668	3.2695999	-1.0553789
15.3666668	3.3183999	-0.8835731
15.3666668	3.3671999	-0.9449320
15.3666668	3.4159999	-0.9387960
15.3666668	3.4647999	-0.9878836
15.3666668	3.5135999	-0.8222136
15.3666668	3.5623999	-0.9326606
15.3666668	3.6111999	-0.9387960
15.3666668	3.6599998	-0.9387960
15.3666668	3.7087998	-0.9387960
15.3666668	3.7575998	-0.9142528
15.3666668	3.8063998	-0.9081168
15.3666668	3.8551998	-0.7853980
15.3666668	3.9039998	-0.6381359
15.3666668	3.9527998	-0.6442719
15.3666668	4.0016003	-0.5583692
15.3666668	4.0503998	-0.5583692
15.3666668	4.0991998	-0.7485828
15.3666668	4.1479998	-0.5890489
15.3666668	4.1967998	-0.6013203
15.3666668	4.2455997	-0.5276895
15.3666668	4.2943997	-0.6810875

15.3666668	4.3431997	-0.7424469
15.3666668	4.3919997	-0.5706410
15.3666668	4.4407997	-0.5706410
15.3666668	4.4895997	-0.6810875
15.3666668	4.5383997	-0.6872234
15.3666668	4.5871997	-0.6933594
15.3666668	4.6359997	-0.5460973
15.3666668	4.6847997	-0.6013203
15.3666668	4.7336001	-0.6504078
15.3666668	4.7824001	-0.7117672
15.3666668	4.8312001	-0.4908738
15.3666668	4.8800001	-0.6933594
15.3666668	4.9288001	-0.3067961
15.3666668	4.9776001	-0.6381359
15.3666668	5.0264001	-0.6381359
15.3666668	5.0752001	-0.6381359
15.3666668	5.1240001	-0.5890489
15.3666668	5.1728001	-0.7301750
15.3666668	5.2216001	-0.7485828
15.3666668	5.2704000	-0.7301750
15.3666668	5.3192000	-0.7179031
15.3666668	5.3680000	613586.0000000
15.3666668	5.4168000	613586.0000000
15.3666668	5.4656000	613586.0000000
15.3666668	5.5144000	613586.0000000
15.3666668	5.5632000	613586.0000000
15.3666668	5.6120000	613586.0000000
15.3666668	5.6608000	613586.0000000
15.3666668	5.7096000	613586.0000000
15.3666668	5.7584000	613586.0000000
15.3666668	5.8072000	613586.0000000
15.3666668	5.8559999	613586.0000000
15.3666668	5.9047999	613586.0000000
15.3666668	5.9535999	613586.0000000
15.3666668	6.0023999	613586.0000000
15.3833332	0.2928000	-4.2337871
15.3833332	0.3416000	-4.2706027
15.3833332	0.3904000	-3.4422529
15.3833332	0.4392000	-3.9208550
15.3833332	0.4880000	-4.2215152
15.3833332	0.5368000	-4.7737484
15.3833332	0.5856000	-4.4730883
15.3833332	0.6344000	-3.8840394
15.3833332	0.6832000	-2.4973209
15.3833332	0.7320000	-2.1107578
15.3833332	0.7808000	-1.6996508
15.3833332	0.8296000	-1.5401168
15.3833332	0.8784000	-1.5401168
15.3833332	0.9272000	-1.4787574
15.3833332	0.9760000	-1.1965051
15.3833332	1.0247999	-1.2026410
15.3833332	1.0735999	-1.1719613
15.3833332	1.1223999	-1.3008156
15.3833332	1.1711999	-1.3008156
15.3833332	1.2200000	-1.3130875
15.3833332	1.2688000	-2.1414371
15.3833332	1.3176000	-2.7673013

15.3833332	1.3664000	-2.7611654
15.3833332	1.4152000	-2.8102529
15.3833332	1.4640000	-2.3991461
15.3833332	1.5128000	-2.0248547
15.3833332	1.5616000	-1.7732821
15.3833332	1.6104000	-1.6137476
15.3833332	1.6592000	-1.6198835
15.3833332	1.7079999	-1.3989906
15.3833332	1.7567999	-1.3928547
15.3833332	1.8055999	-1.4910293
15.3833332	1.8543999	-1.4603496
15.3833332	1.9031999	-1.4173985
15.3833332	1.9519999	-1.4235344
15.3833332	2.0007999	-1.5401168
15.3833332	2.0495999	-1.6014762
15.3833332	2.0983999	-1.5033011
15.3833332	2.1471999	-1.4296703
15.3833332	2.1959999	-1.5585246
15.3833332	2.2447999	-1.4542136
15.3833332	2.2935998	-1.4112625
15.3833332	2.3423998	-1.4051266
15.3833332	2.3912001	-1.3376312
15.3833332	2.4400001	-1.3499031
15.3833332	2.4888000	-1.3683109
15.3833332	2.5376000	-1.3744469
15.3833332	2.5864000	-1.3130875
15.3833332	2.6352000	-1.3805828
15.3833332	2.6840000	-1.2210488
15.3833332	2.7328000	-1.2333207
15.3833332	2.7816000	-1.2824078
15.3833332	2.8304000	-1.2762718
15.3833332	2.8792000	-1.2394567
15.3833332	2.9280000	-1.2026410
15.3833332	2.9768000	-1.1842332
15.3833332	3.0256000	-1.2824078
15.3833332	3.0743999	-1.2333207
15.3833332	3.1231999	-1.2087770
15.3833332	3.1719999	-1.2149129
15.3833332	3.2207999	-1.0983305
15.3833332	3.2695999	-1.0001554
15.3833332	3.3183999	-0.9940195
15.3833332	3.3671999	-0.9081168
15.3833332	3.4159999	-0.8344855
15.3833332	3.4647999	-0.7915339
15.3833332	3.5135999	-0.7853980
15.3833332	3.5623999	-1.1106019
15.3833332	3.6111999	-1.0369711
15.3833332	3.6599998	-0.9326606
15.3833332	3.7087998	-0.9387960
15.3833332	3.7575998	-0.8713012
15.3833332	3.8063998	-0.7424469
15.3833332	3.8551998	-0.7485828
15.3833332	3.9039998	-0.7485828
15.3833332	3.9527998	-0.7363110
15.3833332	4.0016003	-0.8038058
15.3833332	4.0503998	-0.6688156
15.3833332	4.0991998	-0.6688156

15.3833332	4.1479998	-0.7792621
15.3833332	4.1967998	-0.7301750
15.3833332	4.2455997	-0.6565437
15.3833332	4.2943997	-0.6565437
15.3833332	4.3431997	-0.8222136
15.3833332	4.3919997	-0.7853980
15.3833332	4.4407997	-0.6074562
15.3833332	4.4895997	-0.6258640
15.3833332	4.5383997	-0.6626797
15.3833332	4.5871997	-0.6872234
15.3833332	4.6359997	-0.6872234
15.3833332	4.6847997	-0.7056313
15.3833332	4.7336001	-0.6442719
15.3833332	4.7824001	-0.7424469
15.3833332	4.8312001	-0.7056313
15.3833332	4.8800001	-0.7117672
15.3833332	4.9288001	-0.7792621
15.3833332	4.9776001	-0.6197281
15.3833332	5.0264001	-0.5829129
15.3833332	5.0752001	-0.3681555
15.3833332	5.1240001	-0.3620195
15.3833332	5.1728001	-0.3558836
15.3833332	5.2216001	-0.3620195
15.3833332	5.2704000	-0.3742914
15.3833332	5.3192000	613586.0000000
15.3833332	5.3680000	613586.0000000
15.3833332	5.4168000	613586.0000000
15.3833332	5.4656000	613586.0000000
15.3833332	5.5144000	613586.0000000
15.3833332	5.5632000	613586.0000000
15.3833332	5.6120000	613586.0000000
15.3833332	5.6608000	613586.0000000
15.3833332	5.7096000	613586.0000000
15.3833332	5.7584000	613586.0000000
15.3833332	5.8072000	613586.0000000
15.3833332	5.8559999	613586.0000000
15.3833332	5.9047999	613586.0000000
15.3833332	5.9535999	613586.0000000
15.3833332	6.0023999	613586.0000000
15.3999996	0.2928000	-4.3074183
15.3999996	0.3416000	-4.3074183
15.3999996	0.3904000	-4.9578261
15.3999996	0.4392000	-4.8903308
15.3999996	0.4880000	-5.1173601
15.3999996	0.5368000	-5.1173601
15.3999996	0.5856000	-4.6264863
15.3999996	0.6344000	-4.4240007
15.3999996	0.6832000	-2.4605052
15.3999996	0.7320000	-1.5094371
15.3999996	0.7808000	-1.4173985
15.3999996	0.8296000	-1.3437672
15.3999996	0.8784000	-1.3437672
15.3999996	0.9272000	-1.1167378
15.3999996	0.9760000	-1.1106019
15.3999996	1.0247999	-1.0062914
15.3999996	1.0735999	-1.0124273
15.3999996	1.1223999	-1.1474175

15.3999996	1.1711999	-1.2701364
15.3999996	1.2200000	-1.2701364
15.3999996	1.2688000	-0.8528934
15.3999996	1.3176000	-1.0799227
15.3999996	1.3664000	-1.0799227
15.3999996	1.4152000	-1.1658254
15.3999996	1.4640000	-1.1596894
15.3999996	1.5128000	-1.1719613
15.3999996	1.5616000	-1.2762718
15.3999996	1.6104000	-1.1412816
15.3999996	1.6592000	-1.3130875
15.3999996	1.7079999	-1.3069515
15.3999996	1.7567999	-1.2701364
15.3999996	1.8055999	-1.4787574
15.3999996	1.8543999	-1.4726214
15.3999996	1.9031999	-1.5339808
15.3999996	1.9519999	-1.4910293
15.3999996	2.0007999	-1.4848933
15.3999996	2.0495999	-1.2701364
15.3999996	2.0983999	-1.4603496
15.3999996	2.1471999	-1.5523887
15.3999996	2.1959999	-1.5462527
15.3999996	2.2447999	-1.4910293
15.3999996	2.2935998	-1.3621750
15.3999996	2.3423998	-1.3621750
15.3999996	2.3912001	-1.2517285
15.3999996	2.4400001	-1.2455926
15.3999996	2.4888000	-1.2517285
15.3999996	2.5376000	-1.2640004
15.3999996	2.5864000	-1.4051266
15.3999996	2.6352000	-1.3928547
15.3999996	2.6840000	-1.4419422
15.3999996	2.7328000	-1.3253593
15.3999996	2.7816000	-1.3928547
15.3999996	2.8304000	-1.3928547
15.3999996	2.8792000	-1.4542136
15.3999996	2.9280000	-1.2885437
15.3999996	2.9768000	-1.2885437
15.3999996	3.0256000	-1.2946796
15.3999996	3.0743999	-1.3499031
15.3999996	3.1231999	-1.3069515
15.3999996	3.1719999	-1.2946796
15.3999996	3.2207999	-1.2885437
15.3999996	3.2695999	-1.2885437
15.3999996	3.3183999	-1.1167378
15.3999996	3.3671999	-1.0737867
15.3999996	3.4159999	-1.0615149
15.3999996	3.4647999	-0.9510679
15.3999996	3.5135999	-1.0185633
15.3999996	3.5623999	-1.0246992
15.3999996	3.6111999	-1.0369711
15.3999996	3.6599998	-1.0431070
15.3999996	3.7087998	-1.0246992
15.3999996	3.7575998	-0.9326606
15.3999996	3.8063998	-0.9265246
15.3999996	3.8551998	-0.9142528
15.3999996	3.9039998	-0.9817476

15.3999996	3.9527998	-0.7424469
15.3999996	4.0016003	-0.7485828
15.3999996	4.0503998	-0.7485828
15.3999996	4.0991998	-0.9572039
15.3999996	4.1479998	-0.9510679
15.3999996	4.1967998	-1.0185633
15.3999996	4.2455997	-1.0185633
15.3999996	4.2943997	-1.0124273
15.3999996	4.3431997	-1.0553789
15.3999996	4.3919997	-1.0431070
15.3999996	4.4407997	-0.8958449
15.3999996	4.4895997	-0.9510679
15.3999996	4.5383997	-0.9510679
15.3999996	4.5871997	-0.9142528
15.3999996	4.6359997	-0.8283496
15.3999996	4.6847997	-1.0860586
15.3999996	4.7336001	-0.8406215
15.3999996	4.7824001	-0.6381359
15.3999996	4.8312001	-0.7731261
15.3999996	4.8800001	-0.7853980
15.3999996	4.9288001	-0.6872234
15.3999996	4.9776001	-0.8467574
15.3999996	5.0264001	-0.9940195
15.3999996	5.0752001	-0.9387960
15.3999996	5.1240001	-0.9265246
15.3999996	5.1728001	-0.9142528
15.3999996	5.2216001	-0.6626797
15.3999996	5.2704000	613586.0000000
15.3999996	5.3192000	613586.0000000
15.3999996	5.3680000	613586.0000000
15.3999996	5.4168000	613586.0000000
15.3999996	5.4656000	613586.0000000
15.3999996	5.5144000	613586.0000000
15.3999996	5.5632000	613586.0000000
15.3999996	5.6120000	613586.0000000
15.3999996	5.6608000	613586.0000000
15.3999996	5.7096000	613586.0000000
15.3999996	5.7584000	613586.0000000
15.3999996	5.8072000	613586.0000000
15.3999996	5.8559999	613586.0000000
15.3999996	5.9047999	613586.0000000
15.3999996	5.9535999	613586.0000000
15.3999996	6.0023999	613586.0000000
15.4166670	0.2928000	-4.2644668
15.4166670	0.3416000	-4.3749132
15.4166670	0.3904000	-4.1417484
15.4166670	0.4392000	-4.1356125
15.4166670	0.4880000	-4.1417484
15.4166670	0.5368000	-2.9575150
15.4166670	0.5856000	-2.7979810
15.4166670	0.6344000	-2.7857091
15.4166670	0.6832000	-2.2334762
15.4166670	0.7320000	-1.2946796
15.4166670	0.7808000	-1.2333207
15.4166670	0.8296000	-1.2333207
15.4166670	0.8784000	-1.2394567
15.4166670	0.9272000	-1.2578645

15.4166670	0.9760000	-1.2578645
15.4166670	1.0247999	-1.2578645
15.4166670	1.0735999	-1.2149129
15.4166670	1.1223999	-0.9387960
15.4166670	1.1711999	-1.0553789
15.4166670	1.2200000	-1.7241945
15.4166670	1.2688000	-1.7241945
15.4166670	1.3176000	-1.2026410
15.4166670	1.3664000	-1.0799227
15.4166670	1.4152000	-1.0799227
15.4166670	1.4640000	-1.0860586
15.4166670	1.5128000	-1.0431070
15.4166670	1.5616000	-1.1842332
15.4166670	1.6104000	-1.1719613
15.4166670	1.6592000	-1.0921946
15.4166670	1.7079999	-1.0921946
15.4166670	1.7567999	-1.3253593
15.4166670	1.8055999	-1.3314953
15.4166670	1.8543999	-1.3253593
15.4166670	1.9031999	-1.2640004
15.4166670	1.9519999	-1.2701364
15.4166670	2.0007999	-1.2701364
15.4166670	2.0495999	-1.2640004
15.4166670	2.0983999	-1.1719613
15.4166670	2.1471999	-1.1780972
15.4166670	2.1959999	-1.3867188
15.4166670	2.2447999	-1.3867188
15.4166670	2.2935998	-1.2578645
15.4166670	2.3423998	-1.2701364
15.4166670	2.3912001	-1.3437672
15.4166670	2.4400001	-1.3499031
15.4166670	2.4888000	-1.4419422
15.4166670	2.5376000	-1.4419422
15.4166670	2.5864000	-1.4419422
15.4166670	2.6352000	-1.2762718
15.4166670	2.6840000	-1.4051266
15.4166670	2.7328000	-1.3989906
15.4166670	2.7816000	-1.3376312
15.4166670	2.8304000	-1.3253593
15.4166670	2.8792000	-1.3560390
15.4166670	2.9280000	-1.4235344
15.4166670	2.9768000	-1.4480777
15.4166670	3.0256000	-1.3376312
15.4166670	3.0743999	-1.3376312
15.4166670	3.1231999	-1.3376312
15.4166670	3.1719999	-1.3376312
15.4166670	3.2207999	-1.2026410
15.4166670	3.2695999	-1.3008156
15.4166670	3.3183999	-1.3069515
15.4166670	3.3671999	-1.2394567
15.4166670	3.4159999	-1.2701364
15.4166670	3.4647999	-1.2885437
15.4166670	3.5135999	-1.0001554
15.4166670	3.5623999	-0.9940195
15.4166670	3.6111999	-1.0983305
15.4166670	3.6599998	-1.0983305
15.4166670	3.7087998	-1.1106019

15.4166670	3.7575998	-0.9756117
15.4166670	3.8063998	-0.9817476
15.4166670	3.8551998	-1.0001554
15.4166670	3.9039998	-0.8467574
15.4166670	3.9527998	-0.8467574
15.4166670	4.0016003	-0.9572039
15.4166670	4.0503998	-0.9572039
15.4166670	4.0991998	-0.7301750
15.4166670	4.1479998	-0.9081168
15.4166670	4.1967998	-0.9081168
15.4166670	4.2455997	-0.9142528
15.4166670	4.2943997	-0.7915339
15.4166670	4.3431997	-0.7669902
15.4166670	4.3919997	-0.8222136
15.4166670	4.4407997	-0.8222136
15.4166670	4.4895997	-0.7117672
15.4166670	4.5383997	-0.5583692
15.4166670	4.5871997	-0.5583692
15.4166670	4.6359997	-0.6197281
15.4166670	4.6847997	-0.6258640
15.4166670	4.7336001	-0.5399613
15.4166670	4.7824001	-0.9019809
15.4166670	4.8312001	-0.7547188
15.4166670	4.8800001	-0.6258640
15.4166670	4.9288001	-0.7608547
15.4166670	4.9776001	-0.7240391
15.4166670	5.0264001	-0.7179031
15.4166670	5.0752001	-0.7792621
15.4166670	5.1240001	-0.8406215
15.4166670	5.1728001	-0.7117672
15.4166670	5.2216001	-1.1412816
15.4166670	5.2704000	-1.0124273
15.4166670	5.3192000	613586.0000000
15.4166670	5.3680000	613586.0000000
15.4166670	5.4168000	613586.0000000
15.4166670	5.4656000	613586.0000000
15.4166670	5.5144000	613586.0000000
15.4166670	5.5632000	613586.0000000
15.4166670	5.6120000	613586.0000000
15.4166670	5.6608000	613586.0000000
15.4166670	5.7096000	613586.0000000
15.4166670	5.7584000	613586.0000000
15.4166670	5.8072000	613586.0000000
15.4166670	5.8559999	613586.0000000
15.4166670	5.9047999	613586.0000000
15.4166670	5.9535999	613586.0000000
15.4166670	6.0023999	613586.0000000
15.4333334	0.2928000	-5.0314569
15.4333334	0.3416000	-3.2459035
15.4333334	0.3904000	-4.1478844
15.4333334	0.4392000	-4.9885054
15.4333334	0.4880000	-4.6878452
15.4333334	0.5368000	-4.8596516
15.4333334	0.5856000	-4.8596516
15.4333334	0.6344000	-4.8596516
15.4333334	0.6832000	-2.6629908
15.4333334	0.7320000	-1.8530488

15.4333334	0.7808000	-1.6137476
15.4333334	0.8296000	-1.5953403
15.4333334	0.8784000	-1.3499031
15.4333334	0.9272000	-1.3499031
15.4333334	0.9760000	-1.3621750
15.4333334	1.0247999	-1.3744469
15.4333334	1.0735999	-1.2026410
15.4333334	1.1223999	-1.3253593
15.4333334	1.1711999	-1.4480777
15.4333334	1.2200000	-1.4419422
15.4333334	1.2688000	-1.3253593
15.4333334	1.3176000	-0.9203887
15.4333334	1.3664000	-0.9203887
15.4333334	1.4152000	-1.1290097
15.4333334	1.4640000	-1.3683109
15.4333334	1.5128000	-1.4787574
15.4333334	1.5616000	-1.3744469
15.4333334	1.6104000	-1.3744469
15.4333334	1.6592000	-1.6198835
15.4333334	1.7079999	-1.4542136
15.4333334	1.7567999	-1.2701364
15.4333334	1.8055999	-1.2762718
15.4333334	1.8543999	-1.2762718
15.4333334	1.9031999	-1.2333207
15.4333334	1.9519999	-1.2333207
15.4333334	2.0007999	-1.2824078
15.4333334	2.0495999	-1.1842332
15.4333334	2.0983999	-1.1842332
15.4333334	2.1471999	-1.2824078
15.4333334	2.1959999	-1.2149129
15.4333334	2.2447999	-1.2026410
15.4333334	2.2935998	-1.1965051
15.4333334	2.3423998	-1.1965051
15.4333334	2.3912001	-1.2087770
15.4333334	2.4400001	-1.2149129
15.4333334	2.4888000	-1.3560390
15.4333334	2.5376000	-1.3437672
15.4333334	2.5864000	-1.3376312
15.4333334	2.6352000	-1.4112625
15.4333334	2.6840000	-1.1903691
15.4333334	2.7328000	-1.1965051
15.4333334	2.7816000	-1.1780972
15.4333334	2.8304000	-1.1719613
15.4333334	2.8792000	-1.2210488
15.4333334	2.9280000	-1.3376312
15.4333334	2.9768000	-1.3499031
15.4333334	3.0256000	-1.2762718
15.4333334	3.0743999	-1.2640004
15.4333334	3.1231999	-1.2701364
15.4333334	3.1719999	-1.3130875
15.4333334	3.2207999	-1.1106019
15.4333334	3.2695999	-1.1106019
15.4333334	3.3183999	-1.2026410
15.4333334	3.3671999	-1.0676508
15.4333334	3.4159999	-1.0185633
15.4333334	3.4647999	-1.0185633
15.4333334	3.5135999	-1.0737867

15.4333334	3.5623999	-1.2762718
15.4333334	3.6111999	-1.2271848
15.4333334	3.6599998	-1.2271848
15.4333334	3.7087998	-1.0308352
15.4333334	3.7575998	-1.0308352
15.4333334	3.8063998	-1.0431070
15.4333334	3.8551998	-0.8774371
15.4333334	3.9039998	-0.8283496
15.4333334	3.9527998	-0.8774371
15.4333334	4.0016003	-0.8099418
15.4333334	4.0503998	-0.7424469
15.4333334	4.0991998	-0.8283496
15.4333334	4.1479998	-0.9326606
15.4333334	4.1967998	-0.8897090
15.4333334	4.2455997	-0.7424469
15.4333334	4.2943997	-0.7485828
15.4333334	4.3431997	-0.6933594
15.4333334	4.3919997	-0.6994953
15.4333334	4.4407997	-0.7240391
15.4333334	4.4895997	-0.7853980
15.4333334	4.5383997	-0.7363110
15.4333334	4.5871997	-0.7240391
15.4333334	4.6359997	-0.7179031
15.4333334	4.6847997	-0.9019809
15.4333334	4.7336001	-0.9081168
15.4333334	4.7824001	-0.9940195
15.4333334	4.8312001	-1.2517285
15.4333334	4.8800001	-0.9572039
15.4333334	4.9288001	-0.8467574
15.4333334	4.9776001	-0.9387960
15.4333334	5.0264001	-0.8222136
15.4333334	5.0752001	-0.5767770
15.4333334	5.1240001	-0.7853980
15.4333334	5.1728001	-0.7731261
15.4333334	5.2216001	613586.0000000
15.4333334	5.2704000	613586.0000000
15.4333334	5.3192000	613586.0000000
15.4333334	5.3680000	613586.0000000
15.4333334	5.4168000	613586.0000000
15.4333334	5.4656000	613586.0000000
15.4333334	5.5144000	613586.0000000
15.4333334	5.5632000	613586.0000000
15.4333334	5.6120000	613586.0000000
15.4333334	5.6608000	613586.0000000
15.4333334	5.7096000	613586.0000000
15.4333334	5.7584000	613586.0000000
15.4333334	5.8072000	613586.0000000
15.4333334	5.8559999	613586.0000000
15.4333334	5.9047999	613586.0000000
15.4333334	5.9535999	613586.0000000
15.4333334	6.0023999	613586.0000000
15.4499998	0.2928000	-4.2890105
15.4499998	0.3416000	-4.2828746
15.4499998	0.3904000	-4.2706027
15.4499998	0.4392000	-4.2460589
15.4499998	0.4880000	-4.3687773
15.4499998	0.5368000	-4.7676125

15.4499998	0.5856000	-4.4362726
15.4499998	0.6344000	-3.3563499
15.4499998	0.6832000	-2.3868742
15.4499998	0.7320000	-1.9573593
15.4499998	0.7808000	-1.9941750
15.4499998	0.8296000	-1.6137476
15.4499998	0.8784000	-1.6014762
15.4499998	0.9272000	-1.6076121
15.4499998	0.9760000	-1.7794180
15.4499998	1.0247999	-1.7978253
15.4499998	1.0735999	-1.6751070
15.4499998	1.1223999	-1.6751070
15.4499998	1.1711999	-1.1780972
15.4499998	1.2200000	-1.7119226
15.4499998	1.2688000	-1.5401168
15.4499998	1.3176000	-1.5401168
15.4499998	1.3664000	-1.4848933
15.4499998	1.4152000	-1.5523887
15.4499998	1.4640000	-1.8898644
15.4499998	1.5128000	-1.7303305
15.4499998	1.5616000	-1.8837285
15.4499998	1.6104000	-1.8407769
15.4499998	1.6592000	-2.0432625
15.4499998	1.7079999	-1.7057867
15.4499998	1.7567999	-1.4296703
15.4499998	1.8055999	-1.4235344
15.4499998	1.8543999	-1.4112625
15.4499998	1.9031999	-1.3376312
15.4499998	1.9519999	-1.2517285
15.4499998	2.0007999	-1.1474175
15.4499998	2.0495999	-1.1412816
15.4499998	2.0983999	-1.2517285
15.4499998	2.1471999	-1.2394567
15.4499998	2.1959999	-1.1903691
15.4499998	2.2447999	-1.2946796
15.4499998	2.2935998	-1.2333207
15.4499998	2.3423998	-1.3867188
15.4499998	2.3912001	-1.3253593
15.4499998	2.4400001	-1.3314953
15.4499998	2.4888000	-1.3314953
15.4499998	2.5376000	-1.3008156
15.4499998	2.5864000	-1.2885437
15.4499998	2.6352000	-1.1596894
15.4499998	2.6840000	-1.1596894
15.4499998	2.7328000	-1.1596894
15.4499998	2.7816000	-1.2333207
15.4499998	2.8304000	-1.3130875
15.4499998	2.8792000	-1.1351457
15.4499998	2.9280000	-1.2210488
15.4499998	2.9768000	-1.2271848
15.4499998	3.0256000	-1.2333207
15.4499998	3.0743999	-1.2640004
15.4499998	3.1231999	-1.1228738
15.4499998	3.1719999	-1.3069515
15.4499998	3.2207999	-1.1842332
15.4499998	3.2695999	-1.0492430
15.4499998	3.3183999	-1.0737867

15.4499998	3.3671999	-1.0921946
15.4499998	3.4159999	-1.1965051
15.4499998	3.4647999	-1.1351457
15.4499998	3.5135999	-1.1290097
15.4499998	3.5623999	-0.9449320
15.4499998	3.6111999	-0.7240391
15.4499998	3.6599998	-0.9756117
15.4499998	3.7087998	-0.9142528
15.4499998	3.7575998	-0.8958449
15.4499998	3.8063998	-0.8835731
15.4499998	3.8551998	-0.8651652
15.4499998	3.9039998	-0.9142528
15.4499998	3.9527998	-0.8406215
15.4499998	4.0016003	-0.8467574
15.4499998	4.0503998	-0.8835731
15.4499998	4.0991998	-0.8958449
15.4499998	4.1479998	-0.7179031
15.4499998	4.1967998	-0.7179031
15.4499998	4.2455997	-0.8406215
15.4499998	4.2943997	-0.6442719
15.4499998	4.3431997	-0.6994953
15.4499998	4.3919997	-0.6994953
15.4499998	4.4407997	-0.6994953
15.4499998	4.4895997	-0.6994953
15.4499998	4.5383997	-0.6872234
15.4499998	4.5871997	-0.6810875
15.4499998	4.6359997	-0.6872234
15.4499998	4.6847997	-0.8713012
15.4499998	4.7336001	-1.0737867
15.4499998	4.7824001	-0.6197281
15.4499998	4.8312001	-0.6135921
15.4499998	4.8800001	-0.5215535
15.4499998	4.9288001	-0.5154176
15.4499998	4.9776001	-0.5031457
15.4499998	5.0264001	-0.5031457
15.4499998	5.0752001	-0.5092816
15.4499998	5.1240001	-0.7915339
15.4499998	5.1728001	613586.0000000
15.4499998	5.2216001	613586.0000000
15.4499998	5.2704000	613586.0000000
15.4499998	5.3192000	613586.0000000
15.4499998	5.3680000	613586.0000000
15.4499998	5.4168000	613586.0000000
15.4499998	5.4656000	613586.0000000
15.4499998	5.5144000	613586.0000000
15.4499998	5.5632000	613586.0000000
15.4499998	5.6120000	613586.0000000
15.4499998	5.6608000	613586.0000000
15.4499998	5.7096000	613586.0000000
15.4499998	5.7584000	613586.0000000
15.4499998	5.8072000	613586.0000000
15.4499998	5.8559999	613586.0000000
15.4499998	5.9047999	613586.0000000
15.4499998	5.9535999	613586.0000000
15.4499998	6.0023999	613586.0000000
15.4666662	0.2928000	-4.6448941
15.4666662	0.3416000	-5.9211659

15.4666662	0.3904000	-0.2577085
15.4666662	0.4392000	-0.2638445
15.4666662	0.4880000	-5.4977875
15.4666662	0.5368000	-5.5100594
15.4666662	0.5856000	-4.3258257
15.4666662	0.6344000	-3.0004666
15.4666662	0.6832000	-2.5648160
15.4666662	0.7320000	-2.5709519
15.4666662	0.7808000	-2.3193791
15.4666662	0.8296000	-2.1291652
15.4666662	0.8784000	-2.4175539
15.4666662	0.9272000	-2.2273402
15.4666662	0.9760000	-1.9389520
15.4666662	1.0247999	-2.1291652
15.4666662	1.0735999	-2.1291652
15.4666662	1.1223999	-1.9696312
15.4666662	1.1711999	-1.3621750
15.4666662	1.2200000	-1.4848933
15.4666662	1.2688000	-1.5830684
15.4666662	1.3176000	-1.6751070
15.4666662	1.3664000	-1.7303305
15.4666662	1.4152000	-1.8714566
15.4666662	1.4640000	-1.8223691
15.4666662	1.5128000	-1.8039613
15.4666662	1.5616000	-1.8039613
15.4666662	1.6104000	-1.8100972
15.4666662	1.6592000	-1.6873789
15.4666662	1.7079999	-1.5892043
15.4666662	1.7567999	-1.5953403
15.4666662	1.8055999	-1.6014762
15.4666662	1.8543999	-1.4787574
15.4666662	1.9031999	-1.4787574
15.4666662	1.9519999	-1.4173985
15.4666662	2.0007999	-1.4051266
15.4666662	2.0495999	-1.4542136
15.4666662	2.0983999	-1.3621750
15.4666662	2.1471999	-1.3253593
15.4666662	2.1959999	-1.1842332
15.4666662	2.2447999	-1.3376312
15.4666662	2.2935998	-1.2824078
15.4666662	2.3423998	-1.2762718
15.4666662	2.3912001	-1.1658254
15.4666662	2.4400001	-1.2271848
15.4666662	2.4888000	-1.2271848
15.4666662	2.5376000	-1.2946796
15.4666662	2.5864000	-1.1106019
15.4666662	2.6352000	-1.1842332
15.4666662	2.6840000	-1.1290097
15.4666662	2.7328000	-1.1351457
15.4666662	2.7816000	-1.1351457
15.4666662	2.8304000	-1.1658254
15.4666662	2.8792000	-1.1780972
15.4666662	2.9280000	-1.1719613
15.4666662	2.9768000	-1.0676508
15.4666662	3.0256000	-1.2271848
15.4666662	3.0743999	-1.2394567
15.4666662	3.1231999	-1.1106019

15.4666662	3.1719999	-1.1290097
15.4666662	3.2207999	-1.0369711
15.4666662	3.2695999	-1.0615149
15.4666662	3.3183999	-1.0308352
15.4666662	3.3671999	-1.0860586
15.4666662	3.4159999	-1.0921946
15.4666662	3.4647999	-1.1044660
15.4666662	3.5135999	-0.9510679
15.4666662	3.5623999	-0.8713012
15.4666662	3.6111999	-0.8713012
15.4666662	3.6599998	-0.9694757
15.4666662	3.7087998	-0.9265246
15.4666662	3.7575998	-0.9265246
15.4666662	3.8063998	-0.9326606
15.4666662	3.8551998	-0.8835731
15.4666662	3.9039998	-0.8958449
15.4666662	3.9527998	-0.9387960
15.4666662	4.0016003	-0.7363110
15.4666662	4.0503998	-0.7363110
15.4666662	4.0991998	-0.7669902
15.4666662	4.1479998	-0.7608547
15.4666662	4.1967998	-0.7731261
15.4666662	4.2455997	-0.6626797
15.4666662	4.2943997	-0.7731261
15.4666662	4.3431997	-0.7669902
15.4666662	4.3919997	-0.7547188
15.4666662	4.4407997	-0.7547188
15.4666662	4.4895997	-0.7363110
15.4666662	4.5383997	-0.5583692
15.4666662	4.5871997	-0.5460973
15.4666662	4.6359997	-0.5951843
15.4666662	4.6847997	-0.8835731
15.4666662	4.7336001	-0.7976699
15.4666662	4.7824001	-0.5706410
15.4666662	4.8312001	-0.4847379
15.4666662	4.8800001	-0.4847379
15.4666662	4.9288001	-0.8099418
15.4666662	4.9776001	-0.7547188
15.4666662	5.0264001	-0.5154176
15.4666662	5.0752001	-0.5031457
15.4666662	5.1240001	-0.5890489
15.4666662	5.1728001	-0.3988352
15.4666662	5.2216001	613586.0000000
15.4666662	5.2704000	613586.0000000
15.4666662	5.3192000	613586.0000000
15.4666662	5.3680000	613586.0000000
15.4666662	5.4168000	613586.0000000
15.4666662	5.4656000	613586.0000000
15.4666662	5.5144000	613586.0000000
15.4666662	5.5632000	613586.0000000
15.4666662	5.6120000	613586.0000000
15.4666662	5.6608000	613586.0000000
15.4666662	5.7096000	613586.0000000
15.4666662	5.7584000	613586.0000000
15.4666662	5.8072000	613586.0000000
15.4666662	5.8559999	613586.0000000
15.4666662	5.9047999	613586.0000000

15.4666662	5.9535999	613586.0000000
15.4666662	6.0023999	613586.0000000
15.4833336	0.2928000	-4.9087386
15.4833336	0.3416000	-4.9394183
15.4833336	0.3904000	-4.9394183
15.4833336	0.4392000	-5.0805445
15.4833336	0.4880000	-4.2460589
15.4833336	0.5368000	-4.4485445
15.4833336	0.5856000	-4.7553406
15.4833336	0.6344000	-2.6875343
15.4833336	0.6832000	-2.4789131
15.4833336	0.7320000	-1.9696312
15.4833336	0.7808000	-2.0678062
15.4833336	0.8296000	-2.0800781
15.4833336	0.8784000	-2.0616703
15.4833336	0.9272000	-2.0432625
15.4833336	0.9760000	-1.9144082
15.4833336	1.0247999	-1.9144082
15.4833336	1.0735999	-1.9757671
15.4833336	1.1223999	-2.3561945
15.4833336	1.1711999	-2.1230292
15.4833336	1.2200000	-1.5339808
15.4833336	1.2688000	-1.5401168
15.4833336	1.3176000	-1.3867188
15.4833336	1.3664000	-1.3744469
15.4833336	1.4152000	-1.3805828
15.4833336	1.4640000	-1.3805828
15.4833336	1.5128000	-1.3805828
15.4833336	1.5616000	-1.4787574
15.4833336	1.6104000	-1.6873789
15.4833336	1.6592000	-1.6935148
15.4833336	1.7079999	-1.6935148
15.4833336	1.7567999	-1.6505632
15.4833336	1.8055999	-1.7426023
15.4833336	1.8543999	-1.7180586
15.4833336	1.9031999	-1.7057867
15.4833336	1.9519999	-1.6812429
15.4833336	2.0007999	-1.2149129
15.4833336	2.0495999	-1.2210488
15.4833336	2.0983999	-1.3008156
15.4833336	2.1471999	-1.3008156
15.4833336	2.1959999	-1.2885437
15.4833336	2.2447999	-1.2885437
15.4833336	2.2935998	-1.3069515
15.4833336	2.3423998	-1.2640004
15.4833336	2.3912001	-1.2824078
15.4833336	2.4400001	-1.2824078
15.4833336	2.4888000	-1.3314953
15.4833336	2.5376000	-1.3314953
15.4833336	2.5864000	-1.3621750
15.4833336	2.6352000	-1.1903691
15.4833336	2.6840000	-1.1965051
15.4833336	2.7328000	-1.1596894
15.4833336	2.7816000	-1.1596894
15.4833336	2.8304000	-1.1719613
15.4833336	2.8792000	-1.2762718
15.4833336	2.9280000	-1.2701364

15.4833336	2.9768000	-1.2578645
15.4833336	3.0256000	-1.2333207
15.4833336	3.0743999	-1.2271848
15.4833336	3.1231999	-1.2824078
15.4833336	3.1719999	-1.2885437
15.4833336	3.2207999	-1.3008156
15.4833336	3.2695999	-1.1474175
15.4833336	3.3183999	-1.1474175
15.4833336	3.3671999	-1.1596894
15.4833336	3.4159999	-1.1658254
15.4833336	3.4647999	-1.1044660
15.4833336	3.5135999	-1.0553789
15.4833336	3.5623999	-1.0983305
15.4833336	3.6111999	-1.2762718
15.4833336	3.6599998	-1.1351457
15.4833336	3.7087998	-1.1842332
15.4833336	3.7575998	-1.1903691
15.4833336	3.8063998	-1.0553789
15.4833336	3.8551998	-0.9387960
15.4833336	3.9039998	-0.9387960
15.4833336	3.9527998	-0.9572039
15.4833336	4.0016003	-0.9694757
15.4833336	4.0503998	-1.0737867
15.4833336	4.0991998	-1.0860586
15.4833336	4.1479998	-1.0246992
15.4833336	4.1967998	-0.9878836
15.4833336	4.2455997	-0.9449320
15.4833336	4.2943997	-0.9510679
15.4833336	4.3431997	-1.2271848
15.4833336	4.3919997	-0.8528934
15.4833336	4.4407997	-0.9694757
15.4833336	4.4895997	-0.8897090
15.4833336	4.5383997	-0.8958449
15.4833336	4.5871997	-0.9019809
15.4833336	4.6359997	-0.8344855
15.4833336	4.6847997	-0.8283496
15.4833336	4.7336001	-0.7485828
15.4833336	4.7824001	-0.7485828
15.4833336	4.8312001	-0.9081168
15.4833336	4.8800001	-0.9081168
15.4833336	4.9288001	-0.9081168
15.4833336	4.9776001	-0.8713012
15.4833336	5.0264001	-0.6258640
15.4833336	5.0752001	-0.6320000
15.4833336	5.1240001	-0.6381359
15.4833336	5.1728001	-0.9019809
15.4833336	5.2216001	613586.0000000
15.4833336	5.2704000	613586.0000000
15.4833336	5.3192000	613586.0000000
15.4833336	5.3680000	613586.0000000
15.4833336	5.4168000	613586.0000000
15.4833336	5.4656000	613586.0000000
15.4833336	5.5144000	613586.0000000
15.4833336	5.5632000	613586.0000000
15.4833336	5.6120000	613586.0000000
15.4833336	5.6608000	613586.0000000
15.4833336	5.7096000	613586.0000000

15.4833336	5.7584000	613586.0000000
15.4833336	5.8072000	613586.0000000
15.4833336	5.8559999	613586.0000000
15.4833336	5.9047999	613586.0000000
15.4833336	5.9535999	613586.0000000
15.4833336	6.0023999	613586.0000000
15.5000000	0.2928000	-5.3750687
15.5000000	0.3416000	-5.2830300
15.5000000	0.3904000	-5.3566608
15.5000000	0.4392000	-5.4977875
15.5000000	0.4880000	-0.0674953
15.5000000	0.5368000	-6.0193410
15.5000000	0.5856000	-4.6755733
15.5000000	0.6344000	-4.6755733
15.5000000	0.6832000	-2.8286607
15.5000000	0.7320000	-1.7794180
15.5000000	0.7808000	-1.7794180
15.5000000	0.8296000	-1.4112625
15.5000000	0.8784000	-1.4112625
15.5000000	0.9272000	-1.4419422
15.5000000	0.9760000	-1.3560390
15.5000000	1.0247999	-1.3008156
15.5000000	1.0735999	-1.0062914
15.5000000	1.1223999	-0.9940195
15.5000000	1.1711999	-1.3008156
15.5000000	1.2200000	-1.5830684
15.5000000	1.2688000	-1.3253593
15.5000000	1.3176000	-1.3376312
15.5000000	1.3664000	-1.0983305
15.5000000	1.4152000	-1.1596894
15.5000000	1.4640000	-1.1412816
15.5000000	1.5128000	-1.1535535
15.5000000	1.5616000	-1.1535535
15.5000000	1.6104000	-1.1535535
15.5000000	1.6592000	-1.4603496
15.5000000	1.7079999	-1.4971652
15.5000000	1.7567999	-1.4848933
15.5000000	1.8055999	-1.3928547
15.5000000	1.8543999	-1.5462527
15.5000000	1.9031999	-1.4848933
15.5000000	1.9519999	-1.5339808
15.5000000	2.0007999	-1.2578645
15.5000000	2.0495999	-1.4787574
15.5000000	2.0983999	-1.5401168
15.5000000	2.1471999	-1.4480777
15.5000000	2.1959999	-1.4235344
15.5000000	2.2447999	-1.4235344
15.5000000	2.2935998	-1.3253593
15.5000000	2.3423998	-1.3253593
15.5000000	2.3912001	-1.2578645
15.5000000	2.4400001	-1.3376312
15.5000000	2.4888000	-1.3253593
15.5000000	2.5376000	-1.2271848
15.5000000	2.5864000	-1.4664855
15.5000000	2.6352000	-1.2824078
15.5000000	2.6840000	-1.2824078
15.5000000	2.7328000	-1.4235344

15.5000000	2.7816000	-1.3867188
15.5000000	2.8304000	-1.3867188
15.5000000	2.8792000	-1.3314953
15.5000000	2.9280000	-1.3192234
15.5000000	2.9768000	-1.4542136
15.5000000	3.0256000	-1.3376312
15.5000000	3.0743999	-1.3376312
15.5000000	3.1231999	-1.3744469
15.5000000	3.1719999	-1.4296703
15.5000000	3.2207999	-1.4910293
15.5000000	3.2695999	-1.3621750
15.5000000	3.3183999	-1.2701364
15.5000000	3.3671999	-1.3683109
15.5000000	3.4159999	-1.2885437
15.5000000	3.4647999	-1.4112625
15.5000000	3.5135999	-1.4051266
15.5000000	3.5623999	-1.4051266
15.5000000	3.6111999	-1.2640004
15.5000000	3.6599998	-1.2824078
15.5000000	3.7087998	-1.2824078
15.5000000	3.7575998	-1.3499031
15.5000000	3.8063998	-1.2210488
15.5000000	3.8551998	-1.2087770
15.5000000	3.9039998	-1.2087770
15.5000000	3.9527998	-1.2026410
15.5000000	4.0016003	-1.1228738
15.5000000	4.0503998	-1.1228738
15.5000000	4.0991998	-1.1474175
15.5000000	4.1479998	-1.0492430
15.5000000	4.1967998	-0.9572039
15.5000000	4.2455997	-0.9387960
15.5000000	4.2943997	-0.9940195
15.5000000	4.3431997	-0.9878836
15.5000000	4.3919997	-0.9756117
15.5000000	4.4407997	-0.9817476
15.5000000	4.4895997	-0.9817476
15.5000000	4.5383997	-0.9756117
15.5000000	4.5871997	-0.9878836
15.5000000	4.6359997	-0.9940195
15.5000000	4.6847997	-1.1719613
15.5000000	4.7336001	-0.8283496
15.5000000	4.7824001	-0.8344855
15.5000000	4.8312001	-0.9449320
15.5000000	4.8800001	-0.8344855
15.5000000	4.9288001	-0.7547188
15.5000000	4.9776001	-0.7608547
15.5000000	5.0264001	-0.7240391
15.5000000	5.0752001	-1.0185633
15.5000000	5.1240001	-1.0124273
15.5000000	5.1728001	613586.0000000
15.5000000	5.2216001	613586.0000000
15.5000000	5.2704000	613586.0000000
15.5000000	5.3192000	613586.0000000
15.5000000	5.3680000	613586.0000000
15.5000000	5.4168000	613586.0000000
15.5000000	5.4656000	613586.0000000
15.5000000	5.5144000	613586.0000000

15.5000000	5.5632000	613586.0000000
15.5000000	5.6120000	613586.0000000
15.5000000	5.6608000	613586.0000000
15.5000000	5.7096000	613586.0000000
15.5000000	5.7584000	613586.0000000
15.5000000	5.8072000	613586.0000000
15.5000000	5.8559999	613586.0000000
15.5000000	5.9047999	613586.0000000
15.5000000	5.9535999	613586.0000000
15.5000000	6.0023999	613586.0000000
15.5166664	0.2928000	-5.6266418
15.5166664	0.3416000	-5.4364281
15.5166664	0.3904000	-6.1543312
15.5166664	0.4392000	-6.1543312
15.5166664	0.4880000	-4.9823699
15.5166664	0.5368000	-4.9823699
15.5166664	0.5856000	-3.7920005
15.5166664	0.6344000	-3.5404277
15.5166664	0.6832000	-1.6321554
15.5166664	0.7320000	-1.6444273
15.5166664	0.7808000	-1.0246992
15.5166664	0.8296000	-1.0246992
15.5166664	0.8784000	-1.5278449
15.5166664	0.9272000	-1.6873789
15.5166664	0.9760000	-1.5462527
15.5166664	1.0247999	-1.4726214
15.5166664	1.0735999	-1.5217090
15.5166664	1.1223999	-1.5278449
15.5166664	1.1711999	-1.6321554
15.5166664	1.2200000	-1.7303305
15.5166664	1.2688000	-0.9081168
15.5166664	1.3176000	-1.0553789
15.5166664	1.3664000	-0.9081168
15.5166664	1.4152000	-1.3314953
15.5166664	1.4640000	-1.2578645
15.5166664	1.5128000	-1.2640004
15.5166664	1.5616000	-1.2701364
15.5166664	1.6104000	-1.2701364
15.5166664	1.6592000	-1.3069515
15.5166664	1.7079999	-1.3130875
15.5166664	1.7567999	-1.0860586
15.5166664	1.8055999	-1.1474175
15.5166664	1.8543999	-1.3008156
15.5166664	1.9031999	-1.2885437
15.5166664	1.9519999	-1.0492430
15.5166664	2.0007999	-1.1596894
15.5166664	2.0495999	-1.1535535
15.5166664	2.0983999	-1.1474175
15.5166664	2.1471999	-1.1842332
15.5166664	2.1959999	-1.1780972
15.5166664	2.2447999	-1.1535535
15.5166664	2.2935998	-1.1535535
15.5166664	2.3423998	-1.3560390
15.5166664	2.3912001	-1.3499031
15.5166664	2.4400001	-1.3499031
15.5166664	2.4888000	-1.4051266
15.5166664	2.5376000	-1.4173985

15.5166664	2.5864000	-1.3437672
15.5166664	2.6352000	-1.3437672
15.5166664	2.6840000	-1.2333207
15.5166664	2.7328000	-1.2394567
15.5166664	2.7816000	-1.3130875
15.5166664	2.8304000	-1.2149129
15.5166664	2.8792000	-1.2026410
15.5166664	2.9280000	-1.3253593
15.5166664	2.9768000	-1.3253593
15.5166664	3.0256000	-1.2517285
15.5166664	3.0743999	-1.2455926
15.5166664	3.1231999	-1.3867188
15.5166664	3.1719999	-1.1842332
15.5166664	3.2207999	-1.2271848
15.5166664	3.2695999	-1.2271848
15.5166664	3.3183999	-1.2394567
15.5166664	3.3671999	-1.1780972
15.5166664	3.4159999	-1.3560390
15.5166664	3.4647999	-1.1167378
15.5166664	3.5135999	-1.2333207
15.5166664	3.5623999	-1.2087770
15.5166664	3.6111999	-1.2087770
15.5166664	3.6599998	-1.2087770
15.5166664	3.7087998	-1.0431070
15.5166664	3.7575998	-1.0860586
15.5166664	3.8063998	-1.2149129
15.5166664	3.8551998	-0.9633398
15.5166664	3.9039998	-1.0860586
15.5166664	3.9527998	-1.0860586
15.5166664	4.0016003	-0.9694757
15.5166664	4.0503998	-1.1351457
15.5166664	4.0991998	-1.0553789
15.5166664	4.1479998	-1.0553789
15.5166664	4.1967998	-1.0369711
15.5166664	4.2455997	-0.9817476
15.5166664	4.2943997	-1.0001554
15.5166664	4.3431997	-0.8713012
15.5166664	4.3919997	-0.8774371
15.5166664	4.4407997	-0.9387960
15.5166664	4.4895997	-0.9203887
15.5166664	4.5383997	-0.8897090
15.5166664	4.5871997	-0.9142528
15.5166664	4.6359997	-0.9694757
15.5166664	4.6847997	-0.9694757
15.5166664	4.7336001	-0.8897090
15.5166664	4.7824001	-0.8835731
15.5166664	4.8312001	-0.8283496
15.5166664	4.8800001	-0.8283496
15.5166664	4.9288001	-0.8283496
15.5166664	4.9776001	-0.7547188
15.5166664	5.0264001	-0.6320000
15.5166664	5.0752001	-0.5829129
15.5166664	5.1240001	613586.0000000
15.5166664	5.1728001	613586.0000000
15.5166664	5.2216001	613586.0000000
15.5166664	5.2704000	613586.0000000
15.5166664	5.3192000	613586.0000000

file:///C/Users/ohya2/Documents/Scientific%20Reports/Ezsnow/Supporting%20Information/FALCONDopplervFig6b.txt[2020/04/14 10:21:08]

15.5166664	5.3680000	613586.0000000
15.5166664	5.4168000	613586.0000000
15.5166664	5.4656000	613586.0000000
15.5166664	5.5144000	613586.0000000
15.5166664	5.5632000	613586.0000000
15.5166664	5.6120000	613586.0000000
15.5166664	5.6608000	613586.0000000
15.5166664	5.7096000	613586.0000000
15.5166664	5.7584000	613586.0000000
15.5166664	5.8072000	613586.0000000
15.5166664	5.8559999	613586.0000000
15.5166664	5.9047999	613586.0000000
15.5166664	5.9535999	613586.0000000
15.5166664	6.0023999	613586.0000000
15.5333338	0.2928000	-4.3503695
15.5333338	0.3416000	-4.3442335
15.5333338	0.3904000	-3.4913404
15.5333338	0.4392000	-3.4913404
15.5333338	0.4880000	-4.6817093
15.5333338	0.5368000	-4.5896707
15.5333338	0.5856000	-4.4669523
15.5333338	0.6344000	-3.1538646
15.5333338	0.6832000	-1.9328160
15.5333338	0.7320000	-1.9328160
15.5333338	0.7808000	-1.5646605
15.5333338	0.8296000	-1.5523887
15.5333338	0.8784000	-1.4358063
15.5333338	0.9272000	-1.4358063
15.5333338	0.9760000	-1.4603496
15.5333338	1.0247999	-1.4664855
15.5333338	1.0735999	-1.4787574
15.5333338	1.1223999	-1.4173985
15.5333338	1.1711999	-1.2271848
15.5333338	1.2200000	-1.5523887
15.5333338	1.2688000	-1.4296703
15.5333338	1.3176000	-1.2640004
15.5333338	1.3664000	-1.2640004
15.5333338	1.4152000	-1.2701364
15.5333338	1.4640000	-1.3805828
15.5333338	1.5128000	-1.3805828
15.5333338	1.5616000	-1.6751070
15.5333338	1.6104000	-1.4173985
15.5333338	1.6592000	-1.4296703
15.5333338	1.7079999	-1.4358063
15.5333338	1.7567999	-1.1106019
15.5333338	1.8055999	-1.1106019
15.5333338	1.8543999	-0.8958449
15.5333338	1.9031999	-1.0799227
15.5333338	1.9519999	-1.0799227
15.5333338	2.0007999	-1.1474175
15.5333338	2.0495999	-1.1412816
15.5333338	2.0983999	-1.1228738
15.5333338	2.1471999	-1.2087770
15.5333338	2.1959999	-1.1535535
15.5333338	2.2447999	-1.1474175
15.5333338	2.2935998	-1.1351457
15.5333338	2.3423998	-1.2946796

15.5333338	2.3912001	-1.3069515
15.5333338	2.4400001	-1.3192234
15.5333338	2.4888000	-1.2701364
15.5333338	2.5376000	-1.2701364
15.5333338	2.5864000	-1.1658254
15.5333338	2.6352000	-1.1719613
15.5333338	2.6840000	-1.1658254
15.5333338	2.7328000	-1.2026410
15.5333338	2.7816000	-1.1474175
15.5333338	2.8304000	-1.2210488
15.5333338	2.8792000	-1.0185633
15.5333338	2.9280000	-1.0799227
15.5333338	2.9768000	-1.0799227
15.5333338	3.0256000	-1.0799227
15.5333338	3.0743999	-1.0860586
15.5333338	3.1231999	-1.0676508
15.5333338	3.1719999	-1.0737867
15.5333338	3.2207999	-0.8160777
15.5333338	3.2695999	-0.8774371
15.5333338	3.3183999	-0.9878836
15.5333338	3.3671999	-0.9940195
15.5333338	3.4159999	-0.9694757
15.5333338	3.4647999	-0.9756117
15.5333338	3.5135999	-0.8713012
15.5333338	3.5623999	-0.9572039
15.5333338	3.6111999	-0.9572039
15.5333338	3.6599998	-0.9572039
15.5333338	3.7087998	-0.7547188
15.5333338	3.7575998	-0.7547188
15.5333338	3.8063998	-0.8038058
15.5333338	3.8551998	-0.7915339
15.5333338	3.9039998	-0.7915339
15.5333338	3.9527998	-0.8651652
15.5333338	4.0016003	-0.6749516
15.5333338	4.0503998	-0.7731261
15.5333338	4.0991998	-0.8528934
15.5333338	4.1479998	-0.8528934
15.5333338	4.1967998	-0.6688156
15.5333338	4.2455997	-0.6749516
15.5333338	4.2943997	-0.6749516
15.5333338	4.3431997	-0.6749516
15.5333338	4.3919997	-0.6749516
15.5333338	4.4407997	-0.8590293
15.5333338	4.4895997	-0.9265246
15.5333338	4.5383997	-0.9081168
15.5333338	4.5871997	-0.9142528
15.5333338	4.6359997	-0.9203887
15.5333338	4.6847997	-1.0369711
15.5333338	4.7336001	-0.8283496
15.5333338	4.7824001	-0.8958449
15.5333338	4.8312001	-0.8528934
15.5333338	4.8800001	-0.6626797
15.5333338	4.9288001	-0.4479222
15.5333338	4.9776001	-0.3190680
15.5333338	5.0264001	-0.6504078
15.5333338	5.0752001	-0.6013203
15.5333338	5.1240001	-0.5951843

15.5333338	5.1728001	-0.9694757
15.5333338	5.2216001	-0.8590293
15.5333338	5.2704000	-0.8590293
15.5333338	5.3192000	613586.0000000
15.5333338	5.3680000	613586.0000000
15.5333338	5.4168000	613586.0000000
15.5333338	5.4656000	613586.0000000
15.5333338	5.5144000	613586.0000000
15.5333338	5.5632000	613586.0000000
15.5333338	5.6120000	613586.0000000
15.5333338	5.6608000	613586.0000000
15.5333338	5.7096000	613586.0000000
15.5333338	5.7584000	613586.0000000
15.5333338	5.8072000	613586.0000000
15.5333338	5.8559999	613586.0000000
15.5333338	5.9047999	613586.0000000
15.5333338	5.9535999	613586.0000000
15.5333338	6.0023999	613586.0000000
15.5500002	0.2928000	-4.6694374
15.5500002	0.3416000	-4.5773988
15.5500002	0.3904000	-4.3442335
15.5500002	0.4392000	-3.8349521
15.5500002	0.4880000	-4.0987968
15.5500002	0.5368000	-3.7306414
15.5500002	0.5856000	-4.2460589
15.5500002	0.6344000	-2.7488937
15.5500002	0.6832000	-2.0187187
15.5500002	0.7320000	-2.0923500
15.5500002	0.7808000	-1.8469129
15.5500002	0.8296000	-1.5094371
15.5500002	0.8784000	-1.4664855
15.5500002	0.9272000	-1.4848933
15.5500002	0.9760000	-1.5401168
15.5500002	1.0247999	-1.4480777
15.5500002	1.0735999	-1.4235344
15.5500002	1.1223999	-1.4173985
15.5500002	1.1711999	-1.2455926
15.5500002	1.2200000	-1.3376312
15.5500002	1.2688000	-1.1106019
15.5500002	1.3176000	-1.2701364
15.5500002	1.3664000	-1.2578645
15.5500002	1.4152000	-1.5769324
15.5500002	1.4640000	-1.5892043
15.5500002	1.5128000	-1.7794180
15.5500002	1.5616000	-1.8591847
15.5500002	1.6104000	-1.2885437
15.5500002	1.6592000	-1.2885437
15.5500002	1.7079999	-1.2701364
15.5500002	1.7567999	-1.2640004
15.5500002	1.8055999	-1.3008156
15.5500002	1.8543999	-1.2946796
15.5500002	1.9031999	-1.2824078
15.5500002	1.9519999	-1.2026410
15.5500002	2.0007999	-1.2087770
15.5500002	2.0495999	-1.0553789
15.5500002	2.0983999	-0.9817476
15.5500002	2.1471999	-1.1228738

15.5500002	2.1959999	-1.1167378
15.5500002	2.2447999	-1.2087770
15.5500002	2.2935998	-1.2026410
15.5500002	2.3423998	-1.1351457
15.5500002	2.3912001	-1.1351457
15.5500002	2.4400001	-1.2517285
15.5500002	2.4888000	-1.1167378
15.5500002	2.5376000	-1.1412816
15.5500002	2.5864000	-1.2885437
15.5500002	2.6352000	-1.1596894
15.5500002	2.6840000	-1.1535535
15.5500002	2.7328000	-1.0185633
15.5500002	2.7816000	-1.1719613
15.5500002	2.8304000	-1.1167378
15.5500002	2.8792000	-1.1228738
15.5500002	2.9280000	-0.9572039
15.5500002	2.9768000	-1.0492430
15.5500002	3.0256000	-1.0308352
15.5500002	3.0743999	-1.0246992
15.5500002	3.1231999	-1.0921946
15.5500002	3.1719999	-1.0553789
15.5500002	3.2207999	-1.0001554
15.5500002	3.2695999	-0.8651652
15.5500002	3.3183999	-0.9510679
15.5500002	3.3671999	-0.8958449
15.5500002	3.4159999	-0.8774371
15.5500002	3.4647999	-0.8713012
15.5500002	3.5135999	-0.8590293
15.5500002	3.5623999	-0.9142528
15.5500002	3.6111999	-0.9019809
15.5500002	3.6599998	-0.8283496
15.5500002	3.7087998	-0.7424469
15.5500002	3.7575998	-0.7669902
15.5500002	3.8063998	-0.7179031
15.5500002	3.8551998	-0.6135921
15.5500002	3.9039998	-0.5951843
15.5500002	3.9527998	-0.7117672
15.5500002	4.0016003	-0.7179031
15.5500002	4.0503998	-0.7179031
15.5500002	4.0991998	-0.5951843
15.5500002	4.1479998	-0.5951843
15.5500002	4.1967998	-0.5706410
15.5500002	4.2455997	-0.5460973
15.5500002	4.2943997	-0.5399613
15.5500002	4.3431997	-0.7301750
15.5500002	4.3919997	-0.4970098
15.5500002	4.4407997	-0.5522332
15.5500002	4.4895997	-0.5522332
15.5500002	4.5383997	-0.5767770
15.5500002	4.5871997	-0.5767770
15.5500002	4.6359997	-0.5951843
15.5500002	4.6847997	-0.6074562
15.5500002	4.7336001	-0.6994953
15.5500002	4.7824001	-0.6994953
15.5500002	4.8312001	-0.4724660
15.5500002	4.8800001	-0.5460973
15.5500002	4.9288001	-0.5460973

15.5500002	4.9776001	-0.5583692
15.5500002	5.0264001	-0.5583692
15.5500002	5.0752001	-0.6442719
15.5500002	5.1240001	-0.6381359
15.5500002	5.1728001	-0.6197281
15.5500002	5.2216001	-0.7363110
15.5500002	5.2704000	-0.8467574
15.5500002	5.3192000	613586.0000000
15.5500002	5.3680000	613586.0000000
15.5500002	5.4168000	613586.0000000
15.5500002	5.4656000	613586.0000000
15.5500002	5.5144000	613586.0000000
15.5500002	5.5632000	613586.0000000
15.5500002	5.6120000	613586.0000000
15.5500002	5.6608000	613586.0000000
15.5500002	5.7096000	613586.0000000
15.5500002	5.7584000	613586.0000000
15.5500002	5.8072000	613586.0000000
15.5500002	5.8559999	613586.0000000
15.5500002	5.9047999	613586.0000000
15.5500002	5.9535999	613586.0000000
15.5500002	6.0023999	613586.0000000
15.5666666	0.2928000	-4.9394183
15.5666666	0.3416000	-4.3933210
15.5666666	0.3904000	-4.4117289
15.5666666	0.4392000	-3.6692822
15.5666666	0.4880000	-3.6815541
15.5666666	0.5368000	-4.5099039
15.5666666	0.5856000	-4.1233406
15.5666666	0.6344000	-3.7060976
15.5666666	0.6832000	-1.8775926
15.5666666	0.7320000	-1.5094371
15.5666666	0.7808000	-1.2394567
15.5666666	0.8296000	-1.5523887
15.5666666	0.8784000	-1.5523887
15.5666666	0.9272000	-1.6935148
15.5666666	0.9760000	-1.6935148
15.5666666	1.0247999	-1.5523887
15.5666666	1.0735999	-1.4787574
15.5666666	1.1223999	-1.4910293
15.5666666	1.1711999	-1.3867188
15.5666666	1.2200000	-1.0308352
15.5666666	1.2688000	-1.0308352
15.5666666	1.3176000	-1.0369711
15.5666666	1.3664000	-1.2824078
15.5666666	1.4152000	-1.2701364
15.5666666	1.4640000	-1.2824078
15.5666666	1.5128000	-1.5401168
15.5666666	1.5616000	-1.5462527
15.5666666	1.6104000	-1.5523887
15.5666666	1.6592000	-1.9880390
15.5666666	1.7079999	-2.0064468
15.5666666	1.7567999	-1.8285050
15.5666666	1.8055999	-1.6873789
15.5666666	1.8543999	-1.7548742
15.5666666	1.9031999	-1.6382914
15.5666666	1.9519999	-1.2885437

15.5666666	2.0007999	-1.4726214
15.5666666	2.0495999	-1.3314953
15.5666666	2.0983999	-1.3376312
15.5666666	2.1471999	-1.2701364
15.5666666	2.1959999	-1.2578645
15.5666666	2.2447999	-1.2026410
15.5666666	2.2935998	-1.2149129
15.5666666	2.3423998	-1.2149129
15.5666666	2.3912001	-1.0615149
15.5666666	2.4400001	-1.2087770
15.5666666	2.4888000	-1.1719613
15.5666666	2.5376000	-1.2455926
15.5666666	2.5864000	-1.1719613
15.5666666	2.6352000	-1.0308352
15.5666666	2.6840000	-1.0369711
15.5666666	2.7328000	-1.0431070
15.5666666	2.7816000	-1.0492430
15.5666666	2.8304000	-1.1290097
15.5666666	2.8792000	-1.1290097
15.5666666	2.9280000	-1.1474175
15.5666666	2.9768000	-1.1535535
15.5666666	3.0256000	-1.0615149
15.5666666	3.0743999	-0.9572039
15.5666666	3.1231999	-1.0185633
15.5666666	3.1719999	-0.9326606
15.5666666	3.2207999	-0.9203887
15.5666666	3.2695999	-0.9019809
15.5666666	3.3183999	-0.9142528
15.5666666	3.3671999	-0.8958449
15.5666666	3.4159999	-0.8958449
15.5666666	3.4647999	-0.8835731
15.5666666	3.5135999	-0.8897090
15.5666666	3.5623999	-0.8958449
15.5666666	3.6111999	-0.8160777
15.5666666	3.6599998	-0.8099418
15.5666666	3.7087998	-0.8038058
15.5666666	3.7575998	-0.8038058
15.5666666	3.8063998	-0.8099418
15.5666666	3.8551998	-0.6688156
15.5666666	3.9039998	-0.6688156
15.5666666	3.9527998	-0.7853980
15.5666666	4.0016003	-0.7915339
15.5666666	4.0503998	-0.6688156
15.5666666	4.0991998	-0.6381359
15.5666666	4.1479998	-0.6320000
15.5666666	4.1967998	-0.6565437
15.5666666	4.2455997	-0.6442719
15.5666666	4.2943997	-0.8038058
15.5666666	4.3431997	-0.8160777
15.5666666	4.3919997	-0.8651652
15.5666666	4.4407997	-0.7424469
15.5666666	4.4895997	-0.7363110
15.5666666	4.5383997	-0.7363110
15.5666666	4.5871997	-0.7485828
15.5666666	4.6359997	-0.6442719
15.5666666	4.6847997	-0.6442719
15.5666666	4.7336001	-0.7301750

15.5666666	4.7824001	-0.6565437
15.5666666	4.8312001	-0.5338254
15.5666666	4.8800001	-0.5154176
15.5666666	4.9288001	-0.4786019
15.5666666	4.9776001	-0.4786019
15.5666666	5.0264001	-0.2454371
15.5666666	5.0752001	-0.2454371
15.5666666	5.1240001	-0.4295144
15.5666666	5.1728001	-0.5338254
15.5666666	5.2216001	-0.5460973
15.5666666	5.2704000	-0.5583692
15.5666666	5.3192000	-0.5706410
15.5666666	5.3680000	-0.5706410
15.5666666	5.4168000	-0.6933594
15.5666666	5.4656000	-0.4970098
15.5666666	5.5144000	613586.0000000
15.5666666	5.5632000	613586.0000000
15.5666666	5.6120000	613586.0000000
15.5666666	5.6608000	613586.0000000
15.5666666	5.7096000	613586.0000000
15.5666666	5.7584000	613586.0000000
15.5666666	5.8072000	613586.0000000
15.5666666	5.8559999	613586.0000000
15.5666666	5.9047999	613586.0000000
15.5666666	5.9535999	613586.0000000
15.5666666	6.0023999	613586.0000000
15.5833330	0.2928000	-5.6757293
15.5833330	0.3416000	-5.6757293
15.5833330	0.3904000	-5.8107195
15.5833330	0.4392000	-0.0122719
15.5833330	0.4880000	-0.0122719
15.5833330	0.5368000	-4.6571655
15.5833330	0.5856000	-4.0006218
15.5833330	0.6344000	-3.5526996
15.5833330	0.6832000	-2.7979810
15.5833330	0.7320000	-2.8654761
15.5833330	0.7808000	-1.3989906
15.5833330	0.8296000	-1.3867188
15.5833330	0.8784000	-1.7180586
15.5833330	0.9272000	-1.8469129
15.5833330	0.9760000	-1.6751070
15.5833330	1.0247999	-1.6812429
15.5833330	1.0735999	-1.7487383
15.5833330	1.1223999	-1.3683109
15.5833330	1.1711999	-1.1290097
15.5833330	1.2200000	-1.4173985
15.5833330	1.2688000	-1.4235344
15.5833330	1.3176000	-1.3989906
15.5833330	1.3664000	-1.4971652
15.5833330	1.4152000	-1.2210488
15.5833330	1.4640000	-1.0676508
15.5833330	1.5128000	-1.0553789
15.5833330	1.5616000	-0.9203887
15.5833330	1.6104000	-0.9203887
15.5833330	1.6592000	-1.1842332
15.5833330	1.7079999	-1.1965051
15.5833330	1.7567999	-1.2210488

15.5833330	1.8055999	-1.2946796
15.5833330	1.8543999	-1.3008156
15.5833330	1.9031999	-1.3253593
15.5833330	1.9519999	-1.2026410
15.5833330	2.0007999	-1.1965051
15.5833330	2.0495999	-1.1842332
15.5833330	2.0983999	-1.1106019
15.5833330	2.1471999	-1.2149129
15.5833330	2.1959999	-1.2333207
15.5833330	2.2447999	-1.2271848
15.5833330	2.2935998	-1.1596894
15.5833330	2.3423998	-1.2762718
15.5833330	2.3912001	-1.2578645
15.5833330	2.4400001	-1.1351457
15.5833330	2.4888000	-1.1965051
15.5833330	2.5376000	-0.9756117
15.5833330	2.5864000	-1.2455926
15.5833330	2.6352000	-1.1596894
15.5833330	2.6840000	-1.1903691
15.5833330	2.7328000	-1.1842332
15.5833330	2.7816000	-1.2333207
15.5833330	2.8304000	-1.2333207
15.5833330	2.8792000	-1.1842332
15.5833330	2.9280000	-1.1719613
15.5833330	2.9768000	-1.1044660
15.5833330	3.0256000	-1.1658254
15.5833330	3.0743999	-1.1658254
15.5833330	3.1231999	-1.0615149
15.5833330	3.1719999	-1.0431070
15.5833330	3.2207999	-1.0369711
15.5833330	3.2695999	-0.9572039
15.5833330	3.3183999	-1.0246992
15.5833330	3.3671999	-1.0124273
15.5833330	3.4159999	-0.8774371
15.5833330	3.4647999	-0.8099418
15.5833330	3.5135999	-0.8160777
15.5833330	3.5623999	-0.7792621
15.5833330	3.6111999	-0.6994953
15.5833330	3.6599998	-0.7117672
15.5833330	3.7087998	-0.7179031
15.5833330	3.7575998	-0.7179031
15.5833330	3.8063998	-0.5890489
15.5833330	3.8551998	-0.7056313
15.5833330	3.9039998	-0.6810875
15.5833330	3.9527998	-0.6626797
15.5833330	4.0016003	-0.6688156
15.5833330	4.0503998	-0.7792621
15.5833330	4.0991998	-0.6197281
15.5833330	4.1479998	-0.6504078
15.5833330	4.1967998	-0.6565437
15.5833330	4.2455997	-0.7363110
15.5833330	4.2943997	-0.7363110
15.5833330	4.3431997	-0.6994953
15.5833330	4.3919997	-0.6933594
15.5833330	4.4407997	-0.6933594
15.5833330	4.4895997	-0.6135921
15.5833330	4.5383997	-0.6872234

15.5833330	4.5871997	-0.4724660
15.5833330	4.6359997	-0.5460973
15.5833330	4.6847997	-0.7792621
15.5833330	4.7336001	-0.7792621
15.5833330	4.7824001	-0.5276895
15.5833330	4.8312001	-0.6135921
15.5833330	4.8800001	-0.6197281
15.5833330	4.9288001	-0.6872234
15.5833330	4.9776001	-0.4479222
15.5833330	5.0264001	-0.3926992
15.5833330	5.0752001	-0.4970098
15.5833330	5.1240001	-0.2086215
15.5833330	5.1728001	-0.1963496
15.5833330	5.2216001	-0.0736313
15.5833330	5.2704000	-0.5522332
15.5833330	5.3192000	-0.6320000
15.5833330	5.3680000	-0.6197281
15.5833330	5.4168000	-0.4111071
15.5833330	5.4656000	-0.7056313
15.5833330	5.5144000	613586.0000000
15.5833330	5.5632000	613586.0000000
15.5833330	5.6120000	613586.0000000
15.5833330	5.6608000	613586.0000000
15.5833330	5.7096000	613586.0000000
15.5833330	5.7584000	613586.0000000
15.5833330	5.8072000	613586.0000000
15.5833330	5.8559999	613586.0000000
15.5833330	5.9047999	613586.0000000
15.5833330	5.9535999	613586.0000000
15.5833330	6.0023999	613586.0000000
15.6000004	0.2928000	-4.2399230
15.6000004	0.3416000	-4.2706027
15.6000004	0.3904000	-2.4052820
15.6000004	0.4392000	-3.0495539
15.6000004	0.4880000	-3.0495539
15.6000004	0.5368000	-3.9699423
15.6000004	0.5856000	-3.9699423
15.6000004	0.6344000	-2.7182140
15.6000004	0.6832000	-1.4603496
15.6000004	0.7320000	-1.4542136
15.6000004	0.7808000	-1.3989906
15.6000004	0.8296000	-1.8162332
15.6000004	0.8784000	-1.6628351
15.6000004	0.9272000	-1.7671461
15.6000004	0.9760000	-1.7855535
15.6000004	1.0247999	-1.7855535
15.6000004	1.0735999	-1.9328160
15.6000004	1.1223999	-1.5646605
15.6000004	1.1711999	-1.5094371
15.6000004	1.2200000	-1.7610102
15.6000004	1.2688000	-1.7548742
15.6000004	1.3176000	-1.5155730
15.6000004	1.3664000	-1.5278449
15.6000004	1.4152000	-1.3928547
15.6000004	1.4640000	-1.1719613
15.6000004	1.5128000	-1.0001554
15.6000004	1.5616000	-1.0062914

15.6000004	1.6104000	-1.1167378
15.6000004	1.6592000	-0.9633398
15.6000004	1.7079999	-0.8897090
15.6000004	1.7567999	-1.0553789
15.6000004	1.8055999	-0.7301750
15.6000004	1.8543999	-0.8099418
15.6000004	1.9031999	-0.7976699
15.6000004	1.9519999	-0.8406215
15.6000004	2.0007999	-1.0369711
15.6000004	2.0495999	-0.9633398
15.6000004	2.0983999	-0.8958449
15.6000004	2.1471999	-1.0553789
15.6000004	2.1959999	-1.0615149
15.6000004	2.2447999	-1.0799227
15.6000004	2.2935998	-1.0492430
15.6000004	2.3423998	-1.0860586
15.6000004	2.3912001	-1.0676508
15.6000004	2.4400001	-1.0737867
15.6000004	2.4888000	-1.0553789
15.6000004	2.5376000	-1.0553789
15.6000004	2.5864000	-1.2026410
15.6000004	2.6352000	-1.2271848
15.6000004	2.6840000	-1.2087770
15.6000004	2.7328000	-1.1719613
15.6000004	2.7816000	-1.1903691
15.6000004	2.8304000	-1.1903691
15.6000004	2.8792000	-1.1412816
15.6000004	2.9280000	-1.1535535
15.6000004	2.9768000	-0.9265246
15.6000004	3.0256000	-1.0369711
15.6000004	3.0743999	-1.0246992
15.6000004	3.1231999	-1.0860586
15.6000004	3.1719999	-1.0062914
15.6000004	3.2207999	-0.9940195
15.6000004	3.2695999	-0.9878836
15.6000004	3.3183999	-0.6994953
15.6000004	3.3671999	-0.6504078
15.6000004	3.4159999	-0.7731261
15.6000004	3.4647999	-0.9142528
15.6000004	3.5135999	-0.9081168
15.6000004	3.5623999	-0.8222136
15.6000004	3.6111999	-0.8222136
15.6000004	3.6599998	-0.8958449
15.6000004	3.7087998	-0.8958449
15.6000004	3.7575998	-0.7792621
15.6000004	3.8063998	-0.7792621
15.6000004	3.8551998	-0.7915339
15.6000004	3.9039998	-0.7608547
15.6000004	3.9527998	-0.7608547
15.6000004	4.0016003	-0.8406215
15.6000004	4.0503998	-0.8713012
15.6000004	4.0991998	-0.8651652
15.6000004	4.1479998	-0.8528934
15.6000004	4.1967998	-0.8958449
15.6000004	4.2455997	-0.8897090
15.6000004	4.2943997	-0.9081168
15.6000004	4.3431997	-0.8528934

15.6000004	4.3919997	-0.7485828
15.6000004	4.4407997	-0.9449320
15.6000004	4.4895997	-0.9449320
15.6000004	4.5383997	-0.9633398
15.6000004	4.5871997	-0.9265246
15.6000004	4.6359997	-0.9142528
15.6000004	4.6847997	-0.8774371
15.6000004	4.7336001	-0.7669902
15.6000004	4.7824001	-0.8467574
15.6000004	4.8312001	-0.6872234
15.6000004	4.8800001	-0.5522332
15.6000004	4.9288001	-0.7976699
15.6000004	4.9776001	-0.8160777
15.6000004	5.0264001	-0.7976699
15.6000004	5.0752001	-0.7424469
15.6000004	5.1240001	-0.7485828
15.6000004	5.1728001	-0.7731261
15.6000004	5.2216001	-0.7976699
15.6000004	5.2704000	-0.5460973
15.6000004	5.3192000	-1.1290097
15.6000004	5.3680000	-1.0062914
15.6000004	5.4168000	-1.0062914
15.6000004	5.4656000	613586.0000000
15.6000004	5.5144000	613586.0000000
15.6000004	5.5632000	613586.0000000
15.6000004	5.6120000	613586.0000000
15.6000004	5.6608000	613586.0000000
15.6000004	5.7096000	613586.0000000
15.6000004	5.7584000	613586.0000000
15.6000004	5.8072000	613586.0000000
15.6000004	5.8559999	613586.0000000
15.6000004	5.9047999	613586.0000000
15.6000004	5.9535999	613586.0000000
15.6000004	6.0023999	613586.0000000
15.6166668	0.2928000	-3.4177094
15.6166668	0.3416000	-3.3318064
15.6166668	0.3904000	-2.4298255
15.6166668	0.4392000	-1.1903691
15.6166668	0.4880000	-3.3993015
15.6166668	0.5368000	-5.5100594
15.6166668	0.5856000	-5.3996124
15.6166668	0.6344000	-4.0128937
15.6166668	0.6832000	-3.1109130
15.6166668	0.7320000	-1.5278449
15.6166668	0.7808000	-1.5339808
15.6166668	0.8296000	-1.1903691
15.6166668	0.8784000	-1.1965051
15.6166668	0.9272000	-1.6321554
15.6166668	0.9760000	-1.4542136
15.6166668	1.0247999	-1.6014762
15.6166668	1.0735999	-1.6198835
15.6166668	1.1223999	-1.7732821
15.6166668	1.1711999	-1.6566992
15.6166668	1.2200000	-1.4603496
15.6166668	1.2688000	-1.4664855
15.6166668	1.3176000	-1.5462527
15.6166668	1.3664000	-1.2578645

15.6166668	1.4152000	-1.2578645
15.6166668	1.4640000	-1.2578645
15.6166668	1.5128000	-1.3376312
15.6166668	1.5616000	-1.3253593
15.6166668	1.6104000	-1.3008156
15.6166668	1.6592000	-1.2885437
15.6166668	1.7079999	-0.9940195
15.6166668	1.7567999	-0.9940195
15.6166668	1.8055999	-0.9326606
15.6166668	1.8543999	-0.8528934
15.6166668	1.9031999	-0.8528934
15.6166668	1.9519999	-0.7363110
15.6166668	2.0007999	-0.9142528
15.6166668	2.0495999	-0.8897090
15.6166668	2.0983999	-0.9633398
15.6166668	2.1471999	-0.8651652
15.6166668	2.1959999	-0.9203887
15.6166668	2.2447999	-0.9756117
15.6166668	2.2935998	-0.9510679
15.6166668	2.3423998	-0.8222136
15.6166668	2.3912001	-1.0799227
15.6166668	2.4400001	-1.0737867
15.6166668	2.4888000	-1.1351457
15.6166668	2.5376000	-1.1351457
15.6166668	2.5864000	-0.9572039
15.6166668	2.6352000	-1.0553789
15.6166668	2.6840000	-1.0492430
15.6166668	2.7328000	-1.0308352
15.6166668	2.7816000	-1.0308352
15.6166668	2.8304000	-1.0308352
15.6166668	2.8792000	-1.0246992
15.6166668	2.9280000	-1.0492430
15.6166668	2.9768000	-1.0369711
15.6166668	3.0256000	-0.8897090
15.6166668	3.0743999	-0.8651652
15.6166668	3.1231999	-0.9510679
15.6166668	3.1719999	-0.8590293
15.6166668	3.2207999	-0.8344855
15.6166668	3.2695999	-0.7363110
15.6166668	3.3183999	-0.7608547
15.6166668	3.3671999	-0.9572039
15.6166668	3.4159999	-0.9572039
15.6166668	3.4647999	-0.8160777
15.6166668	3.5135999	-0.7179031
15.6166668	3.5623999	-0.8160777
15.6166668	3.6111999	-0.6442719
15.6166668	3.6599998	-0.6442719
15.6166668	3.7087998	-0.6258640
15.6166668	3.7575998	-0.5951843
15.6166668	3.8063998	-0.6074562
15.6166668	3.8551998	-0.6135921
15.6166668	3.9039998	-0.5890489
15.6166668	3.9527998	-0.5829129
15.6166668	4.0016003	-0.5583692
15.6166668	4.0503998	-0.4786019
15.6166668	4.0991998	-0.4049711
15.6166668	4.1479998	-0.5890489

15.6166668	4.1967998	-0.4663301
15.6166668	4.2455997	-0.4663301
15.6166668	4.2943997	-0.5645051
15.6166668	4.3431997	-0.4479222
15.6166668	4.3919997	-0.4847379
15.6166668	4.4407997	-0.5767770
15.6166668	4.4895997	-0.4417863
15.6166668	4.5383997	-0.4479222
15.6166668	4.5871997	-0.4233789
15.6166668	4.6359997	-0.3497477
15.6166668	4.6847997	-0.3558836
15.6166668	4.7336001	-0.2761164
15.6166668	4.7824001	-0.3129320
15.6166668	4.8312001	-0.4479222
15.6166668	4.8800001	-0.4479222
15.6166668	4.9288001	-0.4540582
15.6166668	4.9776001	-0.2147574
15.6166668	5.0264001	-0.2883883
15.6166668	5.0752001	-0.2208934
15.6166668	5.1240001	-0.2147574
15.6166668	5.1728001	-0.3681555
15.6166668	5.2216001	-0.2761164
15.6166668	5.2704000	-0.4172430
15.6166668	5.3192000	-0.4111071
15.6166668	5.3680000	-0.4111071
15.6166668	5.4168000	-0.5890489
15.6166668	5.4656000	-0.5829129
15.6166668	5.5144000	613586.0000000
15.6166668	5.5632000	613586.0000000
15.6166668	5.6120000	613586.0000000
15.6166668	5.6608000	613586.0000000
15.6166668	5.7096000	613586.0000000
15.6166668	5.7584000	613586.0000000
15.6166668	5.8072000	613586.0000000
15.6166668	5.8559999	613586.0000000
15.6166668	5.9047999	613586.0000000
15.6166668	5.9535999	613586.0000000
15.6166668	6.0023999	613586.0000000
15.6333332	0.2928000	-3.2827189
15.6333332	0.3416000	-3.2765830
15.6333332	0.3904000	-3.2888548
15.6333332	0.4392000	-3.5465636
15.6333332	0.4880000	-3.2949908
15.6333332	0.5368000	-3.3133986
15.6333332	0.5856000	-3.5649714
15.6333332	0.6344000	-2.7304859
15.6333332	0.6832000	-1.8407769
15.6333332	0.7320000	-1.4910293
15.6333332	0.7808000	-1.4910293
15.6333332	0.8296000	-1.2517285
15.6333332	0.8784000	-1.2946796
15.6333332	0.9272000	-1.3192234
15.6333332	0.9760000	-1.2946796
15.6333332	1.0247999	-1.1842332
15.6333332	1.0735999	-1.0308352
15.6333332	1.1223999	-1.3621750
15.6333332	1.1711999	-1.6628351

15.6333332	1.2200000	-1.6566992
15.6333332	1.2688000	-1.7916894
15.6333332	1.3176000	-1.7978253
15.6333332	1.3664000	-1.5769324
15.6333332	1.4152000	-1.6137476
15.6333332	1.4640000	-1.5339808
15.6333332	1.5128000	-1.6628351
15.6333332	1.5616000	-1.5462527
15.6333332	1.6104000	-1.5585246
15.6333332	1.6592000	-1.3253593
15.6333332	1.7079999	-0.9878836
15.6333332	1.7567999	-0.9817476
15.6333332	1.8055999	-1.2578645
15.6333332	1.8543999	-1.2578645
15.6333332	1.9031999	-1.2640004
15.6333332	1.9519999	-1.1965051
15.6333332	2.0007999	-1.1044660
15.6333332	2.0495999	-1.1474175
15.6333332	2.0983999	-0.9756117
15.6333332	2.1471999	-0.9756117
15.6333332	2.1959999	-0.9449320
15.6333332	2.2447999	-1.1903691
15.6333332	2.2935998	-1.1351457
15.6333332	2.3423998	-1.1965051
15.6333332	2.3912001	-0.9633398
15.6333332	2.4400001	-1.0246992
15.6333332	2.4888000	-1.2210488
15.6333332	2.5376000	-1.0062914
15.6333332	2.5864000	-1.0001554
15.6333332	2.6352000	-1.0676508
15.6333332	2.6840000	-1.0615149
15.6333332	2.7328000	-1.0553789
15.6333332	2.7816000	-0.9326606
15.6333332	2.8304000	-0.8467574
15.6333332	2.8792000	-0.8467574
15.6333332	2.9280000	-0.8160777
15.6333332	2.9768000	-0.7424469
15.6333332	3.0256000	-0.8835731
15.6333332	3.0743999	-0.8835731
15.6333332	3.1231999	-0.8160777
15.6333332	3.1719999	-0.8160777
15.6333332	3.2207999	-0.8344855
15.6333332	3.2695999	-0.8467574
15.6333332	3.3183999	-0.7792621
15.6333332	3.3671999	-0.7731261
15.6333332	3.4159999	-0.7179031
15.6333332	3.4647999	-0.5399613
15.6333332	3.5135999	-0.5399613
15.6333332	3.5623999	-0.7301750
15.6333332	3.6111999	-0.7240391
15.6333332	3.6599998	-0.6749516
15.6333332	3.7087998	-0.7669902
15.6333332	3.7575998	-0.7792621
15.6333332	3.8063998	-0.8528934
15.6333332	3.8551998	-0.6872234
15.6333332	3.9039998	-0.7669902
15.6333332	3.9527998	-0.7056313

15.6333332	4.0016003	-0.7056313
15.6333332	4.0503998	-0.6135921
15.6333332	4.0991998	-0.6074562
15.6333332	4.1479998	-0.7547188
15.6333332	4.1967998	-0.7669902
15.6333332	4.2455997	-0.7731261
15.6333332	4.2943997	-0.5829129
15.6333332	4.3431997	-0.5522332
15.6333332	4.3919997	-0.6135921
15.6333332	4.4407997	-0.6381359
15.6333332	4.4895997	-0.6504078
15.6333332	4.5383997	-0.5460973
15.6333332	4.5871997	-0.2515731
15.6333332	4.6359997	-0.4479222
15.6333332	4.6847997	-0.4540582
15.6333332	4.7336001	-0.4479222
15.6333332	4.7824001	-0.4295144
15.6333332	4.8312001	-0.3865633
15.6333332	4.8800001	-0.2699804
15.6333332	4.9288001	-0.3865633
15.6333332	4.9776001	-0.5031457
15.6333332	5.0264001	-0.4970098
15.6333332	5.0752001	-0.4172430
15.6333332	5.1240001	-0.4295144
15.6333332	5.1728001	-0.4295144
15.6333332	5.2216001	-0.4479222
15.6333332	5.2704000	-0.4540582
15.6333332	5.3192000	-0.4724660
15.6333332	5.3680000	-0.6013203
15.6333332	5.4168000	-0.4663301
15.6333332	5.4656000	-0.4786019
15.6333332	5.5144000	-0.5890489
15.6333332	5.5632000	-0.5890489
15.6333332	5.6120000	613586.0000000
15.6333332	5.6608000	613586.0000000
15.6333332	5.7096000	613586.0000000
15.6333332	5.7584000	613586.0000000
15.6333332	5.8072000	613586.0000000
15.6333332	5.8559999	613586.0000000
15.6333332	5.9047999	613586.0000000
15.6333332	5.9535999	613586.0000000
15.6333332	6.0023999	613586.0000000
15.6499996	0.2928000	-3.4054375
15.6499996	0.3416000	-4.1049328
15.6499996	0.3904000	-4.9087386
15.6499996	0.4392000	-4.4976320
15.6499996	0.4880000	-4.6510296
15.6499996	0.5368000	-3.5281558
15.6499996	0.5856000	-3.5281558
15.6499996	0.6344000	-2.0064468
15.6499996	0.6832000	-2.0248547
15.6499996	0.7320000	-0.9878836
15.6499996	0.7808000	-0.9940195
15.6499996	0.8296000	-0.8835731
15.6499996	0.8784000	-1.2885437
15.6499996	0.9272000	-1.1719613
15.6499996	0.9760000	-1.1658254

15.6499996	1.0247999	-1.0860586
15.6499996	1.0735999	-1.1596894
15.6499996	1.1223999	-1.1106019
15.6499996	1.1711999	-1.2578645
15.6499996	1.2200000	-1.2455926
15.6499996	1.2688000	-1.5033011
15.6499996	1.3176000	-1.5033011
15.6499996	1.3664000	-1.3499031
15.6499996	1.4152000	-1.5217090
15.6499996	1.4640000	-1.6751070
15.6499996	1.5128000	-1.6689711
15.6499996	1.5616000	-1.6689711
15.6499996	1.6104000	-1.4664855
15.6499996	1.6592000	-1.4603496
15.6499996	1.7079999	-0.9510679
15.6499996	1.7567999	-1.1780972
15.6499996	1.8055999	-1.1658254
15.6499996	1.8543999	-1.2210488
15.6499996	1.9031999	-1.2394567
15.6499996	1.9519999	-1.3130875
15.6499996	2.0007999	-1.3253593
15.6499996	2.0495999	-1.3376312
15.6499996	2.0983999	-1.3437672
15.6499996	2.1471999	-1.2762718
15.6499996	2.1959999	-1.2640004
15.6499996	2.2447999	-1.2578645
15.6499996	2.2935998	-1.3069515
15.6499996	2.3423998	-1.2701364
15.6499996	2.3912001	-1.2701364
15.6499996	2.4400001	-1.1167378
15.6499996	2.4888000	-1.1596894
15.6499996	2.5376000	-1.1965051
15.6499996	2.5864000	-1.2455926
15.6499996	2.6352000	-1.2394567
15.6499996	2.6840000	-1.0921946
15.6499996	2.7328000	-1.0921946
15.6499996	2.7816000	-1.0983305
15.6499996	2.8304000	-1.0492430
15.6499996	2.8792000	-1.0492430
15.6499996	2.9280000	-0.9817476
15.6499996	2.9768000	-0.9817476
15.6499996	3.0256000	-0.9817476
15.6499996	3.0743999	-1.0246992
15.6499996	3.1231999	-0.9633398
15.6499996	3.1719999	-0.9694757
15.6499996	3.2207999	-0.9510679
15.6499996	3.2695999	-0.9326606
15.6499996	3.3183999	-0.9081168
15.6499996	3.3671999	-0.9510679
15.6499996	3.4159999	-0.9510679
15.6499996	3.4647999	-0.9387960
15.6499996	3.5135999	-1.0431070
15.6499996	3.5623999	-1.0001554
15.6499996	3.6111999	-1.0001554
15.6499996	3.6599998	-0.9326606
15.6499996	3.7087998	-1.0124273
15.6499996	3.7575998	-1.0246992

15.6499996	3.8063998	-1.0001554
15.6499996	3.8551998	-1.0921946
15.6499996	3.9039998	-0.9633398
15.6499996	3.9527998	-1.0431070
15.6499996	4.0016003	-1.0369711
15.6499996	4.0503998	-1.0124273
15.6499996	4.0991998	-0.9633398
15.6499996	4.1479998	-0.9633398
15.6499996	4.1967998	-0.9694757
15.6499996	4.2455997	-0.9326606
15.6499996	4.2943997	-0.9019809
15.6499996	4.3431997	-1.0553789
15.6499996	4.3919997	-0.8835731
15.6499996	4.4407997	-0.8835731
15.6499996	4.4895997	-1.0860586
15.6499996	4.5383997	-0.9387960
15.6499996	4.5871997	-0.9203887
15.6499996	4.6359997	-0.7301750
15.6499996	4.6847997	-0.7240391
15.6499996	4.7336001	-0.7731261
15.6499996	4.7824001	-0.6135921
15.6499996	4.8312001	-0.6074562
15.6499996	4.8800001	-0.5215535
15.6499996	4.9288001	-0.5154176
15.6499996	4.9776001	-0.7117672
15.6499996	5.0264001	-0.8406215
15.6499996	5.0752001	-0.8590293
15.6499996	5.1240001	-0.8528934
15.6499996	5.1728001	-0.8590293
15.6499996	5.2216001	-0.8835731
15.6499996	5.2704000	-0.7485828
15.6499996	5.3192000	-0.7485828
15.6499996	5.3680000	-0.5829129
15.6499996	5.4168000	-0.5706410
15.6499996	5.4656000	-0.5767770
15.6499996	5.5144000	613586.0000000
15.6499996	5.5632000	613586.0000000
15.6499996	5.6120000	613586.0000000
15.6499996	5.6608000	613586.0000000
15.6499996	5.7096000	613586.0000000
15.6499996	5.7584000	613586.0000000
15.6499996	5.8072000	613586.0000000
15.6499996	5.8559999	613586.0000000
15.6499996	5.9047999	613586.0000000
15.6499996	5.9535999	613586.0000000
15.6499996	6.0023999	613586.0000000
15.6666670	0.2928000	-3.5772433
15.6666670	0.3416000	-3.5772433
15.6666670	0.3904000	-2.8593402
15.6666670	0.4392000	-4.2644668
15.6666670	0.4880000	-4.2399230
15.6666670	0.5368000	-4.4362726
15.6666670	0.5856000	-4.0926609
15.6666670	0.6344000	-3.2397676
15.6666670	0.6832000	-2.3439226
15.6666670	0.7320000	-2.3500586
15.6666670	0.7808000	-1.1474175

15.6666670	0.8296000	-1.3314953
15.6666670	0.8784000	-1.2149129
15.6666670	0.9272000	-1.2149129
15.6666670	0.9760000	-1.2026410
15.6666670	1.0247999	-1.3437672
15.6666670	1.0735999	-1.3499031
15.6666670	1.1223999	-1.2517285
15.6666670	1.1711999	-1.3069515
15.6666670	1.2200000	-1.3008156
15.6666670	1.2688000	-1.1351457
15.6666670	1.3176000	-1.1412816
15.6666670	1.3664000	-1.5953403
15.6666670	1.4152000	-1.5523887
15.6666670	1.4640000	-1.3989906
15.6666670	1.5128000	-1.3989906
15.6666670	1.5616000	-1.3989906
15.6666670	1.6104000	-1.4848933
15.6666670	1.6592000	-0.9510679
15.6666670	1.7079999	-0.9572039
15.6666670	1.7567999	-1.1167378
15.6666670	1.8055999	-0.9203887
15.6666670	1.8543999	-0.8160777
15.6666670	1.9031999	-0.8222136
15.6666670	1.9519999	-0.9756117
15.6666670	2.0007999	-1.0615149
15.6666670	2.0495999	-1.1842332
15.6666670	2.0983999	-1.2455926
15.6666670	2.1471999	-1.2455926
15.6666670	2.1959999	-1.2578645
15.6666670	2.2447999	-1.2701364
15.6666670	2.2935998	-1.1719613
15.6666670	2.3423998	-1.1780972
15.6666670	2.3912001	-1.1719613
15.6666670	2.4400001	-1.1474175
15.6666670	2.4888000	-1.3683109
15.6666670	2.5376000	-1.3683109
15.6666670	2.5864000	-1.3069515
15.6666670	2.6352000	-1.1658254
15.6666670	2.6840000	-1.2701364
15.6666670	2.7328000	-1.2701364
15.6666670	2.7816000	-1.2762718
15.6666670	2.8304000	-1.2149129
15.6666670	2.8792000	-1.2026410
15.6666670	2.9280000	-1.1965051
15.6666670	2.9768000	-1.2149129
15.6666670	3.0256000	-1.0615149
15.6666670	3.0743999	-1.0860586
15.6666670	3.1231999	-1.2333207
15.6666670	3.1719999	-1.1412816
15.6666670	3.2207999	-1.0921946
15.6666670	3.2695999	-1.1167378
15.6666670	3.3183999	-1.1167378
15.6666670	3.3671999	-0.9510679
15.6666670	3.4159999	-0.9633398
15.6666670	3.4647999	-1.0062914
15.6666670	3.5135999	-1.0062914
15.6666670	3.5623999	-1.1167378

15.6666670	3.6111999	-1.0001554
15.6666670	3.6599998	-1.0062914
15.6666670	3.7087998	-1.0431070
15.6666670	3.7575998	-1.1167378
15.6666670	3.8063998	-1.1167378
15.6666670	3.8551998	-1.1167378
15.6666670	3.9039998	-1.1167378
15.6666670	3.9527998	-1.1044660
15.6666670	4.0016003	-1.0185633
15.6666670	4.0503998	-1.0246992
15.6666670	4.0991998	-1.0246992
15.6666670	4.1479998	-1.0124273
15.6666670	4.1967998	-0.9510679
15.6666670	4.2455997	-0.9449320
15.6666670	4.2943997	-1.0062914
15.6666670	4.3431997	-1.0001554
15.6666670	4.3919997	-1.0062914
15.6666670	4.4407997	-0.8958449
15.6666670	4.4895997	-0.8406215
15.6666670	4.5383997	-0.7731261
15.6666670	4.5871997	-0.8467574
15.6666670	4.6359997	-0.8406215
15.6666670	4.6847997	-0.7915339
15.6666670	4.7336001	-0.8897090
15.6666670	4.7824001	-0.6565437
15.6666670	4.8312001	-0.6810875
15.6666670	4.8800001	-0.4970098
15.6666670	4.9288001	-0.4724660
15.6666670	4.9776001	-0.1840777
15.6666670	5.0264001	-0.1840777
15.6666670	5.0752001	-0.1779418
15.6666670	5.1240001	-0.3804274
15.6666670	5.1728001	-0.3804274
15.6666670	5.2216001	-0.6381359
15.6666670	5.2704000	-0.6320000
15.6666670	5.3192000	-0.7301750
15.6666670	5.3680000	-0.7301750
15.6666670	5.4168000	-0.7485828
15.6666670	5.4656000	613586.0000000
15.6666670	5.5144000	613586.0000000
15.6666670	5.5632000	613586.0000000
15.6666670	5.6120000	613586.0000000
15.6666670	5.6608000	613586.0000000
15.6666670	5.7096000	613586.0000000
15.6666670	5.7584000	613586.0000000
15.6666670	5.8072000	613586.0000000
15.6666670	5.8559999	613586.0000000
15.6666670	5.9047999	613586.0000000
15.6666670	5.9535999	613586.0000000
15.6666670	6.0023999	613586.0000000
15.6833334	0.2928000	-4.2399230
15.6833334	0.3416000	-3.1600006
15.6833334	0.3904000	-3.5895152
15.6833334	0.4392000	-3.5772433
15.6833334	0.4880000	-3.4238453
15.6833334	0.5368000	-3.7797287
15.6833334	0.5856000	-4.1172047

15.6833334	0.6344000	-3.6999617
15.6833334	0.6832000	-2.2396121
15.6833334	0.7320000	-2.2396121
15.6833334	0.7808000	-2.0371265
15.6833334	0.8296000	-1.6444273
15.6833334	0.8784000	-1.6260195
15.6833334	0.9272000	-1.6689711
15.6833334	0.9760000	-1.6628351
15.6833334	1.0247999	-1.6382914
15.6833334	1.0735999	-1.3805828
15.6833334	1.1223999	-1.4726214
15.6833334	1.1711999	-1.3744469
15.6833334	1.2200000	-1.3744469
15.6833334	1.2688000	-1.1167378
15.6833334	1.3176000	-1.4787574
15.6833334	1.3664000	-1.4971652
15.6833334	1.4152000	-1.5033011
15.6833334	1.4640000	-1.6505632
15.6833334	1.5128000	-1.3621750
15.6833334	1.5616000	-1.6014762
15.6833334	1.6104000	-0.7301750
15.6833334	1.6592000	-1.1780972
15.6833334	1.7079999	-1.3069515
15.6833334	1.7567999	-1.2026410
15.6833334	1.8055999	-0.8528934
15.6833334	1.8543999	-0.8528934
15.6833334	1.9031999	-0.8774371
15.6833334	1.9519999	-0.9756117
15.6833334	2.0007999	-0.9449320
15.6833334	2.0495999	-0.9449320
15.6833334	2.0983999	-1.0799227
15.6833334	2.1471999	-1.0492430
15.6833334	2.1959999	-1.0799227
15.6833334	2.2447999	-1.0615149
15.6833334	2.2935998	-1.0737867
15.6833334	2.3423998	-1.1535535
15.6833334	2.3912001	-1.1780972
15.6833334	2.4400001	-1.2026410
15.6833334	2.4888000	-0.9387960
15.6833334	2.5376000	-1.2640004
15.6833334	2.5864000	-1.3008156
15.6833334	2.6352000	-1.2333207
15.6833334	2.6840000	-1.0124273
15.6833334	2.7328000	-1.0062914
15.6833334	2.7816000	-0.9940195
15.6833334	2.8304000	-1.0431070
15.6833334	2.8792000	-1.0308352
15.6833334	2.9280000	-0.9694757
15.6833334	2.9768000	-1.0553789
15.6833334	3.0256000	-1.0062914
15.6833334	3.0743999	-0.9142528
15.6833334	3.1231999	-0.9387960
15.6833334	3.1719999	-0.9940195
15.6833334	3.2207999	-0.9940195
15.6833334	3.2695999	-0.9756117
15.6833334	3.3183999	-0.9572039
15.6833334	3.3671999	-0.8651652

15.6833334	3.4159999	-0.8958449
15.6833334	3.4647999	-0.8958449
15.6833334	3.5135999	-0.8835731
15.6833334	3.5623999	-0.9449320
15.6833334	3.6111999	-0.9081168
15.6833334	3.6599998	-1.0369711
15.6833334	3.7087998	-0.9081168
15.6833334	3.7575998	-0.9081168
15.6833334	3.8063998	-0.8897090
15.6833334	3.8551998	-1.0062914
15.6833334	3.9039998	-1.0001554
15.6833334	3.9527998	-1.0001554
15.6833334	4.0016003	-0.9510679
15.6833334	4.0503998	-0.9510679
15.6833334	4.0991998	-0.7547188
15.6833334	4.1479998	-0.8835731
15.6833334	4.1967998	-0.7301750
15.6833334	4.2455997	-0.8222136
15.6833334	4.2943997	-0.8160777
15.6833334	4.3431997	-0.7669902
15.6833334	4.3919997	-0.7117672
15.6833334	4.4407997	-0.9265246
15.6833334	4.4895997	-0.6994953
15.6833334	4.5383997	-0.6810875
15.6833334	4.5871997	-0.6810875
15.6833334	4.6359997	-0.7240391
15.6833334	4.6847997	-0.7363110
15.6833334	4.7336001	-0.8713012
15.6833334	4.7824001	-0.8651652
15.6833334	4.8312001	-0.8528934
15.6833334	4.8800001	-0.8774371
15.6833334	4.9288001	-0.8835731
15.6833334	4.9776001	-0.9265246
15.6833334	5.0264001	-0.6504078
15.6833334	5.0752001	-1.0062914
15.6833334	5.1240001	-0.6381359
15.6833334	5.1728001	-0.4663301
15.6833334	5.2216001	-0.4786019
15.6833334	5.2704000	-0.4356503
15.6833334	5.3192000	-0.4479222
15.6833334	5.3680000	613586.0000000
15.6833334	5.4168000	613586.0000000
15.6833334	5.4656000	613586.0000000
15.6833334	5.5144000	613586.0000000
15.6833334	5.5632000	613586.0000000
15.6833334	5.6120000	613586.0000000
15.6833334	5.6608000	613586.0000000
15.6833334	5.7096000	613586.0000000
15.6833334	5.7584000	613586.0000000
15.6833334	5.8072000	613586.0000000
15.6833334	5.8559999	613586.0000000
15.6833334	5.9047999	613586.0000000
15.6833334	5.9535999	613586.0000000
15.6833334	6.0023999	613586.0000000
15.6999998	0.2928000	-3.7183695
15.6999998	0.3416000	-4.0251656
15.6999998	0.3904000	-3.7920005

15.6999998	0.4392000	-3.7920005
15.6999998	0.4880000	-5.4425640
15.6999998	0.5368000	-4.9087386
15.6999998	0.5856000	-4.9026027
15.6999998	0.6344000	-3.1538646
15.6999998	0.6832000	-2.8286607
15.6999998	0.7320000	-1.5155730
15.6999998	0.7808000	-1.3192234
15.6999998	0.8296000	-1.3314953
15.6999998	0.8784000	-1.5401168
15.6999998	0.9272000	-1.5339808
15.6999998	0.9760000	-1.5339808
15.6999998	1.0247999	-1.5769324
15.6999998	1.0735999	-1.5769324
15.6999998	1.1223999	-1.5401168
15.6999998	1.1711999	-1.5401168
15.6999998	1.2200000	-1.4480777
15.6999998	1.2688000	-1.4603496
15.6999998	1.3176000	-1.2271848
15.6999998	1.3664000	-1.2333207
15.6999998	1.4152000	-1.2394567
15.6999998	1.4640000	-1.6996508
15.6999998	1.5128000	-1.6996508
15.6999998	1.5616000	-1.6873789
15.6999998	1.6104000	-1.5707965
15.6999998	1.6592000	-1.3192234
15.6999998	1.7079999	-1.3130875
15.6999998	1.7567999	-0.9940195
15.6999998	1.8055999	-1.1596894
15.6999998	1.8543999	-1.1412816
15.6999998	1.9031999	-1.0799227
15.6999998	1.9519999	-1.0308352
15.6999998	2.0007999	-1.0553789
15.6999998	2.0495999	-1.0737867
15.6999998	2.0983999	-0.9633398
15.6999998	2.1471999	-0.9633398
15.6999998	2.1959999	-0.9572039
15.6999998	2.2447999	-1.0062914
15.6999998	2.2935998	-1.1228738
15.6999998	2.3423998	-1.1167378
15.6999998	2.3912001	-1.0921946
15.6999998	2.4400001	-1.0860586
15.6999998	2.4888000	-1.1596894
15.6999998	2.5376000	-1.0799227
15.6999998	2.5864000	-1.0799227
15.6999998	2.6352000	-1.0921946
15.6999998	2.6840000	-1.0676508
15.6999998	2.7328000	-1.0615149
15.6999998	2.7816000	-1.0185633
15.6999998	2.8304000	-0.9572039
15.6999998	2.8792000	-0.8774371
15.6999998	2.9280000	-0.8835731
15.6999998	2.9768000	-0.9572039
15.6999998	3.0256000	-0.9449320
15.6999998	3.0743999	-0.9449320
15.6999998	3.1231999	-0.9817476
15.6999998	3.1719999	-0.9081168

15.6999998	3.2207999	-0.9203887
15.6999998	3.2695999	-0.9142528
15.6999998	3.3183999	-0.9265246
15.6999998	3.3671999	-0.8406215
15.6999998	3.4159999	-0.8713012
15.6999998	3.4647999	-0.8038058
15.6999998	3.5135999	-0.9081168
15.6999998	3.5623999	-0.9081168
15.6999998	3.6111999	-0.8160777
15.6999998	3.6599998	-0.9326606
15.6999998	3.7087998	-0.9449320
15.6999998	3.7575998	-0.9449320
15.6999998	3.8063998	-0.9510679
15.6999998	3.8551998	-0.8344855
15.6999998	3.9039998	-0.8406215
15.6999998	3.9527998	-0.9142528
15.6999998	4.0016003	-0.9142528
15.6999998	4.0503998	-0.7240391
15.6999998	4.0991998	-0.7485828
15.6999998	4.1479998	-0.7547188
15.6999998	4.1967998	-0.6626797
15.6999998	4.2455997	-0.7608547
15.6999998	4.2943997	-0.6074562
15.6999998	4.3431997	-0.9326606
15.6999998	4.3919997	-0.5460973
15.6999998	4.4407997	-0.5460973
15.6999998	4.4895997	-0.5460973
15.6999998	4.5383997	-0.5276895
15.6999998	4.5871997	-0.5276895
15.6999998	4.6359997	-0.5399613
15.6999998	4.6847997	-0.5583692
15.6999998	4.7336001	-0.4663301
15.6999998	4.7824001	-0.4049711
15.6999998	4.8312001	-0.2086215
15.6999998	4.8800001	-0.4049711
15.6999998	4.9288001	-0.4233789
15.6999998	4.9776001	-0.5215535
15.6999998	5.0264001	-0.5154176
15.6999998	5.0752001	-0.6749516
15.6999998	5.1240001	-0.4172430
15.6999998	5.1728001	-0.4786019
15.6999998	5.2216001	-0.4970098
15.6999998	5.2704000	-0.6688156
15.6999998	5.3192000	-0.6442719
15.6999998	5.3680000	-0.6442719
15.6999998	5.4168000	-0.4172430
15.6999998	5.4656000	-0.3988352
15.6999998	5.5144000	-0.6442719
15.6999998	5.5632000	-0.4847379
15.6999998	5.6120000	613586.0000000
15.6999998	5.6608000	613586.0000000
15.6999998	5.7096000	-0.8283496
15.6999998	5.7584000	613586.0000000
15.6999998	5.8072000	613586.0000000
15.6999998	5.8559999	613586.0000000
15.6999998	5.9047999	613586.0000000
15.6999998	5.9535999	613586.0000000

15.6999998	6.0023999	613586.0000000
15.7166662	0.2928000	-4.7676125
15.7166662	0.3416000	-4.7553406
15.7166662	0.3904000	-6.1052437
15.7166662	0.4392000	-5.0682726
15.7166662	0.4880000	-5.0682726
15.7166662	0.5368000	-5.3075738
15.7166662	0.5856000	-3.7613211
15.7166662	0.6344000	-2.4727771
15.7166662	0.6832000	-2.4727771
15.7166662	0.7320000	-1.5401168
15.7166662	0.7808000	-1.5339808
15.7166662	0.8296000	-1.0799227
15.7166662	0.8784000	-1.0921946
15.7166662	0.9272000	-1.9021363
15.7166662	0.9760000	-2.0309906
15.7166662	1.0247999	-2.0616703
15.7166662	1.0735999	-2.1414371
15.7166662	1.1223999	-2.0003109
15.7166662	1.1711999	-1.6321554
15.7166662	1.2200000	-1.6382914
15.7166662	1.2688000	-1.3314953
15.7166662	1.3176000	-1.3253593
15.7166662	1.3664000	-1.3192234
15.7166662	1.4152000	-1.3805828
15.7166662	1.4640000	-1.5523887
15.7166662	1.5128000	-1.5339808
15.7166662	1.5616000	-1.2640004
15.7166662	1.6104000	-1.2271848
15.7166662	1.6592000	-1.2210488
15.7166662	1.7079999	-0.6872234
15.7166662	1.7567999	-1.0615149
15.7166662	1.8055999	-1.0615149
15.7166662	1.8543999	-1.4480777
15.7166662	1.9031999	-1.5585246
15.7166662	1.9519999	-1.3253593
15.7166662	2.0007999	-1.1167378
15.7166662	2.0495999	-1.0553789
15.7166662	2.0983999	-0.9940195
15.7166662	2.1471999	-0.9878836
15.7166662	2.1959999	-1.0062914
15.7166662	2.2447999	-1.0246992
15.7166662	2.2935998	-1.0737867
15.7166662	2.3423998	-1.0246992
15.7166662	2.3912001	-0.9756117
15.7166662	2.4400001	-0.9940195
15.7166662	2.4888000	-0.8774371
15.7166662	2.5376000	-0.8958449
15.7166662	2.5864000	-1.0308352
15.7166662	2.6352000	-0.9081168
15.7166662	2.6840000	-0.9019809
15.7166662	2.7328000	-0.8958449
15.7166662	2.7816000	-0.9019809
15.7166662	2.8304000	-0.8344855
15.7166662	2.8792000	-0.8344855
15.7166662	2.9280000	-0.7792621
15.7166662	2.9768000	-0.9203887

15.7166662	3.0256000	-0.9265246
15.7166662	3.0743999	-0.8528934
15.7166662	3.1231999	-0.9265246
15.7166662	3.1719999	-0.8651652
15.7166662	3.2207999	-0.8774371
15.7166662	3.2695999	-0.8835731
15.7166662	3.3183999	-0.9387960
15.7166662	3.3671999	-0.9387960
15.7166662	3.4159999	-0.9081168
15.7166662	3.4647999	-0.9081168
15.7166662	3.5135999	-0.9081168
15.7166662	3.5623999	-0.8467574
15.7166662	3.6111999	-0.9633398
15.7166662	3.6599998	-0.8958449
15.7166662	3.7087998	-0.8713012
15.7166662	3.7575998	-0.8713012
15.7166662	3.8063998	-0.8590293
15.7166662	3.8551998	-0.8528934
15.7166662	3.9039998	-0.8590293
15.7166662	3.9527998	-0.8590293
15.7166662	4.0016003	-0.8713012
15.7166662	4.0503998	-0.9265246
15.7166662	4.0991998	-0.8160777
15.7166662	4.1479998	-0.9878836
15.7166662	4.1967998	-0.9940195
15.7166662	4.2455997	-0.8406215
15.7166662	4.2943997	-0.7485828
15.7166662	4.3431997	-0.7792621
15.7166662	4.3919997	-0.8038058
15.7166662	4.4407997	-0.6504078
15.7166662	4.4895997	-0.6504078
15.7166662	4.5383997	-0.6565437
15.7166662	4.5871997	-0.4663301
15.7166662	4.6359997	-0.4663301
15.7166662	4.6847997	-0.7424469
15.7166662	4.7336001	-0.3436117
15.7166662	4.7824001	-0.4847379
15.7166662	4.8312001	-0.3988352
15.7166662	4.8800001	-0.3926992
15.7166662	4.9288001	-0.6565437
15.7166662	4.9776001	-0.7915339
15.7166662	5.0264001	-0.7853980
15.7166662	5.0752001	-0.7179031
15.7166662	5.1240001	-0.6320000
15.7166662	5.1728001	-0.7301750
15.7166662	5.2216001	-0.8897090
15.7166662	5.2704000	-0.8160777
15.7166662	5.3192000	-0.8283496
15.7166662	5.3680000	-0.8713012
15.7166662	5.4168000	-0.8713012
15.7166662	5.4656000	613586.0000000
15.7166662	5.5144000	-0.6504078
15.7166662	5.5632000	613586.0000000
15.7166662	5.6120000	613586.0000000
15.7166662	5.6608000	-0.8344855
15.7166662	5.7096000	613586.0000000
15.7166662	5.7584000	613586.0000000

15.7166662	5.8072000	613586.0000000
15.7166662	5.8559999	613586.0000000
15.7166662	5.9047999	613586.0000000
15.7166662	5.9535999	613586.0000000
15.7166662	6.0023999	613586.0000000
15.7333336	0.2928000	-4.6264863
15.7333336	0.3416000	-4.6203504
15.7333336	0.3904000	-4.8167000
15.7333336	0.4392000	-4.7123890
15.7333336	0.4880000	-4.4240007
15.7333336	0.5368000	-3.7797287
15.7333336	0.5856000	-3.5833793
15.7333336	0.6344000	-2.7611654
15.7333336	0.6832000	-2.8838840
15.7333336	0.7320000	-1.1535535
15.7333336	0.7808000	-1.1474175
15.7333336	0.8296000	-1.6996508
15.7333336	0.8784000	-1.7364664
15.7333336	0.9272000	-1.9757671
15.7333336	0.9760000	-1.9573593
15.7333336	1.0247999	-1.8714566
15.7333336	1.0735999	-2.0003109
15.7333336	1.1223999	-2.0003109
15.7333336	1.1711999	-1.8407769
15.7333336	1.2200000	-1.8530488
15.7333336	1.2688000	-1.7241945
15.7333336	1.3176000	-1.0676508
15.7333336	1.3664000	-1.0737867
15.7333336	1.4152000	-0.9878836
15.7333336	1.4640000	-0.9940195
15.7333336	1.5128000	-1.0737867
15.7333336	1.5616000	-0.6504078
15.7333336	1.6104000	-0.6504078
15.7333336	1.6592000	-0.9694757
15.7333336	1.7079999	-0.9694757
15.7333336	1.7567999	-0.5399613
15.7333336	1.8055999	-0.7363110
15.7333336	1.8543999	-1.0676508
15.7333336	1.9031999	-1.1596894
15.7333336	1.9519999	-1.1596894
15.7333336	2.0007999	-1.2824078
15.7333336	2.0495999	-1.2824078
15.7333336	2.0983999	-1.0246992
15.7333336	2.1471999	-1.1351457
15.7333336	2.1959999	-0.9817476
15.7333336	2.2447999	-0.9756117
15.7333336	2.2935998	-1.0615149
15.7333336	2.3423998	-1.0185633
15.7333336	2.3912001	-0.9694757
15.7333336	2.4400001	-0.9572039
15.7333336	2.4888000	-0.9265246
15.7333336	2.5376000	-0.9265246
15.7333336	2.5864000	-0.9203887
15.7333336	2.6352000	-0.6994953
15.7333336	2.6840000	-0.7056313
15.7333336	2.7328000	-0.7056313
15.7333336	2.7816000	-0.8774371

15.7333336	2.8304000	-0.8897090
15.7333336	2.8792000	-0.7547188
15.7333336	2.9280000	-0.7547188
15.7333336	2.9768000	-0.7547188
15.7333336	3.0256000	-0.8344855
15.7333336	3.0743999	-0.7853980
15.7333336	3.1231999	-0.8713012
15.7333336	3.1719999	-0.8590293
15.7333336	3.2207999	-0.8099418
15.7333336	3.2695999	-0.8099418
15.7333336	3.3183999	-0.7976699
15.7333336	3.3671999	-0.7547188
15.7333336	3.4159999	-0.7731261
15.7333336	3.4647999	-0.7363110
15.7333336	3.5135999	-0.8099418
15.7333336	3.5623999	-0.7976699
15.7333336	3.6111999	-0.7976699
15.7333336	3.6599998	-0.8713012
15.7333336	3.7087998	-0.8038058
15.7333336	3.7575998	-0.7669902
15.7333336	3.8063998	-0.7117672
15.7333336	3.8551998	-0.7117672
15.7333336	3.9039998	-0.7301750
15.7333336	3.9527998	-0.7792621
15.7333336	4.0016003	-0.7179031
15.7333336	4.0503998	-0.7179031
15.7333336	4.0991998	-0.8222136
15.7333336	4.1479998	-0.6013203
15.7333336	4.1967998	-0.6074562
15.7333336	4.2455997	-0.6013203
15.7333336	4.2943997	-0.5951843
15.7333336	4.3431997	-0.4540582
15.7333336	4.3919997	-0.3129320
15.7333336	4.4407997	-0.3558836
15.7333336	4.4895997	-0.6688156
15.7333336	4.5383997	-0.6688156
15.7333336	4.5871997	-0.3558836
15.7333336	4.6359997	-0.5092816
15.7333336	4.6847997	-0.5706410
15.7333336	4.7336001	-0.5338254
15.7333336	4.7824001	-0.5215535
15.7333336	4.8312001	-0.3374758
15.7333336	4.8800001	-0.3804274
15.7333336	4.9288001	-0.4908738
15.7333336	4.9776001	-0.4908738
15.7333336	5.0264001	-0.4540582
15.7333336	5.0752001	-0.6197281
15.7333336	5.1240001	-0.6197281
15.7333336	5.1728001	-0.6197281
15.7333336	5.2216001	-0.7424469
15.7333336	5.2704000	-0.6074562
15.7333336	5.3192000	-0.6013203
15.7333336	5.3680000	-0.6749516
15.7333336	5.4168000	-0.6565437
15.7333336	5.4656000	-0.6565437
15.7333336	5.5144000	-0.4172430
15.7333336	5.5632000	613586.0000000

15.7333336	5.6120000	613586.0000000
15.7333336	5.6608000	613586.0000000
15.7333336	5.7096000	613586.0000000
15.7333336	5.7584000	613586.0000000
15.7333336	5.8072000	613586.0000000
15.7333336	5.8559999	613586.0000000
15.7333336	5.9047999	613586.0000000
15.7333336	5.9535999	613586.0000000
15.7333336	6.0023999	613586.0000000
15.7500000	0.2928000	-3.8472240
15.7500000	0.3416000	-4.7430687
15.7500000	0.3904000	-4.8964667
15.7500000	0.4392000	-3.8656316
15.7500000	0.4880000	-4.0251656
15.7500000	0.5368000	-4.0251656
15.7500000	0.5856000	-3.8410881
15.7500000	0.6344000	-2.2764277
15.7500000	0.6832000	-1.5033011
15.7500000	0.7320000	-1.4112625
15.7500000	0.7808000	-1.4296703
15.7500000	0.8296000	-1.4296703
15.7500000	0.8784000	-0.6994953
15.7500000	0.9272000	-1.3805828
15.7500000	0.9760000	-1.3069515
15.7500000	1.0247999	-1.1351457
15.7500000	1.0735999	-1.1474175
15.7500000	1.1223999	-1.1351457
15.7500000	1.1711999	-1.1290097
15.7500000	1.2200000	-0.9203887
15.7500000	1.2688000	-0.9019809
15.7500000	1.3176000	-1.1780972
15.7500000	1.3664000	-0.7976699
15.7500000	1.4152000	-0.8038058
15.7500000	1.4640000	-0.8774371
15.7500000	1.5128000	-1.1167378
15.7500000	1.5616000	-1.1228738
15.7500000	1.6104000	-1.1228738
15.7500000	1.6592000	-1.1474175
15.7500000	1.7079999	-0.9019809
15.7500000	1.7567999	-1.0860586
15.7500000	1.8055999	-1.0799227
15.7500000	1.8543999	-0.8099418
15.7500000	1.9031999	-1.1044660
15.7500000	1.9519999	-1.0983305
15.7500000	2.0007999	-1.0062914
15.7500000	2.0495999	-0.8467574
15.7500000	2.0983999	-1.0246992
15.7500000	2.1471999	-1.0308352
15.7500000	2.1959999	-1.0492430
15.7500000	2.2447999	-1.0799227
15.7500000	2.2935998	-1.0185633
15.7500000	2.3423998	-1.0185633
15.7500000	2.3912001	-1.0246992
15.7500000	2.4400001	-1.0124273
15.7500000	2.4888000	-1.0001554
15.7500000	2.5376000	-0.9878836
15.7500000	2.5864000	-0.9326606

15.7500000	2.6352000	-0.8467574
15.7500000	2.6840000	-0.8528934
15.7500000	2.7328000	-0.9326606
15.7500000	2.7816000	-0.6381359
15.7500000	2.8304000	-0.6933594
15.7500000	2.8792000	-0.6933594
15.7500000	2.9280000	-0.7547188
15.7500000	2.9768000	-0.7485828
15.7500000	3.0256000	-0.8528934
15.7500000	3.0743999	-0.7485828
15.7500000	3.1231999	-0.8344855
15.7500000	3.1719999	-0.7792621
15.7500000	3.2207999	-0.7731261
15.7500000	3.2695999	-0.7731261
15.7500000	3.3183999	-0.6442719
15.7500000	3.3671999	-0.6688156
15.7500000	3.4159999	-0.6872234
15.7500000	3.4647999	-0.6749516
15.7500000	3.5135999	-0.7117672
15.7500000	3.5623999	-0.7485828
15.7500000	3.6111999	-0.7608547
15.7500000	3.6599998	-0.7363110
15.7500000	3.7087998	-0.6258640
15.7500000	3.7575998	-0.7301750
15.7500000	3.8063998	-0.6810875
15.7500000	3.8551998	-0.6810875
15.7500000	3.9039998	-0.4172430
15.7500000	3.9527998	-0.4172430
15.7500000	4.0016003	-0.4049711
15.7500000	4.0503998	-0.5276895
15.7500000	4.0991998	-0.5276895
15.7500000	4.1479998	-0.5399613
15.7500000	4.1967998	-0.5583692
15.7500000	4.2455997	-0.5583692
15.7500000	4.2943997	-0.4908738
15.7500000	4.3431997	-0.4847379
15.7500000	4.3919997	-0.4663301
15.7500000	4.4407997	-0.8528934
15.7500000	4.4895997	-0.8467574
15.7500000	4.5383997	-0.7301750
15.7500000	4.5871997	-0.7117672
15.7500000	4.6359997	-0.6994953
15.7500000	4.6847997	-0.5092816
15.7500000	4.7336001	-0.5092816
15.7500000	4.7824001	-0.3681555
15.7500000	4.8312001	-0.3497477
15.7500000	4.8800001	-0.6258640
15.7500000	4.9288001	-0.4540582
15.7500000	4.9776001	-0.4479222
15.7500000	5.0264001	-0.4417863
15.7500000	5.0752001	-0.4479222
15.7500000	5.1240001	-0.5092816
15.7500000	5.1728001	-0.5092816
15.7500000	5.2216001	-0.4540582
15.7500000	5.2704000	-0.5215535
15.7500000	5.3192000	-0.4786019
15.7500000	5.3680000	-0.4786019

15.7500000	5.4168000	-0.3558836
15.7500000	5.4656000	-0.4908738
15.7500000	5.5144000	613586.0000000
15.7500000	5.5632000	613586.0000000
15.7500000	5.6120000	613586.0000000
15.7500000	5.6608000	613586.0000000
15.7500000	5.7096000	613586.0000000
15.7500000	5.7584000	613586.0000000
15.7500000	5.8072000	613586.0000000
15.7500000	5.8559999	613586.0000000
15.7500000	5.9047999	613586.0000000
15.7500000	5.9535999	613586.0000000
15.7500000	6.0023999	613586.0000000
15.7666664	0.2928000	-3.6631463
15.7666664	0.3416000	-3.6570103
15.7666664	0.3904000	-3.6447384
15.7666664	0.4392000	-3.6447384
15.7666664	0.4880000	-3.3808937
15.7666664	0.5368000	-3.3870296
15.7666664	0.5856000	-3.3011267
15.7666664	0.6344000	-3.6263306
15.7666664	0.6832000	-2.8102529
15.7666664	0.7320000	-1.1965051
15.7666664	0.7808000	-1.1965051
15.7666664	0.8296000	-0.4724660
15.7666664	0.8784000	-0.4724660
15.7666664	0.9272000	-0.3804274
15.7666664	0.9760000	-0.4417863
15.7666664	1.0247999	-0.3558836
15.7666664	1.0735999	-0.6013203
15.7666664	1.1223999	-0.8160777
15.7666664	1.1711999	-0.8958449
15.7666664	1.2200000	-1.6873789
15.7666664	1.2688000	-1.6751070
15.7666664	1.3176000	-1.6751070
15.7666664	1.3664000	-1.6689711
15.7666664	1.4152000	-1.5217090
15.7666664	1.4640000	-1.3499031
15.7666664	1.5128000	-1.3560390
15.7666664	1.5616000	-1.1903691
15.7666664	1.6104000	-1.2026410
15.7666664	1.6592000	-1.2087770
15.7666664	1.7079999	-0.7547188
15.7666664	1.7567999	-0.7547188
15.7666664	1.8055999	-0.8590293
15.7666664	1.8543999	-0.8160777
15.7666664	1.9031999	-0.8467574
15.7666664	1.9519999	-0.8467574
15.7666664	2.0007999	-1.0676508
15.7666664	2.0495999	-0.8406215
15.7666664	2.0983999	-0.8467574
15.7666664	2.1471999	-1.0492430
15.7666664	2.1959999	-1.0185633
15.7666664	2.2447999	-0.9940195
15.7666664	2.2935998	-0.9694757
15.7666664	2.3423998	-0.9572039
15.7666664	2.3912001	-0.9572039

15.7666664	2.4400001	-0.9572039
15.7666664	2.4888000	-0.9510679
15.7666664	2.5376000	-0.9756117
15.7666664	2.5864000	-0.8835731
15.7666664	2.6352000	-0.8344855
15.7666664	2.6840000	-0.8344855
15.7666664	2.7328000	-0.8835731
15.7666664	2.7816000	-0.8835731
15.7666664	2.8304000	-0.6872234
15.7666664	2.8792000	-0.6933594
15.7666664	2.9280000	-0.7547188
15.7666664	2.9768000	-0.6320000
15.7666664	3.0256000	-0.6320000
15.7666664	3.0743999	-0.5706410
15.7666664	3.1231999	-0.5706410
15.7666664	3.1719999	-0.5645051
15.7666664	3.2207999	-0.5767770
15.7666664	3.2695999	-0.7056313
15.7666664	3.3183999	-0.5583692
15.7666664	3.3671999	-0.5951843
15.7666664	3.4159999	-0.7117672
15.7666664	3.4647999	-0.6135921
15.7666664	3.5135999	-0.6013203
15.7666664	3.5623999	-0.5890489
15.7666664	3.6111999	-0.5829129
15.7666664	3.6599998	-0.6320000
15.7666664	3.7087998	-0.5338254
15.7666664	3.7575998	-0.5338254
15.7666664	3.8063998	-0.5338254
15.7666664	3.8551998	-0.5338254
15.7666664	3.9039998	-0.5522332
15.7666664	3.9527998	-0.3742914
15.7666664	4.0016003	-0.4601941
15.7666664	4.0503998	-0.4172430
15.7666664	4.0991998	-0.4049711
15.7666664	4.1479998	-0.3988352
15.7666664	4.1967998	-0.4049711
15.7666664	4.2455997	-0.4417863
15.7666664	4.2943997	-0.5338254
15.7666664	4.3431997	-0.6320000
15.7666664	4.3919997	-0.4663301
15.7666664	4.4407997	-0.6258640
15.7666664	4.4895997	-0.6994953
15.7666664	4.5383997	-0.4295144
15.7666664	4.5871997	-0.4970098
15.7666664	4.6359997	-0.4908738
15.7666664	4.6847997	-0.4724660
15.7666664	4.7336001	-0.4724660
15.7666664	4.7824001	-0.4724660
15.7666664	4.8312001	-0.4540582
15.7666664	4.8800001	-0.3926992
15.7666664	4.9288001	-0.3926992
15.7666664	4.9776001	-0.4111071
15.7666664	5.0264001	-0.6013203
15.7666664	5.0752001	-0.5890489
15.7666664	5.1240001	-0.5890489
15.7666664	5.1728001	-0.6258640

15.7666664	5.2216001	-0.6258640
15.7666664	5.2704000	-0.6258640
15.7666664	5.3192000	-0.5460973
15.7666664	5.3680000	-0.5338254
15.7666664	5.4168000	-0.5215535
15.7666664	5.4656000	-0.5276895
15.7666664	5.5144000	613586.0000000
15.7666664	5.5632000	613586.0000000
15.7666664	5.6120000	613586.0000000
15.7666664	5.6608000	613586.0000000
15.7666664	5.7096000	613586.0000000
15.7666664	5.7584000	613586.0000000
15.7666664	5.8072000	613586.0000000
15.7666664	5.8559999	613586.0000000
15.7666664	5.9047999	613586.0000000
15.7666664	5.9535999	-0.5399613
15.7666664	6.0023999	613586.0000000
15.7833338	0.2928000	-4.0926609
15.7833338	0.3416000	-4.1294765
15.7833338	0.3904000	-4.1356125
15.7833338	0.4392000	-2.8102529
15.7833338	0.4880000	-3.1600006
15.7833338	0.5368000	-3.5711074
15.7833338	0.5856000	-2.6507189
15.7833338	0.6344000	-2.7182140
15.7833338	0.6832000	-2.1414371
15.7833338	0.7320000	-1.8100972
15.7833338	0.7808000	-0.9756117
15.7833338	0.8296000	-0.9756117
15.7833338	0.8784000	-0.8160777
15.7833338	0.9272000	-0.8099418
15.7833338	0.9760000	-0.8099418
15.7833338	1.0247999	-0.8099418
15.7833338	1.0735999	-0.8160777
15.7833338	1.1223999	-0.9081168
15.7833338	1.1711999	-0.9019809
15.7833338	1.2200000	-1.2517285
15.7833338	1.2688000	-1.2455926
15.7833338	1.3176000	-1.7426023
15.7833338	1.3664000	-1.6014762
15.7833338	1.4152000	-1.9328160
15.7833338	1.4640000	-1.5646605
15.7833338	1.5128000	-1.5646605
15.7833338	1.5616000	-1.4910293
15.7833338	1.6104000	-1.4910293
15.7833338	1.6592000	-1.7916894
15.7833338	1.7079999	-1.5033011
15.7833338	1.7567999	-1.3130875
15.7833338	1.8055999	-1.3130875
15.7833338	1.8543999	-1.7119226
15.7833338	1.9031999	-1.6812429
15.7833338	1.9519999	-1.4603496
15.7833338	2.0007999	-1.4542136
15.7833338	2.0495999	-0.9510679
15.7833338	2.0983999	-0.9572039
15.7833338	2.1471999	-0.9633398
15.7833338	2.1959999	-0.8713012

15.7833338	2.2447999	-0.8713012
15.7833338	2.2935998	-0.8835731
15.7833338	2.3423998	-1.0246992
15.7833338	2.3912001	-1.0246992
15.7833338	2.4400001	-1.0185633
15.7833338	2.4888000	-0.8283496
15.7833338	2.5376000	-1.0921946
15.7833338	2.5864000	-0.8344855
15.7833338	2.6352000	-0.8835731
15.7833338	2.6840000	-0.8651652
15.7833338	2.7328000	-0.7608547
15.7833338	2.7816000	-0.9019809
15.7833338	2.8304000	-0.8283496
15.7833338	2.8792000	-0.8897090
15.7833338	2.9280000	-0.8835731
15.7833338	2.9768000	-0.8958449
15.7833338	3.0256000	-0.9019809
15.7833338	3.0743999	-0.9081168
15.7833338	3.1231999	-0.9142528
15.7833338	3.1719999	-0.9081168
15.7833338	3.2207999	-0.7363110
15.7833338	3.2695999	-0.7669902
15.7833338	3.3183999	-0.6688156
15.7833338	3.3671999	-0.6626797
15.7833338	3.4159999	-0.6688156
15.7833338	3.4647999	-0.6074562
15.7833338	3.5135999	-0.6013203
15.7833338	3.5623999	-0.6442719
15.7833338	3.6111999	-0.5890489
15.7833338	3.6599998	-0.5829129
15.7833338	3.7087998	-0.5399613
15.7833338	3.7575998	-0.5460973
15.7833338	3.8063998	-0.5031457
15.7833338	3.8551998	-0.5460973
15.7833338	3.9039998	-0.5890489
15.7833338	3.9527998	-0.3620195
15.7833338	4.0016003	-0.3436117
15.7833338	4.0503998	-0.3374758
15.7833338	4.0991998	-0.3865633
15.7833338	4.1479998	-0.3988352
15.7833338	4.1967998	-0.3067961
15.7833338	4.2455997	-0.3497477
15.7833338	4.2943997	-0.3865633
15.7833338	4.3431997	-0.4172430
15.7833338	4.3919997	-0.1963496
15.7833338	4.4407997	-0.5890489
15.7833338	4.4895997	-0.5767770
15.7833338	4.5383997	-0.5890489
15.7833338	4.5871997	-0.6933594
15.7833338	4.6359997	-0.7547188
15.7833338	4.6847997	-0.4663301
15.7833338	4.7336001	-0.4847379
15.7833338	4.7824001	-0.3926992
15.7833338	4.8312001	-0.3988352
15.7833338	4.8800001	-0.4417863
15.7833338	4.9288001	-0.4479222
15.7833338	4.9776001	-0.4540582

15.7833338	5.0264001	-0.4908738
15.7833338	5.0752001	-0.5338254
15.7833338	5.1240001	-0.5276895
15.7833338	5.1728001	-0.5276895
15.7833338	5.2216001	-0.6381359
15.7833338	5.2704000	-0.6381359
15.7833338	5.3192000	-0.5890489
15.7833338	5.3680000	-0.5767770
15.7833338	5.4168000	-0.5951843
15.7833338	5.4656000	613586.0000000
15.7833338	5.5144000	-0.5645051
15.7833338	5.5632000	613586.0000000
15.7833338	5.6120000	613586.0000000
15.7833338	5.6608000	613586.0000000
15.7833338	5.7096000	613586.0000000
15.7833338	5.7584000	613586.0000000
15.7833338	5.8072000	613586.0000000
15.7833338	5.8559999	613586.0000000
15.7833338	5.9047999	613586.0000000
15.7833338	5.9535999	613586.0000000
15.7833338	6.0023999	613586.0000000
15.8000002	0.2928000	-2.5525441
15.8000002	0.3416000	-3.6938257
15.8000002	0.3904000	-2.9206996
15.8000002	0.4392000	-3.1354568
15.8000002	0.4880000	-2.7243500
15.8000002	0.5368000	-3.1047771
15.8000002	0.5856000	-2.7918451
15.8000002	0.6344000	-2.9881947
15.8000002	0.6832000	-1.6382914
15.8000002	0.7320000	-1.1106019
15.8000002	0.7808000	-1.3744469
15.8000002	0.8296000	-1.3621750
15.8000002	0.8784000	-1.3560390
15.8000002	0.9272000	-1.3989906
15.8000002	0.9760000	-1.4112625
15.8000002	1.0247999	-1.3928547
15.8000002	1.0735999	-1.4358063
15.8000002	1.1223999	-1.4542136
15.8000002	1.1711999	-1.4112625
15.8000002	1.2200000	-1.4112625
15.8000002	1.2688000	-1.6321554
15.8000002	1.3176000	-1.6198835
15.8000002	1.3664000	-1.6321554
15.8000002	1.4152000	-1.6505632
15.8000002	1.4640000	-1.6566992
15.8000002	1.5128000	-1.6566992
15.8000002	1.5616000	-1.6566992
15.8000002	1.6104000	-1.3499031
15.8000002	1.6592000	-1.3621750
15.8000002	1.7079999	-1.3621750
15.8000002	1.7567999	-1.0246992
15.8000002	1.8055999	-1.3989906
15.8000002	1.8543999	-1.3437672
15.8000002	1.9031999	-1.5953403
15.8000002	1.9519999	-1.6076121
15.8000002	2.0007999	-1.3437672

15.8000002	2.0495999	-1.3437672
15.8000002	2.0983999	-1.3376312
15.8000002	2.1471999	-1.3376312
15.8000002	2.1959999	-1.1167378
15.8000002	2.2447999	-1.0369711
15.8000002	2.2935998	-1.0185633
15.8000002	2.3423998	-1.0308352
15.8000002	2.3912001	-1.0431070
15.8000002	2.4400001	-0.9449320
15.8000002	2.4888000	-1.0246992
15.8000002	2.5376000	-0.8590293
15.8000002	2.5864000	-0.8590293
15.8000002	2.6352000	-0.8590293
15.8000002	2.6840000	-0.9633398
15.8000002	2.7328000	-0.9694757
15.8000002	2.7816000	-0.9694757
15.8000002	2.8304000	-1.0001554
15.8000002	2.8792000	-0.8651652
15.8000002	2.9280000	-0.9326606
15.8000002	2.9768000	-0.9265246
15.8000002	3.0256000	-0.9203887
15.8000002	3.0743999	-0.9817476
15.8000002	3.1231999	-0.8713012
15.8000002	3.1719999	-0.9142528
15.8000002	3.2207999	-0.8713012
15.8000002	3.2695999	-0.8713012
15.8000002	3.3183999	-0.6933594
15.8000002	3.3671999	-0.6933594
15.8000002	3.4159999	-0.7915339
15.8000002	3.4647999	-0.7731261
15.8000002	3.5135999	-0.7731261
15.8000002	3.5623999	-0.7731261
15.8000002	3.6111999	-0.6013203
15.8000002	3.6599998	-0.6688156
15.8000002	3.7087998	-0.6688156
15.8000002	3.7575998	-0.6197281
15.8000002	3.8063998	-0.5154176
15.8000002	3.8551998	-0.5215535
15.8000002	3.9039998	-0.3988352
15.8000002	3.9527998	-0.4049711
15.8000002	4.0016003	-0.4601941
15.8000002	4.0503998	-0.4847379
15.8000002	4.0991998	-0.4847379
15.8000002	4.1479998	-0.4970098
15.8000002	4.1967998	-0.5829129
15.8000002	4.2455997	-0.5890489
15.8000002	4.2943997	-0.6381359
15.8000002	4.3431997	-0.5276895
15.8000002	4.3919997	-0.4786019
15.8000002	4.4407997	-0.3804274
15.8000002	4.4895997	-0.5522332
15.8000002	4.5383997	-0.5522332
15.8000002	4.5871997	-0.5583692
15.8000002	4.6359997	-0.5215535
15.8000002	4.6847997	-0.3436117
15.8000002	4.7336001	-0.3252039
15.8000002	4.7824001	-0.4233789

15.8000002	4.8312001	-0.4111071
15.8000002	4.8800001	-0.3252039
15.8000002	4.9288001	-0.5951843
15.8000002	4.9776001	-0.5951843
15.8000002	5.0264001	-0.6197281
15.8000002	5.0752001	-0.6135921
15.8000002	5.1240001	-0.4724660
15.8000002	5.1728001	-0.4724660
15.8000002	5.2216001	-0.3313398
15.8000002	5.2704000	-0.4172430
15.8000002	5.3192000	-0.4233789
15.8000002	5.3680000	-0.4233789
15.8000002	5.4168000	-0.4601941
15.8000002	5.4656000	-0.3988352
15.8000002	5.5144000	-0.4049711
15.8000002	5.5632000	-0.4786019
15.8000002	5.6120000	613586.0000000
15.8000002	5.6608000	613586.0000000
15.8000002	5.7096000	613586.0000000
15.8000002	5.7584000	613586.0000000
15.8000002	5.8072000	613586.0000000
15.8000002	5.8559999	613586.0000000
15.8000002	5.9047999	613586.0000000
15.8000002	5.9535999	613586.0000000
15.8000002	6.0023999	613586.0000000
15.8166666	0.2928000	-2.0187187
15.8166666	0.3416000	-2.0187187
15.8166666	0.3904000	-3.8594959
15.8166666	0.4392000	-3.3808937
15.8166666	0.4880000	-3.3318064
15.8166666	0.5368000	-3.3195345
15.8166666	0.5856000	-3.3318064
15.8166666	0.6344000	-2.8593402
15.8166666	0.6832000	-2.7059422
15.8166666	0.7320000	-1.7487383
15.8166666	0.7808000	-1.2946796
15.8166666	0.8296000	-1.2762718
15.8166666	0.8784000	-1.3008156
15.8166666	0.9272000	-1.2455926
15.8166666	0.9760000	-1.3989906
15.8166666	1.0247999	-1.4235344
15.8166666	1.0735999	-1.4480777
15.8166666	1.1223999	-1.4664855
15.8166666	1.1711999	-1.4664855
15.8166666	1.2200000	-1.1290097
15.8166666	1.2688000	-1.0983305
15.8166666	1.3176000	-1.0860586
15.8166666	1.3664000	-1.4051266
15.8166666	1.4152000	-1.3683109
15.8166666	1.4640000	-1.3560390
15.8166666	1.5128000	-1.7916894
15.8166666	1.5616000	-1.7916894
15.8166666	1.6104000	-1.5401168
15.8166666	1.6592000	-1.0983305
15.8166666	1.7079999	-1.4480777
15.8166666	1.7567999	-1.3621750
15.8166666	1.8055999	-1.1535535

15.8166666	1.8543999	-1.1474175
15.8166666	1.9031999	-1.1474175
15.8166666	1.9519999	-1.3621750
15.8166666	2.0007999	-1.4848933
15.8166666	2.0495999	-1.4787574
15.8166666	2.0983999	-1.4603496
15.8166666	2.1471999	-1.4542136
15.8166666	2.1959999	-1.4603496
15.8166666	2.2447999	-1.4173985
15.8166666	2.2935998	-1.1228738
15.8166666	2.3423998	-1.1228738
15.8166666	2.3912001	-0.9878836
15.8166666	2.4400001	-0.9756117
15.8166666	2.4888000	-1.0431070
15.8166666	2.5376000	-1.0983305
15.8166666	2.5864000	-1.0431070
15.8166666	2.6352000	-1.0431070
15.8166666	2.6840000	-0.9510679
15.8166666	2.7328000	-1.0615149
15.8166666	2.7816000	-1.0431070
15.8166666	2.8304000	-0.9203887
15.8166666	2.8792000	-0.8774371
15.8166666	2.9280000	-0.8774371
15.8166666	2.9768000	-0.9510679
15.8166666	3.0256000	-0.9817476
15.8166666	3.0743999	-0.9572039
15.8166666	3.1231999	-0.9572039
15.8166666	3.1719999	-0.8160777
15.8166666	3.2207999	-0.8160777
15.8166666	3.2695999	-0.8160777
15.8166666	3.3183999	-0.8958449
15.8166666	3.3671999	-0.7485828
15.8166666	3.4159999	-0.7547188
15.8166666	3.4647999	-0.7915339
15.8166666	3.5135999	-0.6565437
15.8166666	3.5623999	-0.7056313
15.8166666	3.6111999	-0.6933594
15.8166666	3.6599998	-0.6565437
15.8166666	3.7087998	-0.6626797
15.8166666	3.7575998	-0.6504078
15.8166666	3.8063998	-0.4970098
15.8166666	3.8551998	-0.6320000
15.8166666	3.9039998	-0.6381359
15.8166666	3.9527998	-0.4908738
15.8166666	4.0016003	-0.4908738
15.8166666	4.0503998	-0.4847379
15.8166666	4.0991998	-0.4786019
15.8166666	4.1479998	-0.4847379
15.8166666	4.1967998	-0.6688156
15.8166666	4.2455997	-0.5031457
15.8166666	4.2943997	-0.4295144
15.8166666	4.3431997	-0.4356503
15.8166666	4.3919997	-0.4356503
15.8166666	4.4407997	-0.4417863
15.8166666	4.4895997	-0.4479222
15.8166666	4.5383997	-0.5522332
15.8166666	4.5871997	-0.4908738

15.8166666	4.6359997	-0.4908738
15.8166666	4.6847997	-0.4786019
15.8166666	4.7336001	-0.3558836
15.8166666	4.7824001	-0.3558836
15.8166666	4.8312001	-0.4847379
15.8166666	4.8800001	-0.4970098
15.8166666	4.9288001	-0.6810875
15.8166666	4.9776001	-0.6810875
15.8166666	5.0264001	-0.5890489
15.8166666	5.0752001	-0.5215535
15.8166666	5.1240001	-0.6074562
15.8166666	5.1728001	-0.6074562
15.8166666	5.2216001	-0.5829129
15.8166666	5.2704000	-0.5399613
15.8166666	5.3192000	-0.5460973
15.8166666	5.3680000	-0.5583692
15.8166666	5.4168000	-0.5645051
15.8166666	5.4656000	-0.5645051
15.8166666	5.5144000	-0.5583692
15.8166666	5.5632000	613586.0000000
15.8166666	5.6120000	-0.7117672
15.8166666	5.6608000	613586.0000000
15.8166666	5.7096000	613586.0000000
15.8166666	5.7584000	613586.0000000
15.8166666	5.8072000	613586.0000000
15.8166666	5.8559999	613586.0000000
15.8166666	5.9047999	613586.0000000
15.8166666	5.9535999	613586.0000000
15.8166666	6.0023999	613586.0000000
15.8333330	0.2928000	-3.4299812
15.8333330	0.3416000	-3.8472240
15.8333330	0.3904000	-4.2890105
15.8333330	0.4392000	-3.9883502
15.8333330	0.4880000	-3.4299812
15.8333330	0.5368000	-3.9638064
15.8333330	0.5856000	-2.7918451
15.8333330	0.6344000	-2.8102529
15.8333330	0.6832000	-2.0862141
15.8333330	0.7320000	-1.8714566
15.8333330	0.7808000	-1.2946796
15.8333330	0.8296000	-1.3253593
15.8333330	0.8784000	-1.3314953
15.8333330	0.9272000	-1.3499031
15.8333330	0.9760000	-1.3928547
15.8333330	1.0247999	-1.2946796
15.8333330	1.0735999	-1.4910293
15.8333330	1.1223999	-1.3253593
15.8333330	1.1711999	-1.3805828
15.8333330	1.2200000	-0.9019809
15.8333330	1.2688000	-1.1106019
15.8333330	1.3176000	-0.8897090
15.8333330	1.3664000	-1.1842332
15.8333330	1.4152000	-1.1780972
15.8333330	1.4640000	-1.2455926
15.8333330	1.5128000	-1.5892043
15.8333330	1.5616000	-1.5707965
15.8333330	1.6104000	-1.1596894

15.8333330	1.6592000	-1.3989906
15.8333330	1.7079999	-1.3928547
15.8333330	1.7567999	-1.0001554
15.8333330	1.8055999	-1.0001554
15.8333330	1.8543999	-1.1290097
15.8333330	1.9031999	-1.1290097
15.8333330	1.9519999	-0.9940195
15.8333330	2.0007999	-1.0308352
15.8333330	2.0495999	-1.1228738
15.8333330	2.0983999	-1.1658254
15.8333330	2.1471999	-1.1351457
15.8333330	2.1959999	-1.1351457
15.8333330	2.2447999	-1.1290097
15.8333330	2.2935998	-1.0799227
15.8333330	2.3423998	-1.1351457
15.8333330	2.3912001	-1.0492430
15.8333330	2.4400001	-1.1842332
15.8333330	2.4888000	-1.0492430
15.8333330	2.5376000	-1.0492430
15.8333330	2.5864000	-1.0431070
15.8333330	2.6352000	-1.0185633
15.8333330	2.6840000	-0.9878836
15.8333330	2.7328000	-0.9878836
15.8333330	2.7816000	-1.0369711
15.8333330	2.8304000	-0.8835731
15.8333330	2.8792000	-0.9817476
15.8333330	2.9280000	-0.9878836
15.8333330	2.9768000	-0.9572039
15.8333330	3.0256000	-0.9572039
15.8333330	3.0743999	-0.8897090
15.8333330	3.1231999	-0.8774371
15.8333330	3.1719999	-0.8958449
15.8333330	3.2207999	-0.9572039
15.8333330	3.2695999	-0.8590293
15.8333330	3.3183999	-0.6688156
15.8333330	3.3671999	-0.8222136
15.8333330	3.4159999	-0.6810875
15.8333330	3.4647999	-0.6872234
15.8333330	3.5135999	-0.7056313
15.8333330	3.5623999	-0.7056313
15.8333330	3.6111999	-0.6994953
15.8333330	3.6599998	-0.7179031
15.8333330	3.7087998	-0.7179031
15.8333330	3.7575998	-0.7179031
15.8333330	3.8063998	-0.4970098
15.8333330	3.8551998	-0.5092816
15.8333330	3.9039998	-0.4417863
15.8333330	3.9527998	-0.4847379
15.8333330	4.0016003	-0.4847379
15.8333330	4.0503998	-0.4908738
15.8333330	4.0991998	-0.4908738
15.8333330	4.1479998	-0.4908738
15.8333330	4.1967998	-0.4479222
15.8333330	4.2455997	-0.4479222
15.8333330	4.2943997	-0.3252039
15.8333330	4.3431997	-0.3252039
15.8333330	4.3919997	-0.3252039

15.8333330	4.4407997	-0.4111071
15.8333330	4.4895997	-0.4111071
15.8333330	4.5383997	-0.3988352
15.8333330	4.5871997	-0.5645051
15.8333330	4.6359997	-0.4724660
15.8333330	4.6847997	-0.4663301
15.8333330	4.7336001	-0.4295144
15.8333330	4.7824001	-0.4356503
15.8333330	4.8312001	-0.4172430
15.8333330	4.8800001	-0.7301750
15.8333330	4.9288001	-0.7485828
15.8333330	4.9776001	-0.7485828
15.8333330	5.0264001	-0.6810875
15.8333330	5.0752001	-0.5460973
15.8333330	5.1240001	-0.7179031
15.8333330	5.1728001	-0.5767770
15.8333330	5.2216001	-0.5767770
15.8333330	5.2704000	-0.6933594
15.8333330	5.3192000	-0.7669902
15.8333330	5.3680000	-0.7669902
15.8333330	5.4168000	-0.7669902
15.8333330	5.4656000	-0.7608547
15.8333330	5.5144000	613586.0000000
15.8333330	5.5632000	613586.0000000
15.8333330	5.6120000	613586.0000000
15.8333330	5.6608000	613586.0000000
15.8333330	5.7096000	613586.0000000
15.8333330	5.7584000	613586.0000000
15.8333330	5.8072000	613586.0000000
15.8333330	5.8559999	613586.0000000
15.8333330	5.9047999	613586.0000000
15.8333330	5.9535999	613586.0000000
15.8333330	6.0023999	613586.0000000
15.8500004	0.2928000	-3.6938257
15.8500004	0.3416000	-3.6938257
15.8500004	0.3904000	-3.7490492
15.8500004	0.4392000	-3.7490492
15.8500004	0.4880000	-4.4853601
15.8500004	0.5368000	-4.7123890
15.8500004	0.5856000	-3.5465636
15.8500004	0.6344000	-2.1230292
15.8500004	0.6832000	-2.1291652
15.8500004	0.7320000	-2.1353011
15.8500004	0.7808000	-1.6198835
15.8500004	0.8296000	-1.1780972
15.8500004	0.8784000	-1.1106019
15.8500004	0.9272000	-1.2824078
15.8500004	0.9760000	-1.2087770
15.8500004	1.0247999	-1.1842332
15.8500004	1.0735999	-1.1780972
15.8500004	1.1223999	-1.1780972
15.8500004	1.1711999	-0.9878836
15.8500004	1.2200000	-1.1474175
15.8500004	1.2688000	-1.1474175
15.8500004	1.3176000	-0.9572039
15.8500004	1.3664000	-0.9572039
15.8500004	1.4152000	-1.1351457

15.8500004	1.4640000	-1.3437672
15.8500004	1.5128000	-1.3376312
15.8500004	1.5616000	-1.3437672
15.8500004	1.6104000	-0.8406215
15.8500004	1.6592000	-1.5033011
15.8500004	1.7079999	-1.2210488
15.8500004	1.7567999	-1.2333207
15.8500004	1.8055999	-0.9572039
15.8500004	1.8543999	-0.6013203
15.8500004	1.9031999	-1.0737867
15.8500004	1.9519999	-0.9326606
15.8500004	2.0007999	-0.7240391
15.8500004	2.0495999	-0.7301750
15.8500004	2.0983999	-0.5031457
15.8500004	2.1471999	-0.6442719
15.8500004	2.1959999	-0.5890489
15.8500004	2.2447999	-0.7915339
15.8500004	2.2935998	-0.7976699
15.8500004	2.3423998	-0.8467574
15.8500004	2.3912001	-1.0062914
15.8500004	2.4400001	-1.0001554
15.8500004	2.4888000	-0.8774371
15.8500004	2.5376000	-0.8897090
15.8500004	2.5864000	-0.8590293
15.8500004	2.6352000	-0.7608547
15.8500004	2.6840000	-0.9142528
15.8500004	2.7328000	-0.9265246
15.8500004	2.7816000	-0.9878836
15.8500004	2.8304000	-1.0062914
15.8500004	2.8792000	-1.0124273
15.8500004	2.9280000	-0.9572039
15.8500004	2.9768000	-0.9142528
15.8500004	3.0256000	-0.9081168
15.8500004	3.0743999	-0.9019809
15.8500004	3.1231999	-0.7792621
15.8500004	3.1719999	-0.8038058
15.8500004	3.2207999	-0.8344855
15.8500004	3.2695999	-0.8590293
15.8500004	3.3183999	-0.7485828
15.8500004	3.3671999	-0.5951843
15.8500004	3.4159999	-0.7117672
15.8500004	3.4647999	-0.6933594
15.8500004	3.5135999	-0.6872234
15.8500004	3.5623999	-0.7240391
15.8500004	3.6111999	-0.7240391
15.8500004	3.6599998	-0.6258640
15.8500004	3.7087998	-0.7363110
15.8500004	3.7575998	-0.5338254
15.8500004	3.8063998	-0.5338254
15.8500004	3.8551998	-0.5706410
15.8500004	3.9039998	-0.6381359
15.8500004	3.9527998	-0.4908738
15.8500004	4.0016003	-0.4908738
15.8500004	4.0503998	-0.4786019
15.8500004	4.0991998	-0.3558836
15.8500004	4.1479998	-0.3804274
15.8500004	4.1967998	-0.3742914

15.8500004	4.2455997	-0.6994953
15.8500004	4.2943997	-0.6994953
15.8500004	4.3431997	-0.4786019
15.8500004	4.3919997	-0.4417863
15.8500004	4.4407997	-0.4356503
15.8500004	4.4895997	-0.4970098
15.8500004	4.5383997	-0.4847379
15.8500004	4.5871997	-0.4417863
15.8500004	4.6359997	-0.4417863
15.8500004	4.6847997	-0.3129320
15.8500004	4.7336001	-0.3190680
15.8500004	4.7824001	-0.6013203
15.8500004	4.8312001	-0.4970098
15.8500004	4.8800001	-0.4111071
15.8500004	4.9288001	-0.4049711
15.8500004	4.9776001	-0.6442719
15.8500004	5.0264001	-0.6013203
15.8500004	5.0752001	-0.5890489
15.8500004	5.1240001	-0.4724660
15.8500004	5.1728001	-0.3374758
15.8500004	5.2216001	-0.4049711
15.8500004	5.2704000	-0.4847379
15.8500004	5.3192000	-0.4847379
15.8500004	5.3680000	-0.4970098
15.8500004	5.4168000	-0.5031457
15.8500004	5.4656000	613586.0000000
15.8500004	5.5144000	613586.0000000
15.8500004	5.5632000	613586.0000000
15.8500004	5.6120000	613586.0000000
15.8500004	5.6608000	613586.0000000
15.8500004	5.7096000	613586.0000000
15.8500004	5.7584000	613586.0000000
15.8500004	5.8072000	613586.0000000
15.8500004	5.8559999	613586.0000000
15.8500004	5.9047999	613586.0000000
15.8500004	5.9535999	613586.0000000
15.8500004	6.0023999	613586.0000000
15.8666668	0.2928000	-3.9147191
15.8666668	0.3416000	-3.7981365
15.8666668	0.3904000	-4.1356125
15.8666668	0.4392000	-3.5956511
15.8666668	0.4880000	-3.6079230
15.8666668	0.5368000	-3.3072627
15.8666668	0.5856000	-3.2152238
15.8666668	0.6344000	-2.5034568
15.8666668	0.6832000	-2.3684664
15.8666668	0.7320000	-1.7241945
15.8666668	0.7808000	-1.7303305
15.8666668	0.8296000	-1.2455926
15.8666668	0.8784000	-1.0676508
15.8666668	0.9272000	-1.3928547
15.8666668	0.9760000	-1.3989906
15.8666668	1.0247999	-1.1290097
15.8666668	1.0735999	-1.0615149
15.8666668	1.1223999	-1.2026410
15.8666668	1.1711999	-1.2026410
15.8666668	1.2200000	-1.1903691

15.8666668	1.2688000	-1.2578645
15.8666668	1.3176000	-1.0737867
15.8666668	1.3664000	-1.0799227
15.8666668	1.4152000	-1.3621750
15.8666668	1.4640000	-1.0369711
15.8666668	1.5128000	-1.0369711
15.8666668	1.5616000	-0.9940195
15.8666668	1.6104000	-1.0492430
15.8666668	1.6592000	-1.2026410
15.8666668	1.7079999	-1.2762718
15.8666668	1.7567999	-1.1228738
15.8666668	1.8055999	-1.0553789
15.8666668	1.8543999	-1.0553789
15.8666668	1.9031999	-1.0369711
15.8666668	1.9519999	-1.0308352
15.8666668	2.0007999	-0.9203887
15.8666668	2.0495999	-0.9265246
15.8666668	2.0983999	-0.8590293
15.8666668	2.1471999	-0.9019809
15.8666668	2.1959999	-0.7485828
15.8666668	2.2447999	-0.8590293
15.8666668	2.2935998	-0.8406215
15.8666668	2.3423998	-1.0246992
15.8666668	2.3912001	-0.9449320
15.8666668	2.4400001	-0.8344855
15.8666668	2.4888000	-0.8590293
15.8666668	2.5376000	-0.6749516
15.8666668	2.5864000	-0.6504078
15.8666668	2.6352000	-0.7117672
15.8666668	2.6840000	-0.8651652
15.8666668	2.7328000	-1.0001554
15.8666668	2.7816000	-0.9817476
15.8666668	2.8304000	-0.9940195
15.8666668	2.8792000	-0.9940195
15.8666668	2.9280000	-0.9817476
15.8666668	2.9768000	-0.8897090
15.8666668	3.0256000	-0.8897090
15.8666668	3.0743999	-0.8344855
15.8666668	3.1231999	-0.7976699
15.8666668	3.1719999	-0.7608547
15.8666668	3.2207999	-0.7608547
15.8666668	3.2695999	-0.7547188
15.8666668	3.3183999	-0.6504078
15.8666668	3.3671999	-0.6442719
15.8666668	3.4159999	-0.7547188
15.8666668	3.4647999	-0.7547188
15.8666668	3.5135999	-0.7608547
15.8666668	3.5623999	-0.6810875
15.8666668	3.6111999	-0.6749516
15.8666668	3.6599998	-0.6381359
15.8666668	3.7087998	-0.6320000
15.8666668	3.7575998	-0.5706410
15.8666668	3.8063998	-0.5829129
15.8666668	3.8551998	-0.5890489
15.8666668	3.9039998	-0.5890489
15.8666668	3.9527998	-0.4601941
15.8666668	4.0016003	-0.5276895

15.8666668	4.0503998	-0.5460973
15.8666668	4.0991998	-0.4908738
15.8666668	4.1479998	-0.5645051
15.8666668	4.1967998	-0.5460973
15.8666668	4.2455997	-0.8406215
15.8666668	4.2943997	-0.8406215
15.8666668	4.3431997	-0.7485828
15.8666668	4.3919997	-0.7424469
15.8666668	4.4407997	-0.4970098
15.8666668	4.4895997	-0.5154176
15.8666668	4.5383997	-0.5583692
15.8666668	4.5871997	-0.5767770
15.8666668	4.6359997	-0.5154176
15.8666668	4.6847997	-0.5276895
15.8666668	4.7336001	-0.5215535
15.8666668	4.7824001	-0.4847379
15.8666668	4.8312001	-0.4847379
15.8666668	4.8800001	-0.4908738
15.8666668	4.9288001	-0.6994953
15.8666668	4.9776001	-0.6442719
15.8666668	5.0264001	-0.6565437
15.8666668	5.0752001	-0.6626797
15.8666668	5.1240001	-0.3742914
15.8666668	5.1728001	-0.3804274
15.8666668	5.2216001	-0.3620195
15.8666668	5.2704000	-0.3620195
15.8666668	5.3192000	-0.5767770
15.8666668	5.3680000	-0.5767770
15.8666668	5.4168000	613586.0000000
15.8666668	5.4656000	613586.0000000
15.8666668	5.5144000	613586.0000000
15.8666668	5.5632000	613586.0000000
15.8666668	5.6120000	613586.0000000
15.8666668	5.6608000	613586.0000000
15.8666668	5.7096000	613586.0000000
15.8666668	5.7584000	613586.0000000
15.8666668	5.8072000	613586.0000000
15.8666668	5.8559999	613586.0000000
15.8666668	5.9047999	613586.0000000
15.8666668	5.9535999	613586.0000000
15.8666668	6.0023999	613586.0000000
15.8833332	0.2928000	-3.8840394
15.8833332	0.3416000	-3.3808937
15.8833332	0.3904000	-4.1233406
15.8833332	0.4392000	-2.8347967
15.8833332	0.4880000	-4.3871851
15.8833332	0.5368000	-4.2706027
15.8833332	0.5856000	-4.0987968
15.8833332	0.6344000	-3.5895152
15.8833332	0.6832000	-1.8162332
15.8833332	0.7320000	-1.8100972
15.8833332	0.7808000	-0.4908738
15.8833332	0.8296000	-0.3988352
15.8833332	0.8784000	-1.2885437
15.8833332	0.9272000	-1.2701364
15.8833332	0.9760000	-1.1228738
15.8833332	1.0247999	-1.0431070

15.8833332	1.0735999	-1.3928547
15.8833332	1.1223999	-1.3928547
15.8833332	1.1711999	-1.2333207
15.8833332	1.2200000	-1.4296703
15.8833332	1.2688000	-1.2149129
15.8833332	1.3176000	-1.2087770
15.8833332	1.3664000	-1.1903691
15.8833332	1.4152000	-1.1780972
15.8833332	1.4640000	-1.1596894
15.8833332	1.5128000	-1.1596894
15.8833332	1.5616000	-0.5706410
15.8833332	1.6104000	-1.2701364
15.8833332	1.6592000	-0.2393012
15.8833332	1.7079999	-1.3376312
15.8833332	1.7567999	-0.6565437
15.8833332	1.8055999	-0.6565437
15.8833332	1.8543999	-0.7056313
15.8833332	1.9031999	-0.9265246
15.8833332	1.9519999	-0.9510679
15.8833332	2.0007999	-1.0921946
15.8833332	2.0495999	-0.9940195
15.8833332	2.0983999	-1.1228738
15.8833332	2.1471999	-0.9633398
15.8833332	2.1959999	-0.9694757
15.8833332	2.2447999	-1.0369711
15.8833332	2.2935998	-1.0308352
15.8833332	2.3423998	-1.0431070
15.8833332	2.3912001	-0.9694757
15.8833332	2.4400001	-1.0431070
15.8833332	2.4888000	-1.0185633
15.8833332	2.5376000	-1.0308352
15.8833332	2.5864000	-0.9633398
15.8833332	2.6352000	-0.9572039
15.8833332	2.6840000	-0.9878836
15.8833332	2.7328000	-0.9940195
15.8833332	2.7816000	-0.9572039
15.8833332	2.8304000	-0.9019809
15.8833332	2.8792000	-0.9510679
15.8833332	2.9280000	-0.9572039
15.8833332	2.9768000	-0.9817476
15.8833332	3.0256000	-0.9940195
15.8833332	3.0743999	-0.8897090
15.8833332	3.1231999	-0.8713012
15.8833332	3.1719999	-0.8651652
15.8833332	3.2207999	-0.8590293
15.8833332	3.2695999	-0.7669902
15.8833332	3.3183999	-0.7608547
15.8833332	3.3671999	-0.7485828
15.8833332	3.4159999	-0.6872234
15.8833332	3.4647999	-0.7056313
15.8833332	3.5135999	-0.8160777
15.8833332	3.5623999	-0.6749516
15.8833332	3.6111999	-0.6626797
15.8833332	3.6599998	-0.4786019
15.8833332	3.7087998	-0.6749516
15.8833332	3.7575998	-0.5706410
15.8833332	3.8063998	-0.5583692

15.8833332	3.8551998	-0.5338254
15.8833332	3.9039998	-0.5154176
15.8833332	3.9527998	-0.5338254
15.8833332	4.0016003	-0.4970098
15.8833332	4.0503998	-0.4970098
15.8833332	4.0991998	-0.4970098
15.8833332	4.1479998	-0.4847379
15.8833332	4.1967998	-0.5338254
15.8833332	4.2455997	-0.7056313
15.8833332	4.2943997	-0.7179031
15.8833332	4.3431997	-0.6749516
15.8833332	4.3919997	-0.6688156
15.8833332	4.4407997	-0.6504078
15.8833332	4.4895997	-0.4847379
15.8833332	4.5383997	-0.4049711
15.8833332	4.5871997	-0.4172430
15.8833332	4.6359997	-0.4540582
15.8833332	4.6847997	-0.6933594
15.8833332	4.7336001	-0.7669902
15.8833332	4.7824001	-0.7117672
15.8833332	4.8312001	-0.6994953
15.8833332	4.8800001	-0.7056313
15.8833332	4.9288001	-0.6688156
15.8833332	4.9776001	-0.6258640
15.8833332	5.0264001	-0.6258640
15.8833332	5.0752001	-0.6565437
15.8833332	5.1240001	-0.4847379
15.8833332	5.1728001	-0.5399613
15.8833332	5.2216001	-0.4540582
15.8833332	5.2704000	-0.4417863
15.8833332	5.3192000	-0.5399613
15.8833332	5.3680000	-0.5460973
15.8833332	5.4168000	-0.5460973
15.8833332	5.4656000	613586.0000000
15.8833332	5.5144000	-0.8222136
15.8833332	5.5632000	613586.0000000
15.8833332	5.6120000	613586.0000000
15.8833332	5.6608000	613586.0000000
15.8833332	5.7096000	613586.0000000
15.8833332	5.7584000	613586.0000000
15.8833332	5.8072000	613586.0000000
15.8833332	5.8559999	613586.0000000
15.8833332	5.9047999	613586.0000000
15.8833332	5.9535999	613586.0000000
15.8833332	6.0023999	613586.0000000
15.8999996	0.2928000	-3.7060976
15.8999996	0.3416000	-3.6999617
15.8999996	0.3904000	-3.5895152
15.8999996	0.4392000	-4.8473797
15.8999996	0.4880000	-4.8412437
15.8999996	0.5368000	-4.9332824
15.8999996	0.5856000	-4.1785641
15.8999996	0.6344000	-1.2885437
15.8999996	0.6832000	-1.4296703
15.8999996	0.7320000	-1.4296703
15.8999996	0.7808000	-1.1535535
15.8999996	0.8296000	-1.1535535

15.8999996	0.8784000	-1.1965051
15.8999996	0.9272000	-1.2210488
15.8999996	0.9760000	-1.5707965
15.8999996	1.0247999	-1.5769324
15.8999996	1.0735999	-1.6505632
15.8999996	1.1223999	-1.4542136
15.8999996	1.1711999	-1.2701364
15.8999996	1.2200000	-1.2701364
15.8999996	1.2688000	-1.1596894
15.8999996	1.3176000	-1.5830684
15.8999996	1.3664000	-1.3867188
15.8999996	1.4152000	-1.2885437
15.8999996	1.4640000	-1.3008156
15.8999996	1.5128000	-1.2455926
15.8999996	1.5616000	-0.9387960
15.8999996	1.6104000	-0.9387960
15.8999996	1.6592000	-1.0001554
15.8999996	1.7079999	-1.4173985
15.8999996	1.7567999	-1.0737867
15.8999996	1.8055999	-0.8344855
15.8999996	1.8543999	-0.9756117
15.8999996	1.9031999	-0.9756117
15.8999996	1.9519999	-0.7792621
15.8999996	2.0007999	-0.9203887
15.8999996	2.0495999	-0.9878836
15.8999996	2.0983999	-0.9817476
15.8999996	2.1471999	-0.9081168
15.8999996	2.1959999	-0.9142528
15.8999996	2.2447999	-1.0737867
15.8999996	2.2935998	-1.1228738
15.8999996	2.3423998	-1.0615149
15.8999996	2.3912001	-1.0615149
15.8999996	2.4400001	-1.0737867
15.8999996	2.4888000	-0.9940195
15.8999996	2.5376000	-0.9940195
15.8999996	2.5864000	-0.9817476
15.8999996	2.6352000	-1.0492430
15.8999996	2.6840000	-0.9878836
15.8999996	2.7328000	-0.9817476
15.8999996	2.7816000	-0.8835731
15.8999996	2.8304000	-0.8835731
15.8999996	2.8792000	-1.0001554
15.8999996	2.9280000	-0.9940195
15.8999996	2.9768000	-0.8835731
15.8999996	3.0256000	-0.8528934
15.8999996	3.0743999	-0.7056313
15.8999996	3.1231999	-0.7056313
15.8999996	3.1719999	-0.6135921
15.8999996	3.2207999	-0.6074562
15.8999996	3.2695999	-0.6872234
15.8999996	3.3183999	-0.6872234
15.8999996	3.3671999	-0.6381359
15.8999996	3.4159999	-0.6258640
15.8999996	3.4647999	-0.6197281
15.8999996	3.5135999	-0.7301750
15.8999996	3.5623999	-0.7240391
15.8999996	3.6111999	-0.7240391

15.8999996	3.6599998	-0.7179031
15.8999996	3.7087998	-0.6074562
15.8999996	3.7575998	-0.6320000
15.8999996	3.8063998	-0.6381359
15.8999996	3.8551998	-0.5522332
15.8999996	3.9039998	-0.6872234
15.8999996	3.9527998	-0.6994953
15.8999996	4.0016003	-0.5645051
15.8999996	4.0503998	-0.5706410
15.8999996	4.0991998	-0.6688156
15.8999996	4.1479998	-0.6381359
15.8999996	4.1967998	-0.6258640
15.8999996	4.2455997	-0.5829129
15.8999996	4.2943997	-0.5829129
15.8999996	4.3431997	-0.7424469
15.8999996	4.3919997	-0.6320000
15.8999996	4.4407997	-0.6320000
15.8999996	4.4895997	-0.6320000
15.8999996	4.5383997	-0.5154176
15.8999996	4.5871997	-0.6749516
15.8999996	4.6359997	-0.3742914
15.8999996	4.6847997	-0.5890489
15.8999996	4.7336001	-0.5951843
15.8999996	4.7824001	-0.4786019
15.8999996	4.8312001	-0.4786019
15.8999996	4.8800001	-0.7056313
15.8999996	4.9288001	-0.4786019
15.8999996	4.9776001	-0.4786019
15.8999996	5.0264001	-0.4786019
15.8999996	5.0752001	-0.6197281
15.8999996	5.1240001	-0.4847379
15.8999996	5.1728001	-0.5706410
15.8999996	5.2216001	-0.5706410
15.8999996	5.2704000	-0.5706410
15.8999996	5.3192000	-0.5092816
15.8999996	5.3680000	-0.3865633
15.8999996	5.4168000	-0.3988352
15.8999996	5.4656000	613586.0000000
15.8999996	5.5144000	613586.0000000
15.8999996	5.5632000	613586.0000000
15.8999996	5.6120000	613586.0000000
15.8999996	5.6608000	613586.0000000
15.8999996	5.7096000	613586.0000000
15.8999996	5.7584000	613586.0000000
15.8999996	5.8072000	613586.0000000
15.8999996	5.8559999	613586.0000000
15.8999996	5.9047999	613586.0000000
15.8999996	5.9535999	613586.0000000
15.8999996	6.0023999	613586.0000000
15.9166670	0.2928000	-3.9515345
15.9166670	0.3416000	-3.3993015
15.9166670	0.3904000	-2.7979810
15.9166670	0.4392000	-3.5772433
15.9166670	0.4880000	-3.1170490
15.9166670	0.5368000	-3.9638064
15.9166670	0.5856000	-3.9638064
15.9166670	0.6344000	-1.7057867

15.9166670	0.6832000	-1.7057867
15.9166670	0.7320000	-1.3192234
15.9166670	0.7808000	-1.3253593
15.9166670	0.8296000	-1.3805828
15.9166670	0.8784000	-1.3683109
15.9166670	0.9272000	-1.3744469
15.9166670	0.9760000	-1.3130875
15.9166670	1.0247999	-1.4358063
15.9166670	1.0735999	-1.1842332
15.9166670	1.1223999	-1.3989906
15.9166670	1.1711999	-1.4971652
15.9166670	1.2200000	-1.4971652
15.9166670	1.2688000	-1.2824078
15.9166670	1.3176000	-1.3744469
15.9166670	1.3664000	-1.3805828
15.9166670	1.4152000	-1.3928547
15.9166670	1.4640000	-1.4051266
15.9166670	1.5128000	-1.3560390
15.9166670	1.5616000	-1.3560390
15.9166670	1.6104000	-1.2640004
15.9166670	1.6592000	-1.2394567
15.9166670	1.7079999	-1.2333207
15.9166670	1.7567999	-1.2271848
15.9166670	1.8055999	-1.4542136
15.9166670	1.8543999	-1.4480777
15.9166670	1.9031999	-1.4542136
15.9166670	1.9519999	-1.2087770
15.9166670	2.0007999	-1.2087770
15.9166670	2.0495999	-1.2026410
15.9166670	2.0983999	-1.0860586
15.9166670	2.1471999	-1.0921946
15.9166670	2.1959999	-1.0431070
15.9166670	2.2447999	-0.9572039
15.9166670	2.2935998	-1.0369711
15.9166670	2.3423998	-1.0185633
15.9166670	2.3912001	-1.0246992
15.9166670	2.4400001	-1.0308352
15.9166670	2.4888000	-1.0492430
15.9166670	2.5376000	-1.0062914
15.9166670	2.5864000	-1.0615149
15.9166670	2.6352000	-1.0553789
15.9166670	2.6840000	-0.8835731
15.9166670	2.7328000	-0.9694757
15.9166670	2.7816000	-0.9817476
15.9166670	2.8304000	-0.9449320
15.9166670	2.8792000	-0.9449320
15.9166670	2.9280000	-0.9940195
15.9166670	2.9768000	-0.7669902
15.9166670	3.0256000	-0.8651652
15.9166670	3.0743999	-0.9265246
15.9166670	3.1231999	-0.8897090
15.9166670	3.1719999	-0.9449320
15.9166670	3.2207999	-0.9449320
15.9166670	3.2695999	-0.8897090
15.9166670	3.3183999	-0.7179031
15.9166670	3.3671999	-0.7179031
15.9166670	3.4159999	-0.7547188

15.9166670	3.4647999	-0.6688156
15.9166670	3.5135999	-0.7976699
15.9166670	3.5623999	-0.7853980
15.9166670	3.6111999	-0.7485828
15.9166670	3.6599998	-0.7485828
15.9166670	3.7087998	-0.8835731
15.9166670	3.7575998	-0.7608547
15.9166670	3.8063998	-0.7731261
15.9166670	3.8551998	-0.8283496
15.9166670	3.9039998	-0.7547188
15.9166670	3.9527998	-0.7547188
15.9166670	4.0016003	-0.7669902
15.9166670	4.0503998	-0.7608547
15.9166670	4.0991998	-0.7485828
15.9166670	4.1479998	-0.6197281
15.9166670	4.1967998	-0.7117672
15.9166670	4.2455997	-0.7179031
15.9166670	4.2943997	-0.8283496
15.9166670	4.3431997	-0.8344855
15.9166670	4.3919997	-0.8160777
15.9166670	4.4407997	-0.8713012
15.9166670	4.4895997	-0.6197281
15.9166670	4.5383997	-0.6442719
15.9166670	4.5871997	-0.5890489
15.9166670	4.6359997	-0.5951843
15.9166670	4.6847997	-0.8651652
15.9166670	4.7336001	-0.8958449
15.9166670	4.7824001	-0.6749516
15.9166670	4.8312001	-0.6810875
15.9166670	4.8800001	-0.6626797
15.9166670	4.9288001	-0.6565437
15.9166670	4.9776001	-0.6381359
15.9166670	5.0264001	-0.6320000
15.9166670	5.0752001	-0.7976699
15.9166670	5.1240001	-0.7915339
15.9166670	5.1728001	-0.7547188
15.9166670	5.2216001	-0.6933594
15.9166670	5.2704000	-0.5215535
15.9166670	5.3192000	-0.5276895
15.9166670	5.3680000	-0.6074562
15.9166670	5.4168000	-0.5890489
15.9166670	5.4656000	613586.0000000
15.9166670	5.5144000	613586.0000000
15.9166670	5.5632000	613586.0000000
15.9166670	5.6120000	613586.0000000
15.9166670	5.6608000	613586.0000000
15.9166670	5.7096000	613586.0000000
15.9166670	5.7584000	613586.0000000
15.9166670	5.8072000	613586.0000000
15.9166670	5.8559999	613586.0000000
15.9166670	5.9047999	613586.0000000
15.9166670	5.9535999	613586.0000000
15.9166670	6.0023999	613586.0000000
15.9333334	0.2928000	-4.6019425
15.9333334	0.3416000	-3.3072627
15.9333334	0.3904000	-3.0066025
15.9333334	0.4392000	-3.5772433

15.9333334	0.4880000	-4.4178648
15.9333334	0.5368000	-4.0374374
15.9333334	0.5856000	-3.8410881
15.9333334	0.6344000	-2.8225248
15.9333334	0.6832000	-1.8285050
15.9333334	0.7320000	-1.2087770
15.9333334	0.7808000	-1.1903691
15.9333334	0.8296000	-1.4051266
15.9333334	0.8784000	-1.4112625
15.9333334	0.9272000	-1.4173985
15.9333334	0.9760000	-1.4173985
15.9333334	1.0247999	-1.4112625
15.9333334	1.0735999	-1.1719613
15.9333334	1.1223999	-1.1106019
15.9333334	1.1711999	-1.1842332
15.9333334	1.2200000	-1.3867188
15.9333334	1.2688000	-1.3867188
15.9333334	1.3176000	-1.4235344
15.9333334	1.3664000	-1.2333207
15.9333334	1.4152000	-1.2455926
15.9333334	1.4640000	-1.2578645
15.9333334	1.5128000	-1.2578645
15.9333334	1.5616000	-1.2517285
15.9333334	1.6104000	-1.1965051
15.9333334	1.6592000	-1.1965051
15.9333334	1.7079999	-1.0676508
15.9333334	1.7567999	-1.2824078
15.9333334	1.8055999	-1.2762718
15.9333334	1.8543999	-1.3130875
15.9333334	1.9031999	-1.0921946
15.9333334	1.9519999	-1.0799227
15.9333334	2.0007999	-1.0615149
15.9333334	2.0495999	-1.0737867
15.9333334	2.0983999	-1.0860586
15.9333334	2.1471999	-0.9449320
15.9333334	2.1959999	-1.0553789
15.9333334	2.2447999	-1.0492430
15.9333334	2.2935998	-1.0860586
15.9333334	2.3423998	-1.1228738
15.9333334	2.3912001	-1.1412816
15.9333334	2.4400001	-1.1535535
15.9333334	2.4888000	-1.0676508
15.9333334	2.5376000	-1.0492430
15.9333334	2.5864000	-1.0308352
15.9333334	2.6352000	-1.0001554
15.9333334	2.6840000	-1.0799227
15.9333334	2.7328000	-0.8958449
15.9333334	2.7816000	-1.1535535
15.9333334	2.8304000	-0.9817476
15.9333334	2.8792000	-1.1044660
15.9333334	2.9280000	-1.0983305
15.9333334	2.9768000	-0.9878836
15.9333334	3.0256000	-1.0062914
15.9333334	3.0743999	-0.9142528
15.9333334	3.1231999	-0.9265246
15.9333334	3.1719999	-1.0124273
15.9333334	3.2207999	-0.9694757

15.9333334	3.2695999	-0.8038058
15.9333334	3.3183999	-0.8774371
15.9333334	3.3671999	-0.8835731
15.9333334	3.4159999	-0.7363110
15.9333334	3.4647999	-0.7608547
15.9333334	3.5135999	-0.7547188
15.9333334	3.5623999	-0.7424469
15.9333334	3.6111999	-0.8099418
15.9333334	3.6599998	-0.8099418
15.9333334	3.7087998	-0.8038058
15.9333334	3.7575998	-0.7240391
15.9333334	3.8063998	-0.7301750
15.9333334	3.8551998	-0.8467574
15.9333334	3.9039998	-0.8038058
15.9333334	3.9527998	-0.8160777
15.9333334	4.0016003	-0.8160777
15.9333334	4.0503998	-0.7792621
15.9333334	4.0991998	-0.8222136
15.9333334	4.1479998	-0.6810875
15.9333334	4.1967998	-0.6810875
15.9333334	4.2455997	-0.6872234
15.9333334	4.2943997	-0.7485828
15.9333334	4.3431997	-0.7363110
15.9333334	4.3919997	-0.7301750
15.9333334	4.4407997	-0.7301750
15.9333334	4.4895997	-0.7056313
15.9333334	4.5383997	-0.7179031
15.9333334	4.5871997	-0.7363110
15.9333334	4.6359997	-0.7792621
15.9333334	4.6847997	-0.6872234
15.9333334	4.7336001	-0.8099418
15.9333334	4.7824001	-0.8099418
15.9333334	4.8312001	-0.8099418
15.9333334	4.8800001	-0.8038058
15.9333334	4.9288001	-0.7485828
15.9333334	4.9776001	-0.6810875
15.9333334	5.0264001	-0.6197281
15.9333334	5.0752001	-0.6626797
15.9333334	5.1240001	-0.6749516
15.9333334	5.1728001	-0.6688156
15.9333334	5.2216001	-0.8651652
15.9333334	5.2704000	-0.8651652
15.9333334	5.3192000	-0.8897090
15.9333334	5.3680000	-0.6994953
15.9333334	5.4168000	-0.6810875
15.9333334	5.4656000	613586.0000000
15.9333334	5.5144000	613586.0000000
15.9333334	5.5632000	613586.0000000
15.9333334	5.6120000	613586.0000000
15.9333334	5.6608000	613586.0000000
15.9333334	5.7096000	613586.0000000
15.9333334	5.7584000	613586.0000000
15.9333334	5.8072000	613586.0000000
15.9333334	5.8559999	613586.0000000
15.9333334	5.9047999	613586.0000000
15.9333334	5.9535999	613586.0000000
15.9333334	6.0023999	613586.0000000

15.9499998	0.2928000	-3.7060976
15.9499998	0.3416000	-3.6754181
15.9499998	0.3904000	-3.6692822
15.9499998	0.4392000	-2.9268355
15.9499998	0.4880000	-3.5649714
15.9499998	0.5368000	-3.0863695
15.9499998	0.5856000	-3.0863695
15.9499998	0.6344000	-1.3744469
15.9499998	0.6832000	-1.3683109
15.9499998	0.7320000	-1.6873789
15.9499998	0.7808000	-1.1106019
15.9499998	0.8296000	-1.1106019
15.9499998	0.8784000	-1.4419422
15.9499998	0.9272000	-1.4296703
15.9499998	0.9760000	-1.4173985
15.9499998	1.0247999	-1.3928547
15.9499998	1.0735999	-1.1474175
15.9499998	1.1223999	-1.3069515
15.9499998	1.1711999	-0.9878836
15.9499998	1.2200000	-1.1044660
15.9499998	1.2688000	-1.2824078
15.9499998	1.3176000	-1.2394567
15.9499998	1.3664000	-1.1965051
15.9499998	1.4152000	-1.0676508
15.9499998	1.4640000	-1.1658254
15.9499998	1.5128000	-1.1596894
15.9499998	1.5616000	-1.3130875
15.9499998	1.6104000	-1.5707965
15.9499998	1.6592000	-1.5892043
15.9499998	1.7079999	-1.4726214
15.9499998	1.7567999	-1.4173985
15.9499998	1.8055999	-1.0246992
15.9499998	1.8543999	-1.0737867
15.9499998	1.9031999	-1.0737867
15.9499998	1.9519999	-1.1474175
15.9499998	2.0007999	-1.2271848
15.9499998	2.0495999	-1.0921946
15.9499998	2.0983999	-1.0860586
15.9499998	2.1471999	-1.0001554
15.9499998	2.1959999	-1.0124273
15.9499998	2.2447999	-1.0124273
15.9499998	2.2935998	-0.9510679
15.9499998	2.3423998	-1.0737867
15.9499998	2.3912001	-1.0615149
15.9499998	2.4400001	-1.1044660
15.9499998	2.4888000	-0.8344855
15.9499998	2.5376000	-0.9694757
15.9499998	2.5864000	-0.9817476
15.9499998	2.6352000	-0.9878836
15.9499998	2.6840000	-1.0860586
15.9499998	2.7328000	-0.9572039
15.9499998	2.7816000	-1.0308352
15.9499998	2.8304000	-1.0308352
15.9499998	2.8792000	-1.0246992
15.9499998	2.9280000	-1.0185633
15.9499998	2.9768000	-0.8160777
15.9499998	3.0256000	-0.8283496

15.9499998	3.0743999	-0.9203887
15.9499998	3.1231999	-0.9019809
15.9499998	3.1719999	-0.7853980
15.9499998	3.2207999	-0.8406215
15.9499998	3.2695999	-0.8160777
15.9499998	3.3183999	-0.8099418
15.9499998	3.3671999	-0.6933594
15.9499998	3.4159999	-0.7976699
15.9499998	3.4647999	-0.8467574
15.9499998	3.5135999	-0.8528934
15.9499998	3.5623999	-0.7179031
15.9499998	3.6111999	-0.7669902
15.9499998	3.6599998	-0.8283496
15.9499998	3.7087998	-0.8283496
15.9499998	3.7575998	-0.7301750
15.9499998	3.8063998	-0.7363110
15.9499998	3.8551998	-0.7424469
15.9499998	3.9039998	-0.7485828
15.9499998	3.9527998	-0.6442719
15.9499998	4.0016003	-0.6442719
15.9499998	4.0503998	-0.5890489
15.9499998	4.0991998	-0.5767770
15.9499998	4.1479998	-0.4908738
15.9499998	4.1967998	-0.4908738
15.9499998	4.2455997	-0.5399613
15.9499998	4.2943997	-0.4233789
15.9499998	4.3431997	-0.4172430
15.9499998	4.3919997	-0.4601941
15.9499998	4.4407997	-0.3988352
15.9499998	4.4895997	-0.5092816
15.9499998	4.5383997	-0.3620195
15.9499998	4.5871997	-0.3742914
15.9499998	4.6359997	-0.7179031
15.9499998	4.6847997	-0.7117672
15.9499998	4.7336001	-0.7179031
15.9499998	4.7824001	-0.9326606
15.9499998	4.8312001	-0.8774371
15.9499998	4.8800001	-0.8099418
15.9499998	4.9288001	-0.8222136
15.9499998	4.9776001	-0.8713012
15.9499998	5.0264001	-0.8774371
15.9499998	5.0752001	-0.8774371
15.9499998	5.1240001	-0.7915339
15.9499998	5.1728001	-0.8099418
15.9499998	5.2216001	-0.7240391
15.9499998	5.2704000	-0.7240391
15.9499998	5.3192000	-0.7240391
15.9499998	5.3680000	-0.6135921
15.9499998	5.4168000	613586.0000000
15.9499998	5.4656000	613586.0000000
15.9499998	5.5144000	613586.0000000
15.9499998	5.5632000	613586.0000000
15.9499998	5.6120000	613586.0000000
15.9499998	5.6608000	613586.0000000
15.9499998	5.7096000	613586.0000000
15.9499998	5.7584000	613586.0000000
15.9499998	5.8072000	613586.0000000

15.9499998	5.8559999	613586.0000000
15.9499998	5.9047999	613586.0000000
15.9499998	5.9535999	613586.0000000
15.9499998	6.0023999	613586.0000000
15.9666662	0.2928000	-4.6694374
15.9666662	0.3416000	-4.6633015
15.9666662	0.3904000	-4.0251656
15.9666662	0.4392000	-3.7429132
15.9666662	0.4880000	-4.0435734
15.9666662	0.5368000	-3.4852045
15.9666662	0.5856000	-3.1906800
15.9666662	0.6344000	-2.0555344
15.9666662	0.6832000	-2.0493984
15.9666662	0.7320000	-1.9880390
15.9666662	0.7808000	-1.9144082
15.9666662	0.8296000	-1.3621750
15.9666662	0.8784000	-1.2517285
15.9666662	0.9272000	-1.2455926
15.9666662	0.9760000	-1.0492430
15.9666662	1.0247999	-1.0553789
15.9666662	1.0735999	-1.1474175
15.9666662	1.1223999	-1.1044660
15.9666662	1.1711999	-1.0246992
15.9666662	1.2200000	-1.0308352
15.9666662	1.2688000	-0.7608547
15.9666662	1.3176000	-1.2026410
15.9666662	1.3664000	-1.0799227
15.9666662	1.4152000	-1.0799227
15.9666662	1.4640000	-1.1228738
15.9666662	1.5128000	-0.9265246
15.9666662	1.5616000	-0.9203887
15.9666662	1.6104000	-1.1412816
15.9666662	1.6592000	-0.9756117
15.9666662	1.7079999	-1.5892043
15.9666662	1.7567999	-0.9387960
15.9666662	1.8055999	-1.0124273
15.9666662	1.8543999	-1.1106019
15.9666662	1.9031999	-0.9142528
15.9666662	1.9519999	-0.8958449
15.9666662	2.0007999	-0.8897090
15.9666662	2.0495999	-0.9940195
15.9666662	2.0983999	-0.9081168
15.9666662	2.1471999	-0.9019809
15.9666662	2.1959999	-0.9817476
15.9666662	2.2447999	-0.9817476
15.9666662	2.2935998	-0.9940195
15.9666662	2.3423998	-0.8835731
15.9666662	2.3912001	-0.8958449
15.9666662	2.4400001	-0.9019809
15.9666662	2.4888000	-0.9019809
15.9666662	2.5376000	-0.9940195
15.9666662	2.5864000	-1.0676508
15.9666662	2.6352000	-0.9326606
15.9666662	2.6840000	-0.9326606
15.9666662	2.7328000	-0.9817476
15.9666662	2.7816000	-0.9142528
15.9666662	2.8304000	-0.9142528

15.9666662	2.8792000	-0.8897090
15.9666662	2.9280000	-0.9081168
15.9666662	2.9768000	-0.9878836
15.9666662	3.0256000	-0.8467574
15.9666662	3.0743999	-0.8467574
15.9666662	3.1231999	-0.8406215
15.9666662	3.1719999	-0.7485828
15.9666662	3.2207999	-0.6565437
15.9666662	3.2695999	-0.6688156
15.9666662	3.3183999	-0.6565437
15.9666662	3.3671999	-0.7669902
15.9666662	3.4159999	-0.6749516
15.9666662	3.4647999	-0.6933594
15.9666662	3.5135999	-0.6013203
15.9666662	3.5623999	-0.5890489
15.9666662	3.6111999	-0.6565437
15.9666662	3.6599998	-0.6504078
15.9666662	3.7087998	-0.6135921
15.9666662	3.7575998	-0.6135921
15.9666662	3.8063998	-0.6258640
15.9666662	3.8551998	-0.6197281
15.9666662	3.9039998	-0.5215535
15.9666662	3.9527998	-0.5092816
15.9666662	4.0016003	-0.5092816
15.9666662	4.0503998	-0.6135921
15.9666662	4.0991998	-0.6626797
15.9666662	4.1479998	-0.6258640
15.9666662	4.1967998	-0.5767770
15.9666662	4.2455997	-0.5092816
15.9666662	4.2943997	-0.4417863
15.9666662	4.3431997	-0.6258640
15.9666662	4.3919997	-0.6381359
15.9666662	4.4407997	-0.6565437
15.9666662	4.4895997	-0.7853980
15.9666662	4.5383997	-0.6381359
15.9666662	4.5871997	-0.4356503
15.9666662	4.6359997	-0.4356503
15.9666662	4.6847997	-0.6933594
15.9666662	4.7336001	-0.4970098
15.9666662	4.7824001	-0.5031457
15.9666662	4.8312001	-0.3374758
15.9666662	4.8800001	-0.3558836
15.9666662	4.9288001	-0.4847379
15.9666662	4.9776001	-0.4908738
15.9666662	5.0264001	-0.4970098
15.9666662	5.0752001	-0.7424469
15.9666662	5.1240001	-0.7363110
15.9666662	5.1728001	-0.5829129
15.9666662	5.2216001	-0.7117672
15.9666662	5.2704000	-0.6565437
15.9666662	5.3192000	613586.0000000
15.9666662	5.3680000	613586.0000000
15.9666662	5.4168000	613586.0000000
15.9666662	5.4656000	613586.0000000
15.9666662	5.5144000	613586.0000000
15.9666662	5.5632000	613586.0000000
15.9666662	5.6120000	613586.0000000

15.9666662	5.6608000	613586.0000000
15.9666662	5.7096000	613586.0000000
15.9666662	5.7584000	613586.0000000
15.9666662	5.8072000	613586.0000000
15.9666662	5.8559999	613586.0000000
15.9666662	5.9047999	613586.0000000
15.9666662	5.9535999	613586.0000000
15.9666662	6.0023999	613586.0000000
15.9833336	0.2928000	-3.3379424
15.9833336	0.3416000	-3.0127385
15.9833336	0.3904000	-3.5895152
15.9833336	0.4392000	-3.5895152
15.9833336	0.4880000	-3.6938257
15.9833336	0.5368000	-3.5711074
15.9833336	0.5856000	-2.5464082
15.9833336	0.6344000	-2.0616703
15.9833336	0.6832000	-1.7794180
15.9833336	0.7320000	-0.8651652
15.9833336	0.7808000	-0.8590293
15.9833336	0.8296000	-1.4910293
15.9833336	0.8784000	-1.3008156
15.9833336	0.9272000	-0.8958449
15.9833336	0.9760000	-0.6994953
15.9833336	1.0247999	-0.7056313
15.9833336	1.0735999	-0.9878836
15.9833336	1.1223999	-0.9756117
15.9833336	1.1711999	-1.1535535
15.9833336	1.2200000	-1.1474175
15.9833336	1.2688000	-0.7424469
15.9833336	1.3176000	-0.8774371
15.9833336	1.3664000	-1.2701364
15.9833336	1.4152000	-1.1044660
15.9833336	1.4640000	-1.1044660
15.9833336	1.5128000	-1.0615149
15.9833336	1.5616000	-1.3437672
15.9833336	1.6104000	-1.3314953
15.9833336	1.6592000	-1.3376312
15.9833336	1.7079999	-1.0676508
15.9833336	1.7567999	-0.7792621
15.9833336	1.8055999	-0.7792621
15.9833336	1.8543999	-1.1228738
15.9833336	1.9031999	-1.2885437
15.9833336	1.9519999	-1.0676508
15.9833336	2.0007999	-1.0676508
15.9833336	2.0495999	-1.0062914
15.9833336	2.0983999	-1.0431070
15.9833336	2.1471999	-1.0553789
15.9833336	2.1959999	-1.0062914
15.9833336	2.2447999	-0.9694757
15.9833336	2.2935998	-0.9694757
15.9833336	2.3423998	-1.0492430
15.9833336	2.3912001	-1.0553789
15.9833336	2.4400001	-1.0492430
15.9833336	2.4888000	-1.0062914
15.9833336	2.5376000	-1.0799227
15.9833336	2.5864000	-0.9817476
15.9833336	2.6352000	-0.9756117

15.9833336	2.6840000	-0.9756117
15.9833336	2.7328000	-1.0246992
15.9833336	2.7816000	-1.0246992
15.9833336	2.8304000	-0.9756117
15.9833336	2.8792000	-0.9694757
15.9833336	2.9280000	-0.9940195
15.9833336	2.9768000	-0.8713012
15.9833336	3.0256000	-0.8835731
15.9833336	3.0743999	-0.8897090
15.9833336	3.1231999	-0.8283496
15.9833336	3.1719999	-0.6994953
15.9833336	3.2207999	-0.6565437
15.9833336	3.2695999	-0.7547188
15.9833336	3.3183999	-0.7485828
15.9833336	3.3671999	-0.7485828
15.9833336	3.4159999	-0.7117672
15.9833336	3.4647999	-0.7117672
15.9833336	3.5135999	-0.7056313
15.9833336	3.5623999	-0.7608547
15.9833336	3.6111999	-0.6320000
15.9833336	3.6599998	-0.6442719
15.9833336	3.7087998	-0.6504078
15.9833336	3.7575998	-0.5890489
15.9833336	3.8063998	-0.5829129
15.9833336	3.8551998	-0.5706410
15.9833336	3.9039998	-0.5706410
15.9833336	3.9527998	-0.5767770
15.9833336	4.0016003	-0.5890489
15.9833336	4.0503998	-0.6135921
15.9833336	4.0991998	-0.5031457
15.9833336	4.1479998	-0.4111071
15.9833336	4.1967998	-0.4172430
15.9833336	4.2455997	-0.4601941
15.9833336	4.2943997	-0.8590293
15.9833336	4.3431997	-0.7056313
15.9833336	4.3919997	-0.7117672
15.9833336	4.4407997	-0.5031457
15.9833336	4.4895997	-0.5399613
15.9833336	4.5383997	-0.6565437
15.9833336	4.5871997	-0.6504078
15.9833336	4.6359997	-0.5706410
15.9833336	4.6847997	-0.5767770
15.9833336	4.7336001	-0.5460973
15.9833336	4.7824001	-0.5460973
15.9833336	4.8312001	-0.4540582
15.9833336	4.8800001	-0.4540582
15.9833336	4.9288001	-0.6135921
15.9833336	4.9776001	-0.7853980
15.9833336	5.0264001	-0.7179031
15.9833336	5.0752001	-0.8774371
15.9833336	5.1240001	-0.8590293
15.9833336	5.1728001	-0.8590293
15.9833336	5.2216001	-0.9756117
15.9833336	5.2704000	613586.0000000
15.9833336	5.3192000	613586.0000000
15.9833336	5.3680000	613586.0000000
15.9833336	5.4168000	613586.0000000

15.9833336	5.4656000	613586.0000000
15.9833336	5.5144000	613586.0000000
15.9833336	5.5632000	613586.0000000
15.9833336	5.6120000	613586.0000000
15.9833336	5.6608000	613586.0000000
15.9833336	5.7096000	613586.0000000
15.9833336	5.7584000	613586.0000000
15.9833336	5.8072000	613586.0000000
15.9833336	5.8559999	613586.0000000
15.9833336	5.9047999	613586.0000000
15.9833336	5.9535999	613586.0000000
15.9833336	6.0023999	613586.0000000
16.0000000	0.2928000	-3.4177094
16.0000000	0.3416000	-3.3993015
16.0000000	0.3904000	-3.6754181
16.0000000	0.4392000	-3.1845441
16.0000000	0.4880000	-3.1845441
16.0000000	0.5368000	-3.5097482
16.0000000	0.5856000	-3.4299812
16.0000000	0.6344000	-2.9329712
16.0000000	0.6832000	-1.7671461
16.0000000	0.7320000	-1.7671461
16.0000000	0.7808000	-1.2394567
16.0000000	0.8296000	-1.2517285
16.0000000	0.8784000	-0.7976699
16.0000000	0.9272000	-0.7853980
16.0000000	0.9760000	-0.7301750
16.0000000	1.0247999	-0.8222136
16.0000000	1.0735999	-0.5276895
16.0000000	1.1223999	-0.9387960
16.0000000	1.1711999	-0.9449320
16.0000000	1.2200000	-0.9449320
16.0000000	1.2688000	-0.9387960
16.0000000	1.3176000	-0.9387960
16.0000000	1.3664000	-0.9510679
16.0000000	1.4152000	-1.0553789
16.0000000	1.4640000	-1.0553789
16.0000000	1.5128000	-0.9142528
16.0000000	1.5616000	-0.7853980
16.0000000	1.6104000	-1.6628351
16.0000000	1.6592000	-1.7303305
16.0000000	1.7079999	-0.9449320
16.0000000	1.7567999	-1.1290097
16.0000000	1.8055999	-1.1535535
16.0000000	1.8543999	-1.1842332
16.0000000	1.9031999	-1.1903691
16.0000000	1.9519999	-1.0615149
16.0000000	2.0007999	-1.0492430
16.0000000	2.0495999	-1.0921946
16.0000000	2.0983999	-1.0921946
16.0000000	2.1471999	-1.0185633
16.0000000	2.1959999	-0.9756117
16.0000000	2.2447999	-1.0799227
16.0000000	2.2935998	-1.0246992
16.0000000	2.3423998	-1.0185633
16.0000000	2.3912001	-0.9081168
16.0000000	2.4400001	-0.9081168

16.0000000	2.4888000	-0.9817476
16.0000000	2.5376000	-1.0308352
16.0000000	2.5864000	-1.0246992
16.0000000	2.6352000	-1.0246992
16.0000000	2.6840000	-0.9326606
16.0000000	2.7328000	-0.9449320
16.0000000	2.7816000	-0.7547188
16.0000000	2.8304000	-0.8651652
16.0000000	2.8792000	-0.7485828
16.0000000	2.9280000	-0.7424469
16.0000000	2.9768000	-0.7240391
16.0000000	3.0256000	-0.7363110
16.0000000	3.0743999	-0.7363110
16.0000000	3.1231999	-0.8897090
16.0000000	3.1719999	-0.7301750
16.0000000	3.2207999	-0.7179031
16.0000000	3.2695999	-0.6994953
16.0000000	3.3183999	-0.6994953
16.0000000	3.3671999	-0.6320000
16.0000000	3.4159999	-0.5645051
16.0000000	3.4647999	-0.5645051
16.0000000	3.5135999	-0.6320000
16.0000000	3.5623999	-0.6320000
16.0000000	3.6111999	-0.6442719
16.0000000	3.6599998	-0.6074562
16.0000000	3.7087998	-0.6074562
16.0000000	3.7575998	-0.6013203
16.0000000	3.8063998	-0.6074562
16.0000000	3.8551998	-0.5460973
16.0000000	3.9039998	-0.5522332
16.0000000	3.9527998	-0.5522332
16.0000000	4.0016003	-0.5583692
16.0000000	4.0503998	-0.5767770
16.0000000	4.0991998	-0.5092816
16.0000000	4.1479998	-0.5092816
16.0000000	4.1967998	-0.4540582
16.0000000	4.2455997	-0.4540582
16.0000000	4.2943997	-0.5031457
16.0000000	4.3431997	-0.5031457
16.0000000	4.3919997	-0.5031457
16.0000000	4.4407997	-0.7179031
16.0000000	4.4895997	-0.7179031
16.0000000	4.5383997	-0.4724660
16.0000000	4.5871997	-0.4847379
16.0000000	4.6359997	-0.4786019
16.0000000	4.6847997	-0.2638445
16.0000000	4.7336001	-0.4601941
16.0000000	4.7824001	-0.1963496
16.0000000	4.8312001	-0.2699804
16.0000000	4.8800001	-0.2699804
16.0000000	4.9288001	-0.5399613
16.0000000	4.9776001	-0.3006601
16.0000000	5.0264001	-0.6872234
16.0000000	5.0752001	-0.3742914
16.0000000	5.1240001	-0.5154176
16.0000000	5.1728001	-0.6688156
16.0000000	5.2216001	-0.6994953

16.0000000	5.2704000	-0.5951843
16.0000000	5.3192000	-0.5951843
16.0000000	5.3680000	-0.6013203
16.0000000	5.4168000	-0.6074562
16.0000000	5.4656000	613586.0000000
16.0000000	5.5144000	613586.0000000
16.0000000	5.5632000	613586.0000000
16.0000000	5.6120000	613586.0000000
16.0000000	5.6608000	613586.0000000
16.0000000	5.7096000	613586.0000000
16.0000000	5.7584000	613586.0000000
16.0000000	5.8072000	613586.0000000
16.0000000	5.8559999	613586.0000000
16.0000000	5.9047999	613586.0000000
16.0000000	5.9535999	613586.0000000
16.0000000	6.0023999	613586.0000000
16.0166664	0.2928000	-3.4913404
16.0166664	0.3416000	-3.1600006
16.0166664	0.3904000	-3.5404277
16.0166664	0.4392000	-3.2643113
16.0166664	0.4880000	-2.9084277
16.0166664	0.5368000	-2.9268355
16.0166664	0.5856000	-3.1293209
16.0166664	0.6344000	-1.8469129
16.0166664	0.6832000	-1.6873789
16.0166664	0.7320000	-0.7792621
16.0166664	0.7808000	-1.1903691
16.0166664	0.8296000	-0.7424469
16.0166664	0.8784000	-0.7485828
16.0166664	0.9272000	-0.6872234
16.0166664	0.9760000	-0.8528934
16.0166664	1.0247999	-0.8590293
16.0166664	1.0735999	-0.7731261
16.0166664	1.1223999	-0.7792621
16.0166664	1.1711999	-0.9510679
16.0166664	1.2200000	-1.0431070
16.0166664	1.2688000	-1.0431070
16.0166664	1.3176000	-0.9510679
16.0166664	1.3664000	-1.0492430
16.0166664	1.4152000	-1.0492430
16.0166664	1.4640000	-1.0676508
16.0166664	1.5128000	-1.1044660
16.0166664	1.5616000	-1.2026410
16.0166664	1.6104000	-0.9387960
16.0166664	1.6592000	-0.9387960
16.0166664	1.7079999	-0.9326606
16.0166664	1.7567999	-0.9081168
16.0166664	1.8055999	-1.1842332
16.0166664	1.8543999	-1.1780972
16.0166664	1.9031999	-1.1719613
16.0166664	1.9519999	-1.2333207
16.0166664	2.0007999	-1.2210488
16.0166664	2.0495999	-1.2087770
16.0166664	2.0983999	-1.2087770
16.0166664	2.1471999	-1.2087770
16.0166664	2.1959999	-1.1474175
16.0166664	2.2447999	-1.1658254

16.0166664	2.2935998	-1.0369711
16.0166664	2.3423998	-1.0308352
16.0166664	2.3912001	-1.0737867
16.0166664	2.4400001	-1.0246992
16.0166664	2.4888000	-1.0492430
16.0166664	2.5376000	-0.8897090
16.0166664	2.5864000	-0.9878836
16.0166664	2.6352000	-0.9694757
16.0166664	2.6840000	-0.9756117
16.0166664	2.7328000	-1.0001554
16.0166664	2.7816000	-0.8344855
16.0166664	2.8304000	-0.8774371
16.0166664	2.8792000	-0.8160777
16.0166664	2.9280000	-0.7731261
16.0166664	2.9768000	-0.7731261
16.0166664	3.0256000	-0.7547188
16.0166664	3.0743999	-0.6810875
16.0166664	3.1231999	-0.9510679
16.0166664	3.1719999	-0.7485828
16.0166664	3.2207999	-0.7731261
16.0166664	3.2695999	-0.6872234
16.0166664	3.3183999	-0.6688156
16.0166664	3.3671999	-0.5706410
16.0166664	3.4159999	-0.5522332
16.0166664	3.4647999	-0.6074562
16.0166664	3.5135999	-0.6013203
16.0166664	3.5623999	-0.5583692
16.0166664	3.6111999	-0.5583692
16.0166664	3.6599998	-0.5215535
16.0166664	3.7087998	-0.5276895
16.0166664	3.7575998	-0.4172430
16.0166664	3.8063998	-0.4172430
16.0166664	3.8551998	-0.3497477
16.0166664	3.9039998	-0.5154176
16.0166664	3.9527998	-0.5092816
16.0166664	4.0016003	-0.4233789
16.0166664	4.0503998	-0.4111071
16.0166664	4.0991998	-0.5583692
16.0166664	4.1479998	-0.3681555
16.0166664	4.1967998	-0.3804274
16.0166664	4.2455997	-0.3865633
16.0166664	4.2943997	-0.4786019
16.0166664	4.3431997	-0.3313398
16.0166664	4.3919997	-0.3742914
16.0166664	4.4407997	-0.5338254
16.0166664	4.4895997	-0.5522332
16.0166664	4.5383997	-0.6381359
16.0166664	4.5871997	-0.6381359
16.0166664	4.6359997	-0.4663301
16.0166664	4.6847997	-0.4724660
16.0166664	4.7336001	-0.6074562
16.0166664	4.7824001	-0.4295144
16.0166664	4.8312001	-0.4663301
16.0166664	4.8800001	-0.4724660
16.0166664	4.9288001	-0.4540582
16.0166664	4.9776001	-0.3988352
16.0166664	5.0264001	-0.7179031

16.0166664	5.0752001	-0.0920386
16.0166664	5.1240001	-0.1165824
16.0166664	5.1728001	0.1411262
16.0166664	5.2216001	613586.0000000
16.0166664	5.2704000	613586.0000000
16.0166664	5.3192000	613586.0000000
16.0166664	5.3680000	613586.0000000
16.0166664	5.4168000	613586.0000000
16.0166664	5.4656000	613586.0000000
16.0166664	5.5144000	613586.0000000
16.0166664	5.5632000	613586.0000000
16.0166664	5.6120000	613586.0000000
16.0166664	5.6608000	613586.0000000
16.0166664	5.7096000	613586.0000000
16.0166664	5.7584000	613586.0000000
16.0166664	5.8072000	613586.0000000
16.0166664	5.8559999	613586.0000000
16.0166664	5.9047999	613586.0000000
16.0166664	5.9535999	613586.0000000
16.0166664	6.0023999	613586.0000000
16.0333328	0.2928000	-3.3133986
16.0333328	0.3416000	-3.3256705
16.0333328	0.3904000	-2.6875343
16.0333328	0.4392000	-2.7427578
16.0333328	0.4880000	-3.1354568
16.0333328	0.5368000	-3.1354568
16.0333328	0.5856000	-3.1231849
16.0333328	0.6344000	-2.5525441
16.0333328	0.6832000	-1.2824078
16.0333328	0.7320000	-1.2824078
16.0333328	0.7808000	-1.1903691
16.0333328	0.8296000	-1.1965051
16.0333328	0.8784000	-0.9878836
16.0333328	0.9272000	-0.9694757
16.0333328	0.9760000	-0.9142528
16.0333328	1.0247999	-0.8897090
16.0333328	1.0735999	-0.9081168
16.0333328	1.1223999	-1.0308352
16.0333328	1.1711999	-1.0185633
16.0333328	1.2200000	-1.0185633
16.0333328	1.2688000	-1.2394567
16.0333328	1.3176000	-1.2210488
16.0333328	1.3664000	-1.0308352
16.0333328	1.4152000	-1.0921946
16.0333328	1.4640000	-1.1474175
16.0333328	1.5128000	-1.1474175
16.0333328	1.5616000	-1.1474175
16.0333328	1.6104000	-1.2762718
16.0333328	1.6592000	-1.0492430
16.0333328	1.7079999	-1.0553789
16.0333328	1.7567999	-1.0676508
16.0333328	1.8055999	-0.8651652
16.0333328	1.8543999	-0.9203887
16.0333328	1.9031999	-0.9142528
16.0333328	1.9519999	-1.3069515
16.0333328	2.0007999	-1.2455926
16.0333328	2.0495999	-1.1903691

16.0333328	2.0983999	-1.2149129
16.0333328	2.1471999	-1.2026410
16.0333328	2.1959999	-1.1903691
16.0333328	2.2447999	-1.1106019
16.0333328	2.2935998	-1.0799227
16.0333328	2.3423998	-1.1106019
16.0333328	2.3912001	-1.1228738
16.0333328	2.4400001	-1.1228738
16.0333328	2.4888000	-0.9326606
16.0333328	2.5376000	-0.9940195
16.0333328	2.5864000	-0.9940195
16.0333328	2.6352000	-0.9878836
16.0333328	2.6840000	-0.9019809
16.0333328	2.7328000	-0.7669902
16.0333328	2.7816000	-0.9203887
16.0333328	2.8304000	-0.8713012
16.0333328	2.8792000	-0.8713012
16.0333328	2.9280000	-0.5890489
16.0333328	2.9768000	-0.7976699
16.0333328	3.0256000	-0.8038058
16.0333328	3.0743999	-0.5890489
16.0333328	3.1231999	-0.5890489
16.0333328	3.1719999	-0.7056313
16.0333328	3.2207999	-0.7056313
16.0333328	3.2695999	-0.6688156
16.0333328	3.3183999	-0.5645051
16.0333328	3.3671999	-0.6994953
16.0333328	3.4159999	-0.6381359
16.0333328	3.4647999	-0.6381359
16.0333328	3.5135999	-0.5706410
16.0333328	3.5623999	-0.6933594
16.0333328	3.6111999	-0.6442719
16.0333328	3.6599998	-0.6381359
16.0333328	3.7087998	-0.6810875
16.0333328	3.7575998	-0.7056313
16.0333328	3.8063998	-0.5154176
16.0333328	3.8551998	-0.5276895
16.0333328	3.9039998	-0.3804274
16.0333328	3.9527998	-0.4847379
16.0333328	4.0016003	-0.4847379
16.0333328	4.0503998	-0.6504078
16.0333328	4.0991998	-0.5154176
16.0333328	4.1479998	-0.5154176
16.0333328	4.1967998	-0.3988352
16.0333328	4.2455997	-0.5154176
16.0333328	4.2943997	-0.5154176
16.0333328	4.3431997	-0.4479222
16.0333328	4.3919997	-0.4356503
16.0333328	4.4407997	-0.4172430
16.0333328	4.4895997	-0.4786019
16.0333328	4.5383997	-0.4847379
16.0333328	4.5871997	-0.4847379
16.0333328	4.6359997	-0.3067961
16.0333328	4.6847997	-0.3497477
16.0333328	4.7336001	-0.3620195
16.0333328	4.7824001	-0.4663301
16.0333328	4.8312001	-0.2638445

16.0333328	4.8800001	-0.4295144
16.0333328	4.9288001	-0.4786019
16.0333328	4.9776001	-0.2761164
16.0333328	5.0264001	-0.1043105
16.0333328	5.0752001	-0.1104465
16.0333328	5.1240001	0.0674953
16.0333328	5.1728001	-0.2208934
16.0333328	5.2216001	0.0859027
16.0333328	5.2704000	613586.0000000
16.0333328	5.3192000	613586.0000000
16.0333328	5.3680000	613586.0000000
16.0333328	5.4168000	613586.0000000
16.0333328	5.4656000	-0.2086215
16.0333328	5.5144000	613586.0000000
16.0333328	5.5632000	613586.0000000
16.0333328	5.6120000	613586.0000000
16.0333328	5.6608000	613586.0000000
16.0333328	5.7096000	613586.0000000
16.0333328	5.7584000	613586.0000000
16.0333328	5.8072000	613586.0000000
16.0333328	5.8559999	613586.0000000
16.0333328	5.9047999	613586.0000000
16.0333328	5.9535999	613586.0000000
16.0333328	6.0023999	613586.0000000
16.0499992	0.2928000	-2.6752625
16.0499992	0.3416000	-3.1722724
16.0499992	0.3904000	-3.6263306
16.0499992	0.4392000	-3.0802336
16.0499992	0.4880000	-3.0986414
16.0499992	0.5368000	-2.6998062
16.0499992	0.5856000	-2.7182140
16.0499992	0.6344000	-1.9450879
16.0499992	0.6832000	-1.9450879
16.0499992	0.7320000	-1.4542136
16.0499992	0.7808000	-1.4726214
16.0499992	0.8296000	-1.2271848
16.0499992	0.8784000	-1.1965051
16.0499992	0.9272000	-1.2149129
16.0499992	0.9760000	-1.2087770
16.0499992	1.0247999	-1.1106019
16.0499992	1.0735999	-1.0676508
16.0499992	1.1223999	-1.0737867
16.0499992	1.1711999	-1.1903691
16.0499992	1.2200000	-1.0737867
16.0499992	1.2688000	-0.8590293
16.0499992	1.3176000	-1.2824078
16.0499992	1.3664000	-1.1842332
16.0499992	1.4152000	-1.1780972
16.0499992	1.4640000	-1.1658254
16.0499992	1.5128000	-1.1719613
16.0499992	1.5616000	-1.0062914
16.0499992	1.6104000	-1.2026410
16.0499992	1.6592000	-1.1842332
16.0499992	1.7079999	-1.1842332
16.0499992	1.7567999	-0.9817476
16.0499992	1.8055999	-0.9756117
16.0499992	1.8543999	-1.4112625

16.0499992	1.9031999	-1.4173985
16.0499992	1.9519999	-1.2578645
16.0499992	2.0007999	-1.1965051
16.0499992	2.0495999	-1.1903691
16.0499992	2.0983999	-1.2026410
16.0499992	2.1471999	-1.1965051
16.0499992	2.1959999	-1.1719613
16.0499992	2.2447999	-1.1044660
16.0499992	2.2935998	-1.0983305
16.0499992	2.3423998	-1.1842332
16.0499992	2.3912001	-1.1842332
16.0499992	2.4400001	-1.0983305
16.0499992	2.4888000	-1.0246992
16.0499992	2.5376000	-1.1596894
16.0499992	2.5864000	-1.1474175
16.0499992	2.6352000	-0.9817476
16.0499992	2.6840000	-1.0062914
16.0499992	2.7328000	-1.0062914
16.0499992	2.7816000	-1.0062914
16.0499992	2.8304000	-0.9081168
16.0499992	2.8792000	-0.9203887
16.0499992	2.9280000	-0.8283496
16.0499992	2.9768000	-0.8222136
16.0499992	3.0256000	-0.8099418
16.0499992	3.0743999	-0.7363110
16.0499992	3.1231999	-0.7301750
16.0499992	3.1719999	-0.8467574
16.0499992	3.2207999	-0.8467574
16.0499992	3.2695999	-0.6442719
16.0499992	3.3183999	-0.7363110
16.0499992	3.3671999	-0.7424469
16.0499992	3.4159999	-0.7792621
16.0499992	3.4647999	-0.7669902
16.0499992	3.5135999	-0.7669902
16.0499992	3.5623999	-0.7301750
16.0499992	3.6111999	-0.7731261
16.0499992	3.6599998	-0.7915339
16.0499992	3.7087998	-0.6320000
16.0499992	3.7575998	-0.6933594
16.0499992	3.8063998	-0.5583692
16.0499992	3.8551998	-0.6381359
16.0499992	3.9039998	-0.6381359
16.0499992	3.9527998	-0.6504078
16.0499992	4.0016003	-0.6442719
16.0499992	4.0503998	-0.6442719
16.0499992	4.0991998	-0.6994953
16.0499992	4.1479998	-0.8897090
16.0499992	4.1967998	-0.7424469
16.0499992	4.2455997	-0.9572039
16.0499992	4.2943997	-0.9572039
16.0499992	4.3431997	-0.7363110
16.0499992	4.3919997	-0.7363110
16.0499992	4.4407997	-0.7915339
16.0499992	4.4895997	-0.8406215
16.0499992	4.5383997	-0.8283496
16.0499992	4.5871997	-0.5829129
16.0499992	4.6359997	-0.6872234

16.0499992	4.6847997	-0.6994953
16.0499992	4.7336001	-0.4908738
16.0499992	4.7824001	-0.2822523
16.0499992	4.8312001	-0.6688156
16.0499992	4.8800001	-0.4049711
16.0499992	4.9288001	-0.4111071
16.0499992	4.9776001	-0.7240391
16.0499992	5.0264001	-0.6933594
16.0499992	5.0752001	-0.6013203
16.0499992	5.1240001	-0.3313398
16.0499992	5.1728001	-0.3129320
16.0499992	5.2216001	-0.3129320
16.0499992	5.2704000	613586.0000000
16.0499992	5.3192000	613586.0000000
16.0499992	5.3680000	613586.0000000
16.0499992	5.4168000	-0.7608547
16.0499992	5.4656000	-0.7363110
16.0499992	5.5144000	613586.0000000
16.0499992	5.5632000	613586.0000000
16.0499992	5.6120000	613586.0000000
16.0499992	5.6608000	613586.0000000
16.0499992	5.7096000	613586.0000000
16.0499992	5.7584000	613586.0000000
16.0499992	5.8072000	613586.0000000
16.0499992	5.8559999	613586.0000000
16.0499992	5.9047999	613586.0000000
16.0499992	5.9535999	613586.0000000
16.0499992	6.0023999	613586.0000000
16.0666676	0.2928000	-3.2765830
16.0666676	0.3416000	-4.2583308
16.0666676	0.3904000	-4.0313015
16.0666676	0.4392000	-3.7735929
16.0666676	0.4880000	-3.7920005
16.0666676	0.5368000	-3.7981365
16.0666676	0.5856000	-3.3256705
16.0666676	0.6344000	-2.6139033
16.0666676	0.6832000	-1.6628351
16.0666676	0.7320000	-1.6689711
16.0666676	0.7808000	-1.6689711
16.0666676	0.8296000	-1.3008156
16.0666676	0.8784000	-1.4296703
16.0666676	0.9272000	-1.3560390
16.0666676	0.9760000	-1.3683109
16.0666676	1.0247999	-1.4358063
16.0666676	1.0735999	-1.4358063
16.0666676	1.1223999	-1.2087770
16.0666676	1.1711999	-1.1596894
16.0666676	1.2200000	-1.1780972
16.0666676	1.2688000	-1.1965051
16.0666676	1.3176000	-1.1965051
16.0666676	1.3664000	-1.2578645
16.0666676	1.4152000	-1.2640004
16.0666676	1.4640000	-1.1351457
16.0666676	1.5128000	-1.0860586
16.0666676	1.5616000	-1.2210488
16.0666676	1.6104000	-1.2026410
16.0666676	1.6592000	-1.1412816

16.0666676	1.7079999	-0.9633398
16.0666676	1.7567999	-1.3867188
16.0666676	1.8055999	-1.0124273
16.0666676	1.8543999	-1.0553789
16.0666676	1.9031999	-1.1290097
16.0666676	1.9519999	-1.2394567
16.0666676	2.0007999	-1.1965051
16.0666676	2.0495999	-1.1965051
16.0666676	2.0983999	-1.1596894
16.0666676	2.1471999	-1.1965051
16.0666676	2.1959999	-1.2271848
16.0666676	2.2447999	-1.0246992
16.0666676	2.2935998	-1.0246992
16.0666676	2.3423998	-1.1351457
16.0666676	2.3912001	-1.2026410
16.0666676	2.4400001	-0.9694757
16.0666676	2.4888000	-1.0124273
16.0666676	2.5376000	-1.0615149
16.0666676	2.5864000	-1.1351457
16.0666676	2.6352000	-1.1412816
16.0666676	2.6840000	-1.1535535
16.0666676	2.7328000	-0.9756117
16.0666676	2.7816000	-0.9142528
16.0666676	2.8304000	-1.0001554
16.0666676	2.8792000	-0.9081168
16.0666676	2.9280000	-0.9510679
16.0666676	2.9768000	-0.9633398
16.0666676	3.0256000	-0.9633398
16.0666676	3.0743999	-0.8713012
16.0666676	3.1231999	-0.8713012
16.0666676	3.1719999	-0.8774371
16.0666676	3.2207999	-0.7915339
16.0666676	3.2695999	-0.7853980
16.0666676	3.3183999	-0.7056313
16.0666676	3.3671999	-0.7731261
16.0666676	3.4159999	-0.7669902
16.0666676	3.4647999	-0.6872234
16.0666676	3.5135999	-0.6749516
16.0666676	3.5623999	-0.7792621
16.0666676	3.6111999	-0.7669902
16.0666676	3.6599998	-0.7547188
16.0666676	3.7087998	-0.6688156
16.0666676	3.7575998	-0.6688156
16.0666676	3.8063998	-0.6933594
16.0666676	3.8551998	-0.6872234
16.0666676	3.9039998	-0.7056313
16.0666676	3.9527998	-0.6749516
16.0666676	4.0016003	-0.6749516
16.0666676	4.0503998	-0.7669902
16.0666676	4.0991998	-0.7731261
16.0666676	4.1479998	-0.7117672
16.0666676	4.1967998	-0.7117672
16.0666676	4.2455997	-0.7301750
16.0666676	4.2943997	-0.6994953
16.0666676	4.3431997	-0.6626797
16.0666676	4.3919997	-0.6565437
16.0666676	4.4407997	-0.6504078

16.0666676	4.4895997	-0.4111071
16.0666676	4.5383997	-0.4786019
16.0666676	4.5871997	-0.4908738
16.0666676	4.6359997	-0.6749516
16.0666676	4.6847997	-0.6135921
16.0666676	4.7336001	-0.6258640
16.0666676	4.7824001	-0.4295144
16.0666676	4.8312001	-0.6135921
16.0666676	4.8800001	-0.4172430
16.0666676	4.9288001	-0.4601941
16.0666676	4.9776001	-0.4786019
16.0666676	5.0264001	-0.5092816
16.0666676	5.0752001	-0.3374758
16.0666676	5.1240001	-0.3374758
16.0666676	5.1728001	-0.8344855
16.0666676	5.2216001	-1.2149129
16.0666676	5.2704000	-0.9694757
16.0666676	5.3192000	-0.9387960
16.0666676	5.3680000	-0.9940195
16.0666676	5.4168000	613586.0000000
16.0666676	5.4656000	613586.0000000
16.0666676	5.5144000	613586.0000000
16.0666676	5.5632000	613586.0000000
16.0666676	5.6120000	613586.0000000
16.0666676	5.6608000	613586.0000000
16.0666676	5.7096000	613586.0000000
16.0666676	5.7584000	613586.0000000
16.0666676	5.8072000	613586.0000000
16.0666676	5.8559999	613586.0000000
16.0666676	5.9047999	613586.0000000
16.0666676	5.9535999	613586.0000000
16.0666676	6.0023999	613586.0000000
16.0833340	0.2928000	-3.3195345
16.0833340	0.3416000	-3.3195345
16.0833340	0.3904000	-3.3133986
16.0833340	0.4392000	-3.3011267
16.0833340	0.4880000	-3.0986414
16.0833340	0.5368000	-3.6386025
16.0833340	0.5856000	-2.5341363
16.0833340	0.6344000	-1.0246992
16.0833340	0.6832000	-1.0185633
16.0833340	0.7320000	-1.0062914
16.0833340	0.7808000	-1.0062914
16.0833340	0.8296000	-1.3805828
16.0833340	0.8784000	-1.3989906
16.0833340	0.9272000	-1.3253593
16.0833340	0.9760000	-1.3437672
16.0833340	1.0247999	-1.2455926
16.0833340	1.0735999	-1.2394567
16.0833340	1.1223999	-1.2271848
16.0833340	1.1711999	-1.2271848
16.0833340	1.2200000	-1.3560390
16.0833340	1.2688000	-1.1535535
16.0833340	1.3176000	-1.1535535
16.0833340	1.3664000	-1.1719613
16.0833340	1.4152000	-1.1535535
16.0833340	1.4640000	-1.2455926

16.0833340	1.5128000	-1.0799227
16.0833340	1.5616000	-1.1780972
16.0833340	1.6104000	-1.3499031
16.0833340	1.6592000	-1.2701364
16.0833340	1.7079999	-1.2824078
16.0833340	1.7567999	-1.0983305
16.0833340	1.8055999	-1.3069515
16.0833340	1.8543999	-1.4848933
16.0833340	1.9031999	-1.4848933
16.0833340	1.9519999	-1.2946796
16.0833340	2.0007999	-1.2885437
16.0833340	2.0495999	-1.3253593
16.0833340	2.0983999	-1.2087770
16.0833340	2.1471999	-1.2333207
16.0833340	2.1959999	-1.2026410
16.0833340	2.2447999	-1.1719613
16.0833340	2.2935998	-1.1596894
16.0833340	2.3423998	-1.2087770
16.0833340	2.3912001	-1.0615149
16.0833340	2.4400001	-1.0062914
16.0833340	2.4888000	-0.9142528
16.0833340	2.5376000	-0.9326606
16.0833340	2.5864000	-0.9572039
16.0833340	2.6352000	-0.9633398
16.0833340	2.6840000	-0.9694757
16.0833340	2.7328000	-0.9633398
16.0833340	2.7816000	-0.9142528
16.0833340	2.8304000	-0.7179031
16.0833340	2.8792000	-0.7363110
16.0833340	2.9280000	-0.7608547
16.0833340	2.9768000	-0.7669902
16.0833340	3.0256000	-0.6565437
16.0833340	3.0743999	-0.8651652
16.0833340	3.1231999	-0.8528934
16.0833340	3.1719999	-0.7669902
16.0833340	3.2207999	-0.7669902
16.0833340	3.2695999	-0.8099418
16.0833340	3.3183999	-0.7424469
16.0833340	3.3671999	-0.7179031
16.0833340	3.4159999	-0.7301750
16.0833340	3.4647999	-0.8406215
16.0833340	3.5135999	-0.8406215
16.0833340	3.5623999	-0.8651652
16.0833340	3.6111999	-0.8160777
16.0833340	3.6599998	-0.7363110
16.0833340	3.7087998	-0.6933594
16.0833340	3.7575998	-0.6872234
16.0833340	3.8063998	-0.6749516
16.0833340	3.8551998	-0.8283496
16.0833340	3.9039998	-0.8344855
16.0833340	3.9527998	-0.8344855
16.0833340	4.0016003	-0.6749516
16.0833340	4.0503998	-0.6626797
16.0833340	4.0991998	-0.6442719
16.0833340	4.1479998	-0.6320000
16.0833340	4.1967998	-0.6933594
16.0833340	4.2455997	-0.6074562

16.0833340	4.2943997	-0.6565437
16.0833340	4.3431997	-0.6626797
16.0833340	4.3919997	-0.6688156
16.0833340	4.4407997	-0.7301750
16.0833340	4.4895997	-0.7179031
16.0833340	4.5383997	-0.5460973
16.0833340	4.5871997	-0.8038058
16.0833340	4.6359997	-0.6810875
16.0833340	4.6847997	-0.7608547
16.0833340	4.7336001	-0.4724660
16.0833340	4.7824001	-0.7669902
16.0833340	4.8312001	-0.7669902
16.0833340	4.8800001	-0.8958449
16.0833340	4.9288001	-0.7179031
16.0833340	4.9776001	-0.7179031
16.0833340	5.0264001	-0.6565437
16.0833340	5.0752001	-0.7731261
16.0833340	5.1240001	-0.6565437
16.0833340	5.1728001	-0.6442719
16.0833340	5.2216001	-0.5522332
16.0833340	5.2704000	-0.7853980
16.0833340	5.3192000	-0.6258640
16.0833340	5.3680000	-0.6197281
16.0833340	5.4168000	613586.0000000
16.0833340	5.4656000	613586.0000000
16.0833340	5.5144000	613586.0000000
16.0833340	5.5632000	-0.5522332
16.0833340	5.6120000	613586.0000000
16.0833340	5.6608000	613586.0000000
16.0833340	5.7096000	-0.9142528
16.0833340	5.7584000	613586.0000000
16.0833340	5.8072000	613586.0000000
16.0833340	5.8559999	613586.0000000
16.0833340	5.9047999	613586.0000000
16.0833340	5.9535999	613586.0000000
16.0833340	6.0023999	613586.0000000
16.1000004	0.2928000	-3.9699423
16.1000004	0.3416000	-3.8656316
16.1000004	0.3904000	-2.9513791
16.1000004	0.4392000	-3.4299812
16.1000004	0.4880000	-2.5770879
16.1000004	0.5368000	-2.4420974
16.1000004	0.5856000	-2.7918451
16.1000004	0.6344000	-0.9572039
16.1000004	0.6832000	-0.9572039
16.1000004	0.7320000	-0.8099418
16.1000004	0.7808000	-0.7363110
16.1000004	0.8296000	-0.9940195
16.1000004	0.8784000	-1.1228738
16.1000004	0.9272000	-1.0676508
16.1000004	0.9760000	-1.1596894
16.1000004	1.0247999	-1.1658254
16.1000004	1.0735999	-0.9817476
16.1000004	1.1223999	-1.0737867
16.1000004	1.1711999	-1.1596894
16.1000004	1.2200000	-1.1719613
16.1000004	1.2688000	-1.1228738

16.1000004	1.3176000	-0.8528934
16.1000004	1.3664000	-1.0737867
16.1000004	1.4152000	-1.2455926
16.1000004	1.4640000	-1.2640004
16.1000004	1.5128000	-1.1719613
16.1000004	1.5616000	-1.1596894
16.1000004	1.6104000	-1.1535535
16.1000004	1.6592000	-1.2824078
16.1000004	1.7079999	-1.4051266
16.1000004	1.7567999	-1.3744469
16.1000004	1.8055999	-1.3069515
16.1000004	1.8543999	-1.3253593
16.1000004	1.9031999	-1.3130875
16.1000004	1.9519999	-1.3744469
16.1000004	2.0007999	-1.2210488
16.1000004	2.0495999	-1.1290097
16.1000004	2.0983999	-1.2149129
16.1000004	2.1471999	-1.1474175
16.1000004	2.1959999	-1.2026410
16.1000004	2.2447999	-1.2026410
16.1000004	2.2935998	-1.1903691
16.1000004	2.3423998	-1.1903691
16.1000004	2.3912001	-1.0737867
16.1000004	2.4400001	-1.0799227
16.1000004	2.4888000	-1.0676508
16.1000004	2.5376000	-0.9817476
16.1000004	2.5864000	-0.9940195
16.1000004	2.6352000	-1.0492430
16.1000004	2.6840000	-1.0553789
16.1000004	2.7328000	-0.9940195
16.1000004	2.7816000	-0.9510679
16.1000004	2.8304000	-0.9510679
16.1000004	2.8792000	-0.7485828
16.1000004	2.9280000	-0.8835731
16.1000004	2.9768000	-0.8774371
16.1000004	3.0256000	-0.8590293
16.1000004	3.0743999	-0.8283496
16.1000004	3.1231999	-0.9081168
16.1000004	3.1719999	-0.7976699
16.1000004	3.2207999	-0.8897090
16.1000004	3.2695999	-0.8958449
16.1000004	3.3183999	-0.9019809
16.1000004	3.3671999	-0.7853980
16.1000004	3.4159999	-0.8038058
16.1000004	3.4647999	-0.8651652
16.1000004	3.5135999	-0.8467574
16.1000004	3.5623999	-0.7669902
16.1000004	3.6111999	-0.8528934
16.1000004	3.6599998	-0.8528934
16.1000004	3.7087998	-0.8467574
16.1000004	3.7575998	-0.8467574
16.1000004	3.8063998	-0.8406215
16.1000004	3.8551998	-0.7363110
16.1000004	3.9039998	-0.7363110
16.1000004	3.9527998	-0.6933594
16.1000004	4.0016003	-0.9572039
16.1000004	4.0503998	-0.9633398

16.1000004	4.0991998	-0.5215535
16.1000004	4.1479998	-0.5092816
16.1000004	4.1967998	-1.0308352
16.1000004	4.2455997	-0.7485828
16.1000004	4.2943997	-0.6320000
16.1000004	4.3431997	-0.6320000
16.1000004	4.3919997	-0.6381359
16.1000004	4.4407997	-0.6135921
16.1000004	4.4895997	-0.6135921
16.1000004	4.5383997	-0.6074562
16.1000004	4.5871997	-0.6994953
16.1000004	4.6359997	-0.7547188
16.1000004	4.6847997	-0.7976699
16.1000004	4.7336001	-0.8038058
16.1000004	4.7824001	-0.7976699
16.1000004	4.8312001	-0.8038058
16.1000004	4.8800001	-0.8713012
16.1000004	4.9288001	-0.8713012
16.1000004	4.9776001	-0.6626797
16.1000004	5.0264001	-0.6810875
16.1000004	5.0752001	-0.6749516
16.1000004	5.1240001	-0.6197281
16.1000004	5.1728001	-0.6381359
16.1000004	5.2216001	-0.6504078
16.1000004	5.2704000	-0.5829129
16.1000004	5.3192000	613586.0000000
16.1000004	5.3680000	613586.0000000
16.1000004	5.4168000	613586.0000000
16.1000004	5.4656000	613586.0000000
16.1000004	5.5144000	613586.0000000
16.1000004	5.5632000	613586.0000000
16.1000004	5.6120000	613586.0000000
16.1000004	5.6608000	613586.0000000
16.1000004	5.7096000	613586.0000000
16.1000004	5.7584000	613586.0000000
16.1000004	5.8072000	613586.0000000
16.1000004	5.8559999	613586.0000000
16.1000004	5.9047999	613586.0000000
16.1000004	5.9535999	613586.0000000
16.1000004	6.0023999	613586.0000000
16.1166668	0.2928000	-3.4729326
16.1166668	0.3416000	-3.4729326
16.1166668	0.3904000	-3.4667966
16.1166668	0.4392000	-3.3747578
16.1166668	0.4880000	-3.2090878
16.1166668	0.5368000	-3.3072627
16.1166668	0.5856000	-2.5218644
16.1166668	0.6344000	-2.5218644
16.1166668	0.6832000	-1.8714566
16.1166668	0.7320000	-1.4419422
16.1166668	0.7808000	-1.3008156
16.1166668	0.8296000	-1.0308352
16.1166668	0.8784000	-1.0246992
16.1166668	0.9272000	-1.0308352
16.1166668	0.9760000	-1.0001554
16.1166668	1.0247999	-1.0124273
16.1166668	1.0735999	-0.9081168

16.1166668	1.1223999	-0.8897090
16.1166668	1.1711999	-1.1535535
16.1166668	1.2200000	-1.1535535
16.1166668	1.2688000	-1.0246992
16.1166668	1.3176000	-1.2271848
16.1166668	1.3664000	-1.0308352
16.1166668	1.4152000	-1.1167378
16.1166668	1.4640000	-1.1228738
16.1166668	1.5128000	-1.1228738
16.1166668	1.5616000	-1.1167378
16.1166668	1.6104000	-1.1106019
16.1166668	1.6592000	-1.1106019
16.1166668	1.7079999	-1.2087770
16.1166668	1.7567999	-1.3437672
16.1166668	1.8055999	-1.2701364
16.1166668	1.8543999	-1.1596894
16.1166668	1.9031999	-1.1719613
16.1166668	1.9519999	-1.1842332
16.1166668	2.0007999	-1.3437672
16.1166668	2.0495999	-1.2455926
16.1166668	2.0983999	-1.2517285
16.1166668	2.1471999	-1.1596894
16.1166668	2.1959999	-1.0799227
16.1166668	2.2447999	-1.0799227
16.1166668	2.2935998	-1.1167378
16.1166668	2.3423998	-1.1351457
16.1166668	2.3912001	-1.1412816
16.1166668	2.4400001	-1.1290097
16.1166668	2.4888000	-1.0308352
16.1166668	2.5376000	-1.0308352
16.1166668	2.5864000	-1.0921946
16.1166668	2.6352000	-1.1044660
16.1166668	2.6840000	-1.1167378
16.1166668	2.7328000	-0.9449320
16.1166668	2.7816000	-0.9572039
16.1166668	2.8304000	-0.9572039
16.1166668	2.8792000	-0.9572039
16.1166668	2.9280000	-1.0001554
16.1166668	2.9768000	-1.0062914
16.1166668	3.0256000	-1.0308352
16.1166668	3.0743999	-1.0492430
16.1166668	3.1231999	-1.0553789
16.1166668	3.1719999	-1.0553789
16.1166668	3.2207999	-1.0553789
16.1166668	3.2695999	-1.0062914
16.1166668	3.3183999	-1.0062914
16.1166668	3.3671999	-1.0308352
16.1166668	3.4159999	-1.0308352
16.1166668	3.4647999	-0.9019809
16.1166668	3.5135999	-0.8897090
16.1166668	3.5623999	-0.8774371
16.1166668	3.6111999	-0.7179031
16.1166668	3.6599998	-0.7669902
16.1166668	3.7087998	-0.7792621
16.1166668	3.7575998	-0.8283496
16.1166668	3.8063998	-0.8344855
16.1166668	3.8551998	-0.6872234

16.1166668	3.9039998	-0.7485828
16.1166668	3.9527998	-0.5706410
16.1166668	4.0016003	-0.5890489
16.1166668	4.0503998	-0.5276895
16.1166668	4.0991998	-0.8283496
16.1166668	4.1479998	-0.8283496
16.1166668	4.1967998	-0.7792621
16.1166668	4.2455997	-0.9081168
16.1166668	4.2943997	-0.8713012
16.1166668	4.3431997	-0.8590293
16.1166668	4.3919997	-0.8528934
16.1166668	4.4407997	-0.7301750
16.1166668	4.4895997	-0.7301750
16.1166668	4.5383997	-0.6320000
16.1166668	4.5871997	-0.6320000
16.1166668	4.6359997	-0.7240391
16.1166668	4.6847997	-0.7424469
16.1166668	4.7336001	-0.7240391
16.1166668	4.7824001	-0.7301750
16.1166668	4.8312001	-0.7976699
16.1166668	4.8800001	-0.6872234
16.1166668	4.9288001	-0.7240391
16.1166668	4.9776001	-0.7976699
16.1166668	5.0264001	-0.5706410
16.1166668	5.0752001	-0.4356503
16.1166668	5.1240001	-0.4356503
16.1166668	5.1728001	-0.6810875
16.1166668	5.2216001	-0.5154176
16.1166668	5.2704000	-0.5092816
16.1166668	5.3192000	613586.0000000
16.1166668	5.3680000	-1.1780972
16.1166668	5.4168000	-1.2333207
16.1166668	5.4656000	-1.2210488
16.1166668	5.5144000	-1.2210488
16.1166668	5.5632000	613586.0000000
16.1166668	5.6120000	613586.0000000
16.1166668	5.6608000	613586.0000000
16.1166668	5.7096000	613586.0000000
16.1166668	5.7584000	613586.0000000
16.1166668	5.8072000	613586.0000000
16.1166668	5.8559999	613586.0000000
16.1166668	5.9047999	613586.0000000
16.1166668	5.9535999	613586.0000000
16.1166668	6.0023999	613586.0000000
16.1333332	0.2928000	-2.6813984
16.1333332	0.3416000	-2.1598449
16.1333332	0.3904000	-3.8656316
16.1333332	0.4392000	-4.0067577
16.1333332	0.4880000	-4.0128937
16.1333332	0.5368000	-3.4790685
16.1333332	0.5856000	-2.2150683
16.1333332	0.6344000	-1.7855535
16.1333332	0.6832000	-1.7916894
16.1333332	0.7320000	-1.6198835
16.1333332	0.7808000	-1.3989906
16.1333332	0.8296000	-1.3437672
16.1333332	0.8784000	-0.9694757

16.1333332	0.9272000	-1.0676508
16.1333332	0.9760000	-1.0615149
16.1333332	1.0247999	-1.2087770
16.1333332	1.0735999	-1.2087770
16.1333332	1.1223999	-1.2824078
16.1333332	1.1711999	-1.0983305
16.1333332	1.2200000	-1.0369711
16.1333332	1.2688000	-1.0615149
16.1333332	1.3176000	-1.0676508
16.1333332	1.3664000	-1.5217090
16.1333332	1.4152000	-1.3744469
16.1333332	1.4640000	-1.4112625
16.1333332	1.5128000	-1.4112625
16.1333332	1.5616000	-1.4235344
16.1333332	1.6104000	-1.4296703
16.1333332	1.6592000	-1.6076121
16.1333332	1.7079999	-1.2885437
16.1333332	1.7567999	-1.2885437
16.1333332	1.8055999	-1.2885437
16.1333332	1.8543999	-1.0983305
16.1333332	1.9031999	-1.1044660
16.1333332	1.9519999	-1.2149129
16.1333332	2.0007999	-1.2087770
16.1333332	2.0495999	-1.0799227
16.1333332	2.0983999	-1.0676508
16.1333332	2.1471999	-1.0615149
16.1333332	2.1959999	-1.0246992
16.1333332	2.2447999	-1.0308352
16.1333332	2.2935998	-1.0308352
16.1333332	2.3423998	-1.0246992
16.1333332	2.3912001	-1.0246992
16.1333332	2.4400001	-0.9387960
16.1333332	2.4888000	-1.0983305
16.1333332	2.5376000	-1.0983305
16.1333332	2.5864000	-1.0062914
16.1333332	2.6352000	-0.9449320
16.1333332	2.6840000	-0.9449320
16.1333332	2.7328000	-1.0246992
16.1333332	2.7816000	-0.9019809
16.1333332	2.8304000	-0.8038058
16.1333332	2.8792000	-0.8038058
16.1333332	2.9280000	-0.6994953
16.1333332	2.9768000	-0.7117672
16.1333332	3.0256000	-0.7117672
16.1333332	3.0743999	-0.9449320
16.1333332	3.1231999	-0.9572039
16.1333332	3.1719999	-0.9510679
16.1333332	3.2207999	-1.0553789
16.1333332	3.2695999	-0.9265246
16.1333332	3.3183999	-0.9326606
16.1333332	3.3671999	-0.9817476
16.1333332	3.4159999	-0.9694757
16.1333332	3.4647999	-0.9756117
16.1333332	3.5135999	-0.8835731
16.1333332	3.5623999	-0.8835731
16.1333332	3.6111999	-0.8958449
16.1333332	3.6599998	-0.9019809

16.1333332	3.7087998	-0.7976699
16.1333332	3.7575998	-0.8099418
16.1333332	3.8063998	-0.8283496
16.1333332	3.8551998	-0.9572039
16.1333332	3.9039998	-0.8283496
16.1333332	3.9527998	-0.6688156
16.1333332	4.0016003	-0.6688156
16.1333332	4.0503998	-0.6565437
16.1333332	4.0991998	-0.6504078
16.1333332	4.1479998	-0.7179031
16.1333332	4.1967998	-0.6442719
16.1333332	4.2455997	-0.6013203
16.1333332	4.2943997	-0.8713012
16.1333332	4.3431997	-0.8590293
16.1333332	4.3919997	-0.7792621
16.1333332	4.4407997	-0.7853980
16.1333332	4.4895997	-0.7792621
16.1333332	4.5383997	-0.8283496
16.1333332	4.5871997	-0.8406215
16.1333332	4.6359997	-0.4601941
16.1333332	4.6847997	-0.4601941
16.1333332	4.7336001	-0.2822523
16.1333332	4.7824001	-0.3374758
16.1333332	4.8312001	-0.4111071
16.1333332	4.8800001	-0.3988352
16.1333332	4.9288001	-0.6013203
16.1333332	4.9776001	-0.4970098
16.1333332	5.0264001	-0.6504078
16.1333332	5.0752001	-0.4417863
16.1333332	5.1240001	-0.4417863
16.1333332	5.1728001	-0.7976699
16.1333332	5.2216001	-0.7915339
16.1333332	5.2704000	613586.0000000
16.1333332	5.3192000	613586.0000000
16.1333332	5.3680000	613586.0000000
16.1333332	5.4168000	613586.0000000
16.1333332	5.4656000	613586.0000000
16.1333332	5.5144000	613586.0000000
16.1333332	5.5632000	613586.0000000
16.1333332	5.6120000	613586.0000000
16.1333332	5.6608000	613586.0000000
16.1333332	5.7096000	613586.0000000
16.1333332	5.7584000	613586.0000000
16.1333332	5.8072000	613586.0000000
16.1333332	5.8559999	613586.0000000
16.1333332	5.9047999	613586.0000000
16.1333332	5.9535999	613586.0000000
16.1333332	6.0023999	613586.0000000
16.1499996	0.2928000	-4.3687773
16.1499996	0.3416000	-3.7367773
16.1499996	0.3904000	-3.5772433
16.1499996	0.4392000	-3.0618258
16.1499996	0.4880000	-3.4238453
16.1499996	0.5368000	-2.6261752
16.1499996	0.5856000	-2.6261752
16.1499996	0.6344000	-2.2150683
16.1499996	0.6832000	-2.9329712

16.1499996	0.7320000	-2.9329712
16.1499996	0.7808000	-1.8591847
16.1499996	0.8296000	-1.6076121
16.1499996	0.8784000	-1.6076121
16.1499996	0.9272000	-1.1290097
16.1499996	0.9760000	-1.0983305
16.1499996	1.0247999	-1.0001554
16.1499996	1.0735999	-1.1228738
16.1499996	1.1223999	-1.0676508
16.1499996	1.1711999	-1.5094371
16.1499996	1.2200000	-1.3437672
16.1499996	1.2688000	-1.1351457
16.1499996	1.3176000	-1.1965051
16.1499996	1.3664000	-1.2149129
16.1499996	1.4152000	-1.2946796
16.1499996	1.4640000	-1.3683109
16.1499996	1.5128000	-1.3621750
16.1499996	1.5616000	-1.4787574
16.1499996	1.6104000	-1.2210488
16.1499996	1.6592000	-1.4112625
16.1499996	1.7079999	-1.4112625
16.1499996	1.7567999	-1.3867188
16.1499996	1.8055999	-1.2210488
16.1499996	1.8543999	-1.2271848
16.1499996	1.9031999	-1.2701364
16.1499996	1.9519999	-1.2026410
16.1499996	2.0007999	-1.2026410
16.1499996	2.0495999	-1.0921946
16.1499996	2.0983999	-1.0737867
16.1499996	2.1471999	-1.0737867
16.1499996	2.1959999	-1.1658254
16.1499996	2.2447999	-0.9817476
16.1499996	2.2935998	-0.9817476
16.1499996	2.3423998	-0.9694757
16.1499996	2.3912001	-0.9510679
16.1499996	2.4400001	-1.0185633
16.1499996	2.4888000	-1.0124273
16.1499996	2.5376000	-1.0124273
16.1499996	2.5864000	-1.0062914
16.1499996	2.6352000	-1.0246992
16.1499996	2.6840000	-1.0246992
16.1499996	2.7328000	-1.1535535
16.1499996	2.7816000	-1.1596894
16.1499996	2.8304000	-0.8835731
16.1499996	2.8792000	-0.9817476
16.1499996	2.9280000	-0.9817476
16.1499996	2.9768000	-1.0308352
16.1499996	3.0256000	-0.9756117
16.1499996	3.0743999	-1.0676508
16.1499996	3.1231999	-1.0737867
16.1499996	3.1719999	-0.9940195
16.1499996	3.2207999	-0.9142528
16.1499996	3.2695999	-1.0001554
16.1499996	3.3183999	-0.9878836
16.1499996	3.3671999	-0.9817476
16.1499996	3.4159999	-1.1044660
16.1499996	3.4647999	-0.9081168

16.1499996	3.5135999	-0.9081168
16.1499996	3.5623999	-0.9265246
16.1499996	3.6111999	-0.9265246
16.1499996	3.6599998	-0.8528934
16.1499996	3.7087998	-0.8958449
16.1499996	3.7575998	-0.8160777
16.1499996	3.8063998	-0.8344855
16.1499996	3.8551998	-0.8222136
16.1499996	3.9039998	-0.7485828
16.1499996	3.9527998	-0.7792621
16.1499996	4.0016003	-0.7792621
16.1499996	4.0503998	-0.6872234
16.1499996	4.0991998	-0.7976699
16.1499996	4.1479998	-0.5460973
16.1499996	4.1967998	-0.8222136
16.1499996	4.2455997	-0.8283496
16.1499996	4.2943997	-0.8038058
16.1499996	4.3431997	-0.6258640
16.1499996	4.3919997	-0.6320000
16.1499996	4.4407997	-0.6504078
16.1499996	4.4895997	-0.6565437
16.1499996	4.5383997	-0.6565437
16.1499996	4.5871997	-0.6197281
16.1499996	4.6359997	-0.4601941
16.1499996	4.6847997	-0.7117672
16.1499996	4.7336001	-0.7179031
16.1499996	4.7824001	-0.7179031
16.1499996	4.8312001	-0.5645051
16.1499996	4.8800001	-0.5522332
16.1499996	4.9288001	-0.5522332
16.1499996	4.9776001	-0.5399613
16.1499996	5.0264001	-0.5399613
16.1499996	5.0752001	-0.2208934
16.1499996	5.1240001	-0.0061359
16.1499996	5.1728001	-0.4111071
16.1499996	5.2216001	613586.0000000
16.1499996	5.2704000	-0.5215535
16.1499996	5.3192000	613586.0000000
16.1499996	5.3680000	613586.0000000
16.1499996	5.4168000	613586.0000000
16.1499996	5.4656000	613586.0000000
16.1499996	5.5144000	613586.0000000
16.1499996	5.5632000	613586.0000000
16.1499996	5.6120000	613586.0000000
16.1499996	5.6608000	613586.0000000
16.1499996	5.7096000	613586.0000000
16.1499996	5.7584000	613586.0000000
16.1499996	5.8072000	613586.0000000
16.1499996	5.8559999	613586.0000000
16.1499996	5.9047999	613586.0000000
16.1499996	5.9535999	613586.0000000
16.1499996	6.0023999	613586.0000000
16.1666660	0.2928000	-4.1172047
16.1666660	0.3416000	-3.8717675
16.1666660	0.3904000	-2.7182140
16.1666660	0.4392000	-2.7366219
16.1666660	0.4880000	-3.3133986

16.1666660	0.5368000	-3.3072627
16.1666660	0.5856000	-2.5893598
16.1666660	0.6344000	-1.8346410
16.1666660	0.6832000	-1.8530488
16.1666660	0.7320000	-1.1412816
16.1666660	0.7808000	-0.9572039
16.1666660	0.8296000	-2.0555344
16.1666660	0.8784000	-2.8163888
16.1666660	0.9272000	-2.4850490
16.1666660	0.9760000	-2.4727771
16.1666660	1.0247999	-2.0739422
16.1666660	1.0735999	-1.8162332
16.1666660	1.1223999	-1.6812429
16.1666660	1.1711999	-1.2762718
16.1666660	1.2200000	-1.4051266
16.1666660	1.2688000	-1.4112625
16.1666660	1.3176000	-1.2701364
16.1666660	1.3664000	-1.2455926
16.1666660	1.4152000	-1.2394567
16.1666660	1.4640000	-1.2394567
16.1666660	1.5128000	-1.1842332
16.1666660	1.5616000	-1.1658254
16.1666660	1.6104000	-1.0246992
16.1666660	1.6592000	-1.1474175
16.1666660	1.7079999	-1.0983305
16.1666660	1.7567999	-1.2210488
16.1666660	1.8055999	-1.0124273
16.1666660	1.8543999	-1.0246992
16.1666660	1.9031999	-1.0799227
16.1666660	1.9519999	-1.0124273
16.1666660	2.0007999	-1.0124273
16.1666660	2.0495999	-1.0062914
16.1666660	2.0983999	-0.9817476
16.1666660	2.1471999	-0.9817476
16.1666660	2.1959999	-0.9326606
16.1666660	2.2447999	-0.9326606
16.1666660	2.2935998	-0.8958449
16.1666660	2.3423998	-0.9387960
16.1666660	2.3912001	-1.0001554
16.1666660	2.4400001	-0.9817476
16.1666660	2.4888000	-0.9572039
16.1666660	2.5376000	-1.0553789
16.1666660	2.5864000	-1.0185633
16.1666660	2.6352000	-1.0246992
16.1666660	2.6840000	-0.9817476
16.1666660	2.7328000	-0.9572039
16.1666660	2.7816000	-0.9510679
16.1666660	2.8304000	-1.0553789
16.1666660	2.8792000	-0.9510679
16.1666660	2.9280000	-0.9510679
16.1666660	2.9768000	-1.0062914
16.1666660	3.0256000	-0.8897090
16.1666660	3.0743999	-0.9694757
16.1666660	3.1231999	-0.9878836
16.1666660	3.1719999	-0.9326606
16.1666660	3.2207999	-0.9203887
16.1666660	3.2695999	-0.8958449

16.1666660	3.3183999	-0.9326606
16.1666660	3.3671999	-0.9265246
16.1666660	3.4159999	-0.9081168
16.1666660	3.4647999	-0.9081168
16.1666660	3.5135999	-1.0308352
16.1666660	3.5623999	-0.9387960
16.1666660	3.6111999	-0.9387960
16.1666660	3.6599998	-0.9940195
16.1666660	3.7087998	-0.8590293
16.1666660	3.7575998	-0.8713012
16.1666660	3.8063998	-0.8651652
16.1666660	3.8551998	-0.8528934
16.1666660	3.9039998	-0.8528934
16.1666660	3.9527998	-1.1596894
16.1666660	4.0016003	-0.7731261
16.1666660	4.0503998	-0.7669902
16.1666660	4.0991998	-0.9940195
16.1666660	4.1479998	-0.6933594
16.1666660	4.1967998	-0.6872234
16.1666660	4.2455997	-0.6872234
16.1666660	4.2943997	-0.6872234
16.1666660	4.3431997	-0.7056313
16.1666660	4.3919997	-0.6565437
16.1666660	4.4407997	-0.6504078
16.1666660	4.4895997	-0.6565437
16.1666660	4.5383997	-0.4970098
16.1666660	4.5871997	-0.8897090
16.1666660	4.6359997	-0.8958449
16.1666660	4.6847997	-0.7117672
16.1666660	4.7336001	-0.7117672
16.1666660	4.7824001	-0.5092816
16.1666660	4.8312001	-0.4233789
16.1666660	4.8800001	-0.4233789
16.1666660	4.9288001	-0.4724660
16.1666660	4.9776001	-0.4663301
16.1666660	5.0264001	-0.4663301
16.1666660	5.0752001	-0.4540582
16.1666660	5.1240001	-0.6994953
16.1666660	5.1728001	-0.7240391
16.1666660	5.2216001	-0.5154176
16.1666660	5.2704000	-0.3804274
16.1666660	5.3192000	-0.3804274
16.1666660	5.3680000	613586.0000000
16.1666660	5.4168000	613586.0000000
16.1666660	5.4656000	613586.0000000
16.1666660	5.5144000	613586.0000000
16.1666660	5.5632000	613586.0000000
16.1666660	5.6120000	613586.0000000
16.1666660	5.6608000	613586.0000000
16.1666660	5.7096000	613586.0000000
16.1666660	5.7584000	613586.0000000
16.1666660	5.8072000	613586.0000000
16.1666660	5.8559999	613586.0000000
16.1666660	5.9047999	613586.0000000
16.1666660	5.9535999	613586.0000000
16.1666660	6.0023999	613586.0000000
16.1833324	0.2928000	-4.4055929

16.1833324	0.3416000	-4.4055929
16.1833324	0.3904000	-4.1172047
16.1833324	0.4392000	-3.3870296
16.1833324	0.4880000	-4.7307968
16.1833324	0.5368000	-4.7369328
16.1833324	0.5856000	-4.0926609
16.1833324	0.6344000	-2.4114180
16.1833324	0.6832000	-2.1168938
16.1833324	0.7320000	-2.1168938
16.1833324	0.7808000	-1.6260195
16.1833324	0.8296000	-1.4787574
16.1833324	0.8784000	-1.4603496
16.1833324	0.9272000	-1.4542136
16.1833324	0.9760000	-1.4480777
16.1833324	1.0247999	-1.2455926
16.1833324	1.0735999	-1.2455926
16.1833324	1.1223999	-1.3683109
16.1833324	1.1711999	-1.1903691
16.1833324	1.2200000	-1.1903691
16.1833324	1.2688000	-1.0062914
16.1833324	1.3176000	-1.0124273
16.1833324	1.3664000	-1.2455926
16.1833324	1.4152000	-1.2333207
16.1833324	1.4640000	-1.0737867
16.1833324	1.5128000	-1.2333207
16.1833324	1.5616000	-1.2149129
16.1833324	1.6104000	-1.2762718
16.1833324	1.6592000	-1.1474175
16.1833324	1.7079999	-1.1412816
16.1833324	1.7567999	-0.9633398
16.1833324	1.8055999	-0.9633398
16.1833324	1.8543999	-0.9878836
16.1833324	1.9031999	-0.9940195
16.1833324	1.9519999	-0.7915339
16.1833324	2.0007999	-0.7669902
16.1833324	2.0495999	-0.7731261
16.1833324	2.0983999	-0.7792621
16.1833324	2.1471999	-0.8958449
16.1833324	2.1959999	-0.8774371
16.1833324	2.2447999	-0.8344855
16.1833324	2.2935998	-0.8283496
16.1833324	2.3423998	-1.0001554
16.1833324	2.3912001	-1.0369711
16.1833324	2.4400001	-1.0431070
16.1833324	2.4888000	-1.0492430
16.1833324	2.5376000	-0.9572039
16.1833324	2.5864000	-0.9633398
16.1833324	2.6352000	-0.9142528
16.1833324	2.6840000	-0.9142528
16.1833324	2.7328000	-0.9203887
16.1833324	2.7816000	-0.9940195
16.1833324	2.8304000	-1.0124273
16.1833324	2.8792000	-1.0246992
16.1833324	2.9280000	-1.0124273
16.1833324	2.9768000	-0.8958449
16.1833324	3.0256000	-0.8713012
16.1833324	3.0743999	-0.8713012

16.1833324	3.1231999	-0.8713012
16.1833324	3.1719999	-0.8713012
16.1833324	3.2207999	-0.7669902
16.1833324	3.2695999	-0.8713012
16.1833324	3.3183999	-0.8590293
16.1833324	3.3671999	-0.8467574
16.1833324	3.4159999	-0.8467574
16.1833324	3.4647999	-0.8958449
16.1833324	3.5135999	-0.9019809
16.1833324	3.5623999	-0.8467574
16.1833324	3.6111999	-0.8283496
16.1833324	3.6599998	-0.8283496
16.1833324	3.7087998	-0.7056313
16.1833324	3.7575998	-0.6442719
16.1833324	3.8063998	-0.6504078
16.1833324	3.8551998	-0.6442719
16.1833324	3.9039998	-0.7240391
16.1833324	3.9527998	-0.6320000
16.1833324	4.0016003	-0.5890489
16.1833324	4.0503998	-0.5890489
16.1833324	4.0991998	-0.6013203
16.1833324	4.1479998	-0.4724660
16.1833324	4.1967998	-0.4601941
16.1833324	4.2455997	-0.5890489
16.1833324	4.2943997	-0.5890489
16.1833324	4.3431997	-0.8467574
16.1833324	4.3919997	-0.8467574
16.1833324	4.4407997	-0.5276895
16.1833324	4.4895997	-0.5154176
16.1833324	4.5383997	-0.4970098
16.1833324	4.5871997	-0.7056313
16.1833324	4.6359997	-0.6872234
16.1833324	4.6847997	-0.7547188
16.1833324	4.7336001	-0.7424469
16.1833324	4.7824001	-0.7547188
16.1833324	4.8312001	-0.7792621
16.1833324	4.8800001	-0.8344855
16.1833324	4.9288001	-0.8344855
16.1833324	4.9776001	-0.8038058
16.1833324	5.0264001	-0.6504078
16.1833324	5.0752001	-0.8897090
16.1833324	5.1240001	-0.5092816
16.1833324	5.1728001	-0.8590293
16.1833324	5.2216001	-1.0124273
16.1833324	5.2704000	613586.0000000
16.1833324	5.3192000	613586.0000000
16.1833324	5.3680000	613586.0000000
16.1833324	5.4168000	613586.0000000
16.1833324	5.4656000	613586.0000000
16.1833324	5.5144000	613586.0000000
16.1833324	5.5632000	613586.0000000
16.1833324	5.6120000	613586.0000000
16.1833324	5.6608000	613586.0000000
16.1833324	5.7096000	613586.0000000
16.1833324	5.7584000	613586.0000000
16.1833324	5.8072000	613586.0000000
16.1833324	5.8559999	613586.0000000

16.1833324	5.9047999	613586.0000000
16.1833324	5.9535999	613586.0000000
16.1833324	6.0023999	613586.0000000
16.2000008	0.2928000	-4.4792242
16.2000008	0.3416000	-3.8042724
16.2000008	0.3904000	-3.7735929
16.2000008	0.4392000	-3.8594959
16.2000008	0.4880000	-3.3195345
16.2000008	0.5368000	-4.2951465
16.2000008	0.5856000	-4.2951465
16.2000008	0.6344000	-2.6139033
16.2000008	0.6832000	-1.1842332
16.2000008	0.7320000	-0.8406215
16.2000008	0.7808000	-0.9387960
16.2000008	0.8296000	-1.2946796
16.2000008	0.8784000	-1.2946796
16.2000008	0.9272000	-1.0799227
16.2000008	0.9760000	-0.8897090
16.2000008	1.0247999	-0.9510679
16.2000008	1.0735999	-0.9510679
16.2000008	1.1223999	-0.8344855
16.2000008	1.1711999	-0.8038058
16.2000008	1.2200000	-0.8038058
16.2000008	1.2688000	-0.9203887
16.2000008	1.3176000	-0.9265246
16.2000008	1.3664000	-1.1719613
16.2000008	1.4152000	-1.1167378
16.2000008	1.4640000	-1.0860586
16.2000008	1.5128000	-1.0246992
16.2000008	1.5616000	-1.0246992
16.2000008	1.6104000	-0.9203887
16.2000008	1.6592000	-1.1106019
16.2000008	1.7079999	-1.1351457
16.2000008	1.7567999	-0.8590293
16.2000008	1.8055999	-0.8590293
16.2000008	1.8543999	-0.8958449
16.2000008	1.9031999	-0.7669902
16.2000008	1.9519999	-0.6381359
16.2000008	2.0007999	-0.9203887
16.2000008	2.0495999	-0.9203887
16.2000008	2.0983999	-0.9878836
16.2000008	2.1471999	-0.8774371
16.2000008	2.1959999	-0.8590293
16.2000008	2.2447999	-0.8958449
16.2000008	2.2935998	-0.8897090
16.2000008	2.3423998	-0.8713012
16.2000008	2.3912001	-0.8528934
16.2000008	2.4400001	-0.7424469
16.2000008	2.4888000	-0.8528934
16.2000008	2.5376000	-0.8528934
16.2000008	2.5864000	-0.9326606
16.2000008	2.6352000	-0.9265246
16.2000008	2.6840000	-0.9203887
16.2000008	2.7328000	-0.8528934
16.2000008	2.7816000	-0.9081168
16.2000008	2.8304000	-0.9019809
16.2000008	2.8792000	-0.9019809

16.2000008	2.9280000	-0.9203887
16.2000008	2.9768000	-0.9203887
16.2000008	3.0256000	-0.9019809
16.2000008	3.0743999	-0.9019809
16.2000008	3.1231999	-1.0185633
16.2000008	3.1719999	-0.8099418
16.2000008	3.2207999	-0.8713012
16.2000008	3.2695999	-0.8713012
16.2000008	3.3183999	-0.8835731
16.2000008	3.3671999	-0.8344855
16.2000008	3.4159999	-0.7853980
16.2000008	3.4647999	-0.7853980
16.2000008	3.5135999	-0.7853980
16.2000008	3.5623999	-0.7608547
16.2000008	3.6111999	-0.7485828
16.2000008	3.6599998	-0.5706410
16.2000008	3.7087998	-0.5645051
16.2000008	3.7575998	-0.5767770
16.2000008	3.8063998	-0.5767770
16.2000008	3.8551998	-0.4847379
16.2000008	3.9039998	-0.5522332
16.2000008	3.9527998	-0.6320000
16.2000008	4.0016003	-0.5031457
16.2000008	4.0503998	-0.4233789
16.2000008	4.0991998	-0.4356503
16.2000008	4.1479998	-0.4908738
16.2000008	4.1967998	-0.7117672
16.2000008	4.2455997	-0.7056313
16.2000008	4.2943997	-0.4172430
16.2000008	4.3431997	-0.5276895
16.2000008	4.3919997	-0.7240391
16.2000008	4.4407997	-0.7363110
16.2000008	4.4895997	-0.7363110
16.2000008	4.5383997	-0.7363110
16.2000008	4.5871997	-0.5890489
16.2000008	4.6359997	-0.6074562
16.2000008	4.6847997	-0.6442719
16.2000008	4.7336001	-0.7056313
16.2000008	4.7824001	-0.2945242
16.2000008	4.8312001	-0.8590293
16.2000008	4.8800001	-0.8590293
16.2000008	4.9288001	-0.8467574
16.2000008	4.9776001	-0.6381359
16.2000008	5.0264001	-0.6258640
16.2000008	5.0752001	-0.5767770
16.2000008	5.1240001	-0.2024856
16.2000008	5.1728001	-0.2577085
16.2000008	5.2216001	-0.1349902
16.2000008	5.2704000	-0.1227183
16.2000008	5.3192000	-0.2515731
16.2000008	5.3680000	613586.0000000
16.2000008	5.4168000	613586.0000000
16.2000008	5.4656000	613586.0000000
16.2000008	5.5144000	613586.0000000
16.2000008	5.5632000	-0.9142528
16.2000008	5.6120000	613586.0000000
16.2000008	5.6608000	613586.0000000

16.2000008	5.7096000	613586.0000000
16.2000008	5.7584000	613586.0000000
16.2000008	5.8072000	613586.0000000
16.2000008	5.8559999	613586.0000000
16.2000008	5.9047999	613586.0000000
16.2000008	5.9535999	613586.0000000
16.2000008	6.0023999	613586.0000000
16.2166672	0.2928000	-3.3686218
16.2166672	0.3416000	-3.3624859
16.2166672	0.3904000	-4.0865250
16.2166672	0.4392000	-3.6324666
16.2166672	0.4880000	-3.7858646
16.2166672	0.5368000	-3.7858646
16.2166672	0.5856000	-2.5280004
16.2166672	0.6344000	-0.4295144
16.2166672	0.6832000	-0.4295144
16.2166672	0.7320000	-6.2525058
16.2166672	0.7808000	-0.1472621
16.2166672	0.8296000	-1.0921946
16.2166672	0.8784000	-0.6626797
16.2166672	0.9272000	-0.6565437
16.2166672	0.9760000	-0.6565437
16.2166672	1.0247999	-0.6013203
16.2166672	1.0735999	-0.7853980
16.2166672	1.1223999	-0.7976699
16.2166672	1.1711999	-0.6872234
16.2166672	1.2200000	-0.6074562
16.2166672	1.2688000	-1.0492430
16.2166672	1.3176000	-0.9019809
16.2166672	1.3664000	-1.2578645
16.2166672	1.4152000	-1.2149129
16.2166672	1.4640000	-1.2271848
16.2166672	1.5128000	-1.1474175
16.2166672	1.5616000	-1.2087770
16.2166672	1.6104000	-1.0983305
16.2166672	1.6592000	-1.2824078
16.2166672	1.7079999	-1.1412816
16.2166672	1.7567999	-1.1351457
16.2166672	1.8055999	-0.8344855
16.2166672	1.8543999	-1.0860586
16.2166672	1.9031999	-0.7731261
16.2166672	1.9519999	-0.8590293
16.2166672	2.0007999	-0.8344855
16.2166672	2.0495999	-0.8283496
16.2166672	2.0983999	-1.0246992
16.2166672	2.1471999	-0.8958449
16.2166672	2.1959999	-0.9817476
16.2166672	2.2447999	-0.9633398
16.2166672	2.2935998	-0.9572039
16.2166672	2.3423998	-0.9081168
16.2166672	2.3912001	-0.8651652
16.2166672	2.4400001	-0.8713012
16.2166672	2.4888000	-1.0001554
16.2166672	2.5376000	-1.0369711
16.2166672	2.5864000	-1.0369711
16.2166672	2.6352000	-0.9387960
16.2166672	2.6840000	-0.9142528

16.2166672	2.7328000	-1.1167378
16.2166672	2.7816000	-1.0799227
16.2166672	2.8304000	-1.0921946
16.2166672	2.8792000	-1.0308352
16.2166672	2.9280000	-1.1290097
16.2166672	2.9768000	-1.0860586
16.2166672	3.0256000	-0.9265246
16.2166672	3.0743999	-0.9817476
16.2166672	3.1231999	-1.0062914
16.2166672	3.1719999	-0.9694757
16.2166672	3.2207999	-1.0308352
16.2166672	3.2695999	-0.9572039
16.2166672	3.3183999	-0.8528934
16.2166672	3.3671999	-0.9572039
16.2166672	3.4159999	-0.9572039
16.2166672	3.4647999	-0.9449320
16.2166672	3.5135999	-0.8038058
16.2166672	3.5623999	-0.8222136
16.2166672	3.6111999	-0.7792621
16.2166672	3.6599998	-0.7792621
16.2166672	3.7087998	-0.7915339
16.2166672	3.7575998	-0.6994953
16.2166672	3.8063998	-0.6933594
16.2166672	3.8551998	-0.6013203
16.2166672	3.9039998	-0.5767770
16.2166672	3.9527998	-0.5890489
16.2166672	4.0016003	-0.8283496
16.2166672	4.0503998	-0.5890489
16.2166672	4.0991998	-0.5583692
16.2166672	4.1479998	-0.5645051
16.2166672	4.1967998	-0.5645051
16.2166672	4.2455997	-0.6810875
16.2166672	4.2943997	-0.6810875
16.2166672	4.3431997	-0.7915339
16.2166672	4.3919997	-0.7915339
16.2166672	4.4407997	-0.6933594
16.2166672	4.4895997	-0.6749516
16.2166672	4.5383997	-0.7240391
16.2166672	4.5871997	-0.6626797
16.2166672	4.6359997	-0.4663301
16.2166672	4.6847997	-0.4724660
16.2166672	4.7336001	-0.6872234
16.2166672	4.7824001	-0.5767770
16.2166672	4.8312001	-0.4172430
16.2166672	4.8800001	-0.4295144
16.2166672	4.9288001	-0.5767770
16.2166672	4.9776001	-0.5583692
16.2166672	5.0264001	-0.8774371
16.2166672	5.0752001	-0.7608547
16.2166672	5.1240001	-0.7547188
16.2166672	5.1728001	-0.7608547
16.2166672	5.2216001	-0.5583692
16.2166672	5.2704000	613586.0000000
16.2166672	5.3192000	613586.0000000
16.2166672	5.3680000	613586.0000000
16.2166672	5.4168000	613586.0000000
16.2166672	5.4656000	613586.0000000

16.2166672	5.5144000	613586.0000000
16.2166672	5.5632000	613586.0000000
16.2166672	5.6120000	613586.0000000
16.2166672	5.6608000	613586.0000000
16.2166672	5.7096000	613586.0000000
16.2166672	5.7584000	613586.0000000
16.2166672	5.8072000	613586.0000000
16.2166672	5.8559999	613586.0000000
16.2166672	5.9047999	613586.0000000
16.2166672	5.9535999	613586.0000000
16.2166672	6.0023999	613586.0000000
16.2333336	0.2928000	-2.8470683
16.2333336	0.3416000	-4.1110687
16.2333336	0.3904000	-3.6938257
16.2333336	0.4392000	-3.5465636
16.2333336	0.4880000	-3.5465636
16.2333336	0.5368000	-3.5588355
16.2333336	0.5856000	-2.8961558
16.2333336	0.6344000	-2.8347967
16.2333336	0.6832000	-2.5034568
16.2333336	0.7320000	-1.6382914
16.2333336	0.7808000	-1.5769324
16.2333336	0.8296000	-1.5892043
16.2333336	0.8784000	-1.0185633
16.2333336	0.9272000	-1.0185633
16.2333336	0.9760000	-1.0124273
16.2333336	1.0247999	-0.8835731
16.2333336	1.0735999	-0.8897090
16.2333336	1.1223999	-0.9572039
16.2333336	1.1711999	-1.0431070
16.2333336	1.2200000	-1.0369711
16.2333336	1.2688000	-1.1780972
16.2333336	1.3176000	-1.1780972
16.2333336	1.3664000	-1.2455926
16.2333336	1.4152000	-1.2271848
16.2333336	1.4640000	-1.2333207
16.2333336	1.5128000	-1.2455926
16.2333336	1.5616000	-1.1290097
16.2333336	1.6104000	-1.3437672
16.2333336	1.6592000	-1.3989906
16.2333336	1.7079999	-1.0124273
16.2333336	1.7567999	-1.0124273
16.2333336	1.8055999	-1.2885437
16.2333336	1.8543999	-1.3008156
16.2333336	1.9031999	-1.0676508
16.2333336	1.9519999	-1.1842332
16.2333336	2.0007999	-1.1167378
16.2333336	2.0495999	-0.9142528
16.2333336	2.0983999	-1.1044660
16.2333336	2.1471999	-1.1412816
16.2333336	2.1959999	-1.1412816
16.2333336	2.2447999	-1.1228738
16.2333336	2.2935998	-1.1106019
16.2333336	2.3423998	-1.1596894
16.2333336	2.3912001	-1.1596894
16.2333336	2.4400001	-1.0615149
16.2333336	2.4888000	-1.0737867

16.2333336	2.5376000	-1.1290097
16.2333336	2.5864000	-1.0369711
16.2333336	2.6352000	-1.2455926
16.2333336	2.6840000	-1.2824078
16.2333336	2.7328000	-1.1780972
16.2333336	2.7816000	-1.0369711
16.2333336	2.8304000	-1.1658254
16.2333336	2.8792000	-1.1474175
16.2333336	2.9280000	-1.1412816
16.2333336	2.9768000	-1.0983305
16.2333336	3.0256000	-1.0921946
16.2333336	3.0743999	-1.1044660
16.2333336	3.1231999	-1.2455926
16.2333336	3.1719999	-1.0185633
16.2333336	3.2207999	-1.0185633
16.2333336	3.2695999	-1.0921946
16.2333336	3.3183999	-0.9633398
16.2333336	3.3671999	-1.0124273
16.2333336	3.4159999	-0.9081168
16.2333336	3.4647999	-0.9081168
16.2333336	3.5135999	-0.8283496
16.2333336	3.5623999	-0.8528934
16.2333336	3.6111999	-0.8467574
16.2333336	3.6599998	-0.8897090
16.2333336	3.7087998	-0.8713012
16.2333336	3.7575998	-0.9694757
16.2333336	3.8063998	-0.9142528
16.2333336	3.8551998	-0.9203887
16.2333336	3.9039998	-0.9203887
16.2333336	3.9527998	-0.6504078
16.2333336	4.0016003	-0.6504078
16.2333336	4.0503998	-0.6994953
16.2333336	4.0991998	-0.6565437
16.2333336	4.1479998	-0.6626797
16.2333336	4.1967998	-0.5460973
16.2333336	4.2455997	-0.9572039
16.2333336	4.2943997	-0.9510679
16.2333336	4.3431997	-0.8958449
16.2333336	4.3919997	-0.6688156
16.2333336	4.4407997	-0.6749516
16.2333336	4.4895997	-0.6933594
16.2333336	4.5383997	-0.6933594
16.2333336	4.5871997	-0.6933594
16.2333336	4.6359997	-0.6872234
16.2333336	4.6847997	-0.8958449
16.2333336	4.7336001	-0.5522332
16.2333336	4.7824001	-0.5767770
16.2333336	4.8312001	-0.5951843
16.2333336	4.8800001	-0.7853980
16.2333336	4.9288001	-0.7915339
16.2333336	4.9776001	-0.6994953
16.2333336	5.0264001	-0.3497477
16.2333336	5.0752001	-0.3620195
16.2333336	5.1240001	-0.3620195
16.2333336	5.1728001	613586.0000000
16.2333336	5.2216001	613586.0000000
16.2333336	5.2704000	613586.0000000

16.2333336	5.3192000	613586.0000000
16.2333336	5.3680000	613586.0000000
16.2333336	5.4168000	613586.0000000
16.2333336	5.4656000	613586.0000000
16.2333336	5.5144000	613586.0000000
16.2333336	5.5632000	613586.0000000
16.2333336	5.6120000	613586.0000000
16.2333336	5.6608000	613586.0000000
16.2333336	5.7096000	613586.0000000
16.2333336	5.7584000	613586.0000000
16.2333336	5.8072000	613586.0000000
16.2333336	5.8559999	613586.0000000
16.2333336	5.9047999	613586.0000000
16.2333336	5.9535999	613586.0000000
16.2333336	6.0023999	613586.0000000
16.2500000	0.2928000	-3.7122335
16.2500000	0.3416000	-3.2336316
16.2500000	0.3904000	-3.2336316
16.2500000	0.4392000	-4.0497093
16.2500000	0.4880000	-4.4914961
16.2500000	0.5368000	-3.0127385
16.2500000	0.5856000	-3.0127385
16.2500000	0.6344000	-2.2764277
16.2500000	0.6832000	-1.7732821
16.2500000	0.7320000	-1.2824078
16.2500000	0.7808000	-1.5462527
16.2500000	0.8296000	-0.3620195
16.2500000	0.8784000	-0.9572039
16.2500000	0.9272000	-1.0431070
16.2500000	0.9760000	-1.1474175
16.2500000	1.0247999	-0.9694757
16.2500000	1.0735999	-1.1780972
16.2500000	1.1223999	-1.0553789
16.2500000	1.1711999	-1.0492430
16.2500000	1.2200000	-0.8344855
16.2500000	1.2688000	-1.2762718
16.2500000	1.3176000	-1.2824078
16.2500000	1.3664000	-1.0308352
16.2500000	1.4152000	-1.0308352
16.2500000	1.4640000	-1.0921946
16.2500000	1.5128000	-1.2455926
16.2500000	1.5616000	-1.2517285
16.2500000	1.6104000	-1.3805828
16.2500000	1.6592000	-1.1351457
16.2500000	1.7079999	-1.1290097
16.2500000	1.7567999	-1.1290097
16.2500000	1.8055999	-1.2271848
16.2500000	1.8543999	-1.1167378
16.2500000	1.9031999	-1.4542136
16.2500000	1.9519999	-1.2762718
16.2500000	2.0007999	-1.2026410
16.2500000	2.0495999	-1.1167378
16.2500000	2.0983999	-1.0492430
16.2500000	2.1471999	-1.2333207
16.2500000	2.1959999	-1.2394567
16.2500000	2.2447999	-1.0983305
16.2500000	2.2935998	-1.1167378

16.2500000	2.3423998	-1.1658254
16.2500000	2.3912001	-1.1842332
16.2500000	2.4400001	-1.1290097
16.2500000	2.4888000	-1.2087770
16.2500000	2.5376000	-1.2087770
16.2500000	2.5864000	-1.2762718
16.2500000	2.6352000	-1.1167378
16.2500000	2.6840000	-1.1903691
16.2500000	2.7328000	-1.0553789
16.2500000	2.7816000	-1.2517285
16.2500000	2.8304000	-1.2455926
16.2500000	2.8792000	-1.2517285
16.2500000	2.9280000	-1.3253593
16.2500000	2.9768000	-1.2149129
16.2500000	3.0256000	-1.1044660
16.2500000	3.0743999	-1.1228738
16.2500000	3.1231999	-1.0492430
16.2500000	3.1719999	-1.0431070
16.2500000	3.2207999	-0.8467574
16.2500000	3.2695999	-0.8528934
16.2500000	3.3183999	-1.0124273
16.2500000	3.3671999	-1.0553789
16.2500000	3.4159999	-1.1842332
16.2500000	3.4647999	-1.1842332
16.2500000	3.5135999	-1.0799227
16.2500000	3.5623999	-1.0553789
16.2500000	3.6111999	-1.0492430
16.2500000	3.6599998	-1.0246992
16.2500000	3.7087998	-0.8344855
16.2500000	3.7575998	-0.8222136
16.2500000	3.8063998	-0.7608547
16.2500000	3.8551998	-0.8038058
16.2500000	3.9039998	-0.7363110
16.2500000	3.9527998	-0.9387960
16.2500000	4.0016003	-0.9019809
16.2500000	4.0503998	-0.8897090
16.2500000	4.0991998	-1.1290097
16.2500000	4.1479998	-1.1290097
16.2500000	4.1967998	-0.9940195
16.2500000	4.2455997	-0.9940195
16.2500000	4.2943997	-1.0369711
16.2500000	4.3431997	-0.9510679
16.2500000	4.3919997	-1.0124273
16.2500000	4.4407997	-0.9878836
16.2500000	4.4895997	-0.9817476
16.2500000	4.5383997	-1.2271848
16.2500000	4.5871997	-1.2333207
16.2500000	4.6359997	-0.9387960
16.2500000	4.6847997	-0.8651652
16.2500000	4.7336001	-0.8713012
16.2500000	4.7824001	-0.8835731
16.2500000	4.8312001	-0.8344855
16.2500000	4.8800001	-0.5276895
16.2500000	4.9288001	-0.4847379
16.2500000	4.9776001	-0.6626797
16.2500000	5.0264001	-0.6626797
16.2500000	5.0752001	-0.6688156

16.2500000	5.1240001	-0.6688156
16.2500000	5.1728001	613586.0000000
16.2500000	5.2216001	-0.4356503
16.2500000	5.2704000	613586.0000000
16.2500000	5.3192000	613586.0000000
16.2500000	5.3680000	613586.0000000
16.2500000	5.4168000	-0.3006601
16.2500000	5.4656000	-0.3926992
16.2500000	5.5144000	-0.3926992
16.2500000	5.5632000	613586.0000000
16.2500000	5.6120000	613586.0000000
16.2500000	5.6608000	613586.0000000
16.2500000	5.7096000	613586.0000000
16.2500000	5.7584000	613586.0000000
16.2500000	5.8072000	613586.0000000
16.2500000	5.8559999	613586.0000000
16.2500000	5.9047999	613586.0000000
16.2500000	5.9535999	613586.0000000
16.2500000	6.0023999	613586.0000000
16.2666664	0.2928000	-4.3871851
16.2666664	0.3416000	-3.9944861
16.2666664	0.3904000	-3.7551851
16.2666664	0.4392000	-3.9085832
16.2666664	0.4880000	-3.6017871
16.2666664	0.5368000	-3.6754181
16.2666664	0.5856000	-3.6815541
16.2666664	0.6344000	-1.7119226
16.2666664	0.6832000	-1.7057867
16.2666664	0.7320000	-1.4971652
16.2666664	0.7808000	-1.4910293
16.2666664	0.8296000	-2.1168938
16.2666664	0.8784000	-1.9450879
16.2666664	0.9272000	-1.9573593
16.2666664	0.9760000	-1.5278449
16.2666664	1.0247999	-1.4112625
16.2666664	1.0735999	-1.1780972
16.2666664	1.1223999	-1.1780972
16.2666664	1.1711999	-1.2087770
16.2666664	1.2200000	-1.2026410
16.2666664	1.2688000	-1.5462527
16.2666664	1.3176000	-1.3805828
16.2666664	1.3664000	-1.3621750
16.2666664	1.4152000	-1.3253593
16.2666664	1.4640000	-1.2640004
16.2666664	1.5128000	-1.2517285
16.2666664	1.5616000	-1.3253593
16.2666664	1.6104000	-1.3314953
16.2666664	1.6592000	-1.1228738
16.2666664	1.7079999	-1.1167378
16.2666664	1.7567999	-1.2333207
16.2666664	1.8055999	-1.1106019
16.2666664	1.8543999	-1.2885437
16.2666664	1.9031999	-1.2885437
16.2666664	1.9519999	-1.2026410
16.2666664	2.0007999	-1.2149129
16.2666664	2.0495999	-1.2394567
16.2666664	2.0983999	-1.0246992

16.2666664	2.1471999	-1.1780972
16.2666664	2.1959999	-0.9694757
16.2666664	2.2447999	-0.9694757
16.2666664	2.2935998	-1.2455926
16.2666664	2.3423998	-1.2087770
16.2666664	2.3912001	-1.1965051
16.2666664	2.4400001	-1.1290097
16.2666664	2.4888000	-1.1228738
16.2666664	2.5376000	-1.1658254
16.2666664	2.5864000	-1.1474175
16.2666664	2.6352000	-1.1658254
16.2666664	2.6840000	-1.1412816
16.2666664	2.7328000	-1.1412816
16.2666664	2.7816000	-1.1228738
16.2666664	2.8304000	-1.1290097
16.2666664	2.8792000	-1.1719613
16.2666664	2.9280000	-1.1106019
16.2666664	2.9768000	-1.0983305
16.2666664	3.0256000	-1.0983305
16.2666664	3.0743999	-1.1106019
16.2666664	3.1231999	-1.1167378
16.2666664	3.1719999	-1.0062914
16.2666664	3.2207999	-1.0860586
16.2666664	3.2695999	-0.9817476
16.2666664	3.3183999	-1.0921946
16.2666664	3.3671999	-0.9203887
16.2666664	3.4159999	-0.9326606
16.2666664	3.4647999	-0.9387960
16.2666664	3.5135999	-0.9510679
16.2666664	3.5623999	-1.0369711
16.2666664	3.6111999	-1.0492430
16.2666664	3.6599998	-0.9756117
16.2666664	3.7087998	-0.7117672
16.2666664	3.7575998	-0.7117672
16.2666664	3.8063998	-0.6258640
16.2666664	3.8551998	-0.6320000
16.2666664	3.9039998	-0.5890489
16.2666664	3.9527998	-0.5829129
16.2666664	4.0016003	-0.8958449
16.2666664	4.0503998	-0.8651652
16.2666664	4.0991998	-0.7240391
16.2666664	4.1479998	-0.9019809
16.2666664	4.1967998	-0.7915339
16.2666664	4.2455997	-0.6626797
16.2666664	4.2943997	-0.6626797
16.2666664	4.3431997	-0.8160777
16.2666664	4.3919997	-0.5890489
16.2666664	4.4407997	-0.8467574
16.2666664	4.4895997	-0.8528934
16.2666664	4.5383997	-0.8651652
16.2666664	4.5871997	-0.8651652
16.2666664	4.6359997	-0.7731261
16.2666664	4.6847997	-0.7731261
16.2666664	4.7336001	-0.6749516
16.2666664	4.7824001	-0.6749516
16.2666664	4.8312001	-0.6810875
16.2666664	4.8800001	-0.7915339

16.2666664	4.9288001	-0.7240391
16.2666664	4.9776001	-0.7424469
16.2666664	5.0264001	-0.7792621
16.2666664	5.0752001	-0.9449320
16.2666664	5.1240001	613586.0000000
16.2666664	5.1728001	613586.0000000
16.2666664	5.2216001	613586.0000000
16.2666664	5.2704000	613586.0000000
16.2666664	5.3192000	613586.0000000
16.2666664	5.3680000	613586.0000000
16.2666664	5.4168000	613586.0000000
16.2666664	5.4656000	613586.0000000
16.2666664	5.5144000	613586.0000000
16.2666664	5.5632000	613586.0000000
16.2666664	5.6120000	613586.0000000
16.2666664	5.6608000	613586.0000000
16.2666664	5.7096000	613586.0000000
16.2666664	5.7584000	613586.0000000
16.2666664	5.8072000	613586.0000000
16.2666664	5.8559999	613586.0000000
16.2666664	5.9047999	613586.0000000
16.2666664	5.9535999	613586.0000000
16.2666664	6.0023999	613586.0000000
16.2833328	0.2928000	-3.7429132
16.2833328	0.3416000	-3.7429132
16.2833328	0.3904000	-3.6201947
16.2833328	0.4392000	-3.6938257
16.2833328	0.4880000	-4.1969719
16.2833328	0.5368000	-3.9576705
16.2833328	0.5856000	-2.6139033
16.2833328	0.6344000	-2.3868742
16.2833328	0.6832000	-1.5094371
16.2833328	0.7320000	-1.2210488
16.2833328	0.7808000	-1.2271848
16.2833328	0.8296000	-1.4296703
16.2833328	0.8784000	-1.4296703
16.2833328	0.9272000	-1.5155730
16.2833328	0.9760000	-1.4910293
16.2833328	1.0247999	-1.4910293
16.2833328	1.0735999	-1.5278449
16.2833328	1.1223999	-1.5033011
16.2833328	1.1711999	-1.4848933
16.2833328	1.2200000	-1.3621750
16.2833328	1.2688000	-1.2271848
16.2833328	1.3176000	-1.1412816
16.2833328	1.3664000	-1.1412816
16.2833328	1.4152000	-1.3314953
16.2833328	1.4640000	-1.1842332
16.2833328	1.5128000	-1.2946796
16.2833328	1.5616000	-1.3560390
16.2833328	1.6104000	-1.6198835
16.2833328	1.6592000	-1.5033011
16.2833328	1.7079999	-1.4848933
16.2833328	1.7567999	-1.3069515
16.2833328	1.8055999	-1.2640004
16.2833328	1.8543999	-1.2578645
16.2833328	1.9031999	-1.1965051

16.2833328	1.9519999	-0.9265246
16.2833328	2.0007999	-1.1167378
16.2833328	2.0495999	-1.1106019
16.2833328	2.0983999	-1.0246992
16.2833328	2.1471999	-1.1903691
16.2833328	2.1959999	-1.1167378
16.2833328	2.2447999	-1.1106019
16.2833328	2.2935998	-1.1228738
16.2833328	2.3423998	-1.0431070
16.2833328	2.3912001	-1.0185633
16.2833328	2.4400001	-1.0124273
16.2833328	2.4888000	-1.1965051
16.2833328	2.5376000	-1.2455926
16.2833328	2.5864000	-1.1780972
16.2833328	2.6352000	-1.1780972
16.2833328	2.6840000	-1.1228738
16.2833328	2.7328000	-1.1228738
16.2833328	2.7816000	-1.0799227
16.2833328	2.8304000	-1.0799227
16.2833328	2.8792000	-1.0799227
16.2833328	2.9280000	-1.0369711
16.2833328	2.9768000	-0.9817476
16.2833328	3.0256000	-0.9878836
16.2833328	3.0743999	-1.0124273
16.2833328	3.1231999	-1.0124273
16.2833328	3.1719999	-0.9633398
16.2833328	3.2207999	-0.9326606
16.2833328	3.2695999	-0.9265246
16.2833328	3.3183999	-0.9019809
16.2833328	3.3671999	-0.9019809
16.2833328	3.4159999	-0.9940195
16.2833328	3.4647999	-1.0431070
16.2833328	3.5135999	-1.0983305
16.2833328	3.5623999	-1.0615149
16.2833328	3.6111999	-0.9633398
16.2833328	3.6599998	-0.7976699
16.2833328	3.7087998	-0.7976699
16.2833328	3.7575998	-0.7976699
16.2833328	3.8063998	-0.8038058
16.2833328	3.8551998	-0.8099418
16.2833328	3.9039998	-0.8099418
16.2833328	3.9527998	-0.9265246
16.2833328	4.0016003	-0.9081168
16.2833328	4.0503998	-0.8283496
16.2833328	4.0991998	-0.7669902
16.2833328	4.1479998	-0.6320000
16.2833328	4.1967998	-0.6320000
16.2833328	4.2455997	-0.8528934
16.2833328	4.2943997	-0.8528934
16.2833328	4.3431997	-0.5031457
16.2833328	4.3919997	-0.8344855
16.2833328	4.4407997	-0.6872234
16.2833328	4.4895997	-0.6872234
16.2833328	4.5383997	-0.5460973
16.2833328	4.5871997	-0.5522332
16.2833328	4.6359997	-0.6872234
16.2833328	4.6847997	-0.6933594

16.2833328	4.7336001	-0.4233789
16.2833328	4.7824001	-0.4049711
16.2833328	4.8312001	-0.4724660
16.2833328	4.8800001	-0.2822523
16.2833328	4.9288001	-0.2822523
16.2833328	4.9776001	-0.5706410
16.2833328	5.0264001	-0.3865633
16.2833328	5.0752001	-0.1349902
16.2833328	5.1240001	613586.0000000
16.2833328	5.1728001	613586.0000000
16.2833328	5.2216001	613586.0000000
16.2833328	5.2704000	-1.1412816
16.2833328	5.3192000	-1.0308352
16.2833328	5.3680000	-1.2149129
16.2833328	5.4168000	-1.0431070
16.2833328	5.4656000	-0.5829129
16.2833328	5.5144000	-0.7608547
16.2833328	5.5632000	-0.9265246
16.2833328	5.6120000	-0.8099418
16.2833328	5.6608000	-0.8099418
16.2833328	5.7096000	613586.0000000
16.2833328	5.7584000	613586.0000000
16.2833328	5.8072000	613586.0000000
16.2833328	5.8559999	613586.0000000
16.2833328	5.9047999	613586.0000000
16.2833328	5.9535999	613586.0000000
16.2833328	6.0023999	613586.0000000
16.2999992	0.2928000	-4.0128937
16.2999992	0.3416000	-4.2767386
16.2999992	0.3904000	-3.5158842
16.2999992	0.4392000	-3.2827189
16.2999992	0.4880000	-3.4667966
16.2999992	0.5368000	-3.6263306
16.2999992	0.5856000	-2.2396121
16.2999992	0.6344000	-1.5523887
16.2999992	0.6832000	-1.5523887
16.2999992	0.7320000	-1.3192234
16.2999992	0.7808000	-1.3253593
16.2999992	0.8296000	-1.3008156
16.2999992	0.8784000	-1.6935148
16.2999992	0.9272000	-1.2762718
16.2999992	0.9760000	-1.1167378
16.2999992	1.0247999	-1.1167378
16.2999992	1.0735999	-1.1658254
16.2999992	1.1223999	-1.1658254
16.2999992	1.1711999	-1.0921946
16.2999992	1.2200000	-1.0308352
16.2999992	1.2688000	-1.0308352
16.2999992	1.3176000	-1.2026410
16.2999992	1.3664000	-1.2578645
16.2999992	1.4152000	-1.2578645
16.2999992	1.4640000	-1.2640004
16.2999992	1.5128000	-1.1351457
16.2999992	1.5616000	-1.3805828
16.2999992	1.6104000	-1.1106019
16.2999992	1.6592000	-1.5094371
16.2999992	1.7079999	-1.4971652

16.2999992	1.7567999	-1.2701364
16.2999992	1.8055999	-1.4051266
16.2999992	1.8543999	-1.2640004
16.2999992	1.9031999	-1.1474175
16.2999992	1.9519999	-1.1596894
16.2999992	2.0007999	-1.3376312
16.2999992	2.0495999	-1.2517285
16.2999992	2.0983999	-1.2578645
16.2999992	2.1471999	-1.1658254
16.2999992	2.1959999	-1.3192234
16.2999992	2.2447999	-1.2087770
16.2999992	2.2935998	-1.2087770
16.2999992	2.3423998	-1.2455926
16.2999992	2.3912001	-1.2578645
16.2999992	2.4400001	-1.2701364
16.2999992	2.4888000	-1.2455926
16.2999992	2.5376000	-1.2517285
16.2999992	2.5864000	-1.2271848
16.2999992	2.6352000	-1.2271848
16.2999992	2.6840000	-1.2333207
16.2999992	2.7328000	-1.2271848
16.2999992	2.7816000	-1.1658254
16.2999992	2.8304000	-1.1535535
16.2999992	2.8792000	-1.1351457
16.2999992	2.9280000	-1.2394567
16.2999992	2.9768000	-1.1719613
16.2999992	3.0256000	-1.0431070
16.2999992	3.0743999	-0.9878836
16.2999992	3.1231999	-0.9449320
16.2999992	3.1719999	-0.9510679
16.2999992	3.2207999	-0.9633398
16.2999992	3.2695999	-0.9510679
16.2999992	3.3183999	-0.9572039
16.2999992	3.3671999	-0.9756117
16.2999992	3.4159999	-0.9694757
16.2999992	3.4647999	-0.9694757
16.2999992	3.5135999	-0.9817476
16.2999992	3.5623999	-0.9817476
16.2999992	3.6111999	-0.9694757
16.2999992	3.6599998	-0.9694757
16.2999992	3.7087998	-0.7976699
16.2999992	3.7575998	-0.9019809
16.2999992	3.8063998	-0.9572039
16.2999992	3.8551998	-0.8160777
16.2999992	3.9039998	-0.9510679
16.2999992	3.9527998	-0.9387960
16.2999992	4.0016003	-0.9326606
16.2999992	4.0503998	-1.0615149
16.2999992	4.0991998	-1.0431070
16.2999992	4.1479998	-0.7669902
16.2999992	4.1967998	-0.7792621
16.2999992	4.2455997	-0.9694757
16.2999992	4.2943997	-0.9756117
16.2999992	4.3431997	-0.9817476
16.2999992	4.3919997	-1.0676508
16.2999992	4.4407997	-1.0492430
16.2999992	4.4895997	-0.6933594

16.2999992	4.5383997	-0.6504078
16.2999992	4.5871997	-0.9265246
16.2999992	4.6359997	-0.9265246
16.2999992	4.6847997	-0.6872234
16.2999992	4.7336001	-0.6565437
16.2999992	4.7824001	-0.5767770
16.2999992	4.8312001	-0.7117672
16.2999992	4.8800001	-0.4970098
16.2999992	4.9288001	-0.4970098
16.2999992	4.9776001	-0.4970098
16.2999992	5.0264001	-0.2577085
16.2999992	5.0752001	-0.2515731
16.2999992	5.1240001	613586.0000000
16.2999992	5.1728001	-0.5706410
16.2999992	5.2216001	613586.0000000
16.2999992	5.2704000	613586.0000000
16.2999992	5.3192000	613586.0000000
16.2999992	5.3680000	613586.0000000
16.2999992	5.4168000	613586.0000000
16.2999992	5.4656000	613586.0000000
16.2999992	5.5144000	613586.0000000
16.2999992	5.5632000	613586.0000000
16.2999992	5.6120000	613586.0000000
16.2999992	5.6608000	613586.0000000
16.2999992	5.7096000	613586.0000000
16.2999992	5.7584000	613586.0000000
16.2999992	5.8072000	613586.0000000
16.2999992	5.8559999	613586.0000000
16.2999992	5.9047999	613586.0000000
16.2999992	5.9535999	613586.0000000
16.2999992	6.0023999	613586.0000000
16.3166676	0.2928000	-3.8779035
16.3166676	0.3416000	-3.2274957
16.3166676	0.3904000	-3.2152238
16.3166676	0.4392000	-3.4238453
16.3166676	0.4880000	-3.4238453
16.3166676	0.5368000	-2.7795732
16.3166676	0.5856000	-2.1843886
16.3166676	0.6344000	-1.3867188
16.3166676	0.6832000	-0.7547188
16.3166676	0.7320000	-1.1228738
16.3166676	0.7808000	-1.4971652
16.3166676	0.8296000	-1.4603496
16.3166676	0.8784000	-1.1167378
16.3166676	0.9272000	-1.1106019
16.3166676	0.9760000	-1.0983305
16.3166676	1.0247999	-1.0799227
16.3166676	1.0735999	-1.0676508
16.3166676	1.1223999	-0.9817476
16.3166676	1.1711999	-0.9817476
16.3166676	1.2200000	-1.1965051
16.3166676	1.2688000	-1.2455926
16.3166676	1.3176000	-1.0799227
16.3166676	1.3664000	-1.0860586
16.3166676	1.4152000	-1.2210488
16.3166676	1.4640000	-1.3437672
16.3166676	1.5128000	-1.3437672

16.3166676	1.5616000	-1.3437672
16.3166676	1.6104000	-1.3499031
16.3166676	1.6592000	-1.2087770
16.3166676	1.7079999	-1.3621750
16.3166676	1.7567999	-1.3560390
16.3166676	1.8055999	-1.3744469
16.3166676	1.8543999	-1.2701364
16.3166676	1.9031999	-1.3928547
16.3166676	1.9519999	-1.3253593
16.3166676	2.0007999	-1.4603496
16.3166676	2.0495999	-1.2640004
16.3166676	2.0983999	-1.3560390
16.3166676	2.1471999	-1.4296703
16.3166676	2.1959999	-1.3867188
16.3166676	2.2447999	-1.5094371
16.3166676	2.2935998	-1.3683109
16.3166676	2.3423998	-1.3744469
16.3166676	2.3912001	-1.3683109
16.3166676	2.4400001	-1.4173985
16.3166676	2.4888000	-1.4787574
16.3166676	2.5376000	-1.3314953
16.3166676	2.5864000	-1.3314953
16.3166676	2.6352000	-1.3253593
16.3166676	2.6840000	-1.3805828
16.3166676	2.7328000	-1.3805828
16.3166676	2.7816000	-1.3560390
16.3166676	2.8304000	-1.2946796
16.3166676	2.8792000	-1.2946796
16.3166676	2.9280000	-1.2701364
16.3166676	2.9768000	-1.2149129
16.3166676	3.0256000	-1.2149129
16.3166676	3.0743999	-1.2271848
16.3166676	3.1231999	-1.2517285
16.3166676	3.1719999	-1.1965051
16.3166676	3.2207999	-1.0185633
16.3166676	3.2695999	-1.2455926
16.3166676	3.3183999	-1.2762718
16.3166676	3.3671999	-1.2333207
16.3166676	3.4159999	-1.2517285
16.3166676	3.4647999	-1.2333207
16.3166676	3.5135999	-1.2271848
16.3166676	3.5623999	-1.0185633
16.3166676	3.6111999	-1.0185633
16.3166676	3.6599998	-1.2455926
16.3166676	3.7087998	-1.2946796
16.3166676	3.7575998	-0.8835731
16.3166676	3.8063998	-1.2640004
16.3166676	3.8551998	-0.9081168
16.3166676	3.9039998	-0.9081168
16.3166676	3.9527998	-1.2455926
16.3166676	4.0016003	-1.2517285
16.3166676	4.0503998	-0.9756117
16.3166676	4.0991998	-0.9081168
16.3166676	4.1479998	-1.0983305
16.3166676	4.1967998	-1.0983305
16.3166676	4.2455997	-1.0921946
16.3166676	4.2943997	-0.9142528

16.3166676	4.3431997	-0.9142528
16.3166676	4.3919997	-0.8958449
16.3166676	4.4407997	-1.1351457
16.3166676	4.4895997	-0.8160777
16.3166676	4.5383997	-0.6442719
16.3166676	4.5871997	-0.6320000
16.3166676	4.6359997	-0.6872234
16.3166676	4.6847997	-0.6872234
16.3166676	4.7336001	-0.6749516
16.3166676	4.7824001	-0.6749516
16.3166676	4.8312001	-0.6810875
16.3166676	4.8800001	-0.4724660
16.3166676	4.9288001	-0.2699804
16.3166676	4.9776001	-0.3497477
16.3166676	5.0264001	-0.5890489
16.3166676	5.0752001	-0.5276895
16.3166676	5.1240001	-0.2515731
16.3166676	5.1728001	613586.0000000
16.3166676	5.2216001	613586.0000000
16.3166676	5.2704000	613586.0000000
16.3166676	5.3192000	613586.0000000
16.3166676	5.3680000	613586.0000000
16.3166676	5.4168000	613586.0000000
16.3166676	5.4656000	613586.0000000
16.3166676	5.5144000	613586.0000000
16.3166676	5.5632000	613586.0000000
16.3166676	5.6120000	613586.0000000
16.3166676	5.6608000	613586.0000000
16.3166676	5.7096000	613586.0000000
16.3166676	5.7584000	613586.0000000
16.3166676	5.8072000	613586.0000000
16.3166676	5.8559999	613586.0000000
16.3166676	5.9047999	613586.0000000
16.3166676	5.9535999	613586.0000000
16.3166676	6.0023999	613586.0000000
16.3333340	0.2928000	-3.0127385
16.3333340	0.3416000	-3.5342917
16.3333340	0.3904000	-3.4238453
16.3333340	0.4392000	-3.4361172
16.3333340	0.4880000	-4.3012824
16.3333340	0.5368000	-3.6079230
16.3333340	0.5856000	-2.7918451
16.3333340	0.6344000	-2.7918451
16.3333340	0.6832000	-2.1966605
16.3333340	0.7320000	-1.2026410
16.3333340	0.7808000	-1.4112625
16.3333340	0.8296000	-1.3069515
16.3333340	0.8784000	-1.1658254
16.3333340	0.9272000	-1.1596894
16.3333340	0.9760000	-1.0921946
16.3333340	1.0247999	-1.2271848
16.3333340	1.0735999	-1.2455926
16.3333340	1.1223999	-1.0246992
16.3333340	1.1711999	-0.8528934
16.3333340	1.2200000	-0.8467574
16.3333340	1.2688000	-1.1780972
16.3333340	1.3176000	-1.1412816

16.3333340	1.3664000	-1.2333207
16.3333340	1.4152000	-1.3805828
16.3333340	1.4640000	-1.3744469
16.3333340	1.5128000	-1.3499031
16.3333340	1.5616000	-1.3253593
16.3333340	1.6104000	-1.3928547
16.3333340	1.6592000	-1.2026410
16.3333340	1.7079999	-1.4603496
16.3333340	1.7567999	-1.3744469
16.3333340	1.8055999	-1.3130875
16.3333340	1.8543999	-1.3130875
16.3333340	1.9031999	-1.3192234
16.3333340	1.9519999	-1.3867188
16.3333340	2.0007999	-1.3008156
16.3333340	2.0495999	-1.3130875
16.3333340	2.0983999	-1.3069515
16.3333340	2.1471999	-1.4296703
16.3333340	2.1959999	-1.4051266
16.3333340	2.2447999	-1.4603496
16.3333340	2.2935998	-1.5094371
16.3333340	2.3423998	-1.5217090
16.3333340	2.3912001	-1.5155730
16.3333340	2.4400001	-1.5155730
16.3333340	2.4888000	-1.5094371
16.3333340	2.5376000	-1.3683109
16.3333340	2.5864000	-1.3683109
16.3333340	2.6352000	-1.3683109
16.3333340	2.6840000	-1.3130875
16.3333340	2.7328000	-1.4235344
16.3333340	2.7816000	-1.3192234
16.3333340	2.8304000	-1.3008156
16.3333340	2.8792000	-1.3989906
16.3333340	2.9280000	-1.3928547
16.3333340	2.9768000	-1.3805828
16.3333340	3.0256000	-1.3805828
16.3333340	3.0743999	-1.3008156
16.3333340	3.1231999	-1.3560390
16.3333340	3.1719999	-1.2885437
16.3333340	3.2207999	-1.2517285
16.3333340	3.2695999	-1.2640004
16.3333340	3.3183999	-1.2087770
16.3333340	3.3671999	-1.1965051
16.3333340	3.4159999	-1.2026410
16.3333340	3.4647999	-1.2026410
16.3333340	3.5135999	-1.0369711
16.3333340	3.5623999	-0.8774371
16.3333340	3.6111999	-1.0676508
16.3333340	3.6599998	-1.5585246
16.3333340	3.7087998	-1.3499031
16.3333340	3.7575998	-1.3560390
16.3333340	3.8063998	-1.1965051
16.3333340	3.8551998	-1.2394567
16.3333340	3.9039998	-1.5033011
16.3333340	3.9527998	-1.3928547
16.3333340	4.0016003	-1.3928547
16.3333340	4.0503998	-1.0860586
16.3333340	4.0991998	-1.0921946

16.3333340	4.1479998	-1.1351457
16.3333340	4.1967998	-1.0001554
16.3333340	4.2455997	-1.0369711
16.3333340	4.2943997	-1.0308352
16.3333340	4.3431997	-1.0185633
16.3333340	4.3919997	-0.9878836
16.3333340	4.4407997	-0.9878836
16.3333340	4.4895997	-0.9878836
16.3333340	4.5383997	-0.8897090
16.3333340	4.5871997	-0.8897090
16.3333340	4.6359997	-0.9756117
16.3333340	4.6847997	-0.9756117
16.3333340	4.7336001	-0.9756117
16.3333340	4.7824001	-0.8406215
16.3333340	4.8312001	-0.8467574
16.3333340	4.8800001	-0.8528934
16.3333340	4.9288001	-0.6626797
16.3333340	4.9776001	-0.7301750
16.3333340	5.0264001	-0.8222136
16.3333340	5.0752001	613586.0000000
16.3333340	5.1240001	613586.0000000
16.3333340	5.1728001	613586.0000000
16.3333340	5.2216001	613586.0000000
16.3333340	5.2704000	613586.0000000
16.3333340	5.3192000	613586.0000000
16.3333340	5.3680000	613586.0000000
16.3333340	5.4168000	613586.0000000
16.3333340	5.4656000	613586.0000000
16.3333340	5.5144000	613586.0000000
16.3333340	5.5632000	613586.0000000
16.3333340	5.6120000	613586.0000000
16.3333340	5.6608000	613586.0000000
16.3333340	5.7096000	613586.0000000
16.3333340	5.7584000	613586.0000000
16.3333340	5.8072000	613586.0000000
16.3333340	5.8559999	613586.0000000
16.3333340	5.9047999	613586.0000000
16.3333340	5.9535999	613586.0000000
16.3333340	6.0023999	613586.0000000
16.3500004	0.2928000	-3.9085832
16.3500004	0.3416000	-4.6571655
16.3500004	0.3904000	-4.3258257
16.3500004	0.4392000	-3.6324666
16.3500004	0.4880000	-3.2274957
16.3500004	0.5368000	-3.2397676
16.3500004	0.5856000	-2.7673013
16.3500004	0.6344000	-1.6382914
16.3500004	0.6832000	-1.8653207
16.3500004	0.7320000	-1.4726214
16.3500004	0.7808000	-1.5094371
16.3500004	0.8296000	-1.2026410
16.3500004	0.8784000	-1.6566992
16.3500004	0.9272000	-1.4419422
16.3500004	0.9760000	-1.4419422
16.3500004	1.0247999	-1.3192234
16.3500004	1.0735999	-1.3008156
16.3500004	1.1223999	-1.1596894

16.3500004	1.1711999	-1.0860586
16.3500004	1.2200000	-1.0860586
16.3500004	1.2688000	-0.9817476
16.3500004	1.3176000	-0.9694757
16.3500004	1.3664000	-1.3376312
16.3500004	1.4152000	-1.3621750
16.3500004	1.4640000	-1.2762718
16.3500004	1.5128000	-1.2762718
16.3500004	1.5616000	-1.3253593
16.3500004	1.6104000	-1.1719613
16.3500004	1.6592000	-1.4480777
16.3500004	1.7079999	-1.1842332
16.3500004	1.7567999	-1.5033011
16.3500004	1.8055999	-1.6198835
16.3500004	1.8543999	-1.6076121
16.3500004	1.9031999	-1.3744469
16.3500004	1.9519999	-1.2701364
16.3500004	2.0007999	-1.2946796
16.3500004	2.0495999	-1.3376312
16.3500004	2.0983999	-1.3683109
16.3500004	2.1471999	-1.3683109
16.3500004	2.1959999	-1.3130875
16.3500004	2.2447999	-1.4358063
16.3500004	2.2935998	-1.4358063
16.3500004	2.3423998	-1.3253593
16.3500004	2.3912001	-1.3130875
16.3500004	2.4400001	-1.3744469
16.3500004	2.4888000	-1.3560390
16.3500004	2.5376000	-1.2394567
16.3500004	2.5864000	-1.2824078
16.3500004	2.6352000	-1.2455926
16.3500004	2.6840000	-1.2824078
16.3500004	2.7328000	-1.1842332
16.3500004	2.7816000	-1.2701364
16.3500004	2.8304000	-1.1842332
16.3500004	2.8792000	-1.1044660
16.3500004	2.9280000	-1.1044660
16.3500004	2.9768000	-1.0860586
16.3500004	3.0256000	-1.0799227
16.3500004	3.0743999	-1.0737867
16.3500004	3.1231999	-1.1228738
16.3500004	3.1719999	-1.1167378
16.3500004	3.2207999	-1.1290097
16.3500004	3.2695999	-1.1290097
16.3500004	3.3183999	-1.1474175
16.3500004	3.3671999	-1.1474175
16.3500004	3.4159999	-0.7853980
16.3500004	3.4647999	-0.8160777
16.3500004	3.5135999	-0.8222136
16.3500004	3.5623999	-1.4971652
16.3500004	3.6111999	-1.4112625
16.3500004	3.6599998	-1.3130875
16.3500004	3.7087998	-1.2087770
16.3500004	3.7575998	-0.9449320
16.3500004	3.8063998	-0.9449320
16.3500004	3.8551998	-0.8713012
16.3500004	3.9039998	-1.1658254

16.3500004	3.9527998	-1.1658254
16.3500004	4.0016003	-1.3192234
16.3500004	4.0503998	-1.3130875
16.3500004	4.0991998	-0.9940195
16.3500004	4.1479998	-0.9878836
16.3500004	4.1967998	-0.9081168
16.3500004	4.2455997	-0.9817476
16.3500004	4.2943997	-0.9878836
16.3500004	4.3431997	-0.8160777
16.3500004	4.3919997	-0.8160777
16.3500004	4.4407997	-0.8160777
16.3500004	4.4895997	-0.8160777
16.3500004	4.5383997	-0.7608547
16.3500004	4.5871997	-0.8467574
16.3500004	4.6359997	-0.8344855
16.3500004	4.6847997	-0.7179031
16.3500004	4.7336001	-0.7179031
16.3500004	4.7824001	-0.5645051
16.3500004	4.8312001	-0.5645051
16.3500004	4.8800001	-0.8344855
16.3500004	4.9288001	-0.6504078
16.3500004	4.9776001	-0.7853980
16.3500004	5.0264001	-0.7853980
16.3500004	5.0752001	-0.7853980
16.3500004	5.1240001	613586.0000000
16.3500004	5.1728001	613586.0000000
16.3500004	5.2216001	613586.0000000
16.3500004	5.2704000	613586.0000000
16.3500004	5.3192000	613586.0000000
16.3500004	5.3680000	613586.0000000
16.3500004	5.4168000	613586.0000000
16.3500004	5.4656000	613586.0000000
16.3500004	5.5144000	613586.0000000
16.3500004	5.5632000	613586.0000000
16.3500004	5.6120000	613586.0000000
16.3500004	5.6608000	613586.0000000
16.3500004	5.7096000	613586.0000000
16.3500004	5.7584000	613586.0000000
16.3500004	5.8072000	613586.0000000
16.3500004	5.8559999	613586.0000000
16.3500004	5.9047999	613586.0000000
16.3500004	5.9535999	613586.0000000
16.3500004	6.0023999	613586.0000000
16.3666668	0.2928000	-3.4729326
16.3666668	0.3416000	-2.9636509
16.3666668	0.3904000	-2.9697869
16.3666668	0.4392000	-2.9636509
16.3666668	0.4880000	-3.0495539
16.3666668	0.5368000	-3.5772433
16.3666668	0.5856000	-1.8469129
16.3666668	0.6344000	-1.4848933
16.3666668	0.6832000	-1.4910293
16.3666668	0.7320000	-1.8100972
16.3666668	0.7808000	-1.7487383
16.3666668	0.8296000	-1.6198835
16.3666668	0.8784000	-1.3867188
16.3666668	0.9272000	-1.3989906

16.3666668	0.9760000	-1.2394567
16.3666668	1.0247999	-1.3867188
16.3666668	1.0735999	-1.2271848
16.3666668	1.1223999	-1.3376312
16.3666668	1.1711999	-1.1228738
16.3666668	1.2200000	-1.1290097
16.3666668	1.2688000	-0.9510679
16.3666668	1.3176000	-1.0369711
16.3666668	1.3664000	-1.0369711
16.3666668	1.4152000	-1.2762718
16.3666668	1.4640000	-1.1412816
16.3666668	1.5128000	-1.1535535
16.3666668	1.5616000	-1.1290097
16.3666668	1.6104000	-1.2578645
16.3666668	1.6592000	-1.1290097
16.3666668	1.7079999	-1.1290097
16.3666668	1.7567999	-1.1474175
16.3666668	1.8055999	-1.1719613
16.3666668	1.8543999	-1.0124273
16.3666668	1.9031999	-1.0246992
16.3666668	1.9519999	-1.0308352
16.3666668	2.0007999	-1.1412816
16.3666668	2.0495999	-1.1474175
16.3666668	2.0983999	-1.0860586
16.3666668	2.1471999	-1.0860586
16.3666668	2.1959999	-1.1228738
16.3666668	2.2447999	-1.1596894
16.3666668	2.2935998	-1.1596894
16.3666668	2.3423998	-1.1596894
16.3666668	2.3912001	-1.0983305
16.3666668	2.4400001	-1.1658254
16.3666668	2.4888000	-1.1658254
16.3666668	2.5376000	-1.1658254
16.3666668	2.5864000	-1.2271848
16.3666668	2.6352000	-1.0921946
16.3666668	2.6840000	-1.0369711
16.3666668	2.7328000	-1.0246992
16.3666668	2.7816000	-1.0308352
16.3666668	2.8304000	-1.0983305
16.3666668	2.8792000	-1.0983305
16.3666668	2.9280000	-0.9940195
16.3666668	2.9768000	-0.9878836
16.3666668	3.0256000	-0.8528934
16.3666668	3.0743999	-0.9633398
16.3666668	3.1231999	-0.9572039
16.3666668	3.1719999	-0.9510679
16.3666668	3.2207999	-0.9510679
16.3666668	3.2695999	-0.9510679
16.3666668	3.3183999	-0.9572039
16.3666668	3.3671999	-0.7608547
16.3666668	3.4159999	-0.7731261
16.3666668	3.4647999	-0.7915339
16.3666668	3.5135999	-1.1596894
16.3666668	3.5623999	-1.1535535
16.3666668	3.6111999	-0.7731261
16.3666668	3.6599998	-0.7792621
16.3666668	3.7087998	-0.6872234

16.3666668	3.7575998	-0.6933594
16.3666668	3.8063998	-0.6994953
16.3666668	3.8551998	-0.7976699
16.3666668	3.9039998	-0.6197281
16.3666668	3.9527998	-0.5706410
16.3666668	4.0016003	-0.5767770
16.3666668	4.0503998	-0.5890489
16.3666668	4.0991998	-0.4417863
16.3666668	4.1479998	-0.4295144
16.3666668	4.1967998	-0.4786019
16.3666668	4.2455997	-0.4663301
16.3666668	4.2943997	-0.4847379
16.3666668	4.3431997	-0.3006601
16.3666668	4.3919997	-0.3988352
16.3666668	4.4407997	-0.3988352
16.3666668	4.4895997	-0.3129320
16.3666668	4.5383997	-0.3067961
16.3666668	4.5871997	-0.8406215
16.3666668	4.6359997	-0.7424469
16.3666668	4.6847997	-0.5583692
16.3666668	4.7336001	-0.5522332
16.3666668	4.7824001	-0.2024856
16.3666668	4.8312001	-0.1840777
16.3666668	4.8800001	-0.1718059
16.3666668	4.9288001	0.0061359
16.3666668	4.9776001	0.0000000
16.3666668	5.0264001	613586.0000000
16.3666668	5.0752001	-0.4479222
16.3666668	5.1240001	-0.5031457
16.3666668	5.1728001	-0.3988352
16.3666668	5.2216001	613586.0000000
16.3666668	5.2704000	613586.0000000
16.3666668	5.3192000	613586.0000000
16.3666668	5.3680000	613586.0000000
16.3666668	5.4168000	613586.0000000
16.3666668	5.4656000	613586.0000000
16.3666668	5.5144000	613586.0000000
16.3666668	5.5632000	613586.0000000
16.3666668	5.6120000	613586.0000000
16.3666668	5.6608000	613586.0000000
16.3666668	5.7096000	613586.0000000
16.3666668	5.7584000	613586.0000000
16.3666668	5.8072000	613586.0000000
16.3666668	5.8559999	613586.0000000
16.3666668	5.9047999	613586.0000000
16.3666668	5.9535999	613586.0000000
16.3666668	6.0023999	613586.0000000
16.3833332	0.2928000	-3.5649714
16.3833332	0.3416000	-2.9697869
16.3833332	0.3904000	-3.9699423
16.3833332	0.4392000	-3.7060976
16.3833332	0.4880000	-3.0802336
16.3833332	0.5368000	-3.0679617
16.3833332	0.5856000	-2.3991461
16.3833332	0.6344000	-2.3746023
16.3833332	0.6832000	-2.1782527
16.3833332	0.7320000	-2.1475730

16.3833332	0.7808000	-1.9819031
16.3833332	0.8296000	-1.3437672
16.3833332	0.8784000	-1.6198835
16.3833332	0.9272000	-1.6076121
16.3833332	0.9760000	-1.5830684
16.3833332	1.0247999	-1.5278449
16.3833332	1.0735999	-1.4542136
16.3833332	1.1223999	-1.4664855
16.3833332	1.1711999	-1.4173985
16.3833332	1.2200000	-1.4173985
16.3833332	1.2688000	-1.1351457
16.3833332	1.3176000	-1.1228738
16.3833332	1.3664000	-1.1290097
16.3833332	1.4152000	-1.1351457
16.3833332	1.4640000	-1.3008156
16.3833332	1.5128000	-1.0001554
16.3833332	1.5616000	-1.3560390
16.3833332	1.6104000	-1.3560390
16.3833332	1.6592000	-1.1351457
16.3833332	1.7079999	-1.4112625
16.3833332	1.7567999	-1.2946796
16.3833332	1.8055999	-1.3008156
16.3833332	1.8543999	-1.1351457
16.3833332	1.9031999	-1.1167378
16.3833332	1.9519999	-1.1535535
16.3833332	2.0007999	-1.1719613
16.3833332	2.0495999	-1.1719613
16.3833332	2.0983999	-1.1658254
16.3833332	2.1471999	-1.1474175
16.3833332	2.1959999	-1.1474175
16.3833332	2.2447999	-1.1290097
16.3833332	2.2935998	-1.1106019
16.3833332	2.3423998	-1.0615149
16.3833332	2.3912001	-1.0737867
16.3833332	2.4400001	-1.0799227
16.3833332	2.4888000	-1.0983305
16.3833332	2.5376000	-1.0983305
16.3833332	2.5864000	-1.0983305
16.3833332	2.6352000	-0.9817476
16.3833332	2.6840000	-0.9817476
16.3833332	2.7328000	-0.9756117
16.3833332	2.7816000	-0.9694757
16.3833332	2.8304000	-0.9756117
16.3833332	2.8792000	-0.9817476
16.3833332	2.9280000	-0.9265246
16.3833332	2.9768000	-0.9449320
16.3833332	3.0256000	-0.9449320
16.3833332	3.0743999	-0.8222136
16.3833332	3.1231999	-0.9265246
16.3833332	3.1719999	-0.9265246
16.3833332	3.2207999	-0.9387960
16.3833332	3.2695999	-0.9387960
16.3833332	3.3183999	-0.9387960
16.3833332	3.3671999	-0.8160777
16.3833332	3.4159999	-0.8344855
16.3833332	3.4647999	-0.7424469
16.3833332	3.5135999	-0.7424469

16.3833332	3.5623999	-0.7915339
16.3833332	3.6111999	-0.5951843
16.3833332	3.6599998	-0.5338254
16.3833332	3.7087998	-0.5154176
16.3833332	3.7575998	-0.4724660
16.3833332	3.8063998	-0.6442719
16.3833332	3.8551998	-0.6381359
16.3833332	3.9039998	-0.6258640
16.3833332	3.9527998	-0.8222136
16.3833332	4.0016003	-0.4970098
16.3833332	4.0503998	-0.7363110
16.3833332	4.0991998	-0.7424469
16.3833332	4.1479998	-0.4847379
16.3833332	4.1967998	-0.4663301
16.3833332	4.2455997	-0.4663301
16.3833332	4.2943997	-0.4540582
16.3833332	4.3431997	-0.4479222
16.3833332	4.3919997	-0.3190680
16.3833332	4.4407997	-0.2945242
16.3833332	4.4895997	-0.2883883
16.3833332	4.5383997	-0.3558836
16.3833332	4.5871997	-0.3620195
16.3833332	4.6359997	-0.3681555
16.3833332	4.6847997	-0.4724660
16.3833332	4.7336001	-0.4724660
16.3833332	4.7824001	-0.1963496
16.3833332	4.8312001	-0.1104465
16.3833332	4.8800001	-0.1104465
16.3833332	4.9288001	-0.1840777
16.3833332	4.9776001	-0.0306797
16.3833332	5.0264001	0.0000000
16.3833332	5.0752001	-0.2208934
16.3833332	5.1240001	613586.0000000
16.3833332	5.1728001	613586.0000000
16.3833332	5.2216001	613586.0000000
16.3833332	5.2704000	613586.0000000
16.3833332	5.3192000	613586.0000000
16.3833332	5.3680000	613586.0000000
16.3833332	5.4168000	613586.0000000
16.3833332	5.4656000	613586.0000000
16.3833332	5.5144000	613586.0000000
16.3833332	5.5632000	613586.0000000
16.3833332	5.6120000	613586.0000000
16.3833332	5.6608000	613586.0000000
16.3833332	5.7096000	613586.0000000
16.3833332	5.7584000	613586.0000000
16.3833332	5.8072000	613586.0000000
16.3833332	5.8559999	613586.0000000
16.3833332	5.9047999	613586.0000000
16.3833332	5.9535999	613586.0000000
16.3833332	6.0023999	613586.0000000
16.3999996	0.2928000	-3.8963113
16.3999996	0.3416000	-3.2643113
16.3999996	0.3904000	-2.9084277
16.3999996	0.4392000	-2.9022918
16.3999996	0.4880000	-3.7122335
16.3999996	0.5368000	-3.7735929

16.3999996	0.5856000	-1.7364664
16.3999996	0.6344000	-1.5278449
16.3999996	0.6832000	-1.2210488
16.3999996	0.7320000	-1.2210488
16.3999996	0.7808000	-0.9142528
16.3999996	0.8296000	-0.0920386
16.3999996	0.8784000	-0.2883883
16.3999996	0.9272000	-0.5215535
16.3999996	0.9760000	-1.6689711
16.3999996	1.0247999	-1.9205441
16.3999996	1.0735999	-1.8223691
16.3999996	1.1223999	-1.8223691
16.3999996	1.1711999	-1.8223691
16.3999996	1.2200000	-1.8223691
16.3999996	1.2688000	-1.6996508
16.3999996	1.3176000	-1.5033011
16.3999996	1.3664000	-1.4419422
16.3999996	1.4152000	-1.4358063
16.3999996	1.4640000	-1.5769324
16.3999996	1.5128000	-1.5769324
16.3999996	1.5616000	-1.5892043
16.3999996	1.6104000	-1.5953403
16.3999996	1.6592000	-1.4971652
16.3999996	1.7079999	-1.4480777
16.3999996	1.7567999	-1.3805828
16.3999996	1.8055999	-1.2087770
16.3999996	1.8543999	-1.2578645
16.3999996	1.9031999	-1.3499031
16.3999996	1.9519999	-1.3253593
16.3999996	2.0007999	-1.3192234
16.3999996	2.0495999	-1.2640004
16.3999996	2.0983999	-1.2517285
16.3999996	2.1471999	-1.2640004
16.3999996	2.1959999	-1.2946796
16.3999996	2.2447999	-1.2946796
16.3999996	2.2935998	-1.2517285
16.3999996	2.3423998	-1.3253593
16.3999996	2.3912001	-1.2946796
16.3999996	2.4400001	-1.3253593
16.3999996	2.4888000	-1.2640004
16.3999996	2.5376000	-1.2701364
16.3999996	2.5864000	-1.1965051
16.3999996	2.6352000	-1.1535535
16.3999996	2.6840000	-0.9756117
16.3999996	2.7328000	-1.2149129
16.3999996	2.7816000	-1.2885437
16.3999996	2.8304000	-1.2578645
16.3999996	2.8792000	-1.2149129
16.3999996	2.9280000	-1.2149129
16.3999996	2.9768000	-1.2087770
16.3999996	3.0256000	-1.2149129
16.3999996	3.0743999	-1.2210488
16.3999996	3.1231999	-0.9326606
16.3999996	3.1719999	-1.0553789
16.3999996	3.2207999	-1.0615149
16.3999996	3.2695999	-1.0553789
16.3999996	3.3183999	-1.0185633

16.3999996	3.3671999	-0.9940195
16.3999996	3.4159999	-1.0369711
16.3999996	3.4647999	-0.8528934
16.3999996	3.5135999	-0.8528934
16.3999996	3.5623999	-1.0001554
16.3999996	3.6111999	-0.9817476
16.3999996	3.6599998	-0.9756117
16.3999996	3.7087998	-0.9756117
16.3999996	3.7575998	-1.0369711
16.3999996	3.8063998	-0.7547188
16.3999996	3.8551998	-0.8713012
16.3999996	3.9039998	-0.8651652
16.3999996	3.9527998	-0.8713012
16.3999996	4.0016003	-0.9510679
16.3999996	4.0503998	-0.7424469
16.3999996	4.0991998	-0.7424469
16.3999996	4.1479998	-0.7669902
16.3999996	4.1967998	-0.7669902
16.3999996	4.2455997	-0.6074562
16.3999996	4.2943997	-0.6872234
16.3999996	4.3431997	-0.6810875
16.3999996	4.3919997	-0.6749516
16.3999996	4.4407997	-0.6626797
16.3999996	4.4895997	-0.6749516
16.3999996	4.5383997	-0.9326606
16.3999996	4.5871997	-0.8099418
16.3999996	4.6359997	-0.8528934
16.3999996	4.6847997	-0.7056313
16.3999996	4.7336001	-0.8344855
16.3999996	4.7824001	-0.7301750
16.3999996	4.8312001	-0.7117672
16.3999996	4.8800001	-0.7117672
16.3999996	4.9288001	-0.7179031
16.3999996	4.9776001	-0.5583692
16.3999996	5.0264001	613586.0000000
16.3999996	5.0752001	613586.0000000
16.3999996	5.1240001	613586.0000000
16.3999996	5.1728001	613586.0000000
16.3999996	5.2216001	613586.0000000
16.3999996	5.2704000	613586.0000000
16.3999996	5.3192000	613586.0000000
16.3999996	5.3680000	613586.0000000
16.3999996	5.4168000	613586.0000000
16.3999996	5.4656000	613586.0000000
16.3999996	5.5144000	613586.0000000
16.3999996	5.5632000	613586.0000000
16.3999996	5.6120000	613586.0000000
16.3999996	5.6608000	613586.0000000
16.3999996	5.7096000	613586.0000000
16.3999996	5.7584000	613586.0000000
16.3999996	5.8072000	613586.0000000
16.3999996	5.8559999	613586.0000000
16.3999996	5.9047999	613586.0000000
16.3999996	5.9535999	613586.0000000
16.3999996	6.0023999	613586.0000000
16.4166660	0.2928000	-3.4790685
16.4166660	0.3416000	-3.4913404

16.4166660	0.3904000	-3.8533599
16.4166660	0.4392000	-3.2029519
16.4166660	0.4880000	-2.9697869
16.4166660	0.5368000	-2.9697869
16.4166660	0.5856000	-2.3071072
16.4166660	0.6344000	-2.0003109
16.4166660	0.6832000	-2.0003109
16.4166660	0.7320000	-1.7119226
16.4166660	0.7808000	-1.3130875
16.4166660	0.8296000	-1.3130875
16.4166660	0.8784000	-1.8837285
16.4166660	0.9272000	-1.4358063
16.4166660	0.9760000	-1.5094371
16.4166660	1.0247999	-1.5094371
16.4166660	1.0735999	-1.4971652
16.4166660	1.1223999	-1.6812429
16.4166660	1.1711999	-1.5646605
16.4166660	1.2200000	-1.3069515
16.4166660	1.2688000	-1.3069515
16.4166660	1.3176000	-1.5094371
16.4166660	1.3664000	-1.5278449
16.4166660	1.4152000	-1.5339808
16.4166660	1.4640000	-1.5339808
16.4166660	1.5128000	-1.5217090
16.4166660	1.5616000	-1.5217090
16.4166660	1.6104000	-1.6873789
16.4166660	1.6592000	-1.6076121
16.4166660	1.7079999	-1.4112625
16.4166660	1.7567999	-1.2762718
16.4166660	1.8055999	-1.3314953
16.4166660	1.8543999	-1.4787574
16.4166660	1.9031999	-1.4787574
16.4166660	1.9519999	-1.3805828
16.4166660	2.0007999	-1.2640004
16.4166660	2.0495999	-1.3867188
16.4166660	2.0983999	-1.3499031
16.4166660	2.1471999	-1.3437672
16.4166660	2.1959999	-1.3314953
16.4166660	2.2447999	-1.3314953
16.4166660	2.2935998	-1.3989906
16.4166660	2.3423998	-1.3989906
16.4166660	2.3912001	-1.4173985
16.4166660	2.4400001	-1.4112625
16.4166660	2.4888000	-1.4112625
16.4166660	2.5376000	-1.3130875
16.4166660	2.5864000	-1.2517285
16.4166660	2.6352000	-1.3130875
16.4166660	2.6840000	-1.3008156
16.4166660	2.7328000	-1.1167378
16.4166660	2.7816000	-1.1228738
16.4166660	2.8304000	-1.3130875
16.4166660	2.8792000	-1.1596894
16.4166660	2.9280000	-1.1658254
16.4166660	2.9768000	-1.2824078
16.4166660	3.0256000	-1.1842332
16.4166660	3.0743999	-1.1842332
16.4166660	3.1231999	-0.9449320

16.4166660	3.1719999	-0.9449320
16.4166660	3.2207999	-1.0492430
16.4166660	3.2695999	-1.2946796
16.4166660	3.3183999	-0.9878836
16.4166660	3.3671999	-0.9878836
16.4166660	3.4159999	-1.1044660
16.4166660	3.4647999	-1.1535535
16.4166660	3.5135999	-1.3192234
16.4166660	3.5623999	-1.1106019
16.4166660	3.6111999	-0.8958449
16.4166660	3.6599998	-0.9572039
16.4166660	3.7087998	-0.9633398
16.4166660	3.7575998	-1.2394567
16.4166660	3.8063998	-0.9449320
16.4166660	3.8551998	-0.9449320
16.4166660	3.9039998	-1.0001554
16.4166660	3.9527998	-1.0676508
16.4166660	4.0016003	-1.0062914
16.4166660	4.0503998	-0.9694757
16.4166660	4.0991998	-0.9572039
16.4166660	4.1479998	-0.9203887
16.4166660	4.1967998	-0.9694757
16.4166660	4.2455997	-0.8590293
16.4166660	4.2943997	-0.8528934
16.4166660	4.3431997	-0.8590293
16.4166660	4.3919997	-0.7792621
16.4166660	4.4407997	-0.7853980
16.4166660	4.4895997	-0.8467574
16.4166660	4.5383997	-0.8528934
16.4166660	4.5871997	-0.8222136
16.4166660	4.6359997	-0.8160777
16.4166660	4.6847997	-0.8160777
16.4166660	4.7336001	-0.7301750
16.4166660	4.7824001	-0.7301750
16.4166660	4.8312001	-0.8222136
16.4166660	4.8800001	-0.8283496
16.4166660	4.9288001	-0.9203887
16.4166660	4.9776001	-0.9081168
16.4166660	5.0264001	-0.6749516
16.4166660	5.0752001	613586.0000000
16.4166660	5.1240001	613586.0000000
16.4166660	5.1728001	613586.0000000
16.4166660	5.2216001	613586.0000000
16.4166660	5.2704000	613586.0000000
16.4166660	5.3192000	613586.0000000
16.4166660	5.3680000	613586.0000000
16.4166660	5.4168000	613586.0000000
16.4166660	5.4656000	613586.0000000
16.4166660	5.5144000	613586.0000000
16.4166660	5.5632000	613586.0000000
16.4166660	5.6120000	613586.0000000
16.4166660	5.6608000	613586.0000000
16.4166660	5.7096000	613586.0000000
16.4166660	5.7584000	613586.0000000
16.4166660	5.8072000	613586.0000000
16.4166660	5.8559999	613586.0000000
16.4166660	5.9047999	613586.0000000

16.4166660	5.9535999	613586.0000000
16.4166660	6.0023999	613586.0000000
16.4333324	0.2928000	-3.0311460
16.4333324	0.3416000	-3.1784084
16.4333324	0.3904000	-3.1600006
16.4333324	0.4392000	-2.9943306
16.4333324	0.4880000	-3.5649714
16.4333324	0.5368000	-3.5649714
16.4333324	0.5856000	-3.0618258
16.4333324	0.6344000	-1.5585246
16.4333324	0.6832000	-1.4603496
16.4333324	0.7320000	-1.4603496
16.4333324	0.7808000	-1.0860586
16.4333324	0.8296000	-1.0921946
16.4333324	0.8784000	-1.5646605
16.4333324	0.9272000	-1.5707965
16.4333324	0.9760000	-1.5769324
16.4333324	1.0247999	-1.5585246
16.4333324	1.0735999	-1.3928547
16.4333324	1.1223999	-1.3499031
16.4333324	1.1711999	-1.5646605
16.4333324	1.2200000	-1.5646605
16.4333324	1.2688000	-1.5646605
16.4333324	1.3176000	-1.3989906
16.4333324	1.3664000	-1.3989906
16.4333324	1.4152000	-1.3989906
16.4333324	1.4640000	-1.5523887
16.4333324	1.5128000	-1.5155730
16.4333324	1.5616000	-1.5094371
16.4333324	1.6104000	-1.4173985
16.4333324	1.6592000	-1.4173985
16.4333324	1.7079999	-1.3314953
16.4333324	1.7567999	-1.3314953
16.4333324	1.8055999	-1.3314953
16.4333324	1.8543999	-1.3683109
16.4333324	1.9031999	-1.3683109
16.4333324	1.9519999	-1.3683109
16.4333324	2.0007999	-1.3621750
16.4333324	2.0495999	-1.3560390
16.4333324	2.0983999	-1.1965051
16.4333324	2.1471999	-1.3008156
16.4333324	2.1959999	-1.2455926
16.4333324	2.2447999	-1.2517285
16.4333324	2.2935998	-1.1535535
16.4333324	2.3423998	-1.2885437
16.4333324	2.3912001	-1.1044660
16.4333324	2.4400001	-1.1106019
16.4333324	2.4888000	-1.1167378
16.4333324	2.5376000	-1.1965051
16.4333324	2.5864000	-1.2210488
16.4333324	2.6352000	-1.0983305
16.4333324	2.6840000	-1.1106019
16.4333324	2.7328000	-1.1106019
16.4333324	2.7816000	-1.1044660
16.4333324	2.8304000	-1.0921946
16.4333324	2.8792000	-1.0676508
16.4333324	2.9280000	-1.0553789

16.4333324	2.9768000	-1.0431070
16.4333324	3.0256000	-0.9694757
16.4333324	3.0743999	-0.9694757
16.4333324	3.1231999	-0.9633398
16.4333324	3.1719999	-0.9633398
16.4333324	3.2207999	-0.8958449
16.4333324	3.2695999	-0.8406215
16.4333324	3.3183999	-0.9817476
16.4333324	3.3671999	-0.6197281
16.4333324	3.4159999	-0.5154176
16.4333324	3.4647999	-0.0613594
16.4333324	3.5135999	-0.0736313
16.4333324	3.5623999	-0.2883883
16.4333324	3.6111999	-0.8038058
16.4333324	3.6599998	-0.7976699
16.4333324	3.7087998	-0.7363110
16.4333324	3.7575998	-0.5583692
16.4333324	3.8063998	-0.6381359
16.4333324	3.8551998	-0.6749516
16.4333324	3.9039998	-0.7669902
16.4333324	3.9527998	-0.8038058
16.4333324	4.0016003	-0.7792621
16.4333324	4.0503998	-0.7608547
16.4333324	4.0991998	-0.7608547
16.4333324	4.1479998	-0.6565437
16.4333324	4.1967998	-0.6626797
16.4333324	4.2455997	-0.7117672
16.4333324	4.2943997	-0.7301750
16.4333324	4.3431997	-0.7363110
16.4333324	4.3919997	-0.7179031
16.4333324	4.4407997	-0.7363110
16.4333324	4.4895997	-0.6994953
16.4333324	4.5383997	-0.7056313
16.4333324	4.5871997	-0.6626797
16.4333324	4.6359997	-0.7117672
16.4333324	4.6847997	-0.5583692
16.4333324	4.7336001	-0.5583692
16.4333324	4.7824001	-0.5767770
16.4333324	4.8312001	-0.6381359
16.4333324	4.8800001	-0.6320000
16.4333324	4.9288001	-0.9142528
16.4333324	4.9776001	-0.6810875
16.4333324	5.0264001	-0.7240391
16.4333324	5.0752001	613586.0000000
16.4333324	5.1240001	613586.0000000
16.4333324	5.1728001	613586.0000000
16.4333324	5.2216001	613586.0000000
16.4333324	5.2704000	613586.0000000
16.4333324	5.3192000	613586.0000000
16.4333324	5.3680000	613586.0000000
16.4333324	5.4168000	613586.0000000
16.4333324	5.4656000	613586.0000000
16.4333324	5.5144000	613586.0000000
16.4333324	5.5632000	613586.0000000
16.4333324	5.6120000	613586.0000000
16.4333324	5.6608000	613586.0000000
16.4333324	5.7096000	613586.0000000

16.4333324	5.7584000	613586.0000000
16.4333324	5.8072000	613586.0000000
16.4333324	5.8559999	613586.0000000
16.4333324	5.9047999	613586.0000000
16.4333324	5.9535999	613586.0000000
16.4333324	6.0023999	613586.0000000
16.4500008	0.2928000	-3.4790685
16.4500008	0.3416000	-3.3502142
16.4500008	0.3904000	-3.7981365
16.4500008	0.4392000	-3.6570103
16.4500008	0.4880000	-3.8349521
16.4500008	0.5368000	-3.8349521
16.4500008	0.5856000	-3.3931656
16.4500008	0.6344000	-2.9513791
16.4500008	0.6832000	-0.8590293
16.4500008	0.7320000	-0.9756117
16.4500008	0.7808000	-1.1044660
16.4500008	0.8296000	-1.3805828
16.4500008	0.8784000	-1.3805828
16.4500008	0.9272000	-1.4726214
16.4500008	0.9760000	-1.4358063
16.4500008	1.0247999	-1.4480777
16.4500008	1.0735999	-1.4358063
16.4500008	1.1223999	-1.5278449
16.4500008	1.1711999	-1.5339808
16.4500008	1.2200000	-1.6628351
16.4500008	1.2688000	-1.6628351
16.4500008	1.3176000	-1.6382914
16.4500008	1.3664000	-1.6321554
16.4500008	1.4152000	-1.4296703
16.4500008	1.4640000	-1.4419422
16.4500008	1.5128000	-1.3314953
16.4500008	1.5616000	-1.3253593
16.4500008	1.6104000	-1.4235344
16.4500008	1.6592000	-1.4296703
16.4500008	1.7079999	-1.2946796
16.4500008	1.7567999	-1.3008156
16.4500008	1.8055999	-1.1474175
16.4500008	1.8543999	-1.2517285
16.4500008	1.9031999	-1.1167378
16.4500008	1.9519999	-1.1780972
16.4500008	2.0007999	-1.1719613
16.4500008	2.0495999	-1.2578645
16.4500008	2.0983999	-1.1965051
16.4500008	2.1471999	-1.1658254
16.4500008	2.1959999	-1.1596894
16.4500008	2.2447999	-1.1106019
16.4500008	2.2935998	-1.1044660
16.4500008	2.3423998	-1.1167378
16.4500008	2.3912001	-1.0369711
16.4500008	2.4400001	-0.9878836
16.4500008	2.4888000	-1.0001554
16.4500008	2.5376000	-1.0185633
16.4500008	2.5864000	-1.1474175
16.4500008	2.6352000	-0.9694757
16.4500008	2.6840000	-0.9694757
16.4500008	2.7328000	-1.0676508

16.4500008	2.7816000	-1.0676508
16.4500008	2.8304000	-0.9878836
16.4500008	2.8792000	-0.9878836
16.4500008	2.9280000	-0.9817476
16.4500008	2.9768000	-0.8774371
16.4500008	3.0256000	-0.9142528
16.4500008	3.0743999	-0.9449320
16.4500008	3.1231999	-0.9387960
16.4500008	3.1719999	-0.9142528
16.4500008	3.2207999	-0.9326606
16.4500008	3.2695999	-0.9326606
16.4500008	3.3183999	-0.8038058
16.4500008	3.3671999	-0.7608547
16.4500008	3.4159999	-0.9387960
16.4500008	3.4647999	-0.9510679
16.4500008	3.5135999	-0.9572039
16.4500008	3.5623999	-1.0492430
16.4500008	3.6111999	-0.9019809
16.4500008	3.6599998	-0.7547188
16.4500008	3.7087998	-0.7363110
16.4500008	3.7575998	-0.7363110
16.4500008	3.8063998	-0.7915339
16.4500008	3.8551998	-0.7915339
16.4500008	3.9039998	-0.6994953
16.4500008	3.9527998	-0.7485828
16.4500008	4.0016003	-0.7485828
16.4500008	4.0503998	-0.6442719
16.4500008	4.0991998	-0.5706410
16.4500008	4.1479998	-0.5706410
16.4500008	4.1967998	-0.5951843
16.4500008	4.2455997	-0.4663301
16.4500008	4.2943997	-0.4663301
16.4500008	4.3431997	-0.5092816
16.4500008	4.3919997	-0.4172430
16.4500008	4.4407997	-0.5951843
16.4500008	4.4895997	-0.5951843
16.4500008	4.5383997	-0.4847379
16.4500008	4.5871997	-0.4908738
16.4500008	4.6359997	-0.3620195
16.4500008	4.6847997	-0.1779418
16.4500008	4.7336001	-0.1902137
16.4500008	4.7824001	-0.1902137
16.4500008	4.8312001	-0.3129320
16.4500008	4.8800001	-0.1595340
16.4500008	4.9288001	-0.2577085
16.4500008	4.9776001	-0.2577085
16.4500008	5.0264001	-0.2515731
16.4500008	5.0752001	-0.1288543
16.4500008	5.1240001	613586.0000000
16.4500008	5.1728001	613586.0000000
16.4500008	5.2216001	613586.0000000
16.4500008	5.2704000	613586.0000000
16.4500008	5.3192000	613586.0000000
16.4500008	5.3680000	613586.0000000
16.4500008	5.4168000	613586.0000000
16.4500008	5.4656000	613586.0000000
16.4500008	5.5144000	613586.0000000

16.4500008	5.5632000	613586.0000000
16.4500008	5.6120000	613586.0000000
16.4500008	5.6608000	613586.0000000
16.4500008	5.7096000	613586.0000000
16.4500008	5.7584000	613586.0000000
16.4500008	5.8072000	613586.0000000
16.4500008	5.8559999	613586.0000000
16.4500008	5.9047999	613586.0000000
16.4500008	5.9535999	613586.0000000
16.4500008	6.0023999	613586.0000000
16.4666672	0.2928000	-2.7795732
16.4666672	0.3416000	-2.8961558
16.4666672	0.3904000	-2.9697869
16.4666672	0.4392000	-3.9576705
16.4666672	0.4880000	-3.9453988
16.4666672	0.5368000	-3.2827189
16.4666672	0.5856000	-2.4850490
16.4666672	0.6344000	-2.1107578
16.4666672	0.6832000	-1.9021363
16.4666672	0.7320000	-2.0064468
16.4666672	0.7808000	-2.0125828
16.4666672	0.8296000	-2.0923500
16.4666672	0.8784000	-1.6260195
16.4666672	0.9272000	-1.6321554
16.4666672	0.9760000	-1.6260195
16.4666672	1.0247999	-1.6137476
16.4666672	1.0735999	-1.6137476
16.4666672	1.1223999	-1.6689711
16.4666672	1.1711999	-1.5462527
16.4666672	1.2200000	-1.5523887
16.4666672	1.2688000	-1.4480777
16.4666672	1.3176000	-1.4358063
16.4666672	1.3664000	-1.4173985
16.4666672	1.4152000	-1.1228738
16.4666672	1.4640000	-1.3376312
16.4666672	1.5128000	-1.3253593
16.4666672	1.5616000	-1.3253593
16.4666672	1.6104000	-1.2455926
16.4666672	1.6592000	-1.3008156
16.4666672	1.7079999	-1.3069515
16.4666672	1.7567999	-1.4112625
16.4666672	1.8055999	-1.1290097
16.4666672	1.8543999	-1.1290097
16.4666672	1.9031999	-1.2210488
16.4666672	1.9519999	-1.0615149
16.4666672	2.0007999	-1.1903691
16.4666672	2.0495999	-1.1044660
16.4666672	2.0983999	-1.1535535
16.4666672	2.1471999	-1.1474175
16.4666672	2.1959999	-1.0676508
16.4666672	2.2447999	-1.0124273
16.4666672	2.2935998	-1.0983305
16.4666672	2.3423998	-1.0799227
16.4666672	2.3912001	-1.0860586
16.4666672	2.4400001	-1.0001554
16.4666672	2.4888000	-1.0001554
16.4666672	2.5376000	-0.9326606

16.4666672	2.5864000	-0.8958449
16.4666672	2.6352000	-0.8835731
16.4666672	2.6840000	-0.8835731
16.4666672	2.7328000	-0.9387960
16.4666672	2.7816000	-0.9203887
16.4666672	2.8304000	-0.9142528
16.4666672	2.8792000	-0.9142528
16.4666672	2.9280000	-0.9326606
16.4666672	2.9768000	-0.9387960
16.4666672	3.0256000	-0.8160777
16.4666672	3.0743999	-0.8222136
16.4666672	3.1231999	-0.9019809
16.4666672	3.1719999	-0.9019809
16.4666672	3.2207999	-0.7301750
16.4666672	3.2695999	-0.7117672
16.4666672	3.3183999	-0.6933594
16.4666672	3.3671999	-0.6810875
16.4666672	3.4159999	-0.5583692
16.4666672	3.4647999	-0.6933594
16.4666672	3.5135999	-1.3130875
16.4666672	3.5623999	-1.3130875
16.4666672	3.6111999	-0.6504078
16.4666672	3.6599998	-0.5399613
16.4666672	3.7087998	-0.5338254
16.4666672	3.7575998	-0.5460973
16.4666672	3.8063998	-0.5583692
16.4666672	3.8551998	-0.7117672
16.4666672	3.9039998	-0.6872234
16.4666672	3.9527998	-0.6810875
16.4666672	4.0016003	-0.6381359
16.4666672	4.0503998	-0.6258640
16.4666672	4.0991998	-0.5460973
16.4666672	4.1479998	-0.6810875
16.4666672	4.1967998	-0.6074562
16.4666672	4.2455997	-0.6074562
16.4666672	4.2943997	-0.6565437
16.4666672	4.3431997	-0.6626797
16.4666672	4.3919997	-0.6381359
16.4666672	4.4407997	-0.6320000
16.4666672	4.4895997	-0.5460973
16.4666672	4.5383997	-0.5276895
16.4666672	4.5871997	-0.6381359
16.4666672	4.6359997	-0.6381359
16.4666672	4.6847997	-0.6320000
16.4666672	4.7336001	-0.6320000
16.4666672	4.7824001	-0.6258640
16.4666672	4.8312001	-0.6074562
16.4666672	4.8800001	-0.5890489
16.4666672	4.9288001	-0.5154176
16.4666672	4.9776001	-0.6504078
16.4666672	5.0264001	613586.0000000
16.4666672	5.0752001	613586.0000000
16.4666672	5.1240001	-0.4663301
16.4666672	5.1728001	-0.6320000
16.4666672	5.2216001	-0.5338254
16.4666672	5.2704000	613586.0000000
16.4666672	5.3192000	613586.0000000

16.4666672	5.3680000	613586.0000000
16.4666672	5.4168000	613586.0000000
16.4666672	5.4656000	613586.0000000
16.4666672	5.5144000	613586.0000000
16.4666672	5.5632000	613586.0000000
16.4666672	5.6120000	613586.0000000
16.4666672	5.6608000	613586.0000000
16.4666672	5.7096000	613586.0000000
16.4666672	5.7584000	613586.0000000
16.4666672	5.8072000	613586.0000000
16.4666672	5.8559999	613586.0000000
16.4666672	5.9047999	613586.0000000
16.4666672	5.9535999	613586.0000000
16.4666672	6.0023999	613586.0000000
16.4833336	0.2928000	-3.7797287
16.4833336	0.3416000	-3.6447384
16.4833336	0.3904000	-3.7735929
16.4833336	0.4392000	-3.6324666
16.4833336	0.4880000	-3.6263306
16.4833336	0.5368000	-3.6201947
16.4833336	0.5856000	-1.9696312
16.4833336	0.6344000	-1.9696312
16.4833336	0.6832000	-1.8346410
16.4833336	0.7320000	-1.4664855
16.4833336	0.7808000	-1.3989906
16.4833336	0.8296000	-1.1535535
16.4833336	0.8784000	-1.6137476
16.4833336	0.9272000	-1.6137476
16.4833336	0.9760000	-1.4296703
16.4833336	1.0247999	-1.3989906
16.4833336	1.0735999	-1.6137476
16.4833336	1.1223999	-1.4542136
16.4833336	1.1711999	-1.5217090
16.4833336	1.2200000	-1.2333207
16.4833336	1.2688000	-1.2455926
16.4833336	1.3176000	-1.1412816
16.4833336	1.3664000	-1.1228738
16.4833336	1.4152000	-1.0615149
16.4833336	1.4640000	-1.1535535
16.4833336	1.5128000	-1.1535535
16.4833336	1.5616000	-1.1658254
16.4833336	1.6104000	-1.1780972
16.4833336	1.6592000	-1.2333207
16.4833336	1.7079999	-1.1780972
16.4833336	1.7567999	-1.1719613
16.4833336	1.8055999	-1.3805828
16.4833336	1.8543999	-1.0553789
16.4833336	1.9031999	-1.1658254
16.4833336	1.9519999	-1.1412816
16.4833336	2.0007999	-1.1228738
16.4833336	2.0495999	-1.1780972
16.4833336	2.0983999	-1.1780972
16.4833336	2.1471999	-1.1780972
16.4833336	2.1959999	-0.9878836
16.4833336	2.2447999	-0.9817476
16.4833336	2.2935998	-0.9817476
16.4833336	2.3423998	-1.0185633

16.4833336	2.3912001	-1.0308352
16.4833336	2.4400001	-1.1719613
16.4833336	2.4888000	-1.0737867
16.4833336	2.5376000	-1.0737867
16.4833336	2.5864000	-1.1719613
16.4833336	2.6352000	-1.1780972
16.4833336	2.6840000	-1.1903691
16.4833336	2.7328000	-1.1412816
16.4833336	2.7816000	-1.1412816
16.4833336	2.8304000	-1.0553789
16.4833336	2.8792000	-1.0553789
16.4833336	2.9280000	-1.0615149
16.4833336	2.9768000	-1.0676508
16.4833336	3.0256000	-0.9203887
16.4833336	3.0743999	-0.9203887
16.4833336	3.1231999	-0.8713012
16.4833336	3.1719999	-0.9510679
16.4833336	3.2207999	-0.9572039
16.4833336	3.2695999	-0.9510679
16.4833336	3.3183999	-0.8222136
16.4833336	3.3671999	-0.8160777
16.4833336	3.4159999	-0.8835731
16.4833336	3.4647999	-0.8835731
16.4833336	3.5135999	-0.6994953
16.4833336	3.5623999	-0.7363110
16.4833336	3.6111999	-0.6381359
16.4833336	3.6599998	-0.5951843
16.4833336	3.7087998	-0.7731261
16.4833336	3.7575998	-0.8038058
16.4833336	3.8063998	-0.8038058
16.4833336	3.8551998	-0.8038058
16.4833336	3.9039998	-0.9940195
16.4833336	3.9527998	-0.9326606
16.4833336	4.0016003	-0.9449320
16.4833336	4.0503998	-0.9510679
16.4833336	4.0991998	-0.9265246
16.4833336	4.1479998	-0.9326606
16.4833336	4.1967998	-0.9387960
16.4833336	4.2455997	-0.9326606
16.4833336	4.2943997	-0.9265246
16.4833336	4.3431997	-0.7976699
16.4833336	4.3919997	-0.7731261
16.4833336	4.4407997	-0.7792621
16.4833336	4.4895997	-0.6749516
16.4833336	4.5383997	-0.6626797
16.4833336	4.5871997	-0.7240391
16.4833336	4.6359997	-0.7301750
16.4833336	4.6847997	-0.5583692
16.4833336	4.7336001	-0.5583692
16.4833336	4.7824001	-0.5645051
16.4833336	4.8312001	-0.5645051
16.4833336	4.8800001	-0.4479222
16.4833336	4.9288001	613586.0000000
16.4833336	4.9776001	613586.0000000
16.4833336	5.0264001	-0.1718059
16.4833336	5.0752001	-0.1595340
16.4833336	5.1240001	613586.0000000

16.4833336	5.1728001	613586.0000000
16.4833336	5.2216001	613586.0000000
16.4833336	5.2704000	613586.0000000
16.4833336	5.3192000	613586.0000000
16.4833336	5.3680000	613586.0000000
16.4833336	5.4168000	613586.0000000
16.4833336	5.4656000	613586.0000000
16.4833336	5.5144000	613586.0000000
16.4833336	5.5632000	613586.0000000
16.4833336	5.6120000	613586.0000000
16.4833336	5.6608000	613586.0000000
16.4833336	5.7096000	613586.0000000
16.4833336	5.7584000	613586.0000000
16.4833336	5.8072000	613586.0000000
16.4833336	5.8559999	613586.0000000
16.4833336	5.9047999	613586.0000000
16.4833336	5.9535999	613586.0000000
16.4833336	6.0023999	613586.0000000
16.5000000	0.2928000	-3.6815541
16.5000000	0.3416000	-3.6815541
16.5000000	0.3904000	-4.0190296
16.5000000	0.4392000	-4.3074183
16.5000000	0.4880000	-4.2951465
16.5000000	0.5368000	-3.7797287
16.5000000	0.5856000	-1.6566992
16.5000000	0.6344000	-1.4910293
16.5000000	0.6832000	-1.4910293
16.5000000	0.7320000	-1.6873789
16.5000000	0.7808000	-1.4542136
16.5000000	0.8296000	-1.6382914
16.5000000	0.8784000	-1.6321554
16.5000000	0.9272000	-1.6198835
16.5000000	0.9760000	-1.5769324
16.5000000	1.0247999	-1.6505632
16.5000000	1.0735999	-1.6505632
16.5000000	1.1223999	-1.6505632
16.5000000	1.1711999	-1.6260195
16.5000000	1.2200000	-1.3499031
16.5000000	1.2688000	-1.2578645
16.5000000	1.3176000	-1.1412816
16.5000000	1.3664000	-1.1412816
16.5000000	1.4152000	-1.0124273
16.5000000	1.4640000	-1.0246992
16.5000000	1.5128000	-1.0369711
16.5000000	1.5616000	-1.0737867
16.5000000	1.6104000	-1.0737867
16.5000000	1.6592000	-1.0676508
16.5000000	1.7079999	-1.1412816
16.5000000	1.7567999	-0.9694757
16.5000000	1.8055999	-0.9756117
16.5000000	1.8543999	-1.0308352
16.5000000	1.9031999	-1.0921946
16.5000000	1.9519999	-1.0921946
16.5000000	2.0007999	-1.0860586
16.5000000	2.0495999	-1.0799227
16.5000000	2.0983999	-1.0860586
16.5000000	2.1471999	-1.0860586

16.5000000	2.1959999	-1.0737867
16.5000000	2.2447999	-1.0737867
16.5000000	2.2935998	-1.1842332
16.5000000	2.3423998	-1.1842332
16.5000000	2.3912001	-1.1842332
16.5000000	2.4400001	-1.1044660
16.5000000	2.4888000	-1.2087770
16.5000000	2.5376000	-1.0799227
16.5000000	2.5864000	-1.1351457
16.5000000	2.6352000	-1.1351457
16.5000000	2.6840000	-0.9940195
16.5000000	2.7328000	-1.0062914
16.5000000	2.7816000	-1.0062914
16.5000000	2.8304000	-0.9633398
16.5000000	2.8792000	-0.9633398
16.5000000	2.9280000	-0.9633398
16.5000000	2.9768000	-0.9572039
16.5000000	3.0256000	-0.8283496
16.5000000	3.0743999	-0.8283496
16.5000000	3.1231999	-0.7485828
16.5000000	3.1719999	-0.7485828
16.5000000	3.2207999	-0.7363110
16.5000000	3.2695999	-0.7669902
16.5000000	3.3183999	-0.7853980
16.5000000	3.3671999	-0.7853980
16.5000000	3.4159999	-0.8651652
16.5000000	3.4647999	-0.8958449
16.5000000	3.5135999	-0.9081168
16.5000000	3.5623999	-0.9142528
16.5000000	3.6111999	-0.8160777
16.5000000	3.6599998	-0.6135921
16.5000000	3.7087998	-0.5951843
16.5000000	3.7575998	-0.8651652
16.5000000	3.8063998	-0.8651652
16.5000000	3.8551998	-0.7792621
16.5000000	3.9039998	-0.8467574
16.5000000	3.9527998	-0.7301750
16.5000000	4.0016003	-0.7240391
16.5000000	4.0503998	-0.7179031
16.5000000	4.0991998	-0.7179031
16.5000000	4.1479998	-0.7731261
16.5000000	4.1967998	-0.7424469
16.5000000	4.2455997	-0.6135921
16.5000000	4.2943997	-0.6320000
16.5000000	4.3431997	-0.6381359
16.5000000	4.3919997	-0.6320000
16.5000000	4.4407997	-0.6442719
16.5000000	4.4895997	-0.6442719
16.5000000	4.5383997	-0.6442719
16.5000000	4.5871997	-0.4356503
16.5000000	4.6359997	-0.4295144
16.5000000	4.6847997	-0.6135921
16.5000000	4.7336001	-0.5951843
16.5000000	4.7824001	-0.6565437
16.5000000	4.8312001	-0.6442719
16.5000000	4.8800001	-0.6442719
16.5000000	4.9288001	613586.0000000

16.5000000	4.9776001	-1.0860586
16.5000000	5.0264001	-1.0492430
16.5000000	5.0752001	-0.7056313
16.5000000	5.1240001	-0.6933594
16.5000000	5.1728001	613586.0000000
16.5000000	5.2216001	613586.0000000
16.5000000	5.2704000	613586.0000000
16.5000000	5.3192000	613586.0000000
16.5000000	5.3680000	613586.0000000
16.5000000	5.4168000	613586.0000000
16.5000000	5.4656000	613586.0000000
16.5000000	5.5144000	613586.0000000
16.5000000	5.5632000	613586.0000000
16.5000000	5.6120000	613586.0000000
16.5000000	5.6608000	613586.0000000
16.5000000	5.7096000	613586.0000000
16.5000000	5.7584000	613586.0000000
16.5000000	5.8072000	613586.0000000
16.5000000	5.8559999	613586.0000000
16.5000000	5.9047999	613586.0000000
16.5000000	5.9535999	613586.0000000
16.5000000	6.0023999	613586.0000000
16.5166664	0.2928000	-3.7674570
16.5166664	0.3416000	-3.7613211
16.5166664	0.3904000	-3.6140587
16.5166664	0.4392000	-3.2520394
16.5166664	0.4880000	-3.3747578
16.5166664	0.5368000	-3.3624859
16.5166664	0.5856000	-2.2886994
16.5166664	0.6344000	-2.0984859
16.5166664	0.6832000	-1.7180586
16.5166664	0.7320000	-1.4235344
16.5166664	0.7808000	-1.1842332
16.5166664	0.8296000	-1.1842332
16.5166664	0.8784000	-1.1044660
16.5166664	0.9272000	-1.3621750
16.5166664	0.9760000	-1.3683109
16.5166664	1.0247999	-1.4296703
16.5166664	1.0735999	-1.4051266
16.5166664	1.1223999	-1.2640004
16.5166664	1.1711999	-1.2578645
16.5166664	1.2200000	-1.4787574
16.5166664	1.2688000	-1.4726214
16.5166664	1.3176000	-1.3376312
16.5166664	1.3664000	-1.1965051
16.5166664	1.4152000	-1.2210488
16.5166664	1.4640000	-1.1535535
16.5166664	1.5128000	-1.2149129
16.5166664	1.5616000	-1.2149129
16.5166664	1.6104000	-1.2210488
16.5166664	1.6592000	-1.0676508
16.5166664	1.7079999	-0.7976699
16.5166664	1.7567999	-1.1290097
16.5166664	1.8055999	-1.1044660
16.5166664	1.8543999	-1.1106019
16.5166664	1.9031999	-1.1290097
16.5166664	1.9519999	-1.0062914

16.5166664	2.0007999	-0.9633398
16.5166664	2.0495999	-0.9449320
16.5166664	2.0983999	-0.9203887
16.5166664	2.1471999	-0.9203887
16.5166664	2.1959999	-0.9940195
16.5166664	2.2447999	-1.1412816
16.5166664	2.2935998	-1.1290097
16.5166664	2.3423998	-1.1412816
16.5166664	2.3912001	-1.1412816
16.5166664	2.4400001	-1.1903691
16.5166664	2.4888000	-1.1106019
16.5166664	2.5376000	-1.0860586
16.5166664	2.5864000	-1.0860586
16.5166664	2.6352000	-0.9633398
16.5166664	2.6840000	-1.0553789
16.5166664	2.7328000	-1.1106019
16.5166664	2.7816000	-1.0369711
16.5166664	2.8304000	-1.0246992
16.5166664	2.8792000	-0.8528934
16.5166664	2.9280000	-0.7669902
16.5166664	2.9768000	-0.9203887
16.5166664	3.0256000	-0.8958449
16.5166664	3.0743999	-0.9142528
16.5166664	3.1231999	-0.8160777
16.5166664	3.1719999	-0.8038058
16.5166664	3.2207999	-0.8038058
16.5166664	3.2695999	-0.5583692
16.5166664	3.3183999	-0.6381359
16.5166664	3.3671999	-0.6442719
16.5166664	3.4159999	-0.5031457
16.5166664	3.4647999	-0.4970098
16.5166664	3.5135999	-0.6504078
16.5166664	3.5623999	-0.8222136
16.5166664	3.6111999	-0.5890489
16.5166664	3.6599998	-0.5338254
16.5166664	3.7087998	-0.5215535
16.5166664	3.7575998	-0.5767770
16.5166664	3.8063998	-0.7179031
16.5166664	3.8551998	-0.7056313
16.5166664	3.9039998	-0.7056313
16.5166664	3.9527998	-0.7731261
16.5166664	4.0016003	-0.8344855
16.5166664	4.0503998	-0.6565437
16.5166664	4.0991998	-0.6074562
16.5166664	4.1479998	-0.6135921
16.5166664	4.1967998	-0.6135921
16.5166664	4.2455997	-0.6135921
16.5166664	4.2943997	-0.6135921
16.5166664	4.3431997	-0.6381359
16.5166664	4.3919997	-0.6381359
16.5166664	4.4407997	-0.7547188
16.5166664	4.4895997	-0.6258640
16.5166664	4.5383997	-0.6626797
16.5166664	4.5871997	-0.5031457
16.5166664	4.6359997	-0.5645051
16.5166664	4.6847997	-0.6749516
16.5166664	4.7336001	-0.6994953

16.5166664	4.7824001	-0.5338254
16.5166664	4.8312001	-0.5215535
16.5166664	4.8800001	-0.5215535
16.5166664	4.9288001	-0.4540582
16.5166664	4.9776001	613586.0000000
16.5166664	5.0264001	613586.0000000
16.5166664	5.0752001	613586.0000000
16.5166664	5.1240001	-0.5154176
16.5166664	5.1728001	613586.0000000
16.5166664	5.2216001	613586.0000000
16.5166664	5.2704000	613586.0000000
16.5166664	5.3192000	613586.0000000
16.5166664	5.3680000	613586.0000000
16.5166664	5.4168000	613586.0000000
16.5166664	5.4656000	613586.0000000
16.5166664	5.5144000	613586.0000000
16.5166664	5.5632000	613586.0000000
16.5166664	5.6120000	613586.0000000
16.5166664	5.6608000	613586.0000000
16.5166664	5.7096000	613586.0000000
16.5166664	5.7584000	-1.2210488
16.5166664	5.8072000	613586.0000000
16.5166664	5.8559999	613586.0000000
16.5166664	5.9047999	613586.0000000
16.5166664	5.9535999	613586.0000000
16.5166664	6.0023999	613586.0000000
16.5333328	0.2928000	-3.4483888
16.5333328	0.3416000	-3.6938257
16.5333328	0.3904000	-3.6938257
16.5333328	0.4392000	-3.5158842
16.5333328	0.4880000	-3.2949908
16.5333328	0.5368000	-3.7674570
16.5333328	0.5856000	-1.2578645
16.5333328	0.6344000	-1.2517285
16.5333328	0.6832000	-1.0615149
16.5333328	0.7320000	-0.8406215
16.5333328	0.7808000	-0.7485828
16.5333328	0.8296000	-1.1780972
16.5333328	0.8784000	-1.1780972
16.5333328	0.9272000	-1.1903691
16.5333328	0.9760000	-1.3437672
16.5333328	1.0247999	-1.5401168
16.5333328	1.0735999	-1.5339808
16.5333328	1.1223999	-1.2210488
16.5333328	1.1711999	-1.2210488
16.5333328	1.2200000	-1.1903691
16.5333328	1.2688000	-1.2455926
16.5333328	1.3176000	-1.1351457
16.5333328	1.3664000	-1.1596894
16.5333328	1.4152000	-1.3683109
16.5333328	1.4640000	-1.3621750
16.5333328	1.5128000	-1.3499031
16.5333328	1.5616000	-1.3192234
16.5333328	1.6104000	-1.3130875
16.5333328	1.6592000	-1.3130875
16.5333328	1.7079999	-1.0001554
16.5333328	1.7567999	-1.0431070

16.5333328	1.8055999	-1.1965051
16.5333328	1.8543999	-1.2087770
16.5333328	1.9031999	-1.0062914
16.5333328	1.9519999	-0.9572039
16.5333328	2.0007999	-0.8958449
16.5333328	2.0495999	-1.0308352
16.5333328	2.0983999	-1.1106019
16.5333328	2.1471999	-1.0185633
16.5333328	2.1959999	-0.9633398
16.5333328	2.2447999	-0.9633398
16.5333328	2.2935998	-1.0246992
16.5333328	2.3423998	-0.9203887
16.5333328	2.3912001	-0.9203887
16.5333328	2.4400001	-1.0799227
16.5333328	2.4888000	-1.0921946
16.5333328	2.5376000	-0.9633398
16.5333328	2.5864000	-0.9817476
16.5333328	2.6352000	-1.1780972
16.5333328	2.6840000	-1.0799227
16.5333328	2.7328000	-1.0921946
16.5333328	2.7816000	-0.8590293
16.5333328	2.8304000	-0.9265246
16.5333328	2.8792000	-0.9265246
16.5333328	2.9280000	-0.9203887
16.5333328	2.9768000	-0.9265246
16.5333328	3.0256000	-0.9756117
16.5333328	3.0743999	-0.8467574
16.5333328	3.1231999	-0.8590293
16.5333328	3.1719999	-0.8528934
16.5333328	3.2207999	-0.7485828
16.5333328	3.2695999	-0.7485828
16.5333328	3.3183999	-0.7547188
16.5333328	3.3671999	-0.7056313
16.5333328	3.4159999	-0.8406215
16.5333328	3.4647999	-0.5767770
16.5333328	3.5135999	-0.5767770
16.5333328	3.5623999	-0.6442719
16.5333328	3.6111999	-0.6442719
16.5333328	3.6599998	-0.8528934
16.5333328	3.7087998	-0.8528934
16.5333328	3.7575998	-0.7853980
16.5333328	3.8063998	-0.8528934
16.5333328	3.8551998	-1.0124273
16.5333328	3.9039998	-1.0124273
16.5333328	3.9527998	-1.0062914
16.5333328	4.0016003	-0.7669902
16.5333328	4.0503998	-0.7547188
16.5333328	4.0991998	-0.7608547
16.5333328	4.1479998	-0.7424469
16.5333328	4.1967998	-0.7485828
16.5333328	4.2455997	-0.7608547
16.5333328	4.2943997	-0.7608547
16.5333328	4.3431997	-0.5890489
16.5333328	4.3919997	-0.5951843
16.5333328	4.4407997	-0.6872234
16.5333328	4.4895997	-0.6872234
16.5333328	4.5383997	-0.6135921

16.5333328	4.5871997	-0.6381359
16.5333328	4.6359997	-0.6442719
16.5333328	4.6847997	-0.6381359
16.5333328	4.7336001	-0.5706410
16.5333328	4.7824001	-0.5031457
16.5333328	4.8312001	-0.4049711
16.5333328	4.8800001	-0.4356503
16.5333328	4.9288001	613586.0000000
16.5333328	4.9776001	613586.0000000
16.5333328	5.0264001	-0.3988352
16.5333328	5.0752001	613586.0000000
16.5333328	5.1240001	-0.9940195
16.5333328	5.1728001	613586.0000000
16.5333328	5.2216001	613586.0000000
16.5333328	5.2704000	613586.0000000
16.5333328	5.3192000	613586.0000000
16.5333328	5.3680000	613586.0000000
16.5333328	5.4168000	613586.0000000
16.5333328	5.4656000	613586.0000000
16.5333328	5.5144000	613586.0000000
16.5333328	5.5632000	613586.0000000
16.5333328	5.6120000	613586.0000000
16.5333328	5.6608000	613586.0000000
16.5333328	5.7096000	613586.0000000
16.5333328	5.7584000	613586.0000000
16.5333328	5.8072000	613586.0000000
16.5333328	5.8559999	613586.0000000
16.5333328	5.9047999	613586.0000000
16.5333328	5.9535999	613586.0000000
16.5333328	6.0023999	613586.0000000
16.5499992	0.2928000	-3.1845441
16.5499992	0.3416000	-4.0681171
16.5499992	0.3904000	-4.1969719
16.5499992	0.4392000	-3.5588355
16.5499992	0.4880000	-3.5649714
16.5499992	0.5368000	-3.7858646
16.5499992	0.5856000	-2.4236896
16.5499992	0.6344000	-2.4175539
16.5499992	0.6832000	-1.7548742
16.5499992	0.7320000	-1.1965051
16.5499992	0.7808000	-0.8222136
16.5499992	0.8296000	-0.9019809
16.5499992	0.8784000	-0.9019809
16.5499992	0.9272000	-0.9019809
16.5499992	0.9760000	-1.2394567
16.5499992	1.0247999	-1.2149129
16.5499992	1.0735999	-0.9387960
16.5499992	1.1223999	-1.2333207
16.5499992	1.1711999	-1.2394567
16.5499992	1.2200000	-1.2762718
16.5499992	1.2688000	-1.3560390
16.5499992	1.3176000	-1.3314953
16.5499992	1.3664000	-1.5278449
16.5499992	1.4152000	-1.5278449
16.5499992	1.4640000	-1.5339808
16.5499992	1.5128000	-1.1903691
16.5499992	1.5616000	-1.1535535

16.5499992	1.6104000	-1.1474175
16.5499992	1.6592000	-1.1351457
16.5499992	1.7079999	-1.2271848
16.5499992	1.7567999	-1.2394567
16.5499992	1.8055999	-1.2394567
16.5499992	1.8543999	-1.0737867
16.5499992	1.9031999	-1.0921946
16.5499992	1.9519999	-0.9694757
16.5499992	2.0007999	-1.1596894
16.5499992	2.0495999	-1.0615149
16.5499992	2.0983999	-1.1474175
16.5499992	2.1471999	-1.1044660
16.5499992	2.1959999	-1.0615149
16.5499992	2.2447999	-1.0615149
16.5499992	2.2935998	-0.9633398
16.5499992	2.3423998	-1.1903691
16.5499992	2.3912001	-1.0124273
16.5499992	2.4400001	-1.0921946
16.5499992	2.4888000	-1.0676508
16.5499992	2.5376000	-1.0492430
16.5499992	2.5864000	-1.0431070
16.5499992	2.6352000	-1.0308352
16.5499992	2.6840000	-0.8835731
16.5499992	2.7328000	-0.8835731
16.5499992	2.7816000	-0.8835731
16.5499992	2.8304000	-0.7608547
16.5499992	2.8792000	-0.7608547
16.5499992	2.9280000	-1.1106019
16.5499992	2.9768000	-0.8283496
16.5499992	3.0256000	-0.9142528
16.5499992	3.0743999	-0.9510679
16.5499992	3.1231999	-0.8467574
16.5499992	3.1719999	-0.7608547
16.5499992	3.2207999	-0.7547188
16.5499992	3.2695999	-0.8160777
16.5499992	3.3183999	-0.8835731
16.5499992	3.3671999	-0.8528934
16.5499992	3.4159999	-0.8344855
16.5499992	3.4647999	-1.0860586
16.5499992	3.5135999	-0.6749516
16.5499992	3.5623999	-0.5767770
16.5499992	3.6111999	-0.8038058
16.5499992	3.6599998	-0.7547188
16.5499992	3.7087998	-0.7608547
16.5499992	3.7575998	-0.7608547
16.5499992	3.8063998	-0.6626797
16.5499992	3.8551998	-0.6810875
16.5499992	3.9039998	-0.6872234
16.5499992	3.9527998	-0.8406215
16.5499992	4.0016003	-0.7731261
16.5499992	4.0503998	-0.7731261
16.5499992	4.0991998	-0.7731261
16.5499992	4.1479998	-0.7179031
16.5499992	4.1967998	-0.6933594
16.5499992	4.2455997	-0.6933594
16.5499992	4.2943997	-0.5522332
16.5499992	4.3431997	-0.5645051

16.5499992	4.3919997	-0.6074562
16.5499992	4.4407997	-0.6504078
16.5499992	4.4895997	-0.6381359
16.5499992	4.5383997	-0.6749516
16.5499992	4.5871997	-0.5829129
16.5499992	4.6359997	-0.5522332
16.5499992	4.6847997	-0.4847379
16.5499992	4.7336001	-0.4847379
16.5499992	4.7824001	-0.5583692
16.5499992	4.8312001	-0.5706410
16.5499992	4.8800001	-0.3742914
16.5499992	4.9288001	-0.5706410
16.5499992	4.9776001	-0.3190680
16.5499992	5.0264001	613586.0000000
16.5499992	5.0752001	613586.0000000
16.5499992	5.1240001	613586.0000000
16.5499992	5.1728001	613586.0000000
16.5499992	5.2216001	613586.0000000
16.5499992	5.2704000	613586.0000000
16.5499992	5.3192000	613586.0000000
16.5499992	5.3680000	613586.0000000
16.5499992	5.4168000	613586.0000000
16.5499992	5.4656000	613586.0000000
16.5499992	5.5144000	613586.0000000
16.5499992	5.5632000	613586.0000000
16.5499992	5.6120000	613586.0000000
16.5499992	5.6608000	613586.0000000
16.5499992	5.7096000	613586.0000000
16.5499992	5.7584000	613586.0000000
16.5499992	5.8072000	613586.0000000
16.5499992	5.8559999	613586.0000000
16.5499992	5.9047999	613586.0000000
16.5499992	5.9535999	613586.0000000
16.5499992	6.0023999	613586.0000000
16.5666676	0.2928000	-4.0926609
16.5666676	0.3416000	-4.0803890
16.5666676	0.3904000	-4.1785641
16.5666676	0.4392000	-4.1724281
16.5666676	0.4880000	-3.5158842
16.5666676	0.5368000	-3.5342917
16.5666676	0.5856000	-1.9696312
16.5666676	0.6344000	-1.9634953
16.5666676	0.6832000	-1.4173985
16.5666676	0.7320000	-1.4112625
16.5666676	0.7808000	-1.3376312
16.5666676	0.8296000	-1.7119226
16.5666676	0.8784000	-1.7241945
16.5666676	0.9272000	-1.6751070
16.5666676	0.9760000	-1.6751070
16.5666676	1.0247999	-1.7426023
16.5666676	1.0735999	-1.5892043
16.5666676	1.1223999	-1.5953403
16.5666676	1.1711999	-1.5953403
16.5666676	1.2200000	-1.4971652
16.5666676	1.2688000	-1.5217090
16.5666676	1.3176000	-1.5339808
16.5666676	1.3664000	-1.4603496

16.5666676	1.4152000	-1.4542136
16.5666676	1.4640000	-1.5585246
16.5666676	1.5128000	-1.5585246
16.5666676	1.5616000	-1.5401168
16.5666676	1.6104000	-1.6198835
16.5666676	1.6592000	-1.2824078
16.5666676	1.7079999	-1.3008156
16.5666676	1.7567999	-1.3130875
16.5666676	1.8055999	-1.2394567
16.5666676	1.8543999	-1.0615149
16.5666676	1.9031999	-1.0799227
16.5666676	1.9519999	-1.0860586
16.5666676	2.0007999	-1.1228738
16.5666676	2.0495999	-1.1228738
16.5666676	2.0983999	-1.0246992
16.5666676	2.1471999	-1.0431070
16.5666676	2.1959999	-1.1535535
16.5666676	2.2447999	-1.1412816
16.5666676	2.2935998	-1.0308352
16.5666676	2.3423998	-1.0492430
16.5666676	2.3912001	-1.0799227
16.5666676	2.4400001	-1.1351457
16.5666676	2.4888000	-1.1780972
16.5666676	2.5376000	-0.9756117
16.5666676	2.5864000	-1.0799227
16.5666676	2.6352000	-1.0799227
16.5666676	2.6840000	-1.0185633
16.5666676	2.7328000	-0.8344855
16.5666676	2.7816000	-0.6933594
16.5666676	2.8304000	-0.8897090
16.5666676	2.8792000	-0.7853980
16.5666676	2.9280000	-0.8958449
16.5666676	2.9768000	-0.8897090
16.5666676	3.0256000	-0.9081168
16.5666676	3.0743999	-0.9081168
16.5666676	3.1231999	-0.8344855
16.5666676	3.1719999	-0.6565437
16.5666676	3.2207999	-0.7179031
16.5666676	3.2695999	-0.7179031
16.5666676	3.3183999	-0.7669902
16.5666676	3.3671999	-0.6197281
16.5666676	3.4159999	-0.6197281
16.5666676	3.4647999	-0.6258640
16.5666676	3.5135999	-0.7731261
16.5666676	3.5623999	-0.6565437
16.5666676	3.6111999	-0.6688156
16.5666676	3.6599998	-0.7792621
16.5666676	3.7087998	-0.9081168
16.5666676	3.7575998	-0.7424469
16.5666676	3.8063998	-0.7608547
16.5666676	3.8551998	-0.7608547
16.5666676	3.9039998	-0.7669902
16.5666676	3.9527998	-0.7485828
16.5666676	4.0016003	-0.7424469
16.5666676	4.0503998	-0.6504078
16.5666676	4.0991998	-0.6933594
16.5666676	4.1479998	-0.6749516

16.5666676	4.1967998	-0.6074562
16.5666676	4.2455997	-0.7547188
16.5666676	4.2943997	-0.7547188
16.5666676	4.3431997	-0.7547188
16.5666676	4.3919997	-0.7424469
16.5666676	4.4407997	-0.6933594
16.5666676	4.4895997	-0.6320000
16.5666676	4.5383997	-0.6442719
16.5666676	4.5871997	-0.6688156
16.5666676	4.6359997	-0.7424469
16.5666676	4.6847997	-0.7301750
16.5666676	4.7336001	-0.7301750
16.5666676	4.7824001	-0.7301750
16.5666676	4.8312001	-0.8038058
16.5666676	4.8800001	-1.0001554
16.5666676	4.9288001	-0.9081168
16.5666676	4.9776001	-0.6504078
16.5666676	5.0264001	-0.6504078
16.5666676	5.0752001	613586.0000000
16.5666676	5.1240001	613586.0000000
16.5666676	5.1728001	613586.0000000
16.5666676	5.2216001	613586.0000000
16.5666676	5.2704000	613586.0000000
16.5666676	5.3192000	613586.0000000
16.5666676	5.3680000	613586.0000000
16.5666676	5.4168000	613586.0000000
16.5666676	5.4656000	613586.0000000
16.5666676	5.5144000	613586.0000000
16.5666676	5.5632000	613586.0000000
16.5666676	5.6120000	613586.0000000
16.5666676	5.6608000	613586.0000000
16.5666676	5.7096000	613586.0000000
16.5666676	5.7584000	613586.0000000
16.5666676	5.8072000	613586.0000000
16.5666676	5.8559999	613586.0000000
16.5666676	5.9047999	613586.0000000
16.5666676	5.9535999	613586.0000000
16.5666676	6.0023999	613586.0000000
16.5833340	0.2928000	-3.4054375
16.5833340	0.3416000	-3.6570103
16.5833340	0.3904000	-3.6570103
16.5833340	0.4392000	-3.6017871
16.5833340	0.4880000	-3.6079230
16.5833340	0.5368000	-3.2888548
16.5833340	0.5856000	-2.3623304
16.5833340	0.6344000	-1.2026410
16.5833340	0.6832000	-0.7301750
16.5833340	0.7320000	-1.3499031
16.5833340	0.7808000	-1.3376312
16.5833340	0.8296000	-1.3314953
16.5833340	0.8784000	-1.1228738
16.5833340	0.9272000	-1.1290097
16.5833340	0.9760000	-1.5462527
16.5833340	1.0247999	-1.5462527
16.5833340	1.0735999	-1.6198835
16.5833340	1.1223999	-1.6689711
16.5833340	1.1711999	-1.6628351

16.5833340	1.2200000	-1.3621750
16.5833340	1.2688000	-1.3437672
16.5833340	1.3176000	-1.3437672
16.5833340	1.3664000	-1.3314953
16.5833340	1.4152000	-1.4542136
16.5833340	1.4640000	-1.5401168
16.5833340	1.5128000	-1.5462527
16.5833340	1.5616000	-1.5585246
16.5833340	1.6104000	-1.3314953
16.5833340	1.6592000	-1.3744469
16.5833340	1.7079999	-1.4848933
16.5833340	1.7567999	-1.4971652
16.5833340	1.8055999	-1.3192234
16.5833340	1.8543999	-1.2946796
16.5833340	1.9031999	-1.3376312
16.5833340	1.9519999	-1.3314953
16.5833340	2.0007999	-1.1535535
16.5833340	2.0495999	-1.0737867
16.5833340	2.0983999	-1.3008156
16.5833340	2.1471999	-1.0799227
16.5833340	2.1959999	-1.0921946
16.5833340	2.2447999	-1.0860586
16.5833340	2.2935998	-1.1351457
16.5833340	2.3423998	-0.9633398
16.5833340	2.3912001	-0.9142528
16.5833340	2.4400001	-0.9756117
16.5833340	2.4888000	-0.9756117
16.5833340	2.5376000	-1.0921946
16.5833340	2.5864000	-0.8222136
16.5833340	2.6352000	-0.9510679
16.5833340	2.6840000	-0.9387960
16.5833340	2.7328000	-0.9878836
16.5833340	2.7816000	-0.7547188
16.5833340	2.8304000	-0.9694757
16.5833340	2.8792000	-0.8835731
16.5833340	2.9280000	-0.9633398
16.5833340	2.9768000	-0.8160777
16.5833340	3.0256000	-0.8038058
16.5833340	3.0743999	-0.9142528
16.5833340	3.1231999	-0.9572039
16.5833340	3.1719999	-0.9510679
16.5833340	3.2207999	-0.8774371
16.5833340	3.2695999	-0.8038058
16.5833340	3.3183999	-0.7976699
16.5833340	3.3671999	-0.7915339
16.5833340	3.4159999	-0.4970098
16.5833340	3.4647999	-0.5460973
16.5833340	3.5135999	-0.5460973
16.5833340	3.5623999	-0.5767770
16.5833340	3.6111999	-0.5767770
16.5833340	3.6599998	-0.7056313
16.5833340	3.7087998	-0.7240391
16.5833340	3.7575998	-0.7117672
16.5833340	3.8063998	-0.7179031
16.5833340	3.8551998	-0.6013203
16.5833340	3.9039998	-0.6013203
16.5833340	3.9527998	-0.7117672

16.5833340	4.0016003	-0.6381359
16.5833340	4.0503998	-0.7363110
16.5833340	4.0991998	-1.0615149
16.5833340	4.1479998	-0.7976699
16.5833340	4.1967998	-0.7547188
16.5833340	4.2455997	-0.7485828
16.5833340	4.2943997	-0.7424469
16.5833340	4.3431997	-0.7240391
16.5833340	4.3919997	-0.5154176
16.5833340	4.4407997	-0.4908738
16.5833340	4.4895997	-0.5645051
16.5833340	4.5383997	-0.5460973
16.5833340	4.5871997	-0.5276895
16.5833340	4.6359997	-0.4172430
16.5833340	4.6847997	-0.5031457
16.5833340	4.7336001	-0.3804274
16.5833340	4.7824001	-0.3865633
16.5833340	4.8312001	-0.3742914
16.5833340	4.8800001	-0.4417863
16.5833340	4.9288001	-0.4479222
16.5833340	4.9776001	-0.2270293
16.5833340	5.0264001	-0.2638445
16.5833340	5.0752001	-0.2086215
16.5833340	5.1240001	-0.2024856
16.5833340	5.1728001	613586.0000000
16.5833340	5.2216001	613586.0000000
16.5833340	5.2704000	613586.0000000
16.5833340	5.3192000	613586.0000000
16.5833340	5.3680000	613586.0000000
16.5833340	5.4168000	613586.0000000
16.5833340	5.4656000	613586.0000000
16.5833340	5.5144000	613586.0000000
16.5833340	5.5632000	613586.0000000
16.5833340	5.6120000	613586.0000000
16.5833340	5.6608000	613586.0000000
16.5833340	5.7096000	613586.0000000
16.5833340	5.7584000	613586.0000000
16.5833340	5.8072000	613586.0000000
16.5833340	5.8559999	613586.0000000
16.5833340	5.9047999	613586.0000000
16.5833340	5.9535999	613586.0000000
16.5833340	6.0023999	613586.0000000
16.6000004	0.2928000	-4.0374374
16.6000004	0.3416000	-4.0251656
16.6000004	0.3904000	-4.7246609
16.6000004	0.4392000	-4.6633015
16.6000004	0.4880000	-4.4117289
16.6000004	0.5368000	-2.9759228
16.6000004	0.5856000	-2.5648160
16.6000004	0.6344000	-1.9328160
16.6000004	0.6832000	-1.5830684
16.6000004	0.7320000	-1.5646605
16.6000004	0.7808000	-1.3376312
16.6000004	0.8296000	-1.4173985
16.6000004	0.8784000	-1.3376312
16.6000004	0.9272000	-1.5646605
16.6000004	0.9760000	-1.6321554

16.6000004	1.0247999	-1.6321554
16.6000004	1.0735999	-1.6014762
16.6000004	1.1223999	-1.7303305
16.6000004	1.1711999	-1.7303305
16.6000004	1.2200000	-1.6076121
16.6000004	1.2688000	-1.3499031
16.6000004	1.3176000	-1.3499031
16.6000004	1.3664000	-1.3437672
16.6000004	1.4152000	-1.3499031
16.6000004	1.4640000	-1.3560390
16.6000004	1.5128000	-1.4419422
16.6000004	1.5616000	-1.2271848
16.6000004	1.6104000	-1.2271848
16.6000004	1.6592000	-1.2824078
16.6000004	1.7079999	-1.5217090
16.6000004	1.7567999	-1.3069515
16.6000004	1.8055999	-1.4112625
16.6000004	1.8543999	-1.4173985
16.6000004	1.9031999	-1.3499031
16.6000004	1.9519999	-1.4910293
16.6000004	2.0007999	-1.2701364
16.6000004	2.0495999	-1.2578645
16.6000004	2.0983999	-1.5033011
16.6000004	2.1471999	-0.9878836
16.6000004	2.1959999	-0.9817476
16.6000004	2.2447999	-1.0185633
16.6000004	2.2935998	-1.0308352
16.6000004	2.3423998	-1.1228738
16.6000004	2.3912001	-1.3130875
16.6000004	2.4400001	-1.0246992
16.6000004	2.4888000	-1.0001554
16.6000004	2.5376000	-0.9817476
16.6000004	2.5864000	-0.8283496
16.6000004	2.6352000	-0.8222136
16.6000004	2.6840000	-0.9633398
16.6000004	2.7328000	-1.0308352
16.6000004	2.7816000	-0.9265246
16.6000004	2.8304000	-0.9203887
16.6000004	2.8792000	-0.9142528
16.6000004	2.9280000	-0.9940195
16.6000004	2.9768000	-1.0001554
16.6000004	3.0256000	-1.0001554
16.6000004	3.0743999	-0.9878836
16.6000004	3.1231999	-1.1106019
16.6000004	3.1719999	-0.8344855
16.6000004	3.2207999	-0.8038058
16.6000004	3.2695999	-0.8038058
16.6000004	3.3183999	-0.7424469
16.6000004	3.3671999	-0.7363110
16.6000004	3.4159999	-0.7363110
16.6000004	3.4647999	-0.7179031
16.6000004	3.5135999	-0.9756117
16.6000004	3.5623999	-0.6626797
16.6000004	3.6111999	-0.6381359
16.6000004	3.6599998	-0.6933594
16.6000004	3.7087998	-0.7117672
16.6000004	3.7575998	-0.7179031

16.6000004	3.8063998	-0.7117672
16.6000004	3.8551998	-0.7608547
16.6000004	3.9039998	-0.7669902
16.6000004	3.9527998	-0.7301750
16.6000004	4.0016003	-0.7547188
16.6000004	4.0503998	-0.7731261
16.6000004	4.0991998	-0.8344855
16.6000004	4.1479998	-0.8222136
16.6000004	4.1967998	-0.8283496
16.6000004	4.2455997	-0.7240391
16.6000004	4.2943997	-0.7301750
16.6000004	4.3431997	-0.7301750
16.6000004	4.3919997	-0.7424469
16.6000004	4.4407997	-0.6320000
16.6000004	4.4895997	-0.7485828
16.6000004	4.5383997	-0.6074562
16.6000004	4.5871997	-0.6258640
16.6000004	4.6359997	-0.6258640
16.6000004	4.6847997	-0.5645051
16.6000004	4.7336001	-0.5583692
16.6000004	4.7824001	-0.4417863
16.6000004	4.8312001	-0.4479222
16.6000004	4.8800001	-0.2577085
16.6000004	4.9288001	-0.2699804
16.6000004	4.9776001	613586.0000000
16.6000004	5.0264001	613586.0000000
16.6000004	5.0752001	613586.0000000
16.6000004	5.1240001	613586.0000000
16.6000004	5.1728001	613586.0000000
16.6000004	5.2216001	-0.4601941
16.6000004	5.2704000	613586.0000000
16.6000004	5.3192000	613586.0000000
16.6000004	5.3680000	613586.0000000
16.6000004	5.4168000	613586.0000000
16.6000004	5.4656000	613586.0000000
16.6000004	5.5144000	613586.0000000
16.6000004	5.5632000	613586.0000000
16.6000004	5.6120000	613586.0000000
16.6000004	5.6608000	613586.0000000
16.6000004	5.7096000	613586.0000000
16.6000004	5.7584000	613586.0000000
16.6000004	5.8072000	613586.0000000
16.6000004	5.8559999	613586.0000000
16.6000004	5.9047999	613586.0000000
16.6000004	5.9535999	613586.0000000
16.6000004	6.0023999	613586.0000000
16.6166668	0.2928000	-4.0067577
16.6166668	0.3416000	-4.0128937
16.6166668	0.3904000	-3.4361172
16.6166668	0.4392000	-4.2767386
16.6166668	0.4880000	-3.4545248
16.6166668	0.5368000	-2.3868742
16.6166668	0.5856000	-2.3807383
16.6166668	0.6344000	-1.9512239
16.6166668	0.6832000	-1.5523887
16.6166668	0.7320000	-1.3560390
16.6166668	0.7808000	-1.3499031

16.6166668	0.8296000	-1.5278449
16.6166668	0.8784000	-1.5769324
16.6166668	0.9272000	-1.1474175
16.6166668	0.9760000	-1.6812429
16.6166668	1.0247999	-1.6076121
16.6166668	1.0735999	-1.6076121
16.6166668	1.1223999	-1.6014762
16.6166668	1.1711999	-1.4910293
16.6166668	1.2200000	-1.3376312
16.6166668	1.2688000	-1.4173985
16.6166668	1.3176000	-1.4358063
16.6166668	1.3664000	-1.3805828
16.6166668	1.4152000	-1.4419422
16.6166668	1.4640000	-1.2701364
16.6166668	1.5128000	-1.3437672
16.6166668	1.5616000	-1.0921946
16.6166668	1.6104000	-1.5033011
16.6166668	1.6592000	-1.5033011
16.6166668	1.7079999	-1.4664855
16.6166668	1.7567999	-1.4603496
16.6166668	1.8055999	-1.7426023
16.6166668	1.8543999	-1.1903691
16.6166668	1.9031999	-1.2701364
16.6166668	1.9519999	-1.3130875
16.6166668	2.0007999	-1.3008156
16.6166668	2.0495999	-1.1596894
16.6166668	2.0983999	-1.1596894
16.6166668	2.1471999	-1.1044660
16.6166668	2.1959999	-1.1106019
16.6166668	2.2447999	-1.1167378
16.6166668	2.2935998	-1.2455926
16.6166668	2.3423998	-1.1658254
16.6166668	2.3912001	-1.1290097
16.6166668	2.4400001	-1.0308352
16.6166668	2.4888000	-1.0431070
16.6166668	2.5376000	-1.2210488
16.6166668	2.5864000	-1.0799227
16.6166668	2.6352000	-1.0369711
16.6166668	2.6840000	-1.0615149
16.6166668	2.7328000	-1.0431070
16.6166668	2.7816000	-1.1167378
16.6166668	2.8304000	-1.1290097
16.6166668	2.8792000	-1.1228738
16.6166668	2.9280000	-1.0737867
16.6166668	2.9768000	-0.9572039
16.6166668	3.0256000	-0.9572039
16.6166668	3.0743999	-0.7608547
16.6166668	3.1231999	-0.7792621
16.6166668	3.1719999	-1.1290097
16.6166668	3.2207999	-1.1290097
16.6166668	3.2695999	-1.3437672
16.6166668	3.3183999	-1.3314953
16.6166668	3.3671999	-1.0246992
16.6166668	3.4159999	-1.0492430
16.6166668	3.4647999	-1.0615149
16.6166668	3.5135999	-1.0676508
16.6166668	3.5623999	-1.0492430

16.6166668	3.6111999	-0.9572039
16.6166668	3.6599998	-0.9387960
16.6166668	3.7087998	-0.9081168
16.6166668	3.7575998	-0.8835731
16.6166668	3.8063998	-0.9387960
16.6166668	3.8551998	-0.9387960
16.6166668	3.9039998	-0.9387960
16.6166668	3.9527998	-0.8467574
16.6166668	4.0016003	-0.8406215
16.6166668	4.0503998	-0.8406215
16.6166668	4.0991998	-0.8344855
16.6166668	4.1479998	-0.7056313
16.6166668	4.1967998	-0.6320000
16.6166668	4.2455997	-0.7363110
16.6166668	4.2943997	-0.7424469
16.6166668	4.3431997	-0.7485828
16.6166668	4.3919997	-0.7485828
16.6166668	4.4407997	-0.7547188
16.6166668	4.4895997	-0.7424469
16.6166668	4.5383997	-0.7669902
16.6166668	4.5871997	-0.6749516
16.6166668	4.6359997	-0.7056313
16.6166668	4.6847997	-0.7792621
16.6166668	4.7336001	-0.6565437
16.6166668	4.7824001	-0.5767770
16.6166668	4.8312001	-0.5767770
16.6166668	4.8800001	-0.5767770
16.6166668	4.9288001	-0.5092816
16.6166668	4.9776001	-0.4295144
16.6166668	5.0264001	613586.0000000
16.6166668	5.0752001	-0.4233789
16.6166668	5.1240001	-0.5092816
16.6166668	5.1728001	613586.0000000
16.6166668	5.2216001	613586.0000000
16.6166668	5.2704000	613586.0000000
16.6166668	5.3192000	613586.0000000
16.6166668	5.3680000	613586.0000000
16.6166668	5.4168000	613586.0000000
16.6166668	5.4656000	613586.0000000
16.6166668	5.5144000	613586.0000000
16.6166668	5.5632000	613586.0000000
16.6166668	5.6120000	613586.0000000
16.6166668	5.6608000	613586.0000000
16.6166668	5.7096000	613586.0000000
16.6166668	5.7584000	613586.0000000
16.6166668	5.8072000	613586.0000000
16.6166668	5.8559999	613586.0000000
16.6166668	5.9047999	613586.0000000
16.6166668	5.9535999	613586.0000000
16.6166668	6.0023999	613586.0000000
16.6333332	0.2928000	-3.1722724
16.6333332	0.3416000	-3.3502142
16.6333332	0.3904000	-2.9697869
16.6333332	0.4392000	-2.9636509
16.6333332	0.4880000	-2.9452431
16.6333332	0.5368000	-3.5281558
16.6333332	0.5856000	-2.5218644

16.6333332	0.6344000	-2.7979810
16.6333332	0.6832000	-0.4601941
16.6333332	0.7320000	-0.4601941
16.6333332	0.7808000	-1.2640004
16.6333332	0.8296000	-1.2640004
16.6333332	0.8784000	-0.8283496
16.6333332	0.9272000	-0.9817476
16.6333332	0.9760000	-1.4296703
16.6333332	1.0247999	-1.3744469
16.6333332	1.0735999	-1.3621750
16.6333332	1.1223999	-1.3499031
16.6333332	1.1711999	-1.5033011
16.6333332	1.2200000	-1.2946796
16.6333332	1.2688000	-1.2946796
16.6333332	1.3176000	-1.3376312
16.6333332	1.3664000	-1.2394567
16.6333332	1.4152000	-1.2517285
16.6333332	1.4640000	-1.2517285
16.6333332	1.5128000	-1.2333207
16.6333332	1.5616000	-1.1719613
16.6333332	1.6104000	-1.1658254
16.6333332	1.6592000	-1.0737867
16.6333332	1.7079999	-1.3314953
16.6333332	1.7567999	-1.2333207
16.6333332	1.8055999	-1.3805828
16.6333332	1.8543999	-1.2087770
16.6333332	1.9031999	-1.2149129
16.6333332	1.9519999	-1.2026410
16.6333332	2.0007999	-1.1965051
16.6333332	2.0495999	-1.2210488
16.6333332	2.0983999	-1.2149129
16.6333332	2.1471999	-1.0921946
16.6333332	2.1959999	-1.0185633
16.6333332	2.2447999	-1.1903691
16.6333332	2.2935998	-1.1842332
16.6333332	2.3423998	-1.1228738
16.6333332	2.3912001	-1.1167378
16.6333332	2.4400001	-1.3192234
16.6333332	2.4888000	-0.9940195
16.6333332	2.5376000	-1.2087770
16.6333332	2.5864000	-1.0553789
16.6333332	2.6352000	-0.9940195
16.6333332	2.6840000	-0.9878836
16.6333332	2.7328000	-0.9878836
16.6333332	2.7816000	-1.0921946
16.6333332	2.8304000	-1.1351457
16.6333332	2.8792000	-1.1351457
16.6333332	2.9280000	-1.0860586
16.6333332	2.9768000	-0.8897090
16.6333332	3.0256000	-0.6626797
16.6333332	3.0743999	-0.5767770
16.6333332	3.1231999	-0.5767770
16.6333332	3.1719999	-0.8344855
16.6333332	3.2207999	-0.8590293
16.6333332	3.2695999	-1.0615149
16.6333332	3.3183999	-0.6994953
16.6333332	3.3671999	-0.7547188

16.6333332	3.4159999	-0.9940195
16.6333332	3.4647999	-1.0308352
16.6333332	3.5135999	-1.0308352
16.6333332	3.5623999	-1.0308352
16.6333332	3.6111999	-1.0369711
16.6333332	3.6599998	-1.1596894
16.6333332	3.7087998	-1.0921946
16.6333332	3.7575998	-1.1044660
16.6333332	3.8063998	-1.0492430
16.6333332	3.8551998	-1.0369711
16.6333332	3.9039998	-0.9694757
16.6333332	3.9527998	-0.9633398
16.6333332	4.0016003	-0.9817476
16.6333332	4.0503998	-0.9817476
16.6333332	4.0991998	-0.9817476
16.6333332	4.1479998	-0.9633398
16.6333332	4.1967998	-0.9694757
16.6333332	4.2455997	-0.8099418
16.6333332	4.2943997	-0.9265246
16.6333332	4.3431997	-0.9142528
16.6333332	4.3919997	-0.8528934
16.6333332	4.4407997	-0.8713012
16.6333332	4.4895997	-0.8897090
16.6333332	4.5383997	-0.8344855
16.6333332	4.5871997	-0.7792621
16.6333332	4.6359997	-0.7853980
16.6333332	4.6847997	-0.8222136
16.6333332	4.7336001	-0.8406215
16.6333332	4.7824001	-1.0001554
16.6333332	4.8312001	-0.9633398
16.6333332	4.8800001	-0.7485828
16.6333332	4.9288001	-0.7485828
16.6333332	4.9776001	-0.7117672
16.6333332	5.0264001	-0.9326606
16.6333332	5.0752001	613586.0000000
16.6333332	5.1240001	613586.0000000
16.6333332	5.1728001	613586.0000000
16.6333332	5.2216001	613586.0000000
16.6333332	5.2704000	613586.0000000
16.6333332	5.3192000	613586.0000000
16.6333332	5.3680000	613586.0000000
16.6333332	5.4168000	613586.0000000
16.6333332	5.4656000	613586.0000000
16.6333332	5.5144000	613586.0000000
16.6333332	5.5632000	613586.0000000
16.6333332	5.6120000	613586.0000000
16.6333332	5.6608000	613586.0000000
16.6333332	5.7096000	613586.0000000
16.6333332	5.7584000	613586.0000000
16.6333332	5.8072000	613586.0000000
16.6333332	5.8559999	613586.0000000
16.6333332	5.9047999	-1.2455926
16.6333332	5.9535999	-1.2455926
16.6333332	6.0023999	-1.1106019
16.6499996	0.2928000	-3.9883502
16.6499996	0.3416000	-3.4852045
16.6499996	0.3904000	-3.4913404

16.6499996	0.4392000	-3.3931656
16.6499996	0.4880000	-3.5588355
16.6499996	0.5368000	-2.8777480
16.6499996	0.5856000	-2.5954955
16.6499996	0.6344000	-1.4235344
16.6499996	0.6832000	-1.4235344
16.6499996	0.7320000	-1.2640004
16.6499996	0.7808000	-1.5892043
16.6499996	0.8296000	-1.2333207
16.6499996	0.8784000	-1.4971652
16.6499996	0.9272000	-1.5033011
16.6499996	0.9760000	-1.4664855
16.6499996	1.0247999	-1.4419422
16.6499996	1.0735999	-1.4542136
16.6499996	1.1223999	-1.4419422
16.6499996	1.1711999	-1.3437672
16.6499996	1.2200000	-1.2578645
16.6499996	1.2688000	-1.1842332
16.6499996	1.3176000	-1.1780972
16.6499996	1.3664000	-1.1903691
16.6499996	1.4152000	-1.1780972
16.6499996	1.4640000	-1.1535535
16.6499996	1.5128000	-1.1535535
16.6499996	1.5616000	-1.0308352
16.6499996	1.6104000	-1.0308352
16.6499996	1.6592000	-1.1290097
16.6499996	1.7079999	-1.1351457
16.6499996	1.7567999	-1.1351457
16.6499996	1.8055999	-0.9756117
16.6499996	1.8543999	-0.9510679
16.6499996	1.9031999	-1.0001554
16.6499996	1.9519999	-0.8835731
16.6499996	2.0007999	-0.8835731
16.6499996	2.0495999	-0.8774371
16.6499996	2.0983999	-1.1596894
16.6499996	2.1471999	-0.9510679
16.6499996	2.1959999	-0.9449320
16.6499996	2.2447999	-0.9326606
16.6499996	2.2935998	-1.0369711
16.6499996	2.3423998	-1.0369711
16.6499996	2.3912001	-0.9756117
16.6499996	2.4400001	-1.2087770
16.6499996	2.4888000	-1.1167378
16.6499996	2.5376000	-1.0799227
16.6499996	2.5864000	-1.1412816
16.6499996	2.6352000	-1.1351457
16.6499996	2.6840000	-1.1351457
16.6499996	2.7328000	-1.1106019
16.6499996	2.7816000	-1.1167378
16.6499996	2.8304000	-1.1719613
16.6499996	2.8792000	-0.9694757
16.6499996	2.9280000	-0.7731261
16.6499996	2.9768000	-1.1351457
16.6499996	3.0256000	-0.9756117
16.6499996	3.0743999	-0.8713012
16.6499996	3.1231999	-0.9265246
16.6499996	3.1719999	-1.0062914

16.6499996	3.2207999	-0.7731261
16.6499996	3.2695999	-0.8160777
16.6499996	3.3183999	-0.8160777
16.6499996	3.3671999	-0.7731261
16.6499996	3.4159999	-0.9694757
16.6499996	3.4647999	-1.1658254
16.6499996	3.5135999	-1.0001554
16.6499996	3.5623999	-0.9756117
16.6499996	3.6111999	-1.0308352
16.6499996	3.6599998	-1.0185633
16.6499996	3.7087998	-1.0001554
16.6499996	3.7575998	-1.0185633
16.6499996	3.8063998	-1.0001554
16.6499996	3.8551998	-0.9817476
16.6499996	3.9039998	-0.9817476
16.6499996	3.9527998	-0.9633398
16.6499996	4.0016003	-0.9449320
16.6499996	4.0503998	-0.9449320
16.6499996	4.0991998	-0.9449320
16.6499996	4.1479998	-0.9633398
16.6499996	4.1967998	-0.9633398
16.6499996	4.2455997	-0.9633398
16.6499996	4.2943997	-0.8283496
16.6499996	4.3431997	-0.9265246
16.6499996	4.3919997	-0.9265246
16.6499996	4.4407997	-0.9387960
16.6499996	4.4895997	-0.8897090
16.6499996	4.5383997	-0.7976699
16.6499996	4.5871997	-0.7915339
16.6499996	4.6359997	-0.8713012
16.6499996	4.6847997	-0.8897090
16.6499996	4.7336001	-0.9878836
16.6499996	4.7824001	-0.8038058
16.6499996	4.8312001	-0.8038058
16.6499996	4.8800001	-0.5951843
16.6499996	4.9288001	-0.5767770
16.6499996	4.9776001	-0.5767770
16.6499996	5.0264001	-0.5215535
16.6499996	5.0752001	613586.0000000
16.6499996	5.1240001	613586.0000000
16.6499996	5.1728001	613586.0000000
16.6499996	5.2216001	-1.0001554
16.6499996	5.2704000	613586.0000000
16.6499996	5.3192000	613586.0000000
16.6499996	5.3680000	-0.9142528
16.6499996	5.4168000	-1.1780972
16.6499996	5.4656000	613586.0000000
16.6499996	5.5144000	613586.0000000
16.6499996	5.5632000	613586.0000000
16.6499996	5.6120000	613586.0000000
16.6499996	5.6608000	613586.0000000
16.6499996	5.7096000	613586.0000000
16.6499996	5.7584000	613586.0000000
16.6499996	5.8072000	613586.0000000
16.6499996	5.8559999	613586.0000000
16.6499996	5.9047999	613586.0000000
16.6499996	5.9535999	613586.0000000

16.6499996	6.0023999	613586.0000000
16.6666660	0.2928000	-3.5465636
16.6666660	0.3416000	-4.3687773
16.6666660	0.3904000	-4.5589910
16.6666660	0.4392000	-4.5467191
16.6666660	0.4880000	-3.6017871
16.6666660	0.5368000	-2.7366219
16.6666660	0.5856000	-2.7366219
16.6666660	0.6344000	-1.3253593
16.6666660	0.6832000	-1.3314953
16.6666660	0.7320000	-1.3621750
16.6666660	0.7808000	-1.3192234
16.6666660	0.8296000	-1.3192234
16.6666660	0.8784000	-1.2394567
16.6666660	0.9272000	-1.2517285
16.6666660	0.9760000	-1.3867188
16.6666660	1.0247999	-1.3621750
16.6666660	1.0735999	-1.3253593
16.6666660	1.1223999	-1.4296703
16.6666660	1.1711999	-1.4419422
16.6666660	1.2200000	-1.4480777
16.6666660	1.2688000	-1.5094371
16.6666660	1.3176000	-1.5033011
16.6666660	1.3664000	-1.0860586
16.6666660	1.4152000	-1.0921946
16.6666660	1.4640000	-1.0492430
16.6666660	1.5128000	-1.0431070
16.6666660	1.5616000	-0.9572039
16.6666660	1.6104000	-1.0492430
16.6666660	1.6592000	-1.0492430
16.6666660	1.7079999	-1.0308352
16.6666660	1.7567999	-1.0246992
16.6666660	1.8055999	-1.0185633
16.6666660	1.8543999	-0.7301750
16.6666660	1.9031999	-0.7179031
16.6666660	1.9519999	-0.7792621
16.6666660	2.0007999	-0.6442719
16.6666660	2.0495999	-0.6504078
16.6666660	2.0983999	-0.6504078
16.6666660	2.1471999	-0.9572039
16.6666660	2.1959999	-0.9572039
16.6666660	2.2447999	-0.7669902
16.6666660	2.2935998	-0.7669902
16.6666660	2.3423998	-0.9510679
16.6666660	2.3912001	-0.8222136
16.6666660	2.4400001	-0.6504078
16.6666660	2.4888000	-0.8283496
16.6666660	2.5376000	-0.8406215
16.6666660	2.5864000	-1.1167378
16.6666660	2.6352000	-1.0799227
16.6666660	2.6840000	-0.8774371
16.6666660	2.7328000	-0.8713012
16.6666660	2.7816000	-0.9510679
16.6666660	2.8304000	-0.6688156
16.6666660	2.8792000	-1.0185633
16.6666660	2.9280000	-1.2333207
16.6666660	2.9768000	-1.0737867

16.6666660	3.0256000	-1.0676508
16.6666660	3.0743999	-0.9510679
16.6666660	3.1231999	-0.9633398
16.6666660	3.1719999	-0.9694757
16.6666660	3.2207999	-0.9694757
16.6666660	3.2695999	-1.0246992
16.6666660	3.3183999	-1.0124273
16.6666660	3.3671999	-1.0062914
16.6666660	3.4159999	-1.2271848
16.6666660	3.4647999	-1.2271848
16.6666660	3.5135999	-1.2087770
16.6666660	3.5623999	-1.1044660
16.6666660	3.6111999	-1.1044660
16.6666660	3.6599998	-0.9265246
16.6666660	3.7087998	-0.9203887
16.6666660	3.7575998	-0.9265246
16.6666660	3.8063998	-0.9510679
16.6666660	3.8551998	-0.9510679
16.6666660	3.9039998	-0.8958449
16.6666660	3.9527998	-0.9019809
16.6666660	4.0016003	-0.8774371
16.6666660	4.0503998	-0.8835731
16.6666660	4.0991998	-0.7669902
16.6666660	4.1479998	-0.8344855
16.6666660	4.1967998	-0.6933594
16.6666660	4.2455997	-0.6994953
16.6666660	4.2943997	-0.7731261
16.6666660	4.3431997	-0.8099418
16.6666660	4.3919997	-0.6994953
16.6666660	4.4407997	-0.6933594
16.6666660	4.4895997	-0.7424469
16.6666660	4.5383997	-0.6626797
16.6666660	4.5871997	-0.5706410
16.6666660	4.6359997	-0.5767770
16.6666660	4.6847997	-0.5276895
16.6666660	4.7336001	-0.5276895
16.6666660	4.7824001	-0.4601941
16.6666660	4.8312001	-0.4601941
16.6666660	4.8800001	-0.3742914
16.6666660	4.9288001	613586.0000000
16.6666660	4.9776001	613586.0000000
16.6666660	5.0264001	613586.0000000
16.6666660	5.0752001	613586.0000000
16.6666660	5.1240001	613586.0000000
16.6666660	5.1728001	613586.0000000
16.6666660	5.2216001	613586.0000000
16.6666660	5.2704000	613586.0000000
16.6666660	5.3192000	613586.0000000
16.6666660	5.3680000	613586.0000000
16.6666660	5.4168000	613586.0000000
16.6666660	5.4656000	613586.0000000
16.6666660	5.5144000	613586.0000000
16.6666660	5.5632000	613586.0000000
16.6666660	5.6120000	613586.0000000
16.6666660	5.6608000	613586.0000000
16.6666660	5.7096000	613586.0000000
16.6666660	5.7584000	613586.0000000

16.6666660	5.8072000	613586.0000000
16.6666660	5.8559999	613586.0000000
16.6666660	5.9047999	613586.0000000
16.6666660	5.9535999	613586.0000000
16.6666660	6.0023999	613586.0000000
16.6833324	0.2928000	-4.4301367
16.6833324	0.3416000	-3.9392629
16.6833324	0.3904000	-4.1110687
16.6833324	0.4392000	-4.2706027
16.6833324	0.4880000	-3.9147191
16.6833324	0.5368000	-3.3931656
16.6833324	0.5856000	-2.3316510
16.6833324	0.6344000	-2.5218644
16.6833324	0.6832000	-2.2764277
16.6833324	0.7320000	-1.7487383
16.6833324	0.7808000	-1.3499031
16.6833324	0.8296000	-1.3437672
16.6833324	0.8784000	-1.2762718
16.6833324	0.9272000	-1.7855535
16.6833324	0.9760000	-1.7855535
16.6833324	1.0247999	-1.5646605
16.6833324	1.0735999	-1.6382914
16.6833324	1.1223999	-1.6137476
16.6833324	1.1711999	-1.3437672
16.6833324	1.2200000	-1.4664855
16.6833324	1.2688000	-1.4419422
16.6833324	1.3176000	-1.4296703
16.6833324	1.3664000	-1.4296703
16.6833324	1.4152000	-1.3499031
16.6833324	1.4640000	-1.3069515
16.6833324	1.5128000	-1.2701364
16.6833324	1.5616000	-1.2701364
16.6833324	1.6104000	-1.3437672
16.6833324	1.6592000	-1.3437672
16.6833324	1.7079999	-1.1044660
16.6833324	1.7567999	-0.8283496
16.6833324	1.8055999	-1.0553789
16.6833324	1.8543999	-0.8344855
16.6833324	1.9031999	-0.8344855
16.6833324	1.9519999	-1.0308352
16.6833324	2.0007999	-0.9510679
16.6833324	2.0495999	-0.8528934
16.6833324	2.0983999	-0.8651652
16.6833324	2.1471999	-0.8835731
16.6833324	2.1959999	-0.8590293
16.6833324	2.2447999	-0.9387960
16.6833324	2.2935998	-0.8651652
16.6833324	2.3423998	-0.7669902
16.6833324	2.3912001	-0.8038058
16.6833324	2.4400001	-0.9817476
16.6833324	2.4888000	-0.7301750
16.6833324	2.5376000	-0.7485828
16.6833324	2.5864000	-0.7424469
16.6833324	2.6352000	-0.7485828
16.6833324	2.6840000	-0.7363110
16.6833324	2.7328000	-0.8406215
16.6833324	2.7816000	-0.8467574

16.6833324	2.8304000	-0.8283496
16.6833324	2.8792000	-0.8038058
16.6833324	2.9280000	-0.8222136
16.6833324	2.9768000	-0.9387960
16.6833324	3.0256000	-1.0124273
16.6833324	3.0743999	-1.0124273
16.6833324	3.1231999	-1.0246992
16.6833324	3.1719999	-1.0246992
16.6833324	3.2207999	-0.9142528
16.6833324	3.2695999	-0.9265246
16.6833324	3.3183999	-0.9203887
16.6833324	3.3671999	-0.9326606
16.6833324	3.4159999	-0.9326606
16.6833324	3.4647999	-1.0246992
16.6833324	3.5135999	-0.7853980
16.6833324	3.5623999	-1.0062914
16.6833324	3.6111999	-0.8958449
16.6833324	3.6599998	-0.8590293
16.6833324	3.7087998	-0.8099418
16.6833324	3.7575998	-0.8528934
16.6833324	3.8063998	-0.8467574
16.6833324	3.8551998	-0.8160777
16.6833324	3.9039998	-0.8160777
16.6833324	3.9527998	-0.7976699
16.6833324	4.0016003	-0.8038058
16.6833324	4.0503998	-0.9019809
16.6833324	4.0991998	-0.7547188
16.6833324	4.1479998	-0.7547188
16.6833324	4.1967998	-0.8406215
16.6833324	4.2455997	-0.8467574
16.6833324	4.2943997	-0.8467574
16.6833324	4.3431997	-0.8344855
16.6833324	4.3919997	-0.6872234
16.6833324	4.4407997	-0.6872234
16.6833324	4.4895997	-0.8467574
16.6833324	4.5383997	-0.7363110
16.6833324	4.5871997	-0.5276895
16.6833324	4.6359997	-0.5215535
16.6833324	4.6847997	-0.6565437
16.6833324	4.7336001	-0.5338254
16.6833324	4.7824001	-0.4724660
16.6833324	4.8312001	-0.4417863
16.6833324	4.8800001	613586.0000000
16.6833324	4.9288001	613586.0000000
16.6833324	4.9776001	613586.0000000
16.6833324	5.0264001	613586.0000000
16.6833324	5.0752001	613586.0000000
16.6833324	5.1240001	613586.0000000
16.6833324	5.1728001	613586.0000000
16.6833324	5.2216001	-0.8099418
16.6833324	5.2704000	-0.8160777
16.6833324	5.3192000	-0.7608547
16.6833324	5.3680000	-0.7485828
16.6833324	5.4168000	-0.6626797
16.6833324	5.4656000	-0.6626797
16.6833324	5.5144000	613586.0000000
16.6833324	5.5632000	613586.0000000

16.6833324	5.6120000	613586.0000000
16.6833324	5.6608000	613586.0000000
16.6833324	5.7096000	613586.0000000
16.6833324	5.7584000	613586.0000000
16.6833324	5.8072000	-0.6320000
16.6833324	5.8559999	613586.0000000
16.6833324	5.9047999	-0.7915339
16.6833324	5.9535999	-0.8774371
16.6833324	6.0023999	613586.0000000
16.7000008	0.2928000	-4.3626413
16.7000008	0.3416000	-3.8594959
16.7000008	0.3904000	-3.8533599
16.7000008	0.4392000	-3.7060976
16.7000008	0.4880000	-3.3256705
16.7000008	0.5368000	-2.5893598
16.7000008	0.5856000	-2.1598449
16.7000008	0.6344000	-1.9144082
16.7000008	0.6832000	-1.9266801
16.7000008	0.7320000	-1.5646605
16.7000008	0.7808000	-1.5707965
16.7000008	0.8296000	-1.2762718
16.7000008	0.8784000	-1.2885437
16.7000008	0.9272000	-1.6566992
16.7000008	0.9760000	-1.7732821
16.7000008	1.0247999	-1.5401168
16.7000008	1.0735999	-1.5339808
16.7000008	1.1223999	-1.4051266
16.7000008	1.1711999	-1.4051266
16.7000008	1.2200000	-1.2762718
16.7000008	1.2688000	-1.1780972
16.7000008	1.3176000	-1.1780972
16.7000008	1.3664000	-1.2885437
16.7000008	1.4152000	-1.3989906
16.7000008	1.4640000	-1.3253593
16.7000008	1.5128000	-1.4603496
16.7000008	1.5616000	-1.4787574
16.7000008	1.6104000	-1.4910293
16.7000008	1.6592000	-1.1412816
16.7000008	1.7079999	-1.1596894
16.7000008	1.7567999	-1.1596894
16.7000008	1.8055999	-0.8283496
16.7000008	1.8543999	-0.8283496
16.7000008	1.9031999	-0.8467574
16.7000008	1.9519999	-0.8099418
16.7000008	2.0007999	-0.8099418
16.7000008	2.0495999	-0.6135921
16.7000008	2.0983999	-0.8590293
16.7000008	2.1471999	-0.8467574
16.7000008	2.1959999	-0.9326606
16.7000008	2.2447999	-0.9387960
16.7000008	2.2935998	-1.0185633
16.7000008	2.3423998	-0.9572039
16.7000008	2.3912001	-0.8160777
16.7000008	2.4400001	-1.1044660
16.7000008	2.4888000	-0.8344855
16.7000008	2.5376000	-0.8406215
16.7000008	2.5864000	-0.7608547

16.7000008	2.6352000	-0.7731261
16.7000008	2.6840000	-0.9694757
16.7000008	2.7328000	-1.0431070
16.7000008	2.7816000	-1.0308352
16.7000008	2.8304000	-1.0369711
16.7000008	2.8792000	-1.0553789
16.7000008	2.9280000	-1.1290097
16.7000008	2.9768000	-1.1351457
16.7000008	3.0256000	-0.8406215
16.7000008	3.0743999	-1.0492430
16.7000008	3.1231999	-1.1167378
16.7000008	3.1719999	-1.1351457
16.7000008	3.2207999	-1.1351457
16.7000008	3.2695999	-0.9203887
16.7000008	3.3183999	-0.9081168
16.7000008	3.3671999	-0.9142528
16.7000008	3.4159999	-0.9756117
16.7000008	3.4647999	-0.9940195
16.7000008	3.5135999	-0.9142528
16.7000008	3.5623999	-0.7363110
16.7000008	3.6111999	-0.7731261
16.7000008	3.6599998	-0.7485828
16.7000008	3.7087998	-0.7669902
16.7000008	3.7575998	-0.8406215
16.7000008	3.8063998	-0.7547188
16.7000008	3.8551998	-0.8099418
16.7000008	3.9039998	-0.8160777
16.7000008	3.9527998	-0.7240391
16.7000008	4.0016003	-0.7424469
16.7000008	4.0503998	-0.8528934
16.7000008	4.0991998	-0.7915339
16.7000008	4.1479998	-0.8283496
16.7000008	4.1967998	-0.8222136
16.7000008	4.2455997	-0.8283496
16.7000008	4.2943997	-0.8283496
16.7000008	4.3431997	-0.8283496
16.7000008	4.3919997	-0.8222136
16.7000008	4.4407997	-0.7240391
16.7000008	4.4895997	-0.7424469
16.7000008	4.5383997	-0.6504078
16.7000008	4.5871997	-0.7056313
16.7000008	4.6359997	-0.5522332
16.7000008	4.6847997	-0.6504078
16.7000008	4.7336001	-0.6504078
16.7000008	4.7824001	-0.5092816
16.7000008	4.8312001	-0.5154176
16.7000008	4.8800001	-0.6320000
16.7000008	4.9288001	-0.5767770
16.7000008	4.9776001	-0.5706410
16.7000008	5.0264001	-0.6442719
16.7000008	5.0752001	613586.0000000
16.7000008	5.1240001	613586.0000000
16.7000008	5.1728001	613586.0000000
16.7000008	5.2216001	-0.9019809
16.7000008	5.2704000	-0.8774371
16.7000008	5.3192000	-0.8774371
16.7000008	5.3680000	-1.1351457

16.7000008	5.4168000	-1.0492430
16.7000008	5.4656000	-1.0492430
16.7000008	5.5144000	613586.0000000
16.7000008	5.5632000	613586.0000000
16.7000008	5.6120000	613586.0000000
16.7000008	5.6608000	613586.0000000
16.7000008	5.7096000	613586.0000000
16.7000008	5.7584000	613586.0000000
16.7000008	5.8072000	613586.0000000
16.7000008	5.8559999	613586.0000000
16.7000008	5.9047999	613586.0000000
16.7000008	5.9535999	613586.0000000
16.7000008	6.0023999	613586.0000000
16.7166672	0.2928000	-3.2765830
16.7166672	0.3416000	-3.7981365
16.7166672	0.3904000	-3.3133986
16.7166672	0.4392000	-3.2213597
16.7166672	0.4880000	-3.3011267
16.7166672	0.5368000	-2.9943306
16.7166672	0.5856000	-2.3868742
16.7166672	0.6344000	-1.4480777
16.7166672	0.6832000	-1.4358063
16.7166672	0.7320000	-1.7241945
16.7166672	0.7808000	-1.6505632
16.7166672	0.8296000	-1.6382914
16.7166672	0.8784000	-1.6260195
16.7166672	0.9272000	-1.6014762
16.7166672	0.9760000	-1.5830684
16.7166672	1.0247999	-1.1412816
16.7166672	1.0735999	-1.0860586
16.7166672	1.1223999	-1.1658254
16.7166672	1.1711999	-1.0492430
16.7166672	1.2200000	-1.0369711
16.7166672	1.2688000	-1.3376312
16.7166672	1.3176000	-1.2026410
16.7166672	1.3664000	-1.2578645
16.7166672	1.4152000	-1.2517285
16.7166672	1.4640000	-1.6260195
16.7166672	1.5128000	-1.6198835
16.7166672	1.5616000	-1.6873789
16.7166672	1.6104000	-1.6628351
16.7166672	1.6592000	-0.9449320
16.7166672	1.7079999	-1.2210488
16.7166672	1.7567999	-0.9387960
16.7166672	1.8055999	-0.9572039
16.7166672	1.8543999	-0.9387960
16.7166672	1.9031999	-0.8467574
16.7166672	1.9519999	-0.8590293
16.7166672	2.0007999	-0.8651652
16.7166672	2.0495999	-0.8467574
16.7166672	2.0983999	-1.0001554
16.7166672	2.1471999	-1.0185633
16.7166672	2.1959999	-0.9510679
16.7166672	2.2447999	-0.9694757
16.7166672	2.2935998	-0.9817476
16.7166672	2.3423998	-0.9817476
16.7166672	2.3912001	-1.0983305

16.7166672	2.4400001	-0.8958449
16.7166672	2.4888000	-0.9265246
16.7166672	2.5376000	-0.9265246
16.7166672	2.5864000	-1.0676508
16.7166672	2.6352000	-1.0676508
16.7166672	2.6840000	-1.0737867
16.7166672	2.7328000	-1.0124273
16.7166672	2.7816000	-1.0185633
16.7166672	2.8304000	-1.0308352
16.7166672	2.8792000	-1.1290097
16.7166672	2.9280000	-1.1290097
16.7166672	2.9768000	-1.0062914
16.7166672	3.0256000	-0.9265246
16.7166672	3.0743999	-1.0553789
16.7166672	3.1231999	-1.0553789
16.7166672	3.1719999	-1.0615149
16.7166672	3.2207999	-0.8958449
16.7166672	3.2695999	-1.0062914
16.7166672	3.3183999	-1.0001554
16.7166672	3.3671999	-1.0185633
16.7166672	3.4159999	-0.9387960
16.7166672	3.4647999	-1.2149129
16.7166672	3.5135999	-1.0185633
16.7166672	3.5623999	-1.0124273
16.7166672	3.6111999	-0.8283496
16.7166672	3.6599998	-0.8344855
16.7166672	3.7087998	-0.8283496
16.7166672	3.7575998	-0.7792621
16.7166672	3.8063998	-0.7792621
16.7166672	3.8551998	-0.7792621
16.7166672	3.9039998	-0.7853980
16.7166672	3.9527998	-0.8590293
16.7166672	4.0016003	-0.8099418
16.7166672	4.0503998	-0.8160777
16.7166672	4.0991998	-0.8835731
16.7166672	4.1479998	-0.8099418
16.7166672	4.1967998	-0.8038058
16.7166672	4.2455997	-0.9019809
16.7166672	4.2943997	-0.8835731
16.7166672	4.3431997	-0.8835731
16.7166672	4.3919997	-0.7608547
16.7166672	4.4407997	-0.7731261
16.7166672	4.4895997	-0.6810875
16.7166672	4.5383997	-0.6994953
16.7166672	4.5871997	-0.5092816
16.7166672	4.6359997	-0.5092816
16.7166672	4.6847997	613586.0000000
16.7166672	4.7336001	613586.0000000
16.7166672	4.7824001	-0.7301750
16.7166672	4.8312001	613586.0000000
16.7166672	4.8800001	-0.6381359
16.7166672	4.9288001	-0.3129320
16.7166672	4.9776001	-0.3067961
16.7166672	5.0264001	613586.0000000
16.7166672	5.0752001	613586.0000000
16.7166672	5.1240001	-0.1411262
16.7166672	5.1728001	-0.2945242

16.7166672	5.2216001	-0.2945242
16.7166672	5.2704000	0.0736313
16.7166672	5.3192000	-0.7792621
16.7166672	5.3680000	-0.7792621
16.7166672	5.4168000	-0.4908738
16.7166672	5.4656000	-0.2577085
16.7166672	5.5144000	613586.0000000
16.7166672	5.5632000	613586.0000000
16.7166672	5.6120000	613586.0000000
16.7166672	5.6608000	613586.0000000
16.7166672	5.7096000	613586.0000000
16.7166672	5.7584000	613586.0000000
16.7166672	5.8072000	613586.0000000
16.7166672	5.8559999	613586.0000000
16.7166672	5.9047999	613586.0000000
16.7166672	5.9535999	613586.0000000
16.7166672	6.0023999	613586.0000000
16.7333336	0.2928000	-3.9883502
16.7333336	0.3416000	-3.8717675
16.7333336	0.3904000	-3.6938257
16.7333336	0.4392000	-2.6261752
16.7333336	0.4880000	-3.8288162
16.7333336	0.5368000	-3.4299812
16.7333336	0.5856000	-2.3193791
16.7333336	0.6344000	-1.8162332
16.7333336	0.6832000	-1.8162332
16.7333336	0.7320000	-1.8162332
16.7333336	0.7808000	-1.4726214
16.7333336	0.8296000	-0.5706410
16.7333336	0.8784000	-0.7608547
16.7333336	0.9272000	-1.5646605
16.7333336	0.9760000	-1.5401168
16.7333336	1.0247999	-1.3805828
16.7333336	1.0735999	-1.1965051
16.7333336	1.1223999	-1.1351457
16.7333336	1.1711999	-1.1842332
16.7333336	1.2200000	-1.0921946
16.7333336	1.2688000	-1.1044660
16.7333336	1.3176000	-1.1044660
16.7333336	1.3664000	-1.2640004
16.7333336	1.4152000	-1.1596894
16.7333336	1.4640000	-1.4910293
16.7333336	1.5128000	-1.5155730
16.7333336	1.5616000	-1.5094371
16.7333336	1.6104000	-1.4787574
16.7333336	1.6592000	-1.4787574
16.7333336	1.7079999	-1.4664855
16.7333336	1.7567999	-1.5523887
16.7333336	1.8055999	-1.1965051
16.7333336	1.8543999	-1.1965051
16.7333336	1.9031999	-1.3130875
16.7333336	1.9519999	-0.9694757
16.7333336	2.0007999	-1.0308352
16.7333336	2.0495999	-1.0185633
16.7333336	2.0983999	-1.1535535
16.7333336	2.1471999	-1.0676508
16.7333336	2.1959999	-1.0676508

16.7333336	2.2447999	-0.8160777
16.7333336	2.2935998	-0.9940195
16.7333336	2.3423998	-0.9081168
16.7333336	2.3912001	-1.0369711
16.7333336	2.4400001	-1.0492430
16.7333336	2.4888000	-1.0737867
16.7333336	2.5376000	-1.0737867
16.7333336	2.5864000	-0.9940195
16.7333336	2.6352000	-1.1474175
16.7333336	2.6840000	-1.0492430
16.7333336	2.7328000	-1.0737867
16.7333336	2.7816000	-0.9265246
16.7333336	2.8304000	-0.9878836
16.7333336	2.8792000	-0.9756117
16.7333336	2.9280000	-1.0185633
16.7333336	2.9768000	-1.0431070
16.7333336	3.0256000	-0.8160777
16.7333336	3.0743999	-0.7608547
16.7333336	3.1231999	-0.7608547
16.7333336	3.1719999	-0.9940195
16.7333336	3.2207999	-1.0001554
16.7333336	3.2695999	-0.9142528
16.7333336	3.3183999	-0.9081168
16.7333336	3.3671999	-0.8713012
16.7333336	3.4159999	-0.7853980
16.7333336	3.4647999	-0.5522332
16.7333336	3.5135999	-0.5645051
16.7333336	3.5623999	-0.9019809
16.7333336	3.6111999	-0.8222136
16.7333336	3.6599998	-0.8222136
16.7333336	3.7087998	-0.8222136
16.7333336	3.7575998	-0.8283496
16.7333336	3.8063998	-0.8099418
16.7333336	3.8551998	-0.7976699
16.7333336	3.9039998	-0.8651652
16.7333336	3.9527998	-0.8713012
16.7333336	4.0016003	-0.8528934
16.7333336	4.0503998	-0.8528934
16.7333336	4.0991998	-0.7669902
16.7333336	4.1479998	-0.7424469
16.7333336	4.1967998	-0.7363110
16.7333336	4.2455997	-0.7363110
16.7333336	4.2943997	-0.6749516
16.7333336	4.3431997	-0.6688156
16.7333336	4.3919997	-0.6749516
16.7333336	4.4407997	-0.7424469
16.7333336	4.4895997	-0.6688156
16.7333336	4.5383997	-0.6258640
16.7333336	4.5871997	-0.5031457
16.7333336	4.6359997	-0.4049711
16.7333336	4.6847997	-0.4172430
16.7333336	4.7336001	-0.4540582
16.7333336	4.7824001	-0.3926992
16.7333336	4.8312001	-0.4847379
16.7333336	4.8800001	-0.5706410
16.7333336	4.9288001	-0.5706410
16.7333336	4.9776001	613586.0000000

16.7333336	5.0264001	-0.5522332
16.7333336	5.0752001	-0.4847379
16.7333336	5.1240001	613586.0000000
16.7333336	5.1728001	-0.6810875
16.7333336	5.2216001	-0.5215535
16.7333336	5.2704000	613586.0000000
16.7333336	5.3192000	-0.9019809
16.7333336	5.3680000	613586.0000000
16.7333336	5.4168000	613586.0000000
16.7333336	5.4656000	613586.0000000
16.7333336	5.5144000	613586.0000000
16.7333336	5.5632000	613586.0000000
16.7333336	5.6120000	613586.0000000
16.7333336	5.6608000	613586.0000000
16.7333336	5.7096000	613586.0000000
16.7333336	5.7584000	613586.0000000
16.7333336	5.8072000	613586.0000000
16.7333336	5.8559999	613586.0000000
16.7333336	5.9047999	613586.0000000
16.7333336	5.9535999	613586.0000000
16.7333336	6.0023999	613586.0000000
16.7500000	0.2928000	-3.7490492
16.7500000	0.3416000	-3.8533599
16.7500000	0.3904000	-3.8594959
16.7500000	0.4392000	-3.6508744
16.7500000	0.4880000	-3.6447384
16.7500000	0.5368000	-4.1356125
16.7500000	0.5856000	-2.8532043
16.7500000	0.6344000	-1.6628351
16.7500000	0.6832000	-1.6628351
16.7500000	0.7320000	-1.6566992
16.7500000	0.7808000	-1.6444273
16.7500000	0.8296000	-1.0246992
16.7500000	0.8784000	-1.0246992
16.7500000	0.9272000	-1.5646605
16.7500000	0.9760000	-1.5094371
16.7500000	1.0247999	-1.1780972
16.7500000	1.0735999	-1.1719613
16.7500000	1.1223999	-1.3253593
16.7500000	1.1711999	-1.2271848
16.7500000	1.2200000	-1.0983305
16.7500000	1.2688000	-1.1167378
16.7500000	1.3176000	-1.1412816
16.7500000	1.3664000	-1.3928547
16.7500000	1.4152000	-1.3376312
16.7500000	1.4640000	-1.3192234
16.7500000	1.5128000	-1.3376312
16.7500000	1.5616000	-1.3376312
16.7500000	1.6104000	-1.4910293
16.7500000	1.6592000	-1.6935148
16.7500000	1.7079999	-1.4235344
16.7500000	1.7567999	-1.4051266
16.7500000	1.8055999	-1.1351457
16.7500000	1.8543999	-1.2640004
16.7500000	1.9031999	-1.1719613
16.7500000	1.9519999	-1.1351457
16.7500000	2.0007999	-1.1351457

16.7500000	2.0495999	-1.1412816
16.7500000	2.0983999	-1.3744469
16.7500000	2.1471999	-1.1535535
16.7500000	2.1959999	-0.9878836
16.7500000	2.2447999	-0.9633398
16.7500000	2.2935998	-1.0799227
16.7500000	2.3423998	-1.0431070
16.7500000	2.3912001	-1.0492430
16.7500000	2.4400001	-0.9756117
16.7500000	2.4888000	-0.9694757
16.7500000	2.5376000	-0.9878836
16.7500000	2.5864000	-1.0001554
16.7500000	2.6352000	-0.9940195
16.7500000	2.6840000	-1.0001554
16.7500000	2.7328000	-1.0185633
16.7500000	2.7816000	-0.9633398
16.7500000	2.8304000	-0.9326606
16.7500000	2.8792000	-1.1290097
16.7500000	2.9280000	-1.1965051
16.7500000	2.9768000	-1.2149129
16.7500000	3.0256000	-1.0921946
16.7500000	3.0743999	-0.8774371
16.7500000	3.1231999	-0.8160777
16.7500000	3.1719999	-0.8774371
16.7500000	3.2207999	-0.8713012
16.7500000	3.2695999	-1.0001554
16.7500000	3.3183999	-1.0062914
16.7500000	3.3671999	-0.8897090
16.7500000	3.4159999	-1.1903691
16.7500000	3.4647999	-1.2762718
16.7500000	3.5135999	-1.0246992
16.7500000	3.5623999	-0.9694757
16.7500000	3.6111999	-0.9019809
16.7500000	3.6599998	-0.9142528
16.7500000	3.7087998	-0.9203887
16.7500000	3.7575998	-0.9142528
16.7500000	3.8063998	-0.9142528
16.7500000	3.8551998	-0.9203887
16.7500000	3.9039998	-0.9203887
16.7500000	3.9527998	-1.0369711
16.7500000	4.0016003	-0.9449320
16.7500000	4.0503998	-0.9572039
16.7500000	4.0991998	-1.0983305
16.7500000	4.1479998	-0.9387960
16.7500000	4.1967998	-0.9387960
16.7500000	4.2455997	-0.9817476
16.7500000	4.2943997	-0.7547188
16.7500000	4.3431997	-0.7485828
16.7500000	4.3919997	-0.7485828
16.7500000	4.4407997	-0.8774371
16.7500000	4.4895997	-0.8835731
16.7500000	4.5383997	-0.8835731
16.7500000	4.5871997	-0.8897090
16.7500000	4.6359997	-0.7608547
16.7500000	4.6847997	-0.8344855
16.7500000	4.7336001	-0.7117672
16.7500000	4.7824001	613586.0000000

16.7500000	4.8312001	613586.0000000
16.7500000	4.8800001	-0.4786019
16.7500000	4.9288001	-0.3804274
16.7500000	4.9776001	613586.0000000
16.7500000	5.0264001	613586.0000000
16.7500000	5.0752001	613586.0000000
16.7500000	5.1240001	613586.0000000
16.7500000	5.1728001	613586.0000000
16.7500000	5.2216001	613586.0000000
16.7500000	5.2704000	613586.0000000
16.7500000	5.3192000	613586.0000000
16.7500000	5.3680000	613586.0000000
16.7500000	5.4168000	613586.0000000
16.7500000	5.4656000	613586.0000000
16.7500000	5.5144000	613586.0000000
16.7500000	5.5632000	613586.0000000
16.7500000	5.6120000	613586.0000000
16.7500000	5.6608000	613586.0000000
16.7500000	5.7096000	613586.0000000
16.7500000	5.7584000	613586.0000000
16.7500000	5.8072000	613586.0000000
16.7500000	5.8559999	613586.0000000
16.7500000	5.9047999	613586.0000000
16.7500000	5.9535999	613586.0000000
16.7500000	6.0023999	613586.0000000
16.7666664	0.2928000	-3.8840394
16.7666664	0.3416000	-3.4852045
16.7666664	0.3904000	-4.0435734
16.7666664	0.4392000	-4.0435734
16.7666664	0.4880000	-3.4422529
16.7666664	0.5368000	-3.3686218
16.7666664	0.5856000	-2.9145637
16.7666664	0.6344000	-1.7180586
16.7666664	0.6832000	-1.7241945
16.7666664	0.7320000	-1.5217090
16.7666664	0.7808000	-1.5707965
16.7666664	0.8296000	-1.4542136
16.7666664	0.8784000	-1.5217090
16.7666664	0.9272000	-1.5278449
16.7666664	0.9760000	-1.5217090
16.7666664	1.0247999	-1.5217090
16.7666664	1.0735999	-1.5462527
16.7666664	1.1223999	-1.5523887
16.7666664	1.1711999	-1.3928547
16.7666664	1.2200000	-1.5953403
16.7666664	1.2688000	-1.4358063
16.7666664	1.3176000	-1.3560390
16.7666664	1.3664000	-1.3744469
16.7666664	1.4152000	-1.3130875
16.7666664	1.4640000	-1.3008156
16.7666664	1.5128000	-1.1535535
16.7666664	1.5616000	-1.1474175
16.7666664	1.6104000	-1.2824078
16.7666664	1.6592000	-1.2824078
16.7666664	1.7079999	-1.7119226
16.7666664	1.7567999	-1.7241945
16.7666664	1.8055999	-1.4173985

16.7666664	1.8543999	-1.5462527
16.7666664	1.9031999	-1.3499031
16.7666664	1.9519999	-1.3621750
16.7666664	2.0007999	-1.3621750
16.7666664	2.0495999	-1.3008156
16.7666664	2.0983999	-1.3069515
16.7666664	2.1471999	-1.1474175
16.7666664	2.1959999	-1.1474175
16.7666664	2.2447999	-1.0492430
16.7666664	2.2935998	-1.0308352
16.7666664	2.3423998	-1.0431070
16.7666664	2.3912001	-0.8835731
16.7666664	2.4400001	-0.8897090
16.7666664	2.4888000	-1.1474175
16.7666664	2.5376000	-1.1474175
16.7666664	2.5864000	-1.1535535
16.7666664	2.6352000	-1.1535535
16.7666664	2.6840000	-1.1596894
16.7666664	2.7328000	-1.2210488
16.7666664	2.7816000	-1.1842332
16.7666664	2.8304000	-1.2271848
16.7666664	2.8792000	-1.2149129
16.7666664	2.9280000	-1.2149129
16.7666664	2.9768000	-1.1290097
16.7666664	3.0256000	-1.0983305
16.7666664	3.0743999	-0.9203887
16.7666664	3.1231999	-0.9203887
16.7666664	3.1719999	-0.6565437
16.7666664	3.2207999	-0.6504078
16.7666664	3.2695999	-0.6258640
16.7666664	3.3183999	-0.7731261
16.7666664	3.3671999	-0.7669902
16.7666664	3.4159999	-0.7669902
16.7666664	3.4647999	-1.0860586
16.7666664	3.5135999	-0.8651652
16.7666664	3.5623999	-1.0308352
16.7666664	3.6111999	-0.9878836
16.7666664	3.6599998	-1.1106019
16.7666664	3.7087998	-1.1106019
16.7666664	3.7575998	-1.0001554
16.7666664	3.8063998	-0.9756117
16.7666664	3.8551998	-0.9817476
16.7666664	3.9039998	-1.0185633
16.7666664	3.9527998	-1.0737867
16.7666664	4.0016003	-1.3008156
16.7666664	4.0503998	-1.0737867
16.7666664	4.0991998	-1.0615149
16.7666664	4.1479998	-1.1596894
16.7666664	4.1967998	-1.1167378
16.7666664	4.2455997	-1.0492430
16.7666664	4.2943997	-1.0492430
16.7666664	4.3431997	-1.0124273
16.7666664	4.3919997	-1.0001554
16.7666664	4.4407997	-1.0553789
16.7666664	4.4895997	-0.9756117
16.7666664	4.5383997	-0.9817476
16.7666664	4.5871997	613586.0000000

16.7666664	4.6359997	-0.9694757
16.7666664	4.6847997	613586.0000000
16.7666664	4.7336001	613586.0000000
16.7666664	4.7824001	613586.0000000
16.7666664	4.8312001	613586.0000000
16.7666664	4.8800001	-0.5276895
16.7666664	4.9288001	613586.0000000
16.7666664	4.9776001	-0.8099418
16.7666664	5.0264001	-0.7976699
16.7666664	5.0752001	-0.7240391
16.7666664	5.1240001	-0.7240391
16.7666664	5.1728001	-0.5706410
16.7666664	5.2216001	613586.0000000
16.7666664	5.2704000	613586.0000000
16.7666664	5.3192000	613586.0000000
16.7666664	5.3680000	613586.0000000
16.7666664	5.4168000	613586.0000000
16.7666664	5.4656000	613586.0000000
16.7666664	5.5144000	613586.0000000
16.7666664	5.5632000	613586.0000000
16.7666664	5.6120000	613586.0000000
16.7666664	5.6608000	613586.0000000
16.7666664	5.7096000	613586.0000000
16.7666664	5.7584000	613586.0000000
16.7666664	5.8072000	613586.0000000
16.7666664	5.8559999	613586.0000000
16.7666664	5.9047999	613586.0000000
16.7666664	5.9535999	613586.0000000
16.7666664	6.0023999	613586.0000000
16.7833328	0.2928000	-3.7674570
16.7833328	0.3416000	-3.7797287
16.7833328	0.3904000	-3.2949908
16.7833328	0.4392000	-3.1968160
16.7833328	0.4880000	-3.1968160
16.7833328	0.5368000	-3.3870296
16.7833328	0.5856000	-3.1109130
16.7833328	0.6344000	-2.2580199
16.7833328	0.6832000	-1.9205441
16.7833328	0.7320000	-1.7671461
16.7833328	0.7808000	-1.6996508
16.7833328	0.8296000	-1.8346410
16.7833328	0.8784000	-1.8223691
16.7833328	0.9272000	-1.5769324
16.7833328	0.9760000	-1.5892043
16.7833328	1.0247999	-1.6505632
16.7833328	1.0735999	-1.6505632
16.7833328	1.1223999	-1.8346410
16.7833328	1.1711999	-1.7487383
16.7833328	1.2200000	-1.6566992
16.7833328	1.2688000	-1.4848933
16.7833328	1.3176000	-1.4051266
16.7833328	1.3664000	-1.1903691
16.7833328	1.4152000	-1.0921946
16.7833328	1.4640000	-1.1658254
16.7833328	1.5128000	-1.2640004
16.7833328	1.5616000	-1.2640004
16.7833328	1.6104000	-1.1719613

16.7833328	1.6592000	-1.3376312
16.7833328	1.7079999	-1.3376312
16.7833328	1.7567999	-1.8837285
16.7833328	1.8055999	-1.8837285
16.7833328	1.8543999	-1.8469129
16.7833328	1.9031999	-1.4480777
16.7833328	1.9519999	-1.4603496
16.7833328	2.0007999	-1.3437672
16.7833328	2.0495999	-1.1658254
16.7833328	2.0983999	-1.1658254
16.7833328	2.1471999	-1.2946796
16.7833328	2.1959999	-1.2824078
16.7833328	2.2447999	-1.2394567
16.7833328	2.2935998	-1.2394567
16.7833328	2.3423998	-1.2333207
16.7833328	2.3912001	-1.1535535
16.7833328	2.4400001	-1.2517285
16.7833328	2.4888000	-1.2640004
16.7833328	2.5376000	-1.2824078
16.7833328	2.5864000	-1.3867188
16.7833328	2.6352000	-1.5892043
16.7833328	2.6840000	-1.5462527
16.7833328	2.7328000	-1.2149129
16.7833328	2.7816000	-1.2087770
16.7833328	2.8304000	-1.2517285
16.7833328	2.8792000	-1.3376312
16.7833328	2.9280000	-1.3376312
16.7833328	2.9768000	-1.4112625
16.7833328	3.0256000	-1.1903691
16.7833328	3.0743999	-1.2885437
16.7833328	3.1231999	-1.5155730
16.7833328	3.1719999	-1.5155730
16.7833328	3.2207999	-1.5155730
16.7833328	3.2695999	-1.5707965
16.7833328	3.3183999	-1.5523887
16.7833328	3.3671999	-1.5401168
16.7833328	3.4159999	-1.0246992
16.7833328	3.4647999	-1.0246992
16.7833328	3.5135999	-1.1658254
16.7833328	3.5623999	-1.1719613
16.7833328	3.6111999	-1.1780972
16.7833328	3.6599998	-1.1719613
16.7833328	3.7087998	-1.1106019
16.7833328	3.7575998	-1.1106019
16.7833328	3.8063998	-1.0737867
16.7833328	3.8551998	-1.2210488
16.7833328	3.9039998	-1.2517285
16.7833328	3.9527998	-1.2517285
16.7833328	4.0016003	-1.2578645
16.7833328	4.0503998	-1.1535535
16.7833328	4.0991998	-1.2087770
16.7833328	4.1479998	-1.2026410
16.7833328	4.1967998	-1.1903691
16.7833328	4.2455997	-1.1780972
16.7833328	4.2943997	-1.1658254
16.7833328	4.3431997	-0.9572039
16.7833328	4.3919997	-0.8406215

16.7833328	4.4407997	-0.8467574
16.7833328	4.4895997	-0.8651652
16.7833328	4.5383997	-0.8590293
16.7833328	4.5871997	-0.8528934
16.7833328	4.6359997	-0.8283496
16.7833328	4.6847997	-0.6626797
16.7833328	4.7336001	-0.6565437
16.7833328	4.7824001	-0.6626797
16.7833328	4.8312001	-0.7608547
16.7833328	4.8800001	-0.7608547
16.7833328	4.9288001	613586.0000000
16.7833328	4.9776001	613586.0000000
16.7833328	5.0264001	613586.0000000
16.7833328	5.0752001	-0.6442719
16.7833328	5.1240001	-0.6442719
16.7833328	5.1728001	613586.0000000
16.7833328	5.2216001	613586.0000000
16.7833328	5.2704000	613586.0000000
16.7833328	5.3192000	613586.0000000
16.7833328	5.3680000	613586.0000000
16.7833328	5.4168000	613586.0000000
16.7833328	5.4656000	613586.0000000
16.7833328	5.5144000	613586.0000000
16.7833328	5.5632000	613586.0000000
16.7833328	5.6120000	613586.0000000
16.7833328	5.6608000	613586.0000000
16.7833328	5.7096000	613586.0000000
16.7833328	5.7584000	613586.0000000
16.7833328	5.8072000	613586.0000000
16.7833328	5.8559999	613586.0000000
16.7833328	5.9047999	613586.0000000
16.7833328	5.9535999	613586.0000000
16.7833328	6.0023999	613586.0000000
16.7999992	0.2928000	-3.9147191
16.7999992	0.3416000	-3.5220201
16.7999992	0.3904000	-4.9026027
16.7999992	0.4392000	-3.8656316
16.7999992	0.4880000	-2.9391072
16.7999992	0.5368000	-2.6077673
16.7999992	0.5856000	-2.2457480
16.7999992	0.6344000	-1.8653207
16.7999992	0.6832000	-1.8653207
16.7999992	0.7320000	-1.8714566
16.7999992	0.7808000	-1.3621750
16.7999992	0.8296000	-1.5401168
16.7999992	0.8784000	-1.5401168
16.7999992	0.9272000	-1.5401168
16.7999992	0.9760000	-1.5953403
16.7999992	1.0247999	-1.2824078
16.7999992	1.0735999	-1.4296703
16.7999992	1.1223999	-1.4296703
16.7999992	1.1711999	-1.6382914
16.7999992	1.2200000	-1.5830684
16.7999992	1.2688000	-1.5830684
16.7999992	1.3176000	-1.3192234
16.7999992	1.3664000	-1.3192234
16.7999992	1.4152000	-1.0983305

16.7999992	1.4640000	-0.8651652
16.7999992	1.5128000	-0.8774371
16.7999992	1.5616000	-1.0308352
16.7999992	1.6104000	-1.0308352
16.7999992	1.6592000	-0.8283496
16.7999992	1.7079999	-2.0371265
16.7999992	1.7567999	-1.6076121
16.7999992	1.8055999	-1.2394567
16.7999992	1.8543999	-1.3867188
16.7999992	1.9031999	-1.3314953
16.7999992	1.9519999	-1.1965051
16.7999992	2.0007999	-1.1412816
16.7999992	2.0495999	-1.2578645
16.7999992	2.0983999	-1.1351457
16.7999992	2.1471999	-1.1351457
16.7999992	2.1959999	-1.1351457
16.7999992	2.2447999	-1.2455926
16.7999992	2.2935998	-1.0062914
16.7999992	2.3423998	-1.0799227
16.7999992	2.3912001	-1.0799227
16.7999992	2.4400001	-1.2333207
16.7999992	2.4888000	-1.1719613
16.7999992	2.5376000	-1.1780972
16.7999992	2.5864000	-1.1842332
16.7999992	2.6352000	-1.3376312
16.7999992	2.6840000	-1.3621750
16.7999992	2.7328000	-1.0676508
16.7999992	2.7816000	-1.0737867
16.7999992	2.8304000	-1.1412816
16.7999992	2.8792000	-1.1351457
16.7999992	2.9280000	-1.1351457
16.7999992	2.9768000	-1.1903691
16.7999992	3.0256000	-1.1842332
16.7999992	3.0743999	-1.4603496
16.7999992	3.1231999	-1.5217090
16.7999992	3.1719999	-1.5401168
16.7999992	3.2207999	-1.5462527
16.7999992	3.2695999	-1.1658254
16.7999992	3.3183999	-1.1474175
16.7999992	3.3671999	-1.1474175
16.7999992	3.4159999	-0.8528934
16.7999992	3.4647999	-0.8528934
16.7999992	3.5135999	-0.7485828
16.7999992	3.5623999	-0.8590293
16.7999992	3.6111999	-0.8958449
16.7999992	3.6599998	-0.7363110
16.7999992	3.7087998	-0.7485828
16.7999992	3.7575998	-0.8590293
16.7999992	3.8063998	-0.8590293
16.7999992	3.8551998	-0.8713012
16.7999992	3.9039998	-0.8835731
16.7999992	3.9527998	-0.8897090
16.7999992	4.0016003	-0.9878836
16.7999992	4.0503998	-0.9019809
16.7999992	4.0991998	-0.9081168
16.7999992	4.1479998	-0.9142528
16.7999992	4.1967998	-0.9694757

16.7999992	4.2455997	-0.9817476
16.7999992	4.2943997	-0.8774371
16.7999992	4.3431997	-0.8774371
16.7999992	4.3919997	-0.7853980
16.7999992	4.4407997	-0.6688156
16.7999992	4.4895997	-0.6565437
16.7999992	4.5383997	-0.5767770
16.7999992	4.5871997	-0.5767770
16.7999992	4.6359997	-0.5645051
16.7999992	4.6847997	-0.5399613
16.7999992	4.7336001	-0.6810875
16.7999992	4.7824001	-0.6688156
16.7999992	4.8312001	-0.5460973
16.7999992	4.8800001	-0.5460973
16.7999992	4.9288001	613586.0000000
16.7999992	4.9776001	-0.5031457
16.7999992	5.0264001	613586.0000000
16.7999992	5.0752001	613586.0000000
16.7999992	5.1240001	613586.0000000
16.7999992	5.1728001	613586.0000000
16.7999992	5.2216001	613586.0000000
16.7999992	5.2704000	613586.0000000
16.7999992	5.3192000	613586.0000000
16.7999992	5.3680000	613586.0000000
16.7999992	5.4168000	613586.0000000
16.7999992	5.4656000	613586.0000000
16.7999992	5.5144000	613586.0000000
16.7999992	5.5632000	613586.0000000
16.7999992	5.6120000	613586.0000000
16.7999992	5.6608000	613586.0000000
16.7999992	5.7096000	613586.0000000
16.7999992	5.7584000	613586.0000000
16.7999992	5.8072000	613586.0000000
16.7999992	5.8559999	-0.5951843
16.7999992	5.9047999	613586.0000000
16.7999992	5.9535999	613586.0000000
16.7999992	6.0023999	613586.0000000
16.8166676	0.2928000	-3.9576705
16.8166676	0.3416000	-3.9576705
16.8166676	0.3904000	-3.6999617
16.8166676	0.4392000	-3.7060976
16.8166676	0.4880000	-3.6754181
16.8166676	0.5368000	-3.0066025
16.8166676	0.5856000	-2.7673013
16.8166676	0.6344000	-1.6321554
16.8166676	0.6832000	-1.6198835
16.8166676	0.7320000	-1.6505632
16.8166676	0.7808000	-1.6382914
16.8166676	0.8296000	-1.7487383
16.8166676	0.8784000	-1.4910293
16.8166676	0.9272000	-1.6014762
16.8166676	0.9760000	-1.6260195
16.8166676	1.0247999	-1.9450879
16.8166676	1.0735999	-1.7241945
16.8166676	1.1223999	-1.7732821
16.8166676	1.1711999	-1.7487383
16.8166676	1.2200000	-1.7119226

16.8166676	1.2688000	-1.2394567
16.8166676	1.3176000	-1.2210488
16.8166676	1.3664000	-1.2149129
16.8166676	1.4152000	-1.2149129
16.8166676	1.4640000	-1.3069515
16.8166676	1.5128000	-1.3008156
16.8166676	1.5616000	-1.0921946
16.8166676	1.6104000	-1.5033011
16.8166676	1.6592000	-1.1106019
16.8166676	1.7079999	-2.0309906
16.8166676	1.7567999	-1.7241945
16.8166676	1.8055999	-1.2149129
16.8166676	1.8543999	-0.9633398
16.8166676	1.9031999	-0.9694757
16.8166676	1.9519999	-1.2333207
16.8166676	2.0007999	-1.0062914
16.8166676	2.0495999	-1.0124273
16.8166676	2.0983999	-1.1167378
16.8166676	2.1471999	-1.3437672
16.8166676	2.1959999	-1.0246992
16.8166676	2.2447999	-1.1412816
16.8166676	2.2935998	-1.1535535
16.8166676	2.3423998	-1.1842332
16.8166676	2.3912001	-1.1719613
16.8166676	2.4400001	-1.1719613
16.8166676	2.4888000	-1.2517285
16.8166676	2.5376000	-1.2026410
16.8166676	2.5864000	-1.3376312
16.8166676	2.6352000	-1.2946796
16.8166676	2.6840000	-1.3989906
16.8166676	2.7328000	-1.2946796
16.8166676	2.7816000	-0.9756117
16.8166676	2.8304000	-0.9203887
16.8166676	2.8792000	-0.9265246
16.8166676	2.9280000	-0.9265246
16.8166676	2.9768000	-0.8467574
16.8166676	3.0256000	-0.8467574
16.8166676	3.0743999	-0.6504078
16.8166676	3.1231999	-0.6565437
16.8166676	3.1719999	-0.3681555
16.8166676	3.2207999	-0.3804274
16.8166676	3.2695999	-0.3804274
16.8166676	3.3183999	-0.3742914
16.8166676	3.3671999	-0.2270293
16.8166676	3.4159999	-0.2208934
16.8166676	3.4647999	-0.2393012
16.8166676	3.5135999	-0.5522332
16.8166676	3.5623999	-0.5645051
16.8166676	3.6111999	-0.8160777
16.8166676	3.6599998	-0.7424469
16.8166676	3.7087998	-1.1228738
16.8166676	3.7575998	-1.1351457
16.8166676	3.8063998	-0.9756117
16.8166676	3.8551998	-1.0124273
16.8166676	3.9039998	-1.0124273
16.8166676	3.9527998	-0.9019809
16.8166676	4.0016003	-0.8651652

16.8166676	4.0503998	-0.9081168
16.8166676	4.0991998	-0.8528934
16.8166676	4.1479998	-0.7792621
16.8166676	4.1967998	-0.8528934
16.8166676	4.2455997	-0.8528934
16.8166676	4.2943997	-0.5645051
16.8166676	4.3431997	-0.5645051
16.8166676	4.3919997	-0.6810875
16.8166676	4.4407997	-0.6749516
16.8166676	4.4895997	-0.7240391
16.8166676	4.5383997	-0.7179031
16.8166676	4.5871997	-0.7240391
16.8166676	4.6359997	-0.4724660
16.8166676	4.6847997	-0.4479222
16.8166676	4.7336001	-0.4724660
16.8166676	4.7824001	-0.4356503
16.8166676	4.8312001	-0.4479222
16.8166676	4.8800001	-0.3742914
16.8166676	4.9288001	-0.5399613
16.8166676	4.9776001	613586.0000000
16.8166676	5.0264001	613586.0000000
16.8166676	5.0752001	-0.2638445
16.8166676	5.1240001	-0.2761164
16.8166676	5.1728001	613586.0000000
16.8166676	5.2216001	613586.0000000
16.8166676	5.2704000	0.0981746
16.8166676	5.3192000	613586.0000000
16.8166676	5.3680000	613586.0000000
16.8166676	5.4168000	613586.0000000
16.8166676	5.4656000	613586.0000000
16.8166676	5.5144000	613586.0000000
16.8166676	5.5632000	613586.0000000
16.8166676	5.6120000	613586.0000000
16.8166676	5.6608000	-0.9142528
16.8166676	5.7096000	-0.9081168
16.8166676	5.7584000	613586.0000000
16.8166676	5.8072000	-0.6504078
16.8166676	5.8559999	613586.0000000
16.8166676	5.9047999	613586.0000000
16.8166676	5.9535999	613586.0000000
16.8166676	6.0023999	613586.0000000
16.8333340	0.2928000	-3.4054375
16.8333340	0.3416000	-3.4115734
16.8333340	0.3904000	-3.4115734
16.8333340	0.4392000	-3.8288162
16.8333340	0.4880000	-3.6140587
16.8333340	0.5368000	-3.2827189
16.8333340	0.5856000	-2.4359615
16.8333340	0.6344000	-2.0616703
16.8333340	0.6832000	-2.1843886
16.8333340	0.7320000	-2.0493984
16.8333340	0.7808000	-1.4726214
16.8333340	0.8296000	-1.4787574
16.8333340	0.8784000	-1.5830684
16.8333340	0.9272000	-1.4051266
16.8333340	0.9760000	-1.4542136
16.8333340	1.0247999	-1.5278449

16.8333340	1.0735999	-1.5462527
16.8333340	1.1223999	-1.5523887
16.8333340	1.1711999	-1.4542136
16.8333340	1.2200000	-1.6812429
16.8333340	1.2688000	-1.6873789
16.8333340	1.3176000	-1.7119226
16.8333340	1.3664000	-1.1780972
16.8333340	1.4152000	-1.5155730
16.8333340	1.4640000	-1.5094371
16.8333340	1.5128000	-1.4787574
16.8333340	1.5616000	-1.4848933
16.8333340	1.6104000	-1.5278449
16.8333340	1.6592000	-1.5339808
16.8333340	1.7079999	-1.2455926
16.8333340	1.7567999	-1.2455926
16.8333340	1.8055999	-1.2394567
16.8333340	1.8543999	-0.9203887
16.8333340	1.9031999	-0.9326606
16.8333340	1.9519999	-1.3008156
16.8333340	2.0007999	-1.1658254
16.8333340	2.0495999	-1.2578645
16.8333340	2.0983999	-1.2640004
16.8333340	2.1471999	-1.2701364
16.8333340	2.1959999	-1.2210488
16.8333340	2.2447999	-1.2271848
16.8333340	2.2935998	-1.2333207
16.8333340	2.3423998	-1.1903691
16.8333340	2.3912001	-1.0737867
16.8333340	2.4400001	-1.1106019
16.8333340	2.4888000	-1.0308352
16.8333340	2.5376000	-1.0492430
16.8333340	2.5864000	-1.0799227
16.8333340	2.6352000	-1.0860586
16.8333340	2.6840000	-0.8713012
16.8333340	2.7328000	-0.9326606
16.8333340	2.7816000	-0.8835731
16.8333340	2.8304000	-0.9694757
16.8333340	2.8792000	-0.8590293
16.8333340	2.9280000	-0.7485828
16.8333340	2.9768000	-0.7547188
16.8333340	3.0256000	-0.6320000
16.8333340	3.0743999	-0.1718059
16.8333340	3.1231999	-0.1349902
16.8333340	3.1719999	-0.0245438
16.8333340	3.2207999	-0.0245438
16.8333340	3.2695999	0.0122719
16.8333340	3.3183999	0.0184078
16.8333340	3.3671999	-0.0613594
16.8333340	3.4159999	-0.0613594
16.8333340	3.4647999	-0.5092816
16.8333340	3.5135999	-0.3374758
16.8333340	3.5623999	-0.3374758
16.8333340	3.6111999	-0.3374758
16.8333340	3.6599998	-0.1411262
16.8333340	3.7087998	-0.5767770
16.8333340	3.7575998	-0.5767770
16.8333340	3.8063998	-0.5829129

16.8333340	3.8551998	-0.6197281
16.8333340	3.9039998	-0.7853980
16.8333340	3.9527998	-0.7792621
16.8333340	4.0016003	-0.8897090
16.8333340	4.0503998	-0.8099418
16.8333340	4.0991998	-0.8222136
16.8333340	4.1479998	-0.7056313
16.8333340	4.1967998	-0.8038058
16.8333340	4.2455997	-0.7608547
16.8333340	4.2943997	-0.9817476
16.8333340	4.3431997	-0.8038058
16.8333340	4.3919997	613586.0000000
16.8333340	4.4407997	613586.0000000
16.8333340	4.4895997	613586.0000000
16.8333340	4.5383997	613586.0000000
16.8333340	4.5871997	-0.5890489
16.8333340	4.6359997	-0.4970098
16.8333340	4.6847997	-0.5092816
16.8333340	4.7336001	-0.3804274
16.8333340	4.7824001	-0.3804274
16.8333340	4.8312001	-0.4540582
16.8333340	4.8800001	-0.3681555
16.8333340	4.9288001	-0.3742914
16.8333340	4.9776001	-0.3926992
16.8333340	5.0264001	-0.5951843
16.8333340	5.0752001	613586.0000000
16.8333340	5.1240001	613586.0000000
16.8333340	5.1728001	613586.0000000
16.8333340	5.2216001	613586.0000000
16.8333340	5.2704000	613586.0000000
16.8333340	5.3192000	613586.0000000
16.8333340	5.3680000	613586.0000000
16.8333340	5.4168000	613586.0000000
16.8333340	5.4656000	613586.0000000
16.8333340	5.5144000	613586.0000000
16.8333340	5.5632000	613586.0000000
16.8333340	5.6120000	613586.0000000
16.8333340	5.6608000	613586.0000000
16.8333340	5.7096000	613586.0000000
16.8333340	5.7584000	613586.0000000
16.8333340	5.8072000	613586.0000000
16.8333340	5.8559999	613586.0000000
16.8333340	5.9047999	613586.0000000
16.8333340	5.9535999	613586.0000000
16.8333340	6.0023999	613586.0000000
16.8500004	0.2928000	-3.6386025
16.8500004	0.3416000	-3.7674570
16.8500004	0.3904000	-3.4361172
16.8500004	0.4392000	-4.0497093
16.8500004	0.4880000	-2.7734373
16.8500004	0.5368000	-2.1107578
16.8500004	0.5856000	-2.1046219
16.8500004	0.6344000	-1.6566992
16.8500004	0.6832000	-1.6444273
16.8500004	0.7320000	-1.1290097
16.8500004	0.7808000	-1.1167378
16.8500004	0.8296000	-1.2455926

16.8500004	0.8784000	-1.2210488
16.8500004	0.9272000	-1.4051266
16.8500004	0.9760000	-1.3130875
16.8500004	1.0247999	-1.6628351
16.8500004	1.0735999	-1.4910293
16.8500004	1.1223999	-1.4726214
16.8500004	1.1711999	-1.7426023
16.8500004	1.2200000	-1.7303305
16.8500004	1.2688000	-1.5094371
16.8500004	1.3176000	-1.5094371
16.8500004	1.3664000	-1.5094371
16.8500004	1.4152000	-1.4848933
16.8500004	1.4640000	-1.3376312
16.8500004	1.5128000	-1.8346410
16.8500004	1.5616000	-2.1843886
16.8500004	1.6104000	-1.9757671
16.8500004	1.6592000	-2.0678062
16.8500004	1.7079999	-2.0555344
16.8500004	1.7567999	-2.0432625
16.8500004	1.8055999	-0.6933594
16.8500004	1.8543999	-1.0062914
16.8500004	1.9031999	-1.0124273
16.8500004	1.9519999	-1.0308352
16.8500004	2.0007999	-1.1351457
16.8500004	2.0495999	-1.2271848
16.8500004	2.0983999	-1.1842332
16.8500004	2.1471999	-1.1780972
16.8500004	2.1959999	-1.1780972
16.8500004	2.2447999	-1.3253593
16.8500004	2.2935998	-1.3314953
16.8500004	2.3423998	-1.3437672
16.8500004	2.3912001	-1.2578645
16.8500004	2.4400001	-1.1780972
16.8500004	2.4888000	-1.2885437
16.8500004	2.5376000	-1.2946796
16.8500004	2.5864000	-1.2578645
16.8500004	2.6352000	-1.2394567
16.8500004	2.6840000	-1.2271848
16.8500004	2.7328000	-1.2333207
16.8500004	2.7816000	-1.5033011
16.8500004	2.8304000	-1.4910293
16.8500004	2.8792000	-1.3867188
16.8500004	2.9280000	-1.3867188
16.8500004	2.9768000	-1.3989906
16.8500004	3.0256000	-1.3683109
16.8500004	3.0743999	-1.4848933
16.8500004	3.1231999	-1.4971652
16.8500004	3.1719999	-1.5646605
16.8500004	3.2207999	-1.5707965
16.8500004	3.2695999	-1.4419422
16.8500004	3.3183999	-1.5769324
16.8500004	3.3671999	-1.2026410
16.8500004	3.4159999	-1.1780972
16.8500004	3.4647999	-1.0737867
16.8500004	3.5135999	-0.2208934
16.8500004	3.5623999	0.0429516
16.8500004	3.6111999	0.0368156

16.8500004	3.6599998	0.0368156
16.8500004	3.7087998	-0.3742914
16.8500004	3.7575998	-0.3742914
16.8500004	3.8063998	-0.3865633
16.8500004	3.8551998	-0.5583692
16.8500004	3.9039998	-0.5583692
16.8500004	3.9527998	-0.7301750
16.8500004	4.0016003	-0.6565437
16.8500004	4.0503998	-0.6504078
16.8500004	4.0991998	-0.6320000
16.8500004	4.1479998	-0.5767770
16.8500004	4.1967998	-0.5829129
16.8500004	4.2455997	-0.5767770
16.8500004	4.2943997	-0.6504078
16.8500004	4.3431997	-0.6626797
16.8500004	4.3919997	-0.5829129
16.8500004	4.4407997	-0.6074562
16.8500004	4.4895997	-0.6197281
16.8500004	4.5383997	-0.6197281
16.8500004	4.5871997	-0.5338254
16.8500004	4.6359997	-0.5215535
16.8500004	4.6847997	-0.5276895
16.8500004	4.7336001	-0.4724660
16.8500004	4.7824001	-0.3436117
16.8500004	4.8312001	-0.3804274
16.8500004	4.8800001	-0.3865633
16.8500004	4.9288001	-0.3865633
16.8500004	4.9776001	613586.0000000
16.8500004	5.0264001	613586.0000000
16.8500004	5.0752001	613586.0000000
16.8500004	5.1240001	613586.0000000
16.8500004	5.1728001	613586.0000000
16.8500004	5.2216001	613586.0000000
16.8500004	5.2704000	613586.0000000
16.8500004	5.3192000	613586.0000000
16.8500004	5.3680000	613586.0000000
16.8500004	5.4168000	613586.0000000
16.8500004	5.4656000	613586.0000000
16.8500004	5.5144000	613586.0000000
16.8500004	5.5632000	613586.0000000
16.8500004	5.6120000	613586.0000000
16.8500004	5.6608000	-1.0308352
16.8500004	5.7096000	-1.0308352
16.8500004	5.7584000	-0.8835731
16.8500004	5.8072000	-0.8897090
16.8500004	5.8559999	613586.0000000
16.8500004	5.9047999	613586.0000000
16.8500004	5.9535999	613586.0000000
16.8500004	6.0023999	613586.0000000
16.8666668	0.2928000	-3.2520394
16.8666668	0.3416000	-3.2581754
16.8666668	0.3904000	-3.3133986
16.8666668	0.4392000	-3.3072627
16.8666668	0.4880000	-3.3011267
16.8666668	0.5368000	-3.0802336
16.8666668	0.5856000	-2.2886994
16.8666668	0.6344000	-2.2886994

16.8666668	0.6832000	-1.1474175
16.8666668	0.7320000	-1.1412816
16.8666668	0.7808000	-1.3805828
16.8666668	0.8296000	-1.6566992
16.8666668	0.8784000	-1.0553789
16.8666668	0.9272000	-1.4848933
16.8666668	0.9760000	-1.0921946
16.8666668	1.0247999	-1.0860586
16.8666668	1.0735999	-1.2271848
16.8666668	1.1223999	-1.2271848
16.8666668	1.1711999	-1.2271848
16.8666668	1.2200000	-1.4910293
16.8666668	1.2688000	-1.4726214
16.8666668	1.3176000	-1.7732821
16.8666668	1.3664000	-1.7855535
16.8666668	1.4152000	-1.6689711
16.8666668	1.4640000	-1.7180586
16.8666668	1.5128000	-2.1291652
16.8666668	1.5616000	-1.9021363
16.8666668	1.6104000	-2.0187187
16.8666668	1.6592000	-2.0309906
16.8666668	1.7079999	-1.6873789
16.8666668	1.7567999	-0.9878836
16.8666668	1.8055999	-0.8038058
16.8666668	1.8543999	-0.8038058
16.8666668	1.9031999	-0.9633398
16.8666668	1.9519999	-0.9572039
16.8666668	2.0007999	-1.0553789
16.8666668	2.0495999	-1.3560390
16.8666668	2.0983999	-1.1903691
16.8666668	2.1471999	-1.1903691
16.8666668	2.1959999	-1.1965051
16.8666668	2.2447999	-1.2640004
16.8666668	2.2935998	-1.3989906
16.8666668	2.3423998	-1.3069515
16.8666668	2.3912001	-1.4726214
16.8666668	2.4400001	-1.4787574
16.8666668	2.4888000	-1.3928547
16.8666668	2.5376000	-1.5339808
16.8666668	2.5864000	-1.5217090
16.8666668	2.6352000	-1.5094371
16.8666668	2.6840000	-1.6137476
16.8666668	2.7328000	-1.6076121
16.8666668	2.7816000	-1.6505632
16.8666668	2.8304000	-1.6076121
16.8666668	2.8792000	-1.9266801
16.8666668	2.9280000	-1.9266801
16.8666668	2.9768000	-1.9205441
16.8666668	3.0256000	-2.6077673
16.8666668	3.0743999	-2.6077673
16.8666668	3.1231999	-2.5402722
16.8666668	3.1719999	-2.4359615
16.8666668	3.2207999	-2.4420974
16.8666668	3.2695999	-1.2762718
16.8666668	3.3183999	-1.0246992
16.8666668	3.3671999	-1.5523887
16.8666668	3.4159999	-1.7978253

16.8666668	3.4647999	-1.5401168
16.8666668	3.5135999	-1.5278449
16.8666668	3.5623999	-1.4542136
16.8666668	3.6111999	-1.2087770
16.8666668	3.6599998	-1.2087770
16.8666668	3.7087998	-1.1106019
16.8666668	3.7575998	-1.0799227
16.8666668	3.8063998	-1.0676508
16.8666668	3.8551998	-1.0553789
16.8666668	3.9039998	-1.1167378
16.8666668	3.9527998	-1.1228738
16.8666668	4.0016003	-1.1596894
16.8666668	4.0503998	-1.1596894
16.8666668	4.0991998	-1.1596894
16.8666668	4.1479998	-0.9694757
16.8666668	4.1967998	-1.1044660
16.8666668	4.2455997	-1.0983305
16.8666668	4.2943997	-0.9449320
16.8666668	4.3431997	-1.0799227
16.8666668	4.3919997	-1.0737867
16.8666668	4.4407997	-1.0185633
16.8666668	4.4895997	-1.0185633
16.8666668	4.5383997	-1.0369711
16.8666668	4.5871997	-0.9940195
16.8666668	4.6359997	-0.7853980
16.8666668	4.6847997	-0.7853980
16.8666668	4.7336001	-0.7240391
16.8666668	4.7824001	-0.5829129
16.8666668	4.8312001	-0.5829129
16.8666668	4.8800001	-0.4786019
16.8666668	4.9288001	-0.4786019
16.8666668	4.9776001	-0.5338254
16.8666668	5.0264001	613586.0000000
16.8666668	5.0752001	-1.0246992
16.8666668	5.1240001	-1.0860586
16.8666668	5.1728001	-1.1474175
16.8666668	5.2216001	-1.2149129
16.8666668	5.2704000	613586.0000000
16.8666668	5.3192000	613586.0000000
16.8666668	5.3680000	613586.0000000
16.8666668	5.4168000	-1.0676508
16.8666668	5.4656000	-1.0676508
16.8666668	5.5144000	-1.2517285
16.8666668	5.5632000	613586.0000000
16.8666668	5.6120000	613586.0000000
16.8666668	5.6608000	613586.0000000
16.8666668	5.7096000	613586.0000000
16.8666668	5.7584000	613586.0000000
16.8666668	5.8072000	613586.0000000
16.8666668	5.8559999	613586.0000000
16.8666668	5.9047999	613586.0000000
16.8666668	5.9535999	613586.0000000
16.8666668	6.0023999	613586.0000000
16.8833332	0.2928000	-3.4361172
16.8833332	0.3416000	-3.7920005
16.8833332	0.3904000	-3.5036123
16.8833332	0.4392000	-3.4974763

16.8833332	0.4880000	-3.4974763
16.8833332	0.5368000	-2.5586801
16.8833332	0.5856000	-1.5339808
16.8833332	0.6344000	-1.4112625
16.8833332	0.6832000	-1.3989906
16.8833332	0.7320000	-1.5707965
16.8833332	0.7808000	-1.5462527
16.8833332	0.8296000	-1.5646605
16.8833332	0.8784000	-1.3560390
16.8833332	0.9272000	-1.0124273
16.8833332	0.9760000	-1.0062914
16.8833332	1.0247999	-0.9817476
16.8833332	1.0735999	-1.0124273
16.8833332	1.1223999	-1.0676508
16.8833332	1.1711999	-1.2087770
16.8833332	1.2200000	-1.2149129
16.8833332	1.2688000	-1.2333207
16.8833332	1.3176000	-1.3560390
16.8833332	1.3664000	-1.5217090
16.8833332	1.4152000	-1.5339808
16.8833332	1.4640000	-1.8285050
16.8833332	1.5128000	-1.6137476
16.8833332	1.5616000	-1.5892043
16.8833332	1.6104000	-1.5646605
16.8833332	1.6592000	-1.7855535
16.8833332	1.7079999	-1.7855535
16.8833332	1.7567999	-1.2149129
16.8833332	1.8055999	-1.1903691
16.8833332	1.8543999	-1.1780972
16.8833332	1.9031999	-1.1106019
16.8833332	1.9519999	-1.1167378
16.8833332	2.0007999	-1.1228738
16.8833332	2.0495999	-1.1903691
16.8833332	2.0983999	-1.1842332
16.8833332	2.1471999	-1.3560390
16.8833332	2.1959999	-1.3928547
16.8833332	2.2447999	-1.3867188
16.8833332	2.2935998	-1.1965051
16.8833332	2.3423998	-1.1842332
16.8833332	2.3912001	-1.3192234
16.8833332	2.4400001	-1.3253593
16.8833332	2.4888000	-1.2210488
16.8833332	2.5376000	-1.3130875
16.8833332	2.5864000	-1.3130875
16.8833332	2.6352000	-1.2701364
16.8833332	2.6840000	-1.2640004
16.8833332	2.7328000	-1.2701364
16.8833332	2.7816000	-1.3744469
16.8833332	2.8304000	-1.3008156
16.8833332	2.8792000	-1.4296703
16.8833332	2.9280000	-1.4112625
16.8833332	2.9768000	-1.4235344
16.8833332	3.0256000	-1.4603496
16.8833332	3.0743999	-1.3560390
16.8833332	3.1231999	-1.2149129
16.8833332	3.1719999	-1.2149129
16.8833332	3.2207999	-1.0124273

16.8833332	3.2695999	-1.2394567
16.8833332	3.3183999	-1.2578645
16.8833332	3.3671999	-0.9203887
16.8833332	3.4159999	-1.4051266
16.8833332	3.4647999	-2.0125828
16.8833332	3.5135999	-1.7241945
16.8833332	3.5623999	-1.7487383
16.8833332	3.6111999	-1.7487383
16.8833332	3.6599998	-1.7364664
16.8833332	3.7087998	-1.4787574
16.8833332	3.7575998	-1.3989906
16.8833332	3.8063998	-1.3928547
16.8833332	3.8551998	-1.3376312
16.8833332	3.9039998	-1.3314953
16.8833332	3.9527998	-1.2578645
16.8833332	4.0016003	-1.1535535
16.8833332	4.0503998	-1.1474175
16.8833332	4.0991998	-0.9203887
16.8833332	4.1479998	-0.9817476
16.8833332	4.1967998	-0.9449320
16.8833332	4.2455997	-0.9449320
16.8833332	4.2943997	-0.8344855
16.8833332	4.3431997	-0.8344855
16.8833332	4.3919997	-0.7363110
16.8833332	4.4407997	-0.6810875
16.8833332	4.4895997	-0.6504078
16.8833332	4.5383997	-0.5522332
16.8833332	4.5871997	-0.5522332
16.8833332	4.6359997	-0.5522332
16.8833332	4.6847997	-0.3804274
16.8833332	4.7336001	-0.2945242
16.8833332	4.7824001	-0.2883883
16.8833332	4.8312001	-0.3558836
16.8833332	4.8800001	-0.2883883
16.8833332	4.9288001	-0.5522332
16.8833332	4.9776001	-0.2761164
16.8833332	5.0264001	-0.2761164
16.8833332	5.0752001	-0.2822523
16.8833332	5.1240001	-0.4786019
16.8833332	5.1728001	613586.0000000
16.8833332	5.2216001	613586.0000000
16.8833332	5.2704000	613586.0000000
16.8833332	5.3192000	613586.0000000
16.8833332	5.3680000	613586.0000000
16.8833332	5.4168000	613586.0000000
16.8833332	5.4656000	613586.0000000
16.8833332	5.5144000	613586.0000000
16.8833332	5.5632000	613586.0000000
16.8833332	5.6120000	613586.0000000
16.8833332	5.6608000	613586.0000000
16.8833332	5.7096000	613586.0000000
16.8833332	5.7584000	613586.0000000
16.8833332	5.8072000	613586.0000000
16.8833332	5.8559999	613586.0000000
16.8833332	5.9047999	613586.0000000
16.8833332	5.9535999	613586.0000000
16.8833332	6.0023999	613586.0000000

16.8999996	0.2928000	-2.2825637
16.8999996	0.3416000	-2.2948353
16.8999996	0.3904000	-3.7122335
16.8999996	0.4392000	-3.3808937
16.8999996	0.4880000	-3.3808937
16.8999996	0.5368000	-1.7732821
16.8999996	0.5856000	-1.8285050
16.8999996	0.6344000	-1.8285050
16.8999996	0.6832000	-1.8223691
16.8999996	0.7320000	-1.3499031
16.8999996	0.7808000	-1.4910293
16.8999996	0.8296000	-1.3867188
16.8999996	0.8784000	-1.5401168
16.8999996	0.9272000	-1.5401168
16.8999996	0.9760000	-0.9756117
16.8999996	1.0247999	-0.9817476
16.8999996	1.0735999	-1.0676508
16.8999996	1.1223999	-1.0737867
16.8999996	1.1711999	-1.1658254
16.8999996	1.2200000	-1.2762718
16.8999996	1.2688000	-1.5646605
16.8999996	1.3176000	-1.3744469
16.8999996	1.3664000	-1.4848933
16.8999996	1.4152000	-1.3253593
16.8999996	1.4640000	-1.5278449
16.8999996	1.5128000	-1.7978253
16.8999996	1.5616000	-1.4971652
16.8999996	1.6104000	-1.4910293
16.8999996	1.6592000	-1.4542136
16.8999996	1.7079999	-1.6505632
16.8999996	1.7567999	-1.4848933
16.8999996	1.8055999	-1.4480777
16.8999996	1.8543999	-1.4480777
16.8999996	1.9031999	-1.5033011
16.8999996	1.9519999	-1.4971652
16.8999996	2.0007999	-1.5033011
16.8999996	2.0495999	-1.4419422
16.8999996	2.0983999	-1.4419422
16.8999996	2.1471999	-1.4358063
16.8999996	2.1959999	-1.4787574
16.8999996	2.2447999	-1.3744469
16.8999996	2.2935998	-1.3621750
16.8999996	2.3423998	-1.3805828
16.8999996	2.3912001	-1.2701364
16.8999996	2.4400001	-1.2946796
16.8999996	2.4888000	-1.3560390
16.8999996	2.5376000	-1.3499031
16.8999996	2.5864000	-1.1965051
16.8999996	2.6352000	-1.1719613
16.8999996	2.6840000	-1.1106019
16.8999996	2.7328000	-1.1044660
16.8999996	2.7816000	-1.0921946
16.8999996	2.8304000	-0.9142528
16.8999996	2.8792000	-0.9510679
16.8999996	2.9280000	-0.9572039
16.8999996	2.9768000	-1.0615149
16.8999996	3.0256000	-0.8099418

16.8999996	3.0743999	-0.8038058
16.8999996	3.1231999	-0.5522332
16.8999996	3.1719999	-0.4049711
16.8999996	3.2207999	-0.3804274
16.8999996	3.2695999	-1.0369711
16.8999996	3.3183999	-0.6994953
16.8999996	3.3671999	-0.9449320
16.8999996	3.4159999	-1.5523887
16.8999996	3.4647999	-1.5523887
16.8999996	3.5135999	-1.9389520
16.8999996	3.5623999	-1.4910293
16.8999996	3.6111999	-1.2394567
16.8999996	3.6599998	-1.2026410
16.8999996	3.7087998	-1.0431070
16.8999996	3.7575998	-1.0369711
16.8999996	3.8063998	-0.9387960
16.8999996	3.8551998	-0.9449320
16.8999996	3.9039998	-0.9387960
16.8999996	3.9527998	-0.8897090
16.8999996	4.0016003	-0.8160777
16.8999996	4.0503998	-0.8590293
16.8999996	4.0991998	-0.9019809
16.8999996	4.1479998	-0.6626797
16.8999996	4.1967998	-0.6688156
16.8999996	4.2455997	-0.7915339
16.8999996	4.2943997	-0.8283496
16.8999996	4.3431997	-0.8160777
16.8999996	4.3919997	-0.8774371
16.8999996	4.4407997	-0.7056313
16.8999996	4.4895997	-0.6994953
16.8999996	4.5383997	-0.5645051
16.8999996	4.5871997	-0.5460973
16.8999996	4.6359997	-0.5276895
16.8999996	4.6847997	-0.7117672
16.8999996	4.7336001	-0.7056313
16.8999996	4.7824001	-0.7485828
16.8999996	4.8312001	-0.5031457
16.8999996	4.8800001	-0.5522332
16.8999996	4.9288001	-0.5706410
16.8999996	4.9776001	-0.5276895
16.8999996	5.0264001	613586.0000000
16.8999996	5.0752001	-0.6074562
16.8999996	5.1240001	-0.6013203
16.8999996	5.1728001	613586.0000000
16.8999996	5.2216001	613586.0000000
16.8999996	5.2704000	613586.0000000
16.8999996	5.3192000	613586.0000000
16.8999996	5.3680000	613586.0000000
16.8999996	5.4168000	613586.0000000
16.8999996	5.4656000	613586.0000000
16.8999996	5.5144000	613586.0000000
16.8999996	5.5632000	613586.0000000
16.8999996	5.6120000	613586.0000000
16.8999996	5.6608000	613586.0000000
16.8999996	5.7096000	613586.0000000
16.8999996	5.7584000	613586.0000000
16.8999996	5.8072000	613586.0000000

16.8999996	5.8559999	613586.0000000
16.8999996	5.9047999	613586.0000000
16.8999996	5.9535999	613586.0000000
16.8999996	6.0023999	613586.0000000
16.9166660	0.2928000	-3.5711074
16.9166660	0.3416000	-3.5833793
16.9166660	0.3904000	-4.1233406
16.9166660	0.4392000	-3.9699423
16.9166660	0.4880000	-2.9452431
16.9166660	0.5368000	-2.1782527
16.9166660	0.5856000	-1.5523887
16.9166660	0.6344000	-1.5585246
16.9166660	0.6832000	-1.4173985
16.9166660	0.7320000	-1.3437672
16.9166660	0.7808000	-1.3621750
16.9166660	0.8296000	-1.3683109
16.9166660	0.8784000	-1.3069515
16.9166660	0.9272000	-1.6996508
16.9166660	0.9760000	-1.7732821
16.9166660	1.0247999	-1.7057867
16.9166660	1.0735999	-1.7057867
16.9166660	1.1223999	-1.4787574
16.9166660	1.1711999	-1.4296703
16.9166660	1.2200000	-1.4296703
16.9166660	1.2688000	-1.5585246
16.9166660	1.3176000	-1.5646605
16.9166660	1.3664000	-1.7916894
16.9166660	1.4152000	-1.7855535
16.9166660	1.4640000	-1.7057867
16.9166660	1.5128000	-1.6751070
16.9166660	1.5616000	-1.9941750
16.9166660	1.6104000	-1.4910293
16.9166660	1.6592000	-1.4051266
16.9166660	1.7079999	-2.2580199
16.9166660	1.7567999	-2.0064468
16.9166660	1.8055999	-2.0125828
16.9166660	1.8543999	-2.2457480
16.9166660	1.9031999	-1.9941750
16.9166660	1.9519999	-1.8285050
16.9166660	2.0007999	-1.8285050
16.9166660	2.0495999	-1.6812429
16.9166660	2.0983999	-1.5769324
16.9166660	2.1471999	-1.5646605
16.9166660	2.1959999	-1.4542136
16.9166660	2.2447999	-1.4542136
16.9166660	2.2935998	-1.5523887
16.9166660	2.3423998	-1.5585246
16.9166660	2.3912001	-1.5339808
16.9166660	2.4400001	-1.4787574
16.9166660	2.4888000	-1.4173985
16.9166660	2.5376000	-1.4296703
16.9166660	2.5864000	-1.3437672
16.9166660	2.6352000	-1.4358063
16.9166660	2.6840000	-1.4296703
16.9166660	2.7328000	-1.4173985
16.9166660	2.7816000	-1.0431070
16.9166660	2.8304000	-1.3130875

16.9166660	2.8792000	-1.2087770
16.9166660	2.9280000	-1.0492430
16.9166660	2.9768000	-0.6320000
16.9166660	3.0256000	-0.3497477
16.9166660	3.0743999	-0.3497477
16.9166660	3.1231999	-0.3497477
16.9166660	3.1719999	-0.5092816
16.9166660	3.2207999	-0.7424469
16.9166660	3.2695999	-0.5031457
16.9166660	3.3183999	-0.6504078
16.9166660	3.3671999	-1.1596894
16.9166660	3.4159999	-1.2885437
16.9166660	3.4647999	-0.5522332
16.9166660	3.5135999	-1.5339808
16.9166660	3.5623999	-1.3989906
16.9166660	3.6111999	-1.4971652
16.9166660	3.6599998	-1.4726214
16.9166660	3.7087998	-1.4664855
16.9166660	3.7575998	-1.3253593
16.9166660	3.8063998	-1.2333207
16.9166660	3.8551998	-1.1596894
16.9166660	3.9039998	-1.0308352
16.9166660	3.9527998	-1.1842332
16.9166660	4.0016003	-1.0431070
16.9166660	4.0503998	-1.1719613
16.9166660	4.0991998	-0.9694757
16.9166660	4.1479998	-0.5215535
16.9166660	4.1967998	-0.7485828
16.9166660	4.2455997	-0.8590293
16.9166660	4.2943997	-1.1535535
16.9166660	4.3431997	-0.7179031
16.9166660	4.3919997	-0.8283496
16.9166660	4.4407997	-0.8283496
16.9166660	4.4895997	-0.7240391
16.9166660	4.5383997	-0.8099418
16.9166660	4.5871997	-0.6810875
16.9166660	4.6359997	-0.6810875
16.9166660	4.6847997	-0.6810875
16.9166660	4.7336001	-0.6135921
16.9166660	4.7824001	-0.5154176
16.9166660	4.8312001	-0.5154176
16.9166660	4.8800001	-0.4601941
16.9166660	4.9288001	-0.4601941
16.9166660	4.9776001	-0.3558836
16.9166660	5.0264001	613586.0000000
16.9166660	5.0752001	613586.0000000
16.9166660	5.1240001	613586.0000000
16.9166660	5.1728001	613586.0000000
16.9166660	5.2216001	613586.0000000
16.9166660	5.2704000	-0.6626797
16.9166660	5.3192000	-0.6626797
16.9166660	5.3680000	613586.0000000
16.9166660	5.4168000	613586.0000000
16.9166660	5.4656000	613586.0000000
16.9166660	5.5144000	613586.0000000
16.9166660	5.5632000	613586.0000000
16.9166660	5.6120000	613586.0000000

16.9166660	5.6608000	613586.0000000
16.9166660	5.7096000	613586.0000000
16.9166660	5.7584000	613586.0000000
16.9166660	5.8072000	613586.0000000
16.9166660	5.8559999	613586.0000000
16.9166660	5.9047999	613586.0000000
16.9166660	5.9535999	613586.0000000
16.9166660	6.0023999	613586.0000000
16.9333324	0.2928000	-3.2029519
16.9333324	0.3416000	-2.9513791
16.9333324	0.3904000	-2.9513791
16.9333324	0.4392000	-3.6324666
16.9333324	0.4880000	-3.1538646
16.9333324	0.5368000	-1.7794180
16.9333324	0.5856000	-1.3376312
16.9333324	0.6344000	-1.3314953
16.9333324	0.6832000	-1.4112625
16.9333324	0.7320000	-1.4112625
16.9333324	0.7808000	-1.3928547
16.9333324	0.8296000	-1.0246992
16.9333324	0.8784000	-1.2210488
16.9333324	0.9272000	-0.7117672
16.9333324	0.9760000	-0.7179031
16.9333324	1.0247999	-1.4112625
16.9333324	1.0735999	-1.4603496
16.9333324	1.1223999	-1.4664855
16.9333324	1.1711999	-1.6689711
16.9333324	1.2200000	-1.3683109
16.9333324	1.2688000	-1.3192234
16.9333324	1.3176000	-1.0983305
16.9333324	1.3664000	-1.1106019
16.9333324	1.4152000	-1.2026410
16.9333324	1.4640000	-1.2026410
16.9333324	1.5128000	-1.4296703
16.9333324	1.5616000	-1.4235344
16.9333324	1.6104000	-1.4664855
16.9333324	1.6592000	-1.4603496
16.9333324	1.7079999	-1.6198835
16.9333324	1.7567999	-1.9941750
16.9333324	1.8055999	-2.0003109
16.9333324	1.8543999	-1.9450879
16.9333324	1.9031999	-1.9941750
16.9333324	1.9519999	-1.8407769
16.9333324	2.0007999	-1.6321554
16.9333324	2.0495999	-1.6198835
16.9333324	2.0983999	-1.7364664
16.9333324	2.1471999	-1.5339808
16.9333324	2.1959999	-1.7241945
16.9333324	2.2447999	-1.6751070
16.9333324	2.2935998	-1.6812429
16.9333324	2.3423998	-1.4480777
16.9333324	2.3912001	-1.3805828
16.9333324	2.4400001	-1.3130875
16.9333324	2.4888000	-1.4112625
16.9333324	2.5376000	-1.4173985
16.9333324	2.5864000	-1.4235344
16.9333324	2.6352000	-1.3069515

16.9333324	2.6840000	-1.3008156
16.9333324	2.7328000	-1.1228738
16.9333324	2.7816000	-1.1474175
16.9333324	2.8304000	-1.1412816
16.9333324	2.8792000	-1.1351457
16.9333324	2.9280000	-1.1535535
16.9333324	2.9768000	-1.1535535
16.9333324	3.0256000	-0.7301750
16.9333324	3.0743999	-0.7976699
16.9333324	3.1231999	-0.9633398
16.9333324	3.1719999	-0.9572039
16.9333324	3.2207999	-0.7669902
16.9333324	3.2695999	-0.7731261
16.9333324	3.3183999	-0.7915339
16.9333324	3.3671999	-0.8160777
16.9333324	3.4159999	-0.6626797
16.9333324	3.4647999	-0.9019809
16.9333324	3.5135999	-0.4417863
16.9333324	3.5623999	-0.4663301
16.9333324	3.6111999	-0.4724660
16.9333324	3.6599998	-0.5645051
16.9333324	3.7087998	-0.5092816
16.9333324	3.7575998	-0.6258640
16.9333324	3.8063998	-0.6258640
16.9333324	3.8551998	-0.8897090
16.9333324	3.9039998	-0.8774371
16.9333324	3.9527998	-0.7976699
16.9333324	4.0016003	-0.8467574
16.9333324	4.0503998	-0.7547188
16.9333324	4.0991998	-0.7976699
16.9333324	4.1479998	-0.7915339
16.9333324	4.1967998	-0.6749516
16.9333324	4.2455997	-0.8774371
16.9333324	4.2943997	-0.7669902
16.9333324	4.3431997	-0.7608547
16.9333324	4.3919997	-0.5645051
16.9333324	4.4407997	-0.5890489
16.9333324	4.4895997	-0.5890489
16.9333324	4.5383997	-0.6320000
16.9333324	4.5871997	-0.7731261
16.9333324	4.6359997	-0.7608547
16.9333324	4.6847997	-0.5154176
16.9333324	4.7336001	-0.5951843
16.9333324	4.7824001	613586.0000000
16.9333324	4.8312001	-0.4970098
16.9333324	4.8800001	-0.4970098
16.9333324	4.9288001	-0.2883883
16.9333324	4.9776001	-0.3804274
16.9333324	5.0264001	-0.3926992
16.9333324	5.0752001	613586.0000000
16.9333324	5.1240001	613586.0000000
16.9333324	5.1728001	613586.0000000
16.9333324	5.2216001	613586.0000000
16.9333324	5.2704000	613586.0000000
16.9333324	5.3192000	613586.0000000
16.9333324	5.3680000	613586.0000000
16.9333324	5.4168000	613586.0000000

16.9333324	5.4656000	613586.0000000
16.9333324	5.5144000	613586.0000000
16.9333324	5.5632000	613586.0000000
16.9333324	5.6120000	613586.0000000
16.9333324	5.6608000	613586.0000000
16.9333324	5.7096000	613586.0000000
16.9333324	5.7584000	613586.0000000
16.9333324	5.8072000	613586.0000000
16.9333324	5.8559999	613586.0000000
16.9333324	5.9047999	613586.0000000
16.9333324	5.9535999	613586.0000000
16.9333324	6.0023999	613586.0000000
16.9500008	0.2928000	-3.7920005
16.9500008	0.3416000	-4.0558453
16.9500008	0.3904000	-4.0497093
16.9500008	0.4392000	-4.1785641
16.9500008	0.4880000	-4.1785641
16.9500008	0.5368000	-1.5707965
16.9500008	0.5856000	-1.5892043
16.9500008	0.6344000	-1.5953403
16.9500008	0.6832000	-1.3253593
16.9500008	0.7320000	-1.3621750
16.9500008	0.7808000	-1.4112625
16.9500008	0.8296000	-1.4664855
16.9500008	0.8784000	-1.9819031
16.9500008	0.9272000	-1.3867188
16.9500008	0.9760000	-1.3805828
16.9500008	1.0247999	-1.1719613
16.9500008	1.0735999	-1.1351457
16.9500008	1.1223999	-1.1474175
16.9500008	1.1711999	-1.1719613
16.9500008	1.2200000	-1.1842332
16.9500008	1.2688000	-0.9633398
16.9500008	1.3176000	-1.1044660
16.9500008	1.3664000	-1.3867188
16.9500008	1.4152000	-1.2394567
16.9500008	1.4640000	-1.1719613
16.9500008	1.5128000	-0.8099418
16.9500008	1.5616000	-0.8038058
16.9500008	1.6104000	-0.7976699
16.9500008	1.6592000	-1.8530488
16.9500008	1.7079999	-1.8346410
16.9500008	1.7567999	-2.2027965
16.9500008	1.8055999	-2.2027965
16.9500008	1.8543999	-1.8653207
16.9500008	1.9031999	-1.8653207
16.9500008	1.9519999	-1.9021363
16.9500008	2.0007999	-1.8898644
16.9500008	2.0495999	-1.9021363
16.9500008	2.0983999	-1.7241945
16.9500008	2.1471999	-1.8223691
16.9500008	2.1959999	-1.8285050
16.9500008	2.2447999	-1.9819031
16.9500008	2.2935998	-1.7978253
16.9500008	2.3423998	-1.8469129
16.9500008	2.3912001	-1.8469129
16.9500008	2.4400001	-1.8530488

16.9500008	2.4888000	-1.5953403
16.9500008	2.5376000	-1.6076121
16.9500008	2.5864000	-1.6076121
16.9500008	2.6352000	-1.5278449
16.9500008	2.6840000	-1.4542136
16.9500008	2.7328000	-1.4480777
16.9500008	2.7816000	-1.3805828
16.9500008	2.8304000	-1.3744469
16.9500008	2.8792000	-1.4419422
16.9500008	2.9280000	-1.5094371
16.9500008	2.9768000	-1.8285050
16.9500008	3.0256000	-1.7364664
16.9500008	3.0743999	-1.7303305
16.9500008	3.1231999	-1.7855535
16.9500008	3.1719999	-1.0492430
16.9500008	3.2207999	-2.8961558
16.9500008	3.2695999	-2.6568549
16.9500008	3.3183999	-2.7488937
16.9500008	3.3671999	-0.9756117
16.9500008	3.4159999	-0.9019809
16.9500008	3.4647999	-1.0062914
16.9500008	3.5135999	-1.0124273
16.9500008	3.5623999	-1.0001554
16.9500008	3.6111999	-0.9694757
16.9500008	3.6599998	-1.0185633
16.9500008	3.7087998	-1.0246992
16.9500008	3.7575998	-1.0308352
16.9500008	3.8063998	-1.2946796
16.9500008	3.8551998	-0.9387960
16.9500008	3.9039998	-0.9387960
16.9500008	3.9527998	-1.0308352
16.9500008	4.0016003	-1.1474175
16.9500008	4.0503998	-0.9265246
16.9500008	4.0991998	-0.9203887
16.9500008	4.1479998	-0.8774371
16.9500008	4.1967998	-0.8651652
16.9500008	4.2455997	-0.8651652
16.9500008	4.2943997	-0.8774371
16.9500008	4.3431997	-0.8835731
16.9500008	4.3919997	-0.7547188
16.9500008	4.4407997	-0.7179031
16.9500008	4.4895997	-0.6442719
16.9500008	4.5383997	-0.5522332
16.9500008	4.5871997	-0.6749516
16.9500008	4.6359997	-0.4479222
16.9500008	4.6847997	-0.4479222
16.9500008	4.7336001	-0.4417863
16.9500008	4.7824001	-0.4356503
16.9500008	4.8312001	-0.2638445
16.9500008	4.8800001	-0.3620195
16.9500008	4.9288001	-0.4049711
16.9500008	4.9776001	-0.4111071
16.9500008	5.0264001	-0.3865633
16.9500008	5.0752001	-0.4172430
16.9500008	5.1240001	-0.2945242
16.9500008	5.1728001	613586.0000000
16.9500008	5.2216001	613586.0000000

16.9500008	5.2704000	613586.0000000
16.9500008	5.3192000	613586.0000000
16.9500008	5.3680000	613586.0000000
16.9500008	5.4168000	613586.0000000
16.9500008	5.4656000	613586.0000000
16.9500008	5.5144000	613586.0000000
16.9500008	5.5632000	613586.0000000
16.9500008	5.6120000	613586.0000000
16.9500008	5.6608000	613586.0000000
16.9500008	5.7096000	613586.0000000
16.9500008	5.7584000	613586.0000000
16.9500008	5.8072000	613586.0000000
16.9500008	5.8559999	613586.0000000
16.9500008	5.9047999	613586.0000000
16.9500008	5.9535999	613586.0000000
16.9500008	6.0023999	613586.0000000
16.9666672	0.2928000	-3.9883502
16.9666672	0.3416000	-3.9822142
16.9666672	0.3904000	-3.8901753
16.9666672	0.4392000	-2.9881947
16.9666672	0.4880000	-2.4666412
16.9666672	0.5368000	-2.4727771
16.9666672	0.5856000	-1.8162332
16.9666672	0.6344000	-1.8039613
16.9666672	0.6832000	-1.6689711
16.9666672	0.7320000	-1.2824078
16.9666672	0.7808000	-1.2333207
16.9666672	0.8296000	-1.2333207
16.9666672	0.8784000	-1.3069515
16.9666672	0.9272000	-1.4296703
16.9666672	0.9760000	-1.4971652
16.9666672	1.0247999	-1.0001554
16.9666672	1.0735999	-1.0001554
16.9666672	1.1223999	-1.0001554
16.9666672	1.1711999	-1.1412816
16.9666672	1.2200000	-1.0799227
16.9666672	1.2688000	-0.8713012
16.9666672	1.3176000	-0.8590293
16.9666672	1.3664000	-1.0369711
16.9666672	1.4152000	-0.9572039
16.9666672	1.4640000	-1.0431070
16.9666672	1.5128000	-0.8467574
16.9666672	1.5616000	-0.8467574
16.9666672	1.6104000	-0.7669902
16.9666672	1.6592000	-0.7608547
16.9666672	1.7079999	-1.2578645
16.9666672	1.7567999	-1.9328160
16.9666672	1.8055999	-1.8653207
16.9666672	1.8543999	-1.8714566
16.9666672	1.9031999	-1.8653207
16.9666672	1.9519999	-1.9880390
16.9666672	2.0007999	-1.7916894
16.9666672	2.0495999	-1.7610102
16.9666672	2.0983999	-1.8714566
16.9666672	2.1471999	-1.8285050
16.9666672	2.1959999	-1.8346410
16.9666672	2.2447999	-1.7241945

16.9666672	2.2935998	-1.6137476
16.9666672	2.3423998	-1.6137476
16.9666672	2.3912001	-1.5217090
16.9666672	2.4400001	-1.3192234
16.9666672	2.4888000	-1.3192234
16.9666672	2.5376000	-1.2640004
16.9666672	2.5864000	-1.1965051
16.9666672	2.6352000	-1.0983305
16.9666672	2.6840000	-1.0983305
16.9666672	2.7328000	-1.2824078
16.9666672	2.7816000	-1.1167378
16.9666672	2.8304000	-0.8774371
16.9666672	2.8792000	-0.8406215
16.9666672	2.9280000	-0.5154176
16.9666672	2.9768000	-0.4295144
16.9666672	3.0256000	-0.4295144
16.9666672	3.0743999	-0.4479222
16.9666672	3.1231999	-0.3436117
16.9666672	3.1719999	-0.5522332
16.9666672	3.2207999	-0.9203887
16.9666672	3.2695999	-0.9203887
16.9666672	3.3183999	-1.1044660
16.9666672	3.3671999	-0.7240391
16.9666672	3.4159999	-0.7240391
16.9666672	3.4647999	-0.3313398
16.9666672	3.5135999	0.2638445
16.9666672	3.5623999	0.2577085
16.9666672	3.6111999	-1.0308352
16.9666672	3.6599998	-1.1474175
16.9666672	3.7087998	-1.1474175
16.9666672	3.7575998	-0.9326606
16.9666672	3.8063998	-0.9019809
16.9666672	3.8551998	-1.0001554
16.9666672	3.9039998	-0.8835731
16.9666672	3.9527998	-0.8038058
16.9666672	4.0016003	-0.8038058
16.9666672	4.0503998	-0.8038058
16.9666672	4.0991998	-1.0553789
16.9666672	4.1479998	-0.9878836
16.9666672	4.1967998	-1.0062914
16.9666672	4.2455997	-0.9878836
16.9666672	4.2943997	-0.9817476
16.9666672	4.3431997	-0.7240391
16.9666672	4.3919997	-0.6135921
16.9666672	4.4407997	-0.5951843
16.9666672	4.4895997	-0.6320000
16.9666672	4.5383997	-0.6013203
16.9666672	4.5871997	-0.5829129
16.9666672	4.6359997	-0.5583692
16.9666672	4.6847997	-0.4111071
16.9666672	4.7336001	-0.2761164
16.9666672	4.7824001	-0.2761164
16.9666672	4.8312001	-0.2945242
16.9666672	4.8800001	-0.2883883
16.9666672	4.9288001	613586.0000000
16.9666672	4.9776001	-0.2270293
16.9666672	5.0264001	-0.2086215

16.9666672	5.0752001	613586.0000000
16.9666672	5.1240001	-0.3374758
16.9666672	5.1728001	-0.3374758
16.9666672	5.2216001	-0.3006601
16.9666672	5.2704000	613586.0000000
16.9666672	5.3192000	-0.2761164
16.9666672	5.3680000	613586.0000000
16.9666672	5.4168000	613586.0000000
16.9666672	5.4656000	613586.0000000
16.9666672	5.5144000	613586.0000000
16.9666672	5.5632000	613586.0000000
16.9666672	5.6120000	613586.0000000
16.9666672	5.6608000	613586.0000000
16.9666672	5.7096000	613586.0000000
16.9666672	5.7584000	613586.0000000
16.9666672	5.8072000	613586.0000000
16.9666672	5.8559999	613586.0000000
16.9666672	5.9047999	613586.0000000
16.9666672	5.9535999	613586.0000000
16.9666672	6.0023999	613586.0000000
16.9833336	0.2928000	-4.2337871
16.9833336	0.3416000	-4.2828746
16.9833336	0.3904000	-4.2828746
16.9833336	0.4392000	-3.5833793
16.9833336	0.4880000	-3.5956511
16.9833336	0.5368000	-1.5646605
16.9833336	0.5856000	-1.5339808
16.9833336	0.6344000	-1.5217090
16.9833336	0.6832000	-1.6382914
16.9833336	0.7320000	-1.6198835
16.9833336	0.7808000	-1.3499031
16.9833336	0.8296000	-1.0308352
16.9833336	0.8784000	-1.3560390
16.9833336	0.9272000	-1.1903691
16.9833336	0.9760000	-1.1903691
16.9833336	1.0247999	-1.1535535
16.9833336	1.0735999	-1.4112625
16.9833336	1.1223999	-1.4173985
16.9833336	1.1711999	-1.2087770
16.9833336	1.2200000	-1.2026410
16.9833336	1.2688000	-0.8467574
16.9833336	1.3176000	-0.7485828
16.9833336	1.3664000	-1.0185633
16.9833336	1.4152000	-1.0246992
16.9833336	1.4640000	-0.9019809
16.9833336	1.5128000	-0.8222136
16.9833336	1.5616000	-0.7056313
16.9833336	1.6104000	-0.6626797
16.9833336	1.6592000	-0.9940195
16.9833336	1.7079999	-1.4235344
16.9833336	1.7567999	-1.6382914
16.9833336	1.8055999	-1.8775926
16.9833336	1.8543999	-1.5523887
16.9833336	1.9031999	-1.8162332
16.9833336	1.9519999	-1.7610102
16.9833336	2.0007999	-1.7855535
16.9833336	2.0495999	-1.9389520

16.9833336	2.0983999	-1.9389520
16.9833336	2.1471999	-1.8591847
16.9833336	2.1959999	-1.8407769
16.9833336	2.2447999	-1.8407769
16.9833336	2.2935998	-1.7364664
16.9833336	2.3423998	-1.5892043
16.9833336	2.3912001	-1.5953403
16.9833336	2.4400001	-1.6014762
16.9833336	2.4888000	-1.6382914
16.9833336	2.5376000	-1.4726214
16.9833336	2.5864000	-1.6198835
16.9833336	2.6352000	-1.4848933
16.9833336	2.6840000	-1.4848933
16.9833336	2.7328000	-1.4787574
16.9833336	2.7816000	-1.4726214
16.9833336	2.8304000	-1.4603496
16.9833336	2.8792000	-1.4603496
16.9833336	2.9280000	-1.4971652
16.9833336	2.9768000	-1.4603496
16.9833336	3.0256000	-1.5585246
16.9833336	3.0743999	-1.5646605
16.9833336	3.1231999	-1.5462527
16.9833336	3.1719999	-1.5401168
16.9833336	3.2207999	-1.6505632
16.9833336	3.2695999	-1.2885437
16.9833336	3.3183999	-1.2946796
16.9833336	3.3671999	-1.1658254
16.9833336	3.4159999	-1.1780972
16.9833336	3.4647999	-1.4910293
16.9833336	3.5135999	-1.3744469
16.9833336	3.5623999	-1.6873789
16.9833336	3.6111999	-1.6935148
16.9833336	3.6599998	-1.4787574
16.9833336	3.7087998	-1.4971652
16.9833336	3.7575998	-1.5094371
16.9833336	3.8063998	-1.6076121
16.9833336	3.8551998	-1.4726214
16.9833336	3.9039998	-1.5155730
16.9833336	3.9527998	-1.6260195
16.9833336	4.0016003	-1.6076121
16.9833336	4.0503998	-1.4726214
16.9833336	4.0991998	-1.4664855
16.9833336	4.1479998	-1.3008156
16.9833336	4.1967998	-1.0553789
16.9833336	4.2455997	-1.0553789
16.9833336	4.2943997	-0.9081168
16.9833336	4.3431997	-1.0983305
16.9833336	4.3919997	-1.0615149
16.9833336	4.4407997	-1.0492430
16.9833336	4.4895997	-0.9326606
16.9833336	4.5383997	-0.7301750
16.9833336	4.5871997	-0.6933594
16.9833336	4.6359997	-0.6872234
16.9833336	4.6847997	-0.5767770
16.9833336	4.7336001	-0.5767770
16.9833336	4.7824001	-0.5706410
16.9833336	4.8312001	-0.4356503

16.9833336	4.8800001	-0.4540582
16.9833336	4.9288001	-0.3252039
16.9833336	4.9776001	-0.3252039
16.9833336	5.0264001	-0.4295144
16.9833336	5.0752001	-0.2577085
16.9833336	5.1240001	-0.3129320
16.9833336	5.1728001	-0.3006601
16.9833336	5.2216001	-0.3129320
16.9833336	5.2704000	-0.3252039
16.9833336	5.3192000	613586.0000000
16.9833336	5.3680000	613586.0000000
16.9833336	5.4168000	613586.0000000
16.9833336	5.4656000	613586.0000000
16.9833336	5.5144000	613586.0000000
16.9833336	5.5632000	613586.0000000
16.9833336	5.6120000	613586.0000000
16.9833336	5.6608000	613586.0000000
16.9833336	5.7096000	613586.0000000
16.9833336	5.7584000	613586.0000000
16.9833336	5.8072000	613586.0000000
16.9833336	5.8559999	613586.0000000
16.9833336	5.9047999	613586.0000000
16.9833336	5.9535999	613586.0000000
16.9833336	6.0023999	613586.0000000
17.0000000	0.2928000	-4.1172047
17.0000000	0.3416000	-4.2031074
17.0000000	0.3904000	-3.6324666
17.0000000	0.4392000	-3.6263306
17.0000000	0.4880000	-3.1538646
17.0000000	0.5368000	-1.3499031
17.0000000	0.5856000	-1.3560390
17.0000000	0.6344000	-1.3560390
17.0000000	0.6832000	-1.6628351
17.0000000	0.7320000	-1.9573593
17.0000000	0.7808000	-1.6935148
17.0000000	0.8296000	-1.6996508
17.0000000	0.8784000	-1.6996508
17.0000000	0.9272000	-1.6996508
17.0000000	0.9760000	-1.6873789
17.0000000	1.0247999	-1.5707965
17.0000000	1.0735999	-1.6812429
17.0000000	1.1223999	-1.3928547
17.0000000	1.1711999	-1.4910293
17.0000000	1.2200000	-1.5646605
17.0000000	1.2688000	-1.4419422
17.0000000	1.3176000	-1.4848933
17.0000000	1.3664000	-1.5033011
17.0000000	1.4152000	-1.2210488
17.0000000	1.4640000	-0.9572039
17.0000000	1.5128000	-0.9572039
17.0000000	1.5616000	-0.6933594
17.0000000	1.6104000	-0.8406215
17.0000000	1.6592000	-1.4480777
17.0000000	1.7079999	-1.4419422
17.0000000	1.7567999	-1.2640004
17.0000000	1.8055999	-1.2762718
17.0000000	1.8543999	-1.3805828

17.0000000	1.9031999	-1.3989906
17.0000000	1.9519999	-1.6873789
17.0000000	2.0007999	-1.6382914
17.0000000	2.0495999	-1.6751070
17.0000000	2.0983999	-1.6751070
17.0000000	2.1471999	-1.5707965
17.0000000	2.1959999	-1.5707965
17.0000000	2.2447999	-1.5892043
17.0000000	2.2935998	-1.5707965
17.0000000	2.3423998	-1.5278449
17.0000000	2.3912001	-1.4296703
17.0000000	2.4400001	-1.4173985
17.0000000	2.4888000	-1.3560390
17.0000000	2.5376000	-1.2824078
17.0000000	2.5864000	-1.2824078
17.0000000	2.6352000	-1.1719613
17.0000000	2.6840000	-1.1780972
17.0000000	2.7328000	-1.2026410
17.0000000	2.7816000	-1.2026410
17.0000000	2.8304000	-1.1535535
17.0000000	2.8792000	-0.8897090
17.0000000	2.9280000	-0.8897090
17.0000000	2.9768000	-0.6810875
17.0000000	3.0256000	-0.6749516
17.0000000	3.0743999	-0.6565437
17.0000000	3.1231999	-0.9326606
17.0000000	3.1719999	-0.7485828
17.0000000	3.2207999	-1.2210488
17.0000000	3.2695999	-1.5217090
17.0000000	3.3183999	-1.8469129
17.0000000	3.3671999	-1.8407769
17.0000000	3.4159999	-1.8407769
17.0000000	3.4647999	-0.9694757
17.0000000	3.5135999	-1.3437672
17.0000000	3.5623999	-1.3437672
17.0000000	3.6111999	-1.1658254
17.0000000	3.6599998	-0.9265246
17.0000000	3.7087998	-1.7732821
17.0000000	3.7575998	-1.7855535
17.0000000	3.8063998	-1.7487383
17.0000000	3.8551998	-1.7487383
17.0000000	3.9039998	-1.6689711
17.0000000	3.9527998	-1.6566992
17.0000000	4.0016003	-1.5217090
17.0000000	4.0503998	-1.2824078
17.0000000	4.0991998	-1.2333207
17.0000000	4.1479998	-1.2455926
17.0000000	4.1967998	-1.2026410
17.0000000	4.2455997	-1.0799227
17.0000000	4.2943997	-1.0001554
17.0000000	4.3431997	-0.9387960
17.0000000	4.3919997	-0.9387960
17.0000000	4.4407997	-0.9265246
17.0000000	4.4895997	-0.7976699
17.0000000	4.5383997	-0.8038058
17.0000000	4.5871997	-0.8406215
17.0000000	4.6359997	-0.7240391

17.0000000	4.6847997	-0.7363110
17.0000000	4.7336001	-0.5951843
17.0000000	4.7824001	-0.5767770
17.0000000	4.8312001	-0.5767770
17.0000000	4.8800001	-0.5215535
17.0000000	4.9288001	-0.5154176
17.0000000	4.9776001	-0.6442719
17.0000000	5.0264001	-0.5829129
17.0000000	5.0752001	-0.3926992
17.0000000	5.1240001	-0.4172430
17.0000000	5.1728001	-0.4172430
17.0000000	5.2216001	-0.4172430
17.0000000	5.2704000	-0.4908738
17.0000000	5.3192000	-0.4970098
17.0000000	5.3680000	613586.0000000
17.0000000	5.4168000	613586.0000000
17.0000000	5.4656000	613586.0000000
17.0000000	5.5144000	613586.0000000
17.0000000	5.5632000	613586.0000000
17.0000000	5.6120000	613586.0000000
17.0000000	5.6608000	613586.0000000
17.0000000	5.7096000	613586.0000000
17.0000000	5.7584000	613586.0000000
17.0000000	5.8072000	613586.0000000
17.0000000	5.8559999	613586.0000000
17.0000000	5.9047999	613586.0000000
17.0000000	5.9535999	613586.0000000
17.0000000	6.0023999	613586.0000000
17.0166664	0.2928000	-4.4485445
17.0166664	0.3416000	-5.3750687
17.0166664	0.3904000	-4.6633015
17.0166664	0.4392000	-3.9822142
17.0166664	0.4880000	-2.8716121
17.0166664	0.5368000	-1.5953403
17.0166664	0.5856000	-1.5953403
17.0166664	0.6344000	-1.5892043
17.0166664	0.6832000	-1.5155730
17.0166664	0.7320000	-1.6689711
17.0166664	0.7808000	-1.6628351
17.0166664	0.8296000	-1.3867188
17.0166664	0.8784000	-1.3867188
17.0166664	0.9272000	-1.6689711
17.0166664	0.9760000	-1.7426023
17.0166664	1.0247999	-1.3683109
17.0166664	1.0735999	-1.6321554
17.0166664	1.1223999	-1.6321554
17.0166664	1.1711999	-1.3499031
17.0166664	1.2200000	-1.3744469
17.0166664	1.2688000	-1.1228738
17.0166664	1.3176000	-1.4664855
17.0166664	1.3664000	-1.3867188
17.0166664	1.4152000	-1.2455926
17.0166664	1.4640000	-1.2455926
17.0166664	1.5128000	-1.2455926
17.0166664	1.5616000	-1.3314953
17.0166664	1.6104000	-1.3314953
17.0166664	1.6592000	-1.2885437

17.0166664	1.7079999	-0.7179031
17.0166664	1.7567999	-1.4971652
17.0166664	1.8055999	-1.5033011
17.0166664	1.8543999	-1.5094371
17.0166664	1.9031999	-1.4542136
17.0166664	1.9519999	-1.4726214
17.0166664	2.0007999	-1.4787574
17.0166664	2.0495999	-1.4787574
17.0166664	2.0983999	-1.6566992
17.0166664	2.1471999	-1.6382914
17.0166664	2.1959999	-1.4787574
17.0166664	2.2447999	-1.4726214
17.0166664	2.2935998	-1.5155730
17.0166664	2.3423998	-1.5217090
17.0166664	2.3912001	-1.5094371
17.0166664	2.4400001	-1.6321554
17.0166664	2.4888000	-1.5217090
17.0166664	2.5376000	-1.5217090
17.0166664	2.5864000	-1.4358063
17.0166664	2.6352000	-1.4296703
17.0166664	2.6840000	-1.1535535
17.0166664	2.7328000	-1.1535535
17.0166664	2.7816000	-1.1167378
17.0166664	2.8304000	-1.1167378
17.0166664	2.8792000	-0.9572039
17.0166664	2.9280000	-0.9694757
17.0166664	2.9768000	-0.9878836
17.0166664	3.0256000	-1.1106019
17.0166664	3.0743999	-1.1044660
17.0166664	3.1231999	-1.1780972
17.0166664	3.1719999	-1.1903691
17.0166664	3.2207999	-1.2701364
17.0166664	3.2695999	-1.4358063
17.0166664	3.3183999	-1.4358063
17.0166664	3.3671999	-1.2946796
17.0166664	3.4159999	-1.6873789
17.0166664	3.4647999	-1.6935148
17.0166664	3.5135999	-1.5278449
17.0166664	3.5623999	-1.4480777
17.0166664	3.6111999	-1.2640004
17.0166664	3.6599998	-1.2333207
17.0166664	3.7087998	-1.4419422
17.0166664	3.7575998	-1.4358063
17.0166664	3.8063998	-1.4603496
17.0166664	3.8551998	-1.4664855
17.0166664	3.9039998	-1.4787574
17.0166664	3.9527998	-1.3805828
17.0166664	4.0016003	-1.3437672
17.0166664	4.0503998	-1.3683109
17.0166664	4.0991998	-1.1412816
17.0166664	4.1479998	-1.1290097
17.0166664	4.1967998	-1.1228738
17.0166664	4.2455997	-1.0921946
17.0166664	4.2943997	-0.9756117
17.0166664	4.3431997	-0.7976699
17.0166664	4.3919997	-0.7792621
17.0166664	4.4407997	-0.7792621

17.0166664	4.4895997	-0.8774371
17.0166664	4.5383997	-0.7976699
17.0166664	4.5871997	-0.6933594
17.0166664	4.6359997	-0.6933594
17.0166664	4.6847997	-0.5767770
17.0166664	4.7336001	-0.5706410
17.0166664	4.7824001	-0.4724660
17.0166664	4.8312001	-0.4786019
17.0166664	4.8800001	-0.4479222
17.0166664	4.9288001	-0.6688156
17.0166664	4.9776001	-0.5829129
17.0166664	5.0264001	-0.5890489
17.0166664	5.0752001	-0.4356503
17.0166664	5.1240001	-0.6013203
17.0166664	5.1728001	-0.4663301
17.0166664	5.2216001	-0.4663301
17.0166664	5.2704000	-0.4479222
17.0166664	5.3192000	613586.0000000
17.0166664	5.3680000	613586.0000000
17.0166664	5.4168000	-0.7547188
17.0166664	5.4656000	-0.7547188
17.0166664	5.5144000	613586.0000000
17.0166664	5.5632000	613586.0000000
17.0166664	5.6120000	613586.0000000
17.0166664	5.6608000	-0.7731261
17.0166664	5.7096000	-0.7669902
17.0166664	5.7584000	-0.7669902
17.0166664	5.8072000	613586.0000000
17.0166664	5.8559999	613586.0000000
17.0166664	5.9047999	613586.0000000
17.0166664	5.9535999	613586.0000000
17.0166664	6.0023999	613586.0000000
17.0333328	0.2928000	-4.0987968
17.0333328	0.3416000	-4.1724281
17.0333328	0.3904000	-4.2399230
17.0333328	0.4392000	-4.0435734
17.0333328	0.4880000	-2.5770879
17.0333328	0.5368000	-2.0125828
17.0333328	0.5856000	-1.9328160
17.0333328	0.6344000	-1.9328160
17.0333328	0.6832000	-1.3805828
17.0333328	0.7320000	-1.3805828
17.0333328	0.7808000	-1.3989906
17.0333328	0.8296000	-1.3253593
17.0333328	0.8784000	-1.3069515
17.0333328	0.9272000	-1.3008156
17.0333328	0.9760000	-1.8223691
17.0333328	1.0247999	-1.6382914
17.0333328	1.0735999	-1.5278449
17.0333328	1.1223999	-1.5217090
17.0333328	1.1711999	-1.5769324
17.0333328	1.2200000	-1.4235344
17.0333328	1.2688000	-1.9573593
17.0333328	1.3176000	-1.9573593
17.0333328	1.3664000	-1.9573593
17.0333328	1.4152000	-1.6996508
17.0333328	1.4640000	-1.3989906

17.0333328	1.5128000	-1.5646605
17.0333328	1.5616000	-1.5646605
17.0333328	1.6104000	-1.5707965
17.0333328	1.6592000	-1.7978253
17.0333328	1.7079999	-2.0187187
17.0333328	1.7567999	-1.8960004
17.0333328	1.8055999	-1.5646605
17.0333328	1.8543999	-1.5585246
17.0333328	1.9031999	-1.5646605
17.0333328	1.9519999	-1.5094371
17.0333328	2.0007999	-1.6812429
17.0333328	2.0495999	-1.6689711
17.0333328	2.0983999	-1.6198835
17.0333328	2.1471999	-1.6321554
17.0333328	2.1959999	-1.6505632
17.0333328	2.2447999	-1.6505632
17.0333328	2.2935998	-1.6566992
17.0333328	2.3423998	-1.6566992
17.0333328	2.3912001	-1.6566992
17.0333328	2.4400001	-1.6628351
17.0333328	2.4888000	-1.6628351
17.0333328	2.5376000	-1.4419422
17.0333328	2.5864000	-1.4480777
17.0333328	2.6352000	-1.3989906
17.0333328	2.6840000	-1.5217090
17.0333328	2.7328000	-1.3683109
17.0333328	2.7816000	-1.2946796
17.0333328	2.8304000	-1.3928547
17.0333328	2.8792000	-1.2946796
17.0333328	2.9280000	-1.2946796
17.0333328	2.9768000	-1.3989906
17.0333328	3.0256000	-1.1535535
17.0333328	3.0743999	-1.3805828
17.0333328	3.1231999	-1.3560390
17.0333328	3.1719999	-1.2824078
17.0333328	3.2207999	-1.2885437
17.0333328	3.2695999	-1.2701364
17.0333328	3.3183999	-1.6076121
17.0333328	3.3671999	-2.3009713
17.0333328	3.4159999	-2.2764277
17.0333328	3.4647999	-2.2702918
17.0333328	3.5135999	-1.6014762
17.0333328	3.5623999	-1.3008156
17.0333328	3.6111999	-1.3069515
17.0333328	3.6599998	-1.0369711
17.0333328	3.7087998	-0.8283496
17.0333328	3.7575998	-1.3069515
17.0333328	3.8063998	-1.2394567
17.0333328	3.8551998	-1.1290097
17.0333328	3.9039998	-1.2578645
17.0333328	3.9527998	-1.2824078
17.0333328	4.0016003	-1.0185633
17.0333328	4.0503998	-1.0369711
17.0333328	4.0991998	-0.8897090
17.0333328	4.1479998	-0.8651652
17.0333328	4.1967998	-0.8528934
17.0333328	4.2455997	-0.7485828

17.0333328	4.2943997	-0.7056313
17.0333328	4.3431997	-0.8713012
17.0333328	4.3919997	-0.8713012
17.0333328	4.4407997	-0.8038058
17.0333328	4.4895997	-0.7301750
17.0333328	4.5383997	-0.6565437
17.0333328	4.5871997	-0.6565437
17.0333328	4.6359997	-0.5829129
17.0333328	4.6847997	-0.5890489
17.0333328	4.7336001	-0.6013203
17.0333328	4.7824001	-0.5276895
17.0333328	4.8312001	-0.4847379
17.0333328	4.8800001	-0.4417863
17.0333328	4.9288001	-0.3926992
17.0333328	4.9776001	-0.5031457
17.0333328	5.0264001	-0.2945242
17.0333328	5.0752001	-0.2638445
17.0333328	5.1240001	-0.2515731
17.0333328	5.1728001	-0.2699804
17.0333328	5.2216001	-0.2699804
17.0333328	5.2704000	-0.4663301
17.0333328	5.3192000	-0.1779418
17.0333328	5.3680000	613586.0000000
17.0333328	5.4168000	613586.0000000
17.0333328	5.4656000	613586.0000000
17.0333328	5.5144000	613586.0000000
17.0333328	5.5632000	613586.0000000
17.0333328	5.6120000	613586.0000000
17.0333328	5.6608000	613586.0000000
17.0333328	5.7096000	613586.0000000
17.0333328	5.7584000	613586.0000000
17.0333328	5.8072000	613586.0000000
17.0333328	5.8559999	613586.0000000
17.0333328	5.9047999	613586.0000000
17.0333328	5.9535999	613586.0000000
17.0333328	6.0023999	613586.0000000
17.0499992	0.2928000	-4.0865250
17.0499992	0.3416000	-4.1172047
17.0499992	0.3904000	-4.1294765
17.0499992	0.4392000	-3.5465636
17.0499992	0.4880000	-1.6566992
17.0499992	0.5368000	-1.6689711
17.0499992	0.5856000	-1.4051266
17.0499992	0.6344000	-1.4112625
17.0499992	0.6832000	-1.6014762
17.0499992	0.7320000	-1.6444273
17.0499992	0.7808000	-1.9696312
17.0499992	0.8296000	-1.9696312
17.0499992	0.8784000	-1.8407769
17.0499992	0.9272000	-1.5401168
17.0499992	0.9760000	-1.5339808
17.0499992	1.0247999	-1.4358063
17.0499992	1.0735999	-1.4971652
17.0499992	1.1223999	-1.4910293
17.0499992	1.1711999	-1.4664855
17.0499992	1.2200000	-1.4664855
17.0499992	1.2688000	-1.8039613

17.0499992	1.3176000	-1.7426023
17.0499992	1.3664000	-1.6935148
17.0499992	1.4152000	-1.8407769
17.0499992	1.4640000	-1.8346410
17.0499992	1.5128000	-1.6260195
17.0499992	1.5616000	-1.9082723
17.0499992	1.6104000	-1.8346410
17.0499992	1.6592000	-1.8039613
17.0499992	1.7079999	-1.8039613
17.0499992	1.7567999	-1.4910293
17.0499992	1.8055999	-1.4910293
17.0499992	1.8543999	-1.4787574
17.0499992	1.9031999	-1.4358063
17.0499992	1.9519999	-1.4848933
17.0499992	2.0007999	-1.5401168
17.0499992	2.0495999	-1.5523887
17.0499992	2.0983999	-1.5523887
17.0499992	2.1471999	-1.5523887
17.0499992	2.1959999	-1.6260195
17.0499992	2.2447999	-1.6321554
17.0499992	2.2935998	-1.5646605
17.0499992	2.3423998	-1.6198835
17.0499992	2.3912001	-1.5953403
17.0499992	2.4400001	-1.5830684
17.0499992	2.4888000	-1.5033011
17.0499992	2.5376000	-1.4971652
17.0499992	2.5864000	-1.3437672
17.0499992	2.6352000	-1.3253593
17.0499992	2.6840000	-1.3314953
17.0499992	2.7328000	-1.3314953
17.0499992	2.7816000	-1.3192234
17.0499992	2.8304000	-1.3683109
17.0499992	2.8792000	-1.3499031
17.0499992	2.9280000	-0.7301750
17.0499992	2.9768000	-1.4358063
17.0499992	3.0256000	-1.4358063
17.0499992	3.0743999	-1.3805828
17.0499992	3.1231999	-1.3867188
17.0499992	3.1719999	-1.2578645
17.0499992	3.2207999	-1.7487383
17.0499992	3.2695999	-1.7303305
17.0499992	3.3183999	-1.8162332
17.0499992	3.3671999	-1.4726214
17.0499992	3.4159999	-1.6935148
17.0499992	3.4647999	-1.5462527
17.0499992	3.5135999	-1.5339808
17.0499992	3.5623999	-1.1351457
17.0499992	3.6111999	-1.1351457
17.0499992	3.6599998	-1.3621750
17.0499992	3.7087998	-0.9940195
17.0499992	3.7575998	-0.9940195
17.0499992	3.8063998	-0.6504078
17.0499992	3.8551998	-0.6074562
17.0499992	3.9039998	-0.9019809
17.0499992	3.9527998	-0.8897090
17.0499992	4.0016003	-0.9633398
17.0499992	4.0503998	-1.1719613

17.0499992	4.0991998	-1.1474175
17.0499992	4.1479998	-1.1903691
17.0499992	4.1967998	-1.1106019
17.0499992	4.2455997	-1.1167378
17.0499992	4.2943997	-1.0124273
17.0499992	4.3431997	-0.9326606
17.0499992	4.3919997	-0.8222136
17.0499992	4.4407997	-0.9694757
17.0499992	4.4895997	-0.9817476
17.0499992	4.5383997	-0.9694757
17.0499992	4.5871997	-0.8713012
17.0499992	4.6359997	-0.8713012
17.0499992	4.6847997	-0.9019809
17.0499992	4.7336001	-0.9019809
17.0499992	4.7824001	-0.7915339
17.0499992	4.8312001	-0.7853980
17.0499992	4.8800001	-0.9510679
17.0499992	4.9288001	-0.7363110
17.0499992	4.9776001	-0.7485828
17.0499992	5.0264001	-0.7485828
17.0499992	5.0752001	-0.7179031
17.0499992	5.1240001	-0.7240391
17.0499992	5.1728001	-0.6994953
17.0499992	5.2216001	-0.8160777
17.0499992	5.2704000	-0.8160777
17.0499992	5.3192000	613586.0000000
17.0499992	5.3680000	-0.5338254
17.0499992	5.4168000	-0.5338254
17.0499992	5.4656000	-0.5276895
17.0499992	5.5144000	613586.0000000
17.0499992	5.5632000	613586.0000000
17.0499992	5.6120000	613586.0000000
17.0499992	5.6608000	613586.0000000
17.0499992	5.7096000	613586.0000000
17.0499992	5.7584000	613586.0000000
17.0499992	5.8072000	613586.0000000
17.0499992	5.8559999	613586.0000000
17.0499992	5.9047999	613586.0000000
17.0499992	5.9535999	613586.0000000
17.0499992	6.0023999	613586.0000000
17.0666676	0.2928000	-4.7982922
17.0666676	0.3416000	-4.8167000
17.0666676	0.3904000	-4.4669523
17.0666676	0.4392000	-3.3931656
17.0666676	0.4880000	-2.6813984
17.0666676	0.5368000	-2.2764277
17.0666676	0.5856000	-1.6137476
17.0666676	0.6344000	-1.5953403
17.0666676	0.6832000	-1.5830684
17.0666676	0.7320000	-1.6751070
17.0666676	0.7808000	-1.6812429
17.0666676	0.8296000	-1.8407769
17.0666676	0.8784000	-1.8469129
17.0666676	0.9272000	-1.8530488
17.0666676	0.9760000	-1.5523887
17.0666676	1.0247999	-1.6505632
17.0666676	1.0735999	-1.5953403

17.0666676	1.1223999	-1.5769324
17.0666676	1.1711999	-1.5769324
17.0666676	1.2200000	-1.5646605
17.0666676	1.2688000	-1.5523887
17.0666676	1.3176000	-1.9021363
17.0666676	1.3664000	-1.9082723
17.0666676	1.4152000	-1.6566992
17.0666676	1.4640000	-1.8407769
17.0666676	1.5128000	-1.8407769
17.0666676	1.5616000	-1.8469129
17.0666676	1.6104000	-1.8469129
17.0666676	1.6592000	-2.0739422
17.0666676	1.7079999	-1.7364664
17.0666676	1.7567999	-1.7364664
17.0666676	1.8055999	-1.3253593
17.0666676	1.8543999	-1.3314953
17.0666676	1.9031999	-1.3192234
17.0666676	1.9519999	-1.4296703
17.0666676	2.0007999	-1.6812429
17.0666676	2.0495999	-1.4112625
17.0666676	2.0983999	-1.3499031
17.0666676	2.1471999	-1.5953403
17.0666676	2.1959999	-1.5769324
17.0666676	2.2447999	-1.5707965
17.0666676	2.2935998	-1.4542136
17.0666676	2.3423998	-1.3621750
17.0666676	2.3912001	-1.3744469
17.0666676	2.4400001	-1.3867188
17.0666676	2.4888000	-1.2578645
17.0666676	2.5376000	-1.2701364
17.0666676	2.5864000	-1.2885437
17.0666676	2.6352000	-1.2885437
17.0666676	2.6840000	-1.3008156
17.0666676	2.7328000	-1.3560390
17.0666676	2.7816000	-1.4173985
17.0666676	2.8304000	-1.4173985
17.0666676	2.8792000	-1.4112625
17.0666676	2.9280000	-0.4295144
17.0666676	2.9768000	-1.5033011
17.0666676	3.0256000	-1.3314953
17.0666676	3.0743999	-1.1965051
17.0666676	3.1231999	-1.6137476
17.0666676	3.1719999	-1.6137476
17.0666676	3.2207999	-1.5401168
17.0666676	3.2695999	-1.0799227
17.0666676	3.3183999	-1.0185633
17.0666676	3.3671999	-1.0185633
17.0666676	3.4159999	-1.2455926
17.0666676	3.4647999	-1.6812429
17.0666676	3.5135999	-1.5339808
17.0666676	3.5623999	-1.5339808
17.0666676	3.6111999	-1.7364664
17.0666676	3.6599998	-1.8407769
17.0666676	3.7087998	-1.6382914
17.0666676	3.7575998	-1.3069515
17.0666676	3.8063998	-1.3744469
17.0666676	3.8551998	-1.4542136

17.0666676	3.9039998	-1.3314953
17.0666676	3.9527998	-1.1535535
17.0666676	4.0016003	-1.2701364
17.0666676	4.0503998	-1.0799227
17.0666676	4.0991998	-1.0308352
17.0666676	4.1479998	-1.0246992
17.0666676	4.1967998	-1.0308352
17.0666676	4.2455997	-1.0124273
17.0666676	4.2943997	-1.0001554
17.0666676	4.3431997	-0.9633398
17.0666676	4.3919997	-0.8774371
17.0666676	4.4407997	-0.8774371
17.0666676	4.4895997	-1.0185633
17.0666676	4.5383997	-0.8406215
17.0666676	4.5871997	-0.8406215
17.0666676	4.6359997	-0.7731261
17.0666676	4.6847997	-0.7731261
17.0666676	4.7336001	-1.0676508
17.0666676	4.7824001	-1.0001554
17.0666676	4.8312001	-0.9142528
17.0666676	4.8800001	-0.7363110
17.0666676	4.9288001	-0.7731261
17.0666676	4.9776001	-0.7731261
17.0666676	5.0264001	-0.6013203
17.0666676	5.0752001	-0.6810875
17.0666676	5.1240001	-0.5645051
17.0666676	5.1728001	-0.4172430
17.0666676	5.2216001	-0.4172430
17.0666676	5.2704000	-0.4111071
17.0666676	5.3192000	-0.4233789
17.0666676	5.3680000	-0.3313398
17.0666676	5.4168000	613586.0000000
17.0666676	5.4656000	613586.0000000
17.0666676	5.5144000	613586.0000000
17.0666676	5.5632000	613586.0000000
17.0666676	5.6120000	613586.0000000
17.0666676	5.6608000	613586.0000000
17.0666676	5.7096000	613586.0000000
17.0666676	5.7584000	613586.0000000
17.0666676	5.8072000	613586.0000000
17.0666676	5.8559999	613586.0000000
17.0666676	5.9047999	613586.0000000
17.0666676	5.9535999	613586.0000000
17.0666676	6.0023999	613586.0000000
17.0833340	0.2928000	-2.8838840
17.0833340	0.3416000	-2.8716121
17.0833340	0.3904000	-3.0679617
17.0833340	0.4392000	-2.5648160
17.0833340	0.4880000	-1.7794180
17.0833340	0.5368000	-1.7794180
17.0833340	0.5856000	-1.8653207
17.0833340	0.6344000	-1.9757671
17.0833340	0.6832000	-1.8898644
17.0833340	0.7320000	-1.6812429
17.0833340	0.7808000	-1.6873789
17.0833340	0.8296000	-1.4419422
17.0833340	0.8784000	-1.4358063

17.0833340	0.9272000	-1.5646605
17.0833340	0.9760000	-1.5585246
17.0833340	1.0247999	-1.5523887
17.0833340	1.0735999	-1.5462527
17.0833340	1.1223999	-1.4542136
17.0833340	1.1711999	-1.5155730
17.0833340	1.2200000	-1.5523887
17.0833340	1.2688000	-1.5401168
17.0833340	1.3176000	-1.5401168
17.0833340	1.3664000	-1.5339808
17.0833340	1.4152000	-1.6689711
17.0833340	1.4640000	-1.8346410
17.0833340	1.5128000	-1.9328160
17.0833340	1.5616000	-1.8775926
17.0833340	1.6104000	-1.9880390
17.0833340	1.6592000	-1.9021363
17.0833340	1.7079999	-1.4726214
17.0833340	1.7567999	-1.2517285
17.0833340	1.8055999	-1.2333207
17.0833340	1.8543999	-1.1658254
17.0833340	1.9031999	-1.1842332
17.0833340	1.9519999	-1.3621750
17.0833340	2.0007999	-1.4296703
17.0833340	2.0495999	-1.4173985
17.0833340	2.0983999	-1.4296703
17.0833340	2.1471999	-1.3989906
17.0833340	2.1959999	-1.3928547
17.0833340	2.2447999	-1.4051266
17.0833340	2.2935998	-1.2824078
17.0833340	2.3423998	-1.2762718
17.0833340	2.3912001	-1.2885437
17.0833340	2.4400001	-1.4542136
17.0833340	2.4888000	-1.4664855
17.0833340	2.5376000	-1.3314953
17.0833340	2.5864000	-1.3989906
17.0833340	2.6352000	-1.1228738
17.0833340	2.6840000	-1.0001554
17.0833340	2.7328000	-1.2026410
17.0833340	2.7816000	-0.2393012
17.0833340	2.8304000	-1.1412816
17.0833340	2.8792000	-1.1535535
17.0833340	2.9280000	-1.1535535
17.0833340	2.9768000	-1.1719613
17.0833340	3.0256000	-1.3314953
17.0833340	3.0743999	-1.3499031
17.0833340	3.1231999	-1.1719613
17.0833340	3.1719999	-1.1719613
17.0833340	3.2207999	-1.4358063
17.0833340	3.2695999	-1.4358063
17.0833340	3.3183999	-1.4971652
17.0833340	3.3671999	-2.1168938
17.0833340	3.4159999	-2.1353011
17.0833340	3.4647999	-1.3867188
17.0833340	3.5135999	-1.3867188
17.0833340	3.5623999	-1.4112625
17.0833340	3.6111999	-1.5339808
17.0833340	3.6599998	-1.4910293

17.0833340	3.7087998	-1.1719613
17.0833340	3.7575998	-1.1290097
17.0833340	3.8063998	-1.1228738
17.0833340	3.8551998	-1.2824078
17.0833340	3.9039998	-1.0308352
17.0833340	3.9527998	-1.0737867
17.0833340	4.0016003	-0.9940195
17.0833340	4.0503998	-1.0921946
17.0833340	4.0991998	-1.1044660
17.0833340	4.1479998	-1.1106019
17.0833340	4.1967998	-0.9817476
17.0833340	4.2455997	-0.6749516
17.0833340	4.2943997	-0.6994953
17.0833340	4.3431997	-0.6381359
17.0833340	4.3919997	-0.5951843
17.0833340	4.4407997	-0.5890489
17.0833340	4.4895997	-0.5890489
17.0833340	4.5383997	-0.5522332
17.0833340	4.5871997	-0.5583692
17.0833340	4.6359997	-0.5399613
17.0833340	4.6847997	-0.8283496
17.0833340	4.7336001	-0.6013203
17.0833340	4.7824001	-0.5951843
17.0833340	4.8312001	-0.4847379
17.0833340	4.8800001	-0.4786019
17.0833340	4.9288001	-0.4663301
17.0833340	4.9776001	-0.6320000
17.0833340	5.0264001	-0.7240391
17.0833340	5.0752001	-0.6442719
17.0833340	5.1240001	-0.6565437
17.0833340	5.1728001	-0.6504078
17.0833340	5.2216001	-0.4970098
17.0833340	5.2704000	-0.3374758
17.0833340	5.3192000	613586.0000000
17.0833340	5.3680000	613586.0000000
17.0833340	5.4168000	613586.0000000
17.0833340	5.4656000	613586.0000000
17.0833340	5.5144000	613586.0000000
17.0833340	5.5632000	613586.0000000
17.0833340	5.6120000	613586.0000000
17.0833340	5.6608000	613586.0000000
17.0833340	5.7096000	613586.0000000
17.0833340	5.7584000	613586.0000000
17.0833340	5.8072000	613586.0000000
17.0833340	5.8559999	613586.0000000
17.0833340	5.9047999	613586.0000000
17.0833340	5.9535999	613586.0000000
17.0833340	6.0023999	613586.0000000
17.1000004	0.2928000	-3.6999617
17.1000004	0.3416000	-3.8104084
17.1000004	0.3904000	-3.1170490
17.1000004	0.4392000	-3.5342917
17.1000004	0.4880000	-1.6137476
17.1000004	0.5368000	-1.6260195
17.1000004	0.5856000	-1.2026410
17.1000004	0.6344000	-1.1965051
17.1000004	0.6832000	-1.7732821

17.1000004	0.7320000	-1.4358063
17.1000004	0.7808000	-1.5953403
17.1000004	0.8296000	-1.7119226
17.1000004	0.8784000	-1.3069515
17.1000004	0.9272000	-1.6260195
17.1000004	0.9760000	-1.2946796
17.1000004	1.0247999	-1.3376312
17.1000004	1.0735999	-1.3928547
17.1000004	1.1223999	-1.5278449
17.1000004	1.1711999	-1.5278449
17.1000004	1.2200000	-1.5401168
17.1000004	1.2688000	-1.5953403
17.1000004	1.3176000	-1.5953403
17.1000004	1.3664000	-1.5094371
17.1000004	1.4152000	-1.5155730
17.1000004	1.4640000	-1.9144082
17.1000004	1.5128000	-1.8714566
17.1000004	1.5616000	-1.8775926
17.1000004	1.6104000	-1.7303305
17.1000004	1.6592000	-1.5033011
17.1000004	1.7079999	-1.7978253
17.1000004	1.7567999	-1.4112625
17.1000004	1.8055999	-1.4480777
17.1000004	1.8543999	-1.4971652
17.1000004	1.9031999	-1.3192234
17.1000004	1.9519999	-1.3069515
17.1000004	2.0007999	-1.4848933
17.1000004	2.0495999	-1.4296703
17.1000004	2.0983999	-1.4358063
17.1000004	2.1471999	-1.4358063
17.1000004	2.1959999	-1.4419422
17.1000004	2.2447999	-1.2762718
17.1000004	2.2935998	-1.2824078
17.1000004	2.3423998	-1.2824078
17.1000004	2.3912001	-1.3376312
17.1000004	2.4400001	-1.3499031
17.1000004	2.4888000	-1.2578645
17.1000004	2.5376000	-1.3253593
17.1000004	2.5864000	-1.1412816
17.1000004	2.6352000	-1.1474175
17.1000004	2.6840000	-1.1535535
17.1000004	2.7328000	-1.0983305
17.1000004	2.7816000	-1.0983305
17.1000004	2.8304000	-1.1044660
17.1000004	2.8792000	-1.0860586
17.1000004	2.9280000	-1.2333207
17.1000004	2.9768000	-1.2333207
17.1000004	3.0256000	-1.2517285
17.1000004	3.0743999	-1.0001554
17.1000004	3.1231999	-1.1167378
17.1000004	3.1719999	-0.8774371
17.1000004	3.2207999	-0.8222136
17.1000004	3.2695999	-0.8590293
17.1000004	3.3183999	-1.2946796
17.1000004	3.3671999	-1.3437672
17.1000004	3.4159999	-1.5094371
17.1000004	3.4647999	-1.1965051

17.1000004	3.5135999	-1.3069515
17.1000004	3.5623999	-1.3008156
17.1000004	3.6111999	-1.2946796
17.1000004	3.6599998	-1.2701364
17.1000004	3.7087998	-1.1719613
17.1000004	3.7575998	-1.1842332
17.1000004	3.8063998	-1.2026410
17.1000004	3.8551998	-1.2026410
17.1000004	3.9039998	-1.2026410
17.1000004	3.9527998	-1.2701364
17.1000004	4.0016003	-1.2701364
17.1000004	4.0503998	-1.1596894
17.1000004	4.0991998	-1.1658254
17.1000004	4.1479998	-1.1596894
17.1000004	4.1967998	-1.1535535
17.1000004	4.2455997	-1.0308352
17.1000004	4.2943997	-1.0431070
17.1000004	4.3431997	-1.0921946
17.1000004	4.3919997	-1.0860586
17.1000004	4.4407997	-0.9326606
17.1000004	4.4895997	-0.8651652
17.1000004	4.5383997	-0.7301750
17.1000004	4.5871997	-0.7976699
17.1000004	4.6359997	-0.9694757
17.1000004	4.6847997	-0.9694757
17.1000004	4.7336001	-0.8651652
17.1000004	4.7824001	-0.8713012
17.1000004	4.8312001	-0.6810875
17.1000004	4.8800001	-0.6933594
17.1000004	4.9288001	-0.7485828
17.1000004	4.9776001	-0.7485828
17.1000004	5.0264001	-0.6749516
17.1000004	5.0752001	-0.6749516
17.1000004	5.1240001	-0.7056313
17.1000004	5.1728001	-0.7056313
17.1000004	5.2216001	-0.5706410
17.1000004	5.2704000	-0.8160777
17.1000004	5.3192000	-0.9142528
17.1000004	5.3680000	613586.0000000
17.1000004	5.4168000	613586.0000000
17.1000004	5.4656000	613586.0000000
17.1000004	5.5144000	613586.0000000
17.1000004	5.5632000	613586.0000000
17.1000004	5.6120000	613586.0000000
17.1000004	5.6608000	613586.0000000
17.1000004	5.7096000	613586.0000000
17.1000004	5.7584000	613586.0000000
17.1000004	5.8072000	613586.0000000
17.1000004	5.8559999	613586.0000000
17.1000004	5.9047999	613586.0000000
17.1000004	5.9535999	613586.0000000
17.1000004	6.0023999	613586.0000000
17.1166668	0.2928000	-3.8840394
17.1166668	0.3416000	-3.6754181
17.1166668	0.3904000	-2.7673013
17.1166668	0.4392000	-2.7611654
17.1166668	0.4880000	-2.3991461

17.1166668	0.5368000	-2.1905246
17.1166668	0.5856000	-1.8530488
17.1166668	0.6344000	-1.7426023
17.1166668	0.6832000	-1.7671461
17.1166668	0.7320000	-1.5585246
17.1166668	0.7808000	-1.5523887
17.1166668	0.8296000	-1.4419422
17.1166668	0.8784000	-1.6444273
17.1166668	0.9272000	-1.6505632
17.1166668	0.9760000	-1.5401168
17.1166668	1.0247999	-1.5339808
17.1166668	1.0735999	-1.7548742
17.1166668	1.1223999	-1.6935148
17.1166668	1.1711999	-1.6321554
17.1166668	1.2200000	-1.6382914
17.1166668	1.2688000	-1.7426023
17.1166668	1.3176000	-1.6321554
17.1166668	1.3664000	-1.7916894
17.1166668	1.4152000	-1.7119226
17.1166668	1.4640000	-1.6873789
17.1166668	1.5128000	-1.6873789
17.1166668	1.5616000	-1.8285050
17.1166668	1.6104000	-1.8898644
17.1166668	1.6592000	-1.8039613
17.1166668	1.7079999	-1.5523887
17.1166668	1.7567999	-1.5523887
17.1166668	1.8055999	-1.5462527
17.1166668	1.8543999	-1.5094371
17.1166668	1.9031999	-1.4235344
17.1166668	1.9519999	-1.4235344
17.1166668	2.0007999	-1.5033011
17.1166668	2.0495999	-1.5462527
17.1166668	2.0983999	-1.5523887
17.1166668	2.1471999	-1.5523887
17.1166668	2.1959999	-1.5339808
17.1166668	2.2447999	-1.5401168
17.1166668	2.2935998	-1.6014762
17.1166668	2.3423998	-1.5033011
17.1166668	2.3912001	-1.4173985
17.1166668	2.4400001	-1.5953403
17.1166668	2.4888000	-1.5953403
17.1166668	2.5376000	-1.5033011
17.1166668	2.5864000	-1.4051266
17.1166668	2.6352000	-1.3437672
17.1166668	2.6840000	-1.4296703
17.1166668	2.7328000	-1.4235344
17.1166668	2.7816000	-1.4112625
17.1166668	2.8304000	-1.5094371
17.1166668	2.8792000	-1.8714566
17.1166668	2.9280000	-1.9082723
17.1166668	2.9768000	-1.9021363
17.1166668	3.0256000	-1.9082723
17.1166668	3.0743999	-1.9144082
17.1166668	3.1231999	-1.8100972
17.1166668	3.1719999	-1.8100972
17.1166668	3.2207999	-1.5830684
17.1166668	3.2695999	-1.5830684

17.1166668	3.3183999	-1.3130875
17.1166668	3.3671999	-1.6873789
17.1166668	3.4159999	-1.3130875
17.1166668	3.4647999	-1.3192234
17.1166668	3.5135999	-1.0799227
17.1166668	3.5623999	-1.1903691
17.1166668	3.6111999	-1.1044660
17.1166668	3.6599998	-1.1044660
17.1166668	3.7087998	-1.0369711
17.1166668	3.7575998	-1.0246992
17.1166668	3.8063998	-1.0799227
17.1166668	3.8551998	-0.8774371
17.1166668	3.9039998	-0.8835731
17.1166668	3.9527998	-0.8897090
17.1166668	4.0016003	-0.9142528
17.1166668	4.0503998	-1.0001554
17.1166668	4.0991998	-1.0001554
17.1166668	4.1479998	-0.8467574
17.1166668	4.1967998	-0.8528934
17.1166668	4.2455997	-0.9449320
17.1166668	4.2943997	-0.8590293
17.1166668	4.3431997	-0.9019809
17.1166668	4.3919997	-0.6872234
17.1166668	4.4407997	-0.6810875
17.1166668	4.4895997	-0.8897090
17.1166668	4.5383997	-0.8528934
17.1166668	4.5871997	-0.6197281
17.1166668	4.6359997	-0.7792621
17.1166668	4.6847997	-0.6381359
17.1166668	4.7336001	-0.6197281
17.1166668	4.7824001	-0.6197281
17.1166668	4.8312001	-0.6013203
17.1166668	4.8800001	-0.7117672
17.1166668	4.9288001	-0.6013203
17.1166668	4.9776001	-0.5890489
17.1166668	5.0264001	-0.6565437
17.1166668	5.0752001	-0.6442719
17.1166668	5.1240001	-0.5338254
17.1166668	5.1728001	-0.3374758
17.1166668	5.2216001	-0.5951843
17.1166668	5.2704000	-0.5829129
17.1166668	5.3192000	-0.5829129
17.1166668	5.3680000	-0.5829129
17.1166668	5.4168000	613586.0000000
17.1166668	5.4656000	613586.0000000
17.1166668	5.5144000	613586.0000000
17.1166668	5.5632000	613586.0000000
17.1166668	5.6120000	613586.0000000
17.1166668	5.6608000	613586.0000000
17.1166668	5.7096000	613586.0000000
17.1166668	5.7584000	613586.0000000
17.1166668	5.8072000	613586.0000000
17.1166668	5.8559999	613586.0000000
17.1166668	5.9047999	613586.0000000
17.1166668	5.9535999	613586.0000000
17.1166668	6.0023999	613586.0000000
17.1333332	0.2928000	-4.1540203

17.1333332	0.3416000	-3.5833793
17.1333332	0.3904000	-4.2276511
17.1333332	0.4392000	-3.3318064
17.1333332	0.4880000	-2.5954955
17.1333332	0.5368000	-2.0248547
17.1333332	0.5856000	-2.0309906
17.1333332	0.6344000	-1.7732821
17.1333332	0.6832000	-1.7610102
17.1333332	0.7320000	-1.2087770
17.1333332	0.7808000	-1.1965051
17.1333332	0.8296000	-1.4848933
17.1333332	0.8784000	-1.4910293
17.1333332	0.9272000	-1.3744469
17.1333332	0.9760000	-1.6444273
17.1333332	1.0247999	-1.5278449
17.1333332	1.0735999	-1.5707965
17.1333332	1.1223999	-1.6628351
17.1333332	1.1711999	-1.6566992
17.1333332	1.2200000	-1.7487383
17.1333332	1.2688000	-1.7487383
17.1333332	1.3176000	-1.6751070
17.1333332	1.3664000	-1.6628351
17.1333332	1.4152000	-1.6505632
17.1333332	1.4640000	-1.7732821
17.1333332	1.5128000	-1.8346410
17.1333332	1.5616000	-1.8346410
17.1333332	1.6104000	-1.5953403
17.1333332	1.6592000	-1.5830684
17.1333332	1.7079999	-1.5953403
17.1333332	1.7567999	-1.6505632
17.1333332	1.8055999	-1.7426023
17.1333332	1.8543999	-1.6321554
17.1333332	1.9031999	-1.6751070
17.1333332	1.9519999	-1.6689711
17.1333332	2.0007999	-1.8346410
17.1333332	2.0495999	-1.5401168
17.1333332	2.0983999	-1.5462527
17.1333332	2.1471999	-1.6321554
17.1333332	2.1959999	-1.4910293
17.1333332	2.2447999	-1.4358063
17.1333332	2.2935998	-1.3621750
17.1333332	2.3423998	-1.3499031
17.1333332	2.3912001	-1.5953403
17.1333332	2.4400001	-1.4480777
17.1333332	2.4888000	-1.4603496
17.1333332	2.5376000	-1.5033011
17.1333332	2.5864000	-1.5585246
17.1333332	2.6352000	-1.4051266
17.1333332	2.6840000	-1.3928547
17.1333332	2.7328000	-1.4971652
17.1333332	2.7816000	-1.4726214
17.1333332	2.8304000	-1.4726214
17.1333332	2.8792000	-1.4664855
17.1333332	2.9280000	-1.5339808
17.1333332	2.9768000	-1.5339808
17.1333332	3.0256000	-1.4726214
17.1333332	3.0743999	-1.4910293

17.1333332	3.1231999	-1.4910293
17.1333332	3.1719999	-1.2517285
17.1333332	3.2207999	-1.2517285
17.1333332	3.2695999	-1.2394567
17.1333332	3.3183999	-1.1719613
17.1333332	3.3671999	-1.2762718
17.1333332	3.4159999	-1.4051266
17.1333332	3.4647999	-1.2762718
17.1333332	3.5135999	-1.2578645
17.1333332	3.5623999	-1.0799227
17.1333332	3.6111999	-1.2394567
17.1333332	3.6599998	-1.1474175
17.1333332	3.7087998	-1.1412816
17.1333332	3.7575998	-1.1106019
17.1333332	3.8063998	-1.1044660
17.1333332	3.8551998	-1.0799227
17.1333332	3.9039998	-1.0615149
17.1333332	3.9527998	-1.0369711
17.1333332	4.0016003	-0.9142528
17.1333332	4.0503998	-0.9142528
17.1333332	4.0991998	-0.9265246
17.1333332	4.1479998	-0.9326606
17.1333332	4.1967998	-0.9940195
17.1333332	4.2455997	-1.0369711
17.1333332	4.2943997	-1.0308352
17.1333332	4.3431997	-0.9449320
17.1333332	4.3919997	-1.0062914
17.1333332	4.4407997	-0.8958449
17.1333332	4.4895997	-0.7853980
17.1333332	4.5383997	-0.8651652
17.1333332	4.5871997	-0.8713012
17.1333332	4.6359997	-0.8897090
17.1333332	4.6847997	-0.8958449
17.1333332	4.7336001	-0.7179031
17.1333332	4.7824001	-0.6504078
17.1333332	4.8312001	-0.6565437
17.1333332	4.8800001	-0.8038058
17.1333332	4.9288001	-0.7976699
17.1333332	4.9776001	-0.5338254
17.1333332	5.0264001	-0.8099418
17.1333332	5.0752001	-0.4479222
17.1333332	5.1240001	-0.6258640
17.1333332	5.1728001	-0.6320000
17.1333332	5.2216001	-0.6320000
17.1333332	5.2704000	-0.6749516
17.1333332	5.3192000	-0.6626797
17.1333332	5.3680000	613586.0000000
17.1333332	5.4168000	613586.0000000
17.1333332	5.4656000	613586.0000000
17.1333332	5.5144000	613586.0000000
17.1333332	5.5632000	613586.0000000
17.1333332	5.6120000	613586.0000000
17.1333332	5.6608000	613586.0000000
17.1333332	5.7096000	613586.0000000
17.1333332	5.7584000	613586.0000000
17.1333332	5.8072000	613586.0000000
17.1333332	5.8559999	613586.0000000

17.1333332	5.9047999	613586.0000000
17.1333332	5.9535999	613586.0000000
17.1333332	6.0023999	613586.0000000
17.1499996	0.2928000	-3.9453988
17.1499996	0.3416000	-3.9453988
17.1499996	0.3904000	-3.9453988
17.1499996	0.4392000	-3.5281558
17.1499996	0.4880000	-2.6200392
17.1499996	0.5368000	-2.0678062
17.1499996	0.5856000	-2.0862141
17.1499996	0.6344000	-1.5217090
17.1499996	0.6832000	-1.5094371
17.1499996	0.7320000	-1.2578645
17.1499996	0.7808000	-1.5523887
17.1499996	0.8296000	-1.6935148
17.1499996	0.8784000	-1.8039613
17.1499996	0.9272000	-2.0555344
17.1499996	0.9760000	-1.8837285
17.1499996	1.0247999	-1.9205441
17.1499996	1.0735999	-1.9266801
17.1499996	1.1223999	-1.8714566
17.1499996	1.1711999	-1.7303305
17.1499996	1.2200000	-1.6628351
17.1499996	1.2688000	-1.6505632
17.1499996	1.3176000	-1.7794180
17.1499996	1.3664000	-1.7916894
17.1499996	1.4152000	-1.7364664
17.1499996	1.4640000	-1.8100972
17.1499996	1.5128000	-1.8039613
17.1499996	1.5616000	-1.6260195
17.1499996	1.6104000	-1.6198835
17.1499996	1.6592000	-1.7241945
17.1499996	1.7079999	-1.7180586
17.1499996	1.7567999	-1.3560390
17.1499996	1.8055999	-1.3928547
17.1499996	1.8543999	-1.3867188
17.1499996	1.9031999	-1.5339808
17.1499996	1.9519999	-1.4726214
17.1499996	2.0007999	-1.4480777
17.1499996	2.0495999	-1.4112625
17.1499996	2.0983999	-1.4173985
17.1499996	2.1471999	-1.4112625
17.1499996	2.1959999	-1.5707965
17.1499996	2.2447999	-1.3928547
17.1499996	2.2935998	-1.5953403
17.1499996	2.3423998	-1.5953403
17.1499996	2.3912001	-1.5401168
17.1499996	2.4400001	-1.4726214
17.1499996	2.4888000	-1.4603496
17.1499996	2.5376000	-1.4603496
17.1499996	2.5864000	-1.4173985
17.1499996	2.6352000	-1.5462527
17.1499996	2.6840000	-1.5401168
17.1499996	2.7328000	-1.6566992
17.1499996	2.7816000	-1.6566992
17.1499996	2.8304000	-0.5951843
17.1499996	2.8792000	-1.7057867

17.1499996	2.9280000	-1.6198835
17.1499996	2.9768000	-1.6014762
17.1499996	3.0256000	-1.4848933
17.1499996	3.0743999	-1.4358063
17.1499996	3.1231999	-1.4480777
17.1499996	3.1719999	-1.4542136
17.1499996	3.2207999	-1.3253593
17.1499996	3.2695999	-1.3069515
17.1499996	3.3183999	-1.3069515
17.1499996	3.3671999	-1.3008156
17.1499996	3.4159999	-1.1658254
17.1499996	3.4647999	-1.3499031
17.1499996	3.5135999	-1.3499031
17.1499996	3.5623999	-1.2333207
17.1499996	3.6111999	-1.2333207
17.1499996	3.6599998	-1.1596894
17.1499996	3.7087998	-1.1228738
17.1499996	3.7575998	-0.9387960
17.1499996	3.8063998	-1.0615149
17.1499996	3.8551998	-1.0676508
17.1499996	3.9039998	-0.8344855
17.1499996	3.9527998	-0.8344855
17.1499996	4.0016003	-0.8467574
17.1499996	4.0503998	-0.8406215
17.1499996	4.0991998	-0.9633398
17.1499996	4.1479998	-0.7179031
17.1499996	4.1967998	-0.7117672
17.1499996	4.2455997	-0.7363110
17.1499996	4.2943997	-0.6872234
17.1499996	4.3431997	-0.6994953
17.1499996	4.3919997	-0.7179031
17.1499996	4.4407997	-0.7547188
17.1499996	4.4895997	-0.6994953
17.1499996	4.5383997	-0.6994953
17.1499996	4.5871997	-0.7117672
17.1499996	4.6359997	-0.7117672
17.1499996	4.6847997	-0.6074562
17.1499996	4.7336001	-0.6013203
17.1499996	4.7824001	-0.6442719
17.1499996	4.8312001	-0.5829129
17.1499996	4.8800001	-0.6810875
17.1499996	4.9288001	-0.8038058
17.1499996	4.9776001	-0.8038058
17.1499996	5.0264001	-0.7976699
17.1499996	5.0752001	-0.6749516
17.1499996	5.1240001	-0.6933594
17.1499996	5.1728001	-0.5276895
17.1499996	5.2216001	-0.5399613
17.1499996	5.2704000	-0.5460973
17.1499996	5.3192000	613586.0000000
17.1499996	5.3680000	613586.0000000
17.1499996	5.4168000	613586.0000000
17.1499996	5.4656000	613586.0000000
17.1499996	5.5144000	613586.0000000
17.1499996	5.5632000	613586.0000000
17.1499996	5.6120000	613586.0000000
17.1499996	5.6608000	613586.0000000

17.1499996	5.7096000	613586.0000000
17.1499996	5.7584000	613586.0000000
17.1499996	5.8072000	613586.0000000
17.1499996	5.8559999	613586.0000000
17.1499996	5.9047999	613586.0000000
17.1499996	5.9535999	613586.0000000
17.1499996	6.0023999	613586.0000000
17.1666660	0.2928000	-4.2031074
17.1666660	0.3416000	-4.2092433
17.1666660	0.3904000	-3.5220201
17.1666660	0.4392000	-3.3379424
17.1666660	0.4880000	-2.2273402
17.1666660	0.5368000	-2.3684664
17.1666660	0.5856000	-2.0003109
17.1666660	0.6344000	-2.0003109
17.1666660	0.6832000	-1.9941750
17.1666660	0.7320000	-1.5585246
17.1666660	0.7808000	-1.9819031
17.1666660	0.8296000	-1.8223691
17.1666660	0.8784000	-1.9880390
17.1666660	0.9272000	-1.9880390
17.1666660	0.9760000	-1.9021363
17.1666660	1.0247999	-1.9021363
17.1666660	1.0735999	-1.8960004
17.1666660	1.1223999	-1.9144082
17.1666660	1.1711999	-1.9634953
17.1666660	1.2200000	-1.9144082
17.1666660	1.2688000	-1.6935148
17.1666660	1.3176000	-1.6996508
17.1666660	1.3664000	-1.6996508
17.1666660	1.4152000	-1.7057867
17.1666660	1.4640000	-1.7057867
17.1666660	1.5128000	-1.6260195
17.1666660	1.5616000	-1.6505632
17.1666660	1.6104000	-1.6689711
17.1666660	1.6592000	-1.6935148
17.1666660	1.7079999	-1.5155730
17.1666660	1.7567999	-1.4112625
17.1666660	1.8055999	-1.2578645
17.1666660	1.8543999	-1.2885437
17.1666660	1.9031999	-1.3683109
17.1666660	1.9519999	-1.3805828
17.1666660	2.0007999	-1.3867188
17.1666660	2.0495999	-1.4726214
17.1666660	2.0983999	-1.4726214
17.1666660	2.1471999	-1.5278449
17.1666660	2.1959999	-1.3683109
17.1666660	2.2447999	-1.3683109
17.1666660	2.2935998	-1.4664855
17.1666660	2.3423998	-1.5339808
17.1666660	2.3912001	-1.3867188
17.1666660	2.4400001	-1.5094371
17.1666660	2.4888000	-1.5094371
17.1666660	2.5376000	-1.6505632
17.1666660	2.5864000	-1.5094371
17.1666660	2.6352000	-1.3744469
17.1666660	2.6840000	-1.6566992

17.1666660	2.7328000	-1.5646605
17.1666660	2.7816000	-1.5033011
17.1666660	2.8304000	-1.4787574
17.1666660	2.8792000	-1.3008156
17.1666660	2.9280000	-1.2946796
17.1666660	2.9768000	-1.2517285
17.1666660	3.0256000	-1.1658254
17.1666660	3.0743999	-1.3253593
17.1666660	3.1231999	-0.9449320
17.1666660	3.1719999	-0.9449320
17.1666660	3.2207999	-0.9326606
17.1666660	3.2695999	-1.0124273
17.1666660	3.3183999	-0.8958449
17.1666660	3.3671999	-0.8958449
17.1666660	3.4159999	-0.7792621
17.1666660	3.4647999	-0.7547188
17.1666660	3.5135999	-0.6626797
17.1666660	3.5623999	-0.8099418
17.1666660	3.6111999	-0.9633398
17.1666660	3.6599998	-0.9019809
17.1666660	3.7087998	-0.9510679
17.1666660	3.7575998	-0.9449320
17.1666660	3.8063998	-0.9326606
17.1666660	3.8551998	-0.8038058
17.1666660	3.9039998	-0.8283496
17.1666660	3.9527998	-0.7547188
17.1666660	4.0016003	-0.7424469
17.1666660	4.0503998	-0.6933594
17.1666660	4.0991998	-0.8038058
17.1666660	4.1479998	-0.8958449
17.1666660	4.1967998	-0.7485828
17.1666660	4.2455997	-0.7485828
17.1666660	4.2943997	-0.7301750
17.1666660	4.3431997	-0.7117672
17.1666660	4.3919997	-0.7056313
17.1666660	4.4407997	-0.6810875
17.1666660	4.4895997	-0.6135921
17.1666660	4.5383997	-0.6933594
17.1666660	4.5871997	-0.6933594
17.1666660	4.6359997	-0.5645051
17.1666660	4.6847997	-0.6320000
17.1666660	4.7336001	-0.4663301
17.1666660	4.7824001	-0.5154176
17.1666660	4.8312001	-0.3252039
17.1666660	4.8800001	-0.3252039
17.1666660	4.9288001	-0.4601941
17.1666660	4.9776001	-0.3006601
17.1666660	5.0264001	-0.3804274
17.1666660	5.0752001	-0.4049711
17.1666660	5.1240001	-0.3497477
17.1666660	5.1728001	-0.3558836
17.1666660	5.2216001	-0.4111071
17.1666660	5.2704000	-0.2208934
17.1666660	5.3192000	613586.0000000
17.1666660	5.3680000	613586.0000000
17.1666660	5.4168000	613586.0000000
17.1666660	5.4656000	613586.0000000

17.1666660	5.5144000	613586.0000000
17.1666660	5.5632000	613586.0000000
17.1666660	5.6120000	613586.0000000
17.1666660	5.6608000	613586.0000000
17.1666660	5.7096000	613586.0000000
17.1666660	5.7584000	613586.0000000
17.1666660	5.8072000	613586.0000000
17.1666660	5.8559999	613586.0000000
17.1666660	5.9047999	613586.0000000
17.1666660	5.9535999	613586.0000000
17.1666660	6.0023999	613586.0000000
17.1833324	0.2928000	-3.9699423
17.1833324	0.3416000	-4.0190296
17.1833324	0.3904000	-4.0251656
17.1833324	0.4392000	-2.8900199
17.1833324	0.4880000	-2.5034568
17.1833324	0.5368000	-2.1782527
17.1833324	0.5856000	-1.8960004
17.1833324	0.6344000	-1.7610102
17.1833324	0.6832000	-1.7241945
17.1833324	0.7320000	-2.0125828
17.1833324	0.7808000	-1.5278449
17.1833324	0.8296000	-1.7916894
17.1833324	0.8784000	-2.0923500
17.1833324	0.9272000	-2.0862141
17.1833324	0.9760000	-1.8898644
17.1833324	1.0247999	-1.8898644
17.1833324	1.0735999	-1.8223691
17.1833324	1.1223999	-1.7487383
17.1833324	1.1711999	-1.7487383
17.1833324	1.2200000	-1.7855535
17.1833324	1.2688000	-1.6505632
17.1833324	1.3176000	-1.5462527
17.1833324	1.3664000	-1.5585246
17.1833324	1.4152000	-1.5585246
17.1833324	1.4640000	-1.5278449
17.1833324	1.5128000	-1.6628351
17.1833324	1.5616000	-1.6873789
17.1833324	1.6104000	-1.6260195
17.1833324	1.6592000	-1.6198835
17.1833324	1.7079999	-1.8346410
17.1833324	1.7567999	-1.6444273
17.1833324	1.8055999	-2.0678062
17.1833324	1.8543999	-1.6444273
17.1833324	1.9031999	-1.5707965
17.1833324	1.9519999	-1.4480777
17.1833324	2.0007999	-1.6444273
17.1833324	2.0495999	-1.5462527
17.1833324	2.0983999	-1.4787574
17.1833324	2.1471999	-1.4910293
17.1833324	2.1959999	-1.4419422
17.1833324	2.2447999	-1.4112625
17.1833324	2.2935998	-1.4051266
17.1833324	2.3423998	-1.4971652
17.1833324	2.3912001	-1.5278449
17.1833324	2.4400001	-1.5217090
17.1833324	2.4888000	-1.4296703

17.1833324	2.5376000	-1.6505632
17.1833324	2.5864000	-1.4480777
17.1833324	2.6352000	-0.6135921
17.1833324	2.6840000	-0.6074562
17.1833324	2.7328000	-0.5092816
17.1833324	2.7816000	-1.4787574
17.1833324	2.8304000	-1.2885437
17.1833324	2.8792000	-1.2701364
17.1833324	2.9280000	-1.1596894
17.1833324	2.9768000	-1.1044660
17.1833324	3.0256000	-1.1842332
17.1833324	3.0743999	-1.1842332
17.1833324	3.1231999	-1.1474175
17.1833324	3.1719999	-0.9633398
17.1833324	3.2207999	-0.9203887
17.1833324	3.2695999	-0.9633398
17.1833324	3.3183999	-0.9572039
17.1833324	3.3671999	-1.0185633
17.1833324	3.4159999	-0.7853980
17.1833324	3.4647999	-0.8467574
17.1833324	3.5135999	-0.8774371
17.1833324	3.5623999	-0.9387960
17.1833324	3.6111999	-0.9387960
17.1833324	3.6599998	-0.9817476
17.1833324	3.7087998	-0.9940195
17.1833324	3.7575998	-0.8774371
17.1833324	3.8063998	-0.9694757
17.1833324	3.8551998	-0.8344855
17.1833324	3.9039998	-0.8467574
17.1833324	3.9527998	-0.8467574
17.1833324	4.0016003	-0.7669902
17.1833324	4.0503998	-0.7669902
17.1833324	4.0991998	-0.7731261
17.1833324	4.1479998	-0.7792621
17.1833324	4.1967998	-0.7976699
17.1833324	4.2455997	-0.7056313
17.1833324	4.2943997	-0.6994953
17.1833324	4.3431997	-0.6994953
17.1833324	4.3919997	-0.8897090
17.1833324	4.4407997	-0.6442719
17.1833324	4.4895997	-0.7363110
17.1833324	4.5383997	-0.7424469
17.1833324	4.5871997	-0.6565437
17.1833324	4.6359997	-0.6749516
17.1833324	4.6847997	-0.6749516
17.1833324	4.7336001	-0.7853980
17.1833324	4.7824001	-0.7117672
17.1833324	4.8312001	-0.6933594
17.1833324	4.8800001	-0.5399613
17.1833324	4.9288001	-0.5399613
17.1833324	4.9776001	-0.5399613
17.1833324	5.0264001	-0.6074562
17.1833324	5.0752001	-0.5767770
17.1833324	5.1240001	-0.5767770
17.1833324	5.1728001	-0.5645051
17.1833324	5.2216001	613586.0000000
17.1833324	5.2704000	613586.0000000

17.1833324	5.3192000	613586.0000000
17.1833324	5.3680000	613586.0000000
17.1833324	5.4168000	613586.0000000
17.1833324	5.4656000	613586.0000000
17.1833324	5.5144000	613586.0000000
17.1833324	5.5632000	613586.0000000
17.1833324	5.6120000	613586.0000000
17.1833324	5.6608000	613586.0000000
17.1833324	5.7096000	613586.0000000
17.1833324	5.7584000	613586.0000000
17.1833324	5.8072000	613586.0000000
17.1833324	5.8559999	613586.0000000
17.1833324	5.9047999	613586.0000000
17.1833324	5.9535999	613586.0000000
17.1833324	6.0023999	613586.0000000
17.2000008	0.2928000	-3.6876900
17.2000008	0.3416000	-3.7306414
17.2000008	0.3904000	-3.7060976
17.2000008	0.4392000	-3.5526996
17.2000008	0.4880000	-3.0066025
17.2000008	0.5368000	-2.0616703
17.2000008	0.5856000	-1.7978253
17.2000008	0.6344000	-1.7303305
17.2000008	0.6832000	-1.5278449
17.2000008	0.7320000	-1.5523887
17.2000008	0.7808000	-1.7610102
17.2000008	0.8296000	-1.7610102
17.2000008	0.8784000	-1.7487383
17.2000008	0.9272000	-1.8714566
17.2000008	0.9760000	-1.6076121
17.2000008	1.0247999	-1.6628351
17.2000008	1.0735999	-1.5155730
17.2000008	1.1223999	-1.5523887
17.2000008	1.1711999	-1.7180586
17.2000008	1.2200000	-1.7119226
17.2000008	1.2688000	-1.7364664
17.2000008	1.3176000	-1.5462527
17.2000008	1.3664000	-1.6198835
17.2000008	1.4152000	-1.6137476
17.2000008	1.4640000	-1.6014762
17.2000008	1.5128000	-1.7119226
17.2000008	1.5616000	-1.5769324
17.2000008	1.6104000	-1.4664855
17.2000008	1.6592000	-1.3928547
17.2000008	1.7079999	-1.8346410
17.2000008	1.7567999	-1.8285050
17.2000008	1.8055999	-1.5769324
17.2000008	1.8543999	-1.6628351
17.2000008	1.9031999	-1.6628351
17.2000008	1.9519999	-1.5707965
17.2000008	2.0007999	-1.5585246
17.2000008	2.0495999	-1.3989906
17.2000008	2.0983999	-1.3989906
17.2000008	2.1471999	-1.5769324
17.2000008	2.1959999	-1.4112625
17.2000008	2.2447999	-1.4664855
17.2000008	2.2935998	-1.4664855

17.2000008	2.3423998	-1.4787574
17.2000008	2.3912001	-0.6872234
17.2000008	2.4400001	-0.6810875
17.2000008	2.4888000	-0.6994953
17.2000008	2.5376000	-1.3130875
17.2000008	2.5864000	-1.3253593
17.2000008	2.6352000	-1.3376312
17.2000008	2.6840000	-1.2394567
17.2000008	2.7328000	-1.2394567
17.2000008	2.7816000	-1.2517285
17.2000008	2.8304000	-1.0799227
17.2000008	2.8792000	-1.0553789
17.2000008	2.9280000	-1.0553789
17.2000008	2.9768000	-1.0492430
17.2000008	3.0256000	-1.0553789
17.2000008	3.0743999	-0.9265246
17.2000008	3.1231999	-0.9694757
17.2000008	3.1719999	-0.9694757
17.2000008	3.2207999	-0.9633398
17.2000008	3.2695999	-1.0799227
17.2000008	3.3183999	-1.0921946
17.2000008	3.3671999	-1.0983305
17.2000008	3.4159999	-1.2026410
17.2000008	3.4647999	-1.2087770
17.2000008	3.5135999	-1.0062914
17.2000008	3.5623999	-1.0492430
17.2000008	3.6111999	-1.0369711
17.2000008	3.6599998	-0.9694757
17.2000008	3.7087998	-0.9142528
17.2000008	3.7575998	-0.7669902
17.2000008	3.8063998	-0.7853980
17.2000008	3.8551998	-0.7976699
17.2000008	3.9039998	-0.8099418
17.2000008	3.9527998	-0.7240391
17.2000008	4.0016003	-0.7240391
17.2000008	4.0503998	-0.6197281
17.2000008	4.0991998	-0.5890489
17.2000008	4.1479998	-0.6626797
17.2000008	4.1967998	-0.6688156
17.2000008	4.2455997	-0.7363110
17.2000008	4.2943997	-0.7363110
17.2000008	4.3431997	-0.6872234
17.2000008	4.3919997	-0.4970098
17.2000008	4.4407997	-0.5583692
17.2000008	4.4895997	-0.5276895
17.2000008	4.5383997	-0.4417863
17.2000008	4.5871997	-0.5154176
17.2000008	4.6359997	-0.3926992
17.2000008	4.6847997	-0.3926992
17.2000008	4.7336001	-0.4970098
17.2000008	4.7824001	-0.3804274
17.2000008	4.8312001	-0.3804274
17.2000008	4.8800001	-0.4417863
17.2000008	4.9288001	-0.3190680
17.2000008	4.9776001	-0.2454371
17.2000008	5.0264001	-0.3681555
17.2000008	5.0752001	-0.3681555

17.2000008	5.1240001	-0.3436117
17.2000008	5.1728001	613586.0000000
17.2000008	5.2216001	613586.0000000
17.2000008	5.2704000	613586.0000000
17.2000008	5.3192000	613586.0000000
17.2000008	5.3680000	613586.0000000
17.2000008	5.4168000	613586.0000000
17.2000008	5.4656000	613586.0000000
17.2000008	5.5144000	613586.0000000
17.2000008	5.5632000	613586.0000000
17.2000008	5.6120000	613586.0000000
17.2000008	5.6608000	613586.0000000
17.2000008	5.7096000	613586.0000000
17.2000008	5.7584000	613586.0000000
17.2000008	5.8072000	613586.0000000
17.2000008	5.8559999	613586.0000000
17.2000008	5.9047999	613586.0000000
17.2000008	5.9535999	613586.0000000
17.2000008	6.0023999	613586.0000000
17.2166672	0.2928000	-3.6324666
17.2166672	0.3416000	-4.2767386
17.2166672	0.3904000	-3.6263306
17.2166672	0.4392000	-3.5404277
17.2166672	0.4880000	-2.2150683
17.2166672	0.5368000	-1.8898644
17.2166672	0.5856000	-2.1537089
17.2166672	0.6344000	-1.8162332
17.2166672	0.6832000	-1.8960004
17.2166672	0.7320000	-1.9021363
17.2166672	0.7808000	-1.7548742
17.2166672	0.8296000	-1.8898644
17.2166672	0.8784000	-1.8837285
17.2166672	0.9272000	-1.9021363
17.2166672	0.9760000	-1.9021363
17.2166672	1.0247999	-1.8530488
17.2166672	1.0735999	-1.8530488
17.2166672	1.1223999	-1.6505632
17.2166672	1.1711999	-1.6996508
17.2166672	1.2200000	-1.6873789
17.2166672	1.2688000	-1.6935148
17.2166672	1.3176000	-1.8285050
17.2166672	1.3664000	-1.8285050
17.2166672	1.4152000	-1.7057867
17.2166672	1.4640000	-1.5033011
17.2166672	1.5128000	-1.3989906
17.2166672	1.5616000	-1.4787574
17.2166672	1.6104000	-1.5094371
17.2166672	1.6592000	-1.5033011
17.2166672	1.7079999	-1.4848933
17.2166672	1.7567999	-1.4787574
17.2166672	1.8055999	-1.5585246
17.2166672	1.8543999	-1.5585246
17.2166672	1.9031999	-1.4603496
17.2166672	1.9519999	-1.6996508
17.2166672	2.0007999	-1.5707965
17.2166672	2.0495999	-1.4910293
17.2166672	2.0983999	-1.4664855

17.2166672	2.1471999	-1.4480777
17.2166672	2.1959999	-1.4848933
17.2166672	2.2447999	-1.4848933
17.2166672	2.2935998	-1.4726214
17.2166672	2.3423998	-0.8590293
17.2166672	2.3912001	-0.8651652
17.2166672	2.4400001	-0.7117672
17.2166672	2.4888000	-1.3805828
17.2166672	2.5376000	-1.3437672
17.2166672	2.5864000	-1.3437672
17.2166672	2.6352000	-1.1842332
17.2166672	2.6840000	-1.1842332
17.2166672	2.7328000	-1.2210488
17.2166672	2.7816000	-1.2087770
17.2166672	2.8304000	-1.2149129
17.2166672	2.8792000	-1.1412816
17.2166672	2.9280000	-1.1412816
17.2166672	2.9768000	-1.2517285
17.2166672	3.0256000	-1.2455926
17.2166672	3.0743999	-1.2640004
17.2166672	3.1231999	-1.0921946
17.2166672	3.1719999	-1.1044660
17.2166672	3.2207999	-1.0124273
17.2166672	3.2695999	-1.0124273
17.2166672	3.3183999	-1.0124273
17.2166672	3.3671999	-1.0246992
17.2166672	3.4159999	-0.9633398
17.2166672	3.4647999	-0.9510679
17.2166672	3.5135999	-0.9449320
17.2166672	3.5623999	-0.8222136
17.2166672	3.6111999	-0.8406215
17.2166672	3.6599998	-0.9387960
17.2166672	3.7087998	-0.9449320
17.2166672	3.7575998	-0.9572039
17.2166672	3.8063998	-0.8958449
17.2166672	3.8551998	-0.8958449
17.2166672	3.9039998	-0.8897090
17.2166672	3.9527998	-0.8835731
17.2166672	4.0016003	-0.6626797
17.2166672	4.0503998	-0.7915339
17.2166672	4.0991998	-0.8344855
17.2166672	4.1479998	-0.5583692
17.2166672	4.1967998	-0.5951843
17.2166672	4.2455997	-0.6994953
17.2166672	4.2943997	-0.6258640
17.2166672	4.3431997	-0.6197281
17.2166672	4.3919997	-0.7056313
17.2166672	4.4407997	-0.5522332
17.2166672	4.4895997	-0.6074562
17.2166672	4.5383997	-0.5215535
17.2166672	4.5871997	-0.6197281
17.2166672	4.6359997	-0.6810875
17.2166672	4.6847997	-0.5154176
17.2166672	4.7336001	-0.5215535
17.2166672	4.7824001	-0.3620195
17.2166672	4.8312001	-0.6197281
17.2166672	4.8800001	-0.3252039

17.2166672	4.9288001	-0.3129320
17.2166672	4.9776001	-0.3190680
17.2166672	5.0264001	-0.4172430
17.2166672	5.0752001	-0.4111071
17.2166672	5.1240001	-0.1533980
17.2166672	5.1728001	-0.1533980
17.2166672	5.2216001	613586.0000000
17.2166672	5.2704000	613586.0000000
17.2166672	5.3192000	613586.0000000
17.2166672	5.3680000	613586.0000000
17.2166672	5.4168000	613586.0000000
17.2166672	5.4656000	613586.0000000
17.2166672	5.5144000	613586.0000000
17.2166672	5.5632000	613586.0000000
17.2166672	5.6120000	613586.0000000
17.2166672	5.6608000	613586.0000000
17.2166672	5.7096000	613586.0000000
17.2166672	5.7584000	613586.0000000
17.2166672	5.8072000	613586.0000000
17.2166672	5.8559999	613586.0000000
17.2166672	5.9047999	613586.0000000
17.2166672	5.9535999	613586.0000000
17.2166672	6.0023999	613586.0000000
17.2333336	0.2928000	-3.9331269
17.2333336	0.3416000	-3.9453988
17.2333336	0.3904000	-3.9515345
17.2333336	0.4392000	-3.9576705
17.2333336	0.4880000	-2.6936703
17.2333336	0.5368000	-2.0432625
17.2333336	0.5856000	-2.0432625
17.2333336	0.6344000	-2.0432625
17.2333336	0.6832000	-1.8346410
17.2333336	0.7320000	-1.8346410
17.2333336	0.7808000	-1.6014762
17.2333336	0.8296000	-1.6444273
17.2333336	0.8784000	-1.6812429
17.2333336	0.9272000	-1.6812429
17.2333336	0.9760000	-1.9696312
17.2333336	1.0247999	-1.9696312
17.2333336	1.0735999	-1.8653207
17.2333336	1.1223999	-1.8469129
17.2333336	1.1711999	-1.9328160
17.2333336	1.2200000	-1.7426023
17.2333336	1.2688000	-1.7610102
17.2333336	1.3176000	-1.8100972
17.2333336	1.3664000	-1.8100972
17.2333336	1.4152000	-1.7732821
17.2333336	1.4640000	-1.6566992
17.2333336	1.5128000	-1.6505632
17.2333336	1.5616000	-1.6505632
17.2333336	1.6104000	-1.4787574
17.2333336	1.6592000	-1.4910293
17.2333336	1.7079999	-1.4971652
17.2333336	1.7567999	-1.2394567
17.2333336	1.8055999	-1.6260195
17.2333336	1.8543999	-1.4664855
17.2333336	1.9031999	-1.3437672

17.2333336	1.9519999	-1.3560390
17.2333336	2.0007999	-1.6260195
17.2333336	2.0495999	-1.6321554
17.2333336	2.0983999	-1.5707965
17.2333336	2.1471999	-1.6076121
17.2333336	2.1959999	-1.5155730
17.2333336	2.2447999	-1.5217090
17.2333336	2.2935998	-1.5339808
17.2333336	2.3423998	-1.4419422
17.2333336	2.3912001	-1.4603496
17.2333336	2.4400001	-1.3928547
17.2333336	2.4888000	-0.7669902
17.2333336	2.5376000	-1.2394567
17.2333336	2.5864000	-1.4051266
17.2333336	2.6352000	-1.3008156
17.2333336	2.6840000	-1.3008156
17.2333336	2.7328000	-1.1719613
17.2333336	2.7816000	-0.4908738
17.2333336	2.8304000	-1.2946796
17.2333336	2.8792000	-1.1965051
17.2333336	2.9280000	-1.1044660
17.2333336	2.9768000	-1.0983305
17.2333336	3.0256000	-1.1903691
17.2333336	3.0743999	-0.9817476
17.2333336	3.1231999	-0.9265246
17.2333336	3.1719999	-0.9449320
17.2333336	3.2207999	-0.9817476
17.2333336	3.2695999	-0.9817476
17.2333336	3.3183999	-1.0431070
17.2333336	3.3671999	-0.9081168
17.2333336	3.4159999	-0.9142528
17.2333336	3.4647999	-0.9142528
17.2333336	3.5135999	-0.9142528
17.2333336	3.5623999	-0.8160777
17.2333336	3.6111999	-0.8590293
17.2333336	3.6599998	-0.9940195
17.2333336	3.7087998	-0.8897090
17.2333336	3.7575998	-0.8958449
17.2333336	3.8063998	-0.7179031
17.2333336	3.8551998	-0.6504078
17.2333336	3.9039998	-0.7547188
17.2333336	3.9527998	-0.7547188
17.2333336	4.0016003	-0.7117672
17.2333336	4.0503998	-0.7301750
17.2333336	4.0991998	-0.6504078
17.2333336	4.1479998	-0.6381359
17.2333336	4.1967998	-0.6626797
17.2333336	4.2455997	-0.6749516
17.2333336	4.2943997	-0.6810875
17.2333336	4.3431997	-0.5460973
17.2333336	4.3919997	-0.6197281
17.2333336	4.4407997	-0.6320000
17.2333336	4.4895997	-0.6381359
17.2333336	4.5383997	-0.5706410
17.2333336	4.5871997	-0.5645051
17.2333336	4.6359997	-0.5031457
17.2333336	4.6847997	-0.4970098

17.2333336	4.7336001	-0.5092816
17.2333336	4.7824001	-0.5031457
17.2333336	4.8312001	-0.4417863
17.2333336	4.8800001	-0.5583692
17.2333336	4.9288001	-0.4417863
17.2333336	4.9776001	-0.4417863
17.2333336	5.0264001	-0.3252039
17.2333336	5.0752001	613586.0000000
17.2333336	5.1240001	613586.0000000
17.2333336	5.1728001	613586.0000000
17.2333336	5.2216001	613586.0000000
17.2333336	5.2704000	613586.0000000
17.2333336	5.3192000	613586.0000000
17.2333336	5.3680000	613586.0000000
17.2333336	5.4168000	613586.0000000
17.2333336	5.4656000	613586.0000000
17.2333336	5.5144000	613586.0000000
17.2333336	5.5632000	613586.0000000
17.2333336	5.6120000	613586.0000000
17.2333336	5.6608000	613586.0000000
17.2333336	5.7096000	613586.0000000
17.2333336	5.7584000	613586.0000000
17.2333336	5.8072000	613586.0000000
17.2333336	5.8559999	613586.0000000
17.2333336	5.9047999	613586.0000000
17.2333336	5.9535999	613586.0000000
17.2333336	6.0023999	613586.0000000
17.2500000	0.2928000	-4.4976320
17.2500000	0.3416000	-4.5037680
17.2500000	0.3904000	-3.2827189
17.2500000	0.4392000	-3.3686218
17.2500000	0.4880000	-2.9513791
17.2500000	0.5368000	-2.1291652
17.2500000	0.5856000	-1.9205441
17.2500000	0.6344000	-1.9144082
17.2500000	0.6832000	-1.4358063
17.2500000	0.7320000	-1.4419422
17.2500000	0.7808000	-1.5523887
17.2500000	0.8296000	-1.9021363
17.2500000	0.8784000	-1.9082723
17.2500000	0.9272000	-1.9205441
17.2500000	0.9760000	-1.9144082
17.2500000	1.0247999	-1.8898644
17.2500000	1.0735999	-1.8039613
17.2500000	1.1223999	-1.8223691
17.2500000	1.1711999	-1.8837285
17.2500000	1.2200000	-1.7426023
17.2500000	1.2688000	-1.7057867
17.2500000	1.3176000	-1.6321554
17.2500000	1.3664000	-1.6444273
17.2500000	1.4152000	-1.8346410
17.2500000	1.4640000	-1.6873789
17.2500000	1.5128000	-1.9634953
17.2500000	1.5616000	-1.5339808
17.2500000	1.6104000	-1.6260195
17.2500000	1.6592000	-1.6260195
17.2500000	1.7079999	-1.6628351

17.2500000	1.7567999	-1.6014762
17.2500000	1.8055999	-1.7057867
17.2500000	1.8543999	-1.6996508
17.2500000	1.9031999	-1.5339808
17.2500000	1.9519999	-1.5462527
17.2500000	2.0007999	-1.5646605
17.2500000	2.0495999	-1.6382914
17.2500000	2.0983999	-1.5769324
17.2500000	2.1471999	-0.7669902
17.2500000	2.1959999	-1.3928547
17.2500000	2.2447999	-1.3928547
17.2500000	2.2935998	-1.3867188
17.2500000	2.3423998	-1.4296703
17.2500000	2.3912001	-1.4296703
17.2500000	2.4400001	-1.3805828
17.2500000	2.4888000	-1.1351457
17.2500000	2.5376000	-1.2885437
17.2500000	2.5864000	-1.2946796
17.2500000	2.6352000	-1.3683109
17.2500000	2.6840000	-1.1535535
17.2500000	2.7328000	-1.1596894
17.2500000	2.7816000	-1.1596894
17.2500000	2.8304000	-1.0492430
17.2500000	2.8792000	-0.9878836
17.2500000	2.9280000	-1.1965051
17.2500000	2.9768000	-0.9694757
17.2500000	3.0256000	-0.9817476
17.2500000	3.0743999	-0.8283496
17.2500000	3.1231999	-0.8222136
17.2500000	3.1719999	-0.8222136
17.2500000	3.2207999	-0.8222136
17.2500000	3.2695999	-0.7179031
17.2500000	3.3183999	-0.7363110
17.2500000	3.3671999	-0.7485828
17.2500000	3.4159999	-0.7363110
17.2500000	3.4647999	-0.7424469
17.2500000	3.5135999	-0.7547188
17.2500000	3.5623999	-0.6626797
17.2500000	3.6111999	-0.7056313
17.2500000	3.6599998	-0.8038058
17.2500000	3.7087998	-0.8160777
17.2500000	3.7575998	-0.8099418
17.2500000	3.8063998	-0.6504078
17.2500000	3.8551998	-0.6442719
17.2500000	3.9039998	-0.6442719
17.2500000	3.9527998	-0.6442719
17.2500000	4.0016003	-0.6197281
17.2500000	4.0503998	-0.6504078
17.2500000	4.0991998	-0.6504078
17.2500000	4.1479998	-0.6381359
17.2500000	4.1967998	-0.6258640
17.2500000	4.2455997	-0.6320000
17.2500000	4.2943997	-0.6381359
17.2500000	4.3431997	-0.5706410
17.2500000	4.3919997	-0.5706410
17.2500000	4.4407997	-0.5706410
17.2500000	4.4895997	-0.7056313

17.2500000	4.5383997	-0.5338254
17.2500000	4.5871997	-0.4847379
17.2500000	4.6359997	-0.4908738
17.2500000	4.6847997	-0.4970098
17.2500000	4.7336001	-0.3804274
17.2500000	4.7824001	-0.6810875
17.2500000	4.8312001	-0.3620195
17.2500000	4.8800001	-0.3620195
17.2500000	4.9288001	-0.4356503
17.2500000	4.9776001	-0.4356503
17.2500000	5.0264001	-0.4233789
17.2500000	5.0752001	613586.0000000
17.2500000	5.1240001	613586.0000000
17.2500000	5.1728001	613586.0000000
17.2500000	5.2216001	613586.0000000
17.2500000	5.2704000	613586.0000000
17.2500000	5.3192000	613586.0000000
17.2500000	5.3680000	613586.0000000
17.2500000	5.4168000	613586.0000000
17.2500000	5.4656000	613586.0000000
17.2500000	5.5144000	613586.0000000
17.2500000	5.5632000	613586.0000000
17.2500000	5.6120000	613586.0000000
17.2500000	5.6608000	613586.0000000
17.2500000	5.7096000	613586.0000000
17.2500000	5.7584000	613586.0000000
17.2500000	5.8072000	613586.0000000
17.2500000	5.8559999	613586.0000000
17.2500000	5.9047999	613586.0000000
17.2500000	5.9535999	613586.0000000
17.2500000	6.0023999	613586.0000000
17.2666664	0.2928000	-3.2949908
17.2666664	0.3416000	-3.5588355
17.2666664	0.3904000	-3.5588355
17.2666664	0.4392000	-3.9085832
17.2666664	0.4880000	-2.4420974
17.2666664	0.5368000	-1.8653207
17.2666664	0.5856000	-1.8591847
17.2666664	0.6344000	-1.8530488
17.2666664	0.6832000	-1.7487383
17.2666664	0.7320000	-1.7610102
17.2666664	0.7808000	-1.5769324
17.2666664	0.8296000	-1.9757671
17.2666664	0.8784000	-1.9880390
17.2666664	0.9272000	-1.8530488
17.2666664	0.9760000	-1.8469129
17.2666664	1.0247999	-1.8591847
17.2666664	1.0735999	-1.8591847
17.2666664	1.1223999	-1.8100972
17.2666664	1.1711999	-1.6689711
17.2666664	1.2200000	-1.7364664
17.2666664	1.2688000	-1.7364664
17.2666664	1.3176000	-1.7303305
17.2666664	1.3664000	-1.7487383
17.2666664	1.4152000	-1.5155730
17.2666664	1.4640000	-1.9573593
17.2666664	1.5128000	-1.8653207

17.2666664	1.5616000	-1.8653207
17.2666664	1.6104000	-1.8714566
17.2666664	1.6592000	-1.8837285
17.2666664	1.7079999	-1.9021363
17.2666664	1.7567999	-1.9082723
17.2666664	1.8055999	-1.9266801
17.2666664	1.8543999	-1.7241945
17.2666664	1.9031999	-1.5646605
17.2666664	1.9519999	-1.4910293
17.2666664	2.0007999	-1.4971652
17.2666664	2.0495999	-1.4910293
17.2666664	2.0983999	-1.5339808
17.2666664	2.1471999	-1.5339808
17.2666664	2.1959999	-0.7976699
17.2666664	2.2447999	-1.4726214
17.2666664	2.2935998	-1.4664855
17.2666664	2.3423998	-1.3130875
17.2666664	2.3912001	-1.3130875
17.2666664	2.4400001	-1.3253593
17.2666664	2.4888000	-1.3253593
17.2666664	2.5376000	-1.3253593
17.2666664	2.5864000	-1.1106019
17.2666664	2.6352000	-1.1106019
17.2666664	2.6840000	-1.1167378
17.2666664	2.7328000	-1.1044660
17.2666664	2.7816000	-1.0983305
17.2666664	2.8304000	-1.0246992
17.2666664	2.8792000	-0.9572039
17.2666664	2.9280000	-0.9572039
17.2666664	2.9768000	-0.9449320
17.2666664	3.0256000	-0.9019809
17.2666664	3.0743999	-0.9081168
17.2666664	3.1231999	-1.0001554
17.2666664	3.1719999	-0.9878836
17.2666664	3.2207999	-0.8774371
17.2666664	3.2695999	-1.1351457
17.2666664	3.3183999	-0.8038058
17.2666664	3.3671999	-0.8283496
17.2666664	3.4159999	-1.0062914
17.2666664	3.4647999	-1.0860586
17.2666664	3.5135999	-0.9633398
17.2666664	3.5623999	-0.9510679
17.2666664	3.6111999	-0.8099418
17.2666664	3.6599998	-0.7056313
17.2666664	3.7087998	-0.7117672
17.2666664	3.7575998	-0.9019809
17.2666664	3.8063998	-0.6381359
17.2666664	3.8551998	-0.6258640
17.2666664	3.9039998	-0.7179031
17.2666664	3.9527998	-0.7056313
17.2666664	4.0016003	-0.7547188
17.2666664	4.0503998	-0.7608547
17.2666664	4.0991998	-0.6810875
17.2666664	4.1479998	-0.7240391
17.2666664	4.1967998	-0.7301750
17.2666664	4.2455997	-0.7485828
17.2666664	4.2943997	-0.7485828

17.2666664	4.3431997	-0.7976699
17.2666664	4.3919997	-0.7976699
17.2666664	4.4407997	-0.7240391
17.2666664	4.4895997	-0.7424469
17.2666664	4.5383997	-0.7301750
17.2666664	4.5871997	-0.5890489
17.2666664	4.6359997	-0.7608547
17.2666664	4.6847997	-0.5338254
17.2666664	4.7336001	-0.5338254
17.2666664	4.7824001	-0.5031457
17.2666664	4.8312001	-0.5031457
17.2666664	4.8800001	-0.4970098
17.2666664	4.9288001	-0.5583692
17.2666664	4.9776001	-0.5522332
17.2666664	5.0264001	-0.6626797
17.2666664	5.0752001	613586.0000000
17.2666664	5.1240001	-0.7547188
17.2666664	5.1728001	-0.7547188
17.2666664	5.2216001	613586.0000000
17.2666664	5.2704000	613586.0000000
17.2666664	5.3192000	613586.0000000
17.2666664	5.3680000	613586.0000000
17.2666664	5.4168000	613586.0000000
17.2666664	5.4656000	613586.0000000
17.2666664	5.5144000	613586.0000000
17.2666664	5.5632000	613586.0000000
17.2666664	5.6120000	613586.0000000
17.2666664	5.6608000	613586.0000000
17.2666664	5.7096000	613586.0000000
17.2666664	5.7584000	613586.0000000
17.2666664	5.8072000	613586.0000000
17.2666664	5.8559999	613586.0000000
17.2666664	5.9047999	613586.0000000
17.2666664	5.9535999	613586.0000000
17.2666664	6.0023999	613586.0000000
17.2833328	0.2928000	-3.2090878
17.2833328	0.3416000	-3.8840394
17.2833328	0.3904000	-3.7367773
17.2833328	0.4392000	-3.1477287
17.2833328	0.4880000	-2.4114180
17.2833328	0.5368000	-2.4114180
17.2833328	0.5856000	-1.9880390
17.2833328	0.6344000	-1.9144082
17.2833328	0.6832000	-1.8285050
17.2833328	0.7320000	-1.8407769
17.2833328	0.7808000	-1.6566992
17.2833328	0.8296000	-1.6505632
17.2833328	0.8784000	-1.6628351
17.2833328	0.9272000	-1.8223691
17.2833328	0.9760000	-2.0984859
17.2833328	1.0247999	-1.6689711
17.2833328	1.0735999	-1.8653207
17.2833328	1.1223999	-1.8530488
17.2833328	1.1711999	-1.7610102
17.2833328	1.2200000	-1.7487383
17.2833328	1.2688000	-1.7487383
17.2833328	1.3176000	-1.6873789

17.2833328	1.3664000	-1.7548742
17.2833328	1.4152000	-1.6996508
17.2833328	1.4640000	-1.7180586
17.2833328	1.5128000	-1.8162332
17.2833328	1.5616000	-1.8223691
17.2833328	1.6104000	-1.8775926
17.2833328	1.6592000	-1.8714566
17.2833328	1.7079999	-2.0371265
17.2833328	1.7567999	-2.0248547
17.2833328	1.8055999	-1.3805828
17.2833328	1.8543999	-1.6014762
17.2833328	1.9031999	-1.7303305
17.2833328	1.9519999	-1.2578645
17.2833328	2.0007999	-1.5830684
17.2833328	2.0495999	-1.5953403
17.2833328	2.0983999	-1.5278449
17.2833328	2.1471999	-1.5462527
17.2833328	2.1959999	-1.5523887
17.2833328	2.2447999	-1.5462527
17.2833328	2.2935998	-1.4664855
17.2833328	2.3423998	-1.4848933
17.2833328	2.3912001	-1.4910293
17.2833328	2.4400001	-1.3130875
17.2833328	2.4888000	-1.3192234
17.2833328	2.5376000	-1.3805828
17.2833328	2.5864000	-1.2701364
17.2833328	2.6352000	-1.3928547
17.2833328	2.6840000	-1.0676508
17.2833328	2.7328000	-1.0799227
17.2833328	2.7816000	-1.0921946
17.2833328	2.8304000	-0.9694757
17.2833328	2.8792000	-1.2762718
17.2833328	2.9280000	-1.0062914
17.2833328	2.9768000	-1.0001554
17.2833328	3.0256000	-1.0860586
17.2833328	3.0743999	-1.0799227
17.2833328	3.1231999	-0.8774371
17.2833328	3.1719999	-0.8774371
17.2833328	3.2207999	-0.8774371
17.2833328	3.2695999	-0.9265246
17.2833328	3.3183999	-0.9326606
17.2833328	3.3671999	-0.8160777
17.2833328	3.4159999	-0.8344855
17.2833328	3.4647999	-0.7792621
17.2833328	3.5135999	-0.7792621
17.2833328	3.5623999	-0.7792621
17.2833328	3.6111999	-0.7301750
17.2833328	3.6599998	-0.7179031
17.2833328	3.7087998	-0.8774371
17.2833328	3.7575998	-0.7301750
17.2833328	3.8063998	-0.7363110
17.2833328	3.8551998	-0.7240391
17.2833328	3.9039998	-0.7485828
17.2833328	3.9527998	-0.7608547
17.2833328	4.0016003	-0.7915339
17.2833328	4.0503998	-0.7608547
17.2833328	4.0991998	-0.7547188

17.2833328	4.1479998	-0.6320000
17.2833328	4.1967998	-0.7915339
17.2833328	4.2455997	-0.7547188
17.2833328	4.2943997	-0.7547188
17.2833328	4.3431997	-0.7608547
17.2833328	4.3919997	-0.7608547
17.2833328	4.4407997	-0.6749516
17.2833328	4.4895997	-0.8897090
17.2833328	4.5383997	-0.7056313
17.2833328	4.5871997	-0.6381359
17.2833328	4.6359997	-0.6442719
17.2833328	4.6847997	-0.7117672
17.2833328	4.7336001	-0.7117672
17.2833328	4.7824001	-0.6994953
17.2833328	4.8312001	-0.6994953
17.2833328	4.8800001	-0.6872234
17.2833328	4.9288001	-0.4540582
17.2833328	4.9776001	-0.6442719
17.2833328	5.0264001	-0.5890489
17.2833328	5.0752001	-0.5951843
17.2833328	5.1240001	613586.0000000
17.2833328	5.1728001	613586.0000000
17.2833328	5.2216001	-0.4233789
17.2833328	5.2704000	613586.0000000
17.2833328	5.3192000	613586.0000000
17.2833328	5.3680000	613586.0000000
17.2833328	5.4168000	613586.0000000
17.2833328	5.4656000	613586.0000000
17.2833328	5.5144000	613586.0000000
17.2833328	5.5632000	613586.0000000
17.2833328	5.6120000	613586.0000000
17.2833328	5.6608000	613586.0000000
17.2833328	5.7096000	613586.0000000
17.2833328	5.7584000	613586.0000000
17.2833328	5.8072000	613586.0000000
17.2833328	5.8559999	613586.0000000
17.2833328	5.9047999	613586.0000000
17.2833328	5.9535999	613586.0000000
17.2833328	6.0023999	613586.0000000
17.2999992	0.2928000	-3.1477287
17.2999992	0.3416000	-3.3747578
17.2999992	0.3904000	-3.3870296
17.2999992	0.4392000	-3.2765830
17.2999992	0.4880000	-2.4850490
17.2999992	0.5368000	-2.0248547
17.2999992	0.5856000	-2.0003109
17.2999992	0.6344000	-1.8100972
17.2999992	0.6832000	-1.9144082
17.2999992	0.7320000	-1.8346410
17.2999992	0.7808000	-1.8407769
17.2999992	0.8296000	-1.7487383
17.2999992	0.8784000	-1.7487383
17.2999992	0.9272000	-1.7794180
17.2999992	0.9760000	-1.7794180
17.2999992	1.0247999	-1.7364664
17.2999992	1.0735999	-1.6996508
17.2999992	1.1223999	-1.7303305

17.2999992	1.1711999	-1.7303305
17.2999992	1.2200000	-1.6751070
17.2999992	1.2688000	-1.8469129
17.2999992	1.3176000	-1.8530488
17.2999992	1.3664000	-1.7364664
17.2999992	1.4152000	-1.5892043
17.2999992	1.4640000	-1.8898644
17.2999992	1.5128000	-1.8837285
17.2999992	1.5616000	-1.7978253
17.2999992	1.6104000	-1.7548742
17.2999992	1.6592000	-1.8346410
17.2999992	1.7079999	-1.7487383
17.2999992	1.7567999	-1.5217090
17.2999992	1.8055999	-1.4419422
17.2999992	1.8543999	-0.9633398
17.2999992	1.9031999	-1.1719613
17.2999992	1.9519999	-1.0921946
17.2999992	2.0007999	-1.4848933
17.2999992	2.0495999	-0.8283496
17.2999992	2.0983999	-0.8283496
17.2999992	2.1471999	-0.9265246
17.2999992	2.1959999	-1.3192234
17.2999992	2.2447999	-1.3192234
17.2999992	2.2935998	-1.0860586
17.2999992	2.3423998	-1.0860586
17.2999992	2.3912001	-1.0799227
17.2999992	2.4400001	-0.9265246
17.2999992	2.4888000	-0.9203887
17.2999992	2.5376000	-1.1719613
17.2999992	2.5864000	-1.1658254
17.2999992	2.6352000	-1.1044660
17.2999992	2.6840000	-1.1167378
17.2999992	2.7328000	-0.9694757
17.2999992	2.7816000	-0.9940195
17.2999992	2.8304000	-0.9940195
17.2999992	2.8792000	-0.9940195
17.2999992	2.9280000	-1.0001554
17.2999992	2.9768000	-1.0001554
17.2999992	3.0256000	-1.0062914
17.2999992	3.0743999	-0.9203887
17.2999992	3.1231999	-0.8590293
17.2999992	3.1719999	-0.7056313
17.2999992	3.2207999	-0.8344855
17.2999992	3.2695999	-0.9019809
17.2999992	3.3183999	-0.7792621
17.2999992	3.3671999	-0.6320000
17.2999992	3.4159999	-0.6258640
17.2999992	3.4647999	-0.6135921
17.2999992	3.5135999	-0.6013203
17.2999992	3.5623999	-0.5890489
17.2999992	3.6111999	-0.6626797
17.2999992	3.6599998	-0.5031457
17.2999992	3.7087998	-0.5767770
17.2999992	3.7575998	-0.6933594
17.2999992	3.8063998	-0.6933594
17.2999992	3.8551998	-0.4970098
17.2999992	3.9039998	-0.5829129

17.2999992	3.9527998	-0.5890489
17.2999992	4.0016003	-0.4479222
17.2999992	4.0503998	-0.4479222
17.2999992	4.0991998	-0.5522332
17.2999992	4.1479998	-0.5522332
17.2999992	4.1967998	-0.5031457
17.2999992	4.2455997	-0.9019809
17.2999992	4.2943997	-0.9019809
17.2999992	4.3431997	-0.7669902
17.2999992	4.3919997	-0.7547188
17.2999992	4.4407997	-0.7669902
17.2999992	4.4895997	-0.5460973
17.2999992	4.5383997	-0.5460973
17.2999992	4.5871997	-0.7915339
17.2999992	4.6359997	-0.7976699
17.2999992	4.6847997	-0.6258640
17.2999992	4.7336001	-0.5583692
17.2999992	4.7824001	-0.4847379
17.2999992	4.8312001	-0.4356503
17.2999992	4.8800001	-0.4540582
17.2999992	4.9288001	613586.0000000
17.2999992	4.9776001	-0.5338254
17.2999992	5.0264001	-0.3190680
17.2999992	5.0752001	-0.3067961
17.2999992	5.1240001	-0.2699804
17.2999992	5.1728001	613586.0000000
17.2999992	5.2216001	613586.0000000
17.2999992	5.2704000	613586.0000000
17.2999992	5.3192000	613586.0000000
17.2999992	5.3680000	613586.0000000
17.2999992	5.4168000	613586.0000000
17.2999992	5.4656000	613586.0000000
17.2999992	5.5144000	613586.0000000
17.2999992	5.5632000	613586.0000000
17.2999992	5.6120000	613586.0000000
17.2999992	5.6608000	613586.0000000
17.2999992	5.7096000	613586.0000000
17.2999992	5.7584000	613586.0000000
17.2999992	5.8072000	613586.0000000
17.2999992	5.8559999	613586.0000000
17.2999992	5.9047999	613586.0000000
17.2999992	5.9535999	613586.0000000
17.2999992	6.0023999	613586.0000000
17.3166676	0.2928000	-3.2581754
17.3166676	0.3416000	-3.2520394
17.3166676	0.3904000	-3.1477287
17.3166676	0.4392000	-3.1538646
17.3166676	0.4880000	-2.7550297
17.3166676	0.5368000	-2.0432625
17.3166676	0.5856000	-1.8591847
17.3166676	0.6344000	-1.8591847
17.3166676	0.6832000	-1.8653207
17.3166676	0.7320000	-1.6751070
17.3166676	0.7808000	-1.8714566
17.3166676	0.8296000	-1.8837285
17.3166676	0.8784000	-1.8898644
17.3166676	0.9272000	-1.9696312

17.3166676	0.9760000	-1.9512239
17.3166676	1.0247999	-2.0371265
17.3166676	1.0735999	-1.6812429
17.3166676	1.1223999	-1.6812429
17.3166676	1.1711999	-1.8960004
17.3166676	1.2200000	-1.9512239
17.3166676	1.2688000	-1.8285050
17.3166676	1.3176000	-1.7180586
17.3166676	1.3664000	-1.7487383
17.3166676	1.4152000	-1.6444273
17.3166676	1.4640000	-1.6444273
17.3166676	1.5128000	-1.6812429
17.3166676	1.5616000	-1.6873789
17.3166676	1.6104000	-1.7241945
17.3166676	1.6592000	-1.7303305
17.3166676	1.7079999	-1.0921946
17.3166676	1.7567999	-1.2333207
17.3166676	1.8055999	-1.4726214
17.3166676	1.8543999	-1.4726214
17.3166676	1.9031999	-1.4910293
17.3166676	1.9519999	-1.4910293
17.3166676	2.0007999	-1.3683109
17.3166676	2.0495999	-1.3621750
17.3166676	2.0983999	-1.1044660
17.3166676	2.1471999	-0.8160777
17.3166676	2.1959999	-0.8160777
17.3166676	2.2447999	-0.6688156
17.3166676	2.2935998	-0.6626797
17.3166676	2.3423998	-1.1535535
17.3166676	2.3912001	-1.1535535
17.3166676	2.4400001	-1.1535535
17.3166676	2.4888000	-1.0737867
17.3166676	2.5376000	-1.0615149
17.3166676	2.5864000	-1.1351457
17.3166676	2.6352000	-0.9633398
17.3166676	2.6840000	-0.9756117
17.3166676	2.7328000	-0.9142528
17.3166676	2.7816000	-0.8958449
17.3166676	2.8304000	-0.8897090
17.3166676	2.8792000	-0.8774371
17.3166676	2.9280000	-0.8713012
17.3166676	2.9768000	-0.8651652
17.3166676	3.0256000	-0.8038058
17.3166676	3.0743999	-0.7976699
17.3166676	3.1231999	-0.6749516
17.3166676	3.1719999	-0.7792621
17.3166676	3.2207999	-0.6442719
17.3166676	3.2695999	-0.6258640
17.3166676	3.3183999	-0.6197281
17.3166676	3.3671999	-0.6135921
17.3166676	3.4159999	-0.6320000
17.3166676	3.4647999	-0.5951843
17.3166676	3.5135999	-0.6013203
17.3166676	3.5623999	-0.5338254
17.3166676	3.6111999	-0.6565437
17.3166676	3.6599998	-0.6994953
17.3166676	3.7087998	-0.6135921

17.3166676	3.7575998	-0.5460973
17.3166676	3.8063998	-0.5338254
17.3166676	3.8551998	-0.5154176
17.3166676	3.9039998	-0.5092816
17.3166676	3.9527998	-0.5031457
17.3166676	4.0016003	-0.5583692
17.3166676	4.0503998	-1.0369711
17.3166676	4.0991998	-1.0369711
17.3166676	4.1479998	-0.7853980
17.3166676	4.1967998	-0.7976699
17.3166676	4.2455997	-0.8038058
17.3166676	4.2943997	-0.8283496
17.3166676	4.3431997	-0.7056313
17.3166676	4.3919997	-0.7117672
17.3166676	4.4407997	-0.5460973
17.3166676	4.4895997	-0.7485828
17.3166676	4.5383997	-0.7792621
17.3166676	4.5871997	-0.7485828
17.3166676	4.6359997	-0.5276895
17.3166676	4.6847997	-0.5890489
17.3166676	4.7336001	-0.4111071
17.3166676	4.7824001	-0.4111071
17.3166676	4.8312001	-0.4233789
17.3166676	4.8800001	-0.3865633
17.3166676	4.9288001	613586.0000000
17.3166676	4.9776001	-0.3067961
17.3166676	5.0264001	-0.3067961
17.3166676	5.0752001	613586.0000000
17.3166676	5.1240001	613586.0000000
17.3166676	5.1728001	613586.0000000
17.3166676	5.2216001	613586.0000000
17.3166676	5.2704000	613586.0000000
17.3166676	5.3192000	613586.0000000
17.3166676	5.3680000	613586.0000000
17.3166676	5.4168000	613586.0000000
17.3166676	5.4656000	613586.0000000
17.3166676	5.5144000	613586.0000000
17.3166676	5.5632000	613586.0000000
17.3166676	5.6120000	613586.0000000
17.3166676	5.6608000	613586.0000000
17.3166676	5.7096000	613586.0000000
17.3166676	5.7584000	613586.0000000
17.3166676	5.8072000	613586.0000000
17.3166676	5.8559999	613586.0000000
17.3166676	5.9047999	613586.0000000
17.3166676	5.9535999	613586.0000000
17.3166676	6.0023999	613586.0000000
17.3333340	0.2928000	-3.4790685
17.3333340	0.3416000	-3.5588355
17.3333340	0.3904000	-3.5465636
17.3333340	0.4392000	-2.2396121
17.3333340	0.4880000	-2.2457480
17.3333340	0.5368000	-2.2396121
17.3333340	0.5856000	-1.9021363
17.3333340	0.6344000	-1.8285050
17.3333340	0.6832000	-1.8285050
17.3333340	0.7320000	-1.6198835

17.3333340	0.7808000	-1.8162332
17.3333340	0.8296000	-1.9512239
17.3333340	0.8784000	-1.8591847
17.3333340	0.9272000	-1.9205441
17.3333340	0.9760000	-1.6996508
17.3333340	1.0247999	-1.6996508
17.3333340	1.0735999	-1.5523887
17.3333340	1.1223999	-1.8407769
17.3333340	1.1711999	-1.8837285
17.3333340	1.2200000	-1.6751070
17.3333340	1.2688000	-1.8223691
17.3333340	1.3176000	-1.8285050
17.3333340	1.3664000	-1.8469129
17.3333340	1.4152000	-1.7671461
17.3333340	1.4640000	-1.6812429
17.3333340	1.5128000	-1.7610102
17.3333340	1.5616000	-1.7610102
17.3333340	1.6104000	-1.7610102
17.3333340	1.6592000	-1.7303305
17.3333340	1.7079999	-1.7364664
17.3333340	1.7567999	-1.2762718
17.3333340	1.8055999	-1.4296703
17.3333340	1.8543999	-1.4296703
17.3333340	1.9031999	-1.5830684
17.3333340	1.9519999	-0.8897090
17.3333340	2.0007999	-1.2762718
17.3333340	2.0495999	-0.8222136
17.3333340	2.0983999	-1.2578645
17.3333340	2.1471999	-1.3008156
17.3333340	2.1959999	-1.2885437
17.3333340	2.2447999	-1.2946796
17.3333340	2.2935998	-1.2333207
17.3333340	2.3423998	-1.2394567
17.3333340	2.3912001	-1.2333207
17.3333340	2.4400001	-1.2578645
17.3333340	2.4888000	-1.2578645
17.3333340	2.5376000	-1.2517285
17.3333340	2.5864000	-0.9694757
17.3333340	2.6352000	-1.0431070
17.3333340	2.6840000	-0.8835731
17.3333340	2.7328000	-0.9694757
17.3333340	2.7816000	-0.9449320
17.3333340	2.8304000	-0.8528934
17.3333340	2.8792000	-0.8590293
17.3333340	2.9280000	-0.8651652
17.3333340	2.9768000	-0.8651652
17.3333340	3.0256000	-0.8713012
17.3333340	3.0743999	-0.6933594
17.3333340	3.1231999	-0.8467574
17.3333340	3.1719999	-0.6933594
17.3333340	3.2207999	-0.7853980
17.3333340	3.2695999	-0.7179031
17.3333340	3.3183999	-0.5706410
17.3333340	3.3671999	-0.7117672
17.3333340	3.4159999	-0.6135921
17.3333340	3.4647999	-0.6626797
17.3333340	3.5135999	-0.6626797

17.3333340	3.5623999	-0.6749516
17.3333340	3.6111999	-0.5829129
17.3333340	3.6599998	-0.5829129
17.3333340	3.7087998	-0.5154176
17.3333340	3.7575998	-0.5215535
17.3333340	3.8063998	-0.4724660
17.3333340	3.8551998	-0.7485828
17.3333340	3.9039998	-0.6135921
17.3333340	3.9527998	-0.5338254
17.3333340	4.0016003	-0.7179031
17.3333340	4.0503998	-0.7056313
17.3333340	4.0991998	-0.6994953
17.3333340	4.1479998	-0.6381359
17.3333340	4.1967998	-0.5154176
17.3333340	4.2455997	-0.6197281
17.3333340	4.2943997	-0.5522332
17.3333340	4.3431997	-0.5460973
17.3333340	4.3919997	-0.6565437
17.3333340	4.4407997	-0.4049711
17.3333340	4.4895997	-0.3988352
17.3333340	4.5383997	-0.4172430
17.3333340	4.5871997	-0.4601941
17.3333340	4.6359997	-0.3742914
17.3333340	4.6847997	-0.2945242
17.3333340	4.7336001	-0.3620195
17.3333340	4.7824001	-0.3374758
17.3333340	4.8312001	-0.4049711
17.3333340	4.8800001	-0.2270293
17.3333340	4.9288001	613586.0000000
17.3333340	4.9776001	613586.0000000
17.3333340	5.0264001	613586.0000000
17.3333340	5.0752001	-0.2454371
17.3333340	5.1240001	613586.0000000
17.3333340	5.1728001	613586.0000000
17.3333340	5.2216001	613586.0000000
17.3333340	5.2704000	613586.0000000
17.3333340	5.3192000	613586.0000000
17.3333340	5.3680000	613586.0000000
17.3333340	5.4168000	613586.0000000
17.3333340	5.4656000	613586.0000000
17.3333340	5.5144000	613586.0000000
17.3333340	5.5632000	613586.0000000
17.3333340	5.6120000	613586.0000000
17.3333340	5.6608000	613586.0000000
17.3333340	5.7096000	613586.0000000
17.3333340	5.7584000	613586.0000000
17.3333340	5.8072000	613586.0000000
17.3333340	5.8559999	613586.0000000
17.3333340	5.9047999	613586.0000000
17.3333340	5.9535999	613586.0000000
17.3333340	6.0023999	613586.0000000
17.3500004	0.2928000	-3.6631463
17.3500004	0.3416000	-4.6694374
17.3500004	0.3904000	-4.0865250
17.3500004	0.4392000	-3.3686218
17.3500004	0.4880000	-2.6507189
17.3500004	0.5368000	-2.6445830

17.3500004	0.5856000	-1.9696312
17.3500004	0.6344000	-1.9757671
17.3500004	0.6832000	-1.6628351
17.3500004	0.7320000	-1.6628351
17.3500004	0.7808000	-1.7487383
17.3500004	0.8296000	-1.8407769
17.3500004	0.8784000	-1.8039613
17.3500004	0.9272000	-1.9205441
17.3500004	0.9760000	-1.9205441
17.3500004	1.0247999	-1.6076121
17.3500004	1.0735999	-1.8100972
17.3500004	1.1223999	-1.8039613
17.3500004	1.1711999	-1.8530488
17.3500004	1.2200000	-1.7916894
17.3500004	1.2688000	-1.8837285
17.3500004	1.3176000	-1.8837285
17.3500004	1.3664000	-1.8591847
17.3500004	1.4152000	-1.8407769
17.3500004	1.4640000	-1.7916894
17.3500004	1.5128000	-1.8223691
17.3500004	1.5616000	-1.8223691
17.3500004	1.6104000	-1.8837285
17.3500004	1.6592000	-1.9021363
17.3500004	1.7079999	-1.4296703
17.3500004	1.7567999	-1.4542136
17.3500004	1.8055999	-1.2394567
17.3500004	1.8543999	-1.2210488
17.3500004	1.9031999	-1.2946796
17.3500004	1.9519999	-0.9510679
17.3500004	2.0007999	-0.8897090
17.3500004	2.0495999	-1.0615149
17.3500004	2.0983999	-1.0799227
17.3500004	2.1471999	-1.0921946
17.3500004	2.1959999	-1.3314953
17.3500004	2.2447999	-1.4603496
17.3500004	2.2935998	-1.2394567
17.3500004	2.3423998	-1.4726214
17.3500004	2.3912001	-1.4603496
17.3500004	2.4400001	-1.1228738
17.3500004	2.4888000	-1.0737867
17.3500004	2.5376000	-1.1965051
17.3500004	2.5864000	-1.0615149
17.3500004	2.6352000	-1.0676508
17.3500004	2.6840000	-1.0737867
17.3500004	2.7328000	-0.8774371
17.3500004	2.7816000	-0.9019809
17.3500004	2.8304000	-0.9081168
17.3500004	2.8792000	-1.0246992
17.3500004	2.9280000	-1.0185633
17.3500004	2.9768000	-0.9326606
17.3500004	3.0256000	-0.8713012
17.3500004	3.0743999	-0.9387960
17.3500004	3.1231999	-0.9510679
17.3500004	3.1719999	-0.8406215
17.3500004	3.2207999	-0.8344855
17.3500004	3.2695999	-0.7731261
17.3500004	3.3183999	-0.6626797

17.3500004	3.3671999	-0.6688156
17.3500004	3.4159999	-0.6565437
17.3500004	3.4647999	-0.6626797
17.3500004	3.5135999	-0.6933594
17.3500004	3.5623999	-0.6872234
17.3500004	3.6111999	-0.6872234
17.3500004	3.6599998	-0.6320000
17.3500004	3.7087998	-0.7853980
17.3500004	3.7575998	-0.5338254
17.3500004	3.8063998	-0.7853980
17.3500004	3.8551998	-0.7301750
17.3500004	3.9039998	-0.7363110
17.3500004	3.9527998	-0.9449320
17.3500004	4.0016003	-0.9572039
17.3500004	4.0503998	-0.7731261
17.3500004	4.0991998	-0.7669902
17.3500004	4.1479998	-0.8160777
17.3500004	4.1967998	-0.8590293
17.3500004	4.2455997	-0.7424469
17.3500004	4.2943997	-0.7485828
17.3500004	4.3431997	-0.7424469
17.3500004	4.3919997	-0.7240391
17.3500004	4.4407997	-0.6320000
17.3500004	4.4895997	-0.5031457
17.3500004	4.5383997	-0.4847379
17.3500004	4.5871997	-0.6197281
17.3500004	4.6359997	-0.6197281
17.3500004	4.6847997	-0.6197281
17.3500004	4.7336001	-0.6197281
17.3500004	4.7824001	-0.4540582
17.3500004	4.8312001	-0.5276895
17.3500004	4.8800001	-0.5031457
17.3500004	4.9288001	-0.4786019
17.3500004	4.9776001	-0.4847379
17.3500004	5.0264001	-0.4908738
17.3500004	5.0752001	613586.0000000
17.3500004	5.1240001	613586.0000000
17.3500004	5.1728001	613586.0000000
17.3500004	5.2216001	613586.0000000
17.3500004	5.2704000	613586.0000000
17.3500004	5.3192000	613586.0000000
17.3500004	5.3680000	613586.0000000
17.3500004	5.4168000	613586.0000000
17.3500004	5.4656000	613586.0000000
17.3500004	5.5144000	613586.0000000
17.3500004	5.5632000	613586.0000000
17.3500004	5.6120000	613586.0000000
17.3500004	5.6608000	613586.0000000
17.3500004	5.7096000	613586.0000000
17.3500004	5.7584000	613586.0000000
17.3500004	5.8072000	613586.0000000
17.3500004	5.8559999	613586.0000000
17.3500004	5.9047999	613586.0000000
17.3500004	5.9535999	613586.0000000
17.3500004	6.0023999	613586.0000000
17.3666668	0.2928000	-4.6633015
17.3666668	0.3416000	-4.6633015

17.3666668	0.3904000	-3.5833793
17.3666668	0.4392000	-3.0004666
17.3666668	0.4880000	-2.5709519
17.3666668	0.5368000	-2.0555344
17.3666668	0.5856000	-2.1966605
17.3666668	0.6344000	-1.7610102
17.3666668	0.6832000	-1.9082723
17.3666668	0.7320000	-1.9082723
17.3666668	0.7808000	-1.8469129
17.3666668	0.8296000	-2.1659808
17.3666668	0.8784000	-2.1659808
17.3666668	0.9272000	-1.7426023
17.3666668	0.9760000	-1.7364664
17.3666668	1.0247999	-1.9757671
17.3666668	1.0735999	-1.9389520
17.3666668	1.1223999	-1.9389520
17.3666668	1.1711999	-1.8714566
17.3666668	1.2200000	-1.8653207
17.3666668	1.2688000	-1.8653207
17.3666668	1.3176000	-1.8714566
17.3666668	1.3664000	-1.8469129
17.3666668	1.4152000	-1.8469129
17.3666668	1.4640000	-1.6444273
17.3666668	1.5128000	-1.8100972
17.3666668	1.5616000	-1.8837285
17.3666668	1.6104000	-1.6935148
17.3666668	1.6592000	-1.6935148
17.3666668	1.7079999	-1.8100972
17.3666668	1.7567999	-1.1290097
17.3666668	1.8055999	-1.1228738
17.3666668	1.8543999	-1.2026410
17.3666668	1.9031999	-1.5278449
17.3666668	1.9519999	-1.4235344
17.3666668	2.0007999	-1.5523887
17.3666668	2.0495999	-1.5646605
17.3666668	2.0983999	-1.6014762
17.3666668	2.1471999	-1.6014762
17.3666668	2.1959999	-1.4051266
17.3666668	2.2447999	-1.3253593
17.3666668	2.2935998	-1.3253593
17.3666668	2.3423998	-1.1351457
17.3666668	2.3912001	-1.1535535
17.3666668	2.4400001	-1.1535535
17.3666668	2.4888000	-1.1167378
17.3666668	2.5376000	-1.3683109
17.3666668	2.5864000	-1.1719613
17.3666668	2.6352000	-1.1719613
17.3666668	2.6840000	-1.1044660
17.3666668	2.7328000	-1.0983305
17.3666668	2.7816000	-1.0001554
17.3666668	2.8304000	-0.9940195
17.3666668	2.8792000	-0.9019809
17.3666668	2.9280000	-0.8958449
17.3666668	2.9768000	-1.0308352
17.3666668	3.0256000	-0.9633398
17.3666668	3.0743999	-0.9817476
17.3666668	3.1231999	-0.9817476

17.3666668	3.1719999	-0.8958449
17.3666668	3.2207999	-0.8835731
17.3666668	3.2695999	-0.8713012
17.3666668	3.3183999	-0.9326606
17.3666668	3.3671999	-0.7731261
17.3666668	3.4159999	-0.7731261
17.3666668	3.4647999	-0.7240391
17.3666668	3.5135999	-0.6381359
17.3666668	3.5623999	-0.7240391
17.3666668	3.6111999	-0.9817476
17.3666668	3.6599998	-0.6565437
17.3666668	3.7087998	-1.0246992
17.3666668	3.7575998	-0.7792621
17.3666668	3.8063998	-1.1228738
17.3666668	3.8551998	-0.9449320
17.3666668	3.9039998	-1.0124273
17.3666668	3.9527998	-1.0124273
17.3666668	4.0016003	-1.0001554
17.3666668	4.0503998	-0.9878836
17.3666668	4.0991998	-0.9817476
17.3666668	4.1479998	-0.9817476
17.3666668	4.1967998	-0.8528934
17.3666668	4.2455997	-0.9633398
17.3666668	4.2943997	-0.7056313
17.3666668	4.3431997	-0.6872234
17.3666668	4.3919997	-0.6872234
17.3666668	4.4407997	-0.8467574
17.3666668	4.4895997	-0.6933594
17.3666668	4.5383997	-0.6688156
17.3666668	4.5871997	-0.6565437
17.3666668	4.6359997	-0.6504078
17.3666668	4.6847997	-0.6565437
17.3666668	4.7336001	-0.6074562
17.3666668	4.7824001	-0.6626797
17.3666668	4.8312001	-0.6688156
17.3666668	4.8800001	-0.5951843
17.3666668	4.9288001	-0.5583692
17.3666668	4.9776001	613586.0000000
17.3666668	5.0264001	613586.0000000
17.3666668	5.0752001	613586.0000000
17.3666668	5.1240001	-0.9510679
17.3666668	5.1728001	-0.9387960
17.3666668	5.2216001	-0.9387960
17.3666668	5.2704000	-0.9326606
17.3666668	5.3192000	613586.0000000
17.3666668	5.3680000	613586.0000000
17.3666668	5.4168000	613586.0000000
17.3666668	5.4656000	613586.0000000
17.3666668	5.5144000	613586.0000000
17.3666668	5.5632000	613586.0000000
17.3666668	5.6120000	613586.0000000
17.3666668	5.6608000	613586.0000000
17.3666668	5.7096000	613586.0000000
17.3666668	5.7584000	613586.0000000
17.3666668	5.8072000	613586.0000000
17.3666668	5.8559999	613586.0000000
17.3666668	5.9047999	613586.0000000

17.3666668	5.9535999	613586.0000000
17.3666668	6.0023999	613586.0000000
17.3833332	0.2928000	-4.8596516
17.3833332	0.3416000	-4.8780589
17.3833332	0.3904000	-4.4301367
17.3833332	0.4392000	-3.1784084
17.3833332	0.4880000	-3.3318064
17.3833332	0.5368000	-2.2089324
17.3833332	0.5856000	-2.2089324
17.3833332	0.6344000	-1.9634953
17.3833332	0.6832000	-2.0003109
17.3833332	0.7320000	-1.8591847
17.3833332	0.7808000	-1.6260195
17.3833332	0.8296000	-1.7303305
17.3833332	0.8784000	-1.8346410
17.3833332	0.9272000	-1.9941750
17.3833332	0.9760000	-1.9941750
17.3833332	1.0247999	-1.9082723
17.3833332	1.0735999	-1.8407769
17.3833332	1.1223999	-1.8530488
17.3833332	1.1711999	-1.8346410
17.3833332	1.2200000	-1.7610102
17.3833332	1.2688000	-1.4235344
17.3833332	1.3176000	-1.9144082
17.3833332	1.3664000	-1.8530488
17.3833332	1.4152000	-1.6689711
17.3833332	1.4640000	-1.6751070
17.3833332	1.5128000	-1.6751070
17.3833332	1.5616000	-1.2946796
17.3833332	1.6104000	-1.7487383
17.3833332	1.6592000	-1.0860586
17.3833332	1.7079999	-1.0860586
17.3833332	1.7567999	-1.6260195
17.3833332	1.8055999	-0.9510679
17.3833332	1.8543999	-1.5462527
17.3833332	1.9031999	-1.6689711
17.3833332	1.9519999	-1.6751070
17.3833332	2.0007999	-1.6751070
17.3833332	2.0495999	-1.3744469
17.3833332	2.0983999	-1.3744469
17.3833332	2.1471999	-0.8835731
17.3833332	2.1959999	-1.3130875
17.3833332	2.2447999	-1.3130875
17.3833332	2.2935998	-1.2762718
17.3833332	2.3423998	-1.2701364
17.3833332	2.3912001	-1.3192234
17.3833332	2.4400001	-1.3069515
17.3833332	2.4888000	-1.1719613
17.3833332	2.5376000	-1.1719613
17.3833332	2.5864000	-1.1658254
17.3833332	2.6352000	-1.1658254
17.3833332	2.6840000	-1.0799227
17.3833332	2.7328000	-1.0983305
17.3833332	2.7816000	-1.0492430
17.3833332	2.8304000	-0.9633398
17.3833332	2.8792000	-0.9510679
17.3833332	2.9280000	-1.0553789

17.3833332	2.9768000	-1.0737867
17.3833332	3.0256000	-0.9019809
17.3833332	3.0743999	-0.9142528
17.3833332	3.1231999	-0.8099418
17.3833332	3.1719999	-0.8160777
17.3833332	3.2207999	-0.8283496
17.3833332	3.2695999	-0.8897090
17.3833332	3.3183999	-0.8897090
17.3833332	3.3671999	-0.8835731
17.3833332	3.4159999	-0.8958449
17.3833332	3.4647999	-0.7301750
17.3833332	3.5135999	-0.7608547
17.3833332	3.5623999	-0.6933594
17.3833332	3.6111999	-0.8713012
17.3833332	3.6599998	-1.0246992
17.3833332	3.7087998	-1.0369711
17.3833332	3.7575998	-0.7363110
17.3833332	3.8063998	-0.7363110
17.3833332	3.8551998	-0.7915339
17.3833332	3.9039998	-0.8099418
17.3833332	3.9527998	-0.8222136
17.3833332	4.0016003	-0.9387960
17.3833332	4.0503998	-0.9449320
17.3833332	4.0991998	-0.9510679
17.3833332	4.1479998	-0.8958449
17.3833332	4.1967998	-0.8344855
17.3833332	4.2455997	-0.9878836
17.3833332	4.2943997	-0.6810875
17.3833332	4.3431997	-0.7179031
17.3833332	4.3919997	-0.7363110
17.3833332	4.4407997	-0.7915339
17.3833332	4.4895997	-0.7915339
17.3833332	4.5383997	-0.7608547
17.3833332	4.5871997	-0.7792621
17.3833332	4.6359997	-0.8160777
17.3833332	4.6847997	-0.8222136
17.3833332	4.7336001	-0.6626797
17.3833332	4.7824001	-0.6565437
17.3833332	4.8312001	-0.6197281
17.3833332	4.8800001	-0.6013203
17.3833332	4.9288001	613586.0000000
17.3833332	4.9776001	613586.0000000
17.3833332	5.0264001	613586.0000000
17.3833332	5.0752001	613586.0000000
17.3833332	5.1240001	-1.0492430
17.3833332	5.1728001	-0.9326606
17.3833332	5.2216001	-0.9326606
17.3833332	5.2704000	-0.7731261
17.3833332	5.3192000	-0.7731261
17.3833332	5.3680000	-0.7792621
17.3833332	5.4168000	-0.7056313
17.3833332	5.4656000	613586.0000000
17.3833332	5.5144000	613586.0000000
17.3833332	5.5632000	613586.0000000
17.3833332	5.6120000	613586.0000000
17.3833332	5.6608000	613586.0000000
17.3833332	5.7096000	613586.0000000

17.3833332	5.7584000	613586.0000000
17.3833332	5.8072000	613586.0000000
17.3833332	5.8559999	613586.0000000
17.3833332	5.9047999	613586.0000000
17.3833332	5.9535999	613586.0000000
17.3833332	6.0023999	613586.0000000
17.3999996	0.2928000	-4.7737484
17.3999996	0.3416000	-4.8780589
17.3999996	0.3904000	-3.8165443
17.3999996	0.4392000	-3.0066025
17.3999996	0.4880000	-2.8716121
17.3999996	0.5368000	-2.3193791
17.3999996	0.5856000	-2.2764277
17.3999996	0.6344000	-2.0678062
17.3999996	0.6832000	-2.0678062
17.3999996	0.7320000	-2.1230292
17.3999996	0.7808000	-1.9082723
17.3999996	0.8296000	-2.3132432
17.3999996	0.8784000	-2.0371265
17.3999996	0.9272000	-2.0371265
17.3999996	0.9760000	-1.9021363
17.3999996	1.0247999	-1.9021363
17.3999996	1.0735999	-1.7119226
17.3999996	1.1223999	-2.0125828
17.3999996	1.1711999	-2.0125828
17.3999996	1.2200000	-1.8407769
17.3999996	1.2688000	-1.2701364
17.3999996	1.3176000	-1.6996508
17.3999996	1.3664000	-1.6935148
17.3999996	1.4152000	-1.7855535
17.3999996	1.4640000	-1.5707965
17.3999996	1.5128000	-1.3192234
17.3999996	1.5616000	-1.6321554
17.3999996	1.6104000	-1.1167378
17.3999996	1.6592000	-1.1228738
17.3999996	1.7079999	-1.3805828
17.3999996	1.7567999	-1.0676508
17.3999996	1.8055999	-1.1535535
17.3999996	1.8543999	-1.3989906
17.3999996	1.9031999	-1.3928547
17.3999996	1.9519999	-1.2640004
17.3999996	2.0007999	-1.2640004
17.3999996	2.0495999	-1.2701364
17.3999996	2.0983999	-0.7301750
17.3999996	2.1471999	-1.3314953
17.3999996	2.1959999	-1.3314953
17.3999996	2.2447999	-1.3376312
17.3999996	2.2935998	-1.3376312
17.3999996	2.3423998	-1.2885437
17.3999996	2.3912001	-1.2149129
17.3999996	2.4400001	-1.2271848
17.3999996	2.4888000	-1.2394567
17.3999996	2.5376000	-1.1658254
17.3999996	2.5864000	-1.1351457
17.3999996	2.6352000	-1.0492430
17.3999996	2.6840000	-1.0185633
17.3999996	2.7328000	-1.0431070

17.3999996	2.7816000	-1.0553789
17.3999996	2.8304000	-0.9203887
17.3999996	2.8792000	-0.9203887
17.3999996	2.9280000	-0.9572039
17.3999996	2.9768000	-1.0308352
17.3999996	3.0256000	-0.8467574
17.3999996	3.0743999	-0.8344855
17.3999996	3.1231999	-0.7792621
17.3999996	3.1719999	-0.7792621
17.3999996	3.2207999	-0.8344855
17.3999996	3.2695999	-0.7608547
17.3999996	3.3183999	-0.8344855
17.3999996	3.3671999	-0.7792621
17.3999996	3.4159999	-0.7792621
17.3999996	3.4647999	-0.8406215
17.3999996	3.5135999	-0.8283496
17.3999996	3.5623999	-0.8344855
17.3999996	3.6111999	-0.8406215
17.3999996	3.6599998	-0.8528934
17.3999996	3.7087998	-0.8528934
17.3999996	3.7575998	-0.8897090
17.3999996	3.8063998	-1.0615149
17.3999996	3.8551998	-0.7731261
17.3999996	3.9039998	-0.9817476
17.3999996	3.9527998	-0.8467574
17.3999996	4.0016003	-1.0431070
17.3999996	4.0503998	-1.0369711
17.3999996	4.0991998	-0.6504078
17.3999996	4.1479998	-0.6442719
17.3999996	4.1967998	-0.6381359
17.3999996	4.2455997	-0.8222136
17.3999996	4.2943997	-0.9694757
17.3999996	4.3431997	-0.7547188
17.3999996	4.3919997	-0.5706410
17.3999996	4.4407997	-0.4786019
17.3999996	4.4895997	-0.5522332
17.3999996	4.5383997	-0.6013203
17.3999996	4.5871997	-0.4172430
17.3999996	4.6359997	-0.4233789
17.3999996	4.6847997	613586.0000000
17.3999996	4.7336001	613586.0000000
17.3999996	4.7824001	613586.0000000
17.3999996	4.8312001	613586.0000000
17.3999996	4.8800001	-0.3436117
17.3999996	4.9288001	-0.3190680
17.3999996	4.9776001	613586.0000000
17.3999996	5.0264001	-0.7792621
17.3999996	5.0752001	-0.7792621
17.3999996	5.1240001	-0.7853980
17.3999996	5.1728001	613586.0000000
17.3999996	5.2216001	613586.0000000
17.3999996	5.2704000	613586.0000000
17.3999996	5.3192000	613586.0000000
17.3999996	5.3680000	613586.0000000
17.3999996	5.4168000	613586.0000000
17.3999996	5.4656000	613586.0000000
17.3999996	5.5144000	613586.0000000

17.3999996	5.5632000	613586.0000000
17.3999996	5.6120000	613586.0000000
17.3999996	5.6608000	613586.0000000
17.3999996	5.7096000	613586.0000000
17.3999996	5.7584000	613586.0000000
17.3999996	5.8072000	613586.0000000
17.3999996	5.8559999	613586.0000000
17.3999996	5.9047999	613586.0000000
17.3999996	5.9535999	613586.0000000
17.3999996	6.0023999	613586.0000000
17.4166660	0.2928000	-4.7676125
17.4166660	0.3416000	-4.4178648
17.4166660	0.3904000	-4.3565054
17.4166660	0.4392000	-2.9022918
17.4166660	0.4880000	-2.7979810
17.4166660	0.5368000	-2.4666412
17.4166660	0.5856000	-2.2641559
17.4166660	0.6344000	-2.0371265
17.4166660	0.6832000	-2.0371265
17.4166660	0.7320000	-1.9389520
17.4166660	0.7808000	-1.9389520
17.4166660	0.8296000	-1.9205441
17.4166660	0.8784000	-1.9205441
17.4166660	0.9272000	-1.6628351
17.4166660	0.9760000	-1.6628351
17.4166660	1.0247999	-1.6505632
17.4166660	1.0735999	-1.8407769
17.4166660	1.1223999	-1.8407769
17.4166660	1.1711999	-1.4603496
17.4166660	1.2200000	-1.3376312
17.4166660	1.2688000	-1.6382914
17.4166660	1.3176000	-1.4664855
17.4166660	1.3664000	-1.4664855
17.4166660	1.4152000	-1.1903691
17.4166660	1.4640000	-1.7916894
17.4166660	1.5128000	-1.6014762
17.4166660	1.5616000	-1.0431070
17.4166660	1.6104000	-1.0553789
17.4166660	1.6592000	-1.0553789
17.4166660	1.7079999	-1.1044660
17.4166660	1.7567999	-1.1044660
17.4166660	1.8055999	-1.2701364
17.4166660	1.8543999	-1.1228738
17.4166660	1.9031999	-1.4173985
17.4166660	1.9519999	-1.2946796
17.4166660	2.0007999	-1.1351457
17.4166660	2.0495999	-1.1351457
17.4166660	2.0983999	-1.0921946
17.4166660	2.1471999	-1.0983305
17.4166660	2.1959999	-1.0983305
17.4166660	2.2447999	-1.3621750
17.4166660	2.2935998	-1.3621750
17.4166660	2.3423998	-1.2026410
17.4166660	2.3912001	-1.1412816
17.4166660	2.4400001	-1.1351457
17.4166660	2.4888000	-1.1290097
17.4166660	2.5376000	-1.0860586

17.4166660	2.5864000	-1.0860586
17.4166660	2.6352000	-1.1167378
17.4166660	2.6840000	-0.9940195
17.4166660	2.7328000	-1.0062914
17.4166660	2.7816000	-0.9510679
17.4166660	2.8304000	-0.9572039
17.4166660	2.8792000	-1.0737867
17.4166660	2.9280000	-1.0492430
17.4166660	2.9768000	-0.9510679
17.4166660	3.0256000	-0.8528934
17.4166660	3.0743999	-0.8467574
17.4166660	3.1231999	-0.8467574
17.4166660	3.1719999	-0.7976699
17.4166660	3.2207999	-0.8099418
17.4166660	3.2695999	-0.8467574
17.4166660	3.3183999	-0.8222136
17.4166660	3.3671999	-0.7056313
17.4166660	3.4159999	-0.7056313
17.4166660	3.4647999	-0.7915339
17.4166660	3.5135999	-0.9510679
17.4166660	3.5623999	-0.9633398
17.4166660	3.6111999	-1.0246992
17.4166660	3.6599998	-0.8651652
17.4166660	3.7087998	-0.9019809
17.4166660	3.7575998	-0.9019809
17.4166660	3.8063998	-0.8467574
17.4166660	3.8551998	-0.7915339
17.4166660	3.9039998	-0.8528934
17.4166660	3.9527998	-0.8344855
17.4166660	4.0016003	-1.0369711
17.4166660	4.0503998	-0.7792621
17.4166660	4.0991998	-0.9940195
17.4166660	4.1479998	-0.9081168
17.4166660	4.1967998	613586.0000000
17.4166660	4.2455997	-0.6013203
17.4166660	4.2943997	-0.5583692
17.4166660	4.3431997	-0.7669902
17.4166660	4.3919997	-0.5276895
17.4166660	4.4407997	-0.5399613
17.4166660	4.4895997	-0.4724660
17.4166660	4.5383997	-0.4233789
17.4166660	4.5871997	-0.4724660
17.4166660	4.6359997	-0.4786019
17.4166660	4.6847997	613586.0000000
17.4166660	4.7336001	613586.0000000
17.4166660	4.7824001	613586.0000000
17.4166660	4.8312001	613586.0000000
17.4166660	4.8800001	613586.0000000
17.4166660	4.9288001	613586.0000000
17.4166660	4.9776001	-0.7608547
17.4166660	5.0264001	-0.8283496
17.4166660	5.0752001	-0.8283496
17.4166660	5.1240001	613586.0000000
17.4166660	5.1728001	613586.0000000
17.4166660	5.2216001	613586.0000000
17.4166660	5.2704000	613586.0000000
17.4166660	5.3192000	613586.0000000

17.4166660	5.3680000	613586.0000000
17.4166660	5.4168000	613586.0000000
17.4166660	5.4656000	613586.0000000
17.4166660	5.5144000	613586.0000000
17.4166660	5.5632000	613586.0000000
17.4166660	5.6120000	613586.0000000
17.4166660	5.6608000	613586.0000000
17.4166660	5.7096000	613586.0000000
17.4166660	5.7584000	613586.0000000
17.4166660	5.8072000	613586.0000000
17.4166660	5.8559999	613586.0000000
17.4166660	5.9047999	613586.0000000
17.4166660	5.9535999	613586.0000000
17.4166660	6.0023999	613586.0000000
17.4333324	0.2928000	-4.3871851
17.4333324	0.3416000	-4.1356125
17.4333324	0.3904000	-3.5711074
17.4333324	0.4392000	-3.5711074
17.4333324	0.4880000	-2.6200392
17.4333324	0.5368000	-2.6200392
17.4333324	0.5856000	-2.2027965
17.4333324	0.6344000	-2.0309906
17.4333324	0.6832000	-1.9021363
17.4333324	0.7320000	-2.1782527
17.4333324	0.7808000	-2.1230292
17.4333324	0.8296000	-1.9205441
17.4333324	0.8784000	-1.7794180
17.4333324	0.9272000	-2.3255150
17.4333324	0.9760000	-2.1168938
17.4333324	1.0247999	-1.8039613
17.4333324	1.0735999	-1.8039613
17.4333324	1.1223999	-1.3867188
17.4333324	1.1711999	-1.3130875
17.4333324	1.2200000	-1.3130875
17.4333324	1.2688000	-1.4542136
17.4333324	1.3176000	-1.1290097
17.4333324	1.3664000	-1.5830684
17.4333324	1.4152000	-1.5830684
17.4333324	1.4640000	-1.1412816
17.4333324	1.5128000	-1.1412816
17.4333324	1.5616000	-1.0185633
17.4333324	1.6104000	-0.8897090
17.4333324	1.6592000	-0.8958449
17.4333324	1.7079999	-0.9019809
17.4333324	1.7567999	-1.3744469
17.4333324	1.8055999	-1.1903691
17.4333324	1.8543999	-1.1780972
17.4333324	1.9031999	-1.1719613
17.4333324	1.9519999	-1.0553789
17.4333324	2.0007999	-1.1106019
17.4333324	2.0495999	-1.0308352
17.4333324	2.0983999	-1.0246992
17.4333324	2.1471999	-1.2210488
17.4333324	2.1959999	-1.2149129
17.4333324	2.2447999	-1.1106019
17.4333324	2.2935998	-1.1106019
17.4333324	2.3423998	-1.1106019

17.4333324	2.3912001	-1.0921946
17.4333324	2.4400001	-0.9694757
17.4333324	2.4888000	-0.9633398
17.4333324	2.5376000	-1.0369711
17.4333324	2.5864000	-1.0308352
17.4333324	2.6352000	-1.0308352
17.4333324	2.6840000	-1.0369711
17.4333324	2.7328000	-0.8160777
17.4333324	2.7816000	-0.8222136
17.4333324	2.8304000	-0.8958449
17.4333324	2.8792000	-0.8835731
17.4333324	2.9280000	-0.9019809
17.4333324	2.9768000	-0.8897090
17.4333324	3.0256000	-0.7915339
17.4333324	3.0743999	-0.7853980
17.4333324	3.1231999	-0.7853980
17.4333324	3.1719999	-0.7363110
17.4333324	3.2207999	-0.6933594
17.4333324	3.2695999	-0.8774371
17.4333324	3.3183999	-0.8774371
17.4333324	3.3671999	-0.7485828
17.4333324	3.4159999	-0.6565437
17.4333324	3.4647999	-0.9633398
17.4333324	3.5135999	-0.9694757
17.4333324	3.5623999	-0.6933594
17.4333324	3.6111999	-0.9694757
17.4333324	3.6599998	-0.8897090
17.4333324	3.7087998	-0.8774371
17.4333324	3.7575998	-0.9019809
17.4333324	3.8063998	-0.8958449
17.4333324	3.8551998	-0.8835731
17.4333324	3.9039998	-0.6626797
17.4333324	3.9527998	-0.6626797
17.4333324	4.0016003	-0.6626797
17.4333324	4.0503998	-0.6872234
17.4333324	4.0991998	-0.6872234
17.4333324	4.1479998	-0.5092816
17.4333324	4.1967998	-0.7608547
17.4333324	4.2455997	-0.7608547
17.4333324	4.2943997	-0.6810875
17.4333324	4.3431997	-0.5092816
17.4333324	4.3919997	613586.0000000
17.4333324	4.4407997	613586.0000000
17.4333324	4.4895997	-0.4417863
17.4333324	4.5383997	613586.0000000
17.4333324	4.5871997	613586.0000000
17.4333324	4.6359997	613586.0000000
17.4333324	4.6847997	613586.0000000
17.4333324	4.7336001	613586.0000000
17.4333324	4.7824001	613586.0000000
17.4333324	4.8312001	613586.0000000
17.4333324	4.8800001	613586.0000000
17.4333324	4.9288001	-0.6810875
17.4333324	4.9776001	613586.0000000
17.4333324	5.0264001	613586.0000000
17.4333324	5.0752001	613586.0000000
17.4333324	5.1240001	-0.2454371

17.4333324	5.1728001	-0.2577085
17.4333324	5.2216001	613586.0000000
17.4333324	5.2704000	613586.0000000
17.4333324	5.3192000	613586.0000000
17.4333324	5.3680000	613586.0000000
17.4333324	5.4168000	613586.0000000
17.4333324	5.4656000	613586.0000000
17.4333324	5.5144000	613586.0000000
17.4333324	5.5632000	613586.0000000
17.4333324	5.6120000	613586.0000000
17.4333324	5.6608000	613586.0000000
17.4333324	5.7096000	613586.0000000
17.4333324	5.7584000	613586.0000000
17.4333324	5.8072000	613586.0000000
17.4333324	5.8559999	613586.0000000
17.4333324	5.9047999	613586.0000000
17.4333324	5.9535999	613586.0000000
17.4333324	6.0023999	613586.0000000
17.4500008	0.2928000	-4.2399230
17.4500008	0.3416000	-4.2460589
17.4500008	0.3904000	-3.4361172
17.4500008	0.4392000	-3.4238453
17.4500008	0.4880000	-2.8225248
17.4500008	0.5368000	-2.3439226
17.4500008	0.5856000	-2.2948353
17.4500008	0.6344000	-1.8530488
17.4500008	0.6832000	-1.8530488
17.4500008	0.7320000	-1.7548742
17.4500008	0.7808000	-1.6382914
17.4500008	0.8296000	-1.6382914
17.4500008	0.8784000	-1.5523887
17.4500008	0.9272000	-1.4173985
17.4500008	0.9760000	-1.4173985
17.4500008	1.0247999	-1.3499031
17.4500008	1.0735999	-1.3621750
17.4500008	1.1223999	-1.6505632
17.4500008	1.1711999	-1.5707965
17.4500008	1.2200000	-1.5585246
17.4500008	1.2688000	-1.6382914
17.4500008	1.3176000	-1.6382914
17.4500008	1.3664000	-1.1719613
17.4500008	1.4152000	-1.3805828
17.4500008	1.4640000	-1.3805828
17.4500008	1.5128000	-1.0615149
17.4500008	1.5616000	-1.0431070
17.4500008	1.6104000	-0.9387960
17.4500008	1.6592000	-1.1596894
17.4500008	1.7079999	-1.1535535
17.4500008	1.7567999	-1.1351457
17.4500008	1.8055999	-1.0921946
17.4500008	1.8543999	-1.0799227
17.4500008	1.9031999	-1.0799227
17.4500008	1.9519999	-1.0983305
17.4500008	2.0007999	-0.9449320
17.4500008	2.0495999	-0.9817476
17.4500008	2.0983999	-1.2271848
17.4500008	2.1471999	-1.1903691

17.4500008	2.1959999	-1.0553789
17.4500008	2.2447999	-1.1228738
17.4500008	2.2935998	-1.1167378
17.4500008	2.3423998	-1.1167378
17.4500008	2.3912001	-1.0246992
17.4500008	2.4400001	-1.1290097
17.4500008	2.4888000	-1.1351457
17.4500008	2.5376000	-1.0431070
17.4500008	2.5864000	-1.0369711
17.4500008	2.6352000	-0.9510679
17.4500008	2.6840000	-0.9387960
17.4500008	2.7328000	-0.8958449
17.4500008	2.7816000	-0.8897090
17.4500008	2.8304000	-0.9019809
17.4500008	2.8792000	-0.8406215
17.4500008	2.9280000	-0.7669902
17.4500008	2.9768000	-1.0431070
17.4500008	3.0256000	-0.9633398
17.4500008	3.0743999	-0.8160777
17.4500008	3.1231999	-0.9019809
17.4500008	3.1719999	-0.8958449
17.4500008	3.2207999	-0.9142528
17.4500008	3.2695999	-0.8038058
17.4500008	3.3183999	-0.6994953
17.4500008	3.3671999	-0.9817476
17.4500008	3.4159999	-0.9756117
17.4500008	3.4647999	-0.9756117
17.4500008	3.5135999	-0.8713012
17.4500008	3.5623999	-0.8774371
17.4500008	3.6111999	-1.0369711
17.4500008	3.6599998	-0.9203887
17.4500008	3.7087998	-0.8897090
17.4500008	3.7575998	-0.8467574
17.4500008	3.8063998	-0.8283496
17.4500008	3.8551998	-0.8283496
17.4500008	3.9039998	-0.9878836
17.4500008	3.9527998	-0.9817476
17.4500008	4.0016003	-0.9817476
17.4500008	4.0503998	613586.0000000
17.4500008	4.0991998	613586.0000000
17.4500008	4.1479998	-0.7669902
17.4500008	4.1967998	613586.0000000
17.4500008	4.2455997	613586.0000000
17.4500008	4.2943997	-0.4970098
17.4500008	4.3431997	613586.0000000
17.4500008	4.3919997	613586.0000000
17.4500008	4.4407997	613586.0000000
17.4500008	4.4895997	613586.0000000
17.4500008	4.5383997	613586.0000000
17.4500008	4.5871997	613586.0000000
17.4500008	4.6359997	613586.0000000
17.4500008	4.6847997	613586.0000000
17.4500008	4.7336001	613586.0000000
17.4500008	4.7824001	613586.0000000
17.4500008	4.8312001	613586.0000000
17.4500008	4.8800001	613586.0000000
17.4500008	4.9288001	613586.0000000

17.4500008	4.9776001	613586.0000000
17.4500008	5.0264001	-0.5706410
17.4500008	5.0752001	-0.5583692
17.4500008	5.1240001	-0.5522332
17.4500008	5.1728001	-1.0431070
17.4500008	5.2216001	613586.0000000
17.4500008	5.2704000	613586.0000000
17.4500008	5.3192000	613586.0000000
17.4500008	5.3680000	613586.0000000
17.4500008	5.4168000	613586.0000000
17.4500008	5.4656000	613586.0000000
17.4500008	5.5144000	613586.0000000
17.4500008	5.5632000	613586.0000000
17.4500008	5.6120000	613586.0000000
17.4500008	5.6608000	613586.0000000
17.4500008	5.7096000	613586.0000000
17.4500008	5.7584000	613586.0000000
17.4500008	5.8072000	613586.0000000
17.4500008	5.8559999	613586.0000000
17.4500008	5.9047999	613586.0000000
17.4500008	5.9535999	613586.0000000
17.4500008	6.0023999	613586.0000000
17.4666672	0.2928000	-4.7001171
17.4666672	0.3416000	-4.6326222
17.4666672	0.3904000	-3.3993015
17.4666672	0.4392000	-2.6936703
17.4666672	0.4880000	-2.1905246
17.4666672	0.5368000	-1.9819031
17.4666672	0.5856000	-1.9512239
17.4666672	0.6344000	-1.7487383
17.4666672	0.6832000	-1.7426023
17.4666672	0.7320000	-1.6873789
17.4666672	0.7808000	-1.3130875
17.4666672	0.8296000	-1.2271848
17.4666672	0.8784000	-1.8162332
17.4666672	0.9272000	-1.8162332
17.4666672	0.9760000	-1.6382914
17.4666672	1.0247999	-1.7548742
17.4666672	1.0735999	-1.4848933
17.4666672	1.1223999	-1.3253593
17.4666672	1.1711999	-1.5462527
17.4666672	1.2200000	-1.5462527
17.4666672	1.2688000	-1.4542136
17.4666672	1.3176000	-1.4542136
17.4666672	1.3664000	-1.2824078
17.4666672	1.4152000	-1.2885437
17.4666672	1.4640000	-0.9387960
17.4666672	1.5128000	-0.9387960
17.4666672	1.5616000	-1.1351457
17.4666672	1.6104000	-1.1290097
17.4666672	1.6592000	-0.8651652
17.4666672	1.7079999	-1.1106019
17.4666672	1.7567999	-1.0921946
17.4666672	1.8055999	-1.0860586
17.4666672	1.8543999	-0.8283496
17.4666672	1.9031999	-0.9265246
17.4666672	1.9519999	-0.9265246

17.4666672	2.0007999	-1.2517285
17.4666672	2.0495999	-1.1965051
17.4666672	2.0983999	-1.1965051
17.4666672	2.1471999	-1.1596894
17.4666672	2.1959999	-1.1719613
17.4666672	2.2447999	-1.0799227
17.4666672	2.2935998	-1.0492430
17.4666672	2.3423998	-1.0615149
17.4666672	2.3912001	-1.0308352
17.4666672	2.4400001	-1.0124273
17.4666672	2.4888000	-0.9265246
17.4666672	2.5376000	-0.8958449
17.4666672	2.5864000	-0.9019809
17.4666672	2.6352000	-0.8897090
17.4666672	2.6840000	-0.8222136
17.4666672	2.7328000	-0.8222136
17.4666672	2.7816000	-0.8467574
17.4666672	2.8304000	-0.7240391
17.4666672	2.8792000	-0.7179031
17.4666672	2.9280000	-0.7240391
17.4666672	2.9768000	-0.6749516
17.4666672	3.0256000	-0.8344855
17.4666672	3.0743999	-0.8283496
17.4666672	3.1231999	-0.6810875
17.4666672	3.1719999	-0.8283496
17.4666672	3.2207999	-0.6994953
17.4666672	3.2695999	-0.7240391
17.4666672	3.3183999	-0.5399613
17.4666672	3.3671999	-0.7424469
17.4666672	3.4159999	-0.5522332
17.4666672	3.4647999	-0.7424469
17.4666672	3.5135999	-0.6688156
17.4666672	3.5623999	-0.6626797
17.4666672	3.6111999	-0.6626797
17.4666672	3.6599998	-0.7240391
17.4666672	3.7087998	-0.7240391
17.4666672	3.7575998	-0.7117672
17.4666672	3.8063998	-0.4786019
17.4666672	3.8551998	-0.6626797
17.4666672	3.9039998	-0.6626797
17.4666672	3.9527998	613586.0000000
17.4666672	4.0016003	-0.7424469
17.4666672	4.0503998	-0.6258640
17.4666672	4.0991998	-0.6197281
17.4666672	4.1479998	-0.7608547
17.4666672	4.1967998	-0.7547188
17.4666672	4.2455997	-0.7056313
17.4666672	4.2943997	-0.7669902
17.4666672	4.3431997	613586.0000000
17.4666672	4.3919997	613586.0000000
17.4666672	4.4407997	613586.0000000
17.4666672	4.4895997	613586.0000000
17.4666672	4.5383997	613586.0000000
17.4666672	4.5871997	613586.0000000
17.4666672	4.6359997	613586.0000000
17.4666672	4.6847997	-0.7117672
17.4666672	4.7336001	-0.5092816

17.4666672	4.7824001	-0.5154176
17.4666672	4.8312001	-0.3804274
17.4666672	4.8800001	-0.3436117
17.4666672	4.9288001	-0.3436117
17.4666672	4.9776001	-0.3374758
17.4666672	5.0264001	-0.8774371
17.4666672	5.0752001	613586.0000000
17.4666672	5.1240001	613586.0000000
17.4666672	5.1728001	613586.0000000
17.4666672	5.2216001	613586.0000000
17.4666672	5.2704000	613586.0000000
17.4666672	5.3192000	613586.0000000
17.4666672	5.3680000	613586.0000000
17.4666672	5.4168000	613586.0000000
17.4666672	5.4656000	613586.0000000
17.4666672	5.5144000	613586.0000000
17.4666672	5.5632000	613586.0000000
17.4666672	5.6120000	613586.0000000
17.4666672	5.6608000	613586.0000000
17.4666672	5.7096000	613586.0000000
17.4666672	5.7584000	613586.0000000
17.4666672	5.8072000	613586.0000000
17.4666672	5.8559999	613586.0000000
17.4666672	5.9047999	613586.0000000
17.4666672	5.9535999	613586.0000000
17.4666672	6.0023999	613586.0000000
17.4833336	0.2928000	-4.5283113
17.4833336	0.3416000	-4.3442335
17.4833336	0.3904000	-3.1477287
17.4833336	0.4392000	-2.8532043
17.4833336	0.4880000	-2.2027965
17.4833336	0.5368000	-1.8346410
17.4833336	0.5856000	-1.9573593
17.4833336	0.6344000	-1.5769324
17.4833336	0.6832000	-1.7057867
17.4833336	0.7320000	-1.7119226
17.4833336	0.7808000	-1.6076121
17.4833336	0.8296000	-1.6444273
17.4833336	0.8784000	-1.6382914
17.4833336	0.9272000	-1.2210488
17.4833336	0.9760000	-1.1351457
17.4833336	1.0247999	-1.3621750
17.4833336	1.0735999	-1.1780972
17.4833336	1.1223999	-1.1658254
17.4833336	1.1711999	-1.1719613
17.4833336	1.2200000	-1.1719613
17.4833336	1.2688000	-1.1719613
17.4833336	1.3176000	-1.1965051
17.4833336	1.3664000	-1.1965051
17.4833336	1.4152000	-1.0185633
17.4833336	1.4640000	-1.1658254
17.4833336	1.5128000	-1.1780972
17.4833336	1.5616000	-1.1780972
17.4833336	1.6104000	-1.1780972
17.4833336	1.6592000	-1.1658254
17.4833336	1.7079999	-1.1535535
17.4833336	1.7567999	-0.9387960

17.4833336	1.8055999	-0.9326606
17.4833336	1.8543999	-1.3499031
17.4833336	1.9031999	-1.2149129
17.4833336	1.9519999	-1.2149129
17.4833336	2.0007999	-1.0983305
17.4833336	2.0495999	-1.0615149
17.4833336	2.0983999	-1.0615149
17.4833336	2.1471999	-1.0615149
17.4833336	2.1959999	-1.0062914
17.4833336	2.2447999	-1.0062914
17.4833336	2.2935998	-0.9019809
17.4833336	2.3423998	-0.9142528
17.4833336	2.3912001	-0.9265246
17.4833336	2.4400001	-0.8590293
17.4833336	2.4888000	-0.7731261
17.4833336	2.5376000	-0.7669902
17.4833336	2.5864000	-0.7608547
17.4833336	2.6352000	-0.7547188
17.4833336	2.6840000	-0.8344855
17.4833336	2.7328000	-0.6933594
17.4833336	2.7816000	-0.6258640
17.4833336	2.8304000	-0.6258640
17.4833336	2.8792000	-0.6320000
17.4833336	2.9280000	-0.8344855
17.4833336	2.9768000	-0.6381359
17.4833336	3.0256000	-0.6381359
17.4833336	3.0743999	-0.6197281
17.4833336	3.1231999	-0.6749516
17.4833336	3.1719999	-0.6749516
17.4833336	3.2207999	-0.4724660
17.4833336	3.2695999	-0.5706410
17.4833336	3.3183999	-0.5645051
17.4833336	3.3671999	-0.7669902
17.4833336	3.4159999	-0.7669902
17.4833336	3.4647999	-0.8590293
17.4833336	3.5135999	-0.7608547
17.4833336	3.5623999	-0.8774371
17.4833336	3.6111999	-0.8774371
17.4833336	3.6599998	-0.7976699
17.4833336	3.7087998	-0.7485828
17.4833336	3.7575998	-0.7608547
17.4833336	3.8063998	-0.7608547
17.4833336	3.8551998	-0.6197281
17.4833336	3.9039998	-0.6933594
17.4833336	3.9527998	-0.6933594
17.4833336	4.0016003	-0.6504078
17.4833336	4.0503998	-0.4970098
17.4833336	4.0991998	-0.4970098
17.4833336	4.1479998	-0.6810875
17.4833336	4.1967998	-0.6749516
17.4833336	4.2455997	-0.6197281
17.4833336	4.2943997	-0.5031457
17.4833336	4.3431997	-0.5092816
17.4833336	4.3919997	-0.6749516
17.4833336	4.4407997	-0.6749516
17.4833336	4.4895997	-0.4724660
17.4833336	4.5383997	-0.5645051

17.4833336	4.5871997	-0.7117672
17.4833336	4.6359997	-0.7117672
17.4833336	4.6847997	-0.9940195
17.4833336	4.7336001	-0.9940195
17.4833336	4.7824001	-1.2394567
17.4833336	4.8312001	-1.2517285
17.4833336	4.8800001	-0.9940195
17.4833336	4.9288001	-0.8283496
17.4833336	4.9776001	-0.8283496
17.4833336	5.0264001	-0.6810875
17.4833336	5.0752001	-0.7301750
17.4833336	5.1240001	613586.0000000
17.4833336	5.1728001	613586.0000000
17.4833336	5.2216001	613586.0000000
17.4833336	5.2704000	613586.0000000
17.4833336	5.3192000	613586.0000000
17.4833336	5.3680000	613586.0000000
17.4833336	5.4168000	613586.0000000
17.4833336	5.4656000	613586.0000000
17.4833336	5.5144000	613586.0000000
17.4833336	5.5632000	-1.0246992
17.4833336	5.6120000	613586.0000000
17.4833336	5.6608000	613586.0000000
17.4833336	5.7096000	613586.0000000
17.4833336	5.7584000	613586.0000000
17.4833336	5.8072000	613586.0000000
17.4833336	5.8559999	613586.0000000
17.4833336	5.9047999	613586.0000000
17.4833336	5.9535999	613586.0000000
17.4833336	6.0023999	613586.0000000
17.5000000	0.2928000	-4.7737484
17.5000000	0.3416000	-3.7735929
17.5000000	0.3904000	-3.5281558
17.5000000	0.4392000	-3.5281558
17.5000000	0.4880000	-1.9757671
17.5000000	0.5368000	-1.9819031
17.5000000	0.5856000	-1.7855535
17.5000000	0.6344000	-1.8775926
17.5000000	0.6832000	-1.8714566
17.5000000	0.7320000	-1.5830684
17.5000000	0.7808000	-1.5462527
17.5000000	0.8296000	-1.3744469
17.5000000	0.8784000	-1.1965051
17.5000000	0.9272000	-1.6260195
17.5000000	0.9760000	-1.6137476
17.5000000	1.0247999	-1.1535535
17.5000000	1.0735999	-1.3376312
17.5000000	1.1223999	-1.3437672
17.5000000	1.1711999	-1.0860586
17.5000000	1.2200000	-1.0860586
17.5000000	1.2688000	-1.3867188
17.5000000	1.3176000	-1.1474175
17.5000000	1.3664000	-1.1474175
17.5000000	1.4152000	-1.1228738
17.5000000	1.4640000	-1.2824078
17.5000000	1.5128000	-1.1596894
17.5000000	1.5616000	-1.1658254

17.5000000	1.6104000	-1.1780972
17.5000000	1.6592000	-1.1719613
17.5000000	1.7079999	-0.8590293
17.5000000	1.7567999	-0.8651652
17.5000000	1.8055999	-1.4051266
17.5000000	1.8543999	-1.2578645
17.5000000	1.9031999	-1.1044660
17.5000000	1.9519999	-1.1044660
17.5000000	2.0007999	-1.1167378
17.5000000	2.0495999	-1.1167378
17.5000000	2.0983999	-1.0431070
17.5000000	2.1471999	-1.0124273
17.5000000	2.1959999	-1.0185633
17.5000000	2.2447999	-1.0062914
17.5000000	2.2935998	-0.9326606
17.5000000	2.3423998	-0.8344855
17.5000000	2.3912001	-0.8958449
17.5000000	2.4400001	-0.9817476
17.5000000	2.4888000	-0.9817476
17.5000000	2.5376000	-0.8958449
17.5000000	2.5864000	-0.7056313
17.5000000	2.6352000	-0.7117672
17.5000000	2.6840000	-0.5767770
17.5000000	2.7328000	-0.8467574
17.5000000	2.7816000	-0.8467574
17.5000000	2.8304000	-0.7853980
17.5000000	2.8792000	-0.7179031
17.5000000	2.9280000	-0.7240391
17.5000000	2.9768000	-0.6626797
17.5000000	3.0256000	-0.6626797
17.5000000	3.0743999	-0.7485828
17.5000000	3.1231999	-0.7669902
17.5000000	3.1719999	-0.5092816
17.5000000	3.2207999	-0.5092816
17.5000000	3.2695999	-0.6810875
17.5000000	3.3183999	-0.5767770
17.5000000	3.3671999	-0.5767770
17.5000000	3.4159999	-0.6626797
17.5000000	3.4647999	-0.6626797
17.5000000	3.5135999	-0.4172430
17.5000000	3.5623999	-0.4233789
17.5000000	3.6111999	-0.7363110
17.5000000	3.6599998	-0.7363110
17.5000000	3.7087998	-0.6320000
17.5000000	3.7575998	-0.7056313
17.5000000	3.8063998	-0.7056313
17.5000000	3.8551998	-0.6872234
17.5000000	3.9039998	-0.6688156
17.5000000	3.9527998	-0.5767770
17.5000000	4.0016003	-0.5706410
17.5000000	4.0503998	-0.7240391
17.5000000	4.0991998	-0.6994953
17.5000000	4.1479998	-0.5154176
17.5000000	4.1967998	-0.5276895
17.5000000	4.2455997	-0.5338254
17.5000000	4.2943997	-0.5338254
17.5000000	4.3431997	-0.4724660

17.5000000	4.3919997	-0.5031457
17.5000000	4.4407997	-0.5645051
17.5000000	4.4895997	-0.5645051
17.5000000	4.5383997	-0.5706410
17.5000000	4.5871997	-0.6381359
17.5000000	4.6359997	-0.8713012
17.5000000	4.6847997	-0.7424469
17.5000000	4.7336001	-0.6135921
17.5000000	4.7824001	-0.6074562
17.5000000	4.8312001	-0.6013203
17.5000000	4.8800001	-0.8038058
17.5000000	4.9288001	-0.8222136
17.5000000	4.9776001	613586.0000000
17.5000000	5.0264001	-0.9817476
17.5000000	5.0752001	613586.0000000
17.5000000	5.1240001	-0.9081168
17.5000000	5.1728001	-0.9081168
17.5000000	5.2216001	613586.0000000
17.5000000	5.2704000	613586.0000000
17.5000000	5.3192000	613586.0000000
17.5000000	5.3680000	613586.0000000
17.5000000	5.4168000	613586.0000000
17.5000000	5.4656000	613586.0000000
17.5000000	5.5144000	613586.0000000
17.5000000	5.5632000	613586.0000000
17.5000000	5.6120000	613586.0000000
17.5000000	5.6608000	613586.0000000
17.5000000	5.7096000	613586.0000000
17.5000000	5.7584000	613586.0000000
17.5000000	5.8072000	613586.0000000
17.5000000	5.8559999	613586.0000000
17.5000000	5.9047999	613586.0000000
17.5000000	5.9535999	613586.0000000
17.5000000	6.0023999	613586.0000000
17.5166664	0.2928000	-3.8104084
17.5166664	0.3416000	-3.7613211
17.5166664	0.3904000	-3.5036123
17.5166664	0.4392000	-3.5036123
17.5166664	0.4880000	-2.3193791
17.5166664	0.5368000	-2.0371265
17.5166664	0.5856000	-1.7364664
17.5166664	0.6344000	-1.7303305
17.5166664	0.6832000	-1.8162332
17.5166664	0.7320000	-1.1719613
17.5166664	0.7808000	-1.1719613
17.5166664	0.8296000	-1.4542136
17.5166664	0.8784000	-2.0616703
17.5166664	0.9272000	-1.8162332
17.5166664	0.9760000	-1.6566992
17.5166664	1.0247999	-1.2640004
17.5166664	1.0735999	-1.6382914
17.5166664	1.1223999	-0.9572039
17.5166664	1.1711999	-1.3314953
17.5166664	1.2200000	-1.2333207
17.5166664	1.2688000	-1.2333207
17.5166664	1.3176000	-1.1719613
17.5166664	1.3664000	-1.1842332

17.5166664	1.4152000	-0.8467574
17.5166664	1.4640000	-0.8467574
17.5166664	1.5128000	-1.2087770
17.5166664	1.5616000	-1.1412816
17.5166664	1.6104000	-1.0860586
17.5166664	1.6592000	-1.3192234
17.5166664	1.7079999	-1.4112625
17.5166664	1.7567999	-0.9940195
17.5166664	1.8055999	-1.2333207
17.5166664	1.8543999	-1.2149129
17.5166664	1.9031999	-1.1228738
17.5166664	1.9519999	-1.0737867
17.5166664	2.0007999	-1.0124273
17.5166664	2.0495999	-0.9387960
17.5166664	2.0983999	-0.9510679
17.5166664	2.1471999	-0.9326606
17.5166664	2.1959999	-0.9142528
17.5166664	2.2447999	-0.8958449
17.5166664	2.2935998	-0.9510679
17.5166664	2.3423998	-0.8774371
17.5166664	2.3912001	-0.8958449
17.5166664	2.4400001	-0.9019809
17.5166664	2.4888000	-0.8528934
17.5166664	2.5376000	-0.8222136
17.5166664	2.5864000	-0.7669902
17.5166664	2.6352000	-0.7976699
17.5166664	2.6840000	-0.8222136
17.5166664	2.7328000	-0.8344855
17.5166664	2.7816000	-0.7731261
17.5166664	2.8304000	-0.7853980
17.5166664	2.8792000	-0.6872234
17.5166664	2.9280000	-0.6933594
17.5166664	2.9768000	-0.6381359
17.5166664	3.0256000	-0.6135921
17.5166664	3.0743999	-0.4663301
17.5166664	3.1231999	-0.4663301
17.5166664	3.1719999	-0.6810875
17.5166664	3.2207999	-0.6135921
17.5166664	3.2695999	-0.6074562
17.5166664	3.3183999	-0.6074562
17.5166664	3.3671999	-0.5215535
17.5166664	3.4159999	-0.5276895
17.5166664	3.4647999	-0.7424469
17.5166664	3.5135999	-0.7424469
17.5166664	3.5623999	-0.7424469
17.5166664	3.6111999	-0.8467574
17.5166664	3.6599998	-0.7669902
17.5166664	3.7087998	-0.6504078
17.5166664	3.7575998	-0.5276895
17.5166664	3.8063998	-0.5276895
17.5166664	3.8551998	-0.6810875
17.5166664	3.9039998	-0.7056313
17.5166664	3.9527998	-0.6442719
17.5166664	4.0016003	-0.6442719
17.5166664	4.0503998	-0.6320000
17.5166664	4.0991998	-0.5460973
17.5166664	4.1479998	-0.5399613

17.5166664	4.1967998	-0.4786019
17.5166664	4.2455997	-0.4724660
17.5166664	4.2943997	-0.7853980
17.5166664	4.3431997	-0.7853980
17.5166664	4.3919997	-0.7731261
17.5166664	4.4407997	-0.4049711
17.5166664	4.4895997	-0.5154176
17.5166664	4.5383997	-0.4479222
17.5166664	4.5871997	-0.4479222
17.5166664	4.6359997	-0.4601941
17.5166664	4.6847997	-0.6381359
17.5166664	4.7336001	-0.6197281
17.5166664	4.7824001	-0.3620195
17.5166664	4.8312001	-0.8651652
17.5166664	4.8800001	-0.5460973
17.5166664	4.9288001	613586.0000000
17.5166664	4.9776001	613586.0000000
17.5166664	5.0264001	613586.0000000
17.5166664	5.0752001	613586.0000000
17.5166664	5.1240001	613586.0000000
17.5166664	5.1728001	613586.0000000
17.5166664	5.2216001	613586.0000000
17.5166664	5.2704000	-0.6933594
17.5166664	5.3192000	613586.0000000
17.5166664	5.3680000	-0.9694757
17.5166664	5.4168000	613586.0000000
17.5166664	5.4656000	613586.0000000
17.5166664	5.5144000	613586.0000000
17.5166664	5.5632000	613586.0000000
17.5166664	5.6120000	613586.0000000
17.5166664	5.6608000	613586.0000000
17.5166664	5.7096000	613586.0000000
17.5166664	5.7584000	613586.0000000
17.5166664	5.8072000	613586.0000000
17.5166664	5.8559999	613586.0000000
17.5166664	5.9047999	613586.0000000
17.5166664	5.9535999	613586.0000000
17.5166664	6.0023999	613586.0000000
17.5333328	0.2928000	-3.6631463
17.5333328	0.3416000	-3.9085832
17.5333328	0.3904000	-3.9085832
17.5333328	0.4392000	-2.8225248
17.5333328	0.4880000	-2.1475730
17.5333328	0.5368000	-2.1475730
17.5333328	0.5856000	-1.8407769
17.5333328	0.6344000	-1.7732821
17.5333328	0.6832000	-1.4235344
17.5333328	0.7320000	-1.4235344
17.5333328	0.7808000	-1.4848933
17.5333328	0.8296000	-1.4787574
17.5333328	0.8784000	-1.5523887
17.5333328	0.9272000	-1.4480777
17.5333328	0.9760000	-1.1719613
17.5333328	1.0247999	-1.2333207
17.5333328	1.0735999	-1.2271848
17.5333328	1.1223999	-1.3928547
17.5333328	1.1711999	-1.3867188

17.5333328	1.2200000	-1.1044660
17.5333328	1.2688000	-1.1106019
17.5333328	1.3176000	-1.1044660
17.5333328	1.3664000	-1.0860586
17.5333328	1.4152000	-1.2333207
17.5333328	1.4640000	-1.2271848
17.5333328	1.5128000	-1.1780972
17.5333328	1.5616000	-1.2333207
17.5333328	1.6104000	-1.4112625
17.5333328	1.6592000	-1.2701364
17.5333328	1.7079999	-1.3867188
17.5333328	1.7567999	-1.1658254
17.5333328	1.8055999	-1.1658254
17.5333328	1.8543999	-1.1719613
17.5333328	1.9031999	-1.1106019
17.5333328	1.9519999	-1.1106019
17.5333328	2.0007999	-1.1228738
17.5333328	2.0495999	-1.1780972
17.5333328	2.0983999	-1.0308352
17.5333328	2.1471999	-1.0431070
17.5333328	2.1959999	-0.9694757
17.5333328	2.2447999	-0.9694757
17.5333328	2.2935998	-0.9572039
17.5333328	2.3423998	-0.9694757
17.5333328	2.3912001	-0.9694757
17.5333328	2.4400001	-1.0246992
17.5333328	2.4888000	-0.8406215
17.5333328	2.5376000	-0.8283496
17.5333328	2.5864000	-0.6810875
17.5333328	2.6352000	-0.7731261
17.5333328	2.6840000	-0.7669902
17.5333328	2.7328000	-0.7731261
17.5333328	2.7816000	-0.7915339
17.5333328	2.8304000	-0.8099418
17.5333328	2.8792000	-0.7179031
17.5333328	2.9280000	-0.7117672
17.5333328	2.9768000	-0.8160777
17.5333328	3.0256000	-0.7056313
17.5333328	3.0743999	-0.7056313
17.5333328	3.1231999	-0.7792621
17.5333328	3.1719999	-0.6197281
17.5333328	3.2207999	-0.6258640
17.5333328	3.2695999	-0.6320000
17.5333328	3.3183999	-0.7363110
17.5333328	3.3671999	-0.8528934
17.5333328	3.4159999	-0.9878836
17.5333328	3.4647999	-1.0062914
17.5333328	3.5135999	-0.9019809
17.5333328	3.5623999	-0.9265246
17.5333328	3.6111999	-0.9265246
17.5333328	3.6599998	-0.8406215
17.5333328	3.7087998	-0.7240391
17.5333328	3.7575998	-0.7608547
17.5333328	3.8063998	-0.7669902
17.5333328	3.8551998	-0.7853980
17.5333328	3.9039998	-0.7056313
17.5333328	3.9527998	-0.6381359

17.5333328	4.0016003	-0.6013203
17.5333328	4.0503998	-0.6688156
17.5333328	4.0991998	-0.6810875
17.5333328	4.1479998	-0.6197281
17.5333328	4.1967998	-0.6197281
17.5333328	4.2455997	-0.6258640
17.5333328	4.2943997	-0.6381359
17.5333328	4.3431997	-0.9081168
17.5333328	4.3919997	-0.9081168
17.5333328	4.4407997	-0.7363110
17.5333328	4.4895997	-0.7240391
17.5333328	4.5383997	-0.5706410
17.5333328	4.5871997	-0.5890489
17.5333328	4.6359997	-0.5890489
17.5333328	4.6847997	-0.5890489
17.5333328	4.7336001	613586.0000000
17.5333328	4.7824001	-0.6810875
17.5333328	4.8312001	-0.6074562
17.5333328	4.8800001	-0.6933594
17.5333328	4.9288001	613586.0000000
17.5333328	4.9776001	613586.0000000
17.5333328	5.0264001	-0.6013203
17.5333328	5.0752001	-0.7547188
17.5333328	5.1240001	613586.0000000
17.5333328	5.1728001	613586.0000000
17.5333328	5.2216001	613586.0000000
17.5333328	5.2704000	613586.0000000
17.5333328	5.3192000	613586.0000000
17.5333328	5.3680000	613586.0000000
17.5333328	5.4168000	613586.0000000
17.5333328	5.4656000	613586.0000000
17.5333328	5.5144000	613586.0000000
17.5333328	5.5632000	613586.0000000
17.5333328	5.6120000	613586.0000000
17.5333328	5.6608000	613586.0000000
17.5333328	5.7096000	613586.0000000
17.5333328	5.7584000	613586.0000000
17.5333328	5.8072000	613586.0000000
17.5333328	5.8559999	613586.0000000
17.5333328	5.9047999	613586.0000000
17.5333328	5.9535999	613586.0000000
17.5333328	6.0023999	613586.0000000
17.5499992	0.2928000	-3.5772433
17.5499992	0.3416000	-3.6324666
17.5499992	0.3904000	-3.4422529
17.5499992	0.4392000	-2.8838840
17.5499992	0.4880000	-2.4911849
17.5499992	0.5368000	-1.7855535
17.5499992	0.5856000	-1.4173985
17.5499992	0.6344000	-1.4358063
17.5499992	0.6832000	-1.4542136
17.5499992	0.7320000	-1.4971652
17.5499992	0.7808000	-1.4787574
17.5499992	0.8296000	-1.3744469
17.5499992	0.8784000	-1.3867188
17.5499992	0.9272000	-1.5094371
17.5499992	0.9760000	-1.5033011

17.5499992	1.0247999	-1.2394567
17.5499992	1.0735999	-1.2149129
17.5499992	1.1223999	-1.2640004
17.5499992	1.1711999	-1.1965051
17.5499992	1.2200000	-1.1780972
17.5499992	1.2688000	-1.1412816
17.5499992	1.3176000	-1.1351457
17.5499992	1.3664000	-1.0676508
17.5499992	1.4152000	-0.8590293
17.5499992	1.4640000	-1.0369711
17.5499992	1.5128000	-1.0308352
17.5499992	1.5616000	-1.2333207
17.5499992	1.6104000	-1.3069515
17.5499992	1.6592000	-1.3130875
17.5499992	1.7079999	-1.2455926
17.5499992	1.7567999	-1.2640004
17.5499992	1.8055999	-1.1167378
17.5499992	1.8543999	-1.1106019
17.5499992	1.9031999	-1.0369711
17.5499992	1.9519999	-1.0799227
17.5499992	2.0007999	-1.0799227
17.5499992	2.0495999	-1.0737867
17.5499992	2.0983999	-1.0921946
17.5499992	2.1471999	-1.1106019
17.5499992	2.1959999	-1.0124273
17.5499992	2.2447999	-0.9940195
17.5499992	2.2935998	-0.9940195
17.5499992	2.3423998	-0.9878836
17.5499992	2.3912001	-0.8713012
17.5499992	2.4400001	-0.6749516
17.5499992	2.4888000	-0.9878836
17.5499992	2.5376000	-0.8283496
17.5499992	2.5864000	-0.8406215
17.5499992	2.6352000	-0.7485828
17.5499992	2.6840000	-0.8099418
17.5499992	2.7328000	-0.7915339
17.5499992	2.7816000	-0.6749516
17.5499992	2.8304000	-0.7669902
17.5499992	2.8792000	-0.8160777
17.5499992	2.9280000	-0.8099418
17.5499992	2.9768000	-0.8099418
17.5499992	3.0256000	-0.8099418
17.5499992	3.0743999	-0.8160777
17.5499992	3.1231999	-0.6626797
17.5499992	3.1719999	-1.0001554
17.5499992	3.2207999	-0.9265246
17.5499992	3.2695999	-0.9326606
17.5499992	3.3183999	-0.8774371
17.5499992	3.3671999	-1.0615149
17.5499992	3.4159999	-1.0492430
17.5499992	3.4647999	-0.9142528
17.5499992	3.5135999	-0.8590293
17.5499992	3.5623999	-0.8590293
17.5499992	3.6111999	-0.8222136
17.5499992	3.6599998	-0.9940195
17.5499992	3.7087998	-0.7363110
17.5499992	3.7575998	-0.7363110

17.5499992	3.8063998	-0.7424469
17.5499992	3.8551998	-0.7485828
17.5499992	3.9039998	-0.7424469
17.5499992	3.9527998	-0.7240391
17.5499992	4.0016003	-0.5583692
17.5499992	4.0503998	-0.6258640
17.5499992	4.0991998	-0.6320000
17.5499992	4.1479998	-0.6381359
17.5499992	4.1967998	-0.6994953
17.5499992	4.2455997	-0.7240391
17.5499992	4.2943997	-0.5583692
17.5499992	4.3431997	-0.6565437
17.5499992	4.3919997	-0.6504078
17.5499992	4.4407997	-0.6074562
17.5499992	4.4895997	-0.5890489
17.5499992	4.5383997	-0.7056313
17.5499992	4.5871997	-0.6872234
17.5499992	4.6359997	-0.7485828
17.5499992	4.6847997	-0.5522332
17.5499992	4.7336001	-0.5522332
17.5499992	4.7824001	-0.5583692
17.5499992	4.8312001	-0.7731261
17.5499992	4.8800001	-0.7731261
17.5499992	4.9288001	-0.7547188
17.5499992	4.9776001	-0.7485828
17.5499992	5.0264001	613586.0000000
17.5499992	5.0752001	613586.0000000
17.5499992	5.1240001	613586.0000000
17.5499992	5.1728001	613586.0000000
17.5499992	5.2216001	-1.2087770
17.5499992	5.2704000	613586.0000000
17.5499992	5.3192000	-1.2640004
17.5499992	5.3680000	613586.0000000
17.5499992	5.4168000	613586.0000000
17.5499992	5.4656000	-1.3744469
17.5499992	5.5144000	613586.0000000
17.5499992	5.5632000	613586.0000000
17.5499992	5.6120000	613586.0000000
17.5499992	5.6608000	613586.0000000
17.5499992	5.7096000	613586.0000000
17.5499992	5.7584000	613586.0000000
17.5499992	5.8072000	613586.0000000
17.5499992	5.8559999	613586.0000000
17.5499992	5.9047999	613586.0000000
17.5499992	5.9535999	613586.0000000
17.5499992	6.0023999	613586.0000000
17.5666676	0.2928000	-4.4914961
17.5666676	0.3416000	-3.9883502
17.5666676	0.3904000	-3.4238453
17.5666676	0.4392000	-2.7979810
17.5666676	0.4880000	-2.0432625
17.5666676	0.5368000	-1.9144082
17.5666676	0.5856000	-1.5585246
17.5666676	0.6344000	-1.5585246
17.5666676	0.6832000	-1.4542136
17.5666676	0.7320000	-1.4664855
17.5666676	0.7808000	-1.4480777

17.5666676	0.8296000	-1.7671461
17.5666676	0.8784000	-1.2701364
17.5666676	0.9272000	-1.6873789
17.5666676	0.9760000	-1.4480777
17.5666676	1.0247999	-1.3376312
17.5666676	1.0735999	-1.3499031
17.5666676	1.1223999	-1.2885437
17.5666676	1.1711999	-1.4787574
17.5666676	1.2200000	-1.1228738
17.5666676	1.2688000	-1.0492430
17.5666676	1.3176000	-1.0492430
17.5666676	1.3664000	-1.0553789
17.5666676	1.4152000	-1.0553789
17.5666676	1.4640000	-1.0431070
17.5666676	1.5128000	-1.4051266
17.5666676	1.5616000	-1.1780972
17.5666676	1.6104000	-1.3069515
17.5666676	1.6592000	-1.2517285
17.5666676	1.7079999	-1.2640004
17.5666676	1.7567999	-1.1596894
17.5666676	1.8055999	-1.1535535
17.5666676	1.8543999	-1.2087770
17.5666676	1.9031999	-1.0983305
17.5666676	1.9519999	-1.0799227
17.5666676	2.0007999	-1.1474175
17.5666676	2.0495999	-1.1596894
17.5666676	2.0983999	-1.0431070
17.5666676	2.1471999	-1.0860586
17.5666676	2.1959999	-1.0124273
17.5666676	2.2447999	-0.9940195
17.5666676	2.2935998	-0.9878836
17.5666676	2.3423998	-0.9142528
17.5666676	2.3912001	-0.9142528
17.5666676	2.4400001	-1.0001554
17.5666676	2.4888000	-1.0062914
17.5666676	2.5376000	-0.9387960
17.5666676	2.5864000	-0.9142528
17.5666676	2.6352000	-0.8651652
17.5666676	2.6840000	-0.8590293
17.5666676	2.7328000	-0.7853980
17.5666676	2.7816000	-0.7853980
17.5666676	2.8304000	-0.8038058
17.5666676	2.8792000	-0.8222136
17.5666676	2.9280000	-0.6749516
17.5666676	2.9768000	-0.7424469
17.5666676	3.0256000	-0.7485828
17.5666676	3.0743999	-0.6013203
17.5666676	3.1231999	-0.6074562
17.5666676	3.1719999	-0.8528934
17.5666676	3.2207999	-0.8528934
17.5666676	3.2695999	-0.8590293
17.5666676	3.3183999	-0.9326606
17.5666676	3.3671999	-0.9387960
17.5666676	3.4159999	-0.9387960
17.5666676	3.4647999	-0.7669902
17.5666676	3.5135999	-0.8835731
17.5666676	3.5623999	-0.8651652

17.5666676	3.6111999	-0.7547188
17.5666676	3.6599998	-0.7424469
17.5666676	3.7087998	-0.5645051
17.5666676	3.7575998	-0.6135921
17.5666676	3.8063998	-0.6135921
17.5666676	3.8551998	-0.5276895
17.5666676	3.9039998	-0.5276895
17.5666676	3.9527998	-0.9142528
17.5666676	4.0016003	-0.6013203
17.5666676	4.0503998	-0.5583692
17.5666676	4.0991998	-0.9203887
17.5666676	4.1479998	-0.7853980
17.5666676	4.1967998	-0.5951843
17.5666676	4.2455997	-0.6013203
17.5666676	4.2943997	-0.6197281
17.5666676	4.3431997	-0.6258640
17.5666676	4.3919997	-0.6258640
17.5666676	4.4407997	-0.6074562
17.5666676	4.4895997	-0.5890489
17.5666676	4.5383997	-0.5951843
17.5666676	4.5871997	-0.4908738
17.5666676	4.6359997	-0.8713012
17.5666676	4.6847997	-0.8528934
17.5666676	4.7336001	-0.8406215
17.5666676	4.7824001	-0.7424469
17.5666676	4.8312001	-0.7547188
17.5666676	4.8800001	-0.7792621
17.5666676	4.9288001	-0.7363110
17.5666676	4.9776001	613586.0000000
17.5666676	5.0264001	613586.0000000
17.5666676	5.0752001	613586.0000000
17.5666676	5.1240001	613586.0000000
17.5666676	5.1728001	613586.0000000
17.5666676	5.2216001	613586.0000000
17.5666676	5.2704000	613586.0000000
17.5666676	5.3192000	-0.7853980
17.5666676	5.3680000	-0.9265246
17.5666676	5.4168000	-0.9387960
17.5666676	5.4656000	-0.9387960
17.5666676	5.5144000	-0.8958449
17.5666676	5.5632000	-0.9510679
17.5666676	5.6120000	-0.9510679
17.5666676	5.6608000	-0.8958449
17.5666676	5.7096000	-0.7608547
17.5666676	5.7584000	-0.5645051
17.5666676	5.8072000	-0.3681555
17.5666676	5.8559999	613586.0000000
17.5666676	5.9047999	-0.8467574
17.5666676	5.9535999	613586.0000000
17.5666676	6.0023999	-0.9142528
17.5833340	0.2928000	-3.3686218
17.5833340	0.3416000	-3.5588355
17.5833340	0.3904000	-3.4238453
17.5833340	0.4392000	-2.7182140
17.5833340	0.4880000	-2.0616703
17.5833340	0.5368000	-2.0616703
17.5833340	0.5856000	-1.7241945

17.5833340	0.6344000	-1.6260195
17.5833340	0.6832000	-1.5585246
17.5833340	0.7320000	-1.6076121
17.5833340	0.7808000	-1.6137476
17.5833340	0.8296000	-1.6076121
17.5833340	0.8784000	-1.3437672
17.5833340	0.9272000	-1.3008156
17.5833340	0.9760000	-1.3560390
17.5833340	1.0247999	-1.3867188
17.5833340	1.0735999	-1.3989906
17.5833340	1.1223999	-1.3130875
17.5833340	1.1711999	-1.2455926
17.5833340	1.2200000	-1.1596894
17.5833340	1.2688000	-1.3253593
17.5833340	1.3176000	-1.1719613
17.5833340	1.3664000	-1.1719613
17.5833340	1.4152000	-1.2026410
17.5833340	1.4640000	-1.2087770
17.5833340	1.5128000	-1.2946796
17.5833340	1.5616000	-1.3069515
17.5833340	1.6104000	-1.3192234
17.5833340	1.6592000	-1.3069515
17.5833340	1.7079999	-1.2578645
17.5833340	1.7567999	-1.2578645
17.5833340	1.8055999	-1.0615149
17.5833340	1.8543999	-1.0737867
17.5833340	1.9031999	-1.0124273
17.5833340	1.9519999	-1.1474175
17.5833340	2.0007999	-1.0615149
17.5833340	2.0495999	-1.1412816
17.5833340	2.0983999	-1.0615149
17.5833340	2.1471999	-1.0553789
17.5833340	2.1959999	-1.0492430
17.5833340	2.2447999	-1.0492430
17.5833340	2.2935998	-1.0676508
17.5833340	2.3423998	-1.0676508
17.5833340	2.3912001	-1.0615149
17.5833340	2.4400001	-0.9081168
17.5833340	2.4888000	-0.9081168
17.5833340	2.5376000	-0.8406215
17.5833340	2.5864000	-0.7240391
17.5833340	2.6352000	-0.7424469
17.5833340	2.6840000	-0.9081168
17.5833340	2.7328000	-0.9265246
17.5833340	2.7816000	-0.7117672
17.5833340	2.8304000	-0.7117672
17.5833340	2.8792000	-1.0124273
17.5833340	2.9280000	-1.0124273
17.5833340	2.9768000	-0.9940195
17.5833340	3.0256000	-0.6872234
17.5833340	3.0743999	-0.8344855
17.5833340	3.1231999	-0.8590293
17.5833340	3.1719999	-0.8590293
17.5833340	3.2207999	-0.8528934
17.5833340	3.2695999	-0.7608547
17.5833340	3.3183999	-0.7547188
17.5833340	3.3671999	-0.7547188

17.5833340	3.4159999	-0.7669902
17.5833340	3.4647999	-0.7731261
17.5833340	3.5135999	-0.7669902
17.5833340	3.5623999	-0.7179031
17.5833340	3.6111999	-0.5645051
17.5833340	3.6599998	-0.5645051
17.5833340	3.7087998	-0.5215535
17.5833340	3.7575998	-0.5154176
17.5833340	3.8063998	-0.5645051
17.5833340	3.8551998	-0.6872234
17.5833340	3.9039998	-0.5706410
17.5833340	3.9527998	-0.8528934
17.5833340	4.0016003	-0.8344855
17.5833340	4.0503998	-0.7669902
17.5833340	4.0991998	-0.7056313
17.5833340	4.1479998	-0.6504078
17.5833340	4.1967998	-0.6688156
17.5833340	4.2455997	-0.5645051
17.5833340	4.2943997	-0.5215535
17.5833340	4.3431997	-0.5890489
17.5833340	4.3919997	-0.7117672
17.5833340	4.4407997	-0.6258640
17.5833340	4.4895997	-0.6258640
17.5833340	4.5383997	-0.6074562
17.5833340	4.5871997	-0.6074562
17.5833340	4.6359997	-0.6749516
17.5833340	4.6847997	-0.8590293
17.5833340	4.7336001	-0.8590293
17.5833340	4.7824001	-0.9387960
17.5833340	4.8312001	-0.8222136
17.5833340	4.8800001	-0.9510679
17.5833340	4.9288001	-0.8590293
17.5833340	4.9776001	613586.0000000
17.5833340	5.0264001	613586.0000000
17.5833340	5.0752001	613586.0000000
17.5833340	5.1240001	613586.0000000
17.5833340	5.1728001	613586.0000000
17.5833340	5.2216001	613586.0000000
17.5833340	5.2704000	613586.0000000
17.5833340	5.3192000	613586.0000000
17.5833340	5.3680000	613586.0000000
17.5833340	5.4168000	-1.1044660
17.5833340	5.4656000	-1.1044660
17.5833340	5.5144000	-1.1106019
17.5833340	5.5632000	-1.1719613
17.5833340	5.6120000	-0.6810875
17.5833340	5.6608000	-0.6872234
17.5833340	5.7096000	-0.8344855
17.5833340	5.7584000	-0.8406215
17.5833340	5.8072000	613586.0000000
17.5833340	5.8559999	613586.0000000
17.5833340	5.9047999	613586.0000000
17.5833340	5.9535999	613586.0000000
17.5833340	6.0023999	613586.0000000
17.6000004	0.2928000	-2.9329712
17.6000004	0.3416000	-3.3931656
17.6000004	0.3904000	-4.0435734

17.6000004	0.4392000	-3.0802336
17.6000004	0.4880000	-2.2334762
17.6000004	0.5368000	-1.9941750
17.6000004	0.5856000	-1.5769324
17.6000004	0.6344000	-1.5707965
17.6000004	0.6832000	-1.5646605
17.6000004	0.7320000	-1.6689711
17.6000004	0.7808000	-1.6751070
17.6000004	0.8296000	-1.5953403
17.6000004	0.8784000	-1.5892043
17.6000004	0.9272000	-1.6505632
17.6000004	0.9760000	-1.5462527
17.6000004	1.0247999	-1.5523887
17.6000004	1.0735999	-1.2087770
17.6000004	1.1223999	-1.2210488
17.6000004	1.1711999	-1.4051266
17.6000004	1.2200000	-1.2026410
17.6000004	1.2688000	-1.2087770
17.6000004	1.3176000	-1.4112625
17.6000004	1.3664000	-1.4051266
17.6000004	1.4152000	-1.1167378
17.6000004	1.4640000	-1.3867188
17.6000004	1.5128000	-1.3989906
17.6000004	1.5616000	-1.3437672
17.6000004	1.6104000	-1.4051266
17.6000004	1.6592000	-1.4051266
17.6000004	1.7079999	-1.4112625
17.6000004	1.7567999	-1.4235344
17.6000004	1.8055999	-1.2271848
17.6000004	1.8543999	-1.3376312
17.6000004	1.9031999	-1.2455926
17.6000004	1.9519999	-1.2087770
17.6000004	2.0007999	-1.2149129
17.6000004	2.0495999	-1.2149129
17.6000004	2.0983999	-1.0860586
17.6000004	2.1471999	-1.1965051
17.6000004	2.1959999	-1.2026410
17.6000004	2.2447999	-1.0553789
17.6000004	2.2935998	-1.0369711
17.6000004	2.3423998	-1.2640004
17.6000004	2.3912001	-0.9878836
17.6000004	2.4400001	-0.9878836
17.6000004	2.4888000	-0.9265246
17.6000004	2.5376000	-0.9265246
17.6000004	2.5864000	-1.1106019
17.6000004	2.6352000	-0.9756117
17.6000004	2.6840000	-1.0185633
17.6000004	2.7328000	-1.0308352
17.6000004	2.7816000	-0.9203887
17.6000004	2.8304000	-0.8774371
17.6000004	2.8792000	-0.8713012
17.6000004	2.9280000	-0.8713012
17.6000004	2.9768000	-0.8651652
17.6000004	3.0256000	-0.8528934
17.6000004	3.0743999	-1.0921946
17.6000004	3.1231999	-1.1228738
17.6000004	3.1719999	-1.1412816

17.6000004	3.2207999	-1.1412816
17.6000004	3.2695999	-0.9940195
17.6000004	3.3183999	-0.7669902
17.6000004	3.3671999	-0.8958449
17.6000004	3.4159999	-1.0492430
17.6000004	3.4647999	-0.9694757
17.6000004	3.5135999	-0.7792621
17.6000004	3.5623999	-0.8467574
17.6000004	3.6111999	-0.8283496
17.6000004	3.6599998	-0.8344855
17.6000004	3.7087998	-1.2640004
17.6000004	3.7575998	-0.9756117
17.6000004	3.8063998	-0.9387960
17.6000004	3.8551998	-0.9756117
17.6000004	3.9039998	-0.9694757
17.6000004	3.9527998	-0.9694757
17.6000004	4.0016003	-0.7547188
17.6000004	4.0503998	-0.7547188
17.6000004	4.0991998	-0.7301750
17.6000004	4.1479998	-0.7363110
17.6000004	4.1967998	-0.7424469
17.6000004	4.2455997	-0.6994953
17.6000004	4.2943997	-0.6994953
17.6000004	4.3431997	-0.7853980
17.6000004	4.3919997	-0.7915339
17.6000004	4.4407997	-0.9449320
17.6000004	4.4895997	613586.0000000
17.6000004	4.5383997	613586.0000000
17.6000004	4.5871997	-0.7731261
17.6000004	4.6359997	613586.0000000
17.6000004	4.6847997	-0.7669902
17.6000004	4.7336001	613586.0000000
17.6000004	4.7824001	613586.0000000
17.6000004	4.8312001	613586.0000000
17.6000004	4.8800001	-0.5583692
17.6000004	4.9288001	-0.5583692
17.6000004	4.9776001	-0.9510679
17.6000004	5.0264001	613586.0000000
17.6000004	5.0752001	-0.7056313
17.6000004	5.1240001	-1.3008156
17.6000004	5.1728001	-1.3069515
17.6000004	5.2216001	-1.3069515
17.6000004	5.2704000	-1.6014762
17.6000004	5.3192000	-1.5707965
17.6000004	5.3680000	-1.4542136
17.6000004	5.4168000	-1.4542136
17.6000004	5.4656000	-1.3499031
17.6000004	5.5144000	-1.3376312
17.6000004	5.5632000	-1.3376312
17.6000004	5.6120000	-1.4051266
17.6000004	5.6608000	-1.1903691
17.6000004	5.7096000	-1.3253593
17.6000004	5.7584000	-1.0492430
17.6000004	5.8072000	613586.0000000
17.6000004	5.8559999	613586.0000000
17.6000004	5.9047999	613586.0000000
17.6000004	5.9535999	613586.0000000

17.6000004	6.0023999	613586.0000000
17.6166668	0.2928000	-3.2090878
17.6166668	0.3416000	-3.6201947
17.6166668	0.3904000	-3.0434179
17.6166668	0.4392000	-2.9145637
17.6166668	0.4880000	-2.0800781
17.6166668	0.5368000	-1.7119226
17.6166668	0.5856000	-1.7241945
17.6166668	0.6344000	-1.7303305
17.6166668	0.6832000	-1.6321554
17.6166668	0.7320000	-1.6566992
17.6166668	0.7808000	-1.6628351
17.6166668	0.8296000	-1.7057867
17.6166668	0.8784000	-1.7119226
17.6166668	0.9272000	-1.4419422
17.6166668	0.9760000	-1.4480777
17.6166668	1.0247999	-1.2333207
17.6166668	1.0735999	-1.1106019
17.6166668	1.1223999	-1.1167378
17.6166668	1.1711999	-1.4051266
17.6166668	1.2200000	-1.4051266
17.6166668	1.2688000	-1.4235344
17.6166668	1.3176000	-1.3130875
17.6166668	1.3664000	-1.3253593
17.6166668	1.4152000	-1.2885437
17.6166668	1.4640000	-1.3621750
17.6166668	1.5128000	-1.3867188
17.6166668	1.5616000	-1.3805828
17.6166668	1.6104000	-1.3805828
17.6166668	1.6592000	-1.3744469
17.6166668	1.7079999	-1.3253593
17.6166668	1.7567999	-1.4051266
17.6166668	1.8055999	-1.2762718
17.6166668	1.8543999	-1.3621750
17.6166668	1.9031999	-1.2517285
17.6166668	1.9519999	-1.1474175
17.6166668	2.0007999	-1.1412816
17.6166668	2.0495999	-1.1351457
17.6166668	2.0983999	-1.1044660
17.6166668	2.1471999	-1.1044660
17.6166668	2.1959999	-1.1842332
17.6166668	2.2447999	-1.1658254
17.6166668	2.2935998	-0.9633398
17.6166668	2.3423998	-0.9817476
17.6166668	2.3912001	-1.2087770
17.6166668	2.4400001	-1.2087770
17.6166668	2.4888000	-1.0983305
17.6166668	2.5376000	-1.1044660
17.6166668	2.5864000	-1.2578645
17.6166668	2.6352000	-1.2394567
17.6166668	2.6840000	-1.2885437
17.6166668	2.7328000	-1.0062914
17.6166668	2.7816000	-1.0185633
17.6166668	2.8304000	-0.9203887
17.6166668	2.8792000	-0.9203887
17.6166668	2.9280000	-0.8344855
17.6166668	2.9768000	-0.7792621

17.6166668	3.0256000	-0.8160777
17.6166668	3.0743999	-1.0615149
17.6166668	3.1231999	-1.0799227
17.6166668	3.1719999	-1.0431070
17.6166668	3.2207999	-1.0246992
17.6166668	3.2695999	-0.9203887
17.6166668	3.3183999	-0.6994953
17.6166668	3.3671999	-0.6074562
17.6166668	3.4159999	-0.6504078
17.6166668	3.4647999	-0.8283496
17.6166668	3.5135999	-0.8222136
17.6166668	3.5623999	-1.0737867
17.6166668	3.6111999	-0.7179031
17.6166668	3.6599998	-0.6994953
17.6166668	3.7087998	-1.0921946
17.6166668	3.7575998	-0.8344855
17.6166668	3.8063998	-0.9572039
17.6166668	3.8551998	-0.6381359
17.6166668	3.9039998	-0.8344855
17.6166668	3.9527998	-0.6994953
17.6166668	4.0016003	-0.6994953
17.6166668	4.0503998	-0.7240391
17.6166668	4.0991998	-0.6135921
17.6166668	4.1479998	-0.6074562
17.6166668	4.1967998	-0.5951843
17.6166668	4.2455997	-0.5706410
17.6166668	4.2943997	-0.5338254
17.6166668	4.3431997	-0.7976699
17.6166668	4.3919997	-0.9449320
17.6166668	4.4407997	-0.9449320
17.6166668	4.4895997	-0.9387960
17.6166668	4.5383997	-0.8651652
17.6166668	4.5871997	613586.0000000
17.6166668	4.6359997	613586.0000000
17.6166668	4.6847997	613586.0000000
17.6166668	4.7336001	-1.4603496
17.6166668	4.7824001	613586.0000000
17.6166668	4.8312001	613586.0000000
17.6166668	4.8800001	613586.0000000
17.6166668	4.9288001	-0.9510679
17.6166668	4.9776001	-0.9633398
17.6166668	5.0264001	-0.9449320
17.6166668	5.0752001	-0.9572039
17.6166668	5.1240001	-1.0062914
17.6166668	5.1728001	613586.0000000
17.6166668	5.2216001	-0.8222136
17.6166668	5.2704000	-1.0492430
17.6166668	5.3192000	-1.0431070
17.6166668	5.3680000	613586.0000000
17.6166668	5.4168000	613586.0000000
17.6166668	5.4656000	613586.0000000
17.6166668	5.5144000	613586.0000000
17.6166668	5.5632000	613586.0000000
17.6166668	5.6120000	613586.0000000
17.6166668	5.6608000	613586.0000000
17.6166668	5.7096000	613586.0000000
17.6166668	5.7584000	613586.0000000

17.6166668	5.8072000	613586.0000000
17.6166668	5.8559999	613586.0000000
17.6166668	5.9047999	613586.0000000
17.6166668	5.9535999	613586.0000000
17.6166668	6.0023999	613586.0000000
17.6333332	0.2928000	-3.2397676
17.6333332	0.3416000	-3.8410881
17.6333332	0.3904000	-3.1477287
17.6333332	0.4392000	-2.2702918
17.6333332	0.4880000	-2.0493984
17.6333332	0.5368000	-1.9634953
17.6333332	0.5856000	-1.7303305
17.6333332	0.6344000	-1.8591847
17.6333332	0.6832000	-1.6198835
17.6333332	0.7320000	-1.5707965
17.6333332	0.7808000	-1.4542136
17.6333332	0.8296000	-1.3376312
17.6333332	0.8784000	-1.2333207
17.6333332	0.9272000	-1.2210488
17.6333332	0.9760000	-1.0492430
17.6333332	1.0247999	-1.1290097
17.6333332	1.0735999	-1.0124273
17.6333332	1.1223999	-1.1842332
17.6333332	1.1711999	-1.1842332
17.6333332	1.2200000	-1.1842332
17.6333332	1.2688000	-1.2455926
17.6333332	1.3176000	-1.2517285
17.6333332	1.3664000	-1.1719613
17.6333332	1.4152000	-1.1780972
17.6333332	1.4640000	-1.2640004
17.6333332	1.5128000	-1.3069515
17.6333332	1.5616000	-1.3928547
17.6333332	1.6104000	-1.3744469
17.6333332	1.6592000	-1.3744469
17.6333332	1.7079999	-1.3683109
17.6333332	1.7567999	-1.2087770
17.6333332	1.8055999	-1.2026410
17.6333332	1.8543999	-1.0799227
17.6333332	1.9031999	-1.2271848
17.6333332	1.9519999	-0.9756117
17.6333332	2.0007999	-1.0492430
17.6333332	2.0495999	-1.0246992
17.6333332	2.0983999	-1.0124273
17.6333332	2.1471999	-1.0124273
17.6333332	2.1959999	-1.0246992
17.6333332	2.2447999	-1.0246992
17.6333332	2.2935998	-1.0246992
17.6333332	2.3423998	-0.8958449
17.6333332	2.3912001	-1.0369711
17.6333332	2.4400001	-1.0615149
17.6333332	2.4888000	-1.0799227
17.6333332	2.5376000	-0.9387960
17.6333332	2.5864000	-0.9387960
17.6333332	2.6352000	-0.8283496
17.6333332	2.6840000	-0.8283496
17.6333332	2.7328000	-0.7547188
17.6333332	2.7816000	-0.7792621

17.6333332	2.8304000	-0.7731261
17.6333332	2.8792000	-0.9633398
17.6333332	2.9280000	-0.8038058
17.6333332	2.9768000	-0.8467574
17.6333332	3.0256000	-0.7179031
17.6333332	3.0743999	-0.5399613
17.6333332	3.1231999	-0.3436117
17.6333332	3.1719999	-0.6135921
17.6333332	3.2207999	-0.6197281
17.6333332	3.2695999	-0.6197281
17.6333332	3.3183999	-0.7056313
17.6333332	3.3671999	-0.6197281
17.6333332	3.4159999	-0.5645051
17.6333332	3.4647999	-0.6810875
17.6333332	3.5135999	-0.8222136
17.6333332	3.5623999	-0.5706410
17.6333332	3.6111999	-0.5645051
17.6333332	3.6599998	-0.7240391
17.6333332	3.7087998	-0.7179031
17.6333332	3.7575998	-0.5092816
17.6333332	3.8063998	-0.5338254
17.6333332	3.8551998	-0.4724660
17.6333332	3.9039998	-0.3129320
17.6333332	3.9527998	-0.3129320
17.6333332	4.0016003	-0.2577085
17.6333332	4.0503998	-0.2577085
17.6333332	4.0991998	-0.4663301
17.6333332	4.1479998	-0.6994953
17.6333332	4.1967998	-0.5829129
17.6333332	4.2455997	613586.0000000
17.6333332	4.2943997	-0.6504078
17.6333332	4.3431997	-0.5951843
17.6333332	4.3919997	-0.8160777
17.6333332	4.4407997	-0.7915339
17.6333332	4.4895997	-0.7915339
17.6333332	4.5383997	613586.0000000
17.6333332	4.5871997	613586.0000000
17.6333332	4.6359997	613586.0000000
17.6333332	4.6847997	613586.0000000
17.6333332	4.7336001	613586.0000000
17.6333332	4.7824001	613586.0000000
17.6333332	4.8312001	-0.6810875
17.6333332	4.8800001	613586.0000000
17.6333332	4.9288001	613586.0000000
17.6333332	4.9776001	613586.0000000
17.6333332	5.0264001	613586.0000000
17.6333332	5.0752001	613586.0000000
17.6333332	5.1240001	-0.7424469
17.6333332	5.1728001	613586.0000000
17.6333332	5.2216001	613586.0000000
17.6333332	5.2704000	613586.0000000
17.6333332	5.3192000	613586.0000000
17.6333332	5.3680000	613586.0000000
17.6333332	5.4168000	613586.0000000
17.6333332	5.4656000	613586.0000000
17.6333332	5.5144000	613586.0000000
17.6333332	5.5632000	613586.0000000

17.6333332	5.6120000	613586.0000000
17.6333332	5.6608000	-1.4051266
17.6333332	5.7096000	-1.4051266
17.6333332	5.7584000	-1.0246992
17.6333332	5.8072000	613586.0000000
17.6333332	5.8559999	613586.0000000
17.6333332	5.9047999	613586.0000000
17.6333332	5.9535999	613586.0000000
17.6333332	6.0023999	-0.3926992
17.6499996	0.2928000	-3.2581754
17.6499996	0.3416000	-3.5526996
17.6499996	0.3904000	-3.9392629
17.6499996	0.4392000	-2.9206996
17.6499996	0.4880000	-1.9819031
17.6499996	0.5368000	-1.9757671
17.6499996	0.5856000	-1.4112625
17.6499996	0.6344000	-1.4603496
17.6499996	0.6832000	-1.4112625
17.6499996	0.7320000	-1.4112625
17.6499996	0.7808000	-1.4173985
17.6499996	0.8296000	-1.2455926
17.6499996	0.8784000	-1.2517285
17.6499996	0.9272000	-1.1351457
17.6499996	0.9760000	-1.0185633
17.6499996	1.0247999	-1.0369711
17.6499996	1.0735999	-1.0431070
17.6499996	1.1223999	-0.9387960
17.6499996	1.1711999	-0.9387960
17.6499996	1.2200000	-1.2087770
17.6499996	1.2688000	-1.2640004
17.6499996	1.3176000	-1.1842332
17.6499996	1.3664000	-1.1780972
17.6499996	1.4152000	-1.2210488
17.6499996	1.4640000	-1.1965051
17.6499996	1.5128000	-1.1965051
17.6499996	1.5616000	-1.2762718
17.6499996	1.6104000	-1.3683109
17.6499996	1.6592000	-1.1903691
17.6499996	1.7079999	-1.1842332
17.6499996	1.7567999	-1.1228738
17.6499996	1.8055999	-1.1167378
17.6499996	1.8543999	-1.1167378
17.6499996	1.9031999	-1.1228738
17.6499996	1.9519999	-1.1228738
17.6499996	2.0007999	-1.1412816
17.6499996	2.0495999	-1.1658254
17.6499996	2.0983999	-1.1719613
17.6499996	2.1471999	-1.0737867
17.6499996	2.1959999	-1.0860586
17.6499996	2.2447999	-1.0860586
17.6499996	2.2935998	-1.0553789
17.6499996	2.3423998	-0.9510679
17.6499996	2.3912001	-0.9019809
17.6499996	2.4400001	-0.9019809
17.6499996	2.4888000	-0.8344855
17.6499996	2.5376000	-0.8467574
17.6499996	2.5864000	-0.8467574

17.6499996	2.6352000	-0.8222136
17.6499996	2.6840000	-0.9694757
17.6499996	2.7328000	-0.8038058
17.6499996	2.7816000	-0.8099418
17.6499996	2.8304000	-0.7056313
17.6499996	2.8792000	-1.0308352
17.6499996	2.9280000	-1.0553789
17.6499996	2.9768000	-0.5951843
17.6499996	3.0256000	-0.6749516
17.6499996	3.0743999	-0.6749516
17.6499996	3.1231999	-0.6197281
17.6499996	3.1719999	-0.5276895
17.6499996	3.2207999	-0.5338254
17.6499996	3.2695999	-0.4786019
17.6499996	3.3183999	-0.4724660
17.6499996	3.3671999	-0.5092816
17.6499996	3.4159999	-0.5092816
17.6499996	3.4647999	-0.6258640
17.6499996	3.5135999	-0.6258640
17.6499996	3.5623999	-0.7485828
17.6499996	3.6111999	-0.5583692
17.6499996	3.6599998	-0.5460973
17.6499996	3.7087998	-0.5399613
17.6499996	3.7575998	-0.6135921
17.6499996	3.8063998	-0.6074562
17.6499996	3.8551998	-0.5951843
17.6499996	3.9039998	-0.7363110
17.6499996	3.9527998	-0.4663301
17.6499996	4.0016003	-0.4786019
17.6499996	4.0503998	-0.9142528
17.6499996	4.0991998	-0.9572039
17.6499996	4.1479998	-0.9572039
17.6499996	4.1967998	-0.8528934
17.6499996	4.2455997	-0.9694757
17.6499996	4.2943997	-0.6688156
17.6499996	4.3431997	-0.7731261
17.6499996	4.3919997	-0.6810875
17.6499996	4.4407997	-0.8160777
17.6499996	4.4895997	-0.8222136
17.6499996	4.5383997	613586.0000000
17.6499996	4.5871997	613586.0000000
17.6499996	4.6359997	613586.0000000
17.6499996	4.6847997	613586.0000000
17.6499996	4.7336001	-0.5645051
17.6499996	4.7824001	-0.7976699
17.6499996	4.8312001	-0.7915339
17.6499996	4.8800001	-0.4479222
17.6499996	4.9288001	-0.4479222
17.6499996	4.9776001	-0.4540582
17.6499996	5.0264001	-0.3374758
17.6499996	5.0752001	613586.0000000
17.6499996	5.1240001	613586.0000000
17.6499996	5.1728001	613586.0000000
17.6499996	5.2216001	613586.0000000
17.6499996	5.2704000	613586.0000000
17.6499996	5.3192000	613586.0000000
17.6499996	5.3680000	-1.1535535

17.6499996	5.4168000	-0.8958449
17.6499996	5.4656000	-0.6994953
17.6499996	5.5144000	-0.7056313
17.6499996	5.5632000	-0.8774371
17.6499996	5.6120000	-0.8958449
17.6499996	5.6608000	-0.7485828
17.6499996	5.7096000	-0.7301750
17.6499996	5.7584000	-0.8713012
17.6499996	5.8072000	-0.8713012
17.6499996	5.8559999	-0.8774371
17.6499996	5.9047999	-0.5154176
17.6499996	5.9535999	-0.5031457
17.6499996	6.0023999	613586.0000000
17.6666660	0.2928000	-3.9822142
17.6666660	0.3416000	-4.1294765
17.6666660	0.3904000	-3.2459035
17.6666660	0.4392000	-2.6077673
17.6666660	0.4880000	-1.8346410
17.6666660	0.5368000	-1.5830684
17.6666660	0.5856000	-1.5155730
17.6666660	0.6344000	-1.5033011
17.6666660	0.6832000	-1.5155730
17.6666660	0.7320000	-1.8285050
17.6666660	0.7808000	-1.4051266
17.6666660	0.8296000	-1.4235344
17.6666660	0.8784000	-1.0860586
17.6666660	0.9272000	-1.0308352
17.6666660	0.9760000	-1.0431070
17.6666660	1.0247999	-1.0860586
17.6666660	1.0735999	-1.0676508
17.6666660	1.1223999	-1.0615149
17.6666660	1.1711999	-1.1535535
17.6666660	1.2200000	-1.0185633
17.6666660	1.2688000	-1.0185633
17.6666660	1.3176000	-1.0921946
17.6666660	1.3664000	-1.2762718
17.6666660	1.4152000	-1.2026410
17.6666660	1.4640000	-1.2026410
17.6666660	1.5128000	-1.3192234
17.6666660	1.5616000	-1.3192234
17.6666660	1.6104000	-1.2394567
17.6666660	1.6592000	-1.2394567
17.6666660	1.7079999	-1.2333207
17.6666660	1.7567999	-1.3130875
17.6666660	1.8055999	-1.3192234
17.6666660	1.8543999	-1.3192234
17.6666660	1.9031999	-1.2578645
17.6666660	1.9519999	-1.2517285
17.6666660	2.0007999	-1.0860586
17.6666660	2.0495999	-1.1167378
17.6666660	2.0983999	-1.3314953
17.6666660	2.1471999	-1.2026410
17.6666660	2.1959999	-1.3069515
17.6666660	2.2447999	-1.0124273
17.6666660	2.2935998	-1.0124273
17.6666660	2.3423998	-1.0124273
17.6666660	2.3912001	-0.9326606

17.6666660	2.4400001	-1.2333207
17.6666660	2.4888000	-1.2333207
17.6666660	2.5376000	-1.1780972
17.6666660	2.5864000	-0.8651652
17.6666660	2.6352000	-0.8099418
17.6666660	2.6840000	-0.8651652
17.6666660	2.7328000	-0.8467574
17.6666660	2.7816000	-1.1719613
17.6666660	2.8304000	-0.8651652
17.6666660	2.8792000	-0.8713012
17.6666660	2.9280000	-0.6565437
17.6666660	2.9768000	-0.6749516
17.6666660	3.0256000	-0.6749516
17.6666660	3.0743999	-0.6320000
17.6666660	3.1231999	-0.7547188
17.6666660	3.1719999	-0.7547188
17.6666660	3.2207999	-0.7547188
17.6666660	3.2695999	-0.6626797
17.6666660	3.3183999	-0.6810875
17.6666660	3.3671999	-0.6872234
17.6666660	3.4159999	-0.7179031
17.6666660	3.4647999	-0.7608547
17.6666660	3.5135999	-0.6810875
17.6666660	3.5623999	-0.7608547
17.6666660	3.6111999	-0.7608547
17.6666660	3.6599998	-0.7976699
17.6666660	3.7087998	-0.7976699
17.6666660	3.7575998	-0.6442719
17.6666660	3.8063998	-0.6442719
17.6666660	3.8551998	-0.6258640
17.6666660	3.9039998	-0.6197281
17.6666660	3.9527998	-0.6258640
17.6666660	4.0016003	-0.6135921
17.6666660	4.0503998	-0.6135921
17.6666660	4.0991998	-0.9817476
17.6666660	4.1479998	-0.9142528
17.6666660	4.1967998	-0.9142528
17.6666660	4.2455997	-0.9081168
17.6666660	4.2943997	-0.8344855
17.6666660	4.3431997	-0.8344855
17.6666660	4.3919997	-0.7669902
17.6666660	4.4407997	-0.6749516
17.6666660	4.4895997	-0.7853980
17.6666660	4.5383997	-0.7915339
17.6666660	4.5871997	-1.0062914
17.6666660	4.6359997	-0.7056313
17.6666660	4.6847997	-0.7056313
17.6666660	4.7336001	-0.7976699
17.6666660	4.7824001	-0.6565437
17.6666660	4.8312001	-0.6626797
17.6666660	4.8800001	-0.6626797
17.6666660	4.9288001	-0.8222136
17.6666660	4.9776001	613586.0000000
17.6666660	5.0264001	613586.0000000
17.6666660	5.0752001	613586.0000000
17.6666660	5.1240001	613586.0000000
17.6666660	5.1728001	-1.0369711

17.6666660	5.2216001	-1.0308352
17.6666660	5.2704000	-1.0308352
17.6666660	5.3192000	-1.0799227
17.6666660	5.3680000	-1.1167378
17.6666660	5.4168000	-0.9756117
17.6666660	5.4656000	-0.6135921
17.6666660	5.5144000	-0.5154176
17.6666660	5.5632000	-0.7363110
17.6666660	5.6120000	-0.7853980
17.6666660	5.6608000	-0.7301750
17.6666660	5.7096000	-0.5829129
17.6666660	5.7584000	-0.6626797
17.6666660	5.8072000	-0.7485828
17.6666660	5.8559999	-0.7485828
17.6666660	5.9047999	613586.0000000
17.6666660	5.9535999	613586.0000000
17.6666660	6.0023999	613586.0000000
17.6833324	0.2928000	-4.3012824
17.6833324	0.3416000	-3.5220201
17.6833324	0.3904000	-3.1906800
17.6833324	0.4392000	-2.0248547
17.6833324	0.4880000	-2.0248547
17.6833324	0.5368000	-1.8775926
17.6833324	0.5856000	-1.7548742
17.6833324	0.6344000	-1.9328160
17.6833324	0.6832000	-1.9450879
17.6833324	0.7320000	-1.7978253
17.6833324	0.7808000	-1.3805828
17.6833324	0.8296000	-1.4971652
17.6833324	0.8784000	-1.4971652
17.6833324	0.9272000	-1.2701364
17.6833324	0.9760000	-1.3253593
17.6833324	1.0247999	-1.2210488
17.6833324	1.0735999	-0.9510679
17.6833324	1.1223999	-0.9449320
17.6833324	1.1711999	-1.2271848
17.6833324	1.2200000	-1.0492430
17.6833324	1.2688000	-1.1290097
17.6833324	1.3176000	-1.0737867
17.6833324	1.3664000	-1.1351457
17.6833324	1.4152000	-1.1412816
17.6833324	1.4640000	-1.1719613
17.6833324	1.5128000	-1.3744469
17.6833324	1.5616000	-1.2578645
17.6833324	1.6104000	-1.2333207
17.6833324	1.6592000	-1.2149129
17.6833324	1.7079999	-1.2026410
17.6833324	1.7567999	-1.2701364
17.6833324	1.8055999	-1.3560390
17.6833324	1.8543999	-1.3560390
17.6833324	1.9031999	-1.3560390
17.6833324	1.9519999	-1.1596894
17.6833324	2.0007999	-1.1719613
17.6833324	2.0495999	-1.1842332
17.6833324	2.0983999	-1.1780972
17.6833324	2.1471999	-1.0983305
17.6833324	2.1959999	-1.0799227

17.6833324	2.2447999	-1.0431070
17.6833324	2.2935998	-1.0246992
17.6833324	2.3423998	-1.0001554
17.6833324	2.3912001	-0.9756117
17.6833324	2.4400001	-1.0921946
17.6833324	2.4888000	-0.9203887
17.6833324	2.5376000	-1.1228738
17.6833324	2.5864000	-0.8283496
17.6833324	2.6352000	-0.8467574
17.6833324	2.6840000	-0.9387960
17.6833324	2.7328000	-1.1228738
17.6833324	2.7816000	-0.7485828
17.6833324	2.8304000	-0.7608547
17.6833324	2.8792000	-0.8099418
17.6833324	2.9280000	-0.7608547
17.6833324	2.9768000	-0.7608547
17.6833324	3.0256000	-0.8774371
17.6833324	3.0743999	-0.8713012
17.6833324	3.1231999	-0.9817476
17.6833324	3.1719999	-0.8038058
17.6833324	3.2207999	-0.8038058
17.6833324	3.2695999	-0.9694757
17.6833324	3.3183999	-0.7301750
17.6833324	3.3671999	-0.7179031
17.6833324	3.4159999	-0.7056313
17.6833324	3.4647999	-0.7731261
17.6833324	3.5135999	-0.9019809
17.6833324	3.5623999	-0.7853980
17.6833324	3.6111999	-0.7976699
17.6833324	3.6599998	-0.6442719
17.6833324	3.7087998	-0.5890489
17.6833324	3.7575998	-0.6013203
17.6833324	3.8063998	-0.4908738
17.6833324	3.8551998	-0.5031457
17.6833324	3.9039998	-0.4970098
17.6833324	3.9527998	-0.6381359
17.6833324	4.0016003	-0.8038058
17.6833324	4.0503998	-0.5890489
17.6833324	4.0991998	-0.5890489
17.6833324	4.1479998	-0.8651652
17.6833324	4.1967998	-0.7915339
17.6833324	4.2455997	-0.7792621
17.6833324	4.2943997	-0.5522332
17.6833324	4.3431997	-0.5522332
17.6833324	4.3919997	-0.6074562
17.6833324	4.4407997	613586.0000000
17.6833324	4.4895997	613586.0000000
17.6833324	4.5383997	-0.5583692
17.6833324	4.5871997	-0.3558836
17.6833324	4.6359997	-0.5092816
17.6833324	4.6847997	-0.5154176
17.6833324	4.7336001	-0.4356503
17.6833324	4.7824001	-0.4417863
17.6833324	4.8312001	-0.3436117
17.6833324	4.8800001	-0.3252039
17.6833324	4.9288001	-0.3252039
17.6833324	4.9776001	-0.2208934

17.6833324	5.0264001	613586.0000000
17.6833324	5.0752001	613586.0000000
17.6833324	5.1240001	613586.0000000
17.6833324	5.1728001	-0.4295144
17.6833324	5.2216001	-0.4295144
17.6833324	5.2704000	-0.6994953
17.6833324	5.3192000	-0.8038058
17.6833324	5.3680000	-0.8099418
17.6833324	5.4168000	-0.7669902
17.6833324	5.4656000	-0.5890489
17.6833324	5.5144000	-0.6135921
17.6833324	5.5632000	-0.7363110
17.6833324	5.6120000	-0.7363110
17.6833324	5.6608000	-0.6381359
17.6833324	5.7096000	-0.9326606
17.6833324	5.7584000	-0.6688156
17.6833324	5.8072000	-0.6749516
17.6833324	5.8559999	-0.8283496
17.6833324	5.9047999	-0.5829129
17.6833324	5.9535999	-0.5399613
17.6833324	6.0023999	-0.5338254
17.7000008	0.2928000	-3.4790685
17.7000008	0.3416000	-3.6815541
17.7000008	0.3904000	-3.0679617
17.7000008	0.4392000	-2.2273402
17.7000008	0.4880000	-1.9757671
17.7000008	0.5368000	-1.9819031
17.7000008	0.5856000	-1.8530488
17.7000008	0.6344000	-1.4910293
17.7000008	0.6832000	-1.4848933
17.7000008	0.7320000	-1.6076121
17.7000008	0.7808000	-2.0309906
17.7000008	0.8296000	-1.4419422
17.7000008	0.8784000	-1.2578645
17.7000008	0.9272000	-1.2640004
17.7000008	0.9760000	-1.2701364
17.7000008	1.0247999	-1.1351457
17.7000008	1.0735999	-0.8958449
17.7000008	1.1223999	-1.0860586
17.7000008	1.1711999	-1.0799227
17.7000008	1.2200000	-1.1658254
17.7000008	1.2688000	-1.1596894
17.7000008	1.3176000	-1.1474175
17.7000008	1.3664000	-1.1965051
17.7000008	1.4152000	-1.2333207
17.7000008	1.4640000	-1.2333207
17.7000008	1.5128000	-1.1351457
17.7000008	1.5616000	-1.1351457
17.7000008	1.6104000	-1.1596894
17.7000008	1.6592000	-1.1474175
17.7000008	1.7079999	-1.2517285
17.7000008	1.7567999	-1.2517285
17.7000008	1.8055999	-1.2517285
17.7000008	1.8543999	-1.2640004
17.7000008	1.9031999	-1.3621750
17.7000008	1.9519999	-1.2087770
17.7000008	2.0007999	-1.1842332

17.7000008	2.0495999	-1.1167378
17.7000008	2.0983999	-1.1167378
17.7000008	2.1471999	-1.0431070
17.7000008	2.1959999	-1.0431070
17.7000008	2.2447999	-1.0553789
17.7000008	2.2935998	-1.3744469
17.7000008	2.3423998	-1.2762718
17.7000008	2.3912001	-1.2640004
17.7000008	2.4400001	-1.1535535
17.7000008	2.4888000	-1.2210488
17.7000008	2.5376000	-0.9694757
17.7000008	2.5864000	-1.0737867
17.7000008	2.6352000	-1.0431070
17.7000008	2.6840000	-1.0921946
17.7000008	2.7328000	-0.8590293
17.7000008	2.7816000	-0.6994953
17.7000008	2.8304000	-0.7915339
17.7000008	2.8792000	-0.7792621
17.7000008	2.9280000	-0.6381359
17.7000008	2.9768000	-0.6381359
17.7000008	3.0256000	-0.8160777
17.7000008	3.0743999	-0.7056313
17.7000008	3.1231999	-0.9326606
17.7000008	3.1719999	-0.9326606
17.7000008	3.2207999	-0.7792621
17.7000008	3.2695999	-0.7792621
17.7000008	3.3183999	-0.7424469
17.7000008	3.3671999	-0.8406215
17.7000008	3.4159999	-0.8406215
17.7000008	3.4647999	-0.8406215
17.7000008	3.5135999	-0.7363110
17.7000008	3.5623999	-0.5706410
17.7000008	3.6111999	-0.5767770
17.7000008	3.6599998	-0.5583692
17.7000008	3.7087998	-0.5645051
17.7000008	3.7575998	-0.9142528
17.7000008	3.8063998	-0.9203887
17.7000008	3.8551998	-0.8590293
17.7000008	3.9039998	-0.8406215
17.7000008	3.9527998	-0.9817476
17.7000008	4.0016003	-0.5399613
17.7000008	4.0503998	-0.8835731
17.7000008	4.0991998	-0.7608547
17.7000008	4.1479998	-0.6013203
17.7000008	4.1967998	-0.6688156
17.7000008	4.2455997	-0.7731261
17.7000008	4.2943997	-0.7731261
17.7000008	4.3431997	-0.7731261
17.7000008	4.3919997	-0.5767770
17.7000008	4.4407997	-0.5890489
17.7000008	4.4895997	613586.0000000
17.7000008	4.5383997	613586.0000000
17.7000008	4.5871997	613586.0000000
17.7000008	4.6359997	613586.0000000
17.7000008	4.6847997	613586.0000000
17.7000008	4.7336001	613586.0000000
17.7000008	4.7824001	613586.0000000

17.7000008	4.8312001	-0.2699804
17.7000008	4.8800001	-0.2761164
17.7000008	4.9288001	-0.2761164
17.7000008	4.9776001	613586.0000000
17.7000008	5.0264001	613586.0000000
17.7000008	5.0752001	613586.0000000
17.7000008	5.1240001	613586.0000000
17.7000008	5.1728001	-0.6933594
17.7000008	5.2216001	-0.9694757
17.7000008	5.2704000	-0.9817476
17.7000008	5.3192000	-0.9817476
17.7000008	5.3680000	-0.9817476
17.7000008	5.4168000	-0.8774371
17.7000008	5.4656000	-0.9265246
17.7000008	5.5144000	-0.8467574
17.7000008	5.5632000	-0.8160777
17.7000008	5.6120000	-0.8099418
17.7000008	5.6608000	-0.8283496
17.7000008	5.7096000	-0.8222136
17.7000008	5.7584000	-0.7056313
17.7000008	5.8072000	-0.7117672
17.7000008	5.8559999	-0.6442719
17.7000008	5.9047999	-0.6504078
17.7000008	5.9535999	613586.0000000
17.7000008	6.0023999	-0.6135921
17.7166672	0.2928000	-3.4177094
17.7166672	0.3416000	-3.6876900
17.7166672	0.3904000	-3.1170490
17.7166672	0.4392000	-3.1231849
17.7166672	0.4880000	-2.0555344
17.7166672	0.5368000	-2.0555344
17.7166672	0.5856000	-1.8530488
17.7166672	0.6344000	-1.7916894
17.7166672	0.6832000	-1.6566992
17.7166672	0.7320000	-1.6382914
17.7166672	0.7808000	-1.4971652
17.7166672	0.8296000	-1.2824078
17.7166672	0.8784000	-1.2762718
17.7166672	0.9272000	-1.5462527
17.7166672	0.9760000	-1.3008156
17.7166672	1.0247999	-1.3008156
17.7166672	1.0735999	-1.3253593
17.7166672	1.1223999	-1.3376312
17.7166672	1.1711999	-1.2762718
17.7166672	1.2200000	-1.1658254
17.7166672	1.2688000	-1.1596894
17.7166672	1.3176000	-1.1535535
17.7166672	1.3664000	-1.1535535
17.7166672	1.4152000	-1.1290097
17.7166672	1.4640000	-1.2210488
17.7166672	1.5128000	-1.2087770
17.7166672	1.5616000	-1.2087770
17.7166672	1.6104000	-1.1228738
17.7166672	1.6592000	-1.1842332
17.7166672	1.7079999	-1.3253593
17.7166672	1.7567999	-1.2455926
17.7166672	1.8055999	-1.2455926

17.7166672	1.8543999	-1.4603496
17.7166672	1.9031999	-1.1167378
17.7166672	1.9519999	-1.1106019
17.7166672	2.0007999	-1.0246992
17.7166672	2.0495999	-1.0431070
17.7166672	2.0983999	-1.3008156
17.7166672	2.1471999	-1.2824078
17.7166672	2.1959999	-1.2946796
17.7166672	2.2447999	-1.0737867
17.7166672	2.2935998	-1.1842332
17.7166672	2.3423998	-1.1290097
17.7166672	2.3912001	-1.1228738
17.7166672	2.4400001	-1.1228738
17.7166672	2.4888000	-1.0308352
17.7166672	2.5376000	-0.7179031
17.7166672	2.5864000	-1.1106019
17.7166672	2.6352000	-0.7424469
17.7166672	2.6840000	-0.7117672
17.7166672	2.7328000	-0.7792621
17.7166672	2.7816000	-0.6688156
17.7166672	2.8304000	-0.6872234
17.7166672	2.8792000	-0.6197281
17.7166672	2.9280000	-0.6258640
17.7166672	2.9768000	-0.5645051
17.7166672	3.0256000	-0.5767770
17.7166672	3.0743999	-0.7117672
17.7166672	3.1231999	-0.6872234
17.7166672	3.1719999	-0.6872234
17.7166672	3.2207999	-0.6994953
17.7166672	3.2695999	-0.6258640
17.7166672	3.3183999	-0.6504078
17.7166672	3.3671999	-0.6504078
17.7166672	3.4159999	-0.5338254
17.7166672	3.4647999	-0.5399613
17.7166672	3.5135999	-0.5276895
17.7166672	3.5623999	-1.0737867
17.7166672	3.6111999	-0.9633398
17.7166672	3.6599998	-0.9572039
17.7166672	3.7087998	-0.9572039
17.7166672	3.7575998	-0.8958449
17.7166672	3.8063998	-0.9694757
17.7166672	3.8551998	-0.8467574
17.7166672	3.9039998	-0.9081168
17.7166672	3.9527998	-0.9081168
17.7166672	4.0016003	-0.6626797
17.7166672	4.0503998	-0.7731261
17.7166672	4.0991998	-0.7547188
17.7166672	4.1479998	-0.7792621
17.7166672	4.1967998	-0.7547188
17.7166672	4.2455997	-0.7485828
17.7166672	4.2943997	-0.6381359
17.7166672	4.3431997	-0.5154176
17.7166672	4.3919997	-0.5092816
17.7166672	4.4407997	613586.0000000
17.7166672	4.4895997	-0.6258640
17.7166672	4.5383997	-0.5338254
17.7166672	4.5871997	-0.8651652

17.7166672	4.6359997	-0.8099418
17.7166672	4.6847997	-0.7976699
17.7166672	4.7336001	-0.5767770
17.7166672	4.7824001	-0.5154176
17.7166672	4.8312001	-0.5154176
17.7166672	4.8800001	-0.5829129
17.7166672	4.9288001	613586.0000000
17.7166672	4.9776001	-0.4786019
17.7166672	5.0264001	-0.4847379
17.7166672	5.0752001	-0.5645051
17.7166672	5.1240001	613586.0000000
17.7166672	5.1728001	613586.0000000
17.7166672	5.2216001	613586.0000000
17.7166672	5.2704000	613586.0000000
17.7166672	5.3192000	-0.7117672
17.7166672	5.3680000	-0.7117672
17.7166672	5.4168000	-0.7179031
17.7166672	5.4656000	-0.7669902
17.7166672	5.5144000	-0.6749516
17.7166672	5.5632000	-0.6749516
17.7166672	5.6120000	-0.6994953
17.7166672	5.6608000	-0.8160777
17.7166672	5.7096000	-0.8099418
17.7166672	5.7584000	-0.7240391
17.7166672	5.8072000	-0.7117672
17.7166672	5.8559999	-0.7117672
17.7166672	5.9047999	613586.0000000
17.7166672	5.9535999	613586.0000000
17.7166672	6.0023999	613586.0000000
17.7333336	0.2928000	-4.6264863
17.7333336	0.3416000	-3.6815541
17.7333336	0.3904000	-2.6998062
17.7333336	0.4392000	-2.6813984
17.7333336	0.4880000	-2.4359615
17.7333336	0.5368000	-2.1537089
17.7333336	0.5856000	-1.9205441
17.7333336	0.6344000	-1.9696312
17.7333336	0.6832000	-1.3744469
17.7333336	0.7320000	-1.1903691
17.7333336	0.7808000	-1.2517285
17.7333336	0.8296000	-1.2455926
17.7333336	0.8784000	-1.3008156
17.7333336	0.9272000	-1.1903691
17.7333336	0.9760000	-1.0615149
17.7333336	1.0247999	-1.0553789
17.7333336	1.0735999	-0.9326606
17.7333336	1.1223999	-0.9203887
17.7333336	1.1711999	-0.9572039
17.7333336	1.2200000	-0.9387960
17.7333336	1.2688000	-0.9387960
17.7333336	1.3176000	-1.0369711
17.7333336	1.3664000	-1.0369711
17.7333336	1.4152000	-1.0431070
17.7333336	1.4640000	-0.9694757
17.7333336	1.5128000	-1.0921946
17.7333336	1.5616000	-1.0921946
17.7333336	1.6104000	-1.0921946

17.7333336	1.6592000	-1.0983305
17.7333336	1.7079999	-0.9817476
17.7333336	1.7567999	-1.0369711
17.7333336	1.8055999	-1.0185633
17.7333336	1.8543999	-0.9510679
17.7333336	1.9031999	-0.9387960
17.7333336	1.9519999	-0.9326606
17.7333336	2.0007999	-1.1228738
17.7333336	2.0495999	-1.2701364
17.7333336	2.0983999	-1.1780972
17.7333336	2.1471999	-1.1903691
17.7333336	2.1959999	-1.1903691
17.7333336	2.2447999	-1.3130875
17.7333336	2.2935998	-1.0676508
17.7333336	2.3423998	-1.0737867
17.7333336	2.3912001	-0.9694757
17.7333336	2.4400001	-0.9756117
17.7333336	2.4888000	-0.9756117
17.7333336	2.5376000	-0.9572039
17.7333336	2.5864000	-0.5829129
17.7333336	2.6352000	-0.5767770
17.7333336	2.6840000	-0.7731261
17.7333336	2.7328000	-0.7976699
17.7333336	2.7816000	-0.6749516
17.7333336	2.8304000	-0.6135921
17.7333336	2.8792000	-0.5522332
17.7333336	2.9280000	-0.7669902
17.7333336	2.9768000	-0.5522332
17.7333336	3.0256000	-0.6135921
17.7333336	3.0743999	-0.4970098
17.7333336	3.1231999	-0.4295144
17.7333336	3.1719999	-0.4970098
17.7333336	3.2207999	-0.5031457
17.7333336	3.2695999	-0.3620195
17.7333336	3.3183999	-0.4479222
17.7333336	3.3671999	-0.4479222
17.7333336	3.4159999	-0.3436117
17.7333336	3.4647999	-0.7424469
17.7333336	3.5135999	-0.7547188
17.7333336	3.5623999	-0.6933594
17.7333336	3.6111999	-0.6810875
17.7333336	3.6599998	-0.7056313
17.7333336	3.7087998	-0.7976699
17.7333336	3.7575998	-0.5951843
17.7333336	3.8063998	-0.5338254
17.7333336	3.8551998	-0.5338254
17.7333336	3.9039998	-0.2945242
17.7333336	3.9527998	-0.2945242
17.7333336	4.0016003	-0.4786019
17.7333336	4.0503998	-0.3865633
17.7333336	4.0991998	-0.1840777
17.7333336	4.1479998	-0.4233789
17.7333336	4.1967998	613586.0000000
17.7333336	4.2455997	0.0122719
17.7333336	4.2943997	-0.1288543
17.7333336	4.3431997	613586.0000000
17.7333336	4.3919997	-0.1043105

17.7333336	4.4407997	-0.3190680
17.7333336	4.4895997	-0.3252039
17.7333336	4.5383997	-0.3374758
17.7333336	4.5871997	-0.3374758
17.7333336	4.6359997	-0.2883883
17.7333336	4.6847997	-0.1288543
17.7333336	4.7336001	-0.2024856
17.7333336	4.7824001	-0.3313398
17.7333336	4.8312001	-0.2883883
17.7333336	4.8800001	-0.1656699
17.7333336	4.9288001	-0.1779418
17.7333336	4.9776001	-0.1840777
17.7333336	5.0264001	-0.4356503
17.7333336	5.0752001	-0.4356503
17.7333336	5.1240001	613586.0000000
17.7333336	5.1728001	613586.0000000
17.7333336	5.2216001	613586.0000000
17.7333336	5.2704000	613586.0000000
17.7333336	5.3192000	613586.0000000
17.7333336	5.3680000	-0.4111071
17.7333336	5.4168000	613586.0000000
17.7333336	5.4656000	-0.4479222
17.7333336	5.5144000	-0.4479222
17.7333336	5.5632000	-0.6013203
17.7333336	5.6120000	-0.4908738
17.7333336	5.6608000	-0.5460973
17.7333336	5.7096000	-0.5154176
17.7333336	5.7584000	613586.0000000
17.7333336	5.8072000	613586.0000000
17.7333336	5.8559999	613586.0000000
17.7333336	5.9047999	613586.0000000
17.7333336	5.9535999	613586.0000000
17.7333336	6.0023999	613586.0000000
17.7500000	0.2928000	-4.2828746
17.7500000	0.3416000	-3.9208550
17.7500000	0.3904000	-3.9269910
17.7500000	0.4392000	-2.7059422
17.7500000	0.4880000	-2.3071072
17.7500000	0.5368000	-1.8591847
17.7500000	0.5856000	-1.8407769
17.7500000	0.6344000	-1.5707965
17.7500000	0.6832000	-1.5401168
17.7500000	0.7320000	-1.3253593
17.7500000	0.7808000	-1.1290097
17.7500000	0.8296000	-1.1351457
17.7500000	0.8784000	-1.1290097
17.7500000	0.9272000	-1.2271848
17.7500000	0.9760000	-1.2333207
17.7500000	1.0247999	-1.0308352
17.7500000	1.0735999	-1.0860586
17.7500000	1.1223999	-1.0860586
17.7500000	1.1711999	-1.1044660
17.7500000	1.2200000	-1.0860586
17.7500000	1.2688000	-1.0921946
17.7500000	1.3176000	-1.0185633
17.7500000	1.3664000	-1.1167378
17.7500000	1.4152000	-1.2026410

17.7500000	1.4640000	-1.1780972
17.7500000	1.5128000	-1.0799227
17.7500000	1.5616000	-1.0799227
17.7500000	1.6104000	-1.1044660
17.7500000	1.6592000	-1.0001554
17.7500000	1.7079999	-1.0124273
17.7500000	1.7567999	-1.1106019
17.7500000	1.8055999	-1.1106019
17.7500000	1.8543999	-1.0431070
17.7500000	1.9031999	-1.0431070
17.7500000	1.9519999	-1.3376312
17.7500000	2.0007999	-1.3499031
17.7500000	2.0495999	-1.2946796
17.7500000	2.0983999	-1.1658254
17.7500000	2.1471999	-1.2333207
17.7500000	2.1959999	-1.0983305
17.7500000	2.2447999	-1.1106019
17.7500000	2.2935998	-1.1106019
17.7500000	2.3423998	-0.7669902
17.7500000	2.3912001	-0.9510679
17.7500000	2.4400001	-0.7915339
17.7500000	2.4888000	-0.8283496
17.7500000	2.5376000	-0.8406215
17.7500000	2.5864000	-0.6688156
17.7500000	2.6352000	-0.6872234
17.7500000	2.6840000	-0.6994953
17.7500000	2.7328000	-0.4847379
17.7500000	2.7816000	-0.4847379
17.7500000	2.8304000	-0.5706410
17.7500000	2.8792000	-0.6320000
17.7500000	2.9280000	-0.5276895
17.7500000	2.9768000	-0.5092816
17.7500000	3.0256000	-0.4417863
17.7500000	3.0743999	-0.4172430
17.7500000	3.1231999	-0.4172430
17.7500000	3.1719999	-0.3129320
17.7500000	3.2207999	-0.3865633
17.7500000	3.2695999	-0.4111071
17.7500000	3.3183999	-0.4233789
17.7500000	3.3671999	-0.3742914
17.7500000	3.4159999	-0.3006601
17.7500000	3.4647999	-0.3067961
17.7500000	3.5135999	-0.3129320
17.7500000	3.5623999	-0.6381359
17.7500000	3.6111999	-0.2393012
17.7500000	3.6599998	-0.2454371
17.7500000	3.7087998	-0.2024856
17.7500000	3.7575998	-0.3006601
17.7500000	3.8063998	-0.2331653
17.7500000	3.8551998	-0.2331653
17.7500000	3.9039998	-0.2454371
17.7500000	3.9527998	-0.1533980
17.7500000	4.0016003	-0.1165824
17.7500000	4.0503998	-0.6504078
17.7500000	4.0991998	-0.5890489
17.7500000	4.1479998	-0.4417863
17.7500000	4.1967998	-0.4417863

17.7500000	4.2455997	-0.0122719
17.7500000	4.2943997	-0.3436117
17.7500000	4.3431997	-0.2638445
17.7500000	4.3919997	-0.2393012
17.7500000	4.4407997	-0.2454371
17.7500000	4.4895997	-0.2331653
17.7500000	4.5383997	-0.2208934
17.7500000	4.5871997	-0.2208934
17.7500000	4.6359997	-0.2270293
17.7500000	4.6847997	-0.1288543
17.7500000	4.7336001	-0.1840777
17.7500000	4.7824001	-0.1840777
17.7500000	4.8312001	-0.1902137
17.7500000	4.8800001	-0.1840777
17.7500000	4.9288001	-0.1656699
17.7500000	4.9776001	-0.2086215
17.7500000	5.0264001	-0.3804274
17.7500000	5.0752001	-0.2699804
17.7500000	5.1240001	-0.2699804
17.7500000	5.1728001	-0.2577085
17.7500000	5.2216001	-0.2761164
17.7500000	5.2704000	-0.4295144
17.7500000	5.3192000	-0.4847379
17.7500000	5.3680000	-0.4908738
17.7500000	5.4168000	-0.4908738
17.7500000	5.4656000	-0.6074562
17.7500000	5.5144000	-0.6320000
17.7500000	5.5632000	-0.5399613
17.7500000	5.6120000	-0.5399613
17.7500000	5.6608000	-0.5522332
17.7500000	5.7096000	-0.5522332
17.7500000	5.7584000	613586.0000000
17.7500000	5.8072000	613586.0000000
17.7500000	5.8559999	613586.0000000
17.7500000	5.9047999	613586.0000000
17.7500000	5.9535999	613586.0000000
17.7500000	6.0023999	613586.0000000
17.7666664	0.2928000	-3.9883502
17.7666664	0.3416000	-4.0006218
17.7666664	0.3904000	-3.8288162
17.7666664	0.4392000	-2.6691267
17.7666664	0.4880000	-1.9757671
17.7666664	0.5368000	-1.5892043
17.7666664	0.5856000	-1.3621750
17.7666664	0.6344000	-1.4480777
17.7666664	0.6832000	-1.2087770
17.7666664	0.7320000	-1.2149129
17.7666664	0.7808000	-1.2149129
17.7666664	0.8296000	-1.3805828
17.7666664	0.8784000	-1.1780972
17.7666664	0.9272000	-1.1719613
17.7666664	0.9760000	-1.1719613
17.7666664	1.0247999	-0.9878836
17.7666664	1.0735999	-0.9817476
17.7666664	1.1223999	-0.8897090
17.7666664	1.1711999	-0.8897090
17.7666664	1.2200000	-1.0369711

17.7666664	1.2688000	-1.0185633
17.7666664	1.3176000	-0.9387960
17.7666664	1.3664000	-1.1719613
17.7666664	1.4152000	-1.1780972
17.7666664	1.4640000	-1.1780972
17.7666664	1.5128000	-1.1780972
17.7666664	1.5616000	-1.3008156
17.7666664	1.6104000	-1.2210488
17.7666664	1.6592000	-1.0860586
17.7666664	1.7079999	-1.2885437
17.7666664	1.7567999	-1.0799227
17.7666664	1.8055999	-1.1412816
17.7666664	1.8543999	-1.1412816
17.7666664	1.9031999	-1.3744469
17.7666664	1.9519999	-1.3928547
17.7666664	2.0007999	-1.2762718
17.7666664	2.0495999	-1.2640004
17.7666664	2.0983999	-1.2455926
17.7666664	2.1471999	-1.4112625
17.7666664	2.1959999	-1.3928547
17.7666664	2.2447999	-1.2087770
17.7666664	2.2935998	-1.2271848
17.7666664	2.3423998	-1.2087770
17.7666664	2.3912001	-1.1965051
17.7666664	2.4400001	-0.8958449
17.7666664	2.4888000	-0.8958449
17.7666664	2.5376000	-0.8528934
17.7666664	2.5864000	-0.7731261
17.7666664	2.6352000	-0.7731261
17.7666664	2.6840000	-0.7547188
17.7666664	2.7328000	-0.7547188
17.7666664	2.7816000	-0.6872234
17.7666664	2.8304000	-0.6749516
17.7666664	2.8792000	-0.6994953
17.7666664	2.9280000	-0.6381359
17.7666664	2.9768000	-0.5583692
17.7666664	3.0256000	-0.5522332
17.7666664	3.0743999	-0.6013203
17.7666664	3.1231999	-0.6442719
17.7666664	3.1719999	-0.6197281
17.7666664	3.2207999	-0.5338254
17.7666664	3.2695999	-0.5338254
17.7666664	3.3183999	-0.5645051
17.7666664	3.3671999	-0.5645051
17.7666664	3.4159999	-0.9817476
17.7666664	3.4647999	-0.5460973
17.7666664	3.5135999	-0.5460973
17.7666664	3.5623999	-0.5460973
17.7666664	3.6111999	-0.3926992
17.7666664	3.6599998	-0.3129320
17.7666664	3.7087998	-0.4847379
17.7666664	3.7575998	-0.6626797
17.7666664	3.8063998	-0.9326606
17.7666664	3.8551998	-0.9203887
17.7666664	3.9039998	-0.9203887
17.7666664	3.9527998	-0.7731261
17.7666664	4.0016003	-0.7731261

17.7666664	4.0503998	-0.7731261
17.7666664	4.0991998	-0.4049711
17.7666664	4.1479998	-0.2515731
17.7666664	4.1967998	-0.6810875
17.7666664	4.2455997	-0.6688156
17.7666664	4.2943997	-0.6442719
17.7666664	4.3431997	-0.6381359
17.7666664	4.3919997	-0.6504078
17.7666664	4.4407997	-0.4111071
17.7666664	4.4895997	-0.3804274
17.7666664	4.5383997	-0.4233789
17.7666664	4.5871997	-0.5338254
17.7666664	4.6359997	-0.5522332
17.7666664	4.6847997	-0.4479222
17.7666664	4.7336001	-0.5092816
17.7666664	4.7824001	-0.5092816
17.7666664	4.8312001	-0.5154176
17.7666664	4.8800001	-0.3865633
17.7666664	4.9288001	-0.3865633
17.7666664	4.9776001	-0.5399613
17.7666664	5.0264001	-0.5399613
17.7666664	5.0752001	-0.3190680
17.7666664	5.1240001	-0.2454371
17.7666664	5.1728001	-0.4049711
17.7666664	5.2216001	-0.3129320
17.7666664	5.2704000	-0.6749516
17.7666664	5.3192000	-0.6749516
17.7666664	5.3680000	-0.6872234
17.7666664	5.4168000	-0.7056313
17.7666664	5.4656000	-0.6381359
17.7666664	5.5144000	-0.5583692
17.7666664	5.5632000	613586.0000000
17.7666664	5.6120000	613586.0000000
17.7666664	5.6608000	613586.0000000
17.7666664	5.7096000	613586.0000000
17.7666664	5.7584000	613586.0000000
17.7666664	5.8072000	613586.0000000
17.7666664	5.8559999	613586.0000000
17.7666664	5.9047999	613586.0000000
17.7666664	5.9535999	613586.0000000
17.7666664	6.0023999	613586.0000000
17.7833328	0.2928000	-3.8042724
17.7833328	0.3416000	-3.3256705
17.7833328	0.3904000	-3.2090878
17.7833328	0.4392000	-3.2090878
17.7833328	0.4880000	-2.2457480
17.7833328	0.5368000	-1.9696312
17.7833328	0.5856000	-1.7426023
17.7833328	0.6344000	-1.5707965
17.7833328	0.6832000	-1.3069515
17.7833328	0.7320000	-1.3192234
17.7833328	0.7808000	-1.3008156
17.7833328	0.8296000	-1.2946796
17.7833328	0.8784000	-1.2824078
17.7833328	0.9272000	-1.2762718
17.7833328	0.9760000	-1.5339808
17.7833328	1.0247999	-1.3069515

17.7833328	1.0735999	-1.3192234
17.7833328	1.1223999	-1.2517285
17.7833328	1.1711999	-1.2271848
17.7833328	1.2200000	-1.2149129
17.7833328	1.2688000	-1.2149129
17.7833328	1.3176000	-1.2149129
17.7833328	1.3664000	-1.3069515
17.7833328	1.4152000	-1.2885437
17.7833328	1.4640000	-1.2026410
17.7833328	1.5128000	-1.2026410
17.7833328	1.5616000	-1.1167378
17.7833328	1.6104000	-1.1228738
17.7833328	1.6592000	-1.0246992
17.7833328	1.7079999	-1.0615149
17.7833328	1.7567999	-1.2149129
17.7833328	1.8055999	-1.2149129
17.7833328	1.8543999	-1.6689711
17.7833328	1.9031999	-1.0921946
17.7833328	1.9519999	-1.1658254
17.7833328	2.0007999	-1.5217090
17.7833328	2.0495999	-1.5094371
17.7833328	2.0983999	-1.4358063
17.7833328	2.1471999	-1.4480777
17.7833328	2.1959999	-1.3069515
17.7833328	2.2447999	-1.3008156
17.7833328	2.2935998	-0.9326606
17.7833328	2.3423998	-1.2455926
17.7833328	2.3912001	-1.1290097
17.7833328	2.4400001	-0.9265246
17.7833328	2.4888000	-1.0615149
17.7833328	2.5376000	-0.9756117
17.7833328	2.5864000	-0.9142528
17.7833328	2.6352000	-0.8774371
17.7833328	2.6840000	-0.8222136
17.7833328	2.7328000	-0.6810875
17.7833328	2.7816000	-0.6688156
17.7833328	2.8304000	-0.6626797
17.7833328	2.8792000	-0.6565437
17.7833328	2.9280000	-0.7117672
17.7833328	2.9768000	-0.7240391
17.7833328	3.0256000	-0.7240391
17.7833328	3.0743999	-0.5951843
17.7833328	3.1231999	-0.5951843
17.7833328	3.1719999	-0.6074562
17.7833328	3.2207999	-0.7424469
17.7833328	3.2695999	-0.5951843
17.7833328	3.3183999	-0.5951843
17.7833328	3.3671999	-0.6810875
17.7833328	3.4159999	-0.5829129
17.7833328	3.4647999	-0.9326606
17.7833328	3.5135999	-0.9265246
17.7833328	3.5623999	-0.9142528
17.7833328	3.6111999	-0.6258640
17.7833328	3.6599998	-0.6872234
17.7833328	3.7087998	-0.6933594
17.7833328	3.7575998	-0.6933594
17.7833328	3.8063998	-0.6810875

17.7833328	3.8551998	-0.9326606
17.7833328	3.9039998	-0.9203887
17.7833328	3.9527998	-1.0062914
17.7833328	4.0016003	-1.0185633
17.7833328	4.0503998	-0.7853980
17.7833328	4.0991998	-0.7915339
17.7833328	4.1479998	-0.7976699
17.7833328	4.1967998	-0.3865633
17.7833328	4.2455997	-0.6994953
17.7833328	4.2943997	-0.7547188
17.7833328	4.3431997	-0.7608547
17.7833328	4.3919997	-0.7485828
17.7833328	4.4407997	-0.7363110
17.7833328	4.4895997	-0.5890489
17.7833328	4.5383997	-0.6320000
17.7833328	4.5871997	-0.7240391
17.7833328	4.6359997	-0.5767770
17.7833328	4.6847997	-0.5890489
17.7833328	4.7336001	-0.5890489
17.7833328	4.7824001	-0.5706410
17.7833328	4.8312001	-0.5031457
17.7833328	4.8800001	-0.4970098
17.7833328	4.9288001	-0.3436117
17.7833328	4.9776001	-0.4479222
17.7833328	5.0264001	-0.5645051
17.7833328	5.0752001	-0.5645051
17.7833328	5.1240001	613586.0000000
17.7833328	5.1728001	-0.3681555
17.7833328	5.2216001	613586.0000000
17.7833328	5.2704000	613586.0000000
17.7833328	5.3192000	613586.0000000
17.7833328	5.3680000	613586.0000000
17.7833328	5.4168000	613586.0000000
17.7833328	5.4656000	613586.0000000
17.7833328	5.5144000	613586.0000000
17.7833328	5.5632000	613586.0000000
17.7833328	5.6120000	613586.0000000
17.7833328	5.6608000	613586.0000000
17.7833328	5.7096000	-0.6872234
17.7833328	5.7584000	613586.0000000
17.7833328	5.8072000	613586.0000000
17.7833328	5.8559999	613586.0000000
17.7833328	5.9047999	613586.0000000
17.7833328	5.9535999	613586.0000000
17.7833328	6.0023999	613586.0000000
17.7999992	0.2928000	-4.0067577
17.7999992	0.3416000	-3.1968160
17.7999992	0.3904000	-3.5220201
17.7999992	0.4392000	-1.8960004
17.7999992	0.4880000	-1.8960004
17.7999992	0.5368000	-1.8898644
17.7999992	0.5856000	-1.2701364
17.7999992	0.6344000	-1.2640004
17.7999992	0.6832000	-0.9756117
17.7999992	0.7320000	-1.2824078
17.7999992	0.7808000	-1.4358063
17.7999992	0.8296000	-1.4235344

17.7999992	0.8784000	-1.2455926
17.7999992	0.9272000	-1.2824078
17.7999992	0.9760000	-1.3744469
17.7999992	1.0247999	-1.2824078
17.7999992	1.0735999	-1.2701364
17.7999992	1.1223999	-1.2885437
17.7999992	1.1711999	-1.2946796
17.7999992	1.2200000	-1.3130875
17.7999992	1.2688000	-1.3130875
17.7999992	1.3176000	-1.3253593
17.7999992	1.3664000	-0.9694757
17.7999992	1.4152000	-1.2394567
17.7999992	1.4640000	-1.0983305
17.7999992	1.5128000	-1.0983305
17.7999992	1.5616000	-1.2946796
17.7999992	1.6104000	-1.4848933
17.7999992	1.6592000	-1.2210488
17.7999992	1.7079999	-1.2271848
17.7999992	1.7567999	-1.2271848
17.7999992	1.8055999	-1.2455926
17.7999992	1.8543999	-1.2455926
17.7999992	1.9031999	-1.5462527
17.7999992	1.9519999	-1.4296703
17.7999992	2.0007999	-1.4971652
17.7999992	2.0495999	-1.5462527
17.7999992	2.0983999	-1.3867188
17.7999992	2.1471999	-1.3805828
17.7999992	2.1959999	-1.4971652
17.7999992	2.2447999	-1.3744469
17.7999992	2.2935998	-1.0983305
17.7999992	2.3423998	-1.0921946
17.7999992	2.3912001	-1.0553789
17.7999992	2.4400001	-1.0431070
17.7999992	2.4888000	-0.8713012
17.7999992	2.5376000	-0.8099418
17.7999992	2.5864000	-0.7424469
17.7999992	2.6352000	-0.7485828
17.7999992	2.6840000	-0.7547188
17.7999992	2.7328000	-0.7547188
17.7999992	2.7816000	-0.8099418
17.7999992	2.8304000	-0.8222136
17.7999992	2.8792000	-0.9081168
17.7999992	2.9280000	-0.9142528
17.7999992	2.9768000	-0.8283496
17.7999992	3.0256000	-0.8283496
17.7999992	3.0743999	-0.8406215
17.7999992	3.1231999	-0.7915339
17.7999992	3.1719999	-0.6688156
17.7999992	3.2207999	-0.6565437
17.7999992	3.2695999	-0.6504078
17.7999992	3.3183999	-0.6688156
17.7999992	3.3671999	-0.6749516
17.7999992	3.4159999	-0.6626797
17.7999992	3.4647999	-0.5951843
17.7999992	3.5135999	-0.8283496
17.7999992	3.5623999	-0.5645051
17.7999992	3.6111999	-0.5645051

17.7999992	3.6599998	-0.5583692
17.7999992	3.7087998	-0.7363110
17.7999992	3.7575998	-0.7792621
17.7999992	3.8063998	-0.8406215
17.7999992	3.8551998	-0.8406215
17.7999992	3.9039998	-0.4049711
17.7999992	3.9527998	-0.8467574
17.7999992	4.0016003	-0.4663301
17.7999992	4.0503998	-0.4295144
17.7999992	4.0991998	-0.4295144
17.7999992	4.1479998	-0.6749516
17.7999992	4.1967998	-0.4356503
17.7999992	4.2455997	-0.7485828
17.7999992	4.2943997	-0.7485828
17.7999992	4.3431997	-0.6994953
17.7999992	4.3919997	-0.6933594
17.7999992	4.4407997	-0.6994953
17.7999992	4.4895997	-0.7056313
17.7999992	4.5383997	-0.5829129
17.7999992	4.5871997	-0.5399613
17.7999992	4.6359997	-0.5338254
17.7999992	4.6847997	613586.0000000
17.7999992	4.7336001	-0.3926992
17.7999992	4.7824001	-0.3926992
17.7999992	4.8312001	-0.4663301
17.7999992	4.8800001	-0.5399613
17.7999992	4.9288001	-0.5522332
17.7999992	4.9776001	-0.3190680
17.7999992	5.0264001	613586.0000000
17.7999992	5.0752001	-0.1043105
17.7999992	5.1240001	-0.1104465
17.7999992	5.1728001	613586.0000000
17.7999992	5.2216001	-0.2761164
17.7999992	5.2704000	-0.2822523
17.7999992	5.3192000	613586.0000000
17.7999992	5.3680000	613586.0000000
17.7999992	5.4168000	613586.0000000
17.7999992	5.4656000	613586.0000000
17.7999992	5.5144000	613586.0000000
17.7999992	5.5632000	613586.0000000
17.7999992	5.6120000	613586.0000000
17.7999992	5.6608000	613586.0000000
17.7999992	5.7096000	613586.0000000
17.7999992	5.7584000	-0.5399613
17.7999992	5.8072000	-0.5338254
17.7999992	5.8559999	613586.0000000
17.7999992	5.9047999	613586.0000000
17.7999992	5.9535999	-0.4601941
17.7999992	6.0023999	-0.4663301
17.8166676	0.2928000	-4.4730883
17.8166676	0.3416000	-3.7981365
17.8166676	0.3904000	-3.7920005
17.8166676	0.4392000	-2.2886994
17.8166676	0.4880000	-2.1168938
17.8166676	0.5368000	-1.7180586
17.8166676	0.5856000	-1.7180586
17.8166676	0.6344000	-1.2640004

17.8166676	0.6832000	-1.2455926
17.8166676	0.7320000	-1.2578645
17.8166676	0.7808000	-1.3744469
17.8166676	0.8296000	-1.6996508
17.8166676	0.8784000	-1.6935148
17.8166676	0.9272000	-1.4296703
17.8166676	0.9760000	-1.4296703
17.8166676	1.0247999	-1.5155730
17.8166676	1.0735999	-1.5094371
17.8166676	1.1223999	-1.3928547
17.8166676	1.1711999	-1.3069515
17.8166676	1.2200000	-1.3008156
17.8166676	1.2688000	-1.3560390
17.8166676	1.3176000	-1.3621750
17.8166676	1.3664000	-1.1842332
17.8166676	1.4152000	-1.1842332
17.8166676	1.4640000	-1.1903691
17.8166676	1.5128000	-1.1596894
17.8166676	1.5616000	-1.1351457
17.8166676	1.6104000	-1.2149129
17.8166676	1.6592000	-1.2026410
17.8166676	1.7079999	-1.4542136
17.8166676	1.7567999	-1.2271848
17.8166676	1.8055999	-1.2394567
17.8166676	1.8543999	-1.4358063
17.8166676	1.9031999	-1.4542136
17.8166676	1.9519999	-1.4664855
17.8166676	2.0007999	-1.4358063
17.8166676	2.0495999	-1.4296703
17.8166676	2.0983999	-1.4112625
17.8166676	2.1471999	-1.4112625
17.8166676	2.1959999	-1.3253593
17.8166676	2.2447999	-1.3437672
17.8166676	2.2935998	-1.3560390
17.8166676	2.3423998	-1.0062914
17.8166676	2.3912001	-0.9265246
17.8166676	2.4400001	-0.9142528
17.8166676	2.4888000	-0.8713012
17.8166676	2.5376000	-0.8590293
17.8166676	2.5864000	-0.9203887
17.8166676	2.6352000	-0.9265246
17.8166676	2.6840000	-0.9326606
17.8166676	2.7328000	-0.8835731
17.8166676	2.7816000	-0.7669902
17.8166676	2.8304000	-0.7915339
17.8166676	2.8792000	-0.5092816
17.8166676	2.9280000	-0.5460973
17.8166676	2.9768000	-0.5460973
17.8166676	3.0256000	-0.5522332
17.8166676	3.0743999	-0.5829129
17.8166676	3.1231999	-0.6810875
17.8166676	3.1719999	-0.6626797
17.8166676	3.2207999	-0.6565437
17.8166676	3.2695999	-0.6442719
17.8166676	3.3183999	-1.0062914
17.8166676	3.3671999	-1.0308352
17.8166676	3.4159999	-0.5338254

17.8166676	3.4647999	-0.6872234
17.8166676	3.5135999	-0.6872234
17.8166676	3.5623999	-0.7608547
17.8166676	3.6111999	-0.7731261
17.8166676	3.6599998	-0.3742914
17.8166676	3.7087998	-0.8099418
17.8166676	3.7575998	-0.6504078
17.8166676	3.8063998	-0.7976699
17.8166676	3.8551998	-0.7915339
17.8166676	3.9039998	-0.7792621
17.8166676	3.9527998	-0.7731261
17.8166676	4.0016003	-0.7915339
17.8166676	4.0503998	-0.4847379
17.8166676	4.0991998	-0.4724660
17.8166676	4.1479998	-0.7424469
17.8166676	4.1967998	-0.7363110
17.8166676	4.2455997	-0.6626797
17.8166676	4.2943997	-0.6688156
17.8166676	4.3431997	-0.7056313
17.8166676	4.3919997	-0.7056313
17.8166676	4.4407997	-0.7056313
17.8166676	4.4895997	-0.7056313
17.8166676	4.5383997	-0.7056313
17.8166676	4.5871997	-0.5829129
17.8166676	4.6359997	-0.5767770
17.8166676	4.6847997	-0.5767770
17.8166676	4.7336001	-0.3436117
17.8166676	4.7824001	-0.3436117
17.8166676	4.8312001	-0.2638445
17.8166676	4.8800001	-0.2515731
17.8166676	4.9288001	-0.1349902
17.8166676	4.9776001	-0.1288543
17.8166676	5.0264001	613586.0000000
17.8166676	5.0752001	-0.0429516
17.8166676	5.1240001	-0.1902137
17.8166676	5.1728001	-0.5829129
17.8166676	5.2216001	-0.7179031
17.8166676	5.2704000	-0.7056313
17.8166676	5.3192000	-0.6994953
17.8166676	5.3680000	613586.0000000
17.8166676	5.4168000	613586.0000000
17.8166676	5.4656000	613586.0000000
17.8166676	5.5144000	613586.0000000
17.8166676	5.5632000	-0.7485828
17.8166676	5.6120000	613586.0000000
17.8166676	5.6608000	-0.7731261
17.8166676	5.7096000	-0.6749516
17.8166676	5.7584000	-0.6381359
17.8166676	5.8072000	-0.5583692
17.8166676	5.8559999	-0.5460973
17.8166676	5.9047999	-0.5092816
17.8166676	5.9535999	-0.4970098
17.8166676	6.0023999	613586.0000000
17.8333340	0.2928000	-3.9822142
17.8333340	0.3416000	-3.9822142
17.8333340	0.3904000	-3.4177094
17.8333340	0.4392000	-2.3868742

17.8333340	0.4880000	-1.9389520
17.8333340	0.5368000	-1.7487383
17.8333340	0.5856000	-1.2394567
17.8333340	0.6344000	-1.2455926
17.8333340	0.6832000	-1.2762718
17.8333340	0.7320000	-1.4173985
17.8333340	0.7808000	-1.3621750
17.8333340	0.8296000	-1.3683109
17.8333340	0.8784000	-1.5094371
17.8333340	0.9272000	-1.2271848
17.8333340	0.9760000	-1.3805828
17.8333340	1.0247999	-1.3867188
17.8333340	1.0735999	-1.4296703
17.8333340	1.1223999	-1.3560390
17.8333340	1.1711999	-1.3560390
17.8333340	1.2200000	-1.2087770
17.8333340	1.2688000	-1.4051266
17.8333340	1.3176000	-1.0308352
17.8333340	1.3664000	-1.0921946
17.8333340	1.4152000	-1.1596894
17.8333340	1.4640000	-1.1228738
17.8333340	1.5128000	-1.1044660
17.8333340	1.5616000	-1.1474175
17.8333340	1.6104000	-1.5401168
17.8333340	1.6592000	-1.5339808
17.8333340	1.7079999	-1.4726214
17.8333340	1.7567999	-1.4664855
17.8333340	1.8055999	-1.1719613
17.8333340	1.8543999	-1.5339808
17.8333340	1.9031999	-1.3253593
17.8333340	1.9519999	-1.5585246
17.8333340	2.0007999	-1.3069515
17.8333340	2.0495999	-1.2946796
17.8333340	2.0983999	-1.2885437
17.8333340	2.1471999	-1.3130875
17.8333340	2.1959999	-1.0369711
17.8333340	2.2447999	-0.9633398
17.8333340	2.2935998	-0.9572039
17.8333340	2.3423998	-0.8958449
17.8333340	2.3912001	-0.7853980
17.8333340	2.4400001	-0.7853980
17.8333340	2.4888000	-0.8406215
17.8333340	2.5376000	-0.7976699
17.8333340	2.5864000	-0.7731261
17.8333340	2.6352000	-0.7240391
17.8333340	2.6840000	-0.6749516
17.8333340	2.7328000	-0.7853980
17.8333340	2.7816000	-0.4786019
17.8333340	2.8304000	-0.6688156
17.8333340	2.8792000	-0.6442719
17.8333340	2.9280000	-0.6320000
17.8333340	2.9768000	-0.6442719
17.8333340	3.0256000	-0.5522332
17.8333340	3.0743999	-0.5522332
17.8333340	3.1231999	-0.5522332
17.8333340	3.1719999	-0.9387960
17.8333340	3.2207999	-0.9878836

17.8333340	3.2695999	-0.9878836
17.8333340	3.3183999	-0.9817476
17.8333340	3.3671999	-0.8283496
17.8333340	3.4159999	-0.8958449
17.8333340	3.4647999	-0.9817476
17.8333340	3.5135999	-0.9694757
17.8333340	3.5623999	-0.9694757
17.8333340	3.6111999	-0.8467574
17.8333340	3.6599998	-0.8651652
17.8333340	3.7087998	-0.8651652
17.8333340	3.7575998	-0.6074562
17.8333340	3.8063998	-0.6197281
17.8333340	3.8551998	-0.5215535
17.8333340	3.9039998	-0.5092816
17.8333340	3.9527998	-0.4847379
17.8333340	4.0016003	-0.4847379
17.8333340	4.0503998	-0.6933594
17.8333340	4.0991998	-0.3681555
17.8333340	4.1479998	-0.3681555
17.8333340	4.1967998	-0.6810875
17.8333340	4.2455997	-0.8038058
17.8333340	4.2943997	-0.4724660
17.8333340	4.3431997	-0.2638445
17.8333340	4.3919997	-0.2761164
17.8333340	4.4407997	-0.2024856
17.8333340	4.4895997	-0.2024856
17.8333340	4.5383997	-0.2577085
17.8333340	4.5871997	-0.4049711
17.8333340	4.6359997	-0.3988352
17.8333340	4.6847997	-0.3006601
17.8333340	4.7336001	-0.2577085
17.8333340	4.7824001	-0.2822523
17.8333340	4.8312001	-0.1595340
17.8333340	4.8800001	-0.1595340
17.8333340	4.9288001	-0.1718059
17.8333340	4.9776001	-0.1840777
17.8333340	5.0264001	-0.0552235
17.8333340	5.0752001	-0.0552235
17.8333340	5.1240001	-0.4724660
17.8333340	5.1728001	-0.4663301
17.8333340	5.2216001	-0.3252039
17.8333340	5.2704000	-0.3374758
17.8333340	5.3192000	-0.6013203
17.8333340	5.3680000	-0.4786019
17.8333340	5.4168000	-0.4724660
17.8333340	5.4656000	-0.6381359
17.8333340	5.5144000	-0.6381359
17.8333340	5.5632000	613586.0000000
17.8333340	5.6120000	613586.0000000
17.8333340	5.6608000	613586.0000000
17.8333340	5.7096000	-0.8344855
17.8333340	5.7584000	-0.8406215
17.8333340	5.8072000	-0.8528934
17.8333340	5.8559999	-0.6933594
17.8333340	5.9047999	-0.7608547
17.8333340	5.9535999	613586.0000000
17.8333340	6.0023999	613586.0000000

17.8500004	0.2928000	-4.4669523
17.8500004	0.3416000	-3.7306414
17.8500004	0.3904000	-3.7306414
17.8500004	0.4392000	-2.3930101
17.8500004	0.4880000	-1.7671461
17.8500004	0.5368000	-1.6321554
17.8500004	0.5856000	-1.6505632
17.8500004	0.6344000	-1.5217090
17.8500004	0.6832000	-1.4419422
17.8500004	0.7320000	-1.4235344
17.8500004	0.7808000	-1.1903691
17.8500004	0.8296000	-1.1965051
17.8500004	0.8784000	-1.2946796
17.8500004	0.9272000	-1.2946796
17.8500004	0.9760000	-1.2946796
17.8500004	1.0247999	-1.1719613
17.8500004	1.0735999	-1.1842332
17.8500004	1.1223999	-1.2149129
17.8500004	1.1711999	-1.2210488
17.8500004	1.2200000	-1.1228738
17.8500004	1.2688000	-1.1228738
17.8500004	1.3176000	-1.2271848
17.8500004	1.3664000	-1.0431070
17.8500004	1.4152000	-1.3130875
17.8500004	1.4640000	-1.2333207
17.8500004	1.5128000	-0.9326606
17.8500004	1.5616000	-0.9326606
17.8500004	1.6104000	-1.5217090
17.8500004	1.6592000	-1.3008156
17.8500004	1.7079999	-1.4296703
17.8500004	1.7567999	-1.4358063
17.8500004	1.8055999	-1.3008156
17.8500004	1.8543999	-1.3008156
17.8500004	1.9031999	-1.5217090
17.8500004	1.9519999	-1.4603496
17.8500004	2.0007999	-1.4664855
17.8500004	2.0495999	-1.2394567
17.8500004	2.0983999	-1.3867188
17.8500004	2.1471999	-1.2087770
17.8500004	2.1959999	-1.1535535
17.8500004	2.2447999	-1.1596894
17.8500004	2.2935998	-0.8774371
17.8500004	2.3423998	-0.8651652
17.8500004	2.3912001	-0.8467574
17.8500004	2.4400001	-0.8467574
17.8500004	2.4888000	-0.7117672
17.8500004	2.5376000	-0.7117672
17.8500004	2.5864000	-0.7363110
17.8500004	2.6352000	-0.7301750
17.8500004	2.6840000	-0.7301750
17.8500004	2.7328000	-0.7853980
17.8500004	2.7816000	-0.8713012
17.8500004	2.8304000	-0.5767770
17.8500004	2.8792000	-0.4601941
17.8500004	2.9280000	-0.9019809
17.8500004	2.9768000	-0.4049711
17.8500004	3.0256000	-0.5645051

17.8500004	3.0743999	-0.5645051
17.8500004	3.1231999	-0.9019809
17.8500004	3.1719999	-0.6013203
17.8500004	3.2207999	-0.9878836
17.8500004	3.2695999	-0.9387960
17.8500004	3.3183999	-1.0246992
17.8500004	3.3671999	-1.0124273
17.8500004	3.4159999	-1.0124273
17.8500004	3.4647999	-0.8958449
17.8500004	3.5135999	-0.8897090
17.8500004	3.5623999	-0.8897090
17.8500004	3.6111999	-0.8835731
17.8500004	3.6599998	-0.7731261
17.8500004	3.7087998	-0.9878836
17.8500004	3.7575998	-0.9878836
17.8500004	3.8063998	-0.9878836
17.8500004	3.8551998	-0.5092816
17.8500004	3.9039998	-0.4970098
17.8500004	3.9527998	-0.7792621
17.8500004	4.0016003	-0.2699804
17.8500004	4.0503998	-0.2761164
17.8500004	4.0991998	-0.6749516
17.8500004	4.1479998	-0.6626797
17.8500004	4.1967998	-0.6504078
17.8500004	4.2455997	-0.7915339
17.8500004	4.2943997	-0.6749516
17.8500004	4.3431997	-0.6749516
17.8500004	4.3919997	-0.5154176
17.8500004	4.4407997	-0.5706410
17.8500004	4.4895997	-0.5583692
17.8500004	4.5383997	-0.5031457
17.8500004	4.5871997	-0.5031457
17.8500004	4.6359997	-0.3742914
17.8500004	4.6847997	-0.3006601
17.8500004	4.7336001	-0.1411262
17.8500004	4.7824001	-0.1411262
17.8500004	4.8312001	-0.2822523
17.8500004	4.8800001	-0.3742914
17.8500004	4.9288001	-0.3620195
17.8500004	4.9776001	-0.1656699
17.8500004	5.0264001	-0.4724660
17.8500004	5.0752001	-0.3252039
17.8500004	5.1240001	-0.3313398
17.8500004	5.1728001	-0.3436117
17.8500004	5.2216001	-0.3436117
17.8500004	5.2704000	-0.3436117
17.8500004	5.3192000	-0.3620195
17.8500004	5.3680000	-0.3804274
17.8500004	5.4168000	-0.5276895
17.8500004	5.4656000	-0.5092816
17.8500004	5.5144000	-0.5031457
17.8500004	5.5632000	-0.5154176
17.8500004	5.6120000	-0.5951843
17.8500004	5.6608000	-0.4908738
17.8500004	5.7096000	-0.5399613
17.8500004	5.7584000	-0.5276895
17.8500004	5.8072000	-0.3497477

17.8500004	5.8559999	-0.3313398
17.8500004	5.9047999	613586.0000000
17.8500004	5.9535999	613586.0000000
17.8500004	6.0023999	613586.0000000
17.8666668	0.2928000	-4.6510296
17.8666668	0.3416000	-3.7490492
17.8666668	0.3904000	-3.0127385
17.8666668	0.4392000	-3.0127385
17.8666668	0.4880000	-2.4850490
17.8666668	0.5368000	-1.8837285
17.8666668	0.5856000	-1.7303305
17.8666668	0.6344000	-1.5830684
17.8666668	0.6832000	-1.2333207
17.8666668	0.7320000	-1.2455926
17.8666668	0.7808000	-1.3499031
17.8666668	0.8296000	-1.3560390
17.8666668	0.8784000	-1.3192234
17.8666668	0.9272000	-1.5278449
17.8666668	0.9760000	-1.5462527
17.8666668	1.0247999	-1.0983305
17.8666668	1.0735999	-1.0983305
17.8666668	1.1223999	-1.1044660
17.8666668	1.1711999	-1.1106019
17.8666668	1.2200000	-1.1044660
17.8666668	1.2688000	-1.0983305
17.8666668	1.3176000	-1.0124273
17.8666668	1.3664000	-0.8528934
17.8666668	1.4152000	-0.8528934
17.8666668	1.4640000	-1.0062914
17.8666668	1.5128000	-1.3560390
17.8666668	1.5616000	-1.3130875
17.8666668	1.6104000	-1.2824078
17.8666668	1.6592000	-1.2824078
17.8666668	1.7079999	-1.2026410
17.8666668	1.7567999	-1.2149129
17.8666668	1.8055999	-1.3008156
17.8666668	1.8543999	-1.3192234
17.8666668	1.9031999	-1.0860586
17.8666668	1.9519999	-1.2946796
17.8666668	2.0007999	-1.1903691
17.8666668	2.0495999	-1.1290097
17.8666668	2.0983999	-1.2087770
17.8666668	2.1471999	-1.0001554
17.8666668	2.1959999	-0.8835731
17.8666668	2.2447999	-0.8651652
17.8666668	2.2935998	-0.7179031
17.8666668	2.3423998	-0.7117672
17.8666668	2.3912001	-0.6994953
17.8666668	2.4400001	-0.8283496
17.8666668	2.4888000	-0.6810875
17.8666668	2.5376000	-0.6688156
17.8666668	2.5864000	-0.6565437
17.8666668	2.6352000	-0.6320000
17.8666668	2.6840000	-0.6074562
17.8666668	2.7328000	-0.5215535
17.8666668	2.7816000	-0.6074562
17.8666668	2.8304000	-0.5583692

17.8666668	2.8792000	-0.4970098
17.8666668	2.9280000	-0.5092816
17.8666668	2.9768000	-0.5154176
17.8666668	3.0256000	-0.5031457
17.8666668	3.0743999	-0.5215535
17.8666668	3.1231999	-0.5215535
17.8666668	3.1719999	-0.5031457
17.8666668	3.2207999	-1.0676508
17.8666668	3.2695999	-1.0062914
17.8666668	3.3183999	-0.9510679
17.8666668	3.3671999	-0.9510679
17.8666668	3.4159999	-0.8651652
17.8666668	3.4647999	-0.8713012
17.8666668	3.5135999	-0.5460973
17.8666668	3.5623999	-0.4356503
17.8666668	3.6111999	-0.6688156
17.8666668	3.6599998	-0.6688156
17.8666668	3.7087998	-0.4049711
17.8666668	3.7575998	-0.4295144
17.8666668	3.8063998	-0.5522332
17.8666668	3.8551998	-0.1840777
17.8666668	3.9039998	-0.1779418
17.8666668	3.9527998	-0.4847379
17.8666668	4.0016003	-0.5031457
17.8666668	4.0503998	-0.5031457
17.8666668	4.0991998	-0.7485828
17.8666668	4.1479998	-0.8344855
17.8666668	4.1967998	-0.8160777
17.8666668	4.2455997	-0.7731261
17.8666668	4.2943997	-0.7792621
17.8666668	4.3431997	-0.9142528
17.8666668	4.3919997	-0.8590293
17.8666668	4.4407997	-0.7301750
17.8666668	4.4895997	-0.6749516
17.8666668	4.5383997	-0.6872234
17.8666668	4.5871997	-0.3006601
17.8666668	4.6359997	-0.2822523
17.8666668	4.6847997	-0.3865633
17.8666668	4.7336001	-0.3988352
17.8666668	4.7824001	-0.4049711
17.8666668	4.8312001	-0.3313398
17.8666668	4.8800001	-0.3313398
17.8666668	4.9288001	-0.1349902
17.8666668	4.9776001	-0.1472621
17.8666668	5.0264001	-0.0552235
17.8666668	5.0752001	-0.0245438
17.8666668	5.1240001	-0.1902137
17.8666668	5.1728001	-0.2638445
17.8666668	5.2216001	-0.2638445
17.8666668	5.2704000	-0.2454371
17.8666668	5.3192000	-0.6442719
17.8666668	5.3680000	-0.3926992
17.8666668	5.4168000	-0.5338254
17.8666668	5.4656000	-0.5399613
17.8666668	5.5144000	-0.5399613
17.8666668	5.5632000	-0.6565437
17.8666668	5.6120000	-0.6565437

17.8666668	5.6608000	-0.6442719
17.8666668	5.7096000	-0.5645051
17.8666668	5.7584000	-0.5583692
17.8666668	5.8072000	613586.0000000
17.8666668	5.8559999	613586.0000000
17.8666668	5.9047999	613586.0000000
17.8666668	5.9535999	613586.0000000
17.8666668	6.0023999	-0.5154176
17.8833332	0.2928000	-4.3871851
17.8833332	0.3416000	-3.7429132
17.8833332	0.3904000	-3.3256705
17.8833332	0.4392000	-2.3009713
17.8833332	0.4880000	-1.9757671
17.8833332	0.5368000	-1.9757671
17.8833332	0.5856000	-1.5585246
17.8833332	0.6344000	-1.1167378
17.8833332	0.6832000	-1.2824078
17.8833332	0.7320000	-1.2333207
17.8833332	0.7808000	-1.3192234
17.8833332	0.8296000	-1.3314953
17.8833332	0.8784000	-1.2333207
17.8833332	0.9272000	-1.2149129
17.8833332	0.9760000	-1.0676508
17.8833332	1.0247999	-1.0676508
17.8833332	1.0735999	-1.1780972
17.8833332	1.1223999	-0.8467574
17.8833332	1.1711999	-0.8467574
17.8833332	1.2200000	-1.0308352
17.8833332	1.2688000	-0.8897090
17.8833332	1.3176000	-0.8835731
17.8833332	1.3664000	-0.9326606
17.8833332	1.4152000	-0.9019809
17.8833332	1.4640000	-1.1658254
17.8833332	1.5128000	-1.1596894
17.8833332	1.5616000	-1.2210488
17.8833332	1.6104000	-1.4235344
17.8833332	1.6592000	-1.4235344
17.8833332	1.7079999	-1.4358063
17.8833332	1.7567999	-1.5707965
17.8833332	1.8055999	-1.1106019
17.8833332	1.8543999	-1.1106019
17.8833332	1.9031999	-1.2517285
17.8833332	1.9519999	-1.0921946
17.8833332	2.0007999	-1.1044660
17.8833332	2.0495999	-1.1044660
17.8833332	2.0983999	-1.1044660
17.8833332	2.1471999	-0.9019809
17.8833332	2.1959999	-0.8222136
17.8833332	2.2447999	-0.7363110
17.8833332	2.2935998	-0.7363110
17.8833332	2.3423998	-0.7363110
17.8833332	2.3912001	-0.7240391
17.8833332	2.4400001	-0.7731261
17.8833332	2.4888000	-0.6994953
17.8833332	2.5376000	-0.7608547
17.8833332	2.5864000	-0.7608547
17.8833332	2.6352000	-0.6504078

17.8833332	2.6840000	-0.7056313
17.8833332	2.7328000	-0.6994953
17.8833332	2.7816000	-0.5890489
17.8833332	2.8304000	-0.8651652
17.8833332	2.8792000	-1.0737867
17.8833332	2.9280000	-1.0737867
17.8833332	2.9768000	-0.7056313
17.8833332	3.0256000	-0.7669902
17.8833332	3.0743999	-1.2455926
17.8833332	3.1231999	-1.2333207
17.8833332	3.1719999	-1.1167378
17.8833332	3.2207999	-1.1106019
17.8833332	3.2695999	-0.7301750
17.8833332	3.3183999	-0.7301750
17.8833332	3.3671999	-0.9878836
17.8833332	3.4159999	-0.5890489
17.8833332	3.4647999	-0.6872234
17.8833332	3.5135999	-0.4724660
17.8833332	3.5623999	-0.4908738
17.8833332	3.6111999	-0.2945242
17.8833332	3.6599998	-0.2945242
17.8833332	3.7087998	-0.2945242
17.8833332	3.7575998	-0.4540582
17.8833332	3.8063998	-0.9203887
17.8833332	3.8551998	-0.9265246
17.8833332	3.9039998	-1.3989906
17.8833332	3.9527998	-1.1658254
17.8833332	4.0016003	-1.2026410
17.8833332	4.0503998	-0.3926992
17.8833332	4.0991998	-0.4847379
17.8833332	4.1479998	-0.7608547
17.8833332	4.1967998	-0.7669902
17.8833332	4.2455997	-0.6994953
17.8833332	4.2943997	-0.6933594
17.8833332	4.3431997	-0.4479222
17.8833332	4.3919997	-0.6197281
17.8833332	4.4407997	-0.6688156
17.8833332	4.4895997	-0.7117672
17.8833332	4.5383997	-0.7056313
17.8833332	4.5871997	-0.6749516
17.8833332	4.6359997	-0.6749516
17.8833332	4.6847997	-0.5645051
17.8833332	4.7336001	-0.5767770
17.8833332	4.7824001	-0.3252039
17.8833332	4.8312001	-0.5092816
17.8833332	4.8800001	-0.3926992
17.8833332	4.9288001	-0.4172430
17.8833332	4.9776001	-0.1595340
17.8833332	5.0264001	-0.1595340
17.8833332	5.0752001	-0.4295144
17.8833332	5.1240001	-0.2208934
17.8833332	5.1728001	-0.4970098
17.8833332	5.2216001	-0.4908738
17.8833332	5.2704000	-0.5767770
17.8833332	5.3192000	-0.5645051
17.8833332	5.3680000	-0.5522332
17.8833332	5.4168000	-0.7240391

17.8833332	5.4656000	-0.7363110
17.8833332	5.5144000	-0.6994953
17.8833332	5.5632000	-0.6994953
17.8833332	5.6120000	-0.5951843
17.8833332	5.6608000	-0.4970098
17.8833332	5.7096000	-0.5276895
17.8833332	5.7584000	-0.5276895
17.8833332	5.8072000	-0.5706410
17.8833332	5.8559999	-0.5645051
17.8833332	5.9047999	-0.6994953
17.8833332	5.9535999	-0.6504078
17.8833332	6.0023999	-0.5092816
17.8999996	0.2928000	-3.7060976
17.8999996	0.3416000	-3.7183695
17.8999996	0.3904000	-2.7550297
17.8999996	0.4392000	-2.2764277
17.8999996	0.4880000	-2.1230292
17.8999996	0.5368000	-1.6260195
17.8999996	0.5856000	-1.5585246
17.8999996	0.6344000	-1.4603496
17.8999996	0.6832000	-1.4787574
17.8999996	0.7320000	-1.2885437
17.8999996	0.7808000	-1.3989906
17.8999996	0.8296000	-1.3069515
17.8999996	0.8784000	-1.2517285
17.8999996	0.9272000	-1.2517285
17.8999996	0.9760000	-1.2333207
17.8999996	1.0247999	-0.8528934
17.8999996	1.0735999	-1.0553789
17.8999996	1.1223999	-1.1290097
17.8999996	1.1711999	-1.1780972
17.8999996	1.2200000	-0.8651652
17.8999996	1.2688000	-0.8958449
17.8999996	1.3176000	-1.2824078
17.8999996	1.3664000	-1.1106019
17.8999996	1.4152000	-0.8344855
17.8999996	1.4640000	-0.8283496
17.8999996	1.5128000	-0.8222136
17.8999996	1.5616000	-1.1044660
17.8999996	1.6104000	-1.1044660
17.8999996	1.6592000	-1.3805828
17.8999996	1.7079999	-0.9510679
17.8999996	1.7567999	-1.2149129
17.8999996	1.8055999	-1.2087770
17.8999996	1.8543999	-1.0737867
17.8999996	1.9031999	-1.1596894
17.8999996	1.9519999	-1.1474175
17.8999996	2.0007999	-1.1535535
17.8999996	2.0495999	-0.9081168
17.8999996	2.0983999	-0.8467574
17.8999996	2.1471999	-0.8406215
17.8999996	2.1959999	-0.8958449
17.8999996	2.2447999	-0.7853980
17.8999996	2.2935998	-0.7915339
17.8999996	2.3423998	-0.7792621
17.8999996	2.3912001	-0.6626797
17.8999996	2.4400001	-0.6626797

17.8999996	2.4888000	-0.7976699
17.8999996	2.5376000	-0.7915339
17.8999996	2.5864000	-0.7915339
17.8999996	2.6352000	-0.7792621
17.8999996	2.6840000	-0.7731261
17.8999996	2.7328000	-0.8467574
17.8999996	2.7816000	-0.8344855
17.8999996	2.8304000	-0.7301750
17.8999996	2.8792000	-0.7117672
17.8999996	2.9280000	-0.7301750
17.8999996	2.9768000	-0.7363110
17.8999996	3.0256000	-1.2149129
17.8999996	3.0743999	-0.7915339
17.8999996	3.1231999	-0.7915339
17.8999996	3.1719999	-0.7240391
17.8999996	3.2207999	-0.7240391
17.8999996	3.2695999	-0.7117672
17.8999996	3.3183999	-1.1044660
17.8999996	3.3671999	-1.1106019
17.8999996	3.4159999	-0.7301750
17.8999996	3.4647999	-0.6074562
17.8999996	3.5135999	-0.6074562
17.8999996	3.5623999	-0.6933594
17.8999996	3.6111999	-0.6749516
17.8999996	3.6599998	-0.6565437
17.8999996	3.7087998	-0.8344855
17.8999996	3.7575998	-0.8099418
17.8999996	3.8063998	-0.8774371
17.8999996	3.8551998	-0.6626797
17.8999996	3.9039998	-0.5951843
17.8999996	3.9527998	-0.4111071
17.8999996	4.0016003	-0.4233789
17.8999996	4.0503998	-0.8222136
17.8999996	4.0991998	-0.6749516
17.8999996	4.1479998	-0.6810875
17.8999996	4.1967998	-0.7363110
17.8999996	4.2455997	-0.8467574
17.8999996	4.2943997	-0.7056313
17.8999996	4.3431997	-0.7179031
17.8999996	4.3919997	-0.7179031
17.8999996	4.4407997	-0.5399613
17.8999996	4.4895997	-0.5215535
17.8999996	4.5383997	-0.5154176
17.8999996	4.5871997	-0.2945242
17.8999996	4.6359997	-0.3067961
17.8999996	4.6847997	-0.3681555
17.8999996	4.7336001	-0.3804274
17.8999996	4.7824001	-0.4663301
17.8999996	4.8312001	-0.3988352
17.8999996	4.8800001	-0.3988352
17.8999996	4.9288001	-0.7547188
17.8999996	4.9776001	-0.7608547
17.8999996	5.0264001	-0.7608547
17.8999996	5.0752001	-0.7608547
17.8999996	5.1240001	-0.6565437
17.8999996	5.1728001	-0.9142528
17.8999996	5.2216001	-0.9265246

17.8999996	5.2704000	-0.7424469
17.8999996	5.3192000	-0.7424469
17.8999996	5.3680000	-0.8038058
17.8999996	5.4168000	-0.8774371
17.8999996	5.4656000	-0.8774371
17.8999996	5.5144000	-0.7547188
17.8999996	5.5632000	-0.7853980
17.8999996	5.6120000	-0.7056313
17.8999996	5.6608000	-0.6994953
17.8999996	5.7096000	-0.7485828
17.8999996	5.7584000	-0.7179031
17.8999996	5.8072000	-0.7792621
17.8999996	5.8559999	-0.8222136
17.8999996	5.9047999	613586.0000000
17.8999996	5.9535999	613586.0000000
17.8999996	6.0023999	613586.0000000
17.9166660	0.2928000	-4.1847000
17.9166660	0.3416000	-3.5220201
17.9166660	0.3904000	-3.6324666
17.9166660	0.4392000	-2.6875343
17.9166660	0.4880000	-2.3377867
17.9166660	0.5368000	-1.9573593
17.9166660	0.5856000	-1.5155730
17.9166660	0.6344000	-1.5155730
17.9166660	0.6832000	-1.2701364
17.9166660	0.7320000	-1.2762718
17.9166660	0.7808000	-1.3560390
17.9166660	0.8296000	-1.0185633
17.9166660	0.8784000	-1.2271848
17.9166660	0.9272000	-1.0553789
17.9166660	0.9760000	-1.2762718
17.9166660	1.0247999	-1.0185633
17.9166660	1.0735999	-1.0369711
17.9166660	1.1223999	-1.0185633
17.9166660	1.1711999	-0.9633398
17.9166660	1.2200000	-0.9572039
17.9166660	1.2688000	-0.9510679
17.9166660	1.3176000	-1.1658254
17.9166660	1.3664000	-1.1412816
17.9166660	1.4152000	-1.1228738
17.9166660	1.4640000	-1.1228738
17.9166660	1.5128000	-1.1044660
17.9166660	1.5616000	-1.3253593
17.9166660	1.6104000	-1.3314953
17.9166660	1.6592000	-1.2517285
17.9166660	1.7079999	-1.2885437
17.9166660	1.7567999	-1.3499031
17.9166660	1.8055999	-1.3867188
17.9166660	1.8543999	-1.3867188
17.9166660	1.9031999	-0.7853980
17.9166660	1.9519999	-0.7853980
17.9166660	2.0007999	-0.7976699
17.9166660	2.0495999	-1.0983305
17.9166660	2.0983999	-0.9326606
17.9166660	2.1471999	-0.8958449
17.9166660	2.1959999	-0.8958449
17.9166660	2.2447999	-0.7363110

17.9166660	2.2935998	-0.8038058
17.9166660	2.3423998	-0.5890489
17.9166660	2.3912001	-0.5583692
17.9166660	2.4400001	-0.6872234
17.9166660	2.4888000	-0.6933594
17.9166660	2.5376000	-0.6994953
17.9166660	2.5864000	-0.8590293
17.9166660	2.6352000	-0.8590293
17.9166660	2.6840000	-0.6749516
17.9166660	2.7328000	-0.7117672
17.9166660	2.7816000	-0.7056313
17.9166660	2.8304000	-0.6810875
17.9166660	2.8792000	-0.6994953
17.9166660	2.9280000	-0.6994953
17.9166660	2.9768000	-1.2640004
17.9166660	3.0256000	-1.2578645
17.9166660	3.0743999	-1.2517285
17.9166660	3.1231999	-0.7792621
17.9166660	3.1719999	-0.7853980
17.9166660	3.2207999	-0.7792621
17.9166660	3.2695999	-0.7547188
17.9166660	3.3183999	-0.8283496
17.9166660	3.3671999	-0.9694757
17.9166660	3.4159999	-0.9756117
17.9166660	3.4647999	-0.7363110
17.9166660	3.5135999	-0.9387960
17.9166660	3.5623999	-0.9387960
17.9166660	3.6111999	-0.9142528
17.9166660	3.6599998	-0.8283496
17.9166660	3.7087998	-0.9449320
17.9166660	3.7575998	-0.8651652
17.9166660	3.8063998	-0.8406215
17.9166660	3.8551998	-0.8467574
17.9166660	3.9039998	-0.7240391
17.9166660	3.9527998	-0.6688156
17.9166660	4.0016003	-0.6688156
17.9166660	4.0503998	-0.5276895
17.9166660	4.0991998	-0.5154176
17.9166660	4.1479998	-0.6933594
17.9166660	4.1967998	-0.6872234
17.9166660	4.2455997	-0.6135921
17.9166660	4.2943997	-0.6381359
17.9166660	4.3431997	-0.6688156
17.9166660	4.3919997	-0.5951843
17.9166660	4.4407997	-0.3436117
17.9166660	4.4895997	-0.3926992
17.9166660	4.5383997	-0.4233789
17.9166660	4.5871997	-0.2761164
17.9166660	4.6359997	-0.2822523
17.9166660	4.6847997	-0.2208934
17.9166660	4.7336001	-0.2638445
17.9166660	4.7824001	-0.1840777
17.9166660	4.8312001	-0.1902137
17.9166660	4.8800001	-0.2147574
17.9166660	4.9288001	-0.2638445
17.9166660	4.9776001	-0.1104465
17.9166660	5.0264001	-0.0920386

17.9166660	5.0752001	-0.4601941
17.9166660	5.1240001	-0.4479222
17.9166660	5.1728001	-0.7424469
17.9166660	5.2216001	-0.7976699
17.9166660	5.2704000	-0.7179031
17.9166660	5.3192000	-0.7240391
17.9166660	5.3680000	-0.6994953
17.9166660	5.4168000	-0.6994953
17.9166660	5.4656000	-0.7608547
17.9166660	5.5144000	613586.0000000
17.9166660	5.5632000	613586.0000000
17.9166660	5.6120000	-0.9326606
17.9166660	5.6608000	-0.8344855
17.9166660	5.7096000	-0.8283496
17.9166660	5.7584000	613586.0000000
17.9166660	5.8072000	613586.0000000
17.9166660	5.8559999	613586.0000000
17.9166660	5.9047999	613586.0000000
17.9166660	5.9535999	613586.0000000
17.9166660	6.0023999	-0.8774371
17.9333324	0.2928000	-4.2092433
17.9333324	0.3416000	-3.7551851
17.9333324	0.3904000	-3.2459035
17.9333324	0.4392000	-2.3071072
17.9333324	0.4880000	-2.2580199
17.9333324	0.5368000	-1.8591847
17.9333324	0.5856000	-1.4603496
17.9333324	0.6344000	-1.3805828
17.9333324	0.6832000	-1.3621750
17.9333324	0.7320000	-1.0921946
17.9333324	0.7808000	-1.0246992
17.9333324	0.8296000	-1.0308352
17.9333324	0.8784000	-1.2517285
17.9333324	0.9272000	-1.2455926
17.9333324	0.9760000	-1.0246992
17.9333324	1.0247999	-1.0185633
17.9333324	1.0735999	-1.0185633
17.9333324	1.1223999	-1.0185633
17.9333324	1.1711999	-1.1228738
17.9333324	1.2200000	-1.1596894
17.9333324	1.2688000	-0.8528934
17.9333324	1.3176000	-0.8528934
17.9333324	1.3664000	-1.2455926
17.9333324	1.4152000	-1.1106019
17.9333324	1.4640000	-1.1842332
17.9333324	1.5128000	-1.1903691
17.9333324	1.5616000	-1.1596894
17.9333324	1.6104000	-1.3130875
17.9333324	1.6592000	-1.3192234
17.9333324	1.7079999	-1.0308352
17.9333324	1.7567999	-1.1842332
17.9333324	1.8055999	-1.2026410
17.9333324	1.8543999	-1.2762718
17.9333324	1.9031999	-1.2824078
17.9333324	1.9519999	-0.8467574
17.9333324	2.0007999	-0.8590293
17.9333324	2.0495999	-0.8406215

17.9333324	2.0983999	-0.8528934
17.9333324	2.1471999	-0.8651652
17.9333324	2.1959999	-0.7792621
17.9333324	2.2447999	-0.7547188
17.9333324	2.2935998	-1.1780972
17.9333324	2.3423998	-0.8467574
17.9333324	2.3912001	-0.7117672
17.9333324	2.4400001	-0.7240391
17.9333324	2.4888000	-0.6994953
17.9333324	2.5376000	-0.6994953
17.9333324	2.5864000	-0.6074562
17.9333324	2.6352000	-0.6565437
17.9333324	2.6840000	-0.6565437
17.9333324	2.7328000	-0.7547188
17.9333324	2.7816000	-0.7547188
17.9333324	2.8304000	-0.7669902
17.9333324	2.8792000	-0.8344855
17.9333324	2.9280000	-0.8344855
17.9333324	2.9768000	-1.1412816
17.9333324	3.0256000	-1.1474175
17.9333324	3.0743999	-0.8958449
17.9333324	3.1231999	-0.7915339
17.9333324	3.1719999	-0.7915339
17.9333324	3.2207999	-1.0615149
17.9333324	3.2695999	-1.1535535
17.9333324	3.3183999	-1.1535535
17.9333324	3.3671999	-0.7669902
17.9333324	3.4159999	-0.7669902
17.9333324	3.4647999	-0.8528934
17.9333324	3.5135999	-0.5890489
17.9333324	3.5623999	-0.5890489
17.9333324	3.6111999	-1.0983305
17.9333324	3.6599998	-1.0921946
17.9333324	3.7087998	-0.7669902
17.9333324	3.7575998	-0.4663301
17.9333324	3.8063998	-0.6626797
17.9333324	3.8551998	-0.6872234
17.9333324	3.9039998	-0.7056313
17.9333324	3.9527998	-0.6074562
17.9333324	4.0016003	-0.6074562
17.9333324	4.0503998	-0.8651652
17.9333324	4.0991998	-0.8651652
17.9333324	4.1479998	-0.8590293
17.9333324	4.1967998	-0.8038058
17.9333324	4.2455997	-0.7669902
17.9333324	4.2943997	-0.5031457
17.9333324	4.3431997	-0.4970098
17.9333324	4.3919997	-0.5092816
17.9333324	4.4407997	-0.4049711
17.9333324	4.4895997	-0.4049711
17.9333324	4.5383997	-0.4049711
17.9333324	4.5871997	-0.2699804
17.9333324	4.6359997	-0.1840777
17.9333324	4.6847997	-0.1840777
17.9333324	4.7336001	-0.1595340
17.9333324	4.7824001	-0.1533980
17.9333324	4.8312001	-0.3497477

17.9333324	4.8800001	-0.3436117
17.9333324	4.9288001	-0.3620195
17.9333324	4.9776001	-0.1288543
17.9333324	5.0264001	-0.1533980
17.9333324	5.0752001	-0.3681555
17.9333324	5.1240001	-0.4970098
17.9333324	5.1728001	-0.5767770
17.9333324	5.2216001	-0.4663301
17.9333324	5.2704000	-0.4663301
17.9333324	5.3192000	613586.0000000
17.9333324	5.3680000	-0.8651652
17.9333324	5.4168000	-0.6994953
17.9333324	5.4656000	-0.6994953
17.9333324	5.5144000	-0.6565437
17.9333324	5.5632000	-0.8958449
17.9333324	5.6120000	-0.7731261
17.9333324	5.6608000	-0.7731261
17.9333324	5.7096000	-0.7915339
17.9333324	5.7584000	-0.8283496
17.9333324	5.8072000	-0.8283496
17.9333324	5.8559999	-0.8099418
17.9333324	5.9047999	-0.8406215
17.9333324	5.9535999	613586.0000000
17.9333324	6.0023999	613586.0000000
17.9500008	0.2928000	-3.9822142
17.9500008	0.3416000	-2.9206996
17.9500008	0.3904000	-2.6691267
17.9500008	0.4392000	-2.6691267
17.9500008	0.4880000	-2.2886994
17.9500008	0.5368000	-1.9144082
17.9500008	0.5856000	-1.9144082
17.9500008	0.6344000	-1.7180586
17.9500008	0.6832000	-1.7180586
17.9500008	0.7320000	-1.5585246
17.9500008	0.7808000	-1.5523887
17.9500008	0.8296000	-1.4235344
17.9500008	0.8784000	-1.1167378
17.9500008	0.9272000	-1.1228738
17.9500008	0.9760000	-1.2271848
17.9500008	1.0247999	-1.2149129
17.9500008	1.0735999	-0.9019809
17.9500008	1.1223999	-1.2455926
17.9500008	1.1711999	-0.9510679
17.9500008	1.2200000	-1.2026410
17.9500008	1.2688000	-0.9694757
17.9500008	1.3176000	-0.9081168
17.9500008	1.3664000	-1.0799227
17.9500008	1.4152000	-1.2087770
17.9500008	1.4640000	-1.1290097
17.9500008	1.5128000	-1.1290097
17.9500008	1.5616000	-1.1412816
17.9500008	1.6104000	-1.1535535
17.9500008	1.6592000	-1.0983305
17.9500008	1.7079999	-0.9694757
17.9500008	1.7567999	-1.2578645
17.9500008	1.8055999	-1.2640004
17.9500008	1.8543999	-1.0921946

17.9500008	1.9031999	-1.1535535
17.9500008	1.9519999	-0.9510679
17.9500008	2.0007999	-0.9510679
17.9500008	2.0495999	-0.9756117
17.9500008	2.0983999	-0.7669902
17.9500008	2.1471999	-0.8528934
17.9500008	2.1959999	-0.8467574
17.9500008	2.2447999	-0.8406215
17.9500008	2.2935998	-0.8099418
17.9500008	2.3423998	-0.8344855
17.9500008	2.3912001	-0.8344855
17.9500008	2.4400001	-0.8283496
17.9500008	2.4888000	-0.7792621
17.9500008	2.5376000	-0.8283496
17.9500008	2.5864000	-0.9756117
17.9500008	2.6352000	-0.8774371
17.9500008	2.6840000	-0.8774371
17.9500008	2.7328000	-1.0737867
17.9500008	2.7816000	-0.9081168
17.9500008	2.8304000	-0.7485828
17.9500008	2.8792000	-0.7485828
17.9500008	2.9280000	-0.7240391
17.9500008	2.9768000	-0.7056313
17.9500008	3.0256000	-1.0799227
17.9500008	3.0743999	-1.0737867
17.9500008	3.1231999	-0.9633398
17.9500008	3.1719999	-1.0492430
17.9500008	3.2207999	-0.6872234
17.9500008	3.2695999	-0.6994953
17.9500008	3.3183999	-0.7056313
17.9500008	3.3671999	-0.7056313
17.9500008	3.4159999	-0.7179031
17.9500008	3.4647999	-0.7363110
17.9500008	3.5135999	-0.7240391
17.9500008	3.5623999	-0.6320000
17.9500008	3.6111999	-0.6320000
17.9500008	3.6599998	-0.5583692
17.9500008	3.7087998	-0.5522332
17.9500008	3.7575998	-1.2640004
17.9500008	3.8063998	-1.2640004
17.9500008	3.8551998	-1.2210488
17.9500008	3.9039998	-1.2026410
17.9500008	3.9527998	-1.1965051
17.9500008	4.0016003	-1.0983305
17.9500008	4.0503998	-1.0431070
17.9500008	4.0991998	-1.0553789
17.9500008	4.1479998	-1.1474175
17.9500008	4.1967998	-1.1412816
17.9500008	4.2455997	-1.1167378
17.9500008	4.2943997	-1.0921946
17.9500008	4.3431997	-0.8713012
17.9500008	4.3919997	-0.8590293
17.9500008	4.4407997	-0.8406215
17.9500008	4.4895997	-0.9081168
17.9500008	4.5383997	-0.9142528
17.9500008	4.5871997	-0.9142528
17.9500008	4.6359997	-0.9142528

17.9500008	4.6847997	-0.4540582
17.9500008	4.7336001	-0.7240391
17.9500008	4.7824001	-0.7240391
17.9500008	4.8312001	-0.6320000
17.9500008	4.8800001	-0.6442719
17.9500008	4.9288001	-0.6504078
17.9500008	4.9776001	-0.6442719
17.9500008	5.0264001	-0.7117672
17.9500008	5.0752001	-0.6504078
17.9500008	5.1240001	-1.0124273
17.9500008	5.1728001	-1.0431070
17.9500008	5.2216001	-1.1474175
17.9500008	5.2704000	-1.0860586
17.9500008	5.3192000	-1.2271848
17.9500008	5.3680000	-0.7976699
17.9500008	5.4168000	613586.0000000
17.9500008	5.4656000	-1.2333207
17.9500008	5.5144000	-1.2455926
17.9500008	5.5632000	613586.0000000
17.9500008	5.6120000	613586.0000000
17.9500008	5.6608000	613586.0000000
17.9500008	5.7096000	613586.0000000
17.9500008	5.7584000	613586.0000000
17.9500008	5.8072000	613586.0000000
17.9500008	5.8559999	613586.0000000
17.9500008	5.9047999	613586.0000000
17.9500008	5.9535999	613586.0000000
17.9500008	6.0023999	613586.0000000
17.9666672	0.2928000	-3.7551851
17.9666672	0.3416000	-3.6692822
17.9666672	0.3904000	-3.6692822
17.9666672	0.4392000	-2.5341363
17.9666672	0.4880000	-2.1782527
17.9666672	0.5368000	-1.7794180
17.9666672	0.5856000	-1.7732821
17.9666672	0.6344000	-1.7119226
17.9666672	0.6832000	-1.5646605
17.9666672	0.7320000	-1.3989906
17.9666672	0.7808000	-1.3989906
17.9666672	0.8296000	-1.2578645
17.9666672	0.8784000	-1.1167378
17.9666672	0.9272000	-1.1719613
17.9666672	0.9760000	-1.1842332
17.9666672	1.0247999	-0.8222136
17.9666672	1.0735999	-0.8222136
17.9666672	1.1223999	-1.0921946
17.9666672	1.1711999	-1.0921946
17.9666672	1.2200000	-1.1290097
17.9666672	1.2688000	-0.7792621
17.9666672	1.3176000	-1.0369711
17.9666672	1.3664000	-0.9633398
17.9666672	1.4152000	-1.0124273
17.9666672	1.4640000	-1.0185633
17.9666672	1.5128000	-1.0369711
17.9666672	1.5616000	-1.2701364
17.9666672	1.6104000	-1.0431070
17.9666672	1.6592000	-1.0431070

17.9666672	1.7079999	-1.0553789
17.9666672	1.7567999	-1.2087770
17.9666672	1.8055999	-1.2087770
17.9666672	1.8543999	-0.8528934
17.9666672	1.9031999	-1.1535535
17.9666672	1.9519999	-1.0124273
17.9666672	2.0007999	-1.2701364
17.9666672	2.0495999	-0.9572039
17.9666672	2.0983999	-0.9694757
17.9666672	2.1471999	-0.9572039
17.9666672	2.1959999	-0.9572039
17.9666672	2.2447999	-1.0799227
17.9666672	2.2935998	-1.0799227
17.9666672	2.3423998	-1.0860586
17.9666672	2.3912001	-1.0676508
17.9666672	2.4400001	-0.8713012
17.9666672	2.4888000	-0.8713012
17.9666672	2.5376000	-0.8835731
17.9666672	2.5864000	-0.8713012
17.9666672	2.6352000	-0.7240391
17.9666672	2.6840000	-0.5460973
17.9666672	2.7328000	-0.5522332
17.9666672	2.7816000	-0.6994953
17.9666672	2.8304000	-0.6504078
17.9666672	2.8792000	-1.0615149
17.9666672	2.9280000	-1.0615149
17.9666672	2.9768000	-0.6933594
17.9666672	3.0256000	-0.9326606
17.9666672	3.0743999	-0.9387960
17.9666672	3.1231999	-0.6504078
17.9666672	3.1719999	-0.6504078
17.9666672	3.2207999	-0.7179031
17.9666672	3.2695999	-0.6872234
17.9666672	3.3183999	-1.0737867
17.9666672	3.3671999	-1.0737867
17.9666672	3.4159999	-0.8222136
17.9666672	3.4647999	-0.7547188
17.9666672	3.5135999	-0.7547188
17.9666672	3.5623999	-0.7547188
17.9666672	3.6111999	-0.6565437
17.9666672	3.6599998	-0.9265246
17.9666672	3.7087998	-0.9265246
17.9666672	3.7575998	-0.9142528
17.9666672	3.8063998	-1.1474175
17.9666672	3.8551998	-1.1290097
17.9666672	3.9039998	-0.8099418
17.9666672	3.9527998	-0.8160777
17.9666672	4.0016003	-0.7792621
17.9666672	4.0503998	-0.7240391
17.9666672	4.0991998	-0.7363110
17.9666672	4.1479998	613586.0000000
17.9666672	4.1967998	613586.0000000
17.9666672	4.2455997	-0.9265246
17.9666672	4.2943997	-0.7731261
17.9666672	4.3431997	-0.5951843
17.9666672	4.3919997	-0.6565437
17.9666672	4.4407997	-0.6442719

17.9666672	4.4895997	-0.4540582
17.9666672	4.5383997	-0.4663301
17.9666672	4.5871997	-0.2638445
17.9666672	4.6359997	-0.7179031
17.9666672	4.6847997	-0.7056313
17.9666672	4.7336001	-0.6135921
17.9666672	4.7824001	-0.6074562
17.9666672	4.8312001	-0.4295144
17.9666672	4.8800001	-0.5706410
17.9666672	4.9288001	-0.1411262
17.9666672	4.9776001	-0.4111071
17.9666672	5.0264001	-0.5154176
17.9666672	5.0752001	-0.5215535
17.9666672	5.1240001	-0.5338254
17.9666672	5.1728001	613586.0000000
17.9666672	5.2216001	613586.0000000
17.9666672	5.2704000	613586.0000000
17.9666672	5.3192000	613586.0000000
17.9666672	5.3680000	613586.0000000
17.9666672	5.4168000	613586.0000000
17.9666672	5.4656000	613586.0000000
17.9666672	5.5144000	-0.7485828
17.9666672	5.5632000	-0.6197281
17.9666672	5.6120000	-0.6994953
17.9666672	5.6608000	-0.7179031
17.9666672	5.7096000	-0.7669902
17.9666672	5.7584000	613586.0000000
17.9666672	5.8072000	613586.0000000
17.9666672	5.8559999	-0.9265246
17.9666672	5.9047999	-0.8406215
17.9666672	5.9535999	613586.0000000
17.9666672	6.0023999	613586.0000000
17.9833336	0.2928000	-4.1724281
17.9833336	0.3416000	-3.4115734
17.9833336	0.3904000	-3.2520394
17.9833336	0.4392000	-2.7550297
17.9833336	0.4880000	-2.0125828
17.9833336	0.5368000	-1.5892043
17.9833336	0.5856000	-1.4848933
17.9833336	0.6344000	-1.4910293
17.9833336	0.6832000	-1.1596894
17.9833336	0.7320000	-1.1658254
17.9833336	0.7808000	-1.1535535
17.9833336	0.8296000	-1.1106019
17.9833336	0.8784000	-1.1535535
17.9833336	0.9272000	-0.9265246
17.9833336	0.9760000	-0.9203887
17.9833336	1.0247999	-1.1351457
17.9833336	1.0735999	-1.1167378
17.9833336	1.1223999	-1.0921946
17.9833336	1.1711999	-0.9940195
17.9833336	1.2200000	-1.0369711
17.9833336	1.2688000	-1.0369711
17.9833336	1.3176000	-1.1903691
17.9833336	1.3664000	-0.9510679
17.9833336	1.4152000	-1.1412816
17.9833336	1.4640000	-1.1965051

17.9833336	1.5128000	-1.2026410
17.9833336	1.5616000	-1.2087770
17.9833336	1.6104000	-1.1535535
17.9833336	1.6592000	-1.1719613
17.9833336	1.7079999	-1.1719613
17.9833336	1.7567999	-1.0553789
17.9833336	1.8055999	-1.0615149
17.9833336	1.8543999	-0.9694757
17.9833336	1.9031999	-0.9633398
17.9833336	1.9519999	-1.1474175
17.9833336	2.0007999	-1.1535535
17.9833336	2.0495999	-1.1903691
17.9833336	2.0983999	-1.1780972
17.9833336	2.1471999	-1.0983305
17.9833336	2.1959999	-1.0921946
17.9833336	2.2447999	-0.7608547
17.9833336	2.2935998	-1.0062914
17.9833336	2.3423998	-0.6994953
17.9833336	2.3912001	-1.1106019
17.9833336	2.4400001	-0.7608547
17.9833336	2.4888000	-0.6872234
17.9833336	2.5376000	-0.6810875
17.9833336	2.5864000	-0.6749516
17.9833336	2.6352000	-1.0921946
17.9833336	2.6840000	-1.0921946
17.9833336	2.7328000	-1.1106019
17.9833336	2.7816000	-1.1167378
17.9833336	2.8304000	-1.1167378
17.9833336	2.8792000	-1.0983305
17.9833336	2.9280000	-1.0553789
17.9833336	2.9768000	-1.0185633
17.9833336	3.0256000	-1.0185633
17.9833336	3.0743999	-0.7976699
17.9833336	3.1231999	-0.7792621
17.9833336	3.1719999	-1.0921946
17.9833336	3.2207999	-1.0860586
17.9833336	3.2695999	-1.0676508
17.9833336	3.3183999	-0.8958449
17.9833336	3.3671999	-0.6381359
17.9833336	3.4159999	-0.6381359
17.9833336	3.4647999	-0.6381359
17.9833336	3.5135999	-0.6381359
17.9833336	3.5623999	-0.6320000
17.9833336	3.6111999	-0.6442719
17.9833336	3.6599998	-0.2761164
17.9833336	3.7087998	-0.2761164
17.9833336	3.7575998	-0.3129320
17.9833336	3.8063998	-0.4540582
17.9833336	3.8551998	-0.5706410
17.9833336	3.9039998	-0.7915339
17.9833336	3.9527998	-0.5829129
17.9833336	4.0016003	-0.5890489
17.9833336	4.0503998	-0.5829129
17.9833336	4.0991998	-0.5829129
17.9833336	4.1479998	-0.5767770
17.9833336	4.1967998	-0.8528934
17.9833336	4.2455997	-0.8467574

17.9833336	4.2943997	-0.7179031
17.9833336	4.3431997	-0.7056313
17.9833336	4.3919997	-0.6258640
17.9833336	4.4407997	-0.7179031
17.9833336	4.4895997	-0.4601941
17.9833336	4.5383997	-0.5583692
17.9833336	4.5871997	-0.7363110
17.9833336	4.6359997	-0.7301750
17.9833336	4.6847997	-0.6749516
17.9833336	4.7336001	-0.7117672
17.9833336	4.7824001	-0.6320000
17.9833336	4.8312001	-0.4970098
17.9833336	4.8800001	-0.6565437
17.9833336	4.9288001	-0.6626797
17.9833336	4.9776001	-0.2822523
17.9833336	5.0264001	-0.6013203
17.9833336	5.0752001	-0.4049711
17.9833336	5.1240001	-0.3988352
17.9833336	5.1728001	613586.0000000
17.9833336	5.2216001	-0.6749516
17.9833336	5.2704000	613586.0000000
17.9833336	5.3192000	613586.0000000
17.9833336	5.3680000	613586.0000000
17.9833336	5.4168000	613586.0000000
17.9833336	5.4656000	-0.7485828
17.9833336	5.5144000	-0.7424469
17.9833336	5.5632000	-0.7547188
17.9833336	5.6120000	-0.5951843
17.9833336	5.6608000	-0.6197281
17.9833336	5.7096000	-0.6197281
17.9833336	5.7584000	-0.6135921
17.9833336	5.8072000	613586.0000000
17.9833336	5.8559999	613586.0000000
17.9833336	5.9047999	613586.0000000
17.9833336	5.9535999	613586.0000000
17.9833336	6.0023999	-0.8528934
18.0000000	0.2928000	-3.3011267
18.0000000	0.3416000	-3.1231849
18.0000000	0.3904000	-2.4850490
18.0000000	0.4392000	-2.1905246
18.0000000	0.4880000	-1.9941750
18.0000000	0.5368000	-1.9941750
18.0000000	0.5856000	-1.8714566
18.0000000	0.6344000	-1.8837285
18.0000000	0.6832000	-1.8346410
18.0000000	0.7320000	-1.8530488
18.0000000	0.7808000	-1.6444273
18.0000000	0.8296000	-1.6505632
18.0000000	0.8784000	-1.6014762
18.0000000	0.9272000	-1.3253593
18.0000000	0.9760000	-1.2149129
18.0000000	1.0247999	-1.2517285
18.0000000	1.0735999	-0.8590293
18.0000000	1.1223999	-1.2149129
18.0000000	1.1711999	-1.2271848
18.0000000	1.2200000	-1.1658254
18.0000000	1.2688000	-0.9142528

18.0000000	1.3176000	-0.9142528
18.0000000	1.3664000	-0.9142528
18.0000000	1.4152000	-1.2333207
18.0000000	1.4640000	-1.3192234
18.0000000	1.5128000	-1.2885437
18.0000000	1.5616000	-1.2210488
18.0000000	1.6104000	-0.8099418
18.0000000	1.6592000	-0.9756117
18.0000000	1.7079999	-0.8160777
18.0000000	1.7567999	-0.9510679
18.0000000	1.8055999	-1.1106019
18.0000000	1.8543999	-1.2210488
18.0000000	1.9031999	-0.8774371
18.0000000	1.9519999	-0.8713012
18.0000000	2.0007999	-0.8651652
18.0000000	2.0495999	-1.1228738
18.0000000	2.0983999	-0.8222136
18.0000000	2.1471999	-0.8038058
18.0000000	2.1959999	-0.7853980
18.0000000	2.2447999	-0.7792621
18.0000000	2.2935998	-0.7179031
18.0000000	2.3423998	-0.7731261
18.0000000	2.3912001	-0.8406215
18.0000000	2.4400001	-0.8406215
18.0000000	2.4888000	-0.7853980
18.0000000	2.5376000	-0.8467574
18.0000000	2.5864000	-1.0983305
18.0000000	2.6352000	-1.1228738
18.0000000	2.6840000	-1.1719613
18.0000000	2.7328000	-1.1780972
18.0000000	2.7816000	-1.1903691
18.0000000	2.8304000	-1.1903691
18.0000000	2.8792000	-1.1228738
18.0000000	2.9280000	-1.0492430
18.0000000	2.9768000	-1.0492430
18.0000000	3.0256000	-1.0001554
18.0000000	3.0743999	-1.0062914
18.0000000	3.1231999	-1.0246992
18.0000000	3.1719999	-1.0185633
18.0000000	3.2207999	-1.1106019
18.0000000	3.2695999	-0.6688156
18.0000000	3.3183999	-0.9387960
18.0000000	3.3671999	-0.6626797
18.0000000	3.4159999	-0.7731261
18.0000000	3.4647999	-0.7485828
18.0000000	3.5135999	-0.7485828
18.0000000	3.5623999	-1.1412816
18.0000000	3.6111999	-1.1412816
18.0000000	3.6599998	-0.8713012
18.0000000	3.7087998	-0.8835731
18.0000000	3.7575998	-0.9019809
18.0000000	3.8063998	-0.7240391
18.0000000	3.8551998	-0.7179031
18.0000000	3.9039998	-0.4540582
18.0000000	3.9527998	-0.2577085
18.0000000	4.0016003	-0.2515731
18.0000000	4.0503998	613586.0000000

18.0000000	4.0991998	-1.0921946
18.0000000	4.1479998	-0.8283496
18.0000000	4.1967998	-0.8283496
18.0000000	4.2455997	-0.6565437
18.0000000	4.2943997	-0.6626797
18.0000000	4.3431997	-0.6626797
18.0000000	4.3919997	-0.5890489
18.0000000	4.4407997	-0.5890489
18.0000000	4.4895997	-0.6688156
18.0000000	4.5383997	-0.6688156
18.0000000	4.5871997	-0.6688156
18.0000000	4.6359997	-0.6626797
18.0000000	4.6847997	-0.3804274
18.0000000	4.7336001	-0.3681555
18.0000000	4.7824001	-0.3374758
18.0000000	4.8312001	-0.3374758
18.0000000	4.8800001	-0.3313398
18.0000000	4.9288001	-0.3252039
18.0000000	4.9776001	-0.3129320
18.0000000	5.0264001	-0.2945242
18.0000000	5.0752001	613586.0000000
18.0000000	5.1240001	613586.0000000
18.0000000	5.1728001	613586.0000000
18.0000000	5.2216001	613586.0000000
18.0000000	5.2704000	613586.0000000
18.0000000	5.3192000	613586.0000000
18.0000000	5.3680000	613586.0000000
18.0000000	5.4168000	613586.0000000
18.0000000	5.4656000	613586.0000000
18.0000000	5.5144000	613586.0000000
18.0000000	5.5632000	613586.0000000
18.0000000	5.6120000	-0.9142528
18.0000000	5.6608000	-0.9081168
18.0000000	5.7096000	-0.9142528
18.0000000	5.7584000	-0.7915339
18.0000000	5.8072000	-0.7915339
18.0000000	5.8559999	613586.0000000
18.0000000	5.9047999	613586.0000000
18.0000000	5.9535999	613586.0000000
18.0000000	6.0023999	613586.0000000
18.0166664	0.2928000	-3.7674570
18.0166664	0.3416000	-3.6508744
18.0166664	0.3904000	-3.3133986
18.0166664	0.4392000	-2.9636509
18.0166664	0.4880000	-2.3991461
18.0166664	0.5368000	-2.2027965
18.0166664	0.5856000	-2.1905246
18.0166664	0.6344000	-1.8653207
18.0166664	0.6832000	-1.7180586
18.0166664	0.7320000	-1.4173985
18.0166664	0.7808000	-1.6628351
18.0166664	0.8296000	-1.4173985
18.0166664	0.8784000	-1.4112625
18.0166664	0.9272000	-1.6382914
18.0166664	0.9760000	-1.6321554
18.0166664	1.0247999	-1.6321554
18.0166664	1.0735999	-1.3621750

18.0166664	1.1223999	-1.3437672
18.0166664	1.1711999	-1.3499031
18.0166664	1.2200000	-1.3621750
18.0166664	1.2688000	-1.1780972
18.0166664	1.3176000	-1.2578645
18.0166664	1.3664000	-1.3192234
18.0166664	1.4152000	-1.0492430
18.0166664	1.4640000	-1.0001554
18.0166664	1.5128000	-1.0615149
18.0166664	1.5616000	-0.9878836
18.0166664	1.6104000	-0.9817476
18.0166664	1.6592000	-0.9694757
18.0166664	1.7079999	-1.1535535
18.0166664	1.7567999	-1.1719613
18.0166664	1.8055999	-1.1842332
18.0166664	1.8543999	-1.1842332
18.0166664	1.9031999	-1.1719613
18.0166664	1.9519999	-1.0615149
18.0166664	2.0007999	-0.9940195
18.0166664	2.0495999	-0.8099418
18.0166664	2.0983999	-1.0308352
18.0166664	2.1471999	-1.0308352
18.0166664	2.1959999	-0.8958449
18.0166664	2.2447999	-0.9019809
18.0166664	2.2935998	-0.9081168
18.0166664	2.3423998	-0.9081168
18.0166664	2.3912001	-0.7301750
18.0166664	2.4400001	-0.7179031
18.0166664	2.4888000	-0.8774371
18.0166664	2.5376000	-0.8774371
18.0166664	2.5864000	-0.8774371
18.0166664	2.6352000	-1.2578645
18.0166664	2.6840000	-1.1228738
18.0166664	2.7328000	-1.1228738
18.0166664	2.7816000	-1.1228738
18.0166664	2.8304000	-1.2640004
18.0166664	2.8792000	-0.9756117
18.0166664	2.9280000	-0.8958449
18.0166664	2.9768000	-0.8283496
18.0166664	3.0256000	-0.7792621
18.0166664	3.0743999	-0.7179031
18.0166664	3.1231999	-0.7179031
18.0166664	3.1719999	-1.0676508
18.0166664	3.2207999	-1.0676508
18.0166664	3.2695999	-0.8283496
18.0166664	3.3183999	-0.8344855
18.0166664	3.3671999	-0.8038058
18.0166664	3.4159999	-0.9326606
18.0166664	3.4647999	-1.1044660
18.0166664	3.5135999	-1.1044660
18.0166664	3.5623999	-1.2087770
18.0166664	3.6111999	-1.2087770
18.0166664	3.6599998	-1.0431070
18.0166664	3.7087998	-0.8038058
18.0166664	3.7575998	-0.8038058
18.0166664	3.8063998	-1.0308352
18.0166664	3.8551998	-1.0431070

18.0166664	3.9039998	-0.6749516
18.0166664	3.9527998	-0.7363110
18.0166664	4.0016003	-1.1842332
18.0166664	4.0503998	-1.1658254
18.0166664	4.0991998	-1.1535535
18.0166664	4.1479998	-0.6933594
18.0166664	4.1967998	-0.6872234
18.0166664	4.2455997	-0.4601941
18.0166664	4.2943997	-0.4601941
18.0166664	4.3431997	-0.3129320
18.0166664	4.3919997	-0.3190680
18.0166664	4.4407997	-0.0797672
18.0166664	4.4895997	-0.0797672
18.0166664	4.5383997	-0.3190680
18.0166664	4.5871997	-0.3129320
18.0166664	4.6359997	-0.1840777
18.0166664	4.6847997	-0.1963496
18.0166664	4.7336001	-0.1349902
18.0166664	4.7824001	-0.0736313
18.0166664	4.8312001	-0.1533980
18.0166664	4.8800001	-0.1411262
18.0166664	4.9288001	613586.0000000
18.0166664	4.9776001	613586.0000000
18.0166664	5.0264001	613586.0000000
18.0166664	5.0752001	613586.0000000
18.0166664	5.1240001	613586.0000000
18.0166664	5.1728001	613586.0000000
18.0166664	5.2216001	613586.0000000
18.0166664	5.2704000	613586.0000000
18.0166664	5.3192000	613586.0000000
18.0166664	5.3680000	613586.0000000
18.0166664	5.4168000	-0.9081168
18.0166664	5.4656000	-0.8958449
18.0166664	5.5144000	-0.7669902
18.0166664	5.5632000	-0.7547188
18.0166664	5.6120000	-0.7547188
18.0166664	5.6608000	-0.7547188
18.0166664	5.7096000	613586.0000000
18.0166664	5.7584000	613586.0000000
18.0166664	5.8072000	613586.0000000
18.0166664	5.8559999	613586.0000000
18.0166664	5.9047999	613586.0000000
18.0166664	5.9535999	613586.0000000
18.0166664	6.0023999	613586.0000000
18.0333328	0.2928000	-3.8349521
18.0333328	0.3416000	-3.7183695
18.0333328	0.3904000	-3.2765830
18.0333328	0.4392000	-2.1414371
18.0333328	0.4880000	-2.1414371
18.0333328	0.5368000	-2.0064468
18.0333328	0.5856000	-1.6014762
18.0333328	0.6344000	-1.6689711
18.0333328	0.6832000	-1.8653207
18.0333328	0.7320000	-1.8653207
18.0333328	0.7808000	-2.0248547
18.0333328	0.8296000	-1.9512239
18.0333328	0.8784000	-1.9328160

18.0333328	0.9272000	-1.8653207
18.0333328	0.9760000	-1.7855535
18.0333328	1.0247999	-1.7855535
18.0333328	1.0735999	-1.5401168
18.0333328	1.1223999	-1.5401168
18.0333328	1.1711999	-1.5401168
18.0333328	1.2200000	-1.5401168
18.0333328	1.2688000	-1.5523887
18.0333328	1.3176000	-1.3130875
18.0333328	1.3664000	-1.3192234
18.0333328	1.4152000	-1.0799227
18.0333328	1.4640000	-0.9019809
18.0333328	1.5128000	-1.0492430
18.0333328	1.5616000	-1.0492430
18.0333328	1.6104000	-0.8958449
18.0333328	1.6592000	-1.1412816
18.0333328	1.7079999	-1.1719613
18.0333328	1.7567999	-1.3069515
18.0333328	1.8055999	-1.1412816
18.0333328	1.8543999	-1.1412816
18.0333328	1.9031999	-1.2394567
18.0333328	1.9519999	-1.1412816
18.0333328	2.0007999	-1.1474175
18.0333328	2.0495999	-1.0492430
18.0333328	2.0983999	-1.1596894
18.0333328	2.1471999	-1.0860586
18.0333328	2.1959999	-0.9633398
18.0333328	2.2447999	-1.0308352
18.0333328	2.2935998	-0.8406215
18.0333328	2.3423998	-0.8344855
18.0333328	2.3912001	-0.9387960
18.0333328	2.4400001	-1.0062914
18.0333328	2.4888000	-1.0185633
18.0333328	2.5376000	-1.0124273
18.0333328	2.5864000	-1.1719613
18.0333328	2.6352000	-1.1535535
18.0333328	2.6840000	-1.1474175
18.0333328	2.7328000	-0.9265246
18.0333328	2.7816000	-1.0860586
18.0333328	2.8304000	-1.0799227
18.0333328	2.8792000	-1.0062914
18.0333328	2.9280000	-1.0062914
18.0333328	2.9768000	-0.6933594
18.0333328	3.0256000	-0.7240391
18.0333328	3.0743999	-0.7485828
18.0333328	3.1231999	-0.7301750
18.0333328	3.1719999	-0.7179031
18.0333328	3.2207999	-1.1044660
18.0333328	3.2695999	-0.9203887
18.0333328	3.3183999	-1.0799227
18.0333328	3.3671999	-1.2149129
18.0333328	3.4159999	-1.2455926
18.0333328	3.4647999	-1.1535535
18.0333328	3.5135999	-1.0001554
18.0333328	3.5623999	-1.0062914
18.0333328	3.6111999	-0.8713012
18.0333328	3.6599998	-0.9633398

18.0333328	3.7087998	-0.9694757
18.0333328	3.7575998	-1.0492430
18.0333328	3.8063998	-1.1596894
18.0333328	3.8551998	-0.9510679
18.0333328	3.9039998	-1.1290097
18.0333328	3.9527998	-1.1351457
18.0333328	4.0016003	-1.1412816
18.0333328	4.0503998	-1.0369711
18.0333328	4.0991998	-0.9387960
18.0333328	4.1479998	-1.0983305
18.0333328	4.1967998	-0.9878836
18.0333328	4.2455997	-0.9817476
18.0333328	4.2943997	-0.9817476
18.0333328	4.3431997	-0.9940195
18.0333328	4.3919997	-0.9817476
18.0333328	4.4407997	-0.8835731
18.0333328	4.4895997	-0.8958449
18.0333328	4.5383997	-1.0124273
18.0333328	4.5871997	-0.6872234
18.0333328	4.6359997	-0.4847379
18.0333328	4.6847997	-0.5890489
18.0333328	4.7336001	-0.5829129
18.0333328	4.7824001	-0.4295144
18.0333328	4.8312001	-0.4295144
18.0333328	4.8800001	-0.3804274
18.0333328	4.9288001	-0.3804274
18.0333328	4.9776001	-0.6442719
18.0333328	5.0264001	-0.6381359
18.0333328	5.0752001	-0.6074562
18.0333328	5.1240001	613586.0000000
18.0333328	5.1728001	-0.4111071
18.0333328	5.2216001	613586.0000000
18.0333328	5.2704000	-0.5338254
18.0333328	5.3192000	-0.5154176
18.0333328	5.3680000	-0.7976699
18.0333328	5.4168000	-0.8774371
18.0333328	5.4656000	-0.8590293
18.0333328	5.5144000	-0.8467574
18.0333328	5.5632000	-0.9081168
18.0333328	5.6120000	-0.8099418
18.0333328	5.6608000	-0.8099418
18.0333328	5.7096000	613586.0000000
18.0333328	5.7584000	613586.0000000
18.0333328	5.8072000	613586.0000000
18.0333328	5.8559999	613586.0000000
18.0333328	5.9047999	613586.0000000
18.0333328	5.9535999	613586.0000000
18.0333328	6.0023999	613586.0000000
18.0499992	0.2928000	-3.9822142
18.0499992	0.3416000	-3.9760783
18.0499992	0.3904000	-3.9760783
18.0499992	0.4392000	-2.5157285
18.0499992	0.4880000	-1.8837285
18.0499992	0.5368000	-1.6382914
18.0499992	0.5856000	-1.5585246
18.0499992	0.6344000	-1.5585246
18.0499992	0.6832000	-1.4664855

18.0499992	0.7320000	-1.5892043
18.0499992	0.7808000	-1.5892043
18.0499992	0.8296000	-1.8039613
18.0499992	0.8784000	-1.8039613
18.0499992	0.9272000	-1.8898644
18.0499992	0.9760000	-1.9082723
18.0499992	1.0247999	-1.8346410
18.0499992	1.0735999	-1.8407769
18.0499992	1.1223999	-1.6566992
18.0499992	1.1711999	-1.6444273
18.0499992	1.2200000	-1.6321554
18.0499992	1.2688000	-1.2394567
18.0499992	1.3176000	-1.0553789
18.0499992	1.3664000	-1.0615149
18.0499992	1.4152000	-1.0615149
18.0499992	1.4640000	-1.0983305
18.0499992	1.5128000	-0.9572039
18.0499992	1.5616000	-1.0369711
18.0499992	1.6104000	-0.8406215
18.0499992	1.6592000	-1.0308352
18.0499992	1.7079999	-1.0308352
18.0499992	1.7567999	-1.0308352
18.0499992	1.8055999	-1.0308352
18.0499992	1.8543999	-1.2087770
18.0499992	1.9031999	-1.1228738
18.0499992	1.9519999	-1.1351457
18.0499992	2.0007999	-1.1351457
18.0499992	2.0495999	-1.1965051
18.0499992	2.0983999	-1.1903691
18.0499992	2.1471999	-1.1903691
18.0499992	2.1959999	-1.2210488
18.0499992	2.2447999	-1.2149129
18.0499992	2.2935998	-1.2087770
18.0499992	2.3423998	-0.8344855
18.0499992	2.3912001	-0.8222136
18.0499992	2.4400001	-0.8528934
18.0499992	2.4888000	-1.2701364
18.0499992	2.5376000	-1.1842332
18.0499992	2.5864000	-1.1842332
18.0499992	2.6352000	-0.7056313
18.0499992	2.6840000	-0.7240391
18.0499992	2.7328000	-0.7485828
18.0499992	2.7816000	-0.7792621
18.0499992	2.8304000	-0.7117672
18.0499992	2.8792000	-0.5583692
18.0499992	2.9280000	-0.9142528
18.0499992	2.9768000	-0.8344855
18.0499992	3.0256000	-0.8406215
18.0499992	3.0743999	-0.8406215
18.0499992	3.1231999	-0.8713012
18.0499992	3.1719999	-0.9387960
18.0499992	3.2207999	-0.9449320
18.0499992	3.2695999	-0.9510679
18.0499992	3.3183999	-1.1228738
18.0499992	3.3671999	-1.4726214
18.0499992	3.4159999	-0.9572039
18.0499992	3.4647999	-1.3683109

18.0499992	3.5135999	-1.3130875
18.0499992	3.5623999	-1.2333207
18.0499992	3.6111999	-1.3192234
18.0499992	3.6599998	-1.0185633
18.0499992	3.7087998	-1.1535535
18.0499992	3.7575998	-1.1596894
18.0499992	3.8063998	-1.0369711
18.0499992	3.8551998	-1.0308352
18.0499992	3.9039998	-1.0860586
18.0499992	3.9527998	-0.7056313
18.0499992	4.0016003	-0.7179031
18.0499992	4.0503998	-0.9326606
18.0499992	4.0991998	-0.6074562
18.0499992	4.1479998	-1.1228738
18.0499992	4.1967998	-1.0983305
18.0499992	4.2455997	-0.9633398
18.0499992	4.2943997	-1.0308352
18.0499992	4.3431997	-0.9387960
18.0499992	4.3919997	-0.8958449
18.0499992	4.4407997	-0.8590293
18.0499992	4.4895997	-0.6197281
18.0499992	4.5383997	-0.5522332
18.0499992	4.5871997	-0.5460973
18.0499992	4.6359997	-0.5829129
18.0499992	4.6847997	-0.4172430
18.0499992	4.7336001	-0.4233789
18.0499992	4.7824001	-0.4172430
18.0499992	4.8312001	-0.4847379
18.0499992	4.8800001	-0.4233789
18.0499992	4.9288001	-0.7669902
18.0499992	4.9776001	-0.6565437
18.0499992	5.0264001	613586.0000000
18.0499992	5.0752001	-0.3804274
18.0499992	5.1240001	-0.3865633
18.0499992	5.1728001	-0.7485828
18.0499992	5.2216001	-0.7547188
18.0499992	5.2704000	-0.9203887
18.0499992	5.3192000	-0.8099418
18.0499992	5.3680000	-0.6933594
18.0499992	5.4168000	613586.0000000
18.0499992	5.4656000	-0.7547188
18.0499992	5.5144000	-0.7976699
18.0499992	5.5632000	-0.7915339
18.0499992	5.6120000	-0.8713012
18.0499992	5.6608000	-0.8774371
18.0499992	5.7096000	-0.9817476
18.0499992	5.7584000	-0.9817476
18.0499992	5.8072000	-1.0983305
18.0499992	5.8559999	-1.0983305
18.0499992	5.9047999	613586.0000000
18.0499992	5.9535999	613586.0000000
18.0499992	6.0023999	613586.0000000
18.0666676	0.2928000	-4.8596516
18.0666676	0.3416000	-2.6691267
18.0666676	0.3904000	-1.9205441
18.0666676	0.4392000	-1.9266801
18.0666676	0.4880000	-1.9266801

18.0666676	0.5368000	-1.3192234
18.0666676	0.5856000	-1.4296703
18.0666676	0.6344000	-1.4296703
18.0666676	0.6832000	-1.7180586
18.0666676	0.7320000	-1.6444273
18.0666676	0.7808000	-1.6444273
18.0666676	0.8296000	-1.5217090
18.0666676	0.8784000	-1.5339808
18.0666676	0.9272000	-1.7978253
18.0666676	0.9760000	-1.6996508
18.0666676	1.0247999	-1.6628351
18.0666676	1.0735999	-1.6566992
18.0666676	1.1223999	-1.6505632
18.0666676	1.1711999	-1.3499031
18.0666676	1.2200000	-1.4971652
18.0666676	1.2688000	-1.1412816
18.0666676	1.3176000	-1.3130875
18.0666676	1.3664000	-1.0185633
18.0666676	1.4152000	-0.8283496
18.0666676	1.4640000	-1.2087770
18.0666676	1.5128000	-1.0676508
18.0666676	1.5616000	-0.9326606
18.0666676	1.6104000	-1.0737867
18.0666676	1.6592000	-1.0737867
18.0666676	1.7079999	-1.3621750
18.0666676	1.7567999	-1.1719613
18.0666676	1.8055999	-1.2455926
18.0666676	1.8543999	-1.2394567
18.0666676	1.9031999	-1.2824078
18.0666676	1.9519999	-1.2885437
18.0666676	2.0007999	-0.9203887
18.0666676	2.0495999	-0.8897090
18.0666676	2.0983999	-1.1780972
18.0666676	2.1471999	-1.0676508
18.0666676	2.1959999	-0.9203887
18.0666676	2.2447999	-0.9019809
18.0666676	2.2935998	-0.9019809
18.0666676	2.3423998	-0.8467574
18.0666676	2.3912001	-0.8590293
18.0666676	2.4400001	-1.1106019
18.0666676	2.4888000	-1.1167378
18.0666676	2.5376000	-1.1167378
18.0666676	2.5864000	-1.0492430
18.0666676	2.6352000	-1.1903691
18.0666676	2.6840000	-0.8774371
18.0666676	2.7328000	-0.8774371
18.0666676	2.7816000	-0.8897090
18.0666676	2.8304000	-0.9142528
18.0666676	2.8792000	-0.9142528
18.0666676	2.9280000	-0.8099418
18.0666676	2.9768000	-0.8160777
18.0666676	3.0256000	-1.0492430
18.0666676	3.0743999	-1.0492430
18.0666676	3.1231999	-0.8835731
18.0666676	3.1719999	-0.8897090
18.0666676	3.2207999	-0.8160777
18.0666676	3.2695999	-1.0308352

18.0666676	3.3183999	-0.9081168
18.0666676	3.3671999	-0.9081168
18.0666676	3.4159999	-1.0553789
18.0666676	3.4647999	-0.9694757
18.0666676	3.5135999	-0.9694757
18.0666676	3.5623999	-0.9633398
18.0666676	3.6111999	-0.9510679
18.0666676	3.6599998	-0.7485828
18.0666676	3.7087998	-0.5583692
18.0666676	3.7575998	-0.5522332
18.0666676	3.8063998	-0.8283496
18.0666676	3.8551998	-0.8344855
18.0666676	3.9039998	-1.0860586
18.0666676	3.9527998	-1.1719613
18.0666676	4.0016003	-1.1658254
18.0666676	4.0503998	-1.0553789
18.0666676	4.0991998	-1.0553789
18.0666676	4.1479998	-1.0615149
18.0666676	4.1967998	-0.7424469
18.0666676	4.2455997	-0.7608547
18.0666676	4.2943997	-0.7669902
18.0666676	4.3431997	-0.6442719
18.0666676	4.3919997	-0.1165824
18.0666676	4.4407997	-0.1288543
18.0666676	4.4895997	0.0306797
18.0666676	4.5383997	-0.0552235
18.0666676	4.5871997	-0.1779418
18.0666676	4.6359997	-0.2883883
18.0666676	4.6847997	-0.2270293
18.0666676	4.7336001	-0.3558836
18.0666676	4.7824001	-0.1840777
18.0666676	4.8312001	-0.2086215
18.0666676	4.8800001	-0.3681555
18.0666676	4.9288001	-0.3497477
18.0666676	4.9776001	-0.3988352
18.0666676	5.0264001	-0.3988352
18.0666676	5.0752001	-0.5031457
18.0666676	5.1240001	-0.3742914
18.0666676	5.1728001	-0.4786019
18.0666676	5.2216001	-0.5890489
18.0666676	5.2704000	-0.6013203
18.0666676	5.3192000	-0.6258640
18.0666676	5.3680000	-0.6626797
18.0666676	5.4168000	-0.6565437
18.0666676	5.4656000	613586.0000000
18.0666676	5.5144000	-0.8038058
18.0666676	5.5632000	-0.8099418
18.0666676	5.6120000	613586.0000000
18.0666676	5.6608000	613586.0000000
18.0666676	5.7096000	613586.0000000
18.0666676	5.7584000	613586.0000000
18.0666676	5.8072000	613586.0000000
18.0666676	5.8559999	613586.0000000
18.0666676	5.9047999	613586.0000000
18.0666676	5.9535999	613586.0000000
18.0666676	6.0023999	613586.0000000
18.0833340	0.2928000	-3.5097482

18.0833340	0.3416000	-3.2888548
18.0833340	0.3904000	-2.7120781
18.0833340	0.4392000	-2.0309906
18.0833340	0.4880000	-2.0432625
18.0833340	0.5368000	-1.8100972
18.0833340	0.5856000	-1.5830684
18.0833340	0.6344000	-1.4726214
18.0833340	0.6832000	-1.4664855
18.0833340	0.7320000	-1.8714566
18.0833340	0.7808000	-1.7426023
18.0833340	0.8296000	-1.7426023
18.0833340	0.8784000	-1.4480777
18.0833340	0.9272000	-1.4542136
18.0833340	0.9760000	-1.5278449
18.0833340	1.0247999	-1.4787574
18.0833340	1.0735999	-1.4910293
18.0833340	1.1223999	-1.4910293
18.0833340	1.1711999	-1.3805828
18.0833340	1.2200000	-1.3805828
18.0833340	1.2688000	-1.3192234
18.0833340	1.3176000	-1.2640004
18.0833340	1.3664000	-0.9694757
18.0833340	1.4152000	-1.2578645
18.0833340	1.4640000	-0.9633398
18.0833340	1.5128000	-1.2149129
18.0833340	1.5616000	-1.3621750
18.0833340	1.6104000	-1.3621750
18.0833340	1.6592000	-1.2885437
18.0833340	1.7079999	-1.1842332
18.0833340	1.7567999	-0.9756117
18.0833340	1.8055999	-0.8590293
18.0833340	1.8543999	-1.0676508
18.0833340	1.9031999	-1.1842332
18.0833340	1.9519999	-1.1842332
18.0833340	2.0007999	-0.9019809
18.0833340	2.0495999	-0.9142528
18.0833340	2.0983999	-1.0676508
18.0833340	2.1471999	-0.7608547
18.0833340	2.1959999	-0.8344855
18.0833340	2.2447999	-0.8467574
18.0833340	2.2935998	-0.8467574
18.0833340	2.3423998	-1.3867188
18.0833340	2.3912001	-1.1535535
18.0833340	2.4400001	-1.0921946
18.0833340	2.4888000	-1.0431070
18.0833340	2.5376000	-0.8958449
18.0833340	2.5864000	-0.8897090
18.0833340	2.6352000	-1.1903691
18.0833340	2.6840000	-1.1903691
18.0833340	2.7328000	-1.1842332
18.0833340	2.7816000	-0.8467574
18.0833340	2.8304000	-0.7424469
18.0833340	2.8792000	-1.1228738
18.0833340	2.9280000	-1.3069515
18.0833340	2.9768000	-1.3130875
18.0833340	3.0256000	-1.4726214
18.0833340	3.0743999	-1.0921946

18.0833340	3.1231999	-1.0921946
18.0833340	3.1719999	-1.0799227
18.0833340	3.2207999	-1.1044660
18.0833340	3.2695999	-1.2701364
18.0833340	3.3183999	-1.2762718
18.0833340	3.3671999	-1.2333207
18.0833340	3.4159999	-0.9019809
18.0833340	3.4647999	-0.8160777
18.0833340	3.5135999	-0.9694757
18.0833340	3.5623999	-0.7915339
18.0833340	3.6111999	-0.8713012
18.0833340	3.6599998	-1.0983305
18.0833340	3.7087998	-1.3867188
18.0833340	3.7575998	-1.2517285
18.0833340	3.8063998	-1.2517285
18.0833340	3.8551998	-1.2640004
18.0833340	3.9039998	-1.2578645
18.0833340	3.9527998	-1.2455926
18.0833340	4.0016003	-1.1719613
18.0833340	4.0503998	-1.1658254
18.0833340	4.0991998	-1.0308352
18.0833340	4.1479998	-1.0185633
18.0833340	4.1967998	-1.1290097
18.0833340	4.2455997	-0.5890489
18.0833340	4.2943997	-0.5706410
18.0833340	4.3431997	-0.0245438
18.0833340	4.3919997	-0.5890489
18.0833340	4.4407997	-1.2455926
18.0833340	4.4895997	-1.2394567
18.0833340	4.5383997	-1.0615149
18.0833340	4.5871997	-0.9572039
18.0833340	4.6359997	-0.7179031
18.0833340	4.6847997	-0.4786019
18.0833340	4.7336001	-0.4786019
18.0833340	4.7824001	-0.5460973
18.0833340	4.8312001	-0.3804274
18.0833340	4.8800001	-0.4295144
18.0833340	4.9288001	-0.4540582
18.0833340	4.9776001	-0.4601941
18.0833340	5.0264001	-0.5645051
18.0833340	5.0752001	-0.5645051
18.0833340	5.1240001	-0.8835731
18.0833340	5.1728001	-0.8713012
18.0833340	5.2216001	-0.9756117
18.0833340	5.2704000	-0.9265246
18.0833340	5.3192000	-0.9265246
18.0833340	5.3680000	613586.0000000
18.0833340	5.4168000	613586.0000000
18.0833340	5.4656000	-0.9694757
18.0833340	5.5144000	-0.9572039
18.0833340	5.5632000	-0.9572039
18.0833340	5.6120000	-0.9572039
18.0833340	5.6608000	-1.0001554
18.0833340	5.7096000	613586.0000000
18.0833340	5.7584000	-0.8406215
18.0833340	5.8072000	613586.0000000
18.0833340	5.8559999	613586.0000000

18.0833340	5.9047999	613586.0000000
18.0833340	5.9535999	613586.0000000
18.0833340	6.0023999	613586.0000000
18.1000004	0.2928000	-3.4361172
18.1000004	0.3416000	-2.6016314
18.1000004	0.3904000	-2.2334762
18.1000004	0.4392000	-2.2580199
18.1000004	0.4880000	-1.7732821
18.1000004	0.5368000	-1.8714566
18.1000004	0.5856000	-1.8591847
18.1000004	0.6344000	-1.9512239
18.1000004	0.6832000	-2.0739422
18.1000004	0.7320000	-2.0678062
18.1000004	0.7808000	-1.9205441
18.1000004	0.8296000	-1.8162332
18.1000004	0.8784000	-1.7426023
18.1000004	0.9272000	-1.4603496
18.1000004	0.9760000	-1.4726214
18.1000004	1.0247999	-1.5953403
18.1000004	1.0735999	-1.5523887
18.1000004	1.1223999	-1.6751070
18.1000004	1.1711999	-1.5155730
18.1000004	1.2200000	-1.4296703
18.1000004	1.2688000	-1.4296703
18.1000004	1.3176000	-1.0983305
18.1000004	1.3664000	-1.1596894
18.1000004	1.4152000	-1.0369711
18.1000004	1.4640000	-0.9694757
18.1000004	1.5128000	-1.0431070
18.1000004	1.5616000	-1.1351457
18.1000004	1.6104000	-1.2517285
18.1000004	1.6592000	-1.2517285
18.1000004	1.7079999	-1.1965051
18.1000004	1.7567999	-1.1965051
18.1000004	1.8055999	-1.2026410
18.1000004	1.8543999	-1.0001554
18.1000004	1.9031999	-1.1167378
18.1000004	1.9519999	-0.9203887
18.1000004	2.0007999	-0.9142528
18.1000004	2.0495999	-1.0553789
18.1000004	2.0983999	-1.0553789
18.1000004	2.1471999	-0.7853980
18.1000004	2.1959999	-0.7915339
18.1000004	2.2447999	-0.7424469
18.1000004	2.2935998	-0.8651652
18.1000004	2.3423998	-0.8651652
18.1000004	2.3912001	-1.3008156
18.1000004	2.4400001	-1.3008156
18.1000004	2.4888000	-0.9142528
18.1000004	2.5376000	-0.9142528
18.1000004	2.5864000	-1.0124273
18.1000004	2.6352000	-0.9019809
18.1000004	2.6840000	-0.5154176
18.1000004	2.7328000	-0.2270293
18.1000004	2.7816000	-0.7179031
18.1000004	2.8304000	-0.6013203
18.1000004	2.8792000	-0.7608547

18.1000004	2.9280000	-0.5154176
18.1000004	2.9768000	-0.6320000
18.1000004	3.0256000	-0.6626797
18.1000004	3.0743999	-0.6688156
18.1000004	3.1231999	-0.7547188
18.1000004	3.1719999	-1.1228738
18.1000004	3.2207999	-1.0492430
18.1000004	3.2695999	-1.0492430
18.1000004	3.3183999	-1.3683109
18.1000004	3.3671999	-1.3683109
18.1000004	3.4159999	-1.5585246
18.1000004	3.4647999	-1.2578645
18.1000004	3.5135999	-1.2578645
18.1000004	3.5623999	-0.9940195
18.1000004	3.6111999	-1.0001554
18.1000004	3.6599998	-1.0062914
18.1000004	3.7087998	-1.2210488
18.1000004	3.7575998	-0.8835731
18.1000004	3.8063998	-0.6626797
18.1000004	3.8551998	-0.5645051
18.1000004	3.9039998	-0.8406215
18.1000004	3.9527998	-0.7240391
18.1000004	4.0016003	-0.7240391
18.1000004	4.0503998	-0.7424469
18.1000004	4.0991998	-0.6810875
18.1000004	4.1479998	-0.6810875
18.1000004	4.1967998	-1.2271848
18.1000004	4.2455997	-1.6566992
18.1000004	4.2943997	-1.6751070
18.1000004	4.3431997	-0.8897090
18.1000004	4.3919997	-0.8897090
18.1000004	4.4407997	-0.8897090
18.1000004	4.4895997	-0.8774371
18.1000004	4.5383997	-0.6994953
18.1000004	4.5871997	-0.4663301
18.1000004	4.6359997	-0.4663301
18.1000004	4.6847997	-0.4663301
18.1000004	4.7336001	-0.5767770
18.1000004	4.7824001	-0.5890489
18.1000004	4.8312001	-0.6810875
18.1000004	4.8800001	-0.4295144
18.1000004	4.9288001	-0.6135921
18.1000004	4.9776001	-0.5890489
18.1000004	5.0264001	-0.4970098
18.1000004	5.0752001	-0.5031457
18.1000004	5.1240001	-0.6626797
18.1000004	5.1728001	-0.6626797
18.1000004	5.2216001	-0.6565437
18.1000004	5.2704000	-0.8467574
18.1000004	5.3192000	-0.7608547
18.1000004	5.3680000	-0.6933594
18.1000004	5.4168000	613586.0000000
18.1000004	5.4656000	613586.0000000
18.1000004	5.5144000	613586.0000000
18.1000004	5.5632000	613586.0000000
18.1000004	5.6120000	613586.0000000
18.1000004	5.6608000	613586.0000000

18.1000004	5.7096000	613586.0000000
18.1000004	5.7584000	613586.0000000
18.1000004	5.8072000	613586.0000000
18.1000004	5.8559999	613586.0000000
18.1000004	5.9047999	613586.0000000
18.1000004	5.9535999	613586.0000000
18.1000004	6.0023999	613586.0000000
18.1166668	0.2928000	-3.7920005
18.1166668	0.3416000	-2.4298255
18.1166668	0.3904000	-1.9880390
18.1166668	0.4392000	-1.9082723
18.1166668	0.4880000	-1.9082723
18.1166668	0.5368000	-1.5278449
18.1166668	0.5856000	-1.5155730
18.1166668	0.6344000	-1.5155730
18.1166668	0.6832000	-1.8285050
18.1166668	0.7320000	-1.8285050
18.1166668	0.7808000	-1.5953403
18.1166668	0.8296000	-1.5953403
18.1166668	0.8784000	-1.5033011
18.1166668	0.9272000	-1.4910293
18.1166668	0.9760000	-1.6566992
18.1166668	1.0247999	-1.6198835
18.1166668	1.0735999	-1.6260195
18.1166668	1.1223999	-1.6382914
18.1166668	1.1711999	-1.4787574
18.1166668	1.2200000	-1.4726214
18.1166668	1.2688000	-1.4603496
18.1166668	1.3176000	-1.2578645
18.1166668	1.3664000	-1.3314953
18.1166668	1.4152000	-1.3376312
18.1166668	1.4640000	-1.3437672
18.1166668	1.5128000	-1.2640004
18.1166668	1.5616000	-1.2701364
18.1166668	1.6104000	-1.1412816
18.1166668	1.6592000	-1.2455926
18.1166668	1.7079999	-1.1106019
18.1166668	1.7567999	-1.1228738
18.1166668	1.8055999	-1.4480777
18.1166668	1.8543999	-0.9878836
18.1166668	1.9031999	-0.8897090
18.1166668	1.9519999	-0.9572039
18.1166668	2.0007999	-0.9572039
18.1166668	2.0495999	-1.0124273
18.1166668	2.0983999	-0.8528934
18.1166668	2.1471999	-1.2640004
18.1166668	2.1959999	-0.7976699
18.1166668	2.2447999	-0.7976699
18.1166668	2.2935998	-0.7976699
18.1166668	2.3423998	-1.1719613
18.1166668	2.3912001	-1.1842332
18.1166668	2.4400001	-1.3683109
18.1166668	2.4888000	-1.3008156
18.1166668	2.5376000	-1.2578645
18.1166668	2.5864000	-0.8344855
18.1166668	2.6352000	-0.8344855
18.1166668	2.6840000	-0.9387960

18.1166668	2.7328000	-1.1780972
18.1166668	2.7816000	-0.9326606
18.1166668	2.8304000	-1.0737867
18.1166668	2.8792000	-1.0860586
18.1166668	2.9280000	-1.3744469
18.1166668	2.9768000	-1.1412816
18.1166668	3.0256000	-1.0308352
18.1166668	3.0743999	-0.9940195
18.1166668	3.1231999	-0.9878836
18.1166668	3.1719999	-0.9572039
18.1166668	3.2207999	-1.0860586
18.1166668	3.2695999	-1.0431070
18.1166668	3.3183999	-1.0860586
18.1166668	3.3671999	-1.0737867
18.1166668	3.4159999	-0.9756117
18.1166668	3.4647999	-0.8406215
18.1166668	3.5135999	-0.5890489
18.1166668	3.5623999	-0.5951843
18.1166668	3.6111999	-0.5399613
18.1166668	3.6599998	-0.6013203
18.1166668	3.7087998	-0.7792621
18.1166668	3.7575998	-0.5706410
18.1166668	3.8063998	-0.5706410
18.1166668	3.8551998	-0.5829129
18.1166668	3.9039998	-0.5031457
18.1166668	3.9527998	-0.6320000
18.1166668	4.0016003	-1.2701364
18.1166668	4.0503998	-1.2640004
18.1166668	4.0991998	-0.8590293
18.1166668	4.1479998	-0.8651652
18.1166668	4.1967998	-0.8774371
18.1166668	4.2455997	-0.8774371
18.1166668	4.2943997	-0.9756117
18.1166668	4.3431997	-0.9756117
18.1166668	4.3919997	-0.8344855
18.1166668	4.4407997	-0.9081168
18.1166668	4.4895997	-0.7669902
18.1166668	4.5383997	-0.4049711
18.1166668	4.5871997	-0.2331653
18.1166668	4.6359997	-0.3252039
18.1166668	4.6847997	-0.5092816
18.1166668	4.7336001	-0.3742914
18.1166668	4.7824001	-0.7547188
18.1166668	4.8312001	-0.7485828
18.1166668	4.8800001	-0.6320000
18.1166668	4.9288001	-0.6320000
18.1166668	4.9776001	-0.7731261
18.1166668	5.0264001	-0.7853980
18.1166668	5.0752001	-0.3006601
18.1166668	5.1240001	-0.3129320
18.1166668	5.1728001	-0.6994953
18.1166668	5.2216001	-0.7547188
18.1166668	5.2704000	-0.8283496
18.1166668	5.3192000	-0.8160777
18.1166668	5.3680000	613586.0000000
18.1166668	5.4168000	613586.0000000
18.1166668	5.4656000	613586.0000000

18.1166668	5.5144000	613586.0000000
18.1166668	5.5632000	613586.0000000
18.1166668	5.6120000	613586.0000000
18.1166668	5.6608000	613586.0000000
18.1166668	5.7096000	613586.0000000
18.1166668	5.7584000	-0.9265246
18.1166668	5.8072000	-1.0737867
18.1166668	5.8559999	613586.0000000
18.1166668	5.9047999	-1.1535535
18.1166668	5.9535999	-1.1351457
18.1166668	6.0023999	-1.0860586
18.1333332	0.2928000	-3.7674570
18.1333332	0.3416000	-2.8900199
18.1333332	0.3904000	-1.8775926
18.1333332	0.4392000	-1.8775926
18.1333332	0.4880000	-1.8898644
18.1333332	0.5368000	-1.8775926
18.1333332	0.5856000	-1.2762718
18.1333332	0.6344000	-1.4603496
18.1333332	0.6832000	-1.4971652
18.1333332	0.7320000	-1.6076121
18.1333332	0.7808000	-1.4726214
18.1333332	0.8296000	-1.4664855
18.1333332	0.8784000	-1.4848933
18.1333332	0.9272000	-1.3867188
18.1333332	0.9760000	-1.3130875
18.1333332	1.0247999	-1.4112625
18.1333332	1.0735999	-1.5033011
18.1333332	1.1223999	-1.5094371
18.1333332	1.1711999	-1.5155730
18.1333332	1.2200000	-1.5155730
18.1333332	1.2688000	-1.5033011
18.1333332	1.3176000	-1.6566992
18.1333332	1.3664000	-1.2640004
18.1333332	1.4152000	-1.4910293
18.1333332	1.4640000	-0.9694757
18.1333332	1.5128000	-1.1965051
18.1333332	1.5616000	-1.2946796
18.1333332	1.6104000	-1.0921946
18.1333332	1.6592000	-0.9387960
18.1333332	1.7079999	-0.9572039
18.1333332	1.7567999	-1.1290097
18.1333332	1.8055999	-1.0246992
18.1333332	1.8543999	-1.1965051
18.1333332	1.9031999	-1.1965051
18.1333332	1.9519999	-1.2824078
18.1333332	2.0007999	-1.2701364
18.1333332	2.0495999	-0.9817476
18.1333332	2.0983999	-1.0431070
18.1333332	2.1471999	-1.1351457
18.1333332	2.1959999	-1.4603496
18.1333332	2.2447999	-1.4480777
18.1333332	2.2935998	-1.5769324
18.1333332	2.3423998	-1.5769324
18.1333332	2.3912001	-1.6014762
18.1333332	2.4400001	-1.5953403
18.1333332	2.4888000	-0.9817476

18.1333332	2.5376000	-1.0983305
18.1333332	2.5864000	-0.7547188
18.1333332	2.6352000	-1.0799227
18.1333332	2.6840000	-1.1965051
18.1333332	2.7328000	-0.9817476
18.1333332	2.7816000	-1.0185633
18.1333332	2.8304000	-0.9142528
18.1333332	2.8792000	-0.9019809
18.1333332	2.9280000	-1.0062914
18.1333332	2.9768000	-1.0062914
18.1333332	3.0256000	-0.9019809
18.1333332	3.0743999	-0.9756117
18.1333332	3.1231999	-0.9756117
18.1333332	3.1719999	-0.9878836
18.1333332	3.2207999	-0.9878836
18.1333332	3.2695999	-1.0062914
18.1333332	3.3183999	-0.8528934
18.1333332	3.3671999	-0.8344855
18.1333332	3.4159999	-0.7363110
18.1333332	3.4647999	-0.6258640
18.1333332	3.5135999	-0.6626797
18.1333332	3.5623999	-0.8160777
18.1333332	3.6111999	-0.8713012
18.1333332	3.6599998	-0.7301750
18.1333332	3.7087998	-0.7301750
18.1333332	3.7575998	-0.8590293
18.1333332	3.8063998	-1.2087770
18.1333332	3.8551998	-1.2087770
18.1333332	3.9039998	-1.3621750
18.1333332	3.9527998	-1.3499031
18.1333332	4.0016003	-1.2149129
18.1333332	4.0503998	-0.9019809
18.1333332	4.0991998	-0.8958449
18.1333332	4.1479998	-0.9019809
18.1333332	4.1967998	-0.8038058
18.1333332	4.2455997	-0.7976699
18.1333332	4.2943997	-0.7915339
18.1333332	4.3431997	-0.4970098
18.1333332	4.3919997	-0.5154176
18.1333332	4.4407997	-0.5276895
18.1333332	4.4895997	-0.4417863
18.1333332	4.5383997	-0.0674953
18.1333332	4.5871997	-0.1902137
18.1333332	4.6359997	-0.1902137
18.1333332	4.6847997	-0.1902137
18.1333332	4.7336001	-0.5276895
18.1333332	4.7824001	-0.5276895
18.1333332	4.8312001	-0.5215535
18.1333332	4.8800001	-0.6013203
18.1333332	4.9288001	-0.3558836
18.1333332	4.9776001	-0.4049711
18.1333332	5.0264001	-0.5890489
18.1333332	5.0752001	-0.6749516
18.1333332	5.1240001	-0.5460973
18.1333332	5.1728001	-0.7117672
18.1333332	5.2216001	-0.7117672
18.1333332	5.2704000	613586.0000000

18.1333332	5.3192000	-0.6074562
18.1333332	5.3680000	-0.6074562
18.1333332	5.4168000	-0.7731261
18.1333332	5.4656000	-0.5338254
18.1333332	5.5144000	-0.5338254
18.1333332	5.5632000	-0.5399613
18.1333332	5.6120000	-0.9142528
18.1333332	5.6608000	-0.8467574
18.1333332	5.7096000	-1.0185633
18.1333332	5.7584000	-0.9449320
18.1333332	5.8072000	-1.0369711
18.1333332	5.8559999	-0.9387960
18.1333332	5.9047999	-0.9387960
18.1333332	5.9535999	-1.0185633
18.1333332	6.0023999	-1.0001554
18.1499996	0.2928000	-3.4545248
18.1499996	0.3416000	-3.0802336
18.1499996	0.3904000	-2.1659808
18.1499996	0.4392000	-2.1046219
18.1499996	0.4880000	-1.6996508
18.1499996	0.5368000	-1.6935148
18.1499996	0.5856000	-1.2701364
18.1499996	0.6344000	-1.4787574
18.1499996	0.6832000	-1.7487383
18.1499996	0.7320000	-1.6751070
18.1499996	0.7808000	-1.4419422
18.1499996	0.8296000	-1.4480777
18.1499996	0.8784000	-1.3437672
18.1499996	0.9272000	-1.3069515
18.1499996	0.9760000	-1.2946796
18.1499996	1.0247999	-1.2885437
18.1499996	1.0735999	-1.3008156
18.1499996	1.1223999	-1.3008156
18.1499996	1.1711999	-1.4787574
18.1499996	1.2200000	-1.3867188
18.1499996	1.2688000	-1.4112625
18.1499996	1.3176000	-1.1290097
18.1499996	1.3664000	-1.4173985
18.1499996	1.4152000	-1.5401168
18.1499996	1.4640000	-1.2946796
18.1499996	1.5128000	-1.2946796
18.1499996	1.5616000	-1.2946796
18.1499996	1.6104000	-1.1658254
18.1499996	1.6592000	-1.1596894
18.1499996	1.7079999	-1.0062914
18.1499996	1.7567999	-0.9572039
18.1499996	1.8055999	-0.9572039
18.1499996	1.8543999	-1.0185633
18.1499996	1.9031999	-1.2149129
18.1499996	1.9519999	-0.8774371
18.1499996	2.0007999	-0.8958449
18.1499996	2.0495999	-0.9019809
18.1499996	2.0983999	-0.9694757
18.1499996	2.1471999	-1.0921946
18.1499996	2.1959999	-1.5892043
18.1499996	2.2447999	-1.2885437
18.1499996	2.2935998	-0.9817476

18.1499996	2.3423998	-1.5217090
18.1499996	2.3912001	-1.3130875
18.1499996	2.4400001	-1.3130875
18.1499996	2.4888000	-1.0553789
18.1499996	2.5376000	-0.9081168
18.1499996	2.5864000	-1.2701364
18.1499996	2.6352000	-1.0921946
18.1499996	2.6840000	-1.0615149
18.1499996	2.7328000	-0.9694757
18.1499996	2.7816000	-1.2026410
18.1499996	2.8304000	-0.9019809
18.1499996	2.8792000	-0.9265246
18.1499996	2.9280000	-0.9387960
18.1499996	2.9768000	-1.1106019
18.1499996	3.0256000	-1.1044660
18.1499996	3.0743999	-1.0921946
18.1499996	3.1231999	-1.0921946
18.1499996	3.1719999	-1.0062914
18.1499996	3.2207999	-1.0124273
18.1499996	3.2695999	-1.0124273
18.1499996	3.3183999	-1.1290097
18.1499996	3.3671999	-0.6994953
18.1499996	3.4159999	-0.6074562
18.1499996	3.4647999	-0.2147574
18.1499996	3.5135999	-0.0674953
18.1499996	3.5623999	-0.2515731
18.1499996	3.6111999	-0.5706410
18.1499996	3.6599998	-0.3988352
18.1499996	3.7087998	-0.5092816
18.1499996	3.7575998	-0.2024856
18.1499996	3.8063998	-0.2086215
18.1499996	3.8551998	0.1411262
18.1499996	3.9039998	0.1595340
18.1499996	3.9527998	-0.9510679
18.1499996	4.0016003	-1.0185633
18.1499996	4.0503998	-1.0124273
18.1499996	4.0991998	-0.7424469
18.1499996	4.1479998	-0.4908738
18.1499996	4.1967998	-0.2883883
18.1499996	4.2455997	-0.5031457
18.1499996	4.2943997	-0.5031457
18.1499996	4.3431997	-0.1472621
18.1499996	4.3919997	-0.1288543
18.1499996	4.4407997	-0.3620195
18.1499996	4.4895997	-1.2455926
18.1499996	4.5383997	-1.2394567
18.1499996	4.5871997	-0.8958449
18.1499996	4.6359997	-0.7240391
18.1499996	4.6847997	-0.6381359
18.1499996	4.7336001	-0.7669902
18.1499996	4.7824001	-0.6749516
18.1499996	4.8312001	-0.4233789
18.1499996	4.8800001	-0.3129320
18.1499996	4.9288001	-0.6749516
18.1499996	4.9776001	-0.4540582
18.1499996	5.0264001	-0.7976699
18.1499996	5.0752001	-0.3926992

18.1499996	5.1240001	-0.8344855
18.1499996	5.1728001	-0.8467574
18.1499996	5.2216001	-0.8222136
18.1499996	5.2704000	613586.0000000
18.1499996	5.3192000	-0.8835731
18.1499996	5.3680000	-0.8897090
18.1499996	5.4168000	-0.8958449
18.1499996	5.4656000	-0.8774371
18.1499996	5.5144000	-0.8713012
18.1499996	5.5632000	-0.8651652
18.1499996	5.6120000	613586.0000000
18.1499996	5.6608000	613586.0000000
18.1499996	5.7096000	613586.0000000
18.1499996	5.7584000	613586.0000000
18.1499996	5.8072000	-0.9019809
18.1499996	5.8559999	-0.9019809
18.1499996	5.9047999	-0.8344855
18.1499996	5.9535999	-0.8406215
18.1499996	6.0023999	-0.8344855
18.1666660	0.2928000	-4.0926609
18.1666660	0.3416000	-3.8410881
18.1666660	0.3904000	-2.6813984
18.1666660	0.4392000	-2.1905246
18.1666660	0.4880000	-1.8837285
18.1666660	0.5368000	-1.4480777
18.1666660	0.5856000	-1.4296703
18.1666660	0.6344000	-1.3744469
18.1666660	0.6832000	-1.3744469
18.1666660	0.7320000	-1.5523887
18.1666660	0.7808000	-1.2394567
18.1666660	0.8296000	-1.1658254
18.1666660	0.8784000	-1.0921946
18.1666660	0.9272000	-1.1106019
18.1666660	0.9760000	-0.9510679
18.1666660	1.0247999	-0.9387960
18.1666660	1.0735999	-1.1596894
18.1666660	1.1223999	-1.3928547
18.1666660	1.1711999	-1.3867188
18.1666660	1.2200000	-1.3805828
18.1666660	1.2688000	-1.5339808
18.1666660	1.3176000	-1.5339808
18.1666660	1.3664000	-1.4358063
18.1666660	1.4152000	-1.4296703
18.1666660	1.4640000	-1.4173985
18.1666660	1.5128000	-1.2394567
18.1666660	1.5616000	-1.2578645
18.1666660	1.6104000	-1.2149129
18.1666660	1.6592000	-1.2149129
18.1666660	1.7079999	-1.0062914
18.1666660	1.7567999	-0.9940195
18.1666660	1.8055999	-1.0124273
18.1666660	1.8543999	-1.1044660
18.1666660	1.9031999	-1.1842332
18.1666660	1.9519999	-1.1903691
18.1666660	2.0007999	-1.2517285
18.1666660	2.0495999	-1.2394567
18.1666660	2.0983999	-1.2578645

18.1666660	2.1471999	-1.2762718
18.1666660	2.1959999	-1.2762718
18.1666660	2.2447999	-1.0737867
18.1666660	2.2935998	-0.9510679
18.1666660	2.3423998	-1.2762718
18.1666660	2.3912001	-1.1106019
18.1666660	2.4400001	-1.1106019
18.1666660	2.4888000	-1.3192234
18.1666660	2.5376000	-1.3192234
18.1666660	2.5864000	-0.9326606
18.1666660	2.6352000	-1.0001554
18.1666660	2.6840000	-1.0492430
18.1666660	2.7328000	-1.1965051
18.1666660	2.7816000	-1.1106019
18.1666660	2.8304000	-1.2394567
18.1666660	2.8792000	-1.2394567
18.1666660	2.9280000	-1.2271848
18.1666660	2.9768000	-1.0308352
18.1666660	3.0256000	-1.0369711
18.1666660	3.0743999	-1.0431070
18.1666660	3.1231999	-1.0492430
18.1666660	3.1719999	-1.1412816
18.1666660	3.2207999	-1.1351457
18.1666660	3.2695999	-1.0676508
18.1666660	3.3183999	-1.0676508
18.1666660	3.3671999	-0.7792621
18.1666660	3.4159999	-0.7915339
18.1666660	3.4647999	-1.1965051
18.1666660	3.5135999	-1.1965051
18.1666660	3.5623999	-0.7792621
18.1666660	3.6111999	-0.8897090
18.1666660	3.6599998	-1.0124273
18.1666660	3.7087998	-1.1167378
18.1666660	3.7575998	-0.8222136
18.1666660	3.8063998	-0.8344855
18.1666660	3.8551998	-0.7056313
18.1666660	3.9039998	-0.8713012
18.1666660	3.9527998	-0.8835731
18.1666660	4.0016003	-0.8835731
18.1666660	4.0503998	-0.6933594
18.1666660	4.0991998	-0.6994953
18.1666660	4.1479998	-0.9142528
18.1666660	4.1967998	-0.5154176
18.1666660	4.2455997	-0.6810875
18.1666660	4.2943997	-0.4417863
18.1666660	4.3431997	-0.4417863
18.1666660	4.3919997	-0.9019809
18.1666660	4.4407997	-1.0246992
18.1666660	4.4895997	-0.9081168
18.1666660	4.5383997	-0.9019809
18.1666660	4.5871997	-1.0001554
18.1666660	4.6359997	-0.9940195
18.1666660	4.6847997	-0.8713012
18.1666660	4.7336001	-0.8590293
18.1666660	4.7824001	-0.8590293
18.1666660	4.8312001	-0.6381359
18.1666660	4.8800001	-0.7301750

18.1666660	4.9288001	-0.7485828
18.1666660	4.9776001	-0.7424469
18.1666660	5.0264001	-0.7424469
18.1666660	5.0752001	-0.7424469
18.1666660	5.1240001	-0.8222136
18.1666660	5.1728001	-0.8774371
18.1666660	5.2216001	-0.8590293
18.1666660	5.2704000	-0.8774371
18.1666660	5.3192000	-0.7240391
18.1666660	5.3680000	-0.8467574
18.1666660	5.4168000	613586.0000000
18.1666660	5.4656000	613586.0000000
18.1666660	5.5144000	-0.8774371
18.1666660	5.5632000	-0.8774371
18.1666660	5.6120000	-0.7792621
18.1666660	5.6608000	613586.0000000
18.1666660	5.7096000	613586.0000000
18.1666660	5.7584000	613586.0000000
18.1666660	5.8072000	613586.0000000
18.1666660	5.8559999	613586.0000000
18.1666660	5.9047999	613586.0000000
18.1666660	5.9535999	613586.0000000
18.1666660	6.0023999	-0.7853980
18.1833324	0.2928000	-2.9636509
18.1833324	0.3416000	-2.9636509
18.1833324	0.3904000	-2.5586801
18.1833324	0.4392000	-2.1721168
18.1833324	0.4880000	-2.1659808
18.1833324	0.5368000	-1.6935148
18.1833324	0.5856000	-1.6751070
18.1833324	0.6344000	-1.4664855
18.1833324	0.6832000	-1.5646605
18.1833324	0.7320000	-1.4051266
18.1833324	0.7808000	-1.2394567
18.1833324	0.8296000	-1.2824078
18.1833324	0.8784000	-1.2210488
18.1833324	0.9272000	-1.0799227
18.1833324	0.9760000	-0.8774371
18.1833324	1.0247999	-1.2394567
18.1833324	1.0735999	-1.2333207
18.1833324	1.1223999	-1.2271848
18.1833324	1.1711999	-1.0983305
18.1833324	1.2200000	-1.0369711
18.1833324	1.2688000	-0.9694757
18.1833324	1.3176000	-1.3437672
18.1833324	1.3664000	-1.3437672
18.1833324	1.4152000	-1.2517285
18.1833324	1.4640000	-1.0246992
18.1833324	1.5128000	-1.3130875
18.1833324	1.5616000	-1.0799227
18.1833324	1.6104000	-1.1167378
18.1833324	1.6592000	-1.1167378
18.1833324	1.7079999	-0.9203887
18.1833324	1.7567999	-1.1351457
18.1833324	1.8055999	-1.1351457
18.1833324	1.8543999	-1.3314953
18.1833324	1.9031999	-1.1780972

18.1833324	1.9519999	-1.4726214
18.1833324	2.0007999	-1.4542136
18.1833324	2.0495999	-1.4664855
18.1833324	2.0983999	-1.3744469
18.1833324	2.1471999	-1.3621750
18.1833324	2.1959999	-1.1903691
18.1833324	2.2447999	-1.1412816
18.1833324	2.2935998	-1.1596894
18.1833324	2.3423998	-1.4603496
18.1833324	2.3912001	-1.3314953
18.1833324	2.4400001	-0.8406215
18.1833324	2.4888000	-0.9633398
18.1833324	2.5376000	-0.9633398
18.1833324	2.5864000	-1.1106019
18.1833324	2.6352000	-1.1965051
18.1833324	2.6840000	-1.1780972
18.1833324	2.7328000	-1.2946796
18.1833324	2.7816000	-1.2394567
18.1833324	2.8304000	-1.2517285
18.1833324	2.8792000	-0.8835731
18.1833324	2.9280000	-0.8897090
18.1833324	2.9768000	-0.9694757
18.1833324	3.0256000	-1.0553789
18.1833324	3.0743999	-1.0615149
18.1833324	3.1231999	-1.1412816
18.1833324	3.1719999	-1.0492430
18.1833324	3.2207999	-1.2701364
18.1833324	3.2695999	-1.0369711
18.1833324	3.3183999	-0.9326606
18.1833324	3.3671999	-0.9510679
18.1833324	3.4159999	-1.1903691
18.1833324	3.4647999	-1.2026410
18.1833324	3.5135999	-1.1842332
18.1833324	3.5623999	-1.1780972
18.1833324	3.6111999	-0.7056313
18.1833324	3.6599998	-0.6872234
18.1833324	3.7087998	-1.0615149
18.1833324	3.7575998	-1.1351457
18.1833324	3.8063998	-0.8528934
18.1833324	3.8551998	-0.8528934
18.1833324	3.9039998	-0.8528934
18.1833324	3.9527998	-0.6013203
18.1833324	4.0016003	-0.6442719
18.1833324	4.0503998	-0.8344855
18.1833324	4.0991998	-0.8344855
18.1833324	4.1479998	-0.9019809
18.1833324	4.1967998	-0.6565437
18.1833324	4.2455997	-0.6320000
18.1833324	4.2943997	-0.9510679
18.1833324	4.3431997	-0.6135921
18.1833324	4.3919997	-0.6258640
18.1833324	4.4407997	-0.6442719
18.1833324	4.4895997	-0.6504078
18.1833324	4.5383997	-0.6565437
18.1833324	4.5871997	-0.3558836
18.1833324	4.6359997	-0.3497477
18.1833324	4.6847997	-0.6135921

18.1833324	4.7336001	-0.5890489
18.1833324	4.7824001	-0.5829129
18.1833324	4.8312001	-0.3681555
18.1833324	4.8800001	-0.3742914
18.1833324	4.9288001	-0.3804274
18.1833324	4.9776001	-0.3804274
18.1833324	5.0264001	-0.4356503
18.1833324	5.0752001	-0.4663301
18.1833324	5.1240001	-0.6688156
18.1833324	5.1728001	-0.6749516
18.1833324	5.2216001	-0.6810875
18.1833324	5.2704000	-0.6872234
18.1833324	5.3192000	-0.6565437
18.1833324	5.3680000	613586.0000000
18.1833324	5.4168000	613586.0000000
18.1833324	5.4656000	613586.0000000
18.1833324	5.5144000	613586.0000000
18.1833324	5.5632000	613586.0000000
18.1833324	5.6120000	613586.0000000
18.1833324	5.6608000	613586.0000000
18.1833324	5.7096000	613586.0000000
18.1833324	5.7584000	613586.0000000
18.1833324	5.8072000	613586.0000000
18.1833324	5.8559999	613586.0000000
18.1833324	5.9047999	613586.0000000
18.1833324	5.9535999	613586.0000000
18.1833324	6.0023999	613586.0000000
18.2000008	0.2928000	-3.9147191
18.2000008	0.3416000	-2.8716121
18.2000008	0.3904000	-2.5402722
18.2000008	0.4392000	-1.9328160
18.2000008	0.4880000	-1.7794180
18.2000008	0.5368000	-1.5892043
18.2000008	0.5856000	-1.4173985
18.2000008	0.6344000	-1.0676508
18.2000008	0.6832000	-1.0799227
18.2000008	0.7320000	-1.2701364
18.2000008	0.7808000	-1.2701364
18.2000008	0.8296000	-0.9510679
18.2000008	0.8784000	-0.8590293
18.2000008	0.9272000	-0.5706410
18.2000008	0.9760000	-1.0737867
18.2000008	1.0247999	-1.0308352
18.2000008	1.0735999	-1.0308352
18.2000008	1.1223999	-0.9633398
18.2000008	1.1711999	-0.9756117
18.2000008	1.2200000	-1.0676508
18.2000008	1.2688000	-1.0860586
18.2000008	1.3176000	-0.9326606
18.2000008	1.3664000	-1.2394567
18.2000008	1.4152000	-1.2271848
18.2000008	1.4640000	-1.2271848
18.2000008	1.5128000	-1.1167378
18.2000008	1.5616000	-1.1044660
18.2000008	1.6104000	-1.2026410
18.2000008	1.6592000	-1.0737867
18.2000008	1.7079999	-0.9878836

18.2000008	1.7567999	-0.9878836
18.2000008	1.8055999	-0.9878836
18.2000008	1.8543999	-1.3314953
18.2000008	1.9031999	-1.3621750
18.2000008	1.9519999	-0.9756117
18.2000008	2.0007999	-0.9817476
18.2000008	2.0495999	-1.2762718
18.2000008	2.0983999	-0.9142528
18.2000008	2.1471999	-1.3069515
18.2000008	2.1959999	-1.4112625
18.2000008	2.2447999	-1.4051266
18.2000008	2.2935998	-1.2701364
18.2000008	2.3423998	-1.5830684
18.2000008	2.3912001	-1.4848933
18.2000008	2.4400001	-1.4787574
18.2000008	2.4888000	-1.4358063
18.2000008	2.5376000	-1.4603496
18.2000008	2.5864000	-1.4664855
18.2000008	2.6352000	-1.3867188
18.2000008	2.6840000	-1.0615149
18.2000008	2.7328000	-1.3008156
18.2000008	2.7816000	-1.3130875
18.2000008	2.8304000	-1.3069515
18.2000008	2.8792000	-1.0799227
18.2000008	2.9280000	-1.0369711
18.2000008	2.9768000	-1.1412816
18.2000008	3.0256000	-0.8835731
18.2000008	3.0743999	-0.8897090
18.2000008	3.1231999	-1.0369711
18.2000008	3.1719999	-0.7792621
18.2000008	3.2207999	-0.7669902
18.2000008	3.2695999	-0.7669902
18.2000008	3.3183999	-0.9449320
18.2000008	3.3671999	-0.9449320
18.2000008	3.4159999	-0.9326606
18.2000008	3.4647999	-0.9387960
18.2000008	3.5135999	-0.8897090
18.2000008	3.5623999	-1.1780972
18.2000008	3.6111999	-1.1596894
18.2000008	3.6599998	-1.1412816
18.2000008	3.7087998	-0.9878836
18.2000008	3.7575998	-1.0615149
18.2000008	3.8063998	-1.0676508
18.2000008	3.8551998	-0.7853980
18.2000008	3.9039998	-0.7117672
18.2000008	3.9527998	-0.7117672
18.2000008	4.0016003	-0.9572039
18.2000008	4.0503998	-0.9019809
18.2000008	4.0991998	-1.0124273
18.2000008	4.1479998	-1.0369711
18.2000008	4.1967998	-0.7117672
18.2000008	4.2455997	-0.8958449
18.2000008	4.2943997	-0.8958449
18.2000008	4.3431997	-0.5154176
18.2000008	4.3919997	-0.4663301
18.2000008	4.4407997	-0.8222136
18.2000008	4.4895997	-0.8160777

18.2000008	4.5383997	-0.6933594
18.2000008	4.5871997	-0.6320000
18.2000008	4.6359997	-0.6258640
18.2000008	4.6847997	-0.3681555
18.2000008	4.7336001	-0.5399613
18.2000008	4.7824001	-0.4847379
18.2000008	4.8312001	-0.4356503
18.2000008	4.8800001	-0.4417863
18.2000008	4.9288001	-0.4540582
18.2000008	4.9776001	-0.4540582
18.2000008	5.0264001	-0.4233789
18.2000008	5.0752001	-0.4356503
18.2000008	5.1240001	613586.0000000
18.2000008	5.1728001	613586.0000000
18.2000008	5.2216001	613586.0000000
18.2000008	5.2704000	613586.0000000
18.2000008	5.3192000	613586.0000000
18.2000008	5.3680000	613586.0000000
18.2000008	5.4168000	613586.0000000
18.2000008	5.4656000	-0.6810875
18.2000008	5.5144000	-0.6810875
18.2000008	5.5632000	-0.6810875
18.2000008	5.6120000	-0.6810875
18.2000008	5.6608000	-0.6810875
18.2000008	5.7096000	-0.6872234
18.2000008	5.7584000	613586.0000000
18.2000008	5.8072000	613586.0000000
18.2000008	5.8559999	613586.0000000
18.2000008	5.9047999	613586.0000000
18.2000008	5.9535999	613586.0000000
18.2000008	6.0023999	613586.0000000
18.2166672	0.2928000	-3.4422529
18.2166672	0.3416000	-2.0862141
18.2166672	0.3904000	-1.7610102
18.2166672	0.4392000	-1.7610102
18.2166672	0.4880000	-1.6689711
18.2166672	0.5368000	-1.5769324
18.2166672	0.5856000	-1.6137476
18.2166672	0.6344000	-1.4542136
18.2166672	0.6832000	-1.5646605
18.2166672	0.7320000	-1.2333207
18.2166672	0.7808000	-0.8897090
18.2166672	0.8296000	-0.9203887
18.2166672	0.8784000	-0.7117672
18.2166672	0.9272000	-0.7117672
18.2166672	0.9760000	-0.8713012
18.2166672	1.0247999	-0.9940195
18.2166672	1.0735999	-1.0553789
18.2166672	1.1223999	-1.2578645
18.2166672	1.1711999	-0.9326606
18.2166672	1.2200000	-0.9387960
18.2166672	1.2688000	-1.2517285
18.2166672	1.3176000	-1.2333207
18.2166672	1.3664000	-1.1290097
18.2166672	1.4152000	-1.1412816
18.2166672	1.4640000	-1.1351457
18.2166672	1.5128000	-1.0185633

18.2166672	1.5616000	-1.0185633
18.2166672	1.6104000	-0.9572039
18.2166672	1.6592000	-0.9694757
18.2166672	1.7079999	-1.0246992
18.2166672	1.7567999	-1.6198835
18.2166672	1.8055999	-1.2394567
18.2166672	1.8543999	-1.1290097
18.2166672	1.9031999	-1.1167378
18.2166672	1.9519999	-1.1228738
18.2166672	2.0007999	-1.0860586
18.2166672	2.0495999	-1.0860586
18.2166672	2.0983999	-1.0860586
18.2166672	2.1471999	-1.5462527
18.2166672	2.1959999	-1.4296703
18.2166672	2.2447999	-0.9449320
18.2166672	2.2935998	-1.1780972
18.2166672	2.3423998	-1.2517285
18.2166672	2.3912001	-1.3376312
18.2166672	2.4400001	-1.4910293
18.2166672	2.4888000	-1.4848933
18.2166672	2.5376000	-1.1596894
18.2166672	2.5864000	-1.3130875
18.2166672	2.6352000	-1.3130875
18.2166672	2.6840000	-1.1412816
18.2166672	2.7328000	-1.1351457
18.2166672	2.7816000	-1.1719613
18.2166672	2.8304000	-1.1228738
18.2166672	2.8792000	-1.2517285
18.2166672	2.9280000	-1.2578645
18.2166672	2.9768000	-1.2578645
18.2166672	3.0256000	-1.2517285
18.2166672	3.0743999	-1.2517285
18.2166672	3.1231999	-1.1290097
18.2166672	3.1719999	-0.9756117
18.2166672	3.2207999	-0.9694757
18.2166672	3.2695999	-0.7056313
18.2166672	3.3183999	-0.8713012
18.2166672	3.3671999	-0.8038058
18.2166672	3.4159999	-1.1228738
18.2166672	3.4647999	-1.1228738
18.2166672	3.5135999	-0.9203887
18.2166672	3.5623999	-1.1719613
18.2166672	3.6111999	-1.3560390
18.2166672	3.6599998	-0.9019809
18.2166672	3.7087998	-0.8406215
18.2166672	3.7575998	-0.7363110
18.2166672	3.8063998	-0.7363110
18.2166672	3.8551998	-0.8160777
18.2166672	3.9039998	-1.0860586
18.2166672	3.9527998	-0.8590293
18.2166672	4.0016003	-0.7056313
18.2166672	4.0503998	-0.7363110
18.2166672	4.0991998	-0.6442719
18.2166672	4.1479998	-0.9449320
18.2166672	4.1967998	-0.9878836
18.2166672	4.2455997	-0.9878836
18.2166672	4.2943997	-0.7301750

18.2166672	4.3431997	-0.7301750
18.2166672	4.3919997	-0.7117672
18.2166672	4.4407997	-0.4356503
18.2166672	4.4895997	-0.5645051
18.2166672	4.5383997	-0.4233789
18.2166672	4.5871997	-0.4233789
18.2166672	4.6359997	-0.3436117
18.2166672	4.6847997	-0.6320000
18.2166672	4.7336001	-0.3374758
18.2166672	4.7824001	-0.3497477
18.2166672	4.8312001	-0.6749516
18.2166672	4.8800001	-0.6320000
18.2166672	4.9288001	-0.6135921
18.2166672	4.9776001	-0.6135921
18.2166672	5.0264001	-0.5338254
18.2166672	5.0752001	-0.5460973
18.2166672	5.1240001	-0.5583692
18.2166672	5.1728001	-0.5645051
18.2166672	5.2216001	613586.0000000
18.2166672	5.2704000	613586.0000000
18.2166672	5.3192000	613586.0000000
18.2166672	5.3680000	613586.0000000
18.2166672	5.4168000	613586.0000000
18.2166672	5.4656000	-0.7240391
18.2166672	5.5144000	-0.7424469
18.2166672	5.5632000	-0.7608547
18.2166672	5.6120000	613586.0000000
18.2166672	5.6608000	613586.0000000
18.2166672	5.7096000	613586.0000000
18.2166672	5.7584000	613586.0000000
18.2166672	5.8072000	613586.0000000
18.2166672	5.8559999	613586.0000000
18.2166672	5.9047999	613586.0000000
18.2166672	5.9535999	613586.0000000
18.2166672	6.0023999	613586.0000000
18.2333336	0.2928000	-3.3686218
18.2333336	0.3416000	-1.5953403
18.2333336	0.3904000	-1.5953403
18.2333336	0.4392000	-1.6076121
18.2333336	0.4880000	-1.3376312
18.2333336	0.5368000	-1.3499031
18.2333336	0.5856000	-1.4726214
18.2333336	0.6344000	-1.3499031
18.2333336	0.6832000	-1.3314953
18.2333336	0.7320000	-1.1106019
18.2333336	0.7808000	-1.0615149
18.2333336	0.8296000	-1.0676508
18.2333336	0.8784000	-1.1658254
18.2333336	0.9272000	-0.5951843
18.2333336	0.9760000	-1.4112625
18.2333336	1.0247999	-1.5278449
18.2333336	1.0735999	-1.6505632
18.2333336	1.1223999	-1.3069515
18.2333336	1.1711999	-1.3192234
18.2333336	1.2200000	-1.3192234
18.2333336	1.2688000	-1.2701364
18.2333336	1.3176000	-1.2640004

18.2333336	1.3664000	-0.9326606
18.2333336	1.4152000	-0.9326606
18.2333336	1.4640000	-0.9756117
18.2333336	1.5128000	-1.2762718
18.2333336	1.5616000	-1.2824078
18.2333336	1.6104000	-1.1167378
18.2333336	1.6592000	-1.4910293
18.2333336	1.7079999	-1.1474175
18.2333336	1.7567999	-1.2824078
18.2333336	1.8055999	-1.0246992
18.2333336	1.8543999	-1.0308352
18.2333336	1.9031999	-1.2149129
18.2333336	1.9519999	-1.2087770
18.2333336	2.0007999	-1.3314953
18.2333336	2.0495999	-1.3253593
18.2333336	2.0983999	-1.3253593
18.2333336	2.1471999	-1.3376312
18.2333336	2.1959999	-1.2885437
18.2333336	2.2447999	-1.6751070
18.2333336	2.2935998	-1.3376312
18.2333336	2.3423998	-1.2762718
18.2333336	2.3912001	-1.2762718
18.2333336	2.4400001	-1.2210488
18.2333336	2.4888000	-1.0615149
18.2333336	2.5376000	-1.0615149
18.2333336	2.5864000	-1.0492430
18.2333336	2.6352000	-1.1106019
18.2333336	2.6840000	-1.0983305
18.2333336	2.7328000	-0.9694757
18.2333336	2.7816000	-0.8897090
18.2333336	2.8304000	-0.9081168
18.2333336	2.8792000	-1.0799227
18.2333336	2.9280000	-0.9142528
18.2333336	2.9768000	-0.8897090
18.2333336	3.0256000	-1.0308352
18.2333336	3.0743999	-1.0676508
18.2333336	3.1231999	-0.6994953
18.2333336	3.1719999	-0.7117672
18.2333336	3.2207999	-0.8774371
18.2333336	3.2695999	-0.8835731
18.2333336	3.3183999	-1.1658254
18.2333336	3.3671999	-1.1596894
18.2333336	3.4159999	-1.1842332
18.2333336	3.4647999	-1.0492430
18.2333336	3.5135999	-1.0492430
18.2333336	3.5623999	-0.8222136
18.2333336	3.6111999	-1.2701364
18.2333336	3.6599998	-1.0369711
18.2333336	3.7087998	-0.6135921
18.2333336	3.7575998	-0.6013203
18.2333336	3.8063998	-1.0369711
18.2333336	3.8551998	-0.9572039
18.2333336	3.9039998	-0.8222136
18.2333336	3.9527998	-0.9387960
18.2333336	4.0016003	-0.8344855
18.2333336	4.0503998	-0.6258640
18.2333336	4.0991998	-0.7547188

18.2333336	4.1479998	-0.7424469
18.2333336	4.1967998	-0.7424469
18.2333336	4.2455997	-0.7424469
18.2333336	4.2943997	-0.6442719
18.2333336	4.3431997	-0.7547188
18.2333336	4.3919997	-0.6749516
18.2333336	4.4407997	-0.7853980
18.2333336	4.4895997	-0.6381359
18.2333336	4.5383997	-0.5829129
18.2333336	4.5871997	-0.6013203
18.2333336	4.6359997	-0.3865633
18.2333336	4.6847997	-0.5951843
18.2333336	4.7336001	-0.5215535
18.2333336	4.7824001	-0.5215535
18.2333336	4.8312001	-0.3436117
18.2333336	4.8800001	-0.3497477
18.2333336	4.9288001	-0.4786019
18.2333336	4.9776001	-0.4786019
18.2333336	5.0264001	-0.5767770
18.2333336	5.0752001	-0.6197281
18.2333336	5.1240001	613586.0000000
18.2333336	5.1728001	613586.0000000
18.2333336	5.2216001	613586.0000000
18.2333336	5.2704000	-0.5767770
18.2333336	5.3192000	-0.5767770
18.2333336	5.3680000	-0.5338254
18.2333336	5.4168000	-0.5215535
18.2333336	5.4656000	613586.0000000
18.2333336	5.5144000	-0.8590293
18.2333336	5.5632000	-0.8651652
18.2333336	5.6120000	-0.7056313
18.2333336	5.6608000	-0.7117672
18.2333336	5.7096000	613586.0000000
18.2333336	5.7584000	613586.0000000
18.2333336	5.8072000	613586.0000000
18.2333336	5.8559999	-0.6749516
18.2333336	5.9047999	-0.6688156
18.2333336	5.9535999	613586.0000000
18.2333336	6.0023999	613586.0000000
18.2500000	0.2928000	-1.8469129
18.2500000	0.3416000	-1.8469129
18.2500000	0.3904000	-1.6076121
18.2500000	0.4392000	-1.3008156
18.2500000	0.4880000	-1.3008156
18.2500000	0.5368000	-1.6873789
18.2500000	0.5856000	-1.6873789
18.2500000	0.6344000	-1.2885437
18.2500000	0.6832000	-1.4112625
18.2500000	0.7320000	-1.3499031
18.2500000	0.7808000	-1.3499031
18.2500000	0.8296000	-1.0860586
18.2500000	0.8784000	-0.9878836
18.2500000	0.9272000	-0.9817476
18.2500000	0.9760000	-1.0431070
18.2500000	1.0247999	-1.5339808
18.2500000	1.0735999	-1.4603496
18.2500000	1.1223999	-0.9387960

18.2500000	1.1711999	-1.0492430
18.2500000	1.2200000	-1.0369711
18.2500000	1.2688000	-1.2578645
18.2500000	1.3176000	-1.2517285
18.2500000	1.3664000	-1.2394567
18.2500000	1.4152000	-1.1228738
18.2500000	1.4640000	-1.1228738
18.2500000	1.5128000	-1.3130875
18.2500000	1.5616000	-1.2640004
18.2500000	1.6104000	-1.2701364
18.2500000	1.6592000	-1.2701364
18.2500000	1.7079999	-1.1596894
18.2500000	1.7567999	-1.2885437
18.2500000	1.8055999	-0.9572039
18.2500000	1.8543999	-0.9633398
18.2500000	1.9031999	-0.9940195
18.2500000	1.9519999	-0.9203887
18.2500000	2.0007999	-0.9633398
18.2500000	2.0495999	-1.5707965
18.2500000	2.0983999	-1.5707965
18.2500000	2.1471999	-1.5278449
18.2500000	2.1959999	-1.5278449
18.2500000	2.2447999	-1.3253593
18.2500000	2.2935998	-1.3314953
18.2500000	2.3423998	-1.3192234
18.2500000	2.3912001	-1.3069515
18.2500000	2.4400001	-1.1596894
18.2500000	2.4888000	-1.1719613
18.2500000	2.5376000	-1.0431070
18.2500000	2.5864000	-0.9694757
18.2500000	2.6352000	-0.8406215
18.2500000	2.6840000	-1.0676508
18.2500000	2.7328000	-1.1228738
18.2500000	2.7816000	-1.1106019
18.2500000	2.8304000	-1.1351457
18.2500000	2.8792000	-1.1596894
18.2500000	2.9280000	-1.1535535
18.2500000	2.9768000	-1.0921946
18.2500000	3.0256000	-1.0921946
18.2500000	3.0743999	-1.0062914
18.2500000	3.1231999	-1.0983305
18.2500000	3.1719999	-1.0676508
18.2500000	3.2207999	-1.0553789
18.2500000	3.2695999	-1.0553789
18.2500000	3.3183999	-0.8590293
18.2500000	3.3671999	-0.8651652
18.2500000	3.4159999	-0.7240391
18.2500000	3.4647999	-0.5460973
18.2500000	3.5135999	-0.8222136
18.2500000	3.5623999	-0.7792621
18.2500000	3.6111999	-0.7669902
18.2500000	3.6599998	-0.8774371
18.2500000	3.7087998	-0.8713012
18.2500000	3.7575998	-1.4419422
18.2500000	3.8063998	-0.9203887
18.2500000	3.8551998	-1.6935148
18.2500000	3.9039998	-1.2701364

18.2500000	3.9527998	-1.2517285
18.2500000	4.0016003	-1.2517285
18.2500000	4.0503998	-1.1903691
18.2500000	4.0991998	-1.1780972
18.2500000	4.1479998	-0.9265246
18.2500000	4.1967998	-0.9081168
18.2500000	4.2455997	-0.9019809
18.2500000	4.2943997	-0.6258640
18.2500000	4.3431997	-1.3928547
18.2500000	4.3919997	-1.1842332
18.2500000	4.4407997	-1.1780972
18.2500000	4.4895997	-1.0799227
18.2500000	4.5383997	-0.9081168
18.2500000	4.5871997	-0.9510679
18.2500000	4.6359997	-0.7853980
18.2500000	4.6847997	-0.5645051
18.2500000	4.7336001	-0.3129320
18.2500000	4.7824001	-0.3865633
18.2500000	4.8312001	-0.4049711
18.2500000	4.8800001	-0.2393012
18.2500000	4.9288001	-0.5338254
18.2500000	4.9776001	-0.5706410
18.2500000	5.0264001	-0.4847379
18.2500000	5.0752001	-0.5092816
18.2500000	5.1240001	-0.4540582
18.2500000	5.1728001	-0.5399613
18.2500000	5.2216001	-0.6810875
18.2500000	5.2704000	-0.6749516
18.2500000	5.3192000	-0.7485828
18.2500000	5.3680000	-0.7547188
18.2500000	5.4168000	-0.7853980
18.2500000	5.4656000	-0.8835731
18.2500000	5.5144000	-0.8835731
18.2500000	5.5632000	-0.8958449
18.2500000	5.6120000	-1.0983305
18.2500000	5.6608000	-1.2517285
18.2500000	5.7096000	-1.2455926
18.2500000	5.7584000	-1.2271848
18.2500000	5.8072000	-0.6504078
18.2500000	5.8559999	-1.3683109
18.2500000	5.9047999	-1.4848933
18.2500000	5.9535999	-1.4726214
18.2500000	6.0023999	-1.2026410
18.2666664	0.2928000	-1.5585246
18.2666664	0.3416000	-1.5585246
18.2666664	0.3904000	-1.4542136
18.2666664	0.4392000	-1.5646605
18.2666664	0.4880000	-1.5401168
18.2666664	0.5368000	-1.6198835
18.2666664	0.5856000	-1.6137476
18.2666664	0.6344000	-1.3621750
18.2666664	0.6832000	-1.1842332
18.2666664	0.7320000	-1.1780972
18.2666664	0.7808000	-0.9449320
18.2666664	0.8296000	-1.2885437
18.2666664	0.8784000	-1.3008156
18.2666664	0.9272000	-1.3437672

18.2666664	0.9760000	-1.4542136
18.2666664	1.0247999	-1.3867188
18.2666664	1.0735999	-1.4051266
18.2666664	1.1223999	-1.4173985
18.2666664	1.1711999	-1.5462527
18.2666664	1.2200000	-1.4358063
18.2666664	1.2688000	-1.2455926
18.2666664	1.3176000	-1.2394567
18.2666664	1.3664000	-1.2333207
18.2666664	1.4152000	-1.0983305
18.2666664	1.4640000	-1.0799227
18.2666664	1.5128000	-1.3192234
18.2666664	1.5616000	-1.0308352
18.2666664	1.6104000	-1.0431070
18.2666664	1.6592000	-1.1290097
18.2666664	1.7079999	-1.1228738
18.2666664	1.7567999	-1.1167378
18.2666664	1.8055999	-1.3253593
18.2666664	1.8543999	-1.3192234
18.2666664	1.9031999	-1.3130875
18.2666664	1.9519999	-1.3437672
18.2666664	2.0007999	-1.3437672
18.2666664	2.0495999	-1.0246992
18.2666664	2.0983999	-1.3314953
18.2666664	2.1471999	-1.4358063
18.2666664	2.1959999	-1.2149129
18.2666664	2.2447999	-1.5278449
18.2666664	2.2935998	-1.4480777
18.2666664	2.3423998	-1.3867188
18.2666664	2.3912001	-1.1106019
18.2666664	2.4400001	-1.3130875
18.2666664	2.4888000	-1.3314953
18.2666664	2.5376000	-1.3499031
18.2666664	2.5864000	-1.2271848
18.2666664	2.6352000	-1.0124273
18.2666664	2.6840000	-1.0124273
18.2666664	2.7328000	-1.2455926
18.2666664	2.7816000	-1.2026410
18.2666664	2.8304000	-1.3437672
18.2666664	2.8792000	-1.3376312
18.2666664	2.9280000	-1.1535535
18.2666664	2.9768000	-1.1535535
18.2666664	3.0256000	-1.0246992
18.2666664	3.0743999	-0.9878836
18.2666664	3.1231999	-0.9817476
18.2666664	3.1719999	-0.7301750
18.2666664	3.2207999	-0.9142528
18.2666664	3.2695999	-0.9510679
18.2666664	3.3183999	-0.8835731
18.2666664	3.3671999	-0.8651652
18.2666664	3.4159999	-1.0369711
18.2666664	3.4647999	-1.1903691
18.2666664	3.5135999	-0.8958449
18.2666664	3.5623999	-0.9203887
18.2666664	3.6111999	-0.9326606
18.2666664	3.6599998	-0.9387960
18.2666664	3.7087998	-1.1106019

18.2666664	3.7575998	-1.1167378
18.2666664	3.8063998	-0.7915339
18.2666664	3.8551998	-0.6013203
18.2666664	3.9039998	-0.5951843
18.2666664	3.9527998	-0.3865633
18.2666664	4.0016003	-0.5890489
18.2666664	4.0503998	-0.4786019
18.2666664	4.0991998	-0.4601941
18.2666664	4.1479998	-0.6504078
18.2666664	4.1967998	-0.6626797
18.2666664	4.2455997	-1.1412816
18.2666664	4.2943997	-0.9265246
18.2666664	4.3431997	-0.7792621
18.2666664	4.3919997	-0.7792621
18.2666664	4.4407997	-0.6135921
18.2666664	4.4895997	-0.5645051
18.2666664	4.5383997	-0.5583692
18.2666664	4.5871997	-0.3006601
18.2666664	4.6359997	-0.2822523
18.2666664	4.6847997	-0.5215535
18.2666664	4.7336001	-0.5215535
18.2666664	4.7824001	-0.4970098
18.2666664	4.8312001	-0.5031457
18.2666664	4.8800001	-0.5031457
18.2666664	4.9288001	-0.3313398
18.2666664	4.9776001	-0.5215535
18.2666664	5.0264001	-0.5460973
18.2666664	5.0752001	-0.5460973
18.2666664	5.1240001	-0.5583692
18.2666664	5.1728001	-0.6504078
18.2666664	5.2216001	-0.6197281
18.2666664	5.2704000	-0.6933594
18.2666664	5.3192000	-0.8222136
18.2666664	5.3680000	-0.7485828
18.2666664	5.4168000	-0.7424469
18.2666664	5.4656000	-0.8283496
18.2666664	5.5144000	-0.8222136
18.2666664	5.5632000	-0.8958449
18.2666664	5.6120000	-0.7363110
18.2666664	5.6608000	-0.6749516
18.2666664	5.7096000	-0.5645051
18.2666664	5.7584000	-0.5031457
18.2666664	5.8072000	-0.8099418
18.2666664	5.8559999	-0.9081168
18.2666664	5.9047999	613586.0000000
18.2666664	5.9535999	613586.0000000
18.2666664	6.0023999	613586.0000000
18.2833328	0.2928000	-2.1537089
18.2833328	0.3416000	-2.1414371
18.2833328	0.3904000	-2.3132432
18.2833328	0.4392000	-2.0555344
18.2833328	0.4880000	-1.6689711
18.2833328	0.5368000	-1.7548742
18.2833328	0.5856000	-1.3805828
18.2833328	0.6344000	-1.4726214
18.2833328	0.6832000	-1.5094371
18.2833328	0.7320000	-1.2333207

18.2833328	0.7808000	-1.1903691
18.2833328	0.8296000	-1.1044660
18.2833328	0.8784000	-0.8222136
18.2833328	0.9272000	-1.4358063
18.2833328	0.9760000	-1.3805828
18.2833328	1.0247999	-0.7915339
18.2833328	1.0735999	-0.7301750
18.2833328	1.1223999	-0.8897090
18.2833328	1.1711999	-1.2087770
18.2833328	1.2200000	-1.0983305
18.2833328	1.2688000	-1.0983305
18.2833328	1.3176000	-1.0308352
18.2833328	1.3664000	-1.0431070
18.2833328	1.4152000	-0.9633398
18.2833328	1.4640000	-1.1412816
18.2833328	1.5128000	-1.1596894
18.2833328	1.5616000	-1.1658254
18.2833328	1.6104000	-1.1658254
18.2833328	1.6592000	-0.9449320
18.2833328	1.7079999	-0.9572039
18.2833328	1.7567999	-1.0553789
18.2833328	1.8055999	-1.1596894
18.2833328	1.8543999	-1.1658254
18.2833328	1.9031999	-1.4051266
18.2833328	1.9519999	-1.3989906
18.2833328	2.0007999	-1.3805828
18.2833328	2.0495999	-1.3253593
18.2833328	2.0983999	-1.3376312
18.2833328	2.1471999	-1.2578645
18.2833328	2.1959999	-1.2640004
18.2833328	2.2447999	-1.0737867
18.2833328	2.2935998	-1.0553789
18.2833328	2.3423998	-0.9449320
18.2833328	2.3912001	-1.2762718
18.2833328	2.4400001	-1.1903691
18.2833328	2.4888000	-1.3499031
18.2833328	2.5376000	-1.3437672
18.2833328	2.5864000	-1.2455926
18.2833328	2.6352000	-1.2824078
18.2833328	2.6840000	-1.3621750
18.2833328	2.7328000	-1.3683109
18.2833328	2.7816000	-1.2210488
18.2833328	2.8304000	-1.2640004
18.2833328	2.8792000	-1.2640004
18.2833328	2.9280000	-1.0615149
18.2833328	2.9768000	-1.1290097
18.2833328	3.0256000	-1.1290097
18.2833328	3.0743999	-1.3069515
18.2833328	3.1231999	-1.3069515
18.2833328	3.1719999	-1.3069515
18.2833328	3.2207999	-1.3069515
18.2833328	3.2695999	-1.2087770
18.2833328	3.3183999	-1.1903691
18.2833328	3.3671999	-1.0553789
18.2833328	3.4159999	-1.0553789
18.2833328	3.4647999	-1.2824078
18.2833328	3.5135999	-1.0308352

18.2833328	3.5623999	-0.6749516
18.2833328	3.6111999	-0.6810875
18.2833328	3.6599998	-0.3129320
18.2833328	3.7087998	-0.3190680
18.2833328	3.7575998	-0.5399613
18.2833328	3.8063998	-0.6320000
18.2833328	3.8551998	-0.8344855
18.2833328	3.9039998	-0.8283496
18.2833328	3.9527998	-0.6626797
18.2833328	4.0016003	-0.7179031
18.2833328	4.0503998	-0.5645051
18.2833328	4.0991998	-0.9694757
18.2833328	4.1479998	-0.8713012
18.2833328	4.1967998	-0.8528934
18.2833328	4.2455997	-0.5522332
18.2833328	4.2943997	-0.5399613
18.2833328	4.3431997	-0.3681555
18.2833328	4.3919997	-0.4908738
18.2833328	4.4407997	-0.4847379
18.2833328	4.4895997	-0.4847379
18.2833328	4.5383997	-0.4172430
18.2833328	4.5871997	-0.3252039
18.2833328	4.6359997	-0.2393012
18.2833328	4.6847997	-0.2577085
18.2833328	4.7336001	-0.2577085
18.2833328	4.7824001	-0.2577085
18.2833328	4.8312001	-0.2515731
18.2833328	4.8800001	-0.2515731
18.2833328	4.9288001	-0.3129320
18.2833328	4.9776001	-0.3067961
18.2833328	5.0264001	-0.4601941
18.2833328	5.0752001	-0.4049711
18.2833328	5.1240001	-0.4233789
18.2833328	5.1728001	-0.6258640
18.2833328	5.2216001	-0.6258640
18.2833328	5.2704000	-0.7976699
18.2833328	5.3192000	-0.7792621
18.2833328	5.3680000	-0.7669902
18.2833328	5.4168000	-0.7179031
18.2833328	5.4656000	-0.7179031
18.2833328	5.5144000	-0.7240391
18.2833328	5.5632000	-1.0124273
18.2833328	5.6120000	-1.0369711
18.2833328	5.6608000	-1.0369711
18.2833328	5.7096000	-1.0492430
18.2833328	5.7584000	-1.0492430
18.2833328	5.8072000	-1.0431070
18.2833328	5.8559999	-1.1412816
18.2833328	5.9047999	-1.0062914
18.2833328	5.9535999	613586.0000000
18.2833328	6.0023999	613586.0000000
18.2999992	0.2928000	-1.5401168
18.2999992	0.3416000	-1.6996508
18.2999992	0.3904000	-3.1968160
18.2999992	0.4392000	-2.9329712
18.2999992	0.4880000	-2.3746023
18.2999992	0.5368000	-2.0984859

18.2999992	0.5856000	-1.5953403
18.2999992	0.6344000	-1.1842332
18.2999992	0.6832000	-1.1842332
18.2999992	0.7320000	-1.4051266
18.2999992	0.7808000	-1.2210488
18.2999992	0.8296000	-0.7915339
18.2999992	0.8784000	-0.7240391
18.2999992	0.9272000	-1.2578645
18.2999992	0.9760000	-0.9940195
18.2999992	1.0247999	-0.8528934
18.2999992	1.0735999	-1.1106019
18.2999992	1.1223999	-0.8713012
18.2999992	1.1711999	-0.8651652
18.2999992	1.2200000	-1.1474175
18.2999992	1.2688000	-1.4296703
18.2999992	1.3176000	-1.1535535
18.2999992	1.3664000	-1.2210488
18.2999992	1.4152000	-0.9817476
18.2999992	1.4640000	-1.3008156
18.2999992	1.5128000	-1.0737867
18.2999992	1.5616000	-1.0860586
18.2999992	1.6104000	-1.0860586
18.2999992	1.6592000	-1.0983305
18.2999992	1.7079999	-1.4603496
18.2999992	1.7567999	-1.3499031
18.2999992	1.8055999	-1.0369711
18.2999992	1.8543999	-0.9510679
18.2999992	1.9031999	-1.5278449
18.2999992	1.9519999	-1.4173985
18.2999992	2.0007999	-1.4112625
18.2999992	2.0495999	-1.3989906
18.2999992	2.0983999	-1.2578645
18.2999992	2.1471999	-1.1412816
18.2999992	2.1959999	-1.1474175
18.2999992	2.2447999	-1.5094371
18.2999992	2.2935998	-1.5094371
18.2999992	2.3423998	-1.3008156
18.2999992	2.3912001	-1.2701364
18.2999992	2.4400001	-0.9756117
18.2999992	2.4888000	-0.9633398
18.2999992	2.5376000	-1.2271848
18.2999992	2.5864000	-1.2210488
18.2999992	2.6352000	-0.9940195
18.2999992	2.6840000	-1.1228738
18.2999992	2.7328000	-0.8222136
18.2999992	2.7816000	-0.8222136
18.2999992	2.8304000	-1.0615149
18.2999992	2.8792000	-1.1474175
18.2999992	2.9280000	-0.9142528
18.2999992	2.9768000	-0.9142528
18.2999992	3.0256000	-0.9081168
18.2999992	3.0743999	-1.1596894
18.2999992	3.1231999	-1.0431070
18.2999992	3.1719999	-0.9326606
18.2999992	3.2207999	-1.0001554
18.2999992	3.2695999	-1.0001554
18.2999992	3.3183999	-0.9326606

18.2999992	3.3671999	-0.9387960
18.2999992	3.4159999	-0.9449320
18.2999992	3.4647999	-0.9326606
18.2999992	3.5135999	-0.6688156
18.2999992	3.5623999	-0.6442719
18.2999992	3.6111999	-0.7179031
18.2999992	3.6599998	-0.5645051
18.2999992	3.7087998	-0.5890489
18.2999992	3.7575998	-0.5951843
18.2999992	3.8063998	-0.6013203
18.2999992	3.8551998	-0.6135921
18.2999992	3.9039998	-0.7117672
18.2999992	3.9527998	-0.7669902
18.2999992	4.0016003	-0.7669902
18.2999992	4.0503998	-0.7669902
18.2999992	4.0991998	-0.7547188
18.2999992	4.1479998	-0.6381359
18.2999992	4.1967998	-0.6381359
18.2999992	4.2455997	-0.7731261
18.2999992	4.2943997	-0.6442719
18.2999992	4.3431997	-0.6442719
18.2999992	4.3919997	-0.6810875
18.2999992	4.4407997	-0.6810875
18.2999992	4.4895997	-0.3190680
18.2999992	4.5383997	-0.3190680
18.2999992	4.5871997	-0.3313398
18.2999992	4.6359997	-0.3313398
18.2999992	4.6847997	-0.5399613
18.2999992	4.7336001	-0.5399613
18.2999992	4.7824001	-0.5460973
18.2999992	4.8312001	-0.4970098
18.2999992	4.8800001	-0.5645051
18.2999992	4.9288001	-0.6013203
18.2999992	4.9776001	-0.5031457
18.2999992	5.0264001	-0.5031457
18.2999992	5.0752001	-0.6688156
18.2999992	5.1240001	-0.8099418
18.2999992	5.1728001	-0.8099418
18.2999992	5.2216001	-0.8160777
18.2999992	5.2704000	-0.7179031
18.2999992	5.3192000	-0.7240391
18.2999992	5.3680000	-0.7301750
18.2999992	5.4168000	-0.7976699
18.2999992	5.4656000	-0.9142528
18.2999992	5.5144000	-0.9081168
18.2999992	5.5632000	-0.8651652
18.2999992	5.6120000	-0.9694757
18.2999992	5.6608000	-1.0001554
18.2999992	5.7096000	-1.0001554
18.2999992	5.7584000	613586.0000000
18.2999992	5.8072000	613586.0000000
18.2999992	5.8559999	613586.0000000
18.2999992	5.9047999	613586.0000000
18.2999992	5.9535999	613586.0000000
18.2999992	6.0023999	613586.0000000
18.3166676	0.2928000	-2.2150683
18.3166676	0.3416000	-1.9144082

18.3166676	0.3904000	-1.9144082
18.3166676	0.4392000	-1.9144082
18.3166676	0.4880000	-1.6444273
18.3166676	0.5368000	-1.4971652
18.3166676	0.5856000	-1.5339808
18.3166676	0.6344000	-1.5155730
18.3166676	0.6832000	-1.5217090
18.3166676	0.7320000	-1.3805828
18.3166676	0.7808000	-1.2946796
18.3166676	0.8296000	-1.2026410
18.3166676	0.8784000	-1.4726214
18.3166676	0.9272000	-0.9142528
18.3166676	0.9760000	-0.9326606
18.3166676	1.0247999	-1.5217090
18.3166676	1.0735999	-1.5830684
18.3166676	1.1223999	-1.5892043
18.3166676	1.1711999	-1.3621750
18.3166676	1.2200000	-1.6996508
18.3166676	1.2688000	-1.6935148
18.3166676	1.3176000	-1.3192234
18.3166676	1.3664000	-1.3192234
18.3166676	1.4152000	-1.7487383
18.3166676	1.4640000	-1.4112625
18.3166676	1.5128000	-1.5278449
18.3166676	1.5616000	-1.6076121
18.3166676	1.6104000	-1.1903691
18.3166676	1.6592000	-1.1965051
18.3166676	1.7079999	-1.0492430
18.3166676	1.7567999	-1.0615149
18.3166676	1.8055999	-1.5953403
18.3166676	1.8543999	-1.0369711
18.3166676	1.9031999	-1.5155730
18.3166676	1.9519999	-1.4603496
18.3166676	2.0007999	-1.5094371
18.3166676	2.0495999	-1.3744469
18.3166676	2.0983999	-1.3744469
18.3166676	2.1471999	-1.3805828
18.3166676	2.1959999	-1.3314953
18.3166676	2.2447999	-1.3314953
18.3166676	2.2935998	-1.3744469
18.3166676	2.3423998	-1.2946796
18.3166676	2.3912001	-1.0431070
18.3166676	2.4400001	-1.0431070
18.3166676	2.4888000	-1.1228738
18.3166676	2.5376000	-1.1719613
18.3166676	2.5864000	-1.1780972
18.3166676	2.6352000	-1.0431070
18.3166676	2.6840000	-1.0553789
18.3166676	2.7328000	-1.0185633
18.3166676	2.7816000	-1.0062914
18.3166676	2.8304000	-0.8283496
18.3166676	2.8792000	-1.0431070
18.3166676	2.9280000	-1.0431070
18.3166676	2.9768000	-1.0737867
18.3166676	3.0256000	-1.0676508
18.3166676	3.0743999	-0.9142528
18.3166676	3.1231999	-0.9940195

18.3166676	3.1719999	-0.9940195
18.3166676	3.2207999	-0.8283496
18.3166676	3.2695999	-0.9142528
18.3166676	3.3183999	-0.7915339
18.3166676	3.3671999	-0.8713012
18.3166676	3.4159999	-0.8590293
18.3166676	3.4647999	-0.8590293
18.3166676	3.5135999	-0.7056313
18.3166676	3.5623999	-0.6872234
18.3166676	3.6111999	-0.6381359
18.3166676	3.6599998	-0.7731261
18.3166676	3.7087998	-0.9265246
18.3166676	3.7575998	-0.7117672
18.3166676	3.8063998	-0.9326606
18.3166676	3.8551998	-1.1167378
18.3166676	3.9039998	-0.7669902
18.3166676	3.9527998	-0.9265246
18.3166676	4.0016003	-0.9081168
18.3166676	4.0503998	-0.8897090
18.3166676	4.0991998	-0.9081168
18.3166676	4.1479998	-0.7853980
18.3166676	4.1967998	-0.5645051
18.3166676	4.2455997	-0.5583692
18.3166676	4.2943997	-0.6810875
18.3166676	4.3431997	-0.5092816
18.3166676	4.3919997	-0.6074562
18.3166676	4.4407997	-0.6197281
18.3166676	4.4895997	-0.6381359
18.3166676	4.5383997	-0.2761164
18.3166676	4.5871997	-0.2699804
18.3166676	4.6359997	-0.4111071
18.3166676	4.6847997	-0.3988352
18.3166676	4.7336001	-0.3988352
18.3166676	4.7824001	-0.3988352
18.3166676	4.8312001	-0.4049711
18.3166676	4.8800001	-0.5092816
18.3166676	4.9288001	-0.4417863
18.3166676	4.9776001	-0.4417863
18.3166676	5.0264001	-0.5767770
18.3166676	5.0752001	-0.5154176
18.3166676	5.1240001	-0.5031457
18.3166676	5.1728001	-0.5031457
18.3166676	5.2216001	-0.5399613
18.3166676	5.2704000	-0.4295144
18.3166676	5.3192000	-0.4295144
18.3166676	5.3680000	-0.3497477
18.3166676	5.4168000	-0.3497477
18.3166676	5.4656000	-0.2761164
18.3166676	5.5144000	-0.3497477
18.3166676	5.5632000	-0.6258640
18.3166676	5.6120000	-0.6258640
18.3166676	5.6608000	-0.7056313
18.3166676	5.7096000	-0.6320000
18.3166676	5.7584000	-0.6872234
18.3166676	5.8072000	613586.0000000
18.3166676	5.8559999	613586.0000000
18.3166676	5.9047999	613586.0000000

18.3166676	5.9535999	613586.0000000
18.3166676	6.0023999	613586.0000000
18.3333340	0.2928000	-2.2764277
18.3333340	0.3416000	-2.1843886
18.3333340	0.3904000	-1.7855535
18.3333340	0.4392000	-1.7732821
18.3333340	0.4880000	-1.5339808
18.3333340	0.5368000	-1.5278449
18.3333340	0.5856000	-1.3253593
18.3333340	0.6344000	-1.0369711
18.3333340	0.6832000	-1.1596894
18.3333340	0.7320000	-1.5646605
18.3333340	0.7808000	-1.5646605
18.3333340	0.8296000	-2.2150683
18.3333340	0.8784000	-1.8837285
18.3333340	0.9272000	-2.5954955
18.3333340	0.9760000	-2.4420974
18.3333340	1.0247999	-2.1475730
18.3333340	1.0735999	-2.0739422
18.3333340	1.1223999	-1.9941750
18.3333340	1.1711999	-2.1782527
18.3333340	1.2200000	-2.1659808
18.3333340	1.2688000	-2.2150683
18.3333340	1.3176000	-2.0003109
18.3333340	1.3664000	-2.1966605
18.3333340	1.4152000	-2.0248547
18.3333340	1.4640000	-1.7426023
18.3333340	1.5128000	-1.5892043
18.3333340	1.5616000	-1.4173985
18.3333340	1.6104000	-1.4173985
18.3333340	1.6592000	-1.4235344
18.3333340	1.7079999	-1.6935148
18.3333340	1.7567999	-1.3560390
18.3333340	1.8055999	-1.3560390
18.3333340	1.8543999	-1.3437672
18.3333340	1.9031999	-1.2578645
18.3333340	1.9519999	-1.2640004
18.3333340	2.0007999	-1.2455926
18.3333340	2.0495999	-1.2271848
18.3333340	2.0983999	-1.1965051
18.3333340	2.1471999	-0.9878836
18.3333340	2.1959999	-1.1596894
18.3333340	2.2447999	-1.2026410
18.3333340	2.2935998	-0.9878836
18.3333340	2.3423998	-1.1167378
18.3333340	2.3912001	-1.1106019
18.3333340	2.4400001	-0.9510679
18.3333340	2.4888000	-0.9019809
18.3333340	2.5376000	-0.8222136
18.3333340	2.5864000	-0.8222136
18.3333340	2.6352000	-0.8099418
18.3333340	2.6840000	-0.7915339
18.3333340	2.7328000	-0.7915339
18.3333340	2.7816000	-0.7915339
18.3333340	2.8304000	-0.7915339
18.3333340	2.8792000	-0.8467574
18.3333340	2.9280000	-0.8344855

18.3333340	2.9768000	-0.6197281
18.3333340	3.0256000	-0.7301750
18.3333340	3.0743999	-0.9878836
18.3333340	3.1231999	-0.7547188
18.3333340	3.1719999	-0.7731261
18.3333340	3.2207999	-0.9572039
18.3333340	3.2695999	-0.9633398
18.3333340	3.3183999	-0.8958449
18.3333340	3.3671999	-0.6565437
18.3333340	3.4159999	-0.6504078
18.3333340	3.4647999	-0.6933594
18.3333340	3.5135999	-0.6135921
18.3333340	3.5623999	-0.4172430
18.3333340	3.6111999	-0.4049711
18.3333340	3.6599998	-0.6688156
18.3333340	3.7087998	-0.5399613
18.3333340	3.7575998	-0.5460973
18.3333340	3.8063998	-0.5460973
18.3333340	3.8551998	-0.1656699
18.3333340	3.9039998	-0.1656699
18.3333340	3.9527998	-0.1656699
18.3333340	4.0016003	-0.7608547
18.3333340	4.0503998	-0.7731261
18.3333340	4.0991998	-0.7608547
18.3333340	4.1479998	-0.6504078
18.3333340	4.1967998	-0.6381359
18.3333340	4.2455997	-0.5031457
18.3333340	4.2943997	-0.7853980
18.3333340	4.3431997	-0.5645051
18.3333340	4.3919997	-0.4540582
18.3333340	4.4407997	-0.6135921
18.3333340	4.4895997	-0.4724660
18.3333340	4.5383997	-0.2577085
18.3333340	4.5871997	-0.4663301
18.3333340	4.6359997	-0.5215535
18.3333340	4.6847997	-0.5215535
18.3333340	4.7336001	-0.4601941
18.3333340	4.7824001	-0.4847379
18.3333340	4.8312001	-0.4663301
18.3333340	4.8800001	-0.4663301
18.3333340	4.9288001	-0.4356503
18.3333340	4.9776001	-0.5460973
18.3333340	5.0264001	-0.5460973
18.3333340	5.0752001	-0.6013203
18.3333340	5.1240001	-0.5951843
18.3333340	5.1728001	-0.5890489
18.3333340	5.2216001	-0.5829129
18.3333340	5.2704000	-0.6504078
18.3333340	5.3192000	-0.6442719
18.3333340	5.3680000	-0.7731261
18.3333340	5.4168000	-0.7117672
18.3333340	5.4656000	-0.7117672
18.3333340	5.5144000	-0.3190680
18.3333340	5.5632000	-0.4540582
18.3333340	5.6120000	-0.5276895
18.3333340	5.6608000	-0.5276895
18.3333340	5.7096000	-0.6994953

18.3333340	5.7584000	-0.3865633
18.3333340	5.8072000	-0.4908738
18.3333340	5.8559999	613586.0000000
18.3333340	5.9047999	-0.8713012
18.3333340	5.9535999	-0.7915339
18.3333340	6.0023999	-0.8958449
18.3500004	0.2928000	-0.7056313
18.3500004	0.3416000	-1.1106019
18.3500004	0.3904000	-1.1167378
18.3500004	0.4392000	-1.3192234
18.3500004	0.4880000	-1.3192234
18.3500004	0.5368000	-1.9573593
18.3500004	0.5856000	-1.3989906
18.3500004	0.6344000	-1.3989906
18.3500004	0.6832000	-1.0615149
18.3500004	0.7320000	-1.3499031
18.3500004	0.7808000	-1.3499031
18.3500004	0.8296000	-1.2640004
18.3500004	0.8784000	-1.2701364
18.3500004	0.9272000	-1.6014762
18.3500004	0.9760000	-1.5769324
18.3500004	1.0247999	-1.5892043
18.3500004	1.0735999	-1.1719613
18.3500004	1.1223999	-1.3867188
18.3500004	1.1711999	-1.4051266
18.3500004	1.2200000	-1.3499031
18.3500004	1.2688000	-1.4603496
18.3500004	1.3176000	-1.4726214
18.3500004	1.3664000	-1.4051266
18.3500004	1.4152000	-1.4112625
18.3500004	1.4640000	-1.4664855
18.3500004	1.5128000	-1.4664855
18.3500004	1.5616000	-1.5155730
18.3500004	1.6104000	-1.2210488
18.3500004	1.6592000	-1.2210488
18.3500004	1.7079999	-1.6260195
18.3500004	1.7567999	-1.3744469
18.3500004	1.8055999	-1.3560390
18.3500004	1.8543999	-1.3314953
18.3500004	1.9031999	-1.3314953
18.3500004	1.9519999	-1.0983305
18.3500004	2.0007999	-1.1351457
18.3500004	2.0495999	-0.8160777
18.3500004	2.0983999	-1.1228738
18.3500004	2.1471999	-1.2026410
18.3500004	2.1959999	-1.2701364
18.3500004	2.2447999	-1.1167378
18.3500004	2.2935998	-1.1167378
18.3500004	2.3423998	-1.1106019
18.3500004	2.3912001	-1.0308352
18.3500004	2.4400001	-1.0308352
18.3500004	2.4888000	-0.9878836
18.3500004	2.5376000	-0.9694757
18.3500004	2.5864000	-0.8590293
18.3500004	2.6352000	-0.8467574
18.3500004	2.6840000	-0.8713012
18.3500004	2.7328000	-0.8651652

18.3500004	2.7816000	-0.8160777
18.3500004	2.8304000	-0.9510679
18.3500004	2.8792000	-0.8897090
18.3500004	2.9280000	-0.9265246
18.3500004	2.9768000	-0.8099418
18.3500004	3.0256000	-1.0185633
18.3500004	3.0743999	-1.1351457
18.3500004	3.1231999	-0.6994953
18.3500004	3.1719999	-0.9449320
18.3500004	3.2207999	-0.5829129
18.3500004	3.2695999	-0.5645051
18.3500004	3.3183999	-0.6258640
18.3500004	3.3671999	-0.4970098
18.3500004	3.4159999	-0.6442719
18.3500004	3.4647999	-0.4724660
18.3500004	3.5135999	-0.4786019
18.3500004	3.5623999	-0.7240391
18.3500004	3.6111999	-0.6442719
18.3500004	3.6599998	-0.6504078
18.3500004	3.7087998	-0.6688156
18.3500004	3.7575998	-0.9326606
18.3500004	3.8063998	-0.8651652
18.3500004	3.8551998	-1.0431070
18.3500004	3.9039998	-1.0001554
18.3500004	3.9527998	-0.8406215
18.3500004	4.0016003	-0.7669902
18.3500004	4.0503998	-0.7669902
18.3500004	4.0991998	-0.7731261
18.3500004	4.1479998	-0.9633398
18.3500004	4.1967998	-0.7731261
18.3500004	4.2455997	-0.7792621
18.3500004	4.2943997	-0.5522332
18.3500004	4.3431997	-0.5460973
18.3500004	4.3919997	-0.5276895
18.3500004	4.4407997	-0.4540582
18.3500004	4.4895997	-0.6381359
18.3500004	4.5383997	-0.6872234
18.3500004	4.5871997	-0.3620195
18.3500004	4.6359997	-0.5583692
18.3500004	4.6847997	-0.5460973
18.3500004	4.7336001	-0.5583692
18.3500004	4.7824001	-0.5583692
18.3500004	4.8312001	-0.5583692
18.3500004	4.8800001	-0.5645051
18.3500004	4.9288001	-0.5706410
18.3500004	4.9776001	-0.5706410
18.3500004	5.0264001	-0.4172430
18.3500004	5.0752001	-0.9081168
18.3500004	5.1240001	-0.7915339
18.3500004	5.1728001	-0.8590293
18.3500004	5.2216001	-0.8651652
18.3500004	5.2704000	-0.8467574
18.3500004	5.3192000	-0.8406215
18.3500004	5.3680000	-0.7179031
18.3500004	5.4168000	-0.9326606
18.3500004	5.4656000	-0.9572039
18.3500004	5.5144000	-0.9572039

18.3500004	5.5632000	-0.8467574
18.3500004	5.6120000	-0.6810875
18.3500004	5.6608000	-0.7240391
18.3500004	5.7096000	-0.8713012
18.3500004	5.7584000	-0.9142528
18.3500004	5.8072000	-0.7915339
18.3500004	5.8559999	-0.8099418
18.3500004	5.9047999	-0.8160777
18.3500004	5.9535999	-0.7731261
18.3500004	6.0023999	-0.6258640
18.3666668	0.2928000	-2.5402722
18.3666668	0.3416000	-2.3930101
18.3666668	0.3904000	-2.3807383
18.3666668	0.4392000	-2.1721168
18.3666668	0.4880000	-2.1721168
18.3666668	0.5368000	-1.6382914
18.3666668	0.5856000	-1.6321554
18.3666668	0.6344000	-1.7794180
18.3666668	0.6832000	-1.7916894
18.3666668	0.7320000	-1.8039613
18.3666668	0.7808000	-1.2640004
18.3666668	0.8296000	-1.2640004
18.3666668	0.8784000	-1.0860586
18.3666668	0.9272000	-1.0799227
18.3666668	0.9760000	-0.7853980
18.3666668	1.0247999	-0.9878836
18.3666668	1.0735999	-1.1780972
18.3666668	1.1223999	-1.3683109
18.3666668	1.1711999	-1.3560390
18.3666668	1.2200000	-1.3621750
18.3666668	1.2688000	-1.3069515
18.3666668	1.3176000	-1.0676508
18.3666668	1.3664000	-1.0062914
18.3666668	1.4152000	-1.0124273
18.3666668	1.4640000	-1.0185633
18.3666668	1.5128000	-1.1044660
18.3666668	1.5616000	-1.1044660
18.3666668	1.6104000	-1.2087770
18.3666668	1.6592000	-1.2026410
18.3666668	1.7079999	-0.8528934
18.3666668	1.7567999	-0.9756117
18.3666668	1.8055999	-1.0062914
18.3666668	1.8543999	-0.9633398
18.3666668	1.9031999	-0.9694757
18.3666668	1.9519999	-1.1535535
18.3666668	2.0007999	-1.0860586
18.3666668	2.0495999	-1.1535535
18.3666668	2.0983999	-0.7915339
18.3666668	2.1471999	-0.8222136
18.3666668	2.1959999	-0.8651652
18.3666668	2.2447999	-1.0615149
18.3666668	2.2935998	-1.1044660
18.3666668	2.3423998	-1.1106019
18.3666668	2.3912001	-1.0308352
18.3666668	2.4400001	-0.8528934
18.3666668	2.4888000	-0.8590293
18.3666668	2.5376000	-1.0676508

18.3666668	2.5864000	-0.8958449
18.3666668	2.6352000	-0.8958449
18.3666668	2.6840000	-0.8099418
18.3666668	2.7328000	-0.8099418
18.3666668	2.7816000	-1.0737867
18.3666668	2.8304000	-1.0001554
18.3666668	2.8792000	-0.9633398
18.3666668	2.9280000	-0.9449320
18.3666668	2.9768000	-0.8038058
18.3666668	3.0256000	-0.8038058
18.3666668	3.0743999	-1.0124273
18.3666668	3.1231999	-1.0615149
18.3666668	3.1719999	-1.0553789
18.3666668	3.2207999	-0.8099418
18.3666668	3.2695999	-0.5890489
18.3666668	3.3183999	-0.5951843
18.3666668	3.3671999	-0.6074562
18.3666668	3.4159999	-0.3865633
18.3666668	3.4647999	-0.2086215
18.3666668	3.5135999	-0.3926992
18.3666668	3.5623999	-0.2577085
18.3666668	3.6111999	-0.2270293
18.3666668	3.6599998	-0.2331653
18.3666668	3.7087998	-0.2393012
18.3666668	3.7575998	-0.2577085
18.3666668	3.8063998	-0.7853980
18.3666668	3.8551998	-0.6504078
18.3666668	3.9039998	-0.6504078
18.3666668	3.9527998	-0.5951843
18.3666668	4.0016003	-0.7117672
18.3666668	4.0503998	-0.7056313
18.3666668	4.0991998	-0.7976699
18.3666668	4.1479998	-0.5706410
18.3666668	4.1967998	-0.7915339
18.3666668	4.2455997	-1.0308352
18.3666668	4.2943997	-1.0308352
18.3666668	4.3431997	-0.5399613
18.3666668	4.3919997	-0.8958449
18.3666668	4.4407997	-0.8897090
18.3666668	4.4895997	-1.1965051
18.3666668	4.5383997	-1.1903691
18.3666668	4.5871997	-0.8897090
18.3666668	4.6359997	-0.8958449
18.3666668	4.6847997	-0.6749516
18.3666668	4.7336001	-0.6013203
18.3666668	4.7824001	-0.6074562
18.3666668	4.8312001	-0.6197281
18.3666668	4.8800001	-0.8774371
18.3666668	4.9288001	-0.7363110
18.3666668	4.9776001	-0.9081168
18.3666668	5.0264001	-0.9265246
18.3666668	5.0752001	-0.7915339
18.3666668	5.1240001	-0.9203887
18.3666668	5.1728001	-0.9203887
18.3666668	5.2216001	-0.8651652
18.3666668	5.2704000	-0.8713012
18.3666668	5.3192000	-0.8651652

18.3666668	5.3680000	-0.7424469
18.3666668	5.4168000	-0.7485828
18.3666668	5.4656000	-0.9081168
18.3666668	5.5144000	-0.8590293
18.3666668	5.5632000	-0.7424469
18.3666668	5.6120000	-0.8467574
18.3666668	5.6608000	-0.7853980
18.3666668	5.7096000	-0.8283496
18.3666668	5.7584000	-0.8467574
18.3666668	5.8072000	-0.9019809
18.3666668	5.8559999	-0.6381359
18.3666668	5.9047999	613586.0000000
18.3666668	5.9535999	613586.0000000
18.3666668	6.0023999	613586.0000000
18.3833332	0.2928000	-2.6323111
18.3833332	0.3416000	-2.0003109
18.3833332	0.3904000	-1.9082723
18.3833332	0.4392000	-1.9144082
18.3833332	0.4880000	-1.9144082
18.3833332	0.5368000	-1.9082723
18.3833332	0.5856000	-1.4112625
18.3833332	0.6344000	-1.5094371
18.3833332	0.6832000	-1.2701364
18.3833332	0.7320000	-1.2701364
18.3833332	0.7808000	-1.3805828
18.3833332	0.8296000	-1.5155730
18.3833332	0.8784000	-1.4051266
18.3833332	0.9272000	-1.4112625
18.3833332	0.9760000	-1.4173985
18.3833332	1.0247999	-1.2087770
18.3833332	1.0735999	-1.2026410
18.3833332	1.1223999	-1.3376312
18.3833332	1.1711999	-0.9940195
18.3833332	1.2200000	-0.6504078
18.3833332	1.2688000	-0.7915339
18.3833332	1.3176000	-1.2578645
18.3833332	1.3664000	-0.9572039
18.3833332	1.4152000	-0.8590293
18.3833332	1.4640000	-0.8590293
18.3833332	1.5128000	-0.8528934
18.3833332	1.5616000	-1.3192234
18.3833332	1.6104000	-1.0737867
18.3833332	1.6592000	-1.0676508
18.3833332	1.7079999	-1.1658254
18.3833332	1.7567999	-1.1719613
18.3833332	1.8055999	-0.8713012
18.3833332	1.8543999	-0.8283496
18.3833332	1.9031999	-0.6197281
18.3833332	1.9519999	-0.6135921
18.3833332	2.0007999	-0.5645051
18.3833332	2.0495999	-0.5951843
18.3833332	2.0983999	-0.7179031
18.3833332	2.1471999	-0.8038058
18.3833332	2.1959999	-0.8038058
18.3833332	2.2447999	-0.8958449
18.3833332	2.2935998	-0.6688156
18.3833332	2.3423998	-0.6626797

18.3833332	2.3912001	-0.7976699
18.3833332	2.4400001	-0.5951843
18.3833332	2.4888000	-0.5951843
18.3833332	2.5376000	-0.6688156
18.3833332	2.5864000	-0.4479222
18.3833332	2.6352000	-0.4479222
18.3833332	2.6840000	-0.4417863
18.3833332	2.7328000	-0.3620195
18.3833332	2.7816000	-0.5829129
18.3833332	2.8304000	-0.9081168
18.3833332	2.8792000	-0.9081168
18.3833332	2.9280000	-0.9142528
18.3833332	2.9768000	-0.4601941
18.3833332	3.0256000	-0.6258640
18.3833332	3.0743999	-0.7669902
18.3833332	3.1231999	-0.7669902
18.3833332	3.1719999	-0.9142528
18.3833332	3.2207999	-0.8467574
18.3833332	3.2695999	-0.8283496
18.3833332	3.3183999	-0.8038058
18.3833332	3.3671999	-0.8099418
18.3833332	3.4159999	-0.6994953
18.3833332	3.4647999	-0.4111071
18.3833332	3.5135999	-0.4049711
18.3833332	3.5623999	-0.2454371
18.3833332	3.6111999	-0.5338254
18.3833332	3.6599998	-0.5338254
18.3833332	3.7087998	-0.4663301
18.3833332	3.7575998	-0.7179031
18.3833332	3.8063998	-0.7179031
18.3833332	3.8551998	-0.8099418
18.3833332	3.9039998	-0.4356503
18.3833332	3.9527998	-0.5399613
18.3833332	4.0016003	-0.5276895
18.3833332	4.0503998	-0.3252039
18.3833332	4.0991998	-0.3129320
18.3833332	4.1479998	-0.1718059
18.3833332	4.1967998	-0.1840777
18.3833332	4.2455997	-0.2024856
18.3833332	4.2943997	-0.2024856
18.3833332	4.3431997	-0.2086215
18.3833332	4.3919997	0.0613594
18.3833332	4.4407997	-0.1595340
18.3833332	4.4895997	-0.3558836
18.3833332	4.5383997	-0.3497477
18.3833332	4.5871997	-0.3436117
18.3833332	4.6359997	-0.2822523
18.3833332	4.6847997	-0.3006601
18.3833332	4.7336001	-0.3374758
18.3833332	4.7824001	-0.4724660
18.3833332	4.8312001	-0.5460973
18.3833332	4.8800001	-0.5460973
18.3833332	4.9288001	-0.7301750
18.3833332	4.9776001	-0.7240391
18.3833332	5.0264001	-0.6749516
18.3833332	5.0752001	-0.6749516
18.3833332	5.1240001	-0.8590293

18.3833332	5.1728001	-0.8651652
18.3833332	5.2216001	-0.7792621
18.3833332	5.2704000	-0.7792621
18.3833332	5.3192000	-0.7792621
18.3833332	5.3680000	-0.7915339
18.3833332	5.4168000	-0.8467574
18.3833332	5.4656000	-0.8528934
18.3833332	5.5144000	-0.7792621
18.3833332	5.5632000	-0.7608547
18.3833332	5.6120000	-0.7485828
18.3833332	5.6608000	-0.7056313
18.3833332	5.7096000	-0.7056313
18.3833332	5.7584000	-0.7117672
18.3833332	5.8072000	-0.7363110
18.3833332	5.8559999	-0.7363110
18.3833332	5.9047999	-0.7363110
18.3833332	5.9535999	613586.0000000
18.3833332	6.0023999	-0.3742914
18.3999996	0.2928000	-2.2457480
18.3999996	0.3416000	-1.8775926
18.3999996	0.3904000	-1.7548742
18.3999996	0.4392000	-1.6321554
18.3999996	0.4880000	-1.4112625
18.3999996	0.5368000	-1.3989906
18.3999996	0.5856000	-1.6628351
18.3999996	0.6344000	-1.2149129
18.3999996	0.6832000	-1.7057867
18.3999996	0.7320000	-1.7057867
18.3999996	0.7808000	-1.6014762
18.3999996	0.8296000	-1.6014762
18.3999996	0.8784000	-1.1167378
18.3999996	0.9272000	-1.5707965
18.3999996	0.9760000	-1.3744469
18.3999996	1.0247999	-1.2824078
18.3999996	1.0735999	-1.2946796
18.3999996	1.1223999	-1.4726214
18.3999996	1.1711999	-1.2394567
18.3999996	1.2200000	-1.3744469
18.3999996	1.2688000	-1.3683109
18.3999996	1.3176000	-1.3744469
18.3999996	1.3664000	-1.1106019
18.3999996	1.4152000	-1.1106019
18.3999996	1.4640000	-1.2394567
18.3999996	1.5128000	-0.9387960
18.3999996	1.5616000	-1.1474175
18.3999996	1.6104000	-0.8528934
18.3999996	1.6592000	-0.8528934
18.3999996	1.7079999	-1.0860586
18.3999996	1.7567999	-1.0799227
18.3999996	1.8055999	-0.8038058
18.3999996	1.8543999	-0.7608547
18.3999996	1.9031999	-0.5154176
18.3999996	1.9519999	-0.6994953
18.3999996	2.0007999	-0.8406215
18.3999996	2.0495999	-0.8713012
18.3999996	2.0983999	-0.7608547
18.3999996	2.1471999	-1.0062914

18.3999996	2.1959999	-0.7853980
18.3999996	2.2447999	-0.8713012
18.3999996	2.2935998	-0.8651652
18.3999996	2.3423998	-0.8651652
18.3999996	2.3912001	-0.8713012
18.3999996	2.4400001	-0.8590293
18.3999996	2.4888000	-0.8528934
18.3999996	2.5376000	-0.7608547
18.3999996	2.5864000	-0.7485828
18.3999996	2.6352000	-0.7424469
18.3999996	2.6840000	-0.7731261
18.3999996	2.7328000	-0.4172430
18.3999996	2.7816000	-0.8406215
18.3999996	2.8304000	-0.6872234
18.3999996	2.8792000	-0.9510679
18.3999996	2.9280000	-0.9572039
18.3999996	2.9768000	-0.9019809
18.3999996	3.0256000	-0.9203887
18.3999996	3.0743999	-0.8958449
18.3999996	3.1231999	-1.0431070
18.3999996	3.1719999	-0.9081168
18.3999996	3.2207999	-0.8528934
18.3999996	3.2695999	-0.9326606
18.3999996	3.3183999	-0.9326606
18.3999996	3.3671999	-0.2577085
18.3999996	3.4159999	-0.2699804
18.3999996	3.4647999	-0.8099418
18.3999996	3.5135999	-0.5154176
18.3999996	3.5623999	-0.5645051
18.3999996	3.6111999	-0.6994953
18.3999996	3.6599998	-0.5583692
18.3999996	3.7087998	-0.5399613
18.3999996	3.7575998	-0.5399613
18.3999996	3.8063998	-0.5829129
18.3999996	3.8551998	-0.5829129
18.3999996	3.9039998	-0.1718059
18.3999996	3.9527998	-0.6197281
18.3999996	4.0016003	-0.6565437
18.3999996	4.0503998	-0.6626797
18.3999996	4.0991998	-0.6688156
18.3999996	4.1479998	-0.6197281
18.3999996	4.1967998	-0.6381359
18.3999996	4.2455997	-0.5031457
18.3999996	4.2943997	-0.5522332
18.3999996	4.3431997	-0.6013203
18.3999996	4.3919997	-0.3067961
18.3999996	4.4407997	-0.3006601
18.3999996	4.4895997	-0.4970098
18.3999996	4.5383997	-0.4786019
18.3999996	4.5871997	-0.3926992
18.3999996	4.6359997	-0.4111071
18.3999996	4.6847997	-0.4111071
18.3999996	4.7336001	-0.4111071
18.3999996	4.7824001	-0.5399613
18.3999996	4.8312001	-0.4970098
18.3999996	4.8800001	-0.5154176
18.3999996	4.9288001	-0.6074562

18.3999996	4.9776001	-0.6688156
18.3999996	5.0264001	-0.5276895
18.3999996	5.0752001	-0.4540582
18.3999996	5.1240001	-0.4663301
18.3999996	5.1728001	-0.5399613
18.3999996	5.2216001	-0.5338254
18.3999996	5.2704000	-0.5276895
18.3999996	5.3192000	-0.6810875
18.3999996	5.3680000	-0.4786019
18.3999996	5.4168000	-0.7240391
18.3999996	5.4656000	-0.7301750
18.3999996	5.5144000	-0.5767770
18.3999996	5.5632000	-0.5767770
18.3999996	5.6120000	-0.6381359
18.3999996	5.6608000	-0.5338254
18.3999996	5.7096000	-0.5522332
18.3999996	5.7584000	-0.6933594
18.3999996	5.8072000	-0.6135921
18.3999996	5.8559999	-0.4847379
18.3999996	5.9047999	-0.5460973
18.3999996	5.9535999	-0.6197281
18.3999996	6.0023999	-0.6197281
18.4166660	0.2928000	-2.4359615
18.4166660	0.3416000	-2.4420974
18.4166660	0.3904000	-2.1353011
18.4166660	0.4392000	-1.7548742
18.4166660	0.4880000	-1.7610102
18.4166660	0.5368000	-1.5278449
18.4166660	0.5856000	-1.3560390
18.4166660	0.6344000	-1.2087770
18.4166660	0.6832000	-1.2762718
18.4166660	0.7320000	-1.6689711
18.4166660	0.7808000	-1.6689711
18.4166660	0.8296000	-1.3683109
18.4166660	0.8784000	-1.0921946
18.4166660	0.9272000	-1.2578645
18.4166660	0.9760000	-1.2578645
18.4166660	1.0247999	-1.3130875
18.4166660	1.0735999	-1.3008156
18.4166660	1.1223999	-0.8713012
18.4166660	1.1711999	-0.8713012
18.4166660	1.2200000	-1.0921946
18.4166660	1.2688000	-0.8467574
18.4166660	1.3176000	-1.0676508
18.4166660	1.3664000	-1.0553789
18.4166660	1.4152000	-0.9142528
18.4166660	1.4640000	-0.9081168
18.4166660	1.5128000	-1.1658254
18.4166660	1.5616000	-1.4419422
18.4166660	1.6104000	-1.4419422
18.4166660	1.6592000	-1.0246992
18.4166660	1.7079999	-1.0246992
18.4166660	1.7567999	-1.0369711
18.4166660	1.8055999	-1.0431070
18.4166660	1.8543999	-0.9878836
18.4166660	1.9031999	-0.9817476
18.4166660	1.9519999	-0.7608547

18.4166660	2.0007999	-0.7056313
18.4166660	2.0495999	-0.7240391
18.4166660	2.0983999	-0.7424469
18.4166660	2.1471999	-0.6258640
18.4166660	2.1959999	-0.7976699
18.4166660	2.2447999	-0.8222136
18.4166660	2.2935998	-0.9326606
18.4166660	2.3423998	-0.9572039
18.4166660	2.3912001	-0.7301750
18.4166660	2.4400001	-0.7301750
18.4166660	2.4888000	-0.7424469
18.4166660	2.5376000	-0.8344855
18.4166660	2.5864000	-0.8344855
18.4166660	2.6352000	-0.9817476
18.4166660	2.6840000	-0.8528934
18.4166660	2.7328000	-0.7485828
18.4166660	2.7816000	-0.9387960
18.4166660	2.8304000	-0.9326606
18.4166660	2.8792000	-0.9019809
18.4166660	2.9280000	-0.8958449
18.4166660	2.9768000	-0.9387960
18.4166660	3.0256000	-0.9694757
18.4166660	3.0743999	-0.9081168
18.4166660	3.1231999	-0.8406215
18.4166660	3.1719999	-0.9203887
18.4166660	3.2207999	-0.9203887
18.4166660	3.2695999	-0.7915339
18.4166660	3.3183999	-0.7976699
18.4166660	3.3671999	-0.8038058
18.4166660	3.4159999	-0.8160777
18.4166660	3.4647999	-0.7424469
18.4166660	3.5135999	-0.5092816
18.4166660	3.5623999	-0.5092816
18.4166660	3.6111999	-0.4049711
18.4166660	3.6599998	-0.2822523
18.4166660	3.7087998	0.0368156
18.4166660	3.7575998	-0.7301750
18.4166660	3.8063998	-0.4847379
18.4166660	3.8551998	-1.2578645
18.4166660	3.9039998	-1.2578645
18.4166660	3.9527998	-1.2640004
18.4166660	4.0016003	-0.5276895
18.4166660	4.0503998	-0.9387960
18.4166660	4.0991998	-0.9387960
18.4166660	4.1479998	-0.9387960
18.4166660	4.1967998	-0.9326606
18.4166660	4.2455997	-0.5829129
18.4166660	4.2943997	-0.7608547
18.4166660	4.3431997	-0.7485828
18.4166660	4.3919997	-0.6688156
18.4166660	4.4407997	-0.4295144
18.4166660	4.4895997	-0.3374758
18.4166660	4.5383997	-0.3190680
18.4166660	4.5871997	-0.3252039
18.4166660	4.6359997	-0.4908738
18.4166660	4.6847997	-0.4970098
18.4166660	4.7336001	-0.6197281

18.4166660	4.7824001	-0.6197281
18.4166660	4.8312001	-0.6381359
18.4166660	4.8800001	-0.6442719
18.4166660	4.9288001	-0.6504078
18.4166660	4.9776001	-0.6442719
18.4166660	5.0264001	-0.6135921
18.4166660	5.0752001	-0.6749516
18.4166660	5.1240001	-0.6381359
18.4166660	5.1728001	613586.0000000
18.4166660	5.2216001	613586.0000000
18.4166660	5.2704000	613586.0000000
18.4166660	5.3192000	613586.0000000
18.4166660	5.3680000	613586.0000000
18.4166660	5.4168000	-0.7056313
18.4166660	5.4656000	-0.7117672
18.4166660	5.5144000	-0.7792621
18.4166660	5.5632000	-0.7731261
18.4166660	5.6120000	-0.7731261
18.4166660	5.6608000	-0.6135921
18.4166660	5.7096000	-0.6135921
18.4166660	5.7584000	613586.0000000
18.4166660	5.8072000	-0.4847379
18.4166660	5.8559999	613586.0000000
18.4166660	5.9047999	613586.0000000
18.4166660	5.9535999	-0.5645051
18.4166660	6.0023999	-0.5583692
18.4333324	0.2928000	-2.5893598
18.4333324	0.3416000	-2.6936703
18.4333324	0.3904000	-2.3930101
18.4333324	0.4392000	-2.0003109
18.4333324	0.4880000	-1.3376312
18.4333324	0.5368000	-0.9326606
18.4333324	0.5856000	-1.7978253
18.4333324	0.6344000	-2.0003109
18.4333324	0.6832000	-1.7610102
18.4333324	0.7320000	-1.7487383
18.4333324	0.7808000	-1.1903691
18.4333324	0.8296000	-1.2578645
18.4333324	0.8784000	-1.2517285
18.4333324	0.9272000	-1.5830684
18.4333324	0.9760000	-1.5892043
18.4333324	1.0247999	-1.5401168
18.4333324	1.0735999	-1.5401168
18.4333324	1.1223999	-1.1474175
18.4333324	1.1711999	-1.1351457
18.4333324	1.2200000	-1.2210488
18.4333324	1.2688000	-1.2149129
18.4333324	1.3176000	-1.2271848
18.4333324	1.3664000	-1.0615149
18.4333324	1.4152000	-1.0615149
18.4333324	1.4640000	-1.0615149
18.4333324	1.5128000	-1.2640004
18.4333324	1.5616000	-1.2087770
18.4333324	1.6104000	-0.9081168
18.4333324	1.6592000	-0.9081168
18.4333324	1.7079999	-0.8897090
18.4333324	1.7567999	-0.8590293

18.4333324	1.8055999	-0.8651652
18.4333324	1.8543999	-0.8713012
18.4333324	1.9031999	-0.8713012
18.4333324	1.9519999	-1.1719613
18.4333324	2.0007999	-1.1719613
18.4333324	2.0495999	-0.7792621
18.4333324	2.0983999	-1.2149129
18.4333324	2.1471999	-1.1044660
18.4333324	2.1959999	-1.0983305
18.4333324	2.2447999	-1.1412816
18.4333324	2.2935998	-0.9019809
18.4333324	2.3423998	-0.9019809
18.4333324	2.3912001	-0.8774371
18.4333324	2.4400001	-0.9387960
18.4333324	2.4888000	-0.9326606
18.4333324	2.5376000	-0.7792621
18.4333324	2.5864000	-0.7792621
18.4333324	2.6352000	-1.0492430
18.4333324	2.6840000	-1.0615149
18.4333324	2.7328000	-1.0737867
18.4333324	2.7816000	-1.1106019
18.4333324	2.8304000	-1.1106019
18.4333324	2.8792000	-0.9510679
18.4333324	2.9280000	-1.0124273
18.4333324	2.9768000	-0.8528934
18.4333324	3.0256000	-0.8713012
18.4333324	3.0743999	-0.8835731
18.4333324	3.1231999	-0.8590293
18.4333324	3.1719999	-0.9449320
18.4333324	3.2207999	-0.4049711
18.4333324	3.2695999	-0.4049711
18.4333324	3.3183999	-0.4786019
18.4333324	3.3671999	-0.3804274
18.4333324	3.4159999	-0.3742914
18.4333324	3.4647999	-0.3804274
18.4333324	3.5135999	-0.5829129
18.4333324	3.5623999	-0.5215535
18.4333324	3.6111999	-0.5215535
18.4333324	3.6599998	-0.5829129
18.4333324	3.7087998	-0.3988352
18.4333324	3.7575998	-0.3988352
18.4333324	3.8063998	-0.3926992
18.4333324	3.8551998	-0.7179031
18.4333324	3.9039998	-0.6258640
18.4333324	3.9527998	-1.1474175
18.4333324	4.0016003	-1.0246992
18.4333324	4.0503998	-0.9572039
18.4333324	4.0991998	-0.6013203
18.4333324	4.1479998	-0.7056313
18.4333324	4.1967998	-0.7179031
18.4333324	4.2455997	-0.8283496
18.4333324	4.2943997	-0.8283496
18.4333324	4.3431997	-0.6013203
18.4333324	4.3919997	-0.4233789
18.4333324	4.4407997	-0.5154176
18.4333324	4.4895997	-0.4172430
18.4333324	4.5383997	-0.4356503

18.4333324	4.5871997	-0.6074562
18.4333324	4.6359997	-0.5890489
18.4333324	4.6847997	-0.7117672
18.4333324	4.7336001	-0.7117672
18.4333324	4.7824001	-0.6074562
18.4333324	4.8312001	-0.6013203
18.4333324	4.8800001	-0.6013203
18.4333324	4.9288001	-0.6013203
18.4333324	4.9776001	-0.4049711
18.4333324	5.0264001	-0.5706410
18.4333324	5.0752001	-0.5767770
18.4333324	5.1240001	-0.5767770
18.4333324	5.1728001	-0.5338254
18.4333324	5.2216001	-0.6688156
18.4333324	5.2704000	-0.6749516
18.4333324	5.3192000	-0.6749516
18.4333324	5.3680000	-0.6013203
18.4333324	5.4168000	-0.5890489
18.4333324	5.4656000	-0.6994953
18.4333324	5.5144000	-0.6933594
18.4333324	5.5632000	-0.6994953
18.4333324	5.6120000	-0.6933594
18.4333324	5.6608000	-0.6381359
18.4333324	5.7096000	-0.6381359
18.4333324	5.7584000	-0.5767770
18.4333324	5.8072000	-0.5890489
18.4333324	5.8559999	-0.7240391
18.4333324	5.9047999	-0.9265246
18.4333324	5.9535999	-0.8406215
18.4333324	6.0023999	-0.8406215
18.4500008	0.2928000	-2.8409326
18.4500008	0.3416000	-2.4911849
18.4500008	0.3904000	-1.7487383
18.4500008	0.4392000	-1.7487383
18.4500008	0.4880000	-1.7487383
18.4500008	0.5368000	-1.7487383
18.4500008	0.5856000	-1.6505632
18.4500008	0.6344000	-1.6566992
18.4500008	0.6832000	-1.3928547
18.4500008	0.7320000	-1.3989906
18.4500008	0.7808000	-1.3683109
18.4500008	0.8296000	-1.4480777
18.4500008	0.8784000	-1.3499031
18.4500008	0.9272000	-1.3437672
18.4500008	0.9760000	-1.5339808
18.4500008	1.0247999	-1.2517285
18.4500008	1.0735999	-1.2517285
18.4500008	1.1223999	-1.1290097
18.4500008	1.1711999	-1.1044660
18.4500008	1.2200000	-1.1903691
18.4500008	1.2688000	-1.4664855
18.4500008	1.3176000	-1.4480777
18.4500008	1.3664000	-1.3192234
18.4500008	1.4152000	-1.3192234
18.4500008	1.4640000	-1.3130875
18.4500008	1.5128000	-1.1412816
18.4500008	1.5616000	-1.1474175

18.4500008	1.6104000	-1.1351457
18.4500008	1.6592000	-1.1351457
18.4500008	1.7079999	-1.1596894
18.4500008	1.7567999	-1.1658254
18.4500008	1.8055999	-1.0860586
18.4500008	1.8543999	-1.0860586
18.4500008	1.9031999	-0.9387960
18.4500008	1.9519999	-0.9387960
18.4500008	2.0007999	-1.0246992
18.4500008	2.0495999	-1.3376312
18.4500008	2.0983999	-1.3376312
18.4500008	2.1471999	-1.3376312
18.4500008	2.1959999	-1.2762718
18.4500008	2.2447999	-1.2762718
18.4500008	2.2935998	-1.2640004
18.4500008	2.3423998	-1.1167378
18.4500008	2.3912001	-1.0983305
18.4500008	2.4400001	-1.2271848
18.4500008	2.4888000	-1.1228738
18.4500008	2.5376000	-1.0615149
18.4500008	2.5864000	-1.2885437
18.4500008	2.6352000	-1.2824078
18.4500008	2.6840000	-1.1228738
18.4500008	2.7328000	-1.1228738
18.4500008	2.7816000	-1.1228738
18.4500008	2.8304000	-1.0431070
18.4500008	2.8792000	-1.0369711
18.4500008	2.9280000	-0.8528934
18.4500008	2.9768000	-1.0246992
18.4500008	3.0256000	-0.9940195
18.4500008	3.0743999	-1.0246992
18.4500008	3.1231999	-1.0431070
18.4500008	3.1719999	-1.0369711
18.4500008	3.2207999	-0.6320000
18.4500008	3.2695999	-0.6320000
18.4500008	3.3183999	-0.7056313
18.4500008	3.3671999	-0.6749516
18.4500008	3.4159999	-0.5215535
18.4500008	3.4647999	-0.5829129
18.4500008	3.5135999	-0.7792621
18.4500008	3.5623999	-0.7915339
18.4500008	3.6111999	-0.8467574
18.4500008	3.6599998	-0.9940195
18.4500008	3.7087998	-0.9326606
18.4500008	3.7575998	-1.0431070
18.4500008	3.8063998	-1.0431070
18.4500008	3.8551998	-0.8222136
18.4500008	3.9039998	-0.7301750
18.4500008	3.9527998	-0.7424469
18.4500008	4.0016003	-0.4724660
18.4500008	4.0503998	-0.4786019
18.4500008	4.0991998	-0.3742914
18.4500008	4.1479998	-0.4847379
18.4500008	4.1967998	-0.4847379
18.4500008	4.2455997	-0.6135921
18.4500008	4.2943997	-0.4601941
18.4500008	4.3431997	-0.7424469

18.4500008	4.3919997	-0.8038058
18.4500008	4.4407997	-0.7976699
18.4500008	4.4895997	-0.9142528
18.4500008	4.5383997	-0.9019809
18.4500008	4.5871997	-0.7853980
18.4500008	4.6359997	-0.7976699
18.4500008	4.6847997	-0.6258640
18.4500008	4.7336001	-0.6258640
18.4500008	4.7824001	-0.6074562
18.4500008	4.8312001	-0.5092816
18.4500008	4.8800001	-0.7056313
18.4500008	4.9288001	-0.6626797
18.4500008	4.9776001	-0.6626797
18.4500008	5.0264001	-0.7240391
18.4500008	5.0752001	-0.7056313
18.4500008	5.1240001	-0.6994953
18.4500008	5.1728001	-0.6994953
18.4500008	5.2216001	-0.6074562
18.4500008	5.2704000	-0.6074562
18.4500008	5.3192000	-0.6135921
18.4500008	5.3680000	-0.7608547
18.4500008	5.4168000	-0.7915339
18.4500008	5.4656000	-0.5767770
18.4500008	5.5144000	-0.6933594
18.4500008	5.5632000	-0.6810875
18.4500008	5.6120000	-0.6810875
18.4500008	5.6608000	-0.6872234
18.4500008	5.7096000	-0.6872234
18.4500008	5.7584000	-0.6688156
18.4500008	5.8072000	-0.7731261
18.4500008	5.8559999	-0.4847379
18.4500008	5.9047999	613586.0000000
18.4500008	5.9535999	-0.5460973
18.4500008	6.0023999	-0.5522332
18.4666672	0.2928000	-2.8102529
18.4666672	0.3416000	-2.7120781
18.4666672	0.3904000	-2.3623304
18.4666672	0.4392000	-2.3623304
18.4666672	0.4880000	-1.5769324
18.4666672	0.5368000	-1.5830684
18.4666672	0.5856000	-1.7180586
18.4666672	0.6344000	-1.6751070
18.4666672	0.6832000	-1.7303305
18.4666672	0.7320000	-1.6137476
18.4666672	0.7808000	-1.5953403
18.4666672	0.8296000	-1.2149129
18.4666672	0.8784000	-1.2394567
18.4666672	0.9272000	-1.2578645
18.4666672	0.9760000	-1.2701364
18.4666672	1.0247999	-1.3867188
18.4666672	1.0735999	-1.0431070
18.4666672	1.1223999	-1.0799227
18.4666672	1.1711999	-1.0124273
18.4666672	1.2200000	-1.5830684
18.4666672	1.2688000	-1.0983305
18.4666672	1.3176000	-1.1842332
18.4666672	1.3664000	-1.5585246

18.4666672	1.4152000	-1.5462527
18.4666672	1.4640000	-1.4664855
18.4666672	1.5128000	-1.4603496
18.4666672	1.5616000	-1.4480777
18.4666672	1.6104000	-1.4419422
18.4666672	1.6592000	-1.5646605
18.4666672	1.7079999	-1.0983305
18.4666672	1.7567999	-1.4848933
18.4666672	1.8055999	-1.4664855
18.4666672	1.8543999	-1.4419422
18.4666672	1.9031999	-1.4542136
18.4666672	1.9519999	-1.6076121
18.4666672	2.0007999	-1.5707965
18.4666672	2.0495999	-1.5339808
18.4666672	2.0983999	-1.5155730
18.4666672	2.1471999	-1.5094371
18.4666672	2.1959999	-1.3928547
18.4666672	2.2447999	-1.3130875
18.4666672	2.2935998	-1.3928547
18.4666672	2.3423998	-1.3928547
18.4666672	2.3912001	-1.3867188
18.4666672	2.4400001	-1.3989906
18.4666672	2.4888000	-1.3130875
18.4666672	2.5376000	-1.2946796
18.4666672	2.5864000	-1.0799227
18.4666672	2.6352000	-1.0308352
18.4666672	2.6840000	-1.2026410
18.4666672	2.7328000	-1.0062914
18.4666672	2.7816000	-0.9878836
18.4666672	2.8304000	-0.9817476
18.4666672	2.8792000	-1.1658254
18.4666672	2.9280000	-0.9940195
18.4666672	2.9768000	-0.9878836
18.4666672	3.0256000	-1.0062914
18.4666672	3.0743999	-0.7669902
18.4666672	3.1231999	-0.7669902
18.4666672	3.1719999	-0.5890489
18.4666672	3.2207999	-0.4970098
18.4666672	3.2695999	-0.9265246
18.4666672	3.3183999	-0.9387960
18.4666672	3.3671999	-1.0124273
18.4666672	3.4159999	-1.0185633
18.4666672	3.4647999	-1.0062914
18.4666672	3.5135999	-1.0492430
18.4666672	3.5623999	-1.0737867
18.4666672	3.6111999	-1.1351457
18.4666672	3.6599998	-1.0676508
18.4666672	3.7087998	-0.8835731
18.4666672	3.7575998	-0.8713012
18.4666672	3.8063998	-0.8713012
18.4666672	3.8551998	-0.6994953
18.4666672	3.9039998	-0.8344855
18.4666672	3.9527998	-0.8344855
18.4666672	4.0016003	-1.0185633
18.4666672	4.0503998	-0.7363110
18.4666672	4.0991998	-0.6933594
18.4666672	4.1479998	-0.6197281

18.4666672	4.1967998	-0.3742914
18.4666672	4.2455997	-0.8528934
18.4666672	4.2943997	-0.8528934
18.4666672	4.3431997	-0.6749516
18.4666672	4.3919997	-0.6749516
18.4666672	4.4407997	-0.8528934
18.4666672	4.4895997	-0.8222136
18.4666672	4.5383997	-0.8467574
18.4666672	4.5871997	-0.8467574
18.4666672	4.6359997	-0.4479222
18.4666672	4.6847997	-0.4540582
18.4666672	4.7336001	-0.8222136
18.4666672	4.7824001	-0.8222136
18.4666672	4.8312001	-0.5767770
18.4666672	4.8800001	-0.5767770
18.4666672	4.9288001	-0.5767770
18.4666672	4.9776001	-0.5706410
18.4666672	5.0264001	-0.6258640
18.4666672	5.0752001	-0.6872234
18.4666672	5.1240001	-0.6933594
18.4666672	5.1728001	-0.6626797
18.4666672	5.2216001	-0.6565437
18.4666672	5.2704000	-0.6504078
18.4666672	5.3192000	-0.6320000
18.4666672	5.3680000	-0.6626797
18.4666672	5.4168000	-0.8099418
18.4666672	5.4656000	-0.8099418
18.4666672	5.5144000	-0.7485828
18.4666672	5.5632000	-0.6933594
18.4666672	5.6120000	-0.6013203
18.4666672	5.6608000	-0.6074562
18.4666672	5.7096000	-0.6135921
18.4666672	5.7584000	-0.4908738
18.4666672	5.8072000	-0.5767770
18.4666672	5.8559999	-0.6013203
18.4666672	5.9047999	-0.6074562
18.4666672	5.9535999	-0.6135921
18.4666672	6.0023999	-0.6197281
18.4833336	0.2928000	-3.6631463
18.4833336	0.3416000	-3.0618258
18.4833336	0.3904000	-2.6445830
18.4833336	0.4392000	-2.4727771
18.4833336	0.4880000	-2.4973209
18.4833336	0.5368000	-2.3071072
18.4833336	0.5856000	-1.5646605
18.4833336	0.6344000	-1.5585246
18.4833336	0.6832000	-1.3253593
18.4833336	0.7320000	-1.2640004
18.4833336	0.7808000	-1.2517285
18.4833336	0.8296000	-1.2517285
18.4833336	0.8784000	-1.1842332
18.4833336	0.9272000	-1.0369711
18.4833336	0.9760000	-1.2149129
18.4833336	1.0247999	-1.2333207
18.4833336	1.0735999	-1.2455926
18.4833336	1.1223999	-1.2640004
18.4833336	1.1711999	-1.0676508

18.4833336	1.2200000	-1.0799227
18.4833336	1.2688000	-1.2333207
18.4833336	1.3176000	-1.2333207
18.4833336	1.3664000	-1.3560390
18.4833336	1.4152000	-1.5339808
18.4833336	1.4640000	-1.4235344
18.4833336	1.5128000	-1.2026410
18.4833336	1.5616000	-1.2087770
18.4833336	1.6104000	-1.7057867
18.4833336	1.6592000	-1.6566992
18.4833336	1.7079999	-1.4480777
18.4833336	1.7567999	-1.4480777
18.4833336	1.8055999	-1.2762718
18.4833336	1.8543999	-1.5094371
18.4833336	1.9031999	-1.7548742
18.4833336	1.9519999	-1.7548742
18.4833336	2.0007999	-1.5401168
18.4833336	2.0495999	-1.7794180
18.4833336	2.0983999	-1.7426023
18.4833336	2.1471999	-1.7671461
18.4833336	2.1959999	-1.5155730
18.4833336	2.2447999	-1.5339808
18.4833336	2.2935998	-1.4848933
18.4833336	2.3423998	-1.4112625
18.4833336	2.3912001	-1.4480777
18.4833336	2.4400001	-1.5217090
18.4833336	2.4888000	-1.3376312
18.4833336	2.5376000	-1.4112625
18.4833336	2.5864000	-1.4235344
18.4833336	2.6352000	-1.3008156
18.4833336	2.6840000	-1.1903691
18.4833336	2.7328000	-1.2210488
18.4833336	2.7816000	-1.2210488
18.4833336	2.8304000	-1.1290097
18.4833336	2.8792000	-1.0553789
18.4833336	2.9280000	-1.1780972
18.4833336	2.9768000	-1.2824078
18.4833336	3.0256000	-1.2824078
18.4833336	3.0743999	-0.9387960
18.4833336	3.1231999	-0.9387960
18.4833336	3.1719999	-1.2333207
18.4833336	3.2207999	-1.2455926
18.4833336	3.2695999	-1.2333207
18.4833336	3.3183999	-1.2394567
18.4833336	3.3671999	-1.1780972
18.4833336	3.4159999	-1.0553789
18.4833336	3.4647999	-0.9572039
18.4833336	3.5135999	-1.0492430
18.4833336	3.5623999	-0.8958449
18.4833336	3.6111999	-1.0185633
18.4833336	3.6599998	-0.7792621
18.4833336	3.7087998	-1.0001554
18.4833336	3.7575998	-0.9081168
18.4833336	3.8063998	-0.8958449
18.4833336	3.8551998	-0.8958449
18.4833336	3.9039998	-0.9081168
18.4833336	3.9527998	-0.9633398

18.4833336	4.0016003	-0.9572039
18.4833336	4.0503998	-1.0185633
18.4833336	4.0991998	-1.0124273
18.4833336	4.1479998	-1.0124273
18.4833336	4.1967998	-0.8160777
18.4833336	4.2455997	-0.8038058
18.4833336	4.2943997	-0.7976699
18.4833336	4.3431997	-0.7915339
18.4833336	4.3919997	-0.8038058
18.4833336	4.4407997	-0.8283496
18.4833336	4.4895997	-0.6749516
18.4833336	4.5383997	-0.6994953
18.4833336	4.5871997	-0.7056313
18.4833336	4.6359997	-0.5399613
18.4833336	4.6847997	-0.7056313
18.4833336	4.7336001	-0.7056313
18.4833336	4.7824001	-0.6442719
18.4833336	4.8312001	-0.8344855
18.4833336	4.8800001	-0.8283496
18.4833336	4.9288001	-0.8406215
18.4833336	4.9776001	-0.8283496
18.4833336	5.0264001	-0.8713012
18.4833336	5.0752001	-0.7669902
18.4833336	5.1240001	-0.8344855
18.4833336	5.1728001	-0.7669902
18.4833336	5.2216001	-0.9142528
18.4833336	5.2704000	-0.9203887
18.4833336	5.3192000	-0.7853980
18.4833336	5.3680000	-0.8406215
18.4833336	5.4168000	-0.8406215
18.4833336	5.4656000	-0.6626797
18.4833336	5.5144000	-0.7853980
18.4833336	5.5632000	-0.7731261
18.4833336	5.6120000	-0.5645051
18.4833336	5.6608000	-0.5583692
18.4833336	5.7096000	-0.5522332
18.4833336	5.7584000	-0.7485828
18.4833336	5.8072000	-0.7424469
18.4833336	5.8559999	-0.6994953
18.4833336	5.9047999	-0.5890489
18.4833336	5.9535999	-0.5645051
18.4833336	6.0023999	-0.5890489
18.5000000	0.2928000	-3.9269910
18.5000000	0.3416000	-3.6324666
18.5000000	0.3904000	-3.1354568
18.5000000	0.4392000	-2.4973209
18.5000000	0.4880000	-1.6628351
18.5000000	0.5368000	-1.6566992
18.5000000	0.5856000	-1.6260195
18.5000000	0.6344000	-1.3867188
18.5000000	0.6832000	-1.2824078
18.5000000	0.7320000	-1.4173985
18.5000000	0.7808000	-1.7057867
18.5000000	0.8296000	-1.3008156
18.5000000	0.8784000	-1.2946796
18.5000000	0.9272000	-0.9142528
18.5000000	0.9760000	-1.3130875

18.5000000	1.0247999	-1.3008156
18.5000000	1.0735999	-1.3989906
18.5000000	1.1223999	-0.9878836
18.5000000	1.1711999	-1.2701364
18.5000000	1.2200000	-1.2578645
18.5000000	1.2688000	-1.2578645
18.5000000	1.3176000	-1.2394567
18.5000000	1.3664000	-1.2394567
18.5000000	1.4152000	-1.2394567
18.5000000	1.4640000	-1.6198835
18.5000000	1.5128000	-1.2210488
18.5000000	1.5616000	-1.5769324
18.5000000	1.6104000	-1.5769324
18.5000000	1.6592000	-1.0553789
18.5000000	1.7079999	-1.0676508
18.5000000	1.7567999	-1.2087770
18.5000000	1.8055999	-1.4235344
18.5000000	1.8543999	-1.4173985
18.5000000	1.9031999	-1.4848933
18.5000000	1.9519999	-1.4910293
18.5000000	2.0007999	-1.6444273
18.5000000	2.0495999	-1.6444273
18.5000000	2.0983999	-1.7548742
18.5000000	2.1471999	-1.7548742
18.5000000	2.1959999	-1.6628351
18.5000000	2.2447999	-1.6689711
18.5000000	2.2935998	-1.5707965
18.5000000	2.3423998	-1.5769324
18.5000000	2.3912001	-1.4787574
18.5000000	2.4400001	-1.4051266
18.5000000	2.4888000	-1.2762718
18.5000000	2.5376000	-1.2824078
18.5000000	2.5864000	-1.3008156
18.5000000	2.6352000	-1.2946796
18.5000000	2.6840000	-1.2885437
18.5000000	2.7328000	-1.0553789
18.5000000	2.7816000	-1.1044660
18.5000000	2.8304000	-0.9940195
18.5000000	2.8792000	-1.0676508
18.5000000	2.9280000	-1.0308352
18.5000000	2.9768000	-0.9694757
18.5000000	3.0256000	-1.3376312
18.5000000	3.0743999	-1.3314953
18.5000000	3.1231999	-1.3192234
18.5000000	3.1719999	-1.3069515
18.5000000	3.2207999	-0.9326606
18.5000000	3.2695999	-1.0799227
18.5000000	3.3183999	-1.0737867
18.5000000	3.3671999	-1.0676508
18.5000000	3.4159999	-1.1780972
18.5000000	3.4647999	-1.1658254
18.5000000	3.5135999	-1.0369711
18.5000000	3.5623999	-1.0369711
18.5000000	3.6111999	-1.0369711
18.5000000	3.6599998	-1.0553789
18.5000000	3.7087998	-1.0308352
18.5000000	3.7575998	-0.8344855

18.5000000	3.8063998	-1.1044660
18.5000000	3.8551998	-0.8099418
18.5000000	3.9039998	-0.9449320
18.5000000	3.9527998	-0.9878836
18.5000000	4.0016003	-0.7669902
18.5000000	4.0503998	-0.7792621
18.5000000	4.0991998	-1.0431070
18.5000000	4.1479998	-0.9510679
18.5000000	4.1967998	-0.8651652
18.5000000	4.2455997	-0.8713012
18.5000000	4.2943997	-0.5338254
18.5000000	4.3431997	-0.5460973
18.5000000	4.3919997	-0.8835731
18.5000000	4.4407997	-0.6994953
18.5000000	4.4895997	-0.9142528
18.5000000	4.5383997	-0.9326606
18.5000000	4.5871997	-0.8344855
18.5000000	4.6359997	-0.8958449
18.5000000	4.6847997	-0.8160777
18.5000000	4.7336001	-0.7915339
18.5000000	4.7824001	-0.7915339
18.5000000	4.8312001	-0.7853980
18.5000000	4.8800001	-0.7915339
18.5000000	4.9288001	-0.9878836
18.5000000	4.9776001	-0.9940195
18.5000000	5.0264001	-1.0553789
18.5000000	5.0752001	-1.0553789
18.5000000	5.1240001	-1.0431070
18.5000000	5.1728001	-0.8897090
18.5000000	5.2216001	-1.0185633
18.5000000	5.2704000	-1.0185633
18.5000000	5.3192000	-1.0001554
18.5000000	5.3680000	-0.9633398
18.5000000	5.4168000	-0.9633398
18.5000000	5.4656000	-0.9449320
18.5000000	5.5144000	-0.9449320
18.5000000	5.5632000	-0.8283496
18.5000000	5.6120000	-0.8283496
18.5000000	5.6608000	-0.8406215
18.5000000	5.7096000	-0.9510679
18.5000000	5.7584000	-0.7547188
18.5000000	5.8072000	-0.7976699
18.5000000	5.8559999	-0.8038058
18.5000000	5.9047999	-0.8038058
18.5000000	5.9535999	-0.8897090
18.5000000	6.0023999	-0.7608547
18.5166664	0.2928000	-4.0681171
18.5166664	0.3416000	-3.5588355
18.5166664	0.3904000	-3.5220201
18.5166664	0.4392000	-2.5893598
18.5166664	0.4880000	-2.1537089
18.5166664	0.5368000	-1.6382914
18.5166664	0.5856000	-1.4296703
18.5166664	0.6344000	-0.7117672
18.5166664	0.6832000	-1.1658254
18.5166664	0.7320000	-1.1658254
18.5166664	0.7808000	-1.3130875

18.5166664	0.8296000	-1.3130875
18.5166664	0.8784000	-1.2210488
18.5166664	0.9272000	-1.1167378
18.5166664	0.9760000	-1.1228738
18.5166664	1.0247999	-1.1228738
18.5166664	1.0735999	-1.2885437
18.5166664	1.1223999	-1.0983305
18.5166664	1.1711999	-1.1658254
18.5166664	1.2200000	-1.1658254
18.5166664	1.2688000	-1.1596894
18.5166664	1.3176000	-1.2578645
18.5166664	1.3664000	-1.0308352
18.5166664	1.4152000	-1.1658254
18.5166664	1.4640000	-1.2087770
18.5166664	1.5128000	-0.9940195
18.5166664	1.5616000	-1.5155730
18.5166664	1.6104000	-1.2087770
18.5166664	1.6592000	-1.5707965
18.5166664	1.7079999	-1.0737867
18.5166664	1.7567999	-1.6382914
18.5166664	1.8055999	-1.6444273
18.5166664	1.8543999	-1.6566992
18.5166664	1.9031999	-1.6873789
18.5166664	1.9519999	-1.7548742
18.5166664	2.0007999	-1.6321554
18.5166664	2.0495999	-1.7119226
18.5166664	2.0983999	-1.6505632
18.5166664	2.1471999	-1.6505632
18.5166664	2.1959999	-1.6505632
18.5166664	2.2447999	-1.6014762
18.5166664	2.2935998	-1.3928547
18.5166664	2.3423998	-1.4542136
18.5166664	2.3912001	-1.4235344
18.5166664	2.4400001	-1.4235344
18.5166664	2.4888000	-1.4112625
18.5166664	2.5376000	-1.3989906
18.5166664	2.5864000	-1.4051266
18.5166664	2.6352000	-1.1719613
18.5166664	2.6840000	-1.3008156
18.5166664	2.7328000	-1.3069515
18.5166664	2.7816000	-1.3130875
18.5166664	2.8304000	-1.3130875
18.5166664	2.8792000	-1.3253593
18.5166664	2.9280000	-1.2026410
18.5166664	2.9768000	-1.1965051
18.5166664	3.0256000	-1.2578645
18.5166664	3.0743999	-1.1780972
18.5166664	3.1231999	-1.1596894
18.5166664	3.1719999	-1.0492430
18.5166664	3.2207999	-1.0369711
18.5166664	3.2695999	-1.0124273
18.5166664	3.3183999	-1.0246992
18.5166664	3.3671999	-1.0369711
18.5166664	3.4159999	-0.9387960
18.5166664	3.4647999	-0.9326606
18.5166664	3.5135999	-0.9326606
18.5166664	3.5623999	-0.9081168

18.5166664	3.6111999	-0.8283496
18.5166664	3.6599998	-1.0001554
18.5166664	3.7087998	-1.0983305
18.5166664	3.7575998	-1.0983305
18.5166664	3.8063998	-0.9694757
18.5166664	3.8551998	-0.6565437
18.5166664	3.9039998	-0.7792621
18.5166664	3.9527998	-0.9387960
18.5166664	4.0016003	-0.8897090
18.5166664	4.0503998	-1.0799227
18.5166664	4.0991998	-0.8222136
18.5166664	4.1479998	-0.8590293
18.5166664	4.1967998	-0.8590293
18.5166664	4.2455997	-0.9694757
18.5166664	4.2943997	-0.9817476
18.5166664	4.3431997	-1.0431070
18.5166664	4.3919997	-1.0921946
18.5166664	4.4407997	-1.1290097
18.5166664	4.4895997	-1.1535535
18.5166664	4.5383997	-1.1658254
18.5166664	4.5871997	-1.1658254
18.5166664	4.6359997	-1.2824078
18.5166664	4.6847997	-1.1903691
18.5166664	4.7336001	-1.1903691
18.5166664	4.7824001	-1.1535535
18.5166664	4.8312001	-1.0553789
18.5166664	4.8800001	-1.1228738
18.5166664	4.9288001	-1.0185633
18.5166664	4.9776001	-1.0246992
18.5166664	5.0264001	-1.0369711
18.5166664	5.0752001	-0.9756117
18.5166664	5.1240001	-1.0369711
18.5166664	5.1728001	-1.0369711
18.5166664	5.2216001	-1.0431070
18.5166664	5.2704000	-1.0001554
18.5166664	5.3192000	-1.0001554
18.5166664	5.3680000	-1.0676508
18.5166664	5.4168000	-0.9633398
18.5166664	5.4656000	-0.9019809
18.5166664	5.5144000	-0.7547188
18.5166664	5.5632000	-0.9142528
18.5166664	5.6120000	-0.8835731
18.5166664	5.6608000	-0.8713012
18.5166664	5.7096000	-0.9326606
18.5166664	5.7584000	-0.9081168
18.5166664	5.8072000	-0.9019809
18.5166664	5.8559999	-0.7731261
18.5166664	5.9047999	-0.7976699
18.5166664	5.9535999	-0.7976699
18.5166664	6.0023999	613586.0000000
18.5333328	0.2928000	-4.0251656
18.5333328	0.3416000	-3.4483888
18.5333328	0.3904000	-2.9513791
18.5333328	0.4392000	-2.3561945
18.5333328	0.4880000	-2.3561945
18.5333328	0.5368000	-1.2824078
18.5333328	0.5856000	-1.1596894

18.5333328	0.6344000	-1.0983305
18.5333328	0.6832000	-1.4358063
18.5333328	0.7320000	-1.4419422
18.5333328	0.7808000	-1.4480777
18.5333328	0.8296000	-1.2762718
18.5333328	0.8784000	-1.2210488
18.5333328	0.9272000	-1.2210488
18.5333328	0.9760000	-1.2149129
18.5333328	1.0247999	-1.2394567
18.5333328	1.0735999	-0.9756117
18.5333328	1.1223999	-1.3437672
18.5333328	1.1711999	-1.2394567
18.5333328	1.2200000	-1.2455926
18.5333328	1.2688000	-1.2885437
18.5333328	1.3176000	-1.1780972
18.5333328	1.3664000	-1.3683109
18.5333328	1.4152000	-1.3621750
18.5333328	1.4640000	-1.1535535
18.5333328	1.5128000	-1.2210488
18.5333328	1.5616000	-1.2210488
18.5333328	1.6104000	-1.5585246
18.5333328	1.6592000	-1.5585246
18.5333328	1.7079999	-1.3621750
18.5333328	1.7567999	-1.5217090
18.5333328	1.8055999	-1.4971652
18.5333328	1.8543999	-1.4848933
18.5333328	1.9031999	-1.3867188
18.5333328	1.9519999	-1.5830684
18.5333328	2.0007999	-1.5830684
18.5333328	2.0495999	-1.5646605
18.5333328	2.0983999	-1.5585246
18.5333328	2.1471999	-1.6566992
18.5333328	2.1959999	-1.6137476
18.5333328	2.2447999	-1.4542136
18.5333328	2.2935998	-1.3683109
18.5333328	2.3423998	-1.3683109
18.5333328	2.3912001	-1.3621750
18.5333328	2.4400001	-1.4542136
18.5333328	2.4888000	-1.3437672
18.5333328	2.5376000	-1.4358063
18.5333328	2.5864000	-1.4358063
18.5333328	2.6352000	-1.3867188
18.5333328	2.6840000	-1.3867188
18.5333328	2.7328000	-1.3253593
18.5333328	2.7816000	-1.5094371
18.5333328	2.8304000	-1.5217090
18.5333328	2.8792000	-1.4603496
18.5333328	2.9280000	-1.3253593
18.5333328	2.9768000	-1.3376312
18.5333328	3.0256000	-1.1596894
18.5333328	3.0743999	-1.1596894
18.5333328	3.1231999	-1.3314953
18.5333328	3.1719999	-1.3253593
18.5333328	3.2207999	-1.3314953
18.5333328	3.2695999	-1.1228738
18.5333328	3.3183999	-1.2271848
18.5333328	3.3671999	-1.2271848

18.5333328	3.4159999	-1.2026410
18.5333328	3.4647999	-1.1351457
18.5333328	3.5135999	-0.8713012
18.5333328	3.5623999	-0.9756117
18.5333328	3.6111999	-0.9756117
18.5333328	3.6599998	-0.9940195
18.5333328	3.7087998	-0.9878836
18.5333328	3.7575998	-0.9756117
18.5333328	3.8063998	-0.7792621
18.5333328	3.8551998	-0.7853980
18.5333328	3.9039998	-0.8160777
18.5333328	3.9527998	-0.8222136
18.5333328	4.0016003	-0.7976699
18.5333328	4.0503998	-0.7976699
18.5333328	4.0991998	-0.5645051
18.5333328	4.1479998	-0.6994953
18.5333328	4.1967998	-0.6872234
18.5333328	4.2455997	-0.7976699
18.5333328	4.2943997	-0.8160777
18.5333328	4.3431997	-0.9081168
18.5333328	4.3919997	-0.8344855
18.5333328	4.4407997	-0.6074562
18.5333328	4.4895997	-0.6074562
18.5333328	4.5383997	-0.5399613
18.5333328	4.5871997	-0.8099418
18.5333328	4.6359997	-0.8344855
18.5333328	4.6847997	-1.0185633
18.5333328	4.7336001	-1.0185633
18.5333328	4.7824001	-0.9142528
18.5333328	4.8312001	-0.9387960
18.5333328	4.8800001	-1.0246992
18.5333328	4.9288001	-1.1044660
18.5333328	4.9776001	-0.9203887
18.5333328	5.0264001	-0.9203887
18.5333328	5.0752001	-0.8897090
18.5333328	5.1240001	-1.0553789
18.5333328	5.1728001	-0.8528934
18.5333328	5.2216001	-0.8528934
18.5333328	5.2704000	-0.8406215
18.5333328	5.3192000	-0.8344855
18.5333328	5.3680000	-0.8467574
18.5333328	5.4168000	-0.8222136
18.5333328	5.4656000	-0.8222136
18.5333328	5.5144000	-0.9633398
18.5333328	5.5632000	-0.8958449
18.5333328	5.6120000	-0.8958449
18.5333328	5.6608000	-0.9633398
18.5333328	5.7096000	-0.8590293
18.5333328	5.7584000	-0.9142528
18.5333328	5.8072000	-0.8099418
18.5333328	5.8559999	-0.8160777
18.5333328	5.9047999	613586.0000000
18.5333328	5.9535999	-0.7853980
18.5333328	6.0023999	-0.7424469
18.5499992	0.2928000	-3.9638064
18.5499992	0.3416000	-3.5036123
18.5499992	0.3904000	-3.5036123

18.5499992	0.4392000	-2.1353011
18.5499992	0.4880000	-2.1353011
18.5499992	0.5368000	-2.0125828
18.5499992	0.5856000	-1.8039613
18.5499992	0.6344000	-1.5462527
18.5499992	0.6832000	-0.8651652
18.5499992	0.7320000	-1.2455926
18.5499992	0.7808000	-1.3560390
18.5499992	0.8296000	-1.3376312
18.5499992	0.8784000	-1.3314953
18.5499992	0.9272000	-1.1228738
18.5499992	0.9760000	-1.2394567
18.5499992	1.0247999	-1.2394567
18.5499992	1.0735999	-1.2394567
18.5499992	1.1223999	-0.8835731
18.5499992	1.1711999	-0.8774371
18.5499992	1.2200000	-1.1596894
18.5499992	1.2688000	-1.1474175
18.5499992	1.3176000	-1.2087770
18.5499992	1.3664000	-1.1780972
18.5499992	1.4152000	-1.2149129
18.5499992	1.4640000	-1.2149129
18.5499992	1.5128000	-1.1596894
18.5499992	1.5616000	-1.0124273
18.5499992	1.6104000	-1.0124273
18.5499992	1.6592000	-1.2885437
18.5499992	1.7079999	-1.2824078
18.5499992	1.7567999	-1.2578645
18.5499992	1.8055999	-1.3130875
18.5499992	1.8543999	-1.4419422
18.5499992	1.9031999	-1.4419422
18.5499992	1.9519999	-1.4542136
18.5499992	2.0007999	-1.3376312
18.5499992	2.0495999	-1.4419422
18.5499992	2.0983999	-1.4419422
18.5499992	2.1471999	-1.4480777
18.5499992	2.1959999	-1.4480777
18.5499992	2.2447999	-1.4480777
18.5499992	2.2935998	-1.5339808
18.5499992	2.3423998	-1.4112625
18.5499992	2.3912001	-1.4480777
18.5499992	2.4400001	-1.4235344
18.5499992	2.4888000	-1.4603496
18.5499992	2.5376000	-1.2271848
18.5499992	2.5864000	-1.2640004
18.5499992	2.6352000	-1.2517285
18.5499992	2.6840000	-1.2455926
18.5499992	2.7328000	-1.3253593
18.5499992	2.7816000	-1.2271848
18.5499992	2.8304000	-1.2394567
18.5499992	2.8792000	-1.2578645
18.5499992	2.9280000	-1.3621750
18.5499992	2.9768000	-1.3621750
18.5499992	3.0256000	-1.2885437
18.5499992	3.0743999	-1.3376312
18.5499992	3.1231999	-1.3192234
18.5499992	3.1719999	-1.1658254

18.5499992	3.2207999	-1.2210488
18.5499992	3.2695999	-1.1596894
18.5499992	3.3183999	-1.1780972
18.5499992	3.3671999	-1.1044660
18.5499992	3.4159999	-0.7792621
18.5499992	3.4647999	-0.7853980
18.5499992	3.5135999	-0.5890489
18.5499992	3.5623999	-0.9019809
18.5499992	3.6111999	-0.8897090
18.5499992	3.6599998	-0.9387960
18.5499992	3.7087998	-0.9265246
18.5499992	3.7575998	-0.9019809
18.5499992	3.8063998	-0.9817476
18.5499992	3.8551998	-1.2824078
18.5499992	3.9039998	-1.2455926
18.5499992	3.9527998	-1.1474175
18.5499992	4.0016003	-1.1596894
18.5499992	4.0503998	-1.0983305
18.5499992	4.0991998	-1.0983305
18.5499992	4.1479998	-0.8283496
18.5499992	4.1967998	-0.8222136
18.5499992	4.2455997	-0.8651652
18.5499992	4.2943997	-0.8897090
18.5499992	4.3431997	-0.8835731
18.5499992	4.3919997	-0.8651652
18.5499992	4.4407997	-0.7669902
18.5499992	4.4895997	-0.8283496
18.5499992	4.5383997	-0.7179031
18.5499992	4.5871997	-0.7608547
18.5499992	4.6359997	-0.7608547
18.5499992	4.6847997	-0.3313398
18.5499992	4.7336001	-0.8713012
18.5499992	4.7824001	-0.8713012
18.5499992	4.8312001	-0.9203887
18.5499992	4.8800001	-0.9633398
18.5499992	4.9288001	-0.8467574
18.5499992	4.9776001	-0.8467574
18.5499992	5.0264001	-0.9449320
18.5499992	5.0752001	-0.9203887
18.5499992	5.1240001	-0.9081168
18.5499992	5.1728001	-0.8958449
18.5499992	5.2216001	-0.8651652
18.5499992	5.2704000	-0.8651652
18.5499992	5.3192000	-1.0369711
18.5499992	5.3680000	-0.9817476
18.5499992	5.4168000	-0.9756117
18.5499992	5.4656000	-0.9694757
18.5499992	5.5144000	-0.8528934
18.5499992	5.5632000	-0.8528934
18.5499992	5.6120000	-0.8651652
18.5499992	5.6608000	-0.7240391
18.5499992	5.7096000	-0.7117672
18.5499992	5.7584000	-0.8038058
18.5499992	5.8072000	-0.7424469
18.5499992	5.8559999	613586.0000000
18.5499992	5.9047999	-0.8038058
18.5499992	5.9535999	-0.8590293

18.5499992	6.0023999	613586.0000000
18.5666676	0.2928000	-3.7060976
18.5666676	0.3416000	-3.8594959
18.5666676	0.3904000	-3.3993015
18.5666676	0.4392000	-2.8900199
18.5666676	0.4880000	-2.8900199
18.5666676	0.5368000	-1.5585246
18.5666676	0.5856000	-0.9387960
18.5666676	0.6344000	-1.0799227
18.5666676	0.6832000	-1.0615149
18.5666676	0.7320000	-1.0246992
18.5666676	0.7808000	-1.0124273
18.5666676	0.8296000	-0.8038058
18.5666676	0.8784000	-0.9756117
18.5666676	0.9272000	-0.8897090
18.5666676	0.9760000	-1.1842332
18.5666676	1.0247999	-1.1842332
18.5666676	1.0735999	-1.1596894
18.5666676	1.1223999	-1.3130875
18.5666676	1.1711999	-1.1351457
18.5666676	1.2200000	-1.1412816
18.5666676	1.2688000	-0.9817476
18.5666676	1.3176000	-0.9081168
18.5666676	1.3664000	-1.1780972
18.5666676	1.4152000	-1.4235344
18.5666676	1.4640000	-1.4235344
18.5666676	1.5128000	-1.0921946
18.5666676	1.5616000	-1.0983305
18.5666676	1.6104000	-1.2946796
18.5666676	1.6592000	-1.3130875
18.5666676	1.7079999	-1.3192234
18.5666676	1.7567999	-1.3008156
18.5666676	1.8055999	-1.2824078
18.5666676	1.8543999	-1.2824078
18.5666676	1.9031999	-1.2885437
18.5666676	1.9519999	-1.4112625
18.5666676	2.0007999	-1.2210488
18.5666676	2.0495999	-1.2210488
18.5666676	2.0983999	-1.3560390
18.5666676	2.1471999	-1.2026410
18.5666676	2.1959999	-1.2087770
18.5666676	2.2447999	-1.2824078
18.5666676	2.2935998	-1.2824078
18.5666676	2.3423998	-1.1780972
18.5666676	2.3912001	-1.1719613
18.5666676	2.4400001	-1.1658254
18.5666676	2.4888000	-1.2087770
18.5666676	2.5376000	-1.2026410
18.5666676	2.5864000	-1.1290097
18.5666676	2.6352000	-1.1903691
18.5666676	2.6840000	-1.0308352
18.5666676	2.7328000	-1.1658254
18.5666676	2.7816000	-1.1535535
18.5666676	2.8304000	-1.1474175
18.5666676	2.8792000	-1.0369711
18.5666676	2.9280000	-1.0308352
18.5666676	2.9768000	-0.9694757

18.5666676	3.0256000	-1.0308352
18.5666676	3.0743999	-1.1658254
18.5666676	3.1231999	-1.1596894
18.5666676	3.1719999	-1.1658254
18.5666676	3.2207999	-1.1719613
18.5666676	3.2695999	-1.1719613
18.5666676	3.3183999	-1.1719613
18.5666676	3.3671999	-1.1780972
18.5666676	3.4159999	-0.8590293
18.5666676	3.4647999	-0.8651652
18.5666676	3.5135999	-0.9387960
18.5666676	3.5623999	-1.0308352
18.5666676	3.6111999	-1.1719613
18.5666676	3.6599998	-1.1965051
18.5666676	3.7087998	-1.1596894
18.5666676	3.7575998	-1.1535535
18.5666676	3.8063998	-0.6933594
18.5666676	3.8551998	-0.5890489
18.5666676	3.9039998	-0.9449320
18.5666676	3.9527998	-0.9387960
18.5666676	4.0016003	-0.9326606
18.5666676	4.0503998	-1.2578645
18.5666676	4.0991998	-1.2394567
18.5666676	4.1479998	-1.1106019
18.5666676	4.1967998	-0.7179031
18.5666676	4.2455997	-0.9878836
18.5666676	4.2943997	-1.2210488
18.5666676	4.3431997	-0.9694757
18.5666676	4.3919997	-0.6197281
18.5666676	4.4407997	-0.6258640
18.5666676	4.4895997	-0.8283496
18.5666676	4.5383997	-1.0001554
18.5666676	4.5871997	-0.4233789
18.5666676	4.6359997	-0.8897090
18.5666676	4.6847997	-0.8774371
18.5666676	4.7336001	-0.7669902
18.5666676	4.7824001	-0.7669902
18.5666676	4.8312001	-0.7669902
18.5666676	4.8800001	-0.7547188
18.5666676	4.9288001	-0.7240391
18.5666676	4.9776001	-0.7301750
18.5666676	5.0264001	-0.6565437
18.5666676	5.0752001	-0.7056313
18.5666676	5.1240001	-0.7056313
18.5666676	5.1728001	-0.7424469
18.5666676	5.2216001	-0.9265246
18.5666676	5.2704000	-0.7547188
18.5666676	5.3192000	-0.7424469
18.5666676	5.3680000	-0.8590293
18.5666676	5.4168000	-0.8528934
18.5666676	5.4656000	-0.8528934
18.5666676	5.5144000	-0.8406215
18.5666676	5.5632000	-0.8283496
18.5666676	5.6120000	-0.8038058
18.5666676	5.6608000	-0.8038058
18.5666676	5.7096000	-0.8038058
18.5666676	5.7584000	-0.7915339

18.5666676	5.8072000	-0.8406215
18.5666676	5.8559999	-0.8406215
18.5666676	5.9047999	-0.8590293
18.5666676	5.9535999	-0.8651652
18.5666676	6.0023999	-0.8099418
18.5833340	0.2928000	-4.8044281
18.5833340	0.3416000	-4.0313015
18.5833340	0.3904000	-3.0986414
18.5833340	0.4392000	-3.0986414
18.5833340	0.4880000	-2.4911849
18.5833340	0.5368000	-2.4727771
18.5833340	0.5856000	-0.9326606
18.5833340	0.6344000	-0.9265246
18.5833340	0.6832000	-1.1167378
18.5833340	0.7320000	-1.1228738
18.5833340	0.7808000	-1.5892043
18.5833340	0.8296000	-1.1474175
18.5833340	0.8784000	-1.3253593
18.5833340	0.9272000	-1.5769324
18.5833340	0.9760000	-1.3253593
18.5833340	1.0247999	-0.9265246
18.5833340	1.0735999	-0.9326606
18.5833340	1.1223999	-1.0185633
18.5833340	1.1711999	-1.0921946
18.5833340	1.2200000	-1.0921946
18.5833340	1.2688000	-1.0921946
18.5833340	1.3176000	-1.1044660
18.5833340	1.3664000	-1.2824078
18.5833340	1.4152000	-1.2824078
18.5833340	1.4640000	-1.2026410
18.5833340	1.5128000	-1.0921946
18.5833340	1.5616000	-1.0983305
18.5833340	1.6104000	-1.2087770
18.5833340	1.6592000	-1.4358063
18.5833340	1.7079999	-1.2087770
18.5833340	1.7567999	-1.2762718
18.5833340	1.8055999	-1.1658254
18.5833340	1.8543999	-1.1474175
18.5833340	1.9031999	-1.2087770
18.5833340	1.9519999	-1.2026410
18.5833340	2.0007999	-1.1596894
18.5833340	2.0495999	-1.1596894
18.5833340	2.0983999	-1.1596894
18.5833340	2.1471999	-1.1535535
18.5833340	2.1959999	-1.1474175
18.5833340	2.2447999	-1.1228738
18.5833340	2.2935998	-1.1596894
18.5833340	2.3423998	-1.1780972
18.5833340	2.3912001	-1.2087770
18.5833340	2.4400001	-1.1351457
18.5833340	2.4888000	-1.1965051
18.5833340	2.5376000	-1.2026410
18.5833340	2.5864000	-1.2087770
18.5833340	2.6352000	-1.0062914
18.5833340	2.6840000	-1.1535535
18.5833340	2.7328000	-1.1535535
18.5833340	2.7816000	-0.9019809

18.5833340	2.8304000	-1.1044660
18.5833340	2.8792000	-1.0492430
18.5833340	2.9280000	-1.0492430
18.5833340	2.9768000	-1.0983305
18.5833340	3.0256000	-1.1106019
18.5833340	3.0743999	-1.1106019
18.5833340	3.1231999	-1.0676508
18.5833340	3.1719999	-1.0062914
18.5833340	3.2207999	-1.1596894
18.5833340	3.2695999	-1.0921946
18.5833340	3.3183999	-1.0921946
18.5833340	3.3671999	-1.0799227
18.5833340	3.4159999	-0.9756117
18.5833340	3.4647999	-1.0062914
18.5833340	3.5135999	-1.0983305
18.5833340	3.5623999	-0.9756117
18.5833340	3.6111999	-0.9756117
18.5833340	3.6599998	-0.9633398
18.5833340	3.7087998	-0.8897090
18.5833340	3.7575998	-0.8835731
18.5833340	3.8063998	-0.7853980
18.5833340	3.8551998	-0.9203887
18.5833340	3.9039998	-0.9387960
18.5833340	3.9527998	-0.6442719
18.5833340	4.0016003	-0.6810875
18.5833340	4.0503998	-0.6933594
18.5833340	4.0991998	-0.6933594
18.5833340	4.1479998	-0.6872234
18.5833340	4.1967998	-0.7669902
18.5833340	4.2455997	-0.7485828
18.5833340	4.2943997	-0.2270293
18.5833340	4.3431997	-0.2208934
18.5833340	4.3919997	-0.4970098
18.5833340	4.4407997	-0.6565437
18.5833340	4.4895997	-0.6626797
18.5833340	4.5383997	-0.8713012
18.5833340	4.5871997	-0.8774371
18.5833340	4.6359997	-1.0431070
18.5833340	4.6847997	-1.0492430
18.5833340	4.7336001	-0.9019809
18.5833340	4.7824001	-0.9019809
18.5833340	4.8312001	-0.9756117
18.5833340	4.8800001	-0.8713012
18.5833340	4.9288001	-0.8406215
18.5833340	4.9776001	-0.7915339
18.5833340	5.0264001	-0.7792621
18.5833340	5.0752001	-0.8038058
18.5833340	5.1240001	-0.7915339
18.5833340	5.1728001	-0.7853980
18.5833340	5.2216001	-1.0124273
18.5833340	5.2704000	-1.0124273
18.5833340	5.3192000	-1.0124273
18.5833340	5.3680000	-1.0553789
18.5833340	5.4168000	-0.9940195
18.5833340	5.4656000	613586.0000000
18.5833340	5.5144000	-0.7792621
18.5833340	5.5632000	-0.9019809

18.5833340	5.6120000	-0.8897090
18.5833340	5.6608000	-0.8528934
18.5833340	5.7096000	-0.8590293
18.5833340	5.7584000	-0.9326606
18.5833340	5.8072000	-0.9081168
18.5833340	5.8559999	-0.8958449
18.5833340	5.9047999	-0.9019809
18.5833340	5.9535999	-1.0124273
18.5833340	6.0023999	-0.8467574
18.6000004	0.2928000	-3.7920005
18.6000004	0.3416000	-3.1477287
18.6000004	0.3904000	-3.1354568
18.6000004	0.4392000	-2.2396121
18.6000004	0.4880000	-1.9696312
18.6000004	0.5368000	-2.3746023
18.6000004	0.5856000	-0.6994953
18.6000004	0.6344000	-1.3130875
18.6000004	0.6832000	-1.2333207
18.6000004	0.7320000	-1.7241945
18.6000004	0.7808000	-1.7057867
18.6000004	0.8296000	-1.3499031
18.6000004	0.8784000	-1.1596894
18.6000004	0.9272000	-1.0185633
18.6000004	0.9760000	-1.0308352
18.6000004	1.0247999	-1.1412816
18.6000004	1.0735999	-1.1290097
18.6000004	1.1223999	-1.0737867
18.6000004	1.1711999	-1.1658254
18.6000004	1.2200000	-1.3744469
18.6000004	1.2688000	-1.2701364
18.6000004	1.3176000	-1.4112625
18.6000004	1.3664000	-1.4051266
18.6000004	1.4152000	-1.2455926
18.6000004	1.4640000	-1.2455926
18.6000004	1.5128000	-1.0737867
18.6000004	1.5616000	-1.0860586
18.6000004	1.6104000	-1.3989906
18.6000004	1.6592000	-1.1842332
18.6000004	1.7079999	-1.4419422
18.6000004	1.7567999	-1.1596894
18.6000004	1.8055999	-1.0799227
18.6000004	1.8543999	-1.0921946
18.6000004	1.9031999	-1.1474175
18.6000004	1.9519999	-1.0676508
18.6000004	2.0007999	-1.0676508
18.6000004	2.0495999	-1.2394567
18.6000004	2.0983999	-1.1044660
18.6000004	2.1471999	-1.1106019
18.6000004	2.1959999	-1.1903691
18.6000004	2.2447999	-1.1290097
18.6000004	2.2935998	-1.1412816
18.6000004	2.3423998	-1.1412816
18.6000004	2.3912001	-0.9878836
18.6000004	2.4400001	-1.0860586
18.6000004	2.4888000	-1.0860586
18.6000004	2.5376000	-1.0921946
18.6000004	2.5864000	-0.9572039

18.6000004	2.6352000	-1.3069515
18.6000004	2.6840000	-1.1842332
18.6000004	2.7328000	-1.2026410
18.6000004	2.7816000	-1.2026410
18.6000004	2.8304000	-1.2762718
18.6000004	2.8792000	-1.1965051
18.6000004	2.9280000	-1.0369711
18.6000004	2.9768000	-1.0553789
18.6000004	3.0256000	-1.1535535
18.6000004	3.0743999	-1.1596894
18.6000004	3.1231999	-0.9633398
18.6000004	3.1719999	-1.0369711
18.6000004	3.2207999	-1.0553789
18.6000004	3.2695999	-1.0676508
18.6000004	3.3183999	-1.0431070
18.6000004	3.3671999	-0.8160777
18.6000004	3.4159999	-1.0860586
18.6000004	3.4647999	-1.0001554
18.6000004	3.5135999	-1.0001554
18.6000004	3.5623999	-0.9878836
18.6000004	3.6111999	-0.9694757
18.6000004	3.6599998	-0.9387960
18.6000004	3.7087998	-0.9817476
18.6000004	3.7575998	-1.0185633
18.6000004	3.8063998	-0.9019809
18.6000004	3.8551998	-0.9019809
18.6000004	3.9039998	-0.9203887
18.6000004	3.9527998	-0.6013203
18.6000004	4.0016003	-0.9019809
18.6000004	4.0503998	-0.6013203
18.6000004	4.0991998	-0.9817476
18.6000004	4.1479998	-0.9387960
18.6000004	4.1967998	-0.7976699
18.6000004	4.2455997	-0.7915339
18.6000004	4.2943997	-0.3926992
18.6000004	4.3431997	-0.6626797
18.6000004	4.3919997	-0.7608547
18.6000004	4.4407997	-0.7547188
18.6000004	4.4895997	-0.7608547
18.6000004	4.5383997	-0.7608547
18.6000004	4.5871997	-0.7669902
18.6000004	4.6359997	-0.7424469
18.6000004	4.6847997	-0.7301750
18.6000004	4.7336001	-0.5829129
18.6000004	4.7824001	-0.9019809
18.6000004	4.8312001	-0.8406215
18.6000004	4.8800001	-0.8406215
18.6000004	4.9288001	-0.7301750
18.6000004	4.9776001	-0.7915339
18.6000004	5.0264001	-0.8283496
18.6000004	5.0752001	-0.8283496
18.6000004	5.1240001	-0.7608547
18.6000004	5.1728001	-0.5276895
18.6000004	5.2216001	-0.6810875
18.6000004	5.2704000	-0.7669902
18.6000004	5.3192000	-0.9572039
18.6000004	5.3680000	-0.9940195

18.6000004	5.4168000	-0.9878836
18.6000004	5.4656000	-0.9694757
18.6000004	5.5144000	-0.9694757
18.6000004	5.5632000	613586.0000000
18.6000004	5.6120000	613586.0000000
18.6000004	5.6608000	613586.0000000
18.6000004	5.7096000	613586.0000000
18.6000004	5.7584000	613586.0000000
18.6000004	5.8072000	613586.0000000
18.6000004	5.8559999	613586.0000000
18.6000004	5.9047999	613586.0000000
18.6000004	5.9535999	613586.0000000
18.6000004	6.0023999	613586.0000000
18.6166668	0.2928000	-4.0374374
18.6166668	0.3416000	-3.9208550
18.6166668	0.3904000	-3.2949908
18.6166668	0.4392000	-3.2029519
18.6166668	0.4880000	-2.3377867
18.6166668	0.5368000	-1.7119226
18.6166668	0.5856000	-2.7795732
18.6166668	0.6344000	-2.7795732
18.6166668	0.6832000	-1.0001554
18.6166668	0.7320000	-0.9387960
18.6166668	0.7808000	-1.0553789
18.6166668	0.8296000	-0.4663301
18.6166668	0.8784000	-0.4663301
18.6166668	0.9272000	-0.7976699
18.6166668	0.9760000	-1.4419422
18.6166668	1.0247999	-1.1965051
18.6166668	1.0735999	-1.4910293
18.6166668	1.1223999	-1.3928547
18.6166668	1.1711999	-1.3867188
18.6166668	1.2200000	-1.3253593
18.6166668	1.2688000	-1.4296703
18.6166668	1.3176000	-1.4173985
18.6166668	1.3664000	-1.1596894
18.6166668	1.4152000	-1.1535535
18.6166668	1.4640000	-1.3253593
18.6166668	1.5128000	-1.3314953
18.6166668	1.5616000	-1.3499031
18.6166668	1.6104000	-1.2762718
18.6166668	1.6592000	-1.2762718
18.6166668	1.7079999	-1.2701364
18.6166668	1.7567999	-1.2701364
18.6166668	1.8055999	-1.3560390
18.6166668	1.8543999	-1.1596894
18.6166668	1.9031999	-1.0431070
18.6166668	1.9519999	-1.0369711
18.6166668	2.0007999	-1.0369711
18.6166668	2.0495999	-1.0860586
18.6166668	2.0983999	-1.0860586
18.6166668	2.1471999	-0.9756117
18.6166668	2.1959999	-0.8406215
18.6166668	2.2447999	-1.0983305
18.6166668	2.2935998	-0.9572039
18.6166668	2.3423998	-1.1903691
18.6166668	2.3912001	-1.1903691

18.6166668	2.4400001	-1.2026410
18.6166668	2.4888000	-1.0615149
18.6166668	2.5376000	-1.0185633
18.6166668	2.5864000	-1.0921946
18.6166668	2.6352000	-1.1965051
18.6166668	2.6840000	-1.1719613
18.6166668	2.7328000	-1.2210488
18.6166668	2.7816000	-1.2394567
18.6166668	2.8304000	-1.2578645
18.6166668	2.8792000	-1.2210488
18.6166668	2.9280000	-1.3130875
18.6166668	2.9768000	-1.3069515
18.6166668	3.0256000	-1.0369711
18.6166668	3.0743999	-1.3253593
18.6166668	3.1231999	-1.2333207
18.6166668	3.1719999	-1.1474175
18.6166668	3.2207999	-1.0983305
18.6166668	3.2695999	-0.8835731
18.6166668	3.3183999	-1.2210488
18.6166668	3.3671999	-1.2271848
18.6166668	3.4159999	-1.1842332
18.6166668	3.4647999	-1.1842332
18.6166668	3.5135999	-1.1842332
18.6166668	3.5623999	-1.1719613
18.6166668	3.6111999	-1.1596894
18.6166668	3.6599998	-1.0676508
18.6166668	3.7087998	-0.9203887
18.6166668	3.7575998	-0.9203887
18.6166668	3.8063998	-0.8528934
18.6166668	3.8551998	-0.8344855
18.6166668	3.9039998	-0.6688156
18.6166668	3.9527998	-0.6872234
18.6166668	4.0016003	-0.9449320
18.6166668	4.0503998	-0.6320000
18.6166668	4.0991998	-0.7424469
18.6166668	4.1479998	-0.6381359
18.6166668	4.1967998	-0.6442719
18.6166668	4.2455997	-0.7608547
18.6166668	4.2943997	-0.7731261
18.6166668	4.3431997	-0.4295144
18.6166668	4.3919997	-0.7179031
18.6166668	4.4407997	-0.7363110
18.6166668	4.4895997	-0.7363110
18.6166668	4.5383997	-0.5215535
18.6166668	4.5871997	-0.5215535
18.6166668	4.6359997	-0.6626797
18.6166668	4.6847997	-0.6626797
18.6166668	4.7336001	-0.9633398
18.6166668	4.7824001	-0.9633398
18.6166668	4.8312001	-0.9572039
18.6166668	4.8800001	-0.9510679
18.6166668	4.9288001	-0.8222136
18.6166668	4.9776001	-1.0921946
18.6166668	5.0264001	-0.9817476
18.6166668	5.0752001	-1.2149129
18.6166668	5.1240001	-0.9817476
18.6166668	5.1728001	-1.0246992

18.6166668	5.2216001	613586.0000000
18.6166668	5.2704000	-0.9203887
18.6166668	5.3192000	-0.7976699
18.6166668	5.3680000	-0.7976699
18.6166668	5.4168000	-0.8099418
18.6166668	5.4656000	-0.8467574
18.6166668	5.5144000	-0.9940195
18.6166668	5.5632000	-0.9694757
18.6166668	5.6120000	613586.0000000
18.6166668	5.6608000	613586.0000000
18.6166668	5.7096000	-1.0185633
18.6166668	5.7584000	613586.0000000
18.6166668	5.8072000	613586.0000000
18.6166668	5.8559999	613586.0000000
18.6166668	5.9047999	613586.0000000
18.6166668	5.9535999	613586.0000000
18.6166668	6.0023999	613586.0000000
18.6333332	0.2928000	-3.5956511
18.6333332	0.3416000	-3.5956511
18.6333332	0.3904000	-3.6140587
18.6333332	0.4392000	-3.3624859
18.6333332	0.4880000	-3.3624859
18.6333332	0.5368000	-2.7857091
18.6333332	0.5856000	-1.3314953
18.6333332	0.6344000	-1.4726214
18.6333332	0.6832000	-1.4664855
18.6333332	0.7320000	-1.3130875
18.6333332	0.7808000	-1.4971652
18.6333332	0.8296000	-1.6751070
18.6333332	0.8784000	-1.3744469
18.6333332	0.9272000	-0.8406215
18.6333332	0.9760000	-1.2640004
18.6333332	1.0247999	-1.0001554
18.6333332	1.0735999	-1.0062914
18.6333332	1.1223999	-1.1474175
18.6333332	1.1711999	-1.3744469
18.6333332	1.2200000	-1.0369711
18.6333332	1.2688000	-1.3867188
18.6333332	1.3176000	-1.3376312
18.6333332	1.3664000	-1.3499031
18.6333332	1.4152000	-1.3499031
18.6333332	1.4640000	-1.2271848
18.6333332	1.5128000	-1.4726214
18.6333332	1.5616000	-1.3130875
18.6333332	1.6104000	-1.2701364
18.6333332	1.6592000	-1.4358063
18.6333332	1.7079999	-1.1658254
18.6333332	1.7567999	-1.3314953
18.6333332	1.8055999	-1.3744469
18.6333332	1.8543999	-1.1596894
18.6333332	1.9031999	-1.1290097
18.6333332	1.9519999	-1.1351457
18.6333332	2.0007999	-1.1351457
18.6333332	2.0495999	-1.1228738
18.6333332	2.0983999	-1.0185633
18.6333332	2.1471999	-1.0062914
18.6333332	2.1959999	-1.0124273

18.6333332	2.2447999	-1.1903691
18.6333332	2.2935998	-1.1965051
18.6333332	2.3423998	-1.1290097
18.6333332	2.3912001	-1.1228738
18.6333332	2.4400001	-1.1044660
18.6333332	2.4888000	-1.0983305
18.6333332	2.5376000	-1.1351457
18.6333332	2.5864000	-1.1474175
18.6333332	2.6352000	-1.0860586
18.6333332	2.6840000	-1.0860586
18.6333332	2.7328000	-1.1535535
18.6333332	2.7816000	-0.9756117
18.6333332	2.8304000	-1.0369711
18.6333332	2.8792000	-1.0369711
18.6333332	2.9280000	-1.1535535
18.6333332	2.9768000	-1.0983305
18.6333332	3.0256000	-1.0492430
18.6333332	3.0743999	-0.9265246
18.6333332	3.1231999	-1.1474175
18.6333332	3.1719999	-1.1228738
18.6333332	3.2207999	-1.1351457
18.6333332	3.2695999	-1.0921946
18.6333332	3.3183999	-1.0860586
18.6333332	3.3671999	-1.0246992
18.6333332	3.4159999	-1.0308352
18.6333332	3.4647999	-1.0369711
18.6333332	3.5135999	-0.9817476
18.6333332	3.5623999	-0.9878836
18.6333332	3.6111999	-0.8467574
18.6333332	3.6599998	-0.8897090
18.6333332	3.7087998	-0.8897090
18.6333332	3.7575998	-0.6749516
18.6333332	3.8063998	-0.6872234
18.6333332	3.8551998	-0.7056313
18.6333332	3.9039998	-0.9265246
18.6333332	3.9527998	-0.9510679
18.6333332	4.0016003	-0.9633398
18.6333332	4.0503998	-0.6504078
18.6333332	4.0991998	-0.6565437
18.6333332	4.1479998	-0.6749516
18.6333332	4.1967998	-0.8835731
18.6333332	4.2455997	-0.7976699
18.6333332	4.2943997	-0.5522332
18.6333332	4.3431997	-0.6810875
18.6333332	4.3919997	-0.6135921
18.6333332	4.4407997	-0.9694757
18.6333332	4.4895997	-0.9572039
18.6333332	4.5383997	-0.9326606
18.6333332	4.5871997	-1.1106019
18.6333332	4.6359997	-0.9817476
18.6333332	4.6847997	-0.9940195
18.6333332	4.7336001	-0.9817476
18.6333332	4.7824001	-0.9878836
18.6333332	4.8312001	-0.8774371
18.6333332	4.8800001	-0.9081168
18.6333332	4.9288001	-0.9449320
18.6333332	4.9776001	-0.8958449

18.6333332	5.0264001	-0.8897090
18.6333332	5.0752001	-0.8835731
18.6333332	5.1240001	-0.9633398
18.6333332	5.1728001	-0.8222136
18.6333332	5.2216001	-0.8038058
18.6333332	5.2704000	-0.7731261
18.6333332	5.3192000	-0.7608547
18.6333332	5.3680000	-0.9878836
18.6333332	5.4168000	-0.9694757
18.6333332	5.4656000	-0.9817476
18.6333332	5.5144000	-1.0062914
18.6333332	5.5632000	-0.7915339
18.6333332	5.6120000	613586.0000000
18.6333332	5.6608000	613586.0000000
18.6333332	5.7096000	613586.0000000
18.6333332	5.7584000	613586.0000000
18.6333332	5.8072000	613586.0000000
18.6333332	5.8559999	613586.0000000
18.6333332	5.9047999	613586.0000000
18.6333332	5.9535999	613586.0000000
18.6333332	6.0023999	613586.0000000
18.6499996	0.2928000	-3.9515345
18.6499996	0.3416000	-3.6263306
18.6499996	0.3904000	-3.1170490
18.6499996	0.4392000	-2.5157285
18.6499996	0.4880000	-2.5157285
18.6499996	0.5368000	-2.3193791
18.6499996	0.5856000	-1.8407769
18.6499996	0.6344000	-1.8346410
18.6499996	0.6832000	-1.6566992
18.6499996	0.7320000	-1.3314953
18.6499996	0.7808000	-1.2087770
18.6499996	0.8296000	-1.1903691
18.6499996	0.8784000	-1.1965051
18.6499996	0.9272000	-0.9940195
18.6499996	0.9760000	-1.0983305
18.6499996	1.0247999	-1.2762718
18.6499996	1.0735999	-1.1903691
18.6499996	1.1223999	-1.1842332
18.6499996	1.1711999	-1.2333207
18.6499996	1.2200000	-1.3683109
18.6499996	1.2688000	-1.2640004
18.6499996	1.3176000	-1.2578645
18.6499996	1.3664000	-1.3621750
18.6499996	1.4152000	-1.3499031
18.6499996	1.4640000	-1.2701364
18.6499996	1.5128000	-1.3130875
18.6499996	1.5616000	-1.0983305
18.6499996	1.6104000	-1.2333207
18.6499996	1.6592000	-1.3008156
18.6499996	1.7079999	-1.0983305
18.6499996	1.7567999	-1.0921946
18.6499996	1.8055999	-1.3069515
18.6499996	1.8543999	-1.1106019
18.6499996	1.9031999	-1.0308352
18.6499996	1.9519999	-1.1719613
18.6499996	2.0007999	-1.1658254

18.6499996	2.0495999	-1.1658254
18.6499996	2.0983999	-1.0124273
18.6499996	2.1471999	-1.0062914
18.6499996	2.1959999	-1.0062914
18.6499996	2.2447999	-1.0062914
18.6499996	2.2935998	-1.0124273
18.6499996	2.3423998	-1.0246992
18.6499996	2.3912001	-1.0431070
18.6499996	2.4400001	-1.0062914
18.6499996	2.4888000	-1.0921946
18.6499996	2.5376000	-0.9878836
18.6499996	2.5864000	-0.9878836
18.6499996	2.6352000	-0.9265246
18.6499996	2.6840000	-0.9081168
18.6499996	2.7328000	-1.0921946
18.6499996	2.7816000	-1.0185633
18.6499996	2.8304000	-1.0799227
18.6499996	2.8792000	-1.0860586
18.6499996	2.9280000	-1.0676508
18.6499996	2.9768000	-0.7485828
18.6499996	3.0256000	-0.9572039
18.6499996	3.0743999	-1.0246992
18.6499996	3.1231999	-1.0246992
18.6499996	3.1719999	-1.0492430
18.6499996	3.2207999	-0.8897090
18.6499996	3.2695999	-0.8528934
18.6499996	3.3183999	-0.9019809
18.6499996	3.3671999	-1.0001554
18.6499996	3.4159999	-0.9940195
18.6499996	3.4647999	-0.9940195
18.6499996	3.5135999	-0.9142528
18.6499996	3.5623999	-0.9142528
18.6499996	3.6111999	-0.8651652
18.6499996	3.6599998	-0.8651652
18.6499996	3.7087998	-0.8467574
18.6499996	3.7575998	-0.8467574
18.6499996	3.8063998	-0.7853980
18.6499996	3.8551998	-0.7853980
18.6499996	3.9039998	-0.9878836
18.6499996	3.9527998	-0.9940195
18.6499996	4.0016003	-1.0062914
18.6499996	4.0503998	-0.6013203
18.6499996	4.0991998	-0.8222136
18.6499996	4.1479998	-0.8038058
18.6499996	4.1967998	-0.7240391
18.6499996	4.2455997	-0.6810875
18.6499996	4.2943997	-0.3742914
18.6499996	4.3431997	-0.6565437
18.6499996	4.3919997	-0.7363110
18.6499996	4.4407997	-0.4786019
18.6499996	4.4895997	-0.8099418
18.6499996	4.5383997	-0.8160777
18.6499996	4.5871997	-0.7179031
18.6499996	4.6359997	-0.7117672
18.6499996	4.6847997	-0.7301750
18.6499996	4.7336001	-0.7976699
18.6499996	4.7824001	-0.6197281

18.6499996	4.8312001	-0.8467574
18.6499996	4.8800001	-0.8283496
18.6499996	4.9288001	-0.8406215
18.6499996	4.9776001	-0.8651652
18.6499996	5.0264001	-0.9878836
18.6499996	5.0752001	-1.0737867
18.6499996	5.1240001	-1.0860586
18.6499996	5.1728001	-1.0799227
18.6499996	5.2216001	-0.9572039
18.6499996	5.2704000	-1.1290097
18.6499996	5.3192000	-1.0124273
18.6499996	5.3680000	-1.0185633
18.6499996	5.4168000	-1.0185633
18.6499996	5.4656000	613586.0000000
18.6499996	5.5144000	613586.0000000
18.6499996	5.5632000	613586.0000000
18.6499996	5.6120000	613586.0000000
18.6499996	5.6608000	613586.0000000
18.6499996	5.7096000	613586.0000000
18.6499996	5.7584000	613586.0000000
18.6499996	5.8072000	613586.0000000
18.6499996	5.8559999	613586.0000000
18.6499996	5.9047999	613586.0000000
18.6499996	5.9535999	613586.0000000
18.6499996	6.0023999	613586.0000000
18.6666660	0.2928000	-4.6203504
18.6666660	0.3416000	-3.8533599
18.6666660	0.3904000	-3.7613211
18.6666660	0.4392000	-3.0679617
18.6666660	0.4880000	-2.6691267
18.6666660	0.5368000	-2.0493984
18.6666660	0.5856000	-1.9941750
18.6666660	0.6344000	-1.9880390
18.6666660	0.6832000	-1.2946796
18.6666660	0.7320000	-0.9510679
18.6666660	0.7808000	-1.1044660
18.6666660	0.8296000	-1.0983305
18.6666660	0.8784000	-1.4051266
18.6666660	0.9272000	-1.3989906
18.6666660	0.9760000	-1.3744469
18.6666660	1.0247999	-1.3744469
18.6666660	1.0735999	-1.3192234
18.6666660	1.1223999	-1.3314953
18.6666660	1.1711999	-1.3744469
18.6666660	1.2200000	-1.3989906
18.6666660	1.2688000	-1.2026410
18.6666660	1.3176000	-1.2149129
18.6666660	1.3664000	-1.0921946
18.6666660	1.4152000	-1.0921946
18.6666660	1.4640000	-1.1780972
18.6666660	1.5128000	-1.1596894
18.6666660	1.5616000	-1.0308352
18.6666660	1.6104000	-1.1596894
18.6666660	1.6592000	-0.9203887
18.6666660	1.7079999	-0.7915339
18.6666660	1.7567999	-0.7731261
18.6666660	1.8055999	-0.9081168

18.6666660	1.8543999	-0.8897090
18.6666660	1.9031999	-1.0001554
18.6666660	1.9519999	-1.0062914
18.6666660	2.0007999	-0.8774371
18.6666660	2.0495999	-0.8774371
18.6666660	2.0983999	-1.0676508
18.6666660	2.1471999	-0.9265246
18.6666660	2.1959999	-0.8222136
18.6666660	2.2447999	-1.1290097
18.6666660	2.2935998	-1.1351457
18.6666660	2.3423998	-0.9756117
18.6666660	2.3912001	-0.9817476
18.6666660	2.4400001	-1.0185633
18.6666660	2.4888000	-0.9449320
18.6666660	2.5376000	-0.9449320
18.6666660	2.5864000	-0.8467574
18.6666660	2.6352000	-0.9940195
18.6666660	2.6840000	-0.9633398
18.6666660	2.7328000	-0.9633398
18.6666660	2.7816000	-0.9449320
18.6666660	2.8304000	-0.9510679
18.6666660	2.8792000	-1.0185633
18.6666660	2.9280000	-1.0185633
18.6666660	2.9768000	-1.0185633
18.6666660	3.0256000	-0.9081168
18.6666660	3.0743999	-0.6320000
18.6666660	3.1231999	-0.7485828
18.6666660	3.1719999	-1.0246992
18.6666660	3.2207999	-0.7424469
18.6666660	3.2695999	-0.7424469
18.6666660	3.3183999	-0.8713012
18.6666660	3.3671999	-0.9142528
18.6666660	3.4159999	-0.9572039
18.6666660	3.4647999	-0.9572039
18.6666660	3.5135999	-0.7424469
18.6666660	3.5623999	-0.7424469
18.6666660	3.6111999	-0.6135921
18.6666660	3.6599998	-0.6258640
18.6666660	3.7087998	-0.6258640
18.6666660	3.7575998	-0.6810875
18.6666660	3.8063998	-0.6504078
18.6666660	3.8551998	-0.6626797
18.6666660	3.9039998	-0.6626797
18.6666660	3.9527998	-0.6626797
18.6666660	4.0016003	-0.6626797
18.6666660	4.0503998	-0.7485828
18.6666660	4.0991998	-0.7485828
18.6666660	4.1479998	-0.8958449
18.6666660	4.1967998	-0.8099418
18.6666660	4.2455997	-0.8038058
18.6666660	4.2943997	-0.7608547
18.6666660	4.3431997	-0.8528934
18.6666660	4.3919997	-0.8713012
18.6666660	4.4407997	-0.8467574
18.6666660	4.4895997	-0.8406215
18.6666660	4.5383997	-0.8099418
18.6666660	4.5871997	-0.8038058

18.6666660	4.6359997	-0.5154176
18.6666660	4.6847997	613586.0000000
18.6666660	4.7336001	-0.3988352
18.6666660	4.7824001	-0.3926992
18.6666660	4.8312001	-0.9203887
18.6666660	4.8800001	-0.8467574
18.6666660	4.9288001	-0.8406215
18.6666660	4.9776001	-0.8344855
18.6666660	5.0264001	-0.8590293
18.6666660	5.0752001	-0.7547188
18.6666660	5.1240001	-0.7485828
18.6666660	5.1728001	-0.8713012
18.6666660	5.2216001	-0.7915339
18.6666660	5.2704000	-0.9510679
18.6666660	5.3192000	-1.0124273
18.6666660	5.3680000	-1.0124273
18.6666660	5.4168000	-0.9081168
18.6666660	5.4656000	613586.0000000
18.6666660	5.5144000	613586.0000000
18.6666660	5.5632000	613586.0000000
18.6666660	5.6120000	613586.0000000
18.6666660	5.6608000	613586.0000000
18.6666660	5.7096000	-0.9081168
18.6666660	5.7584000	-0.9081168
18.6666660	5.8072000	-0.7363110
18.6666660	5.8559999	-0.7363110
18.6666660	5.9047999	613586.0000000
18.6666660	5.9535999	613586.0000000
18.6666660	6.0023999	613586.0000000
18.6833324	0.2928000	-4.0313015
18.6833324	0.3416000	-4.0313015
18.6833324	0.3904000	-4.0313015
18.6833324	0.4392000	-3.0434179
18.6833324	0.4880000	-3.0556898
18.6833324	0.5368000	-2.3255150
18.6833324	0.5856000	-2.3255150
18.6833324	0.6344000	-1.3744469
18.6833324	0.6832000	-1.4235344
18.6833324	0.7320000	-1.4419422
18.6833324	0.7808000	-1.4419422
18.6833324	0.8296000	-1.2946796
18.6833324	0.8784000	-1.3376312
18.6833324	0.9272000	-1.4235344
18.6833324	0.9760000	-0.7915339
18.6833324	1.0247999	-1.0185633
18.6833324	1.0735999	-0.7853980
18.6833324	1.1223999	-1.2640004
18.6833324	1.1711999	-1.3253593
18.6833324	1.2200000	-1.3192234
18.6833324	1.2688000	-1.3130875
18.6833324	1.3176000	-1.2149129
18.6833324	1.3664000	-1.1903691
18.6833324	1.4152000	-1.2885437
18.6833324	1.4640000	-1.2762718
18.6833324	1.5128000	-1.0431070
18.6833324	1.5616000	-0.9633398
18.6833324	1.6104000	-0.9817476

18.6833324	1.6592000	-0.9694757
18.6833324	1.7079999	-0.7363110
18.6833324	1.7567999	-0.8713012
18.6833324	1.8055999	-1.1044660
18.6833324	1.8543999	-1.1106019
18.6833324	1.9031999	-1.1167378
18.6833324	1.9519999	-0.9756117
18.6833324	2.0007999	-1.2455926
18.6833324	2.0495999	-1.2455926
18.6833324	2.0983999	-1.1228738
18.6833324	2.1471999	-1.1228738
18.6833324	2.1959999	-1.1044660
18.6833324	2.2447999	-1.1842332
18.6833324	2.2935998	-1.1780972
18.6833324	2.3423998	-1.0799227
18.6833324	2.3912001	-1.0553789
18.6833324	2.4400001	-1.0001554
18.6833324	2.4888000	-1.0246992
18.6833324	2.5376000	-0.8590293
18.6833324	2.5864000	-1.0492430
18.6833324	2.6352000	-0.8835731
18.6833324	2.6840000	-0.8713012
18.6833324	2.7328000	-0.9756117
18.6833324	2.7816000	-1.0369711
18.6833324	2.8304000	-1.0369711
18.6833324	2.8792000	-0.7792621
18.6833324	2.9280000	-0.7547188
18.6833324	2.9768000	-0.7485828
18.6833324	3.0256000	-1.0062914
18.6833324	3.0743999	-0.9940195
18.6833324	3.1231999	-0.9572039
18.6833324	3.1719999	-0.9572039
18.6833324	3.2207999	-0.9694757
18.6833324	3.2695999	-0.6565437
18.6833324	3.3183999	-0.9387960
18.6833324	3.3671999	-0.8651652
18.6833324	3.4159999	-0.6565437
18.6833324	3.4647999	-0.7179031
18.6833324	3.5135999	-0.5460973
18.6833324	3.5623999	-0.6442719
18.6833324	3.6111999	-0.6442719
18.6833324	3.6599998	-0.6504078
18.6833324	3.7087998	-0.5890489
18.6833324	3.7575998	-0.5890489
18.6833324	3.8063998	-0.5951843
18.6833324	3.8551998	-0.7179031
18.6833324	3.9039998	-0.6994953
18.6833324	3.9527998	-0.7179031
18.6833324	4.0016003	-0.7424469
18.6833324	4.0503998	-0.8344855
18.6833324	4.0991998	-0.8344855
18.6833324	4.1479998	-0.4479222
18.6833324	4.1967998	-0.4540582
18.6833324	4.2455997	-0.3436117
18.6833324	4.2943997	-0.6320000
18.6833324	4.3431997	-0.1902137
18.6833324	4.3919997	-0.3374758

18.6833324	4.4407997	-0.3681555
18.6833324	4.4895997	-0.3558836
18.6833324	4.5383997	-0.6626797
18.6833324	4.5871997	613586.0000000
18.6833324	4.6359997	0.0245438
18.6833324	4.6847997	-0.1963496
18.6833324	4.7336001	-0.1963496
18.6833324	4.7824001	-0.7117672
18.6833324	4.8312001	-0.7056313
18.6833324	4.8800001	-0.6933594
18.6833324	4.9288001	-0.8467574
18.6833324	4.9776001	-0.8467574
18.6833324	5.0264001	-0.7547188
18.6833324	5.0752001	-0.7240391
18.6833324	5.1240001	-0.7117672
18.6833324	5.1728001	613586.0000000
18.6833324	5.2216001	613586.0000000
18.6833324	5.2704000	613586.0000000
18.6833324	5.3192000	613586.0000000
18.6833324	5.3680000	613586.0000000
18.6833324	5.4168000	613586.0000000
18.6833324	5.4656000	613586.0000000
18.6833324	5.5144000	613586.0000000
18.6833324	5.5632000	613586.0000000
18.6833324	5.6120000	613586.0000000
18.6833324	5.6608000	613586.0000000
18.6833324	5.7096000	613586.0000000
18.6833324	5.7584000	613586.0000000
18.6833324	5.8072000	613586.0000000
18.6833324	5.8559999	613586.0000000
18.6833324	5.9047999	613586.0000000
18.6833324	5.9535999	613586.0000000
18.6833324	6.0023999	613586.0000000
18.7000008	0.2928000	-3.9822142
18.7000008	0.3416000	-3.4729326
18.7000008	0.3904000	-3.0618258
18.7000008	0.4392000	-3.0740976
18.7000008	0.4880000	-2.7120781
18.7000008	0.5368000	-2.4911849
18.7000008	0.5856000	-2.1721168
18.7000008	0.6344000	-1.7487383
18.7000008	0.6832000	-1.4787574
18.7000008	0.7320000	-1.1228738
18.7000008	0.7808000	-0.8713012
18.7000008	0.8296000	-0.7608547
18.7000008	0.8784000	-0.7976699
18.7000008	0.9272000	-0.9142528
18.7000008	0.9760000	-0.9142528
18.7000008	1.0247999	-1.2149129
18.7000008	1.0735999	-1.2701364
18.7000008	1.1223999	-1.2701364
18.7000008	1.1711999	-1.1842332
18.7000008	1.2200000	-1.1780972
18.7000008	1.2688000	-1.1658254
18.7000008	1.3176000	-1.2026410
18.7000008	1.3664000	-1.0921946
18.7000008	1.4152000	-1.0921946

18.7000008	1.4640000	-1.0983305
18.7000008	1.5128000	-1.0983305
18.7000008	1.5616000	-1.0921946
18.7000008	1.6104000	-0.9265246
18.7000008	1.6592000	-0.9510679
18.7000008	1.7079999	-0.8774371
18.7000008	1.7567999	-0.8835731
18.7000008	1.8055999	-0.8958449
18.7000008	1.8543999	-1.1351457
18.7000008	1.9031999	-1.0369711
18.7000008	1.9519999	-1.0492430
18.7000008	2.0007999	-1.0615149
18.7000008	2.0495999	-1.2762718
18.7000008	2.0983999	-1.3437672
18.7000008	2.1471999	-1.0553789
18.7000008	2.1959999	-1.0553789
18.7000008	2.2447999	-0.8283496
18.7000008	2.2935998	-0.8651652
18.7000008	2.3423998	-0.8590293
18.7000008	2.3912001	-0.9572039
18.7000008	2.4400001	-0.8467574
18.7000008	2.4888000	-0.9203887
18.7000008	2.5376000	-0.9265246
18.7000008	2.5864000	-0.9265246
18.7000008	2.6352000	-1.0001554
18.7000008	2.6840000	-1.0062914
18.7000008	2.7328000	-0.8528934
18.7000008	2.7816000	-0.8651652
18.7000008	2.8304000	-0.8774371
18.7000008	2.8792000	-1.0308352
18.7000008	2.9280000	-0.7853980
18.7000008	2.9768000	-0.8038058
18.7000008	3.0256000	-0.7301750
18.7000008	3.0743999	-1.0369711
18.7000008	3.1231999	-0.7792621
18.7000008	3.1719999	-0.7853980
18.7000008	3.2207999	-0.8344855
18.7000008	3.2695999	-0.8528934
18.7000008	3.3183999	-0.8774371
18.7000008	3.3671999	-0.7240391
18.7000008	3.4159999	-0.6074562
18.7000008	3.4647999	-0.8222136
18.7000008	3.5135999	-0.6749516
18.7000008	3.5623999	-0.6749516
18.7000008	3.6111999	-0.6872234
18.7000008	3.6599998	-0.6933594
18.7000008	3.7087998	-0.7976699
18.7000008	3.7575998	-0.8835731
18.7000008	3.8063998	-0.8897090
18.7000008	3.8551998	-0.7179031
18.7000008	3.9039998	-0.7915339
18.7000008	3.9527998	-0.7976699
18.7000008	4.0016003	-0.8222136
18.7000008	4.0503998	-0.4233789
18.7000008	4.0991998	-0.5276895
18.7000008	4.1479998	-0.4663301
18.7000008	4.1967998	-0.6688156

18.7000008	4.2455997	-0.5890489
18.7000008	4.2943997	-0.5890489
18.7000008	4.3431997	-0.6013203
18.7000008	4.3919997	-0.6135921
18.7000008	4.4407997	-0.6135921
18.7000008	4.4895997	-0.6872234
18.7000008	4.5383997	-0.8467574
18.7000008	4.5871997	-0.8406215
18.7000008	4.6359997	-0.8528934
18.7000008	4.6847997	-0.9081168
18.7000008	4.7336001	-0.9081168
18.7000008	4.7824001	-0.8835731
18.7000008	4.8312001	-0.8406215
18.7000008	4.8800001	-0.8406215
18.7000008	4.9288001	-0.8406215
18.7000008	4.9776001	-0.8344855
18.7000008	5.0264001	-0.8467574
18.7000008	5.0752001	613586.0000000
18.7000008	5.1240001	613586.0000000
18.7000008	5.1728001	613586.0000000
18.7000008	5.2216001	613586.0000000
18.7000008	5.2704000	613586.0000000
18.7000008	5.3192000	613586.0000000
18.7000008	5.3680000	613586.0000000
18.7000008	5.4168000	613586.0000000
18.7000008	5.4656000	613586.0000000
18.7000008	5.5144000	613586.0000000
18.7000008	5.5632000	613586.0000000
18.7000008	5.6120000	613586.0000000
18.7000008	5.6608000	613586.0000000
18.7000008	5.7096000	-0.8406215
18.7000008	5.7584000	613586.0000000
18.7000008	5.8072000	613586.0000000
18.7000008	5.8559999	613586.0000000
18.7000008	5.9047999	613586.0000000
18.7000008	5.9535999	613586.0000000
18.7000008	6.0023999	613586.0000000
18.7166672	0.2928000	-3.6079230
18.7166672	0.3416000	-3.3747578
18.7166672	0.3904000	-3.1047771
18.7166672	0.4392000	-2.7979810
18.7166672	0.4880000	-2.6813984
18.7166672	0.5368000	-2.6813984
18.7166672	0.5856000	-2.6875343
18.7166672	0.6344000	-2.1782527
18.7166672	0.6832000	-2.1721168
18.7166672	0.7320000	-2.1721168
18.7166672	0.7808000	-1.1842332
18.7166672	0.8296000	-1.1228738
18.7166672	0.8784000	-1.6873789
18.7166672	0.9272000	-2.0739422
18.7166672	0.9760000	-2.0739422
18.7166672	1.0247999	-1.6873789
18.7166672	1.0735999	-0.9817476
18.7166672	1.1223999	-0.9817476
18.7166672	1.1711999	-1.0553789
18.7166672	1.2200000	-1.2333207

18.7166672	1.2688000	-1.2333207
18.7166672	1.3176000	-1.0921946
18.7166672	1.3664000	-1.2026410
18.7166672	1.4152000	-1.2640004
18.7166672	1.4640000	-1.1412816
18.7166672	1.5128000	-0.9449320
18.7166672	1.5616000	-0.9633398
18.7166672	1.6104000	-0.9633398
18.7166672	1.6592000	-0.9633398
18.7166672	1.7079999	-0.9878836
18.7166672	1.7567999	-1.0369711
18.7166672	1.8055999	-1.2333207
18.7166672	1.8543999	-1.2394567
18.7166672	1.9031999	-1.1474175
18.7166672	1.9519999	-0.9510679
18.7166672	2.0007999	-0.9449320
18.7166672	2.0495999	-1.2333207
18.7166672	2.0983999	-1.2517285
18.7166672	2.1471999	-1.2517285
18.7166672	2.1959999	-1.2455926
18.7166672	2.2447999	-1.2824078
18.7166672	2.2935998	-1.0553789
18.7166672	2.3423998	-0.9326606
18.7166672	2.3912001	-1.0983305
18.7166672	2.4400001	-1.1596894
18.7166672	2.4888000	-1.1351457
18.7166672	2.5376000	-1.0553789
18.7166672	2.5864000	-1.0615149
18.7166672	2.6352000	-0.9142528
18.7166672	2.6840000	-0.9203887
18.7166672	2.7328000	-0.9081168
18.7166672	2.7816000	-0.9081168
18.7166672	2.8304000	-0.8651652
18.7166672	2.8792000	-0.9449320
18.7166672	2.9280000	-0.9326606
18.7166672	2.9768000	-1.0246992
18.7166672	3.0256000	-1.2701364
18.7166672	3.0743999	-0.9572039
18.7166672	3.1231999	-0.9387960
18.7166672	3.1719999	-0.9572039
18.7166672	3.2207999	-1.0431070
18.7166672	3.2695999	-1.0369711
18.7166672	3.3183999	-0.6810875
18.7166672	3.3671999	-0.9878836
18.7166672	3.4159999	-0.9510679
18.7166672	3.4647999	-0.8835731
18.7166672	3.5135999	-0.8774371
18.7166672	3.5623999	-0.8774371
18.7166672	3.6111999	-0.9326606
18.7166672	3.6599998	-0.9265246
18.7166672	3.7087998	-0.9203887
18.7166672	3.7575998	-0.9142528
18.7166672	3.8063998	-0.7669902
18.7166672	3.8551998	-0.8160777
18.7166672	3.9039998	-0.8038058
18.7166672	3.9527998	-0.7485828
18.7166672	4.0016003	-0.8467574

18.7166672	4.0503998	-0.8038058
18.7166672	4.0991998	-0.8038058
18.7166672	4.1479998	-0.7485828
18.7166672	4.1967998	-0.5399613
18.7166672	4.2455997	-0.3374758
18.7166672	4.2943997	-0.3865633
18.7166672	4.3431997	-0.3804274
18.7166672	4.3919997	-0.3742914
18.7166672	4.4407997	-0.9940195
18.7166672	4.4895997	-0.7424469
18.7166672	4.5383997	-0.7363110
18.7166672	4.5871997	-0.9940195
18.7166672	4.6359997	-0.9940195
18.7166672	4.6847997	-1.0553789
18.7166672	4.7336001	-1.0492430
18.7166672	4.7824001	-0.9694757
18.7166672	4.8312001	-0.8344855
18.7166672	4.8800001	-0.9449320
18.7166672	4.9288001	-0.9510679
18.7166672	4.9776001	-1.0676508
18.7166672	5.0264001	-1.0860586
18.7166672	5.0752001	-1.0124273
18.7166672	5.1240001	-1.0921946
18.7166672	5.1728001	-0.9387960
18.7166672	5.2216001	-0.9449320
18.7166672	5.2704000	-0.9387960
18.7166672	5.3192000	613586.0000000
18.7166672	5.3680000	613586.0000000
18.7166672	5.4168000	613586.0000000
18.7166672	5.4656000	613586.0000000
18.7166672	5.5144000	613586.0000000
18.7166672	5.5632000	613586.0000000
18.7166672	5.6120000	613586.0000000
18.7166672	5.6608000	613586.0000000
18.7166672	5.7096000	613586.0000000
18.7166672	5.7584000	613586.0000000
18.7166672	5.8072000	613586.0000000
18.7166672	5.8559999	613586.0000000
18.7166672	5.9047999	613586.0000000
18.7166672	5.9535999	613586.0000000
18.7166672	6.0023999	613586.0000000
18.7333336	0.2928000	-4.1049328
18.7333336	0.3416000	-3.7981365
18.7333336	0.3904000	-3.7858646
18.7333336	0.4392000	-3.3379424
18.7333336	0.4880000	-2.8409326
18.7333336	0.5368000	-1.8407769
18.7333336	0.5856000	-1.7610102
18.7333336	0.6344000	-1.3499031
18.7333336	0.6832000	-0.8283496
18.7333336	0.7320000	-0.8344855
18.7333336	0.7808000	-0.7179031
18.7333336	0.8296000	-0.2945242
18.7333336	0.8784000	-1.1167378
18.7333336	0.9272000	-1.1228738
18.7333336	0.9760000	-1.1351457
18.7333336	1.0247999	-0.9817476

18.7333336	1.0735999	-0.9756117
18.7333336	1.1223999	-1.1535535
18.7333336	1.1711999	-1.1474175
18.7333336	1.2200000	-1.1351457
18.7333336	1.2688000	-1.1290097
18.7333336	1.3176000	-1.2517285
18.7333336	1.3664000	-1.2885437
18.7333336	1.4152000	-1.2517285
18.7333336	1.4640000	-1.2455926
18.7333336	1.5128000	-1.2087770
18.7333336	1.5616000	-1.2087770
18.7333336	1.6104000	-1.1290097
18.7333336	1.6592000	-1.0799227
18.7333336	1.7079999	-1.0799227
18.7333336	1.7567999	-1.1596894
18.7333336	1.8055999	-0.8897090
18.7333336	1.8543999	-1.2517285
18.7333336	1.9031999	-1.2455926
18.7333336	1.9519999	-0.9203887
18.7333336	2.0007999	-0.9203887
18.7333336	2.0495999	-1.0431070
18.7333336	2.0983999	-1.0246992
18.7333336	2.1471999	-1.0369711
18.7333336	2.1959999	-1.0431070
18.7333336	2.2447999	-1.0492430
18.7333336	2.2935998	-1.0124273
18.7333336	2.3423998	-1.0185633
18.7333336	2.3912001	-1.0676508
18.7333336	2.4400001	-1.0676508
18.7333336	2.4888000	-1.0676508
18.7333336	2.5376000	-1.0062914
18.7333336	2.5864000	-1.0124273
18.7333336	2.6352000	-1.1842332
18.7333336	2.6840000	-1.1780972
18.7333336	2.7328000	-1.1780972
18.7333336	2.7816000	-1.0553789
18.7333336	2.8304000	-1.0001554
18.7333336	2.8792000	-0.9817476
18.7333336	2.9280000	-1.0062914
18.7333336	2.9768000	-1.0124273
18.7333336	3.0256000	-1.0492430
18.7333336	3.0743999	-1.1903691
18.7333336	3.1231999	-1.1412816
18.7333336	3.1719999	-1.1106019
18.7333336	3.2207999	-1.1106019
18.7333336	3.2695999	-0.9326606
18.7333336	3.3183999	-0.9449320
18.7333336	3.3671999	-0.9510679
18.7333336	3.4159999	-0.9387960
18.7333336	3.4647999	-0.9572039
18.7333336	3.5135999	-0.9878836
18.7333336	3.5623999	-0.8651652
18.7333336	3.6111999	-0.8835731
18.7333336	3.6599998	-1.0431070
18.7333336	3.7087998	-1.0001554
18.7333336	3.7575998	-1.0001554
18.7333336	3.8063998	-0.6994953

18.7333336	3.8551998	-0.8713012
18.7333336	3.9039998	-0.8774371
18.7333336	3.9527998	-0.8774371
18.7333336	4.0016003	-0.8774371
18.7333336	4.0503998	-0.6320000
18.7333336	4.0991998	-0.6933594
18.7333336	4.1479998	-0.7056313
18.7333336	4.1967998	-0.7117672
18.7333336	4.2455997	-0.5890489
18.7333336	4.2943997	-0.6688156
18.7333336	4.3431997	-0.9633398
18.7333336	4.3919997	-0.9756117
18.7333336	4.4407997	-0.6810875
18.7333336	4.4895997	-0.7853980
18.7333336	4.5383997	-0.6074562
18.7333336	4.5871997	-0.6381359
18.7333336	4.6359997	-0.3742914
18.7333336	4.6847997	-0.9081168
18.7333336	4.7336001	-1.0246992
18.7333336	4.7824001	-1.0185633
18.7333336	4.8312001	-1.0553789
18.7333336	4.8800001	-1.1658254
18.7333336	4.9288001	-1.3437672
18.7333336	4.9776001	-1.3437672
18.7333336	5.0264001	613586.0000000
18.7333336	5.0752001	613586.0000000
18.7333336	5.1240001	613586.0000000
18.7333336	5.1728001	613586.0000000
18.7333336	5.2216001	613586.0000000
18.7333336	5.2704000	613586.0000000
18.7333336	5.3192000	613586.0000000
18.7333336	5.3680000	613586.0000000
18.7333336	5.4168000	613586.0000000
18.7333336	5.4656000	613586.0000000
18.7333336	5.5144000	613586.0000000
18.7333336	5.5632000	613586.0000000
18.7333336	5.6120000	613586.0000000
18.7333336	5.6608000	613586.0000000
18.7333336	5.7096000	613586.0000000
18.7333336	5.7584000	613586.0000000
18.7333336	5.8072000	613586.0000000
18.7333336	5.8559999	613586.0000000
18.7333336	5.9047999	613586.0000000
18.7333336	5.9535999	613586.0000000
18.7333336	6.0023999	613586.0000000
18.7500000	0.2928000	-3.4115734
18.7500000	0.3416000	-3.4115734
18.7500000	0.3904000	-2.9513791
18.7500000	0.4392000	-2.5464082
18.7500000	0.4880000	-2.5341363
18.7500000	0.5368000	-2.3132432
18.7500000	0.5856000	-2.1782527
18.7500000	0.6344000	-1.4296703
18.7500000	0.6832000	-1.2824078
18.7500000	0.7320000	-1.5155730
18.7500000	0.7808000	-1.5033011
18.7500000	0.8296000	-1.5033011

18.7500000	0.8784000	-1.5462527
18.7500000	0.9272000	-1.7057867
18.7500000	0.9760000	-1.5278449
18.7500000	1.0247999	-1.5217090
18.7500000	1.0735999	-1.5094371
18.7500000	1.1223999	-1.3805828
18.7500000	1.1711999	-1.3683109
18.7500000	1.2200000	-1.2394567
18.7500000	1.2688000	-1.2149129
18.7500000	1.3176000	-1.3499031
18.7500000	1.3664000	-1.1658254
18.7500000	1.4152000	-1.1658254
18.7500000	1.4640000	-1.1842332
18.7500000	1.5128000	-1.1351457
18.7500000	1.5616000	-1.2210488
18.7500000	1.6104000	-0.9878836
18.7500000	1.6592000	-0.9878836
18.7500000	1.7079999	-1.1535535
18.7500000	1.7567999	-1.1535535
18.7500000	1.8055999	-1.1658254
18.7500000	1.8543999	-1.1596894
18.7500000	1.9031999	-0.9510679
18.7500000	1.9519999	-0.9510679
18.7500000	2.0007999	-1.1474175
18.7500000	2.0495999	-1.2946796
18.7500000	2.0983999	-1.3989906
18.7500000	2.1471999	-1.3008156
18.7500000	2.1959999	-1.3069515
18.7500000	2.2447999	-1.2149129
18.7500000	2.2935998	-1.1351457
18.7500000	2.3423998	-1.1474175
18.7500000	2.3912001	-1.1535535
18.7500000	2.4400001	-1.1658254
18.7500000	2.4888000	-1.2394567
18.7500000	2.5376000	-1.2394567
18.7500000	2.5864000	-1.0492430
18.7500000	2.6352000	-1.0431070
18.7500000	2.6840000	-1.0431070
18.7500000	2.7328000	-1.0246992
18.7500000	2.7816000	-1.2026410
18.7500000	2.8304000	-1.2946796
18.7500000	2.8792000	-1.3008156
18.7500000	2.9280000	-1.3867188
18.7500000	2.9768000	-1.1535535
18.7500000	3.0256000	-1.1535535
18.7500000	3.0743999	-1.0983305
18.7500000	3.1231999	-0.9510679
18.7500000	3.1719999	-0.9510679
18.7500000	3.2207999	-1.0369711
18.7500000	3.2695999	-1.1106019
18.7500000	3.3183999	-1.1106019
18.7500000	3.3671999	-0.7731261
18.7500000	3.4159999	-1.0246992
18.7500000	3.4647999	-1.0246992
18.7500000	3.5135999	-1.0185633
18.7500000	3.5623999	-1.0308352
18.7500000	3.6111999	-0.9510679

18.7500000	3.6599998	-0.9694757
18.7500000	3.7087998	-0.9756117
18.7500000	3.7575998	-0.9449320
18.7500000	3.8063998	-0.9265246
18.7500000	3.8551998	-0.9449320
18.7500000	3.9039998	-0.8590293
18.7500000	3.9527998	-0.8651652
18.7500000	4.0016003	-0.8099418
18.7500000	4.0503998	-0.8099418
18.7500000	4.0991998	-0.5706410
18.7500000	4.1479998	-0.7731261
18.7500000	4.1967998	-1.0369711
18.7500000	4.2455997	-1.0308352
18.7500000	4.2943997	-1.0185633
18.7500000	4.3431997	-0.9142528
18.7500000	4.3919997	-0.9142528
18.7500000	4.4407997	-0.9081168
18.7500000	4.4895997	-0.6442719
18.7500000	4.5383997	-0.6504078
18.7500000	4.5871997	-1.0431070
18.7500000	4.6359997	-0.9940195
18.7500000	4.6847997	-1.0001554
18.7500000	4.7336001	613586.0000000
18.7500000	4.7824001	613586.0000000
18.7500000	4.8312001	-0.7731261
18.7500000	4.8800001	-0.7117672
18.7500000	4.9288001	613586.0000000
18.7500000	4.9776001	613586.0000000
18.7500000	5.0264001	613586.0000000
18.7500000	5.0752001	613586.0000000
18.7500000	5.1240001	613586.0000000
18.7500000	5.1728001	613586.0000000
18.7500000	5.2216001	613586.0000000
18.7500000	5.2704000	613586.0000000
18.7500000	5.3192000	613586.0000000
18.7500000	5.3680000	613586.0000000
18.7500000	5.4168000	613586.0000000
18.7500000	5.4656000	613586.0000000
18.7500000	5.5144000	613586.0000000
18.7500000	5.5632000	613586.0000000
18.7500000	5.6120000	613586.0000000
18.7500000	5.6608000	613586.0000000
18.7500000	5.7096000	613586.0000000
18.7500000	5.7584000	613586.0000000
18.7500000	5.8072000	613586.0000000
18.7500000	5.8559999	613586.0000000
18.7500000	5.9047999	613586.0000000
18.7500000	5.9535999	613586.0000000
18.7500000	6.0023999	613586.0000000
18.7666664	0.2928000	-3.8717675
18.7666664	0.3416000	-3.8717675
18.7666664	0.3904000	-3.2397676
18.7666664	0.4392000	-2.9452431
18.7666664	0.4880000	-2.4114180
18.7666664	0.5368000	-2.3561945
18.7666664	0.5856000	-2.3500586
18.7666664	0.6344000	-2.2764277

18.7666664	0.6832000	-2.4973209
18.7666664	0.7320000	-1.3867188
18.7666664	0.7808000	-1.3805828
18.7666664	0.8296000	-1.3805828
18.7666664	0.8784000	-1.3805828
18.7666664	0.9272000	-1.2946796
18.7666664	0.9760000	-1.2946796
18.7666664	1.0247999	-1.2946796
18.7666664	1.0735999	-1.3008156
18.7666664	1.1223999	-1.3560390
18.7666664	1.1711999	-1.3437672
18.7666664	1.2200000	-1.3314953
18.7666664	1.2688000	-1.2824078
18.7666664	1.3176000	-1.2087770
18.7666664	1.3664000	-1.2762718
18.7666664	1.4152000	-1.2762718
18.7666664	1.4640000	-1.2271848
18.7666664	1.5128000	-1.2210488
18.7666664	1.5616000	-1.3192234
18.7666664	1.6104000	-1.2701364
18.7666664	1.6592000	-1.2210488
18.7666664	1.7079999	-1.3437672
18.7666664	1.7567999	-1.3192234
18.7666664	1.8055999	-1.3130875
18.7666664	1.8543999	-1.3130875
18.7666664	1.9031999	-1.1842332
18.7666664	1.9519999	-1.1842332
18.7666664	2.0007999	-1.0062914
18.7666664	2.0495999	-1.1106019
18.7666664	2.0983999	-1.1106019
18.7666664	2.1471999	-1.1903691
18.7666664	2.1959999	-1.1903691
18.7666664	2.2447999	-1.1228738
18.7666664	2.2935998	-1.2210488
18.7666664	2.3423998	-1.1290097
18.7666664	2.3912001	-1.1044660
18.7666664	2.4400001	-1.0921946
18.7666664	2.4888000	-0.9694757
18.7666664	2.5376000	-1.1290097
18.7666664	2.5864000	-1.2394567
18.7666664	2.6352000	-1.2271848
18.7666664	2.6840000	-1.2333207
18.7666664	2.7328000	-1.1474175
18.7666664	2.7816000	-1.1474175
18.7666664	2.8304000	-1.0676508
18.7666664	2.8792000	-1.0185633
18.7666664	2.9280000	-1.0001554
18.7666664	2.9768000	-0.9878836
18.7666664	3.0256000	-1.1474175
18.7666664	3.0743999	-1.1412816
18.7666664	3.1231999	-1.0124273
18.7666664	3.1719999	-1.2394567
18.7666664	3.2207999	-1.0431070
18.7666664	3.2695999	-0.9081168
18.7666664	3.3183999	-0.9142528
18.7666664	3.3671999	-1.0185633
18.7666664	3.4159999	-1.0001554

18.7666664	3.4647999	-1.0001554
18.7666664	3.5135999	-0.9387960
18.7666664	3.5623999	-0.8222136
18.7666664	3.6111999	-0.9326606
18.7666664	3.6599998	-0.9326606
18.7666664	3.7087998	-0.7363110
18.7666664	3.7575998	-0.7669902
18.7666664	3.8063998	-0.7853980
18.7666664	3.8551998	-0.7915339
18.7666664	3.9039998	-0.7731261
18.7666664	3.9527998	-0.7792621
18.7666664	4.0016003	-0.7976699
18.7666664	4.0503998	-0.8651652
18.7666664	4.0991998	-0.8651652
18.7666664	4.1479998	-0.8590293
18.7666664	4.1967998	-0.8590293
18.7666664	4.2455997	-0.8835731
18.7666664	4.2943997	-0.7179031
18.7666664	4.3431997	-0.5829129
18.7666664	4.3919997	-0.5767770
18.7666664	4.4407997	-0.2270293
18.7666664	4.4895997	0.1963496
18.7666664	4.5383997	0.1963496
18.7666664	4.5871997	0.2147574
18.7666664	4.6359997	0.0859027
18.7666664	4.6847997	-1.0737867
18.7666664	4.7336001	-1.3499031
18.7666664	4.7824001	-1.3008156
18.7666664	4.8312001	-1.3130875
18.7666664	4.8800001	-1.4971652
18.7666664	4.9288001	-1.5339808
18.7666664	4.9776001	-1.4112625
18.7666664	5.0264001	613586.0000000
18.7666664	5.0752001	613586.0000000
18.7666664	5.1240001	613586.0000000
18.7666664	5.1728001	613586.0000000
18.7666664	5.2216001	613586.0000000
18.7666664	5.2704000	613586.0000000
18.7666664	5.3192000	613586.0000000
18.7666664	5.3680000	-2.0862141
18.7666664	5.4168000	613586.0000000
18.7666664	5.4656000	613586.0000000
18.7666664	5.5144000	613586.0000000
18.7666664	5.5632000	613586.0000000
18.7666664	5.6120000	613586.0000000
18.7666664	5.6608000	613586.0000000
18.7666664	5.7096000	613586.0000000
18.7666664	5.7584000	613586.0000000
18.7666664	5.8072000	613586.0000000
18.7666664	5.8559999	613586.0000000
18.7666664	5.9047999	613586.0000000
18.7666664	5.9535999	613586.0000000
18.7666664	6.0023999	613586.0000000
18.7833328	0.2928000	-3.4790685
18.7833328	0.3416000	-3.6447384
18.7833328	0.3904000	-3.6631463
18.7833328	0.4392000	-3.0802336

18.7833328	0.4880000	-2.2948353
18.7833328	0.5368000	-2.2027965
18.7833328	0.5856000	-1.7241945
18.7833328	0.6344000	-1.0308352
18.7833328	0.6832000	-1.0308352
18.7833328	0.7320000	-1.5217090
18.7833328	0.7808000	-1.7057867
18.7833328	0.8296000	-1.7180586
18.7833328	0.8784000	-1.2824078
18.7833328	0.9272000	-1.2824078
18.7833328	0.9760000	-1.4112625
18.7833328	1.0247999	-1.4112625
18.7833328	1.0735999	-1.2210488
18.7833328	1.1223999	-1.1535535
18.7833328	1.1711999	-1.2517285
18.7833328	1.2200000	-1.2701364
18.7833328	1.2688000	-1.2701364
18.7833328	1.3176000	-1.2087770
18.7833328	1.3664000	-1.2087770
18.7833328	1.4152000	-1.2087770
18.7833328	1.4640000	-1.2087770
18.7833328	1.5128000	-1.1842332
18.7833328	1.5616000	-1.1658254
18.7833328	1.6104000	-1.1965051
18.7833328	1.6592000	-1.1351457
18.7833328	1.7079999	-1.2701364
18.7833328	1.7567999	-1.4112625
18.7833328	1.8055999	-1.1228738
18.7833328	1.8543999	-0.9878836
18.7833328	1.9031999	-0.9878836
18.7833328	1.9519999	-1.0001554
18.7833328	2.0007999	-1.0799227
18.7833328	2.0495999	-1.0737867
18.7833328	2.0983999	-1.2271848
18.7833328	2.1471999	-1.2333207
18.7833328	2.1959999	-1.0124273
18.7833328	2.2447999	-1.0185633
18.7833328	2.2935998	-1.0737867
18.7833328	2.3423998	-0.9081168
18.7833328	2.3912001	-0.8958449
18.7833328	2.4400001	-0.9019809
18.7833328	2.4888000	-0.9756117
18.7833328	2.5376000	-0.9817476
18.7833328	2.5864000	-0.9633398
18.7833328	2.6352000	-1.0246992
18.7833328	2.6840000	-1.0185633
18.7833328	2.7328000	-1.1780972
18.7833328	2.7816000	-1.1780972
18.7833328	2.8304000	-1.0246992
18.7833328	2.8792000	-1.0308352
18.7833328	2.9280000	-1.0308352
18.7833328	2.9768000	-0.9756117
18.7833328	3.0256000	-0.9817476
18.7833328	3.0743999	-1.0737867
18.7833328	3.1231999	-0.8958449
18.7833328	3.1719999	-0.8958449
18.7833328	3.2207999	-1.1290097

18.7833328	3.2695999	-1.1228738
18.7833328	3.3183999	-1.0001554
18.7833328	3.3671999	-0.9510679
18.7833328	3.4159999	-0.8958449
18.7833328	3.4647999	-0.8590293
18.7833328	3.5135999	-0.7240391
18.7833328	3.5623999	-0.5767770
18.7833328	3.6111999	-0.6933594
18.7833328	3.6599998	-0.5951843
18.7833328	3.7087998	-0.6013203
18.7833328	3.7575998	-0.6197281
18.7833328	3.8063998	-0.5031457
18.7833328	3.8551998	-0.5031457
18.7833328	3.9039998	-0.5092816
18.7833328	3.9527998	-0.6810875
18.7833328	4.0016003	-0.6872234
18.7833328	4.0503998	-0.7915339
18.7833328	4.0991998	-0.7915339
18.7833328	4.1479998	-0.8099418
18.7833328	4.1967998	-0.8835731
18.7833328	4.2455997	-0.8344855
18.7833328	4.2943997	-0.8406215
18.7833328	4.3431997	-0.8283496
18.7833328	4.3919997	-0.9449320
18.7833328	4.4407997	-0.8222136
18.7833328	4.4895997	-0.9756117
18.7833328	4.5383997	-0.9081168
18.7833328	4.5871997	-0.6320000
18.7833328	4.6359997	-0.9694757
18.7833328	4.6847997	-0.7853980
18.7833328	4.7336001	-0.7669902
18.7833328	4.7824001	-0.7669902
18.7833328	4.8312001	-0.4847379
18.7833328	4.8800001	613586.0000000
18.7833328	4.9288001	613586.0000000
18.7833328	4.9776001	613586.0000000
18.7833328	5.0264001	-0.3742914
18.7833328	5.0752001	-0.3374758
18.7833328	5.1240001	-0.3436117
18.7833328	5.1728001	-0.2638445
18.7833328	5.2216001	613586.0000000
18.7833328	5.2704000	613586.0000000
18.7833328	5.3192000	613586.0000000
18.7833328	5.3680000	613586.0000000
18.7833328	5.4168000	613586.0000000
18.7833328	5.4656000	613586.0000000
18.7833328	5.5144000	613586.0000000
18.7833328	5.5632000	613586.0000000
18.7833328	5.6120000	613586.0000000
18.7833328	5.6608000	613586.0000000
18.7833328	5.7096000	613586.0000000
18.7833328	5.7584000	613586.0000000
18.7833328	5.8072000	613586.0000000
18.7833328	5.8559999	613586.0000000
18.7833328	5.9047999	613586.0000000
18.7833328	5.9535999	613586.0000000
18.7833328	6.0023999	613586.0000000

18.7999992	0.2928000	-3.6140587
18.7999992	0.3416000	-3.6079230
18.7999992	0.3904000	-3.7183695
18.7999992	0.4392000	-3.1968160
18.7999992	0.4880000	-3.1968160
18.7999992	0.5368000	-2.5341363
18.7999992	0.5856000	-1.6321554
18.7999992	0.6344000	-1.0001554
18.7999992	0.6832000	-1.4910293
18.7999992	0.7320000	-1.4787574
18.7999992	0.7808000	-1.3744469
18.7999992	0.8296000	-0.5215535
18.7999992	0.8784000	-0.8651652
18.7999992	0.9272000	-1.0921946
18.7999992	0.9760000	-1.0799227
18.7999992	1.0247999	-1.4419422
18.7999992	1.0735999	-1.4358063
18.7999992	1.1223999	-1.4296703
18.7999992	1.1711999	-1.2946796
18.7999992	1.2200000	-1.3130875
18.7999992	1.2688000	-1.2333207
18.7999992	1.3176000	-1.2640004
18.7999992	1.3664000	-1.1903691
18.7999992	1.4152000	-1.1842332
18.7999992	1.4640000	-1.1228738
18.7999992	1.5128000	-1.1106019
18.7999992	1.5616000	-1.2394567
18.7999992	1.6104000	-1.2578645
18.7999992	1.6592000	-1.0799227
18.7999992	1.7079999	-1.2026410
18.7999992	1.7567999	-1.2087770
18.7999992	1.8055999	-1.2026410
18.7999992	1.8543999	-0.9878836
18.7999992	1.9031999	-1.0553789
18.7999992	1.9519999	-1.2271848
18.7999992	2.0007999	-1.2026410
18.7999992	2.0495999	-1.1903691
18.7999992	2.0983999	-1.3867188
18.7999992	2.1471999	-1.3805828
18.7999992	2.1959999	-1.1167378
18.7999992	2.2447999	-1.1167378
18.7999992	2.2935998	-1.1535535
18.7999992	2.3423998	-1.0676508
18.7999992	2.3912001	-1.1780972
18.7999992	2.4400001	-1.0124273
18.7999992	2.4888000	-1.0124273
18.7999992	2.5376000	-1.0799227
18.7999992	2.5864000	-1.0921946
18.7999992	2.6352000	-1.0799227
18.7999992	2.6840000	-0.9387960
18.7999992	2.7328000	-1.0860586
18.7999992	2.7816000	-1.0431070
18.7999992	2.8304000	-1.1044660
18.7999992	2.8792000	-0.8774371
18.7999992	2.9280000	-0.8958449
18.7999992	2.9768000	-0.8897090
18.7999992	3.0256000	-0.7915339

18.7999992	3.0743999	-1.0308352
18.7999992	3.1231999	-0.9572039
18.7999992	3.1719999	-0.7915339
18.7999992	3.2207999	-1.0001554
18.7999992	3.2695999	-0.8344855
18.7999992	3.3183999	-0.6872234
18.7999992	3.3671999	-0.9326606
18.7999992	3.4159999	-0.7301750
18.7999992	3.4647999	-0.7179031
18.7999992	3.5135999	-0.8528934
18.7999992	3.5623999	-0.8467574
18.7999992	3.6111999	-0.8406215
18.7999992	3.6599998	-0.7240391
18.7999992	3.7087998	-0.7363110
18.7999992	3.7575998	-0.5583692
18.7999992	3.8063998	-0.7301750
18.7999992	3.8551998	-0.7547188
18.7999992	3.9039998	-0.3865633
18.7999992	3.9527998	-0.6504078
18.7999992	4.0016003	-0.6442719
18.7999992	4.0503998	-0.9756117
18.7999992	4.0991998	-0.6810875
18.7999992	4.1479998	-0.7608547
18.7999992	4.1967998	-0.8160777
18.7999992	4.2455997	-0.8713012
18.7999992	4.2943997	-0.8774371
18.7999992	4.3431997	-1.0553789
18.7999992	4.3919997	-1.0615149
18.7999992	4.4407997	-0.7976699
18.7999992	4.4895997	-1.0124273
18.7999992	4.5383997	-0.8590293
18.7999992	4.5871997	-0.8590293
18.7999992	4.6359997	-1.0369711
18.7999992	4.6847997	-0.7853980
18.7999992	4.7336001	-1.0124273
18.7999992	4.7824001	-1.1290097
18.7999992	4.8312001	-1.1351457
18.7999992	4.8800001	-1.2087770
18.7999992	4.9288001	-1.1106019
18.7999992	4.9776001	-1.2394567
18.7999992	5.0264001	-1.2333207
18.7999992	5.0752001	-1.1842332
18.7999992	5.1240001	613586.0000000
18.7999992	5.1728001	613586.0000000
18.7999992	5.2216001	613586.0000000
18.7999992	5.2704000	613586.0000000
18.7999992	5.3192000	613586.0000000
18.7999992	5.3680000	613586.0000000
18.7999992	5.4168000	613586.0000000
18.7999992	5.4656000	613586.0000000
18.7999992	5.5144000	613586.0000000
18.7999992	5.5632000	613586.0000000
18.7999992	5.6120000	613586.0000000
18.7999992	5.6608000	613586.0000000
18.7999992	5.7096000	613586.0000000
18.7999992	5.7584000	613586.0000000
18.7999992	5.8072000	613586.0000000

18.7999992	5.8559999	613586.0000000
18.7999992	5.9047999	-1.2517285
18.7999992	5.9535999	613586.0000000
18.7999992	6.0023999	-1.2762718
18.8166676	0.2928000	-4.3626413
18.8166676	0.3416000	-3.9269910
18.8166676	0.3904000	-3.5342917
18.8166676	0.4392000	-3.6876900
18.8166676	0.4880000	-2.4973209
18.8166676	0.5368000	-2.4973209
18.8166676	0.5856000	-2.2212043
18.8166676	0.6344000	-2.2212043
18.8166676	0.6832000	-1.2271848
18.8166676	0.7320000	-1.2149129
18.8166676	0.7808000	-1.0492430
18.8166676	0.8296000	-1.1290097
18.8166676	0.8784000	-1.4542136
18.8166676	0.9272000	-1.4603496
18.8166676	0.9760000	-1.2762718
18.8166676	1.0247999	-1.2762718
18.8166676	1.0735999	-1.0553789
18.8166676	1.1223999	-1.0615149
18.8166676	1.1711999	-1.1842332
18.8166676	1.2200000	-1.2824078
18.8166676	1.2688000	-1.2210488
18.8166676	1.3176000	-1.2210488
18.8166676	1.3664000	-1.1842332
18.8166676	1.4152000	-1.1535535
18.8166676	1.4640000	-1.1474175
18.8166676	1.5128000	-1.2210488
18.8166676	1.5616000	-1.3437672
18.8166676	1.6104000	-1.0676508
18.8166676	1.6592000	-1.0615149
18.8166676	1.7079999	-1.0615149
18.8166676	1.7567999	-1.0737867
18.8166676	1.8055999	-1.3805828
18.8166676	1.8543999	-1.0615149
18.8166676	1.9031999	-0.9203887
18.8166676	1.9519999	-0.9694757
18.8166676	2.0007999	-1.1290097
18.8166676	2.0495999	-1.1290097
18.8166676	2.0983999	-1.0615149
18.8166676	2.1471999	-1.0615149
18.8166676	2.1959999	-1.2026410
18.8166676	2.2447999	-1.2026410
18.8166676	2.2935998	-1.1842332
18.8166676	2.3423998	-1.1719613
18.8166676	2.3912001	-1.1903691
18.8166676	2.4400001	-1.0553789
18.8166676	2.4888000	-1.2026410
18.8166676	2.5376000	-1.0185633
18.8166676	2.5864000	-1.1474175
18.8166676	2.6352000	-1.1106019
18.8166676	2.6840000	-1.0124273
18.8166676	2.7328000	-1.1842332
18.8166676	2.7816000	-1.1842332
18.8166676	2.8304000	-1.0553789

18.8166676	2.8792000	-1.0615149
18.8166676	2.9280000	-0.9572039
18.8166676	2.9768000	-0.8713012
18.8166676	3.0256000	-0.8835731
18.8166676	3.0743999	-0.9449320
18.8166676	3.1231999	-0.8160777
18.8166676	3.1719999	-0.8283496
18.8166676	3.2207999	-0.8283496
18.8166676	3.2695999	-1.0369711
18.8166676	3.3183999	-1.0246992
18.8166676	3.3671999	-0.9326606
18.8166676	3.4159999	-0.8713012
18.8166676	3.4647999	-0.8774371
18.8166676	3.5135999	-0.9940195
18.8166676	3.5623999	-1.0001554
18.8166676	3.6111999	-0.7853980
18.8166676	3.6599998	-0.7731261
18.8166676	3.7087998	-0.6565437
18.8166676	3.7575998	-0.7179031
18.8166676	3.8063998	-0.7117672
18.8166676	3.8551998	-0.7117672
18.8166676	3.9039998	-0.6872234
18.8166676	3.9527998	-0.6749516
18.8166676	4.0016003	-0.7915339
18.8166676	4.0503998	-0.7915339
18.8166676	4.0991998	-0.6994953
18.8166676	4.1479998	-0.7731261
18.8166676	4.1967998	-0.7731261
18.8166676	4.2455997	-0.6135921
18.8166676	4.2943997	-0.9572039
18.8166676	4.3431997	-0.9940195
18.8166676	4.3919997	-0.9940195
18.8166676	4.4407997	-0.8406215
18.8166676	4.4895997	-0.7792621
18.8166676	4.5383997	-0.7915339
18.8166676	4.5871997	-0.8835731
18.8166676	4.6359997	-0.8897090
18.8166676	4.6847997	-0.8897090
18.8166676	4.7336001	-0.9387960
18.8166676	4.7824001	-0.9449320
18.8166676	4.8312001	-0.7485828
18.8166676	4.8800001	-0.6442719
18.8166676	4.9288001	-0.7240391
18.8166676	4.9776001	613586.0000000
18.8166676	5.0264001	613586.0000000
18.8166676	5.0752001	613586.0000000
18.8166676	5.1240001	613586.0000000
18.8166676	5.1728001	613586.0000000
18.8166676	5.2216001	613586.0000000
18.8166676	5.2704000	613586.0000000
18.8166676	5.3192000	613586.0000000
18.8166676	5.3680000	613586.0000000
18.8166676	5.4168000	613586.0000000
18.8166676	5.4656000	613586.0000000
18.8166676	5.5144000	613586.0000000
18.8166676	5.5632000	613586.0000000
18.8166676	5.6120000	613586.0000000

18.8166676	5.6608000	613586.0000000
18.8166676	5.7096000	613586.0000000
18.8166676	5.7584000	613586.0000000
18.8166676	5.8072000	613586.0000000
18.8166676	5.8559999	613586.0000000
18.8166676	5.9047999	613586.0000000
18.8166676	5.9535999	613586.0000000
18.8166676	6.0023999	613586.0000000
18.8333340	0.2928000	-2.8900199
18.8333340	0.3416000	-2.6629908
18.8333340	0.3904000	-2.6752625
18.8333340	0.4392000	-2.8593402
18.8333340	0.4880000	-2.3193791
18.8333340	0.5368000	-1.9144082
18.8333340	0.5856000	-1.6751070
18.8333340	0.6344000	-1.6751070
18.8333340	0.6832000	-0.9387960
18.8333340	0.7320000	-1.1780972
18.8333340	0.7808000	-1.2149129
18.8333340	0.8296000	-0.8099418
18.8333340	0.8784000	-1.0492430
18.8333340	0.9272000	-1.7364664
18.8333340	0.9760000	-1.1780972
18.8333340	1.0247999	-1.1780972
18.8333340	1.0735999	-1.1780972
18.8333340	1.1223999	-1.1780972
18.8333340	1.1711999	-1.1596894
18.8333340	1.2200000	-1.1474175
18.8333340	1.2688000	-1.1290097
18.8333340	1.3176000	-1.1044660
18.8333340	1.3664000	-1.1044660
18.8333340	1.4152000	-1.2640004
18.8333340	1.4640000	-1.2271848
18.8333340	1.5128000	-1.2271848
18.8333340	1.5616000	-1.2333207
18.8333340	1.6104000	-1.2394567
18.8333340	1.6592000	-1.1474175
18.8333340	1.7079999	-1.2394567
18.8333340	1.7567999	-1.2394567
18.8333340	1.8055999	-1.2394567
18.8333340	1.8543999	-1.2271848
18.8333340	1.9031999	-1.1474175
18.8333340	1.9519999	-1.1412816
18.8333340	2.0007999	-1.1474175
18.8333340	2.0495999	-1.0431070
18.8333340	2.0983999	-1.0431070
18.8333340	2.1471999	-1.0983305
18.8333340	2.1959999	-1.0983305
18.8333340	2.2447999	-1.1412816
18.8333340	2.2935998	-1.1474175
18.8333340	2.3423998	-1.1474175
18.8333340	2.3912001	-1.0737867
18.8333340	2.4400001	-1.1106019
18.8333340	2.4888000	-1.1474175
18.8333340	2.5376000	-1.0369711
18.8333340	2.5864000	-1.0001554
18.8333340	2.6352000	-1.0001554

18.8333340	2.6840000	-1.0001554
18.8333340	2.7328000	-1.0001554
18.8333340	2.7816000	-0.9878836
18.8333340	2.8304000	-0.9878836
18.8333340	2.8792000	-0.9817476
18.8333340	2.9280000	-1.1658254
18.8333340	2.9768000	-0.8897090
18.8333340	3.0256000	-0.8897090
18.8333340	3.0743999	-0.9878836
18.8333340	3.1231999	-1.0001554
18.8333340	3.1719999	-0.9940195
18.8333340	3.2207999	-1.1044660
18.8333340	3.2695999	-0.8713012
18.8333340	3.3183999	-1.0185633
18.8333340	3.3671999	-0.9817476
18.8333340	3.4159999	-1.0001554
18.8333340	3.4647999	-0.9756117
18.8333340	3.5135999	-0.7731261
18.8333340	3.5623999	-0.7363110
18.8333340	3.6111999	-0.9510679
18.8333340	3.6599998	-0.7853980
18.8333340	3.7087998	-0.7792621
18.8333340	3.7575998	-0.7056313
18.8333340	3.8063998	-0.7608547
18.8333340	3.8551998	-0.7547188
18.8333340	3.9039998	-0.6565437
18.8333340	3.9527998	-0.7853980
18.8333340	4.0016003	-0.7853980
18.8333340	4.0503998	-0.7301750
18.8333340	4.0991998	-0.8590293
18.8333340	4.1479998	-0.8897090
18.8333340	4.1967998	-0.7915339
18.8333340	4.2455997	-0.8038058
18.8333340	4.2943997	-0.8528934
18.8333340	4.3431997	-0.8528934
18.8333340	4.3919997	-0.8160777
18.8333340	4.4407997	-0.7056313
18.8333340	4.4895997	-0.7117672
18.8333340	4.5383997	-0.8897090
18.8333340	4.5871997	-0.8528934
18.8333340	4.6359997	-0.8467574
18.8333340	4.6847997	-0.8467574
18.8333340	4.7336001	-0.8283496
18.8333340	4.7824001	-0.7240391
18.8333340	4.8312001	-0.6565437
18.8333340	4.8800001	-0.8835731
18.8333340	4.9288001	613586.0000000
18.8333340	4.9776001	613586.0000000
18.8333340	5.0264001	613586.0000000
18.8333340	5.0752001	-0.9694757
18.8333340	5.1240001	-0.9756117
18.8333340	5.1728001	-0.9878836
18.8333340	5.2216001	-0.9387960
18.8333340	5.2704000	-0.9449320
18.8333340	5.3192000	-0.9449320
18.8333340	5.3680000	-0.9203887
18.8333340	5.4168000	-0.8528934

18.8333340	5.4656000	-0.9019809
18.8333340	5.5144000	613586.0000000
18.8333340	5.5632000	613586.0000000
18.8333340	5.6120000	613586.0000000
18.8333340	5.6608000	613586.0000000
18.8333340	5.7096000	613586.0000000
18.8333340	5.7584000	613586.0000000
18.8333340	5.8072000	613586.0000000
18.8333340	5.8559999	613586.0000000
18.8333340	5.9047999	613586.0000000
18.8333340	5.9535999	613586.0000000
18.8333340	6.0023999	613586.0000000
18.8500004	0.2928000	-3.0250101
18.8500004	0.3416000	-2.8470683
18.8500004	0.3904000	-2.7120781
18.8500004	0.4392000	-2.7059422
18.8500004	0.4880000	-2.6875343
18.8500004	0.5368000	-2.4789131
18.8500004	0.5856000	-2.3930101
18.8500004	0.6344000	-2.0739422
18.8500004	0.6832000	-2.1291652
18.8500004	0.7320000	-1.0553789
18.8500004	0.7808000	-1.0737867
18.8500004	0.8296000	-0.9510679
18.8500004	0.8784000	-0.9510679
18.8500004	0.9272000	-1.3928547
18.8500004	0.9760000	-1.5646605
18.8500004	1.0247999	-1.5217090
18.8500004	1.0735999	-1.4173985
18.8500004	1.1223999	-1.3867188
18.8500004	1.1711999	-1.3928547
18.8500004	1.2200000	-1.3867188
18.8500004	1.2688000	-1.2578645
18.8500004	1.3176000	-1.2087770
18.8500004	1.3664000	-1.2026410
18.8500004	1.4152000	-1.1228738
18.8500004	1.4640000	-1.0860586
18.8500004	1.5128000	-1.0799227
18.8500004	1.5616000	-1.1903691
18.8500004	1.6104000	-1.2640004
18.8500004	1.6592000	-1.2578645
18.8500004	1.7079999	-1.3560390
18.8500004	1.7567999	-1.3499031
18.8500004	1.8055999	-1.0983305
18.8500004	1.8543999	-1.0983305
18.8500004	1.9031999	-1.1044660
18.8500004	1.9519999	-1.0921946
18.8500004	2.0007999	-1.2210488
18.8500004	2.0495999	-1.0921946
18.8500004	2.0983999	-1.1658254
18.8500004	2.1471999	-1.1167378
18.8500004	2.1959999	-1.1228738
18.8500004	2.2447999	-1.2517285
18.8500004	2.2935998	-1.2149129
18.8500004	2.3423998	-1.0799227
18.8500004	2.3912001	-1.1719613
18.8500004	2.4400001	-1.1106019

18.8500004	2.4888000	-1.1106019
18.8500004	2.5376000	-1.3008156
18.8500004	2.5864000	-1.0431070
18.8500004	2.6352000	-1.0431070
18.8500004	2.6840000	-1.2087770
18.8500004	2.7328000	-1.2149129
18.8500004	2.7816000	-1.3560390
18.8500004	2.8304000	-1.0431070
18.8500004	2.8792000	-0.9694757
18.8500004	2.9280000	-0.9510679
18.8500004	2.9768000	-0.8467574
18.8500004	3.0256000	-0.8528934
18.8500004	3.0743999	-1.0983305
18.8500004	3.1231999	-1.0799227
18.8500004	3.1719999	-1.1658254
18.8500004	3.2207999	-1.1535535
18.8500004	3.2695999	-1.2210488
18.8500004	3.3183999	-1.2333207
18.8500004	3.3671999	-0.9203887
18.8500004	3.4159999	-0.7731261
18.8500004	3.4647999	-0.9203887
18.8500004	3.5135999	-0.9265246
18.8500004	3.5623999	-0.9203887
18.8500004	3.6111999	-0.9142528
18.8500004	3.6599998	-0.9203887
18.8500004	3.7087998	-0.8344855
18.8500004	3.7575998	-0.8283496
18.8500004	3.8063998	-0.7669902
18.8500004	3.8551998	-0.7792621
18.8500004	3.9039998	-0.8283496
18.8500004	3.9527998	-0.8283496
18.8500004	4.0016003	-0.5154176
18.8500004	4.0503998	-0.5092816
18.8500004	4.0991998	-0.6074562
18.8500004	4.1479998	-0.9142528
18.8500004	4.1967998	-0.9142528
18.8500004	4.2455997	-0.9203887
18.8500004	4.2943997	-0.9265246
18.8500004	4.3431997	-0.9142528
18.8500004	4.3919997	-0.9203887
18.8500004	4.4407997	-0.9449320
18.8500004	4.4895997	-0.9449320
18.8500004	4.5383997	-0.7853980
18.8500004	4.5871997	-0.7976699
18.8500004	4.6359997	-0.7976699
18.8500004	4.6847997	-0.7792621
18.8500004	4.7336001	-0.7547188
18.8500004	4.7824001	-0.7179031
18.8500004	4.8312001	613586.0000000
18.8500004	4.8800001	613586.0000000
18.8500004	4.9288001	613586.0000000
18.8500004	4.9776001	613586.0000000
18.8500004	5.0264001	-0.7117672
18.8500004	5.0752001	-0.7792621
18.8500004	5.1240001	-0.6688156
18.8500004	5.1728001	-0.6872234
18.8500004	5.2216001	-0.7731261

18.8500004	5.2704000	-0.7669902
18.8500004	5.3192000	-0.7485828
18.8500004	5.3680000	-0.7056313
18.8500004	5.4168000	-0.7056313
18.8500004	5.4656000	613586.0000000
18.8500004	5.5144000	613586.0000000
18.8500004	5.5632000	613586.0000000
18.8500004	5.6120000	613586.0000000
18.8500004	5.6608000	613586.0000000
18.8500004	5.7096000	613586.0000000
18.8500004	5.7584000	613586.0000000
18.8500004	5.8072000	613586.0000000
18.8500004	5.8559999	613586.0000000
18.8500004	5.9047999	613586.0000000
18.8500004	5.9535999	613586.0000000
18.8500004	6.0023999	613586.0000000
18.8666668	0.2928000	-3.6999617
18.8666668	0.3416000	-3.7122335
18.8666668	0.3904000	-3.2152238
18.8666668	0.4392000	-2.5280004
18.8666668	0.4880000	-2.2702918
18.8666668	0.5368000	-2.1168938
18.8666668	0.5856000	-2.1168938
18.8666668	0.6344000	-1.7057867
18.8666668	0.6832000	-1.3437672
18.8666668	0.7320000	-1.3069515
18.8666668	0.7808000	-1.8469129
18.8666668	0.8296000	-1.2946796
18.8666668	0.8784000	-1.4419422
18.8666668	0.9272000	-1.2946796
18.8666668	0.9760000	-1.2946796
18.8666668	1.0247999	-1.3621750
18.8666668	1.0735999	-1.2394567
18.8666668	1.1223999	-1.2578645
18.8666668	1.1711999	-1.2762718
18.8666668	1.2200000	-1.2885437
18.8666668	1.2688000	-1.1474175
18.8666668	1.3176000	-1.1965051
18.8666668	1.3664000	-1.3805828
18.8666668	1.4152000	-1.2394567
18.8666668	1.4640000	-1.2394567
18.8666668	1.5128000	-1.3130875
18.8666668	1.5616000	-1.3314953
18.8666668	1.6104000	-1.3069515
18.8666668	1.6592000	-1.3008156
18.8666668	1.7079999	-1.2946796
18.8666668	1.7567999	-1.2824078
18.8666668	1.8055999	-1.2578645
18.8666668	1.8543999	-1.2578645
18.8666668	1.9031999	-1.2333207
18.8666668	1.9519999	-1.1412816
18.8666668	2.0007999	-1.4235344
18.8666668	2.0495999	-1.2578645
18.8666668	2.0983999	-1.2210488
18.8666668	2.1471999	-1.3192234
18.8666668	2.1959999	-1.1535535
18.8666668	2.2447999	-1.1658254

18.8666668	2.2935998	-1.2149129
18.8666668	2.3423998	-1.3314953
18.8666668	2.3912001	-1.2885437
18.8666668	2.4400001	-1.2701364
18.8666668	2.4888000	-1.2517285
18.8666668	2.5376000	-1.1658254
18.8666668	2.5864000	-1.1780972
18.8666668	2.6352000	-1.1842332
18.8666668	2.6840000	-1.1474175
18.8666668	2.7328000	-1.1474175
18.8666668	2.7816000	-1.1535535
18.8666668	2.8304000	-1.1596894
18.8666668	2.8792000	-1.0492430
18.8666668	2.9280000	-1.0431070
18.8666668	2.9768000	-1.1106019
18.8666668	3.0256000	-1.1167378
18.8666668	3.0743999	-1.1780972
18.8666668	3.1231999	-1.1412816
18.8666668	3.1719999	-0.9694757
18.8666668	3.2207999	-1.2210488
18.8666668	3.2695999	-1.2271848
18.8666668	3.3183999	-1.1167378
18.8666668	3.3671999	-0.9756117
18.8666668	3.4159999	-0.7056313
18.8666668	3.4647999	-0.5154176
18.8666668	3.5135999	-0.5154176
18.8666668	3.5623999	-0.5276895
18.8666668	3.6111999	-0.6074562
18.8666668	3.6599998	-0.6381359
18.8666668	3.7087998	-0.6381359
18.8666668	3.7575998	-0.6197281
18.8666668	3.8063998	-0.8958449
18.8666668	3.8551998	-0.6810875
18.8666668	3.9039998	-0.6013203
18.8666668	3.9527998	-0.7976699
18.8666668	4.0016003	-0.8038058
18.8666668	4.0503998	-1.0676508
18.8666668	4.0991998	-1.2271848
18.8666668	4.1479998	-0.9756117
18.8666668	4.1967998	-0.9940195
18.8666668	4.2455997	-0.7976699
18.8666668	4.2943997	-0.7976699
18.8666668	4.3431997	-0.7240391
18.8666668	4.3919997	-0.6933594
18.8666668	4.4407997	-0.6258640
18.8666668	4.4895997	-0.6320000
18.8666668	4.5383997	-0.9572039
18.8666668	4.5871997	-0.7485828
18.8666668	4.6359997	-0.7424469
18.8666668	4.6847997	-0.7547188
18.8666668	4.7336001	-0.8038058
18.8666668	4.7824001	613586.0000000
18.8666668	4.8312001	613586.0000000
18.8666668	4.8800001	-0.7363110
18.8666668	4.9288001	-0.7240391
18.8666668	4.9776001	613586.0000000
18.8666668	5.0264001	613586.0000000

18.8666668	5.0752001	613586.0000000
18.8666668	5.1240001	613586.0000000
18.8666668	5.1728001	613586.0000000
18.8666668	5.2216001	613586.0000000
18.8666668	5.2704000	613586.0000000
18.8666668	5.3192000	613586.0000000
18.8666668	5.3680000	613586.0000000
18.8666668	5.4168000	613586.0000000
18.8666668	5.4656000	-0.7485828
18.8666668	5.5144000	613586.0000000
18.8666668	5.5632000	613586.0000000
18.8666668	5.6120000	613586.0000000
18.8666668	5.6608000	613586.0000000
18.8666668	5.7096000	613586.0000000
18.8666668	5.7584000	613586.0000000
18.8666668	5.8072000	613586.0000000
18.8666668	5.8559999	613586.0000000
18.8666668	5.9047999	613586.0000000
18.8666668	5.9535999	613586.0000000
18.8666668	6.0023999	613586.0000000
18.8833332	0.2928000	-3.9699423
18.8833332	0.3416000	-3.5772433
18.8833332	0.3904000	-3.0372820
18.8833332	0.4392000	-2.9084277
18.8833332	0.4880000	-2.4605052
18.8833332	0.5368000	-2.1353011
18.8833332	0.5856000	-1.7364664
18.8833332	0.6344000	-1.0308352
18.8833332	0.6832000	-1.3008156
18.8833332	0.7320000	-1.5462527
18.8833332	0.7808000	-1.7364664
18.8833332	0.8296000	-1.4051266
18.8833332	0.8784000	-1.4112625
18.8833332	0.9272000	-1.5830684
18.8833332	0.9760000	-1.3130875
18.8833332	1.0247999	-1.3069515
18.8833332	1.0735999	-1.3069515
18.8833332	1.1223999	-1.2026410
18.8833332	1.1711999	-1.1903691
18.8833332	1.2200000	-1.1965051
18.8833332	1.2688000	-1.2149129
18.8833332	1.3176000	-1.2210488
18.8833332	1.3664000	-1.3253593
18.8833332	1.4152000	-1.3192234
18.8833332	1.4640000	-1.3130875
18.8833332	1.5128000	-1.2946796
18.8833332	1.5616000	-1.2640004
18.8833332	1.6104000	-1.2640004
18.8833332	1.6592000	-1.2517285
18.8833332	1.7079999	-1.2333207
18.8833332	1.7567999	-1.2394567
18.8833332	1.8055999	-1.2946796
18.8833332	1.8543999	-1.1842332
18.8833332	1.9031999	-1.2824078
18.8833332	1.9519999	-1.1596894
18.8833332	2.0007999	-1.1474175
18.8833332	2.0495999	-1.1474175

18.8833332	2.0983999	-1.1719613
18.8833332	2.1471999	-1.0737867
18.8833332	2.1959999	-1.2333207
18.8833332	2.2447999	-1.0921946
18.8833332	2.2935998	-1.3069515
18.8833332	2.3423998	-1.1658254
18.8833332	2.3912001	-1.3560390
18.8833332	2.4400001	-1.3069515
18.8833332	2.4888000	-1.2394567
18.8833332	2.5376000	-1.2885437
18.8833332	2.5864000	-1.2946796
18.8833332	2.6352000	-1.3805828
18.8833332	2.6840000	-1.3130875
18.8833332	2.7328000	-1.1903691
18.8833332	2.7816000	-1.4235344
18.8833332	2.8304000	-1.3192234
18.8833332	2.8792000	-1.1658254
18.8833332	2.9280000	-1.1535535
18.8833332	2.9768000	-1.1412816
18.8833332	3.0256000	-1.1351457
18.8833332	3.0743999	-1.0860586
18.8833332	3.1231999	-1.0492430
18.8833332	3.1719999	-1.0369711
18.8833332	3.2207999	-0.9694757
18.8833332	3.2695999	-0.6135921
18.8833332	3.3183999	-0.5890489
18.8833332	3.3671999	-0.6994953
18.8833332	3.4159999	-0.6994953
18.8833332	3.4647999	-0.7485828
18.8833332	3.5135999	-0.9326606
18.8833332	3.5623999	-0.8283496
18.8833332	3.6111999	-0.8283496
18.8833332	3.6599998	-0.9081168
18.8833332	3.7087998	-0.8958449
18.8833332	3.7575998	-1.0001554
18.8833332	3.8063998	-1.1106019
18.8833332	3.8551998	-1.1044660
18.8833332	3.9039998	-1.0921946
18.8833332	3.9527998	-1.0676508
18.8833332	4.0016003	-0.9449320
18.8833332	4.0503998	-0.8344855
18.8833332	4.0991998	-0.8406215
18.8833332	4.1479998	-0.8528934
18.8833332	4.1967998	-0.8774371
18.8833332	4.2455997	-0.8713012
18.8833332	4.2943997	-1.0001554
18.8833332	4.3431997	-0.9081168
18.8833332	4.3919997	-0.9142528
18.8833332	4.4407997	-1.0983305
18.8833332	4.4895997	-1.0921946
18.8833332	4.5383997	-1.1719613
18.8833332	4.5871997	-0.7915339
18.8833332	4.6359997	-0.7976699
18.8833332	4.6847997	-0.7976699
18.8833332	4.7336001	-0.7240391
18.8833332	4.7824001	-0.4049711
18.8833332	4.8312001	613586.0000000

18.8833332	4.8800001	613586.0000000
18.8833332	4.9288001	613586.0000000
18.8833332	4.9776001	613586.0000000
18.8833332	5.0264001	-0.5767770
18.8833332	5.0752001	613586.0000000
18.8833332	5.1240001	-0.6933594
18.8833332	5.1728001	-0.6872234
18.8833332	5.2216001	-0.6994953
18.8833332	5.2704000	-0.7179031
18.8833332	5.3192000	-0.7179031
18.8833332	5.3680000	-0.7240391
18.8833332	5.4168000	613586.0000000
18.8833332	5.4656000	613586.0000000
18.8833332	5.5144000	613586.0000000
18.8833332	5.5632000	-0.7179031
18.8833332	5.6120000	-0.7179031
18.8833332	5.6608000	-0.7853980
18.8833332	5.7096000	-0.7792621
18.8833332	5.7584000	-0.7853980
18.8833332	5.8072000	-0.7915339
18.8833332	5.8559999	-0.7976699
18.8833332	5.9047999	613586.0000000
18.8833332	5.9535999	613586.0000000
18.8833332	6.0023999	613586.0000000
18.8999996	0.2928000	-4.4669523
18.8999996	0.3416000	-3.6263306
18.8999996	0.3904000	-3.2459035
18.8999996	0.4392000	-3.2397676
18.8999996	0.4880000	-2.8347967
18.8999996	0.5368000	-1.7855535
18.8999996	0.5856000	-1.7732821
18.8999996	0.6344000	-1.2640004
18.8999996	0.6832000	-1.2762718
18.8999996	0.7320000	-1.3560390
18.8999996	0.7808000	-1.7241945
18.8999996	0.8296000	-1.7180586
18.8999996	0.8784000	-1.3744469
18.8999996	0.9272000	-1.3621750
18.8999996	0.9760000	-1.0308352
18.8999996	1.0247999	-1.3008156
18.8999996	1.0735999	-1.2946796
18.8999996	1.1223999	-1.3376312
18.8999996	1.1711999	-1.3376312
18.8999996	1.2200000	-1.2026410
18.8999996	1.2688000	-1.1965051
18.8999996	1.3176000	-1.2210488
18.8999996	1.3664000	-1.2149129
18.8999996	1.4152000	-1.2149129
18.8999996	1.4640000	-1.2149129
18.8999996	1.5128000	-1.3621750
18.8999996	1.5616000	-1.1351457
18.8999996	1.6104000	-1.2333207
18.8999996	1.6592000	-1.2824078
18.8999996	1.7079999	-1.2824078
18.8999996	1.7567999	-1.2824078
18.8999996	1.8055999	-1.1290097
18.8999996	1.8543999	-1.4051266

18.8999996	1.9031999	-1.4112625
18.8999996	1.9519999	-1.2885437
18.8999996	2.0007999	-1.1535535
18.8999996	2.0495999	-1.2026410
18.8999996	2.0983999	-1.2210488
18.8999996	2.1471999	-1.2210488
18.8999996	2.1959999	-1.2149129
18.8999996	2.2447999	-1.2824078
18.8999996	2.2935998	-1.3683109
18.8999996	2.3423998	-1.2946796
18.8999996	2.3912001	-1.2271848
18.8999996	2.4400001	-1.3192234
18.8999996	2.4888000	-1.3314953
18.8999996	2.5376000	-1.3621750
18.8999996	2.5864000	-1.2087770
18.8999996	2.6352000	-1.2149129
18.8999996	2.6840000	-1.3499031
18.8999996	2.7328000	-1.3499031
18.8999996	2.7816000	-1.3499031
18.8999996	2.8304000	-1.3069515
18.8999996	2.8792000	-1.3928547
18.8999996	2.9280000	-1.3867188
18.8999996	2.9768000	-1.0308352
18.8999996	3.0256000	-1.0308352
18.8999996	3.0743999	-1.0246992
18.8999996	3.1231999	-0.9878836
18.8999996	3.1719999	-0.8406215
18.8999996	3.2207999	-0.9081168
18.8999996	3.2695999	-0.8099418
18.8999996	3.3183999	-0.7485828
18.8999996	3.3671999	-0.6626797
18.8999996	3.4159999	-0.8283496
18.8999996	3.4647999	-0.9387960
18.8999996	3.5135999	-0.9326606
18.8999996	3.5623999	-0.9265246
18.8999996	3.6111999	-1.0124273
18.8999996	3.6599998	-1.0124273
18.8999996	3.7087998	-1.0124273
18.8999996	3.7575998	-0.9203887
18.8999996	3.8063998	-0.8774371
18.8999996	3.8551998	-0.8590293
18.8999996	3.9039998	-0.6749516
18.8999996	3.9527998	-0.6749516
18.8999996	4.0016003	-0.8958449
18.8999996	4.0503998	-0.9817476
18.8999996	4.0991998	-0.8160777
18.8999996	4.1479998	-0.8528934
18.8999996	4.1967998	-0.7792621
18.8999996	4.2455997	-0.7669902
18.8999996	4.2943997	-0.7424469
18.8999996	4.3431997	-0.6013203
18.8999996	4.3919997	-0.6994953
18.8999996	4.4407997	-0.7301750
18.8999996	4.4895997	-0.8038058
18.8999996	4.5383997	-0.6013203
18.8999996	4.5871997	-0.2147574
18.8999996	4.6359997	-0.2086215

18.8999996	4.6847997	-0.2393012
18.8999996	4.7336001	613586.0000000
18.8999996	4.7824001	613586.0000000
18.8999996	4.8312001	613586.0000000
18.8999996	4.8800001	613586.0000000
18.8999996	4.9288001	613586.0000000
18.8999996	4.9776001	613586.0000000
18.8999996	5.0264001	-0.5583692
18.8999996	5.0752001	613586.0000000
18.8999996	5.1240001	-0.5829129
18.8999996	5.1728001	-0.5767770
18.8999996	5.2216001	613586.0000000
18.8999996	5.2704000	-0.7056313
18.8999996	5.3192000	-0.7117672
18.8999996	5.3680000	-0.7179031
18.8999996	5.4168000	-0.6749516
18.8999996	5.4656000	613586.0000000
18.8999996	5.5144000	613586.0000000
18.8999996	5.5632000	613586.0000000
18.8999996	5.6120000	613586.0000000
18.8999996	5.6608000	613586.0000000
18.8999996	5.7096000	613586.0000000
18.8999996	5.7584000	613586.0000000
18.8999996	5.8072000	613586.0000000
18.8999996	5.8559999	613586.0000000
18.8999996	5.9047999	613586.0000000
18.8999996	5.9535999	613586.0000000
18.8999996	6.0023999	613586.0000000
18.9166660	0.2928000	-3.4790685
18.9166660	0.3416000	-3.1661365
18.9166660	0.3904000	-3.1661365
18.9166660	0.4392000	-3.1661365
18.9166660	0.4880000	-2.6507189
18.9166660	0.5368000	-2.4727771
18.9166660	0.5856000	-2.0248547
18.9166660	0.6344000	-2.0125828
18.9166660	0.6832000	-1.2271848
18.9166660	0.7320000	-0.8467574
18.9166660	0.7808000	-1.4910293
18.9166660	0.8296000	-1.3867188
18.9166660	0.8784000	-1.3069515
18.9166660	0.9272000	-1.3008156
18.9166660	0.9760000	-1.0737867
18.9166660	1.0247999	-0.9633398
18.9166660	1.0735999	-1.1228738
18.9166660	1.1223999	-1.1106019
18.9166660	1.1711999	-1.1106019
18.9166660	1.2200000	-1.1474175
18.9166660	1.2688000	-1.2640004
18.9166660	1.3176000	-1.0983305
18.9166660	1.3664000	-1.2149129
18.9166660	1.4152000	-1.1290097
18.9166660	1.4640000	-1.3069515
18.9166660	1.5128000	-1.3928547
18.9166660	1.5616000	-1.1842332
18.9166660	1.6104000	-1.1842332
18.9166660	1.6592000	-1.2946796

18.9166660	1.7079999	-1.2946796
18.9166660	1.7567999	-1.2394567
18.9166660	1.8055999	-1.2455926
18.9166660	1.8543999	-1.3989906
18.9166660	1.9031999	-1.1535535
18.9166660	1.9519999	-1.2210488
18.9166660	2.0007999	-1.1903691
18.9166660	2.0495999	-1.1903691
18.9166660	2.0983999	-1.2394567
18.9166660	2.1471999	-1.2149129
18.9166660	2.1959999	-1.2026410
18.9166660	2.2447999	-1.0799227
18.9166660	2.2935998	-1.1535535
18.9166660	2.3423998	-1.3928547
18.9166660	2.3912001	-1.3928547
18.9166660	2.4400001	-1.2640004
18.9166660	2.4888000	-1.2026410
18.9166660	2.5376000	-1.3683109
18.9166660	2.5864000	-1.3683109
18.9166660	2.6352000	-1.4051266
18.9166660	2.6840000	-1.1228738
18.9166660	2.7328000	-1.1106019
18.9166660	2.7816000	-1.1044660
18.9166660	2.8304000	-1.0983305
18.9166660	2.8792000	-1.0921946
18.9166660	2.9280000	-0.9817476
18.9166660	2.9768000	-0.8590293
18.9166660	3.0256000	-0.8651652
18.9166660	3.0743999	-0.9756117
18.9166660	3.1231999	-0.9694757
18.9166660	3.1719999	-0.6442719
18.9166660	3.2207999	-0.8835731
18.9166660	3.2695999	-0.8590293
18.9166660	3.3183999	-0.7853980
18.9166660	3.3671999	-0.7301750
18.9166660	3.4159999	-0.7301750
18.9166660	3.4647999	-0.7976699
18.9166660	3.5135999	-0.7976699
18.9166660	3.5623999	-0.7976699
18.9166660	3.6111999	-0.8713012
18.9166660	3.6599998	-0.8406215
18.9166660	3.7087998	-0.6994953
18.9166660	3.7575998	-0.6933594
18.9166660	3.8063998	-0.6810875
18.9166660	3.8551998	-0.6565437
18.9166660	3.9039998	-0.6442719
18.9166660	3.9527998	-0.6810875
18.9166660	4.0016003	-0.7915339
18.9166660	4.0503998	-0.7424469
18.9166660	4.0991998	-0.7731261
18.9166660	4.1479998	-0.6810875
18.9166660	4.1967998	-0.6994953
18.9166660	4.2455997	-0.7056313
18.9166660	4.2943997	-0.7240391
18.9166660	4.3431997	-0.7853980
18.9166660	4.3919997	-0.7240391
18.9166660	4.4407997	-0.7301750

18.9166660	4.4895997	-0.7363110
18.9166660	4.5383997	-0.3252039
18.9166660	4.5871997	-0.5583692
18.9166660	4.6359997	-0.1656699
18.9166660	4.6847997	-0.1656699
18.9166660	4.7336001	613586.0000000
18.9166660	4.7824001	613586.0000000
18.9166660	4.8312001	613586.0000000
18.9166660	4.8800001	613586.0000000
18.9166660	4.9288001	-0.1472621
18.9166660	4.9776001	-0.4786019
18.9166660	5.0264001	-0.4111071
18.9166660	5.0752001	-0.3067961
18.9166660	5.1240001	-0.3006601
18.9166660	5.1728001	-0.2945242
18.9166660	5.2216001	-0.1227183
18.9166660	5.2704000	-0.1227183
18.9166660	5.3192000	-0.1165824
18.9166660	5.3680000	613586.0000000
18.9166660	5.4168000	-0.4663301
18.9166660	5.4656000	613586.0000000
18.9166660	5.5144000	613586.0000000
18.9166660	5.5632000	-0.7179031
18.9166660	5.6120000	-0.6013203
18.9166660	5.6608000	-0.6074562
18.9166660	5.7096000	-0.6135921
18.9166660	5.7584000	-0.6197281
18.9166660	5.8072000	-0.5583692
18.9166660	5.8559999	-0.5522332
18.9166660	5.9047999	613586.0000000
18.9166660	5.9535999	613586.0000000
18.9166660	6.0023999	613586.0000000
18.9333324	0.2928000	-2.8838840
18.9333324	0.3416000	-2.8838840
18.9333324	0.3904000	-2.7611654
18.9333324	0.4392000	-2.7611654
18.9333324	0.4880000	-2.6445830
18.9333324	0.5368000	-2.2641559
18.9333324	0.5856000	-1.9880390
18.9333324	0.6344000	-1.9880390
18.9333324	0.6832000	-1.5830684
18.9333324	0.7320000	-1.4235344
18.9333324	0.7808000	-1.2394567
18.9333324	0.8296000	-1.2455926
18.9333324	0.8784000	-1.2578645
18.9333324	0.9272000	-1.7671461
18.9333324	0.9760000	-1.7732821
18.9333324	1.0247999	-1.3253593
18.9333324	1.0735999	-1.2640004
18.9333324	1.1223999	-1.0246992
18.9333324	1.1711999	-1.0492430
18.9333324	1.2200000	-1.0676508
18.9333324	1.2688000	-1.0676508
18.9333324	1.3176000	-1.1290097
18.9333324	1.3664000	-1.1290097
18.9333324	1.4152000	-1.2394567
18.9333324	1.4640000	-1.0983305

18.9333324	1.5128000	-1.0983305
18.9333324	1.5616000	-1.2578645
18.9333324	1.6104000	-1.2701364
18.9333324	1.6592000	-1.2701364
18.9333324	1.7079999	-1.2640004
18.9333324	1.7567999	-1.2640004
18.9333324	1.8055999	-1.1290097
18.9333324	1.8543999	-1.1290097
18.9333324	1.9031999	-1.2517285
18.9333324	1.9519999	-1.2517285
18.9333324	2.0007999	-1.1903691
18.9333324	2.0495999	-1.0369711
18.9333324	2.0983999	-1.2762718
18.9333324	2.1471999	-1.2946796
18.9333324	2.1959999	-1.1965051
18.9333324	2.2447999	-1.3928547
18.9333324	2.2935998	-1.3989906
18.9333324	2.3423998	-1.4603496
18.9333324	2.3912001	-1.2026410
18.9333324	2.4400001	-1.1965051
18.9333324	2.4888000	-1.1903691
18.9333324	2.5376000	-1.4296703
18.9333324	2.5864000	-1.3008156
18.9333324	2.6352000	-1.3867188
18.9333324	2.6840000	-1.2640004
18.9333324	2.7328000	-1.2640004
18.9333324	2.7816000	-1.2701364
18.9333324	2.8304000	-1.1474175
18.9333324	2.8792000	-1.1412816
18.9333324	2.9280000	-1.1106019
18.9333324	2.9768000	-1.1044660
18.9333324	3.0256000	-0.8528934
18.9333324	3.0743999	-1.0246992
18.9333324	3.1231999	-0.8590293
18.9333324	3.1719999	-0.8283496
18.9333324	3.2207999	-0.8222136
18.9333324	3.2695999	-0.9142528
18.9333324	3.3183999	-0.8651652
18.9333324	3.3671999	-0.8590293
18.9333324	3.4159999	-0.9326606
18.9333324	3.4647999	-0.9142528
18.9333324	3.5135999	-0.7915339
18.9333324	3.5623999	-0.7915339
18.9333324	3.6111999	-0.5460973
18.9333324	3.6599998	-0.8590293
18.9333324	3.7087998	-0.8590293
18.9333324	3.7575998	-0.6258640
18.9333324	3.8063998	-0.6258640
18.9333324	3.8551998	-0.8651652
18.9333324	3.9039998	-0.7608547
18.9333324	3.9527998	-0.7669902
18.9333324	4.0016003	-0.5951843
18.9333324	4.0503998	-0.6013203
18.9333324	4.0991998	-0.8283496
18.9333324	4.1479998	-0.8160777
18.9333324	4.1967998	-0.7424469
18.9333324	4.2455997	-0.7485828

18.9333324	4.2943997	-0.7424469
18.9333324	4.3431997	-0.7301750
18.9333324	4.3919997	-0.7301750
18.9333324	4.4407997	-0.7301750
18.9333324	4.4895997	-0.3190680
18.9333324	4.5383997	-0.8099418
18.9333324	4.5871997	-0.3742914
18.9333324	4.6359997	-0.2393012
18.9333324	4.6847997	-0.2331653
18.9333324	4.7336001	613586.0000000
18.9333324	4.7824001	613586.0000000
18.9333324	4.8312001	613586.0000000
18.9333324	4.8800001	613586.0000000
18.9333324	4.9288001	-0.2515731
18.9333324	4.9776001	-0.3742914
18.9333324	5.0264001	-0.3497477
18.9333324	5.0752001	-0.3497477
18.9333324	5.1240001	-0.3313398
18.9333324	5.1728001	-0.5031457
18.9333324	5.2216001	-0.5829129
18.9333324	5.2704000	-0.5829129
18.9333324	5.3192000	-0.6197281
18.9333324	5.3680000	-0.5460973
18.9333324	5.4168000	-0.5338254
18.9333324	5.4656000	-0.5154176
18.9333324	5.5144000	-0.4172430
18.9333324	5.5632000	-0.6074562
18.9333324	5.6120000	-0.6442719
18.9333324	5.6608000	-0.6565437
18.9333324	5.7096000	-0.6504078
18.9333324	5.7584000	-0.6565437
18.9333324	5.8072000	-0.6565437
18.9333324	5.8559999	-0.7547188
18.9333324	5.9047999	-0.7485828
18.9333324	5.9535999	-0.8160777
18.9333324	6.0023999	-0.8222136
18.9500008	0.2928000	-3.7674570
18.9500008	0.3416000	-3.0740976
18.9500008	0.3904000	-3.2274957
18.9500008	0.4392000	-2.7734373
18.9500008	0.4880000	-2.2948353
18.9500008	0.5368000	-2.2948353
18.9500008	0.5856000	-2.1843886
18.9500008	0.6344000	-2.1168938
18.9500008	0.6832000	-2.1353011
18.9500008	0.7320000	-1.8653207
18.9500008	0.7808000	-1.4664855
18.9500008	0.8296000	-1.3192234
18.9500008	0.8784000	-1.1412816
18.9500008	0.9272000	-1.4726214
18.9500008	0.9760000	-1.2885437
18.9500008	1.0247999	-1.2824078
18.9500008	1.0735999	-1.2824078
18.9500008	1.1223999	-1.2394567
18.9500008	1.1711999	-1.2210488
18.9500008	1.2200000	-0.9449320
18.9500008	1.2688000	-1.1658254

18.9500008	1.3176000	-1.1658254
18.9500008	1.3664000	-1.2210488
18.9500008	1.4152000	-1.0737867
18.9500008	1.4640000	-1.1044660
18.9500008	1.5128000	-1.1228738
18.9500008	1.5616000	-1.1780972
18.9500008	1.6104000	-1.1596894
18.9500008	1.6592000	-1.1290097
18.9500008	1.7079999	-1.1106019
18.9500008	1.7567999	-1.0492430
18.9500008	1.8055999	-1.0553789
18.9500008	1.8543999	-1.0431070
18.9500008	1.9031999	-1.0308352
18.9500008	1.9519999	-1.2455926
18.9500008	2.0007999	-1.2455926
18.9500008	2.0495999	-1.1351457
18.9500008	2.0983999	-1.3069515
18.9500008	2.1471999	-1.3560390
18.9500008	2.1959999	-1.3805828
18.9500008	2.2447999	-1.3805828
18.9500008	2.2935998	-1.3683109
18.9500008	2.3423998	-1.3192234
18.9500008	2.3912001	-1.3192234
18.9500008	2.4400001	-1.3069515
18.9500008	2.4888000	-1.2517285
18.9500008	2.5376000	-1.3867188
18.9500008	2.5864000	-1.3744469
18.9500008	2.6352000	-1.2885437
18.9500008	2.6840000	-1.1903691
18.9500008	2.7328000	-1.2946796
18.9500008	2.7816000	-1.3008156
18.9500008	2.8304000	-1.0615149
18.9500008	2.8792000	-1.1167378
18.9500008	2.9280000	-1.1167378
18.9500008	2.9768000	-1.0431070
18.9500008	3.0256000	-1.0062914
18.9500008	3.0743999	-0.9387960
18.9500008	3.1231999	-0.9019809
18.9500008	3.1719999	-0.9019809
18.9500008	3.2207999	-0.8897090
18.9500008	3.2695999	-0.8774371
18.9500008	3.3183999	-0.6258640
18.9500008	3.3671999	-0.6258640
18.9500008	3.4159999	-0.4233789
18.9500008	3.4647999	-0.7056313
18.9500008	3.5135999	-0.7547188
18.9500008	3.5623999	-0.7976699
18.9500008	3.6111999	-0.7976699
18.9500008	3.6599998	-0.9694757
18.9500008	3.7087998	-0.6994953
18.9500008	3.7575998	-0.8651652
18.9500008	3.8063998	-0.8038058
18.9500008	3.8551998	-0.9265246
18.9500008	3.9039998	-0.8222136
18.9500008	3.9527998	-0.8283496
18.9500008	4.0016003	-0.8283496
18.9500008	4.0503998	-0.6320000

18.9500008	4.0991998	-0.6320000
18.9500008	4.1479998	-0.6197281
18.9500008	4.1967998	-0.5829129
18.9500008	4.2455997	-0.5951843
18.9500008	4.2943997	-0.5951843
18.9500008	4.3431997	-0.6442719
18.9500008	4.3919997	-0.5092816
18.9500008	4.4407997	-0.5092816
18.9500008	4.4895997	-0.5031457
18.9500008	4.5383997	-0.2761164
18.9500008	4.5871997	-0.2945242
18.9500008	4.6359997	-0.3497477
18.9500008	4.6847997	613586.0000000
18.9500008	4.7336001	613586.0000000
18.9500008	4.7824001	-0.1779418
18.9500008	4.8312001	-0.1963496
18.9500008	4.8800001	-0.3006601
18.9500008	4.9288001	-0.2945242
18.9500008	4.9776001	-0.0736313
18.9500008	5.0264001	-0.5215535
18.9500008	5.0752001	-0.5215535
18.9500008	5.1240001	-0.6258640
18.9500008	5.1728001	-0.7301750
18.9500008	5.2216001	-0.6197281
18.9500008	5.2704000	-0.7915339
18.9500008	5.3192000	-0.7792621
18.9500008	5.3680000	-0.7240391
18.9500008	5.4168000	-0.7301750
18.9500008	5.4656000	-0.7485828
18.9500008	5.5144000	-0.8160777
18.9500008	5.5632000	-0.7547188
18.9500008	5.6120000	-0.7363110
18.9500008	5.6608000	-0.7056313
18.9500008	5.7096000	-0.7056313
18.9500008	5.7584000	-0.7056313
18.9500008	5.8072000	-0.6626797
18.9500008	5.8559999	-0.6135921
18.9500008	5.9047999	613586.0000000
18.9500008	5.9535999	613586.0000000
18.9500008	6.0023999	-0.8283496
18.9666672	0.2928000	-3.3502142
18.9666672	0.3416000	-3.0618258
18.9666672	0.3904000	-3.0556898
18.9666672	0.4392000	-2.9268355
18.9666672	0.4880000	-2.9145637
18.9666672	0.5368000	-2.4114180
18.9666672	0.5856000	-1.3867188
18.9666672	0.6344000	-1.0615149
18.9666672	0.6832000	-1.0369711
18.9666672	0.7320000	-1.2578645
18.9666672	0.7808000	-1.2517285
18.9666672	0.8296000	-1.2578645
18.9666672	0.8784000	-1.5646605
18.9666672	0.9272000	-1.3867188
18.9666672	0.9760000	-1.2149129
18.9666672	1.0247999	-1.2271848
18.9666672	1.0735999	-1.1106019

18.9666672	1.1223999	-1.1106019
18.9666672	1.1711999	-1.1044660
18.9666672	1.2200000	-1.0921946
18.9666672	1.2688000	-1.1780972
18.9666672	1.3176000	-1.1780972
18.9666672	1.3664000	-1.1719613
18.9666672	1.4152000	-1.0492430
18.9666672	1.4640000	-1.2271848
18.9666672	1.5128000	-1.1535535
18.9666672	1.5616000	-1.1167378
18.9666672	1.6104000	-1.0431070
18.9666672	1.6592000	-1.0615149
18.9666672	1.7079999	-1.0553789
18.9666672	1.7567999	-1.0615149
18.9666672	1.8055999	-1.0676508
18.9666672	1.8543999	-1.2149129
18.9666672	1.9031999	-1.2149129
18.9666672	1.9519999	-1.1719613
18.9666672	2.0007999	-1.1842332
18.9666672	2.0495999	-1.5094371
18.9666672	2.0983999	-1.5094371
18.9666672	2.1471999	-1.2455926
18.9666672	2.1959999	-1.2394567
18.9666672	2.2447999	-1.2578645
18.9666672	2.2935998	-1.2578645
18.9666672	2.3423998	-1.3621750
18.9666672	2.3912001	-1.4235344
18.9666672	2.4400001	-1.1290097
18.9666672	2.4888000	-1.2762718
18.9666672	2.5376000	-1.2762718
18.9666672	2.5864000	-1.1903691
18.9666672	2.6352000	-1.1719613
18.9666672	2.6840000	-1.2824078
18.9666672	2.7328000	-1.1596894
18.9666672	2.7816000	-1.0799227
18.9666672	2.8304000	-1.0369711
18.9666672	2.8792000	-1.0492430
18.9666672	2.9280000	-1.0553789
18.9666672	2.9768000	-0.9265246
18.9666672	3.0256000	-0.9633398
18.9666672	3.0743999	-0.9449320
18.9666672	3.1231999	-0.6933594
18.9666672	3.1719999	-0.6872234
18.9666672	3.2207999	-0.8590293
18.9666672	3.2695999	-0.7731261
18.9666672	3.3183999	-0.7731261
18.9666672	3.3671999	-0.8160777
18.9666672	3.4159999	-0.9081168
18.9666672	3.4647999	-0.7915339
18.9666672	3.5135999	-0.8713012
18.9666672	3.5623999	-0.7853980
18.9666672	3.6111999	-0.8038058
18.9666672	3.6599998	-0.7731261
18.9666672	3.7087998	-0.7669902
18.9666672	3.7575998	-0.6626797
18.9666672	3.8063998	-0.8038058
18.9666672	3.8551998	-0.6197281

18.9666672	3.9039998	-0.6197281
18.9666672	3.9527998	-0.6688156
18.9666672	4.0016003	-0.6135921
18.9666672	4.0503998	-0.6197281
18.9666672	4.0991998	-0.4479222
18.9666672	4.1479998	-0.5645051
18.9666672	4.1967998	-0.7056313
18.9666672	4.2455997	-0.5154176
18.9666672	4.2943997	-0.5276895
18.9666672	4.3431997	-0.4663301
18.9666672	4.3919997	-0.4663301
18.9666672	4.4407997	-0.4724660
18.9666672	4.4895997	-0.3681555
18.9666672	4.5383997	-0.3681555
18.9666672	4.5871997	-0.3742914
18.9666672	4.6359997	613586.0000000
18.9666672	4.6847997	613586.0000000
18.9666672	4.7336001	-0.2883883
18.9666672	4.7824001	-0.2945242
18.9666672	4.8312001	-0.4049711
18.9666672	4.8800001	-0.5092816
18.9666672	4.9288001	-0.5092816
18.9666672	4.9776001	-0.5583692
18.9666672	5.0264001	-0.5583692
18.9666672	5.0752001	-0.4601941
18.9666672	5.1240001	-0.5767770
18.9666672	5.1728001	-0.5706410
18.9666672	5.2216001	-0.5706410
18.9666672	5.2704000	-0.5767770
18.9666672	5.3192000	-0.6135921
18.9666672	5.3680000	-0.5460973
18.9666672	5.4168000	-0.5399613
18.9666672	5.4656000	-0.5399613
18.9666672	5.5144000	-0.5031457
18.9666672	5.5632000	-0.5951843
18.9666672	5.6120000	-0.5951843
18.9666672	5.6608000	-0.5460973
18.9666672	5.7096000	-0.5276895
18.9666672	5.7584000	-0.5154176
18.9666672	5.8072000	-0.5215535
18.9666672	5.8559999	-0.5276895
18.9666672	5.9047999	-0.5890489
18.9666672	5.9535999	-0.5890489
18.9666672	6.0023999	-0.5522332
18.9833336	0.2928000	-3.3379424
18.9833336	0.3416000	-2.8163888
18.9833336	0.3904000	-2.8163888
18.9833336	0.4392000	-2.4359615
18.9833336	0.4880000	-2.4727771
18.9833336	0.5368000	-2.4727771
18.9833336	0.5856000	-1.9144082
18.9833336	0.6344000	-1.9021363
18.9833336	0.6832000	-1.7610102
18.9833336	0.7320000	-0.4970098
18.9833336	0.7808000	-1.0737867
18.9833336	0.8296000	-1.2210488
18.9833336	0.8784000	-1.2394567

18.9833336	0.9272000	-1.1228738
18.9833336	0.9760000	-1.1167378
18.9833336	1.0247999	-1.1228738
18.9833336	1.0735999	-1.1780972
18.9833336	1.1223999	-1.1228738
18.9833336	1.1711999	-1.0431070
18.9833336	1.2200000	-1.0492430
18.9833336	1.2688000	-0.9633398
18.9833336	1.3176000	-1.0124273
18.9833336	1.3664000	-1.2087770
18.9833336	1.4152000	-1.1903691
18.9833336	1.4640000	-1.1780972
18.9833336	1.5128000	-1.0676508
18.9833336	1.5616000	-1.1535535
18.9833336	1.6104000	-1.1412816
18.9833336	1.6592000	-1.2026410
18.9833336	1.7079999	-1.0615149
18.9833336	1.7567999	-1.0431070
18.9833336	1.8055999	-1.0553789
18.9833336	1.8543999	-1.0431070
18.9833336	1.9031999	-1.0369711
18.9833336	1.9519999	-1.1965051
18.9833336	2.0007999	-1.1903691
18.9833336	2.0495999	-1.5707965
18.9833336	2.0983999	-1.3621750
18.9833336	2.1471999	-1.3744469
18.9833336	2.1959999	-1.3683109
18.9833336	2.2447999	-1.3560390
18.9833336	2.2935998	-1.4480777
18.9833336	2.3423998	-1.4603496
18.9833336	2.3912001	-1.2762718
18.9833336	2.4400001	-1.3314953
18.9833336	2.4888000	-1.3805828
18.9833336	2.5376000	-1.3989906
18.9833336	2.5864000	-1.4664855
18.9833336	2.6352000	-1.3928547
18.9833336	2.6840000	-1.2824078
18.9833336	2.7328000	-1.3314953
18.9833336	2.7816000	-1.3130875
18.9833336	2.8304000	-1.0860586
18.9833336	2.8792000	-1.1596894
18.9833336	2.9280000	-1.0246992
18.9833336	2.9768000	-1.0001554
18.9833336	3.0256000	-0.8467574
18.9833336	3.0743999	-0.9817476
18.9833336	3.1231999	-0.8528934
18.9833336	3.1719999	-0.8467574
18.9833336	3.2207999	-0.8590293
18.9833336	3.2695999	-0.7853980
18.9833336	3.3183999	-0.7731261
18.9833336	3.3671999	-0.7976699
18.9833336	3.4159999	-0.7424469
18.9833336	3.4647999	-0.7301750
18.9833336	3.5135999	-0.7179031
18.9833336	3.5623999	-0.5460973
18.9833336	3.6111999	-1.0308352
18.9833336	3.6599998	-1.0062914

18.9833336	3.7087998	-0.8835731
18.9833336	3.7575998	-0.9019809
18.9833336	3.8063998	-0.8222136
18.9833336	3.8551998	-0.8222136
18.9833336	3.9039998	-0.8713012
18.9833336	3.9527998	-0.8222136
18.9833336	4.0016003	-0.6933594
18.9833336	4.0503998	-0.8713012
18.9833336	4.0991998	-0.4295144
18.9833336	4.1479998	-0.4233789
18.9833336	4.1967998	-0.4172430
18.9833336	4.2455997	-0.3129320
18.9833336	4.2943997	-0.6320000
18.9833336	4.3431997	-0.6442719
18.9833336	4.3919997	-0.4233789
18.9833336	4.4407997	-0.4233789
18.9833336	4.4895997	-0.3436117
18.9833336	4.5383997	-0.3313398
18.9833336	4.5871997	-0.3252039
18.9833336	4.6359997	-0.5829129
18.9833336	4.6847997	-0.5706410
18.9833336	4.7336001	-0.4970098
18.9833336	4.7824001	-0.4970098
18.9833336	4.8312001	-0.4970098
18.9833336	4.8800001	-0.4970098
18.9833336	4.9288001	-0.4540582
18.9833336	4.9776001	-0.3988352
18.9833336	5.0264001	-0.6074562
18.9833336	5.0752001	-0.4908738
18.9833336	5.1240001	-0.4908738
18.9833336	5.1728001	-0.4970098
18.9833336	5.2216001	-0.5031457
18.9833336	5.2704000	-0.5645051
18.9833336	5.3192000	-0.5399613
18.9833336	5.3680000	-0.5890489
18.9833336	5.4168000	-0.6994953
18.9833336	5.4656000	-0.6320000
18.9833336	5.5144000	-0.6013203
18.9833336	5.5632000	-0.5767770
18.9833336	5.6120000	-0.6320000
18.9833336	5.6608000	-0.6320000
18.9833336	5.7096000	-0.5399613
18.9833336	5.7584000	-0.5399613
18.9833336	5.8072000	613586.0000000
18.9833336	5.8559999	-0.4908738
18.9833336	5.9047999	-0.4908738
18.9833336	5.9535999	613586.0000000
18.9833336	6.0023999	613586.0000000
19.0000000	0.2928000	-2.7734373
19.0000000	0.3416000	-2.5280004
19.0000000	0.3904000	-2.5218644
19.0000000	0.4392000	-2.2518840
19.0000000	0.4880000	-2.2764277
19.0000000	0.5368000	-2.0248547
19.0000000	0.5856000	-1.4480777
19.0000000	0.6344000	-1.5523887
19.0000000	0.6832000	-1.5646605

19.0000000	0.7320000	-1.5217090
19.0000000	0.7808000	-1.5830684
19.0000000	0.8296000	-1.3008156
19.0000000	0.8784000	-0.9019809
19.0000000	0.9272000	-0.9019809
19.0000000	0.9760000	-0.6872234
19.0000000	1.0247999	-0.6626797
19.0000000	1.0735999	-0.6565437
19.0000000	1.1223999	-1.0921946
19.0000000	1.1711999	-1.0921946
19.0000000	1.2200000	-1.0921946
19.0000000	1.2688000	-1.2026410
19.0000000	1.3176000	-1.2271848
19.0000000	1.3664000	-1.3069515
19.0000000	1.4152000	-1.3069515
19.0000000	1.4640000	-1.1903691
19.0000000	1.5128000	-1.1842332
19.0000000	1.5616000	-1.2149129
19.0000000	1.6104000	-1.1535535
19.0000000	1.6592000	-1.1535535
19.0000000	1.7079999	-1.0676508
19.0000000	1.7567999	-1.2026410
19.0000000	1.8055999	-1.2394567
19.0000000	1.8543999	-1.1474175
19.0000000	1.9031999	-1.1474175
19.0000000	1.9519999	-1.1842332
19.0000000	2.0007999	-1.1965051
19.0000000	2.0495999	-1.1965051
19.0000000	2.0983999	-1.3621750
19.0000000	2.1471999	-1.3376312
19.0000000	2.1959999	-1.4173985
19.0000000	2.2447999	-1.2149129
19.0000000	2.2935998	-1.2149129
19.0000000	2.3423998	-1.5094371
19.0000000	2.3912001	-1.5094371
19.0000000	2.4400001	-1.2824078
19.0000000	2.4888000	-1.3744469
19.0000000	2.5376000	-1.2824078
19.0000000	2.5864000	-1.2701364
19.0000000	2.6352000	-1.2640004
19.0000000	2.6840000	-1.2394567
19.0000000	2.7328000	-1.2517285
19.0000000	2.7816000	-1.2578645
19.0000000	2.8304000	-1.1167378
19.0000000	2.8792000	-1.0921946
19.0000000	2.9280000	-1.0431070
19.0000000	2.9768000	-1.0615149
19.0000000	3.0256000	-0.9449320
19.0000000	3.0743999	-0.9510679
19.0000000	3.1231999	-0.8160777
19.0000000	3.1719999	-0.7669902
19.0000000	3.2207999	-0.9572039
19.0000000	3.2695999	-0.8467574
19.0000000	3.3183999	-0.8406215
19.0000000	3.3671999	-0.8038058
19.0000000	3.4159999	-0.8222136
19.0000000	3.4647999	-0.6933594

19.0000000	3.5135999	-0.8406215
19.0000000	3.5623999	-0.6442719
19.0000000	3.6111999	-0.9817476
19.0000000	3.6599998	-0.9878836
19.0000000	3.7087998	-0.8283496
19.0000000	3.7575998	-0.5399613
19.0000000	3.8063998	-0.5399613
19.0000000	3.8551998	-0.9203887
19.0000000	3.9039998	-0.8590293
19.0000000	3.9527998	-0.9878836
19.0000000	4.0016003	-1.0369711
19.0000000	4.0503998	-0.8467574
19.0000000	4.0991998	-0.8590293
19.0000000	4.1479998	-0.7424469
19.0000000	4.1967998	-0.7424469
19.0000000	4.2455997	-0.5154176
19.0000000	4.2943997	-0.6994953
19.0000000	4.3431997	-0.5338254
19.0000000	4.3919997	-0.5338254
19.0000000	4.4407997	-0.5276895
19.0000000	4.4895997	613586.0000000
19.0000000	4.5383997	613586.0000000
19.0000000	4.5871997	613586.0000000
19.0000000	4.6359997	-1.0860586
19.0000000	4.6847997	-1.0246992
19.0000000	4.7336001	-1.0369711
19.0000000	4.7824001	-0.9203887
19.0000000	4.8312001	-0.9203887
19.0000000	4.8800001	-0.9203887
19.0000000	4.9288001	-0.9387960
19.0000000	4.9776001	-0.8713012
19.0000000	5.0264001	-1.0737867
19.0000000	5.0752001	-1.0860586
19.0000000	5.1240001	-1.0062914
19.0000000	5.1728001	613586.0000000
19.0000000	5.2216001	-0.7915339
19.0000000	5.2704000	-0.8344855
19.0000000	5.3192000	-0.8344855
19.0000000	5.3680000	-0.8344855
19.0000000	5.4168000	-0.9081168
19.0000000	5.4656000	-0.7976699
19.0000000	5.5144000	613586.0000000
19.0000000	5.5632000	613586.0000000
19.0000000	5.6120000	613586.0000000
19.0000000	5.6608000	-0.8774371
19.0000000	5.7096000	613586.0000000
19.0000000	5.7584000	613586.0000000
19.0000000	5.8072000	613586.0000000
19.0000000	5.8559999	613586.0000000
19.0000000	5.9047999	613586.0000000
19.0000000	5.9535999	-0.9019809
19.0000000	6.0023999	-0.8958449
19.0166664	0.2928000	-2.6936703
19.0166664	0.3416000	-2.3807383
19.0166664	0.3904000	-2.1537089
19.0166664	0.4392000	-2.2396121
19.0166664	0.4880000	-2.2150683

19.0166664	0.5368000	-2.2089324
19.0166664	0.5856000	-1.8346410
19.0166664	0.6344000	-1.8346410
19.0166664	0.6832000	-1.3192234
19.0166664	0.7320000	-1.1842332
19.0166664	0.7808000	-1.2210488
19.0166664	0.8296000	-1.4235344
19.0166664	0.8784000	-1.4235344
19.0166664	0.9272000	-1.1780972
19.0166664	0.9760000	-1.1903691
19.0166664	1.0247999	-1.1903691
19.0166664	1.0735999	-1.2701364
19.0166664	1.1223999	-1.2701364
19.0166664	1.1711999	-1.1780972
19.0166664	1.2200000	-1.1596894
19.0166664	1.2688000	-1.3499031
19.0166664	1.3176000	-1.2824078
19.0166664	1.3664000	-1.2394567
19.0166664	1.4152000	-1.2333207
19.0166664	1.4640000	-1.3744469
19.0166664	1.5128000	-1.3744469
19.0166664	1.5616000	-1.2149129
19.0166664	1.6104000	-1.2210488
19.0166664	1.6592000	-1.2210488
19.0166664	1.7079999	-1.2210488
19.0166664	1.7567999	-1.4296703
19.0166664	1.8055999	-1.4235344
19.0166664	1.8543999	-1.5155730
19.0166664	1.9031999	-1.5401168
19.0166664	1.9519999	-1.4112625
19.0166664	2.0007999	-1.4112625
19.0166664	2.0495999	-1.3989906
19.0166664	2.0983999	-1.5094371
19.0166664	2.1471999	-1.6260195
19.0166664	2.1959999	-1.4971652
19.0166664	2.2447999	-1.4603496
19.0166664	2.2935998	-1.3867188
19.0166664	2.3423998	-1.5585246
19.0166664	2.3912001	-1.4419422
19.0166664	2.4400001	-1.4235344
19.0166664	2.4888000	-1.3805828
19.0166664	2.5376000	-1.3805828
19.0166664	2.5864000	-1.4296703
19.0166664	2.6352000	-1.2640004
19.0166664	2.6840000	-1.2701364
19.0166664	2.7328000	-1.2762718
19.0166664	2.7816000	-1.2087770
19.0166664	2.8304000	-1.2026410
19.0166664	2.8792000	-1.1965051
19.0166664	2.9280000	-1.0737867
19.0166664	2.9768000	-1.0553789
19.0166664	3.0256000	-0.9878836
19.0166664	3.0743999	-0.9387960
19.0166664	3.1231999	-0.7117672
19.0166664	3.1719999	-0.7056313
19.0166664	3.2207999	-0.8222136
19.0166664	3.2695999	-0.6135921

19.0166664	3.3183999	-0.6135921
19.0166664	3.3671999	-0.4908738
19.0166664	3.4159999	-0.6872234
19.0166664	3.4647999	-0.8283496
19.0166664	3.5135999	-1.1535535
19.0166664	3.5623999	-1.1535535
19.0166664	3.6111999	-1.1535535
19.0166664	3.6599998	-1.1535535
19.0166664	3.7087998	-1.2210488
19.0166664	3.7575998	-1.2149129
19.0166664	3.8063998	-1.2333207
19.0166664	3.8551998	-1.1412816
19.0166664	3.9039998	-1.1412816
19.0166664	3.9527998	-1.0983305
19.0166664	4.0016003	-0.9633398
19.0166664	4.0503998	-0.9510679
19.0166664	4.0991998	-0.8467574
19.0166664	4.1479998	-0.8406215
19.0166664	4.1967998	-0.8222136
19.0166664	4.2455997	-0.4724660
19.0166664	4.2943997	-0.4786019
19.0166664	4.3431997	-0.5154176
19.0166664	4.3919997	-0.6565437
19.0166664	4.4407997	-0.4724660
19.0166664	4.4895997	-0.4601941
19.0166664	4.5383997	-0.5276895
19.0166664	4.5871997	-0.5338254
19.0166664	4.6359997	-0.6626797
19.0166664	4.6847997	-0.4724660
19.0166664	4.7336001	-0.4724660
19.0166664	4.7824001	-0.4295144
19.0166664	4.8312001	-0.5276895
19.0166664	4.8800001	-0.3129320
19.0166664	4.9288001	-0.3988352
19.0166664	4.9776001	-0.4663301
19.0166664	5.0264001	-0.5215535
19.0166664	5.0752001	-0.6074562
19.0166664	5.1240001	-0.6013203
19.0166664	5.1728001	-0.6504078
19.0166664	5.2216001	-0.6442719
19.0166664	5.2704000	-0.7608547
19.0166664	5.3192000	-0.7853980
19.0166664	5.3680000	-0.7424469
19.0166664	5.4168000	-0.7485828
19.0166664	5.4656000	-0.7179031
19.0166664	5.5144000	-0.7363110
19.0166664	5.5632000	-0.8038058
19.0166664	5.6120000	-0.7117672
19.0166664	5.6608000	-0.7363110
19.0166664	5.7096000	613586.0000000
19.0166664	5.7584000	-0.5951843
19.0166664	5.8072000	-0.6013203
19.0166664	5.8559999	-0.7976699
19.0166664	5.9047999	-0.7976699
19.0166664	5.9535999	-0.9633398
19.0166664	6.0023999	613586.0000000
19.0333328	0.2928000	-2.3684664

19.0333328	0.3416000	-2.3684664
19.0333328	0.3904000	-2.3746023
19.0333328	0.4392000	-2.2027965
19.0333328	0.4880000	-2.2396121
19.0333328	0.5368000	-2.0309906
19.0333328	0.5856000	-2.0248547
19.0333328	0.6344000	-1.7548742
19.0333328	0.6832000	-1.6076121
19.0333328	0.7320000	-1.1596894
19.0333328	0.7808000	-1.1596894
19.0333328	0.8296000	-0.0613594
19.0333328	0.8784000	-0.8774371
19.0333328	0.9272000	-0.8713012
19.0333328	0.9760000	-0.8651652
19.0333328	1.0247999	-1.1658254
19.0333328	1.0735999	-1.1658254
19.0333328	1.1223999	-1.1044660
19.0333328	1.1711999	-1.0921946
19.0333328	1.2200000	-1.2271848
19.0333328	1.2688000	-1.2271848
19.0333328	1.3176000	-1.2394567
19.0333328	1.3664000	-1.0185633
19.0333328	1.4152000	-1.1842332
19.0333328	1.4640000	-1.0983305
19.0333328	1.5128000	-1.0921946
19.0333328	1.5616000	-1.1842332
19.0333328	1.6104000	-1.1842332
19.0333328	1.6592000	-1.2640004
19.0333328	1.7079999	-1.2578645
19.0333328	1.7567999	-1.3744469
19.0333328	1.8055999	-1.3867188
19.0333328	1.8543999	-1.3867188
19.0333328	1.9031999	-1.3989906
19.0333328	1.9519999	-1.5094371
19.0333328	2.0007999	-1.5217090
19.0333328	2.0495999	-1.5033011
19.0333328	2.0983999	-1.3008156
19.0333328	2.1471999	-1.4910293
19.0333328	2.1959999	-1.4910293
19.0333328	2.2447999	-1.4051266
19.0333328	2.2935998	-1.2333207
19.0333328	2.3423998	-1.4848933
19.0333328	2.3912001	-1.4726214
19.0333328	2.4400001	-1.4910293
19.0333328	2.4888000	-1.4910293
19.0333328	2.5376000	-1.3499031
19.0333328	2.5864000	-1.3867188
19.0333328	2.6352000	-1.2455926
19.0333328	2.6840000	-1.2517285
19.0333328	2.7328000	-1.2087770
19.0333328	2.7816000	-1.0369711
19.0333328	2.8304000	-1.0308352
19.0333328	2.8792000	-1.0308352
19.0333328	2.9280000	-1.1228738
19.0333328	2.9768000	-0.9449320
19.0333328	3.0256000	-0.9387960
19.0333328	3.0743999	-0.8774371

19.0333328	3.1231999	-0.5890489
19.0333328	3.1719999	-0.6442719
19.0333328	3.2207999	-0.6442719
19.0333328	3.2695999	-1.2455926
19.0333328	3.3183999	-1.2455926
19.0333328	3.3671999	-1.0860586
19.0333328	3.4159999	-1.1412816
19.0333328	3.4647999	-1.0062914
19.0333328	3.5135999	-1.0062914
19.0333328	3.5623999	-1.0431070
19.0333328	3.6111999	-1.0001554
19.0333328	3.6599998	-1.0062914
19.0333328	3.7087998	-1.0124273
19.0333328	3.7575998	-0.7792621
19.0333328	3.8063998	-0.7792621
19.0333328	3.8551998	-0.7853980
19.0333328	3.9039998	-0.8222136
19.0333328	3.9527998	-0.6074562
19.0333328	4.0016003	-0.6135921
19.0333328	4.0503998	-0.6135921
19.0333328	4.0991998	-0.8528934
19.0333328	4.1479998	-0.8528934
19.0333328	4.1967998	-0.8528934
19.0333328	4.2455997	-0.8344855
19.0333328	4.2943997	-0.8344855
19.0333328	4.3431997	-0.8958449
19.0333328	4.3919997	-0.9265246
19.0333328	4.4407997	-0.8713012
19.0333328	4.4895997	-0.7731261
19.0333328	4.5383997	-0.6810875
19.0333328	4.5871997	-0.6749516
19.0333328	4.6359997	-0.5890489
19.0333328	4.6847997	-0.5890489
19.0333328	4.7336001	-0.6013203
19.0333328	4.7824001	-0.6013203
19.0333328	4.8312001	-0.7853980
19.0333328	4.8800001	-0.6626797
19.0333328	4.9288001	-0.6626797
19.0333328	4.9776001	-0.6626797
19.0333328	5.0264001	-0.7117672
19.0333328	5.0752001	-0.7240391
19.0333328	5.1240001	-0.7915339
19.0333328	5.1728001	-0.7240391
19.0333328	5.2216001	-0.7179031
19.0333328	5.2704000	-0.7547188
19.0333328	5.3192000	-0.7547188
19.0333328	5.3680000	-0.7117672
19.0333328	5.4168000	-0.6381359
19.0333328	5.4656000	-0.6320000
19.0333328	5.5144000	-0.6258640
19.0333328	5.5632000	-0.6135921
19.0333328	5.6120000	-0.6135921
19.0333328	5.6608000	-0.6013203
19.0333328	5.7096000	-0.7117672
19.0333328	5.7584000	-0.6258640
19.0333328	5.8072000	-0.6258640
19.0333328	5.8559999	-0.6688156

19.0333328	5.9047999	-0.8038058
19.0333328	5.9535999	-0.8835731
19.0333328	6.0023999	-0.8651652
19.0499992	0.2928000	-2.7979810
19.0499992	0.3416000	-2.8163888
19.0499992	0.3904000	-2.5709519
19.0499992	0.4392000	-2.5709519
19.0499992	0.4880000	-2.1230292
19.0499992	0.5368000	-1.8653207
19.0499992	0.5856000	-1.7732821
19.0499992	0.6344000	-1.7794180
19.0499992	0.6832000	-0.9081168
19.0499992	0.7320000	-0.8590293
19.0499992	0.7808000	-0.8835731
19.0499992	0.8296000	-1.0308352
19.0499992	0.8784000	-1.0921946
19.0499992	0.9272000	-1.2701364
19.0499992	0.9760000	-1.0492430
19.0499992	1.0247999	-1.0308352
19.0499992	1.0735999	-1.2087770
19.0499992	1.1223999	-1.0062914
19.0499992	1.1711999	-1.1780972
19.0499992	1.2200000	-1.1842332
19.0499992	1.2688000	-1.2578645
19.0499992	1.3176000	-1.2578645
19.0499992	1.3664000	-1.2640004
19.0499992	1.4152000	-1.1780972
19.0499992	1.4640000	-1.0983305
19.0499992	1.5128000	-1.4358063
19.0499992	1.5616000	-1.5155730
19.0499992	1.6104000	-1.3253593
19.0499992	1.6592000	-1.3314953
19.0499992	1.7079999	-1.3192234
19.0499992	1.7567999	-1.3253593
19.0499992	1.8055999	-1.1167378
19.0499992	1.8543999	-1.1842332
19.0499992	1.9031999	-1.3130875
19.0499992	1.9519999	-1.4971652
19.0499992	2.0007999	-1.5585246
19.0499992	2.0495999	-1.5585246
19.0499992	2.0983999	-1.2087770
19.0499992	2.1471999	-1.2087770
19.0499992	2.1959999	-1.4480777
19.0499992	2.2447999	-1.4419422
19.0499992	2.2935998	-1.4664855
19.0499992	2.3423998	-1.3130875
19.0499992	2.3912001	-1.3069515
19.0499992	2.4400001	-1.3621750
19.0499992	2.4888000	-1.4296703
19.0499992	2.5376000	-1.3805828
19.0499992	2.5864000	-1.3560390
19.0499992	2.6352000	-1.2517285
19.0499992	2.6840000	-1.4542136
19.0499992	2.7328000	-1.3499031
19.0499992	2.7816000	-1.1842332
19.0499992	2.8304000	-1.1780972
19.0499992	2.8792000	-1.1780972

19.0499992	2.9280000	-1.1106019
19.0499992	2.9768000	-1.0860586
19.0499992	3.0256000	-1.0615149
19.0499992	3.0743999	-1.0676508
19.0499992	3.1231999	-1.0615149
19.0499992	3.1719999	-1.0492430
19.0499992	3.2207999	-1.2762718
19.0499992	3.2695999	-1.1412816
19.0499992	3.3183999	-1.1351457
19.0499992	3.3671999	-1.1290097
19.0499992	3.4159999	-1.1290097
19.0499992	3.4647999	-1.1351457
19.0499992	3.5135999	-0.9694757
19.0499992	3.5623999	-0.9694757
19.0499992	3.6111999	-0.9633398
19.0499992	3.6599998	-0.9633398
19.0499992	3.7087998	-1.0431070
19.0499992	3.7575998	-0.8713012
19.0499992	3.8063998	-0.8651652
19.0499992	3.8551998	-0.7240391
19.0499992	3.9039998	-0.7117672
19.0499992	3.9527998	-0.6626797
19.0499992	4.0016003	-0.6197281
19.0499992	4.0503998	-0.6135921
19.0499992	4.0991998	-0.6074562
19.0499992	4.1479998	-0.7363110
19.0499992	4.1967998	-0.7363110
19.0499992	4.2455997	-0.8651652
19.0499992	4.2943997	-0.7424469
19.0499992	4.3431997	-0.7485828
19.0499992	4.3919997	-0.7485828
19.0499992	4.4407997	-0.7363110
19.0499992	4.4895997	-0.7792621
19.0499992	4.5383997	-0.7179031
19.0499992	4.5871997	-0.7853980
19.0499992	4.6359997	-0.8099418
19.0499992	4.6847997	-0.8099418
19.0499992	4.7336001	-0.8038058
19.0499992	4.7824001	-0.7424469
19.0499992	4.8312001	-0.6258640
19.0499992	4.8800001	-0.6258640
19.0499992	4.9288001	-0.7608547
19.0499992	4.9776001	-0.7363110
19.0499992	5.0264001	-0.7301750
19.0499992	5.0752001	-0.7669902
19.0499992	5.1240001	-0.7424469
19.0499992	5.1728001	-0.7547188
19.0499992	5.2216001	-0.7547188
19.0499992	5.2704000	-0.8897090
19.0499992	5.3192000	-0.7976699
19.0499992	5.3680000	-0.8283496
19.0499992	5.4168000	-0.8344855
19.0499992	5.4656000	-0.6749516
19.0499992	5.5144000	-0.6442719
19.0499992	5.5632000	-0.6442719
19.0499992	5.6120000	-0.7731261
19.0499992	5.6608000	-0.8897090

19.0499992	5.7096000	-0.8038058
19.0499992	5.7584000	-0.8038058
19.0499992	5.8072000	-0.8651652
19.0499992	5.8559999	-0.7976699
19.0499992	5.9047999	-0.7179031
19.0499992	5.9535999	-0.7731261
19.0499992	6.0023999	-1.1228738
19.0666676	0.2928000	-2.9697869
19.0666676	0.3416000	-2.7673013
19.0666676	0.3904000	-2.5218644
19.0666676	0.4392000	-2.5218644
19.0666676	0.4880000	-2.4666412
19.0666676	0.5368000	-2.0616703
19.0666676	0.5856000	-1.9021363
19.0666676	0.6344000	-1.9696312
19.0666676	0.6832000	-1.9757671
19.0666676	0.7320000	-1.2087770
19.0666676	0.7808000	-1.2087770
19.0666676	0.8296000	-0.8467574
19.0666676	0.8784000	-0.8651652
19.0666676	0.9272000	-0.8038058
19.0666676	0.9760000	-1.0737867
19.0666676	1.0247999	-1.3376312
19.0666676	1.0735999	-1.3314953
19.0666676	1.1223999	-1.2394567
19.0666676	1.1711999	-1.1596894
19.0666676	1.2200000	-1.1535535
19.0666676	1.2688000	-1.1903691
19.0666676	1.3176000	-1.2701364
19.0666676	1.3664000	-1.2333207
19.0666676	1.4152000	-1.3867188
19.0666676	1.4640000	-1.4419422
19.0666676	1.5128000	-1.4542136
19.0666676	1.5616000	-1.4910293
19.0666676	1.6104000	-1.4910293
19.0666676	1.6592000	-1.6505632
19.0666676	1.7079999	-1.6505632
19.0666676	1.7567999	-1.6505632
19.0666676	1.8055999	-1.2455926
19.0666676	1.8543999	-1.6137476
19.0666676	1.9031999	-1.2824078
19.0666676	1.9519999	-1.5217090
19.0666676	2.0007999	-1.5953403
19.0666676	2.0495999	-1.5953403
19.0666676	2.0983999	-1.5830684
19.0666676	2.1471999	-1.5585246
19.0666676	2.1959999	-1.5646605
19.0666676	2.2447999	-1.4419422
19.0666676	2.2935998	-1.2578645
19.0666676	2.3423998	-1.2578645
19.0666676	2.3912001	-1.4358063
19.0666676	2.4400001	-1.4480777
19.0666676	2.4888000	-1.3560390
19.0666676	2.5376000	-1.2762718
19.0666676	2.5864000	-1.3683109
19.0666676	2.6352000	-1.3069515
19.0666676	2.6840000	-1.2946796

19.0666676	2.7328000	-1.2333207
19.0666676	2.7816000	-1.2333207
19.0666676	2.8304000	-1.1903691
19.0666676	2.8792000	-1.1965051
19.0666676	2.9280000	-1.1044660
19.0666676	2.9768000	-1.0983305
19.0666676	3.0256000	-1.2026410
19.0666676	3.0743999	-1.2026410
19.0666676	3.1231999	-1.1044660
19.0666676	3.1719999	-1.2271848
19.0666676	3.2207999	-1.0737867
19.0666676	3.2695999	-1.0983305
19.0666676	3.3183999	-1.0615149
19.0666676	3.3671999	-1.0001554
19.0666676	3.4159999	-1.0921946
19.0666676	3.4647999	-0.9387960
19.0666676	3.5135999	-0.9326606
19.0666676	3.5623999	-1.0676508
19.0666676	3.6111999	-1.0124273
19.0666676	3.6599998	-0.9878836
19.0666676	3.7087998	-0.9817476
19.0666676	3.7575998	-0.9756117
19.0666676	3.8063998	-0.9510679
19.0666676	3.8551998	-1.0062914
19.0666676	3.9039998	-1.0246992
19.0666676	3.9527998	-0.9019809
19.0666676	4.0016003	-1.1228738
19.0666676	4.0503998	-1.0676508
19.0666676	4.0991998	-0.9572039
19.0666676	4.1479998	-0.7731261
19.0666676	4.1967998	-0.9633398
19.0666676	4.2455997	-0.9756117
19.0666676	4.2943997	-1.1290097
19.0666676	4.3431997	-0.9878836
19.0666676	4.3919997	-0.9878836
19.0666676	4.4407997	-0.8958449
19.0666676	4.4895997	-0.9019809
19.0666676	4.5383997	-0.8283496
19.0666676	4.5871997	-0.8467574
19.0666676	4.6359997	-0.6994953
19.0666676	4.6847997	-0.6994953
19.0666676	4.7336001	-0.6810875
19.0666676	4.7824001	-0.8099418
19.0666676	4.8312001	-0.8099418
19.0666676	4.8800001	-0.7485828
19.0666676	4.9288001	-0.4970098
19.0666676	4.9776001	-0.7240391
19.0666676	5.0264001	-0.6626797
19.0666676	5.0752001	-0.6749516
19.0666676	5.1240001	-0.5829129
19.0666676	5.1728001	-0.6872234
19.0666676	5.2216001	-0.6872234
19.0666676	5.2704000	-0.6258640
19.0666676	5.3192000	-0.7731261
19.0666676	5.3680000	-0.7056313
19.0666676	5.4168000	-0.6933594
19.0666676	5.4656000	-0.7117672

19.0666676	5.5144000	-0.7240391
19.0666676	5.5632000	-0.7363110
19.0666676	5.6120000	-0.7424469
19.0666676	5.6608000	-0.7424469
19.0666676	5.7096000	-0.7179031
19.0666676	5.7584000	-0.8590293
19.0666676	5.8072000	-0.8467574
19.0666676	5.8559999	613586.0000000
19.0666676	5.9047999	-0.9142528
19.0666676	5.9535999	-1.0799227
19.0666676	6.0023999	-0.8099418
19.0833340	0.2928000	-3.6876900
19.0833340	0.3416000	-2.6875343
19.0833340	0.3904000	-2.5893598
19.0833340	0.4392000	-2.3377867
19.0833340	0.4880000	-1.9573593
19.0833340	0.5368000	-1.9634953
19.0833340	0.5856000	-1.9082723
19.0833340	0.6344000	-1.6382914
19.0833340	0.6832000	-1.6751070
19.0833340	0.7320000	-1.6751070
19.0833340	0.7808000	-1.6444273
19.0833340	0.8296000	-1.2946796
19.0833340	0.8784000	-1.0124273
19.0833340	0.9272000	-1.0124273
19.0833340	0.9760000	-0.9265246
19.0833340	1.0247999	-0.9265246
19.0833340	1.0735999	-0.9265246
19.0833340	1.1223999	-1.1228738
19.0833340	1.1711999	-1.1658254
19.0833340	1.2200000	-1.0246992
19.0833340	1.2688000	-1.1167378
19.0833340	1.3176000	-1.1228738
19.0833340	1.3664000	-1.1290097
19.0833340	1.4152000	-1.1228738
19.0833340	1.4640000	-1.1658254
19.0833340	1.5128000	-1.4235344
19.0833340	1.5616000	-1.4235344
19.0833340	1.6104000	-1.5892043
19.0833340	1.6592000	-1.5892043
19.0833340	1.7079999	-1.4848933
19.0833340	1.7567999	-1.3253593
19.0833340	1.8055999	-1.4235344
19.0833340	1.8543999	-1.4235344
19.0833340	1.9031999	-1.3130875
19.0833340	1.9519999	-1.5585246
19.0833340	2.0007999	-1.5585246
19.0833340	2.0495999	-1.4480777
19.0833340	2.0983999	-1.3683109
19.0833340	2.1471999	-1.3621750
19.0833340	2.1959999	-1.5523887
19.0833340	2.2447999	-1.5523887
19.0833340	2.2935998	-1.4542136
19.0833340	2.3423998	-1.4542136
19.0833340	2.3912001	-1.4358063
19.0833340	2.4400001	-1.4296703
19.0833340	2.4888000	-1.4848933

19.0833340	2.5376000	-1.4787574
19.0833340	2.5864000	-1.2885437
19.0833340	2.6352000	-1.3437672
19.0833340	2.6840000	-1.1351457
19.0833340	2.7328000	-1.1290097
19.0833340	2.7816000	-1.1535535
19.0833340	2.8304000	-1.1474175
19.0833340	2.8792000	-1.0431070
19.0833340	2.9280000	-1.0431070
19.0833340	2.9768000	-1.0308352
19.0833340	3.0256000	-1.1351457
19.0833340	3.0743999	-1.0615149
19.0833340	3.1231999	-0.9449320
19.0833340	3.1719999	-1.1965051
19.0833340	3.2207999	-1.1965051
19.0833340	3.2695999	-1.2087770
19.0833340	3.3183999	-1.1596894
19.0833340	3.3671999	-1.3069515
19.0833340	3.4159999	-1.2087770
19.0833340	3.4647999	-1.2455926
19.0833340	3.5135999	-0.9694757
19.0833340	3.5623999	-0.9694757
19.0833340	3.6111999	-0.9878836
19.0833340	3.6599998	-0.9940195
19.0833340	3.7087998	-0.8160777
19.0833340	3.7575998	-0.7485828
19.0833340	3.8063998	-0.6994953
19.0833340	3.8551998	-0.6994953
19.0833340	3.9039998	-0.6749516
19.0833340	3.9527998	-0.7608547
19.0833340	4.0016003	-0.9694757
19.0833340	4.0503998	-0.9694757
19.0833340	4.0991998	-1.0799227
19.0833340	4.1479998	-1.0983305
19.0833340	4.1967998	-1.1658254
19.0833340	4.2455997	-1.1658254
19.0833340	4.2943997	-0.9694757
19.0833340	4.3431997	-1.0185633
19.0833340	4.3919997	-1.0185633
19.0833340	4.4407997	-0.8958449
19.0833340	4.4895997	-0.9081168
19.0833340	4.5383997	-0.9081168
19.0833340	4.5871997	-0.9142528
19.0833340	4.6359997	-0.7853980
19.0833340	4.6847997	-0.8897090
19.0833340	4.7336001	-0.7179031
19.0833340	4.7824001	-0.8835731
19.0833340	4.8312001	-0.7669902
19.0833340	4.8800001	-0.7669902
19.0833340	4.9288001	-0.8835731
19.0833340	4.9776001	-0.6320000
19.0833340	5.0264001	-0.6381359
19.0833340	5.0752001	-0.7915339
19.0833340	5.1240001	-0.9387960
19.0833340	5.1728001	-0.8222136
19.0833340	5.2216001	-0.8222136
19.0833340	5.2704000	-0.7608547

19.0833340	5.3192000	-0.7669902
19.0833340	5.3680000	-0.7915339
19.0833340	5.4168000	-0.9694757
19.0833340	5.4656000	-0.9203887
19.0833340	5.5144000	-0.7301750
19.0833340	5.5632000	-0.7301750
19.0833340	5.6120000	613586.0000000
19.0833340	5.6608000	-0.7669902
19.0833340	5.7096000	-0.8467574
19.0833340	5.7584000	-0.7853980
19.0833340	5.8072000	613586.0000000
19.0833340	5.8559999	-0.8038058
19.0833340	5.9047999	-0.8160777
19.0833340	5.9535999	-0.8222136
19.0833340	6.0023999	-0.8222136
19.1000004	0.2928000	-3.0740976
19.1000004	0.3416000	-2.9943306
19.1000004	0.3904000	-2.9881947
19.1000004	0.4392000	-2.3255150
19.1000004	0.4880000	-2.0003109
19.1000004	0.5368000	-2.0003109
19.1000004	0.5856000	-1.4112625
19.1000004	0.6344000	-1.3499031
19.1000004	0.6832000	-1.5217090
19.1000004	0.7320000	-1.5278449
19.1000004	0.7808000	-1.2946796
19.1000004	0.8296000	-1.2946796
19.1000004	0.8784000	-0.9081168
19.1000004	0.9272000	-0.9019809
19.1000004	0.9760000	-1.0246992
19.1000004	1.0247999	-1.0246992
19.1000004	1.0735999	-1.0124273
19.1000004	1.1223999	-1.2210488
19.1000004	1.1711999	-0.9878836
19.1000004	1.2200000	-1.1474175
19.1000004	1.2688000	-1.0921946
19.1000004	1.3176000	-1.2946796
19.1000004	1.3664000	-1.3069515
19.1000004	1.4152000	-1.1719613
19.1000004	1.4640000	-1.1658254
19.1000004	1.5128000	-1.3989906
19.1000004	1.5616000	-1.4173985
19.1000004	1.6104000	-1.4480777
19.1000004	1.6592000	-1.4480777
19.1000004	1.7079999	-1.3683109
19.1000004	1.7567999	-1.3437672
19.1000004	1.8055999	-1.3376312
19.1000004	1.8543999	-1.5646605
19.1000004	1.9031999	-1.5646605
19.1000004	1.9519999	-1.4787574
19.1000004	2.0007999	-1.4848933
19.1000004	2.0495999	-1.4787574
19.1000004	2.0983999	-1.5339808
19.1000004	2.1471999	-1.3867188
19.1000004	2.1959999	-1.4173985
19.1000004	2.2447999	-1.3867188
19.1000004	2.2935998	-1.3744469

19.1000004	2.3423998	-1.3437672
19.1000004	2.3912001	-1.3376312
19.1000004	2.4400001	-1.3437672
19.1000004	2.4888000	-1.3805828
19.1000004	2.5376000	-1.3867188
19.1000004	2.5864000	-1.2640004
19.1000004	2.6352000	-1.1903691
19.1000004	2.6840000	-1.1719613
19.1000004	2.7328000	-1.1719613
19.1000004	2.7816000	-1.0799227
19.1000004	2.8304000	-1.0799227
19.1000004	2.8792000	-1.3805828
19.1000004	2.9280000	-1.1658254
19.1000004	2.9768000	-1.1106019
19.1000004	3.0256000	-1.1474175
19.1000004	3.0743999	-0.9326606
19.1000004	3.1231999	-1.2455926
19.1000004	3.1719999	-1.3008156
19.1000004	3.2207999	-1.2455926
19.1000004	3.2695999	-1.1167378
19.1000004	3.3183999	-1.1228738
19.1000004	3.3671999	-1.1965051
19.1000004	3.4159999	-1.1965051
19.1000004	3.4647999	-0.9633398
19.1000004	3.5135999	-0.9633398
19.1000004	3.5623999	-0.9142528
19.1000004	3.6111999	-1.0124273
19.1000004	3.6599998	-0.8958449
19.1000004	3.7087998	-0.8958449
19.1000004	3.7575998	-0.8958449
19.1000004	3.8063998	-0.6320000
19.1000004	3.8551998	-0.6197281
19.1000004	3.9039998	-0.6074562
19.1000004	3.9527998	-0.6197281
19.1000004	4.0016003	-1.0185633
19.1000004	4.0503998	-1.0185633
19.1000004	4.0991998	-1.0185633
19.1000004	4.1479998	-1.0185633
19.1000004	4.1967998	-0.6442719
19.1000004	4.2455997	-0.9633398
19.1000004	4.2943997	-1.0246992
19.1000004	4.3431997	-0.9694757
19.1000004	4.3919997	-0.7976699
19.1000004	4.4407997	-0.8099418
19.1000004	4.4895997	-0.8099418
19.1000004	4.5383997	-0.8160777
19.1000004	4.5871997	-0.6135921
19.1000004	4.6359997	-0.6135921
19.1000004	4.6847997	-0.6381359
19.1000004	4.7336001	-0.6504078
19.1000004	4.7824001	-0.3988352
19.1000004	4.8312001	-0.6688156
19.1000004	4.8800001	-0.7976699
19.1000004	4.9288001	-1.0308352
19.1000004	4.9776001	-1.1167378
19.1000004	5.0264001	-1.1106019
19.1000004	5.0752001	-0.7976699

19.1000004	5.1240001	-0.8222136
19.1000004	5.1728001	-0.7853980
19.1000004	5.2216001	-0.9572039
19.1000004	5.2704000	-0.9633398
19.1000004	5.3192000	-0.8958449
19.1000004	5.3680000	-0.8835731
19.1000004	5.4168000	-0.8958449
19.1000004	5.4656000	-0.9572039
19.1000004	5.5144000	-0.9572039
19.1000004	5.5632000	613586.0000000
19.1000004	5.6120000	613586.0000000
19.1000004	5.6608000	-0.7976699
19.1000004	5.7096000	613586.0000000
19.1000004	5.7584000	613586.0000000
19.1000004	5.8072000	613586.0000000
19.1000004	5.8559999	613586.0000000
19.1000004	5.9047999	-0.9940195
19.1000004	5.9535999	-1.0001554
19.1000004	6.0023999	613586.0000000
19.1166668	0.2928000	-3.9269910
19.1166668	0.3416000	-3.2274957
19.1166668	0.3904000	-2.9881947
19.1166668	0.4392000	-2.1537089
19.1166668	0.4880000	-1.6198835
19.1166668	0.5368000	-1.6198835
19.1166668	0.5856000	-0.9756117
19.1166668	0.6344000	-0.9633398
19.1166668	0.6832000	-0.9572039
19.1166668	0.7320000	-0.9817476
19.1166668	0.7808000	-0.9817476
19.1166668	0.8296000	-0.5645051
19.1166668	0.8784000	-1.6014762
19.1166668	0.9272000	-1.5830684
19.1166668	0.9760000	-1.5830684
19.1166668	1.0247999	-1.6566992
19.1166668	1.0735999	-1.0553789
19.1166668	1.1223999	-1.0492430
19.1166668	1.1711999	-1.0492430
19.1166668	1.2200000	-1.0737867
19.1166668	1.2688000	-1.0737867
19.1166668	1.3176000	-1.3744469
19.1166668	1.3664000	-1.3437672
19.1166668	1.4152000	-1.2885437
19.1166668	1.4640000	-1.3130875
19.1166668	1.5128000	-1.4419422
19.1166668	1.5616000	-1.3560390
19.1166668	1.6104000	-1.4726214
19.1166668	1.6592000	-1.5401168
19.1166668	1.7079999	-1.5523887
19.1166668	1.7567999	-1.5401168
19.1166668	1.8055999	-1.3314953
19.1166668	1.8543999	-1.3314953
19.1166668	1.9031999	-1.5769324
19.1166668	1.9519999	-1.4787574
19.1166668	2.0007999	-1.5033011
19.1166668	2.0495999	-1.4971652
19.1166668	2.0983999	-1.4910293

19.1166668	2.1471999	-1.5953403
19.1166668	2.1959999	-1.3437672
19.1166668	2.2447999	-1.3928547
19.1166668	2.2935998	-1.5033011
19.1166668	2.3423998	-1.3130875
19.1166668	2.3912001	-1.4910293
19.1166668	2.4400001	-1.3499031
19.1166668	2.4888000	-1.3499031
19.1166668	2.5376000	-1.3560390
19.1166668	2.5864000	-1.3560390
19.1166668	2.6352000	-1.2640004
19.1166668	2.6840000	-1.2578645
19.1166668	2.7328000	-1.2394567
19.1166668	2.7816000	-1.2394567
19.1166668	2.8304000	-1.0676508
19.1166668	2.8792000	-1.0676508
19.1166668	2.9280000	-1.0676508
19.1166668	2.9768000	-0.9510679
19.1166668	3.0256000	-0.9572039
19.1166668	3.0743999	-1.0676508
19.1166668	3.1231999	-1.0492430
19.1166668	3.1719999	-1.0553789
19.1166668	3.2207999	-1.0369711
19.1166668	3.2695999	-1.0001554
19.1166668	3.3183999	-1.0676508
19.1166668	3.3671999	-0.9817476
19.1166668	3.4159999	-0.9510679
19.1166668	3.4647999	-0.8283496
19.1166668	3.5135999	-0.8283496
19.1166668	3.5623999	-0.8897090
19.1166668	3.6111999	-1.0860586
19.1166668	3.6599998	-1.0737867
19.1166668	3.7087998	-1.0062914
19.1166668	3.7575998	-1.0615149
19.1166668	3.8063998	-1.2333207
19.1166668	3.8551998	-1.1351457
19.1166668	3.9039998	-0.9878836
19.1166668	3.9527998	-0.9878836
19.1166668	4.0016003	-0.9756117
19.1166668	4.0503998	-0.9756117
19.1166668	4.0991998	-0.8651652
19.1166668	4.1479998	-1.1044660
19.1166668	4.1967998	-1.0246992
19.1166668	4.2455997	-1.0799227
19.1166668	4.2943997	-1.0983305
19.1166668	4.3431997	-0.9142528
19.1166668	4.3919997	-0.8774371
19.1166668	4.4407997	-0.8651652
19.1166668	4.4895997	-0.8713012
19.1166668	4.5383997	-0.7976699
19.1166668	4.5871997	-0.7915339
19.1166668	4.6359997	-0.9203887
19.1166668	4.6847997	-0.9265246
19.1166668	4.7336001	-0.8897090
19.1166668	4.7824001	-0.8958449
19.1166668	4.8312001	-1.0983305
19.1166668	4.8800001	-0.9019809

19.1166668	4.9288001	-1.0553789
19.1166668	4.9776001	-0.5706410
19.1166668	5.0264001	-0.8774371
19.1166668	5.0752001	-0.8651652
19.1166668	5.1240001	-1.0492430
19.1166668	5.1728001	-1.0431070
19.1166668	5.2216001	-0.9387960
19.1166668	5.2704000	-0.9633398
19.1166668	5.3192000	-0.9510679
19.1166668	5.3680000	-0.9572039
19.1166668	5.4168000	-0.9510679
19.1166668	5.4656000	-0.9265246
19.1166668	5.5144000	-0.8283496
19.1166668	5.5632000	-0.8160777
19.1166668	5.6120000	-0.8406215
19.1166668	5.6608000	-0.8406215
19.1166668	5.7096000	-0.8344855
19.1166668	5.7584000	-0.9081168
19.1166668	5.8072000	613586.0000000
19.1166668	5.8559999	-0.9572039
19.1166668	5.9047999	-0.8651652
19.1166668	5.9535999	-0.8590293
19.1166668	6.0023999	-0.8651652
19.1333332	0.2928000	-2.3255150
19.1333332	0.3416000	-2.0187187
19.1333332	0.3904000	-1.8898644
19.1333332	0.4392000	-1.7610102
19.1333332	0.4880000	-2.1168938
19.1333332	0.5368000	-2.1291652
19.1333332	0.5856000	-2.1475730
19.1333332	0.6344000	-2.3868742
19.1333332	0.6832000	-2.3255150
19.1333332	0.7320000	-1.9757671
19.1333332	0.7808000	-1.5892043
19.1333332	0.8296000	-1.5892043
19.1333332	0.8784000	-1.5339808
19.1333332	0.9272000	-1.3437672
19.1333332	0.9760000	-1.5523887
19.1333332	1.0247999	-1.6628351
19.1333332	1.0735999	-1.6628351
19.1333332	1.1223999	-1.4051266
19.1333332	1.1711999	-1.4051266
19.1333332	1.2200000	-1.4848933
19.1333332	1.2688000	-1.5033011
19.1333332	1.3176000	-1.5033011
19.1333332	1.3664000	-1.4419422
19.1333332	1.4152000	-1.1106019
19.1333332	1.4640000	-1.3069515
19.1333332	1.5128000	-1.3683109
19.1333332	1.5616000	-1.2701364
19.1333332	1.6104000	-1.4787574
19.1333332	1.6592000	-1.6382914
19.1333332	1.7079999	-1.6566992
19.1333332	1.7567999	-1.6566992
19.1333332	1.8055999	-1.6566992
19.1333332	1.8543999	-1.4112625
19.1333332	1.9031999	-1.4112625

19.1333332	1.9519999	-1.3989906
19.1333332	2.0007999	-1.4664855
19.1333332	2.0495999	-1.3130875
19.1333332	2.0983999	-1.4664855
19.1333332	2.1471999	-1.4787574
19.1333332	2.1959999	-1.4603496
19.1333332	2.2447999	-1.4542136
19.1333332	2.2935998	-1.4480777
19.1333332	2.3423998	-1.3621750
19.1333332	2.3912001	-1.5278449
19.1333332	2.4400001	-1.5094371
19.1333332	2.4888000	-1.3744469
19.1333332	2.5376000	-1.2210488
19.1333332	2.5864000	-1.3069515
19.1333332	2.6352000	-1.3314953
19.1333332	2.6840000	-1.3376312
19.1333332	2.7328000	-1.1044660
19.1333332	2.7816000	-1.0308352
19.1333332	2.8304000	-1.0369711
19.1333332	2.8792000	-1.0492430
19.1333332	2.9280000	-1.1351457
19.1333332	2.9768000	-0.9449320
19.1333332	3.0256000	-1.0185633
19.1333332	3.0743999	-1.0062914
19.1333332	3.1231999	-1.1535535
19.1333332	3.1719999	-1.1228738
19.1333332	3.2207999	-1.1228738
19.1333332	3.2695999	-1.1903691
19.1333332	3.3183999	-1.0124273
19.1333332	3.3671999	-1.0062914
19.1333332	3.4159999	-1.0185633
19.1333332	3.4647999	-0.9633398
19.1333332	3.5135999	-0.9940195
19.1333332	3.5623999	-0.9572039
19.1333332	3.6111999	-0.9572039
19.1333332	3.6599998	-0.7731261
19.1333332	3.7087998	-0.7485828
19.1333332	3.7575998	-0.8038058
19.1333332	3.8063998	-0.9142528
19.1333332	3.8551998	-0.9142528
19.1333332	3.9039998	-0.8344855
19.1333332	3.9527998	-0.8099418
19.1333332	4.0016003	-0.8099418
19.1333332	4.0503998	-0.8344855
19.1333332	4.0991998	-0.8835731
19.1333332	4.1479998	-0.8835731
19.1333332	4.1967998	-0.8774371
19.1333332	4.2455997	-0.8467574
19.1333332	4.2943997	-0.8467574
19.1333332	4.3431997	-0.8099418
19.1333332	4.3919997	-0.7485828
19.1333332	4.4407997	-0.7485828
19.1333332	4.4895997	-0.8467574
19.1333332	4.5383997	-0.9694757
19.1333332	4.5871997	-0.8897090
19.1333332	4.6359997	-0.7547188
19.1333332	4.6847997	-0.7608547

19.1333332	4.7336001	-0.5645051
19.1333332	4.7824001	-0.5706410
19.1333332	4.8312001	-0.7731261
19.1333332	4.8800001	-0.6626797
19.1333332	4.9288001	-0.8344855
19.1333332	4.9776001	-0.5031457
19.1333332	5.0264001	-0.4908738
19.1333332	5.0752001	-0.7731261
19.1333332	5.1240001	613586.0000000
19.1333332	5.1728001	-1.1780972
19.1333332	5.2216001	-0.9756117
19.1333332	5.2704000	-0.9694757
19.1333332	5.3192000	-0.9633398
19.1333332	5.3680000	-0.9694757
19.1333332	5.4168000	-0.9817476
19.1333332	5.4656000	-0.8774371
19.1333332	5.5144000	-0.8958449
19.1333332	5.5632000	-0.9081168
19.1333332	5.6120000	-1.0185633
19.1333332	5.6608000	-0.8283496
19.1333332	5.7096000	-0.9572039
19.1333332	5.7584000	-0.9510679
19.1333332	5.8072000	-0.9449320
19.1333332	5.8559999	613586.0000000
19.1333332	5.9047999	613586.0000000
19.1333332	5.9535999	613586.0000000
19.1333332	6.0023999	613586.0000000
19.1499996	0.2928000	-2.6077673
19.1499996	0.3416000	-2.0248547
19.1499996	0.3904000	-1.8469129
19.1499996	0.4392000	-1.8407769
19.1499996	0.4880000	-1.8469129
19.1499996	0.5368000	-1.8530488
19.1499996	0.5856000	-1.4787574
19.1499996	0.6344000	-1.4726214
19.1499996	0.6832000	-1.6198835
19.1499996	0.7320000	-1.6137476
19.1499996	0.7808000	-1.6076121
19.1499996	0.8296000	-1.3192234
19.1499996	0.8784000	-1.1167378
19.1499996	0.9272000	-1.2271848
19.1499996	0.9760000	-1.0185633
19.1499996	1.0247999	-1.1351457
19.1499996	1.0735999	-1.1658254
19.1499996	1.1223999	-1.1658254
19.1499996	1.1711999	-1.1658254
19.1499996	1.2200000	-1.1719613
19.1499996	1.2688000	-1.4910293
19.1499996	1.3176000	-1.3805828
19.1499996	1.3664000	-1.3805828
19.1499996	1.4152000	-1.2946796
19.1499996	1.4640000	-1.3683109
19.1499996	1.5128000	-1.3069515
19.1499996	1.5616000	-1.4051266
19.1499996	1.6104000	-1.4358063
19.1499996	1.6592000	-1.5892043
19.1499996	1.7079999	-1.6076121

19.1499996	1.7567999	-1.4664855
19.1499996	1.8055999	-1.4726214
19.1499996	1.8543999	-1.4787574
19.1499996	1.9031999	-1.4910293
19.1499996	1.9519999	-1.4910293
19.1499996	2.0007999	-1.3314953
19.1499996	2.0495999	-1.3314953
19.1499996	2.0983999	-1.4971652
19.1499996	2.1471999	-1.4235344
19.1499996	2.1959999	-1.4480777
19.1499996	2.2447999	-1.3621750
19.1499996	2.2935998	-1.4971652
19.1499996	2.3423998	-1.4971652
19.1499996	2.3912001	-1.2885437
19.1499996	2.4400001	-1.2701364
19.1499996	2.4888000	-1.2824078
19.1499996	2.5376000	-1.2333207
19.1499996	2.5864000	-1.2885437
19.1499996	2.6352000	-1.2026410
19.1499996	2.6840000	-1.0431070
19.1499996	2.7328000	-1.0431070
19.1499996	2.7816000	-1.0369711
19.1499996	2.8304000	-1.2946796
19.1499996	2.8792000	-0.8958449
19.1499996	2.9280000	-0.9142528
19.1499996	2.9768000	-1.0799227
19.1499996	3.0256000	-1.2455926
19.1499996	3.0743999	-1.1842332
19.1499996	3.1231999	-1.1780972
19.1499996	3.1719999	-1.1780972
19.1499996	3.2207999	-1.0124273
19.1499996	3.2695999	-1.0124273
19.1499996	3.3183999	-1.0062914
19.1499996	3.3671999	-1.2640004
19.1499996	3.4159999	-0.9756117
19.1499996	3.4647999	-0.9203887
19.1499996	3.5135999	-0.8651652
19.1499996	3.5623999	-0.9265246
19.1499996	3.6111999	-1.0185633
19.1499996	3.6599998	-1.1290097
19.1499996	3.7087998	-1.1290097
19.1499996	3.7575998	-0.9817476
19.1499996	3.8063998	-0.9694757
19.1499996	3.8551998	-0.9694757
19.1499996	3.9039998	-0.9387960
19.1499996	3.9527998	-0.9203887
19.1499996	4.0016003	-0.9203887
19.1499996	4.0503998	-0.9203887
19.1499996	4.0991998	-0.8713012
19.1499996	4.1479998	-0.7792621
19.1499996	4.1967998	-0.7792621
19.1499996	4.2455997	-0.7976699
19.1499996	4.2943997	-0.8344855
19.1499996	4.3431997	-0.8467574
19.1499996	4.3919997	-0.7792621
19.1499996	4.4407997	-0.9081168
19.1499996	4.4895997	-0.8222136

19.1499996	4.5383997	-0.8835731
19.1499996	4.5871997	-0.7976699
19.1499996	4.6359997	-0.8222136
19.1499996	4.6847997	-0.5522332
19.1499996	4.7336001	-0.6197281
19.1499996	4.7824001	-0.4724660
19.1499996	4.8312001	-0.7301750
19.1499996	4.8800001	-0.7853980
19.1499996	4.9288001	-0.7669902
19.1499996	4.9776001	-0.5890489
19.1499996	5.0264001	-0.2761164
19.1499996	5.0752001	-0.7853980
19.1499996	5.1240001	-0.7853980
19.1499996	5.1728001	-0.7792621
19.1499996	5.2216001	-0.8774371
19.1499996	5.2704000	-0.9878836
19.1499996	5.3192000	-1.0492430
19.1499996	5.3680000	-1.0553789
19.1499996	5.4168000	-1.0676508
19.1499996	5.4656000	-1.1290097
19.1499996	5.5144000	-1.1290097
19.1499996	5.5632000	-1.0369711
19.1499996	5.6120000	-1.0553789
19.1499996	5.6608000	-1.1044660
19.1499996	5.7096000	-1.0676508
19.1499996	5.7584000	-0.9878836
19.1499996	5.8072000	613586.0000000
19.1499996	5.8559999	613586.0000000
19.1499996	5.9047999	613586.0000000
19.1499996	5.9535999	613586.0000000
19.1499996	6.0023999	613586.0000000
19.1666660	0.2928000	-3.4299812
19.1666660	0.3416000	-2.3991461
19.1666660	0.3904000	-2.0187187
19.1666660	0.4392000	-1.9021363
19.1666660	0.4880000	-1.9634953
19.1666660	0.5368000	-2.0248547
19.1666660	0.5856000	-2.0248547
19.1666660	0.6344000	-1.8591847
19.1666660	0.6832000	-2.0984859
19.1666660	0.7320000	-1.3744469
19.1666660	0.7808000	-1.3744469
19.1666660	0.8296000	-1.3437672
19.1666660	0.8784000	-1.1965051
19.1666660	0.9272000	-0.9694757
19.1666660	0.9760000	-1.1290097
19.1666660	1.0247999	-1.1290097
19.1666660	1.0735999	-1.3560390
19.1666660	1.1223999	-1.3560390
19.1666660	1.1711999	-1.1780972
19.1666660	1.2200000	-1.3867188
19.1666660	1.2688000	-1.3867188
19.1666660	1.3176000	-1.4112625
19.1666660	1.3664000	-1.3314953
19.1666660	1.4152000	-1.4848933
19.1666660	1.4640000	-1.4787574
19.1666660	1.5128000	-1.4112625

19.1666660	1.5616000	-1.3805828
19.1666660	1.6104000	-1.4726214
19.1666660	1.6592000	-1.5523887
19.1666660	1.7079999	-1.3376312
19.1666660	1.7567999	-1.3253593
19.1666660	1.8055999	-1.4603496
19.1666660	1.8543999	-1.2149129
19.1666660	1.9031999	-1.4787574
19.1666660	1.9519999	-1.6321554
19.1666660	2.0007999	-1.5953403
19.1666660	2.0495999	-1.4112625
19.1666660	2.0983999	-1.3928547
19.1666660	2.1471999	-1.3744469
19.1666660	2.1959999	-1.2885437
19.1666660	2.2447999	-1.3928547
19.1666660	2.2935998	-1.3867188
19.1666660	2.3423998	-1.3069515
19.1666660	2.3912001	-1.3130875
19.1666660	2.4400001	-1.3253593
19.1666660	2.4888000	-1.2210488
19.1666660	2.5376000	-1.1780972
19.1666660	2.5864000	-1.1658254
19.1666660	2.6352000	-1.1228738
19.1666660	2.6840000	-1.1106019
19.1666660	2.7328000	-1.0983305
19.1666660	2.7816000	-1.1044660
19.1666660	2.8304000	-1.0308352
19.1666660	2.8792000	-0.8958449
19.1666660	2.9280000	-0.8958449
19.1666660	2.9768000	-1.0737867
19.1666660	3.0256000	-1.0124273
19.1666660	3.0743999	-0.9019809
19.1666660	3.1231999	-0.9817476
19.1666660	3.1719999	-0.8406215
19.1666660	3.2207999	-0.8897090
19.1666660	3.2695999	-0.7731261
19.1666660	3.3183999	-0.6872234
19.1666660	3.3671999	-0.7669902
19.1666660	3.4159999	-1.0431070
19.1666660	3.4647999	-0.9878836
19.1666660	3.5135999	-0.9817476
19.1666660	3.5623999	-1.0246992
19.1666660	3.6111999	-1.0124273
19.1666660	3.6599998	-1.0983305
19.1666660	3.7087998	-1.0983305
19.1666660	3.7575998	-0.9081168
19.1666660	3.8063998	-0.8958449
19.1666660	3.8551998	-0.8835731
19.1666660	3.9039998	-0.8835731
19.1666660	3.9527998	-0.8897090
19.1666660	4.0016003	-0.9019809
19.1666660	4.0503998	-0.7424469
19.1666660	4.0991998	-0.8467574
19.1666660	4.1479998	-0.8406215
19.1666660	4.1967998	-0.6504078
19.1666660	4.2455997	-0.6688156
19.1666660	4.2943997	-0.6688156

19.1666660	4.3431997	-0.6749516
19.1666660	4.3919997	-0.5399613
19.1666660	4.4407997	-0.7056313
19.1666660	4.4895997	-0.7792621
19.1666660	4.5383997	-0.7363110
19.1666660	4.5871997	-0.8835731
19.1666660	4.6359997	-0.7608547
19.1666660	4.6847997	-0.8528934
19.1666660	4.7336001	-0.8651652
19.1666660	4.7824001	-0.6320000
19.1666660	4.8312001	-0.2822523
19.1666660	4.8800001	613586.0000000
19.1666660	4.9288001	613586.0000000
19.1666660	4.9776001	-0.7731261
19.1666660	5.0264001	-0.7731261
19.1666660	5.0752001	613586.0000000
19.1666660	5.1240001	613586.0000000
19.1666660	5.1728001	-0.7731261
19.1666660	5.2216001	-1.0615149
19.1666660	5.2704000	613586.0000000
19.1666660	5.3192000	613586.0000000
19.1666660	5.3680000	613586.0000000
19.1666660	5.4168000	613586.0000000
19.1666660	5.4656000	613586.0000000
19.1666660	5.5144000	-1.1228738
19.1666660	5.5632000	-0.9940195
19.1666660	5.6120000	-0.9878836
19.1666660	5.6608000	-0.9940195
19.1666660	5.7096000	-1.0369711
19.1666660	5.7584000	-1.0860586
19.1666660	5.8072000	613586.0000000
19.1666660	5.8559999	613586.0000000
19.1666660	5.9047999	613586.0000000
19.1666660	5.9535999	613586.0000000
19.1666660	6.0023999	613586.0000000
19.1833324	0.2928000	-2.8716121
19.1833324	0.3416000	-2.3746023
19.1833324	0.3904000	-2.3746023
19.1833324	0.4392000	-2.2457480
19.1833324	0.4880000	-1.9450879
19.1833324	0.5368000	-1.9082723
19.1833324	0.5856000	-1.9082723
19.1833324	0.6344000	-2.0800781
19.1833324	0.6832000	-2.0800781
19.1833324	0.7320000	-1.5217090
19.1833324	0.7808000	-1.5217090
19.1833324	0.8296000	-1.5585246
19.1833324	0.8784000	-1.5707965
19.1833324	0.9272000	-1.4419422
19.1833324	0.9760000	-1.5217090
19.1833324	1.0247999	-1.9144082
19.1833324	1.0735999	-1.7487383
19.1833324	1.1223999	-1.2394567
19.1833324	1.1711999	-1.0124273
19.1833324	1.2200000	-1.1474175
19.1833324	1.2688000	-1.6996508
19.1833324	1.3176000	-1.6935148

19.1833324	1.3664000	-1.6689711
19.1833324	1.4152000	-1.5769324
19.1833324	1.4640000	-1.5646605
19.1833324	1.5128000	-1.5523887
19.1833324	1.5616000	-1.4480777
19.1833324	1.6104000	-1.5094371
19.1833324	1.6592000	-1.5462527
19.1833324	1.7079999	-1.3314953
19.1833324	1.7567999	-1.3192234
19.1833324	1.8055999	-1.3069515
19.1833324	1.8543999	-1.3499031
19.1833324	1.9031999	-1.3621750
19.1833324	1.9519999	-1.3744469
19.1833324	2.0007999	-1.4173985
19.1833324	2.0495999	-1.4112625
19.1833324	2.0983999	-1.3560390
19.1833324	2.1471999	-1.3069515
19.1833324	2.1959999	-1.2946796
19.1833324	2.2447999	-1.2885437
19.1833324	2.2935998	-1.3437672
19.1833324	2.3423998	-1.3499031
19.1833324	2.3912001	-1.3008156
19.1833324	2.4400001	-1.2946796
19.1833324	2.4888000	-1.2333207
19.1833324	2.5376000	-1.2578645
19.1833324	2.5864000	-1.2578645
19.1833324	2.6352000	-1.2271848
19.1833324	2.6840000	-1.1106019
19.1833324	2.7328000	-1.0369711
19.1833324	2.7816000	-1.0369711
19.1833324	2.8304000	-1.0431070
19.1833324	2.8792000	-1.0308352
19.1833324	2.9280000	-1.2394567
19.1833324	2.9768000	-1.1412816
19.1833324	3.0256000	-1.0615149
19.1833324	3.0743999	-1.0615149
19.1833324	3.1231999	-1.0860586
19.1833324	3.1719999	-0.8651652
19.1833324	3.2207999	-0.7853980
19.1833324	3.2695999	-0.9265246
19.1833324	3.3183999	-0.9203887
19.1833324	3.3671999	-0.9081168
19.1833324	3.4159999	-0.8835731
19.1833324	3.4647999	-1.0369711
19.1833324	3.5135999	-1.0431070
19.1833324	3.5623999	-0.9203887
19.1833324	3.6111999	-0.8283496
19.1833324	3.6599998	-1.0553789
19.1833324	3.7087998	-1.2026410
19.1833324	3.7575998	-1.1351457
19.1833324	3.8063998	-1.1228738
19.1833324	3.8551998	-0.9510679
19.1833324	3.9039998	-0.8099418
19.1833324	3.9527998	-0.8528934
19.1833324	4.0016003	-0.8467574
19.1833324	4.0503998	-0.8406215
19.1833324	4.0991998	-0.9019809

19.1833324	4.1479998	-0.8958449
19.1833324	4.1967998	-0.8099418
19.1833324	4.2455997	-0.8099418
19.1833324	4.2943997	-0.9510679
19.1833324	4.3431997	-0.8590293
19.1833324	4.3919997	-0.8651652
19.1833324	4.4407997	-1.0553789
19.1833324	4.4895997	-0.8958449
19.1833324	4.5383997	-1.1719613
19.1833324	4.5871997	-1.1719613
19.1833324	4.6359997	-1.1658254
19.1833324	4.6847997	-1.0615149
19.1833324	4.7336001	-0.9633398
19.1833324	4.7824001	-0.9633398
19.1833324	4.8312001	-0.5890489
19.1833324	4.8800001	-0.3988352
19.1833324	4.9288001	-0.4111071
19.1833324	4.9776001	-0.6442719
19.1833324	5.0264001	-0.6442719
19.1833324	5.0752001	613586.0000000
19.1833324	5.1240001	613586.0000000
19.1833324	5.1728001	613586.0000000
19.1833324	5.2216001	613586.0000000
19.1833324	5.2704000	-0.8099418
19.1833324	5.3192000	-0.7179031
19.1833324	5.3680000	-0.7179031
19.1833324	5.4168000	-0.6504078
19.1833324	5.4656000	-0.6504078
19.1833324	5.5144000	-0.6626797
19.1833324	5.5632000	-0.9572039
19.1833324	5.6120000	-0.9510679
19.1833324	5.6608000	-0.8958449
19.1833324	5.7096000	-0.8222136
19.1833324	5.7584000	613586.0000000
19.1833324	5.8072000	613586.0000000
19.1833324	5.8559999	613586.0000000
19.1833324	5.9047999	613586.0000000
19.1833324	5.9535999	613586.0000000
19.1833324	6.0023999	613586.0000000
19.2000008	0.2928000	-3.5895152
19.2000008	0.3416000	-2.4666412
19.2000008	0.3904000	-2.1598449
19.2000008	0.4392000	-2.0187187
19.2000008	0.4880000	-1.6873789
19.2000008	0.5368000	-1.4480777
19.2000008	0.5856000	-1.1290097
19.2000008	0.6344000	-0.9326606
19.2000008	0.6832000	-1.3867188
19.2000008	0.7320000	-1.9205441
19.2000008	0.7808000	-1.9144082
19.2000008	0.8296000	-1.9144082
19.2000008	0.8784000	-1.5830684
19.2000008	0.9272000	-1.5830684
19.2000008	0.9760000	-1.4910293
19.2000008	1.0247999	-1.2394567
19.2000008	1.0735999	-1.3192234
19.2000008	1.1223999	-1.3376312

19.2000008	1.1711999	-1.3376312
19.2000008	1.2200000	-1.3560390
19.2000008	1.2688000	-1.2149129
19.2000008	1.3176000	-1.2149129
19.2000008	1.3664000	-1.5707965
19.2000008	1.4152000	-1.5707965
19.2000008	1.4640000	-1.2946796
19.2000008	1.5128000	-1.3805828
19.2000008	1.5616000	-1.3683109
19.2000008	1.6104000	-1.1965051
19.2000008	1.6592000	-1.1780972
19.2000008	1.7079999	-1.1780972
19.2000008	1.7567999	-1.3805828
19.2000008	1.8055999	-1.3744469
19.2000008	1.8543999	-1.3744469
19.2000008	1.9031999	-1.6076121
19.2000008	1.9519999	-1.6260195
19.2000008	2.0007999	-1.4603496
19.2000008	2.0495999	-1.3314953
19.2000008	2.0983999	-1.1719613
19.2000008	2.1471999	-1.1842332
19.2000008	2.1959999	-1.4726214
19.2000008	2.2447999	-1.3069515
19.2000008	2.2935998	-1.3008156
19.2000008	2.3423998	-1.2824078
19.2000008	2.3912001	-1.1903691
19.2000008	2.4400001	-1.1903691
19.2000008	2.4888000	-1.1167378
19.2000008	2.5376000	-1.3253593
19.2000008	2.5864000	-1.3253593
19.2000008	2.6352000	-1.0615149
19.2000008	2.6840000	-1.1903691
19.2000008	2.7328000	-1.1903691
19.2000008	2.7816000	-1.0308352
19.2000008	2.8304000	-1.0369711
19.2000008	2.8792000	-1.0369711
19.2000008	2.9280000	-1.0124273
19.2000008	2.9768000	-1.0124273
19.2000008	3.0256000	-0.9510679
19.2000008	3.0743999	-0.9326606
19.2000008	3.1231999	-0.8528934
19.2000008	3.1719999	-0.8467574
19.2000008	3.2207999	-0.8528934
19.2000008	3.2695999	-0.7240391
19.2000008	3.3183999	-0.9203887
19.2000008	3.3671999	-1.2578645
19.2000008	3.4159999	-0.9817476
19.2000008	3.4647999	-1.0615149
19.2000008	3.5135999	-1.0492430
19.2000008	3.5623999	-0.9449320
19.2000008	3.6111999	-0.9510679
19.2000008	3.6599998	-1.0246992
19.2000008	3.7087998	-1.0124273
19.2000008	3.7575998	-1.0062914
19.2000008	3.8063998	-0.8897090
19.2000008	3.8551998	-0.8897090
19.2000008	3.9039998	-0.9510679

19.2000008	3.9527998	-0.9510679
19.2000008	4.0016003	-0.9510679
19.2000008	4.0503998	-0.7669902
19.2000008	4.0991998	-0.7424469
19.2000008	4.1479998	-0.6135921
19.2000008	4.1967998	-0.8467574
19.2000008	4.2455997	-0.9142528
19.2000008	4.2943997	-0.9203887
19.2000008	4.3431997	-0.9326606
19.2000008	4.3919997	-0.9878836
19.2000008	4.4407997	-0.8406215
19.2000008	4.4895997	-0.9019809
19.2000008	4.5383997	-0.8344855
19.2000008	4.5871997	-0.7179031
19.2000008	4.6359997	-0.7179031
19.2000008	4.6847997	-0.5399613
19.2000008	4.7336001	-0.5338254
19.2000008	4.7824001	-0.6442719
19.2000008	4.8312001	-0.7792621
19.2000008	4.8800001	-0.7731261
19.2000008	4.9288001	-0.7792621
19.2000008	4.9776001	-0.7608547
19.2000008	5.0264001	-0.7547188
19.2000008	5.0752001	-0.7363110
19.2000008	5.1240001	-0.7485828
19.2000008	5.1728001	-0.6994953
19.2000008	5.2216001	-0.6994953
19.2000008	5.2704000	-0.7424469
19.2000008	5.3192000	-0.7424469
19.2000008	5.3680000	-0.7424469
19.2000008	5.4168000	-0.9265246
19.2000008	5.4656000	-0.8099418
19.2000008	5.5144000	-0.8099418
19.2000008	5.5632000	-0.8038058
19.2000008	5.6120000	-0.8099418
19.2000008	5.6608000	613586.0000000
19.2000008	5.7096000	613586.0000000
19.2000008	5.7584000	613586.0000000
19.2000008	5.8072000	-0.7301750
19.2000008	5.8559999	613586.0000000
19.2000008	5.9047999	-0.7240391
19.2000008	5.9535999	613586.0000000
19.2000008	6.0023999	613586.0000000
19.2166672	0.2928000	-2.7243500
19.2166672	0.3416000	-1.9328160
19.2166672	0.3904000	-2.0862141
19.2166672	0.4392000	-2.0862141
19.2166672	0.4880000	-2.0984859
19.2166672	0.5368000	-1.8223691
19.2166672	0.5856000	-1.2394567
19.2166672	0.6344000	-1.3744469
19.2166672	0.6832000	-1.2946796
19.2166672	0.7320000	-1.4419422
19.2166672	0.7808000	-1.6198835
19.2166672	0.8296000	-1.6137476
19.2166672	0.8784000	-1.6137476
19.2166672	0.9272000	-1.5217090

19.2166672	0.9760000	-1.3744469
19.2166672	1.0247999	-1.3928547
19.2166672	1.0735999	-1.3069515
19.2166672	1.1223999	-1.3130875
19.2166672	1.1711999	-1.1842332
19.2166672	1.2200000	-1.1167378
19.2166672	1.2688000	-1.3130875
19.2166672	1.3176000	-1.1596894
19.2166672	1.3664000	-1.1780972
19.2166672	1.4152000	-1.1535535
19.2166672	1.4640000	-1.1596894
19.2166672	1.5128000	-1.2087770
19.2166672	1.5616000	-1.2149129
19.2166672	1.6104000	-1.1903691
19.2166672	1.6592000	-1.3683109
19.2166672	1.7079999	-1.3621750
19.2166672	1.7567999	-1.3499031
19.2166672	1.8055999	-1.4419422
19.2166672	1.8543999	-1.0246992
19.2166672	1.9031999	-1.0246992
19.2166672	1.9519999	-1.2885437
19.2166672	2.0007999	-1.2885437
19.2166672	2.0495999	-1.1719613
19.2166672	2.0983999	-1.4051266
19.2166672	2.1471999	-1.2087770
19.2166672	2.1959999	-1.2578645
19.2166672	2.2447999	-1.3130875
19.2166672	2.2935998	-1.1290097
19.2166672	2.3423998	-1.1965051
19.2166672	2.3912001	-1.2087770
19.2166672	2.4400001	-1.0369711
19.2166672	2.4888000	-1.0308352
19.2166672	2.5376000	-1.0308352
19.2166672	2.5864000	-1.0492430
19.2166672	2.6352000	-1.0492430
19.2166672	2.6840000	-1.0369711
19.2166672	2.7328000	-0.8835731
19.2166672	2.7816000	-0.8835731
19.2166672	2.8304000	-0.8467574
19.2166672	2.8792000	-0.7547188
19.2166672	2.9280000	-0.7669902
19.2166672	2.9768000	-0.8897090
19.2166672	3.0256000	-0.9203887
19.2166672	3.0743999	-0.9326606
19.2166672	3.1231999	-0.9326606
19.2166672	3.1719999	-0.7792621
19.2166672	3.2207999	-0.8099418
19.2166672	3.2695999	-0.8160777
19.2166672	3.3183999	-0.8099418
19.2166672	3.3671999	-0.7424469
19.2166672	3.4159999	-0.7424469
19.2166672	3.4647999	-0.9449320
19.2166672	3.5135999	-0.9572039
19.2166672	3.5623999	-0.9449320
19.2166672	3.6111999	-0.7301750
19.2166672	3.6599998	-0.8467574
19.2166672	3.7087998	-0.8467574

19.2166672	3.7575998	-0.8528934
19.2166672	3.8063998	-0.8651652
19.2166672	3.8551998	-0.7056313
19.2166672	3.9039998	-0.7117672
19.2166672	3.9527998	-0.6994953
19.2166672	4.0016003	-0.6933594
19.2166672	4.0503998	-0.6994953
19.2166672	4.0991998	-0.8713012
19.2166672	4.1479998	-0.8897090
19.2166672	4.1967998	-0.5706410
19.2166672	4.2455997	-0.5706410
19.2166672	4.2943997	-0.4601941
19.2166672	4.3431997	-0.4663301
19.2166672	4.3919997	-0.7056313
19.2166672	4.4407997	-0.7301750
19.2166672	4.4895997	-0.7240391
19.2166672	4.5383997	-0.7915339
19.2166672	4.5871997	-0.8897090
19.2166672	4.6359997	-0.8897090
19.2166672	4.6847997	-0.8038058
19.2166672	4.7336001	-0.5031457
19.2166672	4.7824001	-0.5031457
19.2166672	4.8312001	-0.5031457
19.2166672	4.8800001	-0.5951843
19.2166672	4.9288001	-0.9449320
19.2166672	4.9776001	-1.0737867
19.2166672	5.0264001	-1.0615149
19.2166672	5.0752001	-1.0492430
19.2166672	5.1240001	-1.0615149
19.2166672	5.1728001	-1.0737867
19.2166672	5.2216001	-1.0737867
19.2166672	5.2704000	-0.9940195
19.2166672	5.3192000	-1.0185633
19.2166672	5.3680000	-0.8774371
19.2166672	5.4168000	-0.7792621
19.2166672	5.4656000	-0.7976699
19.2166672	5.5144000	-0.8406215
19.2166672	5.5632000	-0.8038058
19.2166672	5.6120000	-0.8160777
19.2166672	5.6608000	-0.8160777
19.2166672	5.7096000	-0.8099418
19.2166672	5.7584000	-0.7669902
19.2166672	5.8072000	-0.7547188
19.2166672	5.8559999	-0.7424469
19.2166672	5.9047999	613586.0000000
19.2166672	5.9535999	613586.0000000
19.2166672	6.0023999	613586.0000000
19.2333336	0.2928000	-2.4359615
19.2333336	0.3416000	-2.4420974
19.2333336	0.3904000	-2.0678062
19.2333336	0.4392000	-1.9144082
19.2333336	0.4880000	-1.3008156
19.2333336	0.5368000	-1.4051266
19.2333336	0.5856000	-1.3989906
19.2333336	0.6344000	-1.3989906
19.2333336	0.6832000	-1.2946796
19.2333336	0.7320000	-1.2210488

19.2333336	0.7808000	-0.9878836
19.2333336	0.8296000	-1.0431070
19.2333336	0.8784000	-1.0431070
19.2333336	0.9272000	-0.9019809
19.2333336	0.9760000	-0.7547188
19.2333336	1.0247999	-1.6014762
19.2333336	1.0735999	-1.6014762
19.2333336	1.1223999	-1.3683109
19.2333336	1.1711999	-1.5707965
19.2333336	1.2200000	-1.5769324
19.2333336	1.2688000	-1.2455926
19.2333336	1.3176000	-1.3805828
19.2333336	1.3664000	-1.2885437
19.2333336	1.4152000	-1.2885437
19.2333336	1.4640000	-1.5462527
19.2333336	1.5128000	-1.4480777
19.2333336	1.5616000	-1.2517285
19.2333336	1.6104000	-1.2517285
19.2333336	1.6592000	-1.4726214
19.2333336	1.7079999	-1.3928547
19.2333336	1.7567999	-1.3867188
19.2333336	1.8055999	-1.1351457
19.2333336	1.8543999	-1.3314953
19.2333336	1.9031999	-1.3437672
19.2333336	1.9519999	-1.2087770
19.2333336	2.0007999	-1.3376312
19.2333336	2.0495999	-1.3130875
19.2333336	2.0983999	-1.3069515
19.2333336	2.1471999	-1.1719613
19.2333336	2.1959999	-1.1842332
19.2333336	2.2447999	-1.1842332
19.2333336	2.2935998	-1.1842332
19.2333336	2.3423998	-1.0062914
19.2333336	2.3912001	-1.1903691
19.2333336	2.4400001	-0.9572039
19.2333336	2.4888000	-0.9633398
19.2333336	2.5376000	-1.0369711
19.2333336	2.5864000	-1.0431070
19.2333336	2.6352000	-0.9878836
19.2333336	2.6840000	-0.9878836
19.2333336	2.7328000	-0.9510679
19.2333336	2.7816000	-0.8467574
19.2333336	2.8304000	-0.7669902
19.2333336	2.8792000	-0.7669902
19.2333336	2.9280000	-0.7608547
19.2333336	2.9768000	-0.7608547
19.2333336	3.0256000	-0.6810875
19.2333336	3.0743999	-0.9019809
19.2333336	3.1231999	-0.7608547
19.2333336	3.1719999	-0.8713012
19.2333336	3.2207999	-0.6749516
19.2333336	3.2695999	-0.8713012
19.2333336	3.3183999	-0.8160777
19.2333336	3.3671999	-0.8160777
19.2333336	3.4159999	-0.8160777
19.2333336	3.4647999	-0.6565437
19.2333336	3.5135999	-0.7485828

19.2333336	3.5623999	-0.7485828
19.2333336	3.6111999	-0.6810875
19.2333336	3.6599998	-0.8651652
19.2333336	3.7087998	-0.8467574
19.2333336	3.7575998	-0.8406215
19.2333336	3.8063998	-0.7853980
19.2333336	3.8551998	-0.7976699
19.2333336	3.9039998	-0.7608547
19.2333336	3.9527998	-0.6933594
19.2333336	4.0016003	-0.8958449
19.2333336	4.0503998	-0.9019809
19.2333336	4.0991998	-0.8222136
19.2333336	4.1479998	-0.4663301
19.2333336	4.1967998	-0.4724660
19.2333336	4.2455997	-0.7179031
19.2333336	4.2943997	-0.7117672
19.2333336	4.3431997	-0.7792621
19.2333336	4.3919997	-0.7731261
19.2333336	4.4407997	-0.8897090
19.2333336	4.4895997	-0.8590293
19.2333336	4.5383997	-0.8590293
19.2333336	4.5871997	-0.6504078
19.2333336	4.6359997	-0.6504078
19.2333336	4.6847997	-0.4601941
19.2333336	4.7336001	-0.2515731
19.2333336	4.7824001	-0.2699804
19.2333336	4.8312001	-0.5645051
19.2333336	4.8800001	-0.4540582
19.2333336	4.9288001	-0.4540582
19.2333336	4.9776001	-0.6504078
19.2333336	5.0264001	-0.6320000
19.2333336	5.0752001	-0.8283496
19.2333336	5.1240001	-0.7056313
19.2333336	5.1728001	-0.6994953
19.2333336	5.2216001	-0.6320000
19.2333336	5.2704000	-0.6320000
19.2333336	5.3192000	-0.5706410
19.2333336	5.3680000	-0.5829129
19.2333336	5.4168000	-0.5399613
19.2333336	5.4656000	-0.6381359
19.2333336	5.5144000	-0.5706410
19.2333336	5.5632000	-0.4847379
19.2333336	5.6120000	-0.4847379
19.2333336	5.6608000	-0.6442719
19.2333336	5.7096000	613586.0000000
19.2333336	5.7584000	-0.4172430
19.2333336	5.8072000	-0.4295144
19.2333336	5.8559999	613586.0000000
19.2333336	5.9047999	613586.0000000
19.2333336	5.9535999	613586.0000000
19.2333336	6.0023999	613586.0000000
19.2500000	0.2928000	-2.4911849
19.2500000	0.3416000	-2.5034568
19.2500000	0.3904000	-2.8225248
19.2500000	0.4392000	-1.7916894
19.2500000	0.4880000	-1.7241945
19.2500000	0.5368000	-1.5094371

19.2500000	0.5856000	-1.6566992
19.2500000	0.6344000	-1.6628351
19.2500000	0.6832000	-1.3130875
19.2500000	0.7320000	-1.1412816
19.2500000	0.7808000	-1.1474175
19.2500000	0.8296000	-1.1658254
19.2500000	0.8784000	-1.8653207
19.2500000	0.9272000	-1.5769324
19.2500000	0.9760000	-1.5953403
19.2500000	1.0247999	-1.6260195
19.2500000	1.0735999	-1.6260195
19.2500000	1.1223999	-1.5585246
19.2500000	1.1711999	-1.3253593
19.2500000	1.2200000	-1.3253593
19.2500000	1.2688000	-1.2578645
19.2500000	1.3176000	-1.2333207
19.2500000	1.3664000	-1.3192234
19.2500000	1.4152000	-1.3069515
19.2500000	1.4640000	-1.4358063
19.2500000	1.5128000	-1.3069515
19.2500000	1.5616000	-1.3069515
19.2500000	1.6104000	-1.3314953
19.2500000	1.6592000	-1.3867188
19.2500000	1.7079999	-1.2149129
19.2500000	1.7567999	-1.1167378
19.2500000	1.8055999	-0.9510679
19.2500000	1.8543999	-1.0001554
19.2500000	1.9031999	-1.3008156
19.2500000	1.9519999	-1.3008156
19.2500000	2.0007999	-1.1228738
19.2500000	2.0495999	-1.0860586
19.2500000	2.0983999	-1.0860586
19.2500000	2.1471999	-1.1044660
19.2500000	2.1959999	-1.0246992
19.2500000	2.2447999	-1.0799227
19.2500000	2.2935998	-1.0676508
19.2500000	2.3423998	-1.0431070
19.2500000	2.3912001	-0.8958449
19.2500000	2.4400001	-1.0124273
19.2500000	2.4888000	-0.9326606
19.2500000	2.5376000	-0.7792621
19.2500000	2.5864000	-0.8897090
19.2500000	2.6352000	-0.8774371
19.2500000	2.6840000	-0.8467574
19.2500000	2.7328000	-0.8467574
19.2500000	2.7816000	-0.9326606
19.2500000	2.8304000	-0.9387960
19.2500000	2.8792000	-0.6135921
19.2500000	2.9280000	-0.6013203
19.2500000	2.9768000	-0.5951843
19.2500000	3.0256000	-0.7240391
19.2500000	3.0743999	-0.5338254
19.2500000	3.1231999	-0.8528934
19.2500000	3.1719999	-0.8406215
19.2500000	3.2207999	-0.7056313
19.2500000	3.2695999	-0.6994953
19.2500000	3.3183999	-0.6872234

19.2500000	3.3671999	-0.6872234
19.2500000	3.4159999	-0.5522332
19.2500000	3.4647999	-0.5522332
19.2500000	3.5135999	-0.9633398
19.2500000	3.5623999	-1.0737867
19.2500000	3.6111999	-1.0062914
19.2500000	3.6599998	-0.9265246
19.2500000	3.7087998	-0.8222136
19.2500000	3.7575998	-0.8222136
19.2500000	3.8063998	-0.7792621
19.2500000	3.8551998	-0.7792621
19.2500000	3.9039998	-0.7608547
19.2500000	3.9527998	-0.6381359
19.2500000	4.0016003	-0.5031457
19.2500000	4.0503998	-0.6626797
19.2500000	4.0991998	-0.5951843
19.2500000	4.1479998	-0.6074562
19.2500000	4.1967998	-0.6074562
19.2500000	4.2455997	-0.4663301
19.2500000	4.2943997	-0.4111071
19.2500000	4.3431997	-0.4540582
19.2500000	4.3919997	-0.4540582
19.2500000	4.4407997	-0.5399613
19.2500000	4.4895997	-0.5399613
19.2500000	4.5383997	-0.6013203
19.2500000	4.5871997	-0.5338254
19.2500000	4.6359997	-0.5154176
19.2500000	4.6847997	-0.3558836
19.2500000	4.7336001	-0.3558836
19.2500000	4.7824001	-0.5951843
19.2500000	4.8312001	-0.6074562
19.2500000	4.8800001	-0.6135921
19.2500000	4.9288001	-0.5031457
19.2500000	4.9776001	-0.6933594
19.2500000	5.0264001	-0.8222136
19.2500000	5.0752001	-0.8099418
19.2500000	5.1240001	-1.0799227
19.2500000	5.1728001	-0.9387960
19.2500000	5.2216001	-0.7853980
19.2500000	5.2704000	-0.7669902
19.2500000	5.3192000	-0.7485828
19.2500000	5.3680000	-0.7179031
19.2500000	5.4168000	-0.7179031
19.2500000	5.4656000	-0.7731261
19.2500000	5.5144000	-0.5522332
19.2500000	5.5632000	-0.5460973
19.2500000	5.6120000	-0.5215535
19.2500000	5.6608000	-0.5092816
19.2500000	5.7096000	-0.4479222
19.2500000	5.7584000	-0.3558836
19.2500000	5.8072000	-0.3620195
19.2500000	5.8559999	613586.0000000
19.2500000	5.9047999	-0.3190680
19.2500000	5.9535999	613586.0000000
19.2500000	6.0023999	613586.0000000
19.2666664	0.2928000	-2.5034568
19.2666664	0.3416000	-1.7794180

19.2666664	0.3904000	-1.6505632
19.2666664	0.4392000	-1.4603496
19.2666664	0.4880000	-1.3867188
19.2666664	0.5368000	-1.6137476
19.2666664	0.5856000	-1.2885437
19.2666664	0.6344000	-1.3499031
19.2666664	0.6832000	-1.2762718
19.2666664	0.7320000	-1.3928547
19.2666664	0.7808000	-1.3621750
19.2666664	0.8296000	-1.3621750
19.2666664	0.8784000	-1.4419422
19.2666664	0.9272000	-1.3560390
19.2666664	0.9760000	-1.0062914
19.2666664	1.0247999	-1.2087770
19.2666664	1.0735999	-1.3683109
19.2666664	1.1223999	-1.4419422
19.2666664	1.1711999	-1.3130875
19.2666664	1.2200000	-1.3069515
19.2666664	1.2688000	-1.2885437
19.2666664	1.3176000	-1.3437672
19.2666664	1.3664000	-1.3314953
19.2666664	1.4152000	-1.6689711
19.2666664	1.4640000	-1.5094371
19.2666664	1.5128000	-1.2701364
19.2666664	1.5616000	-1.2824078
19.2666664	1.6104000	-1.4419422
19.2666664	1.6592000	-1.0983305
19.2666664	1.7079999	-1.2701364
19.2666664	1.7567999	-1.3253593
19.2666664	1.8055999	-1.3253593
19.2666664	1.8543999	-1.3376312
19.2666664	1.9031999	-1.3437672
19.2666664	1.9519999	-1.2578645
19.2666664	2.0007999	-1.2578645
19.2666664	2.0495999	-1.2578645
19.2666664	2.0983999	-1.2762718
19.2666664	2.1471999	-1.1290097
19.2666664	2.1959999	-1.1106019
19.2666664	2.2447999	-1.0983305
19.2666664	2.2935998	-1.0737867
19.2666664	2.3423998	-1.0737867
19.2666664	2.3912001	-0.9265246
19.2666664	2.4400001	-0.9265246
19.2666664	2.4888000	-0.9756117
19.2666664	2.5376000	-0.9633398
19.2666664	2.5864000	-0.8835731
19.2666664	2.6352000	-0.8897090
19.2666664	2.6840000	-0.8897090
19.2666664	2.7328000	-0.8897090
19.2666664	2.7816000	-1.0185633
19.2666664	2.8304000	-0.7853980
19.2666664	2.8792000	-0.7731261
19.2666664	2.9280000	-0.7731261
19.2666664	2.9768000	-0.6872234
19.2666664	3.0256000	-0.7240391
19.2666664	3.0743999	-0.7240391
19.2666664	3.1231999	-0.8897090

19.2666664	3.1719999	-0.9019809
19.2666664	3.2207999	-0.6749516
19.2666664	3.2695999	-0.6749516
19.2666664	3.3183999	-0.7424469
19.2666664	3.3671999	-0.6135921
19.2666664	3.4159999	-0.8835731
19.2666664	3.4647999	-0.7915339
19.2666664	3.5135999	-0.9019809
19.2666664	3.5623999	-0.9142528
19.2666664	3.6111999	-0.8406215
19.2666664	3.6599998	-0.8406215
19.2666664	3.7087998	-0.7669902
19.2666664	3.7575998	-0.7669902
19.2666664	3.8063998	-0.5767770
19.2666664	3.8551998	-0.5767770
19.2666664	3.9039998	-0.5767770
19.2666664	3.9527998	-0.5583692
19.2666664	4.0016003	-0.5460973
19.2666664	4.0503998	-0.2331653
19.2666664	4.0991998	-0.2331653
19.2666664	4.1479998	-0.1840777
19.2666664	4.1967998	-0.2883883
19.2666664	4.2455997	-0.6933594
19.2666664	4.2943997	-0.6933594
19.2666664	4.3431997	-0.6994953
19.2666664	4.3919997	-0.4417863
19.2666664	4.4407997	-0.5154176
19.2666664	4.4895997	-0.2208934
19.2666664	4.5383997	-0.2331653
19.2666664	4.5871997	-0.0245438
19.2666664	4.6359997	-0.0122719
19.2666664	4.6847997	-0.0184078
19.2666664	4.7336001	0.0552235
19.2666664	4.7824001	-0.2208934
19.2666664	4.8312001	-0.3497477
19.2666664	4.8800001	-0.5338254
19.2666664	4.9288001	-0.5399613
19.2666664	4.9776001	-0.5460973
19.2666664	5.0264001	-0.7117672
19.2666664	5.0752001	-0.8344855
19.2666664	5.1240001	-1.4787574
19.2666664	5.1728001	-1.4664855
19.2666664	5.2216001	-1.4664855
19.2666664	5.2704000	-1.3560390
19.2666664	5.3192000	-1.3192234
19.2666664	5.3680000	-1.3192234
19.2666664	5.4168000	-1.1535535
19.2666664	5.4656000	-1.0553789
19.2666664	5.5144000	-1.0124273
19.2666664	5.5632000	-1.0124273
19.2666664	5.6120000	-1.0062914
19.2666664	5.6608000	-1.0062914
19.2666664	5.7096000	-0.8406215
19.2666664	5.7584000	613586.0000000
19.2666664	5.8072000	613586.0000000
19.2666664	5.8559999	-0.7424469
19.2666664	5.9047999	-0.6688156

19.2666664	5.9535999	613586.0000000
19.2666664	6.0023999	613586.0000000
19.2833328	0.2928000	-2.5402722
19.2833328	0.3416000	-2.5402722
19.2833328	0.3904000	-2.0371265
19.2833328	0.4392000	-1.9696312
19.2833328	0.4880000	-1.5278449
19.2833328	0.5368000	-1.5278449
19.2833328	0.5856000	-1.6566992
19.2833328	0.6344000	-1.7548742
19.2833328	0.6832000	-1.4664855
19.2833328	0.7320000	-1.2394567
19.2833328	0.7808000	-1.4971652
19.2833328	0.8296000	-1.4542136
19.2833328	0.8784000	-1.4848933
19.2833328	0.9272000	-1.4296703
19.2833328	0.9760000	-1.4235344
19.2833328	1.0247999	-1.4173985
19.2833328	1.0735999	-1.1780972
19.2833328	1.1223999	-1.1842332
19.2833328	1.1711999	-1.3376312
19.2833328	1.2200000	-1.2578645
19.2833328	1.2688000	-1.2517285
19.2833328	1.3176000	-1.2578645
19.2833328	1.3664000	-1.3621750
19.2833328	1.4152000	-1.3621750
19.2833328	1.4640000	-1.3560390
19.2833328	1.5128000	-1.3989906
19.2833328	1.5616000	-1.3805828
19.2833328	1.6104000	-1.2885437
19.2833328	1.6592000	-1.3683109
19.2833328	1.7079999	-1.3437672
19.2833328	1.7567999	-1.3253593
19.2833328	1.8055999	-1.1351457
19.2833328	1.8543999	-1.2333207
19.2833328	1.9031999	-1.3192234
19.2833328	1.9519999	-1.3314953
19.2833328	2.0007999	-1.1780972
19.2833328	2.0495999	-1.3008156
19.2833328	2.0983999	-1.3130875
19.2833328	2.1471999	-1.1719613
19.2833328	2.1959999	-1.0983305
19.2833328	2.2447999	-1.0799227
19.2833328	2.2935998	-1.1412816
19.2833328	2.3423998	-1.2455926
19.2833328	2.3912001	-1.0492430
19.2833328	2.4400001	-1.0553789
19.2833328	2.4888000	-1.1780972
19.2833328	2.5376000	-1.1780972
19.2833328	2.5864000	-1.1228738
19.2833328	2.6352000	-1.1903691
19.2833328	2.6840000	-1.1842332
19.2833328	2.7328000	-0.9817476
19.2833328	2.7816000	-1.0492430
19.2833328	2.8304000	-1.0369711
19.2833328	2.8792000	-1.0308352
19.2833328	2.9280000	-0.9019809

19.2833328	2.9768000	-0.8835731
19.2833328	3.0256000	-0.6810875
19.2833328	3.0743999	-0.7669902
19.2833328	3.1231999	-1.0431070
19.2833328	3.1719999	-0.6320000
19.2833328	3.2207999	-0.7547188
19.2833328	3.2695999	-0.8283496
19.2833328	3.3183999	-0.8160777
19.2833328	3.3671999	-0.9387960
19.2833328	3.4159999	-0.9572039
19.2833328	3.4647999	-0.8958449
19.2833328	3.5135999	-0.8958449
19.2833328	3.5623999	-0.8897090
19.2833328	3.6111999	-1.0001554
19.2833328	3.6599998	-0.9081168
19.2833328	3.7087998	-0.9019809
19.2833328	3.7575998	-0.7608547
19.2833328	3.8063998	-0.8958449
19.2833328	3.8551998	-0.5890489
19.2833328	3.9039998	-0.6872234
19.2833328	3.9527998	-0.6810875
19.2833328	4.0016003	-0.5951843
19.2833328	4.0503998	-0.6074562
19.2833328	4.0991998	-0.3129320
19.2833328	4.1479998	-0.3067961
19.2833328	4.1967998	-0.5215535
19.2833328	4.2455997	-0.5399613
19.2833328	4.2943997	-0.5154176
19.2833328	4.3431997	-0.5154176
19.2833328	4.3919997	-0.5767770
19.2833328	4.4407997	-0.5706410
19.2833328	4.4895997	-0.5767770
19.2833328	4.5383997	-0.5706410
19.2833328	4.5871997	-0.1472621
19.2833328	4.6359997	-0.1533980
19.2833328	4.6847997	0.0674953
19.2833328	4.7336001	-0.0122719
19.2833328	4.7824001	0.1718059
19.2833328	4.8312001	0.1963496
19.2833328	4.8800001	0.2086215
19.2833328	4.9288001	-0.3190680
19.2833328	4.9776001	-0.3190680
19.2833328	5.0264001	-0.1656699
19.2833328	5.0752001	-0.1411262
19.2833328	5.1240001	-0.1349902
19.2833328	5.1728001	-0.1718059
19.2833328	5.2216001	-0.2393012
19.2833328	5.2704000	-0.2393012
19.2833328	5.3192000	-0.3497477
19.2833328	5.3680000	-0.3742914
19.2833328	5.4168000	-0.5276895
19.2833328	5.4656000	-0.5215535
19.2833328	5.5144000	-0.5092816
19.2833328	5.5632000	-0.7301750
19.2833328	5.6120000	-0.7363110
19.2833328	5.6608000	613586.0000000
19.2833328	5.7096000	613586.0000000

19.2833328	5.7584000	613586.0000000
19.2833328	5.8072000	613586.0000000
19.2833328	5.8559999	613586.0000000
19.2833328	5.9047999	613586.0000000
19.2833328	5.9535999	613586.0000000
19.2833328	6.0023999	613586.0000000
19.2999992	0.2928000	-2.5157285
19.2999992	0.3416000	-1.8530488
19.2999992	0.3904000	-1.8285050
19.2999992	0.4392000	-1.8162332
19.2999992	0.4880000	-1.7057867
19.2999992	0.5368000	-1.6996508
19.2999992	0.5856000	-1.6198835
19.2999992	0.6344000	-1.4542136
19.2999992	0.6832000	-1.3437672
19.2999992	0.7320000	-1.5769324
19.2999992	0.7808000	-1.5523887
19.2999992	0.8296000	-1.2946796
19.2999992	0.8784000	-1.3069515
19.2999992	0.9272000	-1.3560390
19.2999992	0.9760000	-1.2149129
19.2999992	1.0247999	-1.2210488
19.2999992	1.0735999	-1.4419422
19.2999992	1.1223999	-1.4480777
19.2999992	1.1711999	-1.4664855
19.2999992	1.2200000	-1.4848933
19.2999992	1.2688000	-1.3253593
19.2999992	1.3176000	-1.3130875
19.2999992	1.3664000	-1.3192234
19.2999992	1.4152000	-1.4910293
19.2999992	1.4640000	-1.4787574
19.2999992	1.5128000	-1.2087770
19.2999992	1.5616000	-1.3805828
19.2999992	1.6104000	-1.4296703
19.2999992	1.6592000	-1.4358063
19.2999992	1.7079999	-1.4358063
19.2999992	1.7567999	-1.2885437
19.2999992	1.8055999	-1.2885437
19.2999992	1.8543999	-1.1351457
19.2999992	1.9031999	-1.1351457
19.2999992	1.9519999	-1.3130875
19.2999992	2.0007999	-1.1965051
19.2999992	2.0495999	-1.0860586
19.2999992	2.0983999	-1.1596894
19.2999992	2.1471999	-1.1167378
19.2999992	2.1959999	-1.2824078
19.2999992	2.2447999	-1.2885437
19.2999992	2.2935998	-1.2087770
19.2999992	2.3423998	-1.2026410
19.2999992	2.3912001	-1.3560390
19.2999992	2.4400001	-1.3560390
19.2999992	2.4888000	-0.9940195
19.2999992	2.5376000	-1.0001554
19.2999992	2.5864000	-1.1965051
19.2999992	2.6352000	-0.9142528
19.2999992	2.6840000	-0.9081168
19.2999992	2.7328000	-1.0308352

19.2999992	2.7816000	-1.0369711
19.2999992	2.8304000	-0.7669902
19.2999992	2.8792000	-0.7731261
19.2999992	2.9280000	-0.7853980
19.2999992	2.9768000	-0.4540582
19.2999992	3.0256000	-0.6872234
19.2999992	3.0743999	-1.2762718
19.2999992	3.1231999	-1.0124273
19.2999992	3.1719999	-1.0921946
19.2999992	3.2207999	-0.9449320
19.2999992	3.2695999	-1.0124273
19.2999992	3.3183999	-1.0185633
19.2999992	3.3671999	-0.7976699
19.2999992	3.4159999	-0.9142528
19.2999992	3.4647999	-1.0124273
19.2999992	3.5135999	-1.0062914
19.2999992	3.5623999	-0.9449320
19.2999992	3.6111999	-0.9510679
19.2999992	3.6599998	-0.7240391
19.2999992	3.7087998	-0.7240391
19.2999992	3.7575998	-0.6626797
19.2999992	3.8063998	-0.6810875
19.2999992	3.8551998	-0.6994953
19.2999992	3.9039998	-0.7056313
19.2999992	3.9527998	-0.7056313
19.2999992	4.0016003	-0.8160777
19.2999992	4.0503998	-0.8160777
19.2999992	4.0991998	-0.5890489
19.2999992	4.1479998	-0.5522332
19.2999992	4.1967998	-0.5460973
19.2999992	4.2455997	-0.5338254
19.2999992	4.2943997	-0.6381359
19.2999992	4.3431997	-0.6381359
19.2999992	4.3919997	-0.6258640
19.2999992	4.4407997	-0.6381359
19.2999992	4.4895997	-0.6688156
19.2999992	4.5383997	-0.7485828
19.2999992	4.5871997	-0.9572039
19.2999992	4.6359997	-1.2333207
19.2999992	4.6847997	-1.2333207
19.2999992	4.7336001	-1.1474175
19.2999992	4.7824001	-0.8160777
19.2999992	4.8312001	-0.7056313
19.2999992	4.8800001	-0.5460973
19.2999992	4.9288001	-0.7792621
19.2999992	4.9776001	-0.6381359
19.2999992	5.0264001	-0.6994953
19.2999992	5.0752001	-0.7363110
19.2999992	5.1240001	-0.7424469
19.2999992	5.1728001	-0.8835731
19.2999992	5.2216001	-0.8099418
19.2999992	5.2704000	-0.6197281
19.2999992	5.3192000	-0.7608547
19.2999992	5.3680000	-0.7485828
19.2999992	5.4168000	-0.8406215
19.2999992	5.4656000	-0.8406215
19.2999992	5.5144000	-0.7301750

19.2999992	5.5632000	-0.7301750
19.2999992	5.6120000	613586.0000000
19.2999992	5.6608000	613586.0000000
19.2999992	5.7096000	613586.0000000
19.2999992	5.7584000	613586.0000000
19.2999992	5.8072000	613586.0000000
19.2999992	5.8559999	613586.0000000
19.2999992	5.9047999	613586.0000000
19.2999992	5.9535999	613586.0000000
19.2999992	6.0023999	613586.0000000
19.3166676	0.2928000	-2.6139033
19.3166676	0.3416000	-2.3193791
19.3166676	0.3904000	-1.5401168
19.3166676	0.4392000	-1.5401168
19.3166676	0.4880000	-1.3621750
19.3166676	0.5368000	-1.3744469
19.3166676	0.5856000	-1.3621750
19.3166676	0.6344000	-1.4971652
19.3166676	0.6832000	-1.1719613
19.3166676	0.7320000	-1.2149129
19.3166676	0.7808000	-1.2149129
19.3166676	0.8296000	-1.2087770
19.3166676	0.8784000	-1.3314953
19.3166676	0.9272000	-1.1842332
19.3166676	0.9760000	-1.3805828
19.3166676	1.0247999	-1.2701364
19.3166676	1.0735999	-1.2455926
19.3166676	1.1223999	-1.2455926
19.3166676	1.1711999	-1.3683109
19.3166676	1.2200000	-1.1658254
19.3166676	1.2688000	-1.1596894
19.3166676	1.3176000	-1.1596894
19.3166676	1.3664000	-1.4235344
19.3166676	1.4152000	-1.3683109
19.3166676	1.4640000	-1.0615149
19.3166676	1.5128000	-1.3069515
19.3166676	1.5616000	-1.0799227
19.3166676	1.6104000	-1.0737867
19.3166676	1.6592000	-1.3560390
19.3166676	1.7079999	-1.3376312
19.3166676	1.7567999	-1.2885437
19.3166676	1.8055999	-1.2271848
19.3166676	1.8543999	-1.0308352
19.3166676	1.9031999	-1.3130875
19.3166676	1.9519999	-1.2455926
19.3166676	2.0007999	-1.2333207
19.3166676	2.0495999	-1.1412816
19.3166676	2.0983999	-1.1351457
19.3166676	2.1471999	-1.1903691
19.3166676	2.1959999	-1.1780972
19.3166676	2.2447999	-1.2640004
19.3166676	2.2935998	-1.2640004
19.3166676	2.3423998	-1.1106019
19.3166676	2.3912001	-1.2210488
19.3166676	2.4400001	-1.2087770
19.3166676	2.4888000	-1.1719613
19.3166676	2.5376000	-0.9572039

19.3166676	2.5864000	-0.9510679
19.3166676	2.6352000	-0.7485828
19.3166676	2.6840000	-0.7547188
19.3166676	2.7328000	-0.9449320
19.3166676	2.7816000	-0.9326606
19.3166676	2.8304000	-0.9756117
19.3166676	2.8792000	-0.8590293
19.3166676	2.9280000	-0.7915339
19.3166676	2.9768000	-1.0185633
19.3166676	3.0256000	-1.0185633
19.3166676	3.0743999	-0.8713012
19.3166676	3.1231999	-0.8713012
19.3166676	3.1719999	-1.1903691
19.3166676	3.2207999	-0.9878836
19.3166676	3.2695999	-0.9817476
19.3166676	3.3183999	-0.9878836
19.3166676	3.3671999	-0.9817476
19.3166676	3.4159999	-1.0737867
19.3166676	3.4647999	-1.0676508
19.3166676	3.5135999	-1.0983305
19.3166676	3.5623999	-1.0983305
19.3166676	3.6111999	-0.9142528
19.3166676	3.6599998	-0.8651652
19.3166676	3.7087998	-0.8528934
19.3166676	3.7575998	-0.7731261
19.3166676	3.8063998	-0.8099418
19.3166676	3.8551998	-0.8099418
19.3166676	3.9039998	-0.8283496
19.3166676	3.9527998	-0.8528934
19.3166676	4.0016003	-0.7853980
19.3166676	4.0503998	-0.7853980
19.3166676	4.0991998	-0.7056313
19.3166676	4.1479998	-0.6994953
19.3166676	4.1967998	-0.6994953
19.3166676	4.2455997	-0.4970098
19.3166676	4.2943997	-0.5031457
19.3166676	4.3431997	-0.6565437
19.3166676	4.3919997	-0.6626797
19.3166676	4.4407997	-0.4172430
19.3166676	4.4895997	-0.6074562
19.3166676	4.5383997	-0.6565437
19.3166676	4.5871997	-0.7056313
19.3166676	4.6359997	-0.4847379
19.3166676	4.6847997	-0.4847379
19.3166676	4.7336001	-0.4417863
19.3166676	4.7824001	-0.4356503
19.3166676	4.8312001	-0.5645051
19.3166676	4.8800001	-0.3926992
19.3166676	4.9288001	-0.3926992
19.3166676	4.9776001	-0.3926992
19.3166676	5.0264001	-0.3926992
19.3166676	5.0752001	-0.4479222
19.3166676	5.1240001	-0.4908738
19.3166676	5.1728001	-0.7485828
19.3166676	5.2216001	-0.7485828
19.3166676	5.2704000	-0.8222136
19.3166676	5.3192000	-0.8528934

19.3166676	5.3680000	-0.8651652
19.3166676	5.4168000	-0.8651652
19.3166676	5.4656000	-0.6688156
19.3166676	5.5144000	-0.4786019
19.3166676	5.5632000	-0.4786019
19.3166676	5.6120000	613586.0000000
19.3166676	5.6608000	613586.0000000
19.3166676	5.7096000	613586.0000000
19.3166676	5.7584000	-0.4233789
19.3166676	5.8072000	613586.0000000
19.3166676	5.8559999	613586.0000000
19.3166676	5.9047999	613586.0000000
19.3166676	5.9535999	613586.0000000
19.3166676	6.0023999	613586.0000000
19.3333340	0.2928000	-1.0615149
19.3333340	0.3416000	-1.0983305
19.3333340	0.3904000	-1.4235344
19.3333340	0.4392000	-1.5401168
19.3333340	0.4880000	-1.5585246
19.3333340	0.5368000	-1.5769324
19.3333340	0.5856000	-1.5830684
19.3333340	0.6344000	-1.4358063
19.3333340	0.6832000	-1.3805828
19.3333340	0.7320000	-1.3744469
19.3333340	0.7808000	-1.3744469
19.3333340	0.8296000	-1.3805828
19.3333340	0.8784000	-1.4051266
19.3333340	0.9272000	-1.3989906
19.3333340	0.9760000	-1.2762718
19.3333340	1.0247999	-1.3008156
19.3333340	1.0735999	-1.3130875
19.3333340	1.1223999	-1.3989906
19.3333340	1.1711999	-1.3989906
19.3333340	1.2200000	-1.4051266
19.3333340	1.2688000	-1.2885437
19.3333340	1.3176000	-1.3437672
19.3333340	1.3664000	-1.3376312
19.3333340	1.4152000	-1.3437672
19.3333340	1.4640000	-1.0799227
19.3333340	1.5128000	-1.2824078
19.3333340	1.5616000	-1.2578645
19.3333340	1.6104000	-1.2578645
19.3333340	1.6592000	-1.2640004
19.3333340	1.7079999	-1.2640004
19.3333340	1.7567999	-1.2640004
19.3333340	1.8055999	-1.3437672
19.3333340	1.8543999	-1.3437672
19.3333340	1.9031999	-1.2210488
19.3333340	1.9519999	-1.2210488
19.3333340	2.0007999	-1.2824078
19.3333340	2.0495999	-1.2946796
19.3333340	2.0983999	-1.1719613
19.3333340	2.1471999	-1.1719613
19.3333340	2.1959999	-1.3069515
19.3333340	2.2447999	-1.1290097
19.3333340	2.2935998	-1.2394567
19.3333340	2.3423998	-1.2271848

19.3333340	2.3912001	-1.2149129
19.3333340	2.4400001	-1.1658254
19.3333340	2.4888000	-1.1596894
19.3333340	2.5376000	-1.2149129
19.3333340	2.5864000	-1.2149129
19.3333340	2.6352000	-1.2210488
19.3333340	2.6840000	-1.2271848
19.3333340	2.7328000	-1.0799227
19.3333340	2.7816000	-1.0246992
19.3333340	2.8304000	-1.0246992
19.3333340	2.8792000	-1.1658254
19.3333340	2.9280000	-1.1658254
19.3333340	2.9768000	-1.1290097
19.3333340	3.0256000	-1.1228738
19.3333340	3.0743999	-1.0983305
19.3333340	3.1231999	-1.1106019
19.3333340	3.1719999	-0.9756117
19.3333340	3.2207999	-0.9694757
19.3333340	3.2695999	-0.8528934
19.3333340	3.3183999	-0.7853980
19.3333340	3.3671999	-1.0124273
19.3333340	3.4159999	-1.0246992
19.3333340	3.4647999	-1.0676508
19.3333340	3.5135999	-1.0737867
19.3333340	3.5623999	-0.9756117
19.3333340	3.6111999	-0.9756117
19.3333340	3.6599998	-0.9756117
19.3333340	3.7087998	-0.8283496
19.3333340	3.7575998	-0.8467574
19.3333340	3.8063998	-0.8283496
19.3333340	3.8551998	-0.8406215
19.3333340	3.9039998	-0.6442719
19.3333340	3.9527998	-0.7608547
19.3333340	4.0016003	-0.6381359
19.3333340	4.0503998	-0.7608547
19.3333340	4.0991998	-0.7731261
19.3333340	4.1479998	-0.7669902
19.3333340	4.1967998	-0.6320000
19.3333340	4.2455997	-0.6197281
19.3333340	4.2943997	-0.6320000
19.3333340	4.3431997	-0.6258640
19.3333340	4.3919997	-0.5583692
19.3333340	4.4407997	-0.5583692
19.3333340	4.4895997	-0.7792621
19.3333340	4.5383997	-0.6872234
19.3333340	4.5871997	-0.4233789
19.3333340	4.6359997	-0.4724660
19.3333340	4.6847997	-0.5645051
19.3333340	4.7336001	-0.4908738
19.3333340	4.7824001	-0.4847379
19.3333340	4.8312001	-0.4479222
19.3333340	4.8800001	-0.4479222
19.3333340	4.9288001	-0.5645051
19.3333340	4.9776001	-0.6381359
19.3333340	5.0264001	-0.5031457
19.3333340	5.0752001	-0.5338254
19.3333340	5.1240001	-0.6013203

19.3333340	5.1728001	-0.5951843
19.3333340	5.2216001	-0.5276895
19.3333340	5.2704000	-0.4786019
19.3333340	5.3192000	-0.4663301
19.3333340	5.3680000	-0.3558836
19.3333340	5.4168000	-0.3558836
19.3333340	5.4656000	-0.3620195
19.3333340	5.5144000	-0.4601941
19.3333340	5.5632000	-0.4540582
19.3333340	5.6120000	-0.4356503
19.3333340	5.6608000	-0.4233789
19.3333340	5.7096000	-0.4172430
19.3333340	5.7584000	-0.4172430
19.3333340	5.8072000	613586.0000000
19.3333340	5.8559999	-0.5522332
19.3333340	5.9047999	613586.0000000
19.3333340	5.9535999	613586.0000000
19.3333340	6.0023999	-0.4295144
19.3500004	0.2928000	-1.8653207
19.3500004	0.3416000	-1.7426023
19.3500004	0.3904000	-1.7487383
19.3500004	0.4392000	-1.4112625
19.3500004	0.4880000	-1.4051266
19.3500004	0.5368000	-1.1474175
19.3500004	0.5856000	-1.5033011
19.3500004	0.6344000	-1.4848933
19.3500004	0.6832000	-1.2762718
19.3500004	0.7320000	-1.3989906
19.3500004	0.7808000	-1.3928547
19.3500004	0.8296000	-1.2333207
19.3500004	0.8784000	-1.4603496
19.3500004	0.9272000	-1.1965051
19.3500004	0.9760000	-1.4664855
19.3500004	1.0247999	-1.5769324
19.3500004	1.0735999	-1.3008156
19.3500004	1.1223999	-1.0369711
19.3500004	1.1711999	-1.0431070
19.3500004	1.2200000	-1.3560390
19.3500004	1.2688000	-1.3560390
19.3500004	1.3176000	-1.3621750
19.3500004	1.3664000	-1.3621750
19.3500004	1.4152000	-1.3560390
19.3500004	1.4640000	-1.4664855
19.3500004	1.5128000	-1.3376312
19.3500004	1.5616000	-1.1719613
19.3500004	1.6104000	-1.1842332
19.3500004	1.6592000	-1.2640004
19.3500004	1.7079999	-1.2762718
19.3500004	1.7567999	-1.4787574
19.3500004	1.8055999	-1.2026410
19.3500004	1.8543999	-1.3008156
19.3500004	1.9031999	-1.1719613
19.3500004	1.9519999	-1.1719613
19.3500004	2.0007999	-1.3928547
19.3500004	2.0495999	-1.2210488
19.3500004	2.0983999	-1.2210488
19.3500004	2.1471999	-1.2455926

19.3500004	2.1959999	-1.3499031
19.3500004	2.2447999	-1.3130875
19.3500004	2.2935998	-1.3008156
19.3500004	2.3423998	-1.3928547
19.3500004	2.3912001	-1.3253593
19.3500004	2.4400001	-1.2578645
19.3500004	2.4888000	-1.3437672
19.3500004	2.5376000	-1.3192234
19.3500004	2.5864000	-1.3130875
19.3500004	2.6352000	-1.2087770
19.3500004	2.6840000	-1.2149129
19.3500004	2.7328000	-1.1044660
19.3500004	2.7816000	-1.1106019
19.3500004	2.8304000	-1.1106019
19.3500004	2.8792000	-1.1167378
19.3500004	2.9280000	-1.0983305
19.3500004	2.9768000	-1.0062914
19.3500004	3.0256000	-1.0615149
19.3500004	3.0743999	-1.1351457
19.3500004	3.1231999	-0.9940195
19.3500004	3.1719999	-1.0492430
19.3500004	3.2207999	-1.0615149
19.3500004	3.2695999	-1.1290097
19.3500004	3.3183999	-1.1290097
19.3500004	3.3671999	-1.1290097
19.3500004	3.4159999	-1.1167378
19.3500004	3.4647999	-0.9878836
19.3500004	3.5135999	-0.9694757
19.3500004	3.5623999	-0.8038058
19.3500004	3.6111999	-0.8160777
19.3500004	3.6599998	-0.8283496
19.3500004	3.7087998	-0.8344855
19.3500004	3.7575998	-0.9081168
19.3500004	3.8063998	-0.8958449
19.3500004	3.8551998	-0.7976699
19.3500004	3.9039998	-0.7179031
19.3500004	3.9527998	-0.7976699
19.3500004	4.0016003	-0.7853980
19.3500004	4.0503998	-0.7424469
19.3500004	4.0991998	-0.7424469
19.3500004	4.1479998	-0.6381359
19.3500004	4.1967998	-0.6504078
19.3500004	4.2455997	-0.4847379
19.3500004	4.2943997	-0.6135921
19.3500004	4.3431997	-0.6135921
19.3500004	4.3919997	-0.5338254
19.3500004	4.4407997	-0.8467574
19.3500004	4.4895997	-0.7363110
19.3500004	4.5383997	-0.7424469
19.3500004	4.5871997	-0.8344855
19.3500004	4.6359997	-0.8651652
19.3500004	4.6847997	-0.6933594
19.3500004	4.7336001	-0.6872234
19.3500004	4.7824001	-0.6381359
19.3500004	4.8312001	-0.6320000
19.3500004	4.8800001	-0.6442719
19.3500004	4.9288001	-0.7301750

19.3500004	4.9776001	-0.7301750
19.3500004	5.0264001	-0.5890489
19.3500004	5.0752001	-0.6994953
19.3500004	5.1240001	-0.7179031
19.3500004	5.1728001	-0.6074562
19.3500004	5.2216001	-0.6258640
19.3500004	5.2704000	-0.5154176
19.3500004	5.3192000	-0.5154176
19.3500004	5.3680000	-0.5031457
19.3500004	5.4168000	-0.6381359
19.3500004	5.4656000	-0.7056313
19.3500004	5.5144000	-0.7117672
19.3500004	5.5632000	-0.6135921
19.3500004	5.6120000	-0.6135921
19.3500004	5.6608000	613586.0000000
19.3500004	5.7096000	613586.0000000
19.3500004	5.7584000	613586.0000000
19.3500004	5.8072000	613586.0000000
19.3500004	5.8559999	-0.4786019
19.3500004	5.9047999	613586.0000000
19.3500004	5.9535999	613586.0000000
19.3500004	6.0023999	613586.0000000
19.3666668	0.2928000	-2.3623304
19.3666668	0.3416000	-2.3500586
19.3666668	0.3904000	-2.0309906
19.3666668	0.4392000	-2.0309906
19.3666668	0.4880000	-1.7364664
19.3666668	0.5368000	-1.7487383
19.3666668	0.5856000	-1.4910293
19.3666668	0.6344000	-1.4787574
19.3666668	0.6832000	-1.4664855
19.3666668	0.7320000	-1.7426023
19.3666668	0.7808000	-1.4419422
19.3666668	0.8296000	-1.4296703
19.3666668	0.8784000	-1.4296703
19.3666668	0.9272000	-1.3253593
19.3666668	0.9760000	-1.0553789
19.3666668	1.0247999	-0.9510679
19.3666668	1.0735999	-1.7303305
19.3666668	1.1223999	-1.6751070
19.3666668	1.1711999	-1.5585246
19.3666668	1.2200000	-1.4296703
19.3666668	1.2688000	-1.2824078
19.3666668	1.3176000	-1.3314953
19.3666668	1.3664000	-1.1842332
19.3666668	1.4152000	-1.2885437
19.3666668	1.4640000	-1.2885437
19.3666668	1.5128000	-1.0676508
19.3666668	1.5616000	-1.1044660
19.3666668	1.6104000	-1.1044660
19.3666668	1.6592000	-1.1044660
19.3666668	1.7079999	-1.2271848
19.3666668	1.7567999	-1.2946796
19.3666668	1.8055999	-1.1535535
19.3666668	1.8543999	-1.2333207
19.3666668	1.9031999	-1.2333207
19.3666668	1.9519999	-1.3683109

19.3666668	2.0007999	-1.1965051
19.3666668	2.0495999	-1.1842332
19.3666668	2.0983999	-1.2640004
19.3666668	2.1471999	-1.3130875
19.3666668	2.1959999	-1.2210488
19.3666668	2.2447999	-1.2701364
19.3666668	2.2935998	-1.1535535
19.3666668	2.3423998	-1.1474175
19.3666668	2.3912001	-1.3928547
19.3666668	2.4400001	-1.3805828
19.3666668	2.4888000	-1.3867188
19.3666668	2.5376000	-1.2026410
19.3666668	2.5864000	-1.2026410
19.3666668	2.6352000	-1.1965051
19.3666668	2.6840000	-1.0860586
19.3666668	2.7328000	-1.1719613
19.3666668	2.7816000	-0.9940195
19.3666668	2.8304000	-1.1290097
19.3666668	2.8792000	-1.1351457
19.3666668	2.9280000	-1.1106019
19.3666668	2.9768000	-1.0983305
19.3666668	3.0256000	-1.2210488
19.3666668	3.0743999	-1.0615149
19.3666668	3.1231999	-1.0799227
19.3666668	3.1719999	-1.3314953
19.3666668	3.2207999	-1.4910293
19.3666668	3.2695999	-1.3253593
19.3666668	3.3183999	-1.1228738
19.3666668	3.3671999	-0.8835731
19.3666668	3.4159999	-0.8835731
19.3666668	3.4647999	-1.0246992
19.3666668	3.5135999	-1.0246992
19.3666668	3.5623999	-1.0246992
19.3666668	3.6111999	-1.0246992
19.3666668	3.6599998	-0.9756117
19.3666668	3.7087998	-0.8897090
19.3666668	3.7575998	-0.9019809
19.3666668	3.8063998	-0.9081168
19.3666668	3.8551998	-0.9081168
19.3666668	3.9039998	-0.8897090
19.3666668	3.9527998	-0.9019809
19.3666668	4.0016003	-0.8958449
19.3666668	4.0503998	-0.7731261
19.3666668	4.0991998	-0.7547188
19.3666668	4.1479998	-0.7547188
19.3666668	4.1967998	-0.7056313
19.3666668	4.2455997	-0.8590293
19.3666668	4.2943997	-0.8222136
19.3666668	4.3431997	-0.8406215
19.3666668	4.3919997	-0.8467574
19.3666668	4.4407997	-0.6258640
19.3666668	4.4895997	-0.6320000
19.3666668	4.5383997	-0.6442719
19.3666668	4.5871997	-0.5767770
19.3666668	4.6359997	-0.5706410
19.3666668	4.6847997	-0.6258640
19.3666668	4.7336001	-0.6258640

19.3666668	4.7824001	-0.6074562
19.3666668	4.8312001	-0.6074562
19.3666668	4.8800001	-0.7179031
19.3666668	4.9288001	-0.5215535
19.3666668	4.9776001	-0.7608547
19.3666668	5.0264001	-0.7424469
19.3666668	5.0752001	-0.7424469
19.3666668	5.1240001	-0.7424469
19.3666668	5.1728001	-0.7363110
19.3666668	5.2216001	-0.7240391
19.3666668	5.2704000	-0.9326606
19.3666668	5.3192000	-0.6933594
19.3666668	5.3680000	-0.6135921
19.3666668	5.4168000	-0.6074562
19.3666668	5.4656000	-0.4970098
19.3666668	5.5144000	-0.4724660
19.3666668	5.5632000	613586.0000000
19.3666668	5.6120000	613586.0000000
19.3666668	5.6608000	-0.6626797
19.3666668	5.7096000	-0.6626797
19.3666668	5.7584000	-0.5767770
19.3666668	5.8072000	-0.5767770
19.3666668	5.8559999	613586.0000000
19.3666668	5.9047999	613586.0000000
19.3666668	5.9535999	613586.0000000
19.3666668	6.0023999	613586.0000000
19.3833332	0.2928000	-2.3561945
19.3833332	0.3416000	-1.5892043
19.3833332	0.3904000	-1.6137476
19.3833332	0.4392000	-1.6321554
19.3833332	0.4880000	-1.5646605
19.3833332	0.5368000	-1.6505632
19.3833332	0.5856000	-1.6444273
19.3833332	0.6344000	-1.5278449
19.3833332	0.6832000	-1.5094371
19.3833332	0.7320000	-1.4296703
19.3833332	0.7808000	-1.4358063
19.3833332	0.8296000	-1.3376312
19.3833332	0.8784000	-1.1228738
19.3833332	0.9272000	-1.2026410
19.3833332	0.9760000	-1.4419422
19.3833332	1.0247999	-1.0492430
19.3833332	1.0735999	-1.0676508
19.3833332	1.1223999	-1.1228738
19.3833332	1.1711999	-1.2517285
19.3833332	1.2200000	-1.4419422
19.3833332	1.2688000	-1.2946796
19.3833332	1.3176000	-1.3867188
19.3833332	1.3664000	-1.4051266
19.3833332	1.4152000	-1.3989906
19.3833332	1.4640000	-1.3928547
19.3833332	1.5128000	-1.0983305
19.3833332	1.5616000	-1.0921946
19.3833332	1.6104000	-1.1412816
19.3833332	1.6592000	-1.1535535
19.3833332	1.7079999	-1.1842332
19.3833332	1.7567999	-1.3867188

19.3833332	1.8055999	-1.3805828
19.3833332	1.8543999	-1.3069515
19.3833332	1.9031999	-1.3192234
19.3833332	1.9519999	-1.3130875
19.3833332	2.0007999	-1.2333207
19.3833332	2.0495999	-1.1412816
19.3833332	2.0983999	-1.2762718
19.3833332	2.1471999	-1.3499031
19.3833332	2.1959999	-1.2455926
19.3833332	2.2447999	-1.1596894
19.3833332	2.2935998	-1.1719613
19.3833332	2.3423998	-1.3314953
19.3833332	2.3912001	-1.2578645
19.3833332	2.4400001	-1.2333207
19.3833332	2.4888000	-1.2210488
19.3833332	2.5376000	-1.1412816
19.3833332	2.5864000	-1.1474175
19.3833332	2.6352000	-1.0492430
19.3833332	2.6840000	-0.9817476
19.3833332	2.7328000	-0.9817476
19.3833332	2.7816000	-0.9878836
19.3833332	2.8304000	-1.0369711
19.3833332	2.8792000	-1.1658254
19.3833332	2.9280000	-1.1535535
19.3833332	2.9768000	-0.9878836
19.3833332	3.0256000	-1.0308352
19.3833332	3.0743999	-1.0308352
19.3833332	3.1231999	-1.0492430
19.3833332	3.1719999	-0.8958449
19.3833332	3.2207999	-0.9756117
19.3833332	3.2695999	-0.9694757
19.3833332	3.3183999	-0.9694757
19.3833332	3.3671999	-0.9019809
19.3833332	3.4159999	-0.8774371
19.3833332	3.4647999	-0.9878836
19.3833332	3.5135999	-0.8160777
19.3833332	3.5623999	-0.8222136
19.3833332	3.6111999	-0.7731261
19.3833332	3.6599998	-0.6994953
19.3833332	3.7087998	-0.7240391
19.3833332	3.7575998	-0.8651652
19.3833332	3.8063998	-0.8528934
19.3833332	3.8551998	-0.8344855
19.3833332	3.9039998	-0.8344855
19.3833332	3.9527998	-0.8344855
19.3833332	4.0016003	-0.7669902
19.3833332	4.0503998	-0.7056313
19.3833332	4.0991998	-0.6810875
19.3833332	4.1479998	-0.7301750
19.3833332	4.1967998	-1.0369711
19.3833332	4.2455997	-0.9449320
19.3833332	4.2943997	-0.8958449
19.3833332	4.3431997	-0.8958449
19.3833332	4.3919997	-0.5890489
19.3833332	4.4407997	-0.6504078
19.3833332	4.4895997	-0.5460973
19.3833332	4.5383997	-0.6872234

19.3833332	4.5871997	-0.6810875
19.3833332	4.6359997	-0.6688156
19.3833332	4.6847997	-0.6626797
19.3833332	4.7336001	-0.5951843
19.3833332	4.7824001	-0.4663301
19.3833332	4.8312001	-0.4663301
19.3833332	4.8800001	-0.6688156
19.3833332	4.9288001	-0.6688156
19.3833332	4.9776001	-0.5583692
19.3833332	5.0264001	-0.5460973
19.3833332	5.0752001	-0.7853980
19.3833332	5.1240001	-0.7915339
19.3833332	5.1728001	-0.7669902
19.3833332	5.2216001	-0.7669902
19.3833332	5.2704000	-0.6810875
19.3833332	5.3192000	-0.6565437
19.3833332	5.3680000	-0.7117672
19.3833332	5.4168000	-0.5829129
19.3833332	5.4656000	-0.5951843
19.3833332	5.5144000	-0.6197281
19.3833332	5.5632000	613586.0000000
19.3833332	5.6120000	613586.0000000
19.3833332	5.6608000	-0.6013203
19.3833332	5.7096000	-0.6135921
19.3833332	5.7584000	-0.5645051
19.3833332	5.8072000	-0.5645051
19.3833332	5.8559999	-0.5645051
19.3833332	5.9047999	-0.4724660
19.3833332	5.9535999	613586.0000000
19.3833332	6.0023999	613586.0000000
19.3999996	0.2928000	-1.8469129
19.3999996	0.3416000	-2.0187187
19.3999996	0.3904000	-1.8162332
19.3999996	0.4392000	-1.8039613
19.3999996	0.4880000	-1.6812429
19.3999996	0.5368000	-1.6812429
19.3999996	0.5856000	-1.5033011
19.3999996	0.6344000	-1.4910293
19.3999996	0.6832000	-1.4910293
19.3999996	0.7320000	-1.1842332
19.3999996	0.7808000	-1.1903691
19.3999996	0.8296000	-1.2517285
19.3999996	0.8784000	-1.3314953
19.3999996	0.9272000	-1.3376312
19.3999996	0.9760000	-1.3376312
19.3999996	1.0247999	-1.3008156
19.3999996	1.0735999	-1.2333207
19.3999996	1.1223999	-1.5217090
19.3999996	1.1711999	-1.5462527
19.3999996	1.2200000	-1.3683109
19.3999996	1.2688000	-1.4296703
19.3999996	1.3176000	-1.4480777
19.3999996	1.3664000	-1.5523887
19.3999996	1.4152000	-1.3130875
19.3999996	1.4640000	-1.3130875
19.3999996	1.5128000	-1.2517285
19.3999996	1.5616000	-1.1903691

19.3999996	1.6104000	-1.2946796
19.3999996	1.6592000	-1.3008156
19.3999996	1.7079999	-1.3130875
19.3999996	1.7567999	-1.3008156
19.3999996	1.8055999	-1.4848933
19.3999996	1.8543999	-1.4051266
19.3999996	1.9031999	-1.0983305
19.3999996	1.9519999	-1.1228738
19.3999996	2.0007999	-1.1290097
19.3999996	2.0495999	-1.2946796
19.3999996	2.0983999	-1.4419422
19.3999996	2.1471999	-1.4603496
19.3999996	2.1959999	-1.3989906
19.3999996	2.2447999	-1.4051266
19.3999996	2.2935998	-1.3989906
19.3999996	2.3423998	-1.2210488
19.3999996	2.3912001	-1.3683109
19.3999996	2.4400001	-1.3683109
19.3999996	2.4888000	-1.3314953
19.3999996	2.5376000	-1.2578645
19.3999996	2.5864000	-1.2640004
19.3999996	2.6352000	-1.2517285
19.3999996	2.6840000	-1.0983305
19.3999996	2.7328000	-1.1351457
19.3999996	2.7816000	-1.1412816
19.3999996	2.8304000	-0.9449320
19.3999996	2.8792000	-0.9449320
19.3999996	2.9280000	-0.8222136
19.3999996	2.9768000	-0.8283496
19.3999996	3.0256000	-0.9940195
19.3999996	3.0743999	-0.9387960
19.3999996	3.1231999	-0.9387960
19.3999996	3.1719999	-0.9449320
19.3999996	3.2207999	-0.8958449
19.3999996	3.2695999	-0.9081168
19.3999996	3.3183999	-0.8713012
19.3999996	3.3671999	-0.8713012
19.3999996	3.4159999	-0.7915339
19.3999996	3.4647999	-0.8467574
19.3999996	3.5135999	-0.7853980
19.3999996	3.5623999	-0.7792621
19.3999996	3.6111999	-0.9817476
19.3999996	3.6599998	-0.7853980
19.3999996	3.7087998	-0.9019809
19.3999996	3.7575998	-0.9019809
19.3999996	3.8063998	-0.9019809
19.3999996	3.8551998	-0.6749516
19.3999996	3.9039998	-0.6135921
19.3999996	3.9527998	-0.7240391
19.3999996	4.0016003	-0.7056313
19.3999996	4.0503998	-0.6013203
19.3999996	4.0991998	-0.5951843
19.3999996	4.1479998	-0.5829129
19.3999996	4.1967998	-0.6258640
19.3999996	4.2455997	-0.6197281
19.3999996	4.2943997	-0.6565437
19.3999996	4.3431997	-0.7179031

19.3999996	4.3919997	-0.8406215
19.3999996	4.4407997	-0.8344855
19.3999996	4.4895997	-0.6994953
19.3999996	4.5383997	-0.6994953
19.3999996	4.5871997	-0.7669902
19.3999996	4.6359997	-0.6994953
19.3999996	4.6847997	-0.6626797
19.3999996	4.7336001	-0.6626797
19.3999996	4.7824001	-0.6565437
19.3999996	4.8312001	-0.6565437
19.3999996	4.8800001	-0.6810875
19.3999996	4.9288001	-0.7976699
19.3999996	4.9776001	-0.8099418
19.3999996	5.0264001	-0.8160777
19.3999996	5.0752001	-0.6688156
19.3999996	5.1240001	-0.6933594
19.3999996	5.1728001	-0.7547188
19.3999996	5.2216001	-0.7424469
19.3999996	5.2704000	-0.6565437
19.3999996	5.3192000	-0.6565437
19.3999996	5.3680000	613586.0000000
19.3999996	5.4168000	-0.6688156
19.3999996	5.4656000	-0.6258640
19.3999996	5.5144000	-0.5767770
19.3999996	5.5632000	-0.5890489
19.3999996	5.6120000	613586.0000000
19.3999996	5.6608000	613586.0000000
19.3999996	5.7096000	-0.5706410
19.3999996	5.7584000	613586.0000000
19.3999996	5.8072000	613586.0000000
19.3999996	5.8559999	613586.0000000
19.3999996	5.9047999	613586.0000000
19.3999996	5.9535999	613586.0000000
19.3999996	6.0023999	613586.0000000
19.4166660	0.2928000	-2.0187187
19.4166660	0.3416000	-2.0187187
19.4166660	0.3904000	-1.4603496
19.4166660	0.4392000	-1.6935148
19.4166660	0.4880000	-1.6812429
19.4166660	0.5368000	-1.6873789
19.4166660	0.5856000	-1.2762718
19.4166660	0.6344000	-1.3499031
19.4166660	0.6832000	-1.3437672
19.4166660	0.7320000	-1.4235344
19.4166660	0.7808000	-1.4296703
19.4166660	0.8296000	-1.4296703
19.4166660	0.8784000	-1.4358063
19.4166660	0.9272000	-1.4296703
19.4166660	0.9760000	-1.2210488
19.4166660	1.0247999	-1.5585246
19.4166660	1.0735999	-1.5585246
19.4166660	1.1223999	-1.3008156
19.4166660	1.1711999	-1.3008156
19.4166660	1.2200000	-1.3192234
19.4166660	1.2688000	-1.3130875
19.4166660	1.3176000	-1.3253593
19.4166660	1.3664000	-1.3805828

19.4166660	1.4152000	-1.4112625
19.4166660	1.4640000	-1.4173985
19.4166660	1.5128000	-1.4173985
19.4166660	1.5616000	-1.3314953
19.4166660	1.6104000	-1.3314953
19.4166660	1.6592000	-1.4726214
19.4166660	1.7079999	-1.4664855
19.4166660	1.7567999	-1.4480777
19.4166660	1.8055999	-1.1903691
19.4166660	1.8543999	-1.1044660
19.4166660	1.9031999	-1.0860586
19.4166660	1.9519999	-1.1535535
19.4166660	2.0007999	-1.1596894
19.4166660	2.0495999	-1.1596894
19.4166660	2.0983999	-1.1535535
19.4166660	2.1471999	-1.1474175
19.4166660	2.1959999	-1.3008156
19.4166660	2.2447999	-1.3744469
19.4166660	2.2935998	-1.2087770
19.4166660	2.3423998	-1.2087770
19.4166660	2.3912001	-1.2455926
19.4166660	2.4400001	-1.1044660
19.4166660	2.4888000	-1.1412816
19.4166660	2.5376000	-1.1474175
19.4166660	2.5864000	-1.2455926
19.4166660	2.6352000	-1.1474175
19.4166660	2.6840000	-1.1658254
19.4166660	2.7328000	-1.0308352
19.4166660	2.7816000	-1.1535535
19.4166660	2.8304000	-1.0799227
19.4166660	2.8792000	-1.0246992
19.4166660	2.9280000	-1.1044660
19.4166660	2.9768000	-1.1167378
19.4166660	3.0256000	-1.0983305
19.4166660	3.0743999	-0.8958449
19.4166660	3.1231999	-0.9019809
19.4166660	3.1719999	-0.7363110
19.4166660	3.2207999	-0.7669902
19.4166660	3.2695999	-0.7853980
19.4166660	3.3183999	-0.7915339
19.4166660	3.3671999	-0.7792621
19.4166660	3.4159999	-0.6688156
19.4166660	3.4647999	-0.6810875
19.4166660	3.5135999	-0.9203887
19.4166660	3.5623999	-0.9203887
19.4166660	3.6111999	-0.9203887
19.4166660	3.6599998	-0.7853980
19.4166660	3.7087998	-0.8038058
19.4166660	3.7575998	-0.9019809
19.4166660	3.8063998	-0.7179031
19.4166660	3.8551998	-0.7179031
19.4166660	3.9039998	-0.6565437
19.4166660	3.9527998	-0.6504078
19.4166660	4.0016003	-0.7056313
19.4166660	4.0503998	-0.6504078
19.4166660	4.0991998	-0.7363110
19.4166660	4.1479998	-0.5154176

19.4166660	4.1967998	-0.6626797
19.4166660	4.2455997	-0.6504078
19.4166660	4.2943997	-0.6749516
19.4166660	4.3431997	-0.6810875
19.4166660	4.3919997	-0.5092816
19.4166660	4.4407997	-0.5031457
19.4166660	4.4895997	-0.4786019
19.4166660	4.5383997	-0.5583692
19.4166660	4.5871997	-0.5522332
19.4166660	4.6359997	-0.5031457
19.4166660	4.6847997	-0.4724660
19.4166660	4.7336001	-0.5338254
19.4166660	4.7824001	-0.3988352
19.4166660	4.8312001	-0.4417863
19.4166660	4.8800001	-0.4417863
19.4166660	4.9288001	-0.5951843
19.4166660	4.9776001	-0.5706410
19.4166660	5.0264001	-0.6626797
19.4166660	5.0752001	-0.6504078
19.4166660	5.1240001	-0.7179031
19.4166660	5.1728001	-0.7363110
19.4166660	5.2216001	-0.7669902
19.4166660	5.2704000	-0.7853980
19.4166660	5.3192000	-0.7669902
19.4166660	5.3680000	-0.7669902
19.4166660	5.4168000	-0.6626797
19.4166660	5.4656000	-0.6381359
19.4166660	5.5144000	-0.6197281
19.4166660	5.5632000	-0.6074562
19.4166660	5.6120000	-0.6074562
19.4166660	5.6608000	-0.6442719
19.4166660	5.7096000	-0.6381359
19.4166660	5.7584000	-0.6872234
19.4166660	5.8072000	-0.6258640
19.4166660	5.8559999	613586.0000000
19.4166660	5.9047999	613586.0000000
19.4166660	5.9535999	613586.0000000
19.4166660	6.0023999	613586.0000000
19.4333324	0.2928000	-2.2027965
19.4333324	0.3416000	-1.9696312
19.4333324	0.3904000	-1.8162332
19.4333324	0.4392000	-1.7180586
19.4333324	0.4880000	-1.4419422
19.4333324	0.5368000	-1.4480777
19.4333324	0.5856000	-1.4726214
19.4333324	0.6344000	-1.5217090
19.4333324	0.6832000	-1.3560390
19.4333324	0.7320000	-1.4051266
19.4333324	0.7808000	-1.4848933
19.4333324	0.8296000	-1.4726214
19.4333324	0.8784000	-1.4235344
19.4333324	0.9272000	-1.2149129
19.4333324	0.9760000	-1.3805828
19.4333324	1.0247999	-1.3805828
19.4333324	1.0735999	-1.2271848
19.4333324	1.1223999	-1.3253593
19.4333324	1.1711999	-1.3683109

19.4333324	1.2200000	-1.3744469
19.4333324	1.2688000	-1.2762718
19.4333324	1.3176000	-1.2946796
19.4333324	1.3664000	-1.2885437
19.4333324	1.4152000	-1.2517285
19.4333324	1.4640000	-1.2394567
19.4333324	1.5128000	-1.4480777
19.4333324	1.5616000	-1.1842332
19.4333324	1.6104000	-1.2149129
19.4333324	1.6592000	-1.1535535
19.4333324	1.7079999	-1.1780972
19.4333324	1.7567999	-1.1351457
19.4333324	1.8055999	-1.2087770
19.4333324	1.8543999	-1.2210488
19.4333324	1.9031999	-1.2210488
19.4333324	1.9519999	-1.2026410
19.4333324	2.0007999	-1.2026410
19.4333324	2.0495999	-1.2762718
19.4333324	2.0983999	-1.2701364
19.4333324	2.1471999	-1.1658254
19.4333324	2.1959999	-1.2517285
19.4333324	2.2447999	-1.2578645
19.4333324	2.2935998	-1.2517285
19.4333324	2.3423998	-1.1965051
19.4333324	2.3912001	-1.2026410
19.4333324	2.4400001	-1.1351457
19.4333324	2.4888000	-1.1290097
19.4333324	2.5376000	-1.0676508
19.4333324	2.5864000	-1.1228738
19.4333324	2.6352000	-1.1351457
19.4333324	2.6840000	-1.1412816
19.4333324	2.7328000	-1.1903691
19.4333324	2.7816000	-1.1290097
19.4333324	2.8304000	-1.1228738
19.4333324	2.8792000	-0.9572039
19.4333324	2.9280000	-0.9572039
19.4333324	2.9768000	-1.1412816
19.4333324	3.0256000	-1.3253593
19.4333324	3.0743999	-1.0369711
19.4333324	3.1231999	-1.2762718
19.4333324	3.1719999	-0.9940195
19.4333324	3.2207999	-1.0001554
19.4333324	3.2695999	-0.8406215
19.4333324	3.3183999	-0.7669902
19.4333324	3.3671999	-0.9203887
19.4333324	3.4159999	-0.8344855
19.4333324	3.4647999	-0.8283496
19.4333324	3.5135999	-0.6872234
19.4333324	3.5623999	-0.6688156
19.4333324	3.6111999	-0.7301750
19.4333324	3.6599998	-0.7179031
19.4333324	3.7087998	-0.6810875
19.4333324	3.7575998	-0.7731261
19.4333324	3.8063998	-0.7056313
19.4333324	3.8551998	-0.7056313
19.4333324	3.9039998	-0.6933594
19.4333324	3.9527998	-0.8344855

19.4333324	4.0016003	-0.6381359
19.4333324	4.0503998	-0.7056313
19.4333324	4.0991998	-0.7056313
19.4333324	4.1479998	-0.7179031
19.4333324	4.1967998	-0.7117672
19.4333324	4.2455997	-0.7485828
19.4333324	4.2943997	-0.7363110
19.4333324	4.3431997	-0.8713012
19.4333324	4.3919997	-0.8774371
19.4333324	4.4407997	-0.5829129
19.4333324	4.4895997	-0.6933594
19.4333324	4.5383997	-0.6994953
19.4333324	4.5871997	-0.7056313
19.4333324	4.6359997	-0.5951843
19.4333324	4.6847997	-0.6504078
19.4333324	4.7336001	-0.6442719
19.4333324	4.7824001	-0.6504078
19.4333324	4.8312001	-0.4601941
19.4333324	4.8800001	-0.7731261
19.4333324	4.9288001	-0.7731261
19.4333324	4.9776001	-0.6381359
19.4333324	5.0264001	-0.6320000
19.4333324	5.0752001	-0.7117672
19.4333324	5.1240001	-0.7976699
19.4333324	5.1728001	-0.6688156
19.4333324	5.2216001	-0.6626797
19.4333324	5.2704000	-0.6442719
19.4333324	5.3192000	-0.6933594
19.4333324	5.3680000	-0.6872234
19.4333324	5.4168000	-0.6749516
19.4333324	5.4656000	-0.6381359
19.4333324	5.5144000	-0.6320000
19.4333324	5.5632000	-0.5706410
19.4333324	5.6120000	-0.5829129
19.4333324	5.6608000	-0.7240391
19.4333324	5.7096000	-0.7301750
19.4333324	5.7584000	-0.5767770
19.4333324	5.8072000	-0.5890489
19.4333324	5.8559999	-0.6626797
19.4333324	5.9047999	613586.0000000
19.4333324	5.9535999	613586.0000000
19.4333324	6.0023999	613586.0000000
19.4500008	0.2928000	-2.2518840
19.4500008	0.3416000	-2.1537089
19.4500008	0.3904000	-2.1168938
19.4500008	0.4392000	-1.6935148
19.4500008	0.4880000	-1.6873789
19.4500008	0.5368000	-1.3928547
19.4500008	0.5856000	-1.3928547
19.4500008	0.6344000	-1.4664855
19.4500008	0.6832000	-1.4603496
19.4500008	0.7320000	-1.4173985
19.4500008	0.7808000	-1.2517285
19.4500008	0.8296000	-1.2455926
19.4500008	0.8784000	-1.0860586
19.4500008	0.9272000	-1.3253593
19.4500008	0.9760000	-1.3314953

19.4500008	1.0247999	-1.2824078
19.4500008	1.0735999	-1.2824078
19.4500008	1.1223999	-1.3192234
19.4500008	1.1711999	-1.3253593
19.4500008	1.2200000	-1.3683109
19.4500008	1.2688000	-1.2946796
19.4500008	1.3176000	-1.2333207
19.4500008	1.3664000	-1.1903691
19.4500008	1.4152000	-1.1903691
19.4500008	1.4640000	-1.2578645
19.4500008	1.5128000	-1.3437672
19.4500008	1.5616000	-1.2333207
19.4500008	1.6104000	-1.2885437
19.4500008	1.6592000	-1.2640004
19.4500008	1.7079999	-1.1106019
19.4500008	1.7567999	-1.3192234
19.4500008	1.8055999	-1.3192234
19.4500008	1.8543999	-1.3437672
19.4500008	1.9031999	-1.1535535
19.4500008	1.9519999	-1.1412816
19.4500008	2.0007999	-1.0737867
19.4500008	2.0495999	-1.0737867
19.4500008	2.0983999	-1.2824078
19.4500008	2.1471999	-1.1842332
19.4500008	2.1959999	-1.1780972
19.4500008	2.2447999	-1.3560390
19.4500008	2.2935998	-1.2885437
19.4500008	2.3423998	-1.3314953
19.4500008	2.3912001	-1.0676508
19.4500008	2.4400001	-1.2210488
19.4500008	2.4888000	-1.3560390
19.4500008	2.5376000	-1.2394567
19.4500008	2.5864000	-1.1044660
19.4500008	2.6352000	-1.1903691
19.4500008	2.6840000	-1.1474175
19.4500008	2.7328000	-1.1780972
19.4500008	2.7816000	-1.1719613
19.4500008	2.8304000	-1.1658254
19.4500008	2.8792000	-1.1412816
19.4500008	2.9280000	-1.2455926
19.4500008	2.9768000	-1.0676508
19.4500008	3.0256000	-1.0676508
19.4500008	3.0743999	-1.0737867
19.4500008	3.1231999	-1.0799227
19.4500008	3.1719999	-1.0799227
19.4500008	3.2207999	-0.9203887
19.4500008	3.2695999	-0.9081168
19.4500008	3.3183999	-0.8467574
19.4500008	3.3671999	-0.6749516
19.4500008	3.4159999	-0.6749516
19.4500008	3.4647999	-0.6749516
19.4500008	3.5135999	-0.8774371
19.4500008	3.5623999	-1.0001554
19.4500008	3.6111999	-0.9633398
19.4500008	3.6599998	-0.9878836
19.4500008	3.7087998	-0.9326606
19.4500008	3.7575998	-0.8835731

19.4500008	3.8063998	-0.8160777
19.4500008	3.8551998	-0.8222136
19.4500008	3.9039998	-0.7731261
19.4500008	3.9527998	-0.7363110
19.4500008	4.0016003	-0.8528934
19.4500008	4.0503998	-0.9387960
19.4500008	4.0991998	-0.9142528
19.4500008	4.1479998	-0.9081168
19.4500008	4.1967998	-0.9142528
19.4500008	4.2455997	-1.0983305
19.4500008	4.2943997	-1.0369711
19.4500008	4.3431997	-0.8344855
19.4500008	4.3919997	-0.6688156
19.4500008	4.4407997	-0.6749516
19.4500008	4.4895997	-0.7485828
19.4500008	4.5383997	-0.7485828
19.4500008	4.5871997	-0.7424469
19.4500008	4.6359997	-0.6626797
19.4500008	4.6847997	-0.6626797
19.4500008	4.7336001	-0.6504078
19.4500008	4.7824001	-0.6504078
19.4500008	4.8312001	-0.6442719
19.4500008	4.8800001	-0.6320000
19.4500008	4.9288001	-0.7301750
19.4500008	4.9776001	-0.7301750
19.4500008	5.0264001	-0.7608547
19.4500008	5.0752001	-0.7853980
19.4500008	5.1240001	-0.8038058
19.4500008	5.1728001	-0.8099418
19.4500008	5.2216001	-0.8651652
19.4500008	5.2704000	-0.8713012
19.4500008	5.3192000	-0.8222136
19.4500008	5.3680000	-0.8160777
19.4500008	5.4168000	-0.8160777
19.4500008	5.4656000	-0.6810875
19.4500008	5.5144000	-0.6994953
19.4500008	5.5632000	-0.6933594
19.4500008	5.6120000	-0.6810875
19.4500008	5.6608000	-0.6810875
19.4500008	5.7096000	-0.7056313
19.4500008	5.7584000	-0.6135921
19.4500008	5.8072000	-0.6197281
19.4500008	5.8559999	-0.7485828
19.4500008	5.9047999	-0.7485828
19.4500008	5.9535999	613586.0000000
19.4500008	6.0023999	613586.0000000
19.4666672	0.2928000	-2.0739422
19.4666672	0.3416000	-1.9328160
19.4666672	0.3904000	-1.5401168
19.4666672	0.4392000	-1.6137476
19.4666672	0.4880000	-1.7057867
19.4666672	0.5368000	-1.6935148
19.4666672	0.5856000	-1.5033011
19.4666672	0.6344000	-1.4848933
19.4666672	0.6832000	-1.4971652
19.4666672	0.7320000	-1.5094371
19.4666672	0.7808000	-1.1106019

19.4666672	0.8296000	-1.1412816
19.4666672	0.8784000	-1.1474175
19.4666672	0.9272000	-1.1903691
19.4666672	0.9760000	-1.3192234
19.4666672	1.0247999	-1.2578645
19.4666672	1.0735999	-1.2762718
19.4666672	1.1223999	-1.2885437
19.4666672	1.1711999	-1.2885437
19.4666672	1.2200000	-1.1106019
19.4666672	1.2688000	-1.1965051
19.4666672	1.3176000	-1.1780972
19.4666672	1.3664000	-1.1658254
19.4666672	1.4152000	-1.1167378
19.4666672	1.4640000	-1.1167378
19.4666672	1.5128000	-1.1535535
19.4666672	1.5616000	-1.1474175
19.4666672	1.6104000	-1.3560390
19.4666672	1.6592000	-1.2701364
19.4666672	1.7079999	-1.2271848
19.4666672	1.7567999	-1.5707965
19.4666672	1.8055999	-1.0860586
19.4666672	1.8543999	-1.1106019
19.4666672	1.9031999	-1.1228738
19.4666672	1.9519999	-1.4419422
19.4666672	2.0007999	-1.4542136
19.4666672	2.0495999	-1.4542136
19.4666672	2.0983999	-1.4358063
19.4666672	2.1471999	-1.3376312
19.4666672	2.1959999	-1.3621750
19.4666672	2.2447999	-1.3683109
19.4666672	2.2935998	-1.3437672
19.4666672	2.3423998	-1.4173985
19.4666672	2.3912001	-1.2640004
19.4666672	2.4400001	-1.3314953
19.4666672	2.4888000	-1.2824078
19.4666672	2.5376000	-1.2824078
19.4666672	2.5864000	-1.2762718
19.4666672	2.6352000	-1.2762718
19.4666672	2.6840000	-1.2455926
19.4666672	2.7328000	-1.1965051
19.4666672	2.7816000	-1.0860586
19.4666672	2.8304000	-1.3130875
19.4666672	2.8792000	-1.1658254
19.4666672	2.9280000	-1.0799227
19.4666672	2.9768000	-1.1965051
19.4666672	3.0256000	-1.2026410
19.4666672	3.0743999	-0.8528934
19.4666672	3.1231999	-1.0246992
19.4666672	3.1719999	-0.9019809
19.4666672	3.2207999	-0.9081168
19.4666672	3.2695999	-0.9633398
19.4666672	3.3183999	-0.9756117
19.4666672	3.3671999	-0.9633398
19.4666672	3.4159999	-0.9572039
19.4666672	3.4647999	-0.6565437
19.4666672	3.5135999	-0.7853980
19.4666672	3.5623999	-0.7731261

19.4666672	3.6111999	-0.8590293
19.4666672	3.6599998	-0.8774371
19.4666672	3.7087998	-0.8897090
19.4666672	3.7575998	-0.8590293
19.4666672	3.8063998	-0.8590293
19.4666672	3.8551998	-0.7669902
19.4666672	3.9039998	-0.6626797
19.4666672	3.9527998	-0.6626797
19.4666672	4.0016003	-0.9572039
19.4666672	4.0503998	-0.9572039
19.4666672	4.0991998	-0.9633398
19.4666672	4.1479998	-0.9633398
19.4666672	4.1967998	-0.9510679
19.4666672	4.2455997	-0.9326606
19.4666672	4.2943997	-1.0553789
19.4666672	4.3431997	-0.8160777
19.4666672	4.3919997	-0.7117672
19.4666672	4.4407997	-0.7056313
19.4666672	4.4895997	-0.7915339
19.4666672	4.5383997	-0.9326606
19.4666672	4.5871997	-0.7179031
19.4666672	4.6359997	-0.7117672
19.4666672	4.6847997	-0.8467574
19.4666672	4.7336001	-0.8344855
19.4666672	4.7824001	-0.8774371
19.4666672	4.8312001	-0.7976699
19.4666672	4.8800001	-0.8099418
19.4666672	4.9288001	-0.6872234
19.4666672	4.9776001	-0.7056313
19.4666672	5.0264001	-0.6994953
19.4666672	5.0752001	-0.6872234
19.4666672	5.1240001	-0.8467574
19.4666672	5.1728001	-0.8467574
19.4666672	5.2216001	-0.8406215
19.4666672	5.2704000	-0.7976699
19.4666672	5.3192000	-0.8038058
19.4666672	5.3680000	-0.8160777
19.4666672	5.4168000	-0.7731261
19.4666672	5.4656000	-0.7792621
19.4666672	5.5144000	-0.7608547
19.4666672	5.5632000	-0.7485828
19.4666672	5.6120000	-0.7424469
19.4666672	5.6608000	-0.7792621
19.4666672	5.7096000	-0.7547188
19.4666672	5.7584000	-0.7056313
19.4666672	5.8072000	-0.7179031
19.4666672	5.8559999	-0.7179031
19.4666672	5.9047999	-0.7792621
19.4666672	5.9535999	-0.7608547
19.4666672	6.0023999	613586.0000000
19.4833336	0.2928000	-1.5401168
19.4833336	0.3416000	-1.9634953
19.4833336	0.3904000	-1.7057867
19.4833336	0.4392000	-1.9266801
19.4833336	0.4880000	-1.7732821
19.4833336	0.5368000	-1.7732821
19.4833336	0.5856000	-1.3683109

19.4833336	0.6344000	-1.4296703
19.4833336	0.6832000	-1.4112625
19.4833336	0.7320000	-0.9940195
19.4833336	0.7808000	-1.0001554
19.4833336	0.8296000	-1.2640004
19.4833336	0.8784000	-1.0983305
19.4833336	0.9272000	-1.1903691
19.4833336	0.9760000	-1.1842332
19.4833336	1.0247999	-0.9387960
19.4833336	1.0735999	-0.9326606
19.4833336	1.1223999	-0.9019809
19.4833336	1.1711999	-1.2087770
19.4833336	1.2200000	-1.2394567
19.4833336	1.2688000	-1.1290097
19.4833336	1.3176000	-1.1290097
19.4833336	1.3664000	-1.3130875
19.4833336	1.4152000	-1.3192234
19.4833336	1.4640000	-1.5585246
19.4833336	1.5128000	-1.3499031
19.4833336	1.5616000	-1.3437672
19.4833336	1.6104000	-1.3376312
19.4833336	1.6592000	-1.1658254
19.4833336	1.7079999	-1.1535535
19.4833336	1.7567999	-1.3560390
19.4833336	1.8055999	-1.4173985
19.4833336	1.8543999	-1.4112625
19.4833336	1.9031999	-1.3253593
19.4833336	1.9519999	-1.3314953
19.4833336	2.0007999	-1.3376312
19.4833336	2.0495999	-1.3560390
19.4833336	2.0983999	-1.3437672
19.4833336	2.1471999	-1.3437672
19.4833336	2.1959999	-1.4419422
19.4833336	2.2447999	-1.4542136
19.4833336	2.2935998	-1.4603496
19.4833336	2.3423998	-1.2149129
19.4833336	2.3912001	-1.4235344
19.4833336	2.4400001	-1.4235344
19.4833336	2.4888000	-1.1412816
19.4833336	2.5376000	-1.1474175
19.4833336	2.5864000	-1.1474175
19.4833336	2.6352000	-1.3008156
19.4833336	2.6840000	-1.3069515
19.4833336	2.7328000	-1.2885437
19.4833336	2.7816000	-1.1167378
19.4833336	2.8304000	-1.1106019
19.4833336	2.8792000	-1.1535535
19.4833336	2.9280000	-1.1474175
19.4833336	2.9768000	-1.1228738
19.4833336	3.0256000	-1.1474175
19.4833336	3.0743999	-1.0431070
19.4833336	3.1231999	-1.1290097
19.4833336	3.1719999	-1.1167378
19.4833336	3.2207999	-1.1106019
19.4833336	3.2695999	-1.0737867
19.4833336	3.3183999	-0.8958449
19.4833336	3.3671999	-0.7301750

19.4833336	3.4159999	-0.9203887
19.4833336	3.4647999	-1.0246992
19.4833336	3.5135999	-1.0737867
19.4833336	3.5623999	-0.9817476
19.4833336	3.6111999	-0.9878836
19.4833336	3.6599998	-0.9572039
19.4833336	3.7087998	-0.9572039
19.4833336	3.7575998	-0.9694757
19.4833336	3.8063998	-0.9694757
19.4833336	3.8551998	-0.9510679
19.4833336	3.9039998	-0.9449320
19.4833336	3.9527998	-0.7915339
19.4833336	4.0016003	-0.8344855
19.4833336	4.0503998	-0.6810875
19.4833336	4.0991998	-0.6872234
19.4833336	4.1479998	-0.8283496
19.4833336	4.1967998	-1.0553789
19.4833336	4.2455997	-1.0553789
19.4833336	4.2943997	-1.0001554
19.4833336	4.3431997	-0.8160777
19.4833336	4.3919997	-0.8160777
19.4833336	4.4407997	-0.8774371
19.4833336	4.4895997	-0.9817476
19.4833336	4.5383997	-0.8406215
19.4833336	4.5871997	-0.8467574
19.4833336	4.6359997	-0.8283496
19.4833336	4.6847997	-0.9081168
19.4833336	4.7336001	-0.7301750
19.4833336	4.7824001	-0.9019809
19.4833336	4.8312001	-0.9326606
19.4833336	4.8800001	-0.9265246
19.4833336	4.9288001	-0.6994953
19.4833336	4.9776001	-0.6994953
19.4833336	5.0264001	-0.7117672
19.4833336	5.0752001	-0.7301750
19.4833336	5.1240001	-0.6442719
19.4833336	5.1728001	-0.6810875
19.4833336	5.2216001	-0.6933594
19.4833336	5.2704000	-0.6872234
19.4833336	5.3192000	-0.7056313
19.4833336	5.3680000	-0.7424469
19.4833336	5.4168000	-0.7363110
19.4833336	5.4656000	-0.7424469
19.4833336	5.5144000	-0.7424469
19.4833336	5.5632000	-0.6810875
19.4833336	5.6120000	-0.6810875
19.4833336	5.6608000	-0.6810875
19.4833336	5.7096000	-0.7179031
19.4833336	5.7584000	-0.7424469
19.4833336	5.8072000	-0.7608547
19.4833336	5.8559999	-0.7669902
19.4833336	5.9047999	-0.7669902
19.4833336	5.9535999	-0.7424469
19.4833336	6.0023999	-0.7424469
19.5000000	0.2928000	-1.5155730
19.5000000	0.3416000	-1.5585246
19.5000000	0.3904000	-1.3069515

19.5000000	0.4392000	-1.3805828
19.5000000	0.4880000	-1.3805828
19.5000000	0.5368000	-1.2087770
19.5000000	0.5856000	-1.0983305
19.5000000	0.6344000	-1.0983305
19.5000000	0.6832000	-1.2762718
19.5000000	0.7320000	-1.2762718
19.5000000	0.7808000	-1.2640004
19.5000000	0.8296000	-1.2578645
19.5000000	0.8784000	-1.3683109
19.5000000	0.9272000	-1.3683109
19.5000000	0.9760000	-1.2824078
19.5000000	1.0247999	-1.3253593
19.5000000	1.0735999	-1.4664855
19.5000000	1.1223999	-1.1780972
19.5000000	1.1711999	-1.1780972
19.5000000	1.2200000	-1.1842332
19.5000000	1.2688000	-1.1719613
19.5000000	1.3176000	-1.2026410
19.5000000	1.3664000	-1.3867188
19.5000000	1.4152000	-1.1658254
19.5000000	1.4640000	-1.2210488
19.5000000	1.5128000	-1.2394567
19.5000000	1.5616000	-1.3989906
19.5000000	1.6104000	-1.2149129
19.5000000	1.6592000	-1.2333207
19.5000000	1.7079999	-1.2394567
19.5000000	1.7567999	-1.2394567
19.5000000	1.8055999	-1.2394567
19.5000000	1.8543999	-1.3376312
19.5000000	1.9031999	-1.3499031
19.5000000	1.9519999	-1.4051266
19.5000000	2.0007999	-1.3314953
19.5000000	2.0495999	-1.3253593
19.5000000	2.0983999	-1.4173985
19.5000000	2.1471999	-1.3928547
19.5000000	2.1959999	-1.3744469
19.5000000	2.2447999	-1.3805828
19.5000000	2.2935998	-1.3805828
19.5000000	2.3423998	-1.3744469
19.5000000	2.3912001	-1.3744469
19.5000000	2.4400001	-1.2762718
19.5000000	2.4888000	-1.2824078
19.5000000	2.5376000	-1.3253593
19.5000000	2.5864000	-1.3253593
19.5000000	2.6352000	-1.2210488
19.5000000	2.6840000	-1.1351457
19.5000000	2.7328000	-1.0983305
19.5000000	2.7816000	-1.1106019
19.5000000	2.8304000	-1.1167378
19.5000000	2.8792000	-1.1167378
19.5000000	2.9280000	-1.1351457
19.5000000	2.9768000	-1.1228738
19.5000000	3.0256000	-1.0308352
19.5000000	3.0743999	-0.8528934
19.5000000	3.1231999	-0.8528934
19.5000000	3.1719999	-0.8467574

19.5000000	3.2207999	-0.9756117
19.5000000	3.2695999	-0.8774371
19.5000000	3.3183999	-0.8713012
19.5000000	3.3671999	-0.5583692
19.5000000	3.4159999	-0.9203887
19.5000000	3.4647999	-0.9265246
19.5000000	3.5135999	-0.9326606
19.5000000	3.5623999	-0.9387960
19.5000000	3.6111999	-0.9449320
19.5000000	3.6599998	-0.8897090
19.5000000	3.7087998	-0.9817476
19.5000000	3.7575998	-1.0001554
19.5000000	3.8063998	-0.8590293
19.5000000	3.8551998	-0.8467574
19.5000000	3.9039998	-0.8406215
19.5000000	3.9527998	-0.8222136
19.5000000	4.0016003	-1.0308352
19.5000000	4.0503998	-1.0308352
19.5000000	4.0991998	-1.0308352
19.5000000	4.1479998	-0.8283496
19.5000000	4.1967998	-0.8283496
19.5000000	4.2455997	-0.8344855
19.5000000	4.2943997	-0.7915339
19.5000000	4.3431997	-0.6749516
19.5000000	4.3919997	-0.6872234
19.5000000	4.4407997	-0.6810875
19.5000000	4.4895997	-0.6810875
19.5000000	4.5383997	-0.6504078
19.5000000	4.5871997	-0.6626797
19.5000000	4.6359997	-0.7056313
19.5000000	4.6847997	-0.6810875
19.5000000	4.7336001	-0.6197281
19.5000000	4.7824001	-0.6013203
19.5000000	4.8312001	-0.6872234
19.5000000	4.8800001	-0.6320000
19.5000000	4.9288001	-0.7669902
19.5000000	4.9776001	-0.7731261
19.5000000	5.0264001	-0.6320000
19.5000000	5.0752001	-0.6258640
19.5000000	5.1240001	-0.7547188
19.5000000	5.1728001	-0.6933594
19.5000000	5.2216001	-0.7424469
19.5000000	5.2704000	-0.6504078
19.5000000	5.3192000	-0.5767770
19.5000000	5.3680000	-0.5890489
19.5000000	5.4168000	-0.5829129
19.5000000	5.4656000	-0.5583692
19.5000000	5.5144000	-0.5890489
19.5000000	5.5632000	-0.6504078
19.5000000	5.6120000	-0.6320000
19.5000000	5.6608000	-0.6810875
19.5000000	5.7096000	-0.7485828
19.5000000	5.7584000	-0.6688156
19.5000000	5.8072000	-0.6688156
19.5000000	5.8559999	-0.6504078
19.5000000	5.9047999	-0.6381359
19.5000000	5.9535999	-0.6197281

19.5000000	6.0023999	-0.6749516
19.5166664	0.2928000	-2.1537089
19.5166664	0.3416000	-1.1535535
19.5166664	0.3904000	-1.0246992
19.5166664	0.4392000	-0.9081168
19.5166664	0.4880000	-0.9081168
19.5166664	0.5368000	-0.9449320
19.5166664	0.5856000	-1.1106019
19.5166664	0.6344000	-1.1106019
19.5166664	0.6832000	-1.2210488
19.5166664	0.7320000	-1.2271848
19.5166664	0.7808000	-1.2271848
19.5166664	0.8296000	-1.2333207
19.5166664	0.8784000	-1.2578645
19.5166664	0.9272000	-0.9694757
19.5166664	0.9760000	-1.1474175
19.5166664	1.0247999	-1.2394567
19.5166664	1.0735999	-1.0860586
19.5166664	1.1223999	-1.1658254
19.5166664	1.1711999	-1.1658254
19.5166664	1.2200000	-1.0369711
19.5166664	1.2688000	-1.0308352
19.5166664	1.3176000	-1.1535535
19.5166664	1.3664000	-1.1658254
19.5166664	1.4152000	-1.4173985
19.5166664	1.4640000	-1.1965051
19.5166664	1.5128000	-1.3253593
19.5166664	1.5616000	-1.3253593
19.5166664	1.6104000	-1.3314953
19.5166664	1.6592000	-1.3314953
19.5166664	1.7079999	-1.2271848
19.5166664	1.7567999	-1.2210488
19.5166664	1.8055999	-1.2210488
19.5166664	1.8543999	-1.1412816
19.5166664	1.9031999	-1.1290097
19.5166664	1.9519999	-1.1412816
19.5166664	2.0007999	-1.0983305
19.5166664	2.0495999	-1.1842332
19.5166664	2.0983999	-1.1903691
19.5166664	2.1471999	-1.2271848
19.5166664	2.1959999	-1.2394567
19.5166664	2.2447999	-1.1412816
19.5166664	2.2935998	-1.1412816
19.5166664	2.3423998	-1.1780972
19.5166664	2.3912001	-1.1842332
19.5166664	2.4400001	-1.1290097
19.5166664	2.4888000	-1.1228738
19.5166664	2.5376000	-1.1228738
19.5166664	2.5864000	-1.1106019
19.5166664	2.6352000	-1.1106019
19.5166664	2.6840000	-1.2578645
19.5166664	2.7328000	-1.0921946
19.5166664	2.7816000	-1.0921946
19.5166664	2.8304000	-1.1780972
19.5166664	2.8792000	-1.1658254
19.5166664	2.9280000	-1.1842332
19.5166664	2.9768000	-0.9633398

19.5166664	3.0256000	-0.9203887
19.5166664	3.0743999	-0.7608547
19.5166664	3.1231999	-0.7485828
19.5166664	3.1719999	-0.9081168
19.5166664	3.2207999	-0.9081168
19.5166664	3.2695999	-1.0983305
19.5166664	3.3183999	-1.0983305
19.5166664	3.3671999	-1.1903691
19.5166664	3.4159999	-1.0676508
19.5166664	3.4647999	-1.0615149
19.5166664	3.5135999	-1.1167378
19.5166664	3.5623999	-1.1167378
19.5166664	3.6111999	-1.2517285
19.5166664	3.6599998	-1.2578645
19.5166664	3.7087998	-1.2455926
19.5166664	3.7575998	-1.2455926
19.5166664	3.8063998	-1.3314953
19.5166664	3.8551998	-1.1167378
19.5166664	3.9039998	-1.0308352
19.5166664	3.9527998	-1.0308352
19.5166664	4.0016003	-1.0308352
19.5166664	4.0503998	-1.0431070
19.5166664	4.0991998	-0.9940195
19.5166664	4.1479998	-0.9510679
19.5166664	4.1967998	-0.9940195
19.5166664	4.2455997	-0.9940195
19.5166664	4.2943997	-0.9940195
19.5166664	4.3431997	-0.9817476
19.5166664	4.3919997	-0.9081168
19.5166664	4.4407997	-0.9081168
19.5166664	4.4895997	-0.8835731
19.5166664	4.5383997	-0.6197281
19.5166664	4.5871997	-0.6197281
19.5166664	4.6359997	-0.7424469
19.5166664	4.6847997	-0.8590293
19.5166664	4.7336001	-0.8835731
19.5166664	4.7824001	-0.8835731
19.5166664	4.8312001	-0.6994953
19.5166664	4.8800001	-0.8283496
19.5166664	4.9288001	-0.8160777
19.5166664	4.9776001	-0.8038058
19.5166664	5.0264001	-0.7915339
19.5166664	5.0752001	-0.7915339
19.5166664	5.1240001	-0.7363110
19.5166664	5.1728001	-0.7424469
19.5166664	5.2216001	-0.8038058
19.5166664	5.2704000	-0.6320000
19.5166664	5.3192000	-0.6872234
19.5166664	5.3680000	-0.6933594
19.5166664	5.4168000	-0.6994953
19.5166664	5.4656000	-0.8713012
19.5166664	5.5144000	-0.6872234
19.5166664	5.5632000	-0.6688156
19.5166664	5.6120000	-0.7731261
19.5166664	5.6608000	-0.6994953
19.5166664	5.7096000	-0.7056313
19.5166664	5.7584000	-0.6688156

19.5166664	5.8072000	-0.6749516
19.5166664	5.8559999	-0.6810875
19.5166664	5.9047999	-0.6688156
19.5166664	5.9535999	-0.7792621
19.5166664	6.0023999	-0.7179031
19.5333328	0.2928000	-1.9205441
19.5333328	0.3416000	-2.0493984
19.5333328	0.3904000	-1.9389520
19.5333328	0.4392000	-1.5953403
19.5333328	0.4880000	-1.5953403
19.5333328	0.5368000	-1.5094371
19.5333328	0.5856000	-1.3008156
19.5333328	0.6344000	-1.3008156
19.5333328	0.6832000	-1.4664855
19.5333328	0.7320000	-1.4664855
19.5333328	0.7808000	-1.5278449
19.5333328	0.8296000	-1.1535535
19.5333328	0.8784000	-1.2149129
19.5333328	0.9272000	-1.2087770
19.5333328	0.9760000	-1.3560390
19.5333328	1.0247999	-1.2026410
19.5333328	1.0735999	-1.1535535
19.5333328	1.1223999	-1.0799227
19.5333328	1.1711999	-1.0737867
19.5333328	1.2200000	-1.0676508
19.5333328	1.2688000	-1.2149129
19.5333328	1.3176000	-1.2149129
19.5333328	1.3664000	-1.5033011
19.5333328	1.4152000	-1.3805828
19.5333328	1.4640000	-1.0124273
19.5333328	1.5128000	-1.2333207
19.5333328	1.5616000	-1.2333207
19.5333328	1.6104000	-1.5646605
19.5333328	1.6592000	-1.5646605
19.5333328	1.7079999	-1.4542136
19.5333328	1.7567999	-1.1780972
19.5333328	1.8055999	-1.1842332
19.5333328	1.8543999	-1.1106019
19.5333328	1.9031999	-1.0246992
19.5333328	1.9519999	-1.0615149
19.5333328	2.0007999	-1.2333207
19.5333328	2.0495999	-1.2333207
19.5333328	2.0983999	-1.1290097
19.5333328	2.1471999	-1.1412816
19.5333328	2.1959999	-1.2087770
19.5333328	2.2447999	-1.2149129
19.5333328	2.2935998	-1.2149129
19.5333328	2.3423998	-1.1412816
19.5333328	2.3912001	-1.2026410
19.5333328	2.4400001	-1.2026410
19.5333328	2.4888000	-1.2026410
19.5333328	2.5376000	-1.3437672
19.5333328	2.5864000	-1.3314953
19.5333328	2.6352000	-1.2149129
19.5333328	2.6840000	-1.2271848
19.5333328	2.7328000	-1.2087770
19.5333328	2.7816000	-1.0124273

19.5333328	2.8304000	-1.0001554
19.5333328	2.8792000	-0.8222136
19.5333328	2.9280000	-0.6626797
19.5333328	2.9768000	-0.6626797
19.5333328	3.0256000	-0.6688156
19.5333328	3.0743999	-0.8835731
19.5333328	3.1231999	-0.8160777
19.5333328	3.1719999	-1.0553789
19.5333328	3.2207999	-0.9756117
19.5333328	3.2695999	-0.9633398
19.5333328	3.3183999	-0.9633398
19.5333328	3.3671999	-0.9633398
19.5333328	3.4159999	-0.8958449
19.5333328	3.4647999	-0.8406215
19.5333328	3.5135999	-0.9142528
19.5333328	3.5623999	-0.9203887
19.5333328	3.6111999	-0.8160777
19.5333328	3.6599998	-1.0308352
19.5333328	3.7087998	-0.8958449
19.5333328	3.7575998	-0.8038058
19.5333328	3.8063998	-0.7976699
19.5333328	3.8551998	-0.8038058
19.5333328	3.9039998	-1.0062914
19.5333328	3.9527998	-1.0124273
19.5333328	4.0016003	-0.9326606
19.5333328	4.0503998	-0.9081168
19.5333328	4.0991998	-0.9081168
19.5333328	4.1479998	-0.7240391
19.5333328	4.1967998	-0.7424469
19.5333328	4.2455997	-0.9081168
19.5333328	4.2943997	-0.8099418
19.5333328	4.3431997	-0.7424469
19.5333328	4.3919997	-0.6381359
19.5333328	4.4407997	-0.8099418
19.5333328	4.4895997	-0.8099418
19.5333328	4.5383997	-0.7240391
19.5333328	4.5871997	-0.7669902
19.5333328	4.6359997	-0.7547188
19.5333328	4.6847997	-0.7485828
19.5333328	4.7336001	-0.7424469
19.5333328	4.7824001	-0.7792621
19.5333328	4.8312001	-0.7547188
19.5333328	4.8800001	-0.7547188
19.5333328	4.9288001	-0.7669902
19.5333328	4.9776001	-0.6258640
19.5333328	5.0264001	-0.5706410
19.5333328	5.0752001	-0.5706410
19.5333328	5.1240001	-0.4847379
19.5333328	5.1728001	-0.4847379
19.5333328	5.2216001	-0.4724660
19.5333328	5.2704000	-0.6749516
19.5333328	5.3192000	-0.5399613
19.5333328	5.3680000	-0.4786019
19.5333328	5.4168000	-0.4601941
19.5333328	5.4656000	-0.4601941
19.5333328	5.5144000	-0.4540582
19.5333328	5.5632000	-0.5215535

19.5333328	5.6120000	-0.5215535
19.5333328	5.6608000	-0.6810875
19.5333328	5.7096000	-0.5829129
19.5333328	5.7584000	-0.5767770
19.5333328	5.8072000	-0.5829129
19.5333328	5.8559999	-0.5890489
19.5333328	5.9047999	-0.5399613
19.5333328	5.9535999	-0.5399613
19.5333328	6.0023999	-0.5338254
19.5499992	0.2928000	-2.0984859
19.5499992	0.3416000	-2.0984859
19.5499992	0.3904000	-1.6935148
19.5499992	0.4392000	-1.6935148
19.5499992	0.4880000	-1.8346410
19.5499992	0.5368000	-1.9266801
19.5499992	0.5856000	-1.6382914
19.5499992	0.6344000	-1.5339808
19.5499992	0.6832000	-1.3928547
19.5499992	0.7320000	-1.3805828
19.5499992	0.7808000	-1.3008156
19.5499992	0.8296000	-1.2885437
19.5499992	0.8784000	-1.3683109
19.5499992	0.9272000	-1.2517285
19.5499992	0.9760000	-1.2701364
19.5499992	1.0247999	-1.2824078
19.5499992	1.0735999	-1.3069515
19.5499992	1.1223999	-1.3192234
19.5499992	1.1711999	-1.3867188
19.5499992	1.2200000	-1.3928547
19.5499992	1.2688000	-1.4603496
19.5499992	1.3176000	-1.3314953
19.5499992	1.3664000	-1.3437672
19.5499992	1.4152000	-1.5094371
19.5499992	1.4640000	-1.3928547
19.5499992	1.5128000	-1.3192234
19.5499992	1.5616000	-1.6689711
19.5499992	1.6104000	-1.6751070
19.5499992	1.6592000	-1.4235344
19.5499992	1.7079999	-1.4971652
19.5499992	1.7567999	-1.5953403
19.5499992	1.8055999	-1.3192234
19.5499992	1.8543999	-1.5646605
19.5499992	1.9031999	-1.5462527
19.5499992	1.9519999	-1.5339808
19.5499992	2.0007999	-1.3805828
19.5499992	2.0495999	-1.3621750
19.5499992	2.0983999	-1.3683109
19.5499992	2.1471999	-1.3621750
19.5499992	2.1959999	-1.3560390
19.5499992	2.2447999	-1.2517285
19.5499992	2.2935998	-1.1474175
19.5499992	2.3423998	-1.1535535
19.5499992	2.3912001	-1.1535535
19.5499992	2.4400001	-1.2946796
19.5499992	2.4888000	-1.1351457
19.5499992	2.5376000	-1.1351457
19.5499992	2.5864000	-1.2394567

19.5499992	2.6352000	-1.1903691
19.5499992	2.6840000	-1.0124273
19.5499992	2.7328000	-1.0124273
19.5499992	2.7816000	-1.0124273
19.5499992	2.8304000	-0.7915339
19.5499992	2.8792000	-0.6994953
19.5499992	2.9280000	-0.7056313
19.5499992	2.9768000	-0.7056313
19.5499992	3.0256000	-0.8528934
19.5499992	3.0743999	-0.7547188
19.5499992	3.1231999	-1.0246992
19.5499992	3.1719999	-0.7485828
19.5499992	3.2207999	-0.9694757
19.5499992	3.2695999	-1.1351457
19.5499992	3.3183999	-0.9510679
19.5499992	3.3671999	-0.8713012
19.5499992	3.4159999	-0.8713012
19.5499992	3.4647999	-0.8283496
19.5499992	3.5135999	-0.8283496
19.5499992	3.5623999	-0.8283496
19.5499992	3.6111999	-0.7547188
19.5499992	3.6599998	-0.7547188
19.5499992	3.7087998	-0.9019809
19.5499992	3.7575998	-1.0737867
19.5499992	3.8063998	-1.0737867
19.5499992	3.8551998	-1.0308352
19.5499992	3.9039998	-0.9326606
19.5499992	3.9527998	-0.9265246
19.5499992	4.0016003	-0.6258640
19.5499992	4.0503998	-0.7301750
19.5499992	4.0991998	-0.7424469
19.5499992	4.1479998	-0.8528934
19.5499992	4.1967998	-0.8406215
19.5499992	4.2455997	-0.6749516
19.5499992	4.2943997	-0.7485828
19.5499992	4.3431997	-0.5522332
19.5499992	4.3919997	-0.6197281
19.5499992	4.4407997	-0.7424469
19.5499992	4.4895997	-0.8713012
19.5499992	4.5383997	-0.8713012
19.5499992	4.5871997	-0.8897090
19.5499992	4.6359997	-0.8835731
19.5499992	4.6847997	-0.7853980
19.5499992	4.7336001	-0.8774371
19.5499992	4.7824001	-0.8774371
19.5499992	4.8312001	-0.7915339
19.5499992	4.8800001	-0.6626797
19.5499992	4.9288001	-0.6626797
19.5499992	4.9776001	-0.4540582
19.5499992	5.0264001	-0.4417863
19.5499992	5.0752001	-0.5031457
19.5499992	5.1240001	-0.5276895
19.5499992	5.1728001	-0.6381359
19.5499992	5.2216001	-0.6320000
19.5499992	5.2704000	-0.6258640
19.5499992	5.3192000	-0.5338254
19.5499992	5.3680000	-0.5031457

19.5499992	5.4168000	-0.6442719
19.5499992	5.4656000	-0.6381359
19.5499992	5.5144000	-0.5399613
19.5499992	5.5632000	-0.5399613
19.5499992	5.6120000	-0.5706410
19.5499992	5.6608000	-0.4417863
19.5499992	5.7096000	-0.6626797
19.5499992	5.7584000	-0.5399613
19.5499992	5.8072000	-0.6013203
19.5499992	5.8559999	-0.5890489
19.5499992	5.9047999	-0.5706410
19.5499992	5.9535999	-0.5706410
19.5499992	6.0023999	-0.5829129
19.5666676	0.2928000	-2.3868742
19.5666676	0.3416000	-2.4482334
19.5666676	0.3904000	-1.3437672
19.5666676	0.4392000	-1.3437672
19.5666676	0.4880000	-1.0001554
19.5666676	0.5368000	-1.0185633
19.5666676	0.5856000	-1.5155730
19.5666676	0.6344000	-1.4173985
19.5666676	0.6832000	-1.5830684
19.5666676	0.7320000	-1.5892043
19.5666676	0.7808000	-1.3805828
19.5666676	0.8296000	-1.4358063
19.5666676	0.8784000	-1.2455926
19.5666676	0.9272000	-1.3437672
19.5666676	0.9760000	-1.3621750
19.5666676	1.0247999	-1.2455926
19.5666676	1.0735999	-1.2517285
19.5666676	1.1223999	-1.2701364
19.5666676	1.1711999	-1.3130875
19.5666676	1.2200000	-1.5646605
19.5666676	1.2688000	-1.3130875
19.5666676	1.3176000	-1.3069515
19.5666676	1.3664000	-1.3805828
19.5666676	1.4152000	-1.2762718
19.5666676	1.4640000	-1.2640004
19.5666676	1.5128000	-1.3683109
19.5666676	1.5616000	-1.3928547
19.5666676	1.6104000	-1.4787574
19.5666676	1.6592000	-1.5094371
19.5666676	1.7079999	-1.3069515
19.5666676	1.7567999	-1.2946796
19.5666676	1.8055999	-1.1044660
19.5666676	1.8543999	-1.2824078
19.5666676	1.9031999	-1.1167378
19.5666676	1.9519999	-1.2271848
19.5666676	2.0007999	-1.2210488
19.5666676	2.0495999	-1.2517285
19.5666676	2.0983999	-1.2517285
19.5666676	2.1471999	-1.3130875
19.5666676	2.1959999	-1.1106019
19.5666676	2.2447999	-1.1167378
19.5666676	2.2935998	-1.1167378
19.5666676	2.3423998	-1.1290097
19.5666676	2.3912001	-1.1167378

19.5666676	2.4400001	-1.1228738
19.5666676	2.4888000	-1.0553789
19.5666676	2.5376000	-1.1412816
19.5666676	2.5864000	-1.1412816
19.5666676	2.6352000	-1.1106019
19.5666676	2.6840000	-1.0799227
19.5666676	2.7328000	-0.9817476
19.5666676	2.7816000	-0.8283496
19.5666676	2.8304000	-0.8222136
19.5666676	2.8792000	-0.7363110
19.5666676	2.9280000	-0.7853980
19.5666676	2.9768000	-0.7915339
19.5666676	3.0256000	-0.7976699
19.5666676	3.0743999	-1.2271848
19.5666676	3.1231999	-1.1535535
19.5666676	3.1719999	-1.1535535
19.5666676	3.2207999	-1.0369711
19.5666676	3.2695999	-0.9142528
19.5666676	3.3183999	-1.1596894
19.5666676	3.3671999	-1.1658254
19.5666676	3.4159999	-1.1228738
19.5666676	3.4647999	-1.1228738
19.5666676	3.5135999	-1.1842332
19.5666676	3.5623999	-1.0431070
19.5666676	3.6111999	-1.0369711
19.5666676	3.6599998	-1.0921946
19.5666676	3.7087998	-1.1044660
19.5666676	3.7575998	-1.0983305
19.5666676	3.8063998	-1.0860586
19.5666676	3.8551998	-1.0860586
19.5666676	3.9039998	-1.1474175
19.5666676	3.9527998	-1.1474175
19.5666676	4.0016003	-0.3742914
19.5666676	4.0503998	-0.4724660
19.5666676	4.0991998	-0.4663301
19.5666676	4.1479998	-0.4847379
19.5666676	4.1967998	-0.8222136
19.5666676	4.2455997	-0.9694757
19.5666676	4.2943997	-0.8897090
19.5666676	4.3431997	-0.8897090
19.5666676	4.3919997	-0.8283496
19.5666676	4.4407997	-0.4847379
19.5666676	4.4895997	-1.0492430
19.5666676	4.5383997	-1.0492430
19.5666676	4.5871997	-0.9572039
19.5666676	4.6359997	-0.9510679
19.5666676	4.6847997	-1.0001554
19.5666676	4.7336001	-1.0185633
19.5666676	4.7824001	-0.8651652
19.5666676	4.8312001	-0.8713012
19.5666676	4.8800001	-0.8651652
19.5666676	4.9288001	-0.7731261
19.5666676	4.9776001	-0.7301750
19.5666676	5.0264001	-0.7301750
19.5666676	5.0752001	-0.4908738
19.5666676	5.1240001	-0.4663301
19.5666676	5.1728001	-0.4417863

19.5666676	5.2216001	-0.7179031
19.5666676	5.2704000	-0.6381359
19.5666676	5.3192000	-0.6320000
19.5666676	5.3680000	-0.6442719
19.5666676	5.4168000	-0.6504078
19.5666676	5.4656000	-0.6504078
19.5666676	5.5144000	-0.6442719
19.5666676	5.5632000	-0.4295144
19.5666676	5.6120000	-0.4233789
19.5666676	5.6608000	-0.3374758
19.5666676	5.7096000	-0.4724660
19.5666676	5.7584000	-0.4663301
19.5666676	5.8072000	-0.4601941
19.5666676	5.8559999	-0.4601941
19.5666676	5.9047999	-0.5338254
19.5666676	5.9535999	-0.5276895
19.5666676	6.0023999	-0.7792621
19.5833340	0.2928000	-1.7610102
19.5833340	0.3416000	-1.4603496
19.5833340	0.3904000	-1.3499031
19.5833340	0.4392000	-1.4848933
19.5833340	0.4880000	-1.4848933
19.5833340	0.5368000	-1.6751070
19.5833340	0.5856000	-1.3560390
19.5833340	0.6344000	-1.4971652
19.5833340	0.6832000	-1.3989906
19.5833340	0.7320000	-1.1903691
19.5833340	0.7808000	-1.3008156
19.5833340	0.8296000	-1.3867188
19.5833340	0.8784000	-1.3437672
19.5833340	0.9272000	-1.2762718
19.5833340	0.9760000	-1.2701364
19.5833340	1.0247999	-1.2701364
19.5833340	1.0735999	-1.2394567
19.5833340	1.1223999	-1.1596894
19.5833340	1.1711999	-1.0615149
19.5833340	1.2200000	-1.2946796
19.5833340	1.2688000	-1.2946796
19.5833340	1.3176000	-1.3437672
19.5833340	1.3664000	-1.3437672
19.5833340	1.4152000	-1.5094371
19.5833340	1.4640000	-1.3560390
19.5833340	1.5128000	-1.3560390
19.5833340	1.5616000	-1.2087770
19.5833340	1.6104000	-1.2824078
19.5833340	1.6592000	-1.4480777
19.5833340	1.7079999	-1.2517285
19.5833340	1.7567999	-1.3314953
19.5833340	1.8055999	-1.3683109
19.5833340	1.8543999	-1.4726214
19.5833340	1.9031999	-1.4603496
19.5833340	1.9519999	-1.2455926
19.5833340	2.0007999	-1.2517285
19.5833340	2.0495999	-1.2640004
19.5833340	2.0983999	-1.2271848
19.5833340	2.1471999	-1.0492430
19.5833340	2.1959999	-1.1842332

19.5833340	2.2447999	-1.0615149
19.5833340	2.2935998	-1.0615149
19.5833340	2.3423998	-1.0676508
19.5833340	2.3912001	-1.0185633
19.5833340	2.4400001	-1.1719613
19.5833340	2.4888000	-1.1780972
19.5833340	2.5376000	-1.1965051
19.5833340	2.5864000	-1.0124273
19.5833340	2.6352000	-1.1658254
19.5833340	2.6840000	-1.0676508
19.5833340	2.7328000	-1.0553789
19.5833340	2.7816000	-1.0553789
19.5833340	2.8304000	-1.0676508
19.5833340	2.8792000	-1.0615149
19.5833340	2.9280000	-0.9817476
19.5833340	2.9768000	-1.0062914
19.5833340	3.0256000	-1.0185633
19.5833340	3.0743999	-1.0431070
19.5833340	3.1231999	-0.8528934
19.5833340	3.1719999	-0.9572039
19.5833340	3.2207999	-0.8958449
19.5833340	3.2695999	-0.8344855
19.5833340	3.3183999	-0.8467574
19.5833340	3.3671999	-0.7056313
19.5833340	3.4159999	-1.0676508
19.5833340	3.4647999	-0.9817476
19.5833340	3.5135999	-0.8958449
19.5833340	3.5623999	-0.9817476
19.5833340	3.6111999	-0.9817476
19.5833340	3.6599998	-0.9817476
19.5833340	3.7087998	-1.0737867
19.5833340	3.7575998	-1.0737867
19.5833340	3.8063998	-1.0860586
19.5833340	3.8551998	-0.9142528
19.5833340	3.9039998	-0.9142528
19.5833340	3.9527998	-0.7240391
19.5833340	4.0016003	-0.8038058
19.5833340	4.0503998	-0.4601941
19.5833340	4.0991998	-0.4601941
19.5833340	4.1479998	-0.4111071
19.5833340	4.1967998	-0.5031457
19.5833340	4.2455997	-0.5767770
19.5833340	4.2943997	-0.5890489
19.5833340	4.3431997	-0.5276895
19.5833340	4.3919997	-0.7056313
19.5833340	4.4407997	-0.7056313
19.5833340	4.4895997	-0.6442719
19.5833340	4.5383997	-0.7485828
19.5833340	4.5871997	-0.7485828
19.5833340	4.6359997	-0.8713012
19.5833340	4.6847997	-0.8835731
19.5833340	4.7336001	-0.8651652
19.5833340	4.7824001	-0.7424469
19.5833340	4.8312001	-0.5951843
19.5833340	4.8800001	-0.5951843
19.5833340	4.9288001	-0.6688156
19.5833340	4.9776001	-0.5645051

19.5833340	5.0264001	-0.5399613
19.5833340	5.0752001	-0.6258640
19.5833340	5.1240001	-0.5829129
19.5833340	5.1728001	-0.5460973
19.5833340	5.2216001	-0.6258640
19.5833340	5.2704000	-0.4724660
19.5833340	5.3192000	-0.6258640
19.5833340	5.3680000	-0.7240391
19.5833340	5.4168000	-0.7240391
19.5833340	5.4656000	-0.7363110
19.5833340	5.5144000	-0.5767770
19.5833340	5.5632000	-0.5706410
19.5833340	5.6120000	-0.5645051
19.5833340	5.6608000	-0.5706410
19.5833340	5.7096000	-0.6933594
19.5833340	5.7584000	-0.6749516
19.5833340	5.8072000	-0.6749516
19.5833340	5.8559999	-0.6749516
19.5833340	5.9047999	-0.5951843
19.5833340	5.9535999	-0.6074562
19.5833340	6.0023999	613586.0000000
19.6000004	0.2928000	-1.5339808
19.6000004	0.3416000	-1.4358063
19.6000004	0.3904000	-1.3560390
19.6000004	0.4392000	-1.1474175
19.6000004	0.4880000	-1.4358063
19.6000004	0.5368000	-1.1228738
19.6000004	0.5856000	-1.4971652
19.6000004	0.6344000	-1.4603496
19.6000004	0.6832000	-1.3683109
19.6000004	0.7320000	-1.3683109
19.6000004	0.7808000	-1.3130875
19.6000004	0.8296000	-1.3130875
19.6000004	0.8784000	-1.1535535
19.6000004	0.9272000	-1.1535535
19.6000004	0.9760000	-1.1903691
19.6000004	1.0247999	-1.2762718
19.6000004	1.0735999	-1.1412816
19.6000004	1.1223999	-1.4235344
19.6000004	1.1711999	-1.4419422
19.6000004	1.2200000	-1.4664855
19.6000004	1.2688000	-1.5769324
19.6000004	1.3176000	-1.3867188
19.6000004	1.3664000	-1.3867188
19.6000004	1.4152000	-1.1228738
19.6000004	1.4640000	-1.2333207
19.6000004	1.5128000	-1.2210488
19.6000004	1.5616000	-1.2271848
19.6000004	1.6104000	-1.4296703
19.6000004	1.6592000	-1.5339808
19.6000004	1.7079999	-1.2824078
19.6000004	1.7567999	-1.1596894
19.6000004	1.8055999	-1.0799227
19.6000004	1.8543999	-1.0615149
19.6000004	1.9031999	-1.1228738
19.6000004	1.9519999	-1.2026410
19.6000004	2.0007999	-1.2087770

19.6000004	2.0495999	-1.1596894
19.6000004	2.0983999	-1.0431070
19.6000004	2.1471999	-1.3499031
19.6000004	2.1959999	-1.3560390
19.6000004	2.2447999	-1.3621750
19.6000004	2.2935998	-1.3683109
19.6000004	2.3423998	-1.1719613
19.6000004	2.3912001	-1.1290097
19.6000004	2.4400001	-1.2087770
19.6000004	2.4888000	-1.2087770
19.6000004	2.5376000	-1.2824078
19.6000004	2.5864000	-1.0246992
19.6000004	2.6352000	-1.0308352
19.6000004	2.6840000	-0.9387960
19.6000004	2.7328000	-0.9449320
19.6000004	2.7816000	-0.9572039
19.6000004	2.8304000	-0.9142528
19.6000004	2.8792000	-0.9572039
19.6000004	2.9280000	-0.8528934
19.6000004	2.9768000	-0.8406215
19.6000004	3.0256000	-0.8222136
19.6000004	3.0743999	-0.8099418
19.6000004	3.1231999	-0.8038058
19.6000004	3.1719999	-0.6381359
19.6000004	3.2207999	-0.9326606
19.6000004	3.2695999	-0.9265246
19.6000004	3.3183999	-0.9633398
19.6000004	3.3671999	-0.8528934
19.6000004	3.4159999	-0.8528934
19.6000004	3.4647999	-0.9694757
19.6000004	3.5135999	-1.0431070
19.6000004	3.5623999	-1.0921946
19.6000004	3.6111999	-1.0983305
19.6000004	3.6599998	-1.1106019
19.6000004	3.7087998	-1.0308352
19.6000004	3.7575998	-1.0369711
19.6000004	3.8063998	-0.9449320
19.6000004	3.8551998	-0.8528934
19.6000004	3.9039998	-0.7608547
19.6000004	3.9527998	-0.7179031
19.6000004	4.0016003	-0.7669902
19.6000004	4.0503998	-0.7731261
19.6000004	4.0991998	-0.4847379
19.6000004	4.1479998	-0.5338254
19.6000004	4.1967998	-0.5706410
19.6000004	4.2455997	-0.7424469
19.6000004	4.2943997	-0.6013203
19.6000004	4.3431997	-0.6135921
19.6000004	4.3919997	-0.6135921
19.6000004	4.4407997	-0.6626797
19.6000004	4.4895997	-0.6626797
19.6000004	4.5383997	-0.6626797
19.6000004	4.5871997	-0.6626797
19.6000004	4.6359997	-0.6626797
19.6000004	4.6847997	-0.6504078
19.6000004	4.7336001	-0.7608547
19.6000004	4.7824001	-0.5276895

19.6000004	4.8312001	-0.5154176
19.6000004	4.8800001	-0.6749516
19.6000004	4.9288001	-0.6810875
19.6000004	4.9776001	-0.4356503
19.6000004	5.0264001	-0.4356503
19.6000004	5.0752001	-0.4356503
19.6000004	5.1240001	-0.5645051
19.6000004	5.1728001	-0.5767770
19.6000004	5.2216001	-0.4908738
19.6000004	5.2704000	-0.5031457
19.6000004	5.3192000	-0.5154176
19.6000004	5.3680000	-0.4970098
19.6000004	5.4168000	-0.6381359
19.6000004	5.4656000	-0.6258640
19.6000004	5.5144000	-0.5583692
19.6000004	5.5632000	-0.4724660
19.6000004	5.6120000	-0.3742914
19.6000004	5.6608000	-0.3742914
19.6000004	5.7096000	-0.3620195
19.6000004	5.7584000	613586.0000000
19.6000004	5.8072000	-0.6013203
19.6000004	5.8559999	-0.6074562
19.6000004	5.9047999	-0.5522332
19.6000004	5.9535999	-0.4663301
19.6000004	6.0023999	-0.4540582
19.6166668	0.2928000	-1.6689711
19.6166668	0.3416000	-1.6689711
19.6166668	0.3904000	-1.7794180
19.6166668	0.4392000	-1.3130875
19.6166668	0.4880000	-1.2578645
19.6166668	0.5368000	-1.0492430
19.6166668	0.5856000	-1.2394567
19.6166668	0.6344000	-1.2946796
19.6166668	0.6832000	-1.5892043
19.6166668	0.7320000	-1.5892043
19.6166668	0.7808000	-1.4848933
19.6166668	0.8296000	-1.4848933
19.6166668	0.8784000	-1.1903691
19.6166668	0.9272000	-1.2946796
19.6166668	0.9760000	-1.2271848
19.6166668	1.0247999	-1.4173985
19.6166668	1.0735999	-1.4112625
19.6166668	1.1223999	-1.3376312
19.6166668	1.1711999	-1.0246992
19.6166668	1.2200000	-1.4971652
19.6166668	1.2688000	-1.1535535
19.6166668	1.3176000	-1.2517285
19.6166668	1.3664000	-1.3253593
19.6166668	1.4152000	-1.2455926
19.6166668	1.4640000	-1.1780972
19.6166668	1.5128000	-1.1842332
19.6166668	1.5616000	-1.3437672
19.6166668	1.6104000	-1.4542136
19.6166668	1.6592000	-1.4358063
19.6166668	1.7079999	-1.4296703
19.6166668	1.7567999	-1.1658254
19.6166668	1.8055999	-1.2087770

19.6166668	1.8543999	-1.1596894
19.6166668	1.9031999	-0.9326606
19.6166668	1.9519999	-0.9387960
19.6166668	2.0007999	-1.0185633
19.6166668	2.0495999	-1.2394567
19.6166668	2.0983999	-1.2149129
19.6166668	2.1471999	-1.2026410
19.6166668	2.1959999	-1.0185633
19.6166668	2.2447999	-1.1903691
19.6166668	2.2935998	-1.2762718
19.6166668	2.3423998	-1.2701364
19.6166668	2.3912001	-1.1780972
19.6166668	2.4400001	-1.1535535
19.6166668	2.4888000	-1.2578645
19.6166668	2.5376000	-1.2455926
19.6166668	2.5864000	-1.0737867
19.6166668	2.6352000	-1.0737867
19.6166668	2.6840000	-1.0860586
19.6166668	2.7328000	-0.9326606
19.6166668	2.7816000	-0.9449320
19.6166668	2.8304000	-0.9510679
19.6166668	2.8792000	-0.8467574
19.6166668	2.9280000	-0.8406215
19.6166668	2.9768000	-0.8344855
19.6166668	3.0256000	-0.9878836
19.6166668	3.0743999	-0.9756117
19.6166668	3.1231999	-0.8160777
19.6166668	3.1719999	-0.8222136
19.6166668	3.2207999	-0.8222136
19.6166668	3.2695999	-0.8099418
19.6166668	3.3183999	-0.9203887
19.6166668	3.3671999	-0.8099418
19.6166668	3.4159999	-0.7301750
19.6166668	3.4647999	-0.7547188
19.6166668	3.5135999	-0.7485828
19.6166668	3.5623999	-0.7056313
19.6166668	3.6111999	-0.7179031
19.6166668	3.6599998	-0.5890489
19.6166668	3.7087998	-0.6013203
19.6166668	3.7575998	-0.8222136
19.6166668	3.8063998	-0.6688156
19.6166668	3.8551998	-0.6688156
19.6166668	3.9039998	-0.7240391
19.6166668	3.9527998	-0.7915339
19.6166668	4.0016003	-0.8590293
19.6166668	4.0503998	-0.9265246
19.6166668	4.0991998	-0.8528934
19.6166668	4.1479998	-0.8713012
19.6166668	4.1967998	-0.7976699
19.6166668	4.2455997	-0.7976699
19.6166668	4.2943997	-0.5031457
19.6166668	4.3431997	-0.5031457
19.6166668	4.3919997	-0.6258640
19.6166668	4.4407997	-0.6197281
19.6166668	4.4895997	-0.6197281
19.6166668	4.5383997	-0.7117672
19.6166668	4.5871997	-0.7240391

19.6166668	4.6359997	-0.6258640
19.6166668	4.6847997	-0.6810875
19.6166668	4.7336001	-0.7853980
19.6166668	4.7824001	-0.7792621
19.6166668	4.8312001	-0.6810875
19.6166668	4.8800001	-0.6810875
19.6166668	4.9288001	-0.6810875
19.6166668	4.9776001	-0.7363110
19.6166668	5.0264001	-0.6197281
19.6166668	5.0752001	-0.5829129
19.6166668	5.1240001	-0.4663301
19.6166668	5.1728001	-0.4479222
19.6166668	5.2216001	-0.6135921
19.6166668	5.2704000	-0.4172430
19.6166668	5.3192000	-0.4847379
19.6166668	5.3680000	-0.4908738
19.6166668	5.4168000	-0.5645051
19.6166668	5.4656000	-0.5645051
19.6166668	5.5144000	-0.5215535
19.6166668	5.5632000	-0.5276895
19.6166668	5.6120000	613586.0000000
19.6166668	5.6608000	613586.0000000
19.6166668	5.7096000	-0.3558836
19.6166668	5.7584000	-0.4724660
19.6166668	5.8072000	-0.5215535
19.6166668	5.8559999	-0.5215535
19.6166668	5.9047999	-0.5154176
19.6166668	5.9535999	-0.4172430
19.6166668	6.0023999	-0.4111071
19.6333332	0.2928000	-1.6321554
19.6333332	0.3416000	-1.6260195
19.6333332	0.3904000	-1.5033011
19.6333332	0.4392000	-1.5033011
19.6333332	0.4880000	-1.3744469
19.6333332	0.5368000	-1.6137476
19.6333332	0.5856000	-1.2578645
19.6333332	0.6344000	-1.3499031
19.6333332	0.6832000	-1.4542136
19.6333332	0.7320000	-1.4848933
19.6333332	0.7808000	-1.2824078
19.6333332	0.8296000	-1.3499031
19.6333332	0.8784000	-1.5278449
19.6333332	0.9272000	-1.4726214
19.6333332	0.9760000	-1.4112625
19.6333332	1.0247999	-1.2640004
19.6333332	1.0735999	-1.4296703
19.6333332	1.1223999	-1.4296703
19.6333332	1.1711999	-1.4419422
19.6333332	1.2200000	-1.4051266
19.6333332	1.2688000	-1.3928547
19.6333332	1.3176000	-1.3744469
19.6333332	1.3664000	-1.3437672
19.6333332	1.4152000	-1.2333207
19.6333332	1.4640000	-1.3621750
19.6333332	1.5128000	-1.2701364
19.6333332	1.5616000	-1.2885437
19.6333332	1.6104000	-1.3008156

19.6333332	1.6592000	-1.3008156
19.6333332	1.7079999	-1.1351457
19.6333332	1.7567999	-1.3683109
19.6333332	1.8055999	-1.4664855
19.6333332	1.8543999	-1.4480777
19.6333332	1.9031999	-0.8651652
19.6333332	1.9519999	-0.8774371
19.6333332	2.0007999	-0.7485828
19.6333332	2.0495999	-1.1719613
19.6333332	2.0983999	-1.0308352
19.6333332	2.1471999	-1.2271848
19.6333332	2.1959999	-1.2946796
19.6333332	2.2447999	-1.0431070
19.6333332	2.2935998	-1.0553789
19.6333332	2.3423998	-1.2026410
19.6333332	2.3912001	-1.2271848
19.6333332	2.4400001	-1.2271848
19.6333332	2.4888000	-1.2149129
19.6333332	2.5376000	-1.0799227
19.6333332	2.5864000	-1.1106019
19.6333332	2.6352000	-1.2210488
19.6333332	2.6840000	-1.1167378
19.6333332	2.7328000	-1.1167378
19.6333332	2.7816000	-1.2087770
19.6333332	2.8304000	-1.2210488
19.6333332	2.8792000	-0.9633398
19.6333332	2.9280000	-0.9694757
19.6333332	2.9768000	-1.0921946
19.6333332	3.0256000	-1.0921946
19.6333332	3.0743999	-1.0983305
19.6333332	3.1231999	-0.7117672
19.6333332	3.1719999	-0.6994953
19.6333332	3.2207999	-1.0615149
19.6333332	3.2695999	-1.0676508
19.6333332	3.3183999	-0.8590293
19.6333332	3.3671999	-0.8713012
19.6333332	3.4159999	-0.8774371
19.6333332	3.4647999	-0.8897090
19.6333332	3.5135999	-0.9510679
19.6333332	3.5623999	-0.9510679
19.6333332	3.6111999	-0.9265246
19.6333332	3.6599998	-0.8099418
19.6333332	3.7087998	-0.7608547
19.6333332	3.7575998	-0.8528934
19.6333332	3.8063998	-0.9510679
19.6333332	3.8551998	-0.9449320
19.6333332	3.9039998	-0.9572039
19.6333332	3.9527998	-1.0308352
19.6333332	4.0016003	-1.0983305
19.6333332	4.0503998	-0.9265246
19.6333332	4.0991998	-0.8590293
19.6333332	4.1479998	-0.8467574
19.6333332	4.1967998	-0.9694757
19.6333332	4.2455997	-0.7547188
19.6333332	4.2943997	-0.7669902
19.6333332	4.3431997	-0.7731261
19.6333332	4.3919997	-0.8160777

19.6333332	4.4407997	-0.8283496
19.6333332	4.4895997	-0.8038058
19.6333332	4.5383997	-0.8038058
19.6333332	4.5871997	-0.8774371
19.6333332	4.6359997	-0.8774371
19.6333332	4.6847997	-0.9387960
19.6333332	4.7336001	-0.8467574
19.6333332	4.7824001	-0.8160777
19.6333332	4.8312001	-0.9878836
19.6333332	4.8800001	-0.5767770
19.6333332	4.9288001	-0.6933594
19.6333332	4.9776001	-0.5092816
19.6333332	5.0264001	-0.5276895
19.6333332	5.0752001	-0.5215535
19.6333332	5.1240001	-0.3558836
19.6333332	5.1728001	-0.6197281
19.6333332	5.2216001	-0.6258640
19.6333332	5.2704000	-0.6258640
19.6333332	5.3192000	613586.0000000
19.6333332	5.3680000	-0.6013203
19.6333332	5.4168000	613586.0000000
19.6333332	5.4656000	-0.6504078
19.6333332	5.5144000	-0.5460973
19.6333332	5.5632000	-0.5890489
19.6333332	5.6120000	-0.4847379
19.6333332	5.6608000	-0.5031457
19.6333332	5.7096000	-0.4970098
19.6333332	5.7584000	-0.5767770
19.6333332	5.8072000	-0.6258640
19.6333332	5.8559999	-0.5706410
19.6333332	5.9047999	-0.4663301
19.6333332	5.9535999	-0.5031457
19.6333332	6.0023999	613586.0000000
19.6499996	0.2928000	-2.1721168
19.6499996	0.3416000	-1.0431070
19.6499996	0.3904000	-1.0553789
19.6499996	0.4392000	-0.9449320
19.6499996	0.4880000	-0.9449320
19.6499996	0.5368000	-1.2210488
19.6499996	0.5856000	-1.2210488
19.6499996	0.6344000	-1.3744469
19.6499996	0.6832000	-1.3744469
19.6499996	0.7320000	-1.2824078
19.6499996	0.7808000	-1.3867188
19.6499996	0.8296000	-1.3867188
19.6499996	0.8784000	-1.4480777
19.6499996	0.9272000	-1.3621750
19.6499996	0.9760000	-1.3621750
19.6499996	1.0247999	-1.3560390
19.6499996	1.0735999	-1.3437672
19.6499996	1.1223999	-1.1903691
19.6499996	1.1711999	-1.4235344
19.6499996	1.2200000	-1.3867188
19.6499996	1.2688000	-1.1290097
19.6499996	1.3176000	-1.1351457
19.6499996	1.3664000	-1.3130875
19.6499996	1.4152000	-1.3376312

19.6499996	1.4640000	-1.3376312
19.6499996	1.5128000	-1.3376312
19.6499996	1.5616000	-1.2517285
19.6499996	1.6104000	-1.1719613
19.6499996	1.6592000	-1.1719613
19.6499996	1.7079999	-1.4726214
19.6499996	1.7567999	-1.4787574
19.6499996	1.8055999	-1.5401168
19.6499996	1.8543999	-1.6873789
19.6499996	1.9031999	-1.4358063
19.6499996	1.9519999	-1.2517285
19.6499996	2.0007999	-1.3253593
19.6499996	2.0495999	-1.2026410
19.6499996	2.0983999	-1.3560390
19.6499996	2.1471999	-1.2455926
19.6499996	2.1959999	-1.2455926
19.6499996	2.2447999	-1.2824078
19.6499996	2.2935998	-1.2885437
19.6499996	2.3423998	-1.3130875
19.6499996	2.3912001	-1.3130875
19.6499996	2.4400001	-1.2271848
19.6499996	2.4888000	-1.0615149
19.6499996	2.5376000	-1.0615149
19.6499996	2.5864000	-1.0492430
19.6499996	2.6352000	-1.0185633
19.6499996	2.6840000	-1.0185633
19.6499996	2.7328000	-0.8774371
19.6499996	2.7816000	-1.0062914
19.6499996	2.8304000	-0.9878836
19.6499996	2.8792000	-0.8467574
19.6499996	2.9280000	-0.8528934
19.6499996	2.9768000	-1.0615149
19.6499996	3.0256000	-0.7117672
19.6499996	3.0743999	-0.7117672
19.6499996	3.1231999	-0.8099418
19.6499996	3.1719999	-0.7608547
19.6499996	3.2207999	-0.8283496
19.6499996	3.2695999	-0.8283496
19.6499996	3.3183999	-0.8528934
19.6499996	3.3671999	-0.8651652
19.6499996	3.4159999	-0.8713012
19.6499996	3.4647999	-0.9510679
19.6499996	3.5135999	-1.0001554
19.6499996	3.5623999	-1.0983305
19.6499996	3.6111999	-0.9878836
19.6499996	3.6599998	-0.9878836
19.6499996	3.7087998	-1.1658254
19.6499996	3.7575998	-1.1596894
19.6499996	3.8063998	-1.0431070
19.6499996	3.8551998	-1.1535535
19.6499996	3.9039998	-0.9633398
19.6499996	3.9527998	-0.9265246
19.6499996	4.0016003	-0.9449320
19.6499996	4.0503998	-0.9387960
19.6499996	4.0991998	-0.9081168
19.6499996	4.1479998	-0.9081168
19.6499996	4.1967998	-0.7853980

19.6499996	4.2455997	-0.7731261
19.6499996	4.2943997	-0.6626797
19.6499996	4.3431997	-0.8344855
19.6499996	4.3919997	-0.8406215
19.6499996	4.4407997	-0.8467574
19.6499996	4.4895997	-0.9694757
19.6499996	4.5383997	-0.9633398
19.6499996	4.5871997	-0.8774371
19.6499996	4.6359997	-0.8099418
19.6499996	4.6847997	-0.8283496
19.6499996	4.7336001	-0.8099418
19.6499996	4.7824001	-0.9510679
19.6499996	4.8312001	-0.8528934
19.6499996	4.8800001	-0.9510679
19.6499996	4.9288001	-0.8099418
19.6499996	4.9776001	-0.9694757
19.6499996	5.0264001	-0.9142528
19.6499996	5.0752001	-0.9203887
19.6499996	5.1240001	-0.7608547
19.6499996	5.1728001	-0.9633398
19.6499996	5.2216001	-0.8590293
19.6499996	5.2704000	-0.8835731
19.6499996	5.3192000	-0.7301750
19.6499996	5.3680000	-0.8099418
19.6499996	5.4168000	-0.8283496
19.6499996	5.4656000	-0.7240391
19.6499996	5.5144000	-0.7301750
19.6499996	5.5632000	-0.7179031
19.6499996	5.6120000	-0.8222136
19.6499996	5.6608000	-0.7056313
19.6499996	5.7096000	-0.7056313
19.6499996	5.7584000	-0.7485828
19.6499996	5.8072000	-0.6442719
19.6499996	5.8559999	-0.6565437
19.6499996	5.9047999	-0.6565437
19.6499996	5.9535999	-0.6626797
19.6499996	6.0023999	-0.6688156
19.6666660	0.2928000	-1.5585246
19.6666660	0.3416000	-1.7057867
19.6666660	0.3904000	-1.7057867
19.6666660	0.4392000	-1.2271848
19.6666660	0.4880000	-1.2210488
19.6666660	0.5368000	-1.2333207
19.6666660	0.5856000	-1.3376312
19.6666660	0.6344000	-1.2885437
19.6666660	0.6832000	-1.3683109
19.6666660	0.7320000	-1.1965051
19.6666660	0.7808000	-1.2026410
19.6666660	0.8296000	-1.2026410
19.6666660	0.8784000	-1.1167378
19.6666660	0.9272000	-1.1228738
19.6666660	0.9760000	-1.4542136
19.6666660	1.0247999	-1.4112625
19.6666660	1.0735999	-1.5094371
19.6666660	1.1223999	-1.4910293
19.6666660	1.1711999	-1.4910293
19.6666660	1.2200000	-1.3867188

19.6666660	1.2688000	-1.2394567
19.6666660	1.3176000	-1.0799227
19.6666660	1.3664000	-1.0860586
19.6666660	1.4152000	-1.1044660
19.6666660	1.4640000	-1.1780972
19.6666660	1.5128000	-1.2333207
19.6666660	1.5616000	-1.2824078
19.6666660	1.6104000	-1.2824078
19.6666660	1.6592000	-1.2578645
19.6666660	1.7079999	-1.3437672
19.6666660	1.7567999	-1.3437672
19.6666660	1.8055999	-1.3437672
19.6666660	1.8543999	-1.3437672
19.6666660	1.9031999	-1.1719613
19.6666660	1.9519999	-0.9633398
19.6666660	2.0007999	-0.9756117
19.6666660	2.0495999	-1.2824078
19.6666660	2.0983999	-1.2333207
19.6666660	2.1471999	-1.1167378
19.6666660	2.1959999	-1.2149129
19.6666660	2.2447999	-1.2271848
19.6666660	2.2935998	-1.1351457
19.6666660	2.3423998	-1.1290097
19.6666660	2.3912001	-1.1290097
19.6666660	2.4400001	-1.0492430
19.6666660	2.4888000	-1.0492430
19.6666660	2.5376000	-0.9633398
19.6666660	2.5864000	-0.9878836
19.6666660	2.6352000	-0.9633398
19.6666660	2.6840000	-0.8713012
19.6666660	2.7328000	-0.8590293
19.6666660	2.7816000	-0.9878836
19.6666660	2.8304000	-0.8958449
19.6666660	2.8792000	-0.9142528
19.6666660	2.9280000	-0.9142528
19.6666660	2.9768000	-0.9019809
19.6666660	3.0256000	-0.8897090
19.6666660	3.0743999	-0.9572039
19.6666660	3.1231999	-0.9449320
19.6666660	3.1719999	-0.7853980
19.6666660	3.2207999	-0.7853980
19.6666660	3.2695999	-0.8590293
19.6666660	3.3183999	-0.8835731
19.6666660	3.3671999	-0.7669902
19.6666660	3.4159999	-0.7669902
19.6666660	3.4647999	-0.6872234
19.6666660	3.5135999	-0.6933594
19.6666660	3.5623999	-0.6994953
19.6666660	3.6111999	-0.6994953
19.6666660	3.6599998	-0.5522332
19.6666660	3.7087998	-0.6810875
19.6666660	3.7575998	-0.6688156
19.6666660	3.8063998	-0.6565437
19.6666660	3.8551998	-0.6565437
19.6666660	3.9039998	-0.4908738
19.6666660	3.9527998	-0.7731261
19.6666660	4.0016003	-0.7853980

19.6666660	4.0503998	-1.0185633
19.6666660	4.0991998	-1.0124273
19.6666660	4.1479998	-0.9510679
19.6666660	4.1967998	-1.0001554
19.6666660	4.2455997	-0.7853980
19.6666660	4.2943997	-0.7853980
19.6666660	4.3431997	-0.8590293
19.6666660	4.3919997	-0.6994953
19.6666660	4.4407997	-0.6994953
19.6666660	4.4895997	-0.6933594
19.6666660	4.5383997	-0.7056313
19.6666660	4.5871997	-0.6749516
19.6666660	4.6359997	-0.6504078
19.6666660	4.6847997	-0.6565437
19.6666660	4.7336001	-0.5890489
19.6666660	4.7824001	-0.5890489
19.6666660	4.8312001	-0.5031457
19.6666660	4.8800001	-0.3804274
19.6666660	4.9288001	0.0306797
19.6666660	4.9776001	-0.4295144
19.6666660	5.0264001	-0.5031457
19.6666660	5.0752001	-0.3988352
19.6666660	5.1240001	-0.3804274
19.6666660	5.1728001	-0.2086215
19.6666660	5.2216001	-0.3681555
19.6666660	5.2704000	-0.3681555
19.6666660	5.3192000	-0.3681555
19.6666660	5.3680000	-0.5215535
19.6666660	5.4168000	-0.5215535
19.6666660	5.4656000	-0.4356503
19.6666660	5.5144000	-0.4233789
19.6666660	5.5632000	-0.4172430
19.6666660	5.6120000	-0.3374758
19.6666660	5.6608000	-0.2883883
19.6666660	5.7096000	613586.0000000
19.6666660	5.7584000	613586.0000000
19.6666660	5.8072000	613586.0000000
19.6666660	5.8559999	613586.0000000
19.6666660	5.9047999	613586.0000000
19.6666660	5.9535999	613586.0000000
19.6666660	6.0023999	613586.0000000
19.6833324	0.2928000	-1.4235344
19.6833324	0.3416000	-1.6382914
19.6833324	0.3904000	-1.4971652
19.6833324	0.4392000	-1.2271848
19.6833324	0.4880000	-1.2210488
19.6833324	0.5368000	-1.2824078
19.6833324	0.5856000	-1.1167378
19.6833324	0.6344000	-1.1719613
19.6833324	0.6832000	-1.1965051
19.6833324	0.7320000	-1.4542136
19.6833324	0.7808000	-1.4726214
19.6833324	0.8296000	-1.3499031
19.6833324	0.8784000	-1.3621750
19.6833324	0.9272000	-1.3744469
19.6833324	0.9760000	-1.3744469
19.6833324	1.0247999	-1.4787574

19.6833324	1.0735999	-1.2762718
19.6833324	1.1223999	-1.3560390
19.6833324	1.1711999	-1.2271848
19.6833324	1.2200000	-1.0676508
19.6833324	1.2688000	-1.0860586
19.6833324	1.3176000	-1.0860586
19.6833324	1.3664000	-1.1228738
19.6833324	1.4152000	-1.0983305
19.6833324	1.4640000	-1.0860586
19.6833324	1.5128000	-1.2640004
19.6833324	1.5616000	-1.2517285
19.6833324	1.6104000	-1.2517285
19.6833324	1.6592000	-1.3499031
19.6833324	1.7079999	-1.1658254
19.6833324	1.7567999	-1.3253593
19.6833324	1.8055999	-1.4112625
19.6833324	1.8543999	-0.9633398
19.6833324	1.9031999	-0.9633398
19.6833324	1.9519999	-1.0553789
19.6833324	2.0007999	-1.1596894
19.6833324	2.0495999	-1.1596894
19.6833324	2.0983999	-1.0860586
19.6833324	2.1471999	-1.1842332
19.6833324	2.1959999	-1.1842332
19.6833324	2.2447999	-1.1596894
19.6833324	2.2935998	-1.0553789
19.6833324	2.3423998	-1.1167378
19.6833324	2.3912001	-1.1106019
19.6833324	2.4400001	-0.9081168
19.6833324	2.4888000	-0.8958449
19.6833324	2.5376000	-0.8344855
19.6833324	2.5864000	-0.8283496
19.6833324	2.6352000	-0.7976699
19.6833324	2.6840000	-0.7915339
19.6833324	2.7328000	-0.8283496
19.6833324	2.7816000	-0.6258640
19.6833324	2.8304000	-0.5829129
19.6833324	2.8792000	-0.3804274
19.6833324	2.9280000	-0.7117672
19.6833324	2.9768000	-0.7117672
19.6833324	3.0256000	-0.5092816
19.6833324	3.0743999	-0.8160777
19.6833324	3.1231999	-0.8038058
19.6833324	3.1719999	-0.5399613
19.6833324	3.2207999	-0.5706410
19.6833324	3.2695999	-0.6688156
19.6833324	3.3183999	-0.6688156
19.6833324	3.3671999	-0.8344855
19.6833324	3.4159999	-0.8467574
19.6833324	3.4647999	-0.5951843
19.6833324	3.5135999	-0.5951843
19.6833324	3.5623999	-0.7117672
19.6833324	3.6111999	-0.5092816
19.6833324	3.6599998	-0.4908738
19.6833324	3.7087998	-0.7117672
19.6833324	3.7575998	-0.7179031
19.6833324	3.8063998	-0.7117672

19.6833324	3.8551998	-0.7117672
19.6833324	3.9039998	-0.7117672
19.6833324	3.9527998	-0.7485828
19.6833324	4.0016003	-0.7485828
19.6833324	4.0503998	-0.6565437
19.6833324	4.0991998	-0.4233789
19.6833324	4.1479998	-0.4295144
19.6833324	4.1967998	-0.4540582
19.6833324	4.2455997	-0.4601941
19.6833324	4.2943997	-0.5399613
19.6833324	4.3431997	-0.4847379
19.6833324	4.3919997	-0.4847379
19.6833324	4.4407997	-0.4847379
19.6833324	4.4895997	-0.3558836
19.6833324	4.5383997	-0.4049711
19.6833324	4.5871997	-0.4049711
19.6833324	4.6359997	-0.4479222
19.6833324	4.6847997	-0.4417863
19.6833324	4.7336001	-0.5092816
19.6833324	4.7824001	-0.5154176
19.6833324	4.8312001	-0.3988352
19.6833324	4.8800001	-0.2883883
19.6833324	4.9288001	-0.2822523
19.6833324	4.9776001	-0.3804274
19.6833324	5.0264001	-0.4540582
19.6833324	5.0752001	-0.2761164
19.6833324	5.1240001	-0.4356503
19.6833324	5.1728001	-0.4295144
19.6833324	5.2216001	-0.4908738
19.6833324	5.2704000	-0.4417863
19.6833324	5.3192000	-0.3067961
19.6833324	5.3680000	-0.3006601
19.6833324	5.4168000	-0.0981746
19.6833324	5.4656000	-0.1104465
19.6833324	5.5144000	-0.1104465
19.6833324	5.5632000	-0.2086215
19.6833324	5.6120000	613586.0000000
19.6833324	5.6608000	613586.0000000
19.6833324	5.7096000	613586.0000000
19.6833324	5.7584000	613586.0000000
19.6833324	5.8072000	613586.0000000
19.6833324	5.8559999	613586.0000000
19.6833324	5.9047999	613586.0000000
19.6833324	5.9535999	613586.0000000
19.6833324	6.0023999	613586.0000000
19.7000008	0.2928000	-1.6076121
19.7000008	0.3416000	-1.3867188
19.7000008	0.3904000	-1.9266801
19.7000008	0.4392000	-1.5769324
19.7000008	0.4880000	-1.6566992
19.7000008	0.5368000	-1.6566992
19.7000008	0.5856000	-1.3805828
19.7000008	0.6344000	-1.0737867
19.7000008	0.6832000	-1.0615149
19.7000008	0.7320000	-1.3376312
19.7000008	0.7808000	-1.2271848
19.7000008	0.8296000	-1.4173985

19.7000008	0.8784000	-1.4173985
19.7000008	0.9272000	-1.4173985
19.7000008	0.9760000	-1.3683109
19.7000008	1.0247999	-1.3069515
19.7000008	1.0735999	-1.3683109
19.7000008	1.1223999	-1.2087770
19.7000008	1.1711999	-1.3192234
19.7000008	1.2200000	-1.4419422
19.7000008	1.2688000	-1.3314953
19.7000008	1.3176000	-1.1596894
19.7000008	1.3664000	-1.0676508
19.7000008	1.4152000	-1.0553789
19.7000008	1.4640000	-1.1842332
19.7000008	1.5128000	-1.1596894
19.7000008	1.5616000	-1.1535535
19.7000008	1.6104000	-0.9387960
19.7000008	1.6592000	-0.9265246
19.7000008	1.7079999	-1.2885437
19.7000008	1.7567999	-1.3008156
19.7000008	1.8055999	-1.1719613
19.7000008	1.8543999	-1.1719613
19.7000008	1.9031999	-1.2640004
19.7000008	1.9519999	-1.2701364
19.7000008	2.0007999	-1.2333207
19.7000008	2.0495999	-1.1965051
19.7000008	2.0983999	-1.2333207
19.7000008	2.1471999	-1.1965051
19.7000008	2.1959999	-1.0983305
19.7000008	2.2447999	-1.3130875
19.7000008	2.2935998	-1.1596894
19.7000008	2.3423998	-1.0983305
19.7000008	2.3912001	-1.0799227
19.7000008	2.4400001	-1.0431070
19.7000008	2.4888000	-1.0431070
19.7000008	2.5376000	-0.8835731
19.7000008	2.5864000	-0.9326606
19.7000008	2.6352000	-1.0737867
19.7000008	2.6840000	-1.0676508
19.7000008	2.7328000	-0.9694757
19.7000008	2.7816000	-0.9572039
19.7000008	2.8304000	-1.0615149
19.7000008	2.8792000	-0.9449320
19.7000008	2.9280000	-0.9081168
19.7000008	2.9768000	-1.0492430
19.7000008	3.0256000	-1.0615149
19.7000008	3.0743999	-1.0676508
19.7000008	3.1231999	-0.9081168
19.7000008	3.1719999	-1.0062914
19.7000008	3.2207999	-0.8283496
19.7000008	3.2695999	-0.8467574
19.7000008	3.3183999	-0.8406215
19.7000008	3.3671999	-0.8406215
19.7000008	3.4159999	-0.6994953
19.7000008	3.4647999	-0.6994953
19.7000008	3.5135999	-0.6442719
19.7000008	3.5623999	-0.7117672
19.7000008	3.6111999	-0.7240391

19.7000008	3.6599998	-0.6749516
19.7000008	3.7087998	-0.6994953
19.7000008	3.7575998	-0.6933594
19.7000008	3.8063998	-0.6872234
19.7000008	3.8551998	-0.6810875
19.7000008	3.9039998	-0.6074562
19.7000008	3.9527998	-0.6626797
19.7000008	4.0016003	-0.5951843
19.7000008	4.0503998	-0.7485828
19.7000008	4.0991998	-0.8038058
19.7000008	4.1479998	-0.6810875
19.7000008	4.1967998	-0.8038058
19.7000008	4.2455997	-0.4724660
19.7000008	4.2943997	-0.4847379
19.7000008	4.3431997	-0.6135921
19.7000008	4.3919997	-0.5399613
19.7000008	4.4407997	-0.5399613
19.7000008	4.4895997	-0.8651652
19.7000008	4.5383997	-0.5399613
19.7000008	4.5871997	-0.5092816
19.7000008	4.6359997	-0.4233789
19.7000008	4.6847997	-0.6504078
19.7000008	4.7336001	-0.6074562
19.7000008	4.7824001	-0.4417863
19.7000008	4.8312001	-0.5460973
19.7000008	4.8800001	-0.5522332
19.7000008	4.9288001	-0.6504078
19.7000008	4.9776001	-0.6626797
19.7000008	5.0264001	-0.8038058
19.7000008	5.0752001	-0.5338254
19.7000008	5.1240001	-0.8651652
19.7000008	5.1728001	-0.6258640
19.7000008	5.2216001	-0.5583692
19.7000008	5.2704000	-0.4295144
19.7000008	5.3192000	-0.3804274
19.7000008	5.3680000	-0.3006601
19.7000008	5.4168000	-0.3006601
19.7000008	5.4656000	613586.0000000
19.7000008	5.5144000	613586.0000000
19.7000008	5.5632000	613586.0000000
19.7000008	5.6120000	613586.0000000
19.7000008	5.6608000	613586.0000000
19.7000008	5.7096000	613586.0000000
19.7000008	5.7584000	613586.0000000
19.7000008	5.8072000	613586.0000000
19.7000008	5.8559999	613586.0000000
19.7000008	5.9047999	613586.0000000
19.7000008	5.9535999	613586.0000000
19.7000008	6.0023999	613586.0000000
19.7166672	0.2928000	-2.3623304
19.7166672	0.3416000	-1.8898644
19.7166672	0.3904000	-1.8960004
19.7166672	0.4392000	-1.8898644
19.7166672	0.4880000	-1.6321554
19.7166672	0.5368000	-1.6076121
19.7166672	0.5856000	-1.3928547
19.7166672	0.6344000	-1.5953403

19.7166672	0.6832000	-1.1106019
19.7166672	0.7320000	-1.3867188
19.7166672	0.7808000	-1.3867188
19.7166672	0.8296000	-1.3805828
19.7166672	0.8784000	-1.4480777
19.7166672	0.9272000	-1.3437672
19.7166672	0.9760000	-1.4910293
19.7166672	1.0247999	-1.4787574
19.7166672	1.0735999	-1.4787574
19.7166672	1.1223999	-1.2762718
19.7166672	1.1711999	-1.1842332
19.7166672	1.2200000	-1.1842332
19.7166672	1.2688000	-1.2026410
19.7166672	1.3176000	-1.1780972
19.7166672	1.3664000	-1.1596894
19.7166672	1.4152000	-1.1596894
19.7166672	1.4640000	-1.2087770
19.7166672	1.5128000	-1.2210488
19.7166672	1.5616000	-1.1780972
19.7166672	1.6104000	-1.1780972
19.7166672	1.6592000	-1.1842332
19.7166672	1.7079999	-1.2946796
19.7166672	1.7567999	-1.2824078
19.7166672	1.8055999	-1.2701364
19.7166672	1.8543999	-1.2578645
19.7166672	1.9031999	-1.2946796
19.7166672	1.9519999	-1.2824078
19.7166672	2.0007999	-1.2762718
19.7166672	2.0495999	-1.2946796
19.7166672	2.0983999	-1.2762718
19.7166672	2.1471999	-1.1351457
19.7166672	2.1959999	-1.1412816
19.7166672	2.2447999	-1.1412816
19.7166672	2.2935998	-1.0185633
19.7166672	2.3423998	-0.9940195
19.7166672	2.3912001	-0.9817476
19.7166672	2.4400001	-1.1780972
19.7166672	2.4888000	-1.0185633
19.7166672	2.5376000	-0.8160777
19.7166672	2.5864000	-1.0001554
19.7166672	2.6352000	-0.9265246
19.7166672	2.6840000	-0.9265246
19.7166672	2.7328000	-0.9081168
19.7166672	2.7816000	-0.9265246
19.7166672	2.8304000	-0.9387960
19.7166672	2.8792000	-0.8958449
19.7166672	2.9280000	-0.9142528
19.7166672	2.9768000	-0.9081168
19.7166672	3.0256000	-0.9081168
19.7166672	3.0743999	-0.6504078
19.7166672	3.1231999	-0.6688156
19.7166672	3.1719999	-0.6688156
19.7166672	3.2207999	-0.7547188
19.7166672	3.2695999	-0.8713012
19.7166672	3.3183999	-0.8406215
19.7166672	3.3671999	-0.8283496
19.7166672	3.4159999	-0.7792621

19.7166672	3.4647999	-0.7179031
19.7166672	3.5135999	-0.8774371
19.7166672	3.5623999	-0.7485828
19.7166672	3.6111999	-0.9142528
19.7166672	3.6599998	-0.9142528
19.7166672	3.7087998	-0.8099418
19.7166672	3.7575998	-0.8038058
19.7166672	3.8063998	-0.8283496
19.7166672	3.8551998	-0.8283496
19.7166672	3.9039998	-0.8406215
19.7166672	3.9527998	-0.8528934
19.7166672	4.0016003	-0.8651652
19.7166672	4.0503998	-0.7363110
19.7166672	4.0991998	-0.7301750
19.7166672	4.1479998	-0.5951843
19.7166672	4.1967998	-0.5951843
19.7166672	4.2455997	-0.7240391
19.7166672	4.2943997	-0.7179031
19.7166672	4.3431997	-0.6688156
19.7166672	4.3919997	-0.6688156
19.7166672	4.4407997	-0.8099418
19.7166672	4.4895997	-0.8099418
19.7166672	4.5383997	-0.6688156
19.7166672	4.5871997	-0.6565437
19.7166672	4.6359997	-0.7240391
19.7166672	4.6847997	-0.5951843
19.7166672	4.7336001	-0.5890489
19.7166672	4.7824001	-0.8958449
19.7166672	4.8312001	-0.8344855
19.7166672	4.8800001	-0.5338254
19.7166672	4.9288001	-0.7547188
19.7166672	4.9776001	-0.4479222
19.7166672	5.0264001	-0.8406215
19.7166672	5.0752001	-0.9142528
19.7166672	5.1240001	-0.6688156
19.7166672	5.1728001	-0.5767770
19.7166672	5.2216001	-0.5645051
19.7166672	5.2704000	-0.7792621
19.7166672	5.3192000	-0.7792621
19.7166672	5.3680000	-0.7731261
19.7166672	5.4168000	-0.7731261
19.7166672	5.4656000	613586.0000000
19.7166672	5.5144000	613586.0000000
19.7166672	5.5632000	613586.0000000
19.7166672	5.6120000	613586.0000000
19.7166672	5.6608000	613586.0000000
19.7166672	5.7096000	613586.0000000
19.7166672	5.7584000	613586.0000000
19.7166672	5.8072000	613586.0000000
19.7166672	5.8559999	613586.0000000
19.7166672	5.9047999	613586.0000000
19.7166672	5.9535999	613586.0000000
19.7166672	6.0023999	613586.0000000
19.7333336	0.2928000	-1.9880390
19.7333336	0.3416000	-1.6137476
19.7333336	0.3904000	-1.5953403
19.7333336	0.4392000	-1.7978253

19.7333336	0.4880000	-1.8469129
19.7333336	0.5368000	-1.6137476
19.7333336	0.5856000	-1.5033011
19.7333336	0.6344000	-1.4480777
19.7333336	0.6832000	-1.5339808
19.7333336	0.7320000	-1.7180586
19.7333336	0.7808000	-1.3560390
19.7333336	0.8296000	-1.3560390
19.7333336	0.8784000	-1.3683109
19.7333336	0.9272000	-1.4787574
19.7333336	0.9760000	-1.5094371
19.7333336	1.0247999	-1.4358063
19.7333336	1.0735999	-1.4480777
19.7333336	1.1223999	-1.4480777
19.7333336	1.1711999	-1.4480777
19.7333336	1.2200000	-1.5155730
19.7333336	1.2688000	-1.6996508
19.7333336	1.3176000	-1.4664855
19.7333336	1.3664000	-1.2271848
19.7333336	1.4152000	-1.2271848
19.7333336	1.4640000	-1.1535535
19.7333336	1.5128000	-1.0369711
19.7333336	1.5616000	-1.0676508
19.7333336	1.6104000	-1.0553789
19.7333336	1.6592000	-1.0431070
19.7333336	1.7079999	-1.1535535
19.7333336	1.7567999	-1.1535535
19.7333336	1.8055999	-1.2946796
19.7333336	1.8543999	-1.1412816
19.7333336	1.9031999	-1.0737867
19.7333336	1.9519999	-1.0615149
19.7333336	2.0007999	-1.0001554
19.7333336	2.0495999	-0.9940195
19.7333336	2.0983999	-1.0062914
19.7333336	2.1471999	-1.0246992
19.7333336	2.1959999	-1.1167378
19.7333336	2.2447999	-0.9265246
19.7333336	2.2935998	-0.9878836
19.7333336	2.3423998	-1.0615149
19.7333336	2.3912001	-1.0553789
19.7333336	2.4400001	-1.1167378
19.7333336	2.4888000	-1.1167378
19.7333336	2.5376000	-1.0062914
19.7333336	2.5864000	-0.9940195
19.7333336	2.6352000	-0.9940195
19.7333336	2.6840000	-0.9940195
19.7333336	2.7328000	-1.0062914
19.7333336	2.7816000	-1.0001554
19.7333336	2.8304000	-1.0062914
19.7333336	2.8792000	-1.2149129
19.7333336	2.9280000	-1.2149129
19.7333336	2.9768000	-1.1044660
19.7333336	3.0256000	-0.9878836
19.7333336	3.0743999	-1.0676508
19.7333336	3.1231999	-0.9510679
19.7333336	3.1719999	-1.0001554
19.7333336	3.2207999	-1.2885437

19.7333336	3.2695999	-1.0615149
19.7333336	3.3183999	-1.0615149
19.7333336	3.3671999	-0.9203887
19.7333336	3.4159999	-0.9203887
19.7333336	3.4647999	-0.8038058
19.7333336	3.5135999	-0.7915339
19.7333336	3.5623999	-0.7853980
19.7333336	3.6111999	-0.7915339
19.7333336	3.6599998	-0.8099418
19.7333336	3.7087998	-0.8344855
19.7333336	3.7575998	-0.8467574
19.7333336	3.8063998	-0.8528934
19.7333336	3.8551998	-0.8528934
19.7333336	3.9039998	-0.7792621
19.7333336	3.9527998	-0.7608547
19.7333336	4.0016003	-0.7363110
19.7333336	4.0503998	-0.8651652
19.7333336	4.0991998	-0.5890489
19.7333336	4.1479998	-0.5154176
19.7333336	4.1967998	-0.4970098
19.7333336	4.2455997	-0.6258640
19.7333336	4.2943997	-0.6258640
19.7333336	4.3431997	-0.6933594
19.7333336	4.3919997	-0.6994953
19.7333336	4.4407997	-0.6994953
19.7333336	4.4895997	-0.7056313
19.7333336	4.5383997	-0.7117672
19.7333336	4.5871997	-0.5890489
19.7333336	4.6359997	-0.5890489
19.7333336	4.6847997	-0.5890489
19.7333336	4.7336001	-0.5399613
19.7333336	4.7824001	-0.5399613
19.7333336	4.8312001	-0.5951843
19.7333336	4.8800001	-0.4356503
19.7333336	4.9288001	-0.5706410
19.7333336	4.9776001	-0.4847379
19.7333336	5.0264001	-0.4970098
19.7333336	5.0752001	-0.4295144
19.7333336	5.1240001	-0.4111071
19.7333336	5.1728001	-0.4111071
19.7333336	5.2216001	-0.4111071
19.7333336	5.2704000	-0.4233789
19.7333336	5.3192000	-0.4417863
19.7333336	5.3680000	-0.3681555
19.7333336	5.4168000	-0.3681555
19.7333336	5.4656000	-0.3620195
19.7333336	5.5144000	613586.0000000
19.7333336	5.5632000	613586.0000000
19.7333336	5.6120000	613586.0000000
19.7333336	5.6608000	613586.0000000
19.7333336	5.7096000	613586.0000000
19.7333336	5.7584000	613586.0000000
19.7333336	5.8072000	613586.0000000
19.7333336	5.8559999	613586.0000000
19.7333336	5.9047999	613586.0000000
19.7333336	5.9535999	613586.0000000
19.7333336	6.0023999	613586.0000000

19.7500000	0.2928000	-2.6323111
19.7500000	0.3416000	-1.9205441
19.7500000	0.3904000	-1.1412816
19.7500000	0.4392000	-1.5707965
19.7500000	0.4880000	-1.4235344
19.7500000	0.5368000	-1.4112625
19.7500000	0.5856000	-1.1290097
19.7500000	0.6344000	-1.2210488
19.7500000	0.6832000	-1.2333207
19.7500000	0.7320000	-1.2578645
19.7500000	0.7808000	-1.3130875
19.7500000	0.8296000	-1.1780972
19.7500000	0.8784000	-1.3499031
19.7500000	0.9272000	-1.1842332
19.7500000	0.9760000	-1.1965051
19.7500000	1.0247999	-1.2026410
19.7500000	1.0735999	-1.3008156
19.7500000	1.1223999	-1.2885437
19.7500000	1.1711999	-1.3253593
19.7500000	1.2200000	-1.3253593
19.7500000	1.2688000	-1.3130875
19.7500000	1.3176000	-1.3008156
19.7500000	1.3664000	-1.1596894
19.7500000	1.4152000	-1.3867188
19.7500000	1.4640000	-1.1474175
19.7500000	1.5128000	-0.9572039
19.7500000	1.5616000	-0.8897090
19.7500000	1.6104000	-1.1044660
19.7500000	1.6592000	-1.1106019
19.7500000	1.7079999	-1.3560390
19.7500000	1.7567999	-1.3437672
19.7500000	1.8055999	-1.3008156
19.7500000	1.8543999	-1.1474175
19.7500000	1.9031999	-1.1412816
19.7500000	1.9519999	-1.0615149
19.7500000	2.0007999	-1.0431070
19.7500000	2.0495999	-1.0308352
19.7500000	2.0983999	-1.0860586
19.7500000	2.1471999	-1.1290097
19.7500000	2.1959999	-1.0615149
19.7500000	2.2447999	-0.9817476
19.7500000	2.2935998	-0.9878836
19.7500000	2.3423998	-1.2333207
19.7500000	2.3912001	-1.0921946
19.7500000	2.4400001	-1.0860586
19.7500000	2.4888000	-0.9142528
19.7500000	2.5376000	-0.9142528
19.7500000	2.5864000	-0.9510679
19.7500000	2.6352000	-0.9572039
19.7500000	2.6840000	-0.9633398
19.7500000	2.7328000	-1.0921946
19.7500000	2.7816000	-1.0921946
19.7500000	2.8304000	-1.1903691
19.7500000	2.8792000	-1.1719613
19.7500000	2.9280000	-0.9633398
19.7500000	2.9768000	-0.9572039
19.7500000	3.0256000	-0.9510679

19.7500000	3.0743999	-1.1412816
19.7500000	3.1231999	-1.0124273
19.7500000	3.1719999	-1.0124273
19.7500000	3.2207999	-1.0799227
19.7500000	3.2695999	-0.9510679
19.7500000	3.3183999	-0.9449320
19.7500000	3.3671999	-1.0185633
19.7500000	3.4159999	-1.0124273
19.7500000	3.4647999	-0.9510679
19.7500000	3.5135999	-0.8958449
19.7500000	3.5623999	-0.8958449
19.7500000	3.6111999	-0.8897090
19.7500000	3.6599998	-0.8897090
19.7500000	3.7087998	-0.9940195
19.7500000	3.7575998	-0.9326606
19.7500000	3.8063998	-0.9387960
19.7500000	3.8551998	-0.9387960
19.7500000	3.9039998	-0.7301750
19.7500000	3.9527998	-0.7179031
19.7500000	4.0016003	-0.7056313
19.7500000	4.0503998	-1.0001554
19.7500000	4.0991998	-0.7976699
19.7500000	4.1479998	-0.7976699
19.7500000	4.1967998	-0.7915339
19.7500000	4.2455997	-0.7363110
19.7500000	4.2943997	-0.7485828
19.7500000	4.3431997	-0.7485828
19.7500000	4.3919997	-0.6626797
19.7500000	4.4407997	-0.6626797
19.7500000	4.4895997	-0.5338254
19.7500000	4.5383997	-0.5338254
19.7500000	4.5871997	-0.5338254
19.7500000	4.6359997	-0.5460973
19.7500000	4.6847997	-0.6688156
19.7500000	4.7336001	-0.6626797
19.7500000	4.7824001	-0.5890489
19.7500000	4.8312001	-0.5399613
19.7500000	4.8800001	-0.6197281
19.7500000	4.9288001	-0.6381359
19.7500000	4.9776001	-0.5338254
19.7500000	5.0264001	-0.4908738
19.7500000	5.0752001	-0.4417863
19.7500000	5.1240001	-0.4663301
19.7500000	5.1728001	-0.4847379
19.7500000	5.2216001	-0.4847379
19.7500000	5.2704000	-0.3497477
19.7500000	5.3192000	-0.3681555
19.7500000	5.3680000	-0.3865633
19.7500000	5.4168000	-0.4908738
19.7500000	5.4656000	613586.0000000
19.7500000	5.5144000	613586.0000000
19.7500000	5.5632000	613586.0000000
19.7500000	5.6120000	613586.0000000
19.7500000	5.6608000	-0.4724660
19.7500000	5.7096000	613586.0000000
19.7500000	5.7584000	613586.0000000
19.7500000	5.8072000	613586.0000000

19.7500000	5.8559999	613586.0000000
19.7500000	5.9047999	613586.0000000
19.7500000	5.9535999	613586.0000000
19.7500000	6.0023999	613586.0000000
19.7666664	0.2928000	-2.0616703
19.7666664	0.3416000	-1.7916894
19.7666664	0.3904000	-2.0125828
19.7666664	0.4392000	-1.6751070
19.7666664	0.4880000	-1.5707965
19.7666664	0.5368000	-1.4358063
19.7666664	0.5856000	-1.4419422
19.7666664	0.6344000	-1.2149129
19.7666664	0.6832000	-1.2149129
19.7666664	0.7320000	-1.3867188
19.7666664	0.7808000	-1.4480777
19.7666664	0.8296000	-1.6137476
19.7666664	0.8784000	-1.5094371
19.7666664	0.9272000	-1.5155730
19.7666664	0.9760000	-1.4296703
19.7666664	1.0247999	-1.5094371
19.7666664	1.0735999	-1.4173985
19.7666664	1.1223999	-1.4419422
19.7666664	1.1711999	-1.4358063
19.7666664	1.2200000	-1.4051266
19.7666664	1.2688000	-1.3314953
19.7666664	1.3176000	-0.9756117
19.7666664	1.3664000	-0.9694757
19.7666664	1.4152000	-0.8344855
19.7666664	1.4640000	-0.9203887
19.7666664	1.5128000	-1.3437672
19.7666664	1.5616000	-1.2701364
19.7666664	1.6104000	-1.4112625
19.7666664	1.6592000	-1.2578645
19.7666664	1.7079999	-1.2455926
19.7666664	1.7567999	-1.5401168
19.7666664	1.8055999	-1.5339808
19.7666664	1.8543999	-1.3683109
19.7666664	1.9031999	-1.2640004
19.7666664	1.9519999	-1.2455926
19.7666664	2.0007999	-1.2394567
19.7666664	2.0495999	-1.2333207
19.7666664	2.0983999	-1.2210488
19.7666664	2.1471999	-1.1842332
19.7666664	2.1959999	-1.1719613
19.7666664	2.2447999	-1.1658254
19.7666664	2.2935998	-1.2640004
19.7666664	2.3423998	-1.2824078
19.7666664	2.3912001	-1.1474175
19.7666664	2.4400001	-1.1719613
19.7666664	2.4888000	-1.1965051
19.7666664	2.5376000	-1.2455926
19.7666664	2.5864000	-1.1167378
19.7666664	2.6352000	-1.1044660
19.7666664	2.6840000	-1.1044660
19.7666664	2.7328000	-1.1167378
19.7666664	2.7816000	-1.1228738
19.7666664	2.8304000	-1.1290097

19.7666664	2.8792000	-1.1351457
19.7666664	2.9280000	-0.8958449
19.7666664	2.9768000	-0.8897090
19.7666664	3.0256000	-0.8897090
19.7666664	3.0743999	-0.8835731
19.7666664	3.1231999	-1.0676508
19.7666664	3.1719999	-1.0676508
19.7666664	3.2207999	-0.8835731
19.7666664	3.2695999	-0.8897090
19.7666664	3.3183999	-0.9633398
19.7666664	3.3671999	-0.9387960
19.7666664	3.4159999	-0.8528934
19.7666664	3.4647999	-0.8590293
19.7666664	3.5135999	-0.9387960
19.7666664	3.5623999	-0.8406215
19.7666664	3.6111999	-0.8099418
19.7666664	3.6599998	-0.9387960
19.7666664	3.7087998	-0.8713012
19.7666664	3.7575998	-0.8774371
19.7666664	3.8063998	-0.8651652
19.7666664	3.8551998	-0.8344855
19.7666664	3.9039998	-1.0001554
19.7666664	3.9527998	-0.9940195
19.7666664	4.0016003	-0.9817476
19.7666664	4.0503998	-0.8222136
19.7666664	4.0991998	-0.5767770
19.7666664	4.1479998	-1.0553789
19.7666664	4.1967998	-0.8774371
19.7666664	4.2455997	-0.9878836
19.7666664	4.2943997	-1.0737867
19.7666664	4.3431997	-1.0615149
19.7666664	4.3919997	-1.1474175
19.7666664	4.4407997	-0.6749516
19.7666664	4.4895997	-0.4111071
19.7666664	4.5383997	-0.4908738
19.7666664	4.5871997	-0.4847379
19.7666664	4.6359997	-0.4601941
19.7666664	4.6847997	-0.3558836
19.7666664	4.7336001	-0.2761164
19.7666664	4.7824001	-0.2945242
19.7666664	4.8312001	-0.2883883
19.7666664	4.8800001	-0.2761164
19.7666664	4.9288001	-0.2024856
19.7666664	4.9776001	-0.2086215
19.7666664	5.0264001	-0.2761164
19.7666664	5.0752001	-0.2761164
19.7666664	5.1240001	-0.3436117
19.7666664	5.1728001	-0.3374758
19.7666664	5.2216001	-0.3742914
19.7666664	5.2704000	-0.3374758
19.7666664	5.3192000	-0.3313398
19.7666664	5.3680000	-0.3313398
19.7666664	5.4168000	-0.3436117
19.7666664	5.4656000	613586.0000000
19.7666664	5.5144000	-0.4356503
19.7666664	5.5632000	-0.4479222
19.7666664	5.6120000	613586.0000000

19.7666664	5.6608000	-0.3742914
19.7666664	5.7096000	-0.3742914
19.7666664	5.7584000	613586.0000000
19.7666664	5.8072000	613586.0000000
19.7666664	5.8559999	-0.4356503
19.7666664	5.9047999	613586.0000000
19.7666664	5.9535999	613586.0000000
19.7666664	6.0023999	613586.0000000
19.7833328	0.2928000	-2.4727771
19.7833328	0.3416000	-2.1966605
19.7833328	0.3904000	-2.2089324
19.7833328	0.4392000	-1.8653207
19.7833328	0.4880000	-1.8591847
19.7833328	0.5368000	-1.7303305
19.7833328	0.5856000	-1.2946796
19.7833328	0.6344000	-1.1535535
19.7833328	0.6832000	-1.1596894
19.7833328	0.7320000	-1.2394567
19.7833328	0.7808000	-1.3560390
19.7833328	0.8296000	-1.5830684
19.7833328	0.8784000	-1.4910293
19.7833328	0.9272000	-1.2762718
19.7833328	0.9760000	-1.2640004
19.7833328	1.0247999	-1.2640004
19.7833328	1.0735999	-1.3253593
19.7833328	1.1223999	-1.3314953
19.7833328	1.1711999	-1.2946796
19.7833328	1.2200000	-1.3008156
19.7833328	1.2688000	-1.3130875
19.7833328	1.3176000	-1.3069515
19.7833328	1.3664000	-1.0124273
19.7833328	1.4152000	-1.2701364
19.7833328	1.4640000	-1.2885437
19.7833328	1.5128000	-1.0737867
19.7833328	1.5616000	-1.3621750
19.7833328	1.6104000	-1.4296703
19.7833328	1.6592000	-1.4787574
19.7833328	1.7079999	-1.4419422
19.7833328	1.7567999	-1.4419422
19.7833328	1.8055999	-1.3314953
19.7833328	1.8543999	-1.3253593
19.7833328	1.9031999	-1.3253593
19.7833328	1.9519999	-1.3867188
19.7833328	2.0007999	-1.1228738
19.7833328	2.0495999	-1.4419422
19.7833328	2.0983999	-1.3376312
19.7833328	2.1471999	-1.3805828
19.7833328	2.1959999	-1.3253593
19.7833328	2.2447999	-1.3253593
19.7833328	2.2935998	-1.3253593
19.7833328	2.3423998	-1.3192234
19.7833328	2.3912001	-1.3867188
19.7833328	2.4400001	-1.3928547
19.7833328	2.4888000	-1.2946796
19.7833328	2.5376000	-1.3069515
19.7833328	2.5864000	-1.3069515
19.7833328	2.6352000	-1.3069515

19.7833328	2.6840000	-1.2271848
19.7833328	2.7328000	-1.2455926
19.7833328	2.7816000	-1.2087770
19.7833328	2.8304000	-1.1351457
19.7833328	2.8792000	-1.2271848
19.7833328	2.9280000	-1.3192234
19.7833328	2.9768000	-1.1965051
19.7833328	3.0256000	-0.9326606
19.7833328	3.0743999	-0.9510679
19.7833328	3.1231999	-0.9387960
19.7833328	3.1719999	-0.7301750
19.7833328	3.2207999	-0.7915339
19.7833328	3.2695999	-0.7853980
19.7833328	3.3183999	-0.8467574
19.7833328	3.3671999	-0.9878836
19.7833328	3.4159999	-0.9878836
19.7833328	3.4647999	-0.9081168
19.7833328	3.5135999	-0.9756117
19.7833328	3.5623999	-0.9756117
19.7833328	3.6111999	-0.9633398
19.7833328	3.6599998	-0.9817476
19.7833328	3.7087998	-0.9694757
19.7833328	3.7575998	-0.8160777
19.7833328	3.8063998	-0.7792621
19.7833328	3.8551998	-0.8897090
19.7833328	3.9039998	-0.8958449
19.7833328	3.9527998	-0.6872234
19.7833328	4.0016003	-0.6135921
19.7833328	4.0503998	-0.6135921
19.7833328	4.0991998	-0.8038058
19.7833328	4.1479998	-1.0001554
19.7833328	4.1967998	-1.0615149
19.7833328	4.2455997	-0.8099418
19.7833328	4.2943997	-0.7363110
19.7833328	4.3431997	-0.7301750
19.7833328	4.3919997	-0.6749516
19.7833328	4.4407997	-0.6749516
19.7833328	4.4895997	-0.5399613
19.7833328	4.5383997	-0.5399613
19.7833328	4.5871997	-0.5460973
19.7833328	4.6359997	-0.4786019
19.7833328	4.6847997	-0.4724660
19.7833328	4.7336001	-0.4172430
19.7833328	4.7824001	-0.4111071
19.7833328	4.8312001	-0.1656699
19.7833328	4.8800001	-0.8467574
19.7833328	4.9288001	-0.8467574
19.7833328	4.9776001	-0.6135921
19.7833328	5.0264001	-0.4356503
19.7833328	5.0752001	-0.4847379
19.7833328	5.1240001	-0.4356503
19.7833328	5.1728001	-0.3374758
19.7833328	5.2216001	-0.3436117
19.7833328	5.2704000	-0.4049711
19.7833328	5.3192000	613586.0000000
19.7833328	5.3680000	-0.5215535
19.7833328	5.4168000	-0.5215535

19.7833328	5.4656000	-0.4049711
19.7833328	5.5144000	-0.4049711
19.7833328	5.5632000	-0.4663301
19.7833328	5.6120000	-0.4601941
19.7833328	5.6608000	-0.5092816
19.7833328	5.7096000	-0.3804274
19.7833328	5.7584000	-0.3804274
19.7833328	5.8072000	-0.3926992
19.7833328	5.8559999	-0.3865633
19.7833328	5.9047999	613586.0000000
19.7833328	5.9535999	613586.0000000
19.7833328	6.0023999	613586.0000000
19.7999992	0.2928000	-2.1537089
19.7999992	0.3416000	-2.1537089
19.7999992	0.3904000	-2.1659808
19.7999992	0.4392000	-1.6137476
19.7999992	0.4880000	-1.4603496
19.7999992	0.5368000	-1.3744469
19.7999992	0.5856000	-1.2885437
19.7999992	0.6344000	-1.2333207
19.7999992	0.6832000	-1.2394567
19.7999992	0.7320000	-1.2455926
19.7999992	0.7808000	-1.3314953
19.7999992	0.8296000	-1.2210488
19.7999992	0.8784000	-1.1780972
19.7999992	0.9272000	-1.2210488
19.7999992	0.9760000	-1.3867188
19.7999992	1.0247999	-1.4296703
19.7999992	1.0735999	-1.4358063
19.7999992	1.1223999	-1.2333207
19.7999992	1.1711999	-1.3621750
19.7999992	1.2200000	-1.4173985
19.7999992	1.2688000	-1.3192234
19.7999992	1.3176000	-1.2333207
19.7999992	1.3664000	-1.2394567
19.7999992	1.4152000	-1.4542136
19.7999992	1.4640000	-1.3130875
19.7999992	1.5128000	-1.3253593
19.7999992	1.5616000	-1.3437672
19.7999992	1.6104000	-1.5033011
19.7999992	1.6592000	-1.5033011
19.7999992	1.7079999	-1.2885437
19.7999992	1.7567999	-1.2701364
19.7999992	1.8055999	-1.2762718
19.7999992	1.8543999	-1.4664855
19.7999992	1.9031999	-1.2333207
19.7999992	1.9519999	-1.1412816
19.7999992	2.0007999	-1.1474175
19.7999992	2.0495999	-1.2640004
19.7999992	2.0983999	-1.2333207
19.7999992	2.1471999	-1.2087770
19.7999992	2.1959999	-1.2149129
19.7999992	2.2447999	-1.2271848
19.7999992	2.2935998	-1.1044660
19.7999992	2.3423998	-1.2578645
19.7999992	2.3912001	-1.3130875
19.7999992	2.4400001	-1.3069515

19.7999992	2.4888000	-1.1535535
19.7999992	2.5376000	-1.1474175
19.7999992	2.5864000	-1.1474175
19.7999992	2.6352000	-1.1535535
19.7999992	2.6840000	-1.0308352
19.7999992	2.7328000	-1.1658254
19.7999992	2.7816000	-1.1842332
19.7999992	2.8304000	-1.0492430
19.7999992	2.8792000	-1.1780972
19.7999992	2.9280000	-1.0737867
19.7999992	2.9768000	-1.1290097
19.7999992	3.0256000	-1.0062914
19.7999992	3.0743999	-0.9756117
19.7999992	3.1231999	-1.0737867
19.7999992	3.1719999	-1.0124273
19.7999992	3.2207999	-0.6565437
19.7999992	3.2695999	-0.7792621
19.7999992	3.3183999	-0.8774371
19.7999992	3.3671999	-0.8835731
19.7999992	3.4159999	-0.9142528
19.7999992	3.4647999	-0.7731261
19.7999992	3.5135999	-0.8344855
19.7999992	3.5623999	-0.8467574
19.7999992	3.6111999	-1.0001554
19.7999992	3.6599998	-0.8774371
19.7999992	3.7087998	-0.8222136
19.7999992	3.7575998	-0.7853980
19.7999992	3.8063998	-0.7853980
19.7999992	3.8551998	-0.8467574
19.7999992	3.9039998	-0.6442719
19.7999992	3.9527998	-0.6442719
19.7999992	4.0016003	-0.6442719
19.7999992	4.0503998	-0.8406215
19.7999992	4.0991998	-0.7731261
19.7999992	4.1479998	-0.7547188
19.7999992	4.1967998	-0.6258640
19.7999992	4.2455997	-0.6381359
19.7999992	4.2943997	-0.6135921
19.7999992	4.3431997	-0.6320000
19.7999992	4.3919997	-0.6320000
19.7999992	4.4407997	-0.6749516
19.7999992	4.4895997	-0.7547188
19.7999992	4.5383997	-0.6442719
19.7999992	4.5871997	-0.4417863
19.7999992	4.6359997	-0.4540582
19.7999992	4.6847997	-0.4111071
19.7999992	4.7336001	-0.4111071
19.7999992	4.7824001	-0.4049711
19.7999992	4.8312001	-0.4049711
19.7999992	4.8800001	-0.3497477
19.7999992	4.9288001	-0.3313398
19.7999992	4.9776001	-0.3252039
19.7999992	5.0264001	-0.5399613
19.7999992	5.0752001	-0.3313398
19.7999992	5.1240001	-0.4049711
19.7999992	5.1728001	-0.4049711
19.7999992	5.2216001	-0.6933594

19.7999992	5.2704000	-0.5829129
19.7999992	5.3192000	-0.5829129
19.7999992	5.3680000	-0.4540582
19.7999992	5.4168000	-0.4540582
19.7999992	5.4656000	-0.4908738
19.7999992	5.5144000	-0.4908738
19.7999992	5.5632000	-0.3252039
19.7999992	5.6120000	-0.6074562
19.7999992	5.6608000	-0.3374758
19.7999992	5.7096000	-0.2822523
19.7999992	5.7584000	613586.0000000
19.7999992	5.8072000	613586.0000000
19.7999992	5.8559999	613586.0000000
19.7999992	5.9047999	613586.0000000
19.7999992	5.9535999	613586.0000000
19.7999992	6.0023999	613586.0000000
19.8166676	0.2928000	-2.2886994
19.8166676	0.3416000	-1.7916894
19.8166676	0.3904000	-1.7855535
19.8166676	0.4392000	-1.4480777
19.8166676	0.4880000	-1.4542136
19.8166676	0.5368000	-1.4358063
19.8166676	0.5856000	-1.6628351
19.8166676	0.6344000	-1.3130875
19.8166676	0.6832000	-1.3376312
19.8166676	0.7320000	-1.3314953
19.8166676	0.7808000	-1.1965051
19.8166676	0.8296000	-1.0860586
19.8166676	0.8784000	-1.0860586
19.8166676	0.9272000	-1.1842332
19.8166676	0.9760000	-1.2026410
19.8166676	1.0247999	-1.1903691
19.8166676	1.0735999	-1.1903691
19.8166676	1.1223999	-1.2701364
19.8166676	1.1711999	-1.3989906
19.8166676	1.2200000	-1.4051266
19.8166676	1.2688000	-1.1903691
19.8166676	1.3176000	-1.3008156
19.8166676	1.3664000	-1.2455926
19.8166676	1.4152000	-1.3130875
19.8166676	1.4640000	-0.9694757
19.8166676	1.5128000	-1.1842332
19.8166676	1.5616000	-1.1351457
19.8166676	1.6104000	-1.1351457
19.8166676	1.6592000	-1.2149129
19.8166676	1.7079999	-1.2149129
19.8166676	1.7567999	-1.2026410
19.8166676	1.8055999	-1.2578645
19.8166676	1.8543999	-1.2149129
19.8166676	1.9031999	-1.1780972
19.8166676	1.9519999	-1.2394567
19.8166676	2.0007999	-1.2946796
19.8166676	2.0495999	-1.2946796
19.8166676	2.0983999	-1.3253593
19.8166676	2.1471999	-1.1290097
19.8166676	2.1959999	-1.2885437
19.8166676	2.2447999	-1.2885437

19.8166676	2.2935998	-1.3499031
19.8166676	2.3423998	-1.3499031
19.8166676	2.3912001	-1.2394567
19.8166676	2.4400001	-1.2824078
19.8166676	2.4888000	-1.0492430
19.8166676	2.5376000	-1.0492430
19.8166676	2.5864000	-0.9633398
19.8166676	2.6352000	-1.0001554
19.8166676	2.6840000	-1.0062914
19.8166676	2.7328000	-1.1596894
19.8166676	2.7816000	-1.1596894
19.8166676	2.8304000	-1.1106019
19.8166676	2.8792000	-1.0983305
19.8166676	2.9280000	-1.0185633
19.8166676	2.9768000	-0.8283496
19.8166676	3.0256000	-0.9265246
19.8166676	3.0743999	-0.7608547
19.8166676	3.1231999	-0.8160777
19.8166676	3.1719999	-0.8160777
19.8166676	3.2207999	-0.6197281
19.8166676	3.2695999	-0.6749516
19.8166676	3.3183999	-0.6810875
19.8166676	3.3671999	-0.6810875
19.8166676	3.4159999	-0.7485828
19.8166676	3.4647999	-0.7424469
19.8166676	3.5135999	-0.7608547
19.8166676	3.5623999	-0.7669902
19.8166676	3.6111999	-0.8038058
19.8166676	3.6599998	-0.7056313
19.8166676	3.7087998	-0.7301750
19.8166676	3.7575998	-0.6074562
19.8166676	3.8063998	-0.6810875
19.8166676	3.8551998	-0.7363110
19.8166676	3.9039998	-0.6810875
19.8166676	3.9527998	-0.6810875
19.8166676	4.0016003	-0.5092816
19.8166676	4.0503998	-0.5092816
19.8166676	4.0991998	-0.6565437
19.8166676	4.1479998	-0.6626797
19.8166676	4.1967998	-0.5706410
19.8166676	4.2455997	-0.5338254
19.8166676	4.2943997	-0.5399613
19.8166676	4.3431997	-0.5031457
19.8166676	4.3919997	-0.5829129
19.8166676	4.4407997	-0.5399613
19.8166676	4.4895997	-0.6197281
19.8166676	4.5383997	-0.5645051
19.8166676	4.5871997	-0.4970098
19.8166676	4.6359997	-0.5031457
19.8166676	4.6847997	-0.4049711
19.8166676	4.7336001	-0.4049711
19.8166676	4.7824001	-0.5154176
19.8166676	4.8312001	-0.5154176
19.8166676	4.8800001	-0.3681555
19.8166676	4.9288001	-0.3681555
19.8166676	4.9776001	-0.3742914
19.8166676	5.0264001	-0.2699804

19.8166676	5.0752001	-0.2883883
19.8166676	5.1240001	-0.1472621
19.8166676	5.1728001	-0.4786019
19.8166676	5.2216001	-0.4908738
19.8166676	5.2704000	-0.5522332
19.8166676	5.3192000	-0.4970098
19.8166676	5.3680000	-0.5031457
19.8166676	5.4168000	-0.4479222
19.8166676	5.4656000	-0.3988352
19.8166676	5.5144000	-0.3252039
19.8166676	5.5632000	-0.3252039
19.8166676	5.6120000	-0.3620195
19.8166676	5.6608000	-0.2822523
19.8166676	5.7096000	-0.2393012
19.8166676	5.7584000	613586.0000000
19.8166676	5.8072000	0.0184078
19.8166676	5.8559999	613586.0000000
19.8166676	5.9047999	613586.0000000
19.8166676	5.9535999	613586.0000000
19.8166676	6.0023999	613586.0000000
19.8333340	0.2928000	-3.3318064
19.8333340	0.3416000	-2.2948353
19.8333340	0.3904000	-1.6566992
19.8333340	0.4392000	-1.6566992
19.8333340	0.4880000	-1.3744469
19.8333340	0.5368000	-1.1842332
19.8333340	0.5856000	-1.3008156
19.8333340	0.6344000	-1.2946796
19.8333340	0.6832000	-1.1535535
19.8333340	0.7320000	-1.1044660
19.8333340	0.7808000	-1.0860586
19.8333340	0.8296000	-1.1596894
19.8333340	0.8784000	-1.4480777
19.8333340	0.9272000	-1.4542136
19.8333340	0.9760000	-1.3008156
19.8333340	1.0247999	-1.1474175
19.8333340	1.0735999	-1.0492430
19.8333340	1.1223999	-1.0799227
19.8333340	1.1711999	-1.2333207
19.8333340	1.2200000	-1.1474175
19.8333340	1.2688000	-0.9756117
19.8333340	1.3176000	-0.9694757
19.8333340	1.3664000	-0.9817476
19.8333340	1.4152000	-0.9878836
19.8333340	1.4640000	-0.9817476
19.8333340	1.5128000	-1.0676508
19.8333340	1.5616000	-1.1535535
19.8333340	1.6104000	-1.2394567
19.8333340	1.6592000	-1.2394567
19.8333340	1.7079999	-1.2455926
19.8333340	1.7567999	-1.2578645
19.8333340	1.8055999	-1.0799227
19.8333340	1.8543999	-0.9940195
19.8333340	1.9031999	-1.1228738
19.8333340	1.9519999	-1.0799227
19.8333340	2.0007999	-1.1290097
19.8333340	2.0495999	-1.1290097

19.8333340	2.0983999	-1.1290097
19.8333340	2.1471999	-1.2394567
19.8333340	2.1959999	-1.2517285
19.8333340	2.2447999	-1.3069515
19.8333340	2.2935998	-1.3069515
19.8333340	2.3423998	-1.4235344
19.8333340	2.3912001	-1.4112625
19.8333340	2.4400001	-1.2885437
19.8333340	2.4888000	-1.1842332
19.8333340	2.5376000	-1.1842332
19.8333340	2.5864000	-1.1903691
19.8333340	2.6352000	-1.0921946
19.8333340	2.6840000	-1.0860586
19.8333340	2.7328000	-1.0615149
19.8333340	2.7816000	-1.0676508
19.8333340	2.8304000	-0.9387960
19.8333340	2.8792000	-0.9387960
19.8333340	2.9280000	-0.7424469
19.8333340	2.9768000	-0.9326606
19.8333340	3.0256000	-0.8897090
19.8333340	3.0743999	-0.8958449
19.8333340	3.1231999	-0.9694757
19.8333340	3.1719999	-0.9694757
19.8333340	3.2207999	-0.9694757
19.8333340	3.2695999	-1.0246992
19.8333340	3.3183999	-0.9756117
19.8333340	3.3671999	-0.8344855
19.8333340	3.4159999	-0.8344855
19.8333340	3.4647999	-0.8344855
19.8333340	3.5135999	-0.8160777
19.8333340	3.5623999	-0.9387960
19.8333340	3.6111999	-0.9387960
19.8333340	3.6599998	-0.9387960
19.8333340	3.7087998	-0.9387960
19.8333340	3.7575998	-0.8528934
19.8333340	3.8063998	-0.8344855
19.8333340	3.8551998	-0.8222136
19.8333340	3.9039998	-0.8651652
19.8333340	3.9527998	-0.7240391
19.8333340	4.0016003	-0.7853980
19.8333340	4.0503998	-0.7669902
19.8333340	4.0991998	-0.7424469
19.8333340	4.1479998	-0.7363110
19.8333340	4.1967998	-0.7792621
19.8333340	4.2455997	-0.6810875
19.8333340	4.2943997	-0.5951843
19.8333340	4.3431997	-0.6013203
19.8333340	4.3919997	-0.5338254
19.8333340	4.4407997	-0.6749516
19.8333340	4.4895997	-0.4663301
19.8333340	4.5383997	-0.4663301
19.8333340	4.5871997	-0.5031457
19.8333340	4.6359997	-0.5092816
19.8333340	4.6847997	-0.5031457
19.8333340	4.7336001	-0.5215535
19.8333340	4.7824001	-0.4724660
19.8333340	4.8312001	-0.4663301

19.8333340	4.8800001	-0.3313398
19.8333340	4.9288001	-0.2945242
19.8333340	4.9776001	-0.3313398
19.8333340	5.0264001	-0.1718059
19.8333340	5.0752001	-0.2393012
19.8333340	5.1240001	-0.1718059
19.8333340	5.1728001	-0.1411262
19.8333340	5.2216001	-0.4540582
19.8333340	5.2704000	-0.4417863
19.8333340	5.3192000	-0.5031457
19.8333340	5.3680000	-0.5031457
19.8333340	5.4168000	-0.5154176
19.8333340	5.4656000	-0.5215535
19.8333340	5.5144000	-0.5399613
19.8333340	5.5632000	-0.4417863
19.8333340	5.6120000	-0.3006601
19.8333340	5.6608000	-0.2945242
19.8333340	5.7096000	-0.2945242
19.8333340	5.7584000	613586.0000000
19.8333340	5.8072000	613586.0000000
19.8333340	5.8559999	613586.0000000
19.8333340	5.9047999	613586.0000000
19.8333340	5.9535999	613586.0000000
19.8333340	6.0023999	613586.0000000
19.8500004	0.2928000	-2.7857091
19.8500004	0.3416000	-2.1168938
19.8500004	0.3904000	-1.5033011
19.8500004	0.4392000	-1.4358063
19.8500004	0.4880000	-1.4358063
19.8500004	0.5368000	-1.2701364
19.8500004	0.5856000	-1.2701364
19.8500004	0.6344000	-1.2640004
19.8500004	0.6832000	-1.1903691
19.8500004	0.7320000	-1.2087770
19.8500004	0.7808000	-1.2271848
19.8500004	0.8296000	-1.0553789
19.8500004	0.8784000	-1.0553789
19.8500004	0.9272000	-1.3069515
19.8500004	0.9760000	-1.4358063
19.8500004	1.0247999	-1.2333207
19.8500004	1.0735999	-1.2333207
19.8500004	1.1223999	-1.1965051
19.8500004	1.1711999	-1.1965051
19.8500004	1.2200000	-1.0921946
19.8500004	1.2688000	-1.1596894
19.8500004	1.3176000	-1.2578645
19.8500004	1.3664000	-1.2578645
19.8500004	1.4152000	-1.2578645
19.8500004	1.4640000	-1.2394567
19.8500004	1.5128000	-1.2333207
19.8500004	1.5616000	-1.0615149
19.8500004	1.6104000	-1.2210488
19.8500004	1.6592000	-1.2271848
19.8500004	1.7079999	-1.2578645
19.8500004	1.7567999	-1.2578645
19.8500004	1.8055999	-1.2578645
19.8500004	1.8543999	-1.2946796

19.8500004	1.9031999	-1.2578645
19.8500004	1.9519999	-1.2578645
19.8500004	2.0007999	-1.1965051
19.8500004	2.0495999	-1.3069515
19.8500004	2.0983999	-1.2578645
19.8500004	2.1471999	-1.2394567
19.8500004	2.1959999	-1.2394567
19.8500004	2.2447999	-1.2394567
19.8500004	2.2935998	-1.1842332
19.8500004	2.3423998	-1.2026410
19.8500004	2.3912001	-1.2333207
19.8500004	2.4400001	-1.2394567
19.8500004	2.4888000	-1.1658254
19.8500004	2.5376000	-1.1412816
19.8500004	2.5864000	-1.2087770
19.8500004	2.6352000	-1.2333207
19.8500004	2.6840000	-1.2640004
19.8500004	2.7328000	-1.2578645
19.8500004	2.7816000	-1.1658254
19.8500004	2.8304000	-1.1658254
19.8500004	2.8792000	-1.1780972
19.8500004	2.9280000	-1.1780972
19.8500004	2.9768000	-1.1842332
19.8500004	3.0256000	-1.1228738
19.8500004	3.0743999	-1.0062914
19.8500004	3.1231999	-0.9633398
19.8500004	3.1719999	-0.8958449
19.8500004	3.2207999	-0.8897090
19.8500004	3.2695999	-0.6872234
19.8500004	3.3183999	-0.4847379
19.8500004	3.3671999	-1.1412816
19.8500004	3.4159999	-0.8897090
19.8500004	3.4647999	-0.8897090
19.8500004	3.5135999	-0.9817476
19.8500004	3.5623999	-0.9817476
19.8500004	3.6111999	-1.0246992
19.8500004	3.6599998	-1.0185633
19.8500004	3.7087998	-1.0615149
19.8500004	3.7575998	-1.1658254
19.8500004	3.8063998	-1.1658254
19.8500004	3.8551998	-1.1535535
19.8500004	3.9039998	-1.1351457
19.8500004	3.9527998	-0.8713012
19.8500004	4.0016003	-0.7915339
19.8500004	4.0503998	-0.8590293
19.8500004	4.0991998	-0.8590293
19.8500004	4.1479998	-1.0308352
19.8500004	4.1967998	-1.0246992
19.8500004	4.2455997	-1.1106019
19.8500004	4.2943997	-1.1044660
19.8500004	4.3431997	-0.8897090
19.8500004	4.3919997	-0.8897090
19.8500004	4.4407997	-0.8283496
19.8500004	4.4895997	-0.7363110
19.8500004	4.5383997	-0.6013203
19.8500004	4.5871997	-0.6504078
19.8500004	4.6359997	-0.6504078

19.8500004	4.6847997	-0.6749516
19.8500004	4.7336001	-0.5706410
19.8500004	4.7824001	-0.5645051
19.8500004	4.8312001	-0.5154176
19.8500004	4.8800001	-0.4540582
19.8500004	4.9288001	-0.4724660
19.8500004	4.9776001	-0.3988352
19.8500004	5.0264001	-0.3804274
19.8500004	5.0752001	-0.3558836
19.8500004	5.1240001	-0.6933594
19.8500004	5.1728001	-0.6320000
19.8500004	5.2216001	-0.6320000
19.8500004	5.2704000	-0.6197281
19.8500004	5.3192000	-0.6013203
19.8500004	5.3680000	-0.6013203
19.8500004	5.4168000	-0.6810875
19.8500004	5.4656000	-0.6688156
19.8500004	5.5144000	-0.6626797
19.8500004	5.5632000	-0.6197281
19.8500004	5.6120000	-0.4417863
19.8500004	5.6608000	-0.4295144
19.8500004	5.7096000	-0.3681555
19.8500004	5.7584000	-0.3681555
19.8500004	5.8072000	-0.3804274
19.8500004	5.8559999	613586.0000000
19.8500004	5.9047999	613586.0000000
19.8500004	5.9535999	613586.0000000
19.8500004	6.0023999	613586.0000000
19.8666668	0.2928000	-2.9759228
19.8666668	0.3416000	-2.0432625
19.8666668	0.3904000	-2.0432625
19.8666668	0.4392000	-1.6014762
19.8666668	0.4880000	-1.2885437
19.8666668	0.5368000	-1.5646605
19.8666668	0.5856000	-1.2824078
19.8666668	0.6344000	-1.2701364
19.8666668	0.6832000	-1.2210488
19.8666668	0.7320000	-1.2762718
19.8666668	0.7808000	-1.4235344
19.8666668	0.8296000	-1.4358063
19.8666668	0.8784000	-1.4419422
19.8666668	0.9272000	-1.4358063
19.8666668	0.9760000	-1.1658254
19.8666668	1.0247999	-1.2210488
19.8666668	1.0735999	-1.4296703
19.8666668	1.1223999	-1.6198835
19.8666668	1.1711999	-1.3744469
19.8666668	1.2200000	-1.2946796
19.8666668	1.2688000	-1.1290097
19.8666668	1.3176000	-0.9510679
19.8666668	1.3664000	-0.6933594
19.8666668	1.4152000	-1.2762718
19.8666668	1.4640000	-1.2762718
19.8666668	1.5128000	-1.1903691
19.8666668	1.5616000	-1.0799227
19.8666668	1.6104000	-1.3499031
19.8666668	1.6592000	-1.3437672

19.8666668	1.7079999	-1.3253593
19.8666668	1.7567999	-1.4419422
19.8666668	1.8055999	-1.3744469
19.8666668	1.8543999	-1.3130875
19.8666668	1.9031999	-1.2701364
19.8666668	1.9519999	-1.2517285
19.8666668	2.0007999	-1.2455926
19.8666668	2.0495999	-1.2455926
19.8666668	2.0983999	-1.2455926
19.8666668	2.1471999	-1.2455926
19.8666668	2.1959999	-1.2701364
19.8666668	2.2447999	-1.2885437
19.8666668	2.2935998	-1.2762718
19.8666668	2.3423998	-1.2701364
19.8666668	2.3912001	-1.1228738
19.8666668	2.4400001	-1.2210488
19.8666668	2.4888000	-1.3989906
19.8666668	2.5376000	-1.4051266
19.8666668	2.5864000	-1.1535535
19.8666668	2.6352000	-1.2578645
19.8666668	2.6840000	-1.3376312
19.8666668	2.7328000	-1.1658254
19.8666668	2.7816000	-1.1780972
19.8666668	2.8304000	-1.2087770
19.8666668	2.8792000	-1.0185633
19.8666668	2.9280000	-1.0431070
19.8666668	2.9768000	-1.0124273
19.8666668	3.0256000	-1.2210488
19.8666668	3.0743999	-1.1965051
19.8666668	3.1231999	-1.1842332
19.8666668	3.1719999	-0.9756117
19.8666668	3.2207999	-0.9817476
19.8666668	3.2695999	-0.9817476
19.8666668	3.3183999	-1.0062914
19.8666668	3.3671999	-1.1044660
19.8666668	3.4159999	-1.0983305
19.8666668	3.4647999	-1.0799227
19.8666668	3.5135999	-1.2701364
19.8666668	3.5623999	-1.2640004
19.8666668	3.6111999	-1.2640004
19.8666668	3.6599998	-1.2824078
19.8666668	3.7087998	-1.2333207
19.8666668	3.7575998	-1.2210488
19.8666668	3.8063998	-1.1412816
19.8666668	3.8551998	-1.1412816
19.8666668	3.9039998	-1.0369711
19.8666668	3.9527998	-1.2394567
19.8666668	4.0016003	-1.0124273
19.8666668	4.0503998	-0.8467574
19.8666668	4.0991998	-0.8344855
19.8666668	4.1479998	-0.6688156
19.8666668	4.1967998	-0.9694757
19.8666668	4.2455997	-1.0124273
19.8666668	4.2943997	-1.1474175
19.8666668	4.3431997	-0.9449320
19.8666668	4.3919997	-0.8528934
19.8666668	4.4407997	-0.8528934

19.8666668	4.4895997	-0.8528934
19.8666668	4.5383997	-0.8344855
19.8666668	4.5871997	-0.6565437
19.8666668	4.6359997	-0.6135921
19.8666668	4.6847997	-0.6197281
19.8666668	4.7336001	-0.6381359
19.8666668	4.7824001	-0.6381359
19.8666668	4.8312001	-0.5276895
19.8666668	4.8800001	-0.4908738
19.8666668	4.9288001	-0.6933594
19.8666668	4.9776001	-0.6933594
19.8666668	5.0264001	-0.3926992
19.8666668	5.0752001	-0.4970098
19.8666668	5.1240001	-0.6074562
19.8666668	5.1728001	-0.6013203
19.8666668	5.2216001	-0.5890489
19.8666668	5.2704000	-0.6872234
19.8666668	5.3192000	-0.6197281
19.8666668	5.3680000	-0.7056313
19.8666668	5.4168000	-0.7179031
19.8666668	5.4656000	-0.6810875
19.8666668	5.5144000	-0.6749516
19.8666668	5.5632000	-0.5767770
19.8666668	5.6120000	-0.5276895
19.8666668	5.6608000	-0.5583692
19.8666668	5.7096000	-0.4908738
19.8666668	5.7584000	613586.0000000
19.8666668	5.8072000	-0.9326606
19.8666668	5.8559999	-0.8222136
19.8666668	5.9047999	-0.8160777
19.8666668	5.9535999	-0.5154176
19.8666668	6.0023999	-0.6810875
19.8833332	0.2928000	-3.1845441
19.8833332	0.3416000	-2.1843886
19.8833332	0.3904000	-1.9450879
19.8833332	0.4392000	-1.4726214
19.8833332	0.4880000	-1.1965051
19.8833332	0.5368000	-1.1965051
19.8833332	0.5856000	-1.6137476
19.8833332	0.6344000	-1.2762718
19.8833332	0.6832000	-1.2762718
19.8833332	0.7320000	-1.1780972
19.8833332	0.7808000	-1.1780972
19.8833332	0.8296000	-1.1780972
19.8833332	0.8784000	-1.3867188
19.8833332	0.9272000	-1.4848933
19.8833332	0.9760000	-1.6321554
19.8833332	1.0247999	-1.6444273
19.8833332	1.0735999	-1.6505632
19.8833332	1.1223999	-1.4542136
19.8833332	1.1711999	-1.2701364
19.8833332	1.2200000	-1.2333207
19.8833332	1.2688000	-1.2210488
19.8833332	1.3176000	-1.2271848
19.8833332	1.3664000	-1.3560390
19.8833332	1.4152000	-1.3560390
19.8833332	1.4640000	-1.1842332

19.8833332	1.5128000	-1.2087770
19.8833332	1.5616000	-1.2271848
19.8833332	1.6104000	-1.2333207
19.8833332	1.6592000	-1.1412816
19.8833332	1.7079999	-1.2824078
19.8833332	1.7567999	-1.2333207
19.8833332	1.8055999	-1.2210488
19.8833332	1.8543999	-1.2149129
19.8833332	1.9031999	-1.2762718
19.8833332	1.9519999	-1.2824078
19.8833332	2.0007999	-1.2946796
19.8833332	2.0495999	-1.2455926
19.8833332	2.0983999	-1.2455926
19.8833332	2.1471999	-1.3192234
19.8833332	2.1959999	-1.1596894
19.8833332	2.2447999	-1.0246992
19.8833332	2.2935998	-1.0308352
19.8833332	2.3423998	-0.9572039
19.8833332	2.3912001	-1.2271848
19.8833332	2.4400001	-1.1965051
19.8833332	2.4888000	-1.1965051
19.8833332	2.5376000	-1.1780972
19.8833332	2.5864000	-1.1780972
19.8833332	2.6352000	-1.1780972
19.8833332	2.6840000	-1.1044660
19.8833332	2.7328000	-1.0983305
19.8833332	2.7816000	-1.0492430
19.8833332	2.8304000	-1.0185633
19.8833332	2.8792000	-1.0246992
19.8833332	2.9280000	-1.0001554
19.8833332	2.9768000	-0.8283496
19.8833332	3.0256000	-0.7485828
19.8833332	3.0743999	-1.0062914
19.8833332	3.1231999	-1.0001554
19.8833332	3.1719999	-0.6258640
19.8833332	3.2207999	-0.7608547
19.8833332	3.2695999	-0.7792621
19.8833332	3.3183999	-0.8038058
19.8833332	3.3671999	-0.9694757
19.8833332	3.4159999	-0.9694757
19.8833332	3.4647999	-0.8160777
19.8833332	3.5135999	-0.7363110
19.8833332	3.5623999	-0.7731261
19.8833332	3.6111999	-0.9510679
19.8833332	3.6599998	-0.9142528
19.8833332	3.7087998	-0.9203887
19.8833332	3.7575998	-0.8406215
19.8833332	3.8063998	-0.9203887
19.8833332	3.8551998	-0.9142528
19.8833332	3.9039998	-0.5951843
19.8833332	3.9527998	-0.5829129
19.8833332	4.0016003	-0.7485828
19.8833332	4.0503998	-0.7424469
19.8833332	4.0991998	-0.8590293
19.8833332	4.1479998	-1.0062914
19.8833332	4.1967998	-0.7547188
19.8833332	4.2455997	-0.7056313

19.8833332	4.2943997	-0.6933594
19.8833332	4.3431997	-0.6933594
19.8833332	4.3919997	-0.6749516
19.8833332	4.4407997	-0.6565437
19.8833332	4.4895997	-0.6504078
19.8833332	4.5383997	-0.5338254
19.8833332	4.5871997	-0.5031457
19.8833332	4.6359997	-0.5399613
19.8833332	4.6847997	-0.4663301
19.8833332	4.7336001	-0.3558836
19.8833332	4.7824001	-0.3497477
19.8833332	4.8312001	-0.3374758
19.8833332	4.8800001	-0.3436117
19.8833332	4.9288001	-0.4970098
19.8833332	4.9776001	-0.5092816
19.8833332	5.0264001	-0.5092816
19.8833332	5.0752001	-0.4908738
19.8833332	5.1240001	-0.4908738
19.8833332	5.1728001	-0.5399613
19.8833332	5.2216001	-0.4663301
19.8833332	5.2704000	-0.4479222
19.8833332	5.3192000	-0.5706410
19.8833332	5.3680000	-0.4908738
19.8833332	5.4168000	-0.3620195
19.8833332	5.4656000	-0.3681555
19.8833332	5.5144000	-0.3865633
19.8833332	5.5632000	-0.3865633
19.8833332	5.6120000	-0.6994953
19.8833332	5.6608000	-0.6074562
19.8833332	5.7096000	-0.6074562
19.8833332	5.7584000	-0.5951843
19.8833332	5.8072000	-0.6197281
19.8833332	5.8559999	-0.6197281
19.8833332	5.9047999	-0.8835731
19.8833332	5.9535999	-0.6381359
19.8833332	6.0023999	-0.6442719
19.8999996	0.2928000	-3.2152238
19.8999996	0.3416000	-1.6505632
19.8999996	0.3904000	-1.6444273
19.8999996	0.4392000	-1.2578645
19.8999996	0.4880000	-1.2701364
19.8999996	0.5368000	-1.0492430
19.8999996	0.5856000	-1.0492430
19.8999996	0.6344000	-1.1474175
19.8999996	0.6832000	-1.1412816
19.8999996	0.7320000	-1.0676508
19.8999996	0.7808000	-1.0676508
19.8999996	0.8296000	-1.0799227
19.8999996	0.8784000	-1.0983305
19.8999996	0.9272000	-1.1044660
19.8999996	0.9760000	-1.1044660
19.8999996	1.0247999	-1.1044660
19.8999996	1.0735999	-1.2455926
19.8999996	1.1223999	-1.2946796
19.8999996	1.1711999	-1.0553789
19.8999996	1.2200000	-1.0492430
19.8999996	1.2688000	-1.0860586

19.8999996	1.3176000	-1.2271848
19.8999996	1.3664000	-1.2455926
19.8999996	1.4152000	-1.3867188
19.8999996	1.4640000	-1.1351457
19.8999996	1.5128000	-1.1351457
19.8999996	1.5616000	-1.2394567
19.8999996	1.6104000	-1.2271848
19.8999996	1.6592000	-1.3192234
19.8999996	1.7079999	-1.3192234
19.8999996	1.7567999	-0.9387960
19.8999996	1.8055999	-0.9387960
19.8999996	1.8543999	-1.1167378
19.8999996	1.9031999	-0.9756117
19.8999996	1.9519999	-0.9756117
19.8999996	2.0007999	-1.1412816
19.8999996	2.0495999	-1.1535535
19.8999996	2.0983999	-1.1535535
19.8999996	2.1471999	-1.1290097
19.8999996	2.1959999	-1.1290097
19.8999996	2.2447999	-1.0860586
19.8999996	2.2935998	-1.0799227
19.8999996	2.3423998	-1.1290097
19.8999996	2.3912001	-0.9326606
19.8999996	2.4400001	-0.9326606
19.8999996	2.4888000	-0.9265246
19.8999996	2.5376000	-0.9694757
19.8999996	2.5864000	-0.9265246
19.8999996	2.6352000	-0.9817476
19.8999996	2.6840000	-0.9449320
19.8999996	2.7328000	-0.9633398
19.8999996	2.7816000	-0.8958449
19.8999996	2.8304000	-0.9019809
19.8999996	2.8792000	-0.7424469
19.8999996	2.9280000	-0.7976699
19.8999996	2.9768000	-1.0124273
19.8999996	3.0256000	-1.0799227
19.8999996	3.0743999	-1.1412816
19.8999996	3.1231999	-1.0124273
19.8999996	3.1719999	-0.9510679
19.8999996	3.2207999	-0.7608547
19.8999996	3.2695999	-1.1351457
19.8999996	3.3183999	-1.0676508
19.8999996	3.3671999	-1.0553789
19.8999996	3.4159999	-0.8528934
19.8999996	3.4647999	-0.8897090
19.8999996	3.5135999	-0.8897090
19.8999996	3.5623999	-0.7608547
19.8999996	3.6111999	-0.8406215
19.8999996	3.6599998	-0.9019809
19.8999996	3.7087998	-0.7976699
19.8999996	3.7575998	-0.6688156
19.8999996	3.8063998	-0.6688156
19.8999996	3.8551998	-0.6013203
19.8999996	3.9039998	-0.6626797
19.8999996	3.9527998	-0.7056313
19.8999996	4.0016003	-0.7179031
19.8999996	4.0503998	-0.8774371

19.8999996	4.0991998	-0.8958449
19.8999996	4.1479998	-0.7976699
19.8999996	4.1967998	-0.7976699
19.8999996	4.2455997	-0.5276895
19.8999996	4.2943997	-0.6074562
19.8999996	4.3431997	-0.6013203
19.8999996	4.3919997	-0.3926992
19.8999996	4.4407997	-0.3313398
19.8999996	4.4895997	-0.5460973
19.8999996	4.5383997	-0.2147574
19.8999996	4.5871997	-0.2086215
19.8999996	4.6359997	-0.0674953
19.8999996	4.6847997	-0.0306797
19.8999996	4.7336001	-0.0245438
19.8999996	4.7824001	-0.0184078
19.8999996	4.8312001	-0.0122719
19.8999996	4.8800001	-0.0184078
19.8999996	4.9288001	-0.2393012
19.8999996	4.9776001	-0.2270293
19.8999996	5.0264001	-0.1533980
19.8999996	5.0752001	-0.2945242
19.8999996	5.1240001	-0.3804274
19.8999996	5.1728001	-0.3804274
19.8999996	5.2216001	-0.3804274
19.8999996	5.2704000	-0.2331653
19.8999996	5.3192000	-0.2270293
19.8999996	5.3680000	-0.2822523
19.8999996	5.4168000	-0.6074562
19.8999996	5.4656000	-0.6258640
19.8999996	5.5144000	-0.6381359
19.8999996	5.5632000	-0.3865633
19.8999996	5.6120000	-0.3865633
19.8999996	5.6608000	-0.3620195
19.8999996	5.7096000	-0.2638445
19.8999996	5.7584000	-0.4049711
19.8999996	5.8072000	-0.3252039
19.8999996	5.8559999	-0.3190680
19.8999996	5.9047999	-0.3804274
19.8999996	5.9535999	-0.3742914
19.8999996	6.0023999	613586.0000000
19.9166660	0.2928000	-2.6507189
19.9166660	0.3416000	-1.6996508
19.9166660	0.3904000	-1.8898644
19.9166660	0.4392000	-1.8960004
19.9166660	0.4880000	-1.6628351
19.9166660	0.5368000	-1.4726214
19.9166660	0.5856000	-1.4848933
19.9166660	0.6344000	-1.4848933
19.9166660	0.6832000	-1.1596894
19.9166660	0.7320000	-1.1596894
19.9166660	0.7808000	-1.4542136
19.9166660	0.8296000	-1.2762718
19.9166660	0.8784000	-1.2578645
19.9166660	0.9272000	-1.4419422
19.9166660	0.9760000	-1.3008156
19.9166660	1.0247999	-1.2640004
19.9166660	1.0735999	-1.1167378

19.9166660	1.1223999	-1.1228738
19.9166660	1.1711999	-1.0676508
19.9166660	1.2200000	-0.9449320
19.9166660	1.2688000	-0.9510679
19.9166660	1.3176000	-1.1412816
19.9166660	1.3664000	-1.0921946
19.9166660	1.4152000	-1.1044660
19.9166660	1.4640000	-1.0921946
19.9166660	1.5128000	-1.0860586
19.9166660	1.5616000	-1.0799227
19.9166660	1.6104000	-1.0185633
19.9166660	1.6592000	-0.9510679
19.9166660	1.7079999	-1.0062914
19.9166660	1.7567999	-1.2946796
19.9166660	1.8055999	-1.1351457
19.9166660	1.8543999	-1.1290097
19.9166660	1.9031999	-1.0062914
19.9166660	1.9519999	-1.0799227
19.9166660	2.0007999	-1.0983305
19.9166660	2.0495999	-1.1719613
19.9166660	2.0983999	-1.0308352
19.9166660	2.1471999	-1.0308352
19.9166660	2.1959999	-1.1965051
19.9166660	2.2447999	-1.4664855
19.9166660	2.2935998	-1.3989906
19.9166660	2.3423998	-1.2946796
19.9166660	2.3912001	-1.2455926
19.9166660	2.4400001	-1.0369711
19.9166660	2.4888000	-0.7669902
19.9166660	2.5376000	-1.2087770
19.9166660	2.5864000	-1.2210488
19.9166660	2.6352000	-1.2210488
19.9166660	2.6840000	-1.0676508
19.9166660	2.7328000	-0.9019809
19.9166660	2.7816000	-0.9019809
19.9166660	2.8304000	-0.9081168
19.9166660	2.8792000	-0.7853980
19.9166660	2.9280000	-1.1106019
19.9166660	2.9768000	-1.1228738
19.9166660	3.0256000	-1.1228738
19.9166660	3.0743999	-0.9756117
19.9166660	3.1231999	-0.9633398
19.9166660	3.1719999	-0.9510679
19.9166660	3.2207999	-0.8160777
19.9166660	3.2695999	-0.8283496
19.9166660	3.3183999	-0.8344855
19.9166660	3.3671999	-0.8651652
19.9166660	3.4159999	-0.7547188
19.9166660	3.4647999	-0.7731261
19.9166660	3.5135999	-0.8038058
19.9166660	3.5623999	-0.9756117
19.9166660	3.6111999	-0.9756117
19.9166660	3.6599998	-0.9878836
19.9166660	3.7087998	-0.8897090
19.9166660	3.7575998	-0.8406215
19.9166660	3.8063998	-0.8406215
19.9166660	3.8551998	-0.9449320

19.9166660	3.9039998	-1.0124273
19.9166660	3.9527998	-0.8835731
19.9166660	4.0016003	-0.9019809
19.9166660	4.0503998	-0.8283496
19.9166660	4.0991998	-0.7056313
19.9166660	4.1479998	-0.7056313
19.9166660	4.1967998	-0.6565437
19.9166660	4.2455997	-0.6565437
19.9166660	4.2943997	-0.5829129
19.9166660	4.3431997	-0.6074562
19.9166660	4.3919997	-0.6197281
19.9166660	4.4407997	-0.4356503
19.9166660	4.4895997	-0.4479222
19.9166660	4.5383997	-0.3681555
19.9166660	4.5871997	-0.3006601
19.9166660	4.6359997	-0.3006601
19.9166660	4.6847997	-0.6258640
19.9166660	4.7336001	-0.3374758
19.9166660	4.7824001	-0.5460973
19.9166660	4.8312001	-0.5522332
19.9166660	4.8800001	-0.4663301
19.9166660	4.9288001	-0.4540582
19.9166660	4.9776001	-0.4111071
19.9166660	5.0264001	-0.3988352
19.9166660	5.0752001	-0.3988352
19.9166660	5.1240001	-0.3988352
19.9166660	5.1728001	-0.3558836
19.9166660	5.2216001	-0.0490875
19.9166660	5.2704000	-0.2638445
19.9166660	5.3192000	-0.3620195
19.9166660	5.3680000	-0.5522332
19.9166660	5.4168000	-0.5522332
19.9166660	5.4656000	-0.6504078
19.9166660	5.5144000	-0.2883883
19.9166660	5.5632000	-0.4049711
19.9166660	5.6120000	-0.4233789
19.9166660	5.6608000	-0.4295144
19.9166660	5.7096000	-0.4417863
19.9166660	5.7584000	-0.5215535
19.9166660	5.8072000	-0.5215535
19.9166660	5.8559999	-0.5154176
19.9166660	5.9047999	-0.7792621
19.9166660	5.9535999	-0.7792621
19.9166660	6.0023999	-0.8038058
19.9333324	0.2928000	-2.2089324
19.9333324	0.3416000	-2.3377867
19.9333324	0.3904000	-1.8775926
19.9333324	0.4392000	-1.3744469
19.9333324	0.4880000	-1.3805828
19.9333324	0.5368000	-1.1903691
19.9333324	0.5856000	-1.0860586
19.9333324	0.6344000	-1.0676508
19.9333324	0.6832000	-1.0062914
19.9333324	0.7320000	-1.0062914
19.9333324	0.7808000	-1.2517285
19.9333324	0.8296000	-1.1167378
19.9333324	0.8784000	-1.3560390

19.9333324	0.9272000	-1.3314953
19.9333324	0.9760000	-1.2271848
19.9333324	1.0247999	-1.2271848
19.9333324	1.0735999	-1.2087770
19.9333324	1.1223999	-1.3989906
19.9333324	1.1711999	-1.3928547
19.9333324	1.2200000	-1.5892043
19.9333324	1.2688000	-1.5769324
19.9333324	1.3176000	-1.4910293
19.9333324	1.3664000	-1.4787574
19.9333324	1.4152000	-1.3744469
19.9333324	1.4640000	-1.1596894
19.9333324	1.5128000	-1.1474175
19.9333324	1.5616000	-1.1474175
19.9333324	1.6104000	-1.1535535
19.9333324	1.6592000	-1.3621750
19.9333324	1.7079999	-1.2885437
19.9333324	1.7567999	-1.2824078
19.9333324	1.8055999	-1.2271848
19.9333324	1.8543999	-1.2333207
19.9333324	1.9031999	-1.0737867
19.9333324	1.9519999	-1.0492430
19.9333324	2.0007999	-1.0737867
19.9333324	2.0495999	-1.0124273
19.9333324	2.0983999	-0.9940195
19.9333324	2.1471999	-0.9387960
19.9333324	2.1959999	-0.9694757
19.9333324	2.2447999	-1.2701364
19.9333324	2.2935998	-1.2701364
19.9333324	2.3423998	-0.8160777
19.9333324	2.3912001	-0.7179031
19.9333324	2.4400001	-1.1228738
19.9333324	2.4888000	-1.1351457
19.9333324	2.5376000	-1.1412816
19.9333324	2.5864000	-1.2087770
19.9333324	2.6352000	-1.3008156
19.9333324	2.6840000	-1.3008156
19.9333324	2.7328000	-0.9940195
19.9333324	2.7816000	-0.9694757
19.9333324	2.8304000	-0.9633398
19.9333324	2.8792000	-0.9694757
19.9333324	2.9280000	-0.9265246
19.9333324	2.9768000	-0.9019809
19.9333324	3.0256000	-1.1228738
19.9333324	3.0743999	-0.9265246
19.9333324	3.1231999	-0.9265246
19.9333324	3.1719999	-0.8651652
19.9333324	3.2207999	-1.0615149
19.9333324	3.2695999	-1.0615149
19.9333324	3.3183999	-1.0799227
19.9333324	3.3671999	-1.0921946
19.9333324	3.4159999	-1.1842332
19.9333324	3.4647999	-1.1780972
19.9333324	3.5135999	-1.0676508
19.9333324	3.5623999	-0.9203887
19.9333324	3.6111999	-0.9203887
19.9333324	3.6599998	-0.9203887

19.9333324	3.7087998	-0.8467574
19.9333324	3.7575998	-0.7792621
19.9333324	3.8063998	-0.7853980
19.9333324	3.8551998	-0.7485828
19.9333324	3.9039998	-0.9142528
19.9333324	3.9527998	-0.5460973
19.9333324	4.0016003	-0.6504078
19.9333324	4.0503998	-0.5215535
19.9333324	4.0991998	-0.4172430
19.9333324	4.1479998	-0.7363110
19.9333324	4.1967998	-0.7363110
19.9333324	4.2455997	-0.9081168
19.9333324	4.2943997	-0.9019809
19.9333324	4.3431997	-0.8344855
19.9333324	4.3919997	-0.8160777
19.9333324	4.4407997	-0.6688156
19.9333324	4.4895997	-0.6320000
19.9333324	4.5383997	-0.6074562
19.9333324	4.5871997	-0.5092816
19.9333324	4.6359997	-0.5092816
19.9333324	4.6847997	-0.5092816
19.9333324	4.7336001	-0.4479222
19.9333324	4.7824001	-0.4540582
19.9333324	4.8312001	-0.6749516
19.9333324	4.8800001	-0.6074562
19.9333324	4.9288001	-0.6197281
19.9333324	4.9776001	-0.5951843
19.9333324	5.0264001	-0.5276895
19.9333324	5.0752001	-0.4847379
19.9333324	5.1240001	-0.5215535
19.9333324	5.1728001	-0.5460973
19.9333324	5.2216001	-0.4663301
19.9333324	5.2704000	-0.7853980
19.9333324	5.3192000	-0.6320000
19.9333324	5.3680000	-0.6074562
19.9333324	5.4168000	-0.5092816
19.9333324	5.4656000	-0.4847379
19.9333324	5.5144000	-0.7853980
19.9333324	5.5632000	-0.5583692
19.9333324	5.6120000	-0.6565437
19.9333324	5.6608000	-0.6074562
19.9333324	5.7096000	-0.7792621
19.9333324	5.7584000	-0.7853980
19.9333324	5.8072000	-0.7117672
19.9333324	5.8559999	-0.7179031
19.9333324	5.9047999	-0.7547188
19.9333324	5.9535999	-0.6504078
19.9333324	6.0023999	-0.6626797
19.9500008	0.2928000	-2.1782527
19.9500008	0.3416000	-1.6198835
19.9500008	0.3904000	-1.4971652
19.9500008	0.4392000	-1.2946796
19.9500008	0.4880000	-1.4480777
19.9500008	0.5368000	-1.4358063
19.9500008	0.5856000	-1.1965051
19.9500008	0.6344000	-1.2026410
19.9500008	0.6832000	-1.2087770

19.9500008	0.7320000	-1.2087770
19.9500008	0.7808000	-1.2578645
19.9500008	0.8296000	-1.3744469
19.9500008	0.8784000	-1.5401168
19.9500008	0.9272000	-1.2885437
19.9500008	0.9760000	-1.3683109
19.9500008	1.0247999	-1.3805828
19.9500008	1.0735999	-1.3805828
19.9500008	1.1223999	-1.1351457
19.9500008	1.1711999	-1.0737867
19.9500008	1.2200000	-1.8591847
19.9500008	1.2688000	-1.4480777
19.9500008	1.3176000	-1.4480777
19.9500008	1.3664000	-1.6566992
19.9500008	1.4152000	-0.9326606
19.9500008	1.4640000	-1.1658254
19.9500008	1.5128000	-1.1535535
19.9500008	1.5616000	-1.1474175
19.9500008	1.6104000	-1.3989906
19.9500008	1.6592000	-1.3928547
19.9500008	1.7079999	-1.5278449
19.9500008	1.7567999	-1.4419422
19.9500008	1.8055999	-1.1351457
19.9500008	1.8543999	-1.1351457
19.9500008	1.9031999	-1.2210488
19.9500008	1.9519999	-1.2087770
19.9500008	2.0007999	-1.1290097
19.9500008	2.0495999	-1.1351457
19.9500008	2.0983999	-1.1412816
19.9500008	2.1471999	-1.2026410
19.9500008	2.1959999	-1.2087770
19.9500008	2.2447999	-1.2210488
19.9500008	2.2935998	-1.2271848
19.9500008	2.3423998	-1.1228738
19.9500008	2.3912001	-1.1228738
19.9500008	2.4400001	-1.1965051
19.9500008	2.4888000	-1.1290097
19.9500008	2.5376000	-1.0983305
19.9500008	2.5864000	-1.0983305
19.9500008	2.6352000	-1.1903691
19.9500008	2.6840000	-1.1167378
19.9500008	2.7328000	-1.0001554
19.9500008	2.7816000	-1.0369711
19.9500008	2.8304000	-0.9633398
19.9500008	2.8792000	-0.9633398
19.9500008	2.9280000	-0.9633398
19.9500008	2.9768000	-0.9756117
19.9500008	3.0256000	-0.8406215
19.9500008	3.0743999	-0.9878836
19.9500008	3.1231999	-0.7669902
19.9500008	3.1719999	-0.7731261
19.9500008	3.2207999	-0.8651652
19.9500008	3.2695999	-0.8713012
19.9500008	3.3183999	-0.9694757
19.9500008	3.3671999	-0.9326606
19.9500008	3.4159999	-0.7669902
19.9500008	3.4647999	-0.9572039

19.9500008	3.5135999	-0.9572039
19.9500008	3.5623999	-0.8713012
19.9500008	3.6111999	-0.9142528
19.9500008	3.6599998	-0.9572039
19.9500008	3.7087998	-0.9510679
19.9500008	3.7575998	-0.9449320
19.9500008	3.8063998	-0.8590293
19.9500008	3.8551998	-0.9633398
19.9500008	3.9039998	-0.8651652
19.9500008	3.9527998	-0.9265246
19.9500008	4.0016003	-0.9265246
19.9500008	4.0503998	-0.8406215
19.9500008	4.0991998	-0.8406215
19.9500008	4.1479998	-0.9572039
19.9500008	4.1967998	-0.8038058
19.9500008	4.2455997	-0.8038058
19.9500008	4.2943997	-0.7608547
19.9500008	4.3431997	-0.6135921
19.9500008	4.3919997	-0.5460973
19.9500008	4.4407997	-0.5460973
19.9500008	4.4895997	-0.5522332
19.9500008	4.5383997	-0.5154176
19.9500008	4.5871997	-0.6320000
19.9500008	4.6359997	-0.6135921
19.9500008	4.6847997	-0.6074562
19.9500008	4.7336001	-0.6074562
19.9500008	4.7824001	-0.6013203
19.9500008	4.8312001	-0.5031457
19.9500008	4.8800001	-0.5031457
19.9500008	4.9288001	-0.4908738
19.9500008	4.9776001	-0.6442719
19.9500008	5.0264001	-0.5829129
19.9500008	5.0752001	-0.5767770
19.9500008	5.1240001	-0.5829129
19.9500008	5.1728001	-0.6933594
19.9500008	5.2216001	-0.4724660
19.9500008	5.2704000	-0.4724660
19.9500008	5.3192000	-0.4786019
19.9500008	5.3680000	-0.4847379
19.9500008	5.4168000	-0.4786019
19.9500008	5.4656000	-0.4908738
19.9500008	5.5144000	-0.5092816
19.9500008	5.5632000	-0.5645051
19.9500008	5.6120000	-0.6810875
19.9500008	5.6608000	-0.6872234
19.9500008	5.7096000	-0.5399613
19.9500008	5.7584000	-0.6320000
19.9500008	5.8072000	-0.6320000
19.9500008	5.8559999	-0.6749516
19.9500008	5.9047999	-0.6749516
19.9500008	5.9535999	-0.5583692
19.9500008	6.0023999	-0.5767770
19.9666672	0.2928000	-2.1905246
19.9666672	0.3416000	-1.9144082
19.9666672	0.3904000	-1.7548742
19.9666672	0.4392000	-1.7057867
19.9666672	0.4880000	-1.1780972

19.9666672	0.5368000	-0.9326606
19.9666672	0.5856000	-0.8958449
19.9666672	0.6344000	-0.9019809
19.9666672	0.6832000	-1.2087770
19.9666672	0.7320000	-1.2026410
19.9666672	0.7808000	-1.2640004
19.9666672	0.8296000	-1.1535535
19.9666672	0.8784000	-1.1596894
19.9666672	0.9272000	-1.0185633
19.9666672	0.9760000	-1.0246992
19.9666672	1.0247999	-0.9265246
19.9666672	1.0735999	-0.7915339
19.9666672	1.1223999	-0.5583692
19.9666672	1.1711999	-0.5583692
19.9666672	1.2200000	-0.4601941
19.9666672	1.2688000	-1.1780972
19.9666672	1.3176000	-1.1719613
19.9666672	1.3664000	-1.1780972
19.9666672	1.4152000	-1.2271848
19.9666672	1.4640000	-1.2149129
19.9666672	1.5128000	-1.3744469
19.9666672	1.5616000	-1.3621750
19.9666672	1.6104000	-1.2824078
19.9666672	1.6592000	-1.2762718
19.9666672	1.7079999	-1.0308352
19.9666672	1.7567999	-0.6933594
19.9666672	1.8055999	-0.7363110
19.9666672	1.8543999	-1.0860586
19.9666672	1.9031999	-1.1106019
19.9666672	1.9519999	-1.0369711
19.9666672	2.0007999	-1.0308352
19.9666672	2.0495999	-0.8897090
19.9666672	2.0983999	-0.8835731
19.9666672	2.1471999	-1.2946796
19.9666672	2.1959999	-1.2946796
19.9666672	2.2447999	-1.1044660
19.9666672	2.2935998	-1.2333207
19.9666672	2.3423998	-1.2333207
19.9666672	2.3912001	-1.2455926
19.9666672	2.4400001	-1.2578645
19.9666672	2.4888000	-1.1719613
19.9666672	2.5376000	-1.0676508
19.9666672	2.5864000	-1.0308352
19.9666672	2.6352000	-1.0369711
19.9666672	2.6840000	-1.1412816
19.9666672	2.7328000	-0.8406215
19.9666672	2.7816000	-0.9081168
19.9666672	2.8304000	-0.9510679
19.9666672	2.8792000	-0.7976699
19.9666672	2.9280000	-0.7976699
19.9666672	2.9768000	-0.8406215
19.9666672	3.0256000	-0.8467574
19.9666672	3.0743999	-0.8467574
19.9666672	3.1231999	-1.3560390
19.9666672	3.1719999	-1.0001554
19.9666672	3.2207999	-1.1228738
19.9666672	3.2695999	-1.0431070

19.9666672	3.3183999	-1.0431070
19.9666672	3.3671999	-0.9694757
19.9666672	3.4159999	-0.9572039
19.9666672	3.4647999	-0.8713012
19.9666672	3.5135999	-0.9449320
19.9666672	3.5623999	-0.8713012
19.9666672	3.6111999	-0.8651652
19.9666672	3.6599998	-0.8344855
19.9666672	3.7087998	-0.8099418
19.9666672	3.7575998	-0.7915339
19.9666672	3.8063998	-0.7915339
19.9666672	3.8551998	-0.5154176
19.9666672	3.9039998	-0.8467574
19.9666672	3.9527998	-1.0124273
19.9666672	4.0016003	-0.7853980
19.9666672	4.0503998	-0.7792621
19.9666672	4.0991998	-0.5890489
19.9666672	4.1479998	-0.6442719
19.9666672	4.1967998	-0.6442719
19.9666672	4.2455997	-0.7669902
19.9666672	4.2943997	-0.6442719
19.9666672	4.3431997	-0.6688156
19.9666672	4.3919997	-0.6749516
19.9666672	4.4407997	-0.4663301
19.9666672	4.4895997	-0.4049711
19.9666672	4.5383997	-0.3313398
19.9666672	4.5871997	-0.5154176
19.9666672	4.6359997	-0.5031457
19.9666672	4.6847997	-0.5460973
19.9666672	4.7336001	-0.5583692
19.9666672	4.7824001	-0.4786019
19.9666672	4.8312001	-0.4847379
19.9666672	4.8800001	-0.5031457
19.9666672	4.9288001	-0.4172430
19.9666672	4.9776001	-0.4663301
19.9666672	5.0264001	-0.4540582
19.9666672	5.0752001	-0.4540582
19.9666672	5.1240001	-0.4233789
19.9666672	5.1728001	-0.4111071
19.9666672	5.2216001	-0.4908738
19.9666672	5.2704000	-0.4908738
19.9666672	5.3192000	-0.5338254
19.9666672	5.3680000	-0.5460973
19.9666672	5.4168000	-0.5522332
19.9666672	5.4656000	-0.5399613
19.9666672	5.5144000	-0.8038058
19.9666672	5.5632000	-0.8222136
19.9666672	5.6120000	-0.7179031
19.9666672	5.6608000	-0.6749516
19.9666672	5.7096000	-0.6320000
19.9666672	5.7584000	-0.6320000
19.9666672	5.8072000	-0.6933594
19.9666672	5.8559999	-0.6381359
19.9666672	5.9047999	-0.6442719
19.9666672	5.9535999	-0.5951843
19.9666672	6.0023999	-0.6810875
19.9833336	0.2928000	-2.4052820

19.9833336	0.3416000	-1.9696312
19.9833336	0.3904000	-1.9819031
19.9833336	0.4392000	-1.7364664
19.9833336	0.4880000	-1.6689711
19.9833336	0.5368000	-1.4787574
19.9833336	0.5856000	-1.4051266
19.9833336	0.6344000	-1.2271848
19.9833336	0.6832000	-1.3253593
19.9833336	0.7320000	-1.3437672
19.9833336	0.7808000	-1.2210488
19.9833336	0.8296000	-1.3560390
19.9833336	0.8784000	-1.3008156
19.9833336	0.9272000	-1.4112625
19.9833336	0.9760000	-1.4112625
19.9833336	1.0247999	-1.4664855
19.9833336	1.0735999	-1.4664855
19.9833336	1.1223999	-1.4112625
19.9833336	1.1711999	-1.2271848
19.9833336	1.2200000	-1.2210488
19.9833336	1.2688000	-1.4910293
19.9833336	1.3176000	-1.5462527
19.9833336	1.3664000	-1.4051266
19.9833336	1.4152000	-1.2394567
19.9833336	1.4640000	-1.2455926
19.9833336	1.5128000	-1.0369711
19.9833336	1.5616000	-1.2333207
19.9833336	1.6104000	-1.2271848
19.9833336	1.6592000	-1.0431070
19.9833336	1.7079999	-0.9142528
19.9833336	1.7567999	-1.1106019
19.9833336	1.8055999	-1.1351457
19.9833336	1.8543999	-1.0921946
19.9833336	1.9031999	-1.1596894
19.9833336	1.9519999	-1.1719613
19.9833336	2.0007999	-1.1658254
19.9833336	2.0495999	-1.1596894
19.9833336	2.0983999	-1.1596894
19.9833336	2.1471999	-1.0921946
19.9833336	2.1959999	-1.1780972
19.9833336	2.2447999	-1.1658254
19.9833336	2.2935998	-1.1044660
19.9833336	2.3423998	-1.0921946
19.9833336	2.3912001	-1.0615149
19.9833336	2.4400001	-1.1719613
19.9833336	2.4888000	-1.1658254
19.9833336	2.5376000	-1.0492430
19.9833336	2.5864000	-1.0492430
19.9833336	2.6352000	-1.0553789
19.9833336	2.6840000	-1.0921946
19.9833336	2.7328000	-1.0308352
19.9833336	2.7816000	-0.9510679
19.9833336	2.8304000	-0.9510679
19.9833336	2.8792000	-0.9756117
19.9833336	2.9280000	-0.8344855
19.9833336	2.9768000	-0.8958449
19.9833336	3.0256000	-0.8897090
19.9833336	3.0743999	-0.9265246

19.9833336	3.1231999	-0.7915339
19.9833336	3.1719999	-0.7976699
19.9833336	3.2207999	-0.7608547
19.9833336	3.2695999	-1.0860586
19.9833336	3.3183999	-1.0860586
19.9833336	3.3671999	-0.9142528
19.9833336	3.4159999	-0.8099418
19.9833336	3.4647999	-0.8099418
19.9833336	3.5135999	-0.8897090
19.9833336	3.5623999	-0.8651652
19.9833336	3.6111999	-0.8651652
19.9833336	3.6599998	-0.8590293
19.9833336	3.7087998	-0.9326606
19.9833336	3.7575998	-0.9510679
19.9833336	3.8063998	-0.9510679
19.9833336	3.8551998	-0.9387960
19.9833336	3.9039998	-0.8651652
19.9833336	3.9527998	-0.8651652
19.9833336	4.0016003	-0.8651652
19.9833336	4.0503998	-0.9326606
19.9833336	4.0991998	-0.9142528
19.9833336	4.1479998	-0.7117672
19.9833336	4.1967998	-0.7240391
19.9833336	4.2455997	-0.7301750
19.9833336	4.2943997	-0.6197281
19.9833336	4.3431997	-0.4479222
19.9833336	4.3919997	-0.4479222
19.9833336	4.4407997	-0.6504078
19.9833336	4.4895997	-0.8344855
19.9833336	4.5383997	-0.6258640
19.9833336	4.5871997	-0.6258640
19.9833336	4.6359997	-0.5829129
19.9833336	4.6847997	-0.6197281
19.9833336	4.7336001	-0.6197281
19.9833336	4.7824001	-0.4847379
19.9833336	4.8312001	-0.4111071
19.9833336	4.8800001	-0.5338254
19.9833336	4.9288001	-0.6013203
19.9833336	4.9776001	-0.6442719
19.9833336	5.0264001	-0.6565437
19.9833336	5.0752001	-0.6442719
19.9833336	5.1240001	-0.6320000
19.9833336	5.1728001	-0.6320000
19.9833336	5.2216001	-0.4970098
19.9833336	5.2704000	-0.4970098
19.9833336	5.3192000	-0.4786019
19.9833336	5.3680000	-0.6626797
19.9833336	5.4168000	-0.6688156
19.9833336	5.4656000	-0.6320000
19.9833336	5.5144000	-0.6320000
19.9833336	5.5632000	-0.6320000
19.9833336	5.6120000	-0.6258640
19.9833336	5.6608000	-0.6135921
19.9833336	5.7096000	-0.7424469
19.9833336	5.7584000	-0.7363110
19.9833336	5.8072000	-0.6442719
19.9833336	5.8559999	-0.6504078

19.9833336	5.9047999	-0.6565437
19.9833336	5.9535999	-0.6197281
19.9833336	6.0023999	-0.5951843
20.0000000	0.2928000	-4.5037680
20.0000000	0.3416000	-2.5341363
20.0000000	0.3904000	-2.5341363
20.0000000	0.4392000	-1.6935148
20.0000000	0.4880000	-1.4971652
20.0000000	0.5368000	-1.3314953
20.0000000	0.5856000	-1.3253593
20.0000000	0.6344000	-1.3130875
20.0000000	0.6832000	-1.3989906
20.0000000	0.7320000	-1.3989906
20.0000000	0.7808000	-1.3376312
20.0000000	0.8296000	-1.3376312
20.0000000	0.8784000	-1.4971652
20.0000000	0.9272000	-1.4910293
20.0000000	0.9760000	-1.8039613
20.0000000	1.0247999	-1.7794180
20.0000000	1.0735999	-1.5892043
20.0000000	1.1223999	-1.8530488
20.0000000	1.1711999	-1.8407769
20.0000000	1.2200000	-1.7855535
20.0000000	1.2688000	-1.6444273
20.0000000	1.3176000	-1.1167378
20.0000000	1.3664000	-1.0369711
20.0000000	1.4152000	-1.2455926
20.0000000	1.4640000	-1.2455926
20.0000000	1.5128000	-1.0369711
20.0000000	1.5616000	-1.0431070
20.0000000	1.6104000	-0.8283496
20.0000000	1.6592000	-0.8406215
20.0000000	1.7079999	-0.9572039
20.0000000	1.7567999	-1.1412816
20.0000000	1.8055999	-1.1351457
20.0000000	1.8543999	-1.1412816
20.0000000	1.9031999	-1.0799227
20.0000000	1.9519999	-1.1412816
20.0000000	2.0007999	-1.1290097
20.0000000	2.0495999	-1.0553789
20.0000000	2.0983999	-1.0615149
20.0000000	2.1471999	-1.1412816
20.0000000	2.1959999	-1.1351457
20.0000000	2.2447999	-1.2087770
20.0000000	2.2935998	-1.2087770
20.0000000	2.3423998	-1.1412816
20.0000000	2.3912001	-1.1412816
20.0000000	2.4400001	-1.1412816
20.0000000	2.4888000	-1.1351457
20.0000000	2.5376000	-1.1351457
20.0000000	2.5864000	-1.0553789
20.0000000	2.6352000	-1.0615149
20.0000000	2.6840000	-1.0615149
20.0000000	2.7328000	-0.9817476
20.0000000	2.7816000	-1.1290097
20.0000000	2.8304000	-0.9081168
20.0000000	2.8792000	-0.9142528

20.0000000	2.9280000	-1.0553789
20.0000000	2.9768000	-0.8897090
20.0000000	3.0256000	-0.7853980
20.0000000	3.0743999	-0.7853980
20.0000000	3.1231999	-0.7608547
20.0000000	3.1719999	-0.7792621
20.0000000	3.2207999	-0.8897090
20.0000000	3.2695999	-0.8958449
20.0000000	3.3183999	-0.9019809
20.0000000	3.3671999	-0.6872234
20.0000000	3.4159999	-0.7056313
20.0000000	3.4647999	-0.7915339
20.0000000	3.5135999	-0.9633398
20.0000000	3.5623999	-0.8467574
20.0000000	3.6111999	-0.9142528
20.0000000	3.6599998	-0.9142528
20.0000000	3.7087998	-0.9081168
20.0000000	3.7575998	-0.9019809
20.0000000	3.8063998	-0.8590293
20.0000000	3.8551998	-0.6565437
20.0000000	3.9039998	-0.8835731
20.0000000	3.9527998	-0.8835731
20.0000000	4.0016003	-0.9081168
20.0000000	4.0503998	-0.9019809
20.0000000	4.0991998	-0.5583692
20.0000000	4.1479998	-0.9817476
20.0000000	4.1967998	-0.7240391
20.0000000	4.2455997	-1.0185633
20.0000000	4.2943997	-0.5583692
20.0000000	4.3431997	-0.5522332
20.0000000	4.3919997	-0.7301750
20.0000000	4.4407997	-0.7547188
20.0000000	4.4895997	-0.7608547
20.0000000	4.5383997	-0.6565437
20.0000000	4.5871997	-0.6749516
20.0000000	4.6359997	-0.6626797
20.0000000	4.6847997	-0.5583692
20.0000000	4.7336001	-0.5767770
20.0000000	4.7824001	-0.5706410
20.0000000	4.8312001	-0.6688156
20.0000000	4.8800001	-0.6135921
20.0000000	4.9288001	-0.6135921
20.0000000	4.9776001	-0.6135921
20.0000000	5.0264001	-0.5522332
20.0000000	5.0752001	-0.5767770
20.0000000	5.1240001	-0.5399613
20.0000000	5.1728001	-0.5583692
20.0000000	5.2216001	-0.5767770
20.0000000	5.2704000	-0.5890489
20.0000000	5.3192000	-0.6626797
20.0000000	5.3680000	-0.6320000
20.0000000	5.4168000	-0.7424469
20.0000000	5.4656000	-0.6320000
20.0000000	5.5144000	-0.6872234
20.0000000	5.5632000	-0.7056313
20.0000000	5.6120000	-0.6933594
20.0000000	5.6608000	-0.7301750

20.0000000	5.7096000	-0.6135921
20.0000000	5.7584000	-0.6074562
20.0000000	5.8072000	-0.6074562
20.0000000	5.8559999	613586.0000000
20.0000000	5.9047999	-0.6749516
20.0000000	5.9535999	-0.5829129
20.0000000	6.0023999	-0.5829129
20.0166664	0.2928000	-2.9022918
20.0166664	0.3416000	-2.6691267
20.0166664	0.3904000	-1.6996508
20.0166664	0.4392000	-1.1106019
20.0166664	0.4880000	-1.0615149
20.0166664	0.5368000	-1.0676508
20.0166664	0.5856000	-1.2701364
20.0166664	0.6344000	-1.1474175
20.0166664	0.6832000	-1.1474175
20.0166664	0.7320000	-1.3008156
20.0166664	0.7808000	-1.3069515
20.0166664	0.8296000	-1.4296703
20.0166664	0.8784000	-1.4296703
20.0166664	0.9272000	-1.3560390
20.0166664	0.9760000	-1.3376312
20.0166664	1.0247999	-1.1780972
20.0166664	1.0735999	-1.4235344
20.0166664	1.1223999	-1.4235344
20.0166664	1.1711999	-1.2026410
20.0166664	1.2200000	-1.3805828
20.0166664	1.2688000	-1.3621750
20.0166664	1.3176000	-1.3621750
20.0166664	1.3664000	-1.3192234
20.0166664	1.4152000	-1.2087770
20.0166664	1.4640000	-0.9510679
20.0166664	1.5128000	-1.0062914
20.0166664	1.5616000	-1.0615149
20.0166664	1.6104000	-1.0615149
20.0166664	1.6592000	-1.1351457
20.0166664	1.7079999	-1.3253593
20.0166664	1.7567999	-1.0431070
20.0166664	1.8055999	-1.0369711
20.0166664	1.8543999	-1.0983305
20.0166664	1.9031999	-1.0921946
20.0166664	1.9519999	-1.3130875
20.0166664	2.0007999	-1.1474175
20.0166664	2.0495999	-1.0308352
20.0166664	2.0983999	-1.0308352
20.0166664	2.1471999	-1.0185633
20.0166664	2.1959999	-1.0185633
20.0166664	2.2447999	-1.0246992
20.0166664	2.2935998	-1.0246992
20.0166664	2.3423998	-1.0553789
20.0166664	2.3912001	-1.0492430
20.0166664	2.4400001	-1.1903691
20.0166664	2.4888000	-1.0062914
20.0166664	2.5376000	-1.0062914
20.0166664	2.5864000	-0.8958449
20.0166664	2.6352000	-0.8897090
20.0166664	2.6840000	-0.8897090

20.0166664	2.7328000	-0.8160777
20.0166664	2.7816000	-0.7179031
20.0166664	2.8304000	-0.7117672
20.0166664	2.8792000	-0.7608547
20.0166664	2.9280000	-0.7608547
20.0166664	2.9768000	-0.7424469
20.0166664	3.0256000	-0.6381359
20.0166664	3.0743999	-0.6381359
20.0166664	3.1231999	-0.8344855
20.0166664	3.1719999	-0.9756117
20.0166664	3.2207999	-0.9265246
20.0166664	3.2695999	-1.0124273
20.0166664	3.3183999	-1.0062914
20.0166664	3.3671999	-1.0001554
20.0166664	3.4159999	-0.8835731
20.0166664	3.4647999	-1.0676508
20.0166664	3.5135999	-0.9449320
20.0166664	3.5623999	-0.7485828
20.0166664	3.6111999	-0.7547188
20.0166664	3.6599998	-1.0369711
20.0166664	3.7087998	-1.0369711
20.0166664	3.7575998	-1.0492430
20.0166664	3.8063998	-0.8099418
20.0166664	3.8551998	-0.7731261
20.0166664	3.9039998	-1.0983305
20.0166664	3.9527998	-1.0983305
20.0166664	4.0016003	-1.0185633
20.0166664	4.0503998	-0.9265246
20.0166664	4.0991998	-0.6688156
20.0166664	4.1479998	-0.6810875
20.0166664	4.1967998	-0.4908738
20.0166664	4.2455997	-0.5583692
20.0166664	4.2943997	-0.7301750
20.0166664	4.3431997	-0.6258640
20.0166664	4.3919997	-0.6258640
20.0166664	4.4407997	-0.7363110
20.0166664	4.4895997	-0.6872234
20.0166664	4.5383997	-0.6565437
20.0166664	4.5871997	-0.5522332
20.0166664	4.6359997	-0.5583692
20.0166664	4.6847997	-0.5460973
20.0166664	4.7336001	-0.4540582
20.0166664	4.7824001	-0.6749516
20.0166664	4.8312001	-0.5951843
20.0166664	4.8800001	-0.5092816
20.0166664	4.9288001	-0.5092816
20.0166664	4.9776001	-0.6933594
20.0166664	5.0264001	-0.5522332
20.0166664	5.0752001	-0.6013203
20.0166664	5.1240001	-0.6688156
20.0166664	5.1728001	-0.6749516
20.0166664	5.2216001	-0.7363110
20.0166664	5.2704000	-0.8344855
20.0166664	5.3192000	-0.8344855
20.0166664	5.3680000	-0.6442719
20.0166664	5.4168000	-0.6442719
20.0166664	5.4656000	-0.6994953

20.0166664	5.5144000	-0.6994953
20.0166664	5.5632000	-0.6320000
20.0166664	5.6120000	-0.6258640
20.0166664	5.6608000	-0.6074562
20.0166664	5.7096000	-0.6504078
20.0166664	5.7584000	-0.5829129
20.0166664	5.8072000	-0.7117672
20.0166664	5.8559999	-0.6381359
20.0166664	5.9047999	-0.5645051
20.0166664	5.9535999	613586.0000000
20.0166664	6.0023999	613586.0000000
20.0333328	0.2928000	-3.5281558
20.0333328	0.3416000	-2.0800781
20.0333328	0.3904000	-2.0800781
20.0333328	0.4392000	-1.4358063
20.0333328	0.4880000	-1.4419422
20.0333328	0.5368000	-1.5953403
20.0333328	0.5856000	-1.5033011
20.0333328	0.6344000	-1.2824078
20.0333328	0.6832000	-1.1351457
20.0333328	0.7320000	-1.1965051
20.0333328	0.7808000	-1.1965051
20.0333328	0.8296000	-1.1780972
20.0333328	0.8784000	-1.3314953
20.0333328	0.9272000	-1.3805828
20.0333328	0.9760000	-1.1903691
20.0333328	1.0247999	-1.1903691
20.0333328	1.0735999	-1.0921946
20.0333328	1.1223999	-1.0983305
20.0333328	1.1711999	-1.0615149
20.0333328	1.2200000	-1.0553789
20.0333328	1.2688000	-1.3192234
20.0333328	1.3176000	-1.3621750
20.0333328	1.3664000	-1.2701364
20.0333328	1.4152000	-0.9081168
20.0333328	1.4640000	-1.0921946
20.0333328	1.5128000	-1.2149129
20.0333328	1.5616000	-0.9081168
20.0333328	1.6104000	-0.9265246
20.0333328	1.6592000	-0.8406215
20.0333328	1.7079999	-0.9633398
20.0333328	1.7567999	-1.2517285
20.0333328	1.8055999	-1.1106019
20.0333328	1.8543999	-1.3130875
20.0333328	1.9031999	-1.3008156
20.0333328	1.9519999	-1.1290097
20.0333328	2.0007999	-1.0615149
20.0333328	2.0495999	-1.0983305
20.0333328	2.0983999	-0.9081168
20.0333328	2.1471999	-0.9142528
20.0333328	2.1959999	-1.0860586
20.0333328	2.2447999	-1.0860586
20.0333328	2.2935998	-1.1474175
20.0333328	2.3423998	-0.9756117
20.0333328	2.3912001	-0.9326606
20.0333328	2.4400001	-0.7853980
20.0333328	2.4888000	-0.7915339

20.0333328	2.5376000	-0.7915339
20.0333328	2.5864000	-0.6504078
20.0333328	2.6352000	-0.7117672
20.0333328	2.6840000	-0.7056313
20.0333328	2.7328000	-0.7547188
20.0333328	2.7816000	-0.6074562
20.0333328	2.8304000	-0.6197281
20.0333328	2.8792000	-0.7301750
20.0333328	2.9280000	-0.8835731
20.0333328	2.9768000	-0.8835731
20.0333328	3.0256000	-1.2885437
20.0333328	3.0743999	-1.2885437
20.0333328	3.1231999	-1.4358063
20.0333328	3.1719999	-1.2210488
20.0333328	3.2207999	-1.1965051
20.0333328	3.2695999	-1.0676508
20.0333328	3.3183999	-0.9572039
20.0333328	3.3671999	-0.9572039
20.0333328	3.4159999	-1.0062914
20.0333328	3.4647999	-0.9449320
20.0333328	3.5135999	-0.9387960
20.0333328	3.5623999	-0.7117672
20.0333328	3.6111999	-0.7179031
20.0333328	3.6599998	-0.7117672
20.0333328	3.7087998	-0.7976699
20.0333328	3.7575998	-0.7117672
20.0333328	3.8063998	-0.7853980
20.0333328	3.8551998	-0.5154176
20.0333328	3.9039998	-0.7853980
20.0333328	3.9527998	-0.7853980
20.0333328	4.0016003	-0.8222136
20.0333328	4.0503998	-0.5522332
20.0333328	4.0991998	-0.5583692
20.0333328	4.1479998	-0.5583692
20.0333328	4.1967998	-0.7117672
20.0333328	4.2455997	-0.5215535
20.0333328	4.2943997	-0.5276895
20.0333328	4.3431997	-0.6197281
20.0333328	4.3919997	-0.6381359
20.0333328	4.4407997	-0.7240391
20.0333328	4.4895997	-0.5890489
20.0333328	4.5383997	-0.5338254
20.0333328	4.5871997	-0.5338254
20.0333328	4.6359997	-0.4295144
20.0333328	4.6847997	-0.5706410
20.0333328	4.7336001	-0.5583692
20.0333328	4.7824001	-0.5460973
20.0333328	4.8312001	-0.5460973
20.0333328	4.8800001	-0.5399613
20.0333328	4.9288001	-0.5399613
20.0333328	4.9776001	-0.5338254
20.0333328	5.0264001	-0.5522332
20.0333328	5.0752001	-0.4786019
20.0333328	5.1240001	-0.5215535
20.0333328	5.1728001	-0.4295144
20.0333328	5.2216001	-0.4908738
20.0333328	5.2704000	-0.4847379

20.0333328	5.3192000	-0.4786019
20.0333328	5.3680000	-0.4786019
20.0333328	5.4168000	-0.3067961
20.0333328	5.4656000	-0.2822523
20.0333328	5.5144000	-0.2883883
20.0333328	5.5632000	-0.2883883
20.0333328	5.6120000	-0.4417863
20.0333328	5.6608000	-0.5338254
20.0333328	5.7096000	-0.5276895
20.0333328	5.7584000	-0.4172430
20.0333328	5.8072000	-0.4295144
20.0333328	5.8559999	-0.3681555
20.0333328	5.9047999	-0.4233789
20.0333328	5.9535999	-0.4663301
20.0333328	6.0023999	-0.3988352
20.0499992	0.2928000	-3.9638064
20.0499992	0.3416000	-3.9638064
20.0499992	0.3904000	-2.5280004
20.0499992	0.4392000	-2.1843886
20.0499992	0.4880000	-1.5707965
20.0499992	0.5368000	-1.3253593
20.0499992	0.5856000	-1.3867188
20.0499992	0.6344000	-0.9572039
20.0499992	0.6832000	-1.0492430
20.0499992	0.7320000	-1.0553789
20.0499992	0.7808000	-1.0860586
20.0499992	0.8296000	-1.0983305
20.0499992	0.8784000	-1.1474175
20.0499992	0.9272000	-1.2087770
20.0499992	0.9760000	-0.9633398
20.0499992	1.0247999	-0.9633398
20.0499992	1.0735999	-1.1167378
20.0499992	1.1223999	-1.1290097
20.0499992	1.1711999	-1.1535535
20.0499992	1.2200000	-1.1596894
20.0499992	1.2688000	-1.0492430
20.0499992	1.3176000	-1.0492430
20.0499992	1.3664000	-1.0185633
20.0499992	1.4152000	-1.0185633
20.0499992	1.4640000	-0.8038058
20.0499992	1.5128000	-1.3989906
20.0499992	1.5616000	-1.4848933
20.0499992	1.6104000	-1.3069515
20.0499992	1.6592000	-1.3069515
20.0499992	1.7079999	-1.3130875
20.0499992	1.7567999	-1.1965051
20.0499992	1.8055999	-1.2087770
20.0499992	1.8543999	-1.2149129
20.0499992	1.9031999	-1.2149129
20.0499992	1.9519999	-1.0860586
20.0499992	2.0007999	-1.2701364
20.0499992	2.0495999	-1.2701364
20.0499992	2.0983999	-1.1596894
20.0499992	2.1471999	-1.2701364
20.0499992	2.1959999	-1.2701364
20.0499992	2.2447999	-1.1719613
20.0499992	2.2935998	-1.1596894

20.0499992	2.3423998	-1.1596894
20.0499992	2.3912001	-1.0737867
20.0499992	2.4400001	-1.0676508
20.0499992	2.4888000	-1.0553789
20.0499992	2.5376000	-0.8099418
20.0499992	2.5864000	-0.7792621
20.0499992	2.6352000	-0.7792621
20.0499992	2.6840000	-0.7792621
20.0499992	2.7328000	-0.7424469
20.0499992	2.7816000	-0.7608547
20.0499992	2.8304000	-0.6381359
20.0499992	2.8792000	-0.6320000
20.0499992	2.9280000	-0.6320000
20.0499992	2.9768000	-0.8958449
20.0499992	3.0256000	-0.8344855
20.0499992	3.0743999	-0.8406215
20.0499992	3.1231999	-0.9387960
20.0499992	3.1719999	-0.9387960
20.0499992	3.2207999	-1.0369711
20.0499992	3.2695999	-1.0369711
20.0499992	3.3183999	-0.8467574
20.0499992	3.3671999	-0.8406215
20.0499992	3.4159999	-0.8283496
20.0499992	3.4647999	-0.7915339
20.0499992	3.5135999	-0.8038058
20.0499992	3.5623999	-0.7424469
20.0499992	3.6111999	-0.7424469
20.0499992	3.6599998	-0.7485828
20.0499992	3.7087998	-0.8590293
20.0499992	3.7575998	-0.9081168
20.0499992	3.8063998	-0.8160777
20.0499992	3.8551998	-0.7853980
20.0499992	3.9039998	-0.7608547
20.0499992	3.9527998	-0.8160777
20.0499992	4.0016003	-1.0124273
20.0499992	4.0503998	-0.7301750
20.0499992	4.0991998	-0.8835731
20.0499992	4.1479998	-0.7179031
20.0499992	4.1967998	-0.7179031
20.0499992	4.2455997	-0.7363110
20.0499992	4.2943997	-0.9019809
20.0499992	4.3431997	-0.7915339
20.0499992	4.3919997	-0.7853980
20.0499992	4.4407997	-0.6994953
20.0499992	4.4895997	-0.6872234
20.0499992	4.5383997	-0.7915339
20.0499992	4.5871997	-0.8835731
20.0499992	4.6359997	-0.8406215
20.0499992	4.6847997	-0.7547188
20.0499992	4.7336001	-0.9019809
20.0499992	4.7824001	-0.9019809
20.0499992	4.8312001	-0.8467574
20.0499992	4.8800001	-0.8467574
20.0499992	4.9288001	-0.8467574
20.0499992	4.9776001	-0.9756117
20.0499992	5.0264001	-0.8958449
20.0499992	5.0752001	-0.8958449

20.0499992	5.1240001	-0.8958449
20.0499992	5.1728001	-0.8958449
20.0499992	5.2216001	-0.8344855
20.0499992	5.2704000	-0.8344855
20.0499992	5.3192000	-0.8344855
20.0499992	5.3680000	-0.9326606
20.0499992	5.4168000	-0.9326606
20.0499992	5.4656000	-0.9081168
20.0499992	5.5144000	-0.7976699
20.0499992	5.5632000	-0.7976699
20.0499992	5.6120000	-0.8099418
20.0499992	5.6608000	-0.7240391
20.0499992	5.7096000	-0.7240391
20.0499992	5.7584000	-0.5890489
20.0499992	5.8072000	-0.5890489
20.0499992	5.8559999	-0.5890489
20.0499992	5.9047999	-0.5767770
20.0499992	5.9535999	613586.0000000
20.0499992	6.0023999	613586.0000000
20.0666676	0.2928000	-2.4727771
20.0666676	0.3416000	-2.3684664
20.0666676	0.3904000	-2.3746023
20.0666676	0.4392000	-1.8285050
20.0666676	0.4880000	-1.8285050
20.0666676	0.5368000	-1.1167378
20.0666676	0.5856000	-0.9694757
20.0666676	0.6344000	-1.2026410
20.0666676	0.6832000	-1.2701364
20.0666676	0.7320000	-1.1474175
20.0666676	0.7808000	-1.1474175
20.0666676	0.8296000	-1.0921946
20.0666676	0.8784000	-1.3253593
20.0666676	0.9272000	-1.4480777
20.0666676	0.9760000	-1.2701364
20.0666676	1.0247999	-1.2640004
20.0666676	1.0735999	-1.1044660
20.0666676	1.1223999	-1.1044660
20.0666676	1.1711999	-0.9572039
20.0666676	1.2200000	-1.1167378
20.0666676	1.2688000	-1.1044660
20.0666676	1.3176000	-1.1044660
20.0666676	1.3664000	-1.1044660
20.0666676	1.4152000	-1.0676508
20.0666676	1.4640000	-1.0492430
20.0666676	1.5128000	-1.3560390
20.0666676	1.5616000	-1.0921946
20.0666676	1.6104000	-1.1412816
20.0666676	1.6592000	-1.2824078
20.0666676	1.7079999	-1.2824078
20.0666676	1.7567999	-1.2517285
20.0666676	1.8055999	-1.1106019
20.0666676	1.8543999	-1.3008156
20.0666676	1.9031999	-1.1719613
20.0666676	1.9519999	-1.2762718
20.0666676	2.0007999	-1.1106019
20.0666676	2.0495999	-1.1842332
20.0666676	2.0983999	-1.1903691

20.0666676	2.1471999	-1.2885437
20.0666676	2.1959999	-1.2824078
20.0666676	2.2447999	-1.2824078
20.0666676	2.2935998	-1.1228738
20.0666676	2.3423998	-1.2394567
20.0666676	2.3912001	-1.1044660
20.0666676	2.4400001	-1.1106019
20.0666676	2.4888000	-1.1351457
20.0666676	2.5376000	-0.9572039
20.0666676	2.5864000	-0.9265246
20.0666676	2.6352000	-0.8344855
20.0666676	2.6840000	-0.9756117
20.0666676	2.7328000	-0.9142528
20.0666676	2.7816000	-0.9019809
20.0666676	2.8304000	-0.8835731
20.0666676	2.8792000	-0.8897090
20.0666676	2.9280000	-0.6320000
20.0666676	2.9768000	-0.6197281
20.0666676	3.0256000	-0.7915339
20.0666676	3.0743999	-0.7792621
20.0666676	3.1231999	-0.7976699
20.0666676	3.1719999	-0.4663301
20.0666676	3.2207999	-0.4908738
20.0666676	3.2695999	-0.4847379
20.0666676	3.3183999	-0.4786019
20.0666676	3.3671999	-0.6810875
20.0666676	3.4159999	-0.7485828
20.0666676	3.4647999	-0.7608547
20.0666676	3.5135999	-0.7608547
20.0666676	3.5623999	-0.7608547
20.0666676	3.6111999	-0.7056313
20.0666676	3.6599998	-0.6258640
20.0666676	3.7087998	-0.6197281
20.0666676	3.7575998	-0.7301750
20.0666676	3.8063998	-0.5645051
20.0666676	3.8551998	-0.5829129
20.0666676	3.9039998	-0.7301750
20.0666676	3.9527998	-0.7301750
20.0666676	4.0016003	-0.7424469
20.0666676	4.0503998	-0.7179031
20.0666676	4.0991998	-0.7424469
20.0666676	4.1479998	-0.7363110
20.0666676	4.1967998	-0.5767770
20.0666676	4.2455997	-0.5951843
20.0666676	4.2943997	-0.5767770
20.0666676	4.3431997	-0.5092816
20.0666676	4.3919997	-0.4479222
20.0666676	4.4407997	-0.5706410
20.0666676	4.4895997	-0.5706410
20.0666676	4.5383997	-0.5767770
20.0666676	4.5871997	-0.5645051
20.0666676	4.6359997	-0.4786019
20.0666676	4.6847997	-0.5522332
20.0666676	4.7336001	-0.5767770
20.0666676	4.7824001	-0.5890489
20.0666676	4.8312001	-0.5890489
20.0666676	4.8800001	-0.5215535

20.0666676	4.9288001	-0.4540582
20.0666676	4.9776001	-0.4663301
20.0666676	5.0264001	-0.4356503
20.0666676	5.0752001	-0.4295144
20.0666676	5.1240001	-0.5154176
20.0666676	5.1728001	-0.4540582
20.0666676	5.2216001	-0.5215535
20.0666676	5.2704000	-0.5829129
20.0666676	5.3192000	-0.6013203
20.0666676	5.3680000	-0.4847379
20.0666676	5.4168000	-0.6381359
20.0666676	5.4656000	-0.4970098
20.0666676	5.5144000	-0.4970098
20.0666676	5.5632000	-0.4970098
20.0666676	5.6120000	-0.5031457
20.0666676	5.6608000	-0.4724660
20.0666676	5.7096000	-0.4908738
20.0666676	5.7584000	-0.5829129
20.0666676	5.8072000	-0.5706410
20.0666676	5.8559999	613586.0000000
20.0666676	5.9047999	-0.4970098
20.0666676	5.9535999	-0.5031457
20.0666676	6.0023999	613586.0000000
20.0833340	0.2928000	-2.6077673
20.0833340	0.3416000	-2.2641559
20.0833340	0.3904000	-2.2273402
20.0833340	0.4392000	-1.8837285
20.0833340	0.4880000	-1.7671461
20.0833340	0.5368000	-1.9696312
20.0833340	0.5856000	-1.9144082
20.0833340	0.6344000	-1.7671461
20.0833340	0.6832000	-1.4419422
20.0833340	0.7320000	-1.4296703
20.0833340	0.7808000	-1.4787574
20.0833340	0.8296000	-1.4787574
20.0833340	0.8784000	-1.4235344
20.0833340	0.9272000	-1.1412816
20.0833340	0.9760000	-1.2701364
20.0833340	1.0247999	-1.0615149
20.0833340	1.0735999	-0.9203887
20.0833340	1.1223999	-0.9081168
20.0833340	1.1711999	-1.2026410
20.0833340	1.2200000	-1.2762718
20.0833340	1.2688000	-1.2762718
20.0833340	1.3176000	-1.2517285
20.0833340	1.3664000	-1.0860586
20.0833340	1.4152000	-1.0860586
20.0833340	1.4640000	-1.2701364
20.0833340	1.5128000	-0.9142528
20.0833340	1.5616000	-1.2824078
20.0833340	1.6104000	-1.2885437
20.0833340	1.6592000	-1.2946796
20.0833340	1.7079999	-1.1228738
20.0833340	1.7567999	-1.1903691
20.0833340	1.8055999	-1.0676508
20.0833340	1.8543999	-1.2578645
20.0833340	1.9031999	-1.2149129

20.0833340	1.9519999	-1.2087770
20.0833340	2.0007999	-1.1903691
20.0833340	2.0495999	-1.3008156
20.0833340	2.0983999	-1.0737867
20.0833340	2.1471999	-1.0737867
20.0833340	2.1959999	-1.0737867
20.0833340	2.2447999	-1.2333207
20.0833340	2.2935998	-1.1903691
20.0833340	2.3423998	-1.0308352
20.0833340	2.3912001	-1.0369711
20.0833340	2.4400001	-1.0062914
20.0833340	2.4888000	-0.9510679
20.0833340	2.5376000	-0.9449320
20.0833340	2.5864000	-0.8344855
20.0833340	2.6352000	-0.8897090
20.0833340	2.6840000	-0.8897090
20.0833340	2.7328000	-0.9203887
20.0833340	2.7816000	-0.9265246
20.0833340	2.8304000	-0.9142528
20.0833340	2.8792000	-0.6872234
20.0833340	2.9280000	-0.3374758
20.0833340	2.9768000	-0.6749516
20.0833340	3.0256000	-0.6320000
20.0833340	3.0743999	-0.7485828
20.0833340	3.1231999	-0.7485828
20.0833340	3.1719999	-0.7424469
20.0833340	3.2207999	-0.8038058
20.0833340	3.2695999	-0.8528934
20.0833340	3.3183999	-0.8344855
20.0833340	3.3671999	-0.8774371
20.0833340	3.4159999	-0.7240391
20.0833340	3.4647999	-0.5645051
20.0833340	3.5135999	-0.7301750
20.0833340	3.5623999	-0.6442719
20.0833340	3.6111999	-0.6504078
20.0833340	3.6599998	-0.6565437
20.0833340	3.7087998	-0.7240391
20.0833340	3.7575998	-0.7240391
20.0833340	3.8063998	-0.5399613
20.0833340	3.8551998	-0.5583692
20.0833340	3.9039998	-0.4540582
20.0833340	3.9527998	-0.6749516
20.0833340	4.0016003	-0.6810875
20.0833340	4.0503998	-0.6197281
20.0833340	4.0991998	-0.6320000
20.0833340	4.1479998	-0.7179031
20.0833340	4.1967998	-0.7240391
20.0833340	4.2455997	-0.6749516
20.0833340	4.2943997	-0.5215535
20.0833340	4.3431997	-0.5767770
20.0833340	4.3919997	-0.5829129
20.0833340	4.4407997	-0.5645051
20.0833340	4.4895997	-0.3926992
20.0833340	4.5383997	-0.4663301
20.0833340	4.5871997	-0.4724660
20.0833340	4.6359997	-0.4724660
20.0833340	4.6847997	-0.4847379

20.0833340	4.7336001	-0.6258640
20.0833340	4.7824001	-0.6320000
20.0833340	4.8312001	-0.6688156
20.0833340	4.8800001	-0.5890489
20.0833340	4.9288001	-0.6565437
20.0833340	4.9776001	-0.6504078
20.0833340	5.0264001	-0.7240391
20.0833340	5.0752001	-0.7179031
20.0833340	5.1240001	-0.5522332
20.0833340	5.1728001	-0.6013203
20.0833340	5.2216001	-0.6688156
20.0833340	5.2704000	-0.5951843
20.0833340	5.3192000	-0.5951843
20.0833340	5.3680000	-0.5399613
20.0833340	5.4168000	-0.4172430
20.0833340	5.4656000	-0.4049711
20.0833340	5.5144000	-0.3988352
20.0833340	5.5632000	-0.5338254
20.0833340	5.6120000	-0.5399613
20.0833340	5.6608000	-0.6258640
20.0833340	5.7096000	-0.5338254
20.0833340	5.7584000	-0.5767770
20.0833340	5.8072000	-0.6320000
20.0833340	5.8559999	613586.0000000
20.0833340	5.9047999	613586.0000000
20.0833340	5.9535999	-0.5706410
20.0833340	6.0023999	613586.0000000
20.1000004	0.2928000	-2.9391072
20.1000004	0.3416000	-2.5095925
20.1000004	0.3904000	-2.3991461
20.1000004	0.4392000	-1.4664855
20.1000004	0.4880000	-1.4664855
20.1000004	0.5368000	-1.9696312
20.1000004	0.5856000	-1.9696312
20.1000004	0.6344000	-1.4112625
20.1000004	0.6832000	-1.4112625
20.1000004	0.7320000	-1.4112625
20.1000004	0.7808000	-1.4419422
20.1000004	0.8296000	-1.1780972
20.1000004	0.8784000	-1.3805828
20.1000004	0.9272000	-1.4726214
20.1000004	0.9760000	-1.3192234
20.1000004	1.0247999	-1.3069515
20.1000004	1.0735999	-1.3867188
20.1000004	1.1223999	-1.5401168
20.1000004	1.1711999	-1.5278449
20.1000004	1.2200000	-1.3867188
20.1000004	1.2688000	-1.3867188
20.1000004	1.3176000	-1.3008156
20.1000004	1.3664000	-1.4726214
20.1000004	1.4152000	-1.3560390
20.1000004	1.4640000	-1.3314953
20.1000004	1.5128000	-1.0615149
20.1000004	1.5616000	-1.2394567
20.1000004	1.6104000	-1.3069515
20.1000004	1.6592000	-1.3008156
20.1000004	1.7079999	-1.3008156

20.1000004	1.7567999	-1.2824078
20.1000004	1.8055999	-1.1719613
20.1000004	1.8543999	-1.1596894
20.1000004	1.9031999	-1.1474175
20.1000004	1.9519999	-1.2824078
20.1000004	2.0007999	-1.2762718
20.1000004	2.0495999	-1.0921946
20.1000004	2.0983999	-1.1965051
20.1000004	2.1471999	-1.0308352
20.1000004	2.1959999	-0.9940195
20.1000004	2.2447999	-1.1412816
20.1000004	2.2935998	-1.1535535
20.1000004	2.3423998	-1.1535535
20.1000004	2.3912001	-1.1780972
20.1000004	2.4400001	-1.0553789
20.1000004	2.4888000	-1.0553789
20.1000004	2.5376000	-0.7547188
20.1000004	2.5864000	-0.8958449
20.1000004	2.6352000	-0.8835731
20.1000004	2.6840000	-0.9572039
20.1000004	2.7328000	-0.9449320
20.1000004	2.7816000	-0.9572039
20.1000004	2.8304000	-1.1412816
20.1000004	2.8792000	-1.0860586
20.1000004	2.9280000	-1.1412816
20.1000004	2.9768000	-0.8651652
20.1000004	3.0256000	-1.0676508
20.1000004	3.0743999	-0.9510679
20.1000004	3.1231999	-1.0615149
20.1000004	3.1719999	-0.9878836
20.1000004	3.2207999	-1.0001554
20.1000004	3.2695999	-0.9940195
20.1000004	3.3183999	-0.7669902
20.1000004	3.3671999	-0.9633398
20.1000004	3.4159999	-0.9265246
20.1000004	3.4647999	-0.9203887
20.1000004	3.5135999	-0.9694757
20.1000004	3.5623999	-0.8835731
20.1000004	3.6111999	-0.9510679
20.1000004	3.6599998	-0.8590293
20.1000004	3.7087998	-1.0737867
20.1000004	3.7575998	-0.8038058
20.1000004	3.8063998	-0.9756117
20.1000004	3.8551998	-0.9633398
20.1000004	3.9039998	-0.9510679
20.1000004	3.9527998	-0.9510679
20.1000004	4.0016003	-0.8590293
20.1000004	4.0503998	-0.7608547
20.1000004	4.0991998	-0.7669902
20.1000004	4.1479998	-0.8222136
20.1000004	4.1967998	-0.8222136
20.1000004	4.2455997	-0.6626797
20.1000004	4.2943997	-0.6626797
20.1000004	4.3431997	-0.7485828
20.1000004	4.3919997	-0.5706410
20.1000004	4.4407997	-0.5031457
20.1000004	4.4895997	-0.6994953

20.1000004	4.5383997	-0.8038058
20.1000004	4.5871997	-0.5154176
20.1000004	4.6359997	-0.7363110
20.1000004	4.6847997	-0.7301750
20.1000004	4.7336001	-0.7792621
20.1000004	4.7824001	-0.7792621
20.1000004	4.8312001	-0.7792621
20.1000004	4.8800001	-0.7424469
20.1000004	4.9288001	-0.7301750
20.1000004	4.9776001	-0.7915339
20.1000004	5.0264001	-0.7915339
20.1000004	5.0752001	-0.5890489
20.1000004	5.1240001	-0.5890489
20.1000004	5.1728001	-0.7363110
20.1000004	5.2216001	-0.6074562
20.1000004	5.2704000	-0.6197281
20.1000004	5.3192000	-0.5829129
20.1000004	5.3680000	-0.5829129
20.1000004	5.4168000	-0.6872234
20.1000004	5.4656000	-0.5767770
20.1000004	5.5144000	-0.5031457
20.1000004	5.5632000	-0.5645051
20.1000004	5.6120000	-0.5276895
20.1000004	5.6608000	-0.5154176
20.1000004	5.7096000	-0.5767770
20.1000004	5.7584000	-0.5829129
20.1000004	5.8072000	-0.5829129
20.1000004	5.8559999	-0.5829129
20.1000004	5.9047999	-0.5706410
20.1000004	5.9535999	-0.5583692
20.1000004	6.0023999	613586.0000000
20.1166668	0.2928000	-3.5036123
20.1166668	0.3416000	-1.2394567
20.1166668	0.3904000	-1.6689711
20.1166668	0.4392000	-2.0248547
20.1166668	0.4880000	-1.2455926
20.1166668	0.5368000	-1.0921946
20.1166668	0.5856000	-1.0921946
20.1166668	0.6344000	-1.0799227
20.1166668	0.6832000	-1.0983305
20.1166668	0.7320000	-1.1228738
20.1166668	0.7808000	-1.0860586
20.1166668	0.8296000	-1.1719613
20.1166668	0.8784000	-1.2333207
20.1166668	0.9272000	-1.2333207
20.1166668	0.9760000	-1.5953403
20.1166668	1.0247999	-1.3621750
20.1166668	1.0735999	-1.2701364
20.1166668	1.1223999	-1.2394567
20.1166668	1.1711999	-1.2517285
20.1166668	1.2200000	-1.1535535
20.1166668	1.2688000	-0.9633398
20.1166668	1.3176000	-0.9694757
20.1166668	1.3664000	-1.0001554
20.1166668	1.4152000	-1.0185633
20.1166668	1.4640000	-1.3683109
20.1166668	1.5128000	-1.3621750

20.1166668	1.5616000	-1.2026410
20.1166668	1.6104000	-1.4235344
20.1166668	1.6592000	-1.2640004
20.1166668	1.7079999	-1.2701364
20.1166668	1.7567999	-1.3069515
20.1166668	1.8055999	-1.1719613
20.1166668	1.8543999	-1.1658254
20.1166668	1.9031999	-1.1658254
20.1166668	1.9519999	-1.2640004
20.1166668	2.0007999	-1.2640004
20.1166668	2.0495999	-1.3499031
20.1166668	2.0983999	-1.2210488
20.1166668	2.1471999	-1.2210488
20.1166668	2.1959999	-1.2210488
20.1166668	2.2447999	-1.2394567
20.1166668	2.2935998	-1.4664855
20.1166668	2.3423998	-1.3805828
20.1166668	2.3912001	-1.4112625
20.1166668	2.4400001	-1.2824078
20.1166668	2.4888000	-1.3928547
20.1166668	2.5376000	-1.1903691
20.1166668	2.5864000	-0.9756117
20.1166668	2.6352000	-1.1106019
20.1166668	2.6840000	-1.1167378
20.1166668	2.7328000	-0.7853980
20.1166668	2.7816000	-0.8406215
20.1166668	2.8304000	-0.6994953
20.1166668	2.8792000	-0.7056313
20.1166668	2.9280000	-0.7117672
20.1166668	2.9768000	-0.5645051
20.1166668	3.0256000	-0.7363110
20.1166668	3.0743999	-0.4663301
20.1166668	3.1231999	-0.8958449
20.1166668	3.1719999	-0.9142528
20.1166668	3.2207999	-0.8406215
20.1166668	3.2695999	-0.8344855
20.1166668	3.3183999	-0.8099418
20.1166668	3.3671999	-0.8958449
20.1166668	3.4159999	-0.8958449
20.1166668	3.4647999	-0.9694757
20.1166668	3.5135999	-0.8958449
20.1166668	3.5623999	-0.8897090
20.1166668	3.6111999	-1.0185633
20.1166668	3.6599998	-1.0185633
20.1166668	3.7087998	-1.1167378
20.1166668	3.7575998	-0.9572039
20.1166668	3.8063998	-0.9940195
20.1166668	3.8551998	-1.0553789
20.1166668	3.9039998	-0.9756117
20.1166668	3.9527998	-0.9817476
20.1166668	4.0016003	-0.9817476
20.1166668	4.0503998	-1.0062914
20.1166668	4.0991998	-1.0001554
20.1166668	4.1479998	-0.9019809
20.1166668	4.1967998	-0.8958449
20.1166668	4.2455997	-0.8099418
20.1166668	4.2943997	-0.8590293

20.1166668	4.3431997	-0.7485828
20.1166668	4.3919997	-0.6749516
20.1166668	4.4407997	-0.8774371
20.1166668	4.4895997	-0.8590293
20.1166668	4.5383997	-0.8713012
20.1166668	4.5871997	-0.8590293
20.1166668	4.6359997	-0.7547188
20.1166668	4.6847997	-0.8406215
20.1166668	4.7336001	-0.9633398
20.1166668	4.7824001	-0.8897090
20.1166668	4.8312001	-0.6872234
20.1166668	4.8800001	-0.6810875
20.1166668	4.9288001	-0.6504078
20.1166668	4.9776001	-0.7117672
20.1166668	5.0264001	-0.7117672
20.1166668	5.0752001	-0.7056313
20.1166668	5.1240001	-0.7179031
20.1166668	5.1728001	-0.8038058
20.1166668	5.2216001	-0.8222136
20.1166668	5.2704000	-0.6258640
20.1166668	5.3192000	-0.7179031
20.1166668	5.3680000	-0.6994953
20.1166668	5.4168000	-0.7056313
20.1166668	5.4656000	-0.5645051
20.1166668	5.5144000	-0.5583692
20.1166668	5.5632000	-0.5522332
20.1166668	5.6120000	-0.6013203
20.1166668	5.6608000	-0.6258640
20.1166668	5.7096000	-0.6258640
20.1166668	5.7584000	613586.0000000
20.1166668	5.8072000	613586.0000000
20.1166668	5.8559999	-0.5951843
20.1166668	5.9047999	-0.5951843
20.1166668	5.9535999	-0.5276895
20.1166668	6.0023999	613586.0000000
20.1333332	0.2928000	-2.4850490
20.1333332	0.3416000	-2.0862141
20.1333332	0.3904000	-1.9328160
20.1333332	0.4392000	-1.2087770
20.1333332	0.4880000	-1.3621750
20.1333332	0.5368000	-1.3621750
20.1333332	0.5856000	-1.2394567
20.1333332	0.6344000	-1.1965051
20.1333332	0.6832000	-1.2455926
20.1333332	0.7320000	-1.2578645
20.1333332	0.7808000	-1.2026410
20.1333332	0.8296000	-0.9817476
20.1333332	0.8784000	-0.9756117
20.1333332	0.9272000	-1.2824078
20.1333332	0.9760000	-1.1780972
20.1333332	1.0247999	-1.3314953
20.1333332	1.0735999	-1.3376312
20.1333332	1.1223999	-1.4358063
20.1333332	1.1711999	-1.5217090
20.1333332	1.2200000	-1.4910293
20.1333332	1.2688000	-1.3008156
20.1333332	1.3176000	-1.1106019

20.1333332	1.3664000	-1.3805828
20.1333332	1.4152000	-1.0369711
20.1333332	1.4640000	-1.1351457
20.1333332	1.5128000	-1.1474175
20.1333332	1.5616000	-1.3253593
20.1333332	1.6104000	-1.3989906
20.1333332	1.6592000	-1.3989906
20.1333332	1.7079999	-1.3989906
20.1333332	1.7567999	-1.3560390
20.1333332	1.8055999	-1.3621750
20.1333332	1.8543999	-1.4542136
20.1333332	1.9031999	-1.2946796
20.1333332	1.9519999	-1.2946796
20.1333332	2.0007999	-1.2394567
20.1333332	2.0495999	-1.5094371
20.1333332	2.0983999	-1.1412816
20.1333332	2.1471999	-1.1412816
20.1333332	2.1959999	-1.0921946
20.1333332	2.2447999	-1.0983305
20.1333332	2.2935998	-1.3989906
20.1333332	2.3423998	-1.2885437
20.1333332	2.3912001	-1.2885437
20.1333332	2.4400001	-1.4664855
20.1333332	2.4888000	-1.2517285
20.1333332	2.5376000	-1.2394567
20.1333332	2.5864000	-1.0860586
20.1333332	2.6352000	-0.8958449
20.1333332	2.6840000	-0.8958449
20.1333332	2.7328000	-1.2087770
20.1333332	2.7816000	-1.0921946
20.1333332	2.8304000	-1.1780972
20.1333332	2.8792000	-1.0062914
20.1333332	2.9280000	-1.1719613
20.1333332	2.9768000	-1.0921946
20.1333332	3.0256000	-1.1965051
20.1333332	3.0743999	-0.9019809
20.1333332	3.1231999	-0.7485828
20.1333332	3.1719999	-0.7547188
20.1333332	3.2207999	-1.0431070
20.1333332	3.2695999	-0.9203887
20.1333332	3.3183999	-0.9694757
20.1333332	3.3671999	-0.9756117
20.1333332	3.4159999	-1.1290097
20.1333332	3.4647999	-0.8160777
20.1333332	3.5135999	-0.8160777
20.1333332	3.5623999	-0.9142528
20.1333332	3.6111999	-1.0921946
20.1333332	3.6599998	-0.8160777
20.1333332	3.7087998	-0.9694757
20.1333332	3.7575998	-0.9633398
20.1333332	3.8063998	-1.0001554
20.1333332	3.8551998	-1.0062914
20.1333332	3.9039998	-1.0062914
20.1333332	3.9527998	-0.9940195
20.1333332	4.0016003	-0.9081168
20.1333332	4.0503998	-0.8406215
20.1333332	4.0991998	-0.7853980

20.1333332	4.1479998	-0.8222136
20.1333332	4.1967998	-0.8344855
20.1333332	4.2455997	-0.6504078
20.1333332	4.2943997	-0.6626797
20.1333332	4.3431997	-0.7485828
20.1333332	4.3919997	-0.6504078
20.1333332	4.4407997	-0.6626797
20.1333332	4.4895997	-0.7608547
20.1333332	4.5383997	-0.7731261
20.1333332	4.5871997	-0.7731261
20.1333332	4.6359997	-0.8774371
20.1333332	4.6847997	-0.8774371
20.1333332	4.7336001	-0.7731261
20.1333332	4.7824001	-0.7731261
20.1333332	4.8312001	-0.7056313
20.1333332	4.8800001	-0.6994953
20.1333332	4.9288001	-0.6994953
20.1333332	4.9776001	-0.6381359
20.1333332	5.0264001	-0.6381359
20.1333332	5.0752001	-0.5522332
20.1333332	5.1240001	-0.6258640
20.1333332	5.1728001	-0.6872234
20.1333332	5.2216001	-0.6504078
20.1333332	5.2704000	-0.6626797
20.1333332	5.3192000	-0.5951843
20.1333332	5.3680000	-0.6135921
20.1333332	5.4168000	-0.6135921
20.1333332	5.4656000	-0.6258640
20.1333332	5.5144000	-0.5215535
20.1333332	5.5632000	-0.7608547
20.1333332	5.6120000	-0.7608547
20.1333332	5.6608000	-0.6504078
20.1333332	5.7096000	-0.7240391
20.1333332	5.7584000	-0.7301750
20.1333332	5.8072000	-0.5276895
20.1333332	5.8559999	-0.5215535
20.1333332	5.9047999	613586.0000000
20.1333332	5.9535999	-0.6013203
20.1333332	6.0023999	-0.6013203
20.1499996	0.2928000	-2.3746023
20.1499996	0.3416000	-1.9634953
20.1499996	0.3904000	-1.7180586
20.1499996	0.4392000	-1.7303305
20.1499996	0.4880000	-1.7180586
20.1499996	0.5368000	-1.7180586
20.1499996	0.5856000	-1.5278449
20.1499996	0.6344000	-1.2455926
20.1499996	0.6832000	-1.3867188
20.1499996	0.7320000	-1.3192234
20.1499996	0.7808000	-1.5707965
20.1499996	0.8296000	-1.2578645
20.1499996	0.8784000	-1.2762718
20.1499996	0.9272000	-1.2701364
20.1499996	0.9760000	-1.2762718
20.1499996	1.0247999	-1.1596894
20.1499996	1.0735999	-0.9878836
20.1499996	1.1223999	-0.9326606

20.1499996	1.1711999	-0.9326606
20.1499996	1.2200000	-1.1903691
20.1499996	1.2688000	-1.2087770
20.1499996	1.3176000	-1.2701364
20.1499996	1.3664000	-1.2824078
20.1499996	1.4152000	-1.1780972
20.1499996	1.4640000	-1.1658254
20.1499996	1.5128000	-1.1658254
20.1499996	1.5616000	-1.2701364
20.1499996	1.6104000	-1.2640004
20.1499996	1.6592000	-1.3805828
20.1499996	1.7079999	-1.3069515
20.1499996	1.7567999	-1.1106019
20.1499996	1.8055999	-1.2149129
20.1499996	1.8543999	-1.2517285
20.1499996	1.9031999	-1.3928547
20.1499996	1.9519999	-1.1412816
20.1499996	2.0007999	-1.1167378
20.1499996	2.0495999	-1.3130875
20.1499996	2.0983999	-1.1044660
20.1499996	2.1471999	-1.0860586
20.1499996	2.1959999	-1.1842332
20.1499996	2.2447999	-1.1842332
20.1499996	2.2935998	-1.1965051
20.1499996	2.3423998	-1.1106019
20.1499996	2.3912001	-1.2026410
20.1499996	2.4400001	-1.1412816
20.1499996	2.4888000	-1.1351457
20.1499996	2.5376000	-1.0983305
20.1499996	2.5864000	-1.2271848
20.1499996	2.6352000	-0.9081168
20.1499996	2.6840000	-1.0553789
20.1499996	2.7328000	-0.9326606
20.1499996	2.7816000	-1.1719613
20.1499996	2.8304000	-1.1842332
20.1499996	2.8792000	-1.0921946
20.1499996	2.9280000	-1.1965051
20.1499996	2.9768000	-1.1842332
20.1499996	3.0256000	-1.0860586
20.1499996	3.0743999	-0.9081168
20.1499996	3.1231999	-0.8651652
20.1499996	3.1719999	-0.7117672
20.1499996	3.2207999	-0.7117672
20.1499996	3.2695999	-0.7669902
20.1499996	3.3183999	-0.6872234
20.1499996	3.3671999	-0.4601941
20.1499996	3.4159999	-0.8467574
20.1499996	3.4647999	-0.8222136
20.1499996	3.5135999	-0.8038058
20.1499996	3.5623999	-0.7976699
20.1499996	3.6111999	-0.8038058
20.1499996	3.6599998	-0.8160777
20.1499996	3.7087998	-0.9817476
20.1499996	3.7575998	-0.9265246
20.1499996	3.8063998	-0.9203887
20.1499996	3.8551998	-0.8467574
20.1499996	3.9039998	-0.9203887

20.1499996	3.9527998	-0.8590293
20.1499996	4.0016003	-0.8651652
20.1499996	4.0503998	-0.8038058
20.1499996	4.0991998	-0.8099418
20.1499996	4.1479998	-0.8590293
20.1499996	4.1967998	-0.8651652
20.1499996	4.2455997	-0.8038058
20.1499996	4.2943997	-0.7915339
20.1499996	4.3431997	-0.7915339
20.1499996	4.3919997	-0.7363110
20.1499996	4.4407997	-0.7056313
20.1499996	4.4895997	-0.7363110
20.1499996	4.5383997	-0.7731261
20.1499996	4.5871997	-0.7731261
20.1499996	4.6359997	-0.8160777
20.1499996	4.6847997	-0.8222136
20.1499996	4.7336001	-0.6933594
20.1499996	4.7824001	-0.8590293
20.1499996	4.8312001	-0.8099418
20.1499996	4.8800001	-0.8160777
20.1499996	4.9288001	-0.8283496
20.1499996	4.9776001	-0.8651652
20.1499996	5.0264001	-0.8651652
20.1499996	5.0752001	-0.7792621
20.1499996	5.1240001	-0.7731261
20.1499996	5.1728001	-0.6810875
20.1499996	5.2216001	-0.6749516
20.1499996	5.2704000	-0.5460973
20.1499996	5.3192000	-0.6258640
20.1499996	5.3680000	-0.6320000
20.1499996	5.4168000	-0.6381359
20.1499996	5.4656000	-0.7117672
20.1499996	5.5144000	-0.7117672
20.1499996	5.5632000	-0.6749516
20.1499996	5.6120000	613586.0000000
20.1499996	5.6608000	613586.0000000
20.1499996	5.7096000	613586.0000000
20.1499996	5.7584000	613586.0000000
20.1499996	5.8072000	613586.0000000
20.1499996	5.8559999	613586.0000000
20.1499996	5.9047999	613586.0000000
20.1499996	5.9535999	613586.0000000
20.1499996	6.0023999	613586.0000000
20.1666660	0.2928000	-2.7673013
20.1666660	0.3416000	-1.4910293
20.1666660	0.3904000	-2.2334762
20.1666660	0.4392000	-2.3316510
20.1666660	0.4880000	-2.7550297
20.1666660	0.5368000	-1.9634953
20.1666660	0.5856000	-1.9450879
20.1666660	0.6344000	-1.9389520
20.1666660	0.6832000	-1.5155730
20.1666660	0.7320000	-1.5892043
20.1666660	0.7808000	-1.5094371
20.1666660	0.8296000	-1.5094371
20.1666660	0.8784000	-1.2455926
20.1666660	0.9272000	-1.3867188

20.1666660	0.9760000	-1.3989906
20.1666660	1.0247999	-1.8285050
20.1666660	1.0735999	-1.6505632
20.1666660	1.1223999	-1.2333207
20.1666660	1.1711999	-1.0860586
20.1666660	1.2200000	-1.0860586
20.1666660	1.2688000	-1.2026410
20.1666660	1.3176000	-1.0799227
20.1666660	1.3664000	-0.9081168
20.1666660	1.4152000	-1.1780972
20.1666660	1.4640000	-1.2087770
20.1666660	1.5128000	-1.1535535
20.1666660	1.5616000	-1.0369711
20.1666660	1.6104000	-1.0124273
20.1666660	1.6592000	-1.3499031
20.1666660	1.7079999	-1.2210488
20.1666660	1.7567999	-1.1596894
20.1666660	1.8055999	-1.2578645
20.1666660	1.8543999	-1.2271848
20.1666660	1.9031999	-0.9203887
20.1666660	1.9519999	-0.9265246
20.1666660	2.0007999	-1.0001554
20.1666660	2.0495999	-1.0799227
20.1666660	2.0983999	-1.0615149
20.1666660	2.1471999	-1.1719613
20.1666660	2.1959999	-1.1658254
20.1666660	2.2447999	-1.2026410
20.1666660	2.2935998	-1.1965051
20.1666660	2.3423998	-0.9203887
20.1666660	2.3912001	-0.8774371
20.1666660	2.4400001	-0.8835731
20.1666660	2.4888000	-1.0369711
20.1666660	2.5376000	-1.0492430
20.1666660	2.5864000	-0.9142528
20.1666660	2.6352000	-0.8467574
20.1666660	2.6840000	-0.6749516
20.1666660	2.7328000	-0.7547188
20.1666660	2.7816000	-0.7608547
20.1666660	2.8304000	-0.9940195
20.1666660	2.8792000	-0.8897090
20.1666660	2.9280000	-0.8651652
20.1666660	2.9768000	-0.8713012
20.1666660	3.0256000	-0.9694757
20.1666660	3.0743999	-0.9449320
20.1666660	3.1231999	-0.6565437
20.1666660	3.1719999	-0.6504078
20.1666660	3.2207999	-0.6504078
20.1666660	3.2695999	-0.8897090
20.1666660	3.3183999	-0.7547188
20.1666660	3.3671999	-0.7547188
20.1666660	3.4159999	-1.0492430
20.1666660	3.4647999	-1.0492430
20.1666660	3.5135999	-0.9572039
20.1666660	3.5623999	-0.9449320
20.1666660	3.6111999	-0.9633398
20.1666660	3.6599998	-0.9633398
20.1666660	3.7087998	-0.9572039

20.1666660	3.7575998	-0.9756117
20.1666660	3.8063998	-0.8283496
20.1666660	3.8551998	-0.9142528
20.1666660	3.9039998	-0.8774371
20.1666660	3.9527998	-0.8283496
20.1666660	4.0016003	-0.8160777
20.1666660	4.0503998	-0.8283496
20.1666660	4.0991998	-0.7117672
20.1666660	4.1479998	-0.8222136
20.1666660	4.1967998	-0.8222136
20.1666660	4.2455997	-0.6872234
20.1666660	4.2943997	-0.6749516
20.1666660	4.3431997	-0.6626797
20.1666660	4.3919997	-0.6442719
20.1666660	4.4407997	-0.6994953
20.1666660	4.4895997	-0.6135921
20.1666660	4.5383997	-0.7056313
20.1666660	4.5871997	-0.6933594
20.1666660	4.6359997	-0.6442719
20.1666660	4.6847997	-0.5706410
20.1666660	4.7336001	-0.6626797
20.1666660	4.7824001	-0.6626797
20.1666660	4.8312001	-0.7608547
20.1666660	4.8800001	-0.7547188
20.1666660	4.9288001	-0.6442719
20.1666660	4.9776001	-0.7056313
20.1666660	5.0264001	-0.6994953
20.1666660	5.0752001	-0.6504078
20.1666660	5.1240001	-0.5829129
20.1666660	5.1728001	-0.5767770
20.1666660	5.2216001	-0.5767770
20.1666660	5.2704000	-0.5767770
20.1666660	5.3192000	-0.4786019
20.1666660	5.3680000	-0.6810875
20.1666660	5.4168000	-0.4847379
20.1666660	5.4656000	-0.5951843
20.1666660	5.5144000	-0.6933594
20.1666660	5.5632000	613586.0000000
20.1666660	5.6120000	613586.0000000
20.1666660	5.6608000	613586.0000000
20.1666660	5.7096000	613586.0000000
20.1666660	5.7584000	613586.0000000
20.1666660	5.8072000	613586.0000000
20.1666660	5.8559999	613586.0000000
20.1666660	5.9047999	613586.0000000
20.1666660	5.9535999	613586.0000000
20.1666660	6.0023999	613586.0000000
20.1833324	0.2928000	-2.4605052
20.1833324	0.3416000	-2.3561945
20.1833324	0.3904000	-1.4664855
20.1833324	0.4392000	-1.4480777
20.1833324	0.4880000	-1.3499031
20.1833324	0.5368000	-1.4726214
20.1833324	0.5856000	-1.4726214
20.1833324	0.6344000	-1.4848933
20.1833324	0.6832000	-1.1903691
20.1833324	0.7320000	-1.3560390

20.1833324	0.7808000	-1.2026410
20.1833324	0.8296000	-1.1903691
20.1833324	0.8784000	-1.3560390
20.1833324	0.9272000	-1.2640004
20.1833324	0.9760000	-1.3928547
20.1833324	1.0247999	-1.3069515
20.1833324	1.0735999	-1.2578645
20.1833324	1.1223999	-1.2640004
20.1833324	1.1711999	-1.5707965
20.1833324	1.2200000	-1.5707965
20.1833324	1.2688000	-1.5094371
20.1833324	1.3176000	-1.5707965
20.1833324	1.3664000	-1.3744469
20.1833324	1.4152000	-1.2701364
20.1833324	1.4640000	-1.2824078
20.1833324	1.5128000	-1.2824078
20.1833324	1.5616000	-1.2394567
20.1833324	1.6104000	-1.3560390
20.1833324	1.6592000	-1.2210488
20.1833324	1.7079999	-1.2149129
20.1833324	1.7567999	-1.0737867
20.1833324	1.8055999	-0.9633398
20.1833324	1.8543999	-0.8774371
20.1833324	1.9031999	-1.1351457
20.1833324	1.9519999	-0.9510679
20.1833324	2.0007999	-1.2455926
20.1833324	2.0495999	-1.2149129
20.1833324	2.0983999	-1.2087770
20.1833324	2.1471999	-1.2455926
20.1833324	2.1959999	-1.2087770
20.1833324	2.2447999	-1.2333207
20.1833324	2.2935998	-1.2333207
20.1833324	2.3423998	-1.1965051
20.1833324	2.3912001	-1.1842332
20.1833324	2.4400001	-1.0737867
20.1833324	2.4888000	-1.0737867
20.1833324	2.5376000	-1.1167378
20.1833324	2.5864000	-1.1167378
20.1833324	2.6352000	-0.9081168
20.1833324	2.6840000	-0.9019809
20.1833324	2.7328000	-0.8897090
20.1833324	2.7816000	-0.8897090
20.1833324	2.8304000	-0.9756117
20.1833324	2.8792000	-0.9694757
20.1833324	2.9280000	-0.7547188
20.1833324	2.9768000	-0.6994953
20.1833324	3.0256000	-0.7792621
20.1833324	3.0743999	-0.7915339
20.1833324	3.1231999	-0.8160777
20.1833324	3.1719999	-0.8038058
20.1833324	3.2207999	-0.7915339
20.1833324	3.2695999	-0.7915339
20.1833324	3.3183999	-0.8835731
20.1833324	3.3671999	-0.8038058
20.1833324	3.4159999	-0.7363110
20.1833324	3.4647999	-0.8958449
20.1833324	3.5135999	-0.8283496

20.1833324	3.5623999	-0.9510679
20.1833324	3.6111999	-0.7424469
20.1833324	3.6599998	-0.7363110
20.1833324	3.7087998	-0.7976699
20.1833324	3.7575998	-0.7976699
20.1833324	3.8063998	-0.7976699
20.1833324	3.8551998	-0.8467574
20.1833324	3.9039998	-0.7547188
20.1833324	3.9527998	-0.7117672
20.1833324	4.0016003	-0.6135921
20.1833324	4.0503998	-0.4295144
20.1833324	4.0991998	-0.7669902
20.1833324	4.1479998	-0.6442719
20.1833324	4.1967998	-0.8038058
20.1833324	4.2455997	-0.8406215
20.1833324	4.2943997	-0.7240391
20.1833324	4.3431997	-0.6197281
20.1833324	4.3919997	-0.6197281
20.1833324	4.4407997	-0.6320000
20.1833324	4.4895997	-0.6381359
20.1833324	4.5383997	-0.6994953
20.1833324	4.5871997	-0.6810875
20.1833324	4.6359997	-0.6626797
20.1833324	4.6847997	-0.6994953
20.1833324	4.7336001	-0.7056313
20.1833324	4.7824001	-0.7056313
20.1833324	4.8312001	-0.7608547
20.1833324	4.8800001	-0.7485828
20.1833324	4.9288001	-0.8406215
20.1833324	4.9776001	-0.7485828
20.1833324	5.0264001	-0.7240391
20.1833324	5.0752001	-0.7117672
20.1833324	5.1240001	-0.7117672
20.1833324	5.1728001	-0.7547188
20.1833324	5.2216001	-0.7669902
20.1833324	5.2704000	-1.0983305
20.1833324	5.3192000	-1.0983305
20.1833324	5.3680000	613586.0000000
20.1833324	5.4168000	613586.0000000
20.1833324	5.4656000	613586.0000000
20.1833324	5.5144000	613586.0000000
20.1833324	5.5632000	613586.0000000
20.1833324	5.6120000	613586.0000000
20.1833324	5.6608000	613586.0000000
20.1833324	5.7096000	613586.0000000
20.1833324	5.7584000	613586.0000000
20.1833324	5.8072000	613586.0000000
20.1833324	5.8559999	613586.0000000
20.1833324	5.9047999	613586.0000000
20.1833324	5.9535999	613586.0000000
20.1833324	6.0023999	613586.0000000
20.2000008	0.2928000	-2.7366219
20.2000008	0.3416000	-1.4296703
20.2000008	0.3904000	-1.4235344
20.2000008	0.4392000	-1.7364664
20.2000008	0.4880000	-2.6507189
20.2000008	0.5368000	-3.0986414

20.2000008	0.5856000	-3.1600006
20.2000008	0.6344000	-2.2212043
20.2000008	0.6832000	-1.7303305
20.2000008	0.7320000	-1.7303305
20.2000008	0.7808000	-1.2087770
20.2000008	0.8296000	-1.1965051
20.2000008	0.8784000	-1.0492430
20.2000008	0.9272000	-1.2271848
20.2000008	0.9760000	-1.1535535
20.2000008	1.0247999	-1.1106019
20.2000008	1.0735999	-1.3560390
20.2000008	1.1223999	-1.3069515
20.2000008	1.1711999	-1.2946796
20.2000008	1.2200000	-1.3499031
20.2000008	1.2688000	-1.3499031
20.2000008	1.3176000	-1.2885437
20.2000008	1.3664000	-1.1412816
20.2000008	1.4152000	-1.1290097
20.2000008	1.4640000	-1.1290097
20.2000008	1.5128000	-1.1351457
20.2000008	1.5616000	-1.3437672
20.2000008	1.6104000	-1.4358063
20.2000008	1.6592000	-1.3008156
20.2000008	1.7079999	-1.3008156
20.2000008	1.7567999	-0.8651652
20.2000008	1.8055999	-1.1106019
20.2000008	1.8543999	-0.9387960
20.2000008	1.9031999	-0.9326606
20.2000008	1.9519999	-1.1044660
20.2000008	2.0007999	-1.1167378
20.2000008	2.0495999	-1.0124273
20.2000008	2.0983999	-1.0799227
20.2000008	2.1471999	-1.0737867
20.2000008	2.1959999	-1.0676508
20.2000008	2.2447999	-1.1965051
20.2000008	2.2935998	-1.2026410
20.2000008	2.3423998	-1.1290097
20.2000008	2.3912001	-1.2455926
20.2000008	2.4400001	-1.2517285
20.2000008	2.4888000	-1.2517285
20.2000008	2.5376000	-1.2394567
20.2000008	2.5864000	-0.9694757
20.2000008	2.6352000	-1.0492430
20.2000008	2.6840000	-0.8590293
20.2000008	2.7328000	-0.8774371
20.2000008	2.7816000	-0.8774371
20.2000008	2.8304000	-0.9081168
20.2000008	2.8792000	-0.9878836
20.2000008	2.9280000	-0.9572039
20.2000008	2.9768000	-1.4910293
20.2000008	3.0256000	-1.3376312
20.2000008	3.0743999	-1.3376312
20.2000008	3.1231999	-0.8774371
20.2000008	3.1719999	-0.6442719
20.2000008	3.2207999	-0.8958449
20.2000008	3.2695999	-0.8344855
20.2000008	3.3183999	-0.7240391

20.2000008	3.3671999	-0.6197281
20.2000008	3.4159999	-0.6197281
20.2000008	3.4647999	-0.9081168
20.2000008	3.5135999	-0.8528934
20.2000008	3.5623999	-0.6074562
20.2000008	3.6111999	-0.9265246
20.2000008	3.6599998	-0.9265246
20.2000008	3.7087998	-1.0431070
20.2000008	3.7575998	-1.0431070
20.2000008	3.8063998	-0.9265246
20.2000008	3.8551998	-0.8160777
20.2000008	3.9039998	-0.7240391
20.2000008	3.9527998	-0.7240391
20.2000008	4.0016003	-0.7056313
20.2000008	4.0503998	-0.7608547
20.2000008	4.0991998	-0.7608547
20.2000008	4.1479998	-0.7608547
20.2000008	4.1967998	-0.7547188
20.2000008	4.2455997	-0.6074562
20.2000008	4.2943997	-0.6565437
20.2000008	4.3431997	-0.6688156
20.2000008	4.3919997	-0.6749516
20.2000008	4.4407997	-0.7363110
20.2000008	4.4895997	-0.6872234
20.2000008	4.5383997	-0.5951843
20.2000008	4.5871997	-0.6381359
20.2000008	4.6359997	-0.6504078
20.2000008	4.6847997	-0.7056313
20.2000008	4.7336001	-0.6565437
20.2000008	4.7824001	-0.6258640
20.2000008	4.8312001	-0.5338254
20.2000008	4.8800001	-0.5338254
20.2000008	4.9288001	-0.5890489
20.2000008	4.9776001	-0.6626797
20.2000008	5.0264001	-0.4724660
20.2000008	5.0752001	-0.6074562
20.2000008	5.1240001	-0.4786019
20.2000008	5.1728001	-0.4847379
20.2000008	5.2216001	-0.4786019
20.2000008	5.2704000	-0.5399613
20.2000008	5.3192000	-0.4786019
20.2000008	5.3680000	613586.0000000
20.2000008	5.4168000	613586.0000000
20.2000008	5.4656000	-0.6749516
20.2000008	5.5144000	-0.6688156
20.2000008	5.5632000	613586.0000000
20.2000008	5.6120000	613586.0000000
20.2000008	5.6608000	613586.0000000
20.2000008	5.7096000	613586.0000000
20.2000008	5.7584000	613586.0000000
20.2000008	5.8072000	613586.0000000
20.2000008	5.8559999	613586.0000000
20.2000008	5.9047999	613586.0000000
20.2000008	5.9535999	613586.0000000
20.2000008	6.0023999	613586.0000000
20.2166672	0.2928000	-1.6444273
20.2166672	0.3416000	-1.3499031

20.2166672	0.3904000	-1.5217090
20.2166672	0.4392000	-1.6505632
20.2166672	0.4880000	-1.6444273
20.2166672	0.5368000	-1.2885437
20.2166672	0.5856000	-1.4051266
20.2166672	0.6344000	-1.4358063
20.2166672	0.6832000	-1.4480777
20.2166672	0.7320000	-1.3314953
20.2166672	0.7808000	-1.5033011
20.2166672	0.8296000	-1.3928547
20.2166672	0.8784000	-1.3805828
20.2166672	0.9272000	-1.3805828
20.2166672	0.9760000	-1.4664855
20.2166672	1.0247999	-1.4603496
20.2166672	1.0735999	-1.2333207
20.2166672	1.1223999	-1.2455926
20.2166672	1.1711999	-1.2517285
20.2166672	1.2200000	-0.9817476
20.2166672	1.2688000	-0.9817476
20.2166672	1.3176000	-1.0001554
20.2166672	1.3664000	-1.2210488
20.2166672	1.4152000	-1.2149129
20.2166672	1.4640000	-1.0553789
20.2166672	1.5128000	-1.0676508
20.2166672	1.5616000	-1.0921946
20.2166672	1.6104000	-1.4296703
20.2166672	1.6592000	-1.3560390
20.2166672	1.7079999	-1.1228738
20.2166672	1.7567999	-1.1228738
20.2166672	1.8055999	-1.1842332
20.2166672	1.8543999	-1.0860586
20.2166672	1.9031999	-1.0431070
20.2166672	1.9519999	-1.0553789
20.2166672	2.0007999	-1.0553789
20.2166672	2.0495999	-1.0492430
20.2166672	2.0983999	-1.0553789
20.2166672	2.1471999	-0.9387960
20.2166672	2.1959999	-0.9449320
20.2166672	2.2447999	-0.7976699
20.2166672	2.2935998	-0.7976699
20.2166672	2.3423998	-0.8651652
20.2166672	2.3912001	-0.8713012
20.2166672	2.4400001	-1.0860586
20.2166672	2.4888000	-1.0737867
20.2166672	2.5376000	-1.0737867
20.2166672	2.5864000	-1.0124273
20.2166672	2.6352000	-1.0185633
20.2166672	2.6840000	-1.0246992
20.2166672	2.7328000	-1.0308352
20.2166672	2.7816000	-1.3376312
20.2166672	2.8304000	-1.1228738
20.2166672	2.8792000	-1.2517285
20.2166672	2.9280000	-1.2394567
20.2166672	2.9768000	-1.3437672
20.2166672	3.0256000	-1.2149129
20.2166672	3.0743999	-0.8467574
20.2166672	3.1231999	-0.6749516

20.2166672	3.1719999	-0.6749516
20.2166672	3.2207999	-0.6688156
20.2166672	3.2695999	-0.4295144
20.2166672	3.3183999	-0.4295144
20.2166672	3.3671999	-0.4663301
20.2166672	3.4159999	-0.3620195
20.2166672	3.4647999	-0.7547188
20.2166672	3.5135999	-0.7547188
20.2166672	3.5623999	-0.2393012
20.2166672	3.6111999	-0.3988352
20.2166672	3.6599998	-0.9817476
20.2166672	3.7087998	-0.9817476
20.2166672	3.7575998	-0.9203887
20.2166672	3.8063998	-0.8406215
20.2166672	3.8551998	-0.8528934
20.2166672	3.9039998	-0.7669902
20.2166672	3.9527998	-0.7608547
20.2166672	4.0016003	-0.7608547
20.2166672	4.0503998	-0.7731261
20.2166672	4.0991998	-0.7056313
20.2166672	4.1479998	-0.7608547
20.2166672	4.1967998	-0.7485828
20.2166672	4.2455997	-0.6626797
20.2166672	4.2943997	-0.6381359
20.2166672	4.3431997	-0.6442719
20.2166672	4.3919997	-0.6442719
20.2166672	4.4407997	-0.5829129
20.2166672	4.4895997	-0.6749516
20.2166672	4.5383997	-0.6810875
20.2166672	4.5871997	-0.7424469
20.2166672	4.6359997	-0.7485828
20.2166672	4.6847997	-0.6442719
20.2166672	4.7336001	-0.4786019
20.2166672	4.7824001	-0.5338254
20.2166672	4.8312001	-0.5338254
20.2166672	4.8800001	-0.5276895
20.2166672	4.9288001	-0.5890489
20.2166672	4.9776001	-0.6994953
20.2166672	5.0264001	-0.7117672
20.2166672	5.0752001	-0.6135921
20.2166672	5.1240001	-0.5767770
20.2166672	5.1728001	-0.5829129
20.2166672	5.2216001	-0.4479222
20.2166672	5.2704000	-0.3865633
20.2166672	5.3192000	-0.3804274
20.2166672	5.3680000	-0.3742914
20.2166672	5.4168000	-0.5092816
20.2166672	5.4656000	-0.2699804
20.2166672	5.5144000	-0.3190680
20.2166672	5.5632000	-0.7363110
20.2166672	5.6120000	-0.5338254
20.2166672	5.6608000	613586.0000000
20.2166672	5.7096000	-0.5645051
20.2166672	5.7584000	613586.0000000
20.2166672	5.8072000	613586.0000000
20.2166672	5.8559999	613586.0000000
20.2166672	5.9047999	613586.0000000

20.2166672	5.9535999	613586.0000000
20.2166672	6.0023999	613586.0000000
20.2333336	0.2928000	-3.6570103
20.2333336	0.3416000	-1.1842332
20.2333336	0.3904000	-1.4112625
20.2333336	0.4392000	-1.1658254
20.2333336	0.4880000	-1.2885437
20.2333336	0.5368000	-1.1596894
20.2333336	0.5856000	-1.1780972
20.2333336	0.6344000	-1.1596894
20.2333336	0.6832000	-1.1535535
20.2333336	0.7320000	-1.0124273
20.2333336	0.7808000	-1.2026410
20.2333336	0.8296000	-0.9019809
20.2333336	0.8784000	-0.9142528
20.2333336	0.9272000	-1.1719613
20.2333336	0.9760000	-1.2824078
20.2333336	1.0247999	-1.2885437
20.2333336	1.0735999	-1.2946796
20.2333336	1.1223999	-1.3008156
20.2333336	1.1711999	-1.3683109
20.2333336	1.2200000	-1.1658254
20.2333336	1.2688000	-1.1290097
20.2333336	1.3176000	-1.1965051
20.2333336	1.3664000	-1.3008156
20.2333336	1.4152000	-1.4603496
20.2333336	1.4640000	-1.4664855
20.2333336	1.5128000	-1.4726214
20.2333336	1.5616000	-1.2517285
20.2333336	1.6104000	-1.2394567
20.2333336	1.6592000	-0.9203887
20.2333336	1.7079999	-1.0553789
20.2333336	1.7567999	-1.2455926
20.2333336	1.8055999	-1.1412816
20.2333336	1.8543999	-1.1412816
20.2333336	1.9031999	-0.9878836
20.2333336	1.9519999	-0.8651652
20.2333336	2.0007999	-1.1412816
20.2333336	2.0495999	-1.0615149
20.2333336	2.0983999	-1.0553789
20.2333336	2.1471999	-1.0431070
20.2333336	2.1959999	-1.2087770
20.2333336	2.2447999	-1.2087770
20.2333336	2.2935998	-1.1412816
20.2333336	2.3423998	-1.1351457
20.2333336	2.3912001	-1.1228738
20.2333336	2.4400001	-0.9203887
20.2333336	2.4888000	-0.9142528
20.2333336	2.5376000	-0.9878836
20.2333336	2.5864000	-1.0062914
20.2333336	2.6352000	-0.8590293
20.2333336	2.6840000	-0.8528934
20.2333336	2.7328000	-0.9510679
20.2333336	2.7816000	-0.8897090
20.2333336	2.8304000	-0.8835731
20.2333336	2.8792000	-1.0246992
20.2333336	2.9280000	-0.9633398

20.2333336	2.9768000	-0.9633398
20.2333336	3.0256000	-0.5951843
20.2333336	3.0743999	-0.6626797
20.2333336	3.1231999	-0.6872234
20.2333336	3.1719999	-0.6872234
20.2333336	3.2207999	-0.7485828
20.2333336	3.2695999	-0.8283496
20.2333336	3.3183999	-0.8283496
20.2333336	3.3671999	-0.6872234
20.2333336	3.4159999	-0.4049711
20.2333336	3.4647999	-0.2822523
20.2333336	3.5135999	-0.4479222
20.2333336	3.5623999	-0.4847379
20.2333336	3.6111999	-1.0001554
20.2333336	3.6599998	-1.0615149
20.2333336	3.7087998	-1.0737867
20.2333336	3.7575998	-1.0185633
20.2333336	3.8063998	-1.0185633
20.2333336	3.8551998	-0.8528934
20.2333336	3.9039998	-0.8590293
20.2333336	3.9527998	-0.8528934
20.2333336	4.0016003	-0.7424469
20.2333336	4.0503998	-0.7424469
20.2333336	4.0991998	-0.7424469
20.2333336	4.1479998	-0.6872234
20.2333336	4.1967998	-0.6381359
20.2333336	4.2455997	-0.6197281
20.2333336	4.2943997	-0.6197281
20.2333336	4.3431997	-0.7117672
20.2333336	4.3919997	-0.7179031
20.2333336	4.4407997	-0.7240391
20.2333336	4.4895997	-0.7056313
20.2333336	4.5383997	-0.6197281
20.2333336	4.5871997	-0.6197281
20.2333336	4.6359997	-0.7179031
20.2333336	4.6847997	-0.5522332
20.2333336	4.7336001	-0.5645051
20.2333336	4.7824001	-0.4908738
20.2333336	4.8312001	-0.4970098
20.2333336	4.8800001	-0.3926992
20.2333336	4.9288001	-0.5399613
20.2333336	4.9776001	-0.5399613
20.2333336	5.0264001	-0.5031457
20.2333336	5.0752001	-0.5399613
20.2333336	5.1240001	-0.5460973
20.2333336	5.1728001	-0.4049711
20.2333336	5.2216001	-0.4172430
20.2333336	5.2704000	-0.4970098
20.2333336	5.3192000	-0.5829129
20.2333336	5.3680000	-0.4049711
20.2333336	5.4168000	-0.3374758
20.2333336	5.4656000	-0.3436117
20.2333336	5.5144000	-0.4295144
20.2333336	5.5632000	-0.4970098
20.2333336	5.6120000	-0.4111071
20.2333336	5.6608000	-0.4540582
20.2333336	5.7096000	-0.4540582

20.2333336	5.7584000	-0.4417863
20.2333336	5.8072000	613586.0000000
20.2333336	5.8559999	-0.4908738
20.2333336	5.9047999	-0.4417863
20.2333336	5.9535999	613586.0000000
20.2333336	6.0023999	613586.0000000
20.2500000	0.2928000	-2.1353011
20.2500000	0.3416000	-1.2824078
20.2500000	0.3904000	-1.6198835
20.2500000	0.4392000	-1.9880390
20.2500000	0.4880000	-1.7119226
20.2500000	0.5368000	-1.7119226
20.2500000	0.5856000	-1.2578645
20.2500000	0.6344000	-1.1780972
20.2500000	0.6832000	-1.5462527
20.2500000	0.7320000	-1.0676508
20.2500000	0.7808000	-1.1719613
20.2500000	0.8296000	-1.1658254
20.2500000	0.8784000	-0.9387960
20.2500000	0.9272000	-1.0369711
20.2500000	0.9760000	-1.0431070
20.2500000	1.0247999	-1.1780972
20.2500000	1.0735999	-0.9878836
20.2500000	1.1223999	-0.9510679
20.2500000	1.1711999	-1.0983305
20.2500000	1.2200000	-1.0431070
20.2500000	1.2688000	-0.9756117
20.2500000	1.3176000	-0.8835731
20.2500000	1.3664000	-0.9081168
20.2500000	1.4152000	-1.3499031
20.2500000	1.4640000	-1.3314953
20.2500000	1.5128000	-1.4787574
20.2500000	1.5616000	-1.4910293
20.2500000	1.6104000	-1.5830684
20.2500000	1.6592000	-1.5830684
20.2500000	1.7079999	-1.3744469
20.2500000	1.7567999	-1.1658254
20.2500000	1.8055999	-1.0431070
20.2500000	1.8543999	-1.0431070
20.2500000	1.9031999	-1.0369711
20.2500000	1.9519999	-0.9940195
20.2500000	2.0007999	-1.2149129
20.2500000	2.0495999	-1.2210488
20.2500000	2.0983999	-1.2149129
20.2500000	2.1471999	-1.1044660
20.2500000	2.1959999	-1.1412816
20.2500000	2.2447999	-1.3683109
20.2500000	2.2935998	-1.3683109
20.2500000	2.3423998	-1.1290097
20.2500000	2.3912001	-1.0246992
20.2500000	2.4400001	-0.9633398
20.2500000	2.4888000	-0.9694757
20.2500000	2.5376000	-0.9756117
20.2500000	2.5864000	-1.1228738
20.2500000	2.6352000	-0.7608547
20.2500000	2.6840000	-0.9326606
20.2500000	2.7328000	-0.7547188

20.2500000	2.7816000	-0.7547188
20.2500000	2.8304000	-0.6688156
20.2500000	2.8792000	-0.6626797
20.2500000	2.9280000	-0.6565437
20.2500000	2.9768000	-0.5276895
20.2500000	3.0256000	-0.5276895
20.2500000	3.0743999	-0.5767770
20.2500000	3.1231999	-0.5767770
20.2500000	3.1719999	-0.5399613
20.2500000	3.2207999	-0.5399613
20.2500000	3.2695999	-0.9940195
20.2500000	3.3183999	-1.5217090
20.2500000	3.3671999	-1.4971652
20.2500000	3.4159999	-1.5830684
20.2500000	3.4647999	-1.5830684
20.2500000	3.5135999	-1.3192234
20.2500000	3.5623999	-1.3008156
20.2500000	3.6111999	-1.3069515
20.2500000	3.6599998	-1.2149129
20.2500000	3.7087998	-1.3744469
20.2500000	3.7575998	-1.2210488
20.2500000	3.8063998	-1.0737867
20.2500000	3.8551998	-1.0062914
20.2500000	3.9039998	-1.0062914
20.2500000	3.9527998	-0.9326606
20.2500000	4.0016003	-0.9326606
20.2500000	4.0503998	-0.9326606
20.2500000	4.0991998	-0.9081168
20.2500000	4.1479998	-0.9019809
20.2500000	4.1967998	-0.9817476
20.2500000	4.2455997	-0.9817476
20.2500000	4.2943997	-0.9572039
20.2500000	4.3431997	-0.9572039
20.2500000	4.3919997	-0.9142528
20.2500000	4.4407997	-0.9633398
20.2500000	4.4895997	-0.9694757
20.2500000	4.5383997	-0.9817476
20.2500000	4.5871997	-0.9449320
20.2500000	4.6359997	-0.9633398
20.2500000	4.6847997	-0.9633398
20.2500000	4.7336001	-0.9449320
20.2500000	4.7824001	-0.9510679
20.2500000	4.8312001	-0.9081168
20.2500000	4.8800001	-1.0737867
20.2500000	4.9288001	-0.8222136
20.2500000	4.9776001	-0.8283496
20.2500000	5.0264001	-0.6197281
20.2500000	5.0752001	-0.6197281
20.2500000	5.1240001	-0.7301750
20.2500000	5.1728001	-0.7301750
20.2500000	5.2216001	-0.5399613
20.2500000	5.2704000	-0.7792621
20.2500000	5.3192000	-0.7731261
20.2500000	5.3680000	-0.6442719
20.2500000	5.4168000	-0.6442719
20.2500000	5.4656000	-0.6442719
20.2500000	5.5144000	-0.7547188

20.2500000	5.5632000	-0.6074562
20.2500000	5.6120000	613586.0000000
20.2500000	5.6608000	613586.0000000
20.2500000	5.7096000	613586.0000000
20.2500000	5.7584000	613586.0000000
20.2500000	5.8072000	-0.8038058
20.2500000	5.8559999	-0.7424469
20.2500000	5.9047999	-0.7547188
20.2500000	5.9535999	613586.0000000
20.2500000	6.0023999	613586.0000000
20.2666664	0.2928000	-2.3377867
20.2666664	0.3416000	-2.3255150
20.2666664	0.3904000	-2.1905246
20.2666664	0.4392000	-2.0064468
20.2666664	0.4880000	-1.4971652
20.2666664	0.5368000	-1.4787574
20.2666664	0.5856000	-0.9326606
20.2666664	0.6344000	-0.8835731
20.2666664	0.6832000	-1.0431070
20.2666664	0.7320000	-1.0431070
20.2666664	0.7808000	-1.1167378
20.2666664	0.8296000	-1.1596894
20.2666664	0.8784000	-1.1596894
20.2666664	0.9272000	-1.1228738
20.2666664	0.9760000	-1.3008156
20.2666664	1.0247999	-1.1535535
20.2666664	1.0735999	-1.3867188
20.2666664	1.1223999	-1.3928547
20.2666664	1.1711999	-1.4726214
20.2666664	1.2200000	-1.4726214
20.2666664	1.2688000	-1.5033011
20.2666664	1.3176000	-1.5155730
20.2666664	1.3664000	-1.4419422
20.2666664	1.4152000	-1.6505632
20.2666664	1.4640000	-1.6505632
20.2666664	1.5128000	-1.6628351
20.2666664	1.5616000	-1.5094371
20.2666664	1.6104000	-1.8100972
20.2666664	1.6592000	-1.3069515
20.2666664	1.7079999	-1.1965051
20.2666664	1.7567999	-1.2824078
20.2666664	1.8055999	-1.0860586
20.2666664	1.8543999	-1.1658254
20.2666664	1.9031999	-1.1903691
20.2666664	1.9519999	-0.9203887
20.2666664	2.0007999	-1.0492430
20.2666664	2.0495999	-1.1842332
20.2666664	2.0983999	-1.1780972
20.2666664	2.1471999	-1.1780972
20.2666664	2.1959999	-1.3008156
20.2666664	2.2447999	-0.8835731
20.2666664	2.2935998	-1.0001554
20.2666664	2.3423998	-0.8283496
20.2666664	2.3912001	-0.5645051
20.2666664	2.4400001	-0.9510679
20.2666664	2.4888000	-0.8099418
20.2666664	2.5376000	-0.7915339

20.2666664	2.5864000	-0.7853980
20.2666664	2.6352000	-0.6074562
20.2666664	2.6840000	-0.5951843
20.2666664	2.7328000	-0.7608547
20.2666664	2.7816000	-0.7669902
20.2666664	2.8304000	-0.4847379
20.2666664	2.8792000	-0.7179031
20.2666664	2.9280000	-0.6504078
20.2666664	2.9768000	-0.6442719
20.2666664	3.0256000	-0.6504078
20.2666664	3.0743999	-0.9387960
20.2666664	3.1231999	-1.1842332
20.2666664	3.1719999	-1.1658254
20.2666664	3.2207999	-1.1658254
20.2666664	3.2695999	-1.3192234
20.2666664	3.3183999	-1.2026410
20.2666664	3.3671999	-0.8774371
20.2666664	3.4159999	-0.8713012
20.2666664	3.4647999	-0.8528934
20.2666664	3.5135999	-0.8344855
20.2666664	3.5623999	-0.7485828
20.2666664	3.6111999	-0.7669902
20.2666664	3.6599998	-0.7792621
20.2666664	3.7087998	-0.7792621
20.2666664	3.7575998	-0.8222136
20.2666664	3.8063998	-0.8283496
20.2666664	3.8551998	-0.8160777
20.2666664	3.9039998	-0.8835731
20.2666664	3.9527998	-0.8774371
20.2666664	4.0016003	-0.8713012
20.2666664	4.0503998	-0.7179031
20.2666664	4.0991998	-0.7853980
20.2666664	4.1479998	-0.7976699
20.2666664	4.1967998	-0.8038058
20.2666664	4.2455997	-0.7792621
20.2666664	4.2943997	-0.7976699
20.2666664	4.3431997	-0.7976699
20.2666664	4.3919997	-0.6749516
20.2666664	4.4407997	-0.6749516
20.2666664	4.4895997	-0.6688156
20.2666664	4.5383997	-0.6688156
20.2666664	4.5871997	-0.6688156
20.2666664	4.6359997	-0.6749516
20.2666664	4.6847997	-0.7301750
20.2666664	4.7336001	-0.7240391
20.2666664	4.7824001	-0.7117672
20.2666664	4.8312001	-0.7056313
20.2666664	4.8800001	-0.7240391
20.2666664	4.9288001	-0.8099418
20.2666664	4.9776001	-1.1290097
20.2666664	5.0264001	-0.9449320
20.2666664	5.0752001	-0.7792621
20.2666664	5.1240001	-0.8160777
20.2666664	5.1728001	-0.8099418
20.2666664	5.2216001	613586.0000000
20.2666664	5.2704000	-0.9756117
20.2666664	5.3192000	-0.9633398

20.2666664	5.3680000	-0.7976699
20.2666664	5.4168000	-0.7915339
20.2666664	5.4656000	-0.7853980
20.2666664	5.5144000	-1.0799227
20.2666664	5.5632000	-0.9878836
20.2666664	5.6120000	613586.0000000
20.2666664	5.6608000	613586.0000000
20.2666664	5.7096000	613586.0000000
20.2666664	5.7584000	613586.0000000
20.2666664	5.8072000	613586.0000000
20.2666664	5.8559999	613586.0000000
20.2666664	5.9047999	613586.0000000
20.2666664	5.9535999	613586.0000000
20.2666664	6.0023999	613586.0000000
20.2833328	0.2928000	-1.6198835
20.2833328	0.3416000	-1.4480777
20.2833328	0.3904000	-1.3867188
20.2833328	0.4392000	-1.6628351
20.2833328	0.4880000	-1.1965051
20.2833328	0.5368000	-0.9387960
20.2833328	0.5856000	-1.0369711
20.2833328	0.6344000	-0.7915339
20.2833328	0.6832000	-1.3130875
20.2833328	0.7320000	-1.3192234
20.2833328	0.7808000	-1.1965051
20.2833328	0.8296000	-1.3069515
20.2833328	0.8784000	-1.2333207
20.2833328	0.9272000	-1.1290097
20.2833328	0.9760000	-1.1351457
20.2833328	1.0247999	-1.1780972
20.2833328	1.0735999	-1.0799227
20.2833328	1.1223999	-1.0799227
20.2833328	1.1711999	-0.9510679
20.2833328	1.2200000	-1.6628351
20.2833328	1.2688000	-1.5033011
20.2833328	1.3176000	-1.4971652
20.2833328	1.3664000	-1.1412816
20.2833328	1.4152000	-1.0799227
20.2833328	1.4640000	-1.3376312
20.2833328	1.5128000	-1.0124273
20.2833328	1.5616000	-1.3192234
20.2833328	1.6104000	-1.2701364
20.2833328	1.6592000	-1.2824078
20.2833328	1.7079999	-1.2824078
20.2833328	1.7567999	-0.8835731
20.2833328	1.8055999	-0.9510679
20.2833328	1.8543999	-0.9817476
20.2833328	1.9031999	-1.0246992
20.2833328	1.9519999	-1.0676508
20.2833328	2.0007999	-1.1474175
20.2833328	2.0495999	-1.1719613
20.2833328	2.0983999	-1.0246992
20.2833328	2.1471999	-1.1044660
20.2833328	2.1959999	-1.0615149
20.2833328	2.2447999	-0.8283496
20.2833328	2.2935998	-0.6565437
20.2833328	2.3423998	-0.7179031

20.2833328	2.3912001	-0.7179031
20.2833328	2.4400001	-1.0001554
20.2833328	2.4888000	-1.1903691
20.2833328	2.5376000	-1.0492430
20.2833328	2.5864000	-1.0431070
20.2833328	2.6352000	-1.0492430
20.2833328	2.6840000	-0.9019809
20.2833328	2.7328000	-1.1658254
20.2833328	2.7816000	-1.1596894
20.2833328	2.8304000	-1.0185633
20.2833328	2.8792000	-0.6442719
20.2833328	2.9280000	-1.8530488
20.2833328	2.9768000	-1.6873789
20.2833328	3.0256000	-1.6444273
20.2833328	3.0743999	-1.6260195
20.2833328	3.1231999	-1.3683109
20.2833328	3.1719999	-1.2210488
20.2833328	3.2207999	-1.2946796
20.2833328	3.2695999	-1.1965051
20.2833328	3.3183999	-1.1412816
20.2833328	3.3671999	-1.1228738
20.2833328	3.4159999	-1.1228738
20.2833328	3.4647999	-1.0001554
20.2833328	3.5135999	-0.9081168
20.2833328	3.5623999	-0.9694757
20.2833328	3.6111999	-0.8958449
20.2833328	3.6599998	-0.8222136
20.2833328	3.7087998	-0.8283496
20.2833328	3.7575998	-0.8038058
20.2833328	3.8063998	-0.7547188
20.2833328	3.8551998	-0.6749516
20.2833328	3.9039998	-0.6381359
20.2833328	3.9527998	-0.6320000
20.2833328	4.0016003	-0.6258640
20.2833328	4.0503998	-0.7915339
20.2833328	4.0991998	-0.7915339
20.2833328	4.1479998	-0.7792621
20.2833328	4.1967998	-0.7056313
20.2833328	4.2455997	-0.7056313
20.2833328	4.2943997	-0.7240391
20.2833328	4.3431997	-0.7608547
20.2833328	4.3919997	-0.8283496
20.2833328	4.4407997	-0.7608547
20.2833328	4.4895997	-0.7547188
20.2833328	4.5383997	-0.7669902
20.2833328	4.5871997	-0.7240391
20.2833328	4.6359997	-0.6504078
20.2833328	4.6847997	-0.6994953
20.2833328	4.7336001	-0.6994953
20.2833328	4.7824001	-0.7792621
20.2833328	4.8312001	-0.8651652
20.2833328	4.8800001	-0.6810875
20.2833328	4.9288001	-0.6688156
20.2833328	4.9776001	-0.6381359
20.2833328	5.0264001	-0.5583692
20.2833328	5.0752001	-0.7179031
20.2833328	5.1240001	-0.5951843

20.2833328	5.1728001	-0.5154176
20.2833328	5.2216001	-0.7363110
20.2833328	5.2704000	-0.7363110
20.2833328	5.3192000	-0.7363110
20.2833328	5.3680000	-0.6994953
20.2833328	5.4168000	-0.7608547
20.2833328	5.4656000	-0.7915339
20.2833328	5.5144000	-0.8099418
20.2833328	5.5632000	-0.8160777
20.2833328	5.6120000	613586.0000000
20.2833328	5.6608000	613586.0000000
20.2833328	5.7096000	613586.0000000
20.2833328	5.7584000	613586.0000000
20.2833328	5.8072000	613586.0000000
20.2833328	5.8559999	613586.0000000
20.2833328	5.9047999	613586.0000000
20.2833328	5.9535999	613586.0000000
20.2833328	6.0023999	613586.0000000
20.2999992	0.2928000	-1.5646605
20.2999992	0.3416000	-1.5585246
20.2999992	0.3904000	-1.5646605
20.2999992	0.4392000	-1.8407769
20.2999992	0.4880000	-1.2394567
20.2999992	0.5368000	-1.2271848
20.2999992	0.5856000	-1.5830684
20.2999992	0.6344000	-1.5830684
20.2999992	0.6832000	-1.4664855
20.2999992	0.7320000	-1.4664855
20.2999992	0.7808000	-1.6566992
20.2999992	0.8296000	-1.6566992
20.2999992	0.8784000	-1.5830684
20.2999992	0.9272000	-1.3437672
20.2999992	0.9760000	-1.0062914
20.2999992	1.0247999	-1.0062914
20.2999992	1.0735999	-1.0124273
20.2999992	1.1223999	-0.8467574
20.2999992	1.1711999	-0.8467574
20.2999992	1.2200000	-0.4970098
20.2999992	1.2688000	-1.0492430
20.2999992	1.3176000	-0.8344855
20.2999992	1.3664000	-0.8222136
20.2999992	1.4152000	-1.0246992
20.2999992	1.4640000	-1.1596894
20.2999992	1.5128000	-1.1842332
20.2999992	1.5616000	-1.3130875
20.2999992	1.6104000	-1.3192234
20.2999992	1.6592000	-1.4910293
20.2999992	1.7079999	-0.8222136
20.2999992	1.7567999	-0.9694757
20.2999992	1.8055999	-0.9756117
20.2999992	1.8543999	-0.9081168
20.2999992	1.9031999	-1.0615149
20.2999992	1.9519999	-1.0615149
20.2999992	2.0007999	-1.0492430
20.2999992	2.0495999	-1.0431070
20.2999992	2.0983999	-0.9203887
20.2999992	2.1471999	-0.8222136

20.2999992	2.1959999	-1.0246992
20.2999992	2.2447999	-1.2087770
20.2999992	2.2935998	-1.2087770
20.2999992	2.3423998	-1.1474175
20.2999992	2.3912001	-0.8774371
20.2999992	2.4400001	-0.8283496
20.2999992	2.4888000	-0.8283496
20.2999992	2.5376000	-1.0308352
20.2999992	2.5864000	-0.9940195
20.2999992	2.6352000	-1.2885437
20.2999992	2.6840000	-1.2946796
20.2999992	2.7328000	-0.9878836
20.2999992	2.7816000	-0.9081168
20.2999992	2.8304000	-1.0860586
20.2999992	2.8792000	-0.9203887
20.2999992	2.9280000	-0.9203887
20.2999992	2.9768000	-0.9203887
20.2999992	3.0256000	-0.4417863
20.2999992	3.0743999	-0.8897090
20.2999992	3.1231999	-1.5033011
20.2999992	3.1719999	-1.5217090
20.2999992	3.2207999	-1.3437672
20.2999992	3.2695999	-1.3621750
20.2999992	3.3183999	-1.2149129
20.2999992	3.3671999	-0.9878836
20.2999992	3.4159999	-0.9633398
20.2999992	3.4647999	-0.9510679
20.2999992	3.5135999	-0.8651652
20.2999992	3.5623999	-0.8835731
20.2999992	3.6111999	-0.8958449
20.2999992	3.6599998	-0.7915339
20.2999992	3.7087998	-0.7915339
20.2999992	3.7575998	-0.6994953
20.2999992	3.8063998	-0.7056313
20.2999992	3.8551998	-0.7547188
20.2999992	3.9039998	-0.7547188
20.2999992	3.9527998	-0.7792621
20.2999992	4.0016003	-0.7669902
20.2999992	4.0503998	-0.6933594
20.2999992	4.0991998	-0.6933594
20.2999992	4.1479998	-0.7608547
20.2999992	4.1967998	-0.6994953
20.2999992	4.2455997	-0.6810875
20.2999992	4.2943997	-0.6810875
20.2999992	4.3431997	-0.6994953
20.2999992	4.3919997	-0.7363110
20.2999992	4.4407997	-0.8099418
20.2999992	4.4895997	-0.7301750
20.2999992	4.5383997	-0.7301750
20.2999992	4.5871997	-0.6504078
20.2999992	4.6359997	-0.6442719
20.2999992	4.6847997	-0.6872234
20.2999992	4.7336001	-0.6504078
20.2999992	4.7824001	-0.6135921
20.2999992	4.8312001	-0.6074562
20.2999992	4.8800001	-0.6013203
20.2999992	4.9288001	-0.5951843

20.2999992	4.9776001	-0.7363110
20.2999992	5.0264001	-0.6749516
20.2999992	5.0752001	-0.5706410
20.2999992	5.1240001	-0.7117672
20.2999992	5.1728001	-0.6442719
20.2999992	5.2216001	-0.6504078
20.2999992	5.2704000	-0.6994953
20.2999992	5.3192000	613586.0000000
20.2999992	5.3680000	613586.0000000
20.2999992	5.4168000	613586.0000000
20.2999992	5.4656000	613586.0000000
20.2999992	5.5144000	613586.0000000
20.2999992	5.5632000	613586.0000000
20.2999992	5.6120000	613586.0000000
20.2999992	5.6608000	613586.0000000
20.2999992	5.7096000	613586.0000000
20.2999992	5.7584000	613586.0000000
20.2999992	5.8072000	613586.0000000
20.2999992	5.8559999	613586.0000000
20.2999992	5.9047999	613586.0000000
20.2999992	5.9535999	613586.0000000
20.2999992	6.0023999	613586.0000000
20.3166676	0.2928000	-1.9389520
20.3166676	0.3416000	-1.7487383
20.3166676	0.3904000	-1.5523887
20.3166676	0.4392000	-1.1167378
20.3166676	0.4880000	-1.2394567
20.3166676	0.5368000	-1.2394567
20.3166676	0.5856000	-1.4112625
20.3166676	0.6344000	-1.3989906
20.3166676	0.6832000	-1.1474175
20.3166676	0.7320000	-1.1474175
20.3166676	0.7808000	-1.0983305
20.3166676	0.8296000	-1.0860586
20.3166676	0.8784000	-1.0799227
20.3166676	0.9272000	-1.1719613
20.3166676	0.9760000	-1.0860586
20.3166676	1.0247999	-1.5462527
20.3166676	1.0735999	-1.5339808
20.3166676	1.1223999	-1.2333207
20.3166676	1.1711999	-1.0676508
20.3166676	1.2200000	-0.9203887
20.3166676	1.2688000	-0.8283496
20.3166676	1.3176000	-0.8222136
20.3166676	1.3664000	-0.5706410
20.3166676	1.4152000	-1.2149129
20.3166676	1.4640000	-1.5707965
20.3166676	1.5128000	-1.3560390
20.3166676	1.5616000	-1.5523887
20.3166676	1.6104000	-1.5523887
20.3166676	1.6592000	-1.4603496
20.3166676	1.7079999	-1.4726214
20.3166676	1.7567999	-1.4112625
20.3166676	1.8055999	-1.3192234
20.3166676	1.8543999	-1.3314953
20.3166676	1.9031999	-1.3376312
20.3166676	1.9519999	-1.5462527

20.3166676	2.0007999	-1.5462527
20.3166676	2.0495999	-1.0308352
20.3166676	2.0983999	-1.0431070
20.3166676	2.1471999	-0.8590293
20.3166676	2.1959999	-1.1535535
20.3166676	2.2447999	-0.8222136
20.3166676	2.2935998	-1.4235344
20.3166676	2.3423998	-1.1965051
20.3166676	2.3912001	-1.1719613
20.3166676	2.4400001	-1.0185633
20.3166676	2.4888000	-0.8897090
20.3166676	2.5376000	-0.9019809
20.3166676	2.5864000	-0.9142528
20.3166676	2.6352000	-1.2087770
20.3166676	2.6840000	-1.0431070
20.3166676	2.7328000	-1.0369711
20.3166676	2.7816000	-1.1228738
20.3166676	2.8304000	-0.7669902
20.3166676	2.8792000	-0.7669902
20.3166676	2.9280000	-0.9572039
20.3166676	2.9768000	-0.9449320
20.3166676	3.0256000	-0.9203887
20.3166676	3.0743999	-0.7669902
20.3166676	3.1231999	-0.7853980
20.3166676	3.1719999	-0.8467574
20.3166676	3.2207999	-0.8406215
20.3166676	3.2695999	-0.9326606
20.3166676	3.3183999	-0.6565437
20.3166676	3.3671999	-1.1719613
20.3166676	3.4159999	-1.1780972
20.3166676	3.4647999	-1.0615149
20.3166676	3.5135999	-0.9633398
20.3166676	3.5623999	-0.9019809
20.3166676	3.6111999	-0.8774371
20.3166676	3.6599998	-0.8774371
20.3166676	3.7087998	-0.7731261
20.3166676	3.7575998	-0.7363110
20.3166676	3.8063998	-0.6749516
20.3166676	3.8551998	-0.6749516
20.3166676	3.9039998	-0.8038058
20.3166676	3.9527998	-0.6013203
20.3166676	4.0016003	-0.8099418
20.3166676	4.0503998	-0.7547188
20.3166676	4.0991998	-0.7669902
20.3166676	4.1479998	-0.7363110
20.3166676	4.1967998	-0.6442719
20.3166676	4.2455997	-0.6442719
20.3166676	4.2943997	-0.6504078
20.3166676	4.3431997	-0.6626797
20.3166676	4.3919997	-0.6688156
20.3166676	4.4407997	-0.6442719
20.3166676	4.4895997	-0.6442719
20.3166676	4.5383997	-0.6258640
20.3166676	4.5871997	-0.5706410
20.3166676	4.6359997	-0.5092816
20.3166676	4.6847997	-0.5031457
20.3166676	4.7336001	-0.5031457

20.3166676	4.7824001	-0.5031457
20.3166676	4.8312001	-0.4724660
20.3166676	4.8800001	-0.3497477
20.3166676	4.9288001	-0.5890489
20.3166676	4.9776001	-0.4970098
20.3166676	5.0264001	-0.5031457
20.3166676	5.0752001	-0.5031457
20.3166676	5.1240001	-0.5092816
20.3166676	5.1728001	-0.5706410
20.3166676	5.2216001	-0.4724660
20.3166676	5.2704000	-0.4724660
20.3166676	5.3192000	613586.0000000
20.3166676	5.3680000	613586.0000000
20.3166676	5.4168000	613586.0000000
20.3166676	5.4656000	613586.0000000
20.3166676	5.5144000	613586.0000000
20.3166676	5.5632000	613586.0000000
20.3166676	5.6120000	613586.0000000
20.3166676	5.6608000	613586.0000000
20.3166676	5.7096000	613586.0000000
20.3166676	5.7584000	613586.0000000
20.3166676	5.8072000	613586.0000000
20.3166676	5.8559999	613586.0000000
20.3166676	5.9047999	613586.0000000
20.3166676	5.9535999	613586.0000000
20.3166676	6.0023999	613586.0000000
20.3333340	0.2928000	-2.7673013
20.3333340	0.3416000	-1.7794180
20.3333340	0.3904000	-1.2640004
20.3333340	0.4392000	-1.1658254
20.3333340	0.4880000	-0.9694757
20.3333340	0.5368000	-1.3130875
20.3333340	0.5856000	-0.6381359
20.3333340	0.6344000	-0.8835731
20.3333340	0.6832000	-0.9019809
20.3333340	0.7320000	-0.9265246
20.3333340	0.7808000	-0.9265246
20.3333340	0.8296000	-1.1658254
20.3333340	0.8784000	-1.1474175
20.3333340	0.9272000	-1.1044660
20.3333340	0.9760000	-1.0921946
20.3333340	1.0247999	-1.0860586
20.3333340	1.0735999	-0.9510679
20.3333340	1.1223999	-0.9572039
20.3333340	1.1711999	-0.9633398
20.3333340	1.2200000	-1.1535535
20.3333340	1.2688000	-1.2026410
20.3333340	1.3176000	-1.3314953
20.3333340	1.3664000	-1.3437672
20.3333340	1.4152000	-1.4664855
20.3333340	1.4640000	-0.9572039
20.3333340	1.5128000	-1.0308352
20.3333340	1.5616000	-0.9633398
20.3333340	1.6104000	-1.2149129
20.3333340	1.6592000	-1.2149129
20.3333340	1.7079999	-1.2210488
20.3333340	1.7567999	-1.2210488

20.3333340	1.8055999	-1.0492430
20.3333340	1.8543999	-1.0492430
20.3333340	1.9031999	-1.2333207
20.3333340	1.9519999	-1.2455926
20.3333340	2.0007999	-1.2578645
20.3333340	2.0495999	-1.0799227
20.3333340	2.0983999	-1.2271848
20.3333340	2.1471999	-1.2210488
20.3333340	2.1959999	-1.2210488
20.3333340	2.2447999	-1.4419422
20.3333340	2.2935998	-1.0860586
20.3333340	2.3423998	-1.0860586
20.3333340	2.3912001	-1.2333207
20.3333340	2.4400001	-1.1290097
20.3333340	2.4888000	-1.1842332
20.3333340	2.5376000	-1.1842332
20.3333340	2.5864000	-1.1290097
20.3333340	2.6352000	-1.1596894
20.3333340	2.6840000	-1.0308352
20.3333340	2.7328000	-0.8160777
20.3333340	2.7816000	-0.8222136
20.3333340	2.8304000	-0.9449320
20.3333340	2.8792000	-1.0431070
20.3333340	2.9280000	-0.7792621
20.3333340	2.9768000	-0.7792621
20.3333340	3.0256000	-0.8835731
20.3333340	3.0743999	-0.8099418
20.3333340	3.1231999	-0.9449320
20.3333340	3.1719999	-0.6013203
20.3333340	3.2207999	-0.7179031
20.3333340	3.2695999	-0.6933594
20.3333340	3.3183999	-0.5890489
20.3333340	3.3671999	-0.8958449
20.3333340	3.4159999	-0.9019809
20.3333340	3.4647999	-0.9449320
20.3333340	3.5135999	-0.9694757
20.3333340	3.5623999	-0.8835731
20.3333340	3.6111999	-0.8897090
20.3333340	3.6599998	-0.7301750
20.3333340	3.7087998	-0.7485828
20.3333340	3.7575998	-0.7915339
20.3333340	3.8063998	-0.7792621
20.3333340	3.8551998	-0.8713012
20.3333340	3.9039998	-0.8835731
20.3333340	3.9527998	-0.7915339
20.3333340	4.0016003	-0.7853980
20.3333340	4.0503998	-0.7853980
20.3333340	4.0991998	-0.7179031
20.3333340	4.1479998	-0.8160777
20.3333340	4.1967998	-0.8038058
20.3333340	4.2455997	-0.8038058
20.3333340	4.2943997	-0.6504078
20.3333340	4.3431997	-0.8160777
20.3333340	4.3919997	-0.7363110
20.3333340	4.4407997	-0.7363110
20.3333340	4.4895997	-0.6994953
20.3333340	4.5383997	-0.6810875

20.3333340	4.5871997	-0.6688156
20.3333340	4.6359997	-0.6749516
20.3333340	4.6847997	-0.6197281
20.3333340	4.7336001	-0.6197281
20.3333340	4.7824001	-0.6013203
20.3333340	4.8312001	-0.5031457
20.3333340	4.8800001	-0.6074562
20.3333340	4.9288001	-0.6504078
20.3333340	4.9776001	-0.6994953
20.3333340	5.0264001	-0.6320000
20.3333340	5.0752001	-0.7669902
20.3333340	5.1240001	-0.5767770
20.3333340	5.1728001	-0.6135921
20.3333340	5.2216001	-0.6749516
20.3333340	5.2704000	613586.0000000
20.3333340	5.3192000	-0.6872234
20.3333340	5.3680000	-0.6872234
20.3333340	5.4168000	613586.0000000
20.3333340	5.4656000	613586.0000000
20.3333340	5.5144000	613586.0000000
20.3333340	5.5632000	613586.0000000
20.3333340	5.6120000	613586.0000000
20.3333340	5.6608000	613586.0000000
20.3333340	5.7096000	613586.0000000
20.3333340	5.7584000	613586.0000000
20.3333340	5.8072000	613586.0000000
20.3333340	5.8559999	613586.0000000
20.3333340	5.9047999	613586.0000000
20.3333340	5.9535999	613586.0000000
20.3333340	6.0023999	613586.0000000
20.3500004	0.2928000	-1.6444273
20.3500004	0.3416000	-1.6444273
20.3500004	0.3904000	-1.8469129
20.3500004	0.4392000	-1.7057867
20.3500004	0.4880000	-1.2333207
20.3500004	0.5368000	-1.2271848
20.3500004	0.5856000	-1.2087770
20.3500004	0.6344000	-0.9510679
20.3500004	0.6832000	-1.0369711
20.3500004	0.7320000	-1.1228738
20.3500004	0.7808000	-1.2271848
20.3500004	0.8296000	-1.0860586
20.3500004	0.8784000	-1.3928547
20.3500004	0.9272000	-1.4051266
20.3500004	0.9760000	-1.3008156
20.3500004	1.0247999	-1.4726214
20.3500004	1.0735999	-1.2762718
20.3500004	1.1223999	-0.7363110
20.3500004	1.1711999	-0.4970098
20.3500004	1.2200000	-0.8713012
20.3500004	1.2688000	-0.8590293
20.3500004	1.3176000	-0.9572039
20.3500004	1.3664000	-1.3069515
20.3500004	1.4152000	-1.3008156
20.3500004	1.4640000	-1.9573593
20.3500004	1.5128000	-1.6812429
20.3500004	1.5616000	-1.6812429

20.3500004	1.6104000	-1.5217090
20.3500004	1.6592000	-1.5339808
20.3500004	1.7079999	-0.8958449
20.3500004	1.7567999	-0.9878836
20.3500004	1.8055999	-1.1965051
20.3500004	1.8543999	-1.3928547
20.3500004	1.9031999	-1.3989906
20.3500004	1.9519999	-1.3867188
20.3500004	2.0007999	-1.2455926
20.3500004	2.0495999	-1.0001554
20.3500004	2.0983999	-1.0553789
20.3500004	2.1471999	-1.0369711
20.3500004	2.1959999	-1.0369711
20.3500004	2.2447999	-1.2394567
20.3500004	2.2935998	-1.2946796
20.3500004	2.3423998	-1.3008156
20.3500004	2.3912001	-1.3008156
20.3500004	2.4400001	-1.1719613
20.3500004	2.4888000	-1.1658254
20.3500004	2.5376000	-1.1719613
20.3500004	2.5864000	-1.1167378
20.3500004	2.6352000	-1.0124273
20.3500004	2.6840000	-1.0124273
20.3500004	2.7328000	-1.0124273
20.3500004	2.7816000	-1.0860586
20.3500004	2.8304000	-0.9633398
20.3500004	2.8792000	-0.9756117
20.3500004	2.9280000	-1.0308352
20.3500004	2.9768000	-0.9633398
20.3500004	3.0256000	-0.9694757
20.3500004	3.0743999	-0.5154176
20.3500004	3.1231999	-0.9510679
20.3500004	3.1719999	-1.0921946
20.3500004	3.2207999	-1.0921946
20.3500004	3.2695999	-0.9019809
20.3500004	3.3183999	-0.9019809
20.3500004	3.3671999	-0.8958449
20.3500004	3.4159999	-0.8958449
20.3500004	3.4647999	-0.8651652
20.3500004	3.5135999	-0.6013203
20.3500004	3.5623999	-0.6013203
20.3500004	3.6111999	-0.4847379
20.3500004	3.6599998	-0.5583692
20.3500004	3.7087998	-0.7547188
20.3500004	3.7575998	-0.7485828
20.3500004	3.8063998	-0.7424469
20.3500004	3.8551998	-0.9633398
20.3500004	3.9039998	-0.9756117
20.3500004	3.9527998	-0.9387960
20.3500004	4.0016003	-0.8651652
20.3500004	4.0503998	-0.8897090
20.3500004	4.0991998	-0.8713012
20.3500004	4.1479998	-0.8713012
20.3500004	4.1967998	-0.8651652
20.3500004	4.2455997	-0.8283496
20.3500004	4.2943997	-0.7117672
20.3500004	4.3431997	-0.8467574

20.3500004	4.3919997	-0.7056313
20.3500004	4.4407997	-0.7424469
20.3500004	4.4895997	-0.7424469
20.3500004	4.5383997	-0.7240391
20.3500004	4.5871997	-0.7179031
20.3500004	4.6359997	-0.7240391
20.3500004	4.6847997	-0.7363110
20.3500004	4.7336001	-0.6810875
20.3500004	4.7824001	-0.6810875
20.3500004	4.8312001	-0.6626797
20.3500004	4.8800001	-0.6013203
20.3500004	4.9288001	-0.6074562
20.3500004	4.9776001	-0.7669902
20.3500004	5.0264001	-0.5092816
20.3500004	5.0752001	-0.6749516
20.3500004	5.1240001	-0.6749516
20.3500004	5.1728001	613586.0000000
20.3500004	5.2216001	-0.8344855
20.3500004	5.2704000	613586.0000000
20.3500004	5.3192000	613586.0000000
20.3500004	5.3680000	613586.0000000
20.3500004	5.4168000	613586.0000000
20.3500004	5.4656000	613586.0000000
20.3500004	5.5144000	613586.0000000
20.3500004	5.5632000	-0.8958449
20.3500004	5.6120000	-0.8958449
20.3500004	5.6608000	613586.0000000
20.3500004	5.7096000	613586.0000000
20.3500004	5.7584000	613586.0000000
20.3500004	5.8072000	613586.0000000
20.3500004	5.8559999	613586.0000000
20.3500004	5.9047999	613586.0000000
20.3500004	5.9535999	613586.0000000
20.3500004	6.0023999	613586.0000000
20.3666668	0.2928000	-2.1721168
20.3666668	0.3416000	-1.6444273
20.3666668	0.3904000	-1.6382914
20.3666668	0.4392000	-1.3437672
20.3666668	0.4880000	-1.4173985
20.3666668	0.5368000	-0.9387960
20.3666668	0.5856000	-1.2026410
20.3666668	0.6344000	-1.2149129
20.3666668	0.6832000	-1.0860586
20.3666668	0.7320000	-1.1474175
20.3666668	0.7808000	-1.1474175
20.3666668	0.8296000	-1.1412816
20.3666668	0.8784000	-1.2149129
20.3666668	0.9272000	-1.2333207
20.3666668	0.9760000	-1.2333207
20.3666668	1.0247999	-1.2333207
20.3666668	1.0735999	-1.7119226
20.3666668	1.1223999	-1.4296703
20.3666668	1.1711999	-1.4112625
20.3666668	1.2200000	-1.4542136
20.3666668	1.2688000	-1.4542136
20.3666668	1.3176000	-1.6751070
20.3666668	1.3664000	-1.6689711

20.3666668	1.4152000	-1.7978253
20.3666668	1.4640000	-1.0860586
20.3666668	1.5128000	-0.9326606
20.3666668	1.5616000	-0.9510679
20.3666668	1.6104000	-1.1780972
20.3666668	1.6592000	-1.1290097
20.3666668	1.7079999	-1.1290097
20.3666668	1.7567999	-1.1412816
20.3666668	1.8055999	-1.0983305
20.3666668	1.8543999	-1.3069515
20.3666668	1.9031999	-1.3376312
20.3666668	1.9519999	-1.6382914
20.3666668	2.0007999	-1.2149129
20.3666668	2.0495999	-1.1903691
20.3666668	2.0983999	-1.2149129
20.3666668	2.1471999	-1.3805828
20.3666668	2.1959999	-1.3744469
20.3666668	2.2447999	-1.1596894
20.3666668	2.2935998	-1.1535535
20.3666668	2.3423998	-1.1535535
20.3666668	2.3912001	-0.9633398
20.3666668	2.4400001	-0.9633398
20.3666668	2.4888000	-1.0615149
20.3666668	2.5376000	-1.0615149
20.3666668	2.5864000	-1.0615149
20.3666668	2.6352000	-1.0799227
20.3666668	2.6840000	-1.1474175
20.3666668	2.7328000	-1.1903691
20.3666668	2.7816000	-1.0676508
20.3666668	2.8304000	-1.1903691
20.3666668	2.8792000	-1.0308352
20.3666668	2.9280000	-0.6565437
20.3666668	2.9768000	-0.8283496
20.3666668	3.0256000	-0.8344855
20.3666668	3.0743999	-1.2824078
20.3666668	3.1231999	-1.2762718
20.3666668	3.1719999	-1.2640004
20.3666668	3.2207999	-0.9142528
20.3666668	3.2695999	-0.6381359
20.3666668	3.3183999	-1.0553789
20.3666668	3.3671999	-0.7179031
20.3666668	3.4159999	-0.7240391
20.3666668	3.4647999	-0.7179031
20.3666668	3.5135999	-0.7179031
20.3666668	3.5623999	-0.7669902
20.3666668	3.6111999	-1.1167378
20.3666668	3.6599998	-1.1167378
20.3666668	3.7087998	-1.0062914
20.3666668	3.7575998	-1.0185633
20.3666668	3.8063998	-1.0431070
20.3666668	3.8551998	-1.0369711
20.3666668	3.9039998	-0.9633398
20.3666668	3.9527998	-0.9756117
20.3666668	4.0016003	-0.9817476
20.3666668	4.0503998	-0.8590293
20.3666668	4.0991998	-0.8713012
20.3666668	4.1479998	-0.7976699

20.3666668	4.1967998	-0.7976699
20.3666668	4.2455997	-0.6074562
20.3666668	4.2943997	-0.6013203
20.3666668	4.3431997	-0.7056313
20.3666668	4.3919997	-0.7117672
20.3666668	4.4407997	-0.7056313
20.3666668	4.4895997	-0.6381359
20.3666668	4.5383997	-0.6442719
20.3666668	4.5871997	-0.6197281
20.3666668	4.6359997	-0.5890489
20.3666668	4.6847997	-0.5890489
20.3666668	4.7336001	-0.4847379
20.3666668	4.7824001	-0.4847379
20.3666668	4.8312001	-0.4724660
20.3666668	4.8800001	-0.4049711
20.3666668	4.9288001	-0.4724660
20.3666668	4.9776001	-0.4601941
20.3666668	5.0264001	-0.5645051
20.3666668	5.0752001	-0.5767770
20.3666668	5.1240001	-0.5951843
20.3666668	5.1728001	-0.7915339
20.3666668	5.2216001	-0.5338254
20.3666668	5.2704000	-0.5276895
20.3666668	5.3192000	-0.2883883
20.3666668	5.3680000	-0.2883883
20.3666668	5.4168000	613586.0000000
20.3666668	5.4656000	-0.8897090
20.3666668	5.5144000	613586.0000000
20.3666668	5.5632000	613586.0000000
20.3666668	5.6120000	613586.0000000
20.3666668	5.6608000	613586.0000000
20.3666668	5.7096000	613586.0000000
20.3666668	5.7584000	613586.0000000
20.3666668	5.8072000	613586.0000000
20.3666668	5.8559999	613586.0000000
20.3666668	5.9047999	613586.0000000
20.3666668	5.9535999	613586.0000000
20.3666668	6.0023999	613586.0000000
20.3833332	0.2928000	-1.9266801
20.3833332	0.3416000	-1.4971652
20.3833332	0.3904000	-1.4971652
20.3833332	0.4392000	-1.4971652
20.3833332	0.4880000	-1.4910293
20.3833332	0.5368000	-1.4848933
20.3833332	0.5856000	-1.5707965
20.3833332	0.6344000	-1.6076121
20.3833332	0.6832000	-1.3008156
20.3833332	0.7320000	-1.3008156
20.3833332	0.7808000	-1.2946796
20.3833332	0.8296000	-0.9449320
20.3833332	0.8784000	-0.9510679
20.3833332	0.9272000	-0.9081168
20.3833332	0.9760000	-1.1106019
20.3833332	1.0247999	-1.0921946
20.3833332	1.0735999	-1.0246992
20.3833332	1.1223999	-1.2701364
20.3833332	1.1711999	-1.2701364

20.3833332	1.2200000	-1.0431070
20.3833332	1.2688000	-0.7853980
20.3833332	1.3176000	-0.6994953
20.3833332	1.3664000	-0.5338254
20.3833332	1.4152000	-0.5276895
20.3833332	1.4640000	-0.8099418
20.3833332	1.5128000	-0.9387960
20.3833332	1.5616000	-1.0921946
20.3833332	1.6104000	-1.1106019
20.3833332	1.6592000	-1.1658254
20.3833332	1.7079999	-1.6566992
20.3833332	1.7567999	-1.2271848
20.3833332	1.8055999	-1.3683109
20.3833332	1.8543999	-1.3744469
20.3833332	1.9031999	-1.2640004
20.3833332	1.9519999	-1.5585246
20.3833332	2.0007999	-1.7241945
20.3833332	2.0495999	-1.7364664
20.3833332	2.0983999	-1.4910293
20.3833332	2.1471999	-1.4910293
20.3833332	2.1959999	-1.4419422
20.3833332	2.2447999	-1.3069515
20.3833332	2.2935998	-1.2210488
20.3833332	2.3423998	-1.1535535
20.3833332	2.3912001	-1.0185633
20.3833332	2.4400001	-1.3069515
20.3833332	2.4888000	-1.4726214
20.3833332	2.5376000	-1.4112625
20.3833332	2.5864000	-1.4358063
20.3833332	2.6352000	-1.4173985
20.3833332	2.6840000	-1.4848933
20.3833332	2.7328000	-1.4296703
20.3833332	2.7816000	-1.1596894
20.3833332	2.8304000	-1.1596894
20.3833332	2.8792000	-0.7915339
20.3833332	2.9280000	-1.0062914
20.3833332	2.9768000	-0.7179031
20.3833332	3.0256000	-0.9142528
20.3833332	3.0743999	-0.8160777
20.3833332	3.1231999	-0.5645051
20.3833332	3.1719999	-0.5645051
20.3833332	3.2207999	-0.8651652
20.3833332	3.2695999	-0.8467574
20.3833332	3.3183999	-0.8283496
20.3833332	3.3671999	-1.1167378
20.3833332	3.4159999	-1.1535535
20.3833332	3.4647999	-1.1535535
20.3833332	3.5135999	-1.1596894
20.3833332	3.5623999	-1.1412816
20.3833332	3.6111999	-1.1167378
20.3833332	3.6599998	-1.1106019
20.3833332	3.7087998	-1.0921946
20.3833332	3.7575998	-1.1780972
20.3833332	3.8063998	-1.1351457
20.3833332	3.8551998	-1.1412816
20.3833332	3.9039998	-1.1167378
20.3833332	3.9527998	-1.1228738

20.3833332	4.0016003	-0.8958449
20.3833332	4.0503998	-0.9633398
20.3833332	4.0991998	-0.8958449
20.3833332	4.1479998	-0.8713012
20.3833332	4.1967998	-0.8467574
20.3833332	4.2455997	-0.7301750
20.3833332	4.2943997	-0.7240391
20.3833332	4.3431997	-0.8651652
20.3833332	4.3919997	-0.8344855
20.3833332	4.4407997	-0.8344855
20.3833332	4.4895997	-0.8651652
20.3833332	4.5383997	-0.8835731
20.3833332	4.5871997	-0.8222136
20.3833332	4.6359997	-0.8283496
20.3833332	4.6847997	-0.6197281
20.3833332	4.7336001	-0.6933594
20.3833332	4.7824001	-0.6013203
20.3833332	4.8312001	-0.6442719
20.3833332	4.8800001	-0.6994953
20.3833332	4.9288001	-0.8897090
20.3833332	4.9776001	-0.6135921
20.3833332	5.0264001	-0.7485828
20.3833332	5.0752001	-0.9326606
20.3833332	5.1240001	613586.0000000
20.3833332	5.1728001	-0.8344855
20.3833332	5.2216001	-0.7669902
20.3833332	5.2704000	-0.8406215
20.3833332	5.3192000	-0.8283496
20.3833332	5.3680000	613586.0000000
20.3833332	5.4168000	-0.4786019
20.3833332	5.4656000	613586.0000000
20.3833332	5.5144000	-0.4233789
20.3833332	5.5632000	613586.0000000
20.3833332	5.6120000	613586.0000000
20.3833332	5.6608000	613586.0000000
20.3833332	5.7096000	613586.0000000
20.3833332	5.7584000	613586.0000000
20.3833332	5.8072000	613586.0000000
20.3833332	5.8559999	613586.0000000
20.3833332	5.9047999	613586.0000000
20.3833332	5.9535999	613586.0000000
20.3833332	6.0023999	613586.0000000
20.3999996	0.2928000	-1.4603496
20.3999996	0.3416000	-1.4603496
20.3999996	0.3904000	-1.2885437
20.3999996	0.4392000	-1.4787574
20.3999996	0.4880000	-1.3683109
20.3999996	0.5368000	-1.2885437
20.3999996	0.5856000	-1.1535535
20.3999996	0.6344000	-1.1842332
20.3999996	0.6832000	-1.4910293
20.3999996	0.7320000	-1.2885437
20.3999996	0.7808000	-1.2824078
20.3999996	0.8296000	-1.1658254
20.3999996	0.8784000	-1.3314953
20.3999996	0.9272000	-1.3253593
20.3999996	0.9760000	-1.5278449

20.3999996	1.0247999	-1.0676508
20.3999996	1.0735999	-1.2271848
20.3999996	1.1223999	-1.1228738
20.3999996	1.1711999	-1.1290097
20.3999996	1.2200000	-1.1290097
20.3999996	1.2688000	-1.1351457
20.3999996	1.3176000	-1.0431070
20.3999996	1.3664000	-1.1658254
20.3999996	1.4152000	-0.8160777
20.3999996	1.4640000	-1.1658254
20.3999996	1.5128000	-1.1596894
20.3999996	1.5616000	-1.4296703
20.3999996	1.6104000	-1.7671461
20.3999996	1.6592000	-1.2578645
20.3999996	1.7079999	-1.5953403
20.3999996	1.7567999	-1.5953403
20.3999996	1.8055999	-1.6873789
20.3999996	1.8543999	-1.5646605
20.3999996	1.9031999	-1.3621750
20.3999996	1.9519999	-1.5646605
20.3999996	2.0007999	-1.2333207
20.3999996	2.0495999	-1.2333207
20.3999996	2.0983999	-1.3130875
20.3999996	2.1471999	-1.1842332
20.3999996	2.1959999	-1.1351457
20.3999996	2.2447999	-1.1965051
20.3999996	2.2935998	-1.1965051
20.3999996	2.3423998	-1.1106019
20.3999996	2.3912001	-1.1167378
20.3999996	2.4400001	-1.3683109
20.3999996	2.4888000	-1.3744469
20.3999996	2.5376000	-1.2455926
20.3999996	2.5864000	-1.0062914
20.3999996	2.6352000	-1.0001554
20.3999996	2.6840000	-0.9817476
20.3999996	2.7328000	-0.9756117
20.3999996	2.7816000	-0.4233789
20.3999996	2.8304000	-0.4295144
20.3999996	2.8792000	-1.6444273
20.3999996	2.9280000	-1.1228738
20.3999996	2.9768000	-0.5645051
20.3999996	3.0256000	-0.8590293
20.3999996	3.0743999	-0.8590293
20.3999996	3.1231999	-1.4910293
20.3999996	3.1719999	-1.0676508
20.3999996	3.2207999	-1.0737867
20.3999996	3.2695999	-1.0799227
20.3999996	3.3183999	-1.0921946
20.3999996	3.3671999	-1.0308352
20.3999996	3.4159999	-1.0369711
20.3999996	3.4647999	-1.1044660
20.3999996	3.5135999	-1.1167378
20.3999996	3.5623999	-1.0308352
20.3999996	3.6111999	-1.1351457
20.3999996	3.6599998	-1.0308352
20.3999996	3.7087998	-1.1290097
20.3999996	3.7575998	-1.0124273

20.3999996	3.8063998	-1.1903691
20.3999996	3.8551998	-1.1535535
20.3999996	3.9039998	-1.0124273
20.3999996	3.9527998	-1.0124273
20.3999996	4.0016003	-0.9878836
20.3999996	4.0503998	-0.9878836
20.3999996	4.0991998	-0.8958449
20.3999996	4.1479998	-0.8651652
20.3999996	4.1967998	-0.7853980
20.3999996	4.2455997	-0.9387960
20.3999996	4.2943997	-0.8344855
20.3999996	4.3431997	-0.7547188
20.3999996	4.3919997	-0.8897090
20.3999996	4.4407997	-0.8406215
20.3999996	4.4895997	-0.9203887
20.3999996	4.5383997	-0.8590293
20.3999996	4.5871997	-0.8099418
20.3999996	4.6359997	-0.8038058
20.3999996	4.6847997	-0.7915339
20.3999996	4.7336001	-0.8283496
20.3999996	4.7824001	-0.7363110
20.3999996	4.8312001	-0.7363110
20.3999996	4.8800001	-0.7669902
20.3999996	4.9288001	-0.7792621
20.3999996	4.9776001	-0.4970098
20.3999996	5.0264001	-0.8590293
20.3999996	5.0752001	-0.6197281
20.3999996	5.1240001	-0.6197281
20.3999996	5.1728001	613586.0000000
20.3999996	5.2216001	613586.0000000
20.3999996	5.2704000	613586.0000000
20.3999996	5.3192000	613586.0000000
20.3999996	5.3680000	613586.0000000
20.3999996	5.4168000	613586.0000000
20.3999996	5.4656000	-0.5767770
20.3999996	5.5144000	613586.0000000
20.3999996	5.5632000	613586.0000000
20.3999996	5.6120000	613586.0000000
20.3999996	5.6608000	613586.0000000
20.3999996	5.7096000	613586.0000000
20.3999996	5.7584000	613586.0000000
20.3999996	5.8072000	613586.0000000
20.3999996	5.8559999	613586.0000000
20.3999996	5.9047999	613586.0000000
20.3999996	5.9535999	613586.0000000
20.3999996	6.0023999	613586.0000000
20.4166660	0.2928000	-1.6566992
20.4166660	0.3416000	-1.5769324
20.4166660	0.3904000	-1.5769324
20.4166660	0.4392000	-1.6382914
20.4166660	0.4880000	-1.6198835
20.4166660	0.5368000	-1.4173985
20.4166660	0.5856000	-1.0860586
20.4166660	0.6344000	-1.3989906
20.4166660	0.6832000	-0.9940195
20.4166660	0.7320000	-0.9265246
20.4166660	0.7808000	-1.1167378

20.4166660	0.8296000	-1.0431070
20.4166660	0.8784000	-0.8713012
20.4166660	0.9272000	-1.1167378
20.4166660	0.9760000	-1.0799227
20.4166660	1.0247999	-1.1842332
20.4166660	1.0735999	-1.3069515
20.4166660	1.1223999	-1.3805828
20.4166660	1.1711999	-1.5953403
20.4166660	1.2200000	-1.5892043
20.4166660	1.2688000	-1.5892043
20.4166660	1.3176000	-1.6935148
20.4166660	1.3664000	-1.7180586
20.4166660	1.4152000	-1.2455926
20.4166660	1.4640000	-1.2578645
20.4166660	1.5128000	-1.2640004
20.4166660	1.5616000	-1.0553789
20.4166660	1.6104000	-1.0308352
20.4166660	1.6592000	-1.0308352
20.4166660	1.7079999	-1.6751070
20.4166660	1.7567999	-1.6566992
20.4166660	1.8055999	-1.1106019
20.4166660	1.8543999	-1.2333207
20.4166660	1.9031999	-1.4235344
20.4166660	1.9519999	-1.3314953
20.4166660	2.0007999	-1.1903691
20.4166660	2.0495999	-1.1842332
20.4166660	2.0983999	-1.2762718
20.4166660	2.1471999	-1.2640004
20.4166660	2.1959999	-1.3621750
20.4166660	2.2447999	-1.3560390
20.4166660	2.2935998	-1.4112625
20.4166660	2.3423998	-1.4112625
20.4166660	2.3912001	-1.3376312
20.4166660	2.4400001	-1.3560390
20.4166660	2.4888000	-1.2455926
20.4166660	2.5376000	-1.2578645
20.4166660	2.5864000	-1.2455926
20.4166660	2.6352000	-1.0492430
20.4166660	2.6840000	-1.0553789
20.4166660	2.7328000	-1.0615149
20.4166660	2.7816000	-1.0553789
20.4166660	2.8304000	-1.0615149
20.4166660	2.8792000	-1.0615149
20.4166660	2.9280000	-0.9940195
20.4166660	2.9768000	-0.9694757
20.4166660	3.0256000	-0.7976699
20.4166660	3.0743999	-0.7976699
20.4166660	3.1231999	-0.9449320
20.4166660	3.1719999	-0.9019809
20.4166660	3.2207999	-1.0737867
20.4166660	3.2695999	-1.0737867
20.4166660	3.3183999	-1.1106019
20.4166660	3.3671999	-1.1167378
20.4166660	3.4159999	-1.1167378
20.4166660	3.4647999	-1.0737867
20.4166660	3.5135999	-0.9940195
20.4166660	3.5623999	-0.9940195

20.4166660	3.6111999	-1.0001554
20.4166660	3.6599998	-0.8897090
20.4166660	3.7087998	-0.9019809
20.4166660	3.7575998	-1.0308352
20.4166660	3.8063998	-1.0124273
20.4166660	3.8551998	-1.0553789
20.4166660	3.9039998	-0.9449320
20.4166660	3.9527998	-0.9510679
20.4166660	4.0016003	-0.7915339
20.4166660	4.0503998	-0.7915339
20.4166660	4.0991998	-0.8099418
20.4166660	4.1479998	-0.8099418
20.4166660	4.1967998	-0.8038058
20.4166660	4.2455997	-0.8038058
20.4166660	4.2943997	-0.7976699
20.4166660	4.3431997	-0.7424469
20.4166660	4.3919997	-0.7608547
20.4166660	4.4407997	-0.6749516
20.4166660	4.4895997	-0.7179031
20.4166660	4.5383997	-0.5951843
20.4166660	4.5871997	-0.5951843
20.4166660	4.6359997	-0.6074562
20.4166660	4.6847997	-0.5399613
20.4166660	4.7336001	-0.6626797
20.4166660	4.7824001	-0.5829129
20.4166660	4.8312001	-0.5951843
20.4166660	4.8800001	-0.7117672
20.4166660	4.9288001	-0.5706410
20.4166660	4.9776001	-0.5522332
20.4166660	5.0264001	-0.5092816
20.4166660	5.0752001	-0.4049711
20.4166660	5.1240001	-0.3742914
20.4166660	5.1728001	-0.3742914
20.4166660	5.2216001	-0.2393012
20.4166660	5.2704000	-0.2577085
20.4166660	5.3192000	-0.4847379
20.4166660	5.3680000	613586.0000000
20.4166660	5.4168000	613586.0000000
20.4166660	5.4656000	613586.0000000
20.4166660	5.5144000	613586.0000000
20.4166660	5.5632000	613586.0000000
20.4166660	5.6120000	613586.0000000
20.4166660	5.6608000	613586.0000000
20.4166660	5.7096000	613586.0000000
20.4166660	5.7584000	613586.0000000
20.4166660	5.8072000	-0.5890489
20.4166660	5.8559999	613586.0000000
20.4166660	5.9047999	613586.0000000
20.4166660	5.9535999	613586.0000000
20.4166660	6.0023999	613586.0000000
20.4333324	0.2928000	-2.3071072
20.4333324	0.3416000	-1.7794180
20.4333324	0.3904000	-1.4664855
20.4333324	0.4392000	-1.2271848
20.4333324	0.4880000	-1.0676508
20.4333324	0.5368000	-1.0676508
20.4333324	0.5856000	-1.2701364

20.4333324	0.6344000	-1.2640004
20.4333324	0.6832000	-1.1842332
20.4333324	0.7320000	-1.0062914
20.4333324	0.7808000	-1.0124273
20.4333324	0.8296000	-1.2640004
20.4333324	0.8784000	-1.0124273
20.4333324	0.9272000	-1.2271848
20.4333324	0.9760000	-1.2333207
20.4333324	1.0247999	-1.4542136
20.4333324	1.0735999	-1.2455926
20.4333324	1.1223999	-1.2517285
20.4333324	1.1711999	-1.3069515
20.4333324	1.2200000	-1.2210488
20.4333324	1.2688000	-1.2210488
20.4333324	1.3176000	-1.1228738
20.4333324	1.3664000	-1.1106019
20.4333324	1.4152000	-0.9019809
20.4333324	1.4640000	-1.0185633
20.4333324	1.5128000	-0.8222136
20.4333324	1.5616000	-1.1535535
20.4333324	1.6104000	-1.1474175
20.4333324	1.6592000	-1.2271848
20.4333324	1.7079999	-1.0676508
20.4333324	1.7567999	-0.9081168
20.4333324	1.8055999	-1.0185633
20.4333324	1.8543999	-1.0185633
20.4333324	1.9031999	-1.2762718
20.4333324	1.9519999	-1.0308352
20.4333324	2.0007999	-1.0431070
20.4333324	2.0495999	-0.9817476
20.4333324	2.0983999	-1.1965051
20.4333324	2.1471999	-1.1965051
20.4333324	2.1959999	-1.1474175
20.4333324	2.2447999	-1.2455926
20.4333324	2.2935998	-1.2210488
20.4333324	2.3423998	-1.2271848
20.4333324	2.3912001	-1.0062914
20.4333324	2.4400001	-1.0308352
20.4333324	2.4888000	-0.9633398
20.4333324	2.5376000	-0.9633398
20.4333324	2.5864000	-1.0185633
20.4333324	2.6352000	-1.0001554
20.4333324	2.6840000	-0.7485828
20.4333324	2.7328000	-0.7363110
20.4333324	2.7816000	-0.7301750
20.4333324	2.8304000	-0.8283496
20.4333324	2.8792000	-0.6320000
20.4333324	2.9280000	-0.6442719
20.4333324	2.9768000	-0.9081168
20.4333324	3.0256000	-0.9081168
20.4333324	3.0743999	-0.8958449
20.4333324	3.1231999	-0.8774371
20.4333324	3.1719999	-0.8774371
20.4333324	3.2207999	-0.7608547
20.4333324	3.2695999	-0.9387960
20.4333324	3.3183999	-0.9387960
20.4333324	3.3671999	-0.7792621

20.4333324	3.4159999	-0.8528934
20.4333324	3.4647999	-0.6933594
20.4333324	3.5135999	-0.7547188
20.4333324	3.5623999	-0.8406215
20.4333324	3.6111999	-0.7853980
20.4333324	3.6599998	-0.7853980
20.4333324	3.7087998	-0.8897090
20.4333324	3.7575998	-0.8467574
20.4333324	3.8063998	-0.8651652
20.4333324	3.8551998	-0.8897090
20.4333324	3.9039998	-0.6258640
20.4333324	3.9527998	-0.6872234
20.4333324	4.0016003	-0.6933594
20.4333324	4.0503998	-0.5890489
20.4333324	4.0991998	-0.5890489
20.4333324	4.1479998	-0.5522332
20.4333324	4.1967998	-0.4908738
20.4333324	4.2455997	-0.4786019
20.4333324	4.2943997	-0.5460973
20.4333324	4.3431997	-0.5399613
20.4333324	4.3919997	-0.4908738
20.4333324	4.4407997	-0.4847379
20.4333324	4.4895997	-0.4786019
20.4333324	4.5383997	-0.4786019
20.4333324	4.5871997	-0.4786019
20.4333324	4.6359997	-0.4786019
20.4333324	4.6847997	-0.4786019
20.4333324	4.7336001	-0.4786019
20.4333324	4.7824001	-0.4663301
20.4333324	4.8312001	-0.4356503
20.4333324	4.8800001	-0.4356503
20.4333324	4.9288001	-0.4847379
20.4333324	4.9776001	-0.4970098
20.4333324	5.0264001	-0.4049711
20.4333324	5.0752001	-0.3006601
20.4333324	5.1240001	-0.5092816
20.4333324	5.1728001	-0.7915339
20.4333324	5.2216001	-0.5522332
20.4333324	5.2704000	-0.4233789
20.4333324	5.3192000	-0.7976699
20.4333324	5.3680000	-0.6810875
20.4333324	5.4168000	-0.5399613
20.4333324	5.4656000	-0.5399613
20.4333324	5.5144000	-0.5338254
20.4333324	5.5632000	613586.0000000
20.4333324	5.6120000	-0.8774371
20.4333324	5.6608000	613586.0000000
20.4333324	5.7096000	613586.0000000
20.4333324	5.7584000	613586.0000000
20.4333324	5.8072000	613586.0000000
20.4333324	5.8559999	613586.0000000
20.4333324	5.9047999	613586.0000000
20.4333324	5.9535999	613586.0000000
20.4333324	6.0023999	613586.0000000
20.4500008	0.2928000	-2.0003109
20.4500008	0.3416000	-2.5648160
20.4500008	0.3904000	-2.5586801

20.4500008	0.4392000	-1.2946796
20.4500008	0.4880000	-1.2946796
20.4500008	0.5368000	-1.3499031
20.4500008	0.5856000	-1.0369711
20.4500008	0.6344000	-1.0431070
20.4500008	0.6832000	-0.9817476
20.4500008	0.7320000	-1.0983305
20.4500008	0.7808000	-1.2271848
20.4500008	0.8296000	-1.2455926
20.4500008	0.8784000	-1.2701364
20.4500008	0.9272000	-1.0185633
20.4500008	0.9760000	-1.0185633
20.4500008	1.0247999	-1.1965051
20.4500008	1.0735999	-1.1167378
20.4500008	1.1223999	-1.1167378
20.4500008	1.1711999	-1.2517285
20.4500008	1.2200000	-1.2455926
20.4500008	1.2688000	-1.1290097
20.4500008	1.3176000	-1.1351457
20.4500008	1.3664000	-1.1780972
20.4500008	1.4152000	-1.1780972
20.4500008	1.4640000	-1.1719613
20.4500008	1.5128000	-1.2824078
20.4500008	1.5616000	-1.2701364
20.4500008	1.6104000	-1.2333207
20.4500008	1.6592000	-0.9756117
20.4500008	1.7079999	-0.9817476
20.4500008	1.7567999	-1.0492430
20.4500008	1.8055999	-1.0246992
20.4500008	1.8543999	-1.0246992
20.4500008	1.9031999	-0.9572039
20.4500008	1.9519999	-0.9572039
20.4500008	2.0007999	-1.1167378
20.4500008	2.0495999	-1.1167378
20.4500008	2.0983999	-1.1474175
20.4500008	2.1471999	-1.2701364
20.4500008	2.1959999	-1.2640004
20.4500008	2.2447999	-1.1903691
20.4500008	2.2935998	-1.1780972
20.4500008	2.3423998	-1.2517285
20.4500008	2.3912001	-1.1228738
20.4500008	2.4400001	-1.1106019
20.4500008	2.4888000	-0.9817476
20.4500008	2.5376000	-1.0737867
20.4500008	2.5864000	-1.0553789
20.4500008	2.6352000	-0.9878836
20.4500008	2.6840000	-0.8774371
20.4500008	2.7328000	-0.8713012
20.4500008	2.7816000	-0.6994953
20.4500008	2.8304000	-1.1228738
20.4500008	2.8792000	-1.1106019
20.4500008	2.9280000	-1.0983305
20.4500008	2.9768000	-0.8099418
20.4500008	3.0256000	-0.8099418
20.4500008	3.0743999	-0.7915339
20.4500008	3.1231999	-0.7853980
20.4500008	3.1719999	-0.7792621

20.4500008	3.2207999	-0.7485828
20.4500008	3.2695999	-0.7485828
20.4500008	3.3183999	-0.7853980
20.4500008	3.3671999	-0.9817476
20.4500008	3.4159999	-0.9265246
20.4500008	3.4647999	-0.9510679
20.4500008	3.5135999	-0.9510679
20.4500008	3.5623999	-0.9878836
20.4500008	3.6111999	-0.7792621
20.4500008	3.6599998	-0.7179031
20.4500008	3.7087998	-0.6933594
20.4500008	3.7575998	-0.7056313
20.4500008	3.8063998	-0.6933594
20.4500008	3.8551998	-0.6872234
20.4500008	3.9039998	-0.7547188
20.4500008	3.9527998	-0.7608547
20.4500008	4.0016003	-0.6626797
20.4500008	4.0503998	-0.5767770
20.4500008	4.0991998	-0.6258640
20.4500008	4.1479998	-0.6074562
20.4500008	4.1967998	-0.7056313
20.4500008	4.2455997	-0.7056313
20.4500008	4.2943997	-0.7056313
20.4500008	4.3431997	-0.6258640
20.4500008	4.3919997	-0.6442719
20.4500008	4.4407997	-0.6565437
20.4500008	4.4895997	-0.6810875
20.4500008	4.5383997	-0.5829129
20.4500008	4.5871997	-0.5829129
20.4500008	4.6359997	-0.6626797
20.4500008	4.6847997	-0.6626797
20.4500008	4.7336001	-0.6320000
20.4500008	4.7824001	-0.5460973
20.4500008	4.8312001	-0.5583692
20.4500008	4.8800001	-0.6381359
20.4500008	4.9288001	-0.6320000
20.4500008	4.9776001	-0.6074562
20.4500008	5.0264001	-0.6074562
20.4500008	5.0752001	-0.5522332
20.4500008	5.1240001	-0.3497477
20.4500008	5.1728001	-0.5092816
20.4500008	5.2216001	-0.5031457
20.4500008	5.2704000	-0.4540582
20.4500008	5.3192000	-0.5031457
20.4500008	5.3680000	-0.5092816
20.4500008	5.4168000	-0.4356503
20.4500008	5.4656000	-0.3374758
20.4500008	5.5144000	-0.3129320
20.4500008	5.5632000	-0.2331653
20.4500008	5.6120000	613586.0000000
20.4500008	5.6608000	613586.0000000
20.4500008	5.7096000	613586.0000000
20.4500008	5.7584000	613586.0000000
20.4500008	5.8072000	-0.1411262
20.4500008	5.8559999	613586.0000000
20.4500008	5.9047999	613586.0000000
20.4500008	5.9535999	613586.0000000

20.4500008	6.0023999	613586.0000000
20.4666672	0.2928000	-0.5338254
20.4666672	0.3416000	-0.5276895
20.4666672	0.3904000	-0.3988352
20.4666672	0.4392000	-1.5278449
20.4666672	0.4880000	-1.1351457
20.4666672	0.5368000	-1.1412816
20.4666672	0.5856000	-1.3805828
20.4666672	0.6344000	-1.4051266
20.4666672	0.6832000	-1.6137476
20.4666672	0.7320000	-1.5339808
20.4666672	0.7808000	-1.4664855
20.4666672	0.8296000	-1.5953403
20.4666672	0.8784000	-1.1474175
20.4666672	0.9272000	-1.1474175
20.4666672	0.9760000	-1.0983305
20.4666672	1.0247999	-0.7485828
20.4666672	1.0735999	-1.0308352
20.4666672	1.1223999	-1.1351457
20.4666672	1.1711999	-1.1351457
20.4666672	1.2200000	-1.2946796
20.4666672	1.2688000	-1.3621750
20.4666672	1.3176000	-1.1228738
20.4666672	1.3664000	-1.3989906
20.4666672	1.4152000	-1.4051266
20.4666672	1.4640000	-1.3253593
20.4666672	1.5128000	-1.1044660
20.4666672	1.5616000	-1.2271848
20.4666672	1.6104000	-1.0983305
20.4666672	1.6592000	-1.0921946
20.4666672	1.7079999	-1.2271848
20.4666672	1.7567999	-1.2333207
20.4666672	1.8055999	-1.3499031
20.4666672	1.8543999	-1.2210488
20.4666672	1.9031999	-1.1658254
20.4666672	1.9519999	-1.1719613
20.4666672	2.0007999	-1.1351457
20.4666672	2.0495999	-1.1351457
20.4666672	2.0983999	-1.1290097
20.4666672	2.1471999	-1.0615149
20.4666672	2.1959999	-1.2026410
20.4666672	2.2447999	-1.1842332
20.4666672	2.2935998	-1.0062914
20.4666672	2.3423998	-1.0124273
20.4666672	2.3912001	-1.0124273
20.4666672	2.4400001	-0.9142528
20.4666672	2.4888000	-0.9081168
20.4666672	2.5376000	-0.6810875
20.4666672	2.5864000	-0.6872234
20.4666672	2.6352000	-0.6688156
20.4666672	2.6840000	-0.6626797
20.4666672	2.7328000	-0.6626797
20.4666672	2.7816000	-0.7117672
20.4666672	2.8304000	-0.7117672
20.4666672	2.8792000	-0.8651652
20.4666672	2.9280000	-1.1780972
20.4666672	2.9768000	-1.1719613

20.4666672	3.0256000	-1.1351457
20.4666672	3.0743999	-1.1228738
20.4666672	3.1231999	-1.0799227
20.4666672	3.1719999	-1.0062914
20.4666672	3.2207999	-1.0062914
20.4666672	3.2695999	-1.0921946
20.4666672	3.3183999	-1.0308352
20.4666672	3.3671999	-0.9449320
20.4666672	3.4159999	-0.9019809
20.4666672	3.4647999	-0.9019809
20.4666672	3.5135999	-1.0492430
20.4666672	3.5623999	-0.8467574
20.4666672	3.6111999	-0.8958449
20.4666672	3.6599998	-0.8774371
20.4666672	3.7087998	-0.8713012
20.4666672	3.7575998	-0.8528934
20.4666672	3.8063998	-0.9265246
20.4666672	3.8551998	-0.7731261
20.4666672	3.9039998	-0.7853980
20.4666672	3.9527998	-0.8528934
20.4666672	4.0016003	-0.9265246
20.4666672	4.0503998	-0.9387960
20.4666672	4.0991998	-0.7301750
20.4666672	4.1479998	-0.7301750
20.4666672	4.1967998	-0.7424469
20.4666672	4.2455997	-0.9265246
20.4666672	4.2943997	-0.9142528
20.4666672	4.3431997	-0.7853980
20.4666672	4.3919997	-0.7976699
20.4666672	4.4407997	-0.7915339
20.4666672	4.4895997	-0.9387960
20.4666672	4.5383997	-0.8958449
20.4666672	4.5871997	-0.9510679
20.4666672	4.6359997	-0.9449320
20.4666672	4.6847997	-0.9510679
20.4666672	4.7336001	-0.8713012
20.4666672	4.7824001	-0.8835731
20.4666672	4.8312001	-1.0124273
20.4666672	4.8800001	-1.0124273
20.4666672	4.9288001	-1.0001554
20.4666672	4.9776001	-0.8590293
20.4666672	5.0264001	-0.8651652
20.4666672	5.0752001	-0.7424469
20.4666672	5.1240001	613586.0000000
20.4666672	5.1728001	-0.8590293
20.4666672	5.2216001	-0.8590293
20.4666672	5.2704000	-0.8160777
20.4666672	5.3192000	-0.8099418
20.4666672	5.3680000	613586.0000000
20.4666672	5.4168000	613586.0000000
20.4666672	5.4656000	613586.0000000
20.4666672	5.5144000	613586.0000000
20.4666672	5.5632000	613586.0000000
20.4666672	5.6120000	613586.0000000
20.4666672	5.6608000	-0.8590293
20.4666672	5.7096000	-0.8590293
20.4666672	5.7584000	613586.0000000

20.4666672	5.8072000	613586.0000000
20.4666672	5.8559999	613586.0000000
20.4666672	5.9047999	613586.0000000
20.4666672	5.9535999	613586.0000000
20.4666672	6.0023999	613586.0000000
20.4833336	0.2928000	-2.3746023
20.4833336	0.3416000	-1.6137476
20.4833336	0.3904000	-1.7671461
20.4833336	0.4392000	-1.6566992
20.4833336	0.4880000	-1.4419422
20.4833336	0.5368000	-1.3621750
20.4833336	0.5856000	-1.0737867
20.4833336	0.6344000	-1.2885437
20.4833336	0.6832000	-1.0308352
20.4833336	0.7320000	-1.0983305
20.4833336	0.7808000	-0.9449320
20.4833336	0.8296000	-1.2210488
20.4833336	0.8784000	-1.4112625
20.4833336	0.9272000	-1.4726214
20.4833336	0.9760000	-1.4480777
20.4833336	1.0247999	-1.2517285
20.4833336	1.0735999	-1.4603496
20.4833336	1.1223999	-1.1535535
20.4833336	1.1711999	-1.1658254
20.4833336	1.2200000	-1.2026410
20.4833336	1.2688000	-1.0799227
20.4833336	1.3176000	-1.0676508
20.4833336	1.3664000	-0.8835731
20.4833336	1.4152000	-1.4910293
20.4833336	1.4640000	-1.6566992
20.4833336	1.5128000	-1.2578645
20.4833336	1.5616000	-1.5339808
20.4833336	1.6104000	-1.4358063
20.4833336	1.6592000	-1.4419422
20.4833336	1.7079999	-1.2640004
20.4833336	1.7567999	-1.2087770
20.4833336	1.8055999	-1.2087770
20.4833336	1.8543999	-1.2885437
20.4833336	1.9031999	-1.2333207
20.4833336	1.9519999	-1.2333207
20.4833336	2.0007999	-1.2271848
20.4833336	2.0495999	-1.2149129
20.4833336	2.0983999	-1.3376312
20.4833336	2.1471999	-1.3928547
20.4833336	2.1959999	-1.3989906
20.4833336	2.2447999	-1.1965051
20.4833336	2.2935998	-1.1842332
20.4833336	2.3423998	-1.2824078
20.4833336	2.3912001	-1.1719613
20.4833336	2.4400001	-1.1044660
20.4833336	2.4888000	-1.0062914
20.4833336	2.5376000	-0.8774371
20.4833336	2.5864000	-0.8651652
20.4833336	2.6352000	-0.8651652
20.4833336	2.6840000	-0.7669902
20.4833336	2.7328000	-0.7547188
20.4833336	2.7816000	-0.6994953

20.4833336	2.8304000	-0.6504078
20.4833336	2.8792000	-0.5706410
20.4833336	2.9280000	-0.7301750
20.4833336	2.9768000	-0.9142528
20.4833336	3.0256000	-0.7669902
20.4833336	3.0743999	-0.7608547
20.4833336	3.1231999	-0.7547188
20.4833336	3.1719999	-0.7424469
20.4833336	3.2207999	-0.7424469
20.4833336	3.2695999	-0.7485828
20.4833336	3.3183999	-0.7853980
20.4833336	3.3671999	-0.9142528
20.4833336	3.4159999	-0.9265246
20.4833336	3.4647999	-0.9265246
20.4833336	3.5135999	-0.7547188
20.4833336	3.5623999	-0.7608547
20.4833336	3.6111999	-0.7915339
20.4833336	3.6599998	-0.7915339
20.4833336	3.7087998	-0.6933594
20.4833336	3.7575998	-0.6872234
20.4833336	3.8063998	-0.6320000
20.4833336	3.8551998	-0.6504078
20.4833336	3.9039998	-0.6626797
20.4833336	3.9527998	-0.6688156
20.4833336	4.0016003	-0.7669902
20.4833336	4.0503998	-0.5522332
20.4833336	4.0991998	-0.5399613
20.4833336	4.1479998	-0.7424469
20.4833336	4.1967998	-0.7363110
20.4833336	4.2455997	-0.7424469
20.4833336	4.2943997	-0.7117672
20.4833336	4.3431997	-0.7301750
20.4833336	4.3919997	-0.7117672
20.4833336	4.4407997	-0.6504078
20.4833336	4.4895997	-0.6565437
20.4833336	4.5383997	-0.6504078
20.4833336	4.5871997	-0.6504078
20.4833336	4.6359997	-0.6504078
20.4833336	4.6847997	-0.6565437
20.4833336	4.7336001	-0.7976699
20.4833336	4.7824001	-0.6626797
20.4833336	4.8312001	-0.6688156
20.4833336	4.8800001	-0.5522332
20.4833336	4.9288001	-0.4970098
20.4833336	4.9776001	-0.5154176
20.4833336	5.0264001	-0.6872234
20.4833336	5.0752001	-1.1290097
20.4833336	5.1240001	613586.0000000
20.4833336	5.1728001	613586.0000000
20.4833336	5.2216001	613586.0000000
20.4833336	5.2704000	613586.0000000
20.4833336	5.3192000	613586.0000000
20.4833336	5.3680000	613586.0000000
20.4833336	5.4168000	-1.0308352
20.4833336	5.4656000	613586.0000000
20.4833336	5.5144000	613586.0000000
20.4833336	5.5632000	613586.0000000

20.4833336	5.6120000	613586.0000000
20.4833336	5.6608000	613586.0000000
20.4833336	5.7096000	613586.0000000
20.4833336	5.7584000	613586.0000000
20.4833336	5.8072000	613586.0000000
20.4833336	5.8559999	613586.0000000
20.4833336	5.9047999	613586.0000000
20.4833336	5.9535999	613586.0000000
20.4833336	6.0023999	613586.0000000
20.5000000	0.2928000	-1.8100972
20.5000000	0.3416000	-1.6812429
20.5000000	0.3904000	-1.4726214
20.5000000	0.4392000	-1.4112625
20.5000000	0.4880000	-1.3989906
20.5000000	0.5368000	-1.4173985
20.5000000	0.5856000	-1.0431070
20.5000000	0.6344000	-1.0369711
20.5000000	0.6832000	-1.0308352
20.5000000	0.7320000	-1.1044660
20.5000000	0.7808000	-1.1228738
20.5000000	0.8296000	-1.3069515
20.5000000	0.8784000	-1.4542136
20.5000000	0.9272000	-1.6444273
20.5000000	0.9760000	-1.3314953
20.5000000	1.0247999	-1.3192234
20.5000000	1.0735999	-1.4173985
20.5000000	1.1223999	-1.5401168
20.5000000	1.1711999	-1.5339808
20.5000000	1.2200000	-1.3192234
20.5000000	1.2688000	-1.3253593
20.5000000	1.3176000	-1.2517285
20.5000000	1.3664000	-1.6505632
20.5000000	1.4152000	-1.4235344
20.5000000	1.4640000	-1.3867188
20.5000000	1.5128000	-1.2394567
20.5000000	1.5616000	-1.2333207
20.5000000	1.6104000	-1.2271848
20.5000000	1.6592000	-1.2271848
20.5000000	1.7079999	-1.2946796
20.5000000	1.7567999	-1.1535535
20.5000000	1.8055999	-1.3314953
20.5000000	1.8543999	-1.3130875
20.5000000	1.9031999	-1.1903691
20.5000000	1.9519999	-1.1903691
20.5000000	2.0007999	-1.4051266
20.5000000	2.0495999	-1.3989906
20.5000000	2.0983999	-1.3989906
20.5000000	2.1471999	-1.4051266
20.5000000	2.1959999	-1.4173985
20.5000000	2.2447999	-1.4358063
20.5000000	2.2935998	-1.4480777
20.5000000	2.3423998	-1.4480777
20.5000000	2.3912001	-1.1842332
20.5000000	2.4400001	-1.1842332
20.5000000	2.4888000	-1.1780972
20.5000000	2.5376000	-0.9817476
20.5000000	2.5864000	-1.0308352

20.5000000	2.6352000	-1.0246992
20.5000000	2.6840000	-1.0308352
20.5000000	2.7328000	-0.9572039
20.5000000	2.7816000	-0.9449320
20.5000000	2.8304000	-0.8467574
20.5000000	2.8792000	-0.3620195
20.5000000	2.9280000	-0.4663301
20.5000000	2.9768000	-0.6565437
20.5000000	3.0256000	-0.8406215
20.5000000	3.0743999	-0.9326606
20.5000000	3.1231999	-0.5583692
20.5000000	3.1719999	-0.5522332
20.5000000	3.2207999	-0.7301750
20.5000000	3.2695999	-0.6810875
20.5000000	3.3183999	-0.8774371
20.5000000	3.3671999	-0.8651652
20.5000000	3.4159999	-0.8835731
20.5000000	3.4647999	-0.8897090
20.5000000	3.5135999	-0.9019809
20.5000000	3.5623999	-0.7485828
20.5000000	3.6111999	-0.7547188
20.5000000	3.6599998	-0.7608547
20.5000000	3.7087998	-0.6688156
20.5000000	3.7575998	-0.6933594
20.5000000	3.8063998	-0.6810875
20.5000000	3.8551998	-0.6320000
20.5000000	3.9039998	-0.6320000
20.5000000	3.9527998	-0.5767770
20.5000000	4.0016003	-0.5767770
20.5000000	4.0503998	-0.6994953
20.5000000	4.0991998	-0.5890489
20.5000000	4.1479998	-0.5829129
20.5000000	4.1967998	-0.5215535
20.5000000	4.2455997	-0.5460973
20.5000000	4.2943997	-0.6381359
20.5000000	4.3431997	-0.5767770
20.5000000	4.3919997	-0.6442719
20.5000000	4.4407997	-0.6504078
20.5000000	4.4895997	-0.7731261
20.5000000	4.5383997	-0.6013203
20.5000000	4.5871997	-0.6565437
20.5000000	4.6359997	-0.6135921
20.5000000	4.6847997	-0.4970098
20.5000000	4.7336001	-0.4540582
20.5000000	4.7824001	-0.4479222
20.5000000	4.8312001	-0.4417863
20.5000000	4.8800001	-0.4417863
20.5000000	4.9288001	-0.4295144
20.5000000	4.9776001	-0.4233789
20.5000000	5.0264001	-0.2883883
20.5000000	5.0752001	-0.2945242
20.5000000	5.1240001	-0.5460973
20.5000000	5.1728001	613586.0000000
20.5000000	5.2216001	613586.0000000
20.5000000	5.2704000	613586.0000000
20.5000000	5.3192000	-0.8713012
20.5000000	5.3680000	613586.0000000

20.5000000	5.4168000	613586.0000000
20.5000000	5.4656000	-0.6933594
20.5000000	5.5144000	613586.0000000
20.5000000	5.5632000	613586.0000000
20.5000000	5.6120000	613586.0000000
20.5000000	5.6608000	613586.0000000
20.5000000	5.7096000	613586.0000000
20.5000000	5.7584000	613586.0000000
20.5000000	5.8072000	613586.0000000
20.5000000	5.8559999	613586.0000000
20.5000000	5.9047999	613586.0000000
20.5000000	5.9535999	613586.0000000
20.5000000	6.0023999	613586.0000000
20.5166664	0.2928000	-1.8714566
20.5166664	0.3416000	-1.6137476
20.5166664	0.3904000	-1.4419422
20.5166664	0.4392000	-1.4480777
20.5166664	0.4880000	-1.4419422
20.5166664	0.5368000	-1.0431070
20.5166664	0.5856000	-1.0431070
20.5166664	0.6344000	-0.8590293
20.5166664	0.6832000	-1.3621750
20.5166664	0.7320000	-1.3989906
20.5166664	0.7808000	-1.3928547
20.5166664	0.8296000	-1.2455926
20.5166664	0.8784000	-1.4971652
20.5166664	0.9272000	-1.3069515
20.5166664	0.9760000	-1.4419422
20.5166664	1.0247999	-1.7057867
20.5166664	1.0735999	-1.4542136
20.5166664	1.1223999	-1.3744469
20.5166664	1.1711999	-1.2455926
20.5166664	1.2200000	-1.1780972
20.5166664	1.2688000	-1.1044660
20.5166664	1.3176000	-1.3192234
20.5166664	1.3664000	-1.3621750
20.5166664	1.4152000	-1.3621750
20.5166664	1.4640000	-1.3069515
20.5166664	1.5128000	-1.1658254
20.5166664	1.5616000	-1.1596894
20.5166664	1.6104000	-1.1044660
20.5166664	1.6592000	-1.1106019
20.5166664	1.7079999	-1.1106019
20.5166664	1.7567999	-1.3437672
20.5166664	1.8055999	-1.2701364
20.5166664	1.8543999	-1.2824078
20.5166664	1.9031999	-1.2824078
20.5166664	1.9519999	-1.3437672
20.5166664	2.0007999	-1.3376312
20.5166664	2.0495999	-1.1780972
20.5166664	2.0983999	-1.3314953
20.5166664	2.1471999	-1.3192234
20.5166664	2.1959999	-1.3192234
20.5166664	2.2447999	-1.3744469
20.5166664	2.2935998	-1.3621750
20.5166664	2.3423998	-1.3499031
20.5166664	2.3912001	-1.3499031

20.5166664	2.4400001	-1.1719613
20.5166664	2.4888000	-1.1903691
20.5166664	2.5376000	-1.1903691
20.5166664	2.5864000	-1.0615149
20.5166664	2.6352000	-1.0553789
20.5166664	2.6840000	-1.0553789
20.5166664	2.7328000	-1.2333207
20.5166664	2.7816000	-1.0431070
20.5166664	2.8304000	-0.9878836
20.5166664	2.8792000	-0.9449320
20.5166664	2.9280000	-0.7608547
20.5166664	2.9768000	-0.7669902
20.5166664	3.0256000	-0.9203887
20.5166664	3.0743999	-0.6688156
20.5166664	3.1231999	-0.6197281
20.5166664	3.1719999	-0.6933594
20.5166664	3.2207999	-1.1658254
20.5166664	3.2695999	-1.1658254
20.5166664	3.3183999	-1.1044660
20.5166664	3.3671999	-0.9326606
20.5166664	3.4159999	-0.8713012
20.5166664	3.4647999	-0.8835731
20.5166664	3.5135999	-0.9326606
20.5166664	3.5623999	-0.9265246
20.5166664	3.6111999	-0.8099418
20.5166664	3.6599998	-0.8099418
20.5166664	3.7087998	-0.9203887
20.5166664	3.7575998	-0.8467574
20.5166664	3.8063998	-0.8590293
20.5166664	3.8551998	-0.8590293
20.5166664	3.9039998	-0.7915339
20.5166664	3.9527998	-0.8038058
20.5166664	4.0016003	-0.7915339
20.5166664	4.0503998	-0.7363110
20.5166664	4.0991998	-0.6626797
20.5166664	4.1479998	-0.6626797
20.5166664	4.1967998	-0.8283496
20.5166664	4.2455997	-0.8283496
20.5166664	4.2943997	-0.8713012
20.5166664	4.3431997	-0.8713012
20.5166664	4.3919997	-0.7915339
20.5166664	4.4407997	-1.0246992
20.5166664	4.4895997	-1.0246992
20.5166664	4.5383997	-1.0308352
20.5166664	4.5871997	-1.0369711
20.5166664	4.6359997	-1.0308352
20.5166664	4.6847997	-1.0246992
20.5166664	4.7336001	-0.7608547
20.5166664	4.7824001	-0.7485828
20.5166664	4.8312001	-0.7363110
20.5166664	4.8800001	-0.6688156
20.5166664	4.9288001	-0.5951843
20.5166664	4.9776001	-0.5951843
20.5166664	5.0264001	613586.0000000
20.5166664	5.0752001	613586.0000000
20.5166664	5.1240001	613586.0000000
20.5166664	5.1728001	613586.0000000

20.5166664	5.2216001	613586.0000000
20.5166664	5.2704000	613586.0000000
20.5166664	5.3192000	613586.0000000
20.5166664	5.3680000	613586.0000000
20.5166664	5.4168000	613586.0000000
20.5166664	5.4656000	613586.0000000
20.5166664	5.5144000	613586.0000000
20.5166664	5.5632000	613586.0000000
20.5166664	5.6120000	613586.0000000
20.5166664	5.6608000	613586.0000000
20.5166664	5.7096000	613586.0000000
20.5166664	5.7584000	613586.0000000
20.5166664	5.8072000	613586.0000000
20.5166664	5.8559999	613586.0000000
20.5166664	5.9047999	613586.0000000
20.5166664	5.9535999	613586.0000000
20.5166664	6.0023999	613586.0000000
20.5333328	0.2928000	-2.1046219
20.5333328	0.3416000	-1.5094371
20.5333328	0.3904000	-1.4480777
20.5333328	0.4392000	-1.4051266
20.5333328	0.4880000	-1.1290097
20.5333328	0.5368000	-1.2762718
20.5333328	0.5856000	-1.1290097
20.5333328	0.6344000	-1.5523887
20.5333328	0.6832000	-1.3744469
20.5333328	0.7320000	-1.5707965
20.5333328	0.7808000	-1.3867188
20.5333328	0.8296000	-1.3989906
20.5333328	0.8784000	-1.4542136
20.5333328	0.9272000	-1.2271848
20.5333328	0.9760000	-1.3867188
20.5333328	1.0247999	-1.3928547
20.5333328	1.0735999	-1.1167378
20.5333328	1.1223999	-1.2333207
20.5333328	1.1711999	-1.0369711
20.5333328	1.2200000	-1.4173985
20.5333328	1.2688000	-1.4112625
20.5333328	1.3176000	-1.4112625
20.5333328	1.3664000	-1.4726214
20.5333328	1.4152000	-1.2087770
20.5333328	1.4640000	-1.2271848
20.5333328	1.5128000	-1.0308352
20.5333328	1.5616000	-1.0246992
20.5333328	1.6104000	-1.1903691
20.5333328	1.6592000	-1.2701364
20.5333328	1.7079999	-1.2762718
20.5333328	1.7567999	-1.1842332
20.5333328	1.8055999	-1.1596894
20.5333328	1.8543999	-1.3560390
20.5333328	1.9031999	-1.3192234
20.5333328	1.9519999	-1.3008156
20.5333328	2.0007999	-1.2210488
20.5333328	2.0495999	-1.2271848
20.5333328	2.0983999	-1.2271848
20.5333328	2.1471999	-1.0308352
20.5333328	2.1959999	-1.0308352

20.5333328	2.2447999	-1.0553789
20.5333328	2.2935998	-1.0737867
20.5333328	2.3423998	-1.0737867
20.5333328	2.3912001	-1.0799227
20.5333328	2.4400001	-1.0308352
20.5333328	2.4888000	-0.9510679
20.5333328	2.5376000	-0.9510679
20.5333328	2.5864000	-0.9449320
20.5333328	2.6352000	-0.8467574
20.5333328	2.6840000	-0.9019809
20.5333328	2.7328000	-0.9019809
20.5333328	2.7816000	-0.7792621
20.5333328	2.8304000	-0.7792621
20.5333328	2.8792000	-1.1412816
20.5333328	2.9280000	-1.1474175
20.5333328	2.9768000	-1.4112625
20.5333328	3.0256000	-1.1596894
20.5333328	3.0743999	-1.1719613
20.5333328	3.1231999	-1.0001554
20.5333328	3.1719999	-1.0246992
20.5333328	3.2207999	-1.0246992
20.5333328	3.2695999	-1.0185633
20.5333328	3.3183999	-0.7301750
20.5333328	3.3671999	-0.7240391
20.5333328	3.4159999	-0.7424469
20.5333328	3.4647999	-0.7424469
20.5333328	3.5135999	-0.7915339
20.5333328	3.5623999	-0.8774371
20.5333328	3.6111999	-0.9019809
20.5333328	3.6599998	-0.9326606
20.5333328	3.7087998	-0.8160777
20.5333328	3.7575998	-0.6626797
20.5333328	3.8063998	-0.6135921
20.5333328	3.8551998	-0.7117672
20.5333328	3.9039998	-0.7485828
20.5333328	3.9527998	-0.7363110
20.5333328	4.0016003	-0.7485828
20.5333328	4.0503998	-0.7608547
20.5333328	4.0991998	-0.6626797
20.5333328	4.1479998	-0.6688156
20.5333328	4.1967998	-0.8774371
20.5333328	4.2455997	-0.8958449
20.5333328	4.2943997	-0.8958449
20.5333328	4.3431997	-0.8958449
20.5333328	4.3919997	-0.8958449
20.5333328	4.4407997	-0.8897090
20.5333328	4.4895997	-0.9081168
20.5333328	4.5383997	-0.9203887
20.5333328	4.5871997	-0.8344855
20.5333328	4.6359997	-0.8283496
20.5333328	4.6847997	-0.6688156
20.5333328	4.7336001	-0.6810875
20.5333328	4.7824001	-0.6381359
20.5333328	4.8312001	-0.5460973
20.5333328	4.8800001	-0.5399613
20.5333328	4.9288001	-0.5276895
20.5333328	4.9776001	613586.0000000

20.5333328	5.0264001	-0.4172430
20.5333328	5.0752001	-0.4172430
20.5333328	5.1240001	613586.0000000
20.5333328	5.1728001	613586.0000000
20.5333328	5.2216001	-0.4847379
20.5333328	5.2704000	613586.0000000
20.5333328	5.3192000	613586.0000000
20.5333328	5.3680000	613586.0000000
20.5333328	5.4168000	613586.0000000
20.5333328	5.4656000	613586.0000000
20.5333328	5.5144000	613586.0000000
20.5333328	5.5632000	613586.0000000
20.5333328	5.6120000	613586.0000000
20.5333328	5.6608000	613586.0000000
20.5333328	5.7096000	613586.0000000
20.5333328	5.7584000	613586.0000000
20.5333328	5.8072000	613586.0000000
20.5333328	5.8559999	613586.0000000
20.5333328	5.9047999	613586.0000000
20.5333328	5.9535999	613586.0000000
20.5333328	6.0023999	613586.0000000
20.5499992	0.2928000	-2.1168938
20.5499992	0.3416000	-1.7671461
20.5499992	0.3904000	-1.7610102
20.5499992	0.4392000	-1.3069515
20.5499992	0.4880000	-1.3253593
20.5499992	0.5368000	-1.5830684
20.5499992	0.5856000	-1.5953403
20.5499992	0.6344000	-1.3130875
20.5499992	0.6832000	-1.5401168
20.5499992	0.7320000	-1.3683109
20.5499992	0.7808000	-1.3744469
20.5499992	0.8296000	-1.4173985
20.5499992	0.8784000	-1.3314953
20.5499992	0.9272000	-1.3069515
20.5499992	0.9760000	-1.3008156
20.5499992	1.0247999	-1.2885437
20.5499992	1.0735999	-1.2762718
20.5499992	1.1223999	-1.2946796
20.5499992	1.1711999	-1.2026410
20.5499992	1.2200000	-1.5094371
20.5499992	1.2688000	-1.5155730
20.5499992	1.3176000	-1.5094371
20.5499992	1.3664000	-1.2701364
20.5499992	1.4152000	-1.1780972
20.5499992	1.4640000	-0.9694757
20.5499992	1.5128000	-1.1965051
20.5499992	1.5616000	-1.2087770
20.5499992	1.6104000	-1.2640004
20.5499992	1.6592000	-1.1228738
20.5499992	1.7079999	-1.1474175
20.5499992	1.7567999	-1.2455926
20.5499992	1.8055999	-1.2824078
20.5499992	1.8543999	-1.2762718
20.5499992	1.9031999	-1.2087770
20.5499992	1.9519999	-1.2455926
20.5499992	2.0007999	-1.1412816

20.5499992	2.0495999	-1.1474175
20.5499992	2.0983999	-1.2271848
20.5499992	2.1471999	-0.9694757
20.5499992	2.1959999	-0.9694757
20.5499992	2.2447999	-0.9756117
20.5499992	2.2935998	-1.0921946
20.5499992	2.3423998	-0.9326606
20.5499992	2.3912001	-1.0737867
20.5499992	2.4400001	-1.0062914
20.5499992	2.4888000	-1.0062914
20.5499992	2.5376000	-0.9449320
20.5499992	2.5864000	-0.9510679
20.5499992	2.6352000	-1.0001554
20.5499992	2.6840000	-1.0001554
20.5499992	2.7328000	-0.8344855
20.5499992	2.7816000	-0.8344855
20.5499992	2.8304000	-0.8774371
20.5499992	2.8792000	-0.8713012
20.5499992	2.9280000	-0.8713012
20.5499992	2.9768000	-0.9019809
20.5499992	3.0256000	-0.9142528
20.5499992	3.0743999	-0.9203887
20.5499992	3.1231999	-0.7547188
20.5499992	3.1719999	-1.1106019
20.5499992	3.2207999	-1.0308352
20.5499992	3.2695999	-0.6688156
20.5499992	3.3183999	-0.8528934
20.5499992	3.3671999	-0.7853980
20.5499992	3.4159999	-0.7240391
20.5499992	3.4647999	-0.5583692
20.5499992	3.5135999	-0.5829129
20.5499992	3.5623999	-0.5829129
20.5499992	3.6111999	-0.5829129
20.5499992	3.6599998	-0.5890489
20.5499992	3.7087998	-0.5951843
20.5499992	3.7575998	-0.7301750
20.5499992	3.8063998	-0.7240391
20.5499992	3.8551998	-0.8897090
20.5499992	3.9039998	-0.8897090
20.5499992	3.9527998	-0.5706410
20.5499992	4.0016003	-0.5767770
20.5499992	4.0503998	-0.5031457
20.5499992	4.0991998	-0.4908738
20.5499992	4.1479998	-0.5215535
20.5499992	4.1967998	-0.4970098
20.5499992	4.2455997	-0.5154176
20.5499992	4.2943997	-0.5645051
20.5499992	4.3431997	-0.5829129
20.5499992	4.3919997	-0.4847379
20.5499992	4.4407997	-0.4847379
20.5499992	4.4895997	-0.4601941
20.5499992	4.5383997	-0.3926992
20.5499992	4.5871997	-0.3497477
20.5499992	4.6359997	-0.4356503
20.5499992	4.6847997	-0.3497477
20.5499992	4.7336001	-0.3742914
20.5499992	4.7824001	-0.2147574

20.5499992	4.8312001	-0.2945242
20.5499992	4.8800001	-0.1533980
20.5499992	4.9288001	-0.1718059
20.5499992	4.9776001	-0.1718059
20.5499992	5.0264001	-0.2883883
20.5499992	5.0752001	-0.2883883
20.5499992	5.1240001	613586.0000000
20.5499992	5.1728001	613586.0000000
20.5499992	5.2216001	613586.0000000
20.5499992	5.2704000	613586.0000000
20.5499992	5.3192000	613586.0000000
20.5499992	5.3680000	613586.0000000
20.5499992	5.4168000	613586.0000000
20.5499992	5.4656000	613586.0000000
20.5499992	5.5144000	613586.0000000
20.5499992	5.5632000	613586.0000000
20.5499992	5.6120000	613586.0000000
20.5499992	5.6608000	613586.0000000
20.5499992	5.7096000	613586.0000000
20.5499992	5.7584000	613586.0000000
20.5499992	5.8072000	613586.0000000
20.5499992	5.8559999	613586.0000000
20.5499992	5.9047999	613586.0000000
20.5499992	5.9535999	613586.0000000
20.5499992	6.0023999	613586.0000000
20.5666676	0.2928000	-2.2027965
20.5666676	0.3416000	-1.4787574
20.5666676	0.3904000	-1.4971652
20.5666676	0.4392000	-1.3867188
20.5666676	0.4880000	-1.5155730
20.5666676	0.5368000	-1.3867188
20.5666676	0.5856000	-1.3867188
20.5666676	0.6344000	-1.3867188
20.5666676	0.6832000	-1.3867188
20.5666676	0.7320000	-1.3683109
20.5666676	0.7808000	-1.4971652
20.5666676	0.8296000	-1.5033011
20.5666676	0.8784000	-1.4112625
20.5666676	0.9272000	-1.4112625
20.5666676	0.9760000	-1.1351457
20.5666676	1.0247999	-1.1228738
20.5666676	1.0735999	-1.2394567
20.5666676	1.1223999	-1.2333207
20.5666676	1.1711999	-1.0369711
20.5666676	1.2200000	-1.5339808
20.5666676	1.2688000	-1.4051266
20.5666676	1.3176000	-1.0676508
20.5666676	1.3664000	-1.0676508
20.5666676	1.4152000	-0.9694757
20.5666676	1.4640000	-0.9694757
20.5666676	1.5128000	-1.2149129
20.5666676	1.5616000	-1.3499031
20.5666676	1.6104000	-1.2517285
20.5666676	1.6592000	-1.1903691
20.5666676	1.7079999	-1.1903691
20.5666676	1.7567999	-1.1965051
20.5666676	1.8055999	-1.1228738

20.5666676	1.8543999	-1.2026410
20.5666676	1.9031999	-1.2026410
20.5666676	1.9519999	-1.2026410
20.5666676	2.0007999	-1.2026410
20.5666676	2.0495999	-1.3376312
20.5666676	2.0983999	-1.2762718
20.5666676	2.1471999	-1.1351457
20.5666676	2.1959999	-1.1535535
20.5666676	2.2447999	-1.1474175
20.5666676	2.2935998	-1.1780972
20.5666676	2.3423998	-1.1044660
20.5666676	2.3912001	-1.1290097
20.5666676	2.4400001	-1.1290097
20.5666676	2.4888000	-0.9449320
20.5666676	2.5376000	-0.9449320
20.5666676	2.5864000	-1.0308352
20.5666676	2.6352000	-1.0308352
20.5666676	2.6840000	-1.0308352
20.5666676	2.7328000	-1.0124273
20.5666676	2.7816000	-0.9633398
20.5666676	2.8304000	-1.0185633
20.5666676	2.8792000	-1.1412816
20.5666676	2.9280000	-0.9756117
20.5666676	2.9768000	-0.8651652
20.5666676	3.0256000	-0.8590293
20.5666676	3.0743999	-0.8713012
20.5666676	3.1231999	-0.8958449
20.5666676	3.1719999	-0.8713012
20.5666676	3.2207999	-1.0983305
20.5666676	3.2695999	-0.9817476
20.5666676	3.3183999	-0.8222136
20.5666676	3.3671999	-0.5092816
20.5666676	3.4159999	-0.5829129
20.5666676	3.4647999	-0.6442719
20.5666676	3.5135999	-0.7240391
20.5666676	3.5623999	-0.7424469
20.5666676	3.6111999	-0.4479222
20.5666676	3.6599998	-0.5154176
20.5666676	3.7087998	-0.5154176
20.5666676	3.7575998	-0.5767770
20.5666676	3.8063998	-0.7301750
20.5666676	3.8551998	-0.8099418
20.5666676	3.9039998	-0.8651652
20.5666676	3.9527998	-0.9633398
20.5666676	4.0016003	-1.0492430
20.5666676	4.0503998	-1.0369711
20.5666676	4.0991998	-0.9510679
20.5666676	4.1479998	-0.9572039
20.5666676	4.1967998	-0.9633398
20.5666676	4.2455997	-1.0001554
20.5666676	4.2943997	-0.9878836
20.5666676	4.3431997	-0.8958449
20.5666676	4.3919997	-0.8160777
20.5666676	4.4407997	-0.7915339
20.5666676	4.4895997	-0.8038058
20.5666676	4.5383997	-0.8099418
20.5666676	4.5871997	-0.8099418

20.5666676	4.6359997	-0.6565437
20.5666676	4.6847997	-0.6810875
20.5666676	4.7336001	-0.6320000
20.5666676	4.7824001	-0.6135921
20.5666676	4.8312001	-0.5338254
20.5666676	4.8800001	-0.5338254
20.5666676	4.9288001	-0.6258640
20.5666676	4.9776001	-0.5276895
20.5666676	5.0264001	-0.5215535
20.5666676	5.0752001	613586.0000000
20.5666676	5.1240001	613586.0000000
20.5666676	5.1728001	613586.0000000
20.5666676	5.2216001	613586.0000000
20.5666676	5.2704000	613586.0000000
20.5666676	5.3192000	613586.0000000
20.5666676	5.3680000	613586.0000000
20.5666676	5.4168000	613586.0000000
20.5666676	5.4656000	613586.0000000
20.5666676	5.5144000	613586.0000000
20.5666676	5.5632000	613586.0000000
20.5666676	5.6120000	613586.0000000
20.5666676	5.6608000	613586.0000000
20.5666676	5.7096000	613586.0000000
20.5666676	5.7584000	613586.0000000
20.5666676	5.8072000	613586.0000000
20.5666676	5.8559999	613586.0000000
20.5666676	5.9047999	613586.0000000
20.5666676	5.9535999	613586.0000000
20.5666676	6.0023999	613586.0000000
20.5833340	0.2928000	-2.3132432
20.5833340	0.3416000	-1.4173985
20.5833340	0.3904000	-1.2087770
20.5833340	0.4392000	-1.3989906
20.5833340	0.4880000	-1.6137476
20.5833340	0.5368000	-1.1780972
20.5833340	0.5856000	-1.3192234
20.5833340	0.6344000	-1.5033011
20.5833340	0.6832000	-1.5094371
20.5833340	0.7320000	-1.3928547
20.5833340	0.7808000	-1.5707965
20.5833340	0.8296000	-1.5278449
20.5833340	0.8784000	-1.2149129
20.5833340	0.9272000	-1.0553789
20.5833340	0.9760000	-1.1719613
20.5833340	1.0247999	-1.1351457
20.5833340	1.0735999	-1.1719613
20.5833340	1.1223999	-1.3499031
20.5833340	1.1711999	-1.3376312
20.5833340	1.2200000	-1.3253593
20.5833340	1.2688000	-1.3376312
20.5833340	1.3176000	-1.2026410
20.5833340	1.3664000	-1.1106019
20.5833340	1.4152000	-1.1351457
20.5833340	1.4640000	-1.1780972
20.5833340	1.5128000	-0.9081168
20.5833340	1.5616000	-1.0308352
20.5833340	1.6104000	-1.3683109

20.5833340	1.6592000	-1.3560390
20.5833340	1.7079999	-1.2885437
20.5833340	1.7567999	-1.2885437
20.5833340	1.8055999	-1.2946796
20.5833340	1.8543999	-1.1596894
20.5833340	1.9031999	-1.1412816
20.5833340	1.9519999	-1.1351457
20.5833340	2.0007999	-1.1290097
20.5833340	2.0495999	-1.1290097
20.5833340	2.0983999	-1.2394567
20.5833340	2.1471999	-1.2149129
20.5833340	2.1959999	-1.2517285
20.5833340	2.2447999	-1.0921946
20.5833340	2.2935998	-1.1596894
20.5833340	2.3423998	-1.3069515
20.5833340	2.3912001	-1.3069515
20.5833340	2.4400001	-1.1044660
20.5833340	2.4888000	-1.1228738
20.5833340	2.5376000	-1.2210488
20.5833340	2.5864000	-1.2578645
20.5833340	2.6352000	-1.1412816
20.5833340	2.6840000	-1.0860586
20.5833340	2.7328000	-1.0983305
20.5833340	2.7816000	-1.0431070
20.5833340	2.8304000	-1.0246992
20.5833340	2.8792000	-0.9572039
20.5833340	2.9280000	-0.9694757
20.5833340	2.9768000	-1.0369711
20.5833340	3.0256000	-1.0676508
20.5833340	3.0743999	-1.0737867
20.5833340	3.1231999	-0.8528934
20.5833340	3.1719999	-0.8651652
20.5833340	3.2207999	-0.9265246
20.5833340	3.2695999	-0.9203887
20.5833340	3.3183999	-0.9756117
20.5833340	3.3671999	-1.2394567
20.5833340	3.4159999	-1.0308352
20.5833340	3.4647999	-1.0983305
20.5833340	3.5135999	-1.0921946
20.5833340	3.5623999	-1.0921946
20.5833340	3.6111999	-1.0676508
20.5833340	3.6599998	-1.0737867
20.5833340	3.7087998	-0.9510679
20.5833340	3.7575998	-1.0799227
20.5833340	3.8063998	-1.0431070
20.5833340	3.8551998	-1.0431070
20.5833340	3.9039998	-0.9265246
20.5833340	3.9527998	-0.9265246
20.5833340	4.0016003	-0.9572039
20.5833340	4.0503998	-0.9572039
20.5833340	4.0991998	-0.6872234
20.5833340	4.1479998	-0.8406215
20.5833340	4.1967998	-0.8467574
20.5833340	4.2455997	-0.8406215
20.5833340	4.2943997	-0.8222136
20.5833340	4.3431997	-0.8344855
20.5833340	4.3919997	-0.7424469

20.5833340	4.4407997	-0.7424469
20.5833340	4.4895997	-0.7424469
20.5833340	4.5383997	-0.7424469
20.5833340	4.5871997	-0.6749516
20.5833340	4.6359997	-0.6994953
20.5833340	4.6847997	-0.7179031
20.5833340	4.7336001	-0.6994953
20.5833340	4.7824001	-0.7424469
20.5833340	4.8312001	-0.7485828
20.5833340	4.8800001	-0.5583692
20.5833340	4.9288001	-0.5583692
20.5833340	4.9776001	-0.5951843
20.5833340	5.0264001	613586.0000000
20.5833340	5.0752001	613586.0000000
20.5833340	5.1240001	613586.0000000
20.5833340	5.1728001	613586.0000000
20.5833340	5.2216001	613586.0000000
20.5833340	5.2704000	613586.0000000
20.5833340	5.3192000	613586.0000000
20.5833340	5.3680000	613586.0000000
20.5833340	5.4168000	613586.0000000
20.5833340	5.4656000	613586.0000000
20.5833340	5.5144000	613586.0000000
20.5833340	5.5632000	-0.8467574
20.5833340	5.6120000	-0.8467574
20.5833340	5.6608000	613586.0000000
20.5833340	5.7096000	613586.0000000
20.5833340	5.7584000	613586.0000000
20.5833340	5.8072000	613586.0000000
20.5833340	5.8559999	613586.0000000
20.5833340	5.9047999	613586.0000000
20.5833340	5.9535999	613586.0000000
20.5833340	6.0023999	613586.0000000
20.6000004	0.2928000	-1.8223691
20.6000004	0.3416000	-0.7547188
20.6000004	0.3904000	-0.7608547
20.6000004	0.4392000	-1.0983305
20.6000004	0.4880000	-1.0921946
20.6000004	0.5368000	-1.3376312
20.6000004	0.5856000	-1.2885437
20.6000004	0.6344000	-1.5033011
20.6000004	0.6832000	-1.5033011
20.6000004	0.7320000	-1.3560390
20.6000004	0.7808000	-1.3560390
20.6000004	0.8296000	-1.2333207
20.6000004	0.8784000	-1.1596894
20.6000004	0.9272000	-1.2333207
20.6000004	0.9760000	-1.0308352
20.6000004	1.0247999	-1.0308352
20.6000004	1.0735999	-1.2824078
20.6000004	1.1223999	-1.2333207
20.6000004	1.1711999	-1.2394567
20.6000004	1.2200000	-1.1167378
20.6000004	1.2688000	-1.1106019
20.6000004	1.3176000	-1.2087770
20.6000004	1.3664000	-1.1780972
20.6000004	1.4152000	-1.2149129

20.6000004	1.4640000	-1.3253593
20.6000004	1.5128000	-1.3314953
20.6000004	1.5616000	-1.4542136
20.6000004	1.6104000	-1.4542136
20.6000004	1.6592000	-1.3867188
20.6000004	1.7079999	-1.5217090
20.6000004	1.7567999	-1.3805828
20.6000004	1.8055999	-1.3744469
20.6000004	1.8543999	-1.3621750
20.6000004	1.9031999	-1.3621750
20.6000004	1.9519999	-1.3560390
20.6000004	2.0007999	-1.2271848
20.6000004	2.0495999	-1.2333207
20.6000004	2.0983999	-1.2333207
20.6000004	2.1471999	-1.1842332
20.6000004	2.1959999	-1.1842332
20.6000004	2.2447999	-1.2517285
20.6000004	2.2935998	-1.2394567
20.6000004	2.3423998	-1.2333207
20.6000004	2.3912001	-1.1596894
20.6000004	2.4400001	-1.1535535
20.6000004	2.4888000	-1.1596894
20.6000004	2.5376000	-1.2517285
20.6000004	2.5864000	-1.1596894
20.6000004	2.6352000	-1.1535535
20.6000004	2.6840000	-1.1535535
20.6000004	2.7328000	-1.1228738
20.6000004	2.7816000	-1.1719613
20.6000004	2.8304000	-1.1780972
20.6000004	2.8792000	-1.1903691
20.6000004	2.9280000	-1.1965051
20.6000004	2.9768000	-1.1351457
20.6000004	3.0256000	-1.1290097
20.6000004	3.0743999	-1.1044660
20.6000004	3.1231999	-1.1842332
20.6000004	3.1719999	-1.1903691
20.6000004	3.2207999	-0.6749516
20.6000004	3.2695999	-0.6626797
20.6000004	3.3183999	-0.9081168
20.6000004	3.3671999	-1.1106019
20.6000004	3.4159999	-1.0001554
20.6000004	3.4647999	-1.0124273
20.6000004	3.5135999	-1.0246992
20.6000004	3.5623999	-1.0860586
20.6000004	3.6111999	-1.0921946
20.6000004	3.6599998	-1.1044660
20.6000004	3.7087998	-0.9817476
20.6000004	3.7575998	-1.0001554
20.6000004	3.8063998	-1.0553789
20.6000004	3.8551998	-0.8590293
20.6000004	3.9039998	-0.8590293
20.6000004	3.9527998	-0.8713012
20.6000004	4.0016003	-0.8590293
20.6000004	4.0503998	-0.8528934
20.6000004	4.0991998	-0.8038058
20.6000004	4.1479998	-0.7240391
20.6000004	4.1967998	-0.7424469

20.6000004	4.2455997	-0.7424469
20.6000004	4.2943997	-0.7240391
20.6000004	4.3431997	-0.7117672
20.6000004	4.3919997	-0.6688156
20.6000004	4.4407997	-0.6320000
20.6000004	4.4895997	-0.6258640
20.6000004	4.5383997	-0.6135921
20.6000004	4.5871997	-0.6810875
20.6000004	4.6359997	-0.6381359
20.6000004	4.6847997	-0.6626797
20.6000004	4.7336001	-0.4908738
20.6000004	4.7824001	-0.4908738
20.6000004	4.8312001	-0.2638445
20.6000004	4.8800001	-0.3558836
20.6000004	4.9288001	-0.2577085
20.6000004	4.9776001	-0.2577085
20.6000004	5.0264001	613586.0000000
20.6000004	5.0752001	613586.0000000
20.6000004	5.1240001	613586.0000000
20.6000004	5.1728001	613586.0000000
20.6000004	5.2216001	-0.2822523
20.6000004	5.2704000	-0.2086215
20.6000004	5.3192000	-0.4724660
20.6000004	5.3680000	-0.4786019
20.6000004	5.4168000	613586.0000000
20.6000004	5.4656000	613586.0000000
20.6000004	5.5144000	613586.0000000
20.6000004	5.5632000	-0.6135921
20.6000004	5.6120000	613586.0000000
20.6000004	5.6608000	613586.0000000
20.6000004	5.7096000	613586.0000000
20.6000004	5.7584000	613586.0000000
20.6000004	5.8072000	613586.0000000
20.6000004	5.8559999	613586.0000000
20.6000004	5.9047999	613586.0000000
20.6000004	5.9535999	613586.0000000
20.6000004	6.0023999	613586.0000000
20.6166668	0.2928000	-1.3069515
20.6166668	0.3416000	-1.3069515
20.6166668	0.3904000	-1.4419422
20.6166668	0.4392000	-1.1290097
20.6166668	0.4880000	-1.1290097
20.6166668	0.5368000	-1.1106019
20.6166668	0.5856000	-1.2885437
20.6166668	0.6344000	-1.2885437
20.6166668	0.6832000	-1.1903691
20.6166668	0.7320000	-1.3744469
20.6166668	0.7808000	-1.2271848
20.6166668	0.8296000	-1.6014762
20.6166668	0.8784000	-1.3867188
20.6166668	0.9272000	-1.4296703
20.6166668	0.9760000	-1.2271848
20.6166668	1.0247999	-1.3253593
20.6166668	1.0735999	-1.3253593
20.6166668	1.1223999	-1.3192234
20.6166668	1.1711999	-1.3008156
20.6166668	1.2200000	-1.3621750

20.6166668	1.2688000	-1.3683109
20.6166668	1.3176000	-1.1106019
20.6166668	1.3664000	-1.1106019
20.6166668	1.4152000	-1.2762718
20.6166668	1.4640000	-1.3008156
20.6166668	1.5128000	-1.3192234
20.6166668	1.5616000	-1.4358063
20.6166668	1.6104000	-1.3437672
20.6166668	1.6592000	-1.3867188
20.6166668	1.7079999	-1.3805828
20.6166668	1.7567999	-1.2640004
20.6166668	1.8055999	-1.2087770
20.6166668	1.8543999	-1.3928547
20.6166668	1.9031999	-1.4787574
20.6166668	1.9519999	-1.4787574
20.6166668	2.0007999	-1.3989906
20.6166668	2.0495999	-1.2026410
20.6166668	2.0983999	-1.1719613
20.6166668	2.1471999	-1.1719613
20.6166668	2.1959999	-1.2333207
20.6166668	2.2447999	-1.1842332
20.6166668	2.2935998	-1.1842332
20.6166668	2.3423998	-1.2026410
20.6166668	2.3912001	-1.2946796
20.6166668	2.4400001	-1.2517285
20.6166668	2.4888000	-1.2455926
20.6166668	2.5376000	-1.2271848
20.6166668	2.5864000	-1.1167378
20.6166668	2.6352000	-1.1167378
20.6166668	2.6840000	-1.1535535
20.6166668	2.7328000	-0.9817476
20.6166668	2.7816000	-0.9817476
20.6166668	2.8304000	-0.8897090
20.6166668	2.8792000	-0.8958449
20.6166668	2.9280000	-1.0369711
20.6166668	2.9768000	-1.0983305
20.6166668	3.0256000	-1.0737867
20.6166668	3.0743999	-0.9694757
20.6166668	3.1231999	-1.2333207
20.6166668	3.1719999	-1.2333207
20.6166668	3.2207999	-0.9203887
20.6166668	3.2695999	-0.6810875
20.6166668	3.3183999	-0.9510679
20.6166668	3.3671999	-0.9510679
20.6166668	3.4159999	-0.7792621
20.6166668	3.4647999	-0.6994953
20.6166668	3.5135999	-0.6933594
20.6166668	3.5623999	-0.8099418
20.6166668	3.6111999	-1.0308352
20.6166668	3.6599998	-1.0246992
20.6166668	3.7087998	-0.8835731
20.6166668	3.7575998	-0.8774371
20.6166668	3.8063998	-0.8713012
20.6166668	3.8551998	-0.8099418
20.6166668	3.9039998	-0.7976699
20.6166668	3.9527998	-0.7915339
20.6166668	4.0016003	-0.5338254

20.6166668	4.0503998	-0.5338254
20.6166668	4.0991998	-0.7853980
20.6166668	4.1479998	-0.7915339
20.6166668	4.1967998	-0.6688156
20.6166668	4.2455997	-0.6749516
20.6166668	4.2943997	-0.6504078
20.6166668	4.3431997	-0.6504078
20.6166668	4.3919997	-0.5460973
20.6166668	4.4407997	-0.5522332
20.6166668	4.4895997	-0.5583692
20.6166668	4.5383997	-0.4908738
20.6166668	4.5871997	-0.4970098
20.6166668	4.6359997	-0.4847379
20.6166668	4.6847997	-0.4847379
20.6166668	4.7336001	-0.3926992
20.6166668	4.7824001	-0.3988352
20.6166668	4.8312001	-0.6810875
20.6166668	4.8800001	-0.2761164
20.6166668	4.9288001	613586.0000000
20.6166668	4.9776001	613586.0000000
20.6166668	5.0264001	613586.0000000
20.6166668	5.0752001	613586.0000000
20.6166668	5.1240001	-0.5829129
20.6166668	5.1728001	-0.5338254
20.6166668	5.2216001	-0.5460973
20.6166668	5.2704000	613586.0000000
20.6166668	5.3192000	613586.0000000
20.6166668	5.3680000	613586.0000000
20.6166668	5.4168000	613586.0000000
20.6166668	5.4656000	613586.0000000
20.6166668	5.5144000	613586.0000000
20.6166668	5.5632000	613586.0000000
20.6166668	5.6120000	613586.0000000
20.6166668	5.6608000	613586.0000000
20.6166668	5.7096000	613586.0000000
20.6166668	5.7584000	613586.0000000
20.6166668	5.8072000	613586.0000000
20.6166668	5.8559999	613586.0000000
20.6166668	5.9047999	613586.0000000
20.6166668	5.9535999	613586.0000000
20.6166668	6.0023999	613586.0000000
20.6333332	0.2928000	-2.1414371
20.6333332	0.3416000	-1.9819031
20.6333332	0.3904000	-1.2271848
20.6333332	0.4392000	-1.3069515
20.6333332	0.4880000	-1.3130875
20.6333332	0.5368000	-1.3928547
20.6333332	0.5856000	-1.3069515
20.6333332	0.6344000	-1.4664855
20.6333332	0.6832000	-1.4603496
20.6333332	0.7320000	-1.0983305
20.6333332	0.7808000	-1.0983305
20.6333332	0.8296000	-1.2517285
20.6333332	0.8784000	-1.1106019
20.6333332	0.9272000	-1.1044660
20.6333332	0.9760000	-1.0062914
20.6333332	1.0247999	-1.4419422

20.6333332	1.0735999	-1.3867188
20.6333332	1.1223999	-1.0737867
20.6333332	1.1711999	-1.3867188
20.6333332	1.2200000	-1.3867188
20.6333332	1.2688000	-1.1965051
20.6333332	1.3176000	-1.0431070
20.6333332	1.3664000	-1.1965051
20.6333332	1.4152000	-1.0921946
20.6333332	1.4640000	-1.0983305
20.6333332	1.5128000	-1.4664855
20.6333332	1.5616000	-1.4051266
20.6333332	1.6104000	-1.4051266
20.6333332	1.6592000	-1.3989906
20.6333332	1.7079999	-1.3928547
20.6333332	1.7567999	-1.3867188
20.6333332	1.8055999	-1.3683109
20.6333332	1.8543999	-1.1965051
20.6333332	1.9031999	-1.2517285
20.6333332	1.9519999	-1.1044660
20.6333332	2.0007999	-1.0860586
20.6333332	2.0495999	-1.2640004
20.6333332	2.0983999	-1.2333207
20.6333332	2.1471999	-1.1658254
20.6333332	2.1959999	-1.1719613
20.6333332	2.2447999	-1.1965051
20.6333332	2.2935998	-1.2394567
20.6333332	2.3423998	-1.2394567
20.6333332	2.3912001	-1.2333207
20.6333332	2.4400001	-1.3192234
20.6333332	2.4888000	-1.3069515
20.6333332	2.5376000	-1.2149129
20.6333332	2.5864000	-1.0860586
20.6333332	2.6352000	-1.0860586
20.6333332	2.6840000	-1.1780972
20.6333332	2.7328000	-1.1780972
20.6333332	2.7816000	-1.0185633
20.6333332	2.8304000	-0.9019809
20.6333332	2.8792000	-0.9019809
20.6333332	2.9280000	-0.8835731
20.6333332	2.9768000	-1.0676508
20.6333332	3.0256000	-1.0431070
20.6333332	3.0743999	-0.8774371
20.6333332	3.1231999	-0.6013203
20.6333332	3.1719999	-1.0737867
20.6333332	3.2207999	-1.0737867
20.6333332	3.2695999	-1.0431070
20.6333332	3.3183999	-1.0308352
20.6333332	3.3671999	-0.8590293
20.6333332	3.4159999	-0.8713012
20.6333332	3.4647999	-0.7179031
20.6333332	3.5135999	-0.7731261
20.6333332	3.5623999	-0.8774371
20.6333332	3.6111999	-0.9019809
20.6333332	3.6599998	-0.9019809
20.6333332	3.7087998	-0.8528934
20.6333332	3.7575998	-0.9203887
20.6333332	3.8063998	-0.9019809

20.6333332	3.8551998	-0.8406215
20.6333332	3.9039998	-0.8222136
20.6333332	3.9527998	-0.7731261
20.6333332	4.0016003	-0.7792621
20.6333332	4.0503998	-0.7608547
20.6333332	4.0991998	-0.7363110
20.6333332	4.1479998	-0.4111071
20.6333332	4.1967998	-0.3620195
20.6333332	4.2455997	-0.5829129
20.6333332	4.2943997	-0.5829129
20.6333332	4.3431997	-0.5829129
20.6333332	4.3919997	-0.5399613
20.6333332	4.4407997	-0.5399613
20.6333332	4.4895997	-0.7485828
20.6333332	4.5383997	-0.6197281
20.6333332	4.5871997	-0.5522332
20.6333332	4.6359997	-0.3926992
20.6333332	4.6847997	613586.0000000
20.6333332	4.7336001	-0.3804274
20.6333332	4.7824001	-0.3988352
20.6333332	4.8312001	-0.3926992
20.6333332	4.8800001	613586.0000000
20.6333332	4.9288001	613586.0000000
20.6333332	4.9776001	-0.6013203
20.6333332	5.0264001	-0.5951843
20.6333332	5.0752001	-0.5890489
20.6333332	5.1240001	613586.0000000
20.6333332	5.1728001	613586.0000000
20.6333332	5.2216001	613586.0000000
20.6333332	5.2704000	613586.0000000
20.6333332	5.3192000	613586.0000000
20.6333332	5.3680000	613586.0000000
20.6333332	5.4168000	613586.0000000
20.6333332	5.4656000	613586.0000000
20.6333332	5.5144000	613586.0000000
20.6333332	5.5632000	613586.0000000
20.6333332	5.6120000	613586.0000000
20.6333332	5.6608000	613586.0000000
20.6333332	5.7096000	613586.0000000
20.6333332	5.7584000	613586.0000000
20.6333332	5.8072000	613586.0000000
20.6333332	5.8559999	613586.0000000
20.6333332	5.9047999	613586.0000000
20.6333332	5.9535999	613586.0000000
20.6333332	6.0023999	613586.0000000
20.6499996	0.2928000	-1.8407769
20.6499996	0.3416000	-1.8346410
20.6499996	0.3904000	-1.5278449
20.6499996	0.4392000	-1.5033011
20.6499996	0.4880000	-1.2455926
20.6499996	0.5368000	-1.2455926
20.6499996	0.5856000	-1.4051266
20.6499996	0.6344000	-1.4112625
20.6499996	0.6832000	-1.3560390
20.6499996	0.7320000	-1.5094371
20.6499996	0.7808000	-1.2701364
20.6499996	0.8296000	-1.3069515

20.6499996	0.8784000	-1.1719613
20.6499996	0.9272000	-1.1596894
20.6499996	0.9760000	-1.3560390
20.6499996	1.0247999	-1.3560390
20.6499996	1.0735999	-1.3928547
20.6499996	1.1223999	-1.4051266
20.6499996	1.1711999	-1.1658254
20.6499996	1.2200000	-1.0737867
20.6499996	1.2688000	-1.1719613
20.6499996	1.3176000	-1.1228738
20.6499996	1.3664000	-1.5217090
20.6499996	1.4152000	-1.5217090
20.6499996	1.4640000	-1.3253593
20.6499996	1.5128000	-1.1535535
20.6499996	1.5616000	-1.3437672
20.6499996	1.6104000	-1.2885437
20.6499996	1.6592000	-1.3376312
20.6499996	1.7079999	-1.3314953
20.6499996	1.7567999	-1.2271848
20.6499996	1.8055999	-1.2149129
20.6499996	1.8543999	-1.2394567
20.6499996	1.9031999	-1.2394567
20.6499996	1.9519999	-1.2026410
20.6499996	2.0007999	-1.2087770
20.6499996	2.0495999	-1.1535535
20.6499996	2.0983999	-1.1719613
20.6499996	2.1471999	-1.1658254
20.6499996	2.1959999	-1.1412816
20.6499996	2.2447999	-1.2517285
20.6499996	2.2935998	-1.2026410
20.6499996	2.3423998	-1.2762718
20.6499996	2.3912001	-1.3069515
20.6499996	2.4400001	-1.2946796
20.6499996	2.4888000	-1.2824078
20.6499996	2.5376000	-1.1719613
20.6499996	2.5864000	-1.3560390
20.6499996	2.6352000	-1.3928547
20.6499996	2.6840000	-1.3376312
20.6499996	2.7328000	-1.3314953
20.6499996	2.7816000	-1.3376312
20.6499996	2.8304000	-1.3376312
20.6499996	2.8792000	-1.2824078
20.6499996	2.9280000	-1.1965051
20.6499996	2.9768000	-1.2087770
20.6499996	3.0256000	-1.1167378
20.6499996	3.0743999	-1.2087770
20.6499996	3.1231999	-1.1290097
20.6499996	3.1719999	-1.1228738
20.6499996	3.2207999	-1.4664855
20.6499996	3.2695999	-1.3560390
20.6499996	3.3183999	-0.9572039
20.6499996	3.3671999	-1.0676508
20.6499996	3.4159999	-1.0553789
20.6499996	3.4647999	-1.0676508
20.6499996	3.5135999	-0.9449320
20.6499996	3.5623999	-0.9572039
20.6499996	3.6111999	-0.9878836

20.6499996	3.6599998	-0.9940195
20.6499996	3.7087998	-0.9694757
20.6499996	3.7575998	-0.9694757
20.6499996	3.8063998	-0.9265246
20.6499996	3.8551998	-0.9203887
20.6499996	3.9039998	-0.8713012
20.6499996	3.9527998	-0.8038058
20.6499996	4.0016003	-0.8222136
20.6499996	4.0503998	-0.6688156
20.6499996	4.0991998	-0.6933594
20.6499996	4.1479998	-0.6872234
20.6499996	4.1967998	-0.6688156
20.6499996	4.2455997	-0.6626797
20.6499996	4.2943997	-0.7608547
20.6499996	4.3431997	-0.7424469
20.6499996	4.3919997	-0.7485828
20.6499996	4.4407997	-0.6810875
20.6499996	4.4895997	-0.7976699
20.6499996	4.5383997	-0.7976699
20.6499996	4.5871997	613586.0000000
20.6499996	4.6359997	613586.0000000
20.6499996	4.6847997	-0.3742914
20.6499996	4.7336001	-0.3742914
20.6499996	4.7824001	-0.7056313
20.6499996	4.8312001	-0.7056313
20.6499996	4.8800001	-0.5522332
20.6499996	4.9288001	-0.5460973
20.6499996	4.9776001	-0.6749516
20.6499996	5.0264001	-0.6810875
20.6499996	5.0752001	-0.6749516
20.6499996	5.1240001	-0.8283496
20.6499996	5.1728001	-0.7301750
20.6499996	5.2216001	-0.7240391
20.6499996	5.2704000	613586.0000000
20.6499996	5.3192000	613586.0000000
20.6499996	5.3680000	613586.0000000
20.6499996	5.4168000	613586.0000000
20.6499996	5.4656000	613586.0000000
20.6499996	5.5144000	613586.0000000
20.6499996	5.5632000	613586.0000000
20.6499996	5.6120000	613586.0000000
20.6499996	5.6608000	613586.0000000
20.6499996	5.7096000	613586.0000000
20.6499996	5.7584000	613586.0000000
20.6499996	5.8072000	613586.0000000
20.6499996	5.8559999	613586.0000000
20.6499996	5.9047999	613586.0000000
20.6499996	5.9535999	613586.0000000
20.6499996	6.0023999	613586.0000000
20.6666660	0.2928000	-1.5401168
20.6666660	0.3416000	-1.6076121
20.6666660	0.3904000	-1.4235344
20.6666660	0.4392000	-1.4419422
20.6666660	0.4880000	-0.9878836
20.6666660	0.5368000	-1.5401168
20.6666660	0.5856000	-1.5462527
20.6666660	0.6344000	-1.6873789

20.6666660	0.6832000	-1.4358063
20.6666660	0.7320000	-1.4296703
20.6666660	0.7808000	-1.2271848
20.6666660	0.8296000	-1.2455926
20.6666660	0.8784000	-1.4358063
20.6666660	0.9272000	-1.4296703
20.6666660	0.9760000	-1.3437672
20.6666660	1.0247999	-1.2149129
20.6666660	1.0735999	-1.2210488
20.6666660	1.1223999	-1.3314953
20.6666660	1.1711999	-1.0860586
20.6666660	1.2200000	-1.2824078
20.6666660	1.2688000	-1.2824078
20.6666660	1.3176000	-1.2946796
20.6666660	1.3664000	-1.4542136
20.6666660	1.4152000	-1.4480777
20.6666660	1.4640000	-1.3683109
20.6666660	1.5128000	-1.3683109
20.6666660	1.5616000	-1.4235344
20.6666660	1.6104000	-1.2333207
20.6666660	1.6592000	-1.2333207
20.6666660	1.7079999	-1.1965051
20.6666660	1.7567999	-1.2149129
20.6666660	1.8055999	-1.2394567
20.6666660	1.8543999	-1.2824078
20.6666660	1.9031999	-1.1474175
20.6666660	1.9519999	-1.1290097
20.6666660	2.0007999	-1.1412816
20.6666660	2.0495999	-1.3130875
20.6666660	2.0983999	-1.2885437
20.6666660	2.1471999	-1.1290097
20.6666660	2.1959999	-1.1351457
20.6666660	2.2447999	-1.0860586
20.6666660	2.2935998	-1.1780972
20.6666660	2.3423998	-1.1780972
20.6666660	2.3912001	-1.3314953
20.6666660	2.4400001	-1.2946796
20.6666660	2.4888000	-1.2394567
20.6666660	2.5376000	-1.2271848
20.6666660	2.5864000	-1.1412816
20.6666660	2.6352000	-1.1351457
20.6666660	2.6840000	-1.1535535
20.6666660	2.7328000	-1.1719613
20.6666660	2.7816000	-1.1351457
20.6666660	2.8304000	-1.1351457
20.6666660	2.8792000	-1.2149129
20.6666660	2.9280000	-1.2271848
20.6666660	2.9768000	-1.2210488
20.6666660	3.0256000	-1.1658254
20.6666660	3.0743999	-1.2087770
20.6666660	3.1231999	-1.2333207
20.6666660	3.1719999	-1.1351457
20.6666660	3.2207999	-1.0553789
20.6666660	3.2695999	-1.2701364
20.6666660	3.3183999	-1.3989906
20.6666660	3.3671999	-1.2455926
20.6666660	3.4159999	-1.2271848

20.6666660	3.4647999	-1.1474175
20.6666660	3.5135999	-0.9756117
20.6666660	3.5623999	-0.7669902
20.6666660	3.6111999	-0.9019809
20.6666660	3.6599998	-0.7301750
20.6666660	3.7087998	-0.8651652
20.6666660	3.7575998	-0.8528934
20.6666660	3.8063998	-0.8590293
20.6666660	3.8551998	-0.8651652
20.6666660	3.9039998	-0.8283496
20.6666660	3.9527998	-0.8222136
20.6666660	4.0016003	-0.6013203
20.6666660	4.0503998	-0.7179031
20.6666660	4.0991998	-0.6688156
20.6666660	4.1479998	-0.6810875
20.6666660	4.1967998	-0.7792621
20.6666660	4.2455997	-0.7179031
20.6666660	4.2943997	-0.7179031
20.6666660	4.3431997	-0.7117672
20.6666660	4.3919997	-0.6994953
20.6666660	4.4407997	-0.7669902
20.6666660	4.4895997	-0.8099418
20.6666660	4.5383997	-0.5829129
20.6666660	4.5871997	613586.0000000
20.6666660	4.6359997	613586.0000000
20.6666660	4.6847997	613586.0000000
20.6666660	4.7336001	613586.0000000
20.6666660	4.7824001	613586.0000000
20.6666660	4.8312001	613586.0000000
20.6666660	4.8800001	-0.6626797
20.6666660	4.9288001	-0.6504078
20.6666660	4.9776001	-0.8774371
20.6666660	5.0264001	613586.0000000
20.6666660	5.0752001	613586.0000000
20.6666660	5.1240001	613586.0000000
20.6666660	5.1728001	613586.0000000
20.6666660	5.2216001	613586.0000000
20.6666660	5.2704000	613586.0000000
20.6666660	5.3192000	613586.0000000
20.6666660	5.3680000	613586.0000000
20.6666660	5.4168000	613586.0000000
20.6666660	5.4656000	613586.0000000
20.6666660	5.5144000	613586.0000000
20.6666660	5.5632000	613586.0000000
20.6666660	5.6120000	613586.0000000
20.6666660	5.6608000	613586.0000000
20.6666660	5.7096000	613586.0000000
20.6666660	5.7584000	613586.0000000
20.6666660	5.8072000	613586.0000000
20.6666660	5.8559999	613586.0000000
20.6666660	5.9047999	613586.0000000
20.6666660	5.9535999	613586.0000000
20.6666660	6.0023999	613586.0000000
20.6833324	0.2928000	-2.1782527
20.6833324	0.3416000	-1.8039613
20.6833324	0.3904000	-1.6566992
20.6833324	0.4392000	-1.6505632

20.6833324	0.4880000	-1.1780972
20.6833324	0.5368000	-1.2455926
20.6833324	0.5856000	-1.2701364
20.6833324	0.6344000	-0.9817476
20.6833324	0.6832000	-0.9940195
20.6833324	0.7320000	-1.0983305
20.6833324	0.7808000	-1.0983305
20.6833324	0.8296000	-1.0799227
20.6833324	0.8784000	-1.0246992
20.6833324	0.9272000	-1.2640004
20.6833324	0.9760000	-1.2517285
20.6833324	1.0247999	-1.2640004
20.6833324	1.0735999	-1.1351457
20.6833324	1.1223999	-1.5830684
20.6833324	1.1711999	-1.2026410
20.6833324	1.2200000	-1.4542136
20.6833324	1.2688000	-1.4542136
20.6833324	1.3176000	-1.5155730
20.6833324	1.3664000	-1.4910293
20.6833324	1.4152000	-1.3805828
20.6833324	1.4640000	-1.0615149
20.6833324	1.5128000	-1.1842332
20.6833324	1.5616000	-1.1842332
20.6833324	1.6104000	-1.4603496
20.6833324	1.6592000	-1.4726214
20.6833324	1.7079999	-1.3867188
20.6833324	1.7567999	-1.3008156
20.6833324	1.8055999	-1.2149129
20.6833324	1.8543999	-1.2026410
20.6833324	1.9031999	-1.1780972
20.6833324	1.9519999	-1.1658254
20.6833324	2.0007999	-1.2026410
20.6833324	2.0495999	-1.2087770
20.6833324	2.0983999	-1.0369711
20.6833324	2.1471999	-1.1474175
20.6833324	2.1959999	-1.1474175
20.6833324	2.2447999	-1.0983305
20.6833324	2.2935998	-1.2149129
20.6833324	2.3423998	-1.2149129
20.6833324	2.3912001	-1.2333207
20.6833324	2.4400001	-1.1474175
20.6833324	2.4888000	-1.1351457
20.6833324	2.5376000	-1.1290097
20.6833324	2.5864000	-1.1719613
20.6833324	2.6352000	-1.1658254
20.6833324	2.6840000	-1.0676508
20.6833324	2.7328000	-1.2762718
20.6833324	2.7816000	-1.2149129
20.6833324	2.8304000	-1.1535535
20.6833324	2.8792000	-1.1474175
20.6833324	2.9280000	-1.0553789
20.6833324	2.9768000	-1.1535535
20.6833324	3.0256000	-1.1351457
20.6833324	3.0743999	-1.1290097
20.6833324	3.1231999	-1.2578645
20.6833324	3.1719999	-1.2578645
20.6833324	3.2207999	-1.3989906

20.6833324	3.2695999	-1.5769324
20.6833324	3.3183999	-1.5892043
20.6833324	3.3671999	-1.1228738
20.6833324	3.4159999	-1.1106019
20.6833324	3.4647999	-1.0983305
20.6833324	3.5135999	-1.0308352
20.6833324	3.5623999	-1.0308352
20.6833324	3.6111999	-1.0308352
20.6833324	3.6599998	-0.8590293
20.6833324	3.7087998	-1.0615149
20.6833324	3.7575998	-0.9572039
20.6833324	3.8063998	-1.0062914
20.6833324	3.8551998	-1.0062914
20.6833324	3.9039998	-0.9265246
20.6833324	3.9527998	-0.8160777
20.6833324	4.0016003	-0.8283496
20.6833324	4.0503998	-0.7853980
20.6833324	4.0991998	-0.8713012
20.6833324	4.1479998	-0.6749516
20.6833324	4.1967998	-0.8160777
20.6833324	4.2455997	-0.8283496
20.6833324	4.2943997	-0.8467574
20.6833324	4.3431997	-0.8406215
20.6833324	4.3919997	-0.8406215
20.6833324	4.4407997	-0.9449320
20.6833324	4.4895997	-0.8467574
20.6833324	4.5383997	-0.8467574
20.6833324	4.5871997	-0.8528934
20.6833324	4.6359997	-0.8038058
20.6833324	4.6847997	613586.0000000
20.6833324	4.7336001	-0.8713012
20.6833324	4.7824001	613586.0000000
20.6833324	4.8312001	613586.0000000
20.6833324	4.8800001	613586.0000000
20.6833324	4.9288001	613586.0000000
20.6833324	4.9776001	613586.0000000
20.6833324	5.0264001	613586.0000000
20.6833324	5.0752001	613586.0000000
20.6833324	5.1240001	613586.0000000
20.6833324	5.1728001	613586.0000000
20.6833324	5.2216001	613586.0000000
20.6833324	5.2704000	613586.0000000
20.6833324	5.3192000	613586.0000000
20.6833324	5.3680000	613586.0000000
20.6833324	5.4168000	613586.0000000
20.6833324	5.4656000	613586.0000000
20.6833324	5.5144000	613586.0000000
20.6833324	5.5632000	613586.0000000
20.6833324	5.6120000	613586.0000000
20.6833324	5.6608000	613586.0000000
20.6833324	5.7096000	613586.0000000
20.6833324	5.7584000	613586.0000000
20.6833324	5.8072000	613586.0000000
20.6833324	5.8559999	613586.0000000
20.6833324	5.9047999	613586.0000000
20.6833324	5.9535999	613586.0000000
20.6833324	6.0023999	613586.0000000

20.7000008	0.2928000	-1.3683109
20.7000008	0.3416000	-1.6198835
20.7000008	0.3904000	-1.3621750
20.7000008	0.4392000	-1.3192234
20.7000008	0.4880000	-1.2271848
20.7000008	0.5368000	-1.7671461
20.7000008	0.5856000	-1.2333207
20.7000008	0.6344000	-1.4603496
20.7000008	0.6832000	-1.4787574
20.7000008	0.7320000	-1.4542136
20.7000008	0.7808000	-1.4235344
20.7000008	0.8296000	-1.5155730
20.7000008	0.8784000	-1.5217090
20.7000008	0.9272000	-1.4664855
20.7000008	0.9760000	-1.4542136
20.7000008	1.0247999	-1.3621750
20.7000008	1.0735999	-1.3621750
20.7000008	1.1223999	-1.3560390
20.7000008	1.1711999	-1.3560390
20.7000008	1.2200000	-1.3560390
20.7000008	1.2688000	-1.3069515
20.7000008	1.3176000	-1.3069515
20.7000008	1.3664000	-1.3192234
20.7000008	1.4152000	-1.3008156
20.7000008	1.4640000	-1.3008156
20.7000008	1.5128000	-1.2087770
20.7000008	1.5616000	-1.2087770
20.7000008	1.6104000	-1.4848933
20.7000008	1.6592000	-1.4910293
20.7000008	1.7079999	-1.4419422
20.7000008	1.7567999	-1.3314953
20.7000008	1.8055999	-1.2885437
20.7000008	1.8543999	-1.3130875
20.7000008	1.9031999	-1.2087770
20.7000008	1.9519999	-1.2149129
20.7000008	2.0007999	-1.3253593
20.7000008	2.0495999	-1.1780972
20.7000008	2.0983999	-1.1780972
20.7000008	2.1471999	-1.2640004
20.7000008	2.1959999	-1.2762718
20.7000008	2.2447999	-1.4419422
20.7000008	2.2935998	-1.4419422
20.7000008	2.3423998	-1.5217090
20.7000008	2.3912001	-1.3376312
20.7000008	2.4400001	-1.3437672
20.7000008	2.4888000	-1.2517285
20.7000008	2.5376000	-1.4358063
20.7000008	2.5864000	-1.1167378
20.7000008	2.6352000	-1.1290097
20.7000008	2.6840000	-1.1351457
20.7000008	2.7328000	-1.2271848
20.7000008	2.7816000	-1.2271848
20.7000008	2.8304000	-1.0308352
20.7000008	2.8792000	-1.0124273
20.7000008	2.9280000	-1.0062914
20.7000008	2.9768000	-0.9940195
20.7000008	3.0256000	-1.0431070

20.7000008	3.0743999	-1.0492430
20.7000008	3.1231999	-1.0553789
20.7000008	3.1719999	-1.0553789
20.7000008	3.2207999	-1.0431070
20.7000008	3.2695999	-0.9019809
20.7000008	3.3183999	-0.9265246
20.7000008	3.3671999	-0.9326606
20.7000008	3.4159999	-0.9326606
20.7000008	3.4647999	-0.6565437
20.7000008	3.5135999	-0.4479222
20.7000008	3.5623999	-0.7424469
20.7000008	3.6111999	-0.8222136
20.7000008	3.6599998	-0.8222136
20.7000008	3.7087998	-0.7485828
20.7000008	3.7575998	-0.7485828
20.7000008	3.8063998	-0.7547188
20.7000008	3.8551998	-0.7485828
20.7000008	3.9039998	-0.7424469
20.7000008	3.9527998	-0.7547188
20.7000008	4.0016003	-0.7669902
20.7000008	4.0503998	-0.9940195
20.7000008	4.0991998	-0.8774371
20.7000008	4.1479998	-1.0185633
20.7000008	4.1967998	-1.0246992
20.7000008	4.2455997	-0.9203887
20.7000008	4.2943997	-0.9203887
20.7000008	4.3431997	-1.1044660
20.7000008	4.3919997	-0.9019809
20.7000008	4.4407997	-1.0431070
20.7000008	4.4895997	-1.0553789
20.7000008	4.5383997	-0.9878836
20.7000008	4.5871997	613586.0000000
20.7000008	4.6359997	613586.0000000
20.7000008	4.6847997	613586.0000000
20.7000008	4.7336001	613586.0000000
20.7000008	4.7824001	613586.0000000
20.7000008	4.8312001	613586.0000000
20.7000008	4.8800001	613586.0000000
20.7000008	4.9288001	613586.0000000
20.7000008	4.9776001	613586.0000000
20.7000008	5.0264001	-0.8099418
20.7000008	5.0752001	613586.0000000
20.7000008	5.1240001	613586.0000000
20.7000008	5.1728001	613586.0000000
20.7000008	5.2216001	613586.0000000
20.7000008	5.2704000	613586.0000000
20.7000008	5.3192000	613586.0000000
20.7000008	5.3680000	613586.0000000
20.7000008	5.4168000	613586.0000000
20.7000008	5.4656000	613586.0000000
20.7000008	5.5144000	613586.0000000
20.7000008	5.5632000	613586.0000000
20.7000008	5.6120000	613586.0000000
20.7000008	5.6608000	613586.0000000
20.7000008	5.7096000	613586.0000000
20.7000008	5.7584000	613586.0000000
20.7000008	5.8072000	613586.0000000

20.7000008	5.8559999	613586.0000000
20.7000008	5.9047999	613586.0000000
20.7000008	5.9535999	613586.0000000
20.7000008	6.0023999	613586.0000000
20.7166672	0.2928000	-1.3928547
20.7166672	0.3416000	-1.3928547
20.7166672	0.3904000	-1.1596894
20.7166672	0.4392000	-1.1719613
20.7166672	0.4880000	-1.3989906
20.7166672	0.5368000	-1.2455926
20.7166672	0.5856000	-1.2517285
20.7166672	0.6344000	-1.5278449
20.7166672	0.6832000	-1.5339808
20.7166672	0.7320000	-1.6566992
20.7166672	0.7808000	-1.3867188
20.7166672	0.8296000	-1.4112625
20.7166672	0.8784000	-1.3130875
20.7166672	0.9272000	-1.2087770
20.7166672	0.9760000	-1.0431070
20.7166672	1.0247999	-1.0615149
20.7166672	1.0735999	-1.4664855
20.7166672	1.1223999	-1.5217090
20.7166672	1.1711999	-1.5278449
20.7166672	1.2200000	-1.4787574
20.7166672	1.2688000	-1.4112625
20.7166672	1.3176000	-1.4173985
20.7166672	1.3664000	-1.4235344
20.7166672	1.4152000	-1.4235344
20.7166672	1.4640000	-1.4051266
20.7166672	1.5128000	-1.2762718
20.7166672	1.5616000	-1.3744469
20.7166672	1.6104000	-1.3805828
20.7166672	1.6592000	-1.3928547
20.7166672	1.7079999	-1.3621750
20.7166672	1.7567999	-1.2271848
20.7166672	1.8055999	-1.3805828
20.7166672	1.8543999	-1.4173985
20.7166672	1.9031999	-1.3192234
20.7166672	1.9519999	-1.2578645
20.7166672	2.0007999	-1.3192234
20.7166672	2.0495999	-1.3253593
20.7166672	2.0983999	-1.3192234
20.7166672	2.1471999	-1.2885437
20.7166672	2.1959999	-1.3130875
20.7166672	2.2447999	-1.3192234
20.7166672	2.2935998	-1.3989906
20.7166672	2.3423998	-1.3989906
20.7166672	2.3912001	-1.3683109
20.7166672	2.4400001	-1.2762718
20.7166672	2.4888000	-1.1965051
20.7166672	2.5376000	-1.0737867
20.7166672	2.5864000	-1.0860586
20.7166672	2.6352000	-1.0921946
20.7166672	2.6840000	-1.2210488
20.7166672	2.7328000	-1.2640004
20.7166672	2.7816000	-1.2701364
20.7166672	2.8304000	-1.2762718

20.7166672	2.8792000	-1.3499031
20.7166672	2.9280000	-1.3499031
20.7166672	2.9768000	-1.2271848
20.7166672	3.0256000	-1.2149129
20.7166672	3.0743999	-1.1228738
20.7166672	3.1231999	-1.0185633
20.7166672	3.1719999	-0.9817476
20.7166672	3.2207999	-1.0308352
20.7166672	3.2695999	-1.1228738
20.7166672	3.3183999	-0.9265246
20.7166672	3.3671999	-0.8283496
20.7166672	3.4159999	-0.8099418
20.7166672	3.4647999	-0.8038058
20.7166672	3.5135999	-0.6197281
20.7166672	3.5623999	-0.5399613
20.7166672	3.6111999	-0.5276895
20.7166672	3.6599998	-0.4111071
20.7166672	3.7087998	-0.2208934
20.7166672	3.7575998	-0.2024856
20.7166672	3.8063998	-0.0613594
20.7166672	3.8551998	0.0306797
20.7166672	3.9039998	0.2331653
20.7166672	3.9527998	0.2393012
20.7166672	4.0016003	0.2761164
20.7166672	4.0503998	613586.0000000
20.7166672	4.0991998	-0.7363110
20.7166672	4.1479998	-0.6074562
20.7166672	4.1967998	-0.6074562
20.7166672	4.2455997	-0.6074562
20.7166672	4.2943997	-0.6688156
20.7166672	4.3431997	-0.6688156
20.7166672	4.3919997	-0.6135921
20.7166672	4.4407997	613586.0000000
20.7166672	4.4895997	613586.0000000
20.7166672	4.5383997	613586.0000000
20.7166672	4.5871997	613586.0000000
20.7166672	4.6359997	613586.0000000
20.7166672	4.6847997	613586.0000000
20.7166672	4.7336001	613586.0000000
20.7166672	4.7824001	613586.0000000
20.7166672	4.8312001	613586.0000000
20.7166672	4.8800001	613586.0000000
20.7166672	4.9288001	613586.0000000
20.7166672	4.9776001	613586.0000000
20.7166672	5.0264001	613586.0000000
20.7166672	5.0752001	613586.0000000
20.7166672	5.1240001	613586.0000000
20.7166672	5.1728001	613586.0000000
20.7166672	5.2216001	613586.0000000
20.7166672	5.2704000	613586.0000000
20.7166672	5.3192000	613586.0000000
20.7166672	5.3680000	613586.0000000
20.7166672	5.4168000	613586.0000000
20.7166672	5.4656000	613586.0000000
20.7166672	5.5144000	613586.0000000
20.7166672	5.5632000	613586.0000000
20.7166672	5.6120000	613586.0000000

20.7166672	5.6608000	613586.0000000
20.7166672	5.7096000	613586.0000000
20.7166672	5.7584000	613586.0000000
20.7166672	5.8072000	613586.0000000
20.7166672	5.8559999	613586.0000000
20.7166672	5.9047999	613586.0000000
20.7166672	5.9535999	613586.0000000
20.7166672	6.0023999	613586.0000000
20.7333336	0.2928000	-1.0062914
20.7333336	0.3416000	-0.9203887
20.7333336	0.3904000	-0.9265246
20.7333336	0.4392000	-1.5462527
20.7333336	0.4880000	-1.7794180
20.7333336	0.5368000	-1.5646605
20.7333336	0.5856000	-1.5707965
20.7333336	0.6344000	-1.4726214
20.7333336	0.6832000	-1.4726214
20.7333336	0.7320000	-1.4848933
20.7333336	0.7808000	-1.4910293
20.7333336	0.8296000	-1.3989906
20.7333336	0.8784000	-1.3069515
20.7333336	0.9272000	-1.3008156
20.7333336	0.9760000	-1.1965051
20.7333336	1.0247999	-1.1903691
20.7333336	1.0735999	-1.1965051
20.7333336	1.1223999	-1.3683109
20.7333336	1.1711999	-1.4112625
20.7333336	1.2200000	-1.3314953
20.7333336	1.2688000	-1.2333207
20.7333336	1.3176000	-1.2271848
20.7333336	1.3664000	-1.2149129
20.7333336	1.4152000	-1.3253593
20.7333336	1.4640000	-1.2455926
20.7333336	1.5128000	-1.2517285
20.7333336	1.5616000	-1.2701364
20.7333336	1.6104000	-1.3253593
20.7333336	1.6592000	-1.2701364
20.7333336	1.7079999	-1.2885437
20.7333336	1.7567999	-1.1351457
20.7333336	1.8055999	-1.2578645
20.7333336	1.8543999	-1.2517285
20.7333336	1.9031999	-1.2640004
20.7333336	1.9519999	-1.2885437
20.7333336	2.0007999	-1.3192234
20.7333336	2.0495999	-1.0799227
20.7333336	2.0983999	-1.1965051
20.7333336	2.1471999	-1.0615149
20.7333336	2.1959999	-1.1535535
20.7333336	2.2447999	-1.1351457
20.7333336	2.2935998	-1.1351457
20.7333336	2.3423998	-1.1044660
20.7333336	2.3912001	-1.2517285
20.7333336	2.4400001	-1.1167378
20.7333336	2.4888000	-1.1290097
20.7333336	2.5376000	-1.1412816
20.7333336	2.5864000	-1.3560390
20.7333336	2.6352000	-1.3314953

20.7333336	2.6840000	-1.3744469
20.7333336	2.7328000	-1.3621750
20.7333336	2.7816000	-1.2271848
20.7333336	2.8304000	-1.2271848
20.7333336	2.8792000	-1.0983305
20.7333336	2.9280000	-0.9387960
20.7333336	2.9768000	-0.9387960
20.7333336	3.0256000	-0.9572039
20.7333336	3.0743999	-0.9633398
20.7333336	3.1231999	-0.9694757
20.7333336	3.1719999	-0.8835731
20.7333336	3.2207999	-0.8958449
20.7333336	3.2695999	-0.9081168
20.7333336	3.3183999	-0.9019809
20.7333336	3.3671999	-0.8835731
20.7333336	3.4159999	-0.8713012
20.7333336	3.4647999	-0.5154176
20.7333336	3.5135999	-0.4479222
20.7333336	3.5623999	-0.4417863
20.7333336	3.6111999	-0.4479222
20.7333336	3.6599998	-0.4233789
20.7333336	3.7087998	-0.3865633
20.7333336	3.7575998	-0.5706410
20.7333336	3.8063998	-1.1044660
20.7333336	3.8551998	-1.1044660
20.7333336	3.9039998	-0.9633398
20.7333336	3.9527998	-1.2394567
20.7333336	4.0016003	-1.2333207
20.7333336	4.0503998	-1.0860586
20.7333336	4.0991998	-1.0737867
20.7333336	4.1479998	-1.0737867
20.7333336	4.1967998	-1.0983305
20.7333336	4.2455997	-0.9081168
20.7333336	4.2943997	-0.9142528
20.7333336	4.3431997	-0.9326606
20.7333336	4.3919997	-0.8160777
20.7333336	4.4407997	-0.9081168
20.7333336	4.4895997	-0.9142528
20.7333336	4.5383997	-0.9142528
20.7333336	4.5871997	613586.0000000
20.7333336	4.6359997	-0.7976699
20.7333336	4.6847997	613586.0000000
20.7333336	4.7336001	-0.6565437
20.7333336	4.7824001	-0.6565437
20.7333336	4.8312001	-0.8406215
20.7333336	4.8800001	613586.0000000
20.7333336	4.9288001	613586.0000000
20.7333336	4.9776001	613586.0000000
20.7333336	5.0264001	613586.0000000
20.7333336	5.0752001	613586.0000000
20.7333336	5.1240001	-0.3374758
20.7333336	5.1728001	613586.0000000
20.7333336	5.2216001	613586.0000000
20.7333336	5.2704000	613586.0000000
20.7333336	5.3192000	613586.0000000
20.7333336	5.3680000	613586.0000000
20.7333336	5.4168000	613586.0000000

20.7333336	5.4656000	613586.0000000
20.7333336	5.5144000	613586.0000000
20.7333336	5.5632000	613586.0000000
20.7333336	5.6120000	613586.0000000
20.7333336	5.6608000	613586.0000000
20.7333336	5.7096000	613586.0000000
20.7333336	5.7584000	613586.0000000
20.7333336	5.8072000	613586.0000000
20.7333336	5.8559999	613586.0000000
20.7333336	5.9047999	613586.0000000
20.7333336	5.9535999	613586.0000000
20.7333336	6.0023999	613586.0000000
20.7500000	0.2928000	-1.4971652
20.7500000	0.3416000	-1.2578645
20.7500000	0.3904000	-1.3253593
20.7500000	0.4392000	-1.4910293
20.7500000	0.4880000	-1.5401168
20.7500000	0.5368000	-1.4787574
20.7500000	0.5856000	-1.8407769
20.7500000	0.6344000	-1.8775926
20.7500000	0.6832000	-1.8653207
20.7500000	0.7320000	-1.8223691
20.7500000	0.7808000	-1.5033011
20.7500000	0.8296000	-1.6321554
20.7500000	0.8784000	-1.3069515
20.7500000	0.9272000	-1.6137476
20.7500000	0.9760000	-1.6321554
20.7500000	1.0247999	-1.5830684
20.7500000	1.0735999	-1.5830684
20.7500000	1.1223999	-1.5953403
20.7500000	1.1711999	-1.4296703
20.7500000	1.2200000	-1.4296703
20.7500000	1.2688000	-1.4358063
20.7500000	1.3176000	-1.3560390
20.7500000	1.3664000	-1.3744469
20.7500000	1.4152000	-1.5646605
20.7500000	1.4640000	-1.5646605
20.7500000	1.5128000	-1.5707965
20.7500000	1.5616000	-1.2885437
20.7500000	1.6104000	-1.3989906
20.7500000	1.6592000	-1.2701364
20.7500000	1.7079999	-1.2762718
20.7500000	1.7567999	-1.2762718
20.7500000	1.8055999	-1.2885437
20.7500000	1.8543999	-1.3008156
20.7500000	1.9031999	-1.1167378
20.7500000	1.9519999	-1.0799227
20.7500000	2.0007999	-1.0799227
20.7500000	2.0495999	-1.0124273
20.7500000	2.0983999	-1.0431070
20.7500000	2.1471999	-1.1228738
20.7500000	2.1959999	-1.0737867
20.7500000	2.2447999	-1.0615149
20.7500000	2.2935998	-1.0553789
20.7500000	2.3423998	-1.0246992
20.7500000	2.3912001	-1.0062914
20.7500000	2.4400001	-1.0062914

20.7500000	2.4888000	-1.2394567
20.7500000	2.5376000	-1.2455926
20.7500000	2.5864000	-1.2762718
20.7500000	2.6352000	-1.2026410
20.7500000	2.6840000	-1.1596894
20.7500000	2.7328000	-1.1596894
20.7500000	2.7816000	-1.0308352
20.7500000	2.8304000	-0.8406215
20.7500000	2.8792000	-0.8406215
20.7500000	2.9280000	-0.8467574
20.7500000	2.9768000	-0.7424469
20.7500000	3.0256000	-0.7179031
20.7500000	3.0743999	-0.7731261
20.7500000	3.1231999	-0.7608547
20.7500000	3.1719999	-0.8038058
20.7500000	3.2207999	-0.8038058
20.7500000	3.2695999	-0.6504078
20.7500000	3.3183999	-0.7608547
20.7500000	3.3671999	-1.1658254
20.7500000	3.4159999	-1.1719613
20.7500000	3.4647999	-1.0308352
20.7500000	3.5135999	-1.2149129
20.7500000	3.5623999	-1.2149129
20.7500000	3.6111999	-1.2394567
20.7500000	3.6599998	-1.2394567
20.7500000	3.7087998	-1.1412816
20.7500000	3.7575998	-1.1412816
20.7500000	3.8063998	-0.9633398
20.7500000	3.8551998	-0.9633398
20.7500000	3.9039998	-0.7792621
20.7500000	3.9527998	-0.7853980
20.7500000	4.0016003	-0.7976699
20.7500000	4.0503998	-0.9142528
20.7500000	4.0991998	-0.7915339
20.7500000	4.1479998	-0.7915339
20.7500000	4.1967998	-0.6749516
20.7500000	4.2455997	-0.5951843
20.7500000	4.2943997	-0.3988352
20.7500000	4.3431997	-0.3926992
20.7500000	4.3919997	-0.4724660
20.7500000	4.4407997	-0.4540582
20.7500000	4.4895997	-0.4479222
20.7500000	4.5383997	-0.1963496
20.7500000	4.5871997	-0.6197281
20.7500000	4.6359997	-0.7485828
20.7500000	4.6847997	613586.0000000
20.7500000	4.7336001	613586.0000000
20.7500000	4.7824001	613586.0000000
20.7500000	4.8312001	613586.0000000
20.7500000	4.8800001	-0.7547188
20.7500000	4.9288001	-0.6749516
20.7500000	4.9776001	-0.8713012
20.7500000	5.0264001	613586.0000000
20.7500000	5.0752001	613586.0000000
20.7500000	5.1240001	613586.0000000
20.7500000	5.1728001	613586.0000000
20.7500000	5.2216001	613586.0000000

20.7500000	5.2704000	613586.0000000
20.7500000	5.3192000	-0.6688156
20.7500000	5.3680000	-0.7424469
20.7500000	5.4168000	-0.8222136
20.7500000	5.4656000	-0.8283496
20.7500000	5.5144000	-0.7363110
20.7500000	5.5632000	-0.8283496
20.7500000	5.6120000	-0.8774371
20.7500000	5.6608000	-0.8774371
20.7500000	5.7096000	613586.0000000
20.7500000	5.7584000	613586.0000000
20.7500000	5.8072000	613586.0000000
20.7500000	5.8559999	613586.0000000
20.7500000	5.9047999	613586.0000000
20.7500000	5.9535999	613586.0000000
20.7500000	6.0023999	613586.0000000
20.7666664	0.2928000	-1.6812429
20.7666664	0.3416000	-1.4787574
20.7666664	0.3904000	-1.0737867
20.7666664	0.4392000	-1.0431070
20.7666664	0.4880000	-0.7669902
20.7666664	0.5368000	-1.5278449
20.7666664	0.5856000	-1.5339808
20.7666664	0.6344000	-1.5278449
20.7666664	0.6832000	-1.5278449
20.7666664	0.7320000	-1.5278449
20.7666664	0.7808000	-1.4051266
20.7666664	0.8296000	-1.3744469
20.7666664	0.8784000	-1.2824078
20.7666664	0.9272000	-1.2394567
20.7666664	0.9760000	-1.2455926
20.7666664	1.0247999	-1.1535535
20.7666664	1.0735999	-1.1535535
20.7666664	1.1223999	-1.1106019
20.7666664	1.1711999	-1.2333207
20.7666664	1.2200000	-1.2333207
20.7666664	1.2688000	-1.3928547
20.7666664	1.3176000	-1.2455926
20.7666664	1.3664000	-1.2333207
20.7666664	1.4152000	-1.2885437
20.7666664	1.4640000	-1.2762718
20.7666664	1.5128000	-1.3314953
20.7666664	1.5616000	-1.3130875
20.7666664	1.6104000	-1.3130875
20.7666664	1.6592000	-1.3130875
20.7666664	1.7079999	-1.3560390
20.7666664	1.7567999	-1.3130875
20.7666664	1.8055999	-1.1167378
20.7666664	1.8543999	-1.1106019
20.7666664	1.9031999	-1.1044660
20.7666664	1.9519999	-1.3805828
20.7666664	2.0007999	-1.3683109
20.7666664	2.0495999	-1.3008156
20.7666664	2.0983999	-1.1658254
20.7666664	2.1471999	-1.1658254
20.7666664	2.1959999	-1.2824078
20.7666664	2.2447999	-1.2946796

20.7666664	2.2935998	-1.2333207
20.7666664	2.3423998	-1.2333207
20.7666664	2.3912001	-1.2824078
20.7666664	2.4400001	-1.3683109
20.7666664	2.4888000	-1.3130875
20.7666664	2.5376000	-1.2517285
20.7666664	2.5864000	-1.1290097
20.7666664	2.6352000	-1.1290097
20.7666664	2.6840000	-1.1290097
20.7666664	2.7328000	-1.0676508
20.7666664	2.7816000	-1.1290097
20.7666664	2.8304000	-1.1535535
20.7666664	2.8792000	-0.9387960
20.7666664	2.9280000	-1.0799227
20.7666664	2.9768000	-1.0124273
20.7666664	3.0256000	-0.9449320
20.7666664	3.0743999	-1.0553789
20.7666664	3.1231999	-1.0860586
20.7666664	3.1719999	-1.0860586
20.7666664	3.2207999	-1.0860586
20.7666664	3.2695999	-1.0737867
20.7666664	3.3183999	-1.2210488
20.7666664	3.3671999	-1.2271848
20.7666664	3.4159999	-1.2333207
20.7666664	3.4647999	-1.2885437
20.7666664	3.5135999	-1.3683109
20.7666664	3.5623999	-1.3130875
20.7666664	3.6111999	-1.3130875
20.7666664	3.6599998	-1.2333207
20.7666664	3.7087998	-1.2087770
20.7666664	3.7575998	-1.0369711
20.7666664	3.8063998	-1.0246992
20.7666664	3.8551998	-1.0246992
20.7666664	3.9039998	-0.9633398
20.7666664	3.9527998	-0.9449320
20.7666664	4.0016003	-1.0492430
20.7666664	4.0503998	-1.0062914
20.7666664	4.0991998	-1.0615149
20.7666664	4.1479998	-0.9081168
20.7666664	4.1967998	-0.9142528
20.7666664	4.2455997	-0.7792621
20.7666664	4.2943997	-0.7608547
20.7666664	4.3431997	-0.9019809
20.7666664	4.3919997	-0.9019809
20.7666664	4.4407997	-0.6320000
20.7666664	4.4895997	-0.6320000
20.7666664	4.5383997	-0.9203887
20.7666664	4.5871997	-1.0431070
20.7666664	4.6359997	-1.0431070
20.7666664	4.6847997	-1.0615149
20.7666664	4.7336001	-1.0615149
20.7666664	4.7824001	-1.0492430
20.7666664	4.8312001	-1.0369711
20.7666664	4.8800001	613586.0000000
20.7666664	4.9288001	613586.0000000
20.7666664	4.9776001	-0.6872234
20.7666664	5.0264001	613586.0000000

20.7666664	5.0752001	613586.0000000
20.7666664	5.1240001	613586.0000000
20.7666664	5.1728001	613586.0000000
20.7666664	5.2216001	613586.0000000
20.7666664	5.2704000	613586.0000000
20.7666664	5.3192000	613586.0000000
20.7666664	5.3680000	-0.7608547
20.7666664	5.4168000	613586.0000000
20.7666664	5.4656000	613586.0000000
20.7666664	5.5144000	613586.0000000
20.7666664	5.5632000	613586.0000000
20.7666664	5.6120000	613586.0000000
20.7666664	5.6608000	613586.0000000
20.7666664	5.7096000	613586.0000000
20.7666664	5.7584000	613586.0000000
20.7666664	5.8072000	613586.0000000
20.7666664	5.8559999	613586.0000000
20.7666664	5.9047999	613586.0000000
20.7666664	5.9535999	613586.0000000
20.7666664	6.0023999	613586.0000000
20.7833328	0.2928000	-1.3437672
20.7833328	0.3416000	-1.7303305
20.7833328	0.3904000	-1.4235344
20.7833328	0.4392000	-1.4235344
20.7833328	0.4880000	-2.2212043
20.7833328	0.5368000	-1.6260195
20.7833328	0.5856000	-1.4603496
20.7833328	0.6344000	-1.4480777
20.7833328	0.6832000	-1.3437672
20.7833328	0.7320000	-1.3560390
20.7833328	0.7808000	-1.5094371
20.7833328	0.8296000	-1.3130875
20.7833328	0.8784000	-1.3253593
20.7833328	0.9272000	-1.1903691
20.7833328	0.9760000	-1.3499031
20.7833328	1.0247999	-1.2578645
20.7833328	1.0735999	-1.2517285
20.7833328	1.1223999	-1.3928547
20.7833328	1.1711999	-1.4112625
20.7833328	1.2200000	-1.4112625
20.7833328	1.2688000	-1.3867188
20.7833328	1.3176000	-1.2271848
20.7833328	1.3664000	-1.1965051
20.7833328	1.4152000	-1.5155730
20.7833328	1.4640000	-1.3499031
20.7833328	1.5128000	-1.3499031
20.7833328	1.5616000	-1.4235344
20.7833328	1.6104000	-1.3621750
20.7833328	1.6592000	-1.4726214
20.7833328	1.7079999	-1.2762718
20.7833328	1.7567999	-1.2762718
20.7833328	1.8055999	-1.2701364
20.7833328	1.8543999	-1.2640004
20.7833328	1.9031999	-1.1535535
20.7833328	1.9519999	-1.2333207
20.7833328	2.0007999	-1.2149129
20.7833328	2.0495999	-1.2087770

20.7833328	2.0983999	-1.3376312
20.7833328	2.1471999	-1.1596894
20.7833328	2.1959999	-1.1596894
20.7833328	2.2447999	-1.2026410
20.7833328	2.2935998	-1.2149129
20.7833328	2.3423998	-1.3008156
20.7833328	2.3912001	-1.1535535
20.7833328	2.4400001	-1.2271848
20.7833328	2.4888000	-1.2394567
20.7833328	2.5376000	-1.0553789
20.7833328	2.5864000	-1.0983305
20.7833328	2.6352000	-1.0921946
20.7833328	2.6840000	-0.9817476
20.7833328	2.7328000	-1.0921946
20.7833328	2.7816000	-0.9633398
20.7833328	2.8304000	-0.9142528
20.7833328	2.8792000	-1.0308352
20.7833328	2.9280000	-1.0431070
20.7833328	2.9768000	-1.2210488
20.7833328	3.0256000	-1.2149129
20.7833328	3.0743999	-1.3130875
20.7833328	3.1231999	-1.3253593
20.7833328	3.1719999	-1.3805828
20.7833328	3.2207999	-1.3805828
20.7833328	3.2695999	-1.3744469
20.7833328	3.3183999	-1.3253593
20.7833328	3.3671999	-1.2517285
20.7833328	3.4159999	-1.2946796
20.7833328	3.4647999	-1.3130875
20.7833328	3.5135999	-1.3192234
20.7833328	3.5623999	-1.2578645
20.7833328	3.6111999	-1.3008156
20.7833328	3.6599998	-1.1903691
20.7833328	3.7087998	-1.1474175
20.7833328	3.7575998	-1.0369711
20.7833328	3.8063998	-1.0431070
20.7833328	3.8551998	-1.1412816
20.7833328	3.9039998	-1.1351457
20.7833328	3.9527998	-1.0001554
20.7833328	4.0016003	-1.1044660
20.7833328	4.0503998	-1.0001554
20.7833328	4.0991998	-1.0001554
20.7833328	4.1479998	-1.0369711
20.7833328	4.1967998	-1.0615149
20.7833328	4.2455997	-1.1842332
20.7833328	4.2943997	-1.0246992
20.7833328	4.3431997	-0.9878836
20.7833328	4.3919997	-0.9878836
20.7833328	4.4407997	-1.2149129
20.7833328	4.4895997	-1.2149129
20.7833328	4.5383997	-1.2517285
20.7833328	4.5871997	-0.9203887
20.7833328	4.6359997	-1.0062914
20.7833328	4.6847997	-0.8958449
20.7833328	4.7336001	-0.8897090
20.7833328	4.7824001	-0.9817476
20.7833328	4.8312001	-0.9817476

20.7833328	4.8800001	-0.7669902
20.7833328	4.9288001	-0.7669902
20.7833328	4.9776001	613586.0000000
20.7833328	5.0264001	613586.0000000
20.7833328	5.0752001	613586.0000000
20.7833328	5.1240001	-0.8406215
20.7833328	5.1728001	613586.0000000
20.7833328	5.2216001	613586.0000000
20.7833328	5.2704000	613586.0000000
20.7833328	5.3192000	-0.8774371
20.7833328	5.3680000	-0.9694757
20.7833328	5.4168000	-0.9694757
20.7833328	5.4656000	613586.0000000
20.7833328	5.5144000	-0.7485828
20.7833328	5.5632000	-0.7547188
20.7833328	5.6120000	-0.7669902
20.7833328	5.6608000	613586.0000000
20.7833328	5.7096000	613586.0000000
20.7833328	5.7584000	613586.0000000
20.7833328	5.8072000	613586.0000000
20.7833328	5.8559999	-0.6320000
20.7833328	5.9047999	-0.6074562
20.7833328	5.9535999	-0.6074562
20.7833328	6.0023999	613586.0000000
20.7999992	0.2928000	-1.9328160
20.7999992	0.3416000	-1.6382914
20.7999992	0.3904000	-1.2578645
20.7999992	0.4392000	-1.2455926
20.7999992	0.4880000	-1.2455926
20.7999992	0.5368000	-1.6444273
20.7999992	0.5856000	-1.4419422
20.7999992	0.6344000	-1.4296703
20.7999992	0.6832000	-1.4173985
20.7999992	0.7320000	-1.4112625
20.7999992	0.7808000	-1.1780972
20.7999992	0.8296000	-1.3376312
20.7999992	0.8784000	-1.1719613
20.7999992	0.9272000	-1.1719613
20.7999992	0.9760000	-1.4173985
20.7999992	1.0247999	-1.4173985
20.7999992	1.0735999	-1.2578645
20.7999992	1.1223999	-1.1903691
20.7999992	1.1711999	-1.1903691
20.7999992	1.2200000	-1.2517285
20.7999992	1.2688000	-1.1719613
20.7999992	1.3176000	-1.3867188
20.7999992	1.3664000	-1.3192234
20.7999992	1.4152000	-1.2210488
20.7999992	1.4640000	-1.2271848
20.7999992	1.5128000	-1.2026410
20.7999992	1.5616000	-1.2026410
20.7999992	1.6104000	-1.2026410
20.7999992	1.6592000	-1.2210488
20.7999992	1.7079999	-1.2087770
20.7999992	1.7567999	-1.2517285
20.7999992	1.8055999	-1.2517285
20.7999992	1.8543999	-1.0676508

20.7999992	1.9031999	-1.0737867
20.7999992	1.9519999	-1.0676508
20.7999992	2.0007999	-0.9694757
20.7999992	2.0495999	-0.9572039
20.7999992	2.0983999	-1.1780972
20.7999992	2.1471999	-1.1596894
20.7999992	2.1959999	-1.1535535
20.7999992	2.2447999	-1.0062914
20.7999992	2.2935998	-1.0492430
20.7999992	2.3423998	-1.0676508
20.7999992	2.3912001	-1.0676508
20.7999992	2.4400001	-1.1228738
20.7999992	2.4888000	-1.1167378
20.7999992	2.5376000	-1.1044660
20.7999992	2.5864000	-1.1106019
20.7999992	2.6352000	-0.9633398
20.7999992	2.6840000	-0.9203887
20.7999992	2.7328000	-0.9265246
20.7999992	2.7816000	-0.9203887
20.7999992	2.8304000	-0.8897090
20.7999992	2.8792000	-0.7976699
20.7999992	2.9280000	-1.1965051
20.7999992	2.9768000	-1.1228738
20.7999992	3.0256000	-1.2210488
20.7999992	3.0743999	-1.3069515
20.7999992	3.1231999	-1.2885437
20.7999992	3.1719999	-0.9203887
20.7999992	3.2207999	-0.8406215
20.7999992	3.2695999	-0.8406215
20.7999992	3.3183999	-0.9756117
20.7999992	3.3671999	-0.9756117
20.7999992	3.4159999	-1.0431070
20.7999992	3.4647999	-1.1474175
20.7999992	3.5135999	-1.2087770
20.7999992	3.5623999	-1.0921946
20.7999992	3.6111999	-1.0124273
20.7999992	3.6599998	-1.0185633
20.7999992	3.7087998	-0.8528934
20.7999992	3.7575998	-0.9203887
20.7999992	3.8063998	-0.9449320
20.7999992	3.8551998	-1.0124273
20.7999992	3.9039998	-1.0124273
20.7999992	3.9527998	-0.9387960
20.7999992	4.0016003	-0.8344855
20.7999992	4.0503998	-0.8038058
20.7999992	4.0991998	-0.8283496
20.7999992	4.1479998	-0.6320000
20.7999992	4.1967998	-0.6504078
20.7999992	4.2455997	-0.6626797
20.7999992	4.2943997	-0.6626797
20.7999992	4.3431997	-0.3374758
20.7999992	4.3919997	-0.7363110
20.7999992	4.4407997	-0.5645051
20.7999992	4.4895997	-0.5706410
20.7999992	4.5383997	-0.5829129
20.7999992	4.5871997	-0.5583692
20.7999992	4.6359997	-0.5706410

20.7999992	4.6847997	-0.7915339
20.7999992	4.7336001	-0.7056313
20.7999992	4.7824001	613586.0000000
20.7999992	4.8312001	613586.0000000
20.7999992	4.8800001	613586.0000000
20.7999992	4.9288001	613586.0000000
20.7999992	4.9776001	613586.0000000
20.7999992	5.0264001	613586.0000000
20.7999992	5.0752001	613586.0000000
20.7999992	5.1240001	-0.3190680
20.7999992	5.1728001	613586.0000000
20.7999992	5.2216001	613586.0000000
20.7999992	5.2704000	613586.0000000
20.7999992	5.3192000	613586.0000000
20.7999992	5.3680000	613586.0000000
20.7999992	5.4168000	-0.0490875
20.7999992	5.4656000	613586.0000000
20.7999992	5.5144000	613586.0000000
20.7999992	5.5632000	-0.1533980
20.7999992	5.6120000	-0.1533980
20.7999992	5.6608000	613586.0000000
20.7999992	5.7096000	613586.0000000
20.7999992	5.7584000	613586.0000000
20.7999992	5.8072000	613586.0000000
20.7999992	5.8559999	613586.0000000
20.7999992	5.9047999	613586.0000000
20.7999992	5.9535999	613586.0000000
20.7999992	6.0023999	613586.0000000
20.8166676	0.2928000	-1.7180586
20.8166676	0.3416000	-1.6628351
20.8166676	0.3904000	-2.0984859
20.8166676	0.4392000	-1.5585246
20.8166676	0.4880000	-1.5769324
20.8166676	0.5368000	-1.5892043
20.8166676	0.5856000	-1.3683109
20.8166676	0.6344000	-1.3253593
20.8166676	0.6832000	-1.4235344
20.8166676	0.7320000	-1.4358063
20.8166676	0.7808000	-1.4910293
20.8166676	0.8296000	-1.4971652
20.8166676	0.8784000	-1.4542136
20.8166676	0.9272000	-1.3499031
20.8166676	0.9760000	-1.3621750
20.8166676	1.0247999	-1.3560390
20.8166676	1.0735999	-1.3437672
20.8166676	1.1223999	-1.3437672
20.8166676	1.1711999	-1.4173985
20.8166676	1.2200000	-1.1965051
20.8166676	1.2688000	-1.3253593
20.8166676	1.3176000	-1.3192234
20.8166676	1.3664000	-1.1474175
20.8166676	1.4152000	-1.2701364
20.8166676	1.4640000	-1.2149129
20.8166676	1.5128000	-1.3560390
20.8166676	1.5616000	-1.3683109
20.8166676	1.6104000	-1.2394567
20.8166676	1.6592000	-1.2517285

20.8166676	1.7079999	-1.2640004
20.8166676	1.7567999	-1.1167378
20.8166676	1.8055999	-1.0676508
20.8166676	1.8543999	-1.0737867
20.8166676	1.9031999	-1.2149129
20.8166676	1.9519999	-1.2333207
20.8166676	2.0007999	-1.2517285
20.8166676	2.0495999	-1.3928547
20.8166676	2.0983999	-1.3314953
20.8166676	2.1471999	-1.3130875
20.8166676	2.1959999	-1.1044660
20.8166676	2.2447999	-1.1903691
20.8166676	2.2935998	-1.1903691
20.8166676	2.3423998	-1.1903691
20.8166676	2.3912001	-1.1596894
20.8166676	2.4400001	-1.1596894
20.8166676	2.4888000	-1.1474175
20.8166676	2.5376000	-0.9326606
20.8166676	2.5864000	-1.0124273
20.8166676	2.6352000	-0.9756117
20.8166676	2.6840000	-0.9756117
20.8166676	2.7328000	-1.0615149
20.8166676	2.7816000	-1.0369711
20.8166676	2.8304000	-1.1167378
20.8166676	2.8792000	-1.0676508
20.8166676	2.9280000	-1.0799227
20.8166676	2.9768000	-1.0921946
20.8166676	3.0256000	-1.0921946
20.8166676	3.0743999	-1.2824078
20.8166676	3.1231999	-1.2701364
20.8166676	3.1719999	-1.2149129
20.8166676	3.2207999	-1.0431070
20.8166676	3.2695999	-1.0553789
20.8166676	3.3183999	-0.8590293
20.8166676	3.3671999	-0.9142528
20.8166676	3.4159999	-1.0246992
20.8166676	3.4647999	-1.0860586
20.8166676	3.5135999	-0.8897090
20.8166676	3.5623999	-1.2210488
20.8166676	3.6111999	-1.2640004
20.8166676	3.6599998	-1.2885437
20.8166676	3.7087998	-1.0676508
20.8166676	3.7575998	-1.0492430
20.8166676	3.8063998	-1.0737867
20.8166676	3.8551998	-1.0799227
20.8166676	3.9039998	-0.9633398
20.8166676	3.9527998	-1.0001554
20.8166676	4.0016003	-1.0001554
20.8166676	4.0503998	-1.1106019
20.8166676	4.0991998	-0.9633398
20.8166676	4.1479998	-0.6504078
20.8166676	4.1967998	-1.0431070
20.8166676	4.2455997	-1.0431070
20.8166676	4.2943997	-0.9326606
20.8166676	4.3431997	-0.9387960
20.8166676	4.3919997	-0.9326606
20.8166676	4.4407997	-0.9142528

20.8166676	4.4895997	-0.9019809
20.8166676	4.5383997	-0.8406215
20.8166676	4.5871997	-0.8406215
20.8166676	4.6359997	613586.0000000
20.8166676	4.6847997	-1.1412816
20.8166676	4.7336001	-1.1658254
20.8166676	4.7824001	-1.1719613
20.8166676	4.8312001	-1.4971652
20.8166676	4.8800001	-0.7731261
20.8166676	4.9288001	-0.7792621
20.8166676	4.9776001	-0.8344855
20.8166676	5.0264001	-1.0921946
20.8166676	5.0752001	613586.0000000
20.8166676	5.1240001	-0.7853980
20.8166676	5.1728001	613586.0000000
20.8166676	5.2216001	613586.0000000
20.8166676	5.2704000	-0.8590293
20.8166676	5.3192000	-0.8651652
20.8166676	5.3680000	613586.0000000
20.8166676	5.4168000	-0.7363110
20.8166676	5.4656000	-0.6872234
20.8166676	5.5144000	-0.6810875
20.8166676	5.5632000	-0.6381359
20.8166676	5.6120000	-0.6504078
20.8166676	5.6608000	-0.6565437
20.8166676	5.7096000	613586.0000000
20.8166676	5.7584000	-0.7240391
20.8166676	5.8072000	613586.0000000
20.8166676	5.8559999	613586.0000000
20.8166676	5.9047999	613586.0000000
20.8166676	5.9535999	613586.0000000
20.8166676	6.0023999	613586.0000000
20.8333340	0.2928000	-0.6688156
20.8333340	0.3416000	-1.0983305
20.8333340	0.3904000	-1.0369711
20.8333340	0.4392000	-1.1596894
20.8333340	0.4880000	-1.3744469
20.8333340	0.5368000	-1.4296703
20.8333340	0.5856000	-1.3621750
20.8333340	0.6344000	-1.3683109
20.8333340	0.6832000	-1.3805828
20.8333340	0.7320000	-1.4112625
20.8333340	0.7808000	-1.4603496
20.8333340	0.8296000	-1.4664855
20.8333340	0.8784000	-1.5217090
20.8333340	0.9272000	-1.6137476
20.8333340	0.9760000	-1.3621750
20.8333340	1.0247999	-1.4603496
20.8333340	1.0735999	-1.4726214
20.8333340	1.1223999	-1.3744469
20.8333340	1.1711999	-1.4419422
20.8333340	1.2200000	-1.2946796
20.8333340	1.2688000	-1.3008156
20.8333340	1.3176000	-1.2210488
20.8333340	1.3664000	-1.4112625
20.8333340	1.4152000	-1.3437672
20.8333340	1.4640000	-1.4603496

20.8333340	1.5128000	-1.2271848
20.8333340	1.5616000	-1.0308352
20.8333340	1.6104000	-1.0431070
20.8333340	1.6592000	-1.0615149
20.8333340	1.7079999	-1.0737867
20.8333340	1.7567999	-1.0860586
20.8333340	1.8055999	-1.0246992
20.8333340	1.8543999	-1.1842332
20.8333340	1.9031999	-1.0062914
20.8333340	1.9519999	-1.1780972
20.8333340	2.0007999	-1.2333207
20.8333340	2.0495999	-1.2762718
20.8333340	2.0983999	-1.2824078
20.8333340	2.1471999	-1.1474175
20.8333340	2.1959999	-1.1535535
20.8333340	2.2447999	-1.2885437
20.8333340	2.2935998	-1.2824078
20.8333340	2.3423998	-1.1719613
20.8333340	2.3912001	-1.2578645
20.8333340	2.4400001	-1.1044660
20.8333340	2.4888000	-1.0983305
20.8333340	2.5376000	-1.1228738
20.8333340	2.5864000	-1.1290097
20.8333340	2.6352000	-1.0185633
20.8333340	2.6840000	-1.1535535
20.8333340	2.7328000	-1.1719613
20.8333340	2.7816000	-1.1903691
20.8333340	2.8304000	-1.1842332
20.8333340	2.8792000	-1.1658254
20.8333340	2.9280000	-1.1719613
20.8333340	2.9768000	-1.1106019
20.8333340	3.0256000	-1.1228738
20.8333340	3.0743999	-0.9756117
20.8333340	3.1231999	-1.1228738
20.8333340	3.1719999	-1.1412816
20.8333340	3.2207999	-1.0737867
20.8333340	3.2695999	-0.9756117
20.8333340	3.3183999	-0.6810875
20.8333340	3.3671999	-0.9265246
20.8333340	3.4159999	-0.6320000
20.8333340	3.4647999	-0.5522332
20.8333340	3.5135999	-0.5522332
20.8333340	3.5623999	-0.8222136
20.8333340	3.6111999	-0.7547188
20.8333340	3.6599998	-0.7547188
20.8333340	3.7087998	-0.4356503
20.8333340	3.7575998	-0.6749516
20.8333340	3.8063998	-0.6810875
20.8333340	3.8551998	-0.9694757
20.8333340	3.9039998	-0.6872234
20.8333340	3.9527998	-0.7608547
20.8333340	4.0016003	-0.7669902
20.8333340	4.0503998	-0.7853980
20.8333340	4.0991998	-0.6810875
20.8333340	4.1479998	-0.6810875
20.8333340	4.1967998	-0.7608547
20.8333340	4.2455997	-0.7608547

20.8333340	4.2943997	-0.7608547
20.8333340	4.3431997	-0.7547188
20.8333340	4.3919997	-0.7485828
20.8333340	4.4407997	-0.4724660
20.8333340	4.4895997	-0.4724660
20.8333340	4.5383997	-0.6135921
20.8333340	4.5871997	-0.6504078
20.8333340	4.6359997	-0.8222136
20.8333340	4.6847997	-0.8222136
20.8333340	4.7336001	-0.8222136
20.8333340	4.7824001	-0.8283496
20.8333340	4.8312001	-0.8344855
20.8333340	4.8800001	-0.8222136
20.8333340	4.9288001	-0.6320000
20.8333340	4.9776001	-0.3436117
20.8333340	5.0264001	-0.3436117
20.8333340	5.0752001	613586.0000000
20.8333340	5.1240001	-0.2761164
20.8333340	5.1728001	-0.2699804
20.8333340	5.2216001	-0.2699804
20.8333340	5.2704000	613586.0000000
20.8333340	5.3192000	-1.0124273
20.8333340	5.3680000	-1.0185633
20.8333340	5.4168000	-0.9694757
20.8333340	5.4656000	-0.8958449
20.8333340	5.5144000	613586.0000000
20.8333340	5.5632000	-0.8713012
20.8333340	5.6120000	613586.0000000
20.8333340	5.6608000	613586.0000000
20.8333340	5.7096000	613586.0000000
20.8333340	5.7584000	613586.0000000
20.8333340	5.8072000	613586.0000000
20.8333340	5.8559999	613586.0000000
20.8333340	5.9047999	613586.0000000
20.8333340	5.9535999	613586.0000000
20.8333340	6.0023999	613586.0000000
20.8500004	0.2928000	-1.9266801
20.8500004	0.3416000	-1.8407769
20.8500004	0.3904000	-1.4542136
20.8500004	0.4392000	-1.4358063
20.8500004	0.4880000	-1.3744469
20.8500004	0.5368000	-1.3744469
20.8500004	0.5856000	-1.4726214
20.8500004	0.6344000	-1.3805828
20.8500004	0.6832000	-1.3069515
20.8500004	0.7320000	-1.3069515
20.8500004	0.7808000	-1.3253593
20.8500004	0.8296000	-1.3314953
20.8500004	0.8784000	-1.4051266
20.8500004	0.9272000	-1.3989906
20.8500004	0.9760000	-1.2701364
20.8500004	1.0247999	-1.3192234
20.8500004	1.0735999	-1.3130875
20.8500004	1.1223999	-1.3253593
20.8500004	1.1711999	-1.3253593
20.8500004	1.2200000	-1.1780972
20.8500004	1.2688000	-1.1780972

20.8500004	1.3176000	-1.2578645
20.8500004	1.3664000	-1.2578645
20.8500004	1.4152000	-1.2578645
20.8500004	1.4640000	-1.3130875
20.8500004	1.5128000	-1.3008156
20.8500004	1.5616000	-1.3069515
20.8500004	1.6104000	-1.1535535
20.8500004	1.6592000	-1.1351457
20.8500004	1.7079999	-1.1412816
20.8500004	1.7567999	-1.3069515
20.8500004	1.8055999	-1.3008156
20.8500004	1.8543999	-1.2087770
20.8500004	1.9031999	-1.2149129
20.8500004	1.9519999	-1.2640004
20.8500004	2.0007999	-1.2271848
20.8500004	2.0495999	-1.4112625
20.8500004	2.0983999	-1.4112625
20.8500004	2.1471999	-1.2333207
20.8500004	2.1959999	-1.2394567
20.8500004	2.2447999	-1.1474175
20.8500004	2.2935998	-1.2210488
20.8500004	2.3423998	-1.2210488
20.8500004	2.3912001	-1.0737867
20.8500004	2.4400001	-1.1290097
20.8500004	2.4888000	-1.1044660
20.8500004	2.5376000	-1.0983305
20.8500004	2.5864000	-1.1412816
20.8500004	2.6352000	-0.9510679
20.8500004	2.6840000	-1.0860586
20.8500004	2.7328000	-1.0860586
20.8500004	2.7816000	-1.0737867
20.8500004	2.8304000	-1.0737867
20.8500004	2.8792000	-0.8651652
20.8500004	2.9280000	-1.1351457
20.8500004	2.9768000	-1.1290097
20.8500004	3.0256000	-0.9756117
20.8500004	3.0743999	-1.1474175
20.8500004	3.1231999	-1.1290097
20.8500004	3.1719999	-1.3560390
20.8500004	3.2207999	-1.2394567
20.8500004	3.2695999	-1.1903691
20.8500004	3.3183999	-0.9694757
20.8500004	3.3671999	-0.9694757
20.8500004	3.4159999	-1.1719613
20.8500004	3.4647999	-1.0983305
20.8500004	3.5135999	-0.9510679
20.8500004	3.5623999	-1.0246992
20.8500004	3.6111999	-0.8528934
20.8500004	3.6599998	-0.7363110
20.8500004	3.7087998	-0.7301750
20.8500004	3.7575998	-0.5951843
20.8500004	3.8063998	-0.4908738
20.8500004	3.8551998	-0.6872234
20.8500004	3.9039998	-0.5890489
20.8500004	3.9527998	-0.5767770
20.8500004	4.0016003	-0.5522332
20.8500004	4.0503998	-0.4908738

20.8500004	4.0991998	-0.5031457
20.8500004	4.1479998	-0.5154176
20.8500004	4.1967998	-0.3681555
20.8500004	4.2455997	-0.5399613
20.8500004	4.2943997	-0.7363110
20.8500004	4.3431997	613586.0000000
20.8500004	4.3919997	-0.8160777
20.8500004	4.4407997	-0.6258640
20.8500004	4.4895997	-0.6258640
20.8500004	4.5383997	-0.5399613
20.8500004	4.5871997	-0.5460973
20.8500004	4.6359997	-0.6320000
20.8500004	4.6847997	-0.6320000
20.8500004	4.7336001	-0.5460973
20.8500004	4.7824001	-0.5706410
20.8500004	4.8312001	-0.5767770
20.8500004	4.8800001	-0.4540582
20.8500004	4.9288001	-0.4540582
20.8500004	4.9776001	-0.4417863
20.8500004	5.0264001	-0.5031457
20.8500004	5.0752001	-0.4356503
20.8500004	5.1240001	-0.2331653
20.8500004	5.1728001	-0.2331653
20.8500004	5.2216001	-0.1902137
20.8500004	5.2704000	-0.1963496
20.8500004	5.3192000	-0.4479222
20.8500004	5.3680000	-0.4479222
20.8500004	5.4168000	-0.4479222
20.8500004	5.4656000	-0.5338254
20.8500004	5.5144000	613586.0000000
20.8500004	5.5632000	613586.0000000
20.8500004	5.6120000	613586.0000000
20.8500004	5.6608000	613586.0000000
20.8500004	5.7096000	613586.0000000
20.8500004	5.7584000	613586.0000000
20.8500004	5.8072000	613586.0000000
20.8500004	5.8559999	613586.0000000
20.8500004	5.9047999	613586.0000000
20.8500004	5.9535999	613586.0000000
20.8500004	6.0023999	613586.0000000
20.8666668	0.2928000	-6.2034183
20.8666668	0.3416000	-1.3805828
20.8666668	0.3904000	-1.4051266
20.8666668	0.4392000	-1.3989906
20.8666668	0.4880000	-1.1842332
20.8666668	0.5368000	-1.1842332
20.8666668	0.5856000	-1.2271848
20.8666668	0.6344000	-1.2149129
20.8666668	0.6832000	-1.1903691
20.8666668	0.7320000	-1.2394567
20.8666668	0.7808000	-1.2762718
20.8666668	0.8296000	-1.2762718
20.8666668	0.8784000	-1.3928547
20.8666668	0.9272000	-1.3805828
20.8666668	0.9760000	-1.3928547
20.8666668	1.0247999	-1.3867188
20.8666668	1.0735999	-1.3560390

20.8666668	1.1223999	-1.3683109
20.8666668	1.1711999	-1.3683109
20.8666668	1.2200000	-1.0737867
20.8666668	1.2688000	-1.0676508
20.8666668	1.3176000	-1.4419422
20.8666668	1.3664000	-1.4419422
20.8666668	1.4152000	-1.3376312
20.8666668	1.4640000	-1.3253593
20.8666668	1.5128000	-1.4112625
20.8666668	1.5616000	-1.4112625
20.8666668	1.6104000	-1.4296703
20.8666668	1.6592000	-1.3008156
20.8666668	1.7079999	-1.1842332
20.8666668	1.7567999	-1.3621750
20.8666668	1.8055999	-1.3805828
20.8666668	1.8543999	-1.3989906
20.8666668	1.9031999	-1.3867188
20.8666668	1.9519999	-1.3928547
20.8666668	2.0007999	-1.3928547
20.8666668	2.0495999	-1.3314953
20.8666668	2.0983999	-1.1535535
20.8666668	2.1471999	-1.1842332
20.8666668	2.1959999	-1.1903691
20.8666668	2.2447999	-1.3376312
20.8666668	2.2935998	-1.3437672
20.8666668	2.3423998	-1.3253593
20.8666668	2.3912001	-1.3683109
20.8666668	2.4400001	-1.3069515
20.8666668	2.4888000	-1.3008156
20.8666668	2.5376000	-1.3008156
20.8666668	2.5864000	-1.3069515
20.8666668	2.6352000	-1.3253593
20.8666668	2.6840000	-1.3253593
20.8666668	2.7328000	-1.3069515
20.8666668	2.7816000	-1.1290097
20.8666668	2.8304000	-1.2517285
20.8666668	2.8792000	-1.2517285
20.8666668	2.9280000	-1.2578645
20.8666668	2.9768000	-1.1290097
20.8666668	3.0256000	-0.7731261
20.8666668	3.0743999	-0.7608547
20.8666668	3.1231999	-0.7117672
20.8666668	3.1719999	-0.7117672
20.8666668	3.2207999	-0.8774371
20.8666668	3.2695999	-0.8958449
20.8666668	3.3183999	-0.8958449
20.8666668	3.3671999	-0.6258640
20.8666668	3.4159999	-0.6320000
20.8666668	3.4647999	-0.3926992
20.8666668	3.5135999	-0.8958449
20.8666668	3.5623999	-0.9019809
20.8666668	3.6111999	-1.0431070
20.8666668	3.6599998	-1.0431070
20.8666668	3.7087998	-1.0124273
20.8666668	3.7575998	-1.0001554
20.8666668	3.8063998	-0.7915339
20.8666668	3.8551998	-0.8651652

20.8666668	3.9039998	-1.1167378
20.8666668	3.9527998	-1.1412816
20.8666668	4.0016003	-1.1474175
20.8666668	4.0503998	-1.0676508
20.8666668	4.0991998	-1.0676508
20.8666668	4.1479998	-0.8099418
20.8666668	4.1967998	-1.1842332
20.8666668	4.2455997	-1.1658254
20.8666668	4.2943997	-1.1658254
20.8666668	4.3431997	-1.1903691
20.8666668	4.3919997	-1.1290097
20.8666668	4.4407997	-1.1228738
20.8666668	4.4895997	-1.1167378
20.8666668	4.5383997	-1.0615149
20.8666668	4.5871997	-1.0431070
20.8666668	4.6359997	-1.0124273
20.8666668	4.6847997	-1.0124273
20.8666668	4.7336001	-1.0185633
20.8666668	4.7824001	-0.8528934
20.8666668	4.8312001	-0.8897090
20.8666668	4.8800001	-0.9510679
20.8666668	4.9288001	-0.8406215
20.8666668	4.9776001	-0.4847379
20.8666668	5.0264001	-0.5031457
20.8666668	5.0752001	-0.5092816
20.8666668	5.1240001	613586.0000000
20.8666668	5.1728001	613586.0000000
20.8666668	5.2216001	-0.3190680
20.8666668	5.2704000	-0.3190680
20.8666668	5.3192000	-0.3436117
20.8666668	5.3680000	613586.0000000
20.8666668	5.4168000	613586.0000000
20.8666668	5.4656000	613586.0000000
20.8666668	5.5144000	613586.0000000
20.8666668	5.5632000	613586.0000000
20.8666668	5.6120000	613586.0000000
20.8666668	5.6608000	613586.0000000
20.8666668	5.7096000	613586.0000000
20.8666668	5.7584000	613586.0000000
20.8666668	5.8072000	613586.0000000
20.8666668	5.8559999	613586.0000000
20.8666668	5.9047999	613586.0000000
20.8666668	5.9535999	613586.0000000
20.8666668	6.0023999	613586.0000000
20.8833332	0.2928000	-2.1046219
20.8833332	0.3416000	-1.5401168
20.8833332	0.3904000	-1.5401168
20.8833332	0.4392000	-0.9633398
20.8833332	0.4880000	-1.4051266
20.8833332	0.5368000	-1.1474175
20.8833332	0.5856000	-1.3069515
20.8833332	0.6344000	-1.1167378
20.8833332	0.6832000	-1.1719613
20.8833332	0.7320000	-1.1719613
20.8833332	0.7808000	-1.1535535
20.8833332	0.8296000	-1.1412816
20.8833332	0.8784000	-1.3805828

20.8833332	0.9272000	-1.3805828
20.8833332	0.9760000	-1.3867188
20.8833332	1.0247999	-1.3253593
20.8833332	1.0735999	-1.3192234
20.8833332	1.1223999	-1.2824078
20.8833332	1.1711999	-1.3008156
20.8833332	1.2200000	-1.3192234
20.8833332	1.2688000	-1.6628351
20.8833332	1.3176000	-1.4051266
20.8833332	1.3664000	-1.3805828
20.8833332	1.4152000	-1.3867188
20.8833332	1.4640000	-1.3928547
20.8833332	1.5128000	-1.2824078
20.8833332	1.5616000	-1.2824078
20.8833332	1.6104000	-1.1719613
20.8833332	1.6592000	-1.2762718
20.8833332	1.7079999	-1.1965051
20.8833332	1.7567999	-1.2701364
20.8833332	1.8055999	-1.2517285
20.8833332	1.8543999	-1.2517285
20.8833332	1.9031999	-1.2578645
20.8833332	1.9519999	-1.3437672
20.8833332	2.0007999	-1.4112625
20.8833332	2.0495999	-1.2885437
20.8833332	2.0983999	-1.2946796
20.8833332	2.1471999	-1.3437672
20.8833332	2.1959999	-1.3192234
20.8833332	2.2447999	-1.2701364
20.8833332	2.2935998	-1.1842332
20.8833332	2.3423998	-1.3069515
20.8833332	2.3912001	-1.3130875
20.8833332	2.4400001	-1.2701364
20.8833332	2.4888000	-1.3621750
20.8833332	2.5376000	-1.3621750
20.8833332	2.5864000	-1.3744469
20.8833332	2.6352000	-1.4971652
20.8833332	2.6840000	-1.2026410
20.8833332	2.7328000	-1.1965051
20.8833332	2.7816000	-0.9326606
20.8833332	2.8304000	-0.8222136
20.8833332	2.8792000	-0.7608547
20.8833332	2.9280000	-0.7669902
20.8833332	2.9768000	-0.7731261
20.8833332	3.0256000	-0.5215535
20.8833332	3.0743999	0.2086215
20.8833332	3.1231999	0.2086215
20.8833332	3.1719999	0.1472621
20.8833332	3.2207999	-0.1104465
20.8833332	3.2695999	0.1349902
20.8833332	3.3183999	0.4970098
20.8833332	3.3671999	0.0797672
20.8833332	3.4159999	-0.1656699
20.8833332	3.4647999	-0.1718059
20.8833332	3.5135999	-0.3313398
20.8833332	3.5623999	-0.3313398
20.8833332	3.6111999	-0.5583692
20.8833332	3.6599998	-0.5645051

20.8833332	3.7087998	-0.6381359
20.8833332	3.7575998	-1.0062914
20.8833332	3.8063998	-0.9878836
20.8833332	3.8551998	-0.8099418
20.8833332	3.9039998	-0.7363110
20.8833332	3.9527998	-0.8467574
20.8833332	4.0016003	-0.8406215
20.8833332	4.0503998	-0.6504078
20.8833332	4.0991998	-0.9449320
20.8833332	4.1479998	-0.9203887
20.8833332	4.1967998	-0.7976699
20.8833332	4.2455997	-0.8099418
20.8833332	4.2943997	-0.7179031
20.8833332	4.3431997	-0.6197281
20.8833332	4.3919997	-0.6565437
20.8833332	4.4407997	-0.6013203
20.8833332	4.4895997	-0.6258640
20.8833332	4.5383997	-0.6381359
20.8833332	4.5871997	-0.6626797
20.8833332	4.6359997	-0.6626797
20.8833332	4.6847997	-0.7853980
20.8833332	4.7336001	-0.7792621
20.8833332	4.7824001	-0.7547188
20.8833332	4.8312001	-0.7485828
20.8833332	4.8800001	-0.7608547
20.8833332	4.9288001	-0.7669902
20.8833332	4.9776001	613586.0000000
20.8833332	5.0264001	-0.6626797
20.8833332	5.0752001	-0.6626797
20.8833332	5.1240001	613586.0000000
20.8833332	5.1728001	613586.0000000
20.8833332	5.2216001	613586.0000000
20.8833332	5.2704000	613586.0000000
20.8833332	5.3192000	-1.4419422
20.8833332	5.3680000	-1.5953403
20.8833332	5.4168000	613586.0000000
20.8833332	5.4656000	613586.0000000
20.8833332	5.5144000	613586.0000000
20.8833332	5.5632000	613586.0000000
20.8833332	5.6120000	613586.0000000
20.8833332	5.6608000	613586.0000000
20.8833332	5.7096000	613586.0000000
20.8833332	5.7584000	613586.0000000
20.8833332	5.8072000	613586.0000000
20.8833332	5.8559999	613586.0000000
20.8833332	5.9047999	613586.0000000
20.8833332	5.9535999	613586.0000000
20.8833332	6.0023999	613586.0000000
20.8999996	0.2928000	-2.0064468
20.8999996	0.3416000	-1.9757671
20.8999996	0.3904000	-1.8100972
20.8999996	0.4392000	-1.7978253
20.8999996	0.4880000	-1.7978253
20.8999996	0.5368000	-1.3805828
20.8999996	0.5856000	-1.2578645
20.8999996	0.6344000	-1.2578645
20.8999996	0.6832000	-1.2149129

20.8999996	0.7320000	-1.2578645
20.8999996	0.7808000	-1.0860586
20.8999996	0.8296000	-1.2026410
20.8999996	0.8784000	-1.2762718
20.8999996	0.9272000	-1.1842332
20.8999996	0.9760000	-1.1780972
20.8999996	1.0247999	-1.1719613
20.8999996	1.0735999	-1.1842332
20.8999996	1.1223999	-1.2026410
20.8999996	1.1711999	-1.2087770
20.8999996	1.2200000	-1.2271848
20.8999996	1.2688000	-1.2455926
20.8999996	1.3176000	-1.2455926
20.8999996	1.3664000	-1.1780972
20.8999996	1.4152000	-1.2517285
20.8999996	1.4640000	-1.1290097
20.8999996	1.5128000	-1.1535535
20.8999996	1.5616000	-1.1658254
20.8999996	1.6104000	-1.1842332
20.8999996	1.6592000	-1.2885437
20.8999996	1.7079999	-1.2885437
20.8999996	1.7567999	-1.2946796
20.8999996	1.8055999	-1.3314953
20.8999996	1.8543999	-1.3314953
20.8999996	1.9031999	-1.3744469
20.8999996	1.9519999	-1.2455926
20.8999996	2.0007999	-1.3989906
20.8999996	2.0495999	-1.3989906
20.8999996	2.0983999	-1.3989906
20.8999996	2.1471999	-1.3989906
20.8999996	2.1959999	-1.3192234
20.8999996	2.2447999	-1.3192234
20.8999996	2.2935998	-1.2455926
20.8999996	2.3423998	-1.2455926
20.8999996	2.3912001	-1.2455926
20.8999996	2.4400001	-1.1903691
20.8999996	2.4888000	-1.1965051
20.8999996	2.5376000	-1.1903691
20.8999996	2.5864000	-1.1903691
20.8999996	2.6352000	-1.5830684
20.8999996	2.6840000	-1.5892043
20.8999996	2.7328000	-1.0921946
20.8999996	2.7816000	-1.0799227
20.8999996	2.8304000	-1.0185633
20.8999996	2.8792000	-1.0124273
20.8999996	2.9280000	-0.7547188
20.8999996	2.9768000	-0.7485828
20.8999996	3.0256000	-0.7424469
20.8999996	3.0743999	-0.4540582
20.8999996	3.1231999	-0.1718059
20.8999996	3.1719999	-0.4540582
20.8999996	3.2207999	-0.7117672
20.8999996	3.2695999	-0.8038058
20.8999996	3.3183999	-0.8713012
20.8999996	3.3671999	-0.8038058
20.8999996	3.4159999	-0.8038058
20.8999996	3.4647999	-0.8160777

20.8999996	3.5135999	-1.2394567
20.8999996	3.5623999	-1.0431070
20.8999996	3.6111999	-1.0369711
20.8999996	3.6599998	-0.7792621
20.8999996	3.7087998	-0.5890489
20.8999996	3.7575998	-0.5890489
20.8999996	3.8063998	-0.4786019
20.8999996	3.8551998	-0.4724660
20.8999996	3.9039998	-0.4540582
20.8999996	3.9527998	-0.4540582
20.8999996	4.0016003	-0.3313398
20.8999996	4.0503998	-0.3067961
20.8999996	4.0991998	-0.2761164
20.8999996	4.1479998	-0.1840777
20.8999996	4.1967998	-0.1902137
20.8999996	4.2455997	0.0000000
20.8999996	4.2943997	0.0368156
20.8999996	4.3431997	0.0306797
20.8999996	4.3919997	0.1779418
20.8999996	4.4407997	0.1718059
20.8999996	4.4895997	0.2699804
20.8999996	4.5383997	0.2699804
20.8999996	4.5871997	0.3067961
20.8999996	4.6359997	0.4479222
20.8999996	4.6847997	0.5522332
20.8999996	4.7336001	0.3742914
20.8999996	4.7824001	0.3681555
20.8999996	4.8312001	0.2761164
20.8999996	4.8800001	0.2761164
20.8999996	4.9288001	0.3006601
20.8999996	4.9776001	0.3620195
20.8999996	5.0264001	0.5890489
20.8999996	5.0752001	0.1104465
20.8999996	5.1240001	0.4970098
20.8999996	5.1728001	0.5092816
20.8999996	5.2216001	0.3988352
20.8999996	5.2704000	0.4111071
20.8999996	5.3192000	613586.0000000
20.8999996	5.3680000	613586.0000000
20.8999996	5.4168000	0.1472621
20.8999996	5.4656000	-0.0122719
20.8999996	5.5144000	613586.0000000
20.8999996	5.5632000	613586.0000000
20.8999996	5.6120000	613586.0000000
20.8999996	5.6608000	613586.0000000
20.8999996	5.7096000	613586.0000000
20.8999996	5.7584000	613586.0000000
20.8999996	5.8072000	613586.0000000
20.8999996	5.8559999	613586.0000000
20.8999996	5.9047999	613586.0000000
20.8999996	5.9535999	613586.0000000
20.8999996	6.0023999	613586.0000000
20.9166660	0.2928000	-2.0739422
20.9166660	0.3416000	-1.2885437
20.9166660	0.3904000	-1.3683109
20.9166660	0.4392000	-1.4480777
20.9166660	0.4880000	-1.4358063

20.9166660	0.5368000	-1.3314953
20.9166660	0.5856000	-1.2394567
20.9166660	0.6344000	-1.2517285
20.9166660	0.6832000	-1.1719613
20.9166660	0.7320000	-1.2946796
20.9166660	0.7808000	-1.4112625
20.9166660	0.8296000	-1.3989906
20.9166660	0.8784000	-1.3867188
20.9166660	0.9272000	-1.3314953
20.9166660	0.9760000	-1.2824078
20.9166660	1.0247999	-1.2517285
20.9166660	1.0735999	-1.3069515
20.9166660	1.1223999	-1.3192234
20.9166660	1.1711999	-1.3805828
20.9166660	1.2200000	-1.3805828
20.9166660	1.2688000	-1.3867188
20.9166660	1.3176000	-1.4358063
20.9166660	1.3664000	-1.4480777
20.9166660	1.4152000	-1.4603496
20.9166660	1.4640000	-1.4848933
20.9166660	1.5128000	-1.3867188
20.9166660	1.5616000	-1.4112625
20.9166660	1.6104000	-1.4173985
20.9166660	1.6592000	-1.4112625
20.9166660	1.7079999	-1.4173985
20.9166660	1.7567999	-1.3314953
20.9166660	1.8055999	-1.3314953
20.9166660	1.8543999	-1.3805828
20.9166660	1.9031999	-1.3805828
20.9166660	1.9519999	-1.4480777
20.9166660	2.0007999	-1.4480777
20.9166660	2.0495999	-1.4480777
20.9166660	2.0983999	-1.5339808
20.9166660	2.1471999	-1.4235344
20.9166660	2.1959999	-1.4910293
20.9166660	2.2447999	-1.2640004
20.9166660	2.2935998	-1.2640004
20.9166660	2.3423998	-1.3069515
20.9166660	2.3912001	-1.3989906
20.9166660	2.4400001	-1.4664855
20.9166660	2.4888000	-1.2885437
20.9166660	2.5376000	-1.4787574
20.9166660	2.5864000	-1.4480777
20.9166660	2.6352000	-1.5585246
20.9166660	2.6840000	-1.5707965
20.9166660	2.7328000	-1.2640004
20.9166660	2.7816000	-1.2578645
20.9166660	2.8304000	-1.3253593
20.9166660	2.8792000	-1.2762718
20.9166660	2.9280000	-1.2762718
20.9166660	2.9768000	-1.3192234
20.9166660	3.0256000	-1.3192234
20.9166660	3.0743999	-1.2517285
20.9166660	3.1231999	-1.1903691
20.9166660	3.1719999	-1.4664855
20.9166660	3.2207999	-1.4664855
20.9166660	3.2695999	-1.4848933

20.9166660	3.3183999	-1.3192234
20.9166660	3.3671999	-1.9819031
20.9166660	3.4159999	-1.9757671
20.9166660	3.4647999	-2.4359615
20.9166660	3.5135999	-2.4359615
20.9166660	3.5623999	-1.4480777
20.9166660	3.6111999	-1.2885437
20.9166660	3.6599998	-1.0983305
20.9166660	3.7087998	-1.2824078
20.9166660	3.7575998	-1.1965051
20.9166660	3.8063998	-1.4848933
20.9166660	3.8551998	-1.4787574
20.9166660	3.9039998	-1.5585246
20.9166660	3.9527998	-1.6137476
20.9166660	4.0016003	-1.6137476
20.9166660	4.0503998	-1.5278449
20.9166660	4.0991998	-1.5401168
20.9166660	4.1479998	-1.6198835
20.9166660	4.1967998	-1.6198835
20.9166660	4.2455997	-1.6137476
20.9166660	4.2943997	-1.5646605
20.9166660	4.3431997	-1.5646605
20.9166660	4.3919997	-1.5094371
20.9166660	4.4407997	-1.5094371
20.9166660	4.4895997	-1.4542136
20.9166660	4.5383997	-1.4726214
20.9166660	4.5871997	-1.3683109
20.9166660	4.6359997	-1.4726214
20.9166660	4.6847997	-1.4480777
20.9166660	4.7336001	-1.3560390
20.9166660	4.7824001	-1.3683109
20.9166660	4.8312001	-1.3744469
20.9166660	4.8800001	-1.2578645
20.9166660	4.9288001	-1.2517285
20.9166660	4.9776001	-1.0001554
20.9166660	5.0264001	-1.1842332
20.9166660	5.0752001	-0.8651652
20.9166660	5.1240001	-0.7915339
20.9166660	5.1728001	613586.0000000
20.9166660	5.2216001	-0.5951843
20.9166660	5.2704000	-0.4786019
20.9166660	5.3192000	613586.0000000
20.9166660	5.3680000	-0.4601941
20.9166660	5.4168000	-0.5215535
20.9166660	5.4656000	613586.0000000
20.9166660	5.5144000	613586.0000000
20.9166660	5.5632000	613586.0000000
20.9166660	5.6120000	613586.0000000
20.9166660	5.6608000	613586.0000000
20.9166660	5.7096000	613586.0000000
20.9166660	5.7584000	613586.0000000
20.9166660	5.8072000	613586.0000000
20.9166660	5.8559999	613586.0000000
20.9166660	5.9047999	613586.0000000
20.9166660	5.9535999	613586.0000000
20.9166660	6.0023999	613586.0000000
20.9333324	0.2928000	-1.8469129

20.9333324	0.3416000	-1.8837285
20.9333324	0.3904000	-1.9512239
20.9333324	0.4392000	-1.9512239
20.9333324	0.4880000	-1.8530488
20.9333324	0.5368000	-1.8469129
20.9333324	0.5856000	-1.8469129
20.9333324	0.6344000	-1.7978253
20.9333324	0.6832000	-1.7978253
20.9333324	0.7320000	-1.6076121
20.9333324	0.7808000	-1.5033011
20.9333324	0.8296000	-1.4296703
20.9333324	0.8784000	-1.5707965
20.9333324	0.9272000	-1.3989906
20.9333324	0.9760000	-1.3867188
20.9333324	1.0247999	-1.4910293
20.9333324	1.0735999	-1.4173985
20.9333324	1.1223999	-1.5462527
20.9333324	1.1711999	-1.6382914
20.9333324	1.2200000	-1.2271848
20.9333324	1.2688000	-1.5830684
20.9333324	1.3176000	-1.5217090
20.9333324	1.3664000	-1.5278449
20.9333324	1.4152000	-1.6382914
20.9333324	1.4640000	-1.5646605
20.9333324	1.5128000	-1.3560390
20.9333324	1.5616000	-1.3560390
20.9333324	1.6104000	-1.5953403
20.9333324	1.6592000	-1.6014762
20.9333324	1.7079999	-1.2701364
20.9333324	1.7567999	-1.4358063
20.9333324	1.8055999	-1.5155730
20.9333324	1.8543999	-1.5033011
20.9333324	1.9031999	-1.4542136
20.9333324	1.9519999	-1.5523887
20.9333324	2.0007999	-1.6628351
20.9333324	2.0495999	-1.6628351
20.9333324	2.0983999	-1.5830684
20.9333324	2.1471999	-1.5830684
20.9333324	2.1959999	-1.6321554
20.9333324	2.2447999	-1.6014762
20.9333324	2.2935998	-1.7426023
20.9333324	2.3423998	-1.7180586
20.9333324	2.3912001	-1.7426023
20.9333324	2.4400001	-1.7487383
20.9333324	2.4888000	-1.7794180
20.9333324	2.5376000	-1.7119226
20.9333324	2.5864000	-1.6812429
20.9333324	2.6352000	-1.5523887
20.9333324	2.6840000	-1.3989906
20.9333324	2.7328000	-1.3928547
20.9333324	2.7816000	-1.2271848
20.9333324	2.8304000	-1.2210488
20.9333324	2.8792000	-1.2210488
20.9333324	2.9280000	-1.0799227
20.9333324	2.9768000	-0.6810875
20.9333324	3.0256000	-0.6135921
20.9333324	3.0743999	-0.4295144

20.9333324	3.1231999	-0.4356503
20.9333324	3.1719999	-0.2699804
20.9333324	3.2207999	-0.8897090
20.9333324	3.2695999	-1.0553789
20.9333324	3.3183999	-1.0615149
20.9333324	3.3671999	-1.2517285
20.9333324	3.4159999	-1.2517285
20.9333324	3.4647999	-1.5707965
20.9333324	3.5135999	-1.2824078
20.9333324	3.5623999	-1.2824078
20.9333324	3.6111999	-1.3314953
20.9333324	3.6599998	-1.7855535
20.9333324	3.7087998	-1.7732821
20.9333324	3.7575998	-1.6628351
20.9333324	3.8063998	-1.5707965
20.9333324	3.8551998	-1.4358063
20.9333324	3.9039998	-1.4419422
20.9333324	3.9527998	-1.3314953
20.9333324	4.0016003	-1.4358063
20.9333324	4.0503998	-1.4235344
20.9333324	4.0991998	-1.2885437
20.9333324	4.1479998	-1.2701364
20.9333324	4.1967998	-1.3130875
20.9333324	4.2455997	-1.3192234
20.9333324	4.2943997	-1.3253593
20.9333324	4.3431997	-1.3253593
20.9333324	4.3919997	-1.2824078
20.9333324	4.4407997	-1.2824078
20.9333324	4.4895997	-1.2824078
20.9333324	4.5383997	-1.2946796
20.9333324	4.5871997	-1.2517285
20.9333324	4.6359997	-1.2394567
20.9333324	4.6847997	-1.2333207
20.9333324	4.7336001	-1.2333207
20.9333324	4.7824001	-1.2394567
20.9333324	4.8312001	613586.0000000
20.9333324	4.8800001	-1.1167378
20.9333324	4.9288001	-1.1044660
20.9333324	4.9776001	-1.0860586
20.9333324	5.0264001	-0.9326606
20.9333324	5.0752001	-1.1719613
20.9333324	5.1240001	-1.0369711
20.9333324	5.1728001	-1.0492430
20.9333324	5.2216001	-1.0737867
20.9333324	5.2704000	613586.0000000
20.9333324	5.3192000	-1.3008156
20.9333324	5.3680000	-1.3621750
20.9333324	5.4168000	-1.3621750
20.9333324	5.4656000	613586.0000000
20.9333324	5.5144000	613586.0000000
20.9333324	5.5632000	613586.0000000
20.9333324	5.6120000	613586.0000000
20.9333324	5.6608000	613586.0000000
20.9333324	5.7096000	613586.0000000
20.9333324	5.7584000	613586.0000000
20.9333324	5.8072000	613586.0000000
20.9333324	5.8559999	613586.0000000

20.9333324	5.9047999	613586.0000000
20.9333324	5.9535999	613586.0000000
20.9333324	6.0023999	613586.0000000
20.9500008	0.2928000	-1.3621750
20.9500008	0.3416000	-1.2026410
20.9500008	0.3904000	-1.5769324
20.9500008	0.4392000	-1.8530488
20.9500008	0.4880000	-1.3560390
20.9500008	0.5368000	-1.3560390
20.9500008	0.5856000	-1.1842332
20.9500008	0.6344000	-1.4296703
20.9500008	0.6832000	-1.4419422
20.9500008	0.7320000	-1.5339808
20.9500008	0.7808000	-1.6382914
20.9500008	0.8296000	-1.5339808
20.9500008	0.8784000	-1.5339808
20.9500008	0.9272000	-1.5339808
20.9500008	0.9760000	-1.5585246
20.9500008	1.0247999	-1.4235344
20.9500008	1.0735999	-1.5462527
20.9500008	1.1223999	-1.4051266
20.9500008	1.1711999	-1.4726214
20.9500008	1.2200000	-1.1842332
20.9500008	1.2688000	-1.4235344
20.9500008	1.3176000	-1.4173985
20.9500008	1.3664000	-1.3744469
20.9500008	1.4152000	-1.3130875
20.9500008	1.4640000	-1.2946796
20.9500008	1.5128000	-1.3192234
20.9500008	1.5616000	-1.3314953
20.9500008	1.6104000	-1.2271848
20.9500008	1.6592000	-1.4542136
20.9500008	1.7079999	-1.4419422
20.9500008	1.7567999	-1.3376312
20.9500008	1.8055999	-1.3499031
20.9500008	1.8543999	-1.3499031
20.9500008	1.9031999	-1.2824078
20.9500008	1.9519999	-1.3192234
20.9500008	2.0007999	-1.3805828
20.9500008	2.0495999	-1.3621750
20.9500008	2.0983999	-1.3621750
20.9500008	2.1471999	-1.4419422
20.9500008	2.1959999	-1.1351457
20.9500008	2.2447999	-1.1412816
20.9500008	2.2935998	-1.3008156
20.9500008	2.3423998	-1.1842332
20.9500008	2.3912001	-1.2026410
20.9500008	2.4400001	-1.2394567
20.9500008	2.4888000	-1.2455926
20.9500008	2.5376000	-1.2394567
20.9500008	2.5864000	-1.2271848
20.9500008	2.6352000	-2.1966605
20.9500008	2.6840000	-1.6137476
20.9500008	2.7328000	-1.1228738
20.9500008	2.7816000	-1.4664855
20.9500008	2.8304000	-1.6014762
20.9500008	2.8792000	-1.6444273

20.9500008	2.9280000	-1.4848933
20.9500008	2.9768000	-1.4910293
20.9500008	3.0256000	-1.4173985
20.9500008	3.0743999	-1.4296703
20.9500008	3.1231999	-1.2087770
20.9500008	3.1719999	-1.2149129
20.9500008	3.2207999	-1.2149129
20.9500008	3.2695999	-1.2149129
20.9500008	3.3183999	-1.2149129
20.9500008	3.3671999	-0.8344855
20.9500008	3.4159999	-0.8406215
20.9500008	3.4647999	-0.8283496
20.9500008	3.5135999	-0.8222136
20.9500008	3.5623999	-0.8467574
20.9500008	3.6111999	-0.7731261
20.9500008	3.6599998	-0.5583692
20.9500008	3.7087998	-0.5522332
20.9500008	3.7575998	-0.8713012
20.9500008	3.8063998	-0.8222136
20.9500008	3.8551998	-0.8713012
20.9500008	3.9039998	-0.9019809
20.9500008	3.9527998	-0.9142528
20.9500008	4.0016003	-0.6933594
20.9500008	4.0503998	-0.7056313
20.9500008	4.0991998	-0.5890489
20.9500008	4.1479998	-0.5767770
20.9500008	4.1967998	-0.5645051
20.9500008	4.2455997	-0.5583692
20.9500008	4.2943997	-0.9449320
20.9500008	4.3431997	-0.6749516
20.9500008	4.3919997	-0.6933594
20.9500008	4.4407997	-0.7547188
20.9500008	4.4895997	-0.6626797
20.9500008	4.5383997	-0.6442719
20.9500008	4.5871997	-0.6258640
20.9500008	4.6359997	-0.4908738
20.9500008	4.6847997	-0.6197281
20.9500008	4.7336001	-0.6013203
20.9500008	4.7824001	-0.5583692
20.9500008	4.8312001	-0.5522332
20.9500008	4.8800001	-0.5829129
20.9500008	4.9288001	-0.5767770
20.9500008	4.9776001	-0.8160777
20.9500008	5.0264001	-0.8222136
20.9500008	5.0752001	-0.8160777
20.9500008	5.1240001	-0.7915339
20.9500008	5.1728001	-0.6504078
20.9500008	5.2216001	-0.5460973
20.9500008	5.2704000	-0.5767770
20.9500008	5.3192000	613586.0000000
20.9500008	5.3680000	-0.5031457
20.9500008	5.4168000	613586.0000000
20.9500008	5.4656000	-0.5645051
20.9500008	5.5144000	-0.5645051
20.9500008	5.5632000	-0.3988352
20.9500008	5.6120000	-0.3865633
20.9500008	5.6608000	-0.3681555

20.9500008	5.7096000	613586.0000000
20.9500008	5.7584000	613586.0000000
20.9500008	5.8072000	613586.0000000
20.9500008	5.8559999	613586.0000000
20.9500008	5.9047999	613586.0000000
20.9500008	5.9535999	613586.0000000
20.9500008	6.0023999	613586.0000000
20.9666672	0.2928000	-1.5953403
20.9666672	0.3416000	-1.6505632
20.9666672	0.3904000	-1.6444273
20.9666672	0.4392000	-1.4051266
20.9666672	0.4880000	-1.4051266
20.9666672	0.5368000	-1.3499031
20.9666672	0.5856000	-1.4419422
20.9666672	0.6344000	-1.4910293
20.9666672	0.6832000	-1.1903691
20.9666672	0.7320000	-1.1842332
20.9666672	0.7808000	-1.1596894
20.9666672	0.8296000	-1.0492430
20.9666672	0.8784000	-1.0676508
20.9666672	0.9272000	-1.1351457
20.9666672	0.9760000	-1.0676508
20.9666672	1.0247999	-1.0676508
20.9666672	1.0735999	-1.2701364
20.9666672	1.1223999	-1.2578645
20.9666672	1.1711999	-1.2762718
20.9666672	1.2200000	-1.2885437
20.9666672	1.2688000	-1.1780972
20.9666672	1.3176000	-1.4235344
20.9666672	1.3664000	-1.2762718
20.9666672	1.4152000	-1.3928547
20.9666672	1.4640000	-1.6444273
20.9666672	1.5128000	-1.4419422
20.9666672	1.5616000	-1.3376312
20.9666672	1.6104000	-1.3376312
20.9666672	1.6592000	-1.3130875
20.9666672	1.7079999	-1.5155730
20.9666672	1.7567999	-1.5155730
20.9666672	1.8055999	-1.1965051
20.9666672	1.8543999	-1.1044660
20.9666672	1.9031999	-1.3560390
20.9666672	1.9519999	-1.1596894
20.9666672	2.0007999	-1.4542136
20.9666672	2.0495999	-1.4603496
20.9666672	2.0983999	-1.3560390
20.9666672	2.1471999	-1.3499031
20.9666672	2.1959999	-1.2762718
20.9666672	2.2447999	-1.1535535
20.9666672	2.2935998	-1.2455926
20.9666672	2.3423998	-1.2946796
20.9666672	2.3912001	-1.3805828
20.9666672	2.4400001	-1.3744469
20.9666672	2.4888000	-1.2149129
20.9666672	2.5376000	-1.2149129
20.9666672	2.5864000	-1.2946796
20.9666672	2.6352000	-1.3069515
20.9666672	2.6840000	-1.6260195

20.9666672	2.7328000	-0.7485828
20.9666672	2.7816000	-0.7301750
20.9666672	2.8304000	-0.7301750
20.9666672	2.8792000	-0.6074562
20.9666672	2.9280000	-0.4970098
20.9666672	2.9768000	-0.4724660
20.9666672	3.0256000	-0.5276895
20.9666672	3.0743999	-0.4847379
20.9666672	3.1231999	-0.4847379
20.9666672	3.1719999	-0.7424469
20.9666672	3.2207999	-0.7424469
20.9666672	3.2695999	-0.6442719
20.9666672	3.3183999	-0.6381359
20.9666672	3.3671999	-0.7853980
20.9666672	3.4159999	-1.0246992
20.9666672	3.4647999	-1.0308352
20.9666672	3.5135999	-1.0369711
20.9666672	3.5623999	-1.0431070
20.9666672	3.6111999	-0.9940195
20.9666672	3.6599998	-0.8774371
20.9666672	3.7087998	-0.7117672
20.9666672	3.7575998	-0.8038058
20.9666672	3.8063998	-0.8222136
20.9666672	3.8551998	-0.8283496
20.9666672	3.9039998	-0.8283496
20.9666672	3.9527998	-0.7731261
20.9666672	4.0016003	-0.8344855
20.9666672	4.0503998	-0.9387960
20.9666672	4.0991998	-0.6442719
20.9666672	4.1479998	-0.6442719
20.9666672	4.1967998	-0.8835731
20.9666672	4.2455997	-0.8774371
20.9666672	4.2943997	-0.9878836
20.9666672	4.3431997	-0.8160777
20.9666672	4.3919997	-0.8038058
20.9666672	4.4407997	-0.8283496
20.9666672	4.4895997	-0.8958449
20.9666672	4.5383997	-0.9633398
20.9666672	4.5871997	-0.9019809
20.9666672	4.6359997	-0.8528934
20.9666672	4.6847997	-0.8528934
20.9666672	4.7336001	-0.8467574
20.9666672	4.7824001	-0.8897090
20.9666672	4.8312001	-0.8958449
20.9666672	4.8800001	-1.0369711
20.9666672	4.9288001	-0.8713012
20.9666672	4.9776001	-0.7853980
20.9666672	5.0264001	-0.7915339
20.9666672	5.0752001	-0.8160777
20.9666672	5.1240001	-0.6626797
20.9666672	5.1728001	-0.6565437
20.9666672	5.2216001	-0.6442719
20.9666672	5.2704000	-0.5890489
20.9666672	5.3192000	-0.6381359
20.9666672	5.3680000	-0.6320000
20.9666672	5.4168000	-0.6135921
20.9666672	5.4656000	-0.7240391

20.9666672	5.5144000	-0.6565437
20.9666672	5.5632000	613586.0000000
20.9666672	5.6120000	613586.0000000
20.9666672	5.6608000	613586.0000000
20.9666672	5.7096000	613586.0000000
20.9666672	5.7584000	613586.0000000
20.9666672	5.8072000	613586.0000000
20.9666672	5.8559999	613586.0000000
20.9666672	5.9047999	613586.0000000
20.9666672	5.9535999	613586.0000000
20.9666672	6.0023999	613586.0000000
20.9833336	0.2928000	-1.7426023
20.9833336	0.3416000	-1.1719613
20.9833336	0.3904000	-1.1658254
20.9833336	0.4392000	-1.1658254
20.9833336	0.4880000	-1.5830684
20.9833336	0.5368000	-1.4787574
20.9833336	0.5856000	-1.5892043
20.9833336	0.6344000	-1.4542136
20.9833336	0.6832000	-1.4480777
20.9833336	0.7320000	-1.2271848
20.9833336	0.7808000	-1.2578645
20.9833336	0.8296000	-1.3376312
20.9833336	0.8784000	-1.3253593
20.9833336	0.9272000	-1.2394567
20.9833336	0.9760000	-1.3069515
20.9833336	1.0247999	-1.2333207
20.9833336	1.0735999	-1.2271848
20.9833336	1.1223999	-1.1044660
20.9833336	1.1711999	-1.0983305
20.9833336	1.2200000	-1.3805828
20.9833336	1.2688000	-1.3253593
20.9833336	1.3176000	-1.3130875
20.9833336	1.3664000	-1.3928547
20.9833336	1.4152000	-1.4480777
20.9833336	1.4640000	-1.4910293
20.9833336	1.5128000	-1.4848933
20.9833336	1.5616000	-1.2762718
20.9833336	1.6104000	-1.2824078
20.9833336	1.6592000	-1.3499031
20.9833336	1.7079999	-1.2762718
20.9833336	1.7567999	-1.2762718
20.9833336	1.8055999	-1.1106019
20.9833336	1.8543999	-1.2640004
20.9833336	1.9031999	-1.2701364
20.9833336	1.9519999	-1.3867188
20.9833336	2.0007999	-1.3867188
20.9833336	2.0495999	-1.4480777
20.9833336	2.0983999	-1.5094371
20.9833336	2.1471999	-1.6566992
20.9833336	2.1959999	-1.5707965
20.9833336	2.2447999	-1.3928547
20.9833336	2.2935998	-1.3928547
20.9833336	2.3423998	-1.3192234
20.9833336	2.3912001	-1.3253593
20.9833336	2.4400001	-1.3560390
20.9833336	2.4888000	-1.4848933

20.9833336	2.5376000	-1.4848933
20.9833336	2.5864000	-1.3989906
20.9833336	2.6352000	-1.3989906
20.9833336	2.6840000	-1.3928547
20.9833336	2.7328000	-1.0553789
20.9833336	2.7816000	-1.0553789
20.9833336	2.8304000	-1.1596894
20.9833336	2.8792000	-1.1106019
20.9833336	2.9280000	-0.6994953
20.9833336	2.9768000	-0.7056313
20.9833336	3.0256000	-0.4970098
20.9833336	3.0743999	-0.5031457
20.9833336	3.1231999	-0.8099418
20.9833336	3.1719999	-0.8038058
20.9833336	3.2207999	-0.5092816
20.9833336	3.2695999	-0.4908738
20.9833336	3.3183999	-0.6565437
20.9833336	3.3671999	-0.7547188
20.9833336	3.4159999	-0.7485828
20.9833336	3.4647999	-0.8528934
20.9833336	3.5135999	-0.7117672
20.9833336	3.5623999	-0.7056313
20.9833336	3.6111999	-0.6626797
20.9833336	3.6599998	-0.8406215
20.9833336	3.7087998	-0.8160777
20.9833336	3.7575998	-0.8651652
20.9833336	3.8063998	-0.6810875
20.9833336	3.8551998	-0.6810875
20.9833336	3.9039998	-0.5399613
20.9833336	3.9527998	-0.5399613
20.9833336	4.0016003	-0.5338254
20.9833336	4.0503998	-0.5215535
20.9833336	4.0991998	-0.7731261
20.9833336	4.1479998	-0.6994953
20.9833336	4.1967998	-0.6688156
20.9833336	4.2455997	-0.6810875
20.9833336	4.2943997	-0.6810875
20.9833336	4.3431997	-0.7792621
20.9833336	4.3919997	-0.6258640
20.9833336	4.4407997	-0.6442719
20.9833336	4.4895997	-0.6994953
20.9833336	4.5383997	-0.6135921
20.9833336	4.5871997	-0.6381359
20.9833336	4.6359997	-0.8590293
20.9833336	4.6847997	-0.8651652
20.9833336	4.7336001	-0.8651652
20.9833336	4.7824001	-0.8651652
20.9833336	4.8312001	-0.5645051
20.9833336	4.8800001	-0.7363110
20.9833336	4.9288001	-0.4663301
20.9833336	4.9776001	-0.7117672
20.9833336	5.0264001	-0.5951843
20.9833336	5.0752001	-0.6013203
20.9833336	5.1240001	-0.6013203
20.9833336	5.1728001	-0.6074562
20.9833336	5.2216001	-0.7363110
20.9833336	5.2704000	613586.0000000

20.9833336	5.3192000	-0.6442719
20.9833336	5.3680000	-0.6504078
20.9833336	5.4168000	-0.5706410
20.9833336	5.4656000	613586.0000000
20.9833336	5.5144000	613586.0000000
20.9833336	5.5632000	613586.0000000
20.9833336	5.6120000	613586.0000000
20.9833336	5.6608000	613586.0000000
20.9833336	5.7096000	613586.0000000
20.9833336	5.7584000	613586.0000000
20.9833336	5.8072000	613586.0000000
20.9833336	5.8559999	613586.0000000
20.9833336	5.9047999	613586.0000000
20.9833336	5.9535999	613586.0000000
20.9833336	6.0023999	613586.0000000
21.0000000	0.2928000	-1.7610102
21.0000000	0.3416000	-1.6260195
21.0000000	0.3904000	-1.6382914
21.0000000	0.4392000	-1.5217090
21.0000000	0.4880000	-1.2824078
21.0000000	0.5368000	-1.2946796
21.0000000	0.5856000	-1.4910293
21.0000000	0.6344000	-1.4726214
21.0000000	0.6832000	-1.4726214
21.0000000	0.7320000	-1.3376312
21.0000000	0.7808000	-1.1474175
21.0000000	0.8296000	-1.1474175
21.0000000	0.8784000	-1.2455926
21.0000000	0.9272000	-1.2394567
21.0000000	0.9760000	-1.0308352
21.0000000	1.0247999	-1.0185633
21.0000000	1.0735999	-0.9940195
21.0000000	1.1223999	-1.8469129
21.0000000	1.1711999	-1.8346410
21.0000000	1.2200000	-1.2333207
21.0000000	1.2688000	-1.2271848
21.0000000	1.3176000	-1.2210488
21.0000000	1.3664000	-1.3008156
21.0000000	1.4152000	-1.1965051
21.0000000	1.4640000	-1.1965051
21.0000000	1.5128000	-1.2394567
21.0000000	1.5616000	-1.2394567
21.0000000	1.6104000	-1.2455926
21.0000000	1.6592000	-1.1474175
21.0000000	1.7079999	-1.2271848
21.0000000	1.7567999	-1.1965051
21.0000000	1.8055999	-1.3253593
21.0000000	1.8543999	-1.1167378
21.0000000	1.9031999	-1.4112625
21.0000000	1.9519999	-1.4112625
21.0000000	2.0007999	-1.3192234
21.0000000	2.0495999	-1.3192234
21.0000000	2.0983999	-1.0615149
21.0000000	2.1471999	-1.0983305
21.0000000	2.1959999	-1.1780972
21.0000000	2.2447999	-1.0369711
21.0000000	2.2935998	-1.0983305

21.0000000	2.3423998	-1.1474175
21.0000000	2.3912001	-1.1780972
21.0000000	2.4400001	-1.1106019
21.0000000	2.4888000	-1.1535535
21.0000000	2.5376000	-1.0676508
21.0000000	2.5864000	-1.0676508
21.0000000	2.6352000	-0.9694757
21.0000000	2.6840000	-1.1351457
21.0000000	2.7328000	-1.1106019
21.0000000	2.7816000	-1.1106019
21.0000000	2.8304000	-1.0615149
21.0000000	2.8792000	-1.0799227
21.0000000	2.9280000	-0.5890489
21.0000000	2.9768000	-0.6565437
21.0000000	3.0256000	-0.5951843
21.0000000	3.0743999	-0.8406215
21.0000000	3.1231999	-0.7240391
21.0000000	3.1719999	-0.7301750
21.0000000	3.2207999	-0.7240391
21.0000000	3.2695999	-0.9694757
21.0000000	3.3183999	-0.6442719
21.0000000	3.3671999	-1.0001554
21.0000000	3.4159999	-1.0001554
21.0000000	3.4647999	-0.4601941
21.0000000	3.5135999	-0.9817476
21.0000000	3.5623999	-1.1903691
21.0000000	3.6111999	-0.8038058
21.0000000	3.6599998	-0.9387960
21.0000000	3.7087998	-0.5399613
21.0000000	3.7575998	-0.6933594
21.0000000	3.8063998	-0.6565437
21.0000000	3.8551998	-0.5767770
21.0000000	3.9039998	-0.5031457
21.0000000	3.9527998	-0.8160777
21.0000000	4.0016003	-0.8099418
21.0000000	4.0503998	-0.8160777
21.0000000	4.0991998	-0.7976699
21.0000000	4.1479998	-0.7853980
21.0000000	4.1967998	-0.8038058
21.0000000	4.2455997	-0.8651652
21.0000000	4.2943997	-0.8222136
21.0000000	4.3431997	-0.8222136
21.0000000	4.3919997	-0.8222136
21.0000000	4.4407997	-0.6994953
21.0000000	4.4895997	-0.7731261
21.0000000	4.5383997	-0.7731261
21.0000000	4.5871997	-0.7608547
21.0000000	4.6359997	-0.7731261
21.0000000	4.6847997	-0.7915339
21.0000000	4.7336001	-0.8467574
21.0000000	4.7824001	-0.7301750
21.0000000	4.8312001	-0.6872234
21.0000000	4.8800001	-0.5092816
21.0000000	4.9288001	613586.0000000
21.0000000	4.9776001	613586.0000000
21.0000000	5.0264001	-0.4786019
21.0000000	5.0752001	613586.0000000

21.0000000	5.1240001	613586.0000000
21.0000000	5.1728001	613586.0000000
21.0000000	5.2216001	-0.7056313
21.0000000	5.2704000	-0.6074562
21.0000000	5.3192000	-0.6074562
21.0000000	5.3680000	613586.0000000
21.0000000	5.4168000	613586.0000000
21.0000000	5.4656000	613586.0000000
21.0000000	5.5144000	613586.0000000
21.0000000	5.5632000	613586.0000000
21.0000000	5.6120000	613586.0000000
21.0000000	5.6608000	613586.0000000
21.0000000	5.7096000	613586.0000000
21.0000000	5.7584000	613586.0000000
21.0000000	5.8072000	613586.0000000
21.0000000	5.8559999	613586.0000000
21.0000000	5.9047999	613586.0000000
21.0000000	5.9535999	613586.0000000
21.0000000	6.0023999	613586.0000000
21.0166664	0.2928000	-1.4726214
21.0166664	0.3416000	-1.1965051
21.0166664	0.3904000	-0.5951843
21.0166664	0.4392000	-1.3805828
21.0166664	0.4880000	-1.3805828
21.0166664	0.5368000	-1.1412816
21.0166664	0.5856000	-1.1474175
21.0166664	0.6344000	-1.1719613
21.0166664	0.6832000	-1.5401168
21.0166664	0.7320000	-1.5339808
21.0166664	0.7808000	-1.3621750
21.0166664	0.8296000	-1.3008156
21.0166664	0.8784000	-1.3253593
21.0166664	0.9272000	-1.3867188
21.0166664	0.9760000	-1.4603496
21.0166664	1.0247999	-1.3008156
21.0166664	1.0735999	-1.3744469
21.0166664	1.1223999	-1.3130875
21.0166664	1.1711999	-1.4664855
21.0166664	1.2200000	-1.3008156
21.0166664	1.2688000	-1.5278449
21.0166664	1.3176000	-1.5217090
21.0166664	1.3664000	-1.3805828
21.0166664	1.4152000	-1.5217090
21.0166664	1.4640000	-1.3560390
21.0166664	1.5128000	-1.4419422
21.0166664	1.5616000	-1.2517285
21.0166664	1.6104000	-1.1658254
21.0166664	1.6592000	-1.4419422
21.0166664	1.7079999	-1.2578645
21.0166664	1.7567999	-1.1903691
21.0166664	1.8055999	-1.1842332
21.0166664	1.8543999	-1.3867188
21.0166664	1.9031999	-1.3744469
21.0166664	1.9519999	-1.3008156
21.0166664	2.0007999	-1.3621750
21.0166664	2.0495999	-1.4112625
21.0166664	2.0983999	-1.2394567

21.0166664	2.1471999	-1.2333207
21.0166664	2.1959999	-1.2394567
21.0166664	2.2447999	-1.1965051
21.0166664	2.2935998	-1.2333207
21.0166664	2.3423998	-1.1106019
21.0166664	2.3912001	-1.1044660
21.0166664	2.4400001	-1.0983305
21.0166664	2.4888000	-1.0983305
21.0166664	2.5376000	-1.0921946
21.0166664	2.5864000	-0.9326606
21.0166664	2.6352000	-0.9817476
21.0166664	2.6840000	-0.9756117
21.0166664	2.7328000	-0.9756117
21.0166664	2.7816000	-0.8774371
21.0166664	2.8304000	-0.8774371
21.0166664	2.8792000	-0.9265246
21.0166664	2.9280000	-0.7976699
21.0166664	2.9768000	-0.8160777
21.0166664	3.0256000	-0.8160777
21.0166664	3.0743999	-0.8099418
21.0166664	3.1231999	-0.7363110
21.0166664	3.1719999	-0.8958449
21.0166664	3.2207999	-0.9019809
21.0166664	3.2695999	-0.8160777
21.0166664	3.3183999	-0.7608547
21.0166664	3.3671999	-0.8283496
21.0166664	3.4159999	-0.8406215
21.0166664	3.4647999	-0.7915339
21.0166664	3.5135999	-0.5890489
21.0166664	3.5623999	-0.5890489
21.0166664	3.6111999	-1.0001554
21.0166664	3.6599998	-1.1228738
21.0166664	3.7087998	-0.9203887
21.0166664	3.7575998	-0.9203887
21.0166664	3.8063998	-0.7976699
21.0166664	3.8551998	-0.9878836
21.0166664	3.9039998	-0.9756117
21.0166664	3.9527998	-0.7424469
21.0166664	4.0016003	-0.7363110
21.0166664	4.0503998	-0.7363110
21.0166664	4.0991998	-0.6749516
21.0166664	4.1479998	-0.9081168
21.0166664	4.1967998	-1.0246992
21.0166664	4.2455997	-0.9694757
21.0166664	4.2943997	-0.9756117
21.0166664	4.3431997	-0.8038058
21.0166664	4.3919997	-0.8099418
21.0166664	4.4407997	-0.8590293
21.0166664	4.4895997	-0.9019809
21.0166664	4.5383997	-0.8344855
21.0166664	4.5871997	-0.7179031
21.0166664	4.6359997	-0.7117672
21.0166664	4.6847997	-0.7056313
21.0166664	4.7336001	-0.7117672
21.0166664	4.7824001	613586.0000000
21.0166664	4.8312001	613586.0000000
21.0166664	4.8800001	613586.0000000

21.0166664	4.9288001	613586.0000000
21.0166664	4.9776001	613586.0000000
21.0166664	5.0264001	613586.0000000
21.0166664	5.0752001	-0.8222136
21.0166664	5.1240001	-0.8222136
21.0166664	5.1728001	613586.0000000
21.0166664	5.2216001	613586.0000000
21.0166664	5.2704000	613586.0000000
21.0166664	5.3192000	613586.0000000
21.0166664	5.3680000	613586.0000000
21.0166664	5.4168000	613586.0000000
21.0166664	5.4656000	613586.0000000
21.0166664	5.5144000	613586.0000000
21.0166664	5.5632000	613586.0000000
21.0166664	5.6120000	613586.0000000
21.0166664	5.6608000	613586.0000000
21.0166664	5.7096000	613586.0000000
21.0166664	5.7584000	-0.4172430
21.0166664	5.8072000	613586.0000000
21.0166664	5.8559999	613586.0000000
21.0166664	5.9047999	613586.0000000
21.0166664	5.9535999	613586.0000000
21.0166664	6.0023999	613586.0000000
21.0333328	0.2928000	-1.9573593
21.0333328	0.3416000	-2.0739422
21.0333328	0.3904000	-1.8960004
21.0333328	0.4392000	-1.4726214
21.0333328	0.4880000	-1.6996508
21.0333328	0.5368000	-1.3314953
21.0333328	0.5856000	-1.2946796
21.0333328	0.6344000	-1.2824078
21.0333328	0.6832000	-1.2701364
21.0333328	0.7320000	-1.3314953
21.0333328	0.7808000	-1.3376312
21.0333328	0.8296000	-1.3437672
21.0333328	0.8784000	-1.3560390
21.0333328	0.9272000	-1.6014762
21.0333328	0.9760000	-1.5830684
21.0333328	1.0247999	-1.4848933
21.0333328	1.0735999	-1.4910293
21.0333328	1.1223999	-1.6076121
21.0333328	1.1711999	-1.2578645
21.0333328	1.2200000	-1.2517285
21.0333328	1.2688000	-1.5585246
21.0333328	1.3176000	-1.5585246
21.0333328	1.3664000	-1.4296703
21.0333328	1.4152000	-1.4296703
21.0333328	1.4640000	-1.3253593
21.0333328	1.5128000	-1.3253593
21.0333328	1.5616000	-1.1106019
21.0333328	1.6104000	-1.1596894
21.0333328	1.6592000	-1.1535535
21.0333328	1.7079999	-1.2271848
21.0333328	1.7567999	-1.2271848
21.0333328	1.8055999	-1.3130875
21.0333328	1.8543999	-1.3130875
21.0333328	1.9031999	-1.1351457

21.0333328	1.9519999	-1.2701364
21.0333328	2.0007999	-1.2701364
21.0333328	2.0495999	-1.2762718
21.0333328	2.0983999	-1.4971652
21.0333328	2.1471999	-1.5094371
21.0333328	2.1959999	-1.4971652
21.0333328	2.2447999	-1.1965051
21.0333328	2.2935998	-1.0308352
21.0333328	2.3423998	-1.1903691
21.0333328	2.3912001	-0.9694757
21.0333328	2.4400001	-0.9633398
21.0333328	2.4888000	-0.9878836
21.0333328	2.5376000	-0.9019809
21.0333328	2.5864000	-1.0001554
21.0333328	2.6352000	-1.0001554
21.0333328	2.6840000	-1.0124273
21.0333328	2.7328000	-1.0246992
21.0333328	2.7816000	-1.1228738
21.0333328	2.8304000	-0.9940195
21.0333328	2.8792000	-1.0001554
21.0333328	2.9280000	-0.8038058
21.0333328	2.9768000	-1.0062914
21.0333328	3.0256000	-0.2208934
21.0333328	3.0743999	-0.3067961
21.0333328	3.1231999	-0.7117672
21.0333328	3.1719999	-0.7179031
21.0333328	3.2207999	-0.6626797
21.0333328	3.2695999	-0.6933594
21.0333328	3.3183999	-0.6933594
21.0333328	3.3671999	-0.5890489
21.0333328	3.4159999	-0.5829129
21.0333328	3.4647999	-0.5951843
21.0333328	3.5135999	-1.0921946
21.0333328	3.5623999	-0.7424469
21.0333328	3.6111999	-0.5890489
21.0333328	3.6599998	-0.5829129
21.0333328	3.7087998	-0.6872234
21.0333328	3.7575998	-0.5645051
21.0333328	3.8063998	-0.6749516
21.0333328	3.8551998	-0.6749516
21.0333328	3.9039998	-0.6074562
21.0333328	3.9527998	-0.6013203
21.0333328	4.0016003	-0.8774371
21.0333328	4.0503998	-0.6197281
21.0333328	4.0991998	-0.9142528
21.0333328	4.1479998	-0.9142528
21.0333328	4.1967998	-0.9019809
21.0333328	4.2455997	-0.9019809
21.0333328	4.2943997	-1.1474175
21.0333328	4.3431997	-1.0124273
21.0333328	4.3919997	-1.0185633
21.0333328	4.4407997	-1.1903691
21.0333328	4.4895997	-1.1106019
21.0333328	4.5383997	-1.0921946
21.0333328	4.5871997	-1.2149129
21.0333328	4.6359997	-1.1719613
21.0333328	4.6847997	-1.3008156

21.0333328	4.7336001	613586.0000000
21.0333328	4.7824001	613586.0000000
21.0333328	4.8312001	613586.0000000
21.0333328	4.8800001	-0.8528934
21.0333328	4.9288001	-0.9694757
21.0333328	4.9776001	613586.0000000
21.0333328	5.0264001	613586.0000000
21.0333328	5.0752001	-0.6626797
21.0333328	5.1240001	-0.6504078
21.0333328	5.1728001	613586.0000000
21.0333328	5.2216001	613586.0000000
21.0333328	5.2704000	613586.0000000
21.0333328	5.3192000	613586.0000000
21.0333328	5.3680000	613586.0000000
21.0333328	5.4168000	613586.0000000
21.0333328	5.4656000	613586.0000000
21.0333328	5.5144000	613586.0000000
21.0333328	5.5632000	613586.0000000
21.0333328	5.6120000	613586.0000000
21.0333328	5.6608000	613586.0000000
21.0333328	5.7096000	613586.0000000
21.0333328	5.7584000	613586.0000000
21.0333328	5.8072000	613586.0000000
21.0333328	5.8559999	613586.0000000
21.0333328	5.9047999	613586.0000000
21.0333328	5.9535999	613586.0000000
21.0333328	6.0023999	613586.0000000
21.0499992	0.2928000	-5.8168554
21.0499992	0.3416000	-0.9878836
21.0499992	0.3904000	-1.4480777
21.0499992	0.4392000	-1.6137476
21.0499992	0.4880000	-0.9142528
21.0499992	0.5368000	-0.9142528
21.0499992	0.5856000	-1.2885437
21.0499992	0.6344000	-1.0983305
21.0499992	0.6832000	-1.1474175
21.0499992	0.7320000	-1.5094371
21.0499992	0.7808000	-1.4296703
21.0499992	0.8296000	-1.3683109
21.0499992	0.8784000	-1.3437672
21.0499992	0.9272000	-1.3376312
21.0499992	0.9760000	-1.5953403
21.0499992	1.0247999	-1.6137476
21.0499992	1.0735999	-1.3744469
21.0499992	1.1223999	-1.3744469
21.0499992	1.1711999	-1.3805828
21.0499992	1.2200000	-1.3867188
21.0499992	1.2688000	-1.3867188
21.0499992	1.3176000	-1.3192234
21.0499992	1.3664000	-1.3805828
21.0499992	1.4152000	-1.2517285
21.0499992	1.4640000	-1.2087770
21.0499992	1.5128000	-1.2946796
21.0499992	1.5616000	-1.2210488
21.0499992	1.6104000	-1.0062914
21.0499992	1.6592000	-1.2271848
21.0499992	1.7079999	-1.2210488

21.0499992	1.7567999	-1.2149129
21.0499992	1.8055999	-1.1780972
21.0499992	1.8543999	-1.2026410
21.0499992	1.9031999	-1.3253593
21.0499992	1.9519999	-1.3314953
21.0499992	2.0007999	-1.3130875
21.0499992	2.0495999	-1.1228738
21.0499992	2.0983999	-1.2087770
21.0499992	2.1471999	-1.2087770
21.0499992	2.1959999	-0.8713012
21.0499992	2.2447999	-0.8651652
21.0499992	2.2935998	-0.8528934
21.0499992	2.3423998	-0.8528934
21.0499992	2.3912001	-0.9265246
21.0499992	2.4400001	-0.9203887
21.0499992	2.4888000	-0.8528934
21.0499992	2.5376000	-1.0369711
21.0499992	2.5864000	-1.0246992
21.0499992	2.6352000	-1.0062914
21.0499992	2.6840000	-0.9326606
21.0499992	2.7328000	-0.7547188
21.0499992	2.7816000	-0.1472621
21.0499992	2.8304000	-0.1472621
21.0499992	2.8792000	-0.4540582
21.0499992	2.9280000	-0.4540582
21.0499992	2.9768000	-0.8099418
21.0499992	3.0256000	-1.0246992
21.0499992	3.0743999	-1.0246992
21.0499992	3.1231999	-0.3436117
21.0499992	3.1719999	-0.3436117
21.0499992	3.2207999	-0.4233789
21.0499992	3.2695999	-0.4786019
21.0499992	3.3183999	-0.8099418
21.0499992	3.3671999	-0.8038058
21.0499992	3.4159999	-0.8835731
21.0499992	3.4647999	-0.6688156
21.0499992	3.5135999	-0.7056313
21.0499992	3.5623999	-0.7608547
21.0499992	3.6111999	-0.7547188
21.0499992	3.6599998	-0.6626797
21.0499992	3.7087998	-0.5645051
21.0499992	3.7575998	-0.5583692
21.0499992	3.8063998	-0.4724660
21.0499992	3.8551998	-0.5645051
21.0499992	3.9039998	-0.4417863
21.0499992	3.9527998	-0.4417863
21.0499992	4.0016003	-0.4049711
21.0499992	4.0503998	-0.9510679
21.0499992	4.0991998	-0.8528934
21.0499992	4.1479998	-0.8590293
21.0499992	4.1967998	-0.8344855
21.0499992	4.2455997	-0.7056313
21.0499992	4.2943997	-0.8099418
21.0499992	4.3431997	-0.7976699
21.0499992	4.3919997	-0.7853980
21.0499992	4.4407997	-0.8160777
21.0499992	4.4895997	-0.9019809

21.0499992	4.5383997	-0.8835731
21.0499992	4.5871997	-0.7669902
21.0499992	4.6359997	-0.7669902
21.0499992	4.6847997	-0.8099418
21.0499992	4.7336001	613586.0000000
21.0499992	4.7824001	613586.0000000
21.0499992	4.8312001	613586.0000000
21.0499992	4.8800001	-0.6197281
21.0499992	4.9288001	-0.6320000
21.0499992	4.9776001	613586.0000000
21.0499992	5.0264001	613586.0000000
21.0499992	5.0752001	613586.0000000
21.0499992	5.1240001	613586.0000000
21.0499992	5.1728001	613586.0000000
21.0499992	5.2216001	613586.0000000
21.0499992	5.2704000	613586.0000000
21.0499992	5.3192000	613586.0000000
21.0499992	5.3680000	613586.0000000
21.0499992	5.4168000	613586.0000000
21.0499992	5.4656000	613586.0000000
21.0499992	5.5144000	613586.0000000
21.0499992	5.5632000	613586.0000000
21.0499992	5.6120000	613586.0000000
21.0499992	5.6608000	613586.0000000
21.0499992	5.7096000	613586.0000000
21.0499992	5.7584000	613586.0000000
21.0499992	5.8072000	613586.0000000
21.0499992	5.8559999	613586.0000000
21.0499992	5.9047999	613586.0000000
21.0499992	5.9535999	613586.0000000
21.0499992	6.0023999	613586.0000000
21.0666676	0.2928000	-0.7731261
21.0666676	0.3416000	-1.3130875
21.0666676	0.3904000	-1.3499031
21.0666676	0.4392000	-1.3621750
21.0666676	0.4880000	-1.2210488
21.0666676	0.5368000	-1.2210488
21.0666676	0.5856000	-1.1351457
21.0666676	0.6344000	-1.1596894
21.0666676	0.6832000	-1.1903691
21.0666676	0.7320000	-1.0553789
21.0666676	0.7808000	-1.0676508
21.0666676	0.8296000	-1.0676508
21.0666676	0.8784000	-1.0860586
21.0666676	0.9272000	-1.1044660
21.0666676	0.9760000	-1.2210488
21.0666676	1.0247999	-1.2026410
21.0666676	1.0735999	-1.2026410
21.0666676	1.1223999	-1.3499031
21.0666676	1.1711999	-1.3621750
21.0666676	1.2200000	-1.3621750
21.0666676	1.2688000	-1.5830684
21.0666676	1.3176000	-1.4664855
21.0666676	1.3664000	-1.4603496
21.0666676	1.4152000	-1.3069515
21.0666676	1.4640000	-1.2701364
21.0666676	1.5128000	-1.1474175

21.0666676	1.5616000	-1.1474175
21.0666676	1.6104000	-1.1351457
21.0666676	1.6592000	-1.1965051
21.0666676	1.7079999	-1.1965051
21.0666676	1.7567999	-1.2026410
21.0666676	1.8055999	-1.1167378
21.0666676	1.8543999	-0.9265246
21.0666676	1.9031999	-0.9265246
21.0666676	1.9519999	-1.0553789
21.0666676	2.0007999	-1.0676508
21.0666676	2.0495999	-1.2149129
21.0666676	2.0983999	-1.2149129
21.0666676	2.1471999	-0.8099418
21.0666676	2.1959999	-0.7547188
21.0666676	2.2447999	-0.7669902
21.0666676	2.2935998	-0.7363110
21.0666676	2.3423998	-0.8038058
21.0666676	2.3912001	-0.7117672
21.0666676	2.4400001	-0.7117672
21.0666676	2.4888000	-0.7179031
21.0666676	2.5376000	-0.6565437
21.0666676	2.5864000	-0.5767770
21.0666676	2.6352000	-0.6688156
21.0666676	2.6840000	-0.6565437
21.0666676	2.7328000	-0.4540582
21.0666676	2.7816000	-0.4540582
21.0666676	2.8304000	-0.4663301
21.0666676	2.8792000	-0.4601941
21.0666676	2.9280000	-0.4601941
21.0666676	2.9768000	-0.8160777
21.0666676	3.0256000	-0.7547188
21.0666676	3.0743999	-0.7547188
21.0666676	3.1231999	-0.9326606
21.0666676	3.1719999	-0.8528934
21.0666676	3.2207999	-0.8713012
21.0666676	3.2695999	-0.7547188
21.0666676	3.3183999	-0.7547188
21.0666676	3.3671999	-0.7915339
21.0666676	3.4159999	-0.7853980
21.0666676	3.4647999	-0.9019809
21.0666676	3.5135999	-0.6688156
21.0666676	3.5623999	-0.7424469
21.0666676	3.6111999	-0.7363110
21.0666676	3.6599998	-0.6013203
21.0666676	3.7087998	-0.5951843
21.0666676	3.7575998	-0.5829129
21.0666676	3.8063998	-0.5154176
21.0666676	3.8551998	-0.5276895
21.0666676	3.9039998	-0.5215535
21.0666676	3.9527998	-0.8651652
21.0666676	4.0016003	-0.8651652
21.0666676	4.0503998	-0.9940195
21.0666676	4.0991998	-0.9203887
21.0666676	4.1479998	-0.9326606
21.0666676	4.1967998	-0.9633398
21.0666676	4.2455997	-1.0799227
21.0666676	4.2943997	-1.0799227

21.0666676	4.3431997	-1.0983305
21.0666676	4.3919997	-1.1106019
21.0666676	4.4407997	-1.2271848
21.0666676	4.4895997	-1.2087770
21.0666676	4.5383997	-1.1903691
21.0666676	4.5871997	-1.1903691
21.0666676	4.6359997	-1.2026410
21.0666676	4.6847997	-1.4112625
21.0666676	4.7336001	-1.2578645
21.0666676	4.7824001	613586.0000000
21.0666676	4.8312001	613586.0000000
21.0666676	4.8800001	613586.0000000
21.0666676	4.9288001	613586.0000000
21.0666676	4.9776001	613586.0000000
21.0666676	5.0264001	613586.0000000
21.0666676	5.0752001	613586.0000000
21.0666676	5.1240001	613586.0000000
21.0666676	5.1728001	-0.7485828
21.0666676	5.2216001	-0.7485828
21.0666676	5.2704000	-0.7485828
21.0666676	5.3192000	613586.0000000
21.0666676	5.3680000	613586.0000000
21.0666676	5.4168000	-0.8528934
21.0666676	5.4656000	-0.8528934
21.0666676	5.5144000	613586.0000000
21.0666676	5.5632000	613586.0000000
21.0666676	5.6120000	613586.0000000
21.0666676	5.6608000	613586.0000000
21.0666676	5.7096000	613586.0000000
21.0666676	5.7584000	613586.0000000
21.0666676	5.8072000	613586.0000000
21.0666676	5.8559999	613586.0000000
21.0666676	5.9047999	613586.0000000
21.0666676	5.9535999	613586.0000000
21.0666676	6.0023999	613586.0000000
21.0833340	0.2928000	-1.1290097
21.0833340	0.3416000	-1.1903691
21.0833340	0.3904000	-1.2517285
21.0833340	0.4392000	-1.2394567
21.0833340	0.4880000	-1.1474175
21.0833340	0.5368000	-1.3805828
21.0833340	0.5856000	-1.1719613
21.0833340	0.6344000	-1.3805828
21.0833340	0.6832000	-1.3253593
21.0833340	0.7320000	-1.4603496
21.0833340	0.7808000	-1.1535535
21.0833340	0.8296000	-1.2824078
21.0833340	0.8784000	-1.4664855
21.0833340	0.9272000	-1.2333207
21.0833340	0.9760000	-1.2333207
21.0833340	1.0247999	-1.2333207
21.0833340	1.0735999	-1.2333207
21.0833340	1.1223999	-0.9694757
21.0833340	1.1711999	-0.9572039
21.0833340	1.2200000	-1.1474175
21.0833340	1.2688000	-1.1658254
21.0833340	1.3176000	-1.2701364

21.0833340	1.3664000	-1.2701364
21.0833340	1.4152000	-1.3192234
21.0833340	1.4640000	-1.2333207
21.0833340	1.5128000	-1.2394567
21.0833340	1.5616000	-1.2517285
21.0833340	1.6104000	-1.1290097
21.0833340	1.6592000	-1.1412816
21.0833340	1.7079999	-1.3560390
21.0833340	1.7567999	-1.2946796
21.0833340	1.8055999	-1.3805828
21.0833340	1.8543999	-1.3744469
21.0833340	1.9031999	-1.0062914
21.0833340	1.9519999	-1.3621750
21.0833340	2.0007999	-1.3744469
21.0833340	2.0495999	-1.3621750
21.0833340	2.0983999	-1.0676508
21.0833340	2.1471999	-1.0860586
21.0833340	2.1959999	-1.2640004
21.0833340	2.2447999	-1.2578645
21.0833340	2.2935998	-1.0431070
21.0833340	2.3423998	-1.0308352
21.0833340	2.3912001	-1.0308352
21.0833340	2.4400001	-1.0369711
21.0833340	2.4888000	-1.0369711
21.0833340	2.5376000	-1.0369711
21.0833340	2.5864000	-1.0001554
21.0833340	2.6352000	-1.1474175
21.0833340	2.6840000	-1.0615149
21.0833340	2.7328000	-0.9449320
21.0833340	2.7816000	-0.9387960
21.0833340	2.8304000	-0.5399613
21.0833340	2.8792000	-0.2147574
21.0833340	2.9280000	-0.9203887
21.0833340	2.9768000	-0.9817476
21.0833340	3.0256000	-1.0185633
21.0833340	3.0743999	-1.0799227
21.0833340	3.1231999	-1.1780972
21.0833340	3.1719999	-1.2149129
21.0833340	3.2207999	-1.0737867
21.0833340	3.2695999	-1.1290097
21.0833340	3.3183999	-1.1290097
21.0833340	3.3671999	-0.9387960
21.0833340	3.4159999	-1.1658254
21.0833340	3.4647999	-0.9081168
21.0833340	3.5135999	-0.8283496
21.0833340	3.5623999	-0.6626797
21.0833340	3.6111999	-0.8897090
21.0833340	3.6599998	-0.9142528
21.0833340	3.7087998	-0.6994953
21.0833340	3.7575998	-0.8099418
21.0833340	3.8063998	-0.8099418
21.0833340	3.8551998	-1.2026410
21.0833340	3.9039998	-1.0308352
21.0833340	3.9527998	-1.0185633
21.0833340	4.0016003	-1.0615149
21.0833340	4.0503998	-1.0615149
21.0833340	4.0991998	-0.9756117

21.0833340	4.1479998	-0.8958449
21.0833340	4.1967998	-1.0308352
21.0833340	4.2455997	-0.9142528
21.0833340	4.2943997	-0.9142528
21.0833340	4.3431997	-0.9142528
21.0833340	4.3919997	-0.8651652
21.0833340	4.4407997	-0.8713012
21.0833340	4.4895997	-0.8713012
21.0833340	4.5383997	-0.8590293
21.0833340	4.5871997	-0.8590293
21.0833340	4.6359997	-0.7976699
21.0833340	4.6847997	-0.6810875
21.0833340	4.7336001	-0.5767770
21.0833340	4.7824001	-0.4601941
21.0833340	4.8312001	613586.0000000
21.0833340	4.8800001	613586.0000000
21.0833340	4.9288001	613586.0000000
21.0833340	4.9776001	613586.0000000
21.0833340	5.0264001	613586.0000000
21.0833340	5.0752001	-0.6626797
21.0833340	5.1240001	613586.0000000
21.0833340	5.1728001	-0.6074562
21.0833340	5.2216001	-0.6320000
21.0833340	5.2704000	613586.0000000
21.0833340	5.3192000	613586.0000000
21.0833340	5.3680000	613586.0000000
21.0833340	5.4168000	613586.0000000
21.0833340	5.4656000	613586.0000000
21.0833340	5.5144000	613586.0000000
21.0833340	5.5632000	613586.0000000
21.0833340	5.6120000	613586.0000000
21.0833340	5.6608000	-0.5583692
21.0833340	5.7096000	-0.5583692
21.0833340	5.7584000	613586.0000000
21.0833340	5.8072000	613586.0000000
21.0833340	5.8559999	613586.0000000
21.0833340	5.9047999	613586.0000000
21.0833340	5.9535999	613586.0000000
21.0833340	6.0023999	613586.0000000
21.1000004	0.2928000	-1.5707965
21.1000004	0.3416000	-1.5707965
21.1000004	0.3904000	-1.5401168
21.1000004	0.4392000	-1.6812429
21.1000004	0.4880000	-1.5339808
21.1000004	0.5368000	-1.3376312
21.1000004	0.5856000	-1.3437672
21.1000004	0.6344000	-1.3437672
21.1000004	0.6832000	-1.2271848
21.1000004	0.7320000	-1.2333207
21.1000004	0.7808000	-1.2271848
21.1000004	0.8296000	-1.3560390
21.1000004	0.8784000	-1.3314953
21.1000004	0.9272000	-1.3314953
21.1000004	0.9760000	-1.1842332
21.1000004	1.0247999	-1.1903691
21.1000004	1.0735999	-1.1842332
21.1000004	1.1223999	-1.0799227

21.1000004	1.1711999	-1.0799227
21.1000004	1.2200000	-1.2087770
21.1000004	1.2688000	-1.2333207
21.1000004	1.3176000	-1.4173985
21.1000004	1.3664000	-1.4112625
21.1000004	1.4152000	-1.4173985
21.1000004	1.4640000	-1.3744469
21.1000004	1.5128000	-1.3744469
21.1000004	1.5616000	-1.2394567
21.1000004	1.6104000	-1.4419422
21.1000004	1.6592000	-1.4051266
21.1000004	1.7079999	-1.5339808
21.1000004	1.7567999	-1.4910293
21.1000004	1.8055999	-1.4910293
21.1000004	1.8543999	-1.3989906
21.1000004	1.9031999	-1.5523887
21.1000004	1.9519999	-1.4296703
21.1000004	2.0007999	-1.4173985
21.1000004	2.0495999	-1.1474175
21.1000004	2.0983999	-1.1535535
21.1000004	2.1471999	-1.3069515
21.1000004	2.1959999	-1.4848933
21.1000004	2.2447999	-1.3253593
21.1000004	2.2935998	-1.3560390
21.1000004	2.3423998	-1.2885437
21.1000004	2.3912001	-1.2885437
21.1000004	2.4400001	-1.3805828
21.1000004	2.4888000	-1.0737867
21.1000004	2.5376000	-1.0553789
21.1000004	2.5864000	-1.0615149
21.1000004	2.6352000	-1.0615149
21.1000004	2.6840000	-1.0615149
21.1000004	2.7328000	-1.0062914
21.1000004	2.7816000	-1.0062914
21.1000004	2.8304000	-0.8590293
21.1000004	2.8792000	-0.7792621
21.1000004	2.9280000	-0.7424469
21.1000004	2.9768000	-0.4970098
21.1000004	3.0256000	-0.5338254
21.1000004	3.0743999	-0.5399613
21.1000004	3.1231999	-0.0306797
21.1000004	3.1719999	0.1718059
21.1000004	3.2207999	-0.4786019
21.1000004	3.2695999	-0.4724660
21.1000004	3.3183999	-0.5338254
21.1000004	3.3671999	-0.5583692
21.1000004	3.4159999	-0.5583692
21.1000004	3.4647999	-0.8099418
21.1000004	3.5135999	-0.7976699
21.1000004	3.5623999	-0.9203887
21.1000004	3.6111999	-0.8958449
21.1000004	3.6599998	-0.7976699
21.1000004	3.7087998	-1.1842332
21.1000004	3.7575998	-0.6810875
21.1000004	3.8063998	-1.1351457
21.1000004	3.8551998	-1.1290097
21.1000004	3.9039998	-1.1351457

21.1000004	3.9527998	-0.8467574
21.1000004	4.0016003	-0.7976699
21.1000004	4.0503998	-0.9878836
21.1000004	4.0991998	-0.9940195
21.1000004	4.1479998	-0.9940195
21.1000004	4.1967998	-0.7792621
21.1000004	4.2455997	-1.0676508
21.1000004	4.2943997	-1.0860586
21.1000004	4.3431997	-1.0737867
21.1000004	4.3919997	-1.0001554
21.1000004	4.4407997	-1.0001554
21.1000004	4.4895997	-1.0185633
21.1000004	4.5383997	-1.0185633
21.1000004	4.5871997	-1.0185633
21.1000004	4.6359997	613586.0000000
21.1000004	4.6847997	613586.0000000
21.1000004	4.7336001	613586.0000000
21.1000004	4.7824001	613586.0000000
21.1000004	4.8312001	613586.0000000
21.1000004	4.8800001	613586.0000000
21.1000004	4.9288001	613586.0000000
21.1000004	4.9776001	613586.0000000
21.1000004	5.0264001	-0.5215535
21.1000004	5.0752001	613586.0000000
21.1000004	5.1240001	-0.9940195
21.1000004	5.1728001	-0.9878836
21.1000004	5.2216001	613586.0000000
21.1000004	5.2704000	613586.0000000
21.1000004	5.3192000	613586.0000000
21.1000004	5.3680000	613586.0000000
21.1000004	5.4168000	613586.0000000
21.1000004	5.4656000	613586.0000000
21.1000004	5.5144000	613586.0000000
21.1000004	5.5632000	613586.0000000
21.1000004	5.6120000	613586.0000000
21.1000004	5.6608000	613586.0000000
21.1000004	5.7096000	613586.0000000
21.1000004	5.7584000	613586.0000000
21.1000004	5.8072000	613586.0000000
21.1000004	5.8559999	613586.0000000
21.1000004	5.9047999	613586.0000000
21.1000004	5.9535999	613586.0000000
21.1000004	6.0023999	613586.0000000
21.1166668	0.2928000	-1.2578645
21.1166668	0.3416000	-1.2333207
21.1166668	0.3904000	-1.2333207
21.1166668	0.4392000	-1.5953403
21.1166668	0.4880000	-1.6076121
21.1166668	0.5368000	-1.5892043
21.1166668	0.5856000	-1.7057867
21.1166668	0.6344000	-1.2762718
21.1166668	0.6832000	-1.2517285
21.1166668	0.7320000	-1.1780972
21.1166668	0.7808000	-1.1965051
21.1166668	0.8296000	-1.1965051
21.1166668	0.8784000	-1.2885437
21.1166668	0.9272000	-1.3376312

21.1166668	0.9760000	-1.3069515
21.1166668	1.0247999	-1.3560390
21.1166668	1.0735999	-1.3499031
21.1166668	1.1223999	-1.3744469
21.1166668	1.1711999	-1.3867188
21.1166668	1.2200000	-1.4051266
21.1166668	1.2688000	-1.5523887
21.1166668	1.3176000	-1.8714566
21.1166668	1.3664000	-1.9144082
21.1166668	1.4152000	-1.8714566
21.1166668	1.4640000	-1.8100972
21.1166668	1.5128000	-1.8039613
21.1166668	1.5616000	-1.7978253
21.1166668	1.6104000	-1.7978253
21.1166668	1.6592000	-1.8162332
21.1166668	1.7079999	-1.6198835
21.1166668	1.7567999	-1.6198835
21.1166668	1.8055999	-1.6076121
21.1166668	1.8543999	-1.6628351
21.1166668	1.9031999	-1.5217090
21.1166668	1.9519999	-1.5278449
21.1166668	2.0007999	-1.5278449
21.1166668	2.0495999	-1.8039613
21.1166668	2.0983999	-1.7119226
21.1166668	2.1471999	-1.7241945
21.1166668	2.1959999	-1.7241945
21.1166668	2.2447999	-1.7241945
21.1166668	2.2935998	-1.6137476
21.1166668	2.3423998	-1.6321554
21.1166668	2.3912001	-1.4971652
21.1166668	2.4400001	-1.4971652
21.1166668	2.4888000	-1.5707965
21.1166668	2.5376000	-1.2271848
21.1166668	2.5864000	-1.2394567
21.1166668	2.6352000	-1.2517285
21.1166668	2.6840000	-1.2455926
21.1166668	2.7328000	-1.2394567
21.1166668	2.7816000	-1.5155730
21.1166668	2.8304000	-1.5523887
21.1166668	2.8792000	-1.5523887
21.1166668	2.9280000	-1.5585246
21.1166668	2.9768000	-1.2885437
21.1166668	3.0256000	-1.3499031
21.1166668	3.0743999	-1.2394567
21.1166668	3.1231999	-1.0001554
21.1166668	3.1719999	-0.9142528
21.1166668	3.2207999	-0.9265246
21.1166668	3.2695999	-0.9326606
21.1166668	3.3183999	-0.9387960
21.1166668	3.3671999	-0.6872234
21.1166668	3.4159999	-0.6933594
21.1166668	3.4647999	-0.7669902
21.1166668	3.5135999	-0.7608547
21.1166668	3.5623999	-0.7547188
21.1166668	3.6111999	-0.5092816
21.1166668	3.6599998	-0.8406215
21.1166668	3.7087998	-0.6565437

21.1166668	3.7575998	-0.7301750
21.1166668	3.8063998	-0.7424469
21.1166668	3.8551998	-0.8222136
21.1166668	3.9039998	-0.7853980
21.1166668	3.9527998	-0.7731261
21.1166668	4.0016003	-0.7731261
21.1166668	4.0503998	-0.6442719
21.1166668	4.0991998	-0.6442719
21.1166668	4.1479998	-0.7608547
21.1166668	4.1967998	-0.7608547
21.1166668	4.2455997	-0.7669902
21.1166668	4.2943997	-0.7669902
21.1166668	4.3431997	-0.7301750
21.1166668	4.3919997	-0.7179031
21.1166668	4.4407997	-0.6565437
21.1166668	4.4895997	-0.6442719
21.1166668	4.5383997	-0.6504078
21.1166668	4.5871997	-0.6565437
21.1166668	4.6359997	-0.6504078
21.1166668	4.6847997	-0.3681555
21.1166668	4.7336001	-0.3620195
21.1166668	4.7824001	-0.3620195
21.1166668	4.8312001	613586.0000000
21.1166668	4.8800001	613586.0000000
21.1166668	4.9288001	613586.0000000
21.1166668	4.9776001	613586.0000000
21.1166668	5.0264001	-0.5215535
21.1166668	5.0752001	-0.5154176
21.1166668	5.1240001	-0.6197281
21.1166668	5.1728001	613586.0000000
21.1166668	5.2216001	-0.6688156
21.1166668	5.2704000	-0.6626797
21.1166668	5.3192000	613586.0000000
21.1166668	5.3680000	613586.0000000
21.1166668	5.4168000	613586.0000000
21.1166668	5.4656000	613586.0000000
21.1166668	5.5144000	-0.5092816
21.1166668	5.5632000	-0.5092816
21.1166668	5.6120000	613586.0000000
21.1166668	5.6608000	613586.0000000
21.1166668	5.7096000	613586.0000000
21.1166668	5.7584000	613586.0000000
21.1166668	5.8072000	613586.0000000
21.1166668	5.8559999	613586.0000000
21.1166668	5.9047999	613586.0000000
21.1166668	5.9535999	613586.0000000
21.1166668	6.0023999	613586.0000000
21.1333332	0.2928000	-1.3928547
21.1333332	0.3416000	-1.5892043
21.1333332	0.3904000	-1.5523887
21.1333332	0.4392000	-1.7057867
21.1333332	0.4880000	-1.6996508
21.1333332	0.5368000	-1.6935148
21.1333332	0.5856000	-1.3744469
21.1333332	0.6344000	-1.3744469
21.1333332	0.6832000	-1.6444273
21.1333332	0.7320000	-1.6321554

21.1333332	0.7808000	-1.6198835
21.1333332	0.8296000	-1.4419422
21.1333332	0.8784000	-1.5892043
21.1333332	0.9272000	-1.5892043
21.1333332	0.9760000	-1.3437672
21.1333332	1.0247999	-1.4787574
21.1333332	1.0735999	-1.4787574
21.1333332	1.1223999	-1.5892043
21.1333332	1.1711999	-1.5892043
21.1333332	1.2200000	-1.6935148
21.1333332	1.2688000	-1.8898644
21.1333332	1.3176000	-1.7364664
21.1333332	1.3664000	-1.7364664
21.1333332	1.4152000	-1.7548742
21.1333332	1.4640000	-1.8469129
21.1333332	1.5128000	-1.8469129
21.1333332	1.5616000	-1.6812429
21.1333332	1.6104000	-1.6505632
21.1333332	1.6592000	-1.5646605
21.1333332	1.7079999	-1.3192234
21.1333332	1.7567999	-1.4542136
21.1333332	1.8055999	-1.6076121
21.1333332	1.8543999	-1.6076121
21.1333332	1.9031999	-1.5339808
21.1333332	1.9519999	-1.5339808
21.1333332	2.0007999	-1.8100972
21.1333332	2.0495999	-1.6137476
21.1333332	2.0983999	-1.6137476
21.1333332	2.1471999	-1.4051266
21.1333332	2.1959999	-1.5155730
21.1333332	2.2447999	-1.3253593
21.1333332	2.2935998	-1.5278449
21.1333332	2.3423998	-1.3130875
21.1333332	2.3912001	-1.3560390
21.1333332	2.4400001	-1.5155730
21.1333332	2.4888000	-1.4051266
21.1333332	2.5376000	-1.3437672
21.1333332	2.5864000	-1.3499031
21.1333332	2.6352000	-1.3499031
21.1333332	2.6840000	-1.2824078
21.1333332	2.7328000	-1.2394567
21.1333332	2.7816000	-1.2455926
21.1333332	2.8304000	-1.2455926
21.1333332	2.8792000	-1.0615149
21.1333332	2.9280000	-0.6565437
21.1333332	2.9768000	-0.2393012
21.1333332	3.0256000	-0.6442719
21.1333332	3.0743999	-0.6381359
21.1333332	3.1231999	-0.6381359
21.1333332	3.1719999	-0.9142528
21.1333332	3.2207999	-1.0799227
21.1333332	3.2695999	-1.0860586
21.1333332	3.3183999	-1.0369711
21.1333332	3.3671999	-1.0431070
21.1333332	3.4159999	-1.0492430
21.1333332	3.4647999	-0.8774371
21.1333332	3.5135999	-0.5890489

21.1333332	3.5623999	-0.5951843
21.1333332	3.6111999	-0.9572039
21.1333332	3.6599998	-0.7792621
21.1333332	3.7087998	-0.7853980
21.1333332	3.7575998	-0.8160777
21.1333332	3.8063998	-0.8038058
21.1333332	3.8551998	-0.9878836
21.1333332	3.9039998	-0.9510679
21.1333332	3.9527998	-0.9081168
21.1333332	4.0016003	-0.9081168
21.1333332	4.0503998	-0.8344855
21.1333332	4.0991998	-0.9081168
21.1333332	4.1479998	-0.8590293
21.1333332	4.1967998	-0.8835731
21.1333332	4.2455997	-0.8099418
21.1333332	4.2943997	-0.8713012
21.1333332	4.3431997	-0.8528934
21.1333332	4.3919997	-0.8651652
21.1333332	4.4407997	-0.8713012
21.1333332	4.4895997	-0.8651652
21.1333332	4.5383997	-0.7853980
21.1333332	4.5871997	-0.7853980
21.1333332	4.6359997	613586.0000000
21.1333332	4.6847997	613586.0000000
21.1333332	4.7336001	613586.0000000
21.1333332	4.7824001	613586.0000000
21.1333332	4.8312001	613586.0000000
21.1333332	4.8800001	613586.0000000
21.1333332	4.9288001	613586.0000000
21.1333332	4.9776001	613586.0000000
21.1333332	5.0264001	-0.6258640
21.1333332	5.0752001	-0.6258640
21.1333332	5.1240001	-0.7485828
21.1333332	5.1728001	613586.0000000
21.1333332	5.2216001	613586.0000000
21.1333332	5.2704000	613586.0000000
21.1333332	5.3192000	613586.0000000
21.1333332	5.3680000	613586.0000000
21.1333332	5.4168000	613586.0000000
21.1333332	5.4656000	613586.0000000
21.1333332	5.5144000	613586.0000000
21.1333332	5.5632000	613586.0000000
21.1333332	5.6120000	613586.0000000
21.1333332	5.6608000	613586.0000000
21.1333332	5.7096000	613586.0000000
21.1333332	5.7584000	613586.0000000
21.1333332	5.8072000	613586.0000000
21.1333332	5.8559999	613586.0000000
21.1333332	5.9047999	613586.0000000
21.1333332	5.9535999	613586.0000000
21.1333332	6.0023999	613586.0000000
21.1499996	0.2928000	-1.5830684
21.1499996	0.3416000	-1.2578645
21.1499996	0.3904000	-1.3376312
21.1499996	0.4392000	-1.3376312
21.1499996	0.4880000	-1.4358063
21.1499996	0.5368000	-1.4296703

21.1499996	0.5856000	-1.2026410
21.1499996	0.6344000	-1.2517285
21.1499996	0.6832000	-1.3499031
21.1499996	0.7320000	-1.3499031
21.1499996	0.7808000	-1.3560390
21.1499996	0.8296000	-1.2885437
21.1499996	0.8784000	-1.2640004
21.1499996	0.9272000	-1.5155730
21.1499996	0.9760000	-1.4051266
21.1499996	1.0247999	-1.3621750
21.1499996	1.0735999	-1.6198835
21.1499996	1.1223999	-1.4235344
21.1499996	1.1711999	-1.4358063
21.1499996	1.2200000	-1.4419422
21.1499996	1.2688000	-1.4542136
21.1499996	1.3176000	-1.4542136
21.1499996	1.3664000	-1.6628351
21.1499996	1.4152000	-1.6444273
21.1499996	1.4640000	-1.6505632
21.1499996	1.5128000	-1.3253593
21.1499996	1.5616000	-1.3253593
21.1499996	1.6104000	-1.3989906
21.1499996	1.6592000	-1.1842332
21.1499996	1.7079999	-1.3989906
21.1499996	1.7567999	-1.2824078
21.1499996	1.8055999	-1.0308352
21.1499996	1.8543999	-1.2640004
21.1499996	1.9031999	-1.3130875
21.1499996	1.9519999	-1.3069515
21.1499996	2.0007999	-1.0369711
21.1499996	2.0495999	-1.0615149
21.1499996	2.0983999	-1.1474175
21.1499996	2.1471999	-1.2149129
21.1499996	2.1959999	-1.2087770
21.1499996	2.2447999	-1.1106019
21.1499996	2.2935998	-1.1044660
21.1499996	2.3423998	-0.8835731
21.1499996	2.3912001	-0.8099418
21.1499996	2.4400001	-0.7301750
21.1499996	2.4888000	-0.7117672
21.1499996	2.5376000	-0.7117672
21.1499996	2.5864000	-0.6197281
21.1499996	2.6352000	-0.6197281
21.1499996	2.6840000	-0.7363110
21.1499996	2.7328000	-0.8651652
21.1499996	2.7816000	-0.8651652
21.1499996	2.8304000	-0.8528934
21.1499996	2.8792000	-0.8528934
21.1499996	2.9280000	-1.0185633
21.1499996	2.9768000	-0.5276895
21.1499996	3.0256000	-0.9387960
21.1499996	3.0743999	-0.9449320
21.1499996	3.1231999	-0.9387960
21.1499996	3.1719999	-0.8222136
21.1499996	3.2207999	-0.7424469
21.1499996	3.2695999	-0.7240391
21.1499996	3.3183999	-0.6872234

21.1499996	3.3671999	-0.5522332
21.1499996	3.4159999	-0.5522332
21.1499996	3.4647999	-0.2699804
21.1499996	3.5135999	-0.1902137
21.1499996	3.5623999	-0.2208934
21.1499996	3.6111999	-0.9265246
21.1499996	3.6599998	-0.9265246
21.1499996	3.7087998	-0.9326606
21.1499996	3.7575998	-0.9265246
21.1499996	3.8063998	-0.7792621
21.1499996	3.8551998	-0.7853980
21.1499996	3.9039998	-0.7853980
21.1499996	3.9527998	-0.8038058
21.1499996	4.0016003	-0.8897090
21.1499996	4.0503998	-0.8222136
21.1499996	4.0991998	-0.8222136
21.1499996	4.1479998	-0.7179031
21.1499996	4.1967998	-0.8774371
21.1499996	4.2455997	-0.8897090
21.1499996	4.2943997	-1.0001554
21.1499996	4.3431997	-0.9572039
21.1499996	4.3919997	-0.9449320
21.1499996	4.4407997	-0.9756117
21.1499996	4.4895997	-0.9572039
21.1499996	4.5383997	-0.9081168
21.1499996	4.5871997	613586.0000000
21.1499996	4.6359997	613586.0000000
21.1499996	4.6847997	613586.0000000
21.1499996	4.7336001	613586.0000000
21.1499996	4.7824001	613586.0000000
21.1499996	4.8312001	613586.0000000
21.1499996	4.8800001	613586.0000000
21.1499996	4.9288001	613586.0000000
21.1499996	4.9776001	-0.5951843
21.1499996	5.0264001	-0.5767770
21.1499996	5.0752001	-0.4601941
21.1499996	5.1240001	-0.4663301
21.1499996	5.1728001	-0.5890489
21.1499996	5.2216001	613586.0000000
21.1499996	5.2704000	613586.0000000
21.1499996	5.3192000	613586.0000000
21.1499996	5.3680000	613586.0000000
21.1499996	5.4168000	613586.0000000
21.1499996	5.4656000	613586.0000000
21.1499996	5.5144000	613586.0000000
21.1499996	5.5632000	613586.0000000
21.1499996	5.6120000	613586.0000000
21.1499996	5.6608000	613586.0000000
21.1499996	5.7096000	613586.0000000
21.1499996	5.7584000	613586.0000000
21.1499996	5.8072000	613586.0000000
21.1499996	5.8559999	613586.0000000
21.1499996	5.9047999	613586.0000000
21.1499996	5.9535999	613586.0000000
21.1499996	6.0023999	613586.0000000
21.1666660	0.2928000	-1.5523887
21.1666660	0.3416000	-1.7364664

21.1666660	0.3904000	-1.7303305
21.1666660	0.4392000	-1.5892043
21.1666660	0.4880000	-1.2824078
21.1666660	0.5368000	-1.2824078
21.1666660	0.5856000	-1.2824078
21.1666660	0.6344000	-1.1351457
21.1666660	0.6832000	-1.5339808
21.1666660	0.7320000	-1.1842332
21.1666660	0.7808000	-1.2026410
21.1666660	0.8296000	-1.2026410
21.1666660	0.8784000	-1.3744469
21.1666660	0.9272000	-1.3744469
21.1666660	0.9760000	-1.4603496
21.1666660	1.0247999	-1.4664855
21.1666660	1.0735999	-1.3192234
21.1666660	1.1223999	-1.4112625
21.1666660	1.1711999	-1.3867188
21.1666660	1.2200000	-1.3867188
21.1666660	1.2688000	-1.3867188
21.1666660	1.3176000	-1.5646605
21.1666660	1.3664000	-1.5707965
21.1666660	1.4152000	-1.5830684
21.1666660	1.4640000	-1.5278449
21.1666660	1.5128000	-1.4910293
21.1666660	1.5616000	-1.4480777
21.1666660	1.6104000	-1.4419422
21.1666660	1.6592000	-1.1106019
21.1666660	1.7079999	-1.2946796
21.1666660	1.7567999	-1.1965051
21.1666660	1.8055999	-1.0185633
21.1666660	1.8543999	-1.0124273
21.1666660	1.9031999	-1.0983305
21.1666660	1.9519999	-1.0860586
21.1666660	2.0007999	-1.0860586
21.1666660	2.0495999	-1.3314953
21.1666660	2.0983999	-1.0860586
21.1666660	2.1471999	-0.9449320
21.1666660	2.1959999	-0.8467574
21.1666660	2.2447999	-1.0431070
21.1666660	2.2935998	-0.9633398
21.1666660	2.3423998	-0.9633398
21.1666660	2.3912001	-0.8283496
21.1666660	2.4400001	-0.8774371
21.1666660	2.4888000	-0.8774371
21.1666660	2.5376000	-0.7792621
21.1666660	2.5864000	-0.6994953
21.1666660	2.6352000	-0.7056313
21.1666660	2.6840000	-0.7117672
21.1666660	2.7328000	-0.7669902
21.1666660	2.7816000	-0.7424469
21.1666660	2.8304000	-0.7301750
21.1666660	2.8792000	-0.5890489
21.1666660	2.9280000	-0.4417863
21.1666660	2.9768000	-0.4356503
21.1666660	3.0256000	-0.4295144
21.1666660	3.0743999	-0.0061359
21.1666660	3.1231999	0.0552235

21.1666660	3.1719999	-0.2883883
21.1666660	3.2207999	-0.7117672
21.1666660	3.2695999	-0.5215535
21.1666660	3.3183999	0.3313398
21.1666660	3.3671999	0.3313398
21.1666660	3.4159999	-0.6565437
21.1666660	3.4647999	-1.5217090
21.1666660	3.5135999	-1.5953403
21.1666660	3.5623999	-1.1658254
21.1666660	3.6111999	-0.9387960
21.1666660	3.6599998	-0.9326606
21.1666660	3.7087998	-0.9449320
21.1666660	3.7575998	-0.7976699
21.1666660	3.8063998	-0.6994953
21.1666660	3.8551998	-0.7056313
21.1666660	3.9039998	-0.7731261
21.1666660	3.9527998	-0.7117672
21.1666660	4.0016003	-0.6994953
21.1666660	4.0503998	-0.6994953
21.1666660	4.0991998	-0.7669902
21.1666660	4.1479998	-0.6197281
21.1666660	4.1967998	-0.6442719
21.1666660	4.2455997	-0.6442719
21.1666660	4.2943997	-0.5951843
21.1666660	4.3431997	-0.4786019
21.1666660	4.3919997	-0.4356503
21.1666660	4.4407997	-0.4356503
21.1666660	4.4895997	-0.4295144
21.1666660	4.5383997	613586.0000000
21.1666660	4.5871997	-0.5460973
21.1666660	4.6359997	613586.0000000
21.1666660	4.6847997	-0.5583692
21.1666660	4.7336001	-0.5583692
21.1666660	4.7824001	613586.0000000
21.1666660	4.8312001	613586.0000000
21.1666660	4.8800001	613586.0000000
21.1666660	4.9288001	613586.0000000
21.1666660	4.9776001	613586.0000000
21.1666660	5.0264001	613586.0000000
21.1666660	5.0752001	-0.5583692
21.1666660	5.1240001	-0.4111071
21.1666660	5.1728001	-0.4111071
21.1666660	5.2216001	613586.0000000
21.1666660	5.2704000	613586.0000000
21.1666660	5.3192000	613586.0000000
21.1666660	5.3680000	613586.0000000
21.1666660	5.4168000	613586.0000000
21.1666660	5.4656000	613586.0000000
21.1666660	5.5144000	613586.0000000
21.1666660	5.5632000	613586.0000000
21.1666660	5.6120000	613586.0000000
21.1666660	5.6608000	613586.0000000
21.1666660	5.7096000	613586.0000000
21.1666660	5.7584000	613586.0000000
21.1666660	5.8072000	613586.0000000
21.1666660	5.8559999	613586.0000000
21.1666660	5.9047999	613586.0000000

21.1666660	5.9535999	613586.0000000
21.1666660	6.0023999	613586.0000000
21.1833324	0.2928000	-1.8407769
21.1833324	0.3416000	-1.5953403
21.1833324	0.3904000	-1.5953403
21.1833324	0.4392000	-1.4787574
21.1833324	0.4880000	-1.2946796
21.1833324	0.5368000	-1.2946796
21.1833324	0.5856000	-1.2824078
21.1833324	0.6344000	-1.5523887
21.1833324	0.6832000	-1.5523887
21.1833324	0.7320000	-1.2026410
21.1833324	0.7808000	-1.2026410
21.1833324	0.8296000	-1.4419422
21.1833324	0.8784000	-1.4603496
21.1833324	0.9272000	-1.4726214
21.1833324	0.9760000	-1.3192234
21.1833324	1.0247999	-1.2946796
21.1833324	1.0735999	-1.3621750
21.1833324	1.1223999	-1.4664855
21.1833324	1.1711999	-1.4726214
21.1833324	1.2200000	-1.3805828
21.1833324	1.2688000	-1.3867188
21.1833324	1.3176000	-1.6628351
21.1833324	1.3664000	-1.6751070
21.1833324	1.4152000	-1.5278449
21.1833324	1.4640000	-1.4419422
21.1833324	1.5128000	-1.3744469
21.1833324	1.5616000	-1.3314953
21.1833324	1.6104000	-1.5033011
21.1833324	1.6592000	-1.1903691
21.1833324	1.7079999	-1.1658254
21.1833324	1.7567999	-1.1596894
21.1833324	1.8055999	-1.1167378
21.1833324	1.8543999	-1.0246992
21.1833324	1.9031999	-0.8283496
21.1833324	1.9519999	-0.8406215
21.1833324	2.0007999	-1.1167378
21.1833324	2.0495999	-1.1106019
21.1833324	2.0983999	-0.9449320
21.1833324	2.1471999	-0.9940195
21.1833324	2.1959999	-1.0124273
21.1833324	2.2447999	-0.8344855
21.1833324	2.2935998	-0.9142528
21.1833324	2.3423998	-0.9142528
21.1833324	2.3912001	-0.7485828
21.1833324	2.4400001	-0.7485828
21.1833324	2.4888000	-0.7731261
21.1833324	2.5376000	-0.9449320
21.1833324	2.5864000	-0.9510679
21.1833324	2.6352000	-0.7179031
21.1833324	2.6840000	-0.7179031
21.1833324	2.7328000	-0.7179031
21.1833324	2.7816000	-0.8958449
21.1833324	2.8304000	-0.8160777
21.1833324	2.8792000	-0.7731261
21.1833324	2.9280000	-0.7731261

21.1833324	2.9768000	-0.6872234
21.1833324	3.0256000	-0.6749516
21.1833324	3.0743999	-0.6504078
21.1833324	3.1231999	-0.7853980
21.1833324	3.1719999	-1.0676508
21.1833324	3.2207999	-1.0492430
21.1833324	3.2695999	-1.0431070
21.1833324	3.3183999	-1.1412816
21.1833324	3.3671999	-0.6810875
21.1833324	3.4159999	-0.3742914
21.1833324	3.4647999	-0.7117672
21.1833324	3.5135999	-1.1474175
21.1833324	3.5623999	-1.0860586
21.1833324	3.6111999	-1.0737867
21.1833324	3.6599998	-1.0553789
21.1833324	3.7087998	-1.0553789
21.1833324	3.7575998	-1.0799227
21.1833324	3.8063998	-0.7915339
21.1833324	3.8551998	-0.7976699
21.1833324	3.9039998	-0.5154176
21.1833324	3.9527998	-0.6504078
21.1833324	4.0016003	-0.6442719
21.1833324	4.0503998	-0.6381359
21.1833324	4.0991998	-0.5645051
21.1833324	4.1479998	-0.5706410
21.1833324	4.1967998	-0.6994953
21.1833324	4.2455997	-0.6933594
21.1833324	4.2943997	-0.6872234
21.1833324	4.3431997	-0.6810875
21.1833324	4.3919997	-0.5399613
21.1833324	4.4407997	-0.3988352
21.1833324	4.4895997	-0.6074562
21.1833324	4.5383997	-0.4970098
21.1833324	4.5871997	-0.4786019
21.1833324	4.6359997	613586.0000000
21.1833324	4.6847997	613586.0000000
21.1833324	4.7336001	613586.0000000
21.1833324	4.7824001	613586.0000000
21.1833324	4.8312001	613586.0000000
21.1833324	4.8800001	-0.4786019
21.1833324	4.9288001	-0.4970098
21.1833324	4.9776001	-0.3313398
21.1833324	5.0264001	613586.0000000
21.1833324	5.0752001	613586.0000000
21.1833324	5.1240001	613586.0000000
21.1833324	5.1728001	613586.0000000
21.1833324	5.2216001	613586.0000000
21.1833324	5.2704000	613586.0000000
21.1833324	5.3192000	613586.0000000
21.1833324	5.3680000	613586.0000000
21.1833324	5.4168000	613586.0000000
21.1833324	5.4656000	613586.0000000
21.1833324	5.5144000	613586.0000000
21.1833324	5.5632000	613586.0000000
21.1833324	5.6120000	613586.0000000
21.1833324	5.6608000	613586.0000000
21.1833324	5.7096000	613586.0000000

21.1833324	5.7584000	613586.0000000
21.1833324	5.8072000	613586.0000000
21.1833324	5.8559999	613586.0000000
21.1833324	5.9047999	613586.0000000
21.1833324	5.9535999	613586.0000000
21.1833324	6.0023999	613586.0000000
21.2000008	0.2928000	-1.6751070
21.2000008	0.3416000	-1.5830684
21.2000008	0.3904000	-1.5707965
21.2000008	0.4392000	-1.3069515
21.2000008	0.4880000	-1.3008156
21.2000008	0.5368000	-1.2946796
21.2000008	0.5856000	-1.5462527
21.2000008	0.6344000	-1.2824078
21.2000008	0.6832000	-1.6076121
21.2000008	0.7320000	-1.6014762
21.2000008	0.7808000	-1.5769324
21.2000008	0.8296000	-1.4173985
21.2000008	0.8784000	-1.5094371
21.2000008	0.9272000	-1.5033011
21.2000008	0.9760000	-1.4971652
21.2000008	1.0247999	-1.4419422
21.2000008	1.0735999	-1.5155730
21.2000008	1.1223999	-1.5155730
21.2000008	1.1711999	-1.5278449
21.2000008	1.2200000	-1.6198835
21.2000008	1.2688000	-1.6689711
21.2000008	1.3176000	-1.5707965
21.2000008	1.3664000	-1.5462527
21.2000008	1.4152000	-1.6137476
21.2000008	1.4640000	-1.5830684
21.2000008	1.5128000	-1.4051266
21.2000008	1.5616000	-1.2946796
21.2000008	1.6104000	-1.4112625
21.2000008	1.6592000	-1.4112625
21.2000008	1.7079999	-1.4051266
21.2000008	1.7567999	-1.4173985
21.2000008	1.8055999	-1.1474175
21.2000008	1.8543999	-1.1474175
21.2000008	1.9031999	-1.2517285
21.2000008	1.9519999	-1.3130875
21.2000008	2.0007999	-1.3069515
21.2000008	2.0495999	-1.4296703
21.2000008	2.0983999	-1.4112625
21.2000008	2.1471999	-1.3989906
21.2000008	2.1959999	-1.2026410
21.2000008	2.2447999	-1.1351457
21.2000008	2.2935998	-1.1780972
21.2000008	2.3423998	-0.8467574
21.2000008	2.3912001	-0.8344855
21.2000008	2.4400001	-0.8283496
21.2000008	2.4888000	-0.6688156
21.2000008	2.5376000	-0.6749516
21.2000008	2.5864000	-0.6749516
21.2000008	2.6352000	-0.7547188
21.2000008	2.6840000	-1.0001554
21.2000008	2.7328000	-0.8528934

21.2000008	2.7816000	-0.9326606
21.2000008	2.8304000	-0.8344855
21.2000008	2.8792000	-0.8160777
21.2000008	2.9280000	-0.9265246
21.2000008	2.9768000	-0.7179031
21.2000008	3.0256000	-0.7179031
21.2000008	3.0743999	-0.7179031
21.2000008	3.1231999	-0.5522332
21.2000008	3.1719999	-0.5522332
21.2000008	3.2207999	-0.5276895
21.2000008	3.2695999	-0.8835731
21.2000008	3.3183999	-0.8835731
21.2000008	3.3671999	-0.8958449
21.2000008	3.4159999	-0.8958449
21.2000008	3.4647999	-0.9265246
21.2000008	3.5135999	-1.2087770
21.2000008	3.5623999	-0.7117672
21.2000008	3.6111999	-0.5706410
21.2000008	3.6599998	-0.5706410
21.2000008	3.7087998	-0.5338254
21.2000008	3.7575998	-0.5338254
21.2000008	3.8063998	-0.9449320
21.2000008	3.8551998	-0.9449320
21.2000008	3.9039998	-0.6872234
21.2000008	3.9527998	-0.6933594
21.2000008	4.0016003	-0.7179031
21.2000008	4.0503998	-0.7669902
21.2000008	4.0991998	-0.8344855
21.2000008	4.1479998	-0.8222136
21.2000008	4.1967998	-0.8283496
21.2000008	4.2455997	-0.8406215
21.2000008	4.2943997	-0.7179031
21.2000008	4.3431997	-0.7240391
21.2000008	4.3919997	-0.7915339
21.2000008	4.4407997	-0.7915339
21.2000008	4.4895997	-0.8528934
21.2000008	4.5383997	-0.8467574
21.2000008	4.5871997	613586.0000000
21.2000008	4.6359997	613586.0000000
21.2000008	4.6847997	-0.4970098
21.2000008	4.7336001	-0.4970098
21.2000008	4.7824001	613586.0000000
21.2000008	4.8312001	613586.0000000
21.2000008	4.8800001	613586.0000000
21.2000008	4.9288001	613586.0000000
21.2000008	4.9776001	613586.0000000
21.2000008	5.0264001	613586.0000000
21.2000008	5.0752001	613586.0000000
21.2000008	5.1240001	613586.0000000
21.2000008	5.1728001	613586.0000000
21.2000008	5.2216001	613586.0000000
21.2000008	5.2704000	613586.0000000
21.2000008	5.3192000	-0.3067961
21.2000008	5.3680000	613586.0000000
21.2000008	5.4168000	613586.0000000
21.2000008	5.4656000	613586.0000000
21.2000008	5.5144000	613586.0000000

21.2000008	5.5632000	613586.0000000
21.2000008	5.6120000	613586.0000000
21.2000008	5.6608000	613586.0000000
21.2000008	5.7096000	613586.0000000
21.2000008	5.7584000	613586.0000000
21.2000008	5.8072000	613586.0000000
21.2000008	5.8559999	613586.0000000
21.2000008	5.9047999	613586.0000000
21.2000008	5.9535999	613586.0000000
21.2000008	6.0023999	613586.0000000
21.2166672	0.2928000	-1.6505632
21.2166672	0.3416000	-1.4051266
21.2166672	0.3904000	-1.3805828
21.2166672	0.4392000	-1.2333207
21.2166672	0.4880000	-1.3989906
21.2166672	0.5368000	-1.3805828
21.2166672	0.5856000	-1.3805828
21.2166672	0.6344000	-1.4787574
21.2166672	0.6832000	-1.3253593
21.2166672	0.7320000	-1.3437672
21.2166672	0.7808000	-1.3989906
21.2166672	0.8296000	-1.3989906
21.2166672	0.8784000	-1.4051266
21.2166672	0.9272000	-1.2640004
21.2166672	0.9760000	-1.5401168
21.2166672	1.0247999	-1.4910293
21.2166672	1.0735999	-1.4848933
21.2166672	1.1223999	-1.6751070
21.2166672	1.1711999	-1.6873789
21.2166672	1.2200000	-1.4664855
21.2166672	1.2688000	-1.5523887
21.2166672	1.3176000	-1.4787574
21.2166672	1.3664000	-1.4726214
21.2166672	1.4152000	-1.4787574
21.2166672	1.4640000	-1.4971652
21.2166672	1.5128000	-1.5462527
21.2166672	1.5616000	-1.4358063
21.2166672	1.6104000	-1.4358063
21.2166672	1.6592000	-1.4235344
21.2166672	1.7079999	-1.5462527
21.2166672	1.7567999	-1.3499031
21.2166672	1.8055999	-1.5155730
21.2166672	1.8543999	-1.5155730
21.2166672	1.9031999	-1.7855535
21.2166672	1.9519999	-1.7916894
21.2166672	2.0007999	-1.5707965
21.2166672	2.0495999	-1.3314953
21.2166672	2.0983999	-1.3376312
21.2166672	2.1471999	-1.1780972
21.2166672	2.1959999	-1.2578645
21.2166672	2.2447999	-1.0737867
21.2166672	2.2935998	-1.3069515
21.2166672	2.3423998	-1.4358063
21.2166672	2.3912001	-1.4296703
21.2166672	2.4400001	-1.3069515
21.2166672	2.4888000	-1.3008156
21.2166672	2.5376000	-1.1596894

21.2166672	2.5864000	-1.1535535
21.2166672	2.6352000	-1.1412816
21.2166672	2.6840000	-1.1351457
21.2166672	2.7328000	-0.9940195
21.2166672	2.7816000	-0.9756117
21.2166672	2.8304000	-0.8283496
21.2166672	2.8792000	-1.0185633
21.2166672	2.9280000	-1.0921946
21.2166672	2.9768000	-1.0921946
21.2166672	3.0256000	-0.8590293
21.2166672	3.0743999	-1.1903691
21.2166672	3.1231999	-0.9940195
21.2166672	3.1719999	-1.1474175
21.2166672	3.2207999	-1.2762718
21.2166672	3.2695999	-1.1535535
21.2166672	3.3183999	-1.6751070
21.2166672	3.3671999	-1.3928547
21.2166672	3.4159999	-1.0676508
21.2166672	3.4647999	-1.0676508
21.2166672	3.5135999	-1.1044660
21.2166672	3.5623999	-1.0001554
21.2166672	3.6111999	-0.9387960
21.2166672	3.6599998	-0.6381359
21.2166672	3.7087998	-0.7915339
21.2166672	3.7575998	-0.4908738
21.2166672	3.8063998	-0.6626797
21.2166672	3.8551998	-0.6626797
21.2166672	3.9039998	-0.8528934
21.2166672	3.9527998	-0.8713012
21.2166672	4.0016003	-0.9387960
21.2166672	4.0503998	-0.7363110
21.2166672	4.0991998	-0.9572039
21.2166672	4.1479998	-0.9572039
21.2166672	4.1967998	-1.0615149
21.2166672	4.2455997	-1.0799227
21.2166672	4.2943997	-0.8590293
21.2166672	4.3431997	-0.8590293
21.2166672	4.3919997	-0.9817476
21.2166672	4.4407997	613586.0000000
21.2166672	4.4895997	-0.6810875
21.2166672	4.5383997	-0.6994953
21.2166672	4.5871997	-0.7056313
21.2166672	4.6359997	613586.0000000
21.2166672	4.6847997	-0.5767770
21.2166672	4.7336001	-0.5031457
21.2166672	4.7824001	-0.4786019
21.2166672	4.8312001	613586.0000000
21.2166672	4.8800001	613586.0000000
21.2166672	4.9288001	613586.0000000
21.2166672	4.9776001	613586.0000000
21.2166672	5.0264001	613586.0000000
21.2166672	5.0752001	-0.3681555
21.2166672	5.1240001	613586.0000000
21.2166672	5.1728001	-0.5215535
21.2166672	5.2216001	-0.4847379
21.2166672	5.2704000	-0.4724660
21.2166672	5.3192000	613586.0000000

21.2166672	5.3680000	613586.0000000
21.2166672	5.4168000	613586.0000000
21.2166672	5.4656000	613586.0000000
21.2166672	5.5144000	613586.0000000
21.2166672	5.5632000	613586.0000000
21.2166672	5.6120000	613586.0000000
21.2166672	5.6608000	613586.0000000
21.2166672	5.7096000	613586.0000000
21.2166672	5.7584000	613586.0000000
21.2166672	5.8072000	613586.0000000
21.2166672	5.8559999	613586.0000000
21.2166672	5.9047999	613586.0000000
21.2166672	5.9535999	613586.0000000
21.2166672	6.0023999	613586.0000000
21.2333336	0.2928000	-1.8223691
21.2333336	0.3416000	-1.8223691
21.2333336	0.3904000	-1.8162332
21.2333336	0.4392000	-1.4419422
21.2333336	0.4880000	-1.3621750
21.2333336	0.5368000	-1.3744469
21.2333336	0.5856000	-1.6382914
21.2333336	0.6344000	-1.6321554
21.2333336	0.6832000	-1.5339808
21.2333336	0.7320000	-1.4971652
21.2333336	0.7808000	-1.3989906
21.2333336	0.8296000	-1.3989906
21.2333336	0.8784000	-1.4051266
21.2333336	0.9272000	-1.4358063
21.2333336	0.9760000	-1.5585246
21.2333336	1.0247999	-1.5523887
21.2333336	1.0735999	-1.5401168
21.2333336	1.1223999	-1.4848933
21.2333336	1.1711999	-1.4787574
21.2333336	1.2200000	-1.3192234
21.2333336	1.2688000	-1.4480777
21.2333336	1.3176000	-1.4542136
21.2333336	1.3664000	-1.3744469
21.2333336	1.4152000	-1.5094371
21.2333336	1.4640000	-1.5094371
21.2333336	1.5128000	-1.5033011
21.2333336	1.5616000	-1.5033011
21.2333336	1.6104000	-1.5033011
21.2333336	1.6592000	-1.5278449
21.2333336	1.7079999	-1.5339808
21.2333336	1.7567999	-1.6505632
21.2333336	1.8055999	-1.7180586
21.2333336	1.8543999	-1.5155730
21.2333336	1.9031999	-1.6566992
21.2333336	1.9519999	-1.6996508
21.2333336	2.0007999	-1.6935148
21.2333336	2.0495999	-1.6812429
21.2333336	2.0983999	-1.6566992
21.2333336	2.1471999	-1.4480777
21.2333336	2.1959999	-1.6260195
21.2333336	2.2447999	-1.6260195
21.2333336	2.2935998	-1.4542136
21.2333336	2.3423998	-1.5830684

21.2333336	2.3912001	-1.5830684
21.2333336	2.4400001	-1.5953403
21.2333336	2.4888000	-1.2762718
21.2333336	2.5376000	-1.1903691
21.2333336	2.5864000	-1.2640004
21.2333336	2.6352000	-1.2517285
21.2333336	2.6840000	-0.9510679
21.2333336	2.7328000	-1.1719613
21.2333336	2.7816000	-1.1535535
21.2333336	2.8304000	-1.0799227
21.2333336	2.8792000	-1.0860586
21.2333336	2.9280000	-1.0062914
21.2333336	2.9768000	-1.0124273
21.2333336	3.0256000	-1.1351457
21.2333336	3.0743999	-0.7853980
21.2333336	3.1231999	-0.6197281
21.2333336	3.1719999	-0.7240391
21.2333336	3.2207999	-0.6565437
21.2333336	3.2695999	-0.7424469
21.2333336	3.3183999	-0.7424469
21.2333336	3.3671999	-0.9510679
21.2333336	3.4159999	-0.9449320
21.2333336	3.4647999	-0.6135921
21.2333336	3.5135999	-0.6074562
21.2333336	3.5623999	-0.5951843
21.2333336	3.6111999	-0.7240391
21.2333336	3.6599998	-0.7976699
21.2333336	3.7087998	-0.7976699
21.2333336	3.7575998	-0.6135921
21.2333336	3.8063998	-0.9326606
21.2333336	3.8551998	-0.9326606
21.2333336	3.9039998	-0.9510679
21.2333336	3.9527998	-0.4540582
21.2333336	4.0016003	-0.7301750
21.2333336	4.0503998	-0.7179031
21.2333336	4.0991998	-0.8897090
21.2333336	4.1479998	-0.8897090
21.2333336	4.1967998	-0.9142528
21.2333336	4.2455997	-0.7179031
21.2333336	4.2943997	-0.7179031
21.2333336	4.3431997	-0.5031457
21.2333336	4.3919997	-0.5829129
21.2333336	4.4407997	-0.5829129
21.2333336	4.4895997	-0.6565437
21.2333336	4.5383997	-0.6197281
21.2333336	4.5871997	-0.5338254
21.2333336	4.6359997	-0.5154176
21.2333336	4.6847997	613586.0000000
21.2333336	4.7336001	613586.0000000
21.2333336	4.7824001	613586.0000000
21.2333336	4.8312001	613586.0000000
21.2333336	4.8800001	613586.0000000
21.2333336	4.9288001	613586.0000000
21.2333336	4.9776001	-0.4724660
21.2333336	5.0264001	-0.4724660
21.2333336	5.0752001	613586.0000000
21.2333336	5.1240001	613586.0000000

21.2333336	5.1728001	613586.0000000
21.2333336	5.2216001	-0.5276895
21.2333336	5.2704000	-0.5215535
21.2333336	5.3192000	613586.0000000
21.2333336	5.3680000	613586.0000000
21.2333336	5.4168000	613586.0000000
21.2333336	5.4656000	-0.6504078
21.2333336	5.5144000	-0.6504078
21.2333336	5.5632000	613586.0000000
21.2333336	5.6120000	613586.0000000
21.2333336	5.6608000	613586.0000000
21.2333336	5.7096000	613586.0000000
21.2333336	5.7584000	613586.0000000
21.2333336	5.8072000	613586.0000000
21.2333336	5.8559999	613586.0000000
21.2333336	5.9047999	613586.0000000
21.2333336	5.9535999	613586.0000000
21.2333336	6.0023999	613586.0000000
21.2500000	0.2928000	-1.6812429
21.2500000	0.3416000	-1.4910293
21.2500000	0.3904000	-1.3744469
21.2500000	0.4392000	-1.3499031
21.2500000	0.4880000	-1.3560390
21.2500000	0.5368000	-1.3376312
21.2500000	0.5856000	-1.4235344
21.2500000	0.6344000	-1.5401168
21.2500000	0.6832000	-1.4051266
21.2500000	0.7320000	-1.5094371
21.2500000	0.7808000	-1.5033011
21.2500000	0.8296000	-1.4358063
21.2500000	0.8784000	-1.4358063
21.2500000	0.9272000	-1.4235344
21.2500000	0.9760000	-1.5585246
21.2500000	1.0247999	-1.6014762
21.2500000	1.0735999	-1.4480777
21.2500000	1.1223999	-1.3499031
21.2500000	1.1711999	-1.3499031
21.2500000	1.2200000	-1.3499031
21.2500000	1.2688000	-1.3560390
21.2500000	1.3176000	-1.3560390
21.2500000	1.3664000	-1.4603496
21.2500000	1.4152000	-1.3683109
21.2500000	1.4640000	-1.3621750
21.2500000	1.5128000	-1.3437672
21.2500000	1.5616000	-1.3130875
21.2500000	1.6104000	-1.4848933
21.2500000	1.6592000	-1.4848933
21.2500000	1.7079999	-1.4910293
21.2500000	1.7567999	-1.4787574
21.2500000	1.8055999	-1.5585246
21.2500000	1.8543999	-1.5646605
21.2500000	1.9031999	-1.3867188
21.2500000	1.9519999	-1.4603496
21.2500000	2.0007999	-1.5278449
21.2500000	2.0495999	-1.4542136
21.2500000	2.0983999	-1.5769324
21.2500000	2.1471999	-1.6505632

21.2500000	2.1959999	-1.6076121
21.2500000	2.2447999	-1.5339808
21.2500000	2.2935998	-1.5401168
21.2500000	2.3423998	-1.4419422
21.2500000	2.3912001	-1.4480777
21.2500000	2.4400001	-1.2946796
21.2500000	2.4888000	-1.6137476
21.2500000	2.5376000	-1.2762718
21.2500000	2.5864000	-1.2946796
21.2500000	2.6352000	-1.2210488
21.2500000	2.6840000	-1.2333207
21.2500000	2.7328000	-1.2271848
21.2500000	2.7816000	-1.0983305
21.2500000	2.8304000	-1.0860586
21.2500000	2.8792000	-0.8774371
21.2500000	2.9280000	-0.4724660
21.2500000	2.9768000	-0.1104465
21.2500000	3.0256000	-0.2147574
21.2500000	3.0743999	-0.2208934
21.2500000	3.1231999	-0.7792621
21.2500000	3.1719999	-0.7179031
21.2500000	3.2207999	-0.7179031
21.2500000	3.2695999	-1.1596894
21.2500000	3.3183999	-1.0921946
21.2500000	3.3671999	-1.1044660
21.2500000	3.4159999	-1.1106019
21.2500000	3.4647999	-0.6565437
21.2500000	3.5135999	-0.7240391
21.2500000	3.5623999	-0.7240391
21.2500000	3.6111999	-0.8160777
21.2500000	3.6599998	-0.6872234
21.2500000	3.7087998	-0.9203887
21.2500000	3.7575998	-0.9019809
21.2500000	3.8063998	-0.9019809
21.2500000	3.8551998	-0.9142528
21.2500000	3.9039998	-0.5399613
21.2500000	3.9527998	-0.5706410
21.2500000	4.0016003	-0.5767770
21.2500000	4.0503998	-0.8774371
21.2500000	4.0991998	-0.8713012
21.2500000	4.1479998	-0.7424469
21.2500000	4.1967998	-0.6135921
21.2500000	4.2455997	-0.5829129
21.2500000	4.2943997	-0.6381359
21.2500000	4.3431997	-0.6872234
21.2500000	4.3919997	-0.6933594
21.2500000	4.4407997	-0.7117672
21.2500000	4.4895997	-0.6320000
21.2500000	4.5383997	-0.5706410
21.2500000	4.5871997	-0.4786019
21.2500000	4.6359997	-0.4724660
21.2500000	4.6847997	-0.4540582
21.2500000	4.7336001	613586.0000000
21.2500000	4.7824001	613586.0000000
21.2500000	4.8312001	613586.0000000
21.2500000	4.8800001	-0.5951843
21.2500000	4.9288001	-0.3620195

21.2500000	4.9776001	613586.0000000
21.2500000	5.0264001	-0.5215535
21.2500000	5.0752001	-0.5583692
21.2500000	5.1240001	-0.5460973
21.2500000	5.1728001	-0.5154176
21.2500000	5.2216001	-0.5092816
21.2500000	5.2704000	-0.7669902
21.2500000	5.3192000	613586.0000000
21.2500000	5.3680000	-0.6749516
21.2500000	5.4168000	-0.6074562
21.2500000	5.4656000	-0.6381359
21.2500000	5.5144000	613586.0000000
21.2500000	5.5632000	613586.0000000
21.2500000	5.6120000	613586.0000000
21.2500000	5.6608000	613586.0000000
21.2500000	5.7096000	613586.0000000
21.2500000	5.7584000	613586.0000000
21.2500000	5.8072000	613586.0000000
21.2500000	5.8559999	613586.0000000
21.2500000	5.9047999	613586.0000000
21.2500000	5.9535999	613586.0000000
21.2500000	6.0023999	613586.0000000
21.2666664	0.2928000	-1.5953403
21.2666664	0.3416000	-1.3928547
21.2666664	0.3904000	-1.3867188
21.2666664	0.4392000	-1.3867188
21.2666664	0.4880000	-1.7855535
21.2666664	0.5368000	-1.4542136
21.2666664	0.5856000	-1.4358063
21.2666664	0.6344000	-1.4358063
21.2666664	0.6832000	-1.3867188
21.2666664	0.7320000	-1.5217090
21.2666664	0.7808000	-1.5217090
21.2666664	0.8296000	-1.5278449
21.2666664	0.8784000	-1.5155730
21.2666664	0.9272000	-1.5585246
21.2666664	0.9760000	-1.5155730
21.2666664	1.0247999	-1.4419422
21.2666664	1.0735999	-1.4419422
21.2666664	1.1223999	-1.1965051
21.2666664	1.1711999	-1.3008156
21.2666664	1.2200000	-1.3008156
21.2666664	1.2688000	-1.3008156
21.2666664	1.3176000	-1.3008156
21.2666664	1.3664000	-1.5646605
21.2666664	1.4152000	-1.4787574
21.2666664	1.4640000	-1.4787574
21.2666664	1.5128000	-1.4848933
21.2666664	1.5616000	-1.2640004
21.2666664	1.6104000	-1.2517285
21.2666664	1.6592000	-1.2578645
21.2666664	1.7079999	-1.3192234
21.2666664	1.7567999	-1.3130875
21.2666664	1.8055999	-1.3069515
21.2666664	1.8543999	-1.1903691
21.2666664	1.9031999	-1.0001554
21.2666664	1.9519999	-1.0124273

21.2666664	2.0007999	-1.1596894
21.2666664	2.0495999	-1.2946796
21.2666664	2.0983999	-1.2946796
21.2666664	2.1471999	-1.3069515
21.2666664	2.1959999	-1.2885437
21.2666664	2.2447999	-1.2824078
21.2666664	2.2935998	-1.1535535
21.2666664	2.3423998	-1.3683109
21.2666664	2.3912001	-1.2210488
21.2666664	2.4400001	-1.2517285
21.2666664	2.4888000	-1.2026410
21.2666664	2.5376000	-1.3008156
21.2666664	2.5864000	-1.1842332
21.2666664	2.6352000	-1.2517285
21.2666664	2.6840000	-1.5033011
21.2666664	2.7328000	-1.3499031
21.2666664	2.7816000	-1.4480777
21.2666664	2.8304000	-1.2701364
21.2666664	2.8792000	-1.3805828
21.2666664	2.9280000	-1.3928547
21.2666664	2.9768000	-1.1658254
21.2666664	3.0256000	-1.1842332
21.2666664	3.0743999	-1.1658254
21.2666664	3.1231999	-1.0431070
21.2666664	3.1719999	-1.0431070
21.2666664	3.2207999	-1.0308352
21.2666664	3.2695999	-0.9019809
21.2666664	3.3183999	-1.1903691
21.2666664	3.3671999	-1.1965051
21.2666664	3.4159999	-1.2026410
21.2666664	3.4647999	-1.1903691
21.2666664	3.5135999	-0.7731261
21.2666664	3.5623999	-0.7669902
21.2666664	3.6111999	-0.7608547
21.2666664	3.6599998	-0.6749516
21.2666664	3.7087998	-0.6749516
21.2666664	3.7575998	-0.6626797
21.2666664	3.8063998	-0.6933594
21.2666664	3.8551998	-0.6749516
21.2666664	3.9039998	-0.8038058
21.2666664	3.9527998	-0.8222136
21.2666664	4.0016003	-0.8283496
21.2666664	4.0503998	-0.8283496
21.2666664	4.0991998	-0.7424469
21.2666664	4.1479998	-0.6442719
21.2666664	4.1967998	-0.6565437
21.2666664	4.2455997	-0.6749516
21.2666664	4.2943997	-0.8160777
21.2666664	4.3431997	-0.7669902
21.2666664	4.3919997	-0.7179031
21.2666664	4.4407997	-0.7179031
21.2666664	4.4895997	-0.7056313
21.2666664	4.5383997	-0.6135921
21.2666664	4.5871997	-0.6135921
21.2666664	4.6359997	-0.5583692
21.2666664	4.6847997	-0.5706410
21.2666664	4.7336001	-0.6381359

21.2666664	4.7824001	-0.6381359
21.2666664	4.8312001	-0.6320000
21.2666664	4.8800001	613586.0000000
21.2666664	4.9288001	-0.3006601
21.2666664	4.9776001	613586.0000000
21.2666664	5.0264001	613586.0000000
21.2666664	5.0752001	613586.0000000
21.2666664	5.1240001	613586.0000000
21.2666664	5.1728001	613586.0000000
21.2666664	5.2216001	613586.0000000
21.2666664	5.2704000	-0.6320000
21.2666664	5.3192000	-0.6197281
21.2666664	5.3680000	-0.5522332
21.2666664	5.4168000	-0.5522332
21.2666664	5.4656000	613586.0000000
21.2666664	5.5144000	613586.0000000
21.2666664	5.5632000	613586.0000000
21.2666664	5.6120000	613586.0000000
21.2666664	5.6608000	613586.0000000
21.2666664	5.7096000	613586.0000000
21.2666664	5.7584000	613586.0000000
21.2666664	5.8072000	613586.0000000
21.2666664	5.8559999	613586.0000000
21.2666664	5.9047999	613586.0000000
21.2666664	5.9535999	613586.0000000
21.2666664	6.0023999	613586.0000000
21.2833328	0.2928000	-1.2210488
21.2833328	0.3416000	-1.3008156
21.2833328	0.3904000	-1.3008156
21.2833328	0.4392000	-1.3008156
21.2833328	0.4880000	-1.2578645
21.2833328	0.5368000	-1.2640004
21.2833328	0.5856000	-1.2578645
21.2833328	0.6344000	-1.2578645
21.2833328	0.6832000	-1.4910293
21.2833328	0.7320000	-1.1719613
21.2833328	0.7808000	-1.2701364
21.2833328	0.8296000	-1.2333207
21.2833328	0.8784000	-1.2333207
21.2833328	0.9272000	-1.6260195
21.2833328	0.9760000	-1.6076121
21.2833328	1.0247999	-1.3744469
21.2833328	1.0735999	-1.5892043
21.2833328	1.1223999	-1.6628351
21.2833328	1.1711999	-1.4235344
21.2833328	1.2200000	-1.2578645
21.2833328	1.2688000	-1.3805828
21.2833328	1.3176000	-1.3314953
21.2833328	1.3664000	-1.0737867
21.2833328	1.4152000	-1.1474175
21.2833328	1.4640000	-1.1535535
21.2833328	1.5128000	-1.2333207
21.2833328	1.5616000	-1.2271848
21.2833328	1.6104000	-1.3008156
21.2833328	1.6592000	-1.3069515
21.2833328	1.7079999	-1.2087770
21.2833328	1.7567999	-1.2333207

21.2833328	1.8055999	-1.2210488
21.2833328	1.8543999	-1.2149129
21.2833328	1.9031999	-1.0799227
21.2833328	1.9519999	-1.1658254
21.2833328	2.0007999	-1.1290097
21.2833328	2.0495999	-1.0737867
21.2833328	2.0983999	-1.1719613
21.2833328	2.1471999	-1.1903691
21.2833328	2.1959999	-1.0369711
21.2833328	2.2447999	-0.9510679
21.2833328	2.2935998	-0.9510679
21.2833328	2.3423998	-0.8774371
21.2833328	2.3912001	-0.8835731
21.2833328	2.4400001	-0.8774371
21.2833328	2.4888000	-0.8283496
21.2833328	2.5376000	-0.8283496
21.2833328	2.5864000	-0.8713012
21.2833328	2.6352000	-1.0124273
21.2833328	2.6840000	-1.0246992
21.2833328	2.7328000	-1.1842332
21.2833328	2.7816000	-0.9142528
21.2833328	2.8304000	-0.9142528
21.2833328	2.8792000	-0.9203887
21.2833328	2.9280000	-1.0492430
21.2833328	2.9768000	-1.0492430
21.2833328	3.0256000	-1.1596894
21.2833328	3.0743999	-1.0185633
21.2833328	3.1231999	-0.8958449
21.2833328	3.1719999	-0.8835731
21.2833328	3.2207999	-0.8774371
21.2833328	3.2695999	-0.7547188
21.2833328	3.3183999	-0.4233789
21.2833328	3.3671999	-0.4356503
21.2833328	3.4159999	-0.5276895
21.2833328	3.4647999	-0.4479222
21.2833328	3.5135999	-0.5338254
21.2833328	3.5623999	0.0122719
21.2833328	3.6111999	0.0184078
21.2833328	3.6599998	0.0368156
21.2833328	3.7087998	-0.3374758
21.2833328	3.7575998	-0.6994953
21.2833328	3.8063998	-0.4356503
21.2833328	3.8551998	-0.6442719
21.2833328	3.9039998	-0.5460973
21.2833328	3.9527998	-0.8099418
21.2833328	4.0016003	-0.7853980
21.2833328	4.0503998	-0.8590293
21.2833328	4.0991998	-0.7363110
21.2833328	4.1479998	-0.7424469
21.2833328	4.1967998	-0.7240391
21.2833328	4.2455997	-0.7179031
21.2833328	4.2943997	-0.8406215
21.2833328	4.3431997	-0.7056313
21.2833328	4.3919997	-0.7731261
21.2833328	4.4407997	-0.6933594
21.2833328	4.4895997	-0.6933594
21.2833328	4.5383997	-0.7240391

21.2833328	4.5871997	-0.7117672
21.2833328	4.6359997	-0.6320000
21.2833328	4.6847997	-0.6320000
21.2833328	4.7336001	-0.6320000
21.2833328	4.7824001	-0.7608547
21.2833328	4.8312001	-0.7669902
21.2833328	4.8800001	613586.0000000
21.2833328	4.9288001	613586.0000000
21.2833328	4.9776001	613586.0000000
21.2833328	5.0264001	613586.0000000
21.2833328	5.0752001	613586.0000000
21.2833328	5.1240001	613586.0000000
21.2833328	5.1728001	-0.7056313
21.2833328	5.2216001	613586.0000000
21.2833328	5.2704000	613586.0000000
21.2833328	5.3192000	613586.0000000
21.2833328	5.3680000	-0.6688156
21.2833328	5.4168000	-0.6565437
21.2833328	5.4656000	613586.0000000
21.2833328	5.5144000	613586.0000000
21.2833328	5.5632000	613586.0000000
21.2833328	5.6120000	613586.0000000
21.2833328	5.6608000	-0.5890489
21.2833328	5.7096000	613586.0000000
21.2833328	5.7584000	613586.0000000
21.2833328	5.8072000	613586.0000000
21.2833328	5.8559999	-0.7117672
21.2833328	5.9047999	613586.0000000
21.2833328	5.9535999	-0.8835731
21.2833328	6.0023999	-0.8774371
21.2999992	0.2928000	-1.5585246
21.2999992	0.3416000	-1.2455926
21.2999992	0.3904000	-1.2946796
21.2999992	0.4392000	-1.3192234
21.2999992	0.4880000	-1.3621750
21.2999992	0.5368000	-1.3069515
21.2999992	0.5856000	-1.3499031
21.2999992	0.6344000	-1.3499031
21.2999992	0.6832000	-1.1290097
21.2999992	0.7320000	-1.1228738
21.2999992	0.7808000	-1.0308352
21.2999992	0.8296000	-1.0553789
21.2999992	0.8784000	-1.2824078
21.2999992	0.9272000	-1.2946796
21.2999992	0.9760000	-1.1596894
21.2999992	1.0247999	-1.1412816
21.2999992	1.0735999	-0.7547188
21.2999992	1.1223999	-0.7547188
21.2999992	1.1711999	-1.2517285
21.2999992	1.2200000	-1.2640004
21.2999992	1.2688000	-1.2087770
21.2999992	1.3176000	-1.2087770
21.2999992	1.3664000	-1.2087770
21.2999992	1.4152000	-1.1780972
21.2999992	1.4640000	-1.1658254
21.2999992	1.5128000	-1.1596894
21.2999992	1.5616000	-1.0615149

21.2999992	1.6104000	-0.9142528
21.2999992	1.6592000	-1.0124273
21.2999992	1.7079999	-1.0369711
21.2999992	1.7567999	-1.0369711
21.2999992	1.8055999	-1.0062914
21.2999992	1.8543999	-0.9142528
21.2999992	1.9031999	-0.9081168
21.2999992	1.9519999	-1.0492430
21.2999992	2.0007999	-1.0369711
21.2999992	2.0495999	-0.9817476
21.2999992	2.0983999	-1.1106019
21.2999992	2.1471999	-1.1474175
21.2999992	2.1959999	-1.0001554
21.2999992	2.2447999	-0.8897090
21.2999992	2.2935998	-1.1351457
21.2999992	2.3423998	-1.1290097
21.2999992	2.3912001	-0.9817476
21.2999992	2.4400001	-0.9694757
21.2999992	2.4888000	-0.9633398
21.2999992	2.5376000	-1.0492430
21.2999992	2.5864000	-0.8222136
21.2999992	2.6352000	-0.9940195
21.2999992	2.6840000	-1.0001554
21.2999992	2.7328000	-0.9203887
21.2999992	2.7816000	-0.8283496
21.2999992	2.8304000	-0.8222136
21.2999992	2.8792000	-0.8222136
21.2999992	2.9280000	-0.8283496
21.2999992	2.9768000	-0.8222136
21.2999992	3.0256000	-0.8651652
21.2999992	3.0743999	-0.6013203
21.2999992	3.1231999	-0.3804274
21.2999992	3.1719999	-0.3926992
21.2999992	3.2207999	-0.5951843
21.2999992	3.2695999	-0.4786019
21.2999992	3.3183999	-0.6197281
21.2999992	3.3671999	-0.4417863
21.2999992	3.4159999	-0.4356503
21.2999992	3.4647999	-0.2883883
21.2999992	3.5135999	-0.1779418
21.2999992	3.5623999	-0.2515731
21.2999992	3.6111999	-0.4111071
21.2999992	3.6599998	-0.8835731
21.2999992	3.7087998	-0.8958449
21.2999992	3.7575998	-0.8958449
21.2999992	3.8063998	-0.8099418
21.2999992	3.8551998	-0.6381359
21.2999992	3.9039998	-0.8467574
21.2999992	3.9527998	-0.8528934
21.2999992	4.0016003	-0.7547188
21.2999992	4.0503998	-0.7424469
21.2999992	4.0991998	-0.8467574
21.2999992	4.1479998	-0.8651652
21.2999992	4.1967998	-0.8774371
21.2999992	4.2455997	-0.8774371
21.2999992	4.2943997	-0.7117672
21.2999992	4.3431997	-0.7056313

21.2999992	4.3919997	-0.6442719
21.2999992	4.4407997	-0.6381359
21.2999992	4.4895997	-0.5829129
21.2999992	4.5383997	-0.5706410
21.2999992	4.5871997	-0.6810875
21.2999992	4.6359997	-0.5890489
21.2999992	4.6847997	-0.6872234
21.2999992	4.7336001	-0.6872234
21.2999992	4.7824001	613586.0000000
21.2999992	4.8312001	613586.0000000
21.2999992	4.8800001	613586.0000000
21.2999992	4.9288001	613586.0000000
21.2999992	4.9776001	-0.3252039
21.2999992	5.0264001	-0.3374758
21.2999992	5.0752001	-0.3620195
21.2999992	5.1240001	-0.5399613
21.2999992	5.1728001	-0.5399613
21.2999992	5.2216001	-0.5522332
21.2999992	5.2704000	-0.5645051
21.2999992	5.3192000	-0.5031457
21.2999992	5.3680000	-0.5338254
21.2999992	5.4168000	-0.5399613
21.2999992	5.4656000	-0.4479222
21.2999992	5.5144000	-0.4417863
21.2999992	5.5632000	-0.6258640
21.2999992	5.6120000	-0.5829129
21.2999992	5.6608000	613586.0000000
21.2999992	5.7096000	613586.0000000
21.2999992	5.7584000	613586.0000000
21.2999992	5.8072000	613586.0000000
21.2999992	5.8559999	613586.0000000
21.2999992	5.9047999	613586.0000000
21.2999992	5.9535999	613586.0000000
21.2999992	6.0023999	613586.0000000
21.3166676	0.2928000	-1.3928547
21.3166676	0.3416000	-1.5155730
21.3166676	0.3904000	-1.5155730
21.3166676	0.4392000	-1.3928547
21.3166676	0.4880000	-1.3928547
21.3166676	0.5368000	-1.3928547
21.3166676	0.5856000	-1.3744469
21.3166676	0.6344000	-1.4480777
21.3166676	0.6832000	-1.4480777
21.3166676	0.7320000	-1.1842332
21.3166676	0.7808000	-1.2640004
21.3166676	0.8296000	-1.1596894
21.3166676	0.8784000	-1.1474175
21.3166676	0.9272000	-1.2701364
21.3166676	0.9760000	-1.2578645
21.3166676	1.0247999	-1.1290097
21.3166676	1.0735999	-1.3253593
21.3166676	1.1223999	-1.3376312
21.3166676	1.1711999	-1.2946796
21.3166676	1.2200000	-1.1412816
21.3166676	1.2688000	-1.1474175
21.3166676	1.3176000	-1.3130875
21.3166676	1.3664000	-1.2455926

21.3166676	1.4152000	-1.1903691
21.3166676	1.4640000	-1.2885437
21.3166676	1.5128000	-1.1412816
21.3166676	1.5616000	-1.1290097
21.3166676	1.6104000	-1.2149129
21.3166676	1.6592000	-0.8651652
21.3166676	1.7079999	-1.1965051
21.3166676	1.7567999	-1.1965051
21.3166676	1.8055999	-1.1167378
21.3166676	1.8543999	-0.9449320
21.3166676	1.9031999	-0.9572039
21.3166676	1.9519999	-0.9633398
21.3166676	2.0007999	-1.1596894
21.3166676	2.0495999	-1.0492430
21.3166676	2.0983999	-1.0492430
21.3166676	2.1471999	-1.3130875
21.3166676	2.1959999	-1.0431070
21.3166676	2.2447999	-1.0553789
21.3166676	2.2935998	-1.0001554
21.3166676	2.3423998	-1.3314953
21.3166676	2.3912001	-1.0983305
21.3166676	2.4400001	-0.9265246
21.3166676	2.4888000	-0.9940195
21.3166676	2.5376000	-1.0246992
21.3166676	2.5864000	-1.0124273
21.3166676	2.6352000	-0.8528934
21.3166676	2.6840000	-0.8528934
21.3166676	2.7328000	-0.6565437
21.3166676	2.7816000	-0.5215535
21.3166676	2.8304000	-0.5215535
21.3166676	2.8792000	-0.7240391
21.3166676	2.9280000	-1.2026410
21.3166676	2.9768000	-1.0860586
21.3166676	3.0256000	-1.0983305
21.3166676	3.0743999	-1.0185633
21.3166676	3.1231999	-1.1412816
21.3166676	3.1719999	-0.7547188
21.3166676	3.2207999	-0.6320000
21.3166676	3.2695999	-1.0860586
21.3166676	3.3183999	-1.1228738
21.3166676	3.3671999	-1.0124273
21.3166676	3.4159999	-1.5217090
21.3166676	3.4647999	-1.4296703
21.3166676	3.5135999	-1.4296703
21.3166676	3.5623999	-1.0615149
21.3166676	3.6111999	-1.1658254
21.3166676	3.6599998	-1.1719613
21.3166676	3.7087998	-1.0185633
21.3166676	3.7575998	-1.0737867
21.3166676	3.8063998	-0.8835731
21.3166676	3.8551998	-0.8713012
21.3166676	3.9039998	-0.6749516
21.3166676	3.9527998	-0.6688156
21.3166676	4.0016003	-0.5951843
21.3166676	4.0503998	-0.4847379
21.3166676	4.0991998	-0.5829129
21.3166676	4.1479998	-0.5951843

21.3166676	4.1967998	-0.6013203
21.3166676	4.2455997	-0.5276895
21.3166676	4.2943997	-0.5338254
21.3166676	4.3431997	-0.5215535
21.3166676	4.3919997	-0.6381359
21.3166676	4.4407997	-0.5092816
21.3166676	4.4895997	-0.6933594
21.3166676	4.5383997	-0.4540582
21.3166676	4.5871997	-0.6074562
21.3166676	4.6359997	-0.7669902
21.3166676	4.6847997	-0.7669902
21.3166676	4.7336001	-0.5706410
21.3166676	4.7824001	-0.5706410
21.3166676	4.8312001	-0.3804274
21.3166676	4.8800001	-0.5645051
21.3166676	4.9288001	-0.5645051
21.3166676	4.9776001	613586.0000000
21.3166676	5.0264001	613586.0000000
21.3166676	5.0752001	-0.2883883
21.3166676	5.1240001	-0.3988352
21.3166676	5.1728001	-0.3988352
21.3166676	5.2216001	-0.4601941
21.3166676	5.2704000	-0.3436117
21.3166676	5.3192000	-0.3558836
21.3166676	5.3680000	-0.4111071
21.3166676	5.4168000	-0.3865633
21.3166676	5.4656000	-0.3865633
21.3166676	5.5144000	613586.0000000
21.3166676	5.5632000	-0.2086215
21.3166676	5.6120000	-0.2086215
21.3166676	5.6608000	613586.0000000
21.3166676	5.7096000	613586.0000000
21.3166676	5.7584000	613586.0000000
21.3166676	5.8072000	613586.0000000
21.3166676	5.8559999	613586.0000000
21.3166676	5.9047999	613586.0000000
21.3166676	5.9535999	613586.0000000
21.3166676	6.0023999	613586.0000000
21.3333340	0.2928000	-1.2946796
21.3333340	0.3416000	-1.8162332
21.3333340	0.3904000	-1.7119226
21.3333340	0.4392000	-1.7057867
21.3333340	0.4880000	-1.2394567
21.3333340	0.5368000	-1.2394567
21.3333340	0.5856000	-1.2455926
21.3333340	0.6344000	-1.3130875
21.3333340	0.6832000	-1.3192234
21.3333340	0.7320000	-1.1658254
21.3333340	0.7808000	-1.1719613
21.3333340	0.8296000	-1.3130875
21.3333340	0.8784000	-1.2394567
21.3333340	0.9272000	-1.2394567
21.3333340	0.9760000	-1.2455926
21.3333340	1.0247999	-1.0431070
21.3333340	1.0735999	-1.4664855
21.3333340	1.1223999	-1.2333207
21.3333340	1.1711999	-1.4173985

21.3333340	1.2200000	-1.4173985
21.3333340	1.2688000	-1.5339808
21.3333340	1.3176000	-1.3253593
21.3333340	1.3664000	-1.4480777
21.3333340	1.4152000	-1.4480777
21.3333340	1.4640000	-1.3989906
21.3333340	1.5128000	-1.4112625
21.3333340	1.5616000	-1.1903691
21.3333340	1.6104000	-1.3069515
21.3333340	1.6592000	-1.3130875
21.3333340	1.7079999	-1.3192234
21.3333340	1.7567999	-1.3192234
21.3333340	1.8055999	-1.2517285
21.3333340	1.8543999	-1.2087770
21.3333340	1.9031999	-1.2640004
21.3333340	1.9519999	-1.2701364
21.3333340	2.0007999	-1.1167378
21.3333340	2.0495999	-1.1167378
21.3333340	2.0983999	-1.0983305
21.3333340	2.1471999	-1.2394567
21.3333340	2.1959999	-1.2517285
21.3333340	2.2447999	-1.2640004
21.3333340	2.2935998	-1.0921946
21.3333340	2.3423998	-0.8958449
21.3333340	2.3912001	-0.9694757
21.3333340	2.4400001	-1.1535535
21.3333340	2.4888000	-1.1351457
21.3333340	2.5376000	-1.1903691
21.3333340	2.5864000	-1.1842332
21.3333340	2.6352000	-1.1780972
21.3333340	2.6840000	-1.1719613
21.3333340	2.7328000	-1.1596894
21.3333340	2.7816000	-0.9940195
21.3333340	2.8304000	-1.0001554
21.3333340	2.8792000	-0.9756117
21.3333340	2.9280000	-1.0001554
21.3333340	2.9768000	-1.0062914
21.3333340	3.0256000	-0.7179031
21.3333340	3.0743999	-1.3192234
21.3333340	3.1231999	-0.0859027
21.3333340	3.1719999	-0.0920386
21.3333340	3.2207999	-0.4233789
21.3333340	3.2695999	-0.6320000
21.3333340	3.3183999	-0.7853980
21.3333340	3.3671999	-0.7853980
21.3333340	3.4159999	-0.7792621
21.3333340	3.4647999	-0.7547188
21.3333340	3.5135999	-0.7547188
21.3333340	3.5623999	-0.7547188
21.3333340	3.6111999	-0.6135921
21.3333340	3.6599998	-0.5276895
21.3333340	3.7087998	-0.3129320
21.3333340	3.7575998	-0.4724660
21.3333340	3.8063998	-0.8835731
21.3333340	3.8551998	-0.8283496
21.3333340	3.9039998	-0.9572039
21.3333340	3.9527998	-0.8467574

21.3333340	4.0016003	-0.8344855
21.3333340	4.0503998	-0.8774371
21.3333340	4.0991998	-0.9694757
21.3333340	4.1479998	-0.9572039
21.3333340	4.1967998	-0.9572039
21.3333340	4.2455997	-1.0062914
21.3333340	4.2943997	-1.0124273
21.3333340	4.3431997	-0.8344855
21.3333340	4.3919997	-0.8774371
21.3333340	4.4407997	-0.7976699
21.3333340	4.4895997	-0.7976699
21.3333340	4.5383997	-0.7976699
21.3333340	4.5871997	-0.8283496
21.3333340	4.6359997	-0.8283496
21.3333340	4.6847997	-0.8344855
21.3333340	4.7336001	-0.7424469
21.3333340	4.7824001	-0.6626797
21.3333340	4.8312001	-0.9142528
21.3333340	4.8800001	-0.8958449
21.3333340	4.9288001	-0.5829129
21.3333340	4.9776001	-0.5399613
21.3333340	5.0264001	-0.5583692
21.3333340	5.0752001	613586.0000000
21.3333340	5.1240001	-0.7547188
21.3333340	5.1728001	-0.8590293
21.3333340	5.2216001	-0.7424469
21.3333340	5.2704000	-0.7301750
21.3333340	5.3192000	-0.6810875
21.3333340	5.3680000	-0.6810875
21.3333340	5.4168000	-0.5829129
21.3333340	5.4656000	-0.5890489
21.3333340	5.5144000	613586.0000000
21.3333340	5.5632000	613586.0000000
21.3333340	5.6120000	613586.0000000
21.3333340	5.6608000	-0.4663301
21.3333340	5.7096000	-0.4663301
21.3333340	5.7584000	-0.4049711
21.3333340	5.8072000	-0.4295144
21.3333340	5.8559999	613586.0000000
21.3333340	5.9047999	613586.0000000
21.3333340	5.9535999	613586.0000000
21.3333340	6.0023999	-0.5215535
21.3500004	0.2928000	-1.7548742
21.3500004	0.3416000	-1.4480777
21.3500004	0.3904000	-1.6935148
21.3500004	0.4392000	-1.6935148
21.3500004	0.4880000	-1.4235344
21.3500004	0.5368000	-1.4235344
21.3500004	0.5856000	-1.4726214
21.3500004	0.6344000	-1.2640004
21.3500004	0.6832000	-1.2701364
21.3500004	0.7320000	-1.3683109
21.3500004	0.7808000	-1.3683109
21.3500004	0.8296000	-1.3314953
21.3500004	0.8784000	-1.3192234
21.3500004	0.9272000	-1.2517285
21.3500004	0.9760000	-1.2578645

21.3500004	1.0247999	-1.2701364
21.3500004	1.0735999	-1.4971652
21.3500004	1.1223999	-1.2824078
21.3500004	1.1711999	-1.2885437
21.3500004	1.2200000	-1.3314953
21.3500004	1.2688000	-1.3314953
21.3500004	1.3176000	-1.2762718
21.3500004	1.3664000	-1.2762718
21.3500004	1.4152000	-1.1351457
21.3500004	1.4640000	-1.2394567
21.3500004	1.5128000	-1.4112625
21.3500004	1.5616000	-1.4051266
21.3500004	1.6104000	-1.3314953
21.3500004	1.6592000	-1.5401168
21.3500004	1.7079999	-1.4480777
21.3500004	1.7567999	-1.4235344
21.3500004	1.8055999	-1.6689711
21.3500004	1.8543999	-1.6566992
21.3500004	1.9031999	-1.6505632
21.3500004	1.9519999	-1.6505632
21.3500004	2.0007999	-1.6628351
21.3500004	2.0495999	-1.2946796
21.3500004	2.0983999	-1.2946796
21.3500004	2.1471999	-1.2885437
21.3500004	2.1959999	-1.4296703
21.3500004	2.2447999	-1.4358063
21.3500004	2.2935998	-1.4358063
21.3500004	2.3423998	-1.4480777
21.3500004	2.3912001	-1.5339808
21.3500004	2.4400001	-1.5339808
21.3500004	2.4888000	-1.5401168
21.3500004	2.5376000	-1.3069515
21.3500004	2.5864000	-1.3499031
21.3500004	2.6352000	-1.3560390
21.3500004	2.6840000	-1.1842332
21.3500004	2.7328000	-1.1842332
21.3500004	2.7816000	-1.3376312
21.3500004	2.8304000	-1.1290097
21.3500004	2.8792000	-1.1228738
21.3500004	2.9280000	-0.9756117
21.3500004	2.9768000	-0.9142528
21.3500004	3.0256000	-0.8038058
21.3500004	3.0743999	-0.8651652
21.3500004	3.1231999	-0.8590293
21.3500004	3.1719999	-1.0737867
21.3500004	3.2207999	-1.0062914
21.3500004	3.2695999	-1.1780972
21.3500004	3.3183999	-1.1719613
21.3500004	3.3671999	-0.7179031
21.3500004	3.4159999	-0.6258640
21.3500004	3.4647999	-0.0859027
21.3500004	3.5135999	-0.0859027
21.3500004	3.5623999	-0.3681555
21.3500004	3.6111999	-0.3558836
21.3500004	3.6599998	-0.5829129
21.3500004	3.7087998	-0.5645051
21.3500004	3.7575998	-0.5706410

21.3500004	3.8063998	-0.6197281
21.3500004	3.8551998	-0.7301750
21.3500004	3.9039998	-0.7301750
21.3500004	3.9527998	-0.8283496
21.3500004	4.0016003	-0.7301750
21.3500004	4.0503998	-0.8283496
21.3500004	4.0991998	-0.8283496
21.3500004	4.1479998	-0.8283496
21.3500004	4.1967998	-0.7424469
21.3500004	4.2455997	-0.8835731
21.3500004	4.2943997	-1.0308352
21.3500004	4.3431997	-0.7915339
21.3500004	4.3919997	-0.7853980
21.3500004	4.4407997	-0.9940195
21.3500004	4.4895997	-0.9756117
21.3500004	4.5383997	-0.8528934
21.3500004	4.5871997	-0.9265246
21.3500004	4.6359997	-0.9265246
21.3500004	4.6847997	-0.9203887
21.3500004	4.7336001	-0.9203887
21.3500004	4.7824001	-0.9203887
21.3500004	4.8312001	-0.7669902
21.3500004	4.8800001	-0.5829129
21.3500004	4.9288001	-0.6626797
21.3500004	4.9776001	-0.6749516
21.3500004	5.0264001	-0.6872234
21.3500004	5.0752001	613586.0000000
21.3500004	5.1240001	-0.4908738
21.3500004	5.1728001	-0.3497477
21.3500004	5.2216001	-0.3681555
21.3500004	5.2704000	-0.4479222
21.3500004	5.3192000	-0.3742914
21.3500004	5.3680000	-0.3497477
21.3500004	5.4168000	-0.3313398
21.3500004	5.4656000	-0.3374758
21.3500004	5.5144000	-0.4601941
21.3500004	5.5632000	613586.0000000
21.3500004	5.6120000	613586.0000000
21.3500004	5.6608000	-0.3436117
21.3500004	5.7096000	613586.0000000
21.3500004	5.7584000	-0.4540582
21.3500004	5.8072000	-0.4479222
21.3500004	5.8559999	-0.4295144
21.3500004	5.9047999	613586.0000000
21.3500004	5.9535999	613586.0000000
21.3500004	6.0023999	613586.0000000
21.3666668	0.2928000	-1.3069515
21.3666668	0.3416000	-1.5033011
21.3666668	0.3904000	-1.3989906
21.3666668	0.4392000	-1.4051266
21.3666668	0.4880000	-1.2885437
21.3666668	0.5368000	-1.2885437
21.3666668	0.5856000	-1.3560390
21.3666668	0.6344000	-1.3560390
21.3666668	0.6832000	-1.1965051
21.3666668	0.7320000	-1.4358063
21.3666668	0.7808000	-1.3621750

21.3666668	0.8296000	-1.3499031
21.3666668	0.8784000	-1.6566992
21.3666668	0.9272000	-1.6566992
21.3666668	0.9760000	-1.5585246
21.3666668	1.0247999	-1.5585246
21.3666668	1.0735999	-1.3683109
21.3666668	1.1223999	-1.3499031
21.3666668	1.1711999	-1.4419422
21.3666668	1.2200000	-1.4848933
21.3666668	1.2688000	-1.4910293
21.3666668	1.3176000	-1.3683109
21.3666668	1.3664000	-1.3621750
21.3666668	1.4152000	-1.3989906
21.3666668	1.4640000	-1.3928547
21.3666668	1.5128000	-1.3928547
21.3666668	1.5616000	-1.2394567
21.3666668	1.6104000	-1.3437672
21.3666668	1.6592000	-1.2824078
21.3666668	1.7079999	-1.2640004
21.3666668	1.7567999	-1.2087770
21.3666668	1.8055999	-1.2210488
21.3666668	1.8543999	-1.7732821
21.3666668	1.9031999	-1.7855535
21.3666668	1.9519999	-1.7119226
21.3666668	2.0007999	-1.7364664
21.3666668	2.0495999	-1.4787574
21.3666668	2.0983999	-1.5033011
21.3666668	2.1471999	-1.5033011
21.3666668	2.1959999	-1.3069515
21.3666668	2.2447999	-1.3130875
21.3666668	2.2935998	-1.4419422
21.3666668	2.3423998	-1.4358063
21.3666668	2.3912001	-1.5892043
21.3666668	2.4400001	-1.5769324
21.3666668	2.4888000	-1.7241945
21.3666668	2.5376000	-1.5953403
21.3666668	2.5864000	-1.4358063
21.3666668	2.6352000	-1.5339808
21.3666668	2.6840000	-1.5217090
21.3666668	2.7328000	-1.3683109
21.3666668	2.7816000	-1.4358063
21.3666668	2.8304000	-1.4542136
21.3666668	2.8792000	-1.4542136
21.3666668	2.9280000	-1.3253593
21.3666668	2.9768000	-1.3253593
21.3666668	3.0256000	-1.5155730
21.3666668	3.0743999	-1.5094371
21.3666668	3.1231999	-1.0921946
21.3666668	3.1719999	-1.0921946
21.3666668	3.2207999	-0.8958449
21.3666668	3.2695999	-0.7915339
21.3666668	3.3183999	-0.8038058
21.3666668	3.3671999	-0.9387960
21.3666668	3.4159999	-0.8038058
21.3666668	3.4647999	-0.9510679
21.3666668	3.5135999	-0.9510679
21.3666668	3.5623999	-1.0062914

21.3666668	3.6111999	-0.9081168
21.3666668	3.6599998	-0.9081168
21.3666668	3.7087998	-0.8038058
21.3666668	3.7575998	-0.8099418
21.3666668	3.8063998	-0.8774371
21.3666668	3.8551998	-0.9694757
21.3666668	3.9039998	-1.0246992
21.3666668	3.9527998	-1.0799227
21.3666668	4.0016003	-0.9878836
21.3666668	4.0503998	-0.9019809
21.3666668	4.0991998	-0.9081168
21.3666668	4.1479998	-0.9878836
21.3666668	4.1967998	-0.9940195
21.3666668	4.2455997	-0.8897090
21.3666668	4.2943997	-1.0799227
21.3666668	4.3431997	-1.0799227
21.3666668	4.3919997	-1.0799227
21.3666668	4.4407997	-1.0921946
21.3666668	4.4895997	-1.1106019
21.3666668	4.5383997	-1.1658254
21.3666668	4.5871997	-1.1596894
21.3666668	4.6359997	-0.9817476
21.3666668	4.6847997	-0.9756117
21.3666668	4.7336001	-0.9940195
21.3666668	4.7824001	-1.2517285
21.3666668	4.8312001	-1.0921946
21.3666668	4.8800001	-0.7547188
21.3666668	4.9288001	-0.7301750
21.3666668	4.9776001	-0.7240391
21.3666668	5.0264001	-0.7240391
21.3666668	5.0752001	-0.7485828
21.3666668	5.1240001	-0.7424469
21.3666668	5.1728001	-0.7485828
21.3666668	5.2216001	-0.5215535
21.3666668	5.2704000	-0.5338254
21.3666668	5.3192000	-0.3865633
21.3666668	5.3680000	-0.3804274
21.3666668	5.4168000	-0.3742914
21.3666668	5.4656000	-0.5215535
21.3666668	5.5144000	-0.5890489
21.3666668	5.5632000	-0.5890489
21.3666668	5.6120000	-0.5215535
21.3666668	5.6608000	-0.4540582
21.3666668	5.7096000	-0.4356503
21.3666668	5.7584000	-0.4233789
21.3666668	5.8072000	613586.0000000
21.3666668	5.8559999	-0.4724660
21.3666668	5.9047999	-0.4540582
21.3666668	5.9535999	-0.4540582
21.3666668	6.0023999	613586.0000000
21.3833332	0.2928000	-1.5953403
21.3833332	0.3416000	-1.7855535
21.3833332	0.3904000	-1.7916894
21.3833332	0.4392000	-1.6689711
21.3833332	0.4880000	-1.2394567
21.3833332	0.5368000	-1.2394567
21.3833332	0.5856000	-1.5278449

21.3833332	0.6344000	-1.6382914
21.3833332	0.6832000	-1.3621750
21.3833332	0.7320000	-1.4358063
21.3833332	0.7808000	-1.5523887
21.3833332	0.8296000	-1.4848933
21.3833332	0.8784000	-1.6076121
21.3833332	0.9272000	-1.6628351
21.3833332	0.9760000	-1.4726214
21.3833332	1.0247999	-1.4787574
21.3833332	1.0735999	-1.6076121
21.3833332	1.1223999	-1.5769324
21.3833332	1.1711999	-1.5707965
21.3833332	1.2200000	-1.5830684
21.3833332	1.2688000	-1.4603496
21.3833332	1.3176000	-1.5892043
21.3833332	1.3664000	-1.7671461
21.3833332	1.4152000	-1.6935148
21.3833332	1.4640000	-1.7241945
21.3833332	1.5128000	-1.4971652
21.3833332	1.5616000	-1.4603496
21.3833332	1.6104000	-1.2946796
21.3833332	1.6592000	-1.3192234
21.3833332	1.7079999	-1.3376312
21.3833332	1.7567999	-1.2824078
21.3833332	1.8055999	-1.3621750
21.3833332	1.8543999	-1.6382914
21.3833332	1.9031999	-1.6321554
21.3833332	1.9519999	-1.4051266
21.3833332	2.0007999	-1.3376312
21.3833332	2.0495999	-1.3008156
21.3833332	2.0983999	-1.3683109
21.3833332	2.1471999	-1.2455926
21.3833332	2.1959999	-1.1106019
21.3833332	2.2447999	-1.2946796
21.3833332	2.2935998	-1.4419422
21.3833332	2.3423998	-1.5462527
21.3833332	2.3912001	-1.5462527
21.3833332	2.4400001	-1.1780972
21.3833332	2.4888000	-1.0676508
21.3833332	2.5376000	-1.0860586
21.3833332	2.5864000	-1.0737867
21.3833332	2.6352000	-1.2517285
21.3833332	2.6840000	-1.2517285
21.3833332	2.7328000	-1.1106019
21.3833332	2.7816000	-1.0860586
21.3833332	2.8304000	-1.0860586
21.3833332	2.8792000	-1.0799227
21.3833332	2.9280000	-1.0676508
21.3833332	2.9768000	-1.0615149
21.3833332	3.0256000	-1.1535535
21.3833332	3.0743999	-1.3499031
21.3833332	3.1231999	-1.4358063
21.3833332	3.1719999	-1.2640004
21.3833332	3.2207999	-1.1351457
21.3833332	3.2695999	-1.3744469
21.3833332	3.3183999	-1.1167378
21.3833332	3.3671999	-1.1106019

21.3833332	3.4159999	-1.1167378
21.3833332	3.4647999	-1.1351457
21.3833332	3.5135999	-1.1903691
21.3833332	3.5623999	-1.0492430
21.3833332	3.6111999	-1.0983305
21.3833332	3.6599998	-0.9878836
21.3833332	3.7087998	-1.0799227
21.3833332	3.7575998	-1.0799227
21.3833332	3.8063998	-1.0369711
21.3833332	3.8551998	-1.0431070
21.3833332	3.9039998	-0.9572039
21.3833332	3.9527998	-0.9878836
21.3833332	4.0016003	-1.0001554
21.3833332	4.0503998	-1.0001554
21.3833332	4.0991998	-0.8344855
21.3833332	4.1479998	-0.8344855
21.3833332	4.1967998	-0.9203887
21.3833332	4.2455997	-0.9817476
21.3833332	4.2943997	-0.9694757
21.3833332	4.3431997	-0.9510679
21.3833332	4.3919997	-1.0062914
21.3833332	4.4407997	-0.9449320
21.3833332	4.4895997	-0.9387960
21.3833332	4.5383997	-0.9265246
21.3833332	4.5871997	-0.9265246
21.3833332	4.6359997	-0.9449320
21.3833332	4.6847997	-0.8651652
21.3833332	4.7336001	-0.8528934
21.3833332	4.7824001	-0.7485828
21.3833332	4.8312001	-0.6442719
21.3833332	4.8800001	-0.6381359
21.3833332	4.9288001	-0.5092816
21.3833332	4.9776001	-0.5092816
21.3833332	5.0264001	-0.7117672
21.3833332	5.0752001	-0.4479222
21.3833332	5.1240001	-0.4540582
21.3833332	5.1728001	-0.3190680
21.3833332	5.2216001	-0.5215535
21.3833332	5.2704000	-0.5645051
21.3833332	5.3192000	-0.4724660
21.3833332	5.3680000	-0.4786019
21.3833332	5.4168000	-0.5951843
21.3833332	5.4656000	-0.6013203
21.3833332	5.5144000	613586.0000000
21.3833332	5.5632000	613586.0000000
21.3833332	5.6120000	613586.0000000
21.3833332	5.6608000	613586.0000000
21.3833332	5.7096000	613586.0000000
21.3833332	5.7584000	613586.0000000
21.3833332	5.8072000	613586.0000000
21.3833332	5.8559999	-0.6565437
21.3833332	5.9047999	-0.5092816
21.3833332	5.9535999	-0.5154176
21.3833332	6.0023999	-0.5645051
21.3999996	0.2928000	-1.7180586
21.3999996	0.3416000	-1.7119226
21.3999996	0.3904000	-1.5892043

21.3999996	0.4392000	-1.4173985
21.3999996	0.4880000	-1.4173985
21.3999996	0.5368000	-1.2455926
21.3999996	0.5856000	-1.4787574
21.3999996	0.6344000	-1.2517285
21.3999996	0.6832000	-1.3499031
21.3999996	0.7320000	-1.2640004
21.3999996	0.7808000	-1.2701364
21.3999996	0.8296000	-1.2762718
21.3999996	0.8784000	-1.2578645
21.3999996	0.9272000	-1.3008156
21.3999996	0.9760000	-1.3069515
21.3999996	1.0247999	-1.3376312
21.3999996	1.0735999	-1.4971652
21.3999996	1.1223999	-1.5033011
21.3999996	1.1711999	-1.3437672
21.3999996	1.2200000	-1.5217090
21.3999996	1.2688000	-1.3867188
21.3999996	1.3176000	-1.4235344
21.3999996	1.3664000	-1.4235344
21.3999996	1.4152000	-1.4542136
21.3999996	1.4640000	-1.3621750
21.3999996	1.5128000	-1.3560390
21.3999996	1.5616000	-1.0676508
21.3999996	1.6104000	-1.0737867
21.3999996	1.6592000	-0.8406215
21.3999996	1.7079999	-1.1658254
21.3999996	1.7567999	-1.1596894
21.3999996	1.8055999	-1.3253593
21.3999996	1.8543999	-1.3314953
21.3999996	1.9031999	-1.3437672
21.3999996	1.9519999	-1.2946796
21.3999996	2.0007999	-1.3008156
21.3999996	2.0495999	-1.2946796
21.3999996	2.0983999	-1.1412816
21.3999996	2.1471999	-1.0799227
21.3999996	2.1959999	-1.0799227
21.3999996	2.2447999	-1.5462527
21.3999996	2.2935998	-1.2026410
21.3999996	2.3423998	-1.2517285
21.3999996	2.3912001	-1.2455926
21.3999996	2.4400001	-1.1780972
21.3999996	2.4888000	-1.1780972
21.3999996	2.5376000	-1.0737867
21.3999996	2.5864000	-1.0737867
21.3999996	2.6352000	-0.9694757
21.3999996	2.6840000	-1.0676508
21.3999996	2.7328000	-1.1167378
21.3999996	2.7816000	-1.0308352
21.3999996	2.8304000	-1.0001554
21.3999996	2.8792000	-1.0124273
21.3999996	2.9280000	-1.1228738
21.3999996	2.9768000	-0.9940195
21.3999996	3.0256000	-0.9817476
21.3999996	3.0743999	-1.2517285
21.3999996	3.1231999	-1.4726214
21.3999996	3.1719999	-1.3683109

21.3999996	3.2207999	-1.2333207
21.3999996	3.2695999	-1.2333207
21.3999996	3.3183999	-1.2394567
21.3999996	3.3671999	-1.3008156
21.3999996	3.4159999	-1.1842332
21.3999996	3.4647999	-1.1965051
21.3999996	3.5135999	-1.0124273
21.3999996	3.5623999	-1.0431070
21.3999996	3.6111999	-1.0431070
21.3999996	3.6599998	-0.9878836
21.3999996	3.7087998	-0.9019809
21.3999996	3.7575998	-1.0676508
21.3999996	3.8063998	-0.9510679
21.3999996	3.8551998	-0.9019809
21.3999996	3.9039998	-0.9081168
21.3999996	3.9527998	-0.7240391
21.3999996	4.0016003	-0.7363110
21.3999996	4.0503998	-0.8835731
21.3999996	4.0991998	-0.8774371
21.3999996	4.1479998	-0.8897090
21.3999996	4.1967998	-0.8467574
21.3999996	4.2455997	-0.8406215
21.3999996	4.2943997	-0.8528934
21.3999996	4.3431997	-0.9265246
21.3999996	4.3919997	-0.8222136
21.3999996	4.4407997	-0.8344855
21.3999996	4.4895997	-0.8222136
21.3999996	4.5383997	-0.9633398
21.3999996	4.5871997	-0.8897090
21.3999996	4.6359997	-0.9081168
21.3999996	4.6847997	-0.7792621
21.3999996	4.7336001	-0.7608547
21.3999996	4.7824001	-0.7608547
21.3999996	4.8312001	-0.7792621
21.3999996	4.8800001	-0.6565437
21.3999996	4.9288001	-0.8222136
21.3999996	4.9776001	-0.6135921
21.3999996	5.0264001	-0.6074562
21.3999996	5.0752001	-0.7240391
21.3999996	5.1240001	-0.7363110
21.3999996	5.1728001	-0.7424469
21.3999996	5.2216001	-0.7301750
21.3999996	5.2704000	-0.6197281
21.3999996	5.3192000	-0.6258640
21.3999996	5.3680000	-0.6258640
21.3999996	5.4168000	-0.7117672
21.3999996	5.4656000	-0.7301750
21.3999996	5.5144000	-0.7301750
21.3999996	5.5632000	-0.7363110
21.3999996	5.6120000	-0.7547188
21.3999996	5.6608000	-0.6258640
21.3999996	5.7096000	613586.0000000
21.3999996	5.7584000	-0.6994953
21.3999996	5.8072000	-0.7301750
21.3999996	5.8559999	-0.7363110
21.3999996	5.9047999	-0.7853980
21.3999996	5.9535999	-0.8099418

21.3999996	6.0023999	-0.9694757
21.4166660	0.2928000	-1.3805828
21.4166660	0.3416000	-1.1719613
21.4166660	0.3904000	-1.2394567
21.4166660	0.4392000	-1.2455926
21.4166660	0.4880000	-1.3499031
21.4166660	0.5368000	-1.5523887
21.4166660	0.5856000	-1.5585246
21.4166660	0.6344000	-1.3008156
21.4166660	0.6832000	-1.2762718
21.4166660	0.7320000	-1.2885437
21.4166660	0.7808000	-1.3253593
21.4166660	0.8296000	-1.3314953
21.4166660	0.8784000	-1.3314953
21.4166660	0.9272000	-1.4358063
21.4166660	0.9760000	-1.4726214
21.4166660	1.0247999	-1.3560390
21.4166660	1.0735999	-1.2824078
21.4166660	1.1223999	-1.1106019
21.4166660	1.1711999	-1.3008156
21.4166660	1.2200000	-1.2210488
21.4166660	1.2688000	-1.1351457
21.4166660	1.3176000	-1.3192234
21.4166660	1.3664000	-1.1412816
21.4166660	1.4152000	-1.1351457
21.4166660	1.4640000	-1.1351457
21.4166660	1.5128000	-0.8835731
21.4166660	1.5616000	-1.0246992
21.4166660	1.6104000	-1.0124273
21.4166660	1.6592000	-0.8160777
21.4166660	1.7079999	-0.8160777
21.4166660	1.7567999	-0.8897090
21.4166660	1.8055999	-1.0124273
21.4166660	1.8543999	-0.8222136
21.4166660	1.9031999	-0.8160777
21.4166660	1.9519999	-0.7301750
21.4166660	2.0007999	-0.7240391
21.4166660	2.0495999	-0.7240391
21.4166660	2.0983999	-0.8590293
21.4166660	2.1471999	-0.8590293
21.4166660	2.1959999	-0.8590293
21.4166660	2.2447999	-0.8590293
21.4166660	2.2935998	-0.8160777
21.4166660	2.3423998	-0.7976699
21.4166660	2.3912001	-0.7363110
21.4166660	2.4400001	-0.6872234
21.4166660	2.4888000	-0.5829129
21.4166660	2.5376000	-0.7240391
21.4166660	2.5864000	-0.7976699
21.4166660	2.6352000	-0.7547188
21.4166660	2.6840000	-0.5460973
21.4166660	2.7328000	-0.5460973
21.4166660	2.7816000	-0.5645051
21.4166660	2.8304000	-0.7179031
21.4166660	2.8792000	-0.7117672
21.4166660	2.9280000	-0.8590293
21.4166660	2.9768000	-0.8160777

21.4166660	3.0256000	-0.8099418
21.4166660	3.0743999	-0.6504078
21.4166660	3.1231999	-0.8713012
21.4166660	3.1719999	-0.8897090
21.4166660	3.2207999	-0.8406215
21.4166660	3.2695999	-0.8467574
21.4166660	3.3183999	-0.7485828
21.4166660	3.3671999	-0.9326606
21.4166660	3.4159999	-0.9326606
21.4166660	3.4647999	-0.8344855
21.4166660	3.5135999	-1.0921946
21.4166660	3.5623999	-0.9633398
21.4166660	3.6111999	-0.7547188
21.4166660	3.6599998	-0.9694757
21.4166660	3.7087998	-0.9326606
21.4166660	3.7575998	-0.9326606
21.4166660	3.8063998	-0.7731261
21.4166660	3.8551998	-0.6872234
21.4166660	3.9039998	-0.6810875
21.4166660	3.9527998	-0.6810875
21.4166660	4.0016003	-0.8222136
21.4166660	4.0503998	-0.7547188
21.4166660	4.0991998	-0.7669902
21.4166660	4.1479998	-0.7853980
21.4166660	4.1967998	-0.7976699
21.4166660	4.2455997	-0.8528934
21.4166660	4.2943997	-0.7240391
21.4166660	4.3431997	-0.7301750
21.4166660	4.3919997	-0.7363110
21.4166660	4.4407997	-0.7853980
21.4166660	4.4895997	-0.7608547
21.4166660	4.5383997	613586.0000000
21.4166660	4.5871997	613586.0000000
21.4166660	4.6359997	-0.5829129
21.4166660	4.6847997	-0.5460973
21.4166660	4.7336001	-0.5522332
21.4166660	4.7824001	-0.5460973
21.4166660	4.8312001	-0.5215535
21.4166660	4.8800001	-0.6197281
21.4166660	4.9288001	-0.4417863
21.4166660	4.9776001	-0.5522332
21.4166660	5.0264001	-0.4847379
21.4166660	5.0752001	-0.4540582
21.4166660	5.1240001	613586.0000000
21.4166660	5.1728001	-0.5031457
21.4166660	5.2216001	-0.4970098
21.4166660	5.2704000	-0.4847379
21.4166660	5.3192000	-0.5645051
21.4166660	5.3680000	-0.5583692
21.4166660	5.4168000	-0.5215535
21.4166660	5.4656000	-0.4111071
21.4166660	5.5144000	-0.5767770
21.4166660	5.5632000	-0.5399613
21.4166660	5.6120000	613586.0000000
21.4166660	5.6608000	613586.0000000
21.4166660	5.7096000	613586.0000000
21.4166660	5.7584000	-0.5276895

21.4166660	5.8072000	-0.5154176
21.4166660	5.8559999	-0.5215535
21.4166660	5.9047999	613586.0000000
21.4166660	5.9535999	-0.7179031
21.4166660	6.0023999	613586.0000000
21.4333324	0.2928000	-1.6444273
21.4333324	0.3416000	-1.6505632
21.4333324	0.3904000	-1.4910293
21.4333324	0.4392000	-1.3744469
21.4333324	0.4880000	-1.3560390
21.4333324	0.5368000	-1.3130875
21.4333324	0.5856000	-1.3069515
21.4333324	0.6344000	-1.2087770
21.4333324	0.6832000	-1.2087770
21.4333324	0.7320000	-1.3437672
21.4333324	0.7808000	-1.3621750
21.4333324	0.8296000	-1.1596894
21.4333324	0.8784000	-1.0799227
21.4333324	0.9272000	-1.1412816
21.4333324	0.9760000	-1.1535535
21.4333324	1.0247999	-1.1474175
21.4333324	1.0735999	-1.2333207
21.4333324	1.1223999	-1.0185633
21.4333324	1.1711999	-1.0308352
21.4333324	1.2200000	-1.0369711
21.4333324	1.2688000	-0.9081168
21.4333324	1.3176000	-0.9203887
21.4333324	1.3664000	-0.9326606
21.4333324	1.4152000	-0.9326606
21.4333324	1.4640000	-0.9265246
21.4333324	1.5128000	-1.2026410
21.4333324	1.5616000	-0.8835731
21.4333324	1.6104000	-0.8958449
21.4333324	1.6592000	-0.8958449
21.4333324	1.7079999	-0.8344855
21.4333324	1.7567999	-0.5767770
21.4333324	1.8055999	-0.5829129
21.4333324	1.8543999	-0.7363110
21.4333324	1.9031999	-0.4601941
21.4333324	1.9519999	-0.4908738
21.4333324	2.0007999	-0.5645051
21.4333324	2.0495999	-0.7853980
21.4333324	2.0983999	-0.7424469
21.4333324	2.1471999	-0.7363110
21.4333324	2.1959999	-0.8774371
21.4333324	2.2447999	-1.1535535
21.4333324	2.2935998	-0.9326606
21.4333324	2.3423998	-0.9756117
21.4333324	2.3912001	-0.8283496
21.4333324	2.4400001	-0.8283496
21.4333324	2.4888000	-0.8467574
21.4333324	2.5376000	-0.8528934
21.4333324	2.5864000	-1.0983305
21.4333324	2.6352000	-1.3192234
21.4333324	2.6840000	-1.2946796
21.4333324	2.7328000	-1.0615149
21.4333324	2.7816000	-0.9694757

21.4333324	2.8304000	-0.9265246
21.4333324	2.8792000	-0.9081168
21.4333324	2.9280000	-0.9081168
21.4333324	2.9768000	-0.7853980
21.4333324	3.0256000	-0.7179031
21.4333324	3.0743999	-0.7976699
21.4333324	3.1231999	-0.7976699
21.4333324	3.1719999	-0.7117672
21.4333324	3.2207999	-0.7240391
21.4333324	3.2695999	-0.7792621
21.4333324	3.3183999	-0.6688156
21.4333324	3.3671999	-1.1351457
21.4333324	3.4159999	-1.1351457
21.4333324	3.4647999	-1.3069515
21.4333324	3.5135999	-1.1596894
21.4333324	3.5623999	-0.9878836
21.4333324	3.6111999	-0.8897090
21.4333324	3.6599998	-0.9326606
21.4333324	3.7087998	-0.9326606
21.4333324	3.7575998	-0.8283496
21.4333324	3.8063998	-0.8344855
21.4333324	3.8551998	-0.8713012
21.4333324	3.9039998	-0.8099418
21.4333324	3.9527998	-0.6381359
21.4333324	4.0016003	-0.6258640
21.4333324	4.0503998	-0.6320000
21.4333324	4.0991998	-0.6381359
21.4333324	4.1479998	-0.6074562
21.4333324	4.1967998	-0.6074562
21.4333324	4.2455997	613586.0000000
21.4333324	4.2943997	-0.4786019
21.4333324	4.3431997	-0.4847379
21.4333324	4.3919997	-0.4786019
21.4333324	4.4407997	613586.0000000
21.4333324	4.4895997	613586.0000000
21.4333324	4.5383997	613586.0000000
21.4333324	4.5871997	-0.3620195
21.4333324	4.6359997	-0.3681555
21.4333324	4.6847997	-0.3620195
21.4333324	4.7336001	613586.0000000
21.4333324	4.7824001	613586.0000000
21.4333324	4.8312001	-0.2699804
21.4333324	4.8800001	-0.3742914
21.4333324	4.9288001	-0.3742914
21.4333324	4.9776001	613586.0000000
21.4333324	5.0264001	-0.4601941
21.4333324	5.0752001	613586.0000000
21.4333324	5.1240001	613586.0000000
21.4333324	5.1728001	-0.5276895
21.4333324	5.2216001	-0.6074562
21.4333324	5.2704000	-0.5706410
21.4333324	5.3192000	-0.8160777
21.4333324	5.3680000	-0.8038058
21.4333324	5.4168000	-0.6994953
21.4333324	5.4656000	-0.6994953
21.4333324	5.5144000	-0.6626797
21.4333324	5.5632000	-0.6565437

21.4333324	5.6120000	-0.6504078
21.4333324	5.6608000	613586.0000000
21.4333324	5.7096000	613586.0000000
21.4333324	5.7584000	-0.5706410
21.4333324	5.8072000	613586.0000000
21.4333324	5.8559999	613586.0000000
21.4333324	5.9047999	613586.0000000
21.4333324	5.9535999	613586.0000000
21.4333324	6.0023999	613586.0000000
21.4500008	0.2928000	-1.8162332
21.4500008	0.3416000	-1.7855535
21.4500008	0.3904000	-1.3192234
21.4500008	0.4392000	-1.3192234
21.4500008	0.4880000	-1.4726214
21.4500008	0.5368000	-1.6198835
21.4500008	0.5856000	-1.3989906
21.4500008	0.6344000	-1.3867188
21.4500008	0.6832000	-1.3744469
21.4500008	0.7320000	-1.3805828
21.4500008	0.7808000	-1.5094371
21.4500008	0.8296000	-1.2640004
21.4500008	0.8784000	-1.4358063
21.4500008	0.9272000	-1.5155730
21.4500008	0.9760000	-1.4358063
21.4500008	1.0247999	-1.4358063
21.4500008	1.0735999	-1.3069515
21.4500008	1.1223999	-1.3253593
21.4500008	1.1711999	-1.3192234
21.4500008	1.2200000	-1.5278449
21.4500008	1.2688000	-1.5339808
21.4500008	1.3176000	-1.5217090
21.4500008	1.3664000	-1.5217090
21.4500008	1.4152000	-1.5585246
21.4500008	1.4640000	-1.7057867
21.4500008	1.5128000	-1.3867188
21.4500008	1.5616000	-1.3989906
21.4500008	1.6104000	-1.2946796
21.4500008	1.6592000	-1.2946796
21.4500008	1.7079999	-1.4480777
21.4500008	1.7567999	-1.3008156
21.4500008	1.8055999	-1.1535535
21.4500008	1.8543999	-1.1106019
21.4500008	1.9031999	-0.6258640
21.4500008	1.9519999	-0.8160777
21.4500008	2.0007999	-0.8099418
21.4500008	2.0495999	-0.8160777
21.4500008	2.0983999	-0.8160777
21.4500008	2.1471999	-0.9817476
21.4500008	2.1959999	-0.9203887
21.4500008	2.2447999	-0.9265246
21.4500008	2.2935998	-1.1106019
21.4500008	2.3423998	-1.1044660
21.4500008	2.3912001	-1.0860586
21.4500008	2.4400001	-1.1780972
21.4500008	2.4888000	-1.1780972
21.4500008	2.5376000	-1.2578645
21.4500008	2.5864000	-1.2394567

21.4500008	2.6352000	-1.0492430
21.4500008	2.6840000	-0.8344855
21.4500008	2.7328000	-1.0062914
21.4500008	2.7816000	-1.0062914
21.4500008	2.8304000	-1.0185633
21.4500008	2.8792000	-0.9633398
21.4500008	2.9280000	-0.8651652
21.4500008	2.9768000	-0.8713012
21.4500008	3.0256000	-0.8099418
21.4500008	3.0743999	-0.7976699
21.4500008	3.1231999	-0.4847379
21.4500008	3.1719999	-1.0001554
21.4500008	3.2207999	-0.9817476
21.4500008	3.2695999	-0.8651652
21.4500008	3.3183999	-0.8344855
21.4500008	3.3671999	-0.9265246
21.4500008	3.4159999	-0.7485828
21.4500008	3.4647999	-0.6504078
21.4500008	3.5135999	-0.6442719
21.4500008	3.5623999	-0.7485828
21.4500008	3.6111999	-0.7547188
21.4500008	3.6599998	-0.7547188
21.4500008	3.7087998	-0.7608547
21.4500008	3.7575998	-0.7608547
21.4500008	3.8063998	-0.5890489
21.4500008	3.8551998	-0.6749516
21.4500008	3.9039998	-0.7915339
21.4500008	3.9527998	-0.6749516
21.4500008	4.0016003	613586.0000000
21.4500008	4.0503998	613586.0000000
21.4500008	4.0991998	-0.8590293
21.4500008	4.1479998	-0.7424469
21.4500008	4.1967998	-0.7547188
21.4500008	4.2455997	613586.0000000
21.4500008	4.2943997	613586.0000000
21.4500008	4.3431997	613586.0000000
21.4500008	4.3919997	613586.0000000
21.4500008	4.4407997	613586.0000000
21.4500008	4.4895997	613586.0000000
21.4500008	4.5383997	613586.0000000
21.4500008	4.5871997	613586.0000000
21.4500008	4.6359997	613586.0000000
21.4500008	4.6847997	613586.0000000
21.4500008	4.7336001	613586.0000000
21.4500008	4.7824001	613586.0000000
21.4500008	4.8312001	613586.0000000
21.4500008	4.8800001	613586.0000000
21.4500008	4.9288001	-0.6933594
21.4500008	4.9776001	-0.7056313
21.4500008	5.0264001	-0.7240391
21.4500008	5.0752001	-0.7424469
21.4500008	5.1240001	613586.0000000
21.4500008	5.1728001	-0.9081168
21.4500008	5.2216001	-0.9694757
21.4500008	5.2704000	-0.8344855
21.4500008	5.3192000	-0.8406215
21.4500008	5.3680000	613586.0000000

21.4500008	5.4168000	613586.0000000
21.4500008	5.4656000	613586.0000000
21.4500008	5.5144000	-0.6872234
21.4500008	5.5632000	-0.6872234
21.4500008	5.6120000	613586.0000000
21.4500008	5.6608000	613586.0000000
21.4500008	5.7096000	613586.0000000
21.4500008	5.7584000	613586.0000000
21.4500008	5.8072000	613586.0000000
21.4500008	5.8559999	613586.0000000
21.4500008	5.9047999	613586.0000000
21.4500008	5.9535999	613586.0000000
21.4500008	6.0023999	613586.0000000
21.4666672	0.2928000	-1.4542136
21.4666672	0.3416000	-1.6505632
21.4666672	0.3904000	-1.3805828
21.4666672	0.4392000	-1.3805828
21.4666672	0.4880000	-1.4664855
21.4666672	0.5368000	-1.6566992
21.4666672	0.5856000	-1.2824078
21.4666672	0.6344000	-1.2762718
21.4666672	0.6832000	-1.3192234
21.4666672	0.7320000	-1.3314953
21.4666672	0.7808000	-1.2946796
21.4666672	0.8296000	-1.2394567
21.4666672	0.8784000	-1.4358063
21.4666672	0.9272000	-1.3499031
21.4666672	0.9760000	-1.3683109
21.4666672	1.0247999	-1.3744469
21.4666672	1.0735999	-1.4603496
21.4666672	1.1223999	-1.4726214
21.4666672	1.1711999	-1.5953403
21.4666672	1.2200000	-1.4971652
21.4666672	1.2688000	-1.4971652
21.4666672	1.3176000	-1.5401168
21.4666672	1.3664000	-1.5401168
21.4666672	1.4152000	-1.7732821
21.4666672	1.4640000	-1.6014762
21.4666672	1.5128000	-1.6076121
21.4666672	1.5616000	-1.6137476
21.4666672	1.6104000	-1.0001554
21.4666672	1.6592000	-1.0553789
21.4666672	1.7079999	-0.9081168
21.4666672	1.7567999	-0.9081168
21.4666672	1.8055999	-1.0369711
21.4666672	1.8543999	-1.0492430
21.4666672	1.9031999	-0.7301750
21.4666672	1.9519999	-0.8160777
21.4666672	2.0007999	-0.8160777
21.4666672	2.0495999	-0.8344855
21.4666672	2.0983999	-1.0369711
21.4666672	2.1471999	-1.0369711
21.4666672	2.1959999	-1.0369711
21.4666672	2.2447999	-1.2517285
21.4666672	2.2935998	-1.2640004
21.4666672	2.3423998	-1.2762718
21.4666672	2.3912001	-1.0676508

21.4666672	2.4400001	-1.0676508
21.4666672	2.4888000	-1.0737867
21.4666672	2.5376000	-1.1842332
21.4666672	2.5864000	-1.0062914
21.4666672	2.6352000	-1.0185633
21.4666672	2.6840000	-1.0308352
21.4666672	2.7328000	-1.1044660
21.4666672	2.7816000	-1.0308352
21.4666672	2.8304000	-1.0431070
21.4666672	2.8792000	-1.0492430
21.4666672	2.9280000	-1.1106019
21.4666672	2.9768000	-0.9694757
21.4666672	3.0256000	-0.9387960
21.4666672	3.0743999	-0.8344855
21.4666672	3.1231999	-0.7915339
21.4666672	3.1719999	-0.7976699
21.4666672	3.2207999	-1.0185633
21.4666672	3.2695999	-1.0246992
21.4666672	3.3183999	-0.9449320
21.4666672	3.3671999	-0.7669902
21.4666672	3.4159999	-0.7731261
21.4666672	3.4647999	-0.8283496
21.4666672	3.5135999	-0.9633398
21.4666672	3.5623999	-1.0062914
21.4666672	3.6111999	-1.0062914
21.4666672	3.6599998	-0.8344855
21.4666672	3.7087998	-0.8344855
21.4666672	3.7575998	-0.8528934
21.4666672	3.8063998	613586.0000000
21.4666672	3.8551998	613586.0000000
21.4666672	3.9039998	613586.0000000
21.4666672	3.9527998	613586.0000000
21.4666672	4.0016003	613586.0000000
21.4666672	4.0503998	613586.0000000
21.4666672	4.0991998	613586.0000000
21.4666672	4.1479998	613586.0000000
21.4666672	4.1967998	613586.0000000
21.4666672	4.2455997	613586.0000000
21.4666672	4.2943997	613586.0000000
21.4666672	4.3431997	613586.0000000
21.4666672	4.3919997	613586.0000000
21.4666672	4.4407997	613586.0000000
21.4666672	4.4895997	613586.0000000
21.4666672	4.5383997	613586.0000000
21.4666672	4.5871997	613586.0000000
21.4666672	4.6359997	613586.0000000
21.4666672	4.6847997	613586.0000000
21.4666672	4.7336001	613586.0000000
21.4666672	4.7824001	613586.0000000
21.4666672	4.8312001	613586.0000000
21.4666672	4.8800001	-1.1351457
21.4666672	4.9288001	613586.0000000
21.4666672	4.9776001	613586.0000000
21.4666672	5.0264001	613586.0000000
21.4666672	5.0752001	613586.0000000
21.4666672	5.1240001	613586.0000000
21.4666672	5.1728001	613586.0000000

21.4666672	5.2216001	613586.0000000
21.4666672	5.2704000	613586.0000000
21.4666672	5.3192000	613586.0000000
21.4666672	5.3680000	613586.0000000
21.4666672	5.4168000	613586.0000000
21.4666672	5.4656000	613586.0000000
21.4666672	5.5144000	613586.0000000
21.4666672	5.5632000	613586.0000000
21.4666672	5.6120000	613586.0000000
21.4666672	5.6608000	613586.0000000
21.4666672	5.7096000	613586.0000000
21.4666672	5.7584000	613586.0000000
21.4666672	5.8072000	613586.0000000
21.4666672	5.8559999	613586.0000000
21.4666672	5.9047999	613586.0000000
21.4666672	5.9535999	613586.0000000
21.4666672	6.0023999	613586.0000000
21.4833336	0.2928000	-1.6260195
21.4833336	0.3416000	-1.6321554
21.4833336	0.3904000	-1.6505632
21.4833336	0.4392000	-1.6321554
21.4833336	0.4880000	-1.7303305
21.4833336	0.5368000	-1.6260195
21.4833336	0.5856000	-1.5830684
21.4833336	0.6344000	-1.3437672
21.4833336	0.6832000	-1.3437672
21.4833336	0.7320000	-1.3437672
21.4833336	0.7808000	-1.4051266
21.4833336	0.8296000	-1.3437672
21.4833336	0.8784000	-1.5953403
21.4833336	0.9272000	-1.4542136
21.4833336	0.9760000	-1.4480777
21.4833336	1.0247999	-1.5339808
21.4833336	1.0735999	-1.4235344
21.4833336	1.1223999	-1.4664855
21.4833336	1.1711999	-1.4726214
21.4833336	1.2200000	-1.5339808
21.4833336	1.2688000	-1.5585246
21.4833336	1.3176000	-1.3867188
21.4833336	1.3664000	-1.3867188
21.4833336	1.4152000	-1.1658254
21.4833336	1.4640000	-1.4542136
21.4833336	1.5128000	-1.4480777
21.4833336	1.5616000	-1.8591847
21.4833336	1.6104000	-1.5830684
21.4833336	1.6592000	-1.2149129
21.4833336	1.7079999	-1.2701364
21.4833336	1.7567999	-1.1106019
21.4833336	1.8055999	-1.0983305
21.4833336	1.8543999	-1.1474175
21.4833336	1.9031999	-1.1412816
21.4833336	1.9519999	-1.1412816
21.4833336	2.0007999	-1.1290097
21.4833336	2.0495999	-1.2762718
21.4833336	2.0983999	-1.2824078
21.4833336	2.1471999	-1.4848933
21.4833336	2.1959999	-1.5953403

21.4833336	2.2447999	-1.3683109
21.4833336	2.2935998	-1.3499031
21.4833336	2.3423998	-1.2394567
21.4833336	2.3912001	-1.2394567
21.4833336	2.4400001	-1.2394567
21.4833336	2.4888000	-1.2333207
21.4833336	2.5376000	-1.2885437
21.4833336	2.5864000	-1.1290097
21.4833336	2.6352000	-1.0001554
21.4833336	2.6840000	-1.0246992
21.4833336	2.7328000	-0.8897090
21.4833336	2.7816000	-1.0185633
21.4833336	2.8304000	-1.0308352
21.4833336	2.8792000	-1.0492430
21.4833336	2.9280000	-1.1167378
21.4833336	2.9768000	-1.2026410
21.4833336	3.0256000	-0.9878836
21.4833336	3.0743999	-0.8835731
21.4833336	3.1231999	-0.8774371
21.4833336	3.1719999	-0.8774371
21.4833336	3.2207999	-0.8897090
21.4833336	3.2695999	-0.8835731
21.4833336	3.3183999	-0.8099418
21.4833336	3.3671999	-0.9940195
21.4833336	3.4159999	-0.9940195
21.4833336	3.4647999	-0.9817476
21.4833336	3.5135999	-1.0001554
21.4833336	3.5623999	-1.0860586
21.4833336	3.6111999	-1.0308352
21.4833336	3.6599998	-1.2762718
21.4833336	3.7087998	-1.2333207
21.4833336	3.7575998	-0.9572039
21.4833336	3.8063998	-0.9510679
21.4833336	3.8551998	-0.9572039
21.4833336	3.9039998	613586.0000000
21.4833336	3.9527998	613586.0000000
21.4833336	4.0016003	613586.0000000
21.4833336	4.0503998	613586.0000000
21.4833336	4.0991998	-0.8713012
21.4833336	4.1479998	613586.0000000
21.4833336	4.1967998	613586.0000000
21.4833336	4.2455997	613586.0000000
21.4833336	4.2943997	613586.0000000
21.4833336	4.3431997	613586.0000000
21.4833336	4.3919997	613586.0000000
21.4833336	4.4407997	613586.0000000
21.4833336	4.4895997	613586.0000000
21.4833336	4.5383997	613586.0000000
21.4833336	4.5871997	613586.0000000
21.4833336	4.6359997	613586.0000000
21.4833336	4.6847997	-0.6994953
21.4833336	4.7336001	-0.7056313
21.4833336	4.7824001	-0.6197281
21.4833336	4.8312001	613586.0000000
21.4833336	4.8800001	613586.0000000
21.4833336	4.9288001	613586.0000000
21.4833336	4.9776001	613586.0000000

21.4833336	5.0264001	613586.0000000
21.4833336	5.0752001	613586.0000000
21.4833336	5.1240001	613586.0000000
21.4833336	5.1728001	613586.0000000
21.4833336	5.2216001	613586.0000000
21.4833336	5.2704000	613586.0000000
21.4833336	5.3192000	613586.0000000
21.4833336	5.3680000	613586.0000000
21.4833336	5.4168000	613586.0000000
21.4833336	5.4656000	613586.0000000
21.4833336	5.5144000	613586.0000000
21.4833336	5.5632000	613586.0000000
21.4833336	5.6120000	613586.0000000
21.4833336	5.6608000	613586.0000000
21.4833336	5.7096000	613586.0000000
21.4833336	5.7584000	613586.0000000
21.4833336	5.8072000	613586.0000000
21.4833336	5.8559999	613586.0000000
21.4833336	5.9047999	613586.0000000
21.4833336	5.9535999	613586.0000000
21.4833336	6.0023999	613586.0000000
21.5000000	0.2928000	-1.8223691
21.5000000	0.3416000	-1.6628351
21.5000000	0.3904000	-1.5707965
21.5000000	0.4392000	-1.4480777
21.5000000	0.4880000	-1.6444273
21.5000000	0.5368000	-1.1965051
21.5000000	0.5856000	-1.5217090
21.5000000	0.6344000	-1.4051266
21.5000000	0.6832000	-1.3805828
21.5000000	0.7320000	-1.3805828
21.5000000	0.7808000	-1.4419422
21.5000000	0.8296000	-1.4480777
21.5000000	0.8784000	-1.4419422
21.5000000	0.9272000	-1.3805828
21.5000000	0.9760000	-1.4358063
21.5000000	1.0247999	-1.4051266
21.5000000	1.0735999	-1.3867188
21.5000000	1.1223999	-1.3805828
21.5000000	1.1711999	-1.4971652
21.5000000	1.2200000	-1.4848933
21.5000000	1.2688000	-1.4848933
21.5000000	1.3176000	-1.6260195
21.5000000	1.3664000	-1.6260195
21.5000000	1.4152000	-1.6260195
21.5000000	1.4640000	-1.7487383
21.5000000	1.5128000	-1.5278449
21.5000000	1.5616000	-1.3008156
21.5000000	1.6104000	-1.0553789
21.5000000	1.6592000	-1.2271848
21.5000000	1.7079999	-1.2271848
21.5000000	1.7567999	-1.2333207
21.5000000	1.8055999	-1.0615149
21.5000000	1.8543999	-1.0615149
21.5000000	1.9031999	-1.3069515
21.5000000	1.9519999	-1.3314953
21.5000000	2.0007999	-1.3253593

21.5000000	2.0495999	-1.3192234
21.5000000	2.0983999	-1.2333207
21.5000000	2.1471999	-1.5646605
21.5000000	2.1959999	-1.4848933
21.5000000	2.2447999	-1.3744469
21.5000000	2.2935998	-1.3867188
21.5000000	2.3423998	-1.3314953
21.5000000	2.3912001	-1.2640004
21.5000000	2.4400001	-1.0983305
21.5000000	2.4888000	-1.1780972
21.5000000	2.5376000	-1.1719613
21.5000000	2.5864000	-1.1719613
21.5000000	2.6352000	-1.2026410
21.5000000	2.6840000	-1.2885437
21.5000000	2.7328000	-1.2885437
21.5000000	2.7816000	-1.2026410
21.5000000	2.8304000	-1.0492430
21.5000000	2.8792000	-1.1167378
21.5000000	2.9280000	-1.1228738
21.5000000	2.9768000	-1.0860586
21.5000000	3.0256000	-1.0983305
21.5000000	3.0743999	-0.9940195
21.5000000	3.1231999	-0.9940195
21.5000000	3.1719999	-0.8958449
21.5000000	3.2207999	-0.7976699
21.5000000	3.2695999	-0.7792621
21.5000000	3.3183999	-0.7853980
21.5000000	3.3671999	-0.7915339
21.5000000	3.4159999	-0.8467574
21.5000000	3.4647999	-0.6872234
21.5000000	3.5135999	-1.0737867
21.5000000	3.5623999	-1.0676508
21.5000000	3.6111999	-1.0860586
21.5000000	3.6599998	-1.0921946
21.5000000	3.7087998	-1.0185633
21.5000000	3.7575998	-1.0308352
21.5000000	3.8063998	613586.0000000
21.5000000	3.8551998	-0.7915339
21.5000000	3.9039998	-0.7853980
21.5000000	3.9527998	-0.7792621
21.5000000	4.0016003	613586.0000000
21.5000000	4.0503998	613586.0000000
21.5000000	4.0991998	613586.0000000
21.5000000	4.1479998	613586.0000000
21.5000000	4.1967998	613586.0000000
21.5000000	4.2455997	-0.6810875
21.5000000	4.2943997	-0.6749516
21.5000000	4.3431997	613586.0000000
21.5000000	4.3919997	613586.0000000
21.5000000	4.4407997	613586.0000000
21.5000000	4.4895997	-0.7117672
21.5000000	4.5383997	-0.7117672
21.5000000	4.5871997	-0.6504078
21.5000000	4.6359997	-0.5460973
21.5000000	4.6847997	-0.7179031
21.5000000	4.7336001	-0.7179031
21.5000000	4.7824001	613586.0000000

21.5000000	4.8312001	613586.0000000
21.5000000	4.8800001	613586.0000000
21.5000000	4.9288001	-0.6381359
21.5000000	4.9776001	613586.0000000
21.5000000	5.0264001	613586.0000000
21.5000000	5.0752001	-0.7608547
21.5000000	5.1240001	-0.7485828
21.5000000	5.1728001	613586.0000000
21.5000000	5.2216001	613586.0000000
21.5000000	5.2704000	613586.0000000
21.5000000	5.3192000	613586.0000000
21.5000000	5.3680000	-0.7547188
21.5000000	5.4168000	613586.0000000
21.5000000	5.4656000	613586.0000000
21.5000000	5.5144000	613586.0000000
21.5000000	5.5632000	613586.0000000
21.5000000	5.6120000	613586.0000000
21.5000000	5.6608000	613586.0000000
21.5000000	5.7096000	613586.0000000
21.5000000	5.7584000	613586.0000000
21.5000000	5.8072000	613586.0000000
21.5000000	5.8559999	613586.0000000
21.5000000	5.9047999	613586.0000000
21.5000000	5.9535999	613586.0000000
21.5000000	6.0023999	613586.0000000
21.5166664	0.2928000	-1.6566992
21.5166664	0.3416000	-1.7732821
21.5166664	0.3904000	-1.4910293
21.5166664	0.4392000	-1.6076121
21.5166664	0.4880000	-1.6076121
21.5166664	0.5368000	-1.6873789
21.5166664	0.5856000	-1.3683109
21.5166664	0.6344000	-1.5892043
21.5166664	0.6832000	-1.4358063
21.5166664	0.7320000	-1.4296703
21.5166664	0.7808000	-1.4112625
21.5166664	0.8296000	-1.4051266
21.5166664	0.8784000	-1.4051266
21.5166664	0.9272000	-1.3867188
21.5166664	0.9760000	-1.3683109
21.5166664	1.0247999	-1.3683109
21.5166664	1.0735999	-1.3621750
21.5166664	1.1223999	-1.3621750
21.5166664	1.1711999	-1.3867188
21.5166664	1.2200000	-1.3499031
21.5166664	1.2688000	-1.2333207
21.5166664	1.3176000	-1.2210488
21.5166664	1.3664000	-1.1290097
21.5166664	1.4152000	-1.1290097
21.5166664	1.4640000	-1.4419422
21.5166664	1.5128000	-1.0185633
21.5166664	1.5616000	-1.0676508
21.5166664	1.6104000	-1.4173985
21.5166664	1.6592000	-1.7057867
21.5166664	1.7079999	-1.4235344
21.5166664	1.7567999	-1.3130875
21.5166664	1.8055999	-1.3192234

21.5166664	1.8543999	-1.3499031
21.5166664	1.9031999	-1.2885437
21.5166664	1.9519999	-1.2271848
21.5166664	2.0007999	-1.2149129
21.5166664	2.0495999	-1.2946796
21.5166664	2.0983999	-1.2455926
21.5166664	2.1471999	-1.2455926
21.5166664	2.1959999	-1.3621750
21.5166664	2.2447999	-1.2087770
21.5166664	2.2935998	-1.2026410
21.5166664	2.3423998	-1.3130875
21.5166664	2.3912001	-1.3008156
21.5166664	2.4400001	-1.1965051
21.5166664	2.4888000	-1.1719613
21.5166664	2.5376000	-1.2455926
21.5166664	2.5864000	-0.9449320
21.5166664	2.6352000	-1.2210488
21.5166664	2.6840000	-0.6810875
21.5166664	2.7328000	-0.6872234
21.5166664	2.7816000	-0.7915339
21.5166664	2.8304000	-0.7915339
21.5166664	2.8792000	-0.7915339
21.5166664	2.9280000	-0.7792621
21.5166664	2.9768000	-0.7608547
21.5166664	3.0256000	-0.7608547
21.5166664	3.0743999	-0.8160777
21.5166664	3.1231999	-0.7669902
21.5166664	3.1719999	-0.8774371
21.5166664	3.2207999	-0.8713012
21.5166664	3.2695999	-0.8222136
21.5166664	3.3183999	-0.8467574
21.5166664	3.3671999	-0.9387960
21.5166664	3.4159999	-0.7301750
21.5166664	3.4647999	-0.7179031
21.5166664	3.5135999	613586.0000000
21.5166664	3.5623999	-0.4786019
21.5166664	3.6111999	-0.6442719
21.5166664	3.6599998	613586.0000000
21.5166664	3.7087998	613586.0000000
21.5166664	3.7575998	613586.0000000
21.5166664	3.8063998	-0.7240391
21.5166664	3.8551998	-0.7731261
21.5166664	3.9039998	-0.7485828
21.5166664	3.9527998	613586.0000000
21.5166664	4.0016003	613586.0000000
21.5166664	4.0503998	613586.0000000
21.5166664	4.0991998	613586.0000000
21.5166664	4.1479998	613586.0000000
21.5166664	4.1967998	-0.6135921
21.5166664	4.2455997	613586.0000000
21.5166664	4.2943997	613586.0000000
21.5166664	4.3431997	613586.0000000
21.5166664	4.3919997	613586.0000000
21.5166664	4.4407997	-0.5092816
21.5166664	4.4895997	-0.5154176
21.5166664	4.5383997	-0.4970098
21.5166664	4.5871997	-0.5338254

21.5166664	4.6359997	-0.5460973
21.5166664	4.6847997	-0.5706410
21.5166664	4.7336001	613586.0000000
21.5166664	4.7824001	613586.0000000
21.5166664	4.8312001	613586.0000000
21.5166664	4.8800001	613586.0000000
21.5166664	4.9288001	613586.0000000
21.5166664	4.9776001	-1.0124273
21.5166664	5.0264001	-0.8835731
21.5166664	5.0752001	613586.0000000
21.5166664	5.1240001	613586.0000000
21.5166664	5.1728001	613586.0000000
21.5166664	5.2216001	613586.0000000
21.5166664	5.2704000	-0.8467574
21.5166664	5.3192000	-0.8344855
21.5166664	5.3680000	613586.0000000
21.5166664	5.4168000	613586.0000000
21.5166664	5.4656000	613586.0000000
21.5166664	5.5144000	613586.0000000
21.5166664	5.5632000	613586.0000000
21.5166664	5.6120000	613586.0000000
21.5166664	5.6608000	613586.0000000
21.5166664	5.7096000	613586.0000000
21.5166664	5.7584000	613586.0000000
21.5166664	5.8072000	613586.0000000
21.5166664	5.8559999	613586.0000000
21.5166664	5.9047999	613586.0000000
21.5166664	5.9535999	613586.0000000
21.5166664	6.0023999	613586.0000000
21.5333328	0.2928000	-1.4051266
21.5333328	0.3416000	-1.3989906
21.5333328	0.3904000	-1.5646605
21.5333328	0.4392000	-1.5646605
21.5333328	0.4880000	-1.4358063
21.5333328	0.5368000	-1.4419422
21.5333328	0.5856000	-1.3314953
21.5333328	0.6344000	-1.3253593
21.5333328	0.6832000	-1.3069515
21.5333328	0.7320000	-1.4848933
21.5333328	0.7808000	-1.3867188
21.5333328	0.8296000	-1.3805828
21.5333328	0.8784000	-1.3867188
21.5333328	0.9272000	-1.2946796
21.5333328	0.9760000	-1.1228738
21.5333328	1.0247999	-1.2946796
21.5333328	1.0735999	-1.3008156
21.5333328	1.1223999	-1.2149129
21.5333328	1.1711999	-1.2210488
21.5333328	1.2200000	-1.2087770
21.5333328	1.2688000	-1.3499031
21.5333328	1.3176000	-1.4296703
21.5333328	1.3664000	-1.4235344
21.5333328	1.4152000	-1.3008156
21.5333328	1.4640000	-1.6566992
21.5333328	1.5128000	-1.2333207
21.5333328	1.5616000	-1.3069515
21.5333328	1.6104000	-1.2946796

21.5333328	1.6592000	-1.2333207
21.5333328	1.7079999	-1.4173985
21.5333328	1.7567999	-1.3499031
21.5333328	1.8055999	-1.2210488
21.5333328	1.8543999	-1.2149129
21.5333328	1.9031999	-1.3130875
21.5333328	1.9519999	-1.2333207
21.5333328	2.0007999	-1.2455926
21.5333328	2.0495999	-1.1351457
21.5333328	2.0983999	-1.2149129
21.5333328	2.1471999	-1.2087770
21.5333328	2.1959999	-1.3805828
21.5333328	2.2447999	-1.3744469
21.5333328	2.2935998	-1.1658254
21.5333328	2.3423998	-1.1719613
21.5333328	2.3912001	-1.1780972
21.5333328	2.4400001	-0.9756117
21.5333328	2.4888000	-1.0369711
21.5333328	2.5376000	-1.2394567
21.5333328	2.5864000	-1.2026410
21.5333328	2.6352000	-1.1903691
21.5333328	2.6840000	-1.0246992
21.5333328	2.7328000	-1.0246992
21.5333328	2.7816000	-0.7731261
21.5333328	2.8304000	-0.7731261
21.5333328	2.8792000	-0.6258640
21.5333328	2.9280000	-0.5583692
21.5333328	2.9768000	-0.5215535
21.5333328	3.0256000	-1.0553789
21.5333328	3.0743999	-0.9019809
21.5333328	3.1231999	-0.9694757
21.5333328	3.1719999	-1.0246992
21.5333328	3.2207999	-1.0246992
21.5333328	3.2695999	-0.9142528
21.5333328	3.3183999	-0.9572039
21.5333328	3.3671999	-0.9572039
21.5333328	3.4159999	-0.9694757
21.5333328	3.4647999	-0.8590293
21.5333328	3.5135999	-0.8528934
21.5333328	3.5623999	-0.7731261
21.5333328	3.6111999	-0.7363110
21.5333328	3.6599998	-0.7608547
21.5333328	3.7087998	-0.8651652
21.5333328	3.7575998	-0.8897090
21.5333328	3.8063998	613586.0000000
21.5333328	3.8551998	613586.0000000
21.5333328	3.9039998	613586.0000000
21.5333328	3.9527998	613586.0000000
21.5333328	4.0016003	613586.0000000
21.5333328	4.0503998	613586.0000000
21.5333328	4.0991998	-0.9387960
21.5333328	4.1479998	-0.9019809
21.5333328	4.1967998	-0.8099418
21.5333328	4.2455997	-0.9633398
21.5333328	4.2943997	-1.0799227
21.5333328	4.3431997	-0.9265246
21.5333328	4.3919997	-1.0308352

21.5333328	4.4407997	-1.0124273
21.5333328	4.4895997	-0.8528934
21.5333328	4.5383997	-0.9081168
21.5333328	4.5871997	-0.8467574
21.5333328	4.6359997	-0.8222136
21.5333328	4.6847997	-0.8099418
21.5333328	4.7336001	-0.7792621
21.5333328	4.7824001	613586.0000000
21.5333328	4.8312001	613586.0000000
21.5333328	4.8800001	613586.0000000
21.5333328	4.9288001	613586.0000000
21.5333328	4.9776001	613586.0000000
21.5333328	5.0264001	613586.0000000
21.5333328	5.0752001	-0.6810875
21.5333328	5.1240001	613586.0000000
21.5333328	5.1728001	613586.0000000
21.5333328	5.2216001	613586.0000000
21.5333328	5.2704000	613586.0000000
21.5333328	5.3192000	613586.0000000
21.5333328	5.3680000	613586.0000000
21.5333328	5.4168000	613586.0000000
21.5333328	5.4656000	613586.0000000
21.5333328	5.5144000	613586.0000000
21.5333328	5.5632000	613586.0000000
21.5333328	5.6120000	613586.0000000
21.5333328	5.6608000	613586.0000000
21.5333328	5.7096000	613586.0000000
21.5333328	5.7584000	613586.0000000
21.5333328	5.8072000	613586.0000000
21.5333328	5.8559999	613586.0000000
21.5333328	5.9047999	613586.0000000
21.5333328	5.9535999	613586.0000000
21.5333328	6.0023999	613586.0000000
21.5499992	0.2928000	-1.7057867
21.5499992	0.3416000	-1.7057867
21.5499992	0.3904000	-1.9573593
21.5499992	0.4392000	-1.4296703
21.5499992	0.4880000	-1.4787574
21.5499992	0.5368000	-1.4848933
21.5499992	0.5856000	-1.3621750
21.5499992	0.6344000	-1.4787574
21.5499992	0.6832000	-1.4848933
21.5499992	0.7320000	-1.3805828
21.5499992	0.7808000	-1.5155730
21.5499992	0.8296000	-1.4664855
21.5499992	0.8784000	-1.4051266
21.5499992	0.9272000	-1.3989906
21.5499992	0.9760000	-1.2455926
21.5499992	1.0247999	-1.3437672
21.5499992	1.0735999	-1.3437672
21.5499992	1.1223999	-1.2762718
21.5499992	1.1711999	-1.2701364
21.5499992	1.2200000	-1.2762718
21.5499992	1.2688000	-1.2946796
21.5499992	1.3176000	-1.3376312
21.5499992	1.3664000	-1.5953403
21.5499992	1.4152000	-1.7426023

21.5499992	1.4640000	-1.7487383
21.5499992	1.5128000	-1.5401168
21.5499992	1.5616000	-1.2578645
21.5499992	1.6104000	-1.2701364
21.5499992	1.6592000	-1.1167378
21.5499992	1.7079999	-1.3867188
21.5499992	1.7567999	-1.3867188
21.5499992	1.8055999	-1.2271848
21.5499992	1.8543999	-1.2149129
21.5499992	1.9031999	-1.3744469
21.5499992	1.9519999	-1.3744469
21.5499992	2.0007999	-1.1412816
21.5499992	2.0495999	-1.2517285
21.5499992	2.0983999	-1.2333207
21.5499992	2.1471999	-1.2394567
21.5499992	2.1959999	-1.2455926
21.5499992	2.2447999	-1.0921946
21.5499992	2.2935998	-1.0921946
21.5499992	2.3423998	-1.0615149
21.5499992	2.3912001	-1.0676508
21.5499992	2.4400001	-0.9694757
21.5499992	2.4888000	-0.9694757
21.5499992	2.5376000	-1.0246992
21.5499992	2.5864000	-1.0246992
21.5499992	2.6352000	-1.0737867
21.5499992	2.6840000	-1.0246992
21.5499992	2.7328000	-0.9633398
21.5499992	2.7816000	-0.9326606
21.5499992	2.8304000	-0.9265246
21.5499992	2.8792000	-0.9940195
21.5499992	2.9280000	-0.9878836
21.5499992	2.9768000	-0.9940195
21.5499992	3.0256000	-1.0553789
21.5499992	3.0743999	-0.8406215
21.5499992	3.1231999	-0.9817476
21.5499992	3.1719999	-1.1903691
21.5499992	3.2207999	-1.3069515
21.5499992	3.2695999	-1.2026410
21.5499992	3.3183999	-1.0615149
21.5499992	3.3671999	-0.9940195
21.5499992	3.4159999	-1.0308352
21.5499992	3.4647999	-0.8651652
21.5499992	3.5135999	-0.9633398
21.5499992	3.5623999	-0.8590293
21.5499992	3.6111999	-0.8590293
21.5499992	3.6599998	-0.3497477
21.5499992	3.7087998	-0.3497477
21.5499992	3.7575998	-0.3558836
21.5499992	3.8063998	-0.4111071
21.5499992	3.8551998	-0.4172430
21.5499992	3.9039998	613586.0000000
21.5499992	3.9527998	613586.0000000
21.5499992	4.0016003	613586.0000000
21.5499992	4.0503998	613586.0000000
21.5499992	4.0991998	613586.0000000
21.5499992	4.1479998	613586.0000000
21.5499992	4.1967998	613586.0000000

21.5499992	4.2455997	-0.6565437
21.5499992	4.2943997	-0.6565437
21.5499992	4.3431997	-0.6504078
21.5499992	4.3919997	-0.6504078
21.5499992	4.4407997	-0.6626797
21.5499992	4.4895997	613586.0000000
21.5499992	4.5383997	-0.7117672
21.5499992	4.5871997	613586.0000000
21.5499992	4.6359997	613586.0000000
21.5499992	4.6847997	613586.0000000
21.5499992	4.7336001	613586.0000000
21.5499992	4.7824001	-0.5460973
21.5499992	4.8312001	613586.0000000
21.5499992	4.8800001	613586.0000000
21.5499992	4.9288001	613586.0000000
21.5499992	4.9776001	613586.0000000
21.5499992	5.0264001	613586.0000000
21.5499992	5.0752001	613586.0000000
21.5499992	5.1240001	613586.0000000
21.5499992	5.1728001	-0.7056313
21.5499992	5.2216001	613586.0000000
21.5499992	5.2704000	613586.0000000
21.5499992	5.3192000	613586.0000000
21.5499992	5.3680000	613586.0000000
21.5499992	5.4168000	613586.0000000
21.5499992	5.4656000	613586.0000000
21.5499992	5.5144000	613586.0000000
21.5499992	5.5632000	613586.0000000
21.5499992	5.6120000	613586.0000000
21.5499992	5.6608000	613586.0000000
21.5499992	5.7096000	613586.0000000
21.5499992	5.7584000	613586.0000000
21.5499992	5.8072000	613586.0000000
21.5499992	5.8559999	613586.0000000
21.5499992	5.9047999	613586.0000000
21.5499992	5.9535999	613586.0000000
21.5499992	6.0023999	613586.0000000
21.5666676	0.2928000	-1.7057867
21.5666676	0.3416000	-1.6873789
21.5666676	0.3904000	-1.6566992
21.5666676	0.4392000	-1.6751070
21.5666676	0.4880000	-1.6689711
21.5666676	0.5368000	-1.7671461
21.5666676	0.5856000	-1.7671461
21.5666676	0.6344000	-1.5769324
21.5666676	0.6832000	-1.5769324
21.5666676	0.7320000	-1.5830684
21.5666676	0.7808000	-1.6812429
21.5666676	0.8296000	-1.4480777
21.5666676	0.8784000	-1.3683109
21.5666676	0.9272000	-1.3621750
21.5666676	0.9760000	-1.4603496
21.5666676	1.0247999	-1.2824078
21.5666676	1.0735999	-1.2885437
21.5666676	1.1223999	-1.1719613
21.5666676	1.1711999	-1.1780972
21.5666676	1.2200000	-1.2026410

21.5666676	1.2688000	-1.2149129
21.5666676	1.3176000	-1.2455926
21.5666676	1.3664000	-1.2640004
21.5666676	1.4152000	-1.2824078
21.5666676	1.4640000	-1.2701364
21.5666676	1.5128000	-1.4480777
21.5666676	1.5616000	-1.2578645
21.5666676	1.6104000	-1.3130875
21.5666676	1.6592000	-1.2578645
21.5666676	1.7079999	-1.3069515
21.5666676	1.7567999	-1.2578645
21.5666676	1.8055999	-1.2578645
21.5666676	1.8543999	-1.1351457
21.5666676	1.9031999	-1.0001554
21.5666676	1.9519999	-0.9940195
21.5666676	2.0007999	-0.9387960
21.5666676	2.0495999	-0.9387960
21.5666676	2.0983999	-1.0431070
21.5666676	2.1471999	-1.0492430
21.5666676	2.1959999	-1.0492430
21.5666676	2.2447999	-1.1903691
21.5666676	2.2935998	-1.2026410
21.5666676	2.3423998	-1.0308352
21.5666676	2.3912001	-0.8713012
21.5666676	2.4400001	-0.8713012
21.5666676	2.4888000	-1.1658254
21.5666676	2.5376000	-1.1167378
21.5666676	2.5864000	-0.9940195
21.5666676	2.6352000	-0.6013203
21.5666676	2.6840000	-0.9019809
21.5666676	2.7328000	-1.0246992
21.5666676	2.7816000	-0.9633398
21.5666676	2.8304000	-0.9572039
21.5666676	2.8792000	-0.9633398
21.5666676	2.9280000	-1.0921946
21.5666676	2.9768000	-1.0860586
21.5666676	3.0256000	-0.9817476
21.5666676	3.0743999	-0.9142528
21.5666676	3.1231999	-0.9142528
21.5666676	3.1719999	-0.7792621
21.5666676	3.2207999	-0.7915339
21.5666676	3.2695999	-0.5276895
21.5666676	3.3183999	-0.6504078
21.5666676	3.3671999	-0.8651652
21.5666676	3.4159999	-0.6258640
21.5666676	3.4647999	-0.6197281
21.5666676	3.5135999	-0.5645051
21.5666676	3.5623999	-0.4663301
21.5666676	3.6111999	613586.0000000
21.5666676	3.6599998	613586.0000000
21.5666676	3.7087998	613586.0000000
21.5666676	3.7575998	613586.0000000
21.5666676	3.8063998	613586.0000000
21.5666676	3.8551998	-1.0492430
21.5666676	3.9039998	613586.0000000
21.5666676	3.9527998	613586.0000000
21.5666676	4.0016003	613586.0000000

21.5666676	4.0503998	613586.0000000
21.5666676	4.0991998	613586.0000000
21.5666676	4.1479998	-1.0185633
21.5666676	4.1967998	-1.0185633
21.5666676	4.2455997	-0.8222136
21.5666676	4.2943997	-0.6013203
21.5666676	4.3431997	-0.5951843
21.5666676	4.3919997	613586.0000000
21.5666676	4.4407997	-0.6994953
21.5666676	4.4895997	-0.6197281
21.5666676	4.5383997	-0.6135921
21.5666676	4.5871997	-0.5645051
21.5666676	4.6359997	613586.0000000
21.5666676	4.6847997	613586.0000000
21.5666676	4.7336001	613586.0000000
21.5666676	4.7824001	613586.0000000
21.5666676	4.8312001	613586.0000000
21.5666676	4.8800001	613586.0000000
21.5666676	4.9288001	613586.0000000
21.5666676	4.9776001	613586.0000000
21.5666676	5.0264001	613586.0000000
21.5666676	5.0752001	613586.0000000
21.5666676	5.1240001	613586.0000000
21.5666676	5.1728001	613586.0000000
21.5666676	5.2216001	613586.0000000
21.5666676	5.2704000	613586.0000000
21.5666676	5.3192000	613586.0000000
21.5666676	5.3680000	613586.0000000
21.5666676	5.4168000	613586.0000000
21.5666676	5.4656000	613586.0000000
21.5666676	5.5144000	613586.0000000
21.5666676	5.5632000	613586.0000000
21.5666676	5.6120000	613586.0000000
21.5666676	5.6608000	613586.0000000
21.5666676	5.7096000	613586.0000000
21.5666676	5.7584000	613586.0000000
21.5666676	5.8072000	613586.0000000
21.5666676	5.8559999	613586.0000000
21.5666676	5.9047999	613586.0000000
21.5666676	5.9535999	613586.0000000
21.5666676	6.0023999	613586.0000000
21.5833340	0.2928000	-1.4910293
21.5833340	0.3416000	-1.5278449
21.5833340	0.3904000	-1.5278449
21.5833340	0.4392000	-1.5339808
21.5833340	0.4880000	-1.3621750
21.5833340	0.5368000	-1.5769324
21.5833340	0.5856000	-1.5769324
21.5833340	0.6344000	-1.4051266
21.5833340	0.6832000	-1.3989906
21.5833340	0.7320000	-1.2946796
21.5833340	0.7808000	-1.3069515
21.5833340	0.8296000	-1.2210488
21.5833340	0.8784000	-1.3744469
21.5833340	0.9272000	-1.3744469
21.5833340	0.9760000	-1.2701364
21.5833340	1.0247999	-1.4480777

21.5833340	1.0735999	-1.4480777
21.5833340	1.1223999	-1.4051266
21.5833340	1.1711999	-1.1719613
21.5833340	1.2200000	-1.1842332
21.5833340	1.2688000	-1.3008156
21.5833340	1.3176000	-1.3253593
21.5833340	1.3664000	-1.4419422
21.5833340	1.4152000	-1.4848933
21.5833340	1.4640000	-1.3989906
21.5833340	1.5128000	-1.4235344
21.5833340	1.5616000	-1.4235344
21.5833340	1.6104000	-1.4910293
21.5833340	1.6592000	-1.6076121
21.5833340	1.7079999	-1.3069515
21.5833340	1.7567999	-1.2210488
21.5833340	1.8055999	-1.1719613
21.5833340	1.8543999	-1.1780972
21.5833340	1.9031999	-1.1965051
21.5833340	1.9519999	-1.2087770
21.5833340	2.0007999	-1.0921946
21.5833340	2.0495999	-1.0921946
21.5833340	2.0983999	-1.2578645
21.5833340	2.1471999	-1.2517285
21.5833340	2.1959999	-1.1596894
21.5833340	2.2447999	-1.1719613
21.5833340	2.2935998	-1.2394567
21.5833340	2.3423998	-1.2394567
21.5833340	2.3912001	-1.2087770
21.5833340	2.4400001	-1.2026410
21.5833340	2.4888000	-1.2271848
21.5833340	2.5376000	-1.1780972
21.5833340	2.5864000	-1.0799227
21.5833340	2.6352000	-1.3069515
21.5833340	2.6840000	-1.2946796
21.5833340	2.7328000	-1.3069515
21.5833340	2.7816000	-1.3683109
21.5833340	2.8304000	-1.2149129
21.5833340	2.8792000	-1.2455926
21.5833340	2.9280000	-1.2517285
21.5833340	2.9768000	-1.2946796
21.5833340	3.0256000	-1.1842332
21.5833340	3.0743999	-1.3499031
21.5833340	3.1231999	-1.3499031
21.5833340	3.1719999	-1.3314953
21.5833340	3.2207999	-1.2149129
21.5833340	3.2695999	-1.2149129
21.5833340	3.3183999	-1.0431070
21.5833340	3.3671999	-1.0431070
21.5833340	3.4159999	-0.9326606
21.5833340	3.4647999	-0.9387960
21.5833340	3.5135999	613586.0000000
21.5833340	3.5623999	613586.0000000
21.5833340	3.6111999	-0.7976699
21.5833340	3.6599998	613586.0000000
21.5833340	3.7087998	613586.0000000
21.5833340	3.7575998	613586.0000000
21.5833340	3.8063998	613586.0000000

21.5833340	3.8551998	613586.0000000
21.5833340	3.9039998	613586.0000000
21.5833340	3.9527998	613586.0000000
21.5833340	4.0016003	613586.0000000
21.5833340	4.0503998	613586.0000000
21.5833340	4.0991998	-1.0553789
21.5833340	4.1479998	-1.0676508
21.5833340	4.1967998	-1.1596894
21.5833340	4.2455997	-1.0799227
21.5833340	4.2943997	-1.0860586
21.5833340	4.3431997	-0.9572039
21.5833340	4.3919997	613586.0000000
21.5833340	4.4407997	613586.0000000
21.5833340	4.4895997	613586.0000000
21.5833340	4.5383997	613586.0000000
21.5833340	4.5871997	613586.0000000
21.5833340	4.6359997	613586.0000000
21.5833340	4.6847997	613586.0000000
21.5833340	4.7336001	-0.8897090
21.5833340	4.7824001	-0.8774371
21.5833340	4.8312001	-0.8590293
21.5833340	4.8800001	613586.0000000
21.5833340	4.9288001	613586.0000000
21.5833340	4.9776001	613586.0000000
21.5833340	5.0264001	613586.0000000
21.5833340	5.0752001	613586.0000000
21.5833340	5.1240001	613586.0000000
21.5833340	5.1728001	613586.0000000
21.5833340	5.2216001	613586.0000000
21.5833340	5.2704000	613586.0000000
21.5833340	5.3192000	613586.0000000
21.5833340	5.3680000	613586.0000000
21.5833340	5.4168000	613586.0000000
21.5833340	5.4656000	613586.0000000
21.5833340	5.5144000	613586.0000000
21.5833340	5.5632000	613586.0000000
21.5833340	5.6120000	613586.0000000
21.5833340	5.6608000	613586.0000000
21.5833340	5.7096000	613586.0000000
21.5833340	5.7584000	613586.0000000
21.5833340	5.8072000	613586.0000000
21.5833340	5.8559999	613586.0000000
21.5833340	5.9047999	613586.0000000
21.5833340	5.9535999	613586.0000000
21.5833340	6.0023999	613586.0000000
21.6000004	0.2928000	-1.6935148
21.6000004	0.3416000	-1.7426023
21.6000004	0.3904000	-1.7487383
21.6000004	0.4392000	-1.5339808
21.6000004	0.4880000	-1.5401168
21.6000004	0.5368000	-1.4726214
21.6000004	0.5856000	-1.4664855
21.6000004	0.6344000	-1.6260195
21.6000004	0.6832000	-1.6137476
21.6000004	0.7320000	-1.4480777
21.6000004	0.7808000	-1.3192234
21.6000004	0.8296000	-1.3989906

21.6000004	0.8784000	-1.3069515
21.6000004	0.9272000	-1.3253593
21.6000004	0.9760000	-1.3253593
21.6000004	1.0247999	-1.3376312
21.6000004	1.0735999	-1.3376312
21.6000004	1.1223999	-1.3069515
21.6000004	1.1711999	-1.4787574
21.6000004	1.2200000	-1.5769324
21.6000004	1.2688000	-1.5830684
21.6000004	1.3176000	-1.5830684
21.6000004	1.3664000	-1.6076121
21.6000004	1.4152000	-1.6260195
21.6000004	1.4640000	-1.6444273
21.6000004	1.5128000	-1.6505632
21.6000004	1.5616000	-1.6505632
21.6000004	1.6104000	-1.5707965
21.6000004	1.6592000	-1.5646605
21.6000004	1.7079999	-1.1167378
21.6000004	1.7567999	-0.9142528
21.6000004	1.8055999	-0.9142528
21.6000004	1.8543999	-1.0124273
21.6000004	1.9031999	-1.2333207
21.6000004	1.9519999	-1.2210488
21.6000004	2.0007999	-1.1965051
21.6000004	2.0495999	-1.2271848
21.6000004	2.0983999	-1.2333207
21.6000004	2.1471999	-1.2333207
21.6000004	2.1959999	-1.2333207
21.6000004	2.2447999	-1.0921946
21.6000004	2.2935998	-1.1044660
21.6000004	2.3423998	-1.3560390
21.6000004	2.3912001	-1.3744469
21.6000004	2.4400001	-1.3499031
21.6000004	2.4888000	-1.3376312
21.6000004	2.5376000	-1.3376312
21.6000004	2.5864000	-1.3192234
21.6000004	2.6352000	-1.3192234
21.6000004	2.6840000	-1.4051266
21.6000004	2.7328000	-1.4051266
21.6000004	2.7816000	-1.2455926
21.6000004	2.8304000	-1.2517285
21.6000004	2.8792000	-1.2517285
21.6000004	2.9280000	-1.3499031
21.6000004	2.9768000	-1.3437672
21.6000004	3.0256000	-1.2394567
21.6000004	3.0743999	-1.2455926
21.6000004	3.1231999	-1.2271848
21.6000004	3.1719999	-1.1842332
21.6000004	3.2207999	-1.0983305
21.6000004	3.2695999	-1.0246992
21.6000004	3.3183999	-0.9449320
21.6000004	3.3671999	-0.9449320
21.6000004	3.4159999	-0.0736313
21.6000004	3.4647999	613586.0000000
21.6000004	3.5135999	-1.0615149
21.6000004	3.5623999	613586.0000000
21.6000004	3.6111999	-1.1842332

21.6000004	3.6599998	613586.0000000
21.6000004	3.7087998	-0.5890489
21.6000004	3.7575998	-0.5522332
21.6000004	3.8063998	-0.5399613
21.6000004	3.8551998	613586.0000000
21.6000004	3.9039998	613586.0000000
21.6000004	3.9527998	613586.0000000
21.6000004	4.0016003	613586.0000000
21.6000004	4.0503998	-1.0983305
21.6000004	4.0991998	-1.0983305
21.6000004	4.1479998	-1.1290097
21.6000004	4.1967998	613586.0000000
21.6000004	4.2455997	613586.0000000
21.6000004	4.2943997	-0.9265246
21.6000004	4.3431997	-0.9326606
21.6000004	4.3919997	-0.9449320
21.6000004	4.4407997	613586.0000000
21.6000004	4.4895997	-0.8222136
21.6000004	4.5383997	-0.7117672
21.6000004	4.5871997	-0.8774371
21.6000004	4.6359997	613586.0000000
21.6000004	4.6847997	613586.0000000
21.6000004	4.7336001	613586.0000000
21.6000004	4.7824001	613586.0000000
21.6000004	4.8312001	613586.0000000
21.6000004	4.8800001	613586.0000000
21.6000004	4.9288001	613586.0000000
21.6000004	4.9776001	613586.0000000
21.6000004	5.0264001	613586.0000000
21.6000004	5.0752001	613586.0000000
21.6000004	5.1240001	613586.0000000
21.6000004	5.1728001	613586.0000000
21.6000004	5.2216001	-0.6626797
21.6000004	5.2704000	-0.6626797
21.6000004	5.3192000	613586.0000000
21.6000004	5.3680000	613586.0000000
21.6000004	5.4168000	613586.0000000
21.6000004	5.4656000	-0.6258640
21.6000004	5.5144000	613586.0000000
21.6000004	5.5632000	613586.0000000
21.6000004	5.6120000	613586.0000000
21.6000004	5.6608000	613586.0000000
21.6000004	5.7096000	613586.0000000
21.6000004	5.7584000	613586.0000000
21.6000004	5.8072000	613586.0000000
21.6000004	5.8559999	613586.0000000
21.6000004	5.9047999	613586.0000000
21.6000004	5.9535999	613586.0000000
21.6000004	6.0023999	613586.0000000
21.6166668	0.2928000	-1.5523887
21.6166668	0.3416000	-1.4664855
21.6166668	0.3904000	-1.7364664
21.6166668	0.4392000	-1.5830684
21.6166668	0.4880000	-1.1842332
21.6166668	0.5368000	-1.3560390
21.6166668	0.5856000	-1.3683109
21.6166668	0.6344000	-1.5462527

21.6166668	0.6832000	-1.5462527
21.6166668	0.7320000	-1.4910293
21.6166668	0.7808000	-1.4910293
21.6166668	0.8296000	-1.5033011
21.6166668	0.8784000	-1.5033011
21.6166668	0.9272000	-1.5033011
21.6166668	0.9760000	-1.5033011
21.6166668	1.0247999	-1.5278449
21.6166668	1.0735999	-1.5401168
21.6166668	1.1223999	-1.3008156
21.6166668	1.1711999	-1.3805828
21.6166668	1.2200000	-1.3560390
21.6166668	1.2688000	-1.6137476
21.6166668	1.3176000	-1.6076121
21.6166668	1.3664000	-1.5707965
21.6166668	1.4152000	-1.5892043
21.6166668	1.4640000	-1.5953403
21.6166668	1.5128000	-1.7487383
21.6166668	1.5616000	-1.6505632
21.6166668	1.6104000	-1.4971652
21.6166668	1.6592000	-1.6444273
21.6166668	1.7079999	-1.5094371
21.6166668	1.7567999	-1.5278449
21.6166668	1.8055999	-1.5278449
21.6166668	1.8543999	-1.2333207
21.6166668	1.9031999	-1.2394567
21.6166668	1.9519999	-1.3560390
21.6166668	2.0007999	-1.3560390
21.6166668	2.0495999	-1.3437672
21.6166668	2.0983999	-1.3253593
21.6166668	2.1471999	-1.5033011
21.6166668	2.1959999	-1.3376312
21.6166668	2.2447999	-1.0185633
21.6166668	2.2935998	-1.0185633
21.6166668	2.3423998	-1.1780972
21.6166668	2.3912001	-1.1780972
21.6166668	2.4400001	-1.1780972
21.6166668	2.4888000	-1.2271848
21.6166668	2.5376000	-1.3314953
21.6166668	2.5864000	-1.3744469
21.6166668	2.6352000	-1.3805828
21.6166668	2.6840000	-1.3989906
21.6166668	2.7328000	-1.2210488
21.6166668	2.7816000	-1.2394567
21.6166668	2.8304000	-1.2026410
21.6166668	2.8792000	-1.3314953
21.6166668	2.9280000	-1.3253593
21.6166668	2.9768000	-1.3130875
21.6166668	3.0256000	-1.5155730
21.6166668	3.0743999	-1.6076121
21.6166668	3.1231999	-1.4971652
21.6166668	3.1719999	-1.2578645
21.6166668	3.2207999	-1.2517285
21.6166668	3.2695999	-1.3683109
21.6166668	3.3183999	-1.3621750
21.6166668	3.3671999	-1.3560390
21.6166668	3.4159999	-1.3621750

21.6166668	3.4647999	-1.1780972
21.6166668	3.5135999	-1.0860586
21.6166668	3.5623999	-1.4603496
21.6166668	3.6111999	-1.7057867
21.6166668	3.6599998	-1.6935148
21.6166668	3.7087998	-1.6935148
21.6166668	3.7575998	-1.6935148
21.6166668	3.8063998	613586.0000000
21.6166668	3.8551998	613586.0000000
21.6166668	3.9039998	613586.0000000
21.6166668	3.9527998	-0.7853980
21.6166668	4.0016003	-0.7853980
21.6166668	4.0503998	-0.6504078
21.6166668	4.0991998	613586.0000000
21.6166668	4.1479998	613586.0000000
21.6166668	4.1967998	-1.1228738
21.6166668	4.2455997	-1.1228738
21.6166668	4.2943997	-1.1596894
21.6166668	4.3431997	-1.1658254
21.6166668	4.3919997	-1.1903691
21.6166668	4.4407997	-1.1903691
21.6166668	4.4895997	-1.2149129
21.6166668	4.5383997	-1.2149129
21.6166668	4.5871997	613586.0000000
21.6166668	4.6359997	613586.0000000
21.6166668	4.6847997	613586.0000000
21.6166668	4.7336001	613586.0000000
21.6166668	4.7824001	613586.0000000
21.6166668	4.8312001	613586.0000000
21.6166668	4.8800001	613586.0000000
21.6166668	4.9288001	613586.0000000
21.6166668	4.9776001	613586.0000000
21.6166668	5.0264001	613586.0000000
21.6166668	5.0752001	613586.0000000
21.6166668	5.1240001	613586.0000000
21.6166668	5.1728001	-1.0737867
21.6166668	5.2216001	-1.0615149
21.6166668	5.2704000	-1.0676508
21.6166668	5.3192000	613586.0000000
21.6166668	5.3680000	613586.0000000
21.6166668	5.4168000	613586.0000000
21.6166668	5.4656000	613586.0000000
21.6166668	5.5144000	613586.0000000
21.6166668	5.5632000	613586.0000000
21.6166668	5.6120000	613586.0000000
21.6166668	5.6608000	613586.0000000
21.6166668	5.7096000	613586.0000000
21.6166668	5.7584000	613586.0000000
21.6166668	5.8072000	613586.0000000
21.6166668	5.8559999	613586.0000000
21.6166668	5.9047999	613586.0000000
21.6166668	5.9535999	613586.0000000
21.6166668	6.0023999	613586.0000000
21.6333332	0.2928000	-1.7978253
21.6333332	0.3416000	-1.7916894
21.6333332	0.3904000	-1.8346410
21.6333332	0.4392000	-1.8223691

21.6333332	0.4880000	-1.8100972
21.6333332	0.5368000	-1.5585246
21.6333332	0.5856000	-1.9205441
21.6333332	0.6344000	-1.4603496
21.6333332	0.6832000	-1.4603496
21.6333332	0.7320000	-1.3621750
21.6333332	0.7808000	-1.2640004
21.6333332	0.8296000	-1.4173985
21.6333332	0.8784000	-1.3621750
21.6333332	0.9272000	-1.1351457
21.6333332	0.9760000	-1.1351457
21.6333332	1.0247999	-1.3008156
21.6333332	1.0735999	-1.3989906
21.6333332	1.1223999	-1.5278449
21.6333332	1.1711999	-1.4726214
21.6333332	1.2200000	-1.4726214
21.6333332	1.2688000	-1.4726214
21.6333332	1.3176000	-1.6566992
21.6333332	1.3664000	-1.6137476
21.6333332	1.4152000	-1.7426023
21.6333332	1.4640000	-1.7426023
21.6333332	1.5128000	-1.7548742
21.6333332	1.5616000	-1.6873789
21.6333332	1.6104000	-1.7119226
21.6333332	1.6592000	-1.4848933
21.6333332	1.7079999	-1.7303305
21.6333332	1.7567999	-1.7303305
21.6333332	1.8055999	-1.4051266
21.6333332	1.8543999	-1.4419422
21.6333332	1.9031999	-1.4358063
21.6333332	1.9519999	-1.4358063
21.6333332	2.0007999	-1.5401168
21.6333332	2.0495999	-1.4848933
21.6333332	2.0983999	-1.7487383
21.6333332	2.1471999	-1.6137476
21.6333332	2.1959999	-1.4419422
21.6333332	2.2447999	-1.6321554
21.6333332	2.2935998	-1.5646605
21.6333332	2.3423998	-1.4971652
21.6333332	2.3912001	-1.4971652
21.6333332	2.4400001	-1.3744469
21.6333332	2.4888000	-1.3744469
21.6333332	2.5376000	-1.3069515
21.6333332	2.5864000	-1.3499031
21.6333332	2.6352000	-1.4112625
21.6333332	2.6840000	-1.4112625
21.6333332	2.7328000	-1.1842332
21.6333332	2.7816000	-1.1658254
21.6333332	2.8304000	-1.1535535
21.6333332	2.8792000	-1.1474175
21.6333332	2.9280000	-1.3437672
21.6333332	2.9768000	-1.4480777
21.6333332	3.0256000	-1.3499031
21.6333332	3.0743999	-1.5339808
21.6333332	3.1231999	-1.5401168
21.6333332	3.1719999	-1.5339808
21.6333332	3.2207999	-1.3069515

21.6333332	3.2695999	-1.3314953
21.6333332	3.3183999	-1.4848933
21.6333332	3.3671999	-0.9817476
21.6333332	3.4159999	-1.0676508
21.6333332	3.4647999	-1.3621750
21.6333332	3.5135999	-1.2087770
21.6333332	3.5623999	613586.0000000
21.6333332	3.6111999	-0.9940195
21.6333332	3.6599998	613586.0000000
21.6333332	3.7087998	613586.0000000
21.6333332	3.7575998	613586.0000000
21.6333332	3.8063998	613586.0000000
21.6333332	3.8551998	613586.0000000
21.6333332	3.9039998	613586.0000000
21.6333332	3.9527998	613586.0000000
21.6333332	4.0016003	-1.1474175
21.6333332	4.0503998	-1.1719613
21.6333332	4.0991998	-1.2762718
21.6333332	4.1479998	-1.2701364
21.6333332	4.1967998	-1.2640004
21.6333332	4.2455997	-1.0983305
21.6333332	4.2943997	-1.0983305
21.6333332	4.3431997	613586.0000000
21.6333332	4.3919997	613586.0000000
21.6333332	4.4407997	613586.0000000
21.6333332	4.4895997	613586.0000000
21.6333332	4.5383997	613586.0000000
21.6333332	4.5871997	613586.0000000
21.6333332	4.6359997	613586.0000000
21.6333332	4.6847997	613586.0000000
21.6333332	4.7336001	613586.0000000
21.6333332	4.7824001	613586.0000000
21.6333332	4.8312001	613586.0000000
21.6333332	4.8800001	613586.0000000
21.6333332	4.9288001	613586.0000000
21.6333332	4.9776001	613586.0000000
21.6333332	5.0264001	613586.0000000
21.6333332	5.0752001	613586.0000000
21.6333332	5.1240001	613586.0000000
21.6333332	5.1728001	613586.0000000
21.6333332	5.2216001	613586.0000000
21.6333332	5.2704000	613586.0000000
21.6333332	5.3192000	613586.0000000
21.6333332	5.3680000	613586.0000000
21.6333332	5.4168000	613586.0000000
21.6333332	5.4656000	613586.0000000
21.6333332	5.5144000	613586.0000000
21.6333332	5.5632000	613586.0000000
21.6333332	5.6120000	613586.0000000
21.6333332	5.6608000	613586.0000000
21.6333332	5.7096000	613586.0000000
21.6333332	5.7584000	613586.0000000
21.6333332	5.8072000	613586.0000000
21.6333332	5.8559999	613586.0000000
21.6333332	5.9047999	613586.0000000
21.6333332	5.9535999	613586.0000000
21.6333332	6.0023999	613586.0000000

21.6499996	0.2928000	-1.9450879
21.6499996	0.3416000	-1.8960004
21.6499996	0.3904000	-1.8960004
21.6499996	0.4392000	-1.6076121
21.6499996	0.4880000	-1.6444273
21.6499996	0.5368000	-1.5585246
21.6499996	0.5856000	-1.5401168
21.6499996	0.6344000	-1.4358063
21.6499996	0.6832000	-1.4173985
21.6499996	0.7320000	-1.3560390
21.6499996	0.7808000	-1.5094371
21.6499996	0.8296000	-1.3499031
21.6499996	0.8784000	-1.3499031
21.6499996	0.9272000	-1.3437672
21.6499996	0.9760000	-1.2885437
21.6499996	1.0247999	-1.3008156
21.6499996	1.0735999	-1.3008156
21.6499996	1.1223999	-1.3008156
21.6499996	1.1711999	-1.3621750
21.6499996	1.2200000	-1.3683109
21.6499996	1.2688000	-1.6444273
21.6499996	1.3176000	-1.6628351
21.6499996	1.3664000	-1.4542136
21.6499996	1.4152000	-1.5892043
21.6499996	1.4640000	-1.5830684
21.6499996	1.5128000	-1.8346410
21.6499996	1.5616000	-1.8223691
21.6499996	1.6104000	-1.5523887
21.6499996	1.6592000	-1.5646605
21.6499996	1.7079999	-1.1780972
21.6499996	1.7567999	-1.1842332
21.6499996	1.8055999	-1.0553789
21.6499996	1.8543999	-1.2517285
21.6499996	1.9031999	-1.2701364
21.6499996	1.9519999	-1.4112625
21.6499996	2.0007999	-1.2455926
21.6499996	2.0495999	-1.1228738
21.6499996	2.0983999	-1.1228738
21.6499996	2.1471999	-1.0737867
21.6499996	2.1959999	-0.9326606
21.6499996	2.2447999	-0.9326606
21.6499996	2.2935998	-1.0246992
21.6499996	2.3423998	-1.0124273
21.6499996	2.3912001	-1.0860586
21.6499996	2.4400001	-1.0860586
21.6499996	2.4888000	-1.1658254
21.6499996	2.5376000	-1.2026410
21.6499996	2.5864000	-1.1658254
21.6499996	2.6352000	-1.2210488
21.6499996	2.6840000	-1.2210488
21.6499996	2.7328000	-1.2087770
21.6499996	2.7816000	-1.2946796
21.6499996	2.8304000	-1.3069515
21.6499996	2.8792000	-1.1167378
21.6499996	2.9280000	-1.2394567
21.6499996	2.9768000	-1.2149129
21.6499996	3.0256000	-1.3376312

21.6499996	3.0743999	-0.9510679
21.6499996	3.1231999	-0.9449320
21.6499996	3.1719999	-0.8099418
21.6499996	3.2207999	-0.8160777
21.6499996	3.2695999	-0.8713012
21.6499996	3.3183999	-0.9694757
21.6499996	3.3671999	-0.6749516
21.6499996	3.4159999	-0.6442719
21.6499996	3.4647999	-0.6381359
21.6499996	3.5135999	-0.9940195
21.6499996	3.5623999	-0.9756117
21.6499996	3.6111999	-0.5460973
21.6499996	3.6599998	613586.0000000
21.6499996	3.7087998	613586.0000000
21.6499996	3.7575998	613586.0000000
21.6499996	3.8063998	613586.0000000
21.6499996	3.8551998	-0.8713012
21.6499996	3.9039998	-0.8835731
21.6499996	3.9527998	-0.8835731
21.6499996	4.0016003	-0.9081168
21.6499996	4.0503998	-1.2578645
21.6499996	4.0991998	-1.2640004
21.6499996	4.1479998	-1.2640004
21.6499996	4.1967998	-1.0676508
21.6499996	4.2455997	-1.0553789
21.6499996	4.2943997	-1.0492430
21.6499996	4.3431997	-1.0492430
21.6499996	4.3919997	-1.0001554
21.6499996	4.4407997	613586.0000000
21.6499996	4.4895997	-0.8467574
21.6499996	4.5383997	613586.0000000
21.6499996	4.5871997	-0.9081168
21.6499996	4.6359997	-1.1106019
21.6499996	4.6847997	613586.0000000
21.6499996	4.7336001	613586.0000000
21.6499996	4.7824001	613586.0000000
21.6499996	4.8312001	613586.0000000
21.6499996	4.8800001	613586.0000000
21.6499996	4.9288001	613586.0000000
21.6499996	4.9776001	613586.0000000
21.6499996	5.0264001	613586.0000000
21.6499996	5.0752001	613586.0000000
21.6499996	5.1240001	613586.0000000
21.6499996	5.1728001	613586.0000000
21.6499996	5.2216001	613586.0000000
21.6499996	5.2704000	613586.0000000
21.6499996	5.3192000	613586.0000000
21.6499996	5.3680000	613586.0000000
21.6499996	5.4168000	613586.0000000
21.6499996	5.4656000	613586.0000000
21.6499996	5.5144000	613586.0000000
21.6499996	5.5632000	613586.0000000
21.6499996	5.6120000	613586.0000000
21.6499996	5.6608000	613586.0000000
21.6499996	5.7096000	613586.0000000
21.6499996	5.7584000	613586.0000000
21.6499996	5.8072000	613586.0000000

21.6499996	5.8559999	613586.0000000
21.6499996	5.9047999	613586.0000000
21.6499996	5.9535999	613586.0000000
21.6499996	6.0023999	613586.0000000
21.6666660	0.2928000	-1.6505632
21.6666660	0.3416000	-1.7119226
21.6666660	0.3904000	-1.5953403
21.6666660	0.4392000	-1.5953403
21.6666660	0.4880000	-1.6014762
21.6666660	0.5368000	-1.4910293
21.6666660	0.5856000	-1.3560390
21.6666660	0.6344000	-1.3069515
21.6666660	0.6832000	-1.4296703
21.6666660	0.7320000	-1.4358063
21.6666660	0.7808000	-1.4358063
21.6666660	0.8296000	-1.4419422
21.6666660	0.8784000	-1.4542136
21.6666660	0.9272000	-1.4603496
21.6666660	0.9760000	-1.3805828
21.6666660	1.0247999	-1.5155730
21.6666660	1.0735999	-1.5401168
21.6666660	1.1223999	-1.5155730
21.6666660	1.1711999	-1.5217090
21.6666660	1.2200000	-1.5217090
21.6666660	1.2688000	-1.6505632
21.6666660	1.3176000	-1.5769324
21.6666660	1.3664000	-1.5646605
21.6666660	1.4152000	-1.8530488
21.6666660	1.4640000	-1.7426023
21.6666660	1.5128000	-1.6689711
21.6666660	1.5616000	-1.6751070
21.6666660	1.6104000	-1.3928547
21.6666660	1.6592000	-1.4910293
21.6666660	1.7079999	-1.3560390
21.6666660	1.7567999	-0.8958449
21.6666660	1.8055999	-1.2885437
21.6666660	1.8543999	-1.2946796
21.6666660	1.9031999	-1.1044660
21.6666660	1.9519999	-0.9940195
21.6666660	2.0007999	-0.8958449
21.6666660	2.0495999	-0.6565437
21.6666660	2.0983999	-0.6626797
21.6666660	2.1471999	-0.7608547
21.6666660	2.1959999	-1.2394567
21.6666660	2.2447999	-1.1780972
21.6666660	2.2935998	-1.1780972
21.6666660	2.3423998	-1.1842332
21.6666660	2.3912001	-1.1903691
21.6666660	2.4400001	-1.1842332
21.6666660	2.4888000	-1.1842332
21.6666660	2.5376000	-1.1842332
21.6666660	2.5864000	-1.2640004
21.6666660	2.6352000	-1.3130875
21.6666660	2.6840000	-1.3130875
21.6666660	2.7328000	-1.3130875
21.6666660	2.7816000	-1.3008156
21.6666660	2.8304000	-1.2885437

21.6666660	2.8792000	-1.4173985
21.6666660	2.9280000	-1.2087770
21.6666660	2.9768000	-0.9326606
21.6666660	3.0256000	-0.8897090
21.6666660	3.0743999	-0.8897090
21.6666660	3.1231999	-0.7056313
21.6666660	3.1719999	-0.5460973
21.6666660	3.2207999	-0.6197281
21.6666660	3.2695999	-0.5645051
21.6666660	3.3183999	-0.5154176
21.6666660	3.3671999	-0.5092816
21.6666660	3.4159999	-0.5092816
21.6666660	3.4647999	-0.6565437
21.6666660	3.5135999	-0.9326606
21.6666660	3.5623999	-0.9326606
21.6666660	3.6111999	613586.0000000
21.6666660	3.6599998	-0.7056313
21.6666660	3.7087998	-0.6933594
21.6666660	3.7575998	-1.0001554
21.6666660	3.8063998	-0.9756117
21.6666660	3.8551998	-0.4601941
21.6666660	3.9039998	-0.6626797
21.6666660	3.9527998	-0.8897090
21.6666660	4.0016003	-0.8897090
21.6666660	4.0503998	-0.9449320
21.6666660	4.0991998	-0.8283496
21.6666660	4.1479998	-0.8283496
21.6666660	4.1967998	-0.8344855
21.6666660	4.2455997	613586.0000000
21.6666660	4.2943997	613586.0000000
21.6666660	4.3431997	613586.0000000
21.6666660	4.3919997	-1.0001554
21.6666660	4.4407997	-0.9019809
21.6666660	4.4895997	-1.1167378
21.6666660	4.5383997	-0.9694757
21.6666660	4.5871997	-0.7485828
21.6666660	4.6359997	-0.8160777
21.6666660	4.6847997	613586.0000000
21.6666660	4.7336001	613586.0000000
21.6666660	4.7824001	613586.0000000
21.6666660	4.8312001	-0.8590293
21.6666660	4.8800001	613586.0000000
21.6666660	4.9288001	613586.0000000
21.6666660	4.9776001	613586.0000000
21.6666660	5.0264001	613586.0000000
21.6666660	5.0752001	613586.0000000
21.6666660	5.1240001	613586.0000000
21.6666660	5.1728001	613586.0000000
21.6666660	5.2216001	613586.0000000
21.6666660	5.2704000	613586.0000000
21.6666660	5.3192000	613586.0000000
21.6666660	5.3680000	613586.0000000
21.6666660	5.4168000	613586.0000000
21.6666660	5.4656000	613586.0000000
21.6666660	5.5144000	613586.0000000
21.6666660	5.5632000	613586.0000000
21.6666660	5.6120000	613586.0000000

21.6666660	5.6608000	613586.0000000
21.6666660	5.7096000	613586.0000000
21.6666660	5.7584000	613586.0000000
21.6666660	5.8072000	613586.0000000
21.6666660	5.8559999	613586.0000000
21.6666660	5.9047999	613586.0000000
21.6666660	5.9535999	613586.0000000
21.6666660	6.0023999	613586.0000000
21.6833324	0.2928000	-1.8530488
21.6833324	0.3416000	-1.8530488
21.6833324	0.3904000	-1.8960004
21.6833324	0.4392000	-1.6935148
21.6833324	0.4880000	-1.8898644
21.6833324	0.5368000	-1.8960004
21.6833324	0.5856000	-1.6137476
21.6833324	0.6344000	-1.4848933
21.6833324	0.6832000	-1.5033011
21.6833324	0.7320000	-1.5033011
21.6833324	0.7808000	-1.4971652
21.6833324	0.8296000	-1.4910293
21.6833324	0.8784000	-1.4910293
21.6833324	0.9272000	-1.5155730
21.6833324	0.9760000	-1.5217090
21.6833324	1.0247999	-1.5217090
21.6833324	1.0735999	-1.5217090
21.6833324	1.1223999	-1.5217090
21.6833324	1.1711999	-1.4971652
21.6833324	1.2200000	-1.4358063
21.6833324	1.2688000	-1.5769324
21.6833324	1.3176000	-1.6137476
21.6833324	1.3664000	-1.5769324
21.6833324	1.4152000	-1.4726214
21.6833324	1.4640000	-1.4664855
21.6833324	1.5128000	-1.5094371
21.6833324	1.5616000	-1.5033011
21.6833324	1.6104000	-1.4726214
21.6833324	1.6592000	-1.4542136
21.6833324	1.7079999	-1.4419422
21.6833324	1.7567999	-1.2271848
21.6833324	1.8055999	-1.2824078
21.6833324	1.8543999	-1.2701364
21.6833324	1.9031999	-1.3928547
21.6833324	1.9519999	-1.3867188
21.6833324	2.0007999	-1.2640004
21.6833324	2.0495999	-1.0553789
21.6833324	2.0983999	-0.9510679
21.6833324	2.1471999	-0.9633398
21.6833324	2.1959999	-0.9694757
21.6833324	2.2447999	-0.9694757
21.6833324	2.2935998	-0.9694757
21.6833324	2.3423998	-0.9633398
21.6833324	2.3912001	-0.9694757
21.6833324	2.4400001	-0.9756117
21.6833324	2.4888000	-1.0124273
21.6833324	2.5376000	-1.1412816
21.6833324	2.5864000	-1.1535535
21.6833324	2.6352000	-1.0676508

21.6833324	2.6840000	-1.1965051
21.6833324	2.7328000	-1.2026410
21.6833324	2.7816000	-1.0921946
21.6833324	2.8304000	-1.1106019
21.6833324	2.8792000	-1.1106019
21.6833324	2.9280000	-0.9572039
21.6833324	2.9768000	-0.9694757
21.6833324	3.0256000	-0.9694757
21.6833324	3.0743999	-0.9572039
21.6833324	3.1231999	-0.8774371
21.6833324	3.1719999	-0.6810875
21.6833324	3.2207999	-0.6872234
21.6833324	3.2695999	-0.6135921
21.6833324	3.3183999	-0.6135921
21.6833324	3.3671999	-0.6381359
21.6833324	3.4159999	-0.6933594
21.6833324	3.4647999	613586.0000000
21.6833324	3.5135999	-0.2454371
21.6833324	3.5623999	-0.0674953
21.6833324	3.6111999	0.1595340
21.6833324	3.6599998	-0.8774371
21.6833324	3.7087998	-0.4295144
21.6833324	3.7575998	-0.4356503
21.6833324	3.8063998	-0.1349902
21.6833324	3.8551998	-0.3129320
21.6833324	3.9039998	-0.9940195
21.6833324	3.9527998	-0.8590293
21.6833324	4.0016003	-0.9203887
21.6833324	4.0503998	-0.9019809
21.6833324	4.0991998	-0.9019809
21.6833324	4.1479998	-0.8958449
21.6833324	4.1967998	-1.0062914
21.6833324	4.2455997	613586.0000000
21.6833324	4.2943997	-1.1903691
21.6833324	4.3431997	-0.8651652
21.6833324	4.3919997	-0.9572039
21.6833324	4.4407997	-0.9633398
21.6833324	4.4895997	-0.7853980
21.6833324	4.5383997	-0.7731261
21.6833324	4.5871997	-0.8467574
21.6833324	4.6359997	-0.8406215
21.6833324	4.6847997	613586.0000000
21.6833324	4.7336001	613586.0000000
21.6833324	4.7824001	-0.6381359
21.6833324	4.8312001	613586.0000000
21.6833324	4.8800001	613586.0000000
21.6833324	4.9288001	613586.0000000
21.6833324	4.9776001	613586.0000000
21.6833324	5.0264001	613586.0000000
21.6833324	5.0752001	613586.0000000
21.6833324	5.1240001	613586.0000000
21.6833324	5.1728001	613586.0000000
21.6833324	5.2216001	613586.0000000
21.6833324	5.2704000	613586.0000000
21.6833324	5.3192000	613586.0000000
21.6833324	5.3680000	613586.0000000
21.6833324	5.4168000	613586.0000000

21.6833324	5.4656000	613586.0000000
21.6833324	5.5144000	613586.0000000
21.6833324	5.5632000	613586.0000000
21.6833324	5.6120000	613586.0000000
21.6833324	5.6608000	613586.0000000
21.6833324	5.7096000	613586.0000000
21.6833324	5.7584000	613586.0000000
21.6833324	5.8072000	613586.0000000
21.6833324	5.8559999	613586.0000000
21.6833324	5.9047999	613586.0000000
21.6833324	5.9535999	613586.0000000
21.6833324	6.0023999	613586.0000000
21.7000008	0.2928000	-1.6689711
21.7000008	0.3416000	-1.5278449
21.7000008	0.3904000	-1.8162332
21.7000008	0.4392000	-1.3805828
21.7000008	0.4880000	-1.3989906
21.7000008	0.5368000	-1.3683109
21.7000008	0.5856000	-1.5585246
21.7000008	0.6344000	-1.4848933
21.7000008	0.6832000	-1.4971652
21.7000008	0.7320000	-1.4848933
21.7000008	0.7808000	-1.4848933
21.7000008	0.8296000	-1.3744469
21.7000008	0.8784000	-1.6198835
21.7000008	0.9272000	-1.3683109
21.7000008	0.9760000	-1.3744469
21.7000008	1.0247999	-1.3805828
21.7000008	1.0735999	-1.3867188
21.7000008	1.1223999	-1.3867188
21.7000008	1.1711999	-1.3683109
21.7000008	1.2200000	-1.5462527
21.7000008	1.2688000	-1.6014762
21.7000008	1.3176000	-1.6076121
21.7000008	1.3664000	-1.5953403
21.7000008	1.4152000	-1.9144082
21.7000008	1.4640000	-1.8530488
21.7000008	1.5128000	-1.9512239
21.7000008	1.5616000	-1.5707965
21.7000008	1.6104000	-1.5830684
21.7000008	1.6592000	-1.4603496
21.7000008	1.7079999	-1.6689711
21.7000008	1.7567999	-1.2149129
21.7000008	1.8055999	-1.3130875
21.7000008	1.8543999	-0.9510679
21.7000008	1.9031999	-0.7056313
21.7000008	1.9519999	-1.0308352
21.7000008	2.0007999	-1.0185633
21.7000008	2.0495999	-1.0001554
21.7000008	2.0983999	-1.0431070
21.7000008	2.1471999	-1.0308352
21.7000008	2.1959999	-1.0369711
21.7000008	2.2447999	-0.9142528
21.7000008	2.2935998	-0.9203887
21.7000008	2.3423998	-0.9265246
21.7000008	2.3912001	-0.9387960
21.7000008	2.4400001	-1.0553789

21.7000008	2.4888000	-1.0615149
21.7000008	2.5376000	-1.2149129
21.7000008	2.5864000	-1.2087770
21.7000008	2.6352000	-1.2026410
21.7000008	2.6840000	-1.2701364
21.7000008	2.7328000	-1.2149129
21.7000008	2.7816000	-1.2149129
21.7000008	2.8304000	-1.2210488
21.7000008	2.8792000	-1.0431070
21.7000008	2.9280000	-0.8283496
21.7000008	2.9768000	-0.9265246
21.7000008	3.0256000	-1.0492430
21.7000008	3.0743999	-0.8651652
21.7000008	3.1231999	-0.8774371
21.7000008	3.1719999	-1.1228738
21.7000008	3.2207999	-1.0676508
21.7000008	3.2695999	-1.0615149
21.7000008	3.3183999	-0.9878836
21.7000008	3.3671999	-0.8344855
21.7000008	3.4159999	-0.7056313
21.7000008	3.4647999	-0.6994953
21.7000008	3.5135999	-1.1658254
21.7000008	3.5623999	-0.8590293
21.7000008	3.6111999	-0.8713012
21.7000008	3.6599998	-0.5645051
21.7000008	3.7087998	-0.6933594
21.7000008	3.7575998	-0.5092816
21.7000008	3.8063998	-0.8099418
21.7000008	3.8551998	-0.7363110
21.7000008	3.9039998	-0.6933594
21.7000008	3.9527998	-0.6994953
21.7000008	4.0016003	-0.7853980
21.7000008	4.0503998	-0.7915339
21.7000008	4.0991998	-0.7915339
21.7000008	4.1479998	-0.9878836
21.7000008	4.1967998	-0.9633398
21.7000008	4.2455997	-0.9633398
21.7000008	4.2943997	-1.0185633
21.7000008	4.3431997	-1.0185633
21.7000008	4.3919997	-0.8897090
21.7000008	4.4407997	-1.0308352
21.7000008	4.4895997	-1.0308352
21.7000008	4.5383997	-1.0921946
21.7000008	4.5871997	613586.0000000
21.7000008	4.6359997	-0.9387960
21.7000008	4.6847997	-0.7669902
21.7000008	4.7336001	613586.0000000
21.7000008	4.7824001	613586.0000000
21.7000008	4.8312001	613586.0000000
21.7000008	4.8800001	613586.0000000
21.7000008	4.9288001	613586.0000000
21.7000008	4.9776001	613586.0000000
21.7000008	5.0264001	613586.0000000
21.7000008	5.0752001	613586.0000000
21.7000008	5.1240001	613586.0000000
21.7000008	5.1728001	613586.0000000
21.7000008	5.2216001	613586.0000000

21.7000008	5.2704000	613586.0000000
21.7000008	5.3192000	613586.0000000
21.7000008	5.3680000	613586.0000000
21.7000008	5.4168000	613586.0000000
21.7000008	5.4656000	613586.0000000
21.7000008	5.5144000	613586.0000000
21.7000008	5.5632000	613586.0000000
21.7000008	5.6120000	613586.0000000
21.7000008	5.6608000	613586.0000000
21.7000008	5.7096000	613586.0000000
21.7000008	5.7584000	613586.0000000
21.7000008	5.8072000	613586.0000000
21.7000008	5.8559999	613586.0000000
21.7000008	5.9047999	613586.0000000
21.7000008	5.9535999	613586.0000000
21.7000008	6.0023999	613586.0000000
21.7166672	0.2928000	-1.6198835
21.7166672	0.3416000	-1.6198835
21.7166672	0.3904000	-1.3989906
21.7166672	0.4392000	-1.4480777
21.7166672	0.4880000	-1.4480777
21.7166672	0.5368000	-1.4664855
21.7166672	0.5856000	-1.4848933
21.7166672	0.6344000	-1.5094371
21.7166672	0.6832000	-1.3928547
21.7166672	0.7320000	-1.3867188
21.7166672	0.7808000	-1.4358063
21.7166672	0.8296000	-1.4173985
21.7166672	0.8784000	-1.3989906
21.7166672	0.9272000	-1.3376312
21.7166672	0.9760000	-1.4542136
21.7166672	1.0247999	-1.4480777
21.7166672	1.0735999	-1.4971652
21.7166672	1.1223999	-1.5033011
21.7166672	1.1711999	-1.2517285
21.7166672	1.2200000	-1.6873789
21.7166672	1.2688000	-1.5707965
21.7166672	1.3176000	-1.9328160
21.7166672	1.3664000	-1.9266801
21.7166672	1.4152000	-1.9266801
21.7166672	1.4640000	-1.9389520
21.7166672	1.5128000	-1.9328160
21.7166672	1.5616000	-1.5339808
21.7166672	1.6104000	-1.5217090
21.7166672	1.6592000	-1.5217090
21.7166672	1.7079999	-1.1780972
21.7166672	1.7567999	-1.2517285
21.7166672	1.8055999	-1.2517285
21.7166672	1.8543999	-1.1842332
21.7166672	1.9031999	-1.1903691
21.7166672	1.9519999	-1.2087770
21.7166672	2.0007999	-1.1535535
21.7166672	2.0495999	-1.1965051
21.7166672	2.0983999	-1.2087770
21.7166672	2.1471999	-1.1290097
21.7166672	2.1959999	-1.0001554
21.7166672	2.2447999	-0.9878836

21.7166672	2.2935998	-0.9878836
21.7166672	2.3423998	-0.9203887
21.7166672	2.3912001	-1.1780972
21.7166672	2.4400001	-1.1780972
21.7166672	2.4888000	-1.2885437
21.7166672	2.5376000	-1.2946796
21.7166672	2.5864000	-1.1596894
21.7166672	2.6352000	-1.1535535
21.7166672	2.6840000	-1.1658254
21.7166672	2.7328000	-1.0737867
21.7166672	2.7816000	-1.0799227
21.7166672	2.8304000	-1.0860586
21.7166672	2.8792000	-0.9449320
21.7166672	2.9280000	-0.8651652
21.7166672	2.9768000	-0.8774371
21.7166672	3.0256000	-0.8897090
21.7166672	3.0743999	-0.7240391
21.7166672	3.1231999	-0.5706410
21.7166672	3.1719999	-0.4356503
21.7166672	3.2207999	-0.4356503
21.7166672	3.2695999	-0.4295144
21.7166672	3.3183999	0.0245438
21.7166672	3.3671999	-0.2393012
21.7166672	3.4159999	-0.7240391
21.7166672	3.4647999	-0.7240391
21.7166672	3.5135999	-0.7240391
21.7166672	3.5623999	-0.7608547
21.7166672	3.6111999	-0.5583692
21.7166672	3.6599998	-0.5767770
21.7166672	3.7087998	-0.8222136
21.7166672	3.7575998	-0.9510679
21.7166672	3.8063998	-0.9756117
21.7166672	3.8551998	-1.0431070
21.7166672	3.9039998	-1.0246992
21.7166672	3.9527998	-1.1535535
21.7166672	4.0016003	-1.2701364
21.7166672	4.0503998	-1.3069515
21.7166672	4.0991998	-1.1965051
21.7166672	4.1479998	-1.2087770
21.7166672	4.1967998	-1.0737867
21.7166672	4.2455997	-0.9756117
21.7166672	4.2943997	-0.9756117
21.7166672	4.3431997	-0.9694757
21.7166672	4.3919997	-1.0553789
21.7166672	4.4407997	-1.0492430
21.7166672	4.4895997	-1.0185633
21.7166672	4.5383997	-0.8283496
21.7166672	4.5871997	613586.0000000
21.7166672	4.6359997	-0.9817476
21.7166672	4.6847997	-0.8038058
21.7166672	4.7336001	-0.7976699
21.7166672	4.7824001	613586.0000000
21.7166672	4.8312001	613586.0000000
21.7166672	4.8800001	613586.0000000
21.7166672	4.9288001	613586.0000000
21.7166672	4.9776001	613586.0000000
21.7166672	5.0264001	613586.0000000

21.7166672	5.0752001	613586.0000000
21.7166672	5.1240001	613586.0000000
21.7166672	5.1728001	613586.0000000
21.7166672	5.2216001	613586.0000000
21.7166672	5.2704000	-0.7363110
21.7166672	5.3192000	613586.0000000
21.7166672	5.3680000	613586.0000000
21.7166672	5.4168000	613586.0000000
21.7166672	5.4656000	613586.0000000
21.7166672	5.5144000	613586.0000000
21.7166672	5.5632000	613586.0000000
21.7166672	5.6120000	613586.0000000
21.7166672	5.6608000	613586.0000000
21.7166672	5.7096000	613586.0000000
21.7166672	5.7584000	613586.0000000
21.7166672	5.8072000	613586.0000000
21.7166672	5.8559999	613586.0000000
21.7166672	5.9047999	613586.0000000
21.7166672	5.9535999	613586.0000000
21.7166672	6.0023999	613586.0000000
21.7333336	0.2928000	-1.6689711
21.7333336	0.3416000	-1.7364664
21.7333336	0.3904000	-1.7426023
21.7333336	0.4392000	-1.6321554
21.7333336	0.4880000	-1.3744469
21.7333336	0.5368000	-1.4971652
21.7333336	0.5856000	-1.4112625
21.7333336	0.6344000	-1.6935148
21.7333336	0.6832000	-1.6382914
21.7333336	0.7320000	-1.3376312
21.7333336	0.7808000	-1.4358063
21.7333336	0.8296000	-1.3989906
21.7333336	0.8784000	-1.4603496
21.7333336	0.9272000	-1.4726214
21.7333336	0.9760000	-1.5155730
21.7333336	1.0247999	-1.5155730
21.7333336	1.0735999	-1.5585246
21.7333336	1.1223999	-1.6137476
21.7333336	1.1711999	-1.6076121
21.7333336	1.2200000	-1.3621750
21.7333336	1.2688000	-1.5155730
21.7333336	1.3176000	-1.6996508
21.7333336	1.3664000	-1.7057867
21.7333336	1.4152000	-1.7180586
21.7333336	1.4640000	-1.6996508
21.7333336	1.5128000	-1.6873789
21.7333336	1.5616000	-1.8039613
21.7333336	1.6104000	-1.6873789
21.7333336	1.6592000	-1.4848933
21.7333336	1.7079999	-1.3192234
21.7333336	1.7567999	-1.5033011
21.7333336	1.8055999	-1.4971652
21.7333336	1.8543999	-1.0860586
21.7333336	1.9031999	-1.0860586
21.7333336	1.9519999	-1.0860586
21.7333336	2.0007999	-1.2762718
21.7333336	2.0495999	-1.1228738

21.7333336	2.0983999	-1.1290097
21.7333336	2.1471999	-1.1412816
21.7333336	2.1959999	-1.1412816
21.7333336	2.2447999	-1.1903691
21.7333336	2.2935998	-1.2394567
21.7333336	2.3423998	-1.2394567
21.7333336	2.3912001	-1.2394567
21.7333336	2.4400001	-1.1167378
21.7333336	2.4888000	-1.1780972
21.7333336	2.5376000	-1.1903691
21.7333336	2.5864000	-1.1903691
21.7333336	2.6352000	-1.0921946
21.7333336	2.6840000	-1.0860586
21.7333336	2.7328000	-1.0676508
21.7333336	2.7816000	-1.0676508
21.7333336	2.8304000	-1.1719613
21.7333336	2.8792000	-1.1719613
21.7333336	2.9280000	-1.3008156
21.7333336	2.9768000	-1.0492430
21.7333336	3.0256000	-0.9510679
21.7333336	3.0743999	-0.9510679
21.7333336	3.1231999	-1.2824078
21.7333336	3.1719999	-1.2517285
21.7333336	3.2207999	-1.2517285
21.7333336	3.2695999	-1.3683109
21.7333336	3.3183999	-1.5892043
21.7333336	3.3671999	-1.0676508
21.7333336	3.4159999	-1.0185633
21.7333336	3.4647999	-1.2824078
21.7333336	3.5135999	-1.2885437
21.7333336	3.5623999	-0.9449320
21.7333336	3.6111999	-0.8160777
21.7333336	3.6599998	-0.8160777
21.7333336	3.7087998	-0.6688156
21.7333336	3.7575998	-0.6688156
21.7333336	3.8063998	-0.5460973
21.7333336	3.8551998	-0.6258640
21.7333336	3.9039998	-0.9265246
21.7333336	3.9527998	-0.8897090
21.7333336	4.0016003	-0.9019809
21.7333336	4.0503998	-0.8774371
21.7333336	4.0991998	-0.8958449
21.7333336	4.1479998	-0.8160777
21.7333336	4.1967998	-0.8099418
21.7333336	4.2455997	-0.8099418
21.7333336	4.2943997	-0.7915339
21.7333336	4.3431997	-0.7976699
21.7333336	4.3919997	-0.7976699
21.7333336	4.4407997	-0.8528934
21.7333336	4.4895997	-0.8467574
21.7333336	4.5383997	-0.8590293
21.7333336	4.5871997	-0.7669902
21.7333336	4.6359997	-0.7547188
21.7333336	4.6847997	-0.7669902
21.7333336	4.7336001	-0.5951843
21.7333336	4.7824001	613586.0000000
21.7333336	4.8312001	613586.0000000

21.7333336	4.8800001	613586.0000000
21.7333336	4.9288001	613586.0000000
21.7333336	4.9776001	613586.0000000
21.7333336	5.0264001	613586.0000000
21.7333336	5.0752001	613586.0000000
21.7333336	5.1240001	613586.0000000
21.7333336	5.1728001	613586.0000000
21.7333336	5.2216001	613586.0000000
21.7333336	5.2704000	613586.0000000
21.7333336	5.3192000	613586.0000000
21.7333336	5.3680000	-0.8713012
21.7333336	5.4168000	-0.9387960
21.7333336	5.4656000	-0.8406215
21.7333336	5.5144000	-0.8283496
21.7333336	5.5632000	613586.0000000
21.7333336	5.6120000	613586.0000000
21.7333336	5.6608000	613586.0000000
21.7333336	5.7096000	613586.0000000
21.7333336	5.7584000	613586.0000000
21.7333336	5.8072000	613586.0000000
21.7333336	5.8559999	613586.0000000
21.7333336	5.9047999	613586.0000000
21.7333336	5.9535999	613586.0000000
21.7333336	6.0023999	613586.0000000
21.7500000	0.2928000	-1.6382914
21.7500000	0.3416000	-1.4173985
21.7500000	0.3904000	-1.3744469
21.7500000	0.4392000	-1.3744469
21.7500000	0.4880000	-1.5830684
21.7500000	0.5368000	-1.5707965
21.7500000	0.5856000	-1.5769324
21.7500000	0.6344000	-1.7978253
21.7500000	0.6832000	-1.5155730
21.7500000	0.7320000	-1.6076121
21.7500000	0.7808000	-1.6076121
21.7500000	0.8296000	-1.4235344
21.7500000	0.8784000	-1.4173985
21.7500000	0.9272000	-1.5155730
21.7500000	0.9760000	-1.3744469
21.7500000	1.0247999	-1.4848933
21.7500000	1.0735999	-1.6137476
21.7500000	1.1223999	-1.5830684
21.7500000	1.1711999	-1.5830684
21.7500000	1.2200000	-1.5830684
21.7500000	1.2688000	-1.6198835
21.7500000	1.3176000	-1.6198835
21.7500000	1.3664000	-1.6260195
21.7500000	1.4152000	-1.5646605
21.7500000	1.4640000	-1.5646605
21.7500000	1.5128000	-1.6321554
21.7500000	1.5616000	-1.6260195
21.7500000	1.6104000	-1.5339808
21.7500000	1.6592000	-1.5462527
21.7500000	1.7079999	-1.4358063
21.7500000	1.7567999	-1.4358063
21.7500000	1.8055999	-1.5830684
21.7500000	1.8543999	-1.1290097

21.7500000	1.9031999	-1.2087770
21.7500000	1.9519999	-1.1412816
21.7500000	2.0007999	-1.1842332
21.7500000	2.0495999	-1.3989906
21.7500000	2.0983999	-1.3867188
21.7500000	2.1471999	-1.3314953
21.7500000	2.1959999	-1.0860586
21.7500000	2.2447999	-1.0860586
21.7500000	2.2935998	-1.3192234
21.7500000	2.3423998	-1.3314953
21.7500000	2.3912001	-1.3437672
21.7500000	2.4400001	-1.4603496
21.7500000	2.4888000	-1.4542136
21.7500000	2.5376000	-1.4542136
21.7500000	2.5864000	-1.3376312
21.7500000	2.6352000	-1.3376312
21.7500000	2.6840000	-1.3253593
21.7500000	2.7328000	-1.1842332
21.7500000	2.7816000	-1.1903691
21.7500000	2.8304000	-1.1228738
21.7500000	2.8792000	-1.0860586
21.7500000	2.9280000	-1.0246992
21.7500000	2.9768000	-1.0308352
21.7500000	3.0256000	-0.7608547
21.7500000	3.0743999	-0.7608547
21.7500000	3.1231999	-1.0492430
21.7500000	3.1719999	-1.0553789
21.7500000	3.2207999	-1.0983305
21.7500000	3.2695999	-0.8774371
21.7500000	3.3183999	-0.5706410
21.7500000	3.3671999	-0.6565437
21.7500000	3.4159999	-0.4786019
21.7500000	3.4647999	-0.6013203
21.7500000	3.5135999	-0.7792621
21.7500000	3.5623999	-0.7792621
21.7500000	3.6111999	-0.6442719
21.7500000	3.6599998	-0.7731261
21.7500000	3.7087998	-0.7731261
21.7500000	3.7575998	-0.9265246
21.7500000	3.8063998	-0.9203887
21.7500000	3.8551998	-0.9203887
21.7500000	3.9039998	-0.8590293
21.7500000	3.9527998	-0.6504078
21.7500000	4.0016003	-0.9572039
21.7500000	4.0503998	-0.8774371
21.7500000	4.0991998	-0.8774371
21.7500000	4.1479998	-0.9694757
21.7500000	4.1967998	-0.9756117
21.7500000	4.2455997	-1.0615149
21.7500000	4.2943997	-0.9387960
21.7500000	4.3431997	-0.8283496
21.7500000	4.3919997	-0.8406215
21.7500000	4.4407997	-0.9326606
21.7500000	4.4895997	-0.9878836
21.7500000	4.5383997	-0.8283496
21.7500000	4.5871997	-0.8160777
21.7500000	4.6359997	-0.7731261

21.7500000	4.6847997	-0.6013203
21.7500000	4.7336001	613586.0000000
21.7500000	4.7824001	613586.0000000
21.7500000	4.8312001	-0.7179031
21.7500000	4.8800001	-0.9940195
21.7500000	4.9288001	-0.9817476
21.7500000	4.9776001	-0.7179031
21.7500000	5.0264001	-0.8467574
21.7500000	5.0752001	-0.8467574
21.7500000	5.1240001	-0.8344855
21.7500000	5.1728001	-0.7363110
21.7500000	5.2216001	-0.7424469
21.7500000	5.2704000	-0.7363110
21.7500000	5.3192000	613586.0000000
21.7500000	5.3680000	613586.0000000
21.7500000	5.4168000	613586.0000000
21.7500000	5.4656000	613586.0000000
21.7500000	5.5144000	613586.0000000
21.7500000	5.5632000	613586.0000000
21.7500000	5.6120000	613586.0000000
21.7500000	5.6608000	613586.0000000
21.7500000	5.7096000	613586.0000000
21.7500000	5.7584000	613586.0000000
21.7500000	5.8072000	613586.0000000
21.7500000	5.8559999	613586.0000000
21.7500000	5.9047999	613586.0000000
21.7500000	5.9535999	613586.0000000
21.7500000	6.0023999	613586.0000000
21.7666664	0.2928000	-1.7364664
21.7666664	0.3416000	-1.8469129
21.7666664	0.3904000	-1.6137476
21.7666664	0.4392000	-1.6137476
21.7666664	0.4880000	-1.6076121
21.7666664	0.5368000	-1.4480777
21.7666664	0.5856000	-1.4480777
21.7666664	0.6344000	-1.4542136
21.7666664	0.6832000	-1.4542136
21.7666664	0.7320000	-1.4419422
21.7666664	0.7808000	-1.4419422
21.7666664	0.8296000	-1.3437672
21.7666664	0.8784000	-1.3989906
21.7666664	0.9272000	-1.5217090
21.7666664	0.9760000	-1.4296703
21.7666664	1.0247999	-1.5830684
21.7666664	1.0735999	-1.5769324
21.7666664	1.1223999	-1.5707965
21.7666664	1.1711999	-1.5155730
21.7666664	1.2200000	-1.5155730
21.7666664	1.2688000	-1.6260195
21.7666664	1.3176000	-1.6198835
21.7666664	1.3664000	-1.6873789
21.7666664	1.4152000	-1.6812429
21.7666664	1.4640000	-1.6873789
21.7666664	1.5128000	-1.6873789
21.7666664	1.5616000	-1.6873789
21.7666664	1.6104000	-1.6812429
21.7666664	1.6592000	-1.4726214

21.7666664	1.7079999	-1.6935148
21.7666664	1.7567999	-1.7794180
21.7666664	1.8055999	-1.7855535
21.7666664	1.8543999	-1.3560390
21.7666664	1.9031999	-1.3560390
21.7666664	1.9519999	-1.4358063
21.7666664	2.0007999	-1.4235344
21.7666664	2.0495999	-1.4051266
21.7666664	2.0983999	-1.4542136
21.7666664	2.1471999	-1.3560390
21.7666664	2.1959999	-1.3499031
21.7666664	2.2447999	-1.3376312
21.7666664	2.2935998	-1.4296703
21.7666664	2.3423998	-1.4173985
21.7666664	2.3912001	-1.4173985
21.7666664	2.4400001	-1.4235344
21.7666664	2.4888000	-1.4787574
21.7666664	2.5376000	-1.4787574
21.7666664	2.5864000	-1.3253593
21.7666664	2.6352000	-1.4480777
21.7666664	2.6840000	-1.3805828
21.7666664	2.7328000	-1.2271848
21.7666664	2.7816000	-1.1965051
21.7666664	2.8304000	-1.2149129
21.7666664	2.8792000	-1.2149129
21.7666664	2.9280000	-0.7608547
21.7666664	2.9768000	-0.7056313
21.7666664	3.0256000	-0.7179031
21.7666664	3.0743999	-0.7485828
21.7666664	3.1231999	-1.0001554
21.7666664	3.1719999	-0.9940195
21.7666664	3.2207999	-0.9142528
21.7666664	3.2695999	-0.7731261
21.7666664	3.3183999	-1.2394567
21.7666664	3.3671999	-1.6382914
21.7666664	3.4159999	-1.7855535
21.7666664	3.4647999	-1.0369711
21.7666664	3.5135999	-1.1106019
21.7666664	3.5623999	-1.1106019
21.7666664	3.6111999	-1.5339808
21.7666664	3.6599998	-1.5217090
21.7666664	3.7087998	-1.5217090
21.7666664	3.7575998	-1.4603496
21.7666664	3.8063998	-1.2701364
21.7666664	3.8551998	-1.3008156
21.7666664	3.9039998	-1.3008156
21.7666664	3.9527998	-1.1290097
21.7666664	4.0016003	-1.0983305
21.7666664	4.0503998	-1.1044660
21.7666664	4.0991998	-1.0983305
21.7666664	4.1479998	-1.0799227
21.7666664	4.1967998	-0.9756117
21.7666664	4.2455997	-0.6872234
21.7666664	4.2943997	-0.8713012
21.7666664	4.3431997	-0.9203887
21.7666664	4.3919997	-0.9387960
21.7666664	4.4407997	-0.9387960

21.7666664	4.4895997	-0.9387960
21.7666664	4.5383997	-0.9203887
21.7666664	4.5871997	-0.8774371
21.7666664	4.6359997	-0.9817476
21.7666664	4.6847997	-1.0185633
21.7666664	4.7336001	-0.9142528
21.7666664	4.7824001	-1.0431070
21.7666664	4.8312001	-1.0369711
21.7666664	4.8800001	-1.1167378
21.7666664	4.9288001	-1.1228738
21.7666664	4.9776001	-1.1167378
21.7666664	5.0264001	-0.9449320
21.7666664	5.0752001	-0.8774371
21.7666664	5.1240001	-0.8590293
21.7666664	5.1728001	-0.8651652
21.7666664	5.2216001	613586.0000000
21.7666664	5.2704000	613586.0000000
21.7666664	5.3192000	613586.0000000
21.7666664	5.3680000	613586.0000000
21.7666664	5.4168000	613586.0000000
21.7666664	5.4656000	613586.0000000
21.7666664	5.5144000	613586.0000000
21.7666664	5.5632000	613586.0000000
21.7666664	5.6120000	613586.0000000
21.7666664	5.6608000	-0.8038058
21.7666664	5.7096000	-0.8038058
21.7666664	5.7584000	613586.0000000
21.7666664	5.8072000	613586.0000000
21.7666664	5.8559999	613586.0000000
21.7666664	5.9047999	613586.0000000
21.7666664	5.9535999	613586.0000000
21.7666664	6.0023999	613586.0000000
21.7833328	0.2928000	-2.1905246
21.7833328	0.3416000	-1.8469129
21.7833328	0.3904000	-1.5033011
21.7833328	0.4392000	-1.5217090
21.7833328	0.4880000	-1.7180586
21.7833328	0.5368000	-1.7180586
21.7833328	0.5856000	-1.5707965
21.7833328	0.6344000	-1.5769324
21.7833328	0.6832000	-1.5953403
21.7833328	0.7320000	-1.5953403
21.7833328	0.7808000	-1.4603496
21.7833328	0.8296000	-1.4358063
21.7833328	0.8784000	-1.3989906
21.7833328	0.9272000	-1.5953403
21.7833328	0.9760000	-1.3744469
21.7833328	1.0247999	-1.3805828
21.7833328	1.0735999	-1.3928547
21.7833328	1.1223999	-1.4971652
21.7833328	1.1711999	-1.5155730
21.7833328	1.2200000	-1.7426023
21.7833328	1.2688000	-1.7487383
21.7833328	1.3176000	-1.6751070
21.7833328	1.3664000	-1.6751070
21.7833328	1.4152000	-1.6873789
21.7833328	1.4640000	-1.8530488

21.7833328	1.5128000	-1.8346410
21.7833328	1.5616000	-1.7855535
21.7833328	1.6104000	-1.5155730
21.7833328	1.6592000	-1.5155730
21.7833328	1.7079999	-1.2946796
21.7833328	1.7567999	-1.3008156
21.7833328	1.8055999	-1.2946796
21.7833328	1.8543999	-1.2885437
21.7833328	1.9031999	-1.2885437
21.7833328	1.9519999	-1.2946796
21.7833328	2.0007999	-1.3499031
21.7833328	2.0495999	-1.4542136
21.7833328	2.0983999	-1.3683109
21.7833328	2.1471999	-1.3008156
21.7833328	2.1959999	-1.3008156
21.7833328	2.2447999	-1.3499031
21.7833328	2.2935998	-1.3376312
21.7833328	2.3423998	-1.4173985
21.7833328	2.3912001	-1.4971652
21.7833328	2.4400001	-1.4787574
21.7833328	2.4888000	-1.4112625
21.7833328	2.5376000	-1.3683109
21.7833328	2.5864000	-1.2517285
21.7833328	2.6352000	-1.1474175
21.7833328	2.6840000	-1.0799227
21.7833328	2.7328000	-1.0799227
21.7833328	2.7816000	-1.0737867
21.7833328	2.8304000	-1.0124273
21.7833328	2.8792000	-1.0062914
21.7833328	2.9280000	-0.6749516
21.7833328	2.9768000	-0.6872234
21.7833328	3.0256000	-0.8897090
21.7833328	3.0743999	-1.2640004
21.7833328	3.1231999	-1.4848933
21.7833328	3.1719999	-1.5033011
21.7833328	3.2207999	-1.4603496
21.7833328	3.2695999	-1.6076121
21.7833328	3.3183999	-1.3989906
21.7833328	3.3671999	-1.5401168
21.7833328	3.4159999	-1.4419422
21.7833328	3.4647999	-1.2087770
21.7833328	3.5135999	-1.2087770
21.7833328	3.5623999	-0.9387960
21.7833328	3.6111999	-0.7853980
21.7833328	3.6599998	-0.7792621
21.7833328	3.7087998	-0.9326606
21.7833328	3.7575998	-0.9387960
21.7833328	3.8063998	-1.0676508
21.7833328	3.8551998	-1.0799227
21.7833328	3.9039998	-1.0860586
21.7833328	3.9527998	-1.2701364
21.7833328	4.0016003	-1.2824078
21.7833328	4.0503998	-1.2824078
21.7833328	4.0991998	-1.2394567
21.7833328	4.1479998	-1.2210488
21.7833328	4.1967998	-1.2271848
21.7833328	4.2455997	-1.0308352

21.7833328	4.2943997	-1.0308352
21.7833328	4.3431997	-0.9572039
21.7833328	4.3919997	-1.2640004
21.7833328	4.4407997	-1.2640004
21.7833328	4.4895997	-1.0799227
21.7833328	4.5383997	-1.0921946
21.7833328	4.5871997	-1.1106019
21.7833328	4.6359997	-1.1290097
21.7833328	4.6847997	-1.1412816
21.7833328	4.7336001	-1.1474175
21.7833328	4.7824001	-1.2394567
21.7833328	4.8312001	-1.2455926
21.7833328	4.8800001	-0.9878836
21.7833328	4.9288001	-1.0983305
21.7833328	4.9776001	-1.0308352
21.7833328	5.0264001	613586.0000000
21.7833328	5.0752001	-0.7547188
21.7833328	5.1240001	613586.0000000
21.7833328	5.1728001	613586.0000000
21.7833328	5.2216001	613586.0000000
21.7833328	5.2704000	-1.0001554
21.7833328	5.3192000	-0.9756117
21.7833328	5.3680000	-0.9081168
21.7833328	5.4168000	613586.0000000
21.7833328	5.4656000	613586.0000000
21.7833328	5.5144000	613586.0000000
21.7833328	5.5632000	613586.0000000
21.7833328	5.6120000	613586.0000000
21.7833328	5.6608000	613586.0000000
21.7833328	5.7096000	613586.0000000
21.7833328	5.7584000	613586.0000000
21.7833328	5.8072000	613586.0000000
21.7833328	5.8559999	613586.0000000
21.7833328	5.9047999	613586.0000000
21.7833328	5.9535999	613586.0000000
21.7833328	6.0023999	613586.0000000
21.7999992	0.2928000	9999.0000000
21.7999992	0.3416000	9999.0000000
21.7999992	0.3904000	9999.0000000
21.7999992	0.4392000	9999.0000000
21.7999992	0.4880000	9999.0000000
21.7999992	0.5368000	9999.0000000
21.7999992	0.5856000	9999.0000000
21.7999992	0.6344000	9999.0000000
21.7999992	0.6832000	9999.0000000
21.7999992	0.7320000	9999.0000000
21.7999992	0.7808000	9999.0000000
21.7999992	0.8296000	9999.0000000
21.7999992	0.8784000	9999.0000000
21.7999992	0.9272000	9999.0000000
21.7999992	0.9760000	9999.0000000
21.7999992	1.0247999	9999.0000000
21.7999992	1.0735999	9999.0000000
21.7999992	1.1223999	9999.0000000
21.7999992	1.1711999	9999.0000000
21.7999992	1.2200000	9999.0000000
21.7999992	1.2688000	9999.0000000

21.7999992	1.3176000	9999.0000000
21.7999992	1.3664000	9999.0000000
21.7999992	1.4152000	9999.0000000
21.7999992	1.4640000	9999.0000000
21.7999992	1.5128000	9999.0000000
21.7999992	1.5616000	9999.0000000
21.7999992	1.6104000	9999.0000000
21.7999992	1.6592000	9999.0000000
21.7999992	1.7079999	9999.0000000
21.7999992	1.7567999	9999.0000000
21.7999992	1.8055999	9999.0000000
21.7999992	1.8543999	9999.0000000
21.7999992	1.9031999	9999.0000000
21.7999992	1.9519999	9999.0000000
21.7999992	2.0007999	9999.0000000
21.7999992	2.0495999	9999.0000000
21.7999992	2.0983999	9999.0000000
21.7999992	2.1471999	9999.0000000
21.7999992	2.1959999	9999.0000000
21.7999992	2.2447999	9999.0000000
21.7999992	2.2935998	9999.0000000
21.7999992	2.3423998	9999.0000000
21.7999992	2.3912001	9999.0000000
21.7999992	2.4400001	9999.0000000
21.7999992	2.4888000	9999.0000000
21.7999992	2.5376000	9999.0000000
21.7999992	2.5864000	9999.0000000
21.7999992	2.6352000	9999.0000000
21.7999992	2.6840000	9999.0000000
21.7999992	2.7328000	9999.0000000
21.7999992	2.7816000	9999.0000000
21.7999992	2.8304000	9999.0000000
21.7999992	2.8792000	9999.0000000
21.7999992	2.9280000	9999.0000000
21.7999992	2.9768000	9999.0000000
21.7999992	3.0256000	9999.0000000
21.7999992	3.0743999	9999.0000000
21.7999992	3.1231999	9999.0000000
21.7999992	3.1719999	9999.0000000
21.7999992	3.2207999	9999.0000000
21.7999992	3.2695999	9999.0000000
21.7999992	3.3183999	9999.0000000
21.7999992	3.3671999	9999.0000000
21.7999992	3.4159999	9999.0000000
21.7999992	3.4647999	9999.0000000
21.7999992	3.5135999	9999.0000000
21.7999992	3.5623999	9999.0000000
21.7999992	3.6111999	9999.0000000
21.7999992	3.6599998	9999.0000000
21.7999992	3.7087998	9999.0000000
21.7999992	3.7575998	9999.0000000
21.7999992	3.8063998	9999.0000000
21.7999992	3.8551998	9999.0000000
21.7999992	3.9039998	9999.0000000
21.7999992	3.9527998	9999.0000000
21.7999992	4.0016003	9999.0000000
21.7999992	4.0503998	9999.0000000

21.7999992	4.0991998	9999.0000000
21.7999992	4.1479998	9999.0000000
21.7999992	4.1967998	9999.0000000
21.7999992	4.2455997	9999.0000000
21.7999992	4.2943997	9999.0000000
21.7999992	4.3431997	9999.0000000
21.7999992	4.3919997	9999.0000000
21.7999992	4.4407997	9999.0000000
21.7999992	4.4895997	9999.0000000
21.7999992	4.5383997	9999.0000000
21.7999992	4.5871997	9999.0000000
21.7999992	4.6359997	9999.0000000
21.7999992	4.6847997	9999.0000000
21.7999992	4.7336001	9999.0000000
21.7999992	4.7824001	9999.0000000
21.7999992	4.8312001	9999.0000000
21.7999992	4.8800001	9999.0000000
21.7999992	4.9288001	9999.0000000
21.7999992	4.9776001	9999.0000000
21.7999992	5.0264001	9999.0000000
21.7999992	5.0752001	9999.0000000
21.7999992	5.1240001	9999.0000000
21.7999992	5.1728001	9999.0000000
21.7999992	5.2216001	9999.0000000
21.7999992	5.2704000	9999.0000000
21.7999992	5.3192000	9999.0000000
21.7999992	5.3680000	9999.0000000
21.7999992	5.4168000	9999.0000000
21.7999992	5.4656000	9999.0000000
21.7999992	5.5144000	9999.0000000
21.7999992	5.5632000	9999.0000000
21.7999992	5.6120000	9999.0000000
21.7999992	5.6608000	9999.0000000
21.7999992	5.7096000	9999.0000000
21.7999992	5.7584000	9999.0000000
21.7999992	5.8072000	9999.0000000
21.7999992	5.8559999	9999.0000000
21.7999992	5.9047999	9999.0000000
21.7999992	5.9535999	9999.0000000
21.7999992	6.0023999	9999.0000000
21.8166676	0.2928000	9999.0000000
21.8166676	0.3416000	9999.0000000
21.8166676	0.3904000	9999.0000000
21.8166676	0.4392000	9999.0000000
21.8166676	0.4880000	9999.0000000
21.8166676	0.5368000	9999.0000000
21.8166676	0.5856000	9999.0000000
21.8166676	0.6344000	9999.0000000
21.8166676	0.6832000	9999.0000000
21.8166676	0.7320000	9999.0000000
21.8166676	0.7808000	9999.0000000
21.8166676	0.8296000	9999.0000000
21.8166676	0.8784000	9999.0000000
21.8166676	0.9272000	9999.0000000
21.8166676	0.9760000	9999.0000000
21.8166676	1.0247999	9999.0000000
21.8166676	1.0735999	9999.0000000

21.8166676	1.1223999	9999.0000000
21.8166676	1.1711999	9999.0000000
21.8166676	1.2200000	9999.0000000
21.8166676	1.2688000	9999.0000000
21.8166676	1.3176000	9999.0000000
21.8166676	1.3664000	9999.0000000
21.8166676	1.4152000	9999.0000000
21.8166676	1.4640000	9999.0000000
21.8166676	1.5128000	9999.0000000
21.8166676	1.5616000	9999.0000000
21.8166676	1.6104000	9999.0000000
21.8166676	1.6592000	9999.0000000
21.8166676	1.7079999	9999.0000000
21.8166676	1.7567999	9999.0000000
21.8166676	1.8055999	9999.0000000
21.8166676	1.8543999	9999.0000000
21.8166676	1.9031999	9999.0000000
21.8166676	1.9519999	9999.0000000
21.8166676	2.0007999	9999.0000000
21.8166676	2.0495999	9999.0000000
21.8166676	2.0983999	9999.0000000
21.8166676	2.1471999	9999.0000000
21.8166676	2.1959999	9999.0000000
21.8166676	2.2447999	9999.0000000
21.8166676	2.2935998	9999.0000000
21.8166676	2.3423998	9999.0000000
21.8166676	2.3912001	9999.0000000
21.8166676	2.4400001	9999.0000000
21.8166676	2.4888000	9999.0000000
21.8166676	2.5376000	9999.0000000
21.8166676	2.5864000	9999.0000000
21.8166676	2.6352000	9999.0000000
21.8166676	2.6840000	9999.0000000
21.8166676	2.7328000	9999.0000000
21.8166676	2.7816000	9999.0000000
21.8166676	2.8304000	9999.0000000
21.8166676	2.8792000	9999.0000000
21.8166676	2.9280000	9999.0000000
21.8166676	2.9768000	9999.0000000
21.8166676	3.0256000	9999.0000000
21.8166676	3.0743999	9999.0000000
21.8166676	3.1231999	9999.0000000
21.8166676	3.1719999	9999.0000000
21.8166676	3.2207999	9999.0000000
21.8166676	3.2695999	9999.0000000
21.8166676	3.3183999	9999.0000000
21.8166676	3.3671999	9999.0000000
21.8166676	3.4159999	9999.0000000
21.8166676	3.4647999	9999.0000000
21.8166676	3.5135999	9999.0000000
21.8166676	3.5623999	9999.0000000
21.8166676	3.6111999	9999.0000000
21.8166676	3.6599998	9999.0000000
21.8166676	3.7087998	9999.0000000
21.8166676	3.7575998	9999.0000000
21.8166676	3.8063998	9999.0000000
21.8166676	3.8551998	9999.0000000

21.8166676	3.9039998	9999.0000000
21.8166676	3.9527998	9999.0000000
21.8166676	4.0016003	9999.0000000
21.8166676	4.0503998	9999.0000000
21.8166676	4.0991998	9999.0000000
21.8166676	4.1479998	9999.0000000
21.8166676	4.1967998	9999.0000000
21.8166676	4.2455997	9999.0000000
21.8166676	4.2943997	9999.0000000
21.8166676	4.3431997	9999.0000000
21.8166676	4.3919997	9999.0000000
21.8166676	4.4407997	9999.0000000
21.8166676	4.4895997	9999.0000000
21.8166676	4.5383997	9999.0000000
21.8166676	4.5871997	9999.0000000
21.8166676	4.6359997	9999.0000000
21.8166676	4.6847997	9999.0000000
21.8166676	4.7336001	9999.0000000
21.8166676	4.7824001	9999.0000000
21.8166676	4.8312001	9999.0000000
21.8166676	4.8800001	9999.0000000
21.8166676	4.9288001	9999.0000000
21.8166676	4.9776001	9999.0000000
21.8166676	5.0264001	9999.0000000
21.8166676	5.0752001	9999.0000000
21.8166676	5.1240001	9999.0000000
21.8166676	5.1728001	9999.0000000
21.8166676	5.2216001	9999.0000000
21.8166676	5.2704000	9999.0000000
21.8166676	5.3192000	9999.0000000
21.8166676	5.3680000	9999.0000000
21.8166676	5.4168000	9999.0000000
21.8166676	5.4656000	9999.0000000
21.8166676	5.5144000	9999.0000000
21.8166676	5.5632000	9999.0000000
21.8166676	5.6120000	9999.0000000
21.8166676	5.6608000	9999.0000000
21.8166676	5.7096000	9999.0000000
21.8166676	5.7584000	9999.0000000
21.8166676	5.8072000	9999.0000000
21.8166676	5.8559999	9999.0000000
21.8166676	5.9047999	9999.0000000
21.8166676	5.9535999	9999.0000000
21.8166676	6.0023999	9999.0000000
21.8333340	0.2928000	9999.0000000
21.8333340	0.3416000	9999.0000000
21.8333340	0.3904000	9999.0000000
21.8333340	0.4392000	9999.0000000
21.8333340	0.4880000	9999.0000000
21.8333340	0.5368000	9999.0000000
21.8333340	0.5856000	9999.0000000
21.8333340	0.6344000	9999.0000000
21.8333340	0.6832000	9999.0000000
21.8333340	0.7320000	9999.0000000
21.8333340	0.7808000	9999.0000000
21.8333340	0.8296000	9999.0000000
21.8333340	0.8784000	9999.0000000

21.8333340	0.9272000	9999.0000000
21.8333340	0.9760000	9999.0000000
21.8333340	1.0247999	9999.0000000
21.8333340	1.0735999	9999.0000000
21.8333340	1.1223999	9999.0000000
21.8333340	1.1711999	9999.0000000
21.8333340	1.2200000	9999.0000000
21.8333340	1.2688000	9999.0000000
21.8333340	1.3176000	9999.0000000
21.8333340	1.3664000	9999.0000000
21.8333340	1.4152000	9999.0000000
21.8333340	1.4640000	9999.0000000
21.8333340	1.5128000	9999.0000000
21.8333340	1.5616000	9999.0000000
21.8333340	1.6104000	9999.0000000
21.8333340	1.6592000	9999.0000000
21.8333340	1.7079999	9999.0000000
21.8333340	1.7567999	9999.0000000
21.8333340	1.8055999	9999.0000000
21.8333340	1.8543999	9999.0000000
21.8333340	1.9031999	9999.0000000
21.8333340	1.9519999	9999.0000000
21.8333340	2.0007999	9999.0000000
21.8333340	2.0495999	9999.0000000
21.8333340	2.0983999	9999.0000000
21.8333340	2.1471999	9999.0000000
21.8333340	2.1959999	9999.0000000
21.8333340	2.2447999	9999.0000000
21.8333340	2.2935998	9999.0000000
21.8333340	2.3423998	9999.0000000
21.8333340	2.3912001	9999.0000000
21.8333340	2.4400001	9999.0000000
21.8333340	2.4888000	9999.0000000
21.8333340	2.5376000	9999.0000000
21.8333340	2.5864000	9999.0000000
21.8333340	2.6352000	9999.0000000
21.8333340	2.6840000	9999.0000000
21.8333340	2.7328000	9999.0000000
21.8333340	2.7816000	9999.0000000
21.8333340	2.8304000	9999.0000000
21.8333340	2.8792000	9999.0000000
21.8333340	2.9280000	9999.0000000
21.8333340	2.9768000	9999.0000000
21.8333340	3.0256000	9999.0000000
21.8333340	3.0743999	9999.0000000
21.8333340	3.1231999	9999.0000000
21.8333340	3.1719999	9999.0000000
21.8333340	3.2207999	9999.0000000
21.8333340	3.2695999	9999.0000000
21.8333340	3.3183999	9999.0000000
21.8333340	3.3671999	9999.0000000
21.8333340	3.4159999	9999.0000000
21.8333340	3.4647999	9999.0000000
21.8333340	3.5135999	9999.0000000
21.8333340	3.5623999	9999.0000000
21.8333340	3.6111999	9999.0000000
21.8333340	3.6599998	9999.0000000

21.8333340	3.7087998	9999.0000000
21.8333340	3.7575998	9999.0000000
21.8333340	3.8063998	9999.0000000
21.8333340	3.8551998	9999.0000000
21.8333340	3.9039998	9999.0000000
21.8333340	3.9527998	9999.0000000
21.8333340	4.0016003	9999.0000000
21.8333340	4.0503998	9999.0000000
21.8333340	4.0991998	9999.0000000
21.8333340	4.1479998	9999.0000000
21.8333340	4.1967998	9999.0000000
21.8333340	4.2455997	9999.0000000
21.8333340	4.2943997	9999.0000000
21.8333340	4.3431997	9999.0000000
21.8333340	4.3919997	9999.0000000
21.8333340	4.4407997	9999.0000000
21.8333340	4.4895997	9999.0000000
21.8333340	4.5383997	9999.0000000
21.8333340	4.5871997	9999.0000000
21.8333340	4.6359997	9999.0000000
21.8333340	4.6847997	9999.0000000
21.8333340	4.7336001	9999.0000000
21.8333340	4.7824001	9999.0000000
21.8333340	4.8312001	9999.0000000
21.8333340	4.8800001	9999.0000000
21.8333340	4.9288001	9999.0000000
21.8333340	4.9776001	9999.0000000
21.8333340	5.0264001	9999.0000000
21.8333340	5.0752001	9999.0000000
21.8333340	5.1240001	9999.0000000
21.8333340	5.1728001	9999.0000000
21.8333340	5.2216001	9999.0000000
21.8333340	5.2704000	9999.0000000
21.8333340	5.3192000	9999.0000000
21.8333340	5.3680000	9999.0000000
21.8333340	5.4168000	9999.0000000
21.8333340	5.4656000	9999.0000000
21.8333340	5.5144000	9999.0000000
21.8333340	5.5632000	9999.0000000
21.8333340	5.6120000	9999.0000000
21.8333340	5.6608000	9999.0000000
21.8333340	5.7096000	9999.0000000
21.8333340	5.7584000	9999.0000000
21.8333340	5.8072000	9999.0000000
21.8333340	5.8559999	9999.0000000
21.8333340	5.9047999	9999.0000000
21.8333340	5.9535999	9999.0000000
21.8333340	6.0023999	9999.0000000
21.8500004	0.2928000	9999.0000000
21.8500004	0.3416000	9999.0000000
21.8500004	0.3904000	9999.0000000
21.8500004	0.4392000	9999.0000000
21.8500004	0.4880000	9999.0000000
21.8500004	0.5368000	9999.0000000
21.8500004	0.5856000	9999.0000000
21.8500004	0.6344000	9999.0000000
21.8500004	0.6832000	9999.0000000

21.8500004	0.7320000	9999.0000000
21.8500004	0.7808000	9999.0000000
21.8500004	0.8296000	9999.0000000
21.8500004	0.8784000	9999.0000000
21.8500004	0.9272000	9999.0000000
21.8500004	0.9760000	9999.0000000
21.8500004	1.0247999	9999.0000000
21.8500004	1.0735999	9999.0000000
21.8500004	1.1223999	9999.0000000
21.8500004	1.1711999	9999.0000000
21.8500004	1.2200000	9999.0000000
21.8500004	1.2688000	9999.0000000
21.8500004	1.3176000	9999.0000000
21.8500004	1.3664000	9999.0000000
21.8500004	1.4152000	9999.0000000
21.8500004	1.4640000	9999.0000000
21.8500004	1.5128000	9999.0000000
21.8500004	1.5616000	9999.0000000
21.8500004	1.6104000	9999.0000000
21.8500004	1.6592000	9999.0000000
21.8500004	1.7079999	9999.0000000
21.8500004	1.7567999	9999.0000000
21.8500004	1.8055999	9999.0000000
21.8500004	1.8543999	9999.0000000
21.8500004	1.9031999	9999.0000000
21.8500004	1.9519999	9999.0000000
21.8500004	2.0007999	9999.0000000
21.8500004	2.0495999	9999.0000000
21.8500004	2.0983999	9999.0000000
21.8500004	2.1471999	9999.0000000
21.8500004	2.1959999	9999.0000000
21.8500004	2.2447999	9999.0000000
21.8500004	2.2935998	9999.0000000
21.8500004	2.3423998	9999.0000000
21.8500004	2.3912001	9999.0000000
21.8500004	2.4400001	9999.0000000
21.8500004	2.4888000	9999.0000000
21.8500004	2.5376000	9999.0000000
21.8500004	2.5864000	9999.0000000
21.8500004	2.6352000	9999.0000000
21.8500004	2.6840000	9999.0000000
21.8500004	2.7328000	9999.0000000
21.8500004	2.7816000	9999.0000000
21.8500004	2.8304000	9999.0000000
21.8500004	2.8792000	9999.0000000
21.8500004	2.9280000	9999.0000000
21.8500004	2.9768000	9999.0000000
21.8500004	3.0256000	9999.0000000
21.8500004	3.0743999	9999.0000000
21.8500004	3.1231999	9999.0000000
21.8500004	3.1719999	9999.0000000
21.8500004	3.2207999	9999.0000000
21.8500004	3.2695999	9999.0000000
21.8500004	3.3183999	9999.0000000
21.8500004	3.3671999	9999.0000000
21.8500004	3.4159999	9999.0000000
21.8500004	3.4647999	9999.0000000

21.8500004	3.5135999	9999.0000000
21.8500004	3.5623999	9999.0000000
21.8500004	3.6111999	9999.0000000
21.8500004	3.6599998	9999.0000000
21.8500004	3.7087998	9999.0000000
21.8500004	3.7575998	9999.0000000
21.8500004	3.8063998	9999.0000000
21.8500004	3.8551998	9999.0000000
21.8500004	3.9039998	9999.0000000
21.8500004	3.9527998	9999.0000000
21.8500004	4.0016003	9999.0000000
21.8500004	4.0503998	9999.0000000
21.8500004	4.0991998	9999.0000000
21.8500004	4.1479998	9999.0000000
21.8500004	4.1967998	9999.0000000
21.8500004	4.2455997	9999.0000000
21.8500004	4.2943997	9999.0000000
21.8500004	4.3431997	9999.0000000
21.8500004	4.3919997	9999.0000000
21.8500004	4.4407997	9999.0000000
21.8500004	4.4895997	9999.0000000
21.8500004	4.5383997	9999.0000000
21.8500004	4.5871997	9999.0000000
21.8500004	4.6359997	9999.0000000
21.8500004	4.6847997	9999.0000000
21.8500004	4.7336001	9999.0000000
21.8500004	4.7824001	9999.0000000
21.8500004	4.8312001	9999.0000000
21.8500004	4.8800001	9999.0000000
21.8500004	4.9288001	9999.0000000
21.8500004	4.9776001	9999.0000000
21.8500004	5.0264001	9999.0000000
21.8500004	5.0752001	9999.0000000
21.8500004	5.1240001	9999.0000000
21.8500004	5.1728001	9999.0000000
21.8500004	5.2216001	9999.0000000
21.8500004	5.2704000	9999.0000000
21.8500004	5.3192000	9999.0000000
21.8500004	5.3680000	9999.0000000
21.8500004	5.4168000	9999.0000000
21.8500004	5.4656000	9999.0000000
21.8500004	5.5144000	9999.0000000
21.8500004	5.5632000	9999.0000000
21.8500004	5.6120000	9999.0000000
21.8500004	5.6608000	9999.0000000
21.8500004	5.7096000	9999.0000000
21.8500004	5.7584000	9999.0000000
21.8500004	5.8072000	9999.0000000
21.8500004	5.8559999	9999.0000000
21.8500004	5.9047999	9999.0000000
21.8500004	5.9535999	9999.0000000
21.8500004	6.0023999	9999.0000000
21.8666668	0.2928000	9999.0000000
21.8666668	0.3416000	9999.0000000
21.8666668	0.3904000	9999.0000000
21.8666668	0.4392000	9999.0000000
21.8666668	0.4880000	9999.0000000

21.8666668	0.5368000	9999.0000000
21.8666668	0.5856000	9999.0000000
21.8666668	0.6344000	9999.0000000
21.8666668	0.6832000	9999.0000000
21.8666668	0.7320000	9999.0000000
21.8666668	0.7808000	9999.0000000
21.8666668	0.8296000	9999.0000000
21.8666668	0.8784000	9999.0000000
21.8666668	0.9272000	9999.0000000
21.8666668	0.9760000	9999.0000000
21.8666668	1.0247999	9999.0000000
21.8666668	1.0735999	9999.0000000
21.8666668	1.1223999	9999.0000000
21.8666668	1.1711999	9999.0000000
21.8666668	1.2200000	9999.0000000
21.8666668	1.2688000	9999.0000000
21.8666668	1.3176000	9999.0000000
21.8666668	1.3664000	9999.0000000
21.8666668	1.4152000	9999.0000000
21.8666668	1.4640000	9999.0000000
21.8666668	1.5128000	9999.0000000
21.8666668	1.5616000	9999.0000000
21.8666668	1.6104000	9999.0000000
21.8666668	1.6592000	9999.0000000
21.8666668	1.7079999	9999.0000000
21.8666668	1.7567999	9999.0000000
21.8666668	1.8055999	9999.0000000
21.8666668	1.8543999	9999.0000000
21.8666668	1.9031999	9999.0000000
21.8666668	1.9519999	9999.0000000
21.8666668	2.0007999	9999.0000000
21.8666668	2.0495999	9999.0000000
21.8666668	2.0983999	9999.0000000
21.8666668	2.1471999	9999.0000000
21.8666668	2.1959999	9999.0000000
21.8666668	2.2447999	9999.0000000
21.8666668	2.2935998	9999.0000000
21.8666668	2.3423998	9999.0000000
21.8666668	2.3912001	9999.0000000
21.8666668	2.4400001	9999.0000000
21.8666668	2.4888000	9999.0000000
21.8666668	2.5376000	9999.0000000
21.8666668	2.5864000	9999.0000000
21.8666668	2.6352000	9999.0000000
21.8666668	2.6840000	9999.0000000
21.8666668	2.7328000	9999.0000000
21.8666668	2.7816000	9999.0000000
21.8666668	2.8304000	9999.0000000
21.8666668	2.8792000	9999.0000000
21.8666668	2.9280000	9999.0000000
21.8666668	2.9768000	9999.0000000
21.8666668	3.0256000	9999.0000000
21.8666668	3.0743999	9999.0000000
21.8666668	3.1231999	9999.0000000
21.8666668	3.1719999	9999.0000000
21.8666668	3.2207999	9999.0000000
21.8666668	3.2695999	9999.0000000

21.8666668	3.3183999	9999.0000000
21.8666668	3.3671999	9999.0000000
21.8666668	3.4159999	9999.0000000
21.8666668	3.4647999	9999.0000000
21.8666668	3.5135999	9999.0000000
21.8666668	3.5623999	9999.0000000
21.8666668	3.6111999	9999.0000000
21.8666668	3.6599998	9999.0000000
21.8666668	3.7087998	9999.0000000
21.8666668	3.7575998	9999.0000000
21.8666668	3.8063998	9999.0000000
21.8666668	3.8551998	9999.0000000
21.8666668	3.9039998	9999.0000000
21.8666668	3.9527998	9999.0000000
21.8666668	4.0016003	9999.0000000
21.8666668	4.0503998	9999.0000000
21.8666668	4.0991998	9999.0000000
21.8666668	4.1479998	9999.0000000
21.8666668	4.1967998	9999.0000000
21.8666668	4.2455997	9999.0000000
21.8666668	4.2943997	9999.0000000
21.8666668	4.3431997	9999.0000000
21.8666668	4.3919997	9999.0000000
21.8666668	4.4407997	9999.0000000
21.8666668	4.4895997	9999.0000000
21.8666668	4.5383997	9999.0000000
21.8666668	4.5871997	9999.0000000
21.8666668	4.6359997	9999.0000000
21.8666668	4.6847997	9999.0000000
21.8666668	4.7336001	9999.0000000
21.8666668	4.7824001	9999.0000000
21.8666668	4.8312001	9999.0000000
21.8666668	4.8800001	9999.0000000
21.8666668	4.9288001	9999.0000000
21.8666668	4.9776001	9999.0000000
21.8666668	5.0264001	9999.0000000
21.8666668	5.0752001	9999.0000000
21.8666668	5.1240001	9999.0000000
21.8666668	5.1728001	9999.0000000
21.8666668	5.2216001	9999.0000000
21.8666668	5.2704000	9999.0000000
21.8666668	5.3192000	9999.0000000
21.8666668	5.3680000	9999.0000000
21.8666668	5.4168000	9999.0000000
21.8666668	5.4656000	9999.0000000
21.8666668	5.5144000	9999.0000000
21.8666668	5.5632000	9999.0000000
21.8666668	5.6120000	9999.0000000
21.8666668	5.6608000	9999.0000000
21.8666668	5.7096000	9999.0000000
21.8666668	5.7584000	9999.0000000
21.8666668	5.8072000	9999.0000000
21.8666668	5.8559999	9999.0000000
21.8666668	5.9047999	9999.0000000
21.8666668	5.9535999	9999.0000000
21.8666668	6.0023999	9999.0000000
21.8833332	0.2928000	-1.4235344

21.8833332	0.3416000	-1.4358063
21.8833332	0.3904000	-1.4358063
21.8833332	0.4392000	-1.6751070
21.8833332	0.4880000	-1.3805828
21.8833332	0.5368000	-1.3683109
21.8833332	0.5856000	-1.4296703
21.8833332	0.6344000	-1.4296703
21.8833332	0.6832000	-1.4419422
21.8833332	0.7320000	-1.4664855
21.8833332	0.7808000	-1.4664855
21.8833332	0.8296000	-1.4664855
21.8833332	0.8784000	-1.4664855
21.8833332	0.9272000	-1.2824078
21.8833332	0.9760000	-1.2946796
21.8833332	1.0247999	-1.3683109
21.8833332	1.0735999	-1.4542136
21.8833332	1.1223999	-1.3683109
21.8833332	1.1711999	-1.2149129
21.8833332	1.2200000	-1.2026410
21.8833332	1.2688000	-1.1412816
21.8833332	1.3176000	-1.1228738
21.8833332	1.3664000	-1.1290097
21.8833332	1.4152000	-1.4051266
21.8833332	1.4640000	-1.3989906
21.8833332	1.5128000	-1.5278449
21.8833332	1.5616000	-1.2517285
21.8833332	1.6104000	-1.2210488
21.8833332	1.6592000	-1.2149129
21.8833332	1.7079999	-1.2210488
21.8833332	1.7567999	-1.2271848
21.8833332	1.8055999	-1.3989906
21.8833332	1.8543999	-1.4051266
21.8833332	1.9031999	-1.2087770
21.8833332	1.9519999	-1.1903691
21.8833332	2.0007999	-1.3008156
21.8833332	2.0495999	-1.2455926
21.8833332	2.0983999	-1.2517285
21.8833332	2.1471999	-1.2885437
21.8833332	2.1959999	-1.2517285
21.8833332	2.2447999	-1.1044660
21.8833332	2.2935998	-1.1106019
21.8833332	2.3423998	-1.2946796
21.8833332	2.3912001	-1.2824078
21.8833332	2.4400001	-1.2578645
21.8833332	2.4888000	-1.2640004
21.8833332	2.5376000	-1.0983305
21.8833332	2.5864000	-1.1167378
21.8833332	2.6352000	-1.0921946
21.8833332	2.6840000	-1.3130875
21.8833332	2.7328000	-0.9878836
21.8833332	2.7816000	-0.9940195
21.8833332	2.8304000	-0.9878836
21.8833332	2.8792000	-0.8958449
21.8833332	2.9280000	-0.7731261
21.8833332	2.9768000	-0.5890489
21.8833332	3.0256000	-0.5031457
21.8833332	3.0743999	-0.3865633

21.8833332	3.1231999	-0.4970098
21.8833332	3.1719999	-0.6442719
21.8833332	3.2207999	-0.6688156
21.8833332	3.2695999	-0.8099418
21.8833332	3.3183999	-0.4540582
21.8833332	3.3671999	-0.9878836
21.8833332	3.4159999	-0.9817476
21.8833332	3.4647999	-0.5645051
21.8833332	3.5135999	-0.7363110
21.8833332	3.5623999	-1.1596894
21.8833332	3.6111999	-1.2517285
21.8833332	3.6599998	-1.2640004
21.8833332	3.7087998	-1.3069515
21.8833332	3.7575998	-1.1658254
21.8833332	3.8063998	-1.2333207
21.8833332	3.8551998	-1.3069515
21.8833332	3.9039998	-1.3130875
21.8833332	3.9527998	-1.3008156
21.8833332	4.0016003	-1.2946796
21.8833332	4.0503998	-1.2946796
21.8833332	4.0991998	-1.0369711
21.8833332	4.1479998	-1.0431070
21.8833332	4.1967998	-0.9940195
21.8833332	4.2455997	-1.0124273
21.8833332	4.2943997	-1.0124273
21.8833332	4.3431997	-1.0062914
21.8833332	4.3919997	-1.0062914
21.8833332	4.4407997	-0.9081168
21.8833332	4.4895997	-0.8897090
21.8833332	4.5383997	-0.8160777
21.8833332	4.5871997	-0.8283496
21.8833332	4.6359997	-0.8344855
21.8833332	4.6847997	-0.8222136
21.8833332	4.7336001	-0.7792621
21.8833332	4.7824001	-0.5522332
21.8833332	4.8312001	613586.0000000
21.8833332	4.8800001	613586.0000000
21.8833332	4.9288001	-0.6135921
21.8833332	4.9776001	-0.5460973
21.8833332	5.0264001	613586.0000000
21.8833332	5.0752001	613586.0000000
21.8833332	5.1240001	613586.0000000
21.8833332	5.1728001	613586.0000000
21.8833332	5.2216001	-0.9081168
21.8833332	5.2704000	-0.7608547
21.8833332	5.3192000	613586.0000000
21.8833332	5.3680000	613586.0000000
21.8833332	5.4168000	613586.0000000
21.8833332	5.4656000	613586.0000000
21.8833332	5.5144000	613586.0000000
21.8833332	5.5632000	613586.0000000
21.8833332	5.6120000	613586.0000000
21.8833332	5.6608000	613586.0000000
21.8833332	5.7096000	613586.0000000
21.8833332	5.7584000	613586.0000000
21.8833332	5.8072000	613586.0000000
21.8833332	5.8559999	613586.0000000

21.8833332	5.9047999	613586.0000000
21.8833332	5.9535999	613586.0000000
21.8833332	6.0023999	613586.0000000
21.8999996	0.2928000	-1.2640004
21.8999996	0.3416000	-1.1412816
21.8999996	0.3904000	-1.1535535
21.8999996	0.4392000	-1.5155730
21.8999996	0.4880000	-1.5155730
21.8999996	0.5368000	-1.4112625
21.8999996	0.5856000	-1.4112625
21.8999996	0.6344000	-1.7671461
21.8999996	0.6832000	-1.5094371
21.8999996	0.7320000	-1.5094371
21.8999996	0.7808000	-1.2885437
21.8999996	0.8296000	-1.1596894
21.8999996	0.8784000	-1.1596894
21.8999996	0.9272000	-1.1535535
21.8999996	0.9760000	-1.0921946
21.8999996	1.0247999	-1.1903691
21.8999996	1.0735999	-1.0676508
21.8999996	1.1223999	-1.2762718
21.8999996	1.1711999	-1.1351457
21.8999996	1.2200000	-1.1228738
21.8999996	1.2688000	-1.2455926
21.8999996	1.3176000	-1.0921946
21.8999996	1.3664000	-1.2087770
21.8999996	1.4152000	-1.1167378
21.8999996	1.4640000	-1.1167378
21.8999996	1.5128000	-1.0799227
21.8999996	1.5616000	-1.1044660
21.8999996	1.6104000	-1.0983305
21.8999996	1.6592000	-1.2701364
21.8999996	1.7079999	-1.2455926
21.8999996	1.7567999	-1.2210488
21.8999996	1.8055999	-1.2885437
21.8999996	1.8543999	-1.2885437
21.8999996	1.9031999	-1.0676508
21.8999996	1.9519999	-1.0676508
21.8999996	2.0007999	-1.1965051
21.8999996	2.0495999	-0.9142528
21.8999996	2.0983999	-0.9142528
21.8999996	2.1471999	-1.0431070
21.8999996	2.1959999	-1.0860586
21.8999996	2.2447999	-1.0737867
21.8999996	2.2935998	-0.9449320
21.8999996	2.3423998	-0.9387960
21.8999996	2.3912001	-0.9326606
21.8999996	2.4400001	-0.9203887
21.8999996	2.4888000	-1.0492430
21.8999996	2.5376000	-0.6872234
21.8999996	2.5864000	-0.8160777
21.8999996	2.6352000	-0.8222136
21.8999996	2.6840000	-0.8467574
21.8999996	2.7328000	-0.8590293
21.8999996	2.7816000	-0.8651652
21.8999996	2.8304000	-0.8713012
21.8999996	2.8792000	-0.6135921

21.8999996	2.9280000	-0.9081168
21.8999996	2.9768000	-0.5031457
21.8999996	3.0256000	-0.4540582
21.8999996	3.0743999	-0.1718059
21.8999996	3.1231999	-0.4970098
21.8999996	3.1719999	-0.9878836
21.8999996	3.2207999	-0.9817476
21.8999996	3.2695999	-0.8344855
21.8999996	3.3183999	-0.8406215
21.8999996	3.3671999	-1.0860586
21.8999996	3.4159999	-1.0246992
21.8999996	3.4647999	-0.7915339
21.8999996	3.5135999	-1.4419422
21.8999996	3.5623999	-1.5094371
21.8999996	3.6111999	-1.4910293
21.8999996	3.6599998	-1.3989906
21.8999996	3.7087998	-1.3989906
21.8999996	3.7575998	-1.2517285
21.8999996	3.8063998	-1.2517285
21.8999996	3.8551998	-1.0921946
[truncated: 376,617 more chars]
